# The Amino Acid Composition of Quadruplex Binding Proteins Reveals a Shared Motif and Predicts New Potential Quadruplex Interactors

**DOI:** 10.3390/molecules23092341

**Published:** 2018-09-13

**Authors:** Václav Brázda, Jiří Červeň, Martin Bartas, Nikol Mikysková, Jan Coufal, Petr Pečinka

**Affiliations:** 1 Institute of Biophysics, Academy of Sciences of the Czech Republic v.v.i., Královopolská 135, 612 65 Brno, Czech Republic; jac@ibp.cz; 2 Department of Biology and Ecology/Institute of Environmental Technologies, Faculty of Science, University of Ostrava, 710 00 Ostrava, Czech Republic; Jiri.Cerven@osu.cz (J.Č.); dutartas@gmail.com (M.B.); mikyskova.nikol@gmail.com (N.M.); Petr.Pecinka@osu.cz (P.P.)

**Keywords:** quadruplex binding proteins, protein-DNA interactions, RG-rich domain, amino acid composition

## Abstract

The importance of local DNA structures in the regulation of basic cellular processes is an emerging field of research. Amongst local non-B DNA structures, G-quadruplexes are perhaps the most well-characterized to date, and their presence has been demonstrated in many genomes, including that of humans. G-quadruplexes are selectively bound by many regulatory proteins. In this paper, we have analyzed the amino acid composition of all seventy-seven described G-quadruplex binding proteins of Homo sapiens. Our comparison with amino acid frequencies in all human proteins and specific protein subsets (e.g., all nucleic acid binding) revealed unique features of quadruplex binding proteins, with prominent enrichment for glycine (G) and arginine (R). Cluster analysis with bootstrap resampling shows similarities and differences in amino acid composition of particular quadruplex binding proteins. Interestingly, we found that all characterized G-quadruplex binding proteins share a 20 amino acid long motif/domain (RGRGR GRGGG SGGSG GRGRG) which is similar to the previously described RG-rich domain (RRGDG RRRGG GGRGQ GGRGR GGGFKG) of the FRM1 G-quadruplex binding protein. Based on this protein fingerprint, we have predicted a new set of potential G-quadruplex binding proteins sharing this interesting domain rich in glycine and arginine residues.

## 1. Introduction

The discovery of the B-DNA structure in 1953 [1] provided an explanation of basic genetic and related biological processes. Although B-DNA is the most abundant DNA structure, later discoveries pointed to the dynamic nature of DNA, which leads to many alternative DNA forms with important functional roles. These structures were originally called “unusual” DNA structures, as it was thought that they were rare [2,3,4,5]. However, it has been demonstrated that non-B structures are common in the genomes of all organisms, and play important roles in the regulation of many biological functions. The energy for formation of these structures usually originates from negative DNA supercoiling, and the binding of various proteins stabilizes different DNA structures. Many DNA structures have been described, but cruciforms, left-handed Z-DNA, triplexes, and quadruplexes are the most well-known [2,6,7].

A tetrameric arrangement of guanines was first described using crystallographic methods in 1962 [8], and many reports have subsequently verified their existence [9,10,11,12]. Due to the accessibility of bioinformatic tools, it has been found that sequences able to form G-quadruplexes are distributed non-randomly throughout the human and other mammalian genomes, and more than 40% of human genes contain G-rich areas with the potential to form G-quadruplexes [13,14,15,16,17,18]. It was also demonstrated that G-rich sequences can contribute to genome instability [19], and they are being studied intensively as potential targets for drug design [20,21,22]. G-quadruplex ligands can regulate protein expression [23]. Recently, G-quadruplexes were found in mitochondrial DNA, with functions similar to nuclear DNA [24]. RNA can also form quadruplex structures, and it was shown that G-quadruplexes could be formed in the majority of mRNA 5′ untranslated regions [25]. G-quadruplexes have been localized in vivo using specific antibodies and/or intercalating fluorescence compounds that bind and stabilize G-quadruplexes in both DNA and RNA [8,26,27].

Protein-DNA interactions are essential for all organisms. Besides proteins that bind to a particular DNA sequence, there is a group of proteins that bind specifically to various local DNA structures, e.g., to single stranded DNA [28], cruciform structures [29], or quadruplexes [30]. For example, the formation of local DNA structures is an important determinant for effective binding of the tumor suppressor protein p53 [31,32]. Imbalance of mutant p53 protein binding to target sites and local DNA structures seems to be an important part of its gain of function during tumorigenesis [33]. As well as other noncanonical DNA structures, G-quadruplexes are also recognized and bound by specific proteins [30]. While a decade ago only a few proteins with quadruplex binding specificity had been characterized, nowadays a database for proteins that interact with G-quadruplexes contains more than 200 quadruplex binding proteins from various organisms (G4IPDB) [34]. Papers focusing on finding novel G-quadruplex binders have been published; for example, SILAC was used as a quantitative proteomic approach [35]. It has been shown that G-quadruplex binding proteins can stabilize or unfold these structures. Specificity for different structures can be through quadruplex strand orientation, loop length, duplex-quadruplex junctions, groove of the G-quartet barrel, and/or a combination of these properties [24,36]. Besides the well-studied role of G-quadruplex binders in transcriptional regulation, it has been found recently that G-quadruplex binding proteins can have other roles. For example, murine Rif1 organizes higher-order chromatin architectures through its ability to bind several quadruplexes at the same time. This protein can form tetra-, octa- and dodecamers, which allows it to hold different numbers of chromatin fibers together through binding to distant G-quadruplex loci [37]. Because of the diverse biological roles of G-quadruplex binding proteins, we expect the repertoire of G-quadruplex interacting proteins to grow in the future.

Although information about G-quadruplex binding proteins has increased rapidly, there are no statistical studies focused on their amino acid composition. Many recent studies used amino acid composition to predict intricate protein functions [38,39,40,41]. Therefore, in this study we performed detailed analyses of the amino acid residue composition of all known human G-quadruplex binding proteins. Interestingly, we found not only typical enrichment and depletion for several amino acid residues in G-quadruplex binding proteins, but we have also shown the presence of a conserved RG-rich domain as a typical feature for G-quadruplex binding proteins.

## 2. Methods

### 2.1. Amino Acid Composition Analyses

All quadruplex binding proteins known to date were obtained from G4IPDB (a database for G-quadruplex structure forming nucleic acid interacting proteins, accessed from curated database http://bsbe.iiti.ac.in/bsbe/ipdb/index.php) [34] and from additional literature resources [30,42,43,44,45,46,47,48,49]. Canonical amino acid sequences of these seventy-seven known quadruplex binding proteins (Supplementary material S1) were obtained from the UNIPROT database [50] (Homo sapiens). These sequences were inserted into ProtParam tool (https://web.expasy.org/protparam/) [51], their amino acid composition was extracted to Excel 2007, and the matrix of amino acid composition of the individual quadruplex binding proteins was constructed. In the first approximation, the computed mean values of amino acid composition were compared with the expected values, which are human mean amino acid frequencies [52], and the relative amino acid enrichments or depletions were computed (Supplementary material S2).

To be able to use statistical methods, we used defined sets of proteins, and the relative enrichment or depletion of particular amino acid residues of the 77 quadruplex binding proteins were computed using the web-based tool, Composition Profiler (http://www.cprofiler.org/) [53]. Using the function sample in R, we randomly sub-sampled 5000 proteins from all 20,141 human canonical protein sequences obtained from UNIPROT Reviewed Database [50] (Supplementary material S3A). We subsequently compared the amino acid composition of the 77 quadruplex binding proteins with these 5000 randomly sampled sequences (Supplementary material S3B), with a set of 15,224 human nucleic acid binding proteins including isoforms (GO:0003676, Supplementary material S3C), and with a set of 2565 Golgi apparatus proteins including isoforms (as an unrelated negative control) (GO:0044431, Supplementary material S3D) obtained from UNIPROT Reviewed Database [50] and from Ensembl Biomart [54].

To obtain 2-mer amino acid compositions of quadruplex binding proteins, ProtrWeb tool (http://protrweb.scbdd.com/) [55] was used. Data are available in Supplementary material S4 (2-mers). We then compared these data with the 2-mer amino acid compositions of 291 nucleic acid binding proteins (randomly subsampled from Supplementary material S3C due to computational limitations of the server). For a longer K-mer search, we inspected the HRaP database (http://bioinfo.protres.ru/hrap/) (Supplementary material S5) [56].

### 2.2. Correlation Analysis of Quadruplex Binding Proteins Amino Acid Composition

To find out if there are significant correlations between particular amino acids within the set of 77 quadruplex binding proteins, we used an amino acid composition matrix of the above 77 quadruplex binding proteins in R package “corrplot” [57]. The complete source code is available in Supplementary material S6.

### 2.3. Cluster Dendrogram Analysis Based on Amino Acid Composition Matrix

A tree diagram was constructed using the R package “pvclust” [58]. Bootstrap resampling (n = 10,000) and average cluster method were used to construct a cluster dendrogram (the choice of the best cluster method was validated through the function seplot). 

### 2.4. Cluster Dendrogram Analysis Based on CLAP Approach

All 77 quadruplex binding proteins sequences were uploaded to the CLAP web server (http://nslab.mbu.iisc.ernet.in/clap/run.html); default parameters were used. CLAP is an alignment free approach that computes local similarities among selected sequences, and allows comparisons of proteins with multiple domains. Such clusters show high functional and domain architectural similarities [59,60,61]. The resulting dendrogram in newick format (Supplementary material S7) was visualized using iTOL [62] (Supplementary material S8).

### 2.5. Motif Scanning of Known Quadruplex Interaction Sequence from FMR1

All 77 quadruplex binding protein sequences were uploaded to the MEME web server (http://meme-suite.org/tools/fimo), and the FIMO tool [63] was used for analysis of motif occurrence from FMR1 (RRGDGRRRGGGGRGQGGRGRGGGFKG). The selected *p*-value threshold was 0.01. All 2106 hits are listed in Supplementary material S9.

### 2.6. De novo Sequence Logo Generation

To investigate whether the 77 quadruplex binding proteins share a common sequence motif, we performed a GLAM2 search. The GLAM2 web tool (http://meme-suite.org/tools/glam2, allocated in MEME Suite v. 5.0.1) allows discovery of novel, gapped motifs in protein sequences [64,65]. Default search parameters were used. Raw results of this analysis are provided in Supplementary materials S10 and S11.

### 2.7. Motif Alignments

For motif alignments and visualization, the free bioinformatic software UGENE was used [66].

### 2.8. Protein Functional Network Analysis

We used the STRING web server (https://string-db.org/) [67,68] with default parameters to investigate whether the selected set of quadruplex binding proteins forms a functionally enriched network.

### 2.9. Prediction of New Quadruplex Binding Proteins

The FIMO tool was used for the prediction of new quadruplex binding proteins [63]. Analysis of motif occurrence (RGRGR GRGGG SGGSG GRGRG) was performed against the set of human nucleic acid binding proteins (Supplementary material S3C). The selected *p*-value threshold was 0.1. The best 100 results were filtered using Excel.

## 3. Results

### 3.1. Amino Acid Residue Composition Analyses—Identifications of Distinct Enrichments and Depletions in Human Quadruplex Binding Proteins

**Figure 1:**
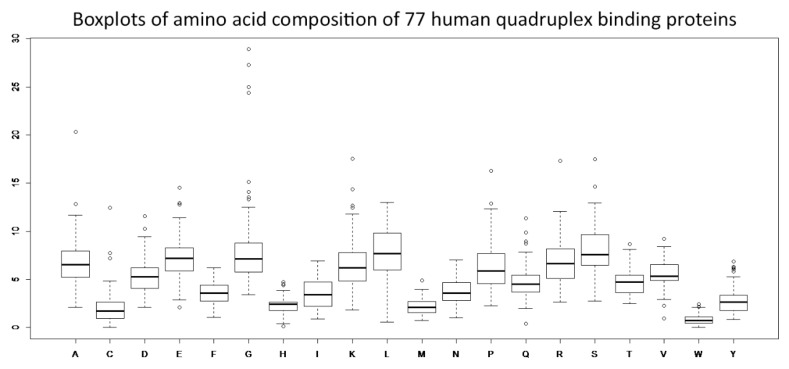
Boxplots of amino acid compositions of 77 quadruplex binding proteins. Thick horizontal lines within boxplots denote mean amino acid composition values. Data within boxes span the interquartile range and whiskers show the lowest and highest values within 1.5 interquartile range. Empty circles denote outliers.

The G4IPDB (a database for G-quadruplex structure forming nucleic acid interacting proteins) has been recently established [34]. This database contains information on 70 human DNA- and RNA-quadruplex binding proteins. All of these proteins have been validated by multiple in vitro and in vivo experiments. In addition to the proteins located in this database, we found seven other human proteins with quadruplex binding preferences in the literature [30,42,43,44,45,46,47,48,49]. Therefore, we included 77 human quadruplex binding proteins in our analyses of amino acid residue composition. We analyzed their amino acid compositions compared to the average amino acid composition of the human proteome by the protParam tool [51,52]. The matrix of the amino acid composition of these quadruplex binding proteins is shown in Supplementary material S2. Detailed statistical characteristics (variance, outliers) are depicted in boxplots (Figure 1).

Based on relative enrichment or a depletion of greater than 10% in comparison to the expected values in the human proteome, the most distinctive enrichments were found for glycine (G), arginine (R), lysine (K) and aspartate (D), while the most notable depletions were detected for tryptophan (W), leucine (L), isoleucine (I), histidine (H), cysteine (C) and threonine (T) (Supplementary material S2). Among the outliers (Figure 1, empty circles) are, for example, FUS with glycine enrichment and SRSF1 with glutamine depletion. All outlier proteins are highlighted in green (enrichment) or red (depletion) in Supplementary material S2.

**Figure 2:**
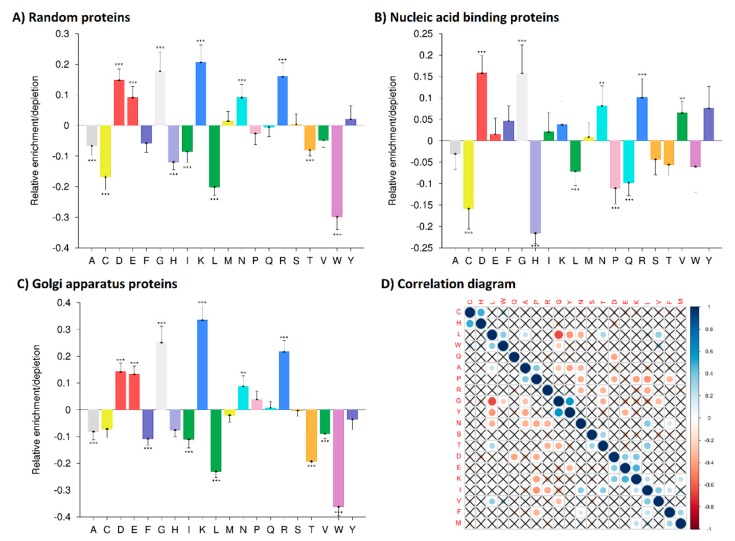
Relative enrichment or depletion of individual amino acids in 77 quadruplex binding proteins versus (**A**) 5000 randomly subsampled human proteins from UNIPROT database, (**B**) 15,224 nucleic acid binding proteins including isoforms from Ensembl Biomart, (**C**) 2565 Golgi apparatus proteins including isoforms from Ensembl Biomart. The analyses were performed using Composition Profiler (10,000 bootstrap iterations with Bonferroni correction for testing multiple hypotheses). Using Bonferroni correction, only values lower than 0.0025 were taken as significant (* *p* < 0.0025; ** *p* < 0.0010; *** *p* < 0.0001). *p*-values are shown in Supplementary material S13. (**D**) Correlation diagram of amino acid content in quadruplex binding proteins. Non-significant correlations (*p*-values > 0.05) are crossed out.

To obtain statistical information about amino acid composition differences, we used the Composition Profiler program. We compared the amino acid compositions of all quadruplex binding proteins with three specific protein groups—first: A random subset of the human proteome (5000 proteins); second: A well-defined group of nucleic acid binding proteins including isoforms (15,224 protein sequences), third: Golgi apparatus proteins including isoforms (2565 sequences), all obtained from the UNIPROT Reviewed Database. The relative enrichments or depletions of quadruplex binding protein amino acid compositions in comparison with these protein groups are shown in Figure 2. Exact *p*-values are listed in Supplementary material S12.

Differences in amino acid composition compared to a random subset of the human proteome were confirmed (Figure 2A). The largest changes were found for lysine (K) (enrichment) and tryptophan (W) (depletion). Statistically significant changes were found for lysine (K), glycine (G), arginine (R), aspartate (D), glutamate (E) and asparagine (N) (enrichments) and for tryptophan (W), leucine (L), cysteine (C), histidine (H), isoleucine (I), threonine (T) and alanine (A) (depletions). Differences of amino acid residue composition compared to a well-defined group of nucleic acid binding proteins including isoforms are depicted below (Figure 2B). Interestingly, even though the overall amino acid residue compositions were similar for DNA binding proteins and quadruplex binding proteins, we observed statistically significant changes for several amino acid residues. The most evident changes were glycine (G) enrichment and histidine (H) depletion in quadruplex binding proteins. Statistically significant changes were also found for aspartate (D), arginine (R), asparagine (N) and valine (V) (enrichments) and for cysteine (C), proline (P), glutamine (Q) and leucine (L) (depletions). Differences of amino acid composition of quadruplex binding proteins compared to Golgi apparatus proteins as an unrelated group were expected, and are shown in Figure 2C. The biggest changes were found for lysine (K) (enrichment) and tryptophan (W) (depletion). Statistically significant changes were found for lysine (K), glycine (G), arginine (R), aspartate (D), glutamate (E) and asparagine (N) (enrichments), and for tryptophan (W), leucine (L), threonine (T), isoleucine (I), phenylalanine (F), alanine (A) and valine (V) (depletions).

2-mer amino acid composition of quadruplex binding proteins showed that the most abundant is GG, followed by SS, EE, and AA, which are also very frequent in DNA-binding proteins (Supplementary material S4). The main difference among DNA-binding proteins and G-quadruplex binding proteins is the abundance of GG in G-quadruplex binding proteins (Supplementary material S4, yellow), depletion of LL for quadruplex-binding proteins (blue), and the particular abundance of GR and RG sequences in quadruplex-binding proteins (orange). Longer K-mer searches did not show any sequences typical for all quadruplex binding proteins (Supplementary material S5).

### 3.2. Correlation Analysis of Human CBPs Amino Acid Composition

The correlation diagram (Figure 2D) demonstrates the relationships between each individual amino acid with all other amino acids in our quadruplex binding protein dataset. Leucine (L) and alanine (A) contents are negatively correlated with glycine (G), tyrosine (Y) and asparagine (N). Proline (P) and arginine (R) content are negatively correlated with asparagine (N) and isoleucine (I). The content of glycine (G) is positively correlated with tyrosine. Finally, the histidine (H) content is positively correlated with cysteine (C). Non-significant correlations (*p*-value > 0.05) are crossed out.

### 3.3. Cluster Analyses

**Figure 3:**
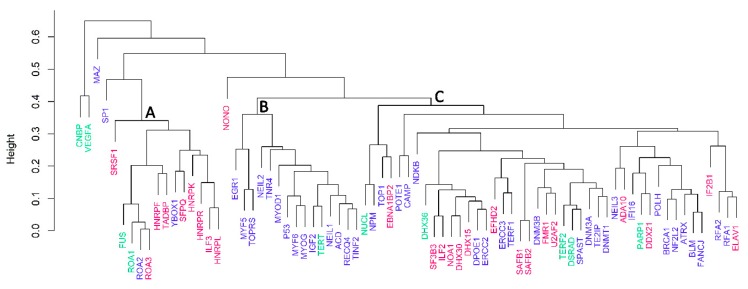
Cluster dendrogram of 77 quadruplex binding proteins based on their amino acid compositions constructed using R package *pvclust* and bootstrap resampling (n = 10,000) with average cluster method. For precise cluster determination, AU values equal to or greater than 95 were chosen as cut-off criterion; the three resulting main clusters (A,B,C) are marked. Protein symbols are highlighted either in red (only RNA quadruplex binding), in blue (only DNA quadruplex binding), or in green (both RNA and DNA quadruplex binding).

To compare the relationship of quadruplex binding proteins according to their amino acid composition, we used statistical clustering (R package pvclust, Figure 3). Based on the cluster dendrogram (Figure 3A), we could clearly discriminate at least three main closely related clusters (**A**—FUS, ROA1, ROA2, ROA3, HNRPF, TADBP, YBOX1, SFPQ, HNRPK, HNRPR, ILF3, HNRPL; **B**—EGR1, MYF5, TOPRS, NEIL2, TNR4, MYOD1, p53, MYF6, MYOG, IGF2, TERT, NEIL1, ACD, RECQ4, TINF2; **C**—NUCL, NPM, TOP1, EBNA1BP2, POTE1, CAMP, NDKB, DHX36, SF3B3, ILF2, NOA1, DHX30, DHX15, DPOE1, ERCC2, EFHD2, ERCC3, TERF1, SAFB1, SAFB2, DNM3B, FMR1, U2AF2, TERF2, DSRAD, SPAST, DNM3A, TE2IP, DNMT1, NEIL3, ADA10, IF16, PARP1, DDX21, POLH, BRCA1, NF2L2, ATRX, BLM, FANCJ, RFA2, RFA1, ELAV1) of proteins supported by approximately unbiased values (AU equal to or greater than 95 is considered to be statistically significant). Most proteins in group A recognize RNA-quadruplexes, whereas proteins in group B are DNA-quadruplex binding proteins, suggesting that amino acid residue composition is important for distinguishing between DNA and RNA quadruplex binding proteins, and implying that different molecular mechanisms of recognition may be involved in various groups of quadruplex binding proteins. Group C contains proteins that bind only DNA or only RNA quadruplexes, or bind to both DNA and RNA quadruplexes. Three proteins, CNBP, VEGFA, and MAZ, were not located in these clusters, and will be discussed below.

### 3.4. Novel Interesting Quadruplex Interaction Motif (NIQI)

**Figure 4:**
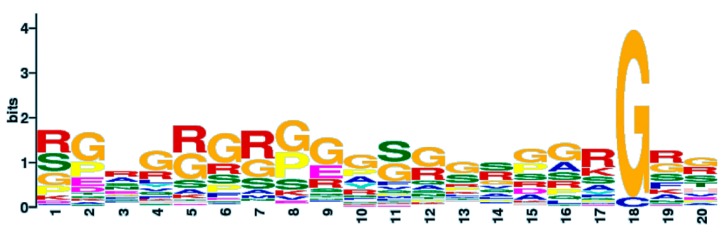
Novel interesting quadruplex interaction motif (NIQI) common to most quadruplex binding proteins.

Due to the relatively large number of quadruplex binding proteins available for analysis, we used their sequences to find out if they share a common motif(s) using GLAM2 software [64,65]. Our results revealed a common RG/rich sequence RGRGRGRGGGSGGSGGRGRG that is shared by quadruplex binding proteins (Figure 4). We propose the name NIQI (Novel Interesting Quadruplex Interaction motif) for this newly found protein motif/domain, in conjunction with its quadruplex binding ability.

**Figure 5:**
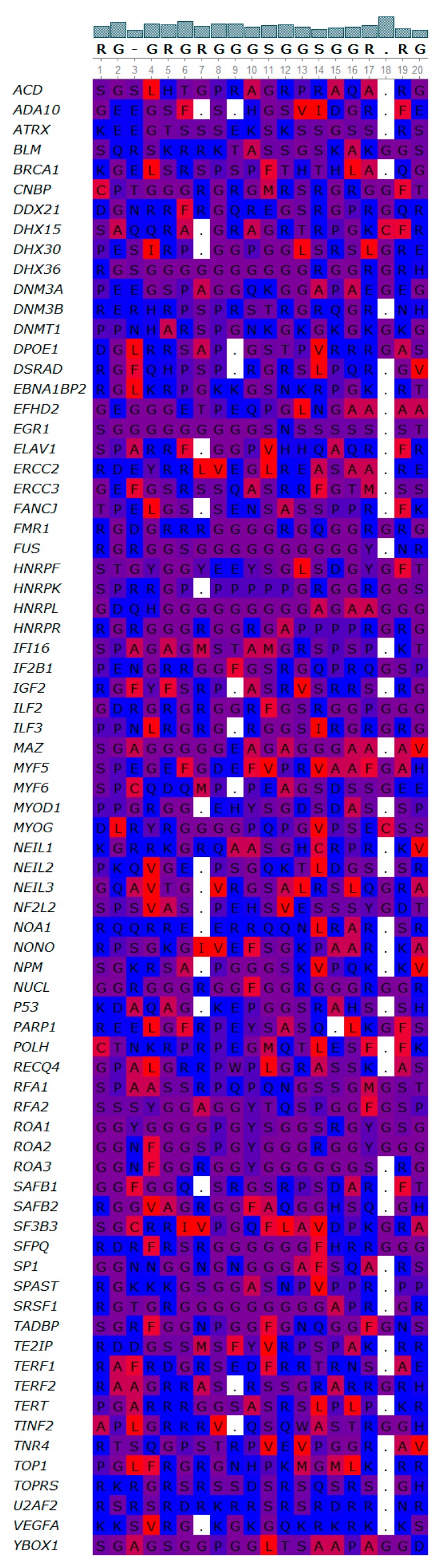
Overlay of NIQI sequence for individual proteins. Gaps are depicted with dots. Colors show hydrophobicity—blue are hydrophilic amino acid residues, red are hydrophobic amino acid residues.

Interestingly, this motif is formed almost exclusively by R and G amino acid residues with a few alternations of S. Using the UGENE software, we display an overlay of this sequence for individual proteins (Figure 5).

The RGRGRGRGGGSGGSGGRGRG motif is very similar (75% in 20 amino acid aligned loci) to a motif derived from the FMR1 protein. The direct interaction of FMR1 protein with quadruplex DNA has recently been shown [69]. The crystal structure of the complex between the human FMR1 RGG peptide bound to G-rich RNA in vitro revealed the importance of the RGG motif for this FMR1 binding to quadruplex RNA. By amino acid composition analysis, we found an enrichment of the R and G residues in other quadruplex binding proteins; therefore, we also analyzed the presence of the RRGDGRRRGGGGRGQGGRGRGGGFKG motif from FMR1 in all quadruplex binding proteins. All quadruplex binding proteins share regions with similarity to this RGG-rich sequence; 55 quadruplex binding proteins with *q* ≤ 0.05 significance, 8 proteins with *q* ≤ 0.1 significance and 14 proteins with *q* > 0.1 significance (Supplementary material S13). The alignment of all sequences using UGENE is shown in Supplementary material S14.

**Figure 6:**
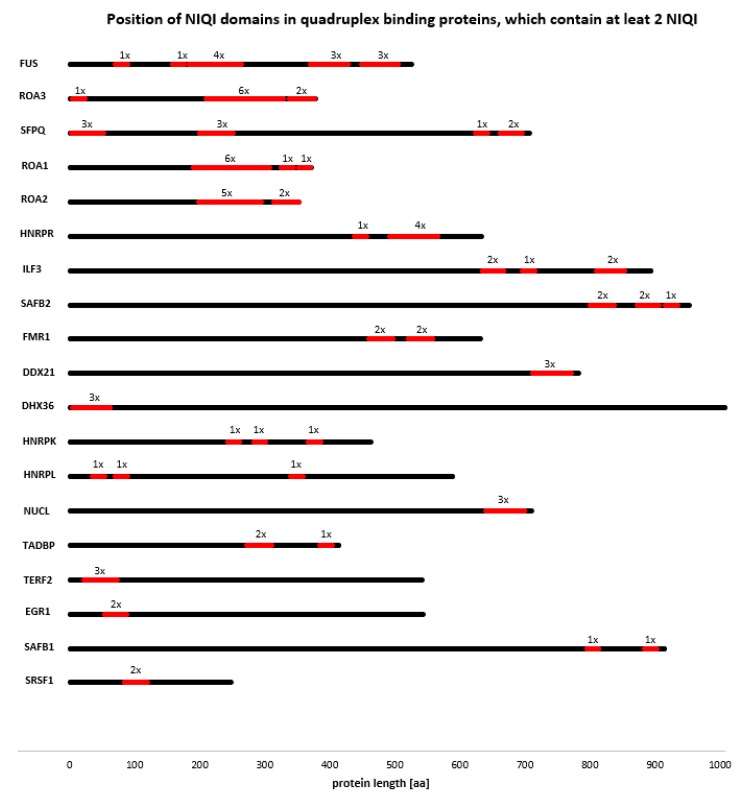
Location of the NIQI motifs in quadruplex binding proteins containing at least two motifs.

**Figure 7:**
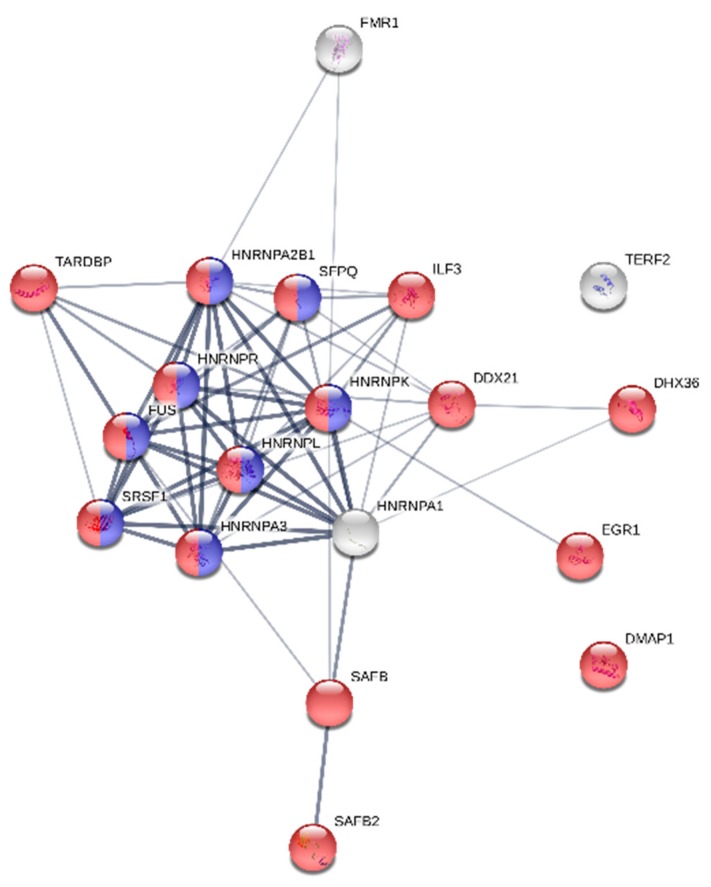
STRING Interaction network of quadruplex binding proteins containing at least two NIQI motifs. Eight quadruplex binding proteins in blue highlighted rings fall into GO:0000398 category (mRNA splicing, via spliceosome) with FDR = 2.12 × 10^−8^. Sixteen quadruplex binding proteins in red highlighted rings fall into GO:0016070 category (RNA metabolic process) with FDR = 1.49 × 10^−7^.

We also found that many quadruplex binding proteins contain two or more NIQI motifs, often repeated two or more times in the same region of the protein (Figure 6). DHX36 and TERF2 contain three NIQI motifs in a row near their N termini. Moreover, proteins containing at least two NIQI motifs form a strong functional interaction network (Figure 7). These results indicate that the RGRGRGRGGGSGGSGGRGRG motif is a common feature of quadruplex binding proteins.

Therefore, we analyzed the entire human proteome for the presence of the NIQI motif at *q* ≤ 0.1 significance. Besides already known quadruplex binding proteins, we found proteins with highly significant NIQI motifs, from which we selected 100 proteins with the best match to the NIQI motif, suggesting that these proteins could be novel quadruplex binding proteins (Supplementary material S15). Interestingly, among these proteins are 22 zinc-finger proteins (Supplementary material S15, green)—a surprising finding given that zinc-finger proteins are abundant in cysteine and histidine residues (which are less represented in quadruplex binding proteins), although these particular zinc finger proteins are abundant in glycine (G) and arginine (R) outside of their zinc finger domains. This group of proteins could theoretically combine binding to the DNA sequence through the zinc finger with binding to quadruplex structures through the NIQI motif. These additional functions could be crucial for determining functional activities within the genome. The second most abundant group in our NIQI predicted proteins are 21 ribonucleoproteins (Supplementary material S15, orange), and the third comprises 15 homeobox proteins. All NIQI-predicted quadruplex binding proteins are listed in Supplementary material S16.

## 4. Discussion

The increasing number of G-quadruplex binding proteins identified in recent years points to the importance of G-quadruplex recognition for important biological processes. The increased data of G-quadruplex proteins allowed a statistically relevant study of amino acid composition in these proteins. It has been demonstrated that the G-quadruplex binding domain depends on the β-spiral structure of the RGG domain in FUS [70]. Moreover, the RGGGGR peptide derived from FMR1 protein stabilizes the transition from G4 to duplex by filling the junction between them with base stacking and Hoogsteen type hydrogen bonds with the double stranded region [71,72]. The RGG/RG motif has been found in more than 1000 human proteins that influence processes including transcription, pre-mRNA splicing, DNA damage signaling, mRNA translation, and apoptosis. They are also associated with several diseases, including neurological and neuromuscular diseases, and cancer [73]. Our results show that the amino acid composition of the known human G-quadruplex binding proteins differs from that of other human proteins, including DNA-binding proteins, with significant enrichments for lysine (K), glycine (G) and arginine (R); 2-mer search showed an abundance of RR, GR, and RG sequences, compared to DNA-binding proteins. Both arginine and glycine are well known disorder-promoting amino acid residues, and their flexibility (especially of glycine amino acid residues) is relatively high [53,74]. From this point of view, we suggest that NIQI regions are likely to be intrinsically disordered to enable arginine residues to interact with, and possibly form, hydrogen bonds with DNA in a wide portfolio of G-quadruplex structures (parallel, antiparallel or mixed types), maybe even differing by the number of guanine tetrads [75]. G-quadruplex binding proteins should therefore be considered a specific group of proteins with unique characteristics.

A cluster dendrogram based on amino acid compositions shows three main clusters of G-quadruplex binding proteins. One group was formed mainly by DNA quadruplex binding proteins, the second by RNA quadruplex binding proteins, and the third by a mix of various proteins including topoisomerases, transcription factors, and both DNA and RNA binding proteins. However, three proteins do not fit into any of these clustered groups. CNBP is a nucleic acid-binding protein that preferentially binds and stabilizes DNA quadruplex formed in the *MYC* gene regulatory region, NHEIII1 [45]. CNBP is extremely rich in cysteine residues (12.43%; 1.59% is the median value in the set of 77 quadruplex binding proteins), and histidines (4.52%; 2.43% is the median of the 77 quadruplex binding proteins). CNBP is a short nucleic acid binding protein (177 aa), and contains seven zinc finger motifs. Tetranucleotide expansion (CCTG) may occur in intron 1 of this gene—5000 repetitions—related to Myotonic Dystrophy type 2 [76]. The second non-classified protein, VEGFA (vascular endothelial growth factor A) preferentially binds DNA and RNA quadruplexes [48,77]. This protein is also rich in cysteines (7.76%) and histidines (4.74%) but does not contain zinc finger domains. Finally, MAZ (Myc-associated zinc finger protein) preferentially binds and unfolds DNA quadruplexes [78]. This protein has an extremely high content of alanine (20.34%; 6.56 is the median value in the set of 77 quadruplex binding proteins) and histidine (4.40%).

G-quadruplexes are widespread in the human genome, and have important roles in diverse biological processes [79,80]. Targeting G-quadruplex structures is, therefore, a promising approach to modify aberrant disease-associated processes. Using GLAM software, we discovered the NIQI motif RGRGRGRGGGSGGSGGRGRG, which is shared by quadruplex binding proteins. The identification of this motif allowed us to predict new potential quadruplex binding proteins. Importantly, it has been shown that synthetically engineered RGG motif specifically binds and stabilizes the G-quadruplexes of human telomerase [81]. Thus, the NIQI motif (or portions thereof) may similarly allow the modification of G-quadruplex stability, or may be used to inhibit binding of endogenous quadruplex binding proteins to modulate transcription, translation or other processes. We found NIQI motifs in several zinc-finger proteins, suggesting that B-DNA binding with G-quadruplex binding could enhance and/or specify the function of some of these proteins. The abundance of ribonucleoproteins and homeobox proteins with highly significant NIQI motifs points to the possible importance of G-quadruplex proteins in human ontogenesis and processes connected to development of human diseases. These data will allow further investigations into the physiological functions of these proteins within the larger family of DNA-binding proteins, such as relative contributions to transcription and DNA repair, and potential roles in inborn errors in DNA metabolism, chromatin remodeling, or transcription.

## 5. Conclusions

In this research, we have analyzed the amino acid residue composition of 77 known human quadruplex-binding proteins. We demonstrated that the composition of these proteins is unique within the human proteome, including specific comparisons to DNA-binding proteins, with significant enrichments (G,D,R,N,V) and depletions (H,C,P,Q,L). We discovered a new 20 amino acid motif (termed NIQI) common to human quadruplex binding proteins, comprised mainly of glycine and arginine residues. Based on this model, we identified additional human proteins that contain highly homologous sequences, and which are therefore potential quadruplex binding proteins. We expect that our findings will contribute to the identification and characterization of G-quadruplex binding proteins, and to the development of tools for the optimization of proteins, peptides, or other small molecules that bind to these structures for potential clinical application.

## Supplementary Materials

The following are available online at http://www.mdpi.com/1420-3049/23/9/2341/s1, Supplementary material S1: Canonical amino acid sequences of seventy-seven known quadruplex binding proteins, Supplementary material S2: Matrix of amino acid composition of the individual quadruplex binding proteins, Supplementary material S3A: All 20,141 human canonical protein sequences obtained from UNIPROT Reviewed Database, Supplementary material S3B: Randomly sub-sampled 5000 proteins from all 20,141 human canonical protein sequences obtained from UNIPROT Reviewed Database, Supplementary material S3C: Set of 15,224 human nucleic acid binding proteins including isoforms, Supplementary material S3D: Set of 2 565 Golgi apparatus proteins including isoforms, Supplementary material S4: 2-mer amino acid compositions of quadruplex binding proteins, Supplementary material S5: Longer K-mer searches, Supplementary material S6: Correlation Analysis of Quadruplex Binding Proteins Amino Acid Composition R source code, Supplementary material S7: CLAP dendrogram in newick format, Supplementary material S8: Dendrogram visualized in iTOL, Supplementary material S9: All 2106 MEME hits, Supplementary material S10: Raw results of GLAM2 analyses (1), Supplementary material S11: Raw results of GLAM2 analyses (2), Supplementary material S12: Composition Profiler exact *p*-values, Supplementary material S13: Shared regions with similarity to FMR1 RGG-rich sequence detailed results, Supplementary material S14: The alignment of all sequences using UGENE, Supplementary material S15: 100 best results of newly predicted quadruplex binding proteins based on presence of NIQI domain (RGRGRGRGGGSGGSGGRGRG), Supplementary material S16: All results of newly predicted quadruplex binding proteins based on presence of NIQI domain (also transcript variants are included) with the q-value lower than 0.1.
