# Supplementary material for: The Amino Acid Composition of Quadruplex Binding Proteins Reveals a Shared Motif and Predicts New Potential Quadruplex Interactors
Source: Molecules. 2018 Sep 13;23(9):2341. doi: 10.3390/molecules23092341 (PMC6225207; doi:10.3390/molecules23092341)
Supplement: Supplementary file 1 [file molecules-23-02341-s001.zip › molecules-355581-supplementary/Supplementary material 1.pdf]

>sp|P62633|CNBP_HUMAN Cellular nucleic acid-binding protein OS=Homo sapiens OX=9606 GN=CNBP PE=1 SV=1
MSSNECFKCGRSGHWARECPTGGGRGRGMRSRGRGGFTSDRGFQFVSSSLPDICYRCGES
GHLAKDCDLQEDACYNCGRGGHIAKDCKEPKREREQCCYNCGKPGHLARDCDHADEQKCY
SCGEFGHIQKDCTKVKCYRCGETGHVAINCSKTSEVNCYRCGESGHLARECTIEATA

>sp|P01344|IGF2_HUMAN Insulin-like growth factor II OS=Homo sapiens OX=9606 GN=IGF2 PE=1 SV=1
MGIPMGKSMLVLLTFLAFASCCIAAYRPSETLCGGELVDTLQFVCGDRGFYFSRPASRVS
RRSRGIVEECCFRSCDLALLETYCATPAKSERDVSTPPTVLPDNFPRYPVGKFFQYDTWK
QSTQRLRRGLPALLRARRGHVLAKELEAFREAKRHRPLIALPTQDPAHGGAPPEMASNRK

>sp|P56270|MAZ_HUMAN Myc-associated zinc finger protein OS=Homo sapiens OX=9606 GN=MAZ PE=1 SV=1
MFPVFPCTLLAPPFPVLGLDSRGVGGLMNSFPPPQGHAQNPLQVGAELQSRFFASQGCAQ
SPFQAAPAPPPTPQAPAAEPLQVDLLPVLAAAQESAAAAAAAAAAAAAVAAAPPAPAAAS
TVDTAALKQPPAPPPPPPPVSAPAAEAAPPASAATIAAAAATAVVAPTSTVAVAPVASAL
EKKTKSKGPYICALCAKEFKNGYNLRRHEAIHTGAKAGRVPSGAMKMPTMVPLSLLSVPQ
LSGAGGGGGEAGAGGGAAAVAAGGVVTTTASGKRIRKNHACEMCGKAFRDVYHLNRHKLS
HSDEKPYQCPVCQQRFKRKDRMSYHVRSHDGAVHKPYNCSHCGKSFSRPDHLNSHVRQVH
STERPFKCEKCEAAFATKDRLRAHTVRHEEKVPCHVCGKMLSSAYISDHMKVHSQGPHHV
CELCNKGTGEVCPMAAAAAAAAAAAAAAVAAPPTAVGSLSGAEGVPVSSQPLPSQPW

>sp|P09651|ROA1_HUMAN Heterogeneous nuclear ribonucleoprotein A1 OS=Homo sapiens OX=9606 GN=HNRNPA1 PE=1 SV=5
MSKSESPKEPEQLRKLFIGGLSFETTDESLRSHFEQWGTLTDCVVMRDPNTKRSRGFGFV
TYATVEEVDAAMNARPHKVDGRVVEPKRAVSREDSQRPGAHLTVKKIFVGGIKEDTEEHH
LRDYFEQYGKIEVIEIMTDRGSGKKRGFAFVTFDDHDSVDKIVIQKYHTVNGHNCEVRKA
LSKQEMASASSSQRGRSGSGNFGGGRGGGFGGNDNFGRGGNFSGRGGFGGSRGGGGYGGS
GDGYNGFGNDGGYGGGGPGYSGGSRGYGSGGQGYGNQGSGYGGSGSYDSYNNGGGGGFGG
GSGSNFGGGGSYNDFGNYNNQSSNFGPMKGGNFGGRSSGPYGGGGQYFAKPRNQGGYGGS
SSSSSYGSGRRF

>sp|P19338|NUCL_HUMAN Nucleolin OS=Homo sapiens OX=9606 GN=NCL PE=1 SV=3
MVKLAKAGKNQGDPKKMAPPPKEVEEDSEDEEMSEDEEDDSSGEEVVIPQKKGKKAAATS
AKKVVVSPTKKVAVATPAKKAAVTPGKKAAATPAKKTVTPAKAVTTPGKKGATPGKALVA
TPGKKGAAIPAKGAKNGKNAKKEDSDEEEDDDSEEDEEDDEDEDEDEDEIEPAAMKAAAA
APASEDEDDEDDEDDEDDDDDEEDDSEEEAMETTPAKGKKAAKVVPVKAKNVAEDEDEEE
DDEDEDDDDDEDDEDDDDEDDEEEEEEEEEEPVKEAPGKRKKEMAKQKAAPEAKKQKVEG
TEPTTAFNLFVGNLNFNKSAPELKTGISDVFAKNDLAVVDVRIGMTRKFGYVDFESAEDL
EKALELTGLKVFGNEIKLEKPKGKDSKKERDARTLLAKNLPYKVTQDELKEVFEDAAEIR
LVSKDGKSKGIAYIEFKTEADAEKTFEEKQGTEIDGRSISLYYTGEKGQNQDYRGGKNST
WSGESKTLVLSNLSYSATEETLQEVFEKATFIKVPQNQNGKSKGYAFIEFASFEDAKEAL
NSCNKREIEGRAIRLELQGPRGSPNARSQPSKTLFVKGLSEDTTEETLKESFDGSVRARI

VTDRETGSSKGFGFVDFNSEEDAKAAKEAMEDGEIDGNKVTLDWAKPKGEGGFGGRGGGR
GGFGGRGGGRGGRGGFGGRGRGGFGGRGGFRGGRGGGGDHKPQGKKTKFE

>sp|P06748|NPM_HUMAN Nucleophosmin OS=Homo sapiens OX=9606 GN=NPM1 PE=1 SV=2
MEDSMDMDMSPLRPQNYLFGCELKADKDYHFKVDNDENEHQLSLRTVSLGAGAKDELHIV
EAEAMNYEGSPIKVTLATLKMSVQPTVSLGGFEITPPVVLRLKCGSGPVHISGQHLVAVE
EDAESEDEEEEDVKLLSISGKRSAPGGGSKVPQKKVKLAADEDDDDDDEEDDDEDDDDDD
FDDEEAEEKAPVKKSIRDTPAKNAQKSNQNGKDSKPSSTPRSKGQESFKKQEKTPKTPKG
PSSVEDIKAKMQASIEKGGSLPKVEAKFINYVKNCFRMTDQEAIQDLWQWRKSL

>sp|P35637|FUS_HUMAN RNA-binding protein FUS OS=Homo sapiens OX=9606 GN=FUS PE=1 SV=1
MASNDYTQQATQSYGAYPTQPGQGYSQQSSQPYGQQSYSGYSQSTDTSGYGQSSYSSYGQ
SQNTGYGTQSTPQGYGSTGGYGSSQSSQSSYGQQSSYPGYGQQPAPSSTSGSYGSSSQSS
SYGQPQSGSYSQQPSYGGQQQSYGQQQSYNPPQGYGQQNQYNSSSGGGGGGGGGGNYGQD
QSSMSSGGGSGGGYGNQDQSGGGGSGGYGQQDRGGRGRGGSGGGGGGGGGGYNRSSGGYE
PRGRGGGRGGRGGMGGSDRGGFNKFGGPRDQGSRHDSEQDNSDNNTIFVQGLGENVTIES
VADYFKQIGIIKTNKKTGQPMINLYTDRETGKLKGEATVSFDDPPSAKAAIDWFDGKEFS
GNPIKVSFATRRADFNRGGGNGRGGRGRGGPMGRGGYGGGGSGGGGRGGFPSGGGGGGGQ
QRAGDWKCPNPTCENMNFSWRNECNQCKAPKPDGPGGGPGGSHMGGNYGDDRRGGRGGYD
RGGYRGRGGDRGGFRGGRGGGDRGGFGPGKMDSRGEHRQDRRERPY

>sp|Q9NUX5|POTE1_HUMAN Protection of telomeres protein 1 OS=Homo sapiens OX=9606 GN=POT1 PE=1 SV=1
MSLVPATNYIYTPLNQLKGGTIVNVYGVVKFFKPPYLSKGTDYCSVVTIVDQTNVKLTCL
LFSGNYEALPIIYKNGDIVRFHRLKIQVYKKETQGITSSGFASLTFEGTLGAPIIPRTSS
KYFNFTTEDHKMVEALRVWASTHMSPSWTLLKLCDVQPMQYFDLTCQLLGKAEVDGASFL
LKVWDGTRTPFPSWRVLIQDLVLEGDLSHIHRLQNLTIDILVYDNHVHVARSLKVGSFLR
IYSLHTKLQSMNSENQTMLSLEFHLHGGTSYGRGIRVLPESNSDVDQLKKDLESANLTAN
QHSDVICQSEPDDSFPSSGSVSLYEVERCQQLSATILTDHQYLERTPLCAILKQKAPQQY
RIRAKLRSYKPRRLFQSVKLHCPKCHLLQEVPHEGDLDIIFQDGATKTPDVKLQNTSLYD
SKIWTTKNQKGRKVAVHFVKNNGILPLSNECLLLIEGGTLSEICKLSNKFNSVIPVRSGH
EDLELLDLSAPFLIQGTIHHYGCKQCSSLRSIQNLNSLVDKTSWIPSSVAEALGIVPLQY
VFVMTFTLDDGTGVLEAYLMDSDKFFQIPASEVLMDDDLQKSVDMIMDMFCPPGIKIDAY
PWLECFIKSYNVTNGTDNQICYQIFDTTVAEDVI

>sp|P54132|BLM_HUMAN Bloom syndrome protein OS=Homo sapiens OX=9606 GN=BLM PE=1 SV=1
MAAVPQNNLQEQLERHSARTLNNKLSLSKPKFSGFTFKKKTSSDNNVSVTNVSVAKTPVL
RNKDVNVTEDFSFSEPLPNTTNQQRVKDFFKNAPAGQETQRGGSKSLLPDFLQTPKEVVC
TTQNTPTVKKSRDTALKKLEFSSSPDSLSTINDWDDMDDFDTSETSKSFVTPPQSHFVRV
STAQKSKKGKRNFFKAQLYTTNTVKTDLPPPSSESEQIDLTEEQKDDSEWLSSDVICIDD
GPIAEVHINEDAQESDSLKTHLEDERDNSEKKKNLEEAELHSTEKVPCIEFDDDDYDTDF
VPPSPEEIISASSSSSKCLSTLKDLDTSDRKEDVLSTSKDLLSKPEKMSMQELNPETSTD
CDARQISLQQQLIHVMEHICKLIDTIPDDKLKLLDCGNELLQQRNIRRKLLTEVDFNKSD
ASLLGSLWRYRPDSLDGPMEGDSCPTGNSMKELNFSHLPSNSVSPGDCLLTTTLGKTGFS

ATRKNLFERPLFNTHLQKSFVSSNWAETPRLGKKNESSYFPGNVLTSTAVKDQNKHTASI
NDLERETQPSYDIDNFDIDDFDDDDDWEDIMHNLAASKSSTAAYQPIKEGRPIKSVSERL
SSAKTDCLPVSSTAQNINFSESIQNYTDKSAQNLASRNLKHERFQSLSFPHTKEMMKIFH
KKFGLHNFRTNQLEAINAALLGEDCFILMPTGGGKSLCYQLPACVSPGVTVVISPLRSLI
VDQVQKLTSLDIPATYLTGDKTDSEATNIYLQLSKKDPIIKLLYVTPEKICASNRLISTL
ENLYERKLLARFVIDEAHCVSQWGHDFRQDYKRMNMLRQKFPSVPVMALTATANPRVQKD
ILTQLKILRPQVFSMSFNRHNLKYYVLPKKPKKVAFDCLEWIRKHHPYDSGIIYCLSRRE
CDTMADTLQRDGLAALAYHAGLSDSARDEVQQKWINQDGCQVICATIAFGMGIDKPDVRF
VIHASLPKSVEGYYQESGRAGRDGEISHCLLFYTYHDVTRLKRLIMMEKDGNHHTRETHF
NNLYSMVHYCENITECRRIQLLAYFGENGFNPDFCKKHPDVSCDNCCKTKDYKTRDVTDD
VKSIVRFVQEHSSSQGMRNIKHVGPSGRFTMNMLVDIFLGSKSAKIQSGIFGKGSAYSRH
NAERLFKKLILDKILDEDLYINANDQAIAYVMLGNKAQTVLNGNLKVDFMETENSSSVKK
QKALVAKVSQREEMVKKCLGELTEVCKSLGKVFGVHYFNIFNTVTLKKLAESLSSDPEVL
LQIDGVTEDKLEKYGAEVISVLQKYSEWTSPAEDSSPGISLSSSRGPGRSAAEELDEEIP
VSSHYFASKTRNERKRKKMPASQRSKRRKTASSGSKAKGGSATCRKISSKTKSSSIIGSS
SASHTSQATSGANSKLGIMAPPKPINRPFLKPSYAFS

>sp|Q9BX63|FANCJ_HUMAN Fanconi anemia group J protein OS=Homo sapiens OX=9606 GN=BRIP1 PE=1 SV=1
MSSMWSEYTIGGVKIYFPYKAYPSQLAMMNSILRGLNSKQHCLLESPTGSGKSLALLCSA
LAWQQSLSGKPADEGVSEKAEVQLSCCCACHSKDFTNNDMNQGTSRHFNYPSTPPSERNG
TSSTCQDSPEKTTLAAKLSAKKQASIYRDENDDFQVEKKRIRPLETTQQIRKRHCFGTEV
HNLDAKVDSGKTVKLNSPLEKINSFSPQKPPGHCSRCCCSTKQGNSQESSNTIKKDHTGK
SKIPKIYFGTRTHKQIAQITRELRRTAYSGVPMTILSSRDHTCVHPEVVGNFNRNEKCME
LLDGKNGKSCYFYHGVHKISDQHTLQTFQGMCKAWDIEELVSLGKKLKACPYYTARELIQ
DADIIFCPYNYLLDAQIRESMDLNLKEQVVILDEAHNIEDCARESASYSVTEVQLRFARD
ELDSMVNNNIRKKDHEPLRAVCCSLINWLEANAEYLVERDYESACKIWSGNEMLLTLHKM
GITTATFPILQGHFSAVLQKEEKISPIYGKEEAREVPVISASTQIMLKGLFMVLDYLFRQ
NSRFADDYKIAIQQTYSWTNQIDISDKNGLLVLPKNKKRSRQKTAVHVLNFWCLNPAVAF
SDINGKVQTIVLTSGTLSPMKSFSSELGVTFTIQLEANHIIKNSQVWVGTIGSGPKGRNL
CATFQNTETFEFQDEVGALLLSVCQTVSQGILCFLPSYKLLEKLKERWLSTGLWHNLELV
KTVIVEPQGGEKTNFDELLQVYYDAIKYKGEKDGALLVAVCRGKVSEGLDFSDDNARAVI
TIGIPFPNVKDLQVELKRQYNDHHSKLRGLLPGRQWYEIQAYRALNQALGRCIRHRNDWG
ALILVDDRFRNNPSRYISGLSKWVRQQIQHHSTFESALESLAEFSKKHQKVLNVSIKDRT
NIQDNESTLEVTSLKYSTPPYLLEAASHLSPENFVEDEAKICVQELQCPKIITKNSPLPS
SIISRKEKNDPVFLEEAGKAEKIVISRSTSPTFNKQTKRVSWSSFNSLGQYFTGKIPKAT
PELGSSENSASSPPRFKTEKMESKTVLPFTDKCESSNLTVNTSFGSCPQSETIISSLKID
ATLTRKNHSEHPLCSEEALDPDIELSLVSEEDKQSTSNRDFETEAEDESIYFTPELYDPE
DTDEEKNDLAETDRGNRLANNSDCILAKDLFEIRTIKEVDSAREVKAEDCIDTKLNGILH
IEESKIDDIDGNVKTTWINELELGKTHEIEIKNFKPSPSKNKGMFPGFK

>sp|Q9H2U1|DHX36_HUMAN ATP-dependent RNA helicase DHX36 OS=Homo sapiens OX=9606 GN=DHX36 PE=1 SV=2
MSYDYHQNWGRDGGPRSSGGGYGGGPAGGHGGNRGSGGGGGGGGGGRGGRGRHPGHLKGR

EIGMWYAKKQGQKNKEAERQERAVVHMDERREEQIVQLLNSVQAKNDKESEAQISWFAPE
DHGYGTEVSTKNTPCSENKLDIQEKKLINQEKKMFRIRNRSYIDRDSEYLLQENEPDGTL
DQKLLEDLQKKKNDLRYIEMQHFREKLPSYGMQKELVNLIDNHQVTVISGETGCGKTTQV
TQFILDNYIERGKGSACRIVCTQPRRISAISVAERVAAERAESCGSGNSTGYQIRLQSRL
PRKQGSILYCTTGIILQWLQSDPYLSSVSHIVLDEIHERNLQSDVLMTVVKDLLNFRSDL
KVILMSATLNAEKFSEYFGNCPMIHIPGFTFPVVEYLLEDVIEKIRYVPEQKEHRSQFKR
GFMQGHVNRQEKEEKEAIYKERWPDYVRELRRRYSASTVDVIEMMEDDKVDLNLIVALIR
YIVLEEEDGAILVFLPGWDNISTLHDLLMSQVMFKSDKFLIIPLHSLMPTVNQTQVFKRT
PPGVRKIVIATNIAETSITIDDVVYVIDGGKIKETHFDTQNNISTMSAEWVSKANAKQRK
GRAGRVQPGHCYHLYNGLRASLLDDYQLPEILRTPLEELCLQIKILRLGGIAYFLSRLMD
PPSNEAVLLSIRHLMELNALDKQEELTPLGVHLARLPVEPHIGKMILFGALFCCLDPVLT
IAASLSFKDPFVIPLGKEKIADARRKELAKDTRSDHLTVVNAFEGWEEARRRGFRYEKDY
CWEYFLSSNTLQMLHNMKGQFAEHLLGAGFVSSRNPKDPESNINSDNEKIIKAVICAGLY
PKVAKIRLNLGKKRKMVKVYTKTDGLVAVHPKSVNVEQTDFHYNWLIYHLKMRTSSIYLY
DCTEVSPYCLLFFGGDISIQKDNDQETIAVDEWIVFQSPARIAHLVKELRKELDILLQEK
IESPHPVDWNDTKSRDCAVLSAIIDLIKTQEKATPRNFPPRFQDGYYS

>sp|P15692|VEGFA_HUMAN Vascular endothelial growth factor A OS=Homo sapiens OX=9606 GN=VEGFA PE=1 SV=2
MNFLLSWVHWSLALLLYLHHAKWSQAAPMAEGGGQNHHEVVKFMDVYQRSYCHPIETLVD
IFQEYPDEIEYIFKPSCVPLMRCGGCCNDEGLECVPTEESNITMQIMRIKPHQGQHIGEM
SFLQHNKCECRPKKDRARQEKKSVRGKGKGQKRKRKKSRYKSWSVYVGARCCLMPWSLPG
PHPCGPCSERRKHLFVQDPQTCKCSCKNTDSRCKARQLELNERTCRCDKPRR

>sp|P08047|SP1_HUMAN Transcription factor Sp1 OS=Homo sapiens OX=9606 GN=SP1 PE=1 SV=3
MSDQDHSMDEMTAVVKIEKGVGGNNGGNGNGGGAFSQARSSSTGSSSSTGGGGQESQPSP
LALLAATCSRIESPNENSNNSQGPSQSGGTGELDLTATQLSQGANGWQIISSSSGATPTS
KEQSGSSTNGSNGSESSKNRTVSGGQYVVAAAPNLQNQQVLTGLPGVMPNIQYQVIPQFQ
TVDGQQLQFAATGAQVQQDGSGQIQIIPGANQQIITNRGSGGNIIAAMPNLLQQAVPLQG
LANNVLSGQTQYVTNVPVALNGNITLLPVNSVSAATLTPSSQAVTISSSGSQESGSQPVT
SGTTISSASLVSSQASSSSFFTNANSYSTTTTTSNMGIMNFTTSGSSGTNSQGQTPQRVS
GLQGSDALNIQQNQTSGGSLQAGQQKEGEQNQQTQQQQILIQPQLVQGGQALQALQAAPL
SGQTFTTQAISQETLQNLQLQAVPNSGPIIIRTPTVGPNGQVSWQTLQLQNLQVQNPQAQ
TITLAPMQGVSLGQTSSSNTTLTPIASAASIPAGTVTVNAAQLSSMPGLQTINLSALGTS
GIQVHPIQGLPLAIANAPGDHGAQLGLHGAGGDGIHDDTAGGEEGENSPDAQPQAGRRTR
REACTCPYCKDSEGRGSGDPGKKKQHICHIQGCGKVYGKTSHLRAHLRWHTGERPFMCTW
SYCGKRFTRSDELQRHKRTHTGEKKFACPECPKRFMRSDHLSKHIKTHQNKKGGPGVALS
VGTLPLDSGAGSEGSGTATPSALITTNMVAMEAICPEGIARLANSGINVMQVADLQSINI
SGNGF

>sp|P38398|BRCA1_HUMAN Breast cancer type 1 susceptibility protein OS=Homo sapiens OX=9606 GN=BRCA1 PE=1 SV=2
MDLSALRVEEVQNVINAMQKILECPICLELIKEPVSTKCDHIFCKFCMLKLLNQKKGPSQ
CPLCKNDITKRSLQESTRFSQLVEELLKIICAFQLDTGLEYANSYNFAKKENNSPEHLKD

EVSIIQSMGYRNRAKRLLQSEPENPSLQETSLSVQLSNLGTVRTLRTKQRIQPQKTSVYI
ELGSDSSEDTVNKATYCSVGDQELLQITPQGTRDEISLDSAKKAACEFSETDVTNTEHHQ
PSNNDLNTTEKRAAERHPEKYQGSSVSNLHVEPCGTNTHASSLQHENSSLLLTKDRMNVE
KAEFCNKSKQPGLARSQHNRWAGSKETCNDRRTPSTEKKVDLNADPLCERKEWNKQKLPC
SENPRDTEDVPWITLNSSIQKVNEWFSRSDELLGSDDSHDGESESNAKVADVLDVLNEVD
EYSGSSEKIDLLASDPHEALICKSERVHSKSVESNIEDKIFGKTYRKKASLPNLSHVTEN
LIIGAFVTEPQIIQERPLTNKLKRKRRPTSGLHPEDFIKKADLAVQKTPEMINQGTNQTE
QNGQVMNITNSGHENKTKGDSIQNEKNPNPIESLEKESAFKTKAEPISSSISNMELELNI
HNSKAPKKNRLRRKSSTRHIHALELVVSRNLSPPNCTELQIDSCSSSEEIKKKKYNQMPV
RHSRNLQLMEGKEPATGAKKSNKPNEQTSKRHDSDTFPELKLTNAPGSFTKCSNTSELKE
FVNPSLPREEKEEKLETVKVSNNAEDPKDLMLSGERVLQTERSVESSSISLVPGTDYGTQ
ESISLLEVSTLGKAKTEPNKCVSQCAAFENPKGLIHGCSKDNRNDTEGFKYPLGHEVNHS
RETSIEMEESELDAQYLQNTFKVSKRQSFAPFSNPGNAEEECATFSAHSGSLKKQSPKVT
FECEQKEENQGKNESNIKPVQTVNITAGFPVVGQKDKPVDNAKCSIKGGSRFCLSSQFRG
NETGLITPNKHGLLQNPYRIPPLFPIKSFVKTKCKKNLLEENFEEHSMSPEREMGNENIP
STVSTISRNNIRENVFKEASSSNINEVGSSTNEVGSSINEIGSSDENIQAELGRNRGPKL
NAMLRLGVLQPEVYKQSLPGSNCKHPEIKKQEYEEVVQTVNTDFSPYLISDNLEQPMGSS
HASQVCSETPDDLLDDGEIKEDTSFAENDIKESSAVFSKSVQKGELSRSPSPFTHTHLAQ
GYRRGAKKLESSEENLSSEDEELPCFQHLLFGKVNNIPSQSTRHSTVATECLSKNTEENL
LSLKNSLNDCSNQVILAKASQEHHLSEETKCSASLFSSQCSELEDLTANTNTQDPFLIGS
SKQMRHQSESQGVGLSDKELVSDDEERGTGLEENNQEEQSMDSNLGEAASGCESETSVSE
DCSGLSSQSDILTTQQRDTMQHNLIKLQQEMAELEAVLEQHGSQPSNSYPSIISDSSALE
DLRNPEQSTSEKAVLTSQKSSEYPISQNPEGLSADKFEVSADSSTSKNKEPGVERSSPSK
CPSLDDRWYMHSCSGSLQNRNYPSQEELIKVVDVEEQQLEESGPHDLTETSYLPRQDLEG
TPYLESGISLFSDDPESDPSEDRAPESARVGNIPSSTSALKVPQLKVAESAQSPAAAHTT
DTAGYNAMEESVSREKPELTASTERVNKRMSMVVSGLTPEEFMLVYKFARKHHITLTNLI
TEETTHVVMKTDAEFVCERTLKYFLGIAGGKWVVSYFWVTQSIKERKMLNEHDFEVRGDV
VNGRNHQGPKRARESQDRKIFRGLEICCYGPFTNMPTDQLEWMVQLCGASVVKELSSFTL
GTGVHPIVVVQPDAWTEDNGFHAIGQMCEAPVVTREWVLDSVALYQCQELDTYLIPQIPH
SHY

>sp|P11387|TOP1_HUMAN DNA topoisomerase 1 OS=Homo sapiens OX=9606 GN=TOP1 PE=1 SV=2
MSGDHLHNDSQIEADFRLNDSHKHKDKHKDREHRHKEHKKEKDREKSKHSNSEHKDSEKK
HKEKEKEKTKHKDGSSEKHKDKHKDRDKEKRKEEKVRASGDAKIKKEKENGFSSPPQIKDEP
EDDGYFVPPKEDIKPLKRPRDEDDADYKPKKIKTEDTKKEKKRKLEEEEDGKLKKPKNKD
KDKKVPEPDNKKKKPKKEEEQKWKWWEEERYPEGIKWKFLEHKGPVFAPPYEPLPENVKF
YYDGKVMKLSPKAEEVATFFAKMLDHEYTTKEIFRKNFFKDWRKEMTNEEKNIITNLSKC
DFTQMSQYFKAQTEARKQMSKEEKLKIKEENEKLLKEYGFCIMDNHKERIANFKIEPPGL
FRGRGNHPKMGMLKRRIMPEDIIINCSKDAKVPSPPPGHKWKEVRHDNKVTWLVSWTENI
QGSIKYIMLNPSSRIKGEKDWQKYETARRLKKCVDKIRNQYREDWKSKEMKVRQRAVALY
FIDKLALRAGNEKEEGETADTVGCCSLRVEHINLHPELDGQEYVVEFDFLGKDSIRYYNK
VPVEKRVFKNLQLFMENKQPEDDLFDRLNTGILNKHLQDLMEGLTAKVFRTYNASITLQQ
QLKELTAPDENIPAKILSYNRANRAVAILCNHQRAPPKTFEKSMMNLQTKIDAKKEQLAD
ARRDLKSAKADAKVMKDAKTKKVVESKKKAVQRLEEQLMKLEVQATDREENKQIALGTSK

LNYLDPRITVAWCKKWGVPIEKIYNKTQREKFAWAIDMADEDYEF

>sp|P09874|PARP1_HUMAN Poly [ADP-ribose] polymerase 1 OS=Homo sapiens OX=9606 GN=PARP1 PE=1 SV=4
MAESSDKLYRVEYAKSGRASCKKCSESIPKDSLRMAIMVQSPMFDGKVPHWYHFSCFWKV
GHSIRHPDVEVDGFSELRWDDQQKVKKTAEAGGVTGKGQDGIGSKAEKTLGDFAAEYAKS
NRSTCKGCMEKIEKGQVRLSKKMVDPEKPQLGMIDRWYHPGCFVKNREELGFRPEYSASQ
LKGFSLLATEDKEALKKQLPGVKSEGKRKGDEVDGVDEVAKKKSKKEKDKDSKLEKALKA
QNDLIWNIKDELKKVCSTNDLKELLIFNKQQVPSGESAILDRVADGMVFGALLPCEECSG
QLVFKSDAYYCTGDVTAWTKCMVKTQTPNRKEWVTPKEFREISYLKKLKVKKQDRIFPPE
TSASVAATPPPSTASAPAAVNSSASADKPLSNMKILTLGKLSRNKDEVKAMIEKLGGKLT
GTANKASLCISTKKEVEKMNKKMEEVKEANIRVVSEDFLQDVSASTKSLQELFLAHILSP
WGAEVKAEPVEVVAPRGKSGAALSKKSKGQVKEEGINKSEKRMKLTLKGGAAVDPDSGLE
HSAHVLEKGGKVFSATLGLVDIVKGTNSYYKLQLLEDDKENRYWIFRSWGRVGTVIGSNK
LEQMPSKEDAIEHFMKLYEEKTGNAWHSKNFTKYPKKFYPLEIDYGQDEEAVKKLTVNPG
TKSKLPKPVQDLIKMIFDVESMKKAMVEYEIDLQKMPLGKLSKRQIQAAYSILSEVQQAV
SQGSSDSQILDLSNRFYTLIPHDFGMKKPPLLNNADSVQAKVEMLDNLLDIEVAYSLLRG
GSDDSSKDPIDVNYEKLKTDIKVVDRDSEEAEIIRKYVKNTHATTHNAYDLEVIDIFKIE
REGECQRYKPFKQLHNRRLLWHGSRTTNFAGILSQGLRIAPPEAPVTGYMFGKGIYFADM
VSKSANYCHTSQGDPIGLILLGEVALGNMYELKHASHISKLPKGKHSVKGLGKTTPDPSA
NISLDGVDVPLGTGISSGVNDTSLLYNEYIVYDIAQVNLKYLLKLKFNFKTSLW

>sp|P04637|P53_HUMAN Cellular tumor antigen p53 OS=Homo sapiens OX=9606 GN=TP53 PE=1 SV=4
MEEPQSDPSVEPPLSQETFSDLWKLLPENNVLSPLPSQAMDDLMLSPDDIEQWFTEDPGP
DEAPRMPEAAPPVAPAPAAPTPAAPAPAPSWPLSSSVPSQKTYQGSYGFRLGFLHSGTAK
SVTCTYSPALNKMFCQLAKTCPVQLWVDSTPPPGTRVRAMAIYKQSQHMTEVVRRCPHHE
RCSDSDGLAPPQHLIRVEGNLRVEYLDDRNTFRHSVVVPYEPPEVGSDCTTIHYNYMCNS
SCMGGMNRRPILTIITLEDSSGNLLGRNSFEVRVCACPGRDRRTEEENLRKKGEPHHELP
PGSTKRALPNNTSSSPQPKKKPLDGEYFTLQIRGRERFEMFRELNEALELKDAQAGKEPG
GSRAHSSHLKSKKGQSTSRHKKLMFKTEGPDSD

>sp|Q16666|IF16_HUMAN Gamma-interferon-inducible protein 16 OS=Homo sapiens OX=9606 GN=IFI16 PE=1 SV=3
MGKKYKNIVLLKGLEVINDYHFRMVKSLLSNDLKLNLKMREEYDKIQIADLMEEKFRGDA
GLGKLIKIFEDIPTLEDLAETLKKEKLKVKGPALSRKRKKEVDATSPAPSTSSTVKTEGA
EATPGAQKRKKSTKEKAGPKGSKVSEEQTQPPSPAGAGMSTAMGRSPSPKTSLSAPPNSS
STENPKTVAKCQVTPRRNVLQKRPVIVKVLSTTKPFEYETPEMEKKIMFHATVATQTQFF
HVKVLNTSLKEKFNGKKIIIISDYLEYDSLLEVNEESTVSEAGPNQTFEVPNKIINRAKE
TLKIDILHKQASGNIVYGVFMLHKKTVNQKTTIYEIQDDRGKMDVVGTGQCHNIPCEEGD
KLQLFCFRLRKKNQMSKLISEMHSFIQIKKKTNPRNNDPKSMKLPQEQRQLPYPSEASTT
FPESHLRTPQMPPTTPSSSFFTKKSEDTISKMNDFMRMQILKEGSHFPGPFMTSIGPAES
HPHTPQMPPSTPSSSFLTTKSEDTISKMNDFMRMQILKEGSHFPGPFMTSIGPAESHPHT
PQMPPSTPSSSFLTTLKPRLKTEPEEVSIEDSAQSDLKEVMVLNATESFVYEPKEQKKMF
HATVATENEVFRVKVFNIDLKEKFTPKKIIAIANYVCRNGFLEVYPFTLVADVNADRNME

IPKGLIRSASVTPKINQLCSQTKGSFVNGVFEVHKKNVRGEFTYYEIQDNTGKMEVVVHG
RLTTINCEEGDKLKLTCFELAPKSGNTGELRSVIHSHIKVIKTRKNKKDILNPDSSMETS
PDFFF

>sp|O94761|RECQ4_HUMAN ATP-dependent DNA helicase Q4 OS=Homo sapiens OX=9606 GN=RECQL4 PE=1 SV=2
MERLRDVRERLQAWERAFRRQRGRRPSQDDVEAAPEETRALYREYRTLKRTTGQAGGGLR
SSESLPAAAEEAPEPRCWGPHLNRAATKSPQSTPGRSRQGSVPDYGQRLKANLKGTLQAG
PALGRRPWPLGRASSKASTPKPPGTGPVPSFAEKVSDEPPQLPEPQPRPGRLQHLQASLS
QRLGSLDPGWLQRCHSEVPDFLGAPKACRPDLGSEESQLLIPGESAVLGPGAGSQGPEAS
AFQEVSIRVGSPQPSSSGGEKRRWNEEPWESPAQVQQESSQAGPPSEGAGAVAVEEDPPG
EPVQAQPPQPCSSPSNPRYHGLSPSSQARAGKAEGTAPLHIFPRLARHDRGNYVRLNMKQ
KHYVRGRALRSRLLRKQAWKQKWRKKGECFGGGGATVTTKESCFLNEQFDHWAAQCPRPA
SEEDTDAVGPEPLVPSPQPVPEVPSLDPTVLPLYSLGPSGQLAETPAEVFQALEQLGHQA
FRPGQERAVMRILSGISTLLVLPTGAGKSLCYQLPALLYSRRSPCLTLVVSPLLSLMDDQ
VSGLPPCLKAACIHSGMTRKQRESVLQKIRAAQVHVLMLTPEALVGAGGLPPAAQLPPVA
FACIDEAHCLSQWSHNFRPCYLRVCKVLRERMGVHCFLGLTATATRRTASDVAQHLAVAE
EPDLHGPAPVPTNLHLSVSMDRDTDQALLTLLQGKRFQNLDSIIIYCNRREDTERIAALL
RTCLHAAWVPGSGGRAPKTTAEAYHAGMCSRERRRVQRAFMQGQLRVVVATVAFGMGLDR
PDVRAVLHLGLPPSFESYVQAVGRAGRDGQPAHCHLFLQPQGEDLRELRRHVHADSTDFL
AVKRLVQRVFPACTCTCTRPPSEQEGAVGGERPVPKYPPQEAEQLSHQAAPGPRRVCMGH
ERALPIQLTVQALDMPEEAIETLLCYLELHPHHWLELLATTYTHCRLNCPGGPAQLQALA
HRCPPLAVCLAQQLPEDPGQGSSSVEFDMVKLVDSMGWELASVRRALCQLQWDHEPRTGV
RRGTGVLVEFSELAFHLRSPGDLTAEEKDQICDFLYGRVQARERQALARLRRTFQAFHSV
AFPSCGPCLEQQDEERSTRLKDLLGRYFEEEEGQEPGGMEDAQGPEPGQARLQDWEDQVR
CDIRQFLSLRPEEKFSSRAVARIFHGIGSPCYPAQVYGQDRRFWRKYLHLSFHALVGLAT
EELLQVAR

>sp|Q07864|DPOE1_HUMAN DNA polymerase epsilon catalytic subunit A OS=Homo sapiens OX=9606 GN=POLE PE=1 SV=5
MSLRSGGRRRADPGADGEASRDDGATSSVSALKRLERSQWTDKMDLRFGFERLKEPGEKT
GWLINMHPTEILDEDKRLGSAVDYYFIQDDGSRFKVALPYKPYFYIATRKGCEREVSSFL
SKKFQGKIAKVETVPKEDLDLPNHLVGLKRNYIRLSFHTVEDLVKVRKEISPAVKKNREQ
DHASDAYTALLSSVLQRGGVITDEEETSKKIADQLDNIVDMREYDVPYHIRLSIDLKIHV
AHWYNVRYRGNAFPVEITRRDDLVERPDPVVLAFDIETTKLPLKFPDAETDQIMMISYMI
DGQGYLITNREIVSEDIEDFEFTPKPEYEGPFCVFNEPDEAHLIQRWFEHVQETKPTIMV
TYNGDFFDWPFVEARAAVHGLSMQQEIGFQKDSQGEYKAPQCIHMDCLRWVKRDSYLPVG
SHNLKAAAKAKLGYDPVELDPEDMCRMATEQPQTLATYSVSDAVATYYLYMKYVHPFIFA
LCTIIPMEPDEVLRKGSGTLCEALLMVQAFHANIIFPNKQEQEFNKLTDDGHVLDSETYV
GGHVEALESGVFRSDIPCRFRMNPAAFDFLLQRVEKTLRHALEEEEKVPVEQVTNFEEVC
DEIKSKLASLKDVPSRIECPLIYHLDVGAMYPNIILTNRLQPSAMVDEATCAACDFNKPG
ANCQRKMAWQWRGEFMPASRSEYHRIQHQLESEKFPPLFPEGPARAFHELSREEQAKYEK
RRLADYCRKAYKKIHITKVEERLTTICQRENSFYVDTVRAFRDRRYEFKGLHKVWKKKLS
AAVEVGDAAEVKRCKNMEVLYDSLQLAHKCILNSFYGYVMRKGARWYSMEMAGIVCFTGA

NIITQARELIEQIGRPLELDTDGIWCVLPNSFPENFVFKTTNVKKPKVTISYPGAMLNIM
VKEGFTNDQYQELAEPSSLTYVTRSENSIFFEVDGPYLAMILPASKEEGKKLKKRYAVFN
EDGSLAELKGFEVKRRGELQLIKIFQSSVFEAFLKGSTLEEVYGSVAKVADYWLDVLYSK
AANMPDSELFELISENRSMSRKLEDYGEQKSTSISTAKRLAEFLGDQMVKDAGLSCRYII
SRKPEGSPVTERAIPLAIFQAEPTVRKHFLRKWLKSSSLQDFDIRAILDWDYYIERLGSA
IQKIITIPAALQQVKNPVPRVKHPDWLHKKLLEKNDVYKQKKISELFTLEGRRQVTMAEA
SEDSPRPSAPDMEDFGLVKLPHPAAPVTVKRKRVLWESQEESQDLTPTVPWQEILGQPPA
LGTSQEEWLVWLRFHKKKWQLQARQRLARRKRQRLESAEGVLRPGAIRDGPATGLGSFLR
RTARSILDLPWQIVQISETSQAGLFRLWALVGSDLHCIRLSIPRVFYVNQRVAKAEEGAS
YRKVNRVLPRSNMVYNLYEYSVPEDMYQEHINEINAELSAPDIEGVYETQVPLLFRALVH
LGCVCVVNKQLVRHLSGWEAETFALEHLEMRSLAQFSYLEPGSIRHIYLYHHAQAHKALF
GIFIPSQRRASVFVLDTVRSNQMPSLGALYSAEHGLLLEKVGPELLPPPKHTFEVRAETD
LKTICRAIQRFLLAYKEERRGPTLIAVQSSWELKRLASEIPVLEEFPLVPICVADKINYG
VLDWQRHGARRMIRHYLNLDTCLSQAFEMSRYFHIPIGNLPEDISTFGSDLFFARHLQRH
NHLLWLSPTARPDLGGKEADDNCLVMEFDDQATVEINSSGCYSTVCVELDLQNLAVNTIL
QSHHVNDMEGADSMGISFDVIQQASLEDMITGGQAASAPASYDETALCSNTFRILKSMVV
GWVKEITQYHNIYADNQVMHFYRWLRSPSSLLHDPALHRTLHNMMKKLFLQLIAEFKRLG
SSVIYANFNRIILCTKKRRVEDAIAYVEYITSSIHSKETFHSLTISFSRCWEFLLWMDPS
NYGGIKGKVSSRIHCGLQDSQKAGGAEDEQENEDDEEERDGEEEEEAEESNVEDLLENNW
NILQFLPQAASCQNYFLMIVSAYIVAVYHCMKDGLRRSAPGSTPVRRRGASQLSQEAEGA
VGALPGMITFSQDYVANELTQSFFTITQKIQKKVTGSRNSTELSEMFPVLPGSHLLLNNP
ALEFIKYVCKVLSLDTNITNQVNKLNRDLLRLVDVGEFSEEAQFRDPCRSYVLPEVICRS
CNFCRDLDLCKDSSFSEDGAVLPQWLCSNCQAPYDSSAIEMTLVEVLQKKLMAFTLQDLV
CLKCRGVKETSMPVYCSCAGDFALTIHTQVFMEQIGIFRNIAQHYGMSYLLETLEWLLQK
NPQLGH

>sp|Q9Y253|POLH_HUMAN DNA polymerase eta OS=Homo sapiens OX=9606 GN=POLH PE=1 SV=1
MATGQDRVVALVDMDCFFVQVEQRQNPHLRNKPCAVVQYKSWKGGGIIAVSYEARAFGVT
RSMWADDAKKLCPDLLLAQVRESRGKANLTKYREASVEVMEIMSRFAVIERASIDEAYVD
LTSAVQERLQKLQGQPISADLLPSTYIEGLPQGPTTAEETVQKEGMRKQGLFQWLDSLQI
DNLTSPDLQLTVGAVIVEEMRAAIERETGFQCSAGISHNKVLAKLACGLNKPNRQTLVSH
GSVPQLFSQMPIRKIRSLGGKLGASVIEILGIEYMGELTQFTESQLQSHFGEKNGSWLYA
MCRGIEHDPVKPRQLPKTIGCSKNFPGKTALATREQVQWWLLQLAQELEERLTKDRNDND
RVATQLVVSIRVQGDKRLSSLRRCCALTRYDAHKMSHDAFTVIKNCNTSGIQTEWSPPLT
MLFLCATKFSASAPSSSTDITSFLSSDPSSLPKVPVTSSEAKTQGSGPAVTATKKATTSL
ESFFQKAAERQKVKEASLSSLTAPTQAPMSNSPSKPSLPFQTSQSTGTEPFFKQKSLLLK
QKQLNNSSVSSPQQNPWSNCKALPNSLPTEYPGCVPVCEGVSKLEESSKATPAEMDLAHN
SQSMHASSASKSVLEVTQKATPNPSLLAAEDQVPCEKCGSLVPVWDMPEHMDYHFALELQ
KSFLQPHSSNPQVVSAVSHQGKRNPKSPLACTNKRPRPEGMQTLESFFKPLTH

>sp|P55265|DSRAD_HUMAN Double-stranded RNA-specific adenosine deaminase OS=Homo sapiens OX=9606 GN=ADAR PE=1 SV=4
MNPRQGYSLSGYYTHPFQGYEHRQLRYQQPGPGSSPSSFLLKQIEFLKGQLPEAPVIGKQ
TPSLPPSLPGLRPRFPVLLASSTRGRQVDIRGVPRGVHLRSQGLQRGFQHPSPRGRSLPQ

RGVDCLSSHFQELSIYQDQEQRILKFLEELGEGKATTAHDLSGKLGTPKKEINRVLYSLA
KKGKLQKEAGTPPLWKIAVSTQAWNQHSGVVRPDGHSQGAPNSDPSLEPEDRNSTSVSED
LLEPFIAVSAQAWNQHSGVVRPDSHSQGSPNSDPGLEPEDSNSTSALEDPLEFLDMAEIK
EKICDYLFNVSDSSALNLAKNIGLTKARDINAVLIDMERQGDVYRQGTTPPIWHLTDKKR
ERMQIKRNTNSVPETAPAAIPETKRNAEFLTCNIPTSNASNNMVTTEKVENGQEPVIKLE
NRQEARPEPARLKPPVHYNGPSKAGYVDFENGQWATDDIPDDLNSIRAAPGEFRAIMEMP
SFYSHGLPRCSPYKKLTECQLKNPISGLLEYAQFASQTCEFNMIEQSGPPHEPRFKFQVV
INGREFPPAEAGSKKVAKQDAAMKAMTILLEEAKAKDSGKSEESSHYSTEKESEKTAESQ
TPTPSATSFFSGKSPVTTLLECMHKLGNSCEFRLLSKEGPAHEPKFQYCVAVGAQTFPSV
SAPSKKVAKQMAAEEAMKALHGEATNSMASDNQPEGMISESLDNLESMMPNKVRKIGELV
RYLNTNPVGGLLEYARSHGFAAEFKLVDQSGPPHEPKFVYQAKVGGRWFPAVCAHSKKQG
KQEAADAALRVLIGENEKAERMGFTEVTPVTGASLRRTMLLLSRSPEAQPKTLPLTGSTF
HDQIAMLSHRCFNTLTNSFQPSLLGRKILAAIIMKKDSEDMGVVVSLGTGNRCVKGDSLS
LKGETVNDCHAEIISRRGFIRFLYSELMKYNSQTAKDSIFEPAKGGEKLQIKKTVSFHLY
ISTAPCGDGALFDKSCSDRAMESTESRHYPVFENPKQGKLRTKVENGEGTIPVESSDIVP
TWDGIRLGERLRTMSCSDKILRWNVLGLQGALLTHFLQPIYLKSVTLGYLFSQGHLTRAI
CCRVTRDGSAFEDGLRHPFIVNHPKVGRVSIYDSKRQSGKTKETSVNWCLADGYDLEILD
GTRGTVDGPRNELSRVSKKNIFLLFKKLCSFRYRRDLLRLSYGEAKKAARDYETAKNYFK
KGLKDMGYGNWISKPQEEKNFYLCPV

>sp|Q16236|NF2L2_HUMAN Nuclear factor erythroid 2-related factor 2 OS=Homo sapiens OX=9606 GN=NFE2L2 PE=1 SV=3
MMDLELPPPGLPSQQDMDLIDILWRQDIDLGVSREVFDFSQRRKEYELEKQKKLEKERQE
QLQKEQEKAFFAQLQLDEETGEFLPIQPAQHIQSETSGSANYSQVAHIPKSDALYFDDCM
QLLAQTFPFVDDNEVSSATFQSLVPDIPGHIESPVFIATNQAQSPETSVAQVAPVDLDGM
QQDIEQVWEELLSIPELQCLNIENDKLVETTMVPSPEAKLTEVDNYHFYSSIPSMEKEVG
NCSPHFLNAFEDSFSSILSTEDPNQLTVNSLNSDATVNTDFGDEFYSAFIAEPSISNSMP
SPATLSHSLSELLNGPIDVSDLSLCKAFNQNHPESTAEFNDSDSGISLNTSPSVASPEHS
VESSSYGDTLLGLSDSEVEELDSAPGSVKQNGPKTPVHSSGDMVQPLSPSQGQSTHVHDA
QCENTPEKELPVSPGHRKTPFTKDKHSSRLEAHLTRDELRAKALHIPFPVEKIINLPVVD
FNEMMSKEQFNEAQLALIRDIRRRGKNKVAAQNCRKRKLENIVELEQDLDHLKDEKEKLL
KEKGENDKSLHLLKKQLSTLYLEVFSMLRDEDGKPYSPSEYSLQQTRDGNVFLVPKSKKP
DVKKN

>sp|P19447|ERCC3_HUMAN General transcription and DNA repair factor IIH helicase subunit XPB OS=Homo sapiens OX=9606 GN=ERCC3 PE=1 SV=1
MGKRDRADRDKKKSRKRHYEDEEDDEEDAPGNDPQEAVPSAAGKQVDESGTKVDEYGAKD
YRLQMPLKDDHTSRPLWVAPDGHIFLEAFSPVYKYAQDFLVAIAEPVCRPTHVHEYKLTA
YSLYAAVSVGLQTSDITEYLRKLSKTGVPDGIMQFIKLCTVSYGKVKLVLKHNRYFVESC
HPDVIQHLLQDPVIRECRLRNSEGEATELITETFTSKSAISKTAESSGGPSTSRVTDPQG
KSDIPMDLFDFYEQMDKDEEEEEETQTVSFEVKQEMIEELQKRCIHLEYPLLAEYDFRND
SVNPDINIDLKPTAVLRPYQEKSLRKMFGNGRARSGVIVLPCGAGKSLVGVTAACTVRKR
CLVLGNSAVSVEQWKAQFKMWSTIDDSQICRFTSDAKDKPIGCSVAISTYSMLGHTTKRS
WEAERVMEWLKTQEWGLMILDEVHTIPAKMFRRVLTIVQAHCKLGLTATLVREDDKIVDL

NFLIGPKLYEANWMELQNNGYIAKVQCAEVWCPMSPEFYREYVAIKTKKRILLYTMNPNK
FRACQFLIKFHERRNDKIIVFADNVFALKEYAIRLNKPYIYGPTSQGERMQILQNFKHNP
KINTIFISKVGDTSFDLPEANVLIQISSHGGSRRQEAQRLGRVLRAKKGMVAEEYNAFFY
SLVSQDTQEMAYSTKRQRFLVDQGYSFKVITKLAGMEEEDLAFSTKEEQQQLLQKVLAAT
DLDAEEEVVAGEFGSRSSQASRRFGTMSSMSGADDTVYMEYHSSRSKAPSKHVHPLFKRF
RK

>sp|P18074|ERCC2_HUMAN General transcription and DNA repair factor IIH helicase subunit XPD OS=Homo sapiens OX=9606 GN=ERCC2 PE=1 SV=1
MKLNVDGLLVYFPYDYIYPEQFSYMRELKRTLDAKGHGVLEMPSGTGKTVSLLALIMAYQ
RAYPLEVTKLIYCSRTVPEIEKVIEELRKLLNFYEKQEGEKLPFLGLALSSRKNLCIHPE
VTPLRFGKDVDGKCHSLTASYVRAQYQHDTSLPHCRFYEEFDAHGREVPLPAGIYNLDDL
KALGRRQGWCPYFLARYSILHANVVVYSYHYLLDPKIADLVSKELARKAVVVFDEAHNID
NVCIDSMSVNLTRRTLDRCQGNLETLQKTVLRIKETDEQRLRDEYRRLVEGLREASAARE
TDAHLANPVLPDEVLQEAVPGSIRTAEHFLGFLRRLLEYVKWRLRVQHVVQESPPAFLSG
LAQRVCIQRKPLRFCAERLRSLLHTLEITDLADFSPLTLLANFATLVSTYAKGFTIIIEP
FDDRTPTIANPILHFSCMDASLAIKPVFERFQSVIITSGTLSPLDIYPKILDFHPVTMAT
FTMTLARVCLCPMIIGRGNDQVAISSKFETREDIAVIRNYGNLLLEMSAVVPDGIVAFFT
SYQYMESTVASWYEQGILENIQRNKLLFIETQDGAETSVALEKYQEACENGRGAILLSVA
RGKVSEGIDFVHHYGRAVIMFGVPYVYTQSRILKARLEYLRDQFQIRENDFLTFDAMRHA
AQCVGRAIRGKTDYGLMVFADKRFARGDKRGKLPRWIQEHLTDANLNLTVDEGVQVAKYF
LRQMAQPFHREDQLGLSLLSLEQLESEETLKRIEQIAQQL

>sp|P54274|TERF1_HUMAN Telomeric repeat-binding factor 1 OS=Homo sapiens OX=9606 GN=TERF1 PE=1 SV=3
MAEDVSSAAPSPRGCADGRDADPTEEQMAETERNDEEQFECQELLECQVQVGAPEEEEEE
EEDAGLVAEAEAVAAGWMLDFLCLSLCRAFRDGRSEDFRRTRNSAEAIIHGLSSLTACQL
RTIYICQFLTRIAAGKTLDAQFENDERITPLESALMIWGSIEKEHDKLHEEIQNLIKIQA
IAVCMENGNFKEAEEVFERIFGDPNSHMPFKSKLLMIISQKDTFHSFFQHFSYNHMMEKI
KSYVNYVLSEKSSTFLMKAAAKVVESKRTRTITSQDKPSGNDVEMETEANLDTRKSVSDK
QSAVTESSEGTVSLLRSHKNLFLSKLQHGTQQQDLNKKERRVGTPQSTKKKKESRRATES
RIPVSKSQPVTPEKHRARKRQAWLWEEDKNLRSGVRKYGEGNWSKILLHYKFNNRTSVML
KDRWRTMKKLKLISSDSED

>sp|Q9BSI4|TINF2_HUMAN TERF1-interacting nuclear factor 2 OS=Homo sapiens OX=9606 GN=TINF2 PE=1 SV=1
MATPLVAGPAALRFAAAASWQVVRGRCVEHFPRVLEFLRSLRAVAPGLVRYRHHERLCMG
LKAKVVVELILQGRPWAQVLKALNHHFPESGPIVRDPKATKQDLRKILEAQETFYQQVKQ
LSEAPVDLASKLQELEQEYGEPFLAAMEKLLFEYLCQLEKALPTPQAQQLQDVLSWMQPG
VSITSSLAWRQYGVDMGWLLPECSVTDSVNLAEPMEQNPPQQQRLALHNPLPKAKPGTHL
PQGPSSRTHPEPLAGRHFNLAPLGRRRVQSQWASTRGGHKERPTVMLFPFRNLGSPTQVI
SKPESKEEHAIYTADLAMGTRAASTGKSKSPCQTLGGRALKENPVDLPATEQKENCLDCY
MDPLRLSLLPPRARKPVCPPSLCSSVITIGDLVLDSDEEENGQGEGKESLENYQKTKFDT
LIPTLCEYLPPSGHGAIPVSSCDCRDSSRPL

>sp|Q96AP0|ACD_HUMAN Adrenocortical dysplasia protein homolog OS=Homo sapiens OX=9606 GN=ACD PE=1 SV=3
MPGRCQSDAAMRVNGPASRAPAGWTSGSLHTGPRAGRPRAQARGVRGRGLLLRPRPAKEL
PLPRKGGAWAPAGNPGPLHPLGVAVGMAGSGRLVLRPWIRELILGSETPSSPRAGQLLEV
LQDAEAAVAGPSHAPDTSDVGATLLVSDGTHSVRCLVTREALDTSDWEEKEFGFRGTEGR
LLLLQDCGVHVQVAEGGAPAEFYLQVDRFSLLPTEQPRLRVPGCNQDLDVQKKLYDCLEE
HLSESTSSNAGLSLSQLLDEMREDQEHQGALVCLAESCLTLEGPCTAPPVTHWAASRCKA
TGEAVYTVPSSMLCISENDQLILSSLGPCQRTQGPELPPPDPALQDLSLTLIASPPSSPS
SSGTPALPGHMSSEESGTSISLLPALSLAAPDPGQRSSSQPSPAICSAPATLTPRSPHAS
RTPSSPLQSCTPSLSPRSHVPSPHQALVTRPQKPSLEFKEFVGLPCKNRPPFPRTGATRG
AQEPCSVWEPPKRHRDGSAFQYEYEPPCTSLCARVQAVRLPPQLMAWALHFLMDAQPGSE
PTPM

>sp|Q9UBP0|SPAST_HUMAN Spastin OS=Homo sapiens OX=9606 GN=SPAST PE=1 SV=1
MNSPGGRGKKKGSGGASNPVPPRPPPPCLAPAPPAAGPAPPPESPHKRNLYYFSYPLFVG
FALLRLVAFHLGLLFVWLCQRFSRALMAAKRSSGAAPAPASASAPAPVPGGEAERVRVFH
KQAFEYISIALRIDEDEKAGQKEQAVEWYKKGIEELEKGIAVIVTGQGEQCERARRLQAK
MMTNLVMAKDRLQLLEKMQPVLPFSKSQTDVYNDSTNLACRNGHLQSESGAVPKRKDPLT
HTSNSLPRSKTVMKTGSAGLSGHHRAPSYSGLSMVSGVKQGSGPAPTTHKGTPKTNRTNK
PSTPTTATRKKKDLKNFRNVDSNLANLIMNEIVDNGTAVKFDDIAGQDLAKQALQEIVIL
PSLRPELFTGLRAPARGLLLFGPPGNGKTMLAKAVAAESNATFFNISAASLTSKYVGEGE
KLVRALFAVARELQPSIIFIDEVDSLLCERREGEHDASRRLKTEFLIEFDGVQSAGDDRV
LVMGATNRPQELDEAVLRRFIKRVYVSLPNEETRLLLLKNLLCKQGSPLTQKELAQLARM
TDGYSGSDLTALAKDAALGPIRELKPEQVKNMSASEMRNIRLSDFTESLKKIKRSVSPQT
LEAYIRWNKDFGDTTV

>sp|P49913|CAMP_HUMAN Cathelicidin antimicrobial peptide OS=Homo sapiens OX=9606 GN=CAMP PE=1 SV=1
MKTQRDGHSLGRWSLVLLLLGLVMPLAIIAQVLSYKEAVLRAIDGINQRSSDANLYRLLD
LDPRPTMDGDPDTPKPVSFTVKETVCPRTTQQSPEDCDFKKDGLVKRCMGTVTLNQARGS
FDISCDKDNKRFALLGDFFRKSKEKIGKEFKRIVQRIKDFLRNLVPRTES

>sp|P22626|ROA2_HUMAN Heterogeneous nuclear ribonucleoproteins A2/B1 OS=Homo sapiens OX=9606 GN=HNRNPA2B1 PE=1 SV=2
MEKTLETVPLERKKREKEQFRKLFIGGLSFETTEESLRNYYEQWGKLTDCVVMRDPASKR
SRGFGFVTFSSMAEVDAAMAARPHSIDGRVVEPKRAVAREESGKPGAHVTVKKLFVGGIK
EDTEEHHLRDYFEEYGKIDTIEIITDRQSGKKRGFGFVTFDDHDPVDKIVLQKYHTINGH
NAEVRKALSRQEMQEVQSSRSGRGGNFGFGDSRGGGGNFGPGPGSNFRGGSDGYGSGRGF
GDGYNGYGGGPGGGNFGGSPGYGGGRGGYGGGGPGYGNQGGGYGGGYDNYGGGNYGSGNY
NDFGNYNQQPSNYGPMKSGNFGGSRNMGGPYGGGNYGPGGSGGSGGYGGRSRY

>sp|P15927|RFA2_HUMAN Replication protein A 32 kDa subunit OS=Homo sapiens OX=9606 GN=RPA2 PE=1 SV=1

MWNSGFESYGSSSYGGAGGYTQSPGGFGSPAPSQAEKKSRARAQHIVPCTISQLLSATLV
DEVFRIGNVEISQVTIVGIIRHAEKAPTNIVYKIDDMTAAPMDVRQWVDTDDTSSENTVV
PPETYVKVAGHLRSFQNKKSLVAFKIMPLEDMNEFTTHILEVINAHMVLSKANSQPSAGR
APISNPGMSEAGNFGGNSFMPANGLTVAQNQVLNLIKACPRPEGLNFQDLKNQLKHMSVS
SIKQAVDFLSNEGHIYSTVDDDHFKSTDAE

>sp|P27694|RFA1_HUMAN Replication protein A 70 kDa DNA-binding subunit OS=Homo sapiens OX=9606 GN=RPA1 PE=1 SV=2
MVGQLSEGAIAAIMQKGDTNIKPILQVINIRPITTGNSPPRYRLLMSDGLNTLSSFMLAT
QLNPLVEEEQLSSNCVCQIHRFIVNTLKDGRRVVILMELEVLKSAEAVGVKIGNPVPYNE
GLGQPQVAPPAPAASPAASSRPQPQNGSSGMGSTVSKAYGASKTFGKAAGPSLSHTSGGT
QSKVVPIASLTPYQSKWTICARVTNKSQIRTWSNSRGEGKLFSLELVDESGEIRATAFNE
QVDKFFPLIEVNKVYYFSKGTLKIANKQFTAVKNDYEMTFNNETSVMPCEDDHHLPTVQF
DFTGIDDLENKSKDSLVDIIGICKSYEDATKITVRSNNREVAKRNIYLMDTSGKVVTATL
WGEDADKFDGSRQPVLAIKGARVSDFGGRSLSVLSSSTIIANPDIPEAYKLRGWFDAEGQ
ALDGVSISDLKSGGVGGSNTNWKTLYEVKSENLGQGDKPDYFSSVATVVYLRKENCMYQA
CPTQDCNKKVIDQQNGLYRCEKCDTEFPNFKYRMILSVNIADFQENQWVTCFQESAEAIL
GQNAAYLGELKDKNEQAFEEVFQNANFRSFIFRVRVKVETYNDESRIKATVMDVKPVDYR
EYGRRLVMSIRRSALM

>sp|P22392|NDKB_HUMAN Nucleoside diphosphate kinase B OS=Homo sapiens OX=9606 GN=NME2 PE=1 SV=1
MANLERTFIAIKPDGVQRGLVGEIIKRFEQKGFRLVAMKFLRASEEHLKQHYIDLKDRPF
FPGLVKYMNSGPVVAMVWEGLNVVKTGRVMLGETNPADSKPGTIRGDFCIQVGRNIIHGS
DSVKSAEKEISLWFKPEELVDYKSCAHDWVYE

>sp|P15172|MYOD1_HUMAN Myoblast determination protein 1 OS=Homo sapiens OX=9606 GN=MYOD1 PE=1 SV=3
MELLSPPLRDVDLTAPDGSLCSFATTDDFYDDPCFDSPDLRFFEDLDPRLMHVGALLKPE
EHSHFPAAVHPAPGAREDEHVRAPSGHHQAGRCLLWACKACKRKTTNADRRKAATMRERR
RLSKVNEAFETLKRCTSSNPNQRLPKVEILRNAIRYIEGLQALLRDQDAAPPGAAAAFYA
PGPLPPGRGGEHYSGDSDASSPRSNCSDGMMDYSGPPSGARRRNCYEGAYYNEAPSEPRP
GKSAAVSSLDCLSSIVERISTESPAAPALLLADVPSESPPRRQEAAAPSEGESSGDPTQS
PDAAPQCPAGANPNPIYQVL

>sp|P13349|MYF5_HUMAN Myogenic factor 5 OS=Homo sapiens OX=9606 GN=MYF5 PE=2 SV=2
MDVMDGCQFSPSEYFYDGSCIPSPEGEFGDEFVPRVAAFGAHKAELQGSDEDEHVRAPTG
HHQAGHCLMWACKACKRKSTTMDRRKAATMRERRRLKKVNQAFETLKRCTTTNPNQRLPK
VEILRNAIRYIESLQELLREQVENYYSLPGQSCSEPTSPTSNCSDGMPECNSPVWSRKSS
TFDSIYCPDVSNVYATDKNSLSSLDCLSNIVDRITSSEQPGLPLQDLASLSPVASTDSQP
ATPGASSSRLIYHVL

>sp|P23409|MYF6_HUMAN Myogenic factor 6 OS=Homo sapiens OX=9606 GN=MYF6 PE=1 SV=1
MMMDLFETGSYFFYLDGENVTLQPLEVAEGSPLYPGSDGTLSPCQDQMPPEAGSDSSGEE

HVLAPPGLQPPHCPGQCLIWACKTCKRKSAPTDRRKAATLRERRRLKKINEAFEALKRRT
VANPNQRLPKVEILRSAISYIERLQDLLHRLDQQEKMQELGVDPFSYRPKQENLEGADFL
RTCSSQWPSVSDHSRGLVITAKEGGASIDSSASSSLRCLSSIVDSISSEERKLPCVEEVV
EK

>sp|P15173|MYOG_HUMAN Myogenin OS=Homo sapiens OX=9606 GN=MYOG PE=1 SV=2
MELYETSPYFYQEPRFYDGENYLPVHLQGFEPPGYERTELTLSPEAPGPLEDKGLGTPEH
CPGQCLPWACKVCKRKSVSVDRRRAATLREKRRLKKVNEAFEALKRSTLLNPNQRLPKVE
ILRSAIQYIERLQALLSSLNQEERDLRYRGGGGPQPGVPSECSSHSASCSPEWGSALEFS
ANPGDHLLTADPTDAHNLHSLTSIVDSITVEDVSVAFPDETMPN

>sp|O14746|TERT_HUMAN Telomerase reverse transcriptase OS=Homo sapiens OX=9606 GN=TERT PE=1 SV=1
MPRAPRCRAVRSLLRSHYREVLPLATFVRRLGPQGWRLVQRGDPAAFRALVAQCLVCVPW
DARPPPAAPSFRQVSCLKELVARVLQRLCERGAKNVLAFGFALLDGARGGPPEAFTTSVR
SYLPNTVTDALRGSGAWGLLLRRVGDDVLVHLLARCALFVLVAPSCAYQVCGPPLYQLGA
ATQARPPPHASGPRRRLGCERAWNHSVREAGVPLGLPAPGARRRGGSASRSLPLPKRPRR
GAAPEPERTPVGQGSWAHPGRTRGPSDRGFCVVSPARPAEEATSLEGALSGTRHSHPSVG
RQHHAGPPSTSRPPRPWDTPCPPVYAETKHFLYSSGDKEQLRPSFLLSSLRPSLTGARRL
VETIFLGSRPWMPGTPRRLPRLPQRYWQMRPLFLELLGNHAQCPYGVLLKTHCPLRAAVT
PAAGVCAREKPQGSVAAPEEEDTDPRRLVQLLRQHSSPWQVYGFVRACLRRLVPPGLWGS
RHNERRFLRNTKKFISLGKHAKLSLQELTWKMSVRDCAWLRRSPGVGCVPAAEHRLREEI
LAKFLHWLMSVYVVELLRSFFYVTETTFQKNRLFFYRKSVWSKLQSIGIRQHLKRVQLRE
LSEAEVRQHREARPALLTSRLRFIPKPDGLRPIVNMDYVVGARTFRREKRAERLTSRVKA
LFSVLNYERARRPGLLGASVLGLDDIHRAWRTFVLRVRAQDPPPELYFVKVDVTGAYDTI
PQDRLTEVIASIIKPQNTYCVRRYAVVQKAAHGHVRKAFKSHVSTLTDLQPYMRQFVAHL
QETSPLRDAVVIEQSSSLNEASSGLFDVFLRFMCHHAVRIRGKSYVQCQGIPQGSILSTL
LCSLCYGDMENKLFAGIRRDGLLLRLVDDFLLVTPHLTHAKTFLRTLVRGVPEYGCVVNL
RKTVVNFPVEDEALGGTAFVQMPAHGLFPWCGLLLDTRTLEVQSDYSSYARTSIRASLTF
NRGFKAGRNMRRKLFGVLRLKCHSLFLDLQVNSLQTVCTNIYKILLLQAYRFHACVLQLP
FHQQVWKNPTFFLRVISDTASLCYSILKAKNAGMSLGAKGAAGPLPSEAVQWLCHQAFLL
KLTRHRVTYVPLLGSLRTAQTQLSRKLPGTTLTALEAAANPALPSDFKTILD

>sp|P43489|TNR4_HUMAN Tumor necrosis factor receptor superfamily member 4 OS=Homo sapiens OX=9606 GN=TNFRSF4 PE=1 SV=1
MCVGARRLGRGPCAALLLLGLGLSTVTGLHCVGDTYPSNDRCCHECRPGNGMVSRCSRSQ
NTVCRPCGPGFYNDVVSSKPCKPCTWCNLRSGSERKQLCTATQDTVCRCRAGTQPLDSYK
PGVDCAPCPPGHFSPGDNQACKPWTNCTLAGKHTLQPASNSSDAICEDRDPPATQPQETQ
GPPARPITVQPTEAWPRTSQGPSTRPVEVPGGRAVAAILGLGLVLGLLGPLAILLALYLL
RRDQRLPPDAHKPPGGGSFRTPIQEEQADAHSTLAKI

>sp|P18146|EGR1_HUMAN Early growth response protein 1 OS=Homo sapiens OX=9606 GN=EGR1 PE=1 SV=1
MAAAKAEMQLMSPLQISDPFGSFPHSPTMDNYPKLEEMMLLSNGAPQFLGAAGAPEGSGS

NSSSSSSSGGGGGGGGGGSNSSSSSSTFNPQADTGEQPYEHLTAESFPDISLNNEKVLVETS
YPSQTTRLPPITYTGRFSLEPAPNSGNTLWPEPLFSLVSGLVSMTNPPASSSSAPSPAAS
SASASQSPPLSCAVPSNDSSPIYSAAPTFPTPNTDIFPEPQSQAFPGSAGTALQYPPPAY
PAAKGGFQVPMIPDYLFPQQQGDLGLGTPDQKPFQGLESRTQQPSLTPLSTIKAFATQSG
SQDLKALNTSYQSQLIKPSRMRKYPNRPSKTPPHERPYACPVESCDRRFSRSDELTRHIR
IHTGQKPFQCRICMRNFSRSDHLTTHIRTHTGEKPFACDICGRKFARSDERKRHTKIHLR
QKDKKADKSVVASSATSSLSSYPSPVATSYPSPVTTSYPSPATTSYPSPVPTSFSSPGSS
TYPSPVHSGFPSPSVATTYSSVPPAFPAQVSSFPSSAVTNSFSASTGLSDMTATFSPRTI
EIC

>sp|P67809|YBOX1_HUMAN Nuclease-sensitive element-binding protein 1 OS=Homo sapiens OX=9606 GN=YBX1 PE=1 SV=3
MSSEAETQQPPAAPPAAPALSAADTKPGTTGSGAGSGGPGGLTSAAPAGGDKKVIATKVL
GTVKWFNVRNGYGFINRNDTKEDVFVHQTAIKKNNPRKYLRSVGDGETVEFDVVEGEKGA
EAANVTGPGGVPVQGSKYAADRNHYRRYPRRRGPPRNYQQNYQNSESGEKNEGSESAPEG
QAQQRRPYRRRRFPPYYMRRPYGRRPQYSNPPVQGEVMEGADNQGAGEQGRPVRQNMYRG
YRPRFRRGPPRQRQPREDGNEEDKENQGDETQGQQPPQRRYRRNFNYRRRRPENPKPQDG
KETKAADPPAENSSAPEAEQGGAE

>sp|Q9NYB0|TE2IP_HUMAN Telomeric repeat-binding factor 2-interacting protein 1 OS=Homo sapiens OX=9606 GN=TERF2IP PE=1 SV=1
MAEAMDLGKDPNGPTHSSTLFVRDDGSSMSFYVRPSPAKRRLSTLILHGGGTVCRVQEPG
AVLLAQPGEALAEASGDFISTQYILDCVERNERLELEAYRLGPASAADTGSEAKPGALAE
GAAEPEPQRHAGRIAFTDADDVAILTYVKENARSPSSVTGNALWKAMEKSSLTQHSWQSL
KDRYLKHLRGQEHKYLLGDAPVSPSSQKLKRKAEEDPEAADSGEPQNKRTPDLPEEEYVK
EEIQENEEAVKKMLVEATREFEEVVVDESPPDFEIHITMCDDDPPTPEEDSETQPDEEEE
EEEEKVSQPEVGAAIKIIRQLMEKFNLDLSTVTQAFLKNSGELEATSAFLASGQRADGYP
IWSRQDDIDLQKDDEDTREALVKKFGAQNVARRIEFRKK

>sp|Q96FI4|NEIL1_HUMAN Endonuclease 8-like 1 OS=Homo sapiens OX=9606 GN=NEIL1 PE=1 SV=3
MPEGPELHLASQFVNEACRALVFGGCVEKSSVSRNPEVPFESSAYRISASARGKELRLIL
SPLPGAQPQQEPLALVFRFGMSGSFQLVPREELPRHAHLRFYTAPPGPRLALCFVDIRRF
GRWDLGGKWQPGRGPCVLQEYQQFRENVLRNLADKAFDRPICEALLDQRFFNGIGNYLRA
EILYRLKIPPFEKARSVLEALQQHRPSPELTLSQKIRTKLQNPDLLELCHSVPKEVVQLG
GKGYGSESGEEDFAAFRAWLRCYGMPGMSSLQDRHGRTIWFQGDPGPLAPKGRKSRKKKS
KATQLSPEDRVEDALPPSKAPSRTRRAKRDLPKRTATQRPEGTSLQQDPEAPTVPKKGRR
KGRQAASGHCRPRKVKADIPSLEPEGTSAS

>sp|Q969S2|NEIL2_HUMAN Endonuclease 8-like 2 OS=Homo sapiens OX=9606 GN=NEIL2 PE=1 SV=3
MPEGPLVRKFHHLVSPFVGQQVVKTGGSSKKLQPASLQSLWLQDTQVHGKKLFLRFDLDE
EMGPPGSSPTPEPPQKEVQKEGAADPKQVGEPSGQKTLDGSSRSAELVPQGEDDSEYLER
DAPAGDAGRWLRVSFGLFGSVWVNDFSRAKKANKRGDWRDPSPRLVLHFGGGGFLAFYNC
QLSWSSSPVVTPTCDILSEKFHRGQALEALGQAQPVCYTLLDQRYFSGLGNIIKNEALYR
AGIHPLSLGSVLSASRREVLVDHVVEFSTAWLQGKFQGRPQHTQVYQKEQCPAGHQVMKE

AFGPEDGLQRLTWWCPQCQPQLSEEPEQCQFS

>sp|Q8TAT5|NEIL3_HUMAN Endonuclease 8-like 3 OS=Homo sapiens OX=9606 GN=NEIL3 PE=1 SV=3
MVEGPGCTLNGEKIRARVLPGQAVTGVRGSALRSLQGRALRLAASTVVVSPQAAALNNDS
SQNVLSLFNGYVYSGVETLGKELFMYFGPKALRIHFGMKGFIMINPLEYKYKNGASPVLE
VQLTKDLICFFDSSVELRNSMESQQRIRMMKELDVCSPEFSFLRAESEVKKQKGRMLGDV
LMDQNVLPGVGNIIKNEALFDSGLHPAVKVCQLTDEQIHHLMKMIRDFSILFYRCRKAGL
ALSKHYKVYKRPNCGQCHCRITVCRFGDNNRMTYFCPHCQKENPQHVDICKLPTRNTIIS
WTSSRVDHVMDSVARKSEEHWTCVVCTLINKPSSKACDACLTSRPIDSVLKSEENSTVFS
HLMKYPCNTFGKPHTEVKINRKTAFGTTTLVLTDFSNKSSTLERKTKQNQILDEEFQNSP
PASVCLNDIQHPSKKTTNDITQPSSKVNISPTISSESKLFSPAHKKPKTAQYSSPELKSC
NPGYSNSELQINMTDGPRTLNPDSPRCSKHNRLCILRVVGKDGENKGRQFYACPLPREAQ
CGFFEWADLSFPFCNHGKRSTMKTVLKIGPNNGKNFFVCPLGKEKQCNFFQWAENGPGIK
IIPGC

>sp|Q9NS56|TOPRS_HUMAN E3 ubiquitin-protein ligase Topors OS=Homo sapiens OX=9606 GN=TOPORS PE=1 SV=1
MGSQPPLGSPLSREEGEAPPPAPASEGRRRSRRVRLRGSCRHRPSFLGCRELAASAPARP
APASSEIMASAAKEFKMDNFSPKAGTSKLQQTVPADASPDSKCPICLDRFDNVSYLDRCL
HKFCFRCVQEWSKNKAECPLCKQPFDSIFHSVRAEDDFKEYVLRPSYNGSFVTPDRRFRY
RTTLTRERNASVYSPSGPVNRRTTTPPDSGVLFEGLGISTRPRDVEIPQFMRQIAVRRPT
TADERSLRKIQEQDIINFRRTLYRAGARVRNIEDGGRYRDISAEFFRRNPACLHRLVPWL
KRELTVLFGAHGSLVNIVQHIIMSNVTRYDLESQAFVSDLRPFLLNRTEHFIHEFISFAR
SPFNMAAFDQHANYDCPAPSYEEGSHSDSSVITISPDEAETQELDINVATVSQAPWDDET
PGPSYSSSEQVHVTMSSLLNTSDSSDEELVTGGATSQIQGVQTNDDLNNDSDDSSDNCVI
VGFVKPLAERTPELVELSSDSEDLGSYEKMETVKTQEQEQSYSSGDSDVSRCSSPHSVLG
KDEQINKGHCDSSTRIKSKKEEKRSTSLSSPRNLNSSVRGDRVYSPYNHRHRKRGRSRSS
DSRSQSRSGHDQKNHRKHHGKKRMKSKRSRSRESSRPRGRRDKKRSRTRDSSWSRRSQTL
SLSSESTSRSRSRSSDHGKRRSRSRNRDRYYLRNNYGSRYKWEYTYYSRNKDRDGYESSY
RRRTLSRAHYSRQSSSPEFRVQSFSERTNARKKNNHSERKYYYYERHRSRSLSSNRSRTA
STGTDRVRNEKPGGKRKYKTRHLEGTNEVAQPSREFASKAKDSHYQKSSSKLDGNYKNES
DTFSDSRSSDRETKHKRRKRKTRSLSVEIVYEGKATDTTKHHKKKKKHKKKHKKHHGDN
ASRSPVVITIDSDSDKDSEVKEDTECDNSGPQDPLQNEFLAPSLEPFETKDVVTIEAEFG
VLDKECDIATLSNNLNNANKTVDNIPPLAASVEQTLDVREESTFVSDLENQPSNIVSLQT
EPSRQLPSPRTSLMSVCLGRDCDMS

>sp|P46100|ATRX_HUMAN Transcriptional regulator ATRX OS=Homo sapiens OX=9606 GN=ATRX PE=1 SV=5
MTAEPMSESKLNTLVQKLHDFLAHSSEESEETSSPPRLAMNQNTDKISGSGSNSDMMENS
KEEGTSSSEKSKSSGSSRSKRKPSIVTKYVESDDEKPLDDETVNEDASNENSENDITMQS
LPKGTVIVQPEPVLNEDKDDFKGPEFRSRSKMKTENLKKRGEDGLHGIVSCTACGQQVNH
FQKDSIYRHPSLQVLICKNCFKYYMSDDISRDSDGMDEQCRWCAEGGNLICCDFCHNAFC
KKCILRNLGRKELSTIMDENNQWYCYICHPEPLLDLVTACNSVFENLEQLLQQNKKKIKV
DSEKSNKVYEHTSRFSPKKTSSNCNGEEKKLDDSCSGSVTYSYSALIVPKEMIKKAKKLI

ETTANMNSSYVKFLKQATDNSEISSATKLRQLKAFKSVLADIKKAHLALEEDLNSEFRAM
DAVNKEKNTKEHKVIDAKFETKARKGEKPCALEKKDISKSEAKLSRKQVDSEHMHQNVPT
EEQRTNKSTGGEHKKSDRKEEPQYEPANTSEDLDMDIVSVPSSVPEDIFENLETAMEVQS
SVDHQGDGSSGTEQEVESSSVKLNISSKDNRGGIKSKTTAKVTKELYVKLTPVSLSNSPI
KGADCQEVPQDKDGYKSCGLNPKLEKCGLGQENSDNEHLVENEVSLLLEESDLRRSPRVK
TTPLRRPTETNPVTSNSDEECNETVKEKQKLSVPVRKKDKRNSSDSAIDNPKPNKLPKSK
QSETVDQNSDSDEMLAILKEVSRMSHSSSSDTDINEIHTNHKTLYDLKTQAGKDDKGKRK
RKSSTSGSDFDTKKGKSAKSSIISKKKRQTQSESSNYDSELEKEIKSMSKIGAARTTKKR
IPNTKDFDSSEDEKHSKKGMDNQGHKNLKTSQEGSSDDAERKQERETFSSAEGTVDKDTT
IMELRDRLPKKQQASASTDGVDKLSGKEQSFTSLEVRKVAETKEKSKHLKTKTCKKVQDG
LSDIAEKFLKKDQSDETSEDDKKQSKKGTEEKKKPSDFKKKVIKMEQQYESSSDGTEKLP
EREEICHFPKGIKQIKNGTTDGEKKSKKIRDKTSKKKDELSDYAEKSTGKGDSCDSSEDK
KSKNGAYGREKKRCKLLGKSSRKRQDCSSSDTEKYSMKEDGCNSSDKRLKRIELRERRNL
SSKRNTKEIQSGSSSSDAEESSEDNKKKKQRTSSKKKAVIVKEKKRNSLRTSTKRKQADI
TSSSSSDIEDDDQNSIGEGSSDEQKIKPVTENLVLSSHTGFCQSSGDEALSKSVPVTVDD
DDDDNDPENRIAKKMLLEEIKANLSSDEDGSSDDEPEEGKKRTGKQNEENPGDEEAKNQV
NSESDSDSEESKKPRYRHRLLRHKLTVSDGESGEEKKTKPKEHKEVKGRNRRKVSSEDSE
DSDFQESGVSEEVSESEDEQRPRTRSAKKAELEENQRSYKQKKKRRRIKVQEDSSSENKS
NSEEEEEEKEEEEEEEEEEEEEEEDENDDSKSPGKGRKKIRKILKDDKLRTETQNALKEE
EERRKRIAERERERERKLREVIEIEDASPTKCPITTKLVLDEDEETKEPLVQVHRNMVIKL
KPHQVDGVQFMWDCCCESVKKTKKSPGSGCILAHCMGLGKTLQVVSFLHTVLLCDKLDFS
TALVVCPLNTALNWMNEFEKWQEGLKDDEKLEVSELATVKRPQERSYMLQRWQEDGGVMI
IGYEMYRNLAQGRNVKSRKLKEIFNKALVDPGPDFVVCDEGHILKNEASAVSKAMNSIRS
RRRIILTGTPLQNNLIEYHCMVNFIKENLLGSIKEFRNRFINPIQNGQCADSTMVDVRVM
KKRAHILYEMLAGCVQRKDYTALTKFLPPKHEYVLAVRMTSIQCKLYQYYLDHLTGVGNN
SEGGRGKAGAKLFQDFQMLSRIWTHPWCLQLDYISKENKGYFDEDSMDEFIASDSDETSM
SLSSDDYTKKKKKGKKGKKDSSSSGSGSDNDVEVIKVWNSRSRGGGEGNVDETGNNPSVS
LKLEESKATSSSNPSSPAPDWYKDFVTDADAEVLEHSGKMVLLFEILRMAEEIGDKVLVF
SQSLISLDLIEDFLELASREKTEDKDKPLIYKGEGKWLRNIDYYRLDGSTTAQSRKKWAE
EFNDETNVRGRLFIISTKAGSLGINLVAANRVIIFDASWNPSYDIQSIFRVYRFGQTKPV
YVYRFLAQGTMEDKIYDRQVTKQSLSFRVVDQQQVERHFTMNELTELYTFEPDLLDDPNS
EKKKKRDTPMLPKDTILAELLQIHKEHIVGYHEHDSLLDHKEEEELTEEERKAAWAEYEA
EKKGLTMRFNIPTGTNLPPVSFNSQTPYIPFNLGALSAMSNQQLEDLINQGREKVVEATN
SVTAVRIQPLEDIISAVWKENMNLSEAQVQALALSRQASQELDVKRREAIYNDVLTKQQM
LISCVQRILMNRRLQQQYNQQQQQMTYQQATLGHLMMPKPPNLIMNPSNYQQIDMRGMY
QPVAGGMQPPPLQRAPPPMRSKNPGPSQGKSM

>sp|Q9Y6K1|DNM3A_HUMAN DNA (cytosine-5)-methyltransferase 3A OS=Homo sapiens OX=9606 GN=DNMT3A PE=1 SV=4
MPAMPSSGPGDTSSSAAEREEDRKDGEEQEEPRGKEERQEPSTTARKVGRPGRKRKHPPV
ESGDTPKDPAVISKSPSMAQDSGASELLPNGDLEKRSEPQPEEGSPAGGQKGGAPAEGEG
AAETLPEASRAVENGCCTPKEGRGAPAEAGKEQKETNIESMKMEGSRGRLRGGLGWESSL
RQRPMPRLTFQAGDPYYISKRKRDEWLARWKREAEKKAKVIAGMNAVEENQGPGESQKVE
EASPPAVQQPTDPASPTVATTPEPVGSDAGDKNATKAGDDEPEYEDGRGFGIGELVWGKL

RGFSWWPGRIVSWWMTGRSRAAEGTRWVMWFGDGKFSVVCVEKLMPLSSFCSAFHQATYN
KQPMYRKAIYEVLQVASSRAGKLFPVCHDSDESDTAKAVEVQNKPMIEWALGGFQPSGPK
GLEPPEEEKNPYKEVYTDMWVEPEAAAYAPPPPAKKPRKSTAEKPKVKEIIDERTRERLV
YEVRQKCRNIEDICISCGSLNVTLEHPLFVGGMCQNCKNCFLECAYQYDDDGYQSYCTIC
CGGREVLMCGNNNCCRCFCVECVDLLVGPGAAQAAIKEDPWNCYMCGHKGTYGLLRRRED
WPSRLQMFFANNHDQEFDPPKVYPPVPAEKRKPIRVLSLFDGIATGLLVLKDLGIQVDRY
IASEVCEDSITVGMVRHQGKIMYVGDVRSVTQKHIQEWGPFDLVIGGSPCNDLSIVNPAR
KGLYEGTGRLFFEFYRLLHDARPKEGDDRPFFWLFENVVAMGVSDKRDISRFLESNPVMI
DAKEVSAAHRARYFWGNLPGMNRPLASTVNDKLELQECLEHGRIAKFSKVRTITTRSNSI
KQGKDQHFPVFMNEKEDILWCTEMERVFGFPVHYTDVSNMSRLARQRLLGRSWSVPVIRH
LFAPLKEYFACV

>sp|Q9UBC3|DNM3B_HUMAN DNA (cytosine-5)-methyltransferase 3B OS=Homo sapiens OX=9606 GN=DNMT3B PE=1 SV=1
MKGDTRHLNGEEDAGGREDSILVNGACSDQSSDSPPILEAIRTPEIRGRRSSSRLSKREV
SSLLSYTQDLTGDGDGEDGDGSDTPVMPKLFRETRTRSESPAVRTRNNNSVSSRERHRPS
PRSTRGRQGRNHVDESPVEFPATRSLRRRATASAGTPWPSPPSSYLTIDLTDDTEDTHGT
PQSSSTPYARLAQDSQQGGMESPQVEADSGDGDSSEYQDGKEFGIGDLVWGKIKGFSWWP
AMVVSWKATSKRQAMSGMRWVQWFGDGKFSEVSADKLVALGLFSQHFNLATFNKLVSYRK
AMYHALEKARVRAGKTFPSSPGDSLEDQLKPMLEWAHGGFKPTGIEGLKPNNTQPVVNKS
KVRRAGSRKLESRKYENKTRRRTADDSATSDYCPAPKRLKTNCYNNGKDRGDEDQSREQM
ASDVANNKSSLEDGCLSCGRKNPVSFHPLFEGGLCQTCRDRFLELFYMYDDDGYQSYCTV
CCEGRELLLCSNTSCCRCFCVECLEVLVGTGTAAEAKLQEPWSCYMCLPQRCHGVLRRRK
DWNVRLQAFFTSDTGLEYEAPKLYPAIPAARRRPIRVLSLFDGIATGYLVLKELGIKVGK
YVASEVCEESIAVGTVKHEGNIKYVNDVRNITKKNIEEWGPFDLVIGGSPCNDLSNVNPA
RKGLYEGTGRLFFEFYHLLNYSRPKEGDDRPFFWMFENVVAMKVGDKRDISRFLECNPVM
IDAIKVSAAHRARYFWGNLPGMNRPVIASKNDKLELQDCLEYNRIAKLKKVQTITTKSNS
IKQGKNQLFPVVMNGKEDVLWCTELERIFGFPVHYTDVSNMGRGARQKLLGRSWSVPVIR
HLFAPLKDYFACE

>sp|P26358|DNMT1_HUMAN DNA (cytosine-5)-methyltransferase 1 OS=Homo sapiens OX=9606 GN=DNMT1 PE=1 SV=2
MPARTAPARVPTLAVPAISLPDDVRRRLKDLERDSLTEKECVKEKLNLLHEFLQTEIKNQ
LCDLETKLRKEELSEEGYLAKVKSLLNKDLSLENGAHAYNREVNGRLENGNQARSEARRV
GMADANSPPKPLSKPRTPRRSKSDGEAKPEPSPSPRITRKSTRQTTITSHFAKGPAKRKP
QEESERAKSDESIKEEDKDQDEKRRRVTSRERVARPLPAEEPERAKSGTRTEKEEERDEK
EEKRLRSQTKEPTPKQKLKEEPDREARAGVQADEDEDGDEKDEKKHRSQPKDLAAKRRPE
EKEPEKVNPQISDEKDEDEKEEKRRKTTPKEPTEKKMARAKTVMNSKTHPPKCIQCGQYL
DDPDLKYGQHPPDAVDEPQMLTNEKLSIFDANESGFESYEALPQHKLTCFSVYCKHGHLC
PIDTGLIEKNIELFFSGSAKPIYDDDPSLEGGVNGKNLGPINEWWITGFDGGEKALIGFS
TSFAEYILMDPSPEYAPIFGLMQEKIYISKIVVEFLQSNSDSTYEDLINKIETTVPPSGL
NLNRFTEDSLLRHAQFVVEQVESYDEAGDSDEQPIFLTPCMRDLIKLAGVTLGQRRAQAR
RQTIRHSTREKDRGPTKATTTKLVYQIFDTFFAEQIEKDDREDKENAFKRRRCGVCEVCQ
QPECGKCKACKDMVKFGGSGRSKQACQERRCPNMAMKEADDDEEVDDNIPEMPSPKKMHQ

GKKKKQNKNRISWVGEAVKTDGKKSYYKKVCIDAETLEVGDCVSVIPDDSSKPLYLARVT
ALWEDSSNGQMFHAHWFCAGTDTVLGATSDPLELFLVDECEDMQLSYIHSKVKVIYKAPS
ENWAMEGGMDPESLLEGDDGKTYFYQLWYDQDYARFESPPKTQPTEDNKFKFCVSCARLA
EMRQKEIPRVLEQLEDLDSRVLYYSATKNGILYRVGDGVYLPPEAFTFNIKLSSPVKRPR
KEPVDEDLYPEHYRKYSDYIKGSNLDAPEPYRIGRIKEIFCPKKSNGRPNETDIKIRVNK
FYRPENTHKSTPASYHADINLLYWSDEEAVVDFKAVQGRCTVEYGEDLPECVQVYSMGGP
NRFYFLEAYNAKSKSFEDPPNHARSPGNKGKGKGKGKGKPKSQACEPSEPEIEIKLPKLR
TLDVFSGCGGLSEGFHQAGISDTLWAIEMWDPAAQAFRLNNPGSTVFTEDCNILLKLVMA
GETTNSRGQRLPQKGDVEMLCGGPPCQGFSGMNRFNSRTYSKFKNSLVVSFLSYCDYYRP
RFFLLENVRNFVSFKRSMVLKLTLRCLVRMGYQCTFGVLQAGQYGVAQTRRRAIILAAAP
GEKLPLFPEPLHVFAPRACQLSVVVDDKKFVSNITRLSSGPFRTITVRDTMSDLPEVRNG
ASALEISYNGEPQSWFQRQLRGAQYQPILRDHICKDMSALVAARMRHIPLAPGSDWRDLP
NIEVRLSDGTMARKLRYTHHDRKNGRSSSGALRGVCSCVEAGKACDPAARQFNTLIPWCL
PHTGNRHNHWAGLYGRLEWDGFFSTTVTNPEPMGKQGRVLHPEQHRVVSVRECARSQGFP
DTYRLFGNILDKHRQVGNAVPPPLAKAIGLEIKLCMLAKARESASAKIKEEEAAKD

>sp|Q06787|FMR1_HUMAN Synaptic functional regulator FMR1 OS=Homo sapiens OX=9606 GN=FMR1 PE=1 SV=1
MEELVVEVRGSNGAFYKAFVKDVHEDSITVAFENNWQPDRQIPFHDVRFPPPVGYNKDIN
ESDEVEVYSRANEKEPCCWWLAKVRMIKGEFYVIEYAACDATYNEIVTIERLRSVNPNKP
ATKDTFHKIKLDVPEDLRQMCAKEAAHKDFKKAVGAFSVTYDPENYQLVILSINEVTSKR
AHMLIDMHFRSLRTKLSLIMRNEEASKQLESSRQLASRFHEQFIVREDLMGLAIGTHGAN
IQQARKVPGVTAIDLDEDTCTFHIYGEDQDAVKKARSFLEFAEDVIQVPRNLVGKVIGKN
GKLIQEIVDKSGVVRVRIEAENEKNVPQEEEIMPPNSLPSNNSRVGPNAPEEKKHLDIKE
NSTHFSQPNSTKVQRVLVASSVVAGESQKPELKAWQGMVPFVFVGTKDSIANATVLLDYH
LNYLKEVDQLRLERLQIDEQLRQIGASSRPPPNRTDKEKSYVTDDGQGMGRGSRPYRNRG
HGRRGPGYTSGTNSEASNASETESDHRDELSDWSLAPTEEERESFLRRGDGRRRGGGGRG
QGGRGRGGGFKGNDDHSRTDNRPRNPREAKGRTTDGSLQIRVDCNNERSVHTKTLQNTSS
EGSRLRTGKDRNQKKEKPDSVDGQQPLVNGVP

>sp|O14672|ADA10_HUMAN Disintegrin and metalloproteinase domain-containing protein 10 OS=Homo sapiens OX=9606 GN=ADAM10 PE=1 SV=1
MVLLRVLILLLSWAAGMGGQYGNPLNKYIRHYEGLSYNVDSLHQKHQRAKRAVSHEDQFL
RLDFHAHGRHFNLRMKRDTSLFSDEFKVETSNKVLDYDTSHIYTGHIYGEEGSFSHGSVI
DGRFEGFIQTRGGTFYVEPAERYIKDRTLPFHSVIYHEDDINYPHKYGPQGGCADHSVFE
RMRKYQMTGVEEVTQIPQEEHAANGPELLRKKRTTSAEKNTCQLYIQTDHLFFKYYGTRE
AVIAQISSHVKAIDTIYQTTDFSGIRNISFMVKRIRINTTADEKDPTNPFRFPNIGVEKF
LELNSEQNHDDYCLAYVFTDRDFDDGVLGLAWVGAPSGSSGGICEKSKLYSDGKKKSLNT
GIITVQNYGSHVPPKVSHITFAHEVGHNFGSPHDSGTECTPGESKNLGQKENGNYIMYAR
ATSGDKLNNNKFSLCSIRNISQVLEKKRNNCFVESGQPICGNGMVEQGEECDCGYSDQCK
DECCFDANQPEGRKCKLKPGKQCSPSQGPCCTAQCAFKSKSEKCRDDSDCAREGICNGFT
ALCPASDPKPNFTDCNRHTQVCINGQCAGSICEKYGLEECTCASSDGKDDKELCHVCCMK
KMDPSTCASTGSVQWSRHFSGRTITLQPGSPCNDFRGYCDVFMRCRLVDADGPLARLKKA
IFSPELYENIAEWIVAHWWAVLLMGIALIMLMAGFIKICSVHTPSSNPKLPPPKPLPGTL

KRRRPPQPIQQPQRQRPRESYQMGHMRR

>tr|A0A024QZ77|A0A024QZ77_HUMAN EF-hand domain family, member D2, isoform CRA_a OS=Homo sapiens OX=9606 GN=EFHD2 PE=2 SV=1
MATDELATKLSRRLQMEGEGGGETPEQPGLNGAAAAAAGAPDEAAEALGSADCELSAKLL
RRADLNQGIGEPQSPSRRVFNPYTEFKEFSRKQIKDMEKMFKQYDAGRDGFIDLMELKLM
MEKLGAPQTHLGLKNMIKEVDEDFDSKLSFREFLLIFRKAAAGELQEDSGLCVLARLSEI
DVSSEGVKGAKSFFEAKVQAINVSSRFEEEIKAEQEERKKQAEEMKQRKAAFKELQSTFK

>sp|Q07955|SRSF1_HUMAN Serine/arginine-rich splicing factor 1 OS=Homo sapiens OX=9606 GN=SRSF1 PE=1 SV=2
MSGGGVIRGPAGNNDCRIYVGNLPPDIRTKDIEDVFYKYGAIRDIDLKNRRGGPPFAFVE
FEDPRDAEDAVYGRDGYDYDGYRLRVEFPRSGRGTGRGGGGGGGGGAPRGRYGPPSRRSE
NRVVVSGLPPSGSWQDLKDHMREAGDVCYADVYRDGTGVVEFVRKEDMTYAVRKLDNTKF
RSHEGETAYIRVKVDGPRSPSYGRSRSRSRSRSRSRSRSNSRSRSYSPRRSRGSPRYSPR
HSRSRSRT

>sp|P26368|U2AF2_HUMAN Splicing factor U2AF 65 kDa subunit OS=Homo sapiens OX=9606 GN=U2AF2 PE=1 SV=4
MSDFDEFERQLNENKQERDKENRHRKRSHSRSRSRDRKRRSRSRDRRNRDQRSASRDRRR
RSKPLTRGAKEEHGGLIRSPRHEKKKKVRKYWDVPPPGFEHITPMQYKAMQAAGQIPATA
LLPTMTPDGLAVTPTPVPVVGSQMTRQARRLYVGNIPFGITEEAMMDFFNAQMRLGGLTQ
APGNPVLAVQINQDKNFAFLEFRSVDETTQAMAFDGIIFQGQSLKIRRPHDYQPLPGMSE
NPSVYVPGVVSTVVPDSAHKLFIGGLPNYLNDDQVKELLTSFGPLKAFNLVKDSATGLSK
GYAFCEYVDINVTDQAIAGLNGMQLGDKKLLVQRASVGAKNATLVSPPSTINQTPVTLQV
PGLMSSQVQMGGHPTEVLCLMNMVLPEELLDDEEYEEIVEDVRDECSKYGLVKSIEIPRP
VDGVEVPGCGKIFVEFTSVFDCQKAMQGLTGRKFANRVVVTKYCDPDSYHRRDFW

>sp|Q8NC60|NOA1_HUMAN Nitric oxide-associated protein 1 OS=Homo sapiens OX=9606 GN=NOA1 PE=1 SV=2
MLPARLPFRLLSLFLRGSAPTAARHGLREPLLERRCAAASSFQHSSSLGRELPYDPVDTE
GFGEGGDMQERFLFPEYILDPEPQPTREKQLQELQQQQEEEERQRQQRREERRQQNLRAR
SREHPVVGHPDPALPPSGVNCSGCGAELHCQDAGVPGYLPREKFLRTAEADGGLARTVCQ
RCWLLSHHRRALRLQVSREQYLELVSAALRRPGPSLVLYMVDLLDLPDALLPDLPALVGP
KQLIVLGNKVDLLPQDAPGYRQRLRERLWEDCARAGLLLAPGHQGPQRPVKDEPQDGENP
NPPNWSRTVVRDVRLISAKTGYGVEELISALQRSWRYRGDVYLVGATNAGKSTLFNTLLE
SDYCTAKGSEAIDRATISPWPGTTLNLLKFPICNPTPYRMFKRHQRLKKDSTQAEEDLSE
QEQNQLNVLKKHGYVVGRVGRTFLYSEEQKDNIPFEFDADSLAFDMENDPVMGTHKSTKQ
VELTAQDVKDAHWFYDTPGITKENCILNLLTEKEVNIVLPTQSIVPRTFVLKPGMVLFLG
AIGRIDFLQGNQSAWFTVVASNILPVHITSLDRADALYQKHAGHTLLQIPMGGKERMAGF
PPLVAEDIMLKEGLGASEAVADIKFSSAGWVSVTPNFKDRLHLRGYTPEGTVLTVRPPLL
PYIVNIKGQRIKKSVAYKTKKPPSLMYNVRKKKGKINV

>sp|P52597|HNRPF_HUMAN Heterogeneous nuclear ribonucleoprotein F OS=Homo sapiens OX=9606 GN=HNRNPF PE=1 SV=3
MMLGPEGGEGFVVKLRGLPWSCSVEDVQNFLSDCTIHDGAAGVHFIYTREGRQSGEAFVE
LGSEDDVKMALKKDRESMGHRYIEVFKSHRTEMDWVLKHSGPNSADSANDGFVRLRGLPF
GCTKEEIVQFFSGLEIVPNGITLPVDPEGKITGEAFVQFASQELAEKALGKHKERIGHRY
IEVFKSSQEEVRSYSDPPLKFMSVQRPGPYDRPGTARRYIGIVKQAGLERMRPGAYSTGY
GGYEEYSGLSDGYGFTTDLFGRDLSYCLSGMYDHRYGDSEFTVQSTTGHCVHMRGLPYKA
TENDIYNFFSPLNPVRVHIEIGPDGRVTGEADVEFATHEEAVAAMSKDRANMQHRYIELF
LNSTTGASNGAYSSQVMQGMGVSAAQATYSGLESQSVSGCYGAGYSGQNSMGGYD

>tr|Q6IB29|Q6IB29_HUMAN EBNA1 binding protein 2 OS=Homo sapiens OX=9606 GN=EBNA1BP2 PE=1 SV=1
MDTPPLSDSESESDESLVTDRELQDAFSRGLLKPGLNVVLEGPKKAVNDVNGLKQCLAEF
KRDLEWVERLDVTLGPVPEIGGSEAPAPQNKDQKAVDPEDDFQREMSFYRQAQAAVLAVL
PRLHQLKVPTKRPTDYFAEMAKSDLQMQKIRQKLQTKQAAMERSEKAKQLRALRKYGKKV
QTEVLQKRQQEKAHMMNAIKKYQKGFSDKLDFLEGDQKPLAQRKKAGAKGQQMRKGPSAK
RRYKNQKFGFGGKKKGSKWNTRESYDDVSSFRAKTAHGRGLKRPGKKGSNKRPGKRTREK
MKNRTH

>sp|P51991|ROA3_HUMAN Heterogeneous nuclear ribonucleoprotein A3 OS=Homo sapiens OX=9606 GN=HNRNPA3 PE=1 SV=2
MEVKPPPGRPQPDSGRRRRRRGEEGHDPKEPEQLRKLFIGGLSFETTDDSLREHFEKWGT
LTDCVVMRDPQTKRSRGFGFVTYSCVEEVDAAMCARPHKVDGRVVEPKRAVSREDSVKPG
AHLTVKKIFVGGIKEDTEEYNLRDYFEKYGKIETIEVMEDRQSGKKRGFAFVTFDDHDTV
DKIVVQKYHTINGHNCEVKKALSKQEMQSAGSQRGRGGGSGNFMGRGGNFGGGGGNFGRG
GNFGGRGGYGGGGGGSRGSYGGGDGGYNGFGGDGGNYGGGPGYSSRGGYGGGGPGYGNQG
GGYGGGGGYDGYNEGGNFGGGNYGGGGNYNDFGNYSGQQQSNYGPMKGGSFGGRSSGSPY
GGGYGSGGGSGGYGSRRF

>sp|P23246|SFPQ_HUMAN Splicing factor, proline- and glutamine-rich OS=Homo sapiens OX=9606 GN=SFPQ PE=1 SV=2
MSRDRFRSRGGGGGGFHRRGGGGGRGGLHDFRSPPPGMGLNQNRGPMGPGPGQSGPKPPI
PPPPPHQQQQQPPPQQPPPQQPPPHQPPPHPQPHQQQQPPPPPQDSSKPVVAQGPGPAPG
VGSAPPASSSAPPATPPTSGAPPGSGPGPTPTPPPAVTSAPPGAPPPTPPSSGVPTTPPQ
AGGPPPPPAAVPGPGPGPKQGPGPGGPKGGKMPGGPKPGGGPGLSTPGGHPKPPHRGGGE
PRGGRQHHPPYHQQHHQGPPPGGPGGRSEEKISDSEGFKANLSLLRRPGEKTYTQRCRLF
VGNLPADITEDEFKRLFAKYGEPGEVFINKGKGFGFIKLESRALAEIAKAELDDTPMRGR
QLRVRFATHAAALSVRNLSPYVSNELLEEAFSQFGPIERAVVIVDDRGRSTGKGIVEFAS
KPAARKAFERCSEGVFLLTTTPRPVIVEPLEQLDDEDGLPEKLAQKNPMYQKERETPPRF
AQHGTFEYEYSQRWKSLDEMEKQQREQVEKNMKDAKDKLESEMEDAYHEHQANLLRQDLM
RRQEELRRMEELHNQEMQKRKEMQLRQEEERRRREEEMMIRQREMEEQMRRQREESYSRM
GYMDPRERDMRMGGGGAMNMGDPYGSGGQKFPPLGGGGGIGYEANPGVPPATMSGSMMGS
DMRTERFGQGGAGPVGGQGPRGMGPGTPAGYGRGREEYEGPNKKPRF

>sp|Q15393|SF3B3_HUMAN Splicing factor 3B subunit 3 OS=Homo sapiens OX=9606 GN=SF3B3 PE=1 SV=4
MFLYNLTLQRATGISFAIHGNFSGTKQQEIVVSRGKILELLRPDPNTGKVHTLLTVEVFG
VIRSLMAFRLTGGTKDYIVVGSDSGRIVILEYQPSKNMFEKIHQETFGKSGCRRIVPGQF
LAVDPKGRAVMISAIEKQKLVYILNRDAAARLTISSPLEAHKANTLVYHVVGVDVGFENP
MFACLEMDYEEADNDPTGEAAANTQQTLTFYELDLGLNHVVRKYSEPLEEHGNFLITVPG
GSDGPSGVLICSENYITYKNFGDQPDIRCPIPRRRNDLDDPERGMIFVCSATHKTKSMFF
FLAQTEQGDIFKITLETDEDMVTEIRLKYFDTVPVAAAMCVLKTGFLFVASEFGNHYLYQ
IAHLGDDDEEPEFSSAMPLEEGDTFFFQPRPLKNLVLVDELDSLSPILFCQIADLANEDT
PQLYVACGRGPRSSLRVLRHGLEVSEMAVSELPGNPNAVWTVRRHIEDEFDAYIIVSFVN
ATLVLSIGETVEEVTDSGFLGTTPTLSCSLLGDDALVQVYPDGIRHIRADKRVNEWKTPG
KKTIVKCAVNQRQVVIALTGGELVYFEMDPSGQLNEYTERKEMSADVVCMSLANVPPGEQ
RSRFLAVGLVDNTVRIISLDPSDCLQPLSMQALPAQPESLCIVEMGGTEKQDELGERGSI
GFLYLNIGLQNGVLLRTVLDPVTGDLSDTRTRYLGSRPVKLFRVRMQGQEAVLAMSSRSW
LSYSYQSRFHLTPLSYETLEFASGFASEQCPEGIVAISTNTLRILALEKLGAVFNQVAFP
LQYTPRKFVIHPESNNLIIIETDHNAYTEATKAQRKQQMAEEMVEAAGEDERELAAEMAA
AFLNENLPESIFGAPKAGNGQWASVIRVMNPIQGNTLDLVQLEQNEAAFSVAVCRFSNTG
EDWYVLVGVAKDLILNPRSVAGGFVYTYKLVNNGEKLEFLHKTPVEEVPAAIAPFQGRVL
IGVGKLLRVYDLGKKKLLRKCENKHIANYISGIQTIGHRVIVSDVQESFIWVRYKRNENQ
LIIFADDTYPRWVTTASLLDYDTVAGADKFGNICVVRLPPNTNDEVDEDPTGNKALWDRG
LLNGASQKAEVIMNYHVGETVLSLQKTTLIPGGSESLVYTTLSGGIGILVPFTSHEDHDF
FQHVEMHLRSEHPPLCGRDHLSFRSYYFPVKNVIDGDLCEQFNSMEPNKQKNVSEELDRT
PPEVSKKLEDIRTRYAF

>sp|Q15717|ELAV1_HUMAN ELAV-like protein 1 OS=Homo sapiens OX=9606 GN=ELAVL1 PE=1 SV=2
MSNGYEDHMAEDCRGDIGRTNLIVNYLPQNMTQDELRSLFSSIGEVESAKLIRDKVAGHS
LGYGFVNYVTAKDAERAINTLNGLRLQSKTIKVSYARPSSEVIKDANLYISGLPRTMTQK
DVEDMFSRFGRIINSRVLVDQTTGLSRGVAFIRFDKRSEAEEAITSFNGHKPPGSSEPIT
VKFAANPNQNKNVALLSQLYHSPARRFGGPVHHQAQRFRFSPMGVDHMSGLSGVNVPGNA
SSGWCIFIYNLGQDADEGILWQMFGPFGAVTNVKVIRDFNTNKCKGFGFVTMTNYEEAAM
AIASLNGYRLGDKILQVSFKTNKSHK

>sp|Q12906|ILF3_HUMAN Interleukin enhancer-binding factor 3 OS=Homo sapiens OX=9606 GN=ILF3 PE=1 SV=3
MRPMRIFVNDDRHVMAKHSSVYPTQEELEAVQNMVSHTERALKAVSDWIDEQEKGSSEQA
ESDNMDVPPEDDSKEGAGEQKTEHMTRTLRGVMRVGLVAKGLLLKGDLDLELVLLCKEKP
TTALLDKVADNLAIQLAAVTEDKYEILQSVDDAAIVIKNTKEPPLSLTIHLTSPVVREEM
EKVLAGETLSVNDPPDVLDRQKCLAALASLRHAKWFQARANGLKSCVIVIRVLRDLCTRV
PTWGPLRGWPLELLCEKSIGTANRPMGAGEALRRVLECLASGIVMPDGSGIYDPCEKEAT
DAIGHLDRQQREDITQSAQHALRLAAFGQLHKVLGMDPLPSKMPKKPKNENPVDYTVQIP
PSTTYAITPMKRPMEEDGEEKSPSKKKKKIQKKEEKAEPPQAMNALMRLNQLKPGLQYKL
VSQTGPVHAPIFTMSVEVDGNSFEASGPSKKTAKLHVAVKVLQDMGLPTGAEGRDSSKGE
DSAEETEAKPAVVAPAPVVEAVSTPSAAFPSDATAEQGPILTKHGKNPVMELNEKRRGLK
YELISETGGSHDKRFVMEVEVDGQKFQGAGSNKKVAKAYAALAALEKLFPDTPLALDANK

KKRAPVPVRGGPKFAAKPHNPGFGMGGPMHNEVPPPPNLRGRGRGGSIRGRGRGRGFGGA
NHGGYMNAGAGYGSYGYGGNSATAGYSQFYSNGGHSGNASGGGGGGGGGSSGYGSYYQGD
NYNSPVPPKHAGKKQPHGGQQKPSYGSGYQSHQGQQQSYNQSPYSNYGPPQGKQKGYNHG
QGSYSYSNSYNSPGGGGGSDYNYESKFNYSGSGGRSGGNSYGSGGASYNPGSHGGYGGGS
GGGSSYQGKQGGYSQSNYNSPGSGQNYSGPPSSYQSSQGGYGRNADHSMNYQYR

>sp|Q15233|NONO_HUMAN Non-POU domain-containing octamer-binding protein OS=Homo sapiens OX=9606 GN=NONO PE=1 SV=4
MQSNKTFNLEKQNHTPRKHHQHHHQQQHHQQQQQQPPPPPIPANGQQASSQNEGLTIDLK
NFRKPGEKTFTQRSRLFVGNLPPDITEEEMRKLFEKYGKAGEVFIHKDKGFGFIRLETRT
LAEIAKVELDNMPLRGKQLRVRFACHSASLTVRNLPQYVSNELLEEAFSVFGQVERAVVI
VDDRGRPSGKGIVEFSGKPAARKALDRCSEGSFLLTTFPRPVTVEPMDQLDDEEGLPEKL
VIKNQQFHKEREQPPRFAQPGSFEYEYAMRWKALIEMEKQQQDQVDRNIKEAREKLEMEM
EAARHEHQVMLMRQDLMRRQEELRRMEELHNQEVQKRKQLELRQEEERRRREEEMRRQQE
EMMRRQQEGFKGTFPDAREQEIRMGQMAMGGAMGINNRGAMPPAPVPAGTPAPPGPATMM
PDGTLGLTPPTTERFGQAATMEGIGAIGGTPPAFNRAAPGAEFAPNKRRRY

>sp|O43390|HNRPR_HUMAN Heterogeneous nuclear ribonucleoprotein R OS=Homo sapiens OX=9606 GN=HNRNPR PE=1 SV=1
MANQVNGNAVQLKEEEEPMDTSSVTHTEHYKTLIEAGLPQKVAERLDEIFQTGLVAYVDL
DERAIDALREFNEEGALSVLQQFKESDLSHVQNKSAFLCGVMKTYRQREKQGSKVQESTK
GPDEAKIKALLERTGYTLDVTTGQRKYGGPPPDSVYSGVQPGIGTEVFVGKIPRDLYEDE
LVPLFEKAGPIWDLRLMMDPLSGQNRGYAFITFCGKEAAQEAVKLCDSYEIRPGKHLGVC
ISVANNRLFVGSIPKNKTKENILEEFSKVTEGLVDVILYHQPDDKKKNRGFCFLEYEDHK
SAAQARRRLMSGKVKVWGNVVTVEWADPVEEPDPEVMAKVKVLFVRNLATTVTEEILEKS
FSEFGKLERVKKLKDYAFVHFEDRGAAVKAMDEMNGKEIEGEEIEIVLAKPPDKKRKERQ
AARQASRSTAYEDYYYHPPPRMPPPIRGRGRGGGRGGYGYPPDYYGYEDYYDDYYGYDYH
DYRGGYEDPYYGYDDGYAVRGRGGGRGGRGAPPPPRGRGAPPPRGRAGYSQRGAPLGPPR
GSRGGRGGPAQQQRGRGSRGSRGNRGGNVGGKRKADGYNQPDSKRRQTNNQQNWGSQPIA
QQPLQQGGDYSGNYGYNNDNQEFYQDTYGQQWK

>sp|Q15554|TERF2_HUMAN Telomeric repeat-binding factor 2 OS=Homo sapiens OX=9606 GN=TERF2 PE=1 SV=3
MAAGAGTAGPASGPGVVRDPAASQPRKRPGREGGEGARRSDTMAGGGGSSDGSGRAAGRR
ASRSSGRARRGRHEPGLGGPAERGAGEARLEEAVNRWVLKFYFHEALRAFRGSRYGDFRQ
IRDIMQALLVRPLGKEHTVSRLLRVMQCLSRIEEGENLDCSFDMEAELTPLESAINVLEM
IKTEFTLTEAVVESSRKLVKEAAVIICIKNKEFEKASKILKKHMSKDPTTQKLRNDLLNI
IREKNLAHPVIQNFSYETFQQKMLRFLESHLDDAEPYLLTMAKKALKSESAASSTGKEDK
QPAPGPVEKPPREPARQLRNPPTTIGMMTLKAAFKTLSGAQDSEAAFAKLDQKDLVLPTQ
ALPASPALKNKRPRKDENESSAPADGEGGSELQPKNKRMTISRLVLEEDSQSTEPSAGLN
SSQEAASAPPSKPTVLNQPLPGEKNPKVPKGKWNSSNGVEEKETWVEEDELFQVQAAPDE
DSTTNITKKQKWTVEESEWVKAGVQKYGEGNWAAISKNYPFVNRTAVMIKDRWRTMKRLG
MN

>sp|P14866|HNRPL_HUMAN Heterogeneous nuclear ribonucleoprotein L OS=Homo sapiens OX=9606 GN=HNRNPL PE=1 SV=2
MSRRLLPRAEKRRRRLEQRQQPDEQRRRSGAMVKMAAAGGGGGGGGRYYGGGSEGGRAPKR
LKTDNAGDQHGGGGGGGGGAGAAGGGGGGGENYDDPHKTPASPVVHIRGLIDGVVEADLVE
ALQEFGPISYVVVMPKKRQALVEFEDVLGACNAVNYAADNQIYIAGHPAFVNYSTSQKIS
RPGDSDDSRSVNSVLLFTILNPIYSITTDVLYTICNPCGPVQRIVIFRKNGVQAMVEFDS
VQSAQRAKASLNGADIYSGCCTLKIEYAKPTRLNVFKNDQDTWDYTNPNLSGQGDPGSNP
NKRQRQPPLLGDHPAEYGGPHGGYHSHYHDEGYGPPPPHYEGRRMGPPVGGHRRGPSRYG
PQYGHPPPPPPPPEYGPHADSPVLMVYGLDQSKMNCDRVFNVFCLYGNVEKVKFMKSKPG
AAMVEMADGYAVDRAITHLNNNFMFGQKLNVCVSKQPAIMPGQSYGLEDGSCSYKDFSES
RNNRFSTPEQAAKNRIQHPSNVLHFFNAPLEVTEENFFEICDELGVKRPSSVKVFSGKSE
RSSSGLLEWESKSDALETLGFLNHYQMKNPNGPYPYTLKLCFSTAQHAS

>sp|Q15424|SAFB1_HUMAN Scaffold attachment factor B1 OS=Homo sapiens OX=9606 GN=SAFB PE=1 SV=4
MAETLSGLGDSGAAGAAALSSASSETGTRRLSDLRVIDLRAELRKRNVDSSGNKSVLMER
LKKAIEDEGGNPDEIEITSEGNKKTSKRSSKGRKPEEEGVEDNGLEENSGDGQEDVETSL
ENLQDIDIMDISVLDEAEIDNGSVADCVEDDDADNLQESLSDSRELVEGEMKELPEQLQE
HAIEDKETINNLDTSSSDFTILQEIEEPSLEPENEKILDILGETCKSEPVKEESSELEQP
FAQDTSSVGPDRKLAEEEDLFDSAHPEEGDLDLASESTAHAQSSKADSLLAVVKREPAEQ
PGDGERTDCEPVGLEPAVEQSSAASELAEASSEELAEAPTEAPSPEARDSKEDGRKFDFD
ACNEVPPAPKESSTSEGADQKMSSPEDDSDTKRLSKEEKGRSSCGRNFWVSGLSSTTRAT
DLKNLFSKYGKVVGAKVVTNARSPGARCYGFVTMSTAEEATKCINHLHKTELHGKMISVE
KAKNEPVGKKTSDKRDSDGKKEKSSNSDRSTNLKRDDKCDRKDDAKKGDDGSGEKSKDQD
DQKPGPSERSRATKSGSRGTERTVVMDKSKGVPVISVKTSGSKERASKSQDRKSASREKR
SVVSFDKVKEPRKSRDSESHSRVRERSEREQRMQAQWEREERERLEIARERLAFQRQRLE
RERMERERLERERMHVEHERRREQERIHREREELRRQQELRYEQERRPAVRRPYDLDRRD
DAYWPEAKRAALDERYHSDFNRQDRFHDFDHRDRGRYPDHSVDRREGSRSMMGEREGQHY
PERHGGPERHGRDSRDGWGGYGSDKRMSEGRGLPPPPRRDWGDHGRREDDRSWQGTADGG
MMDRDHKRWQGGERSMSGHSGPGHMMNRGGMSGRGSFAPGGASRGHPIPHGGMQGGFGGQ
SRGSRPSDARFTRRY

>sp|Q9NZI8|IF2B1_HUMAN Insulin-like growth factor 2 mRNA-binding protein 1 OS=Homo sapiens OX=9606 GN=IGF2BP1 PE=1 SV=2
MNKLYIGNLNESVTPADLEKVFAEHKISYSGQFLVKSGYAFVDCPDEHWAMKAIETFSGK
VELQGKRLEIEHSVPKKQRSRKIQIRNIPPQLRWEVLDSLLAQYGTVENCEQVNTESETA
VVNVTYSNREQTRQAIMKLNGHQLENHALKVSYIPDEQIAQGPENGRRGGFGSRGQPRQG
SPVAAGAPAKQQQVDIPLRLLVPTQYVGAIIGKEGATIRNITKQTQSKIDVHRKENAGAA
EKAISVHSTPEGCSSACKMILEIMHKEAKDTKTADEVPLKILAHNNFVGRLIGKEGRNLK
KVEQDTETKITISSLQDLTLYNPERTITVKGAIENCCRAEQEIMKKVREAYENDVAAMSL
QSHLIPGLNLAAVGLFPASSSAVPPPPSSVTGAAPYSSFMQAPEQEMVQVFIPAQAVGAI
IGKKGQHIKQLSRFASASIKIAPPETPDSKVRMVIITGPPEAQFKAQGRIYGKLKEENFF
GPKEEVKLETHIRVPASAAGRVIGKGGKTVNELQNLTAAEVVVPRDQTPDENDQVIVKII
GHFYASQMAQRKIRDILAQVKQQHQKGQSNQAQARRK

>sp|Q14151|SAFB2_HUMAN Scaffold attachment factor B2 OS=Homo sapiens OX=9606 GN=SAFB2 PE=1 SV=1
MAETLPGSGDSGPGTASLGPGVAETGTRRLSELRVIDLRAELKKRNLDTGGNKSVLMERL
KKAVKEEGQDPDEIGIELEATSKKSAKRCVKGLKMEEEGTEDNGLEDDSRDGQEDMEASL
ENLQNMGMMDMSVLDETEVANSSAPDFGEDGTDGLLDSFCDSKEYVAAQLRQLPAQPPEH
AVDGEGFKNTLETSSLNFKVTPDIEESLLEPENEKILDILGETCKSEPVKEESSELEQPF
AQDTSSVGPDRKLAEEEDLFDSAHPEEGDLDLASESTAHAQSSKADSLLAVVKREPAEQP
GDGERTDCEPVGLEPAVEQSSAASELAEASSEELAEAPTEAPSPEARDSKEDGRKFDFDA
CNEVPPAPKESSTSEGADQKMSSFKEEKDIKPIIKDEKGRVGSGSGRNLWVSGLSSTTRA
TDLKNLFSKYGKVVGAKVVTNARSPGARCYGFVTMSTSDEATKCISHLHRTELHGRMISV
EKAKNEPAGKKLSDRKECEVKKEKLSSVDRHHSVEIKIEKTVIKKEEKIEKKEEKKPEDI
KKEEKDQDELKPGPTNRSRVTKSGSRGMERTVVMDKSKGEPVISVKTTSRSKERSSKSQD
RKSESKEKRDILSFDKIKEQRERERQRQREREIRETERRREREQREREQRLEAFHERKEK
ARLQRERLQLECQRQRLERERMERERLERERMRVERERRKEQERIHREREELRRQQEQLR
YEQERRPGRRPYDLDRRDDAYWPEGKRVAMEDRYRADFPRPDHRFHDFDHRDRGQYQDHA
IDRREGSRPMMGDHRDGQHYGDDRHGHGGPPERHGRDSRDGWGGYGSDKRLSEGRGLPPP
PRGGRDWGEHNQRLEEHQARAWQGAMDAGAASREHARWQGGERGLSGPSGPGHMASRGGV
AGRGGFAQGGHSQGHVVPGGGLEGGGVASQDRGSRVPHPHPHPPPYPHFTRRY

>sp|O43143|DHX15_HUMAN Pre-mRNA-splicing factor ATP-dependent RNA helicase DHX15 OS=Homo sapiens OX=9606 GN=DHX15 PE=1 SV=2
MSKRHRLDLGEDYPSGKKRAGTDGKDRDRDRDREDRSKDRDRERDRGDREREREKEKEKE
LRASTNAMLISAGLPPLKASHSAHSTHSAHSTHSTHSAHSTHAGHAGHTSLPQCINPFTN
LPHTPRYYDILKKRLQLPVWEYKDRFTDILVRHQSFVLVGETGSGKTTQIPQWCVEYMRS
LPGPKRGVACTQPRRVAAMSVAQRVADEMDVMLGQEVGYSIRFEDCSSAKTILKYMTDGM
LLREAMNDPLLERYGVIILDEAHERTLATDILMGVLKEVVRQRSDLKVIVMSATLDAGKF
QIYFDNCPLLTIPGRTHPVEIFYTPEPERDYLEAAIRTVIQIHMCEEEEGDLLLFLTGQE
EIDEACKRIKREVDDLGPEVGDIKIIPLYSTLPPQQQQRIFEPPPPKKQNGAIGRKVVVS
TNIAETSLTIDGVVFVIDPGFAKQKVYNPRIRVESLLVTAISKASAQQRAGRAGRTRPGK
CFRLYTEKAYKTEMQDNTYPEILRSNLGSVVLQLKKLGIDDLVHFDFMDPPAPETLMRAL
ELLNYLAALNDDGDLTELGSMMAEFPLDPQLAKMVIASCDYNCSNEVLSITAMLSVPQCF
VRPTEAKKAADEAKMRFAHIDGDHLTLLNVYHAFKQNHESVQWCYDNFINYRSLMSADNV
RQQLSRIMDRFNLPRRSTDFTSRDYYINIRKALVTGYFMQVAHLERTGHYLTVKDNQVVQ
LHPSTVLDHKPEWVLYNEFVLTTKNYIRTCTDIKPEWLVKIAPQYYDMSNFPQCEAKRQL
DRIIAKLQSKEYSQY

>sp|Q12905|ILF2_HUMAN Interleukin enhancer-binding factor 2 OS=Homo sapiens OX=9606 GN=ILF2 PE=1 SV=2
MRGDRGRGRGGRFGSRGGPGGGFRPFVPHIPFDFYLCEMAFPRVKPAPDETSFSEALLKR
NQDLAPNSAEQASILSLVTKINNVIDNLIVAPGTFEVQIEEVRQVGSYKKGTMTTGHNVA
DLVVILKILPTLEAVAALGNKVVESLRAQDPSEVLTMLTNETGFEISSSDATVKILITTV
PPNLRKLDPELHLDIKVLQSALAAIRHARWFEENASQSTVKVLIRLLKDLRIRFPGFEPL
TPWILDLLGHYAVMNNPTRQPLALNVAYRRCLQILAAGLFLPGSVGITDPCESGNFRVHT

VMTLEQQDMVCYTAQTLVRILSHGGFRKILGQEGDASYLASEISTWDGVIVTPSEKAYEK
PPEKKEGEEEEENTEEPPQGEEEESMETQE

>sp|Q7L2E3|DHX30_HUMAN Putative ATP-dependent RNA helicase DHX30 OS=Homo sapiens OX=9606 GN=DHX30 PE=1 SV=1
MFSLDSFRKDRAQHRQRQCKLPPPRLPPMCVNPTPGGTISRASRDLLKEFPQPKNLLNSV
IGRALGISHAKDKLVYVHTNGPKKKKVTLHIKWPKSVEVEGYGSKKIDAERQAAAAACQL
FKGWGLLGPRNELFDAAKYRVLADRFGSPADSWWRPEPTMPPTSWRQLNPESIRPGGPGG
LSRSLGREEEEDEEEELEEGTIDVTDFLSMTQQDSHAPLRDSRGSSFEMTDDDSAIRALT
QFPLPKNLLAKVIQIATSSSTAKNLMQFHTVGTKTKLSTLTLLWPCPMTFVAKGRRKAEA
ENKAAALACKKLKSLGLVDRNNEPLTHAMYNLASLRELGETQRRPCTIQVPEPILRKIET
FLNHYPVESSWIAPELRLQSDDILPLGKDSGPLSDPITGKPYVPLLEAEEVRLSQSLLEL
WRRRGPVWQEAPQLPVDPHRDTILNAIEQHPVVVISGDTGCGKTTRIPQLLLERYVTEGR
GARCNVIITQPRRISAVSVAQRVSHELGPSLRRNVGFQVRLESKPPSRGGALLFCTVGIL
LRKLQSNPSLEGVSHVIVDEVHERDVNTDFLLILLKGLQRLNPALRLVLMSATGDNERFS
RYFGGCPVIKVPGFMYPVKEHYLEDILAKLGKHQYLHRHRHHESEDECALDLDLVTDLVL
HIDARGEPGGILCFLPGWQEIKGVQQRLQEALGMHESKYLILPVHSNIPMMDQKAIFQQP
PVGVRKIVLATNIAETSITINDIVHVVDSGLHKEERYDLKTKVSCLETVWVSRANVIQRR
GRAGRCQSGFAYHLFPRSRLEKMVPFQVPEILRTPLENLVLQAKIHMPEKTAVEFLSKAV
DSPNIKAVDEAVILLQEIGVLDQREYLTTLGQRLAHISTDPRLAKAIVLAAIFRCLHPLL
VVVSCLTRDPFSSSLQNRAEVDKVKALLSHDSGSDHLAFVRAVAGWEEVLRWQDRSSREN
YLEENLLYAPSLRFIHGLIKQFSENIYEAFLVGKPSDCTLASAQCNEYSEEEELVKGVLM
AGLYPNLIQVRQGKVTRQGKFKPNSVTYRTKSGNILLHKSTINREATRLRSRWLTYFMAV
KSNGSVFVRDSSQVHPLAVLLLTDGDVHIRDDGRRATISLSDSDLLRLEGDSRTVRLLKE
LRRALGRMVERSLRSELAALPPSVQEEHGQLLALLAELLRGPCGSFDVRKTADD

>sp|P61978|HNRPK_HUMAN Heterogeneous nuclear ribonucleoprotein K OS=Homo sapiens OX=9606 GN=HNRNPK PE=1 SV=1
METEQPEETFPNTETNGEFGKRPAEDMEEEQAFKRSRNTDEMVELRILLQSKNAGAVIGK
GGKNIKALRTDYNASVSVPDSSGPERILSISADIETIGEILKKIIPTLEEGLQLPSPTAT
SQLPLESDAVECLNYQHYKGSDFDCELRLLIHQSLAGGIIGVKGAKIKELRENTQTTIKL
FQECCPHSTDRVVLIGGKPDRVVECIKIILDLISESPIKGRAQPYDPNFYDETYDYGGFT
MMFDDRRGRPVGFPMRGRGGFDRMPPGRGGRPMPPSRRDYDDMSPRRGPPPPPPGRGGRG
GSRARNLPLPPPPPPRGGDLMAYDRRGRPGDRYDGMVGFSADETWDSAIDTWSPSEWQMA
YEPQGGSGYDYSYAGGRGSYGDLGGPIITTQVTIPKDLAGSIIGKGGQRIKQIRHESGAS
IKIDEPLEGSEDRIITITGTQDQIQNAQYLLQNSVKQYSGKFF

>sp|Q9NR30|DDX21_HUMAN Nucleolar RNA helicase 2 OS=Homo sapiens OX=9606 GN=DDX21 PE=1 SV=5
MPGKLRSDAGLESDTAMKKGETLRKQTEEKEKKEKPKSDKTEEIAEEEETVFPKAKQVKK
KAEPSEVDMNSPKSKKAKKKEEPSQNDISPKTKSLRKKKEPIEKKVVSSKTKKVTKNEEP
SEEEIDAPKPKKMKKEKEMNGETREKSPKLKNGFPHPEPDCNPSEAASEESNSEIEQEIP
VEQKEGAFSNFPISEETIKLLKGRGVTFLFPIQAKTFHHVYSGKDLIAQARTGTGKTFSF
AIPLIEKLHGELQDRKRGRAPQVLVLAPTRELANQVSKDFSDITKKLSVACFYGGTPYGG

QFERMRNGIDILVGTPGRIKDHIQNGKLDLTKLKHVVLDEVDQMLDMGFADQVEEILSVA
YKKDSEDNPQTLLFSATCPHWVFNVAKKYMKSTYEQVDLIGKKTQKTAITVEHLAIKCHW
TQRAAVIGDVIRVYSGHQGRTIIFCETKKEAQELSQNSAIKQDAQSLHGDIPQKQREITL
KGFRNGSFGVLVATNVAARGLDIPEVDLVIQSSPPKDVESYIHRSGRTGRAGRTGVCICF
YQHKEEYQLVQVEQKAGIKFKRIGVPSATEIIKASSKDAIRLLDSVPPTAISHFKQSAEK
LIEEKGAVEALAAALAHISGATSVDQRSLINSNVGFVTMILQCSIEMPNISYAWKELKEQ
LGEEIDSKVKGMVFLKGKLGVCFDVPTASVTEIQEKWHDSRRWQLSVATEQPELEGPREG
YGGFRGQREGSRGFRGQRDGNRRFRGQREGSRGPRGQRSGGGNKSNRSQNKGQKRSFSKA
FGQ

>sp|Q13148|TADBP_HUMAN TAR DNA-binding protein 43 OS=Homo sapiens OX=9606 GN=TARDBP PE=1 SV=1
MSEYIRVTEDENDEPIEIPSEDDGTVLLSTVTAQFPGACGLRYRNPVSQCMRGVRLVEGI
LHAPDAGWGNLVYVVNYPKDNKRKMDETDASSAVKVKRAVQKTSDLIVLGLPWKTTEQDL
KEYFSTFGEVLMVQVKKDLKTGHSKGFGFVRFTEYETQVKVMSQRHMIDGRWCDCKLPNS
KQSQDEPLRSRKVFVGRCTEDMTEDELREFFSQYGDVMDVFIPKPFRAFAFVTFADDQIA
QSLCGEDLIIKGISVHISNAEPKHNSNRQLERSGRFGGNPGGFGNQGGFGNSRGGGAGLG
NNQGSNMGGGMNFGAFSINPAMMAAAQAALQSSWGMMGMLASQQNQSGPSGNNQNQGNMQ
REPNQAFGSGNNSYSGSNSGAAIGWGSASNAGSGSGFNGGFGSSMDSKSSGWGM
