# Supplementary material for: The Amino Acid Composition of Quadruplex Binding Proteins Reveals a Shared Motif and Predicts New Potential Quadruplex Interactors
Source: Molecules. 2018 Sep 13;23(9):2341. doi: 10.3390/molecules23092341 (PMC6225207; doi:10.3390/molecules23092341)
Supplement: Supplementary file 1 [file molecules-23-02341-s001.zip › molecules-355581-supplementary/Supplementary material 12.pdf]

| 77 QBPs vs. 5 000 randomly selected human proteins | | | 77 QBPs vs. 15 224 human nucleic acid binding proteins including isoforms | | | 77 QBPs vs. 2 565 Golgi apparatus proteins including isoforms | | |
|---|---|---|---|---|---|---|---|---|
| **aa residue** | **change** | **p-value** | **aa residue** | **Change** | **p-value** | **aa residue** | **change** | **p-value** |
| A | Depleted. | 0.000027 | A | Not significant. | 0.047012 | A | Depleted. | 0.000000 |
| C | Depleted. | 0.000000 | C | Depleted. | 0.000000 | C | Not significant. | 0.020112 |
| D | Enriched. | 0.000000 | D | Enriched. | 0.000000 | D | Enriched. | 0.000000 |
| E | Enriched. | 0.000000 | E | Not significant. | 0.275823 | E | Enriched. | 0.000000 |
| F | Not significant. | 0.009811 | F | Not significant. | 0.059237 | F | Depleted. | 0.000001 |
| G | Enriched. | 0.000000 | G | Enriched. | 0.000000 | G | Enriched. | 0.000000 |
| H | Depleted. | 0.000010 | H | Depleted. | 0.000000 | H | Not significant. | 0.006228 |
| I | Depleted. | 0.000044 | I | Not significant. | 0.335044 | I | Depleted. | 0.000000 |
| K | Enriched. | 0.000000 | K | Not significant. | 0.015759 | K | Enriched. | 0.000000 |
| L | Depleted. | 0.000000 | L | Depleted. | 0.000001 | L | Depleted. | 0.000000 |
| M | Not significant. | 0.645290 | M | Not significant. | 0.785506 | M | Not significant. | 0.489690 |
| N | Enriched. | 0.000057 | N | Enriched. | 0.000289 | N | Enriched. | 0.000146 |
| P | Not significant. | 0.099818 | P | Depleted. | 0.000000 | P | Not significant. | 0.040930 |
| Q | Not significant. | 0.750761 | Q | Depleted. | 0.000000 | Q | Not significant. | 0.691177 |
| R | Enriched. | 0.000000 | R | Enriched. | 0.000000 | R | Enriched. | 0.000000 |
| S | Not significant. | 0.798094 | S | Not significant. | 0.002792 | S | Not significant. | 0.870296 |
| T | Depleted. | 0.000018 | T | Not significant. | 0.003142 | T | Depleted. | 0.000000 |
| V | Not significant. | 0.005469 | V | Enriched. | 0.000477 | V | Depleted. | 0.000000 |
| W | Depleted. | 0.000000 | W | Not significant. | 0.183428 | W | Depleted. | 0.000000 |
| Y | Not significant. | 0.462950 | Y | Not significant. | 0.006848 | Y | Not significant. | 0.158704 |
