# Supplementary material for: The Amino Acid Composition of Quadruplex Binding Proteins Reveals a Shared Motif and Predicts New Potential Quadruplex Interactors
Source: Molecules. 2018 Sep 13;23(9):2341. doi: 10.3390/molecules23092341 (PMC6225207; doi:10.3390/molecules23092341)
Supplement: Supplementary file 1 [file molecules-23-02341-s001.zip › molecules-355581-supplementary/Supplementary material 3B - randomly selected 5000 human protein seq.pdf]

>sp|Q9BXJ3|C1QT4_HUMAN
MLPLLLGLLGPAACWALGPTPGPGSSELRSAFSAARTTPLEGTSEMAVTFDKVYVNIGGDFDVATGQFRCRVPGAYFFSFTAGKAPH
KSLSVMLVRNRDEVQALAFDEQRRPGARRAASQSAMLQLDYGDTVWLRLHGAPQYALGAPGATFSGYLVYADADADAPARGPPAPPE
PRSAFSAARTRSLVGSDAGPGPRHQPLAFDTEFVNIGGDFDAAAGVFRCRLPGAYFFSFTLGKLPRKTLSVKLMKNRDEVQAMIYDD
GASRRREMQSQSVMLALRRGDAVWLLSHDHDGYGAYSNHGKYITFSGFLVYPDLAPAAPPGLGASELL
>sp|Q8WTP9|XAGE3_HUMAN
MIWRGRSTYRPRPRRSVPPPELIGPMLEPGDEEPQQEEPPTESRDPAPGQEREEDQGAAETQVPDLEADLQELSQSKTGGECGNGPD
DQGKILPKSEQFKMPEGGDRQPQV
>sp|Q9UKA9|PTBP2_HUMAN
MDGIVTEVAVGVKRGSDELLSGSVLSSPNSNMSSMVVTANGNDSKKFKGEDKMDGAPSRVLHIRKLPGEVTETEVIALGLPFGKVTN
ILMLKGKNQAFLELATEEAAITMVNYYSAVTPHLRNQPIYIQYSNHKELKTDNTLNQRAQAVLQAVTAVQTANTPLSGTTVSESAVT
PAQSPVLRIIIDNMYYPVTLDVLHQIFSKFGAVLKIITFTKNNQFQALLQYGDPVNAQQAKLALDGQNIYNACCTLRIDFSKLVNLN
VKYNNDKSRDYTRPDLPSGDGQPALDPAIAAAFAKETSLLAVPGALSPLAIPNAAAAAAAAAAGRVGMPGVSAGGNTVLLVSNLNEE
MVTPQSLFTLFGVYGDVQRVKILYNKKDSALIQMADGNQSQLAMNHLNGQKMYGKIIRVTLSKHQTVQLPREGLDDQGLTKDFGNSP
LHRFKKPGSKNFQNIFPPSATLHLSNIPPSVAEEDLRTLFANTGGTVKAFKFFQDHKMALLQMATVEEAIQALIDLHNYNLGENHHL
RVSFSKSTI
>sp|Q14703|MBTP1_HUMAN
MKLVNIWLLLLVVLLCGKKHLGDRLEKKSFEKAPCPGCSHLTLKVEFSSTVVEYEYIVAFNGYFTAKARNSFISSALKSSEVDNWRI
IPRNNPSSDYPSDFEVIQIKEKQKAGLLTLEDHPNIKRVTPQRKVFRSLKYAESDPTVPCNETRWSQKWQSSRPLRRASLSLGSGFW
HATGRHSSRRLLRAIPRQVAQTLQADVLWQMGYTGANVRVAVFDTGLSEKHPHFKNVKERTNWTNERTLDDGLGHGTFVAGVIASMR
ECQGFAPDAELHIFRVFTNNQVSYTSWFLDAFNYAILKKIDVLNLSIGGPDFMDHPFVDKVWELTANNVIMVSAIGNDGPLYGTLNN
PADQMDVIGVGGIDFEDNIARFSSRGMTTWELPGGYGRMKPDIVTYGAGVRGSGVKGGCRALSGTSVASPVVAGAVTLLVSTVQKRE
LVNPASMKQALIASARRLPGVNMFEQGHGKLDLLRAYQILNSYKPQASLSPSYIDLTECPYMWPYCSQPIYYGGMPTVVNVTILNGM
GVTGRIVDKPDWQPYLPQNGDNIEVAFSYSSVLWPWSGYLAISISVTKKAASWEGIAQGHVMITVASPAETESKNGAEQTSTVKLPI
KVKIIPTPPRSKRVLWDQYHNLRYPPGYFPRDNLRMKNDPLDWNGDHIHTNFRDMYQHLRSMGYFVEVLGAPFTCFDASQYGTLLMV
DSEEEYFPEEIAKLRRDVDNGLSLVIFSDWYNTSVMRKVKFYDENTRQWWMPDTGGANIPALNELLSVWNMGFSDGLYEGEFTLANH
DMYYASGCSIAKFPEDGVVITQTFKDQGLEVLKQETAVVENVPILGLYQIPAEGGGRIVLYGDSNCLDDSHRQKDCFWLLDALLQYT
SYGVTPPSLSHSGNRQRPPSGAGSVTPERMEGNHLHRYSKVLEAHLGDPKPRPLPACPRLSWAKPQPLNETAPSNLWKHQKLLSIDL
DKVVLPNFRSNRPQVRPLSPGESGAWDIPGGIMPGRYNQEVGQTIPVFAFLGAMVVLAFFVVQINKAKSRPKRRKPRVKRPQLMQQV
HPPKTPSV
>sp|A8MVU1|NCF1C_HUMAN
MYMFLVKWQDLSEKVVYRRFTEIYEFHKTLKEMFPIEAGAINPENRIIPHLPAPKWFDGQRAAENHQGTLTEYCSTLMSLPTKISRC
PHLLDFFKVRPDDLKLPTDNQTKKPETYLMPKDGKSTATDITGPIILQTYRAIADYEKTSGSEMALSTGDVVEVVEKSESGWWFCQM
KAKRGWIPASFLEPLDSPDETEDPEPNYAGEPYVAIKAYTAVEGDEVSLLEGEAVEVIHKLLDGWWVIRKDDVTGYFPSMYLQKSGQ
DVSQAQRQIKRGAPPRRSSIRNAHSIHQRSRKRLSQDAYRRNSVRFLQQRRRQARPGPQSPGSPLEEERQTQRSKPQPAVPPRPSAD
LILNRCSESTKRKLASAV
>sp|Q3SXY7|LRIT3_HUMAN
MHLFACLCIVLSFLEGVGCLCPSQCTCDYHGRNDGSGSRLVLCNDMDMNELPTNLPVDTVKLRIEKTVIRRISAEAFYYLVELQYLW
VTYNSVASIDPSSFYNLKQLHELRLDGNSLAAFPWASLLDMPLLRTLDLHNNKITSVPNEALRYLKNLAYLDLSSNRLTTLPPDFLE
SWTHLVSTPSGVLDLSPSRIILGLQDNPWFCDCHISKMIELSKVVDPAIVLLDPLMTCSEPERLTGILFQRAELEHCLKPSVMTSAT
KIMSALGSNVLLRCDATGFPTPQITWTRSDSSPVNYTVIQESPEEGVRWSIMSLTGISSKDAGDYKCKAKNLAGMSEAVVTVTVLGI
TTTPIPPDTSERTGDHPEWDVQPGSGRSTSVSSASSYLWSSSFSPTSSFSASTLSPPSTASFSLSPFSSSTVSSTTTLSTSISASTT
MANKRSFQLHQGGKRNLKVAKNGSKLPPASTSKKEELALLDQTMLTETNAAIENLRVVSETKESVTLTWNMINTTHNSAVTVLYSKY

GGKDLLLLNADSSKNQVTIDGLEPGGQYMACVCPKGVPPQKDQCITFSTERVEGDDSQWSLLLVVTSTACVVILPLICFLLYKVCKL
QCKSEPFWEDDLAKETYIQFETLFPRSQSVGELWTRSHRDDSEKLLLCSRSSVESQVTFKSEGSRPEYYC
>sp|Q14714|SSPN_HUMAN
MGKNKQPRGQQRQGGPPAADAAGPDDMEPKKGTGAPKECGEEEPRTCCGCRFPLLLALLQLALGIAVTVVGFLMASISSSLLVRDTP
FWAGIIVCLVAYLGLFMLCVSYQVDERTCIQFSMKLLYFLLSALGLTVCVLAVAFAAHHYSQLTQFTCETTLDSCQCKLPSSEPLSR
TFVYRDVTDCTSVTGTFKLFLLIQMILNLVCGLVCLLACFVMWKHRYQVFYVGVRICSLTASEGPQQKI
>sp|Q68DV7|RNF43_HUMAN
MSGGHQLQLAALWPWLLMATLQAGFGRTGLVLAAAVESERSAEQKAIIRVIPLKMDPTGKLNLTLEGVFAGVAEITPAEGKLMQSHP
LYLCNASDDDNLEPGFISIVKLESPRRAPRPCLSLASKARMAGERGASAVLFDITEDRAAAEQLQQPLGLTWPVVLIWGNDAEKLME
FVYKNQKAHVRIELKEPPAWPDYDVWILMTVVGTIFVIILASVLRIRCRPRHSRPDPLQQRTAWAISQLATRRYQASCRQARGEWPD
SGSSCSSAPVCAICLEEFSEGQELRVISCLHEFHRNCVDPWLHQHRTCPLCMFNITEGDSFSQSLGPSRSYQEPGRRLHLIRQHPGH
AHYHLPAAYLLGPSRSAVARPPRPGPFLPSQEPGMGPRHHRFPRAAHPRAPGEQQRLAGAQHPYAQGWGLSHLQSTSQHPAACPVPL
RRARPPDSSGSGESYCTERSGYLADGPASDSSSGPCHGSSSDSVVNCTDISLQGVHGSSSTFCSSLSSDFDPLVYCSPKGDPQRVDM
QPSVTSRPRSLDSVVPTGETQVSSHVHYHRHRHHHYKKRFQWHGRKPGPETGVPQSRPPIPRTQPQPEPPSPDQQVTRSNSAAPSGR
LSNPQCPRALPEPAPGPVDASSICPSTSSLFNLQKSSLSARHPQRKRRGGPSEPTPGSRPQDATVHPACQIFPHYTPSVAYPWSPEA
HPLICGPPGLDKRLLPETPGPCYSNSQPVWLCLTPRQPLEPHPPGEGPSEWSSDTAEGRPCPYPHCQVLSAQPGSEEELEELCEQAV
>sp|Q3KR16|PKHG6_HUMAN
MKAFGPPHEGPLQGLVASRIETYGGRHRASAQSTAGRLYPRGYPVLDPSRRRLQQYVPFARGSGQARGLSPMRLRDPEPEKRHGGHV
GAGLLHSPKLKELTKAHELEVRLHTFSMFGMPRLPPEDRRHWEIGEGGDSGLTIEKSWRELVPGHKEMSQELCHQQEALWELLTTEL
IYVRKLKIMTDLLAAGLLNLQRVGLLMEVSAETLFGNVPSLIRTHRSFWDEVLGPTLEETRASGQPLDPIGLQSGFLTFGQRFHPYV
QYCLRVKQTMAYAREQQETNPLFHAFVQWCEKHKRSGRQMLCDLLIKPHQRITKYPLLLHAVLKRSPEARAQEALNAMIEAVESFLR
HINGQVRQGEEQESLAAAAQRIGPYEVLEPPSDEVEKNLRPFSTLDLTSPMLGVASEHTRQLLLEGPVRVKEGREGKLDVYLFLFSD
VLLVTKPQRKADKAKVIRPPLMLEKLVCQPLRDPNSFLLIHLTEFQCVSSALLVHCPSPTDRAQWLEKTQQAQAALQKLKAEEYVQQ
KRELLTLYRDQDRESPSTRPSTPSLEGSQSSAEGRTPEFSTIIPHLVVTEDTDEDAPLVPDDTSDSGYGTLIPGTPTGSRSPLSRLR
QRALRRDPRLTFSTLELRDIPLRPHPPDPQAPQRRSAPELPEGILKGGSLPQEDPPTWSEEEDGASERGNVVVETLHRARLRGQLPS
SPTHADSAGESPWESSGEEEEEGPLFLKAGHTSLRPMRAEDMLREIREELASQRIEGAEEPRDSRPRKLTRAQLQRMRGPHIIQLDT
PLSASEV
>sp|P78348|ASIC1_HUMAN
MELKAEEEEVGGVQPVSIQAFASSSTLHGLAHIFSYERLSLKRALWALCFLGSLAVLLCVCTERVQYYFHYHHVTKLDEVAASQLTF
PAVTLCNLNEFRFSQVSKNDLYHAGELLALLNNRYEIPDTQMADEKQLEILQDKANFRSFKPKPFNMREFYDRAGHDIRDMLLSCHF
RGEVCSAEDFKVVFTRYGKCYTFNSGRDGRPRLKTMKGGTGNGLEIMLDIQQDEYLPVWGETDETSFEAGIKVQIHSQDEPPFIDQL
GFGVAPGFQTFVACQEQRLIYLPPPWGTCKAVTMDSDLDFFDSYSITACRIDCETRYLVENCNCRMVHMPGDAPYCTPEQYKECADP
ALDFLVEKDQEYCVCEMPCNLTRYGKELSMVKIPSKASAKYLAKKFNKSEQYIGENILVLDIFFEVLNYETIEQKKAYEIAGLLGDI
GGQMGLFIGASILTVLELFDYAYEVIKHKLCRRGKCQKEAKRSSADKGVALSLDDVKRHNPCESLRGHPAGMTYAANILPHHPARGT
FEDFTC
>sp|A8MQ14|ZN850_HUMAN
MNMEGLVMFQDLSIDFSQEEWECLDAAQKDLYRDVMMENYSSLVSLGLSIPKPDVISLLEQGKEPWMVSRDVLGGWCRDSEFRCKTK
DSCLPKEIYEVTSSQWVRMEKCHSLVGSSVRDDWECKGQFQHQDINQERYLEKAIMTYETTPTFCLQTSLTLHHRIHPGEKLYKSTE
CMAFKYGSELTQQQETHTGEKLYKCKECGKAFHHFSYLVKHQRIHTGEKPCACKEYGKAFISGSHLIQHQKMYTDERPHECQESVKA
FRPSAHLIQHWRIHTGDKPYECKECGKSFTSGSTLNQHQQIHTGEKPYHCKQCGKSFTVGSTLIRHQQIHTGEKPYDCKECGKSFAS
GSALIRHQRIHTGEKPYDCKECGKSFTFHSALIRHQRIHTGEKPYDCKECGKSFTFRSGLIGHQAIHTGEKPYDCKECGKSFTAGST
LIQHQRIHTGEKPYDCKECGKSFASGSALLQHQRIHTGEKPYCCKECGKSFTFRSTRNRHQRIHTGEKPYNCKECGKSFASGSALLQ
HQRIHTGEKPYHCKECGKSFTFRSGLIGHQAVHTGEKPYDCKECGKSFTSRSALIQHQRIHTGEKPYHCKECGKSFTVGSTLLQHQQ
IHTGEKPYDCKECGKAFRLRLRLTQHQQIHTGEKPYQCQECGKAFVSVSGLTQHHRIHTGEKPYECPDCGKAFRQRTYLNQHRRIHT

GEKPYECKECGKSFTFCSGLIQHQQNHTDEKPYDGKECGKSFTSHSTLIQHQQIHTGEKPYDCKECGKSFTSHSTLIQHQQIHTGEK
LYDCKECGKSFTSHSTLIQHQPLHTGEKPYHCKECGKSFTLRSALIQHRPVHTGEKRYSCKECGKSFTSRSTLIEHQRIHTGEKPYH
CKECGKSFAFRSAIIQHRRIHTGEKPYDCKECGKAFRRRSKLTQHQRIHTGEKPYRCHECGKAFVRFSGLTKHHSIHTGEKPYECKT
CGKSFRQRTHLTLHQRIHTGDRPYECKECGKSFTCGSELIRHQRTHTGEKPYDCKECGKAFRCPSQLSQHKRIHTGEKTYQCPECGK
AFFYASGLSRHQSVHTGEKPYECKTCGKAFKQLTQLTRHQRIHDLT
>sp|Q96NB1|FOPNL_HUMAN
MATVAELKAVLKDTLEKKGVLGHLKARIRAEVFNALDDDREPRPSLSHENLLINELIREYLEFNKYKYTASVLIAESGQPVVPLDRQ
FLIHELNAFEESKDNTIPLLYGILAHFLRGTKDGIQNAFLKGPSLQPSDPSLGRQPSRRKPMDDHLRKEEQKSTNIEDLHVSQAVNR
>sp|Q13043|STK4_HUMAN
METVQLRNPPRRQLKKLDEDSLTKQPEEVFDVLEKLGEGSYGSVYKAIHKETGQIVAIKQVPVESDLQEIIKEISIMQQCDSPHVVK
YYGSYFKNTDLWIVMEYCGAGSVSDIIRLRNKTLTEDEIATILQSTLKGLEYLHFMRKIHRDIKAGNILLNTEGHAKLADFGVAGQL
TDTMAKRNTVIGTPFWMAPEVIQEIGYNCVADIWSLGITAIEMAEGKPPYADIHPMRAIFMIPTNPPPTFRKPELWSDNFTDFVKQC
LVKSPEQRATATQLLQHPFVRSAKGVSILRDLINEAMDVKLKRQESQQREVDQDDEENSEEDEMDSGTMVRAVGDEMGTVRVASTMT
DGANTMIEHDDTLPSQLGTMVINAEDEEEEGTMKRRDETMQPAKPSFLEYFEQKEKENQINSFGKSVPGPLKNSSDWKIPQDGDYEF
LKSWTVEDLQKRLLALDPMMEQEIEEIRQKYQSKRQPILDAIEAKKRRQQNF
>sp|Q99569|PKP4_HUMAN
MPAPEQASLVEEGQPQTRQEAASTGPGMEPETTATTILASVKEQELQFQRLTRELEVERQIVASQLERCRLGAESPSIASTSSTEKS
FPWRSTDVPNTGVSKPRVSDAVQPNNYLIRTEPEQGTLYSPEQTSLHESEGSLGNSRSSTQMNSYSDSGYQEAGSFHNSQNVSKADN
RQQHSFIGSTNNHVVRNSRAEGQTLVQPSVANRAMRRVSSVPSRAQSPSYVISTGVSPSRGSLRTSLGSGFGSPSVTDPRPLNPSAY
SSTTLPAARAASPYSQRPASPTAIRRIGSVTSRQTSNPNGPTPQYQTTARVGSPLTLTDAQTRVASPSQGQVGSSSPKRSGMTAVPQ
HLGPSLQRTVHDMEQFGQQQYDIYERMVPPRPDSLTGLRSSYASQHSQLGQDLRSAVSPDLHITPIYEGRTYYSPVYRSPNHGTVEL
QGSQTALYRTGSVGIGNLQRTSSQRSTLTYQRNNYALNTTATYAEPYRPIQYRVQECNYNRLQHAVPADDGTTRSPSIDSIQKDPRE
FAWRDPELPEVIHMLQHQFPSVQANAAAYLQHLCFGDNKVKMEVCRLGGIKHLVDLLDHRVLEVQKNACGALRNLVFGKSTDENKIA
MKNVGGIPALLRLLRKSIDAEVRELVTGVLWNLSSCDAVKMTIIRDALSTLTNTVIVPHSGWNNSSFDDDHKIKFQTSLVLRNTTGC
LRNLSSAGEEARKQMRSCEGLVDSLLYVIHTCVNTSDYDSKTVENCVCTLRNLSYRLELEVPQARLLGLNELDDLLGKESPSKDSEP
SCWGKKKKKKKRTPQEDQWDGVGPIPGLSKSPKGVEMLWHPSVVKPYLTLLAESSNPATLEGSAGSLQNLSAGNWKFAAYIRAAVRK
EKGLPILVELLRMDNDRVVSSVATALRNMALDVRNKELIGKYAMRDLVNRLPGGNGPSVLSDETMAAICCALHEVTSKNMENAKALA
DSGGIEKLVNITKGRGDRSSLKVVKAAAQVLNTLWQYRDLRSIYKKDGWNQNHFITPVSTLERDRFKSHPSLSTTNQQMSPIIQSVG
STSSSPALLGIRDPRSEYDRTQPPMQYYNSQGDATHKGLYPGSSKPSPIYISSYSSPAREQNRRLQHQQLYYSQDDSNRKNFDAYRL
YLQSPHSYEDPYFDDRVHFPASTDYSTQYGLKSTTNYVDFYSTKRPSYRAEQYPGSPDSWV
>sp|Q9BQY9|DBND2_HUMAN
MGAGNFLTALEVPVAALAGAASDRRASCERVSPPPPLPHFRLPPLPRSRLPGPVSRPEPGAPLLGCWLQWGAPSPGPLCLLFRLCSC
TCFAPLPAGADMDPNPRAALERQQLRLRERQKFFEDILQPETEFVFPLSHLHLESQRPPIGSISSMEVNVDTLEQVELIDLGDPDAA
DVFLPCEDPPPTPQSSGMDNHLEELSLPVPTSDRTTSRTSSSSSSDSSTNLHSPNPSDDGADTPLAQSDEEEERGDGGAEPGACS
>sp|Q9NR28|DBLOH_HUMAN
MAALKSWLSRSVTSFFRYRQCLCVPVVANFKKRCFSELIRPWHKTVTIGFGVTLCAVPIAQKSEPHSLSSEALMRRAVSLVTDSTST
FLSQTTYALIEAITEYTKAVYTLTSLYRQYTSLLGKMNSEEEDEVWQVIIGARAEMTSKHQEYLKLETTWMTAVGLSEMAAEAAYQT
GADQASITARNHIQLVKLQVEEVHQLSRKAETKLAEAQIEELRQKTQEEGEERAESEQEAYLRED
>sp|Q9Y3L3|3BP1_HUMAN
MMKRQLHRMRQLAQTGSLGRTPETAEFLGEDLLQVEQRLEPAKRAAHNIHKRLQACLQGQSGADMDKRVKKLPLMALSTTMAESFKE
LDPDSSMGKALEMSCAIQNQLARILAEFEMTLERDVLQPLSRLSEEELPAILKHKKSLQKLVSDWNTLKSRLSQATKNSGSSQGLGG
SPGSHSHTTMANKVETLKEEEEELKRKVEQCRDEYLADLYHFVTKEDSYANYFIRLLEIQADYHRRSLSSLDTALAELRENHGQADH
SPSMTATHFPRVYGVSLATHLQELGREIALPIEACVMMLLSEGMKEEGLFRLAAGASVLKRLKQTMASDPHSLEEFCSDPHAVAGAL
KSYLRELPEPLMTFDLYDDWMRAASLKEPGARLQALQEVCSRLPPENLSNLRYLMKFLARLAEEQEVNKMTPSNIAIVLGPNLLWPP

EKEGDQAQLDAASVSSIQVVGVVEALIQSADTLFPGDINFNVSGLFSAVTLQDTVSDRLASEELPSTAVPTPATTPAPAPAPAPAPA
PALASAATKERTESEVPPRPASPKVTRSPPETAAPVEDMARRTKRPAPARPTMPPPQVSGSRSSPPAPPLPPGSGSPGTPQALPRRL
VGSSLRAPTVPPPLPPTPPQPARRQSRRSPASPSPASPGPASPSPVSLSNPAQVDLGAATAEGGAPEAISGVPTPPAIPPQPRPRSL
ASETN
>sp|Q8NC69|KCTD6_HUMAN
MDNGDWGYMMTDPVTLNVGGHLYTTSLTTLTRYPDSMLGAMFGGDFPTARDPQGNYFIDRDGPLFRYVLNFLRTSELTLPLDFKEFD
LLRKEADFYQIEPLIQCLNDPKPLYPMDTFEEVVELSSTRKLSKYSNPVAVIITQLTITTKVHSLLEGISNYFTKWNKHMMDTRDCQ
VSFTFGPCDYHQEVSLRVHLMEYITKQGFTIRNTRVHHMSERANENTVEHNWTFCRLARKTDD
>sp|Q6NSJ2|PHLB3_HUMAN
MGTRSSPEEGTPPPLVPECDVEVQPQGHPEESREQEASEVLAEPSSRGGAEQQAEEEEVGEGSSTESSRDAPEATPPIAMAATPPAS
TSSREGVRGAARRLQGQQLEALTRVALMEQRVKELQRQRKELRIEMEVEVALLRGELAGERVAARREEEQLRELLEQQAASEQRGRQ
QREQEQRRLSQERDRLEGLRQRLRKAQGQLDSQPEDQRERLLQGVQEMREQLDVAQRAYEDLEFQQLERESRQEEEDRDSPGPQVPD
PKVQELQASMAQHRRGALQHRIRVLEEQLKSLGEQMAAESRGLSRKKEEALQALSQERSRLLELNCLQGTPGGDFSEPNPALTKLLF
TQKTDRQLLVLQDAVAHSAATPTSSCLFSVHSSLQGSIGLQRTGSLPRKRGERGSQRGSPRPLSFHCTESLEASALPPAVGDSGRYP
LYQLLNCGRGNSCGAIHPDIAHMERLLQQAMAERERLLKAREGTRRGTEGSSGPAVPAITAPPTPPHPPGPRILDLRQHLEGWGHNP
ENCPHVQVSGCCCRGPLVKMGGRIKTWRKRWFCFDRQARRLAYYADKEETKLKGVIYFQAIEEVYYDHLRCAFKSPNPRLTFCVKTY
ERLFYMVAPSPEAMRIWMDVIVTAADENHAP
>sp|O15194|CTDSL_HUMAN
MDGPAIITQVTNPKEDEGRLPGAGEKASQCNVSLKKQRSRSILSSFFCCFRDYNVEAPPPSSPSVLPPLVEENGGLQKGDQRQVIPI
PSPPAKYLLPEVTVLDYGKKCVVIDLDETLVHSSFKPISNADFIVPVEIDGTIHQVYVLKRPHVDEFLQRMGQLFECVLFTASLAKY
ADPVADLLDRWGVFRARLFRESCVFHRGNYVKDLSRLGRELSKVIIVDNSPASYIFHPENAVPVQSWFDDMTDTELLDLIPFFEGLS
REDDVYSMLHRLCNR
>sp|Q6PHW0|IYD1_HUMAN
MYFLTPILVAILCILVVWIFKNADRSMEKKKGEPRTRAEARPWVDEDLKDSSDLHQAEEDADEWQESEENVEHIPFSHNHYPEKEMV
KRSQEFYELLNKRRSVRFISNEQVPMEVIDNVIRTAGTAPSGAHTEPWTFVVVKDPDVKHKIRKIIEEEEEINYMKRMGHRWVTDLK
KLRTNWIKEYLDTAPILILIFKQVHGFAANGKKKVHYYNEISVSIACGILLAALQNAGLVTVTTTPLNCGPRLRVLLGRPAHEKLLM
LLPVGYPSKEATVPDLKRKPLDQIMVTV
>sp|Q9NQL2|RRAGD_HUMAN
MSQVLGKPQPQDEDDAEEEEEEDELVGLADYGDGPDSSDADPDSGTEEGVLDFSDPFSTEVKPRILLMGLRRSGKSSIQKVVFHKMS
PNETLFLESTNKICREDVSNSSFVNFQIWDFPGQIDFFDPTFDYEMIFRGTGALIFVIDSQDDYMEALARLHLTVTRAYKVNTDINF
EVFIHKVDGLSDDHKIETQRDIHQRANDDLADAGLEKIHLSFYLTSIYDHSIFEAFSKVVQKLIPQLPTLENLLNIFISNSGIEKAF
LFDVVSKIYIATDSTPVDMQTYELCCDMIDVVIDISCIYGLKEDGAGTPYDKESTAIIKLNNTTVLYLKEVTKFLALVCFVREESFE
RKGLIDYNFHCFRKAIHEVFEVRMKVVKSRKVQNRLQKKKRATPNGTPRVLL
>sp|O00629|IMA3_HUMAN
MADNEKLDNQRLKNFKNKGRDLETMRRQRNEVVVELRKNKRDEHLLKRRNVPHEDICEDSDIDGDYRVQNTSLEAIVQNASSDNQGI
QLSAVQAARKLLSSDRNPPIDDLIKSGILPILVHCLERDDNPSLQFEAAWALTNIASGTSEQTQAVVQSNAVPLFLRLLHSPHQNVC
EQAVWALGNIIGDGPQCRDYVISLGVVKPLLSFISPSIPITFLRNVTWVMVNLCRHKDPPPPMETIQEILPALCVLIHHTDVNILVD
TVWALSYLTDAGNEQIQMVIDSGIVPHLVPLLSHQEVKVQTAALRAVGNIVTGTDEQTQVVLNCDALSHFPALLTHPKEKINKEAVW
FLSNITAGNQQQVQAVIDANLVPMIIHLLDKGDFGTQKEAAWAISNLTISGRKDQVAYLIQQNVIPPFCNLLTVKDAQVVQVVLDGL
SNILKMAEDEAETIGNLIEECGGLEKIEQLQNHENEDIYKLAYEIIDQFFSSDDIDEDPSLVPEAIQGGTFGFNSSANVPTEGFQF
>sp|P0DKL9|A14EL_HUMAN
MNEQSEKNNSIQERHTDHSFPEKNCQIGQKQLQQIERQLKCLAFRNPGPQVADFNPETRQQKKKARMSKMNEYFSTKYKIMRKYDKS
GRLICNDADLCDCLEKNCLGCFYPCPKCNSNKCGPECRCNRRWVYDAIVTESGEVISTLPFNVPD
>sp|P81605|DCD_HUMAN

MRFMTLLFLTALAGALVCAYDPEAASAPGSGNPCHEASAAQKENAGEDPGLARQAPKPRKQRSSLLEKGLDGAKKAVGGLGKLGKDA
VEDLESVGKGAVHDVKDVLDSVL
>sp|Q5VTL7|FNDC7_HUMAN
MAGGRETCLPLIGFILICLKMVASAKSAPEIPTIDQAYSKLSNSITVEWATVPGATSYLLTAEDGDTVIETTVANSPGTVTGLKAAT
WYEITIRSISAAGRSQASPPKQAKTVLAAPILEVSSPSSDSILVQWEAVYMAIAFSVSIMRANGLGSIWKENTTNTSLTFTSLEAGT
LYTIKAYAWNANRIPGDDSTCNQRTSPRAPANIQVSFDSGALKASFSWARAEGAFNYTVMALSDSSELTCSTTFSSCTISSLQCGTE
YLISVLASNDAGSSKSSSAMTLKTVACAPGRVTIQEDPPGHLSVAWSSVDLGDYYVVFVKSDDGLEVHCNTSLTQCNFLSECGFTYF
ISVFVYNKAGQSPLGDIFNYTTAPCCPSDINPVLVSSDRVEIVWSPVRGAELYETKAVDGYNMVECNDTTPACTLSALECDTKYNIT
VYSFNEVRGSNMSCTPQFITTAPCSPEIKNVSRDAFSMINVHWRSTNDDATYTVTAQGEKGLYQCSSTGESCTMRGLPCGSVFSVTA
VAETQAGRSLPSYSVPLETVPCCPTGLTVTQITQSVINVSWTIGRVAQTHVAVLESHTGQSKCHTHQNHCLLGCITCGINYTVTLKA
ISATGLTADCSYQSYFSGACCPLGVKLYRLGPNGIRIYWQASRGSANYSTDLYGSKGIFTCTPSAGLSFCDVTEIPCGDVYTVMVSP
VAKTGLKLTFCPKKIYSVTCSGSTLGMVIYRGKRNEE
>sp|P05121|PAI1_HUMAN
MQMSPALTCLVLGLALVFGEGSAVHHPPSYVAHLASDFGVRVFQQVAQASKDRNVVFSPYGVASVLAMLQLTTGGETQQQIQAAMGF
KIDDKGMAPALRHLYKELMGPWNKDEISTTDAIFVQRDLKLVQGFMPHFFRLFRSTVKQVDFSEVERARFIINDWVKTHTKGMISNL
LGKGAVDQLTRLVLVNALYFNGQWKTPFPDSSTHRRLFHKSDGSTVSVPMMAQTNKFNYTEFTTPDGHYYDILELPYHGDTLSMFIA
APYEKEVPLSALTNILSAQLISHWKGNMTRLPRLLVLPKFSLETEVDLRKPLENLGMTDMFRQFQADFTSLSDQEPLHVAQALQKVK
IEVNESGTVASSSTAVIVSARMAPEEIIMDRPFLFVVRHNPTGTVLFMGQVMEP
>sp|Q6IMI6|ST1C3_HUMAN
MAKIEKNAPTMEKKPELFNIMEVDGVPTLILSKEWWEKVCNFQAKPDDLILATYPKSGTTWMHEILDMILNDGDVEKCKRAQTLDRH
AFLELKFPHKEKPDLEFVLEMSSPQLIKTHLPSHLIPPSIWKENCKIVYVARNPKDCLVSYYHFHRMASFMPDPQNLEEFYEKFMSG
KVVGGSWFDHVKGWWAAKDMHRILYLFYEDIKKDPKREIEKILKFLEKDISEEILNKIIYHTSFDVMKQNPMTNYTTLPTSIMDHSI
SPFMRKGMPGDWKNYFTVAQNEEFDKDYQKKMAGSTLTFRTEI
>sp|Q9UN74|PCDA4_HUMAN
MEFSWGSGQESRRLLLLLLLLAAWEAGNGQLHYSVSEEAKHGTFVGRIAQDLGLELAELVPRLFRVASKGRGGLLEVNLQNGILFVN
SRIDREELCRRSAECSIHLEVIVDRPLQVFHVDVEVRDINDNPPVFPATQKNLSIAESRPLDSRFPLEGASDADIGENALLTYRLSP
NEYFSLEKPPDDELVKGLGLILRKSLDREEAPEIFLVLTATDGGKPELTGTVQLLITVLDANDNAPAFDRTIYKVRLLENVPNGTLV
IKLNASDLDEGLNGDIVYSFSNDISPNVKSKFHIDPITGQIIVKGYIDFEESKSYEIIVEGIDKGQLPLSGHCRVIVEVEDNNDNVP
DLEFKSLSLPIREDAPLGTVIALISVSDKDMGVNGLVTCSLTSHVPFKLVSTFKNYYSLVLDSALDRESVSAYELVVTARDGGSPSL
WATASVSVEVADVNDNAPAFAQPEYTVFVKENNPPGCHIFTVSAWDADAQENALVSYSLVERRVGERALSSYVSVHAESGKVYALQP
LDHEELELLQFQVTARDAGVPPLGSNVTLQVFVLDENDNAPALLAPRAGGTGGAVSELVPWSVGVGHVVAKVRAVDADSGYNAWLSY
ELQPGTGGARIPFRVGLYTGEISTTRALDETDAPRHRLLVLVKDHGEPALTATATVLVSLVESGQAPKASSRALVGAVGPDAALVDV
NVYLIIAICAVSSLLVLTLLLYTALRCSALPTEGACAPGKPTLVCSSAVGSWSYSQQRRPRVCSGEGPPKTDLMAFSPSLPDSRDRE
DQLQTTEESFAKPRQPNPDWRYSASLRAGMHSSVHLEEAGILRAGPGGPDQQWPTVSSATPEPEAGEVSPPVGAGVNSNSWTFKYGP
GNPKQSGPGELPDKFIIPGSPAIISIRQEPTNSQIDKSDFITFGKKEETKKKKKKKGNKTQEKKEKGNSTTDNSDQ
>sp|Q9BUK6|MSTO1_HUMAN
MAGGAREVLTLQLGHFAGFVGAHWWNQQDAALGRATDSKEPPGELCPDVLYRTGRTLHGQETYTPRLILMDLKGSLSSLKEEGGLYR
DKQLDAAIAWQGKLTTHKEELYPKNPYLQDFLSAEGVLSSDGVWRVKSIPNGKGSSPLPTATTPKPLIPTEASIRVWSDFLRVHLHP
RSICMIQKYNHDGEAGRLEAFGQGESVLKEPKYQEELEDRLHFYVEECDYLQGFQILCDLHDGFSGVGAKAAELLQDEYSGRGIITW
GLLPGPYHRGEAQRNIYRLLNTAFGLVHLTAHSSLVCPLSLGGSLGLRPEPPVSFPYLHYDATLPFHCSAILATALDTVTVPYRLCS
SPVSMVHLADMLSFCGKKVVTAGAIIPFPLAPGQSLPDSLMQFGGATPWTPLSACGEPSGTRCFAQSVVLRGIDRACHTSQLTPGTP
PPSALHACTTGEEILAQYLQQQQPGVMSSSHLLLTPCRVAPPYPHLFSSCSPPGMVLDGSPKGAAVESIPVFGALCSSSSLHQTLEA
LARDLTKLDLRRWASFMDAGVEHDDVAELLQELQSLAQCYQGGDSLVD
>sp|O95198|KLHL2_HUMAN

METPPLPPACTKQGHQKPLDSKDDNTEKHCPVTVNPWHMKKAFKVMNELRSQNLLCDVTIVAEDMEISAHRVVLAACSPYFHAMFTG
EMSESRAKRVRIKEVDGWTLRMLIDYVYTAEIQVTEENVQVLLPAAGLLQLQDVKKTCCEFLESQLHPVNCLGIRAFADMHACTDLL
NKANTYAEQHFADVVLSEEFLNLGIEQVCSLISSDKLTISSEEKVFEAVIAWVNHDKDVRQEFMARLMEHVRLPLLPREYLVQRVEE
EALVKNSSACKDYLIEAMKYHLLPTEQRILMKSVRTRLRTPMNLPKLMVVVGGQAPKAIRSVECYDFKEERWHQVAELPSRRCRAGM
VYMAGLVFAVGGFNGSLRVRTVDSYDPVKDQWTSVANMRDRRSTLGAAVLNGLLYAVGGFDGSTGLSSVEAYNIKSNEWFHVAPMNT
RRSSVGVGVVGGLLYAVGGYDGASRQCLSTVECYNATTNEWTYIAEMSTRRSGAGVGVLNNLLYAVGGHDGPLVRKSVEVYDPTTNA
WRQVADMNMCRRNAGVCAVNGLLYVVGGDDGSCNLASVEYYNPTTDKWTVVSSCMSTGRSYAGVTVIDKPL
>sp|P54296|MYOM2_HUMAN
MSLVTVPFYQKRHRHFDQSYRNIQTRYLLDEYASKKRASTQASSQKSLSQRSSSQRASSQTSLGGTICRVCAKRVSTQEDEEQENRS
RYQSLVAAYGEAKRQRFLSELAHLEEDVHLARSQARDKLDKYAIQQMMEDKLAWERHTFEERISRAPEILVRLRSHTVWERMSVKLC
FTVQGFPTPVVQWYKDGSLICQAAEPGKYRIESNYGVHTLEINRADFDDTATYSAVATNAHGQVSTNAAVVVRRFRGDEEPFRSVGL
PIGLPLSSMIPYTHFDVQFLEKFGVTFRREGETVTLKCTMLVTPDLKRVQPRAEWYRDDVLLKESKWTKMFFGEGQASLSFSHLHKD
DEGLYTLRIVSRGGVSDHSAFLFVRDADPLVTGAPGAPMDLQCHDANRDYVIVTWKPPNTTTESPVMGYFVDRCEVGTNNWVQCNDA
PVKICKYPVTGLFEGRSYIFRVRAVNSAGISRPSRVSDAVAALDPLDLRRLQAVHLEGEKEIAIYQDDLEGDAQVPGPPTGVHASEI
SRNYVVLSWEPPTPRGKDPLMYFIEKSVVGSGSWQRVNAQTAVRSPRYAVFDLMEGKSYVFRVLSANRHGLSEPSEITSPIQAQDVT
VVPSAPGRVLASRNTKTSVVVQWDRPKHEEDLLGYYVDCCVAGTNLWEPCNHKPIGYNRFVVHGLTTGEQYIFRVKAVNAVGMSENS
QESDVIKVQAALTVPSHPYGITLLNCDGHSMTLGWKVPKFSGGSPILGYYLDKREVHHKNWHEVNSSPSKPTILTVDGLTEGSLYEF
KIAAVNLAGIGEPSDPSEHFKCEAWTMPEPGPAYDLTFCEVRDTSLVMLWKAPVYSGSSPVSGYFVDFREEDAGEWITVNQTTTANR
YLKVSDLQQGKTYVFRVRAVNANGVGKPSDTSEPVLVEARPGTKEISAGVDEQGNIYLGFDCQEMTDASQFTWCKSYEEISDDERFK
IETVGDHSKLYLKNPDKEDLGTYSVSVSDTDGVSSSFVLDPEELERLMALSNEIKNPTIPLKSELAYEIFDKGRVRFWLQAEHLSPD
ASYRFIINDREVSDSEIHRIKCDKATGIIEMVMDRFSIENEGTYTVQIHDGKAKSQSSLVLIGDAFKTVLEEAEFQRKEFLRKQGPH
FAEYLHWDVTEECEVRLVCKVANTKKETVFKWLKDDVLYETETLPNLERGICELLIPKLSKKDHGEYKATLKDDRGQDVSILEIAGK
VYDDMILAMSRVCGKSASPLKVLCTPEGIRLQCFMKYFTDEMKVNWCHKDAKISSSEHMRIGGSEEMAWLQICEPTEKDKGKYTFEI
FDGKDNHQRSLDLSGQAFDEAFAEFQQFKAAAFAEKNRGRLIGGLPDVVTIMEGKTLNLTCTVFGNPDPEVIWFKNDQDIQLSEHFS
VKVEQAKYVSMTIKGVTSEDSGKYSINIKNKYGGEKIDVTVSVYKHGEKIPDMAPPQQAKPKLIPASASAAGQ
>sp|Q5VT03|NTM2D_HUMAN
MFQEPVYFQIFLFQLDSGASGEPGHSLGLTLGFSHCGNCQTAVVSAQPEGMASNGAYPALGPGVTANPGTSLSVFTALPFTTPAPGP
AHGPLLVTAGAPPGGPLVLSNFPSTPLVTEQDGCGPSGAGASNVFVQMRTEVGPVKAAQAQTLVLTQTPLVWQAPGALCGGVVCPPP
LLLAAAPVVPVMAAQVVGGTQACEGGWSQGLPLPPPPPPAAQLPPIVSQGNAGPWPQGAHGEGSLASSQAKAPPDDSCNPRSVYENF
RLWQHYKPLARRHLPQSPDTEALSCFLIPVLRSLARRKPTMTLEEGLWRAMREWQHTSNFDRMIFYEMAEKFLEFEAEEEMQIQKSQ
WMKGPQCLPPPATPRLEPRGPPAPEVVKQPVYLPSKAGPKAPTACLPPPRPQRPVTKARRPPPRPHRRAETKARLPPPRPQRPAETK
VPEEIPPEVVQEYVDIMEELLGPSLGATGEPEKQREEGEVKQPQEEDWTPPDPGLLSYTDKLCSQKDFVTKVEAVIHPQFLEELLSP
DPQMDFLALSQELEQEEGLTLAQLVEKRLLPLKEKQHARAAPSRGTARLDSSSSKFAAGQGAERDVPDPQQGVGMETCPPQTTARDS
QGRGRAHTGMARSKDSVVLLGCQDSPGLRAARPTSPPQDHRPTCPGVGTKDALDLPGGSPVRESHGLAQGSSEEEELPSLAFLLGSQ
HKLLPWWLPQSPVPASGLLSPEKWGPQGTHQSPSAERRGLNLAPSPANKAKKRPLFGSLSPAEKTPHPGPGLRVSGEQSLTWGLGGP
SQSQKRKGDPLVSRKEKKQRCSQ
>sp|Q6ZUJ4|CC062_HUMAN
MHYIKTWSLLGEMSEKLRRCRKELTAAIDRAFEGVSYSQECTGQQRLELSAAPLSFSLPVHRLLCRRHPLAACSSAAPFAAVPCAPE
NENPAFATNHAPVNAKPHALCPERKPLTSKENVLMHSSILAPERESWRTAGEGENWRKENLRKDMERDLKADSNMPLNNSSQEVTKD
LLDMIDHTSIRTIEELAGKIEFENELNHMCGHCQDSPFKEEAWALLMDKSPQKATDADPGSLKQAFDDHNIVETVLDLEEDYNVMTS
FKYQIE
>sp|A8MQ11|PM2P5_HUMAN
MWGRRRKLRRLNDVTISTCHVSAKVGTRLVFDHDGKIIQKTPYPHPRGTTVSVKQLFSTLPVRHKEFQRNIKKKRACFPFAFCRDCQ
FLEGSPAMLPVQPAKLTPRSTPPHPCSLEDNVITVFSSVKNGPGSSR

>sp|A6QL63|BTBDB_HUMAN
MARRGKKPVVRTLEDLTLDSGYGGAADSVRSSNLSLCCSDSHPASPYGGSCWPPLADSMHSRHNSFDTVNTALVEDSEGLDCAGQHC
SRLLPDLDEVPWTLQELEALLLRSRDPRAGPAVPGGLPKDALAKLSTLVSRALVRIAKEAQRLSLRFAKCTKYEIQSAMEIVLSWGL
AAHCTAAALAALSLYNMSSAGGDRLGRGKSARCGLTFSVGRVYRWMVDSRVALRIHEHAAIYLTACMESLFRDIYSRVVASGVPRSC
SGPGSGSGSGPGPSSGPGAAPAADKEREAPGGGAASGGACSAASSASGGSSCCAPPAAAAAAVPPAAAANHHHHHHHALHEAPKFTV
ETLEHTVNNDSEIWGLLQPYQHLICGKNASGVLCLPDSLNLHRDPQRSNKPGELPMFSQSELRTIEQSLLATRVGSIAELSDLVSRA
MHHLQPLNAKHHGNGTPLHHKQGALYWEPEALYTLCYFMHCPQMEWENPNVEPSKVNLQVERPFLVLPPLMEWIRVAVAHAGHRRSF
SMDSDDVRQAARLLLPGVDCEPRQLRADDCFCASRKLDAVAIEAKFKQDLGFRMLNCGRTDLVKQAVSLLGPDGINTMSEQGMTPLM
YACVRGDEAMVQMLLDAGADLNVEVVSTPHKYPSVHPETRHWTALTFAVLHGHIPVVQLLLDAGAKVEGSVEHGEENYSETPLQLAA
AVGNFELVSLLLERGADPLIGTMYRNGISTTPQGDMNSFSQAAAHGHRNVFRKLLAQPEKEKSDILSLEEILAEGTDLAETAPPPLC
ASRNSKAKLRALREAMYHSAEHGYVDVTIDIRSIGVPWTLHTWLESLRIAFQQHRRPLIQCLLKEFKTIQEEEYTEELVTQGLPLMF
EILKASKNEVISQQLCVIFTHCYGPYPIPKLTEIKRKQTSRLDPHFLNNKEMSDVTFLVEGRPFYAHKVLLFTASPRFKALLSSKPT
NDGTCIEIGYVKYSIFQLVMQYLYYGGPESLLIKNNEIMELLSAAKFFQLEALQRHCEIICAKSINTDNCVDIYNHAKFLGVTELSA
YCEGYFLKNMMVLIENEAFKQLLYDKNGEGTGQDVLQDLQRTLAIRIQSIHLSSSKGSVV
>sp|P01570|IFN14_HUMAN
MALPFALMMALVVLSCKSSCSLGCNLSQTHSLNNRRTLMLMAQMRRISPFSCLKDRHDFEFPQEEFDGNQFQKAQAISVLHEMMQQT
FNLFSTKNSSAAWDETLLEKFYIELFQQMNDLEACVIQEVGVEETPLMNEDSILAVKKYFQRITLYLMEKKYSPCAWEVVRAEIMRS
LSFSTNLQKRLRRKD
>sp|O95848|NUD14_HUMAN
MERIEGASVGRCAASPYLRPLTLHYRQNGAQKSWDFMKTHDSVTVLLFNSSRRSLVLVKQFRPAVYAGEVERRFPGSLAAVDQDGPR
ELQPALPGSAGVTVELCAGLVDQPGLSLEEVACKEAWEECGYHLAPSDLRRVATYWSGVGLTGSRQTMFYTEVTDAQRSGPGGGLVE
EGELIEVVHLPLEGAQAFADDPDIPKTLGVIFGVSWFLSQVAPNLDLQ
>sp|Q9H6Z4|RANB3_HUMAN
MADLANEEKPAIAPPVFVFQKDKGQKSPAEQKNLSDSGEEPRGEAEAPHHGTGHPESAGEHALEPPAPAGASASTPPPPAPEAQLPP
FPRELAGRSAGGSSPEGGEDSDREDGNYCPPVKRERTSSLTQFPPSQSEERSSGFRLKPPTLIHGQAPSAGLPSQKPKEQQRSVLRP
AVLQAPQPKALSQTVPSSGTNGVSLPADCTGAVPAASPDTAAWRSPSEAADEVCALEEKEPQKNESSNASEEEACEKKDPATQQAFV
FGQNLRDRVKLINESVDEADMENAGHPSADTPTATNYFLQYISSSLENSTNSADASSNKFVFGQNMSERVLSPPKLNEVSSDANREN
AAAESGSESSSQEATPEKESLAESAAAYTKATARKCLLEKVEVITGEEAESNVLQMQCKLFVFDKTSQSWVERGRGLLRLNDMASTD
DGTLQSRLVMRTQGSLRLILNTKLWAQMQIDKASEKSIRITAMDTEDQGVKVFLISASSKDTGQLYAALHHRILALRSRVEQEQEAK
MPAPEPGAAPSNEEDDSDDDDVLAPSGATAAGAGDEGDGQTTGST
>sp|P49763|PLGF_HUMAN
MPVMRLFPCFLQLLAGLALPAVPPQQWALSAGNGSSEVEVVPFQEVWGRSYCRALERLVDVVSEYPSEVEHMFSPSCVSLLRCTGCC
GDENLHCVPVETANVTMQLLKIRSGDRPSYVELTFSQHVRCECRHSPGRQSPDMPGDFRADAPSFLPPRRSLPMLFRMEWGCALTGS
QSAVWPSSPVPEEIPRMHPGRNGKKQQRKPLREKMKPERCGDAVPRR
>sp|Q9BQ48|RM34_HUMAN
MAVLAGSLLGPTSRSAALLGGRWLQPRAWLGFPDAWGLPTPQQARGKARGNEYQPSNIKRKNKHGWVRRLSTPAGVQVILRRMLKGR
KSLSH
>sp|Q92882|OSTF1_HUMAN
MSKPPPKPVKPGQVKVFRALYTFEPRTPDELYFEEGDIIYITDMSDTNWWKGTSKGRTGLIPSNYVAEQAESIDNPLHEAAKRGNLS
WLRECLDNRVGVNGLDKAGSTALYWACHGGHKDIVEMLFTQPNIELNQQNKLGDTALHAAAWKGYADIVQLLLAKGARTDLRNIEKK
LAFDMATNAACASLLKKKQGTDAVRTLSNAEDYLDDEDSD
>sp|P52746|ZN142_HUMAN
MTDPLLDSQPASSTGEMDGLCPELLLIPPPLSNRGILGPVQSPCPSRDPAPIPTEPGCLLVEATATEEGPGNMEIIVETVAGTLTPG
APGETPAPKLPPGEREPSQEAGTPLPGQETAEEENVEKEEKSDTQKDSQKAVDKGQGAQRLEGDVVSGTESLFKTHMCPECKRCFKK

RTHLVEHLHLHFPDPSLQCPNCQKFFTSKSKLKTHLLRELGEKAHHCPLCHYSAVERNALNRHMASMHEDISNFYSDTYACPVCREE
FRLSQALKEHLKSHTAAAAAEPLPLRCFQEGCSYAAPDRKAFIKHLKETHGVRAVECRHHSCPMLFATAEAMEAHHKSHYAFHCPHC
DFACSNKHLFRKHKKQGHPGSEELRCTFCPFATFNPVAYQDHVGKMHAHEKIHQCPECNFATAHKRVLIRHMLLHTGEKPHKCELCD
FTCRDVSYLSKHMLTHSNTKDYMCTECGYVTKWKHYLRVHMRKHAGDLRYQCNQCSYRCHRADQLSSHKLRHQGKSLMCEVCAFACK
RKYELQKHMASQHHPGTPAPLYPCHYCSYQSRHKQAVLSHENCKHTRLREFHCALCDYRTFSNTTLLFHKRKAHGYVPGDQAWQLRY
ASQEPEGAMQGPTPPPDSEPSNQLSARPEGPGHEPGTVVDPSLDQALPEMSEEVNTGRQEGSEAPHGGDLGGSPSPAEVEEGSCTLH
LEALGVELESVTEPPLEEVTETAPMEFRPLGLEGPDGLEGPELSSFEGIGTSDLSAEENPLLEKPVSEPSTNPPSLEEAPNNWVGTF
KTTPPAETAPLPPLPESESLLKALRRQDKEQAEALVLEGRVQMVVIQGEGRAFRCPHCPFITRREKALNLHSRTGCQGRREPLLCPE
CGASFKQQRGLSTHLLKKCPVLLRKNKGLPRPDSPIPLQPVLPGTQASEDTESGKPPPASQEAELLLPKDAPLELPREPEETEEPLA
TVSGSPVPPAGNSLPTEAPKKHCFDPVPPAGNSSPTEAPKKHHLDPVPPAGNSSPTEALKKHRFEQGKFHCNSCPFLCSRLSSITSH
VAEGCRGGRGGGGKRGTPQTQPDVSPLSNGDSAPPKNGSTESSSGDGDTVLVQKQKGARFSCPTCPFSCQQERALRTHQIRGCPLEE
SGELHCSLCPFTAPAATALRLHQKRRHPTAAPARGPRPHLQCGDCGFTCKQSRCMQQHRRLKHEGVKPHQCPFCDFSTTRRYRLEAH
QSRHTGIGRIPCSSCPQTFGTNSKLRLHRLRVHDKTPTHFCPLCDYSGYLRHDITRHVNSCHQGTPAFACSQCEAQFSSETALKQHA
LRRHPEPAQPAPGSPAETTEGPLHCSRCGLLCPSPASLRGHTRKQHPRLECGACQEAFPSRLALDEHRRQQHFSHRCQLCDFAARER
VGLVKHYLEQHEETSAAVAASDGDGDAGQPPLHCPFCDFTCRHQLVLDHHVKGHGGTRLYKCTDCAYSTKNRQKITWHSRIHTGEKP
YHCHLCPYACADPSRLKYHMRIHKEERKYLCPECGYKCKWVNQLKYHMTKHTGLKPYQCPECEYCTNRADALRVHQETRHREARAFM
CEQCGKAFKTRFLLRTHLRKHSEAKPYVCNVCHRAFRWAAGLRHHALTHTDRHPFFCRLCNYKAKQKFQVVKHVRRHHPDQADPNQG
VGKDPTTTPTVHLHDVQLEDPSPPAPAAPHTGPEG
>sp|Q8NGI2|O52N4_HUMAN
MLTLNKTDLIPASFILNGVPGLEDTQLWISFPFCSMYVVAMVGNCGLLYLIHYEDALHKPMYYFLAMLSFTDLVMCSSTIPKALCIF
WFHLKDIGFDECLVQMFFTHTFTGMESGVLMLMALDRYVAICYPLRYSTILTNPVIAKVGTATFLRGVLLIIPFTFLTKLLPYCRGN
ILPHTYCDHMSVAKLSCGNVKVNAIYGLMVALLIWGFDILCITNSYTMILRAVVSLSSADARQKAFNTCTAHICAIVFSYTPAFFSF
FSHRFGEHIIPPSCHIIVANIYLLLPPTMNPIVYGVKTKQIRDCVIRILSGSKDTKSYSM
>sp|P48775|T23O_HUMAN
MSGCPFLGNNFGYTFKKLPVEGSEEDKSQTGVNRASKGGLIYGNYLHLEKVLNAQELQSETKGNKIHDEHLFIITHQAYELWFKQIL
WELDSVREIFQNGHVRDERNMLKVVSRMHRVSVILKLLVQQFSILETMTALDFNDFREYLSPASGFQSLQFRLLENKIGVLQNMRVP
YNRRHYRDNFKGEENELLLKSEQEKTLLELVEAWLERTPGLEPHGFNFWGKLEKNITRGLEEEFIRIQAKEESEEKEEQVAEFQKQK
EVLLSLFDEKRHEHLLSKGERRLSYRALQGALMIYFYREEPRFQVPFQLLTSLMDIDSLMTKWRYNHVCMVHRMLGSKAGTGGSSGY
HYLRSTVSDRYKVFVDLFNLSTYLIPRHWIPKMNPTIHKFLYTAEYCDSSYFSSDESD
>sp|C9J798|RAS4B_HUMAN
MAKRSSLYIRIVEGKNLPAKDITGSSDPYCIVKVDNEPIIRTATVWKTLCPFWGEEYQVHLPPTFHAVAFYVMDEDALSRDDVIGKV
CLTRDTIASHPKGFSGWAHLTEVDPDEEVQGEIHLRLEVWPGARACRLRCSVLEARDLAPKDRNGTSDPFVRVRYKGRTRETSIVKK
SCYPRWNETFEFELQEGAMEALCVEAWDWDLVSRNDFLGKVVIDVQRLRVVQQEEGWFRLQPDQSKSRRHDEGNLGSLQLEVRLRDE
TVLPSSYYQPLVHLLCHEVKLGMQGPGQLIPLIEETTSTECRQDVATNLLKLFLGQGLAKDFLDLLFQLELSRTSETNTLFRSNSLA
SKSVESFLKVAGMQYLHGVLGPIINKVFEEKKYVELDPSKVEVKDVGCSGLHRPQTEAEVLEQSAQTLRAHLGALLSALSRSVRACP
AVVRATFRQLFRRVRERFPGAQHENVPFIAVTSFLCLRFFSPAIMSPKLFHLRERHADARTSRTLLLLAKAVQNVGNMDTPASRAKE
AWMEPLQPTVHQGVAQLKDFITKLVDIEEKDELDLQRTLSLQAPPVKEGPLFIHRTKGKGPLMSSSFKKLYFSLTTEALSFAKTPSS
KKSALIKLANIRAAEKVEEKSFGGSHVMQVIYTDDAGRPQTAYLQCKCVNELNQWLSALRKVSINNTGLLGSYHPGVFRGDKWSCCH
QKEKTGQGCDKTRSRVTLQEWNDPLDHDLEAQLIYRHLLGVEAMLWERHRELSGGAEAGTVPTSPGKVPEDSLARLLRVLQDLREAH
SSSPAGSPPSEPNCLLELQT
>sp|Q8WWI5|CTL1_HUMAN
MGCCSSASSAAQSSKREWKPLEDRSCTDIPWLLLFILFCIGMGFICGFSIATGAAARLVSGYDSYGNICGQKNTKLEAIPNSGMDHT
QRKYVFFLDPCNLDLINRKIKSVALCVAACPRQELKTLSDVQKFAEINGSALCSYNLKPSEYTTSPKSSVLCPKLPVPASAPIPFFH
RCAPVNISCYAKFAEALITFVSDNSVLHRLISGVMTSKEIILGLCLLSLVLSMILMVIIRYISRVLVWILTILVILGSLGGTGVLWW

LYAKQRRSPKETVTPEQLQIAEDNLRALLIYAISATVFTVILFLIMLVMRKRVALTIALFHVAGKVFIHLPLLVFQPFWTFFALVLF
WVYWIMTLLFLGTTGSPVQNEQGFVEFKISGPLQYMWWYHVVGLIWISEFILACQQMTVAGAVVTYYFTRDKRNLPFTPILASVNRL
IRYHLGTVAKGSFIITLVKIPRMILMYIHSQLKGKENACARCVLKSCICCLWCLEKCLNYLNQNAYTATAINSTNFCTSAKDAFVIL
VENALRVATINTVGDFMLFLGKVLIVCSTGLAGIMLLNYQQDYTVWVLPLIIVCLFAFLVAHCFLSIYEMVVDVLFLCFAIDTKYND
GSPGREFYMDKVLMEFVENSRKAMKEAGKGGVADSRELKPMASGASSA
>sp|Q96RC9|OR8B4_HUMAN
MTLRNSSSVTEFILVGLSEQPELQLPLFLLFLGIYVFTVVGNLGLITLIGINPSLHTPMYFFLFNLSFIDLCYSCVFTPKMLNDFVS
ESIISYVGCMTQLFFFCFFVNSECYVLVSMAYDRYVAICNPLLYMVTMSPRVCFLLMFGSYVVGFAGAMAHTGSMLRLTFCDSNVID
HYLCDVLPLLQLSCTSTHVSELVFFIVVGVITMLSSISIVISYALILSNILCIPSAEGRSKAFSTWGSHIIAVALFFGSGTFTYLTT
SFPGSMNHGRFASVFYTNVVPMLNPSIYSLRNKDDKLALGKTLKRVLF
>sp|Q96JJ6|JPH4_HUMAN
MSPGGKFDFDDGGCYVGGWEAGRAHGYGVCTGPGAQGEYSGCWAHGFESLGVFTGPGGHSYQGHWQQGKREGLGVERKSRWTYRGEW
LGGLKGRSGVWESVSGLRYAGLWKDGFQDGYGTETYSDGGTYQGQWQAGKRHGYGVRQSVPYHQAALLRSPRRTSLDSGHSDPPTPP
PPLPLPGDEGGSPASGSRGGFVLAGPGDADGASSRKRTPAAGGFFRRSLLLSGLRAGGRRSSLGSKRGSLRSEVSSEVGSTGPPGSE
ASGPPAAAPPALIEGSATEVYAGEWRADRRSGFGVSQRSNGLRYEGEWLGNRRHGYGRTTRPDGSREEGKYKRNRLVHGGRVRSLLP
LALRRGKVKEKVDRAVEGARRAVSAARQRQEIAAARAADALLKAVAASSVAEKAVEAARMAKLIAQDLQPMLEAPGRRPRQDSEGSD
TEPLDEDSPGVYENGLTPSEGSPELPSSPASSRQPWRPPACRSPLPPGGDQGPFSSPKAWPEEWGGAGAQAEELAGYEAEDEAGMQG
PGPRDGSPLLGGCSDSSGSLREEEGEDEEPLPPLRAPAGTEPEPIAMLVLRGSSSRGPDAGCLTEELGEPAATERPAQPGAANPLVV
GAVALLDLSLAFLFSQLLT
>sp|P51854|TKTL1_HUMAN
MADAEARAEFPEEARPDRGTLQVLQDMASRLRIHSIRATCSTSSGHPTSCSSSSEIMSVLFFYIMRYKQSDPENPDNDRFVLAKRLS
FVDVATGWLGQGLGVACGMAYTGKYFDRASYRVFCLMSDGESSEGSVWEAMAFASYYSLDNLVAIFDVNRLGHSGALPAEHCINIYQ
RRCEAFGWNTYVVDGRDVEALCQVFWQASQVKHKPTAVVAKTFKGRGTPSIEDAESWHAKPMPRERADAIIKLIESQIQTSRNLDPQ
PPIEDSPEVNITDVRMTSPPDYRVGDKIATRKACGLALAKLGYANNRVVVLDGDTRYSTFSEIFNKEYPERFIECFMAEQNMVSVAL
GCASRGRTIAFASTFAAFLTRAFDHIRIGGLAESNINIIGSHCGVSVGDDGASQMALEDIAMFRTIPKCTIFYPTDAVSTEHAVALA
ANAKGMCFIRTTRPETMVIYTPQERFEIGQAKVLRHCVSDKVTVIGAGITVYEALAAADELSKQDIFIRVIDLFTIKPLDVATIVSS
AKATEGRIITVEDHYPQGGIGEAVCAAVSMDPDIQVHSLAVSGVPQSGKSEELLDMYGISARHIIVAVKCMLLN
>sp|P09669|COX6C_HUMAN
MAPEVLPKPRMRGLLARRLRNHMAVAFVLSLGVAALYKFRVADQRKKAYADFYRNYDVMKDFEEMRKAGIFQSVK
>sp|O15514|RPB4_HUMAN
MAAGGSDPRAGDVEEDASQLIFPKEFETAETLLNSEVHMLLEHRKQQNESAEDEQELSEVFMKTLNYTARFSRFKNRETIASVRSLL
LQKKLHKFELACLANLCPETAEESKALIPSLEGRFEDEELQQILDDIQTKRSFQY
>sp|P27635|RL10_HUMAN
MGRRPARCYRYCKNKPYPKSRFCRGVPDAKIRIFDLGRKKAKVDEFPLCGHMVSDEYEQLSSEALEAARICANKYMVKSCGKDGFHI
RVRLHPFHVIRINKMLSCAGADRLQTGMRGAFGKPQGTVARVHIGQVIMSIRTKLQNKEHVIEALRRAKFKFPGRQKIHISKKWGFT
KFNADEFEDMVAEKRLIPDGCGVKYIPNRGPLDKWRALHS
>sp|Q96EK7|F120B_HUMAN
MGVRGLQGFVGSTCPHICTVVNFKELAEHHRSKYPGCTPTIVVDAMCCLRYWYTPESWICGGQWREYFSALRDFVKTFTAAGIKLIF
FFDGMVEQDKRDEWVKRRLKNNREISRIFHYIKSHKEQPGRNMFFIPSGLAVFTRFALKTLGQETLCSLQEADYEVASYGLQHNCLG
ILGEDTDYLIYDTCPYFSISELCLESLDTVMLCREKLCESLGLCVADLPLLACLLGNDIIPEGMFESFRYKCLSSYTSVKENFDKKG
NIILAVSDHISKVLYLYQGEKKLEEILPLGPNKALFYKGMASYLLPGQKSPWFFQKPKGVITLDKQVISTSSDAESREEVPMCSDAE
SRQEVPMCTGPESRREVPVYTDSEPRQEVPMCSDPEPRQEVPTCTGPESRREVPMCSDPEPRQEVPMCTGPEARQEVPMYTDSEPRQ
EVPMYTDSEPRQEVPMYTGSEPRQEVPMYTGPESRQEVPMYTGPESRQEVLIRTDPESRQEIMCTGHESKQEVPICTDPISKQEDSM
CTHAEINQKLPVATDFEFKLEALMCTNPEIKQEDPTNVGPEVKQQVTMVSDTEILKVARTHHVQAESYLVVYNIMSSGEIECSNTLED

ELDQALPSQAFIYRPIRQRVYSLLLEDCQDVTSTCLAVKEWFVYPGNPLRHPDLVRPLQMTIPGGTPSLKILWLNQEPEIQVRRLDT
LLACFNLSSSREELQAVESPFQALCCLLIYLFVQVDTLCLEDLHAFIAQALCLQGKSTSQLVNLQPDYINPRAVQLGSLLVRGLTTL
VLVNSACGFPWKTSDFMPWNVFDGKLFHQKYLQSEKGYAVEVLLEQNRSRLTKFHNLKAVVCKACMKENRRITGRAHWGSHHAGRWG
RQGSSYHRTGSGYSRSSQGQPWRDQGPGSRQYEHDQWRRY
>sp|Q6IMN6|CAPR2_HUMAN
MEVQVSQASLGFELTSVEKSLREWSRLSREVIAWLCPSSPNFILNFPPPPSASSVSMVQLFSSPFGYQSPSGHSEEEREGNMKSAKP
QVNHSQHGESQRALSPLQSTLSSAASPSQAYETYIENGLICLKHKIRNIEKKKLKLEDYKDRLKSGEHLNPDQLEAVEKYEEVLHNL
EFAKELQKTFSGLSLDLLKAQKKAQRREHMLKLEAEKKKLRTILQVQYVLQNLTQEHVQKDFKGGLNGAVYLPSKELDYLIKFSKLT
CPERNESLSVEDQMEQSSLYFWDLLEGSEKAVVGTTYKHLKDLLSKLLNSGYFESIPVPKNAKEKEVPLEEEMLIQSEKKTQLSKTE
SVKESESLMEFAQPEIQPQEFLNRRYMTEVDYSNKQGEEQPWEADYARKPNLPKRWDMLTEPDGQEKKQESFKSWEASGKHQEVSKP
AVSLEQRKQDTSKLRSTLPEEQKKQEISKSKPSPSQWKQDTPKSKAGYVQEEQKKQETPKLWPVQLQKEQDPKKQTPKSWTPSMQSE
QNTTKSWTTPMCEEQDSKQPETPKSWENNVESQKHSLTSQSQISPKSWGVATASLIPNDQLLPRKLNTEPKDVPKPVHQPVGSSSTL
PKDPVLRKEKLQDLMTQIQGTCNFMQESVLDFDKPSSAIPTSQPPSATPGSPVASKEQNLSSQSDFLQEPLQATSSPVTCSSNACLV
TTDQASSGSETEFMTSETPEAAIPPGKQPSSLASPNPPMAKGSEQGFQSPPASSSSVTINTAPFQAMQTVFNVNAPLPPRKEQEIKE
SPYSPGYNQSFTTASTQTPPQCQLPSIHVEQTVHSQETAANYHPDGTIQVSNGSLAFYPAQTNVFPRPTQPFVNSRGSVRGCTRGGR
LITNSYRSPGGYKGFDTYRGLPSISNGNYSQLQFQAREYSGAPYSQRDNFQQCYKRGGTSGGPRANSRAGWSDSSQVSSPERDNETF
NSGDSGQGDSRSMTPVDVPVTNPAATILPVHVYPLPQQMRVAFSAARTSNLAPGTLDQPIVFDLLLNNLGETFDLQLGRFNCPVNGT
YVFIFHMLKLAVNVPLYVNLMKNEEVLVSAYANDGAPDHETASNHAILQLFQGDQIWLRLHRGAIYGSSWKYSTFSGYLLYQD
>sp|A2RUB6|CCD66_HUMAN
MNLGDGLKLETELLDGKTKLILSPYEHKSKISVKMGNKAKIAKCPLRTKTGHILKSTQDTCIGSEKLLQKKPVGSETSQAKGEKNGM
TFSSTKDLCKQCIDKDCLHIQKEISPATPNMQKTRNTVNTSLVGKQKPHKKHITAENMKSSLVCLTQDQLQQILMTVNQGNRSLSLT
ENGKEAKSQYSLYLNSISNQPKDENIMGLFKKTEMVSSVPAENKSVLNEHQETSKQCEQKIAIENEWKPADIFSTLGERECDRSSLE
AKKAQWRKELDEQVALKKKEKEVSEKWNDPWKKSESDKIIWEKHQILDQSRETVLLEHPFSAVKQELQRKWIEELNKQIEDDRQRKI
EEKIIYSKGEEHDRWAMHFDSLKSYPGSQSQLFSQSTHKQPEYFCVSPDTQELADVSSVCTPTTGSQVEPSEEEHIAKPIKDVVMAN
SKKTNFLRSMTALLDPAQIEERDRRRQKQLEHQKAITAQVEEKRRKKQLEEEQRKKEEQEEELRLAQEREEMQKQYEEDILKQKQKE
EIMTLKTNELFQTMQRAQELAQRLKQEQRIRELAQKGHDTSRLIKNLGVDTIQMEYNASNISNSRHDSDEISGKMNTYMNSTTSKKD
TGVQTDDLNIGIFTNAESHCGSLMERDITNCSSPEISAELIGQFSTKKNKQELTQDKGASLEKENNRCNDQCNQFTRIEKQTKHMKK
YPKRPDWNINKPPKRYIPASEKYPKQLQKQREEKKVRRQMELLHLVEKNNPGHLSQNRGISPEIFHSSHQETESKLRWHLVKKEEEP
LNIHSFSKERSPSSPVPVVKNRTQQTQNTLHLPLKNSSYERENLISGSNQTELSSGISESSHFIPYVRTNEIYYLDPDAPLSGPSTQ
DPQYQNSQDCGQKRQLFDSDCVRDPLLNPNMVKNRDRQQAILKGLSELRQGLLQKQKELESSLLPLAENQEESFGSSF
>sp|Q9H875|PKRI1_HUMAN
MASPAASSVRPPRPKKEPQTLVIPKNAAEEQKLKLERLMKNPDKAVPIPEKMSEWAPRPPPEFVRDVMGSSAGAGSGEFHVYRHLRR
REYQRQDYMDAMAEKQKLDAEFQKRLEKNKIAAEEQTAKRRKKRQKLKEKKLLAKKMKLEQKKQEGPGQPKEQGSSSSAEASGTEEE
EEVPSFTMGR
>sp|Q9P2K1|C2D2A_HUMAN
MNPREEKVKIITEEFIENDEDADMGRQNKNSKVRRQPRKKQPPTAVPKEMVSEKSHLGNPQEPVQEEPKTRLLSMTVRRGPRSLPPI
PSTSRTGFAEFSMRGRMREKLQAARSKAESALLQEIPTPRPRRLRSPSKKELETEFGTEPGKEVERTQQEVDSQSYSRVKFHDSARK
IKPKPQVPPGFPSAEEAYNFFTFNFDPEPEGSEEKPKARHRAGTNQEEEEGEEEEPPAQGGGKEMDEEELLNGDDAEDFLLGLDHVA
DDFVAVRPADYESIHDRLQMEREMLFIPSRQTVPTYKKLPENVQPRFLEDEGLYTGVRPEVARTNQNIMENRLLMQDPERRWFGDDG
RILALPNPIKPFPSRPPVLTQEQSIKAELETLYKKAVKYVHSSQHVIRSGDPPGNFQLDIDISGLIFTHHPCFSREHVLAAKLAQLY
DQYLARHQRNKAKFLTDKLQALRNAVQTGLDPEKPHQSLDTIQKTINEYKSEIRQTRKFRDAEQEKDRTLLKTIIKVWKEMKSLREF
QRFTNTPLKLVLRKEKADQKADEEAYEAEIQAEISELLEEHTEEYAQKMEEYRTSLQQWKAWRKVQRAKKKKRKQAAEEHPGDEIAE
PYPEEDLVKPSPPEPTDRAVIEQEVRERAAQSRRRPWEPTLVPELSLAGSVTPNDQCPRAEVSRREDVKKRSVYLKVLFNNKEVSRT
VSRPLGADFRVHFGQIFNLQIVNWPESLTLQVYETVGHSSPTLLAEVFLPIPETTVVTGRAPTEEVEFSSNQHVTLDHEGVGSGVPF

SFEADGSNQLTLMTSGKVSHSVAWAIGENGIPLIPPLSQQNIGFRSALKKADAISSIGTSGLTDMKKLAKWAAESKLDPNDPNNAPL
MQLISVATSGESYVPDFFRLEQLQQEFNFVSDQELNRSKRFRLLHLRSQEVPEFRNYKQVPVYDREIMEKVFQDYEKRLRDRNVIET
KEHIDTHRAIVAKYLQQVRESVINRFLIAKQYFLLADMIVEEEVPNISILGLSLFKLAEQKRPLRPRRKGRKKVTAQNLSDGDIKLL
VNIVRAYDIPVRKPAVSKFQQPSRSSRMFSEKHAASPSTYSPTHNADYPLGQVLVRPFVEVSFQRTVCHTTTAEGPNPSWNEELELP
FRAPNGDYSTASLQSVKDVVFINIFDEVLHDVLEDDRERGSGIHTRIERHWLGCVKMPFSTIYFQARIDGTFKIDIPPVLLGYSKER
NMILERGFDSVRSLSEGSYITLFITIEPQLVPGESIREKFESQEDEKLLQATEKFQAECALKFPNRQCLTTVIDISGKTVFITRYLK
PLNPPQELLNVYPNNLQATAELVARYVSLIPFLPDTVSFGGICDLWSTSDQFLDLLAGDEEEHAVLLCNYFLSLGKKAWLLMGNAIP
EGPTAYVLTWEQGRYLIWNPCSGHFYGQFDTFCPLKNVGCLIGPDNIWFNIQRYESPLRINFDVTRPKLWKSFFSRSLPYPGLSSVQ
PEELIYQRSDKAAAAELQDRIEKILKEKIMDWRPRHLTRWNRYCTSTLRHFLPLLEKSQGEDVEDDHRAELLKQLGDYRFSGFPLHM
PYSEVKPLIDAVYSTGVHNIDVPNVEFALAVYIHPYPKNVLSVWIYVASLIRNR
>sp|A6NIJ9|O6C70_HUMAN
MKNHTRQIEFILLGLTDNSQLQIVIFLFLLLNCVLSMIGNFTIIALILLDSQLKTPMYFFLRNFSFLEISFTTACIPRFLITIVTRE
KTISCNGCISQLFFYIFLGVTEFFLLAALSYDRYVAICKPLRYMSIMSNKVCYQLVFSSWVTGFLIIFTPLILGLNLDFCASNIIDH
FICDISLILQLSCSDTHLLELIAFLLAVMTLIVTLFLVILSYSYIIKTILKFPSAQQKKKAFSTCSSHMIVVSITYGSCMFIYIKPS
ANERVALSKGVTVLNTSVAPLLNPFIYTLRNQQVKQAFKAVFRKIFSASDK
>sp|O00587|MFNG_HUMAN
MQCRLPRGLAGALLTLLCMGLLCLRYHLNLSPQRVQGTPELSQPNPGPPKLQLHDVFIAVKTTRAFHRLRLELLLDTWVSRTREQTF
VFTDSPDKGLQERLGSHLVVTNCSAEHSHPALSCKMAAEFDTFLASGLRWFCHVDDDNYVNPRALLQLLRAFPLARDVYVGRPSLNR
PIHASEPQPHNRTRLVQFWFATGGAGFCINRKLALKMAPWASGSRFMDTSALIRLPDDCTMGYIIECKLGGRLQPSPLFHSHLETLQ
LLRTAQLPEQVTLSYGVFEGKLNVIKLQGPFSPEEDPSRFRSLHCLLYPDTPWCPQLGAR
>sp|Q8NH03|OR2T3_HUMAN
MCSGNQTSQNQTASTDFTLTGLFAESKHAALLYTVTFLLFLMALTGNALLILLIHSEPRLHTPMYFFISQLALMDLMYLCVTVPKML
VGQVTGDDTISPSGCGIQMFFYLTLAGAEVFLLAAMAYDRYAAVCRPLHYPLLMNQRVCQLLVSACWVLGMVDGLLLTPITMSFPFC
QSRKILSFFCETPALLKLSCSDVSLYKTLMYLCCILMLLAPIMVISSSYTLILHLIHRMNSAAGHRKALATCSSHMIIVLLLFGASF
YTYMLPSSYHTAEQDMMVSAFYTIFTPVLNPLIYSLRNKDVTRALRSMMQSRMNQEK
>sp|Q8WXC6|MYOV2_HUMAN
MKPAVDEMFPEGAGPYVDLDEAGGSTGLLMDLAANEKAVHADFFNDFEDLFDDDDIQ
>sp|Q9NQR4|NIT2_HUMAN
MTSFRLALIQLQISSIKSDNVTRACSFIREAATQGAKIVSLPECFNSPYGAKYFPEYAEKIPGESTQKLSEVAKECSIYLIGGSIPE
EDAGKLYNTCAVFGPDGTLLAKYRKIHLFDIDVPGKITFQESKTLSPGDSFSTFDTPYCRVGLGICYDMRFAELAQIYAQRGCQLLV
YPGAFNLTTGPAHWELLQRSRAVDNQVYVATASPARDDKASYVAWGHSTVVNPWGEVLAKAGTEEAIVYSDIDLKKLAEIRQQIPVF
RQKRSDLYAVEMKKP
>sp|P10153|RNAS2_HUMAN
MVPKLFTSQICLLLLLGLLAVEGSLHVKPPQFTWAQWFETQHINMTSQQCTNAMQVINNYQRRCKNQNTFLLTTFANVVNVCGNPNM
TCPSNKTRKNCHHSGSQVPLIHCNLTTPSPQNISNCRYAQTPANMFYIVACDNRDQRRDPPQYPVVPVHLDRII
>sp|Q02543|RL18A_HUMAN
MKASGTLREYKVVGRCLPTPKCHTPPLYRMRIFAPNHVVAKSRFWYFVSQLKKMKKSSGEIVYCGQVFEKSPLRVKNFGIWLRYDSR
SGTHNMYREYRDLTTAGAVTQCYRDMGARHRARAHSIQIMKVEEIAASKCRRPAVKQFHDSKIKFPLPHRVLRRQHKPRFTTKRPNT
FF
>sp|Q5JSH3|WDR44_HUMAN
MASESDTEEFYDAPEDVHLGGGYPVGSPGKVGLSTFKETENTAYKVGNESPVQELKQDVSKKIIESIIEESQKVLQLEDDSLDSKGK
ELSDQATASPIVARTDLSNIPGLLAIDQVLPEESQKAESQNTFEETELELKKCFPSDETCEKPVDETTKLTQTSSTEQLNVLETETE
VLNKEAVEVKGGGDVLEPVSSDSLSTKDFAAVEEVAPAKPPRHLTPEPDIVASTKKPVPARPPPPTNFPPPRPPPPSRPAPPPRKRK
SELEFETLKTPDIDVPKENITSDSLLTASMASESTVKDSQPSLDLASATSGDKIVTAQENGKAPDGQTVAGEVMGPQRPRSNSGREL

TDEEILASVMIKNLDTGEEIPLSLAEEKLPTGINPLTLHIMRRTKEYVSNDAAQSDDEEKLQSQPTDTDGGRLKQKTTQLKKFLGKS
VKRAKHLAEEYGERAINKVKSVRDEVFHTDQDDPSSSDDEGMPYTRPVKFKAAHGFKGPYDFDQIKVVQDLSGEHMGAVWTMKFSHC
GRLLASAGQDNVVRIWALKNAFDYFNNMRMKYNTEGRVSPSPSQESLSSSKSDTDTGVCSGTDEDPDDKNAPFRQRPFCKYKGHTAD
LLDLSWSKNYFLLSSSMDKTVRLWHISRRECLCCFQHIDFVTAIAFHPRDDRYFLSGSLDGKLRLWNIPDKKVALWNEVDGQTKLIT
AANFCQNGKYAVIGTYDGRCIFYDTEHLKYHTQIHVRSTRGRNKVGRKITGIEPLPGENKILVTSNDSRIRLYDLRDLSLSMKYKGY
VNSSSQIKASFSHDFTYLVSGSEDKYVYIWSTYHDLSKFTSVRRDRNDFWEGIKAHNAVVTSAIFAPNPSLMLSLDVQSEKSEGNEK
SEDAEVLDATPSGIMKTDNTEVLLSADFTGAIKVFVNKRKNVS
>sp|O14772|FPGT_HUMAN
MAAARDPPEVSLREATQRKLRRFSELRGKLVARGEFWDIVAITAADEKQELAYNQQLSEKLKRKELPLGVQYHVFVDPAGAKIGNGG
STLCALQCLEKLYGDKWNSFTILLIHSGGYSQRLPNASALGKIFTALPLGNPIYQMLELKLAMYIDFPLNMNPGILVTCADDIELYS
IGEFEFIRFDKPGFTALAHPSSLTIGTTHGVFVLDPFDDLKHRDLEYRSCHRFLHKPSIEKMYQFNAVCRPGNFCQQDFAGGDIADL
KLDSDYVYTDSLFYMDHKSAKMLLAFYEKIGTLSCEIDAYGDFLQALGPGATVEYTRNTSNVIKEESELVEMRQRIFHLLKGTSLNV
VVLNNSKFYHIGTTEEYLFYFTSDNSLKSELGLQSITFSIFPDIPECSGKTSCIIQSILDSRCSVAPGSVVEYSRLGPDVSVGENCI
ISGSYILTKAALPAHSFVCSLSLKMNRCLKYATMAFGVQDNLKKSVKTLSDIKLLQFFGVCFLSCLDVWNLKVTEELFSGNKTCLSL
WTARIFPVCSSLSDSVITSLKMLNAVKNKSAFSLNSYKLLSIEEMLIYKDVEDMITYREQIFLEISLKSSLM
>sp|Q8WTR4|GDPD5_HUMAN
MVRHQPLQYYEPQLCLSCLTGIYGCRWKRYQRSHDDTTPWERLWFLLLTFTFGLTLTWLYFWWEVHNDYDEFNWYLYNRMGYWSDWP
VPILVTTAAAFAYIAGLLVLALCHIAVGQQMNLHWLHKIGLVVILASTVVAMSAVAQLWEDEWEVLLISLQGTAPFLHVGAVAAVTM
LSWIVAGQFARAERTSSQVTILCTFFTVVFALYLAPLTISSPCIMEKKDLGPKPALIGHRGAPMLAPEHTLMSFRKALEQKLYGLQA
DITISLDGVPFLMHDTTLRRTTNVEEEFPELARRPASMLNWTTLQRLNAGQWFLKTDPFWTASSLSPSDHREAQNQSICSLAELLEL
AKGNATLLLNLRDPPREHPYRSSFINVTLEAVLHSGFPQHQVMWLPSRQRPLVRKVAPGFQQTSGSKEAVASLRRGHIQRLNLRYTQ
VSRQELRDYASWNLSVNLYTVNAPWLFSLLWCAGVPSVTSDNSHALSQVPSPLWIMPPDEYCLMWVTADLVSFTLIVGIFVLQKWRL
GGIRSYNPEQIMLSAAVRRTSRDVSIMKEKLIFSEISDGVEVSDVLSVCSDNSYDTYANSTATPVGPRGGGSHTKTLIERSGR
>sp|P41159|LEP_HUMAN
MHWGTLCGFLWLWPYLFYVQAVPIQKVQDDTKTLIKTIVTRINDISHTQSVSSKQKVTGLDFIPGLHPILTLSKMDQTLAVYQQILT
SMPSRNVIQISNDLENLRDLLHVLAFSKSCHLPWASGLETLDSLGGVLEASGYSTEVVALSRLQGSLQDMLWQLDLSPGC
>sp|Q8IWR1|TRI59_HUMAN
MHNFEEELTCPICYSIFEDPRVLPCSHTFCRNCLENILQASGNFYIWRPLRIPLKCPNCRSITEIAPTGIESLPVNFALRAIIEKYQ
QEDHPDIVTCPEHYRQPLNVYCLLDKKLVCGHCLTIGQHHGHPIDDLQSAYLKEKDTPQKLLEQLTDTHWTDLTHLIEKLKEQKSHS
EKMIQGDKEAVLQYFKELNDTLEQKKKSFLTALCDVGNLINQEYTPQIERMKEIREQQLELMALTISLQEESPLKFLEKVDDVRQHV
QILKQRPLPEVQPVEIYPRVSKILKEEWSRTEIGQIKNVLIPKMKISPKRMSCSWPGKDEKEVEFLKILNIVVVTLISVILMSILFF
NQHIITFLSEITLIWFSEASLSVYQSLSNSLHKVKNILCHIFYLLKEFVWKIVSH
>sp|Q16518|RPE65_HUMAN
MSIQVEHPAGGYKKLFETVEELSSPLTAHVTGRIPLWLTGSLLRCGPGLFEVGSEPFYHLFDGQALLHKFDFKEGHVTYHRRFIRTD
AYVRAMTEKRIVITEFGTCAFPDPCKNIFSRFFSYFRGVEVTDNALVNVYPVGEDYYACTETNFITKINPETLETIKQVDLCNYVSV
NGATAHPHIENDGTVYNIGNCFGKNFSIAYNIVKIPPLQADKEDPISKSEIVVQFPCSDRFKPSYVHSFGLTPNYIVFVETPVKINL
FKFLSSWSLWGANYMDCFESNETMGVWLHIADKKRKKYLNNKYRTSPFNLFHHINTYEDNGFLIVDLCCWKGFEFVYNYLYLANLRE
NWEEVKKNARKAPQPEVRRYVLPLNIDKADTGKNLVTLPNTTATAILCSDETIWLEPEVLFSGPRQAFEFPQINYQKYCGKPYTYAY
GLGLNHFVPDRLCKLNVKTKETWVWQEPDSYPSEPIFVSHPDALEEDDGVVLSVVVSPGAGQKPAYLLILNAKDLSEVARAEVEINI
PVTFHGLFKKS
>sp|O60447|EVI5_HUMAN
MVTNKMTAAFRNPSGKQVATDKVAEKLSSTLSWVKNTVSHTVSQMASQVASPSTSLHTTSSSTTLSTPALSPSSPSQLSPDDLELLA
KLEEQNRLLETDSKSLRSVNGSRRNSGSSLVSSSSASSNLSHLEEDSWILWGRIVNEWEDVRKKKEKQVKELVHKGIPHHFRAIVWQ
LLCSAQSMPIKDQYSELLKMTSPCEKLIRRDIARTYPEHNFFKEKDSLGQEVLFNVMKAYSLVDREVGYCQGSAFIVGLLLMQMPEE

EAFCVFVKLMQDYRLRELFKPSMAELGLCMYQFECMIQEHLPELFVHFQSQSFHTSMYASSWFLTIFLTTFPLPIATRIFDIFMSEG
LEIVFRVGLALLQMNQAELMQLDMEGMLQHFQKVIPHQFDGVPDKLIQAAYQVKYNSKKMKKLEKEYTTIKTKEMEEQVEIKRLRTE
NRLLKQRIETLEKHKCSSNYNEDFVLQLEKELVQARLSEAESQCALKEMQDKVLDIEKRNNSLPDENNIARLQEELIAVKLREAEAI
MGLKELRQQVKDLEEHWQRHLARTTGRWKDPPKKNAMNELQDELMTIRLREAETQAEIREIKQRMMEMETQNQINSNHLRRAEQEVI
SLQEKVQYLSAQNKGLLTQLSEAKRKQAEIECKNKEEVMAVRLREADSIAAVAELRQHIAELEIQKEEGKLQGQLNKSDSNQYIGEL
KDQIAELNHELRCLKGQRGFSGQPPFDGIHIVNHLIGDDESFHSSDEDFIDNSLQETGVGFPLHGKSGSMSLDPAVADGSESETEDS
VLETRESNQVVQKERPPRRRESYSTTV
>sp|Q9NU39|FX4L1_HUMAN
MNLPRAERPRSTPQRSLRDSDGEDGKIDVLGEEEDEDEVEDEEEEASQKFLEQSLQPGLQVARWGGVALPREHIEGGGPSDPSEFGT
EFRAPPRSAAASEDARQPAKPPYSYIALITMAILQSPHKRLTLSGICAFISGRFPYYRRKFPAWQNSIRHNLSLNDCFVKIPREPGH
PGKGTYWSLDPASQDMFDNGSFLRRRKRFKRHQLTPGAHLPHPFPLPAAHAALHNPRPGPLLGAPALPQPVPGAYPNTAPGRRPYAL
LHPHPPRYLLLSAPAYAGAPKKAEGADLATPGTLPVLQPSLGPQPWEEGKGLASPPGGGCISFSIESIMQGVRGAGTGAAQSLSPTA
WSYCPLLQRPSSLSDNFAATAAASGGGLRQRLRSHQGRGAGRAPVGRVGAAAVSGGGRGL
>sp|F8W1W9|NPIB9_HUMAN
MPMRVPEEVTLRNPGSSGRKERPEAGTGSWLGRTRNQVINSLAVYRHRETDFGVGVRDHPGQHGKTPSPQKLDNLIIIIIGFLRRYT
FNILFCTSCLCVSFLKTIFWSRNGHDGSMDVQQRAWRSNRSRQKGLRSICMHTKKRVSSFRGNKIGLKDVITLRRHVETKVRAKIRK
RKVTTKINRHDKINGKRKTARKQKMFQRAQELRRRAEDYHKCKIPPSARKPLCNWVRMAAAEHRHSSGLPYWLYLTAETLKNRMGRQ
PPPPTQQHSITDNSLSLKTPPECLLTPLPPSVDDNIKECPLAPLPPSPLPPSVDDNLKECLFVPLPPSPLPPSVDDNLKECLFVPLP
PSPLPPSVDDNLKTPPLATQEAEVEKPPKPKRWRVDEVEQSPKPKRQREAEAQQLPKPKRRRLSKLRTRHCTQAWAIRINP
>sp|P59534|T2R39_HUMAN
MLGRCFPPDTKEKQQLRMTKLCDPAESELSPFLITLILAVLLAEYLIGIIANGFIMAIHAAEWVQNKAVSTSGRILVFLSVSRIALQ
SLMMLEITISSTSLSFYSEDAVYYAFKISFIFLNFCSLWFAAWLSFFYFVKIANFSYPLFLKLRWRITGLIPWLLWLSVFISFSHSM
FCINICTVYCNNSFPIHSSNSTKKTYLSEINVVGLAFFFNLGIVTPLIMFILTATLLILSLKRHTLHMGSNATGSNDPSMEAHMGAI
KAISYFLILYIFNAVALFIYLSNMFDINSLWNNLCQIIMAAYPASHSILLIQDNPGLRRAWKRLQLRLHLYPKEWTL
>sp|A4D1Z8|GRIFN_HUMAN
MAVQSKAFCAGGLAPGWKLLVQGHADSGEDRFETNFLLETGDIAFHIKPRFSSATVVGNAFQYGRWGPEQVSSIFPLAPGEPFEIEV
SWDAEHFHVYAPEHKVLQFPCRQRPLGATTRVRVLSDHCLAQVELAKRGLSWGDRGY
>sp|Q15651|HMGN3_HUMAN
MPKRKSPENTEGKDGSKVTKQEPTRRSARLSAKPAPPKPEPKPRKTSAKKEPGAKISRGAKGKKEEKQEAGKEGTAPSENGETKAEE
AQKTESVDNEGE
>sp|P32004|L1CAM_HUMAN
MVVALRYVWPLLLCSPCLLIQIPEEYEGHHVMEPPVITEQSPRRLVVFPTDDISLKCEASGKPEVQFRWTRDGVHFKPKEELGVTVY
QSPHSGSFTITGNNSNFAQRFQGIYRCFASNKLGTAMSHEIRLMAEGAPKWPKETVKPVEVEEGESVVLPCNPPPSAEPLRIYWMNS
KILHIKQDERVTMGQNGNLYFANVLTSDNHSDYICHAHFPGTRTIIQKEPIDLRVKATNSMIDRKPRLLFPTNSSSHLVALQGQPLV
LECIAEGFPTPTIKWLRPSGPMPADRVTYQNHNKTLQLLKVGEEDDGEYRCLAENSLGSARHAYYVTVEAAPYWLHKPQSHLYGPGE
TARLDCQVQGRPQPEVTWRINGIPVEELAKDQKYRIQRGALILSNVQPSDTMVTQCEARNRHGLLLANAYIYVVQLPAKILTADNQT
YMAVQGSTAYLLCKAFGAPVPSVQWLDEDGTTVLQDERFFPYANGTLGIRDLQANDTGRYFCLAANDQNNVTIMANLKVKDATQITQ
GPRSTIEKKGSRVTFTCQASFDPSLQPSITWRGDGRDLQELGDSDKYFIEDGRLVIHSLDYSDQGNYSCVASTELDVVESRAQLLVV
GSPGPVPRLVLSDLHLLTQSQVRVSWSPAEDHNAPIEKYDIEFEDKEMAPEKWYSLGKVPGNQTSTTLKLSPYVHYTFRVTAINKYG
PGEPSPVSETVVTPEAAPEKNPVDVKGEGNETTNMVITWKPLRWMDWNAPQVQYRVQWRPQGTRGPWQEQIVSDPFLVVSNTSTFVP
YEIKVQAVNSQGKGPEPQVTIGYSGEDYPQAIPELEGIEILNSSAVLVKWRPVDLAQVKGHLRGYNVTYWREGSQRKHSKRHIHKDH
VVVPANTTSVILSGLRPYSSYHLEVQAFNGRGSGPASEFTFSTPEGVPGHPEALHLECQSNTSLLLRWQPPLSHNGVLTGYVLSYHP
LDEGGKGQLSFNLRDPELRTHNLTDLSPHLRYRFQLQATTKEGPGEAIVREGGTMALSGISDFGNISATAGENYSVVSWVPKEGQCN
FRFHILFKALGEEKGGASLSPQYVSYNQSSYTQWDLQPDTDYEIHLFKERMFRHQMAVKTNGTGRVRLPPAGFATEGWFIGFVSAII

LLLLVLLILCFIKRSKGGKYSVKDKEDTQVDSEARPMKDETFGEYRSLESDNEEKAFGSSQPSLNGDIKPLGSDDSLADYGGSVDVQ
FNEDGSFIGQYSGKKEKEAAGGNDSSGATSPINPAVALE
>sp|Q5T8D3|ACBD5_HUMAN
MFQFHAGSWESWCCCCLIPADRPWDRGQHWQLEMADTRSVHETRFEAAVKVIQSLPKNGSFQPTNEMMLKFYSFYKQATEGPCKLSR
PGFWDPIGRYKWDAWSSLGDMTKEEAMIAYVEEMKKIIETMPMTEKVEELLRVIGPFYEIVEDKKSGRSSDITSVRLEKISKCLEDL
GNVLTSTPNAKTVNGKAESSDSGAESEEEEAQEEVKGAEQSDNDKKMMKKSADHKNLEVIVTNGYDKDGFVQDIQNDIHASSSLNGR
STEEVKPIDENLGQTGKSAVCIHQDINDDHVEDVTGIQHLTSDSDSEVYCDSMEQFGQEESLDSFTSNNGPFQYYLGGHSSQPMENS
GFREDIQVPPGNGNIGNMQVVAVEGKGEVKHGGEDGRNNSGAPHREKRGGETDEFSNVRRGRGHRMQHLSEGTKGRQVGSGGDGERW
GSDRGSRGSLNEQIALVLMRLQEDMQNVLQRLQKLETLTALQAKSSTSTLQTAPQPTSQRPSWWPFEMSPGVLTFAIIWPFIAQWLV
YLYYQRRRRKLN
>sp|Q86WV1|SKAP1_HUMAN
MQAAALPEEIRWLLEDAEEFLAEGLRNENLSAVARDHRDHILRGFQQIKARYYWDFQPQGGDIGQDSSDDNHSGTLGLSLTSDAPFL
SDYQDEGMEDIVKGAQELDNVIKQGYLEKKSKDHSFFGSEWQKRWCVVSRGLFYYYANEKSKQPKGTFLIKGYGVRMAPHLRRDSKK
ESCFELTSQDRRSYEFTATSPAEARDWVDQISFLLKDLSSLTIPYEEDEEEEEKEETYDDIDGFDSPSCGSQCRPTILPGSVGIKEP
TEEKEEEDIYEVLPDEEHDLEEDESGTRRKGVDYASYYQGLWDCHGDQPDELSFQRGDLIRILSKEYNMYGWWVGELNSLVGIVPKE
YLTTAFEVEER
>sp|O43768|ENSA_HUMAN
MSQKQEEENPAEETGEEKQDTQEKEGILPERAEEAKLKAKYPSLGQKPGGSDFLMKRLQKGQKYFDSGDYNMAKAKMKNKQLPSAGP
DKNLVTGDHIPTPQDLPQRKSSLVTSKLAGGQVE
>sp|Q9ULW0|TPX2_HUMAN
MSQVKSSYSYDAPSDFINFSSLDDEGDTQNIDSWFEEKANLENKLLGKNGTGGLFQGKTPLRKANLQQAIVTPLKPVDNTYYKEAEK
ENLVEQSIPSNACSSLEVEAAISRKTPAQPQRRSLRLSAQKDLEQKEKHHVKMKAKRCATPVIIDEILPSKKMKVSNNKKKPEEEGS
AHQDTAEKNASSPEKAKGRHTVPCMPPAKQKFLKSTEEQELEKSMKMQQEVVEMRKKNEEFKKLALAGIGQPVKKSVSQVTKSVDFH
FRTDERIKQHPKNQEEYKEVNFTSELRKHPSSPARVTKGCTIVKPFNLSQGKKRTFDETVSTYVPLAQQVEDFHKRTPNRYHLRSKK
DDINLLPSKSSVTKICRDPQTPVLQTKHRARAVTCKSTAELEAEELEKLQQYKFKARELDPRILEGGPILPKKPPVKPPTEPIGFDL
EIEKRIQERESKKKTEDEHFEFHSRPCPTKILEDVVGVPEKKVLPITVPKSPAFALKNRIRMPTKEDEEEDEPVVIKAQPVPHYGVP
FKPQIPEARTVEICPFSFDSRDKERQLQKEKKIKELQKGEVPKFKALPLPHFDTINLPEKKVKNVTQIEPFCLETDRRGALKAQTWK
HQLEEELRQQKEAACFKARPNTVISQEPFVPKKEKKSVAEGLSGSLVQEPFQLATEKRAKERQELEKRMAEVEAQKAQQLEEARLQE
EEQKKEELARLRRELVHKANPIRKYQGLEIKSSDQPLTVPVSPKFSTRFHC
>sp|Q8WWW8|GAB3_HUMAN
MSAGDAVCTGWLVKSPPERKLQRYAWRKRWFVLRRGRMSGNPDVLEYYRNKHSSKPIRVIDLSECAVWKHVGPSFVRKEFQNNFVFI
VKTTSRTFYLVAKTEQEMQVWVHSISQVCNLGHLEDGADSMESLSYTPSSLQPSSASSLLTAHAASSSLPRDDPNTNAVATEETRSE
SELLFLPDYLVLSNCETGRLHHTSLPTRCDSWSNSDRSLEQASFDDVFVDCLQPLPSSHLVHPSCHGSGAQEVPSSRPQAALIWSRE
INGPPRDHLSSSPLLESSLSSTIQVDKNQGSLPCGAKELDIMSNTPPPRPPKPSHLSERRQEEWSTHSGSKKPECTLVPRRISLSGL
DNMRTWKADVEGQSLRHRDKRLSLNLPCRFSPMYPTASASIEDSYVPMSPQAGASGLGPHCSPDDYIPMNSGSISSPLPELPANLEP
PPVNRDLKPQRKSRPPPLDLRNLSIIREHASLTRTRTVPCSRTSFLSPERNGINSARFFANPVSREDEESYIEMEEHRTASSLSSGA
LTWTKKFSLDYLALDFNSASPAPMQQKLLLSEEQRVDYVQVDEQKTQALQSTKQEWTDERQSKV
>sp|P13761|2B17_HUMAN
MVCLKLPGGSCMAALTVTLMVLSSPLALAGDTQPRFLWQGKYKCHFFNGTERVQFLERLFYNQEEFVRFDSDVGEYRAVTELGRPVA
ESWNSQKDILEDRRGQVDTVCRHNYGVGESFTVQRRVHPEVTVYPAKTQPLQHHNLLVCSVSGFYPGSIEVRWFRNGQEEKAGVVST
GLIQNGDWTFQTLVMLETVPRSGEVYTCQVEHPSVMSPLTVEWRARSESAQSKMLSGVGGFVLGLLFLGAGLFIYFRNQKGHSGLQP
TGFLS
>sp|Q2QD12|RPEL1_HUMAN

MASGCKIGPSILNSDLANLGAKCLQMLDSGADYLHLDVMDGHFVPNITFGHPVVESLRKQLGQDPFFDMHMMVSKPEQWVKPMAVAE
ANQYTFHLEATENPGTLIKDIRENGMKVGLAIKPGTSVEYLAPWANQIDMALVMTVEPGFGEQKFMEDMMPKVHWLRTQFPSLDIEG
DGGVGSDTVHKCAEAGANMTVSGSAIMRSEDPRSVINLLRNICSEAAQKRSLDR
>sp|Q15172|2A5A_HUMAN
MSSSSPPAGAASAAISASEKVDGFTRKSVRKAQRQKRSQGSSQFRSQGSQAELHPLPQLKDATSNEQQELFCQKLQQCCILFDFMDS
VSDLKSKEIKRATLNELVEYVSTNRGVIVESAYSDIVKMISANIFRTLPPSDNPDFDPEEDEPTLEASWPHIQLVYEFFLRFLESPD
FQPSIAKRYIDQKFVQQLLELFDSEDPRERDFLKTVLHRIYGKFLGLRAFIRKQINNIFLRFIYETEHFNGVAELLEILGSIINGFA
LPLKAEHKQFLMKVLIPMHTAKGLALFHAQLAYCVVQFLEKDTTLTEPVIRGLLKFWPKTCSQKEVMFLGEIEEILDVIEPTQFKKI
EEPLFKQISKCVSSSHFQVAERALYFWNNEYILSLIEENIDKILPIMFASLYKISKEHWNPTIVALVYNVLKTLMEMNGKLFDDLTS
SYKAERQREKKKELEREELWKKLEELKLKKALEKQNSAYNMHSILSNTSAE
>sp|Q9NXE8|CWC25_HUMAN
MGGGDLNLKKSWHPQTLRNVEKVWKAEQKHEAERKKIEELQRELREERAREEMQRYAEDVGAVKKKEEKLDWMYQGPGGMVNRDEYL
LGRPIDKYVFEKMEEKEAGCSSETGLLPGSIFAPSGANSLLDMASKIREDPLFIIRKKEEEKKREVLNNPVKMKKIKELLQMSLEKK
EKKKKKEKKKKHKKHKHRSSSSDRSSSEDEHSAGRSQKKMANSSPVLSKVPGYGLQVRNSDRNQGLQGPLTAEQKRGHGMKNHSRSR
SSSHSPPRHASKKSTREAGSRDRRSRSLGRRSRSPRPSKLHNSKVNRRETGQTRSPSPKKEVYQRRHAPGYTRKLSAEELERKRQEM
MENAKWREEERLNILKRHAKDEEREQRLEKLDSRDGKFIHRMKLESASTSSLEDRVKRNIYSLQRTSVALEKNFMKR
>sp|Q9C0D6|FHDC1_HUMAN
MHVMNCVSLVSDKENGNIATAPGFMIGQTPPPAPPPPPPPPPPSPPCSCSREECPSSPPPPPPPPLPGEPPIPPPPPGLPPTTHMNG
YSHLGKKKRMRSFFWKTIPEEQVRGKTNIWTLAARQEHHYQIDTKTIEELFGQQEDTTKSSLPRRGRTLNSSFREAREEITILDAKR
SMNIGIFLKQFKKSPRSIVEDIHQGKSEHYGSETLREFLKFLPESEEVKKLKAFSGDVSKLSLADSFLYGLIQVPNYSLRIEAMVLK
KEFLPSCSSLYTDITVLRTAIKELMSCEELHSILHLVLQAGNIMNAGGYAGNAVGFKLSSLLKLADTKANKPGMNLLHFVAQEAQKK
DTILLNFSEKLHHVQKTARLSLENTEAELHLLFVRTKSLKENIQRDGELCQQMEDFLQFAIEKLRELECWKQELQDEAYTLIDFFCE
DKKTMKLDECFQIFRDFCTKFNKAVKDNHDREAQELRQLQRLKEQEQKQRSWATGELGAFGRSSSENDVELLTKKGAEGLLPFLHPR
PISPSSPSYRPPNTRRSRLSLGPSADRELLTFLESSTGSPEEPNKFHSLPRSSPRQARPTIACLEPAEVRHQDSSFAHKPQASGGQE
EAPNPPSAQAHQLAAAQPENHASAFPRARRQGVSVLRKRYSEPVSLGSAQSPPLSPLALGIKEHELVTGLAQFNLQGSQGMEETSQL
TLSDFSPMELESVGHRGPQSLSASSSSLTPMGRDALGSLSPALEDGKAAPDEPGSAALGSVGSSDPENKDPRPLFCISDTTDCSLTL
DCSEGTDSRPRGGDPEEGGEGDGSMSSGVGEMGDSQVSSNPTSSPPGEAPAPVSVDSEPSCKGGLPRDKPTKRKDVVAPKRGSLKEA
SPGASKPGSARRSQGAVAKSVRTLTASENESMRKVMPITKSSRGAGWRRPELSSRGPSQNPPSSTDTVWSRQNSVRRASTGAEEQRL
PRGSSGSSSTRPGRDVPLQPRGSFKKPSAKPLRNLPRQKPEENKTCRAHSEGPESPKEEPKTPSVPSVPHELPRVPSFARNTVASSS
RSMRTDLPPVAKAPGITRTVSQRQLRVKGDPEDAAPKDSSTLRRASSARAPKKRPESAEGPSANTEAPLKARGAGERASLRRKDSSR
TTLGRILNPLRK
>sp|Q9H0C2|ADT4_HUMAN
MHREPAKKKAEKRLFDASSFGKDLLAGGVAAAVSKTAVAPIERVKLLLQVQASSKQISPEARYKGMVDCLVRIPREQGFFSFWRGNL
ANVIRYFPTQALNFAFKDKYKQLFMSGVNKEKQFWRWFLANLASGGAAGATSLCVVYPLDFARTRLGVDIGKGPEERQFKGLGDCIM
KIAKSDGIAGLYQGFGVSVQGIIVYRASYFGAYDTVKGLLPKPKKTPFLVSFFIAQVVTTCSGILSYPFDTVRRRMMMQSGEAKRQY
KGTLDCFVKIYQHEGISSFFRGAFSNVLRGTGGALVLVLYDKIKEFFHIDIGGR
>sp|Q16762|THTR_HUMAN
MVHQVLYRALVSTKWLAESIRTGKLGPGLRVLDASWYSPGTREARKEYLERHVPGASFFDIEECRDTASPYEMMLPSEAGFAEYVGR
LGISNHTHVVVYDGEHLGSFYAPRVWWMFRVFGHRTVSVLNGGFRNWLKEGHPVTSEPSRPEPAVFKATLDRSLLKTYEQVLENLES
KRFQLVDSRSQGRFLGTEPEPDAVGLDSGHIRGAVNMPFMDFLTEDGFEKGPEELRALFQTKKVDLSQPLIATCRKGVTACHVALAA
YLCGKPDVAVYDGSWSEWFRRAPPESRVSQGKSEKA
>sp|E9PQR5|NPIB8_HUMAN
MVKLSIVLTPQFLSHDQGQLTKELQQHVKSVTCPCEYLRKVINSLAVYRHRETDFGVGVRDHPGQHGKTPSPQKLDNLIIIIIGFLR
CYTFNILFCTSCLCVSFLKTIFWSRNGHDGSMDVQQRAWRSNRSRQKGLRSICMHTKKRVSSFRGNKIGLKDVITLRRHVETKVRAK

IRKRKVTTKINRHDKINGKRKTARKQKMFQRAQELRRRAEDYHKCKIPPSARKPLCNWVRMAAAEHRHSSGLPYWLYLTAETLKNRM
GRQPPPPTQQHSITDNSLSLKTPPECLLTPLPPSVDDNIKECPLAPLPPSPLPPSVDDNLKECLFVPLPPSPLPPSVDDNLKECLFV
PLPPSPLPPSVDDNLKTPPLATQEAEVEKPPKPKRWRVDEVEQSPKPKRQREAEAQQLPKPKRRRLSKLRTRHCTQAWAIRINP
>sp|Q5VY80|RET1L_HUMAN
MAAAAIPALLLCLPLLFLLFGWSRARRDDPHSLCYDITVIPKFRPGPRWCAVQGQVDEKTFLHYDCGNKTVTPVSPLGKKLNVTMAW
KAQNPVLREVVDILTEQLLDIQLENYTPKEPLTLQARMSCEQKAEGHSSGSWQFSIDGQTFLLFDSEKRMWTTVHPGARKMKEKWEN
DKDVAMSFHYISMGDCIGWLEDFLMGMDSTLEPSAGAPLAMSSGTTQLRATATTLILCCLLIILPCFILPGI
>sp|P00338|LDHA_HUMAN
MATLKDQLIYNLLKEEQTPQNKITVVGVGAVGMACAISILMKDLADELALVDVIEDKLKGEMMDLQHGSLFLRTPKIVSGKDYNVTA
NSKLVIITAGARQQEGESRLNLVQRNVNIFKFIIPNVVKYSPNCKLLIVSNPVDILTYVAWKISGFPKNRVIGSGCNLDSARFRYLM
GERLGVHPLSCHGWVLGEHGDSSVPVWSGMNVAGVSLKTLHPDLGTDKDKEQWKEVHKQVVESAYEVIKLKGYTSWAIGLSVADLAE
SIMKNLRRVHPVSTMIKGLYGIKDDVFLSVPCILGQNGISDLVKVTLTSEEEARLKKSADTLWGIQKELQF
>sp|P48050|KCNJ4_HUMAN
MHGHSRNGQAHVPRRKRRNRFVKKNGQCNVYFANLSNKSQRYMADIFTTCVDTRWRYMLMIFSAAFLVSWLFFGLLFWCIAFFHGDL
EASPGVPAAGGPAAGGGGAAPVAPKPCIMHVNGFLGAFLFSVETQTTIGYGFRCVTEECPLAVIAVVVQSIVGCVIDSFMIGTIMAK
MARPKKRAQTLLFSHHAVISVRDGKLCLMWRVGNLRKSHIVEAHVRAQLIKPYMTQEGEYLPLDQRDLNVGYDIGLDRIFLVSPIII
VHEIDEDSPLYGMGKEELESEDFEIVVILEGMVEATAMTTQARSSYLASEILWGHRFEPVVFEEKSHYKVDYSRFHKTYEVAGTPCC
SARELQESKITVLPAPPPPPSAFCYENELALMSQEEEEMEEEAAAAAAVAAGLGLEAGSKEEAGIIRMLEFGSHLDLERMQASLPLD
NISYRRESAI
>sp|P08865|RSSA_HUMAN
MSGALDVLQMKEEDVLKFLAAGTHLGGTNLDFQMEQYIYKRKSDGIYIINLKRTWEKLLLAARAIVAIENPADVSVISSRNTGQRAV
LKFAAATGATPIAGRFTPGTFTNQIQAAFREPRLLVVTDPRADHQPLTEASYVNLPTIALCNTDSPLRYVDIAIPCNNKGAHSVGLM
WWMLAREVLRMRGTISREHPWEVMPDLYFYRDPEEIEKEEQAAAEKAVTKEEFQGEWTAPAPEFTATQPEVADWSEGVQVPSVPIQQ
FPTEDWSAQPATEDWSAAPTAQATEWVGATTDWS
>sp|P55000|SLUR1_HUMAN
MASRWAVQLLLVAAWSMGCGEALKCYTCKEPMTSASCRTITRCKPEDTACMTTLVTVEAEYPFNQSPVVTRSCSSSCVATDPDSIGA
AHLIFCCFRDLCNSEL
>sp|A6NGG3|CI092_HUMAN
MARFQRTKEREEDRCSLNIYYVRNTTQGTAPACVGKRMQPSPTGKGGKCCTVHGLTRKIHNVQPNLQSPILSAACVD
>sp|Q86XD8|ZFAN4_HUMAN
MDNRKEPPFFNDDNMGPFYYRLHFCDTMELFIETLTGTCFELRVSPFETVISVKAKIRRLEGIPICRQHLIWNNMELENDYCLNDYN
ISEGCTLKLVLAMRGGPINTRRVPTDDPLRKMAEYLDSSRVEVWEKTSCSKQVTFLVYQEGDQLNFFPAVDRGDGTLTPLSDSSKKI
DFHLHVLRRKGEHRMSGGSMYNSDTDEDEETEPSSSGQQIIENSITMNKMKLLKAKMKNMNLSKKPKKAVKIKPHPPVAPRPSSGST
APSRHRLLRVLPNIGQSCSPAFGNAYPPEISRNGISSLATQLSAERYISSITGEFLKEDNSWENNTLSHFSSNVKLPPQIPHLELGN
DQELADSVLHLGSSLPRQTKHFLGNLPSSNGNIVLPSEECVTEQSLLPKVGSLASFAEGNADEQSSGLEGACKVNLELLLTNADKGL
KAPEQHLKHVAGVLNGESVETSVLNYRELSPHKNRLLSPLRCSAPMSLHNSLVKPERQSKCFEFGKLQPSSSQSLDVQNITDSSFSR
TTCFQGVKVDSLGKRSDVISKVEARDITEMTNKASKEPVGCVNNISFLASLAGSTSRNRLQSTRGAGRLQNSGTGLSTNLQHFQEEN
FRKSSPQLEHTGVFLSTHGVGMNGNNAAAGKSVGECTTHHLPPVKAPLQTKKKTTNHCFLCGKKTGLASSYECRCGNNFCASHRYAE
THGCTYDYKSAGRRYLHEANPVVNAPKLPKI
>sp|Q9UHP9|SMPX_HUMAN
MNMSKQPVSNVRAIQANINIPMGAFRPGAGQPPRRKECTPEVEEGVPPTSDEEKKPIPGAKKLPGPAVNLSEIQNIKSELKYVPKAE
Q
>sp|Q9P0N9|TBCD7_HUMAN

MTEDSQRNFRSVYYEKVGFRGVEEKKSLEILLKDDRLDTEKLCTFSQRFPLPSMYRALVWKVLLGILPPHHESHAKVMMYRKEQYLD
VLHALKVVRFVSDATPQAEVYLRMYQLESGKLPRSPSFPLEPDDEVFLAIAKAMEEMVEDSVDCYWITRRFVNQLNTKYRDSLPQLP
KAFEQYLNLEDGRLLTHLRMCSAAPKLPYDLWFKRCFAGCLPESSLQRVWDKVVSGSCKILVFVAVEILLTFKIKVMALNSAEKITK
FLENIPQDSSDAIVSKAIDLWHKHCGTPVHSS
>sp|O15371|EIF3D_HUMAN
MAKFMTPVIQDNPSGWGPCAVPEQFRDMPYQPFSKGDRLGKVADWTGATYQDKRYTNKYSSQFGGGSQYAYFHEEDESSFQLVDTAR
TQKTAYQRNRMRFAQRNLRRDKDRRNMLQFNLQILPKSAKQKERERIRLQKKFQKQFGVRQKWDQKSQKPRDSSVEVRSDWEVKEEM
DFPQLMKMRYLEVSEPQDIECCGALEYYDKAFDRITTRSEKPLRSIKRIFHTVTTTDDPVIRKLAKTQGNVFATDAILATLMSCTRS
VYSWDIVVQRVGSKLFFDKRDNSDFDLLTVSETANEPPQDEGNSFNSPRNLAMEATYINHNFSQQCLRMGKERYNFPNPNPFVEDDM
DKNEIASVAYRYRRWKLGDDIDLIVRCEHDGVMTGANGEVSFINIKTLNEWDSRHCNGVDWRQKLDSQRGAVIATELKNNSYKLARW
TCCALLAGSEYLKLGYVSRYHVKDSSRHVILGTQQFKPNEFASQINLSVENAWGILRCVIDICMKLEEGKYLILKDPNKQVIRVYSL
PDGTFSSDEDEEEEEEEEEEEEEEEET
>sp|P07711|CATL1_HUMAN
MNPTLILAAFCLGIASATLTFDHSLEAQWTKWKAMHNRLYGMNEEGWRRAVWEKNMKMIELHNQEYREGKHSFTMAMNAFGDMTSEE
FRQVMNGFQNRKPRKGKVFQEPLFYEAPRSVDWREKGYVTPVKNQGQCGSCWAFSATGALEGQMFRKTGRLISLSEQNLVDCSGPQG
NEGCNGGLMDYAFQYVQDNGGLDSEESYPYEATEESCKYNPKYSVANDTGFVDIPKQEKALMKAVATVGPISVAIDAGHESFLFYKE
GIYFEPDCSSEDMDHGVLVVGYGFESTESDNNKYWLVKNSWGEEWGMGGYVKMAKDRRNHCGIASAASYPTV
>sp|Q96BH3|ESPB1_HUMAN
MTRWSSYLLGWTTFLLYSYESSGGMHEECVFPFTYKGSVYFTCTHIHSLSPWCATRAVYNGQWKYCQSEDYPRCIFPFIYRGKAYNS
CISQGSFLGSLWCSVTSVFDEKQQWKFCETNEYGGNSLRKPCIFPSIYRNNVVSDCMEDESNKLWCPTTENMDKDGKWSFCADTRIS
ALVPGFPCHFPFNYKNKNYFNCTNEGSKENLVWCATSYNYDQDHTWVYC
>sp|Q9NWH9|SLTM_HUMAN
MAAATGAVAASAASGQAEGKKITDLRVIDLKSELKRRNLDITGVKTVLISRLKQAIEEEGGDPDNIELTVSTDTPNKKPTKGKGKKH
EADELSGDASVEDDAFIKDCELENQEAHEQDGNDELKDSEEFGENEEENVHSKELLSAEENKRAHELIEAEGIEDIEKEDIESQEIE
AQEGEDDTFLTAQDGEEEENEKDIAGSGDGTQEVSKPLPSEGSLAEADHTAHEEMEAHTTVKEAEDDNISVTIQAEDAITLDFDGDD
LLETGKNVKITDSEASKPKDGQDAIAQSPEKESKDYEMNANHKDGKKEDCVKGDPVEKEARESSKKAESGDKEKDTLKKGPSSTGAS
GQAKSSSKESKDSKTSSKDDKGSTSSTSGSSGSSTKNIWVSGLSSNTKAADLKNLFGKYGKVLSAKVVTNARSPGAKCYGIVTMSSS
TEVSRCIAHLHRTELHGQLISVEKVKGDPSKKEMKKENDEKSSSRSSGDKKNTSDRSSKTQASVKKEEKRSSEKSEKKESKDTKKIE
GKDEKNDNGASGQTSESIKKSEEKKRISSKSPGHMVILDQTKGDHCRPSRRGRYEKIHGRSKEKERASLDKKRDKDYRRKEILPFEK
MKEQRLREHLVRFERLRRAMELRRRREIAERERRERERIRIIREREERERLQRERERLEIERQKLERERMERERLERERIRIEQERR
KEAERIAREREELRRQQQQLRYEQEKRNSLKRPRDVDHRRDDPYWSENKKLSLDTDARFGHGSDYSRQQNRFNDFDHRERGRFPESS
AVQSSSFERRDRFVGQSEGKKARPTARREDPSFERYPKNFSDSRRNEPPPPRNELRESDRREVRGERDERRTVIIHDRPDITHPRHP
REAGPNPSRPTSWKSEGSMSTDKRETRVERPERSGREVSGHSVRGAPPGNRSSASGYGSREGDRGVITDRGGGSQHYPEERHVVERH
GRDTSGPRKEWHGPPSQGPSYHDTRRMGDGRAGAGMITQHSSNASPINRIVQISGNSMPRGSGSGFKPFKGGPPRRF
>sp|H0YL09|U2Q2L_HUMAN
MGPAVLGQGQEGQPEARACSGLLQPPKRPIVFKEKLTMKTDSLMEEKLECSLWCCLSDPSIPGRCCVLERRIVPWMQQESYSSSSPI
WSVDSDEPNLTSVLERLEDTKENSSVRKETKLFSLFLMNIIFRN
>sp|Q96KF2|PRAC1_HUMAN
MLCAHFSDQGPAHLTTSKSAFLSNKKTSTLKHLLGETRSDGSACNSGISGGRGRKIP
>sp|Q9NSK7|CS012_HUMAN
MERLKSHKPATMTIMVEDIMKLLCSLSGERKMKAAVKHSGKGALVTGAMAFVGGLVGGPPGLAVGGAVGGLLGAWMTSGQFKPVPQI
LMELPPAEQQRLFNEAAAIIRHLEWTDAVQLTALVMGSEALQQQLLAMLVNYVTKELRAEIQYDD
>sp|Q8N3F9|G137C_HUMAN

MRVSVPGPAAAAAPAAGREPSTPGGGSGGGGAVAAASGAAVPGSVQLALSVLHALLYAALFAFAYLQLWRLLLYRERRLSYQSLCLF
LCLLWAALRTTLFSAAFSLSGSLPLLRPPAHLHFFPHWLLYCFPSCLQFSTLCLLNLYLAEVICKVRCATELDRHKILLHLGFIMAS
LLFLVVNLTCAMLVHGDVPENQLKWTVFVRALINDSLFILCAISLVCYICKITKMSSANVYLESKGMSLCQTVVVGSVVILLYSSRA
CYNLVVVTISQDTLESPFNYGWDNLSDKAHVEDISGEEYIVFGMVLFLWEHVPAWSVVLFFRAQRLNQNLAPAGMINSHSYSSRAYF
FDNPRRYDSDDDLPRLGSSREGSLPNSQSLGWYGTMTGCGSSSYTVTPHLNGPMTDTAPLLFTCSNLDLNNHHSLYVTPQN
>sp|Q03933|HSF2_HUMAN
MKQSSNVPAFLSKLWTLVEETHTNEFITWSQNGQSFLVLDEQRFAKEILPKYFKHNNMASFVRQLNMYGFRKVVHIDSGIVKQERDG
PVEFQHPYFKQGQDDLLENIKRKVSSSKPEENKIRQEDLTKIISSAQKVQIKQETIESRLSELKSENESLWKEVSELRAKHAQQQQV
IRKIVQFIVTLVQNNQLVSLKRKRPLLLNTNGAQKKNLFQHIVKEPTDNHHHKVPHSRTEGLKPRERISDDIIIYDVTDDNADEENI
PVIPETNEDVISDPSNCSQYPDIVIVEDDNEDEYAPVIQSGEQNEPARESLSSGSDGSSPLMSSAVQLNGSSSLTSEDPVTMMDSIL
NDNINLLGKVELLDYLDSIDCSLEDFQAMLSGRQFSIDPDLLVDLFTSSVQMNPTDYINNTKSENKGLETTKNNVVQPVSEEGRKSK
SKPDKQLIQYTAFPLLAFLDGNPASSVEQASTTASSEVLSSVDKPIEVDELLDSSLDPEPTQSKLVRLEPLTEAEASEATLFYLCEL
APAPLDSDMPLLDS
>sp|P25021|HRH2_HUMAN
MAPNGTASSFCLDSTACKITITVVLAVLILITVAGNVVVCLAVGLNRRLRNLTNCFIVSLAITDLLLGLLVLPFSAIYQLSCKWSFG
KVFCNIYTSLDVMLCTASILNLFMISLDRYCAVMDPLRYPVLVTPVRVAISLVLIWVISITLSFLSIHLGWNSRNETSKGNHTTSKC
KVQVNEVYGLVDGLVTFYLPLLIMCITYYRIFKVARDQAKRINHISSWKAATIREHKATVTLAAVMGAFIICWFPYFTAFVYRGLRG
DDAINEVLEAIVLWLGYANSALNPILYAALNRDFRTGYQQLFCCRLANRNSHKTSLRSNASQLSRTQSREPRQQEEKPLKLQVWSGT
EVTAPQGATDR
>sp|Q8IWF9|CCD83_HUMAN
MENSGKANKKDTHDGPPKEIKLPTSEALLDYQCQIKEDAVEQFMFQIKTLRKKNQKYHERNSRLKEEQIWHIRHLLKELSEEKAEGL
PVVTREDVEEAMKEKWKFERDQEKNLRDMRMQISNAEKLFLEKLSEKEYWEEYKNVGSERHAKLITSLQNDINTVKENAEKMSEHYK
ITLEDTRKKIIKETLLQLDQKKEWATQNAVKLIDKGSYLEIWENDWLKKEVAIHRKEVEELKNAIHELEAENLVLIDQLSNCRLVDL
KIPRRLYLTQAAGLEVPPEEMSLELPETHIEEKSELQPTEVESRDLMSSSDESTILHLSHENSIEDLQYVKIDKEENSGTEFGDTDM
KYLLYEDEKDFKDYVNLGPLGVKLMSVESKKMPIHFQEKEIPVKLYKDVRSPESHITYKMMKSFL
>sp|Q8IZP1|TBC3A_HUMAN
MDVVEVAGSWWAQEREDIIMKYEKGHRAGLPEDKGPKPFRSYNNNVDHLGIVHETELPPLTAREAKQIRREISRKSKWVDMLGDWEK
YKSSRKLIDRAYKGMPMNIRGPMWSVLLNTEEMKLKNPGRYQIMKEKGKRSSEHIQRIDRDVSGTLRKHIFFRDRYGTKQRELLHIL
LAYEEYNPEVGYCRDLSHIAALFLLYLPEEDAFWALVQLLASERHSLQGFHSPNGGTVQGLQDQQEHVVATSQPKTMGHQDKKDLCG
QCSPLGCLIRILIDGISLGLTLRLWDVYLVEGEQALMPITRIAFKVQQKRLTKTSRCGPWARFCNRFVDTWARDEDTVLKHLRASMK
KLTRKKGDLPPPAKPEQGSSASRPVPASRGGKTLCKGDRQAPPGPPARFPRPIWSASPPRAPRSSTPCPGGAVREDTYPVGTQGVPS
PALAQGGPQGSWRFLQWNSMPRLPTDLDVEGPWFRHYDFRQSCWVRAISQEDQLAPCWQAEHPAERVRSAFAAPSTDSDQGTPFRAR
DEQQCAPTSGPCLCGLHLESSQFPPGF
>sp|P61626|LYSC_HUMAN
MKALIVLGLVLLSVTVQGKVFERCELARTLKRLGMDGYRGISLANWMCLAKWESGYNTRATNYNAGDRSTDYGIFQINSRYWCNDGK
TPGAVNACHLSCSALLQDNIADAVACAKRVVRDPQGIRAWVAWRNRCQNRDVRQYVQGCGV
>sp|P0CL81|GG12G_HUMAN
MSWRGRSTYYWPRPRRYVQPPEMIGPMRPEQFSDEVEPATPEEGEPATQRQDPAAAQEGEDEGASAGQGPKPEAHSQEQGHPQTGCE
CEDGPDGQEMDPPNPEEVKTPEEGEKQSQC
>sp|Q9ULC4|MCTS1_HUMAN
MFKKFDEKENVSNCIQLKTSVIKGIKNQLIEQFPGIEPWLNQIMPKKDPVKIVRCHEHIEILTVNGELLFFRQREGPFYPTLRLLHK
YPFILPHQQVDKGAIKFVLSGANIMCPGLTSPGAKLYPAAVDTIVAIMAEGKQHALCVGVMKMSAEDIEKVNKGIGIENIHYLNDGL
WHMKTYK
>sp|P43308|SSRB_HUMAN

MRLLSFVVLALFAVTQAEEGARLLASKSLLNRYAVEGRDLTLQYNIYNVGSSAALDVELSDDSFPPEDFGIVSGMLNVKWDRIAPAS
NVSHTVVLRPLKAGYFNFTSATITYLAQEDGPVVIGSTSAPGQGGILAQREFDRRFSPHFLDWAAFGVMTLPSIGIPLLLWYSSKRK
YDTPKTKKN
>sp|P10451|OSTP_HUMAN
MRIAVICFCLLGITCAIPVKQADSGSSEEKQLYNKYPDAVATWLNPDPSQKQNLLAPQNAVSSEETNDFKQETLPSKSNESHDHMDD
MDDEDDDDHVDSQDSIDSNDSDDVDDTDDSHQSDESHHSDESDELVTDFPTDLPATEVFTPVVPTVDTYDGRGDSVVYGLRSKSKKF
RRPDIQYPDATDEDITSHMESEELNGAYKAIPVAQDLNAPSDWDSRGKDSYETSQLDDQSAETHSHKQSRLYKRKANDESNEHSDVI
DSQELSKVSREFHSHEFHSHEDMLVVDPKSKEEDKHLKFRISHELDSASSEVN
>sp|Q5VWJ9|SNX30_HUMAN
MAGGPPKALPSTGPHSLRDMPHPLAGSSSEEAVGGDSTPSPDLLMARSFGDKDLILPNGGTPAGTSSPASSSSLLNRLQLDDDIDGE
TRDLFVIVDDPKKHVCTMETYITYRITTKSTRVEFDLPEYSVRRRYQDFDWLRSKLEESQPTHLIPPLPEKFVVKGVVDRFSEEFVE
TRRKALDKFLKRITDHPVLSFNEHFNIFLTAKDLNAYKKQGIALLTRMGESVKHVTGGYKLRTRPLEFAAIGDYLDTFALKLGTIDR
IAQRIIKEEIEYLVELREYGPVYSTWSALEGELAEPLEGVSACIGNCSTALEELTDDMTEDFLPVLREYILYSDSMKSVLKKRDQVQ
AEYEAKLEAVALRKEDRPKVPADVEKCQDRMECFNADLKADMERWQNNKRQDFRQLLMGMADKNIQYYEKCLMAWESIIPLLQEKQE
AK
>sp|Q7Z4B0|CR020_HUMAN
MINLHRLCIIHVVATLLSTLLSLISVAISATCKDEKGKQEMETGQQPSGLSATLTKVKCAKRQKTVVRVRFYMLSMKNKACRKNLSK
GYNQRPEGSKEESHMVVKEKRKGDH
>sp|P61326|MGN_HUMAN
MESDFYLRYYVGHKGKFGHEFLEFEFRPDGKLRYANNSNYKNDVMIRKEAYVHKSVMEELKRIIDDSEITKEDDALWPPPDRVGRQE
LEIVIGDEHISFTTSKIGSLIDVNQSKDPEGLRVFYYLVQDLKCLVFSLIGLHFKIKPI
>sp|Q8N4H5|TOM5_HUMAN
MFRIEGLAPKLDPEEMKRKMREDVISSIRNFLIYVALLRVTPFILKKLDSI
>sp|P09341|GROA_HUMAN
MARAALSAAPSNPRLLRVALLLLLLVAAGRRAAGASVATELRCQCLQTLQGIHPKNIQSVNVKSPGPHCAQTEVIATLKNGRKACLN
PASPIVKKIIEKMLNSDKSN
>sp|Q9H761|CN139_HUMAN
MVQQDVQGLKAQSAGLSISQHLLSGCHGNIIPNPTTLSSRETAIIRRDLGFPRGSWASFLKRLLLSSATRNTRPAHPQHLCGDEGQS
PTVPLPVLMRKGGKKTYPERLQYGHDTFKKPVLWSHDQKPECGEGQQPASEQPARRGVVNLGLVPRHDWLRAQCSTLWNVPWASVAF
LSFAGRGLLPLWQLVS
>sp|P69892|HBG2_HUMAN
MGHFTEEDKATITSLWGKVNVEDAGGETLGRLLVVYPWTQRFFDSFGNLSSASAIMGNPKVKAHGKKVLTSLGDAIKHLDDLKGTFA
QLSELHCDKLHVDPENFKLLGNVLVTVLAIHFGKEFTPEVQASWQKMVTGVASALSSRYH
>sp|Q6P3W2|DJC24_HUMAN
MAVEQMPKKDWYSILGADPSANISDLKQKYQKLILMYHPDKQSTDVPAGTVEECVQKFIEIDQAWKILGNEETKREYDLQRCEDDLR
NVGPVDAQVYLEEMSWNEGDHSFYLSCRCGGKYSVSKDEAEEVSLISCDTCSLIIELLHYN
>sp|Q13615|MTMR3_HUMAN
MDEETRHSLECIQANQIFPRKQLIREDENLQVPFLELHGESTEFVGRAEDAIIALSNYRLHIKFKESLVNVPLQLIESVECRDIFQL
HLTCKDCKVIRCQFSTFEQCQEWLKRLNNAIRPPAKIEDLFSFAYHAWCMEVYASEKEQHGDLCRPGEHVTSRFKNEVERMGFDMNN
AWRISNINEKYKLCGSYPQELIVPAWITDKELESVSSFRSWKRIPAVIYRHQSNGAVIARCGQPEVSWWGWRNADDEHLVQSVAKAC
ASDSRSSGSKLSTRNTSRDFPNGGDLSDVEFDSSLSNASGAESLAIQPQKLLILDARSYAAAVANRAKGGGCECPEYYPNCEVVFMG
MANIHSIRRSFQSLRLLCTQMPDPGNWLSALESTKWLHHLSVLLKSALLVVHAVDQDQRPVLVHCSDGWDRTPQIVALAKLLLDPYY
RTIEGFQVLVEMEWLDFGHKFADRCGHGENSDDLNERCPVFLQWLDCVHQLQRQFPCSFEFNEAFLVKLVQHTYSCLFGTFLCNNAK
ERGEKHTQERTCSVWSLLRAGNKAFKNLLYSSQSEAVLYPVCHVRNLMLWSAVYLPCPSPTTPVDDSCAPYPAPGTSPDDPPLSRLP

KTRSYDNLTTACDNTVPLASRRCSDPSLNEKWQEHRRSLELSSLAGPGEDPLSADSLGKPTRVPGGAELSVAAGVAEGQMENILQEA
TKEESGVEEPAHRAGIEIQEGKEDPLLEKESRRKTPEASAIGLHQDPELGDAALRSHLDMSWPLFSQGISEQQSGLSVLLSSLQVPP
RGEDSLEVPVEQFRIEEIAEGREEAVLPIPVDAKVGYGTSQSCSLLPSQVPFETRGPNVDSSTDMLVEDKVKSVSGPQGHHRSCLVN
SGKDRLPQTMEPSPSETSLVERPQVGSVVHRTSLGSTLSLTRSPCALPLAECKEGLVCNGAPETENRASEQPPGLSTLQMYPTPNGH
CANGEAGRSKDSLSRQLSAMSCSSAHLHSRNLHHKWLHSHSGRPSATSSPDQPSRSHLDDDGMSVYTDTIQQRLRQIESGHQQEVET
LKKQVQELKSRLESQYLTSSLHFNGDFGDEVTSIPDSESNLDQNCLSRCSTEIFSEASWEQVDKQDTEMTRWLPDHLAAHCYACDSA
FWLASRKHHCRNCGNVFCSSCCNQKVPVPSQQLFEPSRVCKSCYSSLHPTSSSIDLELDKPIAATSN
>sp|Q24JP5|T132A_HUMAN
MCARMAGRTTAAPRGPYGPWLCLLVALALDVVRVDCGQAPLDPVYLPAALELLDAPEHFRVQQVGHYPPANSSLSSRSETFLLLQPW
PRAQPLLRASYPPFATQQVVPPRVTEPHQRPVPWDVRAVSVEAAVTPAEPYARVLFHLKGQDWPPGSGSLPCARLHATHPAGTAHQA
CRFQPSLGACVVELELPSHWFSQASTTRAELAYTLEPAAEGPGGCGSGEENDPGEQALPVGGVELRPADPPQYQEVPLDEAVTLRVP
DMPVRPGQLFSATLLLRHNFTASLLTLRIKVKKGLHVTAARPAQPTLWTAKLDRFKGSRHHTTLITCHRAGLTEPDSSPLELSEFLW
VDFVVENSTGGGVAVTRPVTWQLEYPGQAPEAEKDKMVWEILVSERDIRALIPLAKAEELVNTAPLTGVPQHVPVRLVTVDGGGALV
EVTEHVGCESANTQVLQVSEACDAVFVAGKESRGARGVRVDFWWRRLRASLRLTVWAPLLPLRIELTDTTLEQVRGWRVPGPAEGPA
EPAAEASDEAERRARGCHLQYQRAGVRFLAPFAAHPLDGGRRLTHLLGPDWLLDVSHLVAPHARVLDSRVASLEGGRVVVGREPGVT
SIEVRSPLSDSILGEQALAVTDDKVSVLELRVQPVMGISLTLSRGTAHPGEVTATCWAQSALPAPKQEVALSLWLSFSDHTVAPAEL
YDRRDLGLSVSAEEPGAILPAEEQGAQLGVVVSGAGAEGLPLHVALHPPEPCRRGRHRVPLASGTAWLGLPPASTPAPALPSSPAWS
PPATEATMGGKRQVAGSVGGNTGVRGKFERAEEEARKEETEAREEEEEEEEEMVPAPQHVTELELGMYALLGVFCVAIFIFLVNGVV
FVLRYQRKEPPDSATDPTSPQPHNWVWLGTDQEELSRQLDRQSPGPPKGEGSCPCESGGGGEAPTLAPGPPGGTTSSSSTLARKEAG
GRRKRVEFVTFAPAPPAQSPEEPVGAPAVQSILVAGEEDIRWVCEDMGLKDPEELRNYMERIRGSS
>sp|Q9UD71|PPR1B_HUMAN
MDPKDRKKIQFSVPAPPSQLDPRQVEMIRRRRPTPAMLFRLSEHSSPEEEASPHQRASGEGHHLKSKRPNPCAYTPPSLKAVQRIAE
SHLQSISNLNENQASEEEDELGELRELGYPREEDEEEEEDDEEEEEEEDSQAEVLKVIRQSAGQKTTCGQGLEGPWERPPPLDESER
DGGSEDQVEDPALSEPGEEPQRPSPSEPGT
>sp|Q9HCZ1|ZN334_HUMAN
MKMKKFQIPVSFQDLTVNFTQEEWQQLDPAQRLLYRDVMLENYSNLVSVGYHVSKPDVIFKLEQGEEPWIVEEFSNQNYPDIDDALE
KNKEIQDKHLTQTVFFSNKTLITERENVFGKTLNLGMNSVPSRKMPYKCNPGGNSLKTNSEVIVAKKSKENRKIPDGYSGFGKHEKS
HLGMKKYRYNPMRKASNQNENLILHQNIQILKQPFDYNKCGKTFFKRAILITQKGRQTERKPNECNECRKTFSKRSTLIVHQRIHTG
EKPYVCSDCRKTFRVKTSLTRHRRIHTGERPYECSECRKTFIDKSALIVHQKIHGGEKSYECNECGKTFFRKSALAEHFRSHTGEKP
YECKECGNAFSKKSYLVVHQRTHRGEKPNECKECGKTFFCQSALTAHQRIHTGEKPYECSECEKTFFCQSALNVHRRSHTGEKPYEC
SQCGKFLCTKSALIAHQITHRGKKSYECNECGKFFCHKSTLTIHQRTHTGEKHGVFNKCGRISIVKSNCSQCKRMNTKENLYECSEH
GHAVSKNSHLIVHQRTIWERPYECNECGRTYCRKSALTHHQRTHTGQRPYECNECGKTFCQKFSFVEHQRTHTGEKPYECNECGKSF
CHKSAFRVHRRIHTGEKPYECNQCGKTYRRLWTLTEHQKIHTGEKPYECNKCEKTFRHKSNFLLHQKSHKE
>sp|P35573|GDE_HUMAN
MGHSKQIRILLLNEMEKLEKTLFRLEQGYELQFRLGPTLQGKAVTVYTNYPFPGETFNREKFRSLDWENPTEREDDSDKYCKLNLQQ
SGSFQYYFLQGNEKSGGGYIVVDPILRVGADNHVLPLDCVTLQTFLAKCLGPFDEWESRLRVAKESGYNMIHFTPLQTLGLSRSCYS
LANQLELNPDFSRPNRKYTWNDVGQLVEKLKKEWNVICITDVVYNHTAANSKWIQEHPECAYNLVNSPHLKPAWVLDRALWRFSCDV
AEGKYKEKGIPALIENDHHMNSIRKIIWEDIFPKLKLWEFFQVDVNKAVEQFRRLLTQENRRVTKSDPNQHLTIIQDPEYRRFGCTV
DMNIALTTFIPHDKGPAAIEECCNWFHKRMEELNSEKHRLINYHQEQAVNCLLGNVFYERLAGHGPKLGPVTRKHPLVTRYFTFPFE
EIDFSMEESMIHLPNKACFLMAHNGWVMGDDPLRNFAEPGSEVYLRRELICWGDSVKLRYGNKPEDCPYLWAHMKKYTEITATYFQG
VRLDNCHSTPLHVAEYMLDAARNLQPNLYVVAELFTGSEDLDNVFVTRLGISSLIREAMSAYNSHEEGRLVYRYGGEPVGSFVQPCL
RPLMPAIAHALFMDITHDNECPIVHRSAYDALPSTTIVSMACCASGSTRGYDELVPHQISVVSEERFYTKWNPEALPSNTGEVNFQS
GIIAARCAISKLHQELGAKGFIQVYVDQVDEDIVAVTRHSPSIHQSVVAVSRTAFRNPKTSFYSKEVPQMCIPGKIEEVVLEARTIE
RNTKPYRKDENSINGTPDITVEIREHIQLNESKIVKQAGVATKGPNEYIQEIEFENLSPGSVIIFRVSLDPHAQVAVGILRNHLTQF

SPHFKSGSLAVDNADPILKIPFASLASRLTLAELNQILYRCESEEKEDGGGCYDIPNWSALKYAGLQGLMSVLAEIRPKNDLGHPFC
NNLRSGDWMIDYVSNRLISRSGTIAEVGKWLQAMFFYLKQIPRYLIPCYFDAILIGAYTTLLDTAWKQMSSFVQNGSTFVKHLSLGS
VQLCGVGKFPSLPILSPALMDVPYRLNEITKEKEQCCVSLAAGLPHFSSGIFRCWGRDTFIALRGILLITGRYVEARNIILAFAGTL
RHGLIPNLLGEGIYARYNCRDAVWWWLQCIQDYCKMVPNGLDILKCPVSRMYPTDDSAPLPAGTLDQPLFEVIQEAMQKHMQGIQFR
ERNAGPQIDRNMKDEGFNITAGVDEETGFVYGGNRFNCGTWMDKMGESDRARNRGIPATPRDGSAVEIVGLSKSAVRWLLELSKKNI
FPYHEVTVKRHGKAIKVSYDEWNRKIQDNFEKLFHVSEDPSDLNEKHPNLVHKRGIYKDSYGASSPWCDYQLRPNFTIAMVVAPELF
TTEKAWKALEIAEKKLLGPLGMKTLDPDDMVYCGIYDNALDNDNYNLAKGFNYHQGPEWLWPIGYFLRAKLYFSRLMGPETTAKTIV
LVKNVLSRHYVHLERSPWKGLPELTNENAQYCPFSCETQAWSIATILETLYDL
>sp|Q6ZWE6|PKHM3_HUMAN
MEALEVDDISPALEVTEEFFSTLDSNLEKAVQQAEVYGIQEVPELVGHEVLSNITDNGAMRNVTSLGKGGMIWDHCKSRLLETKAQN
VFPAKEQFMVQRGTTPDNLSWMEQKEASTFNFFNICQRRRDRPRSVNDLLDETSTFKPGHARSRSDITQVDWRVVLKTTPLQQQQQQ
QPLLQGPHVTRPSFLLPSPNKIEDAQGNTEHKQTFPNILKKGYLEIRKDHDSYWQSCYAELSPYNLYFYSLDSSGNQNLYATYQLSH
FQSISVLGNLEARMVDTVLYDNTQLQLKAESPWEALDWGQKLWEVVHAAVPGYMGRQNELTISPGLGHHDDYTQNHSFQKKTSGLLP
PSPVLDSSKQYQNILKSGTLYRLTVQNNWKAFTFVLSRAYLMAFQPGKLDEDPLLSYNVDVCLAVQMDNLDGCDSCFQVIFPQDVLR
LRAETRQRAQEWMEALKIAANVARSSEQNLQVTLRNKPKDQMGGHELRKNKRQSVTTSFLSILTTLSLERGLTAQSFKCAGCQRSIG
LSNGKAKVCNYSGWYYCSSCHVDDSFLIPARIVHNWDTSKYKVSKQAKEFLEYVYEEPLIDIQQENAMLYHHAEPLAAVLRLRQRLK
SLRAYLFSCRAAVAEDLRRRIFPREYLLQQIHLYSLADLQQVIEGKLAPFLGKVIKFATSHVYSCSLCSQKGFICEICNNGEILYPF
EDISTSRCESCGAVFHSECKEKSVPCPRCVRRELQKKQKSFWQRLNMDESLEEACTMFELSYQNT
>sp|Q8N697|S15A4_HUMAN
MEGSGGGAGERAPLLGARRAAAAAAAAAGAFAGRRAACGAVLLTELLERAAFYGITSNLVLFLNGAPFCWEGAQASEALLLFMGLTYL
GSPFGGWLADARLGRARAILLSLALYLLGMLAFPLLAAPATRAALCGSARLLNCTAPGPDAAARCCSPATFAGLVLVGLGVATVKAN
ITPFGADQVKDRGPEATRRFFNWFYWSINLGAILSLGGIAYIQQNVSFVTGYAIPTVCVGLAFVVFLCGQSVFITKPPDGSAFTDMF
KILTYSCCSQKRSGERQSNGEGIGVFQQSSKQSLFDSCKMSHGGPFTEEKVEDVKALVKIVPVFLALIPYWTVYFQMQTTYVLQSLH
LRIPEISNITTTPHTLPAAWLTMFDAVLILLLIPLKDKLVDPILRRHGLLPSSLKRIAVGMFFVMCSAFAAGILESKRLNLVKEKTI
NQTIGNVVYHAADLSLWWQVPQYLLIGISEIFASIAGLEFAYSAAPKSMQSAIMGLFFFFSGVGSFVGSGLLALVSIKAIGWMSSHT
DFGNINGCYLNYYFFLLAAIQGATLLLFLIISVKYDHHRDHQRSRANGVPTSRRA
>sp|Q8NE18|NSUN7_HUMAN
MLNSTGELEFSNEEDPEIISQLTSLPLSGGKSSAGVPEKTGYPDSVYVMAANIFQGIRIEKSAQKVLIKYGNEPLRSLSESEDQSFQ
RLSYELAFSALKYQDILETILIDSCIFPSTTIPDHLSSLIIVMLYDFQDRKFQTRVLSDNEEPISEVQEVENLLNSFKIKLAAALAR
CRIKHDALSIYHILPETVRKQELRASTLPLYAWINTCKISPEEVYNNLKRRGYNKVKSVLHIDDKVFAVDQHCYDVLIFPSHLKNDL
INIDLFKDYKLIFQDKSRSLAVHSVKALLNMDDDVLMVNTGSWYTVSHMSILTNNNTSKVFVCGVQSQAKDPDLKTLFTKIGCKNIE
ILHEKFINIESKDHRLQKVKVILLLPRCSGLGVSNPVEFILNEHEDTEFLKDHSQGGISVDKLHVLAQQQYEQLTHAMKFTKAQAVV
YCTCSVFPEENEAVVKKALEFQDLGNKGQPYRLSPPVLPLCSLKEIQLSTDKFFRMEPSEITNGCFLSILTRERDPSETVSVNDVLA
RAAAKGLLDGIELGKSSKREKKKKKSKTSLTKGATTDNGIQMKIAEFLNRETKASANLSETVTKPPLPQKNTAQVGASSQTRKPNKL
APHPAVPAFVKNTCPSRPRERQTHFLRPRPEDRMVALKPIKIVLPPVFMPFSSPQGIRSRMPTQHLYCRWVAPKALVPTCLPTHSLS
RKEEKPKDDTPSSLLRPPRRWL
>sp|O95409|ZIC2_HUMAN
MLLDAGPQFPAIGVGSFARHHHHSAAAAAAAAAAEMQDRELSLAAAQNGFVDSAAAHMGAFKLNPGAHELSPGQSSAFTSQGPGAYPG
SAAAAAAAAALGPHAAHVGSYSGPPFNSTRDFLFRSRGFGDSAPGGGQHGLFGPGAGGLHHAHSDAQGHLLFPGLPEQHGPHGSQNV
LNGQMRLGLPGEVFGRSEQYRQVASPRTDPYSAAQLHNQYGPMNMNMGMNMAAAAAHHHHHHHHHPGAFFRYMRQQCIKQELICKWI
DPEQLSNPKKSCNKTFSTMHELVTHVSVEHVGGPEQSNHVCFWEECPREGKPFKAKYKLVNHIRVHTGEKPFPCPFPGCGKVFARSE
NLKIHKRTHTGEKPFQCEFEGCDRRFANSSDRKKHMHVHTSDKPYLCKMCDKSYTHPSSLRKHMKVHESSPQGSESSPAASSGYESS
TPPGLVSPSAEPQSSSNLSPAAAAAAAAAAAAAAAVSAVHRGGGSGSGGAGGGSGGGSGSGGGGGGAGGGGGGSSGGGSGTAGGHSG
LSSNFNEWYV

>sp|Q15398|DLGP5_HUMAN
MSSSHFASRHRKDISTEMIRTKIAHRKSLSQKENRHKEYERNRHFGLKDVNIPTLEGRILVELDETSQGLVPEKTNVKPRAMKTILG
DQRKQMLQKYKEEKQLQKLKEQREKAKRGIFKVGRYRPDMPCFLLSNQNAVKAEPKKAIPSSVRITRSKAKDQMEQTKIDNESDVRA
IRPGPRQTSEKKVSDKEKKVVQPVMPTSLRMTRSATQAAKQVPRTVSSTTARKPVTRAANENEPEGKVPSKGRPAKNVETKPDKGIS
CKVDSEENTLNSQTNATSGMNPDGVLSKMENLPEINTAKIKGKNSFAPKDFMFQPLDGLKTYQVTPMTPRSANAFLTPSYTWTPLKT
EVDESQATKEILAQKCKTYSTKTIQQDSNKLPCPLGPLTVWHEEHVLNKNEATTKNLNGLPIKEVPSLERNEGRIAQPHHGVPYFRN
ILQSETEKLTSHCFEWDRKLELDIPDDAKDLIRTAVGQTRLLMKERFKQFEGLVDDCEYKRGIKETTCTDLDGFWDMVSFQIEDVIH
KFNNLIKLEESGWQVNNNMNHNMNKNVFRKKVVSGIASKPKQDDAGRIAARNRLAAIKNAMRERIRQEECAETAVSVIPKEVDKIVF
DAGFFRVESPVKLFSGLSVSSEGPSQRLGTPKSVNKAVSQSRNEMGIPQQTTSPENAGPQNTKSEHVKKTLFLSIPESRSSIEDAQC
PGLPDLIEENHVVNKTDLKVDCLSSERMSLPLLAGGVADDINTNKKEGISDVVEGMELNSSITSQDVLMSSPEKNTASQNSILEEGE
TKISQSELFDNKSLTTECHLLDSPGLNCSNPFTQLERRHQEHARHISFGGNLITFSPLQPGEF
>sp|Q96Q27|ASB2_HUMAN
MTRFSYAEYFSLFHSCSAPSRSTAPPESSPARAPMGLFQGVMQKYSSSLFKTSQLAPADPLIKAIKDGDEEALKTMIKEGKNLAEPN
KEGWLPLHEAAYYGQVGCLKVLQRAYPGTIDQRTLQEETAVYLATCRGHLDCLLSLLQAGAEPDISNKSRETPLYKACERKNAEAVK
ILVQHNADTNHRCNRGWTALHESVSRNDLEVMQILVSGGAKVESKNAYGITPLFVAAQSGQLEALRFLAKYGADINTQASDNASALY
EACKNEHEEVVEFLLSQGADANKTNKDGLLPLHIASKKGNYRIVQMLLPVTSRTRIRRSGVSPLHLAAERNHDEVLEALLSARFDVN
TPLAPERARLYEDRRSSALYFAVVNNNVYATELLLQHGADPNRDVISPLLVAIRHGCLRTMQLLLDHGANIDAYIATHPTAFPATIM
FAMKCLSLLKFLMDLGCDGEPCFSCLYGNGPHPPAPQPSSRFNDAPAADKEPSVVQFCEFVSAPEVSRWAGPIIDVLLDYVGNVQLC
SRLKEHIDSFEDWAVIKEKAEPPRPLAHLCRLRVRKAIGKYRIKLLDTLPLPGRLIRYLKYENTQ
>sp|Q9NS86|LANC2_HUMAN
MGETMSKRLKLHLGGEAEMEERAFVNPFPDYEAAAGALLASGAAEETGCVRPPATTDEPGLPFHQDGKIIHNFIRRIQTKIKDLLQQ
MEEGLKTADPHDCSAYTGWTGIALLYLQLYRVTCDQTYLLRSLDYVKRTLRNLNGRRVTFLCGDAGPLAVGAVIYHKLRSDCESQEC
VTKLLQLQRSVVCQESDLPDELLYGRAGYLYALLYLNTEIGPGTVCESAIKEVVNAIIESGKTLSREERKTERCPLLYQWHRKQYVG
AAHGMAGIYYMLMQPAAKVDQETLTEMVKPSIDYVRHKKFRSGNYPSSLSNETDRLVHWCHGAPGVIHMLMQAYKVFKEEKYLKEAM
ECSDVIWQRGLLRKGYGICHGTAGNGYSFLSLYRLTQDKKYLYRACKFAEWCLDYGAHGCRIPDRPYSLFEGMAGAIHFLSDVLGPE
TSRFPAFELDSSKRD
>sp|Q96CM3|RUSD4_HUMAN
MAAPRWSASGPWIRGNGQGCGSLFTLVSKPFCAAAAASTAINAQRLAEKLRAQKREQDTKKEPVSTNAVQRRVQEIVRFTRQLQRVH
PNVLAKALTRGILHQDKNLVVINKPYGLPVHGGPGVQLCITDVLPILAKMLHGHKAEPLHLCHRLDKETTGVMVLAWDKDMAHQVQE
LFRTRQVVKKYWAITVHVPMPSAGVVDIPIVEKEAQGQQQHHKMTLSPSYRMDDGKMVKVRRSRNAQVAVTQYQVLSSTLSSALVEL
QPITGIKHQLRVHLSFGLDCPILGDHKYSDWNRLAPQKLSVGTLKKLGLEQSKARYIPLHLHARQLILPALGSGKEELNLVCKLPRF
FVHSLHRLRLEMPNEDQNENNEAKCLGAQ
>sp|O75382|TRIM3_HUMAN
MAKREDSPGPEVQPMDKQFLVCSICLDRYQCPKVLPCLHTFCERCLQNYIPAQSLTLSCPVCRQTSILPEQGVSALQNNFFISSLME
AMQQAPDGAHDPEDPHPLSVVAGRPLSCPNHEGKTMEFYCEACETAMCGECRAGEHREHGTVLLRDVVEQHKAALQRQLEAVRGRLP
QLSAAIALVGGISQQLQERKAEALAQISAAFEDLEQALQQRKQALVSDLETICGAKQKVLQSQLDTLRQGQEHIGSSCSFAEQALRL
GSAPEVLLVRKHMRERLAALAAQAFPERPHENAQLELVLEVDGLRRSVLNLGALLTTSATAHETVATGEGLRQALVGQPASLTVTTK
DKDGRLVRTGSAELRAEITGPDGTRLPVPVVDHKNGTYELVYTARTEGELLLSVLLYGQPVRGSPFRVRALRPGDLPPSPDDVKRRV
KSPGGPGSHVRQKAVRRPSSMYSTGGKRKDNPIEDELVFRVGSRGREKGEFTNLQGVSAASSGRIVVADSNNQCIQVFSNEGQFKFR
FGVRGRSPGQLQRPTGVAVDTNGDIIVADYDNRWVSIFSPEGKFKTKIGAGRLMGPKGVAVDRNGHIIVVDNKSCCVFTFQPNGKLV
GRFGGRGATDRHFAGPHFVAVNNKNEIVVTDFHNHSVKVYSADGEFLFKFGSHGEGNGQFNAPTGVAVDSNGNIIVADWGNSRIQVF
DSSGSFLSYINTSAEPLYGPQGLALTSDGHVVVADAGNHCFKAYRYLQ
>sp|O00555|CAC1A_HUMAN

MARFGDEMPARYGGGGSGAAAGVVVGSGGGRGAGGSRQGGQPGAQRMYKQSMAQRARTMALYNPIPVRQNCLTVNRSLFLFSEDNVV
RKYAKKITEWPPFEYMILATIIANCIVLALEQHLPDDDKTPMSERLDDTEPYFIGIFCFEAGIKIIALGFAFHKGSYLRNGWNVMDF
VVVLTGILATVGTEFDLRTLRAVRVLRPLKLVSGIPSLQVVLKSIMKAMIPLLQIGLLLFFAILIFAIIGLEFYMGKFHTTCFEEGT
DDIQGESPAPCGTEEPARTCPNGTKCQPYWEGPNNGITQFDNILFAVLTVFQCITMEGWTDLLYNSNDASGNTWNWLYFIPLIIIGS
FFMLNLVLGVLSGEFAKERERVENRRAFLKLRRQQQIERELNGYMEWISKAEEVILAEDETDGEQRHPFDGALRRTTIKKSKTDLLN
PEEAEDQLADIASVGSPFARASIKSAKLENSTFFHKKERRMRFYIRRMVKTQAFYWTVLSLVALNTLCVAIVHYNQPEWLSDFLYYA
EFIFLGLFMSEMFIKMYGLGTRPYFHSSFNCFDCGVIIGSIFEVIWAVIKPGTSFGISVLRALRLLRIFKVTKYWASLRNLVVSLLN
SMKSIISLLFLLFLFIVVFALLGMQLFGGQFNFDEGTPPTNFDTFPAAIMTVFQILTGEDWNEVMYDGIKSQGGVQGGMVFSIYFIV
LTLFGNYTLLNVFLAIAVDNLANAQELTKDEQEEEEAANQKLALQKAKEVAEVSPLSAANMSIAVKEQQKNQKPAKSVWEQRTSEMR
KQNLLASREALYNEMDPDERWKAAYTRHLRPDMKTHLDRPLVVDPQENRNNNTNKSRAAEPTVDQRLGQQRAEDFLRKQARYHDRAR
DPSGSAGLDARRPWAGSQEAELSREGPYGRESDHHAREGSLEQPGFWEGEAERGKAGDPHRRHVHRQGGSRESRSGSPRTGADGEHR
RHRAHRRPGEEGPEDKAERRARHREGSRPARGGEGEGEGPDGGERRRRHRHGAPATYEGDARREDKERRHRRRKENQGSGVPVSGPN
LSTTRPIQQDLGRQDPPLAEDIDNMKNNKLATAESAAPHGSLGHAGLPQSPAKMGNSTDPGPMLAIPAMATNPQNAASRRTPNNPGN
PSNPGPPKTPENSLIVTNPSGTQTNSAKTARKPDHTTVDIPPACPPPLNHTVVQVNKNANPDPLPKKEEEKKEEEEDDRGEDGPKPM
PPYSSMFILSTTNPLRRLCHYILNLRYFEMCILMVIAMSSIALAAEDPVQPNAPRNNVLRYFDYVFTGVFTFEMVIKMIDLGLVLHQ
GAYFRDLWNILDFIVVSGALVAFAFTGNSKGKDINTIKSLRVLRVLRPLKTIKRLPKLKAVFDCVVNSLKNVFNILIVYMLFMFIFA
VVAVQLFKGKFFHCTDESKEFEKDCRGKYLLYEKNEVKARDREWKKYEFHYDNVLWALLTLFTVSTGEGWPQVLKHSVDATFENQGP
SPGYRMEMSIFYVVYFVVFPFFFVNIFVALIIITFQEQGDKMMEEYSLEKNERACIDFAISAKPLTRHMPQNKQSFQYRMWQFVVSP
PFEYTIMAMIALNTIVLMMKFYGASVAYENALRVFNIVFTSLFSLECVLKVMAFGILNYFRDAWNIFDFVTVLGSITDILVTEFGNN
FINLSFLRLFRAARLIKLLRQGYTIRILLWTFVQSFKALPYVCLLIAMLFFIYAIIGMQVFGNIGIDVEDEDSDEDEFQITEHNNFR
TFFQALMLLFRSATGEAWHNIMLSCLSGKPCDKNSGILTRECGNEFAYFYFVSFIFLCSFLMLNLFVAVIMDNFEYLTRDSSILGPH
HLDEYVRVWAEYDPAAWGRMPYLDMYQMLRHMSPPLGLGKKCPARVAYKRLLRMDLPVADDNTVHFNSTLMALIRTALDIKIAKGGA
DKQQMDAELRKEMMAIWPNLSQKTLDLLVTPHKSTDLTVGKIYAAMMIMEYYRQSKAKKLQAMREEQDRTPLMFQRMEPPSPTQEGG
PGQNALPSTQLDPGGALMAHESGLKESPSWVTQRAQEMFQKTGTWSPEQGPPTDMPNSQPNSQSVEMREMGRDGYSDSEHYLPMEGQ
GRAASMPRLPAENQRRRGRPRGNNLSTISDTSPMKRSASVLGPKARRLDDYSLERVPPEENQRHHQRRRDRSHRASERSLGRYTDVD
TGLGTDLSMTTQSGDLPSKERDQERGRPKDRKHRQHHHHHHHHHHPPPPDKDRYAQERPDHGRARARDQRWSRSPSEGREHMAHRQG
SSSVSGSPAPSTSGTSTPRRGRRQLPQTPSTPRPHVSYSPVIRKAGGSGPPQQQQQQQQQQQAVARPGRAATSGPRRYPGPTAEPLA
GDRPPTGGHSSGRSPRMERRVPGPARSESPRACRHGGARWPASGPHVSEGPPGPRHHGYYRGSDYDEADGPGSGGGEEAMAGAYDAP
PPVRHASSGATGRSPRTPRASGPACASPSRHGRRLPNGYYPAHGLARPRGPGSRKGLHEPYSESDDDWC
>sp|O94874|UFL1_HUMAN
MADAWEEIRRLAADFQRAQFAEATQRLSERNCIEIVNKLIAQKQLEVVHTLDGKEYITPAQISKEMRDELHVRGGRVNIVDLQQVIN
VDLIHIENRIGDIIKSEKHVQLVLGQLIDENYLDRLAEEVNDKLQESGQVTISELCKTYDLPGNFLTQALTQRLGRIISGHIDLDNR
GVIFTEAFVARHKARIRGLFSAITRPTAVNSLISKYGFQEQLLYSVLEELVNSGRLRGTVVGGRQDKAVFVPDIYSRTQSTWVDSFF
RQNGYLEFDALSRLGIPDAVSYIKKRYKTTQLLFLKAACVGQGLVDQVEASVEEAISSGTWVDIAPLLPTSLSVEDAAILLQQVMRA
FSKQASTVVFSDTVVVSEKFINDCTELFRELMHQKAEKEMKNNPVHLITEEDLKQISTLESVSTSKKDKKDERRRKATEGSGSMRGG
GGGNAREYKIKKVKKKGRKDDDSDDESQSSHTGKKKPEISFMFQDEIEDFLRKHIQDAPEEFISELAEYLIKPLNKTYLEVVRSVFM
SSTTSASGTGRKRTIKDLQEEVSNLYNNIRLFEKGMKFFADDTQAALTKHLLKSVCTDITNLIFNFLASDLMMAVDDPAAITSEIRK
KILSKLSEETKVALTKLHNSLNEKSIEDFISCLDSAAEACDIMVKRGDKKRERQILFQHRQALAEQLKVTEDPALILHLTSVLLFQF
STHSMLHAPGRCVPQIIAFLNSKIPEDQHALLVKYQGLVVKQLVSQSKKTGQGDYPLNNELDKEQEDVASTTRKELQELSSSIKDLV
LKSRKSSVTEE
>sp|O00444|PLK4_HUMAN
MATCIGEKIEDFKVGNLLGKGSFAGVYRAESIHTGLEVAIKMIDKKAMYKAGMVQRVQNEVKIHCQLKHPSILELYNYFEDSNYVYL
VLEMCHNGEMNRYLKNRVKPFSENEARHFMHQIITGMLYLHSHGILHRDLTLSNLLLTRNMNIKIADFGLATQLKMPHEKHYTLCGT
PNYISPEIATRSAHGLESDVWSLGCMFYTLLIGRPPFDTDTVKNTLNKVVLADYEMPSFLSIEAKDLIHQLLRRNPADRLSLSSVLD

HPFMSRNSSTKSKDLGTVEDSIDSGHATISTAITASSSTSISGSLFDKRRLLIGQPLPNKMTVFPKNKSSTDFSSSGDGNSFYTQWG
NQETSNSGRGRVIQDAEERPHSRYLRRAYSSDRSGTSNSQSQAKTYTMERCHSAEMLSVSKRSGGGENEERYSPTDNNANIFNFFKE
KTSSSSGSFERPDNNQALSNHLCPGKTPFPFADPTPQTETVQQWFGNLQINAHLRKTTEYDSISPNRDFQGHPDLQKDTSKNAWTDT
KVKKNSDASDNAHSVKQQNTMKYMTALHSKPEIIQQECVFGSDPLSEQSKTRGMEPPWGYQNRTLRSITSPLVAHRLKPIRQKTKKA
VVSILDSEEVCVELVKEYASQEYVKEVLQISSDGNTITIYYPNGGRGFPLADRPPSPTDNISRYSFDNLPEKYWRKYQYASRFVQLV
RSKSPKITYFTRYAKCILMENSPGADFEVWFYDGVKIHKTEDFIQVIEKTGKSYTLKSESEVNSLKEEIKMYMDHANEGHRICLALE
SIISEEERKTRSAPFFPIIGRKPGSTSSPKALSPPPSVDSNYPTRERASFNRMVMHSAASPTQAPILNPSMVTNEGLGLTTTASGT
DISSNSLKDCLPKSAQLLKSVFVKNVGWATQLTSGAVWVQFNDGSQLVVQAGVSSISYTSPNGQTTRYGENEKLPDYIKQKLQCLSS
ILLMFSNPTPNFH
>sp|Q9BYN7|ZN341_HUMAN
MAQAIFEALEGMDNQTVLAVQSLLDGQGAVPDPTGQSVNAPPAIQPLDDEDVFLCGKCKKQFNSLPAFMTHKREQCQGNAPALATVS
LATNSIYPPSAAPTAVQQAPTPANRQISTYITVPPSPLIQTLVQGNILVSDDVLMSAMSAFTSLDQPMPQGPPPVQSSLNMHSVPSY
LTQPPPPPPPPPLPPPPPPQPPPPPPQSLGPPGRPNPGGNGVVEVYSAAAPLAGSGTVEIQALGMQPYPPLEVPNQCVEPPVYPTP
TVYSPGKQGFKPKGPNPAAPMTSATGGTVATFDSPATLKTRRAKGARGLPEAAGKPKAQKLKCSYCDKSFTKNFDLQQHIRSHTGEK
PFQCIACGRAFAQKSNVKKHMQTHKVWPPGHSGGTVSRNSVTVQVMALNPSRQEDEESTGLGQPLPGAPQPQALSTAGEEEGDKPES
KQVVLIDSSYLCQFCPSKFSTYFQLKSHMTQHKNEQVYKCVVKSCAQTFPKLDTFLEHIKSHQEELSYRCHLCGKDFPSLYDLGVHQ
YSHSLLPQHSPKKDNAVYKCVKCVNKYSTPEALEHHLQTATHNFPCPHCQKVFPCERYLRRHLPTHGSGGRFKCQVCKKFFRREHYL
KLHAHIHSGEKPYKCSVCESAFNRKDKLKRHMLIHEPFKKYKCPFSTHTGCSKEFNRPDKLKAHILSHSGMKLHKCALCSKSFSRRA
HLAEHQRAHTGNYKFRCAGCAKGFSRHKYLKDHRCRLGPQKDKDLQTRRPPQRRAAPRSCGSGGRKVLTPLPDPLGLEELKDTGAGL
VPEAVPGKPPFAEPDAVLSIVVGGAVGAETELVVPGHAEGLGSNLALAELQAGAEGPCAMLAVPVYIQASE
>sp|Q68CQ1|MROH7_HUMAN
MALSPGANLVFHEDPKMTPSPPSCGAPGLGSGTIPQPHPDMAQVPMLNLLPSPGLALVPDLNDSLSPVSGEASGLVSENTPRPDDSR
AIAPASLQITSSCSGEALDLDSKDVSRPDSQGRLCPASNPILSPSSTEAPRLSSGNHPQSNSEDAFKCLSSKIFKLGQRNSNPSRHE
LNPFIRHHSREGLVLGHCISRPSSKALLIPTSNSSLDLDSNPLLNMGSRNTSKLNLNVAPDSHGTLIPDTNETITLASHNISESVSK
GAFSTTWSTSSKETMNVASSGHSRSDLSVTITQASYVTLIPGSSYGISLHSSTHEPNSTISPPSCMTLILGSNETLSLDSSLLFSDT
STLTLSSQQDDAKDNSIHTVPLEENLESWSEMASIKVGQFPLGFPISNPAGKDAVTLQGIPEGAFDEVTSCLVKVPEKTEGGNNMAL
VENVTTLQKSQDLLEAEGEKKTMIKKIMRQIQEEPLDSLSSSVRKQAMEILTQLSHTQPTLGMRERSELVNVCVHSVFSLPSVQAMQ
EKDEAKAETIQALYHQTLEALQTLLKALFIEDPTPAGLKSILEALGPWMNSGKAHERARAVNTNVSVLNHMLLTLPFFMPLGFPALG
LLLGRLILHIGDPDEEIGCEALDGIIILYTILELQKRARDKEETNKKELYESNKHFLGPYNPVSPCQNILRVIEEFGDFLGPQQIKD
LLLAALEGLKGSSEAPGKDSREMMQLASEVMLSSVLEWYRHRALEVIPEIMQGIYMQLSHIQEPRARQVALLPVSLLASSFMTEVVV
ALLMCPLPLNSNGAEMWRQLILCKPSCDVRDLLDLLLGSLKEKPVTKEGRASIVPLAAASGLCELLSVNSCMGRVRRIYPQLLLALL
IQVHYHIGLNLPGCVAPPKDTKKGAQPSPFVPVRWVVKVVKTLLLRMGCSYETTFLEDQGGWELMEQVESHHRGVALLARAMVQYSC
QELCRILYLLIPLLERGDEKHRITATAFFVELLQMEQVRRIPEEYSLGRMAEGLSHHDPIMKVLSIRGLVILARRSEKTAKVKALLP
SMVKGLKNMDGMLVVEAVHNLKAVFKGRDQKLMDSAVYVEMLQILLPHFSDAREVVRSSCINLYGKVVQKLRAPRTQAMEEQLVSTL
VPLLLTMQEGNSKVSQKCVKTLLRCSYFMAWELPKRAYSRKPWDNQQQTVAKICKCLVNTHRDSAFIFLSQSLEYAKNSRASLRKCS
VMFIGSLVPCMESIMTEDRLNEVKAALDNLRHDPEASVCIYAAQVQDHILASCWQNSWLPHGNSWVCYSATTHRWSPSCENLPTSHQ
RRSWIMQALGSWKMSLKK
>sp|Q6NVV3|NIPA3_HUMAN
MGAQVRLPPGEPCREGYVLSLVCPNSSQAWCEITNVSQLLASPVLYTDLNYSINNLSISANVENKYSLYVGLVLAVSSSIFIGSSFI
LKKKGLLQLASKGFTRAGQGGHSYLKEWLWWVGLLSMGAGEAANFAAYAFAPATLVTPLGALSVLISAILSSYFLNEHLNIHGKIGC
ILSILGSTVMVIHAPQEEEVTSLHEMEMKLRDPGFISFAVIITVISLVLILIVAPKKGQTNILVYISICSLIGAFSVSSVKGLGIAI
KELIEWKPVYKHPLVFVLLAVLVLSVTTQINYLNKALDTFNTSLVTPIYYVFFTSMVVTCSAILFQEWYGMTAGDIIGTLSGFFTII
IGIFLLHAFKNTDITWSELTSTAKKEAVSLNVNENNYVLLENLECSAPGYNDDVTLFSRTDD
>sp|Q9UPN7|PP6R1_HUMAN

MFWKFDLHTSSHLDTLLEREDLSLPELLDEEDVLQECKVVNRKLLDFLLQPPHLQAMVAWVTQEPPDSGEERLRYKYPSVACEILTS
DVPQINDALGADESLLNRLYGFLQSTGSLNPLLASFFSKVMGILINRKTDQLVSFLRKKDDFVDLLLQHIGTSAIMDLLLRLLTCVE
RPQLRQDVVNWLNEEKIVQRLIEQIHPSKDENQHSNASQSLCDIIRLSREQMIQVQDSPEPDQLLATLEKQETIEQLLSNMFEGEQS
QSVIVSGIQVLLTLLEPRRPRSESVTVNSFFSSVDGQLELLAQGALESTVSSVGALHALRPRLSCFHQLLLEPPKLEPLQMTWGMLA
PPLGNTRLHVVKLLASALSANDAALTHELLALDVPNTMLDLFFHYVFNNFLHAQVEGCVSTMLSLGPPPDSSPETPIQNPVVKHLLQ
QCRLVERILTSWEENDRVQCAGGPRKGYMGHLTRVAGALVQNTEKGPNAEQLRQLLKELPSEQQEQWEAFVSGPLAETNKKNMVDLV
NTHHLHSSSDDEDDRLKEFNFPEEAVLQQAFMDFQMQRMTSAFIDHFGFNDEEFGEQEESVNAPFDKTANITFSLNADDENPNANLL
EICYKDRIQQFDDDEEEEDEEEAQGSGESDGEDGAWQGSQLARGARLGQPPGVRSGGSTDSEDEEEEDEEEEEDEEGIGCAARGGAT
PLSYPSPGPQPPGPSWTATFDPVPTDAPTSPRVSGEEELHTGPPAPQGPLSVPQGLPTQSLASPPARDALQLRSQDPTPPSAPQEAT
EGSKVTEPSAPCQALVSIGDLQATFHGIRSAPSSSDSATRDPSTSVPASGAHQPPQTTEGEKSPEPLGLPQSQSAQALTPPPIPNGS
APEGPASPGSQ
>sp|O14828|SCAM3_HUMAN
MAQSRDGGNPFAEPSELDNPFQDPAVIQHRPSRQYATLDVYNPFETREPPPAYEPPAPAPLPPPSAPSLQPSRKLSPTEPKNYGSYS
TQASAAAATAELLKKQEELNRKAEELDRRERELQHAALGGTATRQNNWPPLPSFCPVQPCFFQDISMEIPQEFQKTVSTMYYLWMCS
TLALLLNFLACLASFCVETNNGAGFGLSILWVLLFTPCSFVCWYRPMYKAFRSDSSFNFFVFFFIFFVQDVLFVLQAIGIPGWGFSG
WISALVVPKGNTAVSVLMLLVALLFTGIAVLGIVMLKRIHSLYRRTGASFQKAQQEFAAGVFSNPAVRTAAANAAAGAAENAFRAP
>sp|Q9NP85|PODO_HUMAN
MERRARSSSRESRGRGGRTPHKENKRAKAERSGGGRGRQEAGPEPSGSGRAGTPGEPRAPAATVVDVDEVRGSGEEGTEVVALLESE
RPEEGTKSSGLGACEWLLVLISLLFIIMTFPFSIWFCVKVVQEYERVIIFRLGHLLPGRAKGPGLFFFLPCLDTYHKVDLRLQTLEI
PFHEIVTKDMFIMEIDAICYYRMENASLLLSSLAHVSKAVQFLVQTTMKRLLAHRSLTEILLERKSIAQDAKVALDSVTCIWGIKVE
RIEIKDVRLPAGLQHSLAVEAEAQRQAKVRMIAAEAEKAASESLRMAAEILSGTPAAVQLRYLHTLQSLSTEKPSTVVLPLPFDLLN
CLSSPSNRTQGSLPFPSPSKPVEPLNPKKKDSPML
>sp|Q8NGS9|O13C2_HUMAN
MEWENHTILVEFFLKGLSGHPRLELLFFVLIFIMYVVILLGNGTLILISILDPHLHTPMYFFLGNLSFLDICYTTTSIPSTLVSFLS
ERKTISLSGCAVQMFLGLAMGTTECVLLGMMAFDRYVAICNPLRYPIIMSKDAYVPMAAGSWIIGAVNSAVQSVFVVQLPFCRNNII
NHFTCEILAVMKLACADISDNEFIMLVATTLFILTPLLLIIVSYTLIIVSIFKISSSEGRSKASSTCSAHLTVVIIFYGTILFMYMK
PKSKETLNSDDLDATDKIISMFYGVMTPMMNPLIYSLRNKDVKEAVKHLLNRRFFSK
>sp|P31629|ZEP2_HUMAN
MDTGDTALGQKATSRSGETDKASGRWRQEQSAVIKMSTFGSHEGQRQPQIEPEQIGNTASAQLFGSGKLASPSEVVQQVAEKQYPPH
RPSPYSCQHSLSFPQHSLPQGVMHSTKPHQSLEGPPWLFPGPLPSVASEDLFPFPIHGHSGGYPRKKISSLNPAYSQYSQKSIEQAE
EAHKKEHKPKKPGKYICPYCSRACAKPSVLKKHIRSHTGERPYPCIPCGFSFKTKSNLYKHRKSHAHAIKAGLVPFTESAVSKLDLE
AGFIDVEAEIHSDGEQSTDTDEESSLFAEASDKMSPGPPIPLDIASRGGYHGSLEESLGGPMKVPILIIPKSGIPLPNESSQYIGPD
MLPNPSLNTKADDSHTVKQKLALRLSEKKGQDSEPSLNLLSPHSKGSTDSGYFSRSESAEQQISPPNTNAKSYEEIIFGKYCRLSPR
NALSVTTTSQERAAMGRKGIMEPLPHVNTRLDVKMFEDPVSQLIPSKGDVDPSQTSMLKSTKFNSESRQPQIIPSSIRNEGKLYPAN
FQGSNPVLLEAPVDSSPLIRSNSVPTSSATNLTIPPSLRGSHSFDERMTGSDDVFYPGTVGIPPQRMLRRQAAFELPSVQEGHVEVE
HHGRMLKGISSSSLKEKKLSPGDRVGYDYDVCRKPYKKWEDSETPKQNYRDISCLSSLKHGGEYFMDPVVPLQGVPSMFGTTCENRK
RRKEKSVGDEEDTPMICSSIVSTPVGIMASDYDPKLQMQEGVRSGFAMAGHENLSHGHTERFDPCRPQLQPGSPSLVSEESPSAIDS
DKMSDLGGRKPPGNVISVIQHTNSLSRPNSFERSESAELVACTQDKAPSPSETCDSEISEAPVSPEWAPPGDGAESGGKPSPSQQVQ
QQSYHTQPRLVRQHNIQVPEIRVTEEPDKPEKEKEAQSKEPEKPVEEFQWPQRSETLSQLPAEKLPPKKKRLRLADMEHSSGESSFE
STGTGLSRSPSQESNLSHSSSFSMSFEREETSKLSALPKQDEFGKHSEFLTVPAGSYSLSVPGHHHQKEMRRCSSEQMPCPHPAEVP
EVRSKSFDYGNLSHAPVSGAAASTVSPSRERKKCFLVRQASFSGSPEISQGEVGMDQSVKQEQLEHLHAGLRSGWHHGPPAVLPPLQ
QEDPGKQVAGPCPPLSSGPLHLAQPQIMHMDSQESLRNPLIQPTSYMTSKHLPEQPHLFPHQETIPFSPIQNALFQFQYPTVCMVHL
PAQQPPWWQAHFPHPFAQHPQKSYGKPSFQTEIHSSYPLEHVAEHTGKKPAEYAHTKEQTYPCYSGASGLHPKNLLPKFPSDQSSKS
TETPSEQVLQEDFASANAGSLQSLPGTVVPVRIQTHVPSYGSVMYTSISQILGQNSPAIVICKVDENMTQRTLVTNAAMQGIGFNIA

QVLGQHAGLEKYPIWKAPQTLPLGLESSIPLCLPSTSDSVATLGGSKRMLSPASSLELFMETKQQKRVKEEKMYGQIVEELSAVELT
NSDIKKDLSRPQKPQLVRQGCASEPKDGLQSGSSSFSSLSPSSSQDYPSVSPSSREPFLPSKEMLSGSRAPLPGQKSSGPSESKESS
DELDIDETASDMSMSPQSSSLPAGDGQLEEEGKGHKRPVGMLVRMASAPSGNVADSTLLLTDMADFQQILQFPSLRTTTTVSWCFLN
YTKPNYVQQATFKSSVYASWCISSCNPNPSGLNTKTTLALLRSKQKITAEIYTLAAMHRPGTGKLTSSSAWKQFTQMKPDASFLFGS
KLERKLVGNILKERGKGDIHGDKDIGSKQTEPIRIKIFEGGYKSNEDYVYVRGRGRGKYICEECGIRCKKPSMLKKHIRTHTDVRPY
VCKLCNFAFKTKGNLTKHMKSKAHMKKCLELGVSMTSVDDTETEEAENLEDLHKAAEKHSMSSISTDHQFSDAEESDGEDGDDNDDD
DEDEDDFDDQGDLTPKTRSRSTSPQPPRFSSLPVNVGAVPHGVPSDSSLGHSSLISYLVTLPSIRVTQLMTPSDSCEDTQMTEYQRL
FQSKSTDSEPDKDRLDIPSCMDEECMLPSEPSSSPRDFSPSSHHSSPGYDSSPCRDNSPKRYLIPKGDLSPRRHLSPRRDLSPMRHL
SPRKEAALRREMSQRDVSPRRHLSPRRPVSPGKDITARRDLSPRRERRYMTTIRAPSPRRALYHNPPLSMGQYLQAEPIVLGPPNLR
RGLPQVPYFSLYGDQEGAYEHPGSSLFPEGPNDYVFSHLPLHSQQQVRAPIPMVPVGGIQMVHSMPPALSSLHPSPTLPLPMEGFEE
KKGASGESFSKDPYVLSKQHEKRGPHALQSSGPPSTPSSPRLLMKQSTSEDSLNATEREQEENIQTCTKAIASLRIATEEAALLGPD
QPARVQEPHQNPLGSAHVSIRHFSRPEPGQPCTSATHPDLHDGEKDNFGTSQTPLAHSTFYSKSCVDDKQLDFHSSKELSSSTEESK
DPSSEKSQLH
>sp|Q99748|NRTN_HUMAN
MQRWKAAALASVLCSSVLSIWMCREGLLLSHRLGPALVPLHRLPRTLDARIARLAQYRALLQGAPDAMELRELTPWAGRPPGPRRRA
GPRRRRARARLGARPCGLRELEVRVSELGLGYASDETVLFRYCAGACEAAARVYDLGLRRLRQRRRLRRERVRAQPCCRPTAYEDEV
SFLDAHSRYHTVHELSARECACV
>sp|Q9UM63|PLAL1_HUMAN
MATFPCQLCGKTFLTLEKFTIHNYSHSRERPYKCVQPDCGKAFVSRYKLMRHMATHSPQKSHQCAHCEKTFNRKDHLKNHLQTHDPN
KMAFGCEECGKKYNTMLGYKRHLALHAASSGDLTCGVCALELGSTEVLLDHLKAHAEEKPPSGTKEKKHQCDHCERCFYTRKDVRRH
LVVHTGCKDFLCQFCAQRFGRKDHLTRHTKKTHSQELMKESLQTGDLLSTFHTISPSFQLKAAALPPFPLGASAQNGLASSLPAEVH
SLTLSPPEQAAQPMQPLPESLASLHPSVSPGSPPPPLPNHKYNTTSTSYSPLASLPLKADTKGFCNISLFEDLPLQEPQSPQKLNPG
FDLAKGNAGKVNLPKELPADAVNLTIPASLDLSPLLGFWQLPPPATQNTFGNSTLALGPGESLPHRLSCLGQQQQEPPLAMGTVSLG
QLPLPPIPHVFSAGTGSAILPHFHHAFR
>sp|Q53G59|KLH12_HUMAN
MGGIMAPKDIMTNTHAKSILNSMNSLRKSNTLCDVTLRVEQKDFPAHRIVLAACSDYFCAMFTSELSEKGKPYVDIQGLTASTMEIL
LDFVYTETVHVTVENVQELLPAACLLQLKGVKQACCEFLESQLDPSNCLGIRDFAETHNCVDLMQAAEVFSQKHFPEVVQHEEFILL
SQGEVEKLIKCDEIQVDSEEPVFEAVINWVKHAKKEREESLPNLLQYVRMPLLTPRYITDVIDAEPFIRCSLQCRDLVDEAKKFHLR
PELRSQMQGPRTRARLGANEVLLVVGGFGSQQSPIDVVEKYDPKTQEWSFLPSITRKRRYVASVSLHDRIYVIGGYDGRSRLSSVEC
LDYTADEDGVWYSVAPMNVRRGLAGATTLGDMIYVSGGFDGSRRHTSMERYDPNIDQWSMLGDMQTAREGAGLVVASGVIYCLGGYD
GLNILNSVEKYDPHTGHWTNVTPMATKRSGAGVALLNDHIYVVGGFDGTAHLSSVEAYNIRTDSWTTVTSMTTPRCYVGATVLRGRL
YAIAGYDGNSLLSSIECYDPIIDSWEVVTSMGTQRCDAGVCVLREK
>sp|O96020|CCNE2_HUMAN
MSRRSSRLQAKQQPQPSQTESPQEAQIIQAKKRKTTQDVKKRREEVTKKHQYEIRNCWPPVLSGGISPCIIIETPHKEIGTSDFSRF
TNYRFKNLFINPSPLPDLSWGCSKEVWLNMLKKESRYVHDKHFEVLHSDLEPQMRSILLDWLLEVCEVYTLHRETFYLAQDFFDRFM
LTQKDINKNMLQLIGITSLFIASKLEEIYAPKLQEFAYVTDGACSEEDILRMELIILKALKWELCPVTIISWLNLFLQVDALKDAPK
VLLPQYSQETFIQIAQLLDLCILAIDSLEFQYRILTAAALCHFTSIEVVKKASGLEWDSISECVDWMVPFVNVVKSTSPVKLKTFKK
IPMEDRHNIQTHTNYLAMLEEVNYINTFRKGGQLSPVCNGGIMTPPKSTEKPPGKH
>sp|P29374|ARI4A_HUMAN
MKAADEPAYLTVGTDVSAKYRGAFCEAKIKTVKRLVKVKVLLKQDNTTQLVQDDQVKGPLRVGAIVETRTSDGSFQEAIISKLTDAS
WYTVVFDDGDERTLRRTSLCLKGERHFAESETLDQLPLTNPEHFGTPVIAKKTNRGRRSSLPVTEDEKEEESSEEEDEDKRRLNDEL
LGKVVSVVSATERTEWYPALVISPSCNDDITVKKDQCLVRSFIDSKFYSIARKDIKEVDILNLPESELSTKPGLQKASIFLKTRVVP
DNWKMDISEILESSSSDDEDGPAEENDEEKEKEAKKTEEEVPEEELDPEERDNFLQQLYKFMEDRGTPINKPPVLGYKDLNLFKLFR
LVYHQGGCDNIDSGAVWKQIYMDLGIPILNSAASYNVKTAYRKYLYGFEEYCRSANIQFRTVHHHEPKVKEEKKDLEESMEEALKLD

QEMPLTEVKSEPEENIDSNSESEREEIELKSPRGRRRIARDVNSIKKEIEEEKTEDKLKDNDTENKDVDDDYETAEKKENELLLGRK
NTPKQKEKKIKKQEDSDKDSDEEEEKSQEREETESKCDSEGEEDEEDMEPCLTGTKVKVKYGRGKTQKIYEASIKSTEIDDGEVLYL
VHYYGWNVRYDEWVKADRIIWPLDKGGPKKKQKKKAKNKEDSEKDEKRDEERQKSKRGRPPLKSTLSSNMPYGLSKTANSEGKSDSC
SSDSETEDALEKNLINEELSLKDELEKNENLNDDKLDEENPKISAHILKENDRTQMQPLETLKLEVGENEQIVQIFGNKMEKTEEVK
KEAEKSPKGKGRRSKTKDLSLEIIKISSFGQNEAGSEPHIEAHSLELSSLDNKNFSSATEDEIDQCVKEKKLKRKILGQSSPEKKIR
IENGMEMTNTVSQERTSDCIGSEGMKNLNFEQHFERENEGMPSLIAESNQCIQQLTSERFDSPAEETVNIPLKEDEDAMPLIGPETL
VCHEVDLDDLDEKDKTSIEDVAVESSESNSLVSIPPALPPVVQHNFSVASPLTLSQDESRSVKSESDITIEVDSIAEESQEGLCERE
SANGFETNVASGTCSIIVQERESREKGQKRPSDGNSGLMAKKQKRTPKRTSAAAKNEKNGTGQSSDSEDLPVLDNSSKCTPVKHLNV
SKPQKLARSPARISPHIKDGEKDKHREKHPNSSPRTYKWSFQLNELDNMNSTERISFLQEKLQEIRKYYMSLKSEVATIDRRRKRLK
KKDREVSHAGASMSSASSDTGMSPSSSSPPQNVLAVECR
>sp|Q86X40|LRC28_HUMAN
MASELCKTISVARLEKHKNLFLNYRNLHHFPLELLKDEGLQYLERLYMKRNSLTSLPENLAQKLPNLVELYLHSNNIVVVPEAIGSL
VKLQCLDLSDNALEIVCPEIGRLRALRHLRLANNQLQFLPPEVGDLKELQTLDISTNRLLTLPERLHMCLSLQYLTVDRNRLWYVPR
HLCQLPSLNELSMAGNRLAFLPLDLGRSRELQYVYVDNNIHLKGLPSYLYNKVIGCSGCGAPIQVSEVKLLSFSSGQRTVFLPAEVK
AIGTEHDHVLPLQELAMRGLYHTYHSLLKDLNFLSPISLPRSLLELLHCPLGHCHRCSEPMFTIVYPKLFPLRETPMAGLHQWKTTV
SFVAYCCSTQCLQTFDLLS
>sp|Q9BYR0|KRA47_HUMAN
MVSSCCGSVCSDQGCSQDLCQETCCRPSCCQTTCCRTTCYRPSCCVSSCCRPQCCQSVCCQPTCCRPTCCETTCCHPRCCISSCCRP
SCCMSSCCKPQCCQSVCCQPTCCHPSCCISSCCRPSCCVSRCCRPQCCQSVCCQPTCCRPSCCISSCCRPSCCESSCCRPSCCRPCC
CLRPVCGRVSCHTTCYRPTCVISTCPRPLCCASSCC
>sp|Q9P2X7|DEC1_HUMAN
MTMNVLEAGKWKSIVPAPGEGLLAVLHMMVFTDALHRERSVKWQAGVCYNGGKDFAVSLARPKAAEGIAD
>sp|O43298|ZBT43_HUMAN
MEPGTNSFRVEFPDFSSTILQKLNQQRQQGQLCDVSIVVQGHIFRAHKAVLAASSPYFCDQVLLKNSRRIVLPDVMNPRVFENILLS
SYTGRLVMPAPEIVSYLTAASFLQMWHVVDKCTEVLEGNPTVLCQKLNHGSDHQSPSSSSYNGLVESFELGSGGHTDFPKAQELRDG
ENEEESTKDELSSQLTEHEYLPSNSSTEHDRLSTEMASQDGEEGASDSAEFHYTRPMYSKPSIMAHKRWIHVKPERLEQACEGMDVH
ATYDEHQVTESINTVQTEHTVQPSGVEEDFHIGEKKVEAEFDEQADESNYDEQVDFYGSSMEEFSGERSDGNLIGHRQEAALAAGYS
ENIEMVTGIKEEASHLGFSATDKLYPCQCGKSFTHKSQRDRHMSMHLGLRPYGCGVCGKKFKMKHHLVGHMKIHTGIKPYECNICAK
RFMWRDSFHRHVTSCTKSYEAAKAEQNTTEAN
>sp|P10588|NR2F6_HUMAN
MAMVTGGWGGPGGDTNGVDKAGGYPRAAEDDSASPPGAASDAEPGDEERPGLQVDCVVCGDKSSGKHYGVFTCEGCKSFFKRSIRRN
LSYTCRSNRDCQIDQHHRNQCQYCRLKKCFRVGMRKEAVQRGRIPHSLPGAVAASSGSPPGSALAAVASGGDLFPGQPVSELIAQLL
RAEPYPAAAGRFGAGGGAAGAVLGIDNVCELAARLLFSTVEWARHAPFFPELPVADQVALLRLSWSELFVLNAAQAALPLHTAPLLA
AAGLHAAPMAAERAVAFMDQVRAFQEQVDKLGRLQVDSAEYGCLKAIALFTPDACGLSDPAHVESLQEKAQVALTEYVRAQYPSQPQ
RFGRLLLRLPALRAVPASLISQLFFMRLVGKTPIETLIRDMLLSGSTFNWPYGSGQ
>sp|Q12860|CNTN1_HUMAN
MKMWLLVSHLVIISITTCLAEFTWYRRYGHGVSEEDKGFGPIFEEQPINTIYPEESLEGKVSLNCRARASPFPVYKWRMNNGDVDLT
SDRYSMVGGNLVINNPDKQKDAGIYYCLASNNYGMVRSTEATLSFGYLDPFPPEERPEVRVKEGKGMVLLCDPPYHFPDDLSYRWLL
NEFPVFITMDKRRFVSQTNGNLYIANVEASDKGNYSCFVSSPSITKSVFSKFIPLIPIPERTTKPYPADIVVQFKDVYALMGQNVTL
ECFALGNPVPDIRWRKVLEPMPSTAEISTSGAVLKIFNIQLEDEGIYECEAENIRGKDKHQARIYVQAFPEWVEHINDTEVDIGSDL
YWPCVATGKPIPTIRWLKNGYAYHKGELRLYDVTFENAGMYQCIAENTYGAIYANAELKILALAPTFEMNPMKKKILAAKGGRVIIE
CKPKAAPKPKFSWSKGTEWLVNSSRILIWEDGSLEINNITRNDGGIYTCFAENNRGKANSTGTLVITDPTRIILAPINADITVGENA
TMQCAASFDPALDLTFVWSFNGYVIDFNKENIHYQRNFMLDSNGELLIRNAQLKHAGRYTCTAQTIVDNSSASADLVVRGPPGPPGG
LRIEDIRATSVALTWSRGSDNHSPISKYTIQTKTILSDDWKDAKTDPPIIEGNMEAARAVDLIPWMEYEFRVVATNTLGRGEPSIPS

NRIKTDGAAPNVAPSDVGGGGGRNRELTITWAPLSREYHYGNNFGYIVAFKPFDGEEWKKVTVTNPDTGRYVHKDETMSPSTAFQVK
VKAFNNKGDGPYSLVAVINSAQDAPSEAPTEVGVKVLSSSEISVHWEHVLEKIVESYQIRYWAAHDKEEAANRVQVTSQEYSARLEN
LLPDTQYFIEVGACNSAGCGPPSDMIEAFTKKAPPSQPPRIISSVRSGSRYIITWDHVVALSNESTVTGYKVLYRPDGQHDGKLYST
HKHSIEVPIPRDGEYVVEVRAHSDGGDGVVSQVKISGAPTLSPSLLGLLLPAFGILVYLEF
>sp|Q9UC06|ZNF70_HUMAN
MEVPPATKFGETFAFENRLESQQGLFPGEDLGDPFLQERGLEQMAVIYKEIPLGEQDEENDDYEGNFSLCSSPVQHQSIPPGTRPQD
DELFGQTFLQKSDLSMCQIIHSEEPSPCDCAETDRGDSGPNAPHRTPQPAKPYACRECGKAFSQSSHLLRHLVIHTGEKPYECCECG
KAFSQSSHLLRHQIIHTGEKPYECRECGKAFRQSSALTQHQKIHTGKRPYECRECGKDFSRSSSLRKHERIHTGERPYQCKECGKSF
NQSSGLSQHRKIHTLKKPHECDLCGKAFCHRSHLIRHQRIHTGKKPYKCDECGKAFSQSSNLIEHRKTHTGEKPYKCQKCGKAFSQS
SSLIEHQRIHTGEKPYECCQCGKAFCHSSALIQHQRIHTGKKPYTCECGKAFRHRSALIEHYKTHTREKPYVCNLCGKSFRGSSHLI
RHQKIHSGEKL
>sp|P0C7N8|OR9G9_HUMAN
MQRSNHTVTEFILLGFTTDPGMQLGLFVVFLGVYSLTVVGNSTLIVLICNDSHLHTPMYFVVGNLSFLDLWYSSVYTPKILVICISE
DKSISFAGCLCQFFFSAGLAYSECCLLAAMAYDRYVAISKPLLYAQAMSIKLCALLVAVSYCGGFINSSIITKKTFSFNFCCENIID
DFFCDLLPLVKLACGEKGCYKFLMYFLLASNVICPAVLILASYLFIITSVLRISSSQGRLKAFSTCSSHLTSVTLYYGSILYIYALP
RSSYSFDMDKIVSTFYTEVLPMLNPMIYSLRNKDVKEALKKLLP
>sp|A1L157|TSN11_HUMAN
MAHYKTEQDDWLIIYLKYLLFVFNFFFWVGGAAVLAVGIWTLVEKSGYLSVLASSTFAASAYILIFAGVLVMVTGFLGFGAILWERK
GCLSTYFCLLLVIFLVELVAGVLAHVYYQRLSDELKQHLNRTLAENYGQPGATQITASVDRLQQDFKCCGSNSSADWQHSTYILLRE
AEGRQVPDSCCKTVVVRCGQRAHPSNIYKVEGGCLTKLEQFLADHLLLMGAVGIGVACLQICGMVLTCCLHQRLQRHFY
>sp|Q70EL1|UBP54_HUMAN
MSWKRNYFSGGRGSVQGMFAPRSSTSIAPSKGLSNEPGQNSCFLNSALQVLWHLDIFRRSFRQLTTHKCMGDSCIFCALKGIFNQFQ
CSSEKVLPSDTLRSALAKTFQDEQRFQLGIMDDAAECFENLLMRIHFHIADETKEDICTAQHCISHQKFAMTLFEQCVCTSCGATSD
PLPFIQMVHYISTTSLCNQAICMLERREKPSPSMFGELLQNASTMGDLRNCPSNCGERIRIRRVLMNAPQIITIGLVWDSDHSDLAE
DVIHSLGTCLKLGDLFFRVTDDRAKQSELYLVGMICYYGKHYSTFFFQTKIRKWMYFDDAHVKEIGPKWKDVVTKCIKGHYQPLLLL
YADPQGTPVSTQDLPPQAEFQSYSRTCYDSEDSGREPSISSDTRTDSSTESYPYKHSHHESVVSHFSSDSQGTVIYNVENDSMSQSS
RDTGHLTDSECNQKHTSKKGSLIERKRSSGRVRRKGDEPQASGYHSEGETLKEKQAPRNASKPSSSTNRLRDFKETVSNMIHNRPSL
ASQTNVGSHCRGRGGDQPDKKPPRTLPLHSRDWEIESTSSESKSSSSSKYRPTWRPKRESLNIDSIFSKDKRKHCGYTQLSPFSEDS
AKEFIPDEPSKPPSYDIKFGGPSPQYKRWGPARPGSHLLEQHPRLIQRMESGYESSERNSSSPVSLDAALPESSNVYRDPSAKRSAG
LVPSWRHIPKSHSSSILEVDSTASMGGWTKSQPFSGEEISSKSELDELQEEVARRAQEQELRRKREKELEAAKGFNPHPSRFMDLDE
LQNQGRSDGFERSLQEAESVFEESLHLEQKGDCAAALALCNEAISKLRLALHGASCSTHSRALVDKKLQISIRKARSLQDRMQQQQS
PQQPSQPSACLPTQAGTLSQPTSEQPIPLQVLLSQEAQLESGMDTEFGASSFFHSPASCHESHSSLSPESSAPQHSSPSRSALKLLT
SVEVDNIEPSAFHRQGLPKAPGWTEKNSHHSWEPLDAPEGKLQGSRCDNSSCSKLPPQEGRGIAQEQLFQEKKDPANPSPVMPGIAT
SERGDEHSLGCSPSNSSAQPSLPLYRTCHPIMPVASSFVLHCPDPVQKTNQCLQGQSLKTSLTLKVDRGSEETYRPEFPSTKGLVRS
LAEQFQRMQGVSMRDSTGFKDRSLSGSLRKNSSPSDSKPPFSQGQEKGHWPWAKQQSSLEGGDRPLSWEESTEHSSLALNSGLPNGE
TSSGGQPRLAEPDIYQEKLSQVRDVRSKDLGSSTDLGTSLPLDSWVNITRFCDSQLKHGAPRPGMKSSPHDSHTCVTYPERNHILLH
PHWNQDTEQETSELESLYQASLQASQAGCSGWGQQDTAWHPLSQTGSADGMGRRLHSAHDPGLSKTSTAEMEHGLHEARTVRTSQAT
PCRGLSRECGEDEQYSAENLRRISRSLSGTVVSEREEAPVSSHSFDSSNVRKPLETGHRCSSSSSLPVIHDPSVFLLGPQLYLPQPQ
FLSPDVLMPTMAGEPNRLPGTSRSVQQFLAMCDRGETSQGAKYTGRTLNYQSLPHRSRTDNSWAPWSETNQHIGTRFLTTPGCNPQL
TYTATLPERSKGLQVPHTQSWSDLFHSPSHPPIVHPVYPPSSSLHVPLRSAWNSDPVPGSRTPGPRRVDMPPDDDWRQSSYASHSGH
RRTVGEGFLFVLSDAPRREQIRARVLQHSQW
>sp|P0C7T7|FMAS1_HUMAN
MQLGRSGRRSQKQKHEVDEVYFTLTRVDRSCPYLTVPGPPGAESGAPVRLGLRQRAVSSSRNPNSAGRTPNSYLTQAPVWRRSAPAQ
PPAPQTRHFPGRGADPALGSLPAAGLSGLCARTTGGTAQPPGARDSGASPGAQPQRHRPGCRGPPGSPVIRGPPRCREPGTAHGAQT

PPPTRPNWPRRQPSRGSCALSSRQYGERPPAPPWTQPSVPRRGAATWGQEKAEYATPLGTQEARRASGGRGGVDHGDQGLSPCCGDG
GARGRRRLPTFWPSGLLRLRLDSLGALPPEPSRAGRGRLFGLSVVLLKRNAGGEATPREGGPRRRWLRESCSPLPARHSRRRASWVS
RWMEGAPPRMPSLPL
>sp|P14784|IL2RB_HUMAN
MAAPALSWRLPLLILLLPLATSWASAAVNGTSQFTCFYNSRANISCVWSQDGALQDTSCQVHAWPDRRRWNQTCELLPVSQASWACN
LILGAPDSQKLTTVDIVTLRVLCREGVRWRVMAIQDFKPFENLRLMAPISLQVVHVETHRCNISWEISQASHYFERHLEFEARTLSP
GHTWEEAPLLTLKQKQEWICLETLTPDTQYEFQVRVKPLQGEFTTWSPWSQPLAFRTKPAALGKDTIPWLGHLLVGLSGAFGFIILV
YLLINCRNTGPWLKKVLKCNTPDPSKFFSQLSSEHGGDVQKWLSSPFPSSSFSPGGLAPEISPLEVLERDKVTQLLLQQDKVPEPAS
LSSNHSLTSCFTNQGYFFFHLPDALEIEACQVYFTYDPYSEEDPDEGVAGAPTGSSPQPLQPLSGEDDAYCTFPSRDDLLLFSPSLL
GGPSPPSTAPGGSGAGEERMPPSLQERVPRDWDPQPLGPPTPGVPDLVDFQPPPELVLREAGEEVPDAGPREGVSFPWSRPPGQGEF
RALNARLPLNTDAYLSLQELQGQDPTHLV
>sp|Q49AM3|TTC31_HUMAN
MAPIPKTVGRIKLDCSLRPSCPLEVAAAPKLCKEFGPEDYGEEDIVDFLRRLVESDPQGLHRIHVDGSSGRLQLWHHDYLLGHLDDE
GKSTGQSDRGKGAEGLGTYCGLRKSFLYPPQESEPCPQSPSASATFPSVSDSLLQVAMPQKLLVTEEEANRLAEELVAEEERMKQKA
EKKRLKKKRQKERKRQERLEQYCGEPKASTTSDGDESPPSSPGNPVQGQCGEEEDSLDLSSTFVSLALRKVGDWPLSARREKGLNQE
PQGRGLALQKMGQEEESPPREERPQQSPKVQASPGLLAAALQQSQELAKLGTSFAQNGFYHEAVVLFTQALKLNPQDHRLFGNRSFC
HERLGQPAWALADAQVALTLRPGWPRGLFRLGKALMGLQRFREAAAVFQETLRGGSQPDAARELRSCLLHLTLQGQRGGICAPPLSP
GALQPLPHAELAPSGLPSLRCPRSTALRSPGLSPLLHYPSCHRSHPNQPLSQTQSRRPHPLKPQDPSKGWDILGLGLQHLSQAR
>sp|Q96A83|COQA1_HUMAN
MKLALLLPWACCCLCGSALATGFLYPFSAAALQQHGYPEPGAGSPGSGYASRRHWCHHTVTRTVSCQVQNGSETVVQRVYQSCRWPG
PCANLVSYRTLIRPTYRVSYRTVTVLEWRCCPGFTGSNCDEECMNCTRLSDMSERLTTLEAKVLLLEAAERPSSPDNDLPAPESTPP
TWNEDFLPDAIPLAHPVPRQRRPTGPAGPPGQTGPPGPAGPPGSKGDRGQTGEKGPAGPPGLLGPPGPRGLPGEMGRPGPPGPPGPA
GNPGPSPNSPQGALYSLQPPTDKDNGDSRLASAIVDTVLAGVPGPRGPPGPPGPPGPRGPPGPPGTPGSQGLAGERGTVGPSGEPGV
KGEEGEKAATAEGEGVQQLREALKILAERVLILEHMIGIHDPLASPEGGSGQDAALRANLKMKRGGAQPDGVLAALLGPDPGQKSVD
QASSRK
>sp|P05305|EDN1_HUMAN
MDYLLMIFSLLFVACQGAPETAVLGAELSAVGENGGEKPTPSPPWRLRRSKRCSCSSLMDKECVYFCHLDIIWVNTPEHVVPYGLGS
PRSKRALENLLPTKATDRENRCQCASQKDKKCWNFCQAGKELRAEDIMEKDWNNHKKGKDCSKLGKKCIYQQLVRGRKIRRSSEEHL
RQTRSETMRNSVKSSFHDPKLKGKPSRERYVTHNRAHW
>sp|Q5GH77|XKR3_HUMAN
METVFEEMDEESTGGVSSSKEEIVLGQRLHLSFPFSIIFSTVLYCGEVAFGLYMFEIYRKANDTFWMSFTISFIIVGAILDQIILMF
FNKDLRRNKAALLFWHILLLGPIVRCLHTIRNYHKWLKNLKQEKEETQVSITKRNTMLEREIAFSIRDNFMQQKAFKYMSVIQAFLG
SVPQLILQMYISLTIREWPLNRALLMTFSLLSVTYGAIRCNILAIQISNDDTTIKLPPIEFFCVVMWRFLEVISRVVTLAFFIASLK
LKSLPVLLIIYFVSLLAPWLEFWKSGAHLPGNKENNSNMVGTVLMLFLITLLYAAINFSCWSAVKLQLSDDKIIDGRQRWGHRILHY
SFQFLENVIMILVFRFFGGKTLLNCCDSLIAVQLIISYLLATGFMLLFYQYLYPWQSGKVLPGRTENQPEAPYYYVNIEKTEKNKNK
QLRNYCHSCNRVGYFSIRKSMTCS
>sp|Q4G0Z9|MCMD2_HUMAN
MSNLKMKEAALIYLDRSGGLQKFIDDCKYYNDSKQSYAVYRFKILINPSDVVELDAELGNHILHQPLKAAEVFQSVCFIAVKTLSLI
GQLQTETQINIVLKLTHLPPLPSYGLDLCEFPLDYTSQRFYMMQGIVIAMTTITKYTQGARFLCSDEACPLSKGFQYIRVHVPGATE
SATIRNDFLCNLCASSLQEDRKFRVLGDKQIVEIIATKALRAFQGYSNNQPFRFQSLTIFLRDESVNKMNIGNEYKIIGIPTCVKTS
QTAVCIEANSITFCNSKVPSGISDNFRCLLSLTSSSCWKFTAILANIFASQITPPGTYNLLKLCLLMSLVQTTDRNKELEDCLDILI
ITSDTLLIDRLLNFSINLVPRGIRHLVSTEIFPTLSRNKYGTGAVSIQAGSALLAKGGICFIGDLASHKKDKLEQLQTVLESRSITV
YIPGKKFGEDIDQQMTFPVQCSFWSFVDVDSSSRRNAQKINTLIGQMDCSLIPANLVEAFGLLINCNESSPCHPFLPTVQHTLNKAI

NPEGLFYAASRQFTTEDFEKLLAFAKNLNVEFSLEAERMTHGYYLASRRIRTGSVCGSKLSASALKYLVFLSEAHARLNLRNKVLKE
DVLIAALLFETSLTLKYGATVFCVAPNAVFPFELYNEEYLEQRDLYLTQCQQQLEQFIATYGPGTTIFSSDE
>sp|Q4G112|HSF5_HUMAN
MEALLSTPINPNNFPAKLWRLVNSPRYRSIRWDGRGEGLLIDQPLFEAELLSPPGPGGGGGTAGAGAEPELFKTTSFTSFIRQLNLY
GFRKVVLGGPGGGKPAGNGPLHHFHNPHFRRDQPQLLVHLKRLTSANKAKLAAGLEVPCRPPNRFQRLLITSASAATAPLQHQQPPP
PAGPRPEPHGPVAVGQFHRSFRRDSLSPYSCVSTPSHDHSTYPLKGLDRTPVPHRIWQNSLGMHPGQVETSPTFSDKGVPFPVLQRF
PTEVTYTLQPSTTSVHVQQGPQTMVSSSQKYSNYTPSAQYSQAYYPTAVLQCCSPTHMDALSSCVTPNASSYAHCNYFQNPSMQSSY
PVEFLPSNWPCSTTDENTKTEVNLEAVFQIVDELHSSPKLEMVKVEPVENQCPTSPSYRGQHILANSNNSNPCSASQASQLEPLTPV
GSDIMSFVVGTEQAVACSLPQSPEYIYTIHTAQPVENSTIQESAAIQQAHVKLKEHLNHNPSPSSVVFVQEGPPFSTHQVDANIKCQ
TSSRENILPSEQMGFLISEMGPASKPSEDTGLATPARYREHRSNSQQGKSPDLHLLVDVACKQERFPKEEELKE
>sp|Q8N4P3|MESH1_HUMAN
MGSEAAQLLEAADFAARKHRQQRRKDPEGTPYINHPIGVARILTHEAGITDIVVLQAALLHDTVEDTDTTLDEVELHFGAQVRRLVE
EVTDDKTLPKLERKRLQVEQAPHSSPGAKLVKLADKLYNLRDLNRCTPEGWSEHRVQEYFEWAAQVVKGLQGTNRQLEEALKHLFKQ
RGLTI
>sp|Q96HE7|ERO1A_HUMAN
MGRGWGFLFGLLGAVWLLSSGHGEEQPPETAAQRCFCQVSGYLDDCTCDVETIDRFNNYRLFPRLQKLLESDYFRYYKVNLKRPCPF
WNDISQCGRRDCAVKPCQSDEVPDGIKSASYKYSEEANNLIEECEQAERLGAVDESLSEETQKAVLQWTKHDDSSDNFCEADDIQSP
EAEYVDLLLNPERYTGYKGPDAWKIWNVIYEENCFKPQTIKRPLNPLASGQGTSEENTFYSWLEGLCVEKRAFYRLISGLHASINVH
LSARYLLQETWLEKKWGHNITEFQQRFDGILTEGEGPRRLKNLYFLYLIELRALSKVLPFFERPDFQLFTGNKIQDEENKMLLLEIL
HEIKSFPLHFDENSFFAGDKKEAHKLKEDFRLHFRNISRIMDCVGCFKCRLWGKLQTQGLGTALKILFSEKLIANMPESGPSYEFHL
TRQEIVSLFNAFGRISTSVKELENFRNLLQNIH
>sp|Q9TNN7|1C05_HUMAN
MRVMAPRTLILLLSGALALTETWACSHSMRYFYTAVSRPGRGEPRFIAVGYVDDTQFVQFDSDAASPRGEPRAPWVEQEGPEYWDRE
TQKYKRQAQTDRVNLRKLRGYYNQSEAGSHTLQRMYGCDLGPDGRLLRGYNQFAYDGKDYIALNEDLRSWTAADKAAQITQRKWEAA
REAEQRRAYLEGTCVEWLRRYLENGKKTLQRAEHPKTHVTHHPVSDHEATLRCWALGFYPAEITLTWQRDGEDQTQDTELVETRPAG
DGTFQKWAAVVVPSGEEQRYTCHVQHEGLPEPLTLRWGPSSQPTIPIVGIVAGLAVLAVLAVLGAVMAVVMCRRKSSGGKGGSCSQA
ASSNSAQGSDESLIACKA
>sp|O14908|GIPC1_HUMAN
MPLGLGRRKKAPPLVENEEAEPGRGGLGVGEPGPLGGGGSGGPQMGLPPPPPALRPRLVFHTQLAHGSPTGRIEGFTNVKELYGKIA
EAFRLPTAEVMFCTLNTHKVDMDKLLGGQIGLEDFIFAHVKGQRKEVEVFKSEDALGLTITDNGAGYAFIKRIKEGSVIDHIHLISV
GDMIEAINGQSLLGCRHYEVARLLKELPRGRTFTLKLTEPRKAFDMISQRSAGGRPGSGPQLGTGRGTLRLRSRGPATVEDLPSAFE
EKAIEKVDDLLESYMGIRDTELAATMVELGKDKRNPDELAEALDERLGDFAFPDEFVFDVWGAIGDAKVGRY
>sp|Q5NE16|CATL3_HUMAN
MKMIEQHNQEYREGKHSFTMAMNAFGEMTSEEFRQVVNGFQNQKHRKGKVLQEPLLHDIRKSVDWREKGYVTPVKDQCNWGSVRTDV
RKTEKLVSLSVQTWWTALGFKAMLAAFLENHYFASSMLPTMEAWTLRKPFHMKKSSGDWKVQGHRGASGESLLASGESQQSPEVAQY
SGKHQVQCHLIEEALQMLSGGDEDHDEDKWPHDMRNHLAGEAQV
>sp|Q9HB40|RISC_HUMAN
MELALRRSPVPRWLLLLPLLLGLNAGAVIDWPTEEGKEVWDYVTVRKDAYMFWWLYYATNSCKNFSELPLVMWLQGGPGGSSTGFGN
FEEIGPLDSDLKPRKTTWLQAASLLFVDNPVGTGFSYVNGSGAYAKDLAMVASDMMVLLKTFFSCHKEFQTVPFYIFSESYGGKMAA
GIGLELYKAIQRGTIKCNFAGVALGDSWISPVDSVLSWGPYLYSMSLLEDKGLAEVSKVAEQVLNAVNKGLYREATELWGKAEMIIE
QNTDGVNFYNILTKSTPTSTMESSLEFTQSHLVCLCQRHVRHLQRDALSQLMNGPIRKKLKIIPEDQSWGGQATNVFVNMEEDFMKP
VISIVDELLEAGINVTVYNGQLDLIVDTMGQEAWVRKLKWPELPKFSQLKWKALYSDPKSLETSAFVKSYKNLAFYWILKAGHMVPS
DQGDMALKMMRLVTQQE
>sp|Q687X5|STEA4_HUMAN

MEKTCIDALPLTMNSSEKQETVCIFGTGDFGRSLGLKMLQCGYSVVFGSRNPQKTTLLPSGAEVLSYSEAAKKSGIIIIAIHREHYD
FLTELTEVLNGKILVDISNNLKINQYPESNAEYLAHLVPGAHVVKAFNTISAWALQSGALDASRQVFVCGNDSKAKQRVMDIVRNLG
LTPMDQGSLMAAKEIEKYPLQLFPMWRFPFYLSAVLCVFLFFYCVIRDVIYPYVYEKKDNTFRMAISIPNRIFPITALTLLALVYLP
GVIAAILQLYRGTKYRRFPDWLDHWMLCRKQLGLVALGFAFLHVLYTLVIPIRYYVRWRLGNLTVTQAILKKENPFSTSSAWLSDSY
VALGILGFFLFVLLGITSLPSVSNAVNWREFRFVQSKLGYLTLILCTAHTLVYGGKRFLSPSNLRWYLPAAYVLGLIIPCTVLVIKF
VLIMPCVDNTLTRIRQGWERNSKH
>sp|P55291|CAD15_HUMAN
MDAAFLLVLGLLAQSLCLSLGVPGWRRPTTLYPWRRAPALSRVRRAWVIPPISVSENHKRLPYPLVQIKSDKQQLGSVIYSIQGPGV
DEEPRGVFSIDKFTGKVFLNAMLDREKTDRFRLRAFALDLGGSTLEDPTDLEIVVVDQNDNRPAFLQEAFTGRVLEGAVPGTYVTRA
EATDADDPETDNAALRFSILQQGSPELFSIDELTGEIRTVQVGLDREVVAVYNLTLQVADMSGDGLTATASAIITLDDINDNAPEFT
RDEFFMEAIEAVSGVDVGRLEVEDRDLPGSPNWVARFTILEGDPDGQFTIRTDPKTNEGVLSIVKALDYESCEHYELKVSVQNEAPL
QAAALRAERGQAKVRVHVQDTNEPPVFQENPLRTSLAEGAPPGTLVATFSARDPDTEQLQRLSYSKDYDPEDWLQVDAATGRIQTQH
VLSPASPFLKGGWYRAIVLAQDDASQPRTATGTLSIEILEVNDHAPVLAPPPPGSLCSEPHQGPGLLLGATDEDLPPHGAPFHFQLS
PRLPELGRNWSLSQVNVSHARLRPRHQVPEGLHRLSLLLRDSGQPPQQREQPLNVTVCRCGKDGVCLPGAAALLAGGTGLSLGALVI
VLASALLLLVLVLLVALRARFWKQSRGKGLLHGPQDDLRDNVLNYDEQGGGEEDQDAYDISQLRHPTALSLPLGPPPLRRDAPQGRL
HPQPPRVLPTSPLDIADFINDGLEAADSDPSVPPYDTALIYDYEGDGSVAGTLSSILSSQGDEDQDYDYLRDWGPRFARLADMYGHP
CGLEYGARWDHQAREGLSPGALLPRHRGRTA
>sp|P07814|SYEP_HUMAN
MATLSLTVNSGDPPLGALLAVEHVKDDVSISVEEGKENILHVSENVIFTDVNSILRYLARVATTAGLYGSNLMEHTEIDHWLEFSAT
KLSSCDSFTSTINELNHCLSLRTYLVGNSLSLADLCVWATLKGNAAWQEQLKQKKAPVHVKRWFGFLEAQQAFQSVGTKWDVSTTKA
RVAPEKKQDVGKFVELPGAEMGKVTVRFPPEASGYLHIGHAKAALLNQHYQVNFKGKLIMRFDDTNPEKEKEDFEKVILEDVAMLHI
KPDQFTYTSDHFETIMKYAEKLIQEGKAYVDDTPAEQMKAEREQRIDSKHRKNPIEKNLQMWEEMKKGSQFGQSCCLRAKIDMSSNN
GCMRDPTLYRCKIQPHPRTGNKYNVYPTYDFACPIVDSIEGVTHALRTTEYHDRDEQFYWIIEALGIRKPYIWEYSRLNLNNTVLSK
RKLTWFVNEGLVDGWDDPRFPTVRGVLRRGMTVEGLKQFIAAQGSSRSVVNMEWDKIWAFNKKVIDPVAPRYVALLKKEVIPVNVPE
AQEEMKEVAKHPKNPEVGLKPVWYSPKVFIEGADAETFSEGEMVTFINWGNLNITKIHKNADGKIISLDAKLNLENKDYKKTTKVTW
LAETTHALPIPVICVTYEHLITKPVLGKDEDFKQYVNKNSKHEELMLGDPCLKDLKKGDIIQLQRRGFFICDQPYEPVSPYSCKEAP
CVLIYIPDGHTKEMPTSGSKEKTKVEATKNETSAPFKERPTPSLNNNCTTSEDSLVLYNRVAVQGDVVRELKAKKAPKEDVDAAVKQ
LLSLKAEYKEKTGQEYKPGNPPAEIGQNISSNSSASILESKSLYDEVAAQGEVVRKLKAEKSPKAKINEAVECLLSLKAQYKEKTGK
EYIPGQPPLSQSSDSSPTRNSEPAGLETPEAKVLFDKVASQGEVVRKLKTEKAPKDQVDIAVQELLQLKAQYKSLIGVEYKPVSATG
AEDKDKKKKEKENKSEKQNKPQKQNDGQRKDPSKNQGGGLSSSGAGEGQGPKKQTRLGLEAKKEENLADWYSQVITKSEMIEYHDIS
GCYILRPWAYAIWEAIKDFFDAEIKKLGVENCYFPMFVSQSALEKEKTHVADFAPEVAWVTRSGKTELAEPIAIRPTSETVMYPAYA
KWVQSHRDLPIKLNQWCNVVRWEFKHPQPFLRTREFLWQEGHSAFATMEEAAEEVLQILDLYAQVYEELLAIPVVKGRKTEKEKFAG
GDYTTTIEAFISASGRAIQGGTSHHLGQNFSKMFEIVFEDPKIPGEKQFAYQNSWGLTTRTIGVMTMVHGDNMGLVLPPRVACVQVV
IIPCGITNALSEEDKEALIAKCNDYRRRLLSVNIRVRADLRDNYSPGWKFNHWELKGVPIRLEVGPRDMKSCQFVAVRRDTGEKLTV
AENEAETKLQAILEDIQVTLFTRASEDLKTHMVVANTMEDFQKILDSGKIVQIPFCGEIDCEDWIKKTTARDQDLEPGAPSMGAKSL
CIPFKPLCELQPGAKCVCGKNPAKYYTLFGRSY
>sp|O14843|FFAR3_HUMAN
MDTGPDQSYFSGNHWFVFSVYLLTFLVGLPLNLLALVVFVGKLQRRPVAVDVLLLNLTASDLLLLLFLPFRMVEAANGMHWPLPFIL
CPLSGFIFFTTIYLTALFLAAVSIERFLSVAHPLWYKTRPRLGQAGLVSVACWLLASAHCSVVYVIEFSGDISHSQGTNGTCYLEFR
KDQLAILLPVRLEMAVVLFVVPLIITSYCYSRLVWILGRGGSHRRQRRVAGLLAATLLNFLVCFGPYNVSHVVGYICGESPAWRIYV
TLLSTLNSCVDPFVYYFSSSGFQADFHELLRRLCGLWGQWQQESSMELKEQKGGEEQRADRPAERKTSEHSQGCGTGGQVACAES
>sp|Q03403|TFF2_HUMAN
MGRRDAQLLAALLVLGLCALAGSEKPSPCQCSRLSPHNRTNCGFPGITSDQCFDNGCCFDSSVTGVPWCFHPLPKQESDQCVMEVSD
RRNCGYPGISPEECASRKCCFSNFIFEVPWCFFPKSVEDCHY

>sp|Q8NGX9|OR6P1_HUMAN
MRNLSGGHVEEFVLVGFPTTPPLQLLLFVLFFAIYLLTLLENALIVFTIWLAPSLHRPMYFFLGHLSFLELWYINVTIPRLLAAFLT
QDGRVSYVGCMTQLYFFIALACTECVLLAVMAYDRYLAICGPLLYPSLMPSSLATRLAAASWGSGFFSSMMKLLFISQLSYCGPNII
NHFFCDISPLLNLTCSDKEQAELVDFLLALVMILLPLLAVVSSYTAIIAAILRIPTSRGRHKAFSTCAAHLAVVVIYYSSTLFTYAR
PRAMYTFNHNKIISVLYTIIVPFFNPAIYCLRNKEVKEAFRKTVMGRCHYPRDVQD
>sp|Q6S5H4|POTEB_HUMAN
MVAEVCSMPAASAVKKPFDLRSKMGKWCHHRFPCCRGSGKSNMGTSGDHDDSFMKTLRSKMGKCCHHCFPCCRGSGTSNVGTSGDHD
NSFMKTLRSKMGKWCCHCFPCCRGSGKSNVGTWGDYDDSAFMEPRYHVRREDLDKLHRAAWWGKVPRKDLIVMLRDTDMNKRDKQKR
TALHLASANGNSEVVQLLLDRRCQLNVLDNKKRTALIKAVQCQEDECVLMLLEHGADGNIQDEYGNTALHYAIYNEDKLMAKALLLY
GADIESKNKCGLTPLLLGVHEQKQQVVKFLIKKKANLNALDRYGRTALILAVCCGSASIVNLLLEQNVDVSSQDLSGQTAREYAVSS
HHHVICELLSDYKEKQMLKISSENSNPEQDLKLTSEEESQRLKVSENSQPEKMSQEPEINKDCDREVEEEIKKHGSNPVGLPENLTN
GASAGNGDDGLIPQRKSRKPENQQFPDTENEEYHSDEQNDTQKQLSEEQNTGISQDEILTNKQKQIEVAEKEMNSKLSLSHKKEEDL
LRENSMLREEIAMLRLELDETKHQNQLRENKILEEIESVKEKLLKAIQLNEEALTKTSI
>sp|Q8TEE9|SAP25_HUMAN
MTPLAPWDPKYEAKAGPRPVWGANCSSGASFSGRTLCHPSFWPLYEAASGRGLRPVAPATGHWNGQQAPPDAGFPVVCCEDVFLSDP
LLPRGQRVPLYLSKAPQQMMGSLKLLPPPPIMSARVLPRPSPSRGPSTAWLSGPELIALTGLLQMSQGEPRPSSSAVGPPDHTSDPP
SPCGSPSSSQGADLSLPQTPDTHCP
>sp|Q9NSD7|RL3R1_HUMAN
MQMADAATIATMNKAAGGDKLAELFSLVPDLLEAANTSGNASLQLPDLWWELGLELPDGAPPGHPPGSGGAESADTEARVRILISVV
YWVVCALGLAGNLLVLYLMKSMQGWRKSSINLFVTNLALTDFQFVLTLPFWAVENALDFKWPFGKAMCKIVSMVTSMNMYASVFFLT
AMSVTRYHSVASALKSHRTRGHGRGDCCGRSLGDSCCFSAKALCVWIWALAALASLPSAIFSTTVKVMGEELCLVRFPDKLLGRDRQ
FWLGLYHSQKVLLGFVLPLGIIILCYLLLVRFIADRRAAGTKGGAAVAGGRPTGASARRLSKVTKSVTIVVLSFFLCWLPNQALTTW
SILIKFNAVPFSQEYFLCQVYAFPVSVCLAHSNSCLNPVLYCLVRREFRKALKSLLWRIASPSITSMRPFTATTKPEHEDQGLQAPA
PPHAAAEPDLLYYPPGVVVYSGGRYDLLPSSSAY
>sp|P56178|DLX5_HUMAN
MTGVFDRRVPSIRSGDFQAPFQTSAAMHHPSQESPTLPESSATDSDYYSPTGGAPHGYCSPTSASYGKALNPYQYQYHGVNGSAGSY
PAKAYADYSYASSYHQYGGAYNRVPSATNQPEKEVTEPEVRMVNGKPKKVRKPRTIYSSFQLAALQRRFQKTQYLALPERAELAASL
GLTQTQVKIWFQNKRSKIKKIMKNGEMPPEHSPSSSDPMACNSPQSPAVWEPQGSSRSLSHHPHAHPPTSNQSPASSYLENSASWYT
SAASSINSHLPPPGSLQHPLALASGTLY
>sp|Q9H1M0|N62CL_HUMAN
MQFTSISNSLTSTAAIGLSFTTSTTTTATFTTNTTTTITSGFTVNQNQLLSRGFENLVPYTSTVSVVATPVMTYGHLEGLINEWNLE
LEDQEKYFLLQATQVNAWDHTLIENGEMIRILHGEVNKVKLDQKRLEQELDFILSQQQELEFLLTYLEESTRDQSGLHYLQDADEEH
VEISTRSAEF
>sp|P78318|IGBP1_HUMAN
MAAEDELQLPRLPELFETGRQLLDEVEVATEPAGSRIVQEKVFKGLDLLEKAAEMLSQLDLFSRNEDLEEIASTDLKYLLVPAFQGA
LTMKQVNPSKRLDHLQRAREHFINYLTQCHCYHVAEFELPKTMNNSAENHTANSSMAYPSLVAMASQRQAKIQRYKQKKELEHRLSA
MKSAVESGQADDERVREYYLLHLQRWIDISLEEIESIDQEIKILRERDSSREASTSNSSRQERPPVKPFILTRNMAQAKVFGAGYPS
LPTMTVSDWYEQHRKYGALPDQGIAKAAPEEFRKAAQQQEEQEEKEEEDDEQTLHRAREWDDWKDTHPRGYGNRQNMG
>sp|Q6ZW33|MICLK_HUMAN
MSPPKDPSPSLPLPSSSSHSSSPPSSSSTSVSGNAPDGSSPPQMTASEPLSQVSRGHPSPPTPNFRRRAVAQGAPREIPLYLPHHPK
PEWAEYCLVSPGEDGLSDPAEMTSDECQPAEAPLGDIGSNHRDPHPIWGKDRSWTGQELSPLAGEDREKGSTGARKEEEGGPVLVKE
KLGLKKLVLTQEQKTMLLDWNDSIPESVHLKAGERISQKSAENGRGGRVLKPVRPLLLPRAAGEPLPTQRGAQEKMGTPAEQAQGER
NVPPPKSPLRLIANAIRRSLEPLLSNSEGGKKAWAKQESKTLPAQACTRSFSLRKTNSNKDGDQHSPGRNQSSAFSPPDPALRTHSL
PNRPSKVFPALRSPPCSKIEDVPTLLEKVSLQENFPDASKPPKKRISLFSSLRLKDKSFESFLQESRQRKDIRDLFGSPKRKVLPED

SAQALEKLLQPFKSTSLRQAAPPPPPPPPPPPPPPTAGGADSKNFPLRAQVTEASSSASSTSSSSADEEFDPQLSLQLKEKKTLRRR
KKLEKAMKQLVKQEELKRLYKAQAIQRQLEEVEERQRASEIQGVRLEKALRGEADSGTQDEAQLLQEWFKLVLEKNKLMRYESELLI
MAQELELEDHQSRLEQKLREKMLKEESQKDEKDLNEEQEVFTELMQVIEQRDKLVDSLEEQRIREKAEDQHFESFVFSRGCQLSRT
>sp|P36404|ARL2_HUMAN
MGLLTILKKMKQKERELRLLMLGLDNAGKTTILKKFNGEDIDTISPTLGFNIKTLEHRGFKLNIWDVGGQKSLRSYWRNYFESTDGL
IWVVDSADRQRMQDCQRELQSLLVEERLAGATLLIFANKQDLPGALSSNAIREVLELDSIRSHHWCIQGCSAVTGENLLPGIDWLLD
DISSRIFTAD
>sp|Q86W11|ZSC30_HUMAN
MSGEATVLAYHAPEEQEGLLVVKVEEENYVLDQDFGLQENPWSQEVFRQKFRQFSYSDSTGPREALSRLRELCCQWLRPEVHSKEQI
LELLMLEQFLAILPEELQAWLREHRPENGEEAVTMLEELEKELEEPRQQDTTHGQEMFWQEMTSTGALKSLSLNSPVQPLENQCKTE
TQESQAFQERDGRMVAGKVLMAKQEIVECVASAAMISPGKLPGETHSQRIAEEALGGLDNSKKQKGNAAGNKISQLPSQDRHFSLAT
FNRRIPTEHSVLESHESEGSFSMNSNDITQQSVDTREKLYECFDCGKAFCQSSKLIRHQRIHTGERPYACKECGKAFSLSSDLVRHQ
RIHSGEKPYECCECGKAFRGSSELIRHRRIHTGEKPYECGECGKAFSRSSALIQHKKIHTGDKSYECIACGKAFGRSSILIEHQRIH
TGEKPYECNECGKSFNQSSALTQHQRIHTGEKPYECSECRKTFRHRSGLMQHQRTHTRV
>sp|Q9BZF9|UACA_HUMAN
MKSLKSRLRRQDVPGPASSGAAAASAHAADWNKYDDRLMKAAERGDVEKVTSILAKKGVNPGKLDVEGRSVFHVVTSKGNLECLNAI
LIHGVDITTSDTAGRNALHLAAKYGHALCLQKLLQYNCPTEHADLQGRTALHDAAMADCPSSIQLLCDHGASVNAKDVDGRTPLVLA
TQMSRPTICQLLIDRGADVNSRDKQNRTALMLGCEYGCRDAVEVLIKNGADISLLDALGHDSSYYARIGDNLDILTLLKTASENTNK
GRELWKKGPSLQQRNLTHMQDEVNVKSHQREHQNIQDLEIENEDLKERLRKIQQEQRILLDKVNGLQLQLNEEVMVADDLESEREKL
KSLLAAKEKQHEESLRTIEALKNRFKYFESDHLGSGSHFSNRKEDMLLKQGQMYMADSQCTSPGIPAHMQSRSMLRPLELSLPSQTS
YSENEILKKELEAMRTFCESAKQDRLKLQNELAHKVAECKALALECERVKEDSDEQIKQLEDALKDVQKRMYESEGKVKQMQTHFLA
LKEHLTSEAASGNHRLTEELKDQLKDLKVKYEGASAEVGKLRNQIKQNEMIVEEFKRDEGKLIEENKRLQKELSMCEMEREKKGRKV
TEMEGQAKELSAKLALSIPAEKFENMKSSLSNEVNEKAKKLVEMEREHEKSLSEIRQLKRELENVKAKLAQHVKPEEHEQVKSRLEQ
KSGELGKKITELTLKNQTLQKEIEKVYLDNKLLKEQAHNLTIEMKNHYVPLKVSEDMKKSHDAIIDDLNRKLLDVTQKYTEKKLEME
KLLLENDSLSKDVSRLETVFVPPEKHEKEIIALKSNIVELKKQLSELKKKCGEDQEKIHALTSENTNLKKMMSNQYVPVKTHEEVKM
TLNDTLAKTNRELLDVKKKFEDINQEFVKIKDKNEILKRNLENTQNQIKAEYISLAEHEAKMSSLSQSMRKVQDSNAEILANYRKGQ
EEIVTLHAEIKAQKKELDTIQECIKVKYAPIVSFEECERKFKATEKELKDQLSEQTQKYSVSEEEVKKNKQENDKLKKEIFTLQKDL
RDKTVLIEKSHEMERALSRKTDELNKQLKDLSQKYTEVKNVKEKLVEENAKQTSEILAVQNLLQKQHVPLEQVEALKKSLNGTIENL
KEELKSMQRCYEKEQQTVTKLHQLLENQKNSSVPLAEHLQIKEAFEKEVGIIKASLREKEEESQNKMEEVSKLQSEVQNTKQALKKL
ETREVVDLSKYKATKSDLETQISSLNEKLANLNRKYEEVCEEVLHAKKKEISAKDEKELLHFSIEQEIKDQKERCDKSLTTITELQR
RIQESAKQIEAKDNKITELLNDVERLKQALNGLSQLTYTSGNPTKRQSQLIDTLQHQVKSLEQQLADADRQHQEVIAIYRTHLLSAA
QGHMDEDVQEALLQIIQMRQGLVC
>sp|Q6ICL7|S35E4_HUMAN
MCRCPPEHHDGRMTSAEVGAAAGGAQAAGPPEWPPGSPQALRQPGRARVAMAALVWLLAGASMSSLNKWIFTVHGFGRPLLLSALHM
LVAALACHRGARRPMPGGTRCRVLLLSLTFGTSMACGNVGLRAVPLDLAQLVTTTTPLFTLALSALLLGRRHHPLQLAAMGPLCLGA
ACSLAGEFRTPPTGCGFLLAATCLRGLKSVQQSALLQEERLDAVTLLYATSLPSFCLLAGAALVLEAGVAPPPTAGDSRLWACILLS
CLLSVLYNLASFSLLALTSALTVHVLGNLTVVGNLILSRLLFGSRLSALSYVGIALTLSGMFLYHNCEFVASWAARRGLWRRDQPSK
GL
>sp|P11137|MTAP2_HUMAN
MADERKDEAKAPHWTSAPLTEASAHSHPPEIKDQGGAGEGLVRSANGFPYREDEEGAFGEHGSQGTYSNTKENGINGELTSADRETA
EEVSARIVQVVTAEAVAVLKGEQEKEAQHKDQTAALPLAAEETANLPPSPPPSPASEQTVTVEEDLLTASKMEFHDQQELTPSTAEP
SDQKEKESEKQSKPGEDLKHAALVSQPETTKTYPDKKDMQGTEEEKAPLALFGHTLVASLEDMKQKTEPSLVVPGIDLPKEPPTPKE
QKDWFIEMPTEAKKDEWGLVAPISPGPLTPMREKDVFDDIPKWEGKQFDSPMPSPFQGGSFTLPLDVMKNEIVTETSPFAPAFLQPD
DKKSLQQTSGPATAKDSFKIEEPHEAKPDKMAEAPPSEAMTLPKDAHIPVVEEHVMGKVLEEEKEAINQETVQQRDTFTPSGQEPIL

TEKETELKLEEKTTISDKEAVPKESKPPKPADEEIGIIQTSTEHTFSEQKDQEPTTDMLKQDSFPVSLEQAVTDSAMTSKTLEKAMT
EPSALIEKSSIQELFEMRVDDKDKIEGVGAATSAELDMPFYEDKSGMSKYFETSALKEEATKSIEPGSDYYELSDTRESVHESIDTM
SPMHKNGDKEFQTGKESQPSPPAQEAGYSTLAQSYPSDLPEEPSSPQERMFTIDPKVYGEKRDLHSKNKDDLTLSRSLGLGGRSAIE
QRSMSINLPMSCLDSIALGFNFGRGHDLSPLASDILTNTSGSMDEGDDYLPATTPALEKAPCFPVESKEEEQIEKVKATGEESTQAE
ISCESPFLAKDFYKNGTVMAPDLPEMLDLAGTRSRLASVSADAEVARRKSVPSETVVEDSRTGLPPVTDENHVIVKTDSQLEDLGYC
VFNKYTVPLPSPVQDSENLSGESGTFYEGTDDKVRRDLATDLSLIEVKLAAAGRVKDEFSVDKEASAHISGDKSGLSKEFDQEKKAN
DRLDTVLEKSEEHADSKEHAKKTEEAGDEIETFGLGVTYEQALAKDLSIPTDASSEKAEKGLSSVPEIAEVEPSKKVEQGLDFAVQG
QLDVKISDFGQMASGLNIDDRRATELKLEATQDMTPSSKAPQEADAFMGVESGHMKEGTKVSETEVKEKVAKPDLVHQEAVDKEESY
ESSGEHESLTMESLKADEGKKETSPESSLIQDEIAVKLSVEIPCPPAVSEADLATDERADVQMEFIQGPKEESKETPDISITPSDVA
EPLHETIVSEPAEIQSEEEEIEAQGEYDKLLFRSDTLQITDLGVSGAREEFVETCPSEHKGVIESVVTIEDDFITVVQTTTDEGESG
SHSVRFAALEQPEVERRPSPHDEEEFEVEEAAEAQAEPKDGSPEAPASPEREEVALSEYKTETYDDYKDETTIDDSIMDADSLWVDT
QDDDRSIMTEQLETIPKEEKAEKEARRSSLEKHRKEKPFKTGRGRISTPERKVAKKEPSTVSRDEVRRKKAVYKKAELAKKTEVQAH
SPSRKFILKPAIKYTRPTHLSCVKRKTTAAGGESALAPSVFKQAKDKVSDGVTKSPEKRSSLPRPSSILPPRRGVSGDRDENSFSLN
SSISSSARRTTRSEPIRRAGKSGTSTPTTPGSTAITPGTPPSYSSRTPGTPGTPSYPRTPHTPGTPKSAILVPSEKKVAIIRTPPKS
PATPKQLRLINQPLPDLKNVKSKIGSTDNIKYQPKGGQVQIVTKKIDLSHVTSKCGSLKNIRHRPGGGRVKIESVKLDFKEKAQAKV
GSLDNAHHVPGGGNVKIDSQKLNFREHAKARVDHGAEIITQSPGRSSVASPRRLSNVSSSGSINLLESPQLATLAEDVTAALAKQGL
>sp|Q86SH4|PRNT_HUMAN
MQHSLVFFFAVILHLSHLLHLDASIHPFRLPFSSKPFLLIPMSNTTLPHTAWPLSFLHQTVSTLKAVAVTHSLWHLQIPVDCQACNR
KSKKIYC
>sp|Q8IWA4|MFN1_HUMAN
MAEPVSPLKHFVLAKKAITAIFDQLLEFVTEGSHFVEATYKNPELDRIATEDDLVEMQGYKDKLSIIGEVLSRRHMKVAFFGRTSSG
KSSVINAMLWDKVLPSGIGHITNCFLSVEGTDGDKAYLMTEGSDEKKSVKTVNQLAHALHMDKDLKAGCLVRVFWPKAKCALLRDDL
VLVDSPGTDVTTELDSWIDKFCLDADVFVLVANSESTLMNTEKHFFHKVNERLSKPNIFILNNRWDASASEPEYMEDVRRQHMERCL
HFLVEELKVVNALEAQNRIFFVSAKEVLSARKQKAQGMPESGVALAEGFHARLQEFQNFEQIFEECISQSAVKTKFEQHTIRAKQIL
ATVKNIMDSVNLAAEDKRHYSVEEREDQIDRLDFIRNQMNLLTLDVKKKIKEVTEEVANKVSCAMTDEICRLSVLVDEFCSEFHPNP
DVLKIYKSELNKHIEDGMGRNLADRCTDEVNALVLQTQQEIIENLKPLLPAGIQDKLHTLIPCKKFDLSYNLNYHKLCSDFQEDIVF
PFSLGWSSLVHRFLGPRNAQRVLLGLSEPIFQLPRSLASTPTAPTTPATPDNASQEELMITLVTGLASVTSRTSMGIIIVGGVIWKT
IGWKLLSVSLTMYGALYLYERLSWTTHAKERAFKQQFVNYATEKLRMIVSSTSANCSHQVKQQIATTFARLCQQVDITQKQLEEEIA
RLPKEIDQLEKIQNNSKLLRNKAVQLENELENFTKQFLPSSNEES
>sp|Q8NGV5|O13D1_HUMAN
MYRFTDFDVSNISIYLNHVLFYTTQQAGDLEHMETRNYSAMTEFFLVGLSQYPELQLFLFLLCLIMYMIILLGNSLLIIITILDSRL
HTPMYFFLGNLSFLDICYTSSSIPPMLIIFMSERKSISFIGCALQMVVSLGLGSTECVLLAVMAYDHYVAICNPLRYSIIMNGVLYV
QMAAWSWIIGCLTSLLQTVLTMMLPFCGNNVIDHITCEILALLKLVCSDITINVLIMTVTNIVSLVILLLLIFISYVFILSSILRIN
CAEGRKKAFSTCSAHSIVVILFYGSALFMYMKPKSKNTNTSDEIIGLSYGVVSPMLNPIIYSLRNKEVKEAVKKVLSRHLHLLKM
>sp|P14735|IDE_HUMAN
MRYRLAWLLHPALPSTFRSVLGARLPPPERLCGFQKKTYSKMNNPAIKRIGNHITKSPEDKREYRGLELANGIKVLLISDPTTDKSS
AALDVHIGSLSDPPNIAGLSHFCEHMLFLGTKKYPKENEYSQFLSEHAGSSNAFTSGEHTNYYFDVSHEHLEGALDRFAQFFLCPLF
DESCKDREVNAVDSEHEKNVMNDAWRLFQLEKATGNPKHPFSKFGTGNKYTLETRPNQEGIDVRQELLKFHSAYYSSNLMAVCVLGR
ESLDDLTNLVVKLFSEVENKNVPLPEFPEHPFQEEHLKQLYKIVPIKDIRNLYVTFPIPDLQKYYKSNPGHYLGHLIGHEGPGSLLS
ELKSKGWVNTLVGGQKEGARGFMFFIINVDLTEEGLLHVEDIILHMFQYIQKLRAEGPQEWVFQECKDLNAVAFRFKDKERPRGYTS
KIAGILHYYPLEEVLTAEYLLEEFRPDLIEMVLDKLRPENVRVAIVSKSFEGKTDRTEEWYGTQYKQEAIPDEVIKKWQNADLNGKF
KLPTKNEFIPTNFEILPLEKEATPYPALIKDTAMSKLWFKQDDKFFLPKACLNFEFFSPFAYVDPLHCNMAYLYLELLKDSLNEYAY
AAELAGLSYDLQNTIYGMYLSVKGYNDKQPILLKKIIEKMATFEIDEKRFEIIKEAYMRSLNNFRAEQPHQHAMYYLRLLMTEVAWT
KDELKEALDDVTLPRLKAFIPQLLSRLHIEALLHGNITKQAALGIMQMVEDTLIEHAHTKPLLPSQLVRYREVQLPDRGWFVYQQRN

EVHNNCGIEIYYQTDMQSTSENMFLELFCQIISEPCFNTLRTKEQLGYIVFSGPRRANGIQGLRFIIQSEKPPHYLESRVEAFLITM
EKSIEDMTEEAFQKHIQALAIRRLDKPKKLSAECAKYWGEIISQQYNFDRDNTEVAYLKTLTKEDIIKFYKEMLAVDAPRRHKVSVH
VLAREMDSCPVVGEFPCQNDINLSQAPALPQPEVIQNMTEFKRGLPLFPLVKPHINFMAAKL
>sp|P78368|KC1G2_HUMAN
MDFDKKGGKGETEEGRRMSKAGGGRSSHGIRSSGTSSGVLMVGPNFRVGKKIGCGNFGELRLGKNLYTNEYVAIKLEPIKSRAPQLH
LEYRFYKQLSATEGVPQVYYFGPCGKYNAMVLELLGPSLEDLFDLCDRTFTLKTVLMIAIQLITRMEYVHTKSLIYRDVKPENFLVG
RPGTKRQHAIHIIDFGLAKEYIDPETKKHIPYREHKSLTGTARYMSINTHLGKEQSRRDDLEALGHMFMYFLRGSLPWQGLKADTLK
ERYQKIGDTKRATPIEVLCENFPEEMATYLRYVRRLDFFEKPDYDYLRKLFTDLFDRSGFVFDYEYDWAGKPLPTPIGTVHTDLPSQ
PQLRDKTQPHSKNQALNSTNGELNADDPTAGHSNAPITAPAEVEVADETKCCCFFKRRKRKSLQRHK
>sp|O95251|KAT7_HUMAN
MPRRKRNAGSSSDGTEDSDFSTDLEHTDSSESDGTSRRSARVTRSSARLSQSSQDSSPVRNLQSFGTEEPAYSTRRVTRSQQQPTPV
TPKKYPLRQTRSSGSETEQVVDFSDRETKNTADHDESPPRTPTGNAPSSESDIDISSPNVSHDESIAKDMSLKDSGSDLSHRPKRRR
FHESYNFNMKCPTPGCNSLGHLTGKHERHFSISGCPLYHNLSADECKVRAQSRDKQIEERMLSHRQDDNNRHATRHQAPTERQLRYK
EKVAELRKKRNSGLSKEQKEKYMEHRQTYGNTREPLLENLTSEYDLDLFRRAQARASEDLEKLRLQGQITEGSNMIKTIAFGRYELD
TWYHSPYPEEYARLGRLYMCEFCLKYMKSQTILRRHMAKCVWKHPPGDEIYRKGSISVFEVDGKKNKIYCQNLCLLAKLFLDHKTLY
YDVEPFLFYVMTEADNTGCHLIGYFSKEKNSFLNYNVSCILTMPQYMRQGYGKMLIDFSYLLSKVEEKVGSPERPLSDLGLISYRSY
WKEVLLRYLHNFQGKEISIKEISQETAVNPVDIVSTLQALQMLKYWKGKHLVLKRQDLIDEWIAKEAKRSNSNKTMDPSCLKWTPPK
GT
>sp|Q96M60|F227B_HUMAN
MAGQRTCQRRSSRAGPGKMQEPPKSIEEFLKFQNWDYWPREIHFRDDDKWSCTLKKIKEDSSFVSIYTHLWENVPRIFEALLIMESK
LKEYSLILQNHTSEIFKWKSMISETSSYRKLERYGEFLKKYHKKKKIMLSDEMETEKNIEGCSFTGFKANELTQLPRHLDAEQIYLF
ILKAHNFDERVFKIWKTHFLSEASIALLHDSFWWWFLHKFRPDRENQDCLFDRISESYVTLFMSIPLSRKDAFFQIYPDCLAQAIYA
TFHEAFPESSYLFNDEFKEDLGNNIFLWCSGLKPQKGFWIHWKLKELSTTTIHGSKKAPAKSVKERIADSQEHISTSIDFNIIKILN
NPRAYTLPISKEESRLSRLATKSHYSSTGPEFNRVLFNFGGQSPLILYYLKMHELAGISKAPKKTKIKLTKIFQEPLPAPTYRDVIK
EAKRQFARNQKDFRILQAKATKKPHEVKQDFEKFLHKLRSEAEIERECVASLSSSSSSSPSSTDNYNFEEEEY
>sp|Q5R3F8|PPR29_HUMAN
MLRLGLCAAALLCVCRPGAVRADCWLIEGDKGYVWLAICSQNQPPYETIPQHINSTVHDLRLNENKLKAVLYSSLNRFGNLTDLNLT
KNEISYIEDGAFLGQSSLQVLQLGYNKLSNLTEGMLRGMSRLQFLFVQHNLIEVVTPTAFSECPSLISIDLSSNRLSRLDGATFASL
ASLMVCELAGNPFNCECDLFGFLAWLVVFNNVTKNYDRLQCESPREFAGYPLLVPRPYHSLNAITVLQAKCRNGSLPARPVSHPTPY
STDAQREPDENSGFNPDEILSVEPPASSTTDASAGPAIKLHHVTFTSATLVVIIPHPYSKMYILVQYNNSYFSDVMTLKNKKEIVTL
DKLRAHTEYTFCVTSLRNSRRFNHTCLTFTTRDPVPGDLAPSTSTTTHYIMTILGCLFGMVIVLGAVYYCLRKRRMQEEKQKSVNVK
KTILEMRYGADVDAGSIVHAAQKLGEPPVLPVSRMASIPSMIGEKLPTAKGLEAGLDTPKVATKGNYIEVRTGAGGDGLARPEDDLP
DLENGQGSAAEISTIAKEVDKVNQIINNCIDALKLDSASFLGGGSSSGDPELAFECQSLPAAAAASSATGPGALERPSFLSPPYKES
SHHPLQRQLSADAAVTRKTCSVSSSGSIKSAKVFSLDVPDHPAATGLAKGDSKYIEKGSPLNSPLDRLPLVPAGSGGGSGGGGGIHH
LEVKPAYHCSEHRHSFPALYYEEGADSLSQRVSFLKPLTRSKRDSTYSQLSPRHYYSGYSSSPEYSSESTHKIWERFRPYKKHHREE
VYMAAGHALRKKVQFAKDEDLHDILDYWKGVSAQQKL
>sp|Q711Q0|CJ071_HUMAN
MMQGNKKCTDAFSDSSSIGSVLDDADREVSSLTDRAFRSLCISEDTSFHDSYLAVSPDITRQVFGTFHQRTVGHTQRKSGIWSQLPS
QGTEHSGWAATFQQLPKYVQGEEKYPKTSPPPTPVQRRLEVPVSGLRSSNKPVSKVSTLIKSFDRTESQRCESRPTASKPPALKNPP
KFAPLPENSVNFCFDSAFLTVRRVPAEVSNTHQNSYQPGRKHGEQESSKNPEMACHGSSSFLPAANDTATLCESKFPSPHHKPVTGE
PGRGKGTFLHSENSAFESWNAHQPKLLERKDTAGTVPESKAPKHYGDTTLLREPCPPERTVSPCQVQASCSQEENRLAAGALSTSIP
WGCRDPGAQVFAVEGKAPSSQPDSQEKPAQPPWRKPKTGKKGKESLQDTLEEKTQTNQRGPPLYTKHNPQEQFSENNALDLPVEPNE
HYDPPFNISKLLTPIIPSKHALDSADSQPAERTPSPPGQLNGYQEKEPSECQSRDSYKSKAPSLLFNLKDVRKRVKSTYSSSPLLKV
LDEKTRGKVDGKQEPVSNGVILPNGLEESPPNELSKERPADDPTASHINPQKDPTADPSEPSADSYLTLSTAPTIAKAPFYVNGEAA

ERSSYENKEVEGELEMGPAGSSWCPDSREHRPRKHLSLRLCNRDPEPGGATEKMKTHQLENGLSRSVSQETEPEREAGLQNTHLNQK
FFPGPLSPEEEDVFYSDSQSDFMPSLKGKAKFSTSSSDQSFASFDDQQKMWFTENQREDRRKDVSAGDSQKDEKENVMRKDELQYCA
LSNGHACLENRSQGEALQRERESVSGGRTRKASAEEANFRGSWIGENKGTTFSQAKDLTPSPSSASNRHMLFTIKDNTLRATPVIKP
IMLPLLRTMSLEDSLSSGHKEEELPRPEWGEDPGFCAPENQDILGTSTPTNTRGTRVKCMANEVMEDPGQGSSMARMEASQPAPKGN
FPSMPLVGEGDRVKAPPDAAPGLVASNCKSGSADSGKLAAPWHIPTIALPEGDIEDQPPPWQPENCWEEQTPGFKSHFLSTPRAGPP
GRRLVPSERANSPNPGSPGESSACSPAASNIWEESSQAPGGPELLPEEPNQASPWASSSPARVTRREDLTHALVWEGGSDPLLELSA
EDLRTLSPRGSLLDVATSPAGTSGRLELPAQLERTASKPPAVPPKTEKALRRAKKLASKRRKTDQAQEKHGESQEGKPCPEDLEQTQ
QRPLCPRERPRHNFPVVRSLPPPVHRHSVSGFSEPVGRRPGGPQSLTPLPAYPATQKVLQDPQSGEYFVFDLPLQVKIKTFYDPETG
KYVKVSIPSSEGASPEPPPPDALAAPYVLYPGFQPVPVTALMPLRCSSQLSAPTFLRQGPRASAARARTQSVHESGLQLDPGPHGDC
TPHSAGQRPHGPPQSPGEEGVEAPGLGIISTDDLEDFATEGIS
>sp|Q9NR99|MXRA5_HUMAN
MPKRAHWGALSVVLILLWGHPRVALACPHPCACYVPSEVHCTFRSLASVPAGIAKHVERINLGFNSIQALSETSFAGLTKLELLMIH
GNEIPSIPDGALRDLSSLQVFKFSYNKLRVITGQTLQGLSNLMRLHIDHNKIEFIHPQAFNGLTSLRLLHLEGNLLHQLHPSTFSTF
TFLDYFRLSTIRHLYLAENMVRTLPASMLRNMPLLENLYLQGNPWTCDCEMRWFLEWDAKSRGILKCKKDKAYEGGQLCAMCFSPKK
LYKHEIHKLKDMTCLKPSIESPLRQNRSRSIEEEQEQEEDGGSQLILEKFQLPQWSISLNMTDEHGNMVNLVCDIKKPMDVYKIHLN
QTDPPDIDINATVALDFECPMTRENYEKLWKLIAYYSEVPVKLHRELMLSKDPRVSYQYRQDADEEALYYTGVRAQILAEPEWVMQP
SIDIQLNRRQSTAKKVLLSYYTQYSQTISTKDTRQARGRSWVMIEPSGAVQRDQTVLEGGPCQLSCNVKASESPSIFWVLPDGSILK
APMDDPDSKFSILSSGWLRIKSMEPSDSGLYQCIAQVRDEMDRMVYRVLVQSPSTQPAEKDTVTIGKNPGESVTLPCNALAIPEAHL
SWILPNRRIINDLANTSHVYMLPNGTLSIPKVQVSDSGYYRCVAVNQQGADHFTVGITVTKKGSGLPSKRGRRPGAKALSRVREDIV
EDEGGSGMGDEENTSRRLLHPKDQEVFLKTKDDAINGDKKAKKGRRKLKLWKHSEKEPETNVAEGRRVFESRRRINMANKQINPERW
ADILAKVRGKNLPKGTEVPPLIKTTSPPSLSLEVTPPFPAISPPSASPVQTVTSAEESSADVPLLGEEEHVLGTISSASMGLEHNHN
GVILVEPEVTSTPLEEVVDDLSEKTEEITSTEGDLKGTAAPTLISEPYEPSPTLHTLDTVYEKPTHEETATEGWSAADVGSSPEPTS
SEYEPPLDAVSLAESEPMQYFDPDLETKSQPDEDKMKEDTFAHLTPTPTIWVNDSSTSQLFEDSTIGEPGVPGQSHLQGLTDNIHLV
KSSLSTQDTLLIKKGMKEMSQTLQGGNMLEGDPTHSRSSESEGQESKSITLPDSTLGIMSSMSPVKKPAETTVGTLLDKDTTTATTT
PRQKVAPSSTMSTHPSRRRPNGRRRLRPNKFRHRHKQTPPTTFAPSETFSTQPTQAPDIKISSQVESSLVPTAWVDNTVNTPKQLEM
EKNAEPTSKGTPRRKHGKRPNKHRYTPSTVSSRASGSKPSPSPENKHRNIVTPSSETILLPRTVSLKTEGPYDSLDYMTTTRKIYSS
YPKVQETLPVTYKPTSDGKEIKDDVATNVDKHKSDILVTGESITNAIPTSRSLVSTMGEFKEESSPVGFPGTPTWNPSRTAQPGRLQ
TGIPVTTSGENLTDPPLLKELEDVDFTSEFLSSLTVSTPFHQEEAGSSTTLSSIKVEVASSQAETTTLDQDHLETTVAILLSETRPQ
NHTPTAARMKEPASSSPSTILMSLGQTTTTKPALPSPRISQASRDSKENVFLNYVGNPETEATPVNNEGTQHMSGPNELSTPSSDQD
AFNLSTKLELEKQVFGSRSLPRGPDSQRQDGRVHASHQLTRVPAKPILPTATVRLPEMSTQSASRYFVTSQSPRHWTNKPEITTYPS
GALPENKQFTTPRLSSTTIPLPLHMSKPSIPSKFTDRRTDQFNGYSKVFGNNNIPEARNPVGKPPSPRIPHYSNGRLPFFTNKTLSF
PQLGVTRRPQIPTSPAPVMRERKVIPGSYNRIHSHSTFHLDFGPPAPPLLHTPQTTGSPSTNLQNIPMVSSTQSSISFITSSVQSSG
SFHQSSSKFFAGGPPASKFWSLGEKPQILTKSPQTVSVTAETDTVFPCEATGKPKPFVTWTKVSTGALMTPNTRIQRFEVLKNGTLV
IRKVQVQDRGQYMCTASNLHGLDRMVVLLSVTVQQPQILASHYQDVTVYLGDTIAMECLAKGTPAPQISWIFPDRRVWQTVSPVEGR
ITLHENRTLSIKEASFSDRGVYKCVASNAAGADSLAIRLHVAALPPVIHQEKLENISLPPGLSIHIHCTAKAAPLPSVRWVLGDGTQ
IRPSQFLHGNLFVFPNGTLYIRNLAPKDSGRYECVAANLVGSARRTVQLNVQRAAANARITGTSPRRTDVRYGGTLKLDCSASGDPW
PRILWRLPSKRMIDALFSFDSRIKVFANGTLVVKSVTDKDAGDYLCVARNKVGDDYVVLKVDVVMKPAKIEHKEENDHKVFYGGDLK
VDCVATGLPNPEISWSLPDGSLVNSFMQSDDSGGRTKRYVVFNNGTLYFNEVGMREEGDYTCFAENQVGKDEMRVRVKVVTAPATIR
NKTYLAVQVPYGDVVTVACEAKGEPMPKVTWLSPTNKVIPTSSEKYQIYQDGTLLIQKAQRSDSGNYTCLVRNSAGEDRKTVWIHVN
VQPPKINGNPNPITTVREIAAGGSRKLIDCKAEGIPTPRVLWAFPEGVVLPAPYYGNRITVHGNGSLDIRSLRKSDSVQLVCMARNE
GGEARLILQLTVLEPMEKPIFHDPISEKITAMAGHTISLNCSAAGTPTPSLVWVLPNGTDLQSGQQLQRFYHKADGMLHISGLSSVD
AGAYRCVARNAAGHTERLVSLKVGLKPEANKQYHNLVSIINGETLKLPCTPPGAGQGRFSWTLPNGMHLEGPQTLGRVSLLDNGTLT
VREASVFDRGTYVCRMETEYGPSVTSIPVIVIAYPPRITSEPTPVIYTRPGNTVKLNCMAMGIPKADITWELPDKSHLKAGVQARLY
GNRFLHPQGSLTIQHATQRDAGFYKCMAKNILGSDSKTTYIHVF

>sp|Q02818|NUCB1_HUMAN
MPPSGPRGTLLLLPLLLLLLLRAVLAVPLERGAPNKEETPATESPDTGLYYHRYLQEVIDVLETDGHFREKLQAANAEDIKSGKLSR
ELDFVSHHVRTKLDELKRQEVSRLRMLLKAKMDAEQDPNVQVDHLNLLKQFEHLDPQNQHTFEARDLELLIQTATRDLAQYDAAHHE
EFKRYEMLKEHERRRYLESLGEEQRKEAERKLEEQQRRHREHPKVNVPGSQAQLKEVWEELDGLDPNRFNPKTFFILHDINSDGVLD
EQELEALFTKELEKVYDPKNEEDDMREMEEERLRMREHVMKNVDTNQDRLVTLEEFLASTQRKEFGDTGEGWETVEMHPAYTEEELR
RFEEELAAREAELNAKAQRLSQETEALGRSQGRLEAQKRELQQAVLHMEQRKQQQQQQQGHKAPAAHPEGQLKFHPDTDDVPVPAPA
GDQKEVDTSEKKLLERLPEVEVPQHL
>sp|Q9UM11|FZR_HUMAN
MDQDYERRLLRQIVIQNENTMPRVTEMRRTLTPASSPVSSPSKHGDRFIPSRAGANWSVNFHRINENEKSPSQNRKAKDATSDNGKD
GLAYSALLKNELLGAGIEKVQDPQTEDRRLQPSTPEKKGLFTYSLSTKRSSPDDGNDVSPYSLSPVSNKSQKLLRSPRKPTRKISKI
PFKVLDAPELQDDFYLNLVDWSSLNVLSVGLGTCVYLWSACTSQVTRLCDLSVEGDSVTSVGWSERGNLVAVGTHKGFVQIWDAAAG
KKLSMLEGHTARVGALAWNAEQLSSGSRDRMILQRDIRTPPLQSERRLQGHRQEVCGLKWSTDHQLLASGGNDNKLLVWNHSSLSPV
QQYTEHLAAVKAIAWSPHQHGLLASGGGTADRCIRFWNTLTGQPLQCIDTGSQVCNLAWSKHANELVSTHGYSQNQILVWKYPSLTQ
VAKLTGHSYRVLYLAMSPDGEAIVTGAGDETLRFWNVFSKTRSTKVKWESVSVLNLFTRIR
>sp|Q9Y5Y3|GPR45_HUMAN
MACNSTSLEAYTYLLLNTSNASDSGSTQLPAPLRISLAIVMLLMTVVGFLGNTVVCIIVYQRPAMRSAINLLLATLAFSDIMLSLCC
MPFTAVTLITVRWHFGDHFCRLSATLYWFFVLEGVAILLIISVDRFLIIVQRQDKLNPRRAKVIIAVSWVLSFCIAGPSLTGWTLVE
VPARAPQCVLGYTELPADRAYVVTLVVAVFFAPFGVMLCAYMCILNTVRKNAVRVHNQSDSLDLRQLTRAGLRRLQRQQQVSVDLSF
KTKAFTTILILFVGFSLCWLPHSVYSLLSVFSQRFYCGSSFYATSTCVLWLSYLKSVFNPIVYCWRIKKFREACIELLPQTFQILPK
VPERIRRRIQPSTVYVCNENQSAV
>sp|Q9UQB3|CTND2_HUMAN
MFARKPPGAAPLGAMPVPDQPSSASEKTSSLSPGLNTSNGDGSETETTSAILASVKEQELQFERLTRELEAERQIVASQLERCKLGS
ETGSMSSMSSAEEQFQWQSQDGQKDIEDELTTGLELVDSCIRSLQESGILDPQDYSTGERPSLLSQSALQLNSKPEGSFQYPASYHS
NQTLALGETTPSQLPARGTQARATGQSFSQGTTSRAGHLAGPEPAPPPPPPPREPFAPSLGSAFHLPDAPPAAAAAALYYSSSTLPA
PPRGGSPLAAPQGGSPTKLQRGGSAPEGATYAAPRGSSPKQSPSRLAKSYSTSSPINIVVSSAGLSPIRVTSPPTVQSTISSSPIHQ
LSSTIGTYATLSPTKRLVHASEQYSKHSQELYATATLQRPGSLAAGSRASYSSQHGHLGPELRALQSPEHHIDPIYEDRVYQKPPMR
SLSQSQGDPLPPAHTGTYRTSTAPSSPGVDSVPLQRTGSQHGPQNAAAATFQRASYAAGPASNYADPYRQLQYCPSVESPYSKSGPA
LPPEGTLARSPSIDSIQKDPREFGWRDPELPEVIQMLQHQFPSVQSNAAAYLQHLCFGDNKIKAEIRRQGGIQLLVDLLDHRMTEVH
RSACGALRNLVYGKANDDNKIALKNCGGIPALVRLLRKTTDLEIRELVTGVLWNLSSCDALKMPIIQDALAVLTNAVIIPHSGWENS
PLQDDRKIQLHSSQVLRNATGCLRNVSSAGEEARRRMRECDGLTDALLYVIQSALGSSEIDSKTVENCVCILRNLSYRLAAETSQGQ
HMGTDELDGLLCGEANGKDAESSGCWGKKKKKKKSQDQWDGVGPLPDCAEPPKGIQMLWHPSIVKPYLTLLSECSNPDTLEGAAGAL
QNLAAGSWKWSVYIRAAVRKEKGLPILVELLRIDNDRVVCAVATALRNMALDVRNKELIGKYAMRDLVHRLPGGNNSNNTASKAMSD
DTVTAVCCTLHEVITKNMENAKALRDAGGIEKLVGISKSKGDKHSPKVVKAASQVLNSMWQYRDLRSLYKKDGWSQYHFVASSSTIE
RDRQRPYSSSRTPSISPVRVSPNNRSASAPASPREMISLKERKTDYECTGSNATYHGAKGEHTSRKDAMTAQNTGISTLYRNSYGAP
AEDIKHNQVSAQPVPQEPSRKDYETYQPFQNSTRNYDESFFEDQVHHRPPASEYTMHLGLKSTGNYVDFYSAARPYSELNYETSHYP
ASPDSWV
>sp|Q99622|C10_HUMAN
MASASTQPAALSAEQAKVVLAEVIQAFSAPENAVRMDEARDNACNDMGKMLQFVLPVATQIQQEVIKAYGFSCDGEGVLKFARLVKS
YEAQDPEIASLSGKLKALFLPPMTLPPHGPAAGGSVAAS
>sp|A7KAX9|RHG32_HUMAN
METESESSTLGDDSVFWLESEVIIQVTDCEEEEREEKFRKMKSSVHSEEDDFVPELHRNVHPRERPDWEETLSAMARGADVPEIPGD
LTLKTCGSTASMKVKHVKKLPFTKGHFPKMAECAHFHYENVEFGSIQLSLSEEQNEVMKNGCESKELVYLVQIACQGKSWIVKRSYE
DFRVLDKHLHLCIYDRRFSQLSELPRSDTLKDSPESVTQMLMAYLSRLSAIAGNKINCGPALTWMEIDNKGNHLLVHEESSINTPAV
GAAHVIKRYTARAPDELTLEVGDIVSVIDMPPKVLSTWWRGKHGFQVGLFPGHCVELINQKVPQSVTNSVPKPVSKKHGKLITFLRT

FMKSRPTKQKLKQRGILKERVFGCDLGEHLLNSGFEVPQVLQSCTAFIERYGIVDGIYRLSGVASNIQRLRHEFDSEHVPDLTKEPY
VQDIHSVGSLCKLYFRELPNPLLTYQLYEKFSDAVSAATDEERLIKIHDVIQQLPPPHYRTLEFLMRHLSLLADYCSITNMHAKNLA
IVWAPNLLRSKQIESACFSGTAAFMEVRIQSVVVEFILNHVDVLFSGRISMAMQEGAASLSRPKSLLVSSPSTKLLTLEEAQARTQA
QVNSPIVTENKYIEVGEGPAALQGKFHTIIEFPLERKRPQNKMKKSPVGSWRSFFNLGKSSSVSKRKLQRNESEPSEMKAMALKGGR
AEGTLRSAKSEESLTSLHAVDGDSKLFRPRRPRSSSDALSASFNGEMLGNRCNSYDNLPHDNESEEEGGLLHIPALMSPHSAEDVDL
SPPDIGVASLDFDPMSFQCSPPKAESECLESGASFLDSPGYSKDKPSANKKDAETGSSQCQTPGSTASSEPVSPLQEKLSPFFTLDL
SPTEDKSSKPSSFTEKVVYAFSPKIGRKLSKSPSMSISEPISVTLPPRVSEVIGTVSNTTAQNASSSTWDKCVEERDATNRSPTQIV
KMKTNETVAQEAYESEVQPLDQVAAEEVELPGKEDQSVSSSQSKAVASGQTQTGAVTHDPPQDSVPVSSVSLIPPPPPPKNVARMLA
LALAESAQQASTQSLKRPGTSQAGYTNYGDIAVATTEDNLSSSYSAVALDKAYFQTDRPAEQFHLQNNAPGNCDHPLPETTATGDPT
HSNTTESGEQHHQVDLTGNQPHQAYLSGDPEKARITSVPLDSEKSDDHVSFPEDQSGKNSMPTVSFLDQDQSPPRFYSGDQPPSYLG
ASVDKLHHPLEFADKSPTPPNLPSDKIYPPSGSPEENTSTATMTYMTTTPATAQMSTKEASWDVAEQPTTADFAAATLQRTHRTNRP
LPPPPSQRSAEQPPVVGQVQAATNIGLNNSHKVQGVVPVPERPPEPRAMDDPASAFISDSGAAAAQCPMATAVQPGLPEKVRDGARV
PLLHLRAESVPAHPCGFPAPLPPTRMMESKMIAAIHSSSADATSSSNYHSFVTASSTSVDDALPLPLPVPQPKHASQKTVYSSFARP
DVTTEPFGPDNCLHFNMTPNCQYRPQSVPPHHNKLEQHQVYGARSEPPASMGLRYNTYVAPGRNASGHHSKPCSRVEYVSSLSSSVR
NTCYPEDIPPYPTIRRVQSLHAPPSSMIRSVPISRTEVPPDDEPAYCPRPLYQYKPYQSSQARSDYHVTQLQPYFENGRVHYRYSPY
SSSSSSYYSPDGALCDVDAYGTVQLRPLHRLPNRDFAFYNPRLQGKSLYSYAGLAPRPRANVTGYFSPNDHNVVSMPPAADVKHTYT
SWDLEDMEKYRMQSIRRESRARQKVKGPVMSQYDNMTPAVQDDLGGIYVIHLRSKSDPGKTGLLSVAEGKESRHAAKAISPEGEDRF
YRRHPEAEMDRAHHHGGHGSTQPEKPSLPQKQSSLRSRKLPDMGCSLPEHRAHQEASHRQFCESKNGPPYPQGAGQLDYGSKGIPDT
SEPVSYHNSGVKYAASGQESLRLNHKEVRLSKEMERPWVRQPSAPEKHSRDCYKEEEHLTQSIVPPPKPERSHSLKLHHTQNVERDP
SVLYQYQPHGKRQSSVTVVSQYDNLEDYHSLPQHQRGVFGGGGMGTYVPPGFPHPQSRTYATALGQGAFLPAELSLQHPETQIHAE
>sp|P62324|BTG1_HUMAN
MHPFYTRAATMIGEIAAAVSFISKFLRTKGLTSERQLQTFSQSLQELLAEHYKHHWFPEKPCKGSGYRCIRINHKMDPLIGQAAQRI
GLSSQELFRLLPSELTLWVDPYEVSYRIGEDGSICVLYEASPAGGSTQNSTNVQMVDSRISCKEELLLGRTSPSKNYNMMTVSG
>sp|Q8TBX8|PI42C_HUMAN
MASSSVPPATVSAATAGPGPGFGFASKTKKKHFVQQKVKVFRAADPLVGVFLWGVAHSINELSQVPPPVMLLPDDFKASSKIKVNNH
LFHRENLPSHFKFKEYCPQVFRNLRDRFGIDDQDYLVSLTRNPPSESEGSDGRFLISYDRTLVIKEVSSEDIADMHSNLSNYHQYIV
KCHGNTLLPQFLGMYRVSVDNEDSYMLVMRNMFSHRLPVHRKYDLKGSLVSREASDKEKVKELPTLKDMDFLNKNQKVYIGEEEKKI
FLEKLKRDVEFLVQLKIMDYSLLLGIHDIIRGSEPEEEAPVREDESEVDGDCSLTGPPALVGSYGTSPEGIGGYIHSHRPLGPGEFE
SFIDVYAIRSAEGAPQKEVYFMGLIDILTQYDAKKKAAHAAKTVKHGAGAEISTVHPEQYAKRFLDFITNIFA
>sp|Q68CQ4|DIEXF_HUMAN
MGKRGSRSQSQLLNTLTKKQKKHLRDFGEEHPFYDRVSRKEAKPQICQLSESSDSSDSESDSESEPQQVSGYHRLLATLKNVSEEEE
EDEEEEEEEDSIVDDAEMNDEDGGSDVSVEEEMAAESTESPENVALSADPEGKEDGEEPPGTSQTSPEEFTDAKHESLFSLETNFLE
EESGDNSSLKASQDPFLQHVNKELKEKAIQAVATNPKTTHELKWPILGQLFFSSKFQKLETFKPPKDIDLKSLHLQKPLESTWTKTN
SQFLSGPQKSSSPFTPLQKELFLIMNSYRDLFYPERTALKNGEEIRHVYCLHVINHILKANAQVLGNNSRRRSQKFGVGDDDDFRDQ
GLTRPKVLIVVPFREAALRVVQLFISLLEGDSKKKIIVSNKKRFQGEYGSDPEERPPNLKRPEDYEAVFVGNIDDHFRIGVAILQRS
IRLYAPFYSSDILIASPLGLRTIIGGEGEKKRDFDFLSSIELLIIDQADIYLMQNWEHVLHLMNHMNLLPLDSHGVDFSRVRMWSLN
NWSKYYRQTLLFGALQDAQINSVFNKYCVNMQGQVAVRNVPMTGSISHVLVQLPHVFQRMEAENLASVIDARFNFFVNKILPQYRDA
VMSHTLIYIPSYFDFVRLRNYFKKEELNFTHICEYTQKSGVSRARHFFLQGEKQFLLFTERFHFYKRYTIKGIRNLIFYELPTYPHF
YSEICNMLRATNRGEEATWTCTVLYSKYDAQRLAAVVGVERAAQMLQSNKNVHLFITGEK
>sp|P63010|AP2B1_HUMAN
MTDSKYFTTNKKGEIFELKAELNNEKKEKRKEAVKKVIAAMTVGKDVSSLFPDVVNCMQTDNLELKKLVYLYLMNYAKSQPDMAIMA
VNSFVKDCEDPNPLIRALAVRTMGCIRVDKITEYLCEPLRKCLKDEDPYVRKTAAVCVAKLHDINAQMVEDQGFLDSLRDLIADSNP
MVVANAVAALSEISESHPNSNLLDLNPQNINKLLTALNECTEWGQIFILDCLSNYNPKDDREAQSICERVTPRLSHANSAVVLSAVK
VLMKFLELLPKDSDYYNMLLKKLAPPLVTLLSGEPEVQYVALRNINLIVQKRPEILKQEIKVFFVKYNDPIYVKLEKLDIMIRLASQ

ANIAQVLAELKEYATEVDVDFVRKAVRAIGRCAIKVEQSAERCVSTLLDLIQTKVNYVVQEAIVVIRDIFRKYPNKYESIIATLCEN
LDSLDEPDARAAMIWIVGEYAERIDNADELLESFLEGFHDESTQVQLTLLTAIVKLFLKKPSETQELVQQVLSLATQDSDNPDLRDR
GYIYWRLLSTDPVTAKEVVLSEKPLISEETDLIEPTLLDELICHIGSLASVYHKPPNAFVEGSHGIHRKHLPIHHGSTDAGDSPVGT
TTATNLEQPQVIPSQGDLLGDLLNLDLGPPVNVPQVSSMQMGAVDLLGGGLDSLVGQSFIPSSVPATFAPSPTPAVVSSGLNDLFEL
STGIGMAPGGYVAPKAVWLPAVKAKGLEISGTFTHRQGHIYMEMNFTNKALQHMTDFAIQFNKNSFGVIPSTPLAIHTPLMPNQSID
VSLPLNTLGPVMKMEPLNNLQVAVKNNIDVFYFSCLIPLNVLFVEDGKMERQVFLATWKDIPNENELQFQIKECHLNADTVSSKLQN
NNVYTIAKRNVEGQDMLYQSLKLTNGIWILAELRIQPGNPNYTLSLKCRAPEVSQYIYQVYDSILKN
>sp|Q86SG7|LYG2_HUMAN
MLSSVVFWGLIALIGTSRGSYPFSHSMKPHLHPRLYHGCYGDIMTMKTSGATCDANSVMNCGIRGSEMFAEMDLRAIKPYQTLIKEV
GQRHCVDPAVIAAIISRESHGGSVLQDGWDHRGLKFGLMQLDKQTYHPVGAWDSKEHLSQATGILTERIKAIQKKFPTWSVAQHLKG
GLSAFKSGIEAIATPSDIDNDFVNDIIARAKFYKRQSF
>sp|P48742|LHX1_HUMAN
MVHCAGCKRPILDRFLLNVLDRAWHVKCVQCCECKCNLTEKCFSREGKLYCKNDFFRCFGTKCAGCAQGISPSDLVRRARSKVFHLN
CFTCMMCNKQLSTGEELYIIDENKFVCKEDYLSNSSVAKENSLHSATTGSDPSLSPDSQDPSQDDAKDSESANVSDKEAGSNENDDQ
NLGAKRRGPRTTIKAKQLETLKAAFAATPKPTRHIREQLAQETGLNMRVIQVWFQNRRSKERRMKQLSALGARRHAFFRSPRRMRPL
VDRLEPGELIPNGPFSFYGDYQSEYYGPGGNYDFFPQGPPSSQAQTPVDLPFVPSSGPSGTPLGGLEHPLPGHHPSSEAQRFTDILA
HPPGDSPSPEPSLPGPLHSMSAEVFGPSPPFSSLSVNGGASYGNHLSHPPEMNEAAVW
>sp|O14948|TFEC_HUMAN
MTLDHQIINPTLKWSQPAVPSGGPLVQHAHTTLDSDAGLTENPLTKLLAIGKEDDNAQWHMEDVIEDIIGMESSFKEEGADSPLLMQ
RTLSGSILDVYSGEQGISPINMGLTSASCPSSLPMKREITETDTRALAKERQKKDNHNLIERRRRYNINYRIKELGTLIPKSNDPDM
RWNKGTILKASVEYIKWLQKEQQRARELEHRQKKLEQANRRLLLRIQELEIQARTHGLPTLASLGTVDLGAHVTKQQSHPEQNSVDY
CQQLTVSQGPSPELCDQAIAFSDPLSYFTDLSFSAALKEEQRLDGMLLDDTISPFGTDPLLSATSPAVSKESSRRSSFSSDDGDEL
>sp|Q8NGR4|OR5C1_HUMAN
MNSENLTRAAVAPAEFVLLGITNRWDLRVALFLTCLPVYLVSLLGNMGMALLIRMDARLHTPMYFFLANLSLLDACYSSAIGPKMLV
DLLLPRATIPYTACALQMFVFAGLADTECCLLAAMAYDRYVAIRNPLLYTTAMSQRLCLALLGASGLGGAVSAFVHTTLTFRLSFCR
SRKINSFFCDIPPLLAISCSDTSLNELLLFAICGFIQTATVLAITVSYGFIAGAVIHMRSVEGSRRAASTGGSHLTAVAMMYGTLIF
MYLRPSSSYALDTDKMASVFYTLVIPSLNPLIYSLRNKEVKEALRQTWSRFHCPGQGSQ
>sp|Q01518|CAP1_HUMAN
MADMQNLVERLERAVGRLEAVSHTSDMHRGYADSPSKAGAAPYVQAFDSLLAGPVAEYLKISKEIGGDVQKHAEMVHTGLKLERALL
VTASQCQQPAENKLSDLLAPISEQIKEVITFREKNRGSKLFNHLSAVSESIQALGWVAMAPKPGPYVKEMNDAAMFYTNRVLKEYKD
VDKKHVDWVKAYLSIWTELQAYIKEFHTTGLAWSKTGPVAKELSGLPSGPSAGSCPPPPPPCPPPPPVSTISCSYESASRSSLFAQI
NQGESITHALKHVSDDMKTHKNPALKAQSGPVRSGPKPFSAPKPQTSPSPKRATKKEPAVLELEGKKWRVENQENVSNLVIEDTELK
QVAYIYKCVNTTLQIKGKINSITVDNCKKLGLVFDDVVGIVEIINSKDVKVQVMGKVPTISINKTDGCHAYLSKNSLDCEIVSAKSS
EMNVLIPTEGGDFNEFPVPEQFKTLWNGQKLVTTVTEIAG
>sp|P84157|MXRA7_HUMAN
MEAPAELLAALPALATALALLLAWLLVRRGAAASPEPARAPPEPAPPAEATGAPAPSRPCAPEPAASPAGPEEPGEPAGLGELGEPA
GPGEPEGPGDPAAAPAEAEEQAVEARQEEEQDLDGEKGPSSEGPEEEDGEGFSFKYSPGKLRGNQYKKMMTKEELEEEQRVQKEQLA
AIFKLMKDNKETFGEMSDGDVQEQLRLYDM
>sp|Q9NWY4|HPF1_HUMAN
MVGGGGKRRPGGEGPQCEKTTDVKKSKFCEADVSSDLRKEVENHYKLSLPEDFYHFWKFCEELDPEKPSDSLSASLGLQLVGPYDIL
AGKHKTKKKSTGLNFNLHWRFYYDPPEFQTIIIGDNKTQYHMGYFRDSPDEFPVYVGINEAKKNCIIVPNGDNVFAAVKLFLTKKLR
EITDKKKINLLKNIDEKLTEAARELGYSLEQRTVKMKQRDKKVVTKTFHGAGLVVPVDKNDVGYRELPETDADLKRICKTIVEAASD
EERLKAFAPIQEMMTFVQFANDECDYGMGLELGMDLFCYGSHYFHKVAGQLLPLAYNLLKRNLFAEIIEEHLANRSQENIDQLAA
>sp|P03956|MMP1_HUMAN

MHSFPPLLLLLFWGVVSHSFPATLETQEQDVDLVQKYLEKYYNLKNDGRQVEKRRNSGPVVEKLKQMQEFFGLKVTGKPDAETLKVM
KQPRCGVPDVAQFVLTEGNPRWEQTHLTYRIENYTPDLPRADVDHAIEKAFQLWSNVTPLTFTKVSEGQADIMISFVRGDHRDNSPF
DGPGGNLAHAFQPGPGIGGDAHFDEDERWTNNFREYNLHRVAAHELGHSLGLSHSTDIGALMYPSYTFSGDVQLAQDDIDGIQAIYG
RSQNPVQPIGPQTPKACDSKLTFDAITTIRGEVMFFKDRFYMRTNPFYPEVELNFISVFWPQLPNGLEAAYEFADRDEVRFFKGNKY
WAVQGQNVLHGYPKDIYSSFGFPRTVKHIDAALSEENTGKTYFFVANKYWRYDEYKRSMDPGYPKMIAHDFPGIGHKVDAVFMKDGF
FYFFHGTRQYKFDPKTKRILTLQKANSWFNCRKN
>sp|Q9NZC3|GDE1_HUMAN
MWLWEDQGGLLGPFSFLLLVLLLVTRSPVNACLLTGSLFVLLRVFSFEPVPSCRALQVLKPRDRISAIAHRGGSHDAPENTLAAIRQ
AAKNGATGVELDIEFTSDGIPVLMHDNTVDRTTDGTGRLCDLTFEQIRKLNPAANHRLRNDFPDEKIPTLREAVAECLNHNLTIFFD
VKGHAHKATEALKKMYMEFPQLYNNSVVCSFLPEVIYKMRQTDRDVITALTHRPWSLSHTGDGKPRYDTFWKHFIFVMMDILLDWSM
HNILWYLCGISAFLMQKDFVSPAYLKKWSAKGIQVVGWTVNTFDEKSYYESHLGSSYITDSMVEDCEPHF
>sp|Q8N9T8|KRI1_HUMAN
MPEPRGSSQLRVNAAFAARYNRYREREELQRLKDRYGDRDSSSDSSSESDSSDERVEFDPQQERDFYKTLSLLKKKDPRIYQKDATF
YNRTASSSDSEEDPEALEKQKKVRPMYLKDYERKVILEKAGKYVDEENSDGETSNHRLQETSSQSYVEEQKQLKESFRAFVEDSEDE
DGAGEGGSSLLQKRAKTRQEKAQEEADYIEWLKGQKEIRNPDSLKELTHLKEYWNDPELDEGERFLRDYILNKRYEEEEEEEEDEEE
MEEEEGVHGPPVQLAVDDSSDEGELFLKKQEDFEQKYNFRFEEPDSASVKTYPRSIASSVRRKDERRKEKREETRERKKREKAKKQE
ELKQLKNLKRKEILAKLEKLRKVTGNEMLGLEEGDLEDDFDPAQHDQLMQKCFGDEYYGAVEEEKPQFEEEEGLEDDWNWDTWDGPE
QEGDWSQQELHCEDPNFNMDADYDPSQPRKKKREAPLTGKKKRKSPFAAAVGQEKPVFEPGDKTFEEYLDEYYRLDYEDIIDDLPCR
FKYRTVVPCDFGLSTEEILAADDKELNRWCSLKKTCMYRSEQEELRDKRAYSQKAQNSWKKRQVFKSLCREEAETPAEATGKPQRDE
AGPQRQLPALDGSLMGPESPPAQEEEAPVSPHKKPAPQKRRRAKKARLLGPTVMLGGCEFSRQRLQAFGLNPKRLHFRQLGRQRRKQ
QGPKNSS
>sp|P07101|TY3H_HUMAN
MPTPDATTPQAKGFRRAVSELDAKQAEAIMVRGQGAPGPSLTGSPWPGTAAPAASYTPTPRSPRFIGRRQSLIEDARKEREAAVAAA
AAAVPSEPGDPLEAVAFEEKEGKAVLNLLFSPRATKPSALSRAVKVFETFEAKIHHLETRPAQRPRAGGPHLEYFVRLEVRRGDLAA
LLSGVRQVSEDVRSPAGPKVPWFPRKVSELDKCHHLVTKFDPDLDLDHPGFSDQVYRQRRKLIAEIAFQYRHGDPIPRVEYTAEEIA
TWKEVYTTLKGLYATHACGEHLEAFALLERFSGYREDNIPQLEDVSRFLKERTGFQLRPVAGLLSARDFLASLAFRVFQCTQYIRHA
SSPMHSPEPDCCHELLGHVPMLADRTFAQFSQDIGLASLGASDEEIEKLSTLYWFTVEFGLCKQNGEVKAYGAGLLSSYGELLHCLS
EEPEIRAFDPEAAAVQPYQDQTYQSVYFVSESFSDAKDKLRSYASRIQRPFSVKFDPYTLAIDVLDSPQAVRRSLEGVQDELDTLAH
ALSAIG
>sp|O95476|CNEP1_HUMAN
MMRTQCLLGLRTFVAFAAKLWSFFIYLLRRQIRTVIQYQTVRYDILPLSPVSRNRLAQVKRKILVLDLDETLIHSHHDGVLRPTVRP
GTPPDFILKVVIDKHPVRFFVHKRPHVDFFLEVVSQWYELVVFTASMEIYGSAVADKLDNSRSILKRRYYRQHCTLELGSYIKDLSV
VHSDLSSIVILDNSPGAYRSHPDNAIPIKSWFSDPSDTALLNLLPMLDALRFTADVRSVLSRNLHQHRLW
>sp|P78560|CRADD_HUMAN
MEARDKQVLRSLRLELGAEVLVEGLVLQYLYQEGILTENHIQEINAQTTGLRKTMLLLDILPSRGPKAFDTFLDSLQEFPWVREKLK
KAREEAMTDLPAGDRLTGIPSHILNSSPSDRQINQLAQRLGPEWEPMVLSLGLSQTDIYRCKANHPHNVQSQVVEAFIRWRQRFGKQ
ATFQSLHNGLRAVEVDPSLLLHMLE
>sp|P08686|CP21A_HUMAN
MLLLGLLLLPLLAGARLLWNWWKLRSLHLPPLAPGFLHLLQPDLPIYLLGLTQKFGPIYRLHLGLQDVVVLNSKRTIEEAMVKKWAD
FAGRPEPLTYKLVSKNYPDLSLGDYSLLWKAHKKLTRSALLLGIRDSMEPVVEQLTQEFCERMRAQPGTPVAIEEEFSLLTCSIICY
LTFGDKIKDDNLMPAYYKCIQEVLKTWSHWSIQIVDVIPFLRFFPNPGLRRLKQAIEKRDHIVEMQLRQHKESLVAGQWRDMMDYML
QGVAQPSMEEGSGQLLEGHVHMAAVDLLIGGTETTANTLSWAVVFLLHHPEIQQRLQEELDHELGPGASSSRVPYKDRARLPLLNAT
IAEVLRLRPVVPLALPHRTTRPSSISGYDIPEGTVIIPNLQGAHLDETVWERPHEFWPDRFLEPGKNSRALAFGCGARVCLGEPLAR
LELFVVLTRLLQAFTLLPSGDALPSLQPLPHCSVILKMQPFQVRLQPRGMGAHSPGQNQ

>sp|Q8NH67|O52I2_HUMAN
MCQQILRDCILLIHHLCINRKKVSLVMLGPAYNHTMETPASFLLVGIPGLQSSHLWLAISLSAMYIIALLGNTIIVTAIWMDSTRHE
PMYCFLCVLAAVDIVMASSVVPKMVSIFCSGDSSISFSACFTQMFFVHLATAVETGLLLTMAFDRYVAICKPLHYKRILTPQVMLGM
SMAITIRAIIAITPLSWMVSHLPFCGSNVVVHSYCEHIALARLACADPVPSSLYSLIGSSLMVGSDVAFIAASYILILKAVFGLSSK
TAQLKALSTCGSHVGVMALYYLPGMASIYAAWLGQDVVPLHTQVLLADLYVIIPATLNPIIYGMRTKQLRERIWSYLMHVLFDHSNL
GS
>sp|Q2TAL5|SMTL2_HUMAN
MEPAPDAQEARTVREALGRYEAALEGAVRALHEDMRGLQRGVERRVAEAMRLAGPLARTVADLQRDNQRLQAQLERLTRQVEALGLA
SGMSPVPGTPGTPSPPPAPGVPDRAPRLGSARFASHATFSLSGRGQSLDHDEASESEMRKTSNSCIMENGHQPGAGPGDGPPEIAQN
FSAPDPPRPRPVSLSLRLPHQPVTAITRVSDRFSGETSAAALSPMSAATLGGLNPSPSEVITPWTPSPSEKNSSFTWSVPSSGYGAV
TASKHSNSPPLVTPPQSPVSPQPPAITQVHRQGERRRELVRSQTLPRTSEAQARKALFEKWEQETAAGKGKGEARARLKRSQSFGVA
SASSIKQILLEWCRSKTLGYQHVDLQNFSSSWSDGMAFCALVHSFFPDAFDYNSLSPTQRQKNFELAFTMAENLANCERLIEVEDMM
VMGRKPDPMCVFTYVQSLYNHLRRFE
>sp|Q6ZN08|ZNF66_HUMAN
MGPLQFRDVAIEFSLEEWHCLDMAQRNLYRDVMLENYRNLVFLGIVVSKPDLITHLEQGKKPSTMQRHEMVANPSVLCSHFNQDLWP
EQSIKDSFQKLILRRHKKCGHDNLQLKKGCESVDKCKVHKRGYNGLNQCLTTTQSKMFQCDKHGKVFHQFSNTNRHKIRHTGKNPCK
FTECGKAFNRSSTFTTHKKIHTGEKPYKCIECGKAFNRSSHLTTHKIIHTGEKRYKCEDCGKAFNRSSNLTTHKKIHTGEKPYKCEE
CGKAFKRSSILTTHKRIHTGEKPYKCEECGKVFKYLSSLSTHKIIHTGEKPYKCEECGKAFNWSSHLTTHKRIHTGEKPYKCEECGK
GFKYSSTLTKHKIIHTGEKPYKCEECGEAFKYSCSLTAHKIIHTGKKPYKCEECGKVFKHSSPLSKHKRIHTGEKPYKCEECGKAFS
RSSILTTHKIIHTGEKPYECEDCGKAFNRSSNLTKHKKIHTGEKPYKCEECGKAFKCSSILTTHKRIHTADKPYKCEECGKDFKYSS
TLTRHKKIHTGGKPHKCNKCGKAFISSSNLSRHEIIHMGGNPYKCENVAKP
>sp|O14796|SH21B_HUMAN
MDLPYYHGRLTKQDCETLLLKEGVDGNFLLRDSESIPGVLCLCVSFKNIVYTYRIFREKHGYYRIQTAEGSPKQVFPSLKELISKFE
KPNQGMVVHLLKPIKRTSPSLRWRGLKLELETFVNSNSDYVDVLP
>sp|Q9C0D9|EPT1_HUMAN
MAGYEYVSPEQLAGFDKYKYSAVDTNPLSLYVMHPFWNTIVKVFPTWLAPNLITFSGFLLVVFNFLLMAYFDPDFYASAPGHKHVPD
WVWIVVGILNFVAYTLDGVDGKQARRTNSSTPLGELFDHGLDSWSCVYFVVTVYSIFGRGSTGVSVFVLYLLLWVVLFSFILSHWEK
YNTGILFLPWGYDISQVTISFVYIVTAVVGVEAWYEPFLFNFLYRDLFTAMIIGCALCVTLPMSLLNFFRSYKNNTLKLNSVYEAMV
PLFSPCLLFILSTAWILWSPSDILELHPRVFYFMVGTAFANSTCQLIVCQMSSTRCPTLNWLLVPLFLVVLVVNLGVASYVESILLY
TLTTAFTLAHIHYGVRVVKQLSSHFQIYPFSLRKPNSDULGMEEKNIGL
>sp|Q1A5X7|WHAL1_HUMAN
MMILVFWSNYPYEPVCLASHRNNMEASVPKYKKHLPQLGMQKEMEQDVKRFGQAAWATAIPRLEKLKLMLAQETLQLMRAKELCLNH
KRAEIQGKMEDLPEQEKNINVVDELAIQFYEIQLELYEVKFEILKNKEILLTTQLDSLERLIKDEI
>sp|Q8NG99|OR7G2_HUMAN
MEARNQTAISKFLLLGLIEDPELQPVLFSLFLSMYLVTILGNLLILLAVISDSHLHTPMYFFLSNLSFLDICLSTTTIPKMLVNIQA
QNRSITYSGCLTQICFVLFFAGLENCLLAAMAYDRYVAICHPLRYTVIMNPRLCGLLILLSLLTSVVNALLLSLMVLRLSFCTDLEI
PLFFCELAQVIQLTCSDTLINNILIYFAACIFGGVPLSGIILSYTQITSCVLRMPSASGKHKAVSTCGSHLSIVLLFYGAGLGVYIS
SVVTDSPRKTAVASVMYSVFPQMVNPFIYSLRNKDMKGTLRKFIGRIPSLLWCAICFGFRFLE
>sp|Q15349|KS6A2_HUMAN
MDLSMKKFAVRRFFSVYLRRKSRSKSSSLSRLEEEGVVKEIDISHHVKEGFEKADPSQFELLKVLGQGSYGKVFLVRKVKGSDAGQL
YAMKVLKKATLKVRDRVRSKMERDILAEVNHPFIVKLHYAFQTEGKLYLILDFLRGGDLFTRLSKEVMFTEEDVKFYLAELALALDH
LHSLGIIYRDLKPENILLDEEGHIKITDFGLSKEAIDHDKRAYSFCGTIEYMAPEVVNRRGHTQSADWWSFGVLMFEMLTGSLPFQG
KDRKETMALILKAKLGMPQFLSGEAQSLLRALFKRNPCNRLGAGIDGVEEIKRHPFFVTIDWNTLYRKEIKPPFKPAVGRPEDTFHF
DPEFTARTPTDSPGVPPSANAHHLFRGFSFVASSLIQEPSQQDLHKVPVHPIVQQLHGNNIHFTDGYEIKEDIGVGSYSVCKRCVHK

ATDTEYAVKIIDKSKRDPSEEIEILLRYGQHPNIITLKDVYDDGKFVYLVMELMRGGELLDRILRQRYFSEREASDVLCTITKTMDY
LHSQGVVHRDLKPSNILYRDESGSPESIRVCDFGFAKQLRAGNGLLMTPCYTANFVAPEVLKRQGYDAACDIWSLGILLYTMLAGFT
PFANGPDDTPEEILARIGSGKYALSGGNWDSISDAAKDVVSKMLHVDPHQRLTAMQVLKHPWVVNREYLSPNQLSRQDVHLVKGAMA
ATYFALNRTPQAPRLEPVLSSNLAQRRGMKRLTSTRL
>sp|Q9Y5X4|NR2E3_HUMAN
METRPTALMSSTVAAAAPAAGAASRKESPGRWGLGEDPTGVSPSLQCRVCGDSSSGKHYGIYACNGCSGFFKRSVRRRLIYRCQVGA
GMCPVDKAHRNQCQACRLKKCLQAGMNQDAVQNERQPRSTAQVHLDSMESNTESRPESLVAPPAPAGRSPRGPTPMSAARALGHHFM
ASLITAETCAKLEPEDADENIDVTSNDPEFPSSPYSSSSPCGLDSIHETSARLLFMAVKWAKNLPVFSSLPFRDQVILLEEAWSELF
LLGAIQWSLPLDSCPLLAPPEASAAGGAQGRLTLASMETRVLQETISRFRALAVDPTEFACMKALVLFKPETRGLKDPEHVEALQDQ
SQVMLSQHSKAHHPSQPVRFGKLLLLLPSLRFITAERIELLFFRKTIGNTPMEKLLCDMFKN
>sp|O75449|KTNA1_HUMAN
MSLLMISENVKLAREYALLGNYDSAMVYYQGVLDQMNKYLYSVKDTYLQQKWQQVWQEINVEAKHVKDIMKTLESFKLDSTPLKAAQ
HDLPASEGEVWSMPVPVERRPSPGPRKRQSSQYSDPKSHGNRPSTTVRVHRSSAQNVHNDRGKAVRCREKKEQNKGREEKNKSPAAV
TEPETNKFDSTGYDKDLVEALERDIISQNPNVRWDDIADLVEAKKLLKEAVVLPMWMPEFFKGIRRPWKGVLMVGPPGTGKTLLAKA
VATECKTTFFNVSSSTLTSKYRGESEKLVRLLFEMARFYSPATIFIDEIDSICSRRGTSEEHEASRRVKAELLVQMDGVGGTSENDD
PSKMVMVLAATNFPWDIDEALRRRLEKRIYIPLPSAKGREELLRISLRELELADDVDLASIAENMEGYSGADITNVCRDASLMAMRR
RIEGLTPEEIRNLSKEEMHMPTTMEDFEMALKKVSKSVSAADIERYEKWIFEFGSC
>sp|P48651|PTSS1_HUMAN
MASCVGSRTLSKDDVNYKMHFRMINEQQVEDITIDFFYRPHTITLLSFTIVSLMYFAFTRDDSVPEDNIWRGILSVIFFFLIISVLA
FPNGPFTRPHPALWRMVFGLSVLYFLFLVFLLFLNFEQVKSLMYWLDPNLRYATREADVMEYAVNCHVITWERIISHFDIFAFGHFW
GWAMKALLIRSYGLCWTISITWELTELFFMHLLPNFAECWWDQVILDILLCNGGGIWLGMVVCRFLEMRTYHWASFKDIHTTTGKIK
RAVLQFTPASWTYVRWFDPKSSFQRVAGVYLFMIIWQLTELNTFFLKHIFVFQASHPLSWGRILFIGGITAPTVRQYYAYLTDTQCK
RVGTQCWVFGVIGFLEAIVCIKFGQDLFSKTQILYVVLWLLCVAFTTFLCLYGMIWYAEHYGHREKTYSECEDGTYSPEISWHHRKG
TKGSEDSPPKHAGNNESHSSRRRNRHSKSKVTNGVGKK
>sp|Q9BSR8|YIPF4_HUMAN
MQPPGPPPAYAPTNGDFTFVSSADAEDLSGSIASPDVKLNLGGDFIKESTATTFLRQRGYGWLLEVEDDDPEDNKPLLEELDIDLKD
IYYKIRCVLMPMPSLGFNRQVVRDNPDFWGPLAVVLFFSMISLYGQFRVVSWIITIWIFGSLTIFLLARVLGGEVAYGQVLGVIGYS
LLPLIVIAPVLLVVGSFEVVSTLIKLFGVFWAAYSAASLLVGEEFKTKKPLLIYPIFLLYIYFLSLYTGV
>sp|Q6ZMW3|EMAL6_HUMAN
MADRTAPRCQLRLEWVYGYRGHQCRNNLYYTAGKEVVYFVAGVGVVYNTREHSQKFFLGHNDDIISLALHPDKTLVATGQVGKEPYI
CIWDSYNVQTVSLLKDVHTHGVACLAFDSDGQRLASVGLDAKNTVCIWDWRKGKLLASATGHSDRIFDISWDPYQPNRVVSCGVKHI
KFWTLCGNALTAKRGIFGKTGDLQTILCLACAKEDITYSGALNGDIYVWKGLNLVRTIQGAHSAGIFSMYACEEGFATGGRDGCIRL
WDTDFKPITKIDLRETEQGYKGLSIRSVCWKADRLLAGTQDSEIFEVIVRERDKPMLILQGHCEGELWALALHPKKPLAVTGSDDRS
VRLWSLADHALIARCNMEEAVRSVAFSPDGSQLALGMKDGSFIVLRVRDMTEVVHIKDRKEVIHEMKFSPDGSYLAVGSNDGPVDVY
AVAQRYKKIGECSKSLSFITHIDWSLDSKYLQTNDGAGERLFYRMPSGKPLTSKEEIKGIPWASWTCVKGPEVSGIWPKYTEVTDIN
SVDANYNSSVLVSGDDFGLVKLFKFPCLKRGAKFRKYVGHSAHVTNVRWSHDFQWVLSTGGADHSVFQWRFIPEGVSNGMLETAPQE
GGADSYSEESDSDLSDVPELDSDIEQEAQINYDRQVYKEDLPQLKQQSKEKNHAVPFLKREKAPEDSLKLQFIHGYRGYDCRNNLFY
TQAGEVVYHIAAVAVVYNRQQHSQRLYLGHDDDILSLTIHPVKDYVATGQVGRDAAIHVWDTQTLKCLSLLKGQHQRGVCALDFSAD
GKCLVSVGLDDFHSIVFWDWKKGEKIATTRGHKDKIFVVKCNPHHVDKLVTVGIKHIKFWQQAGGGFTSKRGTFGSVGKLETMMCVS
YGRMEDLVFSGAATGDIFIWKDILLLKTVKAHDGPVFAMYALDKGFVTGGKDGIVELWDDMFERCLKTYAIKRSALSTSSKGLLLED
NPSIRAITLGHGHILVGTKNGEILEIDKSGPMTLLVQGHMEGEVWGLAAHPLLPICATVSDDKTLRIWELSAQHRMLAVRKLKKGGR
CCAFSPDGKALAVGLNDGSFLVVNADTVEDMVSFHHRKEMISDIKFSKDTGKYLAVASHDNFVDIYNVLTSKRVGICKGASSYITHI
DWDSRGKLLQVNSGAREQLFFEAPRGKRHIIRPSEIEKIEWDTWTCVLGPTCEGIWPAHSDITDVNAASLTKDCSLLATGDDFGFVK
LFSYPVKGQHARFKKYVGHSAHVTNVRWLHNDSVLLTVGGADTALMIWTREFVGTQESKLVDSEESDTDVEEDGGYDSDVAREKAID

YTTKIYAVSIREMEGTKPHQQLKEVSVEERPPVSRAAPQPEKLQKNNITKKKKLVEELALDHVFGYRGFDCRNNLHYLNDGADIIFH
TAAAGIVQNLSTGSQSFYLEHTDDILCLTVNQHPKYRNVVATSQIGTTPSIHIWDAMTKHTLSMLRCFHSKGVNYINFSATGKLLVS
VGVDPEHTITVWRWQEGAKVASRGGHLERIFVVEFRPDSDTQFVSVGVKHMKFWTLAGSALLYKKGVIGSLGAAKMQTMLSVAFGAN
NLTFTGAINGDVYVWKDHFLIRLVAKAHTGPVFTMYTTLRDGLIVTGGKERPTKEGGAVKLWDQEMKRCRAFQLETGQLVECVRSVC
RGKGKILVGTKDGEIIEVGEKNAASNILIDGHMEGEIWGLATHPSKDLFISASNDGTARIWDLADKKLLNKVSLGHAARCAAYSPDG
EMVAIGMKNGEFVILLVNSLKVWGKKRDRKSAIQDIRISPDNRFLAVGSSEHTVDFYDLTQGTNLNRIGYCKDIPSFVIQMDFSADG
KYIQVSTGAYKRQVHEVPLGKQVTEAVVIEKITWASWTSVLGDEVIGIWPRNADKADVNCACVTHAGLNIVTGDDFGLVKLFDFPCT
EKFAKHKRYFGHSAHVTNIRFSYDDKYVVSTGGDDCSVFVWRCL
>sp|O60218|AK1BA_HUMAN
MATFVELSTKAKMPIVGLGTWKSPLGKVKEAVKVAIDAGYRHIDCAYVYQNEHEVGEAIQEKIQEKAVKREDLFIVSKLWPTFFERP
LVRKAFEKTLKDLKLSYLDVYLIHWPQGFKSGDDLFPKDDKGNAIGGKATFLDAWEAMEELVDEGLVKALGVSNFSHFQIEKLLNKP
GLKYKPVTNQVECHPYLTQEKLIQYCHSKGITVTAYSPLGSPDRPWAKPEDPSLLEDPKIKEIAAKHKKTAAQVLIRFHIQRNVIVI
PKSVTPARIVENIQVFDFKLSDEEMATILSFNRNWRACNVLQSSHLEDYPFNAEY
>sp|P08118|MSMB_HUMAN
MNVLLGSVVIFATFVTLCNASCYFIPNEGVPGDSTRKCMDLKGNKHPINSEWQTDNCETCTCYETEISCCTLVSTPVGYDKDNCQRI
FKKEDCKYIVVEKKDPKKTCSVSEWII
>sp|Q9NQE7|TSSP_HUMAN
MAVWLAQWLGPLLLVSLWGLLAPASLLRRLGEHIQQFQESSAQGLGLSLGPGAAALPKVGWLEQLLDPFNVSDRRSFLQRYWVNDQH
WVGQDGPIFLHLGGEGSLGPGSVMRGHPAALAPAWGALVISLEHRFYGLSIPAGGLEMAQLRFLSSRLALADVVSARLALSRLFNIS
SSSPWICFGGSYAGSLAAWARLKFPHLIFASVASSAPVRAVLDFSEYNDVVSRSLMSTAIGGSLECRAAVSVAFAEVERRLRSGGAA
QAALRTELSACGPLGRAENQAELLGALQALVGGVVQYDGQTGAPLSVRQLCGLLLGGGGNRSHSTPYCGLRRAVQIVLHSLGQKCLS
FSRAETVAQLRSTEPQLSGVGDRQWLYQTCTEFGFYVTCENPRCPFSQLPALPSQLDLCEQVFGLSALSVAQAVAQTNSYYGGQTPG
ANKVLFVNGDTDPWHVLSVTQALGSSESTLLIRTGSHCLDMAPERPSDSPSLRLGRQNIFQQLQTWLKLAKESQIKGEV
>sp|P36969|GPX4_HUMAN
MSLGRLCRLLKPALLCGALAAPGLAGTMCASRDDWRCARSMHEFSAKDIDGHMVNLDKYRGFVCIVTNVASQUGKTEVNYTQLVDLH
ARYAECGLRILAFPCNQFGKQEPGSNEEIKEFAAGYNVKFDMFSKICVNGDDAHPLWKWMKIQPKGKGILGNAIKWNFTKFLIDKNG
CVVKRYGPMEEPLVIEKDLPHYF
>sp|Q6ZN84|CCD81_HUMAN
MLDTIARALQDLGRQVLPTLPSLSQEEVSIIWGNVSEFVRRQLTLHKGVQIPAFGTFTFIRQKLEVGNNKFILIQRPVFIMVEKLVQ
IHGLKQNKVYTPGEIPIVPLNFVMISLEGPFNRDVVEGCVKETLLFLSRSISMKQNVEFTFKGIGVLMIRDSKVKMRFYKDFLCTMD
GSGALAKALANRPGTVDSVLSSREALRKWPSSVLAFPRIELKEMENKLPMETLVEECGENRERKCKLKDQSDKEEGTRDISSPKRLR
DRQALFPAKVTNVSLLEKFERSESGGKIMTPESLSYPSCLKHDSEMKPQTSPACQDHNKAGQEMCYVCLQRAQRNSLLYYSEERRRE
IEDERLIQQYQMLKDQEALFRHQMKSLATREQNQKNAAYNLGVAEAIRNHKNEKPEFYKSFLFDKRPLSPALNALKQEEYSRSLLKQ
MDNRQENEIKQRQYRELMDRLEQVQLTEELAAQRAKFLKDKMEETQCYKRALDAQIKNKPSRLPPFEPDSSEPIFGKNEGELMVEKQ
KREQNYMKHQLEAAANHKRKAILHQLVDQRRDLQMLQRTQREHLADRTAELERVNRVNQCLQEDWERSAAMKKQRDLEDKAFERASD
KLFLLDQCEKYRRCKQCQRRTSNVGESNLWPLNKFLPGSRLLV
>sp|Q14032|BAAT_HUMAN
MIQLTATPVSALVDEPVHIRATGLIPFQMVSFQASLEDENGDMFYSQAHYRANEFGEVDLNHASSLGGDYMGVHPMGLFWSLKPEKL
LTRLLKRDVMNRPFQVQVKLYDLELIVNNKVASAPKASLTLERWYVAPGVTRIKVREGRLRGALFLPPGEGLFPGVIDLFGGLGGLL
EFRASLLASRGFASLALAYHNYEDLPRKPEVTDLEYFEEAANFLLRHPKVFGSGVGVVSVCQGVQIGLSMAIYLKQVTATVLINGTN
FPFGIPQVYHGQIHQPLPHSAQLISTNALGLLELYRTFETTQVGASQYLFPIEEAQGQFLFIVGEGDKTINSKAHAEQAIGQLKRHG
KNNWTLLSYPGAGHLIEPPYSPLCCASTTHDLRLHWGGEVIPHAAAQEHAWKEIQRFLRKHLIPDVTSQL
>sp|O75888|TNF13_HUMAN

MPASSPFLLAPKGPPGNMGGPVREPALSVALWLSWGAALGAVACAMALLTQQTELQSLRREVSRLQGTGGPSQNGEGYPWQSLPEQS
SDALEAWENGERSRKRRAVLTQKQKKQHSVLHLVPINATSKDDSDVTEVMWQPALRRGRGLQAQGYGVRIQDAGVYLLYSQVLFQDV
TFTMGQVVSREGQGRQETLFRCIRSMPSHPDRAYNSCYSAGVFHLHQGDILSVIIPRARAKLNLSPHGTFLGFVKL
>sp|Q02575|HEN1_HUMAN
MMLNSDTMELDLPPTHSETESGFSDCGGGAGPDGAGPGGPGGGQARGPEPGEPGRKDLQHLSREERRRRRRATAKYRTAHATRERIR
VEAFNLAFAELRKLLPTLPPDKKLSKIEILRLAICYISYLNHVLDV
>sp|Q9BYR3|KRA44_HUMAN
MVNSCCGSVCSDQGCGLENCCRPSYCQTTCCRTTCCRPSCCVSSCCRPQCCQTTCCRTTCCHPSCCVSSCCRPQCCQSVCCQPTCCR
PQCCQTTCCRTTCCRPSCCRPQCCQSVCCQPTCCCPSYCVSSCCRPQCCQTTCCRTTCCRPSCCVSRCYRPHCGQSLCC
>sp|F8WFD2|NPIA3_HUMAN
MFCCLGYEWLSGGCKTWHSAWVINTLADHRHRGTDFGGSPWLLIITVFLRSYKFAISLCTSYLCVSFLKTIFPSQNGHDGSTDVQQR
ARRSNRRRQEGIKIVLEDIFTLWRQVETKVRAKICKMKVTTKVNRHDKINGKRKTAKEHLRKLSMKEREHGEKERQVSEAEENGKLD
MKEIHTYMEMFQRAQALRRRAEDYYRCKITPSARKPLCNRVRMAAAEHRHSSGLPYWPYLTAETLKNRMGHQPPPPTQQHSIIDNSL
SLKTPPECLLTPLPPSALPSADDNLKTPAECLLYPLPPSADDNLKTPPECLLTPLPPSAPPSVDDNLKTPPKCVCSLPFHPQRMIIS
RN
>sp|Q8N9N2|ASCC1_HUMAN
MEVLRPQLIRIDGRNYRKNPVQEQTYQHEEDEEDFYQGSMECADEPCDAYEVEQTPQGFRSTLRAPSLLYNLIHLNTSNDCGFQKIT
LDCQNIYTWKSRHIVGKRGDTRKKIEMETKTSISIPKPGQDGEIVITGQHRNGVISARTRIDVLLDTFRRKQPFTHFLAFFLNEVEV
QEGFLRFQEEVLAKCSMDHGVDSSIFQNPKKLHLTIGMLVLLSEEEIQQTCEMLQQCKEEFINDISGGKPLEVEMAGIEYMNDDPGM
VDVLYAKVHMKDGSNRLQELVDRVLERFQASGLIVKEWNSVKLHATVMNTLFRKDPNAEGRYNLYTAEGKYIFKERESFDGRNILKS
FALLPRLEYNDAISAHCNLCLPGSSDSPASASQVAGITGVSDAYSQSLPGKS
>sp|Q8WXA8|5HT3C_HUMAN
MEGGWPARQSALLCLTVSLLLQGRGDAFTINCSGFDQHGVDPAVFQAVFDRKAFRPFTNYSIPTRVNISFTLSAILGVDAQLQLLTS
FLWMDLVWDNPFINWNPKECVGINKLTVLAENLWLPDIFIVESMDVDQTPSGLTAYISSEGRIKYDKPMRVTSICNLDIFYFPFDQQ
NCTFTFSSFLYTVDSMLLGMDKEVWEITDTSRKVIQTQGEWELLGINKATPKMSMGNNLYDQIMFYVAIRRRPSLYIINLLVPSSFL
VAIDALSFYLPAESENRAPFKITLLLGYNVFLLMMNDLLPASGTPLISVYFALCLSLMVVSLLETVFITYLLHVATTQPPPMPRWLH
SLLLHCTSPGRCCPTAPQKGNKGLGLTLTHLPGPKEPGELAGKKLGPRETEPDGGSGWTKTQLMELWVQFSHAMDTLLFRLYLLFMA
SSILTVIVLWNT
>sp|Q15699|ALX1_HUMAN
MEFLSEKFALKSPPSKNSDFYMGAGGPLEHVMETLDNESFYSKASAGKCVQAFGPLPRAEHHVRLERTSPCQDSSVNYGITKVEGQP
LHTELNRAMDNCNSLRMSPVKGMQEKGELDELGDKCDSNVSSSKKRRHRTTFTSLQLEELEKVFQKTHYPDVYVREQLALRTELTEA
RVQVWFQNRRAKWRKRERYGQIQQAKSHFAATYDISVLPRTDSYPQIQNNLWAGNASGGSVVTSCMLPRDTSSCMTPYSHSPRTDSS
YTGFSNHQNQFSHVPLNNFFTDSLLTGATNGHAFETKPEFERRSSSIAVLRMKAKEHTANISWAM
>sp|Q5MJ08|SPXN4_HUMAN
MEEPTSSTNENKMKSPCESNKRKVDKKKKNLHRASAPEQSLKETEKAKYPTLVFYCRKNKKRNSNQLENNQPTESSTDPIKEKGDLD
ISAGSPQDGGQN
>sp|P49588|SYAC_HUMAN
MDSTLTASEIRQRFIDFFKRNEHTYVHSSATIPLDDPTLLFANAGMNQFKPIFLNTIDPSHPMAKLSRAANTQKCIRAGGKHNDLDD
VGKDVYHHTFFEMLGSWSFGDYFKELACKMALELLTQEFGIPIERLYVTYFGGDEAAGLEADLECKQIWQNLGLDDTKILPGNMKDN
FWEMGDTGPCGPCSEIHYDRIGGRDAAHLVNQDDPNVLEIWNLVFIQYNREADGILKPLPKKSIDTGMGLERLVSVLQNKMSNYDTD
LFVPYFEAIQKGTGARPYTGKVGAEDADGIDMAYRVLADHARTITVALADGGRPDNTGRGYVLRRILRRAVRYAHEKLNASRGFFAT
LVDVVVQSLGDAFPELKKDPDMVKDIINEEEVQFLKTLSRGRRILDRKIQSLGDSKTIPGDTAWLLYDTYGFPVDLTGLIAEEKGLV
VDMDGFEEERKLAQLKSQGKGAGGEDLIMLDIYAIEELRARGLEVTDDSPKYNYHLDSSGSYVFENTVATVMALRREKMFVEEVSTG
QECGVVLDKTCFYAEQGGQIYDEGYLVKVDDSSEDKTEFTVKNAQVRGGYVLHIGTIYGDLKVGDQVWLFIDEPRRRPIMSNHTATH

ILNFALRSVLGEADQKGSLVAPDRLRFDFTAKGAMSTQQIKKAEEIANEMIEAAKAVYTQDCPLAAAKAIQGLRAVFDETYPDPVRV
VSIGVPVSELLDDPSGPAGSLTSVEFCGGTHLRNSSHAGAFVIVTEEAIAKGIRRIVAVTGAEAQKALRKAESLKKCLSVMEAKVKA
QTAPNKDVQREIADLGEALATAVIPQWQKDELRETLKSLKKVMDDLDRASKADVQKRVLEKTKQFIDSNPNQPLVILEMESGASAKA
LNEALKLFKMHSPQTSAMLFTVDNEAGKITCLCQVPQNAANRGLKASEWVQQVSGLMDGKGGGKDVSAQATGKNVGCLQEALQLATS
FAQLRLGDVKN
>sp|Q14953|KI2S5_HUMAN
MLLMVISMACVAFFLLQGAWPHEGFRRKPSLLAHPGPLVKSEETVILQCWSDVMFEHFLLHREGTFNHTLRLIGEHIDGVSKGNFSI
GRMTQDLAGTYRCYGSVTHSPYQLSAPSDPLDIVITGLYEKPSLPAQPGPTVLAGESVTLSCSSRSSYDMYHLSREGEAHERRLPAG
PKVNRTFQADSPLDPATHGGAYRCFGSFRDSPYEWSKSSDPLLVSVTGNSSNSWPSPTEPSSETGNPRHLHVLIGTSVVKLPFTILL
FFLLHRWCSNKKNASVMDQGPAGNRTVNREDSDEQDHQEVSYA
>sp|Q8N402|YV020_HUMAN
MGIHFSCIRGDLKKPSKKRVKREPYSTTMLQVTSLSPINEILRRYSLYTNQHWRHHGFWRKKIQPQEASEEPPAHKDRGGGERPVNA
RVVRVAPLRPGFALCGYAVQDISKEDTVYDICNEDAVDISDEDTVDISNEASVHDISNEAAVCDISNDAVNISNEAAVRDISNDAVD
ICNEAAVHDISNEDTIEDISYEDTVYDITNEDAVRYLCKKDATKEPLTLENDLIVESMSDDEDFAA
>sp|P21266|GSTM3_HUMAN
MSCESSMVLGYWDIRGLAHAIRLLLEFTDTSYEEKRYTCGEAPDYDRSQWLDVKFKLDLDFPNLPYLLDGKNKITQSNAILRYIARK
HNMCGETEEEKIRVDIIENQVMDFRTQLIRLCYSSDHEKLKPQYLEELPGQLKQFSMFLGKFSWFAGEKLTFVDFLTYDILDQNRIF
DPKCLDEFPNLKAFMCRFEALEKIAAYLQSDQFCKMPINNKMAQWGNKPVC
>sp|Q9H2M3|BHMT2_HUMAN
MAPAGRPGAKKGILERLESGEVVIGDGSFLITLEKRGYVKAGLWTPEAVIEHPDAVRQLHMEFLRAGSNVMQTFTFSASEDNMESKW
EDVNAAACDLAREVAGKGDALVAGGICQTSIYKYQKDEARIKKLFRQQLEVFAWKNVDFLIAEYFEHVEEAVWAVEVLKESDRPVAV
TMCIGPEGDMHDITPGECAVRLVKAGASIVGVNCRFGPDTSLKTMELMKEGLEWAGLKAHLMVQPLGFHAPDCGKEGFVDLPEYPFG
LESRVATRWDIQKYAREAYNLGVRYIGGCCGFEPYHIRAIAEELAPERGFLPPASEKHGSWGSGLDMHTKPWIRARARREYWENLLP
ASGRPFCPSLSKPDF
>sp|P54727|RD23B_HUMAN
MQVTLKTLQQQTFKIDIDPEETVKALKEKIESEKGKDAFPVAGQKLIYAGKILNDDTALKEYKIDEKNFVVVMVTKPKAVSTPAPAT
TQQSAPASTTAVTSSTTTTVAQAPTPVPALAPTSTPASITPASATASSEPAPASAAKQEKPAEKPAETPVATSPTATDSTSGDSSRS
NLFEDATSALVTGQSYENMVTEIMSMGYEREQVIAALRASFNNPDRAVEYLLMGIPGDRESQAVVDPPQAASTGAPQSSAVAAAAAT
TTATTTTTSSGGHPLEFLRNQPQFQQMRQIIQQNPSLLPALLQQIGRENPQLLQQISQHQEHFIQMLNEPVQEAGGQGGGGGGGSGG
IAEAGSGHMNYIQVTPQEKEAIERLKALGFPEGLVIQAYFACEKNENLAANFLLQQNFDED
>sp|Q6RVD6|SPAT8_HUMAN
MAPAGMSGAQDNSCLYQEIAPSFQRLPCPRTSSRHFSEAMTCPCGWRPFKGGPGGLKGPVWPAKEENSCSHGRIQRVQRRRVPSASP
LIQKINRRSVLFHPYCWS
>sp|A7E2V4|ZSWM8_HUMAN
MELMFAEWEDGERFSFEDSDRFEEDSLCSFISEAESLCQNWRGWRKQSAGPNSPTGGGGGGGSGGTRMRDGLVIPLVELSAKQVAFH
IPFEVVEKVYPPVPEQLQLRIAFWSFPENEEDIRLYSCLANGSADEFQRGDQLFRMRAVKDPLQIGFHLSATVVPPQMVPPKGAYNV
AVMFDRCRVTSCSCTCGAGAKWCTHVVALCLFRIHNASAVCLRAPVSESLSRLQRDQLQKFAQYLISELPQQILPTAQRLLDELLSS
QSTAINTVCGAPDPTAGPSASDQSTWYLDESTLTDNIKKTLHKFCGPSPVVFSDVNSMYLSSTEPPAAAEWACLLRPLRGREPEGVW
NLLSIVREMFKRRDSNAAPLLEILTDQCLTYEQITGWWYSVRTSASHSSASGHTGRSNGQSEVAAHACASMCDEMVTLWRLAVLDPA
LSPQRRRELCTQLRQWQLKVIENVKRGQHKKTLERLFPGFRPAVEACYFNWEEAYPLPGVTYSGTDRKLALCWARALPSRPGASRSG
GLEESRDRPRPLPTEPAVRPKEPGTKRKGLGEGVPSSQRGPRRLSAEGGDKALHKMGPGGGKAKALGGAGSGSKGSAGGGSKRRLSS
EDSSLEPDLAEMSLDDSSLALGAEASTFGGFPESPPPCPLHGGSRGPSTFLPEPPDTYEEDGGVYFSEGPEPPTASVGPPGLLPGDV
CTQDDLPSTDESGNGLPKTKEAAPAVGEEDDDYQAYYLNAQDGAGGEEEKAEGGAGEEHDLFAGLKPLEQESRMEVLFACAEALHAH
GYSSEASRLTVELAQDLLANPPDLKVEPPPAKGKKNKVSTSRQTWVATNTLSKAAFLLTVLSERPEHHNLAFRVGMFALELQRPPAS

TKALEVKLAYQESEVAALLKKIPLGPSEMSTMRCRAEELREGTLCDYRPVLPLMLASFIFDVLCAPGSRPPSRNWNSETPGDEELGF
EAAVAALGMKTTVSEAEHPLLCEGTRREKGDLALALMITYKDDQAKLKKILDKLLDRESQTHKPQTLSSFYSSSRPTTASQRSPSKH
GGPSAPGALQPLTSGSAGPAQPGSVAGAGPGPTEGFTEKNVPESSPHSPCEGLPSEAALTPRPEGKVPSRLALGSRGGYNGRGWGSP
GRPKKKHTGMASIDSSAPETTSDSSPTLSRRPLRGGWAPTSWGRGQDSDSISSSSSDSLGSSSSSGSRRASASGGARAKTVEVGRYK
GRRPESHAPHVPNQPSEAAAHFYFELAKTVLIKAGGNSSTSIFTHPSSSGGHQGPHRNLHLCAFEIGLYALGLHNFVSPNWLSRTYS
SHVSWITGQAMEIGSAALTILVECWDGHLTPPEVASLADRASRARDSNMVRAAAELALSCLPHAHALNPNEIQRALVQCKEQDNLML
EKACMAVEEAAKGGGVYPEVLFEVAHQWFWLYEQTAGGSSTAREGATSCSASGIRAGGEAGRGMPEGRGGPGTEPVTVAAAAVTAAA
TVVPVISVGSSLYPGPGLGHGHSPGLHPYTALQPHLPCSPQYLTHPAHPAHPMPHMPRPAVFPVPSSAYPQGVHPAFLGAQYPYSVT
PPSLAATAVSFPVPSMAPITVHPYHTEPGLPLPTSVACELWGQGTVSSVHPASTFPAIQGASLPALTTQPSPLVSGGFPPPEEETHS
QPVNPHSLHHLHAAYRVGMLALEMLGRRAHNDHPNNFSRSPPYTDDVKWLLGLAAKLGVNYVHQFCVGAAKGVLSPFVLQEIVMETL
QRLSPAHAHNHLRAPAFHQLVQRCQQAYMQYIHHRLIHLTPADYDDFVNAIRSARSAFCLTPMGMMQFNDILQNLKRSKQTKELWQR
VSLEMATFSP
>sp|O00574|CXCR6_HUMAN
MAEHDYHEDYGFSSFNDSSQEEHQDFLQFSKVFLPCMYLVVFVCGLVGNSLVLVISIFYHKLQSLTDVFLVNLPLADLVFVCTLPFW
AYAGIHEWVFGQVMCKSLLGIYTINFYTSMLILTCITVDRFIVVVKATKAYNQQAKRMTWGKVTSLLIWVISLLVSLPQIIYGNVFN
LDKLICGYHDEAISTVVLATQMTLGFFLPLLTMIVCYSVIIKTLLHAGGFQKHRSLKIIFLVMAVFLLTQMPFNLMKFIRSTHWEYY
AMTSFHYTIMVTEAIAYLRACLNPVLYAFVSLKFRKNFWKLVKDIGCLPYLGVSHQWKSSEDNSKTFSASHNVEATSMFQL
>sp|P78396|CCNA1_HUMAN
METGFPAIMYPGSFIGGWGEEYLSWEGPGLPDFVFQQQPVESEAMHCSNPKSGVVLATVARGPDACQILTRAPLGQDPPQRTVLGLL
TANGQYRRTCGQGITRIRCYSGSENAFPPAGKKALPDCGVQEPPKQGFDIYMDELEQGDRDSCSVREGMAFEDVYEVDTGTLKSDLH
FLLDFNTVSPMLVDSSLLSQSEDISSLGTDVINVTEYAEEIYQYLREAEIRHRPKAHYMKKQPDITEGMRTILVDWLVEVGEEYKLR
AETLYLAVNFLDRFLSCMSVLRGKLQLVGTAAMLLASKYEEIYPPEVDEFVYITDDTYTKRQLLKMEHLLLKVLAFDLTVPTTNQFL
LQYLRRQGVCVRTENLAKYVAELSLLEADPFLKYLPSLIAAAAFCLANYTVNKHFWPETLAAFTGYSLSEIVPCLSELHKAYLDIPH
RPQQAIREKYKASKYLCVSLMEPPAVLLLQ
>sp|Q9BZK8|OCR1_HUMAN
MPVAPSNHCDNQCPHIFSKALVVSVAPSPPRDKPAPYTFTDVSSLCGLQKKCEGGKAMLFTLKRDRFSFLLFVSHC
>sp|Q6S5L8|SHC4_HUMAN
MRERGQDSLAGLVLYVGLFGHPGMLHRAKYSRFRNESITSLDEGSSGGSVGNKGSPQPPHPALAPHLPTEDATLPSQESPTPLCTLI
PRMASMKLANPATLLSLKNFCLGTKEVPRLKLQESRDPGSSGPSSPETSLSRSGTAPPPQQDLVGHRATALTPDSCPLPGPGEPTLR
SRQDRHFLQHLLGMGMNYCVRYMGCVEVLQSMRSLDFGMRTQVTREAISRLCEAVPGANGAIKKRKPPVKFLSTVLGKSNLQFSGMN
IKLTISTCSLTLMNLDNQQIIANHHMQSISFASGGDPDTTDYVAYVAKDPVNQRACHILECHNGMAQDVISTIGQAFELRFKQYLKN
PSLNTSCESEEVHIDSHAEEREDHEYYNEIPGKQPPVGGVSDMRIKVQATEQMAYCPIQCEKLCYLPGNSKCSSVYENCLEQSRAIG
NVHPRGVQSQRDTSLLKHTCRVDLFDDPCYINTQALQSTPGSAGNQRSAQPLGSPWHCGKAPETVQPGATAQPASSHSLPHIKQQLW
SEECYHGKLSRKAAESLLVKDGDFLVRESATSPGQYVLSGLQGGQAKHLLLVDPEGKVRTKDHVFDNVGHLIRYHMDNSLPIISSGS
EVSLKQPVRKDNNPALLHSNK
>sp|P0C7V7|SC11B_HUMAN
MNKWRLYYQVLNFGMIVSSALMIWKGLMVITGSESPIVLLSGSMEPAFHRGYLLFLTNRVEDPIRVGEIAVLRIEGRKIPIVHRVLK
IHEKQNGHIKFLTKGDNNAVDDRGLYKQDQHWLEKKDVVGRARGFVPYIGIGTSLMNDYPKHKYEVLFLLGLFVLVHRE
>sp|P51116|FXR2_HUMAN
MGGLASGGDVEPGLPVEVRGSNGAFYKGFVKDVHEDSVTIFFENNWQSERQIPFGDVRLPPPADYNKEITEGDEVEVYSRANEQEPC
GWWLARVRMMKGDFYVIEYAACDATYNEIVTLERLRPVNPNPLATKGSFFKVTMAVPEDLREACSNENVHKEFKKALGANCIFLNIT
NSELFILSTTEAPVKRASLLGDMHFRSLRTKLLLMSRNEEATKHLETSKQLAAFQEEFTVREDLMGLAIGTHGANIQQARKVPGVT
AIELGEETCTFRIYGETPEACRQARSYLEFSEDSVQVPRNLVGKVIGKNGKVIQEIVDKSGVVRVRVEGDNDKKNPREEGMVPFIFV
GTRENISNAQALLEYHLSYLQEVEQLRLERLQIDEQLRQIGLGFRPPGSGRGSGGSDKAGYSTDESSSSSLHATRTYGGSYGGRGRG

RRTGGPAYGPSSDVSTASETESEKREEPNRAGPGDRDPPTRGEESRRRPTGGRGRGPPPAPRPTSRYNSSSISSVLKDPDSNPYSLL
DTSEPEPPVDSEPGEPPPASARRRRSRRRRTDEDRTVMDGGLESDGPNMTENGLEDESRPQRRNRSRRRRNRGNRTDGSISGDRQPV
TVADYISRAESQSRQRPPLERTKPSEDSLSGQKGDSVSKLPKGPSENGELSAPLELGSMVNGVS
>sp|Q6PGQ7|BORA_HUMAN
MGDVKESKMQITPETPGRIPVLNPFESPSDYSNLHEQTLASPSVFKSTKLPTPGKFRWSIDQLAVINPVEIDPEDIHRQALYLSHSR
IDKDVEDKRQKAIEEFFTKDVIVPSPWTDHEGKQLSQCHSSKCTNINSDSPVGKKLTIHSEKSDAACQTLLSLPVDFNLENILGDYF
RADEFADQSPGNLSSSSLRRKLFLDGNGSISDSLPSASPGSPHSGVQTSLEMFYSIDLSPVKCRSPLQTPSSGQFSSSPIQASAKKY
SLGSITSPSPISSPTFSPIEFQIGETPLSEQRKFTVHSPDASSGTNSNGITNPCIRSPYIDGCSPIKNWSPMRLQMYSGGTQYRTSV
IQIPFTLETQGEDEEDKENIPSTDVSSPAMDAAGIHLRQFSNEASTHGTHLVVTAMSVTQNQSSASEKELALLQDVEREKDNNTVDM
VDPIEIADETTWIKEPVDNGSLPMTDFVSGIAFSIENSHMCMSPLAESSVIPCESSNIQMDSGYNTQNCGSNIMDTVGAESYCKESD
AQTCEVESKSQAFNMKQDHTTQRCWMKTASPFQCSSP
>sp|P49257|LMAN1_HUMAN
MAGSRQRGLRARVRPLFCALLLSLGRFVRGDGVGGDPAVALPHRRFEYKYSFKGPHLVQSDGTVPFWAHAGNAIPSSDQIRVAPSLK
SQRGSVWTKTKAAFENWEVEVTFRVTGRGRIGADGLAIWYAENQGLEGPVFGSADLWNGVGIFFDSFDNDGKKNNPAIVIIGNNGQI
HYDHQNDGASQALASCQRDFRNKPYPVRAKITYYQNTLTVMINNGFTPDKNDYEFCAKVENMIIPAQGHFGISAATGGLADDHDVLS
FLTFQLTEPGKEPPTPDKEISEKEKEKYQEEFEHFQQELDKKKEEFQKGHPDLQGQPAEEIFESVGDRELRQVFEGQNRIHLEIKQL
NRQLDMILDEQRRYVSSLTEEISKRGAGMPGQHGQITQQELDTVVKTQHEILRQVNEMKNSMSETVRLVSGMQHPGSAGGVYETTQH
FIDIKEHLHIVKRDIDNLVQRNMPSNEKPKCPELPPFPSCLSTVHFIIFVVVQTVLFIGYIMYRSQQEAAAKKFF
>sp|Q9BQ04|RBM4B_HUMAN
MVKLFIGNLPREATEQEIRSLFEQYGKVLECDIIKNYGFVHIEDKTAAEDAIRNLHHYKLHGVNINVEASKNKSKASTKLHVGNISP
TCTNQELRAKFEEYGPVIECDIVKDYAFVHMERAEDAVEAIRGLDNTEFQGKRMHVQLSTSRLRTAPGMGDQSGCYRCGKEGHWSKE
CPVDRTGRVADFTEQYNEQYGAVRTPYTMGYGESMYYNDAYGALDYYKRYRVRSYEAVAAAAAASAYNYAEQTMSHLPQVQSTTVTS
HLNSTSVDPYDRHLLPNSGAAATSAAMAAAAATTSSYYGRDRSPLRRAAAMLPTVGEGYGYGPESELSQASAATRNSLYDMARYERE
QYVDRARYSAF
>sp|Q93052|LPP_HUMAN
MSHPSWLPPKSTGEPLGHVPARMETTHSFGNPSISVSTQQPPKKFAPVVAPKPKYNPYKQPGGEGDFLPPPPPPLDDSSALPSISGN
FPPPPPLDEEAFKVQGNPGGKTLEERRSSLDAEIDSLTSILADLECSSPYKPRPPQSSTGSTASPPVSTPVTGHKRMVIPNQPPLTA
TKKSTLKPQPAPQAGPIPVAPIGTLKPQPQPVPASYTTASTSSRPTFNVQVKSAQPSPHYMAAPSSGQIYGSGPQGYNTQPVPVSGQ
CPPPSTRGGMDYAYIPPPGLQPEPGYGYAPNQGRYYEGYYAAGPGYGGRNDSDPTYGQQGHPNTWKREPGYTPPGAGNQNPPGMYPV
TGPKKTYITDPVSAPCAPPLQPKGGHSGQLGPSSVAPSFRPEDELEHLTKKMLYDMENPPADEYFGRCARCGENVVGEGTGCTAMDQ
VFHVDCFTCIICNNKLRGQPFYAVEKKAYCEPCYINTLEQCNVCSKPIMERILRATGKAYHPHCFTCVMCHRSLDGIPFTVDAGGLI
HCIEDFHKKFAPRCSVCKEPIMPAPGQEETVRIVALDRDFHVHCYRCEDCGGLLSEGDNQGCYPLDGHILCKTCNSARIRVLTAKAS
TDL
>sp|Q00059|TFAM_HUMAN
MAFLRSMWGVLSALGRSGAELCTGCGSRLRSPFSFVYLPRWFSSVLASCPKKPVSSYLRFSKEQLPIFKAQNPDAKTTELIRRIAQR
WRELPDSKKKIYQDAYRAEWQVYKEEISRFKEQLTPSQIMSLEKEIMDKHLKRKAMTKKKELTLLGKPKRPRSAYNVYVAERFQEAK
GDSPQEKLKTVKENWKNLSDSEKELYIQHAKEDETRYHNEMKSWEEQMIEVGRKDLLRRTIKKQRKYGAEEC
>sp|Q99466|NOTC4_HUMAN
MQPPSLLLLLLLLLLLCVSVVRPRGLLCGSFPEPCANGGTCLSLSLGQGTCQCAPGFLGETCQFPDPCQNAQLCQNGGSCQALLPAP
LGLPSSPSPLTPSFLCTCLPGFTGERCQAKLEDPCPPSFCSKRGRCHIQASGRPQCSCMPGWTGEQCQLRDFCSANPCVNGGVCLAT
YPQIQCHCPPGFEGHACERDVNECFQDPGPCPKGTSCHNTLGSFQCLCPVGQEGPRCELRAGPCPPRGCSNGGTCQLMPEKDSTFHL
CLCPPGFIGPDCEVNPDNCVSHQCQNGGTCQDGLDTYTCLCPETWTGWDCSEDVDECETQGPPHCRNGGTCQNSAGSFHCVCVSGWG
GTSCEENLDDCIAATCAPGSTCIDRVGSFSCLCPPGRTGLLCHLEDMCLSQPCHGDAQCSTNPLTGSTLCLCQPGYSGPTCHQDLDE
CLMAQQGPSPCEHGGSCLNTPGSFNCLCPPGYTGSRCEADHNECLSQPCHPGSTCLDLLATFHCLCPPGLEGQLCEVETNECASAPC

LNHADCHDLLNGFQCICLPGFSGTRCEEDIDECRSSPCANGGQCQDQPGAFHCKCLPGFEGPRCQTEVDECLSDPCPVGASCLDLPG
AFFCLCPSGFTGQLCEVPLCAPNLCQPKQICKDQKDKANCLCPDGSPGCAPPEDNCTCHHGHCQRSSCVCDVGWTGPECEAELGGCI
SAPCAHGGTCYPQPSGYNCTCPTGYTGPTCSEEMTACHSGPCLNGGSCNPSPGGYYCTCPPSHTGPQCQTSTDYCVSAPCFNGGTCV
NRPGTFSCLCAMGFQGPRCEGKLRPSCADSPCRNRATCQDSPQGPRCLCPTGYTGGSCQTLMDLCAQKPCPRNSHCLQTGPSFHCLC
LQGWTGPLCNLPLSSCQKAALSQGIDVSSLCHNGGLCVDSGPSYFCHCPPGFQGSLCQDHVNPCESRPCQNGATCMAQPSGYLCQCA
PGYDGQNCSKELDACQSQPCHNHGTCTPKPGGFHCACPPGFVGLRCEGDVDECLDQPCHPTGTAACHSLANAFYCQCLPGHTGQWCE
VEIDPCHSQPCFHGGTCEATAGSPLGFICHCPKGFEGPTCSHRAPSCGFHHCHHGGLCLPSPKPGFPPRCACLSGYGGPDCLTPPAP
KGCGPPSPCLYNGSCSETTGLGGPGFRCSCPHSSPGPRCQKPGAKGCEGRSGDGACDAGCSGPGGNWDGGDCSLGVPDPWKGCPSHS
RCWLLFRDGQCHPQCDSEECLFDGYDCETPPACTPAYDQYCHDHFHNGHCEKGCNTAECGWDGGDCRPEDGDPEWGPSLALLVVLSP
PALDQQLFALARVLSLTLRVGLWVRKDRDGRDMVYPYPGARAEEKLGGTRDPTYQERAAPQTQPLGKETDSLSAGFVVVMGVDLSRC
GPDHPASRCPWDPGLLLRFLAAMAAVGALEPLLPGPLLAVHPHAGTAPPANQLPWPVLCSPVAGVILLALGALLVLQLIRRRRREHG
ALWLPPGFTRRPRTQSAPHRRRPPLGEDSIGLKALKPKAEVDEDGVVMCSGPEEGEEVGQAEETGPPSTCQLWSLSGGCGALPQAAM
LTPPQESEMEAPDLDTRGPDGVTPLMSAVCCGEVQSGTFQGAWLGCPEPWEPLLDGGACPQAHTVGTGETPLHLAARFSRPTAARRL
LEAGANPNQPDRAGRTPLHAAVAADAREVCQLLLRSRQTAVDARTEDGTTPLMLAARLAVEDLVEELIAAQADVGARDKWGKTALHW
AAAVNNARAARSLLQAGADKDAQDNREQTPLFLAAREGAVEVAQLLLGLGAARELRDQAGLAPADVAHQRNHWDLLTLLEGAGPPEA
RHKATPGREAGPFPRARTVSVSVPPHGGGALPRCRTLSAGAGPRGGGACLQARTWSVDLAARGGGAYSHCRSLSGVGAGGGPTPRGR
RFSAGMRGPRPNPAIMRGRYGVAAGRGGRVSTDDWPCDWVALGACGSASNIPIPPPCLTPSPERGSPQLDCGPPALQEMPINQGGEG
KK
>sp|O60656|UD19_HUMAN
MACTGWTSPLPLCVCLLLTCGFAEAGKLLVVPMDGSHWFTMRSVVEKLILRGHEVVVVMPEVSWQLGRSLNCTVKTYSTSYTLEDLD
REFKAFAHAQWKAQVRSIYSLLMGSYNDIFDLFFSNCRSLFKDKKLVEYLKESSFDAVFLDPFDNCGLIVAKYFSLPSVVFARGILC
HYLEEGAQCPAPLSYVPRILLGFSDAMTFKERVRNHIMHLEEHLLCHRFFKNALEIASEILQTPVTEYDLYSHTSIWLLRTDFVLDY
PKPVMPNMIFIGGINCHQGKPLPMEFEAYINASGEHGIVVFSLGSMVSEIPEKKAMAIADALGKIPQTVLWRYTGTRPSNLANNTIL
VKWLPQNDLLGHPMTRAFITHAGSHGVYESICNGVPMVMMPLFGDQMDNAKRMETKGAGVTLNVLEMTSEDLENALKAVINDKSYKE
NIMRLSSLHKDRPVEPLDLAVFWVEFVMRHKGAPHLRPAAHDLTWYQYHSLDVIGFLLAVVLTVAFITFKCCAYGYRKCLGKKGRVK
KAHKSKTH
>sp|P05114|HMGN1_HUMAN
MPKRKVSSAEGAAKEEPKRRSARLSAKPPAKVEAKPKKAAAKDKSSDKKVQTKGKRGAKGKQAEVANQETKEDLPAENGETKTEESP
ASDEAGEKEAKSD
>sp|Q9UJM8|HAOX1_HUMAN
MLPRLICINDYEQHAKSVLPKSIYDYYRSGANDEETLADNIAAFSRWKLYPRMLRNVAETDLSTSVLGQRVSMPICVGATAMQRMAH
VDGELATVRACQSLGTGMMLSSWATSSIEEVAEAGPEALRWLQLYIYKDREVTKKLVRQAEKMGYKAIFVTVDTPYLGNRLDDVRNR
FKLPPQLRMKNFETSTLSFSPEENFGDDSGLAAYVAKAIDPSISWEDIKWLRRLTSLPIVAKGILRGDDAREAVKHGLNGILVSNHG
ARQLDGVPATIDVLPEIVEAVEGKVEVFLDGGVRKGTDVLKALALGAKAVFVGRPIVWGLAFQGEKGVQDVLEILKEEFRLAMALSG
CQNVKVIDKTLVRKNPLAVSKI
>sp|Q9HAA7|YG046_HUMAN
MLFGIRILVNTPSPLVTGLHHYNPSIHRDQGECANQWRKGPGSAHLAGLAGRCSLINTPSPLVTGLQRYNPSMDRAQGMCASLEEEA
GLCKPLWAWWELQSHKHSQTSHHRAAGLQSQHAPGSGRVRITGGKV
>sp|Q6P582|MZT2A_HUMAN
MAAQGVGPGPGSAAPPGLEAARQKLALRRKKVLSTEEMELYELAQAAGGGIDPDVFKILVDLLKLNVAPLAVFQMLKSMCAGQRLAS
EPQDPAAVSLPTSSVPETRGRDKGSAALGGVLALAERSNHEGSSQRMPRQPSATRLPKGGGPGKSPTQGST
>sp|A6NCW0|U17L3_HUMAN
MGDDSLYLGGEWQFNHFSKLTSSRPDAAFAEIQRTSLPEKSPLSSETRVDLCDDLAPVARQLAPREKLPLSSRRPAAVGAGLQNMGN
TCYENASLQCLTYTLPLANYMLSREHSQTCQRPKCCMLCTMQAHITWALHSPGHVIQPSQALASGFHRGKQEDVHEFLMFTVDAMKK

ACLPGHKQVDHHSKDTTLIHQIFGGCWRSQIKCLHCHGISDTFDPYLDIALDIQAAQSVKQALEQLVKPEELNGENAYHCGLCLQRA
PASNTLTLHTSAKVLILVLKRFSDVAGNKLAKNVQYPECLDMQPYMSQQNTGPLVYVLYAVLVHAGWSCHDGHYFSYVKAQEGQWYK
MDDAEVTVCSITSVLSQQAYVLFYIQKSEWERHSESVSRGREPRALGAEDTDRRAKQGELKRDHPCLQAPELDEHLVERATQESTLD
HWKFLQEQNKTKPEFNVGKVEGTLPPNALVIHQSKYKCGMKNHHPEQQSSLLNLSSTTRTDQESMNTGTLASLQGRTRRAKGKNKHS
KRALLVCQ
>sp|P46063|RECQ1_HUMAN
MASVSALTEELDSITSELHAVEIQIQELTERQQELIQKKKVLTKKIKQCLEDSDAGASNEYDSSPAAWNKEDFPWSGKVKDILQNVF
KLEKFRPLQLETINVTMAGKEVFLVMPTGGGKSLCYQLPALCSDGFTLVICPLISLMEDQLMVLKQLGISATMLNASSSKEHVKWVH
AEMVNKNSELKLIYVTPEKIAKSKMFMSRLEKAYEARRFTRIAVDEVHCCSQWGHDFRPDYKALGILKRQFPNASLIGLTATATNHV
LTDAQKILCIEKCFTFTASFNRPNLYYEVRQKPSNTEDFIEDIVKLINGRYKGQSGIIYCFSQKDSEQVTVSLQNLGIHAGAYHANL
EPEDKTTVHRKWSANEIQVVVATVAFGMGIDKPDVRFVIHHSMSKSMENYYQESGRAGRDDMKADCILYYGFGDIFRISSMVVMENV
GQQKLYEMVSYCQNISKCRRVLMAQHFDEVWNSEACNKMCDNCCKDSAFERKNITEYCRDLIKILKQAEELNEKLTPLKLIDSWMGK
GAAKLRVAGVVAPTLPREDLEKIIAHFLIQQYLKEDYSFTAYATISYLKIGPKANLLNNEAHAITMQVTKSTQNSFRAESSQTCHSE
QGDKKMEEKNSGNFQKKAANMLQQSGSKNTGAKKRKIDDA
>sp|Q9UHI7|S23A1_HUMAN
MRAQEDLEGRTQHETTRDPSTPLPTEPKFDMLYKIEDVPPWYLCILLGFQHYLTCFSGTIAVPFLLAEALCVGHDQHMVSQLIGTIF
TCVGITTLIQTTVGIRLPLFQASAFAFLVPAKAILALERWKCPPEEEIYGNWSLPLNTSHIWHPRIREVQGAIMVSSVVEVVIGLLG
LPGALLNYIGPLTVTPTVSLIGLSVFQAAGDRAGSHWGISACSILLIILFSQYLRNLTFLLPVYRWGKGLTLLRIQIFKMFPIMLAI
MTVWLLCYVLTLTDVLPTDPKAYGFQARTDARGDIMAIAPWIRIPYPCQWGLPTVTAAAVLGMFSATLAGIIESIGDYYACARLAGA
PPPPVHAINRGIFTEGICCIIAGLLGTGNGSTSSSPNIGVLGITKVGSRRVVQYGAAIMLVLGTIGKFTALFSSLPDPILGGMFCTL
FGMITAVGLSNLQFVDMNSSRNLFVLGFSMFFGLTLPNYLESNPGAINTGILEVDQILIVLLTTEMFVGGCLAFILDNTVPGSPEER
GLIQWKAGAHANSDMSSSLKSYDFPIGMGIVKRITFLKYIPICPVFKGFSSSSKDQIAIPEDTPENTETASVCTKV
>sp|Q8WWY8|LIPH_HUMAN
MLRFYLFISLLCLSRSDAEETCPSFTRLSFHSAVVGTGLNVRLMLYTRKNLTCAQTINSSAFGNLNVTKKTTFIVHGFRPTGSPPVW
MDDLVKGLLSVEDMNVVVVDWNRGATTLIYTHASSKTRKVAMVLKEFIDQMLAEGASLDDIYMIGVSLGAHISGFVGEMYDGWLGRI
TGLDPAGPLFNGKPHQDRLDPSDAQFVDVIHSDTDALGYKEPLGNIDFYPNGGLDQPGCPKTILGGFQYFKCDHQRSVYLYLSSLRE
SCTITAYPCDSYQDYRNGKCVSCGTSQKESCPLLGYYADNWKDHLRGKDPPMTKAFFDTAEESPFCMYHYFVDIITWNKNVRRGDIT
IKLRDKAGNTTESKINHEPTTFQKYHQVSLLARFNQDLDKVAAISLMFSTGSLIGPRYKLRILRMKLRSLAHPERPQLCRYDLVLME
NVETVFQPILCPELQL
>sp|Q3V5L5|MGT5B_HUMAN
MITVNPDGKIMVRRCLVTLRPFRLFVLGIGFFTLCFLMTSLGGQFSARRLGDSPFTIRTEVMGGPESRGVLRKMSDLLELMVKRMDA
LARLENSSELHRAGGDLHFPADRMPPGAGLMERIQAIAQNVSDIAVKVDQILRHSLLLHSKVSEGRRDQCEAPSDPKFPDCSGKVEW
MRARWTSDPCYAFFGVDGTECSFLIYLSEVEWFCPPLPWRNQTAAQRAPKPLPKVQAVFRSNLSHLLDLMGSGKESLIFMKKRTKRL
TAQWALAAQRLAQKLGATQRDQKQILVHIGFLTEESGDVFSPRVLKGGPLGEMVQWADILTALYVLGHGLRVTVSLKELQSNLGVPP
GRGSCPLTMPLPFDLIYTDYHGLQQMKRHMGLSFKKYRCRIRVIDTFGTEPAYNHEEYATLHGYRTNWGYWNLNPKQFMTMFPHTPD
NSFMGFVSEELNETEKRLIKGGKASNMAVVYGKEASIWKLQGKEKFLGILNKYMEIHGTVYYESQRPPEVPAFVKNHGLLPQPEFQQ
LLRKAKLFIGFGFPYEGPAPLEAIANGCIFLQSRFSPPHSSLNHEFFRGKPTSREVFSQHPYAENFIGKPHVWTVDYNNSEEFEAAI
KAIMRTQVDPYLPYEYTCEGMLERIHAYIQHQDFCRAPDPALPEAHAPQSPFVLAPNATHLEWARNTSLAPGAWPPAHALRAWLAVP
GRACTDTCLDHGLICEPSFFPFLNSQDAFLKLQVPCDSTESEMNHLYPAFAQPGQECYLQKEPLLFSCAGSNTKYRRLCPCRDFRKG
QVALCQGCL
>sp|P43351|RAD52_HUMAN
MSGTEEAILGGRDSHPAAGGGSVLCFGQCQYTAEEYQAIQKALRQRLGPEYISSRMAGGGQKVCYIEGHRVINLANEMFGYNGWAHS
ITQQNVDFVDLNNGKFYVGVCAFVRVQLKDGSYHEDVGYGVSEGLKSKALSLEKARKEAVTDGLKRALRSFGNALGNCILDKDYLRS
LNKLPRQLPLEVDLTKAKRQDLEPSVEEARYNSCRPNMALGHPQLQQVTSPSRPSHAVIPADQDCSSRSLSSSAVESEATHQRKLRQ

KQLQQQFRERMEKQQVRVSTPSAEKSEAAPPAPPVTHSTPVTVSEPLLEKDFLAGVTQELIKTLEDNSEKWAVTPDAGDGVVKPSSR
ADPAQTSDTLALNNQMVTQNRTPHSVCHQKPQAKSGSWDLQTYSADQRTTGNWESHRKSQDMKKRKYDPS
>sp|Q93088|BHMT1_HUMAN
MPPVGGKKAKKGILERLNAGEIVIGDGGFVFALEKRGYVKAGPWTPEAAVEHPEAVRQLHREFLRAGSNVMQTFTFYASEDKLENRG
NYVLEKISGQEVNEAACDIARQVADEGDALVAGGVSQTPSYLSCKSETEVKKVFLQQLEVFMKKNVDFLIAEYFEHVEEAVWAVETL
IASGKPVAATMCIGPEGDLHGVPPGECAVRLVKAGASIIGVNCHFDPTISLKTVKLMKEGLEAARLKAHLMSQPLAYHTPDCNKQGF
IDLPEFPFGLEPRVATRWDIQKYAREAYNLGVRYIGGCCGFEPYHIRAIAEELAPERGFLPPASEKHGSWGSGLDMHTKPWVRARAR
KEYWENLRIASGRPYNPSMSKPDGWGVTKGTAELMQQKEATTEQQLKELFEKQKFKSQ
>sp|P09630|HXC6_HUMAN
MNSYFTNPSLSCHLAGGQDVLPNVALNSTAYDPVRHFSTYGAAVAQNRIYSTPFYSPQENVVFSSSRGPYDYGSNSFYQEKDMLSNC
RQNTLGHNTQTSIAQDFSSEQGRTAPQDQKASIQIYPWMQRMNSHSGVGYGADRRRGRQIYSRYQTLELEKEFHFNRYLTRRRRIEI
ANALCLTERQIKIWFQNRRMKWKKESNLTSTLSGGGGGATADSLGGKEEKREETEEEKQKE
>sp|Q5VZF2|MBNL2_HUMAN
MALNVAPVRDTKWLTLEVCRQFQRGTCSRSDEECKFAHPPKSCQVENGRVIACFDSLKGRCSRENCKYLHPPTHLKTQLEINGRNNL
IQQKTAAAMLAQQMQFMFPGTPLHPVPTFPVGPAIGTNTAISFAPYLAPVTPGVGLVPTEILPTTPVIVPGSPPVTVPGSTATQKLL
RTDKLEVCREFQRGNCARGETDCRFAHPADSTMIDTSDNTVTVCMDYIKGRCMREKCKYFHPPAHLQAKIKAAQHQANQAAVAAQAA
AAAATVMAFPPGALHPLPKRQALEKSNGTSAVFNPSVLHYQQALTSAQLQQHAAFIPTGSVLCMTPATSIDNSEIISRNGMECQESA
LRITKHCYCTYYPVSSSIELPQTAC
>sp|Q86VZ6|JAZF1_HUMAN
MTGIAAASFFSNTCRFGGCGLHFPTLADLIEHIEDNHIDTDPRVLEKQELQQPTYVALSYINRFMTDAARREQESLKKKIQPKLSLT
LSSSVSRGNVSTPPRHSSGSLTPPVTPPITPSSSFRSSTPTGSEYDEEEVDYEESDSDESWTTESAISSEAILSSMCMNGGEEKPFA
CPVPGCKKRYKNVNGIKYHAKNGHRTQIRVRKPFKCRCGKSYKTAQGLRHHTINFHPPVSAEIIRKMQQ
>sp|P54922|ADPRH_HUMAN
MEKYVAAMVLSAAGDALGYYNGKWEFLQDGEKIHRQLAQLGGLDALDVGRWRVSDDTVMHLATAEALVEAGKAPKLTQLYYLLAKHY
QDCMEDMDGRAPGGASVHNAMQLKPGKPNGWRIPFNSHEGGCGAAMRAMCIGLRFPHHSQLDTLIQVSIESGRMTHHHPTGYLGALA
SALFTAYAVNSRPPLQWGKGLMELLPEAKKYIVQSGYFVEENLQHWSYFQTKWENYLKLRGILDGESAPTFPESFGVKERDQFYTSL
SYSGWGGSSGHDAPMIAYDAVLAAGDSWKELAHRAFFHGGDSDSTAAIAGCWWGVMYGFKGVSPSNYEKLEYRNRLEETARALYSLG
SKEDTVISL
>sp|P31483|TIA1_HUMAN
MEDEMPKTLYVGNLSRDVTEALILQLFSQIGPCKNCKMIMDTAGNDPYCFVEFHEHRHAAAALAAMNGRKIMGKEVKVNWATTPSSQ
KKDTSSSTVVSTQRSQDHFHVFVGDLSPEITTEDIKAAFAPFGRISDARVVKDMATGKSKGYGFVSFFNKWDAENAIQQMGGQWLGG
RQIRTNWATRKPPAPKSTYESNTKQLSYDEVVNQSSPSNCTVYCGGVTSGLTEQLMRQTFSPFGQIMEIRVFPDKGYSFVRFNSHES
AAHAIVSVNGTTIEGHVVKCYWGKETLDMINPVQQQNQIGYPQPYGQWGQWYGNAQQIGQYMPNGWQVPAYGMYGQAWNQQGFNQTQ
SSAPWMGPNYGVQPPQGQNGSMLPNQPSGYRVAGYETQ
>sp|Q16204|CCDC6_HUMAN
MADSASESDTDGAGGNSSSSAAMQSSCSSTSGGGGGGGGGGGGGKSGGIVISPFRLEELTNRLASLQQENKVLKIELETYKLKCKAL
QEENRDLRKASVTIQARAEQEEEFISNTLFKKIQALQKEKETLAVNYEKEEEFLTNELSRKLMQLQHEKAELEQHLEQEQEFQVNKL
MKKIKKLENDTISKQLTLEQLRREKIDLENTLEQEQEALVNRLWKRMDKLEAEKRILQEKLDQPVSAPPSPRDISMEIDSPENMMRH
IRFLKNEVERLKKQLRAAQLQHSEKMAQYLEEERHMREENLRLQRKLQREMERREALCRQLSESESSLEMDDERYFNEMSAQGLRPR
TVSSPIPYTPSPSSSRPISPGLSYASHTVGFTPPTSLTRAGMSYYNSPGLHVQHMGTSHGITRPSPRRSNSPDKFKRPTPPPSPNTQ
TPVQPPPPPPPPPMQPTVPSAATSQPTPSQHSAHPSSQP
>sp|Q9NPC3|CIP1_HUMAN
MSLCEDMLLCNYRKCRIKLSGYAWVTACSHIFCDQHGSGEFSRSPAICPACNSTLSGKLDIVRTELSPSEEYKAMVLAGLRPEIVLD
ISSRALAFWTYQVHQERLYQEYNFSKAEGHLKQMEKIYTQQIQSKDVELTSMKGEVTSMKKVLEEYKKKFSDISEKLMERNRQYQKL

QGLYDSLRLRNITIANHEGTLEPSMIAQSGVLGFPLGNNSKFPLDNTPVRNRGDGDGDFQFRPFFAGSPTAPEPSNSFFSFVSPSRE
LEQQQVSSRAFKVKRI
>sp|Q8NE79|POPD1_HUMAN
MNYTESSPLRESTAIGFTPELESIIPVPSNKTTCENWREIHHLVFHVANICFAVGLVIPTTLHLHMIFLRGMLTLGCTLYIVWATLY
RCALDIMIWNSVFLGVNILHLSYLLYKKRPVKIEKELSGMYRRLFEPLRVPPDLFRRLTGQFCMIQTLKKGQTYAAEDKTSVDDRLS
ILLKGKMKVSYRGHFLHNIYPCAFIDSPEFRSTQMHKGEKFQVTIIADDNCRFLCWSRERLTYFLESEPFLYEIFRYLIGKDITNKL
YSLNDPTLNDKKAKKLEHQLSLCTQISMLEMRNSIASSSDSDDGLHQFLRGTSSMSSLHVSSPHQRASAKMKPIEEGAEDDDDVFEP
ASPNTLKVHQLP
>sp|Q08378|GOGA3_HUMAN
MDGASAEQDGLQEDRSHSGPSSLPEAPLKPPGPLVPPDQQDKVQCAEVNRASTEGESPDGPGQGGLCQNGPTPPFPDPPSSLDPTTS
PVGPDASPGVAGFHDNLRKSQGTSAEGSVRKEALQSLRLSLPMQETQLCSTDSPLPLEKEEQVRLQARKWLEEQLKQYRVKRQQERS
SQPATKTRLFSTLDPELMLNPENLPRASTLAMTKEYSFLRTSVPRGPKVGSLGLPAHPREKKTSKSSKIRSLADYRTEDSNAGNSGG
NVPAPDSTKGSLKQNRSSAASVVSEISLSPDTDDRLENTSLAGDSVSEVDGNDSDSSSYSSASTRGTYGILSKTVGTQDTPYMVNGQ
EIPADTLGQFPSIKDVLQAAAAEHQDQGQEVNGEVRSRRDSICSSVSLESSAAETQEEMLQVLKEKMRLEGQLEALSLEASQALKEK
AELQAQLAALSTKLQAQVECSHSSQQRQDSLSSEVDTLKQSCWDLERAMTDLQNMLEAKNASLASSNNDLQVAEEQYQRLMAKVEDM
QRSMLSKDNTVHDLRQQMTALQSQLQQVQLERTTLTSKLKASQAEISSLQSVRQWYQQQLALAQEARVRLQGEMAHIQVGQMTQAGL
LEHLKLENVSLSQQLTETQHRSMKEKGRIAAQLQGIEADMLDQEAAFMQIQEAKTMVEEDLQRRLEEFEGERERLQRMADSAASLEQ
QLEQVKLTLLQRDQQLEALQQEHLDLMKQLTLTQEALQSREQSLDALQTHYDELQARLGELQGEAASREDTICLLQNEKIILEAALQ
AAKSGKEELDRGARRLEEGTEETSETLEKLREELAIKSGQVEHLQQETAALKKQMQKIKEQFLQQKVMVEAYRRDATSKDQLISELK
ATRKRLDSELKELRQELMQVHGEKRTAEAELSRLHREVAQVRQHMADLEGHLQSAQKERDEMETHLQSLQFDKEQMVAVTEANEALK
KQIEELQQEARKAITEQKQKMRRLGSDLTSAQKEMKTKHKAYENAVGILSRRLQEALAAKEAADAELGQLRAQGGSSDSSLALHERI
QALEAELQAVSHSKTLLEKELQEVIALTSQELEESREKVLELEDELQESRGFRKKIKRLEESNKKLALELEHEKGKLTGLGQSNAAL
REHNSILETALAKREADLVQLNLQVQAVLQRKEEEDRQMKHLVQALQASLEKEKEKVNSLKEQVAAAKVEAGHNRRHFKAASLELSE
VKKELQAKEHLVQKLQAEADDLQIREGKHSQEIAQFQAELAEARAQLQLLQKQLDEQLSKQPVGNQEMENLKWEVDQKEREIQSLKQ
QLDLTEQQGRKELEGLQQLLQNVKSELEMAQEDLSMTQKDKFMLQAKVSELKNNMKTLLQQNQQLKLDLRRGAAKTRKEPKGEASSS
NPATPIKIPDCPVPASLLEELLRPPPAVSKEPLKNLNSCLQQLKQEMDSLQRQMEEHALTVHESLSSWTPLEPATASPVPPGGHAGP
RGDPQRHSQSRASKEGPGE
>sp|Q9BY76|ANGL4_HUMAN
MSGAPTAGAALMLCAATAVLLSAQGGPVQSKSPRFASWDEMNVLAHGLLQLGQGLREHAERTRSQLSALERRLSACGSACQGTEGST
DLPLAPESRVDPEVLHSLQTQLKAQNSRIQQLFHKVAQQQRHLEKQHLRIQHLQSQFGLLDHKHLDHEVAKPARRKRLPEMAQPVDP
AHNVSRLHRLPRDCQELFQVGERQSGLFEIQPQGSPPFLVNCKMTSDGGWTVIQRRHDGSVDFNRPWEAYKAGFGDPHGEFWLGLEK
VHSITGDRNSRLAVQLRDWDGNAELLQFSVHLGGEDTAYSLQLTAPVAGQLGATTVPPSGLSVPFSTWDQDHDLRRDKNCAKSLSGG
WWFGTCSHSNLNGQYFRSIPQQRQKLKKGIFWKTWRGRYYPLQATTMLIQPMAAEAAS
>sp|Q8NH37|OR4C3_HUMAN
MDIPQNITEFFMLGLSQNSEVQRVLFVVFLLIYVVTVCGNMLIVVTITSSPTLASPVYFFLANLSFIDTFYSSSMAPKLIADSLYEG
RTISYECCMAQLFGAHFLGGVEIILLTVMAYDRYVAICKPLHNTTIMTRHLCAMLVGVAWLGGFLHSLVQLLLVLWLPFCGPNVINH
FACDLYPLLEVACTNTYVIGLLVVANSGLICLLNFLMLAASYIVILYSLRSHSADGRCKALSTCGAHFIVVALFFVPCIFTYVHPFS
TLPIDKNMALFYGILTPMLNPLIYTLRNEEVKNAMRKLFTW
>sp|Q8WX92|NELFB_HUMAN
MFAGLQDLGVANGEDLKETLTNCTEPLKAIEQFQTENGVLLPSLQSALPFLDLHGTPRLEFHQSVFDELRDKLLERVSAIASEGKAE
ERYKKLEDLLEKSFSLVKMPSLQPVVMCVMKHLPKVPEKKLKLVMADKELYRACAVEVKRQIWQDNQALFGDEVSPLLKQYILEKES
ALFSTELSVLHNFFSPSPKTRRQGEVVQRLTRMVGKNVKLYDMVLQFLRTLFLRTRNVHYCTLRAELLMSLHDLDVGEICTVDPCHK
FTWCLDACIRERFVDSKRARELQGFLDGVKKGQEQVLGDLSMILCDPFAINTLALSTVRHLQELVGQETLPRDSPDLLLLLRLLALG
QGAWDMIDSQVFKEPKMEVELITRFLPMLMSFLVDDYTFNVDQKLPAEEKAPVSYPNTLPESFTKFLQEQRMACEVGLYYVLHITKQ

RNKNALLRLLPGLVETFGDLAFGDIFLHLLTGNLALLADEFALEDFCSSLFDGFFLTASPRKENVHRHALRLLIHLHPRVAPSKLEA
LQKALEPTGQSGEAVKELYSQLGEKLEQLDHRKPSPAQAAETPALELPLPSVPAPAPL
>sp|Q9NZM5|GSCR2_HUMAN
MAAGGSGVGGKRSSKSDADSGFLGLRPTSVDPALRRRRRGPRNKKRGWRRLAQEPLGLEVDQFLEDVRLQERTSGGLLSEAPNEKLF
FVDTGSKEKGLTKKRTKVQKKSLLLKKPLRVDLILENTSKVPAPKDVLAHQVPNAKKLRRKEQLWEKLAKQGELPREVRRAQARLLN
PSATRAKPGPQDTVERPFYDLWASDNPLDRPLVGQDEFFLEQTKKKGVKRPARLHTKPSQAPAVEVAPAGASYNPSFEDHQTLLSAA
HEVELQRQKEAEKLERQLALPATEQAATQESTFQELCEGLLEESDGEGEPGQGEGPEAGDAEVCPTPARLATTEKKTEQQRRREKAV
HRLRVQQAALRAARLRHQELFRLRGIKAQVALRLAELARRQRRRQARREAEADKPRRLGRLKYQAPDIDVQLSSELTDSLRTLKPEG
NILRDRFKSFQRRNMIEPRERAKFKRKYKVKLVEKRAFREIQL
>sp|Q8WW18|CQ050_HUMAN
MDKHGVKTPLWKKETEELRAEDAEQEEGKEGSEDEDEDNQRPLEDSATEGEEPPRVAEEGEGRERRSVSYCPLRQESSTQQVALLRR
ADSGFWGWLGPLALLGGLTAPTDRKRSLPEEPCVLEIRRRPPRRGGCACCELLFCKKCRSLHSHPAYVAHCVLDHPDLGKAGAAGNS
>sp|Q96E29|MTEF3_HUMAN
MALSAQQIPRWFNSVKLRSLINAAQLTKRFTRPARTLLHGFSAQPQISSDNCFLQWGFKTYRTSSLWNSSQSTSSSSQENNSAQSSL
LPSMNEQSQKTQNISSFDSELFLEELDELPPLSPMQPISEEEAIQIIADPPLPPASFTLRDYVDHSETLQKLVLLGVDLSKIEKHPE
AANLLLRLDFEKDIKQMLLFLKDVGIEDNQLGAFLTKNHAIFSEDLENLKTRVAYLHSKNFSKADVAQMVRKAPFLLNFSVERLDNR
LGFFQKELELSVKKTRDLVVRLPRLLTGSLEPVKENMKVYRLELGFKHNEIQHMITRIPKMLTANKMKLTETFDFVHNVMSIPHHII
VKFPQVFNTRLFKVKERHLFLTYLGRAQYDPAKPNYISLDKLVSIPDEIFCEEIAKASVQDFEKFLKTL
>sp|Q05BQ5|MBTD1_HUMAN
MFDGYDSCSEDTSSSSSSEESEEEVAPLPSNLPIIKNNGQVYTYPDGKSGMATCEMCGMVGVRDAFYSKTKRFCSVSCSRSYSSNSK
KASILARLQGKPPTKKAKVLQKQPLVAKLAAYAQYQATLQNQAKTKAAVSMEGFSWGNYINSNSFIAAPVTCFKHAPMGTCWGDISE
NVRVEVPNTDCSLPTKVFWIAGIVKLAGYNALLRYEGFENDSGLDFWCNICGSDIHPVGWCAASGKPLVPPRTIQHKYTNWKAFLVK
RLTGAKTLPPDFSQKVSESMQYPFKPCMRVEVVDKRHLCRTRVAVVESVIGGRLRLVYEESEDRTDDFWCHMHSPLIHHIGWSRSIG
HRFKRSDITKKQDGHFDTPPHLFAKVKEVDQSGEWFKEGMKLEAIDPLNLSTICVATIRKVLADGFLMIGIDGSEAADGSDWFCYHA
TSPSIFPVGFCEINMIELTPPRGYTKLPFKWFDYLRETGSIAAPVKLFNKDVPNHGFRVGMKLEAVDLMEPRLICVATVTRIIHRLL
RIHFDGWEEEYDQWVDCESPDLYPVGWCQLTGYQLQPPASQSSRENQSASSKQKKKAKSQQYKGHKKMTTLQLKEELLDGEDYNFLQ
GASDQESNGSANFYIKQEP
>sp|Q8NB15|ZN511_HUMAN
MQLPPALCARLAAGPGAAEPLPVERDPAAGAAPFRFVARPVRFPREHQFFEDGDVQRHLYLQDVIMQVADVPEKPRVPAFACQVAGC
CQVFDALDDYEHHYHTLHGNVCSFCKRAFPSGHLLDAHILEWHDSLFQILSERQDMYQCLVEGCTEKFKTSRDRKDHMVRMHLYPAD
FRFDKPKKSRSPASAEAPGDSGERSEGEAMEICSEPVAASPAPAGERRIYRHRSVSELFLKPVLNMCSVLRILGCTWAAALLILNSE
R
>sp|P50876|R144A_HUMAN
MTTTRYRPTWDLALDPLVSCKLCLGEYPVEQMTTIAQCQCIFCTLCLKQYVELLIKEGLETAISCPDAACPKQGHLQENEIECMVAA
EIMQRYKKLQFEREVLFDPCRTWCPASTCQAVCQLQDVGLQTPQPVQCKACRMEFCSTCKASWHPGQGCPETMPITFLPGETSAAFK
MEEDDAPIKRCPKCKVYIERDEGCAQMMCKNCKHAFCWYCLESLDDDFLLIHYDKGPCRNKLGHSRASVIWHRTQVVGIFAGFGLLL
LVASPFLLLATPFVLCCKCKCSKGDDDPLPT
>sp|Q9P1W9|PIM2_HUMAN
MLTKPLQGPPAPPGTPTPPPGGKDREAFEAEYRLGPLLGKGGFGTVFAGHRLTDRLQVAIKVIPRNRVLGWSPLSDSVTCPLEVALL
WKVGAGGGHPGVIRLLDWFETQEGFMLVLERPLPAQDLFDYITEKGPLGEGPSRCFFGQVVAAIQHCHSRGVVHRDIKDENILIDLR
RGCAKLIDFGSGALLHDEPYTDFDGTRVYSPPEWISRHQYHALPATVWSLGILLYDMVCGDIPFERDQEILEAELHFPAHVSPDCCA
LIRRCLAPKPSSRPSLEEILLDPWMQTPAEDVPLNPSKGGPAPLAWSLLP
>sp|Q96AC1|FERM2_HUMAN

MALDGIRMPDGCYADGTWELSVHVTDLNRDVTLRVTGEVHIGGVMLKLVEKLDVKKDWSDHALWWEKKRTWLLKTHWTLDKYGIQAD
AKLQFTPQHKLLRLQLPNMKYVKVKVNFSDRVFKAVSDICKTFNIRHPEELSLLKKPRDPTKKKKKKLDDQSEDEALELEGPLITPG
SGSIYSSPGLYSKTMTPTYDAHDGSPLSPTSAWFGDSALSEGNPGILAVSQPITSPEILAKMFKPQALLDKAKINQGWLDSSRSLME
QDVKENEALLLRFKYYSFFDLNPKYDAIRINQLYEQAKWAILLEEIECTEEEMMMFAALQYHINKLSIMTSENHLNNSDKEVDEVDA
ALSDLEITLEGGKTSTILGDITSIPELADYIKVFKPKKLTLKGYKQYWCTFKDTSISCYKSKEESSGTPAHQMNLRGCEVTPDVNIS
GQKFNIKLLIPVAEGMNEIWLRCDNEKQYAHWMAACRLASKGKTMADSSYNLEVQNILSFLKMQHLNPDPQLIPEQITTDITPECLV
SPRYLKKYKNKQITARILEAHQNVAQMSLIEAKMRFIQAWQSLPEFGITHFIARFQGGKKEELIGIAYNRLIRMDASTGDAIKTWRF
SNMKQWNVNWEIKMVTVEFADEVRLSFICTEVDCKVVHEFIGGYIFLSTRAKDQNESLDEEMFYKLTSGWV
>sp|Q14695|K0087_HUMAN
MEAWESSQPLLRCEIPCPLPGTDRDGSVSLPGEAASCDLDTLEPEHGNRRVSGNPISVCWAYKVTKVKCWSVRERGGRHIGGPRSTL
KHPAHHGMGKNLATSLPTAASLGLGKGQLLVSIRFMDTTKKRGQSETFNIC
>sp|P43632|KI2S4_HUMAN
MSLMVIIMACVGFFLLQGAWPQEGVHRKPSFLALPGHLVKSEETVILQCWSDVMFEHFLLHREGKFNNTLHLIGEHHDGVSKANFSI
GPMMPVLAGTYRCYGSVPHSPYQLSAPSDPLDMVIIGLYEKPSLSAQPGPTVQAGENVTLSCSSRSSYDMYHLSREGEAHERRLPAV
RSINGTFQADFPLGPATHGGTYRCFGSFRDAPYEWSNSSDPLLVSVTGNPSNSWPSPTEPSSKTGNPRHLHVLIGTSVVKIPFTILL
FFLLHRWCSDKKNAAVMDQEPAGNRTVNSEDSDEQDHQEVSYA
>sp|Q05329|DCE2_HUMAN
MASPGSGFWSFGSEDGSGDSENPGTARAWCQVAQKFTGGIGNKLCALLYGDAEKPAESGGSQPPRAAARKAACACDQKPCSCSKVDV
NYAFLHATDLLPACDGERPTLAFLQDVMNILLQYVVKSFDRSTKVIDFHYPNELLQEYNWELADQPQNLEEILMHCQTTLKYAIKTG
HPRYFNQLSTGLDMVGLAADWLTSTANTNMFTYEIAPVFVLLEYVTLKKMREIIGWPGGSGDGIFSPGGAISNMYAMMIARFKMFPE
VKEKGMAALPRLIAFTSEHSHFSLKKGAAALGIGTDSVILIKCDERGKMIPSDLERRILEAKQKGFVPFLVSATAGTTVYGAFDPLL
AVADICKKYKIWMHVDAAWGGGLLMSRKHKWKLSGVERANSVTWNPHKMMGVPLQCSALLVREEGLMQNCNQMHASYLFQQDKHYDL
SYDTGDKALQCGRHVDVFKLWLMWRAKGTTGFEAHVDKCLELAEYLYNIIKNREGYEMVFDGKPQHTNVCFWYIPPSLRTLEDNEER
MSRLSKVAPVIKARMMEYGTTMVSYQPLGDKVNFFRMVISNPAATHQDIDFLIEEIERLGQDL
>sp|P48788|TNNI2_HUMAN
MGDEEKRNRAITARRQHLKSVMLQIAATELEKEESRREAEKQNYLAEHCPPLHIPGSMSEVQELCKQLHAKIDAAEEEKYDMEVRVQ
KTSKELEDMNQKLFDLRGKFKRPPLRRVRMSADAMLKALLGSKHKVCMDLRANLKQVKKEDTEKERDLRDVGDWRKNIEEKSGMEGR
KKMFESES
>sp|P0CJ79|ZN888_HUMAN
MALPQGLLTFRDVAIEFSQEEWKCLDPAQRTLYRDVMLENYRNLVSLDISSKCMMEFSSIGKGNTEVIHTGTLQRLASHHIGECCFQ
EIEKDIHDFVFQWQEDETNGHEAPMTEIKELTGSTDQYDQRHAGNKPIKYQLGSSFHSHLPELHLARHYRRHTGEKPYKCNECGKTF
SDKSALLVHKTIHTGEKPYKCNECGKVFNQQSNLARHHRVHTGEKPYQCKECDKVFSRKSYLERHRRIHTGEKPYKCKVCDKAFRHD
SHLAQHIVIHTREKPYKCNECGKTFGENSALLVHKTIHTGEKPYKCNECGKVFNQQSNLARHHRLHTGEKPYKCKECDKVFSRKSHL
ERHRRIHTGEKPYKCKVCDKAFRRDSRLAYHHRLHTGEKPYKCNECGKVFRTQSQLACHHRLHTGEKPYKCEECDKVFNIKSHLEIH
RRVHTGEKPYKCRVCDKAFGKAFTSRSHLIRHQRIHTGQKSYKCHQCGKVFSLRSLLAEYQKIYF
>sp|A8K0S8|ME3L2_HUMAN
MARRYDELPHYPSIADGPAALAGFPEAVPAAPGPYGPHRPPQPLPPGLDSDGLKRDKDEIYGHPLFPLLALVFEKCELATCSPRDGA
GAGLGTPRGGDVCSSDSFNEDNTAFAKQVRSERPFFSSNPELDNLMIQAIQVLRFHLLELEKGKMPIDLVIEDRDGGCREDFEDYPA
SCLSLPDQNNIWIRDHEDSGSVHLGTPGPSSGGLASQSGDNSSDQGVGLDTSVASPSSGGEDEDLDQEPRRNKKRGIFPKVATNIMR
AWLFQHLWHPYPSEEQKKQLVQDTGLTILQVNNWFINARRRMVQPMIDQSNRIGQGAAFSPEGQPIGGYTETEPHVAFRPPASVGMS
LNLEGEWHYL
>sp|Q969S9|RRF2M_HUMAN
MLTNLRIFAMSHQTIPSVYINNICCYKIRASLKRLKPHVPLGRNCSSLPGLIGNDIKSLHSIINPPIAKIRNIGIMAHIDAGKTTTT
ERILYYSGYTRSLGDVDDGDTVTDFMAQERERGITIQSAAVTFDWKGYRVNLIDTPGHVDFTLEVERCLRVLDGAVAVFDASAGVEA

QTLTVWRQADKHNIPRICFLNKMDKTGASFKYAVESIREKLKAKPLLLQLPIGEAKTFKGVVDVVMKEKLLWNCNSNDGKDFERKPL
LEMNDPELLKETTEARNALIEQVADLDDEFADLVLEEFSENFDLLPAEKLQTAIHRVTLAQTAVPVLCGSALKNKGIQPLLDAVTMY
LPSPEERNYEFLQWYKDDLCALAFKVLHDKQRGPLVFMRIYSGTIKPQLAIHNINGNCTERISRLLLPFADQHVEIPSLTAGNIALT
VGLKHTATGDTIVSSKSSALAAARRAEREGEKKHRQNNEAERLLLAGVEIPEPVFFCTIEPPSLSKQPDLEHALKCLQREDPSLKVR
LDPDSGQTVLCGMGELHIEIIHDRIKREYGLETYLGPLQVAYRETILNSVRATDTLDRTLGDKRHLVTVEVEARPIETSSVMPVIEF
EYAESINEGLLKVSQEAIENGIHSACLQGPLLGSPIQDVAITLHSLTIHPGTSTTMISACVSRCVQKALKKADKQVLEPLMNLEVTV
ARDYLSPVLADLAQRRGNIQEIQTRQDNKVVIGFVPLAEIMGYSTVLRTLTSGSATFALELSTYQAMNPQDQNTLLNRRSGLT
>sp|Q13434|MKRN4_HUMAN
MAEAAAPGTTVTTSGAGAAAAEAAETAEAVSPTPIPTVTAPSPRAGGGVGGSDGSDGSGGRGDSGAYDGSGACGGSDACDGSGDSSG
DSWTKQVTCRYFKYGICKEGDNCRYSHDLSDRLCGVVCKYFQRGCCVYGDRCRCEHSKPLKQEEATATELTTKSSLAASSSLSSIVG
PLVEMNTNEAESRNSNFATVVAGSEDWANAIEFVPGQPYCGRTVPSCTEAPLQGSVTKEESEEEQTAVETKKQLCPYAAVGQCRYGE
NCVYLHGDLCDMCGLQVLHPMDAAQRSQHIQACIEAHEKDMEFSFAVQRSKDKVCGICMEVVYEKANPNEHRFGILSNCNHTFCLKC
IRKWRSAKEFESRIVKSCPQCRITSNFVIPSEYWVEEKEEKQKLIQKYKEAMSNKACKYFDEGRGSCPFGENCFYKHMYPDGRREEP
QRQQVGTSSRNPGQQRNHFWEFFEEGANSNPFDDEEEAVTFELGEMLLML
>sp|Q9H8W4|PKHF2_HUMAN
MVDRLANSEANTRRISIVENCFGAAGQPLTIPGRVLIGEGVLTKLCRKKPKARQFFLFNDILVYGNIVIQKKKYNKQHIIPLENVTI
DSIKDEGDLRNGWLIKTPTKSFAVYAATATEKSEWMNHINKCVTDLLSKSGKTPSNEHAAVWVPDSEATVCMRCQKAKFTPVNRRHH
CRKCGFVVCGPCSEKRFLLPSQSSKPVRICDFCYDLLSAGDMATCQPARSDSYSQSLKSPLNDMSDDDDDDDSSD
>sp|Q9H7Z7|PGES2_HUMAN
MDPAARVVRALWPGGCALAWRLGGRPQPLLPTQSRAGFAGAAGGPSPVAAARKGSPRLLGAAALALGGALGLYHTARWHLRAQDLHA
ERSAAQLSLSSRLQLTLYQYKTCPFCSKVRAFLDFHALPYQVVEVNPVRRAEIKFSSYRKVPILVAQEGESSQQLNDSSVIISALKT
YLVSGQPLEEIITYYPAMKAVNEQGKEVTEFGNKYWLMLNEKEAQQVYGGKEARTEEMKWRQWADDWLVHLISPNVYRTPTEALASF
DYIVREGKFGAVEGAVAKYMGAAAMYLISKRLKSRHRLQDNVREDLYEAADKWVAAVGKDRPFMGGQKPNLADLAVYGVLRVMEGLD
AFDDLMQHTHIQPWYLRVERAITEASPAH
>sp|O95998|I18BP_HUMAN
MTMRHNWTPDLSPLWVLLLCAHVVTLLVRATPVSQTTTAATASVRSTKDPCPSQPPVFPAAKQCPALEVTWPEVEVPLNGTLSLSCV
ACSRFPNFSILYWLGNGSFIEHLPGRLWEGSTSRERGSTGTQLCKALVLEQLTPALHSTNFSCVLVDPEQVVQRHVVLAQLWAGLRA
TLPPTQEALPSSHSSPQQQG
>sp|Q3SX64|OD3L2_HUMAN
MGTLSCDSTPRLATAPLGRRVTEGQIPETGLRKSCGTATLENGSGPGLYVLPSTVGFINHDCTRVASPAYSLVRRPSEAPPQDTSPG
PIYFLDPKVTRFGRSCTPAYSMQGRAKSRGPEVTPGPGAYSPEKVPPVRHRTPPAFTLGCRLPLKPLDTSAPAPNAYTMPPLWGSQI
FTKPSSPSYTVVGRTPPARPPQDPAEIPGPGQYDSPDANTYRQRLPAFTMLGRPRAPRPLEETPGPGAHCPEQVTVNKARAPAFSMG
IRHSKRASTMAATTPSRPAGHRLPGRCC
>sp|Q8IYR0|CF206_HUMAN
MPPTQAESVIRSIIREIGQECAAHGEIVSETLIAFMVKAVVLDPSNGFNMDRTLMKSDVQNLVKLCMTRLLDTKNPSLDTIKMQVYF
DMNYTNRVEFLEEHHRVLESRLGSVTREITDNRACAKEELESLYRKIISYVLLRSGLGSPTDIKTVREVTAALQSVFPQAELGTFLT
LSKKDKERQLKELTMIVTGIRLFNRDCGKGGEGIDDLPAVLHVAIPATMQHIDYQLETARSQVYRYTAILEKAANDPLMRAELQPYM
LKEALYNIRQYEVFLQIILSDIITGAQEVEMMTKQLGAHLEQLKMTIKSKIAVPTSQVFPIFIALSTLWTSLQDETIVVGVLSNLFT
HIQPFLGAHELYFPERVMQCHLNGATVKTDVCRMKEHMEDRVNVADFRKLEWLFPETTANFDKLLIQYRGFCAYTFAATDGLLLPGN
PAIGILKYKEKYYTFNSKDAAYSFAENPEHYIDIVREKAKKNTELIQLLELHQQFETFIPYSQMRDADKHYIKPITKCESSTQTNTH
ILPPTIVRSYEWNEWELRRKAIKLANLRQKVTHSVQTDLSHLRRENCSQVYPPKDTSTQSMREDSTGVPRPQIYLAGLRGGKSEITD
EVKVNLTRDVDET
>sp|Q8TF27|AGA11_HUMAN

MTIISVTLEIHHHITERDADRSLTILDEQLYSFAFSTVHITKKRNGGGSLNNYSSSIPLTPSTSQEDLYFSVPPTANTPTPICKQSM
GWSNLFTSEKGSDPDKGRKALESHADTIGSGRAIPIKQGMLLKRSGKWLKTWKKKYVTLCSNGVLTYYSSLGDYMKNIHKKEIDLRT
STIKVPGKWPSLATSACAPISSSKSNGLSKDMEALHMSANSDIGLGDSICFSPSISSTTSPKLNLPPSPHANKKKHLKKKSTNNLKD
DGLSSTAEEEEEKFMIVSVTGQTCHFKATTYEERDAWVQAIQSQILASLQSCESSKSKSQLTSQSEAMALQSIQNMRGNSHCVDCET
QNPKWASLNLGVLMCIECSGIHRSLGTRLSRVRSLELDDWPVELRKVMSSIGNDLANSIWEGSSQGQTKPSIESTREEKERWIRSKY
EHKLFLAPLPCTELSLGQHLLRATADEDLRTAILLLAHGSREEVNETCGEGDGCTALHLACRKGNVVLAQLLIWYGVDVMARDAHGN
TALTYARQASSQECINVLLQYGCPDECV
>sp|P14770|GPIX_HUMAN
MPAWGALFLLWATAEATKDCPSPCTCRALETMGLWVDCRGHGLTALPALPARTRHLLLANNSLQSVPPGAFDHLPQLQTLDVTQNPW
HCDCSLTYLRLWLEDRTPEALLQVRCASPSLAAHGPLGRLTGYQLGSCGWQLQASWVRPGVLWDVALVAVAALGLALLAGLLCATTE
ALD
>sp|P35713|SOX18_HUMAN
MQRSPPGYGAQDDPPARRDCAWAPGHGAAADTRGLAAGPAALAAPAAPASPPSPQRSPPRSPEPGRYGLSPAGRGERQAADESRIRR
PMNAFMVWAKDERKRLAQQNPDLHNAVLSKMLGKAWKELNAAEKRPFVEEAERLRVQHLRDHPNYKYRPRRKKQARKARRLEPGLLL
PGLAPPQPPPEPFPAASGSARAFRELPPLGAEFDGLGLPTPERSPLDGLEPGEAAFFPPPAAPEDCALRPFRAPYAPTELSRDPGGC
YGAPLAEALRTAPPAAPLAGLYYGTLGTPGPYPGPLSPPPEAPPLESAEPLGPAADLWADVDLTEFDQYLNCSRTRPDAPGLPYHVA
LAKLGPRAMSCPEESSLISALSDASSAVYYSACISG
>sp|P24539|AT5F1_HUMAN
MLSRVVLSAAATAAPSLKNAAFLGPGVLQATRTFHTGQPHLVPVPPLPEYGGKVRYGLIPEEFFQFLYPKTGVTGPYVLGTGLILYA
LSKEIYVISAETFTALSVLGVMVYGIKKYGPFVADFADKLNEQKLAQLEEAKQASIQHIQNAIDTEKSQQALVQKRHYLFDVQRNNI
AMALEVTYRERLYRVYKEVKNRLDYHISVQNMMRRKEQEHMINWVEKHVVQSISTQQEKETIAKCIADLKLLAKKAQAQPVM
>sp|Q99944|EGFL8_HUMAN
MGSRAELCTLLGGFSFLLLLIPGEGAKGGSLRESQGVCSKQTLVVPLHYNESYSQPVYKPYLTLCAGRRICSTYRTMYRVMWREVRR
EVQQTHAVCCQGWKKRHPGALTCEAICAKPCLNGGVCVRPDQCECAPGWGGKHCHVDVDECRTSITLCSHHCFNTAGSFTCGCPHDL
VLGVDGRTCMEGSPEPPTSASILSVAVREAEKDERALKQEIHELRGRLERLEQWAGQAGAWVRAVLPVPPEELQPEQVAELWGRGDR
IESLSDQVLLLEERLGACSCEDNSLGLGVNHR
>sp|Q8N6M9|ZFN2A_HUMAN
MEFPDLGKHCSEKTCKQLDFLPVKCDACKQDFCKDHFPYAAHKCPFAFQKDVHVPVCPLCNTPIPVKKGQIPDVVVGDHIDRDCDSH
PGKKKEKIFTYRCSKEGCKKKEMLQMVCAQCHGNFCIQHRHPLDHSCRHGSRPTIKAG
>sp|Q92911|SC5A5_HUMAN
MEAVETGERPTFGAWDYGVFALMLLVSTGIGLWVGLARGGQRSAEDFFTGGRRLAALPVGLSLSASFMSAVQVLGVPSEAYRYGLKF
LWMCLGQLLNSVLTALLFMPVFYRLGLTSTYEYLEMRFSRAVRLCGTLQYIVATMLYTGIVIYAPALILNQVTGLDIWASLLSTGII
CTFYTAVGGMKAVVWTDVFQVVVMLSGFWVVLARGVMLVGGPRQVLTLAQNHSRINLMDFNPDPRSRYTFWTFVVGGTLVWLSMYGV
NQAQVQRYVACRTEKQAKLALLINQVGLFLIVSSAACCGIVMFVFYTDCDPLLLGRISAPDQYMPLLVLDIFEDLPGVPGLFLACAY
SGTLSTASTSINAMAAVTVEDLIKPRLRSLAPRKLVIISKGLSLIYGSACLTVAALSSLLGGGVLQGSFTVMGVISGPLLGAFILGM
FLPACNTPGVLAGLGAGLALSLWVALGATLYPPSEQTMRVLPSSAARCVALSVNASGLLDPALLPANDSSRAPSSGMDASRPALADS
FYAISYLYYGALGTLTTVLCGALISCLTGPTKRSTLAPGLLWWDLARQTASVAPKEEVAILDDNLVKGPEELPTGNKKPPGFLPTNE
DRLFFLGQKELEGAGSWTPCVGHDGGRDQQETNL
>sp|Q9Y2L6|FRM4B_HUMAN
MASVFMCGVEDLLFSGSRFVWNLTVSTLRRWYTERLRACHQVLRTWCGLQDVYQMTEGRHCQVHLLDDRRLELLVQPKLLARELLDL
VASHFNLKEKEYFGITFIDDTGQQNWLQLDHRVLDHDLPKKPGPTILHFAVRFYIESISFLKDKTTVELFFLNAKACVHKGQIEVES
ETIFKLAAFILQEAKGDYTSDENARKDLKTLPAFPTKTLQEHPSLAYCEDRVIEHYLKIKGLTRGQAVVQYMKIVEALPTYGVHYYA
VKDKQGLPWWLGISYKGIGQYDIQDKVKPRKLFQWKQLENLYFREKKFAVEVHDPRRISVSRRTFGQSGLFVQTWYANSSLIKSIWV
MAISQHQFYLDRKQSKAKIPSARSLDEIAMDLTETGTQRASKLVTLETKSQFIMASNGSLISSGSQDSEVSEEQKREKILELKKKEK

LLQEKLLKKVEELKKICLREAELTGKMPKEYPLNIGEKPPQVRRRVGTAFKLDDNLLPSEEDPALQELESNFLIQQKLVEAAKKLAN
EPDLCKTVKKKRKQDYTDAMKKLQEIENAINEYRIRCGKKPSQKATVLPEDIIPSESSSLSDTTTYDDPSDAFTFPGQRSSSVPHSP
RILPPKSLGIERIHFRKSSINEQFVDTRQSREMLSTHSSPYKTLERRPQGGRSMPTTPVLTRNAYSSSHLEPESSSQHCRQRSGSLE
SQSHLLSEMDSDKPFFSLSKSQRSSSTEILDDGSSYTSQSSTEYYCVTPVTGPYYTTQTLDTRTRGRRRSKKQNVSTSNSGSMPNLA
QKDSLRNGVYSKSQEPPSSSYYIAGYTPYAECDFYYSGGYVYENDTEGQYSVNPSYRSSAHYGYERQRDYSRSFHEDEVDRVPHNPY
ATLRLPRKAAAKSEHITKNIHKALVAEHLRGWYQRASGQKDQGHSPQTSFDSDRGSQRCLGFAGLQVPCSPSSRASSYSSVSSTNAS
GNWRTQLTIGLSDYETPAHSSYTSCYGNVYNPLPSPSRQYTEISQLDGTDGNQLEDNLESSEQRLFWHEDSKPGTLV
>sp|Q16517|NNAT_HUMAN
MAAVAAASAELLIIGWYIFRVLLQVFLECCIYWVGFAFRNPPGTQPIARSEVFRYSLQKLAYTVSRTGRQVLGERRQRAPN
>sp|Q92536|YLAT2_HUMAN
MEAREPGRPTPTYHLVPNTSQSQVEEDVSSPPQRSSETMQLKKEISLLNGVSLVVGNMIGSGIFVSPKGVLVHTASYGMSLIVWAIG
GLFSVVGALCYAELGTTITKSGASYAYILEAFGGFIAFIRLWVSLLVVEPTGQAIIAITFANYIIQPSFPSCDPPYLACRLLAAACI
CLLTFVNCAYVKWGTRVQDTFTYAKVVALIAIIVMGLVKLCQGHSEHFQDAFEGSSWDMGNLSLALYSALFSYSGWDTLNFVTEEIK
NPERNLPLAIGISMPIVTLIYILTNVAYYTVLNISDVLSSDAVAVTFADQTFGMFSWTIPIAVALSCFGGLNASIFASSRLFFVGSR
EGHLPDLLSMIHIERFTPIPALLFNCTMALIYLIVEDVFQLINYFSFSYWFFVGLSVVGQLYLRWKEPKRPRPLKLSVFFPIVFCIC
SVFLVIVPLFTDTINSLIGIGIALSGVPFYFMGVYLPESRRPLFIRNVLAAITRGTQQLCFCVLTELDVAEEKKDERKTD
>sp|Q9UJU5|FOXD3_HUMAN
MTLSGGGSASDMSGQTVLTAEDVDIDVVGEGDDGLEEKDSDAGCDSPAGPPELRLDEADEVPPAAPHHGQPQPPHQQPLTLPKEAAG
AGAGPGGDVGAPEADGCKGGVGGEEGGASGGGPGAGSGSAGGLAPSKPKNSLVKPPYSYIALITMAILQSPQKKLTLSGICEFISNR
FPYYREKFPAWQNSIRHNLSLNDCFVKIPREPGNPGKGNYWTLDPQSEDMFDNGSFLRRRKRFKRHQQEHLREQTALMMQSFGAYSL
AAAAGAAGPYGRPYGLHPAAAAGAYSHPAAAAAAAAAAALQYPYALPPVAPVLPPAVPLLPSGELGRKAAAFGSQLGPGLQLQLNSL
GAAAAAAGTAGAAGTTASLIKSEPSARPSFSIENIIGGGPAAPGGSAVGAGVAGGTGGSGGGSTAQSFLRPPGTVQSAALMATHQPL
SLSRTTATIAPILSVPLSGQFLQPAASAAAAAAAAAQAKWPAQ
>sp|Q9P0P0|RN181_HUMAN
MASYFDEHDCEPSDPEQETRTNMLLELARSLFNRMDFEDLGLVVDWDHHLPPPAAKTVVENLPRTVIRGSQAELKCPVCLLEFEEEE
TAIEMPCHHLFHSSCILPWLSKTNSCPLCRYELPTDDDTYEEHRRDKARKQQQQHRLENLHGAMYT
>sp|P0C2L3|F163B_HUMAN
MTAGTVVITGGILATVILLCIIAVLCYCRLQYYCCKKDESEEDEEEPDFAVHSHLPPLHSNRNLVLTNGPALYPTASTSFSQKSPQA
RALCRSCSHCEPPTFFLQEPPEEEEDVLNGGERVLYKSVSQEDVELPPGGFGGLQALNPNRLSAMREAFARSRSISTDV
>sp|O95918|OR2H2_HUMAN
MVNQSSTPGFLLLGFSEHPGLERTLFVVVLTSYLLTLVGNTLIILLSALDPKLHSPMYFFLSNLSFLDLCFTTSCVPQMLVNLWGPK
KTISFLDCSVQIFIFLSLGTTECILLTVMAFDRYVAVCQPLHYATIIHPRLCWQLASVAWVIGLVESVVQTPSTLHLPFCPDRQVDD
FVCEVPALIRLSCEDTSYNEIQVAVASVFILVVPLSLILVSYGAITWAVLRINSAKGRRKAFGTCSSHLTVVTLFYSSVIAVYLQPK
NPYAQERGKFFGLFYAVGTPSLNPLIYTLRNKEVTRAFRRLLGKEMGLTQS
>sp|B5MCY1|TDR15_HUMAN
MDSTSFLPTFLDVDLTISHIKCLPKDILVKFQGIKSNECEFDYHVLQREIQHTPKVKNNVEIDEFCLVEERVSGEWQRGRVMEKKNE
LYTVLLIDRGEELRVAGPQIASACGNLFELPPRVVFGIFANILPVGEKWSPKALNYFKSLVGIQVKGYVQAILPLQMFLFEVPKIIS
QALELQLGRLVDGDSFRLIVEMLEEFPQQMPDLLQHKRPELSLGNKDTSLDIQHVLDKLQPSLSVGSTESVKVSSALSPSKFYCQLI
KWTPELENLTAHMTLHYDTVCQETSPTCDNFGLLCVARRRNGQWHRGILQQLLPPNQVKIWFMDYGSSEAIPSIYVKKLKQDFILVP
LFSFPCSLTCLHSPDRDARIFQLSIFKQALLGQVVYAHIDWFNKDECLYYVTLQTQESTVNSKCLLKTVGTQVLCPMSDSKISNILS
ETSVSDVNSFAVESFMGNIEWSIDSLNKKGILKVGFPIKTVQMEIEAAYIAFIAYVLNPSNFWVRTNDHRNEFQEIMKNINKFYDLC
ENDEMILRKPEPGLFCCARYSKDRRFYRAVITEINGYKINVYFLDYGNTDSIPFFDVKILLPEFCELPALAMCCSLAHIFPVEDLWT
KAAIDYFKKLVLNKAILLQVIAKKDDKYTVNIQSVEASENIDVISLMLQAGYAEYFQVELEYFPKSVSEYSMLNSESKNKVNIKKVI
SALLEGPKSKKYHSNNLVENNLSLPKSLAVNISEFKNPFTLSVGPESSWPYKEYIFRPGTVLEVKCSCYYGPGDFSCQLQCKSEDLK

LLMEQIQNYYSIHSDPYEIGQTACVAKYSGKWCRAAVLTQVSKEVDIVFVDYGYQKRVLIEDLCAINPRFLLLESQAFRCCLNHFIE
PVSCKLFSWTRKAFRDLWNFISSSRGLLTCIIYALVIIHPNHLYNLVDLQSSFTSAKEFLMNRGSAQYITLSETFPSLFSLYSYCYS
SFNIQIGSEEEVYISHIYSPQKFYCQLGRNNKDLEMIETKITESVNLQNFPKYDSNKMRVCISKYVEDGLSYRALAIPTDSSSEFQV
YFVDFGNKQLVGENMLRAISAQFPELLFTPMQAIKCFLSDLRDVDIPAEISSWFKDNFLGRSLKAIILSQESDGQLGIELYDGSQYI
NEKIKVLLHAYGKRHCDQACCMEKSNKINENKRFTTSLKGKTGNNYRHNVINKPSPVTYSERKIDQLMHPKNIHARFLKPSVCYKME
PVSKNKMKTSLNDGLKGIKIVPGAAHILENRRVGQKSVKVVSQSFIRALNQTTSQNPYDLIRPQIKDLPQPQIYLNAKVKGYVSNIS
NPANFHIQLAENESVIIRLADALNATARRLRERKSVKPLVGDLVVAEYSGDNAIYRAVIKKILPGNSFEVEFIDYGNSAIVNTSKIY
ELQREFLTVPQLGIHAFLSGVKWNEPDEIWDDKTVDYFTSKVHNKTVYCEFLKKHDQKWEVNMICDEKCVINELLKWKACSKLQKSA
LQMPQVLSQKVRPGDNEMKKGKSNESEGSMNSNQQLFKIPLEEFKLGQLEKAEMLNVSKSGRFYVKLSKNKKILSDLIVLITKEEKK
SPFLSMESIEKGLECLAKSKNTLKWHRSKVEEKYVDDKVLVFLVDCGIYEIVPVCNTKLLSNEIRNIPRQAVPCKWIWFENSKNISF
ECLFADLEINILFLKYLDAVWEVEILVDDLLLLEYLNLNTVPVEENKLRLAEIVYNIESKTPVSSCTIKSFTWVQFQNDRQYSGIAT
AVSDPSDFSIQLEDFFDIMKYLFMLLSDLPETLQTLPQEFIIPGSSCLFKYKSEDQWNRVEISEVSPQSLCLVLVDYGFSFYIRYSE
IINLKVVPEELLNLPRLSYPCILYGILPAKGKHWSEEAKIFFRDFLSKPDLVFQFREYHSETKLKVDVIHEKNNLADILVASGLATY
SKDSPHLDAITATESAKNPI
>sp|P03886|NU1M_HUMAN
MPMANLLLLIVPILIAMAFLMLTERKILGYMQLRKGPNVVGPYGLLQPFADAMKLFTKEPLKPATSTITLYITAPTLALTIALLLWT
PLPMPNPLVNLNLGLLFILATSSLAVYSILWSGWASNSNYALIGALRAVAQTISYEVTLAIILLSTLLMSGSFNLSTLITTQEHLWL
LLPSWPLAMMWFISTLAETNRTPFDLAEGESELVSGFNIEYAAGPFALFFMAEYTNIIMMNTLTTTIFLGTTYDALSPELYTTYFVT
KTLLLTSLFLWIRTAYPRFRYDQLMHLLWKNFLPLTLALLMWYVSMPITISSIPPQT
>sp|Q9BXA7|TSSK1_HUMAN
MDDAAVLKRRGYLLGINLGEGSYAKVKSAYSERLKFNVAIKIIDRKKAPADFLEKFLPREIEILAMLNHCSIIKTYEIFETSHGKVY
IVMELAVQGDLLELIKTRGALHEDEARKKFHQLSLAIKYCHDLDVVHRDLKCDNLLLDKDFNIKLSDFSFSKRCLRDDSGRMALSKT
FCGSPAYAAPEVLQGIPYQPKVYDIWSLGVILYIMVCGSMPYDDSNIKKMLRIQKEHRVNFPRSKHLTGECKDLIYHMLQPDVNRRL
HIDEILSHCWMQPKARGSPSVAINKEGESSRGTEPLWTPEPGSDKKSATKLEPEGEAQPQAQPETKPEGTAMQMSRQSEILGFPSKP
STMETEEGPPQQPPETRAQ
>sp|Q8NG85|OR2L3_HUMAN
MENYNQTSTDFILLGFFPPSRIGLFLFILIVFIFLMALIGNLSMILLIFLDTHLHTPMYFLLSQLSLIDLNYISTIVPKMASDFLSG
NKSISFTGCGIQSFFFSALGGAEALLLASMAYDRYIAICFPLHYPIRMSKRMCVLMITGSWIIGSINACAHTVYVLHIPYCQSRAIN
HFFCDVPAMVTLACMDTWVYEGTVFLSTTIFLVFPFIAISCSYGRVLLAVYHMKSAEGRKKAYLTCSTHLTVVTFYYAPFVYTYLRP
RSLRSPTEDKVLAVFYTTLTPMLNPIIYSLRNKEVMGALTRVSQRICSGKM
>sp|O15405|TOX3_HUMAN
MDVRFYPAAAGDPASLDFAQCLGYYGYSKFGNNNNYMNMAEANNAFFAASEQTFHTPSLGDEEFEIPPITPPPESDPALGMPDVLLP
FQALSDPLPSQGSEFTPQFPPQSLDLPSITISRNLVEQDGVLHSSGLHMDQSHTQVSQYRQDPSLIMRSIVHMTDAARSGVMPPAQL
TTINQSQLSAQLGLNLGGASMPHTSPSPPASKSATPSPSSSINEEDADEANRAIGEKRAAPDSGKKPKTPKKKKKKDPNEPQKPVSA
YALFFRDTQAAIKGQNPNATFGEVSKIVASMWDSLGEEQKQVYKRKTEAAKKEYLKALAAYRASLVSKAAAESAEAQTIRSVQQTLA
STNLTSSLLLNTPLSQHGTVSASPQTLQQSLPRSIAPKPLTMRLPMNQIVTSVTIAANMPSNIGAPLISSMGTTMVGSAPSTQVSPS
VQTQQHQMQLQQQQQQQQQMQQMQQQQLQQHQMHQQIQQQMQQQHFQHHMQQHLQQQQQHLQQQINQQQLQQQLQQRLQLQQLQHM
QHQSQPSPRQHSPVASQITSPIPAIGSPQPASQQHQSQIQSQTQTQVLSQVSIF
>sp|P42167|LAP2B_HUMAN
MPEFLEDPSVLTKDKLKSELVANNVTLPAGEQRKDVYVQLYLQHLTARNRPPLPAGTNSKGPPDFSSDEEREPTPVLGSGAAAAGRS
RAAVGRKATKKTDKPRQEDKDDLDVTELTNEDLLDQLVKYGVNPGPIVGTTRKLYEKKLLKLREQGTESRSSTPLPTISSSAENTRQ
NGSNDSDRYSDNEEDSKIELKLEKREPLKGRAKTPVTLKQRRVEHNQSYSQAGITETEWTSGSSKGGPLQALTRESTRGSRRTPRKR
VETSEHFRIDGPVISESTPIAETIMASSNESLVVNRVTGNFKHASPILPITEFSDIPRRAPKKPLTRAEVGEKTEERRVERDILKEM

FPYEASTPTGISASCRRPIKGAAGRPLELSDFRMEESFSSKYVPKYVPLADVKSEKTKKGRSIPVWIKILLFVVVAVFLFLVYQAME
TNQVNPFSNFLHVDPRKSN
>sp|Q12802|AKP13_HUMAN
MKLNPQQAPLYGDCVVTVLLAEEDKAEDDVVFYLVFLGSTLRHCTSTRKVSSDTLETIAPGHDCCETVKVQLCASKEGLPVFVVAEE
DFHFVQDEAYDAAQFLATSAGNQQALNFTRFLDQSGPPSGDVNSLDKKLVLAFRHLKLPTEWNVLGTDQSLHDAGPRETLMHFAVRL
GLLRLTWFLLQKPGGRGALSIHNQEGATPVSLALERGYHKLHQLLTEENAGEPDSWSSLSYEIPYGDCSVRHHRELDIYTLTSESDS
HHEHPFPGDGCTGPIFKLMNIQQQLMKTNLKQMDSLMPLMMTAQDPSSAPETDGQFLPCAPEPTDPQRLSSSEETESTQCCPGSPVA
QTESPCDLSSIVEEENTDRSCRKKNKGVERKGEEVEPAPIVDSGTVSDQDSCLQSLPDCGVKGTEGLSSCGNRNEETGTKSSGMPTD
QESLSSGDAVLQRDLVMEPGTAQYSSGGELGGISTTNVSTPDTAGEMEHGLMNPDATVWKNVLQGGESTKERFENSNIGTAGASDVH
VTSKPVDKISVPNCAPAASSLDGNKPAESSLAFSNEETSTEKTAETETSRSREESADAPVDQNSVVIPAAAKDKISDGLEPYTLLAA
GIGEAMSPSDLALLGLEEDVMPHQNSETNSSHAQSQKGKSSPICSTTGDDKLCADSACQQNTVTSSGDLVAKLCDNIVSESESTTAR
QPSSQDPPDASHCEDPQAHTVTSDPVRDTQERADFCPFKVVDNKGQRKDVKLDKPLTNMLEVVSHPHPVVPKMEKELVPDQAVISDS
TFSLANSPGSESVTKDDALSFVPSQKEKGTATPELHTATDYRDGPDGNSNEPDTRPLEDRAVGLSTSSTAAELQHGMGNTSLTGLGG
EHEGPAPPAIPEALNIKGNTDSSLQSVGKATLALDSVLTEEGKLLVVSESSAAQEQDKDKAVTCSSIKENALSSGTLQEEQRTPPPG
QDTQQFHEKSISADCAKDKALQLSNSPGASSAFLKAETEHNKEVAPQVSLLTQGGAAQSLVPPGASLATESRQEALGAEHNSSALLP
CLLPDGSDGSDALNCSQPSPLDVGVKNTQSQGKTSACEVSGDVTVDVTGVNALQGMAEPRRENISHNTQDILIPNVLLSQEKNAVLG
LPVALQDKAVTDPQGVGTPEMIPLDWEKGKLEGADHSCTMGDAEEAQIDDEAHPVLLQPVAKELPTDMELSAHDDGAPAGVREVMRA
PPSGRERSTPSLPCMVSAQDAPLPKGADLIEEAASRIVDAVIEQVKAAGALLTEGEACHMSLSSPELGPLTKGLESAFTEKVSTFPP
GESLPMGSTPEEATGSLAGCFAGREEPEKIILPVQGPEPAAEMPDVKAEDEVDFRASSISEEVAVGSIAATLKMKQGPMTQAINREN
WCTIEPCPDAASLLASKQSPECENFLDVGLGRECTSKQGVLKRESGSDSDLFHSPSDDMDSIIFPKPEEEHLACDITGSSSSTDDTA
SLDRHSSHGSDVSLSQILKPNRSRDRQSLDGFYSHGMGAEGRESESEPADPGDVEEEEMDSITEVPANCSVLRSSMRSLSPFRRHSW
GPGKNAASDAEMNHRSSMRVLGDVVRRPPIHRRSFSLEGLTGGAGVGNKPSSSLEVSSANAEELRHPFSGEERVDSLVSLSEEDLES
DQREHRMFDQQICHRSKQQGFNYCTSAISSPLTKSISLMTISHPGLDNSRPFHSTFHNTSANLTESITEENYNFLPHSPSKKDSEWK
SGTKVSRTFSYIKNKMSSSKKSKEKEKEKDKIKEKEKDSKDKEKDKKTVNGHTFSSIPVVGPISCSQCMKPFTNKDAYTCANCSAFV
HKGCRESLASCAKVKMKQPKGSLQAHDTSSLPTVIMRNKPSQPKERPRSAVLLVDETATTPIFANRRSQQSVSLSKSVSIQNITGVG
NDENMSNTWKFLSHSTDSLNKISKVNESTESLTDEGVGTDMNEGQLLGDFEIESKQLEAESWSRIIDSKFLKQQKKDVVKRQEVIYE
LMQTEFHHVRTLKIMSGVYSQGMMADLLFEQQMVEKLFPCLDELISIHSQFFQRILERKKESLVDKSEKNFLIKRIGDVLVNQFSGE
NAERLKKTYGKFCGQHNQSVNYFKDLYAKDKRFQAFVKKKMSSSVVRRLGIPECILLVTQRITKYPVLFQRILQCTKDNEVEQEDLA
QSLSLVKDVIGAVDSKVASYEKKVRLNEIYTKTDSKSIMRMKSGQMFAKEDLKRKKLVRDGSVFLKNAAGRLKEVQAVLLTDILVFL
QEKDQKYIFASLDQKSTVISLKKLIVREVAHEEKGLFLISMGMTDPEMVEVHASSKEERNSWIQIIQDTINTLNRDEDEGIPSENEE
EKKMLDTRARELKEQLHQKDQKILLLLEEKEMIFRDMAECSTPLPEDCSPTHSPRVLFRSNTEEALKGGPLMKSAINEVEILQGLVS
GNLGGTLGPTVSSPIEQDVVGPVSLPRRAETFGGFDSHQMNASKGGEKEEGDDGQDLRRTESDSGLKKGGNANLVFMLKRNSEQVVQ
SVVHLYELLSALQGVVLQQDSYIEDQKLVLSERALTRSLSRPSSLIEQEKQRSLEKQRQDLANLQKQQAQYLEEKRRREREWEARER
ELREREALLAQREEEVQQGQQDLEKEREELQQKKGTYQYDLERLRAAQKQLEREQEQLRREAERLSQRQTERDLCQVSHPHTKLMRI
PSFFPSPEEPPSPSAPSIAKSGSLDSELSVSPKRNSISRTHKDKGPFHILSSTSQTNKGPEGQSQAPASTSASTRLFGLTKPKEKKE
KKKKNKTSRSQPGDGPASEVSAEGEEIFC
>sp|Q6PCB0|VWA1_HUMAN
MLPWTALGLALSLRLALARSGAERGPPASAPRGDLMFLLDSSASVSHYEFSRVREFVGQLVAPLPLGTGALRASLVHVGSRPYTEFP
FGQHSSGEAAQDAVRASAQRMGDTHTGLALVYAKEQLFAEASGARPGVPKVLVWVTDGGSSDPVGPPMQELKDLGVTVFIVSTGRGN
FLELSAAASAPAEKHLHFVDVDDLHIIVQELRGSILDAMRPQQLHATEITSSGFRLAWPPLLTADSGYYVLELVPSAQPGAARRQQL
PGNATDWIWAGLDPDTDYDVALVPESNVRLLRPQILRVRTRPGEAGPGASGPESGAGPAPTQLAALPAPEEAGPERIVISHARPRSL
RVSWAPALGSAAALGYHVQFGPLRGGEAQRVEVPAGRNCTTLQGLAPGTAYLVTVTAAFRSGRESALSAKACTPDGPRPRPRPVPRA
PTPGTASREP
>sp|Q5XLA6|CAR17_HUMAN

MADKVLKEKRKQFIRSVGEGTINGLLGELLETRVLSQEEIEIVKCENATVMDKARALLDSVIRKGAPACQICITYICEEDSHLAGTL
GLSAGPTSGNHLTTQDSQIVLPS
>sp|Q8N888|BCOR2_HUMAN
MKEKLSKKRAEVKGNRSWLEEFLKPSDNEEGPPPKNKVLSNNASSQKPTHSSCIPLLRLPDKQQKVNESIKTDMLCTDKEEECPAAS
LLQKYTNNSEKPSGKRQCKTKHLISQDLRQGFLLTGKCYVENADGKIPLGTCFLLGLI
>sp|Q96LL3|CP092_HUMAN
MGAGVGVAGCTRGHRNWVPSQLPPREIKAGVSLAVVTEFAWVLAPRPKRATASALGTESPRFLDRPDFFDYPDSDQARLLAVAQFIG
EKPIVFINSGSSPGLFHHILVGLLVVAFFFLLFQFCTHINFQKGA
>sp|O60784|TOM1_HUMAN
MDFLLGNPFSSPVGQRIEKATDGSLQSEDWALNMEICDIINETEEGPKDALRAVKKRIVGNKNFHEVMLALTVLETCVKNCGHRFHV
LVASQDFVESVLVRTILPKNNPPTIVHDKVLNLIQSWADAFRSSPDLTGVVTIYEDLRRKGLEFPMTDLDMLSPIHTPQRTVFNSET
QSGQDSVGTDSSQQEDSGQHAAPLPAPPILSGDTPIAPTPEQIGKLRSELEMVSGNVRVMSEMLTELVPTQAEPADLELLQELNRTC
RAMQQRVLELIPQIANEQLTEELLIVNDNLNNVFLRHERFERFRTGQTTKAPSEAEPAADLIDMGPDPAATGNLSSQLAGMNLGSSS
VRAGLQSLEASGRLEDEFDMFALTRGSSLADQRKEVKYEAPQATDGLAGALDARQQSTGAIPVTQACLMEDIEQWLSTDVGNDAEEP
KGVTSEEFDKFLEERAKAADRLPNLSSPSAEGPPGPPSGPAPRKKTQEKDDDMLFAL
>sp|P10415|BCL2_HUMAN
MAHAGRTGYDNREIVMKYIHYKLSQRGYEWDAGDVGAAPPGAAPAPGIFSSQPGHTPHPAASRDPVARTSPLQTPAAPGAAAGPALS
PVPPVVHLTLRQAGDDFSRRYRRDFAEMSSQLHLTPFTARGRFATVVEELFRDGVNWGRIVAFFEFGGVMCVESVNREMSPLVDNIA
LWMTEYLNRHLHTWIQDNGGWDAFVELYGPSMRPLFDFSWLSLKTLLSLALVGACITLGAYLGHK
>sp|P23677|IP3KA_HUMAN
MTLPGGPTGMARPGGARPCSPGLERAPRRSVGELRLLFEARCAAVAAAAAAGEPRARGAKRRGGQVPNGLPRAPPAPVIPQLTVTAE
EPDVPPTSPGPPERERDCLPAAGSSHLQQPRRLSTSSVSSTGSSSLLEDSEDDLLSDSESRSRGNVQLEAGEDVGQKNHWQKIRTMV
NLPVISPFKKRYAWVQLAGHTGSFKAAGTSGLILKRCSEPERYCLARLMADALRGCVPAFHGVVERDGESYLQLQDLLDGFDGPCVL
DCKMGVRTYLEEELTKARERPKLRKDMYKKMLAVDPEAPTEEEHAQRAVTKPRYMQWREGISSSTTLGFRIEGIKKADGSCSTDFKT
TRSREQVLRVFEEFVQGDEEVLRRYLNRLQQIRDTLEVSEFFRRHEVIGSSLLFVHDHCHRAGVWLIDFGKTTPLPDGQILDHRRPW
EEGNREDGYLLGLDNLIGILASLAER
>sp|Q8WUW1|BRK1_HUMAN
MAGQEDPVQREIHQDWANREYIEIITSSIKKIADFLNSFDMSCRSRLATLNEKLTALERRIEYIEARVTKGETLT
>sp|P0DJD8|PEPA3_HUMAN
MKWLLLLGLVALSECIMYKVPLIRKKSLRRTLSERGLLKDFLKKHNLNPARKYFPQWKAPTLVDEQPLENYLDMEYFGTIGIGTPAQ
DFTVVFDTGSSNLWVPSVYCSSLACTNHNRFNPEDSSTYQSTSETVSITYGTGSMTGILGYDTVQVGGISDTNQIFGLSETEPGSFL
YYAPFDGILGLAYPSISSSGATPVFDNIWNQGLVSQDLFSVYLSADDQSGSVVIFGGIDSSYYTGSLNWVPVTVEGYWQITVDSITM
NGEAIACAEGCQAIVDTGTSLLTGPTSPIANIQSDIGASENSDGDMVVSCSAISSLPDIVFTINGVQYPVPPSAYILQSEGSCISGF
QGMNLPTESGELWILGDVFIRQYFTVFDRANNQVGLAPVA
>sp|P06703|S10A6_HUMAN
MACPLDQAIGLLVAIFHKYSGREGDKHTLSKKELKELIQKELTIGSKLQDAEIARLMEDLDRNKDQEVNFQEYVTFLGALALIYNEA
LKG
>sp|Q9Y4E6|WDR7_HUMAN
MAGNSLVLPIVLWGRKAPTHCISAVLLTDDGATIVTGCHDGQICLWDLSVELQINPRALLFGHTASITCLSKACASSDKQYIVSASE
SGEMCLWDVSDGRCIEFTKLACTHTGIQFYQFSVGNQREGRLLCHGHYPEILVVDATSLEVLYSLVSKISPDWISSMSIIRSHRTQE
DTVVALSVTGILKVWIVTSEISDMQDTEPIFEEESKPIYCQNCQSISFCAFTQRSLLVVCSKYWRVFDAGDYSLLCSGPSENGQTWT
GGDFVSSDKVIIWTENGQSYIYKLPASCLPASDSFRSDVGKAVENLIPPVQHILLDRKDKELLICPPVTRFFYGCREYFHKLLIQGD
SSGRLNIWNISDTADKQGSEEGLAMTTSISLQEAFDKLNPCPAGIIDQLSVIPNSNEPLKVTASVYIPAHGRLVCGREDGSIVIVPA
TQTAIVQLLQGEHMLRRGWPPHRTLRGHRNKVTCLLYPHQVSARYDQRYLISGGVDFSVIIWDIFSGEMKHIFCVHGGEITQLLVPP

ENCSARVQHCICSVASDHSVGLLSLREKKCIMLASRHLFPIQVIKWRPSDDYLVVGCSDGSVYVWQMDTGALDRCVMGITAVEILNA
CDEAVPAAVDSLSHPAVNLKQAMTRRSLAALKNMAHHKLQTLATNLLASEASDKGNLPKYSHNSLMVQAIKTNLTDPDIHVLFFDVE
ALIIQLLTEEASRPNTALISPENLQKASGSSDKGGSFLTGKRAAVLFQQVKETIKENIKEHLLDDEEEDEEIMRQRREESDPEYRSS
KSKPLTLLEYNLTMDTAKLFMSCLHAWGLNEVLDEVCLDRLGMLKPHCTVSFGLLSRGGHMSLMLPGYNQPACKLSHGKTEVGRKLP
ASEGVGKGTYGVSRAVTTQHLLSIISLANTLMSMTNATFIGDHMKKGPTRPPRPSTPDLSKARGSPPTSSNIVQGQIKQVAAPVVSA
RSDADHSGSDPPSAPALHTCFLVNEGWSQLAAMHCVMLPDLLGLDKFRPPLLEMLARRWQDRCLEVREAAQALLLAELRRIEQAGRK
EAIDAWAPYLPQYIDHVISPGVTSEAAQTITTAPDASGPEAKVQEEEHDLVDDDITTGCLSSVPQMKKISTSYEERRKQATAIVLLG
VIGAEFGAEIEPPKLLTRPRSSSQIPEGFGLTSGGSNYSLARHTCKALTFLLLQPPSPKLPPHSTIRRTAIDLIGRGFTVWEPYMDV
SAVLMGLLELCADAEKQLANITMGLPLSPAADSARSARHALSLIATARPPAFITTIAKEVHRHTALAANTQSQQNMHTTTLARAKGE
ILRVIEILIEKMPTDVVDLLVEVMDIIMYCLEGSLVKKKGLQECFPAICRFYMVSYYERNHRIAVGARHGSVALYDIRTGKCQTIHG
HKGPITAVAFAPDGRYLATYSNTDSHISFWQMNTSLLGSIGMLNSAPQLRCIKTYQVPPVQPASPGSHNALKLARLIWTSNRNVILM
AHDGKEHRFMV
>sp|O43680|TCF21_HUMAN
MSTGSLSDVEDLQEVEMLECDGLKMDSNKEFVTSNESTEESSNCENGSPQKGRGGLGKRRKAPTKKSPLSGVSQEGKQVQRNAANAR
ERARMRVLSKAFSRLKTTLPWVPPDTKLSKLDTLRLASSYIAHLRQILANDKYENGYIHPVNLTWPFMVAGKPESDLKEVVTASRLC
GTTAS
>sp|P0CG47|UBB_HUMAN
MQIFVKTLTGKTITLEVEPSDTIENVKAKIQDKEGIPPDQQRLIFAGKQLEDGRTLSDYNIQKESTLHLVLRLRGGMQIFVKTLTGK
TITLEVEPSDTIENVKAKIQDKEGIPPDQQRLIFAGKQLEDGRTLSDYNIQKESTLHLVLRLRGGMQIFVKTLTGKTITLEVEPSDT
IENVKAKIQDKEGIPPDQQRLIFAGKQLEDGRTLSDYNIQKESTLHLVLRLRGGC
>sp|Q9H1A4|APC1_HUMAN
MSNFYEERTTMIAARDLQEFVPFGRDHCKHHPNALNLQLRQLQPASELWSSDGAAGLVGSLQEVTIHEKQKESWQLRKGVSEIGEDV
DYDEELYVAGNMVIWSKGSKSQALAVYKAFTVDSPVQQALWCDFIISQDKSEKAYSSNEVEKCICILQSSCINMHSIEGKDYIASLP
FQVANVWPTKYGLLFERSASSHEVPPGSPREPLPTMFSMLHPLDEITPLVCKSGSLFGSSRVQYVVDHAMKIVFLNTDPSIVMTYDA
VQNVHSVWTLRRVKSEEENVVLKFSEQGGTPQNVATSSSLTAHLRSLSKGDSPVTSPFQNYSSIHSQSRSTSSPSLHSRSPSISNMA
ALSRAHSPALGVHSFSGVQRFNISSHNQSPKRHSISHSPNSNSNGSFLAPETEPIVPELCIDHLWTETITNIREKNSQASKVFITSD
LCGQKFLCFLVESQLQLRCVKFQESNDKTQLIFGSVTNIPAKDAAPVEKIDTMLVLEGSGNLVLYTGVVRVGKVFIPGLPAPSLTMS
NTMPRPSTPLDGVSTPKPLSKLLGSLDEVVLLSPVPELRDSSKLHDSLYNEDCTFQQLGTYIHSIRDPVHNRVTLELSNGSMVRITI
PEIATSELVQTCLQAIKFILPKEIAVQMLVKWYNVHSAPGGPSYHSEWNLFVTCLMNMMGYNTDRLAWTRNFDFEGSLSPVIAPKKA
RPSETGSDDDWEYLLNSDYHQNVESHLLNRSLCLSPSEASQMKDEDFSQNLSLDSSTLLFTHIPAIFFVLHLVYEELKLNTLMGEGI
CSLVELLVQLARDLKLGPYVDHYYRDYPTLVRTTGQVCTIDPGQTGFMHHPSFFTSEPPSIYQWVSSCLKGEGMPPYPYLPGICERS
RLVVLSIALYILGDESLVSDESSQYLTRITIAPQKLQVEQEENRFSFRHSTSVSSLAERLVVWMTNVGFTLRDLETLPFGIALPIRD
AIYHCREQPASDWPEAVCLLIGRQDLSKQACEGNLPKGKSVLSSDVPSGTETEEEDDGMNDMNHEVMSLIWSEDLRVQDVRRLLQSA
HPVRVNVVQYPELSDHEFIEEKENRLLQLCQRTMALPVGRGMFTLFSYHPVPTEPLPIPKLNLTGRAPPRNTTVDLNSGNIDVPPNM
TSWASFHNGVAAGLKIAPASQIDSAWIVYNKPKHAELANEYAGFLMALGLNGHLTKLATLNIHDYLTKGHEMTSIGLLLGVSAAKLG
TMDMSITRLLSIHIPALLPPTSTELDVPHNVQVAAVVGIGLVYQGTAHRHTAEVLLAEIGRPPGPEMEYCTDRESYSLAAGLALGMV
CLGHGSNLIGMSDLNVPEQLYQYMVGGHRRFQTGMHREKHKSPSYQIKEGDTINVDVTCPGATLALAMIYLKTNNRSIADWLRAPDT
MYLLDFVKPEFLLLRTLARCLILWDDILPNSKWVDSNVPQIIRENSISLSEIELPCSEDLNLETLSQAHVYIIAGACLSLGFRFAGS
ENLSAFNCLHKFAKDFMTYLSAPNASVTGPHNLETCLSVVLLSLAMVMAGSGNLKVLQLCRFLHMKTGGEMNYGFHLAHHMALGLLF
LGGGRYSLSTSNSSIAALLCALYPHFPAHSTDNRYHLQALRHLYVLAAEPRLLVPVDVDTNTPCYALLEVTYKGTQWYEQTKEELMA
PTLLPELHLLKQIKVKGPRYWELLIDLSKGTQHLKSILSKDGVLYVKLRAGQLSYKEDPMGWQSLLAQTVANRNSEARAFKPETISA
FTSDPALLSFAEYFCKPTVNMGQKQEILDLFSSVLYECVTQETPEMLPAYIAMDQAIRRLGRREMSETSELWQIKLVLEFFSSRSHQ
ERLQNHPKRGLFMNSEFLPVVKCTIDNTLDQWLQVGGDMCVHAYLSGQPLEESQLSMLACFLVYHSVPAPQHLPPIGLEGSTSFAEL
LFKFKQLKMPVRALLRLAPLLLGNPQPMVM

>sp|Q9UGI6|KCNN3_HUMAN
MDTSGHFHDSGVGDLDEDPKCPCPSSGDEQQQQQQQQQQQQPPPPAPPAAPQQPLGPSLQPQPPQLQQQQQQQQQQQQQQQQQQQPP
HPLSQLAQLQSQPVHPGLLHSSPTAFRAPPSSNSTAILHPSSRQGSQLNLNDHLLGHSPSSTATSGPGGGSRHRQASPLVHRRDSNP
FTEIAMSSCKYSGGVMKPLSRLSASRRNLIEAETEGQPLQLFSPSNPPEIVISSREDNHAHQTLLHHPNATHNHQHAGTTASSTTFP
KANKRKNQNIGYKLGHRRALFEKRKRLSDYALIFGMFGIVVMVIETELSWGLYSKDSMFSLALKCLISLSTIILLGLIIAYHTREVQ
LFVIDNGADDWRIAMTYERILYISLEMLVCAIHPIPGEYKFFWTARLAFSYTPSRAEADVDIILSIPMFLRLYLIARVMLLHSKLFT
DASSRSIGALNKINFNTRFVMKTLMTICPGTVLLVFSISLWIIAAWTVRVCERYHDQQDVTSNFLGAMWLISITFLSIGYGDMVPHT
YCGKGVCLLTGIMGAGCTALVVAVVARKLELTKAEKHVHNFMMDTQLTKRIKNAAANVLRETWLIYKHTKLLKKIDHAKVRKHQRKF
LQAIHQLRSVKMEQRKLSDQANTLVDLSKMQNVMYDLITELNDRSEDLEKQIGSLESKLEHLTASFNSLPLLIADTLRQQQQQLLSA
IIEARGVSVAVGTTHTPISDSPIGVSSTSFPTPYTSSSSC
>sp|P25874|UCP1_HUMAN
MGGLTASDVHPTLGVQLFSAGIAACLADVITFPLDTAKVRLQVQGECPTSSVIRYKGVLGTITAVVKTEGRMKLYSGLPAGLQRQIS
SASLRIGLYDTVQEFLTAGKETAPSLGSKILAGLTTGGVAVFIGQPTEVVKVRLQAQSHLHGIKPRYTGTYNAYRIIATTEGLTGLW
KGTTPNLMRSVIINCTELVTYDLMKEAFVKNNILADDVPCHLVSALIAGFCATAMSSPVDVVKTRFINSPPGQYKSVPNCAMKVFTN
EGPTAFFKGLVPSFLRLGSWNVIMFVCFEQLKRELSKSRQTMDCAT
>sp|Q9UGT4|SUSD2_HUMAN
MKPALLPWALLLLATALGPGPGPTADAQESCSMRCGALDGPCSCHPTCSGLGTCCLDFRDFCLEILPYSGSMMGGKDFVVRHFKMSS
PTDASVICRFKDSIQTLGHVDSSGQVHCVSPLLYESGRIPFTVSLDNGHSFPRAGTWLAVHPNKVSMMEKSELVNETRWQYYGTANT
SGNLSLTWHVKSLPTQTITIELWGYEETGMPYSQEWTAKWSYLYPLATHIPNSGSFTFTPKPAPPSYQRWRVGALRIIDSKNYAGQK
DVQALWTNDHALAWHLSDDFREDPVAWARTQCQAWEELEDQLPNFLEELPDCPCTLTQARADSGRFFTDYGCDMEQGSVCTYHPGAV
HCVRSVQASLRYGSGQQCCYTADGTQLLTADSSGGSTPDRGHDWGAPPFRTPPRVPSMSHWLYDVLSFYYCCLWAPDCPRYMQRRPS
NDCRNYRPPRLASAFGDPHFVTFDGTNFTFNGRGEYVLLEAALTDLRVQARAQPGTMSNGTETRGTGLTAVAVQEGNSDVVEVRLAN
RTGGLEVLLNQEVLSFTEQSWMDLKGMFLSVAAGDRVSIMLASGAGLEVSVQGPFLSVSVLLPEKFLTHTHGLLGTLNNDPTDDFTL
HSGRVLPPGTSPQELFLFGANWTVHNASSLLTYDSWFLVHNFLYQPKHDPTFEPLFPSETTLNPSLAQEAAKLCGDDHFCNFDVAAT
GSLSTGTATRVAHQLHQRRMQSLQPVVSCGWLAPPPNGQKEGNRYLAGSTIYFHCDNGYSLAGAETSTCQADGTWSSPTPKCQPGRS
YAVLLGIIFGGLAVVAAVALVYVLLRRRKGNTHVWGAQP
>sp|P30837|AL1B1_HUMAN
MLRFLAPRLLSLQGRTARYSSAAALPSPILNPDIPYNQLFINNEWQDAVSKKTFPTVNPTTGEVIGHVAEGDRADVDRAVKAAREAF
RLGSPWRRMDASERGRLLNLLADLVERDRVYLASLETLDNGKPFQESYALDLDEVIKVYRYFAGWADKWHGKTIPMDGQHFCFTRHE
PVGVCGQIIPWNFPLVMQGWKLAPALATGNTVVMKVAEQTPLSALYLASLIKEAGFPPGVVNIITGYGPTAGAAIAQHVDVDKVAFT
GSTEVGHLIQKAAGDSNLKRVTLELGGKSPSIVLADADMEHAVEQCHEALFFNMGQCCCAGSRTFVEESIYNEFLERTVEKAKQRKV
GNPFELDTQQGPQVDKEQFERVLGYIQLGQKEGAKLLCGGERFGERGFFIKPTVFGGVQDDMRIAKEEIFGPVQPLFKFKKIEEVVE
RANNTRYGLAAAVFTRDLDKAMYFTQALQAGTVWVNTYNIVTCHTPFGGFKESGNGRELGEDGLKAYTEVKTVTIKVPQKNS
>sp|P0C8F1|PATE4_HUMAN
MRKMNTLLLVSLSFLYLKEVMGLKCNTCIYTEGWKCMAGRGTCIAKENELCSTTAYFRGDKHMYSTHMCKYKCREEESSKRGLLRVT
LCCDRNFCNVF
>sp|P54852|EMP3_HUMAN
MSLLLLVVSALHILILILLFVATLDKSWWTLPGKESLNLWYDCTWNNDTKTWACSNVSENGWLKAVQVLMVLSLILCCLSFILFMFQ
LYTMRRGGLFYATGLCQLCTSVAVFTGALIYAIHAEEILEKHPRGGSFGYCFALAWVAFPLALVSGIIYIHLRKRE
>sp|Q9H9A6|LRC40_HUMAN
MSRLKRIAGQDLRAGFKAGGRDCGTSVPQGLLKAARKSGQLNLSGRNLSEVPQCVWRINVDIPEEANQNLSFGATERWWEQTDLTKL
IISNNKLQSLTDDLRLLPALTVLDIHDNQLTSLPSAIRELENLQKLNVSHNKLKILPEEITNLRNLKCLYLQHNELTCISEGFEQLS
NLEDLDLSNNHLTTVPASFSSLSSLVRLNLSSNELKSLPAEINRMKRLKHLDCNSNLLETIPPELAGMESLELLYLRRNKLRFLPEF
PSCSLLKELHVGENQIEMLEAEHLKHLNSILVLDLRDNKLKSVPDEIILLRSLERLDLSNNDISSLPYSLGNLHLKFLALEGNPLRT

IRREIISKGTQEVLKYLRSKIKDDGPSQSESATETAMTLPSESRVNIHAIITLKILDYSDKQATLIPDEVFDAVKSNIVTSINFSKN
QLCEIPKRMVELKEMVSDVDLSFNKLSFISLELCVLQKLTFLDLRNNFLNSLPEEMESLVRLQTINLSFNRFKMLPEVLYRIFTLET
ILISNNQVGSVDPQKMKMMENLTTLDLQNNDLLQIPPELGNCVNLRTLLLDGNPFRVPRAAILMKGTAAILEYLRDRIPT
>sp|Q9UNU6|CP8B1_HUMAN
MVLWGPVLGALLVVIAGYLCLPGMLRQRRPWEPPLDKGTVPWLGHAMAFRKNMFEFLKRMRTKHGDVFTVQLGGQYFTFVMDPLSFG
SILKDTQRKLDFGQYAKKLVLKVFGYRSVQGDHEMIHSASTKHLRGDGLKDLNETMLDSLSFVMLTSKGWSLDASCWHEDSLFRFCY
YILFTAGYLSLFGYTKDKEQDLLQAGELFMEFRKFDLLFPRFVYSLLWPREWLEVGRLQRLFHKMLSVSHSQEKEGISNWLGNMLQF
LREQGVPSAMQDKFNFMMLWASQGNTGPTSFWALLYLLKHPEAIRAVREEATQVLGEARLETKQSFAFKLGALQHTPVLDSVVEETL
RLRAAPTLLRLVHEDYTLKMSSGQEYLFRHGDILALFPYLSVHMDPDIHPEPTVFKYDRFLNPNGSRKVDFFKTGKKIHHYTMPWGS
GVSICPGRFFALSEVKLFILLMVTHFDLELVDPDTPLPHVDPQRWGFGTMQPSHDVRFRYRLHPTE
>sp|Q9NP99|TREM1_HUMAN
MRKTRLWGLLWMLFVSELRAATKLTEEKYELKEGQTLDVKCDYTLEKFASSQKAWQIIRDGEMPKTLACTERPSKNSHPVQVGRIIL
EDYHDHGLLRVRMVNLQVEDSGLYQCVIYQPPKEPHMLFDRIRLVVTKGFSGTPGSNENSTQNVYKIPPTTTKALCPLYTSPRTVTQ
APPKSTADVSTPDSEINLTNVTDIIRVPVFNIVILLAGGFLSKSLVFSVLFAVTLRSFVP
>sp|A6NEH6|TM247_HUMAN
MAAEDREMMEARGAGESCPTFPKMVPGDSKSEGKPRAYLEAESQKPDSSYDYLEEMEACEDGGCQGPLKSLSPKSCRATKGQAGDGP
KPAELPPTPGTERNPEMELEKVRMEFELTRLKYLHEKNQRQRQHEVVMEQLQRERQHEVVMEQLQQEAAPRLFSGGLQNFLLPQNQF
AMFLYCFIFIHIIYVTKEMVFFLFAKHYLFCIAAILLCLIKTFWS
>sp|P20813|CP2B6_HUMAN
MELSVLLFLALLTGLLLLLVQRHPNTHDRLPPGPRPLPLLGNLLQMDRRGLLKSFLRFREKYGDVFTVHLGPRPVVMLCGVEAIREA
LVDKAEAFSGRGKIAMVDPFFRGYGVIFANGNRWKVLRRFSVTTMRDFGMGKRSVEERIQEEAQCLIEELRKSKGALMDPTFLFQSI
TANIICSIVFGKRFHYQDQEFLKMLNLFYQTFSLISSVFGQLFELFSGFLKYFPGAHRQVYKNLQEINAYIGHSVEKHRETLDPSAP
KDLIDTYLLHMEKEKSNAHSEFSHQNLNLNTLSLFFAGTETTSTTLRYGFLLMLKYPHVAERVYREIEQVIGPHRPPELHDRAKMPY
TEAVIYEIQRFSDLLPMGVPHIVTQHTSFRGYIIPKDTEVFLILSTALHDPHYFEKPDAFNPDHFLDANGALKKTEAFIPFSLGKRI
CLGEGIARAELFLFFTTILQNFSMASPVAPEDIDLTPQECGVGKIPPTYQIRFLPR
>sp|Q14103|HNRPD_HUMAN
MSEEQFGGDGAAAAATAAVGGSAGEQEGAMVAATQGAAAAAGSGAGTGGGTASGGTEGGSAESEGAKIDASKNEEDEGHSNSSPRHS
EAATAQREEWKMFIGGLSWDTTKKDLKDYFSKFGEVVDCTLKLDPITGRSRGFGFVLFKESESVDKVMDQKEHKLNGKVIDPKRAKA
MKTKEPVKKIFVGGLSPDTPEEKIREYFGGFGEVESIELPMDNKTNKRRGFCFITFKEEEPVKKIMEKKYHNVGLSKCEIKVAMSKE
QYQQQQQWGSRGGFAGRARGRGGGPSQNWNQGYSNYWNQGYGNYGYNSQGYGGYGGYDYTGYNNYYGYGDYSNQQSGYGKVSRRGGH
QNSYKPY
>sp|Q8N3Z3|GTPB8_HUMAN
MAAPGLRLGAGRLFEMPAVLERLSRYNSTSQAFAEVLRLPKQQLRKLLYPLQEVERFLAPYGRQDLHLRIFDPSPEDIARADNIFTA
TERNRIDYVSSAVRIDHAPDLPRPEVCFIGRSNVGKSSLIKALFSLAPEVEVRVSKKPGHTKKMNFFKVGKHFTVVDMPGYGFRAPE
DFVDMVETYLKERRNLKRTFLLVDSVVGIQKTDNIAIEMCEEFALPYVIVLTKIDKSSKGHLLKQVLQIQKFVNMKTQGCFPQLFPV
SAVTFSGIHLLRCFIASVTGSLD
>sp|A6NH57|ARL5C_HUMAN
MGQLIAKLMSIFGNQEHTVIIVGLDNEGKTTILYRFLTNEVVHMCPTIGSNVEEIILPKTHFFMWDIVRPEALSFIWNTYYSNTEFI
ILVIDSTDRDRLLTTREELYKMLAHEALQDASVLIFANKQDVKDSMRMVEISHFLTLSTIKDHSWHIQGCCALTREGLPARLQWMES
QAAAN
>sp|O60292|SI1L3_HUMAN
MTTYRAIPSDGVDLAASCGARVGDVLPGPHTGDYAPLGFWAQNGSMSQPLGESPATATATATATTRPSPTTPAMPKMGVRARVADWP
PKREALREHSNPSPSQDTDGTKATKMAHSMRSIQNGQPPTSTPASSGSKAFHRLSRRRSKDVEFQDGWPRSPGRAFLPLRHRSSSEI
TLSECDAEDAGEPRGARHTGALPLFREYGSTSSIDVQGMPEQSFFDILNEFRSEQPDARGCQALTELLRADPGPHLMGGGGGAKGDS

HNGQPAKDSLLPLQPTKEKEKARKKPARGLGGGDTVDSSIFRKLRSSKPEGEAGRSPGEADEGRSPPEASRPWVCQKSFAHFDVQSM
LFDLNEAAANRVSVSQRRNTTTGASAASAASAMASLTASRAHSLGGLDPAFTSTEDLNCKENLEQDLGDDNSNDLLLSCPHFRNEIG
GECERNVSFSRASVGSPSSGEGHLAEPALSAYRTNASISVLEVPKEQQRTQSRPRQYSIEHVDLGARYYQDYFVGKEHANYFGVDEK
LGPVAVSIKREKLEDHKEHGPQYQYRIIFRTRELITLRGSILEDATPTATKHGTGRGLPLKDALEYVIPELNIHCLRLALNTPKVTE
QLLKLDEQGLCRKHKVGILYCKAGQSSEEEMYNNEEAGPAFEEFLSLIGEKVCLKGFTKYAAQLDVKTDSTGTHSLYTMYQDYEIMF
HVSTLLPYTPNNRQQLLRKRHIGNDIVTIIFQEPGALPFTPKNIRSHFQHVFIIVRVHNPCTDNVCYSMAVTRSKDAPPFGPPIPSG
TTFRKSDVFRDFLLAKVINAENAAHKSDKFHTMATRTRQEYLKDLAENCVSNTPIDSTGKFNLISLTSKKKEKTKARAGAEQHSAGA
IAWRVVAQDYAQGVEIDCILGISNEFVVLLDLRTKEVVFNCYCGDVIGWTPDSSTLKIFYGRGDHIFLQATEGSVEDIREIVQRLKV
MTSGWETVDMTLRRNGLGQLGFHVKYDGTVAEVEDYGFAWQAGLRQGSRLVEICKVAVVTLTHDQMIDLLRTSVTVKVVIIPPFEDG
TPRRGWPETYDMNTSEPKTEQESITPGGRPPYRSNAPWQWSGPASHNSLPASKWATPTTPGHAQSLSRPLKQTPIVPFRESQPLHSK
RPVSFPETPYTVSPAGADRVPPYRQPSGSFSTPGSATYVRYKPSPERYTAAPHPLLSLDPHFSHDGTSSGDSSSGGLTSQESTMERQ
KPEPLWHVPAQARLSAIAGSSGNKHPSRQDAAGKDSPNRHSKGEPQYSSHSSSNTLSSNASSSHSDDRWFDPLDPLEPEQDPLSKGG
SSDSGIDTTLYTSSPSCMSLAKAPRPAKPHKPPGSMGLCGGGREAAGRSHHADRRREVSPAPAVAGQSKGYRPKLYSSGSSTPTGLA
GGSRDPPRQPSDMGSRVGYPAQVYKTASAETPRPSQLAQPSPFQLSASVPKSFFSKQPVRNKHPTGWKRTEEPPPRPLPFSDPKKQV
DTNTKNVFGQPRLRASLRDLRSPRKNYKSTIEDDLKKLIIMDNLGPEQERDTGQSPQKGLQRTLSDESLCSGRREPSFASPAGLEPG
LPSDVLFTSTCAFPSSTLPARRQHQHPHPPVGPGATPAAGSGFPEKKSTISASELSLADGRDRPLRRLDPGLMPLPDTAAGLEWSSL
VNAAKAYEVQRAVSLFSLNDPALSPDIPPAHSPVHSHLSLERGPPTPRTTPTMSEEPPLDLTGKVYQLEVMLKQLHTDLQKEKQDKV
VLQSEVASLRQNNQRLQEESQAASEQLRKFAEIFCREKKEL
>sp|Q8NA77|TEX19_HUMAN
MCPPVSMRYEEEGMSYLYASWMYQLQHGDQLSICFTCFKAAFLDFKDLLESEDWEEDNWDPELMEHTEAESEQEGSSGMELSWGQSP
GQPVQGGSEAWGPGTLAAAPEGLEDAGLDPHFVPTELWPQEAVPLGLGLEDADWTQGLPWRFEELLTCSHWPSFFPS
>sp|O95407|TNF6B_HUMAN
MRALEGPGLSLLCLVLALPALLPVPAVRGVAETPTYPWRDAETGERLVCAQCPPGTFVQRPCRRDSPTTCGPCPPRHYTQFWNYLER
CRYCNVLCGEREEEARACHATHNRACRCRTGFFAHAGFCLEHASCPPGAGVIAPGTPSQNTQCQPCPPGTFSASSSSSEQCQPHRNC
TALGLALNVPGSSSHDTLCTSCTGFPLSTRVPGAEECERAVIDFVAFQDISIKRLQRLLQALEAPEGWGPTPRAGRAALQLKLRRRL
TELLGAQDGALLVRLLQALRVARMPGLERSVRERFLPVH
>sp|Q8N8H1|ZN321_HUMAN
MMKEFSSTAQGNTEVIHTGTLQRHESHHIRDFCFQEIEKDIHNFEFQWQEEERNGHEAPMTEIKELTGSTDRHDQRHAGNKPIKDQL
GSSFHSHLPELHIFQPEWKIGNQVEKSIINASLILTSQRISCSPKTRISNNYGNNSLHSSLPIQKLGSTHERKIFPM
>sp|A8MUK1|U17L5_HUMAN
MEDDSLYLRGEWQFNHFSKLTSSRPDAAFAEIQRTSLPEKSPLSCETRVDLCDDLAPVARQLAPREKLPLSSRRPAAVGAGLQNMGN
TCYVNASLQCLTYTPPLANYMLSREHSQTCHRHKGCMLCTMQAHITRALHNPGHVIQPSQALAAGFHRGKQEDAHEFLMFTVDAMKK
ACLPGHKQVDHHSKDTTLIHQIFGGYWRSQIKCLHCHGISDTFDPYLDIALDIQAAQSVQQALEQLAKPEELNGENAYHCGVCLQRA
PASKTLTLHTSAKVLILVLKRFSDVTGNKIAKNVQYPECLDMQPYMSQPNTGPLVYVLYAVLVHAGWSCHNGHYFSYVKAQEGQWYK
MDDAEVTASSITSVLSQQAYVLFYIQKSEWERHSESVSRGREPRALGAEDTDRRATQGELKRDHPCLQAPELDEHLVERATQESTLD
HWKFLQEQNKTKPEFNVRKVEGTLPPDVLVIHQSKYKCGMKNHHPEQQSSLLNLSSSTPTHQESMNTGTLASLRGRARRSKGKNKHS
KRALLVCQ
>sp|Q49AR2|CE022_HUMAN
MSDSAGGRAGLRRYPKLPVWVVEDHQEVLPFIYRAIGSKHLPASNVSFLHFDSHPDLLIPVNMPADTVFDKETLFGELSIENWIMPA
VYAGHFSHVIWFHPTWAQQIREGRHHFLVGKDTSTTTIRVTSTDHYFLSDGLYVPEDQLENQKPLQLDVIMVKPYKLCNNQEENDAV
SSAKKPKLALEDSENTASTNCDSSSEGLEKDTATQRSDQTCLEPSCSCSSENQECQTAASTGEILEILKKGKAFVLDIDLDFFSVKN
PFKEMFTQEEYKILQELYQFKKPGTNLTEEDLVDIVDTRIHQLEDLEATFADLCDGDDEETVQRWASNPGMESLVPLVQSLKKRMEV
PDYEMVHQAGLTCDYSELPHHISTEQEIECLIQSVHYLLKNLPNPTLVTIARSSLDDYCPSDQVDTIQEKVLNMLRALYGNLDLQVY
AAESPPS

>sp|Q86W25|NAL13_HUMAN
MNFSVITCPNGGTNQGLLPYLMALDQYQLEEFKLCLEPQQLMDFWSAPQGHFPRIPWANLRAADPLNLSFLLDEHFPKGQAWKVVLG
IFQTMNLTSLCEKVRAEMKENVQTQELQDPTQEDLEMLEAAAGNMQTQGCQDPNQEELDELEEETGNVQAQGCQDPNQEEPEMLEEA
DHRRKYRENMKAELLETWDNISWPKDHVYIRNTSKDEHEELQRLLDPNRTRAQAQTIVLVGRAGVGKTTLAMQAMLHWANGVLFQQR
FSYVFYLSCHKIRYMKETTFAELISLDWPDFDAPIEEFMSQPEKLLFIIDGFEEIIISESRSESLDDGSPCTDWYQELPVTKILHSL
LKKELVPLATLLITIKTWFVRDLKASLVNPCFVQITGFTGDDLRVYFMRHFDDSSEVEKILQQLRKNETLFHSCSAPMVCWTVCSCL
KQPKVRYYDLQSITQTTTSLYAYFFSNLFSTAEVDLADDSWPGQWRALCSLAIEGLWSMNFTFNKEDTEIEGLEVPFIDSLYEFNIL
QKINDCGGCTTFTHLSFQEFFAAMSFVLEEPREFPPHSTKPQEMKMLLQHVLLDKEAYWTPVVLFFFGLLNKNIARELEDTLHCKIS
PRVMEELLKWGEELGKAESASLQFHILRLFHCLHESQEEDFTKKMLGRIFEVDLNILEDEELQASSFCLKHCKRLNKLRLSVSSHIL
ERDLEILETSKFDSRMHAWNSICSTLVTNENLHELDLSNSKLHASSVKGLCLALKNPRCKVQKLTCKSVTPEWVLQDLIIALQGNSK
LTHLNFSSNKLGMTVPLILKALRHSACNLKYLCLEKCNLSAASCQDLALFLTSIQHVTRLCLGFNRLQDDGIKLLCAALTHPKCALE
RLELWFCQLAAPACKHLSDALLQNRSLTHLNLSKNSLRDEGVKFLCEALGRPDGNLQSLNLSGCSFTREGCGELANALSHNHNVKIL
DLGENDLQDDGVKLLCEALKPHRALHTLGLAKCNLTTACCQHLFSVLSSSKSLVNLNLLGNELDTDGVKMLCKALKKSTCRLQKLG
>sp|Q96PG2|M4A10_HUMAN
MKAEATVIPSRCARGLPSWQVLSPVQPWQTSAPQNTTQPKLLAPHQHEKSQKKSSLLKELGAFHITIALLHLVFGGYLASIVKNLHL
VVLKSWYPFWGAASFLISGILAITMKTFSKTYLKMLCLMTNLISLFCVLSGLFVISKDLFLESPFESPIWRMYPNSTVHIQRLELAL
LCFTVLELFLPVPTAVTAWRGDCPSAKNDDACLVPNTPLHLKGLPVEPPPSYQSVIQGDAQHKQHQRLREVKQVAPDTWIVTDGAAI
WTQTAN
>sp|Q16864|VATF_HUMAN
MAGRGKLIAVIGDEDTVTGFLLGGIGELNKNRHPNFLVVEKDTTINEIEDTFRQFLNRDDIGIILINQYIAEMVRHALDAHQQSIPA
VLEIPSKEHPYDAAKDSILRRARGMFTAEDLR
>sp|O95741|CPNE6_HUMAN
MSDPEMGWVPEPPTMTLGASRVELRVSCHGLLDRDTLTKPHPCVLLKLYSDEQWVEVERTEVLRSCSSPVFSRVLALEYFFEEKQPL
QFHVFDAEDGATSPRNDTFLGSTECTLGQIVSQTKVTKPLLLKNGKTAGKSTITIVAEEVSGTNDYVQLTFRAYKLDNKDLFSKSDP
FMEIYKTNEDQSDQLVWRTEVVKNNLNPSWEPFRLSLHSLCSCDVHRPLKFLVYDYDSSGKHDFIGEFTSTFQEMQEGTANPGQEMQ
WDCINPKYRDKKKNYKSSGTVVLAQCTVEKVHTFLDYIMGGCQISFTVAIDFTASNGDPRSSQSLHCLSPRQPNHYLQALRAVGGIC
QDYDSDKRFPAFGFGARIPPNFEVSHDFAINFDPENPECEEISGVIASYRRCLPQIQLYGPTNVAPIINRVAEPAQREQSTGQATKY
SVLLVLTDGVVSDMAETRTAIVRASRLPMSIIIVGVGNADFSDMRLLDGDDGPLRCPRGVPAARDIVQFVPFRDFKDAAPSALAKCV
LAEVPRQVVEYYASQGISPGAPRPCTLATTPSPSP
>sp|O95498|VNN2_HUMAN
MVTSSFPISVAVFALITLQVGTQDSFIAAVYEHAVILPNKTETPVSQEDALNLMNENIDILETAIKQAAEQGARIIVTPEDALYGWK
FTRETVFPYLEDIPDPQVNWIPCQDPHRFGHTPVQARLSCLAKDNSIYVLANLGDKKPCNSRDSTCPPNGYFQYNTNVVYNTEGKLV
ARYHKYHLYSEPQFNVPEKPELVTFNTAFGRFGIFTCFDIFFYDPGVTLVKDFHVDTILFPTAWMNVLPLLTAIEFHSAWAMGMGVN
LLVANTHHVSLNMTGSGIYAPNGPKVYHYDMKTELGKLLLSEVDSHPLSSLAYPTAVNWNAYATTIKPFPVQKNTFRGFISRDGFNF
TELFENAGNLTVCQKELCCHLSYRMLQKEENEVYVLGAFTGLHGRRRREYWQVCTLLKCKTTNLTTCGRPVETASTRFEMFSLSGTF
GTEYVFPEVLLTEIHLSPGKFEVLKDGRLVNKNGSSGPILTVSLFGRWYTKDSLYSSCGTSNSAITYLLIFILLMIIALQNIVML
>sp|P15822|ZEP1_HUMAN
MPRTKQIHPRNLRDKIEEAQKELNGAEVSKKEILQAGVKGTSESLKGVKRKKIVAENHLKKIPKSPLRNPLQAKHKQNTEESSFAVL
HSASESHKKQNYIPVKNGKQFTKQNGETPGIIAEASKSEESVSPKKPLFLQQPSELRRWRSEGADPAKFSDLDEQCDSSSLSSKTRT
DNSECISSHCGTTSPSYTNTAFDVLLKAMEPELSTLSQKGSPCAIKTEKLRPNKTARSPPKLKNSSMDAPNQTSQELVAESQSSCTS
YTVHMSAAQKNEQGAMQSASHLYHQHEHFVPKSNQHNQQLPGCSGFTGSLTNLQNQENAKLEQVYNIAVTSSVGLTSPSSRSQVTPQ
NQQMDSASPLSISPANSTQSPPMPIYNSTHVASVVNQSVEQMCNLLLKDQKPKKQGKYICEYCNRACAKPSVLLKHIRSHTGERPYP
CVTCGFSFKTKSNLYKHKKSHAHTIKLGLVLQPDAGGLFLSHESPKALSIHSDVEDSGESEEEGATDERQHDLGAMELQPVHIIKRM
SNAETLLKSSFTPSSPENVIGDFLLQDRSAESQAVTELPKVVVHHVTVSPLRTDSPKAMDPKPELSSAQKQKDLQVTNVQPLSANMS

QGGVSRLETNENSHQKGDMNPLEGKQDSHVGTVHAQLQRQQATDYSQEQQGKLLSPRSLGSTDSGYFSRSESADQTVSPPTPFARRL
PSTEQDSGRSNGPSAALVTTSTPSALPTGEKALLLPGQMRPPLATKTLEERISKLISDNEALVDDKQLDSVKPRRTSLSRRGSIDSP
KSYIFKDSFQFDLKPVGRRTSSSSDIPKSPFTPTEKSKQVFLLSVPSLDCLPITRSNSMPTTGYSAVPANIIPPPHPLRGSQSFDDK
IGTFYDDVFVSGPNAPVPQSGHPRTLVRQAAIEDSSANESHVLGTGQSLDESHQGCHAAGEAMSVRSKALAQGPHIEKKKSHQGRGT
MFECETCRNRYRKLENFENHKKFYCSELHGPKTKVAMREPEHSPVPGGLQPQILHYRVAGSSGIWEQTPQIRKRRKMKSVGDDEELQ
QNESGTSPKSSEGLQFQNALGCNPSLPKHNVTIRSDQQHKNIQLQNSHIHLVARGPEQTMDPKLSTIMEQQISSAAQDKIELQRHGT
GISVIQHTNSLSRPNSFDKPEPFERASPVSFQELNRTGKSGSLKVIGISQEESHPSRDGSHPHQLALSDALRGELQESSRKSPSERH
VLGQPSRLVRQHNIQVPEILVTEEPDRDLEAQCHDQEKSEKFSWPQRSETLSKLPTEKLPPKKKRLRLAEIEHSSTESSFDSTLSRS
LSRESSLSHTSSFSASLDIEDVSKTEASPKIDFLNKAEFLMIPAGLNTLNVPGCHREMRRTASEQINCTQTSMEVSDLRSKSFDCGS
ITPPQTTPLTELQPPSSPSRVGVTGHVPLLERRRGPLVRQISLNIAPDSHLSPVHPTSFQNTALPSVNAVPYQGPQLTSTSLAEFSA
NTLHSQTQVKDLQAETSNSSSTNVFPVQQLCDINLLNQIHAPPSHQSTQLSLQVSTQGSKPDKNSVLSGSSKSEDCFAPKYQLHCQV
FTSGPSCSSNPVHSLPNQVISDPVGTDHCVTSATLPTKLIDSMSNSHPLLPPELRPLGSQVQKVPSSFMLPIRLQSSVPAYCFATLT
SLPQILVTQDLPNQPICQTNHSVVPISEEQNSVPTLQKGHQNALPNPEKEFLCENVFSEMSQNSSLSESLPITQKISVGRLSPQQES
SASSKRMLSPANSLDIAMEKHQKRAKDENGAVCATDVRPLEALSSRVNEASKQKKPILVRQVCTTEPLDGVMLEKDVFSQPEISNEA
VNLTNVLPADNSSTGCSKFVVIEPISELQEFENIKSSTSLTLTVRSSPAPSENTHISPLKCTDNNQERKSPGVKNQGDKVNIQEQSQ
QPVTSLSLFNIKDTQQLAFPSLKTTTNFTWCYLLRQKSLHLPQKDQKTSAYTDWTVSASNPNPLGLPTKVALALLNSKQNTGKSLYC
QAITTHSKSDLLVYSSKWKSSLSKRALGNQKSTVVEFSNKDASEINSEQDKENSLIKSEPRRIKIFDGGYKSNEEYVYVRGRGRGKY
ICEECGIRCKKPSMLKKHIRTHTDVRPYHCTYCNFSFKTKGNLTKHMKSKAHSKKCVDLGVSVGLIDEQDTEESDEKQRFSYERSGY
DLEESDGPDEDDNENEDDDEDSQAESVLSATPSVTASPQHLPSRSSLQDPVSTDEDVRITDCFSGVHTDPMDVLPRALLTRMTVLST
AQSDYNRKTLSPGKARQRAARDENDTIPSVDTSRSPCHQMSVDYPESEEILRSSMAGKAVAITQSPSSVRLPPAAAEHSPQTAAGMP
SVASPHPDPQEQKQQITLQPTPGLPSPHTHLFSHLPLHSQQQSRTPYNMVPVGGIHVVPAGLTYSTFVPLQAGPVQLTIPAVSVVHR
TLGTHRNTVTEVSGTTNPAGVAELSSVVPCIPIGQIRVPGLQNLSTPGLQSLPSLSMETVNIVGLANTNMAPQVHPPGLALNAVGLQ
VLTANPSSQSSPAPQAHIPGLQILNIALPTLIPSVSQVAVDAQGAPEMPASQSKACETQPKQTSVASANQVSRTESPQGLPTVQREN
AKKVLNPPAPAGDHARLDGLSKMDTEKAASANHVKPKPELTSIQGQPASTSQPLLKAHSEVFTKPSGQQTLSPDRQVPRPTALPRRQ
PTVHFSDVSSDDDEDRLVIAT
>sp|Q12841|FSTL1_HUMAN
MWKRWLALALALVAVAWVRAEEELRSKSKICANVFCGAGRECAVTEKGEPTCLCIEQCKPHKRPVCGSNGKTYLNHCELHRDACLTG
SKIQVDYDGHCKEKKSVSPSASPVVCYQSNRDELRRRIIQWLEAEIIPDGWFSKGSNYSEILDKYFKNFDNGDSRLDSSEFLKFVEQ
NETAINITTYPDQENNKLLRGLCVDALIELSDENADWKLSFQEFLKCLNPSFNPPEKKCALEDETYADGAETEVDCNRCVCACGNWV
CTAMTCDGKNQKGAQTQTEEEMTRYVQELQKHQETAEKTKRVSTKEI
>sp|Q8TAK5|GABP2_HUMAN
MSLVDLGKRLLEAARKGQDDEVRTLMANGAPFTTDWLGTSPLHLAAQYGHYSTAEVLLRAGVSRDARTKVDRTPLHMAAADGHAHIV
ELLVRNGADVNAKDMLKMTALHWATERHHRDVVELLIKYGADVHAFSKFDKSAFDIALEKNNAEILVILQEAMQNQVNVNPERANPV
TDPVSMAAPFIFTSGEVVNLASLISSTNTKTTSGDPHASTVQFSNSTTSVLATLAALAEASVPLSNSHRATANTEEIIEGNSVDSSI
QQVMGSGGQRVITIVTDGVPLGNIQTSIPTGGIGQPFIVTVQDGQQVLTVPAGKVAEETVIKEEEEEEKLPLTKKPRIGEKTNSVEES
KEGNERELLQQQLQEANRRAQEYRHQLLKKEQEAEQYRLKLEAIARQQPNGVDFTMVEEVAEVDAVVVTEGELEERETKVTGSAGTT
EPHTRVSMATVSS
>sp|Q9UL54|TAOK2_HUMAN
MPAGGRAGSLKDPDVAELFFKDDPEKLFSDLREIGHGSFGAVYFARDVRNSEVVAIKKMSYSGKQSNEKWQDIIKEVRFLQKLRHPN
TIQYRGCYLREHTAWLVMEYCLGSASDLLEVHKKPLQEVEIAAVTHGALQGLAYLHSHNMIHRDVKAGNILLSEPGLVKLGDFGSAS
IMAPANSFVGTPYWMAPEVILAMDEGQYDGKVDVWSLGITCIELAERKPPLFNMNAMSALYHIAQNESPVLQSGHWSEYFRNFVDSC
LQKIPQDRPTSEVLLKHRFVLRERPPTVIMDLIQRTKDAVRELDNLQYRKMKKILFQEAPNGPGAEAPEEEEEAEPYMHRAGTLTSL
ESSHSVPSMSISASSQSSSVNSLADASDNEEEEEEEEEEEEEEEGPEAREMAMMQEGEHTVTSHSSIIHRLPGSDNLYDDPYQPEIT
PSPLQPPAAPAPTSTTSSARRRAYCRNRDHFATIRTASLVSRQIQEHEQDSALREQLSGYKRMRRQHQKQLLALESRLRGEREEHSA

RLQRELEAQRAGFGAEAEKLARRHQAIGEKEARAAQAEERKFQQHILGQQKKELAALLEAQKRTYKLRKEQLKEELQENPSTPKREK
AEWLLRQKEQLQQCQAEEEAGLLRRQRQYFELQCRQYKRKMLLARHSLDQDLLREDLNKKQTQKDLECALLLRQHEATRELELRQLQ
AVQRTRAELTRLQHQTELGNQLEYNKRREQELRQKHAAQVRQQPKSLKVRAGQRPPGLPLPIPGALGPPNTGTPIEQQPCSPGQEAV
LDQRMLGEEEEAVGERRILGKEGATLEPKQQRILGEESGAPSPSPQKHGSLVDEEVWGLPEEIEELRVPSLVPQERSIVGQEEAGTW
SLWGKEDESLLDEEFELGWVQGPALTPVPEEEEEEEEGAPIGTPRDPGDGCPSPDIPPEPPPTHLRPCPASQLPGLLSHGLLAGLSF
AVGSSSGLLPLLLLLLLPLLAAQGGGGLQAALLALEVGLVGLGASYLLLCTALHLPSSLFLLLAQGTALGAVLGLSWRRGLMGVPLG
LGAAWLLAWPGLALPLVAMAAGGRWVRQQGPRVRRGISRLWLRVLLRLSPMAFRALQGCGAVGDRGLFALYPKTNKDGFRSRLPVPG
PRRRNPRTTQHPLALLARVWVLCKGWNWRLARASQGLASHLPPWAIHTLASWGLLRGERPTRIPRLLPRSQRQLGPPASRQPLPGTL
AGRRSRTRQSRALPPWR
>sp|Q9UMX1|SUFU_HUMAN
MAELRPSGAPGPTAPPAPGPTAPPAFASLFPPGLHAIYGECRRLYPDQPNPLQVTAIVKYWLGGPDPLDYVSMYRNVGSPSANIPEH
WHYISFGLSDLYGDNRVHEFTGTDGPSGFGFELTFRLKRETGESAPPTWPAELMQGLARYVFQSENTFCSGDHVSWHSPLDNSESRI
QHMLLTEDPQMQPVQTPFGVVTFLQIVGVCTEELHSAQQWNGQGILELLRTVPIAGGPWLITDMRRGETIFEIDPHLQERVDKGIET
DGSNLSGVSAKCAWDDLSRPPEDDEDSRSICIGTQPRRLSGKDTEQIRETLRRGLEINSKPVLPPINPQRQNGLAHDRAPSRKDSLE
SDSSTAIIPHELIRTRQLESVHLKFNQESGALIPLCLRGRLLHGRHFTYKSITGDMAITFVSTGVEGAFATEEHPYAAHGPWLQILL
TEEFVEKMLEDLEDLTSPEEFKLPKEYSWPEKKLKVSILPDVVFDSPLH
>sp|Q96A61|TRI52_HUMAN
MAGYATTPSPMQTLQEEAVCAICLDYFKDPVSISCGHNFCRGCVTQLWSKEDEEDQNEEEDEWEEEEDEEAVGAMDGWDGSIREVLY
RGNADEELFQDQDDDELWLGDSGITNWDNVDYMWDEEEEEEEEDQDYYLGGLRPDLRIDVYREEEILEAYDEDEDEELYPDIHPPPS
LPLPGQFTCPQCRKSFTRRSFRPNLQLANMVQIIRQMCPTPYRGNRSNDQGMCFKHQEALKLFCEVDKEAICVVCRESRSHKQHSVL
PLEEVVQEYQEIKLETTLVGILQIEQESIHSKAYNQ
>sp|P34982|OR1D2_HUMAN
MDGGNQSEGSEFLLLGMSESPEQQRILFWMFLSMYLVTVVGNVLIILAISSDSRLHTPVYFFLANLSFTDLFFVTNTIPKMLVNLQS
HNKAISYAGCLTQLYFLVSLVALDNLILAVMAYDRYVAICCPLHYTTAMSPKLCILLLSLCWVLSVLYGLIHTLLMTRVTFCGSRKI
HYIFCEMYVLLRMACSNIQINHTVLIATGCFIFLIPFGFVIISYVLIIRAILRIPSVSKKYKAFSTCASHLGAVSLFYGTLCMVYLK
PLHTYSVKDSVATVMYAVVTPMMNPFIYSLRNKDMHGALGRLLDKHFKRLT
>sp|Q9NS68|TNR19_HUMAN
MALKVLLEQEKTFFTLLVLLGYLSCKVTCESGDCRQQEFRDRSGNCVPCNQCGPGMELSKECGFGYGEDAQCVTCRLHRFKEDWGFQ
KCKPCLDCAVVNRFQKANCSATSDAICGDCLPGFYRKTKLVGFQDMECVPCGDPPPPYEPHCASKVNLVKIASTASSPRDTALAAVI
CSALATVLLALLILCVIYCKRQFMEKKPSWSLRSQDIQYNGSELSCFDRPQLHEYAHRACCQCRRDSVQTCGPVRLLPSMCCEEACS
PNPATLGCGVHSAASLQARNAGPAGEMVPTFFGSLTQSICGEFSDAWPLMQNPMGGDNISFCDSYPELTGEDIHSLNPELESSTSLD
SNSSQDLVGGAVPVQSHSENFTAATDLSRYNNTLVESASTQDALTMRSQLDQESGAVIHPATQTSLQVRQRLGSL
>sp|P55287|CAD11_HUMAN
MKENYCLQAALVCLGMLCHSHAFAPERRGHLRPSFHGHHEKGKEGQVLQRSKRGWVWNQFFVIEEYTGPDPVLVGRLHSDIDSGDGN
IKYILSGEGAGTIFVIDDKSGNIHATKTLDREERAQYTLMAQAVDRDTNRPLEPPSEFIVKVQDINDNPPEFLHETYHANVPERSNV
GTSVIQVTASDADDPTYGNSAKLVYSILEGQPYFSVEAQTGIIRTALPNMDREAKEEYHVVIQAKDMGGHMGGLSGTTKVTITLTDV
NDNPPKFPQSVYQMSVSEAAVPGEEVGRVKAKDPDIGENGLVTYNIVDGDGMESFEITTDYETQEGVIKLKKPVDFETKRAYSLKVE
AANVHIDPKFISNGPFKDTVTVKISVEDADEPPMFLAPSYIHEVQENAAAGTVVGRVHAKDPDAANSPIRYSIDRHTDLDRFFTINP
EDGFIKTTKPLDREETAWLNITVFAAEIHNRHQEAKVPVAIRVLDVNDNAPKFAAPYEGFICESDQTKPLSNQPIVTISADDKDDTA
NGPRFIFSLPPEIIHNPNFTVRDNRDNTAGVYARRGGFSRQKQDLYLLPIVISDGGIPPMSSTNTLTIKVCGCDVNGALLSCNAEAY
ILNAGLSTGALIAILACIVILLVIVVLFVTLRRQKKEPLIVFEEEDVRENIITYDDEGGGEEDTEAFDIATLQNPDGINGFIPRKDI
KPEYQYMPRPGLRPAPNSVDVDDFINTRIQEADNDPTAPPYDSIQIYGYEGRGSVAGSLSSLESATTDSDLDYDYLQNWGPRFKKLA
DLYGSKDTFDDDS
>sp|Q6ZNG1|ZN600_HUMAN

MMKEVLSTGQGNTEVIHTGTLQRYQSYHIGDFCFQEIEKEIHDIEFQCQEDERNGHEAPMTKIKKLTGSTDQHDHRHAGNKPIKDQL
GSSFYSHLPELHIIQIKGKIGNQFEKSTSDAPSVSTSQRISPRPQIHISNNYGNNSPNSSLLPQKQEVYMREKSFQCNESGKAFNCS
SLLRKHQIPHLGDKQYKCDVCGKLFNHKQYLTCHCRCHTGEKPYKCNECGKSFSQVSSLTCHRRLHTAVKSHKCNECGKIFGQNSAL
VIHKAIHTGEKPYKCNECDKAFNQQSNLARHRRIHTGEKPYKCEECDKVFSRKSTLESHKRIHTGEKPYKCKVCDTAFTWNSQLARH
KRIHTGEKTYKCNECGKTFSHKSSLVCHHRLHGGEKSYKCKVCDKAFAWNSHLVRHTRIHSGGKPYKCNECGKTFGQNSDLLIHKSI
HTGEQPYKYEECEKVFSCGSTLETHKIIHTGEKPYKCKVCDKAFACHSYLAKHTRIHSGEKPYKCNECSKTFRLRSYLASHRRVHSG
EKPYKCNECSKTFSQRSYLHCHRRLHSGEKPYKCNECGKTFSHKPSLVHHRRLHTGEKSYKCTVCDKAFVRNSYLARHTRIHTAEKP
YKCNECGKAFNQQSQLSLHHRIHAGEKLYKCETCDKVFSRKSHLKRHRRIHPGKKPYKCKVCDKTFGSDSHLKQHTGLHTGEKPYKC
NECGKAFSKQSTLIHHQAVHGVGKLD
>sp|Q9NRM1|ENAM_HUMAN
MLVLRCRLGTSFPKLDNLVPKGKMKILLVFLGLLGNSVAMPMHMPRMPGFSSKSEEMMRYNQFNFMNGPHMAHLGPFFGNGLPQQFP
QYQMPMWPQPPPNTWHPRKSSAPKRHNKTDQTQETQKPNQTQSKKPPQKRPLKQPSHNQPQPEEEAQPPQAFPPFGNGLFPYQQPPW
QIPQRLPPPGYGRPPISNEEGGNPYFGYFGYHGFGGRPPYYSEEMFEQDFEKPKEEDPPKAESPGTEPTANSTVTETNSTQPNPKGS
QGGNDTSPTGNSTPGLNTGNNPPAQNGIGPLPAVNASGQGGPGSQIPWRPSQPNIRENHPYPNIRNFPSGRQWYFTGTVMGHRQNRP
FYRNQQVQRGPRWNFFAWERKQVARPGNPVYHKAYPPTSRGNYPNYAGNPANLRRKPQGPNKHPVGTTVAPLGPKPGPVVRNEKIQN
PKEKPLGPKEQIIVPTKNPTSPWRNSQQYEVNKSNYKLPHSEGYMPVPNFNSVDQHENSYYPRGDSRKVPNSDGQTQSQNLPKGIVL
GSRRMPYESETNQSELKHSSYQPAVYPEEIPSPAKEHFPAGRNTWDHQEISPPFKEDPGRQEEHLPHPSHGSRGSVFYPEYNPYDPR
ENSPYLRGNTWDERDDSPNTMGQKESPLYPINTPDQKEIVPYNEEDPVDPTGDEVFPGQNRWGEELSFKGGPTVRHYEGEQYTSNQP
KEYLPYSLDNPSKPREDFYYSEFYPWSPDENFPSYNTASTMPPPIESRGYYVNNAAGPEESTLFPSRNSWDHRIQAQGQRERRPYFN
RNIWDQATHLQKAPARPPDQKGNQPYYSNTPAGLQKNPIWHEGENLNYGMQITRMNSPEREHSSFPNFIPPSYPSGQKEAHLFHLSQ
RGSCCAGSSTGPKDNPLALQDYTPSYGLAPGENQDTSPLYTDGSHTKQTRDIISPTSILPGQRNSSEKRESQNPFRDDVSTLRRNTP
CSIKNQLGQKEIMPFPEASSLQSKNTPCLKNDLGGDGNNILEQVFEDNQLNERTVDLTPEQLVIGTPDEGSNPEGIQSQVQENESER
QQQRPSNILHLPCFGSKLAKHHSSTTGTPSSDGRQSPFDGDSITPTENPNTLVELATEEQFKSINVDPLDADEHSPFEFLQRGTNVQ
DQVQDCLLLQA
>sp|Q6N063|OGFD2_HUMAN
MATVGAPRHFCRCACFCTDNLYVARYGLHVRFRGEQQLRRDYGPILRSRGCVSAKDFQQLLAELEQEVERRQRLGQESAARKALIAS
SYHPARPEVYDSLQDAALAPEFLAVTEYSVSPDADLKGLLQRLETVSEEKRIYRVPVFTAPFCQALLEELEHFEQSDMPKGRPNTMN
NYGVLLHELGLDEPLMTPLRERFLQPLMALLYPDCGGGRLDSHRAFVVKYAPGQDLELGCHYDNAELTLNVALGKVFTGGALYFGGL
FQAPTALTEPLEVEHVVGQGVLHRGGQLHGARPLGTGERWNLVVWLRASAVRNSLCPMCCREPDLVDDEGFGDGFTREEPATVDVCA
LT
>sp|Q9UM13|APC10_HUMAN
MTTPNKTPPGADPKQLERTGTVREIGSQAVWSLSSCKPGFGVDQLRDDNLETYWQSDGSQPHLVNIQFRRKTTVKTLCIYADYKSDE
SYTPSKISVRVGNNFHNLQEIRQLELVEPSGWIHVPLTDNHKKPTRTFMIQIAVLANHQNGRDTHMRQIKIYTPVEESSIGKFPRCT
TIDFMMYRSIR
>sp|O00481|BT3A1_HUMAN
MKMASFLAFLLLNFRVCLLLLQLLMPHSAQFSVLGPSGPILAMVGEDADLPCHLFPTMSAETMELKWVSSSLRQVVNVYADGKEVED
RQSAPYRGRTSILRDGITAGKAALRIHNVTASDSGKYLCYFQDGDFYEKALVELKVAALGSDLHVDVKGYKDGGIHLECRSTGWYPQ
PQIQWSNNKGENIPTVEAPVVADGVGLYAVAASVIMRGSSGEGVSCTIRSSLLGLEKTASISIADPFFRSAQRWIAALAGTLPVLLL
LLGGAGYFLWQQQEEKKTQFRKKKREQELREMAWSTMKQEQSTRVKLLEELRWRSIQYASRGERHSAYNEWKKALFKPADVILDPKT
ANPILLVSEDQRSVQRAKEPQDLPDNPERFNWHYCVLGCESFISGRHYWEVEVGDRKEWHIGVCSKNVQRKGWVKMTPENGFWTMGL
TDGNKYRTLTEPRTNLKLPKPPKKVGVFLDYETGDISFYNAVDGSHIHTFLDVSFSEALYPVFRILTLEPTALTICPA
>sp|Q8NHK3|KI2LB_HUMAN
MSLMVISMACVGFFLLQGAWTHEGGQDKPLLSAWPSAVVPRGGHVTLLCRSRLGFTIFSLYKEDGVPVPELYNKIFWKSILMGPVTP
AHAGTYRCRGSHPRSPIEWSAPSNPLVIVVTGLFGKPSLSAQPGPTVRTGENVALSCSSRSSFDMYHLSREGRAHEPRLPAVPSVDG

TFQADFPLGPATHGGTYTCFSSLHDSPYEWSDPSDPLLVSVTGNSSSSSSSPTEPSSKTGIRRHLHILIGTSVAIILFIILFFFLLH
CCCSNKKNAAVMDQGPAGDRTVNREDSDDQDPQEVTYAQLDHCVFTQTKITSPSQRPKTPPTDTTMYMELPNAKPRSLSPAHKHHSQ
ALRGSSRETTALSQNRVASSHVPAAGI
>sp|Q6U841|S4A10_HUMAN
MEIKDQGAQMEPLLPTRNDEEAVVDRGGTRSILKTHFEKEDLEGHRTLFIGVHVPLGGRKSHRRHRHRGHKHRKRDRERDSGLEDGR
ESPSFDTPSQRVQFILGTEDDDEEHIPHDLFTELDEICWREGEDAEWRETARWLKFEEDVEDGGERWSKPYVATLSLHSLFELRSCI
LNGTVLLDMHANTLEEIADMVLDQQVSSGQLNEDVRHRVHEALMKQHHHQNQKKLTNRIPIVRSFADIGKKQSEPNSMDKNAGQVVS
PQSAPACVENKNDVSRENSTVDFSKGLGGQQKGHTSPCGMKQRHEKGPPHQQEREVDLHFMKKIPPGAEASNILVGELEFLDRTVVA
FVRLSPAVLLQGLAEVPIPTRFLFILLGPLGKGQQYHEIGRSIATLMTDEVFHDVAYKAKDRNDLVSGIDEFLDQVTVLPPGEWDPS
IRIEPPKNVPSQEKRKIPAVPNGTAAHGEAEPHGGHSGPELQRTGRIFGGLILDIKRKAPYFWSDFRDAFSLQCLASFLFLYCACMS
PVITFGGLLGEATEGRISAIESLFGASMTGIAYSLFGGQPLTILGSTGPVLVFEKILFKFCKEYGLSYLSLRASIGLWTATLCIILV
ATDASSLVCYITRFTEEAFASLICIIFIYEALEKLFELSEAYPINMHNDLELLTQYSCNCVEPHNPSNGTLKEWRESNISASDIIWE
NLTVSECKSLHGEYVGRACGHDHPYVPDVLFWSVILFFSTVTLSATLKQFKTSRYFPTKVRSIVSDFAVFLTILCMVLIDYAIGIPS
PKLQVPSVFKPTRDDRGWFVTPLGPNPWWTVIAAIIPALLCTILIFMDQQITAVIINRKEHKLKKGCGYHLDLLMVAVMLGVCSIMG
LPWFVAATVLSITHVNSLKLESECSAPGEQPKFLGIREQRVTGLMIFILMGSSVFMTSILKFIPMPVLYGVFLYMGASSLKGIQFFD
RIKLFWMPAKHQPDFIYLRHVPLRKVHLFTIIQMSCLGLLWIIKVSRAAIVFPMMVLALVFVRKLMDLLFTKRELSWLDDLMPESKK
KKLEDAEKEEEQSMLAMEDEGTVQLPLEGHYRDDPSVINISDEMSKTALWRNLLITADNSKDKESSFPSKSSPS
>sp|O00233|PSMD9_HUMAN
MSDEEARQSGGSSQAGVVTVSDVQELMRRKEEIEAQIKANYDVLESQKGIGMNEPLVDCEGYPRSDVDLYQVRTARHNIICLQNDHK
AVMKQVEEALHQLHARDKEKQARDMAEAHKEAMSRKLGQSESQGPPRAFAKVNSISPGSPASIAGLQVDDEIVEFGSVNTQNFQSLH
NIGSVVQHSEGKPLNVTVIRRGEKHQLRLVPTRWAGKGLLGCNIIPLQR
>sp|O43264|ZW10_HUMAN
MASFVTEVLAHSGRLEKEDLGTRISRLTRRVEEIKGEVCNMISKKYSEFLPSMQSAQGLITQVDKLSEDIDLLKSRIESEVRRDLHV
STGEFTDLKQQLERDSVVLSLLKQLQEFSTAIEEYNCALTEKKYVTGAQRLEEAQKCLKLLKSRKCFDLKILKSLSMELTIQKQNIL
YHLGEEWQKLIVWKFPPSKDTSSLESYLQTELHLYTEQSHKEEKTPMPPISSVLLAFSVLGELHSKLKSFGQMLLKYILRPLASCPS
LHAVIESQPNIVIIRFESIMTNLEYPSPSEVFTKIRLVLEVLQKQLLDLPLDTDLENEKTSTVPLAEMLGDMIWEDLSECLIKNCLV
YSIPTNSSKLQQYEEIIQSTEEFENALKEMRFLKGDTTDLLKYARNINSHFANKKCQDVIVAARNLMTSEIHNTVKIIPDSKINVPE
LPTPDEDNKLEVQKVSNTQYHEVMNLEPENTLDQHSFSLPTCRISESVKKLMELAYQTLLEATTSSDQCAVQLFYSVRNIFHLFHDV
VPTYHKENLQKLPQLAAIHHNNCMYIAHHLLTLGHQFRLRLAPILCDGTATFVDLVPGFRRLGTECFLAQMRAQKGELLERLSSARN
FSNMDDEENYSAASKAVRQVLHQLKRLGIVWQDVLPVNIYCKAMGTLLNTAISEVIGKITALEDISTEDGDRLYSLCKTVMDEGPQV
FAPLSEESKNKKYQEEVPVYVPKWMPFKELMMMLQASLQEIGDRWADGKGPLAAAFSSSEVKALIRALFQNTERRAAALAKIK
>sp|Q9HCI7|MSL2_HUMAN
MNPVNATALYISASRLVLNYDPGDPKAFTEINRLLPYFRQSLSCCVCGHLLQDPIAPTNSTCQHYVCKTCKGKKMMMKPSCSWCKDY
EQFEENKQLSILVNCYKKLCEYITQTTLARDIIEAVDCSSDILALLNDGSLFCEETEKPSDSSFTLCLTHSPLPSTSEPTTDPQASL
SPMSESTLSIAIGSSVINGLPTYNGLSIDRFGINIPSPEHSNTIDVCNTVDIKTEDLSDSLPPVCDTVATDLCSTGIDICSFSEDIK
PGDSLLLSVEEVLRSLETVSNTEVCCPNLQPNLEATVSNGPFLQLSSQSLSHNVFMSTSPALHGLSCTAATPKIAKLNRKRSRSESD
SEKVQPLPISTIIRGPTLGASAPVTVKRESKISLQPIATVPNGGTTPKISKTVLLSTKSMKKSHEHGSKKSHSKTKPGILKKDKAVK
EKIPSHHFMPGSPTKTVYKKPQEKKGCKCGRATQNPSVLTCRGQRCPCYSNRKACLDCICRGCQNSYMANGEKKLEAFAVPEKALEQ
TRLTLGINVTSIAVRNASTSTSVINVTGSPVTTFLAASTHDDKSLDEAIDMRFDC
>sp|Q9HAN9|NMNA1_HUMAN
MENSEKTEVVLLACGSFNPITNMHLRLFELAKDYMNGTGRYTVVKGIISPVGDAYKKKGLIPAYHRVIMAELATKNSKWVEVDTWES
LQKEWKETLKVLRHHQEKLEASDCDHQQNSPTLERPGRKRKWTETQDSSQKKSLEPKTKAVPKVKLLCGADLLESFAVPNLWKSEDI
TQIVANYGLICVTRAGNDAQKFIYESDVLWKHRSNIHVVNEWIANDISSTKIRRALRRGQSIRYLVPDLVQEYIEKHNLYSSESEDR
NAGVILAPLQRNTAEAKT

>sp|Q9UQ13|SHOC2_HUMAN
MSSSLGKEKDSKEKDPKVPSAKEREKEAKASGGFGKESKEKEPKTKGKDAKDGKKDSSAAQPGVAFSVDNTIKRPNPAPGTRKKSSN
AEVIKELNKCREENSMRLDLSKRSIHILPSSIKELTQLTELYLYSNKLQSLPAEVGCLVNLMTLALSENSLTSLPDSLDNLKKLRML
DLRHNKLREIPSVVYRLDSLTTLYLRFNRITTVEKDIKNLSKLSMLSIRENKIKQLPAEIGELCNLITLDVAHNQLEHLPKEIGNCT
QITNLDLQHNELLDLPDTIGNLSSLSRLGLRYNRLSAIPRSLAKCSALEELNLENNNISTLPESLLSSLVKLNSLTLARNCFQLYPV
GGPSQFSTIYSLNMEHNRINKIPFGIFSRAKVLSKLNMKDNQLTSLPLDFGTWTSMVELNLATNQLTKIPEDVSGLVSLEVLILSNN
LLKKLPHGLGNLRKLRELDLEENKLESLPNEIAYLKDLQKLVLTNNQLTTLPRGIGHLTNLTHLGLGENLLTHLPEEIGTLENLEEL
YLNDNPNLHSLPFELALCSKLSIMSIENCPLSHLPPQIVAGGPSFIIQFLKMQGPYRAMV
>sp|O94993|SOX30_HUMAN
MERARPEPPPQPRPLRPAPPPLPVEGTSFWAAAMEPPPSSPTLSAAASATLASSCGEAVASGLQPAVRRLLQVKPEQVLLLPQPQAQ
NEEAAASSAQARLLQFRPDLRLLQPPTASDGATSRPELHPVQPLALHVKAKKQKLGPSLDQSVGPRGAVETGPRASRVVKLEGPGPA
LGYFRGDEKGKLEAEEVMRDSMQGGAGKSPAAIREGVIKTEEPERLLEDCRLGAEPASNGLVHGSAEVILAPTSGAFGPHQQDLRIP
LTLHTVPPGARIQFQGAPPSELIRLTKVPLTPVPTKMQSLLEPSVKIETKDVPLTVLPSDAGIPDTPFSKDRNGHVKRPMNAFMVWA
RIHRPALAKANPAANNAEISVQLGLEWNKLSEEQKKPYYDEAQKIKEKHREEFPGWVYQPRPGKRKRFPLSVSNVFSGTTQNIISTN
PTTVYPYRSPTYSVVIPSLQNPITHPVGETSPAIQLPTPAVQSPSPVTLFQPSVSSAAQVAVQDPSLPVYPALPPQRFTGPSQTDTH
QLHSEATHTVKQPTPVSLESANRISSSASTAHARFATSTIQPPREYSSVSPCPRSAPIPQASPIPHPHVYQPPPLGHPATLFGTPPR
FSFHHPYFLPGPHYFPSSTCPYSRPPFGYGNFPSSMPECLSYYEDRYPKHEGIFSTLNRDYSFRDYSSECTHSENSRSCENMNGTSY
YNSHSHSGEENLNPVPQLDIGTLENVFTAPTSTPSSIQQVNVTDSDEEEEEKVLRDL
>sp|P41221|WNT5A_HUMAN
MKKSIGILSPGVALGMAGSAMSSKFFLVALAIFFSFAQVVIEANSWWSLGMNNPVQMSEVYIIGAQPLCSQLAGLSQGQKKLCHLYQ
DHMQYIGEGAKTGIKECQYQFRHRRWNCSTVDNTSVFGRVMQIGSRETAFTYAVSAAGVVNAMSRACREGELSTCGCSRAARPKDLP
RDWLWGGCGDNIDYGYRFAKEFVDARERERIHAKGSYESARILMNLHNNEAGRRTVYNLADVACKCHGVSGSCSLKTCWLQLADFRK
VGDALKEKYDSAAAMRLNSRGKLVQVNSRFNSPTTQDLVYIDPSPDYCVRNESTGSLGTQGRLCNKTSEGMDGCELMCCGRGYDQFK
TVQTERCHCKFHWCCYVKCKKCTEIVDQFVCK
>sp|Q9BVA6|FICD_HUMAN
MMLIPMASVMAVTEPKWVSVWSRFLWVTLLSMVLGSLLALLLPLGAVEEQCLAVLKGLYLLRSKPDRAQHAATKCTSPSTELSITSR
GATLLVAKTKASPAGKLEARAALNQALEMKRQGKREKAQKLFMHALKMDPDFVDALTEFGIFSEEDKDIIQADYLYTRALTISPYHE
KALVNRDRTLPLVEEIDQRYFSIIDSKVKKVMSIPKGNSALRRVMEETYYHHIYHTVAIEGNTLTLSEIRHILETRYAVPGKSLEEQ
NEVIGMHAAMKYINTTLVSRIGSVTISDVLEIHRRVLGYVDPVEAGRFRTTQVLVGHHIPPHPQDVEKQMQEFVQWLNSEEAMNLHP
VEFAALAHYKLVYIHPFIDGNGRTSRLLMNLILMQAGYPPITIRKEQRSDYYHVLEAANEGDVRPFIRFIAKCTETTLDTLLFATTE
YSVALPEAQPNHSGFKETLPVKP
>sp|P09488|GSTM1_HUMAN
MPMILGYWDIRGLAHAIRLLLEYTDSSYEEKKYTMGDAPDYDRSQWLNEKFKLGLDFPNLPYLIDGAHKITQSNAILCYIARKHNLC
GETEEEKIRVDILENQTMDNHMQLGMICYNPEFEKLKPKYLEELPEKLKLYSEFLGKRPWFAGNKITFVDFLVYDVLDLHRIFEPKC
LDAFPNLKDFISRFEGLEKISAYMKSSRFLPRPVFSKMAVWGNK
>sp|Q03112|EVI1_HUMAN
MKSEDYPHETMAPDIHEERQYRCEDCDQLFESKAELADHQKFPCSTPHSAFSMVEEDFQQKLESENDLQEIHTIQECKECDQVFPDL
QSLEKHMLSHTEEREYKCDQCPKAFNWKSNLIRHQMSHDSGKHYECENCAKVFTDPSNLQRHIRSQHVGARAHACPECGKTFATSSG
LKQHKHIHSSVKPFICEVCHKSYTQFSNLCRHKRMHADCRTQIKCKDCGQMFSTTSSLNKHRRFCEGKNHFAAGGFFGQGISLPGTP
AMDKTSMVNMSHANPGLADYFGANRHPAGLTFPTAPGFSFSFPGLFPSGLYHRPPLIPASSPVKGLSSTEQTNKSQSPLMTHPQILP
ATQDILKALSKHPSVGDNKPVELQPERSSEERPFEKISDQSESSDLDDVSTPSGSDLETTSGSDLESDIESDKEKFKENGKMFKDKV
SPLQNLASINNKKEYSNHSIFSPSLEEQTAVSGAVNDSIKAIASIAEKYFGSTGLVGLQDKKVGALPYPSMFPLPFFPAFSQSMYPF
PDRDLRSLPLKMEPQSPGEVKKLQKGSSESPFDLTTKRKDEKPLTPVPSKPPVTPATSQDQPLDLSMGSRSRASGTKLTEPRKNHVF
GGKKGSNVESRPASDGSLQHARPTPFFMDPIYRVEKRKLTDPLEALKEKYLRPSPGFLFHPQFQLPDQRTWMSAIENMAEKLESFSA

LKPEASELLQSVPSMFNFRAPPNALPENLLRKGKERYTCRYCGKIFPRSANLTRHLRTHTGEQPYRCKYCDRSFSISSNLQRHVRNI
HNKEKPFKCHLCDRCFGQQTNLDRHLKKHENGNMSGTATSSPHSELESTGAILDDKEDAYFTEIRNFIGNSNHGSQSPRNVEERMNG
SHFKDEKALVTSQNSDLLDDEEVEDEVLLDEEDEDNDITGKTGKEPVTSNLHEGNPEDDYEETSALEMSCKTSPVRYKEEEYKSGLS
ALDHIRHFTDSLKMRKMEDNQYSEAELSSFSTSHVPEELKQPLHRKSKSQAYAMMLSLSDKESLHSTSHSSSNVWHSMARAAAESSA
IQSISHV
>sp|O75841|UPK1B_HUMAN
MAKDNSTVRCFQGLLIFGNVIIGCCGIALTAECIFFVSDQHSLYPLLEATDNDDIYGAAWIGIFVGICLFCLSVLGIVGIMKSSRKI
LLAYFILMFIVYAFEVASCITAATQQDFFTPNLFLKQMLERYQNNSPPNNDDQWKNNGVTKTWDRLMLQDNCCGVNGPSDWQKYTSA
FRTENNDADYPWPRQCCVMNNLKEPLNLEACKLGVPGFYHNQGCYELISGPMNRHAWGVAWFGFAILCWTFWVLLGTMFYWSRIEY
>sp|Q9Y548|YIPF1_HUMAN
MAAVDDLQFEEFGNAATSLTANPDATTVNIEDPGETPKHQPGSPRGSGREEDDELLGNDDSDKTELLAGQKKSSPFWTFEYYQTFFD
VDTYQVFDRIKGSLLPIPGKNFVRLYIRSNPDLYGPFWICATLVFAIAISGNLSNFLIHLGEKTYHYVPEFRKVSIAATIIYAYAWL
VPLALWGFLMWRNSKVMNIVSYSFLEIVCVYGYSLFIYIPTAILWIIPQKAVRWILVMIALGISGSLLAMTFWPAVREDNRRVALAT
IVTIVLLHMLLSVGCLAYFFDAPEMDHLPTTTATPNQTVAAAKSS
>sp|P02818|OSTCN_HUMAN
MRALTLLALLALAALCIAGQAGAKPSGAESSKGAAFVSKQEGSEVVKRPRRYLYQWLGAPVPYPDPLEPRREVCELNPDCDELADHI
GFQEAYRRFYGPV
>sp|Q2TAC6|KIF19_HUMAN
MKDSGDSKDQQLMVALRVRPISVAELEEGATLIAHKVDEQMVVLMDPMEDPDDILRAHRSREKSYLFDVAFDFTATQEMVYQATTKS
LIEGVISGYNATVFAYGPTGCGKTYTMLGTDQEPGIYVQTLNDLFRAIEETSNDMEYEVSMSYLEIYNEMIRDLLNPSLGYLELRED
SKGVIQVAGITEVSTINAKEIMQLLMKGNRQRTQEPTAANQTSSRSHAVLQVTVRQRSRVKNILQEVRQGRLFMIDLAGSERASQTQ
NRGQRMKEGAHINRSLLALGNCINALSDKGSNKYINYRDSKLTRLLKDSLGGNSRTVMIAHISPASSAFEESRNTLTYAGRAKNIKT
RVKQNLLNVSYHIAQYTSIIADLRGEIQRLKRKIDEQTGRGQARGRQDRGDIRHIQAEVQLHSGQGEKAGMGQLREQLASAFQEQMD
VRRRLLELENRAMEVQIDTSRHLLTIAGWKHEKSRRALKWREEQRKECYAKDDSEKDSDTGDDQPDILEPPEVAAARESIAALVDEQ
KQLRKQKLALEQRCRELRARGRRLEETLPRRIGSEEQREVLSLLCRVHELEVENTEMQSHALLRDGALRHRHEAVRRLEQHRSLCDE
IIQGQRQIIDDYNLAVPQRLEELYEVYLRELEEGSLEQATIMDQVASRALQDSSLPKITPAGTSLTPDSDLESVKTLSSDAQHLQNS
ALPPLSTESEGHHVFKAGTGAWQAKSSSVPTPPPIQLGSLVTQEAPAQDSLGSWINSSPDSSENLSEIPLSHKERKEILTGTKCIWV
KAARRRSRALGTEGRHLLAPATERSSLSLHSLSEGDDARPPGPLACKRPPSPTLQHAASEDNLSSSTGEAPSRAVGHHGDGPRPWLR
GQKKSLGKKREESLEAKRRKRRSRSFEVTGQGLSHPKTHLLGPHQAERISDHRMPVCRHPAPGIRHLGKVTLPLAKVKLPPSQNTGP
GDSSPLAVPPNPGGGSRRATRGPRLPHGTSTHGKDGCSRHN
>sp|P78562|PHEX_HUMAN
MEAETGSSVETGKKANRGTRIALVVFVGGTLVLGTILFLVSQGLLSLQAKQEYCLKPECIEAAAAILSKVNLSVDPCDNFFRFACDG
WISNNPIPEDMPSYGVYPWLRHNVDLKLKELLEKSISRRRDTEAIQKAKILYSSCMNEKAIEKADAKPLLHILRHSPFRWPVLESNI
GPEGVWSERKFSLLQTLATFRGQYSNSVFIRLYVSPDDKASNEHILKLDQATLSLAVREDYLDNSTEAKSYRDALYKFMVDTAVLLG
ANSSRAEHDMKSVLRLEIKIAEIMIPHENRTSEAMYNKMNISELSAMIPQFDWLGYIKKVIDTRLYPHLKDISPSENVVVRVPQYFK
DLFRILGSERKKTIANYLVWRMVYSRIPNLSRRFQYRWLEFSRVIQGTTTLLPQWDKCVNFIESALPYVVGKMFVDVYFQEDKKEMM
EELVEGVRWAFIDMLEKENEWMDAGTKRKAKEKARAVLAKVGYPEFIMNDTHVNEDLKAIKFSEADYFGNVLQTRKYLAQSDFFWLR
KAVPKTEWFTNPTTVNAFYSASTNQIRFPAGELQKPFFWGTEYPRSLSYGAIGVIVGHEFTHGFDNNGRKYDKNGNLDPWWSTESEE
KFKEKTKCMINQYSNYYWKKAGLNVKGKRTLGENIADNGGLREAFRAYRKWINDRRQGLEEPLLPGITFTNNQLFFLSYAHVRCNSY
RPEAAREQVQIGAHSPPQFRVNGAISNFEEFQKAFNCPPNSTMNRGMDSCRLW
>sp|Q96NU7|HUTI_HUMAN
MASGHSLLLENAQQVVLVCARGERFLARDALRSLAVLEGASLVVGKDGFIKAIGPADVIQRQFSGETFEEIIDCSGKCILPGLVDAH
THPVWAGERVHEFAMKLAGATYMEIHQAGGGIHFTVERTRQATEEELFRSLQQRLQCMMRAGTTLVECKSGYGLDLETELKMLRVIE
RARRELDIGISATYCGAHSVPKGKTATEAADDIINNHLPKLKELGRNGEIHVDNIDVFCEKGVFDLDSTRRILQRGKDIGLQINFHG

DELHPMKAAELGAELGAQAISHLEEVSDEGIVAMATARCSAILLPTTAYMLRLKQPRARKMLDEGVIVALGSDFNPNAYCFSMPMVM
HLACVNMRMSMPEALAAATINAAYALGKSHTHGSLEVGKQGDLIIINSSRWEHLIYQFGGHHELIEYVIAKGKLIYKT
>sp|O75881|CP7B1_HUMAN
MAGEVSAATGRFSLERLGLPGLALAAALLLLALCLLVRRTRRPGEPPLIKGWLPYLGVVLNLRKDPLRFMKTLQKQHGDTFTVLLGG
KYITFILDPFQYQLVIKNHKQLSFRVFSNKLLEKAFSISQLQKNHDMNDELHLCYQFLQGKSLDILLESMMQNLKQVFEPQLLKTTS
WDTAELYPFCSSIIFEITFTTIYGKVIVCDNNKFISELRDDFLKFDDKFAYLVSNIPIELLGNVKSIREKIIKCFSSEKLAKMQGWS
EVFQSRQDVLEKYYVHEDLEIGAHHLGFLWASVANTIPTMFWAMYYLLRHPEAMAAVRDEIDRLLQSTGQKKGSGFPIHLTREQLDS
LICLESSIFEALRLSSYSTTIRFVEEDLTLSSETGDYCVRKGDLVAIFPPVLHGDPEIFEAPEEFRYDRFIEDGKKKTTFFKRGKKL
KCYLMPFGTGTSKCPGRFFALMEIKQLLVILLTYFDLEIIDDKPIGLNYSRLLFGIQYPDSDVLFRYKVKS
>sp|Q8WZ84|OR8D1_HUMAN
MTMENYSMAAQFVLDGLTQQAELQLPLFLLFLGIYVVTVVGNLGMILLIAVSPLLHTPMYYFLSSLSFVDFCYSSVITPKMLVNFLG
KKNTILYSECMVQLFFFVVFVVAEGYLLTAMAYDRYVAICSPLLYNAIMSSWVCSLLVLAAFFLGFLSALTHTSAMMKLSFCKSHII
NHYFCDVLPLLNLSCSNTHLNELLLFIIAGFNTLVPTLAVAVSYAFILYSILHIRSSEGRSKAFGTCSSHLMAVVIFFGSITFMYFK
PPSSNSLDQEKVSSVFYTTVIPMLNPLIYSLRNKDVKKALRKVLVGK
>sp|Q9GZL7|WDR12_HUMAN
MAQLQTRFYTDNKKYAVDDVPFSIPAASEIADLSNIINKLLKDKNEFHKHVEFDFLIKGQFLRMPLDKHMEMENISSEEVVEIEYVE
KYTAPQPEQCMFHDDWISSIKGAEEWILTGSYDKTSRIWSLEGKSIMTIVGHTDVVKDVAWVKKDSLSCLLLSASMDQTILLWEWNV
ERNKVKALHCCRGHAGSVDSIAVDGSGTKFCSGSWDKMLKIWSTVPTDEEDEMEESTNRPRKKQKTEQLGLTRTPIVTLSGHMEAVS
SVLWSDAEEICSASWDHTIRVWDVESGSLKSTLTGNKVFNCISYSPLCKRLASGSTDRHIRLWDPRTKDGSLVSLSLTSHTGWVTSV
KWSPTHEQQLISGSLDNIVKLWDTRSCKAPLYDLAAHEDKVLSVDWTDTGLLLSGGADNKLYSYRYSPTTSHVGA
>sp|Q7Z353|HDX_HUMAN
MNLRSVFTVEQQRILQRYYENGMTNQSKNCFQLILQCAQETKLDFSVVRTWVGNKRRKMSSKNSESGTATTGTSLSAPDITVRNVVN
IARPSSQQSSWTSANNDVIVTGIYSPASSSSRQGTNKHTDTQITEAHKIPIQKTATKNDTEFQLHIPVQRQVAHCKNASLLLGEKTI
ILSRQTSVLNAGNSVFNHAKKNYGNSSVQASEMTVPQKPSVCHRPCKIEPVGIQRSYKPEHTGPALHNLCGQKPTIRDPYCRTQNLE
IREVFSLAVSDYPQRILGGNAPQKPSSAEGNCLSIAMETGDAEDEYAREEELASMRAQIPSYSRFYESGSSLRAENQSTTLPGPGRN
MPNSQMVNIRDMSDNVLYQNRNYHLTPRTSLHTASSTMYSNTNPLRSNFSPHFASSNQLRLSQNQNNYQISGNLTVPWITGCSRKRA
LQDRTQFSDRDLATLKKYWDNGMTSLGSVCREKIEAVATELNVDCEIVRTWIGNRRRKYRLMGIEVPPPRGGPADFSEQPESGSLSA
LTPGEEAGPEVGEDNDRNDEVSICLSEGSSQEEPNEVVPNDARAHKEEDHHAVTTDNVKIEIIDDEESDMISNSEVEQVNSFLDYKN
EEVKFIENELEIQKQKYFKLQTFVRSLILAMKADDKEQQQALLSDLPPELEEMDFNHASLEPDDTSFSVSSLSEKNVSESL
>sp|Q6PIU1|KCNV1_HUMAN
MPSSGRALLDSPLDSGSLTSLDSSVFCSEGEGEPLALGDCFTVNVGGSRFVLSQQALSCFPHTRLGKLAVVVASYRRPGALAAVPSP
LELCDDANPVDNEYFFDRSSQAFRYVLHYYRTGRLHVMEQLCALSFLQEIQYWGIDELSIDSCCRDRYFRRKELSETLDFKKDTEDQ
ESQHESEQDFSQGPCPTVRQKLWNILEKPGSSTAARIFGVISIIFVVVSIINMALMSAELSWLDLQLLEILEYVCISWFTGEFVLRF
LCVRDRCRFLRKVPNIIDLLAILPFYITLLVESLSGSQTTQELENVGRIVQVLRLLRALRMLKLGRHSTGLRSLGMTITQCYEEVGL
LLLFLSVGISIFSTVEYFAEQSIPDTTFTSVPCAWWWATTSMTTVGYGDIRPDTTTGKIVAFMCILSGILVLALPIAIINDRFSACY
FTLKLKEAAVRQREALKKLTKNIATDSYISVNLRDVYARSIMEMLRLKGRERASTRSSGGDDFWF
>sp|Q9UPN9|TRI33_HUMAN
MAENKGGGEAESGGGGSGSAPVTAGAAGPAAQEAEPPLTAVLVEEEEEEGGRAGAEGGAAGPDDGGVAAASSGSAQAASSPAASVGT
GVAGGAVSTPAPAPASAPAPGPSAGPPPGPPASLLDTCAVCQQSLQSRREAEPKLLPCLHSFCLRCLPEPERQLSVPIPGGSNGDIQ
QVGVIRCPVCRQECRQIDLVDNYFVKDTSEAPSSSDEKSEQVCTSCEDNASAVGFCVECGEWLCKTCIEAHQRVKFTKDHLIRKKED
VSESVGASGQRPVFCPVHKQEQLKLFCETCDRLTCRDCQLLEHKEHRYQFLEEAFQNQKGAIENLLAKLLEKKNYVHFAATQVQNRI
KEVNETNKRVEQEIKVAIFTLINEINKKGKSLLQQLENVTKERQMKLLQQQNDITGLSRQVKHVMNFTNWAIASGSSTALLYSKRLI
TFQLRHILKARCDPVPAANGAIRFHCDPTFWAKNVVNLGNLVIESKPAPGYTPNVVVGQVPPGTNHISKTPGQINLAQLRLQHMQQQ
VYAQKHQQLQQMRMQQPPAPVPTTTTTTQQHPRQAAPQMLQQQPPRLISVQTMQRGNMNCGAFQAHQMRLAQNAARIPGIPRHSGPQ

YSMMQPHLQRQHSNPGHAGPFPVVSVHNTTINPTSPTTATMANANRGPTSPSVTAIELIPSVTNPENLPSLPDIPPIQLEDAGSSSL
DNLLSRYISGSHLPPQPTSTMNPSPGPSALSPGSSGLSNSHTPVRPPSTSSTGSRGSCGSSGRTAEKTSLSFKSDQVKVKQEPGTED
EICSFSGGVKQEKTEDGRRSACMLSSPESSLTPPLSTNLHLESELDALASLENHVKIEPADMNESCKQSGLSSLVNGKSPIRSLMHR
SARIGGDGNNKDDDPNEDWCAVCQNGGDLLCCEKCPKVFHLTCHVPTLLSFPSGDWICTFCRDIGKPEVEYDCDNLQHSKKGKTAQG
LSPVDQRKCERLLLYLYCHELSIEFQEPVPASIPNYYKIIKKPMDLSTVKKKLQKKHSQHYQIPDDFVADVRLIFKNCERFNEMMKV
VQVYADTQEINLKADSEVAQAGKAVALYFEDKLTEIYSDRTFAPLPEFEQEEDDGEVTEDSDEDFIQPRRKRLKSDERPVHIK
>sp|Q5T5B0|LCE3E_HUMAN
MSCQQNQKQCQPPPKCPSPKCPPKNPVQCLPPASSGCAPSSGGCGPSSEGGCFLNHHRRHHRCRRQRSNSCDRGSGQQGGGSGCCHG
SGGCC
>sp|Q96P69|GPR78_HUMAN
MGPGEALLAGLLVMVLAVALLSNALVLLCCAYSAELRTRASGVLLVNLSLGHLLLAALDMPFTLLGVMRGRTPSAPGACQVIGFLDT
FLASNAALSVAALSADQWLAVGFPLRYAGRLRPRYAGLLLGCAWGQSLAFSGAALGCSWLGYSSAFASCSLRLPPEPERPRFAAFTA
TLHAVGFVLPLAVLCLTSLQVHRVARRHCQRMDTVTMKALALLADLHPSVRQRCLIQQKRRRHRATRKIGIAIATFLICFAPYVMTR
LAELVPFVTVNAQWGILSKCLTYSKAVADPFTYSLLRRPFRQVLAGMVHRLLKRTPRPASTHDSSLDVAGMVHQLLKRTPRPASTHN
GSVDTENDSCLQQTH
>sp|P27815|PDE4A_HUMAN
MEPPTVPSERSLSLSLPGPREGQATLKPPPQHLWRQPRTPIRIQQRGYSDSAERAERERQPHRPIERADAMDTSDRPGLRTTRMSWP
SSFHGTGTGSGGAGGGSSRRFEAENGPTPSPGRSPLDSQASPGLVLHAGAATSQRRESFLYRSDSDYDMSPKTMSRNSSVTSEAHAE
DLIVTPFAQVLASLRSVRSNFSLLTNVPVPSNKRSPLGGPTPVCKATLSEETCQQLARETLEELDWCLEQLETMQTYRSVSEMASHK
FKRMLNRELTHLSEMSRSGNQVSEYISTTFLDKQNEVEIPSPTMKEREKQQAPRPRPSQPPPPPVPHLQPMSQITGLKKLMHSNSLN
NSNIPRFGVKTDQEELLAQELENLNKWGLNIFCVSDYAGGRSLTCIMYMIFQERDLLKKFRIPVDTMVTYMLTLEDHYHADVAYHNS
LHAADVLQSTHVLLATPALDAVFTDLEILAALFAAAIHDVDHPGVSNQFLINTNSELALMYNDESVLENHHLAVGFKLLQEDNCDIF
QNLSKRQRQSLRKMVIDMVLATDMSKHMTLLADLKTMVETKKVTSSGVLLLDNYSDRIQVLRNMVHCADLSNPTKPLELYRQWTDRI
MAEFFQQGDRERERGMEISPMCDKHTASVEKSQVGFIDYIVHPLWETWADLVHPDAQEILDTLEDNRDWYYSAIRQSPSPPPEEESR
GPGHPPLPDKFQFELTLEEEEEEEISMAQIPCTAQEALTAQGLSGVEEALDATIAWEASPAQESLEVMAQEASLEAELEAVYLTQQA
QSTGSAPVAPDEFSSREEFVVAVSHSSPSALALQSPLLPAWRTLSVSEHAPGLPGLPSTAAEVEAQREHQAAKRACSACAGTFGEDT
SALPAPGGGGSGGDPT
>sp|P33981|TTK_HUMAN
MESEDLSGRELTIDSIMNKVRDIKNKFKNEDLTDELSLNKISADTTDNSGTVNQIMMMANNPEDWLSLLLKLEKNSVPLSDALLNKL
IGRYSQAIEALPPDKYGQNESFARIQVRFAELKAIQEPDDARDYFQMARANCKKFAFVHISFAQFELSQGNVKKSKQLLQKAVERGA
VPLEMLEIALRNLNLQKKQLLSEEEKKNLSASTVLTAQESFSGSLGHLQNRNNSCDSRGQTTKARFLYGENMPPQDAEIGYRNSLRQ
TNKTKQSCPFGRVPVNLLNSPDCDVKTDDSVVPCFMKRQTSRSECRDLVVPGSKPSGNDSCELRNLKSVQNSHFKEPLVSDEKSSEL
IITDSITLKNKTESSLLAKLEETKEYQEPEVPESNQKQWQSKRKSECINQNPAASSNHWQIPELARKVNTEQKHTTFEQPVFSVSKQ
SPPISTSKWFDPKSICKTPSSNTLDDYMSCFRTPVVKNDFPPACQLSTPYGQPACFQQQQHQILATPLQNLQVLASSSANECISVKG
RIYSILKQIGSGGSSKVFQVLNEKKQIYAIKYVNLEEADNQTLDSYRNEIAYLNKLQQHSDKIIRLYDYEITDQYIYMVMECGNIDL
NSWLKKKKSIDPWERKSYWKNMLEAVHTIHQHGIVHSDLKPANFLIVDGMLKLIDFGIANQMQPDTTSVVKDSQVGTVNYMPPEAIK
DMSSSRENGKSKSKISPKSDVWSLGCILYYMTYGKTPFQQIINQISKLHAIIDPNHEIEFPDIPEKDLQDVLKCCLKRDPKQRISIP
ELLAHPYVQIQTHPVNQMAKGTTEEMKYVLGQLVGLNSPNSILKAAKTLYEHYSGGESHNSSSSKTFEKKRGKK
>sp|Q08426|ECHP_HUMAN
MAEYTRLHNALALIRLRNPPVNAISTTLLRDIKEGLQKAVIDHTIKAIVICGAEGKFSAGADIRGFSAPRTFGLTLGHVVDEIQRNE
KPVVAAIQGMAFGGGLELALGCHYRIAHAEAQVGLPEVTLGLLPGARGTQLLPRLTGVPAALDLITSGRRILADEALKLGILDKVVN
SDPVEEAIRFAQRVSDQPLESRRLCNKPIQSLPNMDSIFSEALLKMRRQHPGCLAQEACVRAVQAAVQYPYEVGIKKEEELFLYLLQ
SGQARALQYAFFAERKANKWSTPSGASWKTASARPVSSVGVVGLGTMGRGIVISFARARIPVIAVDSDKNQLATANKMITSVLEKEA
SKMQQSGHPWSGPKPRLTSSVKELGGVDLVIEAVFEEMSLKKQVFAELSAVCKPEAFLCTNTSALDVDEIASSTDRPHLVIGTHFFS

PAHVMKLLEVIPSQYSSPTTIATVMNLSKKIKKIGVVVGNCFGFVGNRMLNPYYNQAYFLLEEGSKPEEVDQVLEEFGFKMGPFRVS
DLAGLDVGWKSRKGQGLTGPTLLPGTPARKRGNRRYCPIPDVLCELGRFGQKTGKGWYQYDKPLGRIHKPDPWLSKFLSRYRKTHHI
EPRTISQDEILERCLYSLINEAFRILGEGIAASPEHIDVVYLHGYGWPRHKGGPMFYASTVGLPTVLEKLQKYYRQNPDIPQLEPSD
YLKKLASQGNPPLKEWQSLAGSPSSKL
>sp|Q9NPH5|NOX4_HUMAN
MAVSWRSWLANEGVKHLCLFIWLSMNVLLFWKTFLLYNQGPEYHYLHQMLGLGLCLSRASASVLNLNCSLILLPMCRTLLAYLRGSQ
KVPSRRTRRLLDKSRTFHITCGVTICIFSGVHVAAHLVNALNFSVNYSEDFVELNAARYRDEDPRKLLFTTVPGLTGVCMVVVLFLM
ITASTYAIRVSNYDIFWYTHNLFFVFYMLLTLHVSGGLLKYQTNLDTHPPGCISLNRTSSQNISLPEYFSEHFHEPFPEGFSKPAEF
TQHKFVKICMEEPRFQANFPQTWLWISGPLCLYCAERLYRYIRSNKPVTIISVMSHPSDVMEIRMVKENFKARPGQYITLHCPSVSA
LENHPFTLTMCPTETKATFGVHLKIVGDWTERFRDLLLPPSSQDSEILPFIQSRNYPKLYIDGPFGSPFEESLNYEVSLCVAGGIGV
TPFASILNTLLDDWKPYKLRRLYFIWVCRDIQSFRWFADLLCMLHNKFWQENRPDYVNIQLYLSQTDGIQKIIGEKYHALNSRLFIG
RPRWKLLFDEIAKYNRGKTVGVFCCGPNSLSKTLHKLSNQNNSYGTRFEYNKESFS
>sp|Q17RW2|COOA1_HUMAN
MHLRAHRTRRGKVSPTAKTKSLLHFIVLCVAGVVVHAQEQGIDILHQLGLGGKDVRHSSPATAVPSASTPLPQGVHLTESGVIFKND
AYIETPFVKILPVNLGQPFTILTGLQSHRVNNAFLFSIRNKNRLQLGVQLLPKKLVVHIRGKQPAVFNYSVHDEQWHSFAITIRNQS
VSMFVECGKKYFSTETIPEVQTFDSNSVFTLGSMNNNSIHFEGIVCQLDIIPSAEASADYCRYVKQQCRQADKYQPETSIPCTTLIP
TKIPEHSPPPKLFAEKVLSEDTFTEGKSIPNIIKNDSETVYKRQEHQISRSQLSSLQSGNVSAVDLTNHGIQAKEMITEEDTQTNFS
LSVTTHRISEAKMNTKEKFSSLLNMSDNITQHDDRVTGLSLFKKMPSILPQIKQDTITNLKKAITANLHTNELMEMQPILNTSLHRV
TNEPSVDNHLDLRKEGEFYPDATYPIENSYETELYDYYYYEDLNTMLEMEYLRGPKGDTGPPGPPGPAGIPGPSGKRGPRGIPGPHG
NPGLPGLPGPKGPKGDPGFSPGQPVPGEKGDQGLSGLMGPPGMQGDKGLKGHPGLPGLPGEQGIPGFAGNIGSPGYPGRQGLAGPEG
NPGPKGAQGFIGSPGEAGQLPGEGERGIPGIRGKKGFKGRQGFPGDFGDRGPAGLDGSPGLVGGTGPPGFPGLRGSVGPVGPIGPAG
IPGPMGLSGNKGLPGIKGDKGEQGTAGELGEPGYPGDKGAVGLPGPPGMRGKSGPSGQTGDPGLQGPSGPPGPEGFPGDIGIPGQNG
PEGPKGLLGNRGPPGPPGLKGTQGEEGPIGAFGELGPRGKPGQKGYAGEPGPEGLKGEVGDQGNIGKIGETGPVGLPGEVGMTGSIG
EKGERGSPGPLGPQGEKGVMGYPGPPGVPGPIGPLGLPGHVGARGPPGSQGPKGQRGSRGPDGLLGEQGIQGAKGEKGDQGKRGPHG
LIGKTGNPGERGFQGKPGLQGLPGSTGDRGLPGEPGLRGLQGDVGPPGEMGMEGPPGTEGESGLQGEPGAKGDVGTAGSVGGTGEPG
LRGEPGAPGEEGLQGKDGLKGVPGGRGLPGEDGEKGEMGLPGIIGPLGRSGQTGLPGPEGIVGIPGQRGRPGKKGDKGQIGPTGEVG
SRGPPGKIGKSGPKGARGTRGAVGHLGLMGPDGEPGIPGYRGHQGQPGPSGLPGPKGEKGYPGEDSTVLGPPGPRGEPGPVGDQGER
GEPGAEGYKGHVGVPGLRGATGQQGPPGEPGDQGEQGLKGERGSEGNKGKKGAPGPSGKPGIPGLQGLLGPKGIQGYHGADGISGNP
GKIGPPGKQGLPGIRGGPGRTGLAGAPGPPGVKGSSGLPGSPGIQGPKGEQGLPGQPGIQGKRGHRGAQGDQGPCGDPGLKGQPGEY
GVQGLTGFQGFPGPKGPEGDAGIVGISGPKGPIGHRGNTGPLGREGIIGPTGRTGPRGEKGFRGETGPQGPRGQPGPPGPPGAPGPR
KQMDINAAIQALIESNTALQMESYQNTEVTLIDHSEEIFKTLNYLSNLLHSIKNPLGTRDNPARICKDLLNCEQKVSDGKYWIDPNL
GCPSDAIEVFCNFSAGGQTCLPPVSVTKLEFGVGKVQMNFLHLLSSEATHIITIHCLNTPRWTSTQTSGPGLPIGFKGWNGQIFKVN
TLLEPKVLSDDCKIQDGSWHKATFLFHTQEPNQLPVIEVQKLPHLKTERKYYIDSSSVCFL
>sp|Q9NP94|S39A2_HUMAN
MEQLLGIKLGCLFALLALTLGCGLTPICFKWFQIDAARGHHRLVLRLLGCISAGVFLGAGFMHMTAEALEEIESQIQKFMVQNRSAS
ERNSSGDADSAHMEYPYGELIISLGFFFVFFLESLALQCCPGAAGGSTVQDEEWGGAHIFELHSHGHLPSPSKGPLRALVLLLSLSF
HSVFEGLAVGLQPTVAATVQLCLAVLAHKGLVVFGVGMRLVHLGTSSRWAVFSILLLALMSPLGLAVGLAVTGGDSEGGRGLAQAVL
EGVAAGTFLYVTFLEILPRELASPEAPLAKWSCVAAGFAFMAFIALWA
>sp|P23919|KTHY_HUMAN
MAARRGALIVLEGVDRAGKSTQSRKLVEALCAAGHRAELLRFPERSTEIGKLLSSYLQKKSDVEDHSVHLLFSANRWEQVPLIKEKL
SQGVTLVVDRYAFSGVAFTGAKENFSLDWCKQPDVGLPKPDLVLFLQLQLADAAKRGAFGHERYENGAFQERALRCFHQLMKDTTLN
WKMVDASKSIEAVHEDIRVLSEDAIRTATEKPLGELWK
>sp|Q96F46|I17RA_HUMAN

MGAARSPPSAVPGPLLGLLLLLLGVLAPGGASLRLLDHRALVCSQPGLNCTVKNSTCLDDSWIHPRNLTPSSPKDLQIQLHFAHTQQ
GDLFPVAHIEWTLQTDASILYLEGAELSVLQLNTNERLCVRFEFLSKLRHHHRRWRFTFSHFVVDPDQEYEVTVHHLPKPIPDGDPN
HQSKNFLVPDCEHARMKVTTPCMSSGSLWDPNITVETLEAHQLRVSFTLWNESTHYQILLTSFPHMENHSCFEHMHHIPAPRPEEFH
QRSNVTLTLRNLKGCCRHQVQIQPFFSSCLNDCLRHSATVSCPEMPDTPEPIPDYMPLWVYWFITGISILLVGSVILLIVCMTWRLA
GPGSEKYSDDTKYTDGLPAADLIPPPLKPRKVWIIYSADHPLYVDVVLKFAQFLLTACGTEVALDLLEEQAISEAGVMTWVGRQKQE
MVESNSKIIVLCSRGTRAKWQALLGRGAPVRLRCDHGKPVGDLFTAAMNMILPDFKRPACFGTYVVCYFSEVSCDGDVPDLFGAAPR
YPLMDRFEEVYFRIQDLEMFQPGRMHRVGELSGDNYLRSPGGRQLRAALDRFRDWQVRCPDWFECENLYSADDQDAPSLDEEVFEEP
LLPPGTGIVKRAPLVREPGSQACLAIDPLVGEEGGAAVAKLEPHLQPRGQPAPQPLHTLVLAAEEGALVAAVEPGPLADGAAVRLAL
AGEGEACPLLGSPGAGRNSVLFLPVDPEDSPLGSSTPMASPDLLPEDVREHLEGLMLSLFEQSLSCQAQGGCSRPAMVLTDPHTPYE
EEQRQSVQSDQGYISRSSPQPPEGLTEMEEEEEEEQDPGKPALPLSPEDLESLRSLQRQLLFRQLQKNSGWDTMGSESEGPSA
>sp|P04350|TBB4A_HUMAN
MREIVHLQAGQCGNQIGAKFWEVISDEHGIDPTGTYHGDSDLQLERINVYYNEATGGNYVPRAVLVDLEPGTMDSVRSGPFGQIFRP
DNFVFGQSGAGNNWAKGHYTEGAELVDAVLDVVRKEAESCDCLQGFQLTHSLGGGTGSGMGTLLISKIREEFPDRIMNTFSVVPSPK
VSDTVVEPYNATLSVHQLVENTDETYCIDNEALYDICFRTLKLTTPTYGDLNHLVSATMSGVTTCLRFPGQLNADLRKLAVNMVPFP
RLHFFMPGFAPLTSRGSQQYRALTVPELTQQMFDAKNMMAACDPRHGRYLTVAAVFRGRMSMKEVDEQMLSVQSKNSSYFVEWIPNN
VKTAVCDIPPRGLKMAATFIGNSTAIQELFKRISEQFTAMFRRKAFLHWYTGEGMDEMEFTEAESNMNDLVSEYQQYQDATAEEGEF
EEEAEEEVA
>sp|Q5K4L6|S27A3_HUMAN
MGVCQRTRAPWKEKSQLERAALGFRKGGSGMFASGWNQTVPIEEAGSMAALLLLPLLLLLPLLLLKLHLWPQLRWLPADLAFAVRAL
CCKRALRARALAAAAADPEGPEGGCSLAWRLAELAQQRAAHTFLIHGSRRFSYSEAERESNRAARAFLRALGWDWGPDGGDSGEGSA
GEGERAAPGAGDAAAGSGAEFAGGDGAARGGGAAAPLSPGATVALLLPAGPEFLWLWFGLAKAGLRTAFVPTALRRGPLLHCLRSCG
ARALVLAPEFLESLEPDLPALRAMGLHLWAAGPGTHPAGISDLLAEVSAEVDGPVPGYLSSPQSITDTCLYIFTSGTTGLPKAARIS
HLKILQCQGFYQLCGVHQEDVIYLALPLYHMSGSLLGIVGCMGIGATVVLKSKFSAGQFWEDCQQHRVTVFQYIGELCRYLVNQPPS
KAERGHKVRLAVGSGLRPDTWERFVRRFGPLQVLETYGLTEGNVATINYTGQRGAVGRASWLYKHIFPFSLIRYDVTTGEPIRDPQG
HCMATSPGEPGLLVAPVSQQSPFLGYAGGPELAQGKLLKDVFRPGDVFFNTGDLLVCDDQGFLRFHDRTGDTFRWKGENVATTEVAE
VFEALDFLQEVNVYGVTVPGHEGRAGMAALVLRPPHALDLMQLYTHVSENLPPYARPRFLRLQESLATTETFKQQKVRMANEGFDPS
TLSDPLYVLDQAVGAYLPLTTARYSALLAGNLRI
>sp|P05976|MYL1_HUMAN
MAPKKDVKKPVAAAAAAPAPAPAPAPAPAPAKPKEEKIDLSAIKIEFSKEQQDEFKEAFLLFDRTGDSKITLSQVGDVLRALGTNPT
NAEVRKVLGNPSNEELNAKKIEFEQFLPMMQAISNNKDQATYEDFVEGLRVFDKEGNGTVMGAELRHVLATLGEKMKEEEVEALMAG
QEDSNGCINYEAFVKHIMSI
>sp|Q8N1I8|CAAS1_HUMAN
MRPEVGREPAALQPRQRPRSDHQLHRSPFTVPPRTPACRSPGPSPPIAVALEVKPQLEDAIGKLAAEAVAVRVFPLAVDDLESDVLV
GRPRVEAQDGEILVVGAGLQEVLGRGALVDEVGVEDVELVSLHDLGRWVVKVVVRLVVLVPLEARVHAVEEARLAWPVLVGPQDGGC
PGSARTAAGKSFHHLWPQGQWASKPRMNEGTPQCACR
>sp|Q6UB99|ANR11_HUMAN
MPKGGCPKAPQQEELPLSSDMVEKQTGKKDKDKVSLTKTPKLERGDGGKEVRERASKRKLPFTAGANGEQKDSDTEKQGPERKRIKK
EPVTRKAGLLFGMGLSGIRAGYPLSERQQVALLMQMTAEESANSPVDTTPKHPSQSTVCQKGTPNSASKTKDKVNKRNERGETRLHR
AAIRGDARRIKELISEGADVNVKDFAGWTALHEACNRGYYDVAKQLLAAGAEVNTKGLDDDTPLHDAANNGHYKVVKLLLRYGGNPQ
QSNRKGETPLKVANSPTMVNLLLGKGTYTSSEESSTESSEEEDAPSFAPSSSVDGNNTDSEFEKGLKHKAKNPEPQKATAPVKDEYE
FDEDDEQDRVPPVDDKHLLKKDYRKETKSNSFISIPKMEVKSYTKNNTIAPKKASHRILSDTSDEEDASVTVGTGEKLRLSAHTILP
GSKTREPSNAKQQKEKNKVKKKRKKETKGREVRFGKRSDKFCSSESESESSESGEDDRDSLGSSGCLKGSPLVLKDPSLFSSLSASS
TSSHGSSAAQKQNPSHTDQHTKHWRTDNWKTISSPAWSEVSSLSDSTRTRLTSESDYSSEGSSVESLKPVRKRQEHRKRASLSEKKS
PFLSSAEGAVPKLDKEGKVVKKHKTKHKHKNKEKGQCSISQELKLKSFTYEYEDSKQKSDKAILLENDLSTENKLKVLKHDRDHFKK

EEKLSKMKLEEKEWLFKDEKSLKRIKDTNKDISRSFREEKDRSNKAEKERSLKEKSPKEEKLRLYKEERKKKSKDRPSKLEKKNDLK
EDKISKEKEKIFKEDKEKLKKEKVYREDSAFDEYCNKNQFLENEDTKFSLSDDQRDRWFSDLSDSSFDFKGEDSWDSPVTDYRDMKS
DSVAKLILETVKEDSKERRRDSRAREKRDYREPFFRKKDRDYLDKNSEKRKEQTEKHKSVPGYLSEKDKKRRESAEAGRDRKDALES
CKERRDGRAKPEEAHREELKECGCESGFKDKSDGDFGKGLEPWERHHPAREKEKKDGPDKERKEKTKPERYKEKSSDKDKSEKSILE
KCQKDKEFDKCFKEKKDTKEKHKDTHGKDKERKASLDQGKEKKEKAFPGIISEDFSEKKDDKKGKEKSWYIADIFTDESEDDRDSCM
GSGFKMGEASDLPRTDGLQEKEEGREAYASDRHRKSSDKQHPERQKDKEPRDRRKDRGAADAGRDKKEKVFEKHKEKKDKESTEKYK
DRKDRASVDSTQDKKNKQKLPEKAEKKHAAEDKAKSKHKEKSDKEHSKERKSSRSADAEKSLLEKLEEEALHEYREDSNDKISEVSS
DSFTDRGQEPGLTAFLEVSFTEPPGDDKPRESACLPEKLKEKERHRHSSSSSKKSHDRERAKKEKAEKKEKGEDYKEGGSRKDSGQY
EKDFLEADAYGVSYNMKADIEDELDKTIELFSTEKKDKNDSEREPSKKIEKELKPYGSSAINILKEKKKREKHREKWRDEKERHRDR
HADGLLRHHRDELLRHHRDEQKPATRDKDSPPRVLKDKSRDEGPRLGDAKLKEKFKDGAEKEKGDPVKMSNGNDKVAPSKDPGKKDA
RPREKLLGDGDLMMTSFERMLSQKDLEIEERHKRHKERMKQMEKLRHRSGDPKLKEKAKPADDGRKKGLDIPAKKPPGLDPPFKDKK
LKESTPIPPAAENKLHPASGADSKDWLAGPHMKEVLPASPRPDQSRPTGVPTPTSVLSCPSYEEVMHTPRTPSCSADDYADLVFDCA
DSQHSTPVPTAPTSACSPSFFDRFSVASSGLSENASQAPARPLSTNLYRSVSVDIRRTPEEEFSVGDKLFRQQSVPAASSYDSPMPP
SMEDRAPLPPVPAEKFACLSPGYYSPDYGLPSPKVDALHCPPAAVVTVTPSPEGVFSSLQAKPSPSPRAELLVPSLEGALPPDLDTS
EDQQATAAIIPPEPSYLEPLDEGPFSAVITEEPVEWAHPSEQALASSLIGGTSENPVSWPVGSDLLLKSPQRFPESPKRFCPADPLH
SAAPGPFSASEAPYPAPPASPAPYALPVAEPGLEDVKDGVDAVPAAISTSEAAPYAPPSGLESFFSNCKSLPEAPLDVAPEPACVAA
VAQVEALGPLENSFLDGSRGLSHLGQVEPVPWADAFAGPEDDLDLGPFSLPELPLQTKDAADGEAEPVEESLAPPEEMPPGAPGVIN
GGDVSTVVAEEPPALPPDQASTRLPAELEPEPSGEPKLDVALEAAVEAETVPEERARGDPDSSVEPAPVPPEQRPLGSGDQGAEAEG
PPAASLCAPDGPAPNTVAQAQAADGAGPEDDTEASRAAAPAEGPPGGIQPEAAEPKPTAEAPKAPRVEEIPQRMTRNRAQMLANQSK
QGPPPSEKECAPTPAPVTRAKARGSEDDDAQAQHPRKRRFQRSTQQLQQQLNTSTQQTREVIQQTLAAIVDAIKLDAIEPYHSDRAN
PYFEYLQIRKKIEEKRKILCCITPQAPQCYAEYVTYTGSYLLDGKPLSKLHIPVIAPPPSLAEPLKELFRQQEAVRGKLRLQHSIER
EKLIVSCEQEILRVHCRAARTIANQAVPFSACTMLLDSEVYNMPLESQGDENKSVRDRFNARQFISWLQDVDDKYDRMKTCLLMRQQ
HEAAALNAVQRMEWQLKVQELDPAGHKSLCVNEVPSFYVPMVDVNDDFVLLPA
>sp|A6NJU9|NPIL5_HUMAN
MVKLSIVLTPQFLSHDQGQLTKELQQHVKSVTCPCEYLRKVINTLADHHHRGTDFGGSPWLHVIIAFPTSYKVVITLWIVYLWVSLL
KTIFWSRNGHDGSTDVQQRAWRSNRRRQEGLRSICMHTKKRVSSFRGNKIGLKDVITLRRHVETKVRAKIRKRKVTTKINHHDKING
KRKTARKQKMFQRAQELRRRAEDYHKCKIPPSARKALCNWVRMAAAEHRHSSGLPYWPYLTAETLKNRMGHQPPPPTQQHSITDNSL
SLKTPPECVLTPLPPSADDNLKTPPECVLTPLPPSADDNLKTPPECLLTPLPPSADDNLKTPPECLLTPLPPSADDNLKTPPECLLT
PLPPSAPPSAPPSADDNLKTRAECLLHPLPPSADDNLKTPSERQLTPLPPSAPPSADDNIKTPAERLRGPLPPSADDNLKTPSERQL
TPLPPSAPPSADDNIKTPAERLRGPLPPSADDNLKTPSERQLTPLPPSAPPSADDNIKTPAERLRGPLPPSADDNLKTPSERQLTPL
PPSAPPSADDNIKTPAERLRGPLPPSADDNLKTPSERQLTPLPPSAPPSADDNIKTPAFHPQRMIISRHLPSVSSLPFHPQLHSQQM
IISRYLLSVCGFRFHHQPMIISRHLPSVSSLPFHPQLHPQQMIISRHLPSVCGGRFHPQRMIISRHLPSVSSLPFHPQLHPQQMIIS
RHLPSVCGGRFHPQRMIISRHLPSVSSLPFHPQLHPQQMIISRHLPSVCGERLRGPLPPSADDNLKTPSERQLTPLPPSAPPSADDN
IKTPAERLRRPLPPSADDNLKTPSERQLTPLPPSAPPSADDNIKTPAERLRGPLPPSADDNLKTPSERQLTPLPPSAPPSADDNIKT
PAERLRGPLPPSADDNLKTPSERQLTPLPPSAPPSADDNIKTPAERLRGPLPPSADDNLKTPSERQLTPLPPSAPPSADDNIKTPAE
RLRGPLPPSADDNLKTPSERQLTPLPPSAPPSADDNIKTPAERLRGPLPPSADDNLKTPPLATQEAEAEKPRKPKRQRAAEMEPPPE
PKRRRVGDVEPSRKPKRRRAADVEPSSPKPKRRRVGDVEPSRKPKRRRAADVEPSSPEPKRRRVGDVEPSRKPKRRRAADVEPSSPE
PKRRRLS
>sp|Q9H6Z9|EGLN3_HUMAN
MPLGHIMRLDLEKIALEYIVPCLHEVGFCYLDNFLGEVVGDCVLERVKQLHCTGALRDGQLAGPRAGVSKRHLRGDQITWIGGNEEG
CEAISFLLSLIDRLVLYCGSRLGKYYVKERSKAMVACYPGNGTGYVRHVDNPNGDGRCITCIYYLNKNWDAKLHGGILRIFPEGKSF
IADVEPIFDRLLFFWSDRRNPHEVQPSYATRYAMTVWYFDAEERAEAKKKFRNLTRKTESALTED
>sp|Q5TGS1|HES3_HUMAN

MEKKRRARINVSLEQLKSLLEKHYSHQIRKRKLEKADILELSVKYMRSLQNSLQGLWPVPRGAEQPSGFRSCLPGVSQLLRRGDEVG
SGLRCPLVPESAAGSTMDSAGLGQEAPALFRPCTPAVWAPAPAAGGPRSPPPLLLLPESLPGSSASVPPPQPASSRCAESPGLGLRV
WRPWGSPGDDLN
>sp|P84103|SRSF3_HUMAN
MHRDSCPLDCKVYVGNLGNNGNKTELERAFGYYGPLRSVWVARNPPGFAFVEFEDPRDAADAVRELDGRTLCGCRVRVELSNGEKRS
RNRGPPPSWGRRPRDDYRRRSPPPRRRSPRRRSFSRSRSRSLSRDRRRERSLSRERNHKPSRSFSRSRSRSRSNERK
>sp|Q01581|HMCS1_HUMAN
MPGSLPLNAEACWPKDVGIVALEIYFPSQYVDQAELEKYDGVDAGKYTIGLGQAKMGFCTDREDINSLCMTVVQNLMERNNLSYDCI
GRLEVGTETIIDKSKSVKTNLMQLFEESGNTDIEGIDTTNACYGGTAAVFNAVNWIESSSWDGRYALVVAGDIAVYATGNARPTGGV
GAVALLIGPNAPLIFERGLRGTHMQHAYDFYKPDMLSEYPIVDGKLSIQCYLSALDRCYSVYCKKIHAQWQKEGNDKDFTLNDFGFM
IFHSPYCKLVQKSLARMLLNDFLNDQNRDKNSIYSGLEAFGDVKLEDTYFDRDVEKAFMKASSELFSQKTKASLLVSNQNGNMYTSS
VYGSLASVLAQYSPQQLAGKRIGVFSYGSGLAATLYSLKVTQDATPGSALDKITASLCDLKSRLDSRTGVAPDVFAENMKLREDTHH
LVNYIPQGSIDSLFEGTWYLVRVDEKHRRTYARRPTPNDDTLDEGVGLVHSNIATEHIPSPAKKVPRLPATAAEPEAAVISNGEH
>sp|Q8NCN5|PDPR_HUMAN
MMFYRLLSIVGRQRASPGWQNWSSARNSTSAAEARSMALPTQAQVVICGGGITGTSVAYHLSKMGWKDIVLLEQGRLAAGSTRFCAG
ILSTARHLTIEQKMADYSNKLYYQLEQETGIQTGYTRTGSIFLAQTQDRLISLKRINAGLNVIGIPSEIISPKKVAELHHLLNVHDL
VGAMHVPEDAVVSSADVALALASAASQNGVQIYDRTSVLHVMVKKGQVTGVETDKGQIECQYFVNCAGQWAYELGLSNEEPVSIPLH
ACEHFYLLTRPLETPLQSSTPTIVDADGRIYIRNWQGGILSGGFEKNPKPIFTEGKNQLEIQNLQEDWDHFEPLLSSLLRRMPELET
LEIMKLVNCPETFTPDMRCIMGESPAVQGYFVLAGMNSAGLSFGGGAGKYLAEWMVHGYPSENVWELDLKRFGALQSSRTFLRHRVM
EVMPLMYDLKVPRWDFQTGRQLRTSPLYDRLDAQGARWMEKHGFERPKYFVPPDKDLLALEQSKTFYKPDWFDIVESEVKCCKEAVC
VIDMSSFTKFEITSTGDQALEVLQYLFSNDLDVPVGHIVHTGMLNEGGGYENDCSIARLNKRSFFMISPTDQQVHCWAWLKKHMPKD
SNLLLEDVTWKYTALNLIGPRAVDVLSELSYAPMTPDHFPSLFCKEMSVGYANGIRVMSMTHTGEPGFMLYIPIEYALHVYNEVMSV
GQKYGIRNAGYYALRSLRIEKFFAFWGQDINNLTTPLECGRESRVKLEKGMDFIGRDALLQQKQNGVYKRLTMFILDDHDSDLDLWP
WWGEPIYRNGQYVGKTTSSAYSYSLERHVCLGFVHNFSEDTGEEQVVTADFINRGEYEIDIAGYRFQAKAKLYPVASLFTQKRRKDD
MELSDLHGK
>sp|A6NC86|PINLY_HUMAN
MRLSRRPETFLLAFVLLCTLLGLGCPLHCEICTAAGSRCHGQMKTCSSDKDTCVLLVGKATSKGKELVHTYKGCIRSQDCYSGVIST
TMGPKDHMVTSSFCCQSDGCNSAFLSVPLTNLTENGLMCPACTASFRDKCMGPMTHCTGKENHCVSLSGHVQAGIFKPRFAMRGCAT
ESMCFTKPGAEVPTGTNVLFLHHIECTHSP
>sp|P20849|CO9A1_HUMAN
MKTCWKIPVFFFVCSFLEPWASAAVKRRPRFPVNSNSNGGNELCPKIRIGQDDLPGFDLISQFQVDKAASRRAIQRVVGSATLQVAY
KLGNNVDFRIPTRNLYPSGLPEEYSFLTTFRMTGSTLKKNWNIWQIQDSSGKEQVGIKINGQTQSVVFSYKGLDGSLQTAAFSNLSS
LFDSQWHKIMIGVERSSATLFVDCNRIESLPIKPRGPIDIDGFAVLGKLADNPQVSVPFELQWMLIHCDPLRPRRETCHELPARITP
SQTTDERGPPGEQGPPGPPGPPGVPGIDGIDGDRGPKGPPGPPGPAGEPGKPGAPGKPGTPGADGLTGPDGSPGSIGSKGQKGEPGV
PGSRGFPGRGIPGPPGPPGTAGLPGELGRVGPVGDPGRRGPPGPPGPPGPRGTIGFHDGDPLCPNACPPGRSGYPGLPGMRGHKGAK
GEIGEPGRQGHKGEEGDQGELGEVGAQGPPGAQGLRGITGIVGDKGEKGARGLDGEPGPQGLPGAPGDQGQRGPPGEAGPKGDRGAE
GARGIPGLPGPKGDTGLPGVDGRDGIPGMPGTKGEPGKPGPPGDAGLQGLPGVPGIPGAKGVAGEKGSTGAPGKPGQMGNSGKPGQQ
GPPGEVGPRGPQGLPGSRGELGPVGSPGLPGKLGSLGSPGLPGLPGPPGLPGMKGDRGVVGEPGPKGEQGASGEEGEAGERGELGDI
GLPGPKGSAGNPGEPGLRGPEGSRGLPGVEGPRGPPGPRGVQGEQGATGLPGVQGPPGRAPTDQHIKQVCMRVIQEHFAEMAASLKR
PDSGATGLPGRPGPPGPPGPPGENGFPGQMGIRGLPGIKGPPGALGLRGPKGDLGEKGERGPPGRGPNGLPGAIGLPGDPGPASYGR
NGRDGERGPPGVAGIPGVPGPPGPPGLPGFCEPASCTMQAGQRAFNKGPDP
>sp|P55055|NR1H2_HUMAN
MSSPTTSSLDTPLPGNGPPQPGAPSSSPTVKEEGPEPWPGGPDPDVPGTDEASSACSTDWVIPDPEEEPERKRKKGPAPKMLGHELC
RVCGDKASGFHYNVLSCEGCKGFFRRSVVRGGARRYACRGGGTCQMDAFMRRKCQQCRLRKCKEAGMREQCVLSEEQIRKKKIRKQQ

QESQSQSQSPVGPQGSSSSASGPGASPGGSEAGSQGSGEGEGVQLTAAQELMIQQLVAAQLQCNKRSFSDQPKVTPWPLGADPQSRD
ARQQRFAHFTELAIISVQEIVDFAKQVPGFLQLGREDQIALLKASTIEIMLLETARRYNHETECITFLKDFTYSKDDFHRAGLQVEF
INPIFEFSRAMRRLGLDDAEYALLIAINIFSADRPNVQEPGRVEALQQPYVEALLSYTRIKRPQDQLRFPRMLMKLVSLRTLSSVHS
EQVFALRLQDKKLPPLLSEIWDVHE
>sp|Q8TDY3|ACTT2_HUMAN
MFNPHALDSPAVIFDNGSGFCKAGLSGEFGPRHMVSSIVGHLKFQAPSAEANQKKYFVGEEALYKQEALQLHSPFERGLITGWDDVE
RLWKHLFEWELGVKPSDQPLLATEPSLNPRENREKMAEVMFENFGVPAFYLSDQAVLALYASACVTGLVVDSGDAVTCTVPIFEGYS
LPHAVTKLHVAGRDITELLMQLLLASGHTFPCQLDKGLVDDIKKKLCYVALEPEKELSRRPEEVLREYKLPDGNIISLGDPLHQAPE
ALFVPQQLGSQSPGLSNMVSSSITKCDTDIQKILFGEIVLSGGTTLFHGLDDRLLKELEQLASKDTPIKITAPPDRWFSTWIGASIV
TSLSSFKQMWVTAADFKEFGTSVVQRRCF
>sp|P63136|POK25_HUMAN
NKSKKRRNRVSFLGAATVEPPKPIPLTWKTEKPVWVNQWPLPKQKLEALHLLANEQLEKGHIEPSFSPWNSPVFVIQKKSGKWRMLT
DLRAVNAVIQPMGPLQPGLPSPAMIPKDWPLIIIDLKDCFFTIPLAEQDCEKFAFTIPAINNKEPATRFQWKVLPQGMLNSPTICQT
FVGRALQPVREKFSDCYIIHYIDDILCAAETKDKLIDCYTFLQAEVANAGLAIASDKIQTSTPFHYLGMQIENRKIKPQKIEIRKDT
LKALNDFQKLLGDINWIRPTLGIPTYAMSNLFSILRGDSDLNSKRMLTPEATKEIKLVEEKIQSAQINRIDPLAPLQLLIFATAHSP
TGIIIQNTDLVEWSFLPHSTVKTFTLYLDQIATLIGQTRLRIIKLCGNDPDKIVVPLTKEQVRQAFINSGAWQIGLANFVGIIDNHY
PKTKIFQFLKLTTWILPKITRREPLENALTVFTDGSSNGKAAYTGPKERVIKTPYQSAQRAELVAVITVLQDFDINIISDSAYVVQA
TRDVETALIKYSMDDQLNQLFNLLQQTVRKRNFPFYITHIRAHTNLPGPLTKANKQADLLVSSALIKAQELHALTHVNAAGLKNKFD
VTWKLAKDIVQHCTQCQVLHLPTQEAGVNPRGLCPNALWQMDVTHVPSFGRLSYVHVTVDTYSHFIWATCHTGESTSHVKKHLLSCF
AVMGVPEKIKTDNGPGYCSKAFQKFLSQWKISHTTGIPYNSQGQAIVERTNRTLKTQLVKQKEGGDSKECTTPQMQLNLALYTLNFL
NIYRNQTTTSAEQHLTGKKNSPHEGKLIWWKDNKNKTWEIGKVITWGRGFACVSPGENQLPVWIPTRHLKFYNEPIRDAKKSTSAET
ETPQSSTVDSQDEQNGDVRRTDEVAIHQEGRAANLGTTKEADAVSYKISREHKGDTNPREYAACSLDDCINGGKSPYACRSSCS
>sp|A6NCQ9|RN222_HUMAN
MSEGESKDSSGSECPVCYEKFRDLEGASRTLSCGHVFCHDCLVKYLLSTRVDGQVQRTLVCPICRYVTFLSKKSSRWPSMLDKSSQT
LAVPVGLPSVPPLDSLGHTNPLAASSPAWRPPPGQARPPGSPGQSAQLPLDLLPSLPRESQIFVISRHGMPLGEQDSVLPRRSLAEL
SEASLAPRSARAFCCRSRALLLITLIAVVAVVAAILPWVLLVRKQA
>sp|Q9NPR9|GP108_HUMAN
MAVSERRGLGRGSPAEWGQRLLLVLLLGGCSGRIHQLALTGEKRADIQLNSFGFYTNGSLEVELSVLRLGLREAEEKSLLVGFSLSR
VRSGRVRSYSTRDFQDCPLQKNSSSFLVLFLINTKDLQVQVRKYGEQKTLFIFPGLLPEAPSKPGLPKPQATVPRKVDGGGTSAASK
PKSTPAVIQGPSGKDKDLVLGLSHLNNSYNFSFHVVIGSQAEEGQYSLNFHNCNNSVPGKEHPFDITVMIREKNPDGFLSAAEMPLF
KLYMVMSACFLAAGIFWVSILCRNTYSVFKIHWLMAALAFTKSISLLFHSINYYFINSQGHPIEGLAVMYYIAHLLKGALLFITIAL
IGSGWAFIKYVLSDKEKKVFGIVIPMQVLANVAYIIIESREEGASDYVLWKEILFLVDLICCGAILFPVVWSIRHLQDASGTDGKVA
VNLAKLKLFRHYYVMVICYVYFTRIIAILLQVAVPFQWQWLYQLLVEGSTLAFFVLTGYKFQPTGNNPYLQLPQEDEEDVQMEQVMT
DSGFREGLSKVNKTASGRELL
>sp|Q9HB63|NET4_HUMAN
MGSCARLLLLWGCTVVAAGLSGVAGVSSRCEKACNPRMGNLALGRKLWADTTCGQNATELYCFYSENTDLTCRQPKCDKCNAAYPHL
AHLPSAMADSSFRFPRTWWQSAEDVHREKIQLDLEAEFYFTHLIVMFKSPRPAAMVLDRSQDFGKTWKPYKYFATNCSATFGLEDDV
VKKGAICTSKYSSPFPCTGGEVIFKALSPPYDTENPYSAKVQEQLKITNLRVQLLKRQSCPCQRNDLNEEPQHFTHYAIYDFIVKGS
CFCNGHADQCIPVHGFRPVKAPGTFHMVHGKCMCKHNTAGSHCQHCAPLYNDRPWEAADGKTGAPNECRTCKCNGHADTCHFDVNVW
EASGNRSGGVCDDCQHNTEGQYCQRCKPGFYRDLRRPFSAPDACKPCSCHPVGSAVLPANSVTFCDPSNGDCPCKPGVAGRRCDRCM
VGYWGFGDYGCRPCDCAGSCDPITGDCISSHTDIDWYHEVPDFRPVHNKSEPAWEWEDAQGFSALLHSGKCECKEQTLGNAKAFCGM
KYSYVLKIKILSAHDKGTHVEVNVKIKKVLKSTKLKIFRGKRTLYPESWTDRGCTCPILNPGLEYLVAGHEDIRTGKLIVNMKSFVQ
HWKPSLGRKVMDILKRECK
>sp|Q96N53|CK037_HUMAN

MTEGLFISCSAVRVKPNRRAGLRRRSPAFLLSANQKTRLFALGSSPRCGPRANGEEASSCAWVSRAPRAACARAKPASRAPEGPVSR
KTRGGEAALASARPATDCLRSGLAVERRRKPNSRPAPGVGSLPGSRPQDPQGAAGRRLSP
>sp|Q6NXE6|ARMC6_HUMAN
MSERCCSRYSSGASIGCTPTSTQAKMVSKRIAQETFDAAVRENIEEFAMGPEEAVKEAVEQFESQGVDLSNIVKTAPKVSADGSQEP
THDILQMLSDLQESVASSRPQEVSAYLTRFCDQCKQDKACRFLAAQKGAYPIIFTAWKLATAGDQGLLLQSLNALSVLTDGQPDLLD
AQGLQLLVATLTQNADEADLTCSGIRCVRHACLKHEQNRQDLVKAGVLPLLTGAITHHGHHTDVVREACWALRVMTFDDDIRVPFGH
AHNHAKMIVQENKGLKVLIEATKAFLDNPGILSELCGTLSRLAIRNEFCQEVVDLGGLSILVSLLADCNDHQMRDQSGVQELVKQVL
STLRAIAGNDDVKDAIVRAGGTESIVAAMTQHLTSPQVCEQSCAALCFLALRKPDNSRIIVEGGGAVAALQAMKAHPQKAGVQKQAC
MLIRNLVAHGQAFSKPILDLGAEALIMQARSAHRDCEDVAKAALRDLGCHVELRELWTGQRGNLAP
>sp|O00591|GBRP_HUMAN
MNYSLHLAFVCLSLFTERMCIQGSQFNVEVGRSDKLSLPGFENLTAGYNKFLRPNFGGEPVQIALTLDIASISSISESNMDYTATIY
LRQRWMDQRLVFEGNKSFTLDARLVEFLWVPDTYIVESKKSFLHEVTVGNRLIRLFSNGTVLYALRITTTVACNMDLSKYPMDTQTC
KLQLESWGYDGNDVEFTWLRGNDSVRGLEHLRLAQYTIERYFTLVTRSQQETGNYTRLVLQFELRRNVLYFILETYVPSTFLVVLSW
VSFWISLDSVPARTCIGVTTVLSMTTLMIGSRTSLPNTNCFIKAIDVYLGICFSFVFGALLEYAVAHYSSLQQMAAKDRGTTKEVEE
VSITNIINSSISSFKRKISFASIEISSDNVDYSDLTMKTSDKFKFVFREKMGRIVDYFTIQNPSNVDHYSKLLFPLIFMLANVFYWA
YYMYF
>sp|Q8N3R9|MPP5_HUMAN
MTTSHMNGHVTEESDSEVKNVDLASPEEHQKHREMAVDCPGDLGTRMMPIRRSAQLERIRQQQEDMRRRREEEGKKQELDLNSSMRL
KKLAQIPPKTGIDNPMFDTEEGIVLESPHYAVKILEIEDLFSSLKHIQHTLVDSQSQEDISLLLQLVQNKDFQNAFKIHNAITVHMN
KASPPFPLISNAQDLAQEVQTVLKPVHHKEGQELTALLNTPHIQALLLAHDKVAEQEMQLEPITDERVYESIGQYGGETVKIVRIEK
ARDIPLGATVRNEMDSVIISRIVKGGAAEKSGLLHEGDEVLEINGIEIRGKDVNEVFDLLSDMHGTLTFVLIPSQQIKPPPAKETVI
HVKAHFDYDPSDDPYVPCRELGLSFQKGDILHVISQEDPNWWQAYREGDEDNQPLAGLVPGKSFQQQREAMKQTIEEDKEPEKSGKL
WCAKKNKKKRKKVLYNANKNDDYDNEEILTYEEMSLYHQPANRKRPIILIGPQNCGQNELRQRLMNKEKDRFASAVPHTTRSRRDQE
VAGRDYHFVSRQAFEADIAAGKFIEHGEFEKNLYGTSIDSVRQVINSGKICLLSLRTQSLKTLRNSDLKPYIIFIAPPSQERLRALL
AKEGKNPKPEELREIIEKTREMEQNNGHYFDTAIVNSDLDKAYQELLRLINKLDTEPQWVPSTWLR
>sp|P37088|SCNNA_HUMAN
MEGNKLEEQDSSPPQSTPGLMKGNKREEQGLGPEPAAPQQPTAEEEALIEFHRSYRELFEFFCNNTTIHGAIRLVCSQHNRMKTAFW
AVLWLCTFGMMYWQFGLLFGEYFSYPVSLNINLNSDKLVFPAVTICTLNPYRYPEIKEELEELDRITEQTLFDLYKYSSFTTLVAGS
RSRRDLRGTLPHPLQRLRVPPPPHGARRARSVASSLRDNNPQVDWKDWKIGFQLCNQNKSDCFYQTYSSGVDAVREWYRFHYINILS
RLPETLPSLEEDTLGNFIFACRFNQVSCNQANYSHFHHPMYGNCYTFNDKNNSNLWMSSMPGINNGLSLMLRAEQNDFIPLLSTVTG
ARVMVHGQDEPAFMDDGGFNLRPGVETSISMRKETLDRLGGDYGDCTKNGSDVPVENLYPSKYTQQVCIHSCFQESMIKECGCAYIF
YPRPQNVEYCDYRKHSSWGYCYYKLQVDFSSDHLGCFTKCRKPCSVTSYQLSAGYSRWPSVTSQEWVFQMLSRQNNYTVNNKRNGVA
KVNIFFKELNYKTNSESPSVTMVTLLSNLGSQWSLWFGSSVLSVVEMAELVFDLLVIMFLMLLRRFRSRYWSPGRGGRGAQEVASTL
ASSPPSHFCPHPMSLSLSQPGPAPSPALTAPPPAYATLGPRPSPGGSAGASSSTCPLGGP
>sp|Q99973|TEP1_HUMAN
MEKLHGHVSAHPDILSLENRCLAMLPDLQPLEKLHQHVSTHSDILSLKNQCLATLPDLKTMEKPHGYVSAHPDILSLENQCLATLSD
LKTMEKPHGHVSAHPDILSLENRCLATLSSLKSTVSASPLFQSLQISHMTQADLYRVNNSNCLLSEPPSWRAQHFSKGLDLSTCPIA
LKSISATETAQEATLGRWFDSEEKKGAETQMPSYSLSLGEEEEVEDLAVKLTSGDSESHPEPTDHVLQEKKMALLSLLCSTLVSEVN
MNNTSDPTLAAIFEICRELALLEPEFILKASLYARQQLNVRNVANNILAIAAFLPACRPHLRRYFCAIVQLPSDWIQVAELYQSLAE
GDKNKLVPLPACLRTAMTDKFAQFDEYQLAKYNPRKHRAKRHPRRPPRSPGMEPPFSHRCFPRYIGFLREEQRKFEKAGDTVSEKKN
PPRFTLKKLVQRLHIHKPAQHVQALLGYRYPSNLQLFSRSRLPGPWDSSRAGKRMKLSRPETWERELSLRGNKASVWEELIENGKLP
FMAMLRNLCNLLRVGISSRHHELILQRLQHAKSVIHSRQFPFRFLNAHDAIDALEAQLRNQALPFPSNITLMRRILTRNEKNRPRRR
FLCHLSRQQLRMAMRIPVLYEQLKREKLRVHKARQWKYDGEMLNRYRQALETAVNLSVKHSLPLLPGRTVLVYLTDANADRLCPKSN
PQGPPLNYALLLIGMMITRAEQVDVVLCGGDTLKTAVLKAEEGILKTAIKLQAQVQEFDENDGWSLNTFGKYLLSLAGQRVPVDRVI

LLGQSMDDGMINVAKQLYWQRVNSKCLFVGILLRRVQYLSTDLNPNDVTLSGCTDAILKFIAEHGASHLLEHVGQMDKIFKIPPPPG
KTGVQSLRPLEEDTPSPLAPVSQQGWRSIRLFISSTFRDMHGERDLLLRSVLPALQARAAPHRISLHGIDLRWGVTEEETRRNRQLE
VCLGEVENAQLFVGILGSRYGYIPPSYNLPDHPHFHWAQQYPSGRSVTEMEVMQFLNRNQRLQPSAQALIYFRDSSFLSSVPDAWKS
DFVSESEEAARRISELKSYLSRQKGITCRRYPCEWGGVAAGRPYVGGLEEFGQLVLQDVWNMIQKLYLQPGALLEQPVSIPDDDLVQ
ATFQQLQKPPSPARPRLLQDTVQRLMLPHGRLSLVTGQSGQGKTAFLASLVSALQAPDGAKVASLVFFHFSGARPDQGLALTLLRRL
CTYLRGQLKEPGALPSTYRSLVWELQQRLLPKSAESLHPGQTQVLIIDGADRLVDQNGQLISDWIPKKLPRCVHLVLSVSSDAGLGE
TLEQSQGAHVLALGPLEASARARLVREELALYGKRLEESPFNNQMRLLLVKRESGRPLYLRLVTDHLRLFTLYEQVSERLRTLPATV
PLLLQHILSTLEKEHGPDVLPQALTALEVTRSGLTVDQLHGVLSVWRTLPKGTKSWEEAVAAGNSGDPYPMGPFACLVQSLRSLLGE
GPLERPGARLCLPDGPLRTAAKRCYGKRPGLEDTAHILIAAQLWKTCDADASGTFRSCPPEALGDLPYHLLQSGNRGLLSKFLTNLH
VVAAHLELGLVSRLLEAHALYASSVPKEEQKLPEADVAVFRTFLRQQASILSQYPRLLPQQAANQPLDSPLCHQASLLSRRWHLQHT
LRWLNKPRTMKNQQSSSLSLAVSSSPTAVAFSTNGQRAAVGTANGTVYLLDLRTWQEEKSVVSGCDGISACLFLSDDTLFLTAFDGL
LELWDLQHGCRVLQTKAHQYQITGCCLSPDCRLLATVCLGGCLKLWDTVRGQLAFQHTYPKSLNCVAFHPEGQVIATGSWAGSISFF
QVDGLKVTKDLGAPGASIRTLAFNVPGGVVAVGRLDSMVELWAWREGARLAAFPAHHGFVAAALFLHAGCQLLTAGEDGKVQVWSGS
LGRPRGHLGSLSLSPALSVALSPDGDRVAVGYRADGIRIYKISSGSQGAQGQALDVAVSALAWLSPKVLVSGAEDGSLQGWALKECS
LQSLWLLSRFQKPVLGLATSQELLASASEDFTVQLWPRQLLTRPHKAEDFPCGTELRGHEGPVSCCSFSTDGGSLATGGRDRSLLCW
DVRTPKTPVLIHSFPACHRDWVTGCAWTKDNLLISCSSDGSVGLWDPESGQRLGQFLGHQSAVSAVAAVEEHVVSVSRDGTLKVWDH
QGVELTSIPAHSGPISHCAAAMEPRAAGQPGSELLVVTVGLDGATRLWHPLLVCQTHTLLGHSGPVRAAAVSETSGLMLTASEDGSV
RLWQVPKEADDTCIPRSSAAVTAVAWAPDGSMAVSGNQAGELILWQEAKAVATAQAPGHIGALIWSSAHTFFVLSADEKISEWQVKL
RKGSAPGNLSLHLNRILQEDLGVLTSLDWAPDGHFLILAKADLKLLCMKPGDAPSEIWSSYTENPMILSTHKEYGIFVLQPKDPGVL
SFLRQKESGEFEERLNFDINLENPSRTLISITQAKPESESSFLCASSDGILWNLAKCSPEGEWTTGNMWQKKANTPETQTPGTDPST
CRESDASMDSDASMDSEPTPHLKTRQRRKIHSGSVTALHVLPELLVTASKDRDVKLWERPSMQLLGLFRCEGSVSCLEPWLGANSTL
QLAVGDVQGNVYFLNWE
>sp|Q9H9B4|SFXN1_HUMAN
MSGELPPNINIKEPRWDQSTFIGRANHFFTVTDPRNILLTNEQLESARKIVHDYRQGIVPPGLTENELWRAKYIYDSAFHPDTGEKM
ILIGRMSAQVPMNMTITGCMMTFYRTTPAVLFWQWINQSFNAVVNYTNRSGDAPLTVNELGTAYVSATTGAVATALGLNALTKHVSP
LIGRFVPFAAVAAANCINIPLMRQRELKVGIPVTDENGNRLGESANAAKQAITQVVVSRILMAAPGMAIPPFIMNTLEKKAFLKRFP
WMSAPIQVGLVGFCLVFATPLCCALFPQKSSMSVTSLEAELQAKIQESHPELRRVYFNKGL
>sp|Q9UBN1|CCG4_HUMAN
MVRCDRGLQMLLTTAGAFAAFSLMAIAIGTDYWLYSSAHICNGTNLTMDDGPPPRRARGDLTHSGLWRVCCIEGIYKGHCFRINHFP
EDNDYDHDSSEYLLRIVRASSVFPILSTILLLLGGLCIGAGRIYSRKNNIVLSAGILFVAAGLSNIIGIIVYISSNTGDPSDKRDED
KKNHYNYGWSFYFGALSFIVAETVGVLAVNIYIEKNKELRFKTKREFLKASSSSPYARMPSYRYRRRRSRSSSRSTEASPSRDVSPM
GLKITGAIPMGELSMYTLSREPLKVTTAASYSPDQEASFLQVHDFFQQDLKEGFHVSMLNRRTTPV
>sp|Q13501|SQSTM_HUMAN
MASLTVKAYLLGKEDAAREIRRFSFCCSPEPEAEAEAAAGPGPCERLLSRVAALFPALRPGGFQAHYRDEDGDLVAFSSDEELTMAM
SYVKDDIFRIYIKEKKECRRDHRPPCAQEAPRNMVHPNVICDGCNGPVVGTRYKCSVCPDYDLCSVCEGKGLHRGHTKLAFPSPFGH
LSEGFSHSRWLRKVKHGHFGWPGWEMGPPGNWSPRPPRAGEARPGPTAESASGPSEDPSVNFLKNVGESVAAALSPLGIEVDIDVEH
GGKRSRLTPVSPESSSTEEKSSSQPSSCCSDPSKPGGNVEGATQSLAEQMRKIALESEGRPEEQMESDNCSGGDDDWTHLSSKEVDP
STGELQSLQMPESEGPSSLDPSQEGPTGLKEAALYPHLPPEADPRLIESLSQMLSMGFSDEGGWLTRLLQTKNYDIGAALDTIQYSK
HPPPL
>sp|Q96MS0|ROBO3_HUMAN
MLRYLLKTLLQMNLFADSLAGDISNSSELLLGFNSSLAALNHTLLPPGDPSLNGSRVGPEDAMPRIVEQPPDLLVSRGEPATLPCRA
EGRPRPNIEWYKNGARVATVREDPRAHRLLLPSGALFFPRIVHGRRARPDEGVYTCVARNYLGAAASRNASLEVAVLRDDFRQSPGN
VVVAVGEPAVLECVPPRGHPEPSVSWRKDGARLKEEEGRITIRGGKLMMSHTLKSDAGMYVCVASNMAGERESAAAEVMVLERPSFL
RRPVNQVVLADAPVTFLCEVKGDPPPRLRWRKEDGELPTGRYEIRSDHSLWIGHVSAEDEGTYTCVAENSVGRAEASGSLSVHVPPQ

LVTQPQDQMAAPGESVAFQCETKGNPPPAIFWQKEGSQVLLFPSQSLQPTGRFSVSPRGQLNITAVQRGDAGYYVCQAVSVAGSILA
KALLEIKGASLDGLPPVILQGPANQTLVLGSSVWLPCRVTGNPQPSVRWKKDGQWLQGDDLQFKTMANGTLYIANVQEMDMGFYSCV
AKSSTGEATWSGWLKMREDWGVSPDPPTEPSSPPGAPSQPVVTEITKNSITLTWKPNPQTGAAVTSYVIEAFSPAAGNTWRTVADGV
QLETHTVSGLQPNTIYLFLVRAVGAWGLSEPSPVSEPVRTQDSSPSRPVEDPWRGQQGLAEVAVRLQEPIVLGPRTLQVSWTVDGPV
QLVQGFRVSWRVAGPEGGSWTMLDLQSPSQQSTVLRGLPPGTQIQIKVQAQGQEGLGAESLSVTRSIPEEAPSGPPQGVAVALGGDG
NSSITVSWEPPLPSQQNGVITEYQIWCLGNESRFHLNRSAAGWARSAMLRGLVPGLLYRTLVAAATSAGVGVPSAPVLVQLPSPPDL
EPGLEVGAGLAVRLARVLREPAFLAGSGAACGALLLGLCAALYWRRKQRKELSHYTASFAYTPAVSFPHSEGLSGASSRPPMGLGPA
PYSWLADSWPHPSRSPSAQEPRGSCCPSNPDPDDRYYNEAGISLYLAQTARGTAAPGEGPVYSTIDPAGEELQTFHGGFPQHPSGDL
GPWSQYAPPEWSQGDSGAKGGKVKLLGKPVQMPSLNWPEALPPPPPSCELSCLEGPEEELEGSSEPEEWCPPMPERSHLTEPSSSGG
CLVTPSRRETPSPTPSYGQQSTATLTPSPPDPPQPPTDMPHLHQMPRRVPLGPSSPLSVSQPMLGIREARPAGLGAGPAASPHLSPS
PAPSTASSAPGRTWQGNGEMTPPLQGPRARFRKKPKALPYRRENSPGDLPPPPLPPPEEEASWALELRAAGSMSSLERERSGERKAV
QAVPLAAQRVLHPDEEAWLPYSRPSFLSRGQGTSTCSTAGSNSSRGSSSSRGSRGPGRSRSRSQSRSQSQRPGQKRREEPR
>sp|Q8WXS8|ATS14_HUMAN
MAPLRALLSYLLPLHCALCAAAGSRTPELHLSGKLSDYGVTVPCSTDFRGRFLSHVVSGPAAASAGSMVVDTPPTLPRHSSHLRVAR
SPLHPGGTLWPGRVGRHSLYFNVTVFGKELHLRLRPNRRLVVPGSSVEWQEDFRELFRQPLRQECVYTGGVTGMPGAAVAISNCDGL
AGLIRTDSTDFFIEPLERGQQEKEASGRTHVVYRREAVQQEWAEPDGDLHNEAFGLGDLPNLLGLVGDQLGDTERKRRHAKPGSYSI
EVLLVVDDSVVRFHGKEHVQNYVLTLMNIVDEIYHDESLGVHINIALVRLIMVGYRQSLSLIERGNPSRSLEQVCRWAHSQQRQDPS
HAEHHDHVVFLTRQDFGPSGYAPVTGMCHPLRSCALNHEDGFSSAFVIAHETGHVLGMEHDGQGNGCADETSLGSVMAPLVQAAFHR
FHWSRCSKLELSRYLPSYDCLLDDPFDPAWPQPPELPGINYSMDEQCRFDFGSGYQTCLAFRTFEPCKQLWCSHPDNPYFCKTKKGP
PLDGTECAPGKWCFKGHCIWKSPEQTYGQDGGWSSWTKFGSCSRSCGGGVRSRSRSCNNPSPAYGGRLCLGPMFEYQVCNSEECPGT
YEDFRAQQCAKRNSYYVHQNAKHSWVPYEPDDDAQKCELICQSADTGDVVFMNQVVHDGTRCSYRDPYSVCARGECVPVGCDKEVGS
MKADDKCGVCGGDNSHCRTVKGTLGKASKQAGALKLVQIPAGARHIQIEALEKSPHRIVVKNQVTGSFILNPKGKEATSRTFTAMGL
EWEDAVEDAKESLKTSGPLPEAIAILALPPTEGGPRSSLAYKYVIHEDLLPLIGSNNVLLEEMDTYEWALKSWAPCSKACGGGIQFT
KYGCRRRRDHHMVQRHLCDHKKRPKPIRRRCNQHPCSQPVWVTEEWGACSRSCGKLGVQTRGIQCLLPLSNGTHKVMPAKACAGDRP
EARRPCLRVPCPAQWRLGAWSQCSATCGEGIQQRQVVCRTNANSLGHCEGDRPDTVQVCSLPACGGNHQNSTVRADVWELGTPEGQW
VPQSEPLHPINKISSTEPCTGDRSVFCQMEVLDRYCSIPGYHRLCCVSCIKKASGPNPGPDPGPTSLPPFSTPGSPLPGPQDPADAA
EPPGKPTGSEDHQHGRATQLPGALDTSSPGTQHPFAPETPIPGASWSISPTTPGGLPWGWTQTPTPVPEDKGQPGEDLRHPGTSLPA
ASPVT
>sp|P40394|ADH7_HUMAN
MFAEIQIQDKDRMGTAGKVIKCKAAVLWEQKQPFSIEEIEVAPPKTKEVRIKILATGICRTDDHVIKGTMVSKFPVIVGHEATGIVE
SIGEGVTTVKPGDKVIPLFLPQCRECNACRNPDGNLCIRSDITGRGVLADGTTRFTCKGKPVHHFMNTSTFTEYTVVDESSVAKIDD
AAPPEKVCLIGCGFSTGYGAAVKTGKVKPGSTCVVFGLGGVGLSVIMGCKSAGASRIIGIDLNKDKFEKAMAVGATECISPKDSTKP
ISEVLSEMTGNNVGYTFEVIGHLETMIDALASCHMNYGTSVVVGVPPSAKMLTYDPMLLFTGRTWKGCVFGGLKSRDDVPKLVTEFL
AKKFDLDQLITHVLPFKKISEGFELLNSGQSIRTVLTF
>sp|Q9H160|ING2_HUMAN
MLGQQQQQLYSSAALLTGERSRLLTCYVQDYLECVESLPHDMQRNVSVLRELDNKYQETLKEIDDVYEKYKKEDDLNQKKRLQQLLQ
RALINSQELGDEKIQIVTQMLELVENRARQMELHSQCFQDPAESERASDKAKMDSSQPERSSRRPRRQRTSESRDLCHMANGIEDCD
DQPPKEKKSKSAKKKKRSKAKQEREASPVEFAIDPNEPTYCLCNQVSYGEMIGCDNEQCPIEWFHFSCVSLTYKPKGKWYCPKCRGD
NEKTMDKSTEKTKKDRRSR
>sp|Q68D06|SLN13_HUMAN
MEANHCSLGVYPSYPDLVIDVGEVTLGEENRKKLQKTQRDQERARVIRAACALLNSGGGVIQMEMANRDERPTEMGLDLEESLRKLI
QYPYLQAFFETKQHGRCFYIFVKSWSGDPFLKDGSFNSRICSLSSSLYCRSGTSVLHMNSRQAFDFLKTKERQSKYNLINEGSPPSK
IMKAVYQNISESNPAYEVFQTDTIEYGEILSFPESPSIEFKQFSTKHIQQYVENIIPEYISAFANTEGGYLFIGVDDKSRKVLGCAK
EQVDPDSLKNVIARAISKLPIVHFCSSKPRVEYSTKIVEVFCGKELYGYLCVIKVKAFCCVVFSEAPKSWMVREKYIRPLTTEEWVE

KMMDADPEFPPDFAEAFESQLSLSDSPSLCRPVYSKKGLEHKADLQQHLFPVPPGHLECTPESLWKELSLQHEGLKELIHKQMRPFS
QGIVILSRSWAVDLNLQEKPGVICDALLIAQNSTPILYTILREQDAEGQDYCTRTAFTLKQKLVNMGGYTGKVCVRAKVLCLSPESS
AEALEAAVSPMDYPASYSLAGTQHMEALLQSLVIVLLGFRSLLSDQLGCEVLNLLTAQQYEIFSRSLRKNRELFVHGLPGSGKTIMA
MKIMEKIRNVFHCEAHRILYVCENQPLRNFISDRNICRAETRETFLREKFEHIQHIVIDEAQNFRTEDGDWYRKAKTITQREKDCPG
VLWIFLDYFQTSHLGHSGLPPLSAQYPREELTRVVRNADEIAEYIQQEMQLIIENPPINIPHGYLAILSEAKWVPGVPGNTKIIKNF
TLEQIVTYVADTCRCFFERGYSPKDVAVLVSTVTEVEQYQSKLLKAMRKKMVVQLSDACDMLGVHIVLDSVRRFSGLERSIVFGIHP
RTADPAILPNILICLASRAKQHLYIFL
>sp|P30679|GNA15_HUMAN
MARSLTWRCCPWCLTEDEKAAARVDQEINRILLEQKKQDRGELKLLLLGPGESGKSTFIKQMRIIHGAGYSEEERKGFRPLVYQNIF
VSMRAMIEAMERLQIPFSRPESKHHASLVMSQDPYKVTTFEKRYAAAMQWLWRDAGIRAYYERRREFHLLDSAVYYLSHLERITEEG
YVPTAQDVLRSRMPTTGINEYCFSVQKTNLRIVDVGGQKSERKKWIHCFENVIALIYLASLSEYDQCLEENNQENRMKESLALFGTI
LELPWFKSTSVILFLNKTDILEEKIPTSHLATYFPSFQGPKQDAEAAKRFILDMYTRMYTGCVDGPEGSKKGARSRRLFSHYTCATD
TQNIRKVFKDVRDSVLARYLDEINLL
>sp|Q6P3V2|Z585A_HUMAN
MPANWTSPQKSSALAPEDHGSSYEGSVSFRDVAIDFSREEWRHLDPSQRNLYRDVMLETYSHLLSVGYQVPEAEVVMLEQGKEPWAL
QGERPRQSCPGEKLWDHNQCRKILSYKQVSSQPQKMYPGEKAYECAKFEKIFTQKSQLKVHLKVLAGEKLYVCIECGKAFVQKPEFI
IHQKTHMREKPFKCNECGKSFFQVSSLFRHQRIHTGEKLYECSQCGKGFSYNSDLSIHEKIHTGERHHECTDCGKAFTQKSTLKMHQ
KIHTGERSYICIECGQAFIQKTHLIAHRRIHTGEKPYECSNCGKSFISKSQLQVHQRVHTRVKPYICTEYGKVFSNNSNLVTHKKVQ
SREKSSICTECGKAFTYRSELIIHQRIHTGEKPYECSDCGKAFTQKSALTVHQRIHTGEKSYICMKCGLAFIQKAHLIAHQIIHTGE
KPHKCGHCGKLFTSKSQLHVHKRIHTGEKPYMCNKCGKAFTNRSNLITHQKTHTGEKSYICSKCGKAFTQRSDLITHQRIHTGEKPY
ECNTCGKAFTQKSHLNIHQKIHTGERQYECHECGKAFNQKSILIVHQKIHTGEKPYVCTECGRAFIRKSNFITHQRIHTGEKPYECS
DCGKSFTSKSQLLVHQPVHTGEKPYVCAECGKAFSGRSNLSKHQKTHTGEKPYICSECGKTFRQKSELITHHRIHTGEKPYECSDCG
KSFTKKSQLQVHQRIHTGEKPYVCAECGKAFTDRSNLNKHQTTHTGDKPYKCGICGKGFVQKSVFSVHQSSHA
>sp|Q9NX04|CA109_HUMAN
MTQDRPLLAVQEALKKCFPVVEEQQGLWQSALRDCQPLLSSLSNLAEQLQAAQNLRFEDVPALRAFPDLKERLRRKQLVAGDIVLDK
LGERLAILLKVRDMVSSHVERVFQIYEQHADTVGIDAVLQPSAVSPSVADMLEWLQDIERHYRKSYLKRKYLLSSIQWGDLANIQAL
PKAWDRISKDEHQDLVQDILLNVSFFLEE
>sp|Q9NYL4|FKB11_HUMAN
MTLRPSLLPLHLLLLLLLSAAVCRAEAGLETESPVRTLQVETLVEPPEPCAEPAAFGDTLHIHYTGSLVDGRIIDTSLTRDPLVIEL
GQKQVIPGLEQSLLDMCVGEKRRAIIPSHLAYGKRGFPPSVPADAVVQYDVELIALIRANYWLKLVKGILPLVGMAMVPALLGLIGY
HLYRKANRPKVSKKKLKEEKRNKSKKK
>sp|O43506|ADA20_HUMAN
MAVGEPLVHIRVTLLLLWFGMFLSISGHSQARPSQYFTSPEVVIPLKVISRGRGAKAPGWLSYSLRFGGQRYIVHMRVNKLLFAAHL
PVFTYTEQHALLQDQPFIQDDCYYHGYVEGVPESLVALSTCSGGFLGMLQINDLVYEIKPISVSATFEHLVYKIDSDDTQFPPMRCG
LTEEKIAHQMELQLSYNFTLKQSSFVGWWTHQRFVELVVVVDNIRYLFSQSNATTVQHEVFNVVNIVDSFYHPLEVDVILTGIDIWT
ASNPLPTSGDLDNVLEDFSIWKNYNLNNRLQHDVAHLFIKDTQGMKLGVAYVKGICQNPFNTGVDVFEDNRLVVFAITLGHELGHNL
GMQHDTQWCVCELQWCIMHAYRKVTTKFSNCSYAQYWDSTISSGLCIQPPPYPGNIFRLKYCGNLVVEEGEECDCGTIRQCAKDPCC
LLNCTLHPGAACAFGICCKDCKFLPSGTLCRQQVGECDLPEWCNGTSHQCPDDVYVQDGISCNVNAFCYEKTCNNHDIQCKEIFGQD
ARSASQSCYQEINTQGNRFGHCGIVGTTYVKCWTPDIMCGRVQCENVGVIPNLIEHSTVQQFHLNDTTCWGTDYHLGMAIPDIGEVK
DGTVCGPEKICIRKKCASMVHLSQACQPKTCNMRGICNNKQHCHCNHEWAPPYCKDKGYGGSADSGPPPKNNMEGLNVMGKLRYLSL
LCLLPLVAFLLFCLHVLFKKRTKSKEDEEG
>sp|Q96G75|RMD5B_HUMAN
MEQCACVERELDKVLQKFLTYGQHCERSLEELLHYVGQLRAELASAALQGTPLSATLSLVMSQCCRKIKDTVQKLASDHKDIHSSVS
RVGKAIDRNFDSEICGVVSDAVWDAREQQQQILQMAIVEHLYQQGMLSVAEELCQESTLNVDLDFKQPFLELNRILEALHEQDLGPA

LEWAVSHRQRLLELNSSLEFKLHRLHFIRLLAGGPAKQLEALSYARHFQPFARLHQREIQVMMGSLVYLRLGLEKSPYCHLLDSSHW
AEICETFTRDACSLLGLSVESPLSVSFASGCVALPVLMNIKAVIEQRQCTGVWNHKDELPIEIELGMKCWYHSVFACPILRQQTSDS
NPPIKLICGHVISRDALNKLINGGKLKCPYCPMEQNPADGKRIIF
>sp|O43502|RA51C_HUMAN
MRGKTFRFEMQRDLVSFPLSPAVRVKLVSAGFQTAEELLEVKPSELSKEVGISKAEALETLQIIRRECLTNKPRYAGTSESHKKCTA
LELLEQEHTQGFIITFCSALDDILGGGVPLMKTTEICGAPGVGKTQLCMQLAVDVQIPECFGGVAGEAVFIDTEGSFMVDRVVDLAT
ACIQHLQLIAEKHKGEEHRKALEDFTLDNILSHIYYFRCRDYTELLAQVYLLPDFLSEHSKVRLVIVDGIAFPFRHDLDDLSLRTRL
LNGLAQQMISLANNHRLAVILTNQMTTKIDRNQALLVPALGESWGHAATIRLIFHWDRKQRLATLYKSPSQKECTVLFQIKPQGFRD
TVVTSACSLQTEGSLSTRKRSRDPEEEL
>sp|P30485|1B47_HUMAN
MRVTAPRTLLLLLWGAVALTETWAGSHSMRYFYTAMSRPGRGEPRFITVGYVDDTLFVRFDSDATSPRKEPRAPWIEQEGPEYWDRE
TQISKTNTQTYREDLRTLLRYYNQSEAGSHTLQRMFGCDVGPDGRLLRGYHQDAYDGKDYIALNEDLSSWTAADTAAQITQRKWEAA
RVAEQLRAYLEGECVEWLRRYLENGKETLQRADPPKTHVTHHPISDHEATLRCWALGFYPAEITLTWQRDGEDQTQDTELVETRPAG
DRTFQKWAAVVVPSGEEQRYTCHVQHEGLPKPLTLRWEPSSQSTVPIVGIVAGLAVLAVVVIGAVVAAVVCRRKSSGGKGGSYSQAA
CSDSAQGSDVSLTA
>sp|Q6PI97|CK088_HUMAN
METGPSEEPSGRKESQEMCPPGLLVFAGSSEQDANLAKQFWISASMYPPSESQLVLRRDSSQRLPVARPRRSRGSENSHSSQSFHLA
SNKNRDIFAEALKIQESEEKVKYLQKAKTREEILQLLRKQREERISKELISLPYKPKAKEHKAKKVVSESDKEDQEEVKTLD
>sp|Q9UGK3|STAP2_HUMAN
MASALRPPRVPKPKGVLPSHYYESFLEKKGPCDRDYKKFWAGLQGLTIYFYNSNRDFQHVEKLNLGAFEKLTDEIPWGSSRDPGTHF
SLILRDQEIKFKVETLECREMWKGFILTVVELRVPTDLTLLPGHLYMMSEVLAKEEARRALETPSCFLKVSRLEAQLLLERYPECGN
LLLRPSGDGADGVSVTTRQMHNGTHVVRHYKVKREGPKYVIDVEQPFSCTSLDAVVNYFVSHTKKALVPFLLDEDYEKVLGYVEADK
ENGENVWVAPSAPGPGPAPCTGGPKPLSPASSQDKLPPLPPLPNQEENYVTPIGDGPAVDYENQDVASSSWPVILKPKKLPKPPAKL
PKPPVGPKPEPKVFNGGLGRKLPVSSAQPLFPTAGLADMTAELQKKLEKRRALEH
>sp|Q5VTH2|FLTOP_HUMAN
MATNYSANQYEKAFSSKYLQNWSPTKPTKESISSHEGYTQIIANDRGHLLPSVPRSKANPWGSFMGTWQMPLKIPPARVTLTSRTTA
GAASLTKWIQKNPDLLKASNGLCPEILGKPHDPDSQKKLRKKSITKTVQQARSPTIIPSSPAANLNSPDELQSSHPSAGHTPGPQRP
AKS
>sp|P0CJ87|DU4L4_HUMAN
MALPTPSDSTLPAEARGRGRRRRLVWTPSQSEALRACFERNPYPGIATRERLAQAIGIPEPRVQIWFQNERSRQLRQHRRESRPWPG
RRGPPEGRRKRTAVTGSQTALLLRAFEKDRFPGIAAREELARETGLPESRIQIWFQNRRARHPGQGGRAPAQAGGLCSAAPGGGHPA
PSWVAFAHTGAWGTGLPAPHVPCAPGALPQGAFVSQAARAAPALQPSQAAPAEGVSQPAPARGDFAYAAPAPPDGALSHPQAPRWPP
HPGKSREDRDPQRDGLPGPCAVAQPGPAQAGPQGQGVLAPPTSQGSPWWGWGRGPQVAGAAWEPQAGEAPPPQPAPPDASARQGQMQ
GIPAPSQALQEPAPWSALPCGLLLDELLASPEFLQQAQPLLETEAPGELEASEEAASLEAPLSEEEYRALLEEL
>sp|O94761|RECQ4_HUMAN
MERLRDVRERLQAWERAFRRQRGRRPSQDDVEAAPEETRALYREYRTLKRTTGQAGGGLRSSESLPAAAEEAPEPRCWGPHLNRAAT
KSPQPTPGRSRQGSVPDYGQRLKANLKGTLQAGPALGRRPWPLGRASSKASTPKPPGTGPVPSFAEKVSDEPPQLPEPQPRPGRLQH
LQASLSQRLGSLDPGWLQRCHSEVPDFLGAPKACRPDLGSEESQLLIPGESAVLGPGAGSQGPEASAFQEVSIRVGSPQPSSSGGEK
RRWNEEPWESPAQVQQESSQAGPPSEGAGAVAVEEDPPGEPVQAQPPQPCSSPSNPRYHGLSPSSQARAGKAEGTAPLHIFPRLARH
DRGNYVRLNMKQKHYVRGRALRSRLLRKQAWKQKWRKKGECFGGGGATVTTKESCFLNEQFDHWAAQCPRPASEEDTDAVGPEPLVP
SPQPVPEVPSLDPTVLPLYSLGPSGQLAETPAEVFQALEQLGHQAFRPGQERAVMRILSGISTLLVLPTGAGKSLCYQLPALLYSRR
SPCLTLVVSPLLSLMDDQVSGLPPCLKAACIHSGMTRKQRESVLQKIRAAQVHVLMLTPEALVGAGGLPPAAQLPPVAFACIDEAHC
LSQWSHNFRPCYLRVCKVLRERMGVHCFLGLTATATRRTASDVAQHLAVAEEPDLHGPAPVPTNLHLSVSMDRDTDQALLTLLQGKR
FQNLDSIIIYCNRREDTERIAALLRTCLHAAWVPGSGGRAPKTTAEAYHAGMCSRERRRVQRAFMQGQLRVVVATVAFGMGLDRPDV

RAVLHLGLPPSFESYVQAVGRAGRDGQPAHCHLFLQPQGEDLRELRRHVHADSTDFLAVKRLVQRVFPACTCTCTRPPSEQEGAVGG
ERPVPKYPPQEAEQLSHQAAPGPRRVCMGHERALPIQLTVQALDMPEEAIETLLCYLELHPHHWLELLATTYTHCRLNCPGGPAQLQ
ALAHRCPPLAVCLAQQLPEDPGQGSSSVEFDMVKLVDSMGWELASVRRALCQLQWDHEPRTGVRRGTGVLVEFSELAFHLRSPGDLT
AEEKDQICDFLYGRVQARERQALARLRRTFQAFHSVAFPSCGPCLEQQDEERSTRLKDLLGRYFEEEEGQEPGGMEDAQGPEPGQAR
LQDWEDQVRCDIRQFLSLRPEEKFSSRAVARIFHGIGSPCYPAQVYGQDRRFWRKYLHLSFHALVGLATEELLQVAR
>sp|Q8IWX7|UN45B_HUMAN
MAEVEAVQLKEEGNRHFQLQDYKAATNSYSQALKLTKDKALLATLYRNRAACGLKTESYVQAASDASRAIDINSSDIKALYRRCQAL
EHLGKLDQAFKDVQRCATLEPRNQNFQEMLRRLNTSIQEKLRVQFSTDSRVQKMFEILLDENSEADKREKAANNLIVLGREEAGAEK
IFQNNGVALLLQLLDTKKPELVLAAVRTLSGMCSGHQARATVILHAVRIDRICSLMAVENEEMSLAVCNLLQAIIDSLSGEDKREHR
GKEEALVLDTKKDLKQITSHLLDMLVSKKVSGQGRDQALNLLNKNVPRKDLAIHDNSRTIYVVDNGLRKILKVVGQVPDLPSCLPLT
DNTRMLASILINKLYDDLRCDPERDHFRKICEEYITGKFDPQDMDKNLNAIQTVSGILQGPFDLGNQLLGLKGVMEMMVALCGSERE
TDQLVAVEALIHASTKLSRATFIITNGVSLLKQIYKTTKNEKIKIRTLVGLCKLGSAGGTDYGLRQFAEGSTEKLAKQCRKWLCNMS
IDTRTRRWAVEGLAYLTLDADVKDDFVQDVPALQAMFELAKAGTSDKTILYSVATTLVNCTNSYDVKEVIPELVQLAKFSKQHVPEE
HPKDKKDFIDMRVKRLLKAGVISALACMVKADSAILTDQTKELLARVFLALCDNPKDRGTIVAQGGGKALIPLALEGTDVGKVKAAH
ALAKIAAVSNPDIAFPGERVYEVVRPLVRLLDTQRDGLQNYEALLGLTNLSGRSDKLRQKIFKERALPDIENYMFENHDQLRQAATE
CMCNMVLHKEVQERFLADGNDRLKLVVLLCGEDDDKVQNAAAGALAMLTAAHKKLCLKMTQVTTQWLEILQRLCLHDQLSVQHRGLV
IAYNLLAADAELAKKLVESELLEILTVVGKQEPDEKKAEVVQTARECLIKCMDYGFIKPVS
>sp|A6NCL1|GEMC1_HUMAN
MNTILPCQDQYFVGGQSYNCPYSTTTSESSVDVSTETWVSFWAAGLLDNRELQQAPQAQESFSDSNFPLPDLCSWEEAQLSSQLYRN
KQLQDTLVQKEEELARLHEENNHLRQYLNSALVKCLEEKAKKLLSSDEFSKAYGKFRKGKRKSKEQRYSPAEIPHPKNAKRNLSSEF
ANCEEQAGPPVDPWVLQTLGLKDLDTIDDTSSANYSALASHPRRVASTFSQFPDDAVDYKNIPREDMPIDYRGDRTTPLHSTATHGE
DFHILSQLSNPPVGLKTLPYYTAHVSPNKTEMAFSTSLSPHCNVKTHSFHQGQAFVRRDEEGGWKFTWVPKQS
>sp|Q8N5B7|CERS5_HUMAN
MATAAQGPLSLLWGWLWSERFWLPENVSWADLEGPADGYGYPRGRHILSVFPLAAGIFFVRLLFERFIAKPCALCIGIEDSGPYQAQ
PNAILEKVFISITKYPDKKRLEGLSKQLDWNVRKIQCWFRHRRNQDKPPTLTKFCESMWRFTFYLCIFCYGIRFLWSSPWFWDIRQC
WHNYPFQPLSSGLYHYYIMELAFYWSLMFSQFTDIKRKDFLIMFVHHLVTIGLISFSYINNMVRVGTLIMCLHDVSDFLLEAAKLAN
YAKYQRLCDTLFVIFSAVFMVTRLGIYPFWILNTTLFESWEIIGPYASWWLLNGLLLTLQLLHVIWSYLIARIALKALIRGKVSKDD
RSDVESSSEEEDVTTCTKSPCDSSSSNGANRVNGHMGGSYWAEE
>sp|P34130|NTF4_HUMAN
MLPLPSCSLPILLLFLLPSVPIESQPPPSTLPPFLAPEWDLLSPRVVLSRGAPAGPPLLFLLEAGAFRESAGAPANRSRRGVSETAP
ASRRGELAVCDAVSGWVTDRRTAVDLRGREVEVLGEVPAAGGSPLRQYFFETRCKADNAEEGGPGAGGGGCRGVDRRHWVSECKAKQ
SYVRALTADAQGRVGWRWIRIDTACVCTLLSRTGRA
>sp|Q9UL12|SARDH_HUMAN
MASLSRALRVAAAHPRQSPTRGMGPCNLSSAAGPTAEKSVPYQRTLKEGQGTSVVAQGPSRPLPSTANVVVIGGGSLGCQTLYHLAK
LGMSGAVLLERERLTSGTTWHTAGLLWQLRPSDVEVELLAHTRRVVSRELEEETGLHTGWIQNGGLFIASNRQRLDEYKRLMSLGKA
YGVESHVLSPAETKTLYPLMNVDDLYGTLYVPHDGTMDPAGTCTTLARAASARGAQVIENCPVTGIRVWTDDFGVRRVAGVETQHGS
IQTPCVVNCAGVWASAVGRMAGVKVPLVAMHHAYVVTERIEGIQNMPNVRDHDASVYLRLQGDALSVGGYEANPIFWEEVSDKFAFG
LFDLDWEVFTQHIEGAINRVPVLEKTGIKSTVCGPESFTPDHKPLMGEAPELRGFFLGCGFNSAGMMLGGGCGQELAHWIIHGRPEK
DMHGYDIRRFHHSLTDHPRWIRERSHESYAKNYSVVFPHDEPLAGRNMRRDPLHEELLGQGCVFQERHGWERPGWFHPRGPAPVLEY
DYYGAYGSRAHEDYAYRRLLADEYTFAFPPHHDTIKKECLACRGAAAVFDMSYFGKFYLVGLDARKAADWLFSADVSRPPGSTVYTC
MLNHRGGTESDLTVSRLAPSHQASPLAPAFEGDGYYLAMGGAVAQHNWSHITTVLQDQKSQCQLIDSSEDLGMISIQGPASRAILQE
VLDADLSNEAFPFSTHKLLRAAGHLVRAMRLSFVGELGWELHIPKASCVPVYRAVMAAGAKHGLINAGYRAIDSLSIEKGYRHWHAD
LRPDDSPLEAGLAFTCKLKSPVPFLGREALEQQRAAGLRRRLVCFTMEDKVPMFGLEAIWRNGQVVGHVRRADFGFAIDKTIAYGYI
HDPSGGPVSLDFVKSGDYALERMGVTYGAQAHLKSPFDPNNKRVKGIY

>sp|Q8TDQ1|CLM1_HUMAN
MPLLTLYLLLFWLSGYSIVTQITGPTTVNGLERGSLTVQCVYRSGWETYLKWWCRGAIWRDCKILVKTSGSEQEVKRDRVSIKDNQK
NRTFTVTMEDLMKTDADTYWCGIEKTGNDLGVTVQVTIDPAPVTQEETSSSPTLTGHHLDNRHKLLKLSVLLPLIFTILLLLLVAAS
LLAWRMMKYQQKAAGMSPEQVLQPLEGDLCYADLTLQLAGTSPQKATTKLSSAQVDQVEVEYVTMASLPKEDISYASLTLGAEDQEP
TYCNMGHLSSHLPGRGPEEPTEYSTISRP
>sp|P19404|NDUV2_HUMAN
MFFSAALRARAAGLTAHWGRHVRNLHKTVMQNGAGGALFVHRDTPENNPDTPFDFTPENYKRIEAIVKNYPEGHKAAAVLPVLDLAQ
RQNGWLPISAMNKVAEVLQVPPMRVYEVATFYTMYNRKPVGKYHIQVCTTTPCMLRNSDSILEAIQKKLGIKVGETTPDKLFTLIEV
ECLGACVNAPMVQINDNYYEDLTAKDIEEIIDELKAGKIPKPGPRSGRFSCEPAGGLTSLTEPPKGPGFGVQAGL
>sp|O95164|UBL3_HUMAN
MSSNVPADMINLRLILVSGKTKEFLFSPNDSASDIAKHVYDNWPMDWEEEQVSSPNILRLIYQGRFLHGNVTLGALKLPFGKTTVMH
LVARETLPEPNSQGQRNREKTGESNCCVIL
>sp|O00203|AP3B1_HUMAN
MSSNSFPYNEQSGGGEATELGQEATSTISPSGAFGLFSSDLKKNEDLKQMLESNKDSAKLDAMKRIVGMIAKGKNASELFPAVVKNV
ASKNIEIKKLVYVYLVRYAEEQQDLALLSISTFQRALKDPNQLIRASALRVLSSIRVPIIVPIMMLAIKEASADLSPYVRKNAAHAI
QKLYSLDPEQKEMLIEVIEKLLKDKSTLVAGSVVMAFEEVCPDRIDLIHKNYRKLCNLLVDVEEWGQVVIIHMLTRYARTQFVSPWK
EGDELEDNGKNFYESDDDQKEKTDKKKKPYTMDPDHRLLIRNTKPLLQSRNAAVVMAVAQLYWHISPKSEAGIISKSLVRLLRSNRE
VQYIVLQNIATMSIQRKGMFEPYLKSFYVRSTDPTMIKTLKLEILTNLANEANISTLLREFQTYVKSQDKQFAAATIQTIGRCATNI
LEVTDTCLNGLVCLLSNRDEIVVAESVVVIKKLLQMQPAQHGEIIKHMAKLLDSITVPVARASILWLIGENCERVPKIAPDVLRKMA
KSFTSEDDLVKLQILNLGAKLYLTNSKQTKLLTQYILNLGKYDQNYDIRDRTRFIRQLIVPNVKSGALSKYAKKIFLAQKPAPLLES
PFKDRDHFQLGTLSHTLNIKATGYLELSNWPEVAPDPSVRNVEVIELAKEWTPAGKAKQENSAKKFYSESEEEEDSSDSSSDSESES
GSESGEQGESGEEGDSNEDSSEDSSSEQDSESGRESGLENKRTAKRNSKAKGKSDSEDGEKENEKSKTSDSSNDESSSIEDSSSDSE
SESEPESESESRRVTKEKEKKTKQDRTPLTKDVSLLDLDDFNPVSTPVALPTPALSPSLMADLEGLHLSTSSSVISVSTPAFVPTKT
HVLLHRMSGKGLAAHYFFPRQPCIFGDKMVSIQITLNNTTDRKIENIHIGEKKLPIGMKMHVFNPIDSLEPEGSITVSMGIDFCDST
QTASFQLCTKDDCFNVNIQPPVGELLLPVAMSEKDFKKEQGVLTGMNETSAVIIAAPQNFTPSVIFQKVVNVANVGAVPSGQDNIHR
FAAKTVHSGSLMLVTVELKEGSTAQLIINTEKTVIGSVLLRELKPVLSQG
>sp|Q9HAZ1|CLK4_HUMAN
MRHSKRTHCPDWDSRESWGHESYRGSHKRKRRSHSSTQENRHCKPHHQFKESDCHYLEARSLNERDYRDRRYVDEYRNDYCEGYVPR
HYHRDIESGYRIHCSKSSVRSRRSSPKRKRNRHCSSHQSRSKSHRRKRSRSIEDDEEGHLICQSGDVLRARYEIVDTLGEGAFGKVV
ECIDHGMDGMHVAVKIVKNVGRYREAARSEIQVLEHLNSTDPNSVFRCVQMLEWFDHHGHVCIVFELLGLSTYDFIKENSFLPFQID
HIRQMAYQICQSINFLHHNKLTHTDLKPENILFVKSDYVVKYNSKMKRDERTLKNTDIKVVDFGSATYDDEHHSTLVSTRHYRAPEV
ILALGWSQPCDVWSIGCILIEYYLGFTVFQTHDSKEHLAMMERILGPIPQHMIQKTRKRKYFHHNQLDWDEHSSAGRYVRRRCKPLK
EFMLCHDEEHEKLFDLVRRMLEYDPTQRITLDEALQHPFFDLLKKK
>sp|P49641|MA2A2_HUMAN
MKLKKQVTVCGAAIFCVAVFSLYLMLDRVQHDPTRHQNGGNFPRSQISVLQNRIEQLEQLLEENHEIISHIKDSVLELTANAEGPPA
MLPYYTVNGSWVVPPEPRPSFFSISPQDCQFALGGRGQKPELQMLTVSEELPFDNVDGGVWRQGFDISYDPHDWDAEDLQVFVVPHS
HNDPGWIKTFDKYYTEQTQHILNSMVSKLQEDPRRRFLWAEVSFFAKWWDNINVQKRAAVRRLVGNGQLEIATGGWVMPDEANSHYF
ALIDQLIEGHQWLERNLGATPRSGWAVDPFGYSSTMPYLLRRANLTSMLIQRVHYAIKKHFAATHSLEFMWRQTWDSDSSTDIFCHM
MPFYSYDVPHTCGPDPKICCQFDFKRLPGGRINCPWKVPPRAITEANVAERAALLLDQYRKKSQLFRSNVLLVPLGDDFRYDKPQEW
DAQFFNYQRLFDFFNSRPNLHVQAQFGTLSDYFDALYKRTGVEPGARPPGFPVLSGDFFSYADREDHYWTGYYTSRPFYKSLDRVLE
AHLRGAEVLYSLAAAHARRSGLAGRYPLSDFTLLTEARRTLGLFQHHDAITGTAKEAVVVDYGVRLLRSLVNLKQVIIHAAHYLVLG
DKETYHFDPEAPFLQVDDTRLSHDALPERTVIQLDSSPRFVVLFNPLEQERFSMVSLLVNSPRVRVLSEEGQPLAVQISAHWSSATE
AVPDVYQVSVPVRLPALGLGVLQLQLGLDGHRTLPSSVRIYLHGRQLSVSRHEAFPLRVIDSGTSDFALSNRYMQVWFSGLTGLLKS
IRRVDEEHEQQVDMQVLVYGTRTSKDKSGAYLFLPDGEAKPYVPKEPPVLRVTEGPFFSEVVAYYEHIHQAVRLYNLPGVEGLSLDI

SSLVDIRDYVNKELALHIHTDIDSQGIFFTDLNGFQVQPRRYLKKLPLQANFYPMPVMAYIQDAQKRLTLHTAQALGVSSLKDGQLE
VILDRRLMQDDNRGLGQGLKDNKRTCNRFRLLLERRTVGSEVQDSHSTSYPSLLSHLTSMYLNAPALALPVARMQLPGPGLRSFHPL
ASSLPCDFHLLNLRTLQAEEDTLPSAETALILHRKGFDCGLEAKNLGFNCTTSQGKVALGSLFHGLDVVFLQPTSLTLLYPLASPSN
STDVYLEPMEIATFRLRLG
>sp|Q8WWL2|SPIR2_HUMAN
MARAGSCGGAAAGAGRPEPWELSLEEVLKAYEQPLNEEQAWAVCFQGCRGLRGSPGRRLRDTGDLLLRGDGSVGAREPEAAEPATMV
VPLASSEAQTVQSLGFAIYRALDWGLDESEERELSPQLERLIDLMANNDSEDSGCGAADEGYGGPEEEEEAEGVPRSVRTFAQAMRL
CAARLTDPRGAQAHYQAVCRALFVETLELRAFLARVREAKEMLQKLREDEPHLETPRAELDSLGHTDWARLWVQLMRELRRGVKLKK
VQEQEFNPLPTEFQLTPFEMLMQDIRARNYKLRKVMVDGDIPPRVKKDAHELILDFIRSRPPLKQVSERRLRPLPPKQRSLHEKILE
EIKQERRLRPVRGEGWAARGFGSLPCILNACSGDAKSTSCINLSVTDAGGSAQRPRPRVLLKAPTLAEMEEMNTSEEEESPCGEVTL
KRDRSFSEHDLAQLRSEVASGLQSATHPPGGTEPPRPRAGSAHVWRPGSRDQGTCPASVSDPSHPLLSNRGSSGDRPEASMTPDAKH
LWLEFSHPVESLALTVEEVMDVRRVLVKAEMEKFLQNKELFSSLKKGKICCCCRAKFPLFSWPPSCLFCKRAVCTSCSIKMKMPSKK
FGHIPVYTLGFESPQRVSAAKTAPIQRRDIFQSLQGPQWQSVEEAFPHIYSHGCVLKDVCSECTSFVADVVRSSRKSVDVLNTTPRR
SRQTQSLYIPNTRTLDFK
>sp|Q99962|SH3G2_HUMAN
MSVAGLKKQFHKATQKVSEKVGGAEGTKLDDDFKEMERKVDVTSRAVMEIMTKTIEYLQPNPASRAKLSMINTMSKIRGQEKGPGYP
QAEALLAEAMLKFGRELGDDCNFGPALGEVGEAMRELSEVKDSLDIEVKQNFIDPLQNLHDKDLREIQHHLKKLEGRRLDFDYKKKR
QGKIPDEELRQALEKFDESKEIAESSMFNLLEMDIEQVSQLSALVQAQLEYHKQAVQILQQVTVRLEERIRQASSQPRREYQPKPRM
SLEFPTGDSTQPNGGLSHTGTPKPSGVQMDQPCCRALYDFEPENEGELGFKEGDIITLTNQIDENWYEGMLHGHSGFFPINYVEILV
ALPH
>sp|Q0P670|CQ074_HUMAN
MENQLWHNTVRCCNQYQESPHDAEDILLLLLGLIVLVNIGINVATMMWHGLQNALDKMIDWATQKNEIQASESPPSGPPDKAQDVHI
HCILDPVQVKMSRPTQYSSFSCHHFSNHHSSSLLRCVRRRRRRHRRCRRRCCNHQQRPQNYRQIPHSHSVFRNPHRSQKMSQLHRVP
FFDQEDPDSYLEEEDNLPFPYPKYPRRGWGGFYQRAGLPSNVGLWGHQGGILASLPPPSLYLSPELRCMPKRVEARSELRLQSYGRH
GSQSRLWGNVEAEQWASSPPPPHRLPPNPSWVPVGHSPYPSVGWMLYDSWDQRRRGTEGFERPPASVSRNARPEAQGCREHHSPQSH
QQSLLGHAYGQSHRSPHPSTEPLGYSSQDPREVRRRAADWAEALPAWRPLTTSASLTVLDEASHQRTPAPSSVLVPHSSQPWPKVQA
ADPAPPPTMFVPLSRNPGGNANYQVYDSLELKRQVQKSRARSSSLPPASTSTLRPSLHRSQTEKLN
>sp|P13994|CC130_HUMAN
MGERKGVNKYYPPDFNPEKHGSLNRYHNSHPLRERARKLSQGILIIRFEMPYNIWCDGCKNHIGMGVRYNAEKKKVGNYYTTPIYRF
RMKCHLCVNYIEMQTDPANCDYVIVSGAQRKEERWDMADNEQVLTTEHEKKQKLETDAMFRLEHGEADRSTLKKALPTLSHIQEAQS
AWKDDFALNSMLRRRFREKKKAIQEEEERDQALQAKASLTIPLVPETEDDRKLAALLKFHTLDSYEDKQKLKRTEIISRSWFPSAPG
SASSSKVSGVLKKLAQSRRTALATSPITVGDLGIVRRRSRDVPESPQHAADTPKSGEPRVPEEAAQDRPMSPGDCPPETTETPKCSS
PRGQEGSRQDKPLSPAGSSQEAADTPDTRHPCSLGSSLVADYSDSESE
>sp|Q969L4|LSM10_HUMAN
MAVSHSVKERTISENSLIILLQGLQGRVTTVDLRDESVAHGRIDNVDAFMNIRLAKVTYTDRWGHQVKLDDLFVTGRNVRYVHIPDD
VNITSTIEQQLQIIHRVRNFGGKGQGRWEFPPKNCK
>sp|A8MVW5|HECA2_HUMAN
MGQDAFMEPFGDTLGVFQCKIYLLLFGACSGLKVTVPSHTVHGVRGQALYLPVHYGFHTPASDIQIIWLFERPHTMPKYLLGSVNKS
VVPDLEYQHKFTMMPPNASLLINPLQFPDEGNYIVKVNIQGNGTLSASQKIQVTVDDPVTKPVVQIHPPSGAVEYVGNMTLTCHVEG
GTRLAYQWLKNGRPVHTSSTYSFSPQNNTLHIAPVTKEDIGNYSCLVRNPVSEMESDIIMPIIYYGPYGLQVNSDKGLKVGEVFTVD
LGEAILFDCSADSHPPNTYSWIRRTDNTTYIIKHGPRLEVASEKVAQKTMDYVCCAYNNITGRQDETHFTVIITSVGLEKLAQKGKS
LSPLASITGISLFLIISMCLLFLWKKYQPYKVIKQKLEGRPETEYRKAQTFSGHEDALDDFGIYEFVAFPDVSGVSRIPSRSVPASD
CVSGQDLHSTVYYEVIQHIPAQQQDHPE
>sp|Q15542|TAF5_HUMAN

MAALAEEQTEVAVKLEPEGPPTLLPPQAGDGAGEGSGGTTNNGPNGGGGNVAASSSTGGDGGTPKPTVAVSAAAPAGAAPVPAAAPD
AGAPHDRQTLLAVLQFLRQSKLREAEEALRREAGLLEEAVAGSGAPGEVDSAGAEVTSALLSRVTASAPGPAAPDPPGTGASGATVV
SGSASGPAAPGKVGSVAVEDQPDVSAVLSAYNQQGDPTMYEEYYSGLKHFIECSLDCHRAELSQLFYPLFVHMYLELVYNQHENEAK
SFFEKFHGDQECYYQDDLRVLSSLTKKEHMKGNETMLDFRTSKFVLRISRDSYQLLKRHLQEKQNNQIWNIVQEHLYIDIFDGMPRS
KQQIDAMVGSLAGEAKREANKSKVFFGLLKEPEIEVPLDDEDEEGENEEGKPKKKKPKKDSIGSKSKKQDPNAPPQNRIPLPELKDS
DKLDKIMNMKETTKRVRLGPDCLPSICFYTFLNAYQGLTAVDVTDDSSLIAGGFADSTVRVWSVTPKKLRSVKQASDLSLIDKESDD
VLERIMDEKTASELKILYGHSGPVYGASFSPDRNYLLSSSEDGTVRLWSLQTFTCLVGYKGHNYPVWDTQFSPYGYYFVSGGHDRVA
RLWATDHYQPLRIFAGHLADVNCTRFHPNSNYVATGSADRTVRLWDVLNGNCVRIFTGHKGPIHSLTFSPNGRFLATGATDGRVLLW
DIGHGLMVGELKGHTDTVCSLRFSRDGEILASGSMDNTVRLWDAIKAFEDLETDDFTTATGHINLPENSQELLLGTYMTKSTPVVHL
HFTRRNLVLAAGAYSPQ
>sp|Q8NCU4|CC191_HUMAN
MLLAPQGRSFSKKRMGLNRWKRFTRKPSPKPTFGPDSVEHWIKRVEKASEFAVSNAFFTRNSDLPRSPWGQITDLKTSEQIEDHDEI
YAEAQELVNDWLDTKLKQELASEEEGDAKNTVSSVTIMPEANGHLKYDKFDDLCGYLEEEEESTTVQKFIDHLLHKNVVDSAMMEDL
GRKENQDKKQQKDPRLTMEMRHKQVKENRLRREKELEYQRIEKTLKKSAFLEAQCLVQEEKKRKALEAKKEEEEIQREMVKLRREII
ERRRTVKAAWKIEKKRQEENSQNSSEKVMFQSTHILPDEEKMVKERKRKLKEVLIQTFKENQQCQKRYFAAWHKLILDHRIKLGKAG
TLSDWKIQLKVLRAWRDYTRFQKLERETQALENDLREENRKQQLATEYNRKQVLRHCFTEWQHWHGAELLKRELALTKEETRKKMDA
LLQAASLGKLSANGLSGISLPEEATAMVGPPVKNGQETAVPPLWEKPPLGSSGCMLSPPLGRTTTGNLQGSLQNVSLSAPGNKQHKT
LGAEPSQQPGSNETLRTTSQKAEPLCLGHFHNRHVFQQQLIEKQKKKLQEQQKTILELKKNLQLAEAQWAAEHALAVTEAQSHLLSK
PREEEPRTCQMLVNSPVASPGTEGRSDSRNSLSGLRRKPKQLMTPHPILKAMEERAIQRAECRRILAEKKKKQEEEKLAQLKAQEEE
RQKREAEEKEAQLERKREEKRLKKMKELEKQKRIKRNQQLEAIAKEHYERVLLRKKGLEPWKRLRMQSKQNIQVAEEHYSLFLQRKY
MLTWFQRSQESLARKMAQADQFYSQILLKRVIQSWLQYVIDLQEEVRKFCVHFLQKKIFRAWFNMVREVKIDSQGKHEIAAEHSDRR
ILWITLRTWKKFVKFMKEERVKEERRQQLRRKVVEILPDFQVPGRYHELYQQSDTWSLSKTSLVNE
>sp|Q9BWW4|SSBP3_HUMAN
MFAKGKGSAVPSDGQAREKLALYVYEYLLHVGAQKSAQTFLSEIRWEKNITLGEPPGFLHSWWCVFWDLYCAAPERRDTCEHSSEAK
AFHDYSAAAAPSPVLGNIPPNDGMPGGPIPPGFFQGPPGSQPSPHAQPPPHNPSSMMGPHSQPFMSPRYAGGPRPPIRMGNQPPGGV
PGTQPLLPNSMDPTRQQGHPNMGGSMQRMNPPRGMGPMGPGPQNYGSGMRPPPNSLGPAMPGINMGPGAGRPWPNPNSANSIPYSSS
SPGTYVGPPGGGGPPGTPIMPSPADSTNSSDNIYTMINPVPPGGSRSNFPMGPGSDGPMGGMGGMEPHHMNGSLGSGDIDGLPKNSP
NNISGISNPPGTPRDDGELGGNFLHSFQNDNYSPSMTMSV
>sp|Q8WUZ0|BCL7C_HUMAN
MAGRTVRAETRSRAKDDIKKVMATIEKVRRWEKRWVTVGDTSLRIFKWVPVVDPQEEERRRAGGGAERSRGRERRGRGASPRGGGPL
ILLDLNDENSNQSFHSEGSLQKGTEPSPGGTPQPSRPVSPAGPPEGVPEEAQPPRLGQERDPGGITAGSTDEPPMLTKEEPVPELLE
AEAPEAYPVFEPVPPVPEAAQGDTEDSEGAPPLKRICPNAPDP
>sp|A8K8V0|ZN785_HUMAN
MGPPLAPRPAHVPGEAGPRRTRESRPGAVSFADVAVYFSPEEWECLRPAQRALYRDVMRETFGHLGALGFSVPKPAFISWVEGEVEA
WSPEAQDPDGESSAAFSRGQGQEAGSRDGNEEKERLKKCPKQKEVAHEVAVKEWWPSVACPEFCNPRQSPMNPWLKDTLTRRLPHSC
PDCGRNFSYPSLLASHQRVHSGERPFSCGQCQARFSQRRYLLQHQFIHTGEKPYPCPDCGRRFRQRGSLAIHRRAHTGEKPYACSDC
KSRFTYPYLLAIHQRKHTGEKPYSCPDCSLRFAYTSLLAIHRRIHTGEKPYPCPDCGRRFTYSSLLLSHRRIHSDSRPFPCVECGKG
FKRKTALEAHRWIHRSCSERRAWQQAVVGRSEPIPVLGGKDPPVHFRHFPDIFQECG
>sp|Q9ULH0|KDIS_HUMAN
MSVLISQSVINYVEEENIPALKALLEKCKDVDERNECGQTPLMIAAEQGNLEIVKELIKNGANCNLEDLDNWTALISASKEGHVHIV
EELLKCGVNLEHRDMGGWTALMWACYKGRTDVVELLLSHGANPSVTGLYSVYPIIWAAGRGHADIVHLLLQNGAKVNCSDKYGTTPL
VWAARKGHLECVKHLLAMGADVDQEGANSMTALIVAVKGGYTQSVKEILKRNPNVNLTDKDGNTALMIASKEGHTEIVQDLLDAGTY
VNIPDRSGDTVLIGAVRGGHVEIVRALLQKYADIDIRGQDNKTALYWAVEKGNATMVRDILQCNPDTEICTKDGETPLIKATKMRNI
EVVELLLDKGAKVSAVDKKGDTPLHIAIRGRSRKLAELLLRNPKDGRLLYRPNKAGETPYNIDCSHQKSILTQIFGARHLSPTETDG

DMLGYDLYSSALADILSEPTMQPPICVGLYAQWGSGKSFLLKKLEDEMKTFAGQQIEPLFQFSWLIVFLTLLLCGGLGLLFAFTVHP
NLGIAVSLSFLALLYIFFIVIYFGGRREGESWNWAWVLSTRLARHIGYLELLLKLMFVNPPELPEQTTKALPVRFLFTDYNRLSSVG
GETSLAEMIATLSDACEREFGFLATRLFRVFKTEDTQGKKKWKKTCCLPSFVIFLFIIGCIISGITLLAIFRVDPKHLTVNAVLISI
ASVVGLAFVLNCRTWWQVLDSLLNSQRKRLHNAASKLHKLKSEGFMKVLKCEVELMARMAKTIDSFTQNQTRLVVIIDGLDACEQDK
VLQMLDTVRVLFSKGPFIAIFASDPHIIIKAINQNLNSVLRDSNINGHDYMRNIVHLPVFLNSRGLSNARKFLVTSATNGDVPCSDT
TGIQEDADRRVSQNSLGEMTKLGSKTALNRRDTYRRRQMQRTITRQMSFDLTKLLVTEDWFSDISPQTMRRLLNIVSVTGRLLRANQ
ISFNWDRLASWINLTEQWPYRTSWLILYLEETEGIPDQMTLKTIYERISKNIPTTKDVEPLLEIDGDIRNFEVFLSSRTPVLVARDV
KVFLPCTVNLDPKLREIIADVRAAREQISIGGLAYPPLPLHEGPPRAPSGYSQPPSVCSSTSFNGPFAGGVVSPQPHSSYYSGMTGP
QHPFYNRPFFAPYLYTPRYYPGGSQHLISRPSVKTSLPRDQNNGLEVIKEDAAEGLSSPTDSSRGSGPAPGPVVLLNSLNVDAVCEK
LKQIEGLDQSMLPQYCTTIKKANINGRVLAQCNIDELKKEMNMNFGDWHLFRSTVLEMRNAESHVVPEDPRFLSESSSGPAPHGEPA
RRASHNELPHTELSSQTPYTLNFSFEELNTLGLDEGAPRHSNLSWQSQTRRTPSLSSLNSQDSSIEISKLTDKVQAEYRDAYREYIA
QMSQLEGGPGSTTISGRSSPHSTYYMGQSSSGGSIHSNLEQEKGKDSEPKPDDGRKSFLMKRGDVIDYSSSGVSTNDASPLDPITEE
DEKSDQSGSKLLPGKKSSERSSLFQTDLKLKGSGLRYQKLPSDEDESGTEESDNTPLLKDDKDRKAEGKVERVPKSPEHSAEPIRTF
IKAKEYLSDALLDKKDSSDSGVRSSESSPNHSLHNEVADDSQLEKANLIELEDDSHSGKRGIPHSLSGLQDPIIARMSICSEDKKSP
SECSLIASSPEENWPACQKAYNLNRTPSTVTLNNNSAPANRANQNFDEMEGIRETSQVILRPSSSPNPTTIQNENLKSMTHKRSQRS
SYTRLSKDPPELHAAASSESTGFGEERESIL
>sp|Q8N6D2|RN182_HUMAN
MASQPPEDTAESQASDELECKICYNRYNLKQRKPKVLECCHRVCAKCLYKIIDFGDSPQGVIVCPFCRFETCLPDDEVSSLPDDNNI
LVNLTCGGKGKKCLPENPTELLLTPKRLASLVSPSHTSSNCLVITIMEVQRESSPSLSSTPVVEFYRPASFDSVTTVSHNWTVWNCT
SLLFQTSIRVLVWLLGLLYFSSLPLGIYLLVSKKVTLGVVFVSLVPSSLVILMVYGFCQCVCHEFLDCMAPPS
>sp|P15586|GNS_HUMAN
MRLLPLAPGRLRRGSPRHLPSCSPALLLLVLGGCLGVFGVAAGTRRPNVVLLLTDDQDEVLGGMTPLKKTKALIGEMGMTFSSAYVP
SALCCPSRASILTGKYPHNHHVVNNTLEGNCSSKSWQKIQEPNTFPAILRSMCGYQTFFAGKYLNEYGAPDAGGLEHVPLGWSYWYA
LEKNSKYYNYTLSINGKARKHGENYSVDYLTDVLANVSLDFLDYKSNFEPFFMMIATPAPHSPWTAAPQYQKAFQNVFAPRNKNFNI
HGTNKHWLIRQAKTPMTNSSIQFLDNAFRKRWQTLLSVDDLVEKLVKRLEFTGELNNTYIFYTSDNGYHTGQFSLPIDKRQLYEFDI
KVPLLVRGPGIKPNQTSKMLVANIDLGPTILDIAGYDLNKTQMDGMSLLPILRGASNLTWRSDVLVEYQGEGRNVTDPTCPSLSPGV
SQCFPDCVCEDAYNNTYACVRTMSALWNLQYCEFDDQEVFVEVYNLTADPDQITNIAKTIDPELLGKMNYRLMMLQSCSGPTCRTPG
VFDPGYRFDPRLMFSNRGSVRTRRFSKHLL
>sp|P53675|CLH2_HUMAN
MAQILPVRFQEHFQLQNLGINPANIGFSTLTMESDKFICIREKVGEQAQVTIIDMSDPMAPIRRPISAESAIMNPASKVIALKAGKT
LQIFNIEMKSKMKAHTMAEEVIFWKWVSVNTVALVTETAVYHWSMEGDSQPMKMFDRHTSLVGCQVIHYRTDEYQKWLLLVGISAQQ
NRVVGAMQLYSVDRKVSQPIEGHAAAFAEFKMEGNAKPATLFCFAVRNPTGGKLHIIEVGQPAAGNQPFVKKAVDVFFPPEAQNDFP
VAMQIGAKHGVIYLITKYGYLHLYDLESGVCICMNRISADTIFVTAPHKPTSGIIGVNKKGQVLSVCVEEDNIVNYATNVLQNPDLG
LRLAVRSNLAGAEKLFVRKFNTLFAQGSYAEAAKVAASAPKGILRTRETVQKFQSIPAQSGQASPLLQYFGILLDQGQLNKLESLEL
CHLVLQQGRKQLLEKWLKEDKLECSEELGDLVKTTDPMLALSVYLRANVPSKVIQCFAETGQFQKIVLYAKKVGYTPDWIFLLRGVM
KISPEQGLQFSRMLVQDEEPLANISQIVDIFMENSLIQQCTSFLLDALKNNRPAEGLLQTWLLEMNLVHAPQVADAILGNKMFTHYD
RAHIAQLCEKAGLLQQALEHYTDLYDIKRAVVHTHLLNPEWLVNFFGSLSVEDSVECLHAMLSANIRQNLQLCVQVASKYHEQLGTQ
ALVELFESFKSYKGLFYFLGSIVNFSQDPDVHLKYIQAACKTGQIKEVERICRESSCYNPERVKNFLKEAKLTDQLPLIIVCDRFGF
VHDLVLYLYRNNLQRYIEIYVQKVNPSRTPAVIGGLLDVDCSEEVIKHLIMAVRGQFSTDELVAEVEKRNRLKLLLPWLESQIQEGC
EEPATHNALAKIYIDSNNSPECFLRENAYYDSSVVGRYCEKRDPHLACVAYERGQCDLELIKVCNENSLFKSEARYLVCRKDPELWA
HVLEETNPSRRQLIDQVVQTALSETRDPEEISVTVKAFMTADLPNELIELLEKIVLDNSVFSEHRNLQNLLILTAIKADRTRVMEYI
SRLDNYDALDIASIAVSSALYEEAFTVFHKFDMNASAIQVLIEHIGNLDRAYEFAERCNEPAVWSQLAQAQLQKDLVKEAINSYIRG
DDPSSYLEVVQSASRSNNWEDLVKFLQMARKKGRESYIETELIFALAKTSRVSELEDFINGPNNAHIQQVGDRCYEEGMYEAAKLLY
SNVSNFARLASTLVHLGEYQAAVDNSRKASSTRTWKEVCFACMDGQEFRFAQLCGLHIVIHADELEELMCYYQDRGYFEELILLLEA

ALGLERAHMGMFTELAILYSKFKPQKMLEHLELFWSRVNIPKVLRAAEQAHLWAELVFLYDKYEEYDNAVLTMMSHPTEAWKEGQFK
DIITKVANVELCYRALQFYLDYKPLLINDLLLVLSPRLDHTWTVSFFSKAGQLPLVKPYLRSVQSHNNKSVNEALNHLLTEEEDYQG
LRASIDAYDNFDNISLAQQLEKHQLMEFRCIAAYLYKGNNWWAQSVELCKKDHLYKDAMQHAAESRDAELAQKLLQWFLEEGKRECF
AACLFTCYDLLRPDMVLELAWRHNLVDLAMPYFIQVMREYLSKVDKLDALESLRKQEEHVTEPAPLVFDFDGHE
>sp|Q8NB66|UN13C_HUMAN
MVANFFKSLILPYIHKLCKGMFTKKLGNTNKNKEYRQQKKDQDFPTAGQTKSPKFSYTFKSTVKKIAKCSSTHNLSTEEDEASKEFS
LSPTFSYRVAIANGLQKNAKVTNSDNEDLLQELSSIESSYSESLNELRSSTENQAQSTHTMPVRRNRKSSSSLAPSEGSSDGERTLH
GLKLGALRKLRKWKKSQECVSSDSELSTMKKSWGIRSKSLDRTVRNPKTNALEPGFSSSGCISQTHDVMEMIFKELQGISQIETELS
ELRGHVNALKHSIDEISSSVEVVQSEIEQLRTGFVQSRRETRDIHDYIKHLGHMGSKASLRFLNVTEERFEYVESVVYQILIDKMGF
SDAPNAIKIEFAQRIGHQRDCPNAKPRPILVYFETPQQRDSVLKKSYKLKGTGIGISTDILTHDIRERKEKGIPSSQTYESMAIKLS
TPEPKIKKNNWQSPDDSDEDLESDLNRNSYAVLSKSELLTKGSTSKPSSKSHSARSKNKTANSSRISNKSDYDKISSQLPESDILEK
QTTTHYADATPLWHSQSDFFTAKLSRSESDFSKLCQSYSEDFSENQFFTRTNGSSLLSSSDRELWQRKQEGTATLYDSPKDQHLNGG
VQGIQGQTETENTETVDSGMSNGMVCASGDRSHYSDSQLSLHEDLSPWKEWNQGADLGLDSSTQEGFDYETNSLFDQQLDVYNKDLE
YLGKCHSDLQDDSESYDLTQDDNSSPCPGLDNEPQGQWVGQYDSYQGANSNELYQNQNQLSMMYRSQSELQSDDSEDAPPKSWHSRL
SIDLSDKTFSFPKFGSTLQRAKSALEVVWNKSTQSLSGYEDSGSSLMGRFRTLSQSTANESSTTLDSDVYTEPYYYKAEDEEDYTEP
VADNETDYVEVMEQVLAKLENRTSITETDEQMQAYDHLSYETPYETPQDEGYDGPADDMVSEEGLEPLNETSAEMEIREDENQNIPE
QPVEITKPKRIRPSFKEAALRAYKKQMAELEEKILAGDSSSVDEKARIVSGNDLDASKFSALQVCGGAGGGLYGIDSMPDLRRKKTL
PIVRDVAMTLAARKSGLSLAMVIRTSLNNEELKMHVFKKTLQALIYPMSSTIPHNFEVWTATTPTYCYECEGLLWGIARQGMKCLEC
GVKCHEKCQDLLNADCLQRAAEKSSKHGAEDKTQTIITAMKERMKIREKNRPEVFEVIQEMFQISKEDFVQFTKAAKQSVLDGTSKW
SAKITITVVSAQGLQAKDKTGSSDPYVTVQVGKNKRRTKTIFGNLNPVWDEKFYFECHNSTDRIKVRVWDEDDDIKSRVKQHFKKES
DDFLGQTIVEVRTLSGEMDVWYNLEKRTDKSAVSGAIRLKINVEIKGEEKVAPYHIQYTCLHENLFHYLTEVKSNGGVKIPEVKGDE
AWKVFFDDASQEIVDEFAMRYGIESIYQAMTHFSCLSSKYMCPGVPAVMSTLLANINAFYAHTTVSTNIQVSASDRFAATNFGREKF
IKLLDQLHNSLRIDLSKYRENFPASNTERLQDLKSTVDLLTSITFFRMKVLELQSPPKASMVVKDCVRACLDSTYKYIFDNCHELYS
QLTDPSKKQDIPREDQGPTTKNLDFWPQLITLMVTIIDEDKTAYTPVLNQFPQELNMGKISAEIMWTLFALDMKYALEEHENQRLCK
STDYMNLHFKVKWFYNEYVRELPAFKDAVPEYSLWFEPFVMQWLDENEDVSMEFLHGALGRDKKDGFQQTSEHALFSCSVVDVFAQL
NQSFEIIKKLECPNPEALSHLMRRFAKTINKVLLQYAAIVSSDFSSHCDKENVPCILMNNIQQLRVQLEKMFESMGGKELDSEASTI
LKELQVKLSGVLDELSVTYGESFQVIIEECIKQMSFELNQMRANGNTTSNKNSAAMDAEIVLRSLMDFLDKTLSLSAKICEKTVLKR
VLKELWKLVLNKIEKQIVLPPLTDQTGPQMIFIAAKDLGQLSKLKEHMIREDARGLTPRQCAIMEVVLATIKQYFHAGGNGLKKNFL
EKSPDLQSLRYALSLYTQTTDALIKKFIDTQTSQSRSSKDAVGQISVHVDITATPGTGDHKVTVKVIAINDLNWQTTAMFRPFVEVC
ILGPNLGDKKRKQGTKTKSNTWSPKYNETFQFILGKENRPGAYELHLSVKDYCFAREDRIIGMTVIQLQNIAEKGSYGAWYPLLKNI
SMDETGLTILRILSQRTSDDVAKEFVRLKSETRSTEESA
>sp|A6NEL2|SWAHB_HUMAN
MARELSQEALLDFLCQAGGRVTNAALLSHFKSFLRDPDASPSQHQHRRELFKGFVNSVAAVRQDPDGTKYVVLKRRYRDLLGEEGLQ
RPREPPAAAPSAGGAAPCSPRGARRGEPPQQQPRRRRREKEPEEEPAGAAARAADAACNGLPGSDSRRAPGKGGGSKGSPGQRPPVP
AAAAAGAQARASCAAAKTQGRCCWECLQNNLAVLPGELGALPHSATAEEKPARALPAQDDRGASREREEGALAEPAPVPAVAHSPPA
TVEAATSRASPPALLPGPAPRGDRPELLTPSSLHYSTLQQQQQRTREWVARHPQVPEARDQGPIRAWSVLPDNFLQLPLEPGSTEPN
SEPPDPCLSSHSLFPVVPDESWESWAGNPSLTVFRSIRCQLSLQDLDDFVDQESDGSEESSSGPKDSPGASEEGLQVVLGTPDRGKL
RNPAGGLSVSRKEGSPSRSPQGLRNRGDGHISQQVPAGANGLAGHPLKPLPWPVPKLRRSLRRSSLAGRAKLSSSDEEYLDEGLLKR
SRRPPRSRKPSKAGTAPSPRVDAGLSLKLAEVKAVVAERGWRHSLWVPSGEGSAALAPHRTSEHKSSLVPLDAREHEWIVKLASGSW
IQVWTLFWEDPQLALHKDFLTGYTALHWIAKHGDLRALQDLVSGAKKAGIVLDVNVRSSCGYTPLHLAAIHGHQGVIKLLVQRLASR
VNVRDSSGKKPWQYLTSNTSGEIWQLLGAPRGKPIFPVYPLVGSSSPTRKAKSKEISRSVTRKTSFAALLKSQHNKWKLANQYEKFH
SPREREEYSD
>sp|Q96T59|CDRTF_HUMAN

MFSCCFPTSRGCCFRNGGSESLFRRCRRRLIPHPRRLSPVVIRRIQVPQDSLGQALAGQATPEIPLGLQLHTVLVQEIQELIEAQTL
APGPCAEVRALPAPAAEPEPAWEEAPPERALELEGAPAKDQTNEELPEITEVPESIKRRLGRRVPAATPAPRGNLLLQAWMRVHSWA
SRLFAPNVLPGTGP
>sp|Q96PT3|DUX5_HUMAN
MPAEVHGSPPASLCPCQSVKFRPGLPEMALLTALDDTLPEEAQGPGRRMILLSTPSQSDALRACFERNLYPGIATKEELAQGIDIPE
PRVQIWFQNERSCQLRQHRRQSRPWPGRRDPQKGRRKRTAITGSQTALLLRAFEKDRFPGIAAREELARETGLPESRIQIWFQNRRA
RHRGQSGRAPTQASIRCNAAPIG
>sp|Q9BQD3|KXDL1_HUMAN
MDLPDSASRVFCGRILSMVNTDDVNAIILAQKNMLDRFEKTNEMLLNFNNLSSARLQQMSERFLHHTRTLVEMKRDLDSIFRRIRTL
KGKLARQHPEAFSHIPEASFLEEEDEDPIPPSTTTTIATSEQSTGSCDTSPDTVSPSLSPGFEDLSHVQPGSPAINGRSQTDDEEMT
GE
>sp|Q96N21|AP4AT_HUMAN
MAAAPPLRDRLSFLHRLPILLKGTSDDDVPCPGYLFEEIAKISHESPGSSQCLLEYLLSRLHSSSGHGKLKVLKILLYLCSHGSSFF
LLILKRNSAFIQEAAAFAGPPDPLHGNSLYQKVRAAAQDLGSTLFSDTVLPLAPSQPLGTPPATGMGSQARPHSTLQGFGYSKEHGR
TAVRHQPGQAGGGWDELDSGPSSQNSSQNSDLSRVSDSGSHSGSDSHSGASREPGDLAERVEVVALSDCQQELSLVRTVTRGPRAFL
SREEAQHFIKACGLLNCEAVLQLLTCHLRGTSECTQLRALCAIASLGSSDLLPQEHILLRTRPWLQELSMGSPGPVTNKATKILRHF
EASCGQLSPARGTSAEPGPTAALPGPSDLLTDAVPLPGSQVFLQPLSSTPVSSRSPAPSSGMPSSPVPTPPPDASPIPAPGDPSEAE
ARLAESRRWRPERIPGGTDSPKRGPSSCAWSRDSLFAGMELVACPRLVGAGAAAGESCPDAPRAPQTSSQRTAAKEPPGSEPSAFAF
LNA
>sp|Q5SQQ9|VAX1_HUMAN
MFGKPDKMDVRCHSDAEAARVSKNAHKESRESKGAEGNLPAAFLKEPQGAFSASGAAEDCNKSKSNSAADPDYCRRILVRDAKGSIR
EIILPKGLDLDRPKRTRTSFTAEQLYRLEMEFQRCQYVVGRERTELARQLNLSETQVKVWFQNRRTKQKKDQGKDSELRSVVSETAA
TCSVLRLLEQGRLLSPPGLPALLPPCATGALGSALRGPSLPALGAGAAAGSAAAAAAAAPGPAGAASPHPPAVGGAPGPGPAGPGGL
HAGAPAAGHSLFSLPVPSLLGSVASRLSSAPLTMAGSLAGNLQELSARYLSSSAFEPYSRTNNKEGAEKKALD
>sp|P02549|SPTA1_HUMAN
MEQFPKETVVESSGPKVLETAEEIQERRQEVLTRYQSFKERVAERGQKLEDSYHLQVFKRDADDLGKWIMEKVNILTDKSYEDPTNI
QGKYQKHQSLEAEVQTKSRLMSELEKTREERFTMGHSAHEETKAHIEELRHLWDLLLELTLEKGDQLLRALKFQQYVQECADILEWI
GDKEAIATSVELGEDWERTEVLHKKFEDFQVELVAKEGRVVEVNQYANECAEENHPDLPLIQSKQNEVNAAWERLRGLALQRQKALS
NAANLQRFKRDVTEAIQWIKEKEPVLTSEDYGKDLVASEGLFHSHKGLERNLAVMSDKVKELCAKAEKLTLSHPSDAPQIQEMKEDL
VSSWEHIRALATSRYEKLQATYWYHRFSSDFDELSGWMNEKTAAINADELPTDVAGGEVLLDRHQQHKHEIDSYDDRFQSADETGQD
LVNANHEASDEVREKMEILDNNWTALLELWDERHRQYEQCLDFHLFYRDSEQVDSWMSRQEAFLENEDLGNSLGSAEALLQKHEDFE
EAFTAQEEKIITVDKTATKLIGDDHYDSENIKAIRDGLLARRDALREKAATRRRLLKESLLLQKLYEDSDDLKNWINKKKKLADDED
YKDIQNLKSRVQKQQVFEKELAVNKTQLENIQKTGQEMIEGGHYASDNVTTRLSEVASLWEELLEATKQKGTQLHEANQQLQFENNA
EDLQRWLEDVEWQVTSEDYGKGLAEVQNRLRKHGLLESAVAARQDQVDILTDLAAYFEEIGHPDSKDIRARQESLVCRFEALKEPLA
TRKKKLLDLLHLQLICRDTEDEEAWIQETEPSATSTYLGKDLIASKKLLNRHRVILENIASHEPRIQEITERGNKMVEEGHFAAEDV
ASRVKSLNQNMESLRARAARRQNDLEANVQFQQYLADLHEAETWIREKEPIVDNTNYGADEEAAGALLKKHEAFLLDLNSFGDSMKA
LRNQANACQQQQAAPVEGVAGEQRVMALYDFQARSPREVTMKKGDVLTLLSSINKDWWKVEAADHQGIVPAVYVRRLAHDEFPMLPQ
RRREEPGNITQRQEQIENQYRSLLDRAEERRRRLLQRYNEFLLAYEAGDMLEWIQEKKAENTGVELDDVWELQKKFDEFQKDLNTNE
PRLRDINKVADDLLFEGLLTPEGAQIRQELNSRWGSLQRLADEQRQLLGSAHAVEVFHREADDTKEQIEKKCQALSAADPGSDLFSV
QALQRRHEGFERDLVPLGDKVTILGETAERLSESHPDATEDLQRQKMELNEAWEDLQGRTKDRKESLNEAQKFYLFLSKARDLQNWI
SSIGGMVSSQELAEDLTGIEILLERHQEHRADMEAEAPTFQALEDFSAELIDSGHHASPEIEKKLQAVKLERDDLEKAWEKRKKILD
QCLELQMFQGNCDQVESWMVARENSLRSDDKSSLDSLEALMKKRDDLDKAITAQEGKITDLEHFAESLIADEHYAKEEIATRLQRVL
DRWKALKAQLIDERTKLGDYANLKQFYRDLEELEEWISEMLPTACDESYKDATNIQRKYLKHQTFAHEVDGRSEQVHGVINLGNSLI
ECSACDGNEEAMKEQLEQLKEHWDHLLERTNDKGKKLNEASRQQRFNTSIRDFEFWLSEAETLLAMKDQARDLASAGNLLKKHQLLE

REMLAREDALKDLNTLAEDLLSSGTFNVDQIVKKKDNVNKRFLNVQELAAAHHEKLKEAYALFQFFQDLDDEESWIEEKLIRVSSQD
YGRDLQGVQNLLKKHKRLEGELVAHEPAIQNVLDMAEKLKDKAAVGQEEIQLRLAQFVEHWEKLKELAKARGLKLEESLEYLQFMQN
AEEEEAWINEKNALAVRGDCGDTLAATQSLLMKHEALENDFAVHETRVQNVCAQGEDILNKVLQEESQNKEISSKIEALNEKTPSLA
KAIAAWKLQLEDDYAFQEFNWKADVVEAWIADKETSLKTNGNGADLGDFLTLLAKQDTLDASLQSFQQERLPEITDLKDKLISAQHN
QSKAIEERYAALLKRWEQLLEASAVHRQKLLEKQLPLQKAEDLFVEFAHKASALNNWCEKMEENLSEPVHCVSLNEIRQLQKDHEDF
LASLARAQADFKCLLELDQQIKALGVPSSPYTWLTVEVLERTWKHLSDIIEEREQELQKEEARQVKNFEMCQEFEQNASTFLQWILE
TRAYFLDGSLLKETGTLESQLEANKRKQKEIQAMKRQLTKIVDLGDNLEDALILDIKYSTIGLAQQWDQLYQLGLRMQHNLEQQIQA
KDIKGVSEETLKEFSTIYKHFDENLTGRLTHKEFRSCLRGLNYYLPMVEEDEHEPKFEKFLDAVDPGRKGYVSLEDYTAFLIDKESE
NIKSSDEIENAFQALAEGKSYITKEDMKQALTPEQVSFCATHMQQYMDPRGRSHLSGYDYVGFTNSYFGN
>sp|Q9UNP4|SIAT9_HUMAN
MRTKAAGCAERRPLQPRTEAAAAPAGRAMPSEYTYVKLRSDCSRPSLQWYTRAQSKMRRPSLLLKDILKCTLLVFGVWILYILKLNY
TTEECDMKKMHYVDPDHVKRAQKYAQQVLQKECRPKFAKTSMALLFEHRYSVDLLPFVQKAPKDSEAESKYDPPFGFRKFSSKVQTL
LELLPEHDLPEHLKAKTCRRCVVIGSGGILHGLELGHTLNQFDVVIRLNSAPVEGYSEHVGNKTTIRMTYPEGAPLSDLEYYSNDLF
VAVLFKSVDFNWLQAMVKKETLPFWVRLFFWKQVAEKIPLQPKHFRILNPVIIKETAFDILQYSEPQSRFWGRDKNVPTIGVIAVVL
ATHLCDEVSLAGFGYDLNQPRTPLHYFDSQCMAAMNFQTMHNVTTETKFLLKLVKEGVVKDLSGGIDREF
>sp|F8VTS6|RFAL1_HUMAN
MAEHFKQIIRCPVCLKDLEEAVQLKCGYACCLQCLNSLQKEPDGEGLLCRFCSVVSQKDDIKPKYKLRALVSIIKELEPKLKSVLTM
NPRMRKFQVDMTFDVDTANNYLIISEDLRSFRSGDLSQNRKEQAERFDTALCVLGTPRFTSGRHYWEVDVGTSQVWDVGVCKESVNR
QGKIELSSEHGFLTVGCREGKVFAASTVPMTPLWVSPQLHRVGIFLDVGMRSIAFYNVSDGCHINTFIEIPVCEPWRPFFAHKRGSQ
DDQSILSICSVINPSTASAPVSSEGK
>sp|Q8WWQ2|HPSE2_HUMAN
MRVLCAFPEAMPSSNSRPPACLAPGALYLALLLHLSLSSQAGDRRPLPVDRAAGLKEKTLILLDVSTKNPVRTVNENFLSLQLDPSI
IHDGWLDFLSSKRLVTLARGLSPAFLRFGGKRTDFLQFQNLRNPAKSRGGPGPDYYLKNYEDDIVRSDVALDKQKGCKIAQHPDVML
ELQREKAAQMHLVLLKEQFSNTYSNLILTARSLDKLYNFADCSGLHLIFALNALRRNPNNSWNSSSALSLLKYSASKKYNISWELGN
EPNNYRTMHGRAVNGSQLGKDYIQLKSLLQPIRIYSRASLYGPNIGRPRKNVIALLDGFMKVAGSTVDAVTWQHCYIDGRVVKVMDF
LKTRLLDTLSDQIRKIQKVVNTYTPGKKIWLEGVVTTSAGGTNNLSDSYAAGFLWLNTLGMLANQGIDVVIRHSFFDHGYNHLVDQN
FNPLPDYWLSLLYKRLIGPKVLAVHVAGLQRKPRPGRVIRDKLRIYAHCTNHHNHNYVRGSITLFIINLHRSRKKIKLAGTLRDKLV
HQYLLQPYGQEGLKSKSVQLNGQPLVMVDDGTLPELKPRPLRAGRTLVIPPVTMGFYVVKNVNALACRYR
>sp|Q8TBF2|PGFS_HUMAN
MSTVDLARVGACILKHAVTGEAVELRSLWREHACVVAGLRRFGCVVCRWIAQDLSSLAGLLDQHGVRLVGVGPEALGLQEFLDGDYF
AGELYLDESKQLYKELGFKRYNSLSILPAALGKPVRDVAAKAKAVGIQGNLSGDLLQSGGLLVVSKGGDKVLLHFVQKSPGDYVPKE
HILQVLGISAEVCASDPPQCDREV
>sp|Q9NXD2|MTMRA_HUMAN
MFSLKPPKPTFRSYLLPPPQTDDKINSEPKIKKLEPVLLPGEIVVNEVNFVRKCIATDTSQYDLWGKLICSNFKISFITDDPMPLQK
FHYRNLLLGEHDVPLTCIEQIVTVNDHKRKQKVLGPNQKLKFNPTELIIYCKDFRIVRFRFDESGPESAKKVCLAIAHYSQPTDLQL
LFAFEYVGKKYHNSANKINGIPSGDGGGGGGGGNGAGGGSSQKTPLFETYSDWDREIKRTGASGWRVCSINEGYMISTCLPEYIVVP
SSLADQDLKIFSHSFVGRRMPLWCWSHSNGSALVRMALIKDVLQQRKIDQRICNAITKSHPQRSDVYKSDLDKTLPNIQEVQAAFVK
LKQLCVNEPFEETEEKWLSSLENTRWLEYVRAFLKHSAELVYMLESKHLSVVLQEEEGRDLSCCVASLVQVMLDPYFRTITGFQSLI
QKEWVMAGYQFLDRCNHLKRSEKESPLFLLFLDATWQLLEQYPAAFEFSETYLAVLYDSTRISLFGTFLFNSPHQRVKQSTEFAISK
NIQLGDEKGLKFPSVWDWSLQFTAKDRTLFHNPFYIGKSTPCIQNGSVKSFKRTKKSYSSTLRGMPSALKNGIISDQELLPRRNSLI
LKPKPDPAQQTDSQNSDTEQYFREWFSKPANLHGVILPRVSGTHIKLWKLCYFRWVPEAQISLGGSITAFHKLSLLADEVDVLSRML
RQQRSGPLEACYGELGQSRMYFNASGPHHTDTSGTPEFLSSSFPFSPVGNLCRRSILGTPLSKFLSGAKIWLSTETLANED
>sp|Q9UID6|ZN639_HUMAN

MNEYPKKRKRKTLHPSRYSDSSGISRIADGFNGIFSDHCYSVCSMRQPDLKYFDNKDDDSDTETSNDLPKFADGIKARNRNQNYLVP
SPVLRILDHTAFSTEKSADIVICDEECDSPESVNQQTQEESPIEVHTAEDVPIAVEVHAISEDYDIETENNSSESLQDQTDEEPPAK
LCKILDKSQALNVTAQQKWPLLRANSSGLYKCELCEFNSKYFSDLKQHMILKHKRTDSNVCRVCKESFSTNMLLIEHAKLHEEDPYI
CKYCDYKTVIFENLSQHIADTHFSDHLYWCEQCDVQFSSSSELYLHFQEHSCDEQYLCQFCEHETNDPEDLHSHVVNEHACKLIELS
DKYNNGEHGQYSLLSKITFDKCKNFFVCQVCGFRSRLHTNVNRHVAIEHTKIFPHVCDDCGKGFSSMLEYCKHLNSHLSEGIYLCQY
CEYSTGQIEDLKIHLDFKHSADLPHKCSDCLMRFGNERELISHLPVHETT
>sp|P56377|AP1S2_HUMAN
MQFMLLFSRQGKLRLQKWYVPLSDKEKKKITRELVQTVLARKPKMCSFLEWRDLKIVYKRYASLYFCCAIEDQDNELITLEIIHRYV
ELLDKYFGSVCELDIIFNFEKAYFILDEFLLGGEVQETSKKNVLKAIEQADLLQEEAETPRSVLEEIGLT
>sp|Q6NZY4|ZCHC8_HUMAN
MAAEVYFGDLELFEPFDHPEESIPKPVHTRFKDDDGDEEDENGVGDAELRERLRQCEETIEQLRAENQELKRKLNILTRPSGILVND
TKLDGPILQILFMNNAISKQYHQEIEEFVSNLVKRFEEQQKNDVEKTSFNLLPQPSSIVLEEDHKVEESCAIKNNKEAFSVVGSVLY
FTNFCLDKLGQPLLNENPQLSEGWEIPKYHQVFSHIVSLEGQEIQVKAKRPKPHCFNCGSEEHQMKDCPMPRNAARISEKRKEYMDA
CGEANNQNFQQRYHAEEVEERFGRFKPGVISEELQDALGVTDKSLPPFIYRMRQLGYPPGWLKEAELENSGLALYDGKDGTDGETEV
GEIQQNKSVTYDLSKLVNYPGFNISTPRGIPDEWRIFGSIPMQACQQKDVFANYLTSNFQAPGVKSGNKRSSSHSSPGSPKKQKNES
NSAGSPADMELDSDMEVPHGSQSSESFQFQPPLPPDTPPLPRGTPPPVFTPPLPKGTPPLTPSDSPQTRTASGAVDEDALTLEELEE
QQRRIWAALEQAESVNSDSDVPVDTPLTGNSVASSPCPNELDLPVPEGKTSEKQTLDEPEVPEIFTKKSEAGHASSPDSEVTSLCQK
EKAELAPVNTEGALLDNGSVVPNCDISNGGSQKLFPADTSPSTATKIHSPIPDMSKFATGITPFEFENMAESTGMYLRIRSLLKNSP
RNQQKNKKASE
>sp|Q9NUM3|S39A9_HUMAN
MDDFISISLLSLAMLVGCYVAGIIPLAVNFSEERLKLVTVLGAGLLCGTALAVIVPEGVHALYEDILEGKHHQASETHNVIASDKAA
EKSVVHEHEHSHDHTQLHAYIGVSLVLGFVFMLLVDQIGNSHVHSTDDPEAARSSNSKITTTLGLVVHAAADGVALGAAASTSQTSV
QLIVFVAIMLHKAPAAFGLVSFLMHAGLERNRIRKHLLVFALAAPVMSMVTYLGLSKSSKEALSEVNATGVAMLFSAGTFLYVATVH
VLPEVGGIGHSHKPDATGGRGLSRLEVAALVLGCLIPLILSVGHQH
>sp|Q92928|RAB1C_HUMAN
MNPGYDCLFKLLLIGDSGVGKSCLLLRFADDPYTESYISTIGVDFKIQTIELDGKTIKLQIWDTAGQERFWTITSSYYRGAHGFLVV
YDVTDQESYANVKQWLQEIDRHASENVNKLLVGNKSDLTTKKVVDNTTAKEFADSLGIPFLETSAKNATNVEQAFMTMAAEIKKQMG
PGAASGGERPNLKIDSTPVKPAGGGCC
>sp|Q8IUD6|RN135_HUMAN
MAGLGLGSAVPVWLAEDDLGCIICQGLLDWPATLPCGHSFCRHCLEALWGARDARRWACPTCRQGAAQQPHLRKNTLLQDLADKYRR
AAREIQAGSDPAHCPCPGSSSLSSAAARPRRRPELQRVAVEKSITEVAQELTELVEHLVDIVRSLQNQRPLSESGPDNELSILGKAF
SSGVDLSMASPKLVTSDTAAGKIRDILHDLEEIQEKLQESVTWKEAPEAQMQGELLEAPSSSSCPLPDQSHPALRRASRFAQWAIHP
TFNLKSLSCSLEVSKDSRTVTVSHRPQPYRWSCERFSTSQVLCSQALSSGKHYWEVDTRNCSHWAVGVASWEMSRDQVLGRTMDSCC
VEWKGTSQLSAWHMVKETVLGSDRPGVVGIWLNLEEGKLAFYSVDNQEKLLYECTISASSPLYPAFWLYGLHPGNYLIIKQVKV
>sp|P0CK97|S35E2_HUMAN
MSSSVKTPALEELVPGSEEKPKGRSPLSWGSLFGHRSEKIVFAKSDGGTDENVLTVTITETTVIESDLGVWSSRALLYLTLWFFFSF
CTLFLNKYILSLLGGEPSMLGAVQMLSTTVIGCVKTLVPCCLYQHKARLSYPPNFLMTMLFVGLMRFATVVLGLVSLKNVAVSFAET
VKSSAPIFTVIMSRMILGEYTGRPSDREEREELQLQPGRGAAASDRRSPVPPSERHGVRPHGENLPGDFQVPQALHRVALSMALPCP
MLPAS
>sp|A6NJS3|IV1U1_HUMAN
MDWNWRILFLVVIAAGAQSQVQLVQSGAEVKKPGASVKVSCKASGYTITSYCMHWVHQVHAQGLEWMGLVCPSDGSTSYAQKFQARV
TITRDTSMSTAYMELSSLRSEDTAMYYCVRDTM
>sp|O75828|CBR3_HUMAN

MSSCSRVALVTGANRGIGLAIARELCRQFSGDVVLTARDVARGQAAVQQLQAEGLSPRFHQLDIDDLQSIRALRDFLRKEYGGLNVL
VNNAAVAFKSDDPMPFDIKAEMTLKTNFFATRNMCNELLPIMKPHGRVVNISSLQCLRAFENCSEDLQERFHSETLTEGDLVDLMKK
FVEDTKNEVHEREGWPNSPYGVSKLGVTVLSRILARRLDEKRKADRILVNACCPGPVKTDMDGKDSIRTVEEGAETPVYLALLPPDA
TEPQGQLVHDKVVQNW
>sp|Q9Y6F7|CDY2_HUMAN
MASQEFEVEAIVDKRQDKNGNTQYLVRWKGYDKQDDTWEPEQHLMNCEKCVHDFNRRQTEKQKKLTWTTTSRIFSNNARRRTSRSTK
ANYSKNSPKTPVTDKHHRSKNCKLFAASKNVRRKAASTLSDTKNMEIINSTIETLAPDSPFDHKKTVSGFQKLEKLDPIAADQQDTV
VFKVTEGKLLRDPLSHPGAEQTGIQNKTQMHPLMSQMSGSVTASMATGSATRKGIVVLIDPLAANGTTDMHTSVPRVKGGQRNITDD
SRGQPFIKKMHFTIRLTESAITYRDIVVKKEDGFTQIVLSTRSTEKNALNTEVIKEMVNALNSAAADDSKLVLFSAAGSVFCCGLDF
GYFVRHLRNDRNTASLEMVDTIKNFVNTFIQFKKPIVVSVNGPAIGLGASILPLCDLVWANEKAWFQTPYTTFGQSPDGCSSITFPK
MMGKASANEMLIAGRKLTAREACAKGLVSQVFLTGTFTQEVMIQIKELASYNAIVLEECKALVRCNIKLELEQANERECEVLRKIWS
SAQGIESMLKYVENKIDEF
>sp|Q0VAQ4|SMAGP_HUMAN
MTSLLTTPSPREELMTTPILQPTEALSPEDGASTALIAVVITVVFLTLLSVVILIFFYLYKNKGSYVTYEPTEGEPSAIVQMESDLA
KGSEKEEYFI
>sp|P55286|CADH8_HUMAN
MPERLAEMLLDLWTPLIILWITLPPCIYMAPMNQSQVLMSGSPLELNSLGEEQRILNRSKRGWVWNQMFVLEEFSGPEPILVGRLHT
DLDPGSKKIKYILSGDGAGTIFQINDVTGDIHAIKRLDREEKAEYTLTAQAVDWETSKPLEPPSEFIIKVQDINDNAPEFLNGPYHA
TVPEMSILGTSVTNVTATDADDPVYGNSAKLVYSILEGQPYFSIEPETAIIKTALPNMDREAKEEYLVVIQAKDMGGHSGGLSGTTT
LTVTLTDVNDNPPKFAQSLYHFSVPEDVVLGTAIGRVKANDQDIGENAQSSYDIIDGDGTALFEITSDAQAQDGIIRLRKPLDFETK
KSYTLKVEAANVHIDPRFSGRGPFKDTATVKIVVEDADEPPVFSSPTYLLEVHENAALNSVIGQVTARDPDITSSPIRFSIDRHTDL
ERQFNINADDGKITLATPLDRELSVWHNITIIATEIRNHSQISRVPVAIKVLDVNDNAPEFASEYEAFLCENGKPGQVIQTVSAMDK
DDPKNGHYFLYSLLPEMVNNPNFTIKKNEDNSLSILAKHNGFNRQKQEVYLLPIIISDSGNPPLSSTSTLTIRVCGCSNDGVVQSCN
VEAYVLPIGLSMGALIAILACIILLLVIVVLFVTLRRHKNEPLIIKDDEDVRENIIRYDDEGGGEEDTEAFDIATLQNPDGINGFLP
RKDIKPDLQFMPRQGLAPVPNGVDVDEFINVRLHEADNDPTAPPYDSIQIYGYEGRGSVAGSLSSLESTTSDSDQNFDYLSDWGPRF
KRLGELYSVGESDKET
>sp|Q15223|NECT1_HUMAN
MARMGLAGAAGRWWGLALGLTAFFLPGVHSQVVQVNDSMYGFIGTDVVLHCSFANPLPSVKITQVTWQKSTNGSKQNVAIYNPSMGV
SVLAPYRERVEFLRPSFTDGTIRLSRLELEDEGVYICEFATFPTGNRESQLNLTVMAKPTNWIEGTQAVLRAKKGQDDKVLVATCTS
ANGKPPSVVSWETRLKGEAEYQEIRNPNGTVTVISRYRLVPSREAHQQSLACIVNYHMDRFKESLTLNVQYEPEVTIEGFDGNWYLQ
RMDVKLTCKADANPPATEYHWTTLNGSLPKGVEAQNRTLFFKGPINYSLAGTYICEATNPIGTRSGQVEVNITEFPYTPSPPEHGRR
AGPVPTAIIGGVAGSILLVLIVVGGIVVALRRRRHTFKGDYSTKKHVYGNGYSKAGIPQHHPPMAQNLQYPDDSDDEKKAGPLGGSS
YEEEEEEEEGGGGGERKVGGPHPKYDEDAKRPYFTVDEAEARQDGYGDRTLGYQYDPEQLDLAENMVSQNDGSFISKKEWYV
>sp|P21108|PRPS3_HUMAN
MPNIKIFSGSSHQDLSQKIADRLGLELGKVVTKKFSNQETCVEIDESVRGEDVYIVQSGCGEINDSLMELLIMINACKIASASRVTA
VIPCFPYARQDKKDKSRSPISAKLVANMLSIAGADHIITMDLHASQIQGFFDIPVDNLYAEPTVLKWIRENIPEWKNCIIVSPDAGG
AKRVTSIADQLNVDFALIHKERKKANEVDCIVLVGDVNDRVAILVDDMADTCVTICLAADKLLSAGATRVYAILTHGIFSGPAISRI
NTACFEAVVVTNTIPQDEKMKHCSKIRVIDISMILAEAIRRTHNGESVSYLFSHVPL
>sp|Q8NGK9|OR5DG_HUMAN
MFLTERNTTSEATFTLLGFSDYLELQIPLFFVFLAVYGFSVVGNLGMIVIIKINPKLHTPMYFFLNHLSFVDFCYSSIIAPMMLVNL
VVEDRTISFSGCLVQFFFFCTFVVTELILFAVMAYDHFVAICNPLLYTVAISQKLCAMLVVVLYAWGVACSLTLACSALKLSFHGFN
TINHFFCELSSLISLSYPDSYLSQLLLFTVATFNEISTLLIILTSYAFIIVTTLKMPSASGHRKVFSTCASHLTAITIFHGTILFLY
CVPNSKNSRHTVKVASVFYTVVIPLLNPLIYSLRNKDVKDAIRKIINTKYFHIKHRHWYPFNFVIEQ
>sp|Q16653|MOG_HUMAN

MASLSRPSLPSCLCSFLLLLLLQVSSSYAGQFRVIGPRHPIRALVGDEVELPCRISPGKNATGMEVGWYRPPFSRVVHLYRNGKDQD
GDQAPEYRGRTELLKDAIGEGKVTLRIRNVRFSDEGGFTCFFRDHSYQEEAAMELKVEDPFYWVSPGVLVLLAVLPVLLLQITVGLI
FLCLQYRLRGKLRAEIENLHRTFDPHFLRVPCWKITLFVIVPVLGPLVALIICYNWLHRRLAGQFLEELRNPF
>sp|Q9NRM7|LATS2_HUMAN
MRPKTFPATTYSGNSRQRLQEIREGLKQPSKSSVQGLPAGPNSDTSLDAKVLGSKDATRQQQQMRATPKFGPYQKALREIRYSLLPF
ANESGTSAAAEVNRQMLQELVNAGCDQEMAGRALKQTGSRSIEAALEYISKMGYLDPRNEQIVRVIKQTSPGKGLMPTPVTRRPSFE
GTGDSFASYHQLSGTPYEGPSFGADGPTALEEMPRPYVDYLFPGVGPHGPGHQHQHPPKGYGASVEAAGAHFPLQGAHYGRPHLLVP
GEPLGYGVQRSPSFQSKTPPETGGYASLPTKGQGGPPGAGLAFPPPAAGLYVPHPHHKQAGPAAHQLHVLGSRSQVFASDSPPQSLL
TPSRNSLNVDLYELGSTSVQQWPAATLARRDSLQKPGLEAPPRAHVAFRPDCPVPSRTNSFNSHQPRPGPPGKAEPSLPAPNTVTAV
TAAHILHPVKSVRVLRPEPQTAVGPSHPAWVPAPAPAPAPAPAPAAEGLDAKEEHALALGGAGAFPLDVEYGGPDRRCPPPPYPKHL
LLRSKSEQYDLDSLCAGMEQSLRAGPNEPEGGDKSRKSAKGDKGGKDKKQIQTSPVPVRKNSRDEEKRESRIKSYSPYAFKFFMEQH
VENVIKTYQQKVNRRLQLEQEMAKAGLCEAEQEQMRKILYQKESNYNRLKRAKMDKSMFVKIKTLGIGAFGEVCLACKVDTHALYAM
KTLRKKDVLNRNQVAHVKAERDILAEADNEWVVKLYYSFQDKDSLYFVMDYIPGGDMMSLLIRMEVFPEHLARFYIAELTLAIESVH
KMGFIHRDIKPDNILIDLDGHIKLTDFGLCTGFRWTHNSKYYQKGSHVRQDSMEPSDLWDDVSNCRCGDRLKTLEQRARKQHQRCLA
HSLVGTPNYIAPEVLLRKGYTQLCDWWSVGVILFEMLVGQPPFLAPTPTETQLKVINWENTLHIPAQVKLSPEARDLITKLCCSADH
RLGRNGADDLKAHPFFSAIDFSSDIRKQPAPYVPTISHPMDTSNFDPVDEESPWNDASEGSTKAWDTLTSPNNKHPEHAFYEFTFRR
FFDDNGYPFRCPKPSGAEASQAESSDLESSDLVDQTEGCQPVYV
>sp|O43283|M3K13_HUMAN
MANFQEHLSCSSSPHLPFSESKTFNGLQDELTAMGNHPSPKLLEDQQEKGMVRTELIESVHSPVTTTVLTSVSEDSRDQFENSVLQL
REHDESETAVSQGNSNTVDGESTSGTEDIKIQFSRSGSGSGGFLEGLFGCLRPVWNIIGKAYSTDYKLQQQDTWEVPFEEISELQWL
GSGAQGAVFLGKFRAEEVAIKKVREQNETDIKHLRKLKHPNIIAFKGVCTQAPCYCIIMEYCAHGQLYEVLRAGRKITPRLLVDWST
GIASGMNYLHLHKIIHRDLKSPNVLVTHTDAVKISDFGTSKELSDKSTKMSFAGTVAWMAPEVIRNEPVSEKVDIWSFGVVLWELLT
GEIPYKDVDSSAIIWGVGSNSLHLPVPSTCPDGFKILMKQTWQSKPRNRPSFRQTLMHLDIASADVLATPQETYFKSQAEWREEVKK
HFEKIKSEGTCIHRLDEELIRRRREELRHALDIREHYERKLERANNLYMELSAIMLQLEMREKELIKREQAVEKKYPGTYKRHPVRP
IIHPNAMEKLMKRKGVPHKSGMQTKRPDLLRSEGIPTTEVAPTASPLSGSPKMSTSSSKSRYRSKPRHRRGNSRGSHSDFAAILKNQ
PAQENSPHPTYLHQAQSQYPSLHHHNSLQQQYQQPPPAMSQSHHPRLNMHGQDIATCANNLRYFGPAAALRSPLSNHAQRQLPGSSP
DLISTAMAADCWRSSEPDKGQAGPWGCCQADAYDPCLQCRPEQYGSLDIPSAEPVGRSPDLSKSPAHNPLLENAQSSEKTEENEFSG
CRSESSLGTSHLGTPPALPRKTRPLQKSGDDSSEEEEGEVDSEVEFPRRQRPHRCISSCQSYSTFSSENFSVSDGEEGNTSDHSNSP
DELADKLEDRLAEKLDDLLSQTPEIPIDISSHSDGLSDKECAVRRVKTQMSLGKLCVEERGYENPMQFEESDCDSSDGECSDATVRT
NKHYSSATW
>sp|Q96DX8|RTP4_HUMAN
MVVDFWTWEQTFQELIQEAKPRATWTLKLDGNLQLDCLAQGWKQYQQRAFGWFRCSSCQRSWASAQVQILCHTYWEHWTSQGQVRMR
LFGQRCQKCSWSQYEMPEFSSDSTMRILSNLVQHILKKYYGNGTRKSPEMPVILEVSLEGSHDTANCEACTLGICGQGLKSCMTKPS
KSLLPHLKTGNSSPGIGAVYLANQAKNQSAEAKEAKGSGYEKLGPSRDPDPLNICVFILLLVFIVVKCFTSE
>sp|Q8N4F0|BPIB2_HUMAN
MAWASRLGLLLALLLPVVGASTPGTVVRLNKAALSYVSEIGKAPLQRALQVTVPHFLDWSGEALQPTRIRILNVHVPRLHLKFIAGF
GVRLLAAANFTFKVFRAPEPLELTLPVELLADTRVTQSSIRTPVVSISACSLFSGHANEFDGSNSTSHALLVLVQKHIKAVLSNKLC
LSISNLVQGVNVHLGTLIGLNPVGPESQIRYSMVSVPTVTSDYISLEVNAVLFLLGKPIILPTDATPFVLPRHVGTEGSMATVGLSQ
QLFDSALLLLQKAGALNLDITGQLRSDDNLLNTSALGRLIPEVARQFPEPMPVVLKVRLGATPVAMLHTNNATLRLQPFVEVLATAS
NSAFQSLFSLDVVVNLRLQLSVSKVKLQGTTSVLGDVQLTVASSNVGFIDTDQVRTLMGTVFEKPLLDHLNALLAMGIALPGVVNLH
YVAPEIFVYEGYVVISSGLFYQS
>sp|Q9P0L1|ZKSC7_HUMAN
MTTAGRGNLGLIPRSTAFQKQEGRLTVKQEPANQTWGQGSSLQKNYPPVCEIFRLHFRQLCYHEMSGPQEALSRLRELCRWWLMPEV
HTKEQILELLVLEQFLSILPGELRTWVQLHHPESGEEAVAVVEDFQRHLSGSEEVSAPAQKQEMHFEETTALGTTKESPPTSPLSGG

SAPGAHLEPPYDPGTHHLPSGDFAQCTSPVPTLPQVGNSGDQAGATVLRMVRPQDTVAYEDLSVDYTQKKWKSLTLSQRALQWNMMP
ENHHSMASLAGENMMKGSELTPKQEFFKGSESSNRTSGGLFGVVPGAAETGDVCEDTFKELEGQTSDEEGSRLENDFLEITDEDKKK
STKDRYDKYKEVGEHPPLSSSPVEHEGVLKGQKSYRCDECGKAFNRSSHLIGHQRIHTGEKPYECNECGKTFRQTSQLIVHLRTHTG
EKPYECSECGKAYRHSSHLIQHQRLHNGEKPYKCNECAKAFTQSSRLTDHQRTHTGEKPYECNECGEAFIRSKSLARHQVLHTGKKP
YKCNECGRAFCSNRNLIDHQRIHTGEKPYECSECGKAFSRSKCLIRHQSLHTGEKPYKCSECGKAFNQNSQLIEHERIHTGEKPFEC
SECGKAFGLSKCLIRHQRLHTGEKPYKCNECGKSFNQNSHLIIHQRIHTGEKPYECNECGKVFSYSSSLMVHQRTHTGEKPYKCNDC
GKAFSDSSQLIVHQRVHTGEKPYECSECGKAFSQRSTFNHHQRTHTGEKSSGLAWSVS
>sp|Q8IYB7|DI3L2_HUMAN
MSHPDYRMNLRPLGTPRGVSAVAGPHDIGASPGDKKSKNRSTRGKKKSIFETYMSKEDVSEGLKRGTLIQGVLRINPKKFHEAFIPS
PDGDRDIFIDGVVARNRALNGDLVVVKLLPEEHWKVVKPESNDKETEAAYESDIPEELCGHHLPQQSLKSYNDSPDVIVEAQFDGSD
SEDGHGITQNVLVDGVKKLSVCVSEKGREDGDAPVTKDETTCISQDTRALSEKSLQRSAKVVYILEKKHSRAATGFLKLLADKNSEL
FRKYALFSPSDHRVPRIYVPLKDCPQDFVARPKDYANTLFICRIVDWKEDCNFALGQLAKSLGQAGEIEPETEGILTEYGVDFSDFS
SEVLECLPQGLPWTIPPEEFSKRRDLRKDCIFTIDPSTARDLDDALSCKPLADGNFKVGVHIADVSYFVPEGSDLDKVAAERATSVY
LVQKVVPMLPRLLCEELCSLNPMSDKLTFSVIWTLTPEGKILDEWFGRTIIRSCTKLSYEHAQSMIESPTEKIPAKELPPISPEHSS
EEVHQAVLNLHGIAKQLRQQRFVDGALRLDQLKLAFTLDHETGLPQGCHIYEYRESNKLVEEFMLLANMAVAHKIHRAFPEQALLRR
HPPPQTRMLSDLVEFCDQMGLPVDFSSAGALNKSLTQTFGDDKYSLARKEVLTNMCSRPMQMALYFCSGLLQDPAQFRHYALNVPLY
THFTSPIRRFADVLVHRLLAAALGYRERLDMAPDTLQKQADHCNDRRMASKRVQELSTSLFFAVLVKESGPLESEAMVMGILKQAFD
VLVLRYGVQKRIYCNALALRSHHFQKVGKKPELTLVWEPEDMEQEPAQQVITIFSLVEVVLQAESTALKYSAILKRPGTQGHLGPEK
EEEESDGEPEDSSTS
>sp|Q8NGN1|OR6T1_HUMAN
MNPENWTQVTSFVLLGFPSSHLIQFLVFLGLMVTYIVTATGKLLIIVLSWIDQRLHIQMYFFLRNFSFLELLLVTVVVPKMLVVILT
GDHTISFVSCIIQSYLYFFLGTTDFFLLAVMSLDRYLAICRPLRYETLMNGHVCSQLVLASWLAGFLWVLCPTVLMASLPFCGPNGI
DHFFRDSWPLLRLSCGDTHLLKLVAFMLSTLVLLGSLALTSVSYACILATVLRAPTAAERRKAFSTCASHLTVVVIIYGSSIFLYIR
MSEAQSKLLNKGASVLSCIITPLLNPFIFTLRNDKVQQALREALGWPRLTAVMKLRVTSQRK
>sp|Q9H4I0|RD21L_HUMAN
MFYTHVLMSKRGPLAKIWLAAHWEKKLTKAHVFECNLEITIEKILSPKVKIALRTSGHLLLGVVRIYNRKAKYLLADCSEAFLKMKM
TFCPGLVDLPKENFEASYNAITLPEEFHDFDTQNMNAIDVSEHFTQNQSRPEEITLRENFDNDLIFQAESFGEESEILRRHSFFDDN
ILLNSSGPLIEHSSGSLTGERSLFYDSGDGFGDEGAAGEMIDNLLQDDQNILLEDMHLNREISLPSEPPNSLAVEPDNSECICVPEN
EKMNETILLSTEEEGFTLDPIDISDIAEKRKGKKRRLLIDPIKELSSKVIHKQLTSFADTLMVLELAPPTQRLMMWKKRGGVHTLLS
TAAQDLIHAELKMLFTKCFLSSGFKLGRKMIQKESVREEVGNQNIVETSMMQEPNYQQELSKPQTWKDVIGGSQHSSHEDTNKNINS
EQDIVEMVSLAAEESSLMNDLFAQEIEYSPVELESLSNEENIETERWNGRILQMLNRLRESNKMGMQSFSLMKLCRNSDRKQAAAKF
YSFLVLKKQLAIELSQSAPYADIIATMGPMFYNI
>sp|Q14624|ITIH4_HUMAN
MKPPRPVRTCSKVLVLLSLLAIHQTTTAEKNGIDIYSLTVDSRVSSRFAHTVVTSRVVNRANTVQEATFQMELPKKAFITNFSMIID
GMTYPGIIKEKAEAQAQYSAAVAKGKSAGLVKATGRNMEQFQVSVSVAPNAKITFELVYEELLKRRLGVYELLLKVRPQQLVKHLQM
DIHIFEPQGISFLETESTFMTNQLVDALTTWQNKTKAHIRFKPTLSQQQKSPEQQETVLDGNLIIRYDVDRAISGGSIQIENGYFVH
YFAPEGLTTMPKNVVFVIDKSGSMSGRKIQQTREALIKILDDLSPRDQFNLIVFSTEATQWRPSLVPASAENVNKARSFAAGIQALG
GTNINDAMLMAVQLLDSSNQEERLPEGSVSLIILLTDGDPTVGETNPRSIQNNVREAVSGRYSLFCLGFGFDVSYAFLEKLALDNGG
LARRIHEDSDSALQLQDFYQEVANPLLTAVTFEYPSNAVEEVTQNNFRLLFKGSEMVVAGKLQDRGPDVLTATVSGKLPTQNITFQT
ESSVAEQEAEFQSPKYIFHNFMERLWAYLTIQQLLEQTVSASDADQQALRNQALNLSLAYSFVTPLTSMVVTKPDDQEQSQVAEKPM
EGESRNRNVHSGSTFFKYYLQGAKIPKPEASFSPRRGWNRQAGAAGSRMNFRPGVLSSRQLGLPGPPDVPDHAAYHPFRRLAILPAS
APPATSNPDPAVSRVMNMKIEETTMTTQTPAPIQAPSAILPLPGQSVERLCVDPRHRQGPVNLLSDPEQGVEVTGQYEREKAGFSWI
EVTFKNPLVWVHASPEHVVVTRNRRSSAYKWKETLFSVMPGLKMTMDKTGLLLLSDPDKVTIGLLFWDGRGEGLRLLLRDTDRFSSH
VGGTLGQFYQEVLWGSPAASDDGRRTLRVQGNDHSATRERRLDYQEGPPGVEISCWSVEL

>sp|Q9NWU5|RM22_HUMAN
MAAAVLGQLGALWIHNLRSRGKLALGVLPQSYIHTSASLDISRKWEKKNKIVYPPQLPGEPRRPAEIYHCRRQIKYSKDKMWYLAKL
IRGMSIDQALAQLEFNDKKGAKIIKEVLLEAQDMAVRDHNVEFRSNLYIAESTSGRGQCLKRIRYHGRGRFGIMEKVYCHYFVKLVE
GPPPPPEPPKTAVAHAKEYIQQLRSRTIVHTL
>sp|Q32NB8|PGPS1_HUMAN
MAVAAAAAAGPVFWRRLLGLLPGRPGLAALLGRLSDRLGRNRDRQRRRSPWLLLAPLLSPAVPQVTSPPCCLCPEGVHRFQWIRNLV
PEFGVSSSHVRVLSSPAEFFELMKGQIRVAKRRVVMASLYLGTGPLEQELVDCLESTLEKSLQAKFPSNLKVSILLDFTRGSRGRKN
SRTMLLPLLRRFPEQVRVSLFHTPHLRGLLRLLIPERFNETIGLQHIKVYLFDNSVILSGANLSDSYFTNRQDRYVFLQDCAEIADF
FTELVDAVGDVSLQLQGDDTVQVVDGMVHPYKGDRAEYCKAANKRVMDVINSARTRQQMLHAQTFHSNSLLTQEDAAAAGDRRPAPD
TWIYPLIQMKPFEIQIDEIVTETLLTEAERGAKVYLTTGYFNLTQAYMDLVLGTRAEYQILLASPEVNGFFGAKGVAGAIPAAYVHI
ERQFFSEVCSLGQQERVQLQEYWRRGWTFHAKGLWLYLAGSSLPCLTLIGSPNFGYRSVHRDLEAQIAIVTENQALQQQLHQEQEQL
YLRSGVVSSATFEQPSRQVKLWVKMVTPLIKNFF
>sp|Q2PZI1|D19L1_HUMAN
MEGRPPPEGRPPPRPRTGRAPRGRRRAVFAAVLHWSHITHLFENDRHFSHLSTLEREMAFRTEMGLYYSYFKTIVEAPSFLNGVWMI
MNDKLTEYPLVINTLKRFNLYPEVILASWYRIYTKIMDLIGIQTKICWTVTRGEGLSPIESCEGLGDPACFYVAVIFILNGLMMALF
FIYGTYLSGSRLGGLVTVLCFFFNHGECTRVMWTPPLRESFSYPFLVLQMLLVTHILRATKLYRGSLIALCISNVFFMLPWQFAQFV
LLTQIASLFAVYVVGYIDICKLRKIIYIHMISLALCFVLMFGNSMLLTSYYASSLVIIWGILAMKPHFLKINVSELSLWVIQGCFWL
FGTVILKYLTSKIFGIADDAHIGNLLTSKFFSYKDFDTLLYTCAAEFDFMEKETPLRYTKTLLLPVVLVVFVAIVRKIISDMWGVLA
KQQTHVRKHQFDHGELVYHALQLLAYTALGILIMRLKLFLTPHMCVMASLICSRQLFGWLFCKVHPGAIVFAILAAMSIQGSANLQT
QWNIVGEFSNLPQEELIEWIKYSTKPDAVFAGAMPTMASVKLSALRPIVNHPHYEDAGLRARTKIVYSMYSRKAAEEVKRELIKLKV
NYYILEESWCVRRSKPGCSMPEIWDVEDPANAGKTPLCNLLVKDSKPHFTTVFQNSVYKVLEVVKE
>sp|P43088|PF2R_HUMAN
MSMNNSKQLVSPAAALLSNTTCQTENRLSVFFSVIFMTVGILSNSLAIAILMKAYQRFRQKSKASFLLLASGLVITDFFGHLINGAI
AVFVYASDKEWIRFDQSNVLCSIFGICMVFSGLCPLLLGSVMAIERCIGVTKPIFHSTKITSKHVKMMLSGVCLFAVFIALLPILGH
RDYKIQASRTWCFYNTEDIKDWEDRFYLLLFSFLGLLALGVSLLCNAITGITLLRVKFKSQQHRQGRSHHLEMVIQLLAIMCVSCIC
WSPFLVTMANIGINGNHSLETCETTLFALRMATWNQILDPWVYILLRKAVLKNLYKLASQCCGVHVISLHIWELSSIKNSLKVAAIS
ESPVAEKSAST
>sp|Q9BUK0|CHCH7_HUMAN
MPSVTQRLRDPDINPCLSESDASTRCLDENNYDRERCSTYFLRYKNCRRFWNSIVMQRRKNGVKPFMPTAAERDEILRAVGNMPY
>sp|P10646|TFPI1_HUMAN
MIYTMKKVHALWASVCLLLNLAPAPLNADSEEDEEHTIITDTELPPLKLMHSFCAFKADDGPCKAIMKRFFFNIFTRQCEEFIYGGC
EGNQNRFESLEECKKMCTRDNANRIIKTTLQQEKPDFCFLEEDPGICRGYITRYFYNNQTKQCERFKYGGCLGNMNNFETLEECKNI
CEDGPNGFQVDNYGTQLNAVNNSLTPQSTKVPSLFEFHGPSWCLTPADRGLCRANENRFYYNSVIGKCRPFKYSGCGGNENNFTSKQ
ECLRACKKGFIQRISKGGLIKTKRKRKKQRVKIAYEEIFVKNM
>sp|Q6ZV60|YL023_HUMAN
MSFSPYSTMITVCVCFNSRVQLTVPSFTAWLRSRYSKALFMVLRRAAQEKDKGVCQGWHCVKKWACKGRIPGQPLQPQPLGPYLRSL
SQHPATQTPRPQARASSRYLELHRSQNRGGSEFKFWFCYCLIACCRDSISSSGKWE
>sp|Q53FA7|QORX_HUMAN
MLAVHFDKPGGPENLYVKEVAKPSPGEGEVLLKVAASALNRADLMQRQGQYDPPPGASNILGLEASGHVAELGPGCQGHWKIGDTAM
ALLPGGGQAQYVTVPEGLLMPIPEGLTLTQAAAIPEAWLTAFQLLHLVGNVQAGDYVLIHAGLSGVGTAAIQLTRMAGAIPLVTAGS
QKKLQMAEKLGAAAGFNYKKEDFSEATLKFTKGAGVNLILDCIGGSYWEKNVNCLALDGRWVLYGLMGGGDINGPLFSKLLFKRGSL
ITSLLRSRDNKYKQMLVNAFTEQILPHFSTEGPQRLLPVLDRIYPVTEIQEAHKYMEANKNIGKIVLELPQ
>sp|O14763|TR10B_HUMAN

MEQRGQNAPAASGARKRHGPGPREARGARPGPRVPKTLVLVVAAVLLLVSAESALITQQDLAPQQRAAPQQKRSSPSEGLCPPGHHI
SEDGRDCISCKYGQDYSTHWNDLLFCLRCTRCDSGEVELSPCTTTRNTVCQCEEGTFREEDSPEMCRKCRTGCPRGMVKVGDCTPWS
DIECVHKESGTKHSGEVPAVEETVTSSPGTPASPCSLSGIIIGVTVAAVVLIVAVFVCKSLLWKKVLPYLKGICSGGGGDPERVDRS
SQRPGAEDNVLNEIVSILQPTQVPEQEMEVQEPAEPTGVNMLSPGESEHLLEPAEAERSQRRRLLVPANEGDPTETLRQCFDDFADL
VPFDSWEPLMRKLGLMDNEIKVAKAEAAGHRDTLYTMLIKWVNKTGRDASVHTLLDALETLGERLAKQKIEDHLLSSGKFMYLEGNA
DSAMS
>sp|P51808|DYLT3_HUMAN
MEEYHRHCDEVGFNAEEAHNIVKECVDGVLGGEDYNHNNINQWTASIVEQSLTHLVKLGKAYKYIVTCAVVQKSAYGFHTASSCFWD
TTSDGTCTVRWENRTMNCIVNVFAIAIVL
>sp|Q6IF36|OR8G2_HUMAN
MVFLSSVETDQRKMSAGNHSSVTEFILAGLSEQPELQLRLFLLFLGIYVVTVVGNLSMITLIGLSSHLHTPMYYFLSGLSFIDLCHS
TIITPKMLVNFVTEKNIISYPECMTQLYFFLIFAIAECHMLAVTAYDRYVAICSPLLYNVIMSYHHCFWLTVGVYVLGILGSTIHTG
FMLRLFLCKTNVINHYFCDLFPLLGLSCSSTYINELLVLVLSAFNILTPALTILASYIFIIASILRIRSTEGRSKAFSTCSSHILAV
AVFFGSAAFMYLQPSSVSSMDQRKVSSVFYTTIVPMLNPQSIA
>sp|O15054|KDM6B_HUMAN
MHRAVDPPGARAAREAFALGGLSCAGAWSSCPPHPPPRSAWLPGGRCSASIGQPPLPAPLPPSHGSSSGHPSKPYYAPGAPTPRPLH
GKLESLHGCVQALLREPAQPGLWEQLGQLYESEHDSEEATRCYHSALRYGGSFAELGPRIGRLQQAQLWNFHTGSCQHRAKVLPPLE
QVWNLLHLEHKRNYGAKRGGPPVKRAAEPPVVQPVPPAALSGPSGEEGLSPGGKRRRGCNSEQTGLPPGLPLPPPPLPPPPPPPPPP
PPPLPGLATSPPFQLTKPGLWSTLHGDAWGPERKGSAPPERQEQRHSLPHPYPYPAPAYTAHPPGHRLVPAAPPGPGPRPPGAESHG
CLPATRPPGSDLRESRVQRSRMDSSVSPAATTACVPYAPSRPPGLPGTTTSSSSSSSSNTGLRGVEPNPGIPGADHYQTPALEVSHH
GRLGPSAHSSRKPFLGAPAATPHLSLPPGPSSPPPPPCPRLLRPPPPPAWLKGPACRAAREDGEILEELFFGTEGPPRPAPPPLPHR
EGFLGPPASRFSVGTQDSHTPPTPPTPTTSSSNSNSGSHSSSPAGPVSFPPPPYLARSIDPLPRPPSPAQNPQDPPLVPLTLALPPA
PPSSCHQNTSGSFRRPESPRPRVSFPKTPEVGPGPPPGPLSKAPQPVPPGVGELPARGPRLFDFPPTPLEDQFEEPAEFKILPDGLA
NIMKMLDESIRKEEEQQQHEAGVAPQPPLKEPFASLQSPFPTDTAPTTTAPAVAVTTTTTTTTTTATQEEEKKPPPALPPPPPLAK
FPPPSQPQPPPPPPPSPASLLKSLASVLEGQKYCYRGTGAAVSTRPGPLPTTQYSPGPPSGATALPPTSAAPSAQGSPQPSASSSSQ
FSTSGGPWARERRAGEEPVPGPMTPTQPPPPLSLPPARSESEVLEEISRACETLVERVGRSATDPADPVDTAEPADSGTERLLPPAQ
AKEEAGGVAAVSGSCKRRQKEHQKEHRRHRRACKDSVGRRPREGRAKAKAKVPKEKSRRVLGNLDLQSEEIQGREKSRPDLGGASKA
KPPTAPAPPSAPAPSAQPTPPSASVPGKKAREEAPGPPGVSRADMLKLRSLSEGPPKELKIRLIKVESGDKETFIASEVEERRLRMA
DLTISHCAADVVRASRNAKVKGKFRESYLSPAQSVKPKINTEEKLPREKLNPPTPSIYLESKRDAFSPVLLQFCTDPRNPITVIRGL
AGSLRLNLGLFSTKTLVEASGEHTVEVRTQVQQPSDENWDLTGTRQIWPCESSRSHTTIAKYAQYQASSFQESLQEEKESEDEESEE
PDSTTGTPPSSAPDPKNHHIIKFGTNIDLSDAKRWKPQLQELLKLPAFMRVTSTGNMLSHVGHTILGMNTVQLYMKVPGSRTPGHQE
NNNFCSVNINIGPGDCEWFAVHEHYWETISAFCDRHGVDYLTGSWWPILDDLYASNIPVYRFVQRPGDLVWINAGTVHWVQATGWCN
NIAWNVGPLTAYQYQLALERYEWNEVKNVKSIVPMIHVSWNVARTVKISDPDLFKMIKFCLLQSMKHCQVQRESLVRAGKKIAYQGR
VKDEPAYYCNECDVEVFNILFVTSENGSRNTYLVHCEGCARRRSAGLQGVVVLEQYRTEELAQAYDAFTLAPASTSR
>sp|P02008|HBAZ_HUMAN
MSLTKTERTIIVSMWAKISTQADTIGTETLERLFLSHPQTKTYFPHFDLHPGSAQLRAHGSKVVAAVGDAVKSIDDIGGALSKLSEL
HAYILRVDPVNFKLLSHCLLVTLAARFPADFTAEAHAAWDKFLSVVSSVLTEKYR
>sp|Q99551|MTEF1_HUMAN
MQSLSLGQTSISKGLNYLTIMAPGNLWHMRNNFLFGSRCWMTRFSAENIFKSVSFRLFGVKCHNTDSEPLKNEDLLKNLLTMGVDID
MARKRQPGVFHRMITNEQDLKMFLLSKGASKEVIASIISRYPRAITRTPENLSKRWDLWRKIVTSDLEIVNILERSPESFFRSNNNL
NLENNIKFLYSVGLTRKCLCRLLTNAPRTFSNSLDLNKQMVEFLQAAGLSLGHNDPADFVRKIIFKNPFILIQSTKRVKANIEFLRS
TFNLNSEELLVLICGPGAEILDLSNDYARRSYANIKEKLFSLGCTEEEVQKFVLSYPDVIFLAEKKFNDKIDCLMEENISISQIIEN
PRVLDSSISTLKSRIKELVNAGCNLSTLNITLLSWSKKRYEAKLKKLSRFA
>sp|P59827|BPIB4_HUMAN

MWMAWCVAALSVVAVCGTSHETNTVLRVTKDVLSNAISGMLQQSDALHSALREVPLGVGDIPYNDFHVRGPPPVYTNGKKLDGIYQY
GHIETNDNTAQLGGKYRYGEILESEGSIRDLRNSGYRSAENAYGGHRGLGRYRAAPVGRLHRRELQPGEIPPGVATGAVGPGGLLGT
GGMLAADGILAGQGGLLGGGGLLGDGGLLGGGGVLGVLGEGGILSTVQGITGLRIVELTLPRVSVRLLPGVGVYLSLYTRVAINGKS
LIGFLDIAVEVNITAKVRLTMDRTGYPRLVIERCDTLLGGIKVKLLRGLLPNLVDNLVNRVLADVLPDLLCPIVDVVLGLVNDQLGL
VDSLIPLGILGSVQYTFSSLPLVTGEFLELDLNTLVGEAGGGLIDYPLGWPAVSPKPMPELPPMGDNTKSQLAMSANFLGSVLTLLQ
KQHALDLDITNGMFEELPPLTTATLGALIPKVFQQYPESCPLIIRIQVLNPPSVMLQKDKALVKVLATAEVMVSQPKDLETTICLID
VDTEFLASFSTEGDKLMIDAKLEKTSLNLRTSNVGNFDIGLMEVLVEKIFDLAFMPAMNAVLGSGVPLPKILNIDFSNADIDVLEDL
LVLSA
>sp|Q9Y653|AGRG1_HUMAN
MTPQSLLQTTLFLLSLLFLVQGAHGRGHREDFRFCSQRNQTHRSSLHYKPTPDLRISIENSEEALTVHAPFPAAHPASRSFPDPRGL
YHFCLYWNRHAGRLHLLYGKRDFLLSDKASSLLCFQHQEESLAQGPPLLATSVTSWWSPQNISLPSAASFTFSFHSPPHTAAHNASV
DMCELKRDLQLLSQFLKHPQKASRRPSAAPASQQLQSLESKLTSVRFMGDMVSFEEDRINATVWKLQPTAGLQDLHIHSRQEEEQSE
IMEYSVLLPRTLFQRTKGRSGEAEKRLLLVDFSSQALFQDKNSSQVLGEKVLGIVVQNTKVANLTEPVVLTFQHQLQPKNVTLQCVF
WVEDPTLSSPGHWSSAGCETVRRETQTSCFCNHLTYFAVLMVSSVEVDAVHKHYLSLLSYVGCVVSALACLVTIAAYLCSRVPLPCR
RKPRDYTIKVHMNLLLAVFLLDTSFLLSEPVALTGSEAGCRASAIFLHFSLLTCLSWMGLEGYNLYRLVVEVFGTYVPGYLLKLSAM
GWGFPIFLVTLVALVDVDNYGPIILAVHRTPEGVIYPSMCWIRDSLVSYITNLGLFSLVFLFNMAMLATMVVQILRLRPHTQKWSHV
LTLLGLSLVLGLPWALIFFSFASGTFQLVVLYLFSIITSFQGFLIFIWYWSMRLQARGGPSPLKSNSDSARLPISSGSTSSSRI
>sp|Q05823|RN5A_HUMAN
MESRDHNNPQEGPTSSSGRRAAVEDNHLLIKAVQNEDVDLVQQLLEGGANVNFQEEEGGWTPLHNAVQMSREDIVELLLRHGADPVL
RKKNGATPFILAAIAGSVKLLKLFLSKGADVNECDFYGFTAFMEAAVYGKVKALKFLYKRGANVNLRRKTKEDQERLRKGGATALMD
AAEKGHVEVLKILLDEMGADVNACDNMGRNALIHALLSSDDSDVEAITHLLLDHGADVNVRGERGKTPLILAVEKKHLGLVQRLLEQ
EHIEINDTDSDGKTALLLAVELKLKKIAELLCKRGASTDCGDLVMTARRNYDHSLVKVLLSHGAKEDFHPPAEDWKPQSSHWGAALK
DLHRIYRPMIGKLKFFIDEKYKIADTSEGGIYLGFYEKQEVAVKTFCEGSPRAQREVSCLQSSRENSHLVTFYGSESHRGHLFVCVT
LCEQTLEACLDVHRGEDVENEEDEFARNVLSSIFKAVQELHLSCGYTHQDLQPQNILIDSKKAAHLADFDKSIKWAGDPQEVKRDLE
DLGRLVLYVVKKGSISFEDLKAQSNEEVVQLSPDEETKDLIHRLFHPGEHVRDCLSDLLGHPFFWTWESRYRTLRNVGNESDIKTRK
SESEILRLLQPGPSEHSKSFDKWTTKINECVMKKMNKFYEKRGNFYQNTVGDLLKFIRNLGEHIDEEKHKKMKLKIGDPSLYFQKTF
PDLVIYVYTKLQNTEYRKHFPQTHSPNKPQCDGAGGASGLASPGC
>sp|Q96SB8|SMC6_HUMAN
MAKRKEENFSSPKNAKRPRQEELEDFDKDGDEDECKGTTLTAAEVGIIESIHLKNFMCHSMLGPFKFGSNVNFVVGNNGSGKSAVLT
ALIVGLGGRAVATNRGSSLKGFVKDGQNSADISITLRNRGDDAFKASVYGNSILIQQHISIDGSRSYKLKSATGSVVSTRKEELIAI
LDHFNIQVDNPVSVLTQEMSKQFLQSKNEGDKYKFFMKATQLEQMKEDYSYIMETKERTKEQIHQGEERLTELKRQCVEKEERFQSI
AGLSTMKTNLESLKHEMAWAVVNEIEKQLNAIRDNIKIGEDRAARLDRKMEEQQVRLNEAEQKYKDIQDKLEKISEETNARAPECMA
LKADVVAKKRAYNEAEVLYNRSLNEYKALKKDDEQLCKRIEELKKSTDQSLEPERLERQKKISWLKERVKAFQNQENSVNQEIEQFQ
QAIEKDKEEHGKIKREELDVKHALSYNQRQLKELKDSKTDRLKRFGPNVPALLEAIDDAYRQGHFTYKPVGPLGACIHLRDPELALA
IESCLKGLLQAYCCHNHADERVLQALMKRFYLPGTSRPPIIVSEFRNEIYDVRHRAAYHPDFPTVLTALEIDNAVVANSLIDMRGIE
TVLLIKNNSVARAVMQSQKPPKNCREAFTADGDQVFAGRYYSSENTRPKFLSRDVDSEISDLENEVENKTAQILNLQQHLSALEKDI
KHNEELLKRCQLHYKELKMKIRKNISEIRELENIEEHQSVDIATLEDEAQENKSKMKMVEEHMEQQKENMEHLKSLKIEAENKYDAI
KFKINQLSELADPLKDELNLADSEVDNQKRGKRHYEEKQKEHLDTLNKKKRELDMKEKELEEKMSQARQICPERIEVEKSASILDKE
INRLRQKIQAEHASHGDREEIMRQYQEARETYLDLDSKVRTLKKFIKLLGEIMEHRFKTYQQFRRCLTLRCKLYFDNLLSQRAYCGK
MNFDHKNETLSISVQPGEGNKAAFNDMRALSGGERSFSTVCFILSLWSIAESPFRCLDEFDVYMDMVNRRIAMDLILKMADSQRFRQ
FILLTPQSMSSLPSSKLIRILRMSDPERGQTTLPFRPVTQEEDDDQR
>sp|O60248|SOX15_HUMAN

MALPGSSQDQAWSLEPPAATAAASSSSGPQEREGAGSPAAPGTLPLEKVKRPMNAFMVWSSAQRRQMAQQNPKMHNSEISKRLGAQW
KLLDEDEKRPFVEEAKRLRARHLRDYPDYKYRPRRKAKSSGAGPSRCGQGRGNLASGGPLWGPGYATTQPSRGFGYRPPSYSTAYLP
GSYGSSHCKLEAPSPCSLPQSDPRLQGELLPTYTHYLPPGSPTPYNPPLAGAPMPLTHL
>sp|Q17RS7|GEN_HUMAN
MGVNDLWQILEPVKQHIPLRNLGGKTIAVDLSLWVCEAQTVKKMMGSVMKPHLRNLFFRISYLTQMDVKLVFVMEGEPPKLKADVIS
KRNQSRYGSSGKSWSQKTGRSHFKSVLRECLHMLECLGIPWVQAAGEAEAMCAYLNAGGHVDGCLTNDGDTFLYGAQTVYRNFTMNT
KDPHVDCYTMSSIKSKLGLDRDALVGLAILLGCDYLPKGVPGVGKEQALKLIQILKGQSLLQRFNRWNETSCNSSPQLLVTKKLAHC
SVCSHPGSPKDHERNGCRLCKSDKYCEPHDYEYCCPCEWHRTEHDRQLSEVENNIKKKACCCEGFPFHEVIQEFLLNKDKLVKVIRY
QRPDLLLFQRFTLEKMEWPNHYACEKLLVLLTHYDMIERKLGSRNSNQLQPIRIVKTRIRNGVHCFEIEWEKPEHYAMEDKQHGEFA
LLTIEEESLFEAAYPEIVAVYQKQKLEIKGKKQKRIKPKENNLPEPDEVMSFQSHMTLKPTCEIFHKQNSKLNSGISPDPTLPQESI
SASLNSLLLPKNTPCLNAQEQFMSSLRPLAIQQIKAVSKSLISESSQPNTSSHNISVIADLHLSTIDWEGTSFSNSPAIQRNTFSHD
LKSEVESELSAIPDGFENIPEQLSCESERYTANIKKVLDEDSDGISPEEHLLSGITDLCLQDLPLKERIFTKLSYPQDNLQPDVNLK
TLSILSVKESCIANSGSDCTSHLSKDLPGIPLQNESRDSKILKGDQLLQEDYKVNTSVPYSVSNTVVKTCNVRPPNTALDHSRKVDM
QTTRKILMKKSVCLDRHSSDEQSAPVFGKAKYTTQRMKHSSQKHNSSHFKESGHNKLSSPKIHIKETEQCVRSYETAENEESCFPDS
TKSSLSSLQCHKKENNSGTCLDSPLPLRQRLKLRFQST
>sp|Q8NFW8|NEUA_HUMAN
MDSVEKGAATSVSNPRGRPSRGRPPKLQRNSRGGQGRGVEKPPHLAALILARGGSKGIPLKNIKHLAGVPLIGWVLRAALDSGAFQS
VWVSTDHDEIENVAKQFGAQVHRRSSEVSKDSSTSLDAIIEFLNYHNEVDIVGNIQATSPCLHPTDLQKVAEMIREEGYDSVFSVVR
RHQFRWSEIQKGVREVTEPLNLNPAKRPRRQDWDGELYENGSFYFAKRHLIEMGYLQGGKMAYYEMRAEHSVDIDVDIDWPIAEQRV
LRYGYFGKEKLKEIKLLVCNIDGCLTNGHIYVSGDQKEIISYDVKDAIGISLLKKSGIEVRLISERACSKQTLSSLKLDCKMEVSVS
DKLAVVDEWRKEMGLCWKEVAYLGNEVSDEECLKRVGLSGAPADACSTAQKAVGYICKCNGGRGAIREFAEHICLLMEKVNNSCQK
>sp|P27352|IF_HUMAN
MAWFALYLLSLLWATAGTSTQTQSSCSVPSAQEPLVNGIQVLMENSVTSSAYPNPSILIAMNLAGAYNLKAQKLLTYQLMSSDNNDL
TIGQLGLTIMALTSSCRDPGDKVSILQRQMENWAPSSPNAEASAFYGPSLAILALCQKNSEATLPIAVRFAKTLLANSSPFNVDTGA
MATLALTCMYNKIPVGSEEGYRSLFGQVLKDIVEKISMKIKDNGIIGDIYSTGLAMQALSVTPEPSKKEWNCKKTTDMILNEIKQGK
FHNPMSIAQILPSLKGKTYLDVPQVTCSPDHEVQPTLPSNPGPGPTSASNITVIYTINNQLRGVELLFNETINVSVKSGSVLLVVLE
EAQRKNPMFKFETTMTSWGLVVSSINNIAENVNHKTYWQFLSGVTPLNEGVADYIPFNHEHITANFTQY
>sp|Q13227|GPS2_HUMAN
MPALLERPKLSNAMARALHRHIMMERERKRQEEEEVDKMMEQKMKEEQERRKKKEMEERMSLEETKEQILKLEEKLLALQEEKHQLF
LQLKKVLHEEEKRRRKEQSDLTTLTSAAYQQSLTVHTGTHLLSMQGSPGGHNRPGTLMAADRAKQMFGPQVLTTRHYVGSAAAFAGT
PEHGQFQGSPGGAYGTAQPPPHYGPTQPAYSPSQQLRAPSAFPAVQYLSQPQPQPYAVHGHFQPTQTGFLQPGGALSLQKQMEHANQ
QTGFSDSSSLRPMHPQALHPAPGLLASPQLPVQMQPAGKSGFAATSQPGPRLPFIQHSQNPRFYHK
>sp|P23443|KS6B1_HUMAN
MRRRRRRDGFYPAPDFRDREAEDMAGVFDIDLDQPEDAGSEDELEEGGQLNESMDHGGVGPYELGMEHCEKFEISETSVNRGPEKIR
PECFELLRVLGKGGYGKVFQVRKVTGANTGKIFAMKVLKKAMIVRNAKDTAHTKAERNILEEVKHPFIVDLIYAFQTGGKLYLILEY
LSGGELFMQLEREGIFMEDTACFYLAEISMALGHLHQKGIIYRDLKPENIMLNHQGHVKLTDFGLCKESIHDGTVTHTFCGTIEYMA
PEILMRSGHNRAVDWWSLGALMYDMLTGAPPFTGENRKKTIDKILKCKLNLPPYLTQEARDLLKKLLKRNAASRLGAGPGDAGEVQA
HPFFRHINWEELLARKVEPPFKPLLQSEEDVSQFDSKFTRQTPVDSPDDSTLSESANQVFLGFTYVAPSVLESVKEKFSFEPKIRSP
RRFIGSPRTPVSPVKFSPGDFWGRGASASTANPQTPVEYPMETSGIEQMDVTMSGEASAPLPIRQPNSGPYKKQAFPMISKRPEHLR
MNL
>sp|P62316|SMD2_HUMAN
MSLLNKPKSEMTPEELQKREEEEFNTGPLSVLTQSVKNNTQVLINCRNNKKLLGRVKAFDRHCNMVLENVKEMWTEVPKSGKGKKKS
KPVNKDRYISKMFLRGDSVIVVLRNPLIAGK
>sp|Q9H1C7|CYTM1_HUMAN

MNQENPPPYPGPGPTAPYPPYPPQPMGPGPMGGPYPPPQGYPYQGYPQYGWQGGPQEPPKTTVYVVEDQRRDELGPSTCLTACWTAL
CCCCLWDMLT
>sp|Q96JB6|LOXL4_HUMAN
MAWSPPATLFLFLLLLGQPPPSRPQSLGTTKLRLVGPESKPEEGRLEVLHQGQWGTVCDDNFAIQEATVACRQLGFEAALTWAHSAK
YGQGEGPIWLDNVRCVGTESSLDQCGSNGWGVSDCSHSEDVGVICHPRRHRGYLSETVSNALGPQGRRLEEVRLKPILASAKQHSPV
TEGAVEVKYEGHWRQVCDQGWTMNNSRVVCGMLGFPSEVPVDSHYYRKVWDLKMRDPKSRLKSLTNKNSFWIHQVTCLGTEPHMANC
QVQVAPARGKLRPACPGGMHAVVSCVAGPHFRPPKTKPQRKGSWAEEPRVRLRSGAQVGEGRVEVLMNRQWGTVCDHRWNLISASVV
CRQLGFGSAREALFGARLGQGLGPIHLSEVRCRGYERTLSDCPALEGSQNGCQHENDAAVRCNVPNMGFQNQVRLAGGRIPEEGLLE
VQVEVNGVPRWGSVCSENWGLTEAMVACRQLGLGFAIHAYKETWFWSGTPRAQEVVMSGVRCSGTELALQQCQRHGPVHCSHGGGRF
LAGVSCMDSAPDLVMNAQLVQETAYLEDRPLSQLYCAHEENCLSKSADHMDWPYGYRRLLRFSTQIYNLGRTDFRPKTGRDSWVWHQ
CHRHYHSIEVFTHYDLLTLNGSKVAEGHKASFCLEDTNCPTGLQRRYACANFGEQGVTVGCWDTYRHDIDCQWVDITDVGPGNYIFQ
VIVNPHYEVAESDFSNNMLQCRCKYDGHRVWLHNCHTGNSYPANAELSLEQEQRLRNNLI
>sp|Q3C1V0|M4A18_HUMAN
MTEQVIGANSVPGIIAPDNVHVIQPSNPVASGNHLQPSEVTTYPISPKVIHCDTGRANLQNLLVVNQNSAAGVQSQPIGYQRQYPVG
TASLQTVPGVIQYTQGTTNLQTWPGDLQNPLNANPGLTHTSNSSQWNTSFASFTSFNPKKFINEEVRTLGAIQILIGLTHIFSAINP
VLYYYPFVTWLSGYPLWGGLSYIVSGSLSVWAAKDPSPCVVNSSISFNIISALFAFAGIFIIITDLSLYYVTTYSKAVSGGLLPFAL
LEFILTCVVSHFGCQATCCRQFENVAVIPTVFSFNPANTTTSPVNATTGPVNAATGPVSATN
>sp|Q13387|JIP2_HUMAN
MADRAEMFSLSTFHSLSPPGCRPPQDISLEEFDDEDLSEITDDCGLGLSYDSDHCEKDSLSLGRSEQPHPICSFQDDFQEFEMIDDN
EEEDDEDEEEEEEEEEGDGEGQEGGDPGSEAPAPGPLIPSPSVEEPHKHRPTTLRLTTLGAQDSLNNNGGFDLVRPASWQETALCSP
APEALRELPGPLPATDTGPGGAQSPVRPGCDCEGNRPAEPPAPGGTSPSSDPGIEADLRSRSSGGRGGRRSSQELSSPGSDSEDAGG
ARLGRMISSISETELELSSDGGSSSSGRSSHLTNSIEEASSPASEPEPPREPPRRPAFLPVGPDDTNSEYESGSESEPDLSEDADSP
WLLSNLVSRMISEGSSPIRCPGQCLSPAPRPPGEPVSPAGGAAQDSQDPEAAAGPGGVELVDMETLCAPPPPAPAAPRPGPAQPGPC
LFLSNPTRDTITPLWAAPGRAARPGRACSAACSEEEDEEDDEEEEDAEDSAGSPGGRGTGPSAPRDASLVYDAVKYTLVVDEHTQLE
LVSLRRCAGLGHDSEEDSGGEASEEEAGAALLGGGQVSGDTSPDSPDLTFSKKFLNVFVNSTSRSSSTESFGLFSCLVNGEEREQTH
RAVFRFIPRHPDELELDVDDPVLVEAEEDDFWFRGFNMRTGERGVFPAFYAHAVPGPAKDLLGSKRSPCWVERFDVQFLGSVEVPCH
QGNGILCAAMQKIATARKLTVHLRPPASCDLEISLRGVKLSLSGGGPEFQRCSHFFQMKNISFCGCHPRNSCYFGFITKHPLLSRFA
CHVFVSQESMRPVAQSVGRAFLEYYQEHLAYACPTEDIYLE
>sp|O75072|FKTN_HUMAN
MSRINKNVVLALLTLTSSAFLLFQLYYYKHYLSTKNGAGLSKSKGSRIGFDSTQWRAVKKFIMLTSNQNVPVFLIDPLILELINKNF
EQVKNTSHGSTSQCKFFCVPRDFTAFALQYHLWKNEEGWFRIAENMGFQCLKIESKDPRLDGIDSLSGTEIPLHYICKLATHAIHLV
VFHERSGNYLWHGHLRLKEHIDRKFVPFRKLQFGRYPGAFDRPELQQVTVDGLEVLIPKDPMHFVEEVPHSRFIECRYKEARAFFQQ
YLDDNTVEAVAFRKSAKELLQLAAKTLNKLGVPFWLSSGTCLGWYRQCNIIPYSKDVDLGIFIQDYKSDIILAFQDAGLPLKHKFGK
VEDSLELSFQGKDDVKLDVFFFYEETDHMWNGGTQAKTGKKFKYLFPKFTLCWTEFVDMKVHVPCETLEYIEANYGKTWKIPVKTWD
WKRSPPNVQPNGIWPISEWDEVIQLY
>sp|Q14541|HNF4G_HUMAN
MNTTDNGVNCLCAICGDRATGKHYGASSCDGCKGFFRRSIRKSHVYSCRFSRQCVVDKDKRNQCRYCRLRKCFRAGMKKEAVQNERD
RISTRRSTFDGSNIPSINTLAQAEVRSRQISVSSPGSSTDINVKKIASIGDVCESMKQQLLVLVEWAKYIPAFCELPLDDQVALLRA
HAGEHLLLGATKRSMMYKDILLLGNNYVIHRNSCEVEISRVANRVLDELVRPFQEIQIDDNEYACLKAIVFFDPDAKGLSDPVKIKN
MRFQVQIGLEDYINDRQYDSRGRFGELLLLLPTLQSITWQMIEQIQFVKLFGMVKIDNLLQEMLLGGASNDGSHLHHPMHPHLSQDP
LTGQTILLGPMSTLVHADQISTPETPLPSPPQGSGQEQYKIAANQASVISHQHLSKQKQL
>sp|Q8IVW8|SPNS2_HUMAN
MMCLECASAAAGGAEEEEADAERRRRRRGAQRGAGGSGCCGARGAGGAGVSAAGDEVQTLSGSVRRAPTGPPGTPGTPGCAATAKGP
GAQQPKPASLGRGRGAAAAILSLGNVLNYLDRYTVAGVLLDIQQHFGVKDRGAGLLQSVFICSFMVAAPIFGYLGDRFNRKVILSCG

IFFWSAVTFSSSFIPQQYFWLLVLSRGLVGIGEASYSTIAPTIIGDLFTKNTRTLMLSVFYFAIPLGSGLGYITGSSVKQAAGDWHW
ALRVSPVLGMITGTLILILVPATKRGHADQLGDQLKARTSWLRDMKALIRNRSYVFSSLATSAVSFATGALGMWIPLYLHRAQVVQK
TAETCNSPPCGAKDSLIFGAITCFTGFLGVVTGAGATRWCRLKTQRADPLVCAVGMLGSAIFICLIFVAAKSSIVGAYICIFVGETL
LFSNWAITADILMYVVIPTRRATAVALQSFTSHLLGDAGSPYLIGFISDLIRQSTKDSPLWEFLSLGYALMLCPFVVVLGGMFFLAT
ALFFVSDRARAEQQVNQLAMPPASVKV
>sp|P0CG34|TB15A_HUMAN
MSDKPDLSEVEKFDRSKLKKTNTEEKNTLPSKETIQQEKECVQTS
>sp|Q96DW6|S2538_HUMAN
MIQNSRPSLLQPQDVGDTVETLMLHPVIKAFLCGSISGTCSTLLFQPLDLLKTRLQTLQPSDHGSRRVGMLAVLLKVVRTESLLGLW
KGMSPSIVRCVPGVGIYFGTLYSLKQYFLRGHPPTALESVMLGVGSRSVAGVCMSPITVIKTRYESGKYGYESIYAALRSIYHSEGH
RGLFSGLTATLLRDAPFSGIYLMFYNQTKNIVPHDQVDATLIPITNFSCGIFAGILASLVTQPADVIKTHMQLYPLKFQWIGQAVTL
IFKDYGLRGFFQGGIPRALRRTLMAAMAWTVYEEMMAKMGLKS
>sp|Q9BV90|SNR25_HUMAN
MDVFQEGLAMVVQDPLLCDLPIQVTLEEVNSQIALEYGQAMTVRVCKMDGEVMPVVVVQSATVLDLKKAIQRYVQLKQEREGGIQHI
SWSYVWRTYHLTSAGEKLTEDRKKLRDYGIRNRDEVSFIKKLRQK
>sp|Q8ND30|LIPB2_HUMAN
MASDASHALEAALEQMDGIIAGTKTGADLSDGTCEPGLASPASYMNPFPVLHLIEDLRLALEMLELPQERAALLSQIPGPTAAYIKE
WFEESLSQVNHHSAASNETYQERLARLEGDKESLILQVSVLTDQVEAQGEKIRDLEVCLEGHQVKLNAAEEMLQQELLSRTSLETQK
LDLMTEVSELKLKLVGMEKEQREQEEKQRKAEELLQELRHLKIKVEELENERNQYEWKLKATKAEVAQLQEQVALKDAEIERLHSQL
SRTAALHSESHTERDQEIQRLKMGMETLLLANEDKDRRIEELTGLLNQYRKVKEIVMVTQGPSERTLSINEEEPEGGFSKWNATNKD
PEELFKQEMPPRCSSPTVGPPPLPQKSLETRAQKKLSCSLEDLRSESVDKCMDGNQPFPVLEPKDSPFLAEHKYPTLPGKLSGATPN
GEAAKSPPTICQPDATGSSLLRLRDTESGWDDTAVVNDLSSTSSGTESGPQSPLTPDGKRNPKGIKKFWGKIRRTQSGNFYTDTLGM
AEFRRGGLRATAGPRLSRTRDSKGQKSDANAPFAQWSTERVCAWLEDFGLAQYVIFARQWVSSGHTLLTATPQDMEKELGIKHPLHR
KKLVLAVKAINTKQEEKSALLDHIWVTRWLDDIGLPQYKDQFHESRVDRRMLQYLTVNDLLFLKVTSQLHHLSIKCAIHVLHVNKFN
PHCLHRRPADESNLSPSEVVQWSNHRVMEWLRSVDLAEYAPNLRGSGVHGGLIILEPRFTGDTLAMLLNIPPQKTLLRRHLTTKFNA
LIGPEAEQEKREKMASPAYTPLTTTAKVRPRKLGFSHFGNIRKKKFDESTDYICPMEPSDGVSDSHRVYSGYRGLSPLDAPELDGLD
QVGQIS
>sp|O75608|LYPA1_HUMAN
MCGNNMSTPLPAIVPAARKATAAVIFLHGLGDTGHGWAEAFAGIRSSHIKYICPHAPVRPVTLNMNVAMPSWFDIIGLSPDSQEDES
GIKQAAENIKALIDQEVKNGIPSNRIILGGFSQGGALSLYTALTTQQKLAGVTALSCWLPLRASFPQGPIGGANRDISILQCHGDCD
PLVPLMFGSLTVEKLKTLVNPANVTFKTYEGMMHSSCQQEMMDVKQFIDKLLPPID
>sp|Q9HC96|CAN10_HUMAN
MRAGRGATPARELFRDAAFPAADSSLFCDLSTPLAQFREDITWRRPQEICATPRLFPDDPREGQVKQGLLGDCWFLCACAALQKSRH
LLDQVIPPGQPSWADQEYRGSFTCRIWQFGRWVEVTTDDRLPCLAGRLCFSRCQREDVFWLPLLEKVYAKVHGSYEHLWAGQVADAL
VDLTGGLAERWNLKGVAGSGGQQDRPGRWEHRTCRQLLHLKDQCLISCCVLSPRAGARELGEFHAFIVSDLRELQGQAGQCILLLRI
QNPWGRRCWQGLWREGGEGWSQVDAAVASELLSQLQEGEFWVEEEEFLREFDELTVGYPVTEAGHLQSLYTERLLCHTRALPGAWVK
GQSAGGCRNNSGFPSNPKFWLRVSEPSEVYIAVLQRSRLHAADWAGRARALVGDSHTSWSPASIPGKHYQAVGLHLWKVEKRRVNLP
RVLSMPPVAGTACHAYDREVHLRCELSPGYYLAVPSTFLKDAPGEFLLRVFSTGRVSLSAIRAVAKNTTPGAALPAGEWGTVQLRGS
WRVGQTAGGSRNFASYPTNPCFPFSVPEGPGPRCVRITLHQHCRPSDTEFHPIGFHIFQVPEGGRSQDAPPLLLQEPLLSCVPHRYA
QEVSRLCLLPAGTYKVVPSTYLPDTEGAFTVTIATRIDRPSIHSQEMLGQFLQEVSIMAVMKT
>sp|Q5SZD4|GLYL3_HUMAN
MLVLNCSTKLLILEKMLKSCFPESLKVYGAVMNINRGNPFQKEVVLDSWPDFKAVITRRQREAETDNLDHYTNAYAVFYKDVRAYRQ
LLEECDVFNWDQVFQIQGLQSELYDVSKAVANSKQLNIKLTSFKAVHFSPVSSLPDTSFLKGPSPRLTYLSVANADLLNRTWSRGGN

EQCLRYIANLISCFPSVCVRDEKGNPVSWSITDQFATMCHGYTLPEHRRKGYSRLVALTLARKLQSRGFPSQGNVLDDNTASISLLK
SLHAEFLPCRFHRLILTPATFSGLPHL
>sp|Q5VUA4|ZN318_HUMAN
MYRSSARSSVSSHRPKDDGGGGPRSGRSSGSSSGPARRSSPPPPPSGSSSRTPARRPRSPSGHRGRRASPSPPRGRRVSPSPPRARR
GSPSPPRGRRLFPPGPAGFRGSSRGESRADYARDGRGDHPGDSGSRRRSPGLCSDSLEKSLRITVGNDHFCVSTPERRRLSDRLGSP
VDNLEDMDRDDLTDDSVFTRSSQCSRGLERYISQEEGPLSPFLGQLDEDYRTKETFLHRSDYSPHISCHDELLRGTERNREKLKGYS
IRSEERSREAKRPRYDDTVKINSMGGDHPSFTSGTRNYRQRRRSPSPRFLDPEFRELDLARRKREEEEERSRSLSQELVGVDGGGTG
CSIPGLSGVLTASEPGYSLHRPEEVSVMPKKSILKKRIEVDIMEPSMQLESFSSSTSSSQDHPLYSGHPSLPLSGAIAAFASEIENK
GTMVETALKEPQGNLYQWGPLPGIPKDNSPLREKFGSFLCHKDNLDLKAEGPERHTDFLLPHERASQDGSGFSRILSMLADSTSTQE
KRRRSFPDIEDEEKFLYGDEEEDLKAESVPKPLGSSESEVMRQKASSLPSSAPAVKLESLEETNPEYAKIHDLLKTIGLDIGVAEIS
QLAARTQERLHGKKPSLRSSADRRSSVDRYFSADHCSSVDHRFSADRCSSVDHCFSADRRSSDPHRLESREAHHSNTHSPEVSHPHP
PSPVDPYLLTKNSPPFLKSDHPVGHISGPEVVGSGFQSSVAVRCMLPSAPSAPIRLPHTAALSQFHMPRASQFAAARIPPNYQGPAI
PPASFDAYRHYMAYAASRWPMYPTSQPSNHPVPEPHRIMPITKQATRSRPNLRVIPTVTPDKPKQKESLRGSIPAAQVPVQVSIPSL
IRYNPEKISDEKNRASQKQKVIEEREKLKNDREARQKKMYYLRTELERLHKQQGEMLRKKRREKDGHKDPLLVEVSRLQDNIMKDIA
ELRQEAEEAEKKQSELDKVAQILGINIFDKSQKSLSDSREPTEKPGKAEKSKSPEKVSSFSNSSSNKESKVNNEKFRTKSPKPAESP
QSATKQLDQPTAAYEYYDAGNHWCKDCNTICGTMFDFFTHMHNKKHTQTLDPYNRPWASKTQSEAKQDAIKRTDKITVPAKGSEFLV
PISGFYCQLCEEFLGDPISGEQHVKGHQHNEKYKKYVDENPLYEERRNLDRQAGLAVVLETERRRQSELKRKLSEKPKEEKKEKKAK
AVKEVKEDDKVSEKLEDQLSEGRNSPEKAENKRNTGIKLQLKEEVKKESPTSSSFGKFSWKKPEKEEEKSSLVTPSISKEEILESSK
DKEDGKTEAGKAKPIKIKLSGKTVVAHTSPWMPVVTTSTQTKIRPNLPIPSTVLRKSCSATMSKPAPLNTFLSIKSSGTTAKPLPVV
KESSADLLLPPDIISKAFGGEEVILKGSPEEKVVLAEKSEPSHLPEQILPPPPPPPPPPPPPVIPHPAAPSAAQANAILAPVKSN
PVVSQTLSPGFVGPNILNPVLPVAIMASAQPAAIPSDETAPGVSESDRDQTLFSVLVRPPPPLSSVFSEQAKKLEKRNSCLATANAK
DLYDIFYSSGGKGAPETKGAPETKLSGGPLANGENSNLSRTKSSDTSSTSPLNSSASQEELHQDEGLVAAPIVSNSEKPIAKTLVAL
GKWSVVEHVGPKSTGSTYGFLQPLTRLCQSRPYETITPKTDTLAIWTSSSFQSDTSRDISPEKSELDLGEPGPPGVEPPPQLLDIQC
KESQKLVEIHLRESVNQDKESQELRKSEDCRESEIETNTELKERVKELSEGIVDEGVSTSIGPHSIDDSNLNHGNRYMWEGEVKQPN
LLMIDKEAEQSNKLMTGSETPSKVVIKLSPQACSFTKAKLDSFLSEARSLLNPQDTPVKISAPELLLHSPARSAMCLTGSPQEQGVS
VVSEEGLENSAPESASRTSRYRSLKLKRERSKDFQVKKIYELAVWDENKKRPETWESPEKPKTEALELQDVHPELTVTIESKALEDF
EATDLKVEELTALGNLGDMPVDFCTTRVSPAHRSPTVLCQKVCEENSVSPIGCNSSDPADFEPIPSFSGFPLDSPKTLVLDFETEGE
RNSPNPRSVRIPSPNILKTGLTENVDRGLGGLEGTHQALDLLAGGMMPEEVKESSQLDKQESLGLELKTINSAGLGPSPCLPDLVDF
VTRTSGVQKDKLCSPLSEPGDPSKCSSLELGPLQLEISNASTTEVAILQVDDDSGDPLNLVKAPVSRSPPREQVIEDNMVPQGMPEQ
ETTVGAIQDHTESSVHN
>sp|P01112|RASH_HUMAN
MTEYKLVVVGAGGVGKSALTIQLIQNHFVDEYDPTIEDSYRKQVVIDGETCLLDILDTAGQEEYSAMRDQYMRTGEGFLCVFAINNT
KSFEDIHQYREQIKRVKDSDDVPMVLVGNKCDLAARTVESRQAQDLARSYGIPYIETSAKTRQGVEDAFYTLVREIRQHKLRKLNPP
DESGPGCMSCKCVLS
>sp|Q8WTQ4|CP078_HUMAN
MSEQQMDLKDLMPTKRKYMWKTAEDRRMSDLTCVLEWLERRQGKKKQAPEKQKPKVVTVLKRNKKKEEKKGKGLMTARGGNRRDTET
SQQALGKRFRKDAASYRSLYGVEQKGKHLSMVPGSYIKDGPKKSDTDIKDAVDPESTQRPNPFRRQSIVLDPMLQEGTFNSQRATFI
RDWSNKMPDMAYERKLKSLMEKSTEPKMETMRMLKPEEVLSCRYLRLSKENIRTLLKLCKDAGMNVDIHPHMVEEDIDAKKVFTGIP
SMAL
>sp|P06899|H2B1J_HUMAN
MPEPAKSAPAPKKGSKKAVTKAQKKDGKKRKRSRKESYSIYVYKVLKQVHPDTGISSKAMGIMNSFVNDIFERIAGEASRLAHYNKR
STITSREIQTAVRLLLPGELAKHAVSEGTKAVTKYTSAK
>sp|P45877|PPIC_HUMAN

MGPGPRLLLPLVLCVGLGALVFSSGAEGFRKRGPSVTAKVFFDVRIGDKDVGRIVIGLFGKVVPKTVENFVALATGEKGYGYKGSKF
HRVIKDFMIQGGDITTGDGTGGVSIYGETFPDENFKLKHYGIGWVSMANAGPDTNGSQFFITLTKPTWLDGKHVVFGKVIDGMTVVH
SIELQATDGHDRPLTNCSIINSGKIDVKTPFVVEIADW
>sp|Q8NI99|ANGL6_HUMAN
MGKPWLRALQLLLLLGASWARAGAPRCTYTFVLPPQKFTGAVCWSGPASTRATPEAANASELAALRMRVGRHEELLRELQRLAAADG
AVAGEVRALRKESRGLSARLGQLRAQLQHEAGPGAGPGADLGAEPAAALALLGERVLNASAEAQRAAARFHQLDVKFRELAQLVTQQ
SSLIARLERLCPGGAGGQQQVLPPPPLVPVVPVRLVGSTSDTSRMLDPAPEPQRDQTQRQQEPMASPMPAGHPAVPTKPVGPWQDCA
EARQAGHEQSGVYELRVGRHVVSVWCEQQLEGGGWTVIQRRQDGSVNFFTTWQHYKAGFGRPDGEYWLGLEPVYQLTSRGDHELLVL
LEDWGGRGARAHYDGFSLEPESDHYRLRLGQYHGDAGDSLSWHNDKPFSTVDRDRDSYSGNCALYQRGGWWYHACAHSNLNGVWHHG
GHYRSRYQDGVYWAEFRGGAYSLRKAAMLIRPLKL
>sp|Q9BSK2|S2533_HUMAN
MATGGQQKENTLLHLFAGGCGGTVGAIFTCPLEVIKTRLQSSRLALRTVYYPQVHLGTISGAGMVRPTSVTPGLFQVLKSILEKEGP
KSLFRGLGPNLVGVAPSRAVYFACYSKAKEQFNGIFVPNSNIVHIFSAGSAAFITNSLMNPIWMVKTRMQLEQKVRGSKQMNTLQCA
RYVYQTEGIRGFYRGLTASYAGISETIICFAIYESLKKYLKEAPLASSANGTEKNSTSFFGLMAAAALSKGCASCIAYPHEVIRTRL
REEGTKYKSFVQTARLVFREEGYLAFYRGLFAQLIRQIPNTAIVLSTYELIVYLLEDRTQ
>sp|Q9Y5W3|KLF2_HUMAN
MALSEPILPSFSTFASPCRERGLQERWPRAEPESGGTDDDLNSVLDFILSMGLDGLGAEAAPEPPPPPPPPAFYYPEPGAPPPYSAP
AGGLVSELLRPELDAPLGPALHGRFLLAPPGRLVKAEPPEADGGGGYGCAPGLTRGPRGLKREGAPGPAASCMRGPGGRPPPPPDTP
PLSPDGPARLPAPGPRASFPPPFGGPGFGAPGPGLHYAPPAPPAFGLFDDAAAAAAALGLAPPAARGLLTPPASPLELLEAKPKRGR
RSWPRKRTATHTCSYAGCGKTYTKSSHLKAHLRTHTGEKPYHCNWDGCGWKFARSDELTRHYRKHTGHRPFQCHLCDRAFSRSDHLA
LHMKRHM
>sp|A1A580|KR231_HUMAN
MSYNCCCGNFSSHSCEGYLCYSGYSRGGSSYPSNLVYSTEPLISQHLPAGFLSLQGLSGDLLGNP
>sp|Q8NAG6|ANKL1_HUMAN
MCSEARLARRLRDALREEEPWAVEELLRCGADPNLVLEDGAAAVHLAAGARHPRGLRCLGALLRQGGDPNARSVEALTPLHVAAAWG
CRRGLELLLSQGADPALRDQDGLRPLDLALQQGHLECARVLQDLDTRTRTRTRIGAETQEPEPAPGTPGLSGPTDETLDSIALQKQP
CRGDNRDIGLEADPGPPSLPVPLETVDKHGSSASPPGHWDYSSDASFVTAVEVSGAEDPASDTPPWAGSLPPTRQGLLHVVHANQRV
PRSQGTEAELNARLQALTLTPPNAAGFQSSPSSMPLLDRSPAHSPPRTPTPGASDCHCLWEHQTSIDSDMATLWLTEDEASSTGGRE
PVGPCRHLPVSTVSDLELLKGLRALGENPHPITPFTRQLYHQQLEEAQIAPGPEFSGHSLELAAALRTGCIPDVQADEDALAQQFEQ
PDPARRWREGVVKSSFTYLLLDPRETQDLPARAFSLTPAERLQTFIRAIFYVGKGTRARPYVHLWEALGHHGRSRKQPHQACPKVRQ
ILDIWASGCGVVSLHCFQHVVAVEAYTREACIVEALGIQTLTNQKQGHCYGVVAGWPPARRRRLGVHLLHRALLVFLAEGERQLHPQ
DIQARG
>sp|P30499|1C01_HUMAN
MRVMEPRTLILLLSGALALTETWACSHSMKYFFTSVSRPGRGEPRFISVGYVDDTQFVRFDSDAASPRGEPRAPWVEQEGPEYWDRE
TQKYNRQAQTDRVSLRNLRGYYNQSEAGSHTLQWMCGCDLGPDGRLLRGYDQYAYDGKDYIALNEDLRSWTAADTAAQITQRKWEAA
REAEERRAYLEGTCVEWLRRYLENGKESLQRAEHPKTHVTHHPVSDHEATLRCWALGFYPAEITLTWQWDGEDQTQDTELVETRPAG
DGTFQKWAAVMVPSGEEQRYTCHVQHEGLPEPLTLRWEPSSQPTIPIVGIVAGLAVLAVLAVLGAVVAVVMCRRKSSGGKGGSCSQA
ASSNSAQGSDESLIASKA
>sp|P04745|AMY1_HUMAN
MKLFWLLFTIGFCWAQYSSNTQQGRTSIVHLFEWRWVDIALECERYLAPKGFGGVQVSPPNENVAIHNPFRPWWERYQPVSYKLCTR
SGNEDEFRNMVTRCNNVGVRIYVDAVINHMCGNAVSAGTSSTCGSYFNPGSRDFPAVPYSGWDFNDGKCKTGSGDIENYNDATQVRD
CRLSGLLDLALGKDYVRSKIAEYMNHLIDIGVAGFRIDASKHMWPGDIKAILDKLHNLNSNWFPEGSKPFIYQEVIDLGGEPIKSSD
YFGNGRVTEFKYGAKLGTVIRKWNGEKMSYLKNWGEGWGFMPSDRALVFVDNHDNQRGHGAGGASILTFWDARLYKMAVGFMLAHPY

GFTRVMSSYRWPRYFENGKDVNDWVGPPNDNGVTKEVTINPDTTCGNDWVCEHRWRQIRNMVNFRNVVDGQPFTNWYDNGSNQVAFG
RGNRGFIVFNNDDWTFSLTLQTGLPAGTYCDVISGDKINGNCTGIKIYVSDDGKAHFSISNSAEDPFIAIHAESKL
>sp|P36894|BMR1A_HUMAN
MPQLYIYIRLLGAYLFIISRVQGQNLDSMLHGTGMKSDSDQKKSENGVTLAPEDTLPFLKCYCSGHCPDDAINNTCITNGHCFAIIE
EDDQGETTLASGCMKYEGSDFQCKDSPKAQLRRTIECCRTNLCNQYLQPTLPPVVIGPFFDGSIRWLVLLISMAVCIIAMIIFSSCF
CYKHYCKSISSRRRYNRDLEQDEAFIPVGESLKDLIDQSQSSGSGSGLPLLVQRTIAKQIQMVRQVGKGRYGEVWMGKWRGEKVAVK
VFFTTEEASWFRETEIYQTVLMRHENILGFIAADIKGTGSWTQLYLITDYHENGSLYDFLKCATLDTRALLKLAYSAACGLCHLHTE
IYGTQGKPAIAHRDLKSKNILIKKNGSCCIADLGLAVKFNSDTNEVDVPLNTRVGTKRYMAPEVLDESLNKNHFQPYIMADIYSFGL
IIWEMARRCITGGIVEEYQLPYYNMVPSDPSYEDMREVVCVKRLRPIVSNRWNSDECLRAVLKLMSECWAHNPASRLTALRIKKTLA
KMVESQDVKI
>sp|U3KPV4|A3LT2_HUMAN
MALKEGLRAWKRIFWRQILLTLGLLGLFLYGLPKFRHLEALIPMGVCPSATMSQLRDNFTGALRPWARPEVLTCTPWGAPIIWDGSF
DPDVAKQEARQQNLTIGLTIFAVGRYLEKYLERFLETAEQHFMAGQSVMYYVFTELPGAVPRVALGPGRRLPVERVARERRWQDVSM
ARMRTLHAALGGLPGREAHFMFCMDVDQHFSGTFGPEALAESVAQLHSWHYHWPSWLLPFERDAHSAAAMAWGQGDFYNHAAVFGGS
VAALRGLTAHCAGGLDWDRARGLEARWHDESHLNKFFWLHKPAKVLSPEFCWSPDIGPRAEIRRPRLLWAPKGYRLLRN
>sp|P50219|MNX1_HUMAN
MEKSKNFRIDALLAVDPPRAASAQSAPLALVTSLAAAASGTGGGGGGGGASGGTSGSCSPASSEPPAAPADRLRAESPSPPRLLAAH
CALLPKPGFLGAGGGGGGTGGGHGGPHHHAHPGAAAAAAAAAAAAAAGGLALGLHPGGAQGGAGLPAQAALYGHPVYGYSAAAAAAA
LAGQHPALSYSYPQVQGAHPAHPADPIKLGAGTFQLDQWLRASTAGMILPKMPDFNSQAQSNLLGKCRRPRTAFTSQQLLELEHQFK
LNKYLSRPKRFEVATSLMLTETQVKIWFQNRRMKWKRSKKAKEQAAQEAEKQKGGGGGAGKGGAEEPGAEELLGPPAPGDKGSGRRL
RDLRDSDPEEDEDEDDEDHFPYSNGASVHAASSDCSSEDDSPPPRPSHQPAPQ
>sp|P63119|VPK21_HUMAN
WASQVSENRPVCKAIIQGKQFEGLVDTGADVSIIALNQWPKNWPKQKAVTGLVGIGTASEVYQSTEILHCLGPDNQESTVQPMITSI
PLNLWGRDLLQQWGAEITMPTPLYSPTSQKIMTKMGYIPGKGLGKNEDGIKVPVEAKINQKREGIGYPF
>sp|Q9NPD7|NRN1_HUMAN
MGLKLNGRYISLILAVQIAYLVQAVRAAGKCDAVFKGFSDCLLKLGDSMANYPQGLDDKTNIKTVCTYWEDFHSCTVTALTDCQEGA
KDMWDKLRKESKNLNIQGSLFELCGSGNGAAGSLLPAFPVLLVSLSAALATWLSF
>sp|B2RBV5|MRUPP_HUMAN
MRPVDADEAREPREEPGSPLSPAPRAGRENLASLERERARAHWRARRKLLEIQSLLDAIKSEVEAEERGARAPAPRPRAEAEERVAR
LCAEAERKAAEAARMGRRIVELHQRIAGCECC
>sp|Q9H361|PABP3_HUMAN
MNPSTPSYPTASLYVGDLHPDVTEAMLYEKFSPAGPILSIRICRDLITSGSSNYAYVNFQHTKDAEHALDTMNFDVIKGKPVRIMWS
QRDPSLRKSGVGNIFVKNLDKSINNKALYDTVSAFGNILSCNVVCDENGSKGYGFVHFETHEAAERAIKKMNGMLLNGRKVFVGQFK
SRKEREAELGARAKEFPNVYIKNFGEDMDDERLKDLFGKFGPALSVKVMTDESGKSKGFGFVSFERHEDAQKAVDEMNGKELNGKQI
YVGRAQKKVERQTELKRTFEQMKQDRITRYQVVNLYVKNLDDGIDDERLRKAFSPFGTITSAKVMMEGGRSKGFGFVCFSSPEEATK
AVTEMNGRIVATKPLYVALAQRKEERQAYLTNEYMQRMASVRAVPNQRAPPSGYFMTAVPQTQNHAAYYPPSQIARLRPSPRWTAQG
ARPHPFQNKPSAIRPGAPRVPFSTMRPASSQVPRVMSTQRVANTSTQTVGPRPAAAAAAAATPAVRTVPRYKYAAGVRNPQQHRNAQ
PQVTMQQLAVHVQGQETLTASRLASAPPQKQKQMLGERLFPLIQAMHPTLAGKITGMLLEIDNSELLYMLESPESLRSKVDEAVAVL
QAHQAKEATQKAVNSATGVPTV
>sp|Q6ZRP0|PR23C_HUMAN
MGSRPCSPSACLAPWWGQQPGGPGPAKRSRLEEPAGPESRAAPSPEDPAGTPAVDALTSMVVLDAGCALRVPLEDVDLVLELAPMSV
LRVSLGGHTLIVIPEVLLSSVDECSGAQGDWSAGLEVDVFLGAHGEDVVVEQEVCASVPEIAAEEEAYEEDADSEFPELWMDSAAGS
AAGLYPSARSMFSPYREGPIRGPCALAPNPSSERRSPRPIFDLEFHLLEPVPSSPLQPLPPSPSPGPHARPELPERPPCKVRRRLFQ
E

>sp|Q9C0B5|ZDHC5_HUMAN
MPAESGKRFKPSKYVPVSAAAIFLVGATTLFFAFTCPGLSLYVSPAVPIYNAIMFLFVLANFSMATFMDPGIFPRAEEDEDKEDDFR
APLYKTVEIKGIQVRMKWCATCRFYRPPRCSHCSVCDNCVEEFDHHCPWVNNCIGRRNYRYFFLFLLSLTAHIMGVFGFGLLYVLYH
IEELSGVRTAVTMAVMCVAGLFFIPVAGLTGFHVVLVARGRTTNEQVTGKFRGGVNPFTNGCCNNVSRVLCSSPAPRYLGRPKKEKT
IVIRPPFLRPEVSDGQITVKIMDNGIQGELRRTKSKGSLEITESQSADAEPPPPPKPDLSRYTGLRTHLGLATNEDSSLLAKDSPPT
PTMYKYRPGYSSSSTSAAMPHSSSAKLSRGDSLKEPTSIAESSRHPSYRSEPSLEPESFRSPTFGKSFHFDPLSSGSRSSSLKSAQG
TGFELGQLQSIRSEGTTSTSYKSLANQTRNGSLSYDSLLTPSDSPDFESVQAGPEPDPPLGYTSPFLSARLAQQREAERHPRLVPTG
PTHREPSPVRYDNLSRHIVASLQEREKLLRQSPPLPGREEEPGLGDSGIQSTPGSGHAPRTSSSSDDSKRSPLGKTPLGRPAVPRFG
KPDGLRGRGVGSPEPGPTAPYLGRSMSYSSQKAQPGVSETEEVALQPLLTPKDEVQLKTTYSKSNGQPKSLGSASPGPGQPPLSSPT
RGGVKKVSGVGGTTYEISV
>sp|Q9NVH6|TMLH_HUMAN
MWYHRLSHLHSRLQDLLKGGVIYPALPQPNFKSLLPLAVHWHHTASKSLTCAWQQHEDHFELKYANTVMRFDYVWLRDHCRSASCYN
SKTHQRSLDTASVDLCIKPKTIRLDETTLFFTWPDGHVTKYDLNWLVKNSYEGQKQKVIQPRILWNAEIYQQAQVPSVDCQSFLETN
EGLKKFLQNFLLYGIAFVENVPPTQEHTEKLAERISLIRETIYGRMWYFTSDFSRGDTAYTKLALDRHTDTTYFQEPCGIQVFHCLK
HEGTGGRTLLVDGFYAAEQVLQKAPEEFELLSKVPLKHEYIEDVGECHNHMIGIGPVLNIYPWNKELYLIRYNNYDRAVINTVPYDV
VHRWYTAHRTLTIELRRPENEFWVKLKPGRVLFIDNWRVLHGRECFTGYRQLCGCYLTRDDVLNTARLLGLQA
>sp|Q5SZI1|LRAD2_HUMAN
MEACCLLQLPQRLLLLGAAALTATALETADLAELCGQTWQGDGLLLRSHAASRRFYFVAPDTDCGLWVQAAAPGDRIRFQFRFFLVY
SLTPAPPALNTSSPAPADPCAPGSYLQFYEGPPGAPRPLGSPLCGLNIPVPVASSGPFLGLRLVTRGRQPRVDFVGEVTSFRLGPCG
AYFRCQNGRCIPSSLVCDPWGMDNCGDGSDQGSWSPADCRGPSPVPSQTGSTDAHTSRSLTPSPALGSAGSLWIAAERSSPAGRDPT
RQDAALEGSTE
>sp|Q71RG6|YH006_HUMAN
MGTGGSLLCGCSLVLSCLCPSASLPDPGNSTWPPGAQAGLPAALALPLPRLPRILFPMAGRPARPSSDFVGCAQGMCCHGRQGTVHI
HTSSVSCWTPCPVTGTGGTAVSRKDRVLPHRRQVSLACVCAVGERAGQLWSQKPVQMARPSARHLLPRGSSPNSQAVLLPSVCPVPW
PPVGPSPGQGEGLSPAFPGVGTDRGDSWALVLQV
>sp|Q71RH2|FA57B_HUMAN
MLTPMVAGGVVFPGLFLLSKNTLQRLPQLRWEEADAVIVSARLVSSVQAIMASTAGYIVSTSCKHIIDDQHWLSSAYTQFAVPYFIY
DIYAMFLCHWHKHQVKGHGGDDGAARAPGSTWAIARGYLHKEFLMVLHHAAMVLVCFPLSVVWRQGKGDFFLGCMLMAEVSTPFVCL
GKILIQYKQQHTLLHKVNGALMLLSFLCCRVLLFPYLYWAYGRHAGLPLLAVPLAIPAHVNLGAALLLAPQLYWFFLICRGACRLFW
PRSRPPPACQAQD
>sp|Q9BT78|CSN4_HUMAN
MAAAVRQDLAQLMNSSGSHKDLAGKYRQILEKAIQLSGAEQLEALKAFVEAMVNENVSLVISRQLLTDFCTHLPNLPDSTAKEIYHF
TLEKIQPRVISFEEQVASIRQHLASIYEKEEDWRNAAQVLVGIPLETGQKQYNVDYKLETYLKIARLYLEDDDPVQAEAYINRASLL
QNESTNEQLQIHYKVCYARVLDYRRKFIEAAQRYNELSYKTIVHESERLEALKHALHCTILASAGQQRSRMLATLFKDERCQQLAAY
GILEKMYLDRIIRGNQLQEFAAMLMPHQKATTADGSSILDRAVIEHNLLSASKLYNNITFEELGALLEIPAAKAEKIASQMITEGRM
NGFIDQIDGIVHFETREALPTWDKQIQSLCFQVNNLLEKISQTAPEWTAQAMEAQMAQ
>sp|P56180|TPTE_HUMAN
MNESPDPTDLAGVIIELGPNDSPQTSEFKGATEEAPAKESPHTSEFKGAARVSPISESVLARLSKFEVEDAENVASYDSKIKKIVHS
IVSSFAFGLFGVFLVLLDVTLILADLIFTDSKLYIPLEYRSISLAIALFFLMDVLLRVFVERRQQYFSDLFNILDTAIIVILLLVDV
VYIFFDIKLLRNIPRWTHLLRLLRLIILLRIFHLFHQKRQLEKLIRRRVSENKRRYTRDGFDLDLTYVTERIIAMSFPSSGRQSFYR
NPIKEVVRFLDKKHRNHYRVYNLCSERAYDPKHFHNRVVRIMIDDHNVPTLHQMVVFTKEVNEWMAQDLENIVAIHCKGGTDRTGTM
VCAFLIASEICSTAKESLYYFGERRTDKTHSEKFQGVKTPSQKRYVAYFAQVKHLYNWNLPPRRILFIKHFIIYSIPRYVRDLKIQI
EMEKKVVFSTISLGKCSVLDNITTDKILIDVFDGLPLYDDVKVQFFYSNLPTYYDNCSFYFWLHTSFIENNRLYLPKNELDNLHKQK
ARRIYPSDFAVEILFGEKMTSSDVVAGSD

>sp|Q9H2H9|S38A1_HUMAN
MMHFKSGLELTELQNMTVPEDDNISNDSNDFTEVENGQINSKFISDRESRRSLTNSHLEKKKCDEYIPGTTSLGMSVFNLSNAIMGS
GILGLAFALANTGILLFLVLLTSVTLLSIYSINLLLICSKETGCMVYEKLGEQVFGTTGKFVIFGATSLQNTGAMLSYLFIVKNELP
SAIKFLMGKEETFSAWYVDGRVLVVIVTFGIILPLCLLKNLGYLGYTSGFSLSCMVFFLIVVIYKKFQIPCIVPELNSTISANSTNA
DTCTPKYVTFNSKTVYALPTIAFAFVCHPSVLPIYSELKDRSQKKMQMVSNISFFAMFVMYFLTAIFGYLTFYDNVQSDLLHKYQSK
DDILILTVRLAVIVAVILTVPVLFFTVRSSLFELAKKTKFNLCRHTVVTCILLVVINLLVIFIPSMKDIFGVVGVTSANMLIFILPS
SLYLKITDQDGDKGTQRIWAALFLGLGVLFSLVSIPLVIYDWACSSSSDEGH
>sp|Q9NQT6|FSCN3_HUMAN
MDETEWIHRHPKAEDLRVGLISWAGTYLTFEACKNTVTATAKSLGRRQTWEILVSNEHETQAVVRLKSVQGLYLLCECDGTVCYGRP
RTSHHGCFLLRFHRNSKWTLQCLISGRYLESNGKDVFCTSHVLSAYHMWTPRPALHVHVILYSPIHRCYARADPTMGRIWVDAAVPC
LEECGFLLHFRDGCYHLETSTHHFLSHVDRLFSQPSSQTAFHMQVRPGGLVALCDGEGGMLYPQGTHLLLGMGCNPMRGEEWFILQH
CPTWVSLRSKTGRFISVIYDGEVRAASERLNRMSLFQFECDSESPTVQLRSANGYYLSQRRHRAVMADGHPLESDTFFRMHWNCGRI
ILQSCRGRFLGIAPNSLLMANVILPGPNEEFGILFANRSFLVLRGRYGYVGSSSGHDLIQCNQDQPDRIHLLPCRPGIYHFQAQGGS
FWSITSFGTFRPWGKFALNFCIELQGSNLLTVLAPNGFYMRADQSGTLLADSEDITRECIWEF
>sp|Q5BN46|CI116_HUMAN
MAEECPRACAEPVAPKATAPPERTSDYYRVSADLPGRFNNPGWFRGYRTQKAVSVYRTSNQAYGSRAPTVHEMPKVFYPNSNKFSQQ
LAAGGMFRNNTLNVYLEKSIVTGPDNCITSCDRLNFHPSYNINRPSICD
>sp|Q9H300|PARL_HUMAN
MAWRGWAQRGWGCGQAWGASVGGRSCEELTAVLTPPQLLGRRFNFFIQQKCGFRKAPRKVEPRRSDPGTSGEAYKRSALIPPVEETV
FYPSPYPIRSLIKPLFFTVGFTGCAFGSAAIWQYESLKSRVQSYFDGIKADWLDSIRPQKEGDFRKEINKWWNNLSDGQRTVTGIIA
ANVLVFCLWRVPSLQRTMIRYFTSNPASKVLCSPMLLSTFSHFSLFHMAANMYVLWSFSSSIVNILGQEQFMAVYLSAGVISNFVSY
VGKVATGRYGPSLGASGAIMTVLAAVCTKIPEGRLAIIFLPMFTFTAGNALKAIIAMDTAGMILGWKFFDHAAHLGGALFGIWYVTY
GHELIWKNREPLVKIWHEIRTNGPKKGGGSK
>sp|Q96E11|RRFM_HUMAN
MALGLKCFRMVHPTFRNYLAASIRPVSEVTLKTVHERQHGHRQYMAYSAVPVRHFATKKAKAKGKGQSQTRVNINAALVEDIINLEE
VNEEMKSVIEALKDNFNKTLNIRTSPGSLDKIAVVTADGKLALNQISQISMKSPQLILVNMASFPECTAAAIKAIRESGMNLNPEVE
GTLIRVPIPQVTREHREMLVKLAKQNTNKAKDSLRKVRTNSMNKLKKSKDTVSEDTIRLIEKQISQMADDTVAELDRHLAVKTKELL
G
>sp|Q9BZD4|NUF2_HUMAN
METLSFPRYNVAEIVIHIRNKILTGADGKNLTKNDLYPNPKPEVLHMIYMRALQIVYGIRLEHFYMMPVNSEVMYPHLMEGFLPFSN
LVTHLDSFLPICRVNDFETADILCPKAKRTSRFLSGIINFIHFREACRETYMEFLWQYKSSADKMQQLNAAHQEALMKLERLDSVPV
EEQEEFKQLSDGIQELQQSLNQDFHQKTIVLQEGNSQKKSNISEKTKRLNELKLSVVSLKEIQESLKTKIVDSPEKLKNYKEKMKDT
VQKLKNARQEVVEKYEIYGDSVDCLPSCQLEVQLYQKKIQDLSDNREKLASILKESLNLEDQIESDESELKKLKTEENSFKRLMIVK
KEKLATAQFKINKKHEDVKQYKRTVIEDCNKVQEKRGAVYERVTTINQEIQKIKLGIQQLKDAAEREKLKSQEIFLNLKTALEKYHD
GIEKAAEDSYAKIDEKTAELKRKMFKMST
>sp|Q14687|GSE1_HUMAN
MKGMSHEPKSPSLGMLSTATRTTATVNPLTPSPLNGALVPSGSPATSSALSAQAAPSSSFAAALRKLAKQAEEPRGSSLSSESSPVS
SPATNHSSPASTPKRVPMGPIIVPPGGHSVPSTPPVVTIAPTKTVNGVWRSESRQDAGSRSSSGGRERLIVEPPLPQEKAGGPAIPS
HLLSTPYPFGLSPSSVVQDSRFPPLNLQRPVHHVVPPSTVTEDYLRSFRPYHTTDDLRMSSLPPLGLDPATAAAYYHPSYLAPHPFP
HPAFRMDDSYCLSALRSPFYPIPTPGSLPPLHPSAMHLHLSGVRYPPELSHSSLAALHSERMSGLSAERLQMDEELRRERERERERE
REREADREREKEREREREKEREQEKEREREKERERELERQREQRAREKELLAAKALEPSFLPVAELHGLRGHATEERGKPSEQLTPT
RAEKLKDAGLQAPKPVQHPLHPVPTPHHTVPSLISNHGIFSLPSSSAATALLIQRTNEEEKWLARQRRLRQEKEDRQSQVSEFRQQV
LEQHLDMGRPPVPAEAEHRPESTTRPGPNRHEPGGRDPPQHFGGPPPLISPKPQLHAAPTALWNPVSLMDNTLETRRAESHSLHSHP
AAFEPSRQAAVPLVKVERVFCPEKAEEGPRKREPAPLDKYQPPPPPPREGGSLEHQPFLPGPGPFLAELEKSTQTILGQQRASLPQA

ATFGELSGPLKPGSPYRPPVPRAPDPAYIYDEFLQQRRRLVSKLDLEERRRREAQEKGYYYDLDDSYDESDEEEVRAHLRCVAEQPP
LKLDTSSEKLEFLQLFGLTTQQQKEELVAQKRRKRRRMLRERSPSPPTIQSKRQTPSPRLALSTRYSPDEMNNSPNFEEKKKFLTIF
NLTHISAEKRKDKERLVEMLRAMKQKALSAAVADSLTNSPRDSPAVSLSEPATQQASLDVEKPVGVAASLSDIPKAAEPGKLEQVRP
QELSRVQELAPASGEKARLSEAPGGKKSLSMLHYIRGAAPKDIPVPLSHSTNGKSKPWEPFVAEEFAHQFHESVLQSTQKALQKHKG
SVAVLSAEQNHKVDTSVHYNIPELQSSSRAPPPQHNGQQEPPTARKGPPTQELDRDSEEEEEEDDEDGEDEEEVPKRKWQGIEAVFE
AYQEHIEEQNLERQVLQTQCRRLEARHYSLSLTAEQLSHSVAELRSQKQKMVSERERLQAELDHLRKCLALPAMHWPRGYLKGYPR
>sp|Q5QNW6|H2B2F_HUMAN
MPDPAKSAPAPKKGSKKAVTKVQKKDGKKRKRSRKESYSVYVYKVLKQVHPDTGISSKAMGIMNSFVNDIFERIAGEASRLAHYNKR
STITSREIQTAVRLLLPGELAKHAVSEGTKAVTKYTSSK
>sp|Q9NR55|BATF3_HUMAN
MSQGLPAAGSVLQRSVAAPGNQPQPQPQQQSPEDDDRKVRRREKNRVAAQRSRKKQTQKADKLHEEYESLEQENTMLRREIGKLTEE
LKHLTEALKEHEKMCPLLLCPMNFVPVPPRPDPVAGCLPR
>sp|Q9HBL6|LRTM1_HUMAN
MKGELLLFSSVIVLLQVVCSCPDKCYCQSSTNFVDCSQQGLAEIPSHLPPQTRTLHLQDNQIHHLPAFAFRSVPWLMTLNLSNNSLS
NLAPGAFHGLQHLQVLNLTQNSLLSLESRLFHSLPQLRELDLSSNNISHLPTSLGETWENLTILAVQQNQLQQLDRALLESMPSVRL
LLLKDNLWKCNCHLLGLKLWLEKFVYKGGLTDGIICESPDTWKGKDLLRIPHELYQPCPLPAPDPVSSQAQWPGSAHGVVLRPPENH
NAGERELLECELKPKPRPANLRHAIATVIITGVVCGIVCLMMLAAAIYGCTYAAITAQYHGGPLAQTNDPGKVEEKERFDSSPA
>sp|P54368|OAZ1_HUMAN
MVKSSLQRILNSHCFAREKEGDKPSATIHASRTMPLLSLHSRGGSSSESSRVSLHCCSNPGPGPRWCSDAPHPPLKIPGGRGNSQRD
HNLSANLFYSDDRLNVTEELTSNDKTRILNVQSRLTDAKRINWRTVLSGGSLYIEIPGGALPEGSKDSFAVLLEFAEEQLRADHVFI
CFHKNREDRAALLRTFSFLGFEIVRPGHPLVPKRPDACFMAYTFERESSGEEEE
>sp|Q7Z5M5|TMC3_HUMAN
MKTSKASQRYRGIRRNASQCYLYQESLLLSNLDDSFSADETGDSNDPEQIFQNIQFQKDLMANIRCRPWTMGQKLRALRQAKNIVLK
FEGRLTRTRGYQAAGAELWRKFARLACNFVVIFIPWEMRIKKIESHFGSGVASYFIFLRWLFGINIVLTIMTGAFIVIPELIAGQPF
GSTARKTIPKEQVSSAQDLDTVWSLGGYLQYSVLFYGYYGRERKIGRAGYRLPLAYFLVGMAVFAYSFIILLKKMAKNSRTSLASAS
NENYTFCWRVFCAWDYLIGNPEAAESKTAAIVNSIREAILEEQEKKKSKNLAVTICLRIIANILVLLSLAGSIYLIYFVVDRSQKLE
QSKKELTLWEKNEVSVVVSLVTMIAPSAFDLIAALEMYHPRTTLRFQLARVLVLYLGNLYSLIIALLDKVNSMSIEEMATKNNTSHW
IDSTTFFATRTAPEEEKWSTSRPGMGLRRNNTWALEETSISAYTMPLIKANKTSLHTQSPQDQCWETYVGQEMLKLSIIDMLFTVAS
ILLIDFFRGLFVRYLSDYWCWDLESKFPEYGEFKIAENVLHLVYNQGMIWMGAFFSPCLPAFNVLKLIGLMYLRSWAVLTCNVPHQQ
VFRASRSNNFYLAMLLFMLFLCMLPTIFAIVRYKPSLNCGPFSGQEKIYDIVSETIEKDFPVWFGSVVGHISSPVVILPAVLLLFML
IYYLQSIARSLKLSNHQLKMQIQNARSEDKKKVAQMVEARIQTQEESTKKLPNDSDLTSQLSSAHSGTPQNNGNVAHFDSGSSKSGR
IETVAQSMPQSPRPGDRAPSSPLPGVPKSRLEHETNRYLHGLCASTSDLHRNRSRTPMTFTTHIEDVHSEPLFRKDFQQINPPHRGP
QASTLLAQGPRPHAPRYYVINECDSYKKKHLNVWPERHFKIDASGDIVELYPRNVRQYASRVPRQPPSPQLSEEEEETPSRDWIKRS
LPPRSLIDLRRAPHFYIGERSESQTRDPEHQGRVHYKSWNEDFEGHLERPAYVPRKPRSRNFQYPQPPLKPRGKPRFEPSLTESDSV
SAASSSDQQNSSADQYLQVTHSQGRFPRSVGQPSRRKAKSGQELTVDLDDLICSDV
>sp|Q8NE01|CNNM3_HUMAN
MAAAVAAAGRLGWLFAALCLGNAAGEAAPGPRVLGFCLEEDGAAGAGWVRGGAARDTPDATFLLRLFGPGFANSSWSWVAPEGAGCR
EEAASPAGEWRALLRLRLRAEAVRPHSALLAVRVEPGGGAAEEAAPPWALGLGAAGLLALAALARGLQLSALALAPAEVQVLRESGS
EAERAAARRLEPARRWAGCALGALLLLASLAQAALAVLLYRAAGQRAVPAVLGSAGLVFLVGEVVPAAVSGRWTLALAPRALGLSRL
AVLLTLPVALPVGQLLELAARPGRLRERVLELARGGGDPYSDLSKGVLRCRTVEDVLTPLEDCFMLDASTVLDFGVLASIMQSGHTR
IPVYEEERSNIVDMLYLKDLAFVDPEDCTPLSTITRFYNHPLHFVFNDTKLDAVLEEFKRGKSHLAIVQKVNNEGEGDPFYEVLGLV
TLEDVIEEIIRSEILDESEDYRDTVVKRKPASLMAPLKRKEEFSLFKVSDDEYKVTISPQLLLATQRFLSREVDVFSPLRISEKVLL
HLLKHPSVNQEVRFDESNRLATHHYLYQRSQPVDYFILILQGRVEVEIGKEGLKFENGAFTYYGVSALTVPSSVHQSPVSSLQPIRH

DLQPDPGDGTHSSAYCPDYTVRALSDLQLIKVTRLQYLNALLATRAQNLPQSPENTDLQVIPGSQTRLLGEKTTTAAGSSHSRPGVP
VEGSPGRNPGV
>sp|P07951|TPM2_HUMAN
MDAIKKKMQMLKLDKENAIDRAEQAEADKKQAEDRCKQLEEEQQALQKKLKGTEDEVEKYSESVKEAQEKLEQAEKKATDAEADVAS
LNRRIQLVEEELDRAQERLATALQKLEEAEKAADESERGMKVIENRAMKDEEKMELQEMQLKEAKHIAEDSDRKYEEVARKLVILEG
ELERSEERAEVAESKCGDLEEELKIVTNNLKSLEAQADKYSTKEDKYEEEIKLLEEKLKEAETRAEFAERSVAKLEKTIDDLEDEVY
AQKMKYKAISEELDNALNDITSL
>sp|Q8NFG4|FLCN_HUMAN
MNAIVALCHFCELHGPRTLFCTEVLHAPLPQGDGNEDSPGQGEQAEEEEGGIQMNSRMRAHSPAEGASVESSSPGPKKSDMCEGCRS
LAAGHPGYISHDKETSIKYVSHQHPSHPQLFSIVRQACVRSLSCEVCPGREGPIFFGDEQHGFVFSHTFFIKDSLARGFQRWYSIIT
IMMDRIYLINSWPFLLGKVRGIIDELQGKALKVFEAEQFGCPQRAQRMNTAFTPFLHQRNGNAARSLTSLTSDDNLWACLHTSFAWL
LKACGSRLTEKLLEGAPTEDTLVQMEKLADLEEESESWDNSEAEEEEKAPVLPESTEGRELTQGPAESSSLSGCGSWQPRKLPVFKS
LRHMRQVLGAPSFRMLAWHVLMGNQVIWKSRDVDLVQSAFEVLRTMLPVGCVRIIPYSSQYEEAYRCNFLGLSPHVQIPPHVLSSEF
AVIVEVHAAARSTLHPVGCEDDQSLSKYEFVVTSGSPVAADRVGPTILNKIEAALTNQNLSVDVVDQCLVCLKEEWMNKVKVLFKFT
KVDSRPKEDTQKLLSILGASEEDNVKLLKFWMTGLSKTYKSHLMSTVRSPTASESRN
>sp|Q9H320|VCX1_HUMAN
MSPKPRASGPPAKATEAGKRKSSSQPSPSDPKKKTTKVAKKGKAVRRGRRGKKGAATKMAAVTAPEAESAPAAPGPSDQPSQELPQH
ELPPEEPVSEGTQHDPLSQEAELEEPLSQESEVEEPLSQESQVEEPLSQESEVEEPLSQESQVEEPLSQESEVEEPLSQESQVEEPL
SQESEMEEPLSQESQVEEPPSQESEMEELPSV
>sp|Q8TCC3|RM30_HUMAN
MAGILRLVVQWPPGRLQTVTKGVESLICTDWIRHKFTRSRIPEKVFQASPEDHEKYGGDPQNPHKLHIVTRIKSTRRRPYWEKDIIK
MLGLEKAHTPQVHKNIPSVNAKLKVVKHLIRIKPLKLPQGLPAEENMSNTCLKSTGELVVQWHLKPVEQKAHES
>sp|Q9NRK6|ABCBA_HUMAN
MRGPPAWPLRLLEPPSPAEPGRLLPVACVWAAASRVPGSLSPFTGLRPARLWGAGPALLWGVGAARRWRSGCRGGGPGASRGVLGLA
RLLGLWARGPGSCRCGAFAGPGAPRLPRARFPGGPAAAAWAGDEAWRRGPAAPPGDKGRLRPAAAGLPEARKLLGLAYPERRRLAAA
VGFLTMSSVISMSAPFFLGKIIDVIYTNPTVDYSDNLTRLCLGLSAVFLCGAAANAIRVYLMQTSGQRIVNRLRTSLFSSILRQEVA
FFDKTRTGELINRLSSDTALLGRSVTENLSDGLRAGAQASVGISMMFFVSPNLATFVLSVVPPVSIIAVIYGRYLRKLTKVTQDSLA
QATQLAEERIGNVRTVRAFGKEMTEIEKYASKVDHVMQLARKEAFARAGFFGATGLSGNLIVLSVLYKGGLLMGSAHMTVGELSSFL
MYAFWVGISIGGLSSFYSELMKGLGAGGRLWELLEREPKLPFNEGVILNEKSFQGALEFKNVHFAYPARPEVPIFQDFSLSIPSGSV
TALVGPSGSGKSTVLSLLLRLYDPASGTISLDGHDIRQLNPVWLRSKIGTVSQEPILFSCSIAENIAYGADDPSSVTAEEIQRVAEV
ANAVAFIRNFPQGFNTVVGEKGVLLSGGQKQRIAIARALLKNPKILLLDEATSALDAENEYLVQEALDRLMDGRTVLVIAHRLSTIK
NANMVAVLDQGKITEYGKHEELLSKPNGIYRKLMNKQSFISA
>sp|A4D1T9|PRS37_HUMAN
MKYVFYLGVLAGTFFFADSSVQKEDPAPYLVYLKSHFNPCVGVLIKPSWVLAPAHCYLPNLKVMLGNFKSRVRDGTEQTINPIQIVR
YWNYSHSAPQDDLMLIKLAKPAMLNPKVQPLTLATTNVRPGTVCLLSGLDWSQENSGRHPDLRQNLEAPVMSDRECQKTEQGKSHRN
SLCVKFVKVFSRIFGEVAVATVICKDKLQGIEVGHFMGGDVGIYTNVYKYVSWIENTAKDK
>sp|Q9Y219|JAG2_HUMAN
MRAQGRGRLPRRLLLLLALWVQAARPMGYFELQLSALRNVNGELLSGACCDGDGRTTRAGGCGHDECDTYVRVCLKEYQAKVTPTGP
CSYGHGATPVLGGNSFYLPPAGAAGDRARARARAGGDQDPGLVVIPFQFAWPRSFTLIVEAWDWDNDTTPNEELLIERVSHAGMINP
EDRWKSLHFSGHVAHLELQIRVRCDENYYSATCNKFCRPRNDFFGHYTCDQYGNKACMDGWMGKECKEAVCKQGCNLLHGGCTVPGE
CRCSYGWQGRFCDECVPYPGCVHGSCVEPWQCNCETNWGGLLCDKDLNYCGSHHPCTNGGTCINAEPDQYRCTCPDGYSGRNCEKAE
HACTSNPCANGGSCHEVPSGFECHCPSGWSGPTCALDIDECASNPCAAGGTCVDQVDGFECICPEQWVGATCQLDANECEGKPCLNA
FSCKNLIGGYYCDCIPGWKGINCHINVNDCRGQCQHGGTCKDLVNGYQCVCPRGFGGRHCELERDECASSPCHSGGLCEDLADGFHC
HCPQGFSGPLCEVDVDLCEPSPCRNGARCYNLEGDYYCACPDDFGGKNCSVPREPCPGGACRVIDGCGSDAGPGMPGTAASGVCGPH

GRCVSQPGGNFSCICDSGFTGTYCHENIDDCLGQPCRNGGTCIDEVDAFRCFCPSGWEGELCDTNPNDCLPDPCHSRGRCYDLVNDF
YCACDDGWKGKTCHSREFQCDAYTCSNGGTCYDSGDTFRCACPPGWKGSTCAVAKNSSCLPNPCVNGGTCVGSGASFSCICRDGWEG
RTCTHNTNDCNPLPCYNGGICVDGVNWFRCECAPGFAGPDCRINIDECQSSPCAYGATCVDEINGYRCSCPPGRAGPRCQEVIGFGR
SCWSRGTPFPHGSSWVEDCNSCRCLDGRRDCSKVWCGWKPCLLAGQPEALSAQCPLGQRCLEKAPGQCLRPPCEAWGECGAEEPPST
PCLPRSGHLDNNCARLTLHFNRDHVPQGTTVGAICSGIRSLPATRAVARDRLLVLLCDRASSGASAVEVAVSFSPARDLPDSSLIQG
AAHAIVAAITQRGNSSLLLAVTEVKVETVVTGGSSTGLLVPVLCGAFSVLWLACVVLCVWWTRKRRKERERSRLPREESANNQWAPL
NPIRNPIERPGGHKDVLYQCKNFTPPPRRADEALPGPAGHAAVREDEEDEDLGRGEEDSLEAEKFLSHKFTKDPGRSPGRPAHWASG
PKVDNRAVRSINEARYAGKE
>sp|Q8NE09|RGS22_HUMAN
MPEKRLTAEPPTITEEEFEDSLATDDFLVDYFNEFLSLPTFSEAIRFNADYGVFEVANDAPQFLEKQLKKILQNQQPRNPIYDVVRK
GKNEVKPVQMNAPDEDETINVNYNIMCLSREEGIKWIKKERLPAFLESDCYFEYRLAKLVSQVRWSKSGMNFTVGSNFSPWIVKKPP
SLPPPATEEDNLVIMKKFYVSLGEASYTQTKDWFALAKQSQQTVSTFSLPCCVPYNKLKSPAISSVSENFIFDDGVHPRTKKDPSKT
NKLISEFEEEEGEEEEVSVSLQDTPSQALLRVYLEKKQDVDESLTMHFSTCEEFLSSYIYFILRGAIQQIVGKPVGETPDYINFNNI
TKVSFDDCFESIHGKNFLSELVQTTKERSEEIEQTSLSSKNESAGPESRADWCISHRTYDIGNRKEFERFKKFIKGTLGERYWWLWM
DIERLKVLKDPGRHQRHLEKMKKCYLVSNGDYYLSAEILSKFKLLDGSQWNEEHLRNIQSEVLKPLLLYWAPRFCVTHSASTKYASA
ELKFWHLRQAKPRKDIDPFPQMATLLPLRPKSCIPQIPEIQKEEFSLSQPPKSPNKSPEVKTATQKPWKRELLYPGSSKDDVIEKGS
KYMSESSKVIHLTSFTDISECLKPQLDRRYAYTEEPRVKTVSDVGALGGSDMENLLQSLYVENRAGFFFTKFCEHSGNKLWKNSVYF
WFDLQAYHQLFYQETLQPFKVCKQAQYLFATYVAPSATLDIGLQQEKKKEIYMKIQPPFEDLFDTAEEYILLLLLLEPWTKMVKSDQI
AYKKVELVEETRQLDSTYFRKLQALHKETFSKKAEDTTCEIGTGILSLSNVSKRTEYWDNVPAEYKHFKFSDLLNNKLEFEHFRQFL
ETHSSSMDLMCWTDIEQFRRITYRDRNQRKAKSIYIKNKYLNKKYFFGPNSPASLYQQNQVMHLSGGWGKILHEQLDAPVLVEIQKH
VQNRLENVWLPLFLASEQFAARQKIKVQMKDIAEELLLQKAEKKIGVWKPVESKWISSSCKIIAFRKALLNPVTSRQFQRFVALKGD
LLENGLLFWQEVQKYKDLCHSHCDESVIQKKITTIINCFINSSIPPALQIDIPVEQAQKIIEHRKELGPYVFREAQMTIFGVLFKFW
PQFCEFRKNLTDENIMSVLERRQEYNKQKKKLAVLEDEKSGKDGIKQYANTSVPAIKTALLSDSFLGLQPYGRQPTWCYSKYIEALE
QERILLKIQEELEKKLFAGLQPLTNFKASSSTMSLKKNMSAHSSQK
>sp|Q6PEY1|TMM88_HUMAN
MADVPGAQRAVPGDGPEPRDPLDCWACAVLVTAQNLLVAAFNLLLLVLVLGTILLPAVTMLGFGFLCHSQFLRSQAPPCTAHLRDPG
FTALLVTGFLLLVPLLVLALASYRRLCLRLRLADCLVPYSRALYRRRRAPQPRQIRASPGSQAVPTSGKVWV
>sp|Q92597|NDRG1_HUMAN
MSREMQDVDLAEVKPLVEKGETITGLLQEFDVQEQDIETLHGSVHVTLCGTPKGNRPVILTYHDIGMNHKTCYNPLFNYEDMQEITQ
HFAVCHVDAPGQQDGAASFPAGYMYPSMDQLAEMLPGVLQQFGLKSIIGMGTGAGAYILTRFALNNPEMVEGLVLINVNPCAEGWMD
WAASKISGWTQALPDMVVSHLFGKEEMQSNVEVVHTYRQHIVNDMNPGNLHLFINAYNSRRDLEIERPMPGTHTVTLQCPALLVVGD
SSPAVDAVVECNSKLDPTKTTLLKMADCGGLPQISQPAKLAEAFKYFVQGMGYMPSASMTRLMRSRTASGSSVTSLDGTRSRSHTSE
GTRSRSHTSEGTRSRSHTSEGAHLDITPNSGAAGNSAGPKSMEVSC
>sp|Q92569|P55G_HUMAN
MYNTVWSMDRDDADWREVMMPYSTELIFYIEMDPPALPPKPPKPMTSAVPNGMKDSSVSLQDAEWYWGDISREEVNDKLRDMPDGTF
LVRDASTKMQGDYTLTLRKGGNNKLIKIYHRDGKYGFSDPLTFNSVVELINHYHHESLAQYNPKLDVKLMYPVSRYQQDQLVKEDNI
DAVGKKLQEYHSQYQEKSKEYDRLYEEYTRTSQEIQMKRTAIEAFNETIKIFEEQCHTQEQHSKEYIERFRREGNEKEIERIMMNYD
KLKSRLGEIHDSKMRLEQDLKNQALDNREIDKKMNSIKPDLIQLRKIRDQHLVWLNHKGVRQKRLNVWLGIKNEDADENYFINEEDE
NLPHYDEKTWFVEDINRVQAEDLLYGKPDGAFLIRESSKKGCYACSVVADGEVKHCVIYSTARGYGFAEPYNLYSSLKELVLHYQQT
SLVQHNDSLNVRLAYPVHAQMPSLCR
>sp|O43613|OX1R_HUMAN
MEPSATPGAQMGVPPGSREPSPVPPDYEDEFLRYLWRDYLYPKQYEWVLIAAYVAVFVVALVGNTLVCLAVWRNHHMRTVTNYFIVN
LSLADVLVTAICLPASLLVDITESWLFGHALCKVIPYLQAVSVSVAVLTLSFIALDRWYAICHPLLFKSTARRARGSILGIWAVSLA
IMVPQAAVMECSSVLPELANRTRLFSVCDERWADDLYPKIYHSCFFIVTYLAPLGLMAMAYFQIFRKLWGRQIPGTTSALVRNWKRP

SDQLGDLEQGLSGEPQPRARAFLAEVKQMRARRKTAKMLMVVLLVFALCYLPISVLNVLKRVFGMFRQASDREAVYACFTFSHWLVY
ANSAANPIIYNFLSGKFREQFKAAFSCCLPGLGPCGSLKAPSPRSSASHKSLSLQSRCSISKISEHVVLTSVTTVLP
>sp|Q96K19|RN170_HUMAN
MAKYQGEVQSLKLDDDSVIEGVSDQVLVAVVVSFALIATLVYALFRNVHQNIHPENQELVRVLREQLQTEQDAPAATRQQFYTDMYC
PICLHQASFPVETNCGHLFCGACIIAYWRYGSWLGAISCPICRQTVTLLLTVFGEDDQSQDVLRLHQDINDYNRRFSGQPRSIMERI
MDLPTLLRHAFREMFSVGGLFWMFRIRIILCLMGAFFYLISPLDFVPEALFGILGFLDDFFVIFLLLIYISIMYREVITQRLTR
>sp|Q902F8|ENK8_HUMAN
MNPSEMQRKAPPRRRRHRNRAPLTHKMNKMVTSEEQMKLPSTKKAEPPTWAQLKKLTQLATKYLENTKVTQTPESMLLAALMIVSMV
VSLPMPAGAAVANYTNWAYVPFPPLIRAVTWMDNPIEVYVNDSVWVPGPIDDRCPAKPEEEGMMINISIGYRYPPICLGRAPGCLMP
AVQNWLVEVPTVSPISRFTYHMVSGMSLRPRVNYLQDFSYQRSLKFRPKGKPCPKEIPKESKNTEVLVWEECVANSAVILQNNEFGT
IIDWAPRGQFYHNCSGQTQSCPSAQVSPAVDSDLTESLDKHKHKKLQSFYPWEWGEKRISTPRPKIVSPVSGPEHPELWRLTVASHH
IRIWSGNQTLETRDRKPFYTVDLNSSLTLPLQSCVKPPYMLVVGNIVIKPDSQTITCENCRLLTCIDSTFNWQHRILLVRAREGVWI
PVSMDRPWEASPSVHILTEVLKGVLNRSKRFIFTLIAVIMGLIAVTATAAVAGVALHSSVQSVNFVNDGQKNSTRLWNSQSSIDQKL
ANQINDLRQTVIWMGDRLMSLEHRFQLQCDWNTSDFCITPQIYNDSEHHWDMVRRHLQGREDNLTLDISKLKEQIFEASKAHLNLVP
GTEAIAGVADGLANLNPVTWVKTIGSTTIINLILILVCLFCLLLVCRCTQQLRRDSDHRERAMMTMAVLSKRKGGNVGKSKRDQIVT
VSV
>sp|Q8N7I0|GVQW1_HUMAN
MPNFLGLHRARSFTVLCFWSTADVLNASGPRRQWHPLASCSSWSRPQSQGSTPGVLCKTGMGREPIPETQCHFANSMCSLHVSVPYF
GNSPNISNFFRWSLALSPRQWCDLGSLQPPSPRFKGFSCLSLPSSWDYRRAPSPANFCILVEMGFHHVGQADLELLTSADLPTSASQ
SAGITGVSHHAWPQTLSLLLY
>sp|Q8NEJ0|DUS18_HUMAN
MTAPSCAFPVQFRQPSVSGLSQITKSLYISNGVAANNKLMLSSNQITMVINVSVEVVNTLYEDIQYMQVPVADSPNSRLCDFFDPIA
DHIHSVEMKQGRTLLHCAAGVSRSAALCLAYLMKYHAMSLLDAHTWTKSCRPIIRPNSGFWEQLIHYEFQLFGKNTVHMVSSPVGMI
PDIYEKEVRLMIPL
>sp|Q9H9P5|UNKL_HUMAN
MPSVSKAAAAALSGSPPQTEKPTHYRYLKEFRTEQCPLFSQHKCAQHRPFTCFHWHFLNQRRRRPLRRRDGTFNYSPDVYCSKYNEA
TGVCPDGDECPYLHRTTGDTERKYHLRYYKTGTCIHETDARGHCVKNGLHCAFAHGPLDLRPPVCDVRELQAQEALQNGQLGGGEGV
PDLQPGVLASQAMIEKILSEDPRWQDANFVLGSYKTEQCPKPPRLCRQGYACPHYHNSRDRRRNPRRFQYRSTPCPSVKHGDEWGEP
SRCDGGDGCQYCHSRTEQQFHPESTKCNDMRQTGYCPRGPFCAFAHVEKSLGMVNEWGCHDLHLTSPSSTGSGGQPGNAKRRDSPAEG
GPRGSEQDSKQNHLAVFAAVHPPAPSVSSSVASSLASSAGSGSSSPTALPAPPARALPLGPASSTVEAVLGSALDLHLSNVNIASLE
KDLEEQDGHDLGAAGPRSLAGSAPVAIPGSLPRAPSLHSPSSASTSPLGSLSQPLPGPVGSSAMTPPQQPPPLRSEPGTLGSAASSY
SPLGLNGVPGSIWDFVSGSFSPSPSPILSAGPPSSSSASPNGAELARVRRQLDEAKRKIRQWEESWQQVKQVCDAWQREAQEAKERA
RVADSDRQLALQKKEEVEAQVIFQLRAKQCVACRERAHGAVLRPCQHHILCEPCAATAPECPYCKGQPLQW
>sp|O95990|F107A_HUMAN
MYSEIQRERADIGGLMARPEYREWNPELIKPKKLLNPVKASRSHQELHRELLMNHRRGLGVDSKPELQRVLEHRRRNQLIKKKKEEL
EAKRLQCPFEQELLRRQQRLNQLEKPPEKEEDHAPEFIKVRENLRRIATLTSEEREL
>sp|Q03518|TAP1_HUMAN
MAELLASAGSACSWDFPRAPPSFPPPAASRGGLGGTRSFRPHRGAESPRPGRDRDGVRVPMASSRCPAPRGCRCLPGASLAWLGTVL
LLLADWVLLRTALPRIFSLLVPTALPLLRVWAVGLSRWAVLWLGACGVLRATVGSKSENAGAQGWLAALKPLAAALGLALPGLALFR
ELISWGAPGSADSTRLLHWGSHPTAFVVSYAAALPAAALWHKLGSLWVPGGQGGSGNPVRRLLGCLGSETRRLSLFLVLVVLSSLGE
MAIPFFTGRLTDWILQDGSADTFTRNLTLMSILTIASAVLEFVGDGIYNNTMGHVHSHLQGEVFGAVLRQETEFFQQNQTGNIMSRV
TEDTSTLSDSLSENLSLFLWYLVRGLCLLGIMLWGSVSLTMVTLITLPLLFLLPKKVGKWYQLLEVQVRESLAKSSQVAIEALSAMP
TVRSFANEEGEAQKFREKLQEIKTLNQKEAVAYAVNSWTTSISGMLLKVGILYIGGQLVTSGAVSSGNLVTFVLYQMQFTQAVEVLL
SIYPRVQKAVGSSEKIFEYLDRTPRCPPSGLLTPLHLEGLVQFQDVSFAYPNRPDVLVLQGLTFTLRPGEVTALVGPNGSGKSTVAA

LLQNLYQPTGGQLLLDGKPLPQYEHRYLHRQVAAVGQEPQVFGRSLQENIAYGLTQKPTMEEITAAAVKSGAHSFISGLPQGYDTEV
DEAGSQLSGGQRQAVALARALIRKPCVLILDDATSALDANSQLQVEQLLYESPERYSRSVLLITQHLSLVEQADHILFLEGGAIREG
GTHQQLMEKKGCYWAMVQAPADAPE
>sp|Q52LA3|LIN52_HUMAN
MGWKMASPTDGTDLEASLLSFEKLDRASPDLWPEQLPGVAEFAASFKSPITSSPPKWMAEIERDDIDMLKELGSLTTANLMEKVRGL
QNLAYQLGLDESREMTRGKFLNILEKPKK
>sp|P0C7W9|F90AE_HUMAN
MMARRDPTSWAKRLVRAQTLQKQRRAPVGPRAPPPDEEDPRLKCKNCGAFGHTARSTRCPMKCWKAALVPATLGKKEGKENLKPWKP
RVEANPGPLNKDKGEKEERPRQQDPQRKALLHMFSGKPPEKPLPNGKGSTEPSDYLRVASGPMPVHTTSKRPRLDPVLADRSATEMS
GRGSVLASLSPLRKASLSSSSSLGPKERQTGAAADMPQPAVRHQGREPLLVVKPTHSRPEGDCREVPQAASKTHGLLQAARPQAQDK
RPAVTSQPCPPAATHSLGLGSNLSFGPGAKRPAQAPIQACLNFPKKPRLGPFQIPESAIQGGELGAPENLQPPPAATELGPSTSPQM
GRRTPAQVPSVDRQPPHSRPCLPTAQACTMSHHPAASHDGAQPLRVLFRRLENGRWSSSLLAAPSFHSPEKPGAFLAQSPHVSEKSE
APCVRVPPSVLYEDLQVSSSSEDSDSDLE
>sp|Q16621|NFE2_HUMAN
MSPCPPQQSRNRVIQLSTSELGEMELTWQEIMSITELQGLNAPSEPSFEPQAPAPYLGPPPPTTYCPCSIHPDSGFPLPPPPYELPA
STSHVPDPPYSYGNMAIPVSKPLSLSGLLSEPLQDPLALLDIGLPAGPPKPQEDPESDSGLSLNYSDAESLELEGTEAGRRRSEYVE
MYPVEYPYSLMPNSLAHSNYTLPAAETPLALEPSSGPVRAKPTARGEAGSRDERRALAMKIPFPTDKIVNLPVDDFNELLARYPLTE
SQLALVRDIRRRGKNKVAAQNCRKRKLETIVQLERELERLTNERERLLRARGEADRTLEVMRQQLTELYRDIFQHLRDESGNSYSPE
EYALQQAADGTIFLVPRGTKMEATD
>sp|Q16623|STX1A_HUMAN
MKDRTQELRTAKDSDDDDDVAVTVDRDRFMDEFFEQVEEIRGFIDKIAENVEEVKRKHSAILASPNPDEKTKEELEELMSDIKKTAN
KVRSKLKSIEQSIEQEEGLNRSSADLRIRKTQHSTLSRKFVEVMSEYNATQSDYRERCKGRIQRQLEITGRTTTSEELEDMLESGNP
AIFASGIIMDSSISKQALSEIETRHSEIIKLENSIRELHDMFMDMAMLVESQGEMIDRIEYNVEHAVDYVERAVSDTKKAVKYQSKA
RRKKIMIIICCVILGIVIASTVGGIFA
>sp|Q96KR7|PHAR3_HUMAN
MAASEDGSGCLVSRGRSQSDPSVLTDSSATSSADAGENPDEMDQTPPARPEYLVSGIRTPPVRRNSKLATLGRIFKPWKWRKKKNEK
LKQTTSALEKKMAGRQGREELIKKGLLEMMEQDAESKTCNPDGGPRSVQSEPPTPKSETLTSEDAQPGSPLATGTDQVSLDKPLSSA
AHLDDAAKMPSASSGEEADAGSLLPTTNELSQALAGADSLDSPPRPLERSVGQLPSPPLLPTPPPKASSKTTKNVTGQATLFQASSM
KSADPSLRGQLSTPTGSPHLTTVHRPLPPSRVIEELHRALATKHRQDSFQGRESKGSPKKRLDVRLSRTSSVERGKEREEAWSFDGA
LENKRTAAKESEENKENLIINSELKDDLLLYQDEEALNDSIISGTLPRKCKKELLAVKLRNRPSKQELEDRNIFPRRTDEERQEIRQ
QIEMKLSKRLSQRPAVEELERRNILKQRNDQTEQEERREIKQRLTRKLNQRPTVDELRDRKILIRFSDYVEVAKAQDYDRRADKPWT
RLSAADKAAIRKELNEYKSNEMEVHASSKHLTRFHRP
>sp|Q8N8Y2|VA0D2_HUMAN
MLEGAELYFNVDHGYLEGLVRGCKASLLTQQDYINLVQCETLEDLKIHLQTTDYGNFLANHTNPLTVSKIDTEMRKRLCGEFEYFRN
HSLEPLSTFLTYMTCSYMIDNVILLMNGALQKKSVKEILGKCHPLGRFTEMEAVNIAETPSDLFNAILIETPLAPFFQDCMSENALD
ELNIELLRNKLYKSYLEAFYKFCKNHGDVTAEVMCPILEFEADRRAFIITLNSFGTELSKEDRETLYPTFGKLYPEGLRLLAQAEDF
DQMKNVADHYGVYKPLFEAVGGSGGKTLEDVFYEREVQMNVLAFNRQFHYGVFYAYVKLKEQEIRNIVWIAECISQRHRTKINSYIP
IL
>sp|Q8TD23|ZN675_HUMAN
MGLLTFRDVAIEFSLEEWQCLDTAQRNLYKNVILENYRNLVFLGIAVSKQDLITCLEQEKEPLTVKRHEMVNEPPVMCSHFAQEFWP
EQNIKDSFEKVTLRRYEKCGNDNFQLKGCKSVDECKLHKGGYNGLNQCLPTMQSKMFQCDKYVKVFNKFSHSDRHKIKHMENKPFKC
KECGRSFCMLSHLTRHERNYTKVNFCKCEECEKAVNQSSKLTKHKRIYTCEKLYKCQECDRTFNQFSNLTEYKKDYAREKPYKCEEC
GKAFNQSSHLTTHKIIHTGEKPYKCEECGKAFNQFSNLTTHKKIHTGEQPYICEECGKAFTQSSTLTTHKRIHTGEKPYKCEECGKA
FNRSSKLTEHKNIHTGEQPYKCEECGKAFNRSSNLTEHRKIHTEEKPYKCKECGKAFKHSSALTTHKRIHTGEKPYKCEECGKAFNR

SSKLTEHKKLHTGKKPYKCEECGKAFIQSSKLTEHKKIHSGEIPYKCEECGKAFKHSSSLTTHKRIHTGEKPYKCEECGKAFSRSSK
LTEHKIIHTGEKPYKCERCDKAFNQSANLTKHKKIHTGEKLQNWNV
>sp|Q9P2A4|ABI3_HUMAN
MAELQQLQEFEIPTGREALRGNHSALLRVADYCEDNYVQATDKRKALEETMAFTTQALASVAYQVGNLAGHTLRMLDLQGAALRQVE
ARVSTLGQMVNMHMEKVARREIGTLATVQRLPPGQKVIAPENLPPLTPYCRRPLNFGCLDDIGHGIKDLSTQLSRTGTLSRKSIKAP
ATPASATLGRPPRIPEPVHLPVVPDGRLSAASSAFSLASAGSAEGVGGAPTPKGQAAPPAPPLPSSLDPPPPPAAVEVFQRPPTLEE
LSPPPPDEELPLPLDLPPPPPLDGDELGLPPPPPGFGPDEPSWVPASYLEKVVTLYPYTSQKDNELSFSEGTVICVTRRYSDGWCEG
VSSEGTGFFPGNYVEPSC
>sp|A6NNV3|SPD16_HUMAN
MQKHYTVAWFLYSAPGVDPSPPCRSLGWKRKKEWSDESEEEPEKELAPEPEETWVVEMLCGLKMKLKQQRVSPILPEHHKDFNSQLA
PGVDPSPPHRSFCWKRKREWWDESEESLEEEPRKVLAPEPEEIWVVEMLCGLKMKLKRRRVSLVLPEHHEAFNRLLEDPVIKRFLAW
DKDLRVSDKYLLAMVIAYFSRAGLPSWQYQRIHFFLALYLANDMEEDDEDPKQNIFYFLYGKTRSRIPLVRNRRFQLCRCMNPRARK
NRSQIALFQKLRFQFFCSMSGRAWVSREELEEIQAYDPEHWVWARDRARLS
>sp|Q9BZ98|TTY12_HUMAN
MIDPETRHKAFLKAWPWQNSTITFVPGLAICHYSSVQVPRRGAILPMLYALCYVKMPSFQHGPGRMYHLTCDWPRKMSLSCHVCRAH
FRD
>sp|P51810|GP143_HUMAN
MASPRLGTFCCPTRDAATQLVLSFQPRAFHALCLGSGGLRLALGLLQLLPGRRPAGPGSPATSPPASVRILRAAAACDLLGCLGMVI
RSTVWLGFPNFVDSVSDMNHTEIWPAAFCVGSAMWIQLLYSACFWWLFCYAVDAYLVIRRSAGLSTILLYHIMAWGLATLLCVEGAA
MLYYPSVSRCERGLDHAIPHYVTMYLPLLLVLVANPILFQKTVTAVASLLKGRQGIYTENERRMGAVIKIRFFKIMLVLIICWLSNI
INESLLFYLEMQTDINGGSLKPVRTAAKTTWFIMGILNPAQGFLLSLAFYGWTGCSLGFQSPRKEIQWESLTTSAAEGAHPSPLMPH
ENPASGKVSQVGGQTSDEALSMLSEGSDASTIEIHTASESCNKNEGDPALPTHGDL
>sp|P02774|VTDB_HUMAN
MKRVLVLLLAVAFGHALERGRDYEKNKVCKEFSHLGKEDFTSLSLVLYSRKFPSGTFEQVSQLVKEVVSLTEACCAEGADPDCYDTR
TSALSAKSCESNSPFPVHPGTAECCTKEGLERKLCMAALKHQPQEFPTYVEPTNDEICEAFRKDPKEYANQFMWEYSTNYGQAPLSL
LVSYTKSYLSMVGSCCTSASPTVCFLKERLQLKHLSLLTTLSNRVCSQYAAYGEKKSRLSNLIKLAQKVPTADLEDVLPLAEDITNI
LSKCCESASEDCMAKELPEHTVKLCDNLSTKNSKFEDCCQEKTAMDVFVCTYFMPAAQLPELPDVELPTNKDVCDPGNTKVMDKYTF
ELSRRTHLPEVFLSKVLEPTLKSLGECCDVEDSTTCFNAKGPLLKKELSSFIDKGQELCADYSENTFTEYKKKLAERLKAKLPDATP
KELAKLVNKRSDFASNCCSINSPPLYCDSEIDAELKNIL
>sp|Q8TDG2|ACTT1_HUMAN
MFNPHALDVPAVIFDNGSGLCKAGLSGEIGPRHVISSVLGHCKFNVPLARLNQKYFVGQEALYKYEALHLHYPIERGLVTGWDDMEK
LWKHLFERELGVKPSQQPVLMTEPSLNPREIREKLAEMMFETFSVPGFYLSNHAVAALYASACVTGLVVDSGDGVTCTVPIFEGYSL
PHAVTKLCMAGRDITEHLTRLLFASGFNFPCILNKAVVNNIKEKLCYIALEPEKELRKSRGEVLGAYRLPDGHVIHFGDELYQVPEV
LFAPDQLGIHSPGLSKMVSSSIMKCDTDIQNKLYADIVLSGGTTLLPGLEERLMKEVEQLASKGTPIKITASPDRCFSAWIGASIMT
SMSSFKQMWVTSADFKEYGTSVVQRRCF
>sp|Q86WZ6|ZN227_HUMAN
MPSQNYDLPQKKQEKMTKFQEAVTFKDVAVVFSREELRLLDLTQRKLYRDVMVENFKNLVAVGHLPFQPDMVSQLEAEEKLWMMETE
TQRSSKHQNKMETLQKFALKYLSNQELSCWQIWKQVASELTRCLQGKSSQLLQGDSIQVSENENNIMNPKGDSSIYIENQEFPFWRT
QHSCGNTYLSESQIQSRGKQIDVKNNLQIHEDFMKKSPFHEHIKTDTEPKPCKGNEYGKIISDGSNQKLPLGEKPHPCGECGRGFSY
SPRLPLHPNVHTGEKCFSQSSHLRTHQRIHPGEKLNRCHESGDCFNKSSFHSYQSNHTGEKSYRCDSCGKGFSSSTGLIIHYRTHTG
EKPYKCEECGKCFSQSSNFQCHQRVHTEEKPYKCEECGKGFGWSVNLRVHQRVHRGEKPYKCEECGKGFTQAAHFHIHQRVHTGEKP
YKCDVCGKGFSHNSPLICHRRVHTGEKPYKCEACGKGFTRNTDLHIHFRVHTGEKPYKCKECGKGFSQASNLQVHQNVHTGEKRFKC
ETCGKGFSQSSKLQTHQRVHTGEKPYRCDVCGKDFSYSSNLKLHQVIHTGEKPYKCEECGKGFSWRSNLHAHQRVHSGEKPYKCEQC
DKSFSQAIDFRVHQRVHTGEKPYKCGVCGKGFSQSSGLQSHQRVHTGEKPYKCDVCGKGFRYSSQFIYHQRGHTGEKPYKCEECGKG

FGRSLNLRHHQRVHTGEKPHICEECGKAFSLPSNLRVHLGVHTREKLFKCEECGKGFSQSARLEAHQRVHTGEKPYKCDICDKDFRH
RSRLTYHQKVHTGKKL
>sp|Q96F83|CN079_HUMAN
MQGRRELGGEPLSDLQEEAASASLRVAPERLSDDSLEWRRTCPDLLLSDGKASISMPREGGSTCTARCPDPGEHSSTWGEFEGFRES
SAKSGQFSQSLELLEGPTEPQPPRTTSAPKECSSHQPCQGGPWVTGTSAVPPSEPILSYENILKCAFQEITVQQAAEDVSTIDHFLE
ISSEEKPGVERVHKLCNESRKLWRALQSIHTTSTSQRLWSESRCQENFFLVLGIDAAQKNLSGGQGHIMEDCDLKEPEGLLTVSSFC
LQHCKALIQTKLSGPPGSKQGRLMTCSRFLKTPSCGGGQHITIPRKRMFTPRKLKLTLFNSDVC
>sp|Q9Y2R0|COA3_HUMAN
MASSGAGDPLDSKRGEAPFAQRIDPTREKLTPEQLHSMRQAELAQWQKVLPRRRTRNIVTGLGIGALVLAIYGYTFYSISQERFLDE
LEDEAKAARARALARASGS
>sp|Q8NHX9|TPC2_HUMAN
MAEPQAESEPLLGGARGGGGDWPAGLTTYRSIQVGPGAAARWDLCIDQAVVFIEDAIQYRSINHRVDASSMWLYRRYYSNVCQRTLS
FTIFLILFLAFIETPSSLTSTADVRYRAAPWEPPCGLTESVEVLCLLVFAADLSVKGYLFGWAHFQKNLWLLGYLVVLVVSLVDWTV
SLSLVCHEPLRIRRLLRPFFLLQNSSMMKKTLKCIRWSLPEMASVGLLLAIHLCLFTMFGMLLFAGGKQDDGQDRERLTYFQNLPES
LTSLLVLLTTANNPDVMIPAYSKNRAYAIFFIVFTVIGSLFLMNLLTAIIYSQFRGYLMKSLQTSLFRRRLGTRAAFEVLSSMVGEG
GAFPQAVGVKPQNLLQVLQKVQLDSSHKQAMMEKVRSYGSVLLSAEEFQKLFNELDRSVVKEHPPRPEYQSPFLQSAQFLFGHYYFD
YLGNLIALANLVSICVFLVLDADVLPAERDDFILGILNCVFIVYYLLEMLLKVFALGLRGYLSYPSNVFDGLLTVVLLVLEISTLAV
YRLPHPGWRPEMVGLLSLWDMTRMLNMLIVFRFLRIIPSMKLMAVVASTVLGLVQNMRAFGGILVVVYYVFAIIGINLFRGVIVALP
GNSSLAPANGSAPCGSFEQLEYWANNFDDFAAALVTLWNLMVVNNWQVFLDAYRRYSGPWSKIYFVLWWLVSSVIWVNLFLALILEN
FLHKWDPRSHLQPLAGTPEATYQMTVELLFRDILEEPGEDELTERLSQHPHLWLCR
>sp|Q9GZZ0|HXD1_HUMAN
MSSYLEYVSCSSSGGVGGDVLSLAPKFCRSDARPVALQPAFPLGNGDGAFVSCLPLAAARPSPSPPAAPARPSVPPPAAPQYAQCTL
EGAYEPGAAPAAAAGGADYGFLGSGPAYDFPGVLGRAADDGGSHVHYATSAVFSGGGSFLLSGQVDYAAFGEPGPFPACLKASADGH
PGAFQTASPAPGTYPKSVSPASGLPAAFSTFEWMKVKRNASKKGKLAEYGAASPSSAIRTNFSTKQLTELEKEFHFNKYLTRARRIE
IANCLHLNDTQVKIWFQNRRMKQKKREREGLLATAIPVAPLQLPLSGTTPTKFIKNPGSPSQSQEPS
>sp|Q8WZA0|LZIC_HUMAN
MASRGKTETSKLKQNLEEQLDRLMQQLQDLEECREELDTDEYEETKKETLEQLSEFNDSLKKIMSGNMTLVDELSGMQLAIQAAISQ
AFKTPEVIRLFAKKQPGQLRTRLAEMDRDLMVGKLERDLYTQQKVEILTALRKLGEKLTADDEAFLSANAGAILSQFEKVSTDLGSG
DKILALASFEVEKTKK
>sp|P02144|MYG_HUMAN
MGLSDGEWQLVLNVWGKVEADIPGHGQEVLIRLFKGHPETLEKFDKFKHLKSEDEMKASEDLKKHGATVLTALGGILKKKGHHEAEI
KPLAQSHATKHKIPVKYLEFISECIIQVLQSKHPGDFGADAQGAMNKALELFRKDMASNYKELGFQG
>sp|O95628|CNOT4_HUMAN
MSRSPDAKEDPVECPLCMEPLEIDDINFFPCTCGYQICRFCWHRIRTDENGLCPACRKPYPEDPAVYKPLSQEELQRIKNEKKQKQN
ERKQKISENRKHLASVRVVQKNLVFVVGLSQRLADPEVLKRPEYFGKFGKIHKVVINNSTSYAGSQGPSASAYVTYIRSEDALRAIQ
CVNNVVVDGRTLKASLGTTKYCSYFLKNMQCPKPDCMYLHELGDEAASFTKEEMQAGKHQEYEQKLLQELYKLNPNFLQLSTGSVDK
NKNKVTPLQRYDTPIDKPSDSLSIGNGDNSQQISNSDTPSPPPGLSKSNPVIPISSSNHSARSPFEGAVTESQSLFSDNFRHPNPIP
SGLPPFPSSPQTSSDWPTAPEPQSLFTSETIPVSSSTDWQAAFGFGSSKQPEDDLGFDPFDVTRKALADLIEKELSVQDQPSLSPTS
LQNSSSHTTTAKGPGSGFLHPAAATNANSLNSTFSVLPQRFPQFQQHRAVYNSFSFPGQAARYPWMAFPRNSIMHLNHTANPTSNSN
FLDLNLPPQHNTGLGGIPVAGEEEVKVSTMPLSTSSHSLQQGQQPTSLHTTVA
>sp|O95644|NFAC1_HUMAN
MPSTSFPVPSKFPLGPAAAVFGRGETLGPAPRAGGTMKSAEEEHYGYASSNVSPALPLPTAHSTLPAPCHNLQTSTPGIIPPADHPS
GYGAALDGGPAGYFLSSGHTRPDGAPALESPRIEITSCLGLYHNNNQFFHDVEVEDVLPSSKRSPSTATLSLPSLEAYRDPSCLSPA
SSLSSRSCNSEASSYESNYSYPYASPQTSPWQSPCVSPKTTDPEEGFPRGLGACTLLGSPRHSPSTSPRASVTEESWLGARSSRPAS

PCNKRKYSLNGRQPPYSPHHSPTPSPHGSPRVSVTDDSWLGNTTQYTSSAIVAAINALTTDSSLDLGDGVPVKSRKTTLEQPPSVAL
KVEPVGEDLGSPPPPADFAPEDYSSFQHIRKGGFCDQYLAVPQHPYQWAKPKPLSPTSYMSPTLPALDWQLPSHSGPYELRIEVQPK
SHHRAHYETEGSRGAVKASAGGHPIVQLHGYLENEPLMLQLFIGTADDRLLRPHAFYQVHRITGKTVSTTSHEAILSNTKVLEIPLL
PENSMRAVIDCAGILKLRNSDIELRKGETDIGRKNTRVRLVFRVHVPQPSGRTLSLQVASNPIECSQRSAQELPLVEKQSTDSYPVV
GGKKMVLSGHNFLQDSKVIFVEKAPDGHHVWEMEAKTDRDLCKPNSLVVEIPPFRNQRITSPVHVSFYVCNGKRKRSQYQRFTYLPA
NVPIIKTEPTDDYEPAPTCGPVSQGLSPLPRPYYSQQLAMPPDPSSCLVAGFPPCPQRSTLMPAAPGVSPKLHDLSPAAYTKGVASP
GHCHLGLPQPAGEAPAVQDVPRPVATHPGSPGQPPPALLPQQVSAPPSSSCPPGLEHSLCPSSPSPPLPPATQEPTCLQPCSPACPP
ATGRPQHLPSTVRRDESPTAGPRLLPEVHEDGSPNLAPIPVTVKREPEELDQLYLDDVNEIIRNDLSSTSTHS
>sp|Q96GX2|A7L3B_HUMAN
MEEISLANLDTNKLEAIAQEIYVDLIEDSCLGFCFEVHRAVKCGYFYLEFAETGSVKDFGIQPVEDKGACRLPLCSLPGEPGNGPDQ
QLQRSPPEFQ
>sp|Q7Z692|CEA19_HUMAN
MEIPMGTQGCFSKSLLLSASILVLWMLQGSQAALYIQKIPEQPQKNQDLLLSVQGVPDTFQDFNWYLGEETYGGTRLFTYIPGIQRP
QRDGSAMGQRDIVGFPNGSMLLRRAQPTDSGTYQVAITINSEWTMKAKTEVQVAEKNKELPSTHLPTNAGILAATIIGSLAAGALLI
SCIAYLLVTRNWRGQSHRLPAPRGQGSLSILCSAVSPVPSVTPSTWMATTEKPELGPAHDAGDNNIYEVMPSPVLLVSPISDTRSIN
PARPLPTPPHLQAEPENHQYQQDLLNPDPAPYCQLVPTS
>sp|Q9UBU3|GHRL_HUMAN
MPSPGTVCSLLLLGMLWLDLAMAGSSFLSPEHQRVQQRKESKKPPAKLQPRALAGWLRPEDGGQAEGAEDELEVRFNAPFDVGIKLS
GVQYQQHSQALGKFLQDILWEEAKEAPADK
>sp|Q8IZ08|GP135_HUMAN
MEEPQPPRPPASMALLGSQHSGAPSAAGPPGGTSSAATAAVLSFSTVATAALGNLSDASGGGTAAAPGGGGLGGSGAAREAGAAVRR
PLGPEAAPLLSHGAAVAAQALVLLLIFLLSSLGNCAVMGVIVKHRQLRTVTNAFILSLSLSDLLTALLCLPAAFLDLFTPPGGSAPA
AAAGPWRGFCAASRFFSSCFGIVSTLSVALISLDRYCAIVRPPREKIGRRRALQLLAGAWLTALGFSLPWELLGAPRELAAAQSFHG
CLYRTSPDPAQLGAAFSVGLVVACYLLPFLLMCFCHYHICKTVRLSDVRVRPVNTYARVLRFFSEVRTATTVLIMIVFVICCWGPYC
FLVLLAAARQAQTMQAPSLLSVVAVWLTWANGAINPVIYAIRNPNISMLLGRNREEGYRTRNVDAFLPSQGPGLQARSRSRLRNRYA
NRLGACNRMSSSNPASGVAGDVAMWARKNPVVLFCREGPPEPVTAVTKQPKSEAGDTSL
>sp|Q8N6N2|TTC9B_HUMAN
MQRGALSPVLMLSAAPEPPPRPPPALSPPGSGPGSGSRHGSARPGPTPEPSGSLGAALDSSLRAAVAFKAEGQRCYREKKFREAIGK
YHRALLQLKAAQGARPSGLPAPAPGPTSSPGPARLSEEQRRLVESTEVECYDSLTACLLQSELVNYERVREYCLKVLEKQQGNFKAT
YRAGIAFYHLGDYARALRYLQEARSREPTDTNVLRYIQLTQLKMNRCSLQREDSGAGSQTRDVIG
>sp|P30876|RPB2_HUMAN
MYDADEDMQYDEDDDEITPDLWQEACWIVISSYFDEKGLVRQQLDSFDEFIQMSVQRIVEDAPPIDLQAEAQHASGEVEEPPRYLLK
FEQIYLSKPTHWERDGAPSPMMPNEARLRNLTYSAPLYVDITKTVIKEGEEQLQTQHQKTFIGKIPIMLRSTYCLLNGLTDRDLCEL
NECPLDPGGYFIINGSEKVLIAQEKMATNTVYVFAKKDSKYAYTGECRSCLENSSRPTSTIWVSMLARGGQGAKKSAIGQRIVATLP
YIKQEVPIIIVFRALGFVSDRDILEHIIYDFEDPEMMEMVKPSLDEAFVIQEQNVALNFIGSRGAKPGVTKEKRIKYAKEVLQKEML
PHVGVSDFCETKKAYFLGYMVHRLLLAALGRRELDDRDHYGNKRLDLAGPLLAFLFRGMFKNLLKEVRIYAQKFIDRGKDFNLELAI
KTRIISDGLKYSLATGNWGDQKKAHQARAGVSQVLNRLTFASTLSHLRRLNSPIGRDGKLAKPRQLHNTLWGMVCPAETPEGHAVGL
VKNLALMAYISVGSQPSPILEFLEEWSMENLEEISPAAIADATKIFVNGCWVGIHKDPEQLMNTLRKLRRQMDIIVSEVSMIRDIRE
REIRIYTDAGRICRPLLIVEKQKLLLKKRHIDQLKEREYNNYSWQDLVASGVVEYIDTLEEETVMLAMTPDDLQEKEVAYCSTYTHC
EIHPSMILGVCASIIPFPDHNQSPRNTYQSAMGKQAMGVYITNFHVRMDTLAHVLYYPQKPLVTTRSMEYLRFRELPAGINSIVAIA
SYTGYNQEDSVIMNRSAVDRGFFRSVFYRSYKEQESKKGFDQEEVFEKPTRETCQGMRHAIYDKLDDDGLIAPGVRVSGDDVIIGKT
VTLPENEDELESTNRRYTKRDCSTFLRTSETGIVDQVMVTLNQEGYKFCKIRVRSVRIPQIGDKFASRHGQKGTCGIQYRQEDMPFT
CEGITPDIIINPHAIPSRMTIGHLIECLQGKVSANKGEIGDATPFNDAVNVQKISNLLSDYGYHLRGNEVLYNGFTGRKITSQIFIG

PTYYQRLKHMVDDKIHSRARGPIQILNRQPMEGRSRDGGLRFGEMERDCQIAHGAAQFLRERLFEASDPYQVHVCNLCGIMAIANTR
THTYECRGCRNKTQISLVRMPYACKLLFQELMSMSIAPRMMSV
>sp|P20929|NEBU_HUMAN
MADDEDYEEVVEYYTEEVVYEEVPGETITKIYETTTTRTSDYEQSETSKPALAQPALAQPASAKPVERRKVIRKKVDPSKFMTPYIA
HSQKMQDLFSPNKYKEKFEKTKGQPYASTTDTPELRRIKKVQDQLSEVKYRMDGDVAKTICHVDEKAKDIEHAKKVSQQVSKVLYKQ
NWEDTKDKYLLPPDAPELVQAVKNTAMFSKKLYTEDWEADKSLFYPYNDSPELRRVAQAQKALSDVAYKKGLAEQQAQFTPLADPPD
IEFAKKVTNQVSKQKYKEDYENKIKGKWSETPCFEVANARMNADNISTRKYQEDFENMKDQIYFMQTETPEYKMNKKAGVAASKVKY
KEDYEKNKGKADYNVLPASENPQLRQLKAAGDALSDKLYKENYEKTKAKSINYCETPKFKLDTVLQNFSSDKKYKDSYLKDILGHYV
GSFEDPYHSHCMKVTAQNSDKNYKAEYEEDRGKGFFPQTITQEYEAIKKLDQCKDHTYKVHPDKTKFTQVTDSPVLLQAQVNSKQLS
DLNYKAKHESEKFKCHIPPDTPAFIQHKVNAYNLSDNLYKQDWEKSKAKKFDIKVDAIPLLAAKANTKNTSDVMYKKDYEKNKGKMI
GVLSINDDPKMLHSLKVAKNQSDRLYKENYEKTKAKSMNYCETPKYQLDTQLKNFSEARYKDLYVKDVLGHYVGSMEDPYHTHCMKV
AAQNSDKSYKAEYEEDKGKCYFPQTITQEYEAIKKLDQCKDHTYKVHPDKTKFTAVTDSPVLLQAQLNTKQLSDLNYKAKHEGEKFK
CHIPADAPQFIQHRVNAYNLSDNVYKQDWEKSKAKKFDIKVDAIPLLAAKANTKNTSDVMYKKDYEKSKGKMIGALSINDDPKMLHS
LKTAKNQSDREYRKDYEKSKTIYTAPLDMLQVTQAKKSQAIASDVDYKHILHSYSYPPDSINVDLAKKAYALQSDVEYKADYNSWMK
GCGWVPFGSLEMEKAKRASDILNEKKYRQHPDTLKFTSIEDAPITVQSKINQAQRSDIAYKAKGEEIIHKYNLPPDLPQFIQAKVNA
YNISENMYKADLKDLSKKGYDLRTDAIPIRAAKAARQAASDVQYKKDYEKAKGKMVGFQSLQDDPKLVHYMNVAKIQSDREYKKDYE
KTKSKYNTPHDMFNVVAAKKAQDVVSNVNYKHSLHHYTYLPDAMDLELSKNMMQIQSDNVYKEDYNNWMKGIGWIPIGSLDVEKVKK
AGDALNEKKYRQHPDTLKFTSIVDSPVMVQAKQNTKQVSDILYKAKGEDVKHKYTMSPDLPQFLQAKCNAYNISDVCYKRDWYDLIA
KGNNVLGDAIPITAAKASRNIASDYKYKEAYEKSKGKHVGFRSLQDDPKLVHYMNVAKLQSDREYKKNYENTKTSYHTPGDMVSITA
AKMAQDVATNVNYKQPLHHYTYLPDAMSLEHTRNVNQIQSDNVYKDEYNSFLKGIGWIPIGSLEVEKVKKAGDALNERKYRQHPDTV
KFTSVPDSMGMVLAQHNTKQLSDLNYKVEGEKLKHKYTIDPELPQFIQAKVNALNMSDAHYKADWKKTIAKGYDLRPDAIPIVAAKS
SRNIASDCKYKEAYEKAKGKQVGFLSLQDDPKLVHYMNVAKIQSDREYKKGYEASKTKYHTPLDMVSVTAAKKSQEVATNANYRQSY
HHYTLLPDALNVEHSRNAMQIQSDNLYKSDFTNWMKGIGWVPIESLEVEKAKKAGEILSEKKYRQHPEKLKFTYAMDTMEQALNKSN
KLNMDKRLYTEKWNKDKTTIHVMPDTPDILLSRVNQITMSDKLYKAGWEEEKKKGYDLRPDAIAIKAARASRDIASDYKYKKAYEQA
KGKHIGFRSLEDDPKLVHFMQVAKMQSDREYKKGYEKSKTSFHTPVDMLSVVAAKKSQEVATNANYRNVIHTYNMLPDAMSFELAKN
MMQIQSDNQYKADYADFMKGIGWLPLGSLEAEKNKKAMEIISEKKYRQHPDTLKYSTLMDSMNMVLAQNNAKIMNEHLYKQAWEADK
TKVHIMPDIPQIILAKANAINMSDKLYKLSLEESKKKGYDLRPDAIPIKAAKASRDIASDYKYKYNYEKGKGKMVGFRSLEDDPKLV
HSMQVAKMQSDREYKKNYENTKTSYHTPADMLSVTAAKDAQANITNTNYKHLIHKYILLPDAMNIELTRNMNRIQSDNEYKQDYNEW
YKGLGWSPAGSLEVEKAKKATEYASDQKYRQHPSNFQFKKLTDSMDMVLAKQNAHTMNKHLYTIDWNKDKTKIHVMPDTPDILQAKQ
NQTLYSQKLYKLGWEEALKKGYDLPVDAISVQLAKASRDIASDYKYKQGYRKQLGHHVGFRSLQDDPKLVLSMNVAKMQSEREYKKD
FEKWKTKFSSPVDMLGVVLAKKCQELVSDVDYKNYLHQWTCLPDQNDVVQAKKVYELQSENLYKSDLEWLRGIGWSPLGSLEAEKNK
RASEIISEKKYRQPPDRNKFTSIPDAMDIVLAKTNAKNRSDRLYREAWDKDKTQIHIMPDTPDIVLAKANLINTSDKLYRMGYEELK
RKGYDLPVDAIPIKAAKASREIASEYKYKEGFRKQLGHHIGARNIEDDPKMMWSMHVAKIQSDREYKKDFEKWKTKFSSPVDMLGVV
LAKKCQTLVSDVDYKNYLHQWTCLPDQSDVIHARQAYDLQSDNLYKSDLQWLKGIGWMTSGSLEDEKNKRATQILSDHVYRQHPDQF
KFSSLMDSIPMVLAKNNAITMNHRLYTEAWDKDKTTVHIMPDTPEVLLAKQNKVNYSEKLYKLGLEEAKRKGYDMRVDAIPIKAAKA
SRDIASEFKYKEGYRKQLGHHIGARAIRDDPKMMWSMHVAKIQSDREYKKDFEKWKTKFSSPVDMLGVVLAKKCQTLVSDVDYKNYL
HQWTCLPDQSDVIHARQAYDLQSDNMYKSDLQWMRGIGWVSIGSLDVEKCKRATEILSDKIYRQPPDRFKFTSVTDSLEQVLAKNNA
ITMNKRLYTEAWDKDKTQIHIMPDTPEIMLARQNKINYSETLYKLANEEAKKKGYDLRSDAIPIVAAKASRDVISDYKYKDGYRKQL
GHHIGARNIEDDPKMMWSMHVAKIQSDREYKKDFEKWKTKFSSPVDMLGVVLAKKCQTLVSDVDYKNYLHEWTCLPDQNDVIHARQA
YDLQSDNIYKSDLQWLRGIGWVPIGSMDVVKCKRAAEILSDNIYRQPPDKLKFTSVTDSLEQVLAKNNALNMNKRLYTEAWDKDKTQ
VHIMPDTPEIMLARQNKINYSESLYRQAMEEAKKEGYDLRSDAIPIVAAKASRDIASDYKYKEAYRKQLGHHIGARAVHDDPKIMWS
LHIAKVQSDREYKKDFEKYKTRYSSPVDMLGIVLAKKCQTLVSDVDYKHPLHEWICLPDQNDIIHARKAYDLQSDNLYKSDLEWMKG
IGWVPIDSLEVVRAKRAGELLSDTIYRQRPETLKFTSITDTPEQVLAKNNALNMNKRLYTEAWDNDKKTIHVMPDTPEIMLAKLNRI
NYSDKLYKLALEESKKEGYDLRLDAIPIQAAKASRDIASDYKYKEGYRKQLGHHIGARNIKDDPKMMWSIHVAKIQSDREYKKEFEK

WKTKFSSPVDMLGVVLAKKCQILVSDIDYKHPLHEWTCLPDQNDVIQARKAYDLQSDAIYKSDLEWLRGIGWVPIGSVEVEKVKRAG
EILSDRKYRQPADQLKFTCITDTPEIVLAKNNALTMSKHLYTEAWDADKTSIHVMPDTPDILLAKSNSANISQKLYTKGWDESKMKD
YDLRADAISIKSAKASRDIASDYKYKEAYEKQKGHHIGAQSIEDDPKIMCAIHAGKIQSEREYKKEFQKWKTKFSSPVDMLSILLAK
KCQTLVTDIDYRNYLHEWTCMPDQNDIIQAKKAYDLQSDALYKADLEWLRGIGWMPQGSPEVLRVKNAQNIFCDSVYRTPVVNLKYT
SIVDTPEVVLAKSNAENISIPKYREVWDKDKTSIHIMPDTPEINLARANALNVSNKLYREGWDEMKAGCDVRLDAIPIQAAKASREI
ASDYKYKLDHEKQKGHYVGTLTARDDNKIRWALIADKLQNEREYRLDWAKWKAKIQSPVDMLSILHSKNSQALVSDMDYRNYLHQWT
CMPDQNDVIQAKKAYELQSDNVYKADLEWLRGIGWMPNDSVSVNHAKHAADIFSEKKYRTKIETLNFTPVDDRVDYVTAKQSGEILD
DIKYRKDWNATKSKYTLTETPLLHTAQEAARILDQYLYKEGWERQKATGYILPPDAVPFVHAHHCNDVQSELKYKAEHVKQKGHYVG
VPTMRDDPKLVWFEHAGQIQNERLYKEDYHKTKAKINIPADMVSVLAAKQGQTLVSDIDYRNYLHQWMCHPDQNDVIQARKAYDLQS
DNVYRADLEWLRGIGWIPLDSVDHVRVTKNQEMMSQIKYKKNALENYPNFRSVVDPPEIVLAKINSVNQSDVKYKETFNKAKGKYTF
SPDTPHISHSKDMGKLYSTILYKGAWEGTKAYGYTLDERYIPIVGAKHADLVNSELKYKETYEKQKGHYLAGKVIGEFPGVVHCLDF
QKMRSALNYRKHYEDTKANVHIPNDMMNHVLAKRCQYILSDLEYRHYFHQWTSLLEEPNVIRVRNAQEILSDNVYKDDLNWLKGIGC
YVWDTPQILHAKKSYDLQSQLQYTAAGKENLQNYNLVTDTPLYVTAVQSGINASEVKYKENYHQIKDKYTTVLETVDYDRTRNLKNL
YSSNLYKEAWDRVKATSYILPSSTLSLTHAKNQKHLASHIKYREEYEKFKALYTLPRSVDDDPNTARCLRVGKLNIDRLYRSVYEKN
KMKIHIVPDMVEMVTAKDSQKKVSEIDYRLRLHEWICHPDLQVNDHVRKVTDQISDIVYKDDLNWLKGIGCYVWDTPEILHAKHAYD
LRDDIKYKAHMLKTRNDYKLVTDTPVYVQAVKSGKQLSDAVYHYDYVHSVRGKVAPTTKTVDLDRALHAYKLQSSNLYKTSLRTLPT
GYRLPGDTPHFKHIKDTRYMSSYFKYKEAYEHTKAYGYTLGPKDVPFVHVRRVNNVTSERLYRELYHKLKDKIHTTPDTPEIRQVKK
TQEAVSELIYKSDFFKMQGHMISLPYTPQVIHCRYVGDITSDIKYKEDLQVLKGFGCFLYDTPDMVRSRHLRKLWSNYLYTDKARKM
RDKYKVVLDTPEYRKVQELKTHLSELVYRAAGKKQKSIFTSVPDTPDLLRAKRGQKLQSQYLYVELATKERPHHHAGNQTTALKHAK
DVKDMVSEKKYKIQYEKMKDKYTPVPDTPILIRAKRAYWNASDLRYKETFQKTKGKYHTVKDALDIVYHRKVTDDISKIKYKENYMS
QLGIWRSIPDRPEHFHHRAVTDTVSDVKYKEDLTWLKGIGCYAYDTPDFTLAEKNKTLYSKYKYKEVFERTKSDFKYVADSPINRHF
KYATQLMNEKKYRADYEQRKDKYHLVVDEPRHLLAKTAGDQISQIKYRKNYEKSKDKFTSIVDTPEHLRTTKVNKQISDILYKLEYN
KAKPRGYTTIHDTPMLLHVRKVKDEVSDLKYKEVYQRNKSNCTIEPDAVHIKAAKDAYKVNTNLDYKKQYEANKAHWKWTPDRPDFL
QAAKSSLQQSDFEYKLDREFLKGCKLSVTDDKNTVLALRNTLIESDLKYKEKHVKERGTCHAVPDTPQILLAKTVSNLVSENKYKDH
VKKHLAQGSYTTLPETRDTVHVKEVTKHVSDTNYKKKFVKEKGKSNYSIMLEPPEVKHAMEVAKKQSDVAYRKDAKENLHYTTVADR
PDIKKATQAAKQASEVEYRAKHRKEGSHGLSMLGRPDIEMAKKAAKLSSQVKYRENFDKEKGKTPKYNPKDSQLYKVMKDANNLASE
VKYKADLKKLHKPVTDMKESLIMNHVLNTSQLASSYQYKKKYEKSKGHYHTIPDNLEQLHLKEATELQSIVKYKEKYEKERGKPMLD
FETPTYITAKESQQMQSGKEYRKDYEESIKGRNLTGLEVTPALLHVKYATKIASEKEYRKDLEESIRGKGLTEMEDTPDMLRAKNAT
QILNEKEYKRDLELEVKGRGLNAMANETPDFMRARNATDIASQIKYKQSAEMEKANFTSVVDTPEIIHAQQVKNLSSQKKYKEDAEK
SMSYYETVLDTPEIQRVRENQKNFSLLQYQCDLKNSKGKITVVQDTPEILRVKENQKNFSSVLYKEDVSPGTAIGKTPEMMRVKQTQ
DHISSVKYKEAIGQGTPIPDLPEVKRVKETQKHISSVMYKENLGTGIPTTVTPEIERVKRNQENFSSVLYKENLGKGIPTPITPEME
RVKRNQENFSSVLYKENMGKGTPLAVTPEMERVKHNQENISSVLYKENVGKATATPVTPEMQRVKRNQENISSVLYKENLGKATPTP
FTPEMERVKRNQENFSSVLYKENMRKATPTPVTPEMERAKRNQENISSVLYSDSFRKQIQGKAAYVLDTPEMRRVRETQRHISTVKY
HEDFEKHKGCFTPVVTDPITERVKKNMQDFSDINYRGIQRKVVEMEQKRNDQDQETITGLRVWRTNPGSVFDYDPAEDNIQSRSLHM
INVQAQRRSREQSRSASALSISGGEEKSEHSEAPDHHLSTYSDGGVFAVSTAYKHAKTTELPQQRSSSVATQQTTVSSIPSHPSTAG
KIFRAMYDYMAADADEVSFKDGDAIINVQAIDEGWMYGTVQRTGRTGMLPANYVEAI
>sp|Q6ZS30|NBEL1_HUMAN
MASRERLFELWMLYCTKKDPDYLKLWLDTFVSSYEQFLDVDFEKLPTRVDDMPPGISLLPDNILQVLRIQLLQCVQKMADGLEEQQQ
ALSILLVKFFIILCRNLSNVEEIGTCSYINYVITMTTLYIQQLKSKKKEKEMADQTCIEEFVIHALAFCESLYDPYRNWRHRISGRI
LSTVEKSRQKYKPASLTVEFVPFFYQCFQESEHLKESLKCCLLHLFGAIVAGGQRNALQAISPATMEVLMRVLADCDSWEDGDPEEV
GRKAELTLKCLTEVVHILLSSNSDQRQVETSTILENYFKLLNSDHSALPNQRRSRQWENRFIALQIKMLNTITAMLDCTDRPVLQAI
FLNSNCFEHLIRLLQNCKVFQGQLDCLAISTIQALTAVMNKSPAAKEVFKERIGYTHMLEVLKSLGQPPLELLKELMNMAVEGDHTS
VGILGISNVQPLLLLIQWLPELQSHDLQIFISDWLKRICCINRQSRTTCVNANMGIRIIETLDLHSSLHQTCAENLIAIHGSLGSQS
VSSEEIRRLLRLLRVDESESVHPYVTPVTRAILTMARKLSLESALQYFNLSHSMAGISVPPIQKWPGSAFSFSAWFCLDQDQLTLGI

ANKGGKRKQLYSFFTGSGMGFEAFITHSGMLVVAVCTKREYATVMLPDHSFCDSLWHNITVVHMPGKRPFGQSFVYIYDNGQQKVSA
PLRFPAMNEPFTSCCIGSAGQRTTTPPPSQIPDPPFSSPITPHRTSFGGILSSASWGGTIEKSKLITKLISAGTQDSEWGCPTSLEG
QLGSVIIFYEPLQPPQVKALYLAGPNCLSPWKCQESDMADLPGNILLYYTAKACKNSICLDLSTNCLHGRLTGNKVVNWDIKDIINC
IGGLNVLFPLLEQISHFSEGGQIPEEKNESTVPESVTPVEGDWLVWTSTKASESRLERNLVATFILIVKHFIQRHPINQGNLIHSHGV
ATLGALLQKVPSTLMDVNVLMAVQLLIEQVSLEKNMQLLQQMYQYLLFDFRIWNRGDFPFRIGHIQYLSTIIKDSRRVFRKKYGVQF
LLDTLRIYYGNGCKYNELSLDDIRTIRTSLYGLIKYFLCKGGSHEEIQSIMGYIAATNEEEQLFGILDVLFSLLRTSPTRGQLFLLL
FEPGNADILYALLLNQKYSDRLREIIFKIMEQMLKCTNVYERSKQHIRLREVGYSGLGLLLNEALVNTSLIKNLTHQIINTDPVINF
KDLLSVVYISHRAHINVRVAICRKVLQILQFQPDAAHQISQQVGWQDTLVRLFLKAKFENGNTLHKHSRAVLMKDNDKNMSTEDTKK
NSDEKTDEEKITSFASANVSSDQWSLEDRHSLDSNTPLFPEDSSVGELSFKSENQEEFWHSNPSHLSLDLSGIDSCEMSDSGSQVPD
SLPSTPSPVESTKSFSVHSDRESSITNDMGFSDDFSLLESQERCEEELLQLLTHILNYVMCKGLEKSDDDTWIERGQVFSALSKPGI
SSELLRPSDEIKLTLLQKMLEWAISENREAKTNPVTAENAFRLVLIIQDFLQSEGLVNSNMWTEKLLEDMMLLFDCLSVCYSESPVW
VKLSQIQIQLLLGFIGRGNLQVCAMASAKLNTLLQTKVIENQDEACYILGKLEHVLSQSIKEQTEIYSFLIPLVRTLVSKIYELLFM
NLHLPSLPFTNGSSSFFEDFQEYCNSNEWQVYIEKYIVPYMKQYEAHTFYDGHENMALYWKDCYEALMVNMHKRDREGGESKLKFQE
LFVEPFNRKARQENLRYNNMLKQLSSQQLATLRRWKAIQLYLTCERGPWAKRKQNPIHWKLANVENYSRMRLKLVPNYNFKTHEEAS
ALRDNLGIQHSQPSSDTLLLEVVKQVKVSDMVEDKLDLPEEDITARVNVDEKEEQDQKEKLVLMEDCELITIIDVIPGRLEITTQHI
YFYDGSIEKEDGVGFDFKWPHSQIREIHLRRYNLRRSALEIFHVDQSNYFLNFKKEVRNKIYSRLLSLHSPNSYYGSRSPQELFKAS
GLTQKWVNREISNFDYLIQINTMAGRTYNDLAQYPVFPWILQDYTSEELDLNNPAVFRDLSKPIGVVNEKNAKAMREKYENFEDPMG
TIDKFHYGTHYSNSAGVMHYLIRVEPFTTLHIQLQSGRFDCADRQFHSIPATWQALMDNPYDVKELIPEFFYFPEFLENQNQFNLGR
LQISKELVNDVILPKWAKSAEDFIYKHRKALESEYVSAHLHEWIDLIFGYKQRGPAAVEALNVFYYCSYEGAVDLDALTDEKERKAL
EGMINNFGQTPCQLLKEPHPPRLSAEEAVQKPTKIDTSTLNLFQHLPELKSFFIEGISDGIPLLKATIPKNQYRSFMSQGSPELLIT
ISMNYVIGTHGWLPYDRNISNYFTFIKDQTVTNPKTQRSINGSFAPGLEITSKLFVVSHDAKLLFSAGYWDNSIQVMSLTKGKIISH
IIRHMDIVTCLATDYCGIHLISGSRDTTCMIWQITQQGGVPVGLASKPFQILYGHTNEVLSVGISTELDMAVSGSRDGTVIIHTIQK
GQYMRTLRPPCESSLFLTIPNLAISWEGHIVVYSSTEEKTTLKDKNALHLFSINGKYLGSQILKEQVSDICIIGEHIVTGSIQGFLS
IRDLHSLNLSINPLAMRLPIHCVCVTKEYSHILVGLEDGKLIVVGVGKPAEMRSGQLSRKFWGSSKRLSQISAGETEYNTQDSK
>sp|Q13361|MFAP5_HUMAN
MSLLGPKVLLFLAAFIITSDWIPLGVNSQRGDDVTQATPETFTEDPNLVNDPATDETVLAVLADIAPSTDDLASLSEKNTTAECWDE
KFTCTRLYSVHRPVKQCIHQLCFTSLRRMYIVNKEICSRLVCKEHEAMKDELCRQMAGLPPRRLRRSNYFRLPPCENVDLQRPNGL
>sp|Q14865|ARI5B_HUMAN
MEPNSLQWVGSPCGLHGPYIFYKAFQFHLEGKPRILSLGDFFFVRCTPKDPICIAELQLLWEERTSRQLLSSSKLYFLPEDTPQGRN
SDHGEDEVIAVSEKVIVKLEDLVKWVHSDFSKWRCGFHAGPVKTEALGRNGQKEALLKYRQSTLNSGLNFKDVLKEKADLGEDEEET
NVIVLSYPQYCRYRSMLKRIQDKPSSILTDQFALALGGIAVVSRNPQILYCRDTFDHPTLIENESICDEFAPNLKGRPRKKKPCPQR
RDSFSGVKDSNNNSDGKAVAKVKCEARSALTKPKNNHNCKKVSNEEKPKVAIGEECRADEQAFLVALYKYMKERKTPIERIPYLGFK
QINLWTMFQAAQKLGGYETITARRQWKHIYDELGGNPGSTSAATCTRRHYERLILPYERFIKGEEDKPLPPIKPRKQENSSQENENK
TKVSGTKRIKHEIPKSKKEKENAPKPQDAAEVSSEQEKEQETLISQKSIPEPLPAADMKKKIEGYQEFSAKPLASRVDPEKDNETDQ
GSNSEKVAEEAGEKGPTPPLPSAPLAPEKDSALVPGASKQPLTSPSALVDSKQESKLCCFTESPESEPQEASFPSFPTTQPPLANQN
ETEDDKLPAMADYIANCTVKVDQLGSDDIHNALKQTPKVLVVQSFDMFKDKDLTGPMNENHGLNYTPLLYSRGNPGIMSPLAKKKLL
SQVSGASLSSSYPYGSPPPLISKKKLIARDDLCSSLSQTHHGQSTDHMAVSRPSVIQHVQSFRSKPSEERKTINDIFKHEKLSRSDP
HRCSFSKHHLNPLADSYVLKQEIQEGKDKLLEKRALPHSHMPSFLADFYSSPHLHSLYRHTEHHLHNEQTSKYPSRDMYRESENSSF
PSHRHQEKLHVNYLTSLHLQDKKSAAAEAPTDDQPTDLSLPKNPHKPTGKVLGLAHSTTGPQESKGISQFQVLGSQSRDCHPKACRV
SPMTMSGPKKYPESLSRSGKPHHVRLENFRKMEGMVHPILHRKMSPQNIGAARPIKRSLEDLDLVIAGKKARAVSPLDPSKEVSGKE
KASEQESEGSKAAHGGHSGGGSEGHKLPLSSPIFPGLYSGSLCNSGLNSRLPAGYSHSLQYLKNQTVLSPLMQPLAFHSLVMQRGIF
TSPTNSQQLYRHLAAATPVGSSYGDLLHNSIYPLAAINPQAAFPSSQLSSVHPSTKL
>sp|A4D1N5|YG018_HUMAN

MSSRRSSLSPWKCPWFVYCFERPISREAGPVVHQSPTVLYLHSELAARQTQGRLLPREPAAEDLRLSLPGGHAALGLFKLQGKDSYP
HPPTVLLGEDLSSSGWNSWVFGILNISRDEEAIGILTTILQLRETESSAVKWTHTKHSRELGP
>sp|P00519|ABL1_HUMAN
MLEICLKLVGCKSKKGLSSSSSCYLEEALQRPVASDFEPQGLSEAARWNSKENLLAGPSENDPNLFVALYDFVASGDNTLSITKGEK
LRVLGYNHNGEWCEAQTKNGQGWVPSNYITPVNSLEKHSWYHGPVSRNAAEYLLSSGINGSFLVRESESSPGQRSISLRYEGRVYHY
RINTASDGKLYVSSESRFNTLAELVHHHSTVADGLITTLHYPAPKRNKPTVYGVSPNYDKWEMERTDITMKHKLGGGQYGEVYEGVW
KKYSLTVAVKTLKEDTMEVEEFLKEAAVMKEIKHPNLVQLLGVCTREPPFYIITEFMTYGNLLDYLRECNRQEVNAVVLLYMATQIS
SAMEYLEKKNFIHRDLAARNCLVGENHLVKVADFGLSRLMTGDTYTAHAGAKFPIKWTAPESLAYNKFSIKSDVWAFGVLLWEIATY
GMSPYPGIDLSQVYELLEKDYRMERPEGCPEKVYELMRACWQWNPSDRPSFAEIHQAFETMFQESSISDEVEKELGKQGVRGAVSTL
LQAPELPTKTRTSRRAAEHRDTTDVPEMPHSKGQGESDPLDHEPAVSPLLPRKERGPPEGGLNEDERLLPKDKKTNLFSALIKKKKK
TAPTPPKRSSSFREMDGQPERRGAGEEEGRDISNGALAFTPLDTADPAKSPKPSNGAGVPNGALRESGGSGFRSPHLWKKSSTLTSS
RLATGEEEGGGSSSKRFLRSCSASCVPHGAKDTEWRSVTLPRDLQSTGRQFDSSTFGGHKSEKPALPRKRAGENRSDQVTRGTVTPP
PRLVKKNEEAADEVFKDIMESSPGSSPPNLTPKPLRRQVTVAPASGLPHKEEAGKGSALGTPAAAEPVTPTSKAGSGAPGGTSKGPA
EESRVRRHKHSSESPGRDKGKLSRLKPAPPPPPAASAGKAGGKPSQSPSQEAAGEAVLGAKTKATSLVDAVNSDAAKPSQPGEGLKK
PVLPATPKPQSAKPSGTPISPAPVPSTLPSASSALAGDQPSSTAFIPLISTRVSLRKTRQPPERIASGAITKGVVLDSTEALCLAIS
RNSEQMASHSAVLEAGKNLYTFCVSYVDSIQQMRNKFAFREAINKLENNLRELQICPATAGSGPAATQDFSKLLSSVKEISDIVQR
>sp|Q96RD9|FCRL5_HUMAN
MLLWVILLVLAPVSGQFARTPRPIIFLQPPWTTVFQGERVTLTCKGFRFYSPQKTKWYHRYLGKEILRETPDNILEVQESGEYRCQA
QGSPLSSPVHLDFSSASLILQAPLSVFEGDSVVLRCRAKAEVTLNNTIYKNDNVLAFLNKRTDFHIPHACLKDNGAYRCTGYKESCC
PVSSNTVKIQVQEPFTRPVLRASSFQPISGNPVTLTCETQLSLERSDVPLRFRFFRDDQTLGLGWSLSPNFQITAMWSKDSGFYWCK
AATMPYSVISDSPRSWIQVQIPASHPVLTLSPEKALNFEGTKVTLHCETQEDSLRTLYRFYHEGVPLRHKSVRCERGASISFSLTTE
NSGNYYCTADNGLGAKPSKAVSLSVTVPVSHPVLNLSSPEDLIFEGAKVTLHCEAQRGSLPILYQFHHEGAALERRSANSAGGVAIS
FSLTAEHSGNYYCTADNGFGPQRSKAVSLSVTVPVSHPVLTLSSAEALTFEGATVTLHCEVQRGSPQILYQFYHEDMPLWSSSTPSV
GRVSFSFSLTEGHSGNYYCTADNGFGPQRSEVVSLFVTVPVSRPILTLRVPRAQAVVGDLLELHCEAPRGSPPILYWFYHEDVTLGS
SSAPSGGEASFNLSLTAEHSGNYSCEANNGLVAQHSDTISLSVIVPVSRPILTFRAPRAQAVVGDLLELHCEALRGSSPILYWFYHE
DVTLGKISAPSGGGASFNLSLTTEHSGIYSCEADNGLEAQRSEMVTLKVAVPVSRPVLTLRAPGTHAAVGDLLELHCEALRGSPLIL
YRFFHEDVTLGNRSSPSGGASLNLSLTAEHSGNYSCEADNGLGAQRSETVTLYITGLTANRSGPFATGVAGGLLSIAGLAAGALLLY
CWLSRKAGRKPASDPARSPSDSDSQEPTYHNVPAWEELQPVYTNANPRGENVVYSEVRIIQEKKKHAVASDPRHLRNKGSPIIYSEV
KVASTPVSGSLFLASSAPHR
>sp|P82279|CRUM1_HUMAN
MALKNINYLLIFYLSFSLLIYIKNSFCNKNNTRCLSNSCQNNSTCKDFSKDNDCSCSDTANNLDKDCDNMKDPCFSNPCQGSATCVN
TPGERSFLCKCPPGYSGTICETTIGSCGKNSCQHGGICHQDPIYPVCICPAGYAGRFCEIDHDECASSPCQNGAVCQDGIDGYSCFC
VPGYQGRHCDLEVDECASDPCKNEATCLNEIGRYTCICPHNYSGVNCELEIDECWSQPCLNGATCQDALGAYFCDCAPGFLGDHCEL
NTDECASQPCLHGGLCVDGENRYSCNCTGSGFTGTHCETLMPLCWSKPCHNNATCEDSVDNYTCHCWPGYTGAQCEIDLNECNSNPC
QSNGECVELSSEKQYGRITGLPSSFSYHEASGYVCICQPGFTGIHCEEDVNECSSNPCQNGGTCENLPGNYTCHCPFDNLSRTFYGG
RDCSDILLGCTHQQCLNNGTCIPHFQDGQHGFSCLCPSGYTGSLCEIATTLSFEGDGFLWVKSGSVTTKGSVCNIALRFQTVQPMAL
LLFRSNRDVFVKLELLSGYIHLSIQVNNQSKVLLFISHNTSDGEWHFVEVIFAEAVTLTLIDDSCKEKCIAKAPTPLESDQSICAFQ
NSFLGGLPVGMTSNGVALLNFYNMPSTPSFVGCLQDIKIDWNHITLENISSGSSLNVKAGCVRKDWCESQPCQSRGRCINLWLSYQC
DCHRPYEGPNCLREYVAGRFGQDDSTGYVIFTLDESYGDTISLSMFVRTLQPSGLLLALENSTYQYIRVWLERGRLAMLTPNSPKLV
VKFVLNDGNVHLISLKIKPYKIELYQSSQNLGFISASTWKIEKGDVIYIGGLPDKQETELNGGFFKGCIQDVRLNNQNLEFFPNPTN
NASLNPVLVNVTQGCAGDNSCKSNPCHNGGVCHSRWDDFSCSCPALTSGKACEEVQWCGFSPCPHGAQCQPVLQGFECIANAVFNGQ
SGQILFRSNGNITRELTNITFGFRTRDANVIILHAEKEPEFLNISIQDSRLFFQLQSGNSFYMLSLTSLQSVNDGTWHEVTLSMTDP
LSQTSRWQMEVDNETPFVTSTIATGSLNFLKDNTDIYVGDRAIDNIKGLQGCLSTIEIGGIYLSYFENVHGFINKPQEEQFLKISTN
SVVTGCLQLNVCNSNPCLHGGNCEDIYSSYHCSCPLGWSGKHCELNIDECFSNPCIHGNCSDRVAAYHCTCEPGYTGVNCEVDIDNC

QSHQCANGATCISHTNGYSCLCFGNFTGKFCRQSRLPSTVCGNEKTNLTCYNGGNCTEFQTELKCMCRPGFTGEWCEKDIDECASDP
CVNGGLCQDLLNKFQCLCDVAFAGERCEVDLADDLISDIFTTIGSVTVALLLILLLAIVASVVTSNKRATQGTYSPSRQEKEGSRVE
MWNLMPPPAMERLI
>sp|Q9Y5U2|TSSC4_HUMAN
MAEAGTGEPSPSVEGEHGTEYDTLPSDTVSLSDSDSDLSLPGGAEVEALSPMGLPGEEDSGPDEPPSPPSGLLPATVQPFHLRGMSS
TFSQRSRDIFDCLEGAARRAPSSVAHTSMSDNGGFKRPLAPSGRSPVEGLGRAHRSPASPRVPPVPDYVAHPERWTKYSLEDVTEVS
EQSNQATALAFLGSQSLAAPTDCVSSFNQDPSSCGEGRVIFTKPVRGVEARHERKRVLGKVGEPGRGGLGNPATDRGEGPVELAHLA
GPGSPEAEEWGSHHGGLQEVEALSGSVHSGSVPGLPPVETVGFHGSRKRSRDHFRNKSSSPEDPGAEV
>sp|Q8IYW5|RN168_HUMAN
MALPKDAIPSLSECQCGICMEILVEPVTLPCNHTLCKPCFQSTVEKASLCCPFCRRRVSSWTRYHTRRNSLVNVELWTIIQKHYPRE
CKLRASGQESEEVADDYQPVRLLSKPGELRREYEEEISKVAAERRASEEEENKASEEYIQRLLAEEEEEEKRQAEKRRRAMEEQLKS
DEELARKLSIDINNFCEGSISASPLNSRKSDPVTPKSEKKSKNKQRNTGDIQKYLTPKSQFGSASHSEAVQEVRKDSVSKDIDSSDR
KSPTGQDTEIEDMPTLSPQISLGVGEQGADSSIESPMPWLCACGAEWYHEGNVKTRPSNHGKELCVLSHERPKTRVPYSKETAVMPC
GRTESGCAPTSGVTQTNGNNTGETENEESCLLISKEISKRKNQESSFEAVKDPCFSAKRRKVSPESSPDQEETEINFTQKLIDLEHL
LFERHKQEEQDRLLALQLQKEVDKEQMVPNRQKGSPDEYHLRATSSPPDKVLNGQRKNPKDGNFKRQTHTKHPTPERGSRDKNRQVS
LKMQLKQSVNRRKMPNSTRDHCKVSKSAHSLQPSISQKSVFQMFQRCTK
>sp|O15353|FOXN1_HUMAN
MVSLPPPQSDVTLPGPTRLEGERQGDLMQAPGLPGSPAPQSKHAGFSCSSFVSDGPPERTPSLPPHSPRIASPGPEQVQGHCPAGPG
PGPFRLSPSDKYPGFGFEEAAASSPGRFLKGSHAPFHPYKRPFHEDVFPEAETTLALKGHSFKTPGPLEAFEEIPVDVAEAEAFLPG
FSAEAWCNGLPYPSQEHGPQVLGSEVKVKPPVLESGAGMFCYQPPLQHMYCSSQPPFHQYSPGGGSYPIPYLGSSHYQYQRMAPQAS
TDGHQPLFPKPIYSYSILIFMALKNSKTGSLPVSEIYNFMTEHFPYFKTAPDGWKNSVRHNLSLNKCFEKVENKSGSSSRKGCLWAL
NPAKIDKMQEELQKWKRKDPIAVRKSMAKPEELDSLIGDKREKLGSPLLGCPPPGLSGSGPIRPLAPPAGLSPPLHSLHPAPGPIPG
KNPLQDLLMGHTPSCYGQTYLHLSPGLAPPGPPQPLFPQPDGHLELRAQPGTPQDSPLPAHTPPSHSAKLLAEPSPARTMHDTLLPD
GDLGTDLDAINPSLTDFDFQGNLWEQLKDDSLALDPLVLVTSSPTSSSMPPPQPPPHCFPPGPCLTETGSGAGDLAAPGSGGSGALG
DLHLTTLYSAFMELEPTPPTAPAGPSVYLSPSSKPVALA
>sp|P28906|CD34_HUMAN
MLVRRGARAGPRMPRGWTALCLLSLLPSGFMSLDNNGTATPELPTQGTFSNVSTNVSYQETTTPSTLGSTSLHPVSQHGNEATTNIT
ETTVKFTSTSVITSVYGNTNSSVQSQTSVISTVFTTPANVSTPETTLKPSLSPGNVSDLSTTSTSLATSPTKPYTSSSPILSDIKAE
IKCSGIREVKLTQGICLEQNKTSSCAEFKKDRGEGLARVLCGEEQADADAGAQVCSLLLAQSEVRPQCLLLVLANRTEISSKLQLMK
KHQSDLKKLGILDFTEQDVASHQSYSQKTLIALVTSGALLAVLGITGYFLMNRRSWSPTGERLGEDPYYTENGGGQGYSSGPGTSPE
AQGKASVNRGAQENGTGQATSRNGHSARQHVVADTEL
>sp|Q9NUV7|SPTC3_HUMAN
MANPGGGAVCNGKLHNHKKQSNGSQSRNCTKNGIVKEAQQNGKPHFYDKLIVESFEEAPLHVMVFTYMGYGIGTLFGYLRDFLRNWG
IEKCNAAVERKEQKDFVPLYQDFENFYTRNLYMRIRDNWNRPICSAPGPLFDLMERVSDDYNWTFRFTGRVIKDVINMGSYNFLGLA
AKYDESMRTIKDVLEVYGTGVASTRHEMGTLDKHKELEDLVAKFLNVEAAMVFGMGFATNSMNIPALVGKGCLILSDELNHTSLVLG
ARLSGATIRIFKHNNTQSLEKLLRDAVIYGQPRTRRAWKKILILVEGVYSMEGSIVHLPQIIALKKKYKAYLYIDEAHSIGAVGPTG
RGVTEFFGLDPHEVDVLMGTFTKSFGASGGYIAGRKDLVDYLRVHSHSAVYASSMSPPIAEQIIRSLKLIMGLDGTTQGLQRVQQLA
KNTRYFRQRLQEMGFIIYGNENASVVPLLLYMPGKVAAFARHMLEKKIGVVVVGFPATPLAEARARFCVSAAHTREMLDTVLEALDE
MGDLLQLKYSRHKKSARPELYDETSFELED
>sp|Q9NQ03|SCRT2_HUMAN
MPRSFLVKKIKGDGFQCSGVPAPTYHPLETAYVLPGARGPPGDNGYAPHRLPPSSYDADQKPGLELAPAEPAYPPAAPEEYSDPESP
QSSLSARYFRGEAAVTDSYSMDAFFISDGRSRRRRGGGGDAGGSGDAGGAGGRAGRAGAQAGGGHRHACAECGKTYATSSNLSRHK
QTHRSLDSQLARKCPTCGKAYVSMPALAMHLLTHNLRHKCGVCGKAFSRPWLLQGHMRSHTGEKPFGCAHCGKAFADRSNLRAHMQT
HSAFKHYRCRQCDKSFALKSYLHKHCEAACAKAAEPPPPTPAGPAS

>sp|Q8TDV2|GP148_HUMAN
MGDELAPCPVGTTAWPALIQLISKTPCMPQAASNTSLGLGDLRVPSSMLYWLFLPSSLLAAATLAVSPLLLVTILRNQRLRQEPHYL
LPANILLSDLAYILLHMLISSSSLGGWELGRMACGILTDAVFAACTSTILSFTAIVLHTYLAVIHPLRYLSFMSHGAAWKAVALIWL
VACCFPTFLIWLSKWQDAQLEEQGASYILPPSMGTQPGCGLLVIVTYTSILCVLFLCTALIANCFWRIYAEAKTSGIWGQGYSRARG
TLLIHSVLITLYVSTGVVFSLDMVLTRYHHIDSGTHTWLLAANSEVLMMLPRAMLTYLYLLRYRQLLGMVRGHLPSRRHQAIFTIS
>sp|Q8IYJ2|CJ067_HUMAN
MMALVRDRRAHYVMSIVIRWVHCFSSSLRGTFGTRWEAMKAKATELRVCCARRKREAREFKPPQMRGSTRLNISDDLKIGFFSTDHA
TQTDSSEILSVKELSSSTQKLAQMMKSLQVDFGFLKQLLQLKFEDRLKEESLSLFTILHDRILEIEKHYQQNEDKMRKSFNQQLADA
IAVIKGMYQQFFEVEEENVSLQDASTVKTNILLRKLKEKEEVIKELKEELDQYKDFGFHKMESFAKETSSPKSNLEKENLEYKVENE
RLLQIISELEEEIQINLKENSGLEDELISMKEMAEKDHKTIQKLMDSRDRLREELHYEKSLVQDVINKQKEDKEMRKKYGSLSVKVA
RSAKGREASLSPWPKSPPSTTALRPHSATMSVSSAGAQKAKMPKKALKEDQAVVEDKHGLESQIEALKANLENEKKKVERFRKEADR
LNKSWEKRFFILRNSFHVLKNEMFTRHTLFRQFAVLADTSFNYIKVKPLLVQSRTTMTAISSSSHCTSSIDGKHVDVVSDQAALQLS
PKGKLSESPKEESLEEPSMRQSSPAETVD
>sp|Q96N66|MBOA7_HUMAN
MSPEEWTYLVVLLISIPIGFLFKKAGPGLKRWGAAAVGLGLTLFTCGPHTLHSLVTILGTWALIQAQPCSCHALALAWTFSYLLFFR
ALSLLGLPTPTPFTNAVQLLLTLKLVSLASEVQDLHLAQRKEMASGFSKGPTLGLLPDVPSLMETLSYSYCYVGIMTGPFFRYRTYL
DWLEQPFPGAVPSLRPLLRRAWPAPLFGLLFLLSSHLFPLEAVREDAFYARPLPARLFYMIPVFFAFRMRFYVAWIAAECGCIAAGF
GAYPVAAKARAGGGPTLQCPPPSSPEKAASLEYDYETIRNIDCYSTDFCVRVRDGMRYWNMTVQWWLAQYIYKSAPARSYVLRSAWT
MLLSAYWHGLHPGYYLSFLTIPLCLAAEGRLESALRGRLSPGGQKAWDWVHWFLKMRAYDYMCMGFVLLSLADTLRYWASIYFCIHF
LALAALGLGLALGGGSPSRRKAASQPTSLAPEKLREE
>sp|Q5T890|ER6L2_HUMAN
MQPGSAPPPGRMDPSAPQPRAETSGKDIWHPGERCLAPSPDNGKLCEASIKSITVDENGKSFAVVLYADFQERKIPLKQLQEVKFVK
DCPRNLIFDDEDLEKPYFPNRKFPSSSVAFKLSDNGDSIPYTINRYLRDYQREGTRFLYGHYIHGGGCILGDDMGLGKTVQVISFLA
AVLHKKGTREDIENNMPEFLLRSMKKEPLSSTAKKMFLIVAPLSVLYNWKDELDTWGYFRVTVLHGNRKDNELIRVKQRKCEIALTT
YETLRLCLDELNSLEWSAVIVDEAHRIKNPKARVTEVMKALKCNVRIGLTGTILQNNMKELWCVMDWAVPGLLGSGTYFKKQFSDPV
EHGQRHTATKRELATGRKAMQRLAKKMSGWFLRRTKTLIKDQLPKKEDRMVYCSLTDFQKAVYQTVLETEDVTLILQSSEPCTCRSG
QKRRNCCYKTNSHGETVKTLYLSYLTVLQKVANHVALLQAASTSKQQETLIKRICDQVFSRFPDFVQKSKDAAFETLSDPKYSGKMK
VLQQLLNHCRKNRDKVLLFSFSTKLLDVLQQYCMASGLDYRRLDGSTKSEERLKIVKEFNSTQDVNICLVSTMAGGLGLNFVGANVV
VLFDPTWNPANDLQAIDRAYRIGQCRDVKVLRLISLGTVEEIMYLRQIYKQQLHCVVVGSENAKRYFEAVQGSKEHQGELFGIHNLF
KFRSQGSCLTKDILEREGQVEAGIMTATTWLKEGPPAHKLEMPRQPDCQECRGTEQAAEPLAKEACDLCSDFSDEEPVGATGIKTAK
NKAPDSSKASSSPGQLTLLQCGFSKLLETKCKAVEDSDGNTASDDESSDEQPTCLSTEAKDAGCEKNQDSLGTSKHQKLDNILNPKE
KHIFYKSEKILEQNISSKSDEKKIKNTDKHCILQNVTESEDSDVICPTQYTTERFPDNSIRFKPPLEGSEDSETEHTVKTRNNDNSR
NTDDKRNGIISKKLSPENTTLKSILKRKGTSDISDESDDIEISSKSRVRKRASSLRFKRIKETKKELHNSPKTMNKTNQVYAANEDH
NSQFIDDYSSSDESLSVSHFSFSKQSHRPRTIRDRTSFSSKLPSHNKKNSTFIPRKPMKCSNEKVVNQEQSYESMDKFLDGVQEVAY
IHSNQNVIGSSKAENHMSRWAAHDVFELKQFSQLPANIAVCSSKTYKEKVDADTLPHTKKGQQPSEGSISLPLYISNPVNQKKKKVY
HTNQTTFIIGETPKGIRRKQFEEMASYFNSSSVNEFAKHITNATSEERQKMLRDFYASQYPEVKEFFVDSVSQFNNSSFEKGEQRTR
KKSDKRESLIKPRLSDSETLSFKDSTNKISQVCSLKTYKRKSVKFQNHISYREEVFFNDAETKKSPVSSTQEIDSGKNSQASEDTVT
SRSLNSESETRERRLENTMKDQQDLTRTGISRKEPLLKLENKKIENPVLENTSVISLLGDTSILDDLFKSHGNSPTQLPKKVLSGPM
EKAKQRPKDFWDILNEQNDESLSKLTDLAVIETLCEKAPLAAPFKRREEPATSLWKSNEKFLWKKFSPSDTDENATNTQSTT
>sp|O15078|CE290_HUMAN
MPPNINWKEIMKVDPDDLPRQEELADNLLISLSKVEVNELKSEKQENVIHLFRITQSLMKMKAQEVELALEEVEKAGEEQAKFENQL
KTKVMKLENELEMAQQSAGGRDTRFLRNEICQLEKQLEQKDRELEDMEKELEKEKKVNEQLALRNEEAENENSKLRRENKRLKKKNE
QLCQDIIDYQKQIDSQKETLLSRRGEDSDYRSQLSKKNYELIQYLDEIQTLTEANEKIEVQNQEMRKNLEESVQEMEKMTDEYNRMK
AIVHQTDNVIDQLKKENDHYQLQVQELTDLLKSKNEEDDPIMVAVNAKVEEWKLILSSKDDEIIEYQQMLHNLREKLKNAQLDADKS

NVMALQQGIQERDSQIKMLTEQVEQYTKEMEKNTCIIEDLKNELQRNKGASTLSQQTHMKIQSTLDILKEKTKEAERTAELAEADAR
EKDKELVEALKRLKDYESGVYGLEDAVVEIKNCKNQIKIRDREIEILTKEINKLELKISDFLDENEALRERVGLEPKTMIDLTEFRN
SKHLKQQQYRAENQILLKEIESLEEERLDLKKKIRQMAQERGKRSATSGLTTEDLNLTENISQGDRISERKLDLLSLKNMSEAQSKN
EFLSRELIEKERDLERSRTVIAKFQNKLKELVEENKQLEEGMKEILQAIKEMQKDPDVKGGETSLIIPSLERLVNAIESKNAEGIFD
ASLHLKAQVDQLTGRNEELRQELRESRKEAINYSQQLAKANLKIDHLEKETSLLRQSEGSNVVFKGIDLPDGIAPSSASIINSQNEY
LIHLLQELENKEKKLKNLEDSLEDYNRKFAVIRHQQSLLYKEYLSEKETWKTESKTIKEEKRKLEDQVQQDAIKVKEYNNLLNALQM
DSDEMKKILAENSRKITVLQVNEKSLIRQYTTLVELERQLRKENEKQKNELLSMEAEVCEKIGCLQRFKEMAIFKIAALQKVVDNSV
SLSELELANKQYNELTAKYRDILQKDNMLVQRTSNLEHLECENISLKEQVESINKELEITKEKLHTIEQAWEQETKLGNESSMDKAK
KSITNSDIVSISKKITMLEMKELNERQRAEHCQKMYEHLRTSLKQMEERNFELETKFAELTKINLDAQKVEQMLRDELADSVSKAVS
DADRQRILELEKNEMELKVEVSKLREISDIARRQVEILNAQQQSRDKEVESLRMQLLDYQAQSDEKSLIAKLHQHNVSLQLSEATAL
GKLESITSKLQKMEAYNLRLEQKLDEKEQALYYARLEGRNRAKHLRQTIQSLRRQFSGALPLAQQEKFSKTMIQLQNDKLKIMQEMK
NSQQEHRNMENKTLEMELKLKGLEELISTLKDTKGAQKVINWHMKIEELRLQELKLNRELVKDKEEIKYLNNIISEYERTISSLEEE
IVQQNKFHEERQMAWDQREVDLERQLDIFDRQQNEILNAAQKFEEATGSIPDPSLPLPNQLEIALRKIKENIRIILETRATCKSLEE
KLKEKESALRLAEQNILSRDKVINELRLRLPATAEREKLIAELGRKEMEPKSHHTLKIAHQTIANMQARLNQKEEVLKKYQRLLEKA
REEQREIVKKHEEDLHILHHRLELQADSSLNKFKQTAWDLMKQSPTPVPTNKHFIRLAEMEQTVAEQDDSLSSLLVKLKKVSQDLER
QREITELKVKEFENIKLQLQENHEDEVKKVKAEVEDLKYLLDQSQKESQCLKSELQAQKEANSRAPTTTMRNLVERLKSQLALKEKQ
QKALSRALLELRAEMTAAAEERIISATSQKEAHLNVQQIVDRHTRELKTQVEDLNENLLKLKEALKTSKNRENSLTDNLNDLNNELQ
KKQKAYNKILREKEEIDQENDELKRQIKRLTSGLQGKPLTDNKQSLIEELQRKVKKLENQLEGKVEEVDLKPMKEKNAKEELIRWEE
GKKWQAKIEGIRNKLKEKEGEVFTLTKQLNTLKDLFAKADKEKLTLQRKLKTTGMTVDQVLGIRALESEKELEELKKRNLDLENDIL
YMRAHQALPRDSVVEDLHLQNRYLQEKLHALEKQFSKDTYSKPSISGIESDDHCQREQELQKENLKLSSENIELKFQLEQANKDLPR
LKNQVRDLKEMCEFLKKEKAEVQRKLGHVRGSGRSGKTIPELEKTIGLMKKVVEKVQRENEQLKKASGILTSEKMANIEQENEKLKA
ELEKLKAHLGHQLSMHYESKTKGTEKIIAENERLRKELKKETDAAEKLRIAKNNLEILNEKMTVQLEETGKRLQFAESRGPQLEGAD
SKSWKSIVVTRMYETKLKELETDIAKKNQSITDLKQLVKEATEREQKVNKYNEDLEQQIKILKHVPEGAETEQGLKRELQVLRLANH
QLDKEKAELIHQIEANKDQSGAESTIPDADQLKEKIKDLETQLKMSDLEKQHLKEEIKKLKKELENFDPSFFEEIEDLKYNYKEEVK
KNILLEEKVKKLSEQLGVELTSPVAASEEFEDEEESPVNFPIY
>sp|P50749|RASF2_HUMAN
MDYSHQTSLVPCGQDKYISKNELLLHLKTYNLYYEGQNLQLRHREEEDEFIVEGLLNISWGLRRPIRLQMQDDNERIRPPPSSSSWH
SGCNLGAQGTTLKPLTVPKVQISEVDAPPEGDQMPSSTDSRGLKPLQEDTPQLMRTRSDVGVRRRGNVRTPSDQRRIRRHRFSINGH
FYNHKTSVFTPAYGSVTNVRINSTMTTPQVLKLLLNKFKIENSAEEFALYVVHTSGEKQKLKATDYPLIARILQGPCEQISKVFLME
KDQVEEVTYDVAQYIKFEMPVLKSFIQKLQEEEDREVKKLMRKYTVLRLMIRQRLEEIAETPATI
>sp|Q92499|DDX1_HUMAN
MAAFSEMGVMPEIAQAVEEMDWLLPTDIQAESIPLILGGGDVLMAAETGSGKTGAFSIPVIQIVYETLKDQQEGKKGKTTIKTGASV
LNKWQMNPYDRGSAFAIGSDGLCCQSREVKEWHGCRATKGLMKGKHYYEVSCHDQGLCRVGWSTMQASLDLGTDKFGFGFGGTGKKS
HNKQFDNYGEEFTMHDTIGCYLDIDKGHVKFSKNGKDLGLAFEIPPHMKNQALFPACVLKNAELKFNFGEEEFKFPPKDGFVALSKA
PDGYIVKSQHSGNAQVTQTKFLPNAPKALIVEPSRELAEQTLNNIKQFKKYIDNPKLRELLIIGGVAARDQLSVLENGVDIVVGTPG
RLDDLVSTGKLNLSQVRFLVLDEADGLLSQGYSDFINRMHNQIPQVTSDGKRLQVIVCSATLHSFDVKKLSEKIMHFPTWVDLKGED
SVPDTVHHVVVPVNPKTDRLWERLGKSHIRTDDVHAKDNTRPGANSPEMWSEAIKILKGEYAVRAIKEHKMDQAIIFCRTKIDCDNL
EQYFIQQGGGPDKKGHQFSCVCLHGDRKPHERKQNLERFKKGDVRFLICTDVAARGIDIHGVPYVINVTLPDEKQNYVHRIGRVGRA
ERMGLAISLVATEKEKVWYHVCSSRGKGCYNTRLKEDGGCTIWYNEMQLLSEIEEHLNCTISQVEPDIKVPVDEFDGKVTYGQKRAA
GGGSYKGHVDILAPTVQELAALEKEAQTSFLHLGYLPNQLFRTF
>sp|P51148|RAB5C_HUMAN
MAGRGGAARPNGPAAGNKICQFKLVLLGESAVGKSSLVLRFVKGQFHEYQESTIGAAFLTQTVCLDDTTVKFEIWDTAGQERYHSLA
PMYYRGAQAAIVVYDITNTDTFARAKNWVKELQRQASPNIVIALAGNKADLASKRAVEFQEAQAYADDNSLLFMETSAKTAMNVNEI
FMAIAKKLPKNEPQNATGAPGRNRGVDLQENNPASRSQCCSN

>sp|Q8NB14|UBP38_HUMAN
MDKILEGLVSSSHPLPLKRVIVRKVVESAEHWLDEAQCEAMFDLTTRLILEGQDPFQRQVGHQVLEAYARYHRPEFESFFNKTFVLG
LLHQGYHSLDRKDVAILDYIHNGLKLIMSCPSVLDLFSLLQVEVLRMVCERPEPQLCARLSDLLTDFVQCIPKGKLSITFCQQLVRT
IGHFQCVSTQERELREYVSQVTKVSNLLQNIWKAEPATLLPSLQEVFASISSTDASFEPSVALASLVQHIPLQMITVLIRSLTTDPN
VKDASMTQALCRMIDWLSWPLAQHVDTWVIALLKGLAAVQKFTILIDVTLLKIELVFNRLWFPLVRPGALAVLSHMLLSFQHSPEAF
HLIVPHVVNLVHSFKNDGLPSSTAFLVQLTELIHCMMYHYSGFPDLYEPILEAIKDFPKPSEEKIKLILNQSAWTSQSNSLASCLSR
LSGKSETGKTGLINLGNTCYMNSVIQALFMATDFRRQVLSLNLNGCNSLMKKLQHLFAFLAHTQREAYAPRIFFEASRPPWFTPRSQ
QDCSEYLRFLLDRLHEEEKILKVQASHKPSEILECSETSLQEVASKAAVLTETPRTSDGEKTLIEKMFGGKLRTHIRCLNCRSTSQK
VEAFTDLSLAFCPSSSLENMSVQDPASSPSIQDGGLMQASVPGPSEEPVVYNPTTAAFICDSLVNEKTIGSPPNEFYCSENTSVPNE
SNKILVNKDVPQKPGGETTPSVTDLLNYFLAPEILTGDNQYYCENCASLQNAEKTMQITEEPEYLILTLLRFSYDQKYHVRRKILDN
VSLPLVLELPVKRITSFSSLSESWSVDVDFTDLSENLAKKLKPSGTDEASCTKLVPYLLSSVVVHSGISSESGHYYSYARNITSTDS
SYQMYHQSEALALASSQSHLLGRDSPSAVFEQDLENKEMSKEWFLFNDSRVTFTSFQSVQKITSRFPKDTAYVLLYKKQHSTNGLSG
NNPTSGLWINGDPPLQKELMDAITKDNKLYLQEQELNARARALQAASASCSFRPNGFDDNDPPGSCGPTGGGGGGGFNTVGRLVF
>sp|Q13351|KLF1_HUMAN
MATAETALPSISTLTALGPFPDTQDDFLKWWRSEEAQDMGPGPPDPTEPPLHVKSEDQPGEEEDDERGADATWDLDLLLTNFSGPEP
GGAPQTCALAPSEASGAQYPPPPETLGAYAGGPGLVAGLLGSEDHSGWVRPALRARAPDAFVGPALAPAPAPEPKALALQPVYPGPG
AGSSGGYFPRTGLSVPAASGAPYGLLSGYPAMYPAPQYQGHFQLFRGLQGPAPGPATSPSFLSCLGPGTVGTGLGGTAEDPGVIAET
APSKRGRRSWARKRQAAHTCAHPGCGKSYTKSSHLKAHLRTHTGEKPYACTWEGCGWRFARSDELTRHYRKHTGQRPFRCQLCPRAF
SRSDHLALHMKRHL
>sp|Q64LD2|WDR25_HUMAN
MTARTLSLMASLVAYDDSDSEAETEHAGSFNATGQQKDTSGVARPPGQDFASGTLDVPKAGAQPTKHGSCEDPGGYRLPLAQLGRSD
WGSCPSQRLQWPGKEPQVTFPIKEPSCSSLWTSHVPASHMPLAAARFKQVKLSRNFPKSSFHAQSESETVGKNGSSFQKKKCEDCVV
PYTPRRLRQRQALSTETGKGKDVEPQGPPAGRAPAPLYVGPGVSEFIQPYLNSHYKETTVPRKVLFHLRGHRGPVNTIQWCPVLSKS
HMLLSTSMDKTFKVWNAVDSGHCLQTYSLHTEAVRAARWAPCGRRILSGGFDFALHLTDLETGTQLFSGRSDFRITTLKFHPKDHNI
FLCGGFSSEMKAWDIRTGKVMRSYKATIQQTLDILFLREGSEFLSSTDASTRDSADRTIIAWDFRTSAKISNQIFHERFTCPSLALH
PREPVFLAQTNGNYLALFSTVWPYRMSRRRRYEGHKVEGYSVGCECSPGGDLLVTGSADGRVLMYSFRTASRACTLQGHTQACVGTT
YHPVLPSVLATCSWGGDMKIWH
>sp|Q9H6D7|HAUS4_HUMAN
MASGDFCSPGEGMEILQQVCSKQLPPCNLSKEDLLQNPYFSKLLLNLSQHVDESGLSLTLAKEQAQAWKEVRLHKTTWLRSEILHRV
IQELLVDYYVKIQDTNVTSEDKKFHETLEQRLLVTELMRLLGPSQEREIPPLLGLEKADLLELMPLSEDFVWMRARLQQEVEEQLKK
KCFTLLCYYDPNSDADSETVKAAKVWKLAEVLVGEQQQCQDAKSQQKEQMLLLEKKSAAYSQVLLRCLTLLQRLLQEHRLKTQSELD
RINAQYLEVKCGAMILKLRMEELKILSDTYTVEKVEVHRLIRDRLEGAIHLQEQDMENSRQVLNSYEVLGEEFDRLVKEYTVLKQAT
ENKRWALQEFSKVYR
>sp|P48058|GRIA4_HUMAN
MRIISRQIVLLFSGFWGLAMGAFPSSVQIGGLFIRNTDQEYTAFRLAIFLHNTSPNASEAPFNLVPHVDNIETANSFAVTNAFCSQY
SRGVFAIFGLYDKRSVHTLTSFCSALHISLITPSFPTEGESQFVLQLRPSLRGALLSLLDHYEWNCFVFLYDTDRGYSILQAIMEKA
GQNGWHVSAICVENFNDVSYRQLLEELDRRQEKKFVIDCEIERLQNILEQIVSVGKHVKGYHYIIANLGFKDISLERFIHGGANVTG
FQLVDFNTPMVIKLMDRWKKLDQREYPGSETPPKYTSALTYDGVLVMAETFRSLRRQKIDISRRGNAGDCLANPAAPWGQGIDMERT
LKQVRIQGLTGNVQFDHYGRRVNYTMDVFELKSTGPRKVGYWNDMDKLVLIQDVPTLGNDTAAIENRTVVVTTIMESPYVMYKKNHE
MFEGNDKYEGYCVDLASEIAKHIGIKYKIAIVPDGKYGARDADTKIWNGMVGELVYGKAEIAIAPLTITLVREEVIDFSKPFMSLGI
SIMIKKPQKSKPGVFSFLDPLAYEIWMCIVFAYIGVSVVLFLVSRFSPYEWHTEEPEDGKEGPSDQPPNEFGIFNSLWFSLGAFMQQ
GCDISPRSLSGRIVGGVWWFFTLIIISSYTANLAAFLTVERMVSPIESAEDLAKQTEIAYGTLDSGSTKEFFRRSKIAVYEKMWTYM
RSAEPSVFTRTTAEGVARVRKSKGKFAFLLESTMNEYIEQRKPCDTMKVGGNLDSKGYGVATPKGSSLRTPVNLAVLKLSEAGVLDK

LKNKWWYDKGECGPKDSGSKDKTSALSLSNVAGVFYILVGGLGLAMLVALIEFCYKSRAEAKRMKLTFSEAIRNKARLSITGSVGEN
GRVLTPDCPKAVHTGTAIRQSSGLAVIASDLP
>sp|Q68CP9|ARID2_HUMAN
MANSTGKAPPDERRKGLAFLDELRQFHHSRGSPFKKIPAVGGKELDLHGLYTRVTTLGGFAKVSEKNQWGEIVEEFNFPRSCSNAAF
ALKQYYLRYLEKYEKVHHFGEDDDEVPPGNPKPQLPIGAIPSSYNYQQHSVSDYLRQSYGLSMDFNSPNDYNKLVLSLLSGLPNEVD
FAINVCTLLSNESKHVMQLEKDPKIITLLLANAGVFDDTLGSFSTVFGEEWKEKTDRDFVKFWKDIVDDNEVRDLISDRNKSHEGTS
GEWIWESLFHPPRKLGINDIEGQRVLQIAVILRNLSFEEGNVKLLAANRTCLRFLLLSAHSHFISLRQLGLDTLGNIAAELLLDPVD
FKTTHLMFHTVTKCLMSRDRFLKMRGMEILGNLCKAEDNGVLICEYVDQDSYREIICHLTLPDVLLVISTLEVLYMLTEMGDVACTK
IAKVEKSIDMLVCLVSMDIQMFGPDALAAVKLIEHPSSSHQMLSEIRPQAIEQVQTQTHVASAPASRAVVAQHVAPPPGIVEIDSEK
FACQWLNAHFEVNPDCSVSRAEMYSEYLSTCSKLARGGILTSTGFYKCLRTVFPNHTVKRVEDSSSNGQAHIHVVGVKRRAIPLPIQ
MYYQQQPVSTSVVRVDSVPDVSPAPSPAGIPHGSQTIGNHFQRTPVANQSSNLTATQMSFPVQGVHTVAQTVSRIPQNPSPHTHQQQ
NAPVTVIQSKAPIPCEVVKATVIQNSIPQTGVPVSIAVGGGPPQSSVVQNHSTGPQPVTVVNSQTLLHHPSVIPQQSPLHTVVPGQI
PSGTPVTVIQQAVPQSHMFGRVQNIPACTSTVSQGQQLITTSPQPVQTSSQQTSAGSQSQDTVIIAPPQYVTTSASNIVSATSVQNF
QVATGQMVTIAGVPSPQASRVGFQNIAPKPLPSQQVSSTVVQQPIQQPQQPTQQSVVIVSQPAQQGQTYAPAIHQIVLANPAALPAG
QTVQLTGQPNITPSSSPSPVPATNNQVPTAMSSSSTPQSQGPPPTVSQMLSVKRQQQQQHSPAPPPQQVQVQVQQPQQVQMQVQPQQ
SNAGVGQPASGESSLIKQLLLPKRGPSTPGGKLILPAPQIPPPNNARAPSPQVVYQVASNQAAGFGVQGQTPAQQLLVGQQNVQLVP
SAMPPSGGVQTVPISNLQILPGPLISNSPATIFQGTSGNQVTITVVPNTSFAPATVSQGNATQLIAPAGITMSGTQTGVGLPVQTLP
ATQASPAGQSSCTTATPPFKGDKIICQKEEEAKEATGLHVHERKIEVMENPSCRRGATNTSNGDTKENEMHVGSLLNGRKYSDSSLP
PSNSGKIQSETNQCSLISNGPSLELGENGASGKQNSEQIDMQDIKSDLRKPLVNGICDFDKGDGSHLSKNIPNHKTSNHVGNGEISP
MEPQGTLDITQQDTAKGDQLERISNGPVLTLGGSSVSSIQEASNAATQQFSGTDLLNGPLASSLNSDVPQQRPSVVVSPHSTTSVIQ
GHQIIAVPDSGSKVSHSPALSSDVRSTNGTAECKTVKRPAEDTDRETVAGIPNKVGVRIVTISDPNNAGCSATMVAVPAGADPSTVA
KVAIESAVQQKQQHPPTYVQNVVPQNTPMPPSPAVQVQGQPNSSQPSPFSGSSQPGDPMRKPGQNFMCLWQSCKKWFQTPSQVFYHA
ATEHGGKDVYPGQCLWEGCEPFQRQRFSFITHLQDKHCSKDALLAGLKQDEPGQAGSQKSSTKQPTVGGTSSTPRAQKAIVNHPSAA
LMALRRGSRNLVFRDFTDEKEGPITKHIRLTAALILKNIGKYSECGRRLLKRHENNLSVLAISNMEASSTLAKCLYELNFTVQSKEQ
EKDSEMLQ
>sp|Q9UIH9|KLF15_HUMAN
MVDHLLPVDENFSSPKCPVGYLGDRLVGRRAYHMLPSPVSEDDSDASSPCSCSSPDSQALCSCYGGGLGTESQDSILDFLLSQATLG
SGGGSGSSIGASSGPVAWGPWRRAAAPVKGEHFCLPEFPLGDPDDVPRPFQPTLEEIEEFLEENMEPGVKEVPEGNSKDLDACSQLS
AGPHKSHLHPGSSGRERCSPPPGGASAGGAQGPGGGPTPDGPIPVLLQIQPVPVKQESGTGPASPGQAPENVKVAQLLVNIQGQTFA
LVPQVVPSSNLNLPSKFVRIAPVPIAAKPVGSGPLGPGPAGLLMGQKFPKNPAAELIKMHKCTFPGCSKMYTKSSHLKAHLRRHTGE
KPFACTWPGCGWRFSRSDELSRHRRSHSGVKPYQCPVCEKKFARSDHLSKHIKVHRFPRSSRSVRSVN
>sp|Q13438|OS9_HUMAN
MAAETLLSSLLGLLLLGLLLPASLTGGVGSLNLEELSEMRYGIEILPLPVMGGQSQSSDVVIVSSKYKQRYECRLPAGAIHFQRERE
EETPAYQGPGIPELLSPMRDAPCLLKTKDWWTYEFCYGRHIQQYHMEDSEIKGEVLYLGYYQSAFDWDDETAKASKQHRLKRYHSQT
YGNGSKCDLNGRPREAEVRFLCDEGAGISGDYIDRVDEPLSCSYVLTIRTPRLCPHPLLRPPPSAAPQAILCHPSLQPEEYMAYVQR
QADSKQYGDKIIEELQDLGPQVWSETKSGVAPQKMAGASPTKDDSKDSDFWKMLNEPEDQAPGGEEVPAEEQDPSPEAADSASGAPN
DFQNNVQVKVIRSPADLIRFIEELKGGTKKGKPNIGQEQPVDDAAEVPQREPEKERGDPERQREMEEEEDEDEDEDEDEDERQLLGE
FEKELEGILLPSDRDRLRSEVKAGMERELENIIQETEKELDPDGLKKESERDRAMLALTSTLNKLIKRLEEKQSPELVKKHKKKRVV
PKKPPPSPQPTEEDPEHRVRVRVTKLRLGGPNQDLTVLEMKRENPQLKQIEGLVKELLEREGLTAAGKIEIKIVRPWAEGTEEGARW
LTDEDTRNLKEIFFNILVPGAEEAQKERQRQKELESNYRRVWGSPGGEGTGDLDEFDF
>sp|Q9BPX5|ARP5L_HUMAN
MARNTLSSRFRRVDIDEFDENKFVDEQEEAAAAAAEPGPDPSEVDGLLRQGDMLRAFHAALRNSPVNTKNQAVKERAQGVVLKVLTN
FKSSEIEQAVQSLDRNGVDLLMKYIYKGFEKPTENSSAVLLQWHEKALAVGGLGSIIRVLTARKTV
>sp|Q08380|LG3BP_HUMAN

MTPPRLFWVWLLVAGTQGVNDGDMRLADGGATNQGRVEIFYRGQWGTVCDNLWDLTDASVVCRALGFENATQALGRAAFGQGSGPIM
LDEVQCTGTEASLADCKSLGWLKSNCRHERDAGVVCTNETRSTHTLDLSRELSEALGQIFDSQRGCDLSISVNVQGEDALGFCGHTV
ILTANLEAQALWKEPGSNVTMSVDAECVPMVRDLLRYFYSRRIDITLSSVKCFHKLASAYGARQLQGYCASLFAILLPQDPSFQMPL
DLYAYAVATGDALLEKLCLQFLAWNFEALTQAEAWPSVPTDLLQLLLPRSDLAVPSELALLKAVDTWSWGERASHEEVEGLVEKIRF
PMMLPEELFELQFNLSLYWSHEALFQKKTLQALEFHTVPFQLLARYKGLNLTEDTYKPRIYTSPTWSAFVTDSSWSARKSQLVYQSR
RGPLVKYSSDYFQAPSDYRYYPYQSFQTPQHPSFLFQDKRVSWSLVYLPTIQSCWNYGFSCSSDELPVLGLTKSGGSDRTIAYENKA
LMLCEGLFVADVTDFEGWKAAIPSALDTNSSKSTSSFPCPAGHFNGFRTVIRPFYLTNSSGVD
>sp|Q6H8Q1|ABLM2_HUMAN
MSAVSQPQAAPSPLEKSPSTAILCNTCGNVCKGEVLRVQDKYFHIKCFVCKACGCDLAEGGFFVRQGEYICTLDYQRLYGTRCFSCD
QFIEGEVVSALGKTYHPDCFVCAVCRLPFPPGDRVTFNGKECMCQKCSLPVSVGSSAHLSQGLRSCGGCGTEIKNGQALVALDKHWH
LGCFKCKSCGKLLNAEYISKDGLPYCEADYHAKFGIRCDSCEKYITGRVLEAGEKHYHPSCALCVRCGQMFAEGEEMYLQGSSIWHP
ACRQAARTEDRNKETRTSSESIISVPASSTSGSPSRVIYAKLGGEILDYRDLAALPKSKAIYDIDRPDMISYSPYISHSAGDRQSYG
EGDQDDRSYKQCRTSSPSSTGSVSLGRYTPTSRSPQHYSRPGSESGRSTPSLSVLSDSKPPPSTYQQAPRHFHVPDTGVKDNIYRKP
PIYRQHAARRSDGEDGSLDQDNRKKSSWLMLKGDADTRTNSPDLDTQSLSHSSGTDRDPLQRMAGDSFHSRFPYSKSDPLPGHGKNG
LDQRNANLAPCGADPDASWGMREYKIYPYDSLIVTNRIRVKLPKDVDRTRLERHLSPEEFQEVFGMSIEEFDRLALWKRNDLKKKAL
LF
>sp|P56199|ITA1_HUMAN
MAPRPRARPGVAVACCWLLTVVLRCCVSFNVDVKNSMTFSGPVEDMFGYTVQQYENEEGKWVLIGSPLVGQPKNRTGDVYKCPVGRG
ESLPCVKLDLPVNTSIPNVTEVKENMTFGSTLVTNPNGGFLACGPLYAYRCGHLHYTTGICSDVSPTFQVVNSIAPVQECSTQLDIV
IVLDGSNSIYPWDSVTAFLNDLLERMDIGPKQTQVGIVQYGENVTHEFNLNKYSSTEEVLVAAKKIVQRGGRQTMTALGIDTARKEA
FTEARGARRGVKKVMVIVTDGESHDNHRLKKVIQDCEDENIQRFSIAILGSYNRGNLSTEKFVEEIKSIASEPTEKHFFNVSDELAL
VTIVKTLGERIFALEATADQSAASFEMEMSQTGFSAHYSQDWVMLGAVGAYDWNGTVVMQKASQIIIPRNTTFNVESTKKNEPLASY
LGYTVNSATASSGDVLYIAGQPRYNHTGQVIIYRMEDGNIKILQTLSGEQIGSYFGSILTTTDIDKDSNTDILLVGAPMYMGTEKEE
QGKVYVYALNQTRFEYQMSLEPIKQTCCSSRQHNSCTTENKNEPCGARFGTAIAAVKDLNLDGFNDIVIGAPLEDDHGGAVYIYHGS
GKTIRKEYAQRIPSGGDGKTLKFFGQSIHGEMDLNGDGLTDVTIGGLGGAALFWSRDVAVVKVTMNFEPNKVNIQKKNCHMEGKETV
CINATVCFDVKLKSKEDTIYEADLQYRVTLDSLRQISRSFFSGTQERKVQRNITVRKSECTKHSFYMLDKHDFQDSVRITLDFNLTD
PENGPVLDDSLPNSVHEYIPFAKDCGNKEKCISDLSLHVATTEKDLLIVRSQNDKFNVSLTVKNTKDSAYNTRTIVHYSPNLVFSGI
EAIQKDSCESNHNITCKVGYPFLRRGEMVTFKILFQFNTSYLMENVTIYLSATSDSEEPPETLSDNVVNISIPVKYEVGLQFYSSAS
EYHISIAANETVPEVINSTEDIGNEINIFYLIRKSGSFPMPELKLSISFPNMTSNGYPVLYPTGLSSSENANCRPHIFEDPFSINSG
KKMTTSTDHLKRGTILDCNTCKFATITCNLTSSDISQVNVSLILWKPTFIKSYFSSLNLTIRGELRSENASLVLSSSNQKRELAIQI
SKDGLPGRVPLWVILLSAFAGLLLLMLLILALWKIGFFKRPLKKKMEK
>sp|Q13268|DHRS2_HUMAN
MLSAVARGYQGWFHPCARLSVRMSSTGIDRKGVLANRVAVVTGSTSGIGFAIARRLARDGAHVVISSRKQQNVDRAMAKLQGEGLSV
AGIVCHVGKAEDREQLVAKALEHCGGVDFLVCSAGVNPLVGSTLGTSEQIWDKILSVNVKSPALLLSQLLPYMENRRGAVILVSSIA
AYNPVVALGVYNVSKTALLGLTRTLALELAPKDIRVNCVVPGIIKTDFSKVFHGNESLWKNFKEHHQLQRIGESEDCAGIVSFLCSP
DASYVNGENIAVAGYSTRL
>sp|P19652|A1AG2_HUMAN
MALSWVLTVLSLLPLLEAQIPLCANLVPVPITNATLDRITGKWFYIASAFRNEEYNKSVQEIQATFFYFTPNKTEDTIFLREYQTRQ
NQCFYNSSYLNVQRENGTVSRYEGGREHVAHLLFLRDTKTLMFGSYLDDEKNWGLSFYADKPETTKEQLGEFYEALDCLCIPRSDVM
YTDWKKDKCEPLEKQHEKERKQEEGES
>sp|Q96AP7|ESAM_HUMAN
MISLPGPLVTNLLRFLFLGLSALAPPSRAQLQLHLPANRLQAVEGGEVVLPAWYTLHGEVSSSQPWEVPFVMWFFKQKEKEDQVLSY
INGVTTSKPGVSLVYSMPSRNLSLRLEGLQEKDSGPYSCSVNVQDKQGKSRGHSIKTLELNVLVPPAPPSCRLQGVPHVGANVTLSC
QSPRSKPAVQYQWDRQLPSFQTFFAPALDVIRGSLSLTNLSSSMAGVYVCKAHNEVGTAQCNVTLEVSTGPGAAVVAGAVVGTLVGL

GLLAGLVLLYHRRGKALEEPANDIKEDAIAPRTLPWPKSSDTISKNGTLSSVTSARALRPPHGPPRPGALTPTPSLSSQALPSPRLP
TTDGAHPQPISPIPGGVSSSGLSRMGAVPVMVPAQSQAGSLV
>sp|O43822|CU002_HUMAN
MKLTRKMVLTRAKASELHSVRKLNCWGSRLTDISICQEMPSLEVITLSVNSISTLEPVSRCQRLSELYLRRNRIPSLAELFYLKGLP
RLRVLWLAENPCCGTSPHRYRMTVLRTLPRLQKLDNQAVTEEELSRALSEGEEITAAPEREGTGHGGPKLCCTLSSLSSAAETGRDP
LDSEEEATSGAQDERGLKPPSRGQFPSLSARDASSSHRGRNVLTAILLLLRELDAEGLEAVQQTVGSRLQALRGEEVQEHAE
>sp|O75369|FLNB_HUMAN
MPVTEKDLAEDAPWKKIQQNTFTRWCNEHLKCVNKRIGNLQTDLSDGLRLIALLEVLSQKRMYRKYHQRPTFRQMQLENVSVALEFL
DRESIKLVSIDSKAIVDGNLKLILGLVWTLILHYSISMPVWEDEGDDDAKKQTPKQRLLGWIQNKIPYLPITNFNQNWQDGKALGAL
VDSCAPGLCPDWESWDPQKPVDNAREAMQQADDWLGVPQVITPEEIIHPDVDEHSVMTYLSQFPKAKLKPGAPLKPKLNPKKARAYG
RGIEPTGNMVKQPAKFTVDTISAGQGDVMVFVEDPEGNKEEAQVTPDSDKNKTYSVEYLPKVTGLHKVTVLFAGQHISKSPFEVSVD
KAQGDASKVTAKGPGLEAVGNIANKPTYFDIYTAGAGVGDIGVEVEDPQGKNTVELLVEDKGNQVYRCVYKPMQPGPHVVKIFFAGD
TIPKSPFVVQVGEACNPNACRASGRGLQPKGVRIRETTDFKVDTKAAGSGELGVTMKGPKGLEELVKQKDFLDGVYAFEYYPSTPGR
YSIAITWGGHHIPKSPFEVQVGPEAGMQKVRAWGPGLHGGIVGRSADFVVESIGSEVGSLGFAIEGPSQAKIEYNDQNDGSCDVKYW
PKEPGEYAVHIMCDDEDIKDSPYMAFIHPATGGYNPDLVRAYGPGLEKSGCIVNNLAEFTVDPKDAGKAPLKIFAQDGEGQRIDIQM
KNRMDGTYACSYTPVKAIKHTIAVVWGGVNIPHSPYRVNIGQGSHPQKVKVFGPGVERSGLKANEPTHFTVDCTEAGEGDVSVGIKC
DARVLSEDEEDVDFDIIHNANDTFTVKYVPPAAGRYTIKVLFASQEIPASPFRVKVDPSHDASKVKAEGPGLSKAGVENGKPTHFTV
YTKGAGKAPLNVQFNSPLPGDAVKDLDIIDNYDYSHTVKYTPTQQGNMQVLVTYGGDPIPKSPFTVGVAAPLDLSKIKLNGLENRVE
VGKDQEFTVDTRGAGGQGKLDVTILSPSRKVVPCLVTPVTGRENSTAKFIPREEGLYAVDVTYDGHPVPGSPYTVEASLPPDPSKVK
AHGPGLEGGLVGKPAEFTIDTKGAGTGGLGLTVEGPCEAKIECSDNGDGTCSVSYLPTKPGEYFVNILFEEVHIPGSPFKADIEMPF
DPSKVVASGPGLEHGKVGEAGLLSVDCSEAGPGALGLEAVSDSGTKAEVSIQNNKDGTYAVTYVPLTAGMYTLTMKYGGELVPHFPA
RVKVEPAVDTSRIKVFGPGIEGKDVFREATTDFTVDSRPLTQVGGDHIKAHIANPSGASTECFVTDNADGTYQVEYTPFEKGLHVVE
VTYDDVPIPNSPFKVAVTEGCQPSRVQAQGPGLKEAFTNKPNVFTVVTRGAGIGGLGITVEGPSESKINCRDNKDGSCSAEYIPFAP
GDYDVNITYGGAHIPGSPFRVPVKDVVDPSKVKIAGPGLGSGVRARVLQSFTVDSSKAGLAPLEVRVLGPRGLVEPVNVVDNGDGTH
TVTYTPSQEGPYMVSVKYADEEIPRSPFKVKVLPTYDASKVTASGPGLSSYGVPASLPVDFAIDARDAGEGLLAVQITDQEGKPKRA
IVHDNKDGTYAVTYIPDKTGRYMIGVTYGGDDIPLSPYRIRATQTGDASKCLATGPGIASTVKTGEEVGFVVDAKTAGKGKVTCTVL
TPDGTEAEADVIENEDGTYDIFYTAAKPGTYVIYVRFGGVDIPNSPFTVMATDGEVTAVEEAPVNACPPGFRPWVTEEAYVPVSDMN
GLGFKPFDLVIPFAVRKGEITGEVHMPSGKTATPEIVDNKDGTVTVRYAPTEVGLHEMHIKYMGSHIPESPLQFYVNYPNSGSVSAY
GPGLVYGVANKTATFTIVTEDAGEGGLDLAIEGPSKAEISCIDNKDGTCTVTYLPTLPGDYSILVKYNDKHIPGSPFTAKITDDSRR
CSQVKLGSAADFLLDISETDLSSLTASIKAPSGRDEPCLLKRLPNNHIGISFIPREVGEHLVSIKKNGNHVANSPVSIMVVQSEIGD
ARRAKVYGRGLSEGRTFEMSDFIVDTRDAGYGGISLAVEGPSKVDIQTEDLEDGTCKVSYFPTVPGVYIVSTKFADEHVPGSPFTVK
ISGEGRVKESITRTSRAPSVATVGSICDLNLKIPEINSSDMSAHVTSPSGRVTEAEIVPMGKNSHCVRFVPQEMGVHTVSVKYRGQH
VTGSPFQFTVGPLGEGGAHKVRAGGPGLERGEAGVPAEFSIWTREAGAGGLSIAVEGPSKAEITFDDHKNGSCGVSYIAQEPGNYEV
SIKFNDEHIPESPYLVPVIAPSDDARRLTVMSLQESGLKVNQPASFAIRLNGAKGKIDAKVHSPSGAVEECHVSELEPDKYAVRFIP
HENGVHTIDVKFNGSHVVGSPFKVRVGEPGQAGNPALVSAYGTGLEGGTTGIQSEFFINTTRAGPGTLSVTIEGPSKVKMDCQETPE
GYKVMYTPMAPGNYLISVKYGGPNHIVGSPFKAKVTGQRLVSPGSANETSSILVESVTRSSTETCYSAIPKASSDASKVTSKGAGLS
KAFVGQKSSFLVDCSKAGSNMLLIGVHGPTTPCEEVSMKHVGNQQYNVTYVVKERGDYVLAVKWGEEHIPGSPFHVTVP
>sp|O60565|GREM1_HUMAN
MSRTAYTVGALLLLLGTLLPAAEGKKKGSQGAIPPPDKAQHNDSEQTQSPQQPGSRNRGRGQGRGTAMPGEEVLESSQEALHVTERK
YLKRDWCKTQPLKQTIHEEGCNSRTIINRFCYGQCNSFYIPRHIRKEEGSFQSCSFCKPKKFTTMMVTLNCPELQPPTKKKRVTRVK
QCRCISIDLD
>sp|P11532|DMD_HUMAN
MLWWEEVEDCYEREDVQKKTFTKWVNAQFSKFGKQHIENLFSDLQDGRRLLDLLEGLTGQKLPKEKGSTRVHALNNVNKALRVLQNN
NVDLVNIGSTDIVDGNHKLTLGLIWNIILHWQVKNVMKNIMAGLQQTNSEKILLSWVRQSTRNYPQVNVINFTTSWSDGLALNALIH

SHRPDLFDWNSVVCQQSATQRLEHAFNIARYQLGIEKLLDPEDVDTTYPDKKSILMYITSLFQVLPQQVSIEAIQEVEMLPRPPKVT
KEEHFQLHHQMHYSQQITVSLAQGYERTSSPKPRFKSYAYTQAAYVTTSDPTRSPFPSQHLEAPEDKSFGSSLMESEVNLDRYQTAL
EEVLSWLLSAEDTLQAQGEISNDVEVVKDQFHTHEGYMMDLTAHQGRVGNILQLGSKLIGTGKLSEDEETEVQEQMNLLNSRWECLR
VASMEKQSNLHRVLMDLQNQKLKELNDWLTKTEERTRKMEEEPLGPDLEDLKRQVQQHKVLQEDLEQEQVRVNSLTHMVVVVDESSG
DHATAALEEQLKVLGDRWANICRWTEDRWVLLQDILLKWQRLTEEQCLFSAWLSEKEDAVNKIHTTGFKDQNEMLSSLQKLAVLKAD
LEKKKQSMGKLYSLKQDLLSTLKNKSVTQKTEAWLDNFARCWDNLVQKLEKSTAQISQAVTTTQPSLTQTTVMETVTTVTTREQILV
KHAQEELPPPPPQKKRQITVDSEIRKRLDVDITELHSWITRSEAVLQSPEFAIFRKEGNFSDLKEKVNAIEREKAEKFRKLQDASRS
AQALVEQMVNEGVNADSIKQASEQLNSRWIEFCQLLSERLNWLEYQNNIIAFYNQLQQLEQMTTTAENWLKIQPTTPSEPTAIKSQL
KICKDEVNRLSDLQPQIERLKIQSIALKEKGQGPMFLDADFVAFTNHFKQVFSDVQAREKELQTIFDTLPPMRYQETMSAIRTWVQQ
SETKLSIPQLSVTDYEIMEQRLGELQALQSSLQEQQSGLYYLSTTVKEMSKKAPSEISRKYQSEFEEIEGRWKKLSSQLVEHCQKLE
EQMNKLRKIQNHIQTLKKWMAEVDVFLKEEWPALGDSEILKKQLKQCRLLVSDIQTIQPSLNSVNEGGQKIKNEAEPEFASRLETEL
KELNTQWDHMCQQVYARKEALKGGLEKTVSLQKDLSEMHEWMTQAEEEYLERDFEYKTPDELQKAVEEMKRAKEEAQQKEAKVKLLT
ESVNSVIAQAPPVAQEALKKELETLTTNYQWLCTRLNGKCKTLEEVWACWHELLSYLEKANKWLNEVEFKLKTTENIPGGAEEISEV
LDSLENLMRHSEDNPNQIRILAQTLTDGGVMDELINEELETFNSRWRELHEEAVRRQKLLEQSIQSAQETEKSLHLIQESLTFIDKQ
LAAYIADKVDAAQMPQEAQKIQSDLTSHEISLEEMKKHNQGKEAAQRVLSQIDVAQKKLQDVSMKFRLFQKPANFEQRLQESKMILD
EVKMHLPALETKSVEQEVVQSQLNHCVNLYKSLSEVKSEVEMVIKTGRQIVQKKQTENPKELDERVTALKLHYNELGAKVTERKQQL
EKCLKLSRKMRKEMNVLTEWLAATDMELTKRSAVEGMPSNLDSEVAWGKATQKEIEKQKVHLKSITEVGEALKTVLGKKETLVEDKL
SLLNSNWIAVTSRAEEWLNLLLEYQKHMETFDQNVDHITKWIIQADTLLDESEKKKPQQKEDVLKRLKAELNDIRPKVDSTRDQAAN
LMANRGDHCRKLVEPQISELNHRFAAISHRIKTGKASIPLKELEQFNSDIQKLLEPLEAEIQQGVNLKEEDFNKDMNEDNEGTVKEL
LQRGDNLQQRITDERKREEIKIKQQLLQTKHNALKDLRSQRRKKALEISHQWYQYKRQADDLLKCLDDIEKKLASLPEPRDERKIKE
IDRELQKKKEELNAVRRQAEGLSEDGAAMAVEPTQIQLSKRWREIESKFAQFRRLNFAQIHTVREETMMVMTEDMPLEISYVPSTYL
TEITHVSQALLEVEQLLNAPDLCAKDFEDLFKQEESLKNIKDSLQQSSGRIDIIHSKKTAALQSATPVERVKLQEALSQLDFQWEKV
NKMYKDRQGRFDRSVEKWRRFHYDIKIFNQWLTEAEQFLRKTQIPENWEHAKYKWYLKELQDGIGQRQTVVRTLNATGEEIIQQSSK
TDASILQEKLGSLNLRWQEVCKQLSDRKKRLEEQKNILSEFQRDLNEFVLWLEEADNIASIPLEPGKEQQLKEKLEQVKLLVEELPL
RQGILKQLNETGGPVLVSAPISPEEQDKLENKLKQTNLQWIKVSRALPEKQGEIEAQIKDLGQLEKKLEDLEEQLNHLLLWLSPIRN
QLEIYNQPNQEGPFDVKETEIAVQAKQPDVEEILSKGQHLYKEKPATQPVKRKLEDLSSEWKAVNRLLQELRAKQPDLAPGLTTIGA
SPTQTVTLVTQPVVTKETAISKLEMPSSLMLEVPALADFNRAWTELTDWLSLLDQVIKSQRVMVGDLEDINEMIIKQKATMQDLEQR
RPQLEELITAAQNLKNKTSNQEARTIITDRIERIQNQWDEVQEHLQNRRQQLNEMLKDSTQWLEAKEEAEQVLGQARAKLESWKEGP
YTVDAIQKKITETKQLAKDLRQWQTNVDVANDLALKLLRDYSADDTRKVHMITENINASWRSIHKRVSEREAALEETHRLLQQFPLD
LEKFLAWLTEAETTANVLQDATRKERLLEDSKGVKELMKQWQDLQGEIEAHTDVYHNLDENSQKILRSLEGSDDAVLLQRRLDNMNF
KWSELRKKSLNIRSHLEASSDQWKRLHLSLQELLVWLQLKDDELSRQAPIGGDFPAVQKQNDVHRAFKRELKTKEPVIMSTLETVRI
FLTEQPLEGLEKLYQEPRELPPEERAQNVTRLLRKQAEEVNTEWEKLNLHSADWQRKIDETLERLQELQEATDELDLKLRQAEVIKG
SWQPVGDLLIDSLQDHLEKVKALRGEIAPLKENVSHVNDLARQLTTLGIQLSPYNLSTLEDLNTRWKLLQVAVEDRVRQLHEAHRDF
GPASQHFLSTSVQGPWERAISPNKVPYYINHETQTTCWDHPKMTELYQSLADLNNVRFSAYRTAMKLRRLQKALCLDLLSLSAACDA
LDQHNLKQNDQPMDILQIINCLTTIYDRLEQEHNNLVNVPLCVDMCLNWLLNVYDTGRTGRIRVLSFKTGIISLCKAHLEDKYRYLF
KQVASSTGFCDQRRLGLLLHDSIQIPRQLGEVASFGGSNIEPSVRSCFQFANNKPEIEAALFLDWMRLEPQSMVWLPVLHRVAAAET
AKHQAKCNICKECPIIGFRYRSLKHFNYDICQSCFFSGRVAKGHKMHYPMVEYCTPTTSGEDVRDFAKVLKNKFRTKRYFAKHPRMG
YLPVQTVLEGDNMETPVTLINFWPVDSAPASSPQLSHDDTHSRIEHYASRLAEMENSNGSYLNDSISPNESIDDEHLLIQHYCQSLN
QDSPLSQPRSPAQILISLESEERGELERILADLEEENRNLQAEYDRLKQQHEHKGLSPLPSPPEMMPTSPQSPRDAELIAEAKLLRQ
HKGRLEARMQILEDHNKQLESQLHRLRQLLEQPQAEAKVNGTTVSSPSTSLQRSDSSQPMLLRVVGSQTSDSMGEEDLLSPPQDTST
GLEEVMEQLNNSFPSSRGRNTPGKPMREDTM
>sp|Q6ZTN6|AN13D_HUMAN
MVQLVLQYRDYQRATQRLAGIPELLNKLRQAPDFYVEMKWEFTSWVPLVSKMCPSDVYRVWKRGESLRVDTSLLGFEHMTWQRGRRS
FIFKGQEAGALVMEVDHDRQVVHVETLGLTLQEPETLLAAMRPSEEHVASRLTSPIVSTHLDTRNVAFERNKCGIWGWRSEKMETVS

GYEAKVYSATNVELVTRTRTEHLSDQDKSRSKAGKTPFQSFLGMAQQHSSHTGAPVQQAASPTNPTAISPEEYFDPNFSLESRNIGR
PIEMSSKVQRFKATLWLSEEHPLSLGDQVTPIIDLMAISNAHFAKLRDFITLRLPPGFPVKIEIPLFHVLNARITFSNLCGCDEPLS
SVWVPAPSSAVAASGNPFPCEVDPTVFEVPNGYSVLGMERNEPLRDEDDDLLQFAIQQSLLEAGTEAEQVTVWEALTNTRPGARPPP
QATVYEEQLQLERALQESLQLSTEPRGPGSPPRTPPAPGPPSFEEQLRLALELSSREQEERERRGQQEEEDLQRILQLSLTEH
>sp|Q6ZSZ6|TSH1_HUMAN
MPRRKQQAPRRSAAYVPEEELKAAEIDEEHVEDDGLSLDIQESEYMCNEETEIKEAQSYQNSPVSSATNQDAGYGSPFSESSDQLAH
FKGSSSREEKEDPQCPDSVSYPQDSLAQIKAVYANLFSESCWSSLALDLKKSGSTTSTNDASQKESSAPTPTPPTCPVSTTGPTTST
PSTSCSSSTSHSSTTSTSSSSGYDWHQAALAKTLQQTSSYGLLPEPSLFSTVQLYRQNNKLYGSVFTGASKFRCKDCSAAYDTLVEL
TVHMNETGHYRDDNRDKDSEKTKRWSKPRKRSLMEMEGKEDAQKVLKCMYCGHSFESLQDLSVHMIKTKHYQKVPLKEPVPAITKLV
PSTKKRALQDLAPPCSPEPAGMAAEVALSESAKDQKAANPYVTPNNRYGYQNGASYTWQFEARKAQILKCMECGSSHDTLQQLTAHM
MVTGHFLKVTTSASKKGKQLVLDPVVEEKIQSIPLPPTTHTRLPASSIKKQPDSPAGSTTSEEKKEPEKEKPPVAGDAEKIKEESED
SLEKFEPSTLYPYLREEDLDDSPKGGLDILKSLENTVSTAISKAQNGAPSWGGYPSIHAAYQLPGTVKPLPAAVQSVQVQPSYAGGV
KSLSSAEHNALLHSPGSLTPPPHKSNVSAMEELVEKVTGKVNIKKEERPPEKEKSSLAKAASPIAKENKDFPKTEEVSGKPQKKGPE
AETGKAKKEGPLDVHTPNGTEPLKAKVTNGCNNLGIIMDHSPEPSFINPLSALQSIMNTHLGKVSKPVSPSLDPLAMLYKISNSMLD
KPVYPATPVKQADAIDRYYYENSDQPIDLTKSKNKPLVSSVADSVASPLRESALMDISDMVKNLTGRLTPKSSTPSTVSEKSDADGS
SFEEALDELSPVHKRKGRQSNWNPQHLLILQAQFASSLRETTEGKYIMSDLGPQERVHISKFTGLSMTTISHWLANVKYQLRRTGGT
KFLKNLDTGHPVFFCNDCASQFRTASTYISHLETHLGFSLKDLSKLPLNQIQEQQNVSKVLTNKTLGPLGATEEDLGSTFQCKLCNR
TFASKHAVKLHLSKTHGKSPEDHLIYVTELEKQ
>sp|Q9P0M9|RM27_HUMAN
MASVVLALRTRTAVTSLLSPTPATALAVRYASKKSGGSSKNLGGKSSGRRQGIKKMEGHYVHAGNIIATQRHFRWHPGAHVGVGKNK
CLYALEEGIVRYTKEVYVPHPRNTEAVDLITRLPKGAVLYKTFVHVVPAKPEGTFKLVAML
>sp|P01699|LV144_HUMAN
MASFPLLLTLLTHCAGSWAQSVLTQPPSASGTPGQRVTISCSGSSSNIGSNTVNWYQQLPGTAPKLLIYSNNQRPSGVPDRFSGSKS
GTSASLAISGLQSEDEADYYCAAWDDSLNG
>sp|Q99707|METH_HUMAN
MSPALQDLSQPEGLKKTLRDEINAILQKRIMVLDGGMGTMIQREKLNEEHFRGQEFKDHARPLKGNNDILSITQPDVIYQIHKEYLL
AGADIIETNTFSSTSIAQADYGLEHLAYRMNMCSAGVARKAAEEVTLQTGIKRFVAGALGPTNKTLSVSPSVERPDYRNITFDELVE
AYQEQAKGLLDGGVDILLIETIFDTANAKAALFALQNLFEEKYAPRPIFISGTIVDKSGRTLSGQTGEGFVISVSHGEPLCIGLNCA
LGAAEMRPFIEIIGKCTTAYVLCYPNAGLPNTFGDYDETPSMMAKHLKDFAMDGLVNIVGGCCGSTPDHIREIAEAVKNCKPRVPPA
TAFEGHMLLSGLEPFRIGPYTNFVNIGERCNVAGSRKFAKLIMAGNYEEALCVAKVQVEMGAQVLDVNMDDGMLDGPSAMTRFCNLI
ASEPDIAKVPLCIDSSNFAVIEAGLKCCQGKCIVNSISLKEGEDDFLEKARKIKKYGAAMVVMAFDEEGQATETDTKIRVCTRAYHL
LVKKLGFNPNDIIFDPNILTIGTGMEEHNLYAINFIHATKVIKETLPGARISGGLSNLSFSFRGMEAIREAMHGVFLYHAIKSGMDM
GIVNAGNLPVYDDIHKELLQLCEDLIWNKDPEATEKLLRYAQTQGTGGKKVIQTDEWRNGPVEERLEYALVKGIEKHIIEDTEEARL
NQKKYPRPLNIIEGPLMNGMKIVGDLFGAGKMFLPQVIKSARVMKKAVGHLIPFMEKEREETRVLNGTVEEEDPYQGTIVLATVKGD
VHDIGKNIVGVVLGCNNFRVIDLGVMTPCDKILKAALDHKADIIGLSGLITPSLDEMIFVAKEMERLAIRIPLLIGGATTSKTHTAV
KIAPRYSAPVIHVLDASKSVVVCSQLLDENLKDEYFEEIMEEYEDIRQDHYESLKERRYLPLSQARKSGFQMDWLSEPHPVKPTFIG
TQVFEDYDLQKLVDYIDWKPFFDVWQLRGKYPNRGFPKIFNDKTVGGEARKVYDDAHNMLNTLISQKKLRARGVVGFWPAQSIQDDI
HLYAEAAVPQAAEPIATFYGLRQQAEKDSASTEPYYCLSDFIAPLHSGIRDYLGLFAVACFGVEELSKAYEDDGDDYSSIMVKALGD
RLAEAFAEELHERVRRELWAYCGSEQLDVADLRRLRYKGIRPAPGYPSQPDHTEKLTMWRLADIEQSTGIRLTESLAMAPASAVSGL
YFSNLKSKYFAVGKISKDQVEDYALRKNISVAEVEKWLGPILGYDTD
>sp|Q6UE05|WBS28_HUMAN
MEALPPVRSSLLGILLQVTRLSVLLVQNRDHLYNFLLLKINLFNHWVSGLAQEARGSCNWQAHLPLGAAACPLGQALWAGLALIQVP
VWLVLQGPRLMWAGMWGSTKGLGLALLSAWEQLGLSVAIWTDLFLSCLHGLMLVALLLVVVTWRVCQKSHCFRLGRQLSKALQVNCV

VRKLLVQLRRLYWWVETMTALTSWHLAYLITWTTCLASHLLQAAFEHTTQLAEAQEVEPQEVSGSSLLPSLSASSDSESGTVLPEQE
TPRE
>sp|Q30167|2B1A_HUMAN
MVCLRLPGGSCMAVLTVTLMVLSSPLALAGDTRPRFLEEVKFECHFFNGTERVRLLERRVHNQEEYARYDSDVGEYRAVTELGRPDA
EYWNSQKDLLERRRAAVDTYCRHNYGVGESFTVQRRVQPKVTVYPSKTQPLQHHNLLVCSVNGFYPGSIEVRWFRNGQEEKTGVVST
GLIQNGDWTFQTLVMLETVPQSGEVYTCQVEHPSVMSPLTVEWRARSESAQSKMLSGVGGFVLGLLFLGAGLFIYFRNQKGHSGLPP
TGFLS
>sp|Q8WXI4|ACO11_HUMAN
MIQNVGNHLRRGLASVFSNRTSRKSALRAGNDSAMADGEGYRNPTEVQMSQLVLPCHTNQRGELSVGQLLKWIDTTACLSAERHAGC
PCVTASMDDIYFEHTISVGQVVNIKAKVNRAFNSSMEVGIQVASEDLCSEKQWNVCKALATFVARREITKVKLKQITPRTEEEKMEH
SVAAERRRMRLVYADTIKDLLANCAIQGDLESRDCSRMVPAEKTRVESVELVLPPHANHQGNTFGGQIMAWMENVATIAASRLCRAH
PTLKAIEMFHFRGPSQVGDRLVLKAIVNNAFKHSMEVGVCVEAYRQEAETHRRHINSAFMTFVVLDADDQPQLLPWIRPQPGDGERR
YREASARKKIRLDRKYIVSCKQTEVPLSVPWDPSNQVYLSYNNVSSLKMLVAKDNWVLSSEISQVRLYTLEDDKFLSFHMEMVVHVD
AAQAFLLLSDLRQRPEWDKHYRSVELVQQVDEDDAIYHVTSPALGGHTKPQDFVILASRRKPCDNGDPYVIALRSVTLPTHRETPEY
RRGETLCSGFCLWREGDQLTKCCWVRVSLTELVSASGFYSWGLESRSKGRRSDGWNGKLAGGHLSTLKAIPVAKINSRFGYLQDT
>sp|A6NKF1|SAC31_HUMAN
MAGRRAQTGSAPPRPAAPHPRPASRAFPQHCRPRDAERPPSPRSPLMPGCELPVGTCPDMCPAAERAQREREHRLHRLEVVPGCRQD
PPRADPQRAVKEYSRPAAGKPRPPPSQLRPPSVLLATVRYLAGEVAESADIARAEVASFVADRLRAVLLDLALQGAGDAEAAVVLEA
ALATLLTVVARLGPDAARGPADPVLLQAQVQEGFGSLRRCYARGAGPHPRQPAFQGLFLLYNLGSVEALHEVLQLPAALRACPPLRK
ALAVDAAFREGNAARLFRLLQTLPYLPSCAVQCHVGHARREALARFARAFSTPKGQTLPLGFMVNLLALDGLREARDLCQAHGLPLD
GEERVVFLRGRYVEEGLPPASTCKVLVESKLRGRTLEEVVMAEEEDEGTDRPGSPA
>sp|Q9H6J7|CK049_HUMAN
MLSPERLALPDYEYLAQRHVLTYMEDAVCQLLENREDISQYGIARFFTEYFNSVCQGTHILFREFSFVQATPHNRVSFLRAFWRCFR
TVGKNGDLLTMKEYHCLLQLLCPDFPLELTQKAARIVLMDDAMDCLMSFSDFLFAFQIQFYYSEFLDSVAAIYEDLLSGKNPNTVIV
PTSSSGQHRQRPALGGAGTLEGVEASLFYQCLENLCDRHKYSCPPPALVKEALSNVQRLTFYGFLMALSKHRGINQALGALPDKGDL
MHDPAMDEELERLLAQVPGLVNSVTASPEASCLPSRTPPRVGSPWRPLHHSRKVDGESDGSTEETDESET
>sp|Q9BZD2|S29A3_HUMAN
MAVVSEDDFQHSSNSTYRTTSSSLRADQEALLEKLLDRPPPGLQRPEDRFCGTYIIFFSLGIGSLLPWNFFITAKEYWMFKLRNSSS
PATGEDPEGSDILNYFESYLAVASTVPSMLCLVANFLLVNRVAVHIRVLASLTVILAIFMVITALVKVDTSSWTRGFFAVTIVCMVI
LSGASTVFSSSIYGMTGSFPMRNSQALISGGAMGGTVSAVASLVDLAASSDVRNSALAFFLTATVFLVLCMGLYLLLSRLEYARYYM
RPVLAAHVFSGEEELPQDSLSAPSVASRFIDSHTPPLRPILKKTASLGFCVTYVFFITSLIYPAICTNIESLNKGSGSLWTTKFFIP
LTTFLLYNFADLCGRQLTAWIQVPGPNSKALPGFVLLRTCLIPLFVLCNYQPRVHLKTVVFQSDVYPALLSSLLGLSNGYLSTLALL
YGPKIVPRELAEATGVVMSFYVCLGLTLGSACSTLLVHLI
>sp|Q2TAZ0|ATG2A_HUMAN
MSRWLWPWSNCVKERVCRYLLHHYLGHFFQEHLSLDQLSLDLYKGSVALRDIHLEIWSVNEVLESMESPLELVEGFVGSIEVAVPWA
ALLTDHCTVRVSGLQLTLQPRRGPAPGAADSQSWASCMTTSLQLAQECLRDGLPEPSEPPQPLEGLEMFAQTIETVLRRIKVTFLDT
VVRVEHSPGDGERGVAVEVRVQRLEYCDEAVRDPSQAPPVDVHQPPAFLHKLLQLAGVRLHYEELPAQEEPPEPPLQIGSCSGYMEL
MVKLKQNEAFPGPKLEVAGQLGSLHLLLTPRQLQQLQELLSAVSLTDHEGLADKLNKSRPLGAEDLWLIEQDLNQQLQAGAVAEPLS
PDPLTNPLLNLDNTDLFFSMAGLTSSVASALSELSLSDVDLASSVRSDMASRRLSAQAHPAGKMAPNPLLDTMRPDSLLKMTLGGVT
LTLLQTSAPSSGPPDLATHFFTEFDATKDGPFGSRDFHHLRPRFQRACPCSHVRLTGTAVQLSWELRTGSRGRRTTSMEVHFGQLEV
LECLWPRGTSEPEYTEILTFPGTLGSQASARPCAHLRHTQILRRVPKSRPRRSVACHCHSELALDLANFQADVELGALDRLAALLRL
ATVPAEPPAGLLTEPLPAMEQQTVFRLSAPRATLRLRFPIADLRPEPDPWAGQAVRAEQLRLELSEPQFRSELSSGPGPPVPTHLEL
TCSDLHGIYEDGGKPPVPCLRVSKALDPKSTGRKYFLPQVVVTVNPQSSSTQWEVAPEKGEELELSVESPCELREPEPSPFSSKRTM
YETEEMVIPGDPEEMRTFQSRTLALSRCSLEVILPSVHIFLPSKEVYESIYNRINNDLLMWEPADLLPTPDPAAQPSGFPGPSGFWH

DSFKMCKSAFKLANCFDLTPDSDSDDEDAHFFSVGASGGPQAAAPEAPSLHLQSTFSTLVTVLKGRITALCETKDEGGKRLEAVHGE
LVLDMEHGTLFSVSQYCGQPGLGYFCLEAEKATLYHRAAVDDYPLPSHLDLPSFAPPAQLAPTIYPSEEGVTERGASGRKGQGRGPH
MLSTAVRIHLDPHKNVKEFLVTLRLHKATLRHYMALPEQSWHSQLLEFLDVLDDPVLGYLPPTVITILHTHLFSCSVDYRPLYLPVR
VLITAETFTLSSNIIMDTSTFLLRFILDDSALYLSDKCEVETLDLRRDYVCVLDVDLLELVIKTWKGSTEGKLSQPLFELRCSNNVV
HVHSCADSCALLVNLLQYVMSTGDLHPPPRPPSPTEIAGQKLSESPASLPSCPPVETALINQRDLADALLDTERSLRELAQPSGGHL
PQASPISVYLFPGERSGAPPPSPPVGGPAGSLGSCSEEKEDEREEEGDGDTLDSDEFCILDAPGLGIPPRDGEPVVTQLHPGPIVVR
DGYFSRPIGSTDLLRAPAHFPVPSTRVVLREVSLVWHLYGGRDFGPHPGHRARTGLSGPRSSPSRCSGPNRPQNSWRTQGGSGRQHH
VLMEIQLSKVSFQHEVYPAEPATGPAAPSQELEERPLSRQVFIVQELEVRDRLASSQINKFLYLHTSERMPRRAHSNMLTIKALHVA
PTTNLGGPECCLRVSLMPLRLNVDQDALFFLKDFFTSLVAGINPVVPGETSAEARPETRAQPSSPLEGQAEGVETTGSQEAPGGGHS
PSPPDQQPIYFREFRFTSEVPIWLDYHGKHVTMDQVGTFAGLLIGLAQLNCSELKLKRLCCRHGLLGVDKVLGYALNEWLQDIRKNQ
LPGLLGGVGPMHSVVQLFQGFRDLLWLPIEQYRKDGRLMRGLQRGAASFGSSTASAALELSNRLVQAIQATAETVYDILSPAAPVSR
SLQDKRSARRLRRGQQPADLREGVAKAYDTVREGILDTAQTICDVASRGHEQKGLTGAVGGVIRQLPPTVVKPLILATEATSSLLGG
MRNQIVPDAHKDHALKWRSDSAQD
>sp|Q9H115|SNAB_HUMAN
MDNAGKEREAVQLMAEAEKRVKASHSFLRGLFGGNTRIEEACEMYTRAANMFKMAKNWSAAGNAFCQAAKLHMQLQSKHDSATSFVD
AGNAYKKADPQEAINCLNAAIDIYTDMGRFTIAAKHHITIAEIYETELVDIEKAIAHYEQSADYYKGEESNSSANKCLLKVAAYAAQ
LEQYQKAIEIYEQVGANTMDNPLLKYSAKDYFFKAALCHFIVDELNAKLALEKYEEMFPAFTDSRECKLLKKLLEAHEEQNSEAYTE
AVKEFDSISRLDQWLTTMLLRIKKSIQGDGEGDGDLK
>sp|Q13442|HAP28_HUMAN
MPKGGRKGGHKGRARQYTSPEEIDAQLQAEKQKAREEEEQKEGGDGAAGDPKKEKKSLDSDESEDEEDDYQQKRKGVEGLIDIENPN
RVAQTTKKVTQLDLDGPKELSRREREEIEKQKAKERYMKMHLAGKTEQAKADLARLAIIRKQREEAARKKEEERKAKDDATLSGKRM
QSLSLNK
>sp|Q8N7W2|BEND7_HUMAN
MEFSERKRSRKSQSFKLVSRDYHHEVYKIPEFSNDVNGEAKETQPIFLGDESMEIKKQITGMRRLLNDSTGRIYQRVGKEGEKLKEE
PQDLDLVWPPRLNSSAEAPQSLHPSSRGVWNELPPQSGQFSGQYGTRSRTFQSQPHPTTSSNGELPVVNSSAGSNCCTCNCQSTLQA
ILQELKTMRKLMQIQAVGTQNRQQPPISLICSQRTAVSRKRNKKKKVPPKTVEPLTVKQKPSGSEMEKKSVVASELSALQAAEHTSP
EESRVLGFGIVLESPSSDPEVQLAEGFDVFMPKSQLDSILSNYTRSGSLLFRKLVCAFFDDKTLANSLPNGKRKRGLNDNRKGLDQN
IVGAIKVFTEKYCTANHVDKLPGPRDWVQILQDQIKLARRRLKRGSEIADSDERLDGIALPPTGACGGPCTVLPGGSAAVTLVLQSS
PQTMSQEKGQMAEPWEEQHLVLLNNLTRDRAETGALSQTSQDFKHHSFLITQVSATLHHQRGIRNFPTPGSAKSLTLHISCLSL
>sp|Q075Z2|BSPH1_HUMAN
MGSLMLLFVETTRNSSACIFPVILNELSSTVETITHFPEVTDGECVFPFHYKNGTYYDCIKSKARHKWCSLNKTYEGYWKFCSAEDF
ANCVFPFWYRRLIYWECTDDGEAFGKKWCSLTKNFNKDRIWKYCE
>sp|O15344|TRI18_HUMAN
METLESELTCPICLELFEDPLLLPCAHSLCFNCAHRILVSHCATNESVESITAFQCPTCRHVITLSQRGLDGLKRNVTLQNIIDRFQ
KASVSGPNSPSETRRERAFDANTMTSAEKVLCQFCDQDPAQDAVKTCVTCEVSYCDECLKATHPNKKPFTGHRLIEPIPDSHIRGLM
CLEHEDEKVNMYCVTDDQLICALCKLVGRHRDHQVAALSERYDKLKQNLESNLTNLIKRNTELETLLAKLIQTCQHVEVNASRQEAK
LTEECDLLIEIIQQRRQIIGTKIKEGKVMRLRKLAQQIANCKQCIERSASLISQAEHSLKENDHARFLQTAKNITERVSMATASSQV
LIPEINLNDTFDTFALDFSREKKLLECLDYLTAPNPPTIREELCTASYDTITVHWTSDDEFSVVSYELQYTIFTGQANVVSLCNSAD
SWMIVPNIKQNHYTVHGLQSGTKYIFMVKAINQAGSRSSEPGKLKTNSQPFKLDPKSAHRKLKVSHDNLTVERDESSSKKSHTPERF
TSQGSYGVAGNVFIDSGRHYWEVVISGSTWYAIGLAYKSAPKHEWIGKNSASWALCRCNNNWVVRHNSKEIPIEPAPHLRRVGILLD
YDNGSIAFYDALNSIHLYTFDVAFAQPVCPTFTVWNKCLTIITGLPIPDHLDCTEQLP
>sp|Q9H1E3|NUCKS_HUMAN

MSRPVRNRKVVDYSQFQESDDADEDYGRDSGPPTKKIRSSPREAKNKRRSGKNSQEDSEDSEDKDVKTKKDDSHSAEDSEDEKEDHK
NVRQQRQAASKAASKQREMLMEDVGSEEEQEEEDEAPFQEKDSGSDEDFLMEDDDDSDYGSSKKKNKKMVKKSKPERKEKKMPKPRL
KATVTPSPVKGKGKVGRPTASKASKEKTPSPKEEDEEPESPPEKKTSTSPPPEKSGDEGSEDEAPSGED
>sp|Q16696|CP2AD_HUMAN
MLASGLLLVTLLACLTVMVLMSVWRQRKSRGKLPPGPTPLPFIGNYLQLNTEQMYNSLMKISERYGPVFTIHLGPRRVVVLCGHDAV
KEALVDQAEEFSGRGEQATFDWLFKGYGVAFSNGERAKQLRRFSIATLRGFGVGKRGIEERIQEEAGFLIDALRGTHGANIDPTFFL
SRTVSNVISSIVFGDRFDYEDKEFLSLLRMMLGSFQFTATSTGQLYEMFSSVMKHLPGPQQQAFKELQGLEDFIAKKVEHNQRTLDP
NSPRDFIDSFLIRMQEEEKNPNTEFYLKNLVMTTLNLFFAGTETVSTTLRYGFLLLMKHPEVEAKVHEEIDRVIGKNRQPKFEDRAK
MPYTEAVIHEIQRFGDMLPMGLAHRVNKDTKFRDFFLPKGTEVFPMLGSVLRDPRFFSNPRDFNPQHFLDKKGQFKKSDAFVPFSIG
KRYCFGEGLARMELFLFFTTIMQNFRFKSPQSPKDIDVSPKHVGFATIPRNYTMSFLPR
>sp|Q93008|USP9X_HUMAN
MTATTRGSPVGGNDNQGQAPDGQSQPPLQQNQTSSPDSSNENSPATPPDEQGQGDAPPQLEDEEPAFPHTDLAKLDDMINRPRWVVP
VLPKGELEVLLEAAIDLSKKGLDVKSEACQRFFRDGLTISFTKILTDEAVSGWKFEIHRCIINNTHRLVELCVAKLSQDWFPLLELL
AMALNPHCKFHIYNGTRPCESVSSSVQLPEDELFARSPDPRSPKGWLVDLLNKFGTLNGFQILHDRFINGSALNVQIIAALIKPFGQ
CYEFLTLHTVKKYFLPIIEMVPQFLENLTDEELKKEAKNEAKNDALSMIIKSLKNLASRVPGQEETVKNLEIFRLKMILRLLQISSF
NGKMNALNEVNKVISSVSYYTHRHGNPEEEEWLTAERMAEWIQQNNILSIVLRDSLHQPQYVEKLEKILRFVIKEKALTLQDLDNIW
AAQAGKHEAIVKNVHDLLAKLAWDFSPEQLDHLFDCFKASWTNASKKQREKLLELIRRLAEDDKDGVMAHKVLNLLWNLAHSDDVPV
DIMDLALSAHIKILDYSCSQDRDTQKIQWIDRFIEELRTNDKWVIPALKQIREICSLFGEAPQNLSQTQRSPHVFYRHDLINQLQHN
HALVTLVAENLATYMESMRLYARDHEDYDPQTVRLGSRYSHVQEVQERLNFLRFLLKDGQLWLCAPQAKQIWKCLAENAVYLCDREA
CFKWYSKLMGDEPDLDPDINKDFFESNVLQLDPSLLTENGMKCFERFFKAVNCREGKLVAKRRAYMMDDLELIGLDYLWRVVIQSND
DIASRAIDLLKEIYTNLGPRLQVNQVVIHEDFIQSCFDRLKASYDTLCVLDGDKDSVNCARQEAVRMVRVLTVLREYINECDSDYHE
ERTILPMSRAFRGKHLSFVVRFPNQGRQVDDLEVWSHTNDTIGSVRRCILNRIKANVAHTKIELFVGGELIDPADDRKLIGQLNLKD
KSLITAKLTQISSNMPSSPDSSSDSSTGSPGNHGNHYSDGPNPEVESCLPGVIMSLHPRYISFLWQVADLGSSLNMPPLRDGARVLM
KLMPPDSTTIEKLRAICLDHAKLGESSLSPSLDSLFFGPSASQVLYLTEVVYALLMPAGAPLADDSSDFQFHFLKSGGLPLVLSMLT
RNNFLPNADMETRRGAYLNALKIAKLLLTAIGYGHVRAVAEACQPGVEGVNPMTQINQVTHDQAVVLQSALQSIPNPSSECMLRNVS
VRLAQQISDEASRYMPDICVIRAIQKIIWASGCGSLQLVFSPNEEITKIYEKTNAGNEPDLEDEQVCCEALEVMTLCFALIPTALDA
LSKEKAWQTFIIDLLLHCHSKTVRQVAQEQFFLMCTRCCMGHRPLLFFITLLFTVLGSTARERAKHSGDYFTLLRHLLNYAYNSNIN
VPNAEVLLNNEIDWLKRIRDDVKRTGETGIEETILEGHLGVTKELLAFQTSEKKFHIGCEKGGANLIKELIDDFIFPASNVYLQYMR
NGELPAEQAIPVCGSPPTINAGFELLVALAVGCVRNLKQIVDSLTEMYYIGTAITTCEALTEWEYLPPVGPRPPKGFVGLKNAGATC
YMNSVIQQLYMIPSIRNGILAIEGTGSDVDDDMSGDEKQDNESNVDPRDDVFGYPQQFEDKPALSKTEDRKEYNIGVLRHLQVIFGH
LAASRLQYYVPRGFWKQFRLWGEPVNLREQHDALEFFNSLVDSLDEALKALGHPAMLSKVLGGSFADQKICQGCPHRYECEESFTTL
NVDIRNHQNLLDSLEQYVKGDLLEGANAYHCEKCNKKVDTVKRLLIKKLPPVLAIQLKRFDYDWERECAIKFNDYFEFPRELDMEPY
TVAGVAKLEGDNVNPESQLIQQSEQSESETAGSTKYRLVGVLVHSGQASGGHYYSYIIQRNGGDGERNRWYKFDDGDVTECKMDDDE
EMKNQCFGGEYMGEVFDHMMKRMSYRRQKRWWNAYILFYERMDTIDQDDELIRYISELAITTRPHQIIMPSAIERSVRKQNVQFMHN
RMQYSMEYFQFMKKLLTCNGVYLNPPPGQDHLLPEAEEITMISIQLAARFLFTTGFHTKKVVRGSASDWYDALCILLRHSKNVRFWF
AHNVLFNVSNRFSEYLLECPSAEVRGAFAKLIVFIAHFSLQDGPCPSPFASPGPSSQAYDNLSLSDHLLRAVLNLLRREVSEHGRHL
QQYFNLFVMYANLGVAEKTQLLKLSVPATFMLVSLDEGPGPPIKYQYAELGKLYSVVSQLIRCCNVSSRMQSSINGNPPLPNPFGDP
NLSQPIMPIQQNVADILFVRTSYVKKIIEDCSNSEETVKLLRFCCWENPQFSSTVLSELLWQVAYSYTYELRPYLDLLLQILLIEDS
WQTHRIHNALKGIPDDRDGLFDTIQRSKNHYQKRAYQCIKCMVALFSNCPVAYQILQGNGDLKRKWTWAVEWLGDELERRPYTGNPQ
YTYNNWSPPVQSNETSNGYFLERSHSARMTLAKACELCPEEVKKATSVQQIEMEESKEPDDQDAPDEHESPPPEDAPLYPHSPGSQY
QQNNHVHGQPYTGPAAHHMNNPQRTGQRAQENYEGSEEVSPPQTKDQ
>sp|P22735|TGM1_HUMAN
MMDGPRSDVGRWGGNPLQPPTTPSPEPEPEPDGRSRRGGGRSFWARCCGCCSCRNAADDDWGPEPSDSRGRGSSSGTRRPGSRGSDS
RRPVSRGSGVNAAGDGTIREGMLVVNGVDLLSSRSDQNRREHHTDEYEYDELIVRRGQPFHMLLLLSRTYESSDRITLELLIGNNPE

VGKGTHVIIPVGKGGSGGWKAQVVKASGQNLNLRVHTSPNAIIGKFQFTVRTQSDAGEFQLPFDPRNEIYILFNPWCPEDIVYVDHE
DWRQEYVLNESGRIYYGTEAQIGERTWNYGQFDHGVLDACLYILDRRGMPYGGRGDPVNVSRVISAMVNSLDDNGVLIGNWSGDYSR
GTNPSAWVGSVEILLSYLRTGYSVPYGQCWVFAGVTTTVLRCLGLATRTVTNFNSAHDTDTSLTMDIYFDENMKPLEHLNHDSVWNF
HVWNDCWMKRPDLPSGFDGWQVVDATPQETSSGIFCCGPCSVESIKNGLVYMKYDTPFIFAEVNSDKVYWQRQDDGSFKIVYVEEKA
IGTLIVTKAISSNMREDITYLYKHPEGSDAERKAVETAAAHGSKPNVYANRGSAEDVAMQVEAQDAVMGQDLMVSVMLINHSSSRRT
VKLHLYLSVTFYTGVSGTIFKETKKEVELAPGASDRVTMPVAYKEYRPHLVDQGAMLLNVSGHVKESGQVLAKQHTFRLRTPDLSLT
LLGAAVVGQECEVQIVFKNPLPVTLTNVVFRLEGSGLQRPKILNVGDIGGNETVTLRQSFVPVRPGPRQLIASLDSPQLSQVHGVIQ
VDVAPAPGDGGFFSDAGGDSHLGETIPMASRGGA
>sp|Q9UKT6|FXL21_HUMAN
MKRNSLSVENKIVQLSGAAKQPKVGFYSSLNQTHTHTVLLDWGSLPHHVVLQIFQYLPLLDRACASSVCRRWNEVFHISDLWRKFEF
ELNQSATSSFKSTHPDLIQQIIKKHFAHLQYVSFKVDSSAESAEAACDILSQLVNCSIQTLGLISTAKPSFMNVSESHFVSALTVVF
INSKSLSSIKIEDTPVDDPSLKILVANNSDTLRLPKMSSCPHVSSDGILCVADRCQGLRELALNYYILTDELFLALSSETHVNLEHL
RIDVVSENPGQIKFHAVKKHSWDALIKHSPRVNVVMHFFLYEEEFETFFKEETPVTHLYFGRSVSKVVLGRVGLNCPRLIELVVCAN
DLQPLDNELICIAEHCTNLTALGLSKCEVSCSAFIRFVRLCERRLTQLSVMEEVLIPDEDYSLDEIHTEVSKYLGRVWFPDVMPLW
>sp|Q96L93|KI16B_HUMAN
MASVKVAVRVRPMNRREKDLEAKFIIQMEKSKTTITNLKIPEGGTGDSGRERTKTFTYDFSFYSADTKSPDYVSQEMVFKTLGTDVV
KSAFEGYNACVFAYGQTGSGKSYTMMGNSGDSGLIPRICEGLFSRINETTRWDEASFRTEVSYLEIYNERVRDLLRRKSSKTFNLRV
REHPKEGPYVEDLSKHLVQNYGDVEELMDAGNINRTTAATGMNDVSSRSHAIFTIKFTQAKFDSEMPCETVSKIHLVDLAGSERADA
TGATGVRLKEGGNINKSLVTLGNVISALADLSQDAANTLAKKKQVFVPYRDSVLTWLLKDSLGGNSKTIMIATISPADVNYGETLST
LRYANRAKNIINKPTINEDANVKLIRELRAEIARLKTLLAQGNQIALLDSPTALSMEEKLQQNEARVQELTKEWTNKWNETQNILKE
QTLALRKEGIGVVLDSELPHLIGIDDDLLSTGIILYHLKEGQTYVGRDDASTEQDIVLHGLDLESEHCIFENIGGTVTLIPLSGSQC
SVNGVQIVEATHLNQGAVILLGRTNMFRFNHPKEAAKLREKRKSGLLSSFSLSMTDLSKSRENLSAVMLYNPGLEFERQQREELEKL
ESKRKLIEEMEEKQKSDKAELERMQQEVETQRKETEIVQLQIRKQEESLKRRSFHIENKLKDLLAEKEKFEEERLREQQEIELQKKR
QEEETFLRVQEELQRLKELNNNEKAEKFQIFQELDQLQKEKDEQYAKLELEKKRLEEQEKEQVMLVAHLEEQLREKQEMIQLLRRGE
VQWVEEEKRDLEGIRESLLRVKEARAGGDEDGEELEKAQLRFFEFKRRQLVKLVNLEKDLVQQKDILKKEVQEEQEILECLKCEHDK
ESRLLEKHDESVTDVTEVPQDFEKIKPVEYRLQYKERQLQYLLQNHLPTLLEEKQRAFEILDRGPLSLDNTLYQVEKEMEEKEEQLA
QYQANANQLQKLQATFEFTANIARQEEKVRKKEKEILESREKQQREALERALARLERRHSALQRHSTLGMEIEEQRQKLASLNSGSR
EQSGLQASLEAEQEALEKDQERLEYEIQQLKQKIYEVDGVQKDHHGTLEGKVASSSLPVSAEKSHLVPLMDARINAYIEEEVQRRLQ
DLHRVISEGCSTSADTMKDNEKLHNGTIQRKLKYERMVSRSLGANPDDLKDPIKISIPRYVLCGQGKDAHFEFEVKITVLDETWTVF
RRYSRFREMHKTLKLKYAELAALEFPPKKLFGNKDERVIAERRSHLEKYLRDFFSVMLQSATSPLHINKVGLTLSKHTICEFSPFFK
KGVFDYSSHGTG
>sp|Q9NTX9|F217B_HUMAN
MNAGPSWNKVQHSKNSSGKRQSKSQVPHASSQPRSSLTAVTQPTEEKLKESISPEARRKRNPLGSRCQGASGNKLFLDFQSMKIIKE
NADEDSASDLSDSERIPIPPSPLTPPDLNLRAEEIDPVYFDLHPGQGHTKPEYYYPNFLPSPFSSWDLRDMALLLNAENKTEAVPRV
GGLLGKYIDRLIQLEWLQVQTVQCEKAKGGKARPPTAPGTSGALKSPGRSKLIASALSKPLPHQEGASKSGPSRKKAFHHEEIHPSH
YAFETSPRPIDVLGGTRFCSQRQTLEMRTEEKKKKSSKSTKLQRWDLSGSGSSSKVETSGHIRVPKQAAVILDSADSCKASKTQAHA
HPRKKGKAESCGHATVSSEKKLKTNGVKQNTYKLK
>sp|P42330|AK1C3_HUMAN
MDSKHQCVKLNDGHFMPVLGFGTYAPPEVPRSKALEVTKLAIEAGFRHIDSAHLYNNEEQVGLAIRSKIADGSVKREDIFYTSKLWS
TFHRPELVRPALENSLKKAQLDYVDLYLIHSPMSLKPGEELSPTDENGKVIFDIVDLCTTWEAMEKCKDAGLAKSIGVSNFNRRQLE
MILNKPGLKYKPVCNQVECHPYFNRSKLLDFCKSKDIVLVAYSALGSQRDKRWVDPNSPVLLEDPVLCALAKKHKRTPALIALRYQL
QRGVVVLAKSYNEQRIRQNVQVFEFQLTAEDMKAIDGLDRNLHYFNSDSFASHPNYPYSDEY
>sp|O94762|RECQ5_HUMAN

MSSHHTTFPFDPERRVRSTLKKVFGFDSFKTPLQESATMAVVKGNKDVFVCMPTGAGKSLCYQLPALLAKGITIVVSPLIALIQDQV
DHLLTLKVRVSSLNSKLSAQERKELLADLEREKPQTKILYITPEMAASSSFQPTLNSLVSRHLLSYLVVDEAHCVSQWGHDFRPDYL
RLGALRSRLGHAPCVALTATATPQVQEDVFAALHLKKPVAIFKTPCFRANLFYDVQFKELISDPYGNLKDFCLKALGQEADKGLSGC
GIVYCRTREACEQLAIELSCRGVNAKAYHAGLKASERTLVQNDWMEEKVPVIVATISFGMGVDKANVRFVAHWNIAKSMAGYYQESG
RAGRDGKPSWCRLYYSRNDRDQVSFLIRKEVAKLQEKRGNKASDKATIMAFDALVTFCEELGCRHAAIAKYFGDALPACAKGCDHCQ
NPTAVRRRLEALERSSSWSKTCIGPSQGNGFDPELYEGGRKGYGDFSRYDEGSGGSGDEGRDEAHKREWNLFYQKQMQLRKGKDPKI
EEFVPPDENCPLKEASSRRIPRLTVKAREHCLRLLEEALSSNRQSTRTADEADLRAKAVELEHETFRNAKVANLYKASVLKKVADIH
RASKDGQPYDMGGSAKSCSAQAEPPEPNEYDIPPASHVYSLKPKRVGAGFPKGSCPFQTATELMETTRIREQAPQPERGGEHEPPSR
PCGLLDEDGSEPLPGPRGEVPGGSAHYGGPSPEKKAKSSSGGSSLAKGRASKKQQLLATAAHKDSQSIARFFCRRVESPALLASAPE
AEGACPSCEGVQGPPMAPEKYTGEEDGAGGHSPAPPQTEECLRERPSTCPPRDQGTPEVQPTPAKDTWKGKRPRSQQENPESQPQKR
PRPSAKPSVVAEVKGSVSASEQGTLNPTAQDPFQLSAPGVSLKEAANVVVKCLTPFYKEGKFASKELFKGFARHLSHLLTQKTSPGR
SVKEEAQNLIRHFFHGRARCESEADWHGLCGPQR
>sp|Q8NI77|KI18A_HUMAN
MSVTEEDLCHHMKVVVRVRPENTKEKAAGFHKVVHVVDKHILVFDPKQEEVSFFHGKKTTNQNVIKKQNKDLKFVFDAVFDETSTQS
EVFEHTTKPILRSFLNGYNCTVLAYGATGAGKTHTMLGSADEPGVMYLTMLHLYKCMDEIKEEKICSTAVSYLEVYNEQIRDLLVNS
GPLAVREDTQKGVVVHGLTLHQPKSSEEILHLLDNGNKNRTQHPTDMNATSSRSHAVFQIYLRQQDKTASINQNVRIAKMSLIDLAG
SERASTSGAKGTRFVEGTNINRSLLALGNVINALADSKRKNQHIPYRNSKLTRLLKDSLGGNCQTIMIAAVSPSSVFYDDTYNTLKY
ANRAKDIKSSLKSNVLNVNNHITQYVKICNEQKAEILLLKEKLKAYEEQKAFTNENDQAKLMISNPQEKEIERFQEILNCLFQNREE
IRQEYLKLEMLLKENELKSFYQQQCHKQIEMMCSEDKVEKATGKRDHRLAMLKTRRSYLEKRREEELKQFDENTNWLHRVEKEMGLL
SQNGHIPKELKKDLHCHHLHLQNKDLKAQIRHMMDLACLQEQQHRQTEAVLNALLPTLRKQYCTLKEAGLSNAAFESDFKEIEHLVE
RKKVVVWADQTAEQPKQNDLPGISVLMTFPQLGPVQPIPCCSSSGGTNLVKIPTEKRTRRKLMPSPLKGQHTLKSPPSQSVQLNDSL
SKELQPIVYTPEDCRKAFQNPSTVTLMKPSSFTTSFQAISSNINSDNCLKMLCEVAIPHNRRKECGQEDLDSTFTICEDIKSSKCKL
PEQESLPNDNKDILQRLDPSSFSTKHSMPVPSMVPSYMAMTTAAKRKRKLTSSTSNSSLTADVNSGFAKRVRQDNSSEKHLQENKPT
MEHKRNICKINPSMVRKFGRNISKGNLR
>sp|Q6TGC4|PADI6_HUMAN
MVSVEGRAMSFQSIIHLSLDSPVHAVCVLGTEICLDLSGCAPQKCQCFTIHGSGRVLIDVANTVISEKEDATIWWPLSDPTYATVKM
TSPSPSVDADKVSVTYYGPNEDAPVGTAVLYLTGIEVSLEVDIYRNGQVEMSSDKQAKKKWIWGPSGWGAILLVNCNPADVGQQLED
KKTKKVIFSEEITNLSQMTLNVQGPSCILKKYRLVLHTSKEESKKARVYWPQKDNSSTFELVLGPDQHAYTLALLGNHLKETFYVEA
IAFPSAEFSGLISYSVSLVEESQDPSIPETVLYKDTVVFRVAPCVFIPCTQVPLEVYLCRELQLQGFVDTVTKLSEKSNSQVASVYE
DPNRLGRWLQDEMAFCYTQAPHKTTSLILDTPQAADLDEFPMKYSLSPGIGYMIQDTEDHKVASMDSIGNLMVSPPVKVQGKEYPLG
RVLIGSSFYPSAEGRAMSKTLRDFLYAQQVQAPVELYSDWLMTGHVDEFMCFIPTDDKNEGKKGFLLLLASPSACYKLFREKQKEGY
GDALLFDELRADQLLSNGREAKTIDQLLADESLKKQNEYVEKCIHLNRDILKTELGLVEQDIIEIPQLFCLEKLTNIPSDQQPKRSF
ARPYFPDLLRMIVMGKNLGIPKPFGPQIKGTCCLEEKICCLLEPLGFKCTFINDFDCYLTEVGDICACANIRRVPFAFKWWKMVP
>sp|Q9Y6C7|L3R2A_HUMAN
MAHHSLNTFYIWHNNVLHTHLVFFLPHLLNQPFSRGSFLIWLLLCWNSWYHLRTLRRQANQANKLSMMLLRVKQSPGTKLCHGDSEL
TSGLLAT
>sp|Q969E1|LEAP2_HUMAN
MWHLKLCAVLMIFLLLLGQIDGSPIPEVSSAKRRPRRMTPFWRGVSLRPIGASCRDDSECITRLCRKRRCSLSVAQE
>sp|O00470|MEIS1_HUMAN
MAQRYDDLPHYGGMDGVGIPSTMYGDPHAARSMQPVHHLNHGPPLHSHQYPHTAHTNAMAPSMGSSVNDALKRDKDAIYGHPLFPLL
ALIFEKCELATCTPREPGVAGGDVCSSESFNEDIAVFAKQIRAEKPLFSSNPELDNLMIQAIQVLRFHLLELEKVHELCDNFCHRYI
SCLKGKMPIDLVIDDREGGSKSDSEDITRSANLTDQPSWNRDHDDTASTRSGGTPGPSSGGHTSHSGDNSSEQGDGLDNSVASPSTG
DDDDPDKDKKRHKKRGIFPKVATNIMRAWLFQHLTHPYPSEEQKKQLAQDTGLTILQVNNWFINARRRIVQPMIDQSNRAVSQGTPY
NPDGQPMGGFVMDGQQHMGIRAPGPMSGMGMNMGMEGQWHYM

>sp|Q8NDY8|TMM52_HUMAN
MARGPLAARGLRLLLPLLPLLPLLPLPQVALGFADGSCDPSDQCPPQARWSSLWHVGLILLAVLLLLLCGVTAGCVRFCCLRKQAQA
QPHLPPARQPCDVAVIPMDSDSPVHSTVTSYSSVQYPLGMRLPLPFGELDLDSMAPPAYSLYTPEPPPSYDEAVKMAKPREEGPALS
QKPSPLLGASGLETTPVPQESGPNTQLPPCSPGAP
>sp|P11387|TOP1_HUMAN
MSGDHLHNDSQIEADFRLNDSHKHKDKHKDREHRHKEHKKEKDREKSKHSNSEHKDSEKKHKEKEKTKHKDGSSEKHKDKHKDRDKE
KRKEEKVRASGDAKIKKEKENGFSSPPQIKDEPEDDGYFVPPKEDIKPLKRPRDEDDADYKPKKIKTEDTKKEKKRKLEEEEDGKLK
KPKNKDKDKKVPEPDNKKKKPKKEEEQKWKWWEEERYPEGIKWKFLEHKGPVFAPPYEPLPENVKFYYDGKVMKLSPKAEEVATFFA
KMLDHEYTTKEIFRKNFFKDWRKEMTNEEKNIITNLSKCDFTQMSQYFKAQTEARKQMSKEEKLKIKEENEKLLKEYGFCIMDNHKE
RIANFKIEPPGLFRGRGNHPKMGMLKRRIMPEDIIINCSKDAKVPSPPPGHKWKEVRHDNKVTWLVSWTENIQGSIKYIMLNPSSRI
KGEKDWQKYETARRLKKCVDKIRNQYREDWKSKEMKVRQRAVALYFIDKLALRAGNEKEEGETADTVGCCSLRVEHINLHPELDGQE
YVVEFDFLGKDSIRYYNKVPVEKRVFKNLQLFMENKQPEDDLFDRLNTGILNKHLQDLMEGLTAKVFRTYNASITLQQQLKELTAPD
ENIPAKILSYNRANRAVAILCNHQRAPPKTFEKSMMNLQTKIDAKKEQLADARRDLKSAKADAKVMKDAKTKKVVESKKKAVQRLEE
QLMKLEVQATDREENKQIALGTSKLNYLDPRITVAWCKKWGVPIEKIYNKTQREKFAWAIDMADEDYEF
>sp|Q9BZ71|PITM3_HUMAN
MAKAGRAGGPPPGGGAPWHLRNVLSDSVESSDDEFFDAREEMAEGKNAILIGMSQWNSNDLVEQIETMGKLDEHQGEGTAPCTSSIL
QEKQRELYRVSLRRQRFPAQGSIEIHEDSEEGCPQRSCKTHVLLLVLHGGNILDTGAGDPSCKAADIHTFSSVLEKVTRAHFPAALG
HILIKFVPCPAICSEAFSLVSHLNPYSHDEGCLSSSQDHVPLAALPLLAISSPQYQDAVATVIERANQVYREFLKSSDGIGFSGQVC
LIGDCVGGLLAFDAICYSAGPSGDSPASSSRKGSISSTQDTPVAVEEDCSLASSKRLSKSNIDISSGLEDEEPKRPLPRKQSDSSTY
DCEAITQHHAFLSSIHSSVLKDESETPAAGGPQLPEVSLGRFDFDVSDFFLFGSPLGLVLAMRRTVLPGLDGFQVRPACSQVYSFFH
CADPSASRLEPLLEPKFHLVPPVSVPRYQRFPLGDGQSLLLADALHTHSPLFLEGSSRDSPPLLDAPASPPQASRFQRPGRRMSEGS
SHSESSESSDSMAPVGASRITAKWWGSKRIDYALYCPDVLTAFPTVALPHLFHASYWESTDVVAFILRQVMRYESVNIKESARLDPA
ALSPANPREKWLRKRTQVKLRNVTANHRANDVIAAEDGPQVLVGRFMYGPLDMVALTGEKVDILVMAEPSSGRWVHLDTEITNSSGR
ITYNVPRPRRLGVGVYPVKMVVRGDQTCAMSYLTVLPRGMECVVFSIDGSFAASVSIMGSDPKVRPGAVDVVRHWQDLGYMILYITG
RPDMQKQRVVSWLSQHNFPQGMIFFSDGLVHDPLRQKAIFLRNLMQECFIKISAAYGSTKDISVYSVLGLPASQIFIVGRPTKKYQT
QCQFLSEGYAAHLAALEASHRSRPKKNNSRMILRKGSFGLHAQPEFLRKRNHLRRTMSVQQPDPPAANPKPERAQSQPESDKDHERP
LPALSWARGPPKFESVP
>sp|Q9H1E5|TMX4_HUMAN
MAGGRCGPQLTALLAAWIAAVAATAGPEEAALPPEQSRVQPMTASNWTLVMEGEWMLKFYAPWCPSCQQTDSEWEAFAKNGEILQIS
VGKVDVIQEPGLSGRFFVTTLPAFFHAKDGIFRRYRGPGIFEDLQNYILEKKWQSVEPLTGWKSPASLTMSGMAGLFSISGKIWHLH
NYFTVTLGIPAWCSYVFFVIATLVFGLFMGLVLVVISECFYVPLPRHLSERSEQNRRSEEAHRAEQLQDAEEEKDDSNEEENKDSLV
DDEEEKEDLGDEDEAEEEEEEDNLAAGVDEERSEANDQGPPGEDGVTREEVEPEEAEEGISEQPCPADTEVVEDSLRQRKSQHADKG
L
>sp|Q9P2K3|RCOR3_HUMAN
MRVGAEYQARIPEFDPGATKYTDKDNGGMLVWSPYHSIPDAKLDEYIAIAKEKHGYNVEQALGMLFWHKHNIEKSLADLPNFTPFPD
EWTVEDKVLFEQAFSFHGKSFHRIQQMLPDKTIASLVKYYYSWKKTRSRTSLMDRQARKLANRHNQGDSDDDVEETHPMDGNDSDYD
PKKEAKKEGNTEQPVQTSKIGLGRREYQSLQHRHHSQRSKCRPPKGMYLTQEDVVAVSCSPNAANTILRQLDMELISLKRQVQNAKQ
VNSALKQKMEGGIEEFKPPESNQKINARWTTEEQLLAVQGVRKYGKDFQAIADVIGNKTVGQVKNFFVNYRRRFNLEEVLQEWEAEQ
GTQASNGDASTLGEETKSASNVPSGKSTDEEEEAQTPQAPRTLGPSPPAPSSTPTPTAPIATLNQPPPLLRPTLPAAPALHRQPPPL
QQQARFIQPRPTLNQPPPPLIRPANSMPPRLNPRPVLSTVGGQQPPSLIGIQTDSQSSLH
>sp|Q8IWZ3|ANKH1_HUMAN
MLTDSGGGGTSFEEDLDSVAPRSAPAGASEPPPPGGVGLGIRTVRLFGEAGPASGVGSSGGGGSGSGTGGGDAALDFKLAAAVLRTG
GGGGASGSDEDEVSEVESFILDQEDLDNPVLKTTSEIFLSSTAEGADLRTVDPETQARLEALLEAAGIGKLSTADGKAFADPEVLRR
LTSSVSCALDEAAAALTRMKAENSHNAGQVDTRSLAEACSDGDVNAVRKLLDEGRSVNEHTEEGESLLCLACSAGYYELAQVLLAMH

ANVEDRGNKGDITPLMAASSGGYLDIVKLLLLHDADVNSQSATGNTALTYACAGGFVDIVKVLLNEGANIEDHNENGHTPLMEAASA
GHVEVARVLLDHGAGINTHSNEFKESALTLACYKGHLDMVRFLLEAGADQEHKTDEMHTALMEACMDGHVEVARLLLDSGAQVNMPA
DSFESPLTLAACGGHVELAALLIERGANLEEVNDEGYTPLMEAAREGHEEMVALLLAQGANINAQTEETQETALTLACCGGFSEVAD
FLIKAGADIELGCSTPLMEASQEGHLELVKYLLASGANVHATTATGDTALTYACENGHTDVADVLLQAGADLEHESEGGRTPLMKAA
RAGHLCTVQFLISKGANVNRATANNDHTVVSLACAGGHLAVVELLLAHGADPTHRLKDGSTMLIEAAKGGHTNVVSYLLDYPNNVLS
VPTTDVSQLPPPSQDQSQVPRVPTHTLAMVVPPQEPDRTSQENSPALLGVQKGTSKQKSSSLQVADQDLLPSFHPYQPLECIVEETE
GKLNELGQRISAIEKAQLKSLELIQGEPLNKDKIEELKKNREEQVQKKKKILKELQKVERQLQMKTQQQFTKEYLETKGQKDTVSLH
QQCSHRGVFPEGEGDGSLPEDHFSELPQVDTILFKDNDVDDEQQSPPSAEQIDFVPVQPLSSPQCNFSSDLGSNGTNSLELQKVSGN
QQIVGQPQIAITGHDQGLLVQEPDGLMVATPAQTLTDTLDDLIAAVSTRVPTGSNSSSQTTECLTPESCSQTTSNVASQSMPPVYPS
VDIDAHTESNHDTALTLACAGGHEELVSVLIARDAKIEHRDKKGFTPLILAATAGHVGVVEILLDKGGDIEAQSERTKDTPLSLACS
GGRQEVVDLLLARGANKEHRNVSDYTPLSLAASGGYVNIIKILLNAGAEINSRTGSKLGISPLMLAAMNGHVPAVKLLLDMGSDINA
QIETNRNTALTLACFQGRAEVVSLLLDRKANVEHRAKTGLTPLMEAASGGYAEVGRVLLDKGADVNAPPVPSSRDTALTIAADKGHY
KFCELLIHRGAHIDVRNKKGNTPLWLASNGGHFDVVQLLVQAGADVDAADNRKITPLMSAFRKGHVKVVQYLVKEVNQFPSDIECMR
YIATITDKELLKKCHQCVETIVKAKDQQAAEANKNASILLKELDLEKSREESRKQALAAKREKRKEKRKKKKEEQKRKQEEDEENKP
KENSELPEDEDEEENDEDVEQEVPIEPPSATTTTTIGISATSATFTNVFGKKRANVVTTPSTNRKNKKNKTKETPPTAHLILPEQHM
SLAQQKADKNKINGEPRGGGAGGNSDSDNLDSTDCNSESSSGGKSQELNFVMDVNSSKYPSLLLHSQEEKTSTATSKTQTRLEGEVT
PNSLSTSYKTVSLPLSSPNIKLNLTSPKRGQKREEGWKEVVRRSKKLSVPASVVSRIMGRGGCNITAIQDVTGAHIDVDKQKDKNGE
RMITIRGGTESTRYAVQLINALIQDPAKELEDLIPKNHIRTPASTKSIHANFSSGVGTTAASSKNAFPLGAPTLVTSQATTLSTFQP
ANKLNKNVPTNVRSSFPVSLPLAYPHPHFALLAAQTMQQIRHPRLPMAQFGGTFSPSPNTWGPFPVRPVNPGNTNSSPKHNNTSRLP
NQNGTVLPSESAGLATASCPITVSSVVAASQQLCVTNTRTPSSVRKQLFACVPKTSPPATVISSVTSTCSSLPSVSSAPITSGQAPT
TFLPASTSQAQLSSQKMESFSAVPPTKEKVSTQDQPMANLCTPSSTANSCSSSASNTPGAPETHPSSSPTPTSSNTQEEAQPSSVSD
LSPMSMPFASNSEPAPLTLTSPRMVAADNQDTSNLPQLAVPAPRVSHRMQPRGSFYSMVPNATIHQDPQSIFVTNPVTLTPPQGPPA
AVQLSSAVNIMNGSQMHINPANKSLPPTFGPATLFNHFSSLFDSSQVPANQGWGDGPLSSRVATDASFTVQSAFLGNSVLGHLENMH
PDNSKAPGFRPPSQRVSTSPVGLPSIDPSGSSPSSSSAPLASFSGIPGTRVFLQGPAPVGTPSFNRQHFSPHPWTSASNSSTSAPPT
LGQPKGVSASQDRKIPPPIGTERLARIRQGGSVAQAPAGTSFVAPVGHSGIWSFGVNAVSEGLSGWSQSVMGNHPMHQQLSDPSTFS
QHQPMERDDSGMVAPSNIFHQPMASGFVDFSKGLPISMYGGTIIPSHPQLADVPGGPLFNGLHNPDPAWNPMIKVIQNSTECTDAQQ
IWPGTWAPHIGNMHLKYVN
>sp|Q15782|CH3L2_HUMAN
MGATTMDQKSLWAGVVVLLLLQGGSAYKLVCYFTNWSQDRQEPGKFTPENIDPFLCSHLIYSFASIENNKVIIKDKSEVMLYQTINS
LKTKNPKLKILLSIGGYLFGSKGFHPMVDSSTSRLEFINSIILFLRNHNFDGLDVSWIYPDQKENTHFTVLIHELAEAFQKDFTKST
KERLLLTAGVSAGRQMIDNSYQVEKLAKDLDFINLLSFDFHGSWEKPLITGHNSPLSKGWQDRGPSSYYNVEYAVGYWIHKGMPSEK
VVMGIPTYGHSFTLASAETTVGAPASGPGAAGPITESSGFLAYYEICQFLKGAKITRLQDQQVPYAVKGNQWVGYDDVKSMETKVQF
LKNLNLGGAMIWSIDMDDFTGKSCNQGPYPLVQAVKRSLGSL
>sp|O60279|SUSD5_HUMAN
MTAEGPSPPARWHRRLPGLWAAALLLLGLPRLSVRADGKFFVLESQNGSQGLQLEAARLSCKSRGAHLASADELRRVVQDCSFAVCT
TGWLADGTLGTTVCSKGSGEQQIMRAVDVRIESNPVPGGTYSALCIKDEEKPCGDPPSFPHTILQGRTGLEMGDELLYVCAPGHIMG
HRETAFTLLCNSCGEWYGLVQACGKDEAEAHIDYEDNFPDDRSVSFRELMEDSRTEADEDRGQGDSSEEAPKQDRLVSISVGRENIA
RDKVFVPTTGLPGAGSSVPADSPGSRLLQKHLFWFPAEAFHKPGLEKEVDDDTKKQFSAGDNHSGVKLVPGEPETKVIYGNTDGPSG
PFVGKNDSKAGDPVVSSSDESWLDGYPVTEGAWRKTEAEEEEDGDRGDGSVGLDENVLVTPDQPILVEVKKPKSSTLTPSEGMTHSS
VLPSQMLDVEALALRPVNASETEGIGDGDLTKYQSTLPWRFITEESPMATLSYELTSSTLEILTVNTVKQTPNHIPSTIMATTQPPV
ETTVPEIQDSFPYLLSEDFFGQEGPGPGASEELHPTLESCVGDGCPGLSRGPVIATIVTVLCLLLLLLAGVGMVWGYRKCQHKSSVYK
LNVGQRQARHYHQQIEMEKV
>sp|Q8TDN6|BRX1_HUMAN

MAATKRKRRGGFAVQAKKPKRNEIDAEPPAKRHATAEEVEEEERDRIPGPVCKGKWKNKERILIFSSRGINFRTRHLMQDLRMLMPH
SKADTKMDRKDKLFVINEVCEMKNCNKCIYFEAKKKQDLYMWLSNSPHGPSAKFLVQNIHTLAELKMTGNCLKGSRPLLSFDPAFDE
LPHYALLKELLIQIFSTPRYHPKSQPFVDHVFTFTILDNRIWFRNFQIIEEDAALVEIGPRFVLNLIKIFQGSFGGPTLYENPHYQS
PNMHRRVIRSITAAKYREKQQVKDVQKLRKKEPKTLLPHDPTADVFVTPAEEKPIEIQWVKPEPKVDLKARKKRIYKRQRKMKQRMD
SGKTK
>sp|Q92585|MAML1_HUMAN
MVLPTCPMAEFALPRHSAVMERLRRRIELCRRHHSTCEARYEAVSPERLELERQHTFALHQRCIQAKAKRAGKHRQPPAATAPAPAA
PAPRLDAADGPEHGRPATHLHDTVKRNLDSATSPQNGDQQNGYGDLFPGHKKTRREAPLGVAISSNGLPPASPLGQSDKPSGADALQ
SSGKHSLGLDSLNKKRLADSSLHLNGGSNPSESFPLSLNKELKQEPVEDLPCMITGTVGSISQSNLMPDLNLNEQEWKELIEELNRS
VPDEDMKDLFNEDFEEKKDPESSGSATQTPLAQDINIKTEFSPAAFEQEQLGSPQVRAGSAGQTFLGPSSAPVSTDSPSLGGSQTLF
HTSGQPRADNPSPNLMPASAQAQNAQRALAGVVLPSQGPGGASELSSAHQLQQIAAKQKREQMLQNPQQATPAPAPGQMSTWQQTGP
SHSSLDVPYPMEKPASPSSYKQDFTNSKLLMMPSVNKSSPRPGGPYLQPSHVNLLSHQPPSNLNQNSANNQGSVLDYGNTKPLSHYK
ADCGQGSPGSGQSKPALMAYLPQQLSHISHEQNSLFLMKPKPGNMPFRSLVPPGQEQNPSSVPVQAQATSVGTQPPAVSVASSHNSS
PYLSSQQQAAVMKQHQLLLDQQKQREQQQKHLQQQQFLQRQQHLLAEQEKQQFQRHLTRPPPQYQDPTQGSFPQQVGQFTGSSAAVP
GMNTLGPSNSSCPRVFPQAGNLMPMGPGHASVSSLPTNSGQQDRGVAQFPGSQNMPQSSLYGMASGITQIVAQPPPQATNGHAHIPR
QTNVGQNTSVSAAYGQNSLGSSGLSQQHNKGTLNPGLTKPPVPRVSPAMGGQNSSWQHQGMPNLSGQTPGNSNVSPFTAASSFHMQQ
QAHLKMSSPQFSQAVPNRPMAPMSSAAAVGSLLPPVSAQQRTSAPAPAPPPTAPQQGLPGLSPAGPELGAFSQSPASQMGGRAGLHC
TQAYPVRTAGQELPFAYSGQPGGSGLSSVAGHTDLIDSLLKNRTSEEWMSDLDDLLGSQ
>sp|A0PJX0|CIB4_HUMAN
MGQCLRYQMHWEDLEEYQALTFLTRNEILCIHDTFLKLCPPGKYYKEATLTMDQVSSLPALRVNPFRDRICRVFSHKGMFSFEDVLG
MASVFSEQACPSLKIEYAFRIYDFNENGFIDEEDLQRIILRLLNSDDMSEDLLMDLTNHVLSESDLDNDNMLSFSEFEHAMAKSPDF
MNSFRIHFWGC
>sp|Q9Y238|DLEC1_HUMAN
METRSSKTRRSLASRTNECQGTMWAPTSPPAGSSSPSQPTWKSSLYSSLAYSEAFHYSFAARPRRLTQLALAQRPEPQLLRLRPSSL
RTQDISHLLTGVFRNLYSAEVIGDEVSASLIKARGSENERHEEFVDQLQQIRELYKQRLDEFEMLERHITQAQARAIAENERVMSQA
GVQDLESLVRLPPVKSVSRWCIDSELLRKHHLISPEDYYTDTVPFHSAPKGISLPGCSKLTFSCEKRSVQKKELNKKLEDSCRKKLA
EFEDELDHTVDSLTWNLTPKAKERTREPLKKASQPRNKNWMNHLRVPQRELDRLLLARMESRNHFLKNPRFFPPNTRYGGKSLVFPP
KKPAPIGEFQSTEPEQSCADTPVFLAKPPIGFFTDYEIGPVYEMVIALQNTTTTSRYLRVLPPSTPYFALGLGMFPGKGGMVAPGMT
CQYIVQFFPDCLGDFDDFILVETQSAHTLLIPLQARRPPPVLTLSPVLDCGYCLIGGVKMTRFICKNVGFSVGRFCIMPKTSWPPLS
FKAIATVGFVEQPPFGILPSVFELAPGHAILVEVLFSPKSLGKAEQTFIIMCDNCQIKELVTIGIGQLIALDLIYISGEKSQPDPGE
LTDLTAQHFIRFEPENLRSTARKQLIIRNATHVELAFYWQIMKPNLQPLMPGETFSMDSIKCYPDKETAFSIMPRKGVLSPHTDHEF
ILSFSPHELRDFHSVLQMVLEEVPEPVSSEAESLGHSSYSVDDVIVLEIEVKGSVEPFQVLLEPYALIIPGENYIGINVKKAFKMWN
NSKSPIRYLWGKISDCHIIEVEPGTGVIEPSEVGDFELNFTGGVPGPTSQDLLCEIEDSPSPVVLHIEAVFKGPALIINVSALQFGL
LRLGQKATNSIQIRNVSQLPATWRMKESPVSLQERPEDVSPFDIEPSSGQLHSLGECRVDITLEALHCQHLETVLELEVENGAWSYL
PVYAEVQKPHVYLQSSQVEVRNLYLGVPTKTTITLINGTLLPTQFHWGKLLGHQAEFCMVTVSPKHGLLGPSEECQLKLELTAHTQE
ELTHLALPCHVSGMKKPLVLGISGKPQGLQVAITISKESSDCSTEQWPGHPKELRLDFGSAVPLRTRVTRQLILTNRSPIRTRFSLK
FEYFGSPQNSLSKKTSLPNMPPALLKTVRMQEHLAKREQLDFMESMLSHGKGAAFFPHFSQGMLGPYQQLCIDITGCANMWGEYWDN
LICTVGDLLPEVIPVHMAAVGCPISSLRTTSYTIDQAQKEPAMRFGTQVSGGDTVTRTLRLNNSSPCDIRLDWETYVPEDKEDRLVE
LLVFYGPPFPLRDQAGNELVCPDTPEGGCLLWSPGPSSSSEFSHETDSSVEGSSSASNRVAQKLISVILQAHEGVPSGHLYCISPKQ
VVVPAGGSSTIYISFTPMVLSPEILHKVECTGYALGFMSLDSKVEREIPGKRHRLQDFAVGPLKLDLHSYVRPAQLSVELDYGGSME
FQCQASDLIPEQPCSGVLSELVTTHHLKLTNTTEIPHYFRLMVSRPFSVSQDGASQDHRAPGPGQKQECEEETASADKQLVLQAQEN
MLVNVSFSLSLSLELLSYQKLPADQTLPGVDIQQSASGEREMVFTQNLLLEYTNQTTQVVPLRAVVAVPELQLSTSWVDFGTCFVSQQR
VREVYLMNLSGCRSYWTMLMGQQEPAKAAVAFRVSPNSGLLEARSANAPPTSIALQVFFTARSSELYESTMVVEGVLGEKSCTLRLR
GQGSYDERYMLPHQP

>sp|Q9H9S0|NANOG_HUMAN
MSVDPACPQSLPCFEASDCKESSPMPVICGPEENYPSLQMSSAEMPHTETVSPLPSSMDLLIQDSPDSSTSPKGKQPTSAEKSVAKK
EDKVPVKKQKTRTVFSSTQLCVLNDRFQRQKYLSLQQMQELSNILNLSYKQVKTWFQNQRMKSKRWQKNNWPKNSNGVTQKASAPTY
PSLYSSYHQGCLVNPTGNLPMWSNQTWNNSTWSNQTQNIQSWSNHSWNTQTWCTQSWNNQAWNSPFYNCGEESLQSCMQFQPNSPAS
DLEAALEAAGEGLNVIQQTTRYFSTPQTMDLFLNYSMNMQPEDV
>sp|Q9UIG8|SO3A1_HUMAN
MQGKKPGGSSGGGRSGELQGDEAQRNKKKKKKVSCFSNIKIFLVSECALMLAQGTVGAYLVSVLTTLERRFNLQSADVGVIASSFEI
GNLALILFVSYFGARGHRPRLIGCGGIVMALGALLSALPEFLTHQYKYEAGEIRWGAEGRDVCAANGSGGDEGPDPDLICRNRTATN
MMYLLLIGAQVLLGIGATPVQPLGVSYIDDHVRRKDSSLYIGILFTMLVFGPACGFILGSFCTKIYVDAVFIDTSNLDITPDDPRWI
GAWWGGFLLCGALLFFSSLLMFGFPQSLPPHSEPAMESEQAMLSEREYERPKPSNGVLRHPLEPDSSASCFQQLRVIPKVTKHLLSN
PVFTCIILAACMEIAVVAGFAAFLGKYLEQQFNLTTSSANQLLGMTAIPCACLGIFLGGLLVKKLSLSALGAIRMAMLVNLVSTACY
VSFLFLGCDTGPVAGVTVPYGNSTAPGSALDPYSPCNNNCECQTDSFTPVCGADGITYLSACFAGCNSTNLTGCACLTTVPAENATV
VPGKCPSPGCQEAFLTFLCVMCICSLIGAMAQTPSVIILIRTVSPELKSYALGVLFLLLRLLGFIPPPLIFGAGIDSTCLFWSTFCG
EQGACVLYDNVVYRYLYVSIAIALKSFAFILYTTTWQCLRKNYKRYIKNHEGGLSTSEFFASTLTLDNLGRDPVPANQTHRTKFIYN
LEDHEWCENMESVL
>sp|A6NKL6|T200C_HUMAN
MIATGGLLRISARKQDPLRPPSQIPKRKRKAKKRRKNDVVVVKGKLKLCSISGLIALCGILVLLVGIAMAVVGYWPKATGTNREGGK
QLPPAGSSHRVPTTANSSSSGSKNRSRSHPRAPGGVNSSSAGAPRSTPPARAASPSSSSTSVGFFFRIFSGYLHSDKLKVFGPLIMG
IGIFLFICANAVLHENRDKKTKIINLRDLYSTVIDVHSLRAKDLAAAAAAAAAAAASSSSSAPAAAPPGAIPLNGFLSYVQSRGLEL
KPGGCGGSGDAFGAAAMLAKGSWPPHPAAPSGGRPRGAASPPDLASSPRCPREPPSLAEAVYSVYRERSGVAGSRRAAAATAAAAAS
SCSSPAPCSPPESWGRQSTASSFVDSSLSAFALLPLQGGRDRGGDAEGASCSWQRPPGERGSQEIPRGELDLSMTNLRGAEGSMRGA
RREPEEPEGAVAARAARGQGGRLPRTGRYAALRRRSTSGLPDYRAPPSPEPPPSPGSADPDSSPLAKAASPSPPLRLEGSPPTRRDS
GSSQSDDPSSSNKGYTPLREAGTSTESVLDAVAGQTRDSAVAAPVLGAEQSSPEGASQEPPTAEQPQPVQRQFTNKEKLIMISRSHA
IGVEEELESTGI
>sp|Q9UNT1|RBL2B_HUMAN
MAEDKTKPSELDQGKYDADDNVKIICLGDSAVGKSKLMERFLMDGFQPQQLSTYALTLYKHTATVDGRTILVDFWDTAGQERFQSMH
ASYYHKAHACIMVFDVQRKVTYRNLSTWYTELREFRPEIPCIVVANKIDDINVTQKSFNFAKKFSLPLYFVSAADGTNVVKLFNDAI
RLAVSYKQNSQDFMDEIFQELENFSLEQEEEDVPDQEQSSSIETPSEEAASPHS
>sp|Q3LI83|KR241_HUMAN
MPAGSMSTTGYPGVCSTTSYRTHCYIPVTSSVTLSSSDLSPTFGHCLPSSYQGNLWLLDYCQESYGEAPTCKSPSCEPKTCSTTGCD
PSNSSVPCNSPSAGQVFSVCETTNVSPSPSCSPSTQTNGYVCNCHIPTRNASKACQTLRNGSNCFGQLNCLSKSFQTLNHCRLSTLG
YKSYQNPCFIPSYVSPLCYISNSCQPQSYLVRNYHYSSYRPTSCRPLSYLSRSFRSLSYIPSTFPPLRYLCSGSRPLKCY
>sp|O95395|GCNT3_HUMAN
MVQWKRLCQLHYLWALGCYMLLATVALKLSFRLKCDSDHLGLESRESQSQYCRNILYNFLKLPAKRSINCSGVTRGDQEAVLQAILN
NLEVKKKREPFTDTHYLSLTRDCEHFKAERKFIQFPLSKEEVEFPIAYSMVIHEKIENFERLLRAVYAPQNIYCVHVDEKSPETFKE
AVKAIISCFPNVFIASKLVRVVYASWSRVQADLNCMEDLLQSSVPWKYFLNTCGTDFPIKSNAEMVQALKMLNGRNSMESEVPPKHK
ETRWKYHFEVVRDTLHLTNKKKDPPPYNLTMFTGNAYIVASRDFVQHVLKNPKSQQLIEWVKDTYSPDEHLWATLQRARWMPGSVPN
HPKYDISDMTSIARLVKWQGHEGDIDKGAPYAPCSGIHQRAICVYGAGDLNWMLQNHHLLANKFDPKVDDNALQCLEEYLRYKAIYG
TEL
>sp|Q8N2R8|FA43A_HUMAN
MLPWKKHKFELLAEAPPRQASKPKGYAVSLHYSALSSLARACPEGALSRVGSMFRSKRKKLHITSEDPTYTVLYLGNATTIQARGDG
CTDLAVGKIWSKSEAGRQGTKMKLTVSAQGIRMVHAEERALRRPGHLYLLHRVTYCVADARLPKVFAWVYRHELKHKAVMLRCHAVL
VSKPEKAQAMALLLYQTSANALAEFKRLKRRDDARHQQQELVGAHTIPLVPLRKLLLHGPCCYKPPVERSRSAPKLGSITEDLLGEQ

LEQELQEEEEEEQPEGCPEEEENRAAEGDPAEEEAEAQRALVVAMHFECGDLLDTLENGRGEALGGGGGSLGPGAGPPPLLLGSASD
MKAELSQLISDLGELSFGNDVRTLQADLRVTRLLSGDSTGSESSIEGGGPDATSATAGDSSRQADGASADEPHSG
>sp|Q66LE6|2ABD_HUMAN
MAGAGGGGCPAGGNDFQWCFSQVKGAIDEDVAEADIISTVEFNYSGDLLATGDKGGRVVIFQREQENKSRPHSRGEYNVYSTFQSHE
PEFDYLKSLEIEEKINKIRWLPQQNAAHFLLSTNDKTIKLWKISERDKRAEGYNLKDEDGRLRDPFRITALRVPILKPMDLMVEASP
RRIFANAHTYHINSISVNSDHETYLSADDLRINLWHLEITDRSFNIVDIKPANMEELTEVITAAEFHPHQCNVFVYSSSKGTIRLCD
MRSSALCDRHSKFFEEPEDPSSRSFFSEIISSISDVKFSHSGRYMMTRDYLSVKVWDLNMESRPVETHQVHEYLRSKLCSLYENDCI
FDKFECCWNGSDSAIMTGSYNNFFRMFDRDTRRDVTLEASRESSKPRASLKPRKVCTGGKRRKDEISVDSLDFNKKILHTAWHPVDN
VIAVAATNNLYIFQDKIN
>sp|Q659C4|LAR1B_HUMAN
MENWPTPSELVNTGFQSVLSQGNKKPQNRKEKEEKVEKRSNSDSKENRETKLNGPGENVSEDEAQSSNQRKRANKHKWVPLHLDVVR
SESQERPGSRNSSRCQPEANKPTHNNRRNDTRSWKRDREKRDDQDDVSSVRSEGGNIRGSFRGRGRGRGRGRGRGRGNPRLNFDYSY
GYQEHGERTDQPFQTELNTSMMYYYDDGTGVQVYPVEEALLKEYIKRQIEYYFSVENLERDFFLRGKMDEQGFLPISLIAGFQRVQA
LTTNLNLILEALKDSTEVEIVDEKMRKKIEPEKWPIPGPPPRSVPPTDFSQLIDCPEFVPGQAFCSHTESAPNSPRIGSPLSPKKNS
ETSILQAMSRGLSTSLPDLDSEPWIEVKKRHQPAPVKLRESVSVPEGSLNQLCSSEEPEQEELDFLFDEEIEQIGRKNTFTDWSDND
SDYEIDDQDLNKILIVTQTPPYVKKHPGGDRTGTHMSRAKITSELAKVINDGLYYYEQDLWMEEDENKHTAIKQEVENFKKLNLISK
EQFENLTPELPFEPNQEVPVAPSQSRQGGVQGVLHIPKKDLTDELAQKLFDVSEITSAAMVHSLPTAVPESPRIHPTRTPKTPRTPR
LQDPNKTPRFYPVVKEPKAIDVKSPRKRKTRHSTNPPLECHVGWVMDSRDRGPGTSSVSTSNASPSEGAPLAGSYGCTPHSFPKFQH
PSHELLKENGFTQQVYHKYRRRCLSERKRLGIGQSQEMNTLFRFWSFFLRDHFNKKMYEEFRQLAWEDAKENYRYGLECLFRFYSYG
LEKKFRREIFQDFQEETKKDYESGQLYGLEKFWAYLKYSQSKTQSIDPKLQEYLCSFKRLEDFRVDPPISDEFGRKRHSSTSGEESN
RHRLPPNSSTKPPNAAKPTSTSELQVPINSPRRNISPESSDNSH
>sp|Q99755|PI51A_HUMAN
MASASSGPSSSVGFSSFDPAVPSCTLSSAASGIKRPMASEVLEARQDSYISLVPYASGMPIKKIGHRSVDSSGETTYKKTTSSALKG
AIQLGITHTVGSLSTKPERDVLMQDFYVVESIFFPSEGSNLTPAHHYNDFRFKTYAPVAFRYFRELFGIRPDDYLYSLCSEPLIELC
SSGASGSLFYVSSDDEFIIKTVQHKEAEFLQKLLPGYYMNLNQNPRTLLPKFYGLYCVQAGGKNIRIVVMNNLLPRSVKMHIKYDLK
GSTYKRRASQKEREKPLPTFKDLDFLQDIPDGLFLDADMYNALCKTLQRDCLVLQSFKIMDYSLLMSIHNIDHAQREPLSSETQYSV
DTRRPAPQKALYSTAMESIQGEARRGGTMETDDHMGGIPARNSKGERLLLYIGIIDILQSYRFVKKLEHSWKALVHDGDTVSVHRPG
FYAERFQRFMCNTVFKKIPLKPSPSKKFRSGSSFSRRAGSSGNSCITYQPSVSGEHKAQVTTKAEVEPGVHLGRPDVLPQTPPLEEI
SEGSPIPDPSFSPLVGETLQMLTTSTTLEKLEVAESEFTH
>sp|P63261|ACTG_HUMAN
MEEEIAALVIDNGSGMCKAGFAGDDAPRAVFPSIVGRPRHQGVMVGMGQKDSYVGDEAQSKRGILTLKYPIEHGIVTNWDDMEKIWH
HTFYNELRVAPEEHPVLLTEAPLNPKANREKMTQIMFETFNTPAMYVAIQAVLSLYASGRTTGIVMDSGDGVTHTVPIYEGYALPHA
ILRLDLAGRDLTDYLMKILTERGYSFTTTAEREIVRDIKEKLCYVALDFEQEMATAASSSSLEKSYELPDGQVITIGNERFRCPEAL
FQPSFLGMESCGIHETTFNSIMKCDVDIRKDLYANTVLSGGTTMYPGIADRMQKEITALAPSTMKIKIIAPPERKYSVWIGGSILAS
LSTFQQMWISKQEYDESGPSIVHRKCF
>sp|P02766|TTHY_HUMAN
MASHRLLLLCLAGLVFVSEAGPTGTGESKCPLMVKVLDAVRGSPAINVAVHVFRKAADDTWEPFASGKTSESGELHGLTTEEEFVEG
IYKVEIDTKSYWKALGISPFHEHAEVVFTANDSGPRRYTIAALLSPYSYSTTAVVTNPKE
>sp|O95081|AGFG2_HUMAN
MVMAAKKGPGPGGGVSGGKAEAEAASEVWCRRVRELGGCSQAGNRHCFECAQRGVTYVDITVGSFVCTTCSGLLRGLNPPHRVKSIS
MTTFTEPEVVFLQSRGNEVCRKIWLGLFDARTSLVPDSRDPQKVKEFLQEKYEKKRWYVPPDQVKGPTYTKGSASTPVQGSIPEGKP
LRTLLGDPAPSLSVAASTSSQPVSQSHARTSQARSTQPPPHSSVKKASTDLLADIGGDPFAAPQMAPAFAAFPAFGGQTPSQGGFAN
FDAFSSGPSSSVFGSLPPAGQASFQAQPTPAGSSQGTPFGATPLAPASQPNSLADVGSFLGPGVPAAGVPSSLFGMAGQVPPLQSVT

MGGGGGSSTGLAFGAFTNPFTAPAAQSPLPSTNPFQPNGLAPGPGFGMSSAGPGFPQAVPPTGAFASSFPAPLFPPQTPLVQQQNGS
SFGDLGSAKLGQRPLSQPAGISTNPFMTGPSSSPFASKPPTTNPFL
>sp|Q8IW92|GLBL2_HUMAN
MTTWSLRRRPARTLGLLLLVVLGFLVLRRLDWSTLVPLRLRHRQLGLQAKGWNFMLEDSTFWIFGGSIHYFRVPREYWRDRLLKMKA
CGLNTLTTYVPWNLHEPERGKFDFSGNLDLEAFVLMAAEIGLWVILRPGPYICSEMDLGGLPSWLLQDPGMRLRTTYKGFTEAVDLY
FDHLMSRVVPLQYKRGGPIIAVQVENEYGSYNKDPAYMPYVKKALEDRGIVELLLTSDNKDGLSKGIVQGVLATINLQSTHELQLLT
TFLFNVQGTQPKMVMEYWTGWFDSWGGPHNILDSSEVLKTVSAIVDAGSSINLYMFHGGTNFGFMNGAMHFHDYKSDVTSYDYDAVL
TEAGDYTAKYMKLRDFFGSISGIPLPPPPDLLPKMPYEPLTPVLYLSLWDALKYLGEPIKSEKPINMENLPVNGGNGQSFGYILYET
SITSSGILSGHVHDRGQVFVNTVSIGFLDYKTTKIAVPLIQGYTVLRILVENRGRVNYGENIDDQRKGLIGNLYLNDSPLKNFRIYS
LDMKKSFFQRFGLDKWSSLPETPTLPAFFLGSLSISSTPCDTFLKLEGWEKGVVFINGQNLGRYWNIGPQKTLYLPGPWLSSGINQV
IVFEETMAGPALQFTETPHLGRNQYIK
>sp|O15537|XLRS1_HUMAN
MSRKIEGFLLLLLFGYEATLGLSSTEDEGEDPWYQKACKCDCQGGPNALWSAGATSLDCIPECPYHKPLGFESGEVTPDQITCSNPE
QYVGWYSSWTANKARLNSQGFGCAWLSKFQDSSQWLQIDLKEIKVISGILTQGRCDIDEWMTKYSVQYRTDERLNWIYYKDQTGNNR
VFYGNSDRTSTVQNLLRPPIISRFIRLIPLGWHVRIAIRMELLECVSKCA
>sp|O60449|LY75_HUMAN
MRTGWATPRRPAGLLMLLFWFFDLAEPSGRAANDPFTIVHGNTGKCIKPVYGWIVADDCDETEDKLWKWVSQHRLFHLHSQKCLGLD
ITKSVNELRMFSCDSSAMLWWKCEHHSLYGAARYRLALKDGHGTAISNASDVWKKGGSEESLCDQPYHEIYTRDGNSYGRPCEFPFL
IDGTWHHDCILDEDHSGPWCATTLNYEYDRKWGICLKPENGCEDNWEKNEQFGSCYQFNTQTALSWKEAYVSCQNQGADLLSINSAA
ELTYLKEKEGIAKIFWIGLNQLYSARGWEWSDHKPLNFLNWDPDRPSAPTIGGSSCARMDAESGLWQSFSCEAQLPYVCRKPLNNTV
ELTDVWTYSDTRCDAGWLPNNGFCYLLVNESNSWDKAHAKCKAFSSDLISIHSLADVEVVVTKLHNEDIKEEVWIGLKNINIPTLFQ
WSDGTEVTLTYWDENEPNVPYNKTPNCVSYLGELGQWKVQSCEEKLKYVCKRKGEKLNDASSDKMCPPDEGWKRHGETCYKIYEDEV
PFGTNCNLTITSRFEQEYLNDLMKKYDKSLRKYFWTGLRDVDSCGEYNWATVGGRRRAVTFSNWNFLEPASPGGCVAMSTGKSVGKW
EVKDCRSFKALSICKKMSGPLGPEEASPKPDDPCPEGWQSFPASLSCYKVFHAERIVRKRNWEEAERFCQALGAHLSSFSHVDEIKE
FLHFLTDQFSGQHWLWIGLNKRSPDLQGSWQWSDRTPVSTIIMPNEFQQDYDIRDCAAVKVFHRPWRRGWHFYDDREFIYLRPFACD
TKLEWVCQIPKGRTPKTPDWYNPDRAGIHGPPLIIEGSEYWFVADLHLNYEEAVLYCASNHSFLATITSFVGLKAIKNKIANISGDG
QKWWIRISEWPIDDHFTYSRYPWHRFPVTFGEECLYMSAKTWLIDLGKPTDCSTKLPFICEKYNVSSLEKYSPDSAAKVQCSEQWIP
FQNKCFLKIKPVSLTFSQASDTCHSYGGTLPSVLSQIEQDFITSLLPDMEATLWIGLRWTAYEKINKWTDNRELTYSNFHPLLVSGR
LRIPENFFEEESRYHCALILNLQKSPFTGTWNFTSCSERHFVSLCQKYSEVKSRQTLQNASETVKYLNNLYKIIPKTLTWHSAKREC
LKSNMQLVSITDPYQQAFLSVQALLHNSSLWIGLFSQDDELNFGWSDGKRLHFSRWAETNGQLEDCVVLDTDGFWKTVDCNDNQPGA
ICYYSGNETEKEVKPVDSVKCPSPVLNTPWIPFQNCCYNFIITKNRHMATTQDEVHTKCQKLNPKSHILSIRDEKENNFVLEQLLYF
NYMASWVMLGITYRNKSLMWFDKTPLSYTHWRAGRPTIKNEKFLAGLSTDGFWDIQTFKVIEEAVYFHQHSILACKIEMVDYKEEYN
TTLPQFMPYEDGIYSVIQKKVTWYEALNMCSQSGGHLASVHNQNGQLFLEDIVKRDGFPLWVGLSSHDGSESSFEWSDGSTFDYIPW
KGQTSPGNCVLLDPKGTWKHEKCNSVKDGAICYKPTKSKKLSRLTYSSRCPAAKENGSRWIQYKGHCYKSDQALHSFSEAKKLCSKH
DHSATIVSIKDEDENKFVSRLMRENNNITMRVWLGLSQHSVDQSWSWLDGSEVTFVKWENKSKSGVGRCSMLIASNETWKKVECEHG
FGRVVCKVPLGPDYTAIAIIVATLSILVLMGGLIWFLFQRHRLHLAGFSSVRYAQGVNEDEIMLPSFHD
>sp|Q2WGJ6|KLH38_HUMAN
MDEESLDGLLFKDHDFSSDLLRQLNSLRQSRILTDVSICAGAREIPCHRNVLASSSPYFRAMFCSSFREKSEAKVQLKGIDPPTLDQ
IVSYVYTGEAHIATDNVLPVMEAASMLQFPKLFEACSSYLQSQLAPSNCLGMIRLSEILSCETLKKKAREVALTSFPEVAASADLKE
LCALELRDYLGDDGLCGEEEKVFEALMVWIKHDLQARKRYMQELFKQVRLQYIHPAFFHHFIANDALLQSSPACQIILETAKRQMFS
LCGTTVPDCKLLLHVPPRNSYQDFLILLGGRKDSQQTTRDVLLYSKQTGQWQSLAKLPTRLYKASAITLHRSIYVLGGMAVSSGRSL
VSHNVYIFSLKLNQWRLGEPMLVARYSHRSTAHKNFIFSIGGIGEGQELMGSMERYDSICNVWESMASMPVGVLHPAVAVKDQRLYL
FGGEDIMQNPVRLIQVYHISRNSWFKMETRMIKNVCAPAVVLGERIVIVGGYTRRILAYDPQSNKFVKCADMKDRRMHHGATVMGNK
LYVTGGRRLTTDCNIEDSASFDCYDPETDTWTSQGQLPHKLFDHACLTLQCIPRTSGLP

>sp|Q9P1A6|DLGP2_HUMAN
MGTAQVLPGILQKHCCILPDRNTESQCTLCGEPEEEEAGDLVQPGISFPGPAEEDLDPQYSWSPTQHFNEERYSPAPRSMKGLSGSR
TQPPLCSGHTCGLAPPEDCEHLHHGPDARPPYLLSPADSCPGGRHRCSPRSSVHSECVMMPVVLGDHVSSSTFPRMHYSSHYDTRDD
CAVAHAGAKINRIPANLLDQFEKQLPLHRDGFHTLQYQRTSAAAEQRSESPGRIRHLVHSVQKLFTKSHSLEGSSKSNANGTKADGR
ADDHHHAHHAKHSKRSKSKERKPEGKPRPGMSSWWSSDDNLDSDSTYRTPSVLNRHHLGPVAHCYPDALQSPFGDLSLKTSKSNNDV
KCSACEGLALTPDAKYLKRSSWSTLTVSQAKEAYRKSSLNLDKPLLHQDAKPALRPCHYLQVPQDEWGGYPTGGKDEEIPCRRMRSG
SYIKAMGDEESGESDSSPKTSPKSAILPEPLLKSIGQRPLGEHQTQTYLQAASDVPVGHSLDPAANYNSPKFRSRNQSYMRAVSTLS
QASCVSQVSEAEINGQFESVCESVFSEVESQAMDALDLPGCFRTRSHSYLRAIQAGYSQDDECIPMMTPSDITSTIRSTAAVSYTNY
KKTPPPVPPRTTSKPLISVTAQSSTESTQDAYQDSRAQRMSPWPQDSRGLYNSTDSLDSNKAMNLALETAAAQRHLPESQSSSVRTS
DKAILVSKAEELLKSRCSSIGIQDSEFPEHQPYPRSDVETATDSDTESRGLREYHSVGVQVEDEKRHGRFKRSNSVTAAVQADLELE
GFPGHITTEDKGLQFGSSFQRHSEPSTPTQYSAVRTVRTQGLFSYREDYRTQVDTSTLPPPDPWLEPAIDTVETGRMSPCRRDGSWF
LKLLHAETKRMEGWCKEMEREAEENDLSEEILGKIRSAVGSAQLLMSQKFQQFYWLCQQNMDPSAMPRPTSQDLAGYWDMLQLSIED
VSMKFDELQRLRLNDWKMMESPERKEERKVPPPIPKKPPKGKFPITREKSLDLPDRQRQEARRRLMAAKRAASFRQNSASERADSIE
IYIPEAQTRL
>sp|Q13520|AQP6_HUMAN
MDAVEPGGRGWASMLACRLWKAISRALFAEFLATGLYVFFGVGSVMRWPTALPSVLQIAITFNLVTAMAVQVTWKASGAHANPAVTL
AFLVGSHISLPRAVAYVAAQLVGATVGAALLYGVMPGDIRETLGINVVRNSVSTGQAVAVELLLTLQLVLCVFASTDSRQTSGSPAT
MIGISVALGHLIGIHFTGCSMNPARSFGPAIIIGKFTVHWVFWVGPLMGALLASLIYNFVLFPDTKTLAQRLAILTGTVEVGTGAGA
GAEPLKKESQPGSGAVEMESV
>sp|P08240|SRPRA_HUMAN
MLDFFTIFSKGGLVLWCFQGVSDSCTGPVNALIRSVLLQERGGNNSFTHEALTLKYKLDNQFELVFVVGFQKILTLTYVDKLIDDVH
RLFRDKYRTEIQQQSALSLLNGTFDFQNDFLRLLREAEESSKIRAPTTMKKFEDSEKAKKPVRSMIETRGEKPKEKAKNSKKKGAKK
EGSDGPLATSKPVPAEKSGLPVGPENGVELSKEELIRRKREEFIQKHGRGMEKSNKSTKSDAPKEKGKKAPRVWELGGCANKEVLDY
STPTTNGTPEAALSEDINLIRGTGSGGQLQDLDCSSSDDEGAAQNSTKPSATKGTLGGMFGMLKGLVGSKSLSREDMESVLDKMRDH
LIAKNVAADIAVQLCESVANKLEGKVMGTFSTVTSTVKQALQESLVQILQPQRRVDMLRDIMDAQRRQRPYVVTFCGVNGVGKSTNL
AKISFWLLENGFSVLIAACDTFRAGAVEQLRTHTRRLSALHPPEKHGGRTMVQLFEKGYGKDAAGIAMEAIAFARNQGFDVVLVDTA
GRMQDNAPLMTALAKLITVNTPDLVLFVGEALVGNEAVDQLVKFNRALADHSMAQTPRLIDGIVLTKFDTIDDKVGAAISMTYITSK
PIVFVGTGQTYCDLRSLNAKAVVAALMKA
>sp|Q86XD5|F131B_HUMAN
MDSTSSLHGSSLHRPSTEQTRTDFSWDGINLSMEDTTSILPKLKRNSNAYGIGALAKSSFSGISRSMKDHVTKPTAMGQGRVAHMIE
WQGWGKTPAVQPQHSHESVRRDTDAYSDLSDGEKEARFLAGVMEQFAISEATLMAWSSMDGEDMSVNSTQEPLGCNYSDNYQELMDS
QDALAQAPMDGWPHSYVSQGMYCLGSSDAWEASDQSLIASPATGSYLGPAFDDSQPSLHEMGPSQPASGYSALEPPPLLGGDTDWAP
GVGAVDLARGPAEEEKRPLAPEEEEDAGCRDLESLSPREDPEMSTALSRKVSDVTSSGVQSFDEEEGEANN
>sp|Q13394|MB211_HUMAN
MIAAQAKLVYHLNKYYNEKCQARKAAIAKTIREVCKVVSDVLKEVEVQEPRFISSLNEMDNRYEGLEVISPTEFEVVLYLNQMGVFN
FVDDGSLPGCAVLKLSDGRKRSMSLWVEFITASGYLSARKIRSRFQTLVAQAVDKCSYRDVVKMVADTSEVKLRIRDRYVVQITPAF
KCTGIWPRSAAHWPLPHIPWPGPNRVAEVKAEGFNLLSKECHSLAGKQSSAESDAWVLQFAEAENRLQMGGCRKKCLSILKTLRDRH
LELPGQPLNNYHMKTLVSYECEKHPRESDWDESCLGDRLNGILLQLISCLQCRRCPHYFLPNLDLFQGKPHSALENAAKQTWRLARE
ILTNPKSLEKL
>sp|Q2HXU8|CL12B_HUMAN
MSEEVTYATLTFQDSAGARNNRDGNNLRKRGHPAPSPIWRHAALGLVTLCLMLLIGLVTLGMMFLQISNDINSDSEKLSQLQKTIQQ
QQDNLSQQLGNSNNLSMEEEFLKSQISSVLKRQEQMAIKLCQELIIHTSDHRCNPCPKMWQWYQNSCYYFTTNEEKTWANSRKDCID
KNSTLVKIDSLEEKDFLMSQPLLMFSFFWLGLSWDSSGRSWFWEDGSVPSPSLFSTKELDQINGSKGCAYFQKGNIYISRCSAEIFW
ICEKTAAPVKTEDLD

>sp|Q9UHI6|DDX20_HUMAN
MAAAFEASGALAAVATAMPAEHVAVQVPAPEPTPGPVRILRTAQDLSSPRTRTGDVLLAEPADFESLLLSRPVLEGLRAAGFERPSP
VQLKAIPLGRCGLDLIVQAKSGTGKTCVFSTIALDSLVLENLSTQILILAPTREIAVQIHSVITAIGIKMEGLECHVFIGGTPLSQD
KTRLKKCHIAVGSPGRIKQLIELDYLNPGSIRLFILDEADKLLEEGSFQEQINWIYSSLPASKQMLAVSATYPEFLANALTKYMRDP
TFVRLNSSDPSLIGLKQYYKVVNSYPLAHKVFEEKTQHLQELFSRIPFNQALVFSNLHSRAQHLADILSSKGFPAECISGNMNQNQR
LDAMAKLKHFHCRVLISTDLTSRGIDAEKVNLVVNLDVPLDWETYMHRIGRAGRFGTLGLTVTYCCRGEEENMMMRIAQKCNINLLP
LPDPIPSGLMEECVDWDVEVKAAVHTYGIASVPNQPLKKQIQKIERTLQIQKAHGDHMASSRNNSVSGLSVKSKNNTKQKLPVKSHS
ECGIIEKATSPKELGCDRQSEEQMKNSVQTPVENSTNSQHQVKEALPVSLPQIPCLSSFKIHQPYTLTFAELVEDYEHYIKEGLEKP
VEIIRHYTGPGDQTVNPQNGFVRNKVIEQRVPVLASSSQSGDSESDSDSYSSRTSSQSKGNKSYLEGSSDNQLKDSESTPVDDRISL
EQPPNGSDTPNPEKYQESPGIQMKTRLKEGASQRAKQSRRNLPRRSSFRLQTEAQEDDWYDCHREIRLSFSDTYQDYEEYWRAYYRA
WQEYYAAASHSYYWNAQRHPSWMAAYHMNTIYLQEMMHSNQ
>sp|P01701|LV151_HUMAN
MTCSPLLLTLLIHCTGSWAQSVLTQPPSVSAAPGQKVTISCSGSSSNIGNNYVSWYQQLPGTAPKLLIYDNNKRPSGIPDRFSGSKS
GTSATLGITGLQTGDEADYYCGTWDSSLSA
>sp|O60613|SEP15_HUMAN
MAAGPSGCLVPAFGLRLLLATVLQAVSAFGAEFSSEACRELGFSSNLLCSSCDLLGQFNLLQLDPDCRGCCQEEAQFETKKLYAGAI
LEVCGUKLGRFPQVQAFVRSDKPKLFRGLQIKYVRGSDPVLKLLDDNGNIAEELSILKWNTDSVEEFLSEKLERI
>sp|P13725|ONCM_HUMAN
MGVLLTQRTLLSLVLALLFPSMASMAAIGSCSKEYRVLLGQLQKQTDLMQDTSRLLDPYIRIQGLDVPKLREHCRERPGAFPSEETL
RGLGRRGFLQTLNATLGCVLHRLADLEQRLPKAQDLERSGLNIEDLEKLQMARPNILGLRNNIYCMAQLLDNSDTAEPTKAGRGASQ
PPTPTPASDAFQRKLEGCRFLHGYHRFMHSVGRVFSKWGESPNRSRRHSPHQALRKGVRRTRPSRKGKRLMTRGQLPR
>sp|P17931|LEG3_HUMAN
MADNFSLHDALSGSGNPNPQGWPGAWGNQPAGAGGYPGASYPGAYPGQAPPGAYPGQAPPGAYPGAPGAYPGAPAPGVYPGPPSGPG
AYPSSGQPSATGAYPATGPYGAPAGPLIVPYNLPLPGGVVPRMLITILGTVKPNANRIALDFQRGNDVAFHFNPRFNENNRRVIVCN
TKLDNNWGREERQSVFPFESGKPFKIQVLVEPDHFKVAVNDAHLLQYNHRVKKLNEISKLGISGDIDLTSASYTMI
>sp|P50222|MEOX2_HUMAN
MEHPLFGCLRSPHATAQGLHPFSQSSLALHGRSDHMSYPELSTSSSSCIIAGYPNEEGMFASQHHRGHHHHHHHHHHHHQQQQHQA
LQTNWHLPQMSSPPSAARHSLCLQPDSGGPPELGSSPPVLCSNSSSLGSSTPTGAACAPGDYGRQALSPAEAEKRSGGKRKSDSSDS
QEGNYKSEVNSKPRKERTAFTKEQIRELEAEFAHHNYLTRLRRYEIAVNLDLTERQVKVWFQNRRMKWKRVKGGQQGAAAREKELVN
VKKGTLLPSELSGIGAATLQQTGDSIANEDSHDSDHSSEHAHL
>sp|O95897|NOE2_HUMAN
MWPLTVPPPLLLLLCSGLAGQTLFQNPEEGWQLYTSAQAPDGKCICTAVIPAQSTCSRDGRSRELRQLMEKVQNVSQSMEVLELRTY
RDLQYVRGMETLMRSLDARLRAADGSLSAKSFQELKDRMTELLPLSSVLEQYKADTRTIVRLREEVRNLSGSLAAIQEEMGAYGYED
LQQRVMALEARLHACAQKLGCGKLTGVSNPITVRAMGSRFGSWMTDTMAPSADSRVWYMDGYYKGRRVLEFRTLGDFIKGQNFIQHL
LPQPWAGTGHVVYNGSLFYNKYQSNVVVKYHFRSRSVLVQRSLPGAGYNNTFPYSWGGFSDMDFMVDESGLWAVYTTNQNAGNIVVS
RLDPHTLEVMRSWDTGYPKRSAGEAFMICGVLYVTNSHLAGAKVYFAYFTNTSSYEYTDVPFHNQYSHISMLDYNPRERALYTWNNG
HQVLYNVTLFHVISTSGDP
>sp|Q9NRR8|C42S1_HUMAN
MSEFWHKLGCCVVEKPQPKKKRRRIDRTMIGEPMNFVHLTHIGSGEMGAGDGLAMTGAVQEQMRSKGNRDRPWSNSRGL
>sp|P17181|INAR1_HUMAN
MMVVLLGATTLVLVAVAPWVLSAAAGGKNLKSPQKVEVDIIDDNFILRWNRSDESVGNVTFSFDYQKTGMDNWIKLSGCQNITSTKC
NFSSLKLNVYEEIKLRIRAEKENTSSWYEVDSFTPFRKAQIGPPEVHLEAEDKAIVIHISPGTKDSVMWALDGLSFTYSLVIWKNSS
GVEERIENIYSRHKIYKLSPETTYCLKVKAALLTSWKIGVYSPVHCIKTTVENELPPPENIEVSVQNQNYVLKWDYTYANMTFQVQW
LHAFLKRNPGNHLYKWKQIPDCENVKTTQCVFPQNVFQKGIYLLRVQASDGNNTSFWSEEIKFDTEIQAFLLPPVFNIRSLSDSFHI

YIGAPKQSGNTPVIQDYPLIYEIIFWENTSNAERKIIEKKTDVTVPNLKPLTVYCVKARAHTMDEKLNKSSVFSDAVCEKTKPGNTS
KIWLIVGICIALFALPFVIYAAKVFLRCINYVFFPSLKPSSSIDEYFSEQPLKNLLLSTSEEQIEKCFIIENISTIATVEETNQTDE
DHKKYSSQTSQDSGNYSNEDESESKTSEELQQDFV
>sp|Q8NGY1|O10Z1_HUMAN
MGQTNVTSWRDFVFLGFSSSGELQLLLFALFLSLYLVTLTSNVFIIIAIRLDSHLHTPMYLFLSFLSFSETCYTLGIIPRMLSGLAG
GDQAISYVGCAAQMFFSASWACTNCFLLAAMGFDRYVAICAPLHYASHMNPTLCAQLVITSFLTGYLFGLGMTLVIFHLSFCSSHEI
QHFFCDTPPVLSLACGDTGPSELRIFILSLLVLLVSFFFITISYAYILAAILRIPSAEGQKKAFSTCASHLTVVIIHYGCASFVYLR
PKASYSLERDQLIAMTYTVVTPLLNPIVYSLRNRAIQTALRNAFRGRLLGKG
>sp|P60520|GBRL2_HUMAN
MKWMFKEDHSLEHRCVESAKIRAKYPDRVPVIVEKVSGSQIVDIDKRKYLVPSDITVAQFMWIIRKRIQLPSEKAIFLFVDKTVPQS
SLTMGQLYEKEKDEDGFLYVAYSGENTFGF
>sp|P26715|NKG2A_HUMAN
MDNQGVIYSDLNLPPNPKRQQRKPKGNKNSILATEQEITYAELNLQKASQDFQGNDKTYHCKDLPSAPEKLIVGILGIICLILMASV
VTIVVIPSTLIQRHNNSSLNTRTQKARHCGHCPEEWITYSNSCYYIGKERRTWEESLLACTSKNSSLLSIDNEEEMKFLSIISPSSW
IGVFRNSSHHPWVTMNGLAFKHEIKDSDNAELNCAVLQVNRLKSAQCGSSIIYHCKHKL
>sp|Q8NH87|OR9G1_HUMAN
MQRSNHTVTEFILLGFTTDPGMQLGLFVVFLGVYSLTVVGNSTLIVLICNDSCLHTPMYFFTGNLSFLDLWYSSVYTPKILVTCISE
DKSISFAGCLCQFFFSAGLAYSECYLLAAVAYDRYVAISKPLLYAQAMSIKLCALLVAVSYCGGFINSSIITKKTFSFNFCRENIID
DFFCDLLPLVELACGEKGGYKIMMYFLLASNVICPAVLILASYLFIITSVLRISSSKGYLKAFSTCSSHLTSVTLYYGSILYIYALP
RSSYSFDMDKIVSTFYTVVFPMLNLMIYSLRNKDVKEALKKLLP
>sp|P24386|RAE1_HUMAN
MADTLPSEFDVIVIGTGLPESIIAAACSRSGRRVLHVDSRSYYGGNWASFSFSGLLSWLKEYQENSDIVSDSPVWQDQILENEEAIA
LSRKDKTIQHVEVFCYASQDLHEDVEEAGALQKNHALVTSANSTEAADSAFLPTEDESLSTMSCEMLTEQTPSSDPENALEVNGAEV
TGEKENHCDDKTCVPSTSAEDMSENVPIAEDTTEQPKKNRITYSQIIKEGRRFNIDLVSKLLYSRGLLIDLLIKSNVSRYAEFKNIT
RILAFREGRVEQVPCSRADVFNSKQLTMVEKRMLMKFLTFCMEYEKYPDEYKGYEEITFYEYLKTQKLTPNLQYIVMHSIAMTSETA
SSTIDGLKATKNFLHCLGRYGNTPFLFPLYGQGELPQCFCRMCAVFGGIYCLRHSVQCLVVDKESRKCKAIIDQFGQRIISEHFLVE
DSYFPENMCSRVQYRQISRAVLITDRSVLKTDSDQQISILTVPAEEPGTFAVRVIELCSSTMTCMKGTYLVHLTCTSSKTAREDLES
VVQKLFVPYTEMEIENEQVEKPRILWALYFNMRDSSDISRSCYNDLPSNVYVCSGPDCGLGNDNAVKQAETLFQEICPNEDFCPPPP
NPEDIILDGDSLQPEASESSAIPEANSETFKESTNLGNLEESSE
>sp|Q12874|SF3A3_HUMAN
METILEQQRRYHEEKERLMDVMAKEMLTKKSTLRDQINSDHRTRAMQDRYMEVSGNLRDLYDDKDGLRKEELNAISGPNEFAEFYNR
LKQIKEFHRKHPNEICVPMSVEFEELLKARENPSEEAQNLVEFTDEEGYGRYLDLHDCYLKYINLKASEKLDYITYLSIFDQLFDIP
KERKNAEYKRYLEMLLEYLQDYTDRVKPLQDQNELFGKIQAEFEKKWENGTFPGWPKETSSALTHAGAHLDLSAFSSWEELASLGLD
RLKSALLALGLKCGGTLEERAQRLFSTKGKSLESLDTSLFAKNPKSKGTKRDTERNKDIAFLEAQIYEYVEILGEQRHLTHENVQRK
QARTGEEREEEEEEQISESESEDEENEIIYNPKNLPLGWDGKPIPYWLYKLHGLNINYNCEICGNYTYRGPKAFQRHFAEWRHAHGM
RCLGIPNTAHFANVTQIEDAVSLWAKLKLQKASERWQPDTEEEYEDSSGNVVNKKTYEDLKRQGLL
>sp|Q5VT79|AXA81_HUMAN
MAWWKAWIEQEGVTVKSSSHFNPDPDAETLYKAMKGIGTNEQAIIDVLTKRSNTQRQQIAKSFKAQFGKDLTETLKSELSGKFERLI
VALMYPPYRYEAKELHDAMKGLGTKEGVIIEILASRTKNQLREIMKAYEEDYGSSLEEDIQADTSGYLERILVCLLQGSRDDVSSFV
DPALALQDAQDLYAAGEKIRGTDEMKFITILCTRSATHLLRVFEEYEKIANKSIEDSIKSETHGSLEEAMLTVVKCTQNLHSYFAER
LYYAMKGAGTRDGTLIRNIVSRSEIDLNLIKCHFKKMYGKTLSSMIMEDTSGDYKNALLSLVGSDP
>sp|P53634|CATC_HUMAN
MGAGPSLLLAALLLLLSGDGAVRCDTPANCTYLDLLGTWVFQVGSSGSQRDVNCSVMGPQEKKVVVYLQKLDTAYDDLGNSGHFTII
YNQGFEIVLNDYKWFAFFKYKEEGSKVTTYCNETMTGWVHDVLGRNWACFTGKKVGTASENVYVNIAHLKNSQEKYSNRLYKYDHNF

VKAINAIQKSWTATTYMEYETLTLGDMIRRSGGHSRKIPRPKPAPLTAEIQQKILHLPTSWDWRNVHGINFVSPVRNQASCGSCYSF
ASMGMLEARIRILTNNSQTPILSPQEVVSCSQYAQGCEGGFPYLIAGKYAQDFGLVEEACFPYTGTDSPCKMKEDCFRYYSSEYHYV
GGFYGGCNEALMKLELVHHGPMAVAFEVYDDFLHYKKGIYHHTGLRDPFNPFELTNHAVLLVGYGTDSASGMDYWIVKNSWGTGWGE
NGYFRIRRGTDECAIESIAVAATPIPKL
>sp|P50150|GBG4_HUMAN
MKEGMSNNSTTSISQARKAVEQLKMEACMDRVKVSQAAADLLAYCEAHVREDPLIIPVPASENPFREKKFFCTIL
>sp|O75718|CRTAP_HUMAN
MEPGRRGAAALLALLCVACALRAGRAQYERYSFRSFPRDELMPLESAYRHALDKYSGEHWAESVGYLEISLRLHRLLRDSEAFCHRN
CSAAPQPEPAAGLASYPELRLFGGLLRRAHCLKRCKQGLPAFRQSQPSREVLADFQRREPYKFLQFAYFKANNLPKAIAAAHTFLLK
HPDDEMMKRNMAYYKSLPGAEDYIKDLETKSYESLFIRAVRAYNGENWRTSITDMELALPDFFKAFYECLAACEGSREIKDFKDFYL
SIADHYVEVLECKIQCEENLTPVIGGYPVEKFVATMYHYLQFAYYKLNDLKNAAPCAVSYLLFDQNDKVMQQNLVYYQYHRDTWGLS
DEHFQPRPEAVQFFNVTTLQKELYDFAKENIMDDDEGEVVEYVDDLLELEETS
>sp|Q53GD3|CTL4_HUMAN
MGGKQRDEDDEAYGKPVKYDPSFRGPIKNRSCTDVICCVLFLLFILGYIVVGIVAWLYGDPRQVLYPRNSTGAYCGMGENKDKPYLL
YFNIFSCILSSNIISVAENGLQCPTPQVCVSSCPEDPWTVGKNEFSQTVGEVFYTKNRNFCLPGVPWNMTVITSLQQELCPSFLLPS
APALGRCFPWTNVTPPALPGITNDTTIQQGISGLIDSLNARDISVKIFEDFAQSWYWILVALGVALVLSLLFILLLRLVAGPLVLVL
ILGVLGVLAYGIYYCWEEYRVLRDKGASISQLGFTTNLSAYQSVQETWLAALIVLAVLEAILLLMLIFLRQRIRIAIALLKEASKAV
GQMMSTMFYPLVTFVLLLICIAYWAMTALYLATSGQPQYVLWASNISSPGCEKVPINTSCNPTAHLVNSSCPGLMCVFQGYSSKGLI
QRSVFNLQIYGVLGLFWTLNWVLALGQCVLAGAFASFYWAFHKPQDIPTFPLISAFIRTLRYHTGSLAFGALILTLVQIARVILEYI
DHKLRGVQNPVARCIMCCFKCCLWCLEKFIKFLNRNAYIMIAIYGKNFCVSAKNAFMLLMRNIVRVVVLDKVTDLLLFFGKLLVVGG
VGVLSFFFFSGRIPGLGKDFKSPHLNYYWLPIMTSILGAYVIASGFFSVFGMCVDTLFLCFLEDLERNNGSLDRPYYMSKSLLKILG
KKNEAPPDNKKRKK
>sp|P54725|RD23A_HUMAN
MAVTITLKTLQQQTFKIRMEPDETVKVLKEKIEAEKGRDAFPVAGQKLIYAGKILSDDVPIRDYRIDEKNFVVVMVTKTKAGQGTSA
PPEASPTAAPESSTSFPPAPTSGMSHPPPAAREDKSPSEESAPTTSPESVSGSVPSSGSSGREEDAASTLVTGSEYETMLTEIMSMG
YERERVVAALRASYNNPHRAVEYLLTGIPGSPEPEHGSVQESQVSEQPATEAAGENPLEFLRDQPQFQNMRQVIQQNPALLPALLQQ
LGQENPQLLQQISRHQEQFIQMLNEPPGELADISDVEGEVGAIGEEAPQMNYIQVTPQEKEAIERLKALGFPESLVIQAYFACEKNE
NLAANFLLSQNFDDE
>sp|Q8IZ21|PHAR4_HUMAN
MEDPFEEADQPTTEPGMVLDSVEAGDTTPPTKRKSKFSGFGKIFKPWKWRKKKSSDKFKETSEVLERKISMRKPREELVKRGVLLED
PEQGGEDPGKPSDAMLKNGHTTPIGNARSSSPVQVEEEPVRLASLRKAIPEEDLKKRLGSTGSQPNSEAESVPENVPKPPLLPPKRP
LSSSHEASEGQAKDATSSGGTARFIISTSITTAPAATTAATSLAKTVNLSVTPSPAPRTLPAAPASTNTTATPSLTHMVPAKQPPIP
PPKPAHRNSNPVIAELSQAINSGTLLSKPSPPLPPKRGIPSTSVPTLESAAAITTKTPSDEREKSTCSMGSELLPMISPRSPSPPLP
THIPPEPPRTPPFPAKTFQVVPEIEFPPSLDLHQEIPQQEDQKKEVPKRILDQNFGEPHIPSRLPPLPLHIRIQQALTSPLPMTPIL
EGSHRAHSLLFENSDSFSEDSSTLGRTRSLPITIEMLKVPDDEEEEEQTCPSTFSEEMTPTSVIPKLPQCLREEEEKESDSDSEGPI
QYRDEEDEDESYQSALANKVKRKDTLAMKLNHRPSEPELNLNSWPCKSKEEWNEIRHQIGNTLIRRLSQRPTPEELEQRNILQPKNE
ADRQAEKREIKRRLTRKLSQRPTVAELLARKILRFNEYVEVTDAQDYDRRADKPWTKLTPADKAAIRKELNEFKSSEMEVHEESKHF
TRYHRP
>sp|Q9BTD8|RBM42_HUMAN
MAGAGPAPGLPGAGGPVVPGPGAGIPGKSGEERLKEMEAEMALFEQEVLGAPVPGIPTAVPAVPTVPTVPTVEAMQVPAAPVIRPII
ATNTYQQVQQTLEARAAAAATVVPPMVGGPPFVGPVGFGPGDRSHLDSPEAREAMFLRRAAVAPQRAPILRPAFVPHVLQRADSALS
SAAAGPRPMALRPPHQALVGPPLPGPPGPPMMLPPMARAPGPPLGSMAALRPPLEEPAAPRELGLGLGLGLKEKEEAVVAAAAGLEE
ASAAVAVGAGGAPAGPAVIGPSLPLALAMPLPEPEPLPLPLEVVRGLLPPLRIPELLSLRPRPRPPRPEPPPGLMALEVPEPLGEDK

KKGKPEKLKRCIRTAAGSSWEDPSLLEWDADDFRIFCGDLGNEVNDDILARAFSRFPSFLKAKVIRDKRTGKTKGYGFVSFKDPSDY
VRAMREMNGKYVGSRPIKLRKSMWKDRNLDVVRKKQKEKKKLGLR
>sp|Q12774|ARHG5_HUMAN
MEAEEAQRGASPPISAIEEFSIIPEAPMRSSQVSALGLEAQEDEDPSYKWREEHRLSATQQSELRDVCDYAIETMPSFPKEGSADVE
PNQESLVAEACDTPEHWEAVPQSLAGRQARTLAPPELWACPIQSEHLDMAPFSSDLGSEEEEVEFWPGLTSLTLGSGQAEEEEETSS
DNSGQTRYYSPCEEHPAETNQNEGSESGTIRQGEELPPEELQESQGLLHPQEVQVLEEQGQQEAGFRGEGTLREDVCADGLLGEEQM
IEQVNDEKGEQKQKQEQVQDVMLGRQGERMGLTGEPEGLNDGEWEQEDMERKAQGQGGPEQGEERKRELQVPEENRADSQDEKSQTF
LGKSEEVTGKQEDHGIKEKGVPVSGQEAKEPESWDGGRLGAVGRARSREEENEHHGPSMPALIAPEDSPHCDLFPGASYLMTQIPGT
QTESRAEELSPAALSPSLEPIRCSHQPISLLGSFLTEESPDKEIDQNSQQEESRLRKGTVSSQGTEVVFASASVTPPRTPDSAPPSP
AEAYPITPASVSARPPVAFPRRETSCAARAPETASAPLSMDDPSPCGTSEMCPAALYGFPSTGTSPPRPPANSTGTVQHLRSDSFPG
SHRTEQTPDLVGMLLSYSHSELPQRPPKPAIYSSVTPRRDRRSGRDYSTVSASPTALSTLKQDSQESISNLERPSSPPSIQPWVSPH
NPAFATESPAYGSSPSFVSMEDVRIHEPLPPPPPQRRDTHPSVVETDGHARVVVPTLKQHSHPPPLALGSGLHAPHKGPLPQASDPA
VARQHRPLPSTPDSSHHAQATPRWRYNKPLPPTPDLPQPHLPPISAPGSSRIYRPLPPLPIIDPPTEPPPLPPKSRGRSRSTRGGHM
NSGGHAKTRPACQDWTVPLPASAGRTSWPPATARSTESFTSTSRSKSEVSPGMAFSNMTNFLCPSSPTTPWTPELQGPTSKDEAGVS
EHPEAPAREPLRRTTPQQGASGPGRSPVGQARQPEKPSHLHLEKASSWPHRRDSGRPPGDSSGQAVAPSEGANKHKGWSRQGLRRPS
ILPEGSSDSRGPAVEKHPGPSDTVVFREKKPKEVMGGFSRRCSKLINSSQLLYQEYSDVVLNKEIQSQQRLESLSETPGPSSPRQPR
KALVSSESYLQRLSMASSGSLWQEIPVVRNSTVLLSMTHEDQKLQEVKFELIVSEASYLRSLNIAVDHFQLSTSLRATLSNQEHQWL
FSRLQDVRDVSATFLSDLEENFENNIFSFQVCDVVLNHAPDFRRVYLPYVTNQTYQERTFQSLMNSNSNFREVLEKLESDPVCQRLS
LKSFLILPFQRITRLKLLLQNILKRTQPGSSEEAEATKAHHALEQLIRDCNNNVQSMRRTEELIYLSQKIEFECKIFPLISQSRWLV
KSGELTALEFSASPGLRRKLNTRPVHLHLFNDCLLLSRPREGSRFLVFDHAPFSSIRGEKCEMKLHGPHKNLFRLFLRQNTQGAQAE
FLFRTETQSEKLRWISALAMPREELDLLECYNSPQVQCLRAYKPRENDELALEKADVVMVTQQSSDGWLEGVRLSDGERGWFPVQQV
EFISNPEVRAQNLKEAHRVKTAKLQLVEQQA
>sp|P62304|RUXE_HUMAN
MAYRGQGQKVQKVMVQPINLIFRYLQNRSRIQVWLYEQVNMRIEGCIIGFDEYMNLVLDDAEEIHSKTKSRKQLGRIMLKGDNITLL
QSVSN
>sp|Q9UKW4|VAV3_HUMAN
MEPWKQCAQWLIHCKVLPTNHRVTWDSAQVFDLAQTLRDGVLLCQLLNNLRAHSINLKEINLRPQMSQFLCLKNIRTFLTACCETFG
MRKSELFEAFDLFDVRDFGKVIETLSRLSRTPIALATGIRPFPTEESINDEDIYKGLPDLIDETLVEDEEDLYDCVYGEDEGGEVYE
DLMKAEEAHQPKCPENDIRSCCLAEIKQTEEKYTETLESIEKYFMAPLKRFLTAAEFDSVFINIPELVKLHRNLMQEIHDSIVNKND
QNLYQVFINYKERLVIYGQYCSGVESAISSLDYISKTKEDVKLKLEECSKRANNGKFTLRDLLVVPMQRVLKYHLLLQELVKHTTDP
TEKANLKLALDAMKDLAQYVNEVKRDNETLREIKQFQLSIENLNQPVLLFGRPQGDGEIRITTLDKHTKQERHIFLFDLAVIVCKRK
GDNYEMKEIIDLQQYKIANNPTTDKENKKWSYGFYLIHTQGQNGLEFYCKTKDLKKKWLEQFEMALSNIRPDYADSNFHDFKMHTFT
RVTSCKVCQMLLRGTFYQGYLCFKCGARAHKECLGRVDNCGRVNSGEQGTLKLPEKRTNGLRRTPKQVDPGLPKMQVIRNYSGTPPP
ALHEGPPLQLQAGDTVELLKGDAHSLFWQGRNLASGEVGFFPSDAVKPCPCVPKPVDYSCQPWYAGAMERLQAETELINRVNSTYLV
RHRTKESGEYAISIKYNNEAKHIKILTRDGFFHIAENRKFKSLMELVEYYKHHSLKEGFRTLDTTLQFPYKEPEHSAGQRGNRAGNS
LLSPKVLGIAIARYDFCARDMRELSLLKGDVVKIYTKMSANGWWRGEVNGRVGWFPSTYVEEDE
>sp|Q9H0X4|F234A_HUMAN
MLDHKDLEAEIHPLKNEERKSQENLGNPSKNEDNVKSAPPQSRLSRCRAAAFFLSLFLCLFVVFVVSFVIPCPDRPASQRMWRIDYS
AAVIYDFLAVDDINGDRIQDVLFLYKNTNSSNNFSRSCVDEGFSSPCTFAAAVSGANGSTLWERPVAQDVALVECAVPQPRGSEAPS
ACILVGRPSSFIAVNLFTGETLWNHSSSFSGNASILSPLLQVPDVDGDGAPDLLVLTQEREEVSGHLYSGSTGHQIGLRGSLGVDGE
SGFLLHVTRTGAHYILFPCASSLCGCSVKGLYEKVTGSGGPFKSDPHWESMLNATTRRMLSHSSGAVRYLMHVPGNAGADVLLVGSE
AFVLLDGQELTPRWTPKAAHVLRKPIFGRYKPDTLAVAVENGTGTDRQILFLDLGTGAVLCSLALPSLPGGPLSASLPTADHRSAFF
FWGLHELGSTSETETGEARHSLYMFHPTLPRVLLELANVSTHIVAFDAVLFEPSRHAAYILLTGPADSEAPGLVSVIKHKVRDLVPS
SRVVRLGEGGPDSDQAIRDRFSRLRYQSEA

>sp|Q13402|MYO7A_HUMAN
MVILQQGDHVWMDLRLGQEFDVPIGAVVKLCDSGQVQVVDDEDNEHWISPQNATHIKPMHPTSVHGVEDMIRLGDLNEAGILRNLLI
RYRDHLIYTYTGSILVAVNPYQLLSIYSPEHIRQYTNKKIGEMPPHIFAIADNCYFNMKRNSRDQCCIISGESGAGKTESTKLILQF
LAAISGQHSWIEQQVLEATPILEAFGNAKTIRNDNSSRFGKYIDIHFNKRGAIEGAKIEQYLLEKSRVCRQALDERNYHVFYCMLEG
MSEDQKKKLGLGQASDYNYLAMGNCITCEGRVDSQEYANIRSAMKVLMFTDTENWEISKLLAAILHLGNLQYEARTFENLDACEVLF
SPSLATAASLLEVNPPDLMSCLTSRTLITRGETVSTPLSREQALDVRDAFVKGIYGRLFVWIVDKINAAIYKPPSQDVKNSRRSIGL
LDIFGFENFAVNSFEQLCINFANEHLQQFFVRHVFKLEQEEYDLESIDWLHIEFTDNQDALDMIANKPMNIISLIDEESKFPKGTDT
TMLHKLNSQHKLNANYIPPKNNHETQFGINHFAGIVYYETQGFLEKNRDTLHGDIIQLVHSSRNKFIKQIFQADVAMGAETRKRSPT
LSSQFKRSLELLMRTLGACQPFFVRCIKPNEFKKPMLFDRHLCVRQLRYSGMMETIRIRRAGYPIRYSFVEFVERYRVLLPGVKPAY
KQGDLRGTCQRMAEAVLGTHDDWQIGKTKIFLKDHHDMLLEVERDKAITDRVILLQKVIRGFKDRSNFLKLKNAATLIQRHWRGHNC
RKNYGLMRLGFLRLQALHRSRKLHQQYRLARQRIIQFQARCRAYLVRKAFRHRLWAVLTVQAYARGMIARRLHQRLRAEYLWRLEAE
KMRLAEEEKLRKEMSAKKAKEEAERKHQERLAQLAREDAERELKEKEAARRKKELLEQMERARHEPVNHSDMVDKMFGFLGTSGGLP
GQEGQAPSGFEDLERGRREMVEEDLDAALPLPDEDEEDLSEYKFAKFAATYFQGTTTHSYTRRPLKQPLLYHDDEGDQLAALAVWIT
ILRFMGDLPEPKYHTAMSDGSEKIPVMTKIYETLGKKTYKRELQALQGEGEAQLPEGQKKSSVRHKLVHLTLKKKSKLTEEVTKRLH
DGESTVQGNSMLEDRPTSNLEKLHFIIGNGILRPALRDEIYCQISKQLTHNPSKSSYARGWILVSLCVGCFAPSEKFVKYLRNFIHG
GPPGYAPYCEERLRRTFVNGTRTQPPSWLELQATKSKKPIMLPVTFMDGTTKTLLTDSATTAKELCNALADKISLKDRFGFSLYIAL
FDKVSSLGSGSDHVMDAISQCEQYAKEQGAQERNAPWRLFFRKEVFTPWHSPSEDNVATNLIYQQVVRGVKFGEYRCEKEDDLAELA
SQQYFVDYGSEMILERLLNLVPTYIPDREITPLKTLEKWAQLAIAAHKKGIYAQRRTDAQKVKEDVVSYARFKWPLLFSRFYEAYKF
SGPSLPKNDVIVAVNWTGVYFVDEQEQVLLELSFPEIMAVSSSRECRVWLSLGCSDLGCAAPHSGWAGLTPAGPCSPCWSCRGAKTT
APSFTLATIKGDEYTFTSSNAEDIRDLVVTFLEGLRKRSKYVVALQDNPNPAGEESGFLSFAKGDLIILDHDTGEQVMNSGWANGIN
ERTKQRGDFPTDSVYVMPTVTMPPREIVALVTMTPDQRQDVVRLLQLRTAEPEVRAKPYTLEEFSYDYFRPPPKHTLSRVMVSKARG
KDRLWSHTREPLKQALLKKLLGSEELSQEACLAFIAVLKYMGDYPSKRTRSVNELTDQIFEGPLKAEPLKDEAYVQILKQLTDNHIR
YSEERGWELLWLCTGLFPPSNILLPHVQRFLQSRKHCPLAIDCLQRLQKALRNGSRKYPPHLVEVEAIQHKTTQIFHKVYFPDDTDE
AFEVESSTKAKDFCQNIATRLLLKSSEGFSLFVKIADKVLSVPENDFFFDFVRHLTDWIKKARPIKDGIVPSLTYQVFFMKKLWTTT
VPGKDPMADSIFHYYQELPKYLRGYHKCTREEVLQLGALIYRVKFEEDKSYFPSIPKLLRELVPQDLIRQVSPDDWKRSIVAYFNKH
AGKSKEEAKLAFLKLIFKWPTFGSAFFEVKQTTEPNFPEILLIAINKYGVSLIDPKTKDILTTHPFTKISNWSSGNTYFHITIGNLV
RGSKLLCETSLGYKMDDLLTSYISQMLTAMSKQRGSRSGK
>sp|Q8NHS9|SPT22_HUMAN
MKRSLNENSARSTAGCLPVPLFNQKKRNRQPLTSNPLKDDSGISTPSDNYDFPPLPTDWAWEAVNPELAPVMKTVDTGQIPHSVSRP
LRSQDSVFNSIQSNTGRSQGGWSYRDGNKNTSLKTWNKNDFKPQCKRTNLVANDGKNSCPVSSGAQQQKQLRIPEPPNLSRNKETEL
LRQTHSSKISGCTMRGLDKNSALQTLKPNFQQNQYKKQMLDDIPEDNTLKETSLYQLQFKEKASSLRIISAVIESMKYWREHAQKTV
LLFEVLAVLDSAVTPGPYYSKTFLMRDGKNTLPCVFYEIDRELPRLIRGRVHRCVGNYDQKKNIFQCVSVRPASVSEQKTFQAFVKI
ADVEMQYYINVMNET
>sp|P00915|CAH1_HUMAN
MASPDWGYDDKNGPEQWSKLYPIANGNNQSPVDIKTSETKHDTSLKPISVSYNPATAKEIINVGHSFHVNFEDNDNRSVLKGGPFSD
SYRLFQFHFHWGSTNEHGSEHTVDGVKYSAELHVAHWNSAKYSSLAEAASKADGLAVIGVLMKVGEANPKLQKVLDALQAIKTKGKR
APFTNFDPSTLLPSSLDFWTYPGSLTHPPLYESVTWIICKESISVSSEQLAQFRSLLSNVEGDNAVPMQHNNRPTQPLKGRTVRASF
>sp|Q9P2C4|TM181_HUMAN
MGAAPSPTQASSRGGGPGPPAPTRAVSSSSRARGGALSALGPSPARPLTTSPAPAPPPRSRPARQQPDPQCWEKRGGAGGDTKGGAA
GPGPGRLRGMDAEYPAFEPPLCSELKHLCRRLREAYRELKEDLTPFKDDRYYRLAPMRLYTLSKRHFVLVFVVFFICFGLTIFVGIR
GPKVIQTSAANFSLNNSKKLKPIQILSNPLSTYNQQLWLTCVVELDQSKETSIKTSFPMTVKVDGVAQDGTTMYIHNKVHNRTRTLT
CAGKCAEIIVAHLGYLNYTQYTVIVGFEHLKLPIKGMNFTWKTYNPAFSRLEIWFRFFFVVLTFIVTCLFAHSLRKFSMRDWGIEQK
WMSVLLPLLLLYNDPFFPLSFLVNSWLPGMLDDLFQSMFLCALLLFWLCVYHGIRVQGERKCLTFYLPKFFIVGLLWLASVTLGIWQ
TVNELHDPMYQYRVDTGNFQGMKVFFMVVAAVYILYLLFLIVRACSELRHMPYVDLRLKFLTALTFVVLVISIAILYLRFGAQVLQD

NFVAELSTHYQNSAEFLSFYGLLNFYLYTLAFVYSPSKNALYESQLKDNPAFSMLNDSDDDVIYGSDYEEMPLQNGQAIRAKYKEES
DSD
>sp|Q9BV44|THUM3_HUMAN
MCDIEEATNQLLDVNLHENQKSVQVTESDLGSESELLVTIGATVPTGFEQTAADEVREKLGSSCKISRDRGKIYFVISVESLAQVHC
LRSVDNLFVVVQEFQDYQFKQTKEEVLKDFEDLAGKLPWSNPLKVWKINASFKKKKAKRKKINQNSSKEKINNGQEVKIDQRNVKKE
FTSHALDSHILDYYENPAIKEDVSTLIGDDLASCKDETDESSKEETEPQVLKFRVTCNRAGEKHCFTSNEAARDFGGAVQDYFKWKA
DMTNFDVEVLLNIHDNEVIVGIALTEESLHRRNITHFGPTTLRSTLAYGMLRLCDPLPYDIIVDPMCGTGAIPIEGATEWSDCFHIA
GDNNPLAVNRAANNIASLLTKSQIKEGKPSWGLPIDAVQWDICNLPLRTGSVDIIVTDLPFGKRMGSKKRNWNLYPACLREMSRVCT
PTTGRAVLLTQDTKCFTKALSGMRHVWRKVDTVWVNVGGLRAAVYVLIRTPQAFVHPSEQDGERGTLWQCKE
>sp|Q13155|AIMP2_HUMAN
MPMYQVKPYHGGGAPLRVELPTCMYRLPNVHGRSYGPAPGAGHVQEESNLSLQALESRQDDILKRLYELKAAVDGLSKMIQTPDADL
DVTNIIQADEPTTLTTNALDLNSVLGKDYGALKDIVINANPASPPLSLLVLHRLLCEHFRVLSTVHTHSSVKSVPENLLKCFGEQNK
KQPRQDYQLGFTLIWKNVPKTQMKFSIQTMCPIEGEGNIARFLFSLFGQKHNAVNATLIDSWVDIAIFQLKEGSSKEKAAVFRSMNS
ALGKSPWLAGNELTVADVVLWSVLQQIGGCSVTVPANVQRWMRSCENLAPFNTALKLLK
>sp|P62906|RL10A_HUMAN
MSSKVSRDTLYEAVREVLHGNQRKRRKFLETVELQISLKNYDPQKDKRFSGTVRLKSTPRPKFSVCVLGDQQHCDEAKAVDIPHMDI
EALKKLNKNKKLVKKLAKKYDAFLASESLIKQIPRILGPGLNKAGKFPSLLTHNENMVAKVDEVKSTIKFQMKKVLCLAVAVGHVKM
TDDELVYNIHLAVNFLVSLLKKNWQNVRALYIKSTMGKPQRLY
>sp|P32238|CCKAR_HUMAN
MDVVDSLLVNGSNITPPCELGLENETLFCLDQPRPSKEWQPAVQILLYSLIFLLSVLGNTLVITVLIRNKRMRTVTNIFLLSLAVSD
LMLCLFCMPFNLIPNLLKDFIFGSAVCKTTTYFMGTSVSVSTFNLVAISLERYGAICKPLQSRVWQTKSHALKVIAATWCLSFTIMT
PYPIYSNLVPFTKNNNQTANMCRFLLPNDVMQQSWHTFLLLILFLIPGIVMMVAYGLISLELYQGIKFEASQKKSAKERKPSTTSSG
KYEDSDGCYLQKTRPPRKLELRQLSTGSSSRANRIRSNSSAANLMAKKRVIRMLIVIVVLFFLCWMPIFSANAWRAYDTASAERRLS
GTPISFILLLSYTSSCVNPIIYCFMNKRFRLGFMATFPCCPNPGPPGARGEVGEEEEGGTTGASLSRFSYSHMSASVPPQ
>sp|P13056|NR2C1_HUMAN
MATIEEIAHQIIEQQMGEIVTEQQTGQKIQIVTALDHNTQGKQFILTNHDGSTPSKVILARQDSTPGKVFLTTPDAAGVNQLFFTTP
DLSAQHLQLLTDNSPDQGPNKVFDLCVVCGDKASGRHYGAVTCEGCKGFFKRSIRKNLVYSCRGSKDCIINKHHRNRCQYCRLQRCI
AFGMKQDSVQCERKPIEVSREKSSNCAASTEKIYIRKDLRSPLTATPTFVTDSESTRSTGLLDSGMFMNIHPSGVKTESAVLMTSDK
AESCQGDLSTLANVVTSLANLGKTKDLSQNSNEMSMIESLSNDDTSLCEFQEMQTNGDVSRAFDTLAKALNPGESTACQSSVAGMEG
SVHLITGDSSINYTEKEGPLLSDSHVAFRLTMPSPMPEYLNVHYIGESASRLLFLSMHWALSIPSFQALGQENSISLVKAYWNELFT
LGLAQCWQVMNVATILATFVNCLHNSLQQDKMSTERRKLLMEHIFKLQEFCNSMVKLCIDGYEYAYLKAIVLFSPDHPSLENMEQIE
KFQEKAYVEFQDYITKTYPDDTYRLSRLLLRLPALRLMNATITEELFFKGLIGNIRIDSVIPHILKMEPADYNSQIIGHSI
>sp|Q8TF63|DCNP1_HUMAN
MHYGAATHIQNSRSHGLETVPGHQRLERGAGGETPEFPGCHSPAPPENFGNELLPLSAPLQGLSEGLYPPGRNKTLPAGVLREGAVQ
FLHRGLCNSNLSSEASARPSGTQDELHSSRRKTGQTRREGARKHLVCSFRLYPFTVHTVSPGNSHLALYQVFKAVKLCPSETSFFLS
RKSLKSSDPWHPPSLSPNSWNRQAGFRAWSSHLISLSLTCSDSQSRRVSSSQQPPLHSLSSHRRAAHVPE
>sp|Q02078|MEF2A_HUMAN
MGRKKIQITRIMDERNRQVTFTKRKFGLMKKAYELSVLCDCEIALIIFNSSNKLFQYASTDMDKVLLKYTEYNEPHESRTNSDIVEA
LNKKEHRGCDSPDPDTSYVLTPHTEEKYKKINEEFDNMMRNHKIAPGLPPQNFSMSVTVPVTSPNALSYTNPGSSLVSPSLAASSTL
TDSSMLSPPQTTLHRNVSPGAPQRPPSTGNAGGMLSTTDLTVPNGAGSSPVGNGFVNSRASPNLIGATGANSLGKVMPTKSPPPPGG
GNLGMNSRKPDLRVVIPPSSKGMMPPLSEEEELELNTQRISSSQATQPLATPVVSVTTPSLPPQGLVYSAMPTAYNTDYSLTSADLS
ALQGFNSPGMLSLGQVSAWQQHHLGQAALSSLVAGGQLSQGSNLSINTNQNISIKSEPISPPRDRMTPSGFQQQQQQQQQQQPPPPP
QPQPQPPQPQPRQEMGRSPVDSLSSSSSSYDGSDREDPRGDFHSPIVLGRPPNTEDRESPSVKRMRMDAWVT
>sp|Q6ZSK4|NTAS1_HUMAN

MKGQEGIRGEGCTDPEIKASPQMWAARFRGMRSRFSPLFSQATEMGPRVSAGWCLSGGGRKVSSLQGDFPPGGFWALSNDSALSLPP
LSLPHPHPLRPPGLGVNEFTQGLHPPLHPAASVFQTCFYRKPHYCSTLRPTTT
>sp|P08048|ZFY_HUMAN
MDEDEFELQPQEPNSFFDGIGADATHMDGDQIVVEIQEAVFVSNIVDSDITVHNFVPDDPDSVVIQDVVEDVVIEEDVQCSDILEEA
DVSENVIIPEQVLDSDVTEEVSLPHCTVPDDVLASDITSTSMSMPEHVLTSESMHVCDIGHVEHMVHDSVVEAEIITDPLTSDIVSE
EVLVADCAPEAVIDASGISVDQQDNDKASCEDYLMISLDDAGKIEHDGSTGVTIDAESEMDPCKVDSTCPEVIKVYIFKADPGEDDL
GGTVDIVESEPENDHGVELLDQNSSIRVPREKMVYMTVNDSQQEDEDLNVAEIADEVYMEVIVGEEDAAVAAAAAAVHEQQIDEDEM
KTFVPIAWAAAYGNNSDGIENRNGTASALLHIDESAGLGRLAKQKPKKKRRPDSRQYQTAIIIGPDGHPLTVYPCMICGKKFKSRGF
LKRHMKNHPEHLAKKKYHCTDCDYTTNKKISLHNHLESHKLTSKAEKAIECDECGKHFSHAGALFTHKMVHKEKGANKMHKCKFCEY
ETAEQGLLNRHLLAVHSKNFPHICVECGKGFRHPSELRKHMRIHTGEKPYQCQYCEYRSADSSNLKTHIKTKHSKEMPFKCDICLLT
FSDTKEVQQHTLVHQESKTHQCLHCDHKSSNSSDLKRHVISVHTKDYPHKCEMCEKGFHRPSELKKHVAVHKGKKMHQCRHCDFKIA
DPFVLSRHILSVHTKDLPFRCKRCRKGFRQQNELKKHMKTHSGRKVYQCEYCEYSTTDASGFKRHVISIHTKDYPHRCEYCKKGFRR
PSEKNQHIMRHHKEVGLP
>sp|Q7L0X0|TRIL_HUMAN
MEAARALRLLLVVCGCLALPPLAEPVCPERCDCQHPQHLLCTNRGLRVVPKTSSLPSPHDVLTYSLGGNFITNITAFDFHRLGQLRR
LDLQYNQIRSLHPKTFEKLSRLEELYLGNNLLQALAPGTLAPLRKLRILYANGNEISRLSRGSFEGLESLVKLRLDGNALGALPDAV
FAPLGNLLYLHLESNRIRFLGKNAFAQLGKLRFLNLSANELQPSLRHAATFAPLRSLSSLILSANNLQHLGPRIFQHLPRLGLLSLR
GNQLTHLAPEAFWGLEALRELRLEGNRLSQLPTALLEPLHSLEALDLSGNELSALHPATFGHLGRLRELSLRNNALSALSGDIFAAS
PALYRLDLDGNGWTCDCRLRGLKRWMGDWHSQGRLLTVFVQCRHPPALRGKYLDYLDDQQLQNGSCADPSPSASLTADRRRQPLPTA
AGEEMTPPAGLAEELPPQPQLQQQGRFLAGVAWDGAARELVGNRSALRLSRRGPGLQQPSPSVAAAAGPAPQSLDLHKKPQRGRPTR
ADPALAEPTPTASPGSAPSPAGDPWQRATKHRLGTEHQERAAQSDGGAGLPPLVSDPCDFNKFILCNLTVEAVGADSASVRWAVREH
RSPRPLGGARFRLLFDRFGQQPKFHRFVYLPESSDSATLRELRGDTPYLVCVEGVLGGRVCPVAPRDHCAGLVTLPEAGSRGGVDYQ
LLTLALLTVNALLVLLALAAWASRWLRRKLRARRKGGAPVHVRHMYSTRRPLRSMGTGVSADFSGFQSHRPRTTVCALSEADLIEFP
CDRFMDSAGGGAGGSLRREDRLLQRFAD
>sp|Q8N7U6|EFHB_HUMAN
MNMEIGHPHEGKDDLGDKRVIMGTKFPMELGIRVGLGKEDSRCGESPVVSNKCEGRMAPPETKFPLSKGLEMGLERQNISRTVMQRG
SLGVDSVSASQGTKPSLLPGRMGLENESLLAGYTHERIIQPPLGRVCGSSQAAGSRRAPLASGPEGVEELVGKPAFVMEPRQEMEKE
STCVLMKPNTEIKLPVEVDIGLTQAEGPDETKNTEPQMGLVIEPPQCQFAQQHEQRKEAGNIESGVEPPDRIRPIYSGKFFDRTPCW
PSAGKVIPVGYRVATCLTEKLPRLITPPEAKKYFNFRYPPAGVERVFYGRANDPQIAPYLTHGIRSKISVLANTLINPQPITTFQQK
IKDKKESIYLSNRRAPLGKSHDQAPGLPKGMDTTNTTFGTAVIKEYSAKDVVNPPKSYEEVFKEGNEGHDLYVVSHNDYYAGEAKNR
KYNPSSFHRCSVYGVPTPHFNDGRAMAKSLYWLHELQMKRGAKFVSKRADDFKEKFQHKLGRVLDPIAETMNVPPDCTFGACLRPEE
YGVGDLIHNRLPDEYLRGKDRQRALIAAVRHHLKKVNYQKFDTLLAAFRHYDKKGDGMIDKDELQEACDQANLSLDDKLLDQLFDYC
DVDNDGFINYLEFANFLNWKDKMLLKEYEERVIIKGRKPDCVNPTEANVEEPEQTLLIKPEDIVLKEAGSTEKTLRTLLRPSDKVSN
YYKTTSSEINAIVGAIPSTCYPICGVPTIRSDIPAPRIRRISDRTNYGEEGSAYSLLYPTIFARKGVFERDFFKTRSKEEIAEILCN
IGVKLSDEEFENVWNLASKKHHRGEVCVENIRNVLDELRHADRIKCKTLM
>sp|Q8NEE6|FXL13_HUMAN
MTPELMIKACSFYTGHLVKTHFCTWRDIARTNENVVLAEKMNRAVTCYNFRLQKSVFHHWHSYMEDQKEKLKNILLRIQQIIYCHKL
TIILTKWRNTARHKSKKKEDELILKHELQLKKWKNRLILKRAAAEESNFPERSSSEVFLVDETLKCDISLLPERAILQIFFYLSLKD
VIICGQVNHAWMLMTQLNSLWNAIDFSSVKNVIPDKYIVSTLQRWRLNVLRLNFRGCLLRPKTFRSVSHCRNLQELNVSDCPTFTDE
SMRHISEGCPGVLCLNLSNTTITNRTMRLLPRHFHNLQNLSLAYCRRFTDKGLQYLNLGNGCHKLIYLDLSGCTQISVQGFRYIANS
CTGIMHLTINDMPTLTDNCVKALVEKCSRITSLVFTGAPHISDCTFRALSACKLRKIRFEGNKRVTDASFKFIDKNYPNLSHIYMAD
CKGITDSSLRSLSPLKQLTVLNLANCVRIGDMGLKQFLDGPASMRIRELNLSNCVRLSDASVMKLSERCPNLNYLSLRNCEHLTAQG
IGYIVNIFSLVSIDLSGTDISNEGLNVLSRHKKLKELSVSECYRITDDGIQAFCKSSLILEHLDVSYCSQLSDMIIKALAIYCINLT

SLSIAGCPKITDSAMEMLSAKCHYLHILDISGCVLLTDQILEDLQIGCKQLRILKMQYCTNISKKAAQRMSSKVQQQEYNTNDPPRW
FGYDREGNPVTELDNITSSKGALELTVKKSTYSSEDQAA
>sp|P60602|ROMO1_HUMAN
MPVAVGPYGQSQPSCFDRVKMGFVMGCAVGMAAGALFGTFSCLRIGMRGRELMGGIGKTMMQSGGTFGTFMAIGMGIRC
>sp|P11277|SPTB1_HUMAN
MTSATEFENVGNQPPYSRINARWDAPDDELDNDNSSARLFERSRIKALADEREVVQKKTFTKWVNSHLARVSCRITDLYKDLRDGRM
LIKLLEVLSGEMLPKPTKGKMRIHCLENVDKALQFLKEQRVHLENMGSHDIVDGNHRLVLGLIWTIILRFQIQDIVVQTQEGRETRS
AKDALLLWCQMKTAGYPHVNVTNFTSSWKDGLAFNALIHKHRPDLIDFDKLKDSNARHNLEHAFNVAERQLGIIPLLDPEDVFTENP
DEKSIITYVVAFYHYFSKMKVLAVEGKRVGKVIDHAIETEKMIEKYSGLASDLLTWIEQTITVLNSRKFANSLTGVQQQLQAFSTYR
TVEKPPKFQEKGNLEVLLFTIQSRMRANNQKVYTPHDGKLVSDINRAWESLEEAEYRRELALRNELIRQEKLEQLARRFDRKAAMRE
TWLSENQRLVAQDNFGYDLAAVEAAKKKHEAIETDTAAYEERVRALEDLAQELEKENYHDQKRITARKDNILRLWSYLQELLQSRRQ
RLETTLALQKLFQDMLHSIDWMDEIKAHLLSAEFGKHLLEVEDLLQKHKLMEADIAIQGDKVKAITAATLKFTEGKGYQPCDPQVIQ
DRISHLEQCFEELSNMAAGRKAQLEQSKRLWKFFWEMDEAESWIKEKEQIYSSLDYGKDLTSVLILQRKHKAFEDELRGLDAHLEQI
FQEAHGMVARKQFGHPQIEARIKEVSAQWDQLKDLAAFCKKNLQDAENFFQFQGDADDLKAWLQDAHRLLSGEDVGQDEGATRALGK
KHKDFLEELEESRGVMEHLEQQAQGFPEEFRDSPDVTHRLQALRELYQQVVAQADLRQQRLQEALDLYTVFGETDACELWMGEKEKW
LAEMEMPDTLEDLEVVQHRFDILDQEMKTLMTQIDGVNLAANSLVESGHPRSREVKQYQDHLNTRWQAFQTLVSERREAVDSALRVH
NYCVDCEETSKWITDKTKVVESTKDLGRDLAGIIAIQRKLSGLERDVAAIQARVDALERESQQLMDSHPEQKEDIGQRQKHLEELWQ
GLQQSLQGQEDLLGEVSQLQAFLQDLDDFQAWLSITQKAVASEDMPESLPEAEQLLQQHAGIKDEIDGHQDSYQRVKESGEKVIQGQ
TDPEYLLLGQRLEGLDTGWNALGRMWESRSHTLAQCLGFQEFQKDAKQAEAILSNQEYTLAHLEPPDSLEAAEAGIRKFEDFLGSME
NNRDKVLSPVDSGNKLVAEGNLYSDKIKEKVQLIEDRHRKNNEKAQEASVLLRDNLELQNFLQNCQELTLWINDKLLTSQDVSYDEA
RNLHNKWLKHQAFVAELASHEGWLENIDAEGKQLMDEKPQFTALVSQKLEALHRLWDELQATTKEKTQHLSAARSSDLRLQTHADLN
KWISAMEDQLRSDDPGKDLTSVNRMLAKLKRVEDQVNVRKEELGELFAQVPSMGEEGGDADLSIEKRFLDLLEPLGRRKKQLESSRA
KLQISRDLEDETLWVEERLPLAQSADYGTNLQTVQLFMKKNQTLQNEILGHTPRVEDVLQRGQQLVEAAEIDCQDLEERLGHLQSSW
DRLREAAAGRLQRLRDANEAQQYYLDADEAEAWIGEQELYVISDEIPKDEEGAIVMLKRHLRQQRAVEDYGRNIKQLASRAQGLLSA
GHPEGEQIIRLQGQVDKHYAGLKDVAEERKRKLENMYHLFQLKRETDDLEQWISEKELVASSPEMGQDFDHVTLLRDKFRDFARETG
AIGQERVDNVNAFIERLIDAGHSEAATIAEWKDGLNEMWADLLELIDTRMQLLAASYDLHRYFYTGAEILGLIDEKHRELPEDVGLD
ASTAESFHRVHTAFERELHLLGVQVQQFQDVATRLQTAYAGEKAEAIQNKEQEVSAAWQALLDACAGRRTQLVDTADKFRFFSMARD
LLSWMESIIRQIETQERPRDVSSVELLMKYHQGINAEIETRSKNFSACLELGESLLQRQHQASEEIREKLQQVMSRRKEMNEKWEAR
WERLRMLLEVCQFSRDASVAEAWLIAQEPYLASGDFGHTVDSVEKLIKRHEAFEKSTASWAERFAALEKPTTLELKERQIAERPAEE
TGPQEEEGETAGEAPVSHHAATERTSPVSLWSRLSSSWESLQPEPSHPY
>sp|P51151|RAB9A_HUMAN
MAGKSSLFKVILLGDGGVGKSSLMNRYVTNKFDTQLFHTIGVEFLNKDLEVDGHFVTMQIWDTAGQERFRSLRTPFYRGSDCCLLTF
SVDDSQSFQNLSNWKKEFIYYADVKEPESFPFVILGNKIDISERQVSTEEAQAWCRDNGDYPYFETSAKDATNVAAAFEEAVRRVLA
TEDRSDHLIQTDTVNLHRKPKPSSSCC
>sp|Q9H4K7|MTG2_HUMAN
MAPARCFSARLRTVFQGVGHWALSTWAGLKPSRLLPQRASPRLLSVGRADLAKHQELPGKKLLSEKKLKRYFVDYRRVLVCGGNGGA
GASCFHSEPRKEFGGPDGGDGGNGGHVILRVDQQVKSLSSVLSRYQGFSGEDGGSKNCFGRSGAVLYIRVPVGTLVKEGGRVVADLS
CVGDEYIAALGGAGGKGNRFFLANNNRAPVTCTPGQPGQQRVLHLELKTVAHAGMVGFPNAGKSSLLRAISNARPAVASYPFTTLKP
HVGIVHYEGHLQIAVADIPGIIRGAHQNRGLGSAFLRHIERCRFLLFVVDLSQPEPWTQVDDLKYELEMYEKGLSARPHAIVANKID
LPEAQANLSQLRDHLGQEVIVLSALTGENLEQLLLHLKVLYDAYAEAELGQGRQPLRW
>sp|Q9BZJ8|GPR61_HUMAN
MESSPIPQSSGNSSTLGRVPQTPGPSTASGVPEVGLRDVASESVALFFMLLLDLTAVAGNAAVMAVIAKTPALRKFVFVFHLCLVDL
LAALTLMPLAMLSSSALFDHALFGEVACRLYLFLSVCFVSLAILSVSAINVERYYYVVHPMRYEVRMTLGLVASVLVGVWVKALAMA
SVPVLGRVSWEEGAPSVPPGCSLQWSHSAYCQLFVVVFAVLYFLLPLLLILVVYCSMFRVARVAAMQHGPLPTWMETPRQRSESLSS

RSTMVTSSGAPQTTPHRTFGGGKAAVVLLAVGGQFLLCWLPYFSFHLYVALSAQPISTGQVESVVTWIGYFCFTSNPFFYGCLNRQI
RGELSKQFVCFFKPAPEEELRLPSREGSIEENFLQFLQGTGCPSESWVSRPLPSPKQEPPAVDFRIPGQIAEETSEFLEQQLTSDII
MSDSYLRPAASPRLES
>sp|Q99665|I12R2_HUMAN
MAHTFRGCSLAFMFIITWLLIKAKIDACKRGDVTVKPSHVILLGSTVNITCSLKPRQGCFHYSRRNKLILYKFDRRINFHHGHSLNS
QVTGLPLGTTLFVCKLACINSDEIQICGAEIFVGVAPEQPQNLSCIQKGEQGTVACTWERGRDTHLYTEYTLQLSGPKNLTWQKQCK
DIYCDYLDFGINLTPESPESNFTAKVTAVNSLGSSSSLPSTFTFLDIVRPLPPWDIRIKFQKASVSRCTLYWRDEGLVLLNRLRYRP
SNSRLWNMVNVTKAKGRHDLLDLKPFTEYEFQISSKLHLYKGSWSDWSESLRAQTPEEEPTGMLDVWYMKRHIDYSRQQISLFWKNL
SVSEARGKILHYQVTLQELTGGKAMTQNITGHTSWTTVIPRTGNWAVAVSAANSKGSSLPTRINIMNLCEAGLLAPRQVSANSEGMD
NILVTWQPPRKDPSAVQEYVVEWRELHPGGDTQVPLNWLRSRPYNVSALISENIKSYICYEIRVYALSGDQGGCSSILGNSKHKAPL
SGPHINAITEEKGSILISWNSIPVQEQMGCLLHYRIYWKERDSNSQPQLCEIPYRVSQNSHPINSLQPRVTYVLWMTALTAAGESSH
GNEREFCLQGKANWMAFVAPSICIAIIMVGIFSTHYFQQKVFVLLAALRPQWCSREIPDPANSTCAKKYPIAEEKTQLPLDRLLIDW
PTPEDPEPLVISEVLHQVTPVFRHPPCSNWPQREKGIQGHQASEKDMMHSASSPPPPRALQAESRQLVDLYKVLESRGSDPKPENPA
CPWTVLPAGDLPTHDGYLPSNIDDLPSHEAPLADSLEELEPQHISLSVFPSSSLHPLTFSCGDKLTLDQLKMRCDSLML
>sp|A6NCE7|MP3B2_HUMAN
MPSEKTFKQRRTFEQRVEDVRLIREQHPTKIPVIIERYKGEKQLPVLDKTKFLVPDHVNMSELIKIIRRRLQLNANQAFFLLVNGHS
MVSVSTPISEVYESEKDEDGFLYMVCASQETFGMKLSV
>sp|Q6IQ20|NAPEP_HUMAN
MDENESNQSLMTSSQYPKEAVRKRQNSARNSGASDSSRFSRKSFKLDYRLEEDVTKSKKGKDGRFVNPWPTWKNPSIPNVLRWLIME
KDHSSVPSSKEELDKELPVLKPYFITNPEEAGVREAGLRVTWLGHATVMVEMDELIFLTDPIFSSRASPSQYMGPKRFRRSPCTISE
LPPIDAVLISHNHYDHLDYNSVIALNERFGNELRWFVPLGLLDWMQKCGCENVIELDWWEENCVPGHDKVTFVFTPSQHWCKRTLMD
DNKVLWGSWSVLGPWNRFFFAGDTGYCPAFEEIGKRFGPFDLAAIPIGAYEPRWFMKYQHVDPEEAVRIHTDVQTKKSMAIHWGTFA
LANEHYLEPPVKLNEALERYGLNAEDFFVLKHGESRYLNNDDENF
>sp|A6NHY2|AKD1B_HUMAN
MDPAGRARGQGATAGGLLLRAAAAAKGLREDLWGAAALPWRSLSRIPKREGLGEEDTAVAGHELLLPNERSFQNAAKSNNLDLMEKL
FEKKVNINVVNNMNRTALHFAVGRNHLSAVDFLLKHKARVDVADKHGLTVIHLAAWSGSLEVMLMLVKAGADQRAKNQDGMSALHFA
TQSNHVRIVEYLIQDLHLKDLNQPDEKGRKPFLLAAERGHVEMIEKLTFLNLHTSEKDKGGNTALHLAAKHGHSPAVQVLLAQWQDI
NEMNELNISSLQIATRNGHASLVNFLLSENVDLHQKVEPKESPLHLVVINNHITVVNSLLSAQHDIDILNQKQQTPLHVAADRGNVE
LVETLLKAGCDLKAVDKQGKTALAVASRSNHSLVVGMLIKAERYYAWREEHHESIRDPSTGFTLTFKQDHSLETRHIRTLLWDLAYH
QLKANEWQRLARSWNFTDDQIRAIEEQWSGNESFREHGHRALLIWLHGTLMTQGDPAKQLYEELVHAGFPKLAEKTRHFKSKTDSNS
KKCVVS
>sp|O43687|AKA7A_HUMAN
MGQLCCFPFSRDEGKISELESSSSAVLQRYSKDIPSWSSGEKNGGEPDDAELVRLSKRLVENAVLKAVQQYLEETQNKNKPGEGSSV
KTEAADQNGNDNENNRK
>sp|Q7Z3J2|CP062_HUMAN
MAVFPWHSRNRNYKAEFASCRLEAVPLEFGDYHPLKPITVTESKTKKVNRKGSTSSTSSSSSSSVVDPLSSVLDGTDPLSMFAATAD
PAALAAAMDSSRRKRDRDDNSVVGSDFEPWTNKRGEILARYTTTEKLSINLFMGSEKGKAGTATLAMSEKVRTRLEELDDFEEGSQK
ELLNLTQQDYVNRIEELNQSLKDAWASDQKVKALKIVIQCSKLLSDTSVIQFYPSKFVLITDILDTFGKLVYERIFSMCVDSRSVLP
DHFSPENANDTAKETCLNWFFKIASIRELIPRFYVEASILKCNKFLSKTGISECLPRLTCMIRGIGDPLVSVYARAYLCRVGMEVAP
HLKETLNKNFFDFLLTFKQIHGDTVQNQLVVQGVELPSYLPLYPPAMDWIFQCISYHAPEALLTEMMERCKKLGNNALLLNSVMSAF
RAEFIATRSMDFIGMIKECDESGFPKHLLFRSLGLNLALADPPESDRLQILNEAWKVITKLKNPQDYINCAEVWVEYTCKHFTKREV
NTVLADVIKHMTPDRAFEDSYPQLQLIIKKVIAHFHDFSVLFSVEKFLPFLDMFQKESVRVEVCKCIMDAFIKHQQEPTKDPVILNA
LLHVCKTMHDSVNALTLEDEKRMLSYLINGFIKMVSFGRDFEQQLSFYVESRSMFCNLEPVLVQLIHSVNRLAMETRKVMKGNHSRK
TAAFVRACVAYCFITIPSLAGIFTRLNLYLHSGQVALANQCLSQADAFFKAAISLVPEVPKMINIDGKMRPSESFLLEFLCNFFSTL

LIVPDHPEHGVLFLVRELLNVIQDYTWEDNSDEKIRIYTCVLHLLSAMSQETYLYHIDKVDSNDSLYGGDSKFLAENNKLCETVMAQ
ILEHLKTLAKDEALKRQSSLGLSFFNSILAHGDLRNNKLNQLSVNLWHLAQRHGCADTRTMVKTLEYIKKQSKQPDMTHLTELALRL
PLQTRT
>sp|Q86UY6|NAA40_HUMAN
MGRKSSKAKEKKQKRLEERAAMDAVCAKVDAANRLGDPLEAFPVFKKYDRNGLNVSIECKRVSGLEPATVDWAFDLTKTNMQTMYEQ
SEWGWKDREKREEMTDDRAWYLIAWENSSVPVAFSHFRFDVECGDEVLYCYEVQLESKVRRKGLGKFLIQILQLMANSTQMKKVMLT
VFKHNHGAYQFFREALQFEIDDSSPSMSGCCGEDCSYEILSRRTKFGDSHHSHAGGHCGGCCH
>sp|Q96LW9|ZSC31_HUMAN
MASTEEQYDLKIVKVEEDPIWDQETHLRGNNFSGQEASRQLFRQFCYQETPGPREALSRLRELCHQWLRPEIHTKEQILELLVLEQF
LTILPEELQAWVREHHPESGEEAVAVVEDLEQELSEPGNQAPDHEHGHSEVLLEDVEHLKVKQEPTDIQLQPMVTQLRYESFCLHQF
QEQDGESIPENQELASKQEILKEMEHLGDSKLQRDVSLDSKYRETCKRDSKAEKQQAHSTGERRHRCNECGKSFTKSSVLIEHQRIH
TGEKPYECEECGKAFSRRSSLNEHRRSHTGEKPYQCKECGKAFSASNGLTRHRRIHTGEKPYECKVCGKAFLLSSCLVQHQRIHTGE
KRYQCRECGKAFIQNAGLFQHLRVHTGEKPYQCSQCSKLFSKRTLLKKHQKIHTGERP
>sp|Q96DA6|TIM14_HUMAN
MASTVVAVGLTIAAAGFAGRYVLQAMKHMEPQVKQVFQSLPKSAFSGGYYRGGFEPKMTKREAALILGVSPTANKGKIRDAHRRIML
LNHPDKGGSPYIAAKINEAKDLLEGQAKK
>sp|O94805|ACL6B_HUMAN
MSGGVYGGDEVGALVFDIGSFSVRAGYAGEDCPKADFPTTVGLLAAEEGGGLELEGDKEKKGKIFHIDTNALHVPRDGAEVMSPLKN
GMIEDWECFRAILDHTYSKHVKSEPNLHPVLMSEAPWNTRAKREKLTELMFEQYNIPAFFLCKTAVLTAFANGRSTGLVLDSGATHT
TAIPVHDGYVLQQGIVKSPLAGDFISMQCRELFQEMAIDIIPPYMIAAKEPVREGAPPNWKKKEKLPQVSKSWHNYMCNEVIQDFQA
SVLQVSDSPYDEQVAAQMPTVHYEMPNGYNTDYGAERLRIPEGLFDPSNVKGLSGNTMLGVGHVVTTSIGMCDIDIRPGLYGSVIVT
GGNTLLQGFTDRLNRELSQKTPPSMRLKLIASNSTMERKFSPWIGGSILASLGTFQQMWISKQEYEEGGKQCVERKCP
>sp|Q96J94|PIWL1_HUMAN
MTGRARARARGRARGQETAQLVGSTASQQPGYIQPRPQPPPAEGELFGRGRQRGTAGGTAKSQGLQISAGFQELSLAERGGRRRDFH
DLGVNTRQNLDHVKESKTGSSGIIVRLSTNHFRLTSRPQWALYQYHIDYNPLMEARRLRSALLFQHEDLIGKCHAFDGTILFLPKRL
QQKVTEVFSKTRNGEDVRITITLTNELPPTSPTCLQFYNIIFRRLLKIMNLQQIGRNYYNPNDPIDIPSHRLVIWPGFTTSILQYEN
SIMLCTDVSHKVLRSETVLDFMFNFYHQTEEHKFQEQVSKELIGLVVLTKYNNKTYRVDDIDWDQNPKSTFKKADGSEVSFLEYYRK
QYNQEITDLKQPVLVSQPKRRRGPGGTLPGPAMLIPELCYLTGLTDKMRNDFNVMKDLAVHTRLTPEQRQREVGRLIDYIHKNDNVQ
RELRDWGLSFDSNLLSFSGRILQTEKIHQGGKTFDYNPQFADWSKETRGAPLISVKPLDNWLLIYTRRNYEAANSLIQNLFKVTPAM
GMQMRKAIMIEVDDRTEAYLRVLQQKVTADTQIVVCLLSSNRKDKYDAIKKYLCTDCPTPSQCVVARTLGKQQTVMAIATKIALQMN
CKMGGELWRVDIPLKLVMIVGIDCYHDMTAGRRSIAGFVASINEGMTRWFSRCIFQDRGQELVDGLKVCLQAALRAWNSCNEYMPSR
IIVYRDGVGDGQLKTLVNYEVPQFLDCLKSIGRGYNPRLTVIVVKKRVNTRFFAQSGGRLQNPLPGTVIDVEVTRPEWYDFFIVSQA
VRSGSVSPTHYNVIYDNSGLKPDHIQRLTYKLCHIYYNWPGVIRVPAPCQYAHKLAFLVGQSIHREPNLSLSNRLYYL
>sp|Q96M89|CC138_HUMAN
MEPRVVKPPGQDLVVESLKSRYGLGGSCPDEYDFSNFYQSKYKRRTLTSPGDLDIYSGDKVGSSLKYSDESKHCRTPLGSLFKHVNV
NCLDDELDSFHDLKKQETEEELIENDYRVSTSKITKQSFKEIEKVALPTNTTSSRPRTECCSDAGDSPLKPVSCPKSKASDKRSLLP
HQISQIYDELFQIHLKLQCETAAQQKFAEELQKRERFLLEREQLLFRHENALSKIKGVEEEVLTRFQIIKEQHDAEVEHLTEVLKEK
NKETKRLRSSFDALKELNDTLKKQLNEASEENRKIDIQAKRVQARLDNLQRKYEFMTIQRLKGSSHAVHEMKSLKQEKAPVSKTYKV
PLNGQVYELLTVFMDWISDHHLSKVKHEESGMDGKKPQLKFASQRNDIQEKCVKLLPLMTEQLQWMPFVNIKLHEPFVKFIYWSLRQ
LDAGAQHSTMTSTLRRLGEDIFKGVVTKGIQDNSPQHSVENKPKTAAFFKSSNLPLRFLSTLIVLKTVTQADYLAQAFDSLCLDLKT
EEGKTLFLEYQAVPVILSHLRISSKGLLSNVIDSLLQMTVESKSLQPFLEACSNSLFFRTCSVLLRAPKLDLQILEKLSIILQKLSK
IKSNKKLFELFTIHLMLQEIQRTTNPEHAFLCINLNSTLFNLGLTKCNSLVSSASP
>sp|Q08ER8|ZN543_HUMAN

MAASAQVSVTFEDVAVTFTQEEWGQLDAAQRTLYQEVMLETCGLLMSLGCPLFKPELIYQLDHRQELWMATKDLSQSSYPGDNTKPK
TTEPTFSHLALPEEVLLQEQLTQGASKNSQLGQSKDQDGPSEMQEVHLKIGIGPQRGKLLEKMSSERDGLGSDDGVCTKITQKQVST
EGDLYECDSHGPVTDALIREEKNSYKCEECGKVFKKNALLVQHERIHTQVKPYECTECGKTFSKSTHLLQHLIIHTGEKPYKCMECG
KAFNRRSHLTRHQRIHSGEKPYKCSECGKAFTHRSTFVLHHRSHTGEKPFVCKECGKAFRDRPGFIRHYIIHTGEKPYECIECGKAF
NRRSYLTWHQQIHTGVKPFECNECGKAFCESADLIQHYIIHTGEKPYKCMECGKAFNRRSHLKQHQRIHTGEKPYECSECGKAFTHC
STFVLHKRTHTGEKPYECKECGKAFSDRADLIRHFSIHTGEKPYECVECGKAFNRSSHLTRHQQIHTGEKPYECIQCGKAFCRSANL
IRHSIIHTGEKPYECSECGKAFNRGSSLTHHQRIHTGRNPTIVTDVGRPFMTAQTSVNIQELLLGKEFLNITTEENLW
>sp|Q9Y286|SIGL7_HUMAN
MLLLLLLPLLWGRERVEGQKSNRKDYSLTMQSSVTVQEGMCVHVRCSFSYPVDSQTDSDPVHGYWFRAGNDISWKAPVATNNPAWAV
QEETRDRFHLLGDPQTKNCTLSIRDARMSDAGRYFFRMEKGNIKWNYKYDQLSVNVTALTHRPNILIPGTLESGCFQNLTCSVPWAC
EQGTPPMISWMGTSVSPLHPSTTRSSVLTLIPQPQHHGTSLTCQVTLPGAGVTTNRTIQLNVSYPPQNLTVTVFQGEGTASTALGNS
SSLSVLEGQSLRLVCAVDSNPPARLSWTWRSLTLYPSQPSNPLVLELQVHLGDEGEFTCRAQNSLGSQHVSLNLSLQQEYTGKMRPV
SGVLLGAVGGAGATALVFLSFCVIFIVVRSCRKKSARPAADVGDIGMKDANTIRGSASQGNLTESWADDNPRHHGLAAHSSGEEREI
QYAPLSFHKGEPQDLSGQEATNNEYSEIKIPK
>sp|P00414|COX3_HUMAN
MTHQSHAYHMVKPSPWPLTGALSALLMTSGLAMWFHFHSMTLLMLGLLTNTLTMYQWWRDVTRESTYQGHHTPPVQKGLRYGMILFI
TSEVFFFAGFFWAFYHSSLAPTPQLGGHWPPTGITPLNPLEVPLLNTSVLLASGVSITWAHHSLMENNRNQMIQALLITILLGLYFT
LLQASEYFESPFTISDGIYGSTFFVATGFHGLHVIIGSTFLTICFIRQLMFHFTSKHHFGFEAAAWYWHFVDVVWLFLYVSIYWWGS
>sp|Q9UIJ5|ZDHC2_HUMAN
MAPSGPGSSARRRCRRVLYWIPVVFITLLLGWSYYAYAIQLCIVSMENTGEQVVCLMAYHLLFAMFVWSYWKTIFTLPMNPSKEFHL
SYAEKDLLEREPRGEAHQEVLRRAAKDLPIYTRTMSGAIRYCDRCQLIKPDRCHHCSVCDKCILKMDHHCPWVNNCVGFSNYKFFLL
FLAYSLLYCLFIAATDLQYFIKFWTNGLPDTQAKFHIMFLFFAAAMFSVSLSSLFGYHCWLVSKNKSTLEAFRSPVFRHGTDKNGFS
LGFSKNMRQVFGDEKKYWLLPIFSSLGDGCSFPTCLVNQDPEQASTPAGLNSTAKNLENHQFPAKPLRESQSHLLTDSQSWTESSIN
PGKCKAGMSNPALTMENET
>sp|P0CW24|PNM6A_HUMAN
MAVTMLQDWCRWMGVNARRGLLILGIPEDCDDAEFQESLEAALRPMGHFTVLGKAFREEDNATAALVELDREVNYALVPREIPGTGG
PWNVVFVPRCSGEEFLGLGRVFHFPEQEGQMVESVAGALGVGLRRVCWLRSIGQAVQPWVEAVRCQSLGVFSGRDQPAPGEESFEVW
LDHTTEMLHVWQGVSERERRRRLLEGLRGTALQLVHALLAENPARTAQDCLAALAQVFGDNESQATIRVKCLTAQQQSGERLSAFVL
RLEVLLQKAMEKEALARASADRVRLRQMLTRAHLTEPLDEALRKLRMAGRSPSFLEMLGLVRESEAWEASLARSVRAQTQEGAGARA
GAQAVARASTKVEAVPGGPGREPEGLLQAGGQEAEELLQEGLKPVLEECDN
>sp|Q96MF4|CC140_HUMAN
MGDECSNPDLLAEPGSSPPWDHGNQRQEAANESNTRVPRVLKAHLGPETAQPTKRSKRNRWRRQSCQGPSPARSGQFLGSADLGLQR
GVLKSAARTCLSEISNSTRASPESAQSTDPGRAARPRTRTLPTPHSFKIGEEAEEMKKKKERKRRKERKKERNFKK
>sp|P48595|SPB10_HUMAN
MDSLATSINQFALELSKKLAESAQGKNIFFSSWSISTSLTIVYLGAKGTTAAQMAQVLQFNRDQGVKCDPESEKKRKMEFNLSNSEE
IHSDFQTLISEILKPNDDYLLKTANAIYGEKTYAFHNKYLEDMKTYFGAEPQPVNFVEASDQIRKDINSWVERQTEGKIQNLLPDDS
VDSTTRMILVNALYFKGIWEHQFLVQNTTEKPFRINETTSKPVQMMFMKKKLHIFHIEKPKAVGLQLYYKSRDLSLLILLPEDINGL
EQLEKAITYEKLNEWTSADMMELYEVQLHLPKFKLEDSYDLKSTLSSMGMSDAFSQSKADFSGMSSARNLFLSNVFHKAFVEINEQG
TEAAAGSGSEIDIRIRVPSIEFNANHPFLFFIRHNKTNTILFYGRLCSP
>sp|Q08431|MFGM_HUMAN
MPRPRLLAALCGALLCAPSLLVALDICSKNPCHNGGLCEEISQEVRGDVFPSYTCTCLKGYAGNHCETKCVEPLGMENGNIANSQIA
ASSVRVTFLGLQHWVPELARLNRAGMVNAWTPSSNDDNPWIQVNLLRRMWVTGVVTQGASRLASHEYLKAFKVAYSLNGHEFDFIHD
VNKKHKEFVGNWNKNAVHVNLFETPVEAQYVRLYPTSCHTACTLRFELLGCELNGCANPLGLKNNSIPDKQITASSSYKTWGLHLFS

WNPSYARLDKQGNFNAWVAGSYGNDQWLQVDLGSSKEVTGIITQGARNFGSVQFVASYKVAYSNDSANWTEYQDPRTGSSKIFPGNW
DNHSHKKNLFETPILARYVRILPVAWHNRIALRLELLGC
>sp|Q02413|DSG1_HUMAN
MDWSFFRVVAMLFIFLVVVEVNSEFRIQVRDYNTKNGTIKWHSIRRQKREWIKFAAACREGEDNSKRNPIAKIHSDCAANQQVTYRI
SGVGIDQPPYGIFVINQKTGEINITSIVDREVTPFFIIYCRALNSMGQDLERPLELRVRVLDINDNPPVFSMATFAGQIEENSNANT
LVMILNATDADEPNNLNSKIAFKIIRQEPSDSPMFIINRNTGEIRTMNNFLDREQYGQYALAVRGSDRDGGADGMSAECECNIKILD
VNDNIPYMEQSSYTIEIQENTLNSNLLEIRVIDLDEEFSANWMAVIFFISGNEGNWFEIEMNERTNVGILKVVKPLDYEAMQSLQLS
IGVRNKAEFHHSIMSQYKLKASAISVTVLNVIEGPVFRPGSKTYVVTGNMGSNDKVGDFVATDLDTGRPSTTVRYVMGNNPADLLAV
DSRTGKLTLKNKVTKEQYNMLGGKYQGTILSIDDNLQRTCTGTININIQSFGNDDRTNTEPNTKITTNTGRQESTSSTNYDTSTTST
DSSQVYSSEPGNGAKDLLSDNVHFGPAGIGLLIMGFLVLGLVPFLMICCDCGGAPRSAAGFEPVPECSDGAIHSWAVEGPQPEPRDI
TTVIPQIPPDNANIIECIDNSGVYTNEYGGREMQDLGGGERMTGFELTEGVKTSGMPEICQEYSGTLRRNSMRECREGGLNMNFMES
YFCQKAYAYADEDEGRPSNDCLLIYDIEGVGSPAGSVGCCSFIGEDLDDSFLDTLGPKFKKLADISLGKESYPDLDPSWPPQSTEPV
CLPQETEPVVSGHPPISPHFGTTTVISESTYPSGPGVLHPKPILDPLGYGNVTVTESYTTSDTLKPSVHVHDNRPASNVVVTERVVG
PISGADLHGMLEMPDLRDGSNVIVTERVIAPSSSLPTSLTIHHPRESSNVVVTERVIQPTSGMIGSLSMHPELANAHNVIVTERVVS
GAGVTGISGTTGISGGIGSSGLVGTSMGAGSGALSGAGISGGGIGLSSLGGTASIGHMRSSSDHHFNQTIGSASPSTARSRITKYST
VQYSK
>sp|Q9H0E3|SP130_HUMAN
MGPPRHPQAGEIEAGGAGGGRRLQVEMSSQQFPRLGAPSTGLSQAPSQIANSGSAGLINPAATVNDESGRDSEVSAREHMSSSSSLQ
SREEKQEPVVVRPYPQVQMLSTHHAVASATPVAVTAPPAHLTPAVPLSFSEGLMKPPPKPTMPSRPIAPAPPSTLSLPPKVPGQVTV
TMESSIPQASAIPVATISGQQGHPSNLHHIMTTNVQMSIIRSNAPGPPLHIGASHLPRGAAAAAVMSSSKVTTVLRPTSQLPNAATA
QPAVQHIIHQPIQSRPPVTTSNAIPPAVVATVSATRAQSPVITTTAAHATDSALSRPTLSIQHPPSAAISIQRPAQSRDVTTRITLP
SHPALGTPKQQLHTMAQKTIFSTGTPVAAATVAPILATNTIPSATTAGSVSHTQAPTSTIVTMTVPSHSSHATAVTTSNIPVAKVVP
QQITHTSPRIQPDYPAERSSLIPISGHRASPNPVAMETRSDNRPSVPVQFQYFLPTYPPSAYPLAAHTYTPITSSVSTIRQYPVSAQ
APNSAITAQTGVGVASTVHLNPMQLMTVDASHARHIQGIQPAPISTQGIQPAPIGTPGIQPAPLGTQGIHSATPINTQGLQPAPMGT
QQPQPEGKTSAVVLADGATIVANPISNPFSAAPAATTVVQTHSQSASTNAPAQGSSPRPSILRKKPATDGAKPKSEIHVSMATPVTV
SMETVSNQNNDQPTIAVPPTAQQPPPTIPTMIAAASPPSQPAVALSTIPGAVPITPPITTIAAAPPPSVTVGGSLSSVLGPPVPEIK
VKEEVEPMDIMRPVSAVPPLATNTVSPSLALLANNLSMPTSDLPPGASPRKKPRKQQHVISTEEGDMMETNSTDDEKSTAKSLLVKA
EKRKSPPKEYIDEEGVRYVPVRPRPPITLLRHYRNPWKAAYHHFQRYSDVRVKEEKKAMLQEIANQKGVSCRAQGWKVHLCAAQLLQ
LTNLEHDVYERLTNLQEGIIPKKKAATDDDLHRINELIQGNMQRCKLVMDQISEARDSMLKVLDHKDRVLKLLNKNGTVKKVSKLKR
KEKV
>sp|P30443|1A01_HUMAN
MAVMAPRTLLLLLSGALALTQTWAGSHSMRYFFTSVSRPGRGEPRFIAVGYVDDTQFVRFDSDAASQKMEPRAPWIEQEGPEYWDQE
TRNMKAHSQTDRANLGTLRGYYNQSEDGSHTIQIMYGCDVGPDGRFLRGYRQDAYDGKDYIALNEDLRSWTAADMAAQITKRKWEAV
HAAEQRRVYLEGRCVDGLRRYLENGKETLQRTDPPKTHMTHHPISDHEATLRCWALGFYPAEITLTWQRDGEDQTQDTELVETRPAG
DGTFQKWAAVVVPSGEEQRYTCHVQHEGLPKPLTLRWELSSQPTIPIVGIIAGLVLLGAVITGAVVAAVMWRRKSSDRKGGSYTQAA
SSDSAQGSDVSLTACKV
>sp|Q6UY01|LRC31_HUMAN
MSQTRKKTSSEGETKPQTSTVNKFLRGSNAESRKEDNDLKTSDSQPSDWIQKTATSETAKPLSSEMEWRSSMEKNEHFLQKLGKKAV
NKCLDLNNCGLTTADMKEMVALLPFLPDLEELDISWNGFVGGTLLSITQQMHLVSKLKILRLGSCRLTTDDVQALGEAFEMIPELEE
LNLSWNSKVGGNLPLILQKFQKGSKIQMIELVDCSLTSEDGTFLGQLLPMLQSLEVLDLSINRDIVGSLNSIAQGLKSTSNLKVLKL
HSCGLSQKSVKILDAAFRYLGELRKLDLSCNKDLGGGFEDSPAQLVMLKHLQVLDLHQCSLTADDVMSLTQVIPLLSNLQELDLSAN
KKMGSSSENLLSRLRFLPALKSLVINNCALESETFTALAEASVHLSALEVFNLSWNKCVGGNLKLLLETLKLSMSLQVLRLSSCSLV
TEDVALLASVIQTGHLAKLQKLDLSYNDSICDAGWTMFCQNVRFLKELIELDISLRPSNFRDCGQWFRHLLYAVTKLPQITEIGMKR
WILPASQEEELECFDQDKKRSIHFDHGGFQ

>sp|P41240|CSK_HUMAN
MSAIQAAWPSGTECIAKYNFHGTAEQDLPFCKGDVLTIVAVTKDPNWYKAKNKVGREGIIPANYVQKREGVKAGTKLSLMPWFHGKI
TREQAERLLYPPETGLFLVRESTNYPGDYTLCVSCDGKVEHYRIMYHASKLSIDEEVYFENLMQLVEHYTSDADGLCTRLIKPKVME
GTVAAQDEFYRSGWALNMKELKLLQTIGKGEFGDVMLGDYRGNKVAVKCIKNDATAQAFLAEASVMTQLRHSNLVQLLGVIVEEKGG
LYIVTEYMAKGSLVDYLRSRGRSVLGGDCLLKFSLDVCEAMEYLEGNNFVHRDLAARNVLVSEDNVAKVSDFGLTKEASSTQDTGKL
PVKWTAPEALREKKFSTKSDVWSFGILLWEIYSFGRVPYPRIPLKDVVPRVEKGYKMDAPDGCPPAVYEVMKNCWHLDAAMRPSFLQ
LREQLEHIKTHELHL
>sp|Q6NTF7|ABC3H_HUMAN
MALLTAETFRLQFNNKRRLRRPYYPRKALLCYQLTPQNGSTPTRGYFENKKKCHAEICFINEIKSMGLDETQCYQVTCYLTWSPCSS
CAWELVDFIKAHDHLNLGIFASRLYYHWCKPQQKGLRLLCGSQVPVEVMGFPKFADCWENFVDHEKPLSFNPYKMLEELDKNSRAIK
RRLERIKIPGVRAQGRYMDILCDAEV
>sp|P33316|DUT_HUMAN
MTPLCPRPALCYHFLTSLLRSAMQNARGARQRAEAAVLSGPGPPLGRAAQHGIPRPLSSAGRLSQGCRGASTVGAAGWKGELPKAGG
SPAPGPETPAISPSKRARPAEVGGMQLRFARLSEHATAPTRGSARAAGYDLYSAYDYTIPPMEKAVVKTDIQIALPSGCYGRVAPRS
GLAAKHFIDVGAGVIDEDYRGNVGVVLFNFGKEKFEVKKGDRIAQLICERIFYPEIEEVQALDDTERGSGGFGSTGKN
>sp|Q0VFX4|YL016_HUMAN
MLKKPSSLEQWEILGTSSGEFRCISRDCPGAGNNNREPSISTRGRTSSSKMVLPHPKVAEEAVGGPQSCKWLSCGLQGTGGGHLEGH
PPRVSQESAPAGHTGISPSSSGVHLIQAKTAGWPQRVSSAEQCLLPIQHVPGADFLHVFTLRLHCGPARNAKLVEALFNSNSSC
>sp|Q9Y5P2|CSAG2_HUMAN
MWMGLIQLVEGVKRKDQGFLEKEFYHKTNIKMRCEFLACWPAFTVLGEAWRDQVDWSRLLRDAGLVKMSRKPRASSPLSNNHPPTPK
RRGSGRHPLNPGPEALSKFPRQPGREKGPIKEVPGTKGSP
>sp|Q96I27|ZN625_HUMAN
MGERLLESKKDHQHGEILTQVPDDMLKKKTPRVKSCGEVSVGHASLNRHHRADTGHKPYEYQEYGQKPYKCTYCKKAFSDLPYFRTH
EWAHTGGKPYDCEECGKSFISRSSIRRHRIMHSGDGPYKCNFCGKALMCLSLYLIHKRTHTGEKPYECKQCGKAFSHSGSLRIHERT
HTGEKPYECSECGKAFHSSTCLHAHKITHTGEKPYECKQCGKAFVSFNSVRYHERTHTGEKPYECKQCGKAFRSASHLRTHGRTHTG
EKPYECKQCGKAFGCASSVKIHERTHTGEKPCSSNTSKGQGEKIA
>sp|Q86UW9|DTX2_HUMAN
MAMAPSPSLVQVYTSPAAVAVWEWQDGLGTWHPYSATVCSFIEQQFVQQKGQRFGLGSLAHSIPLGQADPSLAPYIIDLPSWTQFRQ
DTGTMRAVRRHLFPQHSAPGRGVVWEWLSDDGSWTAYEASVCDYLEQQVARGNQLVDLAPLGYNYTVNYTTHTQTNKTSSFCRSVRR
QAGPPYPVTTIIAPPGHTGVACSCHQCLSGSRTGPVSGRYRHSMTNLPAYPVPQHPPHRTASVFGTHQAFAPYNKPSLSGARSAPRL
NTTNAWGAAPPSLGSQPLYRSSLSHLGPQHLPPGSSTSGAVSASLPSGPSSSPGSVPATVPMQMPKPSRVQQALAGMTSVLMSAIGL
PVCLSRAPQPTSPPASRLASKSHGSVKRLRKMSVKGATPKPEPEPEQVIKNYTEELKVPPDEDCIICMEKLSTASGYSDVTDSKAIG
SLAVGHLTKCSHAFHLLCLLAMYCNGNKDGSLQCPSCKTIYGEKTGTQPQGKMEVLRFQMSLPGHEDCGTILIVYSIPHGIQGPEHP
NPGKPFTARGFPRQCYLPDNAQGRKVLELLKVAWKRRLIFTVGTSSTTGETDTVVWNEIHHKTEMDRNITGHGYPDPNYLQNVLAEL
AAQGVTEDCLEQQ
>sp|Q6PIF6|MYO7B_HUMAN
MSGFRLGDHVWLEPPSTHKTGVAIGGIIKEAKPGKVLVEDDEGKEHWIRAEDFGVLSPMHPNSVQGVDDMIRLGDLNEAGMVHNLLI
RYQQHKIYTYTGSILVAVNPFQVLPLYTLEQVQLYYSRHMGELPPHVFAIANNCYFSMKRNKRDQCCIISGESGAGKTETTKLILQF
LATISGQHSWIEQQVLEANPILEAFGNAKTIRNDNSSRFGKYIDIYFNPSGVIEGARIEQFLLEKSRVCRQAPEERNYHIFYCMLMG
VSAEDKQLLSLGTPSEYHYLTMGNCTSCEGLNDAKDYAHIRSAMKILQFSDSESWDVIKLLAAILHLGNVGFMASVFENLDASDVME
TPAFPTVMKLLEVQHQELRDCLIKHTILIRGEFVTRSLNIAQAADRRDAFVKGIYGHLFLWIVKKINAAIFTPPAQDPKNVRRAIGL
LDIFGFENFENNSFEQLCINFANEHLQQFFVQHVFTMEQEEYRSENISWDYIHYTDNRPTLDLLALKPMSIISLLDEESRFPQGTDL
TMLQKLNSVHANNKAFLQPKNIHDARFGIAHFAGEVYYQAEGFLEKNRDVLSTDILTLVYSSKNKFLREIFNLELAETKLGHGTIRQ
AKAGNHLFKSADSNKRPSTLGSQFKQSLDQLMKILTNCQPYFIRCIKPNEYKKPLLFDRELCLRQLRYSGMMETVHIRKSGFPIRYT

FEEFSQRFGVLLPNAMRMQLQGKLRQMTLGITDVWLRTDKDWKAGKTKIFLRDHQDTLLEVQRSQVLDRAALSIQKVLRGYRYRKEF
LRQRRAAVTLQAWWRGYCNRRNFKLILVGFERLQAIARSQPLARQYQAMRQRTVQLQALCRGYLVRQQVQAKRRAVVVIQAHARGMA
ARRNFQQRKANAPLVIPAEGQKSQGALPAKKRRSIYDTVTDTEMVEKVFGFLPAMIGGQEGQASPHFEDLESKTQKLLEVDLDTVPM
AEEPEEDVDGLAEYTFPKFAVTYFQKSASHTHIRRPLRYPLLYHEDDTDCLAALVIWNVILRFMGDLPEPVLYARSSQQGSSVMRQI
HDTLGREHGAQVPQHSRSAQVASQLNIGEEALEPDGLGADRPMSNLEKVHFIVGYAILRPSLRDEIYCQICKQLSENFKTSSLARGW
ILLSLCLGCFPPSERFMKYLLNFIGQGPATYGPFCAERLRRTYANGVRAEPPTWLELQAVKSKKHIPIQVILATGESLTVPVDSAST
SREMCMHIAHKQGLSDHLGFSLQVAVYDKFWSLGSGRDHMMDAIARCEQMAQERGESQRQSPWRIYFRKEFFTPWHDSREDPVSTEL
IYRQVLRGVWSGEYSFEKEEELVELLARHCYVQLGASAESKAVQELLPSCIPHKLYRTKPPDRWASLVTAACAKAPYTQKQVTPLAV
REQVVDAARLQWPLLFSRLFEVITLSGPRLPKTQLILAVNWKGLCFLDQQEKMLLELSFPEVMGLATNREAQGGQRLLLSTMHEEYE
FVSPSSVAIAELVALFLEGLKERSIFAMALQDRKATDDTTLLAFKKGDLLVLTKKQGLLASENWTLGQNDRTGKTGLVPMACLYTIP
TVTKPSAQLLSLLAMSPEKRKLAAQEGQFTEPRPEEPPKEKLHTLEEFSYEFFRAPEKDMVSMAVLPLARARGHLWAYSCEPLRQPL
LKRVHANVDLWDIACQIFVAILRYMGDYPSRQAWPTLELTDQIFTLALQHPALQDEVYCQILKQLTHNSNRHSEERGWQLLWLCTGL
FPPSKGLLPHAQKFIDTRRGKLLAPDCSRRIQKVLRTGPRKQPPHQVEVEAAEQNVSRICHKIYFPNDTSEMLEVVANTRVRDVCDS
IATRLQLASWEGCSLFIKISDKVISQKEGDFFFDSLREVSDWVKKNKPQKEGAPVTLPYQVYFMRKLWLNISPGKDVNADTILHYHQ
ELPKYLRGFHKCSREDAIHLAGLIYKAQFNNDRSQLASVPKILRELVPENLTRLMSSEEWKKSILLAYDKHKDKTVEEAKVAFLKWI
CRWPTFGSAFFEVKQTSEPSYPDVILIAINRHGVLLIHPKTKDLLTTYPFTKISSWSSGSTYFHMALGSLGRGSRLLCETSLGYKMD
DLLTSYVQQLLSAMNKQRGSKAPALAST
>sp|Q96SA4|SERC2_HUMAN
MGACLGACSLLSCASCLCGSAPCILCSCCPASRNSTVSRLIFTFFLFLGVLVSIIMLSPGVESQLYKLPWVCEEGAGIPTVLQGHID
CGSLLGYRAVYRMCFATAAFFFFFTLLMLCVSSSRDPRAAIQNGFWFFKFLILVGLTVGAFYIPDGSFTNIWFYFGVVGSFLFILIQ
LVLLIDFAHSWNQRWLGKAEECDSRAWYAGLFFFTLLFYLLSIAAVALMFMYYTEPSGCHEGKVFISLNLTFCVCVSIAAVLPKVQD
AQPNSGLLQASVITLYTMFVTWSALSSIPEQKCNPHLPTQLGNETVVAGPEGYETQWWDAPSIVGLIIFLLCTLFISLRSSDHRQVN
SLMQTEECPPMLDATQQQQQVAACEGRAFDNEQDGVTYSYSFFHFCLVLASLHVMMTLTNWYKPGETRKMISTWTAVWVKICASWAG
LLLYLWTLVAPLLLRNRDFS
>sp|P09016|HXD4_HUMAN
MVMSSYMVNSKYVDPKFPPCEEYLQGGYLGEQGADYYGGGAQGADFQPPGLYPRPDFGEQPFGGSGPGPGSALPARGHGQEPGGPGG
HYAAPGEPCPAPPAPPPAPLPGARAYSQSDPKQPPSGTALKQPAVVYPWMKKVHVNSVNPNYTGGEPKRSRTAYTRQQVLELEKEFH
FNRYLTRRRRIEIAHTLCLSERQIKIWFQNRRMKWKKDHKLPNTKGRSSSSSSSSSCSSSVAPSQHLQPMAKDHHTDLTTL
>sp|A6NEV1|PR23A_HUMAN
MGSRPRSPSAFPAPWWGQQPGGPGPAKRLRLEEPAGPEPRVAPSLEDPAGTPAVGALTSIVVLAAGCALRVPLDDVDLVLELPPTSI
LRVSLDGHTLILIPEVLLSSVDERSGAQDDSSAGLEVDVFLGALREDVVVEQEVFCASVPEIAAQEEAYEEDADPEFPELQMDSAAG
SAAGLYSSARSMFSPYREGPIPEPCALAPNPSSEGHSPGPFFDPEFRLLEPVPSSPLQPLPPSPRVGSPGPHAHPPLPKRPPCKARR
RLFQE
>sp|P20337|RAB3B_HUMAN
MASVTDGKTGVKDASDQNFDYMFKLLIIGNSSVGKTSFLFRYADDTFTPAFVSTVGIDFKVKTVYRHEKRVKLQIWDTAGQERYRTI
TTAYYRGAMGFILMYDITNEESFNAVQDWATQIKTYSWDNAQVILVGNKCDMEEERVVPTEKGQLLAEQLGFDFFEASAKENISVRQ
AFERLVDAICDKMSDSLDTDPSMLGSSKNTRLSDTPPLLQQNCSC
>sp|Q5T280|CI114_HUMAN
MAERGRKRPCGPGEHGQRIEWRKWKQQKKEEKKKWKDLKLMKKLERQRAQEEQAKRLEEEEAAAEKEDRGRPYTLSVALPGSILDNA
QSPELRTYLAGQIARACAIFCVDEIVVFDEEGQDAKTVEGEFTGVGKKGQACVQLARILQYLECPQYLRKAFFPKHQDLQFAGLLNP
LDSPHHMRQDEESEFREGIVVDRPTRPGHGSFVNCGMKKEVKIDKNLEPGLRVTVRLNQQQHPDCKTYHGKVVSSQDPRTKAGLYWG
YTVRLASCLSAVFAEAPFQDGYDLTIGTSERGSDVASAQLPNFRHALVVFGGLQGLEAGADADPNLEVAEPSVLFDLYVNTCPGQGS
RTIRTEEAILISLAALQPGLIQAGARHT
>sp|Q8NGG1|OR8J2_HUMAN

MASGNLTWVTEFILVGVSDDPELQIPLFLVFLVLYLLTVAGNLGIITLTSVDPQLQTPMYFFLRHLAIINLCNSTVVAPKMLVNFLV
TKKTISYYGCAAQLGGFLVFIVAEIFTLAAMAYDRYVAIWSPLLYAVVVSPKVCRLLVSLTYLQSLITALTVSSCVFSVSYCSSNII
NHFYCDDVPLLALSCSDTYIPETAVFIFSGTNLLFSMIVVLISYFNIVITILRIRSSEGRQKAFSTCASHMIAVVVFYGTLLFMYLQ
PRSNHSLDTDKMASVFYTLVIPVLNPLIYSLRNKNVKDALKRFLDNPCRSLKLM
>sp|Q9UBP8|KAAG1_HUMAN
MDDDAAPRVEGVPVAVHKHALHDGLRQVAGPGAAAAHLPRWPPPQLAASRREAPPLSQRPHRTQGAGSPPETNEKLTNPQVKEK
>sp|P23219|PGH1_HUMAN
MSRSLLLWFLLFLLLLPPLPVLLADPGAPTPVNPCCYYPCQHQGICVRFGLDRYQCDCTRTGYSGPNCTIPGLWTWLRNSLRPSPSF
THFLLTHGRWFWEFVNATFIREMLMRLVLTVRSNLIPSPPTYNSAHDYISWESFSNVSYYTRILPSVPKDCPTPMGTKGKKQLPDAQ
LLARRFLLRRKFIPDPQGTNLMFAFFAQHFTHQFFKTSGKMGPGFTKALGHGVDLGHIYGDNLERQYQLRLFKDGKLKYQVLDGEMY
PPSVEEAPVLMHYPRGIPPQSQMAVGQEVFGLLPGLMLYATLWLREHNRVCDLLKAEHPTWGDEQLFQTTRLILIGETIKIVIEEYV
QQLSGYFLQLKFDPELLFGVQFQYRNRIAMEFNHLYHWHPLMPDSFKVGSQEYSYEQFLFNTSMLVDYGVEALVDAFSRQIAGRIGG
GRNMDHHILHVAVDVIRESREMRLQPFNEYRKRFGMKPYTSFQELVGEKEMAAELEELYGDIDALEFYPGLLLEKCHPNSIFGESMI
EIGAPFSLKGLLGNPICSPEYWKPSTFGGEVGFNIVKTATLKKLVCLNTKTCPYVSFRVPDASQDDGPAVERPSTEL
>sp|Q9NR34|MA1C1_HUMAN
MLMRKVPGFVPASPWGLRLPQKFLFLLFLSGLVTLCFGALFLLPHSSRLKRLFLAPRTQQPGLEVVAEIAGHAPAREQEPPPNPAPA
APAPGEDDPSSWASPRRRKGGLRRTRPTGPREEATAARGNSIPASRPGDEGVPFRFDFNAFRSRLRHPVLGTRADESQEPQSQVRAQ
REKIKEMMQFAWQSYKRYAMGKNELRPLTKDGYEGNMFGGLSGATVIDSLDTLYLMELKEEFQEAKAWVGESFHLNVSGEASLFEVN
IRYIGGLLSAFYLTGEEVFRIKAIRLGEKLLPAFNTPTGIPKGVVSFKSGNWGWATAGSSSILAEFGSLHLEFLHLTELSGNQVFAE
KVRNIRKVLRKIEKPFGLYPNFLSPVSGNWVQHHVSVGGLGDSFYEYLIKSWLMSGKTDMEAKNMYYEALEAIETYLLNVSPGGLTY
IAEWRGGILDHKMGHLACFSGGMIALGAEDAKEEKRAHYRELAAQITKTCHESYARSDTKLGPEAFWFNSGREAVATQLSESYYILR
PEVVESYMYLWRQTHNPIYREWGWEVVLALEKYCRTEAGFSGIQDVYSSTPNHDNKQQSFFLAETLKYLYLLFSEDDLLSLEDWVFN
TEAHPLPVNHSDSSGRAWGRH
>sp|Q9NQC3|RTN4_HUMAN
MEDLDQSPLVSSSDSPPRPQPAFKYQFVREPEDEEEEEEEEEEDEDEDLEELEVLERKPAAGLSAAPVPTAPAAGAPLMDFGNDFVP
PAPRGPLPAAPPVAPERQPSWDPSPVSSTVPAPSPLSAAAVSPSKLPEDDEPPARPPPPPPASVSPQAEPVWTPPAPAPAAPPSTPA
APKRRGSSGSVDETLFALPAASEPVIRSSAENMDLKEQPGNTISAGQEDFPSVLLETAASLPSLSPLSAASFKEHEYLGNLSTVLPT
EGTLQENVSEASKEVSEKAKTLLIDRDLTEFSELEYSEMGSSFSVSPKAESAVIVANPREEIIVKNKDEEEKLVSNNILHNQQELPT
ALTKLVKEDEVVSSEKAKDSFNEKRVAVEAPMREEYADFKPFERVWEVKDSKEDSDMLAAGGKIESNLESKVDKKCFADSLEQTNHE
KDSESSNDDTSFPSTPEGIKDRSGAYITCAPFNPAATESIATNIFPLLGDPTSENKTDEKKIEEKKAQIVTEKNTSTKTSNPFLVAA
QDSETDYVTTDNLTKVTEEVVANMPEGLTPDLVQEACESELNEVTGTKIAYETKMDLVQTSEVMQESLYPAAQLCPSFEESEATPSP
VLPDIVMEAPLNSAVPSAGASVIQPSSSPLEASSVNYESIKHEPENPPPYEEAMSVSLKKVSGIKEEIKEPENINAALQETEAPYIS
IACDLIKETKLSAEPAPDFSDYSEMAKVEQPVPDHSELVEDSSPDSEPVDLFSDDSIPDVPQKQDETVMLVKESLTETSFESMIEYE
NKEKLSALPPEGGKPYLESFKLSLDNTKDTLLPDEVSTLSKKEKIPLQMEELSTAVYSNDDLFISKEAQIRETETFSDSSPIEIIDE
FPTLISSKTDSFSKLAREYTDLEVSHKSEIANAPDGAGSLPCTELPHDLSLKNIQPKVEEKISFSDDFSKNGSATSKVLLLPPDVSA
LATQAEIESIVKPKVLVKEAEKKLPSDTEKEDRSPSAIFSAELSKTSVVDLLYWRDIKKTGVVFGASLFLLLSLTVFSIVSVTAYIA
LALLSVTISFRIYKGVIQAIQKSDEGHPFRAYLESEVAISEELVQKYSNSALGHVNCTIKELRRLFLVDDLVDSLKFAVLMWVFTYV
GALFNGLTLLILALISLFSVPVIYERHQAQIDHYLGLANKNVKDAMAKIQAKIPGLKRKAE
>sp|P61201|CSN2_HUMAN
MSDMEDDFMCDDEEDYDLEYSEDSNSEPNVDLENQYYNSKALKEDDPKAALSSFQKVLELEGEKGEWGFKALKQMIKINFKLTNFPE
MMNRYKQLLTYIRSAVTRNYSEKSINSILDYISTSKQMDLLQEFYETTLEALKDAKNDRLWFKTNTKLGKLYLEREEYGKLQKILRQ
LHQSCQTDDGEDDLKKGTQLLEIYALEIQMYTAQKNNKKLKALYEQSLHIKSAIPHPLIMGVIRECGGKMHLREGEFEKAHTDFFEA
FKNYDESGSPRRTTCLKYLVLANMLMKSGINPFDSQEAKPYKNDPEILAMTNLVSAYQNNDITEFEKILKTNHSNIMDDPFIREHIE

ELLRNIRTQVLIKLIKPYTRIHIPFISKELNIDVADVESLLVQCILDNTIHGRIDQVNQLLELDHQKRGGARYTALDKWTNQLNSLN
QAVVSKLA
>sp|P05386|RLA1_HUMAN
MASVSELACIYSALILHDDEVTVTEDKINALIKAAGVNVEPFWPGLFAKALANVNIGSLICNVGAGGPAPAAGAAPAGGPAPSTAAA
PAEEKKVEAKKEESEESDDDMGFGLFD
>sp|O60384|ZN861_HUMAN
MWLSTSPYRKGSQCGEAFSQIPGHNLNKKTPPGVKPPESHVCGEVGVGYPSTERHIRDRLGRKPCEYQECRQKAYTCKPCGNAFRFH
HSFHIHERPHSGENLYEC
>sp|O95671|ASML_HUMAN
MVLCPVIGKLLHKRVVLASASPRRQEILSNAGLRFEVVPSKFKEKLDKASFATPYGYAMETAKQKALEVANRLYQKDLRAPDVVIGA
DTIVTVGGLILEKPVDKQDAYRMLSRLSGREHSVFTGVAIVHCSSKDHQLDTRVSEFYEETKVKFSELSEELLWEYVHSGEPMDKAG
GYGIQALGGMLVESVHGDFLNVVGFPLNHFCKQLVKLYYPPRPEDLRRSVKHDSIPAADTFEDLSDVEGGGSEPTQRDAGSRDEKAE
AGEAGQATAEAECHRTRETLPPFPTRLLELIEGFMLSKGLLTACKLKVFDLLKDEAPQKAADIASKVDASACGMERLLDICAAMGLL
EKTEQGYSNTETANVYLASDGEYSLHGFIMHNNDLTWNLFTYLEFAIREGTNQHHRALGKKAEDLFQDAYYQSPETRLRFMRAMHGM
TKLTACQVATAFNLSRFSSACDVGGCTGALARELAREYPRMQVTVFDLPDIIELAAHFQPPGPQAVQIHFAAGDFFRDPLPSAELYV
LCRILHDWPDDKVHKLLSRVAESCKPGAGLLLVETLLDEEKRVAQRALMQSLNMLVQTEGKERSLGEYQCLLELHGFHQVQVVHLGG
VLDAILATKVAP
>sp|Q8N5M9|JAGN1_HUMAN
MASRAGPRAAGTDGSDFQHRERVAMHYQMSVTLKYEIKKLIYVHLVIWLLLVAKMSVGHLRLLSHDQVAMPYQWEYPYLLSILPSLL
GLLSFPRNNISYLVLSMISMGLFSIAPLIYGSMEMFPAAQQLYRHGKAYRFLFGFSAVSIMYLVLVLAVQVHAWQLYYSKKLLDSWF
TSTQEKKHK
>sp|Q8NAN2|MIGA1_HUMAN
MSDCCSAPGISWEAGVGRPAVPGLELQIRRGAMSEETVSESQFSLKTAALRVFDLPLTWYYSLSQIKFSPVAKKLFVVTAVSAISVI
FLAHHFKRKRGKKKGKILPWEPEHLILEYTKRAASDKGSSCSSSRQNLTLSLSSTKDKGSQVCNYANGGLFSKYSGSAQSLASVQSV
NSCHSCACGNSNSWDKADEDDIKLVNIPVTTPENLYLMGMELFEEALRRWEQALTFRNRQAEDEACGSIKLGAGDAIAEENVDDIIS
TEFIHKLEALLQRAYRLQEEFEATLGASDPNSLADDIDKDTDITMKGNVEDFGLRDTLSIASTDSFASAAELAEHREVRHTYSLESL
CHCPFYEEAMHLVEEGKIYSRVLRTEMLECLGDSDFLAKLHCIRQAFQVILSESANRIFLAESGRKILSALIVKARKNPKKFEDVFD
EMIYFLEQTDHWGSTEMELAARGVKNLNFYDVVLDFILMDSFEDLENPPTSIQNVVNNRWLNSSFKETAVASSCWSVLKQKRQQMKI
PDGFFAHFYAICEHISPVLAWGFLGPRNSLYDLCCFFKNQVLLFLKDIFDFEKVRYSSTETLAEDLMQLLIRRTELLMAYLEADALR
HTSSCLSSHGHVMSTGLLEAKVQ
>sp|P80370|DLK1_HUMAN
MTATEALLRVLLLLLAFGHSTYGAECFPACNPQNGFCEDDNVCRCQPGWQGPLCDQCVTSPGCLHGLCGEPGQCICTDGWDGELCDR
DVRACSSAPCANNRTCVSLDDGLYECSCAPGYSGKDCQKKDGPCVINGSPCQHGGTCVDDEGRASHASCLCPPGFSGNFCEIVANSC
TPNPCENDGVCTDIGGDFRCRCPAGFIDKTCSRPVTNCASSPCQNGGTCLQHTQVSYECLCKPEFTGLTCVKKRALSPQQVTRLPSG
YGLAYRLTPGVHELPVQQPEHRILKVSMKELNKKTPLLTEGQAICFTILGVLTSLVVLGTVGIVFLNKCETWVSNLRYNHMLRKKKN
LLLQYNSGEDLAVNIIFPEKIDMTTFSKEAGDEEI
>sp|Q9P0K7|RAI14_HUMAN
MKSLKAKFRKSDTNEWNKNDDRLLQAVENGDAEKVASLLGKKGASATKHDSEGKTAFHLAAAKGHVECLRVMITHGVDVTAQDTTGH
SALHLAAKNSHHECIRKLLQSKCPAESVDSSGKTALHYAAAQGCLQAVQILCEHKSPINLKDLDGNIPLLLAVQNGHSEICHFLLDH
GADVNSRNKSGRTALMLACEIGSSNAVEALIKKGADLNLVDSLGYNALHYSKLSENAGIQSLLLSKISQDADLKTPTKPKQHDQVSK
ISSERSGTPKKRKAPPPPISPTQLSDVSSPRSITSTPLSGKESVFFAEPPFKAEISSIRENKDRLSDSTTGADSLLDISSEADQQDL
LSLLQAKVASLTLHNKELQDKLQAKSPKEAEADLSFDSYHSTQTDLGPSLGKPGETSPPDSKSSPSVLIHSLGKSTTDNDVRIQQLQ
EILQDLQKRLESSEAERKQLQVELQSRRAELVCLNNTEISENSSDLSQKLKETQSKYEEAMKEVLSVQKQMKLGLVSPESMDNYSHF
HELRVTEEEINVLKQDLQNALEESERNKEKVRELEEKLVEREKGTVIKPPVEEYEEMKSSYCSVIENMNKEKAFLFEKYQEAQEEIM

KLKDTLKSQMTQEASDEAEDMKEAMNRMIDELNKQVSELSQLYKEAQAELEDYRKRKSLEDVTAEYIHKAEHEKLMQLTNVSRAKAE
DALSEMKSQYSKVLNELTQLKQLVDAQKENSVSITEHLQVITTLRTAAKEMEEKISNLKEHLASKEVEVAKLEKQLLEEKAAMTDAM
VPRSSYEKLQSSLESEVSVLASKLKESVKEKEKVHSEVVQIRSEVSQVKREKENIQTLLKSKEQEVNELLQKFQQAQEELAEMKRYA
ESSSKLEEDKDKKINEMSKEVTKLKEALNSLSQLSYSTSSSKRQSQQLEALQQQVKQLQNQLAECKKQHQEVISVYRMHLLYAVQGQ
MDEDVQKVLKQILTMCKNQSQKK
>sp|A6NLC8|YE016_HUMAN
METGRQTGVSAEMFAMPRDLKGSKKDGIPEDLDGNLEEPRDQEGELRSEDVMDLTEGDNEASASAPPAAKRRKTDTKGKKERKPTVD
AEEAQRMTTLLSAMSEEQLSRYEVCRRSAFPKACIAGLMRSITGRSVSENVAIAMAGIAKVFVGEVVEEALDVCEMWGEMPPLQPKH
LREAVRRLKPKGLFPNSNYKKIMF
>sp|P42566|EPS15_HUMAN
MAAAAQLSLTQLSSGNPVYEKYYRQVDTGNTGRVLASDAAAFLKKSGLPDLILGKIWDLADTDGKGILNKQEFFVALRLVACAQNGL
EVSLSSLNLAVPPPRFHDTSSPLLISGTSAAELPWAVKPEDKAKYDAIFDSLSPVNGFLSGDKVKPVLLNSKLPVDILGRVWELSDI
DHDGMLDRDEFAVAMFLVYCALEKEPVPMSLPPALVPPSKRKTWVVSPAEKAKYDEIFLKTDKDMDGFVSGLEVREIFLKTGLPSTL
LAHIWSLCDTKDCGKLSKDQFALAFHLISQKLIKGIDPPHVLTPEMIPPSDRASLQKNIIGSSPVADFSAIKELDTLNNEIVDLQRE
KNNVEQDLKEKEDTIKQRTSEVQDLQDEVQRENTNLQKLQAQKQQVQELLDELDEQKAQLEEQLKEVRKKCAEEAQLISSLKAELTS
QESQISTYEEELAKAREELSRLQQETAELEESVESGKAQLEPLQQHLQDSQQEISSMQMKLMEMKDLENHNSQLNWCSSPHSILVNG
ATDYCSLSTSSSETANLNEHVEGQSNLESEPIHQESPARSSPELLPSGVTDENEVTTAVTEKVCSELDNNRHSKEEDPFNVDSSSLT
GPVADTNLDFFQSDPFVGSDPFKDDPFGKIDPFGGDPFKGSDPFASDCFFRQSTDPFATSSTDPFSAANNSSITSVETLKHNDPFAP
GGTVVAASDSATDPFASVFGNESFGGGFADFSTLSKVNNEDPFRSATSSSVSNVVITKNVFEETSVKSEDEPPALPPKIGTPTRPCP
LPPGKRSINKLDSPDPFKLNDPFQPFPGNDSPKEKDPEIFCDPFTSATTTTNKEADPSNFANFSAYPSEEDMIEWAKRESEREEEQR
LARLNQQEQEDLELAIALSKSEISEA
>sp|Q12905|ILF2_HUMAN
MRGDRGRGRGGRFGSRGGPGGGFRPFVPHIPFDFYLCEMAFPRVKPAPDETSFSEALLKRNQDLAPNSAEQASILSLVTKINNVIDN
LIVAPGTFEVQIEEVRQVGSYKKGTMTTGHNVADLVVILKILPTLEAVAALGNKVVESLRAQDPSEVLTMLTNETGFEISSSDATVK
ILITTVPPNLRKLDPELHLDIKVLQSALAAIRHARWFEENASQSTVKVLIRLLKDLRIRFPGFEPLTPWILDLLGHYAVMNNPTRQP
LALNVAYRRCLQILAAGLFLPGSVGITDPCESGNFRVHTVMTLEQQDMVCYTAQTLVRILSHGGFRKILGQEGDASYLASEISTWDG
VIVTPSEKAYEKPPEKKEGEEEEENTEEPPQGEEEESMETQE
>sp|Q8N0V1|ZNAS1_HUMAN
MKDKMWCEDTAQPHRRLPAPPSSSSPTVVFQSHRRLLAPPSSSWTLWVAPRCGCCSPLCPKRVCASLCRVLAVTWSLTKLPFPLSPI
LLSRPGWLPQPSSPGPACAEPSSQEGGDTDLRSYGCFVCGWAWPTLSPRP
>sp|P62851|RS25_HUMAN
MPPKDDKKKKDAGKSAKKDKDPVNKSGGKAKKKKWSKGKVRDKLNNLVLFDKATYDKLCKEVPNYKLITPAVVSERLKIRGSLARAA
LQELLSKGLIKLVSKHRAQVIYTRNTKGGDAPAAGEDA
>sp|Q13698|CAC1S_HUMAN
MEPSSPQDEGLRKKQPKKPVPEILPRPPRALFCLTLENPLRKACISIVEWKPFETIILLTIFANCVALAVYLPMPEDDNNSLNLGLE
KLEYFFLIVFSIEAAMKIIAYGFLFHQDAYLRSGWNVLDFTIVFLGVFTVILEQVNVIQSHTAPMSSKGAGLDVKALRAFRVLRPLR
LVSGVPSLQVVLNSIFKAMLPLFHIALLVLFMVIIYAIIGLELFKGKMHKTCYFIGTDIVATVENEEPSPCARTGSGRRCTINGSEC
RGGWPGPNHGITHFDNFGFSMLTVYQCITMEGWTDVLYWVNDAIGNEWPWIYFVTLILLGSFFILNLVLGVLSGEFTKEREKAKSRG
TFQKLREKQQLDEDLRGYMSWITQGEVMDVEDFREGKLSLDEGGSDTESLYEIAGLNKIIQFIRHWRQWNRIFRWKCHDIVKSKVFY
WLVILIVALNTLSIASEHHNQPLWLTRLQDIANRVLLSLFTTEMLMKMYGLGLRQYFMSIFNRFDCFVVCSGILEILLVESGAMTPL
GISVLRCIRLLRIFKITKYWTSLSNLVASLLNSIRSIASLLLLLFLFIVIFALLGMQLFGGRYDFEDTEVRRSNFDNFPQALISVFQ
VLTGEDWTSMMYNGIMAYGGPSYPGMLVCIYFIILFVCGNYILLNVFLAIAVDNLAEAESLTSAQKAKAEEKKRRKMSKGLPDKSEE
EKSTMAKKLEQKPKGEGIPTTAKLKIDEFESNVNEVKDPYPSADFPGDDEEDEPEIPLSPRPRPLAELQLKEKAVPIPEASSFFIFS
PTNKIRVLCHRIVNATWFTNFILLFILLSSAALAAEDPIRADSMRNQILKHFDIGFTSVFTVEIVLKMTTYGAFLHKGSFCRNYFNM

LDLLVVAVSLISMGLESSAISVVKILRVLRVLRPLRAINRAKGLKHVVQCMFVAISTIGNIVLVTTLLQFMFACIGVQLFKGKFFRC
TDLSKMTEEECRGYYYVYKDGDPMQIELRHREWVHSDFHFDNVLSAMMSLFTVSTFEGWPQLLYKAIDSNAEDVGPIYNNRVEMAIF
FIIYIILIAFFMMNIFVGFVIVTFQEQGETEYKNCELDKNQRQCVQYALKARPLRCYIPKNPYQYQVWYIVTSSYFEYLMFALIMLN
TICLGMQHYNQSEQMNHISDILNVAFTIIFTLEMILKLMAFKARGYFGDPWNVFDFLIVIGSIIDVILSEIDTFLASSGGLYCLGGG
CGNVDPDESARISSAFFRLFRVMRLIKLLSRAEGVRTLLWTFIKSFQALPYVALLIVMLFFIYAVIGMQMFGKIALVDGTQINRNNN
FQTFPQAVLLLFRCATGEAWQEILLACSYGKLCDPESDYAPGEEYTCGTNFAYYYFISFYMLCAFLVINLFVAVIMDNFDYLTRDWS
ILGPHHLDEFKAIWAEYDPEAKGRIKHLDVVTLLRRIQPPLGFGKFCPHRVACKRLVGMNMPLNSDGTVTFNATLFALVRTALKIKT
EGNFEQANEELRAIIKKIWKRTSMKLLDQVIPPIGDDEVTVGKFYATFLIQEHFRKFMKRQEEYYGYRPKKDIVQIQAGLRTIEEEA
APEICRTVSGDLAAEEELERAMVEAAMEEGIFRRTGGLFGQVDNFLERTNSLPPVMANQRPLQFAEIEMEEMESPVFLEDFPQDPRT
NPLARANTNNANANVAYGNSNHSNSHVFSSVHYEREFPEETETPATRGRALGQPCRVLGPHSKPCVEMLKGLLTQRAMPRGQAPPAP
CQCPRVESSMPEDRKSSTPGSLHEETPHSRSTRENTSRCSAPATALLIQKALVRGGLGTLAADANFIMATGQALADACQMEPEEVEI
MATELLKGREAPEGMASSLGCLNLGSSLGSLDQHQGSQETLIPPRL
>sp|P15814|IGLL1_HUMAN
MRPGTGQGGLEAPGEPGPNLRQRWPLLLLGLAVVTHGLLRPTAASQSRALGPGAPGGSSRSSLRSRWGRFLLQRGSWTGPRCWPRGF
QSKHNSVTHVFGSGTQLTVLSQPKATPSVTLFPPSSEELQANKATLVCLMNDFYPGILTVTWKADGTPITQGVEMTTPSKQSNNKYA
ASSYLSLTPEQWRSRRSYSCQVMHEGSTVEKTVAPAECS
>sp|Q8NFA0|UBP32_HUMAN
MGAKESRIGFLSYEEALRRVTDVELKRLKDAFKRTCGLSYYMGQHCFIREVLGDGVPPKVAEVIYCSFGGTSKGLHFNNLIVGLVLL
TRGKDEEKAKYIFSLFSSESGNYVIREEMERMLHVVDGKVPDTLRKCFSEGEKVNYEKFRNWLFLNKDAFTFSRWLLSGGVYVTLTD
DSDTPTFYQTLAGVTHLEESDIIDLEKRYWLLKAQSRTGRFDLETFGPLVSPPIRPSLSEGLFNAFDENRDNHIDFKEISCGLSACC
RGPLAERQKFCFKVFDVDRDGVLSRVELRDMVVALLEVWKDNRTDDIPELHMDLSDIVEGILNAHDTTKMGHLTLEDYQIWSVKNVL
ANEFLNLLFQVCHIVLGLRPATPEEEGQIIRGWLERESRYGLQAGHNWFIISMQWWQQWKEYVKYDANPVVIEPSSVLNGGKYSFGT
AAHPMEQVEDRIGSSLSYVNTTEEKFSDNISTASEASETAGSGFLYSATPGADVCFARQHNTSDNNNQCLLGANGNILLHLNPQKPG
AIDNQPLVTQEPVKATSLTLEGGRLKRTPQLIHGRDYEMVPEPVWRALYHWYGANLALPRPVIKNSKTDIPELELFPRYLLFLRQQP
ATRTQQSNIWVNMGNVPSPNAPLKRVLAYTGCFSRMQTIKEIHEYLSQRLRIKEEDMRLWLYNSENYLTLLDDEDHKLEYLKIQDEQ
HLVIEVRNKDMSWPEEMSFIANSSKIDRHKVPTEKGATGLSNLGNTCFMNSSIQCVSNTQPLTQYFISGRHLYELNRTNPIGMKGHM
AKCYGDLVQELWSGTQKNVAPLKLRWTIAKYAPRFNGFQQQDSQELLAFLLDGLHEDLNRVHEKPYVELKDSDGRPDWEVAAEAWDN
HLRRNRSIVVDLFHGQLRSQVKCKTCGHISVRFDPFNFLSLPLPMDSYMHLEITVIKLDGTTPVRYGLRLNMDEKYTGLKKQLSDLC
GLNSEQILLAEVHGSNIKNFPQDNQKVRLSVSGFLCAFEIPVPVSPISASSPTQTDFSSSPSTNEMFTLTTNGDLPRPIFIPNGMPN
TVVPCGTEKNFTNGMVNGHMPSLPDSPFTGYIIAVHRKMMRTELYFLSSQKNRPSLFGMPLIVPCTVHTRKKDLYDAVWIQVSRLAS
PLPPQEASNHAQDCDDSMGYQYPFTLRVVQKDGNSCAWCPWYRFCRGCKIDCGEDRAFIGNAYIAVDWDPTALHLRYQTSQERVVDE
HESVEQSRRAQAEPINLDSCLRAFTSEEELGENEMYYCSKCKTHCLATKKLDLWRLPPILIIHLKRFQFVNGRWIKSQKIVKFPRES
FDPSAFLVPRDPALCQHKPLTPQGDELSEPRILAREVKKVDAQSSAGEEDVLLSKSPSSLSANIISSPKGSPSSSRKSGTSCPSSKN
SSPNSSPRTLGRSKGRLRLPQIGSKNKLSSSKENLDASKENGAGQICELADALSRGHVLGGSQPELVTPQDHEVALANGFLYEHEAC
GNGYSNGQLGNHSEEDSTDDQREDTRIKPIYNLYAISCHSGILGGGHYVTYAKNPNCKWYCYNDSSCKELHPDEIDTDSAYILFYEQ
QGIDYAQFLPKTDGKKMADTSSMDEDFESDYKKYCVLQ
>sp|Q969U6|FBXW5_HUMAN
MDEGGTPLLPDSLVYQIFLSLGPADVLAAGLVCRQWQAVSRDEFLWREQFYRYYQVARDVPRHPAAMSWYEEFQRLYDTVPCVEVQT
LREHTDQVLHLSFSHSGYQFASCSKDCTVKIWSNDLTISLLHSADMRPYNWSYTQFSQFNKDDSLLLASGVFLGPHNSSSGEIAVIS
LDSFALLSRVRNKPYDVFGCWLTETSLISGNLHRIGDITSCSVLWLNNAFQDVESENVNVVKRLFKIQNLNASTVRTVMVADCSRFD
SPDLLLEAGDPATSPCRIFDLGSDNEEVVAGPAPAHAKEGLRHFLDRVLEGRAQPQLSERMLETKVAELLAQGHTKPPERSATGAKS
KYLIFTTGCLTYSPHQIGIKQILPHQMTTAGPVLGEGRGSDAFFDALDHVIDIHGHIIGMGLSPDNRYLYVNSRAWPNGAVVADPMQ
PPPIAEEIDLLVFDLKTMREVRRALRAHRAYTPNDECFFIFLDVSRDFVASGAEDRHGYIWDRHYNICLARLRHEDVVNSVVFSPQE
QELLLTASDDATIKAWRSPRTMRVLQAPRPRPRTFFSWLASQRR

>sp|O00548|DLL1_HUMAN
MGSRCALALAVLSALLCQVWSSGVFELKLQEFVNKKGLLGNRNCCRGGAGPPPCACRTFFRVCLKHYQASVSPEPPCTYGSAVTPVL
GVDSFSLPDGGGADSAFSNPIRFPFGFTWPGTFSLIIEALHTDSPDDLATENPERLISRLATQRHLTVGEEWSQDLHSSGRTDLKYS
YRFVCDEHYYGEGCSVFCRPRDDAFGHFTCGERGEKVCNPGWKGPYCTEPICLPGCDEQHGFCDKPGECKCRVGWQGRYCDECIRYP
GCLHGTCQQPWQCNCQEGWGGLFCNQDLNYCTHHKPCKNGATCTNTGQGSYTCSCRPGYTGATCELGIDECDPSPCKNGGSCTDLEN
SYSCTCPPGFYGKICELSAMTCADGPCFNGGRCSDSPDGGYSCRCPVGYSGFNCEKKIDYCSSSPCSNGAKCVDLGDAYLCRCQAGF
SGRHCDDNVDDCASSPCANGGTCRDGVNDFSCTCPPGYTGRNCSAPVSRCEHAPCHNGATCHERGHRYVCECARGYGGPNCQFLLPE
LPPGPAVVDLTEKLEGQGGPFPWVAVCAGVILVLMLLLGCAAVVVCVRLRLQKHRPPADPCRGETETMNNLANCQREKDISVSIIGA
TQIKNTNKKADFHGDHSADKNGFKARYPAVDYNLVQDLKGDDTAVRDAHSKRDTKCQPQGSSGEEKGTPTTLRGGEASERKRPDSGC
STSKDTKYQSVYVISEEKDECVIATEV
>sp|P00558|PGK1_HUMAN
MSLSNKLTLDKLDVKGKRVVMRVDFNVPMKNNQITNNQRIKAAVPSIKFCLDNGAKSVVLMSHLGRPDGVPMPDKYSLEPVAVELKS
LLGKDVLFLKDCVGPEVEKACANPAAGSVILLENLRFHVEEEGKGKDASGNKVKAEPAKIEAFRASLSKLGDVYVNDAFGTAHRAHS
SMVGVNLPQKAGGFLMKKELNYFAKALESPERPFLAILGGAKVADKIQLINNMLDKVNEMIIGGGMAFTFLKVLNNMEIGTSLFDEE
GAKIVKDLMSKAEKNGVKITLPVDFVTADKFDENAKTGQATVASGIPAGWMGLDCGPESSKKYAEAVTRAKQIVWNGPVGVFEWEAF
ARGTKALMDEVVKATSRGCITIIGGGDTATCCAKWNTEDKVSHVSTGGGASLELLEGKVLPGVDALSNI
>sp|P49326|FMO5_HUMAN
MTKKRIAVIGGGVSGLSSIKCCVEEGLEPVCFERTDDIGGLWRFQENPEEGRASIYKSVIINTSKEMMCFSDYPIPDHYPNFMHNAQ
VLEYFRMYAKEFDLLKYIRFKTTVCSVKKQPDFATSGQWEVVTESEGKKEMNVFDGVMVCTGHHTNAHLPLESFPGIEKFKGQYFHS
RDYKNPEGFTGKRVIIIGIGNSGGDLAVEISQTAKQVFLSTRRGAWILNRVGDYGYPADVLFSSRLTHFIWKICGQSLANKYLEKKI
NQRFDHEMFGLKPKHRALSQHPTLNDDLPNRIISGLVKVKGNVKEFTETAAIFEDGSREDDIDAVIFATGYSFDFPFLEDSVKVVKN
KISLYKKVFPPNLERPTLAIIGLIQPLGAIMPISELQGRWATQVFKGLKTLPSQSEMMAEISKAQEEIDKRYVESQRHTIQGDYIDT
MEELADLVGVRPNLLSLAFTDPKLALHLLLGPCTPIHYRVQGPGKWDGARKAILTTDDRIRKPLMTRVVERSSSMTSTMTIGKFMLA
LAFFAIIIAYF
>sp|Q8NH56|O52N5_HUMAN
MPLFNSLCWFPTIHVTPPSFILNGIPGLERVHVWISLPLCTMYIIFLVGNLGLVYLIYYEESLHHPMYFFFGHALSLIDLLTCTTTL
PNALCIFWFSLKEINFNACLAQMFFVHGFTGVESGVLMLMALDRYVAICYPLRYATTLTNPIIAKAELATFLRGVLLMIPFPFLVKR
LPFCQSNIISHTYCDHMSVVKLSCASIKVNVIYGLMVALLIGVFDICCISLSYTLILKAAISLSSSDARQKAFSTCTAHISAIIITY
VPAFFTFFAHRFGGHTIPPSLHIIVANLYLLLPPTLNPIVYGVKTKQIRKSVIKFFQGDKGAG
>sp|Q9HAP6|LIN7B_HUMAN
MAALVEPLGLERDVSRAVELLERLQRSGELPPQKLQALQRVLQSRFCSAIREVYEQLYDTLDITGSAEIRAHATAKATVAAFTASEG
HAHPRVVELPKTDEGLGFNIMGGKEQNSPIYISRVIPGGVADRHGGLKRGDQLLSVNGVSVEGEQHEKAVELLKAAQGSVKLVVRYT
PRVLEEMEARFEKMRSARRRQQHQSYSSLESRG
>sp|P0DML2|CSH1_HUMAN
MAPGSRTSLLLAFALLCLPWLQEAGAVQTVPLSRLFDHAMLQAHRAHQLAIDTYQEFEETYIPKDQKYSFLHDSQTSFCFSDSIPTP
SNMEETQQKSNLELLRISLLLIESWLEPVRFLRSMFANNLVYDTSDSDDYHLLKDLEEGIQTLMGRLEDGSRRTGQILKQTYSKFDT
NSHNHDALLKNYGLLYCFRKDMDKVETFLRMVQCRSVEGSCGF
>sp|P23490|LORI_HUMAN
MSYQKKQPTPQPPVDCVKTSGGGGGGGGSGGGGCGFFGGGGSGGGSSGSGCGYSGGGGYSGGGCGGGSSGGGGGGGIGGCGGGSGGS
VKYSGGGGSSGGGSGCFSSGGGGSGCFSSGGGGSSGGGSGCFSSGGGGSSGGGSGCFSSGGGGFSGQAVQCQSYGGVSSGGSSGGGS
GCFSSGGGGGSVCGYSGGGSGCGGGSSGGSGSGYVSSQQVTQTSCAPQPSYGGGSSGGGGSGGSGCFSSGGGGGSSGCGGGSSGIGS
GCIISGGGSVCGGGSSGGGGGGSSVGGSGSGKGVPICHQTQQKQAPTWPSK
>sp|Q8IUW3|SPA2L_HUMAN

MGSSSLSEDYRQCLERELRRGRAGVCGDPSLRAVLWQILVEDFDLHGALQDDALALLTDGLWGRADLAPALRGLARAFELLELAAVH
LYLLPWRKEFTTIKTFSGGYVHVLKGVLSDDLLLKSFQKMGYVRRDSHRLMVTALPPACQLVQVALGCFALRLECEILGEVLAQLGT
SVLPAEELLQARRASGDVASCVAWLQQRLAQDEEPPPLPPRGSPAAYRAPLDLYRDLQEDEGSEDASLYGEPSPGPDSPPAELAYRP
PLWEQSAKLWGTGGRAWEPPAEELPQASSPPYGALEEGLEPEPSAFSFLSLRRELSRPGDLATPESSAAASPRRIRAEGVPASAYRS
VSEPPGYQAHSCLSPGALPTLCCDTCRQLHAAHCAALPACRPGHSLRVLLGDAQRRLWLQRAQMDTLLYNSPGARP
>sp|Q9NQ30|ESM1_HUMAN
MKSVLLLTTLLVPAHLVAAWSNNYAVDCPQHCDSSECKSSPRCKRTVLDDCGCCRVCAAGRGETCYRTVSGMDGMKCGPGLRCQPSN
GEDPFGEEFGICKDCPYGTFGMDCRETCNCQSGICDRGTGKCLKFPFFQYSVTKSSNRFVSLTEHDMASGDGNIVREEVVKENAAGS
PVMRKWLNPR
>sp|A6NHQ2|FBLL1_HUMAN
MKSAASSRGGGGGGRGGGGWGSWGGGRGGGGGAGKGGGGDGGGQGGKGGFGARARGFGGGGRGRGRGGGDGKDRGGGGQRRGGVAKS
KSRRRKGAMVVSVEPHRHEGVFIYRGAEDALVTLNMVPGQSVYGERRVTVTEGGVKQEYRTWNPFRSKLAAAILGGVDQIHIKPKSK
VLYLGAASGTTVSHVSDIIGPDGLVYAVEFSHRAGRDLVNVAKKRTNIIPVLEDARHPLKYRMLIGMVDVIFADVAQPDQSRIVALN
AHTFLRNGGHFLISIKANCIDSTASAEAVFASEVRKLQQENLKPQEQLTLEPYERDHAVVVGVYRPLPKSSSK
>sp|O95214|LERL1_HUMAN
MAGIKALISLSFGGAIGLMFLMLGCALPIYNKYWPLFVLFFYILSPIPYCIARRLVDDTDAMSNACKELAIFLTTGIVVSAFGLPIV
FARAHLIEWGACALVLTGNTVIFATILGFFLVFGSNDDFSWQQW
>sp|O75197|LRP5_HUMAN
MEAAPPGPPWPLLLLLLLLLALCGCPAPAAASPLLLFANRRDVRLVDAGGVKLESTIVVSGLEDAAAVDFQFSKGAVYWTDVSEEAI
KQTYLNQTGAAVQNVVISGLVSPDGLACDWVGKKLYWTDSETNRIEVANLNGTSRKVLFWQDLDQPRAIALDPAHGYMYWTDWGETP
RIERAGMDGSTRKIIVDSDIYWPNGLTIDLEEQKLYWADAKLSFIHRANLDGSFRQKVVEGSLTHPFALTLSGDTLYWTDWQTRSIH
ACNKRTGGKRKEILSALYSPMDIQVLSQERQPFFHTRCEEDNGGCSHLCLLSPSEPFYTCACPTGVQLQDNGRTCKAGAEEVLLLAR
RTDLRRISLDTPDFTDIVLQVDDIRHAIAIDYDPLEGYVYWTDDEVRAIRRAYLDGSGAQTLVNTEINDPDGIAVDWVARNLYWTDT
GTDRIEVTRLNGTSRKILVSEDLDEPRAIALHPVMGLMYWTDWGENPKIECANLDGQERRVLVNASLGWPNGLALDLQEGKLYWGDA
KTDKIEVINVDGTKRRTLLEDKLPHIFGFTLLGDFIYWTDWQRRSIERVHKVKASRDVIIDQLPDLMGLKAVNVAKVVGTNPCADRN
GGCSHLCFFTPHATRCGCPIGLELLSDMKTCIVPEAFLVFTSRAAIHRISLETNNNDVAIPLTGVKEASALDFDVSNNHIYWTDVSL
KTISRAFMNGSSVEHVVEFGLDYPEGMAVDWMGKNLYWADTGTNRIEVARLDGQFRQVLVWRDLDNPRSLALDPTKGYIYWTEWGGK
PRIVRAFMDGTNCMTLVDKVGRANDLTIDYADQRLYWTDLDTNMIESSNMLGQERVVIADDLPHPFGLTQYSDYIYWTDWNLHSIER
ADKTSGRNRTLIQGHLDFVMDILVFHSSRQDGLNDCMHNNGQCGQLCLAIPGGHRCGCASHYTLDPSSRNCSPPTTFLLFSQKSAIS
RMIPDDQHSPDLILPLHGLRNVKAIDYDPLDKFIYWVDGRQNIKRAKDDGTQPFVLTSLSQGQNPDRQPHDLSIDIYSRTLFWTCEA
TNTINVHRLSGEAMGVVLRGDRDKPRAIVVNAERGYLYFTNMQDRAAKIERAALDGTEREVLFTTGLIRPVALVVDNTLGKLFWVDA
DLKRIESCDLSGANRLTLEDANIVQPLGLTILGKHLYWIDRQQQMIERVEKTTGDKRTRIQGRVAHLTGIHAVEEVSLEEFSAHPCA
RDNGGCSHICIAKGDGTPRCSCPVHLVLLQNLLTCGEPPTCSPDQFACATGEIDCIPGAWRCDGFPECDDQSDEEGCPVCSAAQFPC
ARGQCVDLRLRCDGEADCQDRSDEADCDAICLPNQFRCASGQCVLIKQQCDSFPDCIDGSDELMCEITKPPSDDSPAHSSAIGPVIG
IILSLFVMGGVYFVCQRVVCQRYAGANGPFPHEYVSGTPHVPLNFIAPGGSQHGPFTGIACGKSMMSSVSLMGGRGGVPLYDRNHVT
GASSSSSSSTKATLYPPILNPPPSPATDPSLYNMDMFYSSNIPATARPYRPYIIRGMAPPTTPCSTDVCDSDYSASRWKASKYYLDL
NSDSDPYPPPPTPHSQYLSAEDSCPPSPATERSYFHLFPPPPSPCTDSS
>sp|P46091|GPR1_HUMAN
MEDLEETLFEEFENYSYDLDYYSLESDLEEKVQLGVVHWVSLVLYCLAFVLGIPGNAIVIWFTGFKWKKTVTTLWFLNLAIADFIFL
LFLPLYISYVAMNFHWPFGIWLCKANSFTAQLNMFASVFFLTVISLDHYIHLIHPVLSHRHRTLKNSLIVIIFIWLLASLIGGPALY
FRDTVEFNNHTLCYNNFQKHDPDLTLIRHHVLTWVKFIIGYLFPLLTMSICYLCLIFKVKKRSILISSRHFWTILVVVVAFVVCWTP
YHLFSIWELTIHHNSYSHHVMQAGIPLSTGLAFLNSCLNPILYVLISKKFQARFRSSVAEILKYTLWEVSCSGTVSEQLRNSETKNL
CLLETAQ
>sp|Q5T481|RBM20_HUMAN

MVLAAAMSQDADPSGPEQPDRVACSVPGARASPAPSGPRGMQQPPPPPQPPPPPQAGLPQIIQNAAKLLDKNPFSVSNPNPLLPSPA
SLQLAQLQAQLTLHRLKLAQTAVTNNTAAATVLNQVLSKVAMSQPLFNQLRHPSVITGPHGHAGVPQHAAAIPSTRFPSNAIAFSPP
SQTRGPGPSMNLPNQPPSAMVMHPFTGVMPQTPGQPAVILGIGKTGPAPATAGFYEYGKASSGQTYGPETDGQPGFLPSSASTSGSV
TYEGHYSHTGQDGQAAFSKDFYGPNSQGSHVASGFPAEQAGGLKSEVGPLLQGTNSQWESPHGFSGQSKPDLTAGPMWPPPHNQPYE
LYDPEEPTSDRTPPSFGGRLNNSKQGFIGAGRRAKEDQALLSVRPLQAHELNDFHGVAPLHLPHICSICDKKVFDLKDWELHVKGKL
HAQKCLVFSENAGIRCILGSAEGTLCASPNSTAVYNPAGNEDYASNLGTSYVPIPARSFTQSSPTFPLASVGTTFAQRKGAGRVVHI
CNLPEGSCTENDVINLGLPFGKVTNYILMKSTNQAFLEMAYTEAAQAMVQYYQEKSAVINGEKLLIRMSKRYKELQLKKPGKAVAAI
IQDIHSQRERDMFREADRYGPERPRSRSPVSRSLSPRSHTPSFTSCSSSHSPPGPSRADWGNGRDSWEHSPYARREEERDPAPWRDN
GDDKRDRMDPWAHDRKHHPRQLDKAELDERPEGGRPHREKYPRSGSPNLPHSVSSYKSREDGYYRKEPKAKWDKYLKQQQDAPGRSR
RKDEARLRESRHPHPDDSGKEDGLGPKVTRAPEGAKAKQNEKNKTKRTDRDQEGADDRKENTMAENEAGKEEQEGMEESPQSVGRQE
KEAEFSDPENTRTKKEQDWESESEAEGESWYPTNMEELVTVDEVGEEEDFIVEPDIPELEEIVPIDQKDKICPETCLCVTTTLDLDL
AQDFPKEGVKAVGNGAAEISLKSPRELPSASTSCPSDMDVEMPGLNLDAERKPAESETGLSLEDSDCYEKEAKGVESSDVHPAPTVQ
QMSSPKPAEERARQPSPFVDDCKTRGTPEDGACEGSPLEEKASPPIETDLQNQACQEVLTPENSRYVEMKSLEVRSPEYTEVELKQP
LSLPSWEPEDVFSELSIPLGVEFVVPRTGFYCKLCGLFYTSEETAKMSHCRSAVHYRNLQKYLSQLAEEGLKETEGADSPRPEDSGI
VPRFERKKL
>sp|Q5T752|LCE1D_HUMAN
MSCQQSQQQCQPPPKCTPKCTPKCPAPKCPPKCPPVSSCCSVSSGGCCGSSSGGGCGSNSGGCCSSGGGGCCLSHHRRHRSHRRRPQ
SSDCCSQPSGGSSCCGGGSSQHSGGCC
>sp|Q96DF8|DGC14_HUMAN
METPGASASSLLLPAASRPPRKREAGEAGAATSKQRVLDEEEYIEGLQTVIQRDFFPDVEKLQAQKEYLEAEENGDLERMRQIAIKF
GSALGKMSREPPPPYVTPATFETPEVHAGTGVVGNKPRPRGRGLEDGEAGEEEEKEPLPSLDVFLSRYTSEDNASFQEIMEVAKERS
RARHAWLYQAEEEFEKRQKDNLELPSAEHQAIESSQASVETWKYKAKNSLMYYPEGVPDEEQLFKKPRQVVHKNTRFLRDPFSQALS
RCQLQQAAALNAQHKQGKVGPDGKELIPQESPRVGGFGFVATPSPAPGVNESPMMTWGEVENTPLRVEGSETPYVDRTPGPAFKILE
PGRRERLGLKMANEAAAKNRAKKQEALRRVTENLASLTPKGLSPAMSPALQRLVSRTASKYTDRALRASYTPSPARSTHLKTPASGL
QTPTSTPAPGSATRTPLTQDPASITDNLLQLPARRKASDFF
>sp|P47871|GLR_HUMAN
MPPCQPQRPLLLLLLLLACQPQVPSAQVMDFLFEKWKLYGDQCHHNLSLLPPPTELVCNRTFDKYSCWPDTPANTTANISCPWYLPW
HHKVQHRFVFKRCGPDGQWVRGPRGQPWRDASQCQMDGEEIEVQKEVAKMYSSFQVMYTVGYSLSLGALLLALAILGGLSKLHCTRN
AIHANLFASFVLKASSVLVIDGLLRTRYSQKIGDDLSVSTWLSDGAVAGCRVAAVFMQYGIVANYCWLLVEGLYLHNLLGLATLPER
SFFSLYLGIGWGAPMLFVVPWAVVKCLFENVQCWTSNDNMGFWWILRFPVFLAILINFFIFVRIVQLLVAKLRARQMHHTDYKFRLA
KSTLTLIPLLGVHEVVFAFVTDEHAQGTLRSAKLFFDLFLSSFQGLLVAVLYCFLNKEVQSELRRRWHRWRLGKVLWEERNTSNHRA
SSSPGHGPPSKELQFGRGGGSQDSSAETPLAGGLPRLAESPF
>sp|P19801|AOC1_HUMAN
MPALGWAVAAILMLQTAMAEPSPGTLPRKAGVFSDLSNQELKAVHSFLWSKKELRLQPSSTTTMAKNTVFLIEMLLPKKYHVLRFLD
KGERHPVREARAVIFFGDQEHPNVTEFAVGPLPGPCYMRALSPRPGYQSSWASRPISTAEYALLYHTLQEATKPLHQFFLNTTGFSF
QDCHDRCLAFTDVAPRGVASGQRRSWLIIQRYVEGYFLHPTGLELLVDHGSTDAGHWAVEQVWYNGKFYGSPEELARKYADGEVDVV
VLEDPLPGGKGHDSTEEPPLFSSHKPRGDFPSPIHVSGPRLVQPHGPRFRLEGNAVLYGGWSFAFRLRSSSGLQVLNVHFGGERIAY
EVSVQEAVALYGGHTPAGMQTKYLDVGWGLGSVTHELAPGIDCPETATFLDTFHYYDADDPVHYPRALCLFEMPTGVPLRRHFNSNF
KGGFNFYAGLKGQVLVLRTTSTVYNYDYIWDFIFYPNGVMEAKMHATGYVHATFYTPEGLRHGTRLHTHLIGNIHTHLVHYRVDLDV
AGTKNSFQTLQMKLENITNPWSPRHRVVQPTLEQTQYSWERQAAFRFKRKLPKYLLFTSPQENPWGHKRTYRLQIHSMADQVLPPGW
QEEQAITWARYPLAVTKYRESELCSSSIYHQNDPWHPPVVFEQFLHNNENIENEDLVAWVTVGFLHIPHSEDIPNTATPGNSVGFLL
RPFNFFPEDPSLASRDTVIVWPRDNGPNYVQRWIPEDRDCSMPPPFSYNGTYRPV
>sp|Q9NQP4|PFD4_HUMAN

MAATMKKAAAEDVNVTFEDQQKINKFARNTSRITELKEEIEVKKKQLQNLEDACDDIMLADDDCLMIPYQIGDVFISHSQEETQEML
EEAKKNLQEEIDALESRVESIQRVLADLKVQLYAKFGSNINLEADES
>sp|P35914|HMGCL_HUMAN
MAAMRKALPRRLVGLASLRAVSTSSMGTLPKRVKIVEVGPRDGLQNEKNIVSTPVKIKLIDMLSEAGLSVIETTSFVSPKWVPQMGD
HTEVLKGIQKFPGINYPVLTPNLKGFEAAVAAGAKEVVIFGAASELFTKKNINCSIEESFQRFDAILKAAQSANISVRGYVSCALGC
PYEGKISPAKVAEVTKKFYSMGCYEISLGDTIGVGTPGIMKDMLSAVMQEVPLAALAVHCHDTYGQALANTLMALQMGVSVVDSSVA
GLGGCPYAQGASGNLATEDLVYMLEGLGIHTGVNLQKLLEAGNFICQALNRKTSSKVAQATCKL
>sp|Q8NG68|TTL_HUMAN
MYTFVVRDENSSVYAEVSRLLLATGHWKRLRRDNPRFNLMLGERNRLPFGRLGHEPGLVQLVNYYRGADKLCRKASLVKLIKTSPEL
AESCTWFPESYVIYPTNLKTPVAPAQNGIQPPISNSRTDEREFFLASYNRKKEDGEGNVWIAKSSAGAKGEGILISSEASELLDFID
NQGQVHVIQKYLEHPLLLEPGHRKFDIRSWVLVDHQYNIYLYREGVLRTASEPYHVDNFQDKTCHLTNHCIQKEYSKNYGKYEEGNE
MFFKEFNQYLTSALNITLESSILLQIKHIIRNCLLSVEPAISTKHLPYQSFQLFGFDFMVDEELKVWLIEVNGAPACAQKLYAELCQ
GIVDIAISSVFPPPDVEQPQTQPAAFIKL
>sp|P84996|ALEX_HUMAN
MMARPVDPQRSPDPTFRSSTRHSGKLEPMEATAHLLRKQCPSRLNSPAWEASGLHWSSLDSPVGSMQALRPSAQHSWSPEPSVVPDQ
AWEDTALHQKKLCPLSLTSLPREAAVNFSYRSQTLLQEAQVLQGSPELLPRSPKPSGLQRLAPEEATALPLRRLCHLSLMEKDLGTT
AHPRGFPELSHKSTAAASSRQSRPRVRSASLPPRTRLPSGSQAPSAAHPKRLSDLLLTSRAAAPGWRSPDPRSRLAAPPLGSTTLPS
TWTAPQSRLTARPSRSPEPQIRESEQRDPQLRRKQQRWKEPLMPRREEKYPLRGTDPLPPGQPQRIPLPGQPLQPQPILTPGQPQKI
PTPGQHQPILTPGHSQPIPTPGQPLPPQPIPTPGRPLTPQPIPTPGRPLTPQPIQMPGRPLRLPPPLRLLRPGQPMSPQLRQTQGLP
LPQPLLPPGQPKSAGRPLQPLPPGPDARSISDPPAPRSRLPIRLLRGLLARLPGGASPRAAAAAACTTMKGWPAATMTPAETSPTMG
PPDASAGFSIGEIAAAESPSATYSATFSCKPSGAASVDLRVPSPKPRALSRSRRYPWRRSADRCAKKPWRSGPRSAQRRNAVSSSTN
NSRTKRWATCVRTACCF
>sp|Q9BXJ5|C1QT2_HUMAN
MIPWVLLACALPCAADPLLGAFARRDFRKGSPQLVCSLPGPQGPPGPPGAPGPSGMMGRMGFPGKDGQDGHDGDRGDSGEEGPPGRT
GNRGKPGPKGKAGAIGRAGPRGPKGVNGTPGKHGTPGKKGPKGKKGEPGLPGPCSCGSGHTKSAFSVAVTKSYPRERLPIKFDKILM
NEGGHYNASSGKFVCGVPGIYYFTYDITLANKHLAIGLVHNGQYRIRTFDANTGNHDVASGSTILALKQGDEVWLQIFYSEQNGLFY
DPYWTDSLFTGFLIYADQDDPNEV
>sp|Q8NHP1|ARK74_HUMAN
MSRQLSRARPATVLGAMEMGRRMDAPTSAAVTRAFLERGHTEIDTAFLYSDGQSETILGGLGLRMGSSDCRVKIATKANPWIGNSLK
PDSVRSQLETSLKRLQCPUVDLFYLHAPDHSAPVEETLRACHQLHQEGKFVELGLSNYAAWEVAEICTLCKSNGWILPTVYQGMYSA
TTRQVETELFPCLRHFGLRFYAYNPLAGGLLTGKYKYEDKDGKQPVGRFFGTQWAEIYRNHFWKEHHFEGIALVEKALQAAYGASAP
SMTSAALRWMYHHSQLQGAHGDAVILGMSSLEQLEQNLAAAEEGPLEPAVVDAFNQAWHLFAHECPNYFI
>sp|Q9NXC5|MIO_HUMAN
MSGTKPDILWAPHHVDRFVVCDSELSLYHVESTVNSELKAGSLRLSEDSAATLLSINSDTPYMKCVAWYLNYDPECLLAVGQANGRV
VLTSLGQDHNSKFKDLIGKEFVPKHARQCNTLAWNPLDSNWLAAGLDKHRADFSVLIWDICSKYTPDIVPMEKVKLSAGETETTLLV
TKPLYELGQNDACLSLCWLPRDQKLLLAGMHRNLAIFDLRNTSQKMFVNTKAVQGVTVDPYFHDRVASFYEGQVAIWDLRKFEKPVL
TLTEQPKPLTKVAWCPTRTGLLATLTRDSNIIRLYDMQHTPTPIGDETEPTIIERSVQPCDNYIASFAWHPTSQNRMIVVTPNRTMS
DFTVFERISLAWSPITSLMWACGRHLYECTEEENDNSLEKDIATKMRLRALSRYGLDTEQVWRNHILAGNEDPQLKSLWYTLHFMKQ
YTEDMDQKSPGNKGSLVYAGIKSIVKSSLGMVESSRHNWSGLDKQSDIQNLNEERILALQLCGWIKKGTDVDVGPFLNSLVQEGEWE
RAAAVALFNLDIRRAIQILNEGASSEKGDLNLNVVAMALSGYTDEKNSLWREMCSTLRLQLNNPYLCVMFAFLTSETGSYDGVLYEN
KVAVRDRVAFACKFLSDTQLNRYIEKLTNEMKEAGNLEGILLTGLTKDGVDLMESYVDRTGDVQTASYCMLQGSPLDVLKDERVQYW
IENYRNLLDAWRFWHKRAEFDIHRSKLDPSSKPLAQVFVSCNFCGKSISYSCSAVPHQGRGFSQYGVSGSPTKSKVTSCPGCRKPLP
RCALCLINMGTPVSSCPGGTKSDEKVDLSKDKKLAQFNNWFTWCHNCRHGGHAGHMLSWFRDHAECPVSACTCKCMQLDTTGNLVPA
ETVQP

>sp|Q9BXK1|KLF16_HUMAN
MSAAVACVDYFAADVLMAISSGAVVHRGRPGPEGAGPAAGLDVRAARREAASPGTPGPPPPPPAASGPGPGAAAAPHLLAASILADL
RGGPGAAPGGASPASSSSAASSPSSGRAPGAAPSAAAKSHRCPFPDCAKAYYKSSHLKSHLRTHTGERPFACDWQGCDKKFARSDEL
ARHHRTHTGEKRFSCPLCSKRFTRSDHLAKHARRHPGFHPDLLRRPGARSTSPSDSLPCSLAGSPAPSPAPSPAPAGL
>sp|Q9UBG3|CRNN_HUMAN
MPQLLQNINGIIEAFRRYARTEGNCTALTRGELKRLLEQEFADVIVKPHDPATVDEVLRLLDEDHTGTVEFKEFLVLVFKVAQACFK
TLSESAEGACGSQESGSLHSGASQELGEGQRSGTEVGRAGKGQHYEGSSHRQSQQGSRGQNRPGVQTQGQATGSAWVSSYDRQAESQ
SQERISPQIQLSGQTEQTQKAGEGKRNQTTEMRPERQPQTREQDRAHQTGETVTGSGTQTQAGATQTVEQDSSHQTGRTSKQTQEAT
NDQNRGTETHGQGRSQTSQAVTGGHAQIQAGTHTQTPTQTVEQDSSHQTGSTSTQTQESTNGQNRGTEIHGQGRSQTSQAVTGGHTQ
IQAGSHTETVEQDRSQTVSHGGAREQGQTQTQPGSGQRWMQVSNPEAGETVPGGQAQTGASTESGRQEWSSTHPRRCVTEGQGDRQP
TVVGEEWVDDHSRETVILRLDQGNLHTSVSSAQGQDAAQSEEKRGITARELYSYLRSTKP
>sp|P05090|APOD_HUMAN
MVMLLLLLSALAGLFGAAEGQAFHLGKCPNPPVQENFDVNKYLGRWYEIEKIPTTFENGRCIQANYSLMENGKIKVLNQELRADGTV
NQIEGEATPVNLTEPAKLEVKFSWFMPSAPYWILATDYENYALVYSCTCIIQLFHVDFAWILARNPNLPPETVDSLKNILTSNNIDV
KKMTVTDQVNCPKLS
>sp|O75173|ATS4_HUMAN
MSQTGSHPGRGLAGRWLWGAQPCLLLPIVPLSWLVWLLLLLLASLLPSARLASPLPREEEIVFPEKLNGSVLPGSGAPARLLCRLQA
FGETLLLELEQDSGVQVEGLTVQYLGQAPELLGGAEPGTYLTGTINGDPESVASLHWDGGALLGVLQYRGAELHLQPLEGGTPNSAG
GPGAHILRRKSPASGQGPMCNVKAPLGSPSPRPRRAKRFASLSRFVETLVVADDKMAAFHGAGLKRYLLTVMAAAAKAFKHPSIRNP
VSLVVTRLVILGSGEEGPQVGPSAAQTLRSFCAWQRGLNTPEDSDPDHFDTAILFTRQDLCGVSTCDTLGMADVGTVCDPARSCAIV
EDDGLQSAFTAAHELGHVFNMLHDNSKPCISLNGPLSTSRHVMAPVMAHVDPEEPWSPCSARFITDFLDNGYGHCLLDKPEAPLHLP
VTFPGKDYDADRQCQLTFGPDSRHCPQLPPPCAALWCSGHLNGHAMCQTKHSPWADGTPCGPAQACMGGRCLHMDQLQDFNIPQAGG
WGPWGPWGDCSRTCGGGVQFSSRDCTRPVPRNGGKYCEGRRTRFRSCNTEDCPTGSALTFREEQCAAYNHRTDLFKSFPGPMDWVPR
YTGVAPQDQCKLTCQAQALGYYYVLEPRVVDGTPCSPDSSSVCVQGRCIHAGCDRIIGSKKKFDKCMVCGGDGSGCSKQSGSFRKFR
YGYNNVVTIPAGATHILVRQQGNPGHRSIYLALKLPDGSYALNGEYTLMPSPTDVVLPGAVSLRYSGATAASETLSGHGPLAQPLTL
QVLVAGNPQDTRLRYSFFVPRPTPSTPRPTPQDWLHRRAQILEILRRRPWAGRK
>sp|Q8WTX9|ZDHC1_HUMAN
MYKMNICNKPSNKTAPEKSVWTAPAQPSGPSPELQGQRSRRNGWSWPPHPLQIVAWLLYLFFAVIGFGILVPLLPHHWVPAGYACMG
AIFAGHLVVHLTAVSIDPADANVRDKSYAGPLPIFNRSQHAHVIEDLHCNLCNVDVSARSKHCSACNKCVCGFDHHCKWLNNCVGER
NYRLFLHSVASALLGVLLLVLVATYVFVEFFVNPMRLRTNRHFEVLKNHTDVWFVFLPAAPVETQAPAILALAALLILLGLLSTALL
GHLLCFHIYLMWHKLTTYEYIVQHRPPQEAKGVHRELESCPPKMRPIQEMEFYMRTFRHMRPEPPGQAGPAAVNAKHSRPASPDPTP
GRRDCAGPPVQVEWDRKKPLPWRSPLLLLAMWGPQAPPCLCRKRGRGACIKCERLRPRIRRRGLGPPAAAPARRRIPRTPALCTPLA
LPAPTTRRRQSPWTRFQWRRRAWAAPLWPPRGAGADSPRWRGRRVRPPFS
>sp|Q12972|PP1R8_HUMAN
MAAAANSGSSLPLFDCPTWAGKPPPGLHLDVVKGDKLIEKLIIDEKKYYLFGRNPDLCDFTIDHQSCSRVHAALVYHKHLKRVFLID
LNSTHGTFLGHIRLEPHKPQQIPIDSTVSFGASTRAYTLREKPQTLPSAVKGDEKMGGEDDELKGLLGLPEEETELDNLTEFNTAHN
KRISTLTIEEGNLDIQRPKRKRKNSRVTFSEDDEIINPEDVDPSVGRFRNMVQTAVVPVKKKRVEGPGSLGLEESGSRRMQNFAFSG
GLYGGLPPTHSEAGSQPHGIHGTALIGGLPMPYPNLAPDVDLTPVVPSAVNMNPAPNPAVYNPEAVNEPKKKKYAKEAWPGKKPTPS
LLI
>sp|Q9HCE1|MOV10_HUMAN
MPSKFSCRQLREAGQCFESFLVVRGLDMETDRERLRTIYNRDFKISFGTPAPGFSSMLYGMKIANLAYVTKTRVRFFRLDRWADVRF
PEKRRMKLGSDISKHHKSLLAKIFYDRAEYLHGKHGVDVEVQGPHEARDGQLLIRLDLNRKEVLTLRLRNGGTQSVTLTHLFPLCRT
PQFAFYNEDQELPCPLGPGECYELHVHCKTSFVGYFPATVLWELLGPGESGSEGAGTFYIARFLAAVAHSPLAAQLKPMTPFKRTRI
TGNPVVTNRIEEGERPDRAKGYDLELSMALGTYYPPPRLRQLLPMLLQGTSIFTAPKEIAEIKAQLETALKWRNYEVKLRLLLHLEE

LQMEHDIRHYDLESVPMTWDPVDQNPRLLTLEVPGVTESRPSVLRGDHLFALLSSETHQEDPITYKGFVHKVELDRVKLSFSMSLLS
RFVDGLTFKVNFTFNRQPLRVQHRALELTGRWLLWPMLFPVAPRDVPLLPSDVKLKLYDRSLESNPEQLQAMRHIVTGTTRPAPYII
FGPPGTGKTVTLVEAIKQVVKHLPKAHILACAPSNSGADLLCQRLRVHLPSSIYRLLAPSRDIRMVPEDIKPCCNWDAKKGEYVFPA
KKKLQEYRVLITTLITAGRLVSAQFPIDHFTHIFIDEAGHCMEPESLVAIAGLMEVKETGDPGGQLVLAGDPRQLGPVLRSPLTQKH
GLGYSLLERLLTYNSLYKKGPDGYDPQFITKLLRNYRSHPTILDIPNQLYYEGELQACADVVDRERFCRWAGLPRQGFPIIFHGVMG
KDEREGNSPSFFNPEEAATVTSYLKLLLAPSSKKGKARLSPRSVGVISPYRKQVEKIRYCITKLDRELRGLDDIKDLKVGSVEEFQG
QERSVILISTVRSSQSFVQLDLDFNLGFLKNPKRFNVAVTRAKALLIIVGNPLLLGHDPDWKVFLEFCKENGGYTGCPFFPAKLDLQQ
GQNLLQGLSKLSPSTSGPHSHDYLPQEREGEGGLSLQVEPEWRNEL
>sp|Q8N2F6|ARM10_HUMAN
MGGPRGAGWVAAGLLLGAGACYCIYRLTRGRRRGDRELGIRSSKSAGALEEGTSEGQLCGRSARPQTGGTWESQWSKTSQPEDLTDG
SYDDVLNAEQLQKLLYLLESTEDPVIIERALITLGNNAAFSVNQAIIRELGGIPIVANKINHSNQSIKEKALNALNNLSVNVENQIK
IKIYISQVCEDVFSGPLNSAVQLAGLTLLTNMTVTNDHQHMLHSYITDLFQVLLTGNGNTKVQVLKLLLNLSENPAMTEGLLRAQVD
SSFLSLYDSHVAKEILLRVLTLFQNIKNCLKIEGHLAVQPTFTEGSLFFLLHGEECAQKIRALVDHHDAEVKEKVVTIIPKI
>sp|Q8NDV2|GPR26_HUMAN
MNSWDAGLAGLLVGTMGVSLLSNALVLLCLLHSADIRRQAPALFTLNLTCGNLLCTVVNMPLTLAGVVAQRQPAGDRLCRLAAFLDT
FLAANSMLSMAALSIDRWVAVVFPLSYRAKMRLRDAALMVAYTWLHALTFPAAALALSWLGFHQLYASCTLCSRRPDERLRFAVFTG
AFHALSFLLSFVVLCCTYLKVLKVARFHCKRIDVITMQTLVLLVDLHPSVRERCLEEQKRRRQRATKKISTFIGTFLVCFAPYVITR
LVELFSTVPIGSHWGVLSKCLAYSKAASDPFVYSLLRHQYRKSCKEILNRLLHRRSIHSSGLTGDSHSQNILPVSE
>sp|O95825|QORL1_HUMAN
MKGLYFQQSSTDEEITFVFQEKEDLPVTEDNFVKLQVKACALSQINTKLLAEMKMKKDLFPVGREIAGIVLDVGSKVSFFQPDDEVV
GILPLDSEDPGLCEVVRVHEHYLVHKPEKVTWTEAAGSIRDGVRAYTALHYLSHLSPGKSVLIMDGASAFGTIAIQLAHHRGAKVIS
TACSLEDKQCLERFRPPIARVIDVSNGKVHVAESCLEETGGLGVDIVLDAGVRLYSKDDEPAVKLQLLPHKHDIITLLGVGGHWVTT
EENLQLDPPDSHCLFLKGATLAFLNDEVWNLSNVQQGKYLCILKDVMEKLSTGVFRPQLDEPIPLYEAKVSMEAVQKNQGRKKQVVQ
F
>sp|Q5T5A8|LCE3C_HUMAN
MSCQQNQQQCQPPPSCPSPKCPPKSPAQCLPPPSSDCALSSGGCGPSSESGCCLSHHRHFRSHQCRRQRSNSCDRGSGQQGGGSCRG
HGSGGCC
>sp|Q8TCT1|PHOP1_HUMAN
MSGCFPVSGLRCLSRDGRMAAQGAPRFLLTFDFDETIVDENSDDSIVRAAPGQRLPESLRATYREGFYNEYMQRVFKYLGEQGVRPR
DLSAIYEAIPLSPGMSDLLQFVAKQGACFEVILISDANTFGVESSLRAAGHHSLFRRILSNPSGPDARGLLALRPFHTHSCARCPAN
MCKHKVLSDYLRERAHDGVHFERLFYVGDGANDFCPMGLLAGGDVAFPRRGYPMHRLIQEAQKAEPSSFRASVVPWETAADVRLHLQ
QVLKSC
>sp|Q13936|CAC1C_HUMAN
MVNENTRMYIPEENHQGSNYGSPRPAHANMNANAAAGLAPEHIPTPGAALSWQAAIDAARQAKLMGSAGNATISTVSSTQRKRQQYG
KPKKQGSTTATRPPRALLCLTLKNPIRRACISIVEWKPFEIIILLTIFANCVALAIYIPFPEDDSNATNSNLERVEYLFLIIFTVEA
FLKVIAYGLLFHPNAYLRNGWNLLDFIIVVVGLFSAILEQATKADGANALGGKGAGFDVKALRAFRVLRPLRLVSGVPSLQVVLNSI
IKAMVPLLHIALLVLFVIIIYAIIGLELFMGKMHKTCYNQEGIADVPAEDDPSPCALETGHGRQCQNGTVCKPGWDGPKHGITNFDN
FAFAMLTVFQCITMEGWTDVLYWVNDAVGRDWPWIYFVTLIIIGSFFVLNLVLGVLSGEFSKEREKAKARGDFQKLREKQQLEEDLK
GYLDWITQAEDIDPENEDEGMDEEKPRNMSMPTSETESVNTENVAGGDIEGENCGARLAHRISKSKFSRYWRRWNRFCRRKCRAAVK
SNVFYWLVIFLVFLNTLTIASEHYNQPNWLTEVQDTANKALLALFTAEMLLKMYSLGLQAYFVSLFNRFDCFVVCGGILETILVETK
IMSPLGISVLRCVRLLRIFKITRYWNSLSNLVASLLNSVRSIASLLLLLFLFIIIFSLLGMQLFGGKFNFDEMQTRRSTFDNFPQSL
LTVFQILTGEDWNSVMYDGIMAYGGPSFPGMLVCIYFIILFICGNYILLNVFLAIAVDNLADAESLTSAQKEEEEEKERKKLARTAS
PEKKQELVEKPAVGESKEEKIELKSITADGESPPATKINMDDLQPNENEDKSPYPNPETTGEEDEEEPEMPVGPRPRPLSELHLKEK
AVPMPEASAFFIFSSNNRFRLQCHRIVNDTIFTNLILFFILLSSISLAAEDPVQHTSFRNHILFYFDIVFTTIFTIEIALKILGNAD

YVFTSIFTLEIILKMTAYGAFLHKGSFCRNYFNILDLLVVSVSLISFGIQSSAINVVKILRVLRVLRPLRAINRAKGLKHVVQCVFV
AIRTIGNIVIVTTLLQFMFACIGVQLFKGKLYTCSDSSKQTEAECKGNYITYKDGEVDHPIIQPRSWENSKFDFDNVLAAMMALFTV
STFEGWPELLYRSIDSHTEDKGPIYNYRVEISIFFIIYIIIIAFFMMNIFVGFVIVTFQEQGEQEYKNCELDKNQRQCVEYALKARP
LRRYIPKNQHQYKVWYVVNSTYFEYLMFVLILLNTICLAMQHYGQSCLFKIAMNILNMLFTGLFTVEMILKLIAFKPKGYFSDPWNV
FDFLIVIGSIIDVILSETNHYFCDAWNTFDALIVVGSIVDIAITEVNPAEHTQCSPSMNAEENSRISITFFRLFRVMRLVKLLSRGE
GIRTLLWTFIKSFQALPYVALLIVMLFFIYAVIGMQVFGKIALNDTTEINRNNNFQTFPQAVLLLFRCATGEAWQDIMLACMPGKKC
APESEPSNSTEGETPCGSSFAVFYFISFYMLCAFLIINLFVAVIMDNFDYLTRDWSILGPHHLDEFKRIWAEYDPEAKGRIKHLDVV
TLLRRIQPPLGFGKLCPHRVACKRLVSMNMPLNSDGTVMFNATLFALVRTALRIKTEGNLEQANEELRAIIKKIWKRTSMKLLDQVV
PPAGDDEVTVGKFYATFLIQEYFRKFKKRKEQGLVGKPSQRNALSLQAGLRTLHDIGPEIRRAISGDLTAEEELDKAMKEAVSAASE
DDIFRRAGGLFGNHVSYYQSDGRSAFPQTFTTQRPLHINKAGSSQGDTESPSHEKLVDSTFTPSSYSSTGSNANINNANNTALGRLP
RPAGYPSTVSTVEGHGPPLSPAIRVQEVAWKLSSNRERHVPMCEDLELRRDSGSAGTQAHCLLLRKANPSRCHSRESQAAMAGQEET
SQDETYEVKMNHDTEACSEPSLLSTEMLSYQDDENRQLTLPEEDKRDIRQSPKRGFLRSASLGRRASFHLECLKRQKDRGGDISQKT
VLPLHLVHHQALAVAGLSPLLQRSHSPASFPRPFATPPATPGSRGWPPQPVPTLRLEGVESSEKLNSSFPSIHCGSWAETTPGGGGS
SAARRVRPVSLMVPSQAGAPGRQFHGSASSLVEAVLISEGLGQFAQDPKFIEVTTQELADACDMTIEEMESAADNILSGGAPQSPNG
ALLPFVNCRDAGQDRAGGEEDAGCVRARGRPSEEELQDSRVYVSSL
>sp|P25963|IKBA_HUMAN
MFQAAERPQEWAMEGPRDGLKKERLLDDRHDSGLDSMKDEEYEQMVKELQEIRLEPQEVPRGSEPWKQQLTEDGDSFLHLAIIHEEK
ALTMEVIRQVKGDLAFLNFQNNLQQTPLHLAVITNQPEIAEALLGAGCDPELRDFRGNTPLHLACEQGCLASVGVLTQSCTTPHLHS
ILKATNYNGHTCLHLASIHGYLGIVELLVSLGADVNAQEPCNGRTALHLAVDLQNPDLVSLLLKCGADVNRVTYQGYSPYQLTWGRP
STRIQQQLGQLTLENLQMLPESEDEESYDTESEFTEFTEDELPYDDCVFGGQRLTL
>sp|Q96P16|RPR1A_HUMAN
MSAFSEAALEKKLSELSNSQQSVQTLSLWLIHHRKHSRPIVTVWERELRKAKPNRKLTFLYLANDVIQNSKRKGPEFTKDFAPVIVE
AFKHVSSETDESCKKHLGRVLSIWEERSVYENDVLEQLKQALYGDKKPRKRTYEQIKVDENENCSSLGSPSEPPQTLDLVRALQDLE
NAASGDAAVHQRIASLPVEVQEVSLLDKITDKESGERLSKMVEDACMLLADYNGRLAAEIDDRKQLTRMLADFLRCQKEALAEKEHK
LEEYKRKLARVSLVRKELRSRIQSLPDLSRLPNVTGSHMHLPFAGDIYSED
>sp|P61550|ENVT1_HUMAN
MGPEAWVRPLKTAPKPGEAIRLILFIYLSCFFLPVMSSEPSYSFLLTSFTTGRVFANTTWRAGTSKEVSFAVDLCVLFPEPARTHEE
QHNLPVIGAGSVDLAAGFGHSGSQTGCGSSKGAEKGLQNVDFYLCPGNHPDASCRDTYQFFCPDWTCVTLATYSGGSTRSSTLSISR
VPHPKLCTRKNCNPLTITVHDPNAAQWYYGMSWGLRLYIPGFDVGTMFTIQKKILVSWSSPKPIGPLTDLGDPIFQKHPDKVDLTVP
LPFLVPRPQLQQQHLQPSLMSILGGVHHLLNLTQPKLAQDCWLCLKAKPPYYVGLGVEATLKRGPLSCHTRPRALTIGDVSGNASCL
ISTGYNLSASPFQATCNQSLLTSISTSVSYQAPNNTWLACTSGLTRCINGTEPGPLLCVLVHVLPQVYVYSGPEGRQLIAPPELHPR
LHQAVPLLVPLLAGLSIAGSAAIGTAALVQGETGLISLSQQVDADFSNLQSAIDILHSQVESLAEVVLQNCRCLDLLFLSQGGLCAA
LGESCCFYANQSGVIKGTVKKVRENLDRHQQERENNIPWYQSMFNWNPWLTTLITGLAGPLLILLLSLIFGPCILNSFLNFIKQRIA
SVKLTYLKTQYDTLVNN
>sp|P35227|PCGF2_HUMAN
MHRTTRIKITELNPHLMCALCGGYFIDATTIVECLHSFCKTCIVRYLETNKYCPMCDVQVHKTRPLLSIRSDKTLQDIVYKLVPGLF
KDEMKRRRDFYAAYPLTEVPNGSNEDRGEVLEQEKGALSDDEIVSLSIEFYEGARDRDEKKGPLENGDGDKEKTGVRFLRCPAAMTV
MHLAKFLRNKMDVPSKYKVEVLYEDEPLKEYYTLMDIAYIYPWRRNGPLPLKYRVQPACKRLTLATVPTPSEGTNTSGASECESVSD
KAPSPATLPATSSSLPSPATPSHGSPSSHGPPATHPTSPTPPSTASGATTAANGGSLNCLQTPSSTSRGRKMTVNGAPVPPLT
>sp|Q9H208|O10A2_HUMAN
MSFSSLPTEIQSLLFLTFLTIYLVTLMGNCLIILVTLADPMLHSPMYFFLRNLSFLEIGFNLVIVPKMLGTLLAQDTTISFLGCATQ
MYFFFFFGVAECFLLATMAYDRYVAICSPLHYPVIMNQRTRAKLAAASWFPGFPVATVQTTWLFSFPFCGTNKVNHFFCDSPPVLRL
VCADTALFEIYAIVGTILVVMIPCLLILCSYTHIAAAILKIPSAKGKNKAFSTCSSHLLVVSLFYISLSLTYFRPKSNNSPEGKKLL
SLSYTVMTPMLNPIIYSLRNNEVKNALSRTVSKALALRNCIP

>sp|Q9UHD1|CHRD1_HUMAN
MALLCYNRGCGQRFDPETNSDDACTYHPGVPVFHDALKGWSCCKRRTTDFSDFLSIVGCTKGRHNSEKPPEPVKPEVKTTEKKELCE
LKPKFQEHIIQAPKPVEAIKRPSPDEPMTNLELKISASLKQALDKLKLSSGNEENKKEEDNDEIKIGTSCKNGGCSKTYQGLESLEE
VCVYHSGVPIFHEGMKYWSCCRRKTSDFNTFLAQEGCTKGKHMWTKKDAGKKVVPCRHDWHQTGGEVTISVYAKNSLPELSRVEANS
TLLNVHIVFEGEKEFDQNVKLWGVIDVKRSYVTMTATKIEITMRKAEPMQWASLELPAAKKQEKQKDATTD
>sp|Q14773|ICAM4_HUMAN
MGSLFPLSLLFFLAAAYPGVGSALGRRTKRAQSPKGSPLAPSGTSVPFWVRMSPEFVAVQPGKSVQLNCSNSCPQPQNSSLRTPLRQ
GKTLRGPGWVSYQLLDVRAWSSLAHCLVTCAGKTRWATSRITAYKPPHSVILEPPVLKGRKYTLRCHVTQVFPVGYLVVTLRHGSRV
IYSESLERFTGLDLANVTLTYEFAAGPRDFWQPVICHARLNLDGLVVRNSSAPITLMLAWSPAPTALASGSIAALVGILLTVGAAYL
CKCLAMKSQA
>sp|Q6ZP80|TM182_HUMAN
MRLNIAIFFGALFGALGVLLFLVAFGSDYWLLATEVGRCSGEKNIENVTFHHEGFFWRCWFNGIVEENDSNIWKFWYTNQPPSKNCT
HAYLSPYPFMRGEHNSTSYDSAVIYRGFWAVLMLLGVVAVVIASFLIICAAPFASHFLYKAGGGSYIAAGILFSLVVMLYVIWVQAV
ADMESYRNMKMKDCLDFTPSVLYGWSFFLAPAGIFFSLLAGLLFLVVGWHIQIHH
>sp|A0FGR9|ESYT3_HUMAN
MRAEEPCAPGAPSALGAQRTPGPELRLSSQLLPELCTFVVRVLFYLGPVYLAGYLGLSITWLLLGALLWMWWRRNRRGKLGRLAAAF
EFLDNEREFISRELRGQHLPAWIHFPDVERVEWANKIISQTWPYLSMIMESKFREKLEPKIREKSIHLRTFTFTKLYFGQKCPRVNG
VKAHTNTCNRRRVTVDLQICYIGDCEISVELQKIQAGVNGIQLQGTLRVILEPLLVDKPFVGAVTVFFLQKPHLQINWTGLTNLLDA
PGINDVSDSLLEDLIATHLVLPNRVTVPVKKGLDLTNLRFPLPCGVIRVHLLEAEQLAQKDNFLGLRGKSDPYAKVSIGLQHFRSRT
IYRNLNPTWNEVFEFMVYEVPGQDLEVDLYDEDTDRDDFLGSLQICLGDVMTNRVVDEWFVLNDTTSGRLHLRLEWLSLLTDQEVLT
EDHGGLSTAILVVFLESACNLPRNPFDYLNGEYRAKKLSRFARNKVSKDPSSYVKLSVGKKTHTSKTCPHNKDPVWSQVFSFFVHNV
ATERLHLKVLDDDQECALGMLEVPLCQILPYADLTLEQRFQLDHSGLDSLISMRLVLRFLQVEERELGSPYTGPEALKKGPLLIKKV
ATNQGPKAQPQEEGPTDLPCPPDPASDTKDVSRSTTTTTSATTVATEPTSQETGPEPKGKDSAKRFCEPIGEKKSPATIFLTVPGPH
SPGPIKSPRPMKCPASPFAWPPKRLAPSMSSLNSLASSCFDLADISLNIEGGDLRRRQLGEIQLTVRYVCLRRCLSVLINGCRNLTP
CTSSGADPYVRVYLLPERKWACRKKTSVKRKTLEPLFDETFEFFVPMEEVKKRSLDVAVKNSRPLGSHRRKELGKVLIDLSKEDLIK
GFSQWYELTPNGQPRS
>sp|O76082|S22A5_HUMAN
MRDYDEVTAFLGEWGPFQRLIFFLLSASIIPNGFTGLSSVFLIATPEHRCRVPDAANLSSAWRNHTVPLRLRDGREVPHSCRRYRLA
TIANFSALGLEPGRDVDLGQLEQESCLDGWEFSQDVYLSTIVTEWNLVCEDDWKAPLTISLFFVGVLLGSFISGQLSDRFGRKNVLF
VTMGMQTGFSFLQIFSKNFEMFVVLFVLVGMGQISNYVAAFVLGTEILGKSVRIIFSTLGVCIFYAFGYMVLPLFAYFIRDWRMLLV
ALTMPGVLCVALWWFIPESPRWLISQGRFEEAEVIIRKAAKANGIVVPSTIFDPSELQDLSSKKQQSHNILDLLRTWNIRMVTIMSI
MLWMTISVGYFGLSLDTPNLHGDIFVNCFLSAMVEVPAYVLAWLLLQYLPRRYSMATALFLGGSVLLFMQLVPPDLYYLATVLVMVG
KFGVTAAFSMVYVYTAELYPTVVRNMGVGVSSTASRLGSILSPYFVYLGAYDRFLPYILMGSLTILTAILTLFLPESFGTPLPDTID
QMLRVKGMKHRKTPSHTRMLKDGQERPTILKSTAF
>sp|P07203|GPX1_HUMAN
MCAARLAAAAAAAQSVYAFSARPLAGGEPVSLGSLRGKVLLIENVASLUGTTVRDYTQMNELQRRLGPRGLVVLGFPCNQFGHQENA
KNEEILNSLKYVRPGGGFEPNFMLFEKCEVNGAGAHPLFAFLREALPAPSDDATALMTDPKLITWSPVCRNDVAWNFEKFLVGPDGV
PLRRYSRRFQTIDIEPDIEALLSQGPSCA
>sp|Q9BSD7|NTPCR_HUMAN
MARHVFLTGPPGVGKTTLIHKASEVLKSSGVPVDGFYTEEVRQGGRRIGFDVVTLSGTRGPLSRVGLEPPPGKRECRVGQYVVDLTS
FEQLALPVLRNADCSSGPGQRVCVIDEIGKMELFSQLFIQAVRQTLSTPGTIILGTIPVPKGKPLALVEEIRNRKDVKVFNVTKENR
NHLLPDIVTCVQSSRK
>sp|P62314|SMD1_HUMAN

MKLVRFLMKLSHETVTIELKNGTQVHGTITGVDVSMNTHLKAVKMTLKNREPVQLETLSIRGNNIRYFILPDSLPLDTLLVDVEPKV
KSKKREAVAGRGRGRGRGRGRGRGRGRGGPRR
>sp|P68104|EF1A1_HUMAN
MGKEKTHINIVVIGHVDSGKSTTTGHLIYKCGGIDKRTIEKFEKEAAEMGKGSFKYAWVLDKLKAERERGITIDISLWKFETSKYYV
TIIDAPGHRDFIKNMITGTSQADCAVLIVAAGVGEFEAGISKNGQTREHALLAYTLGVKQLIVGVNKMDSTEPPYSQKRYEEIVKEV
STYIKKIGYNPDTVAFVPISGWNGDNMLEPSANMPWFKGWKVTRKDGNASGTTLLEALDCILPPTRPTDKPLRLPLQDVYKIGGIGT
VPVGRVETGVLKPGMVVTFAPVNVTTEVKSVEMHHEALSEALPGDNVGFNVKNVSVKDVRRGNVAGDSKNDPPMEAAGFTAQVIILN
HPGQISAGYAPVLDCHTAHIACKFAELKEKIDRRSGKKLEDGPKFLKSGDAAIVDMVPGKPMCVESFSDYPPLGRFAVRDMRQTVAV
GVIKAVDKKAAGAGKVTKSAQKAQKAK
>sp|O95007|OR6B1_HUMAN
MELENQTRVTKFILVGFPGSLSMRAAMFLIFLVAYILTVAENVIIILLVLQNRPLHKPMYFFLANLSFLETWYISVTVPKLLFSFWS
VNNSISFTLCMIQLYFFIALMCTECVLLAAMAYDRYVAICRPLHYPTIMSHGLCFRLALGSWAIGFGISLAKIYFISCLSFCGPNVI
NHFFCDISPVLNLSCTDMSITELVDFILALVIFLFPLFITVLSYGCILATILCMPTGKQKAFSTCASHLVVVTIFYSAIIFMYARPR
VIHAFNMNKIISIFYAIVTPSLNPFIYCLRNREVKEALKKLAYCQASRSD
>sp|Q8IVF2|AHNK2_HUMAN
MCDCFHMVLPTWPGTPGSVSGRQLQPGEPGAETEDDHSVTEGPADEGIRPRPQGSSPVYEYTTEAADFGLQEDAPGRQGSAGRRRSW
WKRDSGDSRTFFRMSRPEAVQEATEVTLKTEVEAGASGYSVTGGGDQGIFVKQVLKDSSAAKLFNLREGDQLLSTTVFFENIKYEDA
LKILQYSEPYKVQFKIRRQLPAPQDEEWASSDAQHGPQGKEKEDTDVADGCRETPTKTLEGDGDQERLISKPRVGRGRQSQRERLSW
PKFQSIKSKRGPGPQRSHSSSEAYEPRDAHDVSPTSTDTEAQLTVERQEQKAGPGSQRRRKFLNLRFRTGSGQGPSSTGQPGRGFQS
GVGRAGVLEELGPWGDSLEETGAATGSRREERAEQDREVMPAQSMPLPTELGDPRLCEGTPQEGGLRAARLHGKTLEGQAQETAVAQ
RKPRAQPTPGMSREGEGEGLQSLEIGIARLSLRDTTEGGTQIGPPEIRVRVHDLKTPKFAFSTEKEPERERRLSTPQRGKRQDASSK
AGTGLKGEEVEGAGWMPGREPTTHAEAQGDEGDGEEGLQRTRITEEQDKGREDTEGQIRMPKFKIPSLGWSPSKHTKTGREKATEDT
EQGREGEATATADRREQRRTEEGLKDKEDSDSMTNTTKIQLIHDEKRLKKEQILTEKEVATKDSKFKMPKFKMPLFGASAPGKSMEA
SVDVSAPKVEADVSLLSMQGDLKTTDLSVQTPSADLEVQDGQVDVKLPEGPLPEGASLKGHLPKVQRPSLKMPKVDLKGPKLDLKGP
KAEVTAPDVKMSLSSMEVDVQAPRAKLDGARLEGDLSLADKEVTAKDSKFKMPKFKMPSFGVSAPGKSMEDSVDVSAPKVEADVSLS
SMQGDLKATDLSIQPPSADLEVQAGQVDVKLPEGPVPEGAGPKVHLPKVEMPSFKMPKVDLKGPQIDVKGPKLDLKGPKAEVTAPDG
EVSLPSMEVDVQAQKAKLDGAWLEGDLSLADKDVTAKDSKFKMPKFKMPSFGVSAPGKSIKALVDVSAPKVEADLSLPSMQGDLKTT
DLSIQPASTDLKVQADQVDVKLPEGHLPEGAGLKGHLPKVEMPSFKMPKVALKGPQVDVKGPKLDLKSPKAEVTAPDVEVSLPSVEV
DVEAPGAKLDSARLEGELSLADKDVTAKDSRFKMPKFKMPSFGASAPGKSIEASVDVSAPKVEADVSLPSMQGDLKTTDLSIQPPSA
DLEVHAGQVDVKLLEGHVPEGAGFKGHLPKVQMPSLKMPKVDLKGPQVEVRGPKLDLKGHKAEVTAHEVAVSLPSVEVDMQAPGAKL
DGAQLDGDLSLADKDVTAKDSKFKMPKFKMPSFGVSAPGKSIEASVDLSAPKVEADMSLPSMQGDLKTTDLSIQPPSTDLELQAGQL
DVKLPEGPVPEGAGLKGHLPKLQMPSFKVPKVDLKGPEIDIKGPKLDLKDPKVEVTAPDVEVSLPSVEVDVEAPGAKLDGGRLEEDM
SLADKDLTTKDSKFKMPKFKMPSFGVSAPGKSIEASVDVSAPKVEADVSLPSMQGDLKATDLSIQPPSADLEVQAGQVDVKLPEGPV
SEGAGLKGHLPKVQMPSFKMPKVDLKGPQIDVKGPKLDLKGPKVEVTAPDVKMSLSSMEVDVQAPRAKLDGAQLEGDLSLADKAVTA
KDSKFKMPKFKMPSFGVSAPGKSIEASVDVSEPKVEADVSLPSMQGDLKTTDLSIQSPSADLEVQAGQVNVKLPEGPLPEGAGFKGH
LPKVQMPSLKMPKVALKGPQMDVKGPKLDLKGPKAEVMAPDVEVSLPSVEVDVEAPGAKLDSVRLEGDLSLADKDVTAKDSKFKMPK
FKMPSFGVSAPGKSIEASVDVSAPKVEAEVSLPSMQGDLKTTDLCIPLPSADLVVQAGQVDMKLPEGQVPEGAGLKGHLPKVDMPSF
KMPKVDLKGPQTDVKGAKLDLKGPKAEVTAPDVEVSLPSMEVDVQAQKAKLDGARLEGDLSLADKDMTAKDSKFKMPKFKMPSFGVS
APGRSIEASVDVPAPKVEADVSLPSMQGDLKTTDLSIQPPSADLKVQTGQVDVKLPEGHVPEGAGLKGHLPKVEMPSLKMPKVDLKG
PQVDIKGPKLDLKDPKVEMRVPDVEVSLPSMEVDVQAPRAKLDSAHLQGDLTLANKDLTTKDSKFKMPKFKMPSFGVSAPGKSIEAS
VDVSPPKVEADMSLPSMQGDLKTTDLSIQPLSADVKVQAGQVDVKLLEGPVPEEVGLKGHLPKLQMPSFKVPKVDLKGPEIDIKGPK
LDLKDPKVEVTAPDVEVSLPSVEVDVKAPGAKLDGARLEGDMSLADKDVTAKDSKFKMPKFKMLSFGVSALGKSIEASADVSALKVE
ADVSLPSMQGDLKTTDLSVQPPSADLEVQAGQVDVKLPEGPVPEGAGLKGHLPKLQMPSFKMPKVDLKGPQIDVKGPKLDLKGPKTD
VMAPDVEVSQPSVEVDVEAPGAKLDGAWLEGDLSVADKDVTTKDSRFKIPKFKMPSFGVSAPGKSIEASVDVSAPKVEADGSLSSMQ

GDLKATDLSIQPPSADLEVQAGQVDVKLPEGPVPEGAGLKGHLPKVQMPSFKMPEMDLKGPQLDVKGPKLDLKGPKAEVTAPDVEMS
LSSMEVDVQAPRAKLDGARLEGDLSLADKGVTAKDSKFKMPKFKMPSFRVSAPGESIEALVDVSELKVEADMSLPSMQGDLKTTDIS
IQPPSAQLEVQAGQVDVKLPEGHVPEGAGLKGHLPKLQMPSFKMPEVDLKGPQIDVKGPNVDLKGPKAEVTAPDVKMSLSSMEVDVQ
APRAKLDGARLEGDLSLADKGMTAKDSKFKMPKFKMPSFGVSAPGKSIEASVDVSELKVEADGSFPSMQGDLKTTDIRIQPPSAQLE
VQAGQVDVKLPEGHVPEGAGLKGHLPKVQMPSFKMPKVDLKGPQIDVKGPKLDLKGPKAEVTAPDVEVSLPSVEVDVEAPRAKLDGA
RLEGDLSLADKDVTAKDSKFKMPKFKMPSFGVSAPGKSIEVSVDVSAPKVEAEVSLPSMQGDLKTTDISIEPPSAQLEVQAGQVDLK
LPEGHVPEGAGLKGHLPKLQMPSFKMPKVDRKGPQIDVKGPKLDLKGPKTDVTAPDVEVSQPGMEVDVEAPGAKLDGARLEGDLSLA
DKDVTAKDSKFKMPKFKMPSFGVSAPGKSIEVLVDVSAPKVEADLSLPSMQGDLKNTDISIEPPSAQLEVQAGQVDVKLPEGHVLEG
AGLKGHLPKLQMPSFKMPKVDRKGPQIDIKGPKLDLKGPKMDVTAPDVEVSQPSMEVDVEAPGAKLDGARLEGDLSLADKDVTAKDS
KFKMPKFKMPSYRASAPGKSIQASVDVSAPKAEADVSLPSMQGDLKTTDLSIQLPSVDLEVQAGQVDVKLPEGHVPEGAGLKGHLPK
VEMPSFKMPKVDLKSPQVDIKGPKLDLKVPKAEVTVPDVEVSLPSVEVDVQAPRAKLDGARLEGDLSLAEKDVTAKDSKFKMPKFKM
PSFGVSAPGRSIEASLDVSAPKVEADVSLSSMQGDLKATDLSIQPPSADLEVQAVQVDVELLEGPVPEGAGLKGHLPKVEMPSLKTP
KVDLKGPQIDVKGPKLDLKGPKAEVRVPDVEVSLPSVEVDVQAPKAKLDAGRLEGDLSLADKDVTAKDSKFKMPKFKMPSFRVSAPG
KSMEASVDVSAPKVEADVSLPSMQGDLKTTDLSIQPPSADLKVQAGQMDVKLPEGQVPEGAGLKEHLPKVEMPSLKMPKVDLKGPQV
DIKGPKLDLKVSKAEVTAPDVEVSLPSVEVDVQAPRAKLDSAQLEGDLSLADKDVTAKDSKFKMPKFKMPSFGVSAPGKSIEASVHV
SAPKVEADVSLPSMQGDLKTTDLSIQPHSADLTVQARQVDMKLLEGHVPEEAGLKGHLPKVQMPSFKMPKVDLKGPEIDIKGPKLDL
KDPKVEVTAPDVEVSLPSVEVDVEAPGAKLDGARLEGDLSLADKDMTAKDSKFKMPKFKMPSFGVSAPGKSMEASVDVTAPKVEADV
SLPSMQGDLKATDLSVQPPSADLEVQAGQVDVKLPEGPVPEGASLKGHLPKVQMPSFKMPKVDLKGPQIDVKGPKLDLKGPKAEVTA
PDVKMSLSSMEVDVQAPRAKLDGVQLEGDLSLADKDVTAKDSKFKMPKFKMPSFGVSAPGKSMEASVDVSELKAKADVSLPSMQGDL
KTTDLSIQSPSADLEVQAGQVDVKLPEGPLPKGAGLKGHLPKVQMPCLKMPKVALKGPQVDVKGPKLDLKGPKADVMTPVVEVSLPS
MEVDVEAPGAKLDSVRLEGDLSLADKDMTAKDSKFKMPKFKMPSFGVSAPGKSIEASLDVSALKVEADVSLPSMQGDLKTTHLSIQP
PSADLEVQAGQEDVKLPEGPVHEGAGLKGHLPKLQMPSFKVPKVDLKGPQIDVNVPKLDLKGPKVEVTSPNLDVSLPSMEVDIQAPG
AKLDSTRLEGDLSLADKDVTAKDSKFKMPKFKMPSFGMLSPGKSIEVSVDVSAPKMEADMSIPSMQGDLKTTDLRIQAPSADLEVQA
GQVDLKLPEGHMPEVAGLKGHLPKVEMPSFKMPKVDLKGPQVDVKGPKLDLKGPKAEVMAPDVEVSLPSVETDVQAPGSMLDGARLE
GDLSLAHEDVAGKDSKFQGPKLSTSGFEWSSKKVSMSSSEIEGNVTFHEKTSTFPIVESVVHEGDLHDPSRDGNLGLAVGEVGMDSK
FKKLHFKVPKVSFSSTKTPKDSLVPGAKSSIGLSTIPLSSSECSSFELQQVSACSEPSMQMPKVGFAGFPSSRLDLTGPHFESSILS
PCEDVTLTKYQVTVPRAALAPELALEIPSGSQADIPLPKTECSTDLQPPEGVPTSQAESHSGPLNSMIPVSLGQVSFPKFYKPKFVF
SVPQMAVPEGDLHAAVGAPVMSPLSPGERVQCPLPSTQLPSPGTCVSQGPEELVASLQTSVVAPGEAPSEDADHEGKGSPLKMPKIK
LPSFRWSPKKETGPKVDPECSVEDSKLSLVLDKDEVAPQSAIHMDLPPERDGEKGRSTKPGFAMPKLALPKMKASKSGVSLPQRDVD
PSLSSATAGGSFQDTEKASSDGGRGGLGATASATGSEGVNLHRPQVHIPSLGFAKPDLRSSKAKVEVSQPEADLPLPKHDLSTEGDS
RGCGLGDVPVSQPCGEGIAPTPEDPLQPSCRKPDAEVLTVESPEEEAMTKYSQESWFKMPKFRMPSLRRSFRDRGGAGKLEVAQTQA
PAATGGEAAAKVKEFLVSGSNVEAAMSLQLPEADAEVTASESKSSTDILRCDLDSTGLKLHLSTAGMTGDELSTSEVRIHPSKGPLP
FQMPGMRLPETQVLPGEIDETPLSKPGHDLASMEDKTEKWSSQPEGPLKLKASSTDMPSQISVVNVDQLWEDSVLTVKFPKLMVPRF
SFPAPSSEDDVFIPTVREVQCPEANIDTALCKESPGLWGASILKAGAGVPGEQPVDLNLPLEAPPISKVRVHIQGAQVESQEVTIHS
IVTPEFVDLSVPRTFSTQIVRESEIPTSEIQTPSYGFSLLKVKIPEPHTQARVYTTMTQHSRTQEGTEEAPIQATPGVDSISGDLQP
DTGEPFEMISSSVNVLGQQTLTFEVPSGHQLADSCSDEEPAEILEFPPDDSQEATTPLADEGRAPKDKPESKKSGLLWFWLPNIGFS
SSVDETGVDSKNDVQRSAPIQTQPEARPEAELPKKQEKAGWFRFPKLGFSSSPTKKSKSTEDGAELEEQKLQEETITFFDARESFSP
EEKEEGELIGPVGTGLDSRVMVTSAARTELILPEQDRKADDESKGSGLGPNEG
>sp|Q4G0P3|HYDIN_HUMAN
MTSRRLEESMGAVQMGLVNMFKGFQSKVLPPLSPKVVTEEEVNRMLTPSEFLKEMSLTTEQRLAKTRLMCRPQIIELLDMGETTHQK
FSGIDLDQALFQPFPSEIIFQNYTPCEVYEVPLILRNNDKIPRLVKVVEESSPYFKVISPKDIGHKVAPGVPSIFRILFTPEENKDY
AHTLTCVTEREKFIVPIKARGARAILDFPDKLNFSTCPVKYSTQKILLVRNIGNKNAVFHIKTCRPFSIEPAIGTLNVGESMQLEVE
FEPQSVGDHSGRLIVCYDTGEKVFVSLYGAAIDMNIRLDKNSLTIEKTYISLANQRTITIHNRSNIIAHFLWKVFATQQEEDREKYR
ACDDLIKEEKDETDEFFEECITDPLLREHLSVLSRTFANQRRLVQGDSKLFFNNVFTVEPLEGDVWPNSSAEITVYFNPLEAKLYQQ

TIYCDILGREIRLPLRIKGEGMGPKIHFNFELLDIGKVFTGSAHCYEAILYNKGSIDALFNMTPPTSALGACFVFSPKEGIIEPSGV
QAIQISFSSTILGNFEEEFLVNVNGSPEPVKLTIRGCVIGPTFHFNVPALHFGDVSFGFPHTLICSLNNTSLIPMTYKLRIPGDGLG
HKSISYCEQHVDYKRPSWTKEEISSMKPKEFTISPDCGTIRPQGFAAIRVTLCSNTVQKYELALVVDVEGIGEEVLALLITARCVVP
ALHLVNTEVDFGHCFLKYPYEKTLQLANQDDLPGFYEVQPQVCEEVPTVLFSSPTPSGVISPSSTIHIPLVLETQVTGEHRSTVYIS
IFGSQDPPLVCHLKSAGEGPVIYVHPNQVDFGNIYVLKDSSRILNLCNQSFIPAFFQAHMAHKKSLWTIEPNEGMVPPETDVQLALT
ANLNDTLTFKDCVILDIENSSTYRIPVQASGTGSTIVSDKPFAPELNLGAHFSLDTHYYHFKLINKGRRIQQLFWMNDSFRPQAKLS
KKGRVKKGHAHVQPQPSGSQEPRDPQSPVFHLHPASMELYPGQAIDVILEGYSATPRIVKEKLVCHAIIGAQKGKSLVMAVNITCEF
VAPLIQLSTKQLIYRLEKKPNSILKPDYQPLAIKNISTLPVNLLLSTSGPFFICETDKSLLPATPEPIKLEIDEEKNLLIKFDPSYR
NDLNNWVAEEILAIKYVEHPQIDSLDLRGEVHYPNLSFETKELDFGCILNDTELIRYVTITNCSPLVVKFRWFFLVNDEENQIRFVT
LPKKPYSAPVSQMESIPATSEAASPPAILVTVESPEMDLNDFVKTVLVDEDARPEEKELRKTKASSVISDEIKISSTEIERIYSSQS
QVEDQESLQTCEQNEMLSIGIEEVFDILPLFGVLQPHSSHQISFTFYGHANIIAQAKALCEVEEGPTYEITLKGEASLVNYSFDTKD
IHYGLQLFDHVTEREITLTNMGKVGFEFKVLTDHQSSPDNLLPGVPLILPVSGFISSHQEQVLKVYYLPGVPEVFKRSFQIQIAHLD
PENITLSGEGIFPQICLDLPRNLTANEKYEMFLNQARKNTDKEYNKCEMLDHFDIITEEVPEDEPAEVSAHLQMEVERLIVQSYVLE
HQKTTTPDPMDDPCFSHRSRRKLAKIQLPEYILDFGYIILGEVRTHIIKIINTSHFPVSFHADKRVLHETGFSTELDRVKNLPHCET
EIFEVRFDPQGANLPVGSKEVILPIKVVGGPTVHICLQAKVTIPTMTLSRGKVDFATIQCGQCLVETIQLSNHLQVPCEWFVQSQKP
VDKLEKHMPKYLRQKLRAELKPKTRIFEIQPISGVLDPGEKSNVQVKFMPKEEKFYSQTLVFQIAQSAQKLTLLARGQGLEPRLEFS
PSVLDLGPLLLCAPGDEAEVIVKNPCNFPIEFYSLEFDQQYLIEEKILRKLKGYDSYNTLLLPPRNPGEKLPPELYEYFKEIKKSKE
EQMRAKYLENLAQENEEEDITSSDQGTSNSTKRTSLSRGISVTSNLEEWHALLVESKTYLEEEEDEESLEKIIFQTDKLQSIDSHSM
EEVGEVENNPVSKAIARHLGIDISAEGRLAKNRKGIAIIIHGTPLSGKSANAVSVAKYYNAACLSIDSIVLEAVANSNNIPGIRARE
LCIRAAIEQSVKEGEEAAQEAAVGQNVIGQGRLSTDTLGKLASEMTLVAPEIKPGKSVRGSVVITKSKADSHGSGSQKQHHSHQSET
PQISSSPLPPGPIHRWLSVSPSVGGETGLMSCVLPDELLVQILAERIQLSDCYRGVVFDGLDTLFAQNAAAALLCLLKAIGSREHIY
ILNMAQDYAAMKAQEKAKKEQEERKHKGALEKEKERLQNMDEEEYDALTEEEKLTFDRGIQQALRERKKREQERLAKEMQEKKLQQE
LERQKEEDELKRRVKKGKQGPIKEEPPMKKSQAANKQVPPLTKVDVKMETIERKISVREQTMSEKEELNKKKRNMGDVSMHGLPLVQ
DQEDSEGDNSKDPDKQLAPKFKTYELTLKDVQNILMYWDRKQGVQLPPAGMEEAPHEPDDQRQVPLGGRRGRKDRERERLEKERTEK
ERLEREKAERERLEKLRALEERSDWEGEGEEDHEGKKEKDLGVPFLDIQTPDFEGLSWKQALESDKLPKGEQILDILGLGASGPPIP
PPALFSIVSYPVKRPPLTMTDDLEHFVFVIPPSEDISLDEKKEMEIESDFLATTNTTKAQEEQTSSSKGGKQKMKEKIDQVFEIQKD
KRHMALNRKVLSGEPAGTISQLSDTDLDNFNGQHSQEKFTRLNHFRWIVPANGEVTLQVHFSSDEFGNFDQTFNFEILGTCCQYQLY
CRGICTYPYICQDPKVVFPQRKMDMKTNEVIFKKYVMSTETYYFGPLLCGKSRDKYKSSLFPGNMETLTILNTSLMVVEASFYFQND
VKANTYFLEPNTMVLKPNEKQILNVWAYPTSVGVFEDSIVCCINDNPEPAIFQLSCQGIRPELELEPRQLHFDRLLLHRQESRVVLL
RNVTLLPVAWRITSLEHLGDDFTVSLMQGTIPPEAEYGLHLYFQPTKPVNIKKAIRLEVLDAENLLGVVQIENIMVFAEAYDIALDI
TFPKGAEGGLDFGIVRVTEEAKQPLQLKNRGKYEIAFSFSVDSVGISTPNINSMISVQPKKGSLTPTEKPTNVQVFFHAKKEVKIEH
QPVLRCQIIEPNISEGGEIIASIPIKFSANAVYSKYNITPSSVINFGALICGTRKSTTFTIENQGVTDFKFALYKLTGESPIHQKKA
ASHVRHARSRESESFYKTGSSRAAKFSDTIQKEVTTTGQARFAHGMFTVYPGFGSIPSGGQQVINVDCVADAMGKCEEFIAIDISGR
DPAVHPAGILYTLLAEACLPAFVTENNALIFEEHQICTSANLHHILQTIESGGLFVEDENKFIFCNVLVGRQAKARFKISNVGKITC
DVNIVVRPISNKPFARIVDIFEVEPSKMCIASHSHAFATVSFTPQIMQNYQCIFEATLDGLPSTLAKSRGLVFDIAGEGNLPRVTVV
RPVLHNQYGNPLLLFKRLLLGHSEKLPLILKNNGVLPAQLHVDLQDELGVFSLKGRPTTAYIYITEENKPHVKAKKAHTASLVVSPG
DTAEFDVVFHSQKVGRMRGIIHLSVINNQYEETSIHMVGEGYEDDITLDNIHGLVAPTSQEDISISEFTEIIEDNDMEDLVAAALVD
HIQFGDCHIGHSYNASFTVTNHSQVNLIRFEWPVSATIAFSPQMGHLHPGCAKDIVVTMKSDVPINLKNMRIRCKLSRIMFQLPADQ
VPDWDDRMHTVKWVDVPRNMPGTFTTKRKVIETDPEPAHSVLEENYQELQLQISANVDFASYHCQARDVRFKETLVYQTRVFEFDVI
NSGRVQLEFSWVSEDTSKAVSFAKPDHQGSAQKDQLSQGTMHTGSTLDSTMDHWAEGSPQPFSVEPSSGIVPVGKIQKFKVKFSPLD
IGDFESNLFCQIPNLPPGEQGPVLVAKGRSTLPICHFDLKDSDYISGHQRNPELRGSSGGALDPNTRVIEFTTVGIGGKNLRTFTIL
NPTNSTYSFCWISEEIESLQNPAAFTCLTEKGFIHPEKKAEIVFQFTPFHLGITESSWTFLIPEHNITVPFLLVGKTTEPLISLNKS
HLNFSSLLIGREARETVQIINKEEQGFDFSFQDNSRYSEGFSNSLLVCPMEGWIPPLSRFPIDIFFTPKQEGDVNFNLICNVEKKVH
PVTLNVKAEGYTMNVEIKCKDRTGSITLLTPNQTNIINFYEVELNECVQCEFNFINTGKFTFSFQAQLCGSKTLLQYLEFSPIDSTV

DVGQSVHATLSFQPLKKCVLTDLELIIKISHGPTFMCNISGCAVSPAIHFSFTSYNFGTCFIYQAGMPPYKQTLVITNKEETPMSID
CLYTNTTHLEVNSRVDVVKPGNTLEIPITFYPRESINYQELIPFEINGLSQQTVEIKGKGTKMKILVLDPANRIVKLGAVLPGQVVK
RTVSIMNNSLAQLTFNQSILFTIPELQEPKVLTLAPFHNITLKPKEVCKLEVIFAPKKRVPPFSEEVFMECMGLLRPLFLLSGCCQA
LEISLDQEHIPFGPVVYQTQATRRILMMNTGDVGARFKWDIKKFEPHFSISPEEGYITSGMEVSFEVTYHPTEVGKESLCKNILCYI
QGGSPLSLTLSGVCVGPPAVKEVVNFTCQVRSKHTQTILLSNRTNQTWNLHPIFEGEHWEGPEFITLEAHQQNKPYEITYRPRTMNL
ENRKHQGTLFFPLPDGTGWLYALHGTSELPKAVANIYREVPCKTPYTELLPITNWLNKPQRFRVIVEILKPEKPDLSITMKGLDYID
VLSGSKKDYKLNFFSHKEGTYAAKVIFRNEVTNEFLYYNVSFRVIPSGIIKTIEMVTPVRQVASASIKLENPLPYSVTFSTECRMPD
IALPSQFVVPANSEGTFSFEFQPLKAGETFGRLTLHNTDLGYYQYELYLKATPALPEKPVHFQTVLGSSQIILVKFINYTRQRTEYY
CRTDCTDFHAEKLINAAPGGQGGTEASVEVLFEPSHLGETKGILILSSLAGGEYIIPLFGMALPPKPQGPFSIRAGYSIIIPFKNVF
YHMVTFSIIVDNPAFTIRAGESVRPKKINNITVSFEGNPSGSKTPITTKLTVSCPPGEGSETGVKWVYYLKGITL
>sp|Q99440|CD006_HUMAN
MDTQKQIHKTHNSKNQFFTIFFFLSVEFGKEGTRKNFYLLLSIGHYGRKSRRADLGTADTADKTEPECFAASWTFDPNPSVTVSGAH
STAVHQ
>sp|P42575|CASP2_HUMAN
MAAPSAGSWSTFQHKELMAADRGRRILGVCGMHPHHQETLKKNRVVLAKQLLLSELLEHLLEKDIITLEMRELIQAKVGSFSQNVEL
LNLLPKRGPQAFDAFCEALRETKQGHLEDMLLTTLSGLQHVLPPLSCDYDLSLPFPVCESCPLYKKLRLSTDTVEHSLDNKDGPVCL
QVKPCTPEFYQTHFQLAYRLQSRPRGLALVLSNVHFTGEKELEFRSGGDVDHSTLVTLFKLLGYDVHVLCDQTAQEMQEKLQNFAQL
PAHRVTDSCIVALLSHGVEGAIYGVDGKLLQLQEVFQLFDNANCPSLQNKPKMFFIQACRGDETDRGVDQQDGKNHAGSPGCEESDA
GKEKLPKMRLPTRSDMICGYACLKGTAAMRNTKRGSWYIEALAQVFSERACDMHVADMLVKVNALIKDREGYAPGTEFHRCKEMSEY
CSTLCRHLYLFPGHPPT
>sp|Q96NC0|ZMAT2_HUMAN
MASGSGTKNLDFRRKWDKDEYEKLAEKRLTEEREKKDGKPVQPVKRELLRHRDYKVDLESKLGKTIVITKTTPQSEMGGYYCNVCDC
VVKDSINFLDHINGKKHQRNLGMSMRVERSTLDQVKKRFEVNKKKMEEKQKDYDFEERMKELREEEEKAKAYKKEKQKEKKRRAEED
LTFEEDDEMAAVMGFSGFGSTKKSY
>sp|Q8TAV5|CK045_HUMAN
MLTRLVLSAHLSSTTSPPWTHAAISWELDNVLMPSPRIWPQVTPTGRSASVRSEGNTSSLWNFSAGQDVHAIVTRTCESVLSSAVYT
HGCGCVRSATNITCQSSGQQRQAARQEEENSICKAHDSREGRLGYPLSAHQPGSGGPN
>sp|Q69YI7|NAIF1_HUMAN
MAVPAKKRKMNFSEREVEIIVEELELKKHLLVNHFNAGVPLAAKSAAWHGILRRVNAVATCRRELPEVKKKWSDLKTEVRRKVAQVR
AAVEGGEAPGPTEEDGAGGPGTGGGSGGGGPAVAPVLLTPMQQRICNLLGEATIISLPSTTEIHPVALGPSATAAAATVTLTQIPTE
TTYHTLEEGVVEYCTAEAPPPLPPETPVDMMAQHADTSVKPQALKSRIALNSAKLIQEQRVTNLHVKEIAQHLEQQNDLLQMIRRSQ
EVQACAQERQAQAMEGTQAALSVLIQVLRPMIKDFRRYLQSNTANPAPASDPGQVAQNGQPDSIIQ
>sp|Q9NTG1|PKDRE_HUMAN
MRPGPALLLLGVGLSLSVGRLPLPPVPRGAQAAVSGAPGGLLRGAPGLGVRGGRALLSLRPSAVRAGGAVLSGRGSLCFPHGGTGRR
WYCLDLRVLLSAQRLPWPAAPALALVDLQLSARGGRLSLTWSVRLPRSPGRLAWAFRLRLLGPGAARPASPAARVSPRSAAPGPRPQ
QGFVARTECPTDGPARVMLQAVNSSSHRAVESSVSCQINACVIQRVRINTDQKGAPVRLSMQAEATINASVQLDCPAARAIAQYWQV
FSVPAVGQAPDWTQPLDLPQLEIRNSPLFIHIPNNSLQWGVYVFNFTVSITTGNPKMPEVKDSDAVYVWIVRSSLQAVMLGDANITA
NFTEQLILDGSTSSDPDADSPLQGLQFFWYCTTDPRNYGGDRIILGSKEVCHPEQANLKWPWASGPVLTLLPETLKGDHVYFFRMVI
RKDSRTAFSDKRVHVLQGPKAIAHITCIENCERNFIVSDRFSLFLNCTNCASRDFYKWSILSSSGGEMLFDWMGETVTGRNGAYLSI
KAFAFRHFLEAEFSISLYLACWSGVTSVFRHSFIINHGPQIGECKINPAKGIALITKFVVQCSNFRDKHVPLTYKIIVSDLHSVGEI
SSVKENTLGTILYLGPQSTVPPSFLPVGMLASQYGLKIYAQVYDSLGAFSQVTLHATAQAPTDKNSSKTVLNQLLSFTVGPSSLLST
LIQKKDFLPAGYLLYIVASVLNNMKTELPLRDDRVNLRKHLIDQSFLLPVSTLVEIGQVVMTITKLTQKPSEFTWDAQKRATMRVWQ
ANQALQEYQQKDKRFRSEQIEIVSTGILMSLSNILKMTSPHQVVKDPFYVIESLSDTILANKVPGNKTTSMRTPNFNMYVKKVEKWG
INQLFRNEKHCRNCFYPTLNVSSVPGLSANGPISTMFCDFTNDLFPWLNDQENTSVEVSGFRMTGVADNGSVLEITPDVAEVYLVRK

NLTFAAFNLTVGPNSEVDGSLKKTTGGFSFQVDSTVLREVLVHIVTEVMVLFTVLVYTGSQITPTALVATFLVPHDIPPFASQSALF
DPACTVKKARVVCLPVSLLQLIAQHSHSPHCTVSIVLQAPRFVMKLNDKLVRISIFSVQCLDMYGIQSEWREGYCILGEKTSWYEVH
CICKNVVRARRQLGTIGLTGIHLHTHYVMAKVIVIPNPVDLRLNIIKSLHQNPVTLFTVLFIILLYVGLAFWALYRDEMDQHLRGHV
IVLPDNDPYDNLCYLVTIFTGSRWGSGTRANVFVQLRGTVSTSDVHCLSHPHFTTLYRGSINTFLLTTKSDLGDIHSIRVWHNNEGR
SPSWYLSRIKVENLFSRHIWLFICQKWLSVDTTLDRTFHVTHPDERLTRKDFFFIDVSSNLRKNHMWFSIFASVVAKTFNRLQRLSC
CLAMLLSSLLCNIMFFNLNRQEQTESRERKYMRSMMIGIESVLITIPVQLLITFLFTCSQRKPQADLKEVSPQKHPLMSEASEHWEE
YLRKWHAYETAKVHPREVAKPASKGKPRLPKASPKATSKPKHRHRKAQIKTPETLGPNTNSNNNIEDDQDVHSEQHPSQKDLQQLKK
KPRIVLPWWCVYVAWFLVFATSSISSFFIVFYGLTYGYDKSIEWLFASFCSFCQSVLLVQPSKIILLSGFRTNKPKYCKNLSWSTKY
KYTEIRLDGMRMHPEEMQRIHDQIVRIRGTRMYQPLTEDEIRIFKRKKRIKRRALLFLSYILTHFIFLALLLILIVLLRHTDCFYYN
QFIRDRFSMDLATVTKLEDIYRWLNSVLLPLLHNDLNPTFLPESSSKILGLPLMRQVRAKSSEKMCLPAEKFVQNSIRREIHCHPKY
GIDPEDTKNYSGFWNEVDKQAIDESTNGFTYKPQGTQWLYYSYGLLHTYGSGGYALYFFPEQQRFNSTLRLKELQESNWLDEKTWAV
VLELTTFNPDINLFCSISVIFEVSQLGVVNTSISLHSFSLADFDRKASAEIYLYVAILIFFLAYVVDEGCIIMQERASYVRSVYNLL
NFALKCIFTVLIVLFLRKHFLATGIIRFYLSNPEDFIPFHAVSQVDHIMRIILGFLLFLTILKTLRYSRFFYDVRLAQRAIQAALPG
ICHMAFVVSVYFFVYMAFGYLVFGQHEWNYSNLIHSTQTVFSYCVSAFQNTEFSNNRILGVLFLSSFMLVMICVLINLFQAVILSAY
EEMKQPVYEEPSDEVEAMTYLCRKLRTMFSFLTSQSKAKDEPEFFIDMLYGQPEKNSHRYLGLKTRNINGKKMVYLVV
>sp|Q99797|MIPEP_HUMAN
MLCVGRLGGLGARAAALPPRRAGRGSLEAGIRARRVSTSWSPVGAAFNVKPQGSRLDLFGERRGLFGVPELSAPEGFHIAQEKALRK
TELLVDRACSTPPGPQTVLIFDELSDSLCRVADLADFVKIAHPEPAFREAAEEACRSIGTMVEKLNTNVDLYQSLQKLLADKKLVDS
LDPETRRVAELFMFDFEISGIHLDKEKRKRAVDLNVKILDLSSTFLMGTNFPNKIEKHLLPEHIRRNFTSAGDHIIIDGLHAESPDD
LVREAAYKIFLYPNAGQLKCLEELLSSRDLLAKLVGYSTFSHRALQGTIAKNPETVMQFLEKLSDKLSERTLKDFEMIRGMKMKLNP
QNSEVMPWDPPYYSGVIRAERYNIEPSLYCPFFSLGACMEGLNILLNRLLGISLYAEQPAKGEVWSEDVRKLAVVHESEGLLGYIYC
DFFQRADKPHQDCHFTIRGGRLKEDGDYQLPVVVLMLNLPRSSRSSPTLLTPSMMENLFHEMGHAMHSMLGRTRYQHVTGTRCPTDF
AEVPSILMEYFANDYRVVNQFARHYQTGQPLPKNMVSRLCESKKVCAAADMQLQVFYATLDQIYHGKHPLRNSTTDILKETQEKFYG
LPYVPNTAWQLRFSHLVGYGARYYSYLMSRAVASMVWKECFLQDPFNRAAGERYRREMLAHGGGREPMLMVEGMLQKCPSVDDFVSA
LVSDLDLDFETFLMDSE
>sp|Q9Y606|TRUA_HUMAN
MGLQLRALLGAFGRWTLRLGPRPSCSPRMAGNAEPPPAGAACPQDRRSCSGRAGGDRVWEDGEHPAKKLKSGGDEERREKPPKRKIV
LLMAYSGKGYHGMQRNVGSSQFKTIEDDLVSALVRSGCIPENHGEDMRKMSFQRCARTDKGVSAAGQVVSLKVWLIDDILEKINSHL
PSHIRILGLKRVTGGFNSKNRCDARTYCYLLPTFAFAHKDRDVQDETYRLSAETLQQVNRLLACYKGTHNFHNFTSQKGPQDPSACR
YILEMYCEEPFVREGLEFAVIRVKGQSFMMHQIRKMVGLVVAIVKGYAPESVLERSWGTEKVDVPKAPGLGLVLERVHFEKYNQRFG
NDGLHEPLDWAQEEGKVAAFKEEHIYPTIIGTERDERSMAQWLSTLPIHNFSATALTAGGTGAKVPSPLEGSEGDGDTD
>sp|A8MQ03|CRTP1_HUMAN
MDPQEMVVKNPYAHISIPRAHLRPDLGQQLEVASTCSSSSEMQPLPVGPCAPEPTHLLQPTEVPGPKGAKGNQGAAPIQNQQAWQQP
GNPYSSSQRQAGLTYAGPPPAGRGDDIAHHCCCCPCCHCCHCPPFCRCHSCCCCVIS
>sp|P15927|RFA2_HUMAN
MWNSGFESYGSSSYGGAGGYTQSPGGFGSPAPSQAEKKSRARAQHIVPCTISQLLSATLVDEVFRIGNVEISQVTIVGIIRHAEKAP
TNIVYKIDDMTAAPMDVRQWVDTDDTSSENTVVPPETYVKVAGHLRSFQNKKSLVAFKIMPLEDMNEFTTHILEVINAHMVLSKANS
QPSAGRAPISNPGMSEAGNFGGNSFMPANGLTVAQNQVLNLIKACPRPEGLNFQDLKNQLKHMSVSSIKQAVDFLSNEGHIYSTVDD
DHFKSTDAE
>sp|Q86Y07|VRK2_HUMAN
MPPKRNEKYKLPIPFPEGKVLDDMEGNQWVLGKKIGSGGFGLIYLAFPTNKPEKDARHVVKVEYQENGPLFSELKFYQRVAKKDCIK
KWIERKQLDYLGIPLFYGSGLTEFKGRSYRFMVMERLGIDLQKISGQNGTFKKSTVLQLGIRMLDVLEYIHENEYVHGDIKAANLLL
GYKNPDQVYLADYGLSYRYCPNGNHKQYQENPRKGHNGTIEFTSLDAHKGVALSRRSDVEILGYCMLRWLCGKLPWEQNLKDPVAVQ
TAKTNLLDELPQSVLKWAPSGSSCCEIAQFLVCAHSLAYDEKPNYQALKKILNPHGIPLGPLDFSTKGQSINVHTPNSQKVDSQKAA

TKQVNKAHNRLIEKKVHSERSAESCATWKVQKEEKLIGLMNNEAAQESTRRRQKYQESQEPLNEVNSFPQKISYTQFPNSFYEPHQD
FTSPDIFKKSRSPSWYKYTSTVSTGITDLESSTGLWPTISQFTLSEETNADVYYYRIIIPVLLMLVFLALFFL
>sp|Q9Y2M5|KLH20_HUMAN
MEGKPMRRCTNIRPGETGMDVTSRCTLGDPNKLPEGVPQPARMPYISDKHPRQTLEVINLLRKHRELCDVVLVVGAKKIYAHRVILS
ACSPYFRAMFTGELAESRQTEVVIRDIDERAMELLIDFAYTSQITVEEGNVQTLLPAACLLQLAEIQEACCEFLKRQLDPSNCLGIR
AFADTHSCRELLRIADKFTQHNFQEVMESEEFMLLPANQLIDIISSDELNVRSEEQVFNAVMAWVKYSIQERRPQLPQVLQHVRLPL
LSPKFLVGTVGSDPLIKSDEECRDLVDEAKNYLLLPQERPLMQGPRTRPRKPIRCGEVLFAVGGWCSGDAISSVERYDPQTNEWRMV
ASMSKRRCGVGVSVLDDLLYAVGGHDGSSYLNSVERYDPKTNQWSSDVAPTSTCRTSVGVAVLGGFLYAVGGQDGVSCLNIVERYDP
KENKWTRVASMSTRRLGVAVAVLGGFLYAVGGSDGTSPLNTVERYNPQENRWHTIAPMGTRRKHLGCAVYQDMIYAVGGRDDTTELS
SAERYNPRTNQWSPVVAMTSRRSGVGLAVVNGQLMAVGGFDGTTYLKTIEVFDPDANTWRLYGGMNYRRLGGGVGVIKMTHCESHIW
>sp|P60059|SC61G_HUMAN
MDQVMQFVEPSRQFVKDSIRLVKRCTKPDRKEFQKIAMATAIGFAIMGFIGFFVKLIHIPINNIIVGG
>sp|A4D2B8|PM2P1_HUMAN
MVTMCGGHRPENFLHQVLTEFGEELAGEGKSEVGGGAPRSYLQVASAECWAAAPAVHVGEPVHAGGLHTERGADPVIGLYLVHRGGA
CQTPTVGNRQTPTLGIHARPRRRATTSLLTLLLAFGKNAVRCALIGPGSLTSRTRPLTEPLGEKERREVFFPPRPERVEHNVESSRW
EPRRRGACGSRGGNFPSPRGGSGVASLERAESSSTEPAKAIKPIDRKSVHQICSGPVVPSLSTAVKELVENSLDAGATNIDLKLKDY
GVDLIEVSGNGCGVEEENFEGLTLKHHTSKIQEFADLPQVETFGFRGEALSSLCALSDVTISTCHVSAKVGTRLVFDHYGKIIQKTP
YPHPRGMTVSVKQLFSTLPVHHKEFQRNIKKKRACFPFAFCRDCQFPEASPAMLPVQPAELTPRSTPPHPCSLEDNVITVFSSVKNG
PGSSR
>sp|O94832|MYO1D_HUMAN
MAEQESLEFGKADFVLMDTVSMPEFMANLRLRFEKGRIYTFIGEVVVSVNPYKLLNIYGRDTIEQYKGRELYERPPHLFAIADAAYK
AMKRRSKDTCIVISGESGAGKTEASKYIMQYIAAITNPSQRAEVERVKNMLLKSNCVLEAFGNAKTNRNDNSSRFGKYMDINFDFKG
DPIGGHINNYLLEKSRVIVQQPGERSFHSFYQLLQGGSEQMLRSLHLQKSLSSYNYIHVGAQLKSSINDAAEFRVVADAMKVIGFKP
EEIQTVYKILAAILHLGNLKFVVDGDTPLIENGKVVSIIAELLSTKTDMVEKALLYRTVATGRDIIDKQHTEQEASYGRDAFAKAIY
ERLFCWIVTRINDIIEVKNYDTTIHGKNTVIGVLDIYGFEIFDNNSFEQFCINYCNEKLQQLFIQLVLKQEQEEYQREGIPWKHIDY
FNNQIIVDLVEQQHKGIIAILDDACMNVGKVTDEMFLEALNSKLGKHAHFSSRKLCASDKILEFDRDFRIRHYAGDVVYSVIGFIDK
NKDTLFQDFKRLMYNSSNPVLKNMWPEGKLSITEVTKRPLTAATLFKNSMIALVDNLASKEPYYVRCIKPNDKKSPQIFDDERCRHQ
VEYLGLLENVRVRRAGFAFRQTYEKFLHRYKMISEFTWPNHDLPSDKEAVKKLIERCGFQDDVAYGKTKIFIRTPRTLFTLEELRAQ
MLIRIVLFLQKVWRGTLARMRYKRTKAALTIIRYYRRYKVKSYIHEVARRFHGVKTMRDYGKHVKWPSPPKVLRRFEEALQTIFNRW
RASQLIKSIPASDLPQVRAKVAAVEMLKGQRADLGLQRAWEGNYLASKPDTPQTSGTFVPVANELKRKDKYMNVLFSCHVRKVNRFS
KVEDRAIFVTDRHLYKMDPTKQYKVMKTIPLYNLTGLSVSNGKDQLVVFHTKDNKDLIVCLFSKQPTHESRIGELVGVLVNHFKSEK
RHLQVNVTNPVQCSLHGKKCTVSVETRLNQPQPDFTKNRSGFILSVPGN
>sp|Q9H8Y5|ANKZ1_HUMAN
MSPAPDAAPAPASISLFDLSADAPVFQGLSLVSHAPGEALARAPRTSCSGSGERESPERKLLQGPMDISEKLFCSTCDQTFQNHQEQ
REHYKLDWHRFNLKQRLKDKPLLSALDFEKQSSTGDLSSISGSEDSDSASEEDLQTLDRERATFEKLSRPPGFYPHRVLFQNAQGQF
LYAYRCVLGPHQDPPEEAELLLQNLQSRGPRDCVVLMAAAGHFAGAIFQGREVVTHKTFHRYTVRAKRGTAQGLRDARGGPSHSAGA
NLRRYNEATLYKDVRDLLAGPSWAKALEEAGTILLRAPRSGRSLFFGGKGAPLQRGDPRLWDIPLATRRPTFQELQRVLHKLTTLHV
YEEDPREAVRLHSPQTHWKTVREERKKPTEEEIRKICRDEKEALGQNEESPKQGSGSEGEDGFQVELELVELTVGTLDLCESEVLPK
RRRRKRNKKEKSRDQEAGAHRTLLQQTQEEEPSTQSSQAVAAPLGPLLDEAKAPGQPELWNALLAACRAGDVGVLKLQLAPSPADPR
VLSLLSAPLGSGGFTLLHAAAAAGRGSVVRLLLEAGADPTVQDSRARPPYTVAADKSTRNEFRRFMEKNPDAYDYNKAQVPGPLTPE
MEARQATRKREQKAARRQREEQQQRQQEQEEREREEQRRFAALSDREKRALAAERRLAAQLGAPTSPIPDSAIVNTRRCWSCGASLQ
GLTPFHYLDFSFCSTRCLQDHRRQAGRPSS
>sp|A0PJZ3|GXLT2_HUMAN

MKLRSKAAALLLLALAALLLALLSLRAGRAEPPALPARPASAPQRHPAPVPARWPGPGALPGASPGVRRRRPPRPRPRAGRRGAARL
EKLARRPGEPRSFQAVLPPELWIHLAVVACGNRLEETLVMLKSAVLFSHRKIQFHIFTEDSLKPEFDKQLRQWPDSYTKKFEHRIYP
ITFSVGNPQEWKKLFKPCAAQRLFLPVILKDVDSLLYVDTDVLFLRPVDDIWKLLRLFNSTQLAAMAPEHEIPKIGWYSRFARHPFY
GSAGVNSGVMLMNLTRIRSTQFKNSMIPTGLAWEDMLYPLYQKYKNAITWGDQDLLNIIFYFNPECLYVFPCQWNYRPDHCMYGSNC
REAEHEGVSVLHGNRGVYHDDKQPTFRALYEAIRDFPFQDNLFQSMYYPLQLKFLETVHTLCGRIPQVFLKQIEKTMKRAYEKHVII
HVGPNQMH
>sp|Q13106|ZN154_HUMAN
MAAATLRTPTQGTVTFEDVAVHFSWEEWGLLDEAQRCLYRDVMLENLALLTSLDVHHQKQHLGEKHFRSNVGRALFVKTCTFHVSGE
PSTCREVGKDFLAKLGFLHQQAAHTGEQSNSKSDGGAISHRGKTHYNCGEHTKAFSGKHTLVQQQRTLTTERCYICSECGKSFSKSY
SLNDHWRLHTGEKPYECRECGKSFRQSSSLIQHRRVHTAVRPHECDECGKLFSNKSNLIKHRRVHTGERPYECSECGKSFSQRSALL
QHRGVHTGERPYECSECGKFFTYHSSLIKHQKVHSGSRPYECSECGKSFSQNSSLIEHHRVHTGERPYKCSECGKSFSQRSALLQHR
GVHTGERPYECSECGKFFPYSSSLRKHQRVHTGSRPYECSECGKSFTQNSGLIKHRRVHTGEKPYECTECGKSFSHNSSLIKHQRIH
SR
>sp|Q9HD89|RETN_HUMAN
MKALCLLLLPVLGLLVSSKTLCSMEEAINERIQEVAGSLIFRAISSIGLECQSVTSRGDLATCPRGFAVTGCTCGSACGSWDVRAET
TCHCQCAGMDWTGARCCRVQP
>sp|Q07866|KLC1_HUMAN
MYDNMSTMVYIKEDKLEKLTQDEIISKTKQVIQGLEALKNEHNSILQSLLETLKCLKKDDESNLVEEKSNMIRKSLEMLELGLSEAQ
VMMALSNHLNAVESEKQKLRAQVRRLCQENQWLRDELANTQQKLQKSEQSVAQLEEEKKHLEFMNQLKKYDDDISPSEDKDTDSTKE
PLDDLFPNDEDDPGQGIQQQHSSAAAAAQQGGYEIPARLRTLHNLVIQYASQGRYEVAVPLCKQALEDLEKTSGHDHPDVATMLNIL
ALVYRDQNKYKDAANLLNDALAIREKTLGKDHPAVAATLNNLAVLYGKRGKYKEAEPLCKRALEIREKVLGKDHPDVAKQLNNLALL
CQNQGKYEEVEYYYQRALEIYQTKLGPDDPNVAKTKNNLASCYLKQGKFKQAETLYKEILTRAHEREFGSVDDENKPIWMHAEEREE
CKGKQKDGTSFGEYGGWYKACKVDSPTVTTTLKNLGALYRRQGKFEAAETLEEAAMRSRKQGLDNVHKQRVAEVLNDPENMEKRRSR
ESLNVDVVKYESGPDGGEEVSMSVEWNGGVSGRASFCGKRQQQQWPGRRHR
>sp|Q7Z3T8|ZFY16_HUMAN
MDSYFKAAVSDLDKLLDDFEQNPDEQDYLQDVQNAYDSNHCSVSSELASSQRTSLLPKDQECVNSCASSETSYGTNESSLNEKTLKG
LTSIQNEKNVTGLDLLSSVDGGTSDEIQPLYMGRCSKPICDLISDMGNLVHATNSEEDIKKLLPDDFKSNADSLIGLDLSSVSDTPC
VSSTDHDSDTVREQQNDISSELQNREIGGIKELGIKVDTTLSDSYNYSGTENLKDKKIFNQLESIVDFNMSSALTRQSSKMFHAKDK
LQHKSQPCGLLKDVGLVKEEVDVAVITAAECLKEEGKTSALTCSLPKNEDLCLNDSNSRDENFKLPDFSFQEDKTVIKQSAQEDSKS
LDLKDNDVIQDSSSALHVSSKDVPSSLSCLPASGSMCGSLIESKARGDFLPQHEHKDNIQDAVTIHEEIQNSVVLGGEPFKENDLLK
QEKCKSILLQSLIEGMEDRKIDPDQTVIRAESLDGGDTSSTVVESQEGLSGTHVPESSDCCEGFINTFSSNDMDGQDLDYFNIDEGA
KSGPLISDAELDAFLTEQYLQTTNIKSFEENVNDSKSQMNQIDMKGLDDGNINNIYFNAEAGAIGESHGINIICEIVDKQNTIENGL
SLGEKSTIPVQQGLPTSKSEITNQLSVSDINSQSVGGARPKQLFSLPSRTRSSKDLNKPDVPDTIESEPSTADTVVPITCAIDSTAD
PQVSFNSNYIDIESNSEGGSSFVTANEDSVPENTCKEGLVLGQKQPTWVPDSEAPNCMNCQVKFTFTKRRHHCRACGKVFCGVCCNR
KCKLQYLEKEARVCVVCYETISKAQAFERMMSPTGSNLKSNHSDECTTVQPPQENQTSSIPSPATLPVSALKQPGVEGLCSKEQKRV
WFADGILPNGEVADTTKLSSGSKRCSEDFSPLSPDVPMTVNTVDHSHSTTVEKPNNETGDITRNEIIQSPISQVPSVEKLSMNTGNE
GLPTSGSFTLDDDVFAETEEPSSPTGVLVNSNLPIASISDYRLLCDINKYVCNKISLLPNDEDSLPPLLVASGEKGSVPVVEEHPSH
EQIILLLEGESFHPVTFVLNANLLVNVKFIFYSSDKYWYFSTNGLHGLGQAEIIILLLCLPNEDTIPKDIFRLFITIYKDALKGKYI
ENLDNITFTESFLSSKDHGGFLFITPTFQKLDDLSLPSNPFLCGILIQKLEIPWAKVFPMRLMLRLGAEYKAYPAPLTSIRGRKPLF
GEIGHTIMNLLVDLRNYQYTLHNIDQLLIHMEMGKSCIKIPRKKYSDVMKVLNSSNEHVISIGASFSTEADSHLVCIQNDGIYETQA
NSATGHPRKVTGASFVVFNGALKTSSGFLAKSSIVEDGLMVQITPETMNGLRLALREQKDFKITCGKVDAVDLREYVDICWVDAEEK
GNKGVISSVDGISLQGFPSEKIKLEADFETDEKIVKCTEVFYFLKDQDLSILSTSYQFAKEIAMACSAALCPHLKTLKSNGMNKIGL
RVSIDTDMVEFQAGSEGQLLPQHYLNDLDSALIPVIHGGTSNSSLPLEIELVFFIIEHLF
>sp|P52306|GDS1_HUMAN

MDNLSDTLKKLKITAVDKTEDSLEGCLDCLLQALAQNNTETSEKIQASGILQLFASLLTPQSSCKAKVANIIAEVAKNEFMRIPCVD
AGLISPLVQLLNSKDQEVLLQTGRALGNICYDSHEGRSAVDQAGGAQIVIDHLRSLCSITDPANEKLLTVFCGMLMNYSNENDSLQA
QLINMGVIPTLVKLLGIHCQNAALTEMCLVAFGNLAELESSKEQFASTNIAEELVKLFKKQIEHDKREMIFEVLAPLAENDAIKLQL
VEAGLVECLLEIVQQKVDSDKEDDITELKTGSDLMVLLLLGDESMQKLFEGGKGSVFQRVLSWIPSNNHQLQLAGALAIANFARNDA
NCIHMVDNGIVEKLMDLLDRHVEDGNVTVQHAALSALRNLAIPVINKAKMLSAGVTEAVLKFLKSEMPPVQFKLLGTLRMLIDAQAE
AAEQLGKNVKLVERLVEWCEAKDHAGVMGESNRLLSALIRHSKSKDVIKTIVQSGGIKHLVTMATSEHVIMQNEALVALALIAALEL
GTAEKDLESAKLVQILHRLLADERSAPEIKYNSMVLICALMGSECLHKEVQDLAFLDVVSKLRSHENKSVAQQASLTEQRLTVES
>sp|Q5SQH8|CF136_HUMAN
MYQPSRGAARRLGPCLRAYQARPQDQLYPGTLPFPPLWPHSTTTTSPSSPLFWSPLPPRLPTQRLPQVPPLPLPQIQALSSAWVVLP
PGKGEEGPGPELHSGCLDGLRSLFEGPPCPYPGAWIPFQVPGTAHPSPATPSGDPSMEEHLSVMYERLRQELPKLFLQSHDYSLYSL
DVEFINEILNIRTKGRTWYILSLTLCRFLAWNYFAHLRLEVLQLTRHPENWTLQARWRLVGLPVHLLFLRFYKRDKDEHYRTYDAYS
TFYLNSSGLICRHRLDKLMPSHSPPTPVKKLLVGALVALGLSEPEPDLNLCSKP
>sp|Q9UBV4|WNT16_HUMAN
MDRAALLGLARLCALWAALLVLFPYGAQGNWMWLGIASFGVPEKLGCANLPLNSRQKELCKRKPYLLPSIREGARLGIQECGSQFRH
ERWNCMITAAATTAPMGASPLFGYELSSGTKETAFIYAVMAAGLVHSVTRSCSAGNMTECSCDTTLQNGGSASEGWHWGGCSDDVQY
GMWFSRKFLDFPIGNTTGKENKVLLAMNLHNNEAGRQAVAKLMSVDCRCHGVSGSCAVKTCWKTMSSFEKIGHLLKDKYENSIQISD
KTKRKMRRREKDQRKIPIHKDDLLYVNKSPNYCVEDKKLGIPGTQGRECNRTSEGADGCNLLCCGRGYNTHVVRHVERCECKFIWCC
YVRCRRCESMTDVHTCK
>sp|Q01629|IFM2_HUMAN
MNHIVQTFSPVNSGQPPNYEMLKEEQEVAMLGVPHNPAPPMSTVIHIRSETSVPDHVVWSLFNTLFMNTCCLGFIAFAYSVKSRDRK
MVGDVTGAQAYASTAKCLNIWALILGIFMTILLIIIPVLVVQAQR
>sp|Q9UGU0|TCF20_HUMAN
MQSFREQSSYHGNQQSYPQEVHGSSRLEEFSPRQAQMFQNFGGTGGSSGSSGSGSGGGRRGAAAAAAAMASETSGHQGYQGFRKEAG
DFYYMAGNKDPVTTGTPQPPQRRPSGPVQSYGPPQGSSFGNQYGSEGHVGQFQAQHSGLGGVSHYQQDYTGPFSPGSAQYQQQASSQ
QQQQQVQQLRQQLYQSHQPLPQATGQPASSSSHLQPMQRPSTLPSSAAGYQLRVGQFGQHYQSSASSSSSSSFPSPQRFSQSGQSYD
GSYNVNAGSQYEGHNVGSNAQAYGTQSNYSYQPQSMKNFEQAKIPQGTQQGQQQQQPQQQQHPSQHVMQYTNAATKLPLQSQVGQYN
QPEVPVRSPMQFHQNFSPISNPSPAASVVQSPSCSSTPSPLMQTGENLQCGQGSVPMGSRNRILQLMPQLSPTPSMMPSPNSHAAGF
KGFGLEGVPEKRLTDPGLSSLSALSTQVANLPNTVQHMLLSDALTPQKKTSKRPSSSKKADSCTNSEGSSQPEEQLKSPMAESLDGG
CSSSSEDQGERVRQLSGQSTSSDTTYKGGASEKAGSSPAQGAQNEPPRLNASPAAREEATSPGAKDMPLSSDGNPKVNEKTVGVIVS
REAMTGRVEKPGGQDKGSQEDDPAATQRPPSNGGAKETSHASLPQPEPPGGGGSKGNKNGDNNSNHNGEGNGQSGHSAAGPGFTSRT
EPSKSPGSLRYSYKDSFGSAVPRNVSGFPQYPTGQEKGDFTGHGERKGRNEKFPSLLQEVLQGYHHHPDRRYSRSTQEHQGMAGSLE
GTTRPNVLVSQTNELASRGLLNKSIGSLLENPHWGPWERKSSSTAPEMKQINLTDYPIPRKFEIEPQSSAHEPGGSLSERRSVICDI
SPLRQIVRDPGAHSLGHMSADTRIGRNDRLNPTLSQSVILPGGLVSMETKLKSQSGQIKEEDFEQSKSQASFNNKKSGDHCHPPSIK
HESYRGNASPGAATHDSLSDYGPQDSRPTPMRRVPGRVGGREGMRGRSPSQYHDFAEKLKMSPGRSRGPGGDPHHMNPHMTFSERAN
RSSLHTPFSPNSETLASAYHANTRAHAYGDPNAGLNSQLHYKRQMYQQQPEEYKDWSSGSAQGVIAAAQHRQEGPRKSPRQQQFLDR
VRSPLKNDKDGMMYGPPVGTYHDPSAQEAGRCLMSSDGLPNKGMELKHGSQKLQESCWDLSRQTSPAKSSGPPGMSSQKRYGPPHET
DGHGLAEATQSSKPGSVMLRLPGQEDHSSQNPLIMRRRVRSFISPIPSKRQSQDVKNSSTEDKGRLLHSSKEGADKAFNSYAHLSHS
QDIKSIPKRDSSKDLPSPDSRNCPAVTLTSPAKTKILPPRKGRGLKLEAIVQKITSPNIRRSASSNSAEAGGDTVTLDDILSLKSGP
PEGGSVAVQDADIEKRKGEVASDLVSPANQELHVEKPLPRSSEEWRGSVDDKVKTETHAETVTAGKEPPGAMTSTTSQKPGSNQGRP
DGSLGGTAPLIFPDSKNVPPVGILAPEANPKAEEKENDTVTISPKQEGFPPKGYFPSGKKKGRPIGSVNKQKKQQQPPPPPPQPPQI
PEGSADGEPKPKKQRQRRERRKPGAQPRKRKTKQAVPIVEPQEPEIKLKYATQPLDKTDAKNKSFYPYIHVVNKCELGAVCTIINAE
EEEQTKLVRGRKGQRSLTPPPSSTESKALPASSFMLQGPVVTESSVMGHLVCCLCGKWASYRNMGDLFGPFYPQDYAATLPKNPPPK
RATEMQSKVKVRHKSASNGSKTDTEEEEEQQQQQKEQRSLAAHPRFKRRHRSEDCGGGPRSLSRGLPCKKAATEGSSEKTVLDSKPS

VPTTSEGGPELELQIPELPLDSNEFWVHEGCILWANGIYLVCGRLYGLQEALEIAREMKCSHCQEAGATLGCYNKGCSFRYHYPCAI
DADCLLHEENFSVRCPKHKPPLPCPLPPLQNKTAKGSLSTEQSERG
>sp|Q8IZX4|TAF1L_HUMAN
MRPGCDLLLRAAATVTAAIMSDSDSEEDSSGGGPFTLAGILFGNISGAGQLEGESVLDDECKKHLAGLGALGLGSLITELTANEELT
GTGGALVNDEGWIRSTEDAVDYSDINEVAEDESQRHQQTMGSLQPLYHSDYDEDDYDADCEDIDCKLMPPPPPPPGPMKKDKDQDAI
TCVSESGEDIILPSIIAPSFLASEKVDFSSYSDSESEMGPQEATQAESEDGKLTLPLAGIMQHDATKLLPSVTELFPEFRPGKVLRF
LHLFGPGKNVPSVWRSARRKRKKHRELIQEEQIQEVECSVESEVSQKSLWNYDYAPPPPPEQCLADDEITMMVPVESKFSQSTGDVD
KVTDTKPRVAEWRYGPARLWYDMLGVSEDGSGFDYGFKLRKTQHEPVIKSRMMEEFRKLEESNGTDLLADENFLMVTQLHWEDSIIW
DGEDIKHKGTKPQGASLAGWLPSIKTRNVMAYNVQQGFAPTLDDDKPWYSIFPIDNEDLVYGRWEDNIIWDAQAMPRLLEPPVLALD
PNDENLILEIPDEKEEATSNSPSKESKKESSLKKSRILLGKTGVIREEPQQNMSQPEVKDPWNLSNDEYYFPKQQGLRGTFGGNIIQ
HSIPAMELWQPFFPTHMGPIKIRQFHRPPLKKYSFGALSQPGPHSVQPLLKHIKKKAKMREQERQASGGGELFFMRTPQDLTGKDGD
LILAEYSEENGPLMMQVGMATKIKNYYKRKPGKDPGAPDCKYGETVYCHTSPFLGSLHPGQLLQALENNLFRAPVYLHKMPETDFLI
IRTRQGYYIRELVDIFVVGQQCPLFEVPGPNSRRANMHIRDFLQVFIYRLFWKSKDRPRRIRMEDIKKAFPSHSESSIRKRLKLCAD
FKRTGMDSNWWVLKSDFRLPTEEEIRAKVSPEQCCAYYSMIAAKQRLKDAGYGEKSFFAPEEENEEDFQMKIDDEVHAAPWNTTRAF
IAAMKGKCLLEVTGVADPTGCGEGFSYVKIPNKPTQQKDDKEPQAVKKTVTGTDADLRRLSLKNAKQLLRKFGVPEEEIKKLSRWEV
IDVVRTMSTEQAHSGEGPMSKFARGSRFSVAEHQERYKEECQRIFDLQNKVLSSTEVLSTDTDSISAEDSDFEEMGKNIENMLQNKK
TSSQLSREWEEQERKELRRMLLVAGSAASGNNHRDDVTASMTSLKSSATGHCLKIYRTFRDEEGKEYVRCETVRKPAVIDAYVRIRT
TKDEKFIQKFALFDEKHREEMRKERRRIQEQLRRLKRNQEKEKLKGPPEKKPKKMKERPDLKLKCGACGAIGHMRTNKFCPLYYQTN
VPPSKPVAMTEEQEEELEKTVIHNDNEELIKVEGTKIVFGKQLIENVHEVRRKSLVLKFPKQQLPPKKKRRVGTTVHCDYLNIPHKS
IHRRRTDPMVTLSSILESIINDMRDLPNTHPFHTPVNAKVVKDYYKIITRPMDLQTLRENVRKCLYPSREEFREHLELIVKNSATYN
GPKHSLTQISQSMLDLCDEKLKEKEDKLARLEKAINPLLDDDDQVAFSFILDNIVTQKMMAVPDSWPFHHPVNKKFVPDYYKMIVNP
VDLETIRKNISKHKYQSRESFLDDVNLILANSVKYNGPESQYTKTAQEIVNICYQTITEYDEHLTQLEKDICTAKEAALEEAELESL
DPMTPGPYTSQPPDMYDTNTSLSTSRDASVFQDESNLSVLDISTATPEKQMCQGQGRLGEEDSDVDVEGYDDEEEDGKPKPPAPEGG
DGDLADEEEGTVQQPEASVLYEDLLISEGEDDEEDAGSDEEGDNPFSAIQLSESGSDSDVGYGGIRPKQPFMLQHASGEHKDGHGK
>sp|Q9UNF0|PACN2_HUMAN
MSVTYDDSVGVEVSSDSFWEVGNYKRTVKRIDDGHRLCSDLMNCLHERARIEKAYAQQLTEWARRWRQLVEKGPQYGTVEKAWMAFM
SEAERVSELHLEVKASLMNDDFEKIKNWQKEAFHKQMMGGFKETKEAEDGFRKAQKPWAKKLKEVEAAKKAHHAACKEEKLAISREA
NSKADPSLNPEQLKKLQDKIEKCKQDVLKTKEKYEKSLKELDQGTPQYMENMEQVFEQCQQFEEKRLRFFREVLLEVQKHLDLSNVA
GYKAIYHDLEQSIRAADAVEDLRWFRANHGPGMAMNWPQFEEWSADLNRTLSRREKKKATDGVTLTGINQTGDQSLPSKPSSTLNVP
SNPAQSAQSQSSYNPFEDEDDTGSTVSEKDDTKAKNVSSYEKTQSYPTDWSDDESNNPFSSTDANGDSNPFDDDATSGTEVRVRALY
DYEGQEHDELSFKAGDELTKMEDEDEQGWCKGRLDNGQVGLYPANYVEAIQ
>sp|P53999|TCP4_HUMAN
MPKSKELVSSSSSGSDSDSEVDKKLKRKKQVAPEKPVKKQKTGETSRALSSSKQSSSSRDDNMFQIGKMRYVSVRDFKGKVLIDIRE
YWMDPEGEMKPGRKGISLNPEQWSQLKEQISDIDDAVRKL
>sp|Q4KMX7|F106A_HUMAN
MLPSTMFLVHLPLSTNRLHCLRNTSLESYLCSFVHLNHPLHISDRVILISLHEAVRFSFAFSFPRGTLSIAYCLMSSVSTSSEAIMS
TELLANYCHSSLHVCICISSFPNETGNHDSFPGAVVSISDQPTDQCKLAAKELPLRNLLECRFFDCMGEEDLINLGVIGTER
>sp|P32970|CD70_HUMAN
MPEEGSGCSVRRRPYGCVLRAALVPLVAGLVICLVVCIQRFAQAQQQLPLESLGWDVAELQLNHTGPQQDPRLYWQGGPALGRSFLH
GPELDKGQLRIHRDGIYMVHIQVTLAICSSTTASRHHPTTLAVGICSPASRSISLLRLSFHQGCTIASQRLTPLARGDTLCTNLTGT
LLPSRNTDETFFGVQWVRP
>sp|O14756|H17B6_HUMAN
MWLYLAAFVGLYYLLHWYRERQVVSHLQDKYVFITGCDSGFGNLLARQLDARGLRVLAACLTEKGAEQLRGQTSDRLETVTLDVTKM
ESIAAATQWVKEHVGDRGLWGLVNNAGILTPITLCEWLNTEDSMNMLKVNLIGVIQVTLSMLPLVRRARGRIVNVSSILGRVAFFVG

GYCVSKYGVEAFSDILRREIQHFGVKISIVEPGYFRTGMTNMTQSLERMKQSWKEAPKHIKETYGQQYFDALYNIMKEGLLNCSTNL
NLVTDCMEHALTSVHPRTRYSAGWDAKFFFIPLSYLPTSLADYILTRSWPKPAQAV
>sp|Q5VU65|P210L_HUMAN
MTGCPASSRRRGFGLFFFLRLHRLLLLFLVLRGTLANKLNVPQVLLPFGREPGRVPFLLEAQRGCYTWHSTHHDAVTVEPLYENGTL
CSQKAVLIAESTQPIRLSSIILAREIVTDHELRCDVKVDVINSIEIVSRARELYVDDSPLELMVRALDAEGNTFSSLAGMMFEWSIA
QDNESAREELSSKIRILKYSEAEYAPPIYIAEMEKEEKQGDVILVSGIRTGAAVVKVRIHEPFYKKVAAALIRLLVLENIFLIPSHD
IYLLVGTYIKYQVAKMVQGRVTEVKFPLEHYILELQDHRVALNGSHSEKVAILDDKTAMVTASQLGQTNLVFVHKNVHMRSVSGLPN
CTIYVVEPGFLGFTVQPGNRWSLEVGQVYVITVDVFDKSSTKVYISDNLRITYDFPKEYFEEQLTTVNGSYHIVKALKDGVVVINAS
LTSIIYQNKDIQPIKFLIKHQQEVKIYFPIMLTPKFLAFPHHPMGMLYRYKVQVEGGSGNFTWTSSNETVVIVTTKGVVTAGQVRGN
STVLARDVQNPFRYGEIKIHVLKLNKMELLPFHADVEIGQIIEIPIAMYHINKETKEAMAFTDCSHLSLDLNMDKQGVFTLLKEGIQ
RPGPMHCSSTHIAAKSLGHTLVTVSVNECDKYLESSATFAAYEPLKALNPVEVALVTWQSVKEMVFEGGPRPWILEPSRFFLELNAE
KTEKIGIAQVWLPSKRKQNQYIYRIQCLDLGEQVLTFRIGNHPGVLNPSPAVEVLQVRFICAHPASMSVTPVYKVPAGAQPCPLPQH
NKWLIPVSRLRDTVLELAVFDQHRRKFDNFSSLMLEWKSSNETLAHFEDYKSVEMVAKDDGSGQTRLHGHQILKVHQIKGTVLIGVN
FVGYSEKKSPKEISNLPRSVDVELLLVDDVTVVPENATIYNHPDVKETFSLVEGSGYFLVNSSEQGVVTITYMEAESSVELVPLHPG
FFTLEVYDLCLAFLGPATAHLRVSDIQELELDLIDKVEIDKTVLVTVRVLGSSKRPFQNKYFRNMELKLQLASAIVTLTPMEQQDEY
SENYILRATTIGQTTLVAIAKDKMGRKYTSTPRHIEVFPPFRLLPEKMTLIPMNMMQVMSEGGPQPQSIVHFSISNQTVAVVNRRGQ
VTGKIVGTAVVHGTIQTVNEDTGKVIVFSQDEVQIEVVQLRAVRILAAATRLITATKMPVYVMGVTSTQTPFSFSNANPGLTFHWSM
SKRDVLDLVPRHSEVFLQLPVEHNFAMVVHTKAAGRTSIKVTVHCMNSSSGQFEGNLLELSDEVQILVFEKLQLFYPECQPEQILMP
INSQLKLHTNREGAAFVSSRVLKCFPNSSVIEEDGEGLLKAGSIAGTAVLEVTSIEPFGVNQTTITGVQVAPVTYLRVSSQPKLYTA
QGRTLSAFPLGMSLTFTVQFYNSIGEKFHTHNTQLYLALNRDDLLHIGPGNKNYTYMAQAVNRGLTLVGLWDRRHPGMADYIPVAVE
HAIEPDTKLTFVGDIICFSTHLVSQHGEPGIWMISANNILQTDIVTGVGVARSPGTAMIFHDIPGVVKTYREVVVNASSRLMLSYDL
KTYLTNTLNSTVFKLFITTGRNGVNLKGFCTPNQALAITKVLLPATLMLCHVQFSNTLLDIPASKVFQVHSDFSMEKGVYVCIIKVR
PQSEELLQALSVADTSVYGWATLVSERSKNGMQRILIPFIPAFYINQSELVLSHKQDIGEIRVLGVDRVLRKLEVISSSPVLVVAGH
SHSPLTPGLAIYSVRVVNFTSFQQMASPVFINISCVLTSQSEAVVVRAMKDKLGADHCEDSAILKRFTGSYQILLLTLFAVLASTAS
IFLAYNAFLNKIQTVPVVYVPTLGTPQPGFFNSTSSPPHFMSLQPPLAQSRLQHWLWSIRH
>sp|O94818|NOL4_HUMAN
MESERDMYRQFQDWCLRTYGDSGKTKTVTRKKYERIVQLLNGSESSSTDNAKFKFWVKSKGFQLGQPDEVRGGGGGAKQVLYVPVKT
TDGVGVDEKLSLRRVAVVEDFFDIIYSMHVETGPNGEQIRKHAGQKRTYKAISESYAFLPREAVTRFLMSCSECQKRMHLNPDGTDH
KDNGKPPTLVTSMIDYNMPITMAYMKHMKLQLLNSQQDEDESSIESDEFDMSDSTRMSAVNSDLSSNLEERMQSPQNLHGQQDDDSA
AESFNGNETLGHSSIASGGTHSREMGDSNSDGKTGLEQDEQPLNLSDSPLSAQLTSEYRIDDHNSNGKNKYKNLLISDLKMEREARE
NGSKSPAHSYSSYDSGKNESVDRGAEDLSLNRGDEDEDDHEDHDDSEKVNETDGVEAERLKAFNMFVRLFVDENLDRMVPISKQPKE
KIQAIIDSCRRQFPEYQERARKRIRTYLKSCRRMKRSGFEMSRPIPSHLTSAVAESILASACESESRNAAKRMRLERQQDESAPADK
QCKPEATQATYSTSAVPGSQDVLYINGNGTYSYHSYRGLGGGLLNLNDASSSGPTDLSMKRQLATSSGSSSSSNSRPQLSPTEINAV
RQLVAGYRESAAFLLRSADELENLILQQN
>sp|Q14194|DPYL1_HUMAN
MSYQGKKSIPHITSDRLLIKGGRIINDDQSLYADVYLEDGLIKQIGENLIVPGGVKTIEANGRMVIPGGIDVNTYLQKPSQGMTAAD
DFFQGTRAALVGGTTMIIDHVVPEPGSSLLTSFEKWHEAADTKSCCDYSLHVDITSWYDGVREELEVLVQDKGVNSFQVYMAYKDVY
QMSDSQLYEAFTFLKGLGAVILVHAENGDLIAQEQKRILEMGITGPEGHALSRPEELEAEAVFRAITIAGRINCPVYITKVMSKSAA
DIIALARKKGPLVFGEPIAASLGTDGTHYWSKNWAKAAAFVTSPPLSPDPTTPDYLTSLLACGDLQVTGSGHCPYSTAQKAVGKDNF
TLIPEGVNGIEERMTVVWDKAVATGKMDENQFVAVTSTNAAKIFNLYPRKGRIAVGSDADVVIWDPDKLKTITAKSHKSAVEYNIFE
GMECHGSPLVVISQGKIVFEDGNINVNKGMGRFIPRKAFPEHLYQRVKIRNKVFGLQGVSRGMYDGPVYEVPATPKYATPAPSAKSS
PSKHQPPPIRNLHQSNFSLSGAQIDDNNPRRTGHRIVAPPGGRSNITSLG
>sp|Q9UHG0|DCDC2_HUMAN

MSGSSARSSHLSQPVVKSVLVYRNGDPFYAGRRVVIHEKKVSSFEVFLKEVTGGVQAPFGAVRNIYTPRTGHRIRKLDQIQSGGNYV
AGGQEAFKKLNYLDIGEIKKRPMEVVNTEVKPVIHSRINVSARFRKPLQEPCTIFLIANGDLINPASRLLIPRKTLNQWDHVLQMVT
EKITLRSGAVHRLYTLEGKLVESGAELENGQFYVAVGRDKFKKLPYSELLFDKSTMRRPFGQKASSLPPIVGSRKSKGSGNDRHSKS
TVGSSDNSSPQPLKRKGKKEDVNSEKLTKLKQNVKLKNSQETIPNSDEGIFKAGAERSETRGAAEVQEDEDTQVEVPVDQRPAEIVD
EEEDGEKANKDAEQKEDFSGMNGDLEEEGGREATDAPEQVEEILDHSEQQARPARVNGGTDEENGEELQQVNNELQLVLDKERKSQG
AGSGQDEADVDPQRPPRPEVKITSPEENENNQQNKDYAAVA
>sp|A1A4G5|LNP1_HUMAN
MEHKDDDDDDVSFAKWMSSFWGHSWREEDQRGLRERHRLQATSHRKTSLPCPLPVLPRIPSSDCHPRRHSHEDQEFRCRSHVRDYRK
YSEDGSFKEPLESKGRSHSKIEKFSESFERQLCFRTKRSASLGPESRKERNERECLRMEIKSRKKVEEERSSRKEEHGEAHMAPLFE
KGPE
>sp|Q8N475|FSTL5_HUMAN
MFKCWSVVLVLGFIFLESEGRPTKEGGYGLKSYQPLMRLRHKQEKNQESSRVKGFMIQDGPFGSCENKYCGLGRHCVTSRETGQAEC
ACMDLCKRHYKPVCGSDGEFYENHCEVHRAACLKKQKITIVHNEDCFFKGDKCKTTEYSKMKNMLLDLQNQKYIMQENENPNGDDIS
RKKLLVDQMFKYFDADSNGLVDINELTQVIKQEELGKDLFDCTLYVLLKYDDFNADKHLALEEFYRAFQVIQLSLPEDQKLSITAAT
VGQSAVLSCAIQGTLRPPIIWKRNNIILNNLDLEDINDFGDDGSLYITKVTTTHVGNYTCYADGYEQVYQTHIFQVNVPPVIRVYPE
SQAREPGVTASLRCHAEGIPKPQLGWLKNGIDITPKLSKQLTLQANGSEVHISNVRYEDTGAYTCIAKNEAGVDEDISSLFVEDSAR
KTLANILWREEGLGIGNMFYVFYEDGIKVIQPIECEFQRHIKPSEKLLGFQDEVCPKAEGDEVQRCVWASAVNVKDKFIYVAQPTLD
RVLIVDVQSQKVVQAVSTDPVPVKLHYDKSHDQVWVLSWGTLEKTSPTLQVITLASGNVPHHTIHTQPVGKQFDRVDDFFIPTTTLI
ITHMRFGFILHKDEAALQKIDLETMSYIKTINLKDYKCVPQSLAYTHLGGYYFIGCKPDSTGAVSPQVMVDGVTDSVIGFNSDVTGT
PYVSPDGHYLVSINDVKGLVRVQYITIRGEIQEAFDIYTNLHISDLAFQPSFTEAHQYNIYGSSSTQTDVLFVELSSGKVKMIKSLK
EPLKAEEWPWNRKNRQIQDSGLFGQYLMTPSKDSLFILDGRLNKLNCEITEVEKGNTVIWVGDA
>sp|O14668|TMG1_HUMAN
MGRVFLTGEKANSILKRYPRANGFFEEIRQGNIERECKEEFCTFEEAREAFENNEKTKEFWSTYTKAQQGESNRGSDWFQFYLTFPL
IFGLFIILLVIFLIWRCFLRNKTRRQTVTEGHIPFPQHLNIITPPPPPDEVFDSSGLSPGFLGYVVGRSDSVSTRLSNCDPPPTYEE
ATGQVNLQRSETEPHLDPPPEYEDIVNSNSASAIPMVPVVTTIK
>sp|Q13907|IDI1_HUMAN
MPEINTNHLDKQQVQLLAEMCILIDENDNKIGAETKKNCHLNENIEKGLLHRAFSVFLFNTENKLLLQQRSDAKITFPGCFTNTCCS
HPLSNPAELEESDALGVRRAAQRRLKAELGIPLEEVPPEEINYLTRIHYKAQSDGIWGEHEIDYILLVRKNVTLNPDPNEIKSYCYV
SKEELKELLKKAASGEIKITPWFKIIAATFLFKWWDNLNHLNQFVDHEKIYRM
>sp|Q9Y4C4|MFHA1_HUMAN
MAGMDSGNLKTARLWRDAALRARKLRSNLRQLTLTAAGACPGAGADALESPASPQLVLPANLGDIEALNLGNNGLEEVPEGLGSALG
SLRVLVLRRNRFARLPPAVAELGHHLTELDVSHNRLTALGAEVVSALRELRKLNLSHNQLPALPAQLGALAHLEELDVSFNRLAHLP
DSLSCLSRLRTLDVDHNQLTAFPRQLLQLVALEELDVSSNRLRGLPEDISALRALKILWLSGAELGTLPAGFCELASLESLMLDNNG
LQALPAQFSCLQRLKMLNLSSNLFEEFPAALLPLAGLEELYLSRNQLTSVPSLISGLGRLLTLWLDNNRIRYLPDSIVELTGLEELV
LQGNQIAVLPDHFGQLSRVGLWKIKDNPLIQPPYEVCMKGIPYIAAYQKELAHSQPAVQPRLKLLLMGHKAAGKTLLRHCLTEERVE
GCPGGGDKEKCYPPSPPPVSKGIEVTSWTADASRGLRFIVYDLAGDESYEVIQPFFLSPGALYVLVVNLATYEPRHFPTTVGSFLHR
VGARVPHAVVCIVGTHADLCGERELEEKCLDIHRQIALQEKHDAEGLSRLAKVVDEALARDFELRSASPHAAYYGVSDKNLRRRKAH
FQYLLNHRLQILSPVLPVSCRDPRHLRRLRDKLLSVAEHREIFPNLHRVLPRSWQVLEELHFQPPQAQRLWLSWWDSARLGLQAGLT
EDRLQSALSYLHESGKLLYFEDSPALKEHVFHNLTRLIDILNVFFQRDPSLLLHKLLLGTSGEGKAEGESSPPMARSTPSQELLRAT
QLHQYVEGFLLHGLLPAHVIRLLLKPHVQAQQDLQLLLELLEKMGLCYCLNKPKGKPLNGSTAWYKFPCYVQNEVPHAEAWINGTNL
AGQSFVAEQLQIEYSFPFTFPLGLFARYSVQINSHVVHRSDGKFQIFAYRGKVPVVVSYRPARGVLQPDTLSIASHASLPNIWTAWQ
AITPLVEELNVLLQEWPGLHYTVHILCSKCLKRGSPNPHAFPGELLSQPRPEGVAEIICPKNGSERVNVALVYPPTPTVISPCSKKN
VGEKHRNQ
>sp|A0A096LPI5|GVQW2_HUMAN

MQYPFRKLLRPSTESCCVAQARVQWCHLGSLQPPLPGFKQFSCHSLPSSWDYRWNLTLSPRLECSGAISAHCNLCLPDLSDSPASTS
RVAGTTGAHHHAQEPVVIRKM
>sp|Q9H0M0|WWP1_HUMAN
MATASPRSDTSNNHSGRLQLQVTVSSAKLKRKKNWFGTAIYTEVVVDGEITKTAKSSSSSNPKWDEQLTVNVTPQTTLEFQVWSHRT
LKADALLGKATIDLKQALLIHNRKLERVKEQLKLSLENKNGIAQTGELTVVLDGLVIEQENITNCSSSPTIEIQENGDALHENGEPS
ARTTARLAVEGTNGIDNHVPTSTLVQNSCCSYVVNGDNTPSSPSQVAARPKNTPAPKPLASEPADDTVNGESSSFAPTDNASVTGTP
VVSEENALSPNCTSTTVEDPPVQEILTSSENNECIPSTSAELESEARSILEPDTSNSRSSSAFEAAKSRQPDGCMDPVRQQSGNANT
ETLPSGWEQRKDPHGRTYYVDHNTRTTTWERPQPLPPGWERRVDDRRRVYYVDHNTRTTTWQRPTMESVRNFEQWQSQRNQLQGAMQ
QFNQRYLYSASMLAAENDPYGPLPPGWEKRVDSTDRVYFVNHNTKTTQWEDPRTQGLQNEEPLPEGWEIRYTREGVRYFVDHNTRTT
TFKDPRNGKSSVTKGGPQIAYERGFRWKLAHFRYLCQSNALPSHVKINVSRQTLFEDSFQQIMALKPYDLRRRLYVIFRGEEGLDYG
GLAREWFFLLSHEVLNPMYCLFEYAGKNNYCLQINPASTINPDHLSYFCFIGRFIAMALFHGKFIDTGFSLPFYKRMLSKKLTIKDL
ESIDTEFYNSLIWIRDNNIEECGLEMYFSVDMEILGKVTSHDLKLGGSNILVTEENKDEYIGLMTEWRFSRGVQEQTKAFLDGFNEV
VPLQWLQYFDEKELEVMLCGMQEVDLADWQRNTVYRHYTRNSKQIIWFWQFVKETDNEVRMRLLQFVTGTCRLPLGGFAELMGSNGP
QKFCIEKVGKDTWLPRSHTCFNRLDLPPYKSYEQLKEKLLFAIEETEGFGQE
>sp|P60372|KR104_HUMAN
MSVCSSDLSYSSRVCLPGSCDSCSDSWQVDDCPESCCEPPCCAPSCCAPAPCLSLVCTPVSRVSSPCCPVTCEPSPCQSGCTSSCTP
SCCQQSSCQLACCASSPCQQACCVPVCCKTVCCKPVCCVPVCCGDSSCCQQSSCQSACCTSSPCQQACCVPICCKPVCSGISSSCCQ
QSSCVSCVSSPCCQAVCEPSPCQSGCISSCTPSCCQQSSCQPACCTSSSCQQACCVPVCCKTVCCKPVCSEDSSSCCQQSSCQPACC
TSSPCQQACCVPVCCKPVCCKPVGSVPICSGASSLCCQQSSCQPACCTSSQSQQGCCVPVCCKPVSCVPVCSGASSSCCQQSSCQPA
CCTTSCCRPSSSVSLLCRPVCRPACCVPVPSCCAPTSSCQPSCCRPASCVSLL
>sp|Q16774|KGUA_HUMAN
MSGPRPVVLSGPSGAGKSTLLKRLLQEHSGIFGFSVSHTTRNPRPGEENGKDYYFVTREVMQRDIAAGDFIEHAEFSGNLYGTSKVA
VQAVQAMNRICVLDVDLQGVRNIKATDLRPIYISVQPPSLHVLEQRLRQRNTETEESLVKRLAAAQADMESSKEPGLFDVVIINDSL
DQAYAELKEALSEEIKKAQRTGA
>sp|Q9H3Z4|DNJC5_HUMAN
MADQRQRSLSTSGESLYHVLGLDKNATSDDIKKSYRKLALKYHPDKNPDNPEAADKFKEINNAHAILTDATKRNIYDKYGSLGLYVA
EQFGEENVNTYFVLSSWWAKALFVFCGLLTCCYCCCCLCCCFNCCCGKCKPKAPEGEETEFYVSPEDLEAQLQSDEREATDTPIVIQ
PASATETTQLTADSHPSYHTDGFN
>sp|Q03721|KCNC4_HUMAN
MISSVCVSSYRGRKSGNKPPSKTCLKEEMAKGEASEKIIINVGGTRHETYRSTLRTLPGTRLAWLADPDGGGRPETDGGGVGSSGSS
GGGGCEFFFDRHPGVFAYVLNYYRTGKLHCPADVCGPLFEEELTFWGIDETDVEPCCWMTYRQHRDAEEALDIFESPDGGGSGAGPS
DEAGDDERELALQRLGPHEGGAGHGAGSGGCRGWQPRMWALFEDPYSSRAARVVAFASLFFILVSITTFCLETHEAFNIDRNVTEIL
RVGNITSVHFRREVETEPILTYIEGVCVLWFTLEFLVRIVCCPDTLDFVKNLLNIIDFVAILPFYLEVGLSGLSSKAARDVLGFLRV
VRFVRILRIFKLTRHFVGLRVLGHTLRASTNEFLLLIIFLALGVLIFATMIYYAERIGARPSDPRGNDHTDFKNIPIGFWWAVVTMT
TLGYGDMYPKTWSGMLVGALCALAGVLTIAMPVPVIVNNFGMYYSLAMAKQKLPKKRKKHVPRPAQLESPMYCKSEETSPRDSTCSD
TSPPAREEGMIERKRADSKQNGDANAVLSDEEGAGLTQPLASSPTPEERRALRRSTTRDRNKKAAACFLLSTGDYACADGSVRKGTF
VLRDLPLQHSPEAACPPTAGTLFLPH
>sp|A8MXU0|D108A_HUMAN
MRIAVLFFTIFFFMSQVLPAKGKFKEICERPNGSCRDFCLETEIHVGRCLNSRPCCLPLGHQPRIESTTPKKD
>sp|Q9BY07|S4A5_HUMAN
MKVKEEKAGVGKLDHTNHRRRFPDQKECPPIHIGLPVPTYPQRKTDQKGHLSGLQKVHWGLRPDQPQQELTGPGSGASSQDSSMDLI
SRTRSPAAEQLQDILGEEDEAPNPTLFTEMDTLQHDGDQMEWKESARWIKFEEKVEEGGERWSKPHVSTLSLHSLFELRTCLQTGTV
LLDLDSGSLPQIIDDVIEKQIEDGLLRPELRERVSYVLLRRHRHQTKKPIHRSLADIGKSVSTTNRSPARSPGAGPSLHHSTEDLRM
RQSANYGRLCHAQSRSMNDISLTPNTDQRKNKFMKKIPKDSEASNVLVGEVDFLDQPFIAFVRLIQSAMLGGVTEVPVPTRFLFILL

GPSGRAKSYNEIGRAIATLMVDDLFSDVAYKARNREDLIAGIDEFLDEVIVLPPGEWDPNIRIEPPKKVPSADKRKSVFSLAELGQM
NGSVGGGGGAPGGGNGGGGGGGSGGGAGSGGAGGTSSGDDGEMPAMHEIGEELIWTGRFFGGLCLDIKRKLPWFPSDFYDGFHIQSI
SAILFIYLGCITNAITFGGLLGDATDNYQGVMESFLGTAMAGSLFCLFSGQPLIILSSTGPILIFEKLLFDFSKGNGLDYMEFRLWI
GLHSAVQCLILVATDASFIIKYITRFTEEGFSTLISFIFIYDAIKKMIGAFKYYPINMDFKPNFITTYKCECVAPDTVNTTVFNASA
PLAPDTNASLYNLLNLTALDWSLLSKKECLSYGGRLLGNSCKFIPDLALMSFILFFGTYSMTLTLKKFKFSRYFPTKVRALVADFSI
VFSILMFCGIDACFGLETPKLHVPSVIKPTRPDRGWFVAPFGKNPWWVYPASILPALLVTILIFMDQQITAVIVNRKENKLKKAAGY
HLDLFWVGILMALCSFMGLPWYVAATVISIAHIDSLKMETETSAPGEQPQFLGVREQRVTGIIVFILTGISVFLAPILKCIPLPVLY
GVFLYMGVASLNGIQMGTGGSEFKIQKKLTPFWERCKLFLMPAKHQPDHAFLRHVPLRRIHLFTLVQILCLAVLWILKSTVAAIIFP
VMILGLIIVRRLLDFIFSQHDLAWIDNILPEKEKKETDKKRKRKKGAHEDCDEEPQFPPPSVIKIPMESVQSDPQNGIHCIARKRSS
SWSYSL
>sp|O60239|3BP5_HUMAN
MDAALKRSRSEEPAEILPPARDEEEEEEEGMEQGLEEEEEVDPRIQGELEKLNQSTDDINRRETELEDARQKFRSVLVEATVKLDEL
VKKIGKAVEDSKPYWEARRVARQAQLEAQKATQDFQRATEVLRAAKETISLAEQRLLEDDKRQFDSAWQEMLNHATQRVMEAEQTKT
RSELVHKETAARYNAAMGRMRQLEKKLKRAINKSKPYFELKAKYYVQLEQLKKTVDDLQAKLTLAKGEYKMALKNLEMISDEIHERR
RSSAMGPRGCGVGAEGSSTSVEDLPGSKPEPDAISVASEAFEDDSCSNFVSEDDSETQSVSSFSSGPTSPSEMPDQFPAVVRPGSLD
LPSPVSLSEFGMMFPVLGPRSECSGASSPECEVERGDRAEGAENKTSDKANNNRGLSSSSGSGGSSKSQSSTSPEGQALENRMKQLS
LQCSKGRDGIIADIKMVQIG
>sp|P51957|NEK4_HUMAN
MPLAAYCYLRVVGKGSYGEVTLVKHRRDGKQYVIKKLNLRNASSRERRAAEQEAQLLSQLKHPNIVTYKESWEGGDGLLYIVMGFCE
GGDLYRKLKEQKGQLLPENQVVEWFVQIAMALQYLHEKHILHRDLKTQNVFLTRTNIIKVGDLGIARVLENHCDMASTLIGTPYYMS
PELFSNKPYNYKSDVWALGCCVYEMATLKHAFNAKDMNSLVYRIIEGKLPPMPRDYSPELAELIRTMLSKRPEERPSVRSILRQPYI
KRQISFFLEATKIKTSKNNIKNGDSQSKPFATVVSGEAESNHEVIHPQPLSSEGSQTYIMGEGKCLSQEKPRASGLLKSPASLKAHT
CKQDLSNTTELATISSVNIDILPAKGRDSVSDGFVQENQPRYLDASNELGGICSISQVEEEMLQDNTKSSAQPENLIPMWSSDIVTG
EKNEPVKPLQPLIKEQKPKDQSLALSPKLECSGTILAHSNLRLLGSSDSPASASRVAGITGVCHHAQDQVAGECIIEKQGRIHPDLQ
PHNSGSEPSLSRQRRQKRREQTEHRGEKRQVRRDLFAFQESPPRFLPSHPIVGKVDVTSTQKEAENQRRVVTGSVSSSRSSEMSSSK
DRPLSARERRRLKQSQEEMSSSGPSVRKASLSVAGPGKPQEEDQPLPARRLSSDCSVTQERKQIHCLSEDELSSSTSSTDKSDGDYG
EGKGQTNEINALVQLMTQTLKLDSKESCEDVPVANPVSEFKLHRKYRDTLILHGKVAEEAEEIHFKELPSAIMPGSEKIRRLVEVLR
TDVIRGLGVQLLEQVYDLLEEEDEFDREVRLREHMGEKYTTYSVKARQLKFFEENMNF
>sp|A8MTY7|KRA97_HUMAN
MTHCCSPCCQPTCCRTTCWKPTTVTTCSSTPCCQPSCCVSSCCQPCCHPTCCQNTCCRTTCCQPTCVTSCCQPSCCSTPCCQPICCG
SSCCGQTSCGSSCCQPSSCAPIYCRRTCYHPTSVYLPGCLNQSCGSSCCQPCCRPACCETTCCRTTCFQPTCVTSCCQPACC
>sp|Q9NWQ9|CN119_HUMAN
MPLESSSSMPLSFPSLLPSVPHNTNPSPPLMSYITSQEMKCILHWFANWSGPQRERFLEDLVAKAVPEKLQPLLDSLEQLSVSGADR
PPSIFECQLHLWDQWFRGWAEQERNEFVRQLEFSEPDFVAKFYQAVAATAGKD
>sp|Q17RP2|TIGD6_HUMAN
MANKGNKKRRQFSLEEKMKVVGAVDSGKRKGDVAKEFGITPSTLSTFLKDRTKFEEKVREASVGPQRKRMRSALYDDIDKAVFAWFQ
EIHAKNILVTGSVIRKKALNLANMLGYDNFQASVGWLNRFRDRHGIALKAVCREDSDRLMNGLGIDKINEWHAGEIIKLIADYSPDD
IFNADETGVFFQLLPQHTLAAKGDHCRGGKKAKQRLTALFCCNASGTEKMRPLIVGRSASPHCLKNIHSLPCDYRANQWAWMTRDLF
NEWLMQVDARMKRAERRILLLIDNCSAHNMLPHLERIQVGYLPSNCTAVLQPLNLGIIHTMKVLYQSHLLKQILLKLNSSEDQEEVD
IKQAIDMIAAAWWSVKPSTVVKCWQKAGIVPMEFAECDTESAASEPDIAIEKLWHTVAIATCVPNEVNFQDFVTADDDLIISQDTDI
IQDMVAGENTSEAGSEDEGEVSLPEQPKVTITEAISSVQKLRQFLSTCVDIPDAIFGQLNGIDEYLMKRVTQTLIDSKITDFLQTK
>sp|Q8NBJ4|GOLM1_HUMAN
MMGLGNGRRSMKSPPLVLAALVACIIVLGFNYWIASSRSVDLQTRIMELEGRVRRAAAERGAVELKKNEFQGELEKQREQLDKIQSS
HNFQLESVNKLYQDEKAVLVNNITTGERLIRVLQDQLKTLQRNYGRLQQDVLQFQKNQTNLERKFSYDLSQCINQMKEVKEQCEERI

EEVTKKGNEAVASRDLSENNDQRQQLQALSEPQPRLQAAGLPHTEVPQGKGNVLGNSKSQTPAPSSEVVLDSKRQVEKEETNEIQVV
NEEPQRDRLPQEPGREQVVEDRPVGGRGFGGAGELGQTPQVQAALSVSQENPEMEGPERDQLVIPDGQEEEQEAAGEGRNQQKLRGE
DDYNMDENEAESETDKQAALAGNDRNIDVFNVEDQKRDTINLLDQREKRNHTL
>sp|O43175|SERA_HUMAN
MAFANLRKVLISDSLDPCCRKILQDGGLQVVEKQNLSKEELIAELQDCEGLIVRSATKVTADVINAAEKLQVVGRAGTGVDNVDLEA
ATRKGILVMNTPNGNSLSAAELTCGMIMCLARQIPQATASMKDGKWERKKFMGTELNGKTLGILGLGRIGREVATRMQSFGMKTIGY
DPIISPEVSASFGVQQLPLEEIWPLCDFITVHTPLLPSTTGLLNDNTFAQCKKGVRVVNCARGGIVDEGALLRALQSGQCAGAALDV
FTEEPPRDRALVDHENVISCPHLGASTKEAQSRCGEEIAVQFVDMVKGKSLTGVVNAQALTSAFSPHTKPWIGLAEALGTLMRAWAG
SPKGTIQVITQGTSLKNAGNCLSPAVIVGLLKEASKQADVNLVNAKLLVKEAGLNVTTSHSPAAPGEQGFGECLLAVALAGAPYQAV
GLVQGTTPVLQGLNGAVFRPEVPLRRDLPLLLFRTQTSDPAMLPTMIGLLAEAGVRLLSYQTSLVSDGETWHVMGISSLLPSLEAWK
QHVTEAFQFHF
>sp|Q9HA90|EFCC1_HUMAN
MEPVSTGAEAGMEGAGGDPYRRPARRTQWLLSALAHHYGLDRGVENEIVVLATGLDQYLQEVFHHLDCRGAGRLPRADFRALCAVLG
LRAEGATTAGQAAGDGNSRDVTPGDAAAELATDGDSDTDEEARLALRAEPPELTFRQFHARLCGYFGTRAGPRLPRGALSEHIETQI
RLRRPRRRRRPPCAPGPDSGPDCERVARLEEENSSLRELVEDLRAALQSSDARCLALQVGLWKSQASTHEMGHGGPEAAVRELRQAQ
GALAAAEARAGRLRRGQAEVRRRAEEARQVVLRSLHRVRELEALAQQVPGLQRWVRRLEAELQRYRSEDSQLPTPQLANPEPGDKSN
EPEDAGTRDPDPTPEGAWQSDSSSGSRALDEVDEQLFRSVEGQAASDEEEVEEERWQEEKKTPAAEAKTLLARLSSCRGRCDDQTAE
KLMTYFGHFGGANHAHTLGELEACIAMLVEQLRTQGCGGRTLGTSEEEAELQQKVEENEHLRLELQMVETERVRLSLLEEKLVDVLQ
LLQRLRDLNISKRALGKILLSTLDAFRDPTHEGRPSPAAILDALHQALAACQLLRRQPSAPASAAAALTNPLLVSC
>sp|Q15262|PTPRK_HUMAN
MDTTAAAALPAFVALLLLSPWPLLGSAQGQFSAGGCTFDDGPGACDYHQDLYDDFEWVHVSAQEPHYLPPEMPQGSYMIVDSSDHDP
GEKARLQLPTMKENDTHCIDFSYLLYSQKGLNPGTLNILVRVNKGPLANPIWNVTGFTGRDWLRAELAVSTFWPNEYQVIFEAEVSG
GRSGYIAIDDIQVLSYPCDKSPHFLRLGDVEVNAGQNATFQCIATGRDAVHNKLWLQRRNGEDIPVAQTKNINHRRFAASFRLQEVT
KTDQDLYRCVTQSERGSGVSNFAQLIVREPPRPIAPPQLLGVGPTYLLIQLNANSIIGDGPIILKEVEYRMTSGSWTETHAVNAPTY
KLWHLDPDTEYEIRVLLTRPGEGGTGLPGPPLITRTKCAEPMRTPKTLKIAEIQARRIAVDWESLGYNITRCHTFNVTICYHYFRGH
NESKADCLDMDPKAPQHVVNHLPPYTNVSLKMILTNPEGRKESEETIIQTDEDVPGPVPVKSLQGTSFENKIFLNWKEPLDPNGIIT
QYEISYSSIRSFDPAVPVAGPPQTVSNLWNSTHHVFMHLHPGTTYQFFIRASTVKGFGPATAINVTTNISAPTLPDYEGVDASLNET
ATTITVLLRPAQAKGAPISAYQIVVEELHPHRTKREAGAMECYQVPVTYQNAMSGGAPYYFAAELPPGNLPEPAPFTVGDNRTYQGF
WNPPLAPRKGYNIYFQAMSSVEKETKTQCVRIATKAATEEPEVIPDPAKQTDRVVKIAGISAGILVFILLLLVVILIVKKSKLAKKR
KDAMGNTRQEMTHMVNAMDRSYADQSTLHAEDPLSITFMDQHNFSPRYENHSATAESSRLLDVPRYLCEGTESPYQTGQLHPAIRVA
DLLQHINLMKTSDSYGFKEEYESFFEGQSASWDVAKKDQNRAKNRYGNIIAYDHSRVILQPVEDDPSSDYINANYIDGYQRPSHYIA
TQGPVHETVYDFWRMIWQEQSACIVMVTNLVEVGRVKCYKYWPDDTEVYGDFKVTCVEMEPLAEYVVRTFTLERRGYNEIREVKQFH
FTGWPDHGVPYHATGLLSFIRRVKLSNPPSAGPIVVHCSAGAGRTGCYIVIDIMLDMAEREGVVDIYNCVKALRSRRINMVQTEEQY
IFIHDAILEACLCGETAIPVCEFKAAYFDMIRIDSQTNSSHLKDEFQTLNSVTPRLQAEDCSIACLPRNHDKNRFMDMLPPDRCLPF
LITIDGESSNYINAALMDSYRQPAAFIVTQYPLPNTVKDFWRLVYDYGCTSIVMLNEVDLSQGCPQYWPEEGMLRYGPIQVECMSCS
MDCDVINRIFRICNLTRPQEGYLMVQQFQYLGWASHREVPGSKRSFLKLILQVEKWQEECEEGEGRTIIHCLNGGGRSGMFCAIGIV
VEMVKRQNVVDVFHAVKTLRNSKPNMVEAPEQYRFCYDVALEYLESS
>sp|Q9P217|ZSWM5_HUMAN
MADGGEREELLSPSPVSPAKRQCSWPSPQAHHPRGSPGAAGGGAGGVGSSCLVLGARPHLQPDSLLDCAAKTVAEKWAYERVEERFE
RIPEPVQRRIVYWSFPRNEREICMYSSFQYRGGPGAGAAGGAAGASPAEEGPQPPPGAAAPAGSAPGGVAAGASPGLGAGAGAAGCG
GEGLPFRRGIRLLDSGSVENVLQVGFHLSGTVTELATASEPAVTYKVAISFDRCKITSVTCGCGNKDIFYCAHVVALSLYRIRKPDQ
VKLRLPISETLFQMNRDQLQKFIQYLITAHHTEVLPTAQKLADEILSSNSEINQVNGAPDPTAGASIDDENCWHLDEEQVKEQVKLF
LSQGGYCGSGKQLNSMFAKVREMLRMRDSNGARMLTLITEQFVADPRLTLWRQQGTNMTDKCRQLWDELGALWVCIILNPHCKLEEK
SCWLQQLQKWSDLDVCPLEDGNYGHELPNITNALPQSAIHSPDSLSRPRRTVFTRAIEGRELHWQDSHLQRIISSDVYTAPACQRES

ERLLFNSQGQPLWLEHVPTACARVDALRSHGYPKEALRLTVAIINTLRLQQQRQLEIYKHQKKELLQRGTTTITNLEGWVGHPLDPI
DCLFLTLTEACRLNDDGYLEMSDMNESRPPVYQHVPVAAGSPNSSESYLSLALEVALMGLGQQRLMPEGLYAQDKVCRNEEQLLSQL
QELQLDDELVQTLQKQCILLLEGGPFSGLGEVIHRESVPMHTFAKYLFSALLPHDPDLSYKLALRAMRLPVLENSASAGDTSHPHHM
VSVVPSRYPRWFTLGHLESQQCELASTMLTAAKGDTLRLRTILEAIQKHIHSSSLIFKLAQDAFKIATPTDSSTDSTLLNVALELGL
QVMRMTLSTLNWRRREMVRWLVTCATEVGVRALVSILQSWYTLFTPTEATSIVAATAVSHTTILRLSLDYPQREELASCARTLALQC
AMKDPQSCALSALTLCEKDHIAFEAAYQIAIDAAAGGMTHSQLFTIARYMELRGYPLRAFKLASLAMSHLNLAYNQDTHPAINDVLW
ACALSHSLGKNELAALIPLVVKSVHCATVLSDILRRCTVTAPGLAGIPGRRSSGKLMSTDKAPLRQLLDATINAYINTTHSRLTHIS
PRHYGEFIEFLSKARETFLLPQDGHLQFAQFIDNLKQIYKGKKKLMLLVRERFG
>sp|Q8IWA0|WDR75_HUMAN
MVEEENIRVVRCGGSELNFRRAVFSADSKYIFCVSGDFVKVYSTVTEECVHILHGHRNLVTGIQLNPNNHLQLYSCSLDGTIKLWDY
IDGILIKTFIVGCKLHALFTLAQAEDSVFVIVNKEKPDIFQLVSVKLPKSSSQEVEAKELSFVLDYINQSPKCIAFGNEGVYVAAVR
EFYLSVYFFKKKTTSRFTLSSSRNKKHAKNNFTCVACHPTEDCIASGHMDGKIRLWRNFYDDKKYTYTCLHWHHDMVMDLAFSVTGT
SLLSGGRESVLVEWRDATEKNKEFLPRLGATIEHISVSPAGDLFCTSHSDNKIIIIHRNLEASAVIQGLVKDRSIFTGLMIDPRTKA
LVLNGKPGHLQFYSLQSDKQLYNLDIIQQEYINDYGLIQIELTKAAFGCFGNWLATVEQRQEKETELELQMKLWMYNKKTQGFILNT
KINMPHEDCITALCFCNAEKSEQPTLVTASKDGYFKVWILTDDSDIYKKAVGWTCDFVGSYHKYQATNCCFSEDGSLLAVSFEEIVT
IWDSVTWELKCTFCQRAGKIRHLCFGRLTCSKYLLGATENGILCCWNLLSCALEWNAKLNVRVMEPDPNSENIAAISQSSVGSDLFV
FKPSEPRPLYIQKGISREKVQWGVFVPRDVPESFTSEAYQWLNRSQFYFLTKSQSLLTFSTKSPEEKLTPTSKQLLAEESLPTTPFY
FILGKHRQQQDEKLNETLENELVQLPLTENIPAISELLHTPAHVLPSAAFLCSMFVNSLLLSKETKSAKEIPEDVDMEEEKESEDSD
EENDFTEKVQDTSNTGLGEDIIHQLSKSEEKELRKFRKIDYSWIAAL
>sp|Q01973|ROR1_HUMAN
MHRPRRRGTRPPLLALLAALLLAARGAAAQETELSVSAELVPTSSWNISSELNKDSYLTLDEPMNNITTSLGQTAELHCKVSGNPPP
TIRWFKNDAPVVQEPRRLSFRSTIYGSRLRIRNLDTTDTGYFQCVATNGKEVVSSTGVLFVKFGPPPTASPGYSDEYEEDGFCQPYR
GIACARFIGNRTVYMESLHMQGEIENQITAAFTMIGTSSHLSDKCSQFAIPSLCHYAFPYCDETSSVPKPRDLCRDECEILENVLCQ
TEYIFARSNPMILMRLKLPNCEDLPQPESPEAANCIRIGIPMADPINKNHKCYNSTGVDYRGTVSVTKSGRQCQPWNSQYPHTHTFT
ALRFPELNGGHSYCRNPGNQKEAPWCFTLDENFKSDLCDIPACDSKDSKEKNKMEILYILVPSVAIPLAIALLFFFICVCRNNQKSS
SAPVQRQPKHVRGQNVEMSMLNAYKPKSKAKELPLSAVRFMEELGECAFGKIYKGHLYLPGMDHAQLVAIKTLKDYNNPQQWTEFQQ
EASLMAELHHPNIVCLLGAVTQEQPVCMLFEYINQGDLHEFLIMRSPHSDVGCSSDEDGTVKSSLDHGDFLHIAIQIAAGMEYLSSH
FFVHKDLAARNILIGEQLHVKISDLGLSREIYSADYYRVQSKSLLPIRWMPPEAIMYGKFSSDSDIWSFGVVLWEIFSFGLQPYYGF
SNQEVIEMVRKRQLLPCSEDCPPRMYSLMTECWNEIPSRRPRFKDIHVRLRSWEGLSSHTSSTTPSGGNATTQTTSLSASPVSNLSN
PRYPNYMFPSQGITPQGQIAGFIGPPIPQNQRFIPINGYPIPPGYAAFPAAHYQPTGPPRVIQHCPPPKSRSPSSASGSTSTGHVTS
LPSSGSNQEANIPLLPHMSIPNHPGGMGITVFGNKSQKPYKIDSKQASLLGDANIHGHTESMISAEL
>sp|Q8IVI9|NOSTN_HUMAN
MRDPLTDCPYNKVYKNLKEFSQNGENFCKQVTSVLQQRANLEISYAKGLQKLASKLSKALQNTRKSCVSSAWAWASEGMKSTADLHQ
KLGKAIELEAIKPTYQVLNVQEKKRKSLDNEVEKTANLVISNWNQQIKAKKKLMVSTKKHEALFQLVESSKQSMTEKEKRKLLNKLT
KSTEKLEKEDENYYQKNMAGYSTRLKWENTLENCYQSILELEKERIQLLCNNLNQYSQHISLFGQTLTTCHTQIHCAISKIDIEKDI
QAVMEETAILSTENKSEFLLTDYFEEDPNSAMDKERRKSLLKPKLLRLQRDIEKASKDKEGLERMLKTYSSTSSFSDAKSQKDTAAL
MDENNLKLDLLEANSYKLSSMLAELEQRPQPSHPCSNSIFRWREKEHTHSYVKISRPFLMKRLENIVSKASSGGQSNPGSSTPAPGA
AQLSSRLCKALYSFQARQDDELNLEKGDIVIIHEKKEGGWWFGSLNGKKGHFPAAYVEELPSNAGNTATKA
>sp|Q6ZP29|LAAT1_HUMAN
MVWKKLGSRNFSSCPSGSIQWIWDVLGECAQDGWDEASVGLGLISILCFAASTFPQFIKAYKTGNMDQALSLWFLLGWIGGDSCNLI
GSFLADQLPLQTYTAVYYVLADLVMLTLYFYYKFRTRPSLLSAPINSVLLFLMGMACATPLLSAAGPVAAPREAFRGRALLSVESGS
KPFTRQEVIGFVIGSISSVLYLLSRLPQIRTNFLRKSTQGISYSLFALVMLGNTLYGLSVLLKNPEEGQSEGSYLLHHLPWLVGSLG
VLLLDTIISIQFLVYRRSTAASELEPLLPS
>sp|O14718|OPSX_HUMAN

MLRNNLGNSSDSKNEDGSVFSQTEHNIVATYLIMAGMISIISNIIVLGIFIKYKELRTPTNAIIINLAVTDIGVSSIGYPMSAASDL
YGSWKFGYAGCQVYAGLNIFFGMASIGLLTVVAVDRYLTICLPDVGRRMTTNTYIGLILGAWINGLFWALMPIIGWASYAPDPTGAT
CTINWRKNDRSFVSYTMTVIAINFIVPLTVMFYCYYHVTLSIKHHTTSDCTESLNRDWSDQIDVTKMSVIMICMFLVAWSPYSIVCL
WASFGDPKKIPPPMAIIAPLFAKSSTFYNPCIYVVANKKFRRAMLAMFKCQTHQTMPVTSILPMDVSQNPLASGRI
>sp|A8MWD9|RUXGL_HUMAN
MSKAHPPELKKFTDKKFSLKLNGGRHVQGILRGFDPFMNLVIDECVEMATSGQQKNIGMVEIRGNSIIMLEALERV
>sp|Q8NBQ7|AQP11_HUMAN
MSPLLGLRSELQDTCTSLGLMLSVVLLMGLARVVARQQLHRPVAHAFVLEFLATFQLCCCTHELQLLSEQHPAHPTWTLTLVYFFSL
VHGLTLVGTSSNPCGVMMQMMLGGMSPETGAVRLLAQLVSALCSRYCTSALWSLGLTQYHVSERSFACKNPIRVDLLKAVITEAVCS
FLFHSALLHFQEVRTKLRIHLLAALITFLVYAGGSLTGAVFNPALALSLHFMCFDEAFPQFFIVYWLAPSLGILLMILMFSFFLPWL
HNNHTINKKE
>sp|Q9BQE4|SELS_HUMAN
MERQEESLSARPALETEGLRFLHTTVGSLLATYGWYIVFSCILLYVVFQKLSARLRALRQRQLDRAAAAVEPDVVVKRQEALAAARL
KMQEELNAQVEKHKEKLKQLEEEKRRQKIEMWDSMQEGKSYKGNAKKPQEEDSPGPSTSSVLKRKSDRKPLRGGGYNPLSGEGGGAC
SWRPGRRGPSSGGUG
>sp|P35520|CBS_HUMAN
MPSETPQAEVGPTGCPHRSGPHSAKGSLEKGSPEDKEAKEPLWIRPDAPSRCTWQLGRPASESPHHHTAPAKSPKILPDILKKIGDT
PMVRINKIGKKFGLKCELLAKCEFFNAGGSVKDRISLRMIEDAERDGTLKPGDTIIEPTSGNTGIGLALAAAVRGYRCIIVMPEKMS
SEKVDVLRALGAEIVRTPTNARFDSPESHVGVAWRLKNEIPNSHILDQYRNASNPLAHYDTTADEILQQCDGKLDMLVASVGTGGTI
TGIARKLKEKCPGCRIIGVDPEGSILAEPEELNQTEQTTYEVEGIGYDFIPTVLDRTVVDKWFKSNDEEAFTFARMLIAQEGLLCGG
SAGSTVAVAVKAAQELQEGQRCVVILPDSVRNYMTKFLSDRWMLQKGFLKEEDLTEKKPWWWHLRVQELGLSAPLTVLPTITCGHTI
EILREKGFDQAPVVDEAGVILGMVTLGNMLSSLLAGKVQPSDQVGKVIYKQFKQIRLTDTLGRLSHILEMDHFALVVHEQIQYHSTG
KSSQRQMVFGVVTAIDLLNFVAAQERDQK
>sp|Q15125|EBP_HUMAN
MTTNAGPLHPYWPQHLRLDNFVPNDRPTWHILAGLFSVTGVLVVTTWLLSGRAAVVPLGTWRRLSLCWFAVCGFIHLVIEGWFVLYY
EDLLGDQAFLSQLWKEYAKGDSRYILGDNFTVCMETITACLWGPLSLWVVIAFLRQHPLRFILQLVVSVGQIYGDVLYFLTEHRDGF
QHGELGHPLYFWFYFVFMNALWLVLPGVLVLDAVKHLTHAQSTLDAKATKAKSKKN
>sp|Q15418|KS6A1_HUMAN
MPLAQLKEPWPLMELVPLDPENGQTSGEEAGLQPSKDEGVLKEISITHHVKAGSEKADPSHFELLKVLGQGSFGKVFLVRKVTRPDS
GHLYAMKVLKKATLKVRDRVRTKMERDILADVNHPFVVKLHYAFQTEGKLYLILDFLRGGDLFTRLSKEVMFTEEDVKFYLAELALG
LDHLHSLGIIYRDLKPENILLDEEGHIKLTDFGLSKEAIDHEKKAYSFCGTVEYMAPEVVNRQGHSHSADWWSYGVLMFEMLTGSLP
FQGKDRKETMTLILKAKLGMPQFLSTEAQSLLRALFKRNPANRLGSGPDGAEEIKRHVFYSTIDWNKLYRREIKPPFKPAVAQPDDT
FYFDTEFTSRTPKDSPGIPPSAGAHQLFRGFSFVATGLMEDDGKPRAPQAPLHSVVQQLHGKNLVFSDGYVVKETIGVGSYSECKRC
VHKATNMEYAVKVIDKSKRDPSEEIEILLRYGQHPNIITLKDVYDDGKHVYLVTELMRGGELLDKILRQKFFSEREASFVLHTIGKT
VEYLHSQGVVHRDLKPSNILYVDESGNPECLRICDFGFAKQLRAENGLLMTPCYTANFVAPEVLKRQGYDEGCDIWSLGILLYTMLA
GYTPFANGPSDTPEEILTRIGSGKFTLSGGNWNTVSETAKDLVSKMLHVDPHQRLTAKQVLQHPWVTQKDKLPQSQLSHQDLQLVKG
AMAATYSALNSSKPTPQLKPIESSILAQRRVRKLPSTTL
>sp|P0DMV9|HS71B_HUMAN
MAKAAAIGIDLGTTYSCVGVFQHGKVEIIANDQGNRTTPSYVAFTDTERLIGDAAKNQVALNPQNTVFDAKRLIGRKFGDPVVQSDM
KHWPFQVINDGDKPKVQVSYKGETKAFYPEEISSMVLTKMKEIAEAYLGYPVTNAVITVPAYFNDSQRQATKDAGVIAGLNVLRIIN
EPTAAAIAYGLDRTGKGERNVLIFDLGGGTFDVSILTIDDGIFEVKATAGDTHLGGEDFDNRLVNHFVEEFKRKHKKDISQNKRAVR
RLRTACERAKRTLSSSTQASLEIDSLFEGIDFYTSITRARFEELCSDLFRSTLEPVEKALRDAKLDKAQIHDLVLVGGSTRIPKVQK
LLQDFFNGRDLNKSINPDEAVAYGAAVQAAILMGDKSENVQDLLLLDVAPLSLGLETAGGVMTALIKRNSTIPTKQTQIFTTYSDNQ
PGVLIQVYEGERAMTKDNNLLGRFELSGIPPAPRGVPQIEVTFDIDANGILNVTATDKSTGKANKITITNDKGRLSKEEIERMVQEA

EKYKAEDEVQRERVSAKNALESYAFNMKSAVEDEGLKGKISEADKKKVLDKCQEVISWLDANTLAEKDEFEHKRKELEQVCNPIISG
LYQGAGGPGPGGFGAQGPKGGSGSGPTIEEVD
>sp|P01127|PDGFB_HUMAN
MNRCWALFLSLCCYLRLVSAEGDPIPEELYEMLSDHSIRSFDDLQRLLHGDPGEEDGAELDLNMTRSHSGGELESLARGRRSLGSLT
IAEPAMIAECKTRTEVFEISRRLIDRTNANFLVWPPCVEVQRCSGCCNNRNVQCRPTQVQLRPVQVRKIEIVRKKPIFKKATVTLED
HLACKCETVAAARPVTRSPGGSQEQRAKTPQTRVTIRTVRVRRPPKGKHRKFKHTHDKTALKETLGA
>sp|P20774|MIME_HUMAN
MKTLQSTLLLLLLVPLIKPAPPTQQDSRIIYDYGTDNFEESIFSQDYEDKYLDGKNIKEKETVIIPNEKSLQLQKDEAITPLPPKKE
NDEMPTCLLCVCLSGSVYCEEVDIDAVPPLPKESAYLYARFNKIKKLTAKDFADIPNLRRLDFTGNLIEDIEDGTFSKLSLLEELSL
AENQLLKLPVLPPKLTLFNAKYNKIKSRGIKANAFKKLNNLTFLYLDHNALESVPLNLPESLRVIHLQFNNIASITDDTFCKANDTS
YIRDRIEEIRLEGNPIVLGKHPNSFICLKRLPIGSYF
>sp|Q96LT4|SAMD8_HUMAN
MAGPNQLCIRRWTTKHVAVWLKDEGFFEYVDILCNKHRLDGITLLTLTEYDLRSPPLEIKVLGDIKRLMLSVRKLQKIHIDVLEEMG
YNSDSPMGSMTPFISALQSTDWLCNGELSHDCDGPITDLNSDQYQYMNGKNKHSVRRLDPEYWKTILSCIYVFIVFGFTSFIMVIVH
ERVPDMQTYPPLPDIFLDSVPRIPWAFAMTEVCGMILCYIWLLVLLLHKHRSILLRRLCSLMGTVFLLRCFTMFVTSLSVPGQHLQC
TGKIYGSVWEKLHRAFAIWSGFGMTLTGVHTCGDYMFSGHTVVLTMLNFFVTEYTPRSWNFLHTLSWVLNLFGIFFILAAHEHYSID
VFIAFYITTRLFLYYHTLANTRAYQQSRRARIWFPMFSFFECNVNGTVPNEYCWPFSKPAIMKRLIG
>sp|Q99519|NEUR1_HUMAN
MTGERPSTALPDRRWGPRILGFWGGCRVWVFAAIFLLLSLAASWSKAENDFGLVQPLVTMEQLLWVSGRQIGSVDTFRIPLITATPR
GTLLAFAEARKMSSSDEGAKFIALRRSMDQGSTWSPTAFIVNDGDVPDGLNLGAVVSDVETGVVFLFYSLCAHKAGCQVASTMLVWS
KDDGVSWSTPRNLSLDIGTEVFAPGPGSGIQKQREPRKGRLIVCGHGTLERDGVFCLLSDDHGASWRYGSGVSGIPYGQPKQENDFN
PDECQPYELPDGSVVINARNQNNYHCHCRIVLRSYDACDTLRPRDVTFDPELVDPVVAAGAVVTSSGIVFFSNPAHPEFRVNLTLRW
SFSNGTSWRKETVQLWPGPSGYSSLATLEGSMDGEEQAPQLYVLYEKGRNHYTESISVAKISVYGTL
>sp|P02765|FETUA_HUMAN
MKSLVLLLCLAQLWGCHSAPHGPGLIYRQPNCDDPETEEAALVAIDYINQNLPWGYKHTLNQIDEVKVWPQQPSGELFEIEIDTLET
TCHVLDPTPVARCSVRQLKEHAVEGDCDFQLLKLDGKFSVVYAKCDSSPDSAEDVRKVCQDCPLLAPLNDTRVVHAAKAALAAFNAQ
NNGSNFQLEEISRAQLVPLPPSTYVEFTVSGTDCVAKEATEAAKCNLLAEKQYGFCKATLSEKLGGAEVAVTCTVFQTQPVTSQPQP
EGANEAVPTPVVDPDAPPSPPLGAPGLPPAGSPPDSHVLLAAPPGHQLHRAHYDLRHTFMGVVSLGSPSGEVSHPRKTRTVVQPSVG
AAAGPVVPPCPGRIRHFKV
>sp|Q9Y4C1|KDM3A_HUMAN
MVLTLGESWPVLVGRRFLSLSAADGSDGSHDSWDVERVAEWPWLSGTIRAVSHTDVTKKDLKVCVEFDGESWRKRRWIEVYSLLRRA
FLVEHNLVLAERKSPEISERIVQWPAITYKPLLDKAGLGSITSVRFLGDQQRVFLSKDLLKPIQDVNSLRLSLTDNQIVSKEFQALI
VKHLDESHLLKGDKNLVGSEVKIYSLDPSTQWFSATVINGNPASKTLQVNCEEIPALKIVDPSLIHVEVVHDNLVTCGNSARIGAVK
RKSSENNGTLVSKQAKSCSEASPSMCPVQSVPTTVFKEILLGCTAATPPSKDPRQQSTPQAANSPPNLGAKIPQGCHKQSLPEEISS
CLNTKSEALRTKPDVCKAGLLSKSSQIGTGDLKILTEPKGSCTQPKTNTDQENRLESVPQALTGLPKECLPTKASSKAELEIANPPE
LQKHLEHAPSPSDVSNAPEVKAGVNSDSPNNCSGKKVEPSALACRSQNLKESSVKVDNESCCSRSNNKIQNAPSRKSVLTDPAKLKK
LQQSGEAFVQDDSCVNIVAQLPKCRECRLDSLRKDKEQQKDSPVFCRFFHFRRLQFNKHGVLRVEGFLTPNKYDNEAIGLWLPLTKN
VVGIDLDTAKYILANIGDHFCQMVISEKEAMSTIEPHRQVAWKRAVKGVREMCDVCDTTIFNLHWVCPRCGFGVCVDCYRMKRKNCQ
QGAAYKTFSWLKCVKSQIHEPENLMPTQIIPGKALYDVGDIVHSVRAKWGIKANCPCSNRQFKLFSKPASKEDLKQTSLAGEKPTLG
AVLQQNPSVLEPAAVGGEAASKPAGSMKPACPASTSPLNWLADLTSGNVNKENKEKQPTMPILKNEIKCLPPLPPLSKSSTVLHTFN
STILTPVSNNNSGFLRNLLNSSTGKTENGLKNTPKILDDIFASLVQNKTTSDLSKRPQGLTIKPSILGFDTPHYWLCDNRLLCLQDP
NNKSNWNVFRECWKQGQPVMVSGVHHKLNSELWKPESFRKEFGEQEVDLVNCRTNEIITGATVGDFWDGFEDVPNRLKNEKEPMVLK
LKDWPPGEDFRDMMPSRFDDLMANIPLPEYTRRDGKLNLASRLPNYFVRPDLGPKMYNAYGLITPEDRKYGTTNLHLDVSDAANVMV
YVGIPKGQCEQEEEVLKTIQDGDSDELTIKRFIEGKEKPGALWHIYAAKDTEKIREFLKKVSEEQGQENPADHDPIHDQSWYLDRSL

RKRLHQEYGVQGWAIVQFLGDVVFIPAGAPHQVHNLYSCIKVAEDFVSPEHVKHCFWLTQEFRYLSQTHTNHEDKLQVKNVIYHAVK
DAVAMLKASESSFGKP
>sp|Q8TCH9|YV004_HUMAN
MPSTEGFGGLDRLVLPPPPQVLPLLSPARGRIQFSGRKSTGSWVTQMENLLLAQPLPDDCEILCGWRSLLHLTFLIGFAPHFPHWFC
NHHCHQHPPSHLTEVLQSSGELAHQLAPSRCSMRAEGTHRG
>sp|O15297|PPM1D_HUMAN
MAGLYSLGVSVFSDQGGRKYMEDVTQIVVEPEPTAEEKPSPRRSLSQPLPPRPSPAALPGGEVSGKGPAVAAREARDPLPDAGASPA
PSRCCRRRSSVAFFAVCDGHGGREAAQFAREHLWGFIKKQKGFTSSEPAKVCAAIRKGFLACHLAMWKKLAEWPKTMTGLPSTSGTT
ASVVIIRGMKMYVAHVGDSGVVLGIQDDPKDDFVRAVEVTQDHKPELPKERERIEGLGGSVMNKSGVNRVVWKRPRLTHNGPVRRST
VIDQIPFLAVARALGDLWSYDFFSGEFVVSPEPDTSVHTLDPQKHKYIILGSDGLWNMIPPQDAISMCQDQEEKKYLMGEHGQSCAK
MLVNRALGRWRQRMLRADNTSAIVICISPEVDNQGNFTNEDELYLNLTDSPSYNSQETCVMTPSPCSTPPVKSLEEDPWPRVNSKDH
IPALVRSNAFSENFLEVSAEIARENVQGVVIPSKDPEPLEENCAKALTLRIHDSLNNSLPIGLVPTNSTNTVMDQKNLKMSTPGQMK
AQEIERTPPTNFKRTLEESNSGPLMKKHRRNGLSRSSGAQPASLPTTSQRKNSVKLTMRRRLRGQKKIGNPLLHQHRKTVCVC
>sp|Q9BUJ2|HNRL1_HUMAN
MDVRRLKVNELREELQRRGLDTRGLKAELAERLQAALEAEEPDDERELDADDEPGRPGHINEEVETEGGSELEGTAQPPPPGLQPHA
EPGGYSGPDGHYAMDNITRQNQFYDTQVIKQENESGYERRPLEMEQQQAYRPEMKTEMKQGAPTSFLPPEASQLKPDRQQFQSRKRP
YEENRGRGYFEHREDRRGRSPQPPAEEDEDDFDDTLVAIDTYNCDLHFKVARDRSSGYPLTIEGFAYLWSGARASYGVRRGRVCFEM
KINEEISVKHLPSTEPDPHVVRIGWSLDSCSTQLGEEPFSYGYGGTGKKSTNSRFENYGDKFAENDVIGCFADFECGNDVELSFTKN
GKWMGIAFRIQKEALGGQALYPHVLVKNCAVEFNFGQRAEPYCSVLPGFTFIQHLPLSERIRGTVGPKSKAECEILMMVGLPAAGKT
TWAIKHAASNPSKKYNILGTNAIMDKMRVMGLRRQRNYAGRWDVLIQQATQCLNRLIQIAARKKRNYILDQTNVYGSAQRRKMRPFE
GFQRKAIVICPTDEDLKDRTIKRTDEEGKDVPDHAVLEMKANFTLPDVGDFLDEVLFIELQREEADKLVRQYNEEGRKAGPPPEKRF
DNRGGGGFRGRGGGGGFQRYENRGPPGGNRGGFQNRGGGSGGGGNYRGGFNRSGGGGYSQNRWGNNNRDNNNSNNRGSYNRAPQQQP
PPQQPPPPQPPPQQPPPPPSYSPARNPPGASTYNKNSNIPGSSANTSTPTVSSYSPPQPSYSQPPYNQGGYSQGYTAPPPPPPPPPA
YNYGSYGGYNPAPYTPPPPPTAQTYPQPSYNQYQQYAQQWNQYYQNQGQWPPYYGNYDYGSYSGNTQGGTSTQ
>sp|P22103|PNEU_HUMAN
AGEPKLDAGV
>sp|Q9NWR8|MCUB_HUMAN
MLQRGLWPWRTRLLPTPGTWRPARPWPLPPPPQVLRVKLCGNVKYYQSHHYSTVVPPDEITVIYRHGLPLVTLTLPSRKERCQFVVK
PMLSTVGSFLQDLQNEDKGIKTAAIFTADGNMISASTLMDILLMNDFKLVINKIAYDVQCPKREKPSNEHTAEMEHMKSLVHRLFTI
LHLEESQKKREHHLLEKIDHLKEQLQPLEQVKAGIEAHSEAKTSGLLWAGLALLSIQGGALAWLTWWVYSWDIMEPVTYFITFANSM
VFFAYFIVTRQDYTYSAVKSRQFLQFFHKKSKQQHFDVQQYNKLKEDLAKAKESLKQARHSLCLQMQVEELNEKN
>sp|Q8N2H3|PYRD2_HUMAN
MAASGRGLCKAVAASPFPAWRRDNTEARGGLKPEYDAVVIGAGHNGLVAAAYLQRLGVNTAVFERRHVIGGAAVTEEIIPGFKFSRA
SYLLSLLRPQIYTDLELKKHGLRLHLRNPYSFTPMLEEGAGSKVPRCLLLGTDMAENQKQIAQFSQKDAQVFPKYEEFMHRLALAID
PLLDAAPVDMAAFQHGSLLQRMRSLSTLKPLLKAGRILGAQLPRYYEVLTAPITKVLDQWFESEPLKATLATDAVIGAMTSPHTPGS
GYVLLHHVMGGLEGMQGAWGYVQGGMGALSDAIASSATTHGASIFTEKTVAKVQVNSEGCVQGVVLEDGTEVRSKMVLSNTSPQITF
LKLTPQEWLPEEFLERISQLDTRSPVTKINVAVDRLPSFLAAPNAPRGQPLPHHQCSIHLNCEDTLLLHQAFEDAMDGLPSHRPVIE
LCIPSSLDPTLAPPGCHVVSLFTQYMPYTLAGGKAWDEQERDAYADRVFDCIEVYAPGFKDSVVGRDILTPPDLERIFGLPGGNIFH
CAMSLDQLYFARPVPLHSGYRCPLQGLYLCGSGAHPGGGVMGAAGRNAAHVAFRDLKSM
>sp|Q9GZX9|TWSG1_HUMAN
MKLHYVAVLTLAILMFLTWLPESLSCNKALCASDVSKCLIQELCQCRPGEGNCSCCKECMLCLGALWDECCDCVGMCNPRNYSDTPP
TSKSTVEELHEPIPSLFRALTEGDTQLNWNIVSFPVAEELSHHENLVSFLETVNQPHHQNVSVPSNNVHAPYSSDKEHMCTVVYFDD
CMSIHQCKISCESMGASKYRWFHNACCECIGPECIDYGSKTVKCMNCMF
>sp|Q9C075|K1C23_HUMAN

MNSGHSFSQTPSASFHGAGGGWGRPRSFPRAPTVHGGAGGARISLSFTTRSCPPPGGSWGSGRSSPLLGGNGKATMQNLNDRLASYL
EKVRALEEANMKLESRILKWHQQRDPGSKKDYSQYEENITHLQEQIVDGKMTNAQIILLIDNARMAVDDFNLKYENEHSFKKDLEIE
VEGLRRTLDNLTIVTTDLEQEVEGMRKELILMKKHHEQEMEKHHVPSDFNVNVKVDTGPREDLIKVLEDMRQEYELIIKKKHRDLDT
WYKEQSAAMSQEAASPATVQSRQGDIHELKRTFQALEIDLQTQYSTKSALENMLSETQSRYSCKLQDMQEIISHYEEELTQLRHELE
RQNNEYQVLLGIKTHLEKEITTYRRLLEGESEGTREESKSSMKVSATPKIKAITQETINGRLVLCQVNEIQKHA
>sp|P55957|BID_HUMAN
MDCEVNNGSSLRDECITNLLVFGFLQSCSDNSFRRELDALGHELPVLAPQWEGYDELQTDGNRSSHSRLGRIEADSESQEDIIRNIA
RHLAQVGDSMDRSIPPGLVNGLALQLRNTSRSEEDRNRDLATALEQLLQAYPRDMEKEKTMLVLALLLAKKVASHTPSLLRDVFHTT
VNFINQNLRTYVRSLARNGMD
>sp|Q96D21|RHES_HUMAN
MMKTLSSGNCTLSVPAKNSYRMVVLGASRVGKSSIVSRFLNGRFEDQYTPTIEDFHRKVYNIRGDMYQLDILDTSGNHPFPAMRRLS
ILTGDVFILVFSLDNRESFDEVKRLQKQILEVKSCLKNKTKEAAELPMVICGNKNDHGELCRQVPTTEAELLVSGDENCAYFEVSAK
KNTNVDEMFYVLFSMAKLPHEMSPALHRKISVQYGDAFHPRPFCMRRVKEMDAYGMVSPFARRPSVNSDLKYIKAKVLREGQARERD
KCTIQ
>sp|Q9BVM4|GGACT_HUMAN
MALVFVYGTLKRGQPNHRVLRDGAHGSAAFRARGRTLEPYPLVIAGEHNIPWLLHLPGSGRLVEGEVYAVDERMLRFLDDFESCPAL
YQRTVLRVQLLEDRAPGAEEPPAPTAVQCFVYSRATFPPEWAQLPHHDSYDSEGPHGLRYNPRENR
>sp|Q96QU8|XPO6_HUMAN
MASEEASLRALESLMTEFFHDCTTNERKREIEELLNNFAQQIGAWRFCLYFLSSTRNDYVMMYSLTVFENLINKMWLGVPSQDKMEI
RSCLPKLLLAHHKTLPYFIRNKLCKVIVDIGRQDWPMFYHDFFTNILQLIQSPVTTPLGLIMLKTTSEELACPREDLSVARKEELRK
LLLDQVQTVLGLLTGILETVWDKHSVTAATPPPSPTSGESGDLLSNLLQSPSSAKLLNQPIPILDVESEYICSLALECLAHLFSWIP
LSASITPSLLTTIFHFARFGCDIRARKMASVNGSSQNCVSGQERGRLGVLAMSCINELMSKNCVPMEFEEYLLRMFQQTFYLLQKIT
KDNNAHTVKSRLEELDESYIEKFTDFLRLFVSVHLRRIESYSQFPVVEFLTLLFKYTFHQPTHEGYFSCLDIWTLFLDYLTSKIKSR
LGDKEAVLNRYEDALVLLLTEVLNRIQFRYNQAQLEELDDETLDDDQQTEWQRYLRQSLEVVAKVMELLPTHAFSTLFPVLQDNLEV
YLGLQQFIVTSGSGHRLNITAENDCRRLHCSLRDLSSLLQAVGRLAEYFIGDVFAARFNDALTVVERLVKVTLYGSQIKLYNIETAV
PSVLKPDLIDVHAQSLAALQAYSHWLAQYCSEVHRQNTQQFVTLISTTMDAITPLISTKVQDKLLLSACHLLVSLATTVRPVFLISI
PAVQKVFNRITDASALRLVDKAQVLVCRALSNILLLPWPNLPENEQQWPVRSINHASLISALSRDYRNLKPSAVAPQRKMPLDDTKL
IIHQTLSVLEDIVENISGESTKSRQICYQSLQESVQVSLALFPAFIHQSDVTDEMLSFFLTLFRGLRVQMGVPFTEQIIQTFLNMFT
REQLAESILHEGSTGCRVVEKFLKILQVVVQEPGQVFKPFLPSIIALCMEQVYPIIAERPSPDVKAELFELLFRTLHHNWRYFFKST
VLASVQRGIAEEQMENEPQFSAIMQAFGQSFLQPDIHLFKQNLFYLETLNTKQKLYHKKIFRTAMLFQFVNVLLQVLVHKSHDLLQE
EIGIAIYNMASVDFDGFFAAFLPEFLTSCDGVDANQKSVLGRNFKMDRDLPSFTQNVHRLVNDLRYYRLCNDSLPPGTVKL
>sp|P02649|APOE_HUMAN
MKVLWAALLVTFLAGCQAKVEQAVETEPEPELRQQTEWQSGQRWELALGRFWDYLRWVQTLSEQVQEELLSSQVTQELRALMDETMK
ELKAYKSELEEQLTPVAEETRARLSKELQAAQARLGADMEDVCGRLVQYRGEVQAMLGQSTEELRVRLASHLRKLRKRLLRDADDLQ
KRLAVYQAGAREGAERGLSAIRERLGPLVEQGRVRAATVGSLAGQPLQERAQAWGERLRARMEEMGSRTRDRLDEVKEQVAEVRAKL
EEQAQQIRLQAEAFQARLKSWFEPLVEDMQRQWAGLVEKVQAAVGTSAAPVPSDNH
>sp|Q92685|ALG3_HUMAN
MAAGLRKRGRSGSAAQAEGLCKQWLQRAWQERRLLLREPRYTLLVAACLCLAEVGITFWVIHRVAYTEIDWKAYMAEVEGVINGTYD
YTQLQGDTGPLVYPAGFVYIFMGLYYATSRGTDIRMAQNIFAVLYLATLLLVFLIYHQTCKVPPFVFFFMCCASYRVHSIFVLRLFN
DPVAMVLLFLSINLLLAQRWGWGCCFFSLAVSVKMNVLLFAPGLLFLLLTQFGFRGALPKLGICAGLQVVLGLPFLLENPSGYLSRS
FDLGRQFLFHWTVNWRFLPEALFLHRAFHLALLTAHLTLLLLFALCRWHRTGESILSLLRDPSKRKVPPQPLTPNQIVSTLFTSNFI
GICFSRSLHYQFYVWYFHTLPYLLWAMPARWLTHLLRLLVLGLIELSWNTYPSTSCSSAALHICHAVILLQLWLGPQPFPKSTQHSK
KAH
>sp|Q13487|SNPC2_HUMAN

MKPPPRRRAAPARYLGEVTGPATWSAREKRQLVRLLQARQGQPEPDATELARELRGRSEAEIRVFLQQLKGRVAREAIQKVHPGGLQ
GPRRREAQPPAPIEVWTDLAEKITGPLEEALAVAFSQVLTIAATEPVTLLHSKPPKPTQARGKPLLLSAPGGQEDPAPEIPSSAPAA
PSSAPRTPDPAPEKPSESSAGPSTEEDFAVDFEKIYKYLSSVSRSGRSPELSAAESAVVLDLLMSLPEELPLLPCTALVEHMTETYL
RLTAPQPIPAGGSLGPAAEGDGAGSKAPEETPPATEKAEHSELKSPWQAAGICPLNPFLVPLELLGRAATPAR
>sp|Q9Y6B2|EID1_HUMAN
MSEMAELSELYEESSDLQMDVMPGEGDLPQMEVGSGSRELSLRPSRSGAQQLEEEGPMEEEEAQPMAAPEGKRSLANGPNAGEQPGQ
VAGADFESEDEGEEFDDWEDDYDYPEEEQLSGAGYRVSAALEEADKMFLRTREPALDGGFQMHYEKTPFDQLAFIEELFSLMVVNRL
TEELGCDEIIDRE
>sp|Q9H9G7|AGO3_HUMAN
MEIGSAGPAGAQPLLMVPRRPGYGTMGKPIKLLANCFQVEIPKIDVYLYEVDIKPDKCPRRVNREVVDSMVQHFKVTIFGDRRPVYD
GKRSLYTANPLPVATTGVDLDVTLPGEGGKDRPFKVSIKFVSRVSWHLLHEVLTGRTLPEPLELDKPISTNPVHAVDVVLRHLPSMK
YTPVGRSFFSAPEGYDHPLGGGREVWFGFHQSVRPAMWKMMLNIDVSATAFYKAQPVIQFMCEVLDIHNIDEQPRPLTDSHRVKFTK
EIKGLKVEVTHCGTMRRKYRVCNVTRRPASHQTFPLQLENGQTVERTVAQYFREKYTLQLKYPHLPCLQVGQEQKHTYLPLEVCNIV
AGQRCIKKLTDNQTSTMIKATARSAPDRQEEISRLVRSANYETDPFVQEFQFKVRDEMAHVTGRVLPAPMLQYGGRNRTVATPSHGV
WDMRGKQFHTGVEIKMWAIACFATQRQCREEILKGFTDQLRKISKDAGMPIQGQPCFCKYAQGADSVEPMFRHLKNTYSGLQLIIVI
LPGKTPVYAEVKRVGDTLLGMATQCVQVKNVIKTSPQTLSNLCLKINVKLGGINNILVPHQRPSVFQQPVIFLGADVTHPPAGDGKK
PSIAAVVGSMDAHPSRYCATVRVQRPRQEIIQDLASMVRELLIQFYKSTRFKPTRIIFYRDGVSEGQFRQVLYYELLAIREACISLE
KDYQPGITYIVVQKRHHTRLFCADRTERVGRSGNIPAGTTVDTDITHPYEFDFYLCSHAGIQGTSRPSHYHVLWDDNCFTADELQLL
TYQLCHTYVRCTRSVSIPAPAYYAHLVAFRARYHLVDKEHDSAEGSHVSGQSNGRDPQALAKAVQIHQDTLRTMYFA
>sp|Q8N6R0|MET13_HUMAN
MNLLPKSSREFGSVDYWEKFFQQRGKKAFEWYGTYLELCGVLHKYIKPREKVLVIGCGNSELSEQLYDVGYRDIVNIDISEVVIKQM
KECNATRRPQMSFLKMDMTQMEFPDASFQVVLDKGTLDAVLTDEEEKTLQQVDRMLAEVGRVLQVGGRYLCISLAQAHILKKAVGHF
SREGWMVRVHQVANSQDQVLEAEPQFSLPVFAFIMTKFRPVPGSALQIFELCAQEQRKPVRLESAERLAEAVQERQQYAWLCSQLRR
KARLGSVSLDLCDGDTGEPRYTLHVVDSPTVKPSRDNHFAIFIIPQGRETEWLFGMDEGRKQLAASAGFRRLITVALHRGQQYESMD
HIQAELSARVMELAPAGMPTQQQVPFLSVGGDIGVRTVQHQDCSPLSGDYVIEDVQGDDKRYFRRLIFLSNRNVVQSEARLLKDVSH
KAQKKRKKDRKKQRPADAEDLPAAPGQSIDKSYLCCEHHKAMIAGLALLRNPELLLEIPLALLVVGLGGGSLPLFVHDHFPKSCIDA
VEIDPSMLEVATQWFGFSQSDRMKVHIADGLDYIASLAGGGEARPCYDVIMFDVDSKDPTLGMSCPPPAFVEQSFLQKVKSILTPEG
VFILNLVCRDLGLKDSVLAGLKAVFPLLYVRRIEGEVNEILFCQLHPEQKLATPELLETAQALERTLRKPGRGWDDTYVLSDMLKTV
KIV
>sp|O43312|MTSS1_HUMAN
MEAVIEKECSALGGLFQTIISDMKGSYPVWEDFINKAGKLQSQLRTTVVAAAAFLDAFQKVADMATNTRGGTREIGSALTRMCMRHR
SIEAKLRQFSSALIDCLINPLQEQMEEWKKVANQLDKDHAKEYKKARQEIKKKSSDTLKLQKKAKKGRGDIQPQLDSALQDVNDKYL
LLEETEKQAVRKALIEERGRFCTFISMLRPVIEEEISMLGEITHLQTISEDLKSLTMDPHKLPSSSEQVILDLKGSDYSWSYQTPPS
SPSTTMSRKSSVCSSLNSVNSSDSRSSGSHSHSPSSHYRYRSSNLAQQAPVRLSSVSSHDSGFISQDAFQSKSPSPMPPEAPNQLSN
GFSHYSLSSESHVGPTGAGLFPHCLPASRLLPRVTSVHLPDYAHYYTIGPGMFPSSQIPSWKDWAKPGPYDQPLVNTLQRRKEKREP
DPNGGGPTTASGPPAAAEEAQRPRSMTVSAATRPGEEMEACEELALALSRGLQLDTQRSSRDSLQCSSGYSTQTTTPCCSEDTIPSQ
VSDYDYFSVSGDQEADQQEFDKSSTIPRNSDISQSYRRMFQAKRPASTAGLPTTLGPAMVTPGVATIRRTPSTKPSVRRGTIGAGPI
PIKTPVIPVKTPTVPDLPGVLPAPPDGPEERGEHSPESPSVGEGPQGVTSMPSSMWSGQASVNPPLPGPKPSIPEEHRQAIPESEAE
DQEREPPSATVSPGQIPESDPADLSPRDTPQGEDMLNAIRRGVKLKKTTTNDRSAPRFS
>sp|Q6ZNG9|KRBA2_HUMAN
MPSFLVPSLVSSPVLLKLLFSPGPKTIWSLWQQPMLFQEATAFENMTKDWNYLEGSQKDCYRDTMLDSYENTVPQGSFLQLSMMPQR
AGNDPPGVSNASEMEMEISNMREKFLMSVTKLVESKSYNSKVFSKEKYFQTIKEVKEAKEKGKKSSRDYRRAAKYDVISVQGTEKLI
EATHGERDRIRYYVHKEELFDILHDTHLSIGHGGRTRMLKELQGKYGNVTKEVIVLYLTLCKQCHQKNPVPKRGLAPKPMTFKDIDS
TCQVEILDMQSSADGEFKFILYYQDHSTKFIILRPLRTKQAHEVVSVLLDIFTILGTPSVLDSDSGVEFTNQVVHELNELWPDLKIV

SGKYHPGQSQGSLEGASRDVKNMISTWMQSNHSCHWAKGLRFMQMVRNQAFDVSLQQSPFEAMFGYKAKFGLYSSNLPRETVATLQT
EEELEIAEEQLENSLWIRQEERAEIGADRSDMDDDMDPTPEASEPSTSQGTSGLLCW
>sp|P57727|TMPS3_HUMAN
MGENDPPAVEAPFSFRSLFGLDDLKISPVAPDADAVAAQILSLLPLKFFPIIVIGIIALILALAIGLGIHFDCSGKYRCRSSFKCIE
LIARCDGVSDCKDGEDEYRCVRVGGQNAVLQVFTAASWKTMCSDDWKGHYANVACAQLGFPSYVSSDNLRVSSLEGQFREEFVSIDH
LLPDDKVTALHHSVYVREGCASGHVVTLQCTACGHRRGYSSRIVGGNMSLLSQWPWQASLQFQGYHLCGGSVITPLWIITAAHCVYD
LYLPKSWTIQVGLVSLLDNPAPSHLVEKIVYHSKYKPKRLGNDIALMKLAGPLTFNEMIQPVCLPNSEENFPDGKVCWTSGWGATED
GAGDASPVLNHAAVPLISNKICNHRDVYGGIISPSMLCAGYLTGGVDSCQGDSGGPLVCQERRLWKLVGATSFGIGCAEVNKPGVYT
RVTSFLDWIHEQMERDLKT
>sp|O75426|FBX24_HUMAN
MGEKAVPLLRRRRVKRSCPSCGSELGVEEKRGKGNPISIQLFPPELVEHIISFLPVRDLVALGQTCRYFHEVCDGEGVWRRICRRLS
PRLQDQGSGVRPWKRAAILNYTKGLYFQAFGGRRRCLSKSVAPLLAHGYRRFLPTKDHVFILDYVGTLFFLKNALVSTLGQMQWKRA
CRYVVLCRGAKDFASDPRCDTVYRKYLYVLATREPQEVVGTTSSRACDCVEVYLQSSGQRVFKMTFHHSMTFKQIVLVGGQETQRALL
LLTEEGKIYSLVVNETQLDQPRSYTVQLALRKVSHYLPHLRVACMTSNQSSTLYVTDQGGVYFEVHTPGVYRDLFGTLQAFDPLDQQ
MPLALSLPAKILFCALGYNHLGLVDEFGRIFMQGNNRYGQLGTGDKMDRGEPTQVCYLQRPITLWCGLNHSLVLSQSSEFSKELLGC
GCGAGGRLPGWPKGSASFVKLQVKVPLCACALCATRECLYILSSHDIEQHAPYRHLPASRVVGTPEPSLGARAPQDPGGMAQACEEY
LSQIHSCQTLQDRTEKMKEIVGWMPLMAAQKDFFWEALDMLQRAEGGGGGVGPPAPET
>sp|Q9HC23|PROK2_HUMAN
MRSLCCAPLLLLLLLPPLLLTPRAGDAAVITGACDKDSQCGGGMCCAVSIWVKSIRICTPMGKLGDSCHPLTRKNNFGNGRQERRKR
KRSKRKKEVPFFGRRMHHTCPCLPGLACLRTSFNRFICLAQK
>sp|Q5ZPR3|CD276_HUMAN
MLRRRGSPGMGVHVGAALGALWFCLTGALEVQVPEDPVVALVGTDATLCCSFSPEPGFSLAQLNLIWQLTDTKQLVHSFAEGQDQGS
AYANRTALFPDLLAQGNASLRLQRVRVADEGSFTCFVSIRDFGSAAVSLQVAAPYSKPSMTLEPNKDLRPGDTVTITCSSYQGYPEA
EVFWQDGQGVPLTGNVTTSQMANEQGLFDVHSILRVVLGANGTYSCLVRNPVLQQDAHSSVTITPQRSPTGAVEVQVPEDPVVALVG
TDATLRCSFSPEPGFSLAQLNLIWQLTDTKQLVHSFTEGRDQGSAYANRTALFPDLLAQGNASLRLQRVRVADEGSFTCFVSIRDFG
SAAVSLQVAAPYSKPSMTLEPNKDLRPGDTVTITCSSYRGYPEAEVFWQDGQGVPLTGNVTTSQMANEQGLFDVHSVLRVVLGANGT
YSCLVRNPVLQQDAHGSVTITGQPMTFPPEALWVTVGLSVCLIALLVALAFVCWRKIKQSCEEENAGAEDQDGEGEGSKTALQPLKH
SDSKEDDGQEIA
>sp|Q07973|CP24A_HUMAN
MSSPISKSRSLAAFLQQLRSPRQPPRLVTSTAYTSPQPREVPVCPLTAGGETQNAAALPGPTSWPLLGSLLQILWKGGLKKQHDTLV
EYHKKYGKIFRMKLGSFESVHLGSPCLLEALYRTESAYPQRLEIKPWKAYRDYRKEGYGLLILEGEDWQRVRSAFQKKLMKPGEVMK
LDNKINEVLADFMGRIDELCDERGHVEDLYSELNKWSFESICLVLYEKRFGLLQKNAGDEAVNFIMAIKTMMSTFGRMMVTPVELHK
SLNTKVWQDHTLAWDTIFKSVKACIDNRLEKYSQQPSADFLCDIYHQNRLSKKELYAAVTELQLAAVETTANSLMWILYNLSRNPQV
QQKLLKEIQSVLPENQVPRAEDLRNMPYLKACLKESMRLTPSVPFTTRTLDKATVLGEYALPKGTVLMLNTQVLGSSEDNFEDSSQF
RPERWLQEKEKINPFAHLPFGVGKRMCIGRRLAELQLHLALCWIVRKYDIQATDNEPVEMLHSGTLVPSRELPIAFCQR
>sp|P04155|TFF1_HUMAN
MATMENKVICALVLVSMLALGTLAEAQTETCTVAPRERQNCGFPGVTPSQCANKGCCFDDTVRGVPWCFYPNTIDVPPEEECEF
>sp|P17038|ZNF43_HUMAN
MGPLTFMDVAIEFCLEEWQCLDIAQQNLYRNVMLENYRNLVFLGIAVSKPDLITCLEQEKEPWEPMRRHEMVAKPPVMCSHFTQDFW
PEQHIKDPFQKATLRRYKNCEHKNVHLKKDHKSVDECKVHRGGYNGFNQCLPATQSKIFLFDKCVKAFHKFSNSNRHKISHTEKKLF
KCKECGKSFCMLPHLAQHKIIHTRVNFCKCEKCGKAFNCPSIITKHKRINTGEKPYTCEECGKVFNWSSRLTTHKKNYTRYKLYKCE
ECGKAFNKSSILTTHKIIRTGEKFYKCKECAKAFNQSSNLTEHKKIHPGEKPYKCEECGKAFNWPSTLTKHKRIHTGEKPYTCEECG
KAFNQFSNLTTHKRIHTAEKFYKCTECGEAFSRSSNLTKHKKIHTEKKPYKCEECGKAFKWSSKLTEHKLTHTGEKPYKCEECGKAF
NWPSTLTKHNRIHTGEKPYKCEVCGKAFNQFSNLTTHKRIHTAEKPYKCEECGKAFSRSSNLTKHKKIHIEKKPYKCEECGKAFKWS

SKLTEHKITHTGEKPYKCEECGKAFNHFSILTKHKRIHTGEKPYKCEECGKAFTQSSNLTTHKKIHTGEKFYKCEECGKAFTQSSNL
TTHKKIHTGGKPYKCEECGKAFNQFSTLTKHKIIHTEEKPYKCEECGKAFKWSSTLTKHKIIHTGEKPYKCEECGKAFKLSSTLSTH
KIIHTGEKPYKCEKCGKAFNRSSNLIEHKKIHTGEQPYKCEECGKAFNYSSHLNTHKRIHTKEQPYKCKECGKAFNQYSNLTTHNKI
HTGEKLYKPEDVTVILTTPQTFSNIK
>sp|P25440|BRD2_HUMAN
MLQNVTPHNKLPGEGNAGLLGLGPEAAAPGKRIRKPSLLYEGFESPTMASVPALQLTPANPPPPEVSNPKKPGRVTNQLQYLHKVVM
KALWKHQFAWPFRQPVDAVKLGLPDYHKIIKQPMDMGTIKRRLENNYYWAASECMQDFNTMFTNCYIYNKPTDDIVLMAQTLEKIFL
QKVASMPQEEQELVVTIPKNSHKKGAKLAALQGSVTSAHQVPAVSSVSHTALYTPPPEIPTTVLNIPHPSVISSPLLKSLHSAGPPL
LAVTAAPPAQPLAKKKGVKRKADTTTPTPTAILAPGSPASPPGSLEPKAARLPPMRRESGRPIKPPRKDLPDSQQQHQSSKKGKLSE
QLKHCNGILKELLSKKHAAYAWPFYKPVDASALGLHDYHDIIKHPMDLSTVKRKMENRDYRDAQEFAADVRLMFSNCYKYNPPDHDV
VAMARKLQDVFEFRYAKMPDEPLEPGPLPVSTAMPPGLAKSSSESSSEESSSESSSEEEEEEDEEDEEEEESESSDSEEERAHRLAE
LQEQLRAVHEQLAALSQGPISKPKRKREKKEKKKKRKAEKHRGRAGADEDDKGPRAPRPPQPKKSKKASGSGGGSAALGPSGFGPSG
GSGTKLPKKATKTAPPALPTGYDSEEEEESRPMSYDEKRQLSLDINKLPGEKLGRVVHIIQAREPSLRDSNPEEIEIDFETLKPSTL
RELERYVLSCLRKKPRKPYTIKKPVGKTKEELALEKKRELEKRLQDVSGQLNSTKKPPKKANEKTESSSAQQVAVSRLSASSSSSDS
SSSSSSSSSSDTSDSDSG
>sp|Q04826|1B40_HUMAN
MRVTAPRTLLLLLWGAVALTETWAGSHSMRYFHTSVSRPGRGEPRFITVGYVDDTLFVRFDSDATSPRKEPRAPWIEQEGPEYWDRE
TQISKTNTQTYRESLRNLRGYYNQSEAGSHTLQSMYGCDVGPDGRLLRGHNQYAYDGKDYIALNEDLRSWTAADTAAQITQRKWEAA
RVAEQLRAYLEGECVEWLRRYLENGKETLQRADPPKTHVTHHPISDHEATLRCWALGFYPAEITLTWQRDGEDQTQDTELVETRPAG
DRTFQKWAAVVVPSGEEQRYTCHVQHEGLPKPLTLRWEPSSQSTVPIVGIVAGLAVLAVVVIGAVVAAVMCRRKSSGGKGGSYSQAA
CSDSAQGSDVSLTA
>sp|Q9UBD3|XCL2_HUMAN
MRLLILALLGICSLTAYIVEGVGSEVSHRRTCVSLTTQRLPVSRIKTYTITEGSLRAVIFITKRGLKVCADPQATWVRDVVRSMDRK
SNTRNNMIQTKPTGTQQSTNTAVTLTG
>sp|Q96SI9|STRBP_HUMAN
MRSIRSFANDDRHVMVKHSTIYPSPEELEAVQNMVSTVECALKHVSDWLDETNKGTKTEGETEVKKDEAGENYSKDQGGRTLCGVMR
IGLVAKGLLIKDDMDLELVLMCKDKPTETLLNTVKDNLPIQIQKLTEEKYQVEQCVNEASIIIRNTKEPTLTLKVILTSPLIRDELE
KKDGENVSMKDPPDLLDRQKCLNALASLRHAKWFQARANGLKSCVIVLRILRDLCNRVPTWAPLKGWPLELICEKSIGTCNRPLGAG
EALRRVMECLASGILLPGGPGLHDPCERDPTDALSYMTIQQKEDITHSAQHALRLSAFGQIYKVLEMDPLPSSKPFQKYSWSVTDKE
GAGSSALKRPFEDGLGDDKDPNKKMKRNLRKILDSKAIDLMNALMRLNQIRPGLQYKLLSQSGPVHAPVFTMSVDVDGTTYEASGPS
KKTAKLHVAVKVLQAMGYPTGFDADIECMSSDEKSDNESKNETVSSNSSNNTGNSTTETSSTLEVRTQGPILTASGKNPVMELNEKR
RGLKYELISETGGSHDKRFVMEVEVDGQKFRGAGPNKKVAKASAALAALEKLFSGPNAANNKKKKIIPQAKGVVNTAVSAAVQAVRG
RGRGTLTRGAFVGATAAPGYIAPGYGTPYGYSTAAPAYGLPKRMVLLPVMKFPTYPVPHYSFF
>sp|O76080|ZFAN5_HUMAN
MAQETNQTPGPMLCSTGCGFYGNPRTNGMCSVCYKEHLQRQQNSGRMSPMGTASGSNSPTSDSASVQRADTSLNNCEGAAGSTSEKS
RNVPVAALPVTQQMTEMSISREDKITTPKTEVSEPVVTQPSPSVSQPSTSQSEEKAPELPKPKKNRCFMCRKKVGLTGFDCRCGNLF
CGLHRYSDKHNCPYDYKAEAAAKIRKENPVVVAEKIQRI
>sp|Q6YBV0|S36A4_HUMAN
MEAAATPAAAGAARREELDMDVMRPLINEQNFDGTSDEEHEQELLPVQKHYQLDDQEGISFVQTLMHLLKGNIGTGLLGLPLAIKNA
GIVLGPISLVFIGIISVHCMHILVRCSHFLCLRFKKSTLGYSDTVSFAMEVSPWSCLQKQAAWGRSVVDFFLVITQLGFCSVYIVFL
AENVKQVHEGFLESKVFISNSTNSSNPCERRSVDLRIYMLCFLPFIILLVFIRELKNLFVLSFLANVSMAVSLVIIYQYVVRNMPDP
HNLPIVAGWKKYPLFFGTAVFAFEGIGVVLPLENQMKESKRFPQALNIGMGIVTTLYVTLATLGYMCFHDEIKGSITLNLPQDVWLY
QSVKILYSFGIFVTYSIQFYVPAEIIIPGITSKFHTKWKQICEFGIRSFLVSITCAGAILIPRLDIVISFVGAVSSSTLALILPPLV
EILTFSKEHYNIWMVLKNISIAFTGVVGFLLGTYITVEEIIYPTPKVVAGTPQSPFLNLNSTCLTSGLK

>sp|P07438|MT1B_HUMAN
MDPNCSCTTGGSCACAGSCKCKECKCTSCKKCCCSCCPVGCAKCAQGCVCKGSSEKCRCCA
>sp|Q8TDW4|ST7L_HUMAN
MADRGGVGEAAAVGASPASVPGLNPTLGWRERLRAGLAGTGASLWFVAGLGLLYALRIPLRLCENLAAVTVFLNSLTPKFYVALTGT
SSLISGLIFIFEWWYFHKHGTSFIEQVSVSHLQPLMGGTESSISEPGSPSRNRENETSRQNLSECKVWRNPLNLFRGAEYRRYTWVT
GKEPLTYYDMNLSAQDHQTFFTCDTDFLRPSDTVMQKAWRERNPPARIKAAYQALELNNDCATAYVLLAEEEATTIVDAERLFKQAL
KAGETIYRQSQQCQHQSPQHEAQLRRDTNVLVYIKRRLAMCARKLGRIREAVKIMRDLMKEFPPLTMLNIHENLLESLLELQAYPDV
QAVLAKYDDISLPKSAAICYTAALLKTRTVSEKFSPETASRRGLSTAEINAVEAIHRAVEFNPHVPKYLLEMKSLILPPEHILKRGD
SEAIAYAFFHLQHWKRIEGALNLLQCTWEGTFRMIPYPLEKGHLFYPYPSCTETADRELLPTFHHVSVYPKKELPLFIHFTAGFCSS
TAMIAILTHQFPEIMGIFAKAVLGLWCPQPWASSGFEENTQDLKSEDLGLSSG
>sp|Q86UP3|ZFHX4_HUMAN
METCDSPPISRQENGQSTSKLCGTTQLDNEVPEKVAGMEPDRENSSTDDNLKTDERKSEALLGFSVENAAATQVTSAKEIPCNECAT
SFPSLQKYMEHHCPNARLPVLKDDNESEISELEDSDVENLTGEIVYQPDGSAYIIEDSKESGQNAQTGANSKLFSTAMFLDSLASAG
EKSDQSASAPMSFYPQIINTFHIASSLGKPFTADQAFPNTSALAGVGPVLHSFRVYDLRHKREKDYLTSDGSAKNSCVSKDVPNNVD
LSKFDGCVSDGKRKPVLMCFLCKLSFGYIRSFVTHAVHDHRMTLNDEEQKLLSNKCVSAIIQGIGKDKEPLISFLEPKKSTSVYPHF
STTNLIGPDPTFRGLWSAFHVENGDSLPAGFAFLKGSASTSSSAEQPLGITQMPKAEVNLGGLSSLVVNTPITSVSLSHSSSESSKM
SESKDQENNCERPKESNVLHPNGECPVKSEPTEPGDEDEEDAYSNELDDEEVLGELTDSIGNKDFPLLNQSISPLSSSVLKFIEKGT
SSSSATVSDDTEKKKQTAAVRASGSVASNYGISGKDFADASASKDSATAAHPSEIARGDEDSSATPHQHGFTPSTPGTPGPGGDGSP
GSGIECPKCDTVLGSSRSLGGHMTMMHSRNSCKTLKCPKCNWHYKYQQTLEAHMKEKHPEPGGSCVYCKTGQPHPRLARGESYTCGY
KPFRCEVCNYSTTTKGNLSIHMQSDKHLNNVQNLQNGNGEQVFGHSAPAPNTSLSGCGTPSPSKPKQKPTWRCEVCDYETNVARNLR
IHMTSEKHMHNMMLLQQNMKQIQHNLHLGLAPAEAELYQYYLAQNIGLTGMKLENPADPQLMINPFQLDPATAAALAPGLGELSPYI
SDPALKLFQCAVCNKFTSDSLEALSVHVSSERSLPEEEWRAVIGDIYQCKLCNYNTQLKANFQLHCKTDKHMQKYQLVAHIKEGGKS
NEWRLKCIAIGNPVHLKCNACDYYTNSVDKLRLHTTNHRHEAALKLYKHLQKQEGAVNPESCYYYCAVCDYTTKVKLNLVQHVRSVK
HQQTEGLRKLQLHQQGLAPEEDNLSEIFFVKDCPPNELETASLGARTCDDDLTEQHEEAEGAIKPTAVAEDDEKDTSERDNSEGKNS
NKDSVSVAGGTQPLLLAKEEDVATKRSKPTEDNKFCHEQFYQCPYCNYNSRDQSRIQMHVLSQHSVQPVICCPLCQDVLSNKMHLQL
HLTHLHSVSPDCVEKLLMTVPVPDVMMPNSMLLPAAASEKSERDTPAAVTAEGSGKYSGESPMDDKSMAGLEDSKANVEVKNEEQKP
TKEPLEVSEWNKNSSKDVKIPDTLQDQLNEQQKRQPLSVSDRHVYKYRCNHCSLAFKTMQKLQIHSQYHAIRAATMCNLCQRSFRTF
QALKKHLEAGHPELSEAELQQLYASLPVNGELWAESETMSQDDHGLEQEMEREYEVDHEGKASPVGSDSSSIPDDMGSEPKRTLPFR
KGPNFTMEKFLDPSRPYKCTVCKESFTQKNILLVHYNSVSHLHKLKKVLQEASSPVPQETNSNTDNKPYKCSICNVAYSQSSTLEIH
MRSVLHQTKARAAKLEPSGHVAGGHSIAANVNSPGQGMLDSMSLAAVNSKDTHLDAKELNKKQTPDLISAQPAHHPPQSPAQIQMQL
QHELQQQAAFFQPQFLNPAFLPHFPMTPEALLQFQQPQFLFPFYIPGTEFSLGPDLGLPGSATFGMPGMTGMAGSLLEDLKQQIQTQ
HHVGQTQLQILQQQAQQYQATQPQLQPQKQQQPPPPQQQQQQASKLLKQEQSNIVSADCQIMKDVPSYKEAEDISEKPEKPKQEF
ISEGEGLKEGKDTKKQKSLEPSIPPPRIASGARGNAAKALLENFGFELVIQYNENRQKVQKKGKSGEGENTDKLECGTCGKLFSNVL
ILKSHQEHVHGQFFPYAALEKFARQYREAYDKLYPISPSSPETPPPPPPPPPLPPAPPQPSSMGPVKIPNTVSTPLQAPPPTPPPPP
PPPPPPPPPPPPPPPSAPPQVQLPVSLDLPLFPSIMMQPVQHPALPPQLALQLPQMDALSADLTQLCQQQLGLDPNFLRHSQFKRPR
TRITDDQLKILRAYFDINNSPSEEQIQEMAEKSGLSQKVIKHWFRNTLFKERQRNKDSPYNFSNPPITVLEDIRIDPQPTSLEHYKS
DASFSKRSSRTRFTDYQLRVLQDFFDTNAYPKDDEIEQLSTVLNLPTRVIVVWFQNARQKARKSYENQAETKDNEKRELTNERYIRT
SNMQYQCKKCNVVFPRIFDLITHQKKQCYKDEDDDAQDESQTEDSMDATDQVVYKHCTVSGQTDAAKNAAAPAASSGSGTSTPLIPS
PKPEPEKTSPKPEYPAEKPKQSDPSPPSQGTKPALPLASTSSDPPQASTAQPQPQPQPPKQPQLIGRPPSASQTPVPSSPLQISMTS
LQNSLPPQLLQYQCDQCTVAFPTLELWQEHQHMHFLAAQNQFLHSPFLERPMDMPYMIFDPNNPLMTGQLLGSSLTQMPPQASSSHT
TAPTTVAASLKRKLDDKEDNNCSEKEGGNSGEDQHRDKRLRTTITPEQLEILYEKYLLDSNPTRKMLDHIAREVGLKKRVVQVWFQN
TRARERKGQFRAVGPAQSHKRCPFCRALFKAKSALESHIRSRHWNEGKQAGYSLPPSPLISTEDGGESPQKYIYFDYPSLPLTKIDL
SSENELASTVSTPVSKTAELSPKNLLSPSSFKAECSEDVENLNAPPAEAGYDQNKTDFDETSSINTAISDATTGDEGNTEMESTTGS
SGDVKPALSPKEPKTLDTLPKPATTPTTEVCDDKFLFSLTSPSIHFNDKDGDHDQSFYITDDPDDNADRSETSSIADPSSPNPFGSS

NPFKSKSNDRPGHKRFRTQMSNLQLKVLKACFSDYRTPTMQECEMLGNEIGLPKRVVQVWFQNARAKEKKFKINIGKPFMINQGGTE
GTKPECTLCGVKYSARLSIRDHIFSKQHISKVRETVGSQLDREKDYLAPTTVRQLMAQQELDRIKKASDVLGLTVQQPGMMDSSSLH
GISLPTAYPGLPGLPPVLLPGMNGPSSLPGFPQNSNISAGMLGFPTSATSSPALSLSSAPTKPLLQTPPPPPPPPPPPPSSSLSGQQ
TEQQNKESEKKQTKPNKVKKIKEEELEATKPEKHPKKEEKISSALSVLGKVVGETHVDPIQLQALQNAIAGDPASFIGGQFLPYFIP
GFASYFTPQLPGTVQGGYFPPVCGMESLFPYGPTMPQTLAGLSPGALLQQYQQYQQNLQESLQKQQKQQQEQQQKPVQAKTSKVESD
QPQNSNDASETKEDKSTATESTKEEPQLESKSADFSDTYVVPFVKYEFICRKCQMMFTDEDAAVNHQKSFCYFGQPLIDPQETVLRV
PVSKYQCLACDVAISGNEALSQHLQSSLHKEKTIKQAMRNAKEHVRLLPHSVCSPNPNTTSTSQSAASSNNTYPHLSCFSMKSWPNI
LFQASARRAASPPSSPPSLSLPSTVTSSLCSTSGVQTSLPTESCSDESDSELSQKLEDLDNSLEVKAKPASGLDGNFNSIRMDMFSV
>sp|Q8NH48|OR5B3_HUMAN
MENKTEVTQFILLGLTNDSELQVPLFITFPFIYIITLVGNLGIIVLIFWDSCLHNPMYFFLSNLSLVDFCYSSAVTPIVMAGFLIED
KVISYNACAAQMYIFVAFATVENYLLASMAYDRYAAVCKPLHYTTTMTTTVCARLAIGSYLCGFLNASIHTGDTFSLSFCKSNEVHH
FFCDIPAVMVLSCSDRHISELVLIYVVSFNIFIALLVILISYTFIFITILKMHSASVYQKPLSTCASHFIAVGIFYGTIIFMYLQPS
SSHSMDTDKMAPVFYTMVIPMLNPLVYSLRNKEVKSAFKKVVEKAKLSVGWSV
>sp|Q9Y6Z2|CF123_HUMAN
MGTAVGPHHSPAPHDSALPARLLTSDFPYGRSCQIEQVKYSVPDTGLFQHWEGSIPT
>sp|Q5T9G4|ARM12_HUMAN
MGKSIPQYLGQLDIRKSVVSLATGAGAIYLLYKAIKAGIKCKPPLCSNSPICIARLAVERERHGRDSGELRRLLNSLECKQDEYAKS
MILHSITRCVYLLEAEASACTTDDIVLLGYMLDDKDNSVKTQALNTLKAFSGIRKFRLKIQEHSIKVLELISTIWDTELHIAGLRLL
NNLPLPDYVHPQLRRVMPALMEILQSDYILAQVQAVRLLSYLAQKNDLLYDILNCQVHSNFLNLFQPTQSGSLLYEVLVFAERLSEG
RNAPHYHVVKWHYNEQSLHESLFGEESRLADRLLALVIHPEEDVQIQACKVIVSLQYPQDLRARPSSCQPSRSYFKNTE
>sp|Q9NPE2|NGRN_HUMAN
MAVTLSLLLGGRVCAAVTRCGFATRGVAGPGPIGREPDPDSDWEPEERELQEVESTLKRQKQAIRFQKIRRQMEAPGAPPRTLTWEA
MEQIRYLHEEFPESWSVPRLAEGFDVSTDVIRRVLKSKFLPTLEQKLKQDQKVLKKAGLAHSLQHLRGSGNTSKLLPAGHSVSGSLL
MPGHEASSKDPNHSTALKVIESDTHRTNTPRRRKGRNKEIQDLEESFVPVAAPLGHPRELQKYSSDSESPRGTGSGALPSGQKLEEL
KAEEPDNFSSKVVQRGREFFDSNGNFLYRI
>sp|P04196|HRG_HUMAN
MKALIAALLLITLQYSCAVSPTDCSAVEPEAEKALDLINKRRRDGYLFQLLRIADAHLDRVENTTVYYLVLDVQESDCSVLSRKYWN
DCEPPDSRRPSEIVIGQCKVIATRHSHESQDLRVIDFNCTTSSVSSALANTKDSPVLIDFFEDTERYRKQANKALEKYKEENDDFAS
FRVDRIERVARVRGGEGTGYFVDFSVRNCPRHHFPRHPNVFGFCRADLFYDVEALDLESPKNLVINCEVFDPQEHENINGVPPHLGH
PFHWGGHERSSTTKPPFKPHGSRDHHHPHKPHEHGPPPPPDERDHSHGPPLPQGPPPLLPMSCSSCQHATFGTNGAQRHSHNNNSSD
LHPHKHHSHEQHPHGHHPHAHHPHEHDTHRQHPHGHHPHGHHPHGHHPHGHHPHGHHPHCHDFQDYGPCDPPPHNQGHCCHGHGPPP
GHLRRRGPGKGPRPFHCRQIGSVYRLPPLRKGEVLPLPEANFPSFPLPHHKHPLKPDNQPFPQSVSESCPGKFKSGFPQVSMFFTHT
FPK
>sp|Q14055|CO9A2_HUMAN
MAAATASPRSLLVLLQVVVLALAQIRGPPGERGPPGPPGPPGVPGSDGIDGDNGPPGKAGPPGPKGEPGKAGPDGPDGKPGIDGLTG
AKGEPGPMGIPGVKGQPGLPGPPGLPGPGFAGPPGPPGPVGLPGEIGIRGPKGDPGPDGPSGPPGPPGKPGRPGTIQGLEGSADFLC
PTNCPPGMKGPPGLQGVKGHAGKRGILGDPGHQGKPGPKGDVGASGEQGIPGPPGPQGIRGYPGMAGPKGETGPHGYKGMVGAIGAT
GPPGEEGPRGPPGRAGEKGDEGSPGIRGPQGITGPKGATGPPGINGKDGTPGTPGMKGSAGQAGQPGSPGHQGLAGVPGQPGTKGGP
GDQGEPGPQGLPGFSGPPGKEGEPGPRGEIGPQGIMGQKGDQGERGPVGQPGPQGRQGPKGEQGPPGIPGPQGLPGVKGDKGSPGKT
GPRGKVGDPGVAGLPGEKGEKGESGEPGPKGQQGVRGEPGYPGPSGDAGAPGVQGYPGPPGPRGLAGNRGVPGQPGRQGVEGRDATD
QHIVDVALKMLQEQLAEVAVSAKREALGAVGMMGPPGPPGPPGYPGKQGPHGHPGPRGVPGIVGAVGQIGNTGPKGKRGEKGDPGEV
GRGHPGMPGPPGIPGLPGRPGQAINGKDGDRGSPGAPGEAGRPGLPGPVGLPGFCEPAACLGASAYASARLTEPGSIKGP
>sp|Q96QS1|TSN32_HUMAN

MGPWSRVRVAKCQMLVTCFFILLLGLSVATMVTLTYFGAHFAVIRRASLEKNPYQAVHQWAFSAGLSLVGLLTLGAVLSAAATVREA
QGLMAGGFLCFSLAFCAQVQVVFWRLHSPTQVEDAMLDTYDLVYEQAMKGTSHVRRQELAAIQDVFLCCGKKSPFSRLGSTEADLCQ
GEEAAREDCLQGIRSFLRTHQQVASSLTSIGLALTVSALLFSSFLWFAIRCGCSLDRKGKYTLTPRACGRQPQEPSLLRCSQGGPTH
CLHSEAVAIGPRGCSGSLRWLQESDAAPLPLSCHLAAHRALQGRSRGGLSGCPERGLSD
>sp|Q8N9R8|SCAI_HUMAN
MVRGARQPQQPRSRLAPRLTGTVEKPPRKRRSRTEFALKEIMSSGGAEDDIPQGERKTVTDFCYLLDKSKQLFNGLRDLPQYGQKQW
QSYFGRTFDVYTKLWKFQQQHRQVLDNRYGLKRWQIGEIASKIGQLYYHYYLRTSETSYLNEAFSFYSAIRQRSYYSQVNKEDRPEL
VVKKLRYYARFIVVCLLLNKMDVVKDLVKELSDEIEDYTHRFNTEDQVEWNLVLQEVAAFIEADPVMVLNDDNTIVITSNRLAETGA
PLLEQGMIVGQLSLADALIIGNCNNQVKFSELTVDMFRMLQALEREPMNLASQMNKPGMQESADKPTRRENPHKYLLYKPTFSQLYT
FLAASFKELPANSVLLIYLSATGVFPTGRSDSEGPYDFGGVLTNSNRDIINGDAIHKRNQSHKEMHCLHPGDLYPFTRKPLFIIVDS
SNSVAYKNFTNLFGQPLVCLLSPTAYPKALQDQSQRGSLFTLFLNNPLMAFLFVSGLSSMRRGLWEKCQEYLRKINRDIAQLLTHSR
SIDQAFLQFFGDEFLRLLLTRFIFCSATMRMHKIFRETRNYPESYPQLPRDETVENPHLQKHILELASILDVRNVFFENTIDDY
>sp|P62942|FKB1A_HUMAN
MGVQVETISPGDGRTFPKRGQTCVVHYTGMLEDGKKFDSSRDRNKPFKFMLGKQEVIRGWEEGVAQMSVGQRAKLTISPDYAYGATG
HPGIIPPHATLVFDVELLKLE
>sp|P62166|NCS1_HUMAN
MGKSNSKLKPEVVEELTRKTYFTEKEVQQWYKGFIKDCPSGQLDAAGFQKIYKQFFPFGDPTKFATFVFNVFDENKDGRIEFSEFIQ
ALSVTSRGTLDEKLRWAFKLYDLDNDGYITRNEMLDIVDAIYQMVGNTVELPEEENTPEKRVDRIFAMMDKNADGKLTLQEFQEGSK
ADPSIVQALSLYDGLV
>sp|Q9UGQ3|GTR6_HUMAN
MQEPLLGAEGPDYDTFPEKPPPSPGDRARVGTLQNKRVFLATFAAVLGNFSFGYALVYTSPVIPALERSLDPDLHLTKSQASWFGSV
FTLGAAAGGLSAMILNDLLGRKLSIMFSAVPSAAGYALMAGAHGLWMLLLGRTLTGFAGGLTAACIPVYVSEIAPPGVRGALGATPQ
LMAVFGSLSLYALGLLLPWRWLAVAGEAPVLIMILLLSFMPNSPRFLLSRGRDEEALRALAWLRGTDVDVHWEFEQIQDNVRRQSSR
VSWAEARAPHVCRPITVALLMRLLQQLTGITPILVYLQSIFDSTAVLLPPKDDAAIVGAVRLLSVLIAALTMDLAGRKVLLFVSAAI
MFAANLTLGLYIHFGPRPLSPNSTAGLESESWGDLAQPLAAPAGYLTLVPLLATMLFIMGYAVGWGPITWLLMSEVLPLRARGVASG
LCVLASWLTAFVLTKSFLPVVSTFGLQVPFFFFAAICLVSLVFTGCCVPETKGRSLEQIESFFRTGRRSFLR
>sp|Q7Z2E3|APTX_HUMAN
MSNVNLSVSDFWRVMMRVCWLVRQDSRHQRIRLPHLEAVVIGRGPETKITDKKCSRQQVQLKAECNKGYVKVKQVGVNPTSIDSVVI
GKDQEVKLQPGQVLHMVNELYPYIVEFEEEAKNPGLETHRKRKRSGNSDSIERDAAQEAEAGTGLEPGSNSGQCSVPLKKGKDAPIK
KESLGHWSQGLKISMQDPKMQVYKDEQVVVIKDKYPKARYHWLVLPWTSISSLKAVAREHLELLKHMHTVGEKVIVDFAGSSKLRFR
LGYHAIPSMSHVHLHVISQDFDSPCLKNKKHWNSFNTEYFLESQAVIEMVQEAGRVTVRDGMPELLKLPLRCHECQQLLPSIPQLKE
HLRKHWTQ
>sp|Q96D31|CRCM1_HUMAN
MHPEPAPPPSRSSPELPPSGGSTTSGSRRSRRRSGDGEPPGAPPPPPSAVTYPDWIGQSYSEVMSLNEHSMQALSWRKLYLSRAKLK
ASSRTSALLSGFAMVAMVEVQLDADHDYPPGLLIAFSACTTVLVAVHLFALMISTCILPNIEAVSNVHNLNSVKESPHERMHRHIEL
AWAFSTVIGTLLFLAEVVLLCWVKFLPLKKQPGQPRPTSKPPASGAAANVSTSGITPGQAAAIASTTIMVPFGLIFIVFAVHFYRSL
VSHKTDRQFQELNELAEFARLQDQLDHRGDHPLTPGSHYA
>sp|P10265|VPK10_HUMAN
WASQVSENRPVCKAIIQGKQFEGLVDTGADVSIIALNQWPKNWPKQKAVTGLVGIGTASEVYQSMEILHCLGPDNQESTVQPMITSI
PLNLWGRDLLQQWGAEITMPAPLYSPTSQKIMTKMGYIPGKGLGKNEDGIKVPVEAKINQEREGIGYPF
>sp|P24310|CX7A1_HUMAN
MQALRVSQALIRSFSSTARNRFQNRVREKQKLFQEDNDIPLYLKGGIVDNILYRVTMTLCLGGTVYSLYSLGWASFPRN
>sp|Q8WV48|CC107_HUMAN

MAGAVSLLGVVGLLLVSALSGVLGDRANPDLRAHPGNAAHPGSGATEPRRRPPLKDQRERTRAGSLPLGALYTAAVAAFVLYKCLQG
KDETAVLHEEASKQQPLQSEQQLAQLTQQLAQTEQHLNNLMAQLDPLFERVTTLAGAQQELLNMKLWTIHELLQDSKPDKDMEASEP
GEGSGGESAGGGDKVSETGTFLISPHTEASRPLPEDFCLKEDEEEIGDSQAWEEPTNWSTETWNLATSWEVGRGLRRRCSQAVAKGP
SHSLGWEGGTTAEGRLKQSLFS
>sp|P14410|SUIS_HUMAN
MARKKFSGLEISLIVLFVIVTIIAIALIVVLATKTPAVDEISDSTSTPATTRVTTNPSDSGKCPNVLNDPVNVRINCIPEQFPTEGI
CAQRGCCWRPWNDSLIPWCFFVDNHGYNVQDMTTTSIGVEAKLNRIPSPTLFGNDINSVLFTTQNQTPNRFRFKITDPNNRRYEVPH
QYVKEFTGPTVSDTLYDVKVAQNPFSIQVIRKSNGKTLFDTSIGPLVYSDQYLQISTRLPSDYIYGIGEQVHKRFRHDLSWKTWPIF
TRDQLPGDNNNNLYGHQTFFMCIEDTSGKSFGVFLMNSNAMEIFIQPTPIVTYRVTGGILDFYILLGDTPEQVVQQYQQLVGLPAMP
AYWNLGFQLSRWNYKSLDVVKEVVRRNREAGIPFDTQVTDIDYMEDKKDFTYDQVAFNGLPQFVQDLHDHGQKYVIILDPAISIGRR
ANGTTYATYERGNTQHVWINESDGSTPIIGEVWPGLTVYPDFTNPNCIDWWANECSIFHQEVQYDGLWIDMNEVSSFIQGSTKGCNV
NKLNYPPFTPDILDKLMYSKTICMDAVQNWGKQYDVHSLYGYSMAIATEQAVQKVFPNKRSFILTRSTFAGSGRHAAHWLGDNTASW
EQMEWSITGMLEFSLFGIPLVGADICGFVAETTEELCRRWMQLGAFYPFSRNHNSDGYEHQDPAFFGQNSLLVKSSRQYLTIRYTLL
PFLYTLFYKAHVFGETVARPVLHEFYEDTNSWIEDTEFLWGPALLITPVLKQGADTVSAYIPDAIWYDYESGAKRPWRKQRVDMYLP
ADKIGLHLRGGYIIPIQEPDVTTTASRKNPLGLIVALGENNTAKGDFFWDDGETKDTIQNGNYILYTFSVSNNTLDIVCTHSSYQEG
TTLAFQTVKILGLTDSVTEVRVAENNQPMNAHSNFTYDASNQVLLIADLKLNLGRNFSVQWNQIFSENERFNCYPDADLATEQKCTQ
RGCVWRTGSSLSKAPECYFPRQDNSYSVNSARYSSMGITADLQLNTANARIKLPSDPISTLRVEVKYHKNDMLQFKIYDPQKKRYEV
PVPLNIPTTPISTYEDRLYDVEIKENPFGIQIRRRSSGRVIWDSWLPGFAFNDQFIQISTRLPSEYIYGFGEVEHTAFKRDLNWNTW
GMFTRDQPPGYKLNSYGFHPYYMALEEEGNAHGVFLLNSNAMDVTFQPTPALTYRTVGGILDFYMFLGPTPEVATKQYHEVIGHPVM
PAYWALGFQLCRYGYANTSEVRELYDAMVAANIPYDVQYTDIDYMERQLDFTIGEAFQDLPQFVDKIRGEGMRYIIILDPAISGNET
KTYPAFERGQQNDVFVKWPNTNDICWAKVWPDLPNITIDKTLTEDEAVNASRAHVAFPDFFRTSTAEWWAREIVDFYNEKMKFDGLW
IDMNEPSSFVNGTTTNQCRNDELNYPPYFPELTKRTDGLHFRTICMEAEQILSDGTSVLHYDVHNLYGWSQMKPTHDALQKTTGKRG
IVISRSTYPTSGRWGGHWLGDNYARWDNMDKSIIGMMEFSLFGMSYTGADICGFFNNSEYHLCTRWMQLGAFYPYSRNHNIANTRRQ
DPASWNETFAEMSRNILNIRYTLLPYFYTQMHEIHANGGTVIRPLLHEFFDEKPTWDIFKQFLWGPAFMVTPVLEPYVQTVNAYVPN
ARWFDYHTGKDIGVRGQFQTFNASYDTINLHVRGGHILPCQEPAQNTFYSRQKHMKLIVAADDNQMAQGSLFWDDGESIDTYERDLY
LSVQFNLNQTTLTSTILKRGYINKSETRLGSLHVWGKGTTPVNAVTLTYNGNKNSLPFNEDTTNMILRIDLTTHNVTLEEPIEINWS
>sp|Q15126|PMVK_HUMAN
MAPLGGAPRLVLLFSGKRKSGKDFVTEALQSRLGADVCAVLRLSGPLKEQYAQEHGLNFQRLLDTSTYKEAFRKDMIRWGEEKRQAD
PGFFCRKIVEGISQPIWLVSDTRRVSDIQWFREAYGAVTQTVRVVALEQSRQQRGWVFTPGVDDAESECGLDNFGDFDWVIENHGVE
QRLEEQLENLIEFIRSRL
>sp|Q96LZ2|MAGBA_HUMAN
MPRGQKSKLRAREKRRQARGGLEDLIDALDILEEEEESPPSASACLKDVFQSSLDGASNNPHGLREAQSTSTSATAASHTRHPEGVN
DQMEERPICTQDLEATDSFPRGPVDEKVIILVHYLLYKYQMKEPITKADMLRNVTQMSKSQFPVILSRASEHLELIFGLDLKEVEPN
KHIYVLVNKLDLGCDAKLSDETGVPKTGLLMTVLGIIFTNGNCVAEEEVWKVFNTMGLYDGIEHFMFGEPRKLLTKDLVKENYLEYQ
QVPNSDPPRYQFLWGPRAHAETSKMKVLEFLAKVNDTAPSEFSNWYTEALQDEEERARARVAAKARVSATAGARSKVKSSKSSQLQ
>sp|P78383|S35B1_HUMAN
MASSSSLVPDRLRLPLCFLGVFVCYFYYGILQEKITRGKYGEGAKQETFTFALTLVFIQCVINAVFAKILIQFFDTARVDRTRSWLY
AACSISYLGAMVSSNSALQFVNYPTQVLGKSCKPIPVMLLGVTLLKKKYPLAKYLCVLLIVAGVALFMYKPKKVVGIEEHTVGYGEL
LLLLSLTLDGLTGVSQDHMRAHYQTGSNHMMLNINLWSTLLLGMGILFTGELWEFLSFAERYPAIIYNILLFGLTSALGQSFIFMTV
VYFGPLTCSIITTTRKFFTILASVILFANPISPMQWVGTVLVFLGLGLDAKFGKGAKKTSH
>sp|Q6NT52|CGB2_HUMAN
MSTFPVLAEDIPLRERHVKGRVDPHFRAPKMEMFQGLLLLLLLSMGGTWASKEPLRPRCRPINATLAVEKEGCPVCITVNTTICAGY
CPTMTRVLQGVLPALPQVVCNYRDVRFESIRLPGCPRGVNPVVSYAVALSCQCALCRRSTTDCGGPKDHPLTCDDPRFQDSSSSKAP
PPSLPSPSRLPGPSDTPILPQ

>sp|Q9UPQ4|TRI35_HUMAN
MERSPDVSPGPSRSFKEELLCAVCYDPFRDAVTLRCGHNFCRGCVSRCWEVQVSPTCPVCKDRASPADLRTNHTLNNLVEKLLREEA
EGARWTSYRFSRVCRLHRGQLSLFCLEDKELLCCSCQADPRHQGHRVQPVKDTAHDFRAKCRNMEHALREKAKAFWAMRRSYEAIAK
HNQVEAAWLEGRIRQEFDKLREFLRVEEQAILDAMAEETRQKQLLADEKMKQLTEETEVLAHEIERLQMEMKEDDVSFLMKHKSRKR
RLFCTMEPEPVQPGMLIDVCKYLGSLQYRVWKKMLASVESVPFSFDPNTAAGWLSVSDDLTSVTNHGYRVQVENPERFSSAPCLLGS
RVFSQGSHAWEVALGGLQSWRVGVVRVRQDSGAEGHSHSCYHDTRSGFWYVCRTQGVEGDHCVTSDPATSPLVLAIPRRLRVELECE
EGELSFYDAERHCHLYTFHARFGEVRPYFYLGGARGAGPPEPLRICPLHISVKEELDG
>sp|Q9HBM0|VEZA_HUMAN
MTPEFDEEVVFENSPLYQYLQDLGHTDFEICSSLSPKTEKCTTEGQQKPPTRVLPKQGILLKVAETIKSWIFFSQCNKKDDLLHKLD
IGFRLDSLHTILQQEVLLQEDVELIELLDPSILSAGQSQQQENGHLPTLCSLATPNIWDLSMLFAFISLLVMLPTWWIVSSWLVWGV
ILFVYLVIRALRLWRTAKLQVTLKKYSVHLEDMATNSRAFTNLVRKALRLIQETEVISRGFTLVSAACPFNKAGQHPSQHLIGLRKA
VYRTLRANFQAARLATLYMLKNYPLNSESDNVTNYICVVPFKELGLGLSEEQISEEEAHNFTDGFSLPALKVLFQLWVAQSSEFFRR
LALLLSTANSPPGPLLTPALLPHRILSDVTQGLPHAHSACLEELKRSYEFYRYFETQHQSVPQCLSKTQQKSRELNNVHTAVRSLQL
HLKALLNEVIILEDELEKLVCTKETQELVSEAYPILEQKLKLIQPHVQASNNCWEEAISQVDKLLRRNTDKKGKPEIACENPHCTVV
PLKQPTLHIADKDPIPEEQELEAYVDDIDIDSDFRKDDFYYLSQEDKERQKREHEESKRVLQELKSVLGFKASEAERQKWKQLLFSD
HAVLKSLSPVDPVEPISNSEPSMNSDMGKVSKNDTEEESNKSATTDNEISRTEYLCENSLEGKNKDNSSNEVFPQGAEERMCYQCES
EDEPQADGSGLTTAPPTPRDSLQPSIKQRLARLQLSPDFTFTAGLAAEVAARSLSFTTMQEQTFGGEEEEQIIEENKNEIEEK
>sp|P59861|DB131_HUMAN
MRVLFFVFGVLSLMFTVPPARSFISNDECPSEYYHCRLKCNADEHAIRYCADFSICCKLKIIEIDGQKKW
>sp|Q9UJZ1|STML2_HUMAN
MLARAARGTGALLLRGSLLASGRAPRRASSGLPRNTVVLFVPQQEAWVVERMGRFHRILEPGLNILIPVLDRIRYVQSLKEIVINVP
EQSAVTLDNVTLQIDGVLYLRIMDPYKASYGVEDPEYAVTQLAQTTMRSELGKLSLDKVFRERESLNASIVDAINQAADCWGIRCLR
YEIKDIHVPPRVKESMQMQVEAERRKRATVLESEGTRESAINVAEGKKQAQILASEAEKAEQINQAAGEASAVLAKAKAKAEAIRIL
AAALTQHNGDAAASLTVAEQYVSAFSKLAKDSNTILLPSNPGDVTSMVAQAMGVYGALTKAPVPGTPDSLSSGSSRDVQGTDASLDE
ELDRVKMS
>sp|Q8N5C7|DTWD1_HUMAN
MSLNPPIFLKRSEENSSKFVETKQSQTTSIASEDPLQNLCLASQEVLQKAQQSGRSKCLKCGGSRMFYCYTCYVPVENVPIEQIPLV
KLPLKIDIIKHPNETDGKSTAIHAKLLAPEFVNIYTYPCIPEYEEKDHEVALIFPGPQSISIKDISFHLQKRIQNNVRGKNDDPDKP
SFKRKRTEEQEFCDLNDSKCKGTTLKKIIFIDSTWNQTNKIFTDERLQGLLQVELKTRKTCFWRHQKGKPDTFLSTIEAIYYFLVDY
HTDILKEKYRGQYDNLLFFYSFMYQLIKNAKCSGDKETGKLTH
>sp|Q3MHD2|LSM12_HUMAN
MAAPPGEYFSVGSQVSCRTCQEQRLQGEVVAFDYQSKMLALKCPSSSGKPNHADILLINLQYVSEVEIINDRTETPPPLASLNVSKL
ASKARTEKEEKLSQAYAISAGVSLEGQQLFQTIHKTIKDCKWQEKNIVVMEEVVITPPYQVENCKGKEGSALSHVRKIVEKHFRDVE
SQKILQRSQAQQPQKEAALSS
>sp|Q9H175|CSRN2_HUMAN
MDAFTGSGLKRKFDDVDVGSSVSNSDDEISSSDSADSCDSLNPPTTASFTPTSILKRQKQLRRKNVRFDQVTVYYFARRQGFTSVPS
QGGSSLGMAQRHNSVRSYTLCEFAQEQEVNHREILREHLKEEKLHAKKMKLTKNGTVESVEADGLTLDDVSDEDIDVENVEVDDYFF
LQPLPTKRRRALLRASGVHRIDAEEKQELRAIRLSREECGCDCRLYCDPEACACSQAGIKCQVDRMSFPCGCSRDGCGNMAGRIEFN
PIRVRTHYLHTIMKLELESKRQVSRPAAPDEEPSPTASCSLTGAQGSETQDFQEFIAENETAVMHLQSAEELERLKAEEDSSGSSAS
LDSSIESLGVCILEEPLAVPEELCPGLTAPILIQAQLPPGSSVLCFTENSDHPTASTVNSPSYLNSGPLVYYQVEQRPVLGVKGEPG
TEEGSASFPKEKDLNVFSLPVTSLVACSSTDPAALCKSEVGKTPTLEALLPEDCNPEEPENEDFHPSWSPSSLPFRTDNEEGCGMVK
TSQQNEDRPPEDSSLELPLAV
>sp|O00483|NDUA4_HUMAN
MLRQIIGQAKKHPSLIPLFVFIGTGATGATLYLLRLALFNPDVCWDRNNPEPWNKLGPNDQYKFYSVNVDYSKLKKERPDF

>sp|O43526|KCNQ2_HUMAN
MVQKSRNGGVYPGPSGEKKLKVGFVGLDPGAPDSTRDGALLIAGSEAPKRGSILSKPRAGGAGAGKPPKRNAFYRKLQNFLYNVLER
PRGWAFIYHAYVFLLVFSCLVLSVFSTIKEYEKSSEGALYILEIVTIVVFGVEYFVRIWAAGCCCRYRGWRGRLKFARKPFCVIDIM
VLIASIAVLAAGSQGNVFATSALRSLRFLQILRMIRMDRRGGTWKLLGSVVYAHSKELVTAWYIGFLCLILASFLVYLAEKGENDHF
DTYADALWWGLITLTTIGYGDKYPQTWNGRLLAATFTLIGVSFFALPAGILGSGFALKVQEQHRQKHFEKRRNPAAGLIQSAWRFYA
TNLSRTDLHSTWQYYERTVTVPMYSSQTQTYGASRLIPPLNQLELLRNLKSKSGLAFRKDPPPEPSPSKGSPCRGPLCGCCPGRSSQ
KVSLKDRVFSSPRGVAAKGKGSPQAQTVRRSPSADQSLEDSPSKVPKSWSFGDRSRARQAFRIKGAASRQNSEEASLPGEDIVDDKS
CPCEFVTEDLTPGLKVSIRAVCVMRFLVSKRKFKESLRPYDVMDVIEQYSAGHLDMLSRIKSLQSRVDQIVGRGPAITDKDRTKGPA
EAELPEDPSMMGRLGKVEKQVLSMEKKLDFLVNIYMQRMGIPPTETEAYFGAKEPEPAPPYHSPEDSREHVDRHGCIVKIVRSSSST
GQKNFSAPPAAPPVQCPPSTSWQPQSHPRQGHGTSPVGDHGSLVRIPPPPAHERSLSAYGGGNRASMEFLRQEDTPGCRPPEGNLRD
SDTSISIPSVDHEELERSFSGFSISQSKENLDALNSCYAAVAPCAKVRPYIAEGESDTDSDLCTPCGPPPRSATGEGPFGDVGWAGP
RK
>sp|Q9UI08|EVL_HUMAN
MSEQSICQARASVMVYDDTSKKWVPIKPGQQGFSRINIYHNTASNTFRVVGVKLQDQQVVINYSIVKGLKYNQATPTFHQWRDARQV
YGLNFASKEEATTFSNAMLFALNIMNSQEGGPSSQRQVQNGPSPDEMDIQRRQVMEQHQQQRQESLERRTSATGPILPPGHPSSAAS
APVSCSGPPPPPPPPVPPPPTGATPPPPPPLPAGGAQGSSHDESSMSGLAAAIAGAKLRRVQRPEDASGGSSPSGTSKSDANRASSG
GGGGGLMEEMNKLLAKRRKAASQSDKPAEKKEDESQMEDPSTSPSPGTRAASQPPNSSEAGRKPWERSNSVEKPVSSILSRTPSVAK
SPEAKSPLQSQPHSRMKPAGSVNDMALDAFDLDRMKQEILEEVVRELHKVKEEIIDAIRQELSGISTT
>sp|Q8IWX8|CHERP_HUMAN
MEMPLPPDDQELRNVIDKLAQFVARNGPEFEKMTMEKQKDNPKFSFLFGGEFYSYYKCKLALEQQQLICKQQTPELEPAATMPPLPQ
PPLAPAAPIPPAQGAPSMDELIQQSQWNLQQQEQHLLALRQEQVTAAVAHAVEQQMQKLLEETQLDMNEFDNLLQPIIDTCTKDAIS
AGKNWMFSNAKSPPHCELMAGHLRNRITADGAHFELRLHLIYLINDVLHHCQRKQARELLAALQKVVVPIYCTSFLAVEEDKQQKIA
RLLQLWEKNGYFDDSIIQQLQSPALGLGQYQATLINEYSSVVQPVQLAFQQQIQTLKTQHEEFVTSLAQQQQQQQQQQQQLQMPQME
AEVKATPPPPAPPPAPAPAPAIPPTTQPDDSKPPIQMPGSSEYEAPGGVQDPAAAGPRGPGPHDQIPPNKPPWFDQPHPVAPWGQQQ
PPEQPPYPHHQGGPPHCPPWNNSHEGMWGEQRGDPGWNGQRDAPWNNQPDAAWNSQFEGPWNSQHEQPPWGGGQREPPFRMQRPPHF
RGPFPPHQQHPQFNQPPHPHNFNRFPPRFMQDDFPPRHPFERPPYPHRFDYPQGDFPAEMGPPHHHPGHRMPHPGINEHPPWAGPQH
PDFGPPPHGFNGQPPHMRRQGPPHINHDDPSLVPNVPYFDLPAGLMAPLVKLEDHEYKPLDPKDIRLPPPMPPSERLLAAVEAFYSP
PSHDRPRNSEGWEQNGLYEFFRAKMRARRRKGQEKRNSGPSRSRSRSKSRGRSSSRSNSRSSKSSGSYSRSRSRSCSRSYSRSRSRS
RSRSRSSRSRSRSQSRSRSKSYSPGRRRRSRSRSPTPPSSAGLGSNSAPPIPDSRLGEENKGHQMLVKMGWSGSGGLGAKEQGIQDP
IKGGDVRDKWDQYKGVGVALDDPYENYRRNKSYSFIARMKARDECK
>sp|Q9BYD5|CNFN_HUMAN
MSYPVTSQPQCATTSCYQTQLSDWHTGLTDCCNDMPVCLCGTFAPLCLACRISDDFGECCCAPYLPGGLHSIRTGMRERYHIQGSVG
HDWAALTFCLPCALCQMARELKIRE
>sp|Q96AT1|K1143_HUMAN
MSKRNQVSYVRPAEPAFLARFKERVGYREGPTVETKRIQPQPPDEDGDHSDKEDEQPQVVVLKKGDLSVEEVMKIKAEIKAAKADEE
PTPADGRIIYRKPVKHPSDEKYSGLTASSKKKKPNEDEVNQDSVKKNSQKQIKNSSLLSFDNEDENE
>sp|Q9BW27|NUP85_HUMAN
MEELDGEPTVTLIPGVNSKKNQMYFDWGPGEMLVCETSFNKKEKSEMVPSCPFIYIIRKDVDVYSQILRKLFNESHGIFLGLQRIDE
ELTGKSRKSQLVRVSKNYRSVIRACMEEMHQVAIAAKDPANGRQFSSQVSILSAMELIWNLCEILFIEVAPAGPLLLHLLDWVRLHV
CEVDSLSADVLGSENPSKHDSFWNLVTILVLQGRLDEARQMLSKEADASPASAGICRIMGDLMRTMPILSPGNTQTLTELELKWQHW
HEECERYLQDSTFATSPHLESLLKIMLGDEAALLEQKELLSNWYHFLVTRLLYSNPTVKPIDLHYYAQSSLDLFLGGESSPEPLDNI
LLAAFEFDIHQVIKECSIALSNWWFVAHLTDLLDHCKLLQSHNLYFGSNMREFLLLEYASGLFAHPSLWQLGVDYFDYCPELGRVSL
ELHIERIPLNTEQKALKVLRICEQRQMTEQVRSICKILAMKAVRNNRLGSALSWSIRAKDAAFATLVSDRFLRDYCERGCFSDLDLI

DNLGPAMMLSDRLTFLGKYREFHRMYGEKRFADAASLLLSLMTSRIAPRSFWMTLLTDALPLLEQKQVIFSAEQTYELMRCLEDLTS
RRPVHGESDTEQLQDDDIETTKVEMLRLSLARNLARAIIREGSLEGS
>sp|P15516|HIS3_HUMAN
MKFFVFALILALMLSMTGADSHAKRHHGYKRKFHEKHHSHRGYRSNYLYDN
>sp|A6NGB7|TM221_HUMAN
MARSYGGRVLAAMTLLGIAAAVLAALGAQLLFQLQAGRAELRGLRAEGLGQELGAGPGLPEDAAGTLLPLAAALAALVLVLGFTCLL
LAALCGHLGAELARGPGPRRSDWFLYDCRLLRHVALGLFCCGISVYLAALSIYALLLFEIETGAAAASILGSGTLVLVAVLTHTLLR
AARAARRGLHELSPPSFEDDLARPAEVSKASPRAQPQQGIHRRTPYSTCPEPGDPFGSMATATAPAALEGGWESSLPASRMHRTLSA
GLGHWDGVTHEMRRMLGHRPGSMGKDSTLV
>sp|Q96G61|NUD11_HUMAN
MKCKPNQTRTYDPEGFKKRAACLCFRSEREDEVLLVSSSRYPDRWIVPGGGMEPEEEPGGAAVREVYEEAGVKGKLGRLLGVFEQNQ
DRKHRTYVYVLTVTELLEDWEDSVSIGRKREWFKVEDAIKVLQCHKPVHAEYLEKLKLGGSPTNGNSMAPSSPDSDP
>sp|Q8TD07|N2DL4_HUMAN
MRRISLTSSPVRLLLFLLLLLIALEIMVGGHSLCFNFTIKSLSRPGQPWCEAQVFLNKNLFLQYNSDNNMVKPLGLLGKKVYATSTW
GELTQTLGEVGRDLRMLLCDIKPQIKTSDPSTLQVEMFCQREAERCTGASWQFATNGEKSLLFDAMNMTWTVINHEASKIKETWKKD
RGLEKYFRKLSKGDCDHWLREFLGHWEAMPEPTVSPVNASDIHWSSSSLPDRWIILGAFILLVLMGIVLICVWWQNGEWQAGLWPLR
TS
>sp|Q9HB19|PKHA2_HUMAN
MPYVDRQNRICGFLDIEEHENSGKFLRRYFILDTQANCLLWYMDNPQNLAMGAGAVGALQLTYISKVSIATPKQKPKTPFCFVINAL
SQRYFLQANDQKDMKDWVEALNQASKITVPKGGGLPMTTEVLKSLAAPPALEKKPQVAYKTEIIGGVVVHTPISQNGGDGQEGSEPG
SHTILRRSQSYIPTSGCRASTGPPLIKSGYCVKQGNVRKSWKRRFFALDDFTICYFKCEQDREPLRTIFLKDVLKTHECLVKSGDLL
MRDNLFEIITSSRTFYVQADSPEDMHSWIKEIGAAVQALKCHPRETSFSRSISLTRPGSSSLSSGPNSILCRGRPPLEEKKALCKAP
SVASSWQPWTPVPQAGEKLLPPGDTSEDSLFTPRPGEGAPPGVLPSSRIRHRSEPQHPKEKPFMFNLDDENIRTSDV
>sp|Q6UWH4|F198B_HUMAN
MTCPDKPGQLINWFICSLCVPRVRKLWSSRRPRTRRNLLLGTACAIYLGFLVSQVGRASLQHGQAAEKGPHRSRDTAEPSFPEIPLD
GTLAPPESQGNGSTLQPNVVYITLRSKRSKPANIRGTVKPKRRKKHAVASAAPGQEALVGPSLQPQEAAREADAVAPGYAQGANLVK
IGERPWRLVRGPGVRAGGPDFLQPSSRESNIRIYSESAPSWLSKDDIRRMRLLADSAVAGLRPVSSRSGARLLVLEGGAPGAVLRCG
PSPCGLLKQPLDMSEVFAFHLDRILGLNRTLPSVSRKAEFIQDGRPCPIILWDASLSSASNDTHSSVKLTWGTYQQLLKQKCWQNGR
VPKPESGCTEIHHHEWSKMALFDFLLQIYNRLDTNCCGFRPRKEDACVQNGLRPKCDDQGSAALAHIIQRKHDPRHLVFIDNKGFFD
RSEDNLNFKLLEGIKEFPASAVSVLKSQHLRQKLLQSLFLDKVYWESQGGRQGIEKLIDVIEHRAKILITYINAHGVKVLPMNE
>sp|Q9BT17|MTG1_HUMAN
MRLTPRALCSAAQAAWRENFPLCGRDVARWFPGHMAKGLKKMQSSLKLVDCIIEVHDARIPLSGRNPLFQETLGLKPHLLVLNKMDL
ADLTEQQKIMQHLEGEGLKNVIFTNCVKDENVKQIIPMVTELIGRSHRYHRKENLEYCIMVIGVPNVGKSSLINSLRRQHLRKGKAT
RVGGEPGITRAVMSKIQVSERPLMFLLDTPGVLAPRIESVETGLKLALCGTVLDHLVGEETMADYLLYTLNKHQRFGYVQHYGLGSA
CDNVERVLKSVAVKLGKTQKVKVLTGTGNVNIIQPNYPAAARDFLQTFRRGLLGSVMLDLDVLRGHPPAETLP
>sp|Q8N5G0|SMI20_HUMAN
MSRNLRTALIFGGFISLIGAAFYPIYFRPLMRLEEYKKEQAINRAGIVQEDVQPPGLKVWSDPFGRK
>sp|P55201|BRPF1_HUMAN
MGVDFDVKTFCHNLRATKPPYECPVETCRKVYKSYSGIEYHLYHYDHDNPPPPQQTPLRKHKKKGRQSRPANKQSPSPSEVSQSPGR
EVMSYAQAQRMVEVDLHGRVHRISIFDNLDVVSEDEEAPEEAPENGSNKENTETPAATPKSGKHKNKEKRKDSNHHHHHNVSASTTP
KLPEVVYRELEQDTPDAPPRPTSYYRYIEKSAEELDEEVEYDMDEEDYIWLDIMNERRKTEGVSPIPQEIFEYLMDRLEKESYFESH
NKGDPNALVDEDAVCCICNDGECQNSNVILFCDMCNLAVHQECYGVPYIPEGQWLCRRCLQSPSRAVDCALCPNKGGAFKQTDDGRW
AHVVCALWIPEVCFANTVFLEPIDSIEHIPPARWKLTCYICKQRGSGACIQCHKANCYTAFHVTCAQQAGLYMKMEPVRETGANGTS
FSVRKTAYCDIHTPPGSARRLPALSHSEGEEDEDEEEDEGKGWSSEKVKKAKAKSRIKMKKARKILAEKRAAAPVVSVPCIPPHRLS

KITNRLTIQRKSQFMQRLHSYWTLKRQSRNGVPLLRRLQTHLQSQRNCDQVGRDSEDKNWALKEQLKSWQRLRHDLERARLLVELIR
KREKLKRETIKVQQIAMEMQLTPFLILLRKTLEQLQEKDTGNIFSEPVPLSEVPDYLDHIKKPMDFFTMKQNLEAYRYLNFDDFEED
FNLIVSNCLKYNAKDTIFYRAAVRLREQGGAVLRQARRQAEKMGIDFETGMHIPHSLAGDEATHHTEDAAEEERLVLLENQKHLPVE
EQLKLLLERLDEVNASKQSVGRSRRAKMIKKEMTALRRKLAHQRETGRDGPERHGPSSRGSLTPHPAACDKDGQTDSAAEESSSQET
SKGLGPNMSSTPAHEVGRRTSVLFSKKNPKTAGPPKRPGRPPKNRESQMTPSHGGSPVGPPQLPIMSSLRQRKRGRSPRPSSSSDSD
SDKSTEDPPMDLPANGFSGGNQPVKKSFLVYRNDCSLPRSSSDSESSSSSSSSAASDRTSTTPSKQGRGKPSFSRGTFPEDSSEDTS
GTENEAYSVGTGRGVGHSMVRKSLGRGAGWLSEDEDSPLDALDLVWAKCRGYPSYPALIIDPKMPREGMFHHGVPIPVPPLEVLKLG
EQMTQEAREHLYLVLFFDNKRTWQWLPRTKLVPLGVNQDLDKEKMLEGRKSNIRKSVQIAYHRALQHRSKVQGEQSSETSDSD
>sp|Q86WI3|NLRC5_HUMAN
MDPVGLQLGNKNLWSCLVRLLTKDPEWLNAKMKFFLPNTDLDSRNETLDPEQRVILQLNKLHVQGSDTWQSFIHCVCMQLEVPLDLE
VLLLSTFGYDDGFTSQLGAEGKSQPESQLHHGLKRPHQSCGSSPRRKQCKKQQLELAKKYLQLLRTSAQQRYRSQIPGSGQPHAFHQ
VYVPPILRRATASLDTPEGAIMGDVKVEDGADVSISDLFNTRVNKGPRVTVLLGKAGMGKTTLAHRLCQKWAEGHLNCFQALFLFEF
RQLNLITRFLTPSELLFDLYLSPESDHDTVFQYLEKNADQVLLIFDGLDEALQPMGPDGPGPVLTLFSHLCNGTLLPGCRVMATSRP
GKLPACLPAEAAMVHMLGFDGPRVEEYVNHFFSAQPSREGALVELQTNGRLRSLCAVPALCQVACLCLHHLLPDHAPGQSVALLPNM
TQLYMQMVLALSPPGHLPTSSLLDLGEVALRGLETGKVIFYAKDIAPPLIAFGATHSLLTSFCVCTGPGHQQTGYAFTHLSLQEFLA
ALHLMASPKVNKDTLTQYVTLHSRWVQRTKARLGLSDHLPTFLAGLASCTCRPFLSHLAQGNEDCVGAKQAAVVQVLKKLATRKLTG
PKVVELCHCVDETQEPELASLTAQSLPYQLPFHNFPLTCTDLATLTNILEHREAPIHLDFDGCPLEPHCPEALVGCGQIENLSFKSR
KCGDAFAEALSRSLPTMGRLQMLGLAGSKITARGISHLVKALPLCPQLKEVSFRDNQLSDQVVLNIVEVLPHLPRLRKLDLSSNSIC
VSTLLCLARVAVTCPTVRMLQAREADLIFLLSPPTETTAELQRAPDLQESDGQRKGAQSRSLTLRLQKCQLQVHDAEALIALLQEGP
HLEEVDLSGNQLEDEGCRLMAEAASQLHIARKLDLSNNGLSVAGVHCVLRAVSACWTLAELHISLQHKTVIFMFAQEPEEQKGPQER
AAFLDSLMLQMPSELPLSSRRMRLTHCGLQEKHLEQLCKALGGSCHLGHLHLDFSGNALGDEGAARLAQLLPGLGALQSLNLSENGL
SLDAVLGLVRCFSTLQWLFRLDISFESQHILLRGDKTSRDMWATGSLPDFPAAAKFLGFRQRCIPRSLCLSECPLEPPSLTRLCATL
KDCPGPLELQLSCEFLSDQSLETLLDCLPQLPQLSLLQLSQTGLSPKSPFLLANTLSLCPRVKKVDLRSLHHATLHFRSNEEEEGVC
CGRFTGCSLSQEHVESLCWLLSKCKDLSQVDLSANLLGDSGLRCLLECLPQVPISGLLDLSHNSISQESALYLLETLPSCPRVREAS
VNLGSEQSFRIHFSREDQAGKTLRLSECSFRPEHVSRLATGLSKSLQLTELTLTQCCLGQKQLAILLSLVGRPAGLFSLRVQEPWAD
RARVLSLLEVCAQASGSVTEISISETQQQLCVQLEFPRQEENPEAVALRLAHCDLGAHHSLLVGQLMETCARLQQLSLSQVNLCEDD
DASSLLLQSLLLSLSELKTFRLTSSCVSTEGLAHLASGLGHCHHLEELDLSNNQFDEEGTKALMRALEGKWMLKRLDLSHLLLNSST
LALLTHRLSQMTCLQSLRLNRNSIGDVGCCHLSEALRAATSLEELDLSHNQIGDAGVQHLATILPGLPELRKIDLSGNSISSAGGVQ
LAESLVLCRRLEELMLGCNALGDPTALGLAQELPQHLRVLHLPFSHLGPGGALSLAQALDGSPHLEEISLAENNLAGGVLRFCMELP
LLRQIDLVSCKIDNQTAKLLTSSFTSCPALEVILLSWNLLGDEAAAELAQVLPQMGRLKRVDLEKNQITALGAWLLAEGLAQGSSIQ
VIRLWNNPIPCDMAQHLKSQEPRLDFAFFDNQPQAPWGT
>sp|O75791|GRAP2_HUMAN
MEAVAKFDFTASGEDELSFHTGDVLKILSNQEEWFKAELGSQEGYVPKNFIDIQFPKWFHEGLSRHQAENLLMGKEVGFFIIRASQS
SPGDFSISVRHEDDVQHFKVMRDNKGNYFLWTEKFPSLNKLVDYYRTNSISRQKQIFLRDRTREDQGHRGNSLDRRSQGGPHLSGAV
GEEIRPSMNRKLSDHPPTLPLQQHQHQPQPPQYAPAPQQLQQPPQQRYLQHHHFHQERRGGSLDINDGHCGTGLGSEMNAALMHRRH
TDPVQLQAAGRVRWARALYDFEALEDDELGFHSGEVVEVLDSSNPSWWTGRLHNKLGLFPANYVAPMTR
>sp|Q16378|PROL4_HUMAN
MLLVLLSVVLLALSSAQSTDNDVNYEDFTFTIPDVEDSSQRPDQGPQRPPPEGLLPRPPGDSGNQDDGPQQRPPKPGGHHRHPPPPP
FQNQQRPPRRGHRQLSLPRFPSVSLQEASSFFQRDRPARHPQEQPLW
>sp|P01011|AACT_HUMAN
MERMLPLLALGLLAAGFCPAVLCHPNSPLDEENLTQENQDRGTHVDLGLASANVDFAFSLYKQLVLKAPDKNVIFSPLSISTALAFL
SLGAHNTTLTEILKGLKFNLTETSEAEIHQSFQHLLRTLNQSSDELQLSMGNAMFVKEQLSLLDRFTEDAKRLYGSEAFATDFQDSA
AAKKLINDYVKNGTRGKITDLIKDLDSQTMMVLVNYIFFKAKWEMPFDPQDTHQSRFYLSKKKWVMVPMMSLHHLTIPYFRDEELSC

TVVELKYTGNASALFILPDQDKMEEVEAMLLPETLKRWRDSLEFREIGELYLPKFSISRDYNLNDILLQLGIEEAFTSKADLSGITG
ARNLAVSQVVHKAVLDVFEEGTEASAATAVKITLLSALVETRTIVRFNRPFLMIIVPTDTQNIFFMSKVTNPKQA
>sp|Q92574|TSC1_HUMAN
MAQQANVGELLAMLDSPMLGVRDDVTAVFKENLNSDRGPMLVNTLVDYYLETSSQPALHILTTLQEPHDKHLLDRINEYVGKAATRL
SILSLLGHVIRLQPSWKHKLSQAPLLPSLLKCLKMDTDVVVLTTGVLVLITMLPMIPQSGKQHLLDFFDIFGRLSSWCLKKPGHVAE
VYLVHLHASVYALFHRLYGMYPCNFVSFLRSHYSMKENLETFEEVVKPMMEHVRIHPELVTGSKDHELDPRRWKRLETHDVVIECAK
ISLDPTEASYEDGYSVSHQISARFPHRSADVTTSPYADTQNSYGCATSTPYSTSRLMLLNMPGQLPQTLSSPSTRLITEPPQATLWS
PSMVCGMTTPPTSPGNVPPDLSHPYSKVFGTTAGGKGTPLGTPATSPPPAPLCHSDDYVHISLPQATVTPPRKEERMDSARPCLHRQ
HHLLNDRGSEEPPGSKGSVTLSDLPGFLGDLASEEDSIEKDKEEAAISRELSEITTAEAEPVVPRGGFDSPFYRDSLPGSQRKTHSA
ASSSQGASVNPEPLHSSLDKLGPDTPKQAFTPIDLPCGSADESPAGDRECQTSLETSIFTPSPCKIPPPTRVGFGSGQPPPYDHLFE
VALPKTAHHFVIRKTEELLKKAKGNTEEDGVPSTSPMEVLDRLIQQGADAHSKELNKLPLPSKSVDWTHFGGSPPSDEIRTLRDQLL
LLHNQLLYERFKRQQHALRNRRLLRKVIKAAALEEHNAAMKDQLKLQEKDIQMWKVSLQKEQARYNQLQEQRDTMVTKLHSQIRQLQ
HDREEFYNQSQELQTKLEDCRNMIAELRIELKKANNKVCHTELLLSQVSQKLSNSESVQQQMEFLNRQLLVLGEVNELYLEQLQNKH
SDTTKEVEMMKAAYRKELEKNRSHVLQQTQRLDTSQKRILELESHLAKKDHLLLEQKKYLEDVKLQARGQLQAAESRYEAQKRITQV
FELEILDLYGRLEKDGLLKKLEEEKAEAAEAAEERLDCCNDGCSDSMVGHNEEASGHNGETKTPRPSSARGSSGSRGGGGSSSSSSE
LSTPEKPPHQRAGPFSSRWETTMGEASASIPTTVGSLPSSKSFLGMKARELFRNKSESQCDEDGMTSSLSESLKTELGKDLGVEAKI
PLNLDGPHPSPPTPDSVGQLHIMDYNETHHEHS
>sp|O76093|FGF18_HUMAN
MYSAPSACTCLCLHFLLLCFQVQVLVAEENVDFRIHVENQTRARDDVSRKQLRLYQLYSRTSGKHIQVLGRRISARGEDGDKYAQLL
VETDTFGSQVRIKGKETEFYLCMNRKGKLVGKPDGTSKECVFIEKVLENNYTALMSAKYSGWYVGFTKKGRPRKGPKTRENQQDVHF
MKRYPKGQPELQKPFKYTTVTKRSRRIRPTHPA
>sp|P37287|PIGA_HUMAN
MACRGGAGNGHRASATLSRVSPGSLYTCRTRTHNICMVSDFFYPNMGGVESHIYQLSQCLIERGHKVIIVTHAYGNRKGIRYLTSGL
KVYYLPLKVMYNQSTATTLFHSLPLLRYIFVRERVTIIHSHSSFSAMAHDALFHAKTMGLQTVFTDHSLFGFADVSSVLTNKLLTVS
LCDTNHIICVSYTSKENTVLRAALNPEIVSVIPNAVDPTDFTPDPFRRHDSITIVVVSRLVYRKGIDLLSGIIPELCQKYPDLNFII
GGEGPKRIILEEVRERYQLHDRVRLLGALEHKDVRNVLVQGHIFLNTSLTEAFCMAIVEAASCGLQVVSTRVGGIPEVLPENLIILC
EPSVKSLCEGLEKAIFQLKSGTLPAPENIHNIVKTFYTWRNVAERTEKVYDRVSVEAVLPMDKRLDRLISHCGPVTGYIFALLAVFN
FLFLIFLRWMTPDSIIDVAIDATGPRGAWTNNYSHSKRGGENNEISETR
>sp|P15407|FOSL1_HUMAN
MFRDFGEPGPSSGNGGGYGGPAQPPAAAQAAQQKFHLVPSINTMSGSQELQWMVQPHFLGPSSYPRPLTYPQYSPPQPRPGVIRALG
PPPGVRRRPCEQISPEEEERRRVRRERNKLAAAKCRNRRKELTDFLQAETDKLEDEKSGLQREIEELQKQKERLELVLEAHRPICKI
PEGAKEGDTGSTSGTSSPPAPCRPVPCISLSPGPVLEPEALHTPTLMTTPSLTPFTPSLVFTYPSTPEPCASAHRKSSSSSGDPSSD
PLGSPTLLAL
>sp|Q96J42|TXD15_HUMAN
MVPAAGRRPPRVMRLLGWWQVLLWVLGLPVRGVEVAEESGRLWSEEQPAHPLQVGAVYLGEEELLHDPMGQDRAAEEANAVLGLDTQ
GDHMVMLSVIPGEAEDKVSSEPSGVTCGAGGAEDSRCNVRESLFSLDGAGAHFPDREEEYYTEPEVAESDAAPTEDSNNTESLKSPK
VNCEERNITGLENFTLKILNMSQDLMDFLNPNGSDCTLVLFYTPWCRFSASLAPHFNSLPRAFPALHFLALDASQHSSLSTRFGTVA
VPNILLFQGAKPMARFNHTDRTLETLKIFIFNQTGIEAKKNVVVTQADQIGPLPSTLIKSVDWLLVFSLFFLISFIMYATIRTESIR
WLIPGQEQEHVE
>sp|O75529|TAF5L_HUMAN
MKRVRTEQIQMAVSCYLKRRQYVDSDGPLKQGLRLSQTAEEMAANLTVQSESGCANIVSAAPCQAEPQQYEVQFGRLRNFLTDSDSQ
HSHEVMPLLYPLFVYLHLNLVQNSPKSTVESFYSRFHGMFLQNASQKDVIEQLQTTQTIQDILSNFKLRAFLDNKYVVRLQEDSYNY
LIRYLQSDNNTALCKVLTLHIHLDVQPAKRTDYQLYASGSSSRSENNGLEPPDMPSPILQNEAALEVLQESIKRVKDGPPSLTTICF
YAFYNTEQLLNTAEISPDSKLLAAGFDNSCIKLWSLRSKKLKSEPHQVDVSRIHLACDILEEEDDEDDNAGTEMKILRGHCGPVYST

RFLADSSGLLSCSEDMSIRYWDLGSFTNTVLYQGHAYPVWDLDISPYSLYFASGSHDRTARLWSFDRTYPLRIYAGHLADVDCVKFH
PNSNYLATGSTDKTVRLWSAQQGNSVRLFTGHRGPVLSLAFSPNGKYLASAGEDQRLKLWDLASGTLYKELRGHTDNITSLTFSPDS
GLIASASMDNSVRVWDIRNTYCSAPADGSSSELVGVYTGQMSNVLSVQFMACNLLLVTGITQENQEH
>sp|Q9NRS4|TMPS4_HUMAN
MLQDPDSDQPLNSLDVKPLRKPRIPMETFRKVGIPIIIALLSLASIIIVVVLIKVILDKYYFLCGQPLHFIPRKQLCDGELDCPLGE
DEEHCVKSFPEGPAVAVRLSKDRSTLQVLDSATGNWFSACFDNFTEALAETACRQMGYSSKPTFRAVEIGPDQDLDVVEITENSQEL
RMRNSSGPCLSGSLVSLHCLACGKSLKTPRVVGVEEASVDSWPWQVSIQYDKQHVCGGSILDPHWVLTAAHCFRKHTDVFNWKVRAG
SDKLGSFPSLAVAKIIIIEFNPMYPKDNDIALMKLQFPLTFSGTVRPICLPFFDEELTPATPLWIIGWGFTKQNGGKMSDILLQASV
QVIDSTRCNADDAYQGEVTEKMMCAGIPEGGVDTCQGDSGGPLMYQSDQWHVVGIVSWGYGCGGPSTPGVYTKVSAYLNWIYNVWKA
EL
>sp|O15040|TCPR2_HUMAN
MASISEPVTFREFCPLYYLLNAIPTKIQKGFRSIVVYLTALDTNGDYIAVGSSIGMLYLYCRHLNQMRKYNFEGKTESITVVKLLSC
FDDLVAAGTASGRVAVFQLVSSLPGRNKQLRRFDVTGIHKNSITALAWSPNGMKLFSGDDKGKIVYSSLDLDQGLCNSQLVLEEPSS
IVQLDYSQKVLLVSTLQRSLLFYTEEKSVRQIGTQPRKSTGKFGACFIPGLCKQSDLTLYASRPGLRLWKADVHGTVQATFILKDAF
AGGVKPFELHPRLESPNSGSCSLPERHLGLVSCFFQEGWVLSWNEYSIYLLDTVNQATVAGLEGSGDIVSVSCTENEIFFLKGDRNI
IRISSRPEGLTSTVRDGLEMSGCSERVHVQQAEKLPGATVSETRLRGSSMASSVASEPRSRSSSLNSTDSGSGLLPPGLQATPELGK
GSQPLSQRFNAISSEDFDQELVVKPIKVKRKKKKKKTEGGSRSTCHSSLESTPCSEFPGDSPQSLNTDLLSMTSSVLGSSVDQLSAE
SPDQESSFNGEVNGVPQENTDPETFNVLEVSGSMPDSLAEEDDIRTEMPHCHHAHGRELLNGAREDVGGSDVTGLGDEPCPADDGPN
STQLPFQEQDSSPGAHDGEDIQPIGPQSTFCEVPLLNSLTVPSSLSWAPSAEQWLPGTRADEGSPVEPSQEQDILTSMEASGHLSTN
LWHAVTDDDTGQKEIPISERVLGSVGGQLTPVSALAASTHKPWLEQPPRDQTLTSSDEEDIYAHGLPSSSSETSVTELGPSCSQQDL
SRLGAEDAGLLKPDQFAESWMGYSGPGYGILSLVVSEKYIWCLDYKGGLFCSALPGAGLRWQKFEDAVQQVAVSPSGALLWKIEQKS
NRAFACGKVTIKGKRHWYEALPQAVFVALSDDTAWIIRTSGDLYLQTGLSVDRPCARAVKVDCPYPLSQITARNNVVWALTEQRALL
YREGVSSFCPEGEQWKCDIVSERQALEPVCITLGDQQTLWALDIHGNLWFRTGIISKKPQGDDDHWWQVSITDYVVFDQCSLFQTII
HATHSVATAAQAPVEKVADKLRMAFWSQQLQCQPSLLGVNNSGVWISSGKNEFHVAKGSLIGTYWNHVVPRGTASATKWAFVLASAA
PTKEGSFLWLCQSSKDLCSVSAQSAQSRPSTVQLPPEAEMRAYAACQDALWALDSLGQVFIRTLSKSCPTGMHWTRLDLSQLGAVKL
TSLACGNQHIWACDSRGGVYFRVGTQPLNPSLMLPAWIMIEPPVQPAGVSLVSVHSSPNDQMLWVLDSRWNVHVRTGITEEMPVGTA
WEHVPGLQACQLALSTRTVWARCPNGDLARRYGVTDKNPAGDYWKKIPGSVSCFTVTASDELWAVGPPGYLLQRLTKTFSHSHGTQK
SSQAAMPHPEDLEDEWEVI
>sp|O95190|OAZ2_HUMAN
MINTQDSSILPLSNCPQLQCCRHIVPGPLWCSDAPHPLSKIPGGRGGGRDPSLSALIYKDEKLTVTQDLPVNDGKPHIVHFQYEVTE
VKVSSWDAVLSSQSLFVEIPDGLLADGSKEGLLALLEFAEEKMKVNYVFICFRKGREDRAPLLKTFSFLGFEIVRPGHPCVPSRPDV
MFMVYPLDQNLSDED
>sp|P13746|1A11_HUMAN
MAVMAPRTLLLLLSGALALTQTWAGSHSMRYFYTSVSRPGRGEPRFIAVGYVDDTQFVRFDSDAASQRMEPRAPWIEQEGPEYWDQE
TRNVKAQSQTDRVDLGTLRGYYNQSEDGSHTIQIMYGCDVGPDGRFLRGYRQDAYDGKDYIALNEDLRSWTAADMAAQITKRKWEAA
HAAEQQRAYLEGRCVEWLRRYLENGKETLQRTDPPKTHMTHHPISDHEATLRCWALGFYPAEITLTWQRDGEDQTQDTELVETRPAG
DGTFQKWAAVVVPSGEEQRYTCHVQHEGLPKPLTLRWELSSQPTIPIVGIIAGLVLLGAVITGAVVAAVMWRRKSSDRKGGSYTQAA
SSDSAQGSDVSLTACKV
>sp|Q13596|SNX1_HUMAN
MASGGGGCSASERLPPPFPGLEPESEGAAGGSEPEAGDSDTEGEDIFTGAAVVSKHQSPKITTSLLPINNGSKENGIHEEQDQEPQD
LFADATVELSLDSTQNNQKKVLAKTLISLPPQEATNSSKPQPTYEELEEEEQEDQFDLTVGITDPEKIGDGMNAYVAYKVTTQTSLP
LFRSKQFAVKRRFSDFLGLYEKLSEKHSQNGFIVPPPPEKSLIGMTKVKVGKEDSSSAEFLEKRRAALERYLQRIVNHPTMLQDPDV
REFLEKEELPRAVGTQTLSGAGLLKMFNKATDAVSKMTIKMNESDIWFEEKLQEVECEEQRLRKLHAVVETLVNHRKELALNTAQFA

KSLAMLGSSEDNTALSRALSQLAEVEEKIEQLHQEQANNDFFLLAELLSDYIRLLAIVRAAFDQRMKTWQRWQDAQATLQKKREAEA
RLLWANKPDKLQQAKDEILEWESRVTQYERDFERISTVVRKEVIRFEKEKSKDFKNHVIKYLETLLYSQQQLAKYWEAFLPEAKAIS
>sp|P15813|CD1D_HUMAN
MGCLLFLLLWALLQAWGSAEVPQRLFPLRCLQISSFANSSWTRTDGLAWLGELQTHSWSNDSDTVRSLKPWSQGTFSDQQWETLQHI
FRVYRSSFTRDVKEFAKMLRLSYPLELQVSAGCEVHPGNASNNFFHVAFQGKDILSFQGTSWEPTQEAPLWVNLAIQVLNQDKWTRE
TVQWLLNGTCPQFVSGLLESGKSELKKQVKPKAWLSRGPSPGPGRLLLVCHVSGFYPKPVWVKWMRGEQEQQGTQPGDILPNADETW
YLRATLDVVAGEAAGLSCRVKHSSLEGQDIVLYWGGSYTSMGLIALAVLACLLFLLIVGFTSRFKRQTSYQGVL
>sp|Q9ULD5|ZN777_HUMAN
MENQRSSPLSFPSVPQEETLRQAPAGLPRETLFQSRVLPPKEIPSLSPTIPRQGSLPQTSSAPKQETSGRMPHVLQKGPSLLCSAAS
EQETSLQGPLASQEGTQYPPPAAAEQEVSLLSHSPHHQEAPVHSPEAPEKDPLTLSPTVPETDMDPLLQSPVSQKDTPFQISSAVQK
EQPLPTAEITRLAVWAAVQAVERKLEAQAMRLLTLEGRTGTNEKKIADCEKTAVEFANHLESKWVVLGTLLQEYGLLQRRLENMENL
LKNRNFWILRLPPGSNGEVPKVPVTFDDVAVHFSEQEWGNLSEWQKELYKNVMRGNYESLVSMDYAISKPDLMSQMERGERPTMQEQ
EDSEEGETPTDPSAAHDGIVIKIEVQTNDEGSESLETPEPLMGQVEEHGFQDSELGDPCGEQPDLDMQEPENTLEESTEGSSEFSEL
KQMLVQQRNCTEGIVIKTEEQDEEEEEEEEDELPQHLQSLGQLSGRYEASMYQTPLPGEMSPEGEESPPPLQLGNPAVKRLAPSVHG
ERHLSENRGASSQQQRNRRGERPFTCMECGKSFRLKINLIIHQRNHIKEGPYECAECEISFRHKQQLTLHQRIHRVRGGCVSPERGP
TFNPKHALKPRPKSPSSGSGGGGPKPYKCPECDSSFSHKSSLTKHQITHTGERPYTCPECKKSFRLHISLVIHQRVHAGKHEVSFIC
SLCGKSFSRPSHLLRHQRTHTGERPFKCPECEKSFSEKSKLTNHCRVHSRERPPPRWSSSFCSL
>sp|P35221|CTNA1_HUMAN
MTAVHAGNINFKWDPKSLEIRTLAVERLLEPLVTQVTTLVNTNSKGPSNKKRGRSKKAHVLAASVEQATENFLEKGDKIAKESQFLK
EELVAAVEDVRKQGDLMKAAAGEFADDPCSSVKRGNMVRAARALLSAVTRLLILADMADVYKLLVQLKVVEDGILKLRNAGNEQDLG
IQYKALKPEVDKLNIMAAKRQQELKDVGHRDQMAAARGILQKNVPILYTASQACLQHPDVAAYKANRDLIYKQLQQAVTGISNAAQA
TASDDASQHQGGGGGELAYALNNFDKQIIVDPLSFSEERFRPSLEERLESIISGAALMADSSCTRDDRRERIVAECNAVRQALQDLL
SEYMGNAGRKERSDALNSAIDKMTKKTRDLRRQLRKAVMDHVSDSFLETNVPLLVLIEAAKNGNEKEVKEYAQVFREHANKLIEVAN
LACSISNNEEGVKLVRMSASQLEALCPQVINAALALAAKPQSKLAQENMDLFKEQWEKQVRVLTDAVDDITSIDDFLAVSENHILED
VNKCVIALQEKDVDGLDRTAGAIRGRAARVIHVVTSEMDNYEPGVYTEKVLEATKLLSNTVMPRFTEQVEAAVEALSSDPAQPMDEN
EFIDASRLVYDGIRDIRKAVLMIRTPEELDDSDFETEDFDVRSRTSVQTEDDQLIAGQSARAIMAQLPQEQKAKIAEQVASFQEEKS
KLDAEVSKWDDSGNDIIVLAKQMCMIMMEMTDFTRGKGPLKNTSDVISAAKKIAEAGSRMDKLGRTIADHCPDSACKQDLLAYLQRI
ALYCHQLNICSKVKAEVQNLGGELVVSGVDSAMSLIQAAKNLMNAVVQTVKASYVASTKYQKSQGMASLNLPAVSWKMKAPEKKPLV
KREKQDETQTKIKRASQKKHVNPVQALSEFKAMDSI
>sp|Q5JT82|KLF17_HUMAN
MYGRPQAEMEQEAGELSRWQAAHQAAQDNENSAPILNMSSSSGSSGVHTSWNQGLPSIQHFPHSAEMLGSPLVSVEAPGQNVNEGGP
QFSMPLPERGMSYCPQATLTPSRMIYCQRMSPPQQEMTIFSGPQLMPVGEPNIPRVARPFGGNLRMPPNGLPVSASTGIPIMSHTGN
PPVPYPGLSTVPSDETLLGPTVPSTEAQAVLPSMAQMLPPQDAHDLGMPPAESQSLLVLGSQDSLVSQPDSQEGPFLPEQPGPAPQT
VEKNSRPQEGTGRRGSSEARPYCCNYENCGKAYTKRSHLVSHQRKHTGERPYSCNWESCSWSFFRSDELRRHMRVHTRYRPYKCDQC
SREFMRSDHLKQHQKTHRPGPSDPQANNNNGEQDSPPAAGP
>sp|Q9HDB9|GAK5_HUMAN
MGQTKSKTKSKYASYLSFIKILLKRGGVRVSTKNLIKLFQIIEQFCPWFPEQGTLDLKDWKRIGEELKQAGRKGNIIPLTVWNDWAI
IKAALEPFQTKEDSVSVSDAPGSCVIDCNEKTGRKSQKETESLHCEYVTEPVMAQSTQNVDYNQLQGVIYPETLKLEGKGPELVGPS
ESKPRGPSPLPAGQVPVTLQPQTQVKENKTQPPVAYQYWPPAELQYLPPPESQYGYPGMPPALQGRAPYPQPPTVRLNPTASRSGQG
GTLHAVIDEARKQGDLEAWRFLVILQLVQAGEETQVGAPARAETRCEPFTMKMLKDIKEGVKQYGSNSPYIRTLLDSIAHGNRLTPY
DWESLAKSSLSSSQYLQFKTWWIDGVQEQVRKNQATKPTVNIDADQLLGTGPNWSTINQQSVMQNEAIEQVRAICLRAWGKIQDPGT
AFPINSIRQGSKEPYPDFVARLQDAAQKSITDDNARKVIVELMAYENANPECQSAIKPLKGKVPAGVDVITEYVKACDGIGGAMHKA
MLMAQAMRGLTLGGQVRTFGKKCYNCGQIGHLKRSCPVLNKQNIINQAITAKNKKPSGLCPKCGKGKHWANQCHSKFDKDGQPLSGN
RKRGQPQAPQQTGAFPVQLFVPQGFQGQQPLQKIPPLQGVSQLQQSNSCPAPQQAAPQ

>sp|Q9NWQ8|PHAG1_HUMAN
MGPAGSLLGSGQMQITLWGSLAAVAIFFVITFLIFLCSSCDREKKPRQHSGDHENLMNVPSDKEMFSRSVTSLATDAPASSEQNGAL
TNGDILSEDSTLTCMQHYEEVQTSASDLLDSQDSTGKPKCHQSRELPRIPPESAVDTMLTARSVDGDQGLGMEGPYEVLKDSSSQEN
MVEDCLYETVKEIKEVAAAAHLEKGHSGKAKSTSASKELPGPQTEGKAEFAEYASVDRNKKCRQSVNVESILGNSCDPEEEAPPPVP
VKLLDENENLQEKEGGEAEESATDTTSETNKRFSSLSYKSREEDPTLTEEEISAMYSSVNKPGQLVNKSGQSLTVPESTYTSIQGDP
QRSPSSCNDLYATVKDFEKTPNSTLPPAGRPSEEPEPDYEAIQTLNREEEKATLGTNGHHGLVPKENDYESISDLQQGRDITRL
>sp|Q9Y6X3|SCC4_HUMAN
MAAQAAAAAQAAAAQAAQAEAADSWYLALLGFAEHFRTSSPPKIRLCVHCLQAVFPFKPPQRIEARTHLQLGSVLYHHTKNSEQARS
HLEKAWLISQQIPQFEDVKFEAASLLSELYCQENSVDAAKPLLRKAIQISQQTPYWHCRLLFQLAQLHTLEKDLVSACDLLGVGAEY
ARVVGSEYTRALFLLSKGMLLLMERKLQEVHPLLTLCGQIVENWQGNPIQKESLRVFFLVLQVTHYLDAGQVKSVKPCLKQLQQCIQ
TISTLHDDEILPSNPADLFHWLPKEHMCVLVYLVTVMHSMQAGYLEKAQKYTDKALMQLEKLKMLDCSPILSSFQVILLEHIIMCRL
VTGHKATALQEISQVCQLCQQSPRLFSNHAAQLHTLLGLYCVSVNCMDNAEAQFTTALRLTNHQELWAFIVTNLASVYIREGNRHQE
VLYSLLERINPDHSFPVSSHCLRAAAFYVRGLFSFFQGRYNEAKRFLRETLKMSNAEDLNRLTACSLVLLGHIFYVLGNHRESNNMV
VPAMQLASKIPDMSVQLWSSALLRDLNKACGNAMDAHEAAQMHQNFSQQLLQDHIEACSLPEHNLITWTDGPPPVQFQAQNGPNTSL
ASLL
>sp|Q7L211|ABHDD_HUMAN
MEKSWMLWNFVERWLIALASWSWALCRISLLPLIVTFHLYGGIILLLLIFISIAGILYKFQDVLLYFPEQPSSSRLYVPMPTGIPHE
NIFIRTKDGIRLNLILIRYTGDNSPYSPTIIYFHGNAGNIGHRLPNALLMLVNLKVNLLLVDYRGYGKSEGEASEEGLYLDSEAVLD
YVMTRPDLDKTKIFLFGRSLGGAVAIHLASENSHRISAIMVENTFLSIPHMASTLFSFFPMRYLPLWCYKNKFLSYRKISQCRMPSL
FISGLSDQLIPPVMMKQLYELSPSRTKRLAIFPDGTHNDTWQCQGYFTALEQFIKEVVKSHSPEEMAKTSSNVTII
>sp|Q01955|CO4A3_HUMAN
MSARTAPRPQVLLLPLLLVLLAAAPAASKGCVCKDKGQCFCDGAKGEKGEKGFPGPPGSPGQKGFTGPEGLPGPQGPKGFPGLPGLT
GSKGVRGISGLPGFSGSPGLPGTPGNTGPYGLVGVPGCSGSKGEQGFPGLPGTLGYPGIPGAAGLKGQKGAPAKEEDIELDAKGDPG
LPGAPGPQGLPGPPGFPGPVGPPGPPGFFGFPGAMGPRGPKGHMGERVIGHKGERGVKGLTGPPGPPGTVIVTLTGPDNRTDLKGEK
GDKGAMGEPGPPGPSGLPGESYGSEKGAPGDPGLQGKPGKDGVPGFPGSEGVKGNRGFPGLMGEDGIKGQKGDIGPPGFRGPTEYYD
TYQEKGDEGTPGPPGPRGARGPQGPSGPPGVPGSPGSSRPGLRGAPGWPGLKGSKGERGRPGKDAMGTPGSPGCAGSPGLPGSPGPP
GPPGDIVFRKGPPGDHGLPGYLGSPGIPGVDGPKGEPGLLCTQCPYIPGPPGLPGLPGLHGVKGIPGRQGAAGLKGSPGSPGNTGLP
GFPGFPGAQGDPGLKGEKGETLQPEGQVGVPGDPGLRGQPGRKGLDGIPGTPGVKGLPGPKGELALSGEKGDQGPPGDPGSPGSPGP
AGPAGPPGYGPQGEPGLQGTQGVPGAPGPPGEAGPRGELSVSTPVPGPPGPPGPPGHPGPQGPPGIPGSLGKCGDPGLPGPDGEPGI
PGIGFPGPPGPKGDQGFPGTKGSLGCPGKMGEPGLPGKPGLPGAKGEPAVAMPGGPGTPGFPGERGNSGEHGEIGLPGLPGLPGTPG
NEGLDGPRGDPGQPGPPGEQGPPGRCIEGPRGAQGLPGLNGLKGQQGRRGKTGPKGDPGIPGLDRSGFPGETGSPGIPGHQGEMGPL
GQRGYPGNPGILGPPGEDGVIGMMGFPGAIGPPGPPGNPGTPGQRGSPGIPGVKGQRGTPGAKGEQGDKGNPGPSEISHVIGDKGEP
GLKGFAGNPGEKGNRGVPGMPGLKGLKGLPGPAGPPGPRGDLGSTGNPGEPGLRGIPGSMGNMGMPGSKGKRGTLGFPGRAGRPGLP
GIHGLQGDKGEPGYSEGTRPGPPGPTGDPGLPGDMGKKGEMGQPGPPGHLGPAGPEGAPGSPGSPGLPGKPGPHGDLGFKGIKGLLG
PPGIRGPPGLPGFPGSPGPMGIRGDQGRDGIPGPAGEKGETGLLRAPPGPRGNPGAQGAKGDRGAPGFPGLPGRKGAMGDAGPRGPT
GIEGFPGPPGLPGAIIPGQTGNRGPPGSRGSPGAPGPPGPPGSHVIGIKGDKGSMGHPGPKGPPGTAGDMGPPGRLGAPGTPGLPGP
RGDPGFQGFPGVKGEKGNPGFLGSIGPPGPIGPKGPPGVRGDPGTLKIISLPGSPGPPGTPGEPGMQGEPGPPGPPGNLGPCGPRGK
PGKDGKPGTPGPAGEKGNKGSKGEPGPAGSDGLPGLKGKRGDSGSPATWTTRGFVFTRHSQTTAIPSCPEGTVPLYSGFSFLFVQGN
QRAHGQDLGTLGSCLQRFTTMPFLFCNVNDVCNFASRNDYSYWLSTPALMPMNMAPITGRALEPYISRCTVCEGPAIAIAVHSQTTD
IPPCPHGWISLWKGFSFIMFTSAGSEGTGQALASPGSCLEEFRASPFLECHGRGTCNYYSNSYSFWLASLNPERMFRKPIPSTVKAG
ELEKIISRCQVCMKKRH
>sp|P27361|MK03_HUMAN
MAAAAAQGGGGGEPRRTEGVGPGVPGEVEMVKGQPFDVGPRYTQLQYIGEGAYGMVSSAYDHVRKTRVAIKKISPFEHQTYCQRTLR
EIQILLRFRHENVIGIRDILRASTLEAMRDVYIVQDLMETDLYKLLKSQQLSNDHICYFLYQILRGLKYIHSANVLHRDLKPSNLLI

NTTCDLKICDFGLARIADPEHDHTGFLTEYVATRWYRAPEIMLNSKGYTKSIDIWSVGCILAEMLSNRPIFPGKHYLDQLNHILGIL
GSPSQEDLNCIINMKARNYLQSLPSKTKVAWAKLFPKSDSKALDLLDRMLTFNPNKRITVEEALAHPYLEQYYDPTDEPVAEEPFTF
AMELDDLPKERLKELIFQETARFQPGVLEAP
>sp|Q5M9Q1|NKAPL_HUMAN
MPPVSRSSYSEDIVGSRRRRRSSSGSPPSPQSRCSSWDGCSRSHSRGREGLRPPWSELDVGALYPFSRSGSRGRLPRFRNYAFASSW
STSYSGYRYHRHCYAEERQSAEDYEKEESHRQRRLKERERIGELGAPEVWGPSPKFPQLDSDEHTPVEDEEEVTHQKSSSSDSNSEE
HRKKKTSRSRNKKKRKNKSSKRKHRKYSDSDSNSESDTNSDSDDDKKRVKAKKKKKKKHKTKKKKNKKTKKESSDSSCKDSEEDLS
EATWMEQPNVADTMDLIGPEAPIIHTSQDEKPLKYGHALLPGEGAAMAEYVKAGKRIPRRGEIGLTSEEIGSFECSGYVMSGSRHRR
MEAVRLRKENQIYSADEKRALASFNQEERRKRESKILASFREMVHKKTKEKDDK
>sp|Q6UWH6|TX261_HUMAN
MWFMYLLSWLSLFIQVAFITLAVAAGLYYLAELIEEYTVATSRIIKYMIWFSTAVLIGLYVFERFPTSMIGVGLFTNLVYFGLLQTF
PFIMLTSPNFILSCGLVVVNHYLAFQFFAEEYYPFSEVLAYFTFCLWIIPFAFFVSLSAGENVLPSTMQPGDDVVSNYFTKGKRGKR
LGILVVFSFIKEAILPSRQKIY
>sp|P03989|1B27_HUMAN
MRVTAPRTLLLLLWGAVALTETWAGSHSMRYFHTSVSRPGRGEPRFITVGYVDDTLFVRFDSDAASPREEPRAPWIEQEGPEYWDRE
TQICKAKAQTDREDLRTLLRYYNQSEAGSHTLQNMYGCDVGPDGRLLRGYHQDAYDGKDYIALNEDLSSWTAADTAAQITQRKWEAA
RVAEQLRAYLEGECVEWLRRYLENGKETLQRADPPKTHVTHHPISDHEATLRCWALGFYPAEITLTWQRDGEDQTQDTELVETRPAG
DRTFQKWAAVVVPSGEEQRYTCHVQHEGLPKPLTLRWEPSSQSTVPIVGIVAGLAVLAVVVIGAVVAAVMCRRKSSGGKGGSYSQAA
CSDSAQGSDVSLTA
>sp|P17812|PYRG1_HUMAN
MKYILVTGGVISGIGKGIIASSVGTILKSCGLHVTSIKIDPYINIDAGTFSPYEHGEVFVLDDGGEVDLDLGNYERFLDIRLTKDNN
LTTGKIYQYVINKERKGDYLGKTVQVVPHITDAIQEWVMRQALIPVDEDGLEPQVCVIELGGTVGDIESMPFIEAFRQFQFKVKREN
FCNIHVSLVPQPSSTGEQKTKPTQNSVRELRGLGLSPDLVVCRCSNPLDTSVKEKISMFCHVEPEQVICVHDVSSIYRVPLLLEEQG
VVDYFLRRLDLPIERQPRKMLMKWKEMADRYDRLLETCSIALVGKYTKFSDSYASVIKALEHSALAINHKLEIKYIDSADLEPITSQ
EEPVRYHEAWQKLCSAHGVLVPGGFGVRGTEGKIQAIAWARNQKKPFLGVCLGMQLAVVEFSRNVLGWQDANSTEFDPTTSHPVVVD
MPEHNPGQMGGTMRLGKRRTLFQTKNSVMRKLYGDADYLEERHRHRFEVNPVWKKCLEEQGLKFVGQDVEGERMEIVELEDHPFFVG
VQYHPEFLSRPIKPSPPYFGLLLASVGRLSHYLQKGCRLSPRDTYSDRSGSSSPDSEITELKFPSINHD
>sp|O00451|GFRA2_HUMAN
MILANVFCLFFFLDETLRSLASPSSLQGPELHGWRPPVDCVRANELCAAESNCSSRYRTLRQCLAGRDRNTMLANKECQAALEVLQE
SPLYDCRCKRGMKKELQCLQIYWSIHLGLTEGEEFYEASPYEPVTSRLSDIFRLASIFSGTGADPVVSAKSNHCLDAAKACNLNDNC
KKLRSSYISICNREISPTERCNRRKCHKALRQFFDRVPSEYTYRMLFCSCQDQACAERRRQTILPSCSYEDKEKPNCLDLRGVCRTD
HLCRSRLADFHANCRASYQTVTSCPADNYQACLGSYAGMIGFDMTPNYVDSSPTGIVVSPWCSCRGSGNMEEECEKFLRDFTENPCL
RNAIQAFGNGTDVNVSPKGPSFQATQAPRVEKTPSLPDDLSDSTSLGTSVITTCTSVQEQGLKANNSKELSMCFTELTTNIIPGSNK
VIKPNSGPSRARPSAALTVLSVLMLKLAL
>sp|O60826|CCD22_HUMAN
MEEADRILIHSLRQAGTAVPPDVQTLRAFTTELVVEAVVRCLRVINPAVGSGLSPLLPLAMSARFRLAMSLAQACMDLGYPLELGYQ
NFLYPSEPDLRDLLLFLAERLPTDASEDADQPAGDSAILLRAIGSQIRDQLALPWVPPHLRTPKLQHLQGSALQKPFHASRLVVPEL
SSRGEPREFQASPLLLPVPTQVPQPVGRVASLLEHHALQLCQQTGRDRPGDEDWVHRTSRLPPQEDTRAQRQRLQKQLTEHLRQSWG
LLGAPIQARDLGELLQAWGAGAKTGAPKGSRFTHSEKFTFHLEPQAQATQVSDVPATSRRPEQVTWAAQEQELESLREQLEGVNRSI
EEVEADMKTLGVSFVQAESECRHSKLSTAEREQALRLKSRAVELLPDGTANLAKLQLVVENSAQRVIHLAGQWEKHRVPLLAEYRHL
RKLQDCRELESSRRLAEIQELHQSVRAAAEEARRKEEVYKQLMSELETLPRDVSRLAYTQRILEIVGNIRKQKEEITKILSDTKELQ
KEINSLSGKLDRTFAVTDELVFKDAKKDDAVRKAYKYLAALHENCSQLIQTIEDTGTIMREVRDLEEQIETELGKKTLSNLEKIRED
YRALRQENAGLLGRVREA
>sp|Q86YQ8|CPNE8_HUMAN

MDSRYNSTAGIGDLNQLSAAIPATRVEVSVSCRNLLDRDTFSKSDPICVLYVQGVGNKEWREFGRTEVIDNTLNPDFVRKFILDYFF
EERENLRFDLYDVDSKSPNLSKHDFLGQVFCTLGEIVGSQGSRLEKPIVGIPGKKCGTIILTAEELNCCRDAVLMQFCANKLDKKDF
FGKSDPFLVFYRSNEDGSFTICHKTEVVKNTLNPVWQAFKISVRALCNGDYDRTIKVEVYDWDRDGSHDFIGEFTTSYRELSRGQSQ
FNVYEVVNPKKKGKKKKYTNSGTVTLLSFLVETEVSFLDYIKGGTQINFTVAIDFTASNGNPAQPTSLHYMNPYQLNAYGMALKAVG
EIVQDYDSDKMFPALGFGAKLPPDGRISHEFALNGNPQNPYCDGIEGVMEAYYRSLKSVQLYGPTNFAPVINHVARYASSVKDGSQY
FVLLIVTDGVISDMAQTKESIVNASKLPMSIIIVGVGPAEFDAMVELDGDDVRVSSRGKYAERDIVQFVPFRDYIDRSGNHILSMAR
LAKDVLAEIPEQFLSYMRARGIKPSPAPPPYTPPTHVLQTQI
>sp|Q15762|CD226_HUMAN
MDYPTLLLALLHVYRALCEEVLWHTSVPFAENMSLECVYPSMGILTQVEWFKIGTQQDSIAIFSPTHGMVIRKPYAERVYFLNSTMA
SNNMTLFFRNASEDDVGYYSCSLYTYPQGTWQKVIQVVQSDSFEAAVPSNSHIVSEPGKNVTLTCQPQMTWPVQAVRWEKIQPRQID
LLTYCNLVHGRNFTSKFPRQIVSNCSHGRWSVIVIPDVTVSDSGLYRCYLQASAGENETFVMRLTVAEGKTDNQYTLFVAGGTVLLL
LFVISITTIIVIFLNRRRRRERRDLFTESWDTQKAPNNYRSPISTSQPTNQSMDDTREDIYVNYPTFSRRPKTRV
>sp|Q99457|NP1L3_HUMAN
MAEADFKMVSEPVAHGVAEEEMASSTSDSGEESDSSSSSSSTSDSSSSSSTSGSSSGSGSSSSSSGSTSSRSRLYRKKRVPEPSRRA
RRAPLGTNFVDRLPQAVRNRVQALRNIQDECDKVDTLFLKAIHDLERKYAELNKPLYDRRFQIINAEYEPTEEECEWNSEDEEFSSD
EEVQDNTPSEMPPLEGEEEENPKENPEVKAEEKEVPKEIPEVKDEEKEVPKEIPEVKAEEKADSKDCMEATPEVKEDPKEVPQVKAD
DKEQPKATEAKARAAVRETHKRVPEERLQDSVDLKRARKGKPKREDPKGIPDYWLIVLKNVDKLGPMIQKYDEPILKFLSDVSLKFS
KPGQPVSYTFEFHFLPNPYFRNEVLVKTYIIKAKPDHNDPFFSWGWEIEDCKGCKIDWRRGKDVTVTTTQSRTTATGEIEIQPRVVP
NASFFNFFSPPEIPMIGKLEPREDAILDEDFEIGQILHDNVILKSIYYYTGEVNGTYYQFGKHYGNKKYRK
>sp|Q96EW2|HBAP1_HUMAN
MAAGSEATTPVIVAAGAGGEEGEHVKPFKPEKAKEIIMSLQQPAIFCNMVFDWPARHWNAKYLSQVLHGKQIRFRMGMKSMSTVPQF
ETTCNYVEATLEEFLTWNCDQSSISGPFRDYDHSKFWAYADYKYFVSLFEDKTDLFQDVKWSDFGFPGRNGQESTLWIGSLGAHTPC
HLDSYGCNLVFQVQGRKRWHLFPPEDTPFLYPTRIPYEESSVFSKINVVNPDLKRFPQFRKAQRHAVTLSPGQVLFVPRHWWHYVES
IDPVTVSINSWIELEEDHLARVEEAITRMLVCALKTAENPQNTRAWLNPTEVEETSHAVNCCYLNAAVSAFFDRCRTSEVVEIQALR
TDGEHMKKEELNVCNHMEVGQTGSQNLTTGTDKPEAASPFGPDLVPVAQRSEEPPSERGGIFGSDGKDFVDKDGEHFGKLHCAKRQQ
IMSNSENAIEEQIASNTTTTPQTFISTDDLLDCLVNPQVTRIVAQLLIQGRSL
>sp|P01241|SOMA_HUMAN
MATGSRTSLLLAFGLLCLPWLQEGSAFPTIPLSRLFDNAMLRAHRLHQLAFDTYQEFEEAYIPKEQKYSFLQNPQTSLCFSESIPTP
SNREETQQKSNLELLRISLLLIQSWLEPVQFLRSVFANSLVYGASDSNVYDLLKDLEEGIQTLMGRLEDGSPRTGQIFKQTYSKFDT
NSHNDDALLKNYGLLYCFRKDMDKVETFLRIVQCRSVEGSCGF
>sp|Q6P047|CH074_HUMAN
MALLTPQGVKEVFQLQRPQGRERLRRLLNWEEFDEQRDSRRSILLDTLYESIIFAVGKGFPWVEVAQVVKFTEELLRETKGCSITEA
VTILGNKLRDYRGHFNTTHLLALCDYFHHTFIRHYKLYQYVLGQDQQVDLTVAHLEVCMPPHPLPLAEGMDRDLWIHEQQVATLTEA
EAQKRADVLLLKEALRLERENSLQKAFAAAAPAQPGQVLERQELESLICQAVHTQMELLQELLQRQIQNTFAILDLKLQKKTLNLNA
PTPIPPPITSHAGQEEALKPQRASKGKKAKARK
>sp|Q13310|PABP4_HUMAN
MNAAASSYPMASLYVGDLHSDVTEAMLYEKFSPAGPVLSIRVCRDMITRRSLGYAYVNFQQPADAERALDTMNFDVIKGKPIRIMWS
QRDPSLRKSGVGNVFIKNLDKSIDNKALYDTFSAFGNILSCKVVCDENGSKGYAFVHFETQEAADKAIEKMNGMLLNDRKVFVGRFK
SRKEREAELGAKAKEFTNVYIKNFGEEVDDESLKELFSQFGKTLSVKVMRDPNGKSKGFGFVSYEKHEDANKAVEEMNGKEISGKII
FVGRAQKKVERQAELKRKFEQLKQERISRYQGVNLYIKNLDDTIDDEKLRKEFSPFGSITSAKVMLEDGRSKGFGFVCFSSPEEATK
AVTEMNGRIVGSKPLYVALAQRKEERKAHLTNQYMQRVAGMRALPANAILNQFQPAAGGYFVPAVPQAQGRPPYYTPNQLAQMRPNP
RWQQGGRPQGFQGMPSAIRQSGPRPTLRHLAPTGSECPDRLAMDFGGAGAAQQGLTDSCQSGGVPTAVQNLAPRAAVAAAAPRAVAP
YKYASSVRSPHPAIQPLQAPQPAVHVQGQEPLTASMLAAAPPQEQKQMLGERLFPLIQTMHSNLAGKITGMLLEIDNSELLHMLESP
ESLRSKVDEAVAVLQAHHAKKEAAQKVGAVAAATS

>sp|A0AVI4|TM129_HUMAN
MDSPEVTFTLAYLVFAVCFVFTPNEFHAAGLTVQNLLSGWLGSEDAAFVPFHLRRTAATLLCHSLLPLGYYVGMCLAASEKRLHALS
QAPEAWRLFLLLAVTLPSIACILIYYWSRDRWACHPLARTLALYALPQSGWQAVASSVNTEFRRIDKFATGAPGARVIVTDTWVMKV
TTYRVHVAQQQDVHLTVTESRQHELSPDSNLPVQLLTIRVASTNPAVQAFDIWLNSTEYGELCEKLRAPIRRAAHVVIHQSLGDLFL
ETFASLVEVNPAYSVPSSQELEACIGCMQTRASVKLVKTCQEAATGECQQCYCRPMWCLTCMGKWFASRQDPLRPDTWLASRVPCPT
CRARFCILDVCTVR
>sp|Q9UL36|ZN236_HUMAN
MGLCGLLERCWLHHDPDGVLTLNAENTNYAYQVPNFHKCEICLLSFPKESQFQRHMRDHERNDKPHRCDQCPQTFNVEFNLTLHKCT
HSGEDPTCPVCNKKFSRVASLKAHIMLHEKEENLICSECGDEFTLQSQLAVHMEEHRQELAGTRQHACKACKKEFETSSELKEHMKT
HYKIRVSSTRSYNRNIDRSGFTYSCPHCGKTFQKPSQLTRHIRIHTGERPFKCSECGKAFNQKGALQTHMIKHTGEKPHACAFCPAA
FSQKGNLQSHVQRVHSEVKNGPTYNCTECSCVFKSLGSLNTHISKMHMGGPQNSTSSTETAHVLTATLFQTLPLQQTEAQATSASSQ
PSSQAVSDVIQQLLELSEPAPVESGQSPQPGQQLSITVGINQDILQQALENSGLSSIPAAAHPNDSCHAKTSAPHAQNPDVSSVSNE
QTDPTDAEQEKEQESPEKLDKKEKKMIKKKSPFLPGSIREENGVRWHVCPYCAKEFRKPSDLVRHIRIHTHEKPFKCPQCFRAFAVK
STLTAHIKTHTGIKAFKCQYCMKSFSTSGSLKVHIRLHTGVRPFACPHCDKKFRTSGHRKTHIASHFKHTELRKMRHQRKPAKVRVG
KTNIPVPDIPLQEPILITDLGLIQPIPKNQFFQSYFNNNFVNEADRPYKCFYCHRAYKKSCHLKQHIRSHTGEKPFKCSQCGRGFVS
AGVLKAHIRTHTGLKSFKCLICNGAFTTGGSLRRHMGIHNDLRPYMCPYCQKTFKTSLNCKKHMKTHRYELAQQLQQHQQAASIDDS
TVDQQSMQASTQMQVEIESDELPQTAEVVAANPEAMLDLEPQHVVGTEEAGLGQQLADQPLEADEDGFVAPQDPLRGHVDQFEEQSP
AQQSFEPAGLPQGFTVTDTYHQQPQFPPVQQLQDSSTLESQALSTSFHQQSLLQAPSSDGMNVTTRLIQESSQEELDLQAQGSQFLE
DNEDQSRRSYRCDYCNKGFKKSSHLKQHVRSHTGEKPYKCKLCGRGFVSSGVLKSHEKTHTGVKAFSCSVCNASFTTNGSLTRHMAT
HMSMKPYKCPFCEEGFRTTVHCKKHMKRHQTVPSAVSATGETEGGDICMEEEEEHSDRNASRKSRPEVITFTEEETAQLAKIRPQES
ATVSEKVLVQSAAEKDRISELRDKQAELQDEPKHANCCTYCPKSFKKPSDLVRHVRIHTGEKPYKCDECGKSFTVKSTLDCHVKTHT
GQKLFSCHVCSNAFSTKGSLKVHMRLHTGAKPFKCPHCELRFRTSGRRKTHMQFHYKPDPKKARKPMTRSSSEGLQPVNLLNSSSTD
PNVFIMNNSVLTGQFDQNLLQPGLVGQAILPASVSAGGDLTVSLTDGSLATLEGIQLQLAANLVGPNVQISGIDAASINNITLQIDP
SILQQTLQQGNLLAQQLTGEPGLAPQNSSLQTSDSTVPASVVIQPISGLSLQPTVTSANLTIGPLSEQDSVLTTNSSGTQDLTQVMT
SQGLVSPSGGPHEITLTINNSSLSQVLAQAAGPTATSSSGSPQEITLTISELNTTSGSLPSTTPMSPSAISTQNLVMSSSGVGGDAS
VTLTLADTQGMLSGGLDTVTLNITSQGQQFPALLTDPSLSGQGGAGSPQVILVSHTPQSASAACEEIAYQVAGVSGNLAPGNQPEKE
GRAHQCLECDRAFSSAAVLMHHSKEVHGRERIHGCPVCRKAFKRATHLKEHMQTHQAGPSLSSQKPRVFKCDTCEKAFAKPSQLERH
SRIHTGERPFHCTLCEKAFNQKSALQVHMKKHTGERPYKCAYCVMGFTQKSNMKLHMKRAHSYAGALQESAGHPEQDGEELSRTLHL
EEVVQEAAGEWQALTHVF
>sp|Q16625|OCLN_HUMAN
MSSRPLESPPPYRPDEFKPNHYAPSNDIYGGEMHVRPMLSQPAYSFYPEDEILHFYKWTSPPGVIRILSMLIIVMCIAIFACVASTL
AWDRGYGTSLLGGSVGYPYGGSGFGSYGSGYGYGYGYGYGYGGYTDPRAAKGFMLAMAAFCFIAALVIFVTSVIRSEMSRTRRYYLS
VIIVSAILGIMVFIATIVYIMGVNPTAQSSGSLYGSQIYALCNQFYTPAATGLYVDQYLYHYCVVDPQEAIAIVLGFMIIVAFALII
FFAVKTRRKMDRYDKSNILWDKEHIYDEQPPNVEEWVKNVSAGTQDVPSPPSDYVERVDSPMAYSSNGKVNDKRFYPESSYKSTPVP
EVVQELPLTSPVDDFRQPRYSSGGNFETPSKRAPAKGRAGRSKRTEQDHYETDYTTGGESCDELEEDWIREYPPITSDQQRQLYKRN
FDTGLQEYKSLQSELDEINKELSRLDKELDDYREESEEYMAAADEYNRLKQVKGSADYKSKKNHCKQLKSKLSHIKKMVGDYDRQKT
>sp|Q96M94|KLH15_HUMAN
MAGDVEGFCSSIHDTSVSAGFRALYEEGLLLDVTLVIEDHQFQAHKALLATQSDYFRIMFTADMRERDQDKIHLKGLTATGFSHVLQ
FMYYGTIELSMNTVHEILQAAMYVQLIEVVKFCCSFLLAKICLENCAEIMRLLDDFGVNIEGVREKLDTFLLDNFVPLMSRPDFLSY
LSFEKLMSYLDNDHLSRFPEIELYEAVQSWLRHDRRRWRHTDTIIQNIRFCLMTPTSVFEKVKTSEFYRYSRQLRYEVDQALNYFQN
VHQQPLLDMKSSRIRSAKPQTTVFRGMIGHSMVNSKILLLKKPRVWWELEGPQVPLRPDCLAIVNNFVFLLGGEELGPDGEFHASSK
VFRYDPRQNSWLQMADMSVPRSEFAVGVIGKFIYAVAGRTRDETFYSTERYDITNDKWEFVDPYPVNKYGHEGTVLNNKLFITGGIT
SSSTSKQVCVFDPSKEGTIEQRTRRTQVVTNCWENKSKMNYARCFHKMISYNGKLYVFGGVCVILRASFESQGCPSTEVYNPETDQW
TILASMPIGRSGHGVTVLDKQIMVLGGLCYNGHYSDSILTFDPDENKWKEDEYPRMPCKLDGLQVCNLHFPDYVLDEVRRCN

>sp|Q96S42|NODAL_HUMAN
MHAHCLPFLLHAWWALLQAGAATVATALLRTRGQPSSPSPLAYMLSLYRDPLPRADIIRSLQAEDVAVDGQNWTFAFDFSFLSQQED
LAWAELRLQLSSPVDLPTEGSLAIEIFHQPKPDTEQASDSCLERFQMDLFTVTLSQVTFSLGSMVLEVTRPLSKWLKHPGALEKQMS
RVAGECWPRPPTPPATNVLLMLYSNLSQEQRQLGGSTLLWEAESSWRAQEGQLSWEWGKRHRRHHLPDRSQLCRKVKFQVDFNLIGW
GSWIIYPKQYNAYRCEGECPNPVGEEFHPTNHAYIQSLLKRYQPHRVPSTCCAPVKTKPLSMLYVDNGRVLLDHHKDMIVEECGCL
>sp|Q8NBB4|ZSCA1_HUMAN
MLPRPKAPASPRRPQTPTPSEQDADPGPASPRDTEAQRLRFRQFQYHVASGPHLALGQLWTLCRQWLRPEARSKEQMLELLVLEQFL
GALPSKMRTWVQSQGPRSCREAASLVEDLTQMCQQEVLVSLDSVEPQDWSFGEEEDGKSPRSQKEPSQASELILDAVAAAPALPEES
EWLETTQLQQSLHTRAEAEAPRAPGLLGSRARLPLKPSIWDEPEDLLAGPSSDLRAEGTVISSPKGPSAQRISPRRRNRNTDQSGRH
QPSLKHTKGGTQEAVAGISVVPRGPRGGRPFQCADCGMVFTWVTHFIEHQKTHREEGPFPCPECGKVFLHNSVLTEHGKIHLLEPPR
KKAPRSKGPRESVPPRDGAQGPVAPRSPKRPFQCSVCGKAFPWMVHLIDHQKLHTAHGHM
>sp|P42701|I12R1_HUMAN
MEPLVTWVVPLLFLFLLSRQGAACRTSECCFQDPPYPDADSGSASGPRDLRCYRISSDRYECSWQYEGPTAGVSHFLRCCLSSGRCC
YFAAGSATRLQFSDQAGVSVLYTVTLWVESWARNQTEKSPEVTLQLYNSVKYEPPLGDIKVSKLAGQLRMEWETPDNQVGAEVQFRH
RTPSSPWKLGDCGPQDDDTESCLCPLEMNVAQEFQLRRRQLGSQGSSWSKWSSPVCVPPENPPQPQVRFSVEQLGQDGRRRLTLKEQ
PTQLELPEGCQGLAPGTEVTYRLQLHMLSCPCKAKATRTLHLGKMPYLSGAAYNVAVISSNQFGPGLNQTWHIPADTHTEPVALNIS
VGTNGTTMYWPARAQSMTYCIEWQPVGQDGGLATCSLTAPQDPDPAGMATYSWSRESGAMGQEKCYYITIFASAHPEKLTLWSTVLS
TYHFGGNASAAGTPHHVSVKNHSLDSVSVDWAPSLLSTCPGVLKEYVVRCRDEDSKQVSEHPVQPTETQVTLSGLRAGVAYTVQVRA
DTAWLRGVWSQPQRFSIEVQVSDWLIFFASLGSFLSILLVGVLGYLGLNRAARHLCPPLPTPCASSAIEFPGGKETWQWINPVDFQE
EASLQEALVVEMSWDKGERTEPLEKTELPEGAPELALDTELSLEDGDRCKAKM
>sp|Q9NPA2|MMP25_HUMAN
MRLRLRLLALLLLLLAPPARAPKPSAQDVSLGVDWLTRYGYLPPPHPAQAQLQSPEKLRDAIKVMQRFAGLPETGRMDPGTVATMRK
PRCSLPDVLGVAGLVRRRRRYALSGSVWKKRTLTWRVRSFPQSSQLSQETVRVLMSYALMAWGMESGLTFHEVDSPQGQEPDILIDF
ARAFHQDSYPFDGLGGTLAHAFFPGEHPISGDTHFDDEETWTFGSKDGEGTDLFAVAVHEFGHALGLGHSSAPNSIMRPFYQGPVGD
PDKYRLSQDDRDGLQQLYGKAPQTPYDKPTRKPLAPPPQPPASPTHSPSFPIPDRCEGNFDAIANIRGETFFFKGPWFWRLQPSGQL
VSPRPARLHRFWEGLPAQVRVVQAAYARHRDGRILLFSGPQFWVFQDRQLEGGARPLTELGLPPGEEVDAVFSWPQNGKTYLVRGRQ
YWRYDEAAARPDPGYPRDLSLWEGAPPSPDDVTVSNAGDTYFFKGAHYWRFPKNSIKTEPDAPQPMGPNWLDCPAPSSGPRAPRPPK
ATPVSETCDCQCELNQAAGRWPAPIPLLLLPLLVGGVASR
>sp|Q7L0Q8|RHOU_HUMAN
MPPQQGDPAFPDRCEAPPVPPRRERGGRGGRGPGEPGGRGRAGGAEGRGVKCVLVGDGAVGKTSLVVSYTTNGYPTEYIPTAFDNFS
AVVSVDGRPVRLQLCDTAGQDEFDKLRPLCYTNTDIFLLCFSVVSPSSFQNVSEKWVPEIRCHCPKAPIILVGTQSDLREDVKVLIE
LDKCKEKPVPEEAAKLCAEEIKAASYIECSALTQKNLKEVFDAAIVAGIQYSDTQQQPKKSKSRTPDKMKNLSKSWWKKYCCFV
>sp|Q7Z7L9|ZSCA2_HUMAN
MMAADIPRVTTPLSSLVQVPQEEDRQEEEVTTMILEDDSWVQEAVLQEDGPESEPFPQSAGKGGPQEEVTRGPQGALGRLRELCRRW
LRPEVHTKEQMLTMLPKEIQAWLQEHRPESSEEAAALVEDLTQTLQDSDFEIQSENGENCNQDMFENESRKIFSEMPEGESAQHSDG
ESDFERDAGIQRLQGHSPGEDHGEVVSQDREVGQLIGLQGTYLGEKPYECPQCGKTFSRKSHLITHERTHTGEKYYKCDECGKSFSD
GSNFSRHQTTHTGEKPYKCRDCGKSFSRSANLITHQRIHTGEKPFQCAECGKSFSRSPNLIAHQRTHTGEKPYSCPECGKSFGNRSS
LNTHQGIHTGEKPYECKECGESFSYNSNLIRHQRIHTGEKPYKCTDCGQRFSQSSALITHRRTHTGEKPYQCSECGKSFSRSSNLAT
HRRTHMVEKPYKCGVCGKSFSQSSSLIAHQGMHTGEKPYECLTCGESFSWSSNLLKHQRIHTGEKPYKCSECGKCFSQRSQLVVHQR
THTGEKPYKCLMCGKSFSRGSILVMHQRAHLGDKPYRCPECGKGFSWNSVLIIHQRIHTGEKPYKCPECGKGFSNSSNFITHQRTHM
KEKLY
>sp|Q9Y320|TMX2_HUMAN
MAVLAPLIALVYSVPRLSRWLAQPYYLLSALLSAAFLLVRKLPPLCHGLPTQREDGNPCDFDWREVEILMFLSAIVMMKNRRSITVE
QHIGNIFMFSKVANTILFFRLDIRMGLLYITLCIVFLMTCKPPLYMGPEYIKYFNDKTIDEELERDKRVTWIVEFFANWSNDCQSFA

PIYADLSLKYNCTGLNFGKVDVGRYTDVSTRYKVSTSPLTKQLPTLILFQGGKEAMRRPQIDKKGRAVSWTFSEENVIREFNLNELY
QRAKKLSKAGDNIPEEQPVASTPTTVSDGENKKDK
>sp|Q8N987|NECA1_HUMAN
MEDSQETSPSSNNSSEELSSALHLSKGMSIFLDILRRADKNDDGKLSFEEFKAYFADGVLSGEELHELFHTIDTHNTNNLDTEELCE
YFSQHLGEYENVLAALEDLNLSILKAMGKTKKDYQEASNLEQFVTRFLLKETLNQLQSLQNSLECAMETTEEQTRQERQGPAKPEVL
SIQWPGKRSSRRVQRHNSFSPNSPQFNVSGPGLLEEDNQWMTQINRLQKLIDRLEKKDLKLEPPEEEIIEGNTKSHIMLVQRQMSVI
EEDLEEFQLALKHYVESASSQSGCLRISIQKLSNESRYMIYEFWENSSVWNSHLQTNYSKTFQRSNVDFLETPELTSTMLVPASWWI
LNN
>sp|Q9NW82|WDR70_HUMAN
MERSGPSEVTGSDASGPDPQLAVTMGFTGFGKKARTFDLEAMFEQTRRTAVERSRKTLEAREKEEEMNREKELRRQNEDIEPTSSRS
NVVRDCSKSSSRDTSSSESEQSSDSSDDELIGPPLPPKMVGKPVNFMEEDILGPLPPPLNEEEEEAEEEEEEEEEEENPVHKIPDSH
EITLKHGTKTVSALGLDPSGARLVTGGYDYDVKFWDFAGMDASFKAFRSLQPCECHQIKSLQYSNTGDMILVVSGSSQAKVIDRDGF
EVMECIKGDQYIVDMANTKGHTAMLHTGSWHPKIKGEFMTCSNDATVRTWEVENPKKQKSVFKPRTMQGKKVIPTTCTYSRDGNLIA
AACQNGSIQIWDRNLTVHPKFHYKQAHDSGTDTSCVTFSYDGNVLASRGGDDSLKLWDIRQFNKPLFSASGLPTMFPMTDCCFSPDD
KLIVTGTSIQRGCGSGKLVFFERRTFQRVYEIDITDASVVRCLWHPKLNQIMVGTGNGLAKVYYDPNKSQRGAKLCVVKTQRKAKQA
ETLTQDYIITPHALPMFREPRQRSTRKQLEKDRLDPLKSHKPEPPVAGPGRGGRVGTHGGTLSSYIVKNIALDKTDDSNPREAILRH
AKAAEDSPYWVSPAYSKTQPKTMFAQVESDDEEAKNEPEWKKRKI
>sp|Q6XD76|ASCL4_HUMAN
METRKPAERLALPYSLRTAPLGVPGTLPGLPRRDPLRVALRLDAACWEWARSGCARGWQYLPVPLDSAFEPAFLRKRNERERQRVRC
VNEGYARLRDHLPRELADKRLSKVETLRAAIDYIKHLQELLERQAWGLEGAAGAVPQRRAECNSDGESKASSAPSPSSEPEEGGS
>sp|Q9ULW2|FZD10_HUMAN
MQRPGPRLWLVLQVMGSCAAISSMDMERPGDGKCQPIEIPMCKDIGYNMTRMPNLMGHENQREAAIQLHEFAPLVEYGCHGHLRFFL
CSLYAPMCTEQVSTPIPACRVMCEQARLKCSPIMEQFNFKWPDSLDCRKLPNKNDPNYLCMEAPNNGSDEPTRGSGLFPPLFRPQRP
HSAQEHPLKDGGPGRGGCDNPGKFHHVEKSASCAPLCTPGVDVYWSREDKRFAVVWLAIWAVLCFFSSAFTVLTFLIDPARFRYPER
PIIFLSMCYCVYSVGYLIRLFAGAESIACDRDSGQLYVIQEGLESTGCTLVFLVLYYFGMASSLWWVVLTLTWFLAAGKKWGHEAIE
ANSSYFHLAAWAIPAVKTILILVMRRVAGDELTGVCYVGSMDVNALTGFVLIPLACYLVIGTSFILSGFVALFHIRRVMKTGGENTD
KLEKLMVRIGLFSVLYTVPATCVIACYFYERLNMDYWKILAAQHKCKMNNQTKTLDCLMAASIPAVEIFMVKIFMLLVVGITSGMWI
WTSKTLQSWQQVCSRRLKKKSRRKPASVITSGGIYKKAQHPQKTHHGKYEIPAQSPTCV
>sp|Q5VV52|ZN691_HUMAN
MSLCSPTHSAEMSLFLQGPEEMLPLSSEGSEMGSEKEQSPEPHLPEEGEGGKPWRVDDSEGSWIPPGEKEHGQESLSDELQETHPKK
PWQKVTVRARELGDPIAHPRHEADEKPFICAQCGKTFNNTSNLRTHQRIHTGEKPYKCSECGKSFSRSSNRIRHERIHLEEKHYKCP
KCQESFRRRSDLTTHQQDHLGKRPYRCDICGKSFSQSATLAVHHRTHLEPAPYICCECGKSFSNSSSFGVHHRTHTGERPYECTECG
RTFSDISNFGAHQRTHRGEKPYRCTVCGKHFSRSSNLKPFIPLWRMVLYSL
>sp|Q96FV2|SCRN2_HUMAN
MASSSPDSPCSCDCFVSVPPASAIPAVIFAKNSDRPRDEVQEVVFVPAGTHTPGSRLQCTYIEVEQVSKTHAVILSRPSWLWGAEMG
ANEHGVCIGNEAVWTKEPVGEGEALLGMDLLRLALERSSSAQEALHVITGLLEHYGQGGNCLEDAAPFSYHSTFLLADRTEAWVLET
AGRLWAAQRIQEGARNISNQLSIGTDISAQHPELRTHAQAKGWWDGQGAFDFAQIFSLTQQPVRMEAAKARFQAGRELLRQRQGGIT
AEVMMGILRDKESGICMDSGGFRTTASMVSVLPQDPTQPCVHFLTATPDPSRSVFKPFIFGMGVAQAPQVLSPTFGAQDPVRTLPRF
QTQVDRRHTLYRGHQAALGLMERDQDRGQQLQQKQQDLEQEGLEATQGLLAGEWAPPLWELGSLFQAFVKRESQAYA
>sp|Q8NG41|NPB_HUMAN
MARSATLAAAALALCLLLAPPGLAWYKPAAGHSSYSVGRAAGLLSGLRRSPYARRSQPYRGAEPPGGAGASPELQLHPRLRSLAVCV
QDVAPNLQRCERLPDGRGTYQCKANVFLSLRAADCLAA
>sp|P22557|HEM0_HUMAN

MVTAAMLLQCCPVLARGPTSLLGKVVKTHQFLFGIGRCPILATQGPNCSQIHLKATKAGGDSPSWAKGHCPFMLSELQDGKSKIVQK
AAPEVQEDVKAFKTDLPSSLVSVSLRKPFSGPQEQEQISGKVTHLIQNNMPGNYVFSYDQFFRDKIMEKKQDHTYRVFKTVNRWADA
YPFAQHFSEASVASKDVSVWCSNDYLGMSRHPQVLQATQETLQRHGAGAGGTRNISGTSKFHVELEQELAELHQKDSALLFSSCFVA
NDSTLFTLAKILPGCEIYSDAGNHASMIQGIRNSGAAKFVFRHNDPDHLKKLLEKSNPKIPKIVAFETVHSMDGAICPLEELCDVSH
QYGALTFVDEVHAVGLYGSRGAGIGERDGIMHKIDIISGTLGKAFGCVGGYIASTRDLVDMVRSYAAGFIFTTSLPPMVLSGALESV
RLLKGEEGQALRRAHQRNVKHMRQLLMDRGLPVIPCPSHIIPIRVGNAALNSKLCDLLLSKHGIYVQAINYPTVPRGEELLRLAPSP
HHSPQMMEDFVEKLLLAWTAVGLPLQDVSVAACNFCRRPVHFELMSEWERSYFGNMGPQYVTTYA
>sp|P30048|PRDX3_HUMAN
MAAAVGRLLRASVARHVSAIPWGISATAALRPAACGRTSLTNLLCSGSSQAKLFSTSSSCHAPAVTQHAPYFKGTAVVNGEFKDLSL
DDFKGKYLVLFFYPLDFTFVCPTEIVAFSDKANEFHDVNCEVVAVSVDSHFSHLAWINTPRKNGGLGHMNIALLSDLTKQISRDYGV
LLEGSGLALRGLFIIDPNGVIKHLSVNDLPVGRSVEETLRLVKAFQYVETHGEVCPANWTPDSPTIKPSPAASKEYFQKVNQ
>sp|P06746|DPOLB_HUMAN
MSKRKAPQETLNGGITDMLTELANFEKNVSQAIHKYNAYRKAASVIAKYPHKIKSGAEAKKLPGVGTKIAEKIDEFLATGKLRKLEK
IRQDDTSSSINFLTRVSGIGPSAARKFVDEGIKTLEDLRKNEDKLNHHQRIGLKYFGDFEKRIPREEMLQMQDIVLNEVKKVDSEYI
ATVCGSFRRGAESSGDMDVLLTHPSFTSESTKQPKLLHQVVEQLQKVHFITDTLSKGETKFMGVCQLPSKNDEKEYPHRRIDIRLIP
KDQYYCGVLYFTGSDIFNKNMRAHALEKGFTINEYTIRPLGVTGVAGEPLPVDSEKDIFDYIQWKYREPKDRSE
>sp|O76031|CLPX_HUMAN
MPSCGACTCGAAAVRLITSSLASAQRGISGGRIHMSVLGRLGTFETQILQRAPLRSFTETPAYFASKDGISKDGSGDGNKKSASEGS
SKKSGSGNSGKGGNQLRCPKCGDLCTHVETFVSSTRFVKCEKCHHFFVVLSEADSKKSIIKEPESAAEAVKLAFQQKPPPPPKKIYN
YLDKYVVGQSFAKKVLSVAVYNHYKRIYNNIPANLRQQAEVEKQTSLTPRELEIRRREDEYRFTKLLQIAGISPHGNALGASMQQQV
NQQIPQEKRGGEVLDSSHDDIKLEKSNILLLGPTGSGKTLLAQTLAKCLDVPFAICDCTTLTQAGYVGEDIESVIAKLLQDANYNVE
KAQQGIVFLDEVDKIGSVPGIHQLRDVGGEGVQQGLLKLLEGTIVNVPEKNSRKLRGETVQVDTTNILFVASGAFNGLDRIISRRKN
EKYLGFGTPSNLGKGRRAAAAADLANRSGESNTHQDIEEKDRLLRHVEARDLIEFGMIPEFVGRLPVVVPLHSLDEKTLVQILTEPR
NAVIPQYQALFSMDKCELNVTEDALKAIARLALERKTGARGLRSIMEKLLLEPMFEVPNSDIVCVEVDKEVVEGKKEPGYIRAPTKE
SSEEEYDSGVEEEGWPRQADAANS
>sp|Q5TDP6|LGSN_HUMAN
MNNEEDLLQEDSTRDEGNETEANSMNTLRRTRKKVTKPYVCSTEVGETDMSNSNDCMRDSSQILTPPQLSSRMKHIRQAMAKNRLQF
VRFEATDLHGVSRSKTIPAHFFQEKVSHGVCMPRGYLEVIPNPKDNEMNNIRATCFNSDIVLMPELSTFRVLPWADRTARVICDTFT
VTGEPLLTSPRYIAKRQLSHLQASGFSLLSAFIYDFCIFGVPEILNSKIISFPALTFLNNHDQPFMQELVDGLYHTGANVESFSSST
RPGQMEISFLPEFGISSADNAFTLRTGVKEVARKYNYIASFFIETGFCDSGILSHSLWDVDRKKNMFCSTSGTEQLTITGKKWLAGL
LKHSAALSCLMAPSVSCRKRYSKDRKDLKKSVPTTWGYNDNSCIFNIKCHGEKGTRIENKLGSATANPYLVLAATVAAGLDGLHSSN
EVLAGPDESTDFYQVEPSEIPLKLEDALVALEEDQCLRQALGETFIRYFVAMKKYELENEEIAAERNKFLEYFI
>sp|Q8NH40|OR6S1_HUMAN
MSPDGNHSSDPTEFVLAGLPNLNSARVELFSVFLLVYLLNLTGNVLIVGVVRADTRLQTPMYFFLGNLSCLEILLTSVIIPKMLSNF
LSRQHTISFAACITQFYFYFFLGASEFLLLAVMSADRYLAICHPLRYPLLMSGAVCFRVALACWVGGLVPVLGPTVAVALLPFCKQG
AVVQHFFCDSGPLLRLACTNTKKLEETDFVLASLVIVSSLLITAVSYGLIVLAVLSIPSASGRQKAFSTCTSHLIVVTLFYGSAIFL
YVRPSQSGSVDTNWAVTVITTFVTPLLNPFIYALRNEQVKEALKDMFRKVVAGVLGNLLLDKCLSEKAVK
>sp|Q9Y2W2|WBP11_HUMAN
MGRRSTSSTKSGKFMNPTDQARKEARKRELKKNKKQRMMVRAAVLKMKDPKQIIRDMEKLDEMEFNPVQQPQLNEKVLKDKRKKLRE
TFERILRLYEKENPDIYKELRKLEVEYEQKRAQLSQYFDAVKNAQHVEVESIPLPDMPHAPSNILIQDIPLPGAQPPSILKKTSAYG
PPTRAVSILPLLGHGVPRLPPGRKPPGPPPGPPPPQVVQMYGRKVGFALDLPPRRRDEDMLYSPELAQRGHDDDVSSTSEDDGYPED
MDQDKHDDSTDDSDTDKSDGESDGDEFVHRDNGERDNNEEKKSGLSVRFADMPGKSRKKKKNMKELTPLQAMMLRMAGQEIPEEGRE
VEEFSEDDDEDDSDDSEAEKQSQKQHKEESHSDGTSTASSQQQAPPQSVPPSQIQAPPMPGPPPLGPPPAPPLRPPGPPTGLPPGPP
PGAPPFLRPPGMPGLRGPLPRLLPPGPPPGRPPGPPPGPPPGLPPGPPPRGPPPRLPPPAPPGIPPPRPGMMRPPLVPPLGPAPPGL

FPPAPLPNPGVLSAPPNLIQRPKADDTSAATIEKKATATISAKPQITNPKAEITRFVPTALRVRRENKGATAAPQRKSEDDSAVPLA
KAAPKSGPSVPVSVQTKDDVYEAFMKEMEGLL
>sp|Q9BPY8|HOP_HUMAN
MSAETASGPTEDQVEILEYNFNKVDKHPDSTTLCLIAAEAGLSEEETQKWFKQRLAKWRRSEGLPSECRSVTD
>sp|Q9Y263|PLAP_HUMAN
MTSGATRYRLSCSLRGHELDVRGLVCCAYPPGAFVSVSRDRTTRLWAPDSPNRSFTEMHCMSGHSNFVSCVCIIPSSDIYPHGLIAT
GGNDHNICIFSLDSPMPLYILKGHKNTVCSLSSGKFGTLLSGSWDTTAKVWLNDKCMMTLQGHTAAVWAVKILPEQGLMLTGSADKT
VKLWKAGRCERTFSGHEDCVRGLAILSETEFLSCANDASIRRWQITGECLEVYYGHTNYIYSISVFPNCRDFVTTAEDRSLRIWKHG
ECAQTIRLPAQSIWCCCVLDNGDIVVGASDGIIRVFTESEDRTASAEEIKAFEKELSHATIDSKTGDLGDINAEQLPGREHLNEPGT
REGQTRLIRDGEKVEAYQWSVSEGRWIKIGDVVGSSGANQQTSGKVLYEGKEFDYVFSIDVNEGGPSYKLPYNTSDDPWLTAYNFLQ
KNDLNPMFLDQVAKFIIDNTKGQMLGLGNPSFSDPFTGGGRYVPGSSGSSNTLPTADPFTGAGRYVPGSASMGTTMAGVDPFTGNSA
YRSAASKTMNIYFPKKEAVTFDQANPTQILGKLKELNGTAPEEKKLTEDDLILLEKILSLICNSSSEKPTVQQLQILWKAINCPEDI
VFPALDILRLSIKHPSVNENFCNEKEGAQFSSHLINLLNPKGKPANQLLALRTFCNCFVGQAGQKLMMSQRESLMSHAIELKSGSNK
NIHIALATLALNYSVCFHKDHNIEGKAQCLSLISTILEVVQDLEATFRLLVALGTLISDDSNAVQLAKSLGVDSQIKKYSSVSEPAK
VSECCRFILNLL
>sp|Q9BZ97|TTY13_HUMAN
MKTQDDGVLPPYDVNQLLGWDLNLSLFLGLCLMLLLAGSCLPSPGITGLSHGSNREDR
>sp|A8MVJ9|HPF1L_HUMAN
MVGGGWKRRPGAGAGPQCEKTVDVKKSKFCEADVSSDLRKEVENHYTLSLPEDFYHFWKFCEELDSEKPADPLSASLGLQLVDPYNI
LAGKHKMKKKSTVPNFNLHWRFYYDPPEFQTIIIRDKLSATWGISDRDSPDELPVYVGINEAKKNCIIVPNGDNVFAAVKLYLMKKL
KEVTDKKKTNLFKNVDEKLTETARELGYSLEQRTMKMKQRDKKVVTKTFHGTGLVPPVDKNVVGYRELPETDADLKRICKTIVEAAS
DDERRKAFAPIQEMMTFVQFANDECDYGMGLELGMDLFCYGSHYFHKVAGQLLPLAYNLLKRNLFAEIMKDHLANRRKENIDQFAA
>sp|Q9NZH7|IL36B_HUMAN
MNPQREAAPKSYAIRDSRQMVWVLSGNSLIAAPLSRSIKPVTLHLIACRDTEFSDKEKGNMVYLGIKGKDLCLFCAEIQGKPTLQLK
LQGSQDNIGKDTCWKLVGIHTCINLDVRESCFMGTLDQWGIGVGRKKWKSSFQHHHLRKKDKDFSSMRTNIGMPGRM
>sp|Q3SYB3|FX4L6_HUMAN
MNLPRAERLRSTPQRSLRDSDGEDGKIDVLGEEEDEDEVEDEEEAASQQFLEQSLQPGLQVARWGGVALPREHIEGGGGPSDPSEFG
TKFRAPPRSAAASEDARQPAKPPYSYIALITMAILQNPHKRLTLSGICAFISGRFPYYRRKFPAWQNSIRHNLSLNDCFVKIPREPG
HPGKGNYWSLDPASQDMFDNGSFLRRRKRFKRHQLTPGAHLPHPFPLPAAHAALHNPHPGPLLGAPAPPQPVPGAYPNTAPGRRPYA
LLHPHPLRYLLLSAPVYAGAPKKAEGAALATPAPFPCCSPHLVLSLGRRARVWRRHREADASLSALRVLCKGSGERVQGLRRVCPRP
RGATATCSSDHQACCIPRPLPLCCKCPPPPLLGQFCSNSSSIRRRTAPTAALPPRARCWAGTCRPRRPC
>sp|Q8WUB8|PHF10_HUMAN
MAAAAGPGAALSPRPCDSDPATPGAQSPKDDNEDNSNDGTQPSKRRRMGSGDSSRSCETSSQDLGFSYYPAENLIEYKWPPDETGEY
YMLQEQVSEYLGVTSFKRKYPDLERRDLSHKEKLYLRELNVITETQCTLGLTALRSDEVIDLMIKEYPAKHAEYSVILQEKERQRIT
DHYKEYSQMQQQNTQKVEASKVPEYIKKAAKKAAEFNSNLNRERMEERRAYFDLQTHVIQVPQGKYKVLPTERTKVSSYPVALIPGQ
FQEYYKRYSPDELRYLPLNTALYEPPLDPELPALDSDGDSDDGEDGRGDEKRKNKGTSDSSSGNVSEGESPPDSQEDSFQGRQKSKD
KAATPRKDGPKRSVLSKSVPGYKPKVIPNAICGICLKGKESNKKGKAESLIHCSQCENSGHPSCLDMTMELVSMIKTYPWQCMECKT
CIICGQPHHEEEMMFCDMCDRGYHTFCVGLGAIPSGRWICDCCQRAPPTPRKVGRRGKNSKEG
>sp|O00625|PIR_HUMAN
MGSSKKVTLSVLSREQSEGVGARVRRSIGRPELKNLDPFLLFDEFKGGRPGGFPDHPHRGFETVSYLLEGGSMAHEDFCGHTGKMNP
GDLQWMTAGRGILHAEMPCSEEPAHGLQLWVNLRSSEKMVEPQYQELKSEEIPKPSKDGVTVAVISGEALGIKSKVYTRTPTLYLDF
KLDPGAKHSQPIPKGWTSFIYTISGDVYIGPDDAQQKIEPHHTAVLGEGDSVQVENKDPKRSHFVLIAGEPLREPVIQHGPFVMNTN
EEISQAILDFRNAKNGFERAKTWKSKIGN
>sp|Q8N9W8|FA71D_HUMAN

MKKNTSKTTMRINKQDALCTPHSHDPRDLQNMLDGGEYAPFVSPPMLESNFIQVNRRGESIYLHNRANWVTVGICFSSSTHKIPNVM
LLAHLTPGAQKDTETLFKSLLTSPPAEKLVLTRFLPLQFVTLSVHDAENMSLKVKLVSGRAYYLQLCTSAYKQDTLFSQWVALISLL
NQEKAKVSKVSEVSSLSGITNSTDITGSMDVTDVTTFTAILTPYMYAGTGPEHVRDSIDFPEFTDITDITDVTDLPENEVPEVPDVR
IVTEVIEVREATEVTDSSDITNCSGVTVVFENNDLIRAKQEEKEKLKNILKPGCLQDTKSKSELKESSKHVTISNITLTFEGKRYFQ
TTLTPVESEANTSKEMKDKTSEEKMPDFQSTALKAEESRSLRTESNTSVLSPHIKSPSNFLKLVPHLSAPFSRE
>sp|Q8WZ69|CK040_HUMAN
MALVQALVPREREPKLSILQMDRGDPQHSSHWCPEREKVKLLTLKPRETSKNILINFYRAFNLDKDVFIHQANHPLTVPSSVVMGDN
GHTLAEDDKRPCFRVLPCYLERVSSGISISWISAPLPVGAMKHQLLCDLMDLITLSFWLAGQCMSLKATNMQHCKCSIATSDWAIEL
DRTDYKTLPSEYSILALLQVFAGKNCMDRVLLHVDVNYLKSLP
>sp|Q8N1W1|ARG28_HUMAN
MELSCSEAPLYGQMMIYAKFDKNVYLPEDAEFYFTYDGSHQRHVMIAERIEDNVLQSSVPGHGLQETVTVSVCLCSEGYSPVTMGSG
SVTYVDNMACRLARLLVTQANRLTACSHQTLLTPFALTAGALPALDEELVLALTHLELPLEWTVLGSSSLEVSSHRESLLHLAMRWG
LAKLSQFFLCLPGGVQALALPNEEGATPLDLALREGHSKLVEDVTNFQGRWSPSFSRVQLSEEASLHYIHSSETLTLTLNHTAEHLL
EADIKLFRKYFWDRAFLVKAFEPEARPEERTAMPSSGAETEEEIKNSVSSRSAAEKEDIKRVKSLVVQHNEHEDQHSLDLDRSFDIL
KKSKPPSTLLAAGRLSDMLNGGDEVYANCMVIDQVGDLDISYINIEGITATTSPESRGCTLWPQSSKHTLPTETSPSVYPLSENVEG
TAHTEAQQSFMSPSSSCASNLNLSFGWHGFEKEQSHLKKRSSSLDALDADSEGEGHSEPSHICYTPGSQSSSRTGIPSGDELDSFET
NTEPDFNISRAESLPLSSNLQSKESLLSGVRSRSYSCSSPKISLGKTRLVRELTVCSSSEEQRAYSLSEPPRENRIQEEEWDKYIIP
AKSESEKYKVSRTFSFLMNRMTSPRNKSKTKSKDAKDKEKLNRHQFAPGTFSGVLQCLVCDKTLLGKESLQCSNCNANVHKGCKDAA
PACTKKFQEKYNKNKPQTILGNSSFRDIPQPGLSLHPSSSVPVGLPTGRRETVGQVHPLSRSVPGTTLESFRRSATSLESESDHNSC
RSRSHSDELLQSMGSSPSTESFIMEDVVDSSLWSDLSSDAQEFEAESWSLVVDPSFCNRQEKDVIKRQDVIFELMQTEMHHIQTLFI
MSEIFRKGMKEELQLDHSTVDKIFPCLDELLEIHRHFFYSMKERRQESCAGSDRNFVIDRIGDILVQQFSEENASKMKKIYGEFCCH
HKEAVNLFKELQQNKKFQNFIKLRNSNLLARRRGIPECILLVTQRITKYPVLVERILQYTKERTEEHKDLRKALCLIKDMIATVDLK
VNEYEKNQKWLEILNKIENKTYTKLKNGHVFRKQALMSEERTLLYDGLVYWKTATGRFKDILALLLTDVLLFLQEKDQKYIFAAVDQ
KPSVISLQKLIAREVANEERGMFLISASSAGPEMYEIHTNSKEERNNWMRRIQQAVESCPEEKGGRTSESDEDKRKAEARVAKIQQC
QEILTNQDQQICAYLEEKLHIYAELGELSGFEDVHLEPHLLIKPDPGEPPQAASLLAAALKEAESLQVAVKASQMGAVSQSCEDSCG
DSVLADTLSSHDVPGSPTASLVTGGREGRGCSDVDPGIQGVVTDLAVSDAGEKVECRNFPGSSQSEIIQAIQNLTRLLYSLQAALTI
QDSHIEIHRLVLQQQEGLSLGHSILRGGPLQDQKSRDADRQHEELANVHQLQHQLQQEQRRWLRRCEQQQRAQATRESWLQERERЕC
QSQEELLLRSRGELDLQLQEYQHSLERLREGQRLVEREQARMRAQQSLLGHWKHGRQRSLPAVLLPGGPEVMELNRSESLCHENSFF
INEALVQMSFNTFNKLNPSVIHQDATYPTTQSHSDLVRTSEHQVDLKVDPSQPSNVSHKLWTAAGSGHQILPFHESSKDSCKNDLDT
SHTESPTPHDSNSHRPQLQAFITEAKLNLPTRTMTRQDGETGDGAKENIVYL
>sp|Q93100|KPBB_HUMAN
MAGAAGLTAEVSWKVLERRARTKRSGSVYEPLKSINLPRPDNETLWDKLDHYYRIVKSTLLLYQSPTTGLFPTKTCGGDQKAKIQDS
LYCAAGAWALALAYRRIDDDKGRTHELEHSAIKCMRGILYCYMRQADKVQQFKQDPRPTTCLHSVFNVHTGDELLSYEEYGHLQINA
VSLYLLYLVEMISSGLQIIYNTDEVSFIQNLVFCVERVYRVPDFGVWERGSKYNNGSTELHSSSVGLAKAALEAINGFNLFGNQGCS
WSVIFVDLDAHNRNRQTLCSLLPRESRSHNTDAALLPCISYPAFALDDEVLFSQTLDKVVRKLKGKYGFKRFLRDGYRTSLEDPNRC
YYKPAEIKLFDGIECEFPIFFLYMMIDGVFRGNPKQVQEYQDLLTPVLHHTTEGYPVVPKYYYVPADFVEYEKNNPGSQKRFPSNCG
RDGKLFLWGQALYIIAKLLADELISPKDIDPVQRYVPLKDQRNVSMRFSNQGPLENDLVVHVALIAESQRLQVFLNTYGIQTQTPQQ
VEPIQIWPQQELVKAYLQLGINEKLGLSGRPDRPIGCLGTSKIYRILGKTVVCYPIIFDLSDFYMSQDVFLLIDDIKNALQFIKQYW
KMHGRPLFLVLIREDNIRGSRFNPILDMLAALKKGIIGGVKVHVDRLQTLISGAVVEQLDFLRISDTEELPEFKSFEELEPPKHSKV
KRQSSTPSAPELGQQPDVNISEWKDKPTHEILQKLNDCSCLASQAILLGILLKREGPNFITKEGTVSDHIERVYRRAGSQKLWLAVR
YGAAFTQKFSSSIAPHITTFLVHGKQVTLGAFGHEEEVISNPLSPRVIQNIIYYKCNTHDEREAVIQQELVIHIGWIISNNPELFSG
MLKIRIGWIIHAMEYELQIRGGDKPALDLYQLSPSEVKQLLLDILQPQQNGRCWLNRRQIDGSLNRTPTGFYDRVWQILERTPNGII
VAGKHLPQQPTLSDMTMYEMNFSLLVEDTLGNIDQPQYRQIVVELLMVVSIVLERNPELEFQDKVDLDRLVKEAFNEFQKDQSRLKE
IEKQDDMTSFYNTPPLGKRGTCSYLTKAVMNLLLEGEVKPNNDDPCLIS

>sp|Q8IZK6|MCLN2_HUMAN
MARQPYRFPQARIPERGSGVFRLTVRNAMAHRDSEMKEECLREDLKFYFMSPCEKYRARRQIPWKLGLQILKIVMVTTQLVRFGLSN
QLVVAFKEDNTVAFKHLFLKGYSGTDEDDYSCSVYTQEDAYESIFFAINQYHQLKDITLGTLGYGENEDNRIGLKVCKQHYKKGTMF
PSNETLNIDNDVELDCVQLDLQDLSKKPPDWKNSSFFRLEFYRLLQVEISFHLKGIDLQTIHSRELPDCYVFQNTIIFDNKAHSGKI
KIYFDSDAKIEECKDLNIFGSTQKNAQYVLVFDAFVIVICLASLILCTRSIVLALRLRKRFLNFFLEKYKRPVCDTDQWEFINGWYV
LVIISDLMTIIGSILKMEIKAKNLTNYDLCSIFLGTSTLLVWVGVIRYLGYFQAYNVLILTMQASLPKVLRFCACAGMIYLGYTFCG
WIVLGPYHDKFENLNTVAECLFSLVNGDDMFATFAQIQQKSILVWLFSRLYLYSFISLFIYMILSLFIALITDSYDTIKKFQQNGFP
ETDLQEFLKECSSKEEYQKESSAFLSCICCRRRKRSDDHLIPIS
>sp|Q8N0U8|VKORL_HUMAN
MAAPVLLRVSVPRWERVARYAVCAAGILLSIYAYHVEREKERDPEHRALCDLGPWVKCSAALASRWGRGFGLLGSIFGKDGVLNQPN
SVFGLIFYILQLLLGMTASAVAALILMTSSIMSVVGSLYLAYILYFVLKEFCIICIVTYVLNFLLLIINYKRLVYLNEAWKRQLQPK
QD
>sp|Q6UX07|DHR13_HUMAN
MEALLLGAGLLLGAYVLVYYNLVKAPPCGGMGNLRGRTAVVTGANSGIGKMTALELARRGARVVLACRSQERGEAAAFDLRQESGNN
EVIFMALDLASLASVRAFATAFLSSEPRLDILIHNAGISSCGRTREAFNLLLRVNHIGPFLLTHLLLPCLKACAPSRVVVVASAAHC
RGRLDFKRLDRPVVGWRQELRAYADTKLANVLFARELANQLEATGVTCYAAHPGPVNSELFLRHVPGWLRPLLRPLAWLVLRAPRGG
AQTPLYCALQEGIEPLSGRYFANCHVEEVPPAARDDRAAHRLWEASKRLAGLGPGEDAEPDEDPQSEDSEAPSSLSTPHPEEPTVSQ
PYPSPQSSPDLSKMTHRIQAKVEPEIQLS
>sp|P61565|ENK21_HUMAN
MHPSEMQRKAPPRRRRHRNRAPLTHKMNKMVTSEQMKLPSTKKAEPPTWAQLKKLTQLATKYLENTKVTQTPESMLLAALMIVSMVV
SLPMPAGAAAANYTNWAYVPFPPLIRAVTWMDNPIEVYVNDSVWVHGPIDDRCPAKPEEEGMMINISIGYHYPPICLGRAPGCLMPA
VQNWLVEVPTVSPISRFTYNMVSGMSLRPRVNYLQDFSYQRSLKFRPKGKPCPKEIPKESKNTEVLVWEECVANSVVILQNNEFGTI
IDWAPRGQFYHNCSGQTQSCPSAQVSPAVDSDLTESLDKHKHKKLQSFYPWEWGEKGISTPRPKIISPVSGPEHPELWRLTVASHHI
RIWSGNQTLETRDRKPFYTVDLNSSLTVPLQSCVKPPYMLVVGNIVIKPDSQTITCENCRLLTCIDSTFNWQHRILLVRAREGVWIP
VSMDRPWEASPSIHILTEVLKGVLNRSKRFIFTLIAVIMGLIAVTAMAAVAGVALHSFVQSVNFVNDWQKNSTRLWNSQSSIDQKLA
NQINDLRQTVIWMGDRLMSLEHRFQLQCDWNTSDFCITPQIYNESEHHWDMVRRHLQGREDNLTLDISKLKEQIFEASKAHLNLVPG
TEAIAGVADGLANLNPVTWVKTIGSTTIINLILILVCLFCLLLVCRCTQQLRRDSDHRERAMMTMVVLSKRKGGNVGKSKRDQIVTV
SV
>sp|P68871|HBB_HUMAN
MVHLTPEEKSAVTALWGKVNVDEVGGEALGRLLVVYPWTQRFFESFGDLSTPDAVMGNPKVKAHGKKVLGAFSDGLAHLDNLKGTFA
TLSELHCDKLHVDPENFRLLGNVLVCVLAHHFGKEFTPPVQAAYQKVVAGVANALAHKYH
>sp|A5YM72|CRNS1_HUMAN
MLLCLSPAWLMKVPAPGQPGEAALLVSKAVSFHPGGLTFLDDFVPPRRATYFLAGLGLGPGRGREAAELARDLTCPTGASAELARLL
EDRLLTRQLLAQQGGVAVPATLAFTYKPPGLLRGGDASLGLRLVELSGKEGQETLVKEEVEAFLRSEALGDILQVAVKLSGWRWRGR
QAWRLHPRAELGAVVDTVLALLEKLEEEESVLVEAVYPPAQLPCSDGPSPGPGLAVRICAVVCRTQGDRPLLSKVVCGVGRGDRPLR
HHNSLPRTLEVALAQCGLGEEAQVAAVRQRVKAAAEAALAAVLALEAGLSAEQRGGRRAHTDFLGVDFALTAAGGVLTPVALELNGG
LCLEACGALEGLWAAPRLGPAADEAVAAPLVETMLRRSARCLMEGKQLLVVGAGGVSKKFVWEAARDYGLQLHLVESDPNHFASQLV
QTFIHFDMTEHRRDEENARLLAELVRARGLKLDGCFSYWDDCLVLTALLCQELGLPCSSPAAMRLAKQKSLTQLHLLHHHGPPWPAP
SLHAVPCCPLESEADVERAVHQVPLPGVMKLEFGAGAVGVRLVEDAPQCHEHFSRITRDLQGEADHPGIGLGWGNAMLLMEFVEGTE
HDVDLVLFGGRLLAAFVSDNGPTRLPGFTETAACMPTGLAPEQEAQMVQAAFRCCLGCGLLDGVFNVELKLTGAGPRLIEINPRMGG
FYLRDWILELYGVDLLLAAVMVACGLRPALPTRPRARGHLVGVMCLVSQHLQALSSTASRETLQALHDRGLLRLNLLEEALVPGEYE
EPYCSVACAGPSPTEARLRLLGLCQGLGIDGPSYPVAHFLSHFK
>sp|O95155|UBE4B_HUMAN

MEELSADEIRRRRLARLAGGQTSQPTTPLTSPQRENPPGPPIAASAPGPSQSLGLNVHNMTPATSPIGASGVAHRSQSSEGVSSLSS
SPSNSLETQSQSLSRSQSMDIDGVSCEKSMSQVDVDSGIENMEVDENDRREKRSLSDKEPSSGPEVSEEQALQLVCKIFRVSWKDRD
RDVIFLSSLSAQFKQNPKEVFSDFKDLIGQILMEVLMMSTQTRDENPFASLTATSQPIAAAARSPDRNLLLNTGSNPGTSPMFCSVA
SFGASSLSSLYESSPAPTPSFWSSVPVMGPSLASPSRAASQLAVPSTPLSPHSAASGTAAGSQPSSPRYRPYTVTHPWASSGVSILS
SSPSPPALASSPQAVPASSSRQRPSSTGPPLPPASPSATSRRPSSLRISPSLGASGGASNWDSYSDHFTIETCKETDMLNYLIECFD
RVGIEEKKAPKMCSQPAVSQLLSNIRSQCISHTALVLQGSLTQPRSLQQPSFLVPYMLCRNLPYGFIQELVRTTHQDEEVFKQIFIP
ILQGLALAAKECSLDSDYFKYPLMALGELCETKFGKTHPVCNLVASLRLWLPKSLSPGCGRELQRLSYLGAFFSFSVFAEDDVKVVE
KYFSGPAITLENTRVVSQSLQHYLELGRQELFKILHSILLNGETREAALSYMAAVVNANMKKAQMQTDDRLVSTDGFMLNFLWVLQQ
LSTKIKLETVDPTYIFHPRCRITLPNDETRVNATMEDVNDWLTELYGDQPPFSEPKFPTECFFLTLHAHHLSILPSCRRYIRRLRAI
RELNRTVEDLKNNESQWKDSPLATRHREMLKRCKTQLKKLVRCKACADAGLLDESFLRRCLNFYGLLIQLLLRILDPAYPDITLPLN
SDVPKVFAALPEFYVEDVAEFLFFIVQYSPQALYEPCTQDIVMFLVVMLCNQNYIRNPYLVAKLVEVMFMTNPAVQPRTQKFFEMIE
NHPLSTKLLVPSLMKFYTDVEHTGATSEFYDKFTIRYHISTIFKSLWQNIAHHGTFMEEFNSGKQFVRYINMLINDTTFLLDESLES
LKRIHEVQEEMKNKEQWDQLPRDQQQARQSQLAQDERVSRSYLALATETVDMFHILTKQVQKPFLRPELGPRLAAMLNFNLQQLCGP
KCRDLKVENPEKYGFEPKKLLDQLTDIYLQLDCARFAKAIADDQRSYSKELFEEVISKMRKAGIKSTIAIEKFKLLAEKVEEIVAKN
ARAEIDYSDAPDEFRDPLMDTLMTDPVRLPSGTIMDRSIILRHLLNSPTDPFNRQTLTESMLEPVPELKEQIQAWMREKQNSDH
>sp|Q01780|EXOSX_HUMAN
MAPPSTREPRVLSATSATKSDGEMVLPGFPDADSFVKFALGSVVAVTKASGGLPQFGDEYDFYRSFPGFQAFCETQGDRLLQCMSRV
MQYHGCRSNIKDRSKVTELEDKFDLLVDANDVILERVGILLDEASGVNKNQQPVLPAGLQVPKTVVSSWNRKAAEYGKKAKSETFRL
LHAKNIIRPQLKFREKIDNSNTPFLPKIFIKPNAQKPLPQALSKERRERPQDRPEDLDVPPALADFIHQQRTQQVEQDMFAHPYQYE
LNHFTPADAVLQKPQPQLYRPIEETPCHFISSLDELVELNEKLLNCQEFAVDLEHHSYRSFLGLTCLMQISTRTEDFIIDTLELRSD
MYILNESLTDPAIVKVFHGADSDIEWLQKDFGLYVVNMFDTHQAARLLNLGRHSLDHLLKLYCNVDSNKQYQLADWRIRPLPEEMLS
YARDDTHYLLYIYDKMRLEMWERGNGQPVQLQVVWQRSRDICLKKFIKPIFTDESYLELYRKQKKHLNTQQLTAFQLLFAWRDKTAR
REDESYGYVLPNHMMLKIAEELPKEPQGIIACCNPVPPLVRQQINEMHLLIQQAREMPLLKSEVAAGVKKSGPLPSAERLENVLFGP
HDCSHAPPDGYPIIPTSGSVPVQKQASLFPDEKEDNLLGTTCLIATAVITLFNEPSAEDSKKGPLTVAQKKAQNIMESFENPFRMFL
PSLGHRAPVSQAAKFDPSTKIYEISNRWKLAQVQVQKDSKEAVKKKAAEQTAAREQAKEACKAAAEQAISVRQQVVLENAAKKRERA
TSDPRTTEQKQEKKRLKISKKPKDPEPPEKEFTPYDYSQSDFKAFAGNSKSKVSSQFDPNKQTPSGKKCIAAKKIKQSVGNKSMSFP
TGKSDRGFRYNWPQR
>sp|Q14690|RRP5_HUMAN
MANLEESFPRGGTRKIHKPEKAFQQSVEQDNLFDISTEEGSTKRKKSQKGPAKTKKLKIEKRESSKSAREKFEILSVESLCEGMRIL
GCVKEVNELELVISLPNGLQGFVQVTEICDAYTKKLNEQVTQEQPLKDLLHLPELFSPGMLVRCVVSSLGITDRGKKSVKLSLNPKN
VNRVLSAEALKPGMLLTGTVSSLEDHGYLVDIGVDGTRAFLPLLKAQEYIRQKNKGAKLKVGQYLNCIVEKVKGNGGVVSLSVGHSE
VSTAIATEQQSWNLNNLLPGLVVKAQVQKVTPFGLTLNFLTFFTGVVDFMHLDPKKAGTYFSNQAVRACILCVHPRTRVVHLSLRPI
FLQPGRPLTRLSCQNLGAVLDDVPVQGFFKKAGATFRLKDGVLAYARLSHLSDSKNVFNPEAFKPGNTHKCRIIDYSQMDELALLSL
RTSIIEAQYLRYHDIEPGAVVKGTVLTIKSYGMLVKVGEQMRGLVPPMHLADILMKNPEKKYHIGDEVKCRVLLCDPEAKKLMMTLK
KTLIESKLPVITCYADAKPGLQTHGFIIRVKDYGCIVKFYNNVQGLVPKHELSTEYIPDPERVFYTGQVVKVVVLNCEPSKERMLLS
FKLSSDPEPKKEPAGHSQKKGKAINIGQLVDVKVLEKTKDGLEVAVLPHNIRAFLPTSHLSDHVANGPLLHHWLQAGDILHRVLCLS
QSEGRVLLCRKPALVSTVEGGQDPKNFSEIHPGMLLIGFVKSIKDYGVFIQFPSGLSGLAPKAIMSDKFVTSTSDHFVEGQTVAAKV
TNVDEEKQRMLLSLRLSDCGLGDLAITSLLLLNQCLEELQGVRSLMSNRDSVLIQTLAEMTPGMFLDLVVQEVLEDGSVVFSGGPVP
DLVLKASRYHRAGQEVESGQKKKVVILNVDLLKLEVHVSLHQDLVNRKARKLRKGSEHQAIVQHLEKSFAIASLVETGHLAAFSLTS
HLNDTFRFDSEKLQVGQGVSLTLKTTEPGVTGLLLAVEGPAAKRTMRPTQKDSETVDEDEEVDPALTVGTIKKHTLSIGDMVTGTVK
SIKPTHVVVTLEDGIIGCIHASHILDDVPEGTSPTTKLKVGKTVTARVIGGRDMKTFKYLPISHPRFVRTIPELSVRPSELEDGHTA
LNTHSVSPMEKIKQYQAGQTVTCFLKKYNVVKKWLEVEIAPDIRGRIPLLLTSLSFKVLKHPDKKFRVGQALRATVVGPDSSKTLLC
LSLTGPHKLEEGEVAMGRVVKVTPNEGLTVSFPFGKIGTVSIFHMSDSYSETPLEDFVPQKVVRCYILSTADNVLTLSLRSSRTNPE
TKSKVEDPEINSIQDIKEGQLLRGYVGSIQPHGVFFRLGPSVVGLARYSHVSQHSPSKKALYNKHLPEGKLLTARVLRLNHQKNLVE

LSFLPGDTGKPDVLSASLEGQLTKQEERKTEAEERDQKGEKKNQKRNEKKNQKGQEEVEMPSKEKQQPQKPQAQKRGGRECRESGSE
QERVSKKPKKAGLSEEDDSLVDVYYREGKEEAEETNVLPKEKQTKPAEAPRLQLSSGFAWNVGLDSLTPALPPLAESSDSEEDEKPH
QATIKKSKKERELEKQKAEKELSRIEEALMDPGRQPESADDFDRLVLSSPNSSILWLQYMAFHLQATEIEKARAVAERALKTISFRE
EQEKLNVWVALLNLENMYGSQESLTKVFERAVQYNEPLKVFLHLADIYAKSEKFQEAGELYNRMLKRFRQEKAVWIKYGAFLLRRSQ
AAASHRVLQRALECLPSKEHVDVIAKFAQLEFQLGDAERAKAIFENTLSTYPKRTDVWSVYIDMTIKHGSQKDVRDIFERVIHLSLA
PKRMKFFFKRYLDYEKQHGTEKDVQAVKAKALEYVEAKSSVLED
>sp|Q7Z7G8|VP13B_HUMAN
MLESYVTPILMSYVNRYIKNLKPSDLQLSLWGGDVVLSKLELKLDVLEQELKLPFTFLSGHIHELRIHVPWTKLGSEPVVITINTME
CILKLKDGIQDDHESCGSNSTNRSTAESTKSSIKPRRMQQAAPTDPDLPPGYVQSLIRRVVNNVNIVINNLILKYVEDDIVLSVNIT
SAECYTVGELWDRAFMDISATDLVLRKVINFSDCTVCLDKRNASGKIEFYQDPLLYKCSFRTRLHFTYENLNSKMPSVIKIHTLVES
LKLSITDQQLPMFIRIMQLGIALYYGEIGNFKEGEIEDLTCHNKDMLGNITGSEDETRIDMQYPAQHKGQELYSQQDEEQPQGWVSW
AWSFVPAIVSYDDGEEDFVGNDPASTMHQQKAQTLKDPIVSIGFYCTKATVTFKLTEMQVESSYYSPQKVKSKEVLCWEQEGTTVEA
LMMGEPFFDCQIGFVGCRAMCLKGIMGVKDFEENMNRSETEACFFICGDNLSTKGFTYLTNSLFDYRSPENNGTRAEFILDSTHHKE
TYTEIAGMQRFGAFYMDYLYTMENTSGKGSTNQQDFSSGKSEDLGTVQEKSTKSLVIGPLDFRLDSSAVHRILKMIVCALEHEYEPY
SRLKSDIKDENETILNPEEVALLEEYIPTRHTSVTLLKCTCTISMAEFNLLDHLLPVIMGEKNSSNFMNTTNFQSLRPLPSIRILVD
KINLEHSVPMYAEQLVHVVSSLTQPSDNLLHYCYVHCYLKIFGFQAGLTSLDCSGSYCLPVPVIPSFSTALYGKLLKLPTCWTKRSQ
IAITEGIFELPNLTIQATRAQTLLLQAIYQSWSHLGNVSSSAVIEALINEIFLSIGVKSKNPLPTLEGSIQNVELKYCSTSLVKCAS
GTMGSIKICAKAPVDSGKEKLIPLLQGPSDTKDLHSTKWLNESRKPESLLAPDLMAFTIQVPQYIDYCHNSGAVLLCSIQGLAVNID
PILYTWLIYQPQKRTSRHMQQQPVVAVPLVMPVCRRKEDEVSIGSAPLAKQQSYQASEYASSPVKTKTVTESRPLSVPVKAMLNISE
SCRSPEERMKEFIGIVWNAVKHLTLQLEVQSCCVFIPNDSLPSPSTIVSGDIPGTVRSWYHGQTSMPGTLVLCLPQIKIISAGHKYM
EPLQEIPFVIPRPILEEGDAFPWTISLHNFSIYTLLGKQVTLCLVEPMGCTSTLAVTSQKLLATGPDTRHSFVVCLHVDLESLEIKC
SNPQVQLFYELTDIMNKVWNKIQKRGNLNLSPTSPETMAGPVPTSPVRSSIGTAPPDTSTCSPSADIGTTTEGDSIQAGEESPFSDS
VTLEQTTSNIGGTSGRVSLWMQWVLPKITIKLFAPDPENKGTEVCMVSELEDLSASIDVQDVYTKVKCKIESFNIDHYRSSLGEECW
SLGQCGGVFLSCTDKLNRRTLLVRPISKQDPFSNCSGFFPSTTTKLLDGTHQQHGFLSLTYTKAVTKNVRHKLTSRNERRSFHKLSE
GLMDGSPHFLHEILLSAQAFDIVLYFPLLNAIASIFQAKLPKTQKEKRKSPGQPMRTHTLTSRNLPLIYVNTSVIRIFIPKTEEMQP
TVEANQAAKEDTVVLKIGSVAMAPQADNPLGRSVLRKDIYQRALNLGILRDPGSEIEDRQYQIDLQSINIGTAQWHQLKPEKESVSG
GVVTETERNSQNPALEWNMASSIRRHQERRAILTPVLTDFSVRITGAPAVIFTKVVSPENLHTEEILVCGHSLEVNITTNLDFFLSV
AQVQLLHQLIVANMTGLEPSNKAAEISKQEQKKVDIFDGGMAETSSRYSGAQDSGIGSDSVKIRIVQIEQHSGASQHRIARPSRQSS
IVKNLNFIPFDIFITASRISLMTYSCMALSKSKSQEQKNNEKTDKSSLNLPEVDSDVAKPNQACISTVTAEDLLRSSISFPSGKKIG
VLSLESLHASTRSSARQALGITIVRQPGRRGTGDLQLEPFLYFIVSQPSLLLSCHHRKQRVEVSIFDAVLKGVASDYKCIDPGKTLP
EALDYCTVWLQTVPGEIDSKSGIPPSFITLQIKDFLNGPADVNLDISKPLKANLSFTKLDQINLFLKKIKNAHSLAHSEETSAMSNT
MVNKDDLPVSKYYRGKLSKPKIHGDGVQKISAQENMWRAVSCFQKISVQTTQIVISMETVPHTSKPCLLASLSNLNGSLSVKATQKV
PGIILGSSFLLSINDFLLKTSLKERSRILIGPCCATANLEAKWCKHSGNPGPEQSIPKISIDLRGGLLQVFWGQEHLNCLVLLHELL
NGYLNEEGNFEVQVSEPVPQMSSPVEKNQTFKSEQSSDDLRTGLFQYVQDAESLKLPGVYEVLFYNETEDCPGMMLWRYPEPRVLTL
VRITPVPFNTTEDPDISTADLGDVLQVPCSLEYWDELQKVFVAFREFNLSESKVCELQLPDINLVNDQKKLVSSDLWRIVLNSSQNG
ADDQSSASESGSQSTCDPLVTPTALAACTRVDSCFTPWFVPSLCVSFQFAHLEFHLCHHLDQLGTAAPQYLQPFVSDRNMPSELEYM
IVSFREPHMYLRQWNNGSVCQEIQFLAQADCKLLECRNVTMQSVVKPFSIFGQMAVSSDVVEKLLDCTVIVDSVFVNLGQHVVHSLN
TAIQAWQQNKCPEVEELVFSHFVICNDTQETLRFGQVDTDENILLASLHSHQYSWRSHKSPQLLHICIEGWGNWRWSEPFSVDHAGT
FIRTIQYRGRTASLIIKVQQLNGVQKQIIICGRQIICSYLSQSIELKVVQHYIGQDGQAVVREHFDCLTAKQKLPSYILENNELTEL
CVKAKGDEDWSRDVCLESKAPEYSIVIQVPSSNSSIIYVWCTVLTLEPNSQVQQRMIVFSPLFIMRSHLPDPIIIHLEKRSLGLSET
QIIPGKGQEKPLQNIEPDLVHHLTFQAREEYDPSDCAVPISTSLIKQIATKVHPGGTVNQILDEFYGPEKSLQPIWPYNKKDSDRNE
QLSQWDSPMRVKLSIWKPYVRTLLIELLPWALLINESKWDLWLFEGEKIVLQVPAGKIIIPPNFQEAFQIGIYWANTNTVHKSVAIK
LVHNLTSPKWKDGGNGEVVTLDEEAFVDTEIRLGAFPGHQKLCQFCISSMVQQGIQIIIQIEDKTTIINNTPYQIFYKPQLSVCNPHS
GKEYFRVPDSATFSICPGGEQPAMKSSSLPCWDLMPDISQSVLDASLLQKQIMLGFSPAPGADSSQCWSLPAIVRPEFPRQSVAVPL

GNFRENGFCTRAIVLTYQEHLGVTYLTLSEDPSPRVIIHNRCPVKMLIKENIKDIPKFEVYCKKIPSECSIHHELYHQISSYPDCKT
KDLLPSLLLRVEPLDEVTTEWSDAIDINSQGTQVVFLTGFGYVYVDVVHQCGTVFITVAPEGKAGPILTNTNRAPEKIVTFKMFITQ
LSLAVFDDLTHHKASAELLRLTLDNIFLCVAPGAGPLPGEEPVAALFELYCVEICCGDLQLDNQLYNKSNFHFAVLVCQGEKAEPIQ
CSKMQSLLISNKELEEYKEKCFIKLCITLNEGKSILCDINEFSFELKPARLYVEDTFVYYIKTLFDTYLPNSRLAGHSTHLSGGKQV
LPMQVTQHARALVNPVKLRKLVIQPVNLLVSIHASLKLYIASDHTPLSFSVFERGPIFTTARQLVHALAMHYAAGALFRAGWVVGSL
DILGSPASLVRSIGNGVADFFRLPYEGLTRGPGAFVSGVSRGTTSFVKHISKGTLTSITNLATSLARNMDRLSLDEEHYNRQEEWRR
QLPESLGEGLRQGLSRLGISLLGAIAGIVDQPMQNFQKTSEAQASAGHKAKGVISGVGKGIMGVFTKPIGGAAELVSQTGYGILHGA
GLSQLPKQRHQPSDLHADQAPNSHVKYVWKMLQSLGRPEVHMALDVVLVRGSGQEHEGCLLLTSEVLFVVSVSEDTQQQAFPVTEID
CAQDSKQNNLLTVQLKQPRVACDVEVDGVRERLSEQQYNRLVDYITKTSCHLAPSCSSMQIPCPVVAAEPPPSTVKTYHYLVDPHFA
QVFLSKFTMVKNKALRKGFP
>sp|P58166|INHBE_HUMAN
MRLPDVQLWLVLLWALVRAQGTGSVCPSCGGSKLAPQAERALVLELAKQQILDGLHLTSRPRITHPPPQAALTRALRRLQPGSVAPG
NGEEVISFATVTDSTSAYSSLLTFHLSTPRSHHLYHARLWLHVLPTLPGTLCLRIFRWGPRRRRQGSRTLLAEHHITNLGWHTLTLP
SSGLRGEKSGVLKLQLDCRPLEGNSTVTGQPRRLLDTAGHQQPFLELKIRANEPGAGRARRRTPTCEPATPLCCRRDHYVDFQELGW
RDWILQPEGYQLNYCSGQCPPHLAGSPGIAASFHSAVFSLLKANNPWPASTSCCVPTARRPLSLLYLDHNGNVVKTDVPDMVVEACG
CS
>sp|O43463|SUV91_HUMAN
MAENLKGCSVCCKSSWNQLQDLCRLAKLSCPALGISKRNLYDFEVEYLCDYKKIREQEYYLVKWRGYPDSESTWEPRQNLKCVRILK
QFHKDLERELLRRHHRSKTPRHLDPSLANYLVQKAKQRRALRRWEQELNAKRSHLGRITVENEVDLDGPPRAFVYINEYRVGEGITL
NQVAVGCECQDCLWAPTGGCCPGASLHKFAYNDQGQVRLRAGLPIYECNSRCRCGYDCPNRVVQKGIRYDLCIFRTDDGRGWGVRTL
EKIRKNSFVMEYVGEIITSEEAERRGQIYDRQGATYLFDLDYVEDVYTVDAAYYGNISHFVNHSCDPNLQVYNVFIDNLDERLPRIA
FFATRTIRAGEELTFDYNMQVDPVDMESTRMDSNFGLAGLPGSPKKRVRIECKCGTESCRKYLF
>sp|Q8WU39|MZB1_HUMAN
MRLSLPLLLLLLGAWAIPGGLGDRAPLTATAPQLDDEEMYSAHMPAHLRCDACRAVAYQMWQNLAKAETKLHTSNSGGRRELSELVY
TDVLDRSCSRNWQDYGVREVDQVKRLTGPGLSEGPEPSISVMVTGGPWPTRLSRTCLHYLGEFGEDQIYEAHQQGRGALEALLCGGP
QGACSEKVSATREEL
>sp|Q6L8G9|KRA56_HUMAN
MGCCGCSGGCGSGCGGCGSGCGGCGSSCCVPICCCKPVCCCVPACSCTSCGSCGGSKGCCGSCGGSKGGCGSCGGSKGGCGSCGCSQ
CSCCKPCYCSSGCGSSCCQSSCCKPCCSQASCCVPICCQCKI
>sp|P49662|CASP4_HUMAN
MAEGNHRKKPLKVLESLGKDFLTGVLDNLVEQNVLNWKEEEKKKYYDAKTEDKVRVMADSMQEKQRMAGQMLLQTFFNIDQISPNKK
AHPNMEAGPPESGESTDALKLCPHEEFLRLCKERAEEIYPIKERNNRTRLALIICNTEFDHLPPRNGADFDITGMKELLEGLDYSVD
VEENLTARDMESALRAFATRPEHKSSDSTFLVLMSHGILEGICGTVHDEKKPDVLLYDTIFQIFNNRNCLSLKDKPKVIIVQACRGA
NRGELWVRDSPASLEVASSQSSENLEEDAVYKTHVEKDFIAFCSSTPHNVSWRDSTMGSIFITQLITCFQKYSWCCHLEEVFRKVQQ
SFETPRAKAQMPTIERLSMTRYFYLFPGN
>sp|Q52LJ0|FA98B_HUMAN
MRGPEPGPQPTMEGDVLDTLEALGYKGPLLEEQALTKAAEGGLSSPEFSELCIWLGSQIKSLCNLEESITSAGRDDLESFQLEISGF
LKEMACPYSVLISGDIKDRLKKKEDCLKLLLFLSTELQASQILQNKKHKNSQLDKNSEVYQEVQAMFDTLGIPKSTTSDIPHMLNQV
ESKVKDILSKVQKNHVGKPLLKMDLNSEQAEQLERINDALSCEYECRRRMLMKRLDVTVQSFGWSDRAKVKTDDIARIYQPKRYALS
PKTTITMAHLLAAREDLSKIIRTSSGTSREKTACAINKVGVSFSTVENELMISYLMFLQILVYFSFMSW
>sp|Q6IEZ7|OR2T5_HUMAN
MANITRMANHTGKLDFILMGLFRRSKHPALLSVVIFVVFLKALSGNAVLILLIHCDAHLHSPMYFFISQLSLMDMAYISVTVPKMLL
DQVMGVNKVSAPECGMQMFLYLTLAGSEFFLLATMAYDRYVAICHPLRYPVLMNHRVCLFLASGCWFLGSVDGFMLTPITMSFPFCR

SWEIHHFFCEVPAVTILSCSDTSLYETLMYLCCVLMLLIPVTIISSSYLLILLTVHRMNSAEGRKKAFATCSSHLTVVILFYGAAVY
TYMLPSSYHTPEKDMMVSVFYTILTPVLNPLIYSLRNKDVMGALKKMLTVRFVL
>sp|Q13542|4EBP2_HUMAN
MSSSAGSGHQPSQSRAIPTRTVAISDAAQLPHDYCTTPGGTLFSTTPGGTRIIYDRKFLLDRRNSPMAQTPPCHLPNIPGVTSPGTL
IEDSKVEVNNLNNLNNHDRKHAVGDDAQFEMDI
>sp|Q96DZ9|CKLF5_HUMAN
MLSARDRRDRHPEEGVVAELQGFAVDKAFLTSHKGILLETELALTLIIFICFTASISAYMAAALLEFFITLAFLFLYATQYYQRFDR
INWPCLLQGHGQSGGPHPLDLLSHSAKVQPQPWPGLTPPGWHTPAAVPWVPAPAPGFWSWLLWFICFHSLGSSDFLRCVSAIIIFLV
VSFAAVTSRDGAAIAAFVFGIILVSIFAYDAFKIYRTEMAPGASQGDQQ
>sp|Q9NP87|DPOLM_HUMAN
MLPKRRRARVGSPSGDAASSTPPSTRFPGVAIYLVEPRMGRSRRAFLTGLARSKGFRVLDACSSEATHVVMEETSAEEAVSWQERRM
AAAPPGCTPPALLDISWLTESLGAGQPVPVECRHRLEVAGPRKGPLSPAWMPAYACQRPTPLTHHNTGLSEALEILAEAAGFEGSEG
RLLTFCRAASVLKALPSPVTTLSQLQGLPHFGEHSSRVVQELLEHGVCEEVERVRRSERYQTMKLFTQIFGVGVKTADRWYREGLRT
LDDLREQPQKLTQQQKAGLQHHQDLSTPVLRSDVDALQQVVEEAVGQALPGATVTLTGGFRRGKLQGHDVDFLITHPKEGQEAGLLP
RVMCRLQDQGLILYHQHQHSCCESPTRLAQQSHMDAFERSFCIFRLPQPPGAAVGGSTRPCPSWKAVRVDLVVAPVSQFPFALLGWT
GSKLFQRELRRFSRKEKGLWLNSHGLFDPEQKTFFQAASEEDIFRHLGLEYLPPEQRNA
>sp|Q9ULR3|PPM1H_HUMAN
MLTRVKSAVANFMGGIMAGSSGSEHGGGSCGGSDLPLRFPYGRPEFLGLSQDEVECSADHIARPILILKETRRLPWATGYAEVINAG
KSTHNEDQASCEVLTVKKKAGAVTSTPNRNSSKRRSSLPNGEGLQLKENSESEGVSCHYWSLFDGHAGSGAAVVASRLLQHHITEQL
QDIVDILKNSAVLPPTCLGEEPENTPANSRTLTRAASLRGGVGAPGSPSTPPTRFFTEKKIPHECLVIGALESAFKEMDLQIERERS
SYNISGGCTALIVICLLGKLYVANAGDSRAIIIRNGEIIPMSSEFTPETERQRLQYLAFMQPHLLGNEFTHLEFPRRVQRKELGKKM
LYRDFNMTGWAYKTIEDEDLKFPLIYGEGKKARVMATIGVTRGLGDHDLKVHDSNIYIKPFLSSAPEVRIYDLSKYDHGSDDVLILA
TDGLWDVLSNEEVAEAITQFLPNCDPDDPHRYTLAAQDLVMRARGVLKDRGWRISNDRLGSGDDISVYVIPLIHGNKLS
>sp|Q8N954|GPT11_HUMAN
MAEEEDYMSDSFINVQEDIRPGLPMLRQIREARRKEEKQQEANLKNRQKSLKEEEQERRDIGLKNALGCENKGFALLQKMGYKSGQA
LGKSGGGIVEPIPLNIKTGKSGIGHEASLKRKAEEKLESYRKKIHMKNQAEEKAAEQFRMRLKNKQDEMKLEGDLRRSQRACQQLDV
QKNIQVPREAWYWLRLEEETEEDEEEKEQDEDEYKSEDLSVLEKLQILTSYLREEHLYCIWCGTAYEDKEDLSSNCPGPTSADHD
>sp|Q96B23|CR025_HUMAN
MKMEEAVGKVEELIESEAPPKASEQETAKEEDGSVELESQVQKDGVADSTVISSMPCLLMELRRDSSESQLASTESDKPTTGRVYES
DSSNHCMLSPSSSGHLADSDTLSSAEENEPSQAETAVEGDPSGVSGATVGRKSRRSRSESETSTMAAKKNRQSSDKQNGRVAKVKGH
RSQKHKERIRLLRQKREAAARKKYNLLQDSSTSDSDLTCDSSTSSSDDDEEVSGSSKTITAEIPDGPPVVAHYDMSDTNSDPEVVNV
DNLLAAAVVQEHSNSVGGQDTGATWRTSGLLEELNAEAGHLDPGFLASDKTSGNAPLNEEINIASSDSEVEIVGVQEHARCVHPRGG
VIQSVSSWKHGSGTQYVSTRQTQSWTAVTPQQTWASPAEVVDLTLDEDSRRKYLL
>sp|Q8IUK8|CBLN2_HUMAN
MQAPGRGPLGLRLMMPGRRGALREPGGCGSCLGVALALLLLLLPACCPVRAQNDTEPIVLEGKCLVVCDSSPSADGAVTSSLGISVR
SGSAKVAFSATRSTNHEPSEMSNRTMTIYFDQVLVNIGNHFDLASSIFVAPRKGIYSFSFHVVKVYNRQTIQVSLMQNGYPVISAFA
GDQDVTREAASNGVLLLMEREDKVHLKLERGNLMGGWKYSTFSGFLVFPL
>sp|Q9UKN5|PRDM4_HUMAN
MHHRMNEMNLSPVGMEQLTSSSVSNALPVSGSHLGLAASPTHSAIPAPGLPVAIPNLGPSLSSLPSALSLMLPMGIGDRGVMCGLPE
RNYTLPPPPYPHLESSYFRTILPGILSYLADRPPPQYIHPNSINVDGNTALSITNNPSALDPYQSNGNVGLEPGIVSIDSRSVNTHG
AQSLHPSDGHEVALDTAITMENVSRVTSPISTDGMAEELTMDGVAGEHSQIPNGSRSHEPLSVDSVSNNLAADAVGHGGVIPMHGNG
LELPVVMETDHIASRVNGMSDSALSDSIHTVAMSTNSVSVALSTSHNLASLESVSLHEVGLSLEPVAVSSITQEVAMGTGHVDVSSD
SLSFVSPSLQMEDSNSNKENMATLFTIWCTLCDRAYPSDCPEHGPVTFVPDTPIESRARLSLPKQLVLRQSIVGAEVGVWTGETIPV
RTCFGPLIGQQSHSMEVAEWTDKAVNHIWKIYHNGVLEFCIITTDENECNWMMFVRKARNREEQNLVAYPHDGKIFFCTSQDIPPEN

ELLFYYSRDYAQQIGVPEHPDVHLCNCGKECNSYTEFKAHLTSHIHNHLPTQGHSGSHGPSHSKERKWKCSMCPQAFISPSKLHVHF
MGHMGMKPHKCDFCSKAFSDPSNLRTHLKIHTGQKNYRCTLCDKSFTQKAHLESHMVIHTGEKNLKCDYCDKLFMRRQDLKQHVLIH
TQERQIKCPKCDKLFLRTNHLKKHLNSHEGKRDYVCEKCTKAYLTKYHLTRHLKTCKGPTSSSSAPEEEEEDDSEEEDLADSVGTED
CRINSAVYSADESLSAHK
>sp|Q9Y3F4|STRAP_HUMAN
MAMRQTPLTCSGHTRPVVDLAFSGITPYGYFLISACKDGKPMLRQGDTGDWIGTFLGHKGAVWGATLNKDATKAATAAADFTAKVWD
AVSGDELMTLAHKHIVKTVDFTQDSNYLLTGGQDKLLRIYDLNKPEAEPKEISGHTSGIKKALWCSEDKQILSADDKTVRLWDHATM
TEVKSLNFNMSVSSMEYIPEGEILVITYGRSIAFHSAVSLDPIKSFEAPATINSASLHPEKEFLVAGGEDFKLYKYDYNSGEELESY
KGHFGPIHCVRFSPDGELYASGSEDGTLRLWQTVVGKTYGLWKCVLPEEDSGELAKPKIGFPETTEEELEEIASENSDCIFPSAPDV
KA
>sp|O95391|SLU7_HUMAN
MSATVVDAVNAAPLSGSKEMSLEEPKKMTREDWRKKKELEEQRKLGNAPAEVDEEGKDINPHIPQYISSVPWYIDPSKRPTLKHQRP
QPEKQKQFSSSGEWYKRGVKENSIITKYRKGACENCGAMTHKKKDCFERPRRVGAKFTGTNIAPDEHVQPQLMFDYDGKRDRWNGYN
PEEHMKIVEEYAKVDLAKRTLKAQKLQEELASGKLVEQANSPKHQWGEEEPNSQMEKDHNSEDEDEDKYADDIDMPGQNFDSKRRIT
VRNLRIREDIAKYLRNLDPNSAYYDPKTRAMRENPYANAGKNPDEVSYAGDNFVRYTGDTISMAQTQLFAWEAYDKGSEVHLQADPT
KLELLYKSFKVKKEDFKEQQKESILEKYGGQEHLDAPPAELLLAQTEDYVEYSRHGTVIKGQERAVACSKYEEDVKIHNHTHIWGSY
WKEGRWGYKCCHSFFKYSYCTGEAGKEIVNSEECIINEITGEESVKKPQTLMELHQEKLKEEKKKKKKKKKKHRKSSSDSDDEEKKH
EKLKKALNAEEARLLHVKETMQIDERKRPYNSMYETREPTEEEMEAYRMKRQRPDDPMASFLGQ
>sp|P10266|POK10_HUMAN
NKSRKRRNRVSFLGAVTVEPPKPIPLTWKTEKPVWVNQWPLPKQKLEALHLLANEQLEKGHIEPSFSPWNSPVFVIQKKSGKWHTLT
DLRAVNAVIQPMGPLQPGLPSPAMIPKDWPLIIIDLKDCFFTIPLAEQDCEKFAFTIPAINNKEPATRFQWKVLPQGMLNSPTICQT
FVGRALQPVREKFSDCYIIHYIDDILCAAETKDKLIDCYTFLQAEVANAGLAIASDKIQTSTPFHYLGMQIENRKIKPQKIEIRKDT
LKTLNDFQKLLGDINWIRPTLGIPTYAMSNLFSILRGDSDLNSQRILTPEATKEIKLVEEKIQSAQINRIDPLAPLQLLIFATAHSP
TGIIIQNTDLVEWSFLPHSTVKTFTLYLDQIATLIGQTRLRITKLCGNDPDKIVVPLTKEQVRQAFINSGAWQIGLANFVGLIDNHY
PKTKIFQFLKLTTWILPKITRREPLENALTVFTDGSSNGKAAYTGPKERVIKTPYQSAQRDELVAVITVLQDFDQPINIISDSAYVV
QATRDVETALIKYSMDDQLNQLFNLLQQTVRKRNFPFYITYIRAHTNLPGPLTKANEQADLLVSSALIKAQELHALTHVNAAGLKNK
FDVTWKQAKDIVQHCTQCQVLHLPTQEAGVNPRGLCPNALWQMDVTHVPSFGRLSYVHVTVDTYSHFIWATCQTGESTSHVKKHLLS
CFAVMGVPEKIKTDNGPGYCSKAFQKFLSQWKISHTTGIPYNSQGQAIVERTNRTLKTQLVKQKEGGDSKECTTPQMQLNLALYTLN
FLNIYRNQTTTSAEQHLTGKKNSPHEGKLIWWKDNKNKTWEIGKVITWGRGFACVSPGENQLPVWLPTRHLKFYNEPIGDAKKRAST
EMVTPVTWMDNPIEVYVNDSIWVPGPIDDRCPAKPEEEGMMINISIGYRYPPICLGRAPGCLMPAVQNWLVEVPTVSPISRFTYHMV
SGMSLRPRVNYLQDFSYQRSLKFRPKGKPCPKEIPKESKNTEVLVWEECVANSAVIL
>sp|Q8NDX5|PHC3_HUMAN
MDTEPNPGTSSVSTTTSSTTTTTITTSSSRMQQPQISVYSGSDRHAVQVIQQALHRPPSSAAQYLQQMYAAQQQHLMLHTAALQQQH
LSSSQLQSLAAVQASLSSGRPSTSPTGSVTQQSSMSQTSINLSTSPTPAQLISRSQASSSTSGSITQQTMLLGSTSPTLTASQAQMY
LRAQMLIFTPATTVAAVQSDIPVVSSSSSSSCQSAATQVQNLTLRSQKLGVLSSSQNGPPKSTSQTQSLTICHNKTTVTSSKISQRD
PSPESNKKGESPSLESRSTAVTRTSSIHQLIAPASYSPIQPHSLIKHQQIPLHSPPSKVSHHQLILQQQQQIQPITLQNSTQDPPP
SQHCIPLQNHGLPPAPSNAQSQHCSPIQSHPSPLTVSPNQSQSAQQSVVVSPPPPHSPSQSPTIIIHPQALIQPHPLVSSALQPGPN
LQQSTANQVQATAQLNLPSHLPLPASPVVHIGPVQQSALVSPGQQIVSPSHQQYSSLQSSPIPIASPPQMSTSPPAQIPPLPLQSMQ
SLQVQPEILSQGQVLVQNALVSEEELPAAEALVQLPFQTLPPPQTVAVNLQVQPPAPVDPPVVYQVEDVCEEEMPEESDECVRMDRT
PPPPTLSPAAITVGRGEDLTSEHPLLEQVELPAVASVSASVIKSPSDPSHVSVPPPPLLLPAATTRSNSTSMHSSIPSIENKPPQAI
VKPQILTHVIEGFVIQEGLEPFPVSRSSLLIEQPVKKRPLLDNQVINSVCVQPELQNNTKHADNSSDTEMEDMIAEETLEEMDSELL
KCEFCGKMGYANEFLRSKRFCTMSCAKRYNVSCSKKFALSRWNRKPDNQSLGHRGRRPSGPDGAAREHILRQLPITYPSAEEDLASH
EDSVPSAMTTRLRRQSERERERELRDVRIRKMPENSDLLPVAQTEPSIWTVDDVWAFIHSLPGCQDIADEFRAQEIDGQALLLLKED
HLMSAMNIKLGPALKICARINSLKES

>sp|P20333|TNR1B_HUMAN
MAPVAVWAALAVGLELWAAAHALPAQVAFTPYAPEPGSTCRLREYYDQTAQMCCSKCSPGQHAKVFCTKTSDTVCDSCEDSTYTQLW
NWVPECLSCGSRCSSDQVETQACTREQNRICTCRPGWYCALSKQEGCRLCAPLRKCRPGFGVARPGTETSDVVCKPCAPGTFSNTTS
STDICRPHQICNVVAIPGNASMDAVCTSTSPTRSMAPGAVHLPQPVSTRSQHTQPTPEPSTAPSTSFLLPMGPSPPAEGSTGDFALP
VGLIVGVTALGLLIIGVVNCVIMTQVKKKPLCLQREAKVPHLPADKARGTQGPEQQHLLITAPSSSSSSLESSASALDRRAPTRNQP
QAPGVEASGAGEARASTGSSDSSPGGHGTQVNVTCIVNVCSSSDHSSQCSSQASSTMGDTDSSPSESPKDEQVPFSKEECAFRSQLE
TPETLLGSTEEKPLPLGVPDAGMKPS
>sp|P30038|AL4A1_HUMAN
MLLPAPALRRALLSRPWTGAGLRWKHTSSLKVANEPVLAFTQGSPERDALQKALKDLKGRMEAIPCVVGDEEVWTSDVQYQVSPFNH
GHKVAKFCYADKSLLNKAIEAALAARKEWDLKPIADRAQIFLKAADMLSGPRRAEILAKTMVGQGKTVIQAEIDAAAELIDFFRFNA
KYAVELEGQQPISVPPSTNSTVYRGLEGFVAAISPFNFTAIGGNLAGAPALMGNVVLWKPSDTAMLASYAVYRILREAGLPPNIIQF
VPADGPLFGDTVTSSEHLCGINFTGSVPTFKHLWKQVAQNLDRFHTFPRLAGECGGKNFHFVHRSADVESVVSGTLRSAFEYGGQKC
SACSRLYVPHSLWPQIKGRLLEEHSRIKVGDPAEDFGTFFSAVIDAKSFARIKKWLEHARSSPSLTILAGGKCDDSVGYFVEPCIVE
SKDPQEPIMKEEIFGPVLSVYVYPDDKYKETLQLVDSTTSYGLTGAVFSQDKDVVQEATKVLRNAAGNFYINDKSTGSIVGQQPFGG
ARASGTNDKPGGPHYILRWTSPQVIKETHKPLGDWSYAYMQ
>sp|Q3C1V1|CK091_HUMAN
MPKGRRGSHSPTMSQRSAPPLYFPSLYDRGISSSPLSDFNIWKKLFVPLKAGGAPVGGAAGARSLSQALPAPAPPPPPPPGLGPSSE
RPWPSPWPSGLASIPYEPLRFFYSPPPGPEVVASPLVPCPSTPRLASASHPEELCELEIRIKELELLTITGDGFDSQSYTFLKALKD
EKLQGLKTKQPGKKSASLS
>sp|Q9BSG5|RTBDN_HUMAN
MDCRVHMRPIGLTWVLQLTLAWILLEACGGSRPLQARSQQHHGLAADLGKGKLHLAGPCCPSEMDTTETSGPGNHPERCGVPSPECE
SFLEHLQRALRSRFRLRLLGVRQAQPLCEELCQAWFANCEDDITCGPTWLPLSEKRGCEPSCLTYGQTFADGTDLCRSALGHALPVA
APGARHCFNISISAVPRPRPGRRGREAPSRRSRSPRTSILDAAGSGSGSGSGSGP
>sp|Q96RP8|KCNA7_HUMAN
MEPRCPPPCGCCERLVLNVAGLRFETRARTLGRFPDTLLGDPARRGRFYDDARREYFFDRHRPSFDAVLYYYQSGGRLRRPAHVPLD
VFLEEVAFYGLGAAALARLREDEGCPVPPERPLPRRAFARQLWLLFEFPESSQAARVLAVVSVLVILVSIVVFCLETLPDFRDDRDG
TGLAAAAAAGPFPAPLNGSSQMPGNPPRLPFNDPFFVVETLCICWFSFELLVRLLVCPSKAIFFKNVMNLIDFVAILPYFVALGTEL
ARQRGVGQQAMSLAILRVIRLVRVFRIFKLSRHSKGLQILGQTLRASMRELGLLIFFLFIGVVLFSSAVYFAEVDRVDSHFTSIPES
FWWAVVTMTTVGYGDMAPVTVGGKIVGSLCAIAGVLTISLPVPVIVSNFSYFYHRETEGEEAGMFSHVDMQPCGPLEGKANGGLVDG
EVPELPPPLWAPPGKHLVTEV
>sp|P02538|K2C6A_HUMAN
MASTSTTIRSHSSSRRGFSANSARLPGVSRSGFSSVSVSRSRGSGGLGGACGGAGFGSRSLYGLGGSKRISIGGGSCAISGGYGSRA
GGSYGFGGAGSGFGFGGGAGIGFGLGGGAGLAGGFGGPGFPVCPPGGIQEVTVNQSLLTPLNLQIDPTIQRVRAEEREQIKTLNNKF
ASFIDKVRFLEQQNKVLETKWTLLQEQGTKTVRQNLEPLFEQYINNLRRQLDSIVGERGRLDSELRGMQDLVEDFKNKYEDEINKRT
AAENEFVTLKKDVDAAYMNKVELQAKADTLTDEINFLRALYDAELSQMQTHISDTSVVLSMDNNRNLDLDSIIAEVKAQYEEIAQRS
RAEAESWYQTKYEELQVTAGRHGDDLRNTKQEIAEINRMIQRLRSEIDHVKKQCANLQAAIADAEQRGEMALKDAKNKLEGLEDALQ
KAKQDLARLLKEYQELMNVKLALDVEIATYRKLLEGEECRLNGEGVGQVNISVVQSTVSSGYGGASGVGSGLGLGGGSSYSYGSGLG
VGGGFSSSSGRAIGGGLSSVGGGSSTIKYTTTSSSSRKSYKH
>sp|Q8WW01|SEN15_HUMAN
MEERGDSEPTPGCSGLGPGGVRGFGDGGGAPSWAPEDAWMGTHPKYLEMMELDIGDATQVYVAFLVYLDLMESKSWHEVNCVGLPEL
QLICLVGTEIEGEGLQTVVPTPITASLSHNRIREILKASRKLQGDPDLPMSFTLAIVESDSTIVYYKLTDGFMLPDPQNISLRR
>sp|P32754|HPPD_HUMAN
MTTYSDKGAKPERGRFLHFHSVTFWVGNAKQAASFYCSKMGFEPLAYRGLETGSREVVSHVIKQGKIVFVLSSALNPWNKEMGDHLV
KHGDGVKDIAFEVEDCDYIVQKARERGAKIMREPWVEQDKFGKVKFAVLQTYGDTTHTLVEKMNYIGQFLPGYEAPAFMDPLLPKLP

KCSLEMIDHIVGNQPDQEMVSASEWYLKNLQFHRFWSVDDTQVHTEYSSLRSIVVANYEESIKMPINEPAPGKKKSQIQEYVDYNGG
AGVQHIALKTEDIITAIRHLRERGLEFLSVPSTYYKQLREKLKTAKIKVKENIDALEELKILVDYDEKGYLLQIFTKPVQDRPTLFL
EVIQRHNHQGFGAGNFNSLFKAFEEEQNLRGNLTNMETNGVVPGM
>sp|Q9HCK8|CHD8_HUMAN
MADPIMDLFDDPNLFGLDSLTDDSFNQVTQDPIEEALGLPSSLDSLDQMNQDGGGGDVGNSSASELVPPPEETAPTELSKESTAPAP
ESITLHDYTTQPASQEQPAQPVLQTSTPTSGLLQVSKSQEILSQGNPFMGVSATAVSSSSAGGQPPQSAPKIVILKAPPSSSVTGAH
VAQIQAQGITSTAQPLVAGTANGGKVTFTKVLTGTPLRPGVSIVSGNTVLAAKVPGNQAAVQRIVQPSRPVKQLVLQPVKGSAPAGN
PGATGPPLKPAVTLTSTPTQGESKRITLVLQQPQSGGPQGHRHVVLGSLPGKIVLQGNQLAALTQAKNAQGQPAKVVTIQLQVQQPQ
QKIQIVPQPPSSQPQPQQPPSTQPVTLSSVQQAQIMGPGQSPGQRLSVPVKVVLQPQAGSSQGASSGLSVVKVLSASEVAALSSPAS
SAPHSGGKTGMEENRRLEHQKKQEKANRIVAEAIARARARGEQNIPRVLNEDELPSVRPEEEGEKKRRKKSAGERLKEEKPKKSKTS
GASKTKGKSKLNTITPVVGKKRKRNTSSDNSDVEVMPAQSPREDEESSIQKRRSNRQVKRKKYTEDLDIKITDDEEEEEVDVTGPIK
PEPILPEPVQEPDGETLPSMQFFVENPSEEDAAIVDKVLSMRIVKKELPSGQYTEAEEFFVKYKNYSYLHCEWATISQLEKDKRIHQ
KLKRFKTKMAQMRHFFHEDEEPFNPDYVEVDRILDESHSIDKDNGEPVIYYLVKWCSLPYEDSTWELKEDVDEGKIREFKRIQSRHP
ELKRVNRPQASAWKKLELSHEYKNRNQLREYQLEGVNWLLFNWYNRQNCILADEMGLGKTIQSIAFLQEVYNVGIHGPFLVIAPLST
ITNWEREFNTWTEMNTIVYHGSLASRQMIQQYEMYCKDSRGRLIPGAYKFDALITTFEMILSDCPELREIEWRCVIIDEAHRLKNRN
CKLLDSLKHMDLEHKVLLTGTPLQNTVEELFSLLHFLEPSQFPSESEFLKDFGDLKTEEQVQKLQAILKPMMLRRLKEDVEKNLAPK
QETIIEVELTNIQKKYYRAILEKNFSFLSKGAGHTNMPNLLNTMMELRKCCNHPYLINGAEEKILTEFREACHIIPHDFHLQAMVRS
AGKLVLIDKLLPKLKAGGHKVLIFSQMVRCLDILEDYLIQRRYLYERIDGRVRGNLRQAAIDRFSKPDSDRFVFLLCTRAGGLGINL
TAADTCIIFDSDWNPQNDLQAQARCHRIGQSKAVKVYRLITRNSYEREMFDKASLKLGLDKAVLQSMSGRDGNITGIQQFSKKEIED
LLRKGAYAAIMEEDDEGSKFCEEDIDQILLRRTTTITIESEGKGSTFAKASFVASENRTDISLDDPNFWQKWAKKADLDMDLLNSKN
NLVIDTPRVRKQTRHFSTLKDDDLVEFSDLESEDDERPRSRRHDRHHAYGRTDCFRVEKHLLVYGWGRWRDILSHGRFKRRMTERDV
ETICRAILVYCLLHYRGDENIKGFIWDLISPAENGKTKELQNHSGLSIPVPRGRKGKKVKSQSTFDIHKADWIRKYNPDTLFQDESY
KKHLKHQCNKVLLRVRMLYYLRQEVIGDQAEKVLGGAIASEIDIWFPVVDQLEVPTTWWDSEADKSLLIGVFKHGYEKYNTMRADPA
LCFLEKAGRPDDKAIAAEHRVLDNFSDIVEGVDFDKDCEDPEYKPLQGPPKDQDDEGDPLMMMDEEISVIDGDEAQVTQQPGHLFWP
PGSALTARLRRLVTAYQRSYKREQMKIEAAERGDRRRRRCEAAFKLKEIARREKQQRWTRREQTDFYRVVSTFGVEYDPDTMQFHWD
RFRTFARLDKKTDESLTKYFHGFVAMCRQVCRLPPAAGDEPPDPNLFIEPITEERASRTLYRIELLRRLREQVLCHPLLEDRLALCQ
PPGPELPKWWEPVRHDGELLRGAARHGVSQTDCNIMQDPDFSFLAARMNYMQNHQAGAPAPSLSRCSTPLLHQQYTSRTASPLPLRP
DAPVEKSPEETATQVPSLESLTLKLEHEVVARSRPTPQDYEMRVSPSDTTPLVSRSVPPVKLEDEDDSDSELDLSKLSPSSSSSSSS
SSSSSSTDESEDEKEEKLTDQSRSKLYDEESLLSLTMSQDGFPNEDGEQMTPELLLLQERQRASEWPKDRVLINRIDLVCQAVLSGK
WPSSRRSQEMVTGGILGPGNHLLDSPSLTPGEYGDSPVPTPRSSSAASMAEEEASAVSTAAAQFTKLRRGMDEKEFTVQIKDEEGLK
LTFQKHKLMANGVMGDGHPLFHKKKGNRKKLVELEVECMEEPNHLDVDLETRIPVINKVDGTLLVGEDAPRRAELEMWLQGHPEFAV
DPRFLAYMEDRRKQKWQRCKKNNKAELNCLGMEPVQTANSRNGKKGHHTETVFNRVLPGPIAPESSKKRARRMRPDLSKMMALMQGG
STGSLSLHNTFQHSSSGLQSVSSLGHSSATSASLPFMPFVMGGAPSSPHVDSSTMLHHHHHHPHPHHHHHHHPGLRAPGYPSSPVTT
ASGTTLRLPPLQPEEDDDEDEEDDDDLSQGYDSSERDFSLIDDPMMPANSDSSEDADD
>sp|P24394|IL4RA_HUMAN
MGWLCSGLLFPVSCLVLLQVASSGNMKVLQEPTCVSDYMSISTCEWKMNGPTNCSTELRLLYQLVFLLSEAHTCIPENNGGAGCVCH
LLMDDVVSADNYTLDLWAGQQLLWKGSFKPSEHVKPRAPGNLTVHTNVSDTLLLTWSNPYPPDNYLYNHLTYAVNIWSENDPADFRI
YNVTYLEPSLRIAASTLKSGISYRARVRAWAQCYNTTWSEWSPSTKWHNSYREPFEQHLLLGVSVSCIVILAVCLLCYVSITKIKKE
WWDQIPNPARSRLVAIIIQDAQGSQWEKRSRGQEPAKCPHWKNCLTKLLPCFLEHNMKRDEDPHKAAKEMPFQGSGKSAWCPVEISK
TVLWPESISVVRCVELFEAPVECEEEEEVEEEKGSFCASPESSRDDFQEGREGIVARLTESLFLDLLGEENGGFCQQDMGESCLLPP
SGSTSAHMPWDEFPSAGPKEAPPWGKEQPLHLEPSPPASPTQSPDNLTCTETPLVIAGNPAYRSFSNSLSQSPCPRELGPDPLLARH
LEEVEPEMPCVPQLSEPTTVPQPEPETWEQILRRNVLQHGAAAAPVSAPTSGYQEFVHAVEQGGTQASAVVGLGPPGEAGYKAFSSL
LASSAVSPEKCGFGASSGEEGYKPFQDLIPGCPGDPAPVPVPLFTFGLDREPPRSPQSSHLPSSSPEHLGLEPGEKVEDMPKPPLPQ

EQATDPLVDSLGSGIVYSALTCHLCGHLKQCHGQEDGGQTPVMASPCCGCCCGDRSSPPTTPLRAPDPSPGGVPLEASLCPASLAPS
GISEKSKSSSSFHPAPGNAQSSSQTPKIVNFVSVGPTYMRVS
>sp|P28330|ACADL_HUMAN
MAARLLRGSLRVLGGHRAPRQLPAARCSHSGGEERLETPSAKKLTDIGIRRIFSPEHDIFRKSVRKFFQEEVIPHHSEWEKAGEVSR
EVWEKAGKQGLLGVNIAEHLGGIGGDLYSAAIVWEEQAYSNCSGPGFSIHSGIVMSYITNHGSEEQIKHFIPQMTAGKCIGAIAMTE
PGAGSDLQGIKTNAKKDGSDWILNGSKVFISNGSLSDVVIVVAVTNHEAPSPAHGISLFLVENGMKGFIKGRKLHKMGLKAQDTAEL
FFEDIRLPASALLGEENKGFYYIMKELPQERLLIADVAISASEFMFEETRNYVKQRKAFGKTVAHLQTVQHKLAELKTHICVTRAFV
DNCLQLHEAKRLDSATACMAKYWASELQNSVAYDCVQLHGGWGYMWEYPIAKAYVDARVQPIYGGTNEIMKELIAREIVFDK
>sp|Q6UX68|XKR5_HUMAN
MHARLLGLSALLQAAEQSARLYTVAYYFTTGRLLWGWLALAVLLPGFLVQALSYLWFRADGHPGHCSLVMLHLLQLGVWKRHWDAAL
TSLQKELEAPHRGWLQLQEADLSALRLLEALLQTGPHLLLQTYVFLASDFTDIVPGVSTLFSWSSLSWALVSYTRFMGFMKPGHLAM
PWAALFCQQLWRMGMLGTRVLSLVLFYKAYHFWVFVVAGAHWLVMTFWLVAQQSDIIDSTCHWRLFNLLVGAVYILCYLSFWDSPSR
NRMVTFYMVMLLENIILLLLATDFLQGASWTSLQTIAGVLSGFLIGSVSLVIYYSLLHPKSTDIWQGCLRKSCGIAGGDKTERRDSP
RATDLAGKRTESSGSCQGASYEPTILGKPPTPEQVPPEAGLGTQVAVEDSFLSHHHWLWVKLALKTGNVSKINAAFGDNSPAYCPPA
WGLSQQDYLQRKALSAQQELPSSSRDPSTLENSSAFEGVPKAEADPLETSSYVSFASDQQDEAPTQNPAATQGEGTPKEGADAVSGT
QGKGTGGQQRGGEGQQSSTLYFSATAEVATSSQQEGSPATLQTAHSGRRLGKSSPAQPASPHPVGLAPFPDTMADISPILGTGPCRG
FCPSAGFPGRTLSISELEEPLEPKRELSHHAAVGVWVSLPQLRTAHEPCLTSTPKSESIQTDCSCREQMKQEPSFFI
>sp|P28845|DHI1_HUMAN
MAFMKKYLLPILGLFMAYYYYSANEEFRPEMLQGKKVIVTGASKGIGREMAYHLAKMGAHVVVTARSKETLQKVVSHCLELGAASAH
YIAGTMEDMTFAEQFVAQAGKLMGGLDMLILNHITNTSLNLFHDDIHHVRKSMEVNFLSYVVLTVAALPMLKQSNGSIVVVSSLAGK
VAYPMVAAYSASKFALDGFFSSIRKEYSVSRVNVSITLCVLGLIDTETAMKAVSGIVHMQAAPKEECALEIIKGGALRQEEVYYDSS
LWTTLLIRNPCRKILEFLYSTSYNMDRFINK
>sp|A4D0T7|YG055_HUMAN
MTSVSTQLSLVLMSLLLVLPVVEAVEAGDAIALLLGVVLSITGICACLGVYARKRNGQM
>sp|P51608|MECP2_HUMAN
MVAGMLGLREEKSEDQDLQGLKDKPLKFKKVKKDKKEEKEGKHEPVQPSAHHSAEPAEAGKAETSEGSGSAPAVPEASASPKQRRSI
IRDRGPMYDDPTLPEGWTRKLKQRKSGRSAGKYDVYLINPQGKAFRSKVELIAYFEKVGDTSLDPNDFDFTVTGRGSPSRREQKPPK
KPKSPKAPGTGRGRGRPKGSGTTRPKAATSEGVQVKRVLEKSPGKLLVKMPFQTSPGGKAEGGGATTSTQVMVIKRPGRKRKAEADP
QAIPKKRGRKPGSVVAAAAAEAKKKAVKESSIRSVQETVLPIKKRKTRETVSIEVKEVVKPLLVSTLGEKSGKGLKTCKSPGRKSKE
SSPKGRSSSASSPPKKEHHHHHHHSESPKAPVPLLPPLPPPPPEPESSEDPTSPPEPQDLSSSVCKEEKMPRGGSLESDGCPKEPAK
TQPAVATAATAAEKYKHRGEGERKDIVSSSMPRPNREEPVDSRTPVTERVS
>sp|A6NDL7|MT21E_HUMAN
MIHFNLLETQLTPLASFQEIMKKTSIYHLPSFHYLMDSEAQEDTREAYDDKQVVTEIMARCFIPTLITTTSWESFHFIGHEIRITEA
MDCYGAVVWPSALVLCYFLETNAKQYNMVDKNVIEIGAGTGLVSIVASLLGAHVTATDLPELLGNLQYNISRNTKMKSKHLPQVKEL
SWGVALDTNFPRSSNNFDYILAADVVYAHPFLEELLITFDHLCKETTIILWAMKFRLEKENKFVDRFKELFDLEEISSFPSLNIKLY
KAVKKNRRSV
>sp|Q6UXP7|F151B_HUMAN
MAASAGGPGSWSENILEYFLRNSQITAEDGAEITWYHAANHKAQTNEALKSTAHMIEADVLLPSDGSEHSQPIMAHPPETNSDNTLQ
EWLTEVMKSNKGIKLDFKSLAVVEPSMMLLENVKRHLKRPVWINADILPGPNGNSKVIDAKPFLDTVISFFPDVTFSLGWTTGWHPE
KVNEGYSWTMVKEMEYICNELSQPVTFPVRAALVRQSCSQLLWLLKKSNRYSLTIWTGKNDNYSVEDLLYIRDHFDKKQVFYDILEP
QNHEFKQAIGIKVNL
>sp|Q9Y6J6|KCNE2_HUMAN
MSTLSNFTQTLEDVFRRIFITYMDNWRQNTTAEQEALQAKVDAENFYYVILYLMVMIGMFSFIIVAILVSTVKSKRREHSNDPYHQY
IVEDWQEKYKSQILNLEESKATIHENIGAAGFKMSP

>sp|Q7RTZ1|OVCH2_HUMAN
MLISRNKLILLLGIVFFERGKSATLSLPKAPSCGQSLVKVQPWNYFNIFSRILGGSQVEKGSYPWQVSLKQRQKHICGGSIVSPQWV
ITAAHCIANRNIVSTLNVTAGEYDLSQTDPGEQTLTIETVIIHPHFSTKKPMDYDIALLKMAGAFQFGHFVGPICLPELREQFEAGF
ICTTAGWGRLTEGGVLSQVLQEVNLPILTWEECVAALLTLKRPISGKTFLCTGFPDGGRDACQGDSGGSLMCRNKKGAWTLAGVTSW
GLGCGRGWRNNVRKSDQGSPGIFTDISKVLPWIHEHIQTGNRRKSSRAWCSEQDVIVSGAEGKLHFPESLHLYYESKQRCVWTLLVP
EEMHVLLSFSHLDVESCHHSYLSMYSLEDRPIGKFCGESLPSSILIGSNSLRLKFVSDATDNAARFNLTYKALKPNYIPDSGCSYLT
VLFEEGLIQSLNYPENYSDKANCDWIFQASKHHLIKLSFQSLEIEESGDCTSDYVTVHSDVERKKEIARLCGYDVPTPVLSPSSIML
ISFHSDENGTCRGFQATVSFIPKAGKKIELPTLWFPVLILVM
>sp|P0DI80|SMIM6_HUMAN
MDQLVFKETIWNDAFWQNPWDQGGLAVIILFITAVLLLILFAIVFGLLTSTENTQCEAGEEE
>sp|Q8IW50|F219A_HUMAN
MMEEIDRFQVPTAHSEMQPLDPAAASISDGDCDAREGESVAMNYKPSPLQVKLEKQRELARKGSLKNGSMGSPVNQQPKKNNVMART
RLVVPNKGYSSLDQSPDEKPLVALDTDSDDDFDMSRYSSSGYSSAEQINQDLNIQLLKDGYRLDEIPDDEDLDLIPPKSVNPTCMCC
QATSSTACHIQ
>sp|Q96EP9|NTCP4_HUMAN
MDGNDNVTLLFAPLLRDNYTLAPNASSLGPGTDLALAPASSAGPGPGLSLGPGPSFGFSPGPTPTPEPTTSGLAGGAASHGPSPFPR
PWAPHALPFWDTPLNHGLNVFVGAALCITMLGLGCTVDVNHFGAHVRRPVGALLAALCQFGLLPLLAFLLALAFKLDEVAAVAVLLC
GCCPGGNLSNLMSLLVDGDMNLSIIMTISSTLLALVLMPLCLWIYSWAWINTPIVQLLPLGTVTLTLCSTLIPIGLGVFIRYKYSRV
ADYIVKVSLWSLLVTLVVLFIMTGTMLGPELLASIPAAVYVIAIFMPLAGYASGYGLATLFHLPPNCKRTVCLETGSQNVQLCTAIL
KLAFPPQFIGSMYMFPLLYALFQSAEAGIFVLIYKMYGSEMLHKRDPLDEDEDTDISYKKLKEEEMADTSYGTVKAENIIMMETAQT
SL
>sp|Q9NWZ3|IRAK4_HUMAN
MNKPITPSTYVRCLNVGLIRKLSDFIDPQEGWKKLAVAIKKPSGDDRYNQFHIRRFEALLQTGKSPTSELLFDWGTTNCTVGDLVDL
LIQNEFFAPASLLLPDAVPKTANTLPSKEAITVQQKQMPFCDKDRTLMTPVQNLEQSYMPPDSSSPENKSLEVSDTRFHSFSFYELK
NVTNNFDERPISVGGNKMGEGGFGVVYKGYVNNTTVAVKKLAAMVDITTEELKQQFDQEIKVMAKCQHENLVELLGFSSDGDDLCLV
YVYMPNGSLLDRLSCLDGTPPLSWHMRCKIAQGAANGINFLHENHHIHRDIKSANILLDEAFTAKISDFGLARASEKFAQTVMTSRI
VGTTAYMAPEALRGEITPKSDIYSFGVVLLEIITGLPAVDEHREPQLLLDIKEEIEDEEKTIEDYIDKKMNDADSTSVEAMYSVASQ
CLHEKKNKRPDIKKVQQLLQEMTAS
>sp|Q96QR1|SG3A1_HUMAN
MKLAALLGLCVALSCSSAAAFLVGSAKPVAQPVAALESAAEAGAGTLANPLGTLNPLKLLLSSLGIPVNHLIEGSQKCVAELGPQAV
GAVKALKALLGALTVFG
>sp|Q9Y6M0|TEST_HUMAN
MGARGALLLALLLARAGLRKPESQEAAPLSGPCGRRVITSRIVGGEDAELGRWPWQGSLRLWDSHVCGVSLLSHRWALTAAHCFETY
SDLSDPSGWMVQFGQLTSMPSFWSLQAYYTRYFVSNIYLSPRYLGNSPYDIALVKLSAPVTYTKHIQPICLQASTFEFENRTDCWVT
GWGYIKEDEALPSPHTLQEVQVAIINNSMCNHLFLKYSFRKDIFGDMVCAGNAQGGKDACFGDSGGPLACNKNGLWYQIGVVSWGVG
CGRPNRPGVYTNISHHFEWIQKLMAQSGMSQPDPSWPLLFFPLLWALPLLGPV
>sp|Q96BT7|ALKB8_HUMAN
MDSNHQSNYKLSKTEKKFLRKQIKAKHTLLRHEGIETVSYATQSLVVANGGLGNGVSRNQLLPVLEKCGLVDALLMPPNKPYSFARY
RTTEESKRAYVTLNGKEVVDDLGQKITLYLNFVEKVQWKELRPQALPPGLMVVEEIISSEEEKMLLESVDWTEDTDNQNSQKSLKHR
RVKHFGYEFHYENNNVDKDKPLSGGLPDICESFLEKWLRKGYIKHKPDQMTINQYEPGQGIPAHIDTHSAFEDEIVSLSLGSEIVMD
FKHPDGIAVPVMLPRRSLLVMTGESRYLWTHGITCRKFDTVQASESLKSGIITSDVGDLTLSKRGLRTSFTFRKVRQTPCNCSYPLV
CDSQRKETPPSFPESDKEASRLEQEYVHQVYEEIAGHFSSTRHTPWPHIVEFLKALPSGSIVADIGCGNGKYLGINKELYMIGCDRS
QNLVDICRERQFQAFVCDALAVPVRSGSCDACISIAVIHHFATAERRVAALQEIVRLLRPGGKALIYVWAMEQEYNKQKSKYLRGNR

NSQGKKEEMNSDTSVQRSLVEQMRDMGSRDSASSVPRINDSQEGGCNSRQVSNSKLPVHVNRTSFYSQDVLVPWHLKGNPDKGKPVE
PFGPIGSQDPSPVFHRYYHVFREGELEGACRTVSDVRILQSYYDQGNWCVILQKA
>sp|Q695T7|S6A19_HUMAN
MVRLVLPNPGLDARIPSLAELETIEQEEASSRPKWDNKAQYMLTCLGFCVGLGNVWRFPYLCQSHGGGAFMIPFLILLVLEGIPLLY
LEFAIGQRLRRGSLGVWSSIHPALKGLGLASMLTSFMVGLYYNTIISWIMWYLFNSFQEPLPWSDCPLNENQTGYVDECARSSPVDY
FWYRETLNISTSISDSGSIQWWMLLCLACAWSVLYMCTIRGIETTGKAVYITSTLPYVVLTIFLIRGLTLKGATNGIVFLFTPNVTE
LAQPDTWLDAGAQVFFSFSLAFGGLISFSSYNSVHNNCEKDSVIVSIINGFTSVYVAIVVYSVIGFRATQRYDDCFSTNILTLINGF
DLPEGNVTQENFVDMQQRCNASDPAAYAQLVFQTCDINAFLSEAVEGTGLAFIVFTEAITKMPLSPLWSVLFFIMLFCLGLSSMFGN
MEGVVVPLQDLRVIPPKWPKEVLTGLICLGTFLIGFIFTLNSGQYWLSLLDSYAGSIPLLIIAFCEMFSVVYVYGVDRFNKDIEFMI
GHKPNIFWQVTWRVVSPLLMLIIFLFFFVVEVSQELTYSIWDPGYEEFPKSQKISYPNWVYVVVVIVAGVPSLTIPGYAIYKLIRNH
CQKPGDHQGLVSTLSTASMNGDLKY
>sp|Q92854|SEM4D_HUMAN
MRMCTPIRGLLMALAVMFGTAMAFAPIPRITWEHREVHLVQFHEPDIYNYSALLLSEDKDTLYIGAREAVFAVNALNISEKQHEVYW
KVSEDKKAKCAEKGKSKQTECLNYIRVLQPLSATSLYVCGTNAFQPACDHLNLTSFKFLGKNEDGKGRCPFDPAHSYTSVMVDGELY
SGTSYNFLGSEPIISRNSSHSPLRTEYAIPWLNEPSFVFADVIRKSPDSPDGEDDRVYFFFTEVSVEYEFVFRVLIPRIARVCKGDQ
GGLRTLQKKWTSFLKARLICSRPDSGLVFNVLRDVFVLRSPGLKVPVFYALFTPQLNNVGLSAVCAYNLSTAEEVFSHGKYMQSTTV
EQSHTKWVRYNGPVPKPRPGACIDSEARAANYTSSLNLPDKTLQFVKDHPLMDDSVTPIDNRPRLIKKDVNYTQIVVDRTQALDGTV
YDVMFVSTDRGALHKAISLEHAVHIIEETQLFQDFEPVQTLLLSSKKGNRFVYAGSNSGVVQAPLAFCGKHGTCEDCVLARDPYCAW
SPPTATCVALHQTESPSRGLIQEMSGDASVCPDKSKGSYRQHFFKHGGTAELKCSQKSNLARVFWKFQNGVLKAESPKYGLMGRKNL
LIFNLSEGDSGVYQCLSEERVKNKTVFQVVAKHVLEVKVVPKPVVAPTLSVVQTEGSRIATKVLVASTQGSSPPTPAVQATSSGAIT
LPPKPAPTGTSCEPKIVINTVPQLHSEKTMYLKSSDNRLLMSLFLFFFVLFLCLFFYNCYKGYLPRQCLKFRSALLIGKKKPKSDFC
DREQSLKETLVEPGSFSQQNGEHPKPALDTGYETEQDTITSKVPTDREDSQRIDDLSARDKPFDVKCELKFADSDADGD
>sp|Q92506|DHB8_HUMAN
MASQLQNRLRSALALVTGAGSGIGRAVSVRLAGEGATVAACDLDRAAAQETVRLLGGPGSKEGPPRGNHAAFQADVSEARAARCLLE
QVQACFSRPPSVVVSCAGITQDEFLLHMSEDDWDKVIAVNLKGTFLVTQAAAQALVSNGCRGSIINISSIVGKVGNVGQTNYAASKA
GVIGLTQTAARELGRHGIRCNSVLPGFIATPMTQKVPQKVVDKITEMIPMGHLGDPEDVADVVAFLASEDSGYITGTSVEVTGGLFM
>sp|Q9BRD0|BUD13_HUMAN
MAAAPPLSKAEYLKRYLSGADAGVDRGSESGRKRRKKRPKPGGAGGKGMRIVDDDVSWTAISTTKLEKEEEEDDGDLPVVAEFVDER
PEEVKQMEAFRSSAKWKLLGGHNEDLPSNRHFRHDTPDSSPRRVRHGTPDPSPRKDRHDTPDPSPRRARHDTPDPSPLRGARHDSDT
SPPRRIRHDSSDTSPPRRARHDSPDPSPPRRPQHNSSGASPRRVRHDSPDPSPPRRARHGSSDISSPRRVHNNSPDTSRRTLGSSDT
QQLRRARHDSPDLAPNVTYSLPRTKSGKAPERASSKTSPHWKESGASHLSFPKNSKYEYDPDISPPRKKQAKSHFGDKKQLDSKGDC
QKATDSDLSSPRHKQSPGHQDSDSDLSPPRNRPRHRSSDSDLSPPRRRQRTKSSDSDLSPPRRSQPPGKKAAHMYSGAKTGLVLTDI
QREQQELKEQDQETMAFEAEFQYAETVFRDKSGRKRNLKLERLEQRRKAEKDSERDELYAQWGKGLAQSRQQQQNVEDAMKEMQKPL
ARYIDDEDLDRMLREQEREGDPMANFIKKNKAKENKNKKVRPRYSGPAPPPNRFNIWPGYRWDGVDRSNGFEQKRFARLASKKAVEE
LAYKWSVEDM
>sp|Q9UKL4|CXD2_HUMAN
MGEWTILERLLEAAVQQHSTMIGRILLTVVVIFRILIVAIVGETVYDDEQTMFVCNTLQPGCNQACYDRAFPISHIRYWVFQIIMVC
TPSLCFITYSVHQSAKQRERRYSTVFLALDRDPPESIGGPGGTGGGGSGGGKREDKKLQNAIVNGVLQNTENTSKETEPDCLEVKEL
TPHPSGLRTASKSKLRRQEGISRFYIIQVVFRNALEIGFLVGQYFLYGFSVPGLYECNRYPCIKEVECYVSRPTEKTVFLVFMFAVS
GICVVLNLAELNHLGWRKIKLAVRGAQAKRKSIYEIRNKDLPRVSVPNFGRTQSSDSAYV
>sp|Q6ZQT0|YD023_HUMAN
MFARLCPVSETFGRLCPVSETFARLCPVSETFARLCPVSETFARLCPVSETFGRLCPVSEMFGRLSPVSETFGRLCPVSETFGRLCP
VSEMFARLCPVSETFGRLSPVSEMFGRLCPVSEMFGRLCPVSEMFGRLCPVIT
>sp|Q8NH57|O52P1_HUMAN

MESPNHTDVDPSVFFLLGIPGLEQFHLWLSLPVCGLGTATIVGNITILVVVATEPVLHKPVYLFLCMLSTIDLAASVSTVPKLLAIF
WCGAGHISASACLAQMFFIHAFCMMESTVLLAMAFDRYVAICHPLRYATILTDTIIAHIGVAAVVRGSLLMLPCPFLIGRLNFCQSH
VILHTYCEHMAVVKLACGDTRPNRVYGLTAALLVIGVDLFCIGLSYALSAQAVLRLSSHEARSKALGTCGSHVCVILISYTPALFSF
FTHRFGHHVPVHIHILLANVYLLLPPALNPVVYGVKTKQIRKRVVRVFQSGQGMGIKASE
>sp|Q96PE2|ARHGH_HUMAN
MADGAPRPQLYRSVSFKLLERWSGGPGLREEDTDTPGLRRRASCRPTTAARGQPSRRVSKLASGPLAAPAQPRPLRSLSPSVRQLSR
RFDAPRLDDGSAGTRDGGVLPAAAEEAAEGPARGAWPSVTEMRKLFGGPGSRRPSADSESPGTPSPDGAAWEPPARESRQPPTPPPR
TCFPLAGLRSARPLTGPETEGRLRRPQQQQERAQRPADGLHSWHIFSQPQAGARASCSSSSIAASYPVSRSRAASSSEEEEEGPPQL
PGAQSPAYHGGHSSGSDDDRDGEGGHRWGGRPGLRPGSSLLDQDCRPDSDGLNLSSMNSAGVSGSPEPPTSPRAPREEGLREWGSGS
PPCVPGPQEGLRPMSDSVGGAFRVAKVSFPSYLASPAGSRGSSRYSSTETLKDDDLWSSRGSGGWGVYRSPSFGAGEGLLRSQARTR
AKGPGGTSRALRDGGFEPEKSRQRKSLSNPDIASETLTLLSFLRSDLSELRVRKPGGSSGDRGSNPLDGRDSPSAGGPVGQLEPIPI
PAPASPGTRPTLKDLTATLRRAKSFTCSEKPMARRLPRTSALKSSSSELLLTGPGAEEDPLPLIVQDQYVQEARQVFEKIQRMGAQQ
DDGSDAPPGSPDWAGDVTRGQRSQEELSGPESSLTDEGIGADPEPPVAAFCGLGTTGMWRPLSSSSAQTNHHGPGTEDSLGGWALVS
PETPPTPGALRRRRKVPPSGSGGSELSNGEAGEAYRSLSDPIPQRHRAATSEEPTGFSVDSNLLGSLSPKTGLPATSAMDEGLTSGH
SDWSVGSEESKGYQEVIQSIVQGPGTLGRVVDDRIAGKAPKKKSLSDPSRRGELAGPGFEGPGGEPIREVEPMLPPSSSEPILVEQR
AEPEEPGATRSRAQSERALPEALPPPATAHRNFHLDPKLADILSPRLIRRGSKKRPARSSHQELRRDEGSQDQTGSLSRARPSSRHV
RHASVPATFMPIVVPEPPTSVGPPVAVPEPIGFPTRAHPTLQAPSLEDVTKQYMLNLHSGEVPAPVPVDMPCLPLAAPPSAEAKPPE
AARPADEPTPASKCCSKPQVDMRKHVAMTLLDTEQSYVESLRTLMQGYMQPLKQPENSVLCDPSLVDEIFDQIPELLEHHEQFLEQV
RHCMQTWHAQQKVGALLVQSFSKDVLVNIYSAYIDNFLNAKDAVRVAKEARPAFLKFLEQSMRENKEKQALSDLMIKPVQRIPRYEL
LVKDLLKHTPEDHPDHPLLLEAQRNIKQVAERINKGVRSAEEAERHARVLQEIEAHIEGMEDLQAPLRRFLRQEMVIEVKAIGGKKD
RSLFLFTDLIVCTTLKRKSGSLRRSSMSLYTAASVIDTASKYKMLWKLPLEDADIIKGASQATNRENIQKAISRLDEDLTTLGQMSK
LSESLGFPHQSLDDALRDLSAAMHRDLSEKQALCYALSFPPTKLELCATRPEGTDSYIFEFPHPDARLGFEQAFDEAKRKLASSKSC
LDPEFLKAIPIMKTRSGMQFSCAAPTLNSCPEPSPEVWVCNSDGYVGQVCLLSLRAEPDVEACIAVCSARILCIGAVPGLQPRCHRE
PPPSLRSPPETAPEPAGPELDVEAAADEEAATLAEPGPQPCLHISIAGSGLEMTPGLGEGDPRPELVPFDSDSDDESSPSPSGTLQS
QASRSTISSSFGNEETPSSKEATAETTSSEEEQEPGFLPLSGSFGPGGPCGTSPMDGRALRRSSHGSFTRGSLEDLLSVDPEAYQSS
VWLGTEDGCVHVYQSSDSIRDRRNSMKLQHAASVTCILYLNNQVFVSLANGELVVYQREAGHFWDPQNFKSVTLGTQGSPITKMVSV
GGRLWCGCQNRVLVLSPDTLQLEHMFYVGQDSSRCVACMVDSSLGVWVTLKGSAHVCLYHPDTFEQLAEVDVTPPVHRMLAGSDAII
RQHKAACLRITALLVCEELLWVGTSAGVVLTMPTSPGTVSCPRAPLSPTGLGQGHTGHVRFLAAVQLPDGFNLLCPTPPPPPDTGPE
KLPSLEHRDSPWHRGPAPARPKMLVISGGDGYEDFRLSSGGGSSSETVGRDDSTNHLLLWRV
>sp|Q9BWE0|REPI1_HUMAN
MLERRCRGPLAMGLAQPRLLSGPSQESPQTLGKESRGLRQQGTSVAQSGAQAPGRAHRCAHCRRHFPGWVALWLHTRRCQARLPLPC
PECGRRFRHAPFLALHRQVHAAATPDLGFACHLCGQSFRGWVALVLHLRAHSAAKRPIACPKCERRFWRRKQLRAHLRRCHPPAPEA
RPFICGNCGRSFAQWDQLVAHKRVHVAEALEEAAAKALGPRPRGRPAVTAPRPGGDAVDRPFQCACCGKRFRHKPNLIAHRRVHTGE
RPHQCPECGKRFTNKPYLTSHRRIHTGEKPYPCKECGRRFRHKPNLLSHSKIHKRSEGSAQAAPGPGSPQLPAGPQESAAEPTPAVP
LKPAQEPPPGAPPEHPQDPIEAPPSLYSCDDCGRSFRLERFLRAHQRQHTGERPFTCAECGKNFGKKTHLVAHSRVHSGERPFACEE
CGRRFSQGSHLAAHRRDHAPDRPFVCPDCGKAFRHKPYLAAHRRIHTGEKPYVCPDCGKAFSQKSNLVSHRRIHTGERPYACPDCDR
SFSQKSNLITHRKSHIRDGAFCCAICGQTFDDEERLLAHQKKHDV
>sp|Q96AQ6|PBIP1_HUMAN
MASCPDSDNSWVLAGSESLPVETLGPASRMDPESERALQAPHSPSKTDGKELAGTMDGEGTLFQTESPQSGSILTEETEVKGTLEGD
VCGVEPPGPGDTVVQGDLQETTVVTGLGPDTQDLEGQSPPQSLPSTPKAAWIREEGRCSSSDDDTDVDMEGLRRRRGREAGPPQPMV
PLAVENQAGGEGAGGELGISLNMCLLGALVLLGLGVLLFSGGLSESETGPMEEVERQVLPDPEVLEAVGDRQDGLREQLQAPVPPDS
VPSLQNMGLLLDKLAKENQDIRLLQAQLQAQKEELQSLMHQPKGLEEENAQLRGALQQGEAFQRALESELQQLRARLQGLEADCVRG
PDGVCLSGGRGPQGDKAIREQGPREQEPELSFLKQKEQLEAEAQALRQELERQRRLLGSVQQDLERSLQDASRGDPAHAGLAELGHR
LAQKLQGLENWGQDPGVSANASKAWHQKSHFQNSREWSGKEKWWDGQRDRKAEHWKHKKEESGRERKKNWGGQEDREPAGRWKEGRP

RVEESGSKKEGKRQGPKEPPRKSGSFHSSGEKQKQPRWREGTKDSHDPLPSWAELLRPKYRAPQGCSGVDECARQEGLTFFGTELAP
VRQQELASLLRTYLARLPWAGQLTKELPLSPAFFGEDGIFRHDRLRFRDFVDALEDSLEEVAVQQTGDDDEVDDFEDFIFSHFFGDK
ALKKRSGKKDKHSQSPRAAGPREGHSHSHHHHHRG
>sp|Q92887|MRP2_HUMAN
MLEKFCNSTFWNSSFLDSPEADLPLCFEQTVLVWIPLGYLWLLAPWQLLHVYKSRTKRSSTTKLYLAKQVFVGFLLILAAIELALVL
TEDSGQATVPAVRYTNPSLYLGTWLLVLLIQYSRQWCVQKNSWFLSLFWILSILCGTFQFQTLIRTLLQGDNSNLAYSCLFFISYGF
QILILIFSAFSENNESSNNPSSIASFLSSITYSWYDSIILKGYKRPLTLEDVWEVDEEMKTKTLVSKFETHMKRELQKARRALQRRQ
EKSSQQNSGARLPGLNKNQSQSQDALVLEDVEKKKKKSGTKKDVPKSWLMKALFKTFYMVLLKSFLLKLVNDIFTFVSPQLLKLLIS
FASDRDTYLWIGYLCAILLFTAALIQSFCLQCYFQLCFKLGVKVRTAIMASVYKKALTLSNLARKEYTVGETVNLMSVDAQKLMDVT
NFMHMLWSSVLQIVLSIFFLWRELGPSVLAGVGVMVLVIPINAILSTKSKTIQVKNMKNKDKRLKIMNEILSGIKILKYFAWEPSFR
DQVQNLRKKELKNLLAFSQLQCVVIFVFQLTPVLVSVVTFSVYVLVDSNNILDAQKAFTSITLFNILRFPLSMLPMMISSMLQASVS
TERLEKYLGGDDLDTSAIRHDCNFDKAMQFSEASFTWEHDSEATVRDVNLDIMAGQLVAVIGPVGSGKSSLISAMLGEMENVHGHIT
IKGTTAYVPQQSWIQNGTIKDNILFGTEFNEKRYQQVLEACALLPDLEMLPGGDLAEIGEKGINLSGGQKQRISLARATYQNLDIYL
LDDPLSAVDAHVGKHIFNKVLGPNGLLKGKTRLLVTHSMHFLPQVDEIVVLGNGTIVEKGSYSALLAKKGEFAKNLKTFLRHTGPEE
EATVHDGSEEEDDDYGLISSVEEIPEDAASITMRRENSFRRTLSRSSRSNGRHLKSLRNSLKTRNVNSLKEDEELVKGQKLIKKEFI
ETGKVKFSIYLEYLQAIGLFSIFFIILAFVMNSVAFIGSNLWLSAWTSDSKIFNSTDYPASQRDMRVGVYGALGLAQGIFVFIAHFW
SAFGFVHASNILHKQLLNNILRAPMRFFDTTPTGRIVNRFAGDISTVDDTLPQSLRSWITCFLGIISTLVMICMATPVFTIIVIPLG
IIYVSVQMFYVSTSRQLRRLDSVTRSPIYSHFSETVSGLPVIRAFEHQQRFLKHNEVRIDTNQKCVFSWITSNRWLAIRLELVGNLT
VFFSALMMVIYRDTLSGDTVGFVLSNALNITQTLNWLVRMTSEIETNIVAVERITEYTKVENEAPWVTDKRPPPDWPSKGKIQFNNY
QVRYRPELDLVLRGITCDIGSMEKIGVVGRTGAGKSSLTNCLFRILEAAGGQIIIDGVDIASIGLHDLREKLTIIPQDPILFSGSLR
MNLDPFNNYSDEEIWKALELAHLKSFVASLQLGLSHEVTEAGGNLSIGQRQLLCLGRALLRKSKILVLDEATAAVDLETDNLIQTTI
QNEFAHCTVITIAHRLHTIMDSDKVMVLDNGKIIECGSPEELLQIPGPFYFMAKEAGIENVNSTKF
>sp|Q96HA4|CA159_HUMAN
MALRHLALLAGLLVGVASKSMENTVTRNSTAVINTQAEGTLSPPGLSSLPVVREWALTHTAQLPECCVDVVGVNASCPGASLCGPGC
YRRWNADGSASCVRCGNGTLPAYNGSECRSFAGPGAPFPMNRSSGTPGRPHPGAPRVAASLFLGTFFISSGLILSVAGFFYLKRSSK
LPRACYRRNKAPALQPGEAAAMIPPPQSSGNSSCRIPLWGFPSLGQSQGALWVCPQTGLPGSGSRPPLPGSPGDPPTRQGQGRIWLV
PPALDLSWIWPAPPARPPLIPVTSMLFPVPETWGLQERRTHHDRADPQYLLLLEVQLHPRTDAAGLRQALLSSHRFSGAGSGGPKSQ
PVRKPRYVRRERPLDRATDPAAFPGEARISNV
>sp|Q8NAP3|ZBT38_HUMAN
MTVMSLSRDLKDDFHSDTVLSILNEQRIRGILCDVTIIVEDTKFKAHSNVLAASSLYFKNIFWSHTICISSHVLELDDLKAEVFTEI
LNYIYSSTVVVKRQETVTDLAAAGKKLGISFLEDLTDRNFSNSPGPYVFCITEKGVVKEEKNEKRHEEPAITNGPRITNAFSIIETE
NSNNMFSPLDLRASFKKVSDSMRTASLCLERTDVCHEAEPVRTLAEHSYAVSSVAEAYRSQPVREHDGSSPGNTGKENCEALAAKPK
TCRKPKTFSIPQDSDSATENIPPPPVSNLEVNQERSPQPAAVLTRSKSPNNEGDVHFSREDENQSSDVPGPPAAEVPPLVYNCSCCS
KAFDSSTLLSAHMQLHKPTQEPLVCKYCNKQFTTLNRLDRHEQICMRSSHMPIPGGNQRFLENYPTIGQNGGSFTGPEPLLSENRIG
EFSSTGSTLPDTDHMVKFVNGQMLYSCVVCKRSYVTLSSLRRHANVHSWRRTYPCHYCNKVFALAEYRTRHEIWHTGERRYQCIFCL
ETFMTYYILKNHQKSFHAIDHRLSISKKTANGGLKPSVYPYKLYRLLPMKCKRAPYKSYRNSSYENARENSQMNESAPGTYVVQNPH
SSELPTLNFQDTVNTLTNSPAIPLETSACQDIPTSANVQNAEGTKWGEEALKMDLDNNFYSTEVSVSSTENAVSSDLRAGDVPVLSL
SNSSENAASVISYSGSAPSVIVHSSQFSSVIMHSNAIAAMTSSNHRAFSDPAVSQSLKDDSKPEPDKVGRFASRPKSIKEKKKTTSH
TRGEIPEESNYVADPGGSLSKTTNIAEETSKIETYIAKPALPGTSTNSNVAPLCQITVKIGNEAIVKRHILGSKLFYKRGRRPKYQM
QEEPLPQGNDPEPSGDSPLGLCQSECMEMSEVFDDASDQDSTDKPWRPYYNYKPKKKSRQLKKMRKVNWRKEHGNRSPSHKCKYPAE
LDCAVGKAPQDKPFEEEETKEMPKLQCELCDGDKAVGAGNQGRPHRHLTSRPYACELCAKQFQSPSTLKMHMRCHTGEKPYQCKTCG
RCFSVQGNLQKHERIHLGLKEFVCQYCNKAFTLNETLKIHERIHTGEKRYHCQFCFQRFLYLSTKRNHEQRHIREHNGKGYACFQCP
KICKTAAALGMHQKKHLFKSPSQQEKIGDVCHENSNPLENQHFIGSEDNDQKDNIQTGVENVVL
>sp|Q3B8N5|PROX2_HUMAN

MDPNSILLSPQPQICSHLAEACTEGERSSSPPELDRDSPFPWSQVPSSSPTDPEWFGDEHIQAKRARVETIVRGMCLSPNPLVPGNA
QAGVSPRCPKKARERKRKQNLPTPQGLLMPAPAWDQGNRKGGPRVREQLHLLKQQLRHLQEHILQAAKPRDTAQGPGGCGTGKGPLS
AKQGNGCGPRPWVVDGDHQQGTSKDLSGAEKHQESEKPSFLPSGAPASLEILRKELTRAVSQAVDSVLQKVLLDPPGHLTQLGRSFQ
GQVAEGRSEPSPPVGGACKDPLALAALPRRVQLQAGVPVGNLSLAKRLDSPRYPIPPRMTPKPCQDPPANFPLTAPSHIQENQILSQ
LLGHRYNNGHWSSSPPQDSSSQRHPSSEPALRPWRTTKPQPLVLSQQQCPLPFTSAHLESLPLLPSVKMEQRGLHAVMEALPFSLLH
IQEGLNPGHLKKAKLMFFFTRYPSSNLLKVYFPDVQFNRCITSQMIKWFSNFREFYYIQMEKSARQAISDGVTNPKMLVVLRNSELF
QALNMHYNKGNDFEVPDCFLEIASLTLQEFFRAVSAGRDSDPSWKKPIYKIISKLDSDIPEIFKSSSYPQ
>sp|O43462|MBTP2_HUMAN
MIPVSLVVVVVGGWTVVYLTDLVLKSSVYFKHSYEDWLENNGLSISPFHIRWQTAVFNRAFYSWGRRKARMLYQWFNFGMVFGVIAM
FSSFFLLGKTLMQTLAQMMADSPSSYSSSSSSSSSSSSSSSSSSSSSSSSSLHNEQVLQVVVPGINLPVNQLTYFFTAVLISGVVHEIG
HGIAAIREQVRFNGFGIFLFIIYPGAFVDLFTTHLQLISPVQQLRIFCAGIWHNFVLALLGILALVLLPVILLPFYYTGVGVLITEV
AEDSPAIGPRGLFVGDLVTHLQDCPVTNVQDWNECLDTIAYEPQIGYCISASTLQQLSFPVRAYKRLDGSTECCNNHSLTDVCFSYR
NNFNKRLHTCLPARKAVEATQVCRTNKDCKKSSSSSFCIIPSLETHTRLIKVKHPPQIDMLYVGHPLHLHYTVSITSFIPRFNFLSI
DLPVVVETFVKYLISLSGALAIVNAVPCFALDGQWILNSFLDATLTSVIGDNDVKDLIGFFILLGGSVLLAANVTLGLWMVTAR
>sp|Q8NCQ7|PRCA1_HUMAN
MWVRTTLTIERWTKEKTEPKARSWDESLSDVNRLPSWERGHLLAGVASSTDVSTFSEGGDCKEPDKCCWRHKQCTGHIIYPFASDCV
RHSLHLHSVNHCNCNSRLKDSSEDSSSSRGAGPTCSHVIESPCFELTPEEEHVERFRYGWCKSYRPVSVAVIHHPLYHECGADDLNE
EEEEEEEESKPPIPTQVGPATASPDLGTSMATGTPDSTAPITIWRSESPTGKGQGSKVIKKVKKKKEKEKDKEEMDEKAKLKKKAKK
GQLTKKKSPVKLEPSPPDVSRSLSARQLARMSESSPESREELESEDSYNGRGQGELSSEDIVESSSPRKRENTVQAKKTGAKPSQAR
KVNKRKSPPGSNPNLS
>sp|Q96K31|CH076_HUMAN
MDSGCWLFGGEFEDSVFEERPERRSGPPASYCAKLCEPQWFYEETESSDDVEVLTLKKFKGDLAYRRQEYQKALQEYSSISEKLSST
NFAMKRDVQEGQARCLAHLGRHMEALEIAANLENKATNTDHLTTVLYLQLAICSSLQNLEKTIFCLQKLISLHPFNPWNWGKLAEAY
LNLGPALSAALASSQKQHSFTSSDKTIKSFFPHSGKDCLLCFPETLPESSLFSVEANSSNSQKNEKALTNIQNCMAEKRETVLIETQ
LKACASFIRTRLLLQFTQPQQTSFALERNLRTQQEIEDKMKGFSFKEDTLLLIAEVMGEDIPEKIKDEVHPEVKCVGSVALTALVTV
SSEEFEDKWFRKIKDHFCPFENQFHTEIQILA
>sp|P30874|SSR2_HUMAN
MDMADEPLNGSHTWLSIPFDLNGSVVSTNTSNQTEPYYDLTSNAVLTFIYFVVCIIGLCGNTLVIYVILRYAKMKTITNIYILNLAI
ADELFMLGLPFLAMQVALVHWPFGKAICRVVMTVDGINQFTSIFCLTVMSIDRYLAVVHPIKSAKWRRPRTAKMITMAVWGVSLLVI
LPIMIYAGLRSNQWGRSSCTINWPGESGAWYTGFIIYTFILGFLVPLTIICLCYLFIIIKVKSSGIRVGSSKRKKSEKKVTRMVSIV
VAVFIFCWLPFYIFNVSSVSMAISPTPALKGMFDFVVVLTYANSCANPILYAFLSDNFKKSFQNVLCLVKVSGTDDGERSDSKQDKS
RLNETTETQRTLLNGDLQTSI
>sp|Q9NQZ7|ENTP7_HUMAN
MARISFSYLCPASWYFTVPTVSPFLRQRVAFLGLFFISCLLLLMLIIDFRHWSASLPRDRQYERYLARVGELEATDTEDPNLNYGLV
VDCGSSGSRIFVYFWPRHNGNPHDLLDIKQMRDRNSQPVVKKIKPGISAMADTPEHASDYLRPLLSFAAAHVPVKKHKETPLYILCT
AGMRLLPERKQLAILADLVKDLPLEFDFLFSQSQAEVISGKQEGVYAWIGINFVLGRFDHEDESDAEATQELAAGRRRTVGILDMGG
ASLQIAYEVPTSTSVLPAKQEEAAKILLAEFNLGCDVQHTEHVYRVYVTTFLGFGGNFARQRYEDLVLNETLNKNRLLGQKTGLSPD
NPFLDPCLPVGLTDVVERNSQVLHVRGRGDWVSCGAMLSPLLARSNTSQASLNGIYQSPIDFNNSEFYGFSEFFYCTEDVLRIGGRY
HGPTFAKAAQDYCGMAWSVLTQRFKNGLFSSHADEHRLKYQCFKSAWMYQVLHEGFHFPYDYPNLRTAQLVYDREVQWTLGAILYKT
RFLPLRDLRQEGVRQAHGSWFRLSFVYNHYLFFACILVVLLAIFLYLLRLRRIHHRQTRASAPLDLLWLEEVVPMMGVQVGP
>sp|O43920|NDUS5_HUMAN
MPFLDIQKRFGLNIDRWLTIQSGEQPYKMAGRCHAFEKEWIECAHGIGYTRAEKECKIEYDDFVECLLRQKTMRRAGTIRKQRDKLI
KEGKYTPPPHHIGKGEPRP
>sp|P02750|A2GL_HUMAN

MSSWSRQRPKSPGGIQPHVSRTLFLLLLLAASAWGVTLSPKDCQVFRSDHGSSISCQPPAEIPGYLPADTVHLAVEFFNLTHLPANL
LQGASKLQELHLSSNGLESLSPEFLRPVPQLRVLDLTRNALTGLPPGLFQASATLDTLVLKENQLEVLEVSWLHGLKALGHLDLSGN
RLRKLPPGLLANFTLLRTLDLGENQLETLPPDLLRGPLQLERLHLEGNKLQVLGKDLLLPQPDLRYLFLNGNKLARVAAGAFQGLRQ
LDMLDLSNNSLASVPEGLWASLGQPNWDMRDGFDISGNPWICDQNLSDLYRWLQAQKDKMFSQNDTRCAGPEAVKGQTLLAVAKSQ
>sp|P11150|LIPC_HUMAN
MDTSPLCFSILLVLCIFIQSSALGQSLKPEPFGRRAQAVETNKTLHEMKTRFLLFGETNQGCQIRINHPDTLQECGFNSSLPLVMII
HGWSVDGVLENWIWQMVAALKSQPAQPVNVGLVDWITLAHDHYTIAVRNTRLVGKEVAALLRWLEESVQLSRSHVHLIGYSLGAHVS
GFAGSSIGGTHKIGRITGLDAAGPLFEGSAPSNRLSPDDANFVDAIHTFTREHMGLSVGIKQPIGHYDFYPNGGSFQPGCHFLELYR
HIAQHGFNAITQTIKCSHERSVHLFIDSLLHAGTQSMAYPCGDMNSFSQGLCLSCKKGRCNTLGYHVRQEPRSKSKRLFLVTRAQSP
FKVYHYQFKIQFINQTETPIQTTFTMSLLGTKEKMQKIPITLGKGIASNKTYSFLITLDVDIGELIMIKFKWENSAVWANVWDTVQT
IIPWSTGPRHSGLVLKTIRVKAGETQQRMTFCSENTDDLLLRPTQEKIFVKCEIKSKTSKRKIR
>sp|Q9UN75|PCDAC_HUMAN
MVIIGPRGPGSQRLLLSLLLLAAWEVGSGQLHYSVYEEAKHGTFVGRIAQDLGLELAELVPRLFRVASKRHGDLLEVNLQNGILFVN
SRIDREKLCGRSAECSIHLEVIVDRPLQVFHVDVEVKDINDNPPVFREREQKVPVSESAPLDSHFPLEGASDADIGVNSLLTYALSL
NENFELKIKTKKDKSILPELVLRKLLDREQTPKLNLLLMVIDGGKPELTGSVQIQITVLDVNDNGPAFDKPSYKVVLSENVQNDTRV
IQLNASDPDEGLNGEISYGIKMILPVSEKCMFSINPDTGEIRIYGELDFEENNAYEIQVNAIDKGIPSMAGHSMVLVEVLDVNDNVP
EVMVTSLSLPVQEDAQVGTVIALISVSDRDSGANGQVICSLTPHVPFKLVSTYKNYYSLVLDSALDRESVSAYELVVTARDGGSPSL
WATARVSVEVADVNDNAPAFAQPEYTVFVKENNPPGCHIFTVSAWDADAQKNALVSYSLVERRVGEHALSSYVSVHAESGKVYALQP
LDHEELELLQFQVSARDAGVPPLGSNVTLQVFVLDENDNAPALLATPAGSAGGAVSELVPRSVGAGHVVAKVRAVDADSGYNAWLSY
ELQPAAVGAHIPFHVGLYTGEISTTRILDEADAPRHRLLVLVKDHGEPALTSTATVLVSLVENGQAPKTSSRASVGAVDPEAALVDI
NVYLIIAICAVSSLLVLTLLLYTALRCSAPPTVSRCAPGKPTLVCSSAVGSWSYSQQRRQRVCSAESPPKTDLMAFSPSLQLSREDC
LNPPSEPRQPNPDWRYSASLRAGMHSSVHLEEAGILRAGPGGPDQQWPTVSSATPEPEAGEVSPPVGAGVNSNSWTFKYGPGNPKQS
GPGELPDKFIIPGSPAIISIRQEPTNSQIDKSDFITFGKKEETKKKKKKKKGNKTQEKKEKGNSTTDNSDQ
>sp|Q9NWN3|FBX34_HUMAN
MHLKPYWKLQKKEHPPEVSRETQRTPMNHQKAVNDETCKASHITSSVFPSASLGKASSRKPFGILSPNVLCSMSGKSPVESSLNVKT
KKNAPSATIHQGEEEGPLDIWAVVKPGNTKEKIAFFASHQCSNRIGSMKIKSSWDIDGRATKRRKKSGDLKKAKVQVERMREVNSRC
YQPEPFACGIEHCSVHYVSDSGDGVYAGRPLSVIQMVAFLEQRASALLASCSKNCTNSPAIVRFSGQSRGVPAVSESYSAPGACEEP
TERGNLEVGEPQSEPVRVLDMVAKLESECLKRQGQREPGSLSRNNSFRRNVGRVLLANSTQADEGKTKKGVLEAPDTQVNPVGSVSV
DCGPSRADRCSPKEDQAWDGASQDCPPLPAGVSFHIDSAELEPGSQTAVKNSNRYDVEMTDELVGLPFSSHTYSQASELPTDAVDCM
SRELVSLTSRNPDQRKESLCISITVSKVDKDQPSILNSCEDPVPGMLFFLPPGQHLSDYSQLNESTTKESSEASQLEDAAGGDSASE
EKSGSAEPFVLPASSVESTLPVLEASSWKKQVSHDFLETRFKIQQLLEPQQYMAFLPHHIMVKIFRLLPTKSLVALKCTCCYFKFII
EYYNIRPADSRWVRDPRYREDPCKQCKKKYVKGDVSLCRWHPKPYCQALPYGPGYWMCCHRSQKGFPGCKLGLHDNHWVPACHSFNR
AIHKKAKGTEAEEEY
>sp|Q8NA97|FEAS1_HUMAN
MDILPYLHMSHGKCPLLVRGKGEMEGEALLSCLAMNSLGEQEACLDLGSKTPSLEISSNNQERPTNREETGIICPERLFIYSSKDSS
KRLPGGLCIKNKTTCVPVQLHPSSFPKCQEAIVSPQARVKLLNKIKMDTAL
>sp|Q49AM1|MTEF2_HUMAN
MLWKLLLRSQSCRLCSFRKMRSPPKYRPFLACFTYTTDKQSSKENTRTVEKLYKCSVDIRKIRRLKGWVLLEDETYVEEIANILQEL
GADETAVASILERCPEAIVCSPTAVNTQRKLWQLVCKNEEELIKLIEQFPESFFTIKDQENQKLNVQFFQELGLKNVVISRLLTAAP
NVFHNPVEKNKQMVRILQESYLDVGGSEANMKVWLLKLLSQNPFILLNSPTAIKETLEFLQEQGFTSFEILQLLSKLKGFLFQLCPR
SIQNSISFSKNAFKCTDHDLKQLVLKCPALLYYSVPVLEERMQGLLREGISIAQIRETPMVLELTPQIVQYRIRKLNSSGYRIKDGH
LANLNGSKKEFEANFGKIQAKKVRPLFNPVAPLNVEE
>sp|A6NLU0|RFPLA_HUMAN

MAEHFKQIIRCPVCLKDLEEAVQLKCGYACCLQCLNSLQKEPDGEGLLCRFCSVVSQKDDIKPKYKLRALVSIIKELEPKLKSVLTM
NPRMRKFQVDMTFDVDTANNYLIISEDLRSFRSGDLSQNRKEQAERFDTALCVLGTPRFTSGRHYWEVDVGTSQVWDVGVCKESVNR
QGKIVLSSEHGFLTVGCREGKVFAASTVPMTPLWVSPQLHRVGIFLDVGMRSIAFYNVSDGCHIYTFIEIPVCEPWRPFFAHKRGSQ
DDQSILSICSVINPSAASAPVSSEGK
>sp|Q6NTF9|RHBD2_HUMAN
MAASGPGCRSWCLCPEVPSATFFTALLSLLVSGPRLFLLQQPLAPSGLTLKSEALRNWQVYRLVTYIFVYENPISLLCGAIIIWRFA
GNFERTVGTVRHCFFTVIFAIFSAIIFLSFEAVSSLSKLGEVEDARGFTPVAFAMLGVTTVRSRMRRALVFGMVVPSVLVPWLLLGA
SWLIPQTSFLSNVCGLSIGLAYGLTYCYSIDLSERVALKLDQTFPFSLMRRISVFKYVSGSSAERRAAQSRKLNPVPGSYPTQSCHP
HLSPSHPVSQTQHASGQKLASWPSCTPGHMPTLPPYQPASGLCYVQNHFGPNPTSSSVYPASAGTSLGIQPPTPVNSPGTVYSGALG
TPGAAGSKESSRVPMP
>sp|Q658K8|EF1DL_HUMAN
MATNFLVGEKIWFHKFKYGDAERRFYEQMNGPVAGASLQEASMILHDIARARENIPKSLAGSLGPGASSGPSGDHSELVVRIASLEV
DNQRDLAERAGEELARPLGHSPADPAHVSHAPSGAPGQEASHTSRG
>sp|O43597|SPY2_HUMAN
MEARAQSGNGSQPLLQTPRDGGRQRGEPDPRDALTQQVHVLSLDQIRAIRNTNEYTEGPTVVPRPGLKPAPRPSTQHKHERLHGLPE
HRQPPRLQHSQVHSSARAPLSRSISTVSSGSRSSTRTSTSSSSSEQRLLGSSFSSGPVADGIIRVQPKSELKPGELKPLSKEDLGLH
AYRCEDCGKCKCKECTYPRPLPSDWICDKQCLCSAQNVIDYGTCVCCVKGLFYHCSNDDEDNCADNPCSCSQSHCCTRWSAMGVMSL
FLPCLWCYLPAKGCLKLCQGCYDRVNRPGCRCKNSNTVCCKVPTVPPRNFEKPT
>sp|Q13608|PEX6_HUMAN
MALAVLRVLEPFPTETPPLAVLLPPGGPWPAAELGLVLALRPAGESPAGPALLVAALEGPDAGTEEQGPGPPQLLVSRALLRLLALG
SGAWVRARAVRRPPALGWALLGTSLGPGLGPRVGPLLVRRGETLPVPGPRVLETRPALQGLLGPGTRLAVTELRGRARLCPESGDSS
RPPPPPVVSSFAVSGTVRRLQGVLGGTGDSLGVSRSCLRGLGLFQGEWVWVAQARESSNTSQPHLARVQVLEPRWDLSDRLGPGSGP
LGEPLADGLALVPATLAFNLGCDPLEMGELRIQRYLEGSIAPEDKGSCSLLPGPPFARELHIEIVSSPHYSTNGNYDGVLYRHFQIP
RVVQEGDVLCVPTIGQVEILEGSPEKLPRWREMFFKVKKTVGEAPDGPASAYLADTTHTSLYMVGSTLSPVPWLPSEESTLWSSLSP
PGLEALVSELCAVLKPRLQPGGALLTGTSSVLLRGPPGCGKTTVVAAACSHLGLHLLKVPCSSLCAESSGAVETKLQAIFSRARRCR
PAVLLLTAVDLLGRDRDGLGEDARVMAVLRHLLLNEDPLNSCPPLMVVATTSRAQDLPADVQTAFPHELEVPALSEGQRLSILRALT
AHLPLGQEVNLAQLARRCAGFVVGDLYALLTHSSRAACTRIKNSGLAGGLTEEDEGELCAAGFPLLAEDFGQALEQLQTAHSQAVGA
PKIPSVSWHDVGGLQEVKKEILETIQLPLEHPELLSLGLRRSGLLLHGPPGTGKTLLAKAVATECSLTFLSVKGPELINMYVGQSEE
NVREVFARARAAAPCIIFFDELDSLAPSRGRSGDSGGVMDRVVSQLLAELDGLHSTQDVFVIGATNRPDLLDPALLRPGRFDKLVFV
GANEDRASQLRVLSAITRKFKLEPSVSLVNVLDCCPPQLTGADLYSLCSDAMTAALKRRVHDLEEGLEPGSSALMLTMEDLLQAAAR
LQPSVSEQELLRYKRIQRKFAAC
>sp|Q76L83|ASXL2_HUMAN
MREKGRRKKGRTWAEAAKTVLEKYPNTPMSHKEILQVIQREGLKEIRSGTSPLACLNAMLHTNSRGEEGIFYKVPGRMGVYTLKKDV
PDGVKELSEGSEESSDGQSDSQSSENSSSSSDGGSNKEGKKSRWKRKVSSSSPQSGCPSPTIPAGKVISPSQKHSKKALKQALKQQQ
QKKQQQQCRPSISISSNQHLSLKTVKAASDSVPAKPATWEGKQSDGQTGSPQNSNSSFSSSVKVENTLLGLGKKSFQRSERLHTRQM
KRTKCADIDVETPDSILVNTNLRALINKHTFSVLPGDCQQRLLLLLPEVDRQVGPDGLMKLNGSALNNEFFTSAAQGWKERLSEGEF
TPEMQVRIRQEIEKEKKVEPWKEQFFESYYGQSSGLSLEDSKKLTASPSDPKVKKTPAEQPKSMPVSEASLIRIVPVVSQSECKEEA
LQMSSPGRKEECESQGEVQPNFSTSSEPLLSSALNTHELSSILPIKCPKDEDLLEQKPVTSAEQESEKNHLTTASNYNKSESQESLV
TSPSKPKSPGVEKPIVKPTAGAGPQETNMKEPLATLVDQSPESLKRKSSLTQEEAPVSWEKRPRVTENRQHQQPFQVSPQPFLNRGD
RIQVRKVPPLKIPVSRISPMPFHPSQVSPRARFPVSITSPNRTGARTLADIKAKAQLVKAQRAAAAAAAAAAAAASVGGTIPGPGPG
GGQGPGEGGEGQTARGGSPGSDRVSETGKGPTLELAGTGSRGGTRELLPCGPETQPQSETKTTPSQAQPHSVSGAQLQQTPPVPPTP
AVSGACTSVPSPAHIEKLDNEKLNPTRATATVASVSHPQGPSSCRQEKAPSPTGPALISGASPVHCAADGTVELKAGPSKNIPNPSA
SSKTDASVPVAVTPSPLTSLLTTATLEKLPVPQVSATTAPAGSAPPSSTLPAASSLKTPGTSLNMNGPTLRPTSSIPANNPLVTQLL
QGKDVPMEQILPKPLTKVEMKTVPLTAKEERGMGALIATNTTENSTREEVNERQSHPATQQQLGKTLQSKQLPQVPRPLQLFSAKEL

RDSSIDTHQYHEGLSKATQDQILQTLIQRVRRQNLLSVVPPSQFNFAHSGFQLEDISTSQRFMLGFAGRRTSKPAMAGHYLLNISTY
GRGSESFRRTHSVNPEDRFCLSSPTEALKMGYTDCKNATGESSSSKEDDTDEESTGDEQESVTVKEEPQVSQSAGKGDTSSGPHSRE
TLSTSDCLASKNVKAEIPLNEQTTLSKENYLFTRGQTFDEKTLARDLIQAAQKQMAHAVRGKAIRSSPELFSSTVLPLPADSPTHQP
LLLPPLQTPKLYGSPTQIGPSYRGMINVSTSSDMDHNSAVPGSQVSSNVGDVMSFSVTVTTIPASQAMNPSSHGQTIPVQAFSEENS
IEGTPSKCYCRLKAMIMCKGCGAFCHDDCIGPSKLCVSCLVVR
>sp|Q96FX8|PERP_HUMAN
MIRCGLACERCRWILPLLLLSAIAFDIIALAGRGWLQSSDHGQTSSLWWKCSQEGGGSGSYEEGCQSLMEYAWGRAAAAMLFCGFII
LVICFILSFFALCGPQMLVFLRVIGGLLALAAVFQIISLVIYPVKYTQTFTLHANPAVTYIYNWAYGFGWAATIILIGCAFFFCCLP
NYEDDLLGNAKPRYFYTSA
>sp|P14416|DRD2_HUMAN
MDPLNLSWYDDDLERQNWSRPFNGSDGKADRPHYNYYATLLTLLIAVIVFGNVLVCMAVSREKALQTTTNYLIVSLAVADLLVATLV
MPWVVYLEVVGEWKFSRIHCDIFVTLDVMMCTASILNLCAISIDRYTAVAMPMLYNTRYSSKRRVTVMISIVWVLSFTISCPLLFGL
NNADQNECIIANPAFVVYSSIVSFYVPFIVTLLVYIKIYIVLRRRRKRVNTKRSSRAFRAHLRAPLKGNCTHPEDMKLCTVIMKSNG
SFPVNRRRVEAARRAQELEMEMLSSTSPPERTRYSPIPPSHHQLTLPDPSHHGLHSTPDSPAKPEKNGHAKDHPKIAKIFEIQTMPN
GKTRTSLKTMSRRKLSQQKEKKATQMLAIVLGVFIICWLPFFITHILNIHCDCNIPPVLYSAFTWLGYVNSAVNPIIYTTFNIEFRK
AFLKILHC
>sp|Q96JW4|S41A2_HUMAN
MTNSKGRSITDKTSGGPSSGGGFVDWTLRLNTIQSDKFLNLLLSMVPVIYQKNQEDRHKKANGIWQDGLSTAVQTFSNRSEQHMEYH
SFSEQSFHANNGHASSSCSQKYDDYANYNYCDGRETSETTAMLQDEDISSDGDEDAIVEVTPKLPKESSGIMALQILVPFLLAGFGT
VSAGMVLDIVQHWEVFRKVTEVFILVPALLGLKGNLEMTLASRLSTAVNIGKMDSPIEKWNLIIGNLALKQVQATVVGFLAAVAAII
LGWIPEGKYYLDHSILLCSSSVATAFIASLLQGIIMVGVIVGSKKTGINPDNVATPIAASFGDLITLAILAWISQGLYSCLETYYYI
SPLVGVFFLALTPIWIIIAAKHPATRTVLHSGWEPVITAMVISSIGGLILDTTVSDPNLVGIVVYTPVINGIGGNLVAIQASRISTY
LHLHSIPGELPDEPKGCYYPFRTFFGPGVNNKSAQVLLLLVIPGHLIFLYTIHLMKSGHTSLTIIFIVVYLFGAVLQVFTLLWIADW
MVHHFWRKGKDPDSFSIPYLTALGDLLGTALLALSFHFLWLIGDRDGDVGD
>sp|O75665|OFD1_HUMAN
MMAQSNMFTVADVLSQDELRKKLYQTFKDRGILDTLKTQLRNQLIHELMHPVLSGELQPRSISVEGSSLLIGASNSLVADHLQRCGY
EYSLSVFFPESGLAKEKVFTMQDLLQLIKINPTSSLYKSLVSGSDKENQKGFLMHFLKELAEYHQAKESCNMETQTSSTFNRDSLAE
KLQLIDDQFADAYPQRIKFESLEIKLNEYKREIEEQLRAEMCQKLKFFKDTEIAKIKMEAKKKYEKELTMFQNDFEKACQAKSEALV
LREKSTLERIHKHQEIETKEIYAQRQLLLKDMDLLRGREAELKQRVEAFELNQKLQEEKHKSITEALRRQEQNIKSFEETYDRKLKN
ELLKYQLELKDDYIIRTNRLIEDERKNKEKAVHLQEELIAINSKKEELNQSVNRVKELELELESVKAQSLAITKQNHMLNEKVKEMS
DYSLLKEEKLELLAQNKLLKQQLEESRNENLRLLNRLAQPAPELAVFQKELRKAEKAIVVEHEEFESCRQALHKQLQDEIEHSAQLK
AQILGYKASVKSLTTQVADLKLQLKQTQTALENEVYCNPKQSVIDRSVNGLINGNVVPCNGEISGDFLNNPFKQENVLARMVASRIT
NYPTAWVEGSSPDSDLEFVANTKARVKELQQEAERLEKAFRSYHRRVIKNSAKSPLAAKSPPSLHLLEAFKNITSSSPERHIFGEDR
VVSEQPQVGTLEERNDVVEALTGSAASRLRGGTSSRRLSSTPLPKAKRSLESEMYLEGLGRSHIASPSPCPDRMPLPSPTESRHSLS
IPPVSSPPEQKVGLYRRQTELQDKSEFSDVDKLAFKDNEEFESSFESAGNMPRQLEMGGLSPAGDMSHVDAAAAAVPLSYQHPSVDQ
KQIEEQKEEEKIREQQVKERRQREERRQSNLQEVLERERRELEKLYQERKMIEESLKIKIKKELEMENELEMSNQEIKDKSAHSENP
LEKYMKIIQQEQDQESADKSSKKMVQEGSLVDTLQSSDKVESLTGFSHEELDDSW
>sp|O60809|PRA10_HUMAN
MSLQAPSRLLELAGQSLLRNQFLTIFTLDELPREVFPLMFMEAFSMRRFEALKLMVQAWPFLRLPLGSLMKTPHLETLQAVLRGLDT
LVAQKVRPRRWKLQVLDLRDVDENFWTIWSGARVLSCSPEAMSKRQTVEDCPRMGERQPLKVFIDLCLKESTLDECLSYLFGWIHYR
RGLVHLCCSKVQNYSMPTSSFRNLLERIYPDSIQELEVWKKCSLNKTGKFAPYLSQMSNLRELFLAFGYERELYVSVQWPCIPDLDS
PFLCLYYPQMLYIKKISNIKEHLEHLLRYLKNPLGAFIFSDAYLTDRDMECLSQYPSLSQLKELRLIHILMWTTNLEPLGVLLEKVA
ATLKTLVLKDCRIQDPQLRVLLPALSHCSQLTTFNFHGNETSMNALKDLLRHTRGLSKLGLELYPAPLESLDYKGHVNWEILTPIRA
ELMRTLREVRQPKRIFFGPVPCPNCGSWPSEKVDFHLCS

>sp|Q6A162|K1C40_HUMAN
MTSDCSSTHCSPESCGTASGCAPASSCSVETACLPGTCATSRCQTPSFLSRSRGLTGCLLPCYFTGSCNSPCLVGNCAWCEDGVFTS
NEKETMQFLNDRLASYLEKVRSLEETNAELESRIQEQCEQDIPMVCPDYQRYFNTIEDLQQKILCTKAENSRLAVQLDNCKLATDDF
KSKYESELSLRQLLEADISSLHGILEELTLCKSDLEAHVESLKEDLLCLKKNHEEEVNLLREQLGDRLSVELDTAPTLDLNRVLDEM
RCQCETVLANNRREAEEWLAVQTEELNQQQLSSAEQLQGCQMEILELKRTASALEIELQAQQSLTESLECTVAETEAQYSSQLAQIQ
CLIDNLENQLAEIRCDLERQNQEYQVLLDVKARLEGEINTYWGLLDSEDSRLSCSPCSTTCTSSNTCEPCSAYVICTVENCCL
>sp|O94782|UBP1_HUMAN
MPGVIPSESNGLSRGSPSKKNRLSLKFFQKKETKRALDFTDSQENEEKASEYRASEIDQVVPAAQSSPINCEKRENLLPFVGLNNLG
NTCYLNSILQVLYFCPGFKSGVKHLFNIISRKKEALKDEANQKDKGNCKEDSLASYELICSLQSLIISVEQLQASFLLNPEKYTDEL
ATQPRRLLNTLRELNPMYEGYLQHDAQEVLQCILGNIQETCQLLKKEEVKNVAELPTKVEEIPHPKEEMNGINSIEMDSMRHSEDFK
EKLPKGNGKRKSDTEFGNMKKKVKLSKEHQSLEENQRQTRSKRKATSDTLESPPKIIPKYISENESPRPSQKKSRVKINWLKSATKQ
PSILSKFCSLGKITTNQGVKGQSKENECDPEEDLGKCESDNTTNGCGLESPGNTVTPVNVNEVKPINKGEEQIGFELVEKLFQGQLV
LRTRCLECESLTERREDFQDISVPVQEDELSKVEESSEISPEPKTEMKTLRWAISQFASVERIVGEDKYFCENCHHYTEAERSLLFD
KMPEVITIHLKCFAASGLEFDCYGGGLSKINTPLLTPLKLSLEEWSTKPTNDSYGLFAVVMHSGITISSGHYTASVKVTDLNSLELD
KGNFVVDQMCEIGKPEPLNEEEARGVVENYNDEEVSIRVGGNTQPSKVLNKKNVEAIGLLGGQKSKADYELYNKASNPDKVASTAFA
ENRNSETSDTTGTHESDRNKESSDQTGINISGFENKISYVVQSLKEYEGKWLLFDDSEVKVTEEKDFLNSLSPSTSPTSTPYLLFYK
KL
>sp|Q9NRJ2|GSAS1_HUMAN
MTHPLPHDSHTSGAPPLVNKSRDLANGPPFFSPLQSLWEEFLHLLFMGTLLFYRIATKALRGKATLLKSHSKQPAQPGWEPGIRAPS
PVPASSLQDHSRLTSLSRTGKEQRRTLSLIRKTSGTPTESTVATAAASTTEVPSRLPWAARAGFKRTTGVCIALPT
>sp|Q9NYL9|TMOD3_HUMAN
MALPFRKDLEKYKDLDEDELLGNLSETELKQLETVLDDLDPENALLPAGFRQKNQTSKSTTGPFDREHLLSYLEKEALEHKDREDYV
PYTGEKKGKIFIPKQKPVQTFTEEKVSLDPELEEALTSASDTELCDLAAILGMHNLITNTKFCNIMGSSNGVDQEHFSNVVKGEKIL
PVFDEPPNPTNVEESLKRTKENDAHLVEVNLNNIKNIPIPTLKDFAKALETNTHVKCFSLAATRSNDPVATAFAEMLKVNKTLKSLN
VESNFITGVGILALIDALRDNETLAELKIDNQRQQLGTAVELEMAKMLEENTNILKFGYQFTQQGPRTRAANAITKNNDLVRKRRVE
GDHQ
>sp|Q14242|SELPL_HUMAN
MPLQLLLLLILLGPGNSLQLWDTWADEAEKALGPLLARDRRQATEYEYLDYDFLPETEPPEMLRNSTDTTPLTGPGTPESTTVEPAA
RRSTGLDAGGAVTELTTELANMGNLSTDSAAMEIQTTQPAATEAQTTQPVPTEAQTTPLAATEAQTTRLTATEAQTTPLAATEAQTT
PPAATEAQTTQPTGLEAQTTAPAAMEAQTTAPAAMEAQTTPPAAMEAQTTQTTAMEAQTTAPEATEAQTTQPTATEAQTTPLAAMEA
LSTEPSATEALSMEPTTKRGLFIPFSVSSVTHKGIPMAASNLSVNYPVGAPDHISVKQCLLAILILALVATIFFVCTVVLAVRLSRK
GHMYPVRNYSPTEMVCISSLLPDGGEGPSATANGGLSKAKSPGLTPEPREDREGDDLTLHSFLP
>sp|Q92543|SNX19_HUMAN
MKTETVPPFQETPAGSSCHLNNLLSSRKLMAVGVLLGWLLVIHLLVNVWLLCLLSALLVVLGGWLGSSLAGVASGRLHLERFIPLAT
CPPCPEAERQLEREINRTIQMIIRDFVLSWYRSVSQEPAFEEEMEAAMKGLVQELRRRMSVMDSHAVAQSVLTLCGCHLQSYIQAKE
ATAGKNGPVEPSHLWEAYCRATAPHPAVHSPSAEVTYTRGVVNLLLQGLVPKPHLETRTGRHVVVELITCNVILPLISRLSDPDWIH
LVLVGIFSKARDPAPCPASAPEQPSVPTSLPLIAEVEQLPEGRASPVAAPVFLSYSEPEGSAGPSPEVEEGHEAVEGDLGGMCEERK
VGNNSSHFLQPNVRGPLFLCEDSELESPLSELGKETIMLMTPGSFLSDRIQDALCALESSQALEPKDGEASEGAEAEEGPGTETETG
LPVSTLNSCPEIHIDTADKEIEQGDVTASVTALLEGPEKTCPSRPSCLEKDLTNDVSSLDPTLPPVLLSSSPPGPLSSATFSFEPLS
SPDGPVIIQNLRITGTITAREHSGTGFHPYTLYTVKYETALDGENSSGLQQLAYHTVNRRYREFLNLQTRLEEKPDLRKFIKNVKGP
KKLFPDLPLGNMDSDRVEARKSLLESFLKQLCAIPEIANSEEVQEFLALNTDARIAFVKKPFMVSRIDKMVVSAIVDTLKTAFPRSE
PQSPTEELSEAETESKPQTEGKKASKSRLRFSSSKISPALSVTEAQDKILYCLQEGNVESETLSMSAMESFIEKQTKLLEMQPTKAP
EKDPEQPPKGRVDSCVSDAAVPAQDPSNSDPGTETELADTALDLLLLLLTEQWKWLCTENMQKFLRLIFGTLVQRWLEVQVANLTSP

QRWVQYLLLLQESIWPGGVLPKFPRPVRTQEQKLAAEKQALQSLMGVLPDLVVEILGVNKCRLSWGLVLESLQQPLINRHLIYCLGD
IILEFLDLSASVEESAATTSASDTPGNSKRMGVSS
>sp|Q9ULT0|TTC7A_HUMAN
MAAKGAHGSYLKVESELERCRAEGHWDRMPELVRQLQTLSMPGGGGNRRGSPSAAFTFPDTDDFGKLLLAEALLEQCLKENHAKIKD
SMPLLEKNEPKMSEAKNYLSSILNHGRLSPQYMCEAMLILGKLHYVEGSYRDAISMYARAGIDDMSMENKPLYQMRLLSEAFVIKGL
SLERLPNSIASRFRLTEREEEVITCFERASWIAQVFLQELEKTTNNSTSRHLKGCHPLDYELTYFLEAALQSAYVKNLKKGNIVKGM
RELREVLRTVETKATQNFKVMAAKHLAGVLLHSLSEECYWSPLSHPLPEFMGKEESSFATQALRKPHLYEGDNLYCPKDNIEEALLL
LLISESMATRDVVLSRVPEQEEDRTVSLQNAAAIYDLLSITLGRRGQYVMLSECLERAMKFAFGEFHLWYQVALSMVACGKSAYAVS
LLRECVKLRPSDPTVPLMAAKVCIGSLRWLEEAEHFAMMVISLGEEAGEFLPKGYLALGLTYSLQATDATLKSKQDELHRKALQTLE
RAQQLAPSDPQVILYVSLQLALVRQISSAMEQLQEALKVRKDDAHALHLLALLFSAQKHHQHALDVVNMAITEHPENFNLMFTKVKL
EQVLKGPEEALVTCRQVLRLWQTLYSFSQLGGLEKDGSFGEGLTMKKQSGMHLTLPDAHDADSGSRRASSIAASRLEEAMSELTMPS
SVLKQGPMQLWTTLEQIWLQAAELFMEQQHLKEAGFCIQEAAGLFPTSHSVLYMRGRLAEVKGNLEEAKQLYKEALTVNPDGVRIMH
SLGLMLSRLGHKSLAQKVLRDAVERQSTCHEAWQGLGEVLQAQGQNEAAVDCFLTALELEASSPVLPFSIIPREL
>sp|Q9NT22|EMIL3_HUMAN
MGRRRLLVWLCAVAALLSGAQARGTPLLARPAPPGASRYSLYTTGWRPRLRPGPHKALCAYVVVHRNVTCILQEGAESYVKAEYRQCR
WGPKCPGTVTYRTVLRPKYKVGYKTVTDLAWRCCPGFTGKRCPEHLTDHGAASPQLEPEPQIPSGQLDPGPRPPSYSRAAPSPHGRK
GPGLFGERLERLEGDVQRLAQTYGTLSGLVASHEDPNRMTGGPRAPAVPVGFGVIPEGLVGPGDRARGPLTPPLDEILSKVTEVSNT
LQTKVQLLDKVHGLALGHEAHLQRLREAPPSPLTSLALLEEYVDRRLHRLWGSLLDGFEQKLQGVQSECDLRVQEVRRQCEEGQAAS
RRLHQSLDGRELALRQELSQLGSQLQGLSVSGRGSCCGQLALINARMDGLERALQAVTETQRGPGAPAGDELTRLSAAMLEGGVDGL
LEGLETLNGTEGGARGCCLRLDMGGWGVGGFGTMLEERVQSLEERLATLAGELSHDSASPGRSARPLVQTELAVLEQRLVSLETSCT
PSTTSAILDSLVAEVKAWQSRSEALLRQVASHAALLQQLNGTVAEVQGQLAEGTGSSLQGEITLLKVNLNSVSKSLTGLSDSVSQYS
DAFLAANTSLDERERKVEAEVQAIQEQVSSQGSRLQAGHRQVLNLRGELEQLKAGVAKVASGLSRCQDTAQKLQHTVGHFDQRVAQV
EGACRRLGLLAAGLDSLPTEPLRPREGLWSHVDQLNRTLAQHTQDIARLRDDLLDCQAQLAEQVRPGQAN
>sp|Q6ZSR6|YP007_HUMAN
MLVETGFCRVGQAGLELLSSSDKAAGLDLPKCWDYRHEPPRLAPLLIFNPHPSTVLSCNCEYNSFFEFCDSLQQIVIPERVLGTPRH
IFPLPLLFSHFLWSKLKEAPACLLQGSSEHTEIICDLISSSKQFIKKFLSNKPSALHGGDADENDFLQLITRLQKLLFKSLSMYVCV
HIHQHTHACPQLSCLHQNQDEELFYCQN
>sp|Q8IV36|HID1_HUMAN
MGSTDSKLNFRKAVIQLTTKTQPVEATDDAFWDQFWADTATSVQDVFALVPAAEIRAVREESPSNLATLCYKAVEKLVQGAESGCHS
EKEKQIVLNCSRLLTRVLPYIFEDPDWRGFFWSTVPGAGRGGQGEEDDEHARPLAESLLLAIADLLFCPDFTVQSHRRSTVDSAEDV
HSLDSCEYIWEAGVGFAHSPQPNYIHDMNRMELLKLLLTCFSEAMYLPPAPESGSTNPWVQFFCSTENRHALPLFTSLLNTVCAYDP
VGYGIPYNHLLFSDYREPLVEEAAQVLIVTLDHDSASSASPTVDGTTTGTAMDDADPPGPENLFVNYLSRIHREEDFQFILKGIARL
LSNPLLQTYLPNSTKKIQFHQELLVLFWKLCDFNKKFLFFVLKSSDVLDILVPILFFLNDARADQSRVGLMHIGVFILLLLSGERNF
GVRLNKPYSIRVPMDIPVFTGTHADLLIVVFHKIITSGHQRLQPLFDCLLTIVVNVSPYLKSLSMVTANKLLHLLEAFSTTWFLFSA
AQNHHLVFFLLEVFNNIIQYQFDGNSNLVYAIIRKRSIFHQLANLPTDPPTIHKALQRRRRTPEPLSRTGSQEGTSMEGSRPAAPAE
PGTLKTSLVATPGIDKLTEKSQVSEDGTLRSLEPEPQQSLEDGSPAKGEPSQAWREQRRPSTSSASGQWSPTPEWVLSWKSKLPLQT
IMRLLQVLVPQVEKICIDKGLTDESEILRFLQHGTLVGLLPVPHPILIRKYQANSGTAMWFRTYMWGVIYLRNVDPPVWYDTDVKLF
EIQRV
>sp|Q6ZSZ5|ARHGI_HUMAN
MVTVGTNILPSRPAASANTAREDAALFSRRIPPRHKNGAAQPGAAPGPGAPGANMGNAHSKSGDRHSALPGRPELSFYGSFPRKWSE
NVFLDNELLTSKILSVLRPQSERGFRAGDLRYPTHFLSTNSVLASVTASLKEHPRGTLLSDGSPALSRNVGMTVSQKGGPQPTPSPA
GPGTQLGPITGEMDEADSAFLKFKQTADDSLSLTSPNTESIFVEDPYTASLRSEIESDGHEFEAESWSLAVDAAYAKKQKREVVKRQ
DVLYELMQTEVHHVRTLKIMLKVYSRALQEELQFSSKAIGRLFPCADDLLETHSHFLARLKERRQESLEEGSDRNYVIQKIGDLLVQ
QFSGENGERMKEKYGVFCSGHNEAVSHYKLLLQQNKKFQNLIKKIGNFSIVRRLGVQECILLVTQRITKYPVLVERIIQNTEAGTED

YEDLTQALNLIKDIISQVDAKVSECEKGQRLREIAGKMDLKSSSKLKNGLTFRKEDMLQRQLHLEGMLCWKTTSGRLKDILAILLTD
VLLLLQEKDQKYVFASVDSKPPVISLQKLIVREVANEEKAMFLISASLQGPEMYEIYTSSKEDRNAWMAHIQRAVESCPDEEEGPFS
LPEEERKVVEARATRLRDFQERLSMKDQLIAQSLLEKQQIYLEMAEMGGLEDLPQPRGLFRGGDPSETLQGELILKSAMSEIEGIQS
LICRQLGSANGQAEDGGSSTGPPRRAETFAGYDCTNSPTKNGSFKKKVSSTDPRPRDWRGPPNSPDLKLSDSDIPGSSEESPQVVEA
PGTESDPRLPTVLESELVQRIQTLSQLLLNLQAVIAHQDSYVETQRAAIQEREKQFRLQSTRGNLLLEQERQRNFEKQREERAALEK
LQSQLRHEQQRWERERQWQHQELERAGARLQEREGEARQLRERLEQERAELERQRQAYQHDLERLREAQRAVERERERLELLRRLKK
QNTAPGALPPDTLAEAQPPSHPPSFNGEGLEGPRVSMLPSGVGPEYAERPEVARRDSAPTENRLAKSDVPIQLLSATNQFQRQAAVQ
QQIPTKLAASTKGGKDKGGKSRGSQRWESSASFDLKQQLLLNKLMGKDESTSRNRRSLSPILPGRHSPAPPPDPGFPAPSPPPADSP
SEGFSLKAGGTALLPGPPAPSPLPATPLSAKEDASKEDVIFF
>sp|Q9NQW5|PRDM7_HUMAN
MSPERSQEESPEGDTERTERKPMVKDAFKDISIYFTKEEWAEMGDWEKTRYRNVKMNYNALITVGLRATRPAFMCHRRQAIKLQVDD
TEDSDEEWTPRQQVKPPWMAFRGEQSKHQKGMPKASFNNESSLRELSGTPNLLNTSDSEQAQKPVSPPGEASTSGQHSRLKLELRRK
ETEGKMYSLRERKGHAYKEISEPQDDDYLYCEMCQNFFIDSCAAHGPPTFVKDSAVDKGHPNRSALSLPPGLRIGPSGIPQAGLGVW
NEASDLPLGLHFGPYEGRITEDEEAANSGYSWLITKGRNCYEYVDGKDKSSANWMRYVNCARDDEEQNLVAFQYHRQIFYRTCRVIR
PGCELLVWSGDEYGQELGIRSSIEPAESLGQAVNCWSGMGMSMARNWASSGAASGRKSSWQGENQSQRSIHVPHAVWPFQVKNFSVN
MWNAITPLRTSQDHLQENFSNQRIPAQGIRIRSGNILIHAAVMTKPKVKRSKKGPNS
>sp|Q9C0B7|TNG6_HUMAN
MAARQAVGSGAQETCGLDRILEALKLLLSPGGSGSSSLQVTKHDVLLATLKSNLSALEDKFLKDPQWKNLKLLRDEIADKAEWPQNS
VDVTWSFTSQTLLLLLCLKETMIRLAANFNPGKPNPRTPEVAPALSPDALSISQQKTVQFVLQFVVTLGICPYLMPGVGVPLRYRTE
FGAVVQDVVCFDAAPDATRRLYTSCKALLNVAQHTSLGSLIFCHHFGDIAAGLCQLGFCPTKRKLLTPAEEVLTEEERTLSRGALRD
MLDQVYQPLAVRELLILQGGPPQSCTDVKTQMRCRAPAWLRRLCGQLLSERLMRPNGVQAVVRGILEGAGAGAAGGSDAEVTAADWK
KCDLIAKILASCPQQSLSPENYYRDICPQVLDLFHFQDKLTARQFQRVATTTFITLSRERPHLAAKYLLQPVLAPLHRCLNTAELSE
SDMVPGTILVTEEELSRCIEDVFKVYVVGNEPLTVLMDSLLPVLGVLFLLYCFTKQSVSHIRSLCQEILLWILGKLERKKAIASLKG
FAGLDKAVPSLHSLCQFRVATQGGIMITIKEAISDEDEDEALYQKVSSEQGRVEHLGDLLSHCQECGLAGDFFIFCLKELTHVASEN
ETELKTEPFSSKSLLELEQHQTLLVEGQERKLLVLQLMAVLCERMSEQIFTNVTQVVDFVAATLQRACASLAHQAESTVESQTLSMS
MGLVAVMLGGAVQLKSSDFAVLKQLLPLLEKVSNTYPDPVIQELAVDLRITISTHGAFATEAVSMAAQSTLNRKDLEGKIEEQQQTS
HERPTDVAHSHLEQQQSHETAPQTGLQSNAPIIPQGVNEPSTTTSQKSGSVTTEQLQEVLLSAYDPQIPTRAAALRTLSHWIEQREA
KALEMQEKLLKIFLENLEHEDTFVYLSAIQGVALLSDVYPEKILPDLLAQYDSSKDKHTPETRMKVGEVLMRIVRALGDMVSKYREP
LIHTFLRGVRDPDGAHRASSLANLGELCQRLDFLLGSVVHEVTACLIAVAKTDGEVQVRRAAIHVVVLLLRGLSQKATEVLSAVLKD
LYHLLKHVVCLEPDDVAKLHAQLALEELDDIMKNFLFPPQKLEKKIMVLP
>sp|Q8TD57|DYH3_HUMAN
MGATGRLELTLAAPPHPGPAFQRSKARETQGEEEGSEMQIAKSDSIHHMSHSQGQPELPPLPASANEEPSGLYQTVMSHSFYPPLMQ
RTSWTLAAPFKEQHHHRGPSDSIANNYSLMAQDLKLKDLLKVYQPATISVPRDRTGQGLPSSGNRSSSEPMRKKTKFSSRNKEDSTR
IKLAFKTSIFSPMKKEVKTSLTFPGSRPMSPEQQLDVMLQQEMEMESKEKKPSESDLERYYYYLTNGIRKDMIAPEEGEVMVRISKL
ISNTLLTSPFLEPLMVVLVQEKENDYYCSLMKSIVDYILMDPMERKRLFIESIPRLFPQRVIRAPVPWHSVYRSAKKWNEEHLHTVN
PMMLRLKELWFAEFRDLRFVRTAEILAGKLPLQPQEFWDVIQKHCLEAHQTLLNKWIPTCAQLFTSRKEHWIHFAPKSNYDSSRNIE
EYFASVASFMSLQLRELVIKSLEDLVSLFMIHKDGNDFKEPYQEMKFFIPQLIMIKLEVSEPIIVFNPSFDGCWELIRDSFLEIIKN
SNGIPKLKYIPLKFSFTAAAADRQCVKAAEPGEPSMHAAATAMAELKGYNLLLGTVNAEEKLVSDFLIQTFKVFQKNQVGPCKYLNV
YKKYVDLLDNTAEQNIAAFLKENHDIDDFVTKINAIKKRRNEIASMNITVPLAMFCLDATALNHDLCERAQNLKDHLIQFQVDVNRD
TNTSICNQYSHIADKVSEVPANTKELVSLIEFLKKSSAVTVFKLRRQLRDASERLEFLMDYADLPYQIEDIFDNSRNLLLHKRDQAE
MDLIKRCSEFELRLEGYHRELESFRKREVMTTEEMKHNVEKLNELSKNLNRAFAEFELINKEEELLEKEKSTYPLLQAMLKNKVPYE
QLWSTAYEFSIKSEEWMNGPLFLLNAEQIAEEIGNMWRTTYKLIKTLSDVPAPRRLAENVKIKIDKFKQYIPILSISCNPGMKDRHW
QQISEIVGYEIKPTETTCLSNMLEFGFGKFVEKLEPIGAAASKEYSLEKNLDRMKLDWVNVTFSFVKYRDTDTNILCAIDDIQMLLD
DHVIKTQTMCGSPFIKPIEAECRKWEEKLIRIQDNLDAWLKCQATWLYLEPIFSSEDIIAQMPEEGRKFGIVDSYWKSLMSQAVKDN

RILVAADQPRMAEKLQEANFLLEDIQKGLNDYLEKKRLFFPRFFFLSNDELLEILSETKDPLRVQPHLKKCFEGIAKLEFTDNLEIV
GMISSEKETVPFIQKIYPANAKGMVEKWLQQVEQMMLASMREVIGLGIEAYVKVPRNHWVLQWPGQVVICVSSIFWTQEVSQALAEN
TLLDFLKKSNDQIAQIVQLVRGKLSSGARLTLGALTVIDVHARDVVAKLSEDRVSDLNDFQWISQLRYYWVAKDVQVQIITTEALYG
YEYLGNSPRLVITPLTDRCYRTLMGALKLNLGGAPEGPAGTGKTETTKDLAKALAKQCVVFNCSDGLDYKAMGKFFKGLAQAGAWAC
FDEFNRIEVEVLSVVAQQILSIQQAIIRKLKTFIFEGTELSLNPTCAVFITMNPGYAGRAELPDNLKALFRTVAMMVPDYALIGEIS
LYSMGFLDSRSLAQKIVATYRLCSEQLSSQHHYDYGMRAVKSVLTAAGNLKLKYPEENESVLLLRALLDVNLAKFLAQDVPLFQGII
SDLFPGVVLPKPDYEVFLKVLNDNIKKMKLQPVPWFIGKIIQIYEMMLVRHGYMIVGDPMGGKTSAYKVLAAALGDLHAANQMEEFA
VEYKIINPKAITMGQLYGCFDQVSHEWMDGVLANAFREQASSLSDDRKWIIFDGPVDAIWIENMNTVLDDNKKLCLMSGEIIQMNSK
MSLIFEPADLEQASPATVSRCGMIYMEPHQLGWKPLKDSYMDTLPSSLTKEHKELVNDMFMWLVQPCLEFGRLHCKFVVQTSPIHLA
FSMMRLYSSLLDEIRAVEEEEMELGEGLSSQQIFLWLQGLFLFSLVWTVAGTINADSRKKFDVFFRNLIMGMDDNHPRPKSVKLTKN
NIFPERGSIYDFYFIKQASGHWETWTQYITKEEEKVPAGAKVSELIIPTMETARQSFFLKTYLDHEIPMLFVGPTGTGKSAITNNFL
LHLPKNTYLPNCINFSARTSANQTQDIIMSKLDRRRKGLFGPPIGKKAVVFVDDLNMPAKEVYGAQPPIELLRQWIDHGYWFDKKDT
TRLDIVDMLLVTAMGPPGGGRNDITGRFTRHLNIISINAFEDDILTKIFSSIVDWHFGKGFDVMFLRYGKMLVQATKTIYRDAVENF
LPTPSKSHYVFNLRDFSRVIQGVLLCPHTHLQDVEKCIRLWIHEVYRVFYDRLIDKEDRQVFFNMVKETTSNCFKQTIEKVLIHLSP
TGKIVDDNIRSLFFGDYFKPESDQKIYDEITDLKQLTVVMEHYLEEFNNISKAPMSLVMFRFAIEHISRICRVLKQDKGHLLLVGIG
GSGRQSAAKLSTFMNAYELYQIEITKNYAGNDWREDLKKIILQVGVATKSTVFLFADNQIKDESFVEDINMLLNTGDVPNIFPADEK
ADIVEKMQTAARTQGEKVEVTPLSMYNFFIERVINKISFSLAMSPIGDAFRNRLRMFPSLINCCTIDWFQSWPTDALELVANKFLED
VELDDNIRVEVVSMCKYFQESVKKLSLDYYNKLRRHNYVTPTSYLELILTFKTLLNSKRQEVAMMRNRYLTGLQKLDFAASQVAVMQ
RELTALQPQLILTSEETAKMMVKIEAETREADGKKLLVQADEKEANVAAAIAQGIKNECEGDLAEAMPALEAALAALDTLNPADISL
VKSMQNPPGPVKLVMESICIMKGMKPERKPDPSGSGKMIEDYWGVSKKILGDLKFLESLKTYDKDNIPPLTMKRIRERFINHPEFQP
AVIKNVSSACEGLCKWVRAMEVYDRVAKVVAPKRERLREAEGKLAAQMQKLNQKRAELKLVVDRLQALNDDFEEMNTKKKDLEENIE
ICSQKLVRAEKLISGLGGEKDRWTEAARQLGIRYTNLTGDVLLSSGTVAYLGAFTVDYRVQCQNQWLAECKDKVIPGFSDFSLSHTL
GDPIKIRAWQIAGLPVDSFSIDNGIIVSNSRRWALMIDPHGQANKWIKNMEKANKLAVIKFSDSNYMRMLENALQLGTPVLIENIGE
ELDASIEPILLKATFKQQGVEYMRLGENIIEYSRDFKLYITTRLRNPHYLPEVAVKVCLLNFMITPLGLQDQLLGIVAAKEKPELEE
KKNQLIVESAKNKKHLKEIEDKILEVLSMSKGNILEDETAIKVLSSSKVLSEEISEKQKVASMTETQIDETRMGYKPVAVHSATIFF
CISDLANIEPMYQYSLTWFINLYMHSLTHSTKSEELNLRIKYIIDHFTLSIYNNVCRSLFEKDKLLFSLLLTIGIMKQKKEITEEVW
YFLLTGGIALDNPYPNPAPQWLSEKAWAEIVRASALPKLHGLMEHLEQNLGEWKLIYDSAWPHEEQLPGSWKFSQGLEKMVILRCLR
PDKMVPAVREFIAEHMGKLYIEAPTFDLQGSYNDSSCCAPLIFVLSPSADPMAGLLKFADDLGMGGTRTQTISLGQGQGPIAAKMIN
NAIKDGTWVVLQNCHLAASWMPTLEKICEEVIVPESTNARFRLWLTSYPSEKFPVSILQNGIKMTNEPPKGLRANLLRSYLNDPISD
PVFFQSCAKAVMWQKMLFGLCFFHAVVQERRNFGPLGWNIPYEFNESDLRISMWQIQMFLNDYKEVPFDALTYLTGECNYGGRVTDD
KDRRLLLSLLSMFYCKEIEEDYYSLAPGDTYYIPPHGSYQSYIDYLRNLPITAHPEVFGLHENADITKDNQETNQLFEGVLLTLPRQ
SGGSGKSPQEVVEELAQDILSKLPRDFDLEEVMKLYPVVYEESMNTVLRQELIRFNRLTKVVRRSLINLGRAIKGQVLMSSELEEVF
NSMLVGKVPAMWAAKSYPSLKPLGGYVADLLARLTFFQEWIDKGPPVVFWISGFYFTQSFLTGVSQNYARKYTIPIDHIGFEFEVTP
QETVMENNPEDGAYIKGLFLEGARWDRKTMQIGESLPKILYDPLPIIWLKPGESAMFLHQDIYVCPVYKTSARRGTLSTTGHSTNYV
LSIELPTDMPQKHWINRGVASLCQLDN
>sp|Q6UXH9|PAMR1_HUMAN
MELGCWTQLGLTFLQLLLISSLPREYTVINEACPGAEWNIMCRECCEYDQIECVCPGKREVVGYTIPCCRNEENECDSCLIHPGCTI
FENCKSCRNGSWGGTLDDFYVKGFYCAECRAGWYGGDCMRCGQVLRAPKGQILLESYPLNAHCEWTIHAKPGFVIQLRFVMLSLEFD
YMCQYDYVEVRDGDNRDGQIIKRVCGNERPAPIQSIGSSLHVLFHSDGSKNFDGFHAIYEEITACSSSPCFHDGTCVLDKAGSYKCA
CLAGYTGQRCENLLEERNCSDPGGPVNGYQKITGGPGLINGRHAKIGTVVSFFCNNSYVLSGNEKRTCQQNGEWSGKQPICIKACRE
PKISDLVRRRVLPMQVQSRETPLHQLYSAAFSKQKLQSAPTKKPALPFGDLPMGYQHLHTQLQYECISPFYRRLGSSRRTCLRTGKW
SGRAPSCIPICGKIENITAPKTQGLRWPWQAAIYRRTSGVHDGSLHKGAWFLVCSGALVNERTVVVAAHCVTDLGKVTMIKTADLKV
VLGKFYRDDDRDEKTIQSLQISAIILHPNYDPILLDADIAILKLLDKARISTRVQPICLAASRDLSTSFQESHITVAGWNVLADVRS

PGFKNDTLRSGVVSVVDSLLCEEQHEDHGIPVSVTDNMFCASWEPTAPSDICTAETGGIAAVSFPGRASPEPRWHLMGLVSWSYDKT
CSHRLSTAFTKVLPFKDWIERNMK
>sp|Q7Z6I8|CE024_HUMAN
MMHPVASSNPAFCGPGKPSCLNEDAMRAADQFDIYSSQQSKYSHTVNHKPMVCQRQDPLNETHLQTTSGRSIEIKDELKKKKNLNRS
GKRGRPSGTTKSAGYRTSTGRPLGTTKAAGFKTSPGRPLGTTKAAGYKVSPGRPPGSIKALSRLADLGYGCGTAAFPYPMMHGRAVH
GVEETSSEVKPPNE
>sp|Q8N6Q8|MET25_HUMAN
MAASCPLPVTPDLPTLRAKLQGLLQFLRDALSISNAHTVDFYTESVWEELVDLPPETVLAALRKSASETEALPSETRPLVEAEWEAG
MTDFPKIFCETSQKLVSVEAFALAAKYYSVQNLGICTPFEQLLVALRGNQNQRIGENQKAVEFMNMKKSHEVQAMSELISSIADYYG
IKQVIDLGSGKGYLSSFLSLKYGLKVYGIDSSNTNTHGAEERNRKLKKHWKLCHAQSRLDVNGLALKMAKERKVQNKVKNKADTEEV
FNNSPTNQEKMPTSAILPDFSGSVISNIRNQMETLHSQPHQEENLCFENSFSLINLLPINAVEPTSSQQIPNRETSEANKERRKMTS
KSSESNIYSPLTSFITADSELHDIIKDLEDCLMVGLHTCGDLAPNTLRIFTSNSEIKGVCSVGCCYHLLSEEFENQHKERTQEKWGF
PMCHYLKEERWCCGRNARMSACLALERVAAGQGLPTESLFYRAVLQDIIKDCYGITKCDRHVGKIYSKCSSFLDYVRRSLKKLGLDE
SKLPEKIIMNYYEKYKPRMNELEAFNMLKVVLAPCIETLILLDRLCYLKEQEDIAWSALVKLFDPVKSPRCYAVIALKKQQ
>sp|Q9NP62|GCM1_HUMAN
MEPDDFDSEDKEILSWDINDVKLPQNVKKTDWFQEWPDSYAKHIYSSEDKNAQRHLSSWAMRNTNNHNSRILKKSCLGVVVCGRDCL
AEEGRKIYLRPAICDKARQKQQRKRCPNCDGPLKLIPCRGHGGFPVTNFWRHDGRFIFFQSKGEHDHPKPETKLEAEARRAMKKVNT
APSSVSLSLKGSTETRSLPGETQSQGSLPLTWSFQEGVQLPGSYSGHLIANTPQQNSLNDCFSFSKSYGLGGITDLTDQTSTVDPMK
LYEKRKLSSSRTYSSGDLLPPSASGVYSDHGDLQAWSKNAALGRNHLADNCYSNYPFPLTSWPCSFSPSQNSSEPFYQQLPLEPPAA
KTGCPPLWPNPAGNLYEEKVHVDFNSYVQSPAYHSPQEDPFLFTYASHPHQQYSLPSKSSKWDFEEEMTYLGLDHCNNDMLLNLCPL
R
>sp|P57771|RGS8_HUMAN
MAALLMPRRNKGMRTRLGCLSHKSDSCSDFTAILPDKPNRALKRLSTEEATRWADSFDVLLSHKYGVAAFRAFLKTEFSEENLEFWL
ACEEFKKTRSTAKLVSKAHRIFEEFVDVQAPREVNIDFQTREATRKNLQEPSLTCFDQAQGKVHSLMEKDSYPRFLRSKMYLDLLSQ
SQRRLS
>sp|Q9NUR3|TM74B_HUMAN
MPPAQGYEFAAAKGPRDELGPSFPMASPPGLELKTLSNGPQAPRRSAPLGPVAPTREGVENACFSSEEHETHFQNPGNTRLGSSPSP
PGGVSSLPRSQRDDLSLHSEEGPALEPVSRPVDYGFVSALVFLVSGILLVVTAYAIPREARVNPDTVTAREMERLEMYYARLGSHLD
RCIIAGLGLLTVGGMLLSVLLMVSLCKGELYRRRTFVPGKGSRKTYGSINLRMRQLNGDGGQALVENEVVQVSETSHTLQRS
>sp|Q5JTC6|AMER1_HUMAN
METQKDEAAQAKGAAASGSTREQTAEKGAKNKAAEATEGPTSEPSSSGPGRLKKTAMKLFGGKKGICTLPSFFGGGRSKGSGKGSSK
KGLSKSKTHDGLSEAAHGPEDVVSEGTGFSLPLPELPCQFPSSQSAHGALETGSRCKTSVAGATEKAVAEKFPSMPKPKKGLKGFFS
SIRRHRKSKVTGAEQSEPGAKGPERVRARPHEHVSSAPQVPCFEETFQAPRKENANPQDAPGPKVSPTPEPSPPATEKMACKDPEKP
MEACASAHVQPKPAPEASSLEEPHSPETGEKVVAGEVNPPNGPVGDPLSLLFGDVTSLKSFDSLTGCGDIIAEQDMDSMTDSMASGG
QRANRDGTKRSSCLVTYQGGGEEMALPDDDDEEEEEEEEVELEEEEEEVKEEEEDDDLEYLWETAQMYPRPNMNLGYHPTTSPGHHG
YMLLDPVRSYPGLAPGELLTPQSDQQESAPNSDEGYYDSTTPGFEDDSGEALGLVRRDCLPRDSYSGDALYEFYEPDDSLENSPPGD
DCLYDLHGRSSEMFDPFLNFEPFLSSRPPGAMETEEERLVTIQKQLLYWELRREQLEAQEARAREAHAREAHAREAYTREAYGREAY
AREAHTWEAHGREARTREAQAREVRCRETQVRETQARQEKPVLEYQMRPLGPSVMGLAAGVSGTSQISHRGITSAFPTTASSEPDWR
DFRPLEKRYEGTCSKKDQSTCLMQLFQSDAMFEPDMQEANFGGSPRRAYPTYSPPEDPEEEEVEKEGNATVSFSQALVEFTSNGNLF
SSMSCSSDSDSSFTQNLPELPPMVTFDIADVERDGEGKCEENPEFHNDEDLAASLEAFELGYYHKHAFNNYHSRFYQGLPWGVSSLP
RYLGLPGLHPRPPPAAMALNRRSRSLDTAETLEMELSNSHLVQGYLESDELQAQQEDSDEEDEEEEEGEWSRDSPLSLYTEPPGAYD
WPAWAPCPLPVGPGPAWISPNQLDRPSSQSPYRQATCCIPPMTMSISLSVPESRAPGESGPQLARPSHLHLPMGPCYNLQPQASQSM
RARPRDVLLPVDEPSCSSSSGGFSPSPLPQAKPVGITHGIPQLPRVRPEHPQPQPTHYGPSSLDLSKERAEQGASLATSYSSTAMNG
NLAK

>sp|Q9BU20|RSG1_HUMAN
MARPPVPGSVVVPNWHESAEGKEYLACILRKNRRRVFGLLERPVLLPPVSIDTASYKIFVSGKSGVGKTALVAKLAGLEVPVVHHET
TGIQTTVVFWPAKLQASSRVVMFRFEFWDCGESALKKFDHMLLACMENTDAFLFLFSFTDRASFEDLPGQLARIAGEAPGVVRMVIG
SKFDQYMHTDVPERDLTAFRQAWELPLLRVKSVPGRRLADGRTLDGRAGLADVAHILNGLAEQLWHQDQVAAGLLPNPPESAPE
>sp|Q8IYP9|ZDH23_HUMAN
MTQKGSMKPVKKKKTEEPELEPLCCCEYIDRNGEKNHVATCLCDCQDLDEGCDRWITCKSLQPETCERIMDTISDRLRIPWLRGAKK
VNISIIPPLVLLPVFLHVASWHFLLGVVVLTSLPVLALWYYYLTHRRKEQTLFFLSLGLFSLGYMYYVFLQEVVPKGRVGPVQLAVL
TCGLFLILLALHRAKKNPGYLSNPASGDRSLSSSQLECLSRKGQEKTKGFPGADMSGSLNNRTTKDDPKGSSKMPAGSPTKAKEDWC
AKCQLVRPARAWHCRICGICVRRMDHHCVWINSCVGESNHQAFILALLIFLLTSVYGITLTLDTICRDRSVFTALFYCPGVYANYSS
ALSFTCVWYSVIITAGMAYIFLIQLINISYNVTEREVQQALRQKTGRRLLCGLIVDTGLLG
>sp|Q96BA8|CR3L1_HUMAN
MDAVLEPFPADRLFPGSSFLDLGDLNESDFLNNAHFPEHLDHFTENMEDFSNDLFSSFFDDPVLDEKSPLLDMELDSPTPGIQAEHS
YSLSGDSAPQSPLVPIKMEDTTQDAEHGAWALGHKLCSIMVKQEQSPELPVDPLAAPSAMAAAAAMATTPLLGLSPLSRLPIPHQAP
GEMTQLPVIKAEPLEVNQFLKVTPEDLVQMPPTPPSSHGSDSDGSQSPRSLPPSSPVRPMARSSTAISTSPLLTAPHKLQGTSGPLL
LTEEEKRTLIAEGYPIPTKLPLTKAEEKALKRVRRKIKNKISAQESRRKKKEYVECLEKKVETFTSENNELWKKVETLENANRTLLQ
QLQKLQTLVTNKISRPYKMAATQTGTCLMVAALCFVLVLGSLVPCLPEFSSGSQTVKEDPLAADGVYTASQMPSRSLLFYDDGAGLW
EDGRSTLLPMEPPDGWEINPGGPAEQRPRDHLQHDHLDSTHETTKYLSEAWPKDGGNGTSPDFSHSKEWFHDRDLGPNTTIKLS
>sp|Q6JQN1|ACD10_HUMAN
MCVRSCFQSPRLQWVWRTAFLKHTQRRHQGSHRWTHLGGSTYRAVIFDMGGVLIPSPGRVAAEWEVQNRIPSGTILKALMEGGENGP
WMRFMRAEITAEGFLREFGRLCSEMLKTSVPVDSFFSLLTSERVAKQFPVMTEAITQIRAKGLQTAVLSNNFYLPNQKSFLPLDRKQ
FDVIVESCMEGICKPDPRIYKLCLEQLGLQPSESIFLDDLGTNLKEAARLGIHTIKVNDPETAVKELEALLGFTLRVGVPNTRPVKK
TMEIPKDSLQKYLKDLLGIQTTGPLELLQFDHGQSNPTYYIRLANRDLVLRKKPPGTLLPSAHAIEREFRIMKALANAGVPVPNVLD
LCEDSSVIGTPFYVMEYCPGLIYKDPSLPGLEPSHRRAIYTAMNTVLCKIHSVDLQAVGLEDYGKQGDYIPRQVRTWVKQYRASETS
TIPAMERLIEWLPLHLPRQQRTTVVHGDFRLDNLVFHPEEPEVLAVLDWELSTLGDPLADVAYSCLAHYLPSSFPVLRGINDCDLTQ
LGIPAAEEYFRMYCLQMGLPPTENWNFYMAFSFFRVAAILQGVYKRSLTGQASSTYAEQTGKLTEFVSNLAWDFAVKEGFRVFKEMP
FTNPLTRSYHTWARPQSQWCPTGSRSYSSVPEASPAHTSRGGLVISPESLSPPVRELYHRLKHFMEQRVYPAEPELQSHQASAARWS
PSPLIEDLKEKAKAEGLWNLFLPLEADPEKKYGAGLTNVEYAHLCELMGTSLYAPEVCNCSAPDTGNMELLVRYGTEAQKARWLIPL
LEGKARSCFAMTEPQVASSDATNIEASIREEDSFYVINGHKWWITGILDPRCQLCVFMGKTDPHAPRHRQQSVLLVPMDTPGIKIIR
PLTVYGLEDAPGGHGEVRFEHVRVPKENMVLGPGRGFEIAQGRLGPGRIHHCMRLIGFSERALALMKARVKSRLAFGKPLVEQGTVL
ADIAQSRVEIEQARLLVLRAAHLMDLAGNKAAALDIAMIKMVAPSMASRVIDRAIQAFGAAGLSSDYPLAQFFTWARALRFADGPDE
VHRATVAKLELKHRI
>sp|Q15375|EPHA7_HUMAN
MVFQTRYPSWIILCYIWLLRFAHTGEAQAAKEVLLLDSKAQQTELEWISSPPNGWEEISGLDENYTPIRTYQVCQVMEPNQNNWLRT
NWISKGNAQRIFVELKFTLRDCNSLPGVLGTCKETFNLYYYETDYDTGRNIRENLYVKIDTIAADESFTQGDLGERKMKLNTEVREI
GPLSKKGFYLAFQDVGACIALVSVKVYYKKCWSIIENLAIFPDTVTGSEFSSLVEVRGTCVSSAEEEAENAPRMHCSAEGEWLVPIG
KCICKAGYQQKGDTCEPCGRGFYKSSSQDLQCSRCPTHSFSDKEGSSRCECEDGYYRAPSDPPYVACTRPPSAPQNLIFNINQTTVS
LEWSPPADNGGRNDVTYRILCKRCSWEQGECVPCGSNIGYMPQQTGLEDNYVTVMDLLAHANYTFEVEAVNGVSDLSRSQRLFAAVS
ITTGQAAPSQVSGVMKERVLQRSVELSWQEPEHPNGVITEYEIKYYEKDQRERTYSTVKTKSTSASINNLKPGTVYVFQIRAFTAAG
YGNYSPRLDVATLEEATGKMFEATAVSSEQNPVIIIAVVAVAGTIILVFMVFGFIIGRRHCGYSKADQEGDEELYFHFKFPGTKTYI
DPETYEDPNRAVHQFAKELDASCIKIERVIGAGEFGEVCSGRLKLPGKRDVAVAIKTLKVGYTEKQRRDFLCEASIMGQFDHPNVVH
LEGVVTRGKPVMIVIEFMENGALDAFLRKHDGQFTVIQLVGMLRGIAAGMRYLADMGYVHRDLAARNILVNSNLVCKVSDFGLSRVI
EDDPEAVYTTTGGKIPVRWTAPEAIQYRKFTSASDVWSYGIVMWEVMSYGERPYWDMSNQDVIKAIEEGYRLPAPMDCPAGLHQLML
DCWQKERAERPKFEQIVGILDKMIRNPNSLKTPLGTCSRPISPLLDQNTPDFTTFCSVGEWLQAIKMERYKDNFTAAGYNSLESVAR
MTIEDVMSLGITLVGHQKKIMSSIQTMRAQMLHLHGTGIQV

>sp|Q16629|SRSF7_HUMAN
MSRYGRYGGETKVYVGNLGTGAGKGELERAFSYYGPLRTVWIARNPPGFAFVEFEDPRDAEDAVRGLDGKVICGSRVRVELSTGMPR
RSRFDRPPARRPFDPNDRCYECGEKGHYAYDCHRYSRRRRSRSRSRSHSRSRGRRYSRSRSRSRGRRSRSASPRRSRSISLRRSRSA
SLRRSRSGSIKGSRYFQSPSRSRSRSRSISRPRSSRSKSRSPSPKRSRSPSGSPRRSASPERMD
>sp|Q00604|NDP_HUMAN
MRKHVLAASFSMLSLLVIMGDTDSKTDSSFIMDSDPRRCMRHHYVDSISHPLYKCSSKMVLLARCEGHCSQASRSEPLVSFSTVLKQ
PFRSSCHCCRPQTSKLKALRLRCSGGMRLTATYRYILSCHCEECNS
>sp|Q14416|GRM2_HUMAN
MGSLLALLALLLLWGAVAEGPAKKVLTLEGDLVLGGLFPVHQKGGPAEDCGPVNEHRGIQRLEAMLFALDRINRDPHLLPGVRLGAH
ILDSCSKDTHALEQALDFVRASLSRGADGSRHICPDGSYATHGDAPTAITGVIGGSYSDVSIQVANLLRLFQIPQISYASTSAKLSD
KSRYDYFARTVPPDFFQAKAMAEILRFFNWTYVSTVASEGDYGETGIEAFELEARARNICVATSEKVGRAMSRAAFEGVVRALLQKP
SARVAVLFTRSEDARELLAASQRLNASFTWVASDGWGALESVVAGSEGAAEGAITIELASYPISDFASYFQSLDPWNNSRNPWFREF
WEQRFRCSFRQRDCAAHSLRAVPFEQESKIMFVVNAVYAMAHALHNMHRALCPNTTRLCDAMRPVNGRRLYKDFVLNVKFDAPFRPA
DTHNEVRFDRFGDGIGRYNIFTYLRAGSGRYRYQKVGYWAEGLTLDTSLIPWASPSAGPLPASRCSEPCLQNEVKSVQPGEVCCWLC
IPCQPYEYRLDEFTCADCGLGYWPNASLTGCFELPQEYIRWGDAWAVGPVTIACLGALATLFVLGVFVRHNATPVVKASGRELCYIL
LGGVFLCYCMTFIFIAKPSTAVCTLRRLGLGTAFSVCYSALLTKTNRIARIFGGAREGAQRPRFISPASQVAICLALISGQLLIVVA
WLVVEAPGTGKETAPERREVVTLRCNHRDASMLGSLAYNVLLIALCTLYAFKTRKCPENFNEAKFIGFTMYTTCIIWLAFLPIFYVT
SSDYRVQTTTMCVSVSLSGSVVLGCLFAPKLHIILFQPQKNVVSHRAPTSRFGSAAARASSSLGQGSGSQFVPTVCNGREVVDSTTS
SL
>sp|O15037|KHNYN_HUMAN
MPTWGARPASPDRFAVSAEAENKVREQQPHVERIFSVGVSVLPKDCPDNPHIWLQLEGPKENASRAKEYLKGLCSPELQDEIHYPPK
LHCIFLGAQGFFLDCLAWSTSAHLVPRAPGSLMISGLTEAFVMAQSRVEELAERLSWDFTPGPSSGASQCTGVLRDFSALLQSPGDA
HREALLQLPLAVQEELLSLVQEASSGQGPGALASWEGRSSALLGAQCQGVRAPPSDGRESLDTGSMGPGDCRGARGDTYAVEKEGGK
QGGPREMDWGWKELPGEEAWEREVALRPQSVGGGARESAPLKGKALGKEEIALGGGGFCVHREPPGAHGSCHRAAQSRGASLLQRLH
NGNASPPRVPSPPPAPEPPWHCGDRGDCGDRGDVGDRGDKQQGMARGRGPQWKRGARGGNLVTGTQRFKEALQDPFTLCLANVPGQP
DLRHIVIDGSNVAMVHGLQHYFSSRGIAIAVQYFWDRGHRDITVFVPQWRFSKDAKVRESHFLQKLYSLSLLSLTPSRVMDGKRISS
YDDRFMVKLAEETDGIIVSNDQFRDLAEESEKWMAIIRERLLPFTFVGNLFMVPDDPLGRNGPTLDEFLKKPARTQGSSKAQHPSRG
FAEHGKQQQGREEEKGSGGIRKTRETERLRRQLLEVFWGQDHKVDFILQREPYCRDINQLSEALLSLNF
>sp|Q8NGR5|OR1L4_HUMAN
METKNYSSSTSGFILLGLSSNPKLQKPLFAIFLIMYLLTAVGNVLIILAIYSDPRLHTPMYFFLSNLSFMDICFTTVIVPKMLVNFL
SETKIISYVGCLIQMYFFMAFGNTDSYLLASMAIDRLVAICNPLHYDVVMKPWHCLLMLLGSCSISHLHSLFRVLLMSRLSFCASHI
IKHFFCDTQPVLKLSCSDTSSSQMVVMTETLAVIVTPFLCTIFSYLQIIVTVLRIPSAAGKWKAFSTCGSHLTVVVLFYGSVIYVYF
RPLSMYSVMKGRVATVMYTVVTPMLNPFIYSLRNKDMKRGLKKLRHRIYS
>sp|Q6ZN04|MEX3B_HUMAN
MPSSLFADLERNGSGGGGGGSSGGGGETLDDQRALQLALDQLSLLGLDSDEGASLYDSEPRKKSVNMTECVPVPSSEHVAEIVGRQGC
KIKALRAKTNTYIKTPVRGEEPVFVVTGRKEDVAMARREIISAAEHFSMIRASRNKNTALNGAVPGPPNLPGQTTIQVRVPYRVVGL
VVGPKGATIKRIQQQTHTYIVTPSRDKEPVFEVTGMPENVDRAREEIEAHIALRTGGIIELTDENDFHANGTDVGFDLHHGSGGSGP
GSLWSKPTPSITPTPGRKPFSSYRNDSSSSLGSASTDSYFGGGTSSSAAATQRLADYSPPSPALSFAHNGNNNNNGNGYTYTAGGEA
SVPSPDGCPELQPTFDPAPAPPPGAPLIWAQFERSPGGGPAAPVSSSCSSSASSSASSSSVVFPGGGASAPSNANLGLLVHRRLHPG
TSCPRLSPPLHMAPGAGEHHLARRVRSDPGGGGLAYAAYANGLGAQLPGLQPSDTSGSSSSSSSSSSSSSSSSGLRRKGSRDCSVCF
ESEVIAALVPCGHNLFCMECANRICEKSEPECPVCHTAVTQAIRIFS
>sp|P26358|DNMT1_HUMAN
MPARTAPARVPTLAVPAISLPDDVRRRLKDLERDSLTEKECVKEKLNLLHEFLQTEIKNQLCDLETKLRKEELSEEGYLAKVKSLLN
KDLSLENGAHAYNREVNGRLENGNQARSEARRVGMADANSPPKPLSKPRTPRRSKSDGEAKPEPSPSPRITRKSTRQTTITSHFAKG

PAKRKPQEESERAKSDESIKEEDKDQDEKRRRVTSRERVARPLPAEEPERAKSGTRTEKEEERDEKEEKRLRSQTKEPTPKQKLKEE
PDREARAGVQADEDEDGDEKDEKKHRSQPKDLAAKRRPEEKEPEKVNPQISDEKDEDEKEEKRRKTTPKEPTEKKMARAKTVMNSKT
HPPKCIQCGQYLDDPDLKYGQHPPDAVDEPQMLTNEKLSIFDANESGFESYEALPQHKLTCFSVYCKHGHLCPIDTGLIEKNIELFF
SGSAKPIYDDDPSLEGGVNGKNLGPINEWWITGFDGGEKALIGFSTSFAEYILMDPSPEYAPIFGLMQEKIYISKIVVEFLQSNSDS
TYEDLINKIETTVPPSGLNLNRFTEDSLLRHAQFVVEQVESYDEAGDSDEQPIFLTPCMRDLIKLAGVTLGQRRAQARRQTIRHSTR
EKDRGPTKATTTKLVYQIFDTFFAEQIEKDDREDKENAFKRRRCGVCEVCQQPECGKCKACKDMVKFGGSGRSKQACQERRCPNMAM
KEADDDEEVDDNIPEMPSPKKMHQGKKKKQNKNRISWVGEAVKTDGKKSYYKKVCIDAETLEVGDCVSVIPDDSSKPLYLARVTALW
EDSSNGQMFHAHWFCAGTDTVLGATSDPLELFLVDECEDMQLSYIHSKVKVIYKAPSENWAMEGGMDPESLLEGDDGKTYFYQLWYD
QDYARFESPPKTQPTEDNKFKFCVSCARLAEMRQKEIPRVLEQLEDLDSRVLYYSATKNGILYRVGDGVYLPPEAFTFNIKLSSPVK
RPRKEPVDEDLYPEHYRKYSDYIKGSNLDAPEPYRIGRIKEIFCPKKSNGRPNETDIKIRVNKFYRPENTHKSTPASYHADINLLYW
SDEEAVVDFKAVQGRCTVEYGEDLPECVQVYSMGGPNRFYFLEAYNAKSKSFEDPPNHARSPGNKGKGKGKGKGKPKSQACEPSEPE
IEIKLPKLRTLDVFSGCGGLSEGFHQAGISDTLWAIEMWDPAAQAFRLNNPGSTVFTEDCNILLKLVMAGETTNSRGQRLPQKGDVE
MLCGGPPCQGFSGMNRFNSRTYSKFKNSLVVSFLSYCDYYRPRFFLLENVRNFVSFKRSMVLKLTLRCLVRMGYQCTFGVLQAGQYG
VAQTRRRAIILAAAPGEKLPLFPEPLHVFAPRACQLSVVVDDKKFVSNITRLSSGPFRTITVRDTMSDLPEVRNGASALEISYNGEP
QSWFQRQLRGAQYQPILRDHICKDMSALVAARMRHIPLAPGSDWRDLPNIEVRLSDGTMARKLRYTHHDRKNGRSSSGALRGVCSCV
EAGKACDPAARQFNTLIPWCLPHTGNRHNHWAGLYGRLEWDGFFSTTVTNPEPMGKQGRVLHPEQHRVVSVRECARSQGFPDTYRLF
GNILDKHRQVGNAVPPPLAKAIGLEIKLCMLAKARESASAKIKEEEAAKD
>sp|Q9NZ52|GGA3_HUMAN
MAEAEGESLESWLNKATNPSNRQEDWEYIIGFCDQINKELEGPQIAVRLLAHKIQSPQEWEALQALTVLEACMKNCGRRFHNEVGKF
RFLNELIKVVSPKYLGDRVSEKVKTKVIELLYSWTMALPEEAKIKDAYHMLKRQGIVQSDPPIPVDRTLIPSPPPRPKNPVFDDEEK
SKLLAKLLKSKNPDDLQEANKLIKSMVKEDEARIQKVTKRLHTLEEVNNNVRLLSEMLLHYSQEDSSDGDRELMKELFDQCENKRRT
LFKLASETEDNDNSLGDILQASDNLSRVINSYKTIIEGQVINGEVATLTLPDSEGNSQCSNQGTLIDLAELDTTNSLSSVLAPAPTP
PSSGIPILPPPPQASGPPRSRSSSQAEATLGPSSTSNALSWLDEELLCLGLADPAPNVPPKESAGNSQWHLLQREQSDLDFFSPRPG
TAACGASDAPLLQPSAPSSSSSQAPLPPPFPAPVVPASVPAPSAGSSLFSTGVAPALAPKVEPAVPGHHGLALGNSALHHLDALDQL
LEEAKVTSGLVKPTTSPLIPTTTPARPLLPFSTGPGSPLFQPLSFQSQGSPPKGPELSLASIHVPLESIKPSSALPVTAYDKNGFRI
LFHFAKECPPGRPDVLVVVVSMLNTAPLPVKSIVLQAAVPKSMKVKLQPPSGTELSPFSPIQPPAAITQVMLLANPLKEKVRLRYKL
TFALGEQLSTEVGEVDQFPPVEQWGNL
>sp|Q96RI8|TAAR6_HUMAN
MSSNSSLLVAVQLCYANVNGSCVKIPFSPGSRVILYIVFGFGAVLAVFGNLLVMISILHFKQLHSPTNFLVASLACADFLVGVTVMP
FSMVRTVESCWYFGRSFCTFHTCCDVAFCYSSLFHLCFISIDRYIAVTDPLVYPTKFTVSVSGICISVSWILPLMYSGAVFYTGVYD
DGLEELSDALNCIGGCQTVVNQNWVLTDFLSFFIPTFIMIILYGNIFLVARRQAKKIENTGSKTESSSESYKARVARRERKAAKTLG
VTVVAFMISWLPYSIDSLIDAFMGFITPACIYEICCWCAYYNSAMNPLIYALFYPWFRKAIKVIVTGQVLKNSSATMNLFSEHI
>sp|Q9NP77|SSU72_HUMAN
MPSSPLRVAVVCSSNQNRSMEAHNILSKRGFSVRSFGTGTHVKLPGPAPDKPNVYDFKTTYDQMYNDLLRKDKELYTQNGILHMLDR
NKRIKPRPERFQNCKDLFDLILTCEERVYDQVVEDLNSREQETCQPVHVVNVDIQDNHEEATLGAFLICELCQCIQHTEDMENEIDE
LLQEFEEKSGRTFLHTVCFY
>sp|Q8TAA9|VANG1_HUMAN
MDTESTYSGYSYYSSHSKKSHRQGERTRERHKSPRNKDGRGSEKSVTIQPPTGEPLLGNDSTRTEEVQDDNWGETTTAITGTSEHSI
SQEDIARISKDMEDSVGLDCKRYLGLTVASFLGLLVFLTPIAFILLPPILWRDELEPCGTICEGLFISMAFKLLILLIGTWALFFRK
RRADMPRVFVFRALLLVLIFLFVVSYWLFYGVRILDSRDRNYQGIVQYAVSLVDALLFIHYLAIVLLELRQLQPMFTLQVVRSTDGE
SRFYSLGHLSIQRAALVVLENYYKDFTIYNPNLLTASKFRAAKHMAGLKVYNVDGPSNNATGQSRAMIAAAARRRDSSHNELYYEEA
EHERRVKKRKARLVVAVEEAFIHIQRLQAEEQQKAPGEVMDPREAAQAIFPSMARALQKYLRITRQQNYHSMESILQHLAFCITNGM
TPKAFLERYLSAGPTLQYDKDRWLSTQWRLVSDEAVTNGLRDGIVFVLKCLDFSLVVNVKKIPFIILSEEFIDPKSHKFVLRLQSET
SV

>sp|O95486|SC24A_HUMAN
MSQPGIPASGGAPASLQAQNGAALASGSPYTNGPVQNALLSSQESVSQGYNFQLPGSYPHPIPAKTLNPVSGQSNYGGSQGSGQTLN
RPPVASNPVTPSLHSGPAPRMPLPASQNPATTPMPSSSFLPEANLPPPLNWQYNYPSTASQTNHCPRASSQPTVSGNTSLTTNHQYV
SSGYPSLQNSFIKSGPSVPPLVNPPLPTTFQPGAPHGPPPAGGPPPVRALTPLTSSYRDVPQPLFNSAVNQEGITSNTNNGSMVVHS
SYDEIEGGGLLATPQLTNKNPKMSRSVGYSYPSLPPGYQNTTPPGATGVPPSSLNYPSGPQAFTQTPLGANHLTTSMSGLSLQPEGL
RVVNLLQERNMLPSTPLKPPVPNLHEDIQKLNCNPELFRCTLTSIPQTQALLNKAKLPLGLLLHPFKDLVQLPVVTSSTIVRCRSCR
TYINPFVSFLDQRRWKCNLCYRVNDVPEEFLYNPLTRVYGEPHRRPEVQNATIEFMAPSEYMLRPPQPPVYLFVFDVSHNAVETGYL
NSVCQSLLDNLDLLPGNTRTKIGFITFDSTIHFYGLQESLSQPQMLIVSDIEDVFIPMPENLLVNLNESKELVQDLLKTLPQMFTKT
LETQSALGPALQAAFKLMSPTGGRMSVFQTQLPTLGVGALKPREEPNHRSSAKDIHMTPSTDFYKKLALDCSGQQVAVDLFLLSGQY
SDLASLGCISRYSAGSVYYYPSYHHQHNPVQVQKLQKELQRYLTRKIGFEAVMRIRCTKGLSIHTFHGNFFVRSTDLLSLPNVNPDA
GYAVQMSVEESLTDTQLVSFQSALLYTSSKGERRIRVHTLCLPVVSTLNDVFLGADVQAISGLLANMAVDRSMTASLSDARDALVNA
VIDSLSAYRSSVLSNQQPGLMVPFSLRLFPLFVLALLKQKSFQTGTNARLDERIFAMCQVKNQPLVYLMLTTHPSLYRVDNLSDEGA
LNISDRTIPQPPILQLSVEKLSRDGAFLMDAGSVLMLWVGKNCTQNFLSQVLGVQNYASIPQPMTDLPELDTPESARIIAFISWLRE
QRPFFPILYVIRDESPMKANFLQNMIEDRTESALSYYEFLLHIQQQVNK
>sp|O75795|UDB17_HUMAN
MSLKWMSVFLLMQLSCYFSSGSCGKVLVWPTEYSHWINMKTILEELVQRGHEVIVLTSSASILVNASKSSAIKLEVYPTSLTKNDLE
DFFMKMFDRWTYSISKNTFWSYFSQLQELCWEYSDYNIKLCEDAVLNKKLMRKLQESKFDVLLADAVNPCGELLAELLNIPFLYSLR
FSVGYTVEKNGGGFLFPPSYVPVVMSELSDQMIFMERIKNMIYMLYFDFWFQAYDLKKWDQFYSEVLGRPTTLFETMGKAEMWLIRT
YWDFEFPRPFLPNVDFVGGLHCKPAKPLPKEMEEFVQSSGENGIVVFSLGSMISNMSEESANMIASALAQIPQKVLWRFDGKKPNTL
GSNTRLYKWLPQNDLLGHPKTKAFITHGGTNGIYEAIYHGIPMVGIPLFADQHDNIAHMKAKGAALSVDIRTMSSRDLLNALKSVIN
DPIYKENIMKLSRIHHDQPVKPLDRAVFWIEFVMRHKGAKHLRVAAHNLTWIQYHSLDVIAFLLACVATMIFMITKCCLFCFRKLAK
TGKKKKRD
>sp|A2A2V5|SRTM1_HUMAN
MSEPDTSSGFSGSVENGTFLELFPTSLSTSVDPSSGHLSNVYIYVSIFLSLLAFLLLLLIIALQRLKNIISSSSSYPEYPSDAGSSF
TNLEVCSISSQRSTFSNLSS
>sp|O14686|KMT2D_HUMAN
MDSQKLAGEDKDSEPAADGPAASEDPSATESDLPNPHVGEVSVLSSGSPRLQETPQDCSGGPVRRCALCNCGEPSLHGQRELRRFEL
PFDWPRCPVVSPGGSPGPNEAVLPSEDLSQIGFPEGLTPAHLGEPGGSCWAHHWCAAWSAGVWGQEGPELCGVDKAIFSGISQRCSH
CTRLGASIPCRSPGCPRLYHFPCATASGSFLSMKTLQLLCPEHSEGAAYLEEARCAVCEGPGELCDLFFCTSCGHHYHGACLDTALT
ARKRAGWQCPECKVCQACRKPGNDSKMLVCETCDKGYHTFCLKPPMEELPAHSWKCKACRVCRACGAGSAELNPNSEWFENYSLCHR
CHKAQGGQTIRSVAEQHTPVCSRFSPPEPGDTPTDEPDALYVACQGQPKGGHVTSMQPKEPGPLQCEAKPLGKAGVQLEPQLEAPLN
EEMPLLPPPEESPLSPPPEESPTSPPPEASRLSPPPEELPASPLPEALHLSRPLEESPLSPPPEESPLSPPPESSPFSPLEESPLSP
PEESPPSPALETPLSPPPEASPLSPPFEESPLSPPPEELPTSPPPEASRLSPPPEESPMSPPPEESPMSPPPEASRLFPPFEESPLS
PPPEESPLSPPPEASRLSPPPEDSPMSPPPEESPMSPPPEVSRLSPLPVVSRLSPPPEESPLSPPPEESPTSPPPEASRLSPPPEDS
PTSPPPEDSPASPPPEDSLMSLPLEESPLLPLPEEPQLCPRSEGPHLSPRPEEPHLSPRPEEPHLSPQAEEPHLSPQPEEPCLCAVP
EEPHLSPQAEGPHLSPQPEELHLSPQTEEPHLSPVPEEPCLSPQPEESHLSPQSEEPCLSPRPEESHLSPELEKPPLSPRPEKPPEE
PGQCPAPEELPLFPPPGEPSLSPLLGEPALSEPGEPPLSPLPEELPLSPSGEPSLSPQLMPPDPLPPPLSPIITAAAPPALSPLGEL
EYPFGAKGDSDPESPLAAPILETPISPPPEANCTDPEPVPPMILPPSPGSPVGPASPILMEPLPPQCSPLLQHSLVPQNSPPSQCSP
PALPLSVPSPLSPIGKVVGVSDEAELHEMETEKVSEPECPALEPSATSPLPSPMGDLSCPAPSPAPALDDFSGLGEDTAPLDGIDAP
GSQPEPGQTPGSLASELKGSPVLLDPEELAPVTPMEVYPECKQTAGQGSPCEEQEEPRAPVAPTPPTLIKSDIVNEISNLSQGDASA
SFPGSEPLLGSPDPEGGGSLSMELGVSTDVSPARDEGSLRLCTDSLPETDDSLLCDAGTAISGGKAEGEKGRRRSSPARSRIKQGRS
SSFPGRRRPRGGAHGGRGRGRARLKSTASSIETLVVADIDSSPSKEEEEEDDDTMQNTVVLFSNTDKFVLMQDMCVVCGSFGRGAEG
HLLACSQCSQCYHPYCVNSKITKVMLLKGWRCVECIVCEVCGQASDPSRLLLCDDCDISYHTYCLDPPLLTVPKGGWKCKWCVSCMQ
CGAASPGFHCEWQNSYTHCGPCASLVTCPICHAPYVEEDLLIQCRHCERWMHAGCESLFTEDDVEQAADEGFDCVSCQPYVVKPVAP

VAPPELVPMKVKEPEPQYFRFEGVWLTETGMALLRNLTMSPLHKRRQRRGRLGLPGEAGLEGSEPSDALGPDDKKDGDLDTDELLKG
EGGVEHMECEIKLEGPVSPDVEPGKEETEESKKRKRKPYRPGIGGFMVRQRKSHTRTKKGPAAQAEVLSGDGQPDEVIPADLPAEGA
VEQSLAEGDEKKKQQRRGRKKSKLEDMFPAYLQEAFFGKELLDLSRKALFAVGVGRPSFGLGTPKAKGDGGSERKELPTSQKGDDGP
DIADEESRGLEGKADTPGPEDGGVKASPVPSDPEKPGTPGEGMLSSDLDRISTEELPKMESKDLQQLFKDVLGSEREQHLGCGTPGL
EGSRTPLQRPFLQGGLPLGNLPSSSPMDSYPGLCQSPFLDSRERGGFFSPEPGEPDSPWTGSGGTTPSTPTTPTTEGEGDGLSYNQR
SLQRWEKDEELGQLSTISPVLYANINFPNLKQDYPDWSSRCKQIMKLWRKVPAADKAPYLQKAKDNRAAHRINKVQKQAESQINKQT
KVGDIARKTDRPALHLRIPPQPGALGSPPPAAAPTIFIGSPTTPAGLSTSADGFLKPPAGSVPGPDSPGELFLKLPPQVPAQVPSQD
PFGLAPAYPLEPRFPTAPPTYPPYPSPTGAPAQPPMLGASSRPGAGQPGEFHTTPPGTPRHQPSTPDPFLKPRCPSLDNLAVPESPG
VGGGKASEPLLSPPPFGESRKALEVKKEELGASSPSYGPPNLGFVDSPSSGTHLGGLELKTPDVFKAPLTPRASQVEPQSPGLGLRP
QEPPPAQALAPSPPSHPDIFRPGSYTDPYAQPPLTPRPQPPPPESCCALPPRSLPSDPFSRVPASPQSQSSSQSPLTPRPLSAEAFC
PSPVTPRFQSPDPYSRPPSRPQSRDPFAPLHKPPRPQPPEVAFKAGSLAHTSLGAGGFPAALPAGPAGELHAKVPSGQPPNFVRSPG
TGAFVGTPSPMRFTFPQAVGEPSLKPPVPQPGLPPPHGINSHFGPGPTLGKPQSTNYTVATGNFHPSGSPLGPSSGSTGESYGLSPL
RPPSVLPPPAPDGSLPYLSHGASQRSGITSPVEKREDPGTGMGSSLATAELPGTQDPGMSGLSQTELEKQRQRQRLRELLIRQQIQR
NTLRQEKETAAAAAGAVGPPGSWGAEPSSPAFEQLSRGQTPFAGTQDKSSLVGLPPSKLSGPILGPGSFPSDDRLSRPPPPATPSSM
DVNSRQLVGGSQAFYQRAPYPGSLPLQQQQQQLWQQQQATAATSMRFAMSARFPSTPGPELGRQALGSPLAGISTRLPGPGEPVPGP
AGPAQFIELRHNVQKGLGPGGTPFPGQGPPQRPRFYPVSEDPHRLAPEGLRGLAVSGLPPQKPSAPPAPELNNSLHPTPHTKGPTLP
TGLELVNRPPSSTELGRPNPLALEAGKLPCEDPELDDDFDAHKALEDDEELAHLGLGVDVAKGDDELGTLENLETNDPHLDDLLNGD
EFDLLAYTDPELDTGDKKDIFNEHLRLVESANEKAEREALLRGVEPGPLGPEERPPPAADASEPRLASVLPEVKPKVEEGGRHPSPC
QFTIATPKVEPAPAANSLGLGLKPGQSMMGSRDTRMGTGPFSSSGHTAEKASFGATGGPPAHLLTPSPLSGPGGSSLLEKFELESGA
LTLPGGPAASGDELDKMESSLVASELPLLIEDLLEHEKKELQKKQQLSAQLQPAQQQQQQQQQHSLLSAPGPAQAMSLPHEGSSPSL
AGSQQQLSLGLAGARQPGLPQPLMPTQPPAHALQQRLAPSMAMVSNQGHMLSGQHGGQAGLVPQQSSQPVLSQKPMGTMPPSMCMKP
QQLAMQQQLANSFFPDTDLDKFAAEDIIDPIAKAKMVALKGIKKVMAQGSIGVAPGMNRQQVSLLAQRLSGGPSSDLQNHVAAGSGQ
ERSAGDPSQPRPNPPTFAQGVINEADQRQYEEWLFHTQQLLQMQLKVLEEQIGVHRKSRKALCAKQRTAKKAGREFPEADAEKLKLV
TEQQSKIQKQLDQVRKQQKEHTNLMAEYRNKQQQQQQQQQQQQQQHSAVLALSPSQSPRLLTKLPGQLLPGHGLQPPQGPPGGQAGG
LRLTPGGMALPGQPGGPFLNTALAQQQQQQHSGGAGSLAGPSGGFFPGNLALRSLGPDSRLLQERQLQLQQQRMQLAQKLQQQQQQQ
QQQQHLLGQVAIQQQQQQGPGVQTNQALGPKPQGLMPPSSHQGLLVQQLSPQPPQGPQGMLGPAQVAVLQQQHPGALGPQGPHRQVL
MTQSRVLSSPQLAQQGQGLMGHRLVTAQQQQQQQQHQQQGSMAGLSHLQQSLMSHSGQPKLSAQPMGSLQQLQQQQQLQQQQQLQQQ
QQQQLQQQQQLQQQQLQQQQQQQQQLQQQQQQQQLQQQQQQLQQQQQQQQQQFQQQQQQQQMGLLNQSRTLLSPQQQQQQVALGPGMP
AKPLQHFSSPGALGPTLLLTGKEQNTVDPAVSSEATEGPSTHQGGPLAIGTTPESMATEPGEVKPSLSGDSQLLLVQPQPQPQPSSL
QLQPPLRLPGQQQQQVSLLHTAGGGSHGQLGSGSSSEASSVPHLLAQPSVSLGDQPGSMTQNLLGPQQPMLERPMQNNTGPQPPKPG
PVLQSGQGLPGVGIMPTVGQLRAQLQGVLAKNPQLRHLSPQQQQQLQALLMQRLQQSQAVRQTPPYQEPGTQTSPLQGLLGCQPQL
GGFPGPQTGPLQELGAGPRPQGPPRLPAPPGALSTGPVLGPVHPTPPPSSPQEPKRPSQLPSPSSQLPTEAQLPPTHPGTPKPQGPT
LEPPPGRVSPAAAQLADTLFSKGLGPWDPPDNLAETQKPEQSSLVPGHLDQVNGQVVPEASQLSIKQEPREEPCALGAQSVKREANG
EPIGAPGTSNHLLLAGPRSEAGHLLLQKLLRAKNVQLSTGRGSEGLRAEINGHIDSKLAGLEQKLQGTPSNKEDAAARKPLTPKPKR
VQKASDRLVSSRKKLRKEDGVRASEALLKQLKQELSLLPLTEPAITANFSLFAPFGSGCPVNGQSQLRGAFGSGALPTGPDYYSQLL
TKNNLSNPPTPPSSLPPTPPPSVQQKMVNGVTPSEELGEHPKDAASARDSERALRDTSEVKSLDLLAALPTPPHNQTEDVRMESDED
SDSPDSIVPASSPESILGEEAPRFPHLGSGRWEQEDRALSPVIPLIPRASIPVFPDTKPYGALGLEVPGKLPVTTWEKGKGSEVSVM
LTVSAAAAKNLNGVMVAVAELLSMKIPNSYEVLFPESPARAGTEPKKGEAEGPGGKEKGLEGKSPDTGPDWLKQFDAVLPGYTLKSQ
LDILSLLKQESPAPEPPTQHSYTYNVSNLDVRQLSAPPPEEPSPPPSPLAPSPASPPTEPLVELPTEPLAEPPVPSPLPLASSPESA
RPKPRARPPEEGEDSRPPRLKKWKGVRWKRLRLLLTIQKGSGRQEDEREVAEFMEQLGTALRPDKVPRDMRRCCFCHEEGDGATDGP
ARLLNLDLDLWVHLNCALWSTEVYETQGGALMNVEVALHRGLLTKCSLCQRTGATSSCNRMRCPNVYHFACAIRAKCMFFKDKTMLC
PMHKIKGPCEQELSSFAVFRRVYIERDEVKQIASIIQRGERLHMFRVGGLVFHAIGQLLPHQMADFHSATALYPVGYEATRIYWSLR
TNNRRCCYRCSIGENNGRPEFVIKVIEQGLEDLVFTDASPQAVWNRIIEPVAAMRKEADMLRLFPEYLKGEELFGLTVHAVLRIAES
LPGVESCQNYLFRYGRHPLMELPLMINPTGCARSEPKILTHYKRPHTLNSTSMSKAYQSTFTGETNTPYSKQFVHSKSSQYRRLRTE

WKNNVYLARSRIQGLGLYAAKDLEKHTMVIEYIGTIIRNEVANRREKIYEEQNRGIYMFRINNEHVIDATLTGGPARYINHSCAPNC
VAEVVTFDKEDKIIIISSRRIPKGEELTYDYQFDFEDDQHKIPCHCGAWNCRKWMN
>sp|Q5T5A4|CA194_HUMAN
MPPTRDPFQQPTLDNDDSYLGELRASKKLPYKNPTHLAQQQEPWSRLNSTPTITSMRRDAYYFDPEIPKDDLDFRLAALYNHHTGTF
KNKSEILLNQKTTQDTYRTKIQFPGEFLTPPTPPITFLANIRHWINPKKESIHSIQGSIVSPHTAATNGGYSRKKDGGFFST
>sp|Q4ADV7|RIC1_HUMAN
MYFLSGWPKRLLCPLGSPAEAPFHVQSDPQRAFFAVLAAARLSIWYSRPSVLIVTYKEPAKSSTQFGSYKQAEWRPDSTMIAVSTAN
GYILFFHITSTRGDKYLYEPVYPKGSPQMKGTPHFKEEQCAPALNLEMRKILDLQAPIMSLQSVLEDLLVATSDGLLHLIHWEGMTN
GRKAINLCTVPFSVDLQSSRVGSFLGFTDVHIRDMEYCATLDGFAVVFNDGKVGFITPVSSRFTAEQLHGVWPQDVVDGTCVAVNNK
YRLMAFGCVSGSVQVYTIDNSTGAMLLSHKLELTAKQYPDIWNKTGAVKLMRWSPDNSVVIVTWEYGGLSLWSVFGAQLICTLGGDF
AYRSDGTKKDPLKINSMSWGAEGYHLWVISGFGSQNTEIESDLRSVVKQPSILLFQFIKSVLTVNPCMSNQEQVLLQGEDRLYLNCG
EASQTQNPRSSSTHSEHKPSREKSPFADGGLESQGLSTLLGHRHWHVVQISSTYLESNWPIRFSAIDKLGQNIAVVGKFGFAHYSLL
TKKWKLFGNITQEQNMIVTGGLAWWNDFMVLACYNINDRQEELRVYLRTSNLDNAFAHVTKAQAETLLLSVFQDMVIVFRADCSICL
YSIERKSDGPNTTAGIQVLQEVSMSRYIPHPFLVVSVTLTSVSTENGITLKMPQQARGAESIMLNLAGQLIMMQRDRSGPQIREKDS
NPNNQRKLLPFCPPVVLAQSVENVWTTCRANKQKRHLLEALWLSCGGAGMKVWLPLFPRDHRKPHSFLSQRIMLPFHINIYPLAVLF
EDALVLGAVNDTLLYDSLYTRNNAREQLEVLFPFCVVERTSQIYLHHILRQLLVRNLGEQALLLAQSCATLPYFPHVLELMLHEVLE
EEATSREPIPDPLLPTVAKFITEFPLFLQTVVHCARKTEYALWNYLFAAVGNPKDLFEECLMAQDLDTAASYLIILQNMEVPAVSRQ
HATLLFNTALEQGKWDLCRHMIRFLKAIGSGESETPPSTPTAQEPSSSGGFEFFRNRSISLSQSAENVPASKFSLQKTLSMPSGPSG
KRWSKDSDCAENMYIDMMLWRHARRLLEDVRLKDLGCFAAQLGFELISWLCKERTRAARVDNFVIALKRLHKDFLWPLPIIPASSIS
SPFKNGKYRTVGEQLLKSQSADPFLNLEMDAGISNIQRSQSWLSNIGPTHHEIDTASSHGPQMQDAFLSPLSNKGDECSIGSATDLT
ESSSMVDGDWTMVDENFSTLSLTQSELEHISMELASKGPHKSQVQLRYLLHIFMEAGCLDWCIVIGLILRESSIINQILVITQSSEV
DGEMLQNIKTGLHAVDRWASTDCPGYKPFLNIIKPQLQKLSEITEEQVQPDAFQPITMGKTPEQTSPRAEESRGSSSHGSIPQGEVG
SSNMVSRKEEDTAQAEEEEPFQDGTYDCSVS
>sp|Q9Y471|CMAH_HUMAN
MDENNGLLLLELNPPNPWDLQPRSPEELAFGEVQITYLTHACMDLKLGDKRMVFDPWLIGPAFARGWWLLHEPPSDWLERLCQADLI
YISHLHSDHLSYPTLKKLAGRRPDIPIYVGNTERPVFWNLNQSGVQLTNINVVPFGIWQQVDKNLRFMILMDGVHPEMDTCIIVEYK
GHKILNIVDCTRPNGGRLPMKVALMMSDFAGGASGFPMTFSGGKFTEEWKAQFIKTERKKLLNYKARLVKNLQPRIYCPFAGYFVES
HPSDKYIKETNTKNDPNELNNLIKKNSDVITWTPRPGATLDLGRMLKDRTDSKGIIEPPEGTKIYKDSWDFEPYLEILNAALGDEIF
LHSSWIKEYFTWAGFKDYNLVVRMIETDEDFNPFPGGYDYLVDFLDLSFPKERPQREHPYEEIHSRVDVIRHVVKNGLLWDELYIGF
QTRLQRDPDIYHHLFWNHFQIKLPLTPPNWKSFLMCCEQNGPVILQFSTERTNEPNRNKFSVENKA
>sp|P00740|FA9_HUMAN
MQRVNMIMAESPGLITICLLGYLLSAECTVFLDHENANKILNRPKRYNSGKLEEFVQGNLERECMEEKCSFEEAREVFENTERTTEF
WKQYVDGDQCESNPCLNGGSCKDDINSYECWCPFGFEGKNCELDVTCNIKNGRCEQFCKNSADNKVVCSCTEGYRLAENQKSCEPAV
PFPCGRVSVSQTSKLTRAETVFPDVDYVNSTEAETILDNITQSTQSFNDFTRVVGGEDAKPGQFPWQVVLNGKVDAFCGGSIVNEKW
IVTAAHCVETGVKITVVAGEHNIEETEHTEQKRNVIRIIPHHNYNAAINKYNHDIALLELDEPLVLNSYVTPICIADKEYTNIFLKF
GSGYVSGWGRVFHKGRSALVLQYLRVPLVDRATCLRSTKFTIYNNMFCAGFHEGGRDSCQGDSGGPHVTEVEGTSFLTGIISWGEEC
AMKGKYGIYTKVSRYVNWIKEKTKLT
>sp|Q08AD1|CAMP2_HUMAN
MGDAADPREMRKTFIVPAIKPFDHYDFSRAKIACNLAWLVAKAFGTENVPEELQEPFYTDQYDQEHIKPPVVNLLLSAELYCRAGSL
ILKSDAAKPLLGHDAVIQALAQKGLYVTDQEKLVTERDLHKKPIQMSAHLAMIDTLMMAYTVEMVSIEKVIACAQQYSAFFQATDLP
YDIEDAVMYWINKVNEHLKDIMEQEQKLKEHHTVEAPGGQKSPSKWFWKLVPARYRKEQTLLKQLPCIPLVENLLKDGTDGCALAAL
IHFYCPDVVRLEDICLKETMSLADSLYNLQLIQEFCQEYLNQCCHFTLEDMLYAASSIKSNYLVFMAELFWWFEVVKPSFVQPRVVR
PQGAEPVKDMPSIPVLNAAKRNVLDSSSDFPSSGEGATFTQSHHHLPSRYSRPQAHSSASGGIRRSSSMSYVDGFIGTWPKEKRSSV
HGVSFDISFDKEDSVQRSTPNRGITRSISNEGLTLNNSHVSKHIRKNLSFKPINGEEEAESIEEELNIDSHSDLKSCVPLNTNELNS

NENIHYKLPNGALQNRILLDEFGNQIETPSIEEALQIIHDTEKSPHTPQPDQIANGFFLHSQEMSILNSNIKLNQSSPDNVTDTKGA
LSPITDNTEVDTGIHVPSEDIPETMDEDSSLRDYTVSLDSDMDDASKFLQDYDIRTGNTREALSPCPSTVSTKSQPGSSASSSSGVK
MTSFAEQKFRKLNHTDGKSSGSSSQKTTPEGSELNIPHVVAWAQIPEETGLPQGRDTTQLLASEMVHLRMKLEEKRRAIEAQKKKME
AAFTKQRQKMGRTAFLTVVKKKGDGISPLREEAAGAEDEKVYTDRAKEKESQKTDGQRSKSLADIKESMENPQAKWLKSPTTPIDPE
KQWNLASPSEETLNEGEILEYTKSIEKLNSSLHFLQQEMQRLSLQQEMLMQMREQQSWVISPPQPSPQKQIRDFKPSKQAGLSSAIA
PFSSDSPRPTHPSPQSSNRKSASFSVKSQRTPRPNELKITPLNRTLTPPRSVDSLPRLRRFSPSQVPIQTRSFVCFGDDGEPQLKES
KPKEEVKKEELESKGTLEQRGHNPEEKEIKPFESTVSEVLSLPVTETVCLTPNEDQLNQPTEPPPKPVFPPTAPKNVNLIEVSLSDL
KPPEKADVPVEKYDGESDKEQFDDDQKVCCGFFFKDDQKAENDMAMKRAALLEKRLRREKETQLRKQQLEAEMEHKKEETRRKTEEE
RQKKEDERARREFIRQEYMRRKQLKLMEDMDTVIKPRPQVVKQKKQRPKSIHRDHIESPKTPIKGPPVSSLSLASLNTGDNESVHSG
KRTPRSESVEGFLSPSRCGSRNGEKDWENASTTSSVASGTEYTGPKLYKEPSAKSNKHIIQNALAHCCLAGKVNEGQKKKILEEMEK
SDANNFLILFRDSGCQFRSLYTYCPETEEINKLTGIGPKSITKKMIEGLYKYNSDRKQFSHIPAKTLSASVDAITIHSHLWQTKRPV
TPKKLLPTKA
>sp|O43374|RASL2_HUMAN
MAKRSSLYIRIVEGKNLPAKDITGSSDPYCIVKVDNEPIIRTATVWKTLCPFWGEEYQVHLPPTFHAVAFYVMDEDALSRDDVIGKV
CLTRDTIASHPKGFSGWAHLTEVDPDEEVQGEIHLRLEVWPGARACRLRCSVLEARDLAPKDRNGTSDPFVRVRYKGRTRETSIVKK
SCYPRWNETFEFELQEGAMEALCVEAWDWDLVSRNDFLGKVVIDVQRLRVVQQEEGWFRLQPDQSKSRRHDEGNLGSLQLEVRLRDE
TVLPSSYYQPLVHLLCHEVKLGMQGPGQLIPLIEETTSTECRQDVATNLLKLFLGQGLAKDFLDLLFQLELSRTSETNTLFRSNSLA
SKSMESFLKVAGMQYLHGVLGPIINKVFEEKKYVELDPSKVEVKDVGCSGLHRPQTEAEVLEQSAQTLRAHLGALLSALSRSVRACP
AVVRATFRQLFRRVRERFPGAQHENVPFIAVTSFLCLRFFSPAIMSPKLFHLRERHADARTSRTLLLLAKAVQNVGNMDTPASRAKE
AWMEPLQPTVRQGVAQLKDFITKLVDIEEKDELDLQRTLSLQAPPVKEGPLFIHRTKGKGPLMSSSFKKLYFSLTTEALSFAKTPSS
KKSALIKLANIRAAEKVEEKSFGGSHVMQVIYTDDAGRPQTAYLQCKCVNELNQWLSALRKVSINNTGLLGSYHPGVFRGDKWSCCH
QKEKTGQGCDKTRSRVTLQEWNDPLDHDLEAQLIYRHLLGVEAMLWERHRELSGGAEAGTVPTSPGKVPEDSLARLLRVLQDLREAH
SSSPAGSPPSEPNCLLELQT
>sp|Q8N6U8|GP161_HUMAN
MSLNSSLSCRKELSNLTEEEGGEGGVIITQFIAIIVITIFVCLGNLVIVVTLYKKSYLLTLSNKFVFSLTLSNFLLSVLVLPFVVTS
SIRREWIFGVVWCNFSALLYLLISSASMLTLGVIAIDRYYAVLYPMVYPMKITGNRAVMALVYIWLHSLIGCLPPLFGWSSVEFDEF
KWMCVAAWHREPGYTAFWQIWCALFPFLVMLVCYGFIFRVARVKARKVHCGTVVIVEEDAQRTGRKNSSTSTSSSGSRRNAFQGVVY
SANQCKALITILVVLGAFMVTWGPYMVVIASEALWGKSSVSPSLETWATWLSFASAVCHPLIYGLWNKTVRKELLGMCFGDRYYREP
FVQRQRTSRLFSISNRITDLGLSPHLTALMAGGQPLGHSSSTGDTGFSCSQDSGTDMMLLEDYTSDDNPPSHCTCPPKRRSSVTFED
EVEQIKEAAKNSILHVKAEVHKSLDSYAASLAKAIEAEAKINLFGEEALPGVLVTARTVPGGGFGGRRGSRTLVSQRLQLQSIEEGD
VLAAEQR
>sp|O95759|TBCD8_HUMAN
MWLKPEEVLLKNALKLWVTQKSSCYFILQRRRGHGEGGGRLTGRLVGALDAVLDSNARVAPFRILLQVPGSQVYSPIACGATLEEIN
QHWDWLEQNLLHTLSVFDNKDDIASFVKGKVKALIAEETSSRLAEQEEEPEKFREALVKFEARFNFPEAEKLVTYYSCCCWKGRVPR
QGWLYLSINHLCFYSFFLGKELKLVVPWVDIQKLERTSNVFLTDTIRITTQNKERDFSMFLNLDEVFKVMEQLADVTLRRLLDNEVF
DLDPDLQEPSQITKRDLEARAQNEFFRAFFRLPRKEKLHAVVDCSLWTPFSRCHTTGRMFASDSYICFASREDGCCKIILPLREVVS
IEKMEDTSLLPHPIIVSIRSKVAFQFIELRDRDSLVEALLARLKQVHANHPVHYDTSADDDMASLVFHSTSMCSDHRFGDLEMMSSQ
NSEESEKEKSPLMHPDALVTAFQQSGSQSPDSRMSREQIKISLWNDHFVEYGRTVCMFRTEKIRKLVAMGIPESLRGRLWLLFSDAV
TDLASHPGYYGNLVEESLGKCCLVTEEIERDLHRSLPEHPAFQNETGIAALRRVLTAYAHRNPKIGYCQSMNILTSVLLLYTKEEEA
FWLLVAVCERMLPDYFNHRVIGAQVDQSVFEELIKGHLPELAEHMNDLSALASVSLSWFLTLFLSIMPLESAVNVVDCFFYDGIKAI
FQLGLAVLEANAEDLCSSKDDGQALMILSRFLDHIKNEDSPGPPVGSHHAFFSDDQEPYPVTDISDLIRDSYEKFGDQSVEQIEHLR
YKHRIRVLQGHEDTTKQNVLRVVIPEVSILPEDLEELYDLFKREHMMSCYWEQPRPMASRHDPSRPYAEQYRIDARQFAHLFQLVSP
WTCGAHTEILAERTFRLLDDNMDQLIEFKAFVSCLDIMYNGEMNEKIKLLYRLHIPPALTENDRDSQSPLRNPLLSTSRPLVFGKPN
GDAVDYQKQLKQMIKDLAKEKDKTEKELPKMSQREFIQFCKTLYSMFHEDPEENDLYQAIATVTTLLLQIGEVGQRGSSSGSCSQEC

GEELRASAPSPEDSVFADTGKTPQDSQAFPEAAERDWTVSLEHILASLLTEQSLVNFFEKPLDMKSKLENAKINQYNLKTFEMSHQS
QSELKLSNL
>sp|Q3ZAQ7|VMA21_HUMAN
MERPDKAALNALQPPEFRNESSLASTLKTLLFFTALMITVPIGLYFTTKSYIFEGALGMSNRDSYFYAAIVAVVAVHVVLALFVYVA
WNEGSRQWREGKQD
>sp|Q9GZQ4|NMUR2_HUMAN
MSGMEKLQNASWIYQQKLEDPFQKHLNSTEEYLAFLCGPRRSHFFLPVSVVYVPIFVVGVIGNVLVCLVILQHQAMKTPTNYYLFSL
AVSDLLVLLLGMPLEVYEMWRNYPFLFGPVGCYFKTALFETVCFASILSITTVSVERYVAILHPFRAKLQSTRRRALRILGIVWGFS
VLFSLPNTSIHGIKFHYFPNGSLVPGSATCTVIKPMWIYNFIIQVTSFLFYLLPMTVISVLYYLMALRLKKDKSLEADEGNANIQRP
CRKSVNKMLFVLVLVFAICWAPFHIDRLFFSFVEEWSESLAAVFNLVHVVSGVFFYLSSAVNPIIYNLLSRRFQAAFQNVISSFHKQ
WHSQHDPQLPPAQRNIFLTECHFVELTEDIGPQFPCQSSMHNSHLPAALSSEQMSRTNYQSFHFNKT
>sp|P15309|PPAP_HUMAN
MRAAPLLLARAASLSLGFLFLLFFWLDRSVLAKELKFVTLVFRHGDRSPIDTFPTDPIKESSWPQGFGQLTQLGMEQHYELGEYIRK
RYRKFLNESYKHEQVYIRSTDVDRTLMSAMTNLAALFPPEGVSIWNPILLWQPIPVHTVPLSEDQLLYLPFRNCPRFQELESETLKS
EEFQKRLHPYKDFIATLGKLSGLHGQDLFGIWSKVYDPLYCESVHNFTLPSWATEDTMTKLRELSELSLLSLYGIHKQKEKSRLQGG
VLVNEILNHMKRATQIPSYKKLIMYSAHDTTVSGLQMALDVYNGLLPPYASCHLTELYFEKGEYFVEMYYRNETQHEPYPLMLPGCS
PSCPLERFAELVGPVIPQDWSTECMTTNSHQGTEDSTD
>sp|Q92637|FCGRB_HUMAN
MWFLTTLLLWVPVDGQVDTTKAVITLQPPWVSVFQEETVTLHCEVLHLPGSSSTQWFLNGTATQTSTPSYRITSASVNDSGEYRCQR
GLSGRSDPIQLEIHRGWLLLQVSSRVFMEGEPLALRCHAWKDKLVYNVLYYRNGKAFKFFHWNSNLTILKTNISHNGTYHCSGMGKH
RYTSAGISQYTVKGLQLPTPVWFHVLFYLAVGIMFLVNTVLWVTIRKELKRKKKWNLEISLDSGHEKKVISSLQEDRHLEEELKCQE
QKEEQLQEGVHRKEPQGAT
>sp|Q5JSJ4|INT6L_HUMAN
MPILLFLIDTSASMNQRTDLGTSYLDIAKGAVELFLKLRARDPASRGDRYMLVTYDEPPYCIKAGWKENHATFMSELKNLQASGLTT
LGQALRSSFDLLNLNRLISGIDNYGQGRNPFFLEPSILITITDGNKLTSTAGVQEELHLPLNSPLPGSELTKEPFRWDQRLFALVLR
LPGVASTEPEQLGSVPTDESAITQMCEVTGGRSYCVRTQRMLNQCLESLVQKVQSGVVINFEKTGPDPLPIGEDGLMDSSRPSNSFA
AQPWHSCHKLIYVRPNSKTGVPVGHWPIPESFWPDQNLPSLPPRTSHPVVRFSCVDCEPMVIDKLPFDKYELEPSPLTQYILERKSP
HTCWQVFVTSSGKYNELGYPFGYLKASTTLTCVNLFVMPYNYPVLLPLLDDLFKVHKLKPNLKWRQAFDSYLKTLPPYYLLTKLESE
RILASVGKKPPQEIGIKVKNHSGGGMSLTHNKNFRKLLKEITGETALRLTELNTKEFAGFQIGLLNKDLKPQTYRNAYDIPRRGLLD
QLTRMRSNLLKTHKFIVGQDEDSLHSVPVAQMGNYQEYLKTLASPLREIDPDQPKRLHTFGNPFKQDKKGMMIDEADEFVAGPQNKV
KRPGEPNSPMSSKRRRSMSLLLRKPQTPPTVTNHVGGKGPPSASWFPSYPNLIKPTLVHTDATIIHDGHEEKMENGQITPDGFLSKS
APSELINMTGDLMPPNQVDSLSDDFTSLSKDGLIQKPGSNAFVGGAKNCSLSVDDQKDPVASTLGAMPNTLQITPAMAQGINADIKH
QLMKEVRKFGRKYERIFILLEEVQGPLEMKKQFVEFTIKEAARFKRRVLIQYLEKVLEKINSHHLHNNISHINSRSSC
>sp|Q5T764|IFT1B_HUMAN
MSEESDGKLIEDSLIQLRCHFTWKLLIEAPEIPDLENRIWEEIQFLDTKYNVGIHNLLAYVKHLKGQNEEALVSLKKAEDLIQKEHA
NQADIRSLVTWGNFAWVYYHMGRLAEAQTYLDKVENTCKKFANPSRYRMECPEVDCEEGWALAKCGGKNYERAKTCFEKALEGNPEN
PEFNTGYAITVYRLDKFNTASGRNKAFSLHVLKRAVRLNPDDVYIRVLLALKLQDEGQEAEGEKYIEEALTSISSQAYVFQYAAKFY
RRKGSVDKALELLKMALETTPTSAFLHHQMGLCYRAQMIQIKEATNWQPRGQDRETVDRLVQLAICKFEKTIMLKRTFEMAYVDLAE
TYAEIGHHRKAEEHFQKGLRMKIFEDQLKQEIHYHYGRFQEHHGKSQDKAITHYLKGLKIEKMSHSREKLLNALEKLAKRCIHQNVR
VVESVSLLGLIHKLKGEVSDALLCYERALRLAADLNPIF
>sp|P17813|EGLN_HUMAN
MDRGTLPLAVALLLASCSLSPTSLAETVHCDLQPVGPERGEVTYTTSQVSKGCVAQAPNAILEVHVLFLEFPTGPSQLELTLQASKQ
NGTWPREVLLVLSVNSSVFLHLQALGIPLHLAYNSSLVTFQEPPGVNTTELPSFPKTQILEWAAERGPITSAAELNDPQSILLRLGQ
AQGSLSFCMLEASQDMGRTLEWRPRTPALVRGCHLEGVAGHKEAHILRVLPGHSAGPRTVTVKVELSCAPGDLDAVLILQGPPYVSW

LIDANHNMQIWTTGEYSFKIFPEKNIRGFKLPDTPQGLLGEARMLNASIVASFVELPLASIVSLHASSCGGRLQTSPAPIQTTPPKD
TCSPELLMSLIQTKCADDAMTLVLKKELVAHLKCTITGLTFWDPSCEAEDRGDKFVLRSAYSSCGMQVSASMISNEAVVNILSSSSP
QRKKVHCLNMDSLSFQLGLYLSPHFLQASNTIEPGQQSFVQVRVSPSVSEFLLQLDSCHLDLGPEGGTVELIQGRAAKGNCVSLLSP
SPEGDPRFSFLLHFYTVPIPKTGTLSCTVALRPKTGSQDQEVHRTVFMRLNIISPDLSGCTSKGLVLPAVLGITFGAFLIGALLTAA
LWYIYSHTRSPSKREPVVAVAAPASSESSSTNHSIGSTQSTPCSTSSMA
>sp|P49760|CLK2_HUMAN
MPHPRRYHSSERGSRGSYREHYRSRKHKRRRSRSWSSSSDRTRRRRREDSYHVRSRSSYDDRSSDRRVYDRRYCGSYRRNDYSRDRG
DAYYDTDYRHSYEYQRENSSYRSQRSSRRKHRRRRRRSRTFSRSSSQHSSRRAKSVEDDAEGHLIYHVGDWLQERYEIVSTLGEGTF
GRVVQCVDHRRGGARVALKIIKNVEKYKEAARLEINVLEKINEKDPDNKNLCVQMFDWFDYHGHMCISFELLGLSTFDFLKDNNYLP
YPIHQVRHMAFQLCQAVKFLHDNKLTHTDLKPENILFVNSDYELTYNLEKKRDERSVKSTAVRVVDFGSATFDHEHHSTIVSTRHYR
APEVILELGWSQPCDVWSIGCIIFEYYVGFTLFQTHDNREHLAMMERILGPIPSRMIRKTRKQKYFYRGRLDWDENTSAGRYVRENC
KPLRRYLTSEAEEHHQLFDLIESMLEYEPAKRLTLGEALQHPFFARLRAEPPNKLWDSSRDISR
>sp|Q13428|TCOF_HUMAN
MAEARKRRELLPLIYHHLLRAGYVRAAREVKEQSGQKCFLAQPVTLLDIYTHWQQTSELGRKRKAEEDAALQAKKTRVSDPISTSES
SEEEEEAEAETAKATPRLASTNSSVLGADLPSSMKEKAKAETEKAGKTGNSMPHPATGKTVANLLSGKSPRKSAEPSANTTLVSETE
EEGSVPAFGAAAKPGMVSAGQADSSSEDTSSSSDETDVEGKPSVKPAQVKASSVSTKESPARKAAPAPGKVGDVTPQVKGGALPPAK
RAKKPEEESESSEEGSESEEEAPAGTRSQVKASEKILQVRAASAPAKGTPGKGATPAPPGKAGAVASQTKAGKPEEDSESSSEESSD
SEEETPAAKALLQAKASGKTSQVGAASAPAKESPRKGAAPAPPGKTGPAVAKAQAGKREEDSQSSSEESDSEEEAPAQAKPSGKAPQ
VRAASAPAKESPRKGAAPAPPRKTGPAAAQVQVGKQEEDSRSSSEESDSDREALAAMNAAQVKPLGKSPQVKPASTMGMGPLGKGAG
PVPPGKVGPATPSAQVGKWEEDSESSSEESSDSSDGEVPTAVAPAQEKSLGNILQAKPTSSPAKGPPQKAGPVAVQVKAEKPMDNSE
SSEESSDSADSEEAPAAMTAAQAKPALKIPQTKACPKKTNTTASAKVAPVRVGTQAPRKAGTATSPAGSSPAVAGGTQRPAEDSSSS
EESDSEEEKTGLAVTVGQAKSVGKGLQVKAASVPVKGSLGQGTAPVLPGKTGPTVTQVKAEKQEDSESSEEESDSEEAAASPAQVKT
SVKKTQAKANPAAARAPSAKGTISAPGKVVTAAAQAKQRSPSKVKPPVRNPQNSTVLARGPASVPSVGKAVATAAQAQTGPEEDSGS
SEEESDSEEEAETLAQVKPSGKTHQIRAALAPAKESPRKGAAPTPPGKTGPSAAQAGKQDDSGSSSEESDSDGEAPAAVTSAQVIKP
PLIFVDPNRSPAGPAATPAQAQAASTPRKARASESTARSSSSESEDEDVIPATQCLTPGIRTNVVTMPTAHPRIAPKASMAGASSSK
ESSRISDGKKQEGPATQVSKKNPASLPLTQAALKVLAQKASEAQPPVARTQPSSGVDSAVGTLPATSPQSTSVQAKGTNKLRKPKLP
EVQQATKAPESSDDSEDSSDSSSGSEEDGEGPQGAKSAHTLGPTPSRTETLVEETAAESSEDDVVAPSQSLLSGYMTPGLTPANSQA
SKATPKLDSSPSVSSTLAAKDDPDGKQEAKPQQAAGMLSPKTGGKEAASGTTPQKSRKPKKGAGNPQASTLALQSNITQCLLGQPWP
LNEAQVQASVVKVLTELLEQERKKVVDTTKESSRKGWESRKRKLSGDQPAARTPRSKKKKKLGAGEGGEASVSPEKTSTTSKGKAKR
DKASGDVKEKKGKGSLGSQGAKDEPEEELQKGMGTVEGGDQSNPKSKKEKKKSDKRKKDKEKKEKKKKAKKASTKDSESPSQKKKKK
KKKTAEQTV
>sp|O15315|RA51B_HUMAN
MGSKKLKRVGLSQELCDRLSRHQILTCQDFLCLSPLELMKVTGLSYRGVHELLCMVSRACAPKMQTAYGIKAQRSADFSPAFLSTTL
SALDEALHGGVACGSLTEITGPPGCGKTQFCIMMSILATLPTNMGGLEGAVVYIDTESAFSAERLVEIAESRFPRYFNTEEKLLLTS
SKVHLYRELTCDEVLQRIESLEEEIISKGIKLVILDSVASVVRKEFDAQLQGNLKERNKFLAREASSLKYLAEEFSIPVILTNQITT
HLSGALASQADLVSPADDLSLSEGTSGSSCVIAALGNTWSHSVNTRLILQYLDSERRQILIAKSPLAPFTSFVYTIKEEGLVLQETT
FCSVTQAELNWAPEILPPQPPEQLGLQMCHHTQLIF
>sp|P18847|ATF3_HUMAN
MMLQHPGQVSASEVSASAIVPCLSPPGSLVFEDFANLTPFVKEELRFAIQNKHLCHRMSSALESVTVSDRPLGVSITKAEVAPEEDE
RKKRRRERNKIAAAKCRNKKKEKTECLQKESEKLESVNAELKAQIEELKNEKQHLIYMLNLHRPTCIVRAQNGRTPEDERNLFIQQI
KEGTLQS
>sp|P14902|I23O1_HUMAN
MAHAMENSWTISKEYHIDEEVGFALPNPQENLPDFYNDWMFIAKHLPDLIESGQLRERVEKLNMLSIDHLTDHKSQRLARLVLGCIT
MAYVWGKGHGDVRKVLPRNIAVPYCQLSKKLELPPILVYADCVLANWKKKDPNKPLTYENMDVLFSFRDGDCSKGFFLVSLLVEIAA

ASAIKVIPTVFKAMQMQERDTLLKALLEIASCLEKALQVFHQIHDHVNPKAFFSVLRIYLSGWKGNPQLSDGLVYEGFWEDPKEFAG
GSAGQSSVFQCFDVLLGIQQTAGGGHAAQFLQDMRRYMPPAHRNFLCSLESNPSVREFVLSKGDAGLREAYDACVKALVSLRSYHLQ
IVTKYILIPASQQPKENKTSEDPSKLEAKGTGGTDLMNFLKTVRSTTEKSLLKEG
>sp|P46379|BAG6_HUMAN
MEPNDSTSTAVEEPDSLEVLVKTLDSQTRTFIVGAQMNVKEFKEHIAASVSIPSEKQRLIYQGRVLQDDKKLQEYNVGGKVIHLVER
APPQTHLPSGASSGTGSASATHGGGSPPGTRGPGASVHDRNANSYVMVGTFNLPSDGSAVDVHINMEQAPIQSEPRVRLVMAQHMIR
DIQTLLSRMETLPYLQCRGGPQPQHSQPPPQPPAVTPEPVALSSQTSEPVESEAPPREPMEAEEVEERAPAQNPELTPGPAPAGPTP
APETNAPNHPSPAEYVEVLQELQRLESRLQPFLQRYYEVLGAAATTDYNNNHEGREEDQRLINLVGESLRLLGNTFVALSDLRCNLA
CTPPRHLHVVRPMSHYTTPMVLQQAAIPIQINVGTTVTMTGNGTRPPPTPNAEAPPPGPGQASSVAPSSTNVESSAEGAPPPGPAPP
PATSHPRVIRISHQSVEPVVMMHMNIQDSGTQPGGVPSAPTGPLGPPGHGQTLGQQVPGFPTAPTRVVIARPTPPQARPSHPGGPPV
SGTLQGAGLGTNASLAQMVSGLVGQLLMQPVLVAQGTPGMAPPPAPATASASAGTTNTATTAGPAPGGPAQPPPTPQPSMADLQFSQ
LLGNLLGPAGPGAGGSGVASPTITVAMPGVPAFLQGMTDFLQATQTAPPPPPPPPPPPPAPEQQTMPPPGSPSGGAGSPGGLGLESL
SPEFFTSVVQGVLSSLLGSLGARAGSSESIAAFIQRLSGSSNIFEPGADGALGFFGALLSLLCQNFSMVDVVMLLHGHFQPLQRLQP
QLRSFFHQHYLGGQEPTPSNIRMATHTLITGLEEYVRESFSLVQVQPGVDIIRTNLEFLQEQFNSIAAHVLHCTDSGFGARLLELCN
QGLFECLALNLHCLGGQQMELAAVINGRIRRMSRGVNPSLVSWLTTMMGLRLQVVLEHMPVGPDAILRYVRRVGDPPQPLPEEPMEV
QGAERASPEPQRENASPAPGTTAEEAMSRGPPPAPEGGSRDEQDGASAETEPWAAAVPPEWVPIIQQDIQSQRKVKPQPPLSDAYLS
GMPAKRRKTMQGEGPQLLLSEAVSRAAKAAGARPLTSPESLSRDLEAPEVQESYRQQLRSDIQKRLQEDPNYSPQRFPNAQRAFADD
P
>sp|P19957|ELAF_HUMAN
MRASSFLIVVVFLIAGTLVLEAAVTGVPVKGQDTVKGRVPFNGQDPVKGQVSVKGQDKVKAQEPVKGPVSTKPGSCPIILIRCAMLN
PPNRCLKDTDCPGIKKCCEGSCGMACFVPQ
>sp|Q9Y6C5|PTC2_HUMAN
MTRSPPLRELPPSYTPPARTAAPQILAGSLKAPLWLRAYFQGLLFSLGCGIQRHCGKVLFLGLLAFGALALGLRMAIIETNLEQLWV
EVGSRVSQELHYTKEKLGEEAAYTSQMLIQTARQEGENILTPEALGLHLQAALTASKVQVSLYGKSWDLNKICYKSGVPLIENGMIE
RMIEKLFPCVILTPLDCFWEGAKLQGGSAYLPGRPDIQWTNLDPEQLLEELGPFASLEGFRELLDKAQVGQAYVGRPCLHPDDLHCP
PSAPNHHSRQAPNVAHELSGGCHGFSHKFMHWQEELLLGGMARDPQGELLRAEALQSTFLLMSPRQLYEHFRGDYQTHDIGWSEEQA
STVLQAWQRRFVQLAQEALPENASQQIHAFSSTTLDDILHAFSEVSAARVVGGYLLMLAYACVTMLRWDCAQSQGSVGLAGVLLVAL
AVASGLGLCALLGITFNAATTQVLPFLALGIGVDDVFLLAHAFTEALPGTPLQERMGECLQRTGTSVVLTSINNMAAFLMAALVPIP
ALRAFSLQAAIVVGCTFVAVMLVFPAILSLDLRRRHCQRLDVLCCFSSPCSAQVIQILPQELGDGTVPVGIAHLTATVQAFTHCEAS
SQHVVTILPPQAHLVPPPSDPLGSELFSPGGSTRDLLGQEEETRQKAACKSLPCARWNLAHFARYQFAPLLLQSHAKAIVLVLFGAL
LGLSLYGATLVQDGLALTDVVPRGTKEHAFLSAQLRYFSLYEVALVTQGGFDYAHSQRALFDLHQRFSSLKAVLPPPATQAPRTWLH
YYRNWLQGIQAAFDQDWASGRITRHSYRNGSEDGALAYKLLIQTGDAQEPLDFSQLTTRKLVDREGLIPPELFYMGLTVWVSSDPLG
LAASQANFYPPPPEWLHDKYDTTGENLRIPPAQPLEFAQFPFLLRGLQKTADFVEAIEGARAACAEAGQAGVHAYPSGSPFLFWEQY
LGLRRCFLLAVCILLVCTFLVCALLLLNPWTAGLIVLVLAMMTVELFGIMGFLGIKLSAIPVVILVASVGIGVEFTVHVALGFLTTQ
GSRNLRAAHALEHTFAPVTDGAISTLLGLLMLAGSHFDFIVRYFFAALTVLTLLGLLHGLVLLPVLLSILGPPPEVIQMYKESPEIL
SPPAPQGGGLRWGASSSLPQSFARVTTSMTVAIHPPPLPGAYIHPAPDEPPWSPAATSSGNLSSRGPGPATG
>sp|P00390|GSHR_HUMAN
MALLPRALSAGAGPSWRRAARAFRGFLLLLPEPAALTRALSRAMACRQEPQPQGPPPAAGAVASYDYLVIGGGSGGLASARRAAELG
ARAAVVESHKLGGTCVNVGCVPKKVMWNTAVHSEFMHDHADYGFPSCEGKFNWRVIKEKRDAYVSRLNAIYQNNLTKSHIEIIRGHA
AFTSDPKPTIEVSGKKYTAPHILIATGGMPSTPHESQIPGASLGITSDGFFQLEELPGRSVIVGAGYIAVEMAGILSALGSKTSLMI
RHDKVLRSFDSMISTNCTEELENAGVEVLKFSQVKEVKKTLSGLEVSMVTAVPGRLPVMTMIPDVDCLLWAIGRVPNTKDLSLNKLG
IQTDDKGHIIVDEFQNTNVKGIYAVGDVCGKALLTPVAIAAGRKLAHRLFEYKEDSKLDYNNIPTVVFSHPPIGTVGLTEDEAIHKY
GIENVKTYSTSFTPMYHAVTKRKTKCVMKMVCANKEEKVVGIHMQGLGCDEMLQGFAVAVKMGATKADFDNTVAIHPTSSEELVTLR
>sp|Q96QE2|MYCT_HUMAN

MSRKASENVEYTLRSLSSLMGERRRKQPEPDAASAAGECSLLAAAESSTSLQSAGAGGGGVGDLERAARRQFQQDETPAFVYVVAVF
SALGGFLFGYDTGVVSGAMLLLKRQLSLDALWQELLVSSTVGAAAVSALAGGALNGVFGRRAAILLASALFTAGSAVLAAANNKETL
LAGRLVVGLGIGIASMTVPVYIAEVSPPNLRGRLVTINTLFITGGQFFASVVDGAFSYLQKDGWRYMLGLAAVPAVIQFFGFLFLPE
SPRWLIQKGQTQKARRILSQMRGNQTIDEEYDSIKNNIEEEEKEVGSAGPVICRMLSYPPTRRALIVGCGLQMFQQLSGINTIMYYS
ATILQMSGVEDDRLAIWLASVTAFTNFIFTLVGVWLVEKVGRRKLTFGSLAGTTVALIILALGFVLSAQVSPRITFKPIAPSGQNAT
CTRYSYCNECMLDPDCGFCYKMNKSTVIDSSCVPVNKASTNEAAWGRCENETKFKTEDIFWAYNFCPTPYSWTALLGLILYLVFFAP
GMGPMPWTVNSEIYPLWARSTGNACSSGINWIFNVLVSLTFLHTAEYLTYYGAFFLYAGFAAVGLLFIYGCLPETKGKKLEEIESLF
DNRLCTCGTSDSDEGRYIEYIRVKGSNYHLSDNDASDVE
>sp|Q9BXR5|TLR10_HUMAN
MRLIRNIYIFCSIVMTAEGDAPELPEERELMTNCSNMSLRKVPADLTPATTTLDLSYNLLFQLQSSDFHSVSKLRVLILCHNRIQQL
DLKTFEFNKELRYLDLSNNRLKSVTWYLLAGLRYLDLSFNDFDTMPICEEAGNMSHLEILGLSGAKIQKSDFQKIAHLHLNTVFLGF
RTLPHYEEGSLPILNTTKLHIVLPMDTNFWVLLRDGIKTSKILEMTNIDGKSQFVSYEMQRNLSLENAKTSVLLLNKVDLLWDDLFL
ILQFVWHTSVEHFQIRNVTFGGKAYLDHNSFDYSNTVMRTIKLEHVHFRVFYIQQDKIYLLLTKMDIENLTISNAQMPHMLFPNYPT
KFQYLNFANNILTDELFKRTIQLPHLKTLILNGNKLETLSLVSCFANNTPLEHLDLSQNLLQHKNDENCSWPETVVNMNLSYNKLSD
SVFRCLPKSIQILDLNNNQIQTVPKETIHLMALRELNIAFNFLTDLPGCSHFSRLSVLNIEMNFILSPSLDFVQSCQEVKTLNAGRN
PFRCTCELKNFIQLETYSEVMMVGWSDSYTCEYPLNLRGTRLKDVHLHELSCNTALLIVTIVVIMLVLGLAVAFCCLHFDLPWYLRM
LGQCTQTWHRVRKTTQEQLKRNVRFHAFISYSEHDSLWVKNELIPNLEKEDGSILICLYESYFDPGKSISENIVSFIEKSYKSIFVL
SPNFVQNEWCHYEFYFAHHNLFHENSDHIILILLEPIPFYCIPTRYHKLKALLEKKAYLEWPKDRRKCGLFWANLRAAINVNVLATR
EMYELQTFTELNEESRGSTISLMRTDCL
>sp|Q6NXR4|TTI2_HUMAN
MELDSALEAPSQEDSNLSEELSHSAFGQAFSKILHCLARPEARRGNVKDAVLKDLGDLIEATEFDRLFEGTGARLRGMPETLGQVAK
ALEKYAAPSKEEEGGGDGHSEAAEKAAQVGLLFLKLLGKVETAKNSLVGPAWQTGLHHLAGPVYIFAITHSLEQPWTTPRSREVARE
VLTSLLQVTECGSVAGFLHGENEDEKGRLSVILGLLKPDLYKESWKNNPAIKHVFSWTLQQVTRPWLSQHLERVLPASLVISDDYQT
ENKILGVHCLHHIVLNVPAADLLQYNRAQVLYHAISNHLYTPEHHLIQAVLLCLLDLFPILEKTLHWKGDGARPTTHCDEVLRLILT
HMEPEHRLLLRRTYARNLPAFVNRLGILTVRHLKRLERVIIGYLEVYDGPEEEARLKILETLKLLMQHTWPRVSCRLVVLLKALLKL
ICDVARDPNLTPESVKSALLQEATDCLILLDRCSQGRVKGLLAKIPQSCEDRKVVNYIRKVQQVSEGAPYNGT
>sp|P49959|MRE11_HUMAN
MSTADALDDENTFKILVATDIHLGFMEKDAVRGNDTFVTLDEILRLAQENEVDFILLGGDLFHENKPSRKTLHTCLELLRKYCMGDR
PVQFEILSDQSVNFGFSKFPWVNYQDGNLNISIPVFSIHGNHDDPTGADALCALDILSCAGFVNHFGRSMSVEKIDISPVLLQKGST
KIALYGLGSIPDERLYRMFVNKKVTMLRPKEDENSWFNLFVIHQNRSKHGSTNFIPEQFLDDFIDLVIWGHEHECKIAPTKNEQQLF
YISQPGSSVVTSLSPGEAVKKHVGLLRIKGRKMNMHKIPLHTVRQFFMEDIVLANHPDIFNPDNPKVTQAIQSFCLEKIEEMLENAE
RERLGNSHQPEKPLVRLRVDYSGGFEPFSVLRFSQKFVDRVANPKDIIHFFRHREQKEKTGEEINFGKLITKPSEGTTLRVEDLVKQ
YFQTAEKNVQLSLLTERGMGEAVQEFVDKEEKDAIEELVKYQLEKTQRFLKERHIDALEDKIDEEVRRFRETRQKNTNEEDDEVREA
MTRARALRSQSEESASAFSADDLMSIDLAEQMANDSDDSISAATNKGRGRGRGRRGGRGQNSASRGGSQRGRADTGLETSTRSRNSK
TAVSASRNMSIIDAFKSTRQQPSRNVTTKNYSEVIEVDESDVEEDIFPTTSKTDQRWSSTSSSKIMSQSQVSKGVDFESSEDDDDDP
FMNTSSLRRNRR
>sp|Q8N3Z0|PRS35_HUMAN
MENMLLWLIFFTPGWTLIDGSEMEWDFMWHLRKVPRIVSERTFHLTSPAFEADAKMMVNTVCGIECQKELPTPSLSELEDYLSYETV
FENGTRTLTRVKVQDLVLEPTQNITTKGVSVRRKRQVYGTDSRFSILDKRFLTNFPFSTAVKLSTGCSGILISPQHVLTAAHCVHDG
KDYVKGSKKLRVGLLKMRNKSGGKKRRGSKRSRREASGGDQREGTREHLRERAKGGRRRKKSGRGQRIAEGRPSFQWTRVKNTHIPK
GWARGGMGDATLDYDYALLELKRAHKKKYMELGISPTIKKMPGGMIHFSGFDNDRADQLVYRFCSVSDESNDLLYQYCDAESGSTGS
GVYLRLKDPDKKNWKRKIIAVYSGHQWVDVHGVQKDYNVAVRITPLKYAQICLWIHGNDANCAYG
>sp|Q8NGZ4|OR2G3_HUMAN

MGLGNESSLMDFILLGFSDHPRLEAVLFVFVLFFYLLTLVGNFTIIIISYLDPPLHTPMYFFLSNLSLLDICFTTSLAPQTLVNLQR
PKKTITYGGCVAQLYISLALGSTECILLADMALDRYIAVCKPLHYVVIMNPRLCQQLASISWLSGLASSLIHATFTLQLPLCGNHRL
DHFICEVPALLKLACVDTTVNELVLFVVSVLFVVIPPALISISYGFITQAVLRIKSVEARHKAFSTCSSHLTVVIIFYGTIIYVYLQ
PSDSYAQDQGKFISLFYTMVTPTLNPIIYTLRNKDMKEALRKLLSGKL
>sp|Q9HBF5|ST20_HUMAN
MARSRLTATSVSQVQENGFVKKLEPKSGWMTFLEVTGKICEMLFCPEAILLTRKDTPYCETGLIFLTLTKTIANTYFYF
>sp|P27169|PON1_HUMAN
MAKLIALTLLGMGLALFRNHQSSYQTRLNALREVQPVELPNCNLVKGIETGSEDLEILPNGLAFISSGLKYPGIKSFNPNSPGKILL
MDLNEEDPTVLELGITGSKFDVSSFNPHGISTFTDEDNAMYLLVVNHPDAKSTVELFKFQEEEKSLLHLKTIRHKLLPNLNDIVAVG
PEHFYGTNDHYFLDPYLQSWEMYLGLAWSYVVYYSPSEVRVVAEGFDFANGINISPDGKYVYIAELLAHKIHVYEKHANWTLTPLKS
LDFNTLVDNISVDPETGDLWVGCHPNGMKIFFYDSENPPASEVLRIQNILTEEPKVTQVYAENGTVLQGSTVASVYKGKLLIGTVFH
KALYCEL
>sp|Q8N9Y4|F181A_HUMAN
MPLEERRSSGERNDAAPTNHRRPGEKRASTAKQVSSVPFLGAAGHQQSLPSSWKASCSGPLVMASDSDVKMLLNFVNLASSDIKAAL
DKSAPCRRSVDHRKYLQKQLKRFSQKYSRLPRGLPGRAAEPYLKRGSEDRPRRLLLDLGPDSSPGGGGGCKEKVLRNPYREECLAKE
QLPQRQHPEAAQPGQVPMRKRQLPASFWEEPRPTHSYHVGLEGGLGPREGPPYEGKKNCKGLEPLGPETTLVSMSPRALAEKEPLKM
PGVSLVGRVNAWSCCPFQYHGQPIYPGPLGALPQSPVPSLGLWRKSPAFPGELAHLCKDVDGLGQKVCRPVVLKPIPTKPAVPPPIF
NVFGYL
>sp|Q9BYT1|S17A9_HUMAN
MQPPPDEARRDMAGDTQWSRPECQAWTGTLLLGTCLLYCARSSMPICTVSMSQDFGWNKKEAGIVLSSFFWGYCLTQVVGGHLGDRI
GGEKVILLSASAWGSITAVTPLLAHLSSAHLAFMTFSRILMGLLQGVYFPALTSLLSQKVRESERAFTYSIVGAGSQFGTLLTGAVG
SLLLEWYGWQSIFYFSGGLTLLWVWYVYRYLLSEKDLILALGVLAQSRPVSRHNRVPWRRLFRKPAVWAAVVSQLSAACSFFILLSW
LPTFFEETFPDAKGWIFNVVPWLVAIPASLFSGFLSDHLINQGYRAITVRKLMQGMGLGLSSVFALCLGHTSSFCESVVFASASIGL
QTFNHSGISVNIQDLAPSCAGFLFGVANTAGALAGVVGVCLGGYLMETTGSWTCLFNLVAIISNLGLCTFLVFGQAQRVDLSSTHED
L
>sp|Q9BY41|HDAC8_HUMAN
MEEPEEPADSGQSLVPVYIYSPEYVSMCDSLAKIPKRASMVHSLIEAYALHKQMRIVKPKVASMEEMATFHTDAYLQHLQKVSQEGD
DDHPDSIEYGLGYDCPATEGIFDYAAAIGGATITAAQCLIDGMCKVAINWSGGWHHAKKDEASGFCYLNDAVLGILRLRRKFERILY
VDLDLHHGDGVEDAFSFTSKVMTVSLHKFSPGFFPGTGDVSDVGLGKGRYYSVNVPIQDGIQDEKYYQICESVLKEVYQAFNPKAVV
LQLGADTIAGDPMCSFNMTPVGIGKCLKYILQWQLATLILGGGGYNLANTARCWTYLTGVILGKTLSSEIPDHEFFTAYGPDYVLEI
TPSCRPDRNEPHRIQQILNYIKGNLKHVV
>sp|Q9BQ65|USB1_HUMAN
MSAAPLVGYSSSGSEDESEDGMRTRPGDGSHRRGQSPLPRQRFPVPDSVLNMFPGTEEGPEDDSTKHGGRVRTFPHERGNWATHVYV
PYEAKEEFLDLLDVLLPHAQTYVPRLVRMKVFHLSLSQSVVLRHHWILPFVQALKARMTSFHRFFFTANQVKIYTNQEKTRTFIGLE
VTSGHAQFLDLVSEVDRVMEEFNLTTFYQDPSFHLSLAWCVGDARLQLEGQCLQELQAIVDGFEDAEVLLRVHTEQVRCKSGNKFFS
MPLK
>sp|Q9NXJ0|M4A12_HUMAN
MMSSKPTSHAEVNETIPNPYPPSSFMAPGFQQPLGSINLENQAQGAQRAQPYGITSPGIFASSQPGQGNIQMINPSVGTAVMNFKEE
AKALGVIQIMVGLMHIGFGIVLCLISFSFREVLGFASTAVIGGYPFWGGLSFIISGSLSVSASKELSRCLVKGSLGMNIVSSILAFI
GVILLLVDMCINGVAGQDYWAVLSGKGISATLMIFSLLEFFVACATAHFANQANTTTNMSVLVIPNMYESNPVTPASSSAPPRCNNY
SANAPK
>sp|Q9P0T7|TMEM9_HUMAN

MKLLSLVAVVGCLLVPPAEANKSSEDIRCKCICPPYRNISGHIYNQNVSQKDCNCLHVVEPMPVPGHDVEAYCLLCECRYEERSTTT
IKVIIVIYLSVVGALLLYMAFLMLVDPLIRKPDAYTEQLHNEEENEDARSMAAAAASLGGPRANTVLERVEGAQQRWKLQVQEQRKT
VFDRHKMLS
>sp|Q8TED0|UTP15_HUMAN
MAGYKPVAIQTYPILGEKITQDTLYWNNYKTPVQIKEFGAVSKVDFSPQPPYNYAVTASSRIHIYGRYSQEPIKTFSRFKDTAYCAT
FRQDGRLLVAGSEDGGVQLFDISGRAPLRQFEGHTKAVHTVDFTADKYHVVSGADDYTVKLWDIPNSKEILTFKEHSDYVRCGCASK
LNPDLFITGSYDHTVKMFDARTSESVLSVEHGQPVESVLLFPSGGLLVSAGGRYVKVWDMLKGGQLLVSLKNHHKTVTCLCLSSSGQ
RLLSGSLDRKVKVYSTTSYKVVHSFDYAASILSLALAHEDETIVVGMTNGILSVKHRKSEAKKESLPRRRRPAYRTFIKGKNYMKQR
DDILINRPAKKHLELYDRDLKHFRISKALDRVLDPTCTIKTPEITVSIIKELNRRGVLANALAGRDEKEISHVLNFLIRNLSQPRFA
PVLINAAEIIIDIYLPVIGQSPVVDKKFLLLQGLVEKEIDYQRELLETLGMMDMLFATMRRKEGTSVLEHTSDGFPENKKIES
>sp|B2RU33|POTEC_HUMAN
MVTEVCSMPAASAVKKPFDLRSKMGKWFHHRFPCCKGSGKSNMGTSGDHDDSFMKMLRSKMGKCCHHCFPCCRGSGTSNVGTSGDHD
NSFMKTLRSKMGKWCCHCFPCCRGSGKSNVGAWGDYDDSAFMEPRYHVRREDLDKLHRAAWWGKVPRKDLIVMLRDTDMNKRDKQKR
TALHLASANGNSEVVQLLLDRRCQLNVLDNKKRTALIKAVQCQEDECVLMLLEHGADQNIPDEYGNTTLHYAVHNEDKLMAKALLLY
GADIESKNKCGLTPLLLGVHEQKQQVVKFLIKKKANLNALDRYGRTALILAVCCGSASIVNLLLEQNVDVSSQDLSGQTAREYAVSS
HHHVICELLSDYKEKQMLKISSENSNPEQDLKLTSEEESQRLKVSENSQPEKMSQEPEINKDCDREVEEEIKKHGSNPVGLPENLTN
GASAGNGDDGLIPQRRSRKPENQQFPDTENEEYHSDEQNDTRKQLSEEQNTGISQDEILTNKQKQIEVAEKKMNSELSLSHKKEEDL
LRENSMLQEEIAMLISGDWN
>sp|P26717|NKG2C_HUMAN
MSKQRGTFSEVSLAQDPKRQQRKPKGNKSSISGTEQEIFQVELNLQNPSLNHQGIDKIYDCQGLLPPPEKLTAEVLGIICIVLMATV
LKTIVLIPFLEQNNSSPNTRTQKARHCGHCPEEWITYSNSCYYIGKERRTWEESLLACTSKNSSLLSIDNEEEMKFLASILPSSWIG
VFRNSSHHPWVTINGLAFKHKIKDSDNAELNCAVLQVNRLKSAQCGSSMIYHCKHKL
>sp|P24557|THAS_HUMAN
MEALGFLKLEVNGPMVTVALSVALLALLKWYSTSAFSRLEKLGLRHPKPSPFIGNLTFFRQGFWESQMELRKLYGPLCGYYLGRRMF
IVISEPDMIKQVLVENFSNFTNRMASGLEFKSVADSVLFLRDKRWEEVRGALMSAFSPEKLNEMVPLISQACDLLLAHLKRYAESGD
AFDIQRCYCNYTTDVVASVAFGTPVDSWQAPEDPFVKHCKRFFEFCIPRPILVLLLSFPSIMVPLARILPNKNRDELNGFFNKLIRN
VIALRDQQAAEERRRDFLQMVLDARHSASPMGVQDFDIVRDVFSSTGCKPNPSRQHQPSPMARPLTVDEIVGQAFIFLIAGYEIITN
TLSFATYLLATNPDCQEKLLREVDVFKEKHMAPEFCSLEEGLPYLDMVIAETLRMYPPAFRFTREAAQDCEVLGQRIPAGAVLEMAV
GALHHDPEHWPSPETFNPERFTAEARQQHRPFTYLPFGAGPRSCLGVRLGLLEVKLTLLHVLHKFRFQACPETQVPLQLESKSALGP
KNGVYIKIVSR
>sp|Q32M84|BTBDG_HUMAN
MIMSNTHKARLERRVTGSTNRWRLPKQPFSGDLLSLSQMCKALSIDFEEALRNPDRLCISQIQKFFFENFKNKDIQSGEADVILECL
GFKWELHQPQLFQSETLAKLYLKALAQGTTHPLRELEELLRAQSPKKTKEKSPAKRIIISLKINDPLVTKVAFATALKNLYMSEVEI
NLEDLLGVLASAHILQFSGLFQRCVDVMIARLKPSTIKKFYEAGCKYKEEQLTTGCEKWLEMNLVPLGGTQIHLHKIPQDLLHKVLK
SPRLFTFSEFHLLKTMLLWVFLQLNYKIQAIPTYETVMTFFKSFPENCCFLDRDIGRSLRPLFLCLRLHGITKGKDLEVLRHLNFFP
ESWLDQVTVNHYHALENGGDMVHLKDLNTQAVRFGLLFNQENTTYSKTIALYGFFFKIKGLKHDTTSYSFYMQRIKHTDLESPSAVY
EHNHVSLRAARLVKYEIRAEALVDGKWQEFRTNQIKQKFGLTTSSCKSHTLKIQTVGIPIYVSFAFIFPAS
>sp|Q9NXR8|ING3_HUMAN
MLYLEDYLEMIEQLPMDLRDRFTEMREMDLQVQNAMDQLEQRVSEFFMNAKKNKPEWREEQMASIKKDYYKALEDADEKVQLANQIY
DLVDRHLRKLDQELAKFKMELEADNAGITEILERRSLELDTPSQPVNNHHAHSHTPVEKRKYNPTSHHTTTDHIPEKKFKSEALLST
LTSDASKENTLGCRNNNSTASSNNAYNVNSSQPLGSYNIGSLSSGTGAGAITMAAAQAVQATAQMKEGRRTSSLKASYEAFKNNDFQ
LGKEFSMARETVGYSSSSALMTTLTQNASSSAADSRSGRKSKNNNKSSSQQSSSSSSSSSLSSCSSSSTVVQEISQQTTVVPESDSN
SQVDWTYDPNEPRYCICNQVSYGEMVGCDNQDCPIEWFHYGCVGLTEAPKGKWYCPQCTAAMKRRGSRHK
>sp|Q96RD6|PANX2_HUMAN

MHHLLEQSADMATALLAGEKLRELILPGAQDDKAGALAALLLQLKLELPFDRVVTIGTVLVPILLVTLVFTKNFAEEPIYCYTPHNF
TRDQALYARGYCWTELRDALPGVDASLWPSLFEHKFLPYALLAFAAIMYVPALGWEFLASTRLTSELNFLLQEIDNCYHRAAEGRAP
KIEKQIQSKGPGITEREKREIIENAEKEKSPEQNLFEKYLERRGRSNFLAKLYLARHVLILLLSAVPISYLCTYYATQKQNEFTCAL
GASPDGAAGAGPAVRVSCKLPSVQLQRIIAGVDIVLLCVMNLIILVNLIHLFIFRKSNFIFDKLHKVGIKTRRQWRRSQFCDINILA
MFCNENRDHIKSLNRLDFITNESDLMYDNVVRQLLAALAQSNHDATPTVRDSGVQTVDPSANPAEPDGAAEPPVVKRPRKKMKWIPT
SNPLPQPFKEPLAIMRVENSKAEKPKPARRKTATDTLIAPLLDRSAHHYKGGGGDPGPGPAPAPAPPPAPDKKHARHFSLDVHPYIL
GTKKAKAEAVPAALPASRSQEGGFLSQAEDCGLGLAPAPIKDAPLPEKEIPYPTEPARAGLPSGGPFHVRSPPAAPAVAPLTPASLG
KAEPLTILSRNATHPLLHINTLYEAREEEDGGPRLPQDVGDLIAIPAPQQILIATFDEPRTVVSTVEF
>sp|P01764|HV323_HUMAN
MEFGLSWLFLVAILKGVQCEVQLVESGGGLVQPGGSLRLSCAASGFTFSSYAMSWVRQAPGKGLEWVSAISGSGGSTYYADSVKGRF
TISRDNSKNTLYLQMNSLRAEDTAVYYCAK
>sp|Q8N0Y7|PGAM4_HUMAN
MAAYKLVLIRHGESTWNLENRFSCWYDADLSPAGHEEAKRGGQALRDAGYEFDICLTSVQKRVIRTLWTVLDAIDQMWLPVVRTWRL
NERHYGGLTGLNKAETAAKHGEAQVKIWRRSYDVPPPPMEPDHPFYSNISKDRRYADLTEDQLPSYESPKDTIARALPFWNEEIVPQ
IKEGKRVLIAAHGNSLQGIAKHVEGLSEEAIMELNLPTGIPIVYELDKNLKPIKPMQFLGDEETVCKAIEAVAAQGKAKK
>sp|Q6YI46|TMM64_HUMAN
MRSPGGILLQALPRLLQHAALPGLAELPARWALPRGAGGDGPADRLPRGGGASAAAAAAAASGALLGAYLERHGPPEASELPEPGGA
LAGGPGSGGGGVVVGVAEVRNWRCCCLGSTCWCRSLVLVCVLAALCFASLALVRRYLHHLLLWVESLDSLLGVLLFVVGFIVVSFPC
GWGYIVLNVAAGYLYGFVLGMGLMMVGVLIGTFIAHVVCKRLLTAWVAARIQSSEKLSAVIRVVEGGSGLKVVALARLTPIPFGLQN
AVFSITDLSLPNYLMASSVGLLPTQLLNSYLGTTLRTMEDVIAEQSVSGYFVFCLQIIISIGLMFYVVHRAQVELNAAIVACEMELK
SSLVKGNQPNTSGSSFYNKRTLTFSGGGINVV
>sp|Q9NX01|TXN4B_HUMAN
MSFLLPKLTSKKEVDQAIKSTAEKVLVLRFGRDEDPVCLQLDDILSKTSSDLSKMAAIYLVDVDQTAVYTQYFDISYIPSTVFFFNG
QHMKVDYGSPDHTKFVGSFKTKQDFIDLIEVIYRGAMRGKLIVQSPIDPKNIPKYDLLYQDI
>sp|Q5BKZ1|ZN326_HUMAN
MDFEDDYTHSACRNTYQGFNGMDRDYGPGSYGGMDRDYGHGSYGGQRSMDSYLNQSYGMDNHSGGGGGSRFGPYESYDSRSSLGGRD
LYRSGYGFNEPEQSRFGGSYGGRFESSYRNSLDSFGGRNQGGSSWEAPYSRSKLRPGFMEDRGRENYSSYSSFSSPHMKPAPVGSRG
RGTPAYPESTFGSRNYDAFGGPSTGRGRGRGHMGDFGSIHRPGIVVDYQNKSTNVTVAAARGIKRKMMQPFNKPSGTFIKKPKLAKP
MEKISLSKSPTKTDPKNEEEEKRRIEARREKQRRRREKNSEKYGDGYRMAFTCSFCKFRTFEEKDIELHLESSSHQETLDHIQKQTK
FDKVVMEFLHECMVNKFKKTSIRKQQTNNQTEVVKIIEKDVMEGVTVDDHMMKVETVHCSACSVYIPALHSSVQQHLKSPDHIKGKQ
AYKEQIKRESVLTATSILNNPIVKARYERFVKGENPFEIQDHSQDQQIEGDEEDEEKIDEPIEEEEDEDEEEEAEEVGEVEEVEEVE
EVREGGIEGEGNIQGVGEGGEVGVVGEVEGVGEVEEVEELEEETAKEEPADFPVEQPEEN
>sp|P39656|OST48_HUMAN
MGYFRCARAGSFGRRRKMEPSTAARAWALFWLLLPLLGAVCASGPRTLVLLDNLNVRETHSLFFRSLKDRGFELTFKTADDPSLSLI
KYGEFLYDNLIIFSPSVEDFGGNINVETISAFIDGGGSVLVAASSDIGDPLRELGSECGIEFDEEKTAVIDHHNYDISDLGQHTLIV
ADTENLLKAPTIVGKSSLNPILFRGVGMVADPDNPLVLDILTGSSTSYSFFPDKPITQYPHAVGKNTLLIAGLQARNNARVIFSGSL
DFFSDSFFNSAVQKAAPGSQRYSQTGNYELAVALSRWVFKEEGVLRVGPVSHHRVGETAPPNAYTVTDLVEYSIVIQQLSNGKWVPF
DGDDIQLEFVRIDPFVRTFLKKKGGKYSVQFKLPDVYGVFQFKVDYNRLGYTHLYSSTQVSVRPLQHTQYERFIPSAYPYYASAFSM
MLGLFIFSIVFLHMKEKEKSD
>sp|P02743|SAMP_HUMAN
MNKPLLWISVLTSLLEAFAHTDLSGKVFVFPRESVTDHVNLITPLEKPLQNFTLCFRAYSDLSRAYSLFSYNTQGRDNELLVYKERV
GEYSLYIGRHKVTSKVIEKFPAPVHICVSWESSSGIAEFWINGTPLVKKGLRQGYFVEAQPKIVLGQEQDSYGGKFDRSQSFVGEIG
DLYMWDSVLPPENILSAYQGTPLPANILDWQALNYEIRGYVIIKPLVWV
>sp|O95630|STABP_HUMAN

MSDHGDVSLPPEDRVRALSQLGSAVEVNEDIPPRRYFRSGVEIIRMASIYSEEGNIEHAFILYNKYITLFIEKLPKHRDYKSAVIPE
KKDTVKKLKEIAFPKAEELKAELLKRYTKEYTEYNEEKKKEAEELARNMAIQQELEKEKQRVAQQKQQQLEQEQFHAFEEMIRNQEL
EKERLKIVQEFGKVDPGLGGPLVPDLEKPSLDVFPTLTVSSIQPSDCHTTVRPAKPPVVDRSLKPGALSNSESIPTIDGLRHVVVPG
RLCPQFLQLASANTARGVETCGILCGKLMRNEFTITHVLIPKQSAGSDYCNTENEEELFLIQDQQGLITLGWIHTHPTQTAFLSSVD
LHTHCSYQMMLPESVAIVCSPKFQETGFFKLTDHGLEEISSCRQKGFHPHSKDPPLFCSCSHVTVVDRAVTITDLR
>sp|Q9UJS0|CMC2_HUMAN
MAAAKVALTKRADPAELRTIFLKYASIEKNGEFFMSPNDFVTRYLNIFGESQPNPKTVELLSGVVDQTKDGLISFQEFVAFESVLCA
PDALFMVAFQLFDKAGKGEVTFEDVKQVFGQTTIHQHIPFNWDSEFVQLHFGKERKRHLTYAEFTQFLLEIQLEHAKQAFVQRDNAR
TGRVTAIDFRDIMVTIRPHVLTPFVEECLVAAAGGTTSHQVSFSYFNGFNSLLNNMELIRKIYSTLAGTRKDVEVTKEEFVLAAQKF
GQVTPMEVDILFQLADLYEPRGRMTLADIERIAPLEEGTLPFNLAEAQRQKASGDSARPVLLQVAESAYRFGLGSVAGAVGATAVYP
IDLVKTRMQNQRSTGSFVGELMYKNSFDCFKKVLRYEGFFGLYRGLLPQLLGVAPEKAIKLTVNDFVRDKFMHKDGSVPLAAEILAG
GCAGGSQVIFTNPLEIVKIRLQVAGEITTGPRVSALSVVRDLGFFGIYKGAKACFLRDIPFSAIYFPCYAHVKASFANEDGQVSPGS
LLLAGAIAGMPAASLVTPADVIKTRLQVAARAGQTTYSGVIDCFRKILREEGPKALWKGAGARVFRSSPQFGVTLLTYELLQRWFYI
DFGGVKPMGSEPVPKSRINLPAPNPDHVGGYKLAVATFAGIENKFGLYLPLFKPSVSTSKAIGGGP
>sp|Q9P0L2|MARK1_HUMAN
MSARTPLPTVNERDTENHTSVDGYTEPHIQPTKSSSRQNIPRCRNSITSATDEQPHIGNYRLQKTIGKGNFAKVKLARHVLTGREVA
VKIIDKTQLNPTSLQKLFREVRIMKILNHPNIVKLFEVIETEKTLYLVMEYASGGEVFDYLVAHGRMKEKEARAKFRQIVSAVQYCH
QKYIVHRDLKAENLLLDGDMNIKIADFGFSNEFTVGNKLDTFCGSPPYAAPELFQGKKYDGPEVDVWSLGVILYTLVSGSLPFDGQN
LKELRERVLRGKYRIPFYMSTDCENLLKKLLVLNPIKRGSLEQIMKDRWMNVGHEEEELKPYTEPDPDFNDTKRIDIMVTMGFARDE
INDALINQKYDEVMATYILLGRKPPEFEGGESLSSGNLCQRSRPSSDLNNSTLQSPAHLKVQRSISANQKQRRFSDHAGPSIPPAVS
YTKRPQANSVESEQKEEWDKDVARKLGSTTVGSKSEMTASPLVGPERKKSSTIPSNNVYSGGSMARRNTYVCERTTDRYVALQNGKD
SSLTEMSVSSISSAGSSVASAVPSARPRHQKSMSTSGHPIKVTLPTIKDGSEAYRPGTTQRVPAASPSAHSISTATPDRTRFPRGSS
SRSTFHGEQLRERRSVAYNGPPASPSHETGAFAHARRGTSTGIISKITSKFVRRDPSEGEASGRTDTSRSTSGEPKERDKEEGKDSK
PRSLRFTWSMKTTSSMDPNDMMREIRKVLDANNCDYEQKERFLLFCVHGDARQDSLVQWEMEVCKLPRLSLNGVRFKRISGTSIAFK
NIASKIANELKL
>sp|Q14CS0|UBX2B_HUMAN
MAEGGGPEPGEQERRSSGPRPPSARDLQLALAELYEDEVKCKSSKSNRPKATVFKSPRTPPQRFYSSEHEYSGLNIVRPSTGKIVNE
LFKEAREHGAVPLNEATRASGDDKSKSFTGGGYRLGSSFCKRSEYIYGENQLQDVQILLKLWSNGFSLDDGELRPYNEPTNAQFLES
VKRGEIPLELQRLVHGGQVNLDMEDHQDQEYIKPRLRFKAFSGEGQKLGSLTPEIVSTPSSPEEEDKSILNAVVLIDDSVPTTKIQI
RLADGSRLIQRFNSTHRILDVRNFIVQSRPEFAALDFILVTSFPNKELTDESLTLLEADILNTVLLQQLK
>sp|Q5JVG8|ZN506_HUMAN
MGPLQFRDVAIEFSLEEWHCLDAAQRNLYRDVMLENYRNLIFLGIVVSKPNLITCLEQGKKPLTMKRHEMIAKPPVMYSHFAQDLWS
EQSIKDSFQKVILRRYEKCRHDNLQLKKGCESVDECPVHKRGYNGLKQCLATTQRKIFQCDEYVKFLHKFSNSNKHKIRDTGKKSFK
CIEYGKTFNQSSTRTTYKKIDAGEKRYKCEECGKAYKQSSHLTTHKKIHTGEKPYKCEECGKAYKQSCNLTTHKIIHTGEKPYRCRE
CGKAFNHPATLFSHKKIHTGEKPYKCDKCGKAFISSSTLTKHEIIHTGEKPYKCEECGKAFNRSSNLTKHKRIHTGDVPYKCDECGK
TFTWYSSLSKHKRAHTGEKPYKCEECGKAFTAFSTLTEHKIIHTGEKPYKCEECGKAFNWSSALNKHKKIHIRQKPCIVKNVENLLN
VPQPLISIR
>sp|P07954|FUMH_HUMAN
MYRALRLLARSRPLVRAPAAALASAPGLGGAAVPSFWPPNAARMASQNSFRIEYDTFGELKVPNDKYYGAQTVRSTMNFKIGGVTER
MPTPVIKAFGILKRAAAEVNQDYGLDPKIANAIMKAADEVAEGKLNDHFPLVVWQTGSGTQTNMNVNEVISNRAIEMLGGELGSKIP
VHPNDHVNKSQSSNDTFPTAMHIAAAIEVHEVLLPGLQKLHDALDAKSKEFAQIIKIGRTHTQDAVPLTLGQEFSGYVQQVKYAMTR
IKAAMPRIYELAAGGTAVGTGLNTRIGFAEKVAAKVAALTGLPFVTAPNKFEALAAHDALVELSGAMNTTACSLMKIANDIRFLGSG
PRSGLGELILPENEPGSSIMPGKVNPTQCEAMTMVAAQVMGNHVAVTVGGSNGHFELNVFKPMMIKNVLHSARLLGDASVSFTENCV
VGIQANTERINKLMNESLMLVTALNPHIGYDKAAKIAKTAHKNGSTLKETAIELGYLTAEQFDEWVKPKDMLGPK

>sp|Q9H7T3|CJ095_HUMAN
MERSNAATKCGEEPRSGSRRLPKAEGDKSGSAGAPSKNSSRLGGRPCMCTAGRRPNRASGRRRRSCSPAPTWPPLCCYPQSRPTASA
AGPGACMRASGRPHGNTTASTAPPRHPRPRRPGGPALRPTPRPCAGPAPPPASRDCRCRRPRRWPRAGRRGRRAGACKPSCAGAAWS
ARGAPLCSYRTSCAGSCGARTAPTPAPTCASPSAAASSCCRRRRACSSPTTAWSGACGAGPTAATAAQPGKPRSAAAPGRARA
>sp|Q96DP5|FMT_HUMAN
MRVLVRRCWGPPLAHGARRGRPSPQWRALARLGWEDCRDSRVREKPPWRVLFFGTDQFAREALRALHAARENKEEELIDKLEVVTMP
SPSPKGLPVKQYAVQSQLPVYEWPDVGSGEYDVGVVASFGRLLNEALILKFPYGILNVHPSCLPRWRGPAPVIHTVLHGDTVTGVTI
MQIRPKRFDVGPILKQETVPVPPKSTAKELEAVLSRLGANMLISVLKNLPESLSNGRQQPMEGATYAPKISAGTSCIKWEEQTSEQI
FRLYRAIGNIIPLQTLWMANTIKLLDLVEVNSSVLADPKLTGQALIPGSVIYHKQSQILLVYCKDGWIGVRSVMLKKSLTATDFYNG
YLHPWYQKNSQAQPSQCRFQTLRLPTKKKQKKTVAMQQCIE
>sp|A4D1U4|LCHN_HUMAN
MVEQGDAAPLLRWAEGPAVSLPQAPQPQAGGWGRGGGGGARPAAEPPRRREPEEPAAPEVLLQPGRLELGDVEEDQVVAVFVVTFDP
RSGNMVEWCLPQDIDLEGVEFKSMASGSHKIQSDFIYFRKGPFFGLACFANMPVESELERGARMKSVGILSPSYTLLYRYMHFLENQ
VRHQLEMPGHYSHLAAFYEDKKGVLHAGPGRGSSLPPVYWLPSIHRYMYPEMKITHPAGCMSQFIKFFGEQILILWKFALLRKRILI
FSPPPVGVVCYRVYCCCCLANVSLPGIGGTIPESKPFFYVNVADIESLEVEVSYVACTTEKIFEEKRELYDVYVDNQNVKTHHDHLQ
PLLKINSADREKYRRLNEQRQMLLYSQEVEEDYNPCEEDLFVLFFLEQNNRIFQTLLEVSASQDKTLTAEHARGMGLDPQGDRSFLL
DLLEAYGIDVMLVIDNPCCP
>sp|Q5XXA6|ANO1_HUMAN
MRVNEKYSTLPAEDRSVHIINICAIEDIGYLPSEGTLLNSLSVDPDAECKYGLYFRDGRRKVDYILVYHHKRPSGNRTLVRRVQHSD
TPSGARSVKQDHPLPGKGASLDAGSGEPPMDYHEDDKRFRREEYEGNLLEAGLELERDEDTKIHGVGFVKIHAPWNVLCREAEFLKL
KMPTKKMYHINETRGLLKKINSVLQKITDPIQPKVAEHRPQTMKRLSYPFSREKQHLFDLSDKDSFFDSKTRSTIVYEILKRTTCTK
AKYSMGITSLLANGVYAAAYPLHDGDYNGENVEFNDRKLLYEEWARYGVFYKYQPIDLVRKYFGEKIGLYFAWLGVYTQMLIPASIV
GIIVFLYGCATMDENIPSMEMCDQRHNITMCPLCDKTCSYWKMSSACATARASHLFDNPATVFFSVFMALWAATFMEHWKRKQMRLN
YRWDLTGFEEEEEAVKDHPRAEYEARVLEKSLKKESRNKEKRRHIPEESTNKWKQRVKTAMAGVKLTDKVKLTWRDRFPAYLTNLVS
IIFMIAVTFAIVLGVIIYRISMAAALAMNSSPSVRSNIRVTVTATAVIINLVVIILLDEVYGCIARWLTKIEVPKTEKSFEERLIFK
AFLLKFVNSYTPIFYVAFFKGRFVGRPGDYVYIFRSFRMEECAPGGCLMELCIQLSIIMLGKQLIQNNLFEIGIPKMKKLIRYLKLK
QQSPPDHEECVKRKQRYEVDYNLEPFAGLTPEYMEMIIQFGFVTLFVASFPLAPLFALLNNIIEIRLDAKKFVTELRRPVAVRAKDI
GIWYNILRGIGKLAVIINAFVISFTSDFIPRLVYLYMYSKNGTMHGFVNHTLSSFNVSDFQNGTAPNDPLDLGYEVQICRYKDYREP
PWSENKYDISKDFWAVLAARLAFVIVFQNLVMFMSDFVDWVIPDIPKDISQQIHKEKVLMVELFMREEQDKQQLLETWMEKERQKDE
PPCNHHNTKACPDSLGSPAPSHAYHGGVL
>sp|Q96S16|JMJD8_HUMAN
MVTGGAHSRSRGGLPSASRRGLFRGGPKEVTSGVRPGSRPLPRRRSCRPMAGGWAQGSRRAARLPEGGRLMAPASRLLALWALAAVA
LPGSGAEGDGGWRPGGPGAVAEEERCTVERRADLTYAEFVQQYAFVRPVILQGLTDNSRFRALCSRDRLLASFGDRVVRLSTANTYS
YHKVDLPFQEYVEQLLHPQDPTSLGNDTLYFFGDNNFTEWASLFRHYSPPPFGLLGTAPAYSFGIAGAGSGVPFHWHGPGYSEVIYG
RKRWFLYPPEKTPEFHPNKTTLAWLRDTYPALPPSARPLECTIRAGEVLYFPDRWWHATLNLDTSVFISTFLG
>sp|Q5SRR4|LY65C_HUMAN
MRFMAGPAGSQSLGPLCFHSSPQALYTVLLIVLVMMSLVFGKFVPVNWEPPQPLPFPKYLRCYRCLLETKELGCLLGSDICLTPAGS
SCITLHKKNSSGSDVMVSDCRSKEQMSDCSNTRTSPVSGFWIFSQYCFLDFCNDPQNRGLYTP
>sp|Q6ZRI0|OTOG_HUMAN
MGVLASALCWLLCVWLPWGEQAAESLRVQRLGERVVDSGRSGARGMRNVKGMRNGPAQTRVSSSSSHQEATLAMGDKATVVGGQQAE
APDSVAMSSWERRLHRAKCAPSYLFSCFNGGECVHPAFCDCRRFNATGPRCQMVYNAGPERDSICRAWGQHHVETFDGLYYYLSGKG
SYTLVGRHEPEGQSFSIQVHNDPQCGSSPYTCSRAVSLFFVGEQEIHLAKEVTHGGMRVQLPHVMGSARLQQLAGYVIVRHQSAFTL
AWDGASAVYIKMSPELLGWTHGLCGNNNADPKDDLVTSSGKLTDDVVEFVHSWQEQAPNQPPGPTTSSLPRPPCLQQNPGTMQGVYE
QCEALLRPPFDACHAYVSPLPFTASCTSDLCQSMGDVATWCRALAEYARACAQAGRPLQGWRTQLRQCTVHCKEKAFTYNECIACCP

ASCHPRASCVDSEIACVDGCYCPNGLIFEDGGCVAPAECPCEFHGTLYPPGSVVKEDCNTCTCTSGKWECSTAVCPAECSVTGDIHF
TTFDGRRYTFPATCQYILAKSRSSGTFTVTLQNAPCGLNQDGACVQSVSVILHQDPRRQVTLTQAGDVLLFDQYKIIPPYTDDAFEI
RRLSSVFLRVRTNVGVRVLYDREGLRLYLQVDQRWVEDTVGLCGTFNGNTQDDFLSPVGVPESTPQLFGNSWKTLSACSPLVSGSPL
DPCDVHLQAASYSVQACSVLTGEMFAPCSAFLSPVPYFEQCRRDACRCGQPCLCATLAHYAHLCRRHGLPVDFRARLPACALSCEAS
KEYSPCVAPCGRTCQDLASPEACGVDGGDDLSRDECVEGCACPPDTYLDTQADLCVPRNQCSCHFQGVDYPPGDSDIPSLGHCHCKD
GVMSCDSRAPAAACPAGQVFVNCSDLHTDLELSRERTCEQQLLNLSVSARGPCLSGCACPQGLLRHGDACFLPEECPCTWKGKEYFP
GDQVMSPCHTCVCQRGSFQCTLHPCASTCTAYGDRHYRTFDGLPFDFVGACKVHLVKSTSDVSFSVIVENVNCYSSGMICRKFISIN
VGNSLIVFDDDSGNPSPESFLDDKQEVHTWRVGFFTLVHFPQEHITLLWDQRTTVHVQAGPQWQGQLAGLCGNFDLKTINEMRTPEN
LELTNPQEFGSSWAAVECPDTLDPRDMCVLNPLREPFAKKECSILLSEVFEICHPVVDVTWFYSNCLTDTCGCSQGGDCECFCASVS
AYAHQCCQHGVAVDWRTPRLCPYDCDFFNKVLGKGPYQLSSLAAGGALVGMKAVGDDIVLVRTEDVAPADIVSFLLTAALYKAKAHD
PDVVSLEAADRPNFFLHVTANGSLELAKWQGRDTFQQHASFLLHRGTRQAGLVALESLAKPSSFLYVSGAVLALRLYEHTEVFRRGT
LFRLLDAKPSGAAYPICEWRYDACASPCFQTCRDPRAASCRDVPRVEGCVPVCPTPQVLDEVTQRCVYLEDCVEPAVWVPTEALGNE
TLPPSQGLPTPSDEEPQLSQESPRTPTHRPALTPAAPLTTALNPPVTATEEPVVSPGPTQTTLQQPLELTASQLPAGPTESPASKGV
TASLLAIPHTPESSSLPVALQTPTPGMVSGAMETTRVTVIFAGSPNITVSSRSPPAPRFPLMTKAVTVRGHGSLPVRTTPPQPSLTA
SPSSRPVASPGAISRSPTSSGSHKAVLTPAVTKVISRTGVPQPTQAQSASSPSTPLTVAGTAAEQVPVSPLATRSLEIVLSTEKGEA
GHSQPMGSPASPQPHPLPSAPPRPAQHTTMATRSPALPPETPAAASLSTATDGLAATPFMSLESTRPSQLLSGLPPDTSLPLAKVGT
SAPVATPGPKASVITTPLQPQATTLPAQTLSPVLPFTPAAMTQAHPPTHIAPPAAGTAPGLLLGATLPTSGVLPVAEGTASMVSVVP
RKSTTGKVAILSKQVSLPTSMYGSAEGGPTELTPATSHPLTPLVAEPEGAQAGTALPVPTSYALSRVSARTAPQDSMLVLLPQLAEA
HGTSAGPHLAAEPVDEATTEPSGRSAPALSIVEGLAEALATTTEANTSTTCVPIAEQDCVRHICLEGGQLIRVNQSQHCPQGAAPPRC
GILGLAVRVGGDRCCPLWECACRCSIFPDLSFVTFDGSHVALFKEAIYILSQSPDEMLTVHVLDCKSANLGHLNWPPFCLVMLNMTH
LAHQVTIDRFNRKVTVDLQPVWPPVSRYGFRIEDTGHMYMILTPSDIQIQWLHSSGLMIVEASKTSKAQGHGLCGICDGDAANDLTL
KDGSVVGGAEDPAPFLDSWQVPSSLTSVGQTRFRPDSCATTDCSPCLRMVSNRTFSACHRFVPPESFCELWIRDTKYVQQPCVALTV
YVAMCHKFHVCIEWRRSDYCPFLCSSDSTYQACVTACEPPKTCQDGILGPLDPEHCQVLGEGCVCSEGTILHRRHSALCIPEAKCAC
TDSMGVPRALGETWNSSLSGCCQHQCQAPDTIVPVDLGCPSPRPESCLRFGEVALLLPTKDPCCLGTVCVCNQTLCEGLAPTCRPGH
RLLTHFQEDSCCPSYSCECDPDLCEAELVPSCRQDQILITGRLGDSCCTSYFCACGDCPDSIPECQEGEALTVHRNTTELCCPLYQC
VCENFRCPQVQCGLGTALVEVWSPDRCCPYKSCECDCDTIPVPRCHLWEKSQLDEEFMHSVENVCGCAKYECVKAPVCLSRELGVMQ
PGQTVVELSADGVCHTSRCTTVLDPLTNFYQINTTSVLCDIHCEANQEYEHPRDLAACCGSCRNVSCLFTFPNGTTSLFLPGASWIA
DCARHHCSSTPLGAVLVRSPISCPPLNETECAKVGGSVVPSLEGCCRTCKEDGRSCKKVTIRMTIRKNECRSSTPVNLVSCDGRCPS
ASIYNYNINTYARFCKCCREVGLQRRSVQLFCATNATWVPYTVQEPTDCACQWS
>sp|Q86YV6|MYLK4_HUMAN
MLKVKRLEEFNTCYNSNQLEKMAFFQCREEVEKVKCFLEKNSGDQDSRSGHNEAKEVWSNADLTERMPVKSKRTSALAVDIPAPPAP
FDHRIVTAKQGAVNSFYTVSKTEILGGGRFGQVHKCEETATGLKLAAKIIKTRGMKDKEEVKNEISVMNQLDHANLIQLYDAFESKN
DIVLVMEYVDGGELFDRIIDESYNLTELDTILFMKQICEGIRHMHQMYILHLDLKPENILCVNRDAKQIKIIDFGLARRYKPREKLK
VNFGTPEFLAPEVVNYDFVSFPTDMWSVGVIAYMLLSGLSPFLGDNDAETLNNILACRWDLEDEEFQDISEEAKEFISKLLIKEKSW
RISASEALKHPWLSDHKLHSRLNAQKKKNRGSDAQDFVTK
>sp|Q9Y450|HBS1L_HUMAN
MARHRNVRGYNYDEDFEDDDLYGQSVEDDYCISPSTAAQFIYSRRDKPSVEPVEEYDYEDLKESSNSVSNHQLSGFDQARLYSCLDH
MREVLGDAVPDEILIEAVLKNKFDVQKALSGVLEQDRVQSLKDKNEATVSTGKIAKGKPVDSQTSRSESEIVPKVAKMTVSGKKQTM
GFEVPGVSSEENGHSFHTPQKGPPIEDAIASSDVLETASKSANPPHTIQASEEQSSTPAPVKKSGKLRQQIDVKAELEKRQGGKQLL
NLVVIGHVDAGKSTLMGHMLYLLGNINKRTMHKYEQESKKAGKASFAYAWVLDETGEERERGVTMDVGMTKFETTTKVITLMDAPGH
KDFIPNMITGAAQADVAVLVVDASRGEFEAGFETGGQTREHGLLVRSLGVTQLAVAVNKMDQVNWQQERFQEITGKLGHFLKQAGFK
ESDVGFIPTSGLSGENLITRSQSSELTKWYKGLCLLEQIDSFKPPQRSIDKPFRLCVSDVFKDQGSGFCITGKIEAGYIQTGDRLLA
MPPNETCTVKGITLHDEPVDWAAAGDHVSLTLVGMDIIKINVGCIFCGPKVPIKACTRFRARILIFNIEIPITKGFPVLLHYQTVSE
PAVIKRLISVLNKSTGEVTKKKPKFLTKGQNALVELQTQRPIALELYKDFKELGRFMLRYGGSTIAAGVVTEIKE

>sp|Q9Y3B7|RM11_HUMAN
MSKLGRAARGLRKPEVGGVIRAIVRAGLAMPGPPLGPVLGQRGVSINQFCKEFNERTKDIKEGIPLPTKILVKPDRTFEIKIGQPTV
SYFLKAAAGIEKGARQTGKEVAGLVTLKHVYEIARIKAQDEAFALQDVPLSSVVRSIIGSARSLGIRVVKDLSSEELAAFQKERAIF
LAAQKEADLAAQEEAAKK
>sp|Q9UBI9|HDC_HUMAN
MPNPKNSKGGRKNKRANSSGDEQENGAGALAAAGAAGAAAGGALAAAAGCGAAAAGAPGAGGAAGAGGAGTGAANAAAAAGAAAAGD
AKNEAPCATPLICSFGRPVDLEKDDYQKVVCNNEHCPCSTWMHLQCFYEWESSILVQFNCIGRARSWNEKQCRQNMWTKKGYDLAFR
FCSCRCGQGHLKKDTDWYQVKRMQDEKKKKSGSEKNTGRPPGEAAEEAKKCRPPNKPQKGPSHDLPRRHSMDRQNSQEKAVGAAAYG
ARSPGGSPGQSPPTGYSILSPAHFSGPRSSRYLGEFLKNAIHLEPHKKAMAGGHVFRNAHFDYSPAGLAVHRGGHFDTPVQFLRRLD
LSELLTHIPRHKLNTFHVRMEDDAQVGQGEDLRKFILAALSASHRNVVNCALCHRALPVFEQFPLVDGTLFLSPSRHDEIEYDVPCH
LQGRLMHLYAVCVDCLEGVHKIICIKCKSRWDGSWHQLGTMYTYDILAASPCCQARLNCKHCGKPVIDVRIGMQYFSEYSNVQQCPH
CGNLDYHFVKPFSSFKVLEAY
>sp|Q9Y4Z2|NGN3_HUMAN
MTPQPSGAPTVQVTRETERSFPRASEDEVTCPTSAPPSPTRTRGNCAEAEEGGCRGAPRKLRARRGGRSRPKSELALSKQRRSRRKK
ANDRERNRMHNLNSALDALRGVLPTFPDDAKLTKIETLRFAHNYIWALTQTLRIADHSLYALEPPAPHCGELGSPGGSPGDWGSLYS
PVSQAGSLSPAASLEERPGLLGATFSACLSPGSLAFSDFL
>sp|Q8N8L2|ZN491_HUMAN
MGERLFESAEGSQCGETFTQVPEDMLNKKTLPGVKSCESGTCGEIFMGYSSFNRNIRTDTGHQPHKCQKFLEKPYKHKQRRKALSHS
HCFRTHERPHTREKPFDCKECEKSFISPASIRRYMVTHSGDGPYKCKFCGKALDCLSLYLTHERTHTGEKRYECKQCGKAFSWHSSV
RIHERTHTGEKPYECKECGKSFNFSSSFRRHERTHTGEKPYKCKECGKAFNCPSSFHRHERTHTGEKPYECKLYGKALSRLISFRRH
MRMHTGERPHKCKICGKAFYSPSSFQRHERSHTGEKPYKCKQCGKAFTCSTSFQYHERTHTGEKPDGCKQCGKAFRSAKYIRIHGRT
HTGEKPYECKQCGKAFHCVSSFHRHERTHAGEKPYECKHCGKAFTCSIYIRIHERIHTGEKPYQCKECGKAFIRSSYCRKHERTHTI
NI
>sp|P29728|OAS2_HUMAN
MGNGESQLSSVPAQKLGWFIQEYLKPYEECQTLIDEMVNTICDVLQEPEQFPLVQGVAIGGSYGRKTVLRGNSDGTLVLFFSDLKQF
QDQKRSQRDILDKTGDKLKFCLFTKWLKNNFEIQKSLDGFTIQVFTKNQRISFEVLAAFNALSLNDNPSPWIYRELKRSLDKTNASP
GEFAVCFTELQQKFFDNRPGKLKDLILLIKHWHQQCQKKIKDLPSLSPYALELLTVYAWEQGCRKDNFDIAEGVRTVLELIKCQEKL
CIYWMVNYNFEDETIRNILLHQLQSARPVILDPVDPTNNVSGDKICWQWLKKEAQTWLTSPNLDNELPAPSWNVLPAPLFTTPGHLL
DKFIKEFLQPNKCFLEQIDSAVNIIRTFLKENCFRQSTAKIQIVRGGSTAKGTALKTGSDADLVVFHNSLKSYTSQKNERHKIVKEI
HEQLKAFWREKEEELEVSFEPPKWKAPRVLSFSLKSKVLNESVSFDVLPAFNALGQLSSGSTPSPEVYAGLIDLYKSSDLPGGEFST
CFTVLQRNFIRSRPTKLKDLIRLVKHWYKECERKLKPKGSLPPKYALELLTIYAWEQGSGVPDFDTAEGFRTVLELVTQYQQLCIFW
KVNYNFEDETVRKFLLSQLQKTRPVILDPAEPTGDVGGGDRWCWHLLAKEAKEWLSSPCFKDGTGNPIPPWKVPTMQTPGSCGARIH
PIVNEMFSSRSHRILNNNSKRNF
>sp|O95168|NDUB4_HUMAN
MSFPKYKPSSLRTLPETLDPAEYNISPETRRAQAERLAIRAQLKREYLLQYNDPNRRGLIENPALLRWAYARTINVYPNFRPTPKNS
LMGALCGFGPLIFIYYIIKTERDRKEKLIQEGKLDRTFHLSY
>sp|Q13323|BIK_HUMAN
MSEVRPLSRDILMETLLYEQLLEPPTMEVLGMTDSEEDLDPMEDFDSLECMEGSDALALRLACIGDEMDVSLRAPRLAQLSEVAMHS
LGLAFIYDQTEDIRDVLRSFMDGFTTLKENIMRFWRSPNPGSWVSCEQVLLALLLLLALLLPLLSGGLHLLLK
>sp|Q96B96|TM159_HUMAN
MAKEEPQSISRDLQELQKKLSLLIDSFQNNSKVVAFMKSPVGQYLDSHPFLAFTLLVFIVMSAVPVGFFLLIVVLTTLAALLGVIIL
EGLVISVGGFSLLCILCGLGFVSLAMSGMMIASYVVVSSLISCWFSPRPLTQQNTSCDFLPAMKSAEFEGLYQE
>sp|Q86TU6|CN070_HUMAN

MGIGTGHTSMNKGGKDVTLLELSVEKRRWRINMETSKIILEKMQSDDVLDGNRERSNEREGRDSLSEKLKSKQNLKDEEKLRYIKTG
KSIQVEGTVRAKALRWVQ
>sp|Q8TBK6|ZCH10_HUMAN
MATPMHRLIARRQAFDTELQPVKTFWILIQPSIVISEANKQHVRCQKCLEFGHWTYECTGKRKYLHRPSRTAELKKALKEKENRLLL
QQSIGETNVERKAKKKRSKSVTSSSSSSSDSSASDSSSESEETSTSSSSEDSDTDESSSSSSSSASSTTSSSSSDSDSDSSSSSSSS
TSTDSSSDDEPPKKKKKK
>sp|Q9NRL2|BAZ1A_HUMAN
MPLLHRKPFVRQKPPADLRPDEEVFYCKVTNEIFRHYDDFFERTILCNSLVWSCAVTGRPGLTYQEALESEKKARQNLQSFPEPLII
PVLYLTSLTHRSRLHEICDDIFAYVKDRYFVEETVEVIRNNGARLQCRILEVLPPSHQNGFANGHVNSVDGETIIISDSDDSETQSC
SFQNGKKKDAIDPLLFKYKVQPTKKELHESAIVKATQISRRKHLFSRDKLKLFLKQHCEPQDGVIKIKASSLSTYKIAEQDFSYFFP
DDPPTFIFSPANRRRGRPPKRIHISQEDNVANKQTLASYRSKATKERDKLLKQEEMKSLAFEKAKLKREKADALEAKKKEKEDKEKK
REELKKIVEEERLKKKEEKERLKVEREKEREKLREEKRKYVEYLKQWSKPREDMECDDLKELPEPTPVKTRLPPEIFGDALMVLEFL
NAFGELFDLQDEFPDGVTLEVLEEALVGNDSEGPLCELLFFFLTAIFQAIAEEEEEVAKEQLTDADTKDLTEALDEDADPTKSALSA
VASLAAAWPQLHQGCSLKSLDLDSCTLSEILRLHILASGADVTSANAKYRYQKRGGFDATDDACMELRLSNPSLVKKLSSTSVYDLT
PGEKMKILHALCGKLLTLVSTRDFIEDYVDILRQAKQEFRELKAEQHRKEREEAAARIRKRKEEKLKEQEQKMKEKQEKLKEDEQRN
STADISIGEEEREDFDTSIESKDTEQKELDQDMVTEDEDDPGSHKRGRRGKRGQNGFKEFTRQEQINCVTREPLTADEEEALKQEHQ
RKEKELLEKIQSAIACTNIFPLGRDRMYRRYWIFPSIPGLFIEEDYSGLTEDMLLPRPSSFQNNVQSQDPQVSTKTGEPLMSESTSN
IDQGPRDHSVQLPKPVHKPNRWCFYSSCEQLDQLIEALNSRGHRESALKETLLQEKSRICAQLARFSEEKFHFSDKPQPDSKPTYSR
GRSSNAYDPSQMCAEKQLELRLRDFLLDIEDRIYQGTLGAIKVTDRHIWRSALESGRYELLSEENKENGIIKTVNEDVEEMEIDEQT
KVIVKDRLLGIKTETPSTVSTNASTPQSVSSVVHYLAMALFQIEQGIERRFLKAPLDASDSGRSYKTVLDRWRESLLSSASLSQVFL
HLSTLDRSVIWSKSILNARCKICRKKGDAENMVLCDGCDRGHHTYCVRPKLKTVPEGDWFCPECRPKQRSRRLSSRQRPSLESDEDV
EDSMGGEDDEVDGDEEEGQSEEEEYEVEQDEDDSQEEEEVSLPKRGRPQVRLPVKTRGKLSSSFSSRGQQQEPGRYPSRSQQSTPKT
TVSSKTGRSLRKINSAPPTETKSLRIASRSTRHSHGPLQADVFVELLSPRRKRRGRKSANNTPENSPNFPNFRVIATKSSEQSRSVN
IASKLSLQESESKRRCRKRQSPEPSPVTLGRRSSGRQGGVHELSAFEQLVVELVRHDDSWPFLKLVSKIQVPDYYDIIKKPIALNII
REKVNKCEYKLASEFIDDIELMFSNCFEYNPRNTSEAKAGTRLQAFFHIQAQKLGLHVTPSNVDQVSTPPAAKKSRI
>sp|O94810|RGS11_HUMAN
MAAGPAPPPGRPRAQMPHLRKMERVVVSMQDPDQGVKMRSQRLLVTVIPHAVTGSDVVQWLAQKFCVSEEEALHLGAVLVQHGYIYP
LRDPRSLMLRPDETPYRFQTPYFWTSTLRPAAELDYAIYLAKKNIRKRGTLVDYEKDCYDRLHKKINHAWDLVLMQAREQLRAAKQR
SKGDRLVIACQEQTYWLVNRPPPGAPDVLEQGPGRGSCAASRVLMTKSADFHKREIEYFRKALGRTRVKSSVCLEAYLSFCGQRGPH
DPLVSGCLPSNPWISDNDAYWVMNAPTVAAPTKLRVERWGFSFRELLEDPVGRAHFMDFLGKEFSGENLSFWEACEELRYGAQAQVP
TLVDAVYEQFLAPGAAHWVNIDSRTMEQTLEGLRQPHRYVLDDAQLHIYMLMKKDSYPRFLKSDMYKALLAEAGIPLEMKRRVFPFT
WRPRHSSPSPALLPTPVEPTAACGPGGGDGVA
>sp|Q6P1M3|L2GL2_HUMAN
MRRFLRPGHDPVRERLKRDLFQFNKTVEHGFPHQPSALGYSPSLRILAIGTRSGAIKLYGAPGVEFMGLHQENNAVTQIHLLPGQCQ
LVTLLDDNSLHLWSLKVKGGASELQEDESFTLRGPPGAAPSATQITVVLPHSSCELLYLGTESGNVFVVQLPAFRALEDRTISSDAV
LQRLPEEARHRRVFEMVEALQEHPRDPNQILIGYSRGLVVIWDLQGSRVLYHFLSSQQLENIWWQRDGRLLVSCHSDGSYCQWPVSS
EAQQPEPLRSLVPYGPFPCKAITRILWLTTRQGLPFTIFQGGMPRASYGDRHCISVIHDGQQTAFDFTSRVIGFTVLTEADPAATFD
DPYALVVLAEEELVVIDLQTAGWPPVQLPYLASLHCSAITCSHHVSNIPLKLWERIIAAGSRQNAHFSTMEWPIDGGTSLTPAPPQR
DLLLTGHEDGTVRFWDASGVCLRLLYKLSTVRVFLTDTDPNENFSAQGEDEWPPLRKVGSFDPYSDDPRLGIQKIFLCKYSGYLAVA
GTAGQVLVLELNDEAAEQAVEQVEADLLQDQEGYRWKGHERLAARSGPVRFEPGFQPFVLVQCQPPAVVTSLALHSEWRLVAFGTSH
GFGLFDHQQRRQVFVKCTLHPSDQLALEGPLSRVKSLKKSLRQSFRRMRRSRVSSRKRHPAGPPGEAQEGSAKAERPGLQNMELAPV
QRKIEARSAEDSFTGFVRTLYFADTYLKDSSRHCPSLWAGTNGGTIYAFSLRVPPAERRMDEPVRAEQAKEIQLMHRAPVVGILVLD
GHSVPLPEPLEVAHDLSKSPDMQGSHQLLVVSEEQFKVFTLPKVSAKLKLKLTALEGSRVRRVSVAHFGSRRAEDYGEHHLAVLTNL

GDIQVVSLPLLKPQVRYSCIRREDVSGIASCVFTKYGQGFYLISPSEFERFSLSTKWLVEPRCLVDSAETKNHRPGNGAGPKKAPSR
ARNSGTQSDGEEKQPGLVMERALLSDERVLKEIQSTLEGDRGSGNWRSHRAAVGCSLSNGGAE
>sp|Q9NXE4|NSMA3_HUMAN
MAFPHLQQPSFLLASLKADSINKPFAQQCQDLVKVIEDFPAKELHTIFPWLVESIFGSLDGVLVGWNLRCLQGRVNPVEYSIVMEFL
DPGGPMMKLVYKLQAEDYKFDFPVSYLPGPVKASIQECILPDSPLYHNKVQFTPTGGLGLNLALNPFEYYIFFFALSLITQKPLPVS
LHVRTSDCAYFILVDRYLSWFLPTEGSVPPPLSSSPGGTSPSPPPRTPAIPFASYGLHHTSLLKRHISHQTSVNADPASHEIWRSET
LLQVFVEMWLHHYSLEMYQKMQSPHAKLEVLHYRLSVSSALYSPAQPSLQALHAYQESFTPTEEHVLVVRLLLKHLHAFANSLKPEQ
ASPSAHSHATSPLEEFKRAAVPRFVQQKLYLFLQHCFGHWPLDASFRAVLEMWLSYLQPWRYAPDKQAPGSDSQPRCVSEKWAPFVQ
ENLLMYTKLFVGFLNRALRTDLVSPKHALMVFRVAKVFAQPNLAEMIQKGEQLFLEPELVIPHRQHRLFTAPTFTGSFLSPWPPAVT
DASFKVKSHVYSLEGQDCKYTPMFGPEARTLVLRLAQLITQAKHTAKSISDQCAESPAGHSFLSWLGFSSMDTNGSYTANDLDEMGQ
DSVRKTDEYLEKALEYLRQIFRLSEAQLRQFTLALGTTQDENGKKQLPDCIVGEDGLILTPLGRYQIINGLRRFEIEYQGDPELQPI
RSYEIASLVRTLFRLSSAINHRFAGQMAALCSRDDFLGSFCRYHLTEPGLASRHLLSPVGRRQVAGHTRGPRLSLRFLGSYRTLVSL
LLAFFVASLFCVGPLPCTLLLTLGYVLYASAMTLLTERGKLHQP
>sp|Q9UPM6|LHX6_HUMAN
MAQPGSGCKATTRCLEGTAPPAMAQSDAEALAGALDKDEGQASPCTPSTPSVCSPPSAASSVPSAGKNICSSCGLEILDRYLLKVNN
LIWHVRCLECSVCRTSLRQQNSCYIKNKEIFCKMDYFSRFGTKCARCGRQIYASDWVRRARGNAYHLACFACFSCKRQLSTGEEFGL
VEEKVLCRIHYDTMIENLKRAAENGNGLTLEGAVPSEQDSQPKPAKRARTSFTAEQLQVMQAQFAQDNNPDAQTLQKLADMTGLSRR
VIQVWFQNCRARHKKHTPQHPVPPSGAPPSRLPSALSDDIHYTPFSSPERARMVTLHGYIESQVQCGQVHCRLPYTAPPVHLKADMD
GPLSNRGEKVILFQY
>sp|Q9Y6D0|SELK_HUMAN
MVYISNGQVLDSRSQSPWRLSLITDFFWGIAEFVVLFFKTLLQQDVKKRRSYGNSSDSRYDDGRGPPGNPPRRMGRINHLRGPSPPP
MAGGUGR
>sp|P62683|GAK21_HUMAN
MGQTKSKIKSKYASYLSFIKILLKRGGVKVSTKNLIKLFQIIEQFCPWFPEQGTLDLKDWKRIGKELKQAGRKGNIIPLTVWNDWAI
IKAALEPFQTEEDSISVSDAPGSCIIDCNENTRKKSQKETEGLHCEYAAEPVMAQSTQNVDYNQLQEVIYPETLKLEGKGPELVGPS
ESKPRGTSPLPAGQVPVTLQPQTQVKENKTQPPVAYQYWPPAELQYRPPPESQYGYPGMPPAPQGRAPYPQPPTRRLNPTAPPSRQG
SELHEIIDKSRKEGDTEAWQFPVMLEPMPPGEGAQEGEPPTVEARYKSFSIKMLKDMKEGVKQYGPNSPYMRTLLDSIAHGHRLIPY
DWEILAKSSLLPSQFLQFKTWWIDGVQEQVQRNRAANPPVNIDADQLLGIGQNWSTISQQALMQNEAIEQVRAICLRAWEKIQDPGS
TCPSFNTVRQSSKEPYPDFVARLQDVAQKSIADEKARKVIVELMAYENANPECQSAIKPLKGKVPAGSDVISEYVKACDGIGGAMHK
AMLMAQAITGVVLGGQVRTFGGKCYNCGQIGHLKKNCPVLNKQNITIQATTTGREPPDLCPRCKKGKHWASQCRSKFDKNGQPLSGN
EQRGQPQAPQQTGAFPIQPFVPQGFQGQQPPLSQVFQGISQLPQYNNCPPPQAAVQQ
>sp|O15393|TMPS2_HUMAN
MALNSGSPPAIGPYYENHGYQPENPYPAQPTVVPTVYEVHPAQYYPSPVPQYAPRVLTQASNPVVCTQPKSPSGTVCTSKTKKALCI
TLTLGTFLVGAALAAGLLWKFMGSKCSNSGIECDSSGTCINPSNWCDGVSHCPGGEDENRCVRLYGPNFILQVYSSQRKSWHPVCQD
DWNENYGRAACRDMGYKNNFYSSQGIVDDSGSTSFMKLNTSAGNVDIYKKLYHSDACSSKAVVSLRCIACGVNLNSSRQSRIVGGES
ALPGAWPWQVSLHVQNVHVCGGSIITPEWIVTAAHCVEKPLNNPWHWTAFAGILRQSFMFYGAGYQVEKVISHPNYDSKTKNNDIAL
MKLQKPLTFNDLVKPVCLPNPGMMLQPEQLCWISGWGATEEKGKTSEVLNAAKVLLIETQRCNSRYVYDNLITPAMICAGFLQGNVD
SCQGDSGGPLVTSKNNIWWLIGDTSWGSGCAKAYRPGVYGNVMVFTDWIYRQMRADG
>sp|Q9Y2U8|MAN1_HUMAN
MAAAAASAPQQLSDEELFSQLRRYGLSPGPVTESTRPVYLKKLKKLREEEQQQHRSGGRGNKTRNSNNNNTAAATVAAAGPAAAAAA
GMGVRPVSGDLSYLRTPGGLCRISASGPESLLGGPGGASAAPAAGSKVLLGFSSDESDVEASPRDQAGGGGRKDRASLQYRGLKAPP
APLAASEVTNSNSAERRKPHSWWGARRPAGPELQTPPGKDGAVEDEEGEGEDGEERDPETEEPLWASRTVNGSRLVPYSCRENYSDS
EEEDDDDVASSRQVLKDDSLSRHRPRRTHSKPLPPLTAKSAGGRLETSVQGGGGLAMNDRAAAAGSLDRSRNLEEAAAAEQGGGCDQ
VDSSPVPRYRVNAKKLTPLLPPPLTDMDSTLDSSTGSLLKTNNHIGGGAFSVDSPRIYSNSLPPSAAVAASSSLRINHANHTGSNHT

YLKNTYNKPKLSEPEEELLQQFKREEVSPTGSFSAHYLSMFLLTAACLFFLILGLTYLGMRGTGVSEDGELSIENPFGETFGKIQES
EKTLMMNTLYKLHDRLAQLAGDHECGSSSQRTLSVQEAAAYLKDLGPEYEGIFNTSLQWILENGKDVGIRCVGFGPEEELTNITDVQ
FLQSTRPLMSFWCRFRRAFVTVTHRLLLLCLGVVMVCVVLRYMKYRWTKEEEETRQMYDMVVKIIDVLRSHNEACQENKDLQPYMPI
PHVRDSLIQPHDRKKMKKVWDRAVDFLAANESRVRTETRRIGGADFLVWRWIQPSASCDKILVIPSKVWQGQAFHLDRRNSPPNSLT
PCLKIRNMFDPVMEIGDQWHLAIQEAILEKCSDNDGIVHIAVDKNSREGCVYVKCLSPEYAGKAFKALHGSWFDGKLVTVKYLRLDR
YHHRFPQALTSNTPLKPSNKHMNSMSHLRLRTGLTNSQGSS
>sp|Q96PV6|LENG8_HUMAN
MAANVGDQRSTDWSSQYSMVAGAGRENGMETPMHENPEWEKARQALASISKSGAAGGSAKSSSNGPVASAQYVSQAEASALQQQQYY
QWYQQYNYAYPYSYYYPMPPVPGMDESMSYQAPPQQLPSAQPPQPSNPPHGAHTLNSGPQPGTAPATQHSQAGPATGQAYGPHTYTE
PAKPKKGQQLWNRMKPAPGTGGLKFNIQKRPFAVTTQSFGSNAEGQHSGFGPQPNPEKVQNHSGSSARGNLSGKPDDWPQDMKEYVE
RCFTACESEEDKDRTEKLLKEVLQARLQDGSAYTIDWSREPLPGLTREPVAESPKKKRWEAASSLHPPRGAGSATRGGGAPSQRGTP
GAGGAGRARGNSFTKFGNRNVFMKDNSSSSSTDSRSRSSSRSPTRHFRRSDSHSDSDSSYSGNECHPVGRRNPPPKGRGGRGAHMDR
GRGRAQRGKRHDLAPTKRSRKKMAALECEDPERELKKQKRAARFQHGHSRRLRLEPLVLQMSSLESSGADPDWQELQIVGTCPDITK
HYLRLTCAPDPSTVRPVAVLKKSLCMVKCHWKEKQDYAFACEQMKSIRQDLTVQGIRTEFTVEVYETHARIALEKGDHEEFNQCQTQ
LKSLYAENLPGNVGEFTAYRILYYIFTKNSGDITTELAYLTRELKADPCVAHALALRTAWALGNYHRFFRLYCHAPCMSGYLVDKFA
DRERKVALKAMIKTYVVPSSLLPLLFPSFRLVSSCIKLSPCPLLPLPPVFLLIFFSPFPPSLSAFFPWFSTGKTVPLPPSSAM
>sp|Q8N766|EMC1_HUMAN
MAAEWASRFWLWATLLIPAAAVYEDQVGKFDWRQQYVGKVKFASLEFSPGSKKLVVATEKNVIAALNSRTGEILWRHVDKGTAEGAV
DAMLLHGQDVITVSNGGRIMRSWETNIGGLNWEITLDSGSFQALGLVGLQESVRYIAVLKKTTLALHHLSSGHLKWVEHLPESDSIH
YQMVYSYGSGVVWALGVVPFSHVNIVKFNVEDGEIVQQVRVSTPWLQHLSGACGVVDEAVLVCPDPSSRSLQTLALETEWELRQIPL
QSLDLEFGSGFQPRVLPTQPNPVDASRAQFFLHLSPSHYALLQYHYGTLSLLKNFPQTALVSFATTGEKTVAAVMACRNEVQKSSSS
EDGSMGSFSEKSSSKDSLACFNQTYTINLYLVETGRRLLDTTITFSLEQSGTRPERLYIQVFLKKDDSVGYRALVQTEDHLLLFLQQ
LAGKVVLWSREESLAEVVCLEMVDLPLTGAQAELEGEFGKKADGLLGMFLKRLSSQLILLQAWTSHLWKMFYDARKPRSQIKNEINI
DTLARDEFNLQKMMVMVTASGKLFGIESSSGTILWKQYLPNVKPDSSFKLMVQRTTAHFPHPPQCTLLVKDKESGMSSLYVFNPIFG
KWSQVAPPVLKRPILQSLLLPVMDQDYAKVLLLIDDEYKVTAFPATRNVLRQLHELAPSIFFYLVDAEQGRLCGYRLRKDLTTELSW
ELTIPPEVQRIVKVKGKRSSEHVHSQGRVMGDRSVLYKSLNPNLLAVVTESTDAHHERTFIGIFLIDGVTGRIIHSSVQKKAKGPVH
IVHSENWVVYQYWNTKARRNEFTVLELYEGTEQYNATAFSSLDRPQLPQVLQQSYIFPSSISAMEATITERGITSRHLLIGLPSGAI
LSLPKALLDPRRPEIPTEQSREENLIPYSPDVQIHAERFINYNQTVSRMRGIYTAPSGLESTCLVVAYGLDIYQTRVYPSKQFDVLK
DDYDYVLISSVLFGLVFATMITKRLAQVKLLNRAWR
>sp|Q6P2M8|KCC1B_HUMAN
MLLLKKHTEDISSVYEIRERLGSGAFSEVVLAQERGSAHLVALKCIPKKALRGKEALVENEIAVLRRISHPNIVALEDVHESPSHLY
LAMELVTGGELFDRIMERGSYTEKDASHLVGQVLGAVSYLHSLGIVHRDLKPENLLYATPFEDSKIMVSDFGLSKIQAGNMLGTACG
TPGYVAPELLEQKPYGKAVDVWALGVISYILLCGYPPFYDESDPELFSQILRASYEFDSPFWDDISESAKDFIRHLLERDPQKRFTC
QQALRHLWISGDTAFDRDILGSVSEQIRKNFARTHWKRAFNATSFLRHIRKLGQIPEGEGASEQGMARHSHSGLRAGQPPKW
>sp|Q9H898|ZMAT4_HUMAN
MKSSDIDQDLFTDSYCKVCSAQLISESQRVAHYESRKHASKVRLYYMLHPRDGGCPAKRLRSENGSDADMVDKNKCCTLCNMSFTSA
VVADSHYQGKIHAKRLKLLLGEKTPLKTTATPLSPLKPPRMDTAPVVASPYQRRDSDRYCGLCAAWFNNPLMAQQHYDGKKHKKNAA
RVALLEQLGTTLDMGELRGLRRNYRCTICSVSLNSIEQYHAHLKGSKHQTNLKNK
>sp|P52961|NAR1_HUMAN
MQMPAMMSLLLVSVGLMEALQAQSHPITRRDLFSQEIQLDMALASFDDQYAGCAAAMTAALPDLNHTEFQANQVYADSWTLASSQWQ
ERQARWPEWSLSPTRPSPPPLGFRDEHGVALLAYTANSPLHKEFNAAVREAGRSRAHYLHHFSFKTLHFLLTEALQLLGSGQRPPRC
HQVFRGVHGLRFRPAGPRATVRLGGFASASLKHVAAQQFGEDTFFGIWTCLGAPIKGYSFFPGEEEVLIPPFETFQVINASRLAQGP
ARIYLRALGKHSTYNCEYIKDKKCKSGPCHLDNSAMGQSPLSAVWSLLLLLWFLVVRAFPDGPGLL
>sp|P53609|PGTB1_HUMAN

MAATEDERLAGSGEGERLDFLRDRHVRFFQRCLQVLPERYSSLETSRLTIAFFALSGLDMLDSLDVVNKDDIIEWIYSLQVLPTEDR
SNLNRCGFRGSSYLGIPFNPSKAPGTAHPYDSGHIAMTYTGLSCLVILGDDLSRVNKEACLAGLRALQLEDGSFCAVPEGSENDMRF
VYCASCICYMLNNWSGMDMKKAITYIRRSMSYDNGLAQGAGLESHGGSTFCGIASLCLMGKLEEVFSEKELNRIKRWCIMRQQNGYH
GRPNKPVDTCYSFWVGATLKLLKIFQYTNFEKNRNYILSTQDRLVGGFAKWPDSHPDALHAYFGICGLSLMEESGICKVHPALNVST
RTSERLLDLHQSWKTKDSKQCSENVHIST
>sp|P15374|UCHL3_HUMAN
MEGQRWLPLEANPEVTNQFLKQLGLHPNWQFVDVYGMDPELLSMVPRPVCAVLLLFPITEKYEVFRTEEEEKIKSQGQDVTSSVYFM
KQTISNACGTIGLIHAIANNKDKMHFESGSTLKKFLEESVSMSPEERARYLENYDAIRVTHETSAHEGQTEAPSIDEKVDLHFIALV
HVDGHLYELDGRKPFPINHGETSDETLLEDAIEVCKKFMERDPDELRFNAIALSAA
>sp|Q8ND24|RN214_HUMAN
MAASEVAGVVANAPSPPESSSLCASKSDEGLPDGLSTKDSAQKQKNSPLLSVSSQTITKENNRNVHLEHSEQNPGSSAGDTSAAHQV
VLGENLIATALCLSGSGSQSDLKDVASTAGEEGDTSLRESLHPVTRSLKAGCHTKQLASRNCSEEKSPQTSILKEGNRDTSLDFRPV
VSPANGVEGVRVDQDDDQDSSSLKLSQNIAVQTDFKTADSEVNTDQDIEKNLDKMMTERTLLKERYQEVLDKQRQVENQLQVQLKQL
QQRREEEMKNHQEILKAIQDVTIKREETKKKIEKEKKEFLQKEQDLKAEIEKLCEKGRREVWEMELDRLKNQDGEINRNIMEETERA
WKAEILSLESRKELLVLKLEEAEKEAELHLTYLKSTPPTLETVRSKQEWETRLNGVRIMKKNVRDQFNSHIQLVRNGAKLSSLPQIP
TPTLPPPPSETDFMLQVFQPSPSLAPRMPFSIGQVTMPMVMPSADPRSLSFPILNPALSQPSQPSSPLPGSHGRNSPGLGSLVSPHG
PHMPPAASIPPPPGLGGVKASAETPRPQPVDKLEKILEKLLTRFPQCNKAQMTNILQQIKTARTTMAGLTMEELIQLVAARLAEHER
VAASTQPLGRIRALFPAPLAQISTPMFLPSAQVSYPGRSSHAPATCKLCLMCQKLVQPSELHPMACTHVLHKECIKFWAQTNTNDTC
PFCPTLK
>sp|Q9NYY1|IL20_HUMAN
MKASSLAFSLLSAAFYLLWTPSTGLKTLNLGSCVIATNLQEIRNGFSEIRGSVQAKDGNIDIRILRRTESLQDTKPANRCCLLRHLL
RLYLDRVFKNYQTPDHYTLRKISSLANSFLTIKKDLRLCHAHMTCHCGEEAMKKYSQILSHFEKLEPQAAVVKALGELDILLQWMEE
TE
>sp|H0Y354|FA72C_HUMAN
MSTNICSFKDRCVSILCCKFCKQVLSSRGMKAVLLADTEIDLFSTDIPPTNAVDFTGRCYFTKICKCKLKDIACLKCGNIVVYHVIV
PCSSCLLSCNNRHFWMFHSQAVYDINRLDSTGVNVLLRGNLPEIEESTDEDVLNISAEECIR
>sp|Q9UPQ8|DOLK_HUMAN
MTRECPSPAPGPGAPLSGSVLAEAAVVFAVVLSIHATVWDRYSWCAVALAVQAFYVQYKWDRLLQQGSAVFQFRMSANSGLLPASMV
MPLLGLVMKERCQTAGNPFFERFGIVVAATGMAVALFSSVLALGITRPVPTNTCVILGLAGGVIIYIMKHSLSVGEVIEVLEVLLIF
VYLNMILLYLLPRCFTPGEALLVLGGISFVLNQLIKRSLTLVESQGDPVDFFLLVVVVGMVLMGIFFSTLFVFMDSGTWASSIFFHL
MTCVLSLGVVLPWLHRLIRRNPLLWLLQFLFQTDTRIYLLAYWSLLATLACLVVLYQNAKRSSSESKKHQAPTIARKYFHLIVVATY
IPGIIFDRPLLYVAATVCLAVFIFLEYVRYFRIKPLGHTLRSFLSLFLDERDSGPLILTHIYLLLGMSLPIWLIPRPCTQKGSLGGA
RALVPYAGVLAVGVGDTVASIFGSTMGEIRWPGTKKTFEGTMTSIFAQIISVALILIFDSGVDLNYSYAWILGSISTVSLLEAYTTQ
IDNLLLPLYLLILLMA
>sp|Q8TBY0|RBM46_HUMAN
MNEENIDGTNGCSKVRTGIQNEAALLALMEKTGYNMVQENGQRKFGGPPPGWEGPPPPRGCEVFVGKIPRDMYEDELVPVFERAGKI
YEFRLMMEFSGENRGYAFVMYTTKEEAQLAIRILNNYEIRPGKFIGVCVSLDNCRLFIGAIPKEKKKEEILDEMKKVTEGVVDVIVY
PSATDKTKNRGFAFVEYESHRAAAMARRKLIPGTFQLWGHTIQVDWADPEKEVDEETMQRVKVLYVRNLMISTTEETIKAEFNKFKP
GAVERVKKLRDYAFVHFFNREDAVAAMSVMNGKCIDGASIEVTLAKPVNKENTWRQHLNGQISPNSENLIVFANKEESHPKTLGKLP
TLPARLNGQHSPSPPEVERCTYPFYPGTKLTPISMYSLKSNHFNSAVMHLDYYCNKNNWAPPEYYLYSTTSQDGKVLLVYKIVIPAI
ANGSQSYFMPDKLCTTLEDAKELAAQFTLLHLDYNFHRSSINSLSPVSATLSSGTPSVLPYTSRPYSYPGYPLSPTISLANGSHVGQ
RLCISNQASFF
>sp|Q8TBN0|R3GEF_HUMAN

MWSGPPQPDQGLPPPLAAVPVPWKSTDPCQGHRESPGALVETSAGEEAQGQEGPAAAQLDVLRLRSSSMEIREKGSEFLKEELHRAQ
KELKLKDEECERLSKVREQLEQELEELTASLFEEAHKMVREANMKQAASEKQLKEARGKIDMLQAEVTALKTLVITSTPASPNRELH
PQLLSPTKAGPRKGHSRHKSTSSTLCPAVCPAAGHTLTPDREGKEVDTILFAEFQAWRESPTLDKTCPFLERVYREDVGPCLDFTMQ
ELSVLVRAAVEDNTLTIEPVASQTLPTVKVAEVDCSSTNTCALSGLTRTCRHRIRLGDSKSHYYISPSSRARITAVCNFFTYIRYIQ
QGLVRQDAEPMFWEIMRLRKEMSLAKLGFFPQEA
>sp|Q9H074|PAIP1_HUMAN
MSDGFDRAPGAGRGRSRGLGRGGGGPEGGGFPNGAGPAERARHQPPQPKAPGFLQPPPLRQPRTTPPPGAQCEVPASPQRPSRPGAL
PEQTRPLRAPPSSQDKIPQQNSESAMAKPQVVVAPVLMSKLSVNAPEFYPSGYSSSYTESYEDGCEDYPTLSEYVQDFLNHLTEQPG
SFETEIEQFAETLNGCVTTDDALQELVELIYQQATSIPNFSYMGARLCNYLSHHLTISPQSGNFRQLLLQRCRTEYEVKDQAAKGDE
VTRKRFHAFVLFLGELYLNLEIKGTNGQVTRADILQVGLRELLNALFSNPMDDNLICAVKLLKLTGSVLEDAWKEKGKMDMEEIIQR
IENVVLDANCSRDVKQMLLKLVELRSSNWGRVHATSTYREATPENDPNYFMNEPTFYTSDGVPFTAADPDYQEKYQELLEREDFFPD
YEENGTDLSGAGDPYLDDIDDEMDPEIEEAYEKFCLESERKRKQ
>sp|P28472|GBRB3_HUMAN
MWGLAGGRLFGIFSAPVLVAVVCCAQSVNDPGNMSFVKETVDKLLKGYDIRLRPDFGGPPVCVGMNIDIASIDMVSEVNMDYTLTMY
FQQYWRDKRLAYSGIPLNLTLDNRVADQLWVPDTYFLNDKKSFVHGVTVKNRMIRLHPDGTVLYGLRITTTAACMMDLRRYPLDEQN
CTLEIESYGYTTDDIEFYWRGGDKAVTGVERIELPQFSIVEHRLVSRNVVFATGAYPRLSLSFRLKRNIGYFILQTYMPSILITILS
WVSFWINYDASAARVALGITTVLTMTTINTHLRETLPKIPYVKAIDMYLMGCFVFVFLALLEYAFVNYIFFGRGPQRQKKLAEKTAK
AKNDRSKSESNRVDAHGNILLTSLEVHNEMNEVSGGIGDTRNSAISFDNSGIQYRKQSMPREGHGRFLGDRSLPHKKTHLRRRSSQL
KIKIPDLTDVNAIDRWSRIVFPFTFSLFNLVYWLYYVN
>sp|P50336|PPOX_HUMAN
MGRTVVVLGGGISGLAASYHLSRAPCPPKVVLVESSERLGGWIRSVRGPNGAIFELGPRGIRPAGALGARTLLLVSELGLDSEVLPV
RGDHPAAQNRFLYVGGALHALPTGLRGLLRPSPPFSKPLFWAGLRELTKPRGKEPDETVHSFAQRRLGPEVASLAMDSLCRGVFAGN
SRELSIRSCFPSLFQAEQTHRSILLGLLLGAGRTPQPDSALIRQALAERWSQWSLRGGLEMLPQALETHLTSRGVSVLRGQPVCGLS
LQAEGRWKVSLRDSSLEADHVISAIPASVLSELLPAEAAPLARALSAITAVSVAVVNLQYQGAHLPVQGFGHLVPSSEDPGVLGIVY
DSVAFPEQDGSPPGLRVTVMLGGSWLQTLEASGCVLSQELFQQRAQEAAATQLGLKEMPSHCLVHLHKNCIPQYTLGHWQKLESARQ
FLTAHRLPLTLAGASYEGVAVNDCIESGRQAAVSVLGTEPNS
>sp|P60604|UB2G2_HUMAN
MAGTALKRLMAEYKQLTLNPPEGIVAGPMNEENFFEWEALIMGPEDTCFEFGVFPAILSFPLDYPLSPPKMRFTCEMFHPNIYPDGR
VCISILHAPGDDPMGYESSAERWSPVQSVEKILLSVVSMLAEPNDESGANVDASKMWRDDREQFYKIAKQIVQKSLGL
>sp|Q13011|ECH1_HUMAN
MAAGIVASRRLRDLLTRRLTGSNYPGLSISLRLTGSSAQEEASGVALGEAPDHSYESLRVTSAQKHVLHVQLNRPNKRNAMNKVFWR
EMVECFNKISRDADCRAVVISGAGKMFTAGIDLMDMASDILQPKGDDVARISWYLRDIITRYQETFNVIERCPKPVIAAVHGGCIGG
GVDLVTACDIRYCAQDAFFQVKEVDVGLAADVGTLQRLPKVIGNQSLVNELAFTARKMMADEALGSGLVSRVFPDKEVMLDAALALA
AEISSKSPVAVQSTKVNLLYSRDHSVAESLNYVASWNMSMLQTQDLVKSVQATTENKELKTVTFSKL
>sp|O14521|DHSD_HUMAN
MAVLWRLSAVCGALGGRALLLRTPVVRPAHISAFLQDRPIPEWCGVQHIHLSPSHHSGSKAASLHWTSERVVSVLLLGLLPAAYLNP
CSAMDYSLAAALTLHGHWGLGQVVTDYVHGDALQKAAKAGLLALSALTFAGLCYFNYHDVGICKAVAMLWKL
>sp|Q9HCK1|ZDBF2_HUMAN
MQKRQGYCSYCRVQYNNLEQHLFSAQHRSLTRQSRRQICTSSLMERFLQDVLQHHPYHCQESSSTQDETHVNTGSSSEVVHLDDAFS
EEEEEDEDKVEDEDATEERPSEVSEPIEELHSRPHKSQEGTQEVSVRPSVIQKLEKGQQQPLEFVHKIGASVRKCNLVDIGQATNNR
SNLVRPPVICNAPASCLPESSNDRPVTANTTSLPPAAHLDSVSKCDPNKVEKYLEQPDGASRNPVPSSHVETTSFSYQKHKESNRKS
LRMNSDKLVLWKDVKSQGKTLSAGLKFHERMGTKGSLRVKSPSKLAVNPNKTDMPSNKGIFEDTIAKNHEEFFSNMDCTQEEKHLVF
NKTAFWEQKCSVSSEMKFDCISLQSASDQPQETAQDLSLWKEEQIDQEDNYESRGSEMSFDCSSSFHSLTDQSKVSAKEVNLSKEVR
TDVQYKNNKSYVSKISSDCDDILHLVTNQSQMIVKEISLQNARHISLVDQSYESSSSETNFDCDASPQSTSDYPQQSVTEVNLPKEV

HIGLVDKNYGSSSSEVSADSVFPLQSVVDRPPVAVTETKLRKKAHTSLVDNYGSSCSETSFDCDVSLESVVDHPQLTVKGRNLKGRQ
VHLKHKKRKPSSAKAHLDCDVSLGTVADESQRAVEKINLLKEKNADLMDMNCESHGPEMGFQADAQLADQSQVAEIERQKVDVDLEN
KSVQSSRSSLSSDSPASLYHSAHDEPQEALDEVNLKELNIDMEVRSYDCSSSELTFDSDPPLLSVTEQSHLDAEGKERHIDLEDESC
ESDSSEITFDSDIPLYSVIDQPEVAVYEEETVDLESKSNESCVSEITFDSDIPLHSGNDHPEVAVKEVIQKEEYIHLERKNDEPSGS
EISSDSHAPLHSVTNSPEVAVKKLNPQKEEQVHLENKENEPIDSEVSLDYNIIFHSVTGRSEDPIKEISLHTKEHMYLENKSVFETS
LDSDVPLQAATHKPEVIVKETWLQREKHAEFQGRSTEFSGSKTSLDSGVPHYSVTEPQVAVNKINRKKQYVLENKNDKCSGSEIILD
SNVPPQSMTDQPQLAFLKEKHVNLKDKNSKSGDSKITFDSEQLQEAVKKIDQWKEEVIGLKNKINEPSTYKLIHHPDVSVQSVADQP
KVAIKHVNLGNENHMYLEVKNSQYSCSEMNLDSGFLGQSIVNRPQITILEQEHIELEGKHNQCCGSEVSFDSDDPLQSVADRLRETV
KEISLWKDEEVDTEDRRNEAKGFEIMYDSDVLQPVAGQPEEVVKEVSLWKEHVDLENKIVKPTDSRINFDSHEPLQSVTNKIPGANK
EINLLREEHVCLDDKGYVPSDSEIIYVSNIPLQSVIKQPHILEEEHASLEDKSSNSYSPEESSDSNDSFQAAADELQKPVKEINLWK
EDHIYLEDKSYKLGDFDVSYASHIPVQFVTDQSSVPVKEINLQKKDHNDLENKNCEVCGSEIKCHSCVHLQSEVDQPQVSYKEADLQ
KEEHVVMEEKTDQPSDSEMMYDSDVPFQIVVNQFPGSVKETHLPKVVLVDLVPGDSDYEVISDDIPLQLVTDPPQLTVKDISCINTE
CIDIEDKSCDFFGSEVRCNCKASTPSMTNQCKETFKIINRKKDYIILGEPSCQSCGSEMNFNVDASDQSMTYESQGPDEKMVKYIDS
EDKSCGYNGSKGKFNLEDTSHRTTHRLQKAHKEASLRKDPRNAGLKGKSCQSSASAVDFGASSKSALHRRADKKKRSKLKHRDLEVS
CEPDGFEMNFQCAPPLPSDTDQPQETVKKRHPCKKVSSDLKEKNHDSQSSSVLKVDSVRNLKKAKDVIEDNPDEPVLEALPHVPPSF
VGKTWSQIMREDDIKINALVKEFREGRFHCYFDDDCETKKVSSKGKKKVTWADLQGKEDTAPTQAVSESDDIVCGISDIDDLSVALD
KPCHRHPPAERPPKQKGRVASQCQTAKISHSTQTSCKNYPVMKRKIIRQEEDPPKSKCSRLQDDRKTKKKVKIGTVEFPASCTKVLK
PMQPKALVCVLSSLNIKLKEGEGLPFPKMRHHSWDNDIRFICKYKRNIFDYYEPLIKQIVISPPLSVIVPEFERRNWVKIHFNRSNQ
NSSAGDNDADGQGSASAPLMAVPARYGFNSHQGTSDSSLFLEESKVLHARELPKKRNFQLTFLNHDVVKISPKSVRNKLLESQSKKK
IHGKRVTTSSNKLGFPKKVYKPIILQQKPRKASEKQSIWIRTKPSDIIRKYISKYSVFLRHRYQSRSAFLGRYLKKKKSVVSRLKKA
KRTAKVLLNSSVPPAGAEELSSAMANPPPKRPVRASCRVARRRKKTDESYHGRQKGPSTPVRAYDLRSSSCLQQRERMMTRLANKLR
GNEVK
>sp|Q9NZN3|EHD3_HUMAN
MFSWLGTDDRRRKDPEVFQTVSEGLKKLYKSKLLPLEEHYRFHEFHSPALEDADFDNKPMVLLVGQYSTGKTTFIRYLLEQDFPGMR
IGPEPTTDSFIAVMQGDMEGIIPGNALVVDPKKPFRKLNAFGNAFLNRFVCAQLPNPVLESISVIDTPGILSGEKQRISRGYDFAAV
LEWFAERVDRIILLFDAHKLDISDEFSEVIKALKNHEDKMRVVLNKADQIETQQLMRVYGALMWSLGKIVNTPEVIRVYIGSFWSHP
LLIPDNRKLFEAEEQDLFRDIQSLPRNAALRKLNDLIKRARLAKVHAYIISSLKKEMPSVFGKDNKKKELVNNLAEIYGRIEREHQI
SPGDFPNLKRMQDQLQAQDFSKFQPLKSKLLEVVDDMLAHDIAQLMVLVRQEESQRPIQMVKGGAFEGTLHGPFGHGYGEGAGEGID
DAEWVVARDKPMYDEIFYTLSPVDGKITGANAKKEMVRSKLPNSVLGKIWKLADIDKDGMLDDDEFALANHLIKVKLEGHELPNELP
AHLLPPSKRKVAE
>sp|P85298|RHG08_HUMAN
MAGQDPALSTSHPFYDVARHGILQVAGDDRFGRRVVTFSCCRMPPSHELDHQRLLEYLKYTLDQYVENDYTIVYFHYGLNSRNKPSL
GWLQSAYKEFDRKDGDLTMWPRLVSNSKLKRSSHLSLPKYWDYRYKKNLKALYVVHPTSFIKVLWNILKPLISHKFGKKVIYFNYLS
ELHEHLKYDQLVIPPEVLRYDEKLQSLHEGRTPPPTKTPPPRPPLPTQQFGVSLQYLKDKNQGELIPPVLRFTVTYLREKGLRTEGL
FRRSASVQTVREIQRLYNQGKPVNFDDYGDIHIPAVILKTFLRELPQPLLTFQAYEQILGITCVESSLRVTGCRQILRSLPEHNYVV
LRYLMGFLHAVSRESIFNKMNSSNLACVFGLNLIWPSQGVSSLSALVPLNMFTELLIEYYEKIFSTPEAPGEHGLAPWEQGSRAAPL
QEAVPRTQATGLTKPTLPPSPLMAARRRL
>sp|Q9UM00|TMCO1_HUMAN
MSTMFADTLLIVFISVCTALLAEGITWVLVYRTDKYKRLKAEVEKQSKKLEKKKETITESAGRQQKKKIERQEEKLKNNNRDLSMVR
MKSMFAIGFCFTALMGMFNSIFDGRVVAKLPFTPLSYIQGLSHRNLLGDDTTDCSFIFLYILCTMSIRQNIQKILGLAPSRAATKQA
GGFLGPPPPSGKFS
>sp|Q6AWC2|WWC2_HUMAN
MPRRAGSGQLPLPRGWEEARDYDGKVFYIDHNTRRTSWIDPRDRLTKPLSFADCVGDELPWGWEAGFDPQIGVYYIDHINKTTQIED
PRKQWRGEQEKMLKDYLSVAQDALRTQKELYHVKEQRLALALDEYVRLNDAYKEKSSSHTSLFSGSSSSTKYDPDILKAEISTTRLR

VKKLKRELSQMKQELLYKEQGFETLQQIDKKMSGGQSGYELSEAKAILTELKSIRKAISSGEKEKQDLMQSLAKLQERFHLDQNIGR
SEPDLRCSPVNSHLCLSRQTLDAGSQTSISGDIGVRSRSNLAEKVRLSLQYEEAKRSMANLKIELSKLDSEAWPGALDIEKEKLMLI
NEKEELLKELQFVTPQKRTQDELERLEAERQRLEEELLSVRGTPSRALAERLRLEERRKELLQKLEETTKLTTYLHSQLKSLSASTL
SMSSGSSLGSLASSRGSLNTSSRGSLNSLSSTELYYSSQSDQIDVDYQYKLDFLLQEKSGYIPSGPITTIHENEVVKSPSQPGQSGL
CGVAAAATGHTPPLAEAPKSVASLSSRSSLSSLSPPGSPLVLEGTFPMSSSHDASLHQFTADFEDCELSSHFADISLIENQILLDSD
SGGASQSLSEDKDLNECAREPLYEGTADVEKSLPKRRVIHLLGEKTTCVSAAVSDESVAGDSGVYEAFVKQPSEMEDVTYSEEDVAI
VETAQVQIGLRYNAKSSSFMVIIAQLRNLHAFLIPHTSKVYFRVAVLPSSTDVSCLFRTKVHPPTESILFNDVFRVAISQTALQQKT
LRVDLCSVSKHRREECLAGTQISLADLPFSSEVFTLWYNLLPSKQMPCKKNEENEDSVFQPNQPLVDSIDLDAVSALLARTSAELLA
VEQELAQEEEEESGQEEPRGPDGDWLTMLREASDEIVAEKEAEVKLPEDSSCTEDLSSCTSVPEMNEDGNRKESNCAKDLRSQPPTR
IPTLVDKETNTDEAANDNMAVRPKERSSLSSRQHPFVRSSVIVRSQTFSPGERNQYICRLNRSDSDSSTLAKKSLFVRNSTERRSLR
VKRTVCQSVLRRTTQECPVRTSLDLELDLQASLTRQSRLNDELQALRDLRQKLEELKAQGETDLPPGVLEDERFQRLLKQAEKQAEQ
SKEEQKQGLNAEKLMRQVSKDVCRLREQSQKVPRQVQSFREKIAYFTRAKISIPSLPADDV
>sp|A4D1E9|GTPBA_HUMAN
MVHCSCVLFRKYGNFIDKLRLFTRGGSGGMGYPRLGGEGGKGGDVWVVAQNRMTLKQLKDRYPRKRFVAGVGANSKISALKGSKGKD
CEIPVPVGISVTDENGKIIGELNKENDRILVAQGGLGGKLLTNFLPLKGQKRIIHLDLKLIADVGLVGFPNAGKSSLLSCVSHAKPA
IADYAFTTLKPELGKIMYSDFKQISVADLPGLIEGAHMNKGMGHKFLKHIERTRQLLFVVDISGFQLSSHTQYRTAFETIILLTKEL
ELYKEELQTKPALLAVNKMDLPDAQDKFHELMSQLQNPKDFLHLFEKNMIPERTVEFQHIIPISAVTGEGIEELKNCIRKSLDEQAN
QENDALHKKQLLNLWISDTMSSTEPPSKHAVTTSKMDII
>sp|Q86UE6|LRRT1_HUMAN
MDFLLLGLCLYWLLRRPSGVVLCLLGACFQMLPAAPSGCPQLCRCEGRLLYCEALNLTEAPHNLSGLLGLSLRYNSLSELRAGQFTG
LMQLTWLYLDHNHICSVQGDAFQKLRRVKELTLSSNQITQLPNTTFRPMPNLRSVDLSYNKLQALAPDLFHGLRKLTTLHMRANAIQ
FVPVRIFQDCRSLKFLDIGYNQLKSLARNSFAGLFKLTELHLEHNDLVKVNFAHFPRLISLHSLCLRRNKVAIVVSSLDWVWNLEKM
DLSGNEIEYMEPHVFETVPHLQSLQLDSNRLTYIEPRILNSWKSLTSITLAGNLWDCGRNVCALASWLNNFQGRYDGNLQCASPEYA
QGEDVLDAVYAFHLCEDGAEPTSGHLLSAVTNRSDLGPPASSATTLADGGEGQHDGTFEPATVALPGGEHAENAVQIHKVVTGTMAL
IFSFLIVVLVLYVSWKCFPASLRQLRQCFVTQRRKQKQKQTMHQMAAMSAQEYYVDYKPNHIEGALVIINEYGSCTCHQQPARECEV
>sp|O60519|CRBL2_HUMAN
MDDSKVVGGKVKKPGKRGRKPAKIDLKAKLERSRQSARECRARKKLRYQYLEELVSSRERAICALREELEMYKQWCMAMDQGKIPSE
IKALLTGEEQNKSQQNSSRHTKAGKTDANSNSW
>sp|Q86VK4|ZN410_HUMAN
MLSDELESKPELLVQFVQNTSIPLGQGLVESEAKDITCLSLLPVTEASECSRLMLPDDTTNHSNSSKEVPSSAVLRSLRVNVGPDGE
ETRAQTVQKSPEFLSTSESSSLLQDLQPSDSTSFILLNLTRAGLGSSAEHLVFVQDEAEDSGNDFLSSESTDSSIPWFLRVQELAHD
SLIAATRAQLAKNAKTSSNGENVHLGSGDGQSKDSGPLPQVEKKLKCTVEGCDRTFVWPAHFKYHLKTHRNDRSFICPAEGCGKSFY
VLQRLKVHMRTHNGEKPFMCHESGCGKQFTTAGNLKNHRRIHTGEKPFLCEAQGCGRSFAEYSSLRKHLVVHSGEKPHQCQVCGKTF
SQSGSRNVHMRKHHLQLGAAGSQEQEQTAEPLMGSSLLEEASVPSKNLVSMNSQPSLGGESLNLPNTNSILGVDDEVLAEGSPRSLS
SVPDVTHHLVTMQSGRQSYEVSVLTAVNPQELLNQGDLTERRT
>sp|Q99567|NUP88_HUMAN
MAAAEGPVGDGELWQTWLPNHVVFLRLREGLKNQSPTEAEKPASSSLPSSPPPQLLTRNVVFGLGGELFLWDGEDSSFLVVRLRGPS
GGGEEPALSQYQRLLCINPPLFEIYQVLLSPTQHHVALIGIKGLMVLELPKRWGKNSEFEGGKSTVNCSTTPVAERFFTSSTSLTLK
HAAWYPSEILDPHVVLLTSDNVIRIYSLREPQTPTNVIILSEAEEESLVLNKGRAYTASLGETAVAFDFGPLAAVPKTLFGQNGKDE
VVAYPLYILYENGETFLTYISLLHSPGNIGKLLGPLPMHPAAEDNYGYDACAVLCLPCVPNILVIATESGMLYHCVVLEGEEEDDHT
SEKSWDSRIDLIPSLYVFECVELELALKLASGEDDPFDSDFSCPVKLHRDPKCPSRYHCTHEAGVHSVGLTWIHKLHKFLGSDEEDK
DSLQELSTEQKCFVEHILCTKPLPCRQPAPIRGFWIVPDILGPTMICITSTYECLIWPLLSTVHPASPPLLCTREDVEVAESPLRVL
AETPDSFEKHIRSILQRSVANPAFLKASEKDIAPPPEECLQLLSRATQVFREQYILKQDLAKEEIQRRVKLLCDQKKKQLEDLSYCR

EERKSLREMAERLADKYEEAKEKQEDIMNRMKKLLHSFHSELPVLSDSERDMKKELQLIPDQLRHLGNAIKQVTMKKDYQQQKMEKV
LSLPKPTIILSAYQRKCIQSILKEEGEHIREMVKQINDIRNHVNF
>sp|P98170|XIAP_HUMAN
MTFNSFEGSKTCVPADINKEEEFVEEFNRLKTFANFPSGSPVSASTLARAGFLYTGEGDTVRCFSCHAAVDRWQYGDSAVGRHRKVS
PNCRFINGFYLENSATQSTNSGIQNGQYKVENYLGSRDHFALDRPSETHADYLLRTGQVVDISDTIYPRNPAMYSEEARLKSFQNWP
DYAHLTPRELASAGLYYTGIGDQVQCFCCGGKLKNWEPCDRAWSEHRRHFPNCFFVLGRNLNIRSESDAVSSDRNFPNSTNLPRNPS
MADYEARIFTFGTWIYSVNKEQLARAGFYALGEGDKVKCFHCGGGLTDWKPSEDPWEQHAKWYPGCKYLLEQKGQEYINNIHLTHSL
EECLVRTTEKTPSLTRRIDDTIFQNPMVQEAIRMGFSFKDIKKIMEEKIQISGSNYKSLEVLVADLVNAQKDSMQDESSQTSLQKEI
STEEQLRRLQEEKLCKICMDRNIAIVFVPCGHLVTCKQCAEAVDKCPMCYTVITFKQKIFMS
>sp|P19827|ITIH1_HUMAN
MDGAMGPRGLLLCMYLVSLLILQAMPALGSATGRSKSSEKRQAVDTAVDGVFIRSLKVNCKVTSRFAHYVVTSQVVNTANEAREVAF
DLEIPKTAFISDFAVTADGNAFIGDIKDKVTAWKQYRKAAISGENAGLVRASGRTMEQFTIHLTVNPQSKVTFQLTYEEVLKRNHMQ
YEIVIKVKPKQLVHHFEIDVDIFEPQGISKLDAQASFLPKELAAQTIKKSFSGKKGHVLFRPTVSQQQSCPTCSTSLLNGHFKVTYD
VSRDKICDLLVANNHFAHFFAPQNLTNMNKNVVFVIDISGSMRGQKVKQTKEALLKILGDMQPGDYFDLVLFGTRVQSWKGSLVQAS
EANLQAAQDFVRGFSLDEATNLNGGLLRGIEILNQVQESLPELSNHASILIMLTDGDPTEGVTDRSQILKNVRNAIRGRFPLYNLGF
GHNVDFNFLEVMSMENNGRAQRIYEDHDATQQLQGFYSQVAKPLLVDVDLQYPQDAVLALTQNHHKQYYEGSEIVVAGRIADNKQSS
FKADVQAHGEGQEFSITCLVDEEEMKKLLRERGHMLENHVERLWAYLTIQELLAKRMKVDREERANLSSQALQMSLDYGFVTPLTSM
SIRGMADQDGLKPTIDKPSEDSPPLEMLGPRRTFVLSALQPSPTHSSSNTQRLPDRVTGVDTDPHFIIHVPQKEDTLCFNINEEPGV
ILSLVQDPNTGFSVNGQLIGNKARSPGQHDGTYFGRLGIANPATDFQLEVTPQNITLNPGFGGPVFSWRDQAVLRQDGVVVTINKKR
NLVVSVDDGGTFEVVLHRVWKGSSVHQDFLGFYVLDSHRMSARTHGLLGQFFHPIGFEVSDIHPGSDPTKPDATMVVRNRRLTVTRG
LQKDYSKDPWHGAEVSCWFIHNNGAGLIDGAYTDYIVPDIF
>sp|Q9NZD1|GPC5D_HUMAN
MYKDCIESTGDYFLLCDAEGPWGIILESLAILGIVVTILLLLAFLFLMRKIQDCSQWNVLPTQLLFLLSVLGLFGLAFAFIIELNQQ
TAPVRYFLFGVLFALCFSCLLAHASNLVKLVRGCVSFSWTTILCIAIGCSLLQIIIATEYVTLIMTRGMMFVNMTPCQLNVDFVVLL
VYVLFLMALTFFVSKATFCGPCENWKQHGRLIFITVLFSIIIWVVWISMLLRGNPQFQRQPQWDDPVVCIALVTNAWVFLLLYIVPE
LCILYRSCRQECPLQGNACPVTAYQHSFQVENQELSRARDSDGAEEDVALTSYGTPIQPQTVDPTQECFIPQAKLSPQQDAGGV
>sp|Q9NZ20|PA2G3_HUMAN
MGVQAGLFGMLGFLGVALGGSPALRWYRTSCHLTKAVPGNPLGYLSFLAKDAQGLALIHARWDAHRRLQSCSWEDEPELTAAYGALC
AHETAWGSFIHTPGPELQRALATLQSQWEACRALEESPAGARKKRAAGQSGVPGGGHQREKRGWTMPGTLWCGVGDSAGNSSELGVF
QGPDLCCREHDRCPQNISPLQYNYGIRNYRFHTISHCDCDTRFQQCLQNQHDSISDIVGVAFFNVLEIPCFVLEEQEACVAWYWWGG
CRMYGTVPLARLQPRTFYNASWSSRATSPTPSSRSPAPPKPRQKQHLRKGPPHQKGSKRPSKANTTALQDPMVSPRLDVAPTGLQGP
QGGLKPQGARWVCRSFRRHLDQCEHQIGPREIEFQLLNSAQEPLFHCNCTRRLARFLRLHSPPEVTNMLWELLGTTCFKLAPPLDCV
EGKNCSRDPRAIRVSARHLRRLQQRRHQLQDKGTDERQPWPSEPLRGPMSFYNQCLQLTQAARRPDRQQKSWSQ
>sp|P43686|PRS6B_HUMAN
MEEIGILVEKAQDEIPALSVSRPQTGLSFLGPEPEDLEDLYSRYKKLQQELEFLEVQEEYIKDEQKNLKKEFLHAQEEVKRIQSIPL
VIGQFLEAVDQNTAIVGSTTGSNYYVRILSTIDRELLKPNASVALHKHSNALVDVLPPEADSSIMMLTSDQKPDVMYADIGGMDIQK
QEVREAVELPLTHFELYKQIGIDPPRGVLMYGPPGCGKTMLAKAVAHHTTAAFIRVVGSEFVQKYLGEGPRMVRDVFRLAKENAPAI
IFIDEIDAIATKRFDAQTGADREVQRILLELLNQMDGFDQNVNVKVIMATNRADTLDPALLRPGRLDRKIEFPLPDRRQKRLIFSTI
TSKMNLSEEVDLEDYVARPDKISGADINSICQESGMLAVRENRYIVLAKDFEKAYKTVIKKDEQEHEFYK
>sp|Q8N6C7|PGSF1_HUMAN
MPGMRLVCRLAHGHFPRKGQRRRSLTVWKAETSRADCLGAPNIRTAPLGRSEKRTAICFSTGAQDSSQRAPFRLQNPGQLLQLGMHS
LHLHPELPTTDPAFFCKLHFIKGNDPYCLTISHVKSVLTFS
>sp|O60832|DKC1_HUMAN

MADAEVIILPKKHKKKKERKSLPEEDVAEIQHAEEFLIKPESKVAKLDTSQWPLLLKNFDKLNVRTTHYTPLACGSNPLKREIGDYI
RTGFINLDKPSNPSSHEVVAWIRRILRVEKTGHSGTLDPKVTGCLIVCIERATRLVKSQQSAGKEYVGIVRLHNAIEGGTQLSRALE
TLTGALFQRPPLIAAVKRQLRVRTIYESKMIEYDPERRLGIFWVSCEAGTYIRTLCVHLGLLLGVGGQMQELRRVRSGVMSEKDHMV
TMHDVLDAQWLYDNHKDESYLRRVVYPLEKLLTSHKRLVMKDSAVNAICYGAKIMLPGVLRYEDGIEVNQEIVVITTKGEAICMAIA
LMTTAVISTCDHGIVAKIKRVIMERDTYPRKWGLGPKASQKKLMIKQGLLDKHGKPTDSTPATWKQEYVDYSESAKKEVVAEVVKAP
QVVAEAAKTAKRKRESESESDETPPAAPQLIKKEKKKSKKDKKAKAGLESGAEPGDGDSDTTKKKKKKKKAKEVELVSE
>sp|P32942|ICAM3_HUMAN
MATMVPSVLWPRACWTLLVCCLLTPGVQGQEFLLRVEPQNPVLSAGGSLFVNCSTDCPSSEKIALETSLSKELVASGMGWAAFNLSN
VTGNSRILCSVYCNGSQITGSSNITVYRLPERVELAPLPPWQPVGQNFTLRCQVEDGSPRTSLTVVLLRWEEELSRQPAVEEPAEVT
ATVLASRDDHGAPFSCRTELDMQPQGLGLFVNTSAPRQLRTFVLPVTPPRLVAPRFLEVETSWPVDCTLDGLFPASEAQVYLALGDQ
MLNATVMNHGDTLTATATATARADQEGAREIVCNVTLGGERREARENLTVFSFLGPIVNLSEPTAHEGSTVTVSCMAGARVQVTLDG
VPAAAPGQPAQLQLNATESDDGRSFFCSATLEVDGEFLHRNSSVQLRVLYGPKIDRATCPQHLKWKDKTRHVLQCQARGNPYPELRC
LKEGSSREVPVGIPFFVNVTHNGTYQCQASSSRGKYTLVVVMDIEAGSSHFVPVFVAVLLTLGVVTIVLALMYVFREHQRSGSYHVR
EESTYLPLTSMQPTEAMGEEPSRAE
>sp|O15296|LX15B_HUMAN
MAEFRVRVSTGEAFGAGTWDKVSVSIVGTRGESPPLPLDNLGKEFTAGAEEDFQVTLPEDVGRVLLLRVHKAPPVLPLLGPLAPDAW
FCRWFQLTPPRGGHLLFPCYQWLEGAGTLVLQEGTAKVSWADHHPVLQQQRQEELQARQEMYQWKAYNPGWPHCLDEKTVEDLELNI
KYSTAKNANFYLQAGSAFAEMKIKGLLDRKGLWRSLNEMKRIFNFRRTPAAEHAFEHWQEDAFFASQFLNGLNPVLIRRCHYLPKNF
PVTDAMVASVLGPGTSLQAELEKGSLFLVDHGILSGIQTNVINGKPQFSAAPMTLLYQSPGCGPLLPLAIQLSQTPGPNSPIFLPTD
DKWDWLLAKTWVRNAEFSFHEALTHLLHSHLLPEVFTLATLRQLPHCHPLFKLLIPHTRYTLHINTLARELLIVPGQVVDRSTGIGI
EGFSELIQRNMKQLNYSLLCLPEDIRTRGVEDIPGYYYRDDGMQIWGAVERFVSEIIGIYYPSDESVQDDRELQAWVREIFSKGFLN
QESSGIPSSLETREALVQYVTMVIFTCSAKHAAVSAGQFDSCAWMPNLPPSMQLPPPTSKGLATCEGFIATLPPVNATCDVILALWL
LSKEPGDQRPLGTYPDEHFTEEAPRRSIATFQSRLAQISRGIQERNQGLVLPYTYLDPPLIENSVSI
>sp|Q9BRT6|LLPH_HUMAN
MAKSLRSKWKRKMRAEKRKKNAPKEASRLKSILKLDGDVLMKDVQEIATVVVPKPKHCQEKMQCEVKDEKDDMKMETDIKRNKKTLL
DQHGQYPIWMNQRQRKRLKAKREKRKGKSKAKAVKVAKGLAW
>sp|P36952|SPB5_HUMAN
MDALQLANSAFAVDLFKQLCEKEPLGNVLFSPICLSTSLSLAQVGAKGDTANEIGQVLHFENVKDVPFGFQTVTSDVNKLSSFYSLK
LIKRLYVDKSLNLSTEFISSTKRPYAKELETVDFKDKLEETKGQINNSIKDLTDGHFENILADNSVNDQTKILVVNAAYFVGKWMKK
FSESETKECPFRVNKTDTKPVQMMNMEATFCMGNIDSINCKIIELPFQNKHLSMFILLPKDVEDESTGLEKIEKQLNSESLSQWTNP
STMANAKVKLSIPKFKVEKMIDPKACLENLGLKHIFSEDTSDFSGMSETKGVALSNVIHKVCLEITEDGGDSIEVPGARILQHKDEL
NADHPFIYIIRHNKTRNIIFFGKFCSP
>sp|Q96NJ6|ZFP3_HUMAN
MGTENKEVIPKEEISEESEPHGSLLEKFPKVVYQGHEFGAGCEEDMLEGHSRESMEEVIEQMSPQERDFPSGLMIFKKSPSSEKDRE
NNESERGCSPSPNLVTHQGDTTEGVSAFATSGQNFLEILESNKTQRSSVGEKPHTCKECGKAFNQNSHLIQHMRVHSGEKPFECKEC
GKTFGTNSSLRRHLRIHAGEKPFACNECGKAFIQSSHLIHHHRIHTGERPYKCEECGKAFSQNSALILHQRIHTGEKPYECNECGKT
FRVSSQLIQHQRIHTEERYHECNECGKAFKHSSGLIRHQKIHTGEKPYLCNECGKGFGQSSELIRHQRIHTGDKPYECNECGKTFGQ
NSEIIRHIRIHTGEKPYVCKECGKAFRGNSELLRHERIHTGEKPYECFECGKAFRRTSHLIVHQRIHTGEKPHQCNECARTFWDNSE
LLLHQKIHIGEKPYECSECEKTFSQHSQLIIHQRIHTGEKPYECQECQKTFSRSSHLLRHQSVHCME
>sp|Q9HD33|RM47_HUMAN
MAAAGLALLCRRVSSALKSSRSLITPQVPACTGFFLSLLPKSTPNVTSFHQYRLLHTTLSRKGLEEFFDDPKNWGQEKVKSGAAWTC
QQLRNKSNEDLHKLWYVLLKERNMLLTLEQEAKRQRLPMPSPERLDKVVDSMDALDKVVQEREDALRLLQTGQERARPGAWRRDIFG
RIIWHKFKQWVIPWHLNKRYNRKRFFALPYVDHFLRLEREKRARIKARKENLERKKAKILLKKFPHLAEAQKSSLV
>sp|Q7RTR2|NLRC3_HUMAN

MRKQEVRTGREAGQGHGTGSPAEQVKALMDLLAGKGSQGSQAPQALDRTPDAPLGPCSNDSRIQRHRKALLSKVGGGPELGGPWHRL
ASLLLVEGLTDLQLREHDFTQVEATRGGGHPARTVALDRLFLPLSRVSVPPRVSITIGVAGMGKTTLVRHFVRLWAHGQVGKDFSLV
LPLTFRDLNTHEKLCADRLICSVFPHVGEPSLAVAVPARALLILDGLDECRTPLDFSNTVACTDPKKEIPVDHLITNIIRGNLFPEV
SIWITSRPSASGQIPGGLVDRMTEIRGFNEEEIKVCLEQMFPEDQALLGWMLSQVQADRALYLMCTVPAFCRLTGMALGHLWRSRTG
PQDAELWPPRTLCELYSWYFRMALSGEGQEKGKASPRIEQVAHGGRKMVGTLGRLAFHGLLKKKYVFYEQDMKAFGVDLALLQGAPC
SCFLQREETLASSVAYCFTHLSLQEFVAAAYYYGASRRAIFDLFTESGVSWPRLGFLTHFRSAAQRAMQAEDGRLDVFLRFLSGLLS
PRVNALLAGSLLAQGEHQAYRTQVAELLQGCLRPDAAVCARAINVLHCLHELQHTELARSVEEAMESGALARLTGPAHRAALAYLLQ
VSDACAQEANLSLSLSQGVLQSLLPQLLYCRKLRLDTNQFQDPVMELLGSVLSGKDCRIQKISLAENQISNKGAKALARSLLVNRSL
TSLDLRGNSIGPQGAKALADALKINRTLTSLSLQGNTVRDDGARSMAEALASNRTLSMLHLQKNSIGPMGAQRMADALKQNRSLKEL
MFSSNSIGDGGAKALAEALKVNQGLESLDLQSNSISDAGVAALMGALCTNQTLLSLSLRENSISPEGAQAIAHALCANSTLKNLDLT
ANLLHDQGARAIAVAVRENRTLTSLHLQWNFIQAGAAQALGQALQLNRSLTSLDLQENAIGDDGACAVARALKVNTALTALYLQVAS
IGASGAQVLGEALAVNRTLEILDLRGNAIGVAGAKALANALKVNSSLRRLNLQENSLGMDGAICIATALSGNHRLQHINLQGNHIGD
SGARMISEAIKTNAPTCTVEM
>sp|Q3KNS6|ZN829_HUMAN
MPHSPLISIPHVWCHPEEEERMHDELLQAVSKGPVMFRDVSIDFSQEEWECLDADQMNLYKEVMLENFSNLVSVGLSNSKPAVISLL
EQGKEPWMVDRELTRGLCSDLESMCETKILSLKKRHFSQVIITREDMSTFIQPTFLIPPQKTMSEEKPWECKICGKTFNQNSQFIQH
QRIHFGEKHYESKEYGKSFSRGSLVTRHQRIHTGKKPYECKECGKAFSCSSYFSQHQRIHTGEKPYECKECGKAFKYCSNLNDHQRI
HTGEKPYECKVCGKAFTKSSQLFLHLRIHTGEKPYECKECGKAFTQHSRLIQHQRMHTGEKPYECKQCGKAFNSASTLTNHHRIHAG
EKLYECEECRKAFIQSSELIQHQRIHTDEKPYECNECGKAFNKGSNLTRHQRIHTGEKPYDCKECGKAFGSRSDLIRHEGIHTG
>sp|O43561|LAT_HUMAN
MEEAILVPCVLGLLLLPILAMLMALCVHCHRLPGSYDSTSSDSLYPRGIQFKRPHTVAPWPPAYPPVTSYPPLSQPDLLPIPRSPQP
LGGSHRTPSSRRDSDGANSVASYENEGASGIRGAQAGWGVWGPSWTRLTPVSLPPEPACEDADEDEDDYHNPGYLVVLPDSTPATST
AAPSAPALSTPGIRDSAFSMESIDDYVNVPESGESAEASLDGSREYVNVSQELHPGAAKTEPAALSSQEAEEVEEEGAPDYENLQEL
N
>sp|P62875|RPAB5_HUMAN
MIIPVRCFTCGKIVGNKWEAYLGLLQAEYTEGDALDALGLKRYCCRRMLLAHVDLIEKLLNYAPLEK
>sp|Q8N4S7|PAQR4_HUMAN
MAFLAGPRLLDWASSPPHLQFNKFVLTGYRPASSGSGCLRSLFYLHNELGNIYTHGLALLGFLVLVPMTMPWGQLGKDGWLGGTHCV
ACLAPPAGSVLYHLFMCHQGGSAVYARLLALDMCGVCLVNTLGALPIIHCTLACRPWLRPAALVGYTVLSGVAGWRALTAPSTSARL
RAFGWQAAARLLVFGARGVGLGSGAPGSLPCYLRMDALALLGGLVNVARLPERWGPGRFDYWGNSHQIMHLLSVGSILQLHAGVVPD
LLWAAHHACPRD
>sp|Q9Y2Y9|KLF13_HUMAN
MAAAAYVDHFAAECLVSMSSRAVVHGPREGPESRPEGAAVAATPTLPRVEERRDGKDSASLFVVARILADLNQQAPAPAPAERREGA
AARKARTPCRLPPPAPEPTSPGAEGAAAAPPSPAWSEPEPEAGLEPEREPGPAGSGEPGLRQRVRRGRSRADLESPQRKHKCHYAGC
EKVYGKSSHLKAHLRTHTGERPFACSWQDCNKKFARSDELARHYRTHTGEKKFSCPICEKRFMRSDHLTKHARRHANFHPGMLQRRG
GGSRTGSLSDYSRSDASSPTISPASSP
>sp|Q5T6V5|CI064_HUMAN
MDGLLNPRESSKFIAENSRDVFIDSGGVRRVAELLLAKAAGPELRVEGWKALHELNPRAADEAAVNWVFVTDTLNFSFWSEQDEHKC
VVRYRGKTYSGYWSLCAAVNRALDEGIPITSASYYATVTLDQVRNILRSDTDVSMPLVEERHRILNETGKILLEKFGGSFLNCVRES
ENSAQKLMHLVVESFPSYRDVTLFEGKRVSFYKRAQILVADTWSVLEGKGDGCFKDISSITMFADYRLPQVLAHLGALKYSDDLLKK
LLKGEMLSYGDRQEVEIRGCSLWCVELIRDCLLELIEQKGEKPNGEINSILLDYYLWDYAHDHREDMKGIPFHRIRCIYY
>sp|Q6ZW35|F231D_HUMAN
MGSSKGLWKEKPSAHTSECFSTTACPVACILLVWNSQTPAGLQSLCTGRHPSLSARAQWAGPRASREEGTFWTEPVGQERRLIRSGS
SQNESQEDQGADLISHEGLKADNQRESSTWANEVEDRRPQCTSALNLTPSHLHPPHPLTTFFRNVIGIKIPPGLVAMGGTVA

>sp|P0C7M3|SFTA3_HUMAN
MRAGFSDFQLIRDQVLFLQDQAQRLTEWLQLSGFENPVSESTTLCLREREKRIPTCVAVCVPSPGTVHTALLHPTTLSQSRSSSEAK
MLIIHTA
>sp|Q9HA77|SYCM_HUMAN
MLRTTRGPGLGPPLLQAALGLGRAGWHWPAGRAASGGRGRAWLQPTGRETGVQVYNSLTGRKEPLIVAHAEAASWYSCGPTVYDHAH
LGHACSYVRFDIIRRILTKVFGCSIVMVMGITDVDDKIIKRANEMNISPASLASLYEEDFKQDMAALKVLPPTVYLRVTENIPQIIS
FIEGIIARGNAYSTAKGNVYFDLKSRGDKYGKLVGVVPGPVGEPADSDKRHASDFALWKAAKPQEVFWASPWGPGRPGWHIECSAIA
SMVFGSQLDIHSGGIDLAFPHHENEIAQCEVFHQCEQWGNYFLHSGHLHAKGKEEKMSKSLKNYITIKDFLKTFSPDVFRFFCLRSS
YRSAIDYSDSAMLQAQQLLLGLGSFLEDARAYMKGQLACGSVREAMLWERLSSTKRAVKAALADDFDTPRVVDAILGLAHHGNGQLR
ASLKEPEGPRSPAVFGAIISYFEQFFETVGISLANQQYVSGDGSEATLHGVVDELVRFRQKVRQFALAMPEATGDARRQQLLERQPL
LEACDTLRRGLTAHGINIKDRSSTTSTWELLDQRTKDQKSAG
>sp|Q9NQW8|CNGB3_HUMAN
MFKSLTKVNKVKPIGENNENEQSSRRNEEGSHPSNQSQQTTAQEENKGEEKSLKTKSTPVTSEEPHTNIQDKLSKKNSSGDLTTNPD
PQNAAEPTGTVPEQKEMDPGKEGPNSPQNKPPAAPVINEYADAQLHNLVKRMRQRTALYKKKLVEGDLSSPEASPQTAKPTAVPPVK
ESDDKPTEHYYRLLWFKVKKMPLTEYLKRIKLPNSIDSYTDRLYLLWLLLVTLAYNWNCCFIPLRLVFPYQTADNIHYWLIADIICD
IIYLYDMLFIQPRLQFVRGGDIIVDSNELRKHYRTSTKFQLDVASIIPFDICYLFFGFNPMFRANRMLKYTSFFEFNHHLESIMDKA
YIYRVIRTTGYLLFILHINACVYYWASNYEGIGTTRWVYDGEGNEYLRCYYWAVRTLITIGGLPEPQTLFEIVFQLLNFFSGVFVFS
SLIGQMRDVIGAATANQNYFRACMDDTIAYMNNYSIPKLVQKRVRTWYEYTWDSQRMLDESDLLKTLPTTVQLALAIDVNFSIISKV
DLFKGCDTQMIYDMLLRLKSVLYLPGDFVCKKGEIGKEMYIIKHGEVQVLGGPDGTKVLVTLKAGSVFGEISLLAAGGGNRRTANVV
AHGFANLLTLDKKTLQEILVHYPDSERILMKKARVLLKQKAKTAEATPPRKDLALLFPPKEETPKLFKTLLGGTGKASLARLLKLKR
EQAAQKKENSEGGEEEGKENEDKQKENEDKQKENEDKGKENEDKDKGREPEEKPLDRPECTASPIAVEEEPHSVRRTVLPRGTSRQS
LIISMAPSAEGGEEVLTIEVKEKAKQ
>sp|Q8IWW8|HOT_HUMAN
MAAAARARVAYLLRQLQRAACQCPTHSHTYSQAPGLSPSGKTTDYAFEMAVSNIRYGAAVTKEVGMDLKNMGAKNVCLMTDKNLSKL
PPVQVAMDSLVKNGIPFTVYDNVRVEPTDSSFMEAIEFAQKGAFDAYVAVGGGSTMDTCKAANLYASSPHSDFLDYVSAPIGKGKPV
SVPLKPLIAVPTTSGTGSETTGVAIFDYEHLKVKIGITSRAIKPTLGLIDPLHTLHMPARVVANSGFDVLCHALESYTTLPYHLRSP
CPSNPITRPAYQGSNPISDIWAIHALRIVAKYLKRAVRNPDDLEARSHMHLASAFAGIGFGNAGVHLCHGMSYPISGLVKMYKAKDY
NVDHPLVPHGLSVVLTSPAVFTFTAQMFPERHLEMAEILGADTRTARIQDAGLVLADTLRKFLFDLDVDDGLAAVGYSKADIPALVK
GTLPQERVTKLAPCPQSEEDLAALFEASMKLY
>sp|Q9UHY1|NRBP_HUMAN
MSEGESQTVLSSGSDPKVESSSSAPGLTSVSPPVTSTTSAASPEEEEESEDESEILEESPCGRWQKRREEVNQRNVPGIDSAYLAMD
TEEGVEVVWNEVQFSERKNYKLQEEKVRAVFDNLIQLEHLNIVKFHKYWADIKENKARVIFITEYMSSGSLKQFLKKTKKNHKTMNE
KAWKRWCTQILSALSYLHSCDPPIIHGNLTCDTIFIQHNGLIKIGSVAPDTINNHVKTCREEQKNLHFFAPEYGEVTNVTTAVDIYS
FGMCALEMAVLEIQGNGESSYVPQEAISSAIQLLEDPLQREFIQKCLQSEPARRPTARELLFHPALFEVPSLKLLAAHCIVGHQHMI
PENALEEITKNMDTSAVLAEIPAGPGREPVQTLYSQSPALELDKFLEDVRNGIYPLTAFGLPRPQQPQQEEVTSPVVPPSVKTPTPE
PAEVETRKVVLMQCNIESVEEGVKHHLTLLLKLEDKLNRHLSCDLMPNENIPELAAELVQLGFISEADQSRLTSLLEETLNKFNFAR
NSTLNSAAVTVSS
>sp|O95070|YIF1A_HUMAN
MAYHSGYGAHGSKHRARAAPDPPPLFDDTSGGYSSQPGGYPATGADVAFSVNHLLGDPMANVAMAYGSSIASHGKDMVHKELHRFVS
VSKLKYFFAVDTAYVAKKLGLLVFPYTHQNWEVQYSRDAPLPPRQDLNAPDLYIPTMAFITYVLLAGMALGIQKRFSPEVLGLCAST
ALVWVVMEVLALLLGLYLATVRSDLSTFHLLAYSGYKYVGMILSVLTGLLFGSDGYYVALAWTSSALMYFIVRSLRTAALGPDSMGG
PVPRQRLQLYLTLGAAAFQPLIIYWLTFHLVR
>sp|Q14692|BMS1_HUMAN

MEAKDQKKHRKKNSGPKAAKKKKRLLQDLQLGDEEDARKRNPKAFAVQSAVRMARSFHRTQDLKTKKHHIPVVDRTPLEPPPIVVVV
MGPPKVGKSTLIQCLIRNFTRQKLTEIRGPVTIVSGKKRRLTIIECGCDINMMIDLAKVADLVLMLIDASFGFEMETFEFLNICQVH
GFPKIMGVLTHLDSFKHNKQLKKTKKRLKHRFWTEVYPGAKLFYLSGMVHGEYQNQEIHNLGRFITVMKFRPLTWQTSHPYILADRM
EDLTNPEDIRTNIKCDRKVSLYGYLRGAHLKNKSQIHMPGVGDFAVSDISFLPDPCALPEQQKKRCLNEKEKLVYAPLSGVGGVLYD
KDAVYVDLGGSHVFQDEVGPTHELVQSLISTHSTIDAKMASSRVTLFSDSKPLGSEDIDNQGLMMPKEEKQMDLNTGRMRRKAIFGD
EDESGDSDDEEDDEMSEDDGLENGSSDEEAEEEENAEMTDQYMAVKGIKRRKLELEEDSEMDLPAFADSDDDLERSSAEEGEAEEAD
ESSEEEDCTAGEKGISGSKAAGEGSKAGLSPANCQSDRVNLEKSLLMKKAALPTFDSGHCTAEEVFASEDESEESSSLSAEEEDSEN
EEAIRKKLSKPSQVSSGQKLGPQNFIDETSDIENLLKEEEDYKEENNDSKETSGALKWKEDLSRKAAEAFLRQQQAAPNLRKLIYGT
VTEDNEEEDDDTLEELGGLFRVNQPDRECKHKADSLDCSRFLVEAPHDWDLEEVMNSIRDCFVTGKWEDDKDAAKVLAEDEELYGDF
EDLETGDVHKGKSGPNTQNEDIEKEVKEEIDPDEEESAKKKHLDKKRKLKEMFDAEYDEGESTYFDDLKGEMQKQAQLNRAEFEDQD
DEARVQYEGFRPGMYVRIEIENVPCEFVQNFDPHYPIILGGLGNSEGNVGYVQMRLKKHRWYKKILKSRDPIIFSVGWRRFQTIPLY
YIEDHNGRQRLLKYTPQHMHCGAAFWGPITPQGTGFLAIQSVSGIMPDFRIAATGVVLDLDKSIKIVKKLKLTGFPYKIFKNTSFIK
GMFNSALEVAKFEGAVIRTVSGIRGQIKKALRAPEGAFRASFEDKLLMSDIVFMRTWYPVSIPAFYNPVTSLLKPVGEKDTWSGMRT
TGQLRLAHGVRLKANKDSLYKPILRQKKHFNSLHIPKALQKALPFKNKPKTQAKAGKVPKDRRRPAVIREPHERKILALLDALSTVH
SQKMKKAKEQRHLHNKEHFRAKQKEEEEKLKRQKDLRKKLFRIQGQKERRNQKSSLKGAEGQLQ
>sp|Q96R45|OR2A7_HUMAN
MGDNITSITEFLLLGFPVGPRIQMLLFGLFSLFYVFTLLGNGTILGLISLDSRLHAPMYFFLSHLAVVDIAYACNTVPRMLVNLLHP
AKPISFAGRMMQTFLFSTFAVTECLLLVVMSYDLYVAICHPLRYLAIMTWRVCITLAVTSWTTGVLLSLIHLVLLLPLPFCRPQKIY
HFFCEILAVLKLACADTHINENMVLAGAISGLVGPLSTIVVSYMCILCAILQIQSREVQRKAFCTCFSHLCVIGLFYGTAIIMYVGP
RYGNPKEQKKYLLLFHSLFNPMLNPLICSLRNSEVKNTLKRVLGVERAL
>sp|Q96HB5|CC120_HUMAN
MEVKGQLISSPTFNAPAALFGEAAPQVKSERLRGLLDRQRTLQEALSLKLQELRKVCLQEAELTGQLPPECPLEPGERPQLVRRRPP
TARAYPPPHPNQAHHSLCPAEELALEALEREVSVQQQIAAAARRLALAPDLSTEQRRRRRQVQADALRRLHELEEQLRDVRARLGLP
VLPLPQPLPLSTGSVITTQGVCLGMRLAQLSQEDVVLHSESSSLSESGASHDNEEPHGCFSLAERPSPPKAWDQLRAVSGGSPERRT
PWKPPPSDLYGDLKSRRNSVASPTSPTRSLPRSASSFEGRSVPATPVLTRGAGPQLCKPEGLHSRQWSGSQDSQMGFPRADPASDRA
SLFVARTRRSNSSEALLVDRAAGGGAGSPPAPLAPSASGPPVCKSSEVLYERPQPTPAFSSRTAGPPDPPRAARPSSAAPASRGAPR
LPPVCGDFLLDYSLDRGLPRSGGGTGWGELPPAAEVPGPLSRRDGLLTMLPGPPPVYAADSNSPLLRTKDPHTRATRTKPCGLPPEA
AEGPEVHPNPLLWMPPPTRIPSAGERSGHKNLALEGLRDWYIRNSGLAAGPQRRPVLPSVGPPHPPFLHARCYEVGQALYGAPSQAP
LPHSRSFTAPPVSGRYGGCFY
>sp|Q9BZR8|B2L14_HUMAN
MCSTSGCDLEEIPLDDDDLNTIEFKILAYYTRHHVFKSTPALFSPKLLRTRSLSQRGLGNCSANESWTEVSWPCRNSQSSEKAINLG
KKKSSWKAFFGVVEKEDSQSTPAKVSAQGQRTLEYQDSHSQQWSRCLSNVEQCLEHEAVDPKVISIANRVAEIVYSWPPPQATQAGG
FKSKEIFVTEGLSFQLQGHVPVASSSKKDEEEQILAKIVELLKYSGDQLERKLKKDKALMGHFQDGLSYSVFKTITDQVLMGVDPRG
ESEVKAQGFKAALVIDVTAKLTAIDNHPMNRVLGFGTKYLKENFSPWIQQHGGWEKILGISHEEVD
>sp|Q969X6|UTP4_HUMAN
MGEFKVHRVRFFNYVPSGIRCVAYNNQSNRLAVSRTDGTVEIYNLSANYFQEKFFPGHESRATEALCWAEGQRLFSAGLNGEIMEYD
LQALNIKYAMDAFGGPIWSMAASPSGSQLLVGCEDGSVKLFQITPDKIQFERNFDRQKSRILSLSWHPSGTHIAAGSIDYISVFDVK
SGSAVHKMIVDRQYMGVSKRKCIVWGVAFLSDGTIISVDSAGKVQFWDSATGTLVKSHLIANADVQSIAVADQEDSFVVGTAEGTVF
HFQLVPVTSNSSEKQWVRTKPFQHHTHDVRTVAHSPTALISGGTDTHLVFRPLMEKVEVKNYDAALRKITFPHRCLISCSKKRQLLL
FQFAHHLELWRLGSTVATGKNGDTLPLSKNADHLLHLKTKGPENIICSCISPCGSWIAYSTVSRFFLYRLNYEHDNISLKRVSKMPA
FLRSALQILFSEDSTKLFVASNQGALHIVQLSGGSFKHLHAFQPQSGTVEAMCLLAVSPDGNWLAASGTSAGVHVYNVKQLKLHCTV
PAYNFPVTAMAIAPNTNNLVIAHSDQQVFEYSIPDKQYTDWSRTVQKQGFHHLWLQRDTPITHISFHPKRPMHILLHDAYMFCIIDK
SLPLPNDKTLLYNPFPPTNESDVIRRRTAHAFKISKIYKPLLFMDLLDERTLVAVERPLDDIIAQLPPPIKKKKFGT
>sp|Q3MJ16|PA24E_HUMAN

MLLRQMHARVSHSLPDPCQAEDSRPSATCALKAPQTSWDGLLREGLSPCHLLTVRVIRMKNVRQADMLSQTDCFVSLWLPTASQKKL
RTRTISNCPNPEWNESFNFQIQSRVKNVLELSVCDEDTVTPDDHLLTVLYDLTKLCFRKKTHVKFPLNPQGMEELEVEFLLEESPSP
PETLVTNGVLVVIIFLGSCSSRGHGWLLLSGEQDQGRKQWAQLVKDLLVMVNESFENTQRVRPCLEPCCPTSACFQTAACFHYPKYF
QSQVHVEVPKSHWSCGLCCRSRKKGPISQPLDCLSDGQVMTLPVGGGESLGSPETLDVRLGFSLCPAELEFLQKRKVVVAKALKQVL
QLEEDLQEDEVPLIAIMATGGGTRSMTSMYGHLLGLQKLNLLDCASYITGLSGATWTMATLYRDPDWSSKNLEPAIFEARRHVVKDK
LPSLFPDQLRKFQEELRQRSQEGYRVTFTDFWGLLIETCLGDERNECKLSDQRAALSCGQNPLPIYLTINVKDDVSNQDFREWFEFS
PYEVGLQKYGAFIPSELFGSEFFMGRLVKRIPESRICYMLGLWSSIFSLNLLDAWNLSHTSEEFFHRWTREKVQDIEDEPILPEIPK
CDANILETTVVIPGSWLSNSFREILTHRSFVSEFHNFLSGLQLHTNYLQNGQFSRWKDTVLDGFPNQLTESANHLCLLDTAFFVNSS
YPPLLRPERKADLIIHLNYCAGSQTKPLKQTCEYCTVQNIPFPKYELPDENENLKECYLMENPQEPDAPIVTFFPLINDTFRKYKAP
GVERSPEELEQGQVDIYGPKTPYATKELTYTEATFDKLVKLSEYNILNNKDTLLQALRLAVEKKKRLKGQCPS
>sp|Q5YKI7|GGNB1_HUMAN
MKEEDSSFKLCVPGIVALQSPPNKAFRSTDTVGFLESELKKLLGMQQESRLWKLGSQEGRELLTRPEITVVEGEGYEVQRRLRHLPS
PISVAQCLLLEEKGEMGNWPPE
>sp|Q8IUC0|KR131_HUMAN
MSYNCCSGNFSSRSCGGYLHYPASSCGFSYPSNQVYSTDLCSPSTCQLGSSLYRGCQQTCWEPTSCQTSYVESSPCQTSCYRPRTSL
LCSPCQTTYSGSLGFGSSSCRSLGYGSRSCYSVGCGSSGFRSLGYGGCGFPSLGYGVGFCRPTYLASRSCQSSCYRPTCGSGFYY
>sp|Q6VN20|RBP10_HUMAN
MAAATADPGAGNPQPGDSSGGGAGGGLPSPGEQELSRRLQRLYPAVNQQETPLPRSWSPKDKYNYIGLSQGNLRVHYKGHGKNHKDA
ASVRATHPIPAACGIYYFEVKIVSKGRDGYMGIGLSAQGVNMNRLPGWDKHSYGYHGDDGHSFCSSGTGQPYGPTFTTGDVIGCCVN
LINGTCFYTKNGHSLGIAFTDLPANLYPTVGLQTPGEIVDANFGQQPFLFDIEDYMREWRAKVQGTVHCFPISARLGEWQAVLQNMV
SSYLVHHGYCATATAFARMTETPIQEEQASIKNRQKIQKLVLEGRVGEAIETTQRFYPGLLEHNPNLLFMLKCRQFVEMVNGTDSEV
RSLSSRSPKSQDSYPGSPSLSPRHGPSSSHMHNTGADSPSCSNGVASTKSKQNHSKYPAPSSSSSSSSSSSSSSPSSVNYSESNSTD
STKSQHHSSTSNQETSDSEMEMEAEHYPNGVLGSMSTRIVNGAYKHEDLQTDESSMDDRHPRRQLCGGNQAATERIILFGRELQALS
EQLGREYGKNLAHTEMLQDAFSLLAYSDPWSCPVGQQLDPIQREPVCAALNSAILESQNLPKQPPLMLALGQASECLRLMARAGLGS
CSFARVDDYLH
>sp|O95235|KI20A_HUMAN
MSQGILSPPAGLLSDDDVVVSPMFESTAADLGSVVRKNLLSDCSVVSTSLEDKQQVPSEDSMEKVKVYLRVRPLLPSELERQEDQGC
VRIENVETLVLQAPKDSFALKSNERGIGQATHRFTFSQIFGPEVGQASFFNLTVKEMVKDVLKGQNWLIYTYGVTNSGKTHTIQGTI
KDGGILPRSLALIFNSLQGQLHPTPDLKPLLSNEVIWLDSKQIRQEEMKKLSLLNGGLQEEELSTSLKRSVYIESRIGTSTSFDSGI
AGLSSISQCTSSSQLDETSHRWAQPDTAPLPVPANIRFSIWISFFEIYNELLYDLLEPPSQQRKRQTLRLCEDQNGNPYVKDLNWIH
VQDAEEAWKLLKVGRKNQSFASTHLNQNSSRSHSIFSIRILHLQGEGDIVPKISELSLCDLAGSERCKDQKSGERLKEAGNINTSLH
TLGRCIAALRQNQQNRSKQNLVPFRDSKLTRVFQGFFTGRGRSCMIVNVNPCASTYDETLHVAKFSAIASQLVHAPPMQLGFPSLHS
FIKEHSLQVSPSLEKGAKADTGLDDDIENEADISMYGKEELLQVVEAMKTLLLKERQEKLQLEMHLRDEICNEMVEQMQQREQWCSE
HLDTQKELLEEMYEEKLNILKESLTSFYQEEIQERDEKIEELEALLQEARQQSVAHQQSGSELALRRSQRLAASASTQQLQEVKAKL
QQCKAELNSTTEELHKYQKMLEPPPSAKPFTIDVDKKLEEGQKNIRLLRTELQKLGESLQSAERACCHSTGAGKLRQALTTCDDILI
KQDQTLAELQNNMVLVKLDLRKKAACIAEQYHTVLKLQGQVSAKKRLGTNQENQQPNQQPPGKKPFLRNLLPRTPTCQSSTDCSPYA
RILRSRRSPLLKSGPFGKKY
>sp|P48551|INAR2_HUMAN
MLLSQNAFIFRSLNLVLMVYISLVFGISYDSPDYTDESCTFKISLRNFRSILSWELKNHSIVPTHYTLLYTIMSKPEDLKVVKNCAN
TTRSFCDLTDEWRSTHEAYVTVLEGFSGNTTLFSCSHNFWLAIDMSFEPPEFEIVGFTNHINVMVKFPSIVEEELQFDLSLVIEEQS
EGIVKKHKPEIKGNMSGNFTYIIDKLIPNTNYCVSVYLEHSDEQAVIKSPLKCTLLPPGQESESAESAKIGGIITVFLIALVLTSTI
VTLKWIGYICLRNSLPKVLNFHNFLAWPFPNLPPLEAMDMVEVIYINRKKKVWDYNYDDESDSDTEAAPRTSGGGYTMHGLTVRPLG
QASATSTESQLIDPESEEEPDLPEVDVELPTMPKDSPQQLELLSGPCERRKSPLQDPFPEEDYSSTEGSGGRITFNVDLNSVFLRVL
DDEDSDDLEAPLMLSSHLEEMVDPEDPDNVQSNHLLASGEGTQPTFPSPSSEGLWSEDAPSDQSDTSESDVDLGDGYIMR

>sp|Q9H0R4|HDHD2_HUMAN
MAACRALKAVLVDLSGTLHIEDAAVPGAQEALKRLRGASVIIRFVTNTTKESKQDLLERLRKLEFDISEDEIFTSLTAARSLLERKQ
VRPMLLVDDRALPDFKGIQTSDPNAVVMGLAPEHFHYQILNQAFRLLLDGAPLIAIHKARYYKRKDGLALGPGPFVTALEYATDTKA
TVVGKPEKTFFLEALRGTGCEPEEAVMIGDDCRDDVGGAQDVGMLGILVKTGKYRASDEEKINPPPYLTCESFPHAVDHILQHLL
>sp|Q9UK10|ZN225_HUMAN
MTTLKEAVTFKDVAVVFTEEELRLLDLAQRKLYREVMLENFRNLLSVGHQSLHRDTFHFLKEEKFWMMETATQREGNLGGKIQMEME
TVSESGTHEGLFSHQTWEQISSDLTRFQDSMVNSFQFSKQDDMPCQVDAGLSIIHVRQKPSEGRTCKKSFSDVSVLDLHQQLQSREK
SHTCDECGKSFCYSSALRIHQRVHMGEKLYNCDVCGKEFNQSSHLQIHQRIHTGEKPFKCEQCGKGFSRRSGLYVHRKLHTGVKPHI
CEKCGKAFIHDSQLQEHQRIHTGEKPFKCDICCKSFRSRANLNRHSMVHMREKPFRCDTCGKSFGLKSALNSHRMVHTGEKRYKCEE
CGKRFIYRQDLYKHQIDHTGEKPYNCKECGKSFRWASGLSRHVRVHSGETTFKCEECGKGFYTNSQRYSHQRAHSGEKPYRCEECGK
GYKRRLDLDFHQRVHRGEKPYNCKECGKSFGWASCLLNHQRIHSGEKPFKCEECGKRFTQNSQLYTHRRVHSGEKPFKCEECGKRFT
QNSQLYSHRRVHTGVKPYKCEECGKGFNSKFNLDMHQRVHTGERPYNCKECGKSFSRASSILNHKRLHGDEKPFKCEECGKRFTENS
QLHSHQRVHTGEKPYKCEKCGKSFRWASTHLTHQRLHSREKLLQCEDCGKSIVHSSCLKDQQRDQSGEKTSKCEDCGKRYKRRLNLD
TLLSLFLNDT
>sp|Q96CB9|NSUN4_HUMAN
MAALTLRGVRELLKRVDLATVPRRHRYKKKWAATEPKFPAVRLALQNFDMTYSVQFGDLWPSIRVSLLSEQKYGALVNNFAAWDHVS
AKLEQLSAKDFVNEAISHWELQSEGGQSAAPSPASWACSPNLRCFTFDRGDISRFPPARPGSLGVMEYYLMDAASLLPVLALGLQPG
DIVLDLCAAPGGKTLALLQTGCCRNLAANDLSPSRIARLQKILHSYVPEEIRDGNQVRVTSWDGRKWGELEGDTYDRVLVDVPCTTD
RHSLHEEENNIFKRSRKKERQILPVLQVQLLAAGLLATKPGGHVVYSTCSLSHLQNEYVVQGAIELLANQYSIQVQVEDLTHFRRVF
MDTFCFFSSCQVGELVIPNLMANFGPMYFCKMRRLT
>sp|Q9Y6D9|MD1L1_HUMAN
MEDLGENTMVLSTLRSLNNFISQRVEGGSGLDISTSAPGSLQMQYQQSMQLEERAEQIRSKSHLIQVEREKMQMELSHKRARVELER
AASTSARNYEREVDRNQELLTRIRQLQEREAGAEEKMQEQLERNRQCQQNLDAASKRLREKEDSLAQAGETINALKGRISELQWSVM
DQEMRVKRLESEKQELQEQLDLQHKKCQEANQKIQELQASQEARADHEQQIKDLEQKLSLQEQDAAIVKNMKSELVRLPRLERELKQ
LREESAHLREMRETNGLLQEELEGLQRKLGRQEKMQETLVGLELENERLLAKLQSWERLDQTMGLSIRTPEDLSRFVVELQQRELAL
KDKNSAVTSSARGLEKARQQLQEELRQVSGQLLEERKKRETHEALARRLQKRVLLLTKERDGMRAILGSYDSELTPAEYSPQLTRRM
REAEDMVQKVHSHSAEMEAQLSQALEELGGQKQRADMLEMELKMLKSQSSSAEQSFLFSREEADTLRLKVEELEGERSRLEEEKRML
EAQLERRALQGDYDQSRTKVLHMSLNPTSVARQRLREDHSQLQAECERLRGLLRAMERGGTVPADLEAAAASLPSSKEVAELKKQVE
SAELKNQRLKEVFQTKIQEFRKACYTLTGYQIDITTENQYRLTSLYAEHPGDCLIFKATSPSGSKMQLLETEFSHTVGELIEVHLRR
QDSIPAFLSSLTLELFSRQTVA
>sp|Q12986|NFX1_HUMAN
MAEAPPVSGTFKFNTDAAEFIPQEKKNSGLNCGTQRRLDSNRIGRRNYSSPPPCHLSRQVPYDEISAVHQHSYHPSGSKPKSQQTSF
QSSPCNKSPKSHGLQNQPWQKLRNEKHHIRVKKAQSLAEQTSDTAGLESSTRSESGTDLREHSPSESEKEVVGADPRGAKPKKATQF
VYSYGRGPKVKGKLKCEWSNRTTPKPEDAGPESTKPVGVFHPDSSEASSRKGVLDGYGARRNEQRRYPQKRPPWEVEGARPRPGRNP
PKQEGHRHTNAGHRNNMGPIPKDDLNERPAKSTCDSENLAVINKSSRRVDQEKCTVRRQDPQVVSPFSRGKQNHVLKNVETHTGSLI
EQLTTEKYECMVCCELVRVTAPVWSCQSCYHVFHLNCIKKWARSPASQADGQSGWRCPACQNVSAHVPNTYTCFCGKVKNPEWSRNE
IPHSCGEVCRKKQPGQDCPHSCNLLCHPGPCPPCPAFMTKTCECGRTRHTVRCGQAVSVHCSNPCENILNCGQHQCAELCHGGQCQP
CQIILNQVCYCGSTSRDVLCGTDVGKSDGFGDFSCLKICGKDLKCGNHTCSQVCHPQPCQQCPRLPQLVRCCPCGQTPLSQLLELGS
SSRKTCMDPVPSCGKVCGKPLPCGSLDFIHTCEKLCHEGDCGPCSRTSVISCRCSFRTKELPCTSLKSEDATFMCDKRCNKKRLCGR
HKCNEICCVDKEHKCPLICGRKLRCGLHRCEEPCHRGNCQTCWQASFDELTCHCGASVIYPPVPCGTRPPECTQTCARVHECDHPVY
HSCHSEEKCPPCTFLTQKWCMGKHEFRSNIPCHLVDISCGLPCSATLPCGMHKCQRLCHKGECLVDEPCKQPCTTPRADCGHPCMAP
CHTSSPCPVTACKAKVELQCECGRRKEMVICSEASSTYQRIAAISMASKITDMQLGGSVEISKLITKKEVHQARLECDEECSALERK
KRLAEAFHISEDSDPFNIRSSGSKFSDSLKEDARKDLKFVSDVEKEMETLVEAVNKGKNSKKSHSFPPMNRDHRRIIHDLAQVYGLE
SVSYDSEPKRNVVVTAIRGKSVCPPTTLTGVLEREMQARPPPPIPHHRHQSDKNPGSSNLQKITKEPIIDYFDVQD

>sp|Q86YW5|TRML1_HUMAN
MGLTLLLLLLLGLEGQGIVGSLPEVLQAPVGSSILVQCHYRLQDVKAQKVWCRFLPEGCQPLVSSAVDRRAPAGRRTFLTDLGGGLL
QVEMVTLQEEDAGEYGCMVDGARGPQILHRVSLNILPPEEEEETHKIGSLAENAFSDPAGSANPLEPSQDEKSIPLIWGAVLLVGLL
VAAVVLFAVMAKRKQGNRLGVCGRFLSSRVSGMNPSSVVHHVSDSGPAAELPLDVPHIRLDSPPSFDNTTYTSLPLDSPSGKPSLPA
PSSLPPLPPKVLVCSKPVTYATVIFPGGNKGGGTSCGPAQNPPNNQTPSS
>sp|Q12996|CSTF3_HUMAN
MSGDGATEQAAEYVPEKVKKAEKKLEENPYDLDAWSILIREAQNQPIDKARKTYERLVAQFPSSGRFWKLYIEAEIKAKNYDKVEKL
FQRCLMKVLHIDLWKCYLSYVRETKGKLPSYKEKMAQAYDFALDKIGMEIMSYQIWVDYINFLKGVEAVGSYAENQRITAVRRVYQR
GCVNPMINIEQLWRDYNKYEEGINIHLAKKMIEDRSRDYMNARRVAKEYETVMKGLDRNAPSVPPQNTPQEAQQVDMWKKYIQWEKS
NPLRTEDQTLITKRVMFAYEQCLLVLGHHPDIWYEAAQYLEQSSKLLAEKGDMNNAKLFSDEAANIYERAISTLLKKNMLLYFAYAD
YEESRMKYEKVHSIYNRLLAIEDIDPTLVYIQYMKFARRAEGIKSGRMIFKKAREDTRTRHHVYVTAALMEYYCSKDKSVAFKIFEL
GLKKYGDIPEYVLAYIDYLSHLNEDNNTRVLFERVLTSGSLPPEKSGEIWARFLAFESNIGDLASILKVEKRRFTAFKEEYEGKETA
LLVDRYKFMDLYPCSASELKALGYKDVSRAKLAAIIPDPVVAPSIVPVLKDEVDRKPEYPKPDTQQMIPFQPRHLAPPGLHPVPGGV
FPVPPAAVVLMKLLPPPICFQGPFVQVDELMEIFRRCKIPNTVEEAVRIITGGAPELAVEGNGPVESNAVLTKAVKRPNEDSDEDEE
KGAVVPPVHDIYRARQQKRIR
>sp|Q13733|AT1A4_HUMAN
MGLWGKKGTVAPHDQSPRRRPKKGLIKKKMVKREKQKRNMEELKKEVVMDDHKLTLEELSTKYSVDLTKGHSHQRAKEILTRGGPNT
VTPPPTTPEWVKFCKQLFGGFSLLLWTGAILCFVAYSIQIYFNEEPTKDNLYLSIVLSVVVIVTGCFSYYQEAKSSKIMESFKNMVP
QQALVIRGGEKMQINVQEVVLGDLVEIKGGDRVPADLRLISAQGCKVDNSSLTGESEPQSRSPDFTHENPLETRNICFFSTNCVEGT
ARGIVIATGDSTVMGRIASLTSGLAVGQTPIAAEIEHFIHLITVVAVFLGVTFFALSLLLGYGWLEAIIFLIGIIVANVPEGLLATV
TVCLTLTAKRMARKNCLVKNLEAVETLGSTSTICSDKTGTLTQNRMTVAHMWFDMTVYEADTTEEQTGKTFTKSSDTWFMLARIAGL
CNRADFKANQEILPIAKRATTGDASESALLKFIEQSYSSVAEMREKNPKVAEIPFNSTNKYQMSIHLREDSSQTHVLMMKGAPERIL
EFCSTFLLNGQEYSMNDEMKEAFQNAYLELGGLGERVLGFCFLNLPSSFSKGFPFNTDEINFPMDNLCFVGLISMIDPPRAAVPDAV
SKCRSAGIKVIMVTGDHPITAKAIAKGVGIISEGTETAEEVAARLKIPISKVDASAAKAIVVHGAELKDIQSKQLDQILQNHPEIVF
ARTSPQQKLIIVEGCQRLGAVVAVTGDGVNDSPALKKADIGIAMGISGSDVSKQAADMILLDDNFASIVTGVEEGRLIFDNLKKSIM
YTLTSNIPEITPFLMFIILGIPLPLGTITILCIDLGTDMVPAISLAYESAESDIMKRLPRNPKTDNLVNHRLIGMAYGQIGMIQALA
GFFTYFVILAENGFRPVDLLGIRLHWEDKYLNDLEDSYGQQWTYEQRKVVEFTCQTAFFVTIVVVQWADLIISKTRRNSLFQQGMRN
KVLIFGILEETLLAAFLSYTPGMDVALRMYPLKITWWLCAIPYSILIFVYDEIRKLLIRQHPDGWVERETYY
>sp|Q99697|PITX2_HUMAN
METNCRKLVSACVQLGVQPAAVECLFSKDSEIKKVEFTDSPESRKEAASSKFFPRQHPGANEKDKSQQGKNEDVGAEDPSKKKRQRR
QRTHFTSQQLQELEATFQRNRYPDMSTREEIAVWTNLTEARVRVWFKNRRAKWRKRERNQQAELCKNGFGPQFNGLMQPYDDMYPGY
SYNNWAAKGLTSASLSTKSFPFFNSMNVNPLSSQSMFSPPNSISSMSMSSSMVPSAVTGVPGSSLNSLNNLNNLSSPSLNSAVPTPA
CPYAPPTPPYVYRDTCNSSLASLRLKAKQHSSFGYASVQNPASNLSACQYAVDRPV
>sp|Q8N136|DAW1_HUMAN
MKLKSLLLRYYPPGIMLEYEKHGELKTKSIDLLDLGPSTDVSALVEEIQKAEPLLTASRTEQVKLLIQRLQEKLGQNSNHTFYLFKV
LKAHILPLTNVALNKSGSCFITGSYDRTCKLWDTASGEELNTLEGHRNVVYAIAFNNPYGDKIATGSFDKTCKLWSVETGKCYHTFR
GHTAEIVCLSFNPQSTLVATGSMDTTAKLWDIQNGEEVYTLRGHSAEIISLSFNTSGDRIITGSFDHTVVVWDADTGRKVNILIGHC
AEISSASFNWDCSLILTGSMDKTCKLWDATNGKCVATLTGHDDEILDSCFDYTGKLIATASADGTARIFSAATRKCIAKLEGHEGEI
SKISFNPQGNHLLTGSSDKTARIWDAQTGQCLQVLEGHTDEIFSCAFNYKGNIVITGSKDNTCRIWR
>sp|Q7Z6K5|ARPIN_HUMAN
MSRIYHDGALRNKAVQSVRLPGAWDPAAHQGGNGVLLEGELIDVSRHSILDTHGRKERYYVLYIRPSHIHRRKFDAKGNEIEPNFSA
TRKVNTGFLMSSYKVEAKGDTDRLTPEALKGLVNKPELLALTESLTPDHTVAFWMPESEMEVMELELGAGVRLKTRGDGPFLDSLAK
LEAGTVTKCNFTGDGKTGASWTDNIMAQKCSKGAAAEIREQGDGAEDEEWDD
>sp|Q9Y6Q6|TNR11_HUMAN

MAPRARRRRPLFALLLLCALLARLQVALQIAPPCTSEKHYEHLGRCCNKCEPGKYMSSKCTTTSDSVCLPCGPDEYLDSWNEEDKCL
LHKVCDTGKALVAVVAGNSTTPRRCACTAGYHWSQDCECCRRNTECAPGLGAQHPLQLNKDTVCKPCLAGYFSDAFSSTDKCRPWTN
CTFLGKRVEHHGTEKSDAVCSSSLPARKPPNEPHVYLPGLIILLLFASVALVAAIIFGVCYRKKGKALTANLWHWINEACGRLSGDK
ESSGDSCVSTHTANFGQQGACEGVLLLTLEEKTFPEDMCYPDQGGVCQGTCVGGGPYAQGEDARMLSLVSKTEIEEDSFRQMPTEDE
YMDRPSQPTDQLLFLTEPGSKSTPPFSEPLEVGENDSLSQCFTGTQSTVGSESCNCTEPLCRTDWTPMSSENYLQKEVDSGHCPHWA
ASPSPNWADVCTGCRNPPGEDCEPLVGSPKRGPLPQCAYGMGLPPEEEASRTEARDQPEDGADGRLPSSARAGAGSGSSPGGQSPAS
GNVTGNSNSTFISSGQVMNFKGDIIVVYVSQTSQEGAAAAAEPMGRPVQEETLARRDSFAGNGPRFPDPCGGPEGLREPEKASRPVQ
EQGGAKA
>sp|Q6UWV2|MPZL3_HUMAN
MQQRGAAGSRGCALFPLLGVLFFQGVYIVFSLEIRADAHVRGYVGEKIKLKCTFKSTSDVTDKLTIDWTYRPPSSSHTVSIFHYQSF
QYPTTAGTFRDRISWVGNVYKGDASISISNPTIKDNGTFSCAVKNPPDVHHNIPMTELTVTERGFGTMLSSVALLSILVFVPSAVVV
ALLLVRMGRKAAGLKKRSRSGYKKSSIEVSDDTDQEEEEACMARLCVRCAECLDSDYEETY
>sp|O43699|SIGL6_HUMAN
MQGAQEASASEMLPLLLPLLWAGALAQERRFQLEGPESLTVQEGLCVLVPCRLPTTLPASYYGYGYWFLEGADVPVATNDPDEEVQE
ETRGRFHLLWDPRRKNCSLSIRDARRRDNAAYFFRLKSKWMKYGYTSSKLSVRVMALTHRPNISIPGTLESGHPSNLTCSVPWVCEQ
GTPPIFSWMSAAPTSLGPRTTQSSVLTITPRPQDHSTNLTCQVTFPGAGVTMERTIQLNVSYAPQKVAISIFQGNSAAFKILQNTSS
LPVLEGQALRLLCDADGNPPAHLSWFQGFPALNATPISNTGVLELPQVGSAEEGDFTCRAQHPLGSLQISLSLFVHWKPEGRAGGVL
GAVWGASITTLVFLCVCFIFRVKTRRKKAAQPVQNTDDVNPVMVSGSRGHQHQFQTGIVSDHPAEAGPISEDEQELHYAVLHFHKVQ
PQEPKVTDTEYSEIKIHK
>sp|P0CG29|GST2_HUMAN
MGLELFLDLVSQPSRAVYIFAKKNGIPLELRTVDLVKGQHKSKEFLQINSLGKLPTLKDGDFILTESSAILIYLSCKYQTPDHWYPS
DLQARARVHEYLGWHADCIRGTFGIPLWVQVLGPLIGVQVPKEKVERNRTAMDQALQWLEDKFLGDRPFLAGQQVTLADLMALEELM
QPVALGYELFEGRPRLAAWRGRVEAFLGAELCQEAHSIILSILEQAAKKTLPTPSPEAYQAMLLRIARIP
>sp|Q6UXP9|Y0001_HUMAN
MIFMQILEPQEVPSFLMICQRRSPAMHRTCTDHAPLAIAQVWLWVSLAKAGSNRRGPGRAEGTFFSLLAALHAAQHFPNLPTAPGGA
SQSNIVSPELTPKPTTALKHAECLLDLNSHSLYRKPRPKAAVYLNLSLPLKSVHRLSLKKSFGFGKRDFENNSVFIVDSGGTCAGLL
PGYIGWC
>sp|Q99965|ADAM2_HUMAN
MWRVLFLLSGLGGLRMDSNFDSLPVQITVPEKIRSIIKEGIESQASYKIVIEGKPYTVNLMQKNFLPHNFRVYSYSGTGIMKPLDQD
FQNFCHYQGYIEGYPKSVVMVSTCTGLRGVLQFENVSYGIEPLESSVGFEHVIYQVKHKKADVSLYNEKDIESRDLSFKLQSVEPQQ
DFAKYIEMHVIVEKQLYNHMGSDTTVVAQKVFQLIGLTNAIFVSFNITIILSSLELWIDENKIATTGEANELLHTFLRWKTSYLVLR
PHDVAFLLVYREKSNYVGATFQGKMCDANYAGGVVLHPRTISLESLAVILAQLLSLSMGITYDDINKCQCSGAVCIMNPEAIHFSGV
KIFSNCSFEDFAHFISKQKSQCLHNQPRLDPFFKQQAVCGNAKLEAGEECDCGTEQDCALIGETCCDIATCRFKAGSNCAEGPCCEN
CLFMSKERMCRPSFEECDLPEYCNGSSASCPENHYVQTGHPCGLNQWICIDGVCMSGDKQCTDTFGKEVEFGPSECYSHLNSKTDVS
GNCGISDSGYTQCEADNLQCGKLICKYVGKFLLQIPRATIIYANISGHLCIAVEFASDHADSQKMWIKDGTSCGSNKVCRNQRCVSS
SYLGYDCTTDKCNDRGVCNNKKHCHCSASYLPPDCSVQSDLWPGGSIDSGNFPPVAIPARLPERRYIENIYHSKPMRWPFFLFIPFF
IIFCVLIAIMVKVNFQRKKWRTEDYSSDEQPESESEPKG
>sp|A8MTL0|IQCF5_HUMAN
MGPEEKTIMTERSAAVFIQAWWRGMLVRRTLLHAALRAWIIQCWWRQVLEKLLAKRRRMVLEFYVQQEWAAVRLQSWVRMWCVRQRY
CRLLNAVRIIQVYWRWHSCHSRVFIEGHYELKENQLNIQLEISLGLQACKVQQCIPLPLKE
>sp|A5LHX3|PSB11_HUMAN
MALQDVCKWQSPDTQGPSPHLPRAGGWAVPRGCDPQTFLQIHGPRLAHGTTTLAFRFRHGVIAAADTRSSCGSYVACPASCKVIPVH
QHLLGTTSGTSADCATWYRVLQRELRLRELREGQLPSVASAAKLLSAMMSQYRGLDLCVATALCGWDRSGPELFYVYSDGTRLQGDI

FSVGSGSPYAYGVLDRGYRYDMSTQEAYALARCAVAHATHRDAYSGGSVDLFHVRESGWEHVSRSDACVLYVELQKLLEPEPEEDAS
HAHPEPATAHRAAEDRELSVGPGEVTPGDSRMPAGTETV
>sp|Q07325|CXCL9_HUMAN
MKKSGVLFLLGIILLVLIGVQGTPVVRKGRCSCISTNQGTIHLQSLKDLKQFAPSPSCEKIEIIATLKNGVQTCLNPDSADVKELIK
KWEKQVSQKKKQKNGKKHQKKKVLKVRKSQRSRQKKTT
>sp|Q13162|PRDX4_HUMAN
MEALPLLAATTPDHGRHRRLLLLPLLLFLLPAGAVQGWETEERPRTREEECHFYAGGQVYPGEASRVSVADHSLHLSKAKISKPAPY
WEGTAVIDGEFKELKLTDYRGKYLVFFFYPLDFTFVCPTEIIAFGDRLEEFRSINTEVVACSVDSQFTHLAWINTPRRQGGLGPIRI
PLLSDLTHQISKDYGVYLEDSGHTLRGLFIIDDKGILRQITLNDLPVGRSVDETLRLVQAFQYTDKHGEVCPAGWKPGSETIIPDPA
GKLKYFDKLN
>sp|Q9BYR2|KRA45_HUMAN
MVSSCCGSVSSEQSCGLENCCRPSCCQTTCCRTTCCRPSCCKPQCCQSVCYQPTCCHPSCCISSCCRPYCCESSCCRPCCCQTTCCR
TTCCRTTCCCPSCCVSSCCRPQCCQSVCCQPTCCRPSCCISSCCHPSCCESSCCRPCCCVRPVCGRVSCHTTCYRPTCVISTCPRPL
CCASSCC
>sp|Q9P1Z2|CACO1_HUMAN
MEESPLSRAPSRGGVNFLNVARTYIPNTKVECHYTLPPGTMPSASDWIGIFKVEAACVRDYHTFVWSSVPESTTDGSPIHTSVQFQA
SYLPKPGAQLYQFRYVNRQGQVCGQSPPFQFREPRPMDELVTLEEADGGSDILLVVPKATVLQNQLDESQQERNDLMQLKLQLEGQV
TELRSRVQELERALATARQEHTELMEQYKGISRSHGEITEERDILSRQQGDHVARILELEDDIQTISEKVLTKEVELDRLRDTVKAL
TREQEKLLGQLKEVQADKEQSEAELQVAQQENHHLNLDLKEAKSWQEEQSAQAQRLKDKVAQMKDTLGQAQQRVAELEPLKEQLRGA
QELAASSQQKATLLGEELASAAAARDRTIAELHRSRLEVAEVNGRLAELGLHLKEEKCQWSKERAGLLQSVEAEKDKILKLSAEILR
LEKAVQEERTQNQVFKTELAREKDSSLVQLSESKRELTELRSALRVLQKEKEQLQEEKQELLEYMRKLEARLEKVADEKWNEDATTE
DEEAAVGLSCPAALTDSEDESPEDMRLPPYGLCERGDPGSSPAGPREASPLVVISQPAPISPHLSGPAEDSSSDSEAEDEKSVLMAA
VQSGGEEANLLLPELGSAFYDMASGFTVGTLSETSTGGPATPTWKECPICKERFPAESDKDALEDHMDGHFFFSTQDPFTFE
>sp|Q0P140|YA037_HUMAN
MQDVHTKSIACDGDLLPVLQENSISFQMLSLEMSGSFHSSPALENATITILHVSLLSFFRGIQAPCRGSPLLVTDSPGG
>sp|P78563|RED1_HUMAN
MDIEDEENMSSSSTDVKENRNLDNVSPKDGSTPGPGEGSQLSNGGGGGPGRKRPLEEGSNGHSKYRLKKRRKTPGPVLPKNALMQLN
EIKPGLQYTLLSQTGPVHAPLFVMSVEVNGQVFEGSGPTKKKAKLHAAEKALRSFVQFPNASEAHLAMGRTLSVNTDFTSDQADFPD
TLFNGFETPDKAEPPFYVGSNGDDSFSSSGDLSLSASPVPASLAQPPLPVLPPFPPPSGKNPVMILNELRPGLKYDFLSESGESHAK
SFVMSVVVDGQFFEGSGRNKKLAKARAAQSALAAIFNLHLDQTPSRQPIPSEGLQLHLPQVLADAVSRLVLGKFGDLTDNFSSPHAR
RKVLAGVVMTTGTDVKDAKVISVSTGTKCINGEYMSDRGLALNDCHAEIISRRSLLRFLYTQLELYLNNKDDQKRSIFQKSERGGFR
LKENVQFHLYISTSPCGDARIFSPHEPILEGSRSYTQAGVQWCNHGSLQPRPPGLLSDPSTSTFQGAGTTEPADRHPNRKARGQLRT
KIESGEGTIPVRSNASIQTWDGVLQGERLLTMSCSDKIARWNVVGIQGSLLSIFVEPIYFSSIILGSLYHGDHLSRAMYQRISNIED
LPPLYTLNKPLLSGISNAEARQPGKAPNFSVNWTVGDSAIEVINATTGKDELGRASRLCKHALYCRWMRVHGKVPSHLLRSKITKPN
VYHESKLAAKEYQAAKARLFTAFIKAGLGAWVEKPTEQDQFSLTP
>sp|P63302|SELW_HUMAN
MALAVRVVYCGAUGYKSKYLQLKKKLEDEFPGRLDICGEGTPQATGFFEVMVAGKLIHSKKKGDGYVDTESKFLKLVAAIKAALAQG
>sp|P21127|CD11B_HUMAN
MGDEKDSWKVKTLDEILQEKKRRKEQEEKAEIKRLKNSDDRDSKRDSLEEGELRDHRMEITIRNSPYRREDSMEDRGEEDDSLAIKP
PQQMSRKEKVHHRKDEKRKEKRRHRSHSAEGGKHARVKEKEREHERRKRHREEQDKARREWERQKRREMAREHSRRERDRLEQLERK
RERERKMREQQKEQREQKERERRAEERRKEREARREVSAHHRTMREDYSDKVKASHWSRSPPRPPRERFELGDGRKPGEARPARAQK
PAQLKEEKMEERDLLSDLQDISDSERKTSSAESSSAESGSGSEEEEEEEEEEEEEEGSTSEESEEEEEEEEEEEEEETGSNSEEASEQS
AEEVSEEEMSEDEERENENHLLVVPESRFDRDSGESEEAEEEVGEGTPQSSALTEGDYVPDSPALSPIELKQELPKYLPALQGCRSV
EEFQCLNRIEEGTYGVVYRAKDKKTDEIVALKRLKMEKEKEGFPITSLREINTILKAQHPNIVTVREIVVGSNMDKIYIVMNYVEHD

LKSLMETMKQPFLPGEVKTLMIQLLRGVKHLHDNWILHRDLKTSNLLLSHAGILKVGDFGLAREYGSPLKAYTPVVVTLWYRAPELL
LGAKEYSTAVDMWSVGCIFGELLTQKPLFPGKSEIDQINKVFKDLGTPSEKIWPGYSELPAVKKMTFSEHPYNNLRKRFGALLSDQG
FDLMNKFLTYFPGRRISAEDGLKHEYFRETPLPIDPSMFPTWPAKSEQQRVKRGTSPRPPEGGLGYSQLGDDDLKETGFHLTTTNQG
ASAAGPGFSLKF
>sp|A8MVM7|YD021_HUMAN
MDKIRHTEADIFKNGSKRMIATVPLRHSIRDRKPSLHFLHSLASSSSLIYRNALLHKSYKLHLQKNKSQKEKHRHSKMKIAYKDTPR
NRLSRNAKKCLEDNKLVPISEVSLDPIISSNPLLRWWATSASNDSLLEELNNRFEQITNAWVQVSGDEAENCIHKKREHIENDHFKV
ASPLETCLLELEVSPVKMLFQKKYDLNELCTWFMQTTETQSLSLVRKANARNPLEVINTRGIKLGTKYSDFNASPFRKHFKKFALSS
PSKSAEKLHILHKVANSPLLNVKSNLAIARLKRTEFKRLHHERWKREGKLHNHGTVDWNSKRRNLRFFCQNQFLNKTEGETNADIPL
QGKSIVDNQCVLPPEIRGDLQQRVVMPDFKIHASFENKFKSEAKENGTNCSQKDFQKGPRLENVCPNSWRSKTLKDCRIFLRKLNCL
EHRNTFKLNTIIYSPESTDSGNTHQTHMEESKRFTLRSHSARQNSFKKQSKEIENANTNNPSADEFADHLGNSKLSKCVNFDKNPDS
FEVLSNLNKRKRPPWKITEMSTKRHKRQSCNSGQMANYFSKSLVSKIFGQPNFLAPSMKLVKLGAAKCTSALPHLLICPGFHTNKNI
YSRDIEIIFKIYYFNNVDVISFCIW
>sp|Q96LX7|CCD17_HUMAN
MDSHSGEPALLPCGTCDMVFRSSALLATHTQRFCIGHPTQEMTFGAQASVATEPQRAAVVPQEHQGVPQEPQGLPDQQASRSALKRL
TEEVQWLRLSLQEMRPWITEVPRVFAGPWTRSEARPQSPMSEAVGSPSERLRALFRTRARRVAEMEAQSRALQLRGEELSRRLQVVA
CTRGGMSRLFGLEQEIRELQAEAGRTRGALEVLGARIQELQAEPGNPLSSRREAELYSPVQKANPGTLAAEIRALREAYIRDGGRDP
GVLGQIWQLQVEASALELQRSQTRRGRAGATSGELPVVEAENRRLEAEILALQMQRGRAPLGPQDLRLLGDASLQPKGRRDPPLLPP
PVAPPLPPLPGFSEPQLPGTMTRNLGLDSHFLLPTSDMLGPAPYDPGAGLVIFYDFLRGLEASWIWVQLRTGLARDGRDTGRTTALP
PALCLPPPPAPGPMGNCAILASRQPVPRLPPSSSVSLVCELQVWQGLAWARAPQPKAWVSLGLFDQDQRVLSGRWRLPLRALPLDPS
LSLGQLNGIPQAGQAELFLRLVNARDAAVQTLAEINPASVHEYQYPPPVSSTSSLEASFLTPAVGFADPPPRTEEPLSGVKDRDEGL
GPHHSSDLPPVSF
>sp|Q676U5|A16L1_HUMAN
MSSGLRAADFPRWKRHISEQLRRRDRLQRQAFEEIILQYNKLLEKSDLHSVLAQKLQAEKHDVPNRHEISPGHDGTWNDNQLQEMAQ
LRIKHQEELTELHKKRGELAQLVIDLNNQMQRKDREMQMNEAKIAECLQTISDLETECLDLRTKLCDLERANQTLKDEYDALQITFT
ALEGKLRKTTEENQELVTRWMAEKAQEANRLNAENEKDSRRRQARLQKELAEAAKEPLPVEQDDDIEVIVDETSDHTEETSPVRAIS
RAATKRLSQPAGGLLDSITNIFGRRSVSSFPVPQDNVDTHPGSGKEVRVPATALCVFDAHDGEVNAVQFSPGSRLLATGGMDRRVKL
WEVFGEKCEFKGSLSGSNAGITSIEFDSAGSYLLAASNDFASRIWTVDDYRLRHTLTGHSGKVLSAKFLLDNARIVSGSHDRTLKLW
DLRSKVCIKTVFAGSSCNDIVCTEQCVMSGHFDKKIRFWDIRSESIVREMELLGKITALDLNPERTELLSCSRDDLLKVIDLRTNAI
KQTFSAPGFKCGSDWTRVVFSPDGSYVAAGSAEGSLYIWSVLTGKVEKVLSKQHSSSINAVAWSPSGSHVVSVDKGCKAVLWAQY
>sp|Q8N111|CEND_HUMAN
MESRGKSASSPKPDTKVPQVTTEAKVPPAADGKAPLTKPSKKEAPAEKQQPPAAPTTAPAKKTSAKADPALLNNHSNLKPAPTVPSS
PDATPEPKGPGDGAEEDEAASGGPGGRGPWSCENFNPLLVAGGVAVAAIALILGVAFLVRKK
>sp|P28908|TNR8_HUMAN
MRVLLAALGLLFLGALRAFPQDRPFEDTCHGNPSHYYDKAVRRCCYRCPMGLFPTQQCPQRPTDCRKQCEPDYYLDEADRCTACVTC
SRDDLVEKTPCAWNSSRVCECRPGMFCSTSAVNSCARCFFHSVCPAGMIVKFPGTAQKNTVCEPASPGVSPACASPENCKEPSSGTI
PQAKPTPVSPATSSASTMPVRGGTRLAQEAASKLTRAPDSPSSVGRPSSDPGLSPTQPCPEGSGDCRKQCEPDYYLDEAGRCTACVS
CSRDDLVEKTPCAWNSSRTCECRPGMICATSATNSCARCVPYPICAAETVTKPQDMAEKDTTFEAPPLGTQPDCNPTPENGEAPAST
SPTQSLLVDSQASKTLPIPTSAPVALSSTGKPVLDAGPVLFWVILVLVVVVGSSAFLLCHRRACRKRIRQKLHLCYPVQTSQPKLEL
VDSRPRRSSTQLRSGASVTEPVAEERGLMSQPLMETCHSVGAAYLESLPLQDASPAGGPSSPRDLPEPRVSTEHTNNKIEKIYIMKA
DTVIVGTVKAELPEGRGLAGPAEPELEEELEADHTPHYPEQETEPPLGSCSDVMLSVEEEGKEDPLPTAASGK
>sp|Q9BVL4|SELO_HUMAN
MAVYRAALGASLAAARLLPLGRCSPSPAPRSTLSGAAMEPAPRWLAGLRFDNRALRALPVEAPPPGPEGAPSAPRPVPGACFTRVQP
TPLRQPRLVALSEPALALLGLGAPPAREAEAEAALFFSGNALLPGAEPAAHCYCGHQFGQFAGQLGDGAAMYLGEVCTATGERWELQ

LKGAGPTPFSRQADGRKVLRSSIREFLCSEAMFHLGVPTTRAGACVTSESTVVRDVFYDGNPKYEQCTVVLRVASTFIRFGSFEIFK
SADEHTGRAGPSVGRNDIRVQLLDYVISSFYPEIQAAHASDSVQRNAAFFREVTRRTARMVAEWQCVGFCHGVLNTDNMSILGLTID
YGPFGFLDRYDPDHVCNASDNTGRYAYSKQPEVCRWNLRKLAEALQPELPLELGEAILAEEFDAEFQRHYLQKMRRKLGLVQVELEE
DGALVSKLLETMHLTGADFTNTFYLLSSFPVELESPGLAEFLARLMEQCASLEELRLAFRPQMDPRQLSMMLMLAQSNPQLFALMGT
RAGIARELERVEQQSRLEQLSAAELQSRNQGHWADWLQAYRARLDKDLEGAGDAAAWQAEHVRVMHANNPKYVLRNYIAQNAIEAAE
RGDFSEVRRVLKLLETPYHCEAGAATDAEATEADGADGRQRSYSSKPPLWAAELCVTUSS
>sp|P52848|NDST1_HUMAN
MPALACLRRLCRHVSPQAVLFLLFIFCLFSVFISAYYLYGWKRGLEPSADAPEPDCGDPPPVAPSRLLPLKPVQAATPSRTDPLVLV
FVESLYSQLGQEVVAILESSRFKYRTEIAPGKGDMPTLTDKGRGRFALIIYENILKYVNLDAWNRELLDKYCVAYGVGIIGFFKANE
NSLLSAQLKGFPLFLHSNLGLKDCSINPKSPLLYVTRPSEVEKGVLPGEDWTVFQSNHSTYEPVLLAKTRSSESIPHLGADAGLHAA
LHATVVQDLGLHDGIQRVLFGNNLNFWLHKLVFVDAVAFLTGKRLSLPLDRYILVDIDDIFVGKEGTRMKVEDVKALFDTQNELRAH
IPNFTFNLGYSGKFFHTGTNAEDAGDDLLLSYVKEFWWFPHMWSHMQPHLFHNQSVLAEQMALNKKFAVEHGIPTDMGYAVAPHHSG
VYPVHVQLYEAWKQVWSIRVTSTEEYPHLKPARYRRGFIHNGIMVLPRQTCGLFTHTIFYNEYPGGSSELDKIINGGELFLTVLLNP
ISIFMTHLSNYGNDRLGLYTFKHLVRFLHSWTNLRLQTLPPVQLAQKYFQIFSEEKDPLWQDPCEDKRHKDIWSKEKTCDRFPKLLI
IGPQKTGTTALYLFLGMHPDLSSNYPSSETFEEIQFFNGHNYHKGIDWYMEFFPIPSNTTSDFYFEKSANYFDSEVAPRRAAALLPK
AKVLTILINPADRAYSWYQHQRAHDDPVALKYTFHEVITAGSDASSKLRALQNRCLVPGWYATHIERWLSAYHANQILVLDGKLLRT
EPAKVMDMVQKFLGVTNTIDYHKTLAFDPKKGFWCQLLEGGKTKCLGKSKGRKYPEMDLDSRAFLKDYYRDHNIELSKLLYKMGQTL
PTWLREDLQNTR
>sp|Q8TAD2|IL17D_HUMAN
MLVAGFLLALPPSWAAGAPRAGRRPARPRGCADRPEELLEQLYGRLAAGVLSAFHHTLQLGPREQARNASCPAGGRPADRRFRPPTN
LRSVSPWAYRISYDPARYPRYLPEAYCLCRGCLTGLFGEEDVRFRSAPVYMPTVVLRRTPACAGGRSVYTEAYVTIPVGCTCVPEPE
KDADSINSSIDKQGAKLLLGPNDAPAGP
>sp|Q96PD7|DGAT2_HUMAN
MKTLIAAYSGVLRGERQAEADRSQRSHGGPALSREGSGRWGTGSSILSALQDLFSVTWLNRSKVEKQLQVISVLQWVLSFLVLGVAC
SAILMYIFCTDCWLIAVLYFTWLVFDWNTPKKGGRRSQWVRNWAVWRYFRDYFPIQLVKTHNLLTTRNYIFGYHPHGIMGLGAFCNF
STEATEVSKKFPGIRPYLATLAGNFRMPVLREYLMSGGICPVSRDTIDYLLSKNGSGNAIIIVVGGAAESLSSMPGKNAVTLRNRKG
FVKLALRHGADLVPIYSFGENEVYKQVIFEEGSWGRWVQKKFQKYIGFAPCIFHGRGLFSSDTWGLVPYSKPITTVVGEPITIPKLE
HPTQQDIDLYHTMYMEALVKLFDKHKTKFGLPETEVLEVN
>sp|O96028|NSD2_HUMAN
MEFSIKQSPLSVQSVVKCIKMKQAPEILGSANGKTPSCEVNRECSVFLSKAQLSSSLQEGVMQKFNGHDALPFIPADKLKDLTSRVF
NGEPGAHDAKLRFESQEMKGIGTPPNTTPIKNGSPEIKLKITKTYMNGKPLFESSICGDSAADVSQSEENGQKPENKARRNRKRSIK
YDSLLEQGLVEAALVSKISSPSDKKIPAKKESCPNTGRDKDHLLKYNVGDLVWSKVSGYPWWPCMVSADPLLHSYTKLKGQKKSARQ
YHVQFFGDAPERAWIFEKSLVAFEGEGQFEKLCQESAKQAPTKAEKIKLLKPISGKLRAQWEMGIVQAEEAASMSVEERKAKFTFLY
VGDQLHLNPQVAKEAGIAAESLGEMAESSGVSEEAAENPKSVREECIPMKRRRRAKLCSSAETLESHPDIGKSTPQKTAEADPRRGV
GSPPGRKKTTVSMPRSRKGDAASQFLVFCQKHRDEVVAEHPDASGEEIEELLRSQWSLLSEKQRARYNTKFALVAPVQAEEDSGNVN
GKKRNHTKRIQDPTEDAEAEDTPRKRLRTDKHSLRKRDTITDKTARTSSYKAMEAASSLKSQAATKNLSDACKPLKKRNRASTAASS
ALGFSKSSSPSASLTENEVSDSPGDEPSESPYESADETQTEVSVSSKKSERGVTAKKEYVCQLCEKPGSLLLCEGPCCGAFHLACLG
LSRRPEGRFTCSECASGIHSCFVCKESKTDVKRCVVTQCGKFYHEACVKKYPLTVFESRGFRCPLHSCVSCHASNPSNPRPSKGKMM
RCVRCPVAYHSGDACLAAGCSVIASNSIICTAHFTARKGKRHHAHVNVSWCFVCSKGGSLLCCESCPAAFHPDCLNIEMPDGSWFCN
DCRAGKKLHFQDIIWVKLGNYRWWPAEVCHPKNVPPNIQKMKHEIGEFPVFFFGSKDYYWTHQARVFPYMEGDRGSRYQGVRGIGRV
FKNALQEAEARFREIKLQREARETQESERKPPPYKHIKVNKPYGKVQIYTADISEIPKCNCKPTDENPCGFDSECLNRMLMFECHPQ
VCPAGEFCQNQCFTKRQYPETKIIKTDGKGWGLVAKRDIRKGEFVNEYVGELIDEEECMARIKHAHENDITHFYMLTIDKDRIIDAG
PKGNYSRFMNHSCQPNCETLKWTVNGDTRVGLFAVCDIPAGTELTFNYNLDCLGNEKTVCRCGASNCSGFLGDRPKTSTTLSSEEKG

KKTKKKTRRRRAKGEGKRQSEDECFRCGDGGQLVLCDRKFCTKAYHLSCLGLGKRPFGKWECPWHHCDVCGKPSTSFCHLCPNSFCK
EHQDGTAFSCTPDGRSYCCEHDLGAASVRSTKTEKPPPEPGKPKGKRRRRRGWRRVTEGK
>sp|Q9UI46|DNAI1_HUMAN
MIPASAKAPHKQPHKQSISIGRGTRKRDEDSGTEVGEGTDEWAQSKATVRPPDQLELTDAELKEEFTRILTANNPHAPQNIVRYSFK
EGTYKPIGFVNQLAVHYTQVGNLIPKDSDEGRRQHYRDELVAGSQESVKVISETGNLEEDEEPKELETEPGSQTDVPAAGAAEKVTE
EELMTPKQPKERKLTNQFNFSERASQTYNNPVRDRECQTEPPPRTNFSATANQWEIYDAYVEELEKQEKTKEKEKAKTPVAKKSGKM
AMRKLTSMESQTDDLIKLSQAAKIMERMVNQNTYDDIAQDFKYYDDAADEYRDQVGTLLPLWKFQNDKAKRLSVTALCWNPKYRDLF
AVGYGSYDFMKQSRGMLLLYSLKNPSFPEYMFSSNSGVMCLDIHVDHPYLVAVGHYDGNVAIYNLKKPHSQPSFCSSAKSGKHSDPV
WQVKWQKDDMDQNLNFFSVSSDGRIVSWTLVKRKLVHIDVIKLKVEGSTTEVPEGLQLHPVGCGTAFDFHKEIDYMFLVGTEEGKIY
KCSKSYSSQFLDTYDAHNMSVDTVSWNPYHTKVFMSCSSDWTVKIWDHTIKTPMFIYDLNSAVGDVAWAPYSSTVFAAVTTDGKAHI
FDLAINKYEAICNQPVAAKKNRLTHVQFNLIHPIIIVGDDRGHIISLKLSPNLRKMPKEKKGQEVQKGPAVEIAKLDKLLNLVREVK
IKT
>sp|O14957|QCR10_HUMAN
MVTRFLGPRYRELVKNWVPTAYTWGAVGAVGLVWATDWRLILDWVPYINGKFKKDN
>sp|P0C7Q2|ARMS2_HUMAN
MLRLYPGPMVTEAEGKGGPEMASLSSSVVPVSFISTLRESVLDPGVGGEGASDKQRSKLSLSHSMIPAAKIHTELCLPAFFSPAGTQ
RRFQQPQHHLTLSIIHTAAR
>sp|P62308|RUXG_HUMAN
MSKAHPPELKKFMDKKLSLKLNGGRHVQGILRGFDPFMNLVIDECVEMATSGQQNNIGMVVIRGNSIIMLEALERV
>sp|Q16842|SIA4B_HUMAN
MKCSLRVWFLSVAFLLVFIMSLLFTYSHHSMATLPYLDSGALDGTHRVKLVPGYAGLQRLSKERLSGKSCACRRCMGDAGASDWFDS
HFDGNISPVWTRENMDLPPDVQRWWMMLQPQFKSHNTNEVLEKLFQIVPGENPYRFRDPHQCRRCAVVGNSGNLRGSGYGQDVDGHN
FIMRMNQAPTVGFEQDVGSRTTHHFMYPESAKNLPANVSFVLVPFKVLDLLWIASALSTGQIRFTYAPVKSFLRVDKEKVQIYNPAF
FKYIHDRWTEHHGRYPSTGMLVLFFALHVCDEVNVYGFGADSRGNWHHYWENNRYAGEFRKTGVHDADFEAHIIDMLAKASKIEVYR
GN
>sp|P23515|OMGP_HUMAN
MEYQILKMSLCLFILLFLTPGILCICPLQCICTERHRHVDCSGRNLSTLPSGLQENIIHLNLSYNHFTDLHNQLTQYTNLRTLDISN
NRLESLPAHLPRSLWNMSAANNNIKLLDKSDTAYQWNLKYLDVSKNMLEKVVLIKNTLRSLEVLNLSSNKLWTVPTNMPSKLHIVDL
SNNSLTQILPGTLINLTNLTHLYLHNNKFTFIPDQSFDQLFQLQEITLYNNRWSCDHKQNITYLLKWMMETKAHVIGTPCSTQISSL
KEHNMYPTPSGFTSSLFTVSGMQTVDTINSLSVVTQPKVTKIPKQYRTKETTFGATLSKDTTFTSTDKAFVPYPEDTSTETINSHEA
AAATLTIHLQDGMVTNTSLTSSTKSSPTPMTLSITSGMPNNFSEMPQQSTTLNLWREETTTNVKTPLPSVANAWKVNASFLLLLNVV
VMLAV
>sp|Q93070|NAR4_HUMAN
MGPLINRCKKILLPTTVPPATMRIWLLGGLLPFLLLLSGLQRPTEGSEVAIKIDFDFAPGSFDDQYQGCSKQVMEKLTQGDYFTKDI
EAQKNYFRMWQKAHLAWLNQGKVLPQNMTTTHAVAILFYTLNSNVHSDFTRAMASVARTPQQYERSFHFKYLHYYLTSAIQLLRKDS
IMENGTLCYEVHYRTKDVHFNAYTGATIRFGQFLSTSLLKEEAQEFGNQTLFTIFTCLGAPVQYFSLKKEVLIPPYELFKVINMSYH
PRGNWLQLRSTGNLSTYNCQLLKASSKKCIPDPIAIASLSFLTSVIIFSKSRV
>sp|P0C7M6|IQCF3_HUMAN
MGSKCCKGGPDEDAVERQRRQKLLLAQLHHRKRVKAAGQIQAWWRGVLVRRTLLVAALRAWMIQCWWRTLVQRRIRQRRQALLRVYV
IQEQATVKLQSCIRMWQCRQCYRQMCNALCLFQVPESSLAFQTDGFLQVQYAIPSKQPEFHIEILSI
>sp|O43511|S26A4_HUMAN
MAAPGGRSEPPQLPEYSCSYMVSRPVYSELAFQQQHERRLQERKTLRESLAKCCSCSRKRAFGVLKTLVPILEWLPKYRVKEWLLSD
VISGVSTGLVATLQGMAYALLAAVPVGYGLYSAFFPILTYFIFGTSRHISVGPFPVVSLMVGSVVLSMAPDEHFLVSSSNGTVLNTT
MIDTAARDTARVLIASALTLLVGIIQLIFGGLQIGFIVRYLADPLVGGFTTAAAFQVLVSQLKIVLNVSTKNYNGVLSIIYTLVEIF

QNIGDTNLADFTAGLLTIVVCMAVKELNDRFRHKIPVPIPIEVIVTIIATAISYGANLEKNYNAGIVKSIPRGFLPPELPPVSLFSE
MLAASFSIAVVAYAIAVSVGKVYATKYDYTIDGNQEFIAFGISNIFSGFFSCFVATTALSRTAVQESTGGKTQVAGIISAAIVMIAI
LALGKLLEPLQKSVLAAVVIANLKGMFMQLCDIPRLWRQNKIDAVIWVFTCIVSIILGLDLGLLAGLIFGLLTVVLRVQFPSWNGLG
SIPSTDIYKSTKNYKNIEEPQGVKILRFSSPIFYGNVDGFKKCIKSTVGFDAIRVYNKRLKALRKIQKLIKSGQLRATKNGIISDAV
STNNAFEPDEDIEDLEELDIPTKEIEIQVDWNSELPVKVNVPKVPIHSLVLDCGAISFLDVVGVRSLRVIVKEFQRIDVNVYFASLQ
DYVIEKLEQCGFFDDNIRKDTFFLTVHDAILYLQNQVKSQEGQGSILETITLIQDCKDTLELIETELTEEELDVQDEAMRTLAS
>sp|O95347|SMC2_HUMAN
MHIKSIILEGFKSYAQRTEVNGFDPLFNAITGLNGSGKSNILDSICFLLGISNLSQVRASNLQDLVYKNGQAGITKASVSITFDNSD
KKQSPLGFEVHDEITVTRQVVIGGRNKYLINGVNANNTRVQDLFCSVGLNVNNPHFLIMQGRITKVLNMKPPEILSMIEEAAGTRMY
EYKKIAAQKTIEKKEAKLKEIKTILEEEITPTIQKLKEERSSYLEYQKVMREIEHLSRLYIAYQFLLAEDTKVRSAEELKEMQDKVI
KLQEELSENDKKIKALNHEIEELEKRKDKETGGILRSLEDALAEAQRVNTKSQSAFDLKKKNLACEESKRKELEKNMVEDSKTLAAK
EKEVKKITDGLHALQEASNKDAEALAAAQQHFNAVSAGLSSNEDGAEATLAGQMMACKNDISKAQTEAKQAQMKLKHAQQELKNKQA
EVKKMDSGYRKDQEALEAVKRLKEKLEAEMKKLNYEENKEESLLEKRRQLSRDIGRLKETYEALLARFPNLRFAYKDPEKNWNRNCV
KGLVASLISVKDTSATTALELVAGERLYNVVVDTEVTGKKLLERGELKRRYTIIPLNKISARCIAPETLRVAQNLVGPDNVHVALSL
VEYKPELQKAMEFVFGTTFVCDNMDNAKKVAFDKRIMTRTVTLGGDVFDPHGTLSGGARSQAASILTKFQELKDVQDELRIKENELR
ALEEELAGLKNTAEKYRQLKQQWEMKTEEADLLQTKLQQSSYHKQQEELDALKKTIEESEETLKNTKEIQRKAEEKYEVLENKMKNA
EAERERELKDAQKKLDCAKTKADASSKKMKEKQQEVEAITLELEELKREHTSYKQQLEAVNEAIKSYESQIEVMAAEVAKNKESVNK
AQEEVTKQKEVITAQDTVIKAKYAEVAKHKEQNNDSQLKIKELDHNISKHKREAEDGAAKVSKMLKDYDWINAERHLFGQPNSAYDF
KTNNPKEAGQRLQKLQEMKEKLGRNVNMRAMNVLTEAEERYNDLMKKKRIVENDKSKILTTIEDLDQKKNQALNIAWQKVNKDFGSI
FSTLLPGANAMLAPPEGQTVLDGLEFKVALGNTWKENLTELSGGQRSLVALSLILSMLLFKPAPIYILDEVDAALDLSHTQNIGQML
RTHFTHSQFIVVSLKEGMFNNANVLFKTKFVDGVSTVARFTQCQNGKISKEAKSKAKPPKGAHVEV
>sp|Q08174|PCDH1_HUMAN
MDSGAGGRRCPEAALLILGPPRMEHLRHSPGPGGQRLLLPSMLLALLLLLAPSPGHATRVVYKVPEEQPPNTLIGSLAADYGFPDVG
HLYKLEVGAPYLRVDGKTGDIFTTETSIDREGLRECQNQLPGDPCILEFEVSITDLVQNGSPRLLEGQIEVQDINDNTPNFASPVIT
LAIPENTNIGSLFPIPLASDRDAGPNGVASYELQAGPEAQELFGLQVAEDQEEKQPQLIVMGNLDRERWDSYDLTIKVQDGGSPPRA
SSALLRVTVLDTNDNAPKFERPSYEAELSENSPIGHSVIQVKANDSDQGANAEIEYTFHQAPEVVRRLLRLDRNTGLITVQGPVDRE
DLSTLRFSVLAKDRGTNPKSARAQVVVTVKDMNDNAPTIEIRGIGLVTHQDGMANISEDVAEETAVALVQVSDRDEGENAAVTCVVA
GDVPFQLRQASETGSDSKKKYFLQTTTPLDYEKVKDYTIEIVAVDSGNPPLSSTNSLKVQVVDVNDNAPVFTQSVTEVAFPENNKPG
EVIAEITASDADSGSNAELVYSLEPEPAAKGLFTISPETGEIQVKTSLDREQRESYELKVVAADRGSPSLQGTATVLVNVLDCNDND
PKFMLSGYNFSVMENMPALSPVGMVTVIDGDKGENAQVQLSVEQDNGDFVIQNGTGTILSSLSFDREQQSTYTFQLKAVDGGVPPRS
AYVGVTINVLDENDNAPYITAPSNTSHKLLTPQTRLGETVSQVAAEDFDSGVNAELIYSIAGGNPYGLFQIGSHSGAITLEKEIERR
HHGLHRLVVKVSDRGKPPRYGTALVHLYVNETLANRTLLETLLGHSLDTPLDIDIAGDPEYERSKQRGNILFGVVAGVVAVALLIAL
AVLVRYCRQREAKSGYQAGKKETKDLYAPKPSGKASKGNKSKGKKSKSPKPVKPVEDEDEAGLQKSLKFNLMSDAPGDSPRIHLPLN
YPPGSPDLGRHYRSNSPLPSIQLQPQSPSASKKHQVVQDLPPANTFVGTGDTTSTGSEQYSDYSYRTNPPKYPSKQVGQPFQLSTPQ
PLPHPYHGAIWTEVWE
>sp|Q86YJ6|THNS2_HUMAN
MWYVSTRGVAPRVNFEGALFSGYAPDGGLFMPEELPQLDRGTLCQWSTLSYPGLVKELCALFIGSELLPKDELNDLIDRAFSRFRHR
EVVHLSRLRNGLNVLELWHGVTYAFKDLSLSCTTQFLQYFLEKREKHVTVVVGTSGDTGSAAIESVQGAKNMDIIVLLPKGHCTKIQ
ELQMTTVLKQNVHVFGVEGNSDELDEPIKTVFADVAFVKKHNLMSLNSINWSRVLVQMAHHFFAYFQCTPSLDTHPLPLVEVVVPTG
AAGNLAAGYIAQKIGLPIRLVVAVNRNDIIHRTVQQGDFSLSEAVKSTLASAMDIQVPYNMERVFWLLSGSDSQVTRALMEQFERTQ
SVNLPKELHSKLSEAVTSVSVSDEAITQTMGRCWDENQYLLCPHSAVAVNYHYQQIDRQQPSTPRCCLAPASAAKFPEAVLAAGLTP
ETPAEIVALEHKETRCTLMRRGDNWMLMLRDTIEDLSRQWRSHALNTSQ
>sp|Q8N3R3|TCAIM_HUMAN

MFCHLRPMRRLCLEKIFPHWFPFSRALSGAEAVNALRPFYFAVHPDFFGQHPVEREINENSLKRLSVYLENLQKPGFKSLKPTQLTF
YVRETDQSSSDGQEPFSTSGFRAVKFTLHTRDLLSTVLYILNSCSLSVEHIQSLNTNMHTQPLKEAKRMPDRPIKWDKSYYSFTGFK
DPDEDLEQVSRVETTLTSWLDNNGKSAVKKLKNSLPLRKELDRLKDELSHQLQLSDIRWQRSWGIAHRCSQLHSLSRLAQQNLETLK
KAKGCTIIFTDRSGMSAVGHVMLGTMDVHHHWTKLFERLPSYFDLQRRLMILEDQISYLLGGIQVVYIEELQPVLTLEEYYSLLDVF
YNRLLKSRILFHPRSLRGLQMILNSDRYAPSLHELGHFNIPTLCDPANLQWFILTKAQQARENMKRKEELKVIENELIQASTKKFSL
EKLYKEPSISSIQMVDCCKRLLEQSLPYLHGMHLCISHFYSVMQDGDLCIPWNWKNGEAIK
>sp|Q6XZB0|LIPI_HUMAN
MRVYIFLCLMCWVRSDNKRPCLEFSQLSVKDSFRDLFIPRIETILMMYTRNNLNCAEPLFEQNNSLNVNFNTQKKTVWLIHGYRPVG
SIPLWLQNFVRILLNEEDMNVIVVDWSRGATTFIYNRAVKNTRKVAVSLSVHIKNLLKHGASLDNFHFIGVSLGAHISGFVGKIFHG
QLGRITGLDPAGPRFSRKPPYSRLDYTDAKFVDVIHSDSNGLGIQEPLGHIDFYPNGGNKQPGCPKSIFSGIQFIKCNHQRAVHLFM
ASLETNCNFISFPCRSYKDYKTSLCVDCDCFKEKSCPRLGYQAKLFKGVLKERMEGRPLRTTVFLDTSGTYPFCTYYFVLSIIVPDK
TMMDGSFSFKLLNQLGMIEEPRLYEKNKPFYKLQEVKILAQFYNDFVNISSIGLTYFQSSNLQCSTCTYKIQRLMLKSLTYPERPPL
CRYNIVLKDREEVFLNPNTCTPKNT
>sp|Q9Y580|RBM7_HUMAN
MGAAAAEADRTLFVGNLETKVTEELLFELFHQAGPVIKVKIPKDKDGKPKQFAFVNFKHEVSVPYAMNLLNGIKLYGRPIKIQFRSG
SSHAPQDVSLSYPQHHVGNSSPTSTSPSRYERTMDNMTSSAQIIQRSFSSPENFQRQAVMNSALRQMSYGGKFGSSPLDQSGFSPSV
QSHSHSFNQSSSSQWRQGTPSSQRKVRMNSYPYLADRHYSREQRYTDHGSDHHYRGKRDDFFYEDRNHDDWSHDYDNRRDSSRDGKW
RSSRH
>sp|Q07000|1C15_HUMAN
MRVMAPRTLLLLLSGALALTETWACSHSMRYFYTAVSRPGRGEPHFIAVGYVDDTQFVRFDSDAASPRGEPRAPWVEQEGPEYWDRE
TQNYKRQAQTDRVNLRKLRGYYNQSEAGSHIIQRMYGCDLGPDGRLLRGHDQLAYDGKDYIALNEDLRSWTAADTAAQITQRKWEAA
REAEQLRAYLEGTCVEWLRRYLENGKETLQRAEHPKTHVTHHPVSDHEATLRCWALGFYPAEITLTWQRDGEDQTQDTELVETRPAG
DGTFQKWAAVVVPSGEEQRYTCHVQHEGLPEPLTLRWEPSSQPTIPIVGIVAGLAVLAVLAVLGAVMAVVMCRRKSSGGKGGSCSQA
ASSNSAQGSDESLIACKA
>sp|P39059|COFA1_HUMAN
MAPRRNNGQCWCLLMLLSVSTPLPAVTQTRGATETASQGHLDLTQLIGVPLPSSVSFVTGYGGFPAYSFGPGANVGRPARTLIPSTF
FRDFAISVVVKPSSTRGGVLFAITDAFQKVIYLGLRLSGVEDGHQRIILYYTEPGSHVSQEAAAFSVPVMTHRWNRFAMIVQGEEVT
LLVNCEEHSRIPFQRSSQALAFESSAGIFMGNAGATGLERFTGSLQQLTVHPDPRTPEELCDPEESSASGETSGLQEADGVAEILEA
VTYTQASPKEAKVEPINTPPTPSSPFEDMELSGEPVPEGTLETTNMSIIQHSSPKQGSGEILNDTLEGVHSVDGDPITDSGSGAGAF
LDIAEEKNLAATAAGLAEVPISTAGEAEASSVPTGGPTLSMSTENPEEGVTPGPDNEERLAATAAGEAEALASMPGEVEASGVAPGE
LDLSMSAQSLGEEATVGPSSEDSLTTAAAATEVSLSTFEDEEASGVPTDGLAPLTATMAPERAVTSGPGDEEDLAAATTEEPLITAG
GEESGSPPPDGPPLPLPTVAPERWITPAQREHVGMKGQAGPKGEKGDAGEELPGPPEPSGPVGPTAGAEAEGSGLGWGSDVGSGSGD
LVGSEQLLRGPPGPPGPPGLPGIPGKPGTDVFMGPPGSPGEDGPAGEPGPPGPEGQPGVDGATGLPGMKGEKGARGPNGSVGEKGDP
GNRGLPGPPGKKGQAGPPGVMGPPGPPGPPGPPGPGCTMGLGFEDTEGSGSTQLLNEPKLSRPTAAIGLKGEKGDRGPKGERGMDGA
SIVGPPGPRGPPGHIKVLSNSLINITHGFMNFSDIPELVGPPGPDGLPGLPGFPGPRGPKGDTGLPGFPGLKGEQGEKGEPGAILTE
DIPLERLMGKKGEPGMHGAPGPMGPKGPPGHKGEFGLPGRPGRPGLNGLKGTKGDPGVIMQGPPGLPGPPGPPGPPGAVINIKGAIF
PIPVRPHCKMPVDTAHPGSPELITFHGVKGEKGSWGLPGSKGEKGDQGAQGPPGPPLDLAYLRHFLNNLKGENGDKGFKGEKGEKGD
INGSFLMSGPPGLPGNPGPAGQKGETVVGPQGPPGAPGLPGPPGFGRPGDPGPPGPPGPPGPPAILGAAVALPGPPGPPGQPGLPGS
RNLVTAFSNMDDMLQKAHLVIEGTFIYLRDSTEFFIRVRDGWKKLQLGELIPIPADSPPPPALSSNPHQLLPPPNPISSANYEKPAL
HLAALNMPFSGDIRADFQCFKQARAAGLLSTYRAFLSSHLQDLSTIVRKAERYSLPIVNLKGQVLFNNWDSIFSGHGGQFNMHIPIY
SFDGRDIMTDPSWPQKVIWHGSSPHGVRLVDNYCEAWRTADTAVTGLASPLSTGKILDQKAYSCANRLIVLCIENSFMTDARK
>sp|Q13356|PPIL2_HUMAN
MGKRQHQKDKMYITCAEYTHFYGGKKPDLPQTNFRRLPFDHCSLSLQPFVYPVCTPDGIVFDLLNIVPWLKKYGTNPSNGEKLDGRS
LIKLNFSKNSEGKYHCPVLFTVFTNNTHIVAVRTTGNVYAYEAVEQLNIKAKNFRDLLTDEPFSRQDIITLQDPTNLDKFNVSNFYH

VKNNMKIIDPDEEKAKQDPSYYLKNTNAETRETLQELYKEFKGDEILAATMKAPEKKKVDKLNAAHYSTGKVSASFTSTAMVPETTH
EAAAIDEDVLRYQFVKKKGYVRLHTNKGDLNLELHCDLTPKTCENFIRLCKKHYYDGTIFHRSIRNFVIQGGDPTGTGTGGESYWGK
PFKDEFRPNLSHTGRGILSMANSGPNSNRSQFFITFRSCAYLDKKHTIFGRVVGGFDVLTAMENVESDPKTDRPKEEIRIDATTVFV
DPYEEADAQIAQERKTQLKVAPETKVKSSQPQAGSQGPQTFRQGVGKYINPAATKRAAEEEPSTSATVPMSKKKPSRGFGDFSSW
>sp|O75410|TACC1_HUMAN
MAFSPWQILSPVQWAKWTWSAVRGGAAGEDEAGGPEGDPEEEDSQAETKSLSFSSDSEGNFETPEAETPIRSPFKESCDPSLGLAGP
GAKSQESQEADEQLVAEVVEKCSSKTCSKPSENEVPQQAIDSHSVKNFREEPEHDFSKISIVRPFSIETKDSTDISAVLGTKAAHGC
VTAVSGKALPSSPPDALQDEAMTEGSMGVTLEASAEADLKAGNSCPELVPSRRSKLRKPKPVPLRKKAIGGEFSDTNAAVEGTPLPK
ASYHFSPEELDENTSPLLGDARFQKSPPDLKETPGTLSSDTNDSGVELGEESRSSPLKLEFDFTEDTGNIEARKALPRKLGRKLGST
LTPKIQKDGISKSAGLEQPTDPVARDGPLSQTSSKPDPSQWESPSFNPFGSHSVLQNSPPLSSEGSYHFDPDNFDESMDPFKPTTTL
TSSDFCSPTGNHVNEILESPKKAKSRLITSGCKVKKHETQSLALDACSRDEGAVISQISDISNRDGHATDEEKLASTSCGQKSAGAE
VKGEPEEDLEYFECSNVPVSTINHAFSSSEAGIEKETCQKMEEDGSTVLGLLESSAEKAPVSVSCGGESPLDGICLSESDKTAVLTL
IREEIITKEIEANEWKKKYEETRQEVLEMRKIVAEYEKTIAQMIEDEQRTSMTSQKSFQQLTMEKEQALADLNSVERSLSDLFRRYE
NLKGVLEGFKKNEEALKKCAQDYLARVKQEEQRYQALKIHAEEKLDKANEEIAQVRTKAKAESAALHAGLRKEQMKVESLERALQQK
NQEIEELTKICDELIAKLGKTD
>sp|A8K5M9|CO062_HUMAN
METWRKGSFRNASFFKQLSLGRPRRLRRQSSVLSQASTAGGDHEEYSNREVIRELQGRPDGRRLPLWGDEQPRATLLAPPKPPRLYR
ESSSCPNILEPPPAYTAAYSATLPSALSLSSALHQHSEKGLVDTPCFQRTPTPDLSDPFLSFKVDLGISLLEEVLQMLREQFPSEPS
F
>sp|A6NEH8|ZNAS2_HUMAN
MGVWGKKHLRLTHAPSPTFSHLPPSPQAGEPGIDGISLAVVCAAFIRIHPARRHPARNGSRLACTPTPRPNAGLEVVSSARVAASLP
SEGGWTSGAPRSSGLSLPGSAWQPPPLPVLRKPAWPGSPAVKNESKFPNRGSRNFPRRRLPPAPVSGEPPERCKLAREIRWRLWKAH
EGWGGGAKRPLGDPAWSGVKR
>sp|Q68CK6|ACS2B_HUMAN
MHWLRKVQGLCTLWGTQMSSRTLYINSRQLVSLQWGHQEVPAKFNFASDVLDHWADMEKAGKRLPSPALWWVNGKGKELMWNFRELS
ENSQQAANILSGACGLQRGDRVAVMLPRVPEWWLVILGCIRAGLIFMPGTIQMKSTDILYRLQMSKAKAIVAGDEVIQEVDTVASEC
PSLRIKLLVSEKSCDGWLNFKKLLNEASTTHHCVETGSQEASAIYFTSGTSGLPKMAEHSYSSLGLKAKMDAGWTGLQASDIMWTIS
DTGWILNILGSLLESWTLGACTFVHLLPKFDPLVILKTLSSYPIKSMMGAPIVYRMLLQQDLSSYKFPHLQNCLAGGESLLPETLEN
WRAQTGLDIREFYGQTETGLTCMVSKTMKIKPGYMGTAASCYDVQVIDDKGNVLPPGTEGDIGIRVKPIRPIGIFSGYVENPDKTAA
NIRGDFWLLGDRGIKDEDGYFQFMGRADDIINSSGYRIGPSEVENALMKHPAVVETAVISSPDPVRGEVVKAFVILASQFLSHDPEQ
LTKELQQHVKSVTAPYKYPRKIEFVLNLPKTVTGKIQRTKLRDKEWKMSGKARAQ
>sp|Q96RU3|FNBP1_HUMAN
MSWGTELWDQFDNLEKHTQWGIDILEKYIKFVKERTEIELSYAKQLRNLSKKYQPKKNSKEEEEYKYTSCKAFISNLNEMNDYAGQH
EVISENMASQIIVDLARYVQELKQERKSNFHDGRKAQQHIETCWKQLESSKRRFERDCKEADRAQQYFEKMDADINVTKADVEKARQ
QAQIRHQMAEDSKADYSSILQKFNHEQHEYYHTHIPNIFQKIQEMEERRIVRMGESMKTYAEVDRQVIPIIGKCLDGIVKAAESIDQ
KNDSQLVIEAYKSGFEPPGDIEFEDYTQPMKRTVSDNSLSNSRGEGKPDLKFGGKSKGKLWPFIKKNKLMSLLTSPHQPPPPPPASA
SPSAVPNGPQSPKQQKEPLSHRFNEFMTSKPKIHCFRSLKRGLSLKLGATPEDFSNLPPEQRRKKLQQKVDELNKEIQKEMDQRDAI
TKMKDVYLKNPQMGDPASLDHKLAEVSQNIEKLRVETQKFEAWLAEVEGRLPARSEQARRQSGLYDSQNPPTVNNCAQDRESPDGSY
TEEQSQESEMKVLATDFDDEFDDEEPLPAIGTCKALYTFEGQNEGTISVVEGETLYVIEEDKGDGWTRIRRNEDEEGYVPTSYVEVC
LDKNAKDS
>sp|P01037|CYTN_HUMAN
MAQYLSTLLLLLATLAVALAWSPKEEDRIIPGGIYNADLNDEWVQRALHFAISEYNKATKDDYYRRPLRVLRARQQTVGGVNYFFDV
EVGRTICTKSQPNLDTCAFHEQPELQKKQLCSFEIYEVPWENRRSLVKSRCQES
>sp|P27540|ARNT_HUMAN

MAATTANPEMTSDVPSLGPAIASGNSGPGIQGGGAIVQRAIKRRPGLDFDDDGEGNSKFLRCDDDQMSNDKERFARSDDEQSSADKE
RLARENHSEIERRRRNKMTAYITELSDMVPTCSALARKPDKLTILRMAVSHMKSLRGTGNTSTDGSYKPSFLTDQELKHLILEAADG
FLFIVSCETGRVVYVSDSVTPVLNQPQSEWFGSTLYDQVHPDDVDKLREQLSTSENALTGRILDLKTGTVKKEGQQSSMRMCMGSRR
SFICRMRCGSSSVDPVSVNRLSFVRNRCRNGLGSVKDGEPHFVVVHCTGYIKAWPPAGVSLPDDDPEAGQGSKFCLVAIGRLQVTSS
PNCTDMSNVCQPTEFISRHNIEGIFTFVDHRCVATVGYQPQELLGKNIVEFCHPEDQQLLRDSFQQVVKLKGQVLSVMFRFRSKNQE
WLWMRTSSFTFQNPYSDEIEYIICTNTNVKNSSQEPRPTLSNTIQRPQLGPTANLPLEMGSGQLAPRQQQQQTELDMVPGRDGLASY
NHSQVVQPVTTTGPEHSKPLEKSDGLFAQDRDPRFSEIYHNINADQSKGISSSTVPATQQLFSQGNTFPPTPRPAENFRNSGLAPPV
TIVQPSASAGQMLAQISRHSNPTQGATPTWTPTTRSGFSAQQVATQATAKTRTSQFGVGSFQTPSSFSSMSLPGAPTASPGAAAYPS
LTNRGSNFAPETGQTAGQFQTRTAEGVGVWPQWQGQQPHHRSSSSEQHVQQPPAQQPGQPEVFQEMLSMLGDQSNSYNNEEFPDLTM
FPPFSE
>sp|P51677|CCR3_HUMAN
MTTSLDTVETFGTTSYYDDVGLLCEKADTRALMAQFVPPLYSLVFTVGLLGNVVVVMILIKYRRLRIMTNIYLLNLAISDLLFLVTL
PFWIHYVRGHNWVFGHGMCKLLSGFYHTGLYSEIFFIILLTIDRYLAIVHAVFALRARTVTFGVITSIVTWGLAVLAALPEFIFYET
EELFEETLCSALYPEDTVYSWRHFHTLRMTIFCLVLPLLVMAICYTGIIKTLLRCPSKKKYKAIRLIFVIMAVFFIFWTPYNVAILL
SSYQSILFGNDCERSKHLDLVMLVTEVIAYSHCCMNPVIYAFVGERFRKYLRHFFHRHLLMHLGRYIPFLPSEKLERTSSVSPSTAE
PELSIVF
>sp|Q2NL98|VMAC_HUMAN
MSAPPALQIREANAHLAAVHRRAAELEARLDAAERTVHAQAERLALHDQQLRAALDELGRAKDREIATLQEQLMTSEATVHSLQATV
HQRDELIRQLQPRAELLQDICRRRPPLAGLLDALAEAERLGPLPASDPGHPPPGGPGPPLDNSTGEEADRDHLQPAVFGTTV
>sp|O43826|G6PT1_HUMAN
MAAQGYGYYRTVIFSAMFGGYSLYYFNRKTFSFVMPSLVEEIPLDKDDLGFITSSQSAAYAISKFVSGVLSDQMSARWLFSSGLLLV
GLVNIFFAWSSTVPVFAALWFLNGLAQGLGWPPCGKVLRKWFEPSQFGTWWAILSTSMNLAGGLGPILATILAQSYSWRSTLALSGA
LCVVVSFLCLLLIHNEPADVGLRNLDPMPSEGKKGSLKEESTLQELLLSPYLWVLSTGYLVVFGVKTCCTDWGQFFLIQEKGQSALV
GSSYMSALEVGGLVGSIAAGYLSDRAMAKAGLSNYGNPRHGLLLFMMAGMTVSMYLFRVTVTSDSPKLWILVLGAVFGFSSYGPIAL
FGVIANESAPPNLCGTSHAIVGLMANVGGFLAGLPFSTIAKHYSWSTAFWVAEVICAASTAAFFLLRNIRTKMGRVSKKAE
>sp|Q96BR6|ZN669_HUMAN
MVSGLRLASRSGEEGWLKPAVARLGPPRHRLRNLRTESPWRSRGSVLFCSGPGRAGRAAEPLHPVCTCGRHFRRPEPCREPLASPIQ
DSVAFEDVAVNFTQEEWALLDSSQKNLYREVMQETCRNLASVGSQWKDQNIEDHFEKPGKDIRNHIVQRLCESKEDGQYGEVVSQIP
NLDLNENISTGLKPCECSICGKVFVRHSLLNRHILAHSGYKPYGEKQYKCEQCGKFFVSVPGVRRHMIMHSGNPAYKCTICGKAFYF
LNSVERHQRTHTGEKPYKCKQCGKAFTVSGSCLIHERTHTGEKPYECKECGKTFRFSCSFKTHERTHTGERPYKCTKCDKAFSCSTS
LRYHGSIHTGERPYECKQCGKAFSRLSSLCNHRSTHTGEKPYECKQCDQAFSRLSSLHLHERIHTGEKPYECKKCGKAYTRSSHLTR
HERSHDIEAGCSDSAYNPSTLGGQGVWIA
>sp|P04198|MYCN_HUMAN
MPSCSTSTMPGMICKNPDLEFDSLQPCFYPDEDDFYFGGPDSTPPGEDIWKKFELLPTPPLSPSRGFAEHSSEPPSWVTEMLLENEL
WGSPAEEDAFGLGGLGGLTPNPVILQDCMWSGFSAREKLERAVSEKLQHGRGPPTAGSTAQSPGAGAASPAGRGHGGAAGAGRAGAA
LPAELAHPAAECVDPAVVFPFPVNKREPAPVPAAPASAPAAGPAVASGAGIAAPAGAPGVAPPRPGGRQTSGGDHKALSTSGEDTLS
DSDDEDDEEEDEEEEIDVVTVEKRRSSSNTKAVTTFTITVRPKNAALGPGRAQSSELILKRCLPIHQQHNYAAPSPYVESEDAPPQK
KIKSEASPRPLKSVIPPKAKSLSPRNSDSEDSERRRNHNILERQRRNDLRSSFLTLRDHVPELVKNEKAAKVVILKKATEYVHSLQA
EEHQLLLEKEKLQARQQQLLKKIEHARTC
>sp|Q09019|DMWD_HUMAN
MAAGGAEGGSGPGAAMGDCAEIKSQFRTREGFYKLLPGDGAARRSGPASAQTPVPPQPPQPPPGPASASGPGAAGPASSPPPAGPGP
GPALPAVRLSLVRLGEPDSAGAGEPPATPAGLGSGGDRVCFNLGRELYFYPGCCRRGSQRSIDLNKPIDKRIYKGTQPTCHDFNQFT
AATETISLLVGFSAGQVQYLDLIKKDTSKLFNEERLIDKTKVTYLKWLPESESLFLASHASGHLYLYNVSHPCASAPPQYSLLKQGE
GFSVYAAKSKAPRNPLAKWAVGEGPLNEFAFSPDGRHLACVSQDGCLRVFHFDSMLLRGLMKSYFGGLLCVCWSPDGRYVVTGGEDD

LVTVWSFTEGRVVARGHGHKSWVNAVAFDPYTTRAEEAATAAGADGERSGEEEEEEPEAAGTGSAGGAPLSPLPKAGSITYRFGSAG
QDTQFCLWDLTEDVLYPHPPLARTRTLPGTPGTTPPAASSSRGGEPGPGPLPRSLSRSNSLPHPAGGGKAGGPGVAAEPGTPFSIGR
FATLTLQERRDRGAEKEHKRYHSLGNISRGGSGGSGSGGEKPSGPVPRSRLDPAKVLGTALCPRIHEVPLLEPLVCKKIAQERLTVL
LFLEDCIITACQEGLICTWARPGKAFTDEETEAQTGEGSWPRSPSKSVVEGISSQPGNSPSGTVV
>sp|P14854|CX6B1_HUMAN
MAEDMETKIKNYKTAPFDSRFPNQNQTRNCWQNYLDFHRCQKAMTAKGGDISVCEWYQRVYQSLCPTSWVTDWDEQRAEGTFPGKI
>sp|Q5VVC0|CA146_HUMAN
MAESGKEKIKWTTTIIISSSLKSYEVATALENRSHKVRYSDSVENGSIIFSLSGVAFLLMDTKECLLSTEEIFLAKIEKFINIHQNS
FLVLSAALHGPEEWKLMFRIQQRFLGCNLRILPVHNTVNAINLMCTIAKTTSKPYIDSICYRMITAKAYIIEQSPVWKTLQKIKLNS
DSVNPN
>sp|P31151|S10A7_HUMAN
MSNTQAERSIIGMIDMFHKYTRRDDKIEKPSLLTMMKENFPNFLSACDKKGTNYLADVFEKKDKNEDKKIDFSEFLSLLGDIATDYH
KQSHGAAPCSGGSQ
>sp|O00213|APBB1_HUMAN
MSVPSSLSQSAINANSHGGPALSLPLPLHAAHNQLLNAKLQATAVGPKDLRSAMGEGGGPEPGPANAKWLKEGQNQLRRAATAHRDQ
NRNVTLTLAEEASQEPEMAPLGPKGLIHLYSELELSAHNAANRGLRGPGLIISTQEQGPDEGEEKAAGEAEEEEEDDDDEEEEEDLS
SPPGLPEPLESVEAPPRPQALTDGPREHSKSASLLFGMRNSAASDEDSSWATLSQGSPSYGSPEDTDSFWNPNAFETDSDLPAGWMR
VQDTSGTYYWHIPTGTTQWEPPGRASPSQGSSPQEESQLTWTGFAHGEGFEDGEFWKDEPSDEAPMELGLKEPEEGTLTFPAQSLSP
EPLPQEEEKLPPRNTNPGIKCFAVRSLGWVEMTEEELAPGRSSVAVNNCIRQLSYHKNNLHDPMSGGWGEGKDLLLQLEDETLKLVE
PQSQALLHAQPIISIRVWGVGRDSGRERDFAYVARDKLTQMLKCHVFRCEAPAKNIATSLHEICSKIMAERRNARCLVNGLSLDHSK
LVDVPFQVEFPAPKNELVQKFQVYYLGNVPVAKPVGVDVINGALESVLSSSSREQWTPSHVSVAPATLTILHQQTEAVLGECRVRFL
SFLAVGRDVHTFAFIMAAGPASFCCHMFWCEPNAASLSEAVQAACMLRYQKCLDARSQASTSCLPAPPAESVARRVGWTVRRGVQSL
WGSLKPKRLGAHTP
>sp|Q7Z3D4|LYSM3_HUMAN
MAGRHQNRSFPLPGVQSSGQVHAFGNCSDSDILEEDAEVYELRSRGKEKVRRSTSRDRLDDIIVLTKDIQEGDTLNAIALQYCCTVA
DIKRVNNLISDQDFFALRSIKIPVKKFSSLTETLCPPKGRQTSRHSSVQYSSEQQEILPANDSLAYSDSAGSFLKEVDRDIEQIVKC
TDNKRENLNEVVSALTAQQMRFEPDNKNTQRKDPYYGADWGIGWWTAVVIMLIVGIITPVFYLLYYEILAKVDVSHHSTVDSSHLHS
KITPPSQQREMENGIVPTKGIHFSQQDDHKLYSQDSQSPAAQQET
>sp|P62312|LSM6_HUMAN
MSLRKQTPSDFLKQIIGRPVVVKLNSGVDYRGVLACLDGYMNIALEQTEEYVNGQLKNKYGDAFIRGNNVLYISTQKRRM
>sp|Q9UBC5|MYO1A_HUMAN
MPLLEGSVGVEDLVLLEPLVEESLLKNLQLRYENKEIYTYIGNVVISVNPYQQLPIYGPEFIAKYQDYTFYELKPHIYALANVAYQS
LRDRDRDQCILITGESGSGKTEASKLVMSYVAAVCGKGEQVNSVKEQLLQSNPVLEAFGNAKTIRNNNSSRFGKYMDIEFDFKGSPL
GGVITNYLLEKSRLVKQLKGERNFHIFYQLLAGADEQLLKALKLERDTTGYAYLNHEVSRVDGMDDASSFRAVQSAMAVIGFSEEEI
RQVLEVTSMVLKLGNVLVADEFQASGIPASGIRDGRGVREIGEMVGLNSEEVERALCSRTMETAKEKVVTALNVMQAQYARDALAKN
IYSRLFDWIVNRINESIKVGIGEKKKVMGVLDIYGFEILEDNSFEQFVINYCNEKLQQVFIEMTLKEEQEEYKREGIPWTKVDYFDN
GIICKLIEHNQRGILAMLDEECLRPGVVSDSTFLAKLNQLFSKHGHYESKVTQNAQRQYDHTMGLSCFRICHYAGKVTYNVTSFIDK
NNDLLFRDLLQAMWKAQHPLLRSLFPEGNPKQASLKRPPTAGAQFKSSVAILMKNLYSKSPNYIRCIKPNEHQQRGQFSSDLVATQA
RYLGLLENVRVRRAGYAHRQGYGPFLERYRLLSRSTWPHWNGGDREGVEKVLGELSMSSGELAFGKTKIFIRSPKTLFYLEEQRRLR
LQQLATLIQKIYRGWRCRTHYQLMRKSQILISSWFRGNMQKKCYGKIKASVLLIQAFVRGWKARKNYRKYFRSEAALTLADFIYKSM
VQKFLLGLKNNLPSTNVLDKTWPAAPYKCLSTANQELQQLFYQWKCKRFRDQLSPKQVEILREKLCASELFKGKKASYPQSVPIPFC
GDYIGLQGNPKLQKLKGGEEGPVLMAEAVKKVNRGNGKTSSRILLLTKGHVILTDTKKSQAKIVIGLDNVAGVSVTSLKDGLFSLHL
SEMSSVGSKGDFLLVSEHVIELLTKMYRAVLDATQRQLTVTVTEKFSVRFKENSVAVKVVQGPAGGDNSKLRYKKKGSHCLEVTVQ
>sp|Q86WS5|TMPSC_HUMAN

MRLGLLSVALLFVGSSHLYSDHYSPSGRHRLGPSPEPAASSQQAEAVRKRLRRRREGGAHAEDCGTAPLKDVLQGSRIIGGTEAQAG
AWPWVVSLQIKYGRVLVHVCGGTLVRERWVLTAAHCTKDASDPLMWTAVIGTNNIHGRYPHTKKIKIKAIIIHPNFILESYVNDIAL
FHLKKAVRYNDYIQPICLPFDVFQILDGNTKCFISGWGRTKEEGNATNILQDAEVHYISREMCNSERSYGGIIPNTSFCAGDEDGAF
DTCRGDSGGPLMCYLPEYKRFFVMGITSYGHGCGRRGFPGVYIGPSFYQKWLTEHFFHASTQGILTINILRGQILIALCFVILLATT
>sp|Q07654|TFF3_HUMAN
MAARALCMLGLVLALLSSSSAEEYVGLSANQCAVPAKDRVDCGYPHVTPKECNNRGCCFDSRIPGVPWCFKPLQEAECTF
>sp|P01584|IL1B_HUMAN
MAEVPELASEMMAYYSGNEDDLFFEADGPKQMKCSFQDLDLCPLDGGIQLRISDHHYSKGFRQAASVVVAMDKLRKMLVPCPQTFQE
NDLSTFFPFIFEEEPIFFDTWDNEAYVHDAPVRSLNCTLRDSQQKSLVMSGPYELKALHLQGQDMEQQVVFSMSFVQGEESNDKIPV
ALGLKEKNLYLSCVLKDDKPTLQLESVDPKNYPKKKMEKRFVFNKIEINNKLEFESAQFPNWYISTSQAENMPVFLGGTKGGQDITD
FTMQFVSS
>sp|P22301|IL10_HUMAN
MHSSALLCCLVLLTGVRASPGQGTQSENSCTHFPGNLPNMLRDLRDAFSRVKTFFQMKDQLDNLLLKESLLEDFKGYLGCQALSEMI
QFYLEEVMPQAENQDPDIKAHVNSLGENLKTLRLRLRRCHRFLPCENKSKAVEQVKNAFNKLQEKGIYKAMSEFDIFINYIEAYMTM
KIRN
>sp|Q96DR7|ARHGQ_HUMAN
MDGESEVDFSSNSITPLWRRRSIPQPHQVLGRSKPRPQSYQSPNGLLITDFPVEDGGTLLAAQIPAQVPTASDSRTVHRSPLLLGAQ
RRAVANGGTASPEYRAASPRLRRPKSPKLPKAVPGGSPKSPANGAVTLPAPPPPPVLRPPRTPNAPAPCTPEEDLTGLTASPVPSPT
ANGLAANNDSPGSGSQSGRKAKDPERGLFPGPQKSSSEQKLPLQRLPSQENELLENPSVVLSTNSPAALKVGKQQIIPKSLASEIKI
SKSNNQNVEPHKRLLKVRSMVEGLGGPLGHAGEESEVDNDVDSPGSLRRGLRSTSYRRAVVSGFDFDSPTSSKKKNRMSQPVLKVVM
EDKEKFSSLGRIKKKMLKGQGTFDGEENAVLYQNYKEKALDIDSDEESEPKEQKSDEKIVIHHKPLRSTWSQLSAVKRKGLSQTVSQ
EERKRQEAIFEVISSEHSYLLSLEILIRMFKNSKELSDTMTKTERHHLFSNITDVCEASKKFFIELEARHQNNIFIDDISDIVEKHT
ASTFDPYVKYCTNEVYQQRTLQKLLATNPSFKEVLSRIESHEDCRNLPMISFLILPMQRVTRLPLLMDTICQKTPKDSPKYEVCKRA
LKEVSKLVRLCNEGARKMERTEMMYTINSQLEFKIKPFPLVSSSRWLVKRGELTAYVEDTVLFSRRTSKQQVYFFLFNDVLIITKKK
SEESYNVNDYSLRDQLLVESCDNEELNSSPGKNSSTMLYSRQSSASHLFTLTVLSNHANEKVEMLLGAETQSERARWITALGHSSGK
PPADRTSLTQVEIVRSFTAKQPDELSLQVADVVLIYQRVSDGWYEGERLRDGERGWFPMECAKEITCQATIDKNVERMGRLLGLETN
V
>sp|A6NMZ7|CO6A6_HUMAN
MMLLILFLVIICSHISVNQDSGPEYADVVFLVDSSDRLGSKSFPFVKMFITKMISSLPIEADKYRVALAQYSDKLHSEFHLSTFKGR
SPMLNHLRKNFGFIGGSLQIGKALQEAHRTYFSAPANGRDKKQFPPILVVLASSESEDNVEEASKALRKDGVKIISVGVQKASEENL
KAMATSQFHFNLRTVRDLSMFSQNMTHIIKDVIKYKEGAVDDIFVEACQGPSMADVVFLLDMSINGSEENFDYLKGFLEESVSALDI
KENCMRVGLVAYSNETKVINSLSMGINKSEVLQHIQNLSPRTGKAYTGAAIKKLRKEVFSARNGSRKNQGVPQIAVLVTHRDSEDNV
TKAAVNLRREGVTIFTLGIEGASDTQLEKIASHPAEQYVSKLKTFADLAAHNQTFLKKLRNQITHTVSVFSERTETLKSGCVDTEEA
DIYLLIDGSGSTQATDFHEMKTFLSEVVGMFNIAPHKVRVGAVQYADSWDLEFEINKYSNKQDLGKAIENIRQMGGNTNTGAALNFT
LSLLQKAKKQRGNKVPCHLVVLTNGMSKDSILEPANRLREEHIRVYAIGIKEANQTQLREIAGEEKRVYYVHDFDALKDIRNQVVQE
ICTEEACKEMKADIMFLVDSSGSIGPENFSKMKTFMKNLVSKSQIGPDRVQIGVVQFSDINKEEFQLNRFMSQSDISNAIDQMAHIG
QTTLTGSALSFVSQYFSPTKGARPNIRKFLILITDGEAQDIVKEPAVVLRQEGVIIYSVGVFGSNVTQLEEISGRPEMVFYVENFDI
LQRIEDDLVFGICSPREECKRIEVLDVVFVIDSSGSIDYDEYNIMKDFMIGLVKKADVGKNQVRFGALKYADDPEVLFYLDDFGTKL
EVISVLQNDQAMGGSTYTAEALGFSDHMFTEARGSRLNKGVPQVLIVITDGESHDADKLNATAKALRDKGILVLAVGIDGANPVELL
AMAGSSDKYFFVETFGGLKGIFSDVTASVCNSSKVDCEIDKVDLVFLMDGSTSIQPNDFKKMKEFLASVVQDFDVSLNRVRIGAAQF
SDTYHPEFPLGTFIGEKEISFQIENIKQIFGNTHIGAALREVEHYFRPDMGSRINTGTPQVLLVLTDGQSQDEVAQAAEALRHRGID
IYSVGIGDVDDQQLIQITGTAEKKLTVHNFDELKKVNKRIVRNICTTAGESNCFVDVVVGFDVSTQEKGQTLLEGQPWMETYLQDIL
RAISSLNGVSCEVGTETQVSVAFQVTNAMEKYSPKFEIYSENILNSLKDITVKGPSLLNANLLDSLWDTFQNKSAARGKVVLLFSDG
LDDDVEKLEQKSDELRKEGLNALITVALDGPADSSDLADLPYIEFGKGFEYRTQLSIGMRELGSRLSKQLVNVAERTCCCLFCKCIG

GDGTMGDPGPPGKRGPPGFKGSEGYLGEEGIAGERGAPGPVGEQGTKGCYGTKGPKGNRGLNGQEGEVGENGIDGLNGEQGDNGLPG
RKGEKGDEGSQGSPGKRGTPGDRGAKGLRGDPGAPGVDSSIEGPTGLKGERGRQGRRGWPGPPGTPGSRRKTAAHGRRGHTGPQGTA
GIPGPDGLEGSLGLKGPQGPRGEAGVKGEKGGVGSKGPQGPPGPGGEAGNQGRLGSQGNKGEPGDLGEKGAVGFPGPRGLQGNDGSP
GYGSVGRKGAKGQEGFPGESGPKGEIGDPGGPGETGLKGARGKMISAGLPGEMGSPGEPGPPGRKGVKGAKGLASFSTCELIQYVRD
RSPGRHGKPECPVHPTELVFALDHSRDVTEQEFERMKEMMAFLVRDIKVRENSCPVGAHIAILSYNSHARHLVRFSDAYKKSQLLRE
IETIPYERSSASREIGRAMRFISRNVFKRTLPGAHTRKIATFFSSGQSADAHSITTAAMEFGALEIIPVVITFSNVPSVRRAFAIDD
TGTFQVIVVPSGADYIPALERLQRCTFCYDVCKPDASCDQARPPPVQSYMDAAFLLDASRNMGSAEFEDIRAFLGALLDHFEITPEP
ETSVTGDRVALLSHAPPDFLPNTQKSPVRAEFNLTTYRSKRLMKRHVHESVKQLNGDAFIGHALQWTLDNVFLSTPNLRRNKVIFVI
SAGETSHLDGEILKKESLRAKCQGYALFVFSLGPIWDDKELEDLASHPLDHHLVQLGRIHKPDHSYGVKFVKSFINSIRRAINKYPP
INLKIKCNRLNSIDPKQPPRPFRSFVPGPLKATLKEDVLQKAKFFQDKKYLSRVARSGRDDAIQNFMRSTSHTFKNGRMIESAPKQH
D
>sp|P0C7H8|KRA23_HUMAN
MTGSCCGSTLSSLSYGGGCCQPCCCRDPCCCRPVTCQTTVCRPVTCVPRCTRPICEPCRRPVCCDPCSLQEGCCRPITCCPSSCTAV
VCRPCCWATTCCQPVSVQSPCCRPPCGQPTPCSTTCRTSSC
>sp|H3BQW9|F229A_HUMAN
MLPSSTPGPGHATETCPAPPGPERSPAARAPAAASSLGPVSTAGRAPRGLDMSAQEPPQGRRFPIEAGDSRGLAAAPESQDSPEAVA
TEHNPVRPLRRCPGCHCLTLLHVPIDVYLAMGGSPRARAT
>sp|O14802|RPC1_HUMAN
MVKEQFRETDVAKKISHICFGMKSPEEMRQQAHIQVVSKNLYSQDNQHAPLLYGVLDHRMGTSEKDRPCETCGKNLADCLGHYGYID
LELPCFHVGYFRAVIGILQMICKTCCHIMLSQEEKKQFLDYLKRPGLTYLQKRGLKKKISDKCRKKNICHHCGAFNGTVKKCGLLKI
IHEKYKTNKKVVDPIVSNFLQSFETAIEHNKEVEPLLGRAQENLNPLVVLNLFKRIPAEDVPLLLMNPEAGKPSDLILTRLLVPPLC
IRPSVVSDLKSGTNEDDLTMKLTEIIFLNDVIKKHRISGAKTQMIMEDWDFLQLQCALYINSELSGIPLNMAPKKWTRGFVQRLKGK
QGRFRGNLSGKRVDFSGRTVISPDPNLRIDEVAVPVHVAKILTFPEKVNKANINFLRKLVQNGPEVHPGANFIQQRHTQMKRFLKYG
NREKMAQELKYGDIVERHLIDGDVVLFNRQPSLHKLSIMAHLARVKPHRTFRFNECVCTPYNADFDGDEMNLHLPQTEEAKAEALVL
MGTKANLVTPRNGEPLIAAIQDFLTGAYLLTLKDTFFDRAKACQIIASILVGKDEKIKVRLPPPTILKPVTLWTGKQIFSVILRPSD
DNPVRANLRTKGKQYCGKGEDLCANDSYVTIQNSELMSGSMDKGTLGSGSKNNIFYILLRDWGQNEAADAMSRLARLAPVYLSNRGF
SIGIGDVTPGQGLLKAKYELLNAGYKKCDEYIEALNTGKLQQQPGCTAEETLEALILKELSVIRDHAGSACLRELDKSNSPLTMALC
GSKGSFINISQMIACVGQQAISGSRVPDGFENRSLPHFEKHSKLPAAKGFVANSFYSGLTPTEFFFHTMAGREGLVDTAVKTAETGY
MQRRLVKSLEDLCSQYDLTVRSSTGDIIQFIYGGDGLDPAAMEGKDEPLEFKRVLDNIKAVFPCPSEPALSKNELILTTESIMKKSE
FLCCQDSFLQEIKKFIKGVSEKIKKTRDKYGINDNGTTEPRVLYQLDRITPTQVEKFLETCRDKYMRAQMEPGSAVGALCAQSIGEP
GTQMTLKTFHFAGVASMNITLGVPRIKEIINASKAISTPIITAQLDKDDDADYARLVKGRIEKTLLGEISEYIEEVFLPDDCFILVK
LSLERIRLLRLEVNAETVRYSICTSKLRVKPGDVAVHGEAVVCVTPRENSKSSMYYVLQFLKEDLPKVVVQGIPEVSRAVIHIDEQS
GKEKYKLLVEGDNLRAVMATHGVKGTRTTSNNTYEVEKTLGIEAARTTIINEIQYTMVNHGMSIDRRHVMLLSDLMTYKGEVLGITR
FGLAKMKESVLMLASFEKTADHLFDAAYFGQKDSVCGVSECIIMGIPMNIGTGLFKLLHKADRDPNPPKRPLIFDTNEFHIPLVT
>sp|Q969N2|PIGT_HUMAN
MAAAMPLALLVLLLLGPGGWCLAEPPRDSLREELVITPLPSGDVAATFQFRTRWDSELQREGVSHYRLFPKALGQLISKYSLRELHL
SFTQGFWRTRYWGPPFLQAPSGAELWVWFQDTVTDVDKSWKELSNVLSGIFCASLNFIDSTNTVTPTASFKPLGLANDTDHYFLRYA
VLPREVVCTENLTPWKKLLPCSSKAGLSVLLKADRLFHTSYHSQAVHIRPVCRNARCTSISWELRQTLSVVFDAFITGQGKKDWSLF
RMFSRTLTEPCPLASESRVYVDITTYNQDNETLEVHPPPTTTYQDVILGTRKTYAIYDLLDTAMINNSRNLNIQLKWKRPPENEAPP
VPFLHAQRYVSGYGLQKGELSTLLYNTHPYRAFPVLLLDTVPWYLRLYVHTLTITSKGKENKPSYIHYQPAQDRLQPHLLEMLIQLP
ANSVTKVSIQFERALLKWTEYTPDPNHGFYVSPSVLSALVPSMVAAKPVDWEESPLFNSLFPVSDGSNYFVRLYTEPLLVNLPTPDF
SMPYNVICLTCTVVAVCYGSFYNLLTRTFHIEEPRTGGLAKRLANLIRRARGVPPL
>sp|Q8IUH4|ZDH13_HUMAN

MEGPGLGSQCRNHSHGPHPPGFGRYGICAHENKELANAREALPLIEDSSNCDIVKATQYGIFERCKELVEAGYDVRQPDKENVSLLH
WAAINNRLDLVKFYISKGAVVDQLGGDLNSTPLHWAIRQGHLPMVILLLQHGADPTLIDGEGFSSIHLAVLFQHMPIIAYLISKGQS
VNMTDVNGQTPLMLSAHKVIGPEPTGFLLKFNPSLNVVDKIHQNTPLHWAVAAGNVNAVDKLLEAGSSLDIQNVKGETPLDMALQNK
NQLIIHMLKTEAKMRANQKFRLWRWLQKCELFLLLMLSVITMWAIGYILDFNSDSWLLKGCLLVTLFFLTSLFPRFLVGYKNLVYLP
TAFLLSSVFWIFMTWFILFFPDLAGAPFYFSFIFSIVAFLYFFYKTWATDPGFTKASEEEKKVNIITLAETGSLDFRTFCTSCLIRK
PLRSLHCHVCNCCVARYDQHCLWTGRCIGFGNHHYYIFFLFFLSMVCGWIIYGSFIYLSSHCATTFKEDGLWTYLNQIVACSPWVLY
ILMLATFHFSWSTFLLLNQLFQIAFLGLTSHERISLQKQSKHMKQTLSLRKTPYNLGFMQNLADFFQCGCFGLVKPCVVDWTSQYTM
VFHPAREKVLRSV
>sp|O14559|RHG33_HUMAN
MVARSTDSLDGPGEGSVQPLPTAGGPSVKGKPGKRLSAPRGPFPRLADCAHFHYENVDFGHIQLLLSPDREGPSLSGENELVFGVQV
TCQGRSWPVLRSYDDFRSLDAHLHRCIFDRRFSCLPELPPPPEGARAAQMLVPLLLQYLETLSGLVDSNLNCGPVLTWMELDNHGRR
LLLSEEASLNIPAVAAAHVIKRYTAQAPDELSFEVGDIVSVIDMPPTEDRSWWRGKRGFQVGFFPSECVELFTERPGPGLKADADGP
PCGIPAPQGISSLTSAVPRPRGKLAGLLRTFMRSRPSRQRLRQRGILRQRVFGCDLGEHLSNSGQDVPQVLRCCSEFIEAHGVVDGI
YRLSGVSSNIQRLRHEFDSERIPELSGPAFLQDIHSVSSLCKLYFRELPNPLLTYQLYGKFSEAMSVPGEEERLVRVHDVIQQLPPP
HYRTLEYLLRHLARMARHSANTSMHARNLAIVWAPNLLRSMELESVGMGGAAAFREVRVQSVVVEFLLTHVDVLFSDTFTSAGLDPA
GRCLLPRPKSLAGSCPSTRLLTLEEAQARTQGRLGTPTEPTTPKAPASPAERRKGERGEKQRKPGGSSWKTFFALGRGPSVPRKKPL
PWLGGTRAPPQPSGSRPDTVTLRSAKSEESLSSQASGAGLQRLHRLRRPHSSSDAFPVGPAPAGSCESLSSSSSSESSSSESSSSSS
ESSAAGLGALSGSPSHRTSAWLDDGDELDFSPPRCLEGLRGLDFDPLTFRCSSPTPGDPAPPASPAPPAPASAFPPRVTPQAISPRG
PTSPASPAALDISEPLAVSVPPAVLELLGAGGAPASATPTPALSPGRSLRPHLIPLLLRGAEAPLTDACQQEMCSKLRGAQGPLGPD
MESPLPPPPLSLLRPGGAPPPPPKNPARLMALALAERAQQVAEQQSQQECGGTPPASQSPFHRSLSLEVGGEPLGTSGSGPPPNSLA
HPGAWVPGPPPYLPRQQSDGSLLRSQRPMGTSRRGLRGPAQVSAQLRAGGGGRDAPEAAAQSPCSVPSQVPTPGFFSPAPRECLPPF
LGVPKPGLYPLGPPSFQPSSPAPVWRSSLGPPAPLDRGENLYYEIGASEGSPYSGPTRSWSPFRSMPPDRLNASYGMLGQSPPLHRS
PDFLLSYPPAPSCFPPDHLGYSAPQHPARRPTPPEPLYVNLALGPRGPSPASSSSSSPPAHPRSRSDPGPPVPRLPQKQRAPWGPRT
PHRVPGPWGPPEPLLLYRAAPPAYGRGGELHRGSLYRNGGQRGEGAGPPPPYPTPSWSLHSEGQTRSYC
>sp|P49863|GRAK_HUMAN
MTKFSSFSLFFLIVGAYMTHVCFNMEIIGGKEVSPHSRPFMASIQYGGHHVCGGVLIDPQWVLTAAHCQYRFTKGQSPTVVLGAHSL
SKNEASKQTLEIKKFIPFSRVTSDPQSNDIMLVKLQTAAKLNKHVKMLHIRSKTSLRSGTKCKVTGWGATDPDSLRPSDTLREVTVT
VLSRKLCNSQSYYNGDPFITKDMVCAGDAKGQKDSCKGDSGGPLICKGVFHAIVSGGHECGVATKPGIYTLLTKKYQTWIKSNLVPP
HTN
>sp|Q9UHD2|TBK1_HUMAN
MQSTSNHLWLLSDILGQGATANVFRGRHKKTGDLFAIKVFNNISFLRPVDVQMREFEVLKKLNHKNIVKLFAIEEETTTRHKVLIME
FCPCGSLYTVLEEPSNAYGLPESEFLIVLRDVVGGMNHLRENGIVHRDIKPGNIMRVIGEDGQSVYKLTDFGAARELEDDEQFVSLY
GTEEYLHPDMYERAVLRKDHQKKYGATVDLWSIGVTFYHAATGSLPFRPFEGPRRNKEVMYKIITGKPSGAISGVQKAENGPIDWSG
DMPVSCSLSRGLQVLLTPVLANILEADQEKCWGFDQFFAETSDILHRMVIHVFSLQQMTAHKIYIHSYNTATIFHELVYKQTKIISS
NQELIYEGRRLVLEPGRLAQHFPKTTEENPIFVVSREPLNTIGLIYEKISLPKVHPRYDLDGDASMAKAITGVVCYACRIASTLLLY
QELMRKGIRWLIELIKDDYNETVHKKTEVVITLDFCIRNIEKTVKVYEKLMKINLEAAELGEISDIHTKLLRLSSSQGTIETSLQDI
DSRLSPGGSLADAWAHQEGTHPKDRNVEKLQVLLNCMTEIYYQFKKDKAERRLAYNEEQIHKFDKQKLYYHATKAMTHFTDECVKKY
EAFLNKSEEWIRKMLHLRKQLLSLTNQCFDIEEEVSKYQEYTNELQETLPQKMFTASSGIKHTMTPIYPSSNTLVEMTLGMKKLKEE
MEGVVKELAENNHILERFGSLTMDGGLRNVDCL
>sp|Q99062|CSF3R_HUMAN
MARLGNCSLTWAALIILLLPGSLEECGHISVSAPIVHLGDPITASCIIKQNCSHLDPEPQILWRLGAELQPGGRQQRLSDGTQESII
TLPHLNHTQAFLSCCLNWGNSLQILDQVELRAGYPPAIPHNLSCLMNLTTSSLICQWEPGPETHLPTSFTLKSFKSRGNCQTQGDSI
LDCVPKDGQSHCCIPRKHLLLYQNMGIWVQAENALGTSMSPQLCLDPMDVVKLEPPMLRTMDPSPEAAPPQAGCLQLCWEPWQPGLH
INQKCELRHKPQRGEASWALVGPLPLEALQYELCGLLPATAYTLQIRCIRWPLPGHWSDWSPSLELRTTERAPTVRLDTWWRQRQLD

PRTVQLFWKPVPLEEDSGRIQGYVVSWRPSGQAGAILPLCNTTELSCTFHLPSEAQEVALVAYNSAGTSRPTPVVFSESRGPALTRL
HAMARDPHSLWVGWEPPNPWPQGYVIEWGLGPPSASNSNKTWRMEQNGRATGFLLKENIRPFQLYEIIVTPLYQDTMGPSQHVYAYS
QEMAPSHAPELHLKHIGKTWAQLEWVPEPPELGKSPLTHYTIFWTNAQNQSFSAILNASSRGFVLHGLEPASLYHIHLMAASQAGAT
NSTVLTLMTLTPEGSELHIILGLFGLLLLLTCLCGTAWLCCSPNRKNPLWPSVPDPAHSSLGSWVPTIMEEDAFQLPGLGTPPITKL
TVLEEDEKKPVPWESHNSSETCGLPTLVQTYVLQGDPRAVSTQPQSQSGTSDQVLYGQLLGSPTSPGPGHYLRCDSTQPLLAGLTPS
PKSYENLWFQASPLGTLVTPAPSQEDDCVFGPLLNFPLLQGIRVHGMEALGSF
>sp|P59510|ATS20_HUMAN
MWVAKWLTGLLYHLSLFITRSWEVDFHPRQEALVRTLTSYEVVIPERVNEFGEVFPQSHHFSRQKRSSEALEPMPFRTHYRFTAYGQ
LFQLNLTADASFLAAGYTEVHLGTPERGAWESDAGPSDLRHCFYRGQVNSQEDYKAVVSLCGGLTGTFKGQNGEYFLEPIMKADGNE
YEDGHNKPHLIYRQDLNNSFLQTLKYCSVSESQIKETSLPFHTYSNMNEDLNVMKERVLGHTSKNVPLKDERRHSRKKRLISYPRYI
EIMVTADAKVVSAHGSNLQNYILTLMSIVATIYKDPSIGNLIHIVVVKLVMIHREEEGPVINFDGATTLKNFCSWQQTQNDLDDVHP
SHHDTAVLITREDICSSKEKCNMLGLSYLGTICDPLQSCFINEEKGLISAFTIAHELGHTLGVQHDDNPRCKEMKVTKYHVMAPALS
FHMSPWSWSNCSRKYVTEFLDTGYGECLLDKPDEEIYNLPSELPGSRYDGNKQCELAFGPGSQMCPHINICMHLWCTSTEKLHKGCF
TQHVPPADGTDCGPGMHCRHGLCVNKETETRPVNGEWGPWEPYSSCSRTCGGGIESATRRCNRPEPRNGGNYCVGRRMKFRSCNTDS
CPKGTQDFREKQCSDFNGKHLDISGIPSNVRWLPRYSGIGTKDRCKLYCQVAGTNYFYLLKDMVEDGTPCGTETHDICVQGQCMAAG
CDHVLNSSAKIDKCGVCGGDNSSCKTITGVFNSSHYGYNVVVKIPAGATNVDIRQYSYSGQPDDSYLALSDAEGNFLFNGNFLLSTS
KKEINVQGTRTVIEYSGSNNAVERINSTNRQEKEILIEVLCVGNLYNPDVHYSFNIPLEERSDMFTWDPYGPWEGCTKMCQGLQRRN
ITCIHKSDHSVVSDKECDHLPLPSFVTQSCNTDCELRWHVIGKSECSSQCGQGYRTLDIHCMKYSIHEGQTVQVDDHYCGDQLKPPT
QELCHGNCVFTRWHYSEWSQCSRSCGGGERSRESYCMNNFGHRLADNECQELSRVTRENCNEFSCPSWAASEWSECLVTCGKGTKQR
QVWCQLNVDHLSDGFCNSSTKPESLSPCELHTCASWQVGPWGPCTTTCGHGYQMRDVKCVNELASAVLEDTECHEASRPSDRQSCVL
TPCSFISKLETALLPTVLIKKMAQWRHGSWTPCSVSCGRGTQARYVSCRDALDRIADESYCAHLPRPAEIWDCFTPCGEWQAGDWSP
CSASCGHGKTTRQVLCMNYHQPIDENYCDPEVRPLMEQECSLAACPPAHSHFPSSPVQPSYYLSTNLPLTQKLEDNENQVVHPSVRG
NQWRTGPWGSCSSSCSGGLQHRAVVCQDENGQSASYCDAASKPPELQQCGPGPCPQWNYGNWGECSQTCGGGIKSRLVICQFPNGQI
LEDHNCEIVNKPPSVIQCHMHACPADVSWHQEPWTSCSASCGKGRKYREVFCIDQFQRKLEDTNCSQVQKPPTHKACRSVRCPSWKA
NSWNECSVTCGSGVQQRDVYCRLKGVGQVVEEMCDQSTRPCSQRRCWSQDCVQHKGMERGRLNCSTSCERKDSHQRMECTDNQIRQV
NEIVYNSSTISLTSKNCRNPPCNYIVVTADSSQCANNCGFSYRQRITYCTEIPSTKKHKLHRLRPIVYQECPVVPSSQVYQCINSCL
HLATWKVGKWSKCSVTCGIGIMKRQVKCITKHGLSSDLCLNHLKPGAQKKCYANDCKSFTTCKEIQVKNHIRKDGDYYLNIKGRIIK
IYCADMYLENPKEYLTLVQGEENFSEVYGFRLKNPYQCPFNGSRREDCECDNGHLAAGYTVFSKIRIDLTSMQIKTTDLLFSKTIFG
NAVPFATAGDCYSAFRCPQGQFSINLSGTGMKISSTAKWLTQGSYTSVSIRRSEDGTRFFGKCGGYCGKCLPHMTTGLPIQVI
>sp|Q9UEU0|VTI1B_HUMAN
MASSAASSEHFEKLHEIFRGLHEDLQGVPERLLGTAGTEEKKKLIRDFDEKQQEANETLAEMEEELRYAPLSFRNPMMSKLRNYRKD
LAKLHREVRSTPLTATPGGRGDMKYGIYAVENEHMNRLQSQRAMLLQGTESLNRATQSIERSHRIATETDQIGSEIIEELGEQRDQL
ERTKSRLVNTSENLSKSRKILRSMSRKVTTNKLLLSIIILLELAILGGLVYYKFFRSH
>sp|P38484|INGR2_HUMAN
MRPTLLWSLLLLLGVFAAAAAAPPDPLSQLPAPQHPKIRLYNAEQVLSWEPVALSNSTRPVVYQVQFKYTDSKWFTADIMSIGVNCT
QITATECDFTAASPSAGFPMDFNVTLRLRAELGALHSAWVTMPWFQHYRNVTVGPPENIEVTPGEGSLIIRFSSPFDIADTSTAFFC
YYVHYWEKGGIQQVKGPFRSNSISLDNLKPSRVYCLQVQAQLLWNKSNIFRVGHLSNISCYETMADASTELQQVILISVGTFSLLSV
LAGACFFLVLKYRGLIKYWFHTPPSIPLQIEEYLKDPTQPILEALDKDSSPKDDVWDSVSIISFPEKEQEDVLQTL
>sp|Q96PH6|DB118_HUMAN
MKLLLLALPMLVLLPQVIPAYSGEKKCWNRSGHCRKQCKDGEAVKDTCKNLRACCIPSNEDHRRVPATSPTPLSDSTPGIIDDILTV
RFTTDYFEVSSKKDMVEESEAGRGTETSLPNVHHSS
>sp|Q8IYT1|FA71A_HUMAN
MNADFLLPYYTAQSGSSMSMFNTTMGKLQRQLYKGEYDIFKYAPIFESDFIQITKRGEVIDVHNRVRMVTMGIARTSPILPLPDVML
LARPATGCEEYAGHGQATKRKKRKAAKNLELTRLLPLRFVRISVQDHEKQQLRLKFATGRSCYLQLCPALDTRDDLFAYWEKLIYLL

RPPMESNSSTCGIPAEDMMWMPVFQEDRRSLGAVNLQGKGDQDQVSIQSLHMVSEVCGATSAAYAGGEGLQNDFNKPTNVLNASIPK
TSTELAEEPATGGIKEAAAAGAAAGAATGTVAGALSVAAANSAPGQVSAAIAGAATIGAGGNKGNMALAGTASMAPNSTKVAVAGAA
GKSSEHVSSASMSLSREGSVSLAIAGVVLTSRTAAEADMDAAAGPPVSTRQSKSSLSGQHGRERTQASAEGCKEGRERREKDRALGR
SSHRRRTGESRHKTRGDKIAQKSSSRSSFSHRANRDDKKEKGCGNPGSSRHRDSHKGVSHTPISKESRTSHKSGRSLWTTSSGSSKG
LGRVSSFLRNVRANLTTKVVGTPHGRDVNVMAKMAERSTNVAIAETAEGGQGLETVGSMTPDIMETVTFEAH
>sp|Q99829|CPNE1_HUMAN
MAHCVTLVQLSISCDHLIDKDIGSKSDPLCVLLQDVGGGSWAELGRTERVRNCSSPEFSKTLQLEYRFETVQKLRFGIYDIDNKTPE
LRDDDFLGGAECSLGQIVSSQVLTLPLMLKPGKPAGRGTITVSAQELKDNRVVTMEVEARNLDKKDFLGKSDPFLEFFRQGDGKWHL
VYRSEVIKNNLNPTWKRFSVPVQHFCGGNPSTPIQVQCSDYDSDGSHDLIGTFHTSLAQLQAVPAEFECIHPEKQQKKKSYKNSGTI
RVKICRVETEYSFLDYVMGGCQINFTVGVDFTGSNGDPSSPDSLHYLSPTGVNEYLMALWSVGSVVQDYDSDKLFPAFGFGAQVPPD
WQVSHEFALNFNPSNPYCAGIQGIVDAYRQALPQVRLYGPTNFAPIINHVARFAAQAAHQGTASQYFMLLLLTDGAVTDVEATREAV
VRASNLPMSVIIVGVGGADFEAMEQLDADGGPLHTRSGQAAARDIVQFVPYRRFQNAPREALAQTVLAEVPTQLVSYFRAQGWAPLK
PLPPSAKDPAQAPQA
>sp|Q96HE9|PRR11_HUMAN
MPKFKQRRRKLKAKAERLFKKKEASHFQSKLITPPPPPPSPERVGISSIDISQSRSWLTSSWNFNFPNIRDAIKLWTNRVWSIYSWC
QNCITQSLEVLKDTIFPSRICHRELYSVKQQFCILESKLCKLQEALKTISESSSCPSCGQTCHMSGKLTNVPACVLITPGDSKAVLP
PTLPQPASHFPPPPPPPPLPPPPPPLAPVLLRKPSLAKALQAGPLKKDGPMQITVKDLLTVKLKKTQSLDEKRKLIPSPKARNPLVT
VSDLQHVTLKPNSKVLSTRVTNVLITPGKSQMDLRKLLRKVDVERSPGGTPLTNKENMETGTGLTPVMTQALRRKFQLAHPRSPTPT
LPLSTSSFDEQN
>sp|Q9GZR5|ELOV4_HUMAN
MGLLDSEPGSVLNVVSTALNDTVEFYRWTWSIADKRVENWPLMQSPWPTLSISTLYLLFVWLGPKWMKDREPFQMRLVLIIYNFGMV
LLNLFIFRELFMGSYNAGYSYICQSVDYSNNVHEVRIAAALWWYFVSKGVEYLDTVFFILRKKNNQVSFLHVYHHCTMFTLWWIGIK
WVAGGQAFFGAQLNSFIHVIMYSYYGLTAFGPWIQKYLWWKRYLTMLQLIQFHVTIGHTALSLYTDCPFPKWMHWALIAYAISFIFL
FLNFYIRTYKEPKKPKAGKTAMNGISANGVSKSEKQLMIENGKKQKNGKAKGD
>sp|Q8TES7|FBF1_HUMAN
MAPKTKKGCKVTLPEKPVKLASHTRDTTGVSQMFPSSKARTKSLLGDDVFSTMAGLEEADAEVSGISEADPQALLQAMKDLDGMDAD
ILGLKKSNSAPSKKAAKDPGKGELPNHPKPAGGAIPTKKSLPSPSSSGHQNRRFSSEDLEDPLRGLLSYDEGGITKQPPVTQSKTAS
DKSPSTVRDQGPSIPLTPGDTPIRKKEELLFDDGDDIMATLGFGDSPKAEKRQIGDQEGPRPARSTLDELLGRGMATKLLARPGTGE
HREFKLDKKYQRPQDSEDMWGDEDFTFGAYQPTVVSSEGRQSRRQSVSRFFADSGADPKGEPGSKQSPPMASSPIQPRKGGADWLGL
KDEDLDLFPASPTREAHRESSVPVTPSVPPPASQHSTPAGLPPSRAKPPTEGAGSPAKASQASKLRASKEEKEDWLSHALSRKKSQG
LAREQHAGTSEGLHLAGTAGHPPSGSQPLTSTQGLEHAAAGGSSGTTARERPCVRPGVSGSPVTQNHAASALPTGSPKRGTAPGDLS
ATEPATCFPSTQKPTEPSVPVQPLLPESLARSLLPSTEYQKQLLAAQVQLQCSPAELQAELLHSQARLAELEAQVRKLELERAQHEL
LLGSLQQQHQADLELIESAHRSRIKVLETSYQQREERLRRENEELSARYLSQCQEAEQARAELTAQHQRRLAAIAQEKDQEMERLRE
LQRASILDMRRDHEEQLQRLKLLKDREVDAATSATSHTRSLNSIIHQMEKFSSSLHELSSRVEASHLTTSQERELGIRQRDEQLRAL
QERLGQQQRDMEEERSRQQEVIGKMEARLNEQSRLLEQERWRVTAEQSKAESMQRALEEQRKVTAQQMAMERAELERAKSALLEEQK
SVMLKCGEERRRLAAEWAEFSAQQKLSKERAEREAERALQVDTQREGTLISLAKQAELKIRASELRAEEKQLAAERAALEQERQELR
LEKERINATALRVKLRAEEVESMSKVASEKYEEGERALREAQQVQAEQQARLQAVQQQQERLRKQEQHMHQEHLSLAQQRLQLDRAR
QDLPSSLVGLFPRAQGPAASSQSALMPPAPTTRWCSQPPTGLDPSPLHLHARLALLRHMAEQDRDFLENEQFFLETLKKGSYNLTSH
SA
>sp|Q99966|CITE1_HUMAN
MPTTSRPALDVKGGTSPAKEDANQEMSSVAYSNLAVKDRKAVAILHYPGVASNGTKASGAPTSSSGSPIGSPTTTPPTKPPSFNLHP
APHLLASMHLQKLNSQYQGMAAATPGQPGEAGPLQNWDFGAQAGGAESLSPSAGAQSPAIIDSDPVDEEVLMSLVVELGLDRANELP
ELWLGQNEFDFTADFPSSC
>sp|O95521|PRAM1_HUMAN

MSIQAPPRLLELAGQSLLRDQALSISAMEELPRVLYLPLFMEAFSRRHFQTLTVMVQAWPFTCLPLGSLMKTLHLETLKALLEGLHM
LLTQKDRPRRWKLQVLDLRDVDENFWARWPGAWALSCFPETTSKRQTAEDCPRMGEHQPLKVFIDICLKEIPQDECLRYLFQWVYQR
RGLVHLCCSKLVNYLTPIKYLRKSLKIIYLNSIQELEIRNMSWPRLIRKLRCYLKEMKNLRKLVFSRCHHYTSDNELEGRLVAKFSS
VFLRLEHLQLLKIKLITFFSGHLEQLIRCLQNPLENLELTYGYLLEEDMKCLSQYPSLGYLKHLNLSYVLLFRISLEPLGALLEKIA
ASLKTLILEGCQIHYSQLSAILPGLSRCSQLTTFYFGRNCMSIDALKDLLRHTSGLSKLSLETYPAPEESLNSLVRVNWEIFTPLRA
ELMCTLREVRQPKRIFIGPTPCPSCGSSPSEELELHLCC
>sp|Q02763|TIE2_HUMAN
MDSLASLVLCGVSLLLSGTVEGAMDLILINSLPLVSDAETSLTCIASGWRPHEPITIGRDFEALMNQHQDPLEVTQDVTREWAKKVV
WKREKASKINGAYFCEGRVRGEAIRIRTMKMRQQASFLPATLTMTVDKGDNVNISFKKVLIKEEDAVIYKNGSFIHSVPRHEVPDIL
EVHLPHAQPQDAGVYSARYIGGNLFTSAFTRLIVRRCEAQKWGPECNHLCTACMNNGVCHEDTGECICPPGFMGRTCEKACELHTFG
RTCKERCSGQEGCKSYVFCLPDPYGCSCATGWKGLQCNEACHPGFYGPDCKLRCSCNNGEMCDRFQGCLCSPGWQGLQCEREGIQRM
TPKIVDLPDHIEVNSGKFNPICKASGWPLPTNEEMTLVKPDGTVLHPKDFNHTDHFSVAIFTIHRILPPDSGVWVCSVNTVAGMVEK
PFNISVKVLPKPLNAPNVIDTGHNFAVINISSEPYFGDGPIKSKKLLYKPVNHYEAWQHIQVTNEIVTLNYLEPRTEYELCVQLVRR
GEGGEGHPGPVRRFTTASIGLPPPRGLNLLPKSQTTLNLTWQPIFPSSEDDFYVEVERRSVQKSDQQNIKVPGNLTSVLLNNLHPRE
QYVVRARVNTKAQGEWSEDLTAWTLSDILPPQPENIKISNITHSSAVISWTILDGYSISSITIRYKVQGKNEDQHVDVKIKNATITQ
YQLKGLEPETAYQVDIFAENNIGSSNPAFSHELVTLPESQAPADLGGGKMLLIAILGSAGMTCLTVLLAFLIILQLKRANVQRRMAQ
AFQNVREEPAVQFNSGTLALNRKVKNNPDPTIYPVLDWNDIKFQDVIGEGNFGQVLKARIKKDGLRMDAAIKRMKEYASKDDHRDFA
GELEVLCKLGHHPNIINLLGACEHRGYLYLAIEYAPHGNLLDFLRKSRVLETDPAFAIANSTASTLSSQQLLHFAADVARGMDYLSQ
KQFIHRDLAARNILVGENYVAKIADFGLSRGQEVYVKKTMGRLPVRWMAIESLNYSVYTTNSDVWSYGVLLWEIVSLGGTPYCGMTC
AELYEKLPQGYRLEKPLNCDDEVYDLMRQCWREKPYERPSFAQILVSLNRMLEERKTYVNTTLYEKFTYAGIDCSAEEAA
>sp|P78385|KRT83_HUMAN
MTCGFNSIGCGFRPGNFSCVSACGPRPSRCCITAAPYRGISCYRGLTGGFGSHSVCGGFRAGSCGRSFGYRSGGVCGPSPPCITTVS
VNESLLTPLNLEIDPNAQCVKQEEKEQIKSLNSRFAAFIDKVRFLEQQNKLLETKLQFYQNRECCQSNLEPLFAGYIETLRREAECV
EADSGRLASELNHVQEVLEGYKKKYEEEVALRATAENEFVALKKDVDCAYLRKSDLEANVEALIQEIDFLRRLYEEEIRILQSHISD
TSVVVKLDNSRDLNMDCIVAEIKAQYDDIATRSRAEAESWYRSKCEEMKATVIRHGETLRRTKEEINELNRMIQRLTAEVENAKCQN
SKLEAAVAQSEQQGEAALSDARCKLAELEGALQKAKQDMACLIREYQEVMNSKLGLDIEIATYRRLLEGEEQRLCEGVEAVNVCVSS
SRGGVVCGDLCVSGSRPVTGSVCSAPCNGNLVVSTGLCKPCGQLNTTCGGGSCGQGRH
>sp|P61566|ENK24_HUMAN
MVTPVTWMDNPIEVYVNDSEWVPGPTDDRCPAKPEEEGMMINISIGYRYPPICLGTAPGCLMPAVQNWLVEVPIVSPISRFTYHMVS
GMSLRPRVNYLQDFPYQRSLKFRPKGKPCPKEIPKESKNTEVLVWEECVANSAVILQNNEFGTIIDWAPRGQFYHNCSGQTQSCPSA
QVSPAVDSDLTESLDKHKHKKLQSFYPWEWGEKGISTPRPKIISPVSGPEHPELWRLTVASHHIRIWSGNQTLETRDRKPFYTVDLN
SSLTLPLQSCVKPPYMLVVGNIVIKPDSQTITCENCRLLTCIDSTFNWQHRILLVRAREGVWILVSMDRPWEASPSVHILTEVLKGV
LNRSKRFIFTLIAVIMGLIAVTATGAVAGVALHSSVQSVNFVNDWQKNSTRLWNSQSSIDQKLANQINDLRQTVIWMGDRLMSLEHR
FQLQCDWNTSDFCITPQIYNESEHHWDMVRHHLQGREDNLTLDISKLKEQIFEASKAHLNLVPGTEAIAGVADGLANLNPVTWVKTI
GSTTIINLILILVCLFCLLLVCRCTQQLRRDSDHRERAMMTMAVLSKRKGGNVGKSKRDQIVTVSV
>sp|Q96A32|MLRS_HUMAN
MAPKRAKRRTVEGGSSSVFSMFDQTQIQEFKEAFTVIDQNRDGIIDKEDLRDTFAAMGRLNVKNEELDAMMKEASGPINFTVFLTMF
GEKLKGADPEDVITGAFKVLDPEGKGTIKKKFLEELLTTQCDRFSQEEIKNMWAAFPPDVGGNVDYKNICYVITHGDAKDQE
>sp|Q6ZWK4|CA186_HUMAN
MLTEVMEVWHGLVIAVVSLFLQACFLTAINYLLSRHMAHKSEQILKAASLQVPRPSPGHHHPPAVKEMKETQTERDIPMSDSLYRHD
SDTPSDSLDSSCSSPPACQATEDVDYTQVVFSDPGELKNDSPLDYENIKEITDYVNVNPERHKPSFWYFVNPALSEPAEYDQVAM
>sp|Q8ND94|LRN4L_HUMAN

MLGSPCLLWLLAVTFLVPRAQPLAPQDFEEEEADETETAWPPLPAVPCDYDHCRHLQVPCKELQRVGPAACLCPGLSSPAQPPDPPR
MGEVRIAAEEGRAVVHWCAPFSPVLHYWLLLWDGSEAAQKGPPLNATVRRAELKGLKPGGIYVVCVVAANEAGASRVPQAGGEGLEG
ADIPAFGPCSRLAVPPNPRTLVHAAVGVGTALALLSCAALVWHFCLRDRWGCPRRAAARAAGAL
>sp|O95096|NKX22_HUMAN
MSLTNTKTGFSVKDILDLPDTNDEEGSVAEGPEEENEGPEPAKRAGPLGQGALDAVQSLPLKNPFYDSSDNPYTRWLASTEGLQYSL
HGLAAGAPPQDSSSKSPEPSADESPDNDKETPGGGGDAGKKRKRRVLFSKAQTYELERRFRQQRYLSAPEREHLASLIRLTPTQVKI
WFQNHRYKMKRARAEKGMEVTPLPSPRRVAVPVLVRDGKPCHALKAQDLAAATFQAGIPFSAYSAQSLQHMQYNAQYSSASTPQYPT
AHPLVQAQQWTW
>sp|Q15053|K0040_HUMAN
MHYVHVHRVTTQPRNKPQTKCPSGGQSQGPRGQFLDTVLAAMCPIAMLLTADPGMPPTCLWHTPHAKHKEHLSIHLNMVPKCVHMHV
THTHTNSGSRYVGKYILLIKWSLAMYFVQGSTLSTVTKMSHGKALPDSDTYIQFPNQQGPHTPSIP
>sp|P51795|CLCN5_HUMAN
MDFLEEPIPGVGTYDDFNTIDWVREKSRDRDRHREITNKSKESTWALIHSVSDAFSGWLLMLLIGLLSGSLAGLIDISAHWMTDLKE
GICTGGFWFNHEHCCWNSEHVTFEERDKCPEWNSWSQLIISTDEGAFAYIVNYFMYVLWALLFAFLAVSLVKVFAPYACGSGIPEIK
TILSGFIIRGYLGKWTLVIKTITLVLAVSSGLSLGKEGPLVHVACCCGNILCHCFNKYRKNEAKRREVLSAAAAAGVSVAFGAPIGG
VLFSLEEVSYYFPLKTLWRSFFAALVAAFTLRSINPFGNSRLVLFYVEFHTPWHLFELVPFILLGIFGGLWGALFIRTNIAWCRKRK
TTQLGKYPVIEVLVVTAITAILAFPNEYTRMSTSELISELFNDCGLLDSSKLCDYENRFNTSKGGELPDRPAGVGVYSAMWQLALTL
ILKIVITIFTFGMKIPSGLFIPSMAVGAIAGRLLGVGMEQLAYYHQEWTVFNSWCSQGADCITPGLYAMVGAAACLGGVTRMTVSLV
VIMFELTGGLEYIVPLMAAAMTSKWVADALGREGIYDAHIRLNGYPFLEAKEEFAHKTLAMDVMKPRRNDPLLTVLTQDSMTVEDVE
TIISETTYSGFPVVVSRESQRLVGFVLRRDLIISIENARKKQDGVVSTSIIYFTEHSPPLPPYTPPTLKLRNILDLSPFTVTDLTPM
EIVVDIFRKLGLRQCLVTHNGRLLGIITKKDVLKHIAQMANQDPDSILFN
>sp|Q5T7W0|ZN618_HUMAN
MNQPGGAAAPQADGASAAGRKSTASRERLKRSQKSTKVEGPEPVPAEASLSAEQGTMTEVKVKTELPDDYIQEVIWQGEAKEEKKAV
SKDGTSDVPAEICVVIGGVRNQQTLDGKAPEGSPHGGSVRSRYSGTWIFDQALRYASGSYECGICGKKYKYYNCFQTHVRAHRDTEA
TSGEGASQSNNFRYTCDICGKKYKYYSCFQEHRDLHAVDVFSVEGAPENRADPFDQGVVATDEVKEEPPEPFQKIGPKTGNYTCEFC
GKQYKYYTPYQEHVALHAPISTAPGWEPPDDPDTGSECSHPEVSPSPRFVAAKTQTNQSGKKAPASVVRCATLLHRTPPATQTQTFR
TPNSGSPASKATAAESAFSRRVEGKAQNHFEETNSSSQNSSEPYTCGACGIQFQFYNNLLEHMQSHAADNENNIASNQSRSPPAVVE
EKWKPQAQRNSANNTTTSGLTPNSMIPEKERQNIAERLLRVMCADLGALSVVSGKEFLKLAQTLVDSGARYGAFSVTEILGNFNTLA
LKHLPRMYNQVKVKVTCALGSNACLGIGVTCHSQSVGPDSCYILTAYQAEGNHIKSYVLGVKGADIRDSGDLVHHWVQNVLSEFVMS
EIRTVYYVTDCRVSTSAFSKAGMCLRCSACALNSVVQSVLSKRTLQARSMHEVIELLNVCEDLAGSTGLAKETFGSLEETSPPPCWNS
VTDSLLLVHERYEQICEFYSRAKKMNLIQSLNKHLLSNLAAILTPVKQAVIELSNESQPTLQLVLPTYVRLEKLFTAKANDAGTVSK
LCHLFLEALKENFKVHPAHKVAMILDPQQKLRPVPPYQHEEIIGKVCELINEVKESWAEEADFEPAAKKPRSAAVENPAAQEDDRLG
KNEVYDYLQEPLFQATPDLFQYWSCVTQKHTKLAKLAFWLLAVPAVGARSGCVNMCEQALLIKRRRLLSPEDMNKLMFLKSNML
>sp|Q96B36|AKTS1_HUMAN
MASGRPEELWEAVVGAAERFRARTGTELVLLTAAPPPPPRPGPCAYAAHGRGALAEAARRCLHDIALAHRAATAARPPAPPPAPQPP
SPTPSPPRPTLAREDNEEDEDEPTETETSGEQLGISDNGGLFVMDEDATLQDLPPFCESDPESTDDGSLSEETPAGPPTCSVPPASA
LPTQQYAKSLPVSVPVWGFKEKRTEARSSDEENGPPSSPDLDRIAASMRALVLREAEDTQVFGDLPRPRLNTSDFQKLKRKY
>sp|Q92845|KIFA3_HUMAN
MQGEDARYLKRKVKGGNIDVHPSEKALIVHYEVEATILGEMGDPMLGERKECQKIIRLKSLNANTDITSLARKVVEECKLIHPSKLN
EVEQLLYYLQNRRDSLSGKEKKEKSSKPKDPPPFEGMEIDEVANINDMDEYIELLYEDIPDKVRGSALILQLARNPDNLEELLLNET
ALGALARVLREDWKQSVELATNIIYIFFCFSSFSQFHGLITHYKIGALCMNIIDHELKRHELWQEELSKKKKAVDEDPENQTLRKDY
EKTFKKYQGLVVKQEQLLRVALYLLLNLAEDTRTELKMRNKNIVHMLVKALDRDNFELLILVVSFLKKLSIFMENKNDMVEMDIVEK
LVKMIPCEHEDLLNITLRLLLNLSFDTGLRNKMVQVGLLPKLTALLGNDNYKQIAMCVLYHISMDDRFKSMFAYTDCIPQLMKMLFE
CSDERIDLELISFCINLAANKRNVQLICEGNGLKMLMKRALKFKDPLLMKMIRNISQHDGPTKNLFIDYVGDLAAQISNDEEEEFVI

ECLGTLANLTIPDLDWELVLKEYKLVPYLKDKLKPGAAEDDLVLEVVIMIGTVSMDDSCAALLAKSGIIPALIELLNAQQEDDEFVC
QIIYVFYQMVFHQATRDVIIKETQAPAYLIDLMHDKNNEIRKVCDNTLDIIAEYDEEWAKKIQSEKFRWHNSQWLEMVESRQMDESE
QYLYGDDRIEPYIHEGDILERPDLFYNSDGLIASEGAISPDFFNDYHLQNGDVVGQHSFPGSLGMDGFGQPVGILGRPATAYGFRPD
EPYYYGYGS
>sp|A2A3N6|PIPSL_HUMAN
MASEVPYASGMPIKKIGHRSVDSSGGTTSSALKGAIQLGITHTVGSLSTKPESDVLMQDFHMVESIFFPSEGSNLTPAHHYNAFRFK
TYAPVAFRYFWELFGIRPDDYLYSLCSEPLIELCSSGASGSLFYVSSDDEFIVKTVRHKEAEFLQKLLPGYYINLNQNPRTLLPKFY
GLYCVQTGGKNIRIVVMNNLLPRSVKMHIKYDLKGSTYRRRASQKEREKPLPTFKDLDFLQDIPDGLFLDADVHNALCKTLQRDCLV
LQSFKIMDYSLLMSIHNIDHAQREPLSSETQYSVDTRRPAPQKALYSTAMESIQGEARRGGTMETDDHMGGIPARNSKGERLLLYIG
IIDILQSYRFVKKLEHSWKALIHDGDTVSVHRPGFYAEWFQRFMCNTVFKKIPLKPSPSKKLRSGSSFSQRAGSSGNSCITYQPLVS
GEHKAQVTTKAEVEPGVHLGCPDVLPQTPPLEEISEGSPTPDPSFSPLVEETLQMLTTSVDNSEYMGNGDFLPTRLQAQQDAVNTVC
HSKTRSNPENNVGLITLDNDCEVLTTLTPDTGRILSKLHTVQPKGKITFCMGIHVAHLALKHRQGNNHKIRIIAFVGNPVEDNEKNL
VKLAKCLKKEKVNVDIINFGEEEVNTEKLTAFVNTLNGKDGTGSHLVTVPPGPSLADALISFPILAGEGGAMMGLGASDFEFGVDPS
ADPELALVLRVFMEEQRQRQEEEARQAAAASAAEAGIATTGTEDSDDALLKMTISQQEFGHTGLPDLSSMTEEEKIVCAMQMSLQGA
EFGLAESADIDASSAMDTSEPAKEEDDYDVMQDPEFLQSVLENLPGVDPNNEAIRNAVGSLASQATKDSKKDKKEEDKK
>sp|Q02383|SEMG2_HUMAN
MKSIILFVLSLLLILEKQAAVMGQKGGSKGQLPSGSSQFPHGQKGQHYFGQKDQQHTKSKGSFSIQHTYHVDINDHDWTRKSQQYDL
NALHKATKSKQHLGGSQQLLNYKQEGRDHDKSKGHFHMIVIHHKGGQAHHGTQNPSQDQGNSPSGKGLSSQCSNTEKRLWVHGLSKE
QASASGAQKGRTQGGSQSSYVLQTEELVVNKQQRETKNSHQNKGHYQNVVDVREEHSSKLQTSLHPAHQDRLQHGPKDIFTTQDELL
VYNKNQHQTKNLSQDQEHGRKAHKISYPSSRTEERQLHHGEKSVQKDVSKGSISIQTEEKIHGKSQNQVTIHSQDQEHGHKENKISY
QSSSTEERHLNCGEKGIQKGVSKGSISIQTEEQIHGKSQNQVRIPSQAQEYGHKENKISYQSSSTEERRLNSGEKDVQKGVSKGSIS
IQTEEKIHGKSQNQVTIPSQDQEHGHKENKMSYQSSSTEERRLNYGGKSTQKDVSQSSISFQIEKLVEGKSQIQTPNPNQDQWSGQN
AKGKSGQSADSKQDLLSHEQKGRYKQESSESHNIVITEHEVAQDDHLTQQYNEDRNPIST
>sp|Q14314|FGL2_HUMAN
MKLANWYWLSSAVLATYGFLVVANNETEEIKDERAKDVCPVRLESRGKCEEAGECPYQVSLPPLTIQLPKQFSRIEEVFKEVQNLKE
IVNSLKKSCQDCKLQADDNGDPGRNGLLLPSTGAPGEVGDNRVRELESEVNKLSSELKNAKEEINVLHGRLEKLNLVNMNNIENYVD
SKVANLTFVVNSLDGKCSKCPSQEQIQSRPVQHLIYKDCSDYYAIGKRSSETYRVTPDPKNSSFEVYCDMETMGGGWTVLQARLDGS
TNFTRTWQDYKAGFGNLRREFWLGNDKIHLLTKSKEMILRIDLEDFNGVELYALYDQFYVANEFLKYRLHVGNYNGTAGDALRFNKH
YNHDLKFFTTPDKDNDRYPSGNCGLYYSSGWWFDACLSANLNGKYYHQKYRGVRNGIFWGTWPGVSEAHPGGYKSSFKEAKMMIRPK
HFKP
>sp|P01160|ANF_HUMAN
MSSFSTTTVSFLLLLAFQLLGQTRANPMYNAVSNADLMDFKNLLDHLEEKMPLEDEVVPPQVLSEPNEEAGAALSPLPEVPPWTGEV
SPAQRDGGALGRGPWDSSDRSALLKSKLRALLTAPRSLRRSSCFGGRMDRIGAQSGLGCNSFRYRR
>sp|Q6ZN28|MACC1_HUMAN
MLITERKHFRSGRIAQSMSEANLIDMEAGKLSKSCNITECQDPDLLHNWPDAFTLRGNNASKVANPFWNQLSASNPFLDDITQLRNN
RKRNNISILKEDPFLFCREIENGNSFDSSGDELDVHQLLRQTSSRNSGRSKSVSELLDILDDTAHAHQSIHNSDQILLHDLEWLKND
REAYKMAWLSQRQLARSCLDLNTISQSPGWAQTQLAEVTIACKVNHQGGSVQLPESDITVHVPQGHVAVGEFQEVSLRAFLDPPHML
NHDLSCTVSPLLEIMLGNLNTMEALLLEMKIGAEVRKDPFSQVMTEMVCLHSLGKEGPFKVLSNCYIYKDTIQVKLIDLSQVMYLVV
AAQAKALPSPAATIWDYIHKTTSIGIYGPKYIHPSFTVVLTVCGHNYMPGQLTISDIKKGGKNISPVVFQLWGKQSFLLDKPQDLSI
SIFSCDPDFEVKTEGERKEIKQKQLEAGEVVHQQFLFSLVEHREMHLFDFCVQVEPPNGEPVAQFSITTPDPTPNLKRLSNLPGYLQ
KKEEIKSAPLSPKILVKYPTFQDKTLNFSNYGVTLKAVLRQSKIDYFLEYFKGDTIALLGEGKVKAIGQSKVKEWYVGVLRGKIGLV
HCKNVKVISKEQVMFMSDSVFTTRNLLEQIVLPLKKLTYIYSVVLTLVSEKVYDWKVLADVLGYSHLSLEDFDQIQADKESEKVSYV
IKKLKEDCHTERNTRKFLYELIVALLKMDCQELVARLIQEAAVLTSAVKLGKGWRELAEKLVRLTKQQMEAYEIPHRGNTGDVAVEM
MWKPAYDFLYTWSAHYGNNYRDVLQDLQSALDRMKNPVTKHWRELTGVLILVNSLEVLRVTAFSTSEEV

>sp|Q96BJ3|AIDA_HUMAN
MSEVTRSLLQRWGASFRRGADFDSWGQLVEAIDEYQILARHLQKEAQAQHNNSEFTEEQKKTIGKIATCLELRSAALQSTQSQEEFK
LEDLKKLEPILKNILTYNKEFPFDVQPVPLRRILAPGEEENLEFEEDEEEGGAGAGSPDSFPARVPGTLLPRLPSEPGMTLLTIRIE
KIGLKDAGQCIDPYITVSVKDLNGIDLTPVQDTPVASRKEDTYVHFNVDIELQKHVEKLTKGAAIFFEFKHYKPKKRFTSTKCFAFM
EMDEIKPGPIVIELYKKPTDFKRKKLQLLTKKPLYLHLHQTLHKE
>sp|Q3C1V9|YK041_HUMAN
MTPPPPSPLLGTQVKEDRADYKEFQDFSSLPDTHNIAWDHSFYPFQEEEEHGVKGVESVLEKGVLDEGVLEAWGCCRRCRHAGWNRS
QPSPELAGAVIHARAPAVGCRQRSGHLHVRLKQRLLERPCQEPLGKKYQLELPPLYERARKPEGSKNLPADGQGLRAVRADAALPVW
PGGPGRPGPHAPEAGAEGQHQGQWPPADTRHLRTPKWPYKVATEEKPEAEEAEKKRQAKVQEKRLPPWKKRTLNQVHRVPWGQHRAC
QVMLLVSTKQLQRYLHFEKPAKPANMGQDPDCFGKSEGELATCLCGGESVVGETEAPGVRPDPRPEKDAKPAVSGCQEESWLQSEYD
EMHPREEMNVPSLRSGSGCQVSGSPLAKGLHPLQPCHPHYMMWECCLFTLTLGTQACFEVCARRLKLAHFCPDTSLWSCGGQSPPVL
CQLLDIISVSRDKVIGSHEKKPSSNPNQDDFHSSEYAYRCGIAEAVGLPSIPVHPIGYYDAEKLLEYESLLLQVSEPVNPELSAGPI
TGLLDRLSGFRDHGPPGSSCRSQRKGNGVETKDQAHRNRDPRGFQGGVLTARLFKMQVSLELFCGHQEHYLTRPVLAPGYPVHLGKA
GTHWEQGPNMPTPEQHGTWGNRHLFAVLPLLFYTSLEIMSIGYSVVMAGRNVWQPTKGQMPHQPEQSCQTLALPMTIRHSWEGGAIR
ESSGWVSWKCHLKATEASQNPCVRATALRVGCSAVTYGVLGQAQGSAPWTSAFKTFSAGIAGLERHAWETM
>sp|Q9BT81|SOX7_HUMAN
MASLLGAYPWPEGLECPALDAELSDGQSPPAVPRPPGDKGSESRIRRPMNAFMVWAKDERKRLAVQNPDLHNAELSKMLGKSWKALT
LSQKRPYVDEAERLRLQHMQDYPNYKYRPRRKKQAKRLCKRVDPGFLLSSLSRDQNALPEKRSGSRGALGEKEDRGEYSPGTALPSL
RGCYHEGPAGGGGGGTPSSVDTYPYGLPTPPEMSPLDVLEPEQTFFSSPCQEEHGHPRRIPHLPGHPYSPEYAPSPLHCSHPLGSLA
LGQSPGVSMMSPVPGCPPSPAYYSPATYHPLHSNLQAHLGQLSPPPEHPGFDALDQLSQVELLGDMDRNEFDQYLNTPGHPDSATGA
MALSGHVPVSQVTPTGPTETSLISVLADATATYYNSYSVS
>sp|Q9P2S2|NRX2A_HUMAN
MASGSRWRPTPPPLLLLLLLALAARADGLEFGGGPGQWARYARWAGAASSGELSFSLRTNATRALLLYLDDGGDCDFLELLLVDGRL
RLRFTLSCAEPATLQLDTPVADDRWHMVLLTRDARRTALAVDGEARAAEVRSKRREMQVASDLFVGGIPPDVRLSALTLSTVKYEPP
FRGLLANLKLGERPPALLGSQGLRGATADPLCAPARNPCANGGLCTVLAPGEVGCDCSHTGFGGKFCSEEEHPMEGPAHLTLNSEVG
SLLFSEGGAGRGGAGDVHQPTKGKEEFVATFKGNEFFCYDLSHNPIQSSTDEITLAFRTLQRNGLMLHTGKSADYVNLSLKSGAVWL
VINLGSGAFEALVEPVNGKFNDNAWHDVRVTRNLRQHAGIGHAMVNKLHYLVTISVDGILTTTGYTQEDYTMLGSDDFFYIGGSPNT
ADLPGSPVSNNFMGCLKDVVYKNNDFKLELSRLAKEGDPKMKLQGDLSFRCEDVAALDPVTFESPEAFVALPRWSAKRTGSISLDFR
TTEPNGLLLFSQGRRAGGGAGSHSSAQRADYFAMELLDGHLYLLLDMGSGGIKLRASSRKVNDGEWCHVDFQRDGRKGSISVNSRST
PFLATGDSEILDLESELYLGGLPEGGRVDLPLPPEVWTAALRAGYVGCVRDLFIDGRSRDLRGLAEAQGAVGVAPFCSRETLKQCAS
APCRNGGVCREGWNRFICDCIGTGFLGRVCEREATVLSYDGSMYMKIMLPNAMHTEAEDVSLRFMSQRAYGLMMATTSRESADTLRL
ELDGGQMKLTVNLDCLRVGCAPSKGPETLFAGHKLNDNEWHTVRVVRRGKSLQLSVDNVTVEGQMAGAHMRLEFHNIETGIMTERRF
ISVVPSNFIGHLSGLVFNGQPYMDQCKDGDITYCELNARFGLRAIVADPVTFKSRSSYLALATLQAYASMHLFFQFKTTAPDGLLLF
NSGNGNDFIVIELVKGYIHYVFDLGNGPSLMKGNSDKPVNDNQWHNVVVSRDPGNVHTLKIDSRTVTQHSNGARNLDLKGELYIGGL
SKNMFSNLPKLVASRDGFQGCLASVDLNGRLPDLIADALHRIGQVERGCDGPSTTCTEESCANQGVCLQQWDGFTCDCTMTSYGGPV
CNDPGTTYIFGKGGALITYTWPPNDRPSTRMDRLAVGFSTHQRSAVLVRVDSASGLGDYLQLHIDQGTVGVIFNVGTDDITIDEPNA
IVSDGKYHVVRFTRSGGNATLQVDSWPVNERYPAGNFDNERLAIARQRIPYRLGRVVDEWLLDKGRQLTIFNSQAAIKIGGRDQGRP
FQGQVSGLYYNGLKVLALAAESDPNVRTEGHLRLVGEGPSVLLSAETTATTLLADMATTIMETTTTMATTTTRRGRSPTLRDSTTQN
TDDLLVASAECPSDDEDLEECEPSTGGELILPIITEDSLDPPPVATRSPFVPPPPTFYPFLTGVGATQDTLPPPAARRPPSGGPCQA
ERDDSDCEEPIEASGFASGEVFDSSLPPTDDEDFYTTFPLVTDRTTLLSPRKPAPRPNLRTDGATGAPGVLFAPSAPAPNLPAGKMN
HRDPLQPLLENPPLGPGAPTSFEPRRPPPLRPGVTSAPGFPHLPTANPTGPGERGPPGAVEVIRESSSTTGMVVGIVAAAALCILIL
LYAMYKYRNRDEGSYQVDQSRNYISNSAQSNGAVVKEKAPAAPKTPSKAKKNKDKEYYV
>sp|Q9BR10|SPT25_HUMAN

MSYFRTPQTHPGPLPSGQGGAASPGLSLGLCSPVEPVVVASGGTGPLSQKAEQVAPAAQAWGPALAMPQARGCPGGTSWETLRKEYS
RNCHKFPHVRQLESLGWDNGYSRSRAPDLGGPSRPRPLMLCGLSPRVLPVPSEAVGKEASSQPDICILTLAMMIAGIPTVPVPGVRE
EDLIWAAQAFMMAHPEPEGAVEGARWEQAHAHTASGKMPLVRSKRGQPPGSCL
>sp|Q96EK5|KBP_HUMAN
MANVPWAEVCEKFQAALALSRVELHKNPEKEPYKSKYSARALLEEVKALLGPAPEDEDERPEAEDGPGAGDHALGLPAEVVEPEGPV
AQRAVRLAVIEFHLGVNHIDTEELSAGEEHLVKCLRLLRRYRLSHDCISLCIQAQNNLGILWSEREEIETAQAYLESSEALYNQYMK
EVGSPPLDPTERFLPEEEKLTEQERSKRFEKVYTHNLYYLAQVYQHLEMFEKAAHYCHSTLKRQLEHNAYHPIEWAINAATLSQFYI
NKLCFMEARHCLSAANVIFGQTGKISATEDTPEAEGEVPELYHQRKGEIARCWIKYCLTLMQNAQLSMQDNIGELDLDKQSELRALR
KKELDEEESIRKKAVQFGTGELCDAISAVEEKVSYLRPLDFEEARELFLLGQHYVFEAKEFFQIDGYVTDHIEVVQDHSALFKVLAF
FETDMERRCKMHKRRIAMLEPLTVDLNPQYYLLVNRQIQFEIAHAYYDMMDLKVAIADRLRDPDSHIVKKINNLNKSALKYYQLFLD
SLRDPNKVFPEHIGEDVLRPAMLAKFRVARLYGKIITADPKKELENLATSLEHYKFIVDYCEKHPEAAQEIEVELELSKEMVSLLPT
KMERFRTKMALT
>sp|Q6UX46|F150B_HUMAN
MRGPGHPLLLGLLLVLGAAGRGRGGAEPREPADGQALLRLVVELVQELRKHHSAEHKGLQLLGRDCALGRAEAAGLGPSPEQRVEIV
PRDLRMKDKFLKHLTGPLYFSPKCSKHFHRLYHNTRDCTIPAYYKRCARLLTRLAVSPVCMEDKQ
>sp|P05546|HEP2_HUMAN
MKHSLNALLIFLIITSAWGGSKGPLDQLEKGGETAQSADPQWEQLNNKNLSMPLLPADFHKENTVTNDWIPEGEEDDDYLDLEKIFS
EDDDYIDIVDSLSVSPTDSDVSAGNILQLFHGKSRIQRLNILNAKFAFNLYRVLKDQVNTFDNIFIAPVGISTAMGMISLGLKGETH
EQVHSILHFKDFVNASSKYEITTIHNLFRKLTHRLFRRNFGYTLRSVNDLYIQKQFPILLDFKTKVREYYFAEAQIADFSDPAFISK
TNNHIMKLTKGLIKDALENIDPATQMMILNCIYFKGSWVNKFPVEMTHNHNFRLNEREVVKVSMMQTKGNFLAANDQELDCDILQLE
YVGGISMLIVVPHKMSGMKTLEAQLTPRVVERWQKSMTNRTREVLLPKFKLEKNYNLVESLKLMGIRMLFDKNGNMAGISDQRIAID
LFKHQGTITVNEEGTQATTVTTVGFMPLSTQVRFTVDRPFLFLIYEHRTSCLLFMGRVANPSRS
>sp|P14138|EDN3_HUMAN
MEPGLWLLFGLTVTSAAGFVPCSQSGDAGRRGVSQAPTAARSEGDCEETVAGPGEETVAGPGEGTVAPTALQGPSPGSPGQEQAAEG
APEHHRSRRCTCFTYKDKECVYYCHLDIIWINTPEQTVPYGLSNYRGSFRGKRSAGPLPGNLQLSHRPHLRCACVGRYDKACLHFCT
QTLDVSSNSRTAEKTDKEEEGKVEVKDQQSKQALDLHHPKLMPGSGLALAPSTCPRCLFQEGAP
>sp|Q9P0J1|PDP1_HUMAN
MPAPTQLFFPLIRNCELSRIYGTACYCHHKHLCCSSSYIPQSRLRYTPHPAYATFCRPKENWWQYTQGRRYASTPQKFYLTPPQVNS
ILKANEYSFKVPEFDGKNVSSILGFDSNQLPANAPIEDRRSAATCLQTRGMLLGVFDGHAGCACSQAVSERLFYYIAVSLLPHETLL
EIENAVESGRALLPILQWHKHPNDYFSKEASKLYFNSLRTYWQELIDLNTGESTDIDVKEALINAFKRLDNDISLEAQVGDPNSFLN
YLVLRVAFSGATACVAHVDGVDLHVANTGDSRAMLGVQEEDGSWSAVTLSNDHNAQNERELERLKLEHPKSEAKSVVKQDRLLGLLM
PFRAFGDVKFKWSIDLQKRVIESGPDQLNDNEYTKFIPPNYHTPPYLTAEPEVTYHRLRPQDKFLVLATDGLWETMHRQDVVRIVGE
YLTGMHHQQPIAVGGYKVTLGQMHGLLTERRTKMSSVFEDQNAATHLIRHAVGNNEFGTVDHERLSKMLSLPEELARMYRDDITIIV
VQFNSHVVGAYQNQE
>sp|P31942|HNRH3_HUMAN
MDWVMKHNGPNDASDGTVRLRGLPFGCSKEEIVQFFQGLEIVPNGITLTMDYQGRSTGEAFVQFASKEIAENALGKHKERIGHRYIE
IFRSSRSEIKGFYDPPRRLLGQRPGPYDRPIGGRGGYYGAGRGSMYDRMRRGGDGYDGGYGGFDDYGGYNNYGYGNDGFDDRMRDGR
GMGGHGYGGAGDASSGFHGGHFVHMRGLPFRATENDIANFFSPLNPIRVHIDIGADGRATGEADVEFVTHEDAVAAMSKDKNNMQHR
YIELFLNSTPGGGSGMGGSGMGGYGRDGMDNQGGYGSVGRMGMGNNYSGGYGTPDGLGGYGRGGGGSGGYYGQGGMSGGGWRGMY
>sp|Q8NCQ2|CNAS1_HUMAN
MGRAGPRSTADTRPRATVITTRRPRPWQKPTSPRRLHRRRPRGQPASENAAEPSQVAISKGKSPDYANGGKAAMDSRASDAINKSKV
DTSASNPSQRRPSRLRGKRSLALPPRLECLFPSSCGSRRATSRPGFPPLEGSHLGCQLLPL
>sp|Q9NS73|MBIP1_HUMAN

MAAATELNRPSSGDRNLERRCRPNLSREVLYEIFRSLHTLVGQLDLRDDVVKITIDWNKLQSLSAFQPALLFSALEQHILYLQPFLA
KLQSPIKEENTTAVEEIGRTEMGNKNEVNDKFSIGDLQEEEKHKESDLRDVKKTQIHFDPEVVQIKAGKAEIDRRISAFIERKQAEI
NENNVREFCNVIDCNQENSCARTDAIFTPYPGFKSHVKVSRVVNTYGPQTRPEGIPGSGHKPNSMLRDCGNQAVEERLQNIEAHLRL
QTGGPVPRDIYQRIKKLEDKILELEGISPEYFQSVSFSGKRRKVQPPQQNYSLAELDEKISALKQALLRKSREAESMATHHLP
>sp|Q5T2D2|TRML2_HUMAN
MAPAFLLLLLLWPQGCVSGPSADSVYTKVRLLEGETLSVQCSYKGYKNRVEGKVWCKIRKKKCEPGFARVWVKGPRYLLQDDAQAKV
VNITMVALKLQDSGRYWCMRNTSGILYPLMGFQLDVSPAPQTERNIPFTHLDNILKSGTVTTGQAPTSGPDAPFTTGVMVFTPGLIT
LPRLLASTRPASKTGYSFTATSTTSQGPRRTMGSQTVTASPSNARDSSAGPESISTKSGDLSTRSPTTGLCLTSRSLLNRLPSMPSI
RHQDVYSTVLGVVLTLLVLMLIMVYGFWKKRHMASYSMCSDPSTRDPPGRPEPYVEVYLI
>sp|Q96A46|MFRN2_HUMAN
MELEGRGAGGVAGGPAAGPGRSPGESALLDGWLQRGVGRGAGGGEAGACRPPVRQDPDSGPDYEALPAGATVTTHMVAGAVAGILEH
CVMYPIDCVKTRMQSLQPDPAARYRNVLEALWRIIRTEGLWRPMRGLNVTATGAGPAHALYFACYEKLKKTLSDVIHPGGNSHIANG
AAGCVATLLHDAAMNPAEVVKQRMQMYNSPYHRVTDCVRAVWQNEGAGAFYRSYTTQLTMNVPFQAIHFMTYEFLQEHFNPQRRYNP
SSHVLSGACAGAVAAAATTPLDVCKTLLNTQESLALNSHITGHITGMASAFRTVYQVGGVTAYFRGVQARVIYQIPSTAIAWSVYEF
FKYLITKRQEEWRAGK
>sp|Q9NVP2|ASF1B_HUMAN
MAKVSVLNVAVLENPSPFHSPFRFEISFECSEALADDLEWKIIYVGSAESEEFDQILDSVLVGPVPAGRHMFVFQADAPNPSLIPET
DAVGVTVVLITCTYHGQEFIRVGYYVNNEYLNPELRENPPMKPDFSQLQRNILASNPRVTRFHINWDNNMDRLEAIETQDPSLGCGL
PLNCTPIKGLGLPGCIPGLLPENSMDCI
>sp|Q6WRX3|ZY11A_HUMAN
MVHFLHPGHTPRNIVPPDAQKDALGCCVVQEEASPYTLVNICLNVLIANLEKLCSERPDGTLCLPEHWSFPQEVAERFLRVMTWQGK
LTDRTASIFRGNQMKLKLVNIQKAKISTAAFIKAFCRHKLIELNATAVHADLPVPDIISGLCSNRWIQQNLQCLLLDSTSIPQNSRL
LFFSQLTGLRILSVFNVCFHTEDLANVSQLPRLESLDISNTLVTDISALLTCKDRLKSLTMHYLKCLAMTKSQILAVIRELKCLLHL
DISDHRQLKSDLAFHLLQQKDILPNVVSLDISGGNCITDEAVELFIRLRPAMQFVGLLATDAGSSDFFTTKQGLRVAGGASMSQISE
ALSRYRNRSCFVKEALHRLFTETFSMEVTMPAILKLVAIGMRNHPLDLRVQFTASACALNLTRQGLAKGMPVRLLSEVTCLLFKALK
NFPHYQQLQKNCLLSLTNSRILVDVPFDRFDAAKFVMRWLCKHENPKMQTMAVSVTSILALQLSPEQTAQLEELFMAVKELLAIVKQ
KTTENLDDVTFLFTLKALWNLTDGSPAACKHFIENQGLQIFIQVLETFSESAIQSKVLGLLNNIAEVRELSSKLVTEDVLKHINSLL
CSREMEVSYFAAGIIAHLTSDRQLWISRDFQRRTLLQDLHATIQNWPSSSCKMTALVTYRSFKTFFPLLGNFSQPEVQLWALWAMYH
VCSKNPSKYCKMLVEEEGLQLLCDIQEHSEATPKAQQIAASILDDFRMHFMNYQRPTLCQMPF
>sp|A0A087WTH1|TM265_HUMAN
MEDEEKAVEILGNTEAAHPPSPIRCCWLRLRCLAATSIICGCSCLGVMALVFAIKAEERHKAGRSEEAVRWGARARKLILASFAVWL
AVLILGPLLLWLLSYAIAQAE
>sp|C9JC47|F157A_HUMAN
MGPLFTTIPGAHSGPMRPLPKKHVEPMAVRQLLLGNSTMIRHTCPMSVPLSRQVKEVAAQKPSEDIYKNWQQQQQQQQQQQQQQLDL
LFHQRIQISLWPRKQKRRKTEQHSHPFVKKAFRFSAGSGCGRPSSNKMLRSMGGGQRPTGLGSEFFRLLHDLHLLAFAMKRIWIHRR
GEATARPRAPEHPAPPATAVRGRDAASQNLKRRPGSGTDGLRLQGAEPSRLLRTYAGGAVIPTGTPERAQPPPPQDPLGRRRWLSRN
TWGPWPGTTQPPSPQLLRNDWGSCGFMVPEAARGKVFQDSQEGAHIRRETVSKSVCAEPWRHQRARDPAPTNFPLRCQKQRGASASS
GQHEGRVNLVFFIGSPTVIAVPDLQCPTKYSGMLY
>sp|Q9H299|SH3L3_HUMAN
MSGLRVYSTSVTGSREIKSQQSEVTRILDGKRIQYQLVDISQDNALRDEMRALAGNPKATPPQIVNGDQYCGDYELFVEAVEQNTLQ
EFLKLA
>sp|Q8NFN8|GP156_HUMAN
MEPEINCSELCDSFPGQELDRRPLHDLCKTTITSSHHSSKTISSLSPVLLGIVWTFLSCGLLLILFFLAFTIHCRKNRIVKMSSPNL
NIVTLLGSCLTYSSAYLFGIQDVLVGSSMETLIQTRLSMLCIGTSLVFGPILGKSWRLYKVFTQRVPDKRVIIKDLQLLGLVAALLM

ADVILLMTWVLTDPIQCLQILSVSMTVTGKDVSCTSTSTHFCASRYSDVWIALIWGCKGLLLLYGAYLAGLTGHVSSPPVNQSLTIM
VGVNLLVLAAGLLFVVTRYLHSWPNLVFGLTSGGIFVCTTTINCFIFIPQLKQWKAFEEENQTIRRMAKYFSTPNKSFHTQYGEEEN
CHPRGEKSSMERLLTEKNAVIESLQEQVNNAKEKIVRLMSAECTYDLPEGAAPPASSPNKDVQAVASVHTLAAAQGPSGHLSDFQND
PGMAARDSQCTSGPSSYAQSLEGPGKDSSFSPGKEEKISDSKDFSDHLDSGCSQKPWTEQSLGPERGDQVPMNPSQSLLPERGGSDP
QRQRHLENSEEPPERRSRVSSVIREKLQEVLQDLGLGPEASLSTAPSCHQQTWKNSAAFSPQKMPLSKELGFSPYMVRRRRAAQRAR
SHFPGSAPSSVGHRANRTVPGAHSRLHVQNGDSPSLAPQTTDSRVRRPSSRKPSLPSDPQDRPGTLEGSKQSQTEPEGARGSKAAFL
RQPSGSGRAPSPAAPCLSKASPDLPEQWQLWPPVPSGCASLSSQHSYFDTESSSSDEFFCRCHRPYCEICFQSSSDSSDSGTSDTDP
EPTGGLASWEKLWARSKPIVNFKDDLKPTLV
>sp|Q8TE59|ATS19_HUMAN
MRLTHICCCCLLYQLGFLSNGIVSELQFAPDREEWEVVFPALWRREPVDPAGGSGGSADPGWVRGVGGGGSARAQAAGSSREVRSVA
PVPLEEPVEGRSESRLRPPPPSEGEEDEELESQELPRGSSGAAALSPGAPASWQPPPPPQPPPSPPPAQHAEPDGDEVLLRIPAFSR
DLYLLLRRDGRFLAPRFAVEQRPNPGPGPTGAASAPQPPAPPDAGCFYTGAVLRHPGSLASFSTCGGGLMGFIQLNEDFIFIEPLND
TMAITGHPHRVYRQKRSMEEKVTEKSALHSHYCGIISDKGRPRSRKIAESGRGKRYSYKLPQEYNIETVVVADPAMVSYHGADAARR
FILTILNMVFNLFQHKSLSVQVNLRVIKLILLHETPPELYIGHHGEKMLESFCKWQHEEFGKKNDIHLEMSTNWGEDMTSVDAAILI
TRKDFCVHKDEPCDTVGIAYLSGMCSEKRKCIIAEDNGLNLAFTIAHEMGHNMGINHDNDHPSCADGLHIMSGEWIKGQNLGDVSWS
RCSKEDLERFLRSKASNCLLQTNPQSVNSVMVPSKLPGMTYTADEQCQILFGPLASFCQEMQHVICTGLWCKVEGEKECRTKLDPPM
DGTDCDLGKWCKAGECTSRTSAPEHLAGEWSLWSPCSRTCSAGISSRERKCPGLDSEARDCNGPRKQYRICENPPCPAGLPGFRDWQ
CQAYSVRTSSPKHILQWQAVLDEEKPCALFCSPVGKEQPILLSEKVMDGTSCGYQGLDICANGRCQKVGCDGLLGSLAREDHCGVCN
GNGKSCKIIKGDFNHTRGAGYVEVLVIPAGARRIKVVEEKPAHSYLALRDAGKQSINSDWKIEHSGAFNLAGTTVHYVRRGLWEKIS
AKGPTTAPLHLLVLLFQDQNYGLHYEYTIPSDPLPENQSSKAPEPLFMWTHTSWEDCDATCGGGERKTTVSCTKIMSKNISIVDNEK
CKYLTKPEPQIRKCNEQPCQTRWMMTEWTPCSRTCGKGMQSRQVACTQQLSNGTLIRARERDCIGPKPASAQRCEGQDCMTVWEAGV
WSECSVKCGKGIRHRTVRCTNPRKKCVLSTRPREAEDCEDYSKCYVWRMGDWSKCSITCGKGMQSRVIQCMHKITGRHGNECFSSEK
PAAYRPCHLQPCNEKINVNTITSPRLAALTFKCLGDQWPVYCRVIREKNLCQDMRWYQRCCETCRDFYAQKLQQKS
>sp|Q96RA2|OR7D2_HUMAN
MEAGNQTGFLEFILLGLSEDPELQPFIFGLFLSMYLVTVLGNLLIILAISSDSHLHTPMYFFLSNLSWVDICFSTCIVPKMLVNIQT
ENKAISYMDCLTQVYFSMFFPILDTLLLTVMAYDRFVAVCHPLHYMIIMNPHLCGLLVFVTWLIGVMTSLLHISLMMHLIFCKDFEI
PHFFCELTYILQLACSDTFLNSTLIYFMTGVLGVFPLLGIIFSYSRIASSIRKMSSSGGKQKALSTCGSHLSVVSLFYGTGIGVHFT
SAVTHSSQKISVASVMYTVVTPMLNPFIYSLRNKDVKGALGSLLSRAASCL
>sp|Q96N23|CFA54_HUMAN
MAAQGSPSSSPSDDSTTSGSLPELPPTSTATSRSPPESKGSSRSSLLQWTCPEDSLPLAVFYGPLDAKNPLLASCEKEIQELLGFMR
KKKALATTEEEKHEFRRRCATSLFNIWTKYAPRLPADYYNEKLLKVGDSLCQMKEYKLALLQCYGRYLQQFNTNFDENKVDVTQFKA
TFFPKGFKDKTAGLTFHALSGKNMCNYQLVCDSDENLKNKESVVQCLHILSSLRLIMQVALPQEHLCWIIFNGTIYIYTICRKLMVI
GQSSKALEYLLWASMCMESLVPLLSLRYLTWRATLYTAVCQCCYDCHAGIHGEAFARRALAKIDELRQLELMSSSKSQEESRRYFRE
ATMKMAVMIFKRGVFESRRKNKAVFRPKIRINLREVQTLSWPRTVTERLLDEMFDSTASQFLAVLEALSDSNRRILQTGPIVTDEVE
IHDVVSELFMAGKELLIMSNIGADGMLDFPKTSLLELMIGRKDVISVDAAVKFIKLAFTYEEWSLFESSAVHLIYFLQRQDDPESKK
AEKDLTLLIAMEPLINVKRNKGLIFPLENYKEGQSTQIYLKKIAVHDTCLKTCGYSEDIFHLAATLYVCVCTAPQDVQPDKEIVVDT
IMFLWQKCKLGIQRLNISRNDYAKFTQKISTNKWIYLLWQINEVIHCYKMEDIDIVVVAEVTLRLSEILESLGSPGRKFKQSLDVPL
REGTNKFPGAPKGITEILPILQKNPVEQLLFAYKLLDRAIGGINLNCMLTSLPNGSSVIDHCYAKRTHHIDGDTYKPLASNSFMMDL
HLELIQAQHRIAVVLLDKLQVLQTPTVSKDISTKGPEKLKQSGSTDCFTELNIMNKIKKNTLSKAIYLMQKALLIFEKDATSTSSWE
LLMEAYSLIQRIEAEQNALYSYQKYLESSKRKKSRVPPPPILLSRTHCSVTLKPAPFTSEVKVSWYCILGCKAEGSYGKVRLNNNHL
PNSGEAIPADGKSVFEVKGLETNEKYVFAVAAYSNNGKLVGGAIGETTKPILVYPPLSTITARMFLTQVAYQVGNYELAKKVFSPVW
DYFVASPLQDEQSVICLSNIITITQRRLHSDILAETSSILLYLFLRNIFVTSDIKIKEENLFCDNIKGNEIFPSQQIARLIECERVL
VALELSNFLNDSSYALQAVTQCYGLLAPIIYHNIVLVPVVQILIKCIVVLQGLPSIVCSKKHTASFESIQHMIACCIFYITKILRSW
REYDLAVMIINYGKKMLDITPGCKSLFDGSNEQEEMPEEDSSKKSLKTKKPQQILLPEKINEQLALLETHLLKLTKQYVTSELSGGE

DPIFLYPVVLNWSVKGAVKEVMKFKQKPRFLEFFTQVMLKCMNEEKFHLMVEVTTPVHDFLKRRNESLLGLIKVKYKDSALNKKANK
SLKFKAAVMEIGRSAEMQQRIRSKKKETLRDFIFKNPAISEMVAHERNRRTSVRKAAQRYLMDYLNPLILSYVKRKRFHRLSLEEMP
WRAQMNLYLAGAHFNLVLQKLWECTKMKFGTSHMMVSFRSCDPNMFSLYNSGTVLPTRKLTVENYKAMLDFLLTAKKRKANLPSDAE
EFSTFINSIMSDENMSKTQTVYDSDSQSGSSAKEKDRGANLCVMDHFMKIFLYCRRAMVLAHRGGYWTLLQNCCRALWNFTQELQIL
LKQAVDLDKTFPISQDGFLCTSVLPFYLGAELLIDMLIQLQNTSSIKPIEDKGEFSVPSCYGNIKNDNGGSSLTFEHPLDDVNVVDL
KWIHDFVLKSLEVLYQVEKWETLVSLAIQFNTVSHERYTEQVTPLLVYAQRQLLLRIQKFKGPDITQQPCARYEAEYGEKITCRNFI
GKQLKINSSTIEATSNCTDLLKMLISSEYSRAKALVCVPVDVTDTLRCFRETLEKSKYHNRSIRHSRKLLSLFLAQTQDVLQASNQR
SLKVQALHSLGSLLIFAEKKRAAFKCWCQALDDIFRKPDVLHTWKEFGPSLTNVTNSHSPPGFKDYSEEFLSRVGIWGCLQGAVISA
KIAQFIKSLNVEKKTDCCILSALLFQGLLRTTLPHPKAERCYAQYEITQLLPGIELFSDRYRADICSVIASLYYIIRELHFVRQNLI
VLPLLALYQYFVSGICQDITRNLEARILKIEVLIDLRFFSEAFYEISQIFYGKNMPCPIPAGYKATGKMKIFQSFDSGKLLTSKENI
QAIDELRNKGLPAVLVTIGQPHLLNKFNFVKAYFFLSVAATINCVPENKFKTVITNKSKPNLPNLEEIYSKDDGSSFYNLTKLKDEI
TLSMLKSMLLMEAEDRLNFLLSEVEQKTLSQCSAGELEIVVEARLQLAAVALQRHRAAYSAAIVFSTLTLLQDSKLFEKKVVQDDTE
NPVSPGTSVTENKDDSEFLDPISLNAREYFNIHLWLRCRLALVTAFVAQIHGIGIVKEDDMTDCLSLINEVCMEAKSAGDTELQAEF
LTQAVILGLQEKHLKADIMTNLQDIIHLLEGNEFISPQSRLTLARSLVLLDDLTKAEKFKESPSSKTGKLNLLTRAHSILTEQMLAF
GETIEFRSSNTKYANPLQPLKNIYLPHVMLLAKIKMRIGHTVAKQVYYKNKRKDPSKWLPALHLFDVALKLCRTTAVEEHEVEAEIL
FQKGKIERQILMEEKSPSFQLESLYEAIQLSLKNDQNSGLIRDSYLEMALLYFHLKKPKIKISGSPLTLKPPLRRSSSVKETSANKF
EMYSSLAWIAIRAAAQVSEAVLAINLLIGKKNTRMHKVNQVALPNIPEFAALDLLSSYTDYLLDNYQVLFQTSCTFLYQNDDVCDSA
DGRKKTQTKVDITWILLLRYYIHLQRINNLSKLLASATPVSGISLPDDTLLTSLYNSELILRQKEVHFFLKKFLQLYSSSCIDEFPK
ELLCQLENPPLSEKDLRESSAKLYRDSSVQSILSFKPVSGSSCVDITPIEMVTQASNKELCFQWYIPPLDRPPKETEPMVLLLYAYN
LKPLKISDVRHSTYNSTCVGSLWIPLNRVIAIHEKLSNLAQIAELSLPAAPEITSNENIYEVEVEEESVDNEMEDMIIQCCSEIASL
FLNDKEPTPLSEVPFDISLPSIFNLERLFDLANGCILSGGSLFNWIVSIIP
>sp|Q8WV37|ZN480_HUMAN
MLCDEKAQKRRKRKAKESGMALPQGHLTFRDVAIEFSQAEWKCLDPAQRALYKDVMLENYRNLVSLGISLPDLNINSMLEQRREPWS
GESEVKIAKNSDGRECIKGVNTGSSYALGSNAEDKPIKKQLGVSFHLHLSELELFPDERVINGCNQVENFINHSSSVSCLQEMSSSV
KTPIFNRNDFDDSSFLPQEQKVHLREKPYECNEHSKVFRVSSSLTKHQVIHTVEKPYKCNSCGKVFSRNSHLAEHCRIHTGEKPYKC
NVCGKVFSYNSNFARHQRIHTREKPYECNECGKVFSNNSYLARHQRIHAEEKPYKCNECGKGFSHKSSLANHWRIYTGEKPYKCDEC
GKAFYRIALLVRHQKIHTGEKPYKCNECGKVFIQNSHLAQHWRIHTGEKPYKCNECGKVFNQLSNLARHRRIHTGEKPYKCNECGKA
FSEYSGLSAHLVIHTGEKPYKCSECGKAFRHKLSLTNHQRIHTGERPYKCNECGKVFNRIAHLARHRKIHTGEKPYKCNECGKAFSR
ISYLAQHWTIHMG
>sp|Q9H992|MARH7_HUMAN
MESKPSRIPRRISVQPSSSLSARMMSGSRGSSLNDTYHSRDSSFRLDSEYQSTSASASASPFQSAWYSESEITQGARSRSQNQQRDH
DSKRPKLSCTNCTTSAGRNVGNGLNTLSDSSWRHSQVPRSSSMVLGSFGTDLMRERRDLERRTDSSISNLMDYSHRSGDFTTSSYVQ
DRVPSYSQGARPKENSMSTLQLNTSSTNHQLPSEHQTILSSRDSRNSLRSNFSSRESESSRSNTQPGFSYSSSRDEAPIISNSERVV
SSQRPFQESSDNEGRRTTRRLLSRIASSMSSTFFSRRSSQDSLNTRSLNSENSYVSPRILTASQSRSNVPSASEVPDNRASEASQGF
RFLRRRWGLSSLSHNHSSESDSENFNQESEGRNTGPWLSSSLRNRCTPLFSRRRREGRDESSRIPTSDTSSRSHIFRRESNEVVHLE
AQNDPLGAAANRPQASAASSSATTGGSTSDSAQGGRNTGISGILPGSLFRFAVPPALGSNLTDNVMITVDIIPSGWNSADGKSDKTK
SAPSRDPERLQKIKESLLLEDSEEEEGDLCRICQMAAASSSNLLIEPCKCTGSLQYVHQDCMKKWLQAKINSGSSLEAVTTCELCKE
KLELNLEDFDIHELHRAHANEQAEYEFISSGLYLVVLLHLCEQSFSDMMGNTNEPSTRVRFINLARTLQAHMEDLETSEDDSEEDGD
HNRTFDIA
>sp|P50591|TNF10_HUMAN
MAMMEVQGGPSLGQTCVLIVIFTVLLQSLCVAVTYVYFTNELKQMQDKYSKSGIACFLKEDDSYWDPNDEESMNSPCWQVKWQLRQL
VRKMILRTSEETISTVQEKQQNISPLVRERGPQRVAAHITGTRGRSNTLSSPNSKNEKALGRKINSWESSRSGHSFLSNLHLRNGEL
VIHEKGFYYIYSQTYFRFQEEIKENTKNDKQMVQYIYKYTSYPDPILLMKSARNSCWSKDAEYGLYSIYQGGIFELKENDRIFVSVT
NEHLIDMDHEASFFGAFLVG

>sp|Q9BZG2|PPAT_HUMAN
MAGLGFWGHPAGPLLLLLLLVLPPRALPEGPLVFVALVFRHGDRAPLASYPMDPHKEVASTLWPRGLGQLTTEGVRQQLELGRFLRS
RYEAFLSPEYRREEVYIRSTDFDRTLESAQANLAGLFPEAAPGSPEARWRPIPVHTVPVAEDKLLRFPMRSCPRYHELLREATEAAE
YQEALEGWTGFLSRLENFTGLSLVGEPLRRAWKVLDTLMCQQAHGLPLPAWASPDVLRTLAQISALDIGAHVGPPRAAEKAQLTGGI
LLNAILANFSRVQRLGLPLKMVMYSAHDSTLLALQGALGLYDGHTPPYAACLGFEFRKHLGNPAKDGGNVTVSLFYRNDSAHLPLPL
SLPGCPAPCPLGRFYQLTAPARPPAHGVSCHGPYEAAIPPAPVVPLLAGAVAVLVALSLGLGLLAWRPGCLRALGGPV
>sp|O60243|H6ST1_HUMAN
MRRRRAGGRTMVERASKFVLVVAGSVCFMLILYQYAGPGLSLGAPGGRAPPDDLDLFPTPDPHYEKKYYFPVRELERSLRFDMKGDD
VIVFLHIQKTGGTTFGRHLVQNVRLEVPCDCRPGQKKCTCYRPNRRETWLFSRFSTGWSCGLHADWTELTNCVPGVLDRRDSAALRT
PRKFYYITLLRDPVSRYLSEWRHVQRGATWKTSLHMCDGRTPTPEELPPCYEGTDWSGCTLQEFMDCPYNLANNRQVRMLADLSLVG
CYNLSFIPEGKRAQLLLESAKKNLRGMAFFGLTEFQRKTQYLFERTFNLKFIRPFMQYNSTRAGGVEVDEDTIRRIEELNDLDMQLY
DYAKDLFQQRYQYKRQLERREQRLRSREERLLHRAKEALPREDADEPGRVPTEDYMSHIIEKW
>sp|Q9C099|LRCC1_HUMAN
MEAAAAVVAAEAEVENEDGDSSCGDVCFMDKGLQSISELSLDSTLHAVNLHCNNISKIEAIDHIWNLQHLDLSSNQISRIEGLNTLT
KLCTLNLSCNLITKVEGLEELINLTRLNVSYNHIDDLSGLIPLHGIKHKLRYIDLHSNRIDSIHHLLQCMVGLHFLTNLILEKDGDD
NPVCRLPGYRAVILQTLPQLRILDCKNIFGEPVNLTEINSSQLQCLEGLLDNLVSSDSPLNISEDEIIDRMPVITAPIDELVPLEQF
ASTPSDAVLTSFMSVCQSSEPEKNNHENDLQNEIKLQKLDDQILQLLNETSNSIDNVLEKDPRPKRDTDITSESDYGNRKECNRKVP
RRSKIPYDAKTIQTIKHHNKNYNSFVSCNRKMKPPYLKELYVSSSLANCPMLQESEKPKTEIIKVDQSHSEDNTYQSLVEQLDQERE
KRWRAEQAENKLMDYIDELHKHANEKEDIHSLALLTTDRLKEIIFRERNSKGQLEVMVHKLQNEIKKLTVELMKAKDQQEDHLKHLR
TLEKTLEKMERQKRQQQAAQIRLIQEVELKASAADREIYLLRTSLHREREQAQQLHQLLALKEQEHRKELETREFFTDADFQDALAK
EIAKEEKKHEQMIKEYQEKIDVLSQQYMDLENEFRIALTVEARRFQDVKDGFENVATELAKSKHALIWAQRKENESSSLIKDLTCMV
KEQKTKLAEVSKLKQETAANLQNQINTLEILIEDDKQKSIQIELLKHEKVQLISELAAKESLIFGLRTERKVWGHELAQQGSSLAQN
RGKLEAQIESLSRENECLRKTNESDSDALRIKCKIIDDQTETIRKLKDCLQEKDEHIKRLQEKITEIEKCTQEQLDEKSSQLDEVLE
KLERHNERKEKLKQQLKGKEVELEEIRKAYSTLNRKWHDKGELLCHLETQVKEVKEKFENKEKKLKAERDKSIELQKNAMEKLHSMD
DAFKRQVDAIVEAHQAEIAQLANEKQKCIDSANLKVHQIEKEMRELLEETCKNKKTMEAKIKQLAFALNEIQQDM
>sp|P17029|ZKSC1_HUMAN
MMTAESREATGLSPQAAQEKDGIVIVKVEEEDEEDHMWGQDSTLQDTPPPDPEIFRQRFRRFCYQNTFGPREALSRLKELCHQWLRP
EINTKEQILELLVLEQFLSILPKELQVWLQEYRPDSGEEAVTLLEDLELDLSGQQVPGQVHGPEMLARGMVPLDPVQESSSFDLHHE
ATQSHFKHSSRKPRLLQSRALPAAHIPAPPHEGSPRDQAMASALFTADSQAMVKIEDMAVSLILEEWGCQNLARRNLSRDNRQENYG
SAFPQGGENRNENEESTSKAETSEDSASRGETTGRSQKEFGEKRDQEGKTGERQQKNPEEKTRKEKRDSGPAIGKDKKTITGERGPR
EKGKGLGRSFSLSSNFTTPEEVPTGTKSHRCDECGKCFTRSSSLIRHKIIHTGEKPYECSECGKAFSLNSNLVLHQRIHTGEKPHEC
NECGKAFSHSSNLILHQRIHSGEKPYECNECGKAFSQSSDLTKHQRIHTGEKPYECSECGKAFNRNSYLILHRRIHTREKPYKCTKC
GKAFTRSSTLTLHHRIHARERASEYSPASLDAFGAFLKSCV
>sp|Q9UGR2|Z3H7B_HUMAN
MERQKRKADIEKGLQFIQSTLPLKQEEYEAFLLKLVQNLFAEGNDLFREKDYKQALVQYMEGLNVADYAASDQVALPRELLCKLHVN
RAACYFTMGLYEKALEDSEKALGLDSESIRALFRKARALNELGRHKEAYECSSRCSLALPHDESVTQLGQELAQKLGLRVRKAYKRP
QELETFSLLSNGTAAGVADQGTSNGLGSIDDIETGNVPDTREQVEIGAPRDCYVDPRGSPALLPSTPTMPLFPHVLDLLAPLDSSRT
LPSTDSLDDFSDGDVFGPELDTLLDSLSLVQGGLSGSGVPSELPQLIPVFPGGTPLLPPVVGGSIPVSSPLPPASFGLVMDPSKKLA
ASVLDALDPPGPTLDPLDLLPYSETRLDALDSFGSTRGSLDKPDSFMEETNSQDHRPPSGAQKPAPSPEPCMPNTALLIKNPLAATH
EFKQACQLCYPKTGPRAGDYTYREGLEHKCKRDILLGRLRSSEDQTWKRIRPRPTKTSFVGSYYLCKDMINKQDCKYGDNCTFAYHQ
EEIDVWTEERKGTLNRDLLFDPLGGVKRGSLTIAKLLKEHQGIFTFLCEICFDSKPRIISKGTKDSPSVCSNLAAKHSFYNNKCLVH
IVRSTSLKYSKIRQFQEHFQFDVCRHEVRYGCLREDSCHFAHSFIELKVWLLQQYSGMTHEDIVQESKKYWQQMEAHAGKASSSMGA
PRTHGPSTFDLQMKFVCGQCWRNGQVVEPDKDLKYCSAKARHCWTKERRVLLVMSKAKRKWVSVRPLPSIRNFPQQYDLCIHAQNGR
KCQYVGNCSFAHSPEERDMWTFMKENKILDMQQTYDMWLKKHNPGKPGEGTPISSREGEKQIQMPTDYADIMMGYHCWLCGKNSNSK

KQWQQHIQSEKHKEKVFTSDSDASGWAFRFPMGEFRLCDRLQKGKACPDGDKCRCAHGQEELNEWLDRREVLKQKLAKARKDMLLCP
RDDDFGKYNFLLQEDGDLAGATPEAPAAAATATTGE
>sp|P10745|RET3_HUMAN
MMREWVLLMSVLLCGLAGPTHLFQPSLVLDMAKVLLDNYCFPENLLGMQEAIQQAIKSHEILSISDPQTLASVLTAGVQSSLNDPRL
VISYEPSTPEPPPQVPALTSLSEEELLAWLQRGLRHEVLEGNVGYLRVDSVPGQEVLSMMGEFLVAHVWGNLMGTSALVLDLRHCTG
GQVSGIPYIISYLHPGNTILHVDTIYNRPSNTTTEIWTLPQVLGERYGADKDVVVLTSSQTRGVAEDIAHILKQMRRAIVVGERTGG
GALDLRKLRIGESDFFFTVPVSRSLGPLGGGSQTWEGSGVLPCVGTPAEQALEKALAILTLRSALPGVVHCLQEVLKDYYTLVDRVP
TLLQHLASMDFSTVVSEEDLVTKLNAGLQAASEDPRLLVRAIGPTETPSWPAPDAAAEDSPGVAPELPEDEAIRQALVDSVFQVSVL
PGNVGYLRFDSFADASVLGVLAPYVLRQVWEPLQDTEHLIMDLRHNPGGPSSAVPLLLSYFQGPEAGPVHLFTTYDRRTNITQEHFS
HMELPGPRYSTQRGVYLLTSHRTATAAEEFAFLMQSLGWATLVGEITAGNLLHTRTVPLLDTPEGSLALTVPVLTFIDNHGEAWLGG
GVVPDAIVLAEEALDKAQEVLEFHQSLGALVEGTGHLLEAHYARPEVVGQTSALLRAKLAQGAYRTAVDLESLASQLTADLQEVSGD
HRLLVFHSPGELVVEEAPPPPPAVPSPEELTYLIEALFKTEVLPGQLGYLRFDAMAELETVKAVGPQLVRLVWQQLVDTAALVIDLR
YNPGSYSTAIPLLCSYFFEAEPRQHLYSVFDRATSKVTEVWTLPQVAGQRYGSHKDLYILMSHTSGSAAEAFAHTMQDLQRATVIGE
PTAGGALSVGIYQVGSSPLYASMPTQMAMSATTGKAWDLAGVEPDITVPMSEALSIAQDIVALRAKVPTVLQTAGKLVADNYASAEL
GAKMATKLSGLQSRYSRVTSEVALAEILGADLQMLSGDPHLKAAHIPENAKDRIPGIVPMQIPSPEVFEELIKFSFHTNVLEDNIGY
LRFDMFGDGELLTQVSRLLVEHIWKKIMHTDAMIIDMRFNIGGPTSSIPILCSYFFDEGPPVLLDKIYSRPDDSVSELWTHAQVVGE
RYGSKKSMVILTSSVTAGTAEEFTYIMKRLGRALVIGEVTSGGCQPPQTYHVDDTNLYLTIPTARSVGASDGSSWEGVGVTPHVVVP
AEEALARAKEMLQHNQLRVKRSPGLQDHL
>sp|Q86UE3|ZN546_HUMAN
MQVDPPLHGPPNDFLIFQIIPLHSLSIMPRFLWILCFSMEETQGELTSSCGSKTMANVSLAFRDVSIDLSQEEWECLDAVQRDLYKD
VMLENYSNLVSLGYTIPKPDVITLLEQEKEPWIVMREGTRNWFTDLEYKYITKNLLSEKNVCKIYLSQLQTGEKSKNTIHEDTIFRN
GLQCKHEFERQERHQMGCVSQMLIQKQISHPLHPKIHAREKSYECKECRKAFRQQSYLIQHLRIHTGERPYKCMECGKAFCRVGDLR
VHHTIHAGERPYECKECGKAFRLHYHLTEHQRIHSGVKPYECKECGKAFSRVRDLRVHQTIHAGERPYECKECGKAFRLHYQLTEHQ
RIHTGERPYECKVCGKTFRVQRHISQHQKIHTGVKPYKCNECGKAFSHGSYLVQHQKIHTGEKPYECKECGKSFSFHAELARHRRIH
TGEKPYECRECGKAFRLQTELTRHHRTHTGEKPYECKECGKAFICGYQLTLHLRTHTGEIPYECKECGKTFSSRYHLTQHYRIHTGE
KPYICNECGKAFRLQGELTRHHRIHTCEKPYECKECGKAFIHSNQFISHQRIHTSESTYICKECGKIFSRRYNLTQHFKIHTGEKPY
ICNECGKAFRFQTELTQHHRIHTGEKPYKCTECGKAFIRSTHLTQHHRIHTGEKPYECTECGKTFSRHYHLTQHHRGHTGEKPYICN
ECGNAFICSYRLTLHQRIHTGELPYECKECGKTFSRRYHLTQHFRLHTGEKPYSCKECGNAFRLQAELTRHHIVHTGEKPYKCKECG
KAFSVNSELTRHHRIHTGEKPYQCKECGKAFIRSDQLTLHQRNHISEEVLCIM
>sp|Q9UGM5|FETUB_HUMAN
MGLLLPLALCILVLCCGAMSPPQLALNPSALLSRGCNDSDVLAVAGFALRDINKDRKDGYVLRLNRVNDAQEYRRGGLGSLFYLTLD
VLETDCHVLRKKAWQDCGMRIFFESVYGQCKAIFYMNNPSRVLYLAAYNCTLRPVSKKKIYMTCPDCPSSIPTDSSNHQVLEAATES
LAKYNNENTSKQYSLFKVTRASSQWVVGPSYFVEYLIKESPCTKSQASSCSLQSSDSVPVGLCKGSLTRTHWEKFVSVTCDFFESQA
PATGSENSAVNQKPTNLPKVEESQQKNTPPTDSPSKAGPRGSVQYLPDLDDKNSQEKGPQEAFPVHLDLTTNPQGETLDISFLFLEP
MEEKLVVLPFPKEKARTAECPGPAQNASPLVLPP
>sp|P06315|KV502_HUMAN
MGSQVHLLSFLLLWISDTRAETTLTQSPAFMSATPGDKVNISCKASQDIDDDMNWYQQKPGEAAIFIIQEATTLVPGIPPRFSGSGY
GTDFTLTINNIESEDAAYYFCLQHDNFP
>sp|Q86VZ5|SMS1_HUMAN
MLSASTMKEVVYWSPKKVADWLLENAMPEYCEPLEHFTGQDLINLTQEDFKKPPLCRVSSDNGQRLLDMIETLKMEHHLEAHKNGHA
NGHLNIGVDIPTPDGSFSIKIKPNGMPNGYRKEMIKIPMPELERSQYPMEWGKTFLAFLYALSCFVLTTVMISVVHERVPPKEVQPP
LPDTFFDHFNRVQWAFSICEINGMILVGLWLIQWLLLKYKSIISRRFFCIVGTLYLYRCITMYVTTLPVPGMHFNCSPKLFGDWEAQ
LRRIMKLIAGGGLSITGSHNMCGDYLYSGHTVMLTLTYLFIKEYSPRRLWWYHWICWLLSVVGIFCILLAHDHYTVDVVVAYYITTR
LFWWYHTMANQQVLKEASQMNLLARVWWYRPFQYFEKNVQGIVPRSYHWPFPWPVVHLSRQVKYSRLVNDT

>sp|A7XYQ1|SOBP_HUMAN
MAEMEKEGRPPENKRSRKPAHPVKREINEEMKNFAENTMNELLGWYGYDKVELKDGEDIEFRSYPTDGESRQHISVLKENSLPKPKL
PEDSVISPYNISTGYSGLATGNGLSDSPAGSKDHGSVPIIVPLIPPPFIKPPAEDDVSNVQIMCAWCQKVGIKRYSLSMGSEVKSFC
SEKCFAACRRAYFKRNKARDEDGHAENFPQQHYAKETPRLAFKNNCELLVCDWCKHIRHTKEYLDFGDGERRLQFCSAKCLNQYKMD
IFYKETQANLPAGLCSTLHPPMENKAEGTGVQLLTPDSWNIPLTDARRKAPSPVATAGQSQGPGPSASTTVSPSDTANCSVTKIPTP
VPKSIPISETPNIPPVSVQPPASIGPPLGVPPRSPPMVMTNRGPVPLPIFMEQQIMQQIRPPFIRGPPHHASNPNSPLSNPMLPGIG
PPPGGPRNLGPTSSPMHRPMLSPHIHPPSTPTMPGNPPGLLPPPPPGAPLPSLPFPPVSMMPNGPMPVPQMMNFGLPSLAPLVPPPT
LLVPYPVIVPLPVPIPIPIPIPHVSDSKPPNGFSSNGENFIPNAPGDSAAAGGKPSGHSLSPRDSKQGSSKSADSPPGCSGQALSLA
PTPAEHGRSEVVDLTRRAGSPPGPPGAGGQLGFPGVLQGPQDGVIDLTVGHRARLHNVIHRALHAHVKAEREPSAAERRTCGGCRDG
HCSPPAAGDPGPGAPAGPEAAAACNVIVNGTRGAAAEGAKSAEPPPEQPPPPPPPAPPKKLLSPEEPAVSELESVKENNCASNCHLD
GEAAKKLMGEEALAGGDKSDPNLNNPADEDHAYALRMLPKTGCVIQPVPKPAEKAAMAPCIISSPMLSAGPEDLEPPLKRRCLRIRN
QNK
>sp|Q9H5H4|ZN768_HUMAN
MEREALPWGLEPQDVQSSDEMRSPEGYLRGNMSENEEEEISQQEGSGDYEVEEIPFGLEPQSPGFEPQSPEFEPQSPRFEPESPGFE
SRSPGLVPPSPEFAPRSPESDSQSPEFESQSPRYEPQSPGYEPRSPGYEPRSPGYESESSRYESQNTELKTQSPEFEAQSSKFQEGA
EMLLNPEEKSPLNISVGVHPLDSFTQGFGEQPTGDLPIGPPFEMPTGALLSTPQFEMLQNPLGLTGALRGPGRRGGRARGGQGPRPN
ICGICGKSFGRGSTLIQHQRIHTGEKPYKCEVCSKAFSQSSDLIKHQRTHTGERPYKCPRCGKAFADSSYLLRHQRTHSGQKPYKCP
HCGKAFGDSSYLLRHQRTHSHERPYSCTECGKCYSQNSSLRSHQRVHTGQRPFSCGICGKSFSQRSALIPHARSHAREKPFKCPECG
KRFGQSSVLAIHARTHLPGRTYSCPDCGKTFNRSSTLIQHQRSHTGERPYRCAVCGKGFCRSSTLLQHHRVHSGERPYKCDDCGKAF
SQSSDLIRHQRTHAAGRR
>sp|P17010|ZFX_HUMAN
MDEDGLELQQEPNSFFDATGADGTHMDGDQIVVEVQETVFVSDVVDSDITVHNFVPDDPDSVVIQDVIEDVVIEDVQCPDIMEEADV
SETVIIPEQVLDSDVTEEVSLAHCTVPDDVLASDITSASMSMPEHVLTGDSIHVSDVGHVGHVGHVEHVVHDSVVEAEIVTDPLTTD
VVSEEVLVADCASEAVIDANGIPVDQQDDDKGNCEDYLMISLDDAGKIEHDGSSGMTMDTESEIDPCKVDGTCPEVIKVYIFKADPG
EDDLGGTVDIVESEPENDHGVELLDQNSSIRVPREKMVYMTVNDSQPEDEDLNVAEIADEVYMEVIVGEEDAAAAAAAAAVHEQQMD
DNEIKTFMPIAWAAAYGNNSDGIENRNGTASALLHIDESAGLGRLAKQKPKKRRRPDSRQYQTAIIIGPDGHPLTVYPCMICGKKFK
SRGFLKRHMKNHPEHLAKKKYRCTDCDYTTNKKISLHNHLESHKLTSKAEKAIECDECGKHFSHAGALFTHKMVHKEKGANKMHKCK
FCEYETAEQGLLNRHLLAVHSKNFPHICVECGKGFRHPSELKKHMRIHTGEKPYQCQYCEYRSADSSNLKTHVKTKHSKEMPFKCDI
CLLTFSDTKEVQQHALIHQESKTHQCLHCDHKSSNSSDLKRHIISVHTKDYPHKCDMCDKGFHRPSELKKHVAAHKGKKMHQCRHCD
FKIADPFVLSRHILSVHTKDLPFRCKRCRKGFRQQSELKKHMKTHSGRKVYQCEYCEYSTTDASGFKRHVISIHTKDYPHRCEYCKK
GFRRPSEKNQHIMRHHKEVGLP
>sp|Q8NH55|O52E5_HUMAN
MLHTNNTQFHPSTFLVVGVPGLEDVHVWIGFPFFAVYLTALLGNIIILFVIQTEQSLHQPMFYFLAMLAGTDLGLSTATIPKMLGIF
WFNLGEIAFGACITQMYTIHICTGLESVVLTVTGIDRYIAICNPLRYSMILTNKVIAILGIVIIVRTLVFVTPFTFLILRLPFCGVR
IIPHTYCEHMGLAKLACASINVIYGLIAFSVGYIDISVIGFSYVQILRAVFHLPAWDARPKALSTCGSHVCVMLAFYLPALFSFMTH
RFGHNIPHYIHILLANLYVVFPPALNSVIYGVKTKQIREQVLRILNPKSFWHFDPKRIFHNNSVRQ
>sp|Q7Z5P9|MUC19_HUMAN
MKLILWYLVVALWCFFKDVEALLYRQKSDGKIAASRSGGFSYGSSSSGDLDRKKPLFSLEFGSPGETEDKSRQRQDAGSPKSEDTPA
GGFFNSSSSSGDSDRTKPFFSLGLGAPGKAEDKSGDSQDAGGSKSEDTPPGGFFYGSSSSGDSDKKKPLFSFEFGATGEDEDKSRER
WDAGNSRSEDSPADSTNTRYGAGFSSSGASLDVGFGWGISDEKGLEVSKADGRETRGSGSAGGETIVFGPDAGSSVGTGSSGLKLGA
GKGDAAFGFEVSDSNSFGDTGISSKTVEGNQTSSSGGSVSIDLGDTSFRSENQFVGGGSLNSISNLWDSGQEGFGINEIGGNGMSGS
VSAEAGFKGFGSDSSSSGDSSARNGFENSSGISEDSGVILGSSDQHEVELSRTGGNRKRSSDPDEAGNLSPGSDVSDSGGNTWSSDS
GSGGGGVTSSSEYSTSGPLNTPEKGSHIPEATPKYSETNAIIGEISTWSKGAYKSFNGRIFFFESSCPYTFCRHCIESGGDFNIEIK
RNNDSEIEKITVLIDNNDVSIFGDTILVNGESVQIPYNNKLIHIKKYGEHNVLNSRRGILTLMWDKNNKLSLTLHKQYPTCGLCGNF

NSTPGQDINEHIANSKIPGDCPNAVGKSYEVCEDGIQHCNKIIGTYFEKCGKVAALSNDYKMICIDEYCQTRDKTSTCDTYSELSRL
CASDGPGTFESWRSDSDVVCGTQRCPEQHIYKECGPSNPATCSNVAPFQDSECVSGCTCPEGYLLDDIGEKGKCVLKAECPCESSGT
VYQPGEVREGPCGSQCTCQDAKWSCTEALCPGRCKVEGSSLTTFDGVKYNFPGNCHFLAVHNEDWSISVELRPCPSGQTGTCLNSVT
LLLNSSVPVDKYVFNSDGTVTNDKIRNQGYYYSDKIQIFNASSSYLQVETYFHVKLQIQIVPVMQLYVSMPPNQFTDTVGLCGSYNN
KAEDDFMSSQNILEKTSQAFANSWEMMSCPKGNPSSCISIEKEKFAERHCGILLDSSGPLASCHPIVNPKPYHEECKKYTCTCENSQ
DCLCTILGNYVKACAEKETYIVGWRTGLCEHSCPSGLVFKYNVKACNSSCRSLSERDRSCDVEDVPVDGCTCPDAMYQNNEGNCVLK
SQCDCYINDEVMQPGKLIHIDDNKCVCRDGILLCQIPIDLTLQNCSGGAEYVDCSDPKAQRRTNRTCSTRNIPVFDENLPCKRGCFC
PEGMVRNSKGICVFPNDCPCSFGGREYDEGSVTSVGCNECTCIKGSWSCTQNECQTICHIYGEGHVRTFDGKSYSFDGLCQYSFLED
YCGHENGTFRILTESVPCCEDGLTCSRKIIVAFQDQNIVLQDGKVTAVKSTESKKCELNANAYSIHTVGLYLILKFQNGIIVIWDKN
TRLSVILDPNWNGKVCGLCGNNNGDLKDDFTTRYSSVASGALEFGNSWKTSQECSDTVAQTFPCDSNPYCKAWAVRKCEILRDSTFR
DCHNKVDPSAYHDACIEEACACDMEGKYLGFCTAVAMYAEACSAVGVCVSWRKPNLCPVYCDYYNAPGECRWHYEPCGTVTAKTCKD
QLVGQKFSSLLEGCYAKCPDSAPYLDENTMKCVSLSECSCFYNDVIPAGGVIEDNCGRTCYCIAGQLECSETAPTNSTFAVSTTTAT
TILSTGAAITLVTGGPSTAASIPAITTSSSETTGTTLGPLTEPFTTGITETSVPIISTSGNAGMTGVVSPTVTGASGMAGTTGGVDA
ATTGAASENTSERAGTPRVSGETPAVGGGSTPGEAGPGATVSGSTGVSAGSITASPGASATSSESSKSGTTGPSVGGKTGATSSEAT
SSEGMSGVTGQSLGSTAGSDSEITAKTSFTGSSPPGKLTRPSPGSPGHFSGGTTEWGNVATTGAAGENTSGALGSTEGSVEATTSAG
SGNTAGTSGTGDTGPGNTAVSGTPVVSPGATPGAPGSSTPGEADIGNTSFGKSGTPTVSAASTTSSPVSKHTDAASATAVTISGSKP
GTPGTPGGATSGGKITSGWSSSGTSTGASNTPGATGSSTGQTDTSGPSAKVTGNYGQSSEIPGTIKSSSDVSGTMGQSDTTSGPSVA
VTRTSEQSSGVTVASEPSVGVSGTTGPLAEISGTTRPLVSGLRTTGSSAEGSGTTGPSSRESVTTRPLAEGSGTSGQSVTGSRATGL
SATELGTTVSFTGGLGTSRSSARETRTTGPSADGSGTTGPSVVRSGTTRLSVGVTRATESSPGVTGTTTPSAEESRTTGPSVLVTGT
TGQSGQGSGTTGKSFIESGPSVVGSGTTGPTSAGLGTTAPSTRRSSTTKPSVGRTGTTGQSGAESGTTEPSARVAGVTGTSAEVSGR
IEPSATESSTSRPLGETTGTTIPSMEGSEATGPSVIGSETTRLSVIGSGTTGTSSGGSGATRSSGGGMGTTGQSTARSETTGPLFGL
TGTFGQSATVTGTSSNSAGVTTPEKSPGVAMTTGLLVEGSATTQPRILESETTESSAGVIVTSGQSARVTGATGPSAGETGTTEPST
EGSVAAVLFVIGSETTRPLDIGSGTTGTLSGGSSTTRSSDGTTGTTRKSTARSETTGLSGLTGTSGQLAGVTGTSSKSAGVTVTSEK
SAGVAVITGSFVERPVTTGPPLLESETTRPSGGVTVTSGQSARVTETVGASAGVTGTTGPSTEGSGATGPSVVGSGTTRPLAGESGT
TESSAGVTGTRPSSSRESATTGPSDEGSGTTGLSAGVTVTSGQSVRKTGTTGAPAGVTETTRPSVVKSGTTGPSVIGTRTTGTSSGG
SGATRSSGGETETTGQSAVKSGTTESFTRLTRTSGQSAGMTGTSAQSAGVALTSPFVEGLVTTGSSTVGLETTRPSAVGSGKTGPPV
VKAQTTGPSAGVTVTSGQSARMTGASGPSVGVTGTTGPASKGLGTIRPSVVGLETTELSAEGSGTTGPPIVGETTVPSAGVTVTSGY
SDRVTGATEPLAGVTGTIKPSVAGSVTTGPSVTGVETTAKTTSGGLSTTISSVGGTGTTGQSPERSGTTGPFTGLTGTSAQSAGVTM
TSIQSAGVLVTTGLNVDGLGTTGKALIGSGTTGLSAEATGTIGPSTEGLEKTGPSITGSGTTRPLVTESWTAGTSSGGHSTTSPSVR
GTETTGQSAAESVTTGPVTGYTETSGPSAGVTVTPRQSPTVTQTTGSSAAVSGTTVQSLTVSGTTRPSSGQTEITGSSVKESGTTES
SAVRSGTTGPTAGVTGTNGPSSAGVTGITGSSPGVTGTTGSSPGVTGTTGSSARSGTSIPSVGKTGTTRTSVEESRTTRPSAGITGT
NGLSAEVTGTTGPLAGVTGTTGPSAGVTRTTGLSAGETGTTGLSPGVTRTTRSSAGLTGKTGLSAGVTGKTGLSAEVTGTTRLSAGV
TGTTGPSPGVTGTTGTPAGVTGTTELSAGVTGKTGLSSEVTETTGLSYGVKRTIGLSAGSTGTSGQSAGVAGTTTLSAEVTGTTRPS
AGVTGTTGLSAEVTEITGISAVVTGTTGPSAGVTETTGSSAGVAGTTRLSAGVTGITGLSAGVTGTTGLSTEVTGTTGPSAGATGTT
GLSVGVTGITGLSDVVTETTGSSARSGTGIPSVGETRTTSTSVEESRTTRPSAGIMGTNGLPAEVTGTTEPLAGGTGTTGILAGVTG
TTGLSAGETGKIGSSAGVTGKTGSSARVTGKTGPSAEVTGKTGLSAGVTGTTGLSPGVTGTSGLSAEVTGTTGPSAEATGLPGVSAG
VTGTTGSLAGGTGTIGLSAGVTGTTGSSAGVTGTTGLSAGVTGIAGLSAGVTGITGPSAGVTGTTTVSAGVTGTTGLSAEATEITGL
SAGVTGTTGLSAGVTETIRLSAGVTGTIRSSAGVTGITGLSAGVTGTTGPSAGVTGSTGLLAGVTETTGQSAKVTGTTGQSVGVTGT
TRSSGGVTGITGLSAGVTGTNGLSAVTGMTGLSAEVTGTTGLSVGVTGIAGLSAGVTGITGPSAGITGTTTISAGVTGTSGLSAEAT
GITGLSAGVTGKTGLSAGVTETIGLSAEATGTIGSSPGVTGTTGSSTGVTGITGLSAGVTGTTGLSTEVTGTTGPSAGVTRTTGLSA
GVTGITGLSAIVTETTGSSARSGTSIPSVGETGTTRTSVEESRTTRPSAGITGTNGLSAEVTGTIGPLAGGTGTTGLSAGVTGTVGS
SAVVTGTTGLSAGVTGTTGPSAEETGATGPSAEVTETTGPSAGVTGTGRLSAEVTGTTGPSAEVTGLPGESAEVTGTIGSPAGVTGT
TQLSAVVTGITGLSAEVTGTTGLSAGVTGITGLSAEVTRTTGLSAGVTGTIGLSAGVTGTTRPSAGVTGTTGQSAEVTGTTEPSAGL
TETTGSSTGVTGATGPLAGVTGTTGISTEVTGTTGPSARVTGTTVLSAGVTGITGLSAIVTETTGSSARSGTSTPSVGETGTTRTSV

EESRATRPSAGITGTNGQSAEVTWITGPLAGVTGTTGISAGVTGTTGLSAGVTGTIGSSAVVTGINGLSAGVTGTTGPSAEETGATG
PSAEVTGTTGPSAEETGATGPSAEVTGTTGPSGGVTGTNGLSAEVTGTTGPSAEVTGLPGVSAGVTGTIGSPAAVTGTIRPSAVVTG
ITGLSAEVTGTTGLSAWVTGIAGLSAGVTETIGSSAGVTGTNGLSAEATGTTGPSAGVTGTTGLSAGVTGTAGLSARVTESTGLSAG
VTGTTGLSAGVTGTTGPSAGITGTNGLSAEVTGTTGPLAGVTGTIGLSAGVTGIAGLSAGVTESTGLSAGVTGTIRSSAVVTGINGL
SAGVTGTTGPSAEETGATGPSAEVTGTTGPSGGVTGTSGISAEVTGTTGPSAEVTGLPGVSAGVTGTIGSPAAVTGTTRPSAVVTGI
SGLSAEVTGTTGLSAGVTETIGSSAGVTGTNGLSAEATETTGPSAGVTGTTGLSAGVTGTTGPSAGIAGTNGLSAGVTGTTGLSARV
TESTGLSAGVTGTIGSSAVVTETTRLSSGVTGTIGPSAEETGATGLSAEVTGTTGSLAEVTGTTGLSAGVTGTIGSSAVVTGTTGLS
AGITGTNGLSAEVTGTAGPLAGVTGTTGLSAGVTGTTGLSAGVTETTGQSAGVTESTGLSPGVTGTIGSSAVVTGIKGLSAGVTGTT
GPSAEETGATGPSAEVTGTTGPSGGVTGTSVLSVEVTGTTGPSAEVTGLPGVSAGLTGTIGSPAAVRGTTWPSAVVTGISGLSGEVT
GTTGLSAGVTGIGGLSAGVTGTIGSSAGVTGTNALSAEATGTTGPSAGVTGTTGLSAGVTGTTGLSAGVTGTIRSSAVVTETTGLSA
GVTGTTGPSAGIAGTNGLSAEVTGTTGLSAGMTGTTGLSARVTESTGLSAGVTGTIGSSAVVTETTRLSAGVTGTIGPSAEETGATG
LSAEVTRTTGSLAGVTGTTGPSAVVTGKTELSAEVTGTTELSAEVTEKTGPSAEVTGKTGLSAGVMETTGPSAEVTGTTGSSAGVTG
TTGPSAGVTGTTGPSAEATGLPGVSAGVTGTIGSPAGVTGTARLSAVVTGISGLSAEVTGTTGLSTGVTGIAGHSAAVTGITRPSAG
VTGTTTVSAGVTGTIGLSAEATGITLPSAGVTETTGLSAGVTETIGLSAGVTGTIGSSAGVTEITGLSAGVTGTTGPSAGVTGSTVL
SAGVTATTGQSVGVTGTTGPSAGVTGTTGLSAGVTGIAGLSAGVTGITGPSAGVTGTTTVSAGVTGTTGLSAEATEITGLSAGVTGT
TGLSAGVTGIAGLSAGVTETIGSSAGVTGTNGLSAEATGKTGPSAGVTGTTGLSAGVTGTTGLSAGVTETIGLSAGVTGTIGSSAGV
KGTTGQSAEVTGATGQSVGVTGTTRSSGGVTGITGLSAGLRGTTVSSAKAGTSIPLTGKTGTTRTSVEESTTTGPSAGITGTNGLSA
EMTGTNELSAGVTGTIGSSAGVTGTTGLSVEATVTTGLSAGVTGTTVPLAGVTWTPGPSAGVTGIAALSAGVTGKSGLSAGVTGKTG
LSAGVTGTTGPSAEATGKTGLSAGVTGITGPFAEVTGTTGLSAGVIGTTGSSAEVTGITGLSAGVTGKTRSSAGVTGTTGLSAKSGT
SIPSAGKTGTTKTSVEESRTTRPSAGITATTGVPAATSPGAEGESIASTSVATGAIPRSTIAPGSTTTGTTGVTTGTTLAPRSFNIG
TSGGISGKTLKPGSYVSEATTATGTPGAGPSGGTTISSPEVSTISEVSNTGITGVGSETSIETGISNTATTGVAPGTTLAPGSSSTE
ATTSIGGSASTRGGIATEATGSTRGVRTTGSEAPEGTSGEFSGTTISSGGFHTEATTLTGGRGSIGTESRAESTTSLPQSAKTRGGI
LTEATSSTGRIRATGSEAPGGTSRKFSGTTISSGGSHTEATTLAGGRDSTESEFRTATIGVVPATTVAPGSSKTEATTFLGVSGTTS
VGRATGATTSIAGSDTSQAEHPGGTSGEFPGTTITSGDSHTEATALTGSRGSIGTESTVETTTYIGESGTTRGGLATATTGAFSGKT
LEPGNDNTEATGSTGGIRATRTEAPGGTSGEFPGTTFTSGGSHTEATTFTGGKGSTGTESRAATTRAAPGTTLVPGSSNTGATASPG
GSATTRGRITTATTGAFSGKTLESENDNTEATSSTRGVRTTRSEAPGGTSGEFPGTRITSGGSYTATTRAAPGTTLAPGSSNTGATA
SLGGSAMTRGRITTATTGAFSGKTLEPGNNNTEATSSTRGVRTTRSEAPGEATTLTGDRSSTGSESRTATTGVAPGTTVAPGSSKTE
ATTFLGVSGTTNIGRATGATTSIVGSDTSQAERPGGTTVVSPGASSTSQSSRPGTSVTPDSSASESETVTTKEFSGTTAISRTSHTG
TPAASGGQATGSLTATTGVAPGTTVAPGSSNTEATTSVGERETTKAEIITGDTGELSGTTIISENSTTAGITAATGKQAGTSEVAPS
TTVAPGSFSTAATTSPGASGTTGVTTTTKTTTSLGGSGTTGAEIKSATTGAPGSRTGTAGVPSATTVSPGSSNSEATTSVGESGKTG
AETITEATTSTEGTGTSGTGFKTGTSEVAPATTVAPGSFSTAATTSPGASGMTGVTTTTKTTTSLGGSGTTGAKIKLVGTTTTAPES
RTAGVPSGTRVTPGSSNSEATTSVEESRITRAEVITEATTFSGGSGATRAGLPRGTTGEFSGTNFISGSSNTEATTSTEGTGTSGTG
FKIAGITSAPGKQAGTSGVSLATTVAPGSFSTATTSSGASGITRAGPTSETTTSLGGSGTTGAEIKSAAVPSGTTVAPGSSNSEATT
SVGENGKTRGEIITDTTEGTSGKVLEPGSAHTEATTFPGGSGTTRAGPPGGTTGELSRMTIIPGSSNTEATTSTKGTGTSGTGFKTG
TSWVAPGTTVSPGSFSTATISPGASRTTGAAPAAETTTSLEGGGTTGAEIKSGATSGVPGSKTGTAGVPSATTIAPGSSNSEATTSL
GESGKTRVETITGTTEGKTLAAGSAHTEATTFSGGSGSTRAGPLGGASGTSGGYVPGRETEPTTSIEETGTSRTIFKTVGITSAPGR
QAGTSVVAPSTTVAPGSFSTAATTSPGASGMTGVRTTSKTTTSLGGTGTTRTEIKSGATTGAPGIKTDIMGESSRTTILSGSSNTEA
TNSIEETGTSGTGFKTAGITAAPGKQAGTSGVAPGTTVAPGSFSTAATTSPGASGVTGTGPTAETTTFLGGSSTTGAEIKSGATTGA
PGSKTGTAKVLSGTTVASGSSNSEATTFSGITEAVTVPSKNGSMTTALGSQLSSSQTVIPGSSGTISHTTVAPGSSVTGTTTGASDD
QVTGSKTGTTGVALSTTVAPGSSSTEATTSTGVHRTTVVGQKTGATTRGSAKQGTRSTIEATTSFRGTGTTGSGMNTGTTGVVSGNT
ISPSSFNTEATSGTSERPNPGSEIGTTGIVSGTTVAPGSSNTEATTSLGNGGTTEAGSKIVTTGITTGTTIVPGSFNTKATTSTDVG
VATGVGMATGITNIISGRSQPTGSKTGYTVTGSGTTALPGGFRTGNTPGSTGVTSSQEGTTVVSSGITGIPETSISGPSKEASDKTT
APGPPTTVTASTGVKETSETGVQTGSTLVTAGVPTRPQVSQPETTVVATREVETENKTECLASLPPAPVCHGPLGEEKSPGDIWTAN
CHRGTCTDAKTIDCKPEECPSPPTCKTGEKLVKFQSNDTCCEIGYCEPRTCLFNNTDYEIGASFDDPSNPCVSYSCKDTGFAAVVQD

CPKQTWCAEANRIYDSKKCCYTCKNNCRSSLVNVTVIYSGCKKRVQMAKCTGECEKTAKYNYDILLLEHSCLCCREENYELRDIVLD
CPDGSTIPYQYKHITTCSCLDICQLYTTFMYS
>sp|Q8NGL7|OR4P4_HUMAN
MEKSNNSTLFILLGFSQNKNIEVLCFVLFLFCYIAIWMGNLLIMISITCTQLIHQPMYFFLNYLSLSDLCYTSTVTPKLMVDLLAER
KTISYNNCMIQLFTTHFFGGIEIFILTGMAYDRYVAICKPLHYTIIMSRQKCNTIIIVCCTGGFIHSASQFLLTIFVPFCGPNEIDH
YFCDVYPLLKLACSNIHMIGLLVIANSGLIALVTFVVLLLSYVFILYTIRAYSAERRSKALATCSSHVIVVVLFFAPALFIYIRPVT
TFSEDKVFALFYTIIAPMFNPLIYTLRNTEMKNAMRKVWCCQILLKRNQLF
>sp|P04201|MAS_HUMAN
MDGSNVTSFVVEEPTNISTGRNASVGNAHRQIPIVHWVIMSISPVGFVENGILLWFLCFRMRRNPFTVYITHLSIADISLLFCIFIL
SIDYALDYELSSGHYYTIVTLSVTFLFGYNTGLYLLTAISVERCLSVLYPIWYRCHRPKYQSALVCALLWALSCLVTTMEYVMCIDR
EEESHSRNDCRAVIIFIAILSFLVFTPLMLVSSTILVVKIRKNTWASHSSKLYIVIMVTIIIFLIFAMPMRLLYLLYYEYWSTFGNL
HHISLLFSTINSSANPFIYFFVGSSKKKRFKESLKVVLTRAFKDEMQPRRQKDNCNTVTVETVV
>sp|Q8NA69|CS045_HUMAN
MATGALLPCSRPCPMSRLDFLKASHFSLGPDLRLHEGTMRTTSHRDFAYPAATREPPSLQPPPALLFPMDPRWDREERVSEAHRAFP
PPSTPPWELLQAQARERTLAMQAGNLHLHEDAHAGIGLSNAHAAYGWPELPARTRERIRGARLIFDRDSLPPGDRDKLRIPPTTHQA
LFPPHDARPQPRAPSCHLGGPNTLKWDYTRQDGTSYQRQFQALPGPPALRCKRASSGVELGDCKISYGSTCSEQKQAYRPQDLPEDR
YDKAQATAHIHCVNIRPGDGLFRDRTTKAEHFYAREPEPFVLHHDQTPESHILKGNWCPGPGSLDTFMQYFYGQPPPPTQPPSRHVP
HEKLQSHVTLGEPKLLKRFFKTTMGSDYCPSEWRQVQKAPNLHLQQSYLPRGTGEFDFLTMNQKMLKPHRTPPAPVTEEMLQRCKYS
HMEPPLGGLRFFSTQYKDEFPFKYQGPAALRLKNPQEGFVPLGTPHQRGCREKIDPLVPQPPMYLCPSQQ
>sp|O75175|CNOT3_HUMAN
MADKRKLQGEIDRCLKKVSEGVEQFEDIWQKLHNAANANQKEKYEADLKKEIKKLQRLRDQIKTWVASNEIKDKRQLIDNRKLIETQ
MERFKVVERETKTKAYSKEGLGLAQKVDPAQKEKEEVGQWLTNTIDTLNMQVDQFESEVESLSVQTRKKKGDKDKQDRIEGLKRHIE
KHRYHVRMLETILRMLDNDSILVDAIRKIKDDVEYYVDSSQDPDFEENEFLYDDLDLEDIPQALVATSPPSHSHMEDEIFNQSSSTP
TSTTSSSPIPPSPANCTTENSEDDKKRGRSTDSEVSQSPAKNGSKPVHSNQHPQSPAVPPTYPSGPPPAASALSTTPGNNGVPAPAA
PPSALGPKASPAPSHNSGTPAPYAQAVAPPAPSGPSTTQPRPPSVQPSGGGGGGSGGGGSSSSSNSSAGGGAGKQNGATSYSSVVAD
SPAEVALSSSGGNNASSQALGPPSGPHNPPPSTSKEPSAAAPTGAGGVAPGSGNNSGGPSLLVPLPVNPPSSPTPSFSDAKAAGALL
NGPPQFSTAPEIKAPEPLSSLKSMAERAAISSGIEDPVPTLHLTERDIILSSTSAPPASAQPPLQLSEVNIPLSLGVCPLGPVPLTK
EQLYQQAMEEAAWHHMPHPSDSERIRQYLPRNPCPTPPYHHQMPPPHSDTVEFYQRLSTETLFFIFYYLEGTKAQYLAAKALKKQSW
RFHTKYMMWFQRHEEPKTITDEFEQGTYIYFDYEKWGQRKKEGFTFEYRYLEDRDLQ
>sp|Q2NKJ3|CTC1_HUMAN
MAAGRAQVPSSEQAWLEDAQVFIQKTLCPAVKEPNVQLTPLVIDCVKTVWLSQGRNQGSTLPLSYSFVSVQDLKTHQRLPCCSHLSW
SSSAYQAWAQEAGPNGNPLPREQLLLLGTLTDLSADLEQECRNGSLYVRDNTGVLSCELIDLDLSWLGHLFLFPRWSYLPPARWNSS
GEGHLELWDAPVPVFPLTISPGPVTPIPVLYPESASCLLRLRNKLRGVQRNLAGSLVRLSALVKSKQKAYFILSLGRSHPAVTHVSI
IVQVPAQLVWHRALRPGTAYVLTELRVSKIRGQRQHVWMTSQSSRLLLLKPECVQELELELEGPLLEADPKPLPMPSNSEDKKDPES
LVRYSRLLSYSGAVTGVLNEPAGLYELDGQLGLCLAYQQFRGLRRVMRPGVCLQLQDVHLLQSVGGGTRRPVLAPCLRGAVLLQSFS
RQKPGAHSSRQAYGASLYEQLVWERQLGLPLYLWATKALEELACKLCPHVLRHHQFLQHSSPGSPSLGLQLLAPTLDLLAPPGSPVR
NAHNEILEEPHHCPLQKYTRLQTPSSFPTLATLKEEGQRKAWASFDPKALLPLPEASYLPSCQLNRRLAWSWLCLLPSAFCPAQVLL
GVLVASSHKGCLQLRDQSGSLPCLLLAKHSQPLSDPRLIGCLVRAERFQLIVERDVRSSFPSWKELSMPGFIQKQQARVYVQFFLAD
ALILPVPRPCLHSATPSTPQTDPTGPEGPHLGQSRLFLLCHKEALMKRNFCVPPGASPEVPKPALSFYVLGSWLGGTQRKEGTGWGL
PEPQGNDDNDQKVHLIFFGSSVRWFEFLHPGQVYRLIAPGPATPMLFEKDGSSCISRRPLELAGCASCLTVQDNWTLELESSQDIQD
VLDANKSLPESSLTDLLSDNFTDSLVSFSAEILSRTLCEPLVASLWMKLGNTGAMRRCVKLTVALETAECEFPPHLDVYIEDPHLPP
SLGLLPGARVHFSQLEKRVSRSHNVYCCFRSSTYVQVLSFPPETTISIPLPHIYLAELLQGGQSPFQATASCHIVSVFSLQLFWVCA
YCTSICRQGKCTRLGSTCPTQTAISQAIIRLLVEDGTAEAVVTCRNHHVAAALGLCPREWASLLDFVQVPGRVVLQFAGPGAQLESS
ARVDEPMTMFLWTLCTSPSVLRPIVLSFELERKPSKIVPLEPPRLQRFQCGELPFLTHVNPRLRLSCLSIRESEYSSSLGILASSC

>sp|P59537|T2R43_HUMAN
MITFLPIIFSSLVVVTFVIGNFANGFIALVNSIEWFKRQKISFADQILTALAVSRVGLLWVLLLNWYSTVLNPAFNSVEVRTTAYNI
WAVINHFSNWLATTLSIFYLLKIANFSNFIFLHLKRRVKSVILVMLLGPLLFLACHLFVINMNEIVRTKEFEGNMTWKIKLKSAMYF
SNMTVTMVANLVPFTLTLLSFMLLICSLCKHLKKMQLHGKGSQDPSTKVHIKALQTVISFLLLCAIYFLSIMISVWSFGSLENKPVF
MFCKAIRFSYPSIHPFILIWGNKKLKQTFLSVFWQMRYWVKGEKTSSP
>sp|Q96DY7|MTBP_HUMAN
MDRYLLLVIWGEGKFPSAASREAEHGPEVSSGEGTENQPDFTAANVYHLLKRSISASINPEDSTFPACSVGGIPGSKKWFFAVQAIY
GFYQFCSSDWQEIHFDTEKDKIEDVLQTNIEECLGAVECFEEEDSNSRESLSLADLYEEAAENLHQLSDKLPAPGRAMVDIILLLSD
KDPPKLKDYLPTVGALKHLREWYSAKITIAGNHCEINCQKIAEYLSANVVSLEDLRNVIDSKELWRGKIQIWERKFGFEISFPEFCL
KGVTLKNFSTSNLNTDFLAKKIIPSKDKNILPKVFHYYGPALEFVQMIKLSDLPSCYMSDIEFELGLTNSTKQNSVLLLEQISSLCS
KVGALFVLPCTISNILIPPPNQLSSRKWKEYIAKKPKTISVPDVEVKGECSSYYLLLQGNGNRRCKATLIHSANQINGSFALNLIHG
KMKTKTEEAKLSFPFDLLSLPHFSGEQIVQREKQLANVQVLALEECLKRRKLAKQPETVSVAELKSLLVLTRKHFLDYFDAVIPKMI
LRKMDKIKTFNILNDFSPVEPNSSSLMETNPLEWPERHVLQNLETFEKTKQKMRTGSLPHSSEQLLGHKEGPRDSITLLDAKELLKY
FTSDGLPIGDLQPLPIQKGEKTFVLTPELSPGKLQVLPFEKASVCHYHGIEYCLDDRKALERDGGFSELQSRLIRYETQTTCTRESF
PVPTVLSPLPSPVVSSDPGSVPDGEVLQNELRTEVSRLKRRSKDLNCLYPRKRLVKSESSESLLSQTTGNSNHYHHHVTSRKPQTER
SLPVTCPLVPIPSCETPKLATKTSSGQKSMHESKTSRQIKESRSQKHTRILKEVVTETLKKHSITETHECFTACSQRLFEISKFYLK
DLKTSRGLFEEMKKTANNNAVQVIDWVLEKTSKK
>sp|Q8IW03|SIAH3_HUMAN
MLFFTQCFGAVLDLIHLRFQHYKAKRVFSAAGQLVCVVNPTHNLKYVSSRRAVTQSAPEQGSFHPHHLSHHHCHHRHHHHLRHHAHP
HHLHHQEAGLHANPVTPCLCMCPLFSCQWEGRLEVVVPHLRQIHRVDILQGAEIVFLATDMHLPAPADWIIMHSCLGHHFLLVLRKQ
ERHEGHPQFFATMMLIGTPTQADCFTYRLELNRNHRRLKWEATPRSVLECVDSVITDGDCLVLNTSLAQLFSDNGSLAIGIAITATE
VLPSEAEM
>sp|P08174|DAF_HUMAN
MTVARPSVPAALPLLGELPRLLLLVLLCLPAVWGDCGLPPDVPNAQPALEGRTSFPEDTVITYKCEESFVKIPGEKDSVICLKGSQW
SDIEEFCNRSCEVPTRLNSASLKQPYITQNYFPVGTVVEYECRPGYRREPSLSPKLTCLQNLKWSTAVEFCKKKSCPNPGEIRNGQI
DVPGGILFGATISFSCNTGYKLFGSTSSFCLISGSSVQWSDPLPECREIYCPAPPQIDNGIIQGERDHYGYRQSVTYACNKGFTMIG
EHSIYCTVNNDEGEWSGPPPECRGKSLTSKVPPTVQKPTTVNVPTTEVSPTSQKTTTKTTTPNAQATRSTPVSRTTKHFHETTPNKG
SGTTSGTTRLLSGHTCFTLTGLLGTLVTMGLLT
>sp|Q9BZM5|N2DL2_HUMAN
MAAAAATKILLCLPLLLLLSGWSRAGRADPHSLCYDITVIPKFRPGPRWCAVQGQVDEKTFLHYDCGNKTVTPVSPLGKKLNVTTAW
KAQNPVLREVVDILTEQLRDIQLENYTPKEPLTLQARMSCEQKAEGHSSGSWQFSFDGQIFLLFDSEKRMWTTVHPGARKMKEKWEN
DKVVAMSFHYFSMGDCIGWLEDFLMGMDSTLEPSAGAPLAMSSGTTQLRATATTLILCCLLIILPCFILPGI
>sp|A6NCS4|NKX26_HUMAN
MLLSPVTSTPFSVKDILRLERERSCPAASPHPRVRKSPENFQYLRMDAEPRGSEVHNAGGGGGDRKLDGSEPPGGPCEAVLEMDAER
MGEPQPGLNAASPLGGGTRVPERGVGNSGDSVRGGRSEQPKARQRRKPRVLFSQAQVLALERRFKQQRYLSAPEREHLASALQLTST
QVKIWFQNRRYKCKRQRQDKSLELAGHPLTPRRVAVPVLVRDGKPCLGPGPGAPAFPSPYSAAVSPYSCYGGYSGAPYGAGYGTCYA
GAPSGPAPHTPLASAGFGHGGQNATPQGHLAATLQGVRAW
>sp|Q969J2|ZKSC4_HUMAN
MAREPRKNAALDAQSAEDQTGLLTVKVEKEEASALTAEVRAPCSPARGPERSRQRFRGFRYPEAAGPREALSRLRELCGQWLQPEMH
SKEQILELLVLEQFLTILPGNLQSWVREQHPESGEEVVVLLEYLERQLDEPAPQVPVGDQGQELLCCKMALLTQTQGSQSSQCQPMK
ALFKHESLGSQPLHDRVLQVPGLAQGGCCREDAMVASRLTPGSQGLLKMEDVALTLTPGWTQLDSSQVNLYRDEKQENHSSLVSLGG
EIQTKSRDLPPVKKLPEKEHGKICHLREDIAQIPTHAEAGEQEGRLQRKQKNAIGSRRHYCHECGKSFAQSSGLTKHRRIHTGEKPY
ECEDCGKTFIGSSALVIHQRVHTGEKPYECEECGKVFSHSSNLIKHQRTHTGEKPYECDDCGKTFSQSCSLLEHHKIHTGEKPYQCN

MCGKAFRRNSHLLRHQRIHGDKNVQNPEHGESWESQGRTESQWENTEAPVSYKCNECERSFTRNRSLIEHQKIHTGEKPYQCDTCGK
GFTRTSYLVQHQRSHVGKKTLSQ
>sp|Q8NGY9|OR2L8_HUMAN
MENYNQTSTDFILLGLFPPSRIDLFFFILIVFIFLMALIGNLSMILLIFLDTHLHTPMYFLLSQLSLIDLNYISTIVPKMASDFLHG
NKSISFTGCGIQSFFFLALGGAEALLLASMAYDRYIAICFPLHYLIRMSKRVCVLMITGSWIIGSINACAHTVYVLHIPYCRSRAIN
HFFCDVPAMVTLACMDTWVYEGTVFLSATIFLVFPFIGISCSYGQVLFAVYHMKSAEGRKKAYLTCSTHLTVVTFYYAPFVYTYLRP
RSLRSPTEDKVLAVFYTILTPMLNPIIYSLRNKEVMGALTRVSQRICSVKM
>sp|O15085|ARHGB_HUMAN
MSVRLPQSIDRLSSLSSLGDSAPERKSPSHHRQPSDASETTGLVQRCVIIQKDQHGFGFTVSGDRIVLVQSVRPGGAAMKAGVKEGD
RIIKVNGTMVTNSSHLEVVKLIKSGAYVALTLLGSSPSSMGISGLQQDPSPAGAPRITSVIPSPPPPPPLPPPQRITGPKPLQDPEV
QKHATQILRNMLRQEEKELQDILPLYGDTSQRPSEGRLSLDSQEGDSGLDSGTERFPSLSESLMNRNSVLSDPGLDSPRTSPVIMAR
VAQHHRRQGSDAAVPSTGDQGVDQSPKPLIIGPEEDYDPGYFNNESDIIFQDLEKLKSRPAHLGVFLRYIFSQADPSPLLFYLCAEV
YQQASPKDSRSLGKDIWNIFLEKNAPLRVKIPEMLQAEIDSRLRNSEDARGVLCEAQEAAMPEIQEQIHDYRTKRTLGLGSLYGEND
LLDLDGDPLRERQVAEKQLAALGDILSKYEEDRSAPMDFALNTYMSHAGIRLREARPSNTAEKAQSAPDKDKWLPFFPKTKKSSNSK
KEKDALEDKKRNPILKYIGKPKSSSQSTFHIPLSPVEVKPGNVRNIIQHFENNQQYDAPEPGTQRLSTGSFPEDLLESDSSRSEIRL
GRSESLKGREEMKRSRKAENVPRSRSDVDMDAAAEATRLHQSASSSTSSLSTRSLENPTPPFTPKMGRRSIESPSLGFCTDTLLPHL
LEDDLGQLSDLEPEPDAQNWQHTVGKDVVAGLTQREIDRQEVINELFVTEASHLRTLRVLDLIFYQRMKKENLMPREELARLFPNLP
ELIEIHNSWCEAMKKLREEGPIIKEISDLMLARFDGPAREELQQVAAQFCSYQSIALELIKTKQRKESRFQLFMQEAESHPQCRRLQ
LRDLIISEMQRLTKYPLLLESIIKHTEGGTSEHEKLCRARDQCREILKYVNEAVKQTENRHRLEGYQKRLDATALERASNPLAAEFK
SLDLTTRKMIHEGPLTWRISKDKTLDLHVLLLEDLLVLLQKQDEKLLLKCHSKTAVGSSDSKQTFSPVLKLNAVLIRSVATDKRAFF
IICTSKLGPPQIYELVALTSSDKNTWMELLEEAVRNATRHPGAAPMPVHPPPPGPREPAQQGPTPSRVELDDSDVFHGEPEPEELPG
GTGSQQRVQGKHQVLLEDPEQEGSAEEEELGVLPCPSTSLDGENRGIRTRNPIHLAFPGPLFMEGLADSALEDVENLRHLILWSLLP
GHTMETQAAQEPEDDLTPTPSVISVTSHPWDPGSPGQAPPGGEGDNTQLAGLEGERPEQEDMGLCSLEHLPPRTRNSGIWESPELDR
NLAEDASSTEAAGGYKVVRKAEVAGSKVVPALPESGQSEPGPPEVEGGTKATGNCFYVSMPSGPPDSSTDHSEAPMSPPQPDSLPAG
QTEPQPQLQGGNDDPRRPSRSPPSLALRDVGMIFHTIEQLTLKLNRLKDMELAHRELLKSLGGESSGGTTPVGSFHTEAARWTDGSL
SPPAKEPLASDSRNSHELGPCPEDGSDAPLEDSTADAAASPGP
>sp|P54317|LIPR2_HUMAN
MLPPWTLGLLLLATVRGKEVCYGQLGCFSDEKPWAGTLQRPVKLLPWSPEDIDTRFLLYTNENPNNFQLITGTEPDTIEASNFQLDR
KTRFIIHGFLDKAEDSWPSDMCKKMFEVEKVNCICVDWRHGSRAMYTQAVQNIRVVGAETAFLIQALSTQLGYSLEDVHVIGHSLGA
HTAAEAGRRLGGRVGRITGLDPAGPCFQDEPEEVRLDPSDAVFVDVIHTDSSPIVPSLGFGMSQKVGHLDFFPNGGKEMPGCKKNVL
STITDIDGIWEGIGGFVSCNHLRSFEYYSSSVLNPDGFLGYPCASYDEFQESKCFPCPAEGCPKMGHYADQFKGKTSAVEQTFFLNT
GESGNFTSWRYKVSVTLSGKEKVNGYIRIALYGSNENSKQYEIFKGSLKPDASHTCAIDVDFNVGKIQKVKFLWNKRGINLSEPKLG
ASQITVQSGEDGTEYNFCSSDTVEENVLQSLYPC
>sp|Q9Y6X4|F169A_HUMAN
MAFPVDMLENCSHEELENSAEDYMSDLRCGDPENPECFSLLNITIPISLSNVGFVPLYGGDQTQKILALFAPEDSLTAVALYLADQW
WAIDDIVKTSVPSREGLKQVSTLGERVVLYVLNRIIYRKQEMERNEIPFLCHSSTDYAKILWKKGEAIGFYSVKPTGSICASFLTQS
YQLPVLDTMFLRKKYRGKDFGLHMLEDFVDSFTEDALGLRYPLSSLMYTACKQYFEKYPGDHELLWEVEGVGHWYQRIPVTRALQRE
ALKILALSQNEPKRPMSGEYGPASVPEYEARTEDNQSSEMQLTIDSLKDAFASTSEGHDKTSVSTHTRSGNLKRPKIGKRFQDSEFS
SSQGEDEKTSQTSLTASINKLESTARPSESSEEFLEEEPEQRGIEFEDESSDRDARPALETQPQQEKQDGEKESELEPMNGEIMDDS
LKTSLITEEEDSTSEVLDEELKLQPFNSSEDSTNLVPLVVESSKPPEVDAPDKTPRIPDSEMLMDEGTSDEKGHMEEKLSLLPRKKA
HLGSSDNVATMSNEERSDGGFPNSVIAEFSEEPVSENLSPNTTSSLEDQGEEGVSEPQETSTALPQSSLIEVELEDVPFSQNAGQKN
QSEEQSEASSEQLDQFTQSAEKAVDSSSEEIEVEVPVVDRRNLRRKAKGHKGPAKKKAKLT
>sp|P38571|LICH_HUMAN

MKMRFLGLVVCLVLWTLHSEGSGGKLTAVDPETNMNVSEIISYWGFPSEEYLVETEDGYILCLNRIPHGRKNHSDKGPKPVVFLQHG
LLADSSNWVTNLANSSLGFILADAGFDVWMGNSRGNTWSRKHKTLSVSQDEFWAFSYDEMAKYDLPASINFILNKTGQEQVYYVGHS
QGTTIGFIAFSQIPELAKRIKMFFALGPVASVAFCTSPMAKLGRLPDHLIKDLFGDKEFLPQSAFLKWLGTHVCTHVILKELCGNLC
FLLCGFNERNLNMSRVDVYTTHSPAGTSVQNMLHWSQAVKFQKFQAFDWGSSAKNYFHYNQSYPPTYNVKDMLVPTAVWSGGHDWLA
DVYDVNILLTQITNLVFHESIPEWEHLDFIWGLDAPWRLYNKIINLMRKYQ
>sp|Q9ULV8|CBLC_HUMAN
MALAVAPWGRQWEEARALGRAVRMLQRLEEQCVDPRLSVSPPSLRDLLPRTAQLLREVAHSRRAAGGGGPGGPGGSGDFLLIYLANL
EAKSRQVAALLPPRGRRSANDELFRAGSRLRRQLAKLAIIFSHMHAELHALFPGGKYCGHMYQLTKAPAHTFWRESCGARCVLPWAE
FESLLGTCHPVEPGCTALALRTTIDLTCSGHVSIFEFDVFTRLFQPWPTLLKNWQLLAVNHPGYMAFLTYDEVQERLQACRDKPGSY
IFRPSCTRLGQWAIGYVSSDGSILQTIPANKPLSQVLLEGQKDGFYLYPDGKTHNPDLTELGQAEPQQRIHVSEEQLQLYWAMDSTF
ELCKICAESNKDVKIEPCGHLLCSCCLAAWQHSDSQTCPFCRCEIKGWEAVSIYQFHGQATAEDSGNSSDQEGRELELGQVPLSAPP
LPPRPDLPPRKPRNAQPKVRLLKGNSPPAALGPQDPAPA
>sp|Q13415|ORC1_HUMAN
MAHYPTRLKTRKTYSWVGRPLLDRKLHYQTYREMCVKTEGCSTEIHIQIGQFVLIEGDDDENPYVAKLLELFEDDSDPPPKKRARVQ
WFVRFCEVPACKRHLLGRKPGAQEIFWYDYPACDSNINAETIIGLVRVIPLAPKDVVPTNLKNEKTLFVKLSWNEKKFRPLSSELFA
ELNKPQESAAKCQKPVRAKSKSAESPSWTPAEHVAKRIESRHSASKSRQTPTHPLTPRARKRLELGNLGNPQMSQQTSCASLDSPGR
IKRKVAFSEITSPSKRSQPDKLQTLSPALKAPEKTRETGLSYTEDDKKASPEHRIILRTRIAASKTIDIREERTLTPISGGQRSSVV
PSVILKPENIKKRDAKEAKAQNEATSTPHRIRRKSSVLTMNRIRQQLRFLGNSKSDQEEKEILPAAEISDSSSDEEEASTPPLPRRA
PRTVSRNLRSSLKSSLHTLTKVPKKSLKPRTPRCAAPQIRSRSLAAQEPASVLEEARLRLHVSAVPESLPCREQEFQDIYNFVESKL
LDHTGGCMYISGVPGTGKTATVHEVIRCLQQAAQANDVPPFQYIEVNGMKLTEPHQVYVQILQKLTGQKATANHAAELLAKQFCTRG
SPQETTVLLVDELDLLWTHKQDIMYNLFDWPTHKEARLVVLAIANTMDLPERIMMNRVSSRLGLTRMCFQPYTYSQLQQILRSRLKH
LKAFEDDAIQLVARKVAALSGDARRCLDICRRATEICEFSQQKPDSPGLVTIAHSMEAVDEMFSSSYITAIKNSSVLEQSFLRAILA
EFRRSGLEEATFQQIYSQHVALCRMEGLPYPTMSETMAVCSHLGSCRLLLVEPSRNDLLLRVRLNVSQDDVLYALKDE
>sp|Q13275|SEM3F_HUMAN
MLVAGLLLWASLLTGAWPSFPTQDHLPATPRVRLSFKELKATGTAHFFNFLLNTTDYRILLKDEDHDRMYVGSKDYVLSLDLHDINR
EPLIIHWAASPQRIEECVLSGKDVNGECGNFVRLIQPWNRTHLYVCGTGAYNPMCTYVNRGRRAQATPWTQTQAVRGRGSRATDGAL
RPMPTAPRQDYIFYLEPERLESGKGKCPYDPKLDTASALINEELYAGVYIDFMGTDAAIFRTLGKQTAMRTDQYNSRWLNDPSFIHA
ELIPDSAERNDDKLYFFFRERSAEAPQSPAVYARIGRICLNDDGGHCCLVNKWSTFLKARLVCSVPGEDGIETHFDELQDVFVQQTQ
DVRNPVIYAVFTSSGSVFRGSAVCVYSMADIRMVFNGPFAHKEGPNYQWMPFSGKMPYPRPGTCPGGTFTPSMKSTKDYPDEVINFM
RSHPLMYQAVYPLQRRPLVVRTGAPYRLTTIAVDQVDAADGRYEVLFLGTDRGTVQKVIVLPKDDQELEELMLEEVEVFKDPAPVKT
MTISSKRQQLYVASAVGVTHLSLHRCQAYGAACADCCLARDPYCAWDGQACSRYTASSKRRSRRQDVRHGNPIRQCRGFNSNANKNA
VESVQYGVAGSAAFLECQPRSPQATVKWLFQRDPGDRRREIRAEDRFLRTEQGLLLRALQLSDRGLYSCTATENNFKHVVTRVQLHV
LGRDAVHAALFPPLSMSAPPPPGAGPPTPPYQELAQLLAQPEVGLIHQYCQGYWRHVPPSPREAPGAPRSPEPQDQKKPRNRRHHPP
DT
>sp|Q7RTU4|BHA09_HUMAN
MLRGAPGLGLTARKGAEDSAEDLGGPCPEPGGDSGVLGANGASCSRGEAEEPAGRRRARPVRSKARRMAANVRERKRILDYNEAFNA
LRRALRHDLGGKRLSKIATLRRAIHRIAALSLVLRASPAPRGPCGHLECHGPAARGDTGDTGASPPPPAGPSLARPDAARPSVPSAP
RCASCPPHAPLARPSAVAEGPGLAQASGGSWRRCPGASSAGPPPWPRGYLRSAPGMGHPRS
>sp|P63131|VPK7_HUMAN
WASQVSENRPVCKAIIQGKQFEGLVDTGADVSIIALNQWPKNWPKQKAVTGLVGIGTASEVYQSTEILHCLGPDNQESTVQPMITSI
PLNLWGRDLLQQWGAEITMPAPLYSPTSQKIMTKMGYIPGKGLGKNEDGIKVPVEAKINQEREGIGYPF
>sp|Q7Z2K6|ERMP1_HUMAN
MEWGSESAAVRRHRVGVERREGAAAAPPPEREARAQEPLVDGCSGGGRTRKRSPGGSGGASRGAGTGLSEVRAALGLALYLIALRTL
VQLSLQQLVLRGAAGHRGEFDALQARDYLEHITSIGPRTTGSPENEILTVHYLLEQIKLIEVQSNSLHKISVDVQRPTGSFSIDFLG

GFTSYYDNITNVVVKLEPRDGAQHAVLANCHFDSVANSPGASDDAVSCSVMLEVLRVLSTSSEALHHAVIFLFNGAEENVLQASHGF
ITQHPWASLIRAFINLEAAGVGGKELVFQTGPENPWLVQAYVSAAKHPFASVVAQEVFQSGIIPSDTDFRIYRDFGNIPGIDLAFIE
NGYIYHTKYDTADRILTDSIQRAGDNILAVLKHLATSDMLAAASKYRHGNMVFFDVLGLFVIAYPSRIGSIINYMVVMGVVLYLGKK
FLQPKHKTGNYKKDFLCGLGITLISWFTSLVTVLIIAVFISLIGQSLSWYNHFYVSVCLYGTATVAKIILIHTLAKRFYYMNASAQY
LGEVFFDISLFVHCCFLVTLTYQGLCSAFISAVWVAFPLLTKLCVHKDFKQHGAQGKFIAFYLLGMFIPYLYALYLIWAVFEMFTPI
LGRSGSEIPPDVVLASILAGCTMILSSYFINFIYLAKSTKKTMLTLTLVCAITFLLVCSGTFFPYSSNPANPKPKRVFLQHMTRTFH
DLEGNAVKRDSGIWINGFDYTGISHITPHIPEINDSIRAHCEENAPLCGFPWYLPVHFLIRKNWYLPAPEVSPRNPPHFRLISKEQT
PWDSIKLTFEATGPSHMSFYVRAHKGSTLSQWSLGNGTPVTSKGGDYFVFYSHGLQASAWQFWIEVQVSEEHPEGMVTVAIAAHYLS
GEDKRSPQLDALKEKFPDWTFPSAWVCTYDLFVF
>sp|Q14507|EP3A_HUMAN
MTSSLKIWGILLALLCILCRLCVYSNNIYWREFIKLHYLSPSREFKEYKCDVLMREKEALKGKSFHMFIYSLWFKIQRACINEKGSD
RYRNAYVWAPGALKVLECHWEKYNNRYTESRSFSYIEFHCGVDGYVDNIEDLRIIEPISN
>sp|Q9UBC1|IKBL1_HUMAN
MSNPSPQVPEEEASTSVCRPKSSMASTSRRQRRERRFRRYLSAGRLVRAQALLQRHPGLDVDAGQPPPLHRACARHDAPALCLLLRL
GADPAHQDRHGDTALHAAARQGPDAYTDFFLPLLSRCPSAMGIKNKDGETPGQILGWGPPWDSAEEEEEDDASKEREWRQKLQGELE
DEWQEVMGRFEGDASHETQEPESFSAWSDRLAREHAQKCQQQQREAEGSCRPPRAEGSSQSWRQQEEEQRLFRERARAKEEELRESR
ARRAQEALGDREPKPTRAGPREEHPRGAGRGSLWRFGDVPWPCPGGGDPEAMAAALVARGPPLEEQGALRRYLRVQQVRWHPDRFLQ
RFRSQIETWELGRVMGAVTALSQALNRHAEALK
>sp|Q6UWV6|ENPP7_HUMAN
MRGLAVLLTVALATLLAPGAGAPVQSQGSQNKLLLVSFDGFRWNYDQDVDTPNLDAMARDGVKARYMTPAFVTMTSPCHFTLVTGKY
IENHGVVHNMYYNTTSKVKLPYHATLGIQRWWDNGSVPIWITAQRQGLRAGSFFYPGGNVTYQGVAVTRSRKEGIAHNYKNETEWRA
NIDTVMAWFTEEDLDLVTLYFGEPDSTGHRYGPESPERREMVRQVDRTVGYLRESIARNHLTDRLNLIITSDHGMTTVDKRAGDLVE
FHKFPNFTFRDIEFELLDYGPNGMLLPKEGRLEKVYDALKDAHPKLHVYKKEAFPEAFHYANNPRVTPLLMYSDLGYVIHGRINVQF
NNGEHGFDNKDMDMKTIFRAVGPSFRAGLEVEPFESVHVYELMCRLLGIVPEANDGHLATLLPMLHTESALPPDGRPTLLPKGRSAL
PPSSRPLLVMGLLGTVILLSEVA
>sp|Q9H078|CLPB_HUMAN
MLGSLVLRRKALAPRLLLRLLRSPTLRGHGGASGRNVTTGSLGEPQWLRVATGGRPGTSPALFSGRGAATGGRQGGRFDTKCLAAAT
WGRLPGPEETLPGQDSWNGVPSRAGLGMCALAAALVVHCYSKSPSNKDAALLEAARANNMQEVSRLLSEGADVNAKHRLGWTALMVA
AINRNNSVVQVLLAAGADPNLGDDFSSVYKTAKEQGIHSLEDGGQDGASRHITNQWTSALEFRRWLGLPAGVLITREDDFNNRLNNR
ASFKGCTALHYAVLADDYRTVKELLDGGANPLQRNEMGHTPLDYAREGEVMKLLRTSEAKYQEKQRKREAEERRRFPLEQRLKEHII
GQESAIATVGAAIRRKENGWYDEEHPLVFLFLGSSGIGKTELAKQTAKYMHKDAKKGFIRLDMSEFQERHEVAKFIGSPPGYVGHEE
GGQLTKKLKQCPNAVVLFDEVDKAHPDVLTIMLQLFDEGRLTDGKGKTIDCKDAIFIMTSNVASDEIAQHALQLRQEALEMSRNRIA
ENLGDVQISDKITISKNFKENVIRPILKAHFRRDEFLGRINEIVYFLPFCHSELIQLVNKELNFWAKRAKQRHNITLLWDREVADVL
VDGYNVHYGARSIKHEVERRVVNQLAAAYEQDLLPGGCTLRITVEDSDKQLLKSPELPSPQAEKRLPKLRLEIIDKDSKTRRLDIRA
PLHPEKVCNTI
>sp|P22090|RS4Y1_HUMAN
MARGPKKHLKRVAAPKHWMLDKLTGVFAPRPSTGPHKLRECLPLIVFLRNRLKYALTGDEVKKICMQRFIKIDGKVRVDVTYPAGFM
DVISIEKTGEHFRLVYDTKGRFAVHRITVEEAKYKLCKVRKITVGVKGIPHLVTHDARTIRYPDPVIKVNDTVQIDLGTGKIINFIK
FDTGNLCMVIGGANLGRVGVITNRERHPGSFDVVHVKDANGNSFATRLSNIFVIGNGNKPWISLPRGKGIRLTVAEERDKRLATKQS
SG
>sp|Q04844|ACHE_HUMAN
MARAPLGVLLLLGLLGRGVGKNEELRLYHHLFNNYDPGSRPVREPEDTVTISLKVTLTNLISLNEKEETLTTSVWIGIDWQDYRLNY
SKDDFGGIETLRVPSELVWLPEIVLENNIDGQFGVAYDANVLVYEGGSVTWLPPAIYRSVCAVEVTYFPFDWQNCSLIFRSQTYNAE
EVEFTFAVDNDGKTINKIDIDTEAYTENGEWAIDFCPGVIRRHHGGATDGPGETDVIYSLIIRRKPLFYVINIIVPCVLISGLVLLA

YFLPAQAGGQKCTVSINVLLAQTVFLFLIAQKIPETSLSVPLLGRFLIFVMVVATLIVMNCVIVLNVSQRTPTTHAMSPRLRHVLLE
LLPRLLGSPPPPEAPRAASPPRRASSVGLLLRAEELILKKPRSELVFEGQRHRQGTWTAAFCQSLGAAAPEVRCCVDAVNFVAESTR
DQEATGEEVSDWVRMGNALDNICFWAALVLFSVGSSLIFLGAYFNRVPDLPYAPCIQP
>sp|Q86T23|CROL1_HUMAN
MLQAKKTEVAEALTKAEAGRMELELSVTKLRAEEASLQDSLSKLSALNESLAQDKLDLNCLVTQLEEEKAMLQGRQRQAEQEATVAP
AEQEWLEELWLEQEVARQGLEGSL
>sp|Q9H582|ZN644_HUMAN
MRSFLQQDVNKTKSRLNVLNGLANNMDDLKINTDITGAKEELLDDNNFISDKESGVHKPKDCQTSFQKNNTLTLPEELSKDKSENAL
SGGQSSLFIHAGAPTVSSENFILPKGAAVNGPVSHSSLTKTSNMNKGSVSLTTGQPVDQPTTESCSTLKVAADLQLSTPQKASQHQV
LFLLSDVAHAKNPTHSNKKLPTSASVGCDIQNSVGSNIKSDGTLINQVEVGEDGEDLLVKDDCVNTVTGISSGTDGFRSENDTNWDP
QKEFIQFLMTNEETVDKAPPHSKIGLEKKRKRKMDVSKITRYTEDCFSDSNCVPNKSKMQEVDFLEQNEELQAVDSQKYALSKVKPE
STDEDLESVDAFQHLIYNPDKCGEESSPVHTSTFLSNTLKKKCEESDSESPATFSTEEPSFYPCTKCNVNFREKKHLHRHMMYHLDG
NSHFRHLNVPRPYACRECGRTFRDRNSLLKHMIIHQERRQKLMEEIRELKELQDEGRSARLQCPQCVFGTNCPKTFVQHAKTHEKDK
RYYCCEECNFMAVTENELECHRGIAHGAVVKCPMVTSDIAQRKTQKKTFMKDSVVGSSKKSATYICKMCPFTTSAKSVLKKHTEYLH
SSSCVDSFGSPLGLDKRKNDILEEPVDSDSTKTLTKQQSTTFPKNSALKQDVKRTFGSTSQSSSFSKIHKRPHRIQKARKSIAQSGV
NMCNQNSSPHKNVTIKSSVDQKPKYFHQAAKEKSNAKANSHYLYRHKYENYRMIKKSGESYPVHFKKEEASSLNSLHLFSSSSNSHN
NFISDPHKPDAKRPESFKDHRRVAVKRVIKESKKESSVGGEDLDSYPDFLHKMTVVVLQKLNSAEKKDSYETEDESSWDNVELGDYT
TQAIEDETYSDINQEHVNLFPLFKSKVEGQEPGENATLSYDQNDGFYFEYYEDTGSNNFLHEIHDPQHLETADASLSKHSSVFHWTD
LSLEKKSCPYCPATFETGVGLSNHVRGHLHRAGLSYEARHVVSPEQIATSDKMQHFKRTGTGTPVKRVRKAIEKSETTSEHTCQLCG
GWFDTKIGLSNHVRGHLKRLGKTKWDAHKSPICVLNEMMQNEEKYEKILKALNSRRIIPRPFVAQKLASSDDFISQNVIPLEAYRNG
LKTEALSVSASEEEGLNFLNEYDETKPELPSGKKNQSLTLIELLKNKRMGEERNSAISPQKIHNQTARKRFVQKCVLPLNEDSPLMY
QPQKMDLTMHSALDCKQKKSRSRSGSKKKMLTLPHGADEVYILRCRFCGLVFRGPLSVQEDWIKHLQRHIVNANLPRTGAGMVEVTS
LLKKPASITETSFSLLMAEAAS
>sp|Q9NPG1|FZD3_HUMAN
MAMTWIVFSLWPLTVFMGHIGGHSLFSCEPITLRMCQDLPYNTTFMPNLLNHYDQQTAALAMEPFHPMVNLDCSRDFRPFLCALYAP
ICMEYGRVTLPCRRLCQRAYSECSKLMEMFGVPWPEDMECSRFPDCDEPYPRLVDLNLAGEPTEGAPVAVQRDYGFWCPRELKIDPD
LGYSFLHVRDCSPPCPNMYFRREELSFARYFIGLISIICLSATLFTFLTFLIDVTRFRYPERPIIFYAVCYMMVSLIFFIGFLLEDR
VACNASIPAQYKASTVTQGSHNKACTMLFMILYFFTMAGSVWWVILTITWFLAAVPKWGSEAIEKKALLFHASAWGIPGTLTIILLA
MNKIEGDNISGVCFVGLYDVDALRYFVLAPLCLYVVVGVSLLLAGIISLNRVRIEIPLEKENQDKLVKFMIRIGVFSILYLVPLLVV
IGCYFYEQAYRGIWETTWIQERCREYHIPCPYQVTQMSRPDLILFLMKYLMALIVGIPSVFWVGSKKTCFEWASFFHGRRKKEIVNE
SRQVLQEPDFAQSLLRDPNTPIIRKSRGTSTQGTSTHASSTQLAMVDDQRSKAGSIHSKVSSYHGSLHRSRDGRYTPCSYRGMEERL
PHGSMSRLTDHSRHSSSHRLNEQSRHSSIRDLSNNPMTHITHGTSMNRVIEEDGTSA
>sp|Q9H6R6|ZDHC6_HUMAN
MGTFCSVIKFENLQELKRLCHWGPIIALGVIAICSTMAMIDSVLWYWPLHTTGGSVNFIMLINWTVMILYNYFNAMFVGPGFVPLGW
KPEISQDTMYLQYCKVCQAYKAPRSHHCRKCNRCVMKMDHHCPWINNCCGYQNHASFTLFLLLAPLGCIHAAFIFVMTMYTQLYHRL
SFGWNTVKIDMSAARRDPLPIVPFGLAAFATTLFALGLALGTTIAVGMLFFIQMKIILRNKTSIESWIEEKAKDRIQYYQLDEVFVF
PYDMGSRWRNFKQVFTWSGVPEGDGLEWPVREGCHQYSLTIEQLKQKADKRVRSVRYKVIEDYSGACCPLNKGIKTFFTSPCTEEPR
IQLQKGEFILATRGLRYWLYGDKILDDSFIEGVSRIRGWFPRKCVEKCPCDAETDQAPEGEKKNR
>sp|P54803|GALC_HUMAN
MAEWLLSASWQRRAKAMTAAAGSAGRAAVPLLLCALLAPGGAYVLDDSDGLGREFDGIGAVSGGGATSRLLVNYPEPYRSQILDYLF
KPNFGASLHILKVEIGGDGQTTDGTEPSHMHYALDENYFRGYEWWLMKEAKKRNPNITLIGLPWSFPGWLGKGFDWPYVNLQLTAYY
VVTWIVGAKRYHDLDIDYIGIWNERSYNANYIKILRKMLNYQGLQRVKIIASDNLWESISASMLLDAELFKVVDVIGAHYPGTHSAK
DAKLTGKKLWSSEDFSTLNSDMGAGCWGRILNQNYINGYMTSTIAWNLVASYYEQLPYGRCGLMTAQEPWSGHYVVESPVWVSAHTT
QFTQPGWYYLKTVGHLEKGGSYVALTDGLGNLTIIIETMSHKHSKCIRPFLPYFNVSQQFATFVLKGSFSEIPELQVWYTKLGKTSE

RFLFKQLDSLWLLDSDGSFTLSLHEDELFTLTTLTTGRKGSYPLPPKSQPFPSTYKDDFNVDYPFFSEAPNFADQTGVFEYFTNIED
PGEHHFTLRQVLNQRPITWAADASNTISIIGDYNWTNLTIKCDVYIETPDTGGVFIAGRVNKGGILIRSARGIFFWIFANGSYRVTG
DLAGWIIYALGRVEVTAKKWYTLTLTIKGHFTSGMLNDKSLWTDIPVNFPKNGWAAIGTHSFEFAQFDNFLVEATR
>sp|Q9H6N6|MYH16_HUMAN
MGLKVIQQNVHKFLQLRFWGWWKLYNKVKPLLNVARQEEEMKAKEEELRKAMAQTQELVNKVKELEEKTATLSQEKNDLTIQLQAEQ
ENLMDAEERLTWMMKTKMDLESQISDMRERLEEEEGMAASLSAAKRKLEGELSDLKRDLEGLETTLAKTEKEKQALDHKVRTLTGDL
SLREDSITKLQKEKRALEELHQKTLDDLQAEEDKVNHLTKNNSKLSTQIHELEDNWEQEKKIRAEVEKARRKAESDLKMTIDNLNEM
ERSKLDLEEVVKKRDLEINSVNSKYEDEQSLNSTLQRKLKEHQDRIEELEEELEAERAMRAKIEQNRKREAELLKLRRELEEAALQS
EATASTLRKKHVDSMAELTEHVESLQRVKSKLEKDKQVMKAEIDDLNASMETIQKSKMNAEAHVRKLEDSLSEANAKVAELERNQAE
INAIRTRLQAENSELSREYEESQSRLNQILRIKTSLTSQVDDYKRQLDEESKSRSTAVVSLANTKHDLDLVKEQLEEEQGGKSELQR
LVSKLNTEVTTWRTKYETDAIQRTEELEETKRKLAARLQEAEEAAETAQARAASLEKNKQRLQAEVEDLTIDLEKANAAAAALDKKQ
RLFDKMLAEWQQKCEELQVEVDSSQKECRMYMTESFKIKTAYEESLEHLESVKKENKTLQEEIKDLIDQLGEGGRSVHELQKLKKKL
EMEKEELQVALEEAESSLEVEESKVIRIQLELAQVKADIDRRIHEKEEEFEATRKNHQRAIESLQASLEAEAKGRAEALRLKKKMET
DLNEMEIQLDHANKNNSELVKTLKRLQQQIKDLQVQMDEDARQHEELRKQYNLQERRLSLLQTELEEVRSALEGSERSRKLLEQEVV
EITEWHNEINIQNQSLLVVKRKLESDVQRISNEHEELISEFRLTEERAKKAMMDAARMAEELRQEQDHCMHLEKIKKNYEVTIKDLQ
AKMEEAEQLALKGGKRTIMKLEARIKELETELDGEQKQHVETVKTLCKNERRLKELVFQTEEDHKTNQRMQALVEKLQNKLKVYKRQ
IEEAEDQANQTLARYRKTVHELDDAEDRAGMAETALNKLRTRHRVAGKGITSV
>sp|O14773|TPP1_HUMAN
MGLQACLLGLFALILSGKCSYSPEPDQRRTLPPGWVSLGRADPEEELSLTFALRQQNVERLSELVQAVSDPSSPQYGKYLTLENVAD
LVRPSPLTLHTVQKWLLAAGAQKCHSVITQDFLTCWLSIRQAELLLPGAEFHHYVGGPTETHVVRSPHPYQLPQALAPHVDFVGGLH
RFPPTSSLRQRPEPQVTGTVGLHLGVTPSVIRKRYNLTSQDVGSGTSNNSQACAQFLEQYFHDSDLAQFMRLFGGNFAHQASVARVV
GQQGRGRAGIEASLDVQYLMSAGANISTWVYSSPGRHEGQEPFLQWLMLLSNESALPHVHTVSYGDDEDSLSSAYIQRVNTELMKAA
ARGLTLLFASGDSGAGCWSVSGRHQFRPTFPASSPYVTTVGGTSFQEPFLITNEIVDYISGGGFSNVFPRPSYQEEAVTKFLSSSPH
LPPSSYFNASGRAYPDVAALSDGYWVVSNRVPIPWVSGTSASTPVFGGILSLINEHRILSGRPPLGFLNPRLYQQHGAGLFDVTRGC
HESCLDEEVEGQGFCSGPGWDPVTGWGTPNFPALLKTLLNP
>sp|Q15633|TRBP2_HUMAN
MSEEEQGSGTTTGCGLPSIEQMLAANPGKTPISLLQEYGTRIGKTPVYDLLKAEGQAHQPNFTFRVTVGDTSCTGQGPSKKAAKHKA
AEVALKHLKGGSMLEPALEDSSSFSPLDSSLPEDIPVFTAAAAATPVPSVVLTRSPPMELQPPVSPQQSECNPVGALQELVVQKGWR
LPEYTVTQESGPAHRKEFTMTCRVERFIEIGSGTSKKLAKRNAAAKMLLRVHTVPLDARDGNEVEPDDDHFSIGVGSRLDGLRNRGP
GCTWDSLRNSVGEKILSLRSCSLGSLGALGPACCRVLSELSEEQAFHVSYLDIEELSLSGLCQCLVELSTQPATVCHGSATTREAAR
GEAARRALQYLKIMAGSK
>sp|Q9Y2Q9|RT28_HUMAN
MAALCRTRAVAAESHFLRVFLFFRPFRGVGTESGSESGSSNAKEPKTRAGGFASALERHSELLQKVEPLQKGSPKNVESFASMLRHS
PLTQMGPAKDKLVIGRIFHIVENDLYIDFGGKFHCVCRRPEVDGEKYQKGTRVRLRLLDLELTSRFLGATTDTTVLEANAVLLGIQE
SKDSRSKEEHHEK
>sp|O95994|AGR2_HUMAN
MEKIPVSAFLLLVALSYTLARDTTVKPGAKKDTKDSRPKLPQTLSRGWGDQLIWTQTYEEALYKSKTSNKPLMIIHHLDECPHSQAL
KKVFAENKEIQKLAEQFVLLNLVYETTDKHLSPDGQYVPRIMFVDPSLTVRADITGRYSNRLYAYEPADTALLLDNMKKALKLLKTE
L
>sp|P29965|CD40L_HUMAN
MIETYNQTSPRSAATGLPISMKIFMYLLTVFLITQMIGSALFAVYLHRRLDKIEDERNLHEDFVFMKTIQRCNTGERSLSLLNCEEI
KSQFEGFVKDIMLNKEETKKENSFEMQKGDQNPQIAAHVISEASSKTTSVLQWAEKGYYTMSNNLVTLENGKQLTVKRQGLYYIYAQ
VTFCSNREASSQAPFIASLCLKSPGRFERILLRAANTHSSAKPCGQQSIHLGGVFELQPGASVFVNVTDPSQVSHGTGFTSFGLLKL
>sp|Q9UH73|COE1_HUMAN

MFGIQESIQRSGSSMKEEPLGSGMNAVRTWMQGAGVLDANTAAQSGVGLARAHFEKQPPSNLRKSNFFHFVLALYDRQGQPVEIERT
AFVGFVEKEKEANSEKTNNGIHYRLQLLYSNGIRTEQDFYVRLIDSMTKQAIVYEGQDKNPEMCRVLLTHEIMCSRCCDKKSCGNRN
ETPSDPVIIDRFFLKFFLKCNQNCLKNAGNPRDMRRFQVVVSTTVNVDGHVLAVSDNMFVHNNSKHGRRARRLDPSEGTPSYLEHAT
PCIKAISPSEGWTTGGATVIIIGDNFFDGLQVIFGTMLVWSELITPHAIRVQTPPRHIPGVVEVTLSYKSKQFCKGTPGRFIYTALN
EPTIDYGFQRLQKVIPRHPGDPERLPKEVILKRAADLVEALYGMPHNNQEIILKRAADIAEALYSVPRNHNQLPALANTSVHAGMMG
VNSFSGQLAVNVSEASQATNQGFTRNSSSVSPHGYVPSTTPQQTNYNSVTTSMNGYGSAAMSNLGGSPTFLNGSAANSPYAIVPSSP
TMASSTSLPSNCSSSSGIFSFSPANMVSAVKQKSAFAPVVRPQTSPPPTCTSTNGNSLQAISGMIVPPM
>sp|Q86VE0|MYPOP_HUMAN
MASAAAGEAEETTRLRKPRFSFEENQILIREVRAHYPQLYGAQSRRVSVAERRRVWDGIAAKINGITSWKRTGQEVQKRWNDFKRRT
KEKLARVPHSTQGAGPAAEDAFSAEEETIFAILGPGVAAPGAGAGAEEPPAAPSSQPPPPSACPQRYVLSEDRREDRRADTSAHSKA
GSSSPEPWARPSCTPQEGGCPRPKERESPPPSALQPVQLPRLALSPPPPAPPLPPPPPLAQVAPSPPSPPPPPRPPPTLSASDPSLD
FLRAQQETANAIRELAGTLRQGLAKLSEALSALLPLLPGTPVDSLPPPLPPPPPPPPPPRPVLPPPAPKVEITPEPVSVVAAVVDGA
VVAARGVIIAPRSEEGAPRPPPAPLPPHDSPPHKRRKGFPTRKRRGRWKSP
>sp|P06493|CDK1_HUMAN
MEDYTKIEKIGEGTYGVVYKGRHKTTGQVVAMKKIRLESEEEGVPSTAIREISLLKELRHPNIVSLQDVLMQDSRLYLIFEFLSMDL
KKYLDSIPPGQYMDSSLVKSYLYQILQGIVFCHSRRVLHRDLKPQNLLIDDKGTIKLADFGLARAFGIPIRVYTHEVVTLWYRSPEV
LLGSARYSTPVDIWSIGTIFAELATKKPLFHGDSEIDQLFRIFRALGTPNNEVWPEVESLQDYKNTFPKWKPGSLASHVKNLDENGL
DLLSKMLIYDPAKRISGKMALNHPYFNDLDNQIKKM
>sp|Q99616|CCL13_HUMAN
MKVSAVLLCLLLMTAAFNPQGLAQPDALNVPSTCCFTFSSKKISLQRLKSYVITTSRCPQKAVIFRTKLGKEICADPKEKWVQNYMK
HLGRKAHTLKT
>sp|Q9H944|MED20_HUMAN
MGVTCVSQMPVAEGKSVQQTVELLTRKLEMLGAEKQGTFCVDCETYHTAASTLGSQGQTGKLMYVMHNSEYPLSCFALFENGPCLIA
DTNFDVLMVKLKGFFQSAKASKIETRGTRYQYCDFLVKVGTVTMGPSARGISVEVEYGPCVVASDCWSLLLEFLQSFLGSHTPGAPA
VFGNRHDAVYGPADTMVQYMELFNKIRKQQQVPVAGIR
>sp|P12034|FGF5_HUMAN
MSLSFLLLLFFSHLILSAWAHGEKRLAPKGQPGPAATDRNPRGSSSRQSSSSAMSSSSASSSPAASLGSQGSGLEQSSFQWSPSGRR
TGSLYCRVGIGFHLQIYPDGKVNGSHEANMLSVLEIFAVSQGIVGIRGVFSNKFLAMSKKGKLHASAKFTDDCKFRERFQENSYNTY
ASAIHRTEKTGREWYVALNKRGKAKRGCSPRVKPQHISTHFLPRFKQSEQPELSFTVTVPEKKKPPSPIKPKIPLSAPRKNTNSVKY
RLKFRFG
>sp|Q9H246|CA021_HUMAN
MGCASAKHVATVQNEEEAQKGKNYQNGDVFGDEYRIKPVEEVKYMKNGAEEEQKIAARNQENLEKSASSNVRLKTNKEVPGLVHQPR
ANMHISESQQEFFRMLDEKIEKGRDYCSEEEDIT
>sp|Q6NT89|TRNP1_HUMAN
MPGCRISACGPGAQEGTAEQRSPPPPWDPMPSSQPPPPTPTLTPTPTPGQSPPLPDAAGASAGAAEDQELQRWRQGASGIAGLAGPG
GGSGAAAGAGGRALELAEARRRLLEVEGRRRLVSELESRVLQLHRVFLAAELRLAHRAESLSRLSGGVAQAELYLAAHGSRLKKGPR
RGRRGRPPALLASALGLGGCVPWGAGRLRRGHGPEPDSPFRRSPPRGPASPQR
>sp|P98179|RBM3_HUMAN
MSSEEGKLFVGGLNFNTDEQALEDHFSSFGPISEVVVVKDRETQRSRGFGFITFTNPEHASVAMRAMNGESLDGRQIRVDHAGKSAR
GTRGGGFGAHGRGRSYSRGGGDQGYGSGRYYDSRPGGYGYGYGRSRDYNGRNQGGYDRYSGGNYRDNYDN
>sp|P15514|AREG_HUMAN
MRAPLLPPAPVVLSLLILGSGHYAAGLDLNDTYSGKREPFSGDHSADGFEVTSRSEMSSGSEISPVSEMPSSSEPSSGADYDYSEEY
DNEPQIPGYIVDDSVRVEQVVKPPQNKTESENTSDKPKRKKKGGKNGKNRRNRKKKNPCNAEFQNFCIHGECKYIEHLEAVTCKCQQ
EYFGERCGEKSMKTHSMIDSSLSKIALAAIAAFMSAVILTAVAVITVQLRRQYVRKYEGEAEERKKLRQENGNVHAIA

>sp|P0CG00|ZSA5D_HUMAN
MGQCRNWKWGLESFDFSINRRSSAPGQLESASQMRESWRHKAGRRMFSCPEESDPIQALRKLTELCHLWLRPDLHTKEQILDMLVME
QFMISMPQELQVLVKVNGVQSCKDLEDLLRNNRRPKKWSVVNFLGKEYLMQESDVEMAEIPASVRDDPRGVSSQRASSVNEMRPGEG
QASQELQTLPRVPALSRRQEEDFLLPETTVMKSAPKALRPKPTLEKDLNVDREENTGLTSPEPQLPNGPSVVGAKEGKEPKKRASVE
NVDADTPSACVVEREASTHSGSRGDALNLRCPKRSKPDATSISQEGPQGGATPVGNSESPGKPEINSVYSPGPAGAVSHPNGQEAKE
LLPFACGVCNKRFTCNSKLAIHMRSHTGERPFQCNFCERCFTQLSDLRVHQRIHTGEKPYTCDICHKRFNRMFSLKCHKRSHTGEKP
YKCKDCNQVFTYRKNLNEHKLIHSGEKPYKCPKCLRAFRRPETLKYHQKTHQETTAPRECEG
>sp|A1L390|PKHG3_HUMAN
MPVSTSLHQDGSQERPVSLTSTTSSSGSSCDSRSAMEEPSSSEAPAKNGAGSLRSRHLPNSNNNSSSWLNVKGPLSPFNSRAAAGPA
HHKLSYLGRVVREIVETERMYVQDLRSIVEDYLLKIIDTPGLLKPEQVSALFGNIENIYALNSQLLRDLDSCNSDPVAVASCFVERS
QEFDIYTQYCNNYPNSVAALTECMRDKQQAKFFRDRQELLQHSLPLGSYLLKPVQRILKYHLLLQEIAKHFDEEEDGFEVVEDAIDT
MTCVAWYINDMKRRHEHAVRLQEIQSLLINWKGPDLTTYGELVLEGTFRVHRVRNERTFFLFDKTLLITKKRGDHFVYKGNIPCSSL
MLIESTRDSLCFTVTHYKHSKQQYSIQAKTVEEKRNWTHHIKRLILENHHATIPQKAKEAILEMDSYYPNRYRCSPERLKKAWSSQD
EVSTNVRQGRRQSEPTKHLLRQLNEKARAAGMKGKGRRESESSRSSRRPSGRSPTSTEKRMSFESISSLPEVEPDPEAGSEQEVFSA
VEGPSAEETPSDTESPEVLETQLDAHQGLLGMDPPGDMVDFVAAESTEDLKALSSEEEEEMGGAAQEPESLLPPSVLDQASVIAERF
VSSFSRRSSVAQEDSKSSGFGSPRLVSRSSSVLSLEGSEKGLARHGSATDSLSCQLSPEVDISVGVATEDSPSVNGMEPPSPGCPVE
PDRSSCKKKESALSTRDRLLLDKIKSYYENAEHHDAGFSVRRRESLSYIPKGLVRNSISRFNSLPRPDPEPVPPVGSKRQVGSRPTS
WALFELPGPSQAVKGDPPPISDAEFRPSSEIVKIWEGMESSGGSPGKGPGQGQANGFDLHEPLFILEEHELGAITEESATASPESSS
PTEGRSPAHLARELKELVKELSSSTQGELVAPLHPRIVQLSHVMDSHVSERVKNKVYQLARQYSLRIKSNKPVMARPPLQWEKVAPE
RDGKSPTVPCLQEEAGEPLGGKGKRKPVLSLFDYEQLMAQEHSPPKPSSAGEMSPQRFFFNPSAVSQRTTSPGGRPSARSPLSPTET
FSWPDVRELCSKYASRDEARRAGGGRPRGPPVNRSHSVPENMVEPPLSGRVGRCRSLSTKRGRGGGEAAQSPGPLPQSKPDGGETLY
VTADLTLEDNRRVIVMEKGPLPSPTAGLEESSGQGPSSPVALLGQVQDFQQSAECQPKEEGSRDPADPSQQGRVRNLREKFQALNSV
G
>sp|P30989|NTR1_HUMAN
MRLNSSAPGTPGTPAADPFQRAQAGLEEALLAPGFGNASGNASERVLAAPSSELDVNTDIYSKVLVTAVYLALFVVGTVGNTVTAFT
LARKKSLQSLQSTVHYHLGSLALSDLLTLLLAMPVELYNFIWVHHPWAFGDAGCRGYYFLRDACTYATALNVASLSVERYLAICHPF
KAKTLMSRSRTKKFISAIWLASALLAVPMLFTMGEQNRSADGQHAGGLVCTPTIHTATVKVVIQVNTFMSFIFPMVVISVLNTIIAN
KLTVMVRQAAEQGQVCTVGGEHSTFSMAIEPGRVQALRHGVRVLRAVVIAFVVCWLPYHVRRLMFCYISDEQWTPFLYDFYHYFYMV
TNALFYVSSTINPILYNLVSANFRHIFLATLACLCPVWRRRRKRPAFSRKADSVSSNHTLSSNATRETLY
>sp|Q9UQN3|CHM2B_HUMAN
MASLFKKKTVDDVIKEQNRELRGTQRAIIRDRAALEKQEKQLELEIKKMAKIGNKEACKVLAKQLVHLRKQKTRTFAVSSKVTSMST
QTKVMNSQMKMAGAMSTTAKTMQAVNKKMDPQKTLQTMQNFQKENMKMEMTEEMINDTLDDIFDGSDDEEESQDIVNQVLDEIGIEI
SGKMAKAPSAARSLPSASTSKATISDEEIERQLKALGVD
>sp|Q6ZS27|ZN662_HUMAN
MLENYGAVASLAAFPFPKPALISQLERGETPWCSVPRGALDGEAPRGISSGYPFLKPAGISHPEQVEEPLNLKLQGEGPSLICPEGV
LKRKKEDFILKEEIIEEAQDLMVLSSGPQWCGSQELWFGKTCEEKSRLGRWPGYLNGGRMESSTNDIIEVIVKDEMISVEESSGNTD
VNNLLGIHHKILNEQIFYICEECGKCFDQNEDFDQHQKTHNGEKVYGCKECGKAFSFRSHCIAHQRIHSGVKPYECQECAKAFVWKS
NLIRHQRIHTGEKPFECKECGKGFSQNTSLTQHQRIHTGEKPYTCKECGKSFTRNPALLRHQRMHTGEKPYECKDCGKGFMWNSDLS
QHQRVHTGDKPHECTDCGKSFFCKAHLIRHQRIHTGERPYKCNDCGKAFSQNSVLIKHQRRHARDKPYNCQISHLLEH
>sp|Q8NC44|F134A_HUMAN
MASGGGGGNTGAGGGPGMGLSLGLGLGLSLGMSEATSEAEEEAATAEAVGRLATTLWLRLRGWEAVLAAAQRLLVWEKPLHSLVTAA
ALNGLFWLLSSSSLRPFFLLSVSLLAYFLLDLWQPRFLPDVSASSPEEPHSDSEGAGSGARPHLLSVPELCRYLAESWLTFQIHLQE
LLQYKRQNPAQFCVRVCSGCAVLAVLGHYVPGIMISYIVLLSILLWPLVVYHELIQRMYTRLEPLLMQLDYSMKAEANALHHKHDKR
KRQGKNAPPGGDEPLAETESESEAELAGFSPVVDVKKTALALAITDSELSDEEASILESGGFSVSRATTPQLTDVSEDLDQQSLPSE

PEETLSRDLGEGEEGELAPPEDLLGRPQALSRQALDSEEEEEDVAAKETLLRLSSPLHFVNTHFNGAGSPPDGVKCSPGGPVETLSP
ETVSGGLTALPGTLSPPLCLVGSDPAPSPSILPPVPQDSPQPLPAPEEEEALTTEDFELLDQGELEQLNAELGLEPETPPKPPDAPP
LGPDIHSLVQSDQEAQAVAEP
>sp|Q9BQN1|FA83C_HUMAN
MFGGPGPGVLGAQGMAGPLRGRVEELKLPWWRESSPLVLRHSEAARLAADALLERGEAAYLRVISEERELPFLSALDVDYMTSHVRG
GPELSEAQGQEASGPDRLSLLSEVTSGTYFPMASDIDPPDLDLGWPEVPQATGFSPTQAVVHFQRDKAKNIKDLLRFLFSQAHTVVA
VVMDIFTDMELLCDLMEASSRRGVPVYLLLAQEHLRHFLEMCYKMDLNGEHLPNMRVRSTCGDTYCSKAGRRFTGQALEKFVLIDCE
QVVAGSYSFTWLCSQAHTSMVLQLRGRIVEDFDREFRCLYAESQPVEGFCGGEDPLSPRALRPPPVALAFRPDVPSPTSSLPSSTSL
SSIKQSPLMGRSSYLALPGGGDCSDTGVVSSSLGPARREASGQPSLHRQLSDPNHGSPPGLYRANLGKLGAYPWSQSSPALNHNSTS
PLTLAVGSPLLPRSRPLLQFHRGAPALSRFPENGLPGSQEPSPLRGRWVPGTTLETVEEKEKKASPSQSRGQLDLLVPFPRAREVGD
PDSGVTPNSGPLRPGEQAPEDRRLSPSQADSQLDLLSRALGTGGAPELGSLRPGDRALEDRRLSLNQSRGQSDLLMQYPKAQGSRVP
LETNSSARPARRAPDERRQTLGHSQLDLITKFGPFRGEGPGPNGLPISSPARTAGAGSGDEKRLTLGHSKLDLITKYHQLHGARQGT
EPGGPKGGHLNGGNSDLVRDEKRLTLGHSKLDLITKYNKSKFKQLRSRFES
>sp|Q96L92|SNX27_HUMAN
MADEDGEGIHPSAPHRNGGGGGGGGSGLHCAGNGGGGGGGPRVVRIVKSESGYGFNVRGQVSEGGQLRSINGELYAPLQHVSAVLPG
GAADRAGVRKGDRILEVNHVNVEGATHKQVVDLIRAGEKELILTVLSVPPHEADNLDPSDDSLGQSFYDYTEKQAVPISVPRYKHVE
QNGEKFVVYNVYMAGRQLCSKRYREFAILHQNLKREFANFTFPRLPGKWPFSLSEQQLDARRRGLEEYLEKVCSIRVIGESDIMQEF
LSESDENYNGVSDVELRVALPDGTTVTVRVKKNSTTDQVYQAIAAKVGMDSTTVNYFALFEVISHSFVRKLAPNEFPHKLYIQNYTS
AVPGTCLTIRKWLFTTEEEILLNDNDLAVTYFFHQAVDDVKKGYIKAEEKSYQLQKLYEQRKMVMYLNMLRTCEGYNEIIFPHCACD
SRRKGHVITAISITHFKLHACTEEGQLENQVIAFEWDEMQRWDTDEEGMAFCFEYARGEKKPRWVKIFTPYFNYMHECFERVFCELK
WRKENIFQMARSQQRDVAT
>sp|Q3YEC7|RABL6_HUMAN
MFSALKKLVGSDQAPGRDKNIPAGLQSMNQALQRRFAKGVQYNMKIVIRGDRNTGKTALWHRLQGRPFVEEYIPTQEIQVTSIHWSY
KTTDDIVKVEVWDVVDKGKCKKRGDGLKMENDPQEAESEMALDAEFLDVYKNCNGVVMMFDITKQWTFNYILRELPKVPTHVPVCVL
GNYRDMGEHRVILPDDVRDFIDNLDRPPGSSYFRYAESSMKNSFGLKYLHKFFNIPFLQLQRETLLRQLETNQLDMDATLEELSVQQ
ETEDQNYGIFLEMMEARSRGHASPLAANGQSPSPGSQSPVVPAGAVSTGSSSPGTPQPAPQLPLNAAPPSSVPPVPPSEALPPPACP
SAPAPRRSIISRLFGTSPATEAAPPPPEPVPAAEGPATVQSVEDFVPDDRLDRSFLEDTTPARDEKKVGAKAAQQDSDSDGEALGGN
PMVAGFQDDVDLEDQPRGSPPLPAGPVPSQDITLSSEEEAEVAAPTKGPAPAPQQCSEPETKWSSIPASKPRRGTAPTRTAAPPWPG
GVSVRTGPEKRSSTRPPAEMEPGKGEQASSSESDPEGPIAAQMLSFVMDDPDFESEGSDTQRRADDFPVRDDPSDVTDEDEGPAEPP
PPPKLPLPAFRLKNDSDLFGLGLEEAGPKESSEEGKEGKTPSKEKKKKKKKGKEEEEKAAKKKSKHKKSKDKEEGKEERRRRQQRPP
RSRERTAADELEAFLGGGAPGGRHPGGGDYEEL
>sp|P49798|RGS4_HUMAN
MCKGLAGLPASCLRSAKDMKHRLGFLLQKSDSCEHNSSHNKKDKVVICQRVSQEEVKKWAESLENLISHECGLAAFKAFLKSEYSEE
NIDFWISCEEYKKIKSPSKLSPKAKKIYNEFISVQATKEVNLDSCTREETSRNMLEPTITCFDEAQKKIFNLMEKDSYRRFLKSRFY
LDLVNPSSCGAEKQKGAKSSADCASLVPQCA
>sp|Q96S55|WRIP1_HUMAN
MEVSGPEDDPFLSQLHQVQCPVCQQMMPAAHINSHLDRCLLLHPAGHAEPAAGSHRAGERAKGPSPPGAKRRRLSESSALKQPATPT
AAESSEGEGEEGDDGGETESRESYDAPPTPSGARLIPDFPVARSSSPGRKGSGKRPAAAAAAGSASPRSWDEAEAQEEEEAVGDGDG
DGDADADGEDDPGHWDADAAEAATAFGASGGGRPHPRALAAEEIRQMLQGKPLADTMRPDTLQDYFGQSKAVGQDTLLRSLLETNEI
PSLILWGPPGCGKTTLAHIIASNSKKHSIRFVTLSATNAKTNDVRDVIKQAQNEKSFFKRKTILFIDEIHRFNKSQQDTFLPHVECG
TITLIGATTENPSFQVNAALLSRCRVIVLEKLPVEAMVTILMRAINSLGIHVLDSSRPTDPLSHSSNSSSEPAMFIEDKAVDTLAYL
SDGDARAGLNGLQLAVLARLSSRKMFCKKSGQSYSPSRVLITENDVKEGLQRSHILYDRAGEEHYNCISALHKSMRGSDQNASLYWL
ARMLEGGEDPLYVARRLVRFASEDIGLADPSALTQAVAAYQGCHFIGMPECEVLLAQCVVYFARAPKSIEVYSAYNNVKACLRNHQG
PLPPVPLHLRNAPTRLMKDLGYGKGYKYNPMYSEPVDQEYLPEELRGVDFFKQRRC

>sp|A1L190|SYCE3_HUMAN
MDDADPEERNYDNMLKMLSDLNKDLEKLLEEMEKISVQATWMAYDMVVMRTNPTLAESMRRLEDAFVNCKEEMEKNWQELLHETKQR
L
>sp|Q96D15|RCN3_HUMAN
MMWRPSVLLLLLLLRHGAQGKPSPDAGPHGQGRVHQAAPLSDAPHDDAHGNFQYDHEAFLGREVAKEFDQLTPEESQARLGRIVDRM
DRAGDGDGWVSLAELRAWIAHTQQRHIRDSVSAAWDTYDTDRDGRVGWEELRNATYGHYAPGEEFHDVEDAETYKKMLARDERRFRV
ADQDGDSMATREELTAFLHPEEFPHMRDIVIAETLEDLDRNKDGYVQVEEYIADLYSAEPGEEEPAWVQTERQQFRDFRDLNKDGHL
DGSEVGHWVLPPAQDQPLVEANHLLHESDTDKDGRLSKAEILGNWNMFVGSQATNYGEDLTRHHDEL
>sp|O75417|DPOLQ_HUMAN
MNLLRRSGKRRRSESGSDSFSGSGGDSSASPQFLSGSVLSPPPGLGRCLKAAAAGECKPTVPDYERDKLLLANWGLPKAVLEKYHSF
GVKKMFEWQAECLLLGQVLEGKNLVYSAPTSAGKTLVAELLILKRVLEMRKKALFILPFVSVAKEKKYYLQSLFQEVGIKVDGYMGS
TSPSRHFSSLDIAVCTIERANGLINRLIEENKMDLLGMVVVDELHMLGDSHRGYLLELLLTKICYITRKSASCQADLASSLSNAVQI
VGMSATLPNLELVASWLNAELYHTDFRPVPLLESVKVGNSIYDSSMKLVREFEPMLQVKGDEDHVVSLCYETICDNHSVLLFCPSKK
WCEKLADIIAREFYNLHHQAEGLVKPSECPPVILEQKELLEVMDQLRRLPSGLDSVLQKTVPWGVAFHHAGLTFEERDIIEGAFRQG
LIRVLAATSTLSSGVNLPARRVIIRTPIFGGRPLDILTYKQMVGRAGRKGVDTVGESILICKNSEKSKGIALLQGSLKPVRSCLQRR
EGEEVTGSMIRAILEIIVGGVASTSQDMHTYAACTFLAASMKEGKQGIQRNQESVQLGAIEACVMWLLENEFIQSTEASDGTEGKVY
HPTHLGSATLSSSLSPADTLDIFADLQRAMKGFVLENDLHILYLVTPMFEDWTTIDWYRFFCLWEKLPTSMKRVAELVGVEEGFLAR
CVKGKVVARTERQHRQMAIHKRFFTSLVLLDLISEVPLREINQKYGCNRGQIQSLQQSAAVYAGMITVFSNRLGWHNMELLLSQFQK
RLTFGIQRELCDLVRVSLLNAQRARVLYASGFHTVADLARANIVEVEVILKNAVPFKSARKAVDEEEEAVEERRNMRTIWVTGRKGL
TEREAAALIVEEARMILQQDLVEMGVQWNPCALLHSSTCSLTHSESEVKEHTFISQTKSSYKKLTSKNKSNTIFSDSYIKHSPNIVQ
DLNKSREHTSSFNCNFQNGNQEHQTCSIFRARKRASLDINKEKPGASQNEGKTSDKKVVQTFSQKTKKAPLNFNSEKMSRSFRSWKR
RKHLKRSRDSSPLKDSGACRIHLQGQTLSNPSLCEDPFTLDEKKTEFRNSGPFAKNVSLSGKEKDNKTSFPLQIKQNCSWNITLTND
NFVEHIVTGSQSKNVTCQATSVVSEKGRGVAVEAEKINEVLIQNGSKNQNVYMKHHDIHPINQYLRKQSHEQTSTITKQKNIIERQM
PCEAVSSYINRDSNVTINCERIKLNTEENKPSHFQALGDDISRTVIPSEVLPSAGAFSKSEGQHENFLNISRLQEKTGTYTTNKTKN
NHVSDLGLVLCDFEDSFYLDTQSEKIIQQMATENAKLGAKDTNLAAGIMQKSLVQQNSMNSFQKECHIPFPAEQHPLGATKIDHLDL
KTVGTMKQSSDSHGVDILTPESPIFHSPILLEENGLFLKKNEVSVTDSQLNSFLQGYQTQETVKPVILLIPQKRTPTGVEGECLPVP
ETSLNMSDSLLFDSFSDDYLVKEQLPDMQMKEPLPSEVTSNHFSDSLCLQEDLIKKSNVNENQDTHQQLTCSNDESIIFSEMDSVQM
VEALDNVDIFPVQEKNHTVVSPRALELSDPVLDEHHQGDQDGGDQDERAEKSKLTGTRQNHSFIWSGASFDLSPGLQRILDKVSSPL
ENEKLKSMTINFSSLNRKNTELNEEQEVISNLETKQVQGISFSSNNEVKSKIEMLENNANHDETSSLLPRKESNIVDDNGLIPPTPI
PTSASKLTFPGILETPVNPWKTNNVLQPGESYLFGSPSDIKNHDLSPGSRNGFKDNSPISDTSFSLQLSQDGLQLTPASSSSESLSI
IDVASDQNLFQTFIKEWRCKKRFSISLACEKIRSLTSSKTATIGSRFKQASSPQEIPIRDDGFPIKGCDDTLVVGLAVCWGGRDAYY
FSLQKEQKHSEISASLVPPSLDPSLTLKDRMWYLQSCLRKESDKECSVVIYDFIQSYKILLLSCGISLEQSYEDPKVACWLLDPDSQ
EPTLHSIVTSFLPHELPLLEGMETSQGIQSLGLNAGSEHSGRYRASVESILIFNSMNQLNSLLQKENLQDVFRKVEMPSQYCLALLE
LNGIGFSTAECESQKHIMQAKLDAIETQAYQLAGHSFSFTSSDDIAEVLFLELKLPPNREMKNQGSKKTLGSTRRGIDNGRKLRLGR
QFSTSKDVLNKLKALHPLPGLILEWRRITNAITKVVFPLQREKCLNPFLGMERIYPVSQSHTATGRITFTEPNIQNVPRDFEIKMPT
LVGESPPSQAVGKGLLPMGRGKYKKGFSVNPRCQAQMEERAADRGMPFSISMRHAFVPFPGGSILAADYSQLELRILAHLSHDRRLI
QVLNTGADVFRSIAAEWKMIEPESVGDDLRQQAKQICYGIIYGMGAKSLGEQMGIKENDAACYIDSFKSRYTGINQFMTETVKNCKR
DGFVQTILGRRRYLPGIKDNNPYRKAHAERQAINTIVQGSAADIVKIATVNIQKQLETFHSTFKSHGHREGMLQSDQTGLSRKRKLQ
GMFCPIRGGFFILQLHDELLYEVAEEDVVQVAQIVKNEMESAVKLSVKLKVKVKIGASWGELKDFDV
>sp|O95382|M3K6_HUMAN
MAGPCPRSGAERAGSCWQDPLAVALSRGRQLAAPPGRGCARSRPLSVVYVLTREPQPGLEPREGTEAEPLPLRCLREACAQVPRPRP
PPQLRSLPFGTLELGDTAALDAFYNADVVVLEVSSSLVQPSLFYHLGVRESFSMTNNVLLCSQADLPDLQALREDVFQKNSDCVGSY
TLIPYVVTATGRVLCGDAGLLRGLADGLVQAGVGTEALLTPLVGRLARLLEATPTDSCGYFRETIRRDIRQARERFSGPQLRQELAR
LQRRLDSVELLSPDIIMNLLLSYRDVQDYSAIIELVETLQALPTCDVAEQHNVCFHYTFALNRRNRPGDRAKALSVLLPLVQLEGSV

APDLYCMCGRIYKDMFFSSGFQDAGHREQAYHWYRKAFDVEPSLHSGINAAVLLIAAGQHFEDSKELRLIGMKLGCLLARKGCVEKM
QYYWDVGFYLGAQILANDPTQVVLAAEQLYKLNAPIWYLVSVMETFLLYQHFRPTPEPPGGPPRRAHFWLHFLLQSCQPFKTACAQG
DQCLVLVLEMNKVLLPAKLEVRGTDPVSTVTLSLLEPETQDIPSSWTFPVASICGVSASKRDERCCFLYALPPAQDVQLCFPSVGHC
QWFCGLIQAWVTNPDSTAPAEEAEGAGEMLEFDYEYTETGERLVLGKGTYGVVYAGRDRHTRVRIAIKEIPERDSRFSQPLHEEIAL
HRRLRHKNIVRYLGSASQGGYLKIFMEEVPGGSLSSLLRSVWGPLKDNESTISFYTRQILQGLGYLHDNHIVHRDIKGDNVLINTFS
GLLKISDFGTSKRLAGITPCTETFTGTLQYMAPEIIDQGPRGYGKAADIWSLGCTVIEMATGRPPFHELGSPQAAMFQVGMYKVHPP
MPSSLSAEAQAFLLRTFEPDPRLRASAQTLLGDPFLQPGKRSRSPSSPRHAPRPSDAPSASPTPSANSTTQSQTFPCPQAPSQHPPS
PPKRCLSYGGTSQLRVPEEPAAEEPASPEESSGLSLLHQESKRRAMLAAVLEQELPALAENLHQEQKQEQGARLGRNHVEELLRCLG
AHIHTPNRRQLAQELRALQGRLRAQGLGPALLHRPLFAFPDAVKQILRKRQIRPHWMFVLDSLLSRAVRAALGVLGPEVEKEAVSPR
SEELSNEGDSQQSPGQQSPLPVEPEQGPAPLMVQLSLLRAETDRLREILAGKEREYQALVQRALQRLNEEARTYVLAPEPPTALSTD
QGLVQWLQELNVDSGTIQMLLNHSFTLHTLLTYATRDDLIYTRIRGGMVCRIWRAILAQRAGSTPVTSGP
>sp|Q9BZE0|GLIS2_HUMAN
MHSLDEPLDLKLSITKLRAAREKRERTLGVVRPRALHRELGLVDDSPTPGSPGSPPSGFLLNSKFPEKVEGRFSAAPLVDLSLSPPS
GLDSPNGSSSLSPERQGNGDLPPVPSASDFQPLRYLDGVPSSFQFFLPLGSGGALHLPASSFLTPPKDKCLSPDLPLPKQLVCRWAK
CNQLFELLQDLVDHVNDYHVKPEKDAGYCCHWEGCARHGRGFNARYKMLIHIRTHTNEKPHRCPTCSKSFSRLENLKIHNRSHTGEK
PYVCPYEGCNKRYSNSSDRFKHTRTHYVDKPYYCKMPGCHKRYTDPSSLRKHIKAHGHFVSHEQQELLQLRPPPKPPLPAPDGGPYV
SGAQIIIPNPAALFGGPGLPGLPLPLAPGPLDLSALACGNGGGSGGGGGMGPGLPGPVLPLNLAKNPLLPSPFGAGGLGLPVVSLLA
GAAGGKAEGEKGRGSVPTRALGMEGHKTPLERTESSCSRPSPDGLPLLPGTVLDLSTGVNSAASSPEALAPGWVVIPPGSVLLKPAV
VN
>sp|O60674|JAK2_HUMAN
MGMACLTMTEMEGTSTSSIYQNGDISGNANSMKQIDPVLQVYLYHSLGKSEADYLTFPSGEYVAEEICIAASKACGITPVYHNMFAL
MSETERIWYPPNHVFHIDESTRHNVLYRIRFYFPRWYCSGSNRAYRHGISRGAEAPLLDDFVMSYLFAQWRHDFVHGWIKVPVTHET
QEECLGMAVLDMMRIAKENDQTPLAIYNSISYKTFLPKCIRAKIQDYHILTRKRIRYRFRRFIQQFSQCKATARNLKLKYLINLETL
QSAFYTEKFEVKEPGSGPSGEEIFATIIITGNGGIQWSRGKHKESETLTEQDLQLYCDFPNIIDVSIKQANQEGSNESRVVTIHKQD
GKNLEIELSSLREALSFVSLIDGYYRLTADAHHYLCKEVAPPAVLENIQSNCHGPISMDFAISKLKKAGNQTGLYVLRCSPKDFNKY
FLTFAVERENVIEYKHCLITKNENEEYNLSGTKKNFSSLKDLLNCYQMETVRSDNIIFQFTKCCPPKPKDKSNLLVFRTNGVSDVPT
SPTLQRPTHMNQMVFHKIRNEDLIFNESLGQGTFTKIFKGVRREVGDYGQLHETEVLLKVLDKAHRNYSESFFEAASMMSKLSHKHL
VLNYGVCVCGDENILVQEFVKFGSLDTYLKKNKNCINILWKLEVAKQLAWAMHFLEENTLIHGNVCAKNILLIREEDRKTGNPPFIK
LSDPGISITVLPKDILQERIPWVPPECIENPKNLNLATDKWSFGTTLWEICSGGDKPLSALDSQRKLQFYEDRHQLPAPKWAELANL
INNCMDYEPDFRPSFRAIIRDLNSLFTPDYELLTENDMLPNMRIGALGFSGAFEDRDPTQFEERHLKFLQQLGKGNFGSVEMCRYDP
LQDNTGEVVAVKKLQHSTEEHLRDFEREIEILKSLQHDNIVKYKGVCYSAGRRNLKLIMEYLPYGSLRDYLQKHKERIDHIKLLQYT
SQICKGMEYLGTKRYIHRDLATRNILVENENRVKIGDFGLTKVLPQDKEYYKVKEPGESPIFWYAPESLTESKFSVASDVWSFGVVL
YELFTYIEKSKSPPAEFMRMIGNDKQGQMIVFHLIELLKNNGRLPRPDGCPDEIYMIMTECWNNNVNQRPSFRDLALRVDQIRDNMA
G
>sp|Q8NCK7|MOT11_HUMAN
MPAPQRKHRRGGFSHRCFPTPQTAMTPQPAGPPDGGWGWVVAAAAFAINGLSYGLLRSLGLAFPDLAEHFDRSAQDTAWISALALAV
QQAASPVGSALSTRWGARPVVMVGGVLASLGFVFSAFASDLLHLYLGLGLLAGFGWALVFAPALGTLSRYFSRRRVLAVGLALTGNG
ASSLLLAPALQLLLDTFGWRGALLLLGAITLHLTPCGALLLPLVLPGDPPAPPRSPLAALGLSLFTRRAFSIFALGTALVGGGYFVP
YVHLAPHALDRGLGGYGAALVVAVAAMGDAGARLVCGWLADQGWVPLPRLLAVFGALTGLGLWVVGLVPVVGGEESWGGPLLAAAVA
YGLSAGSYAPLVFGVLPGLVGVGGVVQATGLVMMLMSLGGLLGPPLSGFLRDETGDFTASFLLSGSLILSGSFIYIGLPRALPSCGP
ASPPATPPPETGELLPAPQAVLLSPGGPGSTLDTTC
>sp|Q9NQY0|BIN3_HUMAN

MSWIPFKIGQPKKQIVPKTVERDFEREYGKLQQLEEQTRRLQKDMKKSTDADLAMSKSAVKISLDLLSNPLCEQDQDLLNMVTALDT
AMKRMDAFNQEKVNQIQKTVIEPLKKFGSVFPSLNMAVKRREQALQDYRRLQAKVEKYEEKEKTGPVLAKLHQAREELRPVREDFEA
KNRQLLEEMPRFYGSRLDYFQPSFESLIRAQVVYYSEMHKIFGDLSHQLDQPGHSDEQRERENEAKLSELRALSIVADD
>sp|P82970|HMGN5_HUMAN
MPKRKAAGQGDMRQEPKRRSARLSAMLVPVTPEVKPKRTSSSRKMKTKSDMMEENIDTSAQAVAETKQEAVVEEDYNENAKNGEAKI
TEAPASEKEIVEVKEENIEDATEKGGEKKEAVAAEVKNEEEDQKEDEEDQNEEKGEAGKEDKDEKGEEDGKEDKNGNEKGEDAKEKE
DGKKGEDGKGNGEDGKEKGEDEKEEEDRKETGDGKENEDGKEKGDKKEGKDVKVKEDEKEREDGKEDEGGNEEEAGKEKEDLKEEEE
GKEEDEIKEDDGKKEEPQSIV
>sp|Q8NGF9|OR4X2_HUMAN
MTEFIFLVLSPNQEVQRVCFVIFLFLYTAIVLGNFLIVLTVMTSRSLGSPMYFFLSYLSFMEICYSSATAPKLISDLLAERKVISWW
GCMAQLFFLHFFGGTEIFLLTVMAYDHYVAICKPLSYTTIMNWQVCTVLVGIAWVGGFMHSFAQILLIFHLLFCGPNVINHYFCDLV
PLLKLACSDTFLIGLLIVANGGTLSVISFGVLLASYMVILLHLRTWSSEGWCKALSTCGSHFAVVILFFGPCVFNSLRPSTTLPIDK
MVAVFYTVITAILNPVIYSLRNAEMRKAMKRLWIRTLRLNEK
>sp|Q6ZUA9|MROH5_HUMAN
MDRQCSERPYSCTPTGRVSSAVSQNSRISPPVSTSMKDSSCMKVHQDSARRDRWSHPTTILLHKSQSSQATLMLQEHRMFMGEAYSA
ATGFKMLQDMNSADPFHLKYIIKKIKNMAHGSPKLVMETIHDYFIDNPEISSRHKFRLFQTLEMVIGASDVLEETWEKTFTRLALEN
MTKATELEDIYQDAASNMLVAICRHSWRVVAQHLETELLTGVFPHRSLLYVMGVLSSSEELFSQEDKACWEEQLIQMAIKSVPFLST
DVWSKELLWTLTTPSWTQQEQSPEKAFLFTYYGLILQAEKNGATVRRHLQALLETSHQWPKQREGMALTLGLAATRHLDDVWAVLDQ
FGRSRPIRWSLPSSSPKNSEDLRWKWASSTILLAYGQVAAKARAHILPWVDNIVSRMVFYFHYSSWDETLKQSFLTATLMLMGAVSR
SEGAHSYEFFQTSELLQCLMVLMEKEPQDTLCTRSRQQAMHIASSLCKLRPPIDLERKSQLLSTCFRSVFALPLLDALEKHTCLFLE
PPNIQLWPVARERAGWTHQGWGPRAVLHCSEHLQSLYSRTMEALDFMLQSLIMQNPTADELHFLLSHLYIWLASEKAHERQRAVHSC
MILLKFLNHNGYLDPKEDFKRIGQLVGILGMLCQDPDRATQRCSLEGASHLYQLLMCHKTGEALQAESQAPKELSQAHSDGAPLWNS
RDQKATPLGPQEMAKNHIFQLCSFQVIKDIMQQLTLAELSDLIWTAIDGLGSTSPFRVQAASEMLLTAVQEHGAKLEIVSSMAQAIR
LRLCSVHIPQAKEKTLHAITLLARSHTCELVATFLNISIPLDSHTFQLWRALGAGQPTSHLVLTTLLACLQERPLPTGASDSSPCPK
EKTYLRLLAAMNMLHELQFAREFKQAVQEGYPKLFLALLTQMHYVLELNLPSEPQPKQQAQEAAVPSPQSCSTSLEALKSLLSTTGH
WHDFAHLELQGSWELFTTIHTYPKGVGLLARAMVQNHCRQIPAVLRQLLPSLQSPQERERKVAILILTKFLYSPVLLEVLPKQAALT
VLAQGLHDPSPEVRVLSLQGLSNILFHPDKGSLLQGQLRPLLDGFFQSSDQVIVCIMGTVSDTLHRLGAQGTGSQSLGVAISTRSFF
NDERDGIRAAAMALFGDLVAAMADRELSGLRTQVHQSMVPLLLHLKDQCPAVATQAKFTFYRCAVLLRWRLLHTLFCTLAWERGLSA
RHFLWTCLMTRSQEEFSIHLSQALSYLHSHSCHIKTWVTLFIGHTICYHPQAVFQMLNAVDTNLLFRTFEHLRSDPEPSIREFATSQ
LSFLQKVSARPKQ
>sp|Q9P2N4|ATS9_HUMAN
MQFVSWATLLTLLVRDLAEMGSPDAAAAVRKDRLHPRQVKLLETLSEYEIVSPIRVNALGEPFPTNVHFKRTRRSINSATDPWPAFA
SSSSSSTSSQAHYRLSAFGQQFLFNLTANAGFIAPLFTVTLLGTPGVNQTKFYSEEEAELKHCFYKGYVNTNSEHTAVISLCSGMLG
TFRSHDGDYFIEPLQSMDEQEDEEEQNKPHIIYRRSAPQREPSTGRHACDTSEHKNRHSKDKKKTRARKWGERINLAGDVAALNSGL
ATEAFSAYGNKTDNTREKRTHRRTKRFLSYPRFVEVLVVADNRMVSYHGENLQHYILTLMSIVASIYKDPSIGNLINIVIVNLIVIH
NEQDGPSISFNAQTTLKNFCQWQHSKNSPGGIHHDTAVLLTRQDICRAHDKCDTLGLAELGTICDPYRSCSISEDSGLSTAFTIAHE
LGHVFNMPHDDNNKCKEEGVKSPQHVMAPTLNFYTNPWMWSKCSRKYITEFLDTGYGECLLNEPESRPYPLPVQLPGILYNVNKQCE
LIFGPGSQVCPYMMQCRRLWCNNVNGVHKGCRTQHTPWADGTECEPGKHCKYGFCVPKEMDVPVTDGSWGSWSPFGTCSRTCGGGIK
TAIRECNRPEPKNGGKYCVGRRMKFKSCNTEPCLKQKRDFRDEQCAHFDGKHFNINGLLPNVRWVPKYSGILMKDRCKLFCRVAGNT
AYYQLRDRVIDGTPCGQDTNDICVQGLCRQAGCDHVLNSKARRDKCGVCGGDNSSCKTVAGTFNTVHYGYNTVVRIPAGATNIDVRQ
HSFSGETDDDNYLALSSSKGEFLLNGNFVVTMAKREIRIGNAVVEYSGSETAVERINSTDRIEQELLLQVLSVGKLYNPDVRYSFNI
PIEDKPQQFYWNSHGPWQACSKPCQGERKRKLVCTRESDQLTVSDQRCDRLPQPGHITEPCGTDCDLRWHVASRSECSAQCGLGYRT
LDIYCAKYSRLDGKTEKVDDGFCSSHPKPSNREKCSGECNTGGWRYSAWTECSKSCDGGTQRRRAICVNTRNDVLDDSKCTHQEKVT
IQRCSEFPCPQWKSGDWSECLVTCGKGHKHRQVWCQFGEDRLNDRMCDPETKPTSMQTCQQPECASWQAGPWGQCSVTCGQGYQLRA

VKCIIGTYMSVVDDNDCNAATRPTDTQDCELPSCHPPPAAPETRRSTYSAPRTQWRFGSWTPCSATCGKGTRMRYVSCRDENGSVAD
ESACATLPRPVAKEECSVTPCGQWKALDWSSCSVTCGQGRATRQVMCVNYSDHVIDRSECDQDYIPETDQDCSMSPCPQRTPDSGLA
QHPFQNEDYRPRSASPSRTHVLGGNQWRTGPWGACSSTCAGGSQRRVVVCQDENGYTANDCVERIKPDEQRACESGPCPQWAYGNWG
ECTKLCGGGIRTRLVVCQRSNGERFPDLSCEILDKPPDREQCNTHACPHDAAWSTGPWSSCSVSCGRGHKQRNVYCMAKDGSHLESD
YCKHLAKPHGHRKCRGGRCPKWKAGAWSQCSVSCGRGVQQRHVGCQIGTHKIARETECNPYTRPESERDCQGPRCPLYTWRAEEWQE
CTKTCGEGSRYRKVVCVDDNKNEVHGARCDVSKRPVDRESCSLQPCEYVWITGEWSECSVTCGKGYKQRLVSCSEIYTGKENYEYSY
QTTINCPGTQPPSVHPCYLRDCPVSATWRVGNWGSCSVSCGVGVMQRSVQCLTNEDQPSHLCHTDLKPEERKTCRNVYNCELPQNCK
EVKRLKGASEDGEYFLMIRGKLLKIFCAGMHSDHPKEYVTLVHGDSENFSEVYGHRLHNPTECPYNGSRRDDCQCRKDYTAAGFSSF
QKIRIDLTSMQIITTDLQFARTSEGHPVPFATAGDCYSAAKCPQGRFSINLYGTGLSLTESARWISQGNYAVSDIKKSPDGTRVVGK
CGGYCGKCTPSSGTGLEVRVL
>sp|Q8NGE3|O10P1_HUMAN
MAGENHTTLPEFLLLGFSDLKALQGPLFWVVLLVYLVTLLGNSLIILLTQVSPALHSPMYFFLRQLSVVELFYTTDIVPRTLANLGS
PHPQAISFQGCAAQMYVFIVLGISECCLLTAMAYDRYVAICQPLRYSTLLSPRACMAMVGTSWLTGIITATTHASLIFSLPFRSHPI
IPHFLCDILPVLRLASAGKHRSEISVMTATIVFIMIPFSLIVTSYIRILGAILAMASTQSRRKVFSTCSSHLLVVSLFFGTASITYI
RPQAGSSVTTDRVLSLFYTVITPMLNPIIYTLRNKDVRRALRHLVKRQRPSP
>sp|Q15573|TAF1A_HUMAN
MSDFSEELKGPVTDDEEVETSVLSGAGMHFPWLQTYVETVAIGGKRRKDFAQTTSACLSFIQEALLKHQWQQAAEYMYSYFQTLEDS
DSYKRQAAPEIIWKLGSEILFYHPKSNMESFNTFANRMKNIGVMNYLKISLQHALYLLHHGMLKDAKRNLSEAETWRHGENTSSREI
LINLIQAYKGLLQYYTWSEKKMELSKLDKDDYAYNAVAQDVFNHSWKTSANISALIKIPGVWDPFVKSYVEMLEFYGDRDGAQEVLT
NYAYDEKFPSNPNAHIYLYNFLKRQKAPRSKLISVLKILYQIVPSHKLMLEFHTLLRKSEKEEHRKLGLEVLFGVLDFAGCTKNITA
WKYLAKYLKNILMGNHLAWVQEEWNSRKNWWPGFHFSYFWAKSDWKEDTALACEKAFVAGLLLGKGCRYFRYILKQDHQILGKKIKR
MKRSVKKYSIVNPRL
>sp|P07359|GP1BA_HUMAN
MPLLLLLLLLPSPLHPHPICEVSKVASHLEVNCDKRNLTALPPDLPKDTTILHLSENLLYTFSLATLMPYTRLTQLNLDRCELTKLQ
VDGTLPVLGTLDLSHNQLQSLPLLGQTLPALTVLDVSFNRLTSLPLGALRGLGELQELYLKGNELKTLPPGLLTPTPKLEKLSLANN
NLTELPAGLLNGLENLDTLLLQENSLYTIPKGFFGSHLLPFAFLHGNPWLCNCEILYFRRWLQDNAENVYVWKQGVDVKAMTSNVAS
VQCDNSDKFPVYKYPGKGCPTLGDEGDTDLYDYYPEEDTEGDKVRATRTVVKFPTKAHTTPWGLFYSWSTASLDSQMPSSLHPTQES
TKEQTTFPPRWTPNFTLHMESITFSKTPKSTTEPTPSPTTSEPVPEPAPNMTTLEPTPSPTTPEPTSEPAPSPTTPEPTSEPAPSPT
TPEPTSEPAPSPTTPEPTPIPTIATSPTILVSATSLITPKSTFLTTTKPVSLLESTKKTIPELDQPPKLRGVLQGHLESSRNDPFLH
PDFCCLLPLGFYVLGLFWLLFASVVLILLLSWVGHVKPQALDSGQGAALTTATQTTHLELQRGRQVTVPRAWLLFLRGSLPTFRSSL
FLWVRPNGRVGPLVAGRRPSALSQGRGQDLLSTVSIRYSGHSL
>sp|Q8NC54|KCT2_HUMAN
MAAAVPKRMRGPAQAKLLPGSAIQALVGLARPLVLALLLVSAALSSVVSRTDSPSPTVLNSHISTPNVNALTHENQTKPSISQISTT
LPPTTSTKKSGGASVVPHPSPTPLSQEEADNNEDPSIEEEDLLMLNSSPSTAKDTLDNGDYGEPDYDWTTGPRDDDESDDTLEENRG
YMEIEQSVKSFKMPSSNIEEEDSHFFFHLIIFAFCIAVVYITYHNKRKIFLLVQSRKWRDGLCSKTVEYHRLDQNVNEAMPSLKITN
DYIF
>sp|Q13111|CAF1A_HUMAN
MLEELECGAPGARGAATAMDCKDRPAFPVKKLIQARLPFKRLNLVPKGKADDMSDDQGTSVQSKSPDLEASLDTLENNCHVGSDIDF
RPKLVNGKGPLDNFLRNRIETSIGQSTVIIDLTEDSNEQPDSLVDHNKLNSEASPSREAINGQREDTGDQQGLLKAIQNDKLAFPGE
TLSDIPCKTEEEGVGCGGAGRRGDSQECSPRSCPELTSGPRMCPRKEQDSWSEAGGILFKGKVPMVVLQDILAVRPPQIKSLPATPQ
GKNMTPESEVLESFPEEDSVLSHSSLSSPSSTSSPEGPPAPPKQHSSTSPFPTSTPLRRITKKFVKGSTEKNKLRLQRDQERLGKQL
KLRAEREEKEKLKEEAKRAKEEAKKKKEEEKELKEKERREKREKDEKEKAEKQRLKEERRKERQEALEAKLEEKRKKEEEKRLREEE
KRIKAEKAEITRFFQKPKTPQAPKTLAGSCGKFAPFEIKEHMVLAPRRRTAFHPDLCSQLDQLLQQQSGEFSFLKDLKGRQPLRSGP
THVSTRNADIFNSDVVIVERGKGDGVPERRKFGRMKLLQFCENHRPAYWGTWNKKTALIRARDPWAQDTKLLDYEVDSDEEWEEEEP

GESLSHSEGDDDDDMGEDEDEDDGFFVPHGYLSEDEGVTEECADPENHKVRQKLKAKEWDEFLAKGKRFRVLQPVKIGCVWAADRDC
AGDDLKVLQQFAACFLETLPAQEEQTPKASKRERRDEQILAQLLPLLHGNVNGSKVIIREFQEHCRRGLLSNHTGSPRSPSTTYLHT
PTPSEDAAIPSKSRLKRLISENSVYEKRPDFRMCWYVHPQVLQSFQQEHLPVPCQWSYVTSVPSAPKEDSGSVPSTGPSQGTPISLK
RKSAGSMCITQFMKKRRHDGQIGAEDMDGFQADTEEEEEEEGDCMIVDVPDAAEVQAPCGAASGAGGGVGVDTGKATLTSSPLGAS
>sp|Q92664|TF3A_HUMAN
MDPPAVVAESVSSLTIADAFIAAGESSAPTPPRPALPRRFICSFPDCSANYSKAWKLDAHLCKHTGERPFVCDYEGCGKAFIRDYHL
SRHILTHTGEKPFVCAANGCDQKFNTKSNLKKHFERKHENQQKQYICSFEDCKKTFKKHQQLKIHQCQHTNEPLFKCTQEGCGKHFA
SPSKLKRHAKAHEGYVCQKGCSFVAKTWTELLKHVRETHKEEILCEVCRKTFKRKDYLKQHMKTHAPERDVCRCPREGCGRTYTTVF
NLQSHILSFHEESRPFVCEHAGCGKTFAMKQSLTRHAVVHDPDKKKMKLKVKKSREKRSLASHLSGYIPPKRKQGQGLSLCQNGESP
NCVEDKMLSTVAVLTLG
>sp|Q86UU0|BCL9L_HUMAN
MRILANKTRLPHPRRREAPGSPPLSPRGHCPPAPAKPMHPENKLTNHGKTGNGGAQSQHQNVNQGPTCNVGSKGVGAGNHGAKANQI
SPSNSSLKNPQAGVPPFSSLKGKVKRDRSVSVDSGEQREAGTPSLDSEAKEVAPRSKRRCVLERKQPYSGDEWCSGPDSEEDDKPIG
ATHNCNVADPAMAAPQLGPGQTTQLPLSESSVPGAPHGPPPGLRPDAPGGGGGGGGVPGKPPSQFVYVFTTHLANTAAEAVLQGRAD
SILAYHQQNVPRAKLDQAPKVPPTPEPLPLSTPSAGTPQSQPPPLPPPPPPAPGSAPPALPPEGPPEDSSQDLAPNSVGAASTGGGT
GGTHPNTPTATTANNPLPPGGDPSSAPGPALLGEAAAPGNGQRSLVGSEGLSKEQLEHRERSLQTLRDIERLLLRSGETEPFLKGPP
GGAGEGGPPAQAPPPPQQPPTAPPSGLKKYEEPLQSMISQTQSLGGPPLEHEVPGHPPGGDMGQQMNMMIQRLGQDSLTPEQVAWRK
LQEEYYEEKRRKEEQIGLHGSRPLQDMMGMGGMMVRGPPPPYHSKPGDQWPPGMGAQLRGPMDVQDPMQLRGGPPFPGPRFPGNQIQ
RVPGFGGMQSMPMEVPMNAMQRPVRPGMGWTEDLPPMGGPSNFAQNTMPYPGGQGEAERFMTPRVREELLRHQLLEKRSMGMQRPLG
MAGSGMGQSMEMERMMQAHRQMDPAMFPGQMAGGEGLAGTPMGMEFGGGRGLLSPPMGQSGLREVDPPMGPGNLNMNMNVNMNMNMN
LNVQMTPQQQMLMSQKMRGPGDLMGPQGLSPEEMARVRAQNSSGVMGGPQKMLMPSQFPNQGQQGFSGGQGPYQAMSQDMGNTQDMF
SPDQSSMPMSNVGTTRLSHMPLPPASNPPGTVHSAPNRGLGRRPSDLTISINQMGSPGMGHLKSPTLSQVHSPLVTSPSANLKSPQT
PSQMVPLPSANPPGPLKSPQVLGSSLSVRSPTGSPSRLKSPSMAVPSPGWVASPKTAMPSPGVSQNKQPPLNMNSSTTLSNMEQGTL
PPSGPRSSSSAPPANPPSGLMNPSLPFTSSPDPTPSQNPLSLMMTQMSKYAMPSSTPLYHNAIKTIATSDDELLPDRPLLPPPPPPQ
GSGPGISNSQPSQMHLNSAAAQSPMGMNLPGQQPLSHEPPPAMLPSPTPLGSNIPLHPNAQGTGGPPQNSMMMAPGGPDSLNAPCGP
VPSSSQMMPFPPRLQQPHGAMAPTGGGGGGPGLQQHYPSGMALPPEDLPNQPPGPMPPQQHLMGKAMAGRMGDAYPPGVLPGVASVL
NDPELSEVIRPTPTGIPEFDLSRIIPSEKPSSTLQYFPKSENQPPKAQPPNLHLMNLQNMMAEQTPSRPPNLPGQQGVQRGLNMSMC
HPGQMSLLGRTGVPPQQGMVPHGLHQGVMSPPQGLMTQQNFMLMKQRGVGGEVYSQPPHMLSPQGSLMGPPPQQNLMVSHPLRQRSV
SLDSQMGYLPAPGGMANLPF
>sp|Q6V0L0|CP26C_HUMAN
MFPWGLSCLSVLGAAGTALLCAGLLLSLAQHLWTLRWMLSRDRASTLPLPKGSMGWPFFGETLHWLVQGSRFHSSRRERYGTVFKTH
LLGRPVIRVSGAENVRTILLGEHRLVRSQWPQSAHILLGSHTLLGAVGEPHRRRRKVLARVFSRAALERYVPRLQGALRHEVRSWCA
AGGPVSVYDASKALTFRMAARILLGLRLDEAQCATLARTFEQLVENLFSLPLDVPFSGLRKGIRARDQLHRHLEGAISEKLHEDKAA
EPGDALDLIIHSARELGHEPSMQELKESAVELLFAAFFTTASASTSLVLLLLQHPAAIAKIREELVAQGLGRACGCAPGAAGGSEGP
PPDCGCEPDLSLAALGRLRYVDCVVKEVLRLLPPVSGGYRTALRTFELDGYQIPKGWSVMYSIRDTHETAAVYRSPPEGFDPERFGA
AREDSRGASSRFHYIPFGGGARSCLGQELAQAVLQLLAVELVRTARWELATPAFPAMQTVPIVHPVDGLRLFFHPLTPSVAGNGLCL
>sp|Q15269|PWP2_HUMAN
MKFAYRFSNLLGTVYRRGNLNFTCDGNSVISPVGNRVTVFDLKNNKSDTLPLATRYNVKCVGLSPDGRLAIIVDEGGDALLVSLVCR
SVLHHFHFKGSVHSVSFSPDGRKFVVTKGNIAQMYHAPGKKREFNAFVLDKTYFGPYDETTCIDWTDDSRCFVVGSKDMSTWVFGAE
RWDNLIYYALGGHKDAIVACFFESNSLDLYSLSQDGVLCMWQCDTPPEGLRLKPPAGWKADLLQREEEEEEEEDQEGDRETTIRGKA
TPAEEEKTGKVKYSRLAKYFFNKEGDFNNLTAAAFHKKSHLLVTGFASGIFHLHELPEFNLIHSLSISDQSIASVAINSSGDWIAFG
CSGLGQLLVWEWQSESYVLKQQGHFNSMVALAYSPDGQYIVTGGDDGKVKVWNTLSGFCFVTFTEHSSGVTGVTFTATGYVVVTSSM
DGTVRAFDLHRYRNFRTFTSPRPTQFSCVAVDASGEIVSAGAQDSFEIFVWSMQTGRLLDVLSGHEGPISGLCFNPMKSVLASASWD
KTVRLWDMFDSWRTKETLALTSDALAVTFRPDGAELAVATLNSQITFWDPENAVQTGSIEGRHDLKTGRKELDKITAKHAAKGKAFT

ALCYSADGHSILAGGMSKFVCIYHVREQILMKRFEISCNLSLDAMEEFLNRRKMTEFGNLALIDQDAGQEDGVAIPLPGVRKGDMSS
RHFKPEIRVTSLRFSPTGRCWAATTTEGLLIYSLDTRVLFDPFELDTSVTPGRVREALRQQDFTRAILMALRLNESKLVQEALEAVP
RGEIEVVTSSLPELYVEKVLEFLASSFEVSRHLEFYLLWTHKLLMLHGQKLKSRAGTLLPVIQFLQKSIQRHLDDLSKLCSWNHYNM
QYALAVSKQRGTKRSLDPLGSEEEAEASEDDSLHLLGGGGRDSEEEMLA
>sp|P16615|AT2A2_HUMAN
MENAHTKTVEEVLGHFGVNESTGLSLEQVKKLKERWGSNELPAEEGKTLLELVIEQFEDLLVRILLLAACISFVLAWFEEGEETITA
FVEPFVILLILVANAIVGVWQERNAENAIEALKEYEPEMGKVYRQDRKSVQRIKAKDIVPGDIVEIAVGDKVPADIRLTSIKSTTLR
VDQSILTGESVSVIKHTDPVPDPRAVNQDKKNMLFSGTNIAAGKAMGVVVATGVNTEIGKIRDEMVATEQERTPLQQKLDEFGEQLS
KVISLICIAVWIINIGHFNDPVHGGSWIRGAIYYFKIAVALAVAAIPEGLPAVITTCLALGTRRMAKKNAIVRSLPSVETLGCTSVI
CSDKTGTLTTNQMSVCRMFILDRVEGDTCSLNEFTITGSTYAPIGEVHKDDKPVNCHQYDGLVELATICALCNDSALDYNEAKGVYE
KVGEATETALTCLVEKMNVFDTELKGLSKIERANACNSVIKQLMKKEFTLEFSRDRKSMSVYCTPNKPSRTSMSKMFVKGAPEGVID
RCTHIRVGSTKVPMTSGVKQKIMSVIREWGSGSDTLRCLALATHDNPLRREEMHLEDSANFIKYETNLTFVGCVGMLDPPRIEVASS
VKLCRQAGIRVIMITGDNKGTAVAICRRIGIFGQDEDVTSKAFTGREFDELNPSAQRDACLNARCFARVEPSHKSKIVEFLQSFDEI
TAMTGDGVNDAPALKKAEIGIAMGSGTAVAKTASEMVLADDNFSTIVAAVEEGRAIYNNMKQFIRYLISSNVGEVVCIFLTAALGFP
EALIPVQLLWVNLVTDGLPATALGFNPPDLDIMNKPPRNPKEPLISGWLFFRYLAIGCYVGAATVGAAAWWFIAADGGPRVSFYQLS
HFLQCKEDNPDFEGVDCAIFESPYPMTMALSVLVTIEMCNALNSLSENQSLLRMPPWENIWLVGSICLSMSLHFLILYVEPLPLIFQ
ITPLNVTQWLMVLKISLPVILMDETLKFVARNYLEPGKECVQPATKSCSFSACTDGISWPFVLLIMPLVIWVYSTDTNFSDMFWS
>sp|Q96S95|CK2N2_HUMAN
MSEILPYSEDKMGRFGADPEGSDLSFSCRLQDTNSFFAGNQAKRPPKLGQIGRAKRVVIEDDRIDDVLKGMGEKPPSGV
>sp|O14944|EREG_HUMAN
MTAGRRMEMLCAGRVPALLLCLGFHLLQAVLSTTVIPSCIPGESSDNCTALVQTEDNPRVAQVSITKCSSDMNGYCLHGQCIYLVDM
SQNYCRCEVGYTGVRCEHFFLTVHQPLSKEYVALTVILIILFLITVVGSTYYFCRWYRNRKSKEPKKEYERVTSGDPELPQV
>sp|O95625|ZBT11_HUMAN
MSSEESYRAILRYLTNEREPYAPGTEGNVKRKIRKAAACYVVRGGTLYYQRRQRHRKTFAELEVVLQPERRRDLIEAAHLGPGGTHH
TRHQTWHYLSKTYWWRGILKQVKDYIKQCSKCQEKLDRSRPISDVSEMLEELGLDLESGEESNESEDDLSNFTSSPTTASKPAKKKP
VSKHELVFVDTKGVVKRSSPKHCQAVLKQLNEQRLSNQFCDVTLLIEGEEYKAHKSVLSANSEYFRDLFIEKGAVSSHEAVVDLSGF
CKASFLPLLEFAYTSVLSFDFCSMADVAILARHLFMSEVLEICESVHKLMEEKQLTVYKKGEVQTVASTQDLRVQNGGTAPPVASSE
GTTTSLPTELGDCEIVLLVNGELPEAEQNGEVGRQPEPQVSSEAESALSSVGCIADSHPEMESVDLITKNNQTELETSNNRENNTVS
NIHPKLSKENVISSSPEDSGMGNDISAEDICAEDIPKHRQKVDQPLKDQENLVASTAKTDFGPDDDTYRSRLRQRSVNEGAYIRLHK
GMEKKLQKRKAVPKSAVQQVAQKLVQRGKKMKQPKRDAKENTEEASHKCGECGMVFQRRYALIMHKLKHERARDYKCPLCKKQFQYS
ASLRAHLIRHTRKDAPSSSSSNSTSNEASGTSSEKGRTKREFICSICGRTLPKLYSLRIHMLKHTGVKPHACQVCGKTFIYKHGLKL
HQSLHQSQKQFQCELCVKSFVTKRSLQEHMSIHTGESKYLCSVCGKSFHRGSGLSKHFKKHQPKPEVRGYHCTQCEKSFFEARDLRQ
HMNKHLGVKPFQCQFCDKCYSWKKDWYSHVKSHSVTEPYRCNICGKEFYEKALFRRHVKKATHGKKGRAKQNLERVCEKCGRKFTQL
REYRRHMNNHEGVKPFECLTCGVAWADARSLKRHVRTHTGERPYVCPVCSEAYIDARTLRKHMTKFHRDYVPCKIMLEKDTLQFHNQ
GTQVAHAVSILTAGMQEQESSGPQELETVVVTGETMEALEAVAATEEYPSVSTLSDQSIMQVVNYVLAQQQGQKLSEVAEAIQTVKV
EVAHISGGE
>sp|Q6ZUG5|YC006_HUMAN
MPVYCKYQFHKTPVHKTKGEPHGTHVYFQDINVIFLGALHPSDLREYLEGPPMVVEVHDRDRKSEECSQKPVLFGEDPLDSYLNFQA
LISPRETENNPFESQNKMWYPYGIAQVSFADLLLGHKYLNLAVPIHSCEVQPTHCGQDSRRRKVVGLGVPRDGHQHGPMPRGNYLEA
DSQLKLRVDIAVPLRAGARAADPDLGGSQFGRIIFVFDFKKVSLLHSLLQDITMINAKALGLDSYPVRTLQQILSAFKVRVRVQEQQ
HLDVLTGFHLLDGKTHLFILEGLADQGLRQLWENHQSWIPRSEHRKYKVLYNSQLLFRSRLYGDLEAILYHVHLFQPTELLLQQAVF
FLRDTERRRVFQALARIHDICYNSTTLWDVTVRDLLPSSAMIKDLSQEFGMPLSQEELTDEKLFALPPQPAPNLEDYHSRNSTLTLE
IHAHQEPRKRFTYSQDYLSAMVEPLDLKEEEKKAQKKSRQAWLTARGFQVTGLQSDTESSFQDLKLPPIKELNEEWKENSLFANVLE
PVLDRDRWSWDRHHVDFDLYKKPPPFLELLPSPAPKPVTVRKKKGNSPIS

>sp|Q9BTZ2|DHRS4_HUMAN
MHKAGLLGLCARAWNSVRMASSGMTRRDPLANKVALVTASTDGIGFAIARRLAQDGAHVVVSSRKQQNVDQAVATLQGEGLSVTGTV
CHVGKAEDRERLVATAVKLHGGIDILVSNAAVNPFFGSIMDVTEEVWDKTLDINVKAPALMTKAVVPEMEKRGGGSVVIVSSIAAFS
PSPGFSPYNVSKTALLGLTKTLAIELAPRNIRVNCLAPGLIKTSFSRMLWMDKEKEESMKETLRIRRLGEPEDCAGIVSFLCSEDAS
YITGETVVVGGGTPSRL
>sp|P17509|HXB6_HUMAN
MSSYFVNSTFPVTLASGQESFLGQLPLYSSGYADPLRHYPAPYGPGPGQDKGFATSSYYPPAGGGYGRAAPCDYGPAPAFYREKESA
CALSGADEQPPFHPEPRKSDCAQDKSVFGETEEQKCSTPVYPWMQRMNSCNSSSFGPSGRRGRQTYTRYQTLELEKEFHYNRYLTRR
RRIEIAHALCLTERQIKIWFQNRRMKWKKESKLLSASQLSAEEEEEKQAE
>sp|O43824|GTPB6_HUMAN
MWALRAAVRPGLRLSRVGRGRSAPRAAAPSCPARALAAVGRRSPGNLEGPWGGGRGLRADGGRSRTGDDEEEPEDADENAEEELLRG
EPLLPAGTQRVCLVHPDVKWGPGKSQMTRAEWQVAEATALVHTLDGWSVVQTMVVSTKTPDRKLIFGKGNFEHLTEKIRGSPDVTCV
FLNVERMAAPTKKELEAAWGVEVFDRFTVVLHIFRCNARTKEARLQVALAEMPLHRSNLKRDVAHLYRGVGSRYIMGSGESFMQLQQ
RLLREKEAKIRKALDRLRKKRHLLRRQRTRREFPVISVVGYTNCGKTTLIKALTGDAAIQPRDQLFATLDVTAHAGTLPSRMTVLYV
DTIGFLSQLPHGLIESFSATLEDVAHSDLILHVRDVSHPEAELQKCSVLSTLRGLQLPAPLLDSMVEVHNKVDLVPGYSPTEPNVVP
VSALRGHGLQELKAELDAAVLKATGRQILTLRVRLAGAQLSWLYKEATVQEVDVIPEDGAADVRVIISNSAYGKFRKLFPG
>sp|Q8NFR7|CC148_HUMAN
MCAASASPDNLVFHMKNEMRNIKYKPVDYQQLRALTEAKKLASASAKLKIRKAMLTSKLSKEQTLIKQHKQVWWQEYQRLNEVRCKM
ESEIKSLLNEENIGNECLCDLTNFEQELSEQQCTYLKNVINPIQQLRADLKYRQHHTLQHSHPHIEFNSMKVLEEVDFVKKQLKTVF
ERLRLEQQRIENDLSDWSIKILDHSLEEKTNPLSELPIELESLECPYPDLKSSILSEFYKFTQKYQKKLQDFNLQLEDIYRNCQLSE
EDHWIYQAILDQYPGDLFGRRTLYLDMLQRYFPHKSRHDLVEHEKYCDQYRFAIEQQNILISNWNKNKKDFIQKAVLTLTEACATHE
MESMLAKDKKKQQELCADLKAKVRQWRAHQEEVARLEMEISARRREKEEEKEKLWKKKELLQRAEKKKKIKKYWAKKKQKWQEMEMR
DLQRLEELKKLIAEQSLKDRERVKYRQELLERRLMEKKEVALQEAHEDKERARRLEALRKQVAVVAQFDPVRMMSDTMASKARMGIE
IEEEFILQKPLFTLNTYNEQQIISDPRLRFELALREAGLHRTLYAKEILPKISPQKPPRKDMESTVFKI
>sp|O43143|DHX15_HUMAN
MSKRHRLDLGEDYPSGKKRAGTDGKDRDRDRDREDRSKDRDRERDRGDREREREKEKEKELRASTNAMLISAGLPPLKASHSAHSTH
SAHSTHSTHSAHSTHAGHAGHTSLPQCINPFTNLPHTPRYYDILKKRLQLPVWEYKDRFTDILVRHQSFVLVGETGSGKTTQIPQWC
VEYMRSLPGPKRGVACTQPRRVAAMSVAQRVADEMDVMLGQEVGYSIRFEDCSSAKTILKYMTDGMLLREAMNDPLLERYGVIILDE
AHERTLATDILMGVLKEVVRQRSDLKVIVMSATLDAGKFQIYFDNCPLLTIPGRTHPVEIFYTPEPERDYLEAAIRTVIQIHMCEEE
EGDLLLFLTGQEEIDEACKRIKREVDDLGPEVGDIKIIPLYSTLPPQQQQRIFEPPPPKKQNGAIGRKVVVSTNIAETSLTIDGVVF
VIDPGFAKQKVYNPRIRVESLLVTAISKASAQQRAGRAGRTRPGKCFRLYTEKAYKTEMQDNTYPEILRSNLGSVVLQLKKLGIDDL
VHFDFMDPPAPETLMRALELLNYLAALNDDGDLTELGSMMAEFPLDPQLAKMVIASCDYNCSNEVLSITAMLSVPQCFVRPTEAKKA
ADEAKMRFAHIDGDHLTLLNVYHAFKQNHESVQWCYDNFINYRSLMSADNVRQQLSRIMDRFNLPRRSTDFTSRDYYINIRKALVTG
YFMQVAHLERTGHYLTVKDNQVVQLHPSTVLDHKPEWVLYNEFVLTTKNYIRTCTDIKPEWLVKIAPQYYDMSNFPQCEAKRQLDRI
IAKLQSKEYSQY
>sp|Q2T9L4|CO059_HUMAN
MNIRGAPDLGQPSDDPSSGGERERIRQRMKMVIGQLEGILRELKEVAKELREVVSQIDKLTSDFDFELEPDDWTTATVSSTSSSDKA
GMGGPFDLGHLDFMTADILSDSWEFCSFLDVSTPSDSVDGPESTRPGAGPDYRLMNGGTPIPNGPRVETPDSSSEEAFGAGPTVKSQ
LPQRTPGTRERVRFSDKVLYHALCCDDEEGDGEQEVEEEEVGLPPEPAHTEAHAGPHKPSPAPYKSRRSPLTSRHSGSTLAPEQTRR
VTRNSSTQTVSDKSTQTVLPYTATRQKARGKN
>sp|Q96EY7|PTCD3_HUMAN
MAVVSAVRWLGLRSRLGQPLTGRRAGLCEQARSCRFYSGSATLSKVEGTDVTGIEEVVIPKKKTWDKVAVLQALASTVNRDTTAVPY
VFQDDPYLMPASSLESRSFLLAKKSGENVAKFIINSYPKYFQKDIAEPHIPCLMPEYFEPQIKDISEAALKERIELRKVKASVDMFD
QLLQAGTTVSLETTNSLLDLLCYYGDQEPSTDYHFQQTGQSEALEEENDETSRRKAGHQFGVTWRAKNNAERIFSLMPEKNEHSYCT

MIRGMVKHRAYEQALNLYTELLNNRLHADVYTFNALIEATVCAINEKFEEKWSKILELLRHMVAQKVKPNLQTFNTILKCLRRFHVF
ARSPALQVLREMKAIGIEPSLATYHHIIRLFDQPGDPLKRSSFIIYDIMNELMGKRFSPKDPDDDKFFQSAMSICSSLRDLELAYQV
HGLLKTGDNWKFIGPDQHRNFYYSKFFDLICLMEQIDVTLKWYEDLIPSAYFPHSQTMIHLLQALDVANRLEVIPKIWKDSKEYGHT
FRSDLREEILMLMARDKHPPELQVAFADCAADIKSAYESQPIRQTAQDWPATSLNCIAILFLRAGRTQEAWKMLGLFRKHNKIPRSE
LLNELMDSAKVSNSPSQAIEVVELASAFSLPICEGLTQRVMSDFAINQEQKEALSNLTALTSDSDTDSSSDSDSDTSEGK
>sp|P06727|APOA4_HUMAN
MFLKAVVLTLALVAVAGARAEVSADQVATVMWDYFSQLSNNAKEAVEHLQKSELTQQLNALFQDKLGEVNTYAGDLQKKLVPFATEL
HERLAKDSEKLKEEIGKELEELRARLLPHANEVSQKIGDNLRELQQRLEPYADQLRTQVNTQAEQLRRQLTPYAQRMERVLRENADS
LQASLRPHADELKAKIDQNVEELKGRLTPYADEFKVKIDQTVEELRRSLAPYAQDTQEKLNHQLEGLTFQMKKNAEELKARISASAE
ELRQRLAPLAEDVRGNLRGNTEGLQKSLAELGGHLDQQVEEFRRRVEPYGENFNKALVQQMEQLRQKLGPHAGDVEGHLSFLEKDLR
DKVNSFFSTFKEKESQDKTLSLPELEQQQEQQQEQQQEQVQMLAPLES
>sp|P49754|VPS41_HUMAN
MAEAEEQETGSLEESTDESEEEESEEEPKLKYERLSNGVTEILQKDAASCMTVHDKFLALGTHYGKVYLLDVQGNITQKFDVSPVKI
NQISLDESGEHMGVCSEDGKVQVFGLYSGEEFHETFDCPIKIIAVHPHFVRSSCKQFVTGGKKLLLFERSWMNRWKSAVLHEGEGNI
RSVKWRGHLIAWANNMGVKIFDIISKQRITNVPRDDISLRPDMYPCSLCWKDNVTLIIGWGTSVKVCSVKERHASEMRDLPSRYVEI
VSQFETEFYISGLAPLCDQLVVLSYVKEISEKTEREYCARPRLDIIQPLSETCEEISSDALTVRGFQENECRDYHLEYSEGESLFYI
VSPRDVVVAKERDQDDHIDWLLEKKKYEEALMAAEISQKNIKRHKILDIGLAYINHLVERGDYDIAARKCQKILGKNAALWEYEVYK
FKEIGQLKAISPYLPRGDPVLKPLIYEMILHEFLESDYEGFATLIREWPGDLYNNSVIVQAVRDHLKKDSQNKTLLKTLAELYTYDK
NYGNALEIYLTLRHKDVFQLIHKHNLFSSIKDKIVLLMDFDSEKAVDMLLDNEDKISIKKVVEELEDRPELQHVYLHKLFKRDHHKG
QRYHEKQISLYAEYDRPNLLPFLRDSTHCPLEKALEICQQRNFVEETVYLLSRMGNSRSALKMIMEELHDVDKAIEFAKEQDDGELW
EDLILYSIDKPPFITGLLNNIGTHVDPILLIHRIKEGMEIPNLRDSLVKILQDYNLQILLREGCKKILVADSLSLLKKMHRTQMKGV
LVDEENICESCLSPILPSDAAKPFSVVVFHCRHMFHKECLPMPSMNSAAQFCNICSAKNRGPGSAILEMKK
>sp|Q8WXH2|JPH3_HUMAN
MSSGGRFNFDDGGSYCGGWEDGKAHGHGVCTGPKGQGEYTGSWSHGFEVLGVYTWPSGNTYQGTWAQGKRHGIGLESKGKWVYKGEW
THGFKGRYGVRECAGNGAKYEGTWSNGLQDGYGTETYSDGGTYQGQWVGGMRQGYGVRQSVPYGMAAVIRSPLRTSINSLRSEHTNG
TALHPDASPAVAGSPAVSRGGFVLVAHSDSEILKSKKKGLFRRSLLSGLKLRKSESKSSLASQRSKQSSFRSEAGMSTVSSTASDIH
STISLGEAEAELAVIEDDIDATTTETYVGEWKNDKRSGFGVSQRSDGLKYEGEWASNRRHGYGCMTFPDGTKEEGKYKQNILVGGKR
KNLIPLRASKIREKVDRAVEAAERAATIAKQKAEIAASRTSHSRAKAEAALTAAQKAQEEARIARITAKEFSPSFQHRENGLEYQRP
KRQTSCDDIEVLSTGTPLQQESPELYRKGTTPSDLTPDDSPLQSFPTSPAATPPPAPAARNKVAHFSRQVSVDEERGGDIQMLLEGR
AGDCARSSWGEEQAGGSRGVRSGALRGGLLVDDFRTRGSGRKQPGNPKPRERRTESPPVFTWTSHHRASNHSPGGSRLLELQEEKLS
NYRMEMKPLLRMETHPQKRRYSKGGACRGLGDDHRPEDRGFGVQRLRSKAQNKENFRPASSAEPAVQKLASLRLGGAEPRLLRWDLT
FSPPQKSLPVALESDEENGDELKSSTGSAPILVVMVILLNIGVAILFINFFI
>sp|Q9NUY8|TBC23_HUMAN
MAEGEDVPPLPTSSGDGWEKDLEEALEAGGCDLETLRNIIQGRPLPADLRAKVWKIALNVAGKGDSLASWDGILDLPEQNTIHKDCL
QFIDQLSVPEEKAAELLLDIESVITFYCKSRNIKYSTSLSWIHLLKPLVHLQLPRSDLYNCFYAIMNKYIPRDCSQKGRPFHLFRLL
IQYHEPELCSYLDTKKITPDSYALNWLGSLFACYCSTEVTQAIWDGYLQQADPFFIYFLMLIILVNAKEVILTQESDSKEEVIKFLE
NTPSSLNIEDIEDLFSLAQYYCSKTPASFRKDNHHLFGSTLLGIKDDDADLSQALCLAISVSEILQANQLQGEGVRFFVVDCRPAEQ
YNAGHLSTAFHLDSDLMLQNPSEFAQSVKSLLEAQKQSIESGSIAGGEHLCFMGSGREEEDMYMNMVLAHFLQKNKEYVSIASGGFM
ALQQHLADINVDGPENGYGHWIASTSGSRSSINSVDGESPNGSSDRGMKSLVNKMTVALKTKSVNVREKVISFIENTSTPVDRMSFN
LPWPDRSCTERHVSSSDRVGKPYRGVKPVFSIGDEEEYDTDEIDSSSMSDDDRKEVVNIQTWINKPDVKHHFPCKEVKESGHMFPSH
LLVTATHMYCLREIVSRKGLAYIQSRQALNSVVKITSKKKHPELITFKYGNSSASGIEILAIERYLIPNAGDATKAIKQQIMKVLDA
LES
>sp|P56703|WNT3_HUMAN

MEPHLLGLLLGLLLGGTRVLAGYPIWWSLALGQQYTSLGSQPLLCGSIPGLVPKQLRFCRNYIEIMPSVAEGVKLGIQECQHQFRGR
RWNCTTIDDSLAIFGPVLDKATRESAFVHAIASAGVAFAVTRSCAEGTSTICGCDSHHKGPPGEGWKWGGCSEDADFGVLVSREFAD
ARENRPDARSAMNKHNNEAGRTTILDHMHLKCKCHGLSGSCEVKTCWWAQPDFRAIGDFLKDKYDSASEMVVEKHRESRGWVETLRA
KYSLFKPPTERDLVYYENSPNFCEPNPETGSFGTRDRTCNVTSHGIDGCDLLCCGRGHNTRTEKRKEKCHCIFHWCCYVSCQECIRI
YDVHTCK
>sp|P69905|HBA_HUMAN
MVLSPADKTNVKAAWGKVGAHAGEYGAEALERMFLSFPTTKTYFPHFDLSHGSAQVKGHGKKVADALTNAVAHVDDMPNALSALSDL
HAHKLRVDPVNFKLLSHCLLVTLAAHLPAEFTPAVHASLDKFLASVSTVLTSKYR
>sp|P40855|PEX19_HUMAN
MAAAEEGCSVGAEADRELEELLESALDDFDKAKPSPAPPSTTTAPDASGPQKRSPGDTAKDALFASQEKFFQELFDSELASQATAEF
EKAMKELAEEEPHLVEQFQKLSEAAGRVGSDMTSQQEFTSCLKETLSGLAKNATDLQNSSMSEEELTKAMEGLGMDEGDGEGNILPI
MQSIMQNLLSKDVLYPSLKEITEKYPEWLQSHRESLPPEQFEKYQEQHSVMCKICEQFEAETPTDSETTQKARFEMVLDLMQQLQDL
GHPPKELAGEMPPGLNFDLDALNLSGPPGASGEQCLIM
>sp|Q5RGS3|F74A1_HUMAN
MWRELRGCPGGDVETAQRLSQRRRGKSSEAVPEKTWRAQRMSQRRRGESSEAVPEKTWKELRNSETVPEKTWKQLRRCLQEDVERVQ
RLSLLLHLAVFLWIIIAINFSNSGVKSQSSTYLPSGKILK
>sp|Q92576|PHF3_HUMAN
MDIVDTFNHLIPTEHLDDALFLGSNLENEVCEDFSASQNVLEDSLKNMLSDKDPMLGSASNQFCLPVLDSNDPNFQMPCSTVVGLDD
IMDEGVVKESGNDTIDEEELILPNRNLRDKVEENSVRSPRKSPRLMAQEQVRSLRQSTIAKRSNAAPLSNTKKASGKTVSTAKAGVK
QPERSQVKEEVCMSLKPEYHKENRRCSRNSGQIEVVPEVSVSSSHSSVSSCLEMKDEDGLDSKHKCNNPGEIDVPSHELNCSLLSET
CVTIGEKKNEALMECKAKPVGSPLFKFSDKEEHEQNDSISGKTGETVVEEMIATRKVEQDSKETVKLSHEDDHILEDAGSSDISSDA
ACTNPNKTENSLVGLPSCVDEVTECNLELKDTMGIADKTENTLERNKIEPLGYCEDAESNRQLESTEFNKSNLEVVDTSTFGPESNI
LENAICDVPDQNSKQLNAIESTKIESHETANLQDDRNSQSSSVSYLESKSVKSKHTKPVIHSKQNMTTDAPKKIVAAKYEVIHSKTK
VNVKSVKRNTDVPESQQNFHRPVKVRKKQIDKEPKIQSCNSGVKSVKNQAHSVLKKTLQDQTLVQIFKPLTHSLSDKSHAHPGCLKE
PHHPAQTGHVSHSSQKQCHKPQQQAPAMKTNSHVKEELEHPGVEHFKEEDKLKLKKPEKNLQPRQRRSSKSFSLDEPPLFIPDNIAT
IRREGSDHSSSFESKYMWTPSKQCGFCKKPHGNRFMVGCGRCDDWFHGDCVGLSLSQAQQMGEEDKEYVCVKCCAEEDKKTEILDPD
TLENQATVEFHSGDKTMECEKLGLSKHTTNDRTKYIDDTVKHKVKILKRESGEGRNSSDCRDNEIKKWQLAPLRKMGQPVLPRRSSE
EKSEKIPKESTTVTCTGEKASKPGTHEKQEMKKKKVEKGVLNVHPAASASKPSADQIRQSVRHSLKDILMKRLTDSNLKVPEEKAAK
VATKIEKELFSFFRDTDAKYKNKYRSLMFNLKDPKNNILFKKVLKGEVTPDHLIRMSPEELASKELAAWRRRENRHTIEMIEKEQRE
VERRPITKITHKGEIEIESDAPMKEQEAAMEIQEPAANKSLEKPEGSEKQKEEVDSMSKDTTSQHRQHLFDLNCKICIGRMAPPVDD
LSPKKVKVVVGVARKHSDNEAESIADALSSTSNILASEFFEEEKQESPKSTFSPAPRPEMPGTVEVESTFLARLNFIWKGFINMPSV
AKFVTKAYPVSGSPEYLTEDLPDSIQVGGRISPQTVWDYVEKIKASGTKEICVVRFTPVTEEDQISYTLLFAYFSSRKRYGVAANNM
KQVKDMYLIPLGATDKIPHPLVPFDGPGLELHRPNLLLGLIIRQKLKRQHSACASTSHIAETPESAPPIALPPDKKSKIEVSTEEAP
EEENDFFNSFTTVLHKQRNKPQQNLQEDLPTAVEPLMEVTKQEPPKPLRFLPGVLIGWENQPTTLELANKPLPVDDILQSLLGTTGQ
VYDQAQSVMEQNTVKEIPFLNEQTNSKIEKTDNVEVTDGENKEIKVKVDNISESTDKSAEIETSVVGSSSISAGSLTSLSLRGKPPD
VSTEAFLTNLSIQSKQEETVESKEKTLKRQLQEDQENNLQDNQTSNSSPCRSNVGKGNIDGNVSCSENLVANTARSPQFINLKRDPR
QAAGRSQPVTTSESKDGDSCRNGEKHMLPGLSHNKEHLTEQINVEEKLCSAEKNSCVQQSDNLKVAQNSPSVENIQTSQAEQAKPLQ
EDILMQNIETVHPFRRGSAVATSHFEVGNTCPSEFPSKSITFTSRSTSPRTSTNFSPMRPQQPNLQHLKSSPPGFPFPGPPNFPPQS
MFGFPPHLPPPLLPPPGFGFAQNPMVPWPPVVHLPGQPQRMMGPLSQASRYIGPQNFYQVKDIRRPERRHSDPWGRQDQQQLDRPFN
RGKGDRQRFYSDSHHLKRERHEKEWEQESERHRRRDRSQDKDRDRKSREEGHKDKERARLSHGDRGTDGKASRDSRNVDKKPDKPKS
EDYEKDKEREKSKHREGEKDRDRYHKDRDHTDRTKSKR
>sp|Q07065|CKAP4_HUMAN
MPSAKQRGSKGGHGAASPSEKGAHPSGGADDVAKKPPPAPQQPPPPPAPHPQQHPQQHPQNQAHGKGGHRGGGGGGGKSSSSSSASA
AAAAAAASSSASCSRRLGRALNFLFYLALVAAAAFSGWCVHHVLEEVQQVRRSHQDFSRQREELGQGLQGVEQKVQSLQATFGTFES

ILRSSQHKQDLTEKAVKQGESEVSRISEVLQKLQNEILKDLSDGIHVVKDARERDFTSLENTVEERLTELTKSINDNIAIFTEVQKR
SQKEINDMKAKVASLEESEGNKQDLKALKEAVKEIQTSAKSREWDMEALRSTLQTMESDIYTEVRELVSLKQEQQAFKEAADTERLA
LQALTEKLLRSEESVSRLPEEIRRLEEELRQLKSDSHGPKEDGGFRHSEAFEALQQKSQGLDSRLQHVEDGVLSMQVASARQTESLE
SLLSKSQEHEQRLAALQGRLEGLGSSEADQDGLASTVRSLGETQLVLYGDVEELKRSVGELPSTVESLQKVQEQVHTLLSQDQAQAA
RLPPQDFLDRLSSLDNLKASVSQVEADLKMLRTAVDSLVAYSVKIETNENNLESAKGLLDDLRNDLDRLFVKVEKIHEKV
>sp|P53396|ACLY_HUMAN
MSAKAISEQTGKELLYKFICTTSAIQNRFKYARVTPDTDWARLLQDHPWLLSQNLVVKPDQLIKRRGKLGLVGVNLTLDGVKSWLKP
RLGQEATVGKATGFLKNFLIEPFVPHSQAEEFYVCIYATREGDYVLFHHEGGVDVGDVDAKAQKLLVGVDEKLNPEDIKKHLLVHAP
EDKKEILASFISGLFNFYEDLYFTYLEINPLVVTKDGVYVLDLAAKVDATADYICKVKWGDIEFPPPFGREAYPEEAYIADLDAKSG
ASLKLTLLNPKGRIWTMVAGGGASVVYSDTICDLGGVNELANYGEYSGAPSEQQTYDYAKTILSLMTREKHPDGKILIIGGSIANFT
NVAATFKGIVRAIRDYQGPLKEHEVTIFVRRGGPNYQEGLRVMGEVGKTTGIPIHVFGTETHMTAIVGMALGHRPIPNQPPTAAHTA
NFLLNASGSTSTPAPSRTASFSESRADEVAPAKKAKPAMPQDSVPSPRSLQGKSTTLFSRHTKAIVWGMQTRAVQGMLDFDYVCSRD
EPSVAAMVYPFTGDHKQKFYWGHKEILIPVFKNMADAMRKHPEVDVLINFASLRSAYDSTMETMNYAQIRTIAIIAEGIPEALTRKL
IKKADQKGVTIIGPATVGGIKPGCFKIGNTGGMLDNILASKLYRPGSVAYVSRSGGMSNELNNIISRTTDGVYEGVAIGGDRYPGST
FMDHVLRYQDTPGVKMIVVLGEIGGTEEYKICRGIKEGRLTKPIVCWCIGTCATMFSSEVQFGHAGACANQASETAVAKNQALKEAG
VFVPRSFDELGEIIQSVYEDLVANGVIVPAQEVPPPTVPMDYSWARELGLIRKPASFMTSICDERGQELIYAGMPITEVFKEEMGIG
GVLGLLWFQKRLPKYSCQFIEMCLMVTADHGPAVSGAHNTIICARAGKDLVSSLTSGLLTIGDRFGGALDAAAKMFSKAFDSGIIPM
EFVNKMKKEGKLIMGIGHRVKSINNPDMRVQILKDYVRQHFPATPLLDYALEVEKITTSKKPNLILNVDGLIGVAFVDMLRNCGSFT
REEADEYIDIGALNGIFVLGRSMGFIGHYLDQKRLKQGLYRHPWDDISYVLPEHMSM
>sp|O95816|BAG2_HUMAN
MAQAKINAKANEGRFCRSSSMADRSSRLLESLDQLELRVEALREAATAVEQEKEILLEMIHSIQNSQDMRQISDGEREELNLTANRL
MGRTLTVEVSVETIRNPQQQESLKHATRIIDEVVNKFLDDLGNAKSHLMSLYSACSSEVPHGPVDQKFQSIVIGCALEDQKKIKRRL
ETLLRNIENSDKAIKLLEHSKGAGSKTLQQNAESRFN
>sp|Q9UGU5|HMGX4_HUMAN
MAYDDSVKKEDCFDGDHTFEDIGLAAGRSQREKKRSYKDFLREEEEIAAQVRNSSKKKLKDSELYFLGTDTHKKKRKHSSDDYYYGD
ISSLESSQKKKKKSSPQSTDTAMDLLKAITSPLAAGSKPSKKTGEKSSGSSSHSESKKEHHRKKVSGSSGELPLEDGGSHKSKKMKP
LYVNTETLTLREPDGLKMKLILSPKEKGSSSVDEESFQYPSQQATVKKSSKKSARDEQGALLLGHELQSFLKTARKKHKSSSDAHSS
PGPEGCGSDASQFAESHSANLDLSGLEPILVESDSSSGGELEAGELVIDDSYREIKKKKKSKKSKKKKDKEKHKEKRHSKSKRSLGL
SAVPVGEVTVTSGPPPSIPYAGAAAPPLPLPGLHTDGHSEKKKKKEEKDKERERGEKPKKKNMSAYQVFCKEYRVTIVADHPGIDFG
ELSKKLAEVWKQLPEKDKLIWKQKAQYLQHKQNKAEATTVKRKASSSEGSMKVKASSVGVLSPQKKSPPTTMLLPASPAKAPETEPI
DVAAHLQLLGESLSLIGHRLQETEGMVAVSGSLSVLLDSIICALGPLACLTTQLPELNGCPKQVLSNTLDNIAYIMPGL
>sp|Q15691|MARE1_HUMAN
MAVNVYSTSVTSDNLSRHDMLAWINESLQLNLTKIEQLCSGAAYCQFMDMLFPGSIALKKVKFQAKLEHEYIQNFKILQAGFKRMGV
DKIIPVDKLVKGKFQDNFEFVQWFKKFFDANYDGKDYDPVAARQGQETAVAPSLVAPALNKPKKPLTSSSAAPQRPISTQRTAAAPK
AGPGVVRKNPGVGNGDDEAAELMQQVNVLKLTVEDLEKERDFYFGKLRNIELICQENEGENDPVLQRIVDILYATDEGFVIPDEGGP
QEEQEEY
>sp|Q9H340|O51B6_HUMAN
MGLNKSASTFQLTGFPGMEKAHHWIFIPLLAAYISILLGNGTLLFLIRNDHNLHEPMYYFLAMLAATDLGVTLTTMPTVLGVLWLDH
REIGHGACFSQAYFIHTLSVMESGVLLAMAYDCFITIRSPLRYTSILTNTQVMKIGVRVLTRAGLSIMPIVVRLHWFPYCRSHVLSH
AFCLHQDVIKLACADITFNRLYPVVVLFAMVLLDFLIIFFSYILILKTVMGIGSGGERAKALNTCVSHICCILVFYVTVVCLTFIHR
FGKHVPHVVHITMSYIHFLFPPFMNPFIYSIKTKQIQSGILRLFSLPHSRA
>sp|P54793|ARSF_HUMAN
MRPRRPLVFMSLVCALLNTCQAHRVHDDKPNIVLIMVDDLGIGDLGCYGNDTMRTPHIDRLAREGVRLTQHISAASLCSPSRSAFLT
GRYPIRSGMVSSGNRRVIQNLAVPAGLPLNETTLAALLKKQGYSTGLIGKWHQGLNCDSRSDQCHHPYNYGFDYYYGMPFTLVDSCW

PDPSRNTELAFESQLWLCVQLVAIAILTLTFGKLSGWVSVPWLLIFSMILFIFLLGYAWFSSHTSPLYWDCLLMRGHEITEQPMKAE
RAGSIMVKEAISFLERHSKETFLLFFSFLHVHTPLPTTDDFTGTSKHGLYGDNVEEMDSMVGKILDAIDDFGLRNNTLVYFTSDHGG
HLEARRGHAQLGGWNGIYKGGKGMGGWEGGIRVPGIVRWPGKVPAGRLIKEPTSLMDILPTVASVSGGSLPQDRVIDGRDLMPLLQG
NVRHSEHEFLFHYCGSYLHAVRWIPKDDSGSVWKAHYVTPVFQPPASGGCYVTSLCRCFGEQVTYHNPPLLFDLSRDPSESTPLTPA
TEPLHDFVIKKVANALKEHQETIVPVTYQLSELNQGRTWLKPCCGVFPFCLCDKEEEVSQPRGPNEKR
>sp|Q6UXB1|IGFL3_HUMAN
MRPRCCILALVCWITVFLLQCSKGTTDAPVGSGLWLCQPTPRCGNKIYNPSEQCCYDDAILSLKETRRCGSTCTFWPCFELCCPESF
GPQQKFLVKLRVLGMKSQCHLSPISRSCTRNRRHVLYP
>sp|P49759|CLK1_HUMAN
MRHSKRTYCPDWDDKDWDYGKWRSSSSHKRRKRSHSSAQENKRCKYNHSKMCDSHYLESRSINEKDYHSRRYIDEYRNDYTQGCEPG
HRQRDHESRYQNHSSKSSGRSGRSSYKSKHRIHHSTSHRRSHGKSHRRKRTRSVEDDEEGHLICQSGDVLSARYEIVDTLGEGAFGK
VVECIDHKAGGRHVAVKIVKNVDRYCEAARSEIQVLEHLNTTDPNSTFRCVQMLEWFEHHGHICIVFELLGLSTYDFIKENGFLPFR
LDHIRKMAYQICKSVNFLHSNKLTHTDLKPENILFVQSDYTEAYNPKIKRDERTLINPDIKVVDFGSATYDDEHHSTLVSTRHYRAP
EVILALGWSQPCDVWSIGCILIEYYLGFTVFPTHDSKEHLAMMERILGPLPKHMIQKTRKRKYFHHDRLDWDEHSSAGRYVSRRCKP
LKEFMLSQDVEHERLFDLIQKMLEYDPAKRITLREALKHPFFDLLKKSI
>sp|H3BPM6|MKROS_HUMAN
MHCAEAGKALIKFNHCEKYIYSFSVPQCCPLCQQDLGSRKLEDAPVSIANPFTNGHQEKCSFLLRPTQGTFLREYDGRSDLHVGITN
TNGVVYNYSAHGVQRDGEGWEESISIPLLQPNMYGMMEQWDKYLEDFSTSGAWLPHRYEDNHHNCYSYALTFINCVLMAEGRQQLDK
GEFTEKYVVPRTRLASKFITLYRAIREHGFYVTDCPQQQAQPPEGGGLC
>sp|Q14517|FAT1_HUMAN
MGRHLALLLLLLLLFQHFGDSDGSQRLEQTPLQFTHLEYNVTVQENSAAKTYVGHPVKMGVYITHPAWEVRYKIVSGDSENLFKAEE
YILGDFCFLRIRTKGGNTAILNREVKDHYTLIVKALEKNTNVEARTKVRVQVLDTNDLRPLFSPTSYSVSLPENTAIRTSIARVSAT
DADIGTNGEFYYSFKDRTDMFAIHPTSGVIVLTGRLDYLETKLYEMEILAADRGMKLYGSSGISSMAKLTVHIEQANECAPVITAVT
LSPSELDRDPAYAIVTVDDCDQGANGDIASLSIVAGDLLQQFRTVRSFPGSKEYKVKAIGGIDWDSHPFGYNLTLQAKDKGTPPQFS
SVKVIHVTSPQFKAGPVKFEKDVYRAEISEFAPPNTPVVMVKAIPAYSHLRYVFKSTPGKAKFSLNYNTGLISILEPVKRQQAAHFE
LEVTTSDRKASTKVLVKVLGANSNPPEFTQTAYKAAFDENVPIGTTVMSLSAVDPDEGENGYVTYSIANLNHVPFAIDHFTGAVSTS
ENLDYELMPRVYTLRIRASDWGLPYRREVEVLATITLNNLNDNTPLFEKINCEGTIPRDLGVGEQITTVSAIDADELQLVQYQIEAG
NELDFFSLNPNSGVLSLKRSLMDGLGAKVSFHSLRITATDGENFATPLYINITVAASHKLVNLQCEETGVAKMLAEKLLQANKLHNQ
GEVEDIFFDSHSVNAHIPQFRSTLPTGIQVKENQPVGSSVIFMNSTDLDTGFNGKLVYAVSGGNEDSCFMIDMETGMLKILSPLDRE
TTDKYTLNITVYDLGIPQKAAWRLLHVVVVDANDNPPEFLQESYFVEVSEDKEVHSEIIQVEATDKDLGPNGHVTYSIVTDTDTFSI
DSVTGVVNIARPLDRELQHEHSLKIEARDQAREEPQLFSTVVVKVSLEDVNDNPPTFIPPNYRVKVREDLPEGTVIMWLEAHDPDLG
QSGQVRYSLLDHGEGNFDVDKLSGAVRIVQQLDFEKKQVYNLTVRAKDKGKPVSLSSTCYVEVEVVDVNENLHPPVFSSFVEKGTVK
EDAPVGSLVMTVSAHDEDARRDGEIRYSIRDGSGVGVFKIGEETGVIETSDRLDRESTSHYWLTVFATDQGVVPLSSFIEIYIEVED
VNDNAPQTSEPVYYPEIMENSPKDVSVVQIEAFDPDSSSNDKLMYKITSGNPQGFFSIHPKTGLITTTSRKLDREQQDEHILEVTVT
DNGSPPKSTIARVIVKILDENDNKPQFLQKFYKIRLPEREKPDRERNARREPLYHVIATDKDEGPNAEISYSIEDGNEHGKFFIEPK
TGVVSSKRFSAAGEYDILSIKAVDNGRPQKSSTTRLHIEWISKPKPSLEPISFEESFFTFTVMESDPVAHMIGVISVEPPGIPLWFD
ITGGNYDSHFDVDKGTGTIIVAKPLDAEQKSNYNLTVEATDGTTTILTQVFIKVIDTNDHRPQFSTSKYEVVIPEDTAPETEILQIS
AVDQDEKNKLIYTLQSSRDPLSLKKFRLDPATGSLYTSEKLDHEAVHQHTLTVMVRDQDVPVKRNFARIVVNVSDTNDHAPWFTASS
YKGRVYESAAVGSVVLQVTALDKDKGKNAEVLYSIESGNIGNSFMIDPVLGSIKTAKELDRSNQAEYDLMVKATDKGSPPMSEITSV
RIFVTIADNASPKFTSKEYSVELSETVSIGSFVGMVTAHSQSSVVYEIKDGNTGDAFDINPHSGTIITQKALDFETLPIYTLIIQGT
NMAGLSTNTTVLVHLQDENDNAPVFMQAEYTGLISESASINSVVLTDRNVPLVIRAADADKDSNALLVYHIVEPSVHTYFAIDSSTG
AIHTVLSLDYEETSIFHFTVQVHDMGTPRLFAEYAANVTVHVIDINDCPPVFAKPLYEASLLLPTYKGVKVITVNATDADSSAFSQL
IYSITEGNIGEKFSMDYKTGALTVQNTTQLRSRYELTVRASDGRFAGLTSVKINVKESKESHLKFTQDVYSAVVKENSTEAETLAVI
TAIGNPINEPLFYHILNPDRRFKISRTSGVLSTTGTPFDREQQEAFDVVVEVTEEHKPSAVAHVVVKVIVEDQNDNAPVFVNLPYYA

VVKVDTEVGHVIRYVTAVDRDSGRNGEVHYYLKEHHEHFQIGPLGEISLKKQFELDTLNKEYLVTVVAKDGGNPAFSAEVIVPITVM
NKAMPVFEKPFYSAEIAESIQVHSPVVHVQANSPEGLKVFYSITDGDPFSQFTINFNTGVINVIAPLDFEAHPAYKLSIRATDSLTG
AHAEVFVDIIVDDINDNPPVFAQQSYAVTLSEASVIGTSVVQVRATDSDSEPNRGISYQMFGNHSKSHDHFHVDSSTGLISLLRTLD
YEQSRQHTIFVRAVDGGMPTLSSDVIVTVDVTDLNDNPPLFEQQIYEARISEHAPHGHFVTCVKAYDADSSDIDKLQYSILSGNDHK
HFVIDSATGIITLSNLHRHALKPFYSLNLSVSDGVFRSSTQVHVTVIGGNLHSPAFLQNEYEVELAENAPLHTLVMEVKTTDGDSGI
YGHVTYHIVNDFAKDRFYINERGQIFTLEKLDRETPAEKVISVRLMAKDAGGKVAFCTVNVILTDDNDNAPQFRATKYEVNIGSSAA
KGTSVVKVLASDADEGSNADITYAIEADSESVKENLEINKLSGVITTKESLIGLENEFFTFFVRAVDNGSPSKESVVLVYVKILPPE
MQLPKFSEPFYTFTVSEDVPIGTEIDLIRAEHSGTVLYSLVKGNTPESNRDESFVIDRQSGRLKLEKSLDHETTKWYQFSILARCTQ
DDHEMVASVDVSIQVKDANDNSPVFESSPYEAFIVENLPGGSRVIQIRASDADSGTNGQVMYSLDQSQSVEVIESFAINMETGWITT
LKELDHEKRDNYQIKVVASDHGEKIQLSSTAIVDVTVTDVNDSPPRFTAEIYKGTVSEDDPQGGVIAILSTTDADSEEINRQVTYFI
TGGDPLGQFAVETIQNEWKVYVKKPLDREKRDNYLLTITATDGTFSSKAIVEVKVLDANDNSPVCEKTLYSDTIPEDVLPGKLIMQI
SATDADIRSNAEITYTLLGSGAEKFKLNPDTGELKTSTPLDREEQAVYHLLVRATDGGGRFCQASIVLTLEDVNDNAPEFSADPYAI
TVFENTEPGTLLTRVQATDADAGLNRKILYSLIDSADGQFSINELSGIIQLEKPLDRELQAVYTLSLKAVDQGLPRRLTATGTVIVS
VLDINDNPPVFEYREYGATVSEDILVGTEVLQVYAASRDIEANAEITYSIISGNEHGKFSIDSKTGAVFIIENLDYESSHEYYLTVE
ATDGGTPSLSDVATVNVNVTDINDNTPVFSQDTYTTVISEDAVLEQSVITVMADDADGPSNSHIHYSIIDGNQGSSFTIDPVRGEVK
VTKLLDRETISGYTLTVQASDNGSPPRVNTTTVNIDVSDVNDNAPVFSRGNYSVIIQENKPVGFSVLQLVVTDEDSSHNGPPFFFTI
VTGNDEKAFEVNPQGVLLTSSAIKRKEKDHYLLQVKVADNGKPQLSSLTYIDIRVIEESIYPPAILPLEIFITSSGEEYSGGVIGKI
HATDQDVYDTLTYSLDPQMDNLFSVSSTGGKLIAHKKLDIGQYLLNVSVTDGKFTTVADITVHIRQVTQEMLNHTIAIRFANLTPEE
FVGDYWRNFQRALRNILGVRRNDIQIVSLQSSEPHPHLDVLLFVEKPGSAQISTKQLLHKINSSVTDIEEIIGVRILNVFQKLCAGL
DCPWKFCDEKVSVDESVMSTHSTARLSFVTPRHHRAAVCLCKEGRCPPVHHGCEDDPCPEGSECVSDPWEEKHTCVCPSGRFGQCPG
SSSMTLTGNSYVKYRLTENENKLEMKLTMRLRTYSTHAVVMYARGTDYSILEIHHGRLQYKFDCGSGPGIVSVQSIQVNDGQWHAVA
LEVNGNYARLVLDQVHTASGTAPGTLKTLNLDNYVFFGGHIRQQGTRHGRSPQVGNGFRGCMDSIYLNGQELPLNSKPRSYAHIEES
VDVSPGCFLTATEDCASNPCQNGGVCNPSPAGGYYCKCSALYIGTHCEISVNPCSSKPCLYGGTCVVDNGGFVCQCRGLYTGQRCQL
SPYCKDEPCKNGGTCFDSLDGAVCQCDSGFRGERCQSDIDECSGNPCLHGALCENTHGSYHCNCSHEYRGRHCEDAAPNQYVSTPWN
IGLAEGIGIVVFVAGIFLLVVVFVLCRKMISRKKKHQAEPKDKHLGPATAFLQRPYFDSKLNKNIYSDIPPQVPVRPISYTPSIPSD
SRNNLDRNSFEGSAIPEHPEFSTFNPESVHGHRKAVAVCSVAPNLPPPPPSNSPSDSDSIQKPSWDFDYDTKVVDLDPCLSKKPLEE
KPSQPYSARESLSEVQSLSSFQSESCDDNGYHWDTSDWMPSVPLPDIQEFPNYEVIDEQTPLYSADPNAIDTDYYPGGYDIESDFPP
PPEDFPAADELPPLPPEFSNQFESIHPPRDMPAAGSLGSSSRNRQRFNLNQYLPNFYPLDMSEPQTKGTGENSTCREPHAPYPPGYQ
RHFEAPAVESMPMSVYASTASCSDVSACCEVESEVMMSDYESGDDGHFEEVTIPPLDSQQHTEV
>sp|P22607|FGFR3_HUMAN
MGAPACALALCVAVAIVAGASSESLGTEQRVVGRAAEVPGPEPGQQEQLVFGSGDAVELSCPPPGGGPMGPTVWVKDGTGLVPSERV
LVGPQRLQVLNASHEDSGAYSCRQRLTQRVLCHFSVRVTDAPSSGDDEDGEDEAEDTGVDTGAPYWTRPERMDKKLLAVPAANTVRF
RCPAAGNPTPSISWLKNGREFRGEHRIGGIKLRHQQWSLVMESVVPSDRGNYTCVVENKFGSIRQTYTLDVLERSPHRPILQAGLPA
NQTAVLGSDVEFHCKVYSDAQPHIQWLKHVEVNGSKVGPDGTPYVTVLKTAGANTTDKELEVLSLHNVTFEDAGEYTCLAGNSIGFS
HHSAWLVVLPAEEELVEADEAGSVYAGILSYGVGFFLFILVVAAVTLCRLRSPPKKGLGSPTVHKISRFPLKRQVSLESNASMSSNT
PLVRIARLSSGEGPTLANVSELELPADPKWELSRARLTLGKPLGEGCFGQVVMAEAIGIDKDRAAKPVTVAVKMLKDDATDKDLSDL
VSEMEMMKMIGKHKNIINLLGACTQGGPLYVLVEYAAKGNLREFLRARRPPGLDYSFDTCKPPEEQLTFKDLVSCAYQVARGMEYLA
SQKCIHRDLAARNVLVTEDNVMKIADFGLARDVHNLDYYKKTTNGRLPVKWMAPEALFDRVYTHQSDVWSFGVLLWEIFTLGGSPYP
GIPVEELFKLLKEGHRMDKPANCTHDLYMIMRECWHAAPSQRPTFKQLVEDLDRVLTVTSTDEYLDLSAPFEQYSPGGQDTPSSSSS
GDDSVFAHDLLPPAPPSSGGSRT
>sp|O75690|KRA58_HUMAN
MGCCGCSGGCGSGCGGCGSGCGGCGSSCCVPICCCKPVCCCVPACSCSSCGSCGGSKGGRGSCGGSKGDCGSCGGSKGGCGSCGCSQ
CSCYKPCCCSSGCGSSCCQSSCCKPCCSQSSCCKPCSCSSGCGSSCCQSSCCKPCCSQSSCCKPCCCSSGCGSSCCQSSCCKPCCSQ
SSCCVPICCQCKI

>sp|P13726|TF_HUMAN
METPAWPRVPRPETAVARTLLLGWVFAQVAGASGTTNTVAAYNLTWKSTNFKTILEWEPKPVNQVYTVQISTKSGDWKSKCFYTTDT
ECDLTDEIVKDVKQTYLARVFSYPAGNVESTGSAGEPLYENSPEFTPYLETNLGQPTIQSFEQVGTKVNVTVEDERTLVRRNNTFLS
LRDVFGKDLIYTLYYWKSSSSGKKTAKTNTNEFLIDVDKGENYCFSVQAVIPSRTVNRKSTDSPVECMGQEKGEFREIFYIIGAVVF
VVIILVIILAISLHKCRKAGVGQSWKENSPLNVS
>sp|Q7Z5L9|I2BP2_HUMAN
MAAAVAVAAASRRQSCYLCDLPRMPWAMIWDFTEPVCRGCVNYEGADRVEFVIETARQLKRAHGCFPEGRSPPGAAASAAAKPPPLS
AKDILLQQQQQLGHGGPEAAPRAPQALERYPLAAAAERPPRLGSDFGSSRPAASLAQPPTPQPPPVNGILVPNGFSKLEEPPELNRQ
SPNPRRGHAVPPTLVPLMNGSATPLPTALGLGGRAAASLAAVSGTAAASLGSAQPTDLGAHKRPASVSSSAAVEHEQREAAAKEKQP
PPPAHRGPADSLSTAAGAAELSAEGAGKSRGSGEQDWVNRPKTVRDTLLALHQHGHSGPFESKFKKEPALTAGRLLGFEANGANGSK
AVARTARKRKPSPEPEGEVGPPKINGEAQPWLSTSTEGLKIPMTPTSSFVSPPPPTASPHSNRTTPPEAAQNGQSPMAALILVADNA
GGSHASKDANQVHSTTRRNSNSPPSPSSMNQRRLGPREVGGQGAGNTGGLEPVHPASLPDSSLATSAPLCCTLCHERLEDTHFVQCP
SVPSHKFCFPCSRQSIKQQGASGEVYCPSGEKCPLVGSNVPWAFMQGEIATILAGDVKVKKERDS
>sp|P19105|ML12A_HUMAN
MSSKRTKTKTKKRPQRATSNVFAMFDQSQIQEFKEAFNMIDQNRDGFIDKEDLHDMLASLGKNPTDEYLDAMMNEAPGPINFTMFLT
MFGEKLNGTDPEDVIRNAFACFDEEATGTIQEDYLRELLTTMGDRFTDEEVDELYREAPIDKKGNFNYIEFTRILKHGAKDKDD
>sp|Q7RTZ2|U17L1_HUMAN
MGDDSLYLGGEWQFNHFSKLTSSRPDAAFAEIQRTSLPEKSPLSSETRVDLCDDLAPVARQLAPREKLPLSSRRPAAVGAGLQNMGN
TCYENASLQCLTYTLPLANYMLSREHSQTCQRPKCCMLCTMQAHITWALHSPGHVIQPSQALAAGFHRGKQEDVHEFLMFTVDAMKK
ACLPGHKQVDHHCKDTTLIHQIFGGCWRSQIKCLHCHGISDTFDPYLDIALDIQAAQSVKQALEQLVKPEELNGENAYHCGLCLQRA
PASNTLTLHTSAKVLILVLKRFSDVAGNKLAKNVQYPECLDMQPYMSQQNTGPLVYVLYAVLVHAGWSCHDGHYFSYVKAQEVQWYK
MDDAEVTVCSIISVLSQQAYVLFYIQKSEWERHSESVSRGREPRALGAEDTDRRAKQGELKRDHPCLQAPELDEHLVERATQESTLD
HWKFLQEQNKTKPEFNVGKVEGTLPPNALVIHQSKYKCGMKNHHPEQQSSLLNLSSTTRTDQESMNTGTLASLQGRTRRAKGKNKHS
KRALLVCQ
>sp|Q9BWV7|TTLL2_HUMAN
MRGRDLCSSTQSQALGSLRTTTPAFTLNIPSEANHTEQPPAGLGARLQEAGVSIPPRRGRPTPTLEKKKKPHLMAEDEPSGALLKPL
VFRVDETTPAVVQSVLLERGWNKFDKQEQNAEDWNLYWRTSSFRMTEHNSVKPWQQLNHHPGTTKLTRKDCLAKHLKHMRRMYGTSL
YQFIPLTFVMPNDYTKFVAEYFQERQMLGTKHSYWICKPAELSRGRGILIFSDFKDFIFDDMYIVQKYISNPLLIGRYKCDLRIYVC
VTGFKPLTIYVYQEGLVRFATEKFDLSNLQNNYAHLTNSSINKSGASYEKIKEVIGHGCKWTLSRFFSYLRSWDVDDLLLWKKIHRM
VILTILAIAPSVPFAANCFELFGFDILIDDNLKPWLLEVNYSPALTLDCSTDVLVKRKLVHDIIDLIYLNGLRNEGREASNATHGNS
NIDAAKSDRGGLDAPDCLPYDSLSFTSRMYNEDDSVVEKAVSVRPEAAPASQLEGEMSGQDFHLSTREMPQSKPKLRSRHTPHKTLM
PYASLFQSHSCKTKTSPCVLSDRGKAPDPQAGNFVLVFPFNEATLGASRNGLNVKRIIQELQKLMNKQHS
>sp|O60508|PRP17_HUMAN
MSAAIAALAASYGSGSGSESDSDSESSRCPLPAADSLMHLTKSPSSKPSLAVAVDSAPEVAVKEDLETGVHLDPAVKEVQYNPTYET
MFAPEFGPENPFRTQQMAAPRNMLSGYAEPAHINDFMFEQQRRTFATYGYALDPSLDNHQVSAKYIGSVEEAEKNQGLTVFETGQKK
TEKRKKFKENDASNIDGFLGPWAKYVDEKDVAKPSEEEQKELDEITAKRQKKGKQEEEKPGEEKTILHVKEMYDYQGRSYLHIPQDV
GVNLRSTMPPEKCYLPKKQIHVWSGHTKGVSAVRLFPLSGHLLLSCSMDCKIKLWEVYGERRCLRTFIGHSKAVRDICFNTAGTQFL
SAAYDRYLKLWDTETGQCISRFTNRKVPYCVKFNPDEDKQNLFVAGMSDKKIVQWDIRSGEIVQEYDRHLGAVNTIVFVDENRRFVS
TSDDKSLRVWEWDIPVDFKYIAEPSMHSMPAVTLSPNGKWLACQSMDNQILIFGAQNRFRLNKKKIFKGHMVAGYACQVDFSPDMSY
VISGDGNGKLNIWDWKTTKLYSRFKAHDKVCIGAVWHPHETSKVITCGWDGLIKLWD
>sp|Q96P31|FCRL3_HUMAN
MLLWLLLLILTPGREQSGVAPKAVLLLNPPWSTAFKGEKVALICSSISHSLAQGDTYWYHDEKLLKIKHDKIQITEPGNYQCKTRGS
SLSDAVHVEFSPDWLILQALHPVFEGDNVILRCQGKDNKNTHQKVYYKDGKQLPNSYNLEKITVNSVSRDNSKYHCTAYRKFYILDI
EVTSKPLNIQVQELFLHPVLRASSSTPIEGSPMTLTCETQLSPQRPDVQLQFSLFRDSQTLGLGWSRSPRLQIPAMWTEDSGSYWCE

VETVTHSIKKRSLRSQIRVQRVPVSNVNLEIRPTGGQLIEGENMVLICSVAQGSGTVTFSWHKEGRVRSLGRKTQRSLLAELHVLTV
KESDAGRYYCAADNVHSPILSTWIRVTVRIPVSHPVLTFRAPRAHTVVGDLLELHCESLRGSPPILYRFYHEDVTLGNSSAPSGGGA
SFNLSLTAEHSGNYSCDADNGLGAQHSHGVSLRVTVPVSRPVLTLRAPGAQAVVGDLLELHCESLRGSFPILYWFYHEDDTLGNISA
HSGGGASFNLSLTTEHSGNYSCEADNGLGAQHSKVVTLNVTGTSRNRTGLTAAGITGLVLSILVLAAAAALLHYARARRKPGGLSAT
GTSSHSPSECQEPSSSRPSRIDPQEPTHSKPLAPMELEPMYSNVNPGDSNPIYSQIWSIQHTKENSANCPMMHQEHEELTVLYSELK
KTHPDDSAGEASSRGRAHEEDDEENYENVPRVLLASDH
>sp|Q9NXZ1|SAGE1_HUMAN
MQASPLQTSQPTPPEELHAAAYVFTNDGQQMRSDEVNLVATGHQSKKKHSRKSKRHSSSKRRKSMSSWLDKQEDAAVTHSICEERIN
NGQPVADNVLSTAPPWPDATIAHNIREERMENGQSRTDKVLSTAPPQLVHMAAAGIPSMSTRDLHSTVTHNIREERMENGQPQPDNV
LSTGPTGLINMAATPIPAMSARDLYATVTHNVCEQKMENVQPAPDNVLLTLRPRRINMTDTGISPMSTRDPYATITYNVPEEKMEKG
QPQPDNILSTASTGLINVAGAGTPAISTNGLYSTVPHNVCEEKMENDQPQPNNVLSTVQPVIIYLTATGIPGMNTRDQYATITHNVC
EERVVNNQPLPSNALSTVLPGLAYLATADMPAMSTRDQHATIIHNLREEKKDNSQPTPDNVLSAVTPELINLAGAGIPPMSTRDQYA
TVNHHVHEARMENGQRKQDNVLSNVLSGLINMAGASIPAMSSRDLYATITHSVREEKMESGKPQTDKVISNDAPQLGHMAAGGIPSM
STKDLYATVTQNVHEERMENNQPQPSYDLSTVLPGLTYLTVAGIPAMSTRDQYATVTHNVHEEKIKNGQAASDNVFSTVPPAFINMA
ATGVSSMSTRDQYAAVTHNIREEKINNSQPAPGNILSTAPPWLRHMAAAGISSTITRDLYVTATHSVHEEKMTNGQQAPDNSLSTVP
PGCINLSGAGISCRSTRDLYATVIHDIQEEEMENDQTPPDGFLSNSDSPELINMTGHCMPPNALDSFSHDFTSLSKDELLYKPDSNE
FAVGTKNYSVSAGDPPVTVMSLVETVPNTPQISPAMAKKINDDIKYQLMKEVRRFGQNYERIFILLEEVQGSMKVKRQFVEFTIKEA
ARFKKVVLIQQLEKALKEIDSHCHLRKVKHMRKR
>sp|Q9BVR0|HRC23_HUMAN
MHAFCVGQYLEPDQEGVTIPDLGSLSSPLIDTERNLGLLLGLHASYLAMSTPLSPVEIECAKWLQSSIFSGGLQTSQIHYSYNEEKD
EDHCSSPGGTPASKSRLCSHRRALGDHSQAFLQAIADNNIQDHNVKDFLCQIERYCRQCHLTTPIMFPPEHPVEEVGRLLLCCLLKH
EDLGHVALSLVHAGALGIEQVKHRTLPKSVVDVCRVVYQAKCSLIKTHQEQGRSYKEVCAPVIERLRFLFNELRPAVCNDLSIMSKF
KLLSSLPHWRRIAQKIIREPRKKRVPKKPESTDDEEKIGNEESDLEEACILPHSPINVDKRPIAIKSPKDKWQPLLSTVTGVHKYKW
LKQNVQGLYPQSPLLSTIAEFALKEEPVDVEKRKCLLKQLERAEVRLEGIDTILKLYLVSKNFLLPSVPYAMFCGWQRLIPEGIDIG
EPLTDCLKDVDLIPPFNRMLLEVTFGKLYAWAVQNIRNVLVDASAKFKELGIQPVPLQTITNENPSGPSLGTIPQAHFLLVMLSMLT
LQHSANNLDLLLNSGTLALAQTALRLIGPSCDSVEEDMNASAQGASATVLEETRKETAPVQLPVSGPELAAMMKIGTRVMRGVDWKW
GDQDRPPPGLGRVIGELGEDGWIRVQWDTGSTNSYRMGKEGNYDLKLAELPAPAQPSAEDSDTEDDSEAEQTERNIHPTAMMFTSTI
NLLQTLCLSAGVHAEIMQSEATKTLCGLLQMLVYREQHRSWCTLGFVQSIALTLQVCGTLSSLQWITLLMKVVEGHAPFTATSLQRQ
ILAVHLLQAVLPSWDKTERARDMKCLMEKLFDFLGSLLTMCSSDVPLLRESTLRRRRVCPQASLTATHSSTLAEEVVALLHTLHSLT
QWNGLINKYINSQLRSITHSFAGRPSKGAQLEDYFPDSENPEVGGLMAVLAVVGGIDGRLCLGGQVVHDDFGEVTMTRITLKGKITV
QFSDMRTCHVCPLNQLKPLPAVAFNVNNLPFTEPMLSVWAQLVNLAGSKLEKHKIKKSTKQAFAGQVDLDLLRCQQLKLYILKAGRA
LFSHQDKLRQILSQPAVQETGTVHTDDGAVVSPDLGDMSPEGPQPPMILLQQLLASATQPSPVKAIFDKQELEERMSRCCFWRRRTT
KLEQILLFIRRMNSVCEKENTNATASN
>sp|Q8TBA6|GOGA5_HUMAN
MSWFVDLAGKAEDLLNRVDQGAATALSRKDNASNIYSKNTDYTELHQQNTDLIYQTGPKSTYISSAADNIRNQKATILAGTANVKVG
SRTPVEASHPVENASVPRPSSHFVRRKKSEPDDELLFDFLNSSQKEPTGRVEIRKEKGKTPVFQSSQTSSVSSVNPSVTTIKTIEEN
SFGSQTHEAASNSDSSHEGQEESSKENVSSNAACPDHTPTPNDDGKSHELSNLRLENQLLRNEVQSLNQEMASLLQRSKETQEELNK
ARARVEKWNADHSKSDRMTRGLRAQVDDLTEAVAAKDSQLAVLKVRLQEADQLLSTRTEALEALQSEKSRIMQDQSEGNSLQNQALQ
TFQERLHEADATLKREQESYKQMQSEFAARLNKVEMERQNLAEAITLAERKYSDEKKRVDELQQQVKLYKLNLESSKQELIDYKQKA
TRILQSKEKLINSLKEGSGFEGLDSSTASSMELEELRHEKEMQREEIQKLMGQIHQLRSELQDMEAQQVNEAESAREQLQDLHDQIA
GQKASKQELETELERLKQEFHYIEEDLYRTKNTLQSRIKDRDEEIQKLRNQLTNKTLSNSSQSELENRLHQLTETLIQKQTMLESLS
TEKNSLVFQLERLEQQMNSASGSSSNGSSINMSGIDNGEGTRLRNVPVLFNDTETNLAGMYGKVRKAASSIDQFSIRLGIFLRRYPI
ARVFVIIYMALLHLWVMIVLLTYTPEMHHDQPYGK
>sp|Q9Y3A6|TMED5_HUMAN

MGDKIWLPFPVLLLAALPPVLLPGAAGFTPSLDSDFTFTLPAGQKECFYQPMPLKASLEIEYQVLDGAGLDIDFHLASPEGKTLVFE
QRKSDGVHTVETEVGDYMFCFDNTFSTISEKVIFFELILDNMGEQAQEQEDWKKYITGTDILDMKLEDILESINSIKSRLSKSGHIQ
TLLRAFEARDRNIQESNFDRVNFWSMVNLVVMVVVSAIQVYMLKSLFEDKRKSRT
>sp|O43396|TXNL1_HUMAN
MVGVKPVGSDPDFQPELSGAGSRLAVVKFTMRGCGPCLRIAPAFSSMSNKYPQAVFLEVDVHQCQGTAATNNISATPTFLFFRNKVR
IDQYQGADAVGLEEKIKQHLENDPGSNEDTDIPKGYMDLMPFINKAGCECLNESDEHGFDNCLRKDTTFLESDCDEQLLITVAFNQP
VKLYSMKFQGPDNGQGPKYVKIFINLPRSMDFEEAERSEPTQALELTEDDIKEDGIVPLRYVKFQNVNSVTIFVQSNQGEEETTRIS
YFTFIGTPVQATNMNDFKRVVGKKGESH
>sp|Q5T160|SYRM_HUMAN
MACGFRRAIACQLSRVLNLPPENLITSISAVPISQKEEVADFQLSVDSLLEKDNDHSRPDIQVQAKRLAEKLRCDTVVSEISTGQRT
VNFKINRELLTKTVLQQVIEDGSKYGLKSELFSGLPQKKIVVEFSSPNVAKKFHVGHLRSTIIGNFIANLKEALGHQVIRINYLGDW
GMQFGLLGTGFQLFGYEEKLQSNPLQHLFEVYVQVNKEAADDKSVAKAAQEFFQRLELGDVQALSLWQKFRDLSIEEYIRVYKRLGV
YFDEYSGESFYREKSQEVLKLLESKGLLLKTIKGTAVVDLSGNGDPSSICTVMRSDGTSLYATRDLAAAIDRMDKYNFDTMIYVTDK
GQKKHFQQVFQMLKIMGYDWAERCQHVPFGVVQGMKTRRGDVTFLEDVLNEIQLRMLQNMASIKTTKELKNPQETAERVGLAALIIQ
DFKGLLLSDYKFSWDRVFQSRGDTGVFLQYTHARLHSLEETFGCGYLNDFNTACLQEPQSVSILQHLLRFDEVLYKSSQDFQPRHIV
SYLLTLSHLAAVAHKTLQIKDSPPEVAGARLHLFKAVRSVLANGMKLLGITPVCRM
>sp|Q9BRL7|SC22C_HUMAN
MSVIFFACVVRVRDGLPLSASTDFYHTQDFLEWRRRLKSLALRLAQYPGRGSAEGCDFSIHFSSFGDVACMAICSCQCPAAMAFCFL
ETLWWEFTASYDTTCIGLASRPYAFLEFDSIIQKVKWHFNYVSSSQMECSLEKIQEELKLQPPAVLTLEDTDVANGVMNGHTPMHLE
PAPNFRMEPVTALGILSLILNIMCAALNLIRGVHLAEHSLQVAHEEIGNILAFLVPFVACIFQCYLYLFYSPARTMKVVLMLLFICL
GNMYLHGLRNLWQILFHIGVAFLSSYQILTRQLQEKQSDCGV
>sp|Q07001|ACHD_HUMAN
MEGPVLTLGLLAALAVCGSWGLNEEERLIRHLFQEKGYNKELRPVAHKEESVDVALALTLSNLISLKEVEETLTTNVWIEHGWTDNR
LKWNAEEFGNISVLRLPPDMVWLPEIVLENNNDGSFQISYSCNVLVYHYGFVYWLPPAIFRSSCPISVTYFPFDWQNCSLKFSSLKY
TAKEITLSLKQDAKENRTYPVEWIIIDPEGFTENGEWEIVHRPARVNVDPRAPLDSPSRQDITFYLIIRRKPLFYIINILVPCVLIS
FMVNLVFYLPADSGEKTSVAISVLLAQSVFLLLISKRLPATSMAIPLIGKFLLFGMVLVTMVVVICVIVLNIHFRTPSTHVLSEGVK
KLFLETLPELLHMSRPAEDGPSPGALVRRSSSLGYISKAEEYFLLKSRSDLMFEKQSERHGLARRLTTARRPPASSEQAQQELFNEL
KPAVDGANFIVNHMRDQNNYNEEKDSWNRVARTVDRLCLFVVTPVMVVGTAWIFLQGVYNQPPPQPFPGDPYSYNVQDKRFI
>sp|P55061|BI1_HUMAN
MNIFDRKINFDALLKFSHITPSTQQHLKKVYASFALCMFVAAAGAYVHMVTHFIQAGLLSALGSLILMIWLMATPHSHETEQKRLGL
LAGFAFLTGVGLGPALEFCIAVNPSILPTAFMGTAMIFTCFTLSALYARRRSYLFLGGILMSALSLLLLSSLGNVFFGSIWLFQANL
YVGLVVMCGFVLFDTQLIIEKAEHGDQDYIWHCIDLFLDFITVFRKLMMILAMNEKDKKKEKK
>sp|P63215|GBG3_HUMAN
MKGETPVNSTMSIGQARKMVEQLKIEASLCRIKVSKAAADLMTYCDAHACEDPLITPVPTSENPFREKKFFCALL
>sp|Q7Z6G3|NECA2_HUMAN
MCERAARLCRAGAHRLLREPPQQGRALGGLLRWVGARMGEPRESLAPAAPADPGPASPRGGTAVILDIFRRADKNDDGKLSLEEFQL
FFADGVLNEKELEDLFHTIDSDNTNHVDTKELCDYFVDHMGDYEDVLASLETLNHSVLKAMGYTKKVYEGGSNVDQFVTRFLLKETA
NQIQSLLSSVESAVEAIEEQTSQLRQNHIKPSHSAAQTWCGSPTPASAPNHKLMAMEQGKTLPSATEDAKEEGLEAQISRLAELIGR
LESKALWFDLQQRLSDEDGTNMHLQLVRQEMAVCPEQLSEFLDSLRQYLRGTTGVRNCFHITAVRLSDGFTFVIYEFWETEEAWKRH
LQSPLCKAFRHVKVDTLSQPEALSRILVPAAWCTVGRD
>sp|Q17RM4|CC142_HUMAN
MAQASRSGSLPPLVIVPPLRAQPGGTGEEQWERSRTGGLRWEVHCWPSGTSGGTPWWPTPADVSEDYEADAAAWRRGPAGGGPIPPA
LQRLRAVLLRLHREREQLLQARDCAYHLQSAVRLMKTLSPGSPSGGPSPLPQWCRDLQLHPSQGAVLRIGPGETLEPLLLARPIGLA
AQCLEAVIEMQLRALGREPASPGLSSQLAELLFALPAYHTLQRKALSHVPGAARPFPTSRVLRLLTGERGCQVASRLDEALQGSALR

DQLRRRCQEEGDLLPGLLGLVGGVAGSASCGLGLGGAGALWSQYWTLLWAACAQSLDLNLGPWRDPRATAQQLSQALGQASLPQECE
KELASLCHRLLHQSLIWSWDQGFCQALGSALGGQSSLPTSSGTAELLQQLFPPLLDALREPRLRRIFCQPADPAPVALGLCTLQTTL
LWFLGRAQQYLAAWDPASFLLLIQKDLPPLLHEAEALYSLASEESLALEVEQQLGLEIQKLTAQIQLLPEESLSVFSQECHKQAMQG
FKLYMPRGRYWRLRLCPEPPSAPSEYAGLVVRTVLEPVLQGLQGLPPQAQAPALGQALTAIVGAWLDHILTHGIRFSLQGALQLKQD
FGVVRELLEEEQWSLSPDLRQTLLMLSIFQQLDGALLCLLQQPLPKSQVHRRPPCCCACQEVQTTKLPSSCLNSLESLEPPLQPGTS
PAQTGQLQSTLGGRGPSPEGYLVGNQQAWLALRQHQRPRWHLPFFSCLGTSPES
>sp|Q9Y5R2|MMP24_HUMAN
MPRSRGGRAAPGPPPPPPPPGQAPRWSRWRVPGRLLLLLLPALCCLPGAARAAAAAAGAGNRAAVAVAVARADEAEAPFAGQNWLKS
YGYLLPYDSRASALHSAKALQSAVSTMQQFYGIPVTGVLDQTTIEWMKKPRCGVPDHPHLSRRRRNKRYALTGQKWRQKHITYSIHN
YTPKVGELDTRKAIRQAFDVWQKVTPLTFEEVPYHEIKSDRKEADIMIFFASGFHGDSSPFDGEGGFLAHAYFPGPGIGGDTHFDSD
EPWTLGNANHDGNDLFLVAVHELGHALGLEHSSDPSAIMAPFYQYMETHNFKLPQDDLQGIQKIYGPPAEPLEPTRPLPTLPVRRIH
SPSERKHERQPRPPRPPLGDRPSTPGTKPNICDGNFNTVALFRGEMFVFKDRWFWRLRNNRVQEGYPMQIEQFWKGLPARIDAAYER
ADGRFVFFKGDKYWVFKEVTVEPGYPHSLGELGSCLPREGIDTALRWEPVGKTYFFKGERYWRYSEERRATDPGYPKPITVWKGIPQ
APQGAFISKEGYYTYFYKGRDYWKFDNQKLSVEPGYPRNILRDWMGCNQKEVERRKERRLPQDDVDIMVTINDVPGSVNAVAVVIPC
ILSLCILVLVYTIFQFKNKTGPQPVTYYKRPVQEWV
>sp|Q9UNE7|CHIP_HUMAN
MKGKEEKEGGARLGAGGGSPEKSPSAQELKEQGNRLFVGRKYPEAAACYGRAITRNPLVAVYYTNRALCYLKMQQHEQALADCRRAL
ELDGQSVKAHFFLGQCQLEMESYDEAIANLQRAYSLAKEQRLNFGDDIPSALRIAKKKRWNSIEERRIHQESELHSYLSRLIAAERE
RELEECQRNHEGDEDDSHVRAQQACIEAKHDKYMADMDELFSQVDEKRKKRDIPDYLCGKISFELMREPCITPSGITYDRKDIEEHL
QRVGHFDPVTRSPLTQEQLIPNLAMKEVIDAFISENGWVEDY
>sp|P78552|I13R1_HUMAN
MEWPARLCGLWALLLCAGGGGGGGGAAPTETQPPVTNLSVSVENLCTVIWTWNPPEGASSNCSLWYFSHFGDKQDKKIAPETRRSIE
VPLNERICLQVGSQCSTNESEKPSILVEKCISPPEGDPESAVTELQCIWHNLSYMKCSWLPGRNTSPDTNYTLYYWHRSLEKIHQCE
NIFREGQYFGCSFDLTKVKDSSFEQHSVQIMVKDNAGKIKPSFNIVPLTSRVKPDPPHIKNLSFHNDDLYVQWENPQNFISRCLFYE
VEVNNSQTETHNVFYVQEAKCENPEFERNVENTSCFMVPGVLPDTLNTVRIRVKTNKLCYEDDKLWSNWSQEMSIGKKRNSTLYITM
LLIVPVIVAGAIIVLLLYLKRLKIIIFPPIPDPGKIFKEMFGDQNDDTLHWKKYDIYEKQTKEETDSVVLIENLKKASQ
>sp|Q9H920|RN121_HUMAN
MAAVVEVEVGGGAAGERELDEVDMSDLSPEEQWRVEHARMHAKHRGHEAMHAEMVLILIATLVVAQLLLVQWKQRHPRSYNMVTLFQ
MWVVPLYFTVKLHWWRFLVIWILFSAVTAFVTFRATRKPLVQTTPRLVYKWFLLIYKISYATGIVGYMAVMFTLFGLNLLFKIKPED
AMDFGISLLFYGLYYGVLERDFAEMCADYMASTIGFYSESGMPTKHLSDSVCAVCGQQIFVDVSEEGIIENTYRLSCNHVFHEFCIR
GWCIVGKKQTCPYCKEKVDLKRMFSNPWERPHVMYGQLLDWLRYLVAWQPVIIGVVQGINYILGLE
>sp|P52788|SPSY_HUMAN
MAAARHSTLDFMLGAKADGETILKGLQSIFQEQGMAESVHTWQDHGYLATYTNKNGSFANLRIYPHGLVLLDLQSYDGDAQGKEEID
SILNKVEERMKELSQDSTGRVKRLPPIVRGGAIDRYWPTADGRLVEYDIDEVVYDEDSPYQNIKILHSKQFGNILILSGDVNLAESD
LAYTRAIMGSGKEDYTGKDVLILGGGDGGILCEIVKLKPKMVTMVEIDQMVIDGCKKYMRKTCGDVLDNLKGDCYQVLIEDCIPVLK
RYAKEGREFDYVINDLTAVPISTSPEEDSTWEFLRLILDLSMKVLKQDGKYFTQGNCVNLTEALSLYEEQLGRLYCPVEFSKEIVCV
PSYLELWVFYTVWKKAKP
>sp|Q76LX8|ATS13_HUMAN
MHQRHPRARCPPLCVAGILACGFLLGCWGPSHFQQSCLQALEPQAVSSYLSPGAPLKGRPPSPGFQRQRQRQRRAAGGILHLELLVA
VGPDVFQAHQEDTERYVLTNLNIGAELLRDPSLGAQFRVHLVKMVILTEPEGAPNITANLTSSLLSVCGWSQTINPEDDTDPGHADL
VLYITRFDLELPDGNRQVRGVTQLGGACSPTWSCLITEDTGFDLGVTIAHEIGHSFGLEHDGAPGSGCGPSGHVMASDGAAPRAGLA
WSPCSRRQLLSLLSAGRARCVWDPPRPQPGSAGHPPDAQPGLYYSANEQCRVAFGPKAVACTFAREHLDMCQALSCHTDPLDQSSCS
RLLVPLLDGTECGVEKWCSKGRCRSLVELTPIAAVHGRWSSWGPRSPCSRSCGGGVVTRRRQCNNPRPAFGGRACVGADLQAEMCNT
QACEKTQLEFMSQQCARTDGQPLRSSPGGASFYHWGAAVPHSQGDALCRHMCRAIGESFIMKRGDSFLDGTRCMPSGPREDGTLSLC

VSGSCRTFGCDGRMDSQQVWDRCQVCGGDNSTCSPRKGSFTAGRAREYVTFLTVTPNLTSVYIANHRPLFTHLAVRIGGRYVVAGKM
SISPNTTYPSLLEDGRVEYRVALTEDRLPRLEEIRIWGPLQEDADIQVYRRYGEEYGNLTRPDITFTYFQPKPRQAWVWAAVRGPCS
VSCGAGLRWVNYSCLDQARKELVETVQCQGSQQPPAWPEACVLEPCPPYWAVGDFGPCSASCGGGLRERPVRCVEAQGSLLKTLPPA
RCRAGAQQPAVALETCNPQPCPARWEVSEPSSCTSAGGAGLALENETCVPGADGLEAPVTEGPGSVDEKLPAPEPCVGMSCPPGWGH
LDATSAGEKAPSPWGSIRTGAQAAHVWTPAAGSCSVSCGRGLMELRFLCMDSALRVPVQEELCGLASKPGSRREVCQAVPCPARWQY
KLAACSVSCGRGVVRRILYCARAHGEDDGEEILLDTQCQGLPRPEPQEACSLEPCPPRWKVMSLGPCSASCGLGTARRSVACVQLDQ
GQDVEVDEAACAALVRPEASVPCLIADCTYRWHVGTWMECSVSCGDGIQRRRDTCLGPQAQAPVPADFCQHLPKPVTVRGCWAGPCV
GQGTPSLVPHEEAAAPGRTTATPAGASLEWSQARGLLFSPAPQPRRLLPGPQENSVQSSACGRQHLEPTGTIDMRGPGQADCAVAIG
RPLGEVVTLRVLESSLNCSAGDMLLLWGRLTWRKMCRKLLDMTFSSKTNTLVVRQRCGRPGGGVLLRYGSQLAPETFYRECDMQLFG
PWGEIVSPSLSPATSNAGGCRLFINVAPHARIAIHALATNMGAGTEGANASYILIRDTHSLRTTAFHGQQVLYWESESSQAEMEFSE
GFLKAQASLRGQYWTLQSWVPEMQDPQSWKGKEGT
>sp|Q587J7|TDR12_HUMAN
MLQLLVLKIEDPGCFWVIIKGCSPFLDHDVDYQKLNSAMNDFYNSTCQDIEIKPLTLEEGQVCVVYCEELKCWCRAIVKSITSSADQ
YLAECFLVDFAKNIPVKSKNIRVVVESFMQLPYRAKKFSLYCTKPVTLHIDFCRDSTDIVPAKKWDNAAIQYFQNLLKATTQVEARL
CAVEEDTFEVYLYVTIKDEKVCVNDDLVAKNYACYMSPTKNKNLDYLEKPRLNIKSAPSFNKLNPALTLWPMFLQGKDVQGMEDSHG
VNFPAQSLQHTWCKGIVGDLRPTATAQDKAVKCNMDSLRDSPKDKSEKKHHCISLKDTNKRVESSVYWPAKRGITIYADPDVPEASA
LSQKSNEKPLRLTEKKEYDEKNSCVKLLQFLNPDPLRADGISDLQQLQKLKGLQPPVVVLRNKIKPCLTIDSSPLSADLKKALQRNK
FPGPSHTESYSWPPIARGCDVVVISHCESNPLLYLLPVLTVLQTGACYKSLPSRNGPLAVIVCPGWKKAQFIFELLGEYSMSSRPLH
PVLLTIGLHKEEAKNTKLPRGCDVIVTTPYSLLRLLACQSLLFLRLCHLILDEVEVLFLEANEQMFAILDNFKKNIEVEERESAPHQ
IVAVGVHWNKHIEHLIKEFMNDPYIVITAMEEAALYGNVQQVVHLCLECEKTSSLLQALDFIPSQAQKTLIFTCSVAETEIVCKVVE
SSSIFCLKMHKEMIFNLQNVLEQWKKKLSSGSQIILALTDDCVPLLAITDATCVIHFSFPASPKVFGGRLYCMSDHFHAEQGSPAEQ
GDKKAKSVLLLTEKDASHAVGVLRYLERADAKVPAELYEFTAGVLEAKEDKKAGRPLCPYLKAFGFCKDKRICPDRHRINPETDLPR
KLSSQALPSFGYIKIIPFYILNATNYFGRIVDKHMDLYATLNAEMNEYFKDSNKTTVEKVEKFGLYGLAEKTLFHRVQVLEVNQKED
AWALDDILVEFIDEGRTGLVTRDQLLHLPEHFHTLPPQAVEFIVCRVKPADNEIEWNPKVTRYIHHKIVGKLHDAKVILALGNTVWI
DPMVHITNLSSLKTSVIDYNVRAEILSMGMGIDNPEHIEQLKKLREDAKIPACEESLSQTPPRVTGTSPAQDQDHPSEEQGGQGTPP
AEDAACLQSPQPEDTGAEGGAESKTSSENQKPGGYLVFKRWLSSNR
>sp|Q86UR1|NOXA1_HUMAN
MASLGDLVRAWHLGAQAVDRGDWARALHLFSGVPAPPARLCFNAGCVHLLAGDPEAALRAFDQAVTKDTCMAVGFFQRGVANFQLAR
FQEALSDFWLALEQLRGHAAIDYTQLGLRFKLQAWEVLHNVASAQCQLGLWTEAASSLREAMSKWPEGSLNGLDSALDQVQRRGSLP
PRQVPRGEVFRPHRWHLKHLEPVDFLGKAKVVASAIPDDQGWGVRPQQPQGPGANHDARSLIMDSPRAGTHQGPLDAETEVGADRCT
STAYQEQRPQVEQVGKQAPLSPGLPAMGGPGPGPCEDPAGAGGAGAGGSEPLVTVTVQCAFTVALRARRGADLSSLRALLGQALPHQ
AQLGQLSYLAPGEDGHWVPIPEEESLQRAWQDAAACPRGLQLQCRGAGGRPVLYQVVAQHSYSAQGPEDLGFRQGDTVDVLCEVDQA
WLEGHCDGRIGIFPKCFVVPAGPRMSGAPGRLPRSQQGDQP
>sp|P31689|DNJA1_HUMAN
MVKETTYYDVLGVKPNATQEELKKAYRKLALKYHPDKNPNEGEKFKQISQAYEVLSDAKKRELYDKGGEQAIKEGGAGGGFGSPMDI
FDMFFGGGGRMQRERRGKNVVHQLSVTLEDLYNGATRKLALQKNVICDKCEGRGGKKGAVECCPNCRGTGMQIRIHQIGPGMVQQIQ
SVCMECQGHGERISPKDRCKSCNGRKIVREKKILEVHIDKGMKDGQKITFHGEGDQEPGLEPGDIIIVLDQKDHAVFTRRGEDLFMC
MDIQLVEALCGFQKPISTLDNRTIVITSHPGQIVKHGDIKCVLNEGMPIYRRPYEKGRLIIEFKVNFPENGFLSPDKLSLLEKLLPE
RKEVEETDEMDQVELVDFDPNQERRRHYNGEAYEDDEHHPRGGVQCQTS
>sp|P29401|TKT_HUMAN
MESYHKPDQQKLQALKDTANRLRISSIQATTAAGSGHPTSCCSAAEIMAVLFFHTMRYKSQDPRNPHNDRFVLSKGHAAPILYAVWA
EAGFLAEAELLNLRKISSDLDGHPVPKQAFTDVATGSLGQGLGAACGMAYTGKYFDKASYRVYCLLGDGELSEGSVWEAMAFASIYK
LDNLVAILDINRLGQSDPAPLQHQMDIYQKRCEAFGWHAIIVDGHSVEELCKAFGQAKHQPTAIIAKTFKGRGITGVEDKESWHGKP
LPKNMAEQIIQEIYSQIQSKKKILATPPQEDAPSVDIANIRMPSLPSYKVGDKIATRKAYGQALAKLGHASDRIIALDGDTKNSTFS

EIFKKEHPDRFIECYIAEQNMVSIAVGCATRNRTVPFCSTFAAFFTRAFDQIRMAAISESNINLCGSHCGVSIGEDGPSQMALEDLA
MFRSVPTSTVFYPSDGVATEKAVELAANTKGICFIRTSRPENAIIYNNNEDFQVGQAKVVLKSKDDQVTVIGAGVTLHEALAAAELL
KKEKINIRVLDPFTIKPLDRKLILDSARATKGRILTVEDHYYEGGIGEAVSSAVVGEPGITVTHLAVNRVPRSGKPAELLKMFGIDR
DAIAQAVRGLITKA
>sp|Q9NQ69|LHX9_HUMAN
MEIVGCRAEDNSCPFRPPAMLFHGISGGHIQGIMEEMERRSKTEARLAKGAQLNGRDAGMPPLSPEKPALCAGCGGKISDRYYLLAV
DKQWHLRCLKCCECKLALESELTCFAKDGSIYCKEDYYRRFSVQRCARCHLGISASEMVMRARDSVYHLSCFTCSTCNKTLTTGDHF
GMKDSLVYCRAHFETLLQGEYPPQLSYTELAAKSGGLALPYFNGTGTVQKGRPRKRKSPALGVDIVNYNSGCNENEADHLDRDQQPY
PPSQKTKRMRTSFKHHQLRTMKSYFAINHNPDAKDLKQLAQKTGLTKRVLQVWFQNARAKFRRNLLRQENGGVDKADGTSLPAPPSA
DSGALTPPGTATTLTDLTNPTITVVTSVTSNMDSHESGSPSQTTLTNLF
>sp|Q14697|GANAB_HUMAN
MAAVAAVAARRRRSWASLVLAFLGVCLGITLAVDRSNFKTCEESSFCKRQRSIRPGLSPYRALLDSLQLGPDSLTVHLIHEVTKVLL
VLELQGLQKNMTRFRIDELEPRRPRYRVPDVLVADPPIARLSVSGRDENSVELTMAEGPYKIILTARPFRLDLLEDRSLLLSVNARG
LLEFEHQRAPRVSQGSKDPAEGDGAQPEETPRDGDKPEETQGKAEKDEPGAWEETFKTHSDSKPYGPMSVGLDFSLPGMEHVYGIPE
HADNLRLKVTEGGEPYRLYNLDVFQYELYNPMALYGSVPVLLAHNPHRDLGIFWLNAAETWVDISSNTAGKTLFGKMMDYLQGSGET
PQTDVRWMSETGIIDVFLLLGPSISDVFRQYASLTGTQALPPLFSLGYHQSRWNYRDEADVLEVDQGFDDHNLPCDVIWLDIEHADG
KRYFTWDPSRFPQPRTMLERLASKRRKLVAIVDPHIKVDSGYRVHEELRNLGLYVKTRDGSDYEGWCWPGSAGYPDFTNPTMRAWWA
NMFSYDNYEGSAPNLFVWNDMNEPSVFNGPEVTMLKDAQHYGGWEHRDVHNIYGLYVHMATADGLRQRSGGMERPFVLARAFFAGSQ
RFGAVWTGDNTAEWDHLKISIPMCLSLGLVGLSFCGADVGGFFKNPEPELLVRWYQMGAYQPFFRAHAHLDTGRREPWLLPSQHNDI
IRDALGQRYSLLPFWYTLLYQAHREGIPVMRPLWVQYPQDVTTFNIDDQYLLGDALLVHPVSDSGAHGVQVYLPGQGEVWYDIQSYQ
KHHGPQTLYLPVTLSSIPVFQRGGTIVPRWMRVRRSSECMKDDPITLFVALSPQGTAQGELFLDDGHTFNYQTRQEFLLRRFSFSGN
TLVSSSADPEGHFETPIWIERVVIIGAGKPAAVVLQTKGSPESRLSFQHDPETSVLVLRKPGINVASDWSIHLR
>sp|Q9NZ45|CISD1_HUMAN
MSLTSSSSVRVEWIAAVTIAAGTAAIGYLAYKRFYVKDHRNKAMINLHIQKDNPKIVHAFDMEDLGDKAVYCRCWRSKKFPFCDGAH
TKHNEETGDNVGPLIIKKKET
>sp|Q9P2G4|MAP10_HUMAN
MAASLSERLFSLELLVDWVRLEARLLPSPAAAVEQEEEEEEKEQGEASSPRGLCPAVAFRLLDFPTLLVYPPDGPGAPAAEPWPGVI
RFGRGKSCLFRLQPATLHCRLLRTPLATLLLQLPPGRPTPTPQLLGACDISLATAAHRVVGPAASGCSHRHRGRFPLHNRVGERTGD
IALAYRLTDLGSRLLSQLERPLTFTRTGGGAEVSPQTQQERQQLQQPASQPSPKEADKPLGELEIPEAQKDLKEMVKSKAECDNVGS
VENGKTNSVVTCSGAGNGRNVSSLNEEVTELDMETNIFCPPPLYYTNLTQEKPPPAQAKITIEPQMNAPEEMDDASPEKKRVNPPAH
RSCLKHPSSAAHEHPPMLVNPPHIQNIGATNQTCQTEQNRINTIRQLPLLNALLVELSLLYDQPVTSPAHIHPHLAWLYRTEDKKSP
ESSAKSTCRSEAKKDKRSVGGCEKSVSLQYKKNQIENYKEDKYSEKSSGALHKRVPKGRLLYGLTNTLRLRLKLTNPDMLVVHEKRE
LYRKRQSQMLGTKFRIPSSKVKLLSSAEQSQKPQLPEDKYLDSDASFTENSDTSRQISGVFDEPSTSKETKLKYATEKKTVDCSKNR
INNVSLEEVVSPANSIIPERLTPTNILGGNVEMKIQSPCVFQQDAVVDRIVDKEIDIRQVKTTDNDILMADISDKRTGKNSCYENIS
ELKYSDDLSSPCYSEDFCTSEDTSRSFKAHDSSSRTENPKHSQYTSKSSDTGVSKKKNSSDRSSILSPPFSAGSPVHSYRKFHISKT
QDKSLEEASSISASDLSSTHWTEQKENQIDQNSMHNSEITKRAQDISVKTRSSWKSLEKSQSPQTSQVSSYLPSNVSELNVLDSSTS
DHFEEGNDDVGSLNISKQCKDICELVINKLPGYTM
>sp|C9JL84|HHLA1_HUMAN
MLGFLSRGPSMKLCMGLACVLSLWNTVSGIKGEAKKEKGMTFLPTTVSGLREEERKEKGVAFLATTELPARSIDLSALNLTELVNGM
LSRALKDSKKFFSLLSVTSYSSFAFHKFSVAVYNISNLKTVDPAKFPTRYCYCLNNRTNDLSDFTALLVDIIGNSTSYLTEIFKSTS
ILSVNQSNESDCIFICVMTGKSGRNLSDFWEIEEKYPIINYTFTSGLSGVLGAATRGTARTSKPTTKSQKTLPSTSPGHWTQSTPWA
SALRSSPWTETAAPSETEETLNTGRPPELPARATATWFSASHTLPALATRRVARTQWLTADRQTWASISSVPWAQTISEKKPGGSLW
ETRSSPPTTAGTEEAMNTTSLLAPAAEIMATPGSPSQASPTLGAFTHGTQTPSPTKATAPRYPQTGDLSAEWPFTAGEEPVLVPRPH

QVSRCPQPLFKVGAMAAAPLTLAIQRLNPCLMELCQFFQQCLCMSQRSPRTEDMRYCLEYYSWFLKNATYICQRVKRVSHSHTLKQK
CLENICKSV
>sp|Q3LI66|KRA62_HUMAN
MCGSYYGNYYGDHGYGCCGYEGLGYGYGSLRCGYSSCCGYGHGYGSRFFCGCGYGCGSGYYY
>sp|Q9NRR6|INP5E_HUMAN
MPSKAENLRPSEPAPQPPEGRTLQGQLPGAPPAQRAGSPPDAPGSESPALACSTPATPSGEDPPARAAPIAPRPPARPRLERALSLD
DKGWRRRRFRGSQEDLEARNGTSPSRGSVQSEGPGAPAHSCSPPCLSTSLQEIPKSRGVLSSERGSPSSGGNPLSGVASSSPNLPHR
DAAVAGSSPRLPSLLPPRPPPALSLDIASDSLRTANKVDSDLADYKLRAQPLLVRAHSSLGPGRPRSPLACDDCSLRSAKSSFSLLA
PIRSKDVRSRSYLEGSLLASGALLGADELARYFPDRNVALFVATWNMQGQKELPPSLDEFLLPAEADYAQDLYVIGVQEGCSDRREW
ETRLQETLGPHYVLLSSAAHGVLYMSLFIRRDLIWFCSEVECSTVTTRIVSQIKTKGALGISFTFFGTSFLFITSHFTSGDGKVAER
LLDYTRTVQALVLPRNVPDTNPYRSSAADVTTRFDEVFWFGDFNFRLSGGRTVVDALLCQGLVVDVPALLQHDQLIREMRKGSIFKG
FQEPDIHFLPSYKFDIGKDTYDSTSKQRTPSYTDRVLYRSRHKGDICPVSYSSCPGIKTSDHRPVYGLFRVKVRPGRDNIPLAAGKF
DRELYLLGIKRRISKEIQRQQALQSQNSSTICSVS
>sp|P60411|KR109_HUMAN
MAASTMSIRSSAYSDSWQVDDCPESCCEPPCCATSCCAPAPCLTLVCTPVSRVSSPCCQVTCEPSPCQSGCTSSCTPSCCQQSSCQP
AYCTSSPCQQACCVPVCCKPVCCVPVCCGASSCCQQSSYQPACCASSSCQPACCVPVCCKPVCCAPTCSEDSYSCCQQSSCQPACCT
SSPCQQSYCVPVCCKPVCCKPICCVPVCSGASSLCCQQSGCQPACCTTSCCRPSSSVSLLCRPVCRPACCVPVSSCCAPTSSRQPSY
CRQASCVSLLCRPVCSRPACYSFSSGQKSSC
>sp|Q7L0J3|SV2A_HUMAN
MEEGFRDRAAFIRGAKDIAKEVKKHAAKKVVKGLDRVQDEYSRRSYSRFEEEDDDDDFPAPSDGYYRGEGTQDEEEGGASSDATEGH
DEDDEIYEGEYQGIPRAESGGKGERMADGAPLAGVRGGLSDGEGPPGGRGEAQRRKEREELAQQYEAILRECGHGRFQWTLYFVLGL
ALMADGVEVFVVGFVLPSAEKDMCLSDSNKGMLGLIVYLGMMVGAFLWGGLADRLGRRQCLLISLSVNSVFAFFSSFVQGYGTFLFC
RLLSGVGIGGSIPIVFSYFSEFLAQEKRGEHLSWLCMFWMIGGVYAAAMAWAIIPHYGWSFQMGSAYQFHSWRVFVLVCAFPSVFAI
GALTTQPESPRFFLENGKHDEAWMVLKQVHDTNMRAKGHPERVFSVTHIKTIHQEDELIEIQSDTGTWYQRWGVRALSLGGQVWGNF
LSCFGPEYRRITLMMMGVWFTMSFSYYGLTVWFPDMIRHLQAVDYASRTKVFPGERVEHVTFNFTLENQIHRGGQYFNDKFIGLRLK
SVSFEDSLFEECYFEDVTSSNTFFRNCTFINTVFYNTDLFEYKFVNSRLINSTFLHNKEGCPLDVTGTGEGAYMVYFVSFLGTLAVL
PGNIVSALLMDKIGRLRMLAGSSVMSCVSCFFLSFGNSESAMIALLCLFGGVSIASWNALDVLTVELYPSDKRTTAFGFLNALCKLA
AVLGISIFTSFVGITKAAPILFASAALALGSSLALKLPETRGQVLQ
>sp|Q9C0C6|CIPC_HUMAN
MERKNPSRESPRRLSAKVGKGTEMKKVARQLGMAAAESDKDSGFSDGSSECLSSAEQMESEDMLSALGWSREDRPRQNSKTAKNAFP
TLSPMVVMKNVLVKQGSSSSQLQSWTVQPSFEVISAQPQLLFLHPPVPSPVSPCHTGEKKSDSRNYLPILNSYTKIAPHPGKRGLSL
GPEEKGTSGVQKKICTERLGPSLSSSEPTKAGAVPSSPSTPAPPSAKLAEDSALQGVPSLVAGGSPQTLQPVSSSHVAKAPSLTFAS
PASPVCASDSTLHGLESNSPLSPLSANYSSPLWAAEHLCRSPDIFSEQRQSKHRRFQNTLVVLHKSGLLEITLKTKELIRQNQATQV
ELDQLKEQTQLFIEATKSRAPQAWAKLQASLTPGSSNTGSDLEAFSDHPAI
>sp|Q5FYA8|ARSH_HUMAN
MTRNARPNIVLLMADDLGVGDLCCYGNNSVSTPNIDRLASEGVRLTQHLAAASMCTPSRAAFLTGRYPIRSGMVSAYNLNRAFTWLG
GSGGLPTNETTFAKLLQHRGYRTGLIGKWHLGLSCASRNDHCYHPLNHGFHYFYGVPFGLLSDCQASKTPELHRWLRIKLWISTVAL
ALVPFLLLIPKFARWFSVPWKVIFVFALLAFLFFTSWYSSYGFTRRWNCILMRNHEIIQQPMKEEKVASLMLKEALAFIERYKREPF
LLFFSFLHVHTPLISKKKFVGRSKYGRYGDNVEEMDWMVGKILDALDQERLANHTLVYFTSDNGGHLEPLDGAVQLGGWNGIYKGGK
GMGGWEGGIRVPGIFRWPSVLEAGRVINEPTSLMDIYPTLSYIGGGILSQDRVIDGQNLMPLLEGRASHSDHEFLFHYCGVYLHTVR
WHQKDCATVWKAHYVTPKFYPEGTGACYGSGICSCSGDVTYHDPPLLFDISRDPSEALPLNPDNEPLFDSVIKKMEAAIREHRRTLT
PVPQQFSVFNTIWKPWLQPCCGTFPFCGCDKEDDILPMAP
>sp|Q9UNA3|A4GCT_HUMAN

MRKELQLSLSVTLLLVCGFLYQFTLKSSCLFCLPSFKSHQGLEALLSHRRGIVFLETSERMEPPHLVSCSVESAAKIYPEWPVVFFM
KGLTDSTPMPSNSTYPAFSFLSAIDNVFLFPLDMKRLLEDTPLFSWYNQINASAERNWLHISSDASRLAIIWKYGGIYMDTDVISIR
PIPEENFLAAQASRYSSNGIFGFLPHHPFLWECMENFVEHYNSAIWGNQGPELMTRMLRVWCKLEDFQEVSDLRCLNISFLHPQRFY
PISYREWRRYYEVWDTEPSFNVSYALHLWNHMNQEGRAVIRGSNTLVENLYRKHCPRTYRDLIKGPEGSVTGELGPGNK
>sp|Q9BYE4|SPR2G_HUMAN
MSYQQQQCKQPCQPPPVCPTPKCPEPCPPPKCPEPYLPPPCPPEHCPPPPCQDKCPPVQPYPPCQQKYPPKSK
>sp|Q8N7C3|TRIMM_HUMAN
MVCGIQEAAENYRKLFQEILNTSREKLEAAKSILTDEQERMAMIQEEEQNFKKMIESEYSMRLRLLNEECEQNLQRQQECISDLNLR
ETLLNQAIKLATELEEMFQEMLQRLGRVGRENMEKLKESEARASEQVRSLLKLIVELEKKCGEGTLALLKNAKYSLERSKSLLLEHL
EPAHITDLSLCHIRGLSSMFRVLQRHLTLDPETAHPCLALSEDLRTMRLRHGQQDGAGNPERLDFSAMVLAAESFTSGRHYWEVDVE
KATRWQVGIYHGSADAKGSTARASGEKVLLTGSVMGTEWTLWVFPPLKRLFLEKKLDTVGVFLDCEHGQISFYNVTEMSLIYNFSHC
AFQGALRPVFSLCIPNGDTSPDSLTILQHGPSCDATVSP
>sp|P82930|RT34_HUMAN
MARKKVRPRLIAELARRVRALREQLNRPRDSQLYAVDYETLTRPFSGRRLPVRAWADVRRESRLLQLLGRLPLFGLGRLVTRKSWLW
QHDEPCYWRLTRVRPDYTAQNLDHGKAWGILTFKGKTESEAREIEHVMYHDWRLVPKHEEEAFTAFTPAPEDSLASVPYPPLLRAMI
IAERQKNGDTSTEEPMLNVQRIRMEPWDYPAKQEDKGRAKGTPV
>sp|Q9BZL6|KPCD2_HUMAN
MATAPSYPAGLPGSPGPGSPPPPGGLELQSPPPLLPQIPAPGSGVSFHIQIGLTREFVLLPAASELAHVKQLACSIVDQKFPECGFY
GLYDKILLFKHDPTSANLLQLVRSSGDIQEGDLVEVVLSASATFEDFQIRPHALTVHSYRAPAFCDHCGEMLFGLVRQGLKCDGCGL
NYHKRCAFSIPNNCSGARKRRLSSTSLASGHSVRLGTSESLPCTAEELSRSTTELLPRRPPSSSSSSSASSYTGRPIELDKMLLSKV
KVPHTFLIHSYTRPTVCQACKKLLKGLFRQGLQCKDCKFNCHKRCATRVPNDCLGEALINGDVPMEEATDFSEADKSALMDESEDSG
VIPGSHSENALHASEEEEGEGGKAQSSLGYIPLMRVVQSVRHTTRKSSTTLREGWVVHYSNKDTLRKRHYWRLDCKCITLFQNNTTN
RYYKEIPLSEILTVESAQNFSLVPPGTNPHCFEIVTANATYFVGEMPGGTPGGPSGQGAEAARGWETAIRQALMPVILQDAPSAPGH
APHRQASLSISVSNSQIQENVDIATVYQIFPDEVLGSGQFGVVYGGKHRKTGRDVAVKVIDKLRFPTKQESQLRNEVAILQSLRHPG
IVNLECMFETPEKVFVVMEKLHGDMLEMILSSEKGRLPERLTKFLITQILVALRHLHFKNIVHCDLKPENVLLASADPFPQVKLCDF
GFARIIGEKSFRRSVVGTPAYLAPEVLLNQGYNRSLDMWSVGVIMYVSLSGTFPFNEDEDINDQIQNAAFMYPASPWSHISAGAIDL
INNLLQVKMRKRYSVDKSLSHPWLQEYQTWLDLRELEGKMGERYITHESDDVRWEQFAAEHPLPGSGLPTDRDLGGACPPQDHDMQG
LAERISVL
>sp|A2RUC4|TYW5_HUMAN
MAGQHLPVPRLEGVSREQFMQHLYPQRKPLVLEGIDLGPCTSKWTVDYLSQVGGKKEVKIHVAAVAQMDFISKNFVYRTLPFDQLVQ
RAAEEKHKEFFVSEDEKYYLRSLGEDPRKDVADIRKQFPLLKGDIKFPEFFKEEQFFSSVFRISSPGLQLWTHYDVMDNLLIQVTGK
KRVVLFSPRDAQYLYLKGTKSEVLNIDNPDLAKYPLFSKARRYECSLEAGDVLFIPALWFHNVISEEFGVGVNIFWKHLPSECYDKT
DTYGNKDPTAASRAAQILDRALKTLAELPEEYRDFYARRMVLHIQDKAYSKNSE
>sp|P29972|AQP1_HUMAN
MASEFKKKLFWRAVVAEFLATTLFVFISIGSALGFKYPVGNNQTAVQDNVKVSLAFGLSIATLAQSVGHISGAHLNPAVTLGLLLSC
QISIFRALMYIIAQCVGAIVATAILSGITSSLTGNSLGRNDLADGVNSGQGLGIEIIGTLQLVLCVLATTDRRRRDLGGSAPLAIGL
SVALGHLLAIDYTGCGINPARSFGSAVITHNFSNHWIFWVGPFIGGALAVLIYDFILAPRSSDLTDRVKVWTSGQVEEYDLDADDIN
SRVEMKPK
>sp|Q92902|HPS1_HUMAN
MKCVLVATEGAEVLFYWTDQEFEESLRLKFGQSENEEEELPALEDQLSTLLAPVIISSMTMLEKLSDTYTCFSTENGNFLYVLHLFG
ECLFIAINGDHTESEGDLRRKLYVLKYLFEVHFGLVTVDGHLIRKELRPPDLAQRVQLWEHFQSLLWTYSRLREQEQCFAVEALERL
IHPQLCELCIEALERHVIQAVNTSPERGGEEALHAFLLVHSKLLAFYSSHSASSLRPADLLALILLVQDLYPSESTAEDDIQPSPRR
ARSSQNIPVQQAWSPHSTGPTGGSSAETETDSFSLPEEYFTPAPSPGDQSSGSTIWLEGGTPPMDALQIAEDTLQTLVPHCPVPSGP
RRIFLDANVKESYCPLVPHTMYCLPLWQGINLVLLTRSPSAPLALVLSQLMDGFSMLEKKLKEGPEPGASLRSQPLVGDLRQRMDKF

VKNRGAQEIQSTWLEFKAKAFSKSEPGSSWELLQACGKLKRQLCAIYRLNFLTTAPSRGGPHLPQHLQDQVQRLMREKLTDWKDFLL
VKSRRNITMVSYLEDFPGLVHFIYVDRTTGQMVAPSLNCSQKTSSELGKGPLAAFVKTKVWSLIQLARRYLQKGYTTLLFQEGDFYC
SYFLWFENDMGYKLQMIEVPVLSDDSVPIGMLGGDYYRKLLRYYSKNRPTEAVRCYELLALHLSVIPTDLLVQQAGQLARRLWEASR
IPLL
>sp|Q9Y6A1|POMT1_HUMAN
MWGFLKRPVVVTADINLSLVALTGMGLLSRLWRLTYPRAVVFDEVYYGQYISFYMKQIFFLDDSGPPFGHMVLALGGYLGGFDGNFL
WNRIGAEYSSNVPVWSLRLLPALAGALSVPMAYQIVLELHFSHCAAMGAALLMLIENALITQSRLMLLESVLIFFNLLAVLSYLKFF
NCQKHSPFSLSWWFWLTLTGVACSCAVGIKYMGVFTYVLVLGVAAVHAWHLLGDQTLSNVGADVQCCMRPACMGQMQMSQGVCVFCH
LLARAVALLVIPVVLYLLFFYVHLILVFRSGPHDQIMSSAFQASLEGGLARITQGQPLEVAFGSQVTLRNVFGKPVPCWLHSHQDTY
PMIYENGRGSSHQQQVTCYPFKDVNNWWIVKDPRRHQLVVSSPPRPVRHGDMVQLVHGMTTRSLNTHDVAAPLSPHSQEVSCYIDYN
ISMPAQNLWRLEIVNRGSDTDVWKTILSEVRFVHVNTSAVLKLSGAHLPDWGYRQLEIVGEKLSRGYHGSTVWNVEEHRYGASQEQR
ERERELHSPAQVDVSRNLSFMARFSELQWRMLALRSDDSEHKYSSSPLEWVTLDTNIAYWLHPRTSAQIHLLGNIVIWVSGSLALAI
YALLSLWYLLRRRRNVHDLPQDAWLRWVLAGALCAGGWAVNYLPFFLMEKTLFLYHYLPALTFQILLLPVVLQHISDHLCRSQLQRS
IFSALVVAWYSSACHVSNTLRPLTYGDKSLSPHELKALRWKDSWDILIRKH
>sp|Q86TA4|YB049_HUMAN
MLEGVSNEFDHFGISLPLKICLHLGWDEGLVEGKVVRLGQGIGKSICSSCQLFEEAPTQMSTVPSGLPLPILMHLCLLPVCMAHLCP
ASPCYFGATPGSGKFCRLITYSHSSPQLAASLRHRGREVGKDLPYPGLCPLTFHPSFFPPVEGCVSSLPGKLLSPQTIFFQILWLYS
KSSLVL
>sp|H3BQJ8|LY6L_HUMAN
MERLVLTLCTLPLAVASAGCATTPARNLSCYQCFKVSSWTECPPTWCSPLDQVCISNEVVVSFKWSVRVLLSKRCAPRCPNDNMKFE
WSPAPMVQGVITRRCCSWALCNRALTPQEGRWALRGGLLLQVGLSLLRALL
>sp|P67870|CSK2B_HUMAN
MSSSEEVSWISWFCGLRGNEFFCEVDEDYIQDKFNLTGLNEQVPHYRQALDMILDLEPDEELEDNPNQSDLIEQAAEMLYGLIHARY
ILTNRGIAQMLEKYQQGDFGYCPRVYCENQPMLPIGLSDIPGEAMVKLYCPKCMDVYTPKSSRHHHTDGAYFGTGFPHMLFMVHPEY
RPKRPANQFVPRLYGFKIHPMAYQLQLQAASNFKSPVKTIR
>sp|Q9UKX5|ITA11_HUMAN
MDLPRGLVVAWALSLWPGFTDTFNMDTRKPRVIPGSRTAFFGYTVQQHDISGNKWLVVGAPLETNGYQKTGDVYKCPVIHGNCTKLN
LGRVTLSNVSERKDNMRLGLSLATNPKDNSFLACSPLWSHECGSSYYTTGMCSRVNSNFRFSKTVAPALQRCQTYMDIVIVLDGSNS
IYPWVEVQHFLINILKKFYIGPGQIQVGVVQYGEDVVHEFHLNDYRSVKDVVEAASHIEQRGGTETRTAFGIEFARSEAFQKGGRKG
AKKVMIVITDGESHDSPDLEKVIQQSERDNVTRYAVAVLGYYNRRGINPETFLNEIKYIASDPDDKHFFNVTDEAALKDIVDALGDR
IFSLEGTNKNETSFGLEMSQTGFSSHVVEDGVLLGAVGAYDWNGAVLKETSAGKVIPLRESYLKEFPEELKNHGAYLGYTVTSVVSS
RQGRVYVAGAPRFNHTGKVILFTMHNNRSLTIHQAMRGQQIGSYFGSEITSVDIDGDGVTDVLLVGAPMYFNEGRERGKVYVYELRQ
NLFVYNGTLKDSHSYQNARFGSSIASVRDLNQDSYNDVVVGAPLEDNHAGAIYIFHGFRGSILKTPKQRITASELATGLQYFGCSIH
GQLDLNEDGLIDLAVGALGNAVILWSRPVVQINASLHFEPSKINIFHRDCKRSGRDATCLAAFLCFTPIFLAPHFQTTTVGIRYNAT
MDERRYTPRAHLDEGGDRFTNRAVLLSSGQELCERINFHVLDTADYVKPVTFSVEYSLEDPDHGPMLDDGWPTTLRVSVPFWNGCNE
DEHCVPDLVLDARSDLPTAMEYCQRVLRKPAQDCSAYTLSFDTTVFIIESTRQRVAVEATLENRGENAYSTVLNISQSANLQFASLI
QKEDSDGSIECVNEERRLQKQVCNVSYPFFRAKAKVAFRLDFEFSKSIFLHHLEIELAAGSDSNERDSTKEDNVAPLRFHLKYEADV
LFTRSSSLSHYEVKPNSSLERYDGIGPPFSCIFRIQNLGLFPIHGMMMKITIPIATRSGNRLLKLRDFLTDEANTSCNIWGNSTEYR
PTPVEEDLRRAPQLNHSNSDVVSINCNIRLVPNQEINFHLLGNLWLRSLKALKYKSMKIMVNAALQRQFHSPFIFREEDPSRQIVFE
ISKQEDWQVPIWIIVGSTLGGLLLLALLVLALWKLGFFRSARRRREPGLDPTPKVLE
>sp|Q8TD90|MAGE2_HUMAN
MSLVSQNARHCSAEITADYGDGRGEIQATNASGSPTSMLVVDAPQCPQAPINSQCVNTSQAVQDPNDLEVLIDEQSRRLGALRVHDP
LEDRSIALVNFMRMKSQTEGSIQQSEMLEFLREYSDQFPEILRRASAHLDQVFGLNLRVIDPQADTYNLVSKRGFQITDRIAESLDM
PKASLLALVLGHILLNGNRAREASIWDLLLKVDMWDKPQRINNLFGNTRNLLTTDFVCMRFLEYWPVYGTNPLEFEFLWGSRAHREI

TKMEALKFVSDAHDEEPWSWPEEYNKALEGDKTKERSLTAGLEFWSEDTMNDKANDLVQLAISVTEEMLPIHQDELLAHTGKEFEDV
FPNILNRATLILDMFYGLSLIEVDTSEHIYLLVQQPESEEEQVMLESLGRPTQEYVMPILGLIFLMGNRVKEANVWNLLRRFSVDVG
RKHSITRKLMRQRYLECRPLSYSNPVEYELLWGPRAHHETIKMKVLEYMARLYRKRPQNWPEQYREAVEDEEARAKSEATIMFFLDP
T
>sp|O15182|CETN3_HUMAN
MSLALRSELVVDKTKRKKRRELSEEQKQEIKDAFELFDTDKDEAIDYHELKVAMRALGFDVKKADVLKILKDYDREATGKITFEDFN
EVVTDWILERDPHEEILKAFKLFDDDDSGKISLRNLRRVARELGENMSDEELRAMIEEFDKDGDGEINQEEFIAIMTGDI
>sp|P51511|MMP15_HUMAN
MGSDPSAPGRPGWTGSLLGDREEAARPRLLPLLLVLLGCLGLGVAAEDAEVHAENWLRLYGYLPQPSRHMSTMRSAQILASALAEMQ
RFYGIPVTGVLDEETKEWMKRPRCGVPDQFGVRVKANLRRRRKRYALTGRKWNNHHLTFSIQNYTEKLGWYHSMEAVRRAFRVWEQA
TPLVFQEVPYEDIRLRRQKEADIMVLFASGFHGDSSPFDGTGGFLAHAYFPGPGLGGDTHFDADEPWTFSSTDLHGNNLFLVAVHEL
GHALGLEHSSNPNAIMAPFYQWKDVDNFKLPEDDLRGIQQLYGTPDGQPQPTQPLPTVTPRRPGRPDHRPPRPPQPPPPGGKPERPP
KPGPPVQPRATERPDQYGPNICDGDFDTVAMLRGEMFVFKGRWFWRVRHNRVLDNYPMPIGHFWRGLPGDISAAYERQDGRFVFFKG
DRYWLFREANLEPGYPQPLTSYGLGIPYDRIDTAIWWEPTGHTFFFQEDRYWRFNEETQRGDPGYPKPISVWQGIPASPKGAFLSND
AAYTYFYKGTKYWKFDNERLRMEPGYPKSILRDFMGCQEHVEPGPRWPDVARPPFNPHGGAEPGADSAEGDVGDGDGDFGAGVNKDG
GSRVVVQMEEVARTVNVVMVLVPLLLLLCVLGLTYALVQMQRKGAPRVLLYCKRSLQEWV
>sp|Q92733|PRCC_HUMAN
MSLVAYASSDESEPDEAPEPEPEEEEAVAPTSGPALGGLFASLPAPKGPALLPPPPQMLAPAFPPPLLLPPPTGDPRLQPPPPLPFGL
GGFPPPPGVSPAEAAGVGEGLGLGLPSPRGPGLNLPPPIGGAGPPLGLPKPKKRKEPVKIAAPELHKGDSDSEEDEPTKKKTILQGS
SEGTGLSALLPQPKNLTVKETNRLLLPHAFSRKPSDGSPDTKPSRLASKTKTSSLAPVVGTTTTTPSPSAIKAAAKSAALQVTKQIT
QEEDDSDEEVAPENFFSLPEKAEPPGVEPYPYPIPTVPEELPPGTEPEPAFQDDAANAPLEFKMAAGSSGAPWMPKPGDDYSYNQFS
TYGDANAAGAYYQDYYSGGYYPAQDPALVPPQEIAPDASFIDDEAFKRLQGKRNRGREEINFVEIKGDDQLSGAQQWMTKSLTEEKT
MKSFSKKKGEQPTGQQRRKHQITYLIHQAKERELELKNTWSENKLSRRQTQAKYGF
>sp|Q14410|GLPK2_HUMAN
MAAPKTAAVGPLVGAVVQGTNSTRFLVFNSKTAELLSHHKVELTQEFPKEGWVEQDPKEILQSVYECIARTCEKLDELNIDISNIKA
VGVSNQRETTVIWDKLTGEPLYNAVVWLDLRTQTTVEDLSKKIPGNSNFVKSKTGLPLSTYFSAVKLRWMLDNVRNVQKAVEEGRAL
FGTIDSWLIWSLTGGVNGGVHCTDVTNASRTMLFNIHSLEWDKELCDFFEIPMDLLPNVFSSSEIYGLIKTGALEGVPISGCLGDQC
AALVGQMCFQEGQAKNTYGTGCFLLCNTGRKCVFSEHGLLTTVAYKLGREKPAYYALEGSVAIAGAVIRWLRDNLGIIETSGDIERL
AKEVGTSYGCYFVPAFSGLYAPYWEPSARGILCGLTQFTNKCHIAFAALEAVCFQTREILEAMNRDCGIPLRHLQVDGGMTNNKVLM
QLQADILHIPVIKPFMPETTALGAAMAAGAAEGVSVWSLEPQALSVLRMERFEPQIQATESEIRYATWKKAVMKSMGWVTSQSPEGG
DPSIFSSLPLGFFIVSSMVMLIGARYISGVP
>sp|Q8NBF4|YG006_HUMAN
MQLLQVRTTNTLRRLPGTPLMPSAGAVRVVASVQSGHSWWRMWDGPTGAAEAGSCAEMLRTSAPGTSRPNLSFLLGQVIPLAHTGSV
ETLPSEESWGCRQRASPLPPSTAPMAVSASHRGGTGTRTWLVDPTLSRDTSPLGGQSWGSPQPSRGA
>sp|A6NGH8|ANR61_HUMAN
MGNITRKGSRDLVVDSAKSLEDGPSAALHSKLYEAIMREDCTTIEVLLRNHPVNQPITILPNSASNRLLLTQPTESIIPIHLAAKYH
KAQSLLCLLRHGADPEVRDTTGLTTLNLMLLHWPVTSTTWAKPGNRTHRILTDIQNSSITCLRILCAHGAQVNTQGEISNKRSPLHL
AIAYGCYPVLSILTQNGADVNAINEASMTPLHMAANMLNKEMMETLIAYGANVNCAVSSTGNTPLKLAVCTASSKAGRLLGAGVSCI
RLLLTHGAKVNAQDYKGGQTAIHEACFGGREAIINLLLEFEANVNILTRNGESPIYMYLQRSCNVRDTALLARLLYHTYPLRMTNNQG
ILPAGIMLPEFRLLRDTLIKQSQKPLSLQGICKRNIRNIYGEKYKQHLKQFLPVTIWNSVYCCYDLAYTS
>sp|P43629|KI3L1_HUMAN
MSLMVVSMACVGLFLVQRAGPHMGGQDKPFLSAWPSAVVPRGGHVTLRCHYRHRFNNFMLYKEDRIHIPIFHGRIFQESFNMSPVTT
AHAGNYTCRGSHPHSPTGWSAPSNPVVIMVTGNHRKPSLLAHPGPLVKSGERVILQCWSDIMFEHFFLHKEGISKDPSRLVGQIHDG
VSKANFSIGPMMLALAGTYRCYGSVTHTPYQLSAPSDPLDIVVTGPYEKPSLSAQPGPKVQAGESVTLSCSSRSSYDMYHLSREGGA

HERRLPAVRKVNRTFQADFPLGPATHGGTYRCFGSFRHSPYEWSDPSDPLLVSVTGNPSSSWPSPTEPSSKSGNPRHLHILIGTSVV
IILFILLLFFLLHLWCSNKKNAAVMDQEPAGNRTANSEDSDEQDPEEVTYAQLDHCVFTQRKITRPSQRPKTPPTDTILYTELPNAK
PRSKVVSCP
>sp|P41247|PLPL4_HUMAN
MKHINLSFAACGFLGIYHLGAASALCRHGKKLVKDVKAFAGASAGSLVASVLLTAPEKIEECNQFTYKFAEEIRRQSFGAVTPGYDF
MARLRSGMESILPPSAHELAQNRLHVSITNAKTRENHLVSTFSSREDLIKVLLASSFVPIYAGLKLVEYKGQKWVDGGLTNALPILP
VGRTVTISPFSGRLDISPQDKGQLDLYVNIAKQDIMLSLANLVRLNQALFPPSKRKMESLYQCGFDDTVKFLLKENWFE
>sp|Q6UX40|TM107_HUMAN
MGRVSGLVPSRFLTLLAHLVVVITLFWSRDSNIQACLPLTFTPEEYDKQDIQLVAALSVTLGLFAVELAGFLSGVSMFNSTQSLISI
GAHCSASVALSFFIFERWECTTYWYIFVFCSALPAVTEMALFVTVFGLKKKPF
>sp|Q6NUM6|TYW1B_HUMAN
MDPSADTWDLSSPLISLWINRFYIYLGFAVSISLWICVQIVIEMQGFATVLAEAVTSLDLPVAIINLKEYDPDDHLIEEVTSKNVCV
FLVATYTDGLPTESAEWFCKWLEEASIDFRFGKTYLKGMRDAVFGLGNSAYASHFNKVGKNVDKWLWMLGVHRVMSRGEGDCDVVKS
KHGSIEANFRAWKTKFISQLQALQKGERKKSCGGHCKKGKCESHQHGSEEREEGSQEQDELHHRDTKEEEPFESSSEEEFGGEDHQS
LNSIVDVEDLGKIMDHVKKEKREKEQQEEKSGLFRNMGRNEDGERRAMITPALREALTKQVDAPRERSLLQTHILWNESHRCMETTP
SLACANKCVFCWWHHNNPVGTEWLWKMDQPEMILKEAIENHQNMIKQFKGVPGVKAERFEEGMTVKHCALSLVGEPIMYPEINRFLK
LLHQCKISSFLVTNAQFPAEIRNLEPVTQLYVSVDASTKDSLKKIDRPLFKDFWQQFLDSLKALAVKQQRTVYRLTLVKAWNVDELQ
AYAQLVSLGNPDFIEVKGVTYCRESSASSLTMAHVPWHEEVVQFVRELVDLIPEYEIACEHEHSNCLLIAHRKFKIGGEWWTWIDYN
RFQELIQEYEDSGGSKTFSAKDYMARTPHWALFGANERSFDPKDTRHQRKNKSKAISGC
>sp|P23511|NFYA_HUMAN
MEQYTANSNSSTEQIVVQAGQIQQQQQGGVTAVQLQTEAQVASASGQQVQTLQVVQGQPLMVQVSGGQLITSTGQPIMVQAVPGGQG
QTIMQVPVSGTQGLQQIQLVPPGQIQIQGGQAVQVQGQQGQTQQIIIQQPQTAVTAGQTQTQQQIAVQGQQVAQTAEGQTIVYQPVN
ADGTILQQVTVPVSGMITIPAASLAGAQIVQTGANTNTTSSGQGTVTVTLPVAGNVVNSGGMVMMVPGAGSVPAIQRIPLPGAEMLE
EEPLYVNAKQYHRILKRRQARAKLEAEGKIPKERRKYLHESRHRHAMARKRGEGGRFFSPKEKDSPHMQDPNQADEEAMTQIIRVS
>sp|Q8NHJ6|LIRB4_HUMAN
MIPTFTALLCLGLSLGPRTHMQAGPLPKPTLWAEPGSVISWGNSVTIWCQGTLEAREYRLDKEESPAPWDRQNPLEPKNKARFSIPS
MTEDYAGRYRCYYRSPVGWSQPSDPLELVMTGAYSKPTLSALPSPLVTSGKSVTLLCQSRSPMDTFLLIKERAAHPLLHLRSEHGAQ
QHQAEFPMSPVTSVHGGTYRCFSSHGFSHYLLSHPSDPLELIVSGSLEDPRPSPTRSVSTAAGPEDQPLMPTGSVPHSGLRRHWEVL
IGVLVVSILLLSLLLFLLLQHWRQGKHRTLAQRQADFQRPPGAAEPEPKDGGLQRRSSPAADVQGENFCAAVKNTQPEDGVEMDTRQ
SPHDEDPQAVTYAKVKHSRPRREMASPPSPLSGEFLDTKDRQAEEDRQMDTEAAASEAPQDVTYAQLHSFTLRQKATEPPPSQEGAS
PAEPSVYATLAIH
>sp|Q5T1V6|DDX59_HUMAN
MFVPRSLKIKRNANDDGKSCVAKIIKPDPEDLQLDKSRDVPVDAVATEAATIDRHISESCPFPSPGGQLAEVHSVSPEQGAKDSHPS
EEPVKSFSKTQRWAEPGEPICVVCGRYGEYICDKTDEDVCSLECKAKHLLQVKEKEEKSKLSNPQKADSEPESPLNASYVYKEHPFI
LNLQEDQIENLKQQLGILVQGQEVTRPIIDFEHCSLPEVLNHNLKKSGYEVPTPIQMQMIPVGLLGRDILASADTGSGKTAAFLLPV
IMRALFESKTPSALILTPTRELAIQIERQAKELMSGLPRMKTVLLVGGLPLPPQLYRLQQHVKVIIATPGRLLDIIKQSSVELCGVK
IVVVDEADTMLKMGFQQQVLDILENIPNDCQTILVSATIPTSIEQLASQLLHNPVRIITGEKNLPCANVRQIILWVEDPAKKKKLFE
ILNDKKLFKPPVLVFVDCKLGADLLSEAVQKITGLKSISIHSEKSQIERKNILKGLLEGDYEVVVSTGVLGRGLDLISVRLVVNFDM
PSSMDEYVHQIGRVGRLGQNGTAITFINNNSKRLFWDIAKRVKPTGSILPPQLLNSPYLHDQKRKEQQKDKQTQNDLVTGANLMDII
RKHDKSNSQK
>sp|Q7LDI9|GAK6_HUMAN
MGQTKSKIKSKYASYLSFIKILLKRGGVKVSTKNLIKLFQIIEQFCPWFPEQGTLDLKDWKRIGKELKQAGRKGNIIPLTVWNDWAI
IKAALEPFQTEEDSVSVSDAPGSCIIDCNENTRKKSQKETEGLHCEYVAEPVMAQSTQNVDYNQLQEVIYPETLKLEGKGPELVGPS
ESKPRGTSPLPAGQVPVTLQPQKQVKENKTQPPVAYQYWPPAELQYRPPPESQYGYPGMPPAPQGRAPYPQPPTRRLNPTAPPSRQG

SKLHEIIDKSRKEGDTEAWQFPVTLEPMPPGEGAQEGEPPTVEARYKSFSIKKLKDMKEGVKQYGPNSPYMRTLLDSIAHGHRLIPY
DWEILAKSSLSPSQFLQFKTWWIDGVQEQVRRNRAANPPVNIDADQLLGIGQNWSTISQQALMQNEAIEQVRAICLRAWEKIQDPGS
TCPSFNTVRQGSKEPYPDFVARLQDVAQKSIADEKARKVIVELMAYENANPECQSAIKPLKGKVPAGSDVISEYVKACDGIGGAMHK
AMLMAQAITGVVLGGQVRTFGRKCYNCGQIGHLKKNCPVLNKQNITIQATTTGREPPDLCPRCKKGKHWASQCRSKFDKNGQPLSGN
EQRGQPQAPQQTGAFPIQPFVPQGFQGQQPPLSQVFQGISQLPQYNNCPPPQAAVQQ
>sp|O15439|MRP4_HUMAN
MLPVYQEVKPNPLQDANLCSRVFFWWLNPLFKIGHKRRLEEDDMYSVLPEDRSQHLGEELQGFWDKEVLRAENDAQKPSLTRAIIKC
YWKSYLVLGIFTLIEESAKVIQPIFLGKIINYFENYDPMDSVALNTAYAYATVLTFCTLILAILHHLYFYHVQCAGMRLRVAMCHMI
YRKALRLSNMAMGKTTTGQIVNLLSNDVNKFDQVTVFLHFLWAGPLQAIAVTALLWMEIGISCLAGMAVLIILLPLQSCFGKLFSSL
RSKTATFTDARIRTMNEVITGIRIIKMYAWEKSFSNLITNLRKKEISKILRSSCLRGMNLASFFSASKIIVFVTFTTYVLLGSVITA
SRVFVAVTLYGAVRLTVTLFFPSAIERVSEAIVSIRRIQTFLLLDEISQRNRQLPSDGKKMVHVQDFTAFWDKASETPTLQGLSFTV
RPGELLAVVGPVGAGKSSLLSAVLGELAPSHGLVSVHGRIAYVSQQPWVFSGTLRSNILFGKKYEKERYEKVIKACALKKDLQLLED
GDLTVIGDRGTTLSGGQKARVNLARAVYQDADIYLLDDPLSAVDAEVSRHLFELCICQILHEKITILVTHQLQYLKAASQILILKDG
KMVQKGTYTEFLKSGIDFGSLLKKDNEESEQPPVPGTPTLRNRTFSESSVWSQQSSRPSLKDGALESQDTENVPVTLSEENRSEGKV
GFQAYKNYFRAGAHWIVFIFLILLNTAAQVAYVLQDWWLSYWANKQSMLNVTVNGGGNVTEKLDLNWYLGIYSGLTVATVLFGIARS
LLVFYVLVNSSQTLHNKMFESILKAPVLFFDRNPIGRILNRFSKDIGHLDDLLPLTFLDFIQTLLQVVGVVSVAVAVIPWIAIPLVP
LGIIFIFLRRYFLETSRDVKRLESTTRSPVFSHLSSSLQGLWTIRAYKAEERCQELFDAHQDLHSEAWFLFLTTSRWFAVRLDAICA
MFVIIVAFGSLILAKTLDAGQVGLALSYALTLMGMFQWCVRQSAEVENMMISVERVIEYTDLEKEAPWEYQKRPPPAWPHEGVIIFD
NVNFMYSPGGPLVLKHLTALIKSQEKVGIVGRTGAGKSSLISALFRLSEPEGKIWIDKILTTEIGLHDLRKKMSIIPQEPVLFTGTM
RKNLDPFNEHTDEELWNALQEVQLKETIEDLPGKMDTELAESGSNFSVGQRQLVCLARAILRKNQILIIDEATANVDPRTDELIQKK
IREKFAHCTVLTIAHRLNTIIDSDKIMVLDSGRLKEYDEPYVLLQNKESLFYKMVQQLGKAEAAALTETAKQVYFKRNYPHIGHTDH
MVTNTSNGQPSTLTIFETAL
>sp|Q99536|VAT1_HUMAN
MSDEREVAEAATGEDASSPPPKTEAASDPQHPAASEGAAAAAASPPLLRCLVLTGFGGYDKVKLQSRPAAPPAPGPGQLTLRLRACG
LNFADLMARQGLYDRLPPLPVTPGMEGAGVVIAVGEGVSDRKAGDRVMVLNRSGMWQEEVTVPSVQTFLIPEAMTFEEAAALLVNYI
TAYMVLFDFGNLQPGHSVLVHMAAGGVGMAAVQLCRTVENVTVFGTASASKHEALKENGVTHPIDYHTTDYVDEIKKISPKGVDIVM
DPLGGSDTAKGYNLLKPMGKVVTYGMANLLTGPKRNLMALARTWWNQFSVTALQLLQANRAVCGFHLGYLDGEVELVSGVVARLLAL
YNQGHIKPHIDSVWPFEKVADAMKQMQEKKNVGKVLLVPGPEKEN
>sp|P49406|RM19_HUMAN
MAACIAAGHWAAMGLGRSFQAARTLLPPPASIACRVHAGPVRQQSTGPSEPGAFQPPPKPVIVDKHRPVEPERRFLSPEFIPRRGRT
DPLKFQIERKDMLERRKVLHIPEFYVGSILRVTTADPYASGKISQFLGICIQRSGRGLGATFILRNVIEGQGVEICFELYNPRVQEI
QVVKLEKRLDDSLLYLRDALPEYSTFDVNMKPVVQEPNQKVPVNELKVKMKPKPWSKRWERPNFNIKGIRFDLCLTEQQMKEAQKWN
QPWLEFDMMREYDTSKIEAAIWKEIEASKRS
>sp|P00156|CYB_HUMAN
MTPMRKTNPLMKLINHSFIDLPTPSNISAWWNFGSLLGACLILQITTGLFLAMHYSPDASTAFSSIAHITRDVNYGWIIRYLHANGA
SMFFICLFLHIGRGLYYGSFLYSETWNIGIILLLATMATAFMGYVLPWGQMSFWGATVITNLLSAIPYIGTDLVQWIWGGYSVDSPT
LTRFFTFHFILPFIIAALATLHLLFLHETGSNNPLGITSHSDKITFHPYYTIKDALGLLLFLLSLMTLTLFSPDLLGDPDNYTLANP
LNTPPHIKPEWYFLFAYTILRSVPNKLGGVLALLLSILILAMIPILHMSKQQSMMFRPLSQSLYWLLAADLLILTWIGGQPVSYPFT
IIGQVASVLYFTTILILMPTISLIENKMLKWA
>sp|Q9Y303|NAGA_HUMAN
MRGEQGAAGARVLQFTNCRILRGGKLLREDLWVRGGRILDPEKLFFEERRVADERRDCGGRILAPGFIDVQINGGFGVDFSQATEDV
GSGVALVARRILSHGVTSFCPTLVTSPPEVYHKVVPQIPVKSGGPHGAGVLGLHLEGPFISREKRGAHPEAHLRSFEADAFQDLLAT
YGPLDNVRIVTLAPELGRSHEVIRALTARGICVSLGHSVADLRAAEDAVWSGATFITHLFNAMLPFHHRDPGIVGLLTSDRLPAGRC

IFYGMIADGTHTNPAALRIAHRAHPQGLVLVTDAIPALGLGNGRHTLGQQEVEVDGLTAYVAGTKTLSGSIAPMDVCVRHFLQATGC
SMESALEAASLHPAQLLGLEKSKGTLDFGADADFVVLDDSLHVQATYISGELVWQADAARQ
>sp|Q68DY1|ZN626_HUMAN
MGPLQFRDVAIEFSLEEWHCLDTAQRNLYRNVMLENYSNLVFLGITVSKPDLITCLEQGRKPLTMKRNEMIAKPSVMCSHFAQDLWP
EQSMKDSFQKVVLRRYEKCEHDNLQLKKGCISVDECKVHKEGYNELNQCLTTTPRKICQCDKYVKVLHQFPNSNGQKRGHTGKKPFK
YIECGKAFKQFSTLTTHKKIHTGGKPYKCEECGKAFNHSCSLTRHKKIHTGEKPYKCEECGKAFKHSSTLTTHKRNHTGEKPYKCDK
CGKAFMSSSTLSKHEIIHTEKKPYKCEECGKAFNRSSTLTTHKIIHTGEKPYKCEECDKAFKYSYTLTTHKRIHTEDKPYKCEECGK
AFKYSSTLTTHKRIHTGEKPYKCEECGKAFKRSSDLTTHKIIHTGEKPYKCEECGKAFKYSSNLTTHKKIHTGERPYKCEECGKAFN
QSSILTTHRRIHTGEKFYKCEECGKAFKCSSNLTTHKKIHTGERPYKCEECGKAFNQSSILTTHERIILERNSTNVKNVAKPSSGPH
TLLHIR
>sp|Q13409|DC1I2_HUMAN
MSDKSELKAELERKKQRLAQIREEKKRKEEERKKKETDQKKEAVAPVQEESDLEKKRREAEALLQSMGLTPESPIVFSEYWVPPPMS
PSSKSVSTPSEAGSQDSGDGAVGSRTLHWDTDPSVLQLHSDSDLGRGPIKLGMAKITQVDFPPREIVTYTKETQTPVMAQPKEDEEE
DDDVVAPKPPIEPEEEKTLKKDEENDSKAPPHELTEEEKQQILHSEEFLSFFDHSTRIVERALSEQINIFFDYSGRDLEDKEGEIQA
GAKLSLNRQFFDERWSKHRVVSCLDWSSQYPELLVASYNNNEDAPHEPDGVALVWNMKYKKTTPEYVFHCQSAVMSATFAKFHPNLV
VGGTYSGQIVLWDNRSNKRTPVQRTPLSAAAHTHPVYCVNVVGTQNAHNLISISTDGKICSWSLDMLSHPQDSMELVHKQSKAVAVT
SMSFPVGDVNNFVVGSEEGSVYTACRHGSKAGISEMFEGHQGPITGIHCHAAVGAVDFSHLFVTSSFDWTVKLWTTKNNKPLYSFED
NADYVYDVMWSPTHPALFACVDGMGRLDLWNLNNDTEVPTASISVEGNPALNRVRWTHSGREIAVGDSEGQIVIYDVGEQIAVPRND
EWARFGRTLAEINANRADAEEEAATRIPA
>sp|Q8WXA2|PATE1_HUMAN
MDKSLLLELPILLCCFRALSGSLSMRNDAVNEIVAVKNNFPVIEIVQCRMCHLQFPGEKCSRGRGICTATTEEACMVGRMFKRDGNP
WLTFMGCLKNCADVKGIRWSVYLVNFRCCRSHDLCNEDL
>sp|Q9NRW4|DUS22_HUMAN
MGNGMNKILPGLYIGNFKDARDAEQLSKNKVTHILSVHDSARPMLEGVKYLCIPAADSPSQNLTRHFKESIKFIHECRLRGESCLVH
CLAGVSRSVTLVIAYIMTVTDFGWEDALHTVRAGRSCANPNVGFQRQLQEFEKHEVHQYRQWLKEEYGESPLQDAEEAKNILAAPGI
LKFWAFLRRL
>sp|O00165|HAX1_HUMAN
MSLFDLFRGFFGFPGPRSHRDPFFGGMTRDEDDDEEEEEEGGSWGRGNPRFHSPQHPPEEFGFGFSFSPGGGIRFHDNFGFDDLVRD
FNSIFSDMGAWTLPSHPPELPGPESETPGERLREGQTLRDSMLKYPDSHQPRIFGGVLESDARSESPQPAPDWGSQRPFHRFDDVWP
MDPHPRTREDNDLDSQVSQEGLGPVLQPQPKSYFKSISVTKITKPDGIVEERRTVVDSEGRTETTVTRHEADSSPRGDPESPRPPAL
DDAFSILDLFLGRWFRSR
>sp|Q8NGB9|OR4F6_HUMAN
MDEANHSVVSEFVFLGLSDSRKIQLLLFLFFSVFYVSSLMGNLLIVLTVTSDPRLQSPMYFLLANLSIINLVFCSSTAPKMIYDLFR
KHKTISFGGCVVQIFFIHAVGGTEMVLLIAMAFDRYVAICKPLHYLTIMNPQRCILFLVISWIIGIIHSVIQLAFVVDLLFCGPNEL
DSFFCDLPRFIKLACIETYTLGFMVTANSGFISLASFLILIISYIFILVTVQKKSSGGIFKAFSMLSAHVIVVVLVFGPLIFFYIFP
FPTSHLDKFLAIFDAVITPVLNPVIYTFRNKEMMVAMRRRCSQFVNYSKIF
>sp|Q96E52|OMA1_HUMAN
MSFICGLQSAARNHVFFRFNSLSNWRKCNTLASTSRGCHQVQVNHIVNKYQGLGVNQCDRWSFLPGNFHFYSTFNNKRTGGLSSTKS
KEIWRITSKCTVWNDAFSRQLLIKEVTAVPSLSVLHPLSPASIRAIRNFHTSPRFQAAPVPLLLMILKPVQKLFAIIVGRGIRKWWQ
ALPPNKKEVVKENIRKNKWKLFLGLSSFGLLFVVFYFTHLEVSPITGRSKLLLLGKEQFRLLSELEYEAWMEEFKNDMLTEKDARYL
AVKEVLCHLIECNKDVPGISQINWVIHVVDSPIINAFVLPNGQMFVFTGFLNSVTDIHQLSFLLGHEIAHAVLGHAAEKAGMVHLLD
FLGMIFLTMIWAICPRDSLALLCQWIQSKLQEYMFNRPYSRKLEAEADKIGLLLAAKACADIRASSVFWQQMEFVDSLHGQPKMPEW
LSTHPSHGNRVEYLDRLIPQALKIREMCNCPPLSNPDPRLLFKLSTKHFLEESEKEDLNITKKQKMDTLPIQKQEQIPLTYIVEKRT
GS

>sp|Q96NR3|PTHD1_HUMAN
MLRQVLHRGLRTCFSRLGHFIASHPVFFASAPVLISILLGASFSRYQVEESVEHLLAPQHSLAKIERNLVNSLFPVNRSKHRLYSDL
QTPGRYGRVIVTSFQKANMLDQHHTDLILKLHAAVTKIQVPRPGFNYTFAHICILNNDKTCIVDDIVHVLEELKNARATNRTNFAIT
YPITHLKDGRAVYNGHQLGGVTVHSKDRVKSAEAIQLTYYLQSINSLNDMVAERWESSFCDTVRLFQKSNSKVKMYPYTSSSLREDF
QKTSRVSERYLVTSLILVVTMAILCCSMQDCVRSKPWLGLLGLVTISLATLTAAGIINLTGGKYNSTFLGVPFVMLGHGLYGTFEML
SSWRKTREDQHVKERTAAVYADSMLSFSLTTAMYLVTFGIGASPFTNIEAARIFCCNSCIAIFFNYLYVLSFYGSSLVFTGYIENNY
QHSIFCRKVPKPEALQEKPAWYRFLLTARFSEDTAEGEEANTYESHLLVCFLKRYYCDWITNTYVKPFVVLFYLIYISFALMGYLQV
SEGSDLSNIVATATQTIEYTTAQQKYFSNYSPVIGFYIYESIEYWNTSVQEDVLEYTKGFVRISWFESYLNYLRKLNVSTGLPKKNF
TDMLRNSFLKAPQFSHFQEDIIFSKKYNDEVDVVASRMFLVAKTMETNREELYDLLETLRRLSVTSKVKFIVFNPSFVYMDRYASSL
GAPLHNSCISALFLLFFSAFLVADSLINVWITLTVVSVEFGVIGFMTLWKVELDCISVLCLIYGINYTIDNCAPMLSTFVLGKDFTR
TKWVKNALEVHGVAILQSYLCYIVGLIPLAAVPSNLTCTLFRCLFLIAFVTFFHCFAILPVILTFLPPSKKKRKEKKNPENREEIEC
VEMVDIDSTRVVDQITTV
>sp|Q6NUN9|ZN746_HUMAN
MAEAVAAPISPWTMAATIQAMERKIESQAARLLSLEGRTGMAEKKLADCEKTAVEFGNQLEGKWAVLGTLLQEYGLLQRRLENVENL
LRNRNFWILRLPPGSKGESPKEWGKLEDWQKELYKHVMRGNYETLVSLDYAISKPEVLSQIEQGKEPCNWRRPGPKIPDVPVDPSPG
SGPPVPAPDLLMQIKQEGELQLQEQQALGVEAWAAGQPDIGEEPWGLSQLDSGAGDISTDATSGVHSNFSTTIPPTSWQTDLPPHHP
SSACSDGTLKLNTAASTEDVKIVIKTEVQEEEVVATPVHPTDLEAHGTLFGPGQATRFFPSPAQEGAWESQGSSFPSQDPVLGLREP
ARPERDMGELSPAVAQEETPPGDWLFGGVRWGWNFRCKPPVGLNPRTGPEGLPYSSPDNGEAILDPSQAPRPFNEPCKYPGRTKGFG
HKPGLKKHPAAPPGGRPFTCATCGKSFQLQVSLSAHQRSCGAPDGSGPGTGGGGSGSGGGGGGSGGGSARDGSALRCGECGRCFTRP
AHLIRHRMLHTGERPFPCTECEKRFTERSKLIDHYRTHTGVRPFTCTVCGKSFIRKDHLRKHQRNHAAGAKTPARGQPLPTPPAPPD
PFKSPASKGPLASTDLVTDWTCGLSVLGPTDGGDM
>sp|Q15428|SF3A2_HUMAN
MDFQHRPGGKTGSGGVASSSESNRDRRERLRQLALETIDINKDPYFMKNHLGSYECKLCLTLHNNEGSYLAHTQGKKHQTNLARRAA
KEAKEAPAQPAPEKVKVEVKKFVKIGRPGYKVTKQRDSEMGQQSLLFQIDYPEIAEGIMPRHRFMSAYEQRIEPPDRRWQYLLMAAE
PYETIAFKVPSREIDKAEGKFWTHWNRETKQFFLQFHFKMEKPPAPPSLPAGPPGVKRPPPPLMNGLPPRPPLPESLPPPPPGGLPL
PPMPPTGPAPSGPPGPPQLPPPAPGVHPPAPVVHPPASGVHPPAPGVHPPAPGVHPPAPGVHPPTSGVHPPAPGVHPPAPGVHPPAP
GVHPPAPGVHPPAPGVHPPPSAGVHPQAPGVHPAAPAVHPQAPGVHPPAPGMHPQAPGVHPQPPGVHPSAPGVHPQPPGVHPSNPGV
HPPTPMPPMLRPPLPSEGPGNIPPPPPTN
>sp|P26196|DDX6_HUMAN
MSTARTENPVIMGLSSQNGQLRGPVKPTGGPGGGGTQTQQQMNQLKNTNTINNGTQQQAQSMTTTIKPGDDWKKTLKLPPKDLRIKT
SDVTSTKGNEFEDYCLKRELLMGIFEMGWEKPSPIQEESIPIALSGRDILARAKNGTGKSGAYLIPLLERLDLKKDNIQAMVIVPTR
ELALQVSQICIQVSKHMGGAKVMATTGGTNLRDDIMRLDDTVHVVIATPGRILDLIKKGVAKVDHVQMIVLDEADKLLSQDFVQIME
DIILTLPKNRQILLYSATFPLSVQKFMNSHLQKPYEINLMEELTLKGVTQYYAYVTERQKVHCLNTLFSRLQINQSIIFCNSSQRVE
LLAKKISQLGYSCFYIHAKMRQEHRNRVFHDFRNGLCRNLVCTDLFTRGIDIQAVNVVINFDFPKLAETYLHRIGRSGRFGHLGLAI
NLITYDDRFNLKSIEEQLGTEIKPIPSNIDKSLYVAEYHSEPVEDEKP
>sp|Q9Y4C2|TCAF1_HUMAN
MATPSAAFEALMNGVTSWDVPEDAVPCELLLIGEASFPVMVNDMGQVLIAASSYGRGRLVVVSHEDYLVEAQLTPFLLNAVGWLCSS
PGAPIGVHPSLAPLAKILEGSGVDAKVEPEVKDSLGVYCIDAYNETMTEKLVKFMKCGGGLLIGGQAWDWANQGEDERVLFTFPGNL
VTSVAGIYFTDNKGDTSFFKVSKKMPKIPVLVSCEDDLSDDREELLHGISELDISNSDCFPSQLLVHGALAFPLGLDSYHGCVIAAA
RYGRGRVVVTGHKVLFTVGKLGPFLLNAVRWLDGGRRGKVVVQTELRTLSGLLAVGGIDTSIEPNLTSDASVYCFEPVSEVGVKELQ
EFVAEGGGLFVGAQAWWWAFKNPGVSPLARFPGNLLLNPFGISITSQSLNPGPFRTPKAGIRTYHFRSTLAEFQVIMGRKRGNVEKG
WLAKLGPDGAAFLQIPAEEIPAYMSVHRLLRKLLSRYRLPVATRENPVINDCCRGAMLSLATGLAHSGSDLSLLVPEIEDMYSSPYL
RPSESPITVEVNCTNPGTRYCWMSTGLYIPGRQIIEVSLPEAAASADLKIQIGCHTDDLTRASKLFRGPLVINRCCLDKPTKSITCL
WGGLLYIIVPQNSKLGSVPVTVKGAVHAPYYKLGETTLEEWKRRIQENPGPWGELATDNIILTVPTANLRTLENPEPLLRLWDEVMQ

AVARLGAEPFPLRLPQRIVADVQISVGWMHAGYPIMCHLESVQELINEKLIRTKGLWGPVHELGRNQQRQEWEFPPHTTEATCNLWC
VYVHETVLGIPRSRANIALWPPVREKRVRIYLSKGPNVKNWNAWTALETYLQLQEAFGWEPFIRLFTEYRNQTNLPTENVDKMNLWV
KMFSHQVQKNLAPFFEAWAWPIQKEVATSLAYLPEWKENIMKLYLLTQMPH
>sp|O60583|CCNT2_HUMAN
MASGRGASSRWFFTREQLENTPSRRCGVEADKELSCRQQAANLIQEMGQRLNVSQLTINTAIVYMHRFYMHHSFTKFNKNIISSTAL
FLAAKVEEQARKLEHVIKVAHACLHPLEPLLDTKCDAYLQQTQELVILETIMLQTLGFEITIEHPHTDVVKCTQLVRASKDLAQTSY
FMATNSLHLTTFCLQYKPTVIACVCIHLACKWSNWEIPVSTDGKHWWEYVDPTVTLELLDELTHEFLQILEKTPNRLKKIRNWRANQ
AARKPKVDGQVSETPLLGSSLVQNSILVDSVTGVPTNPSFQKPSTSAFPAPVPLNSGNISVQDSHTSDNLSMLATGMPSTSYGLSSH
QEWPQHQDSARTEQLYSQKQETSLSGSQYNINFQQGPSISLHSGLHHRPDKISDHSSVKQEYTHKAGSSKHHGPISTTPGIIPQKMS
LDKYREKRKLETLDLDVRDHYIAAQVEQQHKQGQSQAASSSSVTSPIKMKIPIANTEKYMADKKEKSGSLKLRIPIPPTDKSASKEE
LKMKIKVSSSERHSSSDEGSGKSKHSSPHISRDHKEKHKEHPSSRHHTSSHKHSHSHSGSSSGGSKHSADGIPPTVLRSPVGLSSDG
ISSSSSSSRKRLHVNDASHNHHSKMSKSSKSSGSSSSSSSSVKQYISSHNSVFNHPLPPPPPVTYQVGYGHLSTLVKLDKKPVETNG
PDANHEYSTSSQHMDYKDTFDMLDSLLSAQGMNM
>sp|P07911|UROM_HUMAN
MGQPSLTWMLMVVVASWFITTAATDTSEARWCSECHSNATCTEDEAVTTCTCQEGFTGDGLTCVDLDECAIPGAHNCSANSSCVNTP
GSFSCVCPEGFRLSPGLGCTDVDECAEPGLSHCHALATCVNVVGSYLCVCPAGYRGDGWHCECSPGSCGPGLDCVPEGDALVCADPC
QAHRTLDEYWRSTEYGEGYACDTDLRGWYRFVGQGGARMAETCVPVLRCNTAAPMWLNGTHPSSDEGIVSRKACAHWSGHCCLWDAS
VQVKACAGGYYVYNLTAPPECHLAYCTDPSSVEGTCEECSIDEDCKSNNGRWHCQCKQDFNITDISLLEHRLECGANDMKVSLGKCQ
LKSLGFDKVFMYLSDSRCSGFNDRDNRDWVSVVTPARDGPCGTVLTRNETHATYSNTLYLADEIIIRDLNIKINFACSYPLDMKVSL
KTALQPMVSALNIRVGGTGMFTVRMALFQTPSYTQPYQGSSVTLSTEAFLYVGTMLDGGDLSRFALLMTNCYATPSSNATDPLKYFI
IQDRCPHTRDSTIQVVENGESSQGRFSVQMFRFAGNYDLVYLHCEVYLCDTMNEKCKPTCSGTRFRSGSVIDQSRVLNLGPITRKGV
QATVSRAFSSLGLLKVWLPLLLSATLTLTFQ
>sp|Q86SQ7|SDCG8_HUMAN
MAKSPENSTLEEILGQYQRSLREHASRSIHQLTCALKEGDVTIGEDAPNLSFSTSVGNEDARTAWPELQQSHAVNQLKDLLRQQADK
ESEVSPSRRRKMSPLRSLEHEETNMPTMHDLVHTINDQSQYIHHLEAEVKFCKEELSGMKNKIQVVVLENEGLQQQLKSQRQEETLR
EQTLLDASGNMHNSWITTGEDSGVGETSKRPFSHDNADFGKAASAGEQLELEKLKLTYEEKCEIEESQLKFLRNDLAEYQRTCEDLK
EQLKHKEFLLAANTCNRVGGLCLKCAQHEAVLSQTHTNVHMQTIERLVKERDDLMSALVSVRSSLADTQQREASAYEQVKQVLQISE
EANFEKTKALIQCDQLRKELERQAERLEKELASQQEKRAIEKDMMKKEITKEREYMGSKMLILSQNIAQLEAQVEKVTKEKISAINQ
LEEIQSQLASREMDVTKVCGEMRYQLNKTNMEKDEAEKEHREFRAKTNRDLEIKDQEIEKLRIELDESKQHLEQEQQKAALAREECL
RLTELLGESEHQLHLTRQEKDSIQQSFSKEAKAQALQAQQREQELTQKIQQMEAQHDKTENEQYLLLTSQNTFLTKLKEECCTLAKK
LEQISQKTRSEIAQLSQEKRYTYDKLGKLQRRNEELEEQCVQHGRVHETMKQRLRQLDKHSQATAQQLVQLLSKQNQLLLERQSLSE
EVDRLRTQLPSMPQSDC
>sp|P02686|MBP_HUMAN
MGNHAGKRELNAEKASTNSETNRGESEKKRNLGELSRTTSEDNEVFGEADANQNNGTSSQDTAVTDSKRTADPKNAWQDAHPADPGS
RPHLIRLFSRDAPGREDNTFKDRPSESDELQTIQEDSAATSESLDVMASQKRPSQRHGSKYLATASTMDHARHGFLPRHRDTGILDS
IGRFFGGDRGAPKRGSGKDSHHPARTAHYGSLPQKSHGRTQDENPVVHFFKNIVTPRTPPPSQGKGRGLSLSRFSWGAEGQRPGFGY
GGRASDYKSAHKGFKGVDAQGTLSKIFKLGGRDSRSGSPMARR
>sp|Q13263|TIF1B_HUMAN
MAASAAAASAAAASAASGSPGPGEGSAGGEKRSTAPSAAASASASAAASSPAGGGAEALELLEHCGVCRERLRPEREPRLLPCLHSA
CSACLGPAAPAAANSSGDGGAAGDGTVVDCPVCKQQCFSKDIVENYFMRDSGSKAATDAQDANQCCTSCEDNAPATSYCVECSEPLC
ETCVEAHQRVKYTKDHTVRSTGPAKSRDGERTVYCNVHKHEPLVLFCESCDTLTCRDCQLNAHKDHQYQFLEDAVRNQRKLLASLVK
RLGDKHATLQKSTKEVRSSIRQVSDVQKRVQVDVKMAILQIMKELNKRGRVLVNDAQKVTEGQQERLERQHWTMTKIQKHQEHILRF
ASWALESDNNTALLLSKKLIYFQLHRALKMIVDPVEPHGEMKFQWDLNAWTKSAEAFGKIVAERPGTNSTGPAPMAPPRAPGPLSKQ
GSGSSQPMEVQEGYGFGSGDDPYSSAEPHVSGVKRSRSGEGEVSGLMRKVPRVSLERLDLDLTADSQPPVFKVFPGSTTEDYNLIVI

ERGAAAAATGQPGTAPAGTPGAPPLAGMAIVKEEETEAAIGAPPTATEGPETKPVLMALAEGPGAEGPRLASPSGSTSSGLEVVAPE
GTSAPGGGPGTLDDSATICRVCQKPGDLVMCNQCEFCFHLDCHLPALQDVPGEEWSCSLCHVLPDLKEEDGSLSLDGADSTGVVAKL
SPANQRKCERVLLALFCHEPCRPLHQLATDSTFSLDQPGGTLDLTLIRARLQEKLSPPYSSPQEFAQDVGRMFKQFNKLTEDKADVQ
SIIGLQRFFETRMNEAFGDTKFSAVLVEPPPMSLPGAGLSSQELSGGPGDGP
>sp|Q6PGN9|PSRC1_HUMAN
MEDLEEDVRFIVDETLDFGGLSPSDSREEEDITVLVTPEKPLRRGLSHRSDPNAVAPAPQGVRLSLGPLSPEKLEEILDEANRLAAQ
LEQCALQDRESAGEGLGPRRVKPSPRRETFVLKDSPVRDLLPTVNSLTRSTPSPSSLTPRLRSNDRKGSVRALRATSGKRPSNMKRE
SPTCNLFPASKSPASSPLTRSTPPVRGRAGPSGRAAASEETRAAKLRVSGSGEFVGLTLKFLHPSPPGPPTPIRSVLAPQPSTSNSQ
RLPRPQGAAAKSSSQLPIPSAIPRPASRMPLTSRSVPPGRGALPPDSLSTRKGLPRPSTAGHRVRESGHKVPVSQRLNLPVMGATRS
NLQPPRKVAVPGPTR
>sp|Q6IPR3|TYW3_HUMAN
MDRSAEFRKWKAQCLSKADLSRKGSVDEDVVELVQFLNMRDQFFTTSSCAGRILLLDRGINGFEVQKQNCCWLLVTHKLCVKDDVIV
ALKKANGDATLKFEPFVLHVQCRQLQDAQILHSMAIDSGFRNSGITVGKRGKTMLAVRSTHGLEVPLSHKGKLMVTEEYIDFLLNVA
NQKMEENKKRIERFYNCLQHALERETMTNLHPKIKEKNNSSYIHKKKRNPEKTRAQCITKESDEELENDDDDDLGINVTIFPEDY
>sp|Q96EI5|TCAL4_HUMAN
MEKLYSENEGMASNQGKMENEEQPQDERKPEVTCTLEDKKLENEGKTENKGKTGDEEMLKDKGKPESEGEAKEGKSEREGESEMEGG
SEREGKPEIEGKPESEGEPGSETRAAGKRPAEDDVPRKAKRKTNKGLAHYLKEYKEAIHDMNFSNEDMIREFDNMAKVQDEKRKSKQ
KLGAFLWMQRNLQDPFYPRGPREFRGGCRAPRRDIEDIPYV
>sp|O00300|TR11B_HUMAN
MNNLLCCALVFLDISIKWTTQETFPPKYLHYDEETSHQLLCDKCPPGTYLKQHCTAKWKTVCAPCPDHYYTDSWHTSDECLYCSPVC
KELQYVKQECNRTHNRVCECKEGRYLEIEFCLKHRSCPPGFGVVQAGTPERNTVCKRCPDGFFSNETSSKAPCRKHTNCSVFGLLLT
QKGNATHDNICSGNSESTQKCGIDVTLCEEAFFRFAVPTKFTPNWLSVLVDNLPGTKVNAESVERIKRQHSSQEQTFQLLKLWKHQN
KDQDIVKKIIQDIDLCENSVQRHIGHANLTFEQLRSLMESLPGKKVGAEDIEKTIKACKPSDQILKLLSLWRIKNGDQDTLKGLMHA
LKHSKTYHFPKTVTQSLKKTIRFLHSFTMYKLYQKLFLEMIGNQVQSVKISCL
>sp|Q9Y5S8|NOX1_HUMAN
MGNWVVNHWFSVLFLVVWLGLNVFLFVDAFLKYEKADKYYYTRKILGSTLACARASALCLNFNSTLILLPVCRNLLSFLRGTCSFCS
RTLRKQLDHNLTFHKLVAYMICLHTAIHIIAHLFNFDCYSRSRQATDGSLASILSSLSHDEKKGGSWLNPIQSRNTTVEYVTFTSIA
GLTGVIMTIALILMVTSATEFIRRSYFEVFWYTHHLFIFYILGLGIHGIGGIVRGQTEESMNESHPRKCAESFEMWDDRDSHCRRPK
FEGHPPESWKWILAPVILYICERILRFYRSQQKVVITKVVMHPSKVLELQMNKRGFSMEVGQYIFVNCPSISLLEWHPFTLTSAPEE
DFFSIHIRAAGDWTENLIRAFEQQYSPIPRIEVDGPFGTASEDVFQYEVAVLVGAGIGVTPFASILKSIWYKFQCADHNLKTKKIYF
YWICRETGAFSWFNNLLTSLEQEMEELGKVGFLNYRLFLTGWDSNIVGHAALNFDKATDIVTGLKQKTSFGRPMWDNEFSTIATSHP
KSVVGVFLCGPRTLAKSLRKCCHRYSSLDPRKVQFYFNKENF
>sp|Q9Y6R6|Z780B_HUMAN
MVHGSVTFRDVAIDFSQEEWECLQPDQRTLYRDVMLENYSHLISLGSSISKPDVITLLEQEKEPWIVVSKETSRWYPDLESKYGPEK
ISPENDIFEINLPKHVIKQISKTLGLEAFYFRNDSEYRSRFEGRQGHQEGYINQKIISYEEMPAYTHASPIHNTHKPYECKECGKYF
SCGSNLIQHQSIHTGEKPYKCKECGKAFQLHIQLTRHQKFHTGEKTFECKECGKAFNLPTQLNRHKNIHTVKKLFECKECGKSFNRS
SNLTQHQSIHAGVKPYQCKECGKAFNRGSNLIQHQKIHSNEKPFVCRECEMAFRYHYQLIEHCRIHTGEKPFECKECRKAFTLLTKL
VRHQKIHMGEKPFECRECGKAFSLLNQLNRHKNIHTGEKPFECKECGKSFNRSSNLIQHQSIHADVKPYECKECGKGFNRGANLIQH
QKIHSNEKPFVCRECEMAFRYHYQLIQHCQIHTGGKPFECKECGKAFSLLTQLARHKNIHTGEKPFECKDCGKAFNRGSNLVQHQSI
HTGEKPYECKECGKAFRLHLQLSQHEKTHTGEKPFECKECGKFFRRGSNLNQHRSIHTGKKPFECKECGKAFRLHMHLIRHQKFHTG
EKPFECKECGKAFSLHTQLNHHKNIHTGEKPFKCKECGKSFNRVSNLVQHQSIHAGVKPYECKECGKGFSRVSNLIQHQKTHSSAKP
FVCKECRKTFRYHYQLTEHYRIHTGEKPFECKECGKAFGLLTQLAQHQIIHTGEKPFKCKECGKAFNRGSNLVQPQSIHTGEKPYEC
KECGKAFRLHLQLSLHQKLVQVRNPLNVRNVGQPSDISSNLLNIRKFILG
>sp|Q5TBA9|FRY_HUMAN

MASQQDSGFFEISIKYLLKSWSNTSPVGNGYIKPPVPPASGTHREKGPPTMLPINVDPDSKPGEYVLKSLFVNFTTQAERKIRIIMA
EPLEKPLTKSLQRGEDPQFDQVISSMSSLSEYCLPSILRTLFDWYKRQNGIEDESHEYRPRTSNKSKSDEQQRDYLMERRDLAIDFI
FSLVLIEVLKQIPLHPVIDSLIHDVINLAFKHFKYKEGYLGPNTGNMHIVADLYAEVIGVLAQAKFPAVKKKFMAELKELRHKEQNP
YVVQSIISLIMGMKFFRIKMYPVEDFEASLQFMQECAHYFLEVKDKDIKHALAGLFVEILVPVAAAVKNEVNVPCLRNFVESLYDTT
LELSSRKKHSLALYPLVTCLLCVSQKQLFLNRWHIFLNNCLSNLKNKDPKMARVALESLYRLLWVYMIRIKCESNTATQSRLITIIT
TLFPKGSRGVVPRDMPLNIFVKIIQFIAQERLDFAMKEIIFDFLCVGKPAKAFSLNPERMNIGLRAFLVIADSLQQKDGEPPMPVTG
AVLPSGNTLRVKKTYLSKTLTEEEAKMIGMSLYYSQVRKAVDNILRHLDKEVGRCMMLTNVQMLNKEPEDMITGERKPKIDLFRTCV
AAIPRLLPDGMSKLELIDLLARLSIHMDDELRHIAQNSLQGLLVDFSDWREDVLFGFTNFLLREVNDMHHTLLDSSLKLLLQLLTQW
KLVIQTQGKVYEQANKIRNSELIANGSSHRIQSERGPHCSVLHAVEGFALVLLCSFQVATRKLSVLILKEIRALFIALGQPEDDDRP
MIDVMDQLSSSILESFIHVAVSDSATLPLTHNVDLQWLVEWNAVLVNSHYDVKSPSHVWIFAQSVKDPWVLCLFSFLRQENLPKHCP
TALSYAWPYAFTRLQSVMPLVDPNSPINAKKTSTAGSGDNYVTLWRNYLILCFGVAKPSIMSPGHLRASTPEIMATTPDGTVSYDNK
AIGTPSVGVLLKQLVPLMRLESIEITESLVLGFGRTNSLVFRELVEELHPLMKEALERRPENKKRRERRDLLRLQLLRIFELLADAG
VISDSTNGALERDTLALGALFLEYVDLTRMLLEAENDKEVEILKDIRAHFSAMVANLIQCVPVHHRRFLFPQQSLRHHLFILFSQWA
GPFSIMFTPLDRYSDRNHQITRYQYCALKAMSAVLCCGPVFDNVGLSPDGYLYKWLDNILACQDLRVHQLGCEVVVLLLELNPDQIN
LFNWAIDRCYTGSYQLASGCFKAIATVCGSRNYPFDIVTLLNLVLFKASDTNREIYEISMQLMQILEAKLFVYSKKVAEQRPGSILY
GTHGPLPPLYSVSLALLSCELARMYPELTLPLFSEVSQRFPTTHPNGRQIMLTYLLPWLHNIELVDSRLLLPGSSPSSPEDEVKDRE
GDVTASHGLRGNGWGSPEATSLVLNNLMYMTAKYGDEVPGPEMENAWNALANNEKWSNNLRITLQFLISLCGVSSDTVLLPYIKKVA
IYLCRNNTIQTMEELLFELQQTEPVNPIVQHCDNPPFYRFTASSKASAAASGTTSSSNTVVAGQENFPDAEENKILKESDERFSNVI
RAHTRLESRYSNSSGGSYDEDKNDPISPYTGWLLTITETKQPQPLPMPCTGGCWAPLVDYLPETITPRGPLHRCNIAVIFMTEMVVD
HSVREDWALHLPLLLHAVFLGLDHYRPEVFEHSKKLLLHLLIALSCNSNFHSIASVLLQTREMGEAKTLTVQPAYQPEYLYTGGFDF
LREDQSSPVPDSGLSSSSTSSSISLGGSSGNLPQMTQEVEDVDTAAETDEKANKLIEFLTTRAFGPLWCHEDITPKNQNSKSAEQLT
NFLRHVVSVFKDSKSGFHLEHQLSEVALQTALASSSRHYAGRSFQIFRALKQPLSAHALSDLLSRLVEVIGEHGDEIQGYVMEALLT
LEAAVDNLSDCLKNSDLLTVLSRSSSPDLSSSSKLTASRKSTGQLNMNPGTTSGNTATAERSRHQRSFSVPKKFGVIDRSSDPPRSA
TLDRIQACTQQGLSSKTRSSSSLKDSLTDPSHINHPTNLLATIFWVTVALMESDFEFEYLMALRLLSRLLAHMPLDKAENREKLEKL
QAQLKWADFSGLQQLLLKGFTSLTTTDLTLQLFSLLTPVSKISMVDASHAIGFPLNVLCLLPQLIQHFENPNQFCKDIAERIAQVCL
EEKNPKLSNLAHVMTLYKTHSYTRDCATWVNVVCRYLHEAYADITLNMVTYLAELLEKGLPSVQQPLLQVIYSLLSYMDLSVVPVKQ
FNVEVLKTIEKYVQSVHWREALNILKLVVSRSASLVLPSYQHSDLSKIEIHRVWTSASKELPGKTLDFHFDISETPIIGRRYDELQN
SSGRDGKPRAMAVTRSTSSTSSGSNSNVLVPVSWKRPQYSQKRTKEKLVHVLSLCGQEVGLSKNPSVIFSSCGDLDLLEHQTSLVSS
EDGAREQENMDDTNSEQQFRVFRDFDFLDVELEDGEGESMDNFNWGVRRRSLDSLDKCDMQILEERQLSGSTPSLNKMHHEDSDESS
EEEDLTASQILEHSDLIMTLSPSEETNPMELLTTACDSTPAEPHSFNTRMSSFDASLPDMNNLQISEGSKAEAVREEEDTTVHEDDL
SSSINELPAAFECSDSFSLDMTEGEEKGNRALDQFTLASFGEGDRGVSPPPSPFFSAILAAFQPAACDDAEEAWRSHINQLMCDSDG
SCAVYTFHVFSSLFKNIQKRFCFLTCDAASYLGDNLRGIGSKFVSSSQMLTSCSECPTLFVDAETLLSCGLLDKLKFSVLELQEYLD
TYNNRKEATLSWLANCKATFAGGSRDGVITCQPGDSEEKQLELCQRLYKLHFQLLLLFQSYCKLIGQVHEVSSMPELLNMSRELSDL
KKHLKEASAVIAADPLYSDGAWSEPTFTSTEAAIQSMLECLKNNELGKALRQIRECRSLWPNDIFGSSSDDEVQTLLNIYFRHQTLG
QTGTYALVGSNQSLTEICTKLMELNMEIRDMIRRAQSYRVLTTFLPDSSVSGTSL
>sp|Q969G3|SMCE1_HUMAN
MSKRPSYAPPPTPAPATQMPSTPGFVGYNPYSHLAYNNYRLGGNPGTNSRVTASSGITIPKPPKPPDKPLMPYMRYSRKVWDQVKAS
NPDLKLWEIGKIIGGMWRDLTDEEKQEYLNEYEAEKIEYNESMKAYHNSPAYLAYINAKSRAEAALEEESRQRQSRMEKGEPYMSIQ
PAEDPDDYDDGFSMKHTATARFQRNHRLISEILSESVVPDVRSVVTTARMQVLKRQVQSLMVHQRKLEAELLQIEERHQEKKRKFLE
STDSFNNELKRLCGLKVEVDMEKIAAEIAQAEEQARKRQEEREKEAAEQAERSQSSIVPEEEQAANKGEEKKDDENIPMETEETHLE
ETTESQQNGEEGTSTPEDKESGQEGVDSMAEEGTSDSNTGSESNSATVEEPPTDPIPEDEKKE
>sp|Q96AB3|ISOC2_HUMAN

MAAARPSLGRVLPGSSVLFLCDMQEKFRHNIAYFPQIVSVAARMLKVARLLEVPVMLTEQYPQGLGPTVPELGTEGLRPLAKTCFSM
VPALQQELDSRPQLRSVLLCGIEAQACILNTTLDLLDRGLQVHVVVDACSSRSQVDRLVALARMRQSGAFLSTSEGLILQLVGDAVH
PQFKEIQKLIKEPAPDSGLLGLFQGQNSLLH
>sp|Q8TCC7|S22A8_HUMAN
MTFSEILDRVGSMGHFQFLHVAILGLPILNMANHNLLQIFTAATPVHHCRPPHNASTGPWVLPMGPNGKPERCLRFVHPPNASLPND
TQRAMEPCLDGWVYNSTKDSIVTEWDLVCNSNKLKEMAQSIFMAGILIGGLVLGDLSDRFGRRPILTCSYLLLAASGSGAAFSPTFP
IYMVFRFLCGFGISGITLSTVILNVEWVPTRMRAIMSTALGYCYTFGQFILPGLAYAIPQWRWLQLTVSIPFFVFFLSSWWTPESIR
WLVLSGKSSKALKILRRVAVFNGKKEEGERLSLEELKLNLQKEISLAKAKYTASDLFRIPMLRRMTFCLSLAWFATGFAYYSLAMGV
EEFGVNLYILQIIFGGVDVPAKFITILSLSYLGRHTTQAAALLLAGGAILALTFVPLDLQTVRTVLAVFGKGCLSSSFSCLFLYTSE
LYPTVIRQTGMGVSNLWTRVGSMVSPLVKITGEVQPFIPNIIYGITALLGGSAALFLPETLNQPLPETIEDLENWSLRAKKPKQEPE
VEKASQRIPLQPHGPGLGSS
>sp|Q9P0X4|CAC1I_HUMAN
MAESASPPSSSAAAPAAEPGVTTEQPGPRSPPSSPPGLEEPLDGADPHVPHPDLAPIAFFCLRQTTSPRNWCIKMVCNPWFECVSML
VILLNCVTLGMYQPCDDMDCLSDRCKILQVFDDFIFIFFAMEMVLKMVALGIFGKKCYLGDTWNRLDFFIVMAGMVEYSLDLQNINL
SAIRTVRVLRPLKAINRVPSMRILVNLLLDTLPMLGNVLLLCFFVFFIFGIIGVQLWAGLLRNRCFLEENFTIQGDVALPPYYQPEE
DDEMPFICSLSGDNGIMGCHEIPPLKEQGRECCLSKDDVYDFGAGRQDLNASGLCVNWNRYYNVCRTGSANPHKGAINFDNIGYAWI
VIFQVITLEGWVEIMYYVMDAHSFYNFIYFILLIIVGSFFMINLCLVVIATQFSETKQREHRLMLEQRQRYLSSSTVASYAEPGDCY
EEIFQYVCHILRKAKRRALGLYQALQSRRQALGPEAPAPAKPGPHAKEPRHYHGKTKGQGDEGRHLGSRHCQTLHGPASPGNDHSGR
ELCPQHSPLDATPHTLVQPIPATLASDPASCPCCQHEDGRRPSGLGSTDSGQEGSGSGSSAGGEDEADGDGARSSEDGASSELGKEE
EEEEQADGAVWLCGDVWRETRAKLRGIVDSKYFNRGIMMAILVNTVSMGIEHHEQPEELTNILEICNVVFTSMFALEMILKLAAFGL
FDYLRNPYNIFDSIIVIISIWEIVGQADGGLSVLRTFRLLRVLKLVRFMPALRRQLVVLMKTMDNVATFCMLLMLFIFIFSILGMHI
FGCKFSLRTDTGDTVPDRKNFDSLLWAIVTVFQILTQEDWNVVLYNGMASTSPWASLYFVALMTFGNYVLFNLLVAILVEGFQAEGD
ANRSYSDEDQSSSNIEEFDKLQEGLDSSGDPKLCPIPMTPNGHLDPSLPLGGHLGPAGAAGPAPRLSLQPDPMLVALGSRKSSVMSL
GRMSYDQRSLSSSRSSYYGPWGRSAAWASRRSSWNSLKHKPPSAEHESLLSAERGGGARVCEVAADEGPPRAAPLHTPHAHHIHHGP
HLAHRHRHHRRTLSLDNRDSVDLAELVPAVGAHPRAAWRAAGPAPGHEDCNGRMPSIAKDVFTKMGDRGDRGEDEEEIDYTLCFRVR
KMIDVYKPDWCEVREDWSVYLFSPENRFRVLCQTIIAHKLFDYVVLAFIFLNCITIALERPQIEAGSTERIFLTVSNYIFTAIFVGE
MTLKVVSLGLYFGEQAYLRSSWNVLDGFLVFVSIIDIVVSLASAGGAKILGVLRVLRLLRTLRPLRVISRAPGLKLVVETLISSLKP
IGNIVLICCAFFIIFGILGVQLFKGKFYHCLGVDTRNITNRSDCMAANYRWVHHKYNFDNLGQALMSLFVLASKDGWVNIMYNGLDA
VAVDQQPVTNHNPWMLLYFISFLLIVSFFVLNMFVGVVVENFHKCRQHQEAEEARRREEKRLRRLEKKRRKAQRLPYYATYCHTRLL
IHSMCTSHYLDIFITFIICLNVVTMSLEHYNQPTSLETALKYCNYMFTTVFVLEAVLKLVAFGLRRFFKDRWNQLDLAIVLLSVMGI
TLEEIEINAALPINPTIIRIMRVLRIARVLKLLKMATGMRALLDTVVQALPQVGNLGLLFMLLFFIYAALGVELFGKLVCNDENPCE
GMSRHATFENFGMAFLTLFQVSTGDNWNGIMKDTLRDCTHDERSCLSSLQFVSPLYFVSFVLTAQFVLINVVVAVLMKHLDDSNKEA
QEDAEMDAELELEMAHGLGPGPRLPTGSPGAPGRGPGGAGGGGDTEGGLCRRCYSPAQENLWLDSVSLIIKDSLEGELTIIDNLSGS
IFHHYSSPAGCKKCHHDKQEVQLAETEAFSLNSDRSSSILLGDDLSLEDPTACPPGRKDSKGELDPPEPMRVGDLGECFFPLSSTAV
SPDPENFLCEMEEIPFNPVRSWLKHDSSQAPPSPFSPDASSPLLPMPAEFFHPAVSASQKGPEKGTGTGTLPKIALQGSWASLRSPR
VNCTLLRQATGSDTSLDASPSSSAGSLQTTLEDSLTLSDSPRRALGPPAPAPGPRAGLSPAARRRLSLRGRGLFSLRGLRAHQRSHS
SGGSTSPGCTHHDSMDPSDEEGRGGAGGGGAGSEHSETLSSLSLTSLFCPPPPPPAPGLTPARKFSSTSSLAAPGRPHAAALAHGLA
RSPSWAADRSKDPPGRAPLPMGLGPLAPPPQPLPGELEPGDAASKRKR
>sp|Q96JG6|VPS50_HUMAN
MQKIKSLMTRQGLKSPQESLSDLGAIESLRVPGKEEFRELREQPSDPQAEQELINSIEQVYFSVDSFDIVKYELEKLPPVLNLQELE
AYRDKLKQQQAAVSKKVADLILEKQPAYVKELERVTSLQTGLQLAAVICTNGRRHLNIAKEGFTQASLGLLANQRKRQLLIGLLKSL
RTIKTLQRTDVRLSEMLEEEDYPGAIQLCLECQKAASTFKHYSCISELNSKLQDTLEQIEEQLDVALSKICKNFDINHYTKVQQAYR
LLGKTQTAMDQLHMHFTQAIHNTVFQVVLGYVELCAGNTDTKFQKLQYKDLCTHVTPDSYIPCLADLCKALWEVMLSYYRTMEWHEK
HDNEDTASASEGSNMIGTEETNFDRGYIKKKLEHGLTRIWQDVQLKVKTYLLGTDLSIFKYDDFIFVLDIISRLMQVGEEFCGSKSE

VLQESIRKQSVNYFKNYHRTRLDELRMFLENETWELCPVKSNFSILQLHEFKFMEQSRSPSVSPSKQPVSTSSKTVTLFEQYCSGGN
PFEIQANHKDEETEDVLASNGYESDEQEKSAYQEYDSDSDVPEELKRDYVDEQTGDGPVKSVSRETLKSRKKSDYSLNKVNAPILTN
TTLNVIRLVGKYMQMMNILKPIAFDVIHFMSQLFDYYLYAIYTFFGRNDSLESTGLGLSSSRLRTTLNRIQESLIDLEVSADPTATL
TAAEERKEKVPSPHLSHLVVLTSGDTLYGLAERVVATESLVFLAEQFEFLQPHLDAVMPAVKKPFLQQFYSQTVSTASELRKPIYWI
VAGKALDYEQMLLLMANVKWDVKEIMSQHNIYVDALLKEFEQFNRRLNEVSKRVRIPLPVSNILWEHCIRLANRTIVEGYANVKKCS
NEGRALMQLDFQQFLMKLEKLTDIRPIPDKEFVETYIKAYYLTENDMERWIKEHREYSTKQLTNLVNVCLGSHINKKARQKLLAAID
DIDRPKR
>sp|Q9H9H5|MA6D1_HUMAN
MAWPCISRLCCLARRWNQLDRSDVAVPLTLHGYSDLDSEEPGTGGAASRRGQPPAGARDSGRDVPLTQYQRDFGLWTTPAGPKDPPP
GRGPGAGGRRGKSSAQSSAPPAPGARGVYVLPIGDADAAAAVTTSYRQEFQAWTGVKPSRSTKTKPARVITTHTSGWDSSPGAGFQV
PEVRKKFTPNPSAIFQASAPRILNV
>sp|Q8TBR7|FA57A_HUMAN
MLLTLAGGALFFPGLFALCTWALRRSQPGWSRTDCVMISTRLVSSVHAVLATGSGIVIIRSCDDVITGRHWLAREYVWFLIPYMIYD
SYAMYLCEWCRTRDQNRAPSLTLRNFLSRNRLMITHHAVILFVLVPVAQRLRGDLGDFFVGCIFTAELSTPFVSLGRVLIQLKQQHT
LLYKVNGILTLATFLSCRILLFPFMYWSYGRQQGLSLLQVPFSIPFYCNVANAFLVAPQIYWFCLLCRKAVRLFDTPQAKKDG
>sp|Q49AS3|L37A5_HUMAN
MNRNILEEMLQYLLIDWIVGDQFEIQLNQQLWSLIPNNDVRRLVSHVIRTLKTDCTETHLQLACAKLISRTGLLMKLLSEQQELRTV
SMTAWKPRMNRKSRSRMRS
>sp|Q07075|AMPE_HUMAN
MNFAEREGSKRYCIQTKHVAILCAVVVGVGLIVGLAVGLTRSCDSSGDGGPGTAPAPSHLPSSTASPSGPPAQDQDICPASEDESGQ
WKNFRLPDFVNPVHYDLHVKPLLEEDTYTGTVSISINLSAPTRYLWLHLRETRITRLPELKRPSGDQVQVRRCFEYKKQEYVVVEAE
EELTPSSGDGLYLLTMEFAGWLNGSLVGFYRTTYTENGQVKSIVATDHEPTDARKSFPCFDEPNKKATYTISITHPKEYGALSNMPV
AKEESVDDKWTRTTFEKSVPMSTYLVCFAVHQFDSVKRISNSGKPLTIYVQPEQKHTAEYAANITKSVFDYFEEYFAMNYSLPKLDK
IAIPDFGTGAMENWGLITYRETNLLYDPKESASSNQQRVATVVAHELVHQWFGNIVTMDWWEDLWLNEGFASFFEFLGVNHAETDWQ
MRDQMLLEDVLPVQEDDSLMSSHPIIVTVTTPDEITSVFDGISYSKGSSILRMLEDWIKPENFQKGCQMYLEKYQFKNAKTSDFWAA
LEEASRLPVKEVMDTWTRQMGYPVLNVNGVKNITQKRFLLDPRANPSQPPSDLGYTWNIPVKWTEDNITSSVLFNRSEKEGITLNSS
NPSGNAFLKINPDHIGFYRVNYEVATWDSIATALSLNHKTFSSADRASLIDDAFALARAQLLDYKVALNLTKYLKREENFLPWQRVI
SAVTYIISMFEDDKELYPMIEEYFQGQVKPIADSLGWNDAGDHVTKLLRSSVLGFACKMGDREALNNASSLFEQWLNGTVSLPVNLR
LLVYRYGMQNSGNEISWNYTLEQYQKTSLAQEKEKLLYGLASVKNVTLLSRYLDLLKDTNLIKTQDVFTVIRYISYNSYGKNMAWNW
IQLNWDYLVNRYTLNNRNLGRIVTIAEPFNTELQLWQMESFFAKYPQAGAGEKPREQVLETVKNNIEWLKQHRNTIREWFFNLLESG
>sp|P19876|CXCL3_HUMAN
MAHATLSAAPSNPRLLRVALLLLLLVAASRRAAGASVVTELRCQCLQTLQGIHLKNIQSVNVRSPGPHCAQTEVIATLKNGKKACLN
PASPMVQKIIEKILNKGSTN
>sp|Q15561|TEAD4_HUMAN
MEGTAGTITSNEWSSPTSPEGSTASGGSQALDKPIDNDAEGVWSPDIEQSFQEALAIYPPCGRRKIILSDEGKMYGRNELIARYIKL
RTGKTRTRKQVSSHIQVLARRKAREIQAKLKDQAAKDKALQSMAAMSSAQIISATAFHSSMALARGPGRPAVSGFWQGALPGQAGTS
HDVKPFSQQTYAVQPPLPLPGFESPAGPAPSPSAPPAPPWQGRSVASSKLWMLEFSAFLEQQQDPDTYNKHLFVHIGQSSPSYSDPY
LEAVDIRQIYDKFPEKKGGLKDLFERGPSNAFFLVKFWADLNTNIEDEGSSFYGVSSQYESPENMIITCSTKVCSFGKQVVEKVETE
YARYENGHYSYRIHRSPLCEYMINFIHKLKHLPEKYMMNSVLENFTILQVVTNRDTQETLLCIAYVFEVSASEHGAQHHIYRLVKE
>sp|Q9H9Y2|RPF1_HUMAN
MAKAGDKSSSSGKKSLKRKAAAEELQEAAGAGDGATENGVQPPKAAAFPPGFSISEIKNKQRRHLMFTRWKQQQRKEKLAAKKKLKK
EREALGDKAPPKPVPKTIDNQRVYDETTVDPNDEEVAYDEATDEFASYFNKQTSPKILITTSDRPHGRTVRLCEQLSTVIPNSHVYY
RRGLALKKIIPQCIARDFTDLIVINEDRKTPNGLILSHLPNGPTAHFKMSSVRLRKEIKRRGKDPTEHIPEIILNNFTTRLGHSIGR

MFASLFPHNPQFIGRQVATFHNQRDYIFFRFHRYIFRSEKKVGIQELGPRFTLKLRSLQKGTFDSKYGEYEWVHKPREMDTSRRKFH
L
>sp|Q9HBL0|TENS1_HUMAN
MSVSRTMEDSCELDLVYVTERIIAVSFPSTANEENFRSNLREVAQMLKSKHGGNYLLFNLSERRPDITKLHAKVLEFGWPDLHTPAL
EKICSICKAMDTWLNADPHNVVVLHNKGNRGRIGVVIAAYMHYSNISASADQALDRFAMKRFYEDKIVPIGQPSQRRYVHYFSGLLS
GSIKMNNKPLFLHHVIMHGIPNFESKGGCRPFLRIYQAMQPVYTSGIYNIPGDSQTSVCITIEPGLLLKGDILLKCYHKKFRSPARD
VIFRVQFHTCAIHDLGVVFGKEDLDDAFKDDRFPEYGKVEFVFSYGPEKIQGMEHLENGPSVSVDYNTSDPLIRWDSYDNFSGHRDD
GMEEVVGHTQGPLDGSLYAKVKKKDSLHGSTGAVNATRPTLSATPNHVEHTLSVSSDSGNSTASTKTDKTDEPVPGASSATAALSPQ
EKRELDRLLSGFGLEREKQGAMYHTQHLRSRPAGGSAVPSSGRHVVPAQVHVNGGALASERETDILDDELPNQDGHSAGSMGTLSSL
DGVTNTSEGGYPEALSPLTNGLDKSYPMEPMVNGGGYPYESASRAGPAHAGHTAPMRPSYSAQEGLAGYQREGPHPAWPQPVTTSHY
AHDPSGMFRSQSFSEAEPQLPPAPVRGGSSREAVQRGLNSWQQQQQQQQQPRPPPRQQERAHLESLVASRPSPQPLAETPIPSLPEF
PRAASQQEIEQSIETLNMLMLDLEPASAAAPLHKSQSVPGAWPGASPLSSQPLSGSSRQSHPLTQSRSGYIPSGHSLGTPEPAPRAS
LESVPPGRSYSPYDYQPCLAGPNQDFHSKSPASSSLPAFLPTTHSPPGPQQPPASLPGLTAQPLLSPKEATSDPSRTPEEEPLNLEG
LVAHRVAGVQAREKQPAEPPAPLRRRAASDGQYENQSPEATSPRSPGVRSPVQCVSPELALTIALNPGGRPKEPHLHSYKEAFEEME
GTSPSSPPPSGVRSPPGLAKTPLSALGLKPHNPADILLHPTGVTRRRIQPEEDEGKVVVRLSEEPRSYVESVARTAVAGPRAQDSEP
KSFSAPATQAYGHEIPLRNGTLGGSFVSPSPLSTSSPILSADSTSVGSFPSGESSDQGPRTPTQPLLESGFRSGSLGQPSPSAQRNY
QSSSPLPTVGSSYSSPDYSLQHFSSSPESQARAQFSVAGVHTVPGSPQARHRTVGTNTPPSPGFGWRAINPSMAAPSSPSLSHHQMM
GPPGTGFHGSTVSSPQSSAATTPGSPSLCRHPAGVYQVSGLHNKVATTPGSPSLGRHPGAHQGNLASGLHSNAIASPGSPSLGRHLG
GSGSVVPGSPCLDRHVAYGGYSTPEDRRPTLSRQSSASGYQAPSTPSFPVSPAYYPGLSSPATSPSPDSAAFRQGSPTPALPEKRRM
SVGDRAGSLPNYATINGKVSSPVASGMSSPSGGSTVSFSHTLPDFSKYSMPDNSPETRAKVKFVQDTSKYWYKPEISREQAIALLKD
QEPGAFIIRDSHSFRGAYGLAMKVSSPPPTIMQQNKKGDMTHELVRHFLIETGPRGVKLKGCPNEPNFGSLSALVYQHSIIPLALPC
KLVIPNRDPTDESKDSSGPANSTADLLKQGAACNVLFVNSVDMESLTGPQAISKATSETLAADPTPAATIVHFKVSAQGITLTDNQR
KLFFRRHYPLNTVTFCDLDPQERKWMKTEGGAPAKLFGFVARKQGSTTDNACHLFAELDPNQPASAIVNFVSKVMLNAGQKR
>sp|P51665|PSMD7_HUMAN
MPELAVQKVVVHPLVLLSVVDHFNRIGKVGNQKRVVGVLLGSWQKKVLDVSNSFAVPFDEDDKDDSVWFLDHDYLENMYGMFKKVNA
RERIVGWYHTGPKLHKNDIAINELMKRYCPNSVLVIIDVKPKDLGLPTEAYISVEEVHDDGTPTSKTFEHVTSEIGAEEAEEVGVEH
LLRDIKDTTVGTLSQRITNQVHGLKGLNSKLLDIRSYLEKVATGKLPINHQIIYQLQDVFNLLPDVSLQEFVKAFYLKTNDQMVVVY
LASLIRSVVALHNLINNKIANRDAEKKEGQEKEESKKDRKEDKEKDKDKEKSDVKKEEKKEKK
>sp|Q6UWQ7|IGFL2_HUMAN
MVPRIFAPAYVSVCLLLLCPREVIAPAGSEPWLCQPAPRCGDKIYNPLEQCCYNDAIVSLSETRQCGPPCTFWPCFELCCLDSFGLT
NDFVVKLKVQGVNSQCHSSPISSKCESRRRFP
>sp|P10768|ESTD_HUMAN
MALKQISSNKCFGGLQKVFEHDSVELNCKMKFAVYLPPKAETGKCPALYWLSGLTCTEQNFISKSGYHQSASEHGLVVIAPDTSPRG
CNIKGEDESWDFGTGAGFYVDATEDPWKTNYRMYSYVTEELPQLINANFPVDPQRMSIFGHSMGGHGALICALKNPGKYKSVSAFAP
ICNPVLCPWGKKAFSGYLGTDQSKWKAYDATHLVKSYPGSQLDILIDQGKDDQFLLDGQLLPDNFIAACTEKKIPVVFRLQEGYDHS
YYFIATFITDHIRHHAKYLNA
>sp|Q8TAP8|PPR35_HUMAN
MMMGCGESELKSADGEEAAAVPGPPPEPQVPQLRAPVPEPGLDLSLSPRPDSPQPRHGSPGRRKGRAERRGAARQRRQVRFRLTPPS
PVRSEPQPAVPQELEMPVLKSSLALGLELRAAAGSHFDAAKAVEEQLRKSFQIRCGLEESVSEGLNVPRSKRLFRDLVSLQVPEEQV
LNAALREKLALLPPQARAPHPKEPPGPGPDMTILCDPETLFYESPHLTLDGLPPLRLQLRPRPSEDTFLMHRTLRRWEA
>sp|Q8NC26|ZN114_HUMAN
MSQDSVTFADVAVNFTKEEWTLLDPAQRNLYRDVMLENSRNLAFIDWATPCKTKDATPQPDILPKRTFPEANRVCLTSISSQHSTLR
EDWRCPKTEEPHRQGVNNVKPPAVAPEKDESPVSICEDHEMRNHSKPTCRLVPSQGDSIRQCILTRDSSIFKYNPVLNDSQKTHENN
EDDGVLGWNIQWVPCGRKTELKSSTWTGSQNTVHHIRDEIDTGANRHQRNPFGKAFREDGSLRAHNTHGREKMYDFTQCENTSRNNS

IHAMQMQLYTAETNKKDCQTGATSANAPNSGSHKSHCTGEKTHKCPECGRAFFYQSFLMRHMKIHTGEKPYECGKCGKAFRYSLHLN
KHLRKHVVQKKPYECEECGKVIRESSKYTHIRSHTGEKPYKCKTCGKDFAKSSGLKKHLKTHKDEKPCE
>sp|Q8IXN7|RIMKA_HUMAN
MCSQLWFLTDRRIREDYPQVQILRALRQRCSEQDVRFRAVLMDQIAVTIVGGHLGLQLNQKALTTFPDVVLVRVPTPSVQSDSDITV
LRHLEKLGCRLVNRPQSILNCINKFWTFQELAGHGVPMPDTFSYGGHEDFSKMIDEAEPLGYPVVVKSTRGHRGKAVFLARDKHHLS
DICHLIRHDVPYLFQKYVKESHGKDIRVVVVGGQVIGSMLRCSTDGRMQSNCSLGGVGVKCPLTEQGKQLAIQVSNILGMDFCGIDL
LIMDDGSFVVCEANANVGFLAFDQACNLDVGGIIADYTMSLLPNRQTGKMAVLPGLSSPREKNEPDGCASAQGVAESVYTINSGSTS
SESEPELGEIRDSSASTMGAPPSMLPEPGYNINNRIASELKLK
>sp|Q8WX77|IBPL1_HUMAN
MPRLSLLLPLLLLLLLPLLPPLSPSLGIRDVGGRRPKCGPCRPEGCPAPAPCPAPGISALDECGCCARCLGAEGASCGGRAGGRCGP
GLVCASQAAGAAPEGTGLCVCAQRGTVCGSDGRSYPSVCALRLRARHTPRAHPGHLHKARDGPCEFAPVVVVPPRSVHNVTGAQVGL
SCEVRAVPTPVITWRKVTKSPEGTQALEELPGDHVNIAVQVRGGPSDHEATAWILINPLRKEDEGVYQCHAANMVGEAESHSTVTVL
DLSKYRSFHFPAPDDRM
>sp|Q13595|TRA2A_HUMAN
MSDVEENNFEGRESRSQSKSPTGTPARVKSESRSGSRSPSRVSKHSESHSRSRSKSRSRSRRHSHRRYTRSRSHSHSHRRRSRSRSY
TPEYRRRRSRSHSPMSNRRRHTGSRANPDPNTCLGVFGLSLYTTERDLREVFSRYGPLSGVNVVYDQRTGRSRGFAFVYFERIDDSK
EAMERANGMELDGRRIRVDYSITKRAHTPTPGIYMGRPTHSGGGGGGGGGGGGGGGGGRRRDSYYDRGYDRGYDRYEDYDYRYRRRSP
SPYYSRYRSRSRSRSYSPRRY
>sp|Q96M02|CJ090_HUMAN
MLKLSGEGLRDSYHSRRDQIALKNLQSDVTEAKSDFTKETLASQNTKMISSIVISQMIDENKSRENRASLPLPCAIAQSRAHHAKQS
LANRSGVNIHRAFALLPGRLGIPAPSDERGPEAELPPKEERPCGGPRRGFASITITARRVGPPARALVWGTAGDSLCPKCRAEDTLF
QAPPALANGAHPGRHQRSFACTEFSRNSSVVRLKVPEAHTGLCERRKYWVTHADDKETSFSPDTPLSGKSPLVFSSCVHLRVSQQCP
DSIYYVDKSLSVPIEPPQIASPKMHRSVLSLNLNCSSHRLTADGVDGLVNREPISEALKQELLEGDQDLVGQRWNPGLQESHLKETP
SLRRVHLGTGACPWSGSFPLENTELANVGANQVTVRKGEKDHTTHCHASDHANQLSIHIPGWSYRAVHTKVFSGSSKRQQGEVCMTV
SAPPVEQKPTRHFLPIGDSSPSDDCLSRDLSEPTERRHQSFLKPRILFPGFLCPLQDVCASLQEDNGVQIESKFPKGDYTCCDLVVK
IKECKKSEDPTTPEPSPAAPSPAPRDGAGSPGLSEDCSESQQTPARSLTLQEALEVRKPQFISRSQERLKKLEHMVQQRKAQRKEDL
RQKQSLLPIRTSKKQFTIPHPLSDNLFKPKERCISEKEMHMRSKRIYDNLPEVKKKKEEQRKRVILQSNRLRAEVFKKQLLDQLLQR
NAV
>sp|P12643|BMP2_HUMAN
MVAGTRCLLALLLPQVLLGGAAGLVPELGRRKFAAASSGRPSSQPSDEVLSEFELRLLSMFGLKQRPTPSRDAVVPPYMLDLYRRHS
GQPGSPAPDHRLERAASRANTVRSFHHEESLEELPETSGKTTRRFFFNLSSIPTEEFITSAELQVFREQMQDALGNNSSFHHRINIY
EIIKPATANSKFPVTRLLDTRLVNQNASRWESFDVTPAVMRWTAQGHANHGFVVEVAHLEEKQGVSKRHVRISRSLHQDEHSWSQIR
PLLVTFGHDGKGHPLHKREKRQAKHKQRKRLKSSCKRHPLYVDFSDVGWNDWIVAPPGYHAFYCHGECPFPLADHLNSTNHAIVQTL
VNSVNSKIPKACCVPTELSAISMLYLDENEKVVLKNYQDMVVEGCGCR
>sp|P0CW23|AKAI1_HUMAN
MVFAPGEKPGNEPEEVKLQNASKQIVQNAILQAVQQVSQESQRREERISDNRDHIQLGVGELTKKHEKK
>sp|Q8IY37|DHX37_HUMAN
MGKLRRRYNIKGRQQAGPGPSKGPPEPPPVQLELEDKDTLKGVDASNALVLPGKKKKKTKAPPLSKKEKKPLTKKEKKVLQKILEQK
EKKSQRAEMLQKLSEVQASEAEMRLFYTTSKLGTGNRMYHTKEKADEVVAPGQEKISSLSGAHRKRRRWPSAEEEEEEEEESESELE
EESELDEDPAAEPAEAGVGTTVAPLPPAPAPSSQPVPAGMTVPPPPAAAPPLPRALAKPAVFIPVNRSPEMQEERLKLPILSEEQVI
MEAVAEHPIVIVCGETGSGKTTQVPQFLYEAGFSSEDSIIGVTEPRRVAAVAMSQRVAKEMNLSQRVVSYQIRYEGNVTEETRIKFM
TDGVLLKEIQKDFLLLRYKVVIIDEAHERSVYTDILIGLLSRIVTLRAKRNLPLKLLIMSATLRVEDFTQNPRLFAKPPPVIKVESR
QFPVTVHFNKRTPLEDYSGECFRKVCKIHRMLPAGGILVFLTGQAEVHALCRRLRKAFPPSRARPQEKDDDQKDSVEEMRKFKKSRA
RAKKARAEVLPQINLDHYSVLPAGEGDEDREAEVDEEEGALDSDLDLDLGDGGQDGGEQPDASLPLHVLPLYSLLAPEKQAQVFKPP

PEGTRLCVVATNVAETSLTIPGIKYVVDCGKVKKRYYDRVTGVSSFRVTWVSQASADQRAGRAGRTEPGHCYRLYSSAVFGDFEQFP
PPEITRRPVEDLILQMKALNVEKVINFPFPTPPSVEALLAAEELLIALGALQPPQKAERVKQLQENRLSCPITALGRTMATFPVAPR
YAKMLALSRQHGCLPYAITIVASMTVRELFEELDRPAASDEELTRLKSKRARVAQMKRTWAGQGASLKLGDLMVLLGAVGACEYASC
TPQFCEANGLRYKAMMEIRRLRGQLTTAVNAVCPEAELFVDPKMQPPTESQVTYLRQIVTAGLGDHLARRVQSEEMLEDKWRNAYKT
PLLDDPVFIHPSSVLFKELPEFVVYQEIVETTKMYMKGVSSVEVQWIPALLPSYCQFDKPLEEPAPTYCPERGRVLCHRASVFYRVG
WPLPAIEVDFPEGIDRYKHFARFLLEGQVFRKLASYRSCLLSSPGTMLKTWARLQPRTESLLRALVAEKADCHEALLAAWKKNPKYL
LAEYCEWLPQAMHPDIEKAWPPTTVH
>sp|P48730|KC1D_HUMAN
MELRVGNRYRLGRKIGSGSFGDIYLGTDIAAGEEVAIKLECVKTKHPQLHIESKIYKMMQGGVGIPTIRWCGAEGDYNVMVMELLGP
SLEDLFNFCSRKFSLKTVLLLADQMISRIEYIHSKNFIHRDVKPDNFLMGLGKKGNLVYIIDFGLAKKYRDARTHQHIPYRENKNLT
GTARYASINTHLGIEQSRRDDLESLGYVLMYFNLGSLPWQGLKAATKRQKYERISEKKMSTPIEVLCKGYPSEFATYLNFCRSLRFD
DKPDYSYLRQLFRNLFHRQGFSYDYVFDWNMLKFGASRAADDAERERRDREERLRHSRNPATRGLPSTASGRLRGTQEVAPPTPLTP
TSHTANTSPRPVSGMERERKVSMRLHRGAPVNISSSDLTGRQDTSRMSTSQIPGRVASSGLQSVVHR
>sp|Q96DT0|LEG12_HUMAN
MSQPSGGRAPGTRIYSWSCPTVMSPGEKLDPIPDSFILQPPVFHPVVPYVTTIFGGLHAGKMVMLQGVVPLDAHRFQVDFQCGCSLC
PRPDIAFHFNPRFHTTKPHVICNTLHGGRWQREARWPHLALRRGSSFLILFLFGNEEVKVSVNGQHFLHFRYRLPLSHVDTLGIFGD
ILVEAVGFLNINPFVEGSREYPAGHPFLLMSPRLEVPCSHALPQGLSPGQVIIVRGLVLQEPKHFTVSLRDQAAHAPVTLRASFADR
TLAWISRWGQKKLISAPFLFYPQRFFEVLLLFQEGGLKLALNGQGLGATSMNQQALEQLRELRISGSVQLYCVHS
>sp|Q9NP92|RT30_HUMAN
MAAARCWRPLLRGPRLSLHTAANAAATATETTCQDVAATPVARYPPIVASMTADSKAARLRRIERWQATVHAAESVDEKLRILTKMQ
FMKYMVYPQTFALNADRWYQYFTKTVFLSGLPPPPAEPEPEPEPEPEPALDLAALRAVACDCLLQEHFYLRRRRRVHRYEESEVISL
PFLDQLVSTLVGLLSPHNPALAAAALDYRCPVHFYWVRGEEIIPRGHRRGRIDDLRYQIDDKPNNQIRISKQLAEFVPLDYSVPIEI
PTIKCKPDKLPLFKRQYENHIFVGSKTADPCCYGHTQFHLLPDKLRRERLLRQNCADQIEVVFRANAIASLFAWTGAQAMYQGFWSE
ADVTRPFVSQAVITDGKYFSFFCYQLNTLALTTQADQNNPRKNICWGTQSKPLYETIEDNDVKGFNDDVLLQIVHFLLNRPKEEKSQ
LLEN
>sp|O00443|P3C2A_HUMAN
MAQISSNSGFKECPSSHPEPTRAKDVDKEEALQMEAEALAKLQKDRQVTDNQRGFELSSSTRKKAQVYNKQDYDLMVFPESDSQKRA
LDIDVEKLTQAELEKLLLDDSFETKKTPVLPVTPILSPSFSAQLYFRPTIQRGQWPPGLPGPSTYALPSIYPSTYSKQAAFQNGFNP
RMPTFPSTEPIYLSLPGQSPYFSYPLTPATPFHPQGSLPIYRPVVSTDMAKLFDKIASTSEFLKNGKARTDLEITDSKVSNLQVSPK
SEDISKFDWLDLDPLSKPKVDNVEVLDHEEEKNVSSLLAKDPWDAVLLEERSTANCHLERKVNGKSLSVATVTRSQSLNIRTTQLAK
AQGHISQKDPNGTSSLPTGSSLLQEVEVQNEEMAAFCRSITKLKTKFPYTNHRTNPGYLLSPVTAQRNICGENASVKVSIDIEGFQL
PVTFTCDVSSTVEIIIMQALCWVHDDLNQVDVGSYVLKVCGQEEVLQNNHCLGSHEHIQNCRKWDTEIRLQLLTFSAMCQNLARTAE
DDETPVDLNKHLYQIEKPCKEAMTRHPVEELLDSYHNQVELALQIENQHRAVDQVIKAVRKICSALDGVETLAITESVKKLKRAVNL
PRSKTADVTSLFGGEDTSRSSTRGSLNPENPVQVSINQLTAAIYDLLRLHANSGRSPTDCAQSSKSVKEAWTTTEQLQFTIFAAHGI
SSNWVSNYEKYYLICSLSHNGKDLFKPIQSKKVGTYKNFFYLIKWDELIIFPIQISQLPLESVLHLTLFGILNQSSGSSPDSNKQRK
GPEALGKVSLPLFDFKRFLTCGTKLLYLWTSSHTNSVPGTVTKKGYVMERIVLQVDFPSPAFDIIYTTPQVDRSIIQQHNLETLEND
IKGKLLDILHKDSSLGLSKEDKAFLWEKRYYCFKHPNCLPKILASAPNWKWVNLAKTYSLLHQWPALYPLIALELLDSKFADQEVRS
LAVTWIEAISDDELTDLLPQFVQALKYEIYLNSSLVQFLLSRALGNIQIAHNLYWLLKDALHDVQFSTRYEHVLGALLSVGGKRLRE
ELLKQTKLVQLLGGVAEKVRQASGSARQVVLQRSMERVQSFFQKNKCRLPLKPSLVAKELNIKSCSFFSSNAVPLKVTMVNADPMGE
EINVMFKVGEDLRQDMLALQMIKIMDKIWLKEGLDLRMVIFKCLSTGRDRGMVELVPASDTLRKIQVEYGVTGSFKDKPLAEWLRKY
NPSEEEYEKASENFIYSCAGCCVATYVLGICDRHNDNIMLRSTGHMFHIDFGKFLGHAQMFGSFKRDRAPFVLTSDMAYVINGGEKP
TIRFQLFVDLCCQAYNLIRKQTNLFLNLLSLMIPSGLPELTSIQDLKYVRDALQPQTTDAEATIFFTRLIESSLGSIATKFNFFIHN
LAQLRFSGLPSNDEPILSFSPKTYSFRQDGRIKEVSVFTYHKKYNPDKHYIYVVRILREGQIEPSFVFRTFDEFQELHNKLSIIFPL
WKLPGFPNRMVLGRTHIKDVAAKRKIELNSYLQSLMNASTDVAECDLVCTFFHPLLRDEKAEGIARSADAGSFSPTPGQIGGAVKLS

ISYRNGTLFIMVMHIKDLVTEDGADPNPYVKTYLLPDNHKTSKRKTKISRKTRNPTFNEMLVYSGYSKETLRQRELQLSVLSAESLR
ENFFLGGVTLPLKDFNLSKETVKWYQLTAATYL
>sp|Q6A555|TXND8_HUMAN
MVQIIKDTNEFKTFLTAAGHKLAVVQFSSKRCGPCKRMFPVFHAMSVKYQNVFFANVDVNNSPELAETCHIKTIPTFQMFKKSQKVT
LFSRIKRIICCYRSGFMSNLIFEFCGADAKKLEAKTQELM
>sp|P55771|PAX9_HUMAN
MEPAFGEVNQLGGVFVNGRPLPNAIRLRIVELAQLGIRPCDISRQLRVSHGCVSKILARYNETGSILPGAIGGSKPRVTTPTVVKHI
RTYKQRDPGIFAWEIRDRLLADGVCDKYNVPSVSSISRILRNKIGNLAQQGHYDSYKQHQPTPQPALPYNHIYSYPSPITAAAAKVP
TPPGVPAIPGSVAMPRTWPSSHSVTDILGIRSITDQVSDSSPYHSPKVEEWSSLGRNNFPAAAPHAVNGLEKGALEQEAKYGQAPNG
LPAVGSFVSASSMAPYPTPAQVSPYMTYSAAPSGYVAGHGWQHAGGTSLSPHNCDIPASLAFKGMQAAREGSHSVTASAL
>sp|Q9Y2P0|ZN835_HUMAN
MEGLLSVALQGAELEGNWKHEGQVEDLQENQESCPEPEAVACKGDPAGDSMQERDEFSRIPRTISSPAATQASVPDDSSSRRCSAPG
ESPKERHPDSRQRERGGGPKKPWKCGDCGKAFSYCSAFILHQRIHTGEKPFACPECGKAFSQSVHLTLHQRTHTGEKPYACHECGKA
FSQGSYLASHWRTHTGEKPHRCADCGKAFTRVTHLTQHRRVHTGERPYACAQCAKAFRNRSSLIEHQRIHTGEKPYECSACAKAFRF
SSALIRHQRIHTEEKPYRCGQCAKAFAQIAHLTQHRRVHTGEKPYTCQDCGALFSQSASLAEHRRIHTGEKPYACGQCAKAFTQVSH
LTQHQRTHTGERPYPCHDCGKRFSNRSHLLQHRLVHTGERPYRCLQCGAAFSHVSSLIEHQKIHTGERPYKCGECGKAFSQGSSLAL
HQRTHTGERPYTCPECGKAFSNRSYLIQHHIVHTGEKPYECSGCGKAFSFSSALIRHQRTHADSSGRLCPAPTPDSTPGLSQGGETC
QQGCPGRNPRGPAED
>sp|Q8TBP0|TBC16_HUMAN
MSLGRLLRRASSKASDLLTLTPGGSGSGSPSVLDGEIIYSKNNVCVHPPEGLQGLGEHHPGYLCLYMEKDEMLGATLILAWVPNSRI
QRQDEEALRYITPESSPVRKAPRPRGRRTRSSGASHQPSPTELRPTLTPKDEDILVVAQSVPDRMLASPAPEDEEKLAQGLGVDGAQ
PASQPACSPSGILSTVSPQDVTEEGREPRPEAGEEDGSLELSAEGVSRDSSFDSDSDTFSSPFCLSPISAALAESRGSVFLESDSSP
PSSSDAGLRFPDSNGLLQTPRWDEPQRVCALEQICGVFRVDLGHMRSLRLFFSDEACTSGQLVVASRESQYKVFHFHHGGLDKLSDV
FQQWKYCTEMQLKDQQVAPDKTCMQFSIRRPKLPSSETHPEESMYKRLGVSAWLNHLNELGQVEEEYKLRKAIFFGGIDVSIRGEVW
PFLLRYYSHESTSEEREALRLQKRKEYSEIQQKRLSMTPEEHRAFWRNVQFTVDKDVVRTDRNNQFFRGEDNPNVESMRRILLNYAV
YNPAVGYSQGMSDLVAPILAEVLDESDTFWCFVGLMQNTIFVSSPRDEDMEKQLLYLRELLRLTHVRFYQHLVSLGEDGLQMLFCHR
WLLLCFKREFPEAEALRIWEACWAHYQTDYFHLFICVAIVAIYGDDVIEQQLATDQMLLHFGNLAMHMNGELVLRKARSLLYQFRLL
PRIPCSLHDLCKLCGSGMWDSGSMPAVECTGHHPGSESCPYGGTVEMPSPKSLREGKKGPKTPQDGFGFRR
>sp|P32456|GBP2_HUMAN
MAPEINLPGPMSLIDNTKGQLVVNPEALKILSAITQPVVVVAIVGLYRTGKSYLMNKLAGKKNGFSLGSTVKSHTKGIWMWCVPHPK
KPEHTLVLLDTEGLGDIEKGDNENDSWIFALAILLSSTFVYNSMGTINQQAMDQLHYVTELTDRIKANSSPGNNSVDDSADFVSFFP
AFVWTLRDFTLELEVDGEPITADDYLELSLKLRKGTDKKSKSFNDPRLCIRKFFPKRKCFVFDWPAPKKYLAHLEQLKEEELNPDFI
EQVAEFCSYILSHSNVKTLSGGIPVNGPRLESLVLTYVNAISSGDLPCMENAVLALAQIENSAAVEKAIAHYEQQMGQKVQLPTETL
QELLDLHRDSEREAIEVFMKNSFKDVDQMFQRKLGAQLEARRDDFCKQNSKASSDCCMALLQDIFGPLEEDVKQGTFSKPGGYRLFT
QKLQELKNKYYQVPRKGIQAKEVLKKYLESKEDVADALLQTDQSLSEKEKAIEVERIKAESAEAAKKMLEEIQKKNEEMMEQKEKSY
QEHVKQLTEKMERDRAQLMAEQEKTLALKLQEQERLLKEGFENESKRLQKDIWDIQMRSKSLEPICNIL
>sp|Q05707|COEA1_HUMAN
MKIFQRKMRYWLLPPFLAIVYFCTIVQGQVAPPTRLRYNVISHDSIQISWKAPRGKFGGYKLLVTPTSGGKTNQLNLQNTATKAIIQ
GLMPDQNYTVQIIAYNKDKESKPAQGQFRIKDLEKRKDPKPRVKVVDRGNGSRPSSPEEVKFVCQTPAIADIVILVDGSWSIGRFNF
RLVRHFLENLVTAFDVGSEKTRIGLAQYSGDPRIEWHLNAFSTKDEVIEAVRNLPYKGGNTLTGLALNYIFENSFKPEAGSRTGVSK
IGILITDGKSQDDIIPPSRNLRESGVELFAIGVKNADVNELQEIASEPDSTHVYNVAEFDLMHTVVESLTRTLCSRVEEQDREIKAS
AHAITGPPTELITSEVTARSFMVNWTHAPGNVEKYRVVYYPTRGGKPDEVVVDGTVSSTVLKNLMSLTEYQIAVFAIYAHTASEGLR
GTETTLALPMASDLLLYDVTENSMRVKWDAVPGASGYLILYAPLTEGLAGDEKEMKIGETHTDIELSGLLPNTEYTVTVYAMFGEEA
SDPVTGQETTLALSPPRNLRISNVGSNSARLTWDPTSRQINGYRIVYNNADGTEINEVEVDPITTFPLKGLTPLTEYTIAIFSIYDE

GQSEPLTGVFTTEEVPAQQYLEIDEVTTDSFRVTWHPLSADEGLHKLMWIPVYGGKTEEVVLKEEQDSHVIEGLEPGTEYEVSLLAV
LDDGSESEVVTAVGTTLDSFWTEPATTIVPTTSVTSVFQTGIRNLVVGDETTSSLRVKWDISDSDVQQFRVTYMTAQGDPEEEVIGT
VMVPGSQNNLLLKPLLPDTEYKVTVTPIYTDGEGVSVSAPGKTLPSSGPQNLRVSEEWYNRLRITWDPPSSPVKGYRIVYKPVSVPG
PTLETFVGADINTILITNLLSGMDYNVKIFASQASGFSDALTGMVKTLFLGVTNLQAKHVEMTSLCAHWQVHRHATAYRVVIESLQD
RQKQESTVGGGTTRHCFYGLQPDSEYKISVYTKLQEIEGPSVSIMEKTQSLPTRPPTFPPTIPPAKEVCKAAKADLVFMVDGSWSIG
DENFNKIISFLYSTVGALNKIGTDGTQVAMVQFTDDPRTEFKLNAYKTKETLLDAIKHISYKGGNTKTGKAIKYVRDTLFTAESGTR
RGIPKVIVVITDGRSQDDVNKISREMQLDGYSIFAIGVADADYSELVSIGSKPSARHVFFVDDFDAFKKIEDELITFVCETASATCP
VVHKDGIDLAGFKMMEMFGLVEKDFSSVEGVSMEPGTFNVFPCYQLHKDALVSQPTRYLHPEGLPSDYTISFLFRILPDTPQEPFAL
WEILNKNSDPLVGVILDNGGKTLTYFNYDQSGDFQTVTFEGPEIRKIFYGSFHKLHIVVSETLVKVVIDCKQVGEKAMNASANITSD
GVEVLGKMVRSRGPGGNSAPFQLQMFDIVCSTSWANTDKCCELPGLRDDESCPDLPHSCSCSETNEVALGPAGPPGGPGLRGPKGQQ
GEPGPKGPDGPRGEIGLPGPQGPPGPQGPSGLSIQGMPGMPGEKGEKGDTGLPGPQGIPGGVGSPGRDGSPGQRGLPGKDGSSGPPG
PPGPIGIPGTPGVPGITGSMGPQGALGPPGVPGAKGERGERGDLQSQAMVRSVARQVCEQLIQSHMARYTAILNQIPSHSSSIRTVQ
GPPGEPGRPGSPGAPGEQGPPGTPGFPGNAGVPGTPGERGLTGIKGEKGNPGVGTQGPRGPPGPAGPSGESRPGSPGPPGSPGPRGP
PGHLGVPGPQGPSGQPGYCDPSSCSAYGVRAPHPDQPEFTPVQDELEAMELWGPGV
>sp|Q8N6F7|GCSAM_HUMAN
MGNSLLRENRRQQNTQEMPWNVRMQSPKQRTSRCWDHHIAEGCFCLPWKKILIFEKRQDSQNENERMSSTPIQDNVDQTYSEELCYT
LINHRVLCTRPSGNSAEEYYENVPCKAERPRESLGGTETEYSLLHMPSTDPRHARSPEDEYELLMPHRISSHFLQQPRPLMAPSETQ
FSHL
>sp|Q9UI30|TR112_HUMAN
MKLLTHNLLSSHVRGVGSRGFPLRLQATEVRICPVEFNPNFVARMIPKVEWSAFLEAADNLRLIQVPKGPVEGYEENEEFLRTMHHL
LLEVEVIEGTLQCPESGRMFPISRGIPNMLLSEEETES
>sp|P36915|GNL1_HUMAN
MPRKKPFSVKQKKKQLQDKRERKRGLQDGLRSSSNSRSGSRERREEQTDTSDGESVTHHIRRLNQQPSQGLGPRGYDPNRYRLHFER
DSREEVERRKRAAREQVLQPVSAELLELDIREVYQPGSVLDFPRRPPWSYEMSKEQLMSQEERSFQDYLGKIHGAYSSEKLSYFEHN
LETWRQLWRVLEMSDIVLLITDIRHPVVNFPPALYEYVTGELGLALVLVLNKVDLAPPALVVAWKHYFHQHYPQLHVVLFTSFPRDP
RTPQDPSSVLKKSRRRGRGWTRALGPEQLLRACEAITVGKVDLSSWREKIARDVAGATWGNGSGEEEEEEDGPAVLVEQQTDSAMEP
TGPTQERYKDGVVTIGCVGFPNVGKSSLINGLVGRKVVSVSRTPGHTRYFQTYFLTPSVKLCDCPGLIFPSLLPRQLQVLAGIYPIA
QIQEPYTAVGYLASRIPVQALLHLRHPEAEDPSAEHPWCAWDICEAWAEKRGYKTAKAARNDVYRAANSLLRLAVDGRLSLCFHPPG
YSEQKGTWESHPETTELVVLQGRVGPAGDEEEEEEEELSSSCEEEGEEDRDADEEGEGDEETPTSAPGSSLAGRNPYALLGEDEC
>sp|O60725|ICMT_HUMAN
MAGCAARAPPGSEARLSLATFLLGASVLALPLLTRAGLQGRTGLALYVAGLNALLLLLYRPPRYQIAIRACFLGFVFGCGTLLSFSQ
SSWSHFGWYMCSLSLFHYSEYLVTAVNNPKSLSLDSFLLNHSLEYTVAALSSWLEFTLENIFWPELKQITWLSVTGLLMVVFGECLR
KAAMFTAGSNFNHVVQNEKSDTHTLVTSGVYAWFRHPSYVGWFYWSIGTQVMLCNPICGVSYALTVWRFFRDRTEEEEISLIHFFGE
EYLEYKKRVPTGLPFIKGVKVDL
>sp|I6L899|GOG8R_HUMAN
MAEETQHNKLAAAKKKLKEYWQKNRPRVPAGVNRNRKTNGSIPETATSGGCQPPGDSATGFHREGPTSSATLKDLESPCQERAVVLD
STSVKISRLKNTIKSLKQQKKQVEHQLEEEKKANNERQKAERVLEVQIQTLIIQKEELNTDLYHMERSLRYFEEESKDLAVRLQHSL
QCKGELERALSAVIATEKKKANQLSSCSKAHTEWELEQSLQDQALLKAQLTQLKESFQQLQLERDECAEHIEGERARWHQRMSKMSQ
EICTLKKEKQDMRWVEQLEWSLSKLKNQTAEPLPPEPPAVPSEVELQHLRKELERVAGELQSQVKNNQHISLLNRRQEERIREQEER
LRKQEERLQEQHEKLRQLAKPQSVFEELNNENKSTLQLEQQVKELQEKLGEEHLEVASQQNQQLTAQLSLMALPGEGHGGEHLDSEG
EEAPQPMPSVPEDPESREAMSSFMDHLKEKADLSELLKKQELRFIQYWQERCHQKIHHLLSEPGGRAKDAALGGGHHQAGAQGGDEG
EAAGAAADGIAAYSNYNNGHRKFLAAAHNSADEPGPGAPAPQELGAADKHGDLREVTLTSSAQGEAREDPLLDKPTAQPIVQDHQEH
PGLGSNCCVPLFCWAWLPRRRR
>sp|O14901|KLF11_HUMAN

MHTPDFAGPDDARAVDIMDICESILERKRHDSERSTCSILEQTDMEAVEALVCMSSWGQRSQKGDLLRIRPLTPVSDSGDVTTTVHM
DAATPELPKDFHSLSTLCITPPQSPDLVEPSTRTPVSPQVTDSKACTATDVLQSSAVVARALSGGAERGLLGLEPVPSSPCRAKGTS
VIRHTGESPAACFPTIQTPDCRLSDSREGEEQLLGHFETLQDTHLTDSLLSTNLVSCQPCLHKSGGLLLTDKGQQAGWPGAVQTCSP
KNYENDLPRKTTPLISVSVPAPPVLCQMIPVTGQSSMLPAFLKPPPQLSVGTVRPILAQAAPAPQPVFVGPAVPQGAVMLVLPQGAL
PPPAPCAANVMAAGNTKLLPLAPAPVFITSSQNCVPQVDFSRRRNYVCSFPGCRKTYFKSSHLKAHLRTHTGEKPFNCSWDGCDKKF
ARSDELSRHRRTHTGEKKFVCPVCDRRFMRSDHLTKHARRHMTTKKIPGWQAEVGKLNRIASAESPGSPLVSMPASA
>sp|A6NE01|F186A_HUMAN
MFFKMKNEIDNDPESEKCIKDSTIMRREPQNILSPLMLPNLEIPFSVKDIISRIERAQLHRAREDIDMQLSEIMNNVHRIMTRYTLV
FNSSSERNVSLTEHKKKQRTNFLEKMATYAKTIEIREKTLANILAWLEEWNDVLSEMTLMDVDEHHHWIAQMELLPDTLKAIENNVK
ILSRFSTSFLDEKKKQKKKILSRGTLWKSWKERVIKRPSTARALRPDQMISDQLATNTKVSEIQGMLQELIGTTMFSTLENNAIKYI
SSTIVNLSTALSMLNDELKCVNFQSSTVYAHETSEAEKELSLKIIRDLSNENEMLQQKLQDAEEKCEQLIRSKIVIEQLYAKLSTSS
TLKVLPGPSPQSSRAIIKVGDTEDNMDNILDKELENIVDEVQRKETKDSGIKWDSTISYTAQAERTPDLTELRQQPVASEDISEDST
KDNVSLKKGDFYQEDETDEYQSWKRSHKKATYVYETSGPNLSDNKSGQKVSEAKPSQYYELQVLKKKRKEMKSFSEDKSKSPTEAKR
KHLSLTETKSQGGKSGTSMMMLEQFRKVKRESPFDKRPTAAEIKVEPTTESLDKEGKGEIRSLVEPLSMIQFDDTAEPQKGKIKGKK
HHISSGTITSKEEKTEEKEELTKQVKSHQLVKSLSRVAKETSESTRVLESPDGKSEQSNLEEFQEAIMAFLKQKIDNIGKAFDKKTV
PKEEELLKRAEAEKLGIIKAKMEEYFQKVAETVTKILRKYKDTKKEEQVGEKPIKQKKVVSFMPGLHFQKSPISAKSESSTLLSYES
TDPVINNLIQMILAEIESERDIPTVSTVQKDHKEKEKQRQEQYLQEGQEQMSGMSLKQQLLGERNLLKEHYEKISENWEEKKAWLQM
KEGKQEQQSQKQWQEEEMWKEEQKQATPKQAEQEEKQKQRGQEEEELPKSSLQRLEEGTQKMKTQGLLLEKENGQMRQIQKEAKHLG
PHRRREKGKEKQKPERGLEDLERQIKTKDQMQMKETQPKELEKMVIQTPMTLSPRWKSVLKDVQRSYEGKEFQRNLKTLENLPDEKE
PISITPPPSLQYSLPGALPISGQPLTKCIHLTPQQAQEVGITLTPQQAQAQGITLTLQQAQELGIPLTPQQAQALEILFTPQQAQAL
GIPLTPQQTQVQGITLTPQQDQAPGISLTTQQAQKLGIPLTPQQAQALGIPLTPQQAQELGIPLTPQQAQALRVSLTPQQAQELGIP
LTPQQAQALGITLTLQQAQQLGIPLTPQQAQALGITLTPKQVQELGIPLTPQQAQALGITLTPKQAQELGIPLNPQQAQTLGIPLTP
KQAQALGIPFTPQQAQALGIPLTPQQAQTQEITLTPQQAQALGMPLTTQQAQELGIPLTPQHAQALGMPLTTQQAQELGIPLTPQQA
QALGMPLTTQQAQELGIPLTPQQAQELGIPFTPQQAQAQEITLTPQQAQALGMPLTAQQAQELGITLTPQQAQELGIPLTPQQAQAL
GIPLIPPQAQELGIPLTPQQAQALGILLIPPQAQELGIPLTPQQAQALGIPLIPPQAQELGIPLTPQQVQALGIPLIPPQAQELEIP
LTPQQAQALGIPLTPQQAQELGIPLTPQQAQELGIPLTPQQAQAQGIPLTPQQAQALGISLTPQQAQAQGITLTPQQAQALGVPITP
VNAWVSAVTLTSEQTHALESPMNLEQAQEQLLKLGVPLTLDKAHTLGSPLTLKQVQWSHRPFQKSKASLPTGQSIISRLSPSLRLSL
ASSAPTAEKSSIFGVSSTPLQISRVPLNQGPFAPGKPLEMGILSEPGKLGAPQTLRSSGQTLVYGGQSTSAQFPAPQAPPSPGQLPI
SRAPPTPGQPFIAGVPPTSGQIPSLWAPLSPGQPLVPEASSIPGDLLESGPLTFSEQLQEFQPPATAEQSPYLQAPSTPGQHLATWT
LPGRASSLWIPPTSRHPPTLWPSPAPGKPQKSWSPSVAKKRLAIISSLKSKSVLIHPSAPDFKVAQVPFTTKKFQMSEVSDTSEETQ
ILRDTFAIESFRTFQSHFTKYRTPVYQTPYTDERALLTLMKPTTSPSSLTTLLRTSQISPLEWYQKSRFPPIDKPWILSSVSDTKKP
KVMVPPSSPQELEEKRYFVDVEAQKKNLILLNQAIKTCGLPSQLHTMARTLIIEILHMDTVQLGYLFRKYIAYRLIQHARNNIMKRL
KAIQNTGKGYEARNLHMMLSRLDDYGKKVMQVWTEKQKSLGQKRNQCLKKMIHVFNQLKKIHELNLSQPIPLIIEEKQIPASTTFVQ
KPFLKLLMEEDRTSDICKKFRQQEDQTEAIWNVDLSTSSYPIAEKTSMHSLWAQLGGYPDIPRLLQLEVQSTFRKSLASLQSRVKKI
PK
>sp|Q8NDB6|FA156_HUMAN
MDPLQKRNPASPSKSSPMTAAETSQEGPAPSQPSYSEQPMMGLSNLSPGPGPSQAVPLPEGLLRQRYREEKTLEERRWERLEFLQRK
KAFLRHVRRRHRDHMAPYAVGREARISPLGDRSQNRFRCECRYCQSHRPNLSGIPGESNRAPHPSSWETLVQGLSGLTLSLGTNQPG
PLPEAALQPQETEEKRQRERQQESKIMFQRLLKQWLEEN
>sp|Q99835|SMO_HUMAN
MAAARPARGPELPLLGLLLLLLLGDPGRGAASSGNATGPGPRSAGGSARRSAAVTGPPPPLSHCGRAAPCEPLRYNVCLGSVLPYGA
TSTLLAGDSDSQEEAHGKLVLWSGLRNAPRCWAVIQPLLCAVYMPKCENDRVELPSRTLCQATRGPCAIVERERGWPDFLRCTPDRF
PEGCTNEVQNIKFNSSGQCEVPLVRTDNPKSWYEDVEGCGIQCQNPLFTEAEHQDMHSYIAAFGAVTGLCTLFTLATFVADWRNSNR
YPAVILFYVNACFFVGSIGWLAQFMDGARREIVCRADGTMRLGEPTSNETLSCVIIFVIVYYALMAGVVWFVVLTYAWHTSFKALGT

TYQPLSGKTSYFHLLTWSLPFVLTVAILAVAQVDGDSVSGICFVGYKNYRYRAGFVLAPIGLVLIVGGYFLIRGVMTLFSIKSNHPG
LLSEKAASKINETMLRLGIFGFLAFGFVLITFSCHFYDFFNQAEWERSFRDYVLCQANVTIGLPTKQPIPDCEIKNRPSLLVEKINL
FAMFGTGIAMSTWVWTKATLLIWRRTWCRLTGQSDDEPKRIKKSKMIAKAFSKRHELLQNPGQELSFSMHTVSHDGPVAGLAFDLNE
PSADVSSAWAQHVTKMVARRGAILPQDISVTPVATPVPPEEQANLWLVEAEISPELQKRLGRKKKRRKRKKEVCPLAPPPELHPPAP
APSTIPRLPQLPRQKCLVAAGAWGAGDSCRQGAWTLVSNPFCPEPSPPQDPFLPSAPAPVAWAHGRRQGLGPIHSRTNLMDTELMDA
DSDF
>sp|O15457|MSH4_HUMAN
MLRPEISSTSPSAPAVSPSSGETRSPQGPRYNFGLQETPQSRPSVQVVSASTCPGTSGAAGDRSSSSSSLPCPAPNSRPAQGSYFGN
KRAYAENTVASNFTFGASSSSARDTNYPQTLKTPLSTGNPQRSGYKSWTPQVGYSASSSSAISAHSPSVIVAVVEGRGLARGEIGMA
SIDLKNPQIILSQFADNTTYAKVITKLKILSPLEIIMSNTACAVGNSTKLFTLITENFKNVNFTTIQRKYFNETKGLEYIEQLCIAE
FSTVLMEVQSKYYCLAAVAALLKYVEFIQNSVYAPKSLKICFQGSEQTAMIDSSSAQNLELLINNQDYRNNHTLFGVLNYTKTPGGS
RRLRSNILEPLVDIETINMRLDCVQELLQDEELFFGLQSVISRFLDTEQLLSVLVQIPKQDTVNAAESKITNLIYLKHTLELVDPLK
IAMKNCNTPLLRAYYGSLEDKRFGIILEKIKTVINDDARYMKGCLNMRTQKCYAVRSNINEFLDIARRTYTEIVDDIAGMISQLGEK
YSLPLRTSFSSARGFFIQMTTDCIALPSDQLPSEFIKISKVKNSYSFTSADLIKMNERCQESLREIYHMTYMIVCKLLSEIYEHIHC
LYKLSDTVSMLDMLLSFAHACTLSDYVRPEFTDTLAIKQGWHPILEKISAEKPIANNTYVTEGSNFLIITGPNMSGKSTYLKQIALC
QIMAQIGSYVPAEYSSFRIAKQIFTRISTDDDIETNSSTFMKEMKEIAYILHNANDKSLILIDELGRGTNTEEGIGICYAVCEYLLS
LKAFTLFATHFLELCHIDALYPNVENMHFEVQHVKNTSRNKEAILYTYKLSKGLTEEKNYGLKAAEVSSLPPSIVLDAKEITTQITR
QILQNQRSTPEMERQRAVYHLATRLVQTARNSQLDPDSLRIYLSNLKKKYKEDFPRTEQVPEKTEE
>sp|Q4W5G0|TIGD2_HUMAN
MLGKRKRVVLTIKDKLDIIKKLEEGISFKKLSVVYGIGESTVRDIKKNKERIINYANSSDPTSGVSKRKSMKSSTYEELDRVMIEWF
NQQKTDGIPVSGTICAKQAKFFFDALGMEGDFNASSGWLTRFKQRHGIPKAAGKGTKLKGDETAAREFCGSFQEFVEKENLQPEQIY
GADQTGLFWKCLPSRTLTLETDQSTSGCRSSRERIIIMCCANATGLHKLNLCVVGKAKKPRAFKGTDLSNLPVTYYSQKGAWIEQSV
FRQWFEKYFVPQVQKHLKSKGLLEKAVLLLDFPPARPNEEMLSSDDGRIIVKYLPPNVTSLIQPMSQGVLATVKRYYRAGLLQKYMD
EGNDPKIFWKNLTVLDAIYEVSRAWNMVKSSTITKAWKKLFPGNEENSGMNIDEGAILAANLATVLQNTEECEHVDIENIDQWFDSR
SSDSSCQVLTDSESAEDQTKAAEQKPSSKSRKTELNPEKHISHKAALEWTENLLDYLEQQDDMLLSDKLVLRRLRTIIRKKQKIQNN
KNH
>sp|P40123|CAP2_HUMAN
MANMQGLVERLERAVSRLESLSAESHRPPGNCGEVNGVIAGVAPSVEAFDKLMDSMVAEFLKNSRILAGDVETHAEMVHSAFQAQRA
FLLMASQYQQPHENDVAALLKPISEKIQEIQTFRERNRGSNMFNHLSAVSESIPALGWIAVSPKPGPYVKEMNDAATFYTNRVLKDY
KHSDLRHVDWVKSYLNIWSELQAYIKEHHTTGLTWSKTGPVASTVSAFSVLSSGPGLPPPPPPLPPPGPPPLFENEGKKEESSPSRS
ALFAQLNQGEAITKGLRHVTDDQKTYKNPSLRAQGGGQTQSPTKSHTPSPTSPKSYPSQKHAPVLELEGKKWRVEYQEDRNDLVISET
ELKQVAYIFKCEKSTIQIKGKVNSIIIDNCKKLGLVFDNVVGIVEVINSQDIQIQVMGRVPTISINKTEGCHIYLSEDALDCEIVSA
KSSEMNILIPQDGDYREFPIPEQFKTAWDGSKLITEPAEIMA
>sp|Q9NQT4|EXOS5_HUMAN
MEEETHTDAKIRAENGTGSSPRGPGCSLRHFACEQNLLSRPDGSASFLQGDTSVLAGVYGPAEVKVSKEIFNKATLEVILRPKIGLP
GVAEKSRERLIRNTCEAVVLGTLHPRTSITVVLQVVSDAGSLLACCLNAACMALVDAGVPMRALFCGVACALDSDGTLVLDPTSKQE
KEARAVLTFALDSVERKLLMSSTKGLYSDTELQQCLAAAQAASQHVFRFYRESLQRRYSKS
>sp|P0C7Q3|FA58B_HUMAN
MEGMEDAGEEAGEDAGEDAREGAAAPAARVHFRVARFIMEAGVKLGMQSIPIATACTIYPKFFCETILDAFDPYLIAMSSIYLAGKV
EEQPLWAHDIISVSNRYFNPSSEPLGLDSRLWELRDSIVQRELLMLRVLRFQVSFQHPHKYLLYYLVSLKNWLNCHSWQRTPVAVTA
WALLRDSYHGGLCLRFQAQHIAVVVLYLALQVYGVEVPAEVEAEKLWWQAFSDDLTKPIIDTIVSDLIQIYTIDTEIP
>sp|Q4KMZ8|NKAI1_HUMAN

MGKCSGRCTLVAFCCLQLVAALERQIFDFLGYQWAPILANFLHIMAVILGIFGTVQYRSRYLILYAAWLVLWVGWNAFIICFYLEVG
QLSQDRDFIMTFNTSLHRSWWMENGPGCLVTPVLNSRLALEDHHVISVTGCLLDYPYIEALSSALQIFLALFGFVFACYVSKVFLEE
EDSFDFIGGFDSYGYQAPQKTSHLQLQPLYTSG
>sp|Q9UQM7|KCC2A_HUMAN
MATITCTRFTEEYQLFEELGKGAFSVVRRCVKVLAGQEYAAKIINTKKLSARDHQKLEREARICRLLKHPNIVRLHDSISEEGHHYL
IFDLVTGGELFEDIVAREYYSEADASHCIQQILEAVLHCHQMGVVHRDLKPENLLLASKLKGAAVKLADFGLAIEVEGEQQAWFGFA
GTPGYLSPEVLRKDPYGKPVDLWACGVILYILLVGYPPFWDEDQHRLYQQIKAGAYDFPSPEWDTVTPEAKDLINKMLTINPSKRIT
AAEALKHPWISHRSTVASCMHRQETVDCLKKFNARRKLKGAILTTMLATRNFSGGKSGGNKKSDGVKESSESTNTTIEDEDTKVRKQ
EIIKVTEQLIEAISNGDFESYTKMCDPGMTAFEPEALGNLVEGLDFHRFYFENLWSRNSKPVHTTILNPHIHLMGDESACIAYIRIT
QYLDAGGIPRTAQSEETRVWHRRDGKWQIVHFHRSGAPSVLPH
>sp|Q13557|KCC2D_HUMAN
MASTTTCTRFTDEYQLFEELGKGAFSVVRRCMKIPTGQEYAAKIINTKKLSARDHQKLEREARICRLLKHPNIVRLHDSISEEGFHY
LVFDLVTGGELFEDIVAREYYSEADASHCIQQILESVNHCHLNGIVHRDLKPENLLLASKSKGAAVKLADFGLAIEVQGDQQAWFGF
AGTPGYLSPEVLRKDPYGKPVDMWACGVILYILLVGYPPFWDEDQHRLYQQIKAGAYDFPSPEWDTVTPEAKDLINKMLTINPAKRI
TASEALKHPWICQRSTVASMMHRQETVDCLKKFNARRKLKGAILTTMLATRNFSAAKSLLKKPDGVKESTESSNTTIEDEDVKARKQ
EIIKVTEQLIEAINNGDFEAYTKICDPGLTAFEPEALGNLVEGMDFHRFYFENALSKSNKPIHTIILNPHVHLVGDDAACIAYIRLT
QYMDGSGMPKTMQSEETRVWHRRDGKWQNVHFHRSGSPTVPIKPPCIPNGKENFSGGTSLWQNI
>sp|Q4VXF1|F74A3_HUMAN
MWRELRGCPGGDVETAQRLSRRRRGKSSEAVPEKTWRAQRMSQTRESSEAVPEKTWREFRGCPGEDVERAQKLRDCPGEDMETAQTL
SARRRAESSEAVPEKTWRELKGCPQEDVERVQRLSLLLHLAVFLWIIIAINFSNSGVKSQSSTYLPSGKILK
>sp|Q9NZ53|PDXL2_HUMAN
MGRLLRAARLPPLLSPLLLLLVGGAFLGACVAGSDEPGPEGLTSTSLLDLLLPTGLEPLDSEEPSETMGLGAGLGAPGSGFPSEENE
ESRILQPPQYFWEEEEELNDSSLDLGPTADYVFPDLTEKAGSIEDTSQAQELPNLPSPLPKMNLVEPPWHMPPREEEEEEEEEEERE
KEEVEKQEEEEEEELLPVNGSQEEAKPQVRDFSLTSSSQTPGATKSRHEDSGDQASSGVEVESSMGPSLLLPSVTPTTVTPGDQDST
SQEAEATVLPAAGLGVEFEAPQEASEEATAGAAGLSGQHEEVPALPSFPQTTAPSGAEHPDEDPLGSRTSASSPLAPGDMELTPSSA
TLGQEDLNQQLLEGQAAEAQSRIPWDSTQVICKDWSNLAGKNYIILNMTENIDCEVFRQHRGPQLLALVEEVLPRHGSGHHGAWHIS
LSKPSEKEQHLLMTLVGEQGVVPTQDVLSMLGDIRRSLEEIGIQNYSTTSSCQARASQVRSDYGTLFVVLVVIGAICIIIIALGLLY
NCWQRRLPKLKHVSHGEELRFVENGCHDNPTLDVASDSQSEMQEKHPSLNGGGALNGPGSWGALMGGKRDPEDSDVFEEDTHL
>sp|O75934|SPF27_HUMAN
MAGTGLVAGEVVVDALPYFDQGYEAPGVREAAAALVEEETRRYRPTKNYLSYLTAPDYSAFETDIMRNEFERLAARQPIELLSMKRY
ELPAPSSGQKNDITAWQECVNNSMAQLEHQAVRIENLELMSQHGCNAWKVYNENLVHMIEHAQKELQKLRKHIQDLNWQRKNMQLTA
GSKLREMESNWVSLVSKNYEIERTIVQLENEIYQIKQQHGEANKENIRQDF
>sp|Q86SY8|KTAS1_HUMAN
MEAAGGFQVHFHPSNGDGRFIETSSAADLEVISYSSKVSVTERCFIKVCTVLF
>sp|Q5TB30|DEP1A_HUMAN
MESQGVPPGPYRATKLWNEVTTSFRAGMPLRKHRQHFKKYGNCFTAGEAVDWLYDLLRNNSNFGPEVTRQQTIQLLRKFLKNHVIED
IKGRWGSENVDDNNQLFRFPATSPLKTLPRRYPELRKNNIENFSKDKDSIFKLRNLSRRTPKRHGLHLSQENGEKIKHEIINEDQEN
AIDNRELSQEDVEEVWRYVILIYLQTILGVPSLEEVINPKQVIPQYIMYNMANTSKRGVVILQNKSDDLPHWVLSAMKCLANWPRSN
DMNNPTYVGFERDVFRTIADYFLDLPEPLLTFEYYELFVNILVVCGYITVSDRSSGIHKIQDDPQSSKFLHLNNLNSFKSTECLLLS
LLHREKNKEESDSTERLQISNPGFQERCAKKMQLVNLRNRRVSANDIMGGSCHNLIGLSNMHDLSSNSKPRCCSLEGIVDVPGNSSK
EASSVFHQSFPNIEGQNNKLFLESKPKQEFLLNLHSEENIQKPFSAGFKRTSTLTVQDQEELCNGKCKSKQLCRSQSLLLRSSTRRN
SYINTPVAEIIMKPNVGQGSTSVQTAMESELGESSATINKRLCKSTIELSENSLLPASSMLTGTQSLLQPHLERVAIDALQLCCLLL
PPPNRRKLQLLMRMISRMSQNVDMPKLHDAMGTRSLMIHTFSRCVLCCAEEVDLDELLAGRLVSFLMDHHQEILQVPSYLQTAVEKH

LDYLKKGHIENPGDGLFAPLPTYSYCKQISAQEFDEQKVSTSQAAIAELLENIIKNRSLPLKEKRKKLKQFQKEYPLIYQKRFPTTE
SEAALFGDKPTIKQPMLILRKPKFRSLR
>sp|A6NIM6|S15A5_HUMAN
MSVTGFTITDEKVHLYHSIEKEKTVRHIGDLCSSHSVKKIQVGICLLLVELCERFTFFEVVCNMIPFCTIKLGYHNCQAAILNLCFI
GTSILTPVFVRWLTDVYLGRNKLVYICLFLHFLGTALLSVVAFPLEDFYLGTYHAVNNIPKTEQHRLFYVALLTICLGIGGVRAIVC
PLGAFGLQEYGSQKTMSFFNWFYWLMNLNATIVFLGISYIQHSQAWALVLLIPFMSMLMAVITLHMIYYNLIYQSEKRCSLLTGVGV
LVSALKTCHPQYCHLGRDVTSQLDHAKEKNGGCYSELHVEDTTFFLTLLPLFIFQLLYRMCIMQIPSGYYLQTMNSNLNLDGFLLPI
AVMNAISSLPLLILAPFLEYFSTCLFPSKRVGSFLSTCIIAGNLFAALSVMIAGFFEIHRKHFPAVEQPLSGKVLTVSSMPCFYLIL
QYVLLGVAETLVNPALSVISYRFVPSNVRGTSMNFLTLFNGFGCFTGALLVKLVYLISDGNWFPNTLNKGNLESFFFFLASLTLLNV
LGFCSVSQRYCNLNHFNAQNIRGSNLEETLLLHEKSLKFYGSIQEFSSSIDLWETAL
>sp|Q86V20|FA35A_HUMAN
MSGGSQVHIFWGAPIAPLKITVSEDTASLMSVADPWKKIQLLYSQHSLYLKDEKQHKNLENYKVPESIGSPDLSGHFLANCMNRHVH
VKDDFVRSVSETQNIESQKIHSSRLSDITSSNMQICGFKSTVPHFTEEEKYQKLLSENKIRDEQPKHQPDICGKNFNTNLFQLGHKC
AAVLDLVCSTEKINIGPEVVQRECVPTEYHEIQNQCLGLFSSNAVDKSRSEAAVRKVSDLKISTDTEFLSIITSSQVAFLAQKKDKR
RSPVNKGNVNMETEPKASYGEIRIPEENSIQLDGFTEAYESGQNQAYSLELFSPVCPKTENSRIHINSDKGLEEHTGSQELFSSEDE
LPPNEIRIELCSSGILCSQLNTFHKSAIKRSCTSEDKVGQSEALSRVLQVAKKMKLISNGGDSAVEMDRRNVSEFKSIKKTSLIKNC
DSKSQKYNCLVMVLSPCHVKEINIKFGPNSGSKVPLATVTVIDQSETKKKVFLWRTAAFWAFTVFLGDIILLTDVVIHEDQWIGETV
LQSTFSSQLLNLGSYSSIQPEEYSSVVSEVVLQDLLAYVSSKHSYLRDLPPRQPQRVNSIDFVELEHLQPDVLVHAVLRVVDFTILT
EAVYSYRGQKQKKVMLTVEQAQDQHYALVLWGPGAAWYPQLQRKKGVVLIKAQISELAFPITASQKIALNAHSSLKSIFSSLPNIVY
TGCAKCGLELETDENRIYKQCFSCLPFTMKKIYYRPALMTAIDGRHDVCIRVESKLIEKILLNISADCLNRVIVPSSEITYGMVVAD
LFHSLLAVSAEPCVLKIQSLFVLDENSYPLQQDFSLLDFYPDIVKHGANARL
>sp|Q8IXS6|PALM2_HUMAN
MEMAEAELHKERLQAIAEKRKRQTEIEGKRQQLDEQILLLQHSKSKVLREKWLLQGIPAGTAEEEEARRRQSEEDEFRVKQLEDNIQ
RLEQEIQTLESEESQISAKEQIILEKLKETEKSFKDFQKGFSSTDGAVYAMEINVEKDKQTGETKILSTSTIGPEGVHQKGVKVYDD
GTKVVYEVRSGGTVVENGVHKLSTKDVEELIQKAGQSSLGGGHVSERTVIADGSLSHPKEHMLCKEAKLEMVHKSRKDHSSGNPGQQ
AQAPSAAGPEANLDQPVTMIFMGYQNIEDEEETKKVLGYDETIKAELVLIDEDDEKSLREKTVTDVSTIDGNAAELVSGRPVSDTTE
PSSPEGKEESLATEPAPGTQKKKRCQCCVVM
>sp|Q8IYH5|ZZZ3_HUMAN
MAASRSTRVTRSTVGLNGLDESFCGRTLRNRSIAHPEEISSNSQVRSRSPKKRPEPVPIQKGNNNGRTTDLKQQSTRESWVSPRKRG
LSSSEKDNIERQAIENCERRQTEPVSPVLKRIKRCLRSEAPNSSEEDSPIKSDKESVEQRSTVVDNDADFQGTKRACRCLILDDCEK
REIKKVNVSEEGPLNSAVVEEITGYLAVNGVDDSDSAVINCDDCQPDGNTKQNSIGSYVLQEKSVAENGDTDTQTSMFLDSRKEDSY
IDHKVPCTDSQVQVKLEDHKIVTACLPVEHVNQLTTEPATGPFSETQSSLRDSEEEVDVVGDSSASKEQCKENTNNELDTSLESMPA
SGEPEPSPVLDCVSAQMMSLSEPQEHRYTLRTSPRRAAPTRGSPTKNSSPYRENGQFEENNLSPNETNATVSDNVSQSPTNPGEISQ
NEKGICCDSQNNGSEGVSKPPSEARLNIGHLPSAKESASQHITEEEDDDPDVYYFESDHVALKHNKDYQRLLQTIAVLEAQRSQAVQ
DLESLGRHQREALKNPIGFVEKLQKKADIGLPYPQRVVQLPEIVWDQYTHSLGNFEREFKNRKRHTRRVKLVFDKVGLPARPKSPLD
PKKDGESLSYSMLPLSDGPEGSSSRPQMIRGRLCDDTKPETFNQLWTVEEQKKLEQLLIKYPPEEVESRRWQKIADELGNRTAKQVA
SRVQKYFIKLTKAGIPVPGRTPNLYIYSKKSSTSRRQHPLNKHLFKPSTFMTSHEPPVYMDEDDDRSCFHSHMNTAVEDASDDESIP
IMYRNLPEYKELLQFKKLKKQKLQQMQAESGFVQHVGFKCDNCGIEPIQGVRWHCQDCPPEMSLDFCDSCSDCLHETDIHKEDHQLE
PIYRSETFLDRDYCVSQGTSYNYLDPNYFPANR
>sp|Q9Y6Q5|AP1M2_HUMAN
MSASAVFILDVKGKPLISRNYKGDVAMSKIEHFMPLLVQREEEGALAPLLSHGQVHFLWIKHSNLYLVATTSKNANASLVYSFLYKT
IEVFCEYFKELEEESIRDNFVIVYELLDELMDFGFPQTTDSKILQEYITQQSNKLETGKSRVPPTVTNAVSWRSEGIKYKKNEVFID
VIESVNLLVNANGSVLLSEIVGTIKLKVFLSGMPELRLGLNDRVLFELTGRSKNKSVELEDVKFHQCVRLSRFDNDRTISFIPPDGD

FELMSYRLSTQVKPLIWIESVIEKFSHSRVEIMVKAKGQFKKQSVANGVEISVPVPSDADSPRFKTSVGSAKYVPERNVVIWSIKSF
PGGKEYLMRAHFGLPSVEKEEVEGRPPIGVKFEIPYFTVSGIQVRYMKIIEKSGYQALPWVRYITQSGDYQLRTS
>sp|Q9H0A8|COMD4_HUMAN
MRFRFCGDLDCPDWVLAEISTLAKMSSVKLRLLCSQVLKELLGQGIDYEKILKLTADAKFESGDVKATVAVLSFILSSAAKHSVDGE
SLSSELQQLGLPKEHAASLCRCYEEKQSPLQKHLRVCSLRMNRLAGVGWRVDYTLSSSLLQSVEEPMVHLRLEVAAAPGTPAQPVAM
SLSADKFQVLLAELKQAQTLMSSLG
>sp|P61803|DAD1_HUMAN
MSASVVSVISRFLEEYLSSTPQRLKLLDAYLLYILLTGALQFGYCLLVGTFPFNSFLSGFISCVGSFILAVCLRIQINPQNKADFQG
ISPERAFADFLFASTILHLVVMNFVG
>sp|O00267|SPT5H_HUMAN
MSDSEDSNFSEEEDSERSSDGEEAEVDEERRSAAGSEKEEEPEDEEEEEEEEEYDEEEEEEDDDRPPKKPRHGGFILDEADVDDEYE
DEDQWEDGAEDILEKEEIEASNIDNVVLDEDRSGARRLQNLWRDQREEELGEYYMKKYAKSSVGETVYGGSDELSDDITQQQLLPGV
KDPNLWTVKCKIGEERATAISLMRKFIAYQFTDTPLQIKSVVAPEHVKGYIYVEAYKQTHVKQAIEGVGNLRLGYWNQQMVPIKEMT
DVLKVVKEVANLKPKSWVRLKRGIYKDDIAQVDYVEPSQNTISLKMIPRIDYDRIKARMSLKDWFAKRKKFKRPPQRLFDAEKIRSL
GGDVASDGDFLIFEGNRYSRKGFLFKSFAMSAVITEGVKPTLSELEKFEDQPEGIDLEVVTESTGKEREHNFQPGDNVEVCEGELIN
LQGKILSVDGNKITIMPKHEDLKDMLEFPAQELRKYFKMGDHVKVIAGRFEGDTGLIVRVEENFVILFSDLTMHELKVLPRDLQLCS
ETASGVDVGGQHEWGELVQLDPQTVGVIVRLERETFQVLNMYGKVVTVRHQAVTRKKDNRFAVALDSEQNNIHVKDIVKVIDGPHSG
REGEIRHLFRSFAFLHCKKLVENGGMFVCKTRHLVLAGGSKPRDVTNFTVGGFAPMSPRISSPMHPSAGGQRGGFGSPGGGSGGMSR
GRGRRDNELIGQTVRISQGPYKGYIGVVKDATESTARVELHSTCQTISVDRQRLTTVGSRRPGGMTSTYGRTPMYGSQTPMYGSGSR
TPMYGSQTPLQDGSRTPHYGSQTPLHDGSRTPAQSGAWDPNNPNTPSRAEEEYEYAFDDEPTPSPQAYGGTPNPQTPGYPDPSSPQV
NPQYNPQTPGTPAMYNTDQFSPYAAPSPQGSYQPSPSPQSYHQVAPSPAGYQNTHSPASYHPTPSPMAYQASPSPSPVGYSPMTPGA
PSPGGYNPHTPGSGIEQNSSDWVTTDIQVKVRDTYLDTQVVGQTGVIRSVTGGMCSVYLKDSEKVVSISSEHLEPITPTKNNKVKVI
LGEDREATGVLLSIDGEDGIVRMDLDEQLKILNLRFLGKLLEA
>sp|Q9UJX4|APC5_HUMAN
MASVHESLYFNPMMTNGVVHANVFGIKDWVTPYKIAVLVLLNEMSRTGEGAVSLMERRRLNQLLLPLLQGPDITLSKLYKLIEESCP
QLANSVQIRIKLMAEGELKDMEQFFDDLSDSFSGTEPEVHKTSVVGLFLRHMILAYSKLSFSQVFKLYTALQQYFQNGEKKTVEDAD
MELTSRDEGERKMEKEELDVSVREEEVSCSGPLSQKQAEFFLSQQASLLKNDETKALTPASLQKELNNLLKFNPDFAEAHYLSYLNN
LRVQDVFSSTHSLLHYFDRLILTGAESKSNGEEGYGRSLRYAALNLAALHCRFGHYQQAELALQEAIRIAQESNDHVCLQHCLSWLY
VLGQKRSDSYVLLEHSVKKAVHFGLPYLASLGIQSLVQQRAFAGKTANKLMDALKDSDLLHWKHSLSELIDISIAQKTAIWRLYGRS
TMALQQAQMLLSMNSLEAVNAGVQQNNTESFAVALCHLAELHAEQGCFAAASEVLKHLKERFPPNSQHAQLWMLCDQKIQFDRAMND
GKYHLADSLVTGITALNSIEGVYRKAVVLQAQNQMSEAHKLLQKLLVHCQKLKNTEMVISVLLSVAELYWRSSSPTIALPMLLQALA
LSKEYRLQYLASETVLNLAFAQLILGIPEQALSLLHMAIEPILADGAILDKGRAMFLVAKCQVASAASYDQPKKAEALEAAIENLNE
AKNYFAKVDCKERIRDVVYFQARLYHTLGKTQERNRCAMLFRQLHQELPSHGVPLINHL
>sp|Q7LDG7|GRP2_HUMAN
MAGTLDLDKGCTVEELLRGCIEAFDDSGKVRDPQLVRMFLMMHPWYIPSSQLAAKLLHIYQQSRKDNSNSLQVKTCHLVRYWISAFP
AEFDLNPELAEQIKELKALLDQEGNRRHSSLIDIDSVPTYKWKRQVTQRNPVGQKKRKMSLLFDHLEPMELAEHLTYLEYRSFCKIL
FQDYHSFVTHGCTVDNPVLERFISLFNSVSQWVQLMILSKPTAPQRALVITHFVHVAEKLLQLQNFNTLMAVVGGLSHSSISRLKET
HSHVSPETIKLWEGLTELVTATGNYGNYRRRLAACVGFRFPILGVHLKDLVALQLALPDWLDPARTRLNGAKMKQLFSILEELAMVT
SLRPPVQANPDLLSLLTVSLDQYQTEDELYQLSLQREPRSKSSPTSPTSCTPPPRPPVLEEWTSAAKPKLDQALVVEHIEKMVESVF
RNFDVDGDGHISQEEFQIIRGNFPYLSAFGDLDQNQDGCISREEMVSYFLRSSSVLGGRMGFVHNFQESNSLRPVACRHCKALILGI
YKQGLKCRACGVNCHKQCKDRLSVECRRRAQSVSLEGSAPSPSPMHSHHHRAFSFSLPRPGRRGSRPPEIREEEVQTVEDGVFDIHL
>sp|Q9HCD6|TANC2_HUMAN
MFRNSLKMLLTGGKSSRKNRSSDGGSEEPPDRRQSSVDSRQSRSGQGGISTESDCAFEPDYAVPPLPVSEGDAEQELGPPPSVDEAA
NTLMTRLGFLLGEKVTEVQPGDQYSMEVQDENQTSAITQRISPCSTLTSSTASPPASSPCSTLPPISTNATAKDCSYGAVTSPTSTL

ESRDSGIIATLTSYSENVERTKYAGESSKELGSGGNIKPWQSQKSSMDSCLYRVDENMTASTYSLNKIPERNLETVLSQSVQSIPLY
LMPRPNSVAATSSAHLEDLAYLDEQRHTPLRTSLRMPRQSMGGARTQQDLRVRFAPYRPPDISLKPLLFEVPSITTESVFVGRDWVF
HEIDAQLQSSNASVNQGVVIVGNIGFGKTAIISRLVALSCHGTRMRQIASDSPHASPKHVDANRELPLTQPPSAHSSITSGSCPGTP
EMRRRQEEAMRRLASQVVAYHYCQADNAYTCLVPEFVHNVAALLCRSPQLTAYREQLLREPHLQSMLSLRSCVQDPMASFRRGVLEP
LENLHKERKIPDEDFIILIDGLNEAEFHKPDYGDTIVSFLSKMIGKFPSWLKLIVTVRTSLQEITKLLPFHRIFLDRLEENEAIDQD
LQAYILHRIHSSSEIQNNISLNGKMDNTTFGKLSSHLKTLSQGSYLYLKLTFDLIEKGYLVLKSSSYKVVPVSLSEVYLLQCNMKFP
TQSSFDRVMPLLNVAVASLHPLTDEHIFQAINAGSIEGTLEWEDFQQRMENLSMFLIKRRDMTRMFVHPSFREWLIWREEGEKTKFL
CDPRSGHTLLAFWFSRQEGKLNRQQTIELGHHILKAHIFKGLSKKVGVSSSILQGLWISYSTEGLSMALASLRNLYTPNIKVSRLLI
LGGANINYRTEVLNNAPILCVQSHLGYTEMVALLLEFGANVDASSESGLTPLGYAAAAGYLSIVVLLCKKRAKVDHLDKNGQCALVH
AALRGHLEVVKFLIQCDWTMAGQQQGVFKKSHAIQQALIAAASMGYTEIVSYLLDLPEKDEEEVERAQINSFDSLWGETALTAAAGR
GKLEVCRLLLEQGAAVAQPNRRGAVPLFSTVRQGHWQIVDLLLTHGADVNMADKQGRTPLMMAASEGHLGTVDFLLAQGASIALMDK
EGLTALSWACLKGHLSVVRSLVDNGAATDHADKNGRTPLDLAAFYGDAEVVQFLVDHGAMIEHVDYSGMRPLDRAVGCRNTSVVVTL
LKKGAKIGPATWAMATSKPDIMIILLSKLMEEGDMFYKKGKVKEAAQRYQYALKKFPREGFGEDLKTFRELKVSLLLNLSRCRRKMN
DFGMAEEFATKALELKPKSYEAYYARARAKRSSRQFAAALEDLNEAIKLCPNNREIQRLLLRVEEECRQMQQPQQPPPPPQPQQQLP
EEAEPEPQHEDIYSVQDIFEEEYLEQDVENVSIGLQTEARPSQGLPVIQSPPSSPPHRDSAYISSSPLGSHQVFDFRSSSSVGSPTR
QTYQSTSPALSPTHQNSHYRPSPPHTSPAHQGGSYRFSPPPVGGQGKEYPSPPPSPLRRGPQYRASPPAESMSVYRSQSGSPVRYQQ
ETSVSQLPGRPKSPLSKMAQRPYQMPQLPVAVPQQGLRLQPAKAQIVRSNQPSPAVHSSTVIPTGAYGQVAHSMASKYQSSQGDIGV
SQSRLVYQGSIGGIVGDGRPVQHVQASLSAGAICQHGGLTKEDLPQRPSSAYRGGVRYSQTPQIGRSQSASYYPVCHSKLDLERSSS
QLGSPDVSHLIRRPISVNPNEIKPHPPTPRPLLHSQSVGLRFSPSSNSISSTSNLTPTFRPSSSIQQMEIPLKPAYERSCDELSPVS
PTQGGYPSEPTRSRTTPFMGIIDKTARTQQYPHLHQQNRTWAVSSVDTVLSPTSPGNLPQPESFSPPSSISNIAFYNKTNNAQNGHL
LEDDYYSPHGMLANGSRGDLLERVSQASSYPDVKVARTLPVAQAYQDNLYRQLSRDSRQGQTSPIKPKRPFVESNV
>sp|Q92575|UBXN4_HUMAN
MLWFQGAIPAAIATAKRSGAVFVVFVAGDDEQSTQMAASWEDDKVTEASSNSFVAIKIDTKSEACLQFSQIYPVVCVPSSFFIGDSG
IPLEVIAGSVSADELVTRIHKVRQMHLLKSETSVANGSQSESSVSTPSASFEPNNTCENSQSRNAELCEIPPTSDTKSDTATGGESA
GHATSSQEPSGCSDQRPAEDLNIRVERLTKKLEERREEKRKEEEQREIKKEIERRKTGKEMLDYKRKQEEELTKRMLEERNREKAED
RAARERIKQQIALDRAERAARFAKTKEEVEAAKAAALLAKQAEMEVKRESYARERSTVARIQFRLPDGSSFTNQFPSDAPLEEARQF
AAQTVGNTYGNFSLATMFPRREFTKEDYKKKLLDLELAPSASVVLLPAGRPTASIVHSSSGDIWTLLGTVLYPFLAIWRLISNFLFS
NPPPTQTSVRVTSSEPPNPASSSKSEKREPVRKRVLEKRGDDFKKEGKIYRLRTQDDGEDENNTWNGNSTQQM
>sp|Q8NC24|RELL2_HUMAN
MSEPQPDLEPPQHGLYMLFLLVLVFFLMGLVGFMICHVLKKKGYRCRTSRGSEPDDAQLQPPEDDDMNEDTVERIVRCIIQNEANAE
ALKEMLGDSEGEGTVQLSSVDATSSLQDGAPSHHHTVHLGSAAPCLHCSRSKRPPLVRQGRSKEGKSRPRTGETTVFSVGRFRVTHI
EKRYGLHEHRDGSPTDRSWGSGGGQDPGGGQGSGGGQPKAGMPAMERLPPERPQPQVLASPPVQNGGLRDSSLTPRALEGNPRASAE
PTLRAGGRGPSPGLPTQEANGQPSKPDTSDHQVSLPQGAGSM
>sp|Q9Y5J5|PHLA3_HUMAN
MTAAATATVLKEGVLEKRSGGLLQLWKRKRCVLTERGLQLFEAKGTGGRPKELSFARIKAVECVESTGRHIYFTLVTEGGGEIDFRC
PLEDPGWNAQITLGLVKFKNQQAIQTVRARQSLGTGTLVS
>sp|Q9BXI2|ORNT2_HUMAN
MKSGPGIQAAIDLTAGAAGGTACVLTGQPFDTIKVKMQTFPDLYKGLTDCFLKTYAQVGLRGFYKGTGPALMAYVAENSVLFMCYGF
CQQFVRKVAGMDKQAKLSDLQTAAAGSFASAFAALALCPTELVKCRLQTMYEMEMSGKIAKSHNTIWSVVKGILKKDGPLGFYHGLS
STLLQEVPGYFFFFGGYELSRSFFASGRSKDELGPVHLMLSGGVAGICLWLVVFPVDCIKSRIQVLSMYGKQAGFIGTLLSVVRNEG
IVALYSGLKATMIRAIPANGALFVAYEYSRKMMMKQLEAY
>sp|Q13873|BMPR2_HUMAN
MTSSLQRPWRVPWLPWTILLVSTAAASQNQERLCAFKDPYQQDLGIGESRISHENGTILCSKGSTCYGLWEKSKGDINLVKQGCWSH
IGDPQECHYEECVVTTTPPSIQNGTYRFCCCSTDLCNVNFTENFPPPDTTPLSPPHSFNRDETIIIALASVSVLAVLIVALCFGYRM

LTGDRKQGLHSMNMMEAAASEPSLDLDNLKLLELIGRGRYGAVYKGSLDERPVAVKVFSFANRQNFINEKNIYRVPLMEHDNIARFI
VGDERVTADGRMEYLLVMEYYPNGSLCKYLSLHTSDWVSSCRLAHSVTRGLAYLHTELPRGDHYKPAISHRDLNSRNVLVKNDGTCV
ISDFGLSMRLTGNRLVRPGEEDNAAISEVGTIRYMAPEVLEGAVNLRDCESALKQVDMYALGLIYWEIFMRCTDLFPGESVPEYQMA
FQTEVGNHPTFEDMQVLVSREKQRPKFPEAWKENSLAVRSLKETIEDCWDQDAEARLTAQCAEERMAELMMIWERNKSVSPTVNPMS
TAMQNERNLSHNRRVPKIGPYPDYSSSSYIEDSIHHTDSIVKNISSEHSMSSTPLTIGEKNRNSINYERQQAQARIPSPETSVTSLS
TNTTTTNTTGLTPSTGMTTISEMPYPDETNLHTTNVAQSIGPTPVCLQLTEEDLETNKLDPKEVDKNLKESSDENLMEHSLKQFSGP
DPLSSTSSSLLYPLIKLAVEATGQQDFTQTANGQACLIPDVLPTQIYPLPKQQNLPKRPTSLPLNTKNSTKEPRLKFGSKHKSNLKQ
VETGVAKMNTINAAEPHVVTVTMNGVAGRNHSVNSHAATTQYANGTVLSGQTTNIVTHRAQEMLQNQFIGEDTRLNINSSPDEHEPL
LRREQQAGHDEGVLDRLVDRRERPLEGGRTNSNNNNSNPCSEQDVLAQGVPSTAADPGPSKPRRAQRPNSLDLSATNVLDGSSIQIG
ESTQDGKSGSGEKIKKRVKTPYSLKRWRPSTWVISTESLDCEVNNNGSNRAVHSKSSTAVYLAEGGTATTMVSKDIGMNCL
>sp|Q9UIL4|KIF25_HUMAN
MTWTSGQLQREKQARPGSGAVLAFPDDKDLRVYGPAESQSAVFGDVCPLLTSLLDGYNVCVMAYGQTGSGKSYTMLGRHSDDGPVLP
LDPQSDLGIIPRVAEELFRLILENTSRSPKVEVSIVEVYNNDIFDLLAKDSIAAVSGVKREVVTAKDGRTEVALLASEAVGSASKLM
ELVHGGLQLRAKHPTLVHADSSRSHLIITVTLTTASCSDSTADQACSATLPREQTEAGRAGRSRRASQGALAPQLVPGNPAGHAEQV
QARLQLVDSAGSECVGVSGVTGLALREMACISRSLAALAGVLGALLEHRGHAPYRNSRLTHLLQDCLGGDAKLLVILCISPSQRHLA
QTLQGLGFGIRARQVQRGPARKKPPSSQTEGKRRPD
>sp|Q15020|SART3_HUMAN
MATAAETSASEPEAESKAGPKADGEEDEVKAARTRRKVLSRAVAAATYKTMGPAWDQQEEGVSESDGDEYAMASSAESSPGEYEWEY
DEEEEKNQLEIERLEEQLSINVYDYNCHVDLIRLLRLEGELTKVRMARQKMSEIFPLTEELWLEWLHDEISMAQDGLDREHVYDLFE
KAVKDYICPNIWLEYGQYSVGGIGQKGGLEKVRSVFERALSSVGLHMTKGLALWEAYREFESAIVEAARLEKVHSLFRRQLAIPLYD
MEATFAEYEEWSEDPIPESVIQNYNKALQQLEKYKPYEEALLQAEAPRLAEYQAYIDFEMKIGDPARIQLIFERALVENCLVPDLWI
RYSQYLDRQLKVKDLVLSVHNRAIRNCPWTVALWSRYLLAMERHGVDHQVISVTFEKALNAGFIQATDYVEIWQAYLDYLRRRVDFK
QDSSKELEELRAAFTRALEYLKQEVEERFNESGDPSCVIMQNWARIEARLCNNMQKARELWDSIMTRGNAKYANMWLEYYNLERAHG
DTQHCRKALHRAVQCTSDYPEHVCEVLLTMERTEGSLEDWDIAVQKTETRLARVNEQRMKAAEKEAALVQQEEEKAEQRKRARAEKK
ALKKKKKIRGPEKRGADEDDEKEWGDDEEEQPSKRRRVENSIPAAGETQNVEVAAGPAGKCAAVDVEPPSKQKEKAASLKRDMPKVL
HDSSKDSITVFVSNLPYSMQEPDTKLRPLFEACGEVVQIRPIFSNRGDFRGYCYVEFKEEKSALQALEMDRKSVEGRPMFVSPCVDK
SKNPDFKVFRYSTSLEKHKLFISGLPFSCTKEELEEICKAHGTVKDLRLVTNRAGKPKGLAYVEYENESQASQAVMKMDGMTIKENI
IKVAISNPPQRKVPEKPETRKAPGGPMLLPQTYGARGKGRTQLSLLPRALQRPSAAAPQAENGPAAAPAVAAPAATEAPKMSNADFA
KLFLRK
>sp|P54753|EPHB3_HUMAN
MARARPPPPPSPPPGLLPLLPPLLLLPLLLLPAGCRALEETLMDTKWVTSELAWTSHPESGWEEVSGYDEAMNPIRTYQVCNVRESS
QNNWLRTGFIWRRDVQRVYVELKFTVRDCNSIPNIPGSCKETFNLFYYEADSDVASASSPFWMENPYVKVDTIAPDESFSRLDAGRV
NTKVRSFGPLSKAGFYLAFQDQGACMSLISVRAFYKKCASTTAGFALFPETLTGAEPTSLVIAPGTCIPNAVEVSVPLKLYCNGDGE
WMVPVGACTCATGHEPAAKESQCRPCPPGSYKAKQGEGPCLPCPPNSRTTSPAASICTCHNNFYRADSDSADSACTTVPSPPRGVIS
NVNETSLILEWSEPRDLGGRDDLLYNVICKKCHGAGGASACSRCDDNVEFVPRQLGLTERRVHISHLLAHTRYTFEVQAVNGVSGKS
PLPPRYAAVNITTNQAAPSEVPTLRLHSSSGSSLTLSWAPPERPNGVILDYEMKYFEKSEGIASTVTSQMNSVQLDGLRPDARYVVQ
VRARTVAGYGQYSRPAEFETTSERGSGAQQLQEQLPLIVGSATAGLVFVVAVVVIAIVCLRKQRHGSDSEYTEKLQQYIAPGMKVYI
DPFTYEDPNEAVREFAKEIDVSCVKIEEVIGAGEFGEVCRGRLKQPGRREVFVAIKTLKVGYTERQRRDFLSEASIMGQFDHPNIIR
LEGVVTKSRPVMILTEFMENCALDSFLRLNDGQFTVIQLVGMLRGIAAGMKYLSEMNYVHRDLAARNILVNSNLVCKVSDFGLSRFL
EDDPSDPTYTSSLGGKIPIRWTAPEAIAYRKFTSASDVWSYGIVMWEVMSYGERPYWDMSNQDVINAVEQDYRLPPPMDCPTALHQL
MLDCWVRDRNLRPKFSQIVNTLDKLIRNAASLKVIASAQSGMSQPLLDRTVPDYTTFTTVGDWLDAIKMGRYKESFVSAGFASFDLV
AQMTAEDLLRIGVTLAGHQKKILSSIQDMRLQMNQTLPVQV
>sp|P02679|FIBG_HUMAN

MSWSLHPRNLILYFYALLFLSSTCVAYVATRDNCCILDERFGSYCPTTCGIADFLSTYQTKVDKDLQSLEDILHQVENKTSEVKQLI
KAIQLTYNPDESSKPNMIDAATLKSRKMLEEIMKYEASILTHDSSIRYLQEIYNSNNQKIVNLKEKVAQLEAQCQEPCKDTVQIHDI
TGKDCQDIANKGAKQSGLYFIKPLKANQQFLVYCEIDGSGNGWTVFQKRLDGSVDFKKNWIQYKEGFGHLSPTGTTEFWLGNEKIHL
ISTQSAIPYALRVELEDWNGRTSTADYAMFKVGPEADKYRLTYAYFAGGDAGDAFDGFDFGDDPSDKFFTSHNGMQFSTWDNDNDKF
EGNCAEQDGSGWWMNKCHAGHLNGVYYQGGTYSKASTPNGYDNGIIWATWKTRWYSMKKTTMKIIPFNRLTIGEGQQHHLGGAKQVR
PEHPAETEYDSLYPEDDL
>sp|O15255|CXX1_HUMAN
MGGGRGLLGRETLGPGGGCSGEGPLCYWPPPGSPPAPSLRASLPLEPPRCPLRSCSLPRSACLCSRNSAPGSCCRPWASLWSEPPPS
PSSQPAPPMYIWTLSCAPAASWAPVTHWTDHPLPPLPSPLLPTRLPDDYIILPTDLRCHSHRHPSHPTDRLLLLVIWTHLGGIWAGH
SPWTVIQTAGRPPRDLSPSARPISSPPPETSCVLA
>sp|Q9BVV2|FND11_HUMAN
MSTHVAGLGLDKMKLGNPQSFLDQEEADDQQLLEPEAWKTYTERRNALREFLTSDLSPHLLKRHHARMQLLRKCSYYIEVLPKHLAL
GDQNPLVLPSALFQLIDPWKFQRMKKVGTAQTKIQLLLLGDLLEQLDHGRAELDALLRSPDPRPFLADWALVERRLADVSAVMDSFL
TMMVPGRLHVKHRLVSDVSATKIPHIWLMLSTKMPVVFDRKASAAHQDWARLRWFVTIQPATSEQYELRFRLLDPRTQQECAQCGVI
PVAACTFDVRNLLPNRSYKFTIKRAETSTLVYEPWRDSLTLHTKPEPLEGPALSHSV
>sp|Q9BSJ5|CQ080_HUMAN
MSDNPPRMEVCPYCKKPFKRLKSHLPYCKMIGPTIPTDQKVYQSKPATLPRAKKMKGPIKDLIKAKGKELETENEERNSKLVVDKPE
QTVKTFPLPAVGLERAATTKADKDIKNPIQPSFKMLKNTKPMTTFQEETKAQFYASEKTSPKRELAKDLPKSGESRCNPSEAGASLL
VGSIEPSLSNQDRKYSSTLPNDVQTTSGDLKLDKIDPQRQELLVKLLDVPTGDCHISPKNVSDGVKRVRTLLSNERDSKGRDHLSGV
PTDVTVTETPEKNTESLILSLKMSSLGKIQVMEKQEKGLTLGVETCGSKGNAEKSMSATEKQERTVMSHGCENFNTRDSVTGKESQG
ERPHLSLFIPRETTYQFHSVSQSSSQSLASLATTFLQEKKAEAQNHHCVPDVKALMESPEGQLSLEPKSDSQFQASHTGCQSPLCSA
QRHTPQSPFTNHAAAAGRKTLRSCMGLEWFPELYPGYLGLGVLPGKPQCWNAMTQKPQLISPQGERLSQVSLLERSSTHIRSLEPPA
GLTTSNFSLMRLLGAVQKGWIRCNTTIRKSGFGGITMLFTGYFVLCCSWSFRRLKKLCRPLPWKSTVPPCIGVAKTTGDCRSKTCLD
>sp|Q7RTN6|STRAA_HUMAN
MSFLVSKPERIRRWVSEKFIVEGLRDLELFGEQPPGDTRRKTNDASSESIASFSKQEVMSSFLPEGGCYELLTVIGKGFEDLMTVNL
ARYKPTGEYVTVRRINLEACSNEMVTFLQGELHVSKLFNHPNIVPYRATFIADNELWVVTSFMAYGSAKDLICTHFMDGMNELAIAY
ILQGVLKALDYIHHMGYVHRSVKASHILISVDGKVYLSGLRSNLSMISHGQRQRVVHDFPKYSVKVLPWLSPEVLQQNLQGYDAKSD
IYSVGITACELANGHVPFKDMPATQMLLEKLNGTVPCLLDTSTIPAEELTMSPSRSVANSGLSDSLTTSTPRPSNGDSPSHPYHRTF
SPHFHHFVEQCLQRNPDARPSASTLLNHSFFKQIKRRASEALPELLRPVTPITNFEGSQSQDHSGIFGLVTNLEELEVDDWEF
>sp|Q8NDA2|HMCN2_HUMAN
MPGAPLLRLLTAVSAAVAVAVAGAPGTVMPPTTGDATLAFVFDVTGSMWDELMQVIDGASRILERSLSRRSQAIANYALVPFHDPDI
GPVTLTADPTVFQRELRELYVQGGGDCPEMSVGAIKAAVEVANPGSFIYVFSDARAKDYHKKEELLRLLQLKQSQVVFVLTGDCGDR
THPGYLAYEEIAATSSGQVFHLDKQQVTEVLKWVESAIQASKVHLLSTDHEEEGEHTWRLPFDPSLKEVTISLSGPGPEIEVQDPLG
RILQEDEGLNVLLNIPDSAKVVAFKPEHPGLWSIKVYSSGRHSVRITGVSNIDFRAGFSTQPLLDLNHTLEWPLQGVPISLVINSTG
LKAPGRLDSVELAQSSGKPLLTLPTKPLSNGSTHQLWGGPPFHTPKERFYLKVKGKDHEGNPLLRVSGVSYSGVAPGAPLVSMAPRI
HGYLHQPLLVSCSVHSALPFRLQLRRGEARLGEERHFQESGNSSWEILRASKAEEGTYECTAVSRAGTGRAKAQIVVTDPPPQLVPA
PNVTVSPGETAVLSCRVLGEAPYNLTWVRDWRVLPASTGRVAQLADLSLEISGIIPTDGGRYQCVASNANGVTRASVWLLVREAPQV
SIHTSSQHFSQGVEVKVSCSASGYPTPHISWSRESQALQEDSRIHVDAQGTLIIQGVAPEDAGNYSCQATNEVGTDQETVTLYYTDP
PSVSAVNAVVLVAVGEEAVLVCEASGVPPPRVIWYRGGLEMILAPEGSSSGKLRIPAAQERDAGTYTCRAVNELGDASAEIQLAVGH
APQLTELPRDVTVELGRSALLACRATGRPPPTVTWRRGDGQPLGLRLGAGRGSRSRQPDSGVLFFESVAPEDQAPYVCEARNVFGKV
QAEARLIVTGHAPPQIASSAPTVRVLEGQPVSLPCIVLAGRPLPERHWLKDGRPLPPGSRHSIRADGSLHLDRALQEHAGRYSCVAT
NTAGSQHRDVELVVQVPPRIHPTATHHITNEGVAASLPCVASGVPAPTITWTKETNALTSRGPHYNVSKEGTLLIAQPSAQDAGAYV
CTATNTVGFSSQEMRLSVNTKPRIHMNGSRNADVPLQVTAKAGEEVTLDCEAKGSPPPLVTWTKDSRPVPPITNRYGLLPSGSLRLA
QVQVGDSGHYECTASNPAGSASHRYVLGVQVPPQVQPGPRVLKVLVGEALDLNCVAEGNPEPQLSWSKDGVVLQGRGPQGSVHFAAI

RTSDAGRYRCEASNSAGVDAWEVELRVLEPPHWGADETSGLLERVAGENASLPCPARGTPKPQVTWRKGPSSEPLHGQPGVAVLEEG
SLFLASVSPADSGDYECQATNEVGSTSRRAKLVVYVPPSIREDGRKANVSGMAGQSLTLECDANGFPVPEIVWLKDAQLIPKVGGHR
LLDEGQSLHFPRIQEGDSGLYSCRAENQAGTAQRDFHLLVLTPPSVLGAGAAQEVLGLAGADVELQCWTSGVPTPQVEWTKDRQPVL
PGGPHLQVQEDGQVLRITGSHVGDEGRYQCVAFSPAGQQARDFQLRVHAPPTIWGSNETGEVAVMEDHLVQLLCEARGVPTPNITWF
KDGALLPTSTKVVYTRGGRQLQLGRAQSSDAGVYTCKASNAVGAAEKATRLDVYVPPTIEGAGGRPYVVKAVAGRPVALECVARGHP
SPTLSWHHEGLPVAESNESRLETDGSVLRLESPGEASSGLYSCVASSPAGEAVLQYSVEVQVPPQLLVAEGLGQVTTIVGQPLELPC
QASGSPVPTIQWLQNGRPAEELAGVQVASQGTTLHIDHVELDHSGLFACQATNEAGTAGAEVEVSVHEFPSVSIIGGENITAPFLQP
VTLQCIGDGVPTPSLRWWKDGVALAAFGGNLQIEKVDLRDEGIYTCAATNLAGESKREVALKVLVPPNIEPGPVNKAVLENASVTLE
CLASGVPPPDVSWFKGHQPVSSWMGVTVSVDGRVLRIEQAQLSDAGSYRCVASNVAGSTELRYGLRVNVPPRITLPPSLPGPVLVNT
PVRLTCNATGAPSPTLMWLKDGNPVSPAGTPGLQVFPGGRVLTLASARASDSGRYSCVAVSAVGEDRQDVVLQVHMPPSILGEELNV
SVVANESVALECQSHAMPPPVLSWWKDGRPLEPRPGVHLSADKALLQVDRADVWDAGHYTCEALNQAGHSEKHYNLNVWVAPVFPLR
ESHTLTVREGHPTRLSCECRGVPFPKISWRKDGQPLPGEGAGLQHVSAVGRLLYLGQAQLAQEGTYTCECSNVVGNSSQDLQLEVHV
PPQIAGPREPPTQVSVVQDGVATLECNATGKPPPTVTWERDGQPVGAELGLQLQNQGQSLHVERAQAAHTGRYSCVAENLAGRAERK
FELSVLVPPELIGDLDPLTNITAALHSPLTLLCEAMGIPPPAIRWFRGEEPVSPGEDTYLLAGGWMLKMTQTQEQDSGLYSCLASNE
AGEARRNFSVEVLVPPSIENEDLEEVIKVLDGQTAHLMCNVTGHPQPKLTWFKDGRPLARGDAHHISPDGVLLQVLQANLSSAGHYS
CIAANAVGEKTKHFQLSVLLAPTILGGAEDSADEEVTVTVNNPISLICEALAFPSPNITWMKDGAPFEASRNIQLLPGTHGLQILNA
QKEDAGQYTCVVTNELGEAVKNYHVEVLIPPSISKDDPLAEVGVKEVKTKVNSTLTLECESWAVPPPTIRWYKDGQPVTPSSRLQVL
GEGRLLQIQPTQVSDSGRYLCVATNVAGEDDQDFNVLIQVPPMFQKVGDFSAAFEILSREEEARGGVTEYREIVENNPAYLYCDTNA
IPPPDLTWYREDQPLSAGDEVSVLQGGRVLQIPLVRAENAGRYSCKASNEVGEDWLHYELLVLTPPVILGDTEELVEEVTVNASSTV
SLQCPALGNPVPTISWLQNGLPFSPSPRLQVLEDGQVLQVSTAEVADAASYMCVAENQAGSAEKLFTLRVQVPPRIAGLDLEQVTAI
LNSSVSLPCDVHAHPNPEVTWYKDSQALSLGEEVFLLPGTHTLQLGRARLSDSGMYTCEALNAAGRDQKLVQLSVLVPPAFRQAPRG
PQDAVLVRVGDKAVLSCETDALPEPTVTWYKDGQPLVLAQRTQALRGGQRLEIQEAQVSDKGLYSCKVSNVAGEAVRTFTLTVQVPP
TFENPKTETVSQVAGSPLVLTCDVSGVPAPTVTWLKDRMPVESSAVHGVVSRGGRLQLSRLQPAQAGTYTCVAENTQAEARKDFVVA
VLVAPRIRSSGVAREHHVLEGQEVRLDCEADGQPPPDVAWLKDGSPLGQDMGPHLRFYLDGGSLVLKGLRASDAGAYTCVAHNPAGE
DARLHTVNVLVPPTIKQGADGSGTLVSRPGELVTMVCPVRGSPPIHVSWLKDGLPLPLSQRTLLHGSGHTLRISKVQLADAGIFTCV
AASPAGVADRNFTLQVQVPPVLEPVEFQNDVVVVRGSLVELPCEARGVPLPLVSWMKDGEPLLSQSLEQGPSLQLEAVGAGDSGTYS
CVAVSEAGEARRHFQLTVMEPPHIEDSGQPTELSLTPGAPMELLCDAQGTPQPNITWHKDGQALTRLENNSRATRVLRVENVQVRDA
GLYTCLAESPAGAIEKSFRVRVQAPPNIVGPRGPRFVVGLAPGQLVLECSVEAEPAPKITWHRDGIVLQEDAHTQFPERGRFLQLQA
LSTADSGDYSCTARNAAGSTSVAFRVEIHTVPTIRSGPPAVNVSVNQTALLPCQADGVPAPLVSWRKDRVPLDPRSPRFEILPEGSL
RIQPVLAQDAGHYLCLASNSAGSDRQGRDLRVLALLPCEASGSPKPLVVWWKDGQKLDFRLQQGAYRLLPSNALLLTAPGPQDSAQF
ECVVSNEVGEAHRLYQVTVHVPPTIADDQTDFTVTMMAPVVLTCHSTGIPAPTVSWSKAGAQLGARGSGYRVSPSGALEIGQALPIH
AGRYTCSARNSAGVAHKHVFLTVQASPVVKPLPSVVRAVAEEEVLLPCEASGIPRPTITWQKEGLNVATGVSTQVLPGGQLRIAHAS
PEDAGNYLCIAKNSAGSAMGKTRLVVQVPPVIENGLPDLSTTEGSHAFLPCKARGSPEPNITWDKDGQPVSGAEGKFTIQPSGELLV
KNLEGQDAGTYTCTAENAVGRARRRVHLTILVLPVFTTLPGDRSLRLGDRLWLRCAARGSPTPRIGWTVNDRPVTEGVSEQDGGSTL
QRAAVSREDSGTYVCWAENRVGRTQAVSFVHVKEAPVLQGEAFSYLVEPVGGSIQLDCVVRGDPVPDIHWIKDGLPLRGSHLRHQLQ
NGSLTIRRTERDDAGRYQCLAENEMGVAKKVVILVLQSAPVFQVEPQDMTVRSGDDVALRCQATGEPTPTIEWLQAGQPLRASRRLR
TLPDGSLWLENVETGDAGTYDCVAHNLLGSATARAFLVVRGEPQGSWGSMTGVINGRKFGVATLNTSVMQEAHSGVSSIHSSIRHVP
ANVGPLMRVLVVTIAPIYWALARESGEALNGHSLTGGRFRQESHVEFATGELLTMTQVARGLDPDGLLLLDVVVNGVVPESLADADL
QVQDFEEHYVQTGPGQLFVGSTQRFFQGGLPSFLRCNHSIQYNAARGPQPQLVQHLRASAISSAFDPEAEALRFQLATALQAEENEV
GCPEGFELDSQGAFCVDRDECSGGPSPCSHACLNAPGRFSCTCPTGFALAWDDRNCRDVDECAWDAHLCREGQRCVNLLGSYRCLPD
CGPGFRVADGAGCEDVDECLEGLDDCHYNQLCENTPGGHRCSCPRGYRMQGPSLPCLDVNECLQLPKACAYQCHNLQGSYRCLCPPG
QTLLRDGKACTSLERNGQNVTTVSHRGPLLPWLRPWASIPGTSYHAWVSLRPGPMALSSVGRAWCPPGFIRQNGVCTDLDECRVRNL
CQHACRNTEGSYQCLCPAGYRLLPSGKNCQDINECEEESIECGPGQMCFNTRGSYQCVDTPCPATYRQGPSPGTCFRRCSQDCGTGG

PSTLQYRLLPLPLGVRAHHDVARLTAFSEVGVPANRTELSMLEPDPRSPFALRPLRAGLGAVYTRRALTRAGLYRLTVRAAAPRHQS
VFVLLIAVSPYPY
>sp|Q9BZ81|MAGB5_HUMAN
MTSAGVFNAGSDERANSRDEEYPCSSEVSPSTESSCSNFINIKVGLLEQFLLYKFKMKQRILKEDMLKIVNPRYQNQFAEIHRRASE
HIEVVFAVDLKEVNPTCHLYDLVSKLKLPNNGRIHVGKVLPKTGLLMTFLVVIFLKGNCANKEDTWKFLDMMQIYDGKKYYIYGEPR
KLITQDFVRLTYLEYHQVPCSYPAHYQFLWGPRAYTETSKMKVLEYLAKVNDIAPGAFSSQYEEALQDEEESPSQRCSRNWHYCSGQ
DCLRAKFSSFSQPY
>sp|Q5VST9|OBSCN_HUMAN
MDQPQFSGAPRFLTRPKAFVVSVGKDATLSCQIVGNPTPQVSWEKDQQPVAAGARFRLAQDGDLYRLTILDLALGDSGQYVCRARNA
IGEAFAAVGLQVDAEAACAEQAPHFLLRPTSIRVREGSEATFRCRVGGSPRPAVSWSKDGRRLGEPDGPRVRVEELGEASALRIRAA
RPRDGGTYEVRAENPLGAASAAAALVVDSDAADTASRPGTSTAALLAHLQRRREAMRAEGAPASPPSTGTRTCTVTEGKHARLSCYV
TGEPKPETVWKKDGQLVTEGRRHVVYEDAQENFVLKILFCKQSDRGLYTCTASNLVGQTYSSVLVVVREPAVPFKKRLQDLEVREKE
SATFLCEVPQPSTEAAWFKEETRLWASAKYGIEEEGTERRLTVRNVSADDDAVYICETPEGSRTVAELAVQGNLLRKLPRKTAVRVG
DTAMFCVELAVPVGPVHWLRNQEEVVAGGRVAISAEGTRHTLTISQCCLEDVGQVAFMAGDCQTSTQFCVSAPRKPPLQPPVDPVVK
ARMESSVILSWSPPPHGERPVTIDGYLVEKKKLGTYTWIRCHEAEWVATPELTVADVAEEGNFQFRVSALNSFGQSPYLEFPGTVHL
APKLAVRTPLKAVQAVEGGEVTFSVDLTVASAGEWFLDGQALKASSVYEIHCDRTRHTLTIREVPASLHGAQLKFVANGIESSIRME
VRAAPGLTANKPPAAAAREVLARLHEEAQLLAELSDQAAAVTWLKDGRTLSPGPKYEVQASAGRRVLLVRDVARDDAGLYECVSRGG
RIAYQLSVQGLARFLHKDMAGSCVDAVAGGPAQFECETSEAHVHVHWYKDGMELGHSGERFLQEDVGTRHRLVAATVTRQDEGTYSC
RVGEDSVDFRLRVSEPKVVFAKEQLARRKLQAEAGASATLSCEVAQAQTEVTWYKDGKKLSSSSKVCMEATGCTRRLVVQQAGQADA
GEYSCEAGGQRLSFHLDVKEPKVVFAKDQVAHSEVQAEAGASATLSCEVAQAQTEVMWYKDGKKLSSSLKVHVEAKGCRRRLVVQQA
GKTDAGDYSCEARGGQRVSFRLHITEPKMMFAKEQSVHNEVQAEAGASAMLSCEVAQAQTEVTWYKDGKKLSSSSKVGMEVKGCTRRL
VLPQAGKADAGEYSCEAGGQRVSFHLHITEPKGVFAKEQSVHNEVQAEAGTTAMLSCEVAQPQTEVTWYKDGKKLSSSSKVRMEVKG
CTRRLVVQQVGKADAGEYSCEAGGQRVSFQLHITEPKAVFAKEQLVHNEVRTEAGASATLSCEVAQAQTEVTWYKDGKKLSSSSKVR
IEAAGCMRQLVVQQAGQADAGEYTCEAGGQRLSFHLDVSEPKAVFAKEQLAHRKVQAEAGAIATLSCEVAQAQTEVTWYKDGKKLSS
SSKVRMEAVGCTRRLVVQQACQADTGEYSCEAGGQRLSFSLDVAEPKVVFAKEQPVHREVQAQAGASTTLSCEVAQAQTEVMWYKDG
KKLSFSSKVRMEAVGCTRRLVVQQAGQAVAGEYSCEAGSQRLSFHLHVAEPKAVFAKEQPASREVQAEAGTSATLSCEVAQAQTEVT
WYKDGKKLSSSSKVRMEAVGCTRRLVVQEAGQADAGEYSCKAGDQRLSFHLHVAEPKVVFAKEQPAHREVQAEAGASATLSCEVAQA
QTEVTWYKDGKKLSSSSKVRVEAVGCTRRLVVQQAGQAEAGEYSCEAGGQQLSFRLQVAELEPQISERPCRREPLVVKEHEDIILTA
TLATPSAATVTWLKDGVEIRRSKRHETASQGDTHTLTVHGAQVLDSAIYSCRVGAEGQDFPVQVEEVAAKFCRLLEPVCGELGGTVT
LACELSPACAEVVWRCGNTQLRVGKRFQMVAEGPVRSLTVLGLRAEDAGEYVCESRDDHTSAQLTVSVPRVVKFMSGLSTVVAEEGG
EATFQCVVSPSDVAVVWFRDGALLQPSEKFAISQSGASHSLTISDLVLEDAGQITVEAEGASSSAALRVREAPVLFKKKLEPQTVEE
RSSVTLEVELTRPWPELRWTRNATALAPGKNVEIHAEGARHRLVLHNVGFADRGFFGCETPDDKTQAKLTVEMRQVRLVRGLQAVEA
REQGTATMEVQLSHADVDGSWTRDGLRFQQGPTCHLAVRGPMHTLTLSGLRPEDSGLMVFKAEGVHTSARLVVTELPVSFSRPLQDV
VTTEKEKVTLECELSRPNVDVRWLKDGVELRAGKTMAIAAQGACRSLTIYRCEFADQGVYVCDAHDAQSSASVKVQGRTYTLIYRRV
LAEDAGEIQFVAENAESRAQLRVKELPVTLVRPLRDKIAMEKHRGVLECQVSRASAQVRWFKGSQELQPGPKYELVSDGLYRKLIIS
DVHAEDEDTYTCDAGDVKTSAQFFVEEQSITIVRGLQDVTVMEPAPAWFECETSIPSVRPPKWLLGKTVLQAGGNVGLEQEGTVHRL
MLRRTCSTMTGPVHFTVGKSRSSARLVVSDIPVVLTRPLEPKTGRELQSVVLSCDFRPAPKAVQWYKDDTPLSPSEKFKMSLEGQMA
ELRILRLMPADAGVYRCQAGSAHSSTEVTVEAREVTVTGPLQDAEATEEGWASFSCELSHEDEEVEWSLNGMPLYNDSFHEISHKGR
RHTLVLKSIQRADAGIVRASSLKVSTSARLEVRVKPVVFLKALDDLSAEERGTLALQCEVSDPEAHVVWRKDGVQLGPSDKYDFLHT
AGTRGLVVHDVSPEDAGLYTCHVGSEETRARVRVHDLHVGITKRLKTMEVLEGESCSFECVLSHESASDPAMWTVGGKTVGSSSRFQ
ATRQGRKYILVVREAAPSDAGEVVFSVRGLTSKASLIVRERPAAIIKPLEDQWVAPGEDVELRCELSRAGTPVHWLKDRKAIRKSQK
YDVVCEGTMAMLVIRGASLKDAGEYTCEVEASKSTASLHVEEKANCFTEELTNLQVEEKGTAVFTCKTEHPAATVTWRKGLLELRAS
GKHQPSQEGLTLRLTISALEKADSDTYTCDIGQAQSRAQLLVQGRRVHIIEDLEDVDVQEGSSATFRCRISPANYEPVHWFLDKTPL
HANELNEIDAQPGGYHVLTLRQLALKDSGTIYFEAGDQRASAALRVTEKPSVFSRELTDATITEGEDLTLVCETSTCDIPVCWTKDG

KTLRGSARCQLSHEGHRAQLLITGATLQDSGRYKCEAGGACSSSIVRVHARPVRFQEALKDLEVLEGGAATLRCVLSSVAAPVKWCY
GNNVLRPGDKYSLRQEGAMLELVVRNLRPQDSGRYSCSFGDQTTSATLTVTALPAQFIGKLRNKEATEGATATLRCELSKAAPVEWR
KGSETLRDGDRYCLRQDGAMCELQIRGLAMVDAAEYSCVCGEERTSASLTIRPMPAHFIGRLRHQESIEGATATLRCELSKAAPVEW
RKGRESLRDGDRHSLRQDGAVCELQICGLAVADAGEYSCVCGEERTSATLTVKALPAKFTEGLRNEEAVEGATAMLWCELSKVAPVE
WRKGPENLRDGDRYILRQEGTRCELQICGLAMADAGEYLCVCGQERTSATLTIRALPARFIEDVKNQEAREGATAVLQCELNSAAPV
EWRKGSETLRDGDRYSLRQDGTKCELQIRGLAMADTGEYSCVCGQERTSAMLTVRALPIKFTEGLRNEEATEGATAVLRCELSKMAP
VEWWKGHETLRDGDRHSLRQDGARCELQIRGLVAEDAGEYLCMCGKERTSAMLTVRAMPSKFIEGLRNEEATEGDTATLWCELSKAA
PVEWRKGHETLRDGDRHSLRQDGSRCELQIRGLAVVDAGEYSCVCGQERTSATLTVRALPARFIEDVKNQEAREGATAVLQCELSKA
APVEWRKGSETLRGGDRYSLRQDGTRCELQIHGLSVADTGEYSCVCGQERTSATLTVRAPQPVFREPLQSLQAEEGSTATLQCELSE
PTATVVWSKGGLQLQANGRREPRLQGCTAELVLQDLQREDTGEYTCTCGSQATSATLTVTAAPVRFLRELQHQEVDEGGTAHLCCEL
SRAGASVEWRKGSLQLFPCAKYQMVQDGAAAELLVRGVEQEDAGDYTCDTGHTQSMASLSVRVPRPKFKTRLQSLEQETGDIARLCC
QLSDAESGAVVQWLKEGVELHAGPKYEMRSQGATRELLIHQLEAKDTGEYACVTGGQKTAASLRVTEPEVTIVRGLVDAEVTADEDV
EFSCEVSRAGATGVQWCLQGLPLQSNEVTEVAVRDGRIHTLRLKGVTPEDAGTVSFHLGNHASSAQLTVRAPEVTILEPLQDVQLSE
GQDASFQCRLSRASGQEARWALGGVPLQANEMNDITVEQGTLHLLTLHKVTLEDAGTVSFHVGTCSSEAQLKVTAKNTVVRGLENVE
ALEGGEALFECQLSQPEVAAHTWLLDDEPVHTSENAEVVFFENGLRHLLLLKNLRPQDSCRVTFLAGDMVTSAFLTVRGWRLEILEP
LKNAAVRAGAQACFTCTLSEAVPVGEASWYINGAAVQPDDSDWTVTADGSHHALLLRSAQPHHAGEVTFACRDAVASARLTVLGLPD
PPEDAEVVARSSHTVTLSWAAPMSDGGGGLCGYRVEVKEGATGQWRLCHELVPGPECVVDGLAPGETYRFRVAAVGPVGAGEPVHLP
QTVRLAEPPKPVPPQPSAPESRQVAAGEDVSLELEVVAEAGEVIWHKGMERIQPGGRFEVVSQGRQQMLVIKGFTAEDQGEYHCGLA
QGSICPAAATFQVALSPASVDEAPQPSLPPEAAQEGDLHLLWEALARKRRMSREPTLDSISELPEEDGRSQRLPQEAEEVAPDLSEG
YSTADELARTGDADLSHTSSDDESRAGTPSLVTYLKKAGRPGTSPLASKVGAPAAPSVKPQQQQEPLAAVRPPLGDLSTKDLGDPSM
DKAAVKIQAAFKGYKVRKEMKQQEGPMFSHTFGDTEAQVGDALRLECVVASKADVRARWLKDGVELTDGRHHHIDQLGDGTCSLLIT
GLDRADAGCYTCQVSNKFGQVTHSACVVVSGSESEAESSSGGELDDAFRRAARRLHRLFRTKSPAEVSDEELFLSADEGPAEPEEPA
DWQTYREDEHFICIRFEALTEARQAVTRFQEMFATLGIGVEIKLVEQGPRRVEMCISKETPAPVVPPEPLPSLLTSDAAPVFLTELQ
NQEVQDGYPVSFDCVVTGQPMPSVRWFKDGKLLEEDDHYMINEDQQGGHQLIITAVVPADMGVYRCLAENSMGVSSTKAELRVDLTS
TDYDTAADATESSSYFSAQGYLSSREQEGTESTTDEGQLPQVVEELRDLQVAPGTRLAKFQLKVKGYPAPRLYWFKDGQPLTASAHI
RMTDKKILHTLEIISVTREDSGQYAAYISNAMGAAYSSARLLVRGPDEPEEKPASDVHEQLVPPRMLERFTPKKVKKGSSITFSVKV
EGRPVPTVHWLREEAERGVLWIGPDTPGYTVASSAQQHSLVLLDVGRQHQGTYTCIASNAAGQALCSASLHVSGLPKVEEQEKVKEA
LISTFLQGTTQAISAQGLETASFADLGGQRKEEPLAAKEALGHLSLAEVGTEEFLQKLTSQITEMVSAKITQAKLQVPGGDSDEDSK
TPSASPRHGRSRPSSSIQESSSESEDGDARGEIFDIYVVTADYLPLGAEQDAITLREGQYVEVLDAAHPLRWLVRTKPTKSSPSRQG
WVSPAYLDRRLKLSPEWGAAEAPEFPGEAVSEDEYKARLSSVIQELLSSEQAFVEELQFLQSHHLQHLERCPHVPIAVAGQKAVIFR
NVRDIGRFHSSFLQELQQCDTDDDVAMCFIKNQAAFEQYLEFLVGRVQAESVVVSTAIQEFYKKYAEEALLAGDPSQPPPPPLQHYL
EQPVERVQRYQALLKELIRNKARNRQNCALLEQAYAVVSALPQRAENKLHVSLMENYPGTLQALGEPIRQGHFIVWEGAPGARMPWK
GHNRHVFLFRNHLVICKPRRDSRTDTVSYVFRNMMKLSSIDLNDQVEGDDRAFEVWQEREDSVRKYLLQARTAIIKSSWVKEICGIQ
QRLALPVWRPPDFEEELADCTAELGETVKLACRVTGTPKPVISWYKDGKAVQVDPHHILIEDPDGSCALILDSLTGVDSGQYMCFAA
SAAGNCSTLGKILVQVPPRFVNKVRASPFVEGEDAQFTCTIEGAPYPQIRWYKDGALLTTGNKFQTLSEPRSGLLVLVIRAASKEDL
GLYECELVNRLGSARASAELRIQSPMLQAQEQCHREQLVAAVEDTTLERADQEVTSVLKRLLGPKAPGPSTGDLTGPGPCPRGAPAL
QETGSQPPVTGTSEAPAVPPRVPQPLLHEGPEQEPEAIARAQEWTVPIRMEGAAWPGAGTGELLWDVHSHVVRETTQRTYTYQAIDT
HTARPPSMQVTIEDVQAQTGGTAQFEAIIEGDPQPSVTWYKDSVQLVDSTRLSQQQEGTTYSLVLRHVASKDAGVYTCLAQNTGGQV
LCKAELLVLGGDNEPDSEKQSHRRKLHSFYEVKEEIGRGVFGFVKRVQHKGNKILCAAKFIPLRSRTRAQAYRERDILAALSHPLVT
GLLDQFETRKTLILILELCSSEELLDRLYRKGVVTEAEVKVYIQQLVEGLHYLHSHGVLHLDIKPSNILMVHPAREDIKICDFGFAQ
NITPAELQFSQYGSPEFVSPEIIQQNPVSEASDIWAMGVISYLSLTCSSPFAGESDRATLLNVLEGRVSWSSPMAAHLSEDAKDFIK
ATLQRAPQARPSAAQCLSHPWFLKSMPAEEAHFINTKQLKFLLARSRWQRSLMSYKSILVMRSIPELLRGPPDSPSLGVARHLCRDT
GGSSSSSSSSDNELAPFARAKSLPPSPVTHSPLLHPRGFLRPSASLPEEAEASERSTEAPAPPASPEGAGPPAAQGCVPRHSVIRSL
FYHQAGESPEHGALAPGSRRHPARRRHLLKGGYIAGALPGLREPLMEHRVLEEEAAREEQATLLAKAPSFETALRLPASGTHLAPGH

SHSLEHDSPSTPRPSSEACGEAQRLPSAPSGGAPIRDMGHPQGSKQLPSTGGHPGTAQPERPSPDSPWGQPAPFCHPKQGSAPQEGC
SPHPAVAPCPPGSFPPGSCKEAPLVPSSPFLGQPQAPPAPAKASPPLDSKMGPGDISLPGRPKPGPCSSPGSASQASSSQVSSLRVG
SSQVGTEPGPSLDAEGWTQEAEDLSDSTPTLQRPQEQATMRKFSLGGRGGYAGVAGYGTFAFGGDAGGMLGQGPMWARIAWAVSQSE
EEEQEEARAESQSEEQQEARAESPLPQVSARPVPEVGRAPTRSSPEPTPWEDIGQVSLVQIRDLSGDAEAADTISLDISEVDPAYLN
LSDLYDIKYLPFEFMIFRKVPKSAQPEPPSPMAEEELAEFPEPTWPWPGELGPHAGLEITEESEDVDALLAEAAVGRKRKWSSPSRS
LFHFPGRHLPLDEPAELGLRERVKASVEHISRILKGRPEGLEKEGPPRKKPGLASFRLSGLKSWDRAPTFLRELSDETVVLGQSVTL
ACQVSAQPAAQATWSKDGAPLESSSRVLISATLKNFQLLTILVVVAEDLGVYTCSVSNALGTVTTTGVLRKAERPSSSPCPDIGEVY
ADGVLLVWKPVESYGPVTYIVQCSLEGGSWTTLASDIFDCCYLTSKLSRGGTYTFRTACVSKAGMGPYSSPSEQVLLGGPSHLASEE
ESQGRSAQPLPSTKTFAFQTQIQRGRFSVVRQCWEKASGRALAAKIIPYHPKDKTAVLREYEALKGLRHPHLAQLHAAYLSPRHLVL
ILELCSGPELLPCLAERASYSESEVKDYLWQMLSATQYLHNQHILHLDLRSENMIITEYNLLKVVDLGNAQSLSQEKVLPSDKFKDY
LETMAPELLEGQGAVPQTDIWAIGVTAFIMLSAEYPVSSEGARDLQRGLRKGLVRLSRCYAGLSGGAVAFLRSTLCAQPWGRPCASS
CLQCPWLTEEGPACSRPAPVTFPTARLRVFVRNREKRRALLYKRHNLAQVR
>sp|P0CG35|TB15B_HUMAN
MSDKPDLSEVEKFDRSKLKKTNTEEKNTLPSKETIQQEKECVQTS
>sp|Q86YP4|P66A_HUMAN
MTEEACRTRSQKRALERDPTEDDVESKKIKMERGLLASDLNTDGDMRVTPEPGAGPTQGLLRATEATAMAMGRGEGLVGDGPVDMRT
SHSDMKSERRPPSPDVIVLSDNEQPSSPRVNGLTTVALKETSTEALMKSSPEERERMIKQLKEELRLEEAKLVLLKKLRQSQIQKEA
TAQKPTGSVGSTVTTPPPLVRGTQNIPAGKPSLQTSSARMPGSVIPPPLVRGGQQASSKLGPQASSQVVMPPLVRGAQQIHSIRQHS
STGPPPLLLAPRASVPSVQIQGQRIIQQGLIRVANVPNTSLLVNIPQPTPASLKGTTATSAQANSTPTSVASVVTSAESPASRQAAA
KLALRKQLEKTLLEIPPPKPPAPEMNFLPSAANNEFIYLVGLEEVVQNLLETQGRMSAATVLSREPYMCAQCKTDFTCRWREEKSGA
IMCENCMTTNQKKALKVEHTSRLKAAFVKALQQEQEIEQRLLQQGTAPAQAKAEPTAAPHPVLKQVIKPRRKLAFRSGEARDWSNGA
VLQASSQLSRGSATTPRGVLHTFSPSPKLQNSASATALVSRTGRHSERTVSAGKGSATSNWKKTPLSTGGTLAFVSPSLAVHKSSSA
VDRQREYLLDMIPPRSIPQSATWK
>sp|Q96H72|S39AD_HUMAN
MPGCPCPGCGMAGPRLLFLTALALELLERAGGSQPALRSRGTATACRLDNKESESWGALLSGERLDTWICSLLGSLMVGLSGVFPLL
VIPLEMGTMLRSEAGAWRLKQLLSFALGGLLGNVFLHLLPEAWAYTCSASPGGEGQSLQQQQQLGLWVIAGILTFLALEKMFLDSKE
EGTSQAPNKDPTAAAAALNGGHCLAQPAAEPGLGAVVRSIKVSGYLNLLANTIDNFTHGLAVAASFLVSKKIGLLTTMAILLHEIPH
EVGDFAILLRAGFDRWSAAKLQLSTALGGLLGAGFAICTQSPKGVVGCSPAAEETAAWVLPFTSGGFLYIALVNVLPDLLEEEDPWR
SLQQLLLLCAGIVVMVLFSLFVD
>sp|Q8NG77|O2T12_HUMAN
MEMRNTTPDFILLGLFNHTRAHQVLFMMLLATVLTSLFSNALMILLIHWDHRLHRPMYFLLSQLSLMDMMLVSTTVPKMAADYLTGN
KAISRAGCGVQIFFLPTLGGGECFLLAAMAYDRYAAVCHPLRYPTLMSWQLCLRMTMSSWLLGAADGLLQAVATLSFPYCGAHEIDH
FFCEAPVLVRLACADTSVFENAMYICCVLMLLVPFSLILSSYGLILAAVLLMRSTEARKKAFATCSSHVAVVGLFYGAGIFTYMRPK
SHRSTNHDKVVSAFYTMFTPLLNPLIYSVRNSEVKEALKRWLGTCVNLKHQQNEAHRSR
>sp|O75324|SNN_HUMAN
MSIMDHSPTTGVVTVIVILIAIAALGALILGCWCYLRLQRISQSEDEESIVGDGETKEPFLLVQYSAKGPCVERKAKLMTPNGPEVH
G
>sp|P31274|HXC9_HUMAN
MSATGPISNYYVDSLISHDNEDLLASRFPATGAHPAAARPSGLVPDCSDFPSCSFAPKPAVFSTSWAPVPSQSSVVYHPYGPQPHLG
ADTRYMRTWLEPLSGAVSFPSFPAGGRHYALKPDAYPGRRADCGPGEGRSYPDYMYGSPGELRDRAPQTLPSPEADALAGSKHKEEK
ADLDPSNPVANWIHARSTRKKRCPYTKYQTLELEKEFLFNMYLTRDRRYEVARVLNLTERQVKIWFQNRRMKMKKMNKEKTDKEQS
>sp|Q9UQR1|ZN148_HUMAN
MNIDDKLEGLFLKCGGIDEMQSSRTMVVMGGVSGQSTVSGELQDSVLQDRSMPHQEILAADEVLQESEMRQQDMISHDELMVHEETV
KNDEEQMETHERLPQGLQYALNVPISVKQEITFTDVSEQLMRDKKQIREPVDLQKKKKRKQRSPAKILTINEDGSLGLKTPKSHVCE

HCNAAFRTNYHLQRHVFIHTGEKPFQCSQCDMRFIQKYLLQRHEKIHTGEKPFRCDECGMRFIQKYHMERHKRTHSGEKPYQCEYCL
QYFSRTDRVLKHKRMCHENHDKKLNRCAIKGGLLTSEEDSGFSTSPKDNSLPKKKRQKTEKKSSGMDKESALDKSDLKKDKNDYLPL
YSSSTKVKDEYMVAEYAVEMPHSSVGGSHLEDASGEIHPPKLVLKKINSKRSLKQPLEQNQTISPLSTYEESKVSKYAFELVDKQAL
LDSEGNADIDQVDNLQEGPSKPVHSSTNYDDAMQFLKKKRYLQAASNNSREYALNVGTIASQPSVTQAAVASVIDESTTASILESQA
LNVEIKSNHDKNVIPDEVLQTLLDHYSHKANGQHEISFSVADTEVTSSISINSSEVPEVTPSENVGSSSQASSSDKANMLQEYSKFL
QQALDRTSQNDAYLNSPSLNFVTDNQTLPNQPAFSSIDKQVYATMPINSFRSGMNSPLRTTPDKSHFGLIVGDSQHSFPFSGDETNH
ASATSTQDFLDQVTSQKKAEAQPVHQAYQMSSFEQPFRAPYHGSRAGIATQFSTANGQVNLRGPGTSAEFSEFPLVNVNDNRAGMTS
SPDATTGQTFG
>sp|Q7RTP6|MICA3_HUMAN
MEERKHETMNPAHVLFDRFVQATTCKGTLKAFQELCDHLELKPKDYRSFYHKLKSKLNYWKAKALWAKLDKRGSHKDYKKGKACTNT
KCLIIGAGPCGLRTAIDLSLLGAKVVVIEKRDAFSRNNVLHLWPFTIHDLRGLGAKKFYGKFCAGAIDHISIRQLQLILLKVALILG
IEIHVNVEFQGLIQPPEDQENERIGWRALVHPKTHPVSEYEFEVIIGGDGRRNTLEGFRRKEFRGKLAIAITANFINRNTTAEAKVE
EISGVAFIFNQKFFQELREATGIDLENIVYYKDDTHYFVMTAKKQSLLDKGVILHDYADTELLLSRENVDQEALLSYAREAADFSTQ
QQLPSLDFAINHYGQPDVAMFDFTCMYASENAALVREQNGHQLLVALVGDSLLEPFWPMGTGIARGFLAAMDSAWMVRSWSLGTSPL
EVLAERESIYRLLPQTTPENVSKNFSQYSIDPVTRYPNINVNFLRPSQVRHLYDTGETKDIHLEMESLVNSRTTPKLTRNESVARSS
KLLGWCQRQTDGYAGVNVTDLTMSWKSGLALCAIIHRYRPDLIDFDSLDEQNVEKNNQLAFDIAEKELGISPIMTGKEMASVGEPDK
LSMVMYLTQFYEMFKDSLPSSDTLDLNAEEKAVLIASTRSPISFLSKLGQTISRKRSPKDKKEKDLDGAGKRRKTSQSEEEEAPRGH
RGERPTLVSTLTDRRMDVAVGNQNKVKYMATQLLAKFEENAPAQSIGIRRQGSMKKEFPQNLGGSDTCYFCQKRVYVMERLSAEGKF
FHRSCFKCEYCATTLRLSAYAYDIEDGKFYCKPHYCYRLSGYAQRKRPAVAPLSGKEAKGPLQDGATTDANGRANAVASSTERTPGS
GVNGLEEPSIAKRLRGTPERIELENYRLSLRQAEALQEVPEETQAEHNLSSVLDTGAEEDVASSSSESEMEEEGEEEEEEPRLPPSD
LGGVPWKEAVRIHALLKGKSEEELEASKSFGPGNEEEEEEEEEYEEEEEEDYDEEEEESSEAGNQRLQQVMHAADPLEIQADVHWTH
IREREEEERMAPASESSASGAPLDENDLEEDVDSEPAEIEGEAAEDGDPGDTGAELDDDQHWSDSPSDADRELRLPCPAEGEAELEL
RVSEDEEKLPASPKHQERGPSQATSPIRSPQESALLFIPVHSPSTEGPQLPPVPAATQEKSPEERLFPEPLLPKEKPKADAPSDLKA
VHSPIRSQPVTLPEARTPVSPGSPQPQPPVAASTPPPSPLPICSQPQPSTEATVPSPTQSPIRFQPAPAKTSTPLAPLPVQSQSDTK
DRLGSPLAVDEALRRSDLVEEFWMKSAEIRRSLGLTPVDRSKGPEPSFPTPAFRPVSLKSYSVEKSPQDEGLHLLKPLSIPKRLGLP
KPEGEPLSLPTPRSPSDRELRSAQEERRELSSSSGLGLHGSSSNMKTLGSQSFNTSDSAMLTPPSSPPPPPPPGEEPATLRRKLREA
EPNASVVPPPLPATWMRPPREPAQPPREEVRKSFVESVEEIPFADDVEDTYDDKTEDSSLQEKFFTPPSCWPRPEKPRHPPLAKENG
RLPALEGTLQPQKRGLPLVSAEAKELAEERMRAREKSVKSQALRDAMARQLSRMQQMELASGAPRPRKASSAPSQGKERRPDSPTRP
TLRGSEEPTLKHEATSEEVLSPPSDSGGPDGSFTSSEGSSGKSKKRSSLFSPRRNKKEKKSKGEGRPPEKPSSNLLEEAAAKPKSLW
KSVFSGYKKDKKKKADDKSCPSTPSSGATVDSGKHRVLPVVRAELQLRRQLSFSEDSDLSSDDVLEKSSQKSRREPRTYTEEELNAK
LTRRVQKAARRQAKQEELKRLHRAQIIQRQLQQVEERQRRLEERGVAVEKALRGEAGMGKKDDPKLMQEWFKLVQEKNAMVRYESEL
MIFARELELEDRQSRLQQELRERMAVEDHLKTEEELSEEKQILNEMLEVVEQRDSLVALLEEQRLREREEDKDLEAAMLSKGFSLNW
S
>sp|P51798|CLCN7_HUMAN
MANVSKKVSWSGRDRDDEEAAPLLRRTARPGGGTPLLNGAGPGAARQSPRSALFRVGHMSSVELDDELLDPDMDPPHPFPKEIPHNE
KLLSLKYESLDYDNSENQLFLEEERRINHTAFRTVEIKRWVICALIGILTGLVACFIDIVVENLAGLKYRVIKGNIDKFTEKGGLSF
SLLLWATLNAAFVLVGSVIVAFIEPVAAGSGIPQIKCFLNGVKIPHVVRLKTLVIKVSGVILSVVGGLAVGKEGPMIHSGSVIAAGI
SQGRSTSLKRDFKIFEYFRRDTEKRDFVSAGAAAGVSAAFGAPVGGVLFSLEEGASFWNQFLTWRIFFASMISTFTLNFVLSIYHGN
MWDLSSPGLINFGRFDSEKMAYTIHEIPVFIAMGVVGGVLGAVFNALNYWLTMFRIRYIHRPCLQVIEAVLVAAVTATVAFVLIYSS
RDCQPLQGGSMSYPLQLFCADGEYNSMAAAFFNTPEKSVVSLFHDPPGSYNPLTLGLFTLVYFFLACWTYGLTVSAGVFIPSLLIGA
AWGRLFGISLSYLTGAAIWADPGKYALMGAAAQLGGIVRMTLSLTVIMMEATSNVTYGFPIMLVLMTAKIVGDVFIEGLYDMHIQLQ
SVPFLHWEAPVTSHSLTAREVMSTPVTCLRRREKVGVIVDVLSDTASNHNGFPVVEHADDTQPARLQGLILRSQLIVLLKHKVFVER
SNLGLVQRRLRLKDFRDAYPRFPPIQSIHVSQDERECTMDLSEFMNPSPYTVPQEASLPRVFKLFRALGLRHLVVVDNRNQVVGLVT
RKDLARYRLGKRGLEELSLAQT

>sp|Q8NDZ2|SIMC1_HUMAN
MAPASASGEDLRKLPTMAEVNGEQDFIDLTRETRPRTKDRSGLYVIDLTRAEGENRPIATLDLTLEPVTPSQKEPTSLQTCASLSGK
AVMEGHVDRSSQPTARRIINSDPVDLDLVEENTFVGPPPATSISGGSVYPTEPNCSSATFTGNLSFLASLQLSSDVSSLSPTSNNSR
SSSSSSNQKAPLPCPQQDVSRPPQALPCPLRPLPCPPRASPCPPRASSCPPRALSCPSQTMQCQLPALTHPPQEVPCPRQNIPGPPQ
DSLGLPQDVPGLPQSILHPQDVAYLQDMPRSPGDVPQSPSDVSPSPDAPQSPGGMPHLPGDVLHSPGDMPHSSGDVTHSPRDIPHLP
GDRPDFTQNDVQNRDMPMDISALSSPSCSPSPQSETPLEKVPWLSVMETPARKEISLSEPAKPGSAHVQSRTPQGGLYNRPCLHRLK
YFLRPPVHHLFFQTLIPDKDTRENKGQKLEPIPHRRLRMVTNTIEENFPLGTVQFLMDFVSPQHYPPREIVAHIIQKILLSGSETVD
VLKEAYMLLMKIQQLHPANAKTVEWDWKLLTYVMEEEGQTLPGRVLFLRYVVQTLEDDFQQTLRRQRQHLQQSIANMVLSCDKQPHN
VRDVIKWLVKAVTEDGLTQPPNGNQTSSGTGILKASSSHPSSQPNLTKNTNQLIVCQLQRMLSIAVEVDRTPTCSSNKIAEMMFGFV
LDIPERSQREMFFTTMESHLLRCKVLEIIFLHSCETPTRLPLSLAQALYFLNNSTSLLKCQSDKSQWQTWDELVEHLQFLLSSYQHV
LREHLRSSVIDRKDLIIKRIKPKPQQGDDITVVDVEKQIEAFRSRLIQMLGEPLVPQLQDKVHLLKLLLFYAADLNPDAEPFQKGWS
GS
>sp|Q96LK8|SPT32_HUMAN
MGVTGAHGFPCCGKGSVEVAEMRDDLSQHQIQEEQELEADMLEQKPQLQVDLDLDPDPDPDPELEIGQVPALLESELYPALKLEAEL
DTEANSNEESDFEEPMQLVCKIESVHSNMGLPTPQTFRPWSLNSNCRSFTEENHVSACHHSISAQTSKHLFWANKLIQASEHSLQRA
INMQLNNGSAGQPIRSPLREAIPTNALCSEEQLQIPDAHSAPPTTSSQAPSPLLSSDLPPPIDLTELITFASSLAMASSSRMDLPSL
EHMMKAPPQEALEPSTEPLLTTVEEREPENHAETLPEKPREARAPLKSWSQEDKNFAQSYFDFSKPGIKRATIKGQIQLLQPPATSP
LLQGSKEDSVPPGKEKENPLLVKIHFKLSAPTIPEK
>sp|Q9Y4C0|NRX3A_HUMAN
MSSTLHSVFFTLKVSILLGSLLGLCLGLEFMGLPNQWARYLRWDASTRSDLSFQFKTNVSTGLLLYLDDGGVCDFLCLSLVDGRVQL
RFSMDCAETAVLSNKQVNDSSWHFLMVSRDRLRTVLMLDGEGQSGELQPQRPYMDVVSDLFLGGVPTDIRPSALTLDGVQAMPGFKG
LILDLKYGNSEPRLLGSRGVQMDAEGPCGERPCENGGICFLLDGHPTCDCSTTGYGGKLCSEDVSQDPGLSHLMMSEQAREENVATF
RGSEYLCYDLSQNPIQSSSDEITLSFKTWQRNGLILHTGKSADYVNLALKDGAVSLVINLGSGAFEAIVEPVNGKFNDNAWHDVKVT
RNLRQVTISVDGILTTTGYTQEDYTMLGSDDFFYVGGSPSTADLPGSPVSNNFMGCLKEVVYKNNDIRLELSRLARIADTKMKIYGE
VVFKCENVATLDPINFETPEAYISLPKWNTKRMGSISFDFRTTEPNGLILFTHGKPQERKDARSQKNTKVDFFAVELLDGNLYLLLD
MGSGTIKVKATQKKANDGEWYHVDIQRDGRSGTISVNSRRTPFTASGESEILDLEGDMYLGGLPENRAGLILPTELWTAMLNYGYVG
CIRDLFIDGRSKNIRQLAEMQNAAGVKSSCSRMSAKQCDSYPCKNNAVCKDGWNRFICDCTGTGYWGRTCEREASILSYDGSMYMKI
IMPMVMHTEAEDVSFRFMSQRAYGLLVATTSRDSADTLRLELDGGRVKLMVNLDCIRINCNSSKGPETLYAGQKLNDNEWHTVRVVR
RGKSLKLTVDDDVAEGTMVGDHTRLEFHNIETGIMTEKRYISVVPSSFIGHLQSLMFNGLLYIDLCKNGDIDYCELKARFGLRNIIA
DPVTFKTKSSYLSLATLQAYTSMHLFFQFKTTSPDGFILFNSGDGNDFIAVELVKGYIHYVFDLGNGPNVIKGNSDRPLNDNQWHNV
VITRDNSNTHSLKVDTKVVTQVINGAKNLDLKGDLYMAGLAQGMYSNLPKLVASRDGFQGCLASVDLNGRLPDLINDALHRSGQIER
GCEGPSTTCQEDSCANQGVCMQQWEGFTCDCSMTSYSGNQCNDPGATYIFGKSGGLILYTWPANDRPSTRSDRLAVGFSTTVKDGIL
VRIDSAPGLGDFLQLHIEQGKIGVVFNIGTVDISIKEERTPVNDGKYHVVRFTRNGGNATLQVDNWPVNEHYPTGRQLTIFNTQAQI
AIGGKDKGRLFQGQLSGLYYDGLKVLNMAAENNPNIKINGSVRLVGEVPSILGTTQTTSMPPEMSTTVMETTTTMATTTTRKNRSTA
SIQPTSDDLVSSAECSSDDEDFVECEPSTTGGELVIPLLVEDPLATPPIATRAPSITLPPTFRPLLTIIETTKDSLSMTSEAGLPCL
SDQGSDGCDDDGLVISGYGSGETFDSNLPPTDDEDFYTTFSLVTDKSLSTSIFEGGYKAHAPKWESKDFRPNKVSETSRTTTTSLSP
ELIRFTASSSSGMVPKLPAGKMNNRDLKPQPDIVLLPLPTAYELDSTKLKSPLITSPMFRNVPTANPTEPGIRRVPGASEVIRESSS
TTGMVVGIVAAAALCILILLYAMYKYRNRDEGSYQVDETRNYISNSAQSNGTLMKEKQQSSKSGHKKQKNKDREYYV
>sp|Q9Y305|ACOT9_HUMAN
MRRAALRLCALGKGQLTPGRGLTQGPQNPKKQGIFHIHEVRDKLREIVGASTNWRDHVKAMEERKLLHSFLAKSQDGLPPRRMKDSY
IEVLLPLGSEPELREKYLTVQNTVRFGRILEDLDSLGVLICYMHNKIHSAKMSPLSIVTALVDKIDMCKKSLSPEQDIKFSGHVSWV
GKTSMEVKMQMFQLHGDEFCPVLDATFVMVARDSENKGPAFVNPLIPESPEEEELFRQGELNKGRRIAFSSTSLLKMAPSAEERTTI
HEMFLSTLDPKTISFRSRVLPSNAVWMENSKLKSLEICHPQERNIFNRIFGGFLMRKAYELAWATACSFGGSRPFVVAVDDIMFQKP

VEVGSLLFLSSQVCFTQNNYIQVRVHSEVASLQEKQHTTTNVFHFTFMSEKEVPLVFPKTYGESMLYLDGQRHFNSMSGPATLRKDY
LVEP
>sp|Q9NYQ3|HAOX2_HUMAN
MSLVCLTDFQAHAREQLSKSTRDFIEGGADDSITRDDNIAAFKRIRLRPRYLRDVSEVDTRTTIQGEEISAPICIAPTGFHCLVWPD
GEMSTARAAQAAGICYITSTFASCSLEDIVIAAPEGLRWFQLYVHPDLQLNKQLIQRVESLGFKALVITLDTPVCGNRRHDIRNQLR
RNLTLTDLQSPKKGNAIPYFQMTPISTSLCWNDLSWFQSITRLPIILKGILTKEDAELAVKHNVQGIIVSNHGGRQLDEVLASIDAL
TEVVAAVKGKIEVYLDGGVRTGNDVLKALALGAKCIFLGRPILWGLACKGEHGVKEVLNILTNEFHTSMALTGCRSVAEINRNLVQF
SRL
>sp|Q2NL68|PRSR3_HUMAN
MDRSLPVFSIQDSPFGDAPLGRSHYWPSQSQTWCPKTLSPSRSQRSRLPQAPKALATGPNSPELFEESWPSSSGTPSLPSTTEGQMW
ASPAPTLIDSGDSVVAKYINRFRQAQPTSREERQPAGPTPADFWWLQSDSPDPSSQSAAAGANKPEGRPHTAVPTAVNVTSASHAVA
PLQEIKQNLHTWNSSLLDLETLSLQSRAARLLKRSKASISSSSSLSPSDASTSSFPTSSDGLSPFSETFIPDSSKGLGPRAPASPAP
AQAQTPTPAPAPASSQAPLRPEDDILYQWRQRRKLEQAQGSKGDRAWVPPLTPALRTLAESLKAKALPPAAGSVIRKSEATPSPGAC
LQPEVPLSPAEQATTVKASPPAFQVGSPEALAPPPPAADHAPSEALLAQAALLLQAAEDSDGSEFQDDPVLQVLRAHRAELSRQKRE
ADARLSFLLDQAEDLGSWSPPAGSPPRSPRRLLRREGDSLEARRL
>sp|O75312|ZPR1_HUMAN
MAASGAVEPGPPGAAVAPSPAPAPPPAPDHLFRPISAEDEEQQPTEIESLCMNCYCNGMTRLLLTKIPFFREIIVSSFSCEHCGWNN
TEIQSAGRIQDQGVRYTLSVRALEDMNREVVKTDSAATRIPELDFEIPAFSQKGALTTVEGLITRAISGLEQDQPARRANKDATAER
IDEFIVKLKELKQVASPFTLIIDDPSGNSFVENPHAPQKDDALVITHYNRTRQQEEMLGLQEEAPAEKPEEEDLRNEVLQFSTNCPE
CNAPAQTNMKLVQIPHFKEVIIMATNCENCGHRTNEVKSGGAVEPLGTRITLHITDASDMTRDLLKSETCSVEIPELEFELGMAVLG
GKFTTLEGLLKDIRELVTKNPFTLGDSSNPGQTERLQEFSQKMDQIIEGNMKAHFIMDDPAGNSYLQNVYAPEDDPEMKVERYKRTF
DQNEELGLNDMKTEGYEAGLAPQR
>sp|P60412|KR10B_HUMAN
MAASTMSVCSSAYSDSWQVDDCPESCCEPPCSAPSCCAPAPSLSLVCTPVSCVSSPCCQAACEPSACQSGCTSSCTPSCCQQSSCQP
ACCTSSPCQQACCVPVCCKTVCCKPVCCVPVCCGAASSCCRQSSCQPACCASSSCQPACCVPVCCKPVCCVSTCSEDSSSCCQQSSC
QPACCTSSSYQQACCVPVCCKTVYCKPICCVPVCSRASSSRCQQPSCQPACCTTSCCRPSSSVSLLCHPVCRSTCCVPVSSCCAPTS
SCQSSCCRPASCVSLLCRPASSRLACYSLCSGKKSSC
>sp|Q9UKI2|BORG2_HUMAN
MPAKTPIYLKAANNKKGKKFKLRDILSPDMISPPLGDFRHTIHIGKEGQHDVFGDISFLQGNYELLPGNQEKAHLGQFPGHNEFFRA
NSTSDSVFTETPSPVLKNAISLPTIGGSQALMLPLLSPVTFNSKQESFGPAKLPRLSCEPVMEEKAQEKSSLLENGTVHQGDTSWGS
SGSASQSSQGRDSHSSSLSEQYPDWPAEDMFDHPTPCELIKGKTKSEESLSDLTGSLLSLQLDLGPSLLDEVLNVMDKNK
>sp|Q5CZC0|FSIP2_HUMAN
MELYLGACSKPAKVAVTKTVASVLAADTQQCRDGVHKTHFAGVGPAQLLDLPLGVKLPVIPGSNAVFYTTNFGEKLFRPSYGFNLTD
PYCRLLENQYKSLHDPHLKAYYKRKDILKRLKKGGYITSNNKVVCTLRELNKYRQYLTSLKLDFERNYIKEQRILAKQLHNIPENNQ
IPQHCDVAQVQNWLLKEGTESIKDQERLMRHRYLDMISRKLEQLERTAEEQRLFLMDREERRQREHTRRKLTLRRKIEEEWKTKEML
LLTRMAEDVKREERIEEQQHRNREESDRKKQDLLEKKMAYHLQKMQDTGFNGEDIGKNTFKYRGQDGTHASPKNKKKTSEDIMLVYP
AGDQNTYKETHGHTANAAHQRQNSSNNFTKKNSASVVYQADVQDNGINQKRDGMVSKNSSIFDDRGGINISGQGSIISAQVSPTRNF
SRVSQAFLDPSKEEKETNADWDGRPTKRSSYLCESGPQAHATDPGIFSSPVYTNMQQNLLQNCLQEKVTSEELNIIIQNVMTWVVAT
VTSILYPAITKYEKRLQNNTYPVSDDSILSSDSSSFCSTCSEDFTYRSYTSATTKTFQAEPCAFVVDTSVRRPTTPIKPPPAHVEKT
VVGKTCHIKGQSIISKHKYNKTNLLYSYPKLRSCKSDSHLLASFETGTKKSKDATTETDSLGSSLHCDKTAKAMDEMKNLKNVFVNF
KCYLKGETEVILESILREIMSDLTQAIPSLSSVTAEVFVEQCEREKEILLSNAHIPSVASEIVENMLEKLESAVEKKCVEMFSQDLS
VDIKPSLAASDELLTSSNGKPLKNSMPHTLDPMCDIAEDMVHAILEKLMTLVSFKQNEFLHLKDTNKLSCQQHKTDPICMFLQRAGK
NKSSLESDEASLIVNEEVQNLISNIFSQSSLVAYIEEAINAILGYIQTELNNERIIASEETVVLLQLLEDILFQLHQEPVNESFQKS
RQPRISSPSDTKEKYRLTGTRLSNSPRSGRPFPPINVPGMVLYSDDENEEIDNIVKNVLDSTFKDEKVKSQEQIPNHWFTKGNTCFE

CKRNIKPPTKPGSRSKAAFHDWELKTEPPSTNHEDILKKKLSSNKDISTFSQDQKHQIEKASENIVTSILKEMLKDISSVPFGHLDS
KTGSEASVLVSEKPQGLSHQEWIDQMFSVSEISTVAQEITDSVLNILHKASNYISNTTKSSISSSVHQISLHNSDTEHIVKEAPNKY
PLKTWFDSEKKMKYLSLFDVDPEKPPWLKSGKSEPKPVDDINDKIIRTIFKRLKSFICPKLHMGFKSSLRSQLSKYTAKIVNIVLCA
IQNELELHKENLNLREIDHTKSLTDKGFFANTDKKLESLVTSIDDDILASPLLTCIYDMLLSSENAHQRSISLSSRKPKSATDSVDV
QSILPNRQDKKSFHKYLATPCTHHSVNGGNHIKENAKLQVLERIGETLHEMLSKLLGTHLHSQLSCSQQSREMTNKNQKMAAALQSN
IQLISKAILDYILAKLCGVDMDTSFASCGLKAISESLDIDNPSFASIIEKMAKSTKIISSIVSRRVQEDNKEETKSKAKPVAPVSSK
TPSTKEMHPNKLKAVASDILNMVFAKLEGFANGHLEILGAINDGNKKSNKIGWEYESTNISRDTHEASFLSALYMHAKKVSSAILKV
IQTELNVTSSDLKTSVENPPPETQILKYVVKLILDAVSSDMFNEMESEGGGIETYRYRPTYGSLPGGAESDSFLEDDAYTAKKIIDE
RSPQREEVKTRSLKQWALEKTLNKIEVKLKEPHISPIAPIIRNILNEIFQSTLINQLNVLSLSHSNFNGMPHNVDEPTPQTSVQFMD
KMMDPLLSEADITIVTDNIVRTVFHKLYSAAMTERNVRENRYKTITFSANVSSHEHTYKGKSSVTALDENPCTFQSRFSVADKETKV
NLAEDIVQAILTNLETFATSKVKSLFYSQVNFTVPVALPIQQDHSTLSKALSAKDSYSDEQFSCCSVDHTKSGKTNLCQLSLSKLNT
YALQVARRNLQGIKQELDKERENPFLTHDIGISESIASQIVNALLDIISRKGKCDKNSSDKEIDLDQQKGVIEKLLNETKYRKVLQL
QIQDTIEGILCDIYEKTLFQNNLSFATPTLKCSIADKHSEENSEMFMEGANKIIPKLSVPKSDVILISNDIVNIVLHNLSSAATLVI
NAKNPTSARLPLTFCDTFPKIDCQQPLKGSKTERKTERFSYSRNQKSAYADDNQITVVEKEDTQKSATDSCEENANFITKTIFKRLE
SFATERIDSLITLAFQSKEKSFVIPELENCKQNDSIFYDSSQVESDVNVLKISATETILSQELTDFTFVGRREKLGSTIHLSQARLK
TYADVIASAILKLIKNDLDLEIQKIYPYQNNILFQENIIVSEIVDSMLKMLDDKRSVKEICFNSKENSNFSQLALSNEILLGHKEKE
RSTKQSLFTKYPLEQNQMILENKRQIIVLEEIFMRNGESKNKEKGELLIAVEELLNKLYQRVREVTGHLPPLNETANFISNSKIKTS
DTTQKNSFQSHINSVANDIVESVLGKMYLVVVTSLYENNKSRTEVEISDHNDSLLMKPLRFRETKQAGKISNSPRYAISQAYSYVDS
QNISVMENTLLPYLPLQVKKDLIQMVLNKITNFVSLPLKVSPKDNPKPCFKAHLKTRSKITTLPKFTKKTHLGLSAAKAKSKTKLGP
GEKTLKDSRSKTAIGLSHIMSAGDAKNLLDTKLPTSELKIYAKDIIINILETIVKEFGKVKQTKALPSDQIIAAGKIVNTVLQELYV
TNNCNLAYPMKSSHLRLSQGNIGTGSLPKQQACFYLENVSSQLEHIFPREGIFKKLFDKWQTESNDKENEKCKLLMIAENVLTEISI
KAKELEYSLSLLNLPPLENCESRFYNHFKGASTRAEDTKAQINMFGREIVEMLLEKLQLCFLSQIPTPDSEETLSNSKEHITAKSKY
GFPNKHSLSSLPIYNTKTKDQISVGSSNQIVQEIVETVLNMLESFVDLQFKHISKYEFSEIVKMPIENLSSIQQKLLNKKMLPKLQP
LKMFSDKSESNTINFKENIQNILLRVHSFHSQLLTYAVNIISDMLAVIKNKLDNEISQMEPSSISILKENIVASEIIGTLMDQCTYF
NESLIQNLSRESLFQGAENAYTVNQVELATNMKMFTSKLKEGSLGINPSQVSKTGFVFCSDEDMKEKYRVSSDLPTSVRSSVEDTVK
NSEPTKRPDSETMPSCSTRNKVQDHRPRESNFGSFDQTMKGNSYLPEGSFLQKLLRKASDSTEAALKQVLSFIEMGKGENLRVFHYE
NLKPVVEPNQIQTTISPLKICLAAENIVNTVLSSCGFPSQPHTNENREIMKPFFISKQSSLSEVSGGQKDNEKSLLRMQDKKINYIP
EEENENLEASREDSSFLQKLKKKEYPKIETVKEVEAFTFADHEMGSNEVHLIARHVTTSVVTYLKNFETTVFSEEKMSVSTWSRKKY
ESKQFLRNIYDDSSIYQCCEHLTESVLYHLTSSISDGTKKGREKEKAWEIQEATFSKIISIHSQVFESRSISIGELALCISEIIIKI
LFNNKIIQADIAQKMVAIPTKYTYCPGIVSGGFDDLFQDLLVGVIHVLSKEIEVDYHFESNVRNKSFSMHRNNSVPLCNKINRQASP
RDWQFSTQQIGQLFQKNKLSYLACKLNSLVGNLKTSESKEVVNKVFNIVSDLFSPDECLDTGMDSGKIQRTYFYSSNNEQPNSILTN
NLQLSSKSVFLLNVVCEKLIRILLEECTSTAFPDKGSVSEETSAEECQLLKMLQSVEDGKSDYRKGGMDCECLQVDYMSDLLENVAE
IDQDLLTSDSMLTIISHSLVKSLMDKLSHSIQQAPESLPFANKHLNYRTREIQSSFIKARKSELIELGQSKSSLELRSYDSNSLTVS
LNNPSVVSSKIQAPFNKHCAVKSSSVSPFERQRTKEMDKVAIHNKLHQEGIYAGVYSATFLEGIISELFFNLSMSLWGKNKNITVSW
LNEMNTLFVNNVVNEFNNAQVTVLRNAEERLCFPPVHTETVSKIVDSVYYDVLQQYELKVACGNNPVYDNASIAEQITNGILLEILD
YKLPSCFKEHLIPHSYYPLKPEIILQKLQSNLTEFTSLPRSSSDYSTMLSHSFLEDVIRRLLSQLIPPPITCSSLGKKYLMSSDFNE
MSTCIINKVMSAISKHKIWFTIYDNQYLYTGKNLQKMVDSVYCNILQMSDSLVSIQKSIVSRSPIMIDQIASFIIQEIIENHLQPFL
SGEVLCHPRTPLDPVSTIVTQVLSEVIESHRPQKQSPLDIHLDSFVREIVARLLSKIFSPKHNTEIELKNMTQRIVNSINRHFNKAK
IHILYDDKEQAFFSFNTDIVDELATSVYRNALKQHGLDLAVDKESEDSGIFVENITNLIVAAISDYLLHPLFSGDFSASTYSNSVAE
NIVQDILSNISKSTEPSQSVPLYNTLLPYTFLEDMIRVLLSKLFSSASSLVLNRDTQKDISRVNFNDIASNLVSDIRMKVSQHEIRF
SKEEEETKFIYSEDDIQHLVDSVFANVVQTSGSQESAVQNITSSNDILIDRIAGFIIKHICQKHLQPFVSGKSLSSSDTYFDDERRQ
LFYTSVYSSTFLEDVISGVLRKIFHRVVGIVQTKSIRDSEDELFEKAEELIHLITGEFSKAQVSIIDNTEERLCLPPVERDVVKTIV
DMVYSKVLQEYEMEVVPNKDFLNDTKTLAARITNIILAEIFDFQIHPDLIANLPFKSHSKLSANVLIQRVQYDISKSRFQRQASTMY
TTMLSHSHLEKIVTQLTSQISPLNTSAEQSDTTKSDLSNTVIKLINEIMSIISKHEICIIKYGNKKQSMISAKDIQSMVDSIYADLS

HSNIYQSITKDKKSISDIPVSKIASFIIKEIFNHHIQSFLSEDKTLLLAAVDQTYKLKAIDPKQRELSFIVNSSVFLEEVISELLCK
ILYAFSHNMLVTENPDRVKLKLTRIVTTLVNSIVLEFTTSEILVADNFDKNLCFSERYKEMVQKIVNSVYGKVLDQYKSLIQIHRVI
QSDTICFGRKIYYLLLEEIYDYQVQSLVSGELESSSYSYPQADNIIRNVLNIITKDSHALPPYITVLPHSLLEDMVYRLLGHVFPST
HTENELKEKKFPPDDEFVEAASKLTDEIIKEISEHEIRLSMAEDNAESMQLEPIENLVDSICNNILKTSEFQAEVQKDADKKGCSFL
SKLAGFIMKEIMYHHLQPFLHGEESSFSDLSDYDHVSELAKSGKEKTQPSLYSATFLEDIIIDLVHKFCSLLIITEDSKKNEMAELD
IMGLALKLANSLIREFKKSDIKVLPNAEKMFSFPPIDKETVDKISNFVYEQFIEKCTSHDIQKGDESNIAIGMIAALTQKAISAFRI
QPLFSGDWSSTFFSFLNPDNITQRVQHLPQNTFTQISRCAKENQLSLPDQSYKDTSSTPDCKNMMSTLEINRGTMNRKKSFKTKDTS
VKKGDIQNPVLSSINAIMKSGMINLTSGLATGVTNKKEVDENKVGICTQKHSENVSKVTSTTTVKSKDTQEPNLSETFNNNEIEKKR
NLIPTDKKGKDDEIYTHFSLIIDDTEYEKEVLGSDSEIGYKKKIDNARESSFKKDDKLFQLSSLKSKRNLGTTTDTLEIRIRTSSNE
GRRDSPTQTCRDEEHHSDYEHVQNVIENIFEDVLELSSSPEPAYYSKLSYDQSPPGDNVLNVIQEISRDSAQSVTTKKVSSSTNKNI
SAKEKEEEEREKEKVREEIKSEPSKPDDPQNQRESKPGIFPAKFLEDVITEMVKQLIFSSIPETQIQDRCQNVSDKQNQAKLYDTAM
KLINSLLKEFSDAQIKVFRPDKGNQFPGGKVSSVPKVPPRYKEPTTDEAPSSIKIKSADKMPPMHKMMRKPSSDKIPSIDKTLVNKV
VHSSVCNILNDYGSQDSIWKNINSNGENLARRLTSAVINEIFQRQVNLIFCDEVSVSACLPLESKDVVKKVQKLAQTASKECQTSSP
YTIILPHKFLENVISALFSKIFSTISSTKTKEPEDNLSTELNFLQMKLVSAVATEISQDKYMTIQYVETLQSDDDEIIQLVVQSVYN
NLLPQFGSQEIIQNCVTSGCKILSENIVDLVLREVASNQLQSYFCGELTPHQCVEVENIVEKILKDVFQTTDVPLPKPSHADKLSYN
IIEEIAVKFLSKLLSIFPKVHKERTKSLETDMQKITSKVLNSVQEFISKSKIKLVPPTKESPTVPVADNATIENIVNSIYTSVLKHS
GSYTSVFKDLMGKSNVLSDTIGFLMVNAISNSEFQPQVEEEVSNSELVLEAVKIMEKVIKIIDELKSKEKSSSRKGLTLDAKLLEEV
LALFLAKLIRLPSSSSKDEKNLSKTELNKIASQLSKLVTAEISRSSISLIASDPEEHCLNPENTERIYQVVDSVYSNILQQSGTNKE
FYYDIKDTNTAFPKKVASLIIDGVSSFPLDTINSTISNADLSGELDVNRIVQKAQEHAFNVIPELEQEKLDQNLSEEESPIKIVPHV
GKKPVKIDPKIISEHLAVISIKTQPLEKLKQECLKRTGHSIAELRRASISGRNYSLGSPDLEKRKTERRTSLDKTGRLDVKPLEAVA
RNSFQNIRKPDITKVELLKDVQSKNDLIVRLVAHDIDQVYLENYIKEERDSDEDEVVLTQTFAKEEGIKVFEDQVKEVKKPIQSKLS
PKSTLSTSSLKKFLSLSKCCQTTASANIESTEAISNQVIESKETHVKRAVAELDMATPKTMPETASSSWEEKPQCKKEEKNLVTEPT
HYFIHRIMSSSSYNQEDLISSTGEAEDCHSDPSAKILEESSQEQKPEHGNSVKFITIFERSKDVLGSANPSKEVISETPKPDVSKQG
SKMLTKMSSTLSKVFSQCNTNISRSSSPAHQDEH
>sp|Q5VU57|CBPC6_HUMAN
MAEGSQSAPEAGNDMGNDDAIGGNVSKYIVLPTGYCGQPKKGHLIFDACFESGNLGRVDQVSEFEYDLFIRPDTCNPRFRVWFNFTV
ENVKESQRVIFNIVNFSKTKSLYRDGMAPMVKSTSRPKWQRLPPKNVYYYRCPDHRKNYVMSFAFCFDREEDIYQFAYCYPYTYTRF
QHYLDSLQKRNMDYFFREQLGQSVQQRKLDLLTITSPDNLREGAEQKVVFITGRVHPGETPSSFVCQGIIDFLVSQHPIACVLREYL
VFKIAPMLNPDGVYLGNYRCSLMGFDLNRHWLDPSPWVHPTLHGVKQLIVQMYNDPKTSLEFYIDIHAHSTMMNGFMYGNIFEDEER
FQRQAIFPKLLCQNAEDFSYSSTSFNRDAVKAGTGRRFLGGLLDHTSYCYTLEVSFYSYIISGTTAAVPYTEEAYMKLGRNVARTFL
DYYRLNPVVEKVAIPMPRLRNKEIEVQRRKEKSPPYKHPLLRGPASNYPNSKGDKKSSVNHKDPSTPF
>sp|Q12800|TFCP2_HUMAN
MAWALKLPLADEVIESGLVQDFDASLSGIGQELGAGAYSMSDVLALPIFKQEESSLPPDNENKILPFQYVLCAATSPAVKLHDETLT
YLNQGQSYEIRMLDNRKLGELPEINGKLVKSIFRVVFHDRRLQYTEHQQLEGWRWNRPGDRILDIDIPMSVGIIDPRANPTQLNTVE
FLWDPAKRTSVFIQVHCISTEFTMRKHGGEKGVPFRVQIDTFKENENGEYTEHLHSASCQIKVFKPKGADRKQKTDREKMEKRTPHE
KEKYQPSYETTILTECSPWPEITYVNNSPSPGFNSSHSSFSLGEGNGSPNHQPEPPPPVTDNLLPTTTPQEAQQWLHRNRFSTFTRL
FTNFSGADLLKLTRDDVIQICGPADGIRLFNALKGRMVRPRLTIYVCQESLQLREQQQQQQQQQKHEDGDSNGTFFVYHAIYLEEL
TAVELTEKIAQLFSISPCQISQIYKQGPTGIHVLISDEMIQNFQEEACFILDTMKAETNDSYHIILK
>sp|Q07812|BAX_HUMAN
MDGSGEQPRGGGPTSSEQIMKTGALLLQGFIQDRAGRMGGEAPELALDPVPQDASTKKLSECLKRIGDELDSNMELQRMIAAVDTDS
PREVFFRVAADMFSDGNFNWGRVVALFYFASKLVLKALCTKVPELIRTIMGWTLDFLRERLLGWIQDQGGWDGLLSYFGTPTWQTVT
IFVAGVLTASLTIWKKMG
>sp|O95868|LY66D_HUMAN

MKPQFVGILLSSLLGAALGNRMRCYNCGGSPSSSCKEAVTTCGEGRPQPGLEQIKLPGNPPVTLIHQHPACVAAHHCNQVETESVGD
VTYPAHRDCYLGDLCNSAVASHVAPAGILAAAATALTCLLPGLWSG
>sp|Q8N9R0|CP081_HUMAN
MQYLHCCLQIAPNQEGMVQAGGQGHGLARVVLRAVLSPPCWAPHSPCGSPAATEAGRLMRRLPSVGGRMTAPKTPRFLTRRPPASSP
EDPPLPHPKTPRFLTQRPPASLPRRPRFLTLGPVSSHSSGDLRLWTAHQLPQQGGCPG
>sp|P56749|CLD12_HUMAN
MGCRDVHAATVLSFLCGIASVAGLFAGTLLPNWRKLRLITFNRNEKNLTVYTGLWVKCARYDGSSDCLMYDTTWYSSVDQLDLRVLQ
FALPLSMLIAMGALLLCLIGMCNTAFRSSVPNIKLAKCLVNSAGCHLVAGLLFFLAGTVSLSPSIWVIFYNIHLNKKFEPVFSFDYA
VYVTIASAGGLFMTSLILFIWYCTCKSLPSPFWQPLYSHPPSMHTYSQPYSARSRLSAIEIDIPVVSHTT
>sp|Q9H6X4|TM134_HUMAN
MSAARPQFSIDDAFELSLEDGGPGPESSGVARFGPLHFERRARFEVADEDKQSRLRYQNLENDEDGAQASPEPDGGVGTRDSSRTSI
RSSQWSFSTISSSTQRSYNTCCSWTQHPLIQKNRRVVLASFLLLLLGLVLILVGVGLEATPSPGVSSAIFFVPGFLLLVPGVYHVIF
IYCAVKGHRGFQFFYLPYFEK
>sp|Q969X2|SIA7F_HUMAN
MACSRPPSQCEPTSLPPGPPAGRRHLPLSRRRREMSSNKEQRSAVFVILFALITILILYSSNSANEVFHYGSLRGRSRRPVNLKKWS
ITDGYVPILGNKTLPSRCHQCVIVSSSSHLLGTKLGPEIERAECTIRMNDAPTTGYSADVGNKTTYRVVAHSSVFRVLRRPQEFVNR
TPETVFIFWGPPSKMQKPQGSLVRVIQRAGLVFPNMEAYAVSPGRMRQFDDLFRGETGKDREKSHSWLSTGWFTMVIAVELCDHVHV
YGMVPPNYCSQRPRLQRMPYHYYEPKGPDECVTYIQNEHSRKGNHHRFITEKRVFSSWAQLYGITFSHPSWT
>sp|P26232|CTNA2_HUMAN
MTSATSPIILKWDPKSLEIRTLTVERLLEPLVTQVTTLVNTSNKGPSGKKKGRSKKAHVLAASVEQATQNFLEKGEQIAKESQDLKE
ELVAAVEDVRKQGETMRIASSEFADDPCSSVKRGTMVRAARALLSAVTRLLILADMADVMRLLSHLKIVEEALEAVKNATNEQDLAN
RFKEFGKEMVKLNYVAARRQQELKDPHCRDEMAAARGALKKNATMLYTASQAFLRHPDVAATRANRDYVFKQVQEAIAGISNAAQAT
SPTDEAKGHTGIGELAAALNEFDNKIILDPMTFSEARFRPSLEERLESIISGAALMADSSCTRDDRRERIVAECNAVRQALQDLLSE
YMNNTGRKEKGDPLNIAIDKMTKKTRDLRRQLRKAVMDHISDSFLETNVPLLVLIEAAKSGNEKEVKEYAQVFREHANKLVEVANLA
CSISNNEEGVKLVRMAATQIDSLCPQVINAALTLAARPQSKVAQDNMDVFKDQWEKQVRVLTEAVDDITSVDDFLSVSENHILEDVN
KCVIALQEGDVDTLDRTAGAIRGRAARVIHIINAEMENYEAGVYTEKVLEATKLLSETVMPRFAEQVEVAIEALSANVPQPFEENEF
IDASRLVYDGVRDIRKAVLMIRTPEELEDDSDFEQEDYDVRSRTSVQTEDDQLIAGQSARAIMAQLPQEEKAKIAEQVEIFHQEKSK
LDAEVAKWDDSGNDIIVLAKQMCMIMMEMTDFTRGKGPLKNTSDVINAAKKIAEAGSRMDKLARAVADQCPDSACKQDLLAYLQRIA
LYCHQLNICSKVKAEVQNLGGELIVSGTGVQSTFTTFYEVDCDVIDGGRASQLSTHLPTCAEGAPIGSGSSDSSMLDSATSLIQAAK
NLMNAVVLTVKASYVASTKYQKVYGTAAVNSPVVSWKMKAPEKKPLVKREKPEEFQTRVRRGSQKKHISPVQALSEFKAMDSF
>sp|O60225|SSX5_HUMAN
MNGDDAFVRRPRVGSQIPEKMQKAFDDIAKYFSEKEWEKMKASEKIIYVYMKRKYEAMTKLGFKATLPPFMRNKRVADFQGNDFDND
PNRGNQVEHPQMTFGRLQGIFPKITPEKPAEEGNDSKGVPEASGPQNNGKQLRPSGKLNTSEKVNKTSGPKRGKHAWTHRVRERKQL
VIYEEISDPQEDDE
>sp|P17752|TPH1_HUMAN
MIEDNKENKDHSLERGRASLIFSLKNEVGGLIKALKIFQEKHVNLLHIESRKSKRRNSEFEIFVDCDINREQLNDIFHLLKSHTNVL
SVNLPDNFTLKEDGMETVPWFPKKISDLDHCANRVLMYGSELDADHPGFKDNVYRKRRKYFADLAMNYKHGDPIPKVEFTEEEIKTW
GTVFQELNKLYPTHACREYLKNLPLLSKYCGYREDNIPQLEDVSNFLKERTGFSIRPVAGYLSPRDFLSGLAFRVFHCTQYVRHSSD
PFYTPEPDTCHELLGHVPLLAEPSFAQFSQEIGLASLGASEEAVQKLATCYFFTVEFGLCKQDGQLRVFGAGLLSSISELKHALSGH
AKVKPFDPKITCKQECLITTFQDVYFVSESFEDAKEKMREFTKTIKRPFGVKYNPYTRSIQILKDTKSITSAMNELQHDLDVVSDAL
AKVSRKPSI
>sp|Q92959|SO2A1_HUMAN
MGLLPKLGASQGSDTSTSRAGRCARSVFGNIKVFVLCQGLLQLCQLLYSAYFKSSLTTIEKRFGLSSSSSGLISSLNEISNAILIIF
VSYFGSRVHRPRLIGIGGLFLAAGAFILTLPHFLSEPYQYTLASTGNNSRLQAELCQKHWQDLPPSKCHSTTQNPQKETSSMWGLMV

VAQLLAGIGTVPIQPFGISYVDDFSEPSNSPLYISILFAISVFGPAFGYLLGSVMLQIFVDYGRVNTAAVNLVPGDPRWIGAWWLGL
LISSALLVLTSFPFFFFPRAMPIGAKRAPATADEARKLEEAKSRGSLVDFIKRFPCIFLRLLMNSLFVLVVLAQCTFSSVIAGLSTF
LNKFLEKQYGTSAAYANFLIGAVNLPAAALGMLFGGILMKRFVFSLQAIPRIATTIITISMILCVPLFFMGCSTPTVAEVYPPSTSS
SIHPQSPACRRDCSCPDSIFHPVCGDNGIEYLSPCHAGCSNINMSSATSKQLIYLNCSCVTGGSASAKTGSCPVPCAHFLLPAIFLI
SFVSLIACISHNPLYMMVLRVVNQEEKSFAIGVQFLLMRLLAWLPSPALYGLTIDHSCIRWNSLCLGRRGACAYYDNDALRDRYLGL
QMGYKALGMLLLCFISWRVKKNKEYNVQKAAGLI
>sp|Q58FG0|HS905_HUMAN
MGFHHVGQAGLELLTSGHPALERRPEYLEERRIKEIVKKHSQFIGYPITLFVEKKRNKQVSDAEAEKKEDKRKKKKESNDKPEIEDV
GSDEEEEKKDADKKKKKSKEKYIDQELNKTKPIWTRNPDAITNEEYGEFHQSLTNNWEDHLAVKHFSVEGQLEELKDSRRVMKANQK
HIYYITGETKDQVANSAFVECLQKHGLEVIYMIELIDKYCVQQLKELESKTVVSVAKEGLELPEDEEEKKKQEEKKTKFENLCKIMK
DMLEKKVVKKVVVSNCMEDPQRHTNKIYRMIKLGLGVDEYDPTANDINAAITKEMPPLRGGDDTSRMEEVGGSG
>sp|Q8WXF5|CRGN_HUMAN
MAQRSGKITLYEGKHFTGQKLEVFGDCDNFQDRGFMNRVNSIHVESGAWVCFNHPDFRGQQFILEHGDYPDFFRWNSHSDHMGSCRP
VGMHGEHFRLEIFEGCNFTGQCLEFLEDSPFLQSRGWVKNCVNTIKVYGDGAAWSPRSFGAEDFQLSSSLQSDQGPEEATTKPATTQ
PPFLTANL
>sp|Q6PGP7|TTC37_HUMAN
MSSKEVKTALKSARDAIRNKEYKEALKHCKTVLKQEKNNYNAWVFIGVAAAELEQPDQAQSAYKKAAELEPDQLLAWQGLANLYEKY
NHINAKDDLPGVYQKLLDLYESVDKQKWCDVCKKLVDLYYQEKKHLEVARTWHKLIKTRQEQGAENEELHQLWRKLTQFLAESTEDQ
NNETQQLLFTAFENALGLSDKIPSEDHQVLYRHFIQSLSKFPHESARLKKACEGMINIYPTVQYPLEVLCLHLIESGNLTDEGQQYC
CRLVEMDSKSGPGLIGLGIKALQDKKYEDAVRNLTEGLKESPVCTSGWYHLAEAQVKMHRPKEAVLSCSQALKIVDNLGASGNSLYQ
RNLCLHLKAEALIKLSDYDSSEEAIRTLDQISDADNIPGLLVLKSLAYRNKGSFDEAAKIMEDLLSSYPDLAEVHALEALIHFTKKD
YLQAEKCFQRALEKDTEVAEYHYQLGLTYWFMGEETRKDKTKALTHFLKAARLDTYMGKVFCYLGHYYRDVVGDKNRARGCYRKAFE
LDDTDAESGAAAVDLSVELEDMEMALAILTTVTQKASAGTAKWAWLRRGLYYLKAGQHSQAVADLQAALRADPKDFNCWESLGEAYL
SRGGYTTALKSFTKASELNPESIYSVFKVAAIQQILGKYKEAVAQYQMIIKKKEDYVPALKGLGECHLMMAKAALVDYLDGKAVDYI
EKALEYFTCALQHRADVSCLWKLAGDACTCLYAVAPSKVNVHVLGVLLGQKEGKQVLKKNELLHLGGRCYGRALKLMSTSNTWCDLG
INYYRQAQHLAETGSNMNDLKELLEKSLHCLKKAVRLDSNNHLYWNALGVVACYSGIGNYALAQHCFIKSIQSEQINAVAWTNLGVL
YLTNENIEQAHEAFKMAQSLDPSYLMCWIGQALIAEAVGSYDTMDLFRHTTELNMHTEGALGYAYWVCTTLQDKSNRETELYQYNIL
QMNAIPAAQVILNKYVERIQNYAPAFTMLGYLNEHLQLKKEAANAYQRAILLLQTAEDQDTYNVAIRNYGRLLCSTGEYDKAIQAFK
STPLEVLEDIIGFALALFMKGLYKESSKAYERALSIVESEQDKAHILTALAITEYKQGKTDVAKTLLFKCSILKEPTTESLQALCAL
GLAMQDATLSKAALNELLKHIKHKDSNYQRCLLTSAIYALQGRSVAVQKQISKAVHSNPGDPALWSLLSRVVAQYAQRNAKGGVVAG
NVAHILDSNHGKKALLYTAVNQLAMGSSSAEDEKNTALKTIQKAALLSPGDPAIWAGLMAACHADDKLALVNNTQPKRIDLYLALLS
AVSASIKDEKFFENYNQSLEKWSLSQAVTGLIDTGRISEAETLCTKNLKSNPDQPAVILLLRQVQCKPLLESQKPLPDAVLEELQKT
VMSNSTSVPAWQWLAHVYQSQGMMRAAEMCYRKSLQLASQRGSWSGKLSSLLRLALLALKVCMANISNDHWPSLVQEATTEALKLCF
CPLAVLLQALLQFKRKMGARETRRLLERVVYQPGYPKSIASTARWYLLRHLYAKDDYELIDVLVNNAKTHGDTRALELNQRLSSQ
>sp|Q14774|HLX_HUMAN
MFAAGLAPFYASNFSLWSAAYCSSAGPGGCSFPLDPAAVKKPSFCIADILHAGVGDLGAAPEGLAGASAAALTAHLGSVHPHASFQA
AARSPLRPTPVVAPSEVPAGFPQRLSPLSAAYHHHHPQQQQQQQQPQQQQPPPPPRAGALQPPASGTRVVPNPHHSGSAPAPSSKDL
KFGIDRILSAEFDPKVKEGNTLRDLTSLLTGGRPAGVHLSGLQPSAGQFFASLDPINEASAILSPLNSNPRNSVQHQFQDTFPGPYA
VLTKDTMPQTYKRKRSWSRAVFSNLQRKGLEKRFEIQKYVTKPDRKQLAAMLGLTDAQVKVWFQNRRMKWRHSKEAQAQKDKDKEAG
EKPSGGAPAADGEQDERSPSRSEGEAESESSDSESLDMAPSDTERTEGSERSLHQTTVIKAPVTGALITASSAGSGGSSGGGGNSFS
FSSASSLSSSSTSAGCASSLGGGGASELLPATQPTASSAPKSPEPAQGALGCL
>sp|P14866|HNRPL_HUMAN
MSRRLLPRAEKRRRRLEQRQQPDEQRRRSGAMVKMAAAGGGGGGGRYYGGGSEGGRAPKRLKTDNAGDQHGGGGGGGGGAGAAGGGG
GGENYDDPHKTPASPVVHIRGLIDGVVEADLVEALQEFGPISYVVVMPKKRQALVEFEDVLGACNAVNYAADNQIYIAGHPAFVNYS

TSQKISRPGDSDDSRSVNSVLLFTILNPIYSITTDVLYTICNPCGPVQRIVIFRKNGVQAMVEFDSVQSAQRAKASLNGADIYSGCC
TLKIEYAKPTRLNVFKNDQDTWDYTNPNLSGQGDPGSNPNKRQRQPPLLGDHPAEYGGPHGGYHSHYHDEGYGPPPPHYEGRRMGPP
VGGHRRGPSRYGPQYGHPPPPPPPPPEYGPHADSPVLMVYGLDQSKMNCDRVFNVFCLYGNVEKVKFMKSKPGAAMVEMADGYAVDRA
ITHLNNNFMFGQKLNVCVSKQPAIMPGQSYGLEDGSCSYKDFSESRNNRFSTPEQAAKNRIQHPSNVLHFFNAPLEVTEENFFEICD
ELGVKRPSSVKVFSGKSERSSSGLLEWESKSDALETLGFLNHYQMKNPNGPYPYTLKLCFSTAQHAS
>sp|Q5T5P2|SKT_HUMAN
MEENESQKCEPCLPYSADRRQMQEQGKGNLHVTSPEDAECRRTKERLSNGNSRGSVSKSSRNIPRRHTLGGPRSSKEILGMQTSEMD
RKREAFLEHLKQKYPHHASAIMGHQERLRDQTRSPKLSHSPQPPSLGDPVEHLSETSADSLEAMSEGDAPTPFSRGSRTRASLPVVR
STNQTKERSLGVLYLQYGDETKQLRMPNEITSADTIRALFVSAFPQQLTMKMLESPSVAIYIKDESRNVYYELNDVRNIQDRSLLKV
YNKDPAHAFNHTPKTMNGDMRMQRELVYARGDGPGAPRPGSTAHPPHAIPNSPPSTPVPHSMPPSPSRIPYGGTRSMVVPGNATIPR
DRISSLPVSRPISPSPSAILERRDVKPDEDMSGKNIAMYRNEGFYADPYLYHEGRMSIASSHGGHPLDVPDHIIAYHRTAIRSASAY
CNPSMQAEMHMEQSLYRQKSRKYPDSHLPTLGSKTPPASPHRVSDLRMIDMHAHYNAHGPPHTMQPDRASPSRQAFKKEPGTLVYIE
KPRSAAGLSSLVDLGPPLMEKQVFAYSTATIPKDRETRERMQAMEKQIASLTGLVQSALFKGPITSYSKDASSEKMMKTTANRNHTD
SAGTPHVSGGKMLSALESTVPPSQPPPVGTSAIHMSLLEMRRSVAELRLQLQQMRQLQLQNQELLRAMMKKAELEISGKVMETMKRL
EDPVQRQRVLVEQERQKYLHEEEKIVKKLCELEDFVEDLKKDSTAASRLVTLKDVEDGAFLLRQVGEAVATLKGEFPTLQNKMRAIL
RIEVEAVRFLKEEPHKLDSLLKRVRSMTDVLTMLRRHVTDGLLKGTDAAQAAQYMAMEKATAAEVLKSQEEAAHTSGQPFHSTGAPG
DAKSEVVPLSGMMVRHAQSSPVVIQPSQHSVALLNPAQNLPHVASSPAVPQEATSTLQMSQAPQSPQIPMNGSAMQSLFIEEIHSVS
AKNRAVSIEKAEKKWEEKRQNLDHYNGKEFEKLLEEAQANIMKSIPNLEMPPATGPLPRGDAPVDKVELSEDSPNSEQDLEKLGGKS
PPPPPPPPRRSYLPGSGLTTTRSGDVVYTGRKENITAKASSEDAGPSPQTRATKYPAEEPASAWTPSPPPVTTSSSKDEEEEEEEGD
KIMAELQAFQKCSFMDVNSNSHAEPSRADSHVKDTRSGATVPPKEKKNLEFFHEDVRKSDVEYENGPQMEFQKVTTGAVRPSDPPKW
ERGMENSISDASRTSEYKTEIIMKENSISNMSLLRDSRNYSQETVPKASFGFSGISPLEDEINKGSKISGLQYSIPDTENQTLNYGK
TKEMEKQNTDKCHVSSHTRLTESSVHDFKTEDQEVITTDFGQVVLRPKEARHANVNPNEDGESSSSSPTEENAATDNIAFMITETTV
QVLSSGEVHDIVSQKGEDIQTVNIDARKEMTPRQEGTDNEDPVVCLDKKPVIIIFDEPMDIRSAYKRLSTIFEECDEELERMMMEEK
IEEEEEEENGDSVVQNNNTSQMSHKKVAPGNLRTGQQVETKSQPHSLATETRNPGGQEMNRTELNKFSHVDSPNSECKGEDATDDQF
ESPKKKFKFKFPKKQLAALTQAIRTGTKTGKKTLQVVVYEEEEEDGTLKQHKEAKRFEIARSQPEDTPENTVRRQEQPSIESTSPIS
RTDEIRKNTYRTLDSLEQTIKQLENTISEMSPKALVDTSCSSNRDSVASSSHIAQEASPRPLLVPDEGPTALEPPTSIPSASRKGSS
GAPQTSRMPVPMSAKNRPGTLDKPGKQSKLQDPRQYRQANGSAKKSGGDFKPTSPSLPASKIPALSPSSGKSSSLPSSSGDSSNLPN
PPATKPSIASNPLSPQTGPPAHSASLIPSVSNGSLKFQSLTHTGKGHHLSFSPQSQNGRAPPPLSFSSSPPSPASSVSLNQGAKGTR
TIHTPSLTSYKAQNGSSSKATPSTAKETS
>sp|Q9C0J9|BHE41_HUMAN
MDEGIPHLQERQLLEHRDFIGLDYSSLYMCKPKRSMKRDDTKDTYKLPHRLIEKKRRDRINECIAQLKDLLPEHLKLTTLGHLEKAV
VLELTLKHLKALTALTEQQHQKIIALQNGERSLKSPIQSDLDAFHSGFQTCAKEVLQYLSRFESWTPREPRCVQLINHLHAVATQFL
PTPQLLTQQVPLSKGTGAPSAAGSAAAPCLERAGQKLEPLAYCVPVIQRTQPSAELAAENDTDTDSGYGGEAEARPDREKGKGAGAS
RVTIKQEPPGEDSPAPKRMKLDSRGGGSGGGPGGGAAAAAAALLGPDPAAAAALLRPDAALLSSLVAFGGGGGAPFPQPAAAAAPFC
LPFCFLSPSAAAAYVQPFLDKSGLEKYLYPAAAAAPFPLLYPGIPAPAAAAAAAAAAAAAAAFPCLSSVLSPPPEKAGAAAATLLP
HEVAPLGAPHPQHPHGRTHLPFAGPREPGNPESSAQEDPSQPGKEAP
>sp|Q7Z3J3|RGPD4_HUMAN
MSCSKAYGERYVASVQGSAPSPRKKSTRGFYFAKLYYEAKEYDLAKKYICTYINVREMDPRAHRFLGLLYELEENTEKAVECYRRSV
ELNPTQKDLVLKIAELLCKNDVTDGRAKYWVERAAKLFPGSPAIYKLKEQLLDCEGEDGWNKLFDLIQSELYVRPDDVHVNIRLVEL
YRSTKRLKDAVARCHEAERNIALRSSLEWNSCVVQTLKEYLESLQCLESDKSDWRATNTDLLLAYANLMLLTLSTRDVQESRELLES
FDSALQSAKSSLGGNDELSATFLEMKGHFYMHAGSLLLKMGQHGNNVQWRALSELAALCYLIAFQVPRPKIKLIKGEAGQNLLEMMA
CDRLSQSGHMLLNLSRGKQDFLKVVVETFANKSGQSALYDALFSSQSPKDTSFLGSDDIGNIDVQEPELEDLARYDVGAIRAHNGSL
QHLTWLGLQWNSLPALPGIRKWLKQLFHHLPQETSRLETNAPESICILDLEVFLLGVVYTSHLQLKEKCNSHHSSYQPLCLPLPVCK
QLCTERQKSWWDAVCTLIHRKAVPGNSAKLRLLVQHEINTLRAQEKHGLQPALLVHWAKCLQKMGSGLNSFYDQREYIGRSVHYWKK

VLPLLKIIKKKNSIPEPIDPLFKHFHSVDIQASEIVEYEEDAHVTFAILDAVNGNIEDAMTAFESIKSVVSYWNLALIFHRKAEDIA
NDALSPEEQEECKNYLRKTRGYLIKILDDSDSNLSVVKKLPVPLESVKEMLKSVMQELENYSEGDPLYKNGSLRNADSEIKHSTPSP
TKYSLSPSKSYKYSPKTPPRWAEDQNSLLKMIRQEVKAIKEEMQELKLNSSKSASHHRWPTENYGPDSVPDGYQGSQTFHGAPLTVA
TTGPSVYYSQSPAYNSQYLLRPAANVTPTKGSSNTEFKSTKEGFSIPVSADGFKFGISEPGNQEKESEKPLENDTGFQAQDISGQKN
GRGVIFGQTSSTFTFADVAKSTSGEGFQFGKKDPNFKGFSGAGEKLFSSQCGKMANKANTSGDFEKDDDAYKTEDSDDIHFEPVVQM
PEKVELVIGEEGEKVLYSQGVKLFRFDAEVRQWKERGLGNLKILKNEVNGKPRMLMRREQVLKVCANHWITTTMNLKPLSGSDRAWM
WSASDFSDGDAKLERLAAKFKTPELAEEFKQKFEECQQLLLDIPLQTPHKLVDTGRAAKLIQRAEEMKSGLKDFKTFLTNDQTKVTE
EENKGSGTGAAGASDTTIKPNPENTGPTLEWDNCDLREDALDDSVSSSSVHASPLASSPVRKNLFHFGESTTGSNFSFKSALSPSKS
PAKLNQSGTSVGTDEESDVTQEEERDGQYFEPVVPLPDLVEVSSGEENEKVVFSHRAELYRYDKDVGQWKERGIGDIKILQNYDNKQ
VRIVMRRDQVLKLCANHTITPDMSLQNMKGTERVWVWTACDFADGERKVEHLAVRFKLQDVADSFKKIFDEAKTAQEKDSLITPHVS
RSSTPRESPCGKIAVAVLEETTRERTDVIQGDDVADAASEVEVSSTSETTTKAVVSPPKFVFGSESVKRIFSSEKSKPFAFGNSSAT
GSLFGFSFNASLKSNNSETSSVAQSGSESKVEPKKCELSKNSDIEQSSDSKVKNLSASFPMEESSINYTFKTPEKEPPLWHAEFTKE
ELVQKLSSTTKSADHLNGLLREAEATSAVLMEQIKLLKSEIRRLERNQEQEESAANVEHLKNVLLQFIFLKPGSERERLLPVINTML
QLSPEEKGKLAAVAQGEE
>sp|Q6ZRK6|CCD73_HUMAN
MESNFNTESSSTFTLQSSSETLFSIQLLDFKTSLLEALEELRMRREAEIHYEEQIGKIIVETQELKWQKETLQNQKETLAEQHKEAM
AVFKKQLQMKMCALEEEKGKYQLATEIKEKEIEGLKETLKALQVSKYSLQKKVSEMEQKVQLHLLAKEDYHKQLSEIEKYYATITGQ
FGLVKENHEKLEQNVREAIQSNKRLSALNKKQEAEICSLKKELKKAASDLIKSKVTCQYKMGEENINLTIKEQKFQELQERLNMELE
LNEKINEEITHIQEEKQDIIISFQHMQQLLRQQIQANTEMEAELKVLKENNQTLERDNELQREKVKENEEKFLNLQNEHEKALGTWK
RHAEELNGEINKIKNELSSLKETHIKLQEHYNKLCNQKTFEEDKKFQNVPEVNNENSEMSTEKSENTIIQKYNTEQEIREENMENFC
SDTEYREKEEKKEGSFIEEIIIDDLQLFEKSFKNEIDTVVSQDENQSEISLSKTLSLDKEVISQGGQTSNVTDNRKSVTTEIKDKICL
EKDNGCTEFKSPNNHFVVLDTAIETEKIHLERTRGLDVHHTDVNLEVENNKTSFNSILNETAHNTYHNNNKDVSENEPFKQFRLLPG
TREHALEKEITNSDQTKADLDSSLDIKKNPVPCQKYSLRNSSNVMLDDKQCKIKQIQLLTKKSECSILLSKQTSDFLQVCNDTLEKS
ELTVPCDIVIDHHVSYAAFSANSKLLLKNSDKNVHSMSMLVKPNSSPGGKTMCKNMSDMQNSQFNNCLGYLENTNVNISHLHLNNEN
SHASQAKDVKTAVHMKTCTETEFSNKKNQIDENQVTEATKNDLFLFVSINERQHTLLNNTEKTESLNDIVSGKMFSEGQLEESHSFH
IEPSGDLVNRSGRSTFDLSTSDKKTEKTPVYMNFSDPGPWSKVNHIESQTASSSTPCISLLLKERPLDPSENKKIISMALCKNIGVD
DVGKDIGPDTTSINRVADTLNNWSIHPDPKGEPSEEKNAMAKTFYDSSFPTEHVKTKPLISTPLQSHLQAIKTTKNTSGDDDWQSLI
TNQLNKSENLLSLENDNQPKKRKAEETLEKNNRLK
>sp|Q5SWA1|PR15B_HUMAN
MEPGTGGSRKRLGPRAGFRFWPPFFPRRSQAGSSKFPTPLGPENSGNPTLLSSAQPETRVSYWTKLLSQLLAPLPGLLQKVLIWSQL
FGGMFPTRWLDFAGVYSALRALKGREKPAAPTAQKSLSSLQLDSSDPSVTSPLDWLEEGIHWQYSPPDLKLELKAKGSALDPAAQAF
LLEQQLWGVELLPSSLQSRLYSNRELGSSPSGPLNIQRIDNFSVVSYLLNPSYLDCFPRLEVSYQNSDGNSEVVGFQTLTPESSCLR
EDHCHPQPLSAELIPASWQGCPPLSTEGLPEIHHLRMKRLEFLQQANKGQDLPTPDQDNGYHSLEEEHSLLRMDPKHCRDNPTQFVP
AAGDIPGNTQESTEEKIELLTTEVPLALEEESPSEGCPSSEIPMEKEPGEGRISVVDYSYLEGDLPISARPACSNKLIDYILGGASS
DLETSSDPEGEDWDEEAEDDGFDSDSSLSDSDLEQDPEGLHLWNSFCSVDPYNPQNFTATIQTAARIVPEEPSDSEKDLSGKSDLEN
SSQSGSLPETPEHSSGEEDDWESSADEAESLKLWNSFCNSDDPYNPLNFKAPFQTSGENEKGCRDSKTPSESIVAISECHTLLSCKV
QLLGSQESECPDSVQRDVLSGGRHTHVKRKKVTFLEEVTEYYISGDEDRKGPWEEFARDGCRFQKRIQETEDAIGYCLTFEHRERMF
NRLQGTCFKGLNVLKQC
>sp|Q86UN2|R4RL1_HUMAN
MLRKGCCVELLLLLVAAELPLGGGCPRDCVCYPAPMTVSCQAHNFAAIPEGIPVDSERVFLQNNRIGLLQPGHFSPAMVTLWIYSNN
ITYIHPSTFEGFVHLEELDLGDNRQLRTLAPETFQGLVKLHALYLYKCGLSALPAGVFGGLHSLQYLYLQDNHIEYLQDDIFVDLVN
LSHLFLHGNKLWSLGPGTFRGLVNLDRLLLHENQLQWVHHKAFHDLRRLTTLFLFNNSLSELQGECLAPLGALEFLRLNGNPWDCGC
RARSLWEWLQRFRGSSSAVPCVSPGLRHGQDLKLLRAEDFRNCTGPASPHQIKSHTLTTTDRAARKEHHSPHGPTRSKGHPHGPRPG

HRKPGKNCTNPRNRNQISKAGAGKQAPELPDYAPDYQHKFSFDIMPTARPKRKGKCARRTPIRAPSGVQQASSASSLGASLLAWTLG
LAVTLR
>sp|Q86YD3|TMM25_HUMAN
MALPPGPAALRHTLLLLPALLSSGWGELEPQIDGQTWAERALRENERHAFTCRVAGGPGTPRLAWYLDGQLQEASTSRLLSVGGEAF
SGGTSTFTVTAHRAQHELNCSLQDPRSGRSANASVILNVQFKPEIAQVGAKYQEAQGPGLLVVLFALVRANPPANVTWIDQDGPVTV
NTSDFLVLDAQNYPWLTNHTVQLQLRSLAHNLSVVATNDVGVTSASLPAPGLLATRVEVPLLGIVVAAGLALGTLVGFSTLVACLVC
RKEKKTKGPSRHPSLISSDSNNLKLNNVRLPRENMSLPSNLQLNDLTPDSRAVKPADRQMAQNNSRPELLDPEPGGLLTSQGFIRLP
VLGYIYRVSSVSSDEIWL
>sp|K9M1U5|IFNL4_HUMAN
MRPSVWAAVAAGLWVLCTVIAAAPRRCLLSHYRSLEPRTLAAAKALRDRYEEEALSWGQRNCSFRPRRDPPRPSSCARLRHVARGIA
DAQAVLSGLHRSELLPGAGPILELLAAAGRDVAACLELARPGSSRKVPGAQKRRHKPRRADSPRCRKASVVFNLLRLLTWELRLAAH
SGPCL
>sp|Q13418|ILK_HUMAN
MDDIFTQCREGNAVAVRLWLDNTENDLNQGDDHGFSPLHWACREGRSAVVEMLIMRGARINVMNRGDDTPLHLAASHGHRDIVQKLL
QYKADINAVNEHGNVPLHYACFWGQDQVAEDLVANGALVSICNKYGEMPVDKAKAPLRELLRERAEKMGQNLNRIPYKDTFWKGTTR
TRPRNGTLNKHSGIDFKQLNFLTKLNENHSGELWKGRWQGNDIVVKVLKVRDWSTRKSRDFNEECPRLRIFSHPNVLPVLGACQSPP
APHPTLITHWMPYGSLYNVLHEGTNFVVDQSQAVKFALDMARGMAFLHTLEPLIPRHALNSRSVMIDEDMTARISMADVKFSFQCPG
RMYAPAWVAPEALQKKPEDTNRRSADMWSFAVLLWELVTREVPFADLSNMEIGMKVALEGLRPTIPPGISPHVCKLMKICMNEDPAK
RPKFDMIVPILEKMQDK
>sp|Q8NB91|FANCB_HUMAN
MTSKQAMSSNEQERLLCYNGEVLVFQLSKGNFADKEPTKTPILHVRRMVFDRGTKVFVQKSTGFFTIKEENSHLKIMCCNCVSDFRT
GINLPYIVIEKNKKNNVFEYFLLILHSTNKFEMRLSFKLGYEMKDGLRVLNGPLILWRHVKAFFFISSQTGKVVSVSGNFSSIQWAG
EIENLGMVLLGLKECCLSEEECTQEPSKSDYAIWNTKFCVYSLESQEVLSDIYIIPPAYSSVVTYVHICATEIIKNQLRISLIALTR
KNQLISFQNGTPKNVCQLPFGDPCAVQLMDSGGGNLFFVVSFISNNACAVWKESFQVAAKWEKLSLVLIDDFIGSGTEQVLLLFKDS
LNSDCLTSFKITDLGKINYSSEPSDCNEDDLFEDKQENRYLVVPPLETGLKVCFSSFRELRQHLLLKEKIISKSYKALINLVQGKDD
NTSSAEEKECLVPLCGEEENSVHILDEKLSDNFQDSEQLVEKIWYRVIDDSLVVGVKTTSSLKLSLNDVTLSLLMDQAHDSRFRLLK
CQNRVIKLSTNPFPAPYLMPCEIGLEAKRVTLTPDSKKEESFVCEHPSKKECVQIITAVTSLSPLLTFSKFCCTVLLQIMERESGNC
PKDRYVVCGRVFLSLEDLSTGKYLLTFPKKKPIEHMEDLFALLAAFHKSCFQITSPGYALNSMKVWLLEHMKCEIIKEFPEVYFCER
PGSFYGTLFTWKQRTPFEGILIIYSRNQTVMFQCLHNLIRILPINCFLKNLKSGSENFLIDNMAFTLEKELVTLSSLSSAIAKHESN
FMQRCEVSKGKSSVVAAALSDRRENIHPYRKELQREKKKMLQTNLKVSGALYREITLKVAEVQLKSDFAAQKLSNL
>sp|Q00341|VIGLN_HUMAN
MSSVAVLTQESFAEHRSGLVPQQIKVATLNSEEESDPPTYKDAFPPLPEKAACLESAQEPSGAWGNKIRPIKASVITQVFHVPLEER
KYKDMNQFGEGEQAKICLEIMQRTGAHLELSLAKDQGLSIMVSGKLDAVMKARKDIVARLQTQASATVAIPKEHHRFVIGKNGEKLQ
DLELKTATKIQIPRPDDPSNQIKITGTKEGIEKARHEVLLISAEQDKRAVERLEVEKAFHPFIAGPYNRLVGEIMQETGTRINIPPP
SVNRTEIVFTGEKEQLAQAVARIKKIYEEKKKKTTTIAVEVKKSQHKYVIGPKGNSLQEILERTGVSVEIPPSDSISETVILRGEPE
KLGQALTEVYAKANSFTVSSVAAPSWLHRFIIGKKGQNLAKITQQMPKVHIEFTEGEDKITLEGPTEDVNVAQEQIEGMVKDLINRM
DYVEINIDHKFHRHLIGKSGANINRIKDQYKVSVRIPPDSEKSNLIRIEGDPQGVQQAKRELLELASRMENERTKDLIIEQRFHRTI
IGQKGERIREIRDKFPEVIINFPDPAQKSDIVQLRGPKNEVEKCTKYMQKMVADLVENSYSISVPIFKQFHKNIIGKGGANIKKIRE
ESNTKIDLPAENSNSETIIITGKRANCEAARSRILSIQKDLANIAEVEVSIPAKLHNSLIGTKGRLIRSIMEECGGVHIHFPVEGSG
SDTVVIRGPSSDVEKAKKQLLHLAEEKQTKSFTVDIRAKPEYHKFLIGKGGGKIRKVRDSTGARVIFPAAEDKDQDLITIIGKEDAV
REAQKELEALIQNLDNVVEDSMLVDPKHHRHFVIRRGQVLREIAEEYGGVMVSFPRSGTQSDKVTLKGAKDCVEAAKKRIQEIIEDL
EAQVTLECAIPQKFHRSVMGPKGSRIQQITRDFSVQIKFPDREENAVHSTEPVVQENGDEAGEGREAKDCDPGSPRRCDIIIISGRK
EKCEAAKEALEALVPVTIEVEVPFDLHRYVIGQKGSGIRKMMDEFEVNIHVPAPELQSDIIAITGLAANLDRAKAGLLERVKELQAE
QEDRALRSFKLSVTVDPKYHPKIIGRKGAVITQIRLEHDVNIQFPDKDDGNQPQDQITITGYEKNTEAARDAILRIVGELEQMVSED

VPLDHRVHARIIGARGKAIRKIMDEFKVDIRFPQSGAPDPNCVTVTGLPENVEEAIDHILNLEEEYLADVVDSEALQVYMKPPAHEE
AKAPSRGFVVRDAPWTASSSEKAPDMSSSEEFPSFGAQVAPKTLPWGPKR
>sp|P62955|CCG7_HUMAN
MSHCSSRALTLLSSVFGACGLLLVGIAVSTDYWLYMEEGTVLPQNQTTEVKMALHAGLWRVCFFAGREKGRCVASEYFLEPEINLVT
ENTENILKTVRTATPFPMVSLFLVFTAFVISNIGHIRPQRTILAFVSGIFFILSGLSLVVGLVLYISSINDEVMNRPSSSEQYFHYR
YGWSFAFAASSFLLKEGAGVMSVYLFTKRYAEEEMYRPHPAFYRPRLSDCSDYSGQFLQPEAWRRGRSPSDISSDVSIQMTQNYPPA
IKYPDHLHISTSPC
>sp|P57076|CU059_HUMAN
MVLLHVKRGDESQFLLQAPGSTELEELTVQVARVYNGRLKVQRLCSEMEELAEHGIFLPPNMQGLTDDQIEELKLKDEWGEKCVPSG
GAVFKKDDIGRRNGQAPNEKMKQVLKKTIEEAKAIISKKQVEAGVCVTMEMVKDALDQLRGAVMIVYPMGLPPYDPIRMEFENKEDL
SGTQAGLNVIKEAEAQLWWAAKELRRTKKLSDYVGKNEKTKIIAKIQQRGQGAPAREPIISSEEQKQLMLYYHRRQEELKRLEENDD
DAYLNSPWADNTALKRHFHGVKDIKWRPR
>sp|P11168|GTR2_HUMAN
MTEDKVTGTLVFTVITAVLGSFQFGYDIGVINAPQQVIISHYRHVLGVPLDDRKAINNYVINSTDELPTISYSMNPKPTPWAEEETV
AAAQLITMLWSLSVSSFAVGGMTASFFGGWLGDTLGRIKAMLVANILSLVGALLMGFSKLGPSHILIIAGRSISGLYCGLISGLVPM
YIGEIAPTALRGALGTFHQLAIVTGILISQIIGLEFILGNYDLWHILLGLSGVRAILQSLLLFFCPESPRYLYIKLDEEVKAKQSLK
RLRGYDDVTKDINEMRKEREEASSEQKVSIIQLFTNSSYRQPILVALMLHVAQQFSGINGIFYYSTSIFQTAGISKPVYATIGVGAV
NMVFTAVSVFLVEKAGRRSLFLIGMSGMFVCAIFMSVGLVLLNKFSWMSYVSMIAIFLFVSFFEIGPGPIPWFMVAEFFSQGPRPAA
LAIAAFSNWTCNFIVALCFQYIADFCGPYVFFLFAGVLLAFTLFTFFKVPETKGKSFEEIAAEFQKKSGSAHRPKAAVEMKFLGATE
TV
>sp|Q96LU5|IMP1L_HUMAN
MLRGVLGKTFRLVGYTIQYGCIAHCAFEYVGGVVMCSGPSMEPTIQNSDIVFAENLSRHFYGIQRGDIVIAKSPSDPKSNICKRVIG
LEGDKILTTSPSDFFKSHSYVPMGHVWLEGDNLQNSTDSRCYGPIPYGLIRGRIFFKIWPLSDFGFLRASPNGHRFSDD
>sp|Q9BYR4|KRA43_HUMAN
MVSSCCGSVCSDQSCGQGLGQESCCRPSCCQTTCCRTTCCRPSCCISSCCRPSCCISSCCKPSCCRTTCCRPSCCISSCCRPSCCIS
SCCKPSCCRTTCCRPSCCISSCCRPSCCISSCCKPSCCQTTCCRPSCCISSCYRPQCCQPSCCRPACCISSCCHPSCCVSSCRCPFS
CPTTCCRTTCFHPICCGSSCC
>sp|Q6S8J7|POTEA_HUMAN
MVAEVSPKLAASPMKKPFGFRGKMGKWCCCCFPCCRGSGKNNMGAWRDHDDSAFTEPRYHVRREDLGKLHRAAWWGEVPRADLIVML
RGPGINKRDKKKRTALHLACANGNSEVVSLLLDRQCQLHVFDSKKRTALIKAVQCQEDECALMLLQHGTDPNLPDMYGNTALHYAVY
NEDKLMAKTLLLYGADIESKNKGGLTPLLLAVHGQKQRMVKFLIKKKANLNALDRFGRTALILAVRCGSASIVSLLLQQNIDVFSQD
VFGQTAEDYAVSSHHSIICQLLSDYKENQMPNNSSGNSNPEQDLKLTSEEEPQRLKGSENSQHEKVTQEPDINKDCDREVEEEMQKH
GSNNVGLSENLTDGAAAGNGDGGLVPQRKSRKHENQQFPNTEIEEYHRPEKKSNEKNKVKSQIHSVDNLDDITWPSEIASEDYDLLF
SNYETFTLLIEQLKMDFNDSASLSKIQDAVISEEHLLELKNSHYEQLTVEVEQMENMVHVLQK
>sp|Q8N0U4|F185A_HUMAN
MLAPCSGWELGCFRLCLRQVRLWAGAGRWACWACQARPYSSGGSERWPGSETEVPPPGPGRRTLKEWTLQVSPFGRLRARLPCHLAV
RPLDPLTYPDGDRVLVAVCGVEGGVRGLDGLQVKYDEDLEEMAIVSDTIHPQASVEVNAPLKFGLDIKSSGSGCVKVQSIEGDNCKI
ETEHGTSILQSVKGQKLHVQTKGGKVICLGTVYGNIDIHASDKSAVTIDKLQGSSVTVSTEDGLLKAKYLYTESSFLSSAAGDITLG
SVHGNITLQSKMGNITVDSSSGCLKASTNQGAIDVYVSQLGKVELKSHKGSIIVKVPSSLQAHLQLSGKEVDVNSEVHVQEMAEVRK
DDVVTVTGLMNQASKREKWIKADAPKGTVSFRRQSWFQSLKLQD
>sp|Q6UWY0|ARSK_HUMAN
MLLLWVSVVAALALAVLAPGAGEQRRRAAKAPNVVLVVSDSFDGRLTFHPGSQVVKLPFINFMKTRGTSFLNAYTNSPICCPSRAAM
WSGLFTHLTESWNNFKGLDPNYTTWMDVMERHGYRTQKFGKLDYTSGHHSISNRVEAWTRDVAFLLRQEGRPMVNLIRNRTKVRVME
RDWQNTDKAVNWLRKEAINYTEPFVIYLGLNLPHPYPSPSSGENFGSSTFHTSLYWLEKVSHDAIKIPKWSPLSEMHPVDYYSSYTK

NCTGRFTKKEIKNIRAFYYAMCAETDAMLGEIILALHQLDLLQKTIVIYSSDHGELAMEHRQFYKMSMYEASAHVPLLMMGPGIKAG
LQVSNVVSLVDIYPTMLDIAGIPLPQNLSGYSLLPLSSETFKNEHKVKNLHPPWILSEFHGCNVNASTYMLRTNHWKYIAYSDGASI
LPQLFDLSSDPDELTNVAVKFPEITYSLDQKLHSIINYPKVSASVHQYNKEQFIKWKQSIGQNYSNVIANLRWHQDWQKEPRKYENA
IDQWLKTHMNPRAV
>sp|Q9H6K4|OPA3_HUMAN
MVVGAFPMAKLLYLGIRQVSKPLANRIKEAARRSEFFKTYICLPPAQLYHWVEMRTKMRIMGFRGTVIKPLNEEAAAELGAELLGEA
TIFIVGGGCLVLEYWRHQAQQRHKEEEQRAAWNALRDEVGHLALALEALQAQVQAAPPQGALEELRTELQEVRAQLCNPGRSASHAV
PASKK
>sp|Q8WWN8|ARAP3_HUMAN
MAAPQDLDIAVWLATVHLEQYADTFRRHGLATAGAARGLGHEELKQLGISATGHRKRILRLLQTGTEEGSLDPKSDSAMEPSPSPAP
QAQPPKPVPKPRTVFGGLSGPATTQRPGLSPALGGPGVSRSPEPSPRPPPLPTSSSEQSSALNTVEMMPNSIYFGLDSRGRAQAAQD
KAPDSSQISAPTPALRPTTGTVHIMDPGCLYYGVQPVGTPGAPDRRESRGVCQGRAEHRLSRQDLEAREDAGYASLELPGDSTLLSP
TLETEETSDDLISPYASFSFTADRLTPLLSGWLDKLSPQGNYVFQRRFVQFNGRSLMYFGSDKDPFPKGVIPLTAIEMTRSSKDNKF
QVITGQRVFVFRTESEAQRDMWCSTLQSCLKEQRLLGHPRPPQPPRPLRTGMLELRGHKAKVFAALSPGELALYKSEQAFSLGIGIC
FIELQGCSVRETKSRSFDLLTPHRCFSFTAESGGARQSWAAALQEAVTETLSDYEVAEKIWSNRANRQCADCGSSRPDWAAVNLGVV
ICKQCAGQHRALGSGISKVQSLKLDTSVWSNEIVQLFIVLGNDRANRFWAGTLPPGEGLHPDATPGPRGEFISRKYRLGLFRKPHPQ
YPDHSQLLQALCAAVARPNLLKNMTQLLCVEAFEGEEPWFPPAPDGSCPGLLPSDPSPGVYNEVVVRATYSGFLYCSPVSNKAGPSP
PRRGRDAPPRLWCVLGAALEMFASENSPEPLSLIQPQDIVCLGVSPPPTDPGDRFPFSFELILAGGRIQHFGTDGADSLEAWTSAVG
KWFSPLSCHQLLGPGLLRLGRLWLRSPSHTAPAPGLWLSGFGLLRGDHLFLCSAPGPGPPAPEDMVHLRRLQEISVVSAADTPDKKE
HLVLVETGRTLYLQGEGRLDFTAWNAAIGGAAGGGGTGLQEQQMSRGDIPIIVDACISFVTQHGLRLEGVYRKGGARARSLRLLAEF
RRDARSVKLRPGEHFVEDVTDTLKRFFRELDDPVTSARLLPRWREAAELPQKNQRLEKYKDVIGCLPRVNRRTLATLIGHLYRVQKC
AALNQMCTRNLALLFAPSVFQTDGRGEHEVRVLQELIDGYISVFDIDSDQVAQIDLEVSLITTWKDVQLSQAGDLIMEVYIEQQLPD
NCVTLKVSPTLTAEELTNQVLEMRGTAAGMDLWVTFEIREHGELERPLHPKEKVLEQALQWCQLPEPCSASLLLKKVPLAQAGCLFT
GIRRESPRVGLLRCREEPPRLLGSRFQERFFLLRGRCLLLLKEKKSSKPEREWPLEGAKVYLGIRKKLKPPTPWGFTLILEKMHLYL
SCTDEDEMWDWTTSILKAQHDDQQPVVLRRHSSSDLARQKFGTMPLLPIRGDDSGATLLSANQTLRRLHNRRTLSMFFPMKSSQGSV
EEQEELEEPVYEEPVYEEVGAFPELIQDTSTSFSTTREWTVKPENPLTSQKSLDQPFLSKSSTLGQEERPPEPPPGPPSKSSPQARG
SLEEQLLQELSSLILRKGETTAGLGSPSQPSSPQSPSPTGLPTQTPGFPTQPPCTSSPPSSQPLT
>sp|P78362|SRPK2_HUMAN
MSVNSEKSSSSERPEPQQKAPLVPPPPPPPPPPPPPLPDPTPPEPEEEILGSDDEEQEDPADYCKGGYHPVKIGDLFNGRYHVIRKL
GWGHFSTVWLCWDMQGKRFVAMKVVKSAQHYTETALDEIKLLKCVRESDPSDPNKDMVVQLIDDFKISGMNGIHVCMVFEVLGHHLL
KWIIKSNYQGLPVRCVKSIIRQVLQGLDYLHSKCKIIHTDIKPENILMCVDDAYVRRMAAEATEWQKAGAPPPSGSAVSTAPQQKPI
GKISKNKKKKLKKKQKRQAELLEKRLQEIEELEREAERKIIEENITSAAPSNDQDGEYCPEVKLKTTGLEEAAEAETAKDNGEAEDQ
EEKEDAEKENIEKDEDDVDQELANIDPTWIESPKTNGHIENGPFSLEQQLDDEDDDEEDCPNPEEYNLDEPNAESDYTYSSSYEQFN
GELPNGRHKIPESQFPEFSTSLFSGSLEPVACGSVLSEGSPLTEQEESSPSHDRSRTVSASSTGDLPKAKTRAADLLVNPLDPRNAD
KIRVKIADLGNACWVHKHFTEDIQTRQYRSIEVLIGAGYSTPADIWSTACMAFELATGDYLFEPHSGEDYSRDEDHIAHIIELLGSI
PRHFALSGKYSREFFNRRGELRHITKLKPWSLFDVLVEKYGWPHEDAAQFTDFLIPMLEMVPEKRASAGECLRHPWLNS
>sp|Q15014|MO4L2_HUMAN
MSSRKQGSQPRGQQSAEEENFKKPTRSNMQRSKMRGASSGKKTAGPQQKNLEPALPGRWGGRSAENPPSGSVRKTRKNKQKTPGNGD
GGSTSEAPQPPRKKRARADPTVESEEAFKNRMEVKVKIPEELKPWLVEDWDLVTRQKQLFQLPAKKNVDAILEEYANCKKSQGNVDN
KEYAVNEVVAGIKEYFNVMLGTQLLYKFERPQYAEILLAHPDAPMSQVYGAPHLLRLFVRIGAMLAYTPLDEKSLALLLGYLHDFLK
YLAKNSASLFTASDYKVASAEYHRKAL
>sp|P01906|DQA2_HUMAN

MILNKALLLGALALTAVMSPCGGEDIVADHVASYGVNFYQSHGPSGQYTHEFDGDEEFYVDLETKETVWQLPMFSKFISFDPQSALR
NMAVGKHTLEFMMRQSNSTAATNEVPEVTVFSKFPVTLGQPNTLICLVDNIFPPVVNITWLSNGHSVTEGVSETSFLSKSDHSFFKI
SYLTFLPSADEIYDCKVEHWGLDEPLLKHWEPEIPAPMSELTETLVCALGLSVGLMGIVVGTVFIIQGLRSVGASRHQGLL
>sp|Q9H4Q4|PRD12_HUMAN
MMGSVLPAEALVLKTGLKAPGLALAEVITSDILHSFLYGRWRNVLGEQLFEDKSHHASPKTAFTAEVLAQSFSGEVQKLSSLVLPAE
VIIAQSSIPGEGLGIFSKTWIKAGTEMGPFTGRVIAPEHVDICKNNNLMWEVFNEDGTVRYFIDASQEDHRSWMTYIKCARNEQEQN
LEVVQIGTSIFYKAIEMIPPDQELLVWYGNSHNTFLGIPGVPGLEEDQKKNKHEDFHPADSAAGPAGRMRCVICHRGFNSRSNLRSH
MRIHTLDKPFVCRFCNRRFSQSSTLRNHVRLHTGERPYKCQVCQSAYSQLAGLRAHQKSARHRPPSTALQAHSPALPAPHAHAPALA
AAAAAAAAAAAHHLPAMVL
>sp|P05112|IL4_HUMAN
MGLTSQLLPPLFFLLACAGNFVHGHKCDITLQEIIKTLNSLTEQKTLCTELTVTDIFAASKNTTEKETFCRAATVLRQFYSHHEKDT
RCLGATAQQFHRHKQLIRFLKRLDRNLWGLAGLNSCPVKEANQSTLENFLERLKTIMREKYSKCSS
>sp|P17480|UBF1_HUMAN
MNGEADCPTDLEMAAPKGQDRWSQEDMLTLLECMKNNLPSNDSSKFKTTESHMDWEKVAFKDFSGDMCKLKWVEISNEVRKFRTLTE
LILDAQEHVKNPYKGKKLKKHPDFPKKPLTPYFRFFMEKRAKYAKLHPEMSNLDLTKILSKKYKELPEKKKMKYIQDFQREKQEFER
NLARFREDHPDLIQNAKKSDIPEKPKTPQQLWYTHEKKVYLKVRPDATTKEVKDSLGKQWSQLSDKKRLKWIHKALEQRKEYEEIMR
DYIQKHPELNISEEGITKSTLTKAERQLKDKFDGRPTKPPPNSYSLYCAELMANMKDVPSTERMVLCSQQWKLLSQKEKDAYHKKCD
QKKKDYEVELLRFLESLPEEEQQRVLGEEKMLNINKKQATSPASKKPAQEGGKGGSEKPKRPVSAMFIFSEEKRRQLQEERPELSES
ELTRLLARMWNDLSEKKKAKYKAREAALKAQSERKPGGEREERGKLPESPKRAEEIWQQSVIGDYLARFKNDRVKALKAMEMTWNNM
EKKEKLMWIKKAAEDQKRYERELSEMRAPPAATNSSKKMKFQGEPKKPPMNGYQKFSQELLSNGELNHLPLKERMVEIGSRWQRISQ
SQKEHYKKLAEEQQKQYKVHLDLWVKSLSPQDRAAYKEYISNKRKSMTKLRGPNPKSSRTTLQSKSESEEDDEEDEDDEDEDEEEED
DENGDSSEDGGDSSESSSEDESEDGDENEEDDEDEDDDEDDDEDEDNESEGSSSSSSSSGDSSDSDSN
>sp|Q86XJ0|CAHM3_HUMAN
MDKFRMLFQHFQSSSESVMNGICLLLAAVTVKLYSSFDFNCPCLVHYNALYGLGLLLTPPLALFLCGLLANRQSVVMVEEWRRPAGH
RRKDPGIIRYMCSSVLQRALAAPLVWILLALLDGKCFVCAFSSSVDPEKFLDFANMTPSQVQLFLAKVPCKEDELVRDSPARKAVSR
YLRCLSQAIGWSVTLLLIIAAFLARCLRPCFDQTVFLQRRYWSNYVDLEQKLFDETCCEHARDFAHRCVLHFFASMRSELQARGLRR
GNAGRRLELPAVPEPPAVPEPPEGLDSGSGKAHLRAISSREQVDRLLSTWYSSKPPLDLAASPGLCGGGLSHRAPTLALGTRLSQHT
DV
>sp|O43508|TNF12_HUMAN
MAARRSQRRRGRRGEPGTALLVPLALGLGLALACLGLLLAVVSLGSRASLSAQEPAQEELVAEEDQDPSELNPQTEESQDPAPFLNR
LVRPRRSAPKGRKTRARRAIAAHYEVHPRPGQDGAQAGVDGTVSGWEEARINSSSPLRYNRQIGEFIVTRAGLYYLYCQVHFDEGKA
VYLKLDLLVDGVLALRCLEEFSATAASSLGPQLRLCQVSGLLALRPGSSLRIRTLPWAHLKAAPFLTYFGLFQVH
>sp|P14091|CATE_HUMAN
MKTLLLLLLVLLELGEAQGSLHRVPLRRHPSLKKKLRARSQLSEFWKSHNLDMIQFTESCSMDQSAKEPLINYLDMEYFGTISIGSP
PQNFTVIFDTGSSNLWVPSVYCTSPACKTHSRFQPSQSSTYSQPGQSFSIQYGTGSLSGIIGADQVSAFATQVEGLTVVGQQFGESV
TEPGQTFVDAEFDGILGLGYPSLAVGGVTPVFDNMMAQNLVDLPMFSVYMSSNPEGGAGSELIFGGYDHSHFSGSLNWVPVTKQAYW
QIALDNIQVGGTVMFCSEGCQAIVDTGTSLITGPSDKIKQLQNAIGAAPVDGEYAVECANLNVMPDVTFTINGVPYTLSPTAYTLLD
FVDGMQFCSSGFQGLDIHPPAGPLWILGDVFIRQFYSVFDRGNNRVGLAPAVP
>sp|P27986|P85A_HUMAN
MSAEGYQYRALYDYKKEREEDIDLHLGDILTVNKGSLVALGFSDGQEARPEEIGWLNGYNETTGERGDFPGTYVEYIGRKKISPPTP
KPRPPRPLPVAPGSSKTEADVEQQALTLPDLAEQFAPPDIAPPLLIKLVEAIEKKGLECSTLYRTQSSSNLAELRQLLDCDTPSVDL
EMIDVHVLADAFKRYLLDLPNPVIPAAVYSEMISLAPEVQSSEEYIQLLKKLIRSPSIPHQYWLTLQYLLKHFFKLSQTSSKNLLNA
RVLSEIFSPMLFRFSAASSDNTENLIKVIEILISTEWNERQPAPALPPKPPKPTTVANNGMNNNMSLQDAEWYWGDISREEVNEKLR
DTADGTFLVRDASTKMHGDYTLTLRKGGNNKLIKIFHRDGKYGFSDPLTFSSVVELINHYRNESLAQYNPKLDVKLLYPVSKYQQDQ

VVKEDNIEAVGKKLHEYNTQFQEKSREYDRLYEEYTRTSQEIQMKRTAIEAFNETIKIFEEQCQTQERYSKEYIEKFKREGNEKEIQ
RIMHNYDKLKSRISEIIDSRRRLEEDLKKQAAEYREIDKRMNSIKPDLIQLRKTRDQYLMWLTQKGVRQKKLNEWLGNENTEDQYSL
VEDDEDLPHHDEKTWNVGSSNRNKAENLLRGKRDGTFLVRESSKQGCYACSVVVDGEVKHCVINKTATGYGFAEPYNLYSSLKELVL
HYQHTSLVQHNDSLNVTLAYPVYAQQRR
>sp|Q96N28|PLD3A_HUMAN
MKIWSSEHVFGHPWDTVIQAAMRKYPNPMNPSVLGVDVLQRRVDGRGRLHSLRLLSTEWGLPSLVRAILGTSRTLTYIREHSVVDPV
EKKMELCSTNITLTNLVSVNERLVYTPHPENPEMTVLTQEAIITVKGISLGSYLESLMANTISSNAKKGWAAIEWIIEHSESAVS
>sp|Q9NSA0|S22AB_HUMAN
MAFSKLLEQAGGVGLFQTLQVLTFILPCLMIPSQMLLENFSAAIPGHRCWTHMLDNGSAVSTNMTPKALLTISIPPGPNQGPHQCRR
FRQPQWQLLDPNATATSWSEADTEPCVDGWVYDRSVFTSTIVAKWDLVCSSQGLKPLSQSIFMSGILVGSFIWGLLSYRFGRKPMLS
WCCLQLAVAGTSTIFAPTFVIYCGLRFVAAFGMAGIFLSSLTLMVEWTTTSRRAVTMTVVGCAFSAGQAALGGLAFALRDWRTLQLA
ASVPFFAISLISWWLPESARWLIIKGKPDQALQELRKVARINGHKEAKNLTIEVLMSSVKEEVASAKEPRSVLDLFCVPVLRWRSCA
MLVVNFSLLISYYGLVFDLQSLGRDIFLLQALFGAVDFLGRATTALLLSFLGRRTIQAGSQAMAGLAILANMLVPQDLQTLRVVFAV
LGKGCFGISLTCLTIYKAELFPTPVRMTADGILHTVGRLGAMMGPLILMSRQALPLLPPLLYGVISIASSLVVLFFLPETQGLPLPD
TIQDLESQKSTAAQGNRQEAVTVESTSL
>sp|Q6V702|CD022_HUMAN
MDQEEGLKALDNIVTQFNAYEDFLDSQITTVDLYYLEDETLARQLVELGYRGTGERVKREDFEARKAAIEIARLAERAQQKTLTSAG
KDLQDNFLTALAMREEDNRSGKLSSVIFIRDRNSHGQEISGYIDYAHRLKTEDFEVYFTGKKRLLPRPTDISFYNWDADIAVSNSSP
NYQVIADNPEGLLFRYKRDRKILNVDPKAQPGDNSTRITILTELYVQAVIFDHISRRKT
>sp|O00237|RN103_HUMAN
MWLKLFFLLLYFLVLFVLARFFEAIVWYETGIFATQLVDPVALSFKKLKTILECRGLGYSGLPEKKDVRELVEKSGDLMEGELYSAL
KEEEASESVSSTNFSGEMHFYELVEDTKDGIWLVQVIANDRSPLVGKIHWEKMVKKVSRFGIRTGTFNCSSDPRYCRRRGWVRSTLI
MSVPQTSTSKGKVMLKEYSGRKIEVEHIFKWITAHAASRIKTIYNAEHLKEEWNKSDQYWLKIYLFANLDQPPAFFSALSIKFTGRV
EFIFVNVENWDNKSYMTDIGIYNMPSYILRTPEGIYRYGNHTGEFISLQAMDSFLRSLQPEVNDLFVLSLVLVNLMAWMDLFITQGA
TIKRFVVLISTLGTYNSLLIISWLPVLGFLQLPYLDSFYEYSLKLLRYSNTTTLASWVRADWMFYSSHPALFLSTYLGHGLLIDYFE
KKRRRNNNNDEVNANNLEWLSSLWDWYTSYLFHPIASFQNFPVESDWDEDPDLFLERLAFPDLWLHPLIPTDYIKNLPMWRFKCLGV
QSEEEMSEGSQDTENDSESENTDTLSSEKEVFEDKQSVLHNSPGTASHCDAEACSCANKYCQTSPCERKGRSYGSYNTNEDMEPDWL
TWPADMLHCTECVVCLENFENGCLLMGLPCGHVFHQNCIVMWLAGGRHCCPVCRWPSYKKKQPYAQHQPLSNDVPS
>sp|Q9BTX1|NDC1_HUMAN
MATAVSRPCAGRSRDILWRVLGWRIVASIVWSVLFLPICTTVFIIFSRIDLFHPIQWLSDSFSDLYSSYVIFYFLLLSVVIIIISIF
NVEFYAVVPSIPCSRLALIGKIIHPQQLMHSFIHAAMGMVMAWCAAVITQGQYSFLVVPCTGTNSFGSPAAQTCLNEYHLFFLLTGA
FMGYSYSLLYFVNNMNYLPFPIIQQYKFLRFRRSLLLLVKHSCVESLFLVRNFCILYYFLGYIPKAWISTAMNLHIDEQVHRPLDTV
SGLLNLSLLYHVWLCGVFLLTTWYVSWILFKIYATEAHVFPVQPPFAEGSDECLPKVLNSNPPPIIKYLALQDLMLLSQYSPSRRQE
VFSLSQPGGHPHNWTAISRECLNLLNGMTQKLILYQEAAATNGRVSSSYPVEPKKLNSPEETAFQTPKSSQMPRPSVPPLVKTSLFS
SKLSTPDVVSPFGTPFGSSVMNRMAGIFDVNTCYGSPQSPQLIRRGPRLWTSASDQQMTEFSNPSPSTSISAEGKTMRQPSVIYSWI
QNKREQIKNFLSKRVLIMYFFSKHPEASIQAVFSDAQMHIWALEGLSHLVAASFTEDRFGVVQTTLPAILNTLLTLQEAVDKYFKLP
HASSKPPRISGSLVDTSYKTLRFAFRASLKTAIYRITTTFGEHLNAVQASAEHQKRLQQFLEFKE
>sp|Q96S53|TESK2_HUMAN
MDRSKRNSIAGFPPRVERLEEFEGGGGGEGNVSQVGRVWPSSYRALISAFSRLTRLDDFTCEKIGSGFFSEVFKVRHRASGQVMALK
MNTLSSNRANMLKEVQLMNRLSHPNILRFMGVCVHQGQLHALTEYINSGNLEQLLDSNLHLPWTVRVKLAYDIAVGLSYLHFKGIFH
RDLTSKNCLIKRDENGYSAVVADFGLAEKIPDVSMGSEKLAVVGSPFWMAPEVLRDEPYNEKADVFSYGIILCEIIARIQADPDYLP
RTENFGLDYDAFQHMVGDCPPDFLQLTFNCCNMDPKLRPSFVEIGKTLEEILSRLQEEEQERDRKLQPTARGLLEKAPGVKRLSSLD
DKIPHKSPCPRRTIWLSRSQSDIFSRKPPRTVSVLDPYYRPRDGAARTPKVNPFSARQDLMGGKIKFFDLPSKSVISLVFDLDAPGP

GTMPLADWQEPLAPPIRRWRSLPGSPEFLHQEACPFVGREESLSDGPPPRLSSLKYRVKEIPPFRASALPAAQAHEAMDCSILQEEN
GFGSRPQGTSPCPAGASEEMEVEERPAGSTPATFSTSGIGLQTQGKQDG
>sp|Q9UGK8|SRGEF_HUMAN
MEREPSASEAAPAAAALFAWGANSYGQLGLGHKEDVLLPQQLNDFCKPRSVRRITGGGGHSAVVTDGGDLFVCGLNKDGQLGLGHTE
DIPYFTPCKSLFGCPIQQVACGWDFTIMLTENGQVLSCGSNSFGQLGVPHGPRRCVVPQAIELHKEKVVCIAAGLRHAVAATASGIV
FQWGTGLASCGRRLCPGQTLPLFFTAKEPSRVTGLENSKAMCVLAGSDHSASLTDAGEVYVWGSNKHGQLANEAAFLPVPQKIEAHC
FQNEKVTAIWSGWTHLVAQTETGKMFTWGRADYGQLGRKLETYEGWKLEKQDSFLPCSRPPNSMPSSPHCLTGATEVSCGSEHNLAI
IGGVCYSWGWNEHGMCGDGTEANVWAPKPVQALLSSSGLLVGCGAGHSLALCQLPAHPALVQDPKVTYLSPDAIEDTESQKAMDKER
NWKERQSETSTQSQSDWSRNGGL
>sp|Q12906|ILF3_HUMAN
MRPMRIFVNDDRHVMAKHSSVYPTQEELEAVQNMVSHTERALKAVSDWIDEQEKGSSEQAESDNMDVPPEDDSKEGAGEQKTEHMTR
TLRGVMRVGLVAKGLLLKGDLDLELVLLCKEKPTTALLDKVADNLAIQLAAVTEDKYEILQSVDDAAIVIKNTKEPPLSLTIHLTSP
VVREEMEKVLAGETLSVNDPPDVLDRQKCLAALASLRHAKWFQARANGLKSCVIVIRVLRDLCTRVPTWGPLRGWPLELLCEKSIGT
ANRPMGAGEALRRVLECLASGIVMPDGSGIYDPCEKEATDAIGHLDRQQREDITQSAQHALRLAAFGQLHKVLGMDPLPSKMPKKPK
NENPVDYTVQIPPSTTYAITPMKRPMEEDGEEKSPSKKKKKIQKKEEKAEPPQAMNALMRLNQLKPGLQYKLVSQTGPVHAPIFTMS
VEVDGNSFEASGPSKKTAKLHVAVKVLQDMGLPTGAEGRDSSKGEDSAEETEAKPAVVAPAPVVEAVSTPSAAFPSDATAEQGPILT
KHGKNPVMELNEKRRGLKYELISETGGSHDKRFVMEVEVDGQKFQGAGSNKKVAKAYAALAALEKLFPDTPLALDANKKKRAPVPVR
GGPKFAAKPHNPGFGMGGPMHNEVPPPPNLRGRGRGGSIRGRGRGRGFGGANHGGYMNAGAGYGSYGYGGNSATAGYSQFYSNGGHS
GNASGGGGGGGGGSSGYGSYYQGDNYNSPVPPKHAGKKQPHGGQQKPSYGSGYQSHQGQQQSYNQSPYSNYGPPQGKQKGYNHGQGS
YSYSNSYNSPGGGGGSDYNYESKFNYSGSGGRSGGNSYGSGGASYNPGSHGGYGGGSGGGSSYQGKQGGYSQSNYNSPGSGQNYSGP
PSSYQSSQGGYGRNADHSMNYQYR
>sp|O95149|SPN1_HUMAN
MEELSQALASSFSVSQDLNSTAAPHPRLSQYKSKYSSLEQSERRRRLLELQKSKRLDYVNHARRLAEDDWTGMESEEENKKDDEEMD
IDTVKKLPKHYANQLMLSEWLIDVPSDLGQEWIVVVCPVGKRALIVASRGSTSAYTKSGYCVNRFSSLLPGGNRRNSTAKDYTILDC
IYNEVNQTYYVLDVMCWRGHPFYDCQTDFRFYWMHSKLPEEEGLGEKTKLNPFKFVGLKNFPCTPESLCDVLSMDFPFEVDGLLFYH
KQTHYSPGSTPLVGWLRPYMVSDVLGVAVPAGPLTTKPDYAGHQLQQIMEHKKSQKEGMKEKLTHKASENGHYELEHLSTPKLKGSS
HSPDHPGCLMEN
>sp|O00151|PDLI1_HUMAN
MTTQQIDLQGPGPWGFRLVGGKDFEQPLAISRVTPGSKAALANLCIGDVITAIDGENTSNMTHLEAQNRIKGCTDNLTLTVARSEHK
VWSPLVTEEGKRHPYKMNLASEPQEVLHIGSAHNRSAMPFTASPASSTTARVITNQYNNPAGLYSSENISNFNNALESKTAASGVEA
NSRPLDHAQPPSSLVIDKESEVYKMLQEKQELNEPPKQSTSFLVLQEILESEEKGDPNKPSGFRSVKAPVTKVAASIGNAQKLPMCD
KCGTGIVGVFVKLRDRHRHPECYVCTDCGTNLKQKGHFFVEDQIYCEKHARERVTPPEGYEVVTVFPK
>sp|Q8IY26|PLPP6_HUMAN
MPSPRRSMEGRPLGVSASSSSSSPGSPAHGGGGGGSRFEFQSLLSSRATAVDPTCARLRASESPVHRRGSFPLAAAGPSQSPAPPLP
EEDRMDLNPSFLGIALRSLLAIDLWLSKKLGVCAGESSSWGSVRPLMKLLEISGHGIPWLLGTLYCLCRSDSWAGREVLMNLLFALL
LDLLLVALIKGLVRRRRPAHNQMDMFVTLSVDKYSFPSGHATRAALMSRFILNHLVLAIPLRVLVVLWAFVLGLSRVMLGRHNVTDV
AFGFFLGYMQYSIVDYCWLSPHNAPVLFLLWSQR
>sp|A7MCY6|TBKB1_HUMAN
MESMFEDDISILTQEALGPSEVWLDSPGDPSLGGDMCSASHFALITAYGDIKERLGGLERENATLRRRLKVYEIKYPLISDFGEEHG
FSLYEIKDGSLLEVEKVSLQQRLNQFQHELQKNKEQEEQLGEMIQAYEKLCVEKSDLETELREMRALVETHLRQICGLEQQLRQQQG
LQDAAFSNLSPPPAPAPPCTDLDLHYLALRGGSGLSHAGWPGSTPSVSDLERRRLEEALEAAQGEARGAQLREEQLQAECERLQGEL
KQLQETRAQDLASNQSERDMAWVKRVGDDQVNLALAYTELTEELGRLRELSSLQGRILRTLLQEQARSGGQRHSPLSQRHSPAPQCP
SPSPPARAAPPCPPCQSPVPQRRSPVPPCPSPQQRRSPASPSCPSPVPQRRSPVPPSCQSPSPQRRSPVPPSCPAPQPRPPPPPPPG
ERTLAERAYAKPPSHHVKAGFQGRRSYSELAEGAAYAGASPPWLQAEAATLPKPRAYGSELYGPGRPLSPRRAFEGIRLRFEKQPSE

EDEWAVPTSPPSPEVGTIRCASFCAGFPIPESPAATAYAHAEHAQSWPSINLLMETVGSDIRSCPLCQLGFPVGYPDDALIKHIDSH
LENSKI
>sp|Q8WUA8|TSK_HUMAN
MPWPLLLLLAVSGAQTTRPCFPGCQCEVETFGLFDSFSLTRVDCSGLGPHIMPVPIPLDTAHLDLSSNRLEMVNESVLAGPGYTTLA
GLDLSHNLLTSISPTAFSRLRYLESLDLSHNGLTALPAESFTSSPLSDVNLSHNQLREVSVSAFTTHSQGRALHVDLSHNLIHRLVP
HPTRAGLPAPTIQSLNLAWNRLHAVPNLRDLPLRYLSLDGNPLAVIGPGAFAGLGGLTHLSLASLQRLPELAPSGFRELPGLQVLDL
SGNPKLNWAGAEVFSGLSSLQELDLSGTNLVPLPEALLLHLPALQSVSVGQDVRCRRLVREGTYPRRPGSSPKVALHCVDTRDSAAR
GPTIL
>sp|Q6NUR6|R216L_HUMAN
MEEGNNNEEVIHLNNFHCHRGQDFVIFFWKTQIIQREKTESL
>sp|Q9HCL0|PCD18_HUMAN
MHQMNAKMHFRFVFALLIVSFNHDVLGKNLKYRIYEEQRVGSVIARLSEDVADVLLKLPNPSTVRFRAMQRGNSPLLVVNEDNGEIS
IGATIDREQLCQKNLNCSIEFDVITLPTEHLQLFHIEVEVLDINDNSPQFSRSLIPIEISESAAVGTRIPLDSAFDPDVGENSLHTY
SLSANDFFNIEVRTRTDGAKYAELIVVRELDRELKSSYELQLTASDMGVPQRSGSSILKISISDSNDNSPAFEQQSYIIQLLENSPV
GTLLLDLNATDPDEGANGKIVYSFSSHVSPKIMETFKIDSERGHLTLFKQVDYEITKSYEIDVQAQDLGPNSIPAHCKIIIKVVDVN
DNKPEININLMSPGKEEISYIFEGDPIDTFVALVRVQDKDSGLNGEIVCKLHGHGHFKLQKTYENNYLILTNATLDREKRSEYSLTV
IAEDRGTPSLSTVKHFTVQINDINDNPPHFQRSRYEFVISENNSPGAYITTVTATDPDLGENGQVTYTILESFILGSSITTYVTIDP
SNGAIYALRIFDHEEVSQITFVVEARDGGSPKQLVSNTTVVLTIIDENDNVPVVIGPALRNNTAEITIPKGAESGFHVTRIRAIDRD
SGVNAELSCAIVAGNEENIFIIDPRSCDIHTNVSMDSVPYTEWELSVIIQDKGNPQLHTKVLLKCMIFEYAESVTSTAMTSVSQASL
DVSMIIIISLGAICAVLLVIMVLFATRCNREKKDTRSYNCRVAESTYQHHPKRPSRQIHKGDITLVPTINGTLPIRSHHRSSPSSSP
TLERGQMGSRQSHNSHQSLNSLVTISSNHVPENFSLELTHATPAVEQVSQLLSMLHQGQYQPRPSFRGNKYSRSYRYALQDMDKFSL
KDSGRGDSEAGDSDYDLGRDSPIDRLLGEGFSDLFLTDGRIPAAMRLCTEECRVLGHSDQCWMPPLPSPSSDYRSNMFIPGEEFPTQ
PQQQHPHQSLEDDAQPADSGEKKKSFSTFGKDSPNDEDTGDTSTSSLLSEMSSVFQRLLPPSLDTYSECSEVDRSNSLERRKGPLPA
KTVGYPQGVAAWAASTHFQNPTTNCGPPLGTHSSVQPSSKWLPAMEEIPENYEEDDFDNVLNHLNDGKHELMDASELVAEINKLLQD
VRQS
>sp|Q8N693|ESX1_HUMAN
MESLRGYTHSDIGYRSLAVGEDIEEVNDEKLTVTSLMARGGEDEENTRSKPEYGTEAENNVGTEGSVPSDDQDREGGGGHEPEQQQE
EPPLTKPEQQQEEPPLLELKQEQEEPPQTTVEGPQPAEGPQTAEGPQPPERKRRRRTAFTQFQLQELENFFDESQYPDVVARERLAA
RLNLTEDRVQVWFQNRRAKWKRNQRVLMLRNTATADLAHPLDMFLGGAYYAAPALDPALCVHLVPQLPRPPVLPVPPMPPRPPMVPM
PPRPPIAPMPPMAPVPPGSRMAPVPPGPRMAPVPPWPPMAPVPPWPPMAPVPTGPPMAPVPPGPPMARVPPGPPMARVPPGPPMAPL
PPGPPMAPLPPGPPMAPLPPGPPMAPLPPRSHVPHTGLAPVHITWAPVINSYYACPFF
>sp|P10070|GLI2_HUMAN
METSASATASEKQEAKSGILEAAGFPDPGKKASPLVVAAAAAAAVAAQGVPQHLLPPFHAPLPIDMRHQEGRYHYEPHSVHGVHGPP
ALSGSPVISDISLIRLSPHPAGPGESPFNAPHPYVNPHMEHYLRSVHSSPTLSMISAARGLSPADVAQEHLKERGLFGLPAPGTTPS
DYYHQMTLVAGHPAPYGDLLMQSGGAASAPHLHDYLNPVDVSRFSSPRVTPRLSRKRALSISPLSDASLDLQRMIRTSPNSLVAYIN
NSRSSSAASGSYGHLSAGALSPAFTFPHPINPVAYQQILSQQRGLGSAFGHTPPLIQPSPTFLAQQPMALTSINATPTQLSSSSNCL
SDTNQNKQSSESAVSSTVNPVAIHKRSKVKTEPEGLRPASPLALTQGQVSGHGSCGCALPLSQEQLADLKEDLDRDDCKQEAEVVIY
ETNCHWEDCTKEYDTQEQLVHHINNEHIHGEKKEFVCRWQACTREQKPFKAQYMLVVHMRRHTGEKPHKCTFEGCSKAYSRLENLKT
HLRSHTGEKPYVCEHEGCNKAFSNASDRAKHQNRTHSNEKPYICKIPGCTKRYTDPSSLRKHVKTVHGPDAHVTKKQRNDVHLRTPL
LKENGDSEAGTEPGGPESTEASSTSQAVEDCLHVRAIKTESSGLCQSSPGAQSSCSSEPSPLGSAPNNDSGVEMPGTGPGSLGDLTA
LDDTPPGADTSALAAPSAGGLQLRKHMTTMHRFEQLKKEKLKSLKDSCSWAGPTPHTRNTKLPPLPGSGSILENFSGSGGGGPAGLL
PNPRLSELSASEVTMLSQLQERRDSSTSTVSSAYTVSRRSSGISPYFSSRRSSEASPLGAGRPHNASSADSYDPISTDASRRSSEAS
QCSGGSGLLNLTPAQQYSLRAKYAAATGGPPPTPLPGLERMSLRTRLALLDAPERTLPAGCPRPLGPRRGSDGPTYGHGHAGAAPAF
PHEAPGGGARRASDPVRRPDALSLPRVQRFHSTHNVNPGPLPPCADRRGLRLQSHPSTDGGLARGAYSPRPPSISENVAMEAVAAGV

DGAGPEADLGLPEDDLVLPDDVVQYIKAHASGALDEGTGQVYPTESTGFSDNPRLPSPGLHGQRRMVAADSNVGPSAPMLGGCQLGF
GAPSSLNKNNMPVQWNEVSSGTVDALASQVKPPPFPQGNLAVVQQKPAFGQYPGYSPQGLQASPGGLDSTQPHLQPRSGAPSQGIPR
VNYMQQLRQPVAGSQCPGMTTTMSPHACYGQVHPQLSPSTISGALNQFPQSCSNMPAKPGHLGHPQQTEVAPDPTTMGNRHRELGVP
DSALAGVPPPHPVQSYPQQSHHLAASMSQEGYHQVPSLLPARQPGFMEPQTGPMGVATAGFGLVQPRPPLEPSPTGRHRGVRAVQQQ
LAYARATGHAMAAMPSSQETAEAVPKGAMGNMGSVPPQPPPQDAGGAPDHSMLYYYGQIHMYEQDGGLENLGSCQVMRSQPPQPQAC
QDSIQPQPLPSPGVNQVSSTVDSQLLEAPQIDFDAIMDDGDHSSLFSGALSPSLLHSLSQNSSRLTTPRNSLTLPSIPAGISNMAVG
DMSSMLTSLAEESKFLNMMT
>sp|Q13278|RIG_HUMAN
MPFFSSCLCPSHYSGPSLPSSTSSSLPTGPENQLGFVLLQAMVHHANSSCVRNAFWLQITEKLTPALSIIISVVYLRCPEMVENRIG
FLLNVKDSKTLSVVGPHPKPCIL
>sp|O60443|DFNA5_HUMAN
MFAKATRNFLREVDADGDLIAVSNLNDSDKLQLLSLVTKKKRFWCWQRPKYQFLSLTLGDVLIEDQFPSPVVVESDFVKYEGKFANH
VSGTLETALGKVKLNLGGSSRVESQSSFGTLRKQEVDLQQLIRDSAERTINLRNPVLQQVLEGRNEVLCVLTQKITTMQKCVISEHM
QVEEKCGGIVGIQTKTVQVSATEDGNVTKDSNVVLEIPAATTIAYGVIELYVKLDGQFEFCLLRGKQGGFENKKRIDSVYLDPLVFR
EFAFIDMPDAAHGISSQDGPLSVLKQATLLLERNFHPFAELPEPQQTALSDIFQAVLFDDELLMVLEPVCDDLVSGLSPTVAVLGEL
KPRQQQDLVAFLQLVGCSLQGGCPGPEDAGSKQLFMTAYFLVSALAEMPDSAAALLGTCCKLQIIPTLCHLLRALSDDGVSDLEDPT
LTPLKDTERFGIVQRLFASADISLERLKSSVKAVILKDSKVFPLLLCITLNGLCALGREHS
>sp|Q9BR76|COR1B_HUMAN
MSFRKVVRQSKFRHVFGQPVKNDQCYEDIRVSRVTWDSTFCAVNPKFLAVIVEASGGGAFLVLPLSKTGRIDKAYPTVCGHTGPVLD
IDWCPHNDEVIASGSEDCTVMVWQIPENGLTSPLTEPVVVLEGHTKRVGIIAWHPTARNVLLSAGCDNVVLIWNVGTAEELYRLDSL
HPDLIYNVSWNHNGSLFCSACKDKSVRIIDPRRGTLVAEREKAHEGARPMRAIFLADGKVFTTGFSRMSERQLALWDPENLEEPMAL
QELDSSNGALLPFYDPDTSVVYVCGKGDSSIRYFEITEEPPYIHFLNTFTSKEPQRGMGSMPKRGLEVSKCEIARFYKLHERKCEPI
VMTVPRKSDLFQDDLYPDTAGPEAALEAEEWVSGRDADPILISLREAYVPSKQRDLKISRRNVLSDSRPAMAPGSSHLGAPASTTTA
ADATPSGSLARAGEAGKLEEVMQELRALRALVKEQGDRICRLEEQLGRMENGDA
>sp|O43765|SGTA_HUMAN
MDNKKRLAYAIIQFLHDQLRHGGLSSDAQESLEVAIQCLETAFGVTVEDSDLALPQTLPEIFEAAATGKEMPQDLRSPARTPPSEED
SAEAERLKTEGNEQMKVENFEAAVHFYGKAIELNPANAVYFCNRAAAYSKLGNYAGAVQDCERAICIDPAYSKAYGRMGLALSSLNK
HVEAVAYYKKALELDPDNETYKSNLKIAELKLREAPSPTGGVGSFDIAGLLNNPGFMSMASNLMNNPQIQQLMSGMISGGNNPLGTP
GTSPSQNDLASLIQAGQQFAQQMQQQNPELIEQLRSQIRSRTPSASNDDQQE
>sp|Q76N89|HECW1_HUMAN
MLLHLCSVKNLYQNRFLGLAAMASPSRNSQSRRRCKEPLRYSYNPDQFHNMDLRGGPHDGVTIPRSTSDTDLVTSDSRSTLMVSSSY
YSIGHSQDLVIHWDIKEEVDAGDWIGMYLIDEVLSENFLDYKNRGVNGSHRGQIIWKIDASSYFVEPETKICFKYYHGVSGALRATT
PSVTVKNSAAPIFKSIGADETVQGQGSRRLISFSLSDFQAMGLKKGMFFNPDPYLKISIQPGKHSIFPALPHHGQERRSKIIGNTVN
PIWQAEQFSFVSLPTDVLEIEVKDKFAKSRPIIKRFLGKLSMPVQRLLERHAIGDRVVSYTLGRRLPTDHVSGQLQFRFEITSSIHP
DDEEISLSTEPESAQIQDSPMNNLMESGSGEPRSEAPESSESWKPEQLGEGSVPDGPGNQSIELSRPAEEAAVITEAGDQGMVSVGP
EGAGELLAQVQKDIQPAPSAEELAEQLDLGEEASALLLEDGEAPASTKEEPLEEEATTQSRAGREEEEKEQEEEGDVSTLEQGEGRL
QLRASVKRKSRPCSLPVSELETVIASACGDPETPRTHYIRIHTLLHSMPSAQGGSAAEEEDGAEEESTLKDSSEKDGLSEVDTVAAD
PSALEEDREEPEGATPGTAHPGHSGGHFPSLANGAAQDGDTHPSTGSESDSSPRQGGDHSCEGCDASCCSPSCYSSSCYSTSCYSSS
CYSASCYSPSCYNGNRFASHTRFSSVDSAKISESTVFSSQDDEEEENSAFESVPDSMQSPELDPESTNGAGPWQDELAAPSGHVERS
PEGLESPVAGPSNRREGECPILHNSQPVSQLPSLRPEHHHYPTIDEPLPPNWEARIDSHGRVFYVDHVNRTTTWQRPTAAATPDGMR
RSGSIQQMEQLNRRYQNIQRTIATERSEEDSGSQSCEQAPAGGGGGGGSDSEAESSQSSLDLRREGSLSPVNSQKITLLLQSPAVKF
ITNPEFFTVLHANYSAYRVFTSSTCLKHMILKVRRDARNFERYQHNRDLVNFINMFADTRLELPRGWEIKTDQQGKSFFVDHNSRAT
TFIDPRIPLQNGRLPNHLTHRQHLQRLRSYSAGEASEVSRNRGASLLARPGHSLVAAIRSQHQHESLPLAYNDKIVAFLRQPNIFEM
LQERQPSLARNHTLREKIHYIRTEGNHGLEKLSCDADLVILLSLFEEEIMSYVPLQAAFHPGYSFSPRCSPCSSPQNSPGLQRASAR

APSPYRRDFEAKLRNFYRKLEAKGFGQGPGKIKLIIRRDHLLEGTFNQVMAYSRKELQRNKLYVTFVGEEGLDYSGPSREFFFLLSQ
ELFNPYYGLFEYSANDTYTVQISPMSAFVENHLEWFRFSGRILGLALIHQYLLDAFFTRPFYKALLRLPCDLSDLEYLDEEFHQSLQ
WMKDNNITDILDLTFTVNEEVFGQVTERELKSGGANTQVTEKNKKEYIERMVKWRVERGVVQQTEALVRGFYEVVDSRLVSVFDARE
LELVIAGTAEIDLNDWRNNTEYRGGYHDGHLVIRWFWAAVERFNNEQRLRLLQFVTGTSSVPYEGFAALRGSNGLRRFCIEKWGKIT
SLPRAHTCFNRLDLPPYPSYSMLYEKLLTAVEETSTFGLE
>sp|Q92599|SEPT8_HUMAN
MAATDLERFSNAEPEPRSLSLGGHVGFDSLPDQLVSKSVTQGFSFNILCVGETGIGKSTLMNTLFNTTFETEEASHHEACVRLRPQT
YDLQESNVQLKLTIVDAVGFGDQINKDESYRPIVDYIDAQFENYLQEELKIRRSLFDYHDTRIHVCLYFITPTGHSLKSLDLVTMKK
LDSKVNIIPIIAKADTISKSELHKFKIKIMGELVSNGVQIYQFPTDDEAVAEINAVMNAHLPFAVVGSTEEVKVGNKLVRARQYPWG
VVQVENENHCDFVKLREMLIRVNMEDLREQTHSRHYELYRRCKLEEMGFQDSDGDSQPFSLQETYEAKRKEFLSELQRKEEEMRQMF
VNKVKETELELKEKERELHEKFEHLKRVHQEEKRKVEEKRRELEEETNAFNRRKAAVEALQSQALHATSQQPLRKDKDKKNRSDIGA
HQPGMSLSSSKVMMTKASVEPLNCSSWWPAIQCCSCLVRDATWREGFL
>sp|Q12766|HMGX3_HUMAN
MQRTQPRPCYLNAPQQCPGAERPGRPTAGSHSFLLRPGPLAGSSPFALLDPLQAFEQFVWVRSQARAGLLRLRQGSHAVTRCRPLPV
RREGRRDGSPWRSVVCRYCRCSRQTGASVTTVSLPSSSSSPGLDPRGPRQASVRSLRSEPVLLFLPFRTPYRDSEEGKREGLSRLRA
VCRRAGPRGRGSFSPRDARASPRLHFLVAAVTTGAASRRQRGARVRQPSPSSSRRAKRLRECERRSLHAPPAMDASYDGTEVTVVME
EIEEAYCYTSPGPPKKKKKYKIHGEKTKKPRSAYLLYYYDIYLKVQQELPHLPQSEINKKISESWRLLSVAERSYYLEKAKLEKEGL
DPNSKLSALTAVVPDIPGFRKILPRSDYIIIPKSSLQEDRSCPQLELCVAQNQMSPKGPPLVSNTAPETVPSHAGMAEQCLAVEALA
EEVGALTQSGAVQEIATSEILSQDVLLEDASLEVGESHQPYQTSLVIEETLVNGSPDLPTGSLAVPHPQVGESVSVVTVMRDSSESS
SSAPATQFIMLPLPAYSVVENPTSIKLTTTYTRRGHGTCTSPGCSFTYVTRHKPPKCPTCGNFLGGKWIPKEKPAKVKVELASGVSS
KGSVVKRNQQPVTTEQNSSKENASKLTLENSEAVSQLLNVAPPREVGEESEWEEVIISDAHVLVKEAPGNCGTAVTKTPVVKSGVQP
EVTLGTTDNDSPGADVPTPSEGTSTSSPLPAPKKPTGADLLTPGSRAPELKGRARGKPSLLAAARPMRAILPAPVNVGRGSSMGLPR
ARQAFSLSDKTPSVRTCGLKPSTLKQLGQPIQQPSGPGEVKLPSGPSNRTSQVKVVEVKPDMFPPYKYSCTVTLDLGLATSRGRGKC
KNPSCSYVYTNRHKPRICPSCGVNLAKDRTEKTTKAIEVSSPLPDVLNATEPLSTAQREIQRQSTLQLLRKVLQIPENESELAEVFA
LIHELNSSRLILSNVSEETVTIEQTSWSNYYESPSTQCLLCSSPLFKGGQNSLAGPQECWLLTASRLQTVTAQVKMCLNPHCLALHS
FIDIYTGLFNVGNKLLVSLDLLFAIRNQIKLGEDPRVSINVVLKSVQEQTEKTLTSEELSQLQELLCNGYWAFECLTVRDYNDMICG
ICGVAPKVEMAQRSEENVLALKSVEFTWPEFLGSNEVNVEDFWATMETEVIEQVAFPASIPITKFDASVIAPFFPPLMRGAVVVNTE
KDKNLDVQPVPGSGSALVRLLQEGTCKLDEIGSYSEEKLQHLLRQCGIPFGAEDSKDQLCFSLLALYESVQNGARAIRPPRHFTGGK
IYKVCPHQVVCGSKYLVRGESARDHVDLLASSRHWPPVYVVDMATSVALCADLCYPELTNQMWGRNQGCFSSPTEPPVSVSCPELLD
QHYTVDMTETEHSIQHPVTKTATRRIVHAGLQPNPGDPSAGHHSLALCPELAPYATILASIVDSKPNGVRQRPIAFDNATHYYLYNR
LMDFLTSREIVNRQIHDIVQSCQPGEVVIRDTLYRLGVAQIKTETEEEGEEEEVAAVAE
>sp|P0CF51|TRGC1_HUMAN
DKQLDADVSPKPTIFLPSIAETKLQKAGTYLCLLEKFFPDVIKIHWQEKKSNTILGSQEGNTMKTNDTYMKFSWLTVPEKSLDKEHR
CIVRHENNKNGVDQEIIFPPIKTDVITMDPKDNCSKDANDTLLLQLTNTSAYYMYLLLLLKSVVYFAIITCCLLRRTAFCCNGEKS
>sp|P62685|GAK8_HUMAN
MGQTKSKIKSKYASYLSFIKILLKRGGVKVSTKNLIKLFQIIEQFCPWFPEQGTLDLKDWKRIGKELKQAGRKGNIIPLTVWNDWAI
IKAALEPFQTEEDSISVSDAPGSCLIDCNENTRKKSQKETESLHCEYVAEPVMAQSTQNVDYNQLQEVIYPETLKLEGKGPELVGPS
ESKPRGTSPLPAGQVPVTLQPQKQVKENKTQPPVAYQYWPPAELQYRPPPESQYGYPGMPPAPQGREPYPQPPTRRLNPTAPPSRQG
SELHEIIDKSRKEGDTEAWQFPVTLEPMPPGEGAQEGEPPTVEARYKSFSIKMLKDMKEGVKQYGPNSPYMRTLLDSIAHGHRLIPY
DWEILAKSSLSPSQFLQFKTWWIDGVQEQVRRNRAANPPVNIDADQLLGIGQNWSTISQQALMQNEAIEQVRAICLRAWEKIQDPGS
TCPSFNTVRQGSKEPYPDFVARLQDVAQKSIADEKARKVIVELMAYENANPECQSAIKPLKGKVPAGSDVISEYVKACDGIGGAMHK
AMLMAQAITGVVLGGQVRTFGGKCYNCGQIGHLKKNCPVLNKQNITIQATTTGREPPDLCPRCKKGKHWASQCRSKFDKNGQPLSGN
EQRGQPQAPQQTGAFPIQPFVPQGFQDNNPHCPKCFRE
>sp|P00749|UROK_HUMAN

MRALLARLLLCVLVVSDSKGSNELHQVPSNCDCLNGGTCVSNKYFSNIHWCNCPKKFGGQHCEIDKSKTCYEGNGHFYRGKASTDTM
GRPCLPWNSATVLQQTYHAHRSDALQLGLGKHNYCRNPDNRRRPWCYVQVGLKPLVQECMVHDCADGKKPSSPPEELKFQCGQKTLR
PRFKIIGGEFTTIENQPWFAAIYRRHRGGSVTYVCGGSLISPCWVISATHCFIDYPKKEDYIVYLGRSRLNSNTQGEMKFEVENLIL
HKDYSADTLAHHNDIALLKIRSKEGRCAQPSRTIQTICLPSMYNDPQFGTSCEITGFGKENSTDYLYPEQLKMTVVKLISHRECQQP
HYYGSEVTTKMLCAADPQWKTDSCQGDSGGPLVCSLQGRMTLTGIVSWGRGCALKDKPGVYTRVSHFLPWIRSHTKEENGLAL
>sp|Q14192|FHL2_HUMAN
MTERFDCHHCNESLFGKKYILREESPYCVVCFETLFANTCEECGKPIGCDCKDLSYKDRHWHEACFHCSQCRNSLVDKPFAAKEDQL
LCTDCYSNEYSSKCQECKKTIMPGTRKMEYKGSSWHETCFICHRCQQPIGTKSFIPKDNQNFCVPCYEKQHAMQCVQCKKPITTGGV
TYREQPWHKECFVCTACRKQLSGQRFTARDDFAYCLNCFCDLYAKKCAGCTNPISGLGGTKYISFEERQWHNDCFNCKKCSLSLVGR
GFLTERDDILCPDCGKDI
>sp|Q8TCU4|ALMS1_HUMAN
MEPEDLPWPGELEEEEEEEEEEEEEEEEEAAAAAAANVDDVVVVEEVEEEAGRELDSDSHYGPQHLESIDDEEDEEAKAWLQAHPGRI
LPPLSPPQHRYSEGERTSLEKIVPLTCHVWQQIVYQGNSRTQISDTNVVCLETTAQRGSGDDQKTESWHCLPQEMDSSQTLDTSQTR
FNVRTEDTEVTDFPSLEEGILTQSENQVKEPNRDLFCSPLLVIQDSFASPDLPLLTCLTQDQEFAPDSLFHQSELSFAPLRGIPDKS
EDTEWSSRPSEVSEALFQATAEVASDLASSRFSVSQHPLIGSTAVGSQCPFLPSEQGNNEETISSVDELKIPKDCDRYDDLCSYMSW
KTRKDTQWPENNLADKDQVSVATSFDITDENIATKRSDHFDAARSYGQYWTQEDSSKQAETYLTKGLQGKVESDVITLDGLNENAVV
CSERVAELQRKPTRESEYHSSDLRMLRMSPDTVPKAPKHLKAGDTSKGGIAKVTQSNLKSGITTTPVDSDIGSHLSLSLEDLSQLAV
SSLETTTGQHTDTLNQKTLADTHLTEETLKVTAIPEPADQKTATPTVLSSSHSHRGKPSIFYQQGLPDSHLTEEALKVSAAPGLADQ
TTGMSTLTSTSYSHREKPGTFYQQELPESNLTEEPLEVSAAPGPVEQKTGIPTVSSTSHSHVEDLLFFYRQTLPDGHLTDQALKVSA
VSGPADQKTGTATVLSTPHSHREKPGIFYQQEFADSHQTEETLTKVSATPGPADQKTEIPAVQSSSYSQREKPSILYPQDLADSHLP
EEGLKVSAVAGPADQKTGLPTVPSSAYSHREKLLVFYQQALLDSHLPEEALKVSAVSGPADGKTGTPAVTSTSSASSSLGEKPSAFY
QQTLPNSHLTEEALKVSIVPGPGDQKTGIPSAPSSFYSHREKPIIFSQQTLPDFLFPEEALKVSAVSVLAAQKTGTPTVSSNSHSHS
EKSSVFYQQELPDSDLPRESLKMSAIPGLTDQKTVPTPTVPSGSFSHREKPSIFYQQEWPDSYATEKALKVSTGPGPADQKTEIPAV
QSSSYPQREKPSVLYPQVLSDSHLPEESLKVSAFPGPADQMTDTPAVPSTFYSQREKPGIFYQQTLPESHLPKEALKISVAPGLADQ
KTGTPTVTSTSYSQHREKPSIFHQQALPGTHIPEEAQKVSAVTGPGNQKTWIPRVLSTFYSQREKPGIFYQQTLPGSHIPEEAQKVS
PVLGPADQKTGTPTPTSASYSHTEKPGIFYQQVLPDNHPTEEALKISVASEPVDQTTGTPAVTSTSYSQYREKPSIFYQQSLPSSHL
TEEAKNVSAVPGPADQKTVIPILPSTFYSHTEKPGVFYQQVLPHSHPTEEALKISVASEPVDQTTGTPTVTSTSYSQHTEKPSIFYQ
QSLPGSHLTEEAKNVSAVPGPGDRKTGIPTLPSTFYSHTEKPGSFYQQVLPHSHLPEEALEVSVAPGPVDQTIGTPTVTSPSSSFGE
KPIVIYKQAFPEGHLPEESLKVSVAPGPVGQTTGAPTITSPSYSQHRAKSGSFYQLALLGSQIPEEALRVSSAPGPADQTTGIPTIT
STSYSFGEKPIVNYKQAFPDGHLPEEALKVSIVSGPTEKKTDIPAGPLGSSALGEKPITFYRQALLDSPLNKEVVKVSAAPGPADQK
TETLPVHSTSYSNRGKPVIFYQQTLSDSHLPEEALKVPPVPGPDAQKTETPSVSSSLYSYREKPIVFYQQALPDSELTQEALKVSAV
PQPADQKTGLSTVTSSFYSHTEKPNISYQQELPDSHLTEEALKVSNVPGPADQKTGVSTVTSTSYSHREKPIVSYQRELPHFTEAGL
KILRVPGPADQKTGINILPSNSYPQREHSVISYEQELPDLTEVTLKAIGVPGPADQKTGIQIASSSSYSNREKASIFHQQELPDVTE
EALNVFVVPGQGDRKTEIPTVPLSYYSRREKPSVISQQELPDSHLTEEALKVSPVSIPAEQKTGIPIGLSSSYSHSHKEKLKISTVH
IPDDQKTEFPAATLSSYSQIEKPKISTVIGPNDQKTPSQTAFHSSYSQTVKPNILFQQQLPDRDQSKGILKISAVPELTDVNTGKPV
SLSSSYFHREKSNIFSPQELPGSHVTEDVLKVSTIPGPAGQKTVLPTALPSSFSHREKPDIFYQKDLPDRHLTEDALKISSALGQAD
QITGLQTVPSGTYSHGENHKLVSEHVQRLIDNLNSSDSSVSSNNVLLNSQADDRVVINKPESAGFRDVGSEEIQDAENSAKTLKEIR
TLLMEAENMALKRCNFPAPLARFRDISDISFIQSKKVVCFKEPSSTGVSNGDLLHRQPFTEESPSSRCIQKDIGTQTNLKCRRGIEN
WEFISSTTVRSPLQEAESKVSMALEETLRQYQAAKSVMRSEPEGCSGTIGNKIIIPMMTVIKSDSSSDASDGNGSCSWDSNLPESLE
SVSDVLLNFFPYVSPKTSITDSREEEGVSESEDGGGSSVDSLAAHVKNLLQCESSLNHAKEILRNAEEEESRVRAHAWNMKFNLAHD
CGYSISELNEDDRRKVEEIKAELFGHGRTTDLSKGLQSPRGMGCKPEAVCSHIIIESHEKGCFRTLTSEHPQLDRHPCAFRSAGPSE
MTRGRQNPSSCRAKHVNLSASLDQNNSHFKVWNSLQLKSHSPFQNFIPDEFKISKGLRMPFDEKMDPWLSELVEPAFVPPKEVDFHS
SSQMPSPEPMKKFTTSITFSSHRHSKCISNSSVVKVGVTEGSQCTGASVGVFNSHFTEEQNPPRDLKQKTSSPSSFKMHSNSQDKEV
TILAEGRRQSQKLPVDFERSFQEEKPLERSDFTGSHSEPSTRANCSNFKEIQISDNHTLISMGRPSSTLGVNRSSSRLGVKEKNVTI

TPDLPSCIFLEQRELFEQSKAPRADDHVRKHHSPSPQHQDYVAPDLPSCIFLEQRELFEQCKAPYVDHQMRENHSPLPQGQDSIASD
LPSPISLEQCQSKAPGVDDQMNKHHFPLPQGQDCVVEKNNQHKPKSHISNINVEAKFNTVVSQSAPNHCTLAASASTPPSNRKALSC
VHITLCPKTSSKLDSGTLDERFHSLDAASKARMNSEFNFDLHTVSSRSLEPTSKLLTSKPVAQDQESLGFLGPKSSLDFQVVQPSLP
DSNTITQDLKTIPSQNSQIVTSRQIQVNISDFEGHSNPEGTPVFADRLPEKMKTPLSAFSEKLSSDAVTQITTESPEKTLFSSEIFI
NAEDRGHEIIEPGNQKLRKAPVKFASSSSVQQVTFSRGTDGQPLLLPYKPSGSTKMYYVPQLRQIPPSPDSKSDTTVESSHSGSNDA
IAPDFPAQVLGTRDDDLSATVNIKHKEGIYSKRVVTKASLPVGEKPLQNENADASVQVLITGDENLSDKKQQEIHSTRAVTEAAQAK
EKESLQKDTADSSAAAAAEHSAQVGDPEMKNLPDTKAITQKEEIHRKKTVPEEAWPNNKESLQINIEESECHSEFENTTRSVFRSAK
FYIHHPVHLPSDQDICHESLGKSVFMRHSWKDFFQHHPDKHREHMCLPLPYQNMDKTKTDYTRIKSLSINVNLGNKEVMDTTKSQVR
DYPKHNGQISDPQRDQKVTPEQTTQHTVSLNELWNKYRERQRQQRQPELGDRKELSLVDRLDRLAKILQNPITHSLQVSESTHDDSR
GERSVKEWSGRQQQRNKLQKKKRFKSLEKSHKNTGELKKSKVLSHHRAGRSNQIKIEQIKFDKYILSKQPGFNYISNTSSDCRPSEE
SELLTDTTTNILSGTTSTVESDILTQTDREVALHERSSSVSTIDTARLIQAFGHERVCLSPRRIKLYSSITNQQRRYLEKRSKHSKK
VLNTGHPLVTSEHTRRRHIQVANHVISSDSISSSASSFLSSNSTFCNKQNVHMLNKGIQAGNLEIVNGAKKHTRDVGITFPTPSSSE
AKLEENSDVTSWSEEKREEKMLFTGYPEDRKLKKNKKNSHEGVSWFVPVENVESRSKKENVPNTCGPGISWFEPITKTRPWREPLRE
QNCQGQHLDGRGYLAGPGREAGRDLLRPFVRATLQESLQFHRPDFISRSGERIKRLKLIVQERKLQSMLQTERDALFNIDRERQGHQ
NRMCPLPKRVFLAIQKNKPISKKEMIQRSKRIYEQLPEVQKKREEEKRKSEYKSYRLRAQLYKKRVTNQLLGRKVPWD
>sp|Q8NCG5|CHST4_HUMAN
MLLPKKMKLLLFLVSQMAILALFFHMYSHNISSLSMKAQPERMHVLVLSSWRSGSSFVGQLFGQHPDVFYLMEPAWHVWMTFKQSTA
WMLHMAVRDLIRAVFLCDMSVFDAYMEPGPRRQSSLFQWENSRALCSAPACDIIPQDEIIPRAHCRLLCSQQPFEVVEKACRSYSHV
VLKEVRFFNLQSLYPLLKDPSLNLHIVHLVRDPRAVFRSRERTKGDLMIDSRIVMGQHEQKLKKEDQPYYVMQVICQSQLEIYKTIQ
SLPKALQERYLLVRYEDLARAPVAQTSRMYEFVGLEFLPHLQTWVHNITRGKGMGDHAFHTNARDALNVSQAWRWSLPYEKVSRLQK
ACGDAMNLLGYRHVRSEQEQRNLLLDLLSTWTVPEQIH
>sp|Q6ZRM9|YG024_HUMAN
MASGRWASPGPAWASRRPLQAQVVLKSASPGPAPASQQASSFGSAPAQLPPAFVDPELSPAMLLSPTCLPVACTGPGLAGEQPLQAP
LLPPRGISRPSSGLTAASRDQVPACLPAACVRPSSSVTVACSGPTHASGTLSRGVSPCLTLASLTLREFSVGPCLTLASLTLREVSM
SPCLTLVSLTLRAILPHAGLLRPSSCLCWPFQAQPLPVGGL
>sp|Q9BUX1|CHAC1_HUMAN
MGGAQLELPSGARPGVCVRRSFRAHAGDQPRRPPGPIPVPGTMKQESAAPNTPPTSQSPTPSAQFPRNDGDPQALWIFGYGSLVWRP
DFAYSDSRVGFVRGYSRRFWQGDTFHRGSDKMPGRVVTLLEDHEGCTWGVAYQVQGEQVSKALKYLNVREAVLGGYDTKEVTFYPQD
APDQPLKALAYVATPQNPGYLGPAPEEAIATQILACRGFSGHNLEYLLRLADFMQLCGPQAQDEHLAAIVDAVGTMLPCFCPTEQAL
ALV
>sp|O60285|NUAK1_HUMAN
MEGAAAPVAGDRPDLGLGAPGSPREAVAGATAALEPRKPHGVKRHHHKHNLKHRYELQETLGKGTYGKVKRATERFSGRVVAIKSIR
KDKIKDEQDMVHIRREIEIMSSLNHPHIISIYEVFENKDKIVIIMEYASKGELYDYISERRRLSERETRHFFRQIVSAVHYCHKNGV
VHRDLKLENILLDDNCNIKIADFGLSNLYQKDKFLQTFCGSPLYASPEIVNGRPYRGPEVDSWALGVLLYTLVYGTMPFDGFDHKNL
IRQISSGEYREPTQPSDARGLIRWMLMVNPDRRATIEDIANHWWVNWGYKSSVCDCDALHDSESPLLARIIDWHHRSTGLQADTEAK
MKGLAKPTTSEVMLERQRSLKKSKKENDFAQSGQDAVPESPSKLSSKRPKGILKKRSNSEHRSHSTGFIEGVVGPALPSTFKMEQDL
CRTGVLLPSSPEAEVPGKLSPKQSATMPKKGILKKTQQRESGYYSSPERSESSELLDSNDVMGSSIPSPSPPDPARVTSHSLSCRRK
GILKHSSKYSAGTMDPALVSPEMPTLESLSEPGVPAEGLSRSYSRPSSVISDDSVLSSDSFDLLDLQENRPARQRIRSCVSAENFLQ
IQDFEGLQNRPRPQYLKRYRNRLADSSFSLLTDMDDVTQVYKQALEICSKLN
>sp|O00519|FAAH1_HUMAN
MVQYELWAALPGASGVALACCFVAAAVALRWSGRRTARGAVVRARQRQRAGLENMDRAAQRFRLQNPDLDSEALLALPLPQLVQKLH
SRELAPEAVLFTYVGKAWEVNKGTNCVTSYLADCETQLSQAPRQGLLYGVPVSLKECFTYKGQDSTLGLSLNEGVPAECDSVVVHVL
KLQGAVPFVHTNVPQSMFSYDCSNPLFGQTVNPWKSSKSPGGSSGGEGALIGSGGSPLGLGTDIGGSIRFPSSFCGICGLKPTGNRL
SKSGLKGCVYGQEAVRLSVGPMARDVESLALCLRALLCEDMFRLDPTVPPLPFREEVYTSSQPLRVGYYETDNYTMPSPAMRRAVLE

TKQSLEAAGHTLVPFLPSNIPHALETLSTGGLFSDGGHTFLQNFKGDFVDPCLGDLVSILKLPQWLKGLLAFLVKPLLPRLSAFLSN
MKSRSAGKLWELQHEIEVYRKTVIAQWRALDLDVVLTPMLAPALDLNAPGRATGAVSYTMLYNCLDFPAGVVPVTTVTAEDEAQMEH
YRGYFGDIWDKMLQKGMKKSVGLPVAVQCVALPWQEELCLRFMREVERLMTPEKQSS
>sp|Q6NSX1|CCD70_HUMAN
MATPPFRLIRKMFSFKVSRWMGLACFRSLAASSPSIRQKKLMHKLQEEKAFREEMKIFREKIEDFREEMWTFRGKIHAFRGQILGFW
EEERPFWEEEKTFWKEEKSFWEMEKSFREEEKTFWKKYRTFWKEDKAFWKEDNALWERDRNLLQEDKALWEEEKALWVEERALLEGE
KALWEDKTSLWEEENALWEEERAFWMENNGHIAGEQMLEDGPHNANRGQRLLAFSRGRA
>sp|Q6UXZ0|TMIG1_HUMAN
MAWKSSVIMQMGRFLLLVILFLPREMTSSVLTVNGKTENYILDTTPGSQASLICAVQNHTREEELLWYREEGRVDLKSGNKINSSSV
CVSSISENDNGISFTCRLGRDQSVSVSVVLNVTFPPLLSGNDFQTVEEGSNVKLVCNVKANPQAQMMWYKNSSLLDLEKSRHQIQQT
SESFQLSITKVEKPDNGTYSCIAKSSLKTESLDFHLIVKDKTVGVPIEPIIAACVVIFLTLCFGLIARRKKIMKLCMKDKDPHSETA
L
>sp|Q8N461|FXL16_HUMAN
MSSPGIDGDPKPPCLPRNGLVKLPGQPNGLGAASITKGTPATKNRPCQPPPPPTLPPPSLAAPLSRAALAGGPCTPAGGPASALAPG
HPAERPPLATDEKILNGLFWYFSACEKCVLAQVCKAWRRVLYQPKFWAGLTPVLHAKELYNVLPGGEKEFVNLQGFAARGFEGFCLV
GVSDLDICEFIDNYALSKKGVKAMSLKRSTITDAGLEVMLEQMQGVVRLELSGCNDFTEAGLWSSLSARITSLSVSDCINVADDAIA
AISQLLPNLAELSLQAYHVTDTALAYFTARQGHSTHTLRLLSCWEITNHGVVNVVHSLPNLTALSLSGCSKVTDDGVELVAENLRKL
RSLDLSWCPRITDMALEYVACDLHRLEELVLDRCVRITDTGLSYLSTMSSLRSLYLRWCCQVQDFGLKHLLALGSLRLLSLAGCPLL
TTTGLSGLVQLQELEELELTNCPGATPELFKYFSQHLPRCLVIE
>sp|Q6Q788|APOA5_HUMAN
MASMAAVLTWALALLSAFSATQARKGFWDYFSQTSGDKGRVEQIHQQKMAREPATLKDSLEQDLNNMNKFLEKLRPLSGSEAPRLPQ
DPVGMRRQLQEELEEVKARLQPYMAEAHELVGWNLEGLRQQLKPYTMDLMEQVALRVQELQEQLRVVGEDTKAQLLGGVDEAWALLQ
GLQSRVVHHTGRFKELFHPYAESLVSGIGRHVQELHRSVAPHAPASPARLSRCVQVLSRKLTLKAKALHARIQQNLDQLREELSRAF
AGTGTEEGAGPDPQMLSEEVRQRLQAFRQDTYLQIAAFTRAIDQETEEVQQQLAPPPPGHSAFAPEFQQTDSGKVLSKLQARLDDLW
EDITHSLHDQGHSHLGDP
>sp|Q8NGC0|O5AU1_HUMAN
MTEFHLQSQMPSIRLIFRRLSLGRIKPSQSPRCSTSFMVVPSFSIAEHWRRMKGANLSQGMEFELLGLTTDPQLQRLLFVVFLGMYT
ATLLGNLVMFLLIHVSATLHTPMYSLLKSLSFLDFCYSSTVVPQTLVNFLAKRKVISYFGCMTQMFFYAGFATSECYLIAAMAYDRY
AAICNPLLYSTIMSPEVCASLIVGSYSAGFLNSLIHTGCIFSLKFCGAHVVTHFFCDGPPILSLSCVDTSLCEILLFIFAGFNLLSC
TLTILISYFLILNTILKMSSAQGRFKAFSTCASHLTAICLFFGTTLFMYLRPRSSYSLTQDRTVAVIYTVVIPVLNPLMYSLRNKDV
KKALIKVWGRKTME
>sp|P35625|TIMP3_HUMAN
MTPWLGLIVLLGSWSLGDWGAEACTCSPSHPQDAFCNSDIVIRAKVVGKKLVKEGPFGTLVYTIKQMKMYRGFTKMPHVQYIHTEAS
ESLCGLKLEVNKYQYLLTGRVYDGKMYTGLCNFVERWDQLTLSQRKGLNYRYHLGCNCKIKSCYYLPCFVTSKNECLWTDMLSNFGY
PGYQSKHYACIRQKGGYCSWYRGWAPPDKSIINATDP
>sp|O95755|RAB36_HUMAN
MVIAGASWMLGRAAASPTQTPPTTSTIRVARRSRVALVAMVIAAAGSGGPGRAEPQLSQPSLDCGRMRSSLTPLGPPVSRDRVIASF
PKWYTPEACLQLREHFHGQVSAACQRRNTGTVGLKLSKVVVVGDLYVGKTSLIHRFCKNVFDRDYKATIGVDFEIERFEIAGIPYSL
QIWDTAGQEKFKCIASAYYRGAQVIITAFDLTDVQTLEHTRQWLEDALRENEAGSCFIFLVGTKKDLLSGAACEQAEADAVHLAREM
QAEYWSVSAKTGENVKAFFSRVAALAFEQSVLQDLERQSSARLQVGNGDLIQMEGSPPETQESKRPSSLGCC
>sp|Q96IK0|TM101_HUMAN
MASKIGSRRWMLQLIMQLGSVLLTRCPFWGCFSQLMLYAERAEARRKPDIPVPYLYFDMGAAVLCASFMSFGVKRRWFALGAALQLA
ISTYAAYIGGYVHYGDWLKVRMYSRTVAIIGGFLVLASGAGELYRRKPRSRSLQSTGQVFLGIYLICVAYSLQHSKEDRLAYLNHLP
GGELMIQLFFVLYGILALAFLSGYYVTLAAQILAVLLPPVMLLIDGNVAYWHNTRRVEFWNQMKLLGESVGIFGTAVILATDG

>sp|Q86YR7|MF2L2_HUMAN
MLSCLKEEMPPQELTRRLATVITHVDEIMQQEVRPLMAVEIIEQLHRQFAILSGGRGEDGAPIITFPEFSGFKHIPDEDFLNVMTYL
TSIPSVEAASIGFIVVIDRRRDKWSSVKASLTRIAVAFPGNLQLIFILRPSRFIQRTFTDIGIKYYRNEFKTKVPIIMVNSVSDLHG
YIDKSQLTRELGGTLEYRHGQWVNHRTAIENFALTLKTTAQMLQTFGSCLATAELPRSMLSTEDLLMSHTRQRDKLQDELKLLGKQG
TTLLSCIQEPATKCPNSKLNLNQLENVTTMERLLVQLDETEKAFSHFWSEHHLKLNQCLQLQHFEHDFCKAKLALDNLLEEQAEFTG
IGDSVMHVEQILKEHKKLEEKSQEPLEKAQLLALVGDQLIQSHHYAADAIRPRCVELRHLCDDFINGNKKKWDILGKSLEFHRQLDK
VSQWCEAGIYLLASQAVDKCQSREGVDIALNDIATFLGTVKEYPLLSPKEFYNEFELLLTLDAKAKAQKVLQRLDDVQEIFHKRQVS
LMKLAAKQTRPVQPVAPHPESSPKWVSSKTSQPSTSVPLARPLRTSEEPYTETELNSRGKEDDETKFEVKSEEIFESHHERGNPELE
QQARLGDLSPRRRIIRDLLETEEIYIKEIKSIIDGYITPMDFIWLKHLIPDVLQNNKDFLFGNIRELYEFHNRTFLKELEKCAENPE
LLAHCFLKRKEDLQIYFKYHKNLPRARAIWQECQDCAYFGVCQRQLDHNLPLFKYLKGPSQRLIKYQMLLKGLLDFESPEDMEIDPG
ELGGSAKDGPKRTKDSAFSTELQQALAVIEDLIKSCELAVDLAAVTECPDDIGKLGKLLLHGPFSVWTIHKDRYKMKDLIRFKPSQR
QIYLFERGIVFCKIRMEPGDQGLSPHYSFKKTMKLMTLSIRQLGRGSHRKFEIASRNGLEKYILQAASKEIRDCWFSEISKLLMEQQ
NNIKDQGNPQFEMSTSKGSGAGSGPWIKNMERATTSKEDPASSTGGIKGCSSREFSSMDTFEDCEGAEDMEKESSALSLAGLFQSDD
SHETCSSKSAFLERGESSQGEKEERDEEETATRSTEEERAGASTGRLAPAGATAGFQARALRPRTSAQES
>sp|Q5JXX5|GLRA4_HUMAN
MTTLVPATLSFLLLWTLPGQVLLRVALAKEEVKSGTKGSQPMSPSDFLDKLMGRTSGYDARIRPNFKGPPVNVTCNIFINSFSSITK
TTMDYRVNVFLRQQWNDPRLSYREYPDDSLDLDPSMLDSIWKPDLFFANEKGANFHEVTTDNKLLRIFKNGNVLYSIRLTLILSCLM
DLKNFPMDIQTCTMQLESFGYTMKDLVFEWLEDAPAVQVAEGLTLPQFILRDEKDLGCCTKHYNTGKFTCIEVKFHLERQMGYYLIQ
MYIPSLLIVILSWVSFWINMDAAPARVGLGITTVLTMTTQSSGSRASLPKVSYVKAIDIWMAVCLLFVFAALLEYAAINFVSRQHKE
FIRLRRRQRRQRLEEDIIQESRFYFRGYGLGHCLQARDGGPMEGSGIYSPQPPAPLLREGETTRKLYVD
>sp|P43405|KSYK_HUMAN
MASSGMADSANHLPFFFGNITREEAEDYLVQGGMSDGLYLLRQSRNYLGGFALSVAHGRKAHHYTIERELNGTYAIAGGRTHASPAD
LCHYHSQESDGLVCLLKKPFNRPQGVQPKTGPFEDLKENLIREYVKQTWNLQGQALEQAIISQKPQLEKLIATTAHEKMPWFHGKIS
REESEQIVLIGSKTNGKFLIRARDNNGSYALCLLHEGKVLHYRIDKDKTGKLSIPEGKKFDTLWQLVEHYSYKADGLLRVLTVPCQK
IGTQGNVNFGGRPQLPGSHPATWSAGGIISRIKSYSFPKPGHRKSSPAQGNRQESTVSFNPYEPELAPWAADKGPQREALPMDTEVY
ESPYADPEEIRPKEVYLDRKLLTLEDKELGSGNFGTVKKGYYQMKKVVKTVAVKILKNEANDPALKDELLAEANVMQQLDNPYIVRM
IGICEAESWMLVMEMAELGPLNKYLQQNRHVKDKNIIELVHQVSMGMKYLEESNFVHRDLAARNVLLVTQHYAKISDFGLSKALRAD
ENYYKAQTHGKWPVKWYAPECINYYKFSSKSDVWSFGVLMWEAFSYGQKPYRGMKGSEVTAMLEKGERMGCPAGCPREMYDLMNLCW
TYDVENRPGFAAVELRLRNYYYDVVN
>sp|Q9BRK5|CAB45_HUMAN
MVWPWVAMASRWGPLIGLAPCCLWLLGAVLLMDASARPANHSSTRERVANREENEILPPDHLNGVKLEMDGHLNRGFHQEVFLGKDL
GGFDEDAEPRRSRRKLMVIFSKVDVNTDRKISAKEMQRWIMEKTAEHFQEAMEESKTHFRAVDPDGDGHVSWDEYKVKFLASKGHSE
KEVADAIRLNEELKVDEETQEVLENLKDRWYQADSPPADLLLTEEEFLSFLHPEHSRGMLRFMVKEIVRDLDQDGDKQLSVPEFISL
PVGTVENQQGQDIDDNWVKDRKKEFEELIDSNHDGIVTAEELESYMDPMNEYNALNEAKQMIAVADENQNHHLEPEEVLKYSEFFTG
SKLVDYARSVHEEF
>sp|Q15424|SAFB1_HUMAN
MAETLSGLGDSGAAGAAALSSASSETGTRRLSDLRVIDLRAELRKRNVDSSGNKSVLMERLKKAIEDEGGNPDEIEITSEGNKKTSK
RSSKGRKPEEEGVEDNGLEENSGDGQEDVETSLENLQDIDIMDISVLDEAEIDNGSVADCVEDDDADNLQESLSDSRELVEGEMKEL
PEQLQEHAIEDKETINNLDTSSSDFTILQEIEEPSLEPENEKILDILGETCKSEPVKEESSELEQPFAQDTSSVGPDRKLAEEEDLF
DSAHPEEGDLDLASESTAHAQSSKADSLLAVVKREPAEQPGDGERTDCEPVGLEPAVEQSSAASELAEASSEELAEAPTEAPSPEAR
DSKEDGRKFDFDACNEVPPAPKESSTSEGADQKMSSPEDDSDTKRLSKEEKGRSSCGRNFWVSGLSSTTRATDLKNLFSKYGKVVGA
KVVTNARSPGARCYGFVTMSTAEEATKCINHLHKTELHGKMISVEKAKNEPVGKKTSDKRDSDGKKEKSSNSDRSTNLKRDDKCDRK
DDAKKGDDGSGEKSKDQDDQKPGPSERSRATKSGSRGTERTVVMDKSKGVPVISVKTSGSKERASKSQDRKSASREKRSVVSFDKVK
EPRKSRDSESHSRVRERSEREQRMQAQWEREERERLEIARERLAFQRQRLERERMERERLERERMHVEHERRREQERIHREREELRR

QQELRYEQERRPAVRRPYDLDRRDDAYWPEAKRAALDERYHSDFNRQDRFHDFDHRDRGRYPDHSVDRREGSRSMMGEREGQHYPER
HGGPERHGRDSRDGWGGYGSDKRMSEGRGLPPPPRRDWGDHGRREDDRSWQGTADGGMMDRDHKRWQGGERSMSGHSGPGHMMNRGG
MSGRGSFAPGGASRGHPIPHGGMQGGFGGQSRGSRPSDARFTRRY
>sp|E5RJM6|ANR65_HUMAN
MDSQRPEPREEEEEEQELRWMELDSEEALGTRTEGPSVVQGWGHLLQAVWRGPAGLVTQLLRQGASVEERDHAGRTPLHLAVLRGHA
PLVRLLLQRGAPVGAVDRAGRTALHEAAWHGHSRVAELLLQRGASAAARSGTGLTPLHWAAALGHTLLAARLLEAPGPGPAAAEAED
ARGWTAAHWAAAGGRLAVLELLAAGGAGLDGALLVAAAAGRGAALRFLLARGARVDARDGAGATALGLAAALGRSQDIEVLLGHGAD
PGIRDRHGRSALHRAAARGHLLAVQLLVTQGAEVDARDTLGLTPLHHASREGHVEVAGCLLDRGAQVDATGWLRKTPLHLAAERGHG
PTVGLLLSRGASPTLRTQWAEVAQMPEGDLPQALPELGGGEKECEGIESTG
>sp|Q8NCQ5|FBX15_HUMAN
MATGRGRILQQHWLGLQTLRGPSRGGGAARGRARAFGCRKGPGVKLSAGSAALRCHAGGGQHWESSFSCCSGFLDGMPSEILLKIFS
YLDAVSLLCTGCVSRRFYHLANDNFIWIGIYSTAFSPARSNWKFNSVEKIAMSMSFLSVQDKEAGYWKKEYITKQIASVKAALADIL
KPVNPYTGLPVKTKEALRIFGLGWAIILKEKGGKEYIMEHVDLSINDTSVTVIWYGKKWPCLASLSTLDLCGMTPVFTDWYKTPTKH
RLRWHSLIAKYNLSHLTISTMIGCDRLIRIFCLHPGLLVGVWKKEEELAFVMANLHFHHLVERSTLGSATIPYELPPHSPFLDDSPE
YGLHGYQLHVDLHSGGVFYLCGTFRNLFTKRGNIENGHVKLIVIHLKNNREHLPLIGKVGLSWKTDIFDGCIKSCSMMDVTLLDEHG
KPFWCFSSPVCLRSPATPSDSSSFLGQTYNVDYVDAEGRVHVELVWIRETEEYLIVNLVLYLSIAKINHWFGTEY
>sp|Q52M75|CE027_HUMAN
MTPCLESFCQRAKDCKQNIPLPFLIQDWPLSESKPQGQDFWTPQPLQAAAPSHEFQQVALHPARTRLASRPQGGRLMSSREVGLRAP
RCSCGKRKR
>sp|Q8NH92|OR1S1_HUMAN
MKTFSSFLQIGRNMHQGNQTTITEFILLGFFKQDEHQNLLFVLFLGMYLVTVIGNGLIIVAISLDTYLHTPMYLFLANLSFADISSI
SNSVPKMLVNIQTKSQSISYESCITQMYFSIVFVVIDNLLLGTMAYDHFVAICHPLNYTILMRPRFGILLTVISWFLSNIIALTHTL
LLIQLLFCNHNTLPHFFCDLAPLLKLSCSDTLINELVLFIVGLSVIIFPFTLSFFSYVCIIRAVLRVSSTQGKWKAFSTCGSHLTVV
LLFYGTIVGVYFFPSSTHPEDTDKIGAVLFTVVTPMINPFIYSLRNKDMKGALRKLINRKISSL
>sp|Q9NTJ3|SMC4_HUMAN
MPRKGTQPSTARRREEGPPPPSPDGASSDAEPEPPSGRTESPATAAETASEELDNRSLEEILNSIPPPPPPAMTNEAGAPRLMITHI
VNQNFKSYAGEKILGPFHKRFSCIIGPNGSGKSNVIDSMLFVFGYRAQKIRSKKLSVLIHNSDEHKDIQSCTVEVHFQKIIDKEGDD
YEVIPNSNFYVSRTACRDNTSVYHISGKKKTFKDVGNLLRSHGIDLDHNRFLILQGEVEQIAMMKPKGQTEHDEGMLEYLEDIIGCG
RLNEPIKVLCRRVEILNEHRGEKLNRVKMVEKEKDALEGEKNIAIEFLTLENEIFRKKNHVCQYYIYELQKRIAEMETQKEKIHEDT
KEINEKSNILSNEMKAKNKDVKDTEKKLNKITKFIEENKEKFTQLDLEDVQVREKLKHATSKAKKLEKQLQKDKEKVEEFKSIPAKS
NNIINETTTRNNALEKEKEKEEKKLKEVMDSLKQETQGLQKEKESREKELMGFSKSVNEARSKMDVAQSELDIYLSRHNTAVSQLTK
AKEALIAASETLKERKAAIRDIEGKLPQTEQELKEKEKELQKLTQEETNFKSLVHDLFQKVEEAKSSLAMNRSRGKVLDAIIQEKKS
GRIPGIYGRLGDLGAIDEKYDVAISSCCHALDYIVVDSIDIAQECVNFLKRQNIGVATFIGLDKMAVWAKKMTEIQTPENTPRLFDL
VKVKDEKIRQAFYFALRDTLVADNLDQATRVAYQKDRRWRVVTLQGQIIEQSGTMTGGGSKVMKGRMGSSLVIEISEEEVNKMESQL
QNDSKKAMQIQEQKVQLEERVVKLRHSEREMRNTLEKFTASIQRLIEQEEYLNVQVKELEANVLATAPDKKKQKLLEENVSAFKTEY
DAVAEKAGKVEAEVKRLHNTIVEINNHKLKAQQDKLDKINKQLDECASAITKAQVAIKTADRNLQKAQDSVLRTEKEIKDTEKEVDD
LTAELKSLEDKAAEVVKNTNAAEESLPEIQKEHRNLLQELKVIQENEHALQKDALSIKLKLEQIDGHIAEHNSKIKYWHKEISKISL
HPIEDNPIEEISVLSPEDLEAIKNPDSITNQIALLEARCHEMKPNLGAIAEYKKKEELYLQRVAELDKITYERDSFRQAYEDLRKQR
LNEFMAGFYIITNKLKENYQMLTLGGDAELELVDSLDPFSEGIMFSVRPPKKSWKKIFNLSGGEKTLSSLALVFALHHYKPTPLYFM
DEIDAALDFKNVSIVAFYIYEQTKNAQFIIISLRNNMFEISDRLIGIYKTYNITKSVAVNPKEIASKGLC
>sp|Q86X53|ERIC1_HUMAN
MAAHRKHVFVEKVLQRLFPPVPSGQGKREPQTLAVQNPPKKVTSEKVSQKHAEPLTDTGSETPTARRLYTASGPPEGYVPCWPEPSS
CGSPENASSGDDTEDQDPHDQPKRRRIRKHKSKKKFKNPNNVLIEQAELEKQQSLLQEKSQRQHTDGTTISKNKKRKLKKKQQIKRK
KAAGLAAKAAGVSFMYQPEDSSNEGEGVGEACEEDGVDTSEEDPTLAGEEDVKDTREEDGADASEEDLTRARQEEGADASEEDPTPA

GEEDVKDAREEDGVDTIEEDLTRAGEEDGKDTREEDGADASEEDPTWAGEEEGADSGEEDGADASEEDDTITNEKAHSILNFLKSTQ
EMYFYDGVSRDAASAALADAAEELLDRLASHSMLPSDVSILYHMKTLLLLQDTERLKHALEMFPEHCTMPPDHARVISAFFSYWITH
ILPEKSSD
>sp|P19883|FST_HUMAN
MVRARHQPGGLCLLLLLLCQFMEDRSAQAGNCWLRQAKNGRCQVLYKTELSKEECCSTGRLSTSWTEEDVNDNTLFKWMIFNGGAPN
CIPCKETCENVDCGPGKKCRMNKKNKPRCVCAPDCSNITWKGPVCGLDGKTYRNECALLKARCKEQPELEVQYQGRCKKTCRDVFCP
GSSTCVVDQTNNAYCVTCNRICPEPASSEQYLCGNDGVTYSSACHLRKATCLLGRSIGLAYEGKCIKAKSCEDIQCTGGKKCLWDFK
VGRGRCSLCDELCPDSKSDEPVCASDNATYASECAMKEAACSSGVLLEVKHSGSCNSISEDTEEEEEDEDQDYSFPISSILEW
>sp|P12259|FA5_HUMAN
MFPGCPRLWVLVVLGTSWVGWGSQGTEAAQLRQFYVAAQGISWSYRPEPTNSSLNLSVTSFKKIVYREYEPYFKKEKPQSTISGLLG
PTLYAEVGDIIKVHFKNKADKPLSIHPQGIRYSKLSEGASYLDHTFPAEKMDDAVAPGREYTYEWSISEDSGPTHDDPPCLTHIYYS
HENLIEDFNSGLIGPLLICKKGTLTEGGTQKTFDKQIVLLFAVFDESKSWSQSSSLMYTVNGYVNGTMPDITVCAHDHISWHLLGMS
SGPELFSIHFNGQVLEQNHHKVSAITLVSATSTTANMTVGPEGKWIISSLTPKHLQAGMQAYIDIKNCPKKTRNLKKITREQRRHMK
RWEYFIAAEEVIWDYAPVIPANMDKKYRSQHLDNFSNQIGKHYKKVMYTQYEDESFTKHTVNPNMKEDGILGPIIRAQVRDTLKIVF
KNMASRPYSIYPHGVTFSPYEDEVNSSFTSGRNNTMIRAVQPGETYTYKWNILEFDEPTENDAQCLTRPYYSDVDIMRDIASGLIGL
LLICKSRSLDRRGIQRAADIEQQAVFAVFDENKSWYLEDNINKFCENPDEVKRDDPKFYESNIMSTINGYVPESITTLGFCFDDTVQ
WHFCSVGTQNEILTIHFTGHSFIYGKRHEDTLTLFPMRGESVTVTMDNVGTWMLTSMNSSPRSKKLRLKFRDVKCIPDDDEDSYEIF
EPPESTVMATRKMHDRLEPEDEESDADYDYQNRLAAALGIRSFRNSSLNQEEEEFNLTALALENGTEFVSSNTDIIVGSNYSSPSNI
SKFTVNNLAEPQKAPSHQQATTAGSPLRHLIGKNSVLNSSTAEHSSPYSEDPIEDPLQPDVTGIRLLSLGAGEFKSQEHAKHKGPKV
ERDQAAKHRFSWMKLLAHKVGRHLSQDTGSPSGMRPWEDLPSQDTGSPSRMRPWKDPPSDLLLLKQSNSSKILVGRWHLASEKGSYE
IIQDTDEDTAVNNWLISPQNASRAWGESTPLANKPGKQSGHPKFPRVRHKSLQVRQDGGKSRLKKSQFLIKTRKKKKEKHTHHAPLS
PRTFHPLRSEAYNTFSERRLKHSLVLHKSNETSLPTDLNQTLPSMDFGWIASLPDHNQNSSNDTGQASCPPGLYQTVPPEEHYQTFP
IQDPDQMHSTSDPSHRSSSPELSEMLEYDRSHKSFPTDISQMSPSSEHEVWQTVISPDLSQVTLSPELSQTNLSPDLSHTTLSPELI
QRNLSPALGQMPISPDLSHTTLSPDLSHTTLSLDLSQTNLSPELSQTNLSPALGQMPLSPDLSHTTLSLDFSQTNLSPELSHMTLSP
ELSQTNLSPALGQMPISPDLSHTTLSLDFSQTNLSPELSQTNLSPALGQMPLSPDPSHTTLSLDLSQTNLSPELSQTNLSPDLSEMP
LFADLSQIPLTPDLDQMTLSPDLGETDLSPNFGQMSLSPDLSQVTLSPDISDTTLLPDLSQISPPPDLDQIFYPSESSQSLLLQEFN
ESFPYPDLGQMPSPSSPTLNDTFLSKEFNPLVIVGLSKDGTDYIEIIPKEEVQSSEDDYAEIDYVPYDDPYKTDVRTNINSSRDPDN
IAAWYLRSNNGNRRNYYIAAEEISWDYSEFVQRETDIEDSDDIPEDTTYKKVVFRKYLDSTFTKRDPRGEYEEHLGILGPIIRAEVD
DVIQVRFKNLASRPYSLHAHGLSYEKSSEGKTYEDDSPEWFKEDNAVQPNSSYTYVWHATERSGPESPGSACRAWAYYSAVNPEKDI
HSGLIGPLLICQKGILHKDSNMPMDMREFVLLFMTFDEKKSWYYEKKSRSSWRLTSSEMKKSHEFHAINGMIYSLPGLKMYEQEWVR
LHLLNIGGSQDIHVVHFHGQTLLENGNKQHQLGVWPLLPGSFKTLEMKASKPGWWLLNTEVGENQRAGMQTPFLIMDRDCRMPMGLS
TGIISDSQIKASEFLGYWEPRLARLNNGGSYNAWSVEKLAAEFASKPWIQVDMQKEVIITGIQTQGAKHYLKSCYTTEFYVAYSSNQ
INWQIFKGNSTRNVMYFNGNSDASTIKENQFDPPIVARYIRISPTRAYNRPTLRLELQGCEVNGCSTPLGMENGKIENKQITASSFK
KSWWGDYWEPFRARLNAQGRVNAWQAKANNNKQWLEIDLLKIKKITAIITQGCKSLSSEMYVKSYTIHYSEQGVEWKPYRLKSSMVD
KIFEGNTNTKGHVKNFFNPPIISRFIRVIPKTWNQSIALRLELFGCDIY
>sp|Q9NYF8|BCLF1_HUMAN
MGRSNSRSHSSRSKSRSQSSSRSRSRSHSRKKRYSSRSRSRTYSRSRSRDRMYSRDYRRDYRNNRGMRRPYGYRGRGRGYYQGGGGR
YHRGGYRPVWNRRHSRSPRRGRSRSRSPKRRSVSSQRSRSRSRRSYRSSRSPRSSSSRSSSPYSKSPVSKRRGSQEKQTKKAEGEPQ
EESPLKSKSQEEPKDTFEHDPSESIDEFNKSSATSGDIWPGLSAYDNSPRSPHSPSPIATPPSQSSSCSDAPMLSTVHSAKNTPSQH
SHSIQHSPERSGSGSVGNGSSRYSPSQNSPIHHIPSRRSPAKTIAPQNAPRDESRGRSSFYPDGGDQETAKTGKFLKRFTDEESRVF
LLDRGNTRDKEASKEKGSEKGRAEGEWEDQEALDYFSDKESGKQKFNDSEGDDTEETEDYRQFRKSVLADQGKSFATASHRNTEEEG
LKYKSKVSLKGNRESDGFREEKNYKLKETGYVVERPSTTKDKHKEEDKNSERITVKKETQSPEQVKSEKLKDLFDYSPPLHKNLDAR
EKSTFREESPLRIKMIASDSHRPEVKLKMAPVPLDDSNRPASLTKDRLLASTLVHSVKKEQEFRSIFDHIKLPQASKSTSESFIQHI
VSLVHHVKEQYFKSAAMTLNERFTSYQKATEEHSTRQKSPEIHRRIDISPSTLRKHTRLAGEERVFKEENQKGDKKLRCDSADLRHD

IDRRRKERSKERGDSKGSRESSGSRKQEKTPKDYKEYKSYKDDSKHKREQDHSRSSSSSASPSSPSSREEKESKKEREEEFKTHHEM
KEYSGFAGVSRPRGTFFRIRGRGRARGVFAGTNTGPNNSNTTFQKRPKEEEWDPEYTPKSKKYFLHDDRDDGVDYWAKRGRGRGTFQ
RGRGRFNFKKSGSSPKWTHDKYQGDGIVEDEEETMENNEEKKDRRKEEKE
>sp|Q9BYJ9|YTHD1_HUMAN
MSATSVDTQRTKGQDNKVQNGSLHQKDTVHDNDFEPYLTGQSNQSNSYPSMSDPYLSSYYPPSIGFPYSLNEAPWSTAGDPPIPYLT
TYGQLSNGDHHFMHDAVFGQPGGLGNNIYQHRFNFFPENPAFSAWGTSGSQGQQTQSSAYGSSYTYPPSSLGGTVVDGQPGFHSDTL
SKAPGMNSLEQGMVGLKIGDVSSSAVKTVGSVVSSVALTGVLSGNGGTNVNMPVSKPTSWAAIASKPAKPQPKMKTKSGPVMGGGLP
PPPIKHNMDIGTWDNKGPVPKAPVPQQAPSPQAAPQPQQVAQPLPAQPPALAQPQYQSPQQPPQTRWVAPRNRNAAFGQSGGAGSDS
NSPGNVQPNSAPSVESHPVLEKLKAAHSYNPKEFEWNLKSGRVFIIKSYSEDDIHRSIKYSIWCSTEHGNKRLDSAFRCMSSKGPVY
LLFSVNGSGHFCGVAEMKSPVDYGTSAGVWSQDKWKGKFDVQWIFVKDVPNNQLRHIRLENNDNKPVTNSRDTQEVPLEKAKQVLKI
ISSYKHTTSIFDDFAHYEKRQEEEEVVRKERQSRNKQ
>sp|Q70CQ3|UBP30_HUMAN
MLSSRAEAAMTAADRAIQRFLRTGAAVRYKVMKNWGVIGGIAAALAAGIYVIWGPITERKKRRKGLVPGLVNLGNTCFMNSLLQGLS
ACPAFIRWLEEFTSQYSRDQKEPPSHQYLSLTLLHLLKALSCQEVTDDEVLDASCLLDVLRMYRWQISSFEEQDAHELFHVITSSLE
DERDRQPRVTHLFDVHSLEQQSEITPKQITCRTRGSPHPTSNHWKSQHPFHGRLTSNMVCKHCEHQSPVRFDTFDSLSLSIPAATWG
HPLTLDHCLHHFISSESVRDVVCDNCTKIEAKGTLNGEKVEHQRTTFVKQLKLGKLPQCLCIHLQRLSWSSHGTPLKRHEHVQFNEF
LMMDIYKYHLLGHKPSQHNPKLNKNPGPTLELQDGPGAPTPVLNQPGAPKTQIFMNGACSPSLLPTLSAPMPFPLPVVPDYSSSTYL
FRLMAVVVHHGDMHSGHFVTYRRSPPSARNPLSTSNQWLWVSDDTVRKASLQEVLSSSAYLLFYERVLSRMQHQSQECKSEE
>sp|Q04917|1433F_HUMAN
MGDREQLLQRARLAEQAERYDDMASAMKAVTELNEPLSNEDRNLLSVAYKNVVGARRSSWRVISSIEQKTMADGNEKKLEKVKAYRE
KIEKELETVCNDVLSLLDKFLIKNCNDFQYESKVFYLKMKGDYYRYLAEVASGEKKNSVVEASEAAYKEAFEISKEQMQPTHPIRLG
LALNFSVFYYEIQNAPEQACLLAKQAFDDAIAELDTLNEDSYKDSTLIMQLLRDNLTLWTSDQQDEEAGEGN
>sp|O60330|PCDGC_HUMAN
MIPARLHRDYKGLVLLGILLGTLWETGCTQIRYSVPEELEKGSRVGDISRDLGLEPRELAERGVRIIPRGRTQLFALNPRSGSLVTA
GRIDREELCMGAIKCQLNLDILMEDKVKIYGVEVEVRDINDNAPYFRESELEIKISENAATEMRFPLPHAWDPDIGKNSLQSYELSP
NTHFSLIVQNGADGSKYPELVLKRALDREEKAAHHLVLTASDGGDPVRTGTARIRVMVLDANDNAPAFAQPEYRASVPENLALGTQL
LVVNATDPDEGVNAEVRYSFRYVDDKAAQVFKLDCNSGTISTIGELDHEESGFYQMEVQAMDNAGYSARAKVLITVLDVNDNAPEVV
LTSLASSVPENSPRGTLIALLNVNDQDSEENGQVICFIQGNLPFKLEKSYGNYYSLVTDIVLDREQVPSYNITVTATDRGTPPLSTE
THISLNVADTNDNPPVFPQASYSAYIPENNPRGVSLVSVTAHDPDCEENAQITYSLAENTIQGASLSSYVSINSDTGVLYALSSFDY
EQFRDLQVKVMARDNGHPPLSSNVSLSLFVLDQNDNAPEILYPALPTDGSTGVELAPRSAEPGYLVTKVVAVDRDSGQNAWLSYRLL
KASEPGLFSVGLHTGEVRTARALLDRDALKQSLVVAVQDHGQPPLSATVTLTVAVADSIPQVLADLGSLESPANSETSDLTLYLVVA
VAAVSCVFLAFVILLLALRLRRWHKSRLLQASGGGLTGAPASHFVGVDGVQAFLQTYSHEVSLTTDSRKSHLIFPQPNYADMLVSQE
SFEKSEPLLLSGDSVFSKDSHGLIEQAPPNTDWRFSQAQRPGTSGSQNGDDTGTWPNNQFDTEMLQAMILASASEAADGSSTLGGGA
GTMGLSARYGPQFTLQHVPDYRQNVYIPGSNATLTNAAGKRDGKAPAGGNGNKKKSGKKEKK
>sp|P30556|AGTR1_HUMAN
MILNSSTEDGIKRIQDDCPKAGRHNYIFVMIPTLYSIIFVVGIFGNSLVVIVIYFYMKLKTVASVFLLNLALADLCFLLTLPLWAVY
TAMEYRWPFGNYLCKIASASVSFNLYASVFLLTCLSIDRYLAIVHPMKSRLRRTMLVAKVTCIIIWLLAGLASLPAIIHRNVFFIEN
TNITVCAFHYESQNSTLPIGLGLTKNILGFLFPFLIILTSYTLIWKALKKAYEIQKNKPRNDDIFKIIMAIVLFFFFSWIPHQIFTF
LDVLIQLGIIRDCRIADIVDTAMPITICIAYFNNCLNPLFYGFLGKKFKRYFLQLLKYIPPKAKSHSNLSTKMSTLSYRPSDNVSSS
TKKPAPCFEVE
>sp|Q9NVD7|PARVA_HUMAN
MATSPQKSPSVPKSPTPKSPPSRKKDDSFLGKLGGTLARRKKAKEVSELQEEGMNAINLPLSPIPFELDPEDTMLEENEVRTMVDPN
SRSDPKLQELMKVLIDWINDVLVGERIIVKDLAEDLYDGQVLQKLFEKLESEKLNVAEVTQSEIAQKQKLQTVLEKINETLKLPPRS
IKWNVDSVHAKSLVAILHLLVALSQYFRAPIRLPDHVSIQVVVVQKREGILQSRQIQEEITGNTEALSGRHERDAFDTLFDHAPDKL

NVVKKTLITFVNKHLNKLNLEVTELETQFADGVYLVLLMGLLEGYFVPLHSFFLTPDSFEQKVLNVSFAFELMQDGGLEKPKPRPED
IVNCDLKSTLRVLYNLFTKYRNVE
>sp|Q08462|ADCY2_HUMAN
MWQEAMRRRRYLRDRSEEAAGGGDGLPRSRDWLYESYYCMSQQHPLIVFLLLIVMGSCLALLAVFFALGLEVEDHVAFLITVPTALA
IFFAIFILVCIESVFKKLLRLFSLVIWICLVAMGYLFMCFGGTVSPWDQVSFFLFIIFVVYTMLPFNMRDAIIASVLTSSSHTIVLS
VCLSATPGGKEHLVWQILANVIIFICGNLAGAYHKHLMELALQQTYQDTCNCIKSRIKLEFEKRQQERLLLSLLPAHIAMEMKAEII
QRLQGPKAGQMENTNNFHNLYVKRHTNVSILYADIVGFTRLASDCSPGELVHMLNELFGKFDQIAKENECMRIKILGDCYYCVSGLP
ISLPNHAKNCVKMGLDMCEAIKKVRDATGVDINMRVGVHSGNVLCGVIGLQKWQYDVWSHDVTLANHMEAGGVPGRVHISSVTLEHL
NGAYKVEEGDGDIRDPYLKQHLVKTYFVINPKGERRSPQHLFRPRHTLDGAKMRASVRMTRYLESWGAAKPFAHLHHRDSMTTENGK
ISTTDVPMGQHNFQNRTLRTKSQKKRFEEELNERMIQAIDGINAQKQWLKSEDIQRISLLFYNKVLEKEYRATALPAFKYYVTCACL
IFFCIFIVQILVLPKTSVLGISFGAAFLLLAFILFVCFAGQLLQCSKKASPLLMWLLKSSGIIANRPWPRISLTIITTAIILMMAVF
NMFFLSDSEETIPPTANTTNTSFSASNNQVAILRAQNLFFLPYFIYSCILGLISCSVFLRVNYELKMLIMMVALVGYNTILLHTHAH
VLGDYSQVLFERPGIWKDLKTMGSVSLSIFFITLLVLGRQNEYYCRLDFLWKNKFKKEREEIETMENLNRVLLENVLPAHVAEHFLA
RSLKNEELYHQSYDCVCVMFASIPDFKEFYTESDVNKEGLECLRLLNEIIADFDDLLSKPKFSGVEKIKTIGSTYMAATGLSAVPSQ
EHSQEPERQYMHIGTMVEFAFALVGKLDAINKHSFNDFKLRVGINHGPVIAGVIGAQKPQYDIWGNTVNVASRMDSTGVLDKIQVTE
ETSLVLQTLGYTCTCRGIINVKGKGDLKTYFVNTEMSRSLSQSNVAS
>sp|Q53F19|NCBP3_HUMAN
MAAVRGLRVSVKAEAPAGPALGLPSPEAESGVDRGEPEPMEVEEGELEIVPVRRSLKELIPDTSRRYENKAGSFITGIDVTSKEAIE
KKEQRAKRFHFRSEVNLAQRNVALDRDMMKKAIPKVRLETIYICGVDEMSTQDVFSYFKEYPPAHIEWLDDTSCNVVWLDEMTATRA
LINMSSLPAQDKIRSRDASEDKSAEKRKKDKQEDSSDDDEAEEGEVEDENSSDVELDTLSQVEEESLLRNDLRPANKLAKGNRLFMR
FATKDDKKELGAARRSQYYMKYGNPNYGGMKGILSNSWKRRYHSRRIQRDVIKKRALIGDDVGLTSYKHRHSGLVNVPEEPIEEEEE
EEEEEEEEEEEDQDMDADDRVVVEYHEELPALKQPRERSASRRSSASSSDSDEMDYDLELKMISTPSPKKSMKMTMYADEVESQLKN
IRNSMRADSVSSSNIKNRIGNKLPPEKFADVRHLLDEKRQHSRPRPPVSSTKSDIRQRLGKRPHSPEKAFSSNPVVRREPSSDVHSR
LGVPRQDSKGLYADTREKKSGNLWTRLGSAPKTKEKNTKKVDHRAPGAEEDDSELQRAWGALIKEKEQSRQKKSRLDNLPSLQIEVS
RESSSGSEAES
>sp|Q07687|DLX2_HUMAN
MTGVFDSLVADMHSTQIAASSTYHQHQQPPSGGGAGPGGNSSSSSSLHKPQESPTLPVSTATDSSYYTNQQHPAGGGGGGGSPYAHM
GSYQYQASGLNNVPYSAKSSYDLGYTAAYTSYAPYGTSSSPANNEPEKEDLEPEIRIVNGKPKKVRKPRTIYSSFQLAALQRRFQKT
QYLALPERAELAASLGLTQTQVKIWFQNRRSKFKKMWKSGEIPSEQHPGASASPPCASPPVSAPASWDFGVPQRMAGGGGPGSGGSG
AGSSGSSPSSAASAFLGNYPWYHQTSGSASHLQATAPLLHPTQTPQPHHHHHHHGGGGAPVSAGTIF
>sp|Q8N1L9|BATF2_HUMAN
MHLCGGNGLLTQTDPKEQQRQLKKQKNRAAAQRSRQKHTDKADALHQQHESLEKDNLALRKEIQSLQAELAWWSRTLHVHERLCPMD
CASCSAPGLLGCWDQAEGLLGPGPQGQHGCREQLELFQTPGSCYPAQPLSPGPQPHDSPSLLQCPLPSLSLGPAVVAEPPVQLSPSP
LLFASHTGSSLQGSSSKLSALQPSLTAQTAPPQPLELEHPTRGKLGSSPDNPSSALGLARLQSREHKPALSAATWQGLVVDPSPHPL
LAFPLLSSAQVHF
>sp|Q6ZS46|YF009_HUMAN
MQTCSGKGIKMAFLDVQSSSTPQSLPLLLFSHREGGGRGAGDPGAPAVVPAPVSAPRPASSPARSESRSPPLTLISRHITCCSCESP
GDVLLSGRGGGGGGGGGARTGGGEGEDRRPRPDFSRVTAVAIPGWMEVESPPHPPPQPQVCPTPSQGAPGHGRAGLPEGKGPGGRDW
LRSQSSRCSRATLFGHRAPSPAAPRRGRLPAPGFPSLHSAVSLF
>sp|P50221|MEOX1_HUMAN
MDPAASSCMRSLQPPAPVWGCLRNPHSEGNGASGLPHYPPTPFSFHQKPDFLATATAAYPDFSASCLAATPHSLPQEEHIFTEQHPA
FPQSPNWHFPVSDARRRPNSGPAGGSKEMGTSSLGLVDTTGGPGDDYGVLGSTANETEKKSSRRRKESSDNQENRGKPEGSSKARKE
RTAFTKEQLRELEAEFAHHNYLTRLRRYEIAVNLDLSERQVKVWFQNRRMKWKRVKGGQPISPNGQDPEDGDSTASPSSE
>sp|Q7Z3Z0|K1C25_HUMAN

MSLRLSSASRRSCPRPTTGSLRLYGGGTSFGTGNSCGISGIGSGFSSAFGGSSSGGNTGGGNPCAGFTVNERGLLSGNEKVTMQNLN
DRLASYLDSVHALEEANADLEQKIKGWYEKFGPGSCRGLDHDYSRYFPIIDDLKNQIIASTTSNANAVLQIDNARLTADDFRLKYEN
ELALHQSVEADVNGLRRVLDEITLCRTDLEIQYETLSEEMTYLKKNHKEEMQVLQCAAGGNVNVEMNAAPGVDLTVLLNNMRAEYEA
LAEQNRRDAEAWFNEKSASLQQQISEDVGATTSARNELTEMKRTLQTLEIELQSLLATKHSLECSLTETESNYCAQLAQIQAQIGAL
EEQLHQVRTETEGQKLEYEQLLDIKLHLEKEIETYCLLIGGDDGACKSGGYKSKDYGSGNVGSQVKDPAKAIVVKKVLEEVDQRSKI
LTTRLHSLEEKSQSN
>sp|O14964|HGS_HUMAN
MGRGSGTFERLLDKATSQLLLETDWESILQICDLIRQGDTQAKYAVNSIKKKVNDKNPHVALYALEVMESVVKNCGQTVHDEVANKQ
TMEELKDLLKRQVEVNVRNKILYLIQAWAHAFRNEPKYKVVQDTYQIMKVEGHVFPEFKESDAMFAAERAPDWVDAEECHRCRVQFG
VMTRKHHCRACGQIFCGKCSSKYSTIPKFGIEKEVRVCEPCYEQLNRKAEGKATSTTELPPEYLTSPLSQQSQLPPKRDETALQEEE
ELQLALALSQSEAEEKERLRQKSTYTSYPKAEPMPSASSAPPASSLYSSPVNSSAPLAEDIDPELARYLNRNYWEKKQEEARKSPTP
SAPVPLTEPAAQPGEGHAAPTNVVENPLPETDSQPIPPSGGPFSEPQFHNGESEESHEQFLKALQNAVTTFVNRMKSNHMRGRSITN
DSAVLSLFQSINGMHPQLLELLNQLDERRLYYEGLQDKLAQIRDARGALSALREEHREKLRRAAEEAERQRQIQLAQKLEIMRQKKQ
EYLEVQRQLAIQRLQEQEKERQMRLEQQKQTVQMRAQMPAFPLPYAQLQAMPAAGGVLYQPSGPASFPSTFSPAGSVEGSPMHGVYM
SQPAPAAGPYPSMPSTAADPSMVSAYMYPAGATGAQAAPQAQAGPTASPAYSSYQPTPTAGYQNVASQAPQSLPAISQPPQSSTMGY
MGSQSVSMGYQPYNMQNLMTTLPSQDASLPPQQPYIAGQQPMYQQMAPSGGPPQQQPPVAQQPQAQGPPAQGSEAQLISFD
>sp|Q9GZY8|MFF_HUMAN
MSKGTSSDTSLGRVSRAAFPSPTAAEMAEISRIQYEMEYTEGISQRMRVPEKLKVAPPNADLEQGFQEGVPNASVIMQVPERIVVAG
NNEDVSFSRPADLDLIQSTPFKPLALKTPPRVLTLSERPLDFLDLERPPTTPQNEEIRAVGRLKRERSMSENAVRQNGQLVRNDSLW
HRSDSAPRNKISRFQAPISAPEYTVTPSPQQARVCPPHMLPEDGANLSSARGILSLIQSSTRRAYQQILDVLDENRRPVLRGGSAAA
TSNPHHDNVRYGISNIDTTIEGTSDDLTVVDAASLRRQIIKLNRRLQLLEEENKERAKREMVMYSITVAFWLLNSWLWFRR
>sp|Q9GZW5|SCND2_HUMAN
MAVAVDQQIQTPSVQDLQIVKLEEDSHWEQEISLQGNYPGPETSCQSFWHFRYQEASRPREALLQLQKLCCQWLRPEKCTKEQILEL
LVLEQFPTVLLQEIQIWVRQQHPESGEEAVALVEDLQKEPGRQRLEPRARPSGRTPPAQLRSPWPMTAAGPASRARASETGSTASCA
GRWRTCCAAAAAPSAARSASARTGRSTSSCARAARAPSATEGALTRTPAPRRPLQRRRPGTGPWRPGRQRGAGTAPPGTQPRQRPSP
RPTPRRPRPRLARPRAGQKPWLLVIRSMERVMYVMLIIQMETEDV
>sp|Q14406|CSHL_HUMAN
MAAGSRTSLLLAFALLCLPWLQEAGAVQTVPLSRLFKEAMLQAHRAHQLAIDTYQEFISSWGMEAYITKEQKYSFLHDSQTSFCFSD
SIPTSSNMEETQQKSNLELLHISLLLIESRLEPVRFLRSTFTNNLVYDTSDSDDYHLLKDLEEGIQMLMGRLEDGSHLTGQTLKQTY
SKFDTNSHNHDALLKNYGLLHCFRKDMDKVETFLRMVQCRSVEGSCGF
>sp|Q9UJK0|TSR3_HUMAN
MGRRRAARGPGAEGGRPRHLPTRSLEAFAEEVGAALQASVEPGAADGEGGPGPAALPCTLAMWELGHCDPRRCTGRKLARLGLVRCL
RLGHRFGGLVLSPVGKQYASPADRQLVAQSGVAVIDCSWARLDETPFGKMRGSHLRLLPYLVAANPVNYGRPYRLSCVEAFAATFCI
VGFPDLAVILLRKFKWGKGFLDLNRQLLDKYAACGSPEEVLQAEQEFLANAKESPQEEEIDPFDVDSGREFGNPNRPVASTRLPSDT
DDSDASEDPGPGAERGGASSSCCEEEQTQGRGAEARAPAEVWKGIKKRQRD
>sp|Q8NCM2|KCNH5_HUMAN
MPGGKRGLVAPQNTFLENIVRRSSESSFLLGNAQIVDWPVVYSNDGFCKLSGYHRADVMQKSSTCSFMYGELTDKKTIEKVRQTFDN
YESNCFEVLLYKKNRTPVWFYMQIAPIRNEHEKVVLFLCTFKDITLFKQPIEDDSTKGWTKFARLTRALTNSRSVLQQLTPMNKTEV
VHKHSRLAEVLQLGSDILPQYKQEAPKTPPHIILHYCAFKTTWDWVILILTFYTAIMVPYNVSFKTKQNNIAWLVLDSVVDVIFLVD
IVLNFHTTFVGPGGEVISDPKLIRMNYLKTWFVIDLLSCLPYDIINAFENVDEGISSLFSSLKVVRLLRLGRVARKLDHYLEYGAAV
LVLLVCVFGLVAHWLACIWYSIGDYEVIDEVTNTIQIDSWLYQLALSIGTPYRYNTSAGIWEGGPSKDSLYVSSLYFTMTSLTTIGF
GNIAPTTDVEKMFSVAMMMVGSLLYATIFGNVTTIFQQMYANTNRYHEMLNNVRDFLKLYQVPKGLSERVMDYIVSTWSMSKGIDTE
KVLSICPKDMRADICVHLNRKVFNEHPAFRLASDGCLRALAVEFQTIHCAPGDLIYHAGESVDALCFVVSGSLEVIQDDEVVAILGK
GDVFGDIFWKETTLAHACANVRALTYCDLHIIKREALLKVLDFYTAFANSFSRNLTLTCNLRKRIIFRKISDVKKEEEERLRQKNEV

TLSIPVDHPVRKLFQKFKQQKELRNQGSTQGDPERNQLQVESRSLQNGASITGTSVVTVSQITPIQTSLAYVKTSESLKQNNRDAME
LKPNGGADQKCLKVNSPIRMKNGNGKGWLRLKNNMGAHEEKKEDWNNVTKAESMGLLSEDPKSSDSENSVTKNPLRKTDSCDSGITK
SDLRLDKAGEARSPLEHSPIQADAKHPFYPIPEQALQTTLQEVKHELKEDIQLLSCRMTALEKQVAEILKILSEKSVPQASSPKSQM
PLQVPPQIPCQDIFSVSRPESPESDKDEIHF
>sp|A6NCF6|MA13P_HUMAN
MPHSQKSRHCELEQGLQAPKEAQGLVGVQVAEAEKVNTTASSSPSTLIQGTLEKVSASGTPGTPQSSQRVCSPCTTIKATPWNQSDE
SSRSQEKKDPGASQALMLEKKVDELVKFLSVKYTTKQPITEAEMLKGVIKEHKDHFPPIFMQAHECMEIVFGTDMKEVDPISHSCVL
LKSLDLTYDRRLSDDQGMPKTGLLILTFGVILMEANCASEEKIWEVLNIIRVYAGWKDFIYGEPRKLITRDLVQEKYLECCQVSNSD
PPRYKFPWGPRAHAETTKMKVLEFFSRVSGSDASSFPLLYEEALRDEKEKAQAIIATMGGTTLMASAHSWAKSSSFSCPE
>sp|O43390|HNRPR_HUMAN
MANQVNGNAVQLKEEEEPMDTSSVTHTEHYKTLIEAGLPQKVAERLDEIFQTGLVAYVDLDERAIDALREFNEEGALSVLQQFKESD
LSHVQNKSAFLCGVMKTYRQREKQGSKVQESTKGPDEAKIKALLERTGYTLDVTTGQRKYGGPPPDSVYSGVQPGIGTEVFVGKIPR
DLYEDELVPLFEKAGPIWDLRLMMDPLSGQNRGYAFITFCGKEAAQEAVKLCDSYEIRPGKHLGVCISVANNRLFVGSIPKNKTKEN
ILEEFSKVTEGLVDVILYHQPDDKKKNRGFCFLEYEDHKSAAQARRRLMSGKVKVWGNVVTVEWADPVEEPDPEVMAKVKVLFVRNL
ATTVTEEILEKSFSEFGKLERVKKLKDYAFVHFEDRGAAVKAMDEMNGKEIEGEEIEIVLAKPPDKKRKERQAARQASRSTAYEDYY
YHPPPRMPPPIRGRGRGGGRGGYGYPPDYYGYEDYYDDYYGYDYHDYRGGYEDPYYGYDDGYAVRGRGGGRGGRGAPPPPRGRGAPP
PRGRAGYSQRGAPLGPPRGSRGGRGGPAQQQRGRGSRGSRGNRGGNVGGKRKADGYNQPDSKRRQTNNQQNWGSQPIAQQPLQQGGD
YSGNYGYNNDNQEFYQDTYGQQWK
>sp|Q16537|2A5E_HUMAN
MSSAPTTPPSVDKVDGFSRKSVRKARQKRSQSSSQFRSQGKPIELTPLPLLKDVPSSEQPELFLKKLQQCCVIFDFMDTLSDLKMKE
YKRSTLNELVDYITISRGCLTEQTYPEVVRMVSCNIFRTLPPSDSNEFDPEEDEPTLEASWPHLQLVYEFFIRFLESQEFQPSIAKK
YIDQKFVLQLLELFDSEDPRERDYLKTVLHRIYGKFLGLRAFIRKQINNIFLRFVYETEHFNGVAELLEILGSIINGFALPLKAEHK
QFLVKVLIPLHTVRSLSLFHAQLAYCIVQFLEKDPSLTEPVIRGLMKFWPKTCSQKEVMFLGELEEILDVIEPSQFVKIQEPLFKQI
AKCVSSPHFQVAERALYYWNNEYIMSLIEENSNVILPIMFSSLYRISKEHWNPAIVALVYNVLKAFMEMNSTMFDELTATYKSDRQR
EKKKEKEREELWKKLEDLELKRGLRRDGIIPT
>sp|Q9BVG9|PTSS2_HUMAN
MRRGERRDAGGPRPESPVPAGRASLEEPPDGPSAGQATGPGEGRRSTESEVYDDGTNTFFWRAHTLTVLFILTCTLGYVTLLEETPQ
DTAYNTKRGIVASILVFLCFGVTQAKDGPFSRPHPAYWRFWLCVSVVYELFLIFILFQTVQDGRQFLKYVDPKLGVPLPERDYGGNC
LIYDPDNETDPFHNIWDKLDGFVPAHFLGWYLKTLMIRDWWMCMIISVMFEFLEYSLEHQLPNFSECWWDHWIMDVLVCNGLGIYCG
MKTLEWLSLKTYKWQGLWNIPTYKGKMKRIAFQFTPYSWVRFEWKPASSLRRWLAVCGIILVFLLAELNTFYLKFVLWMPPEHYLVL
LRLVFFVNVGGVAMREIYDFMDDPKPHKKLGPQAWLVAAITATELLIVVKYDPHTLTLSLPFYISQCWTLGSVLALTWTVWRFFLRD
ITLRYKETRWQKWQNKDDQGSTVGNGDQHPLGLDEDLLGPGVAEGEGAPTPN
>sp|Q7L592|NDUF7_HUMAN
MSVLLRSGLGPLCAVARAAIPFIWRGKYFSSGNEPAENPVTPMLRHLMYKIKSTGPITVAEYMKEVLTNPAKGYYVYRDMLGEKGDF
ITSPEISQIFGELLGIWFISEWMATGKSTAFQLVELGPGRGTLVGDILRVFTQLGSVLKNCDISVHLVEVSQKLSEIQALTLTKEKV
PLERNAGSPVYMKGVTKSGIPISWYRDLHDVPKGYSFYLAHEFFDVLPVHKFQKTPQGWREVFVDIDPQVSDKLRFVLAPSATPAEA
FIQHDETRDHVEVCPDAGVIIEELSQRIALTGGAALVADYGHDGTKTDTFRGFCDHKLHDVLIAPGTADLTADVDFSYLRRMAQGKV
ASLGPIKQHTFLKNMGIDVRLKVLLDKSNEPSVRQQLLQGYDMLMNPKKMGERFNFFALLPHQRLQGGRYQRNARQSKPFASVVAGF
SELAWQ
>sp|A6NC98|CC88B_HUMAN
MEGGKGPRLRDFLSGSLATWALGLAGLVGEAEDSEGEEEEEEEEEPPLWLEKRFLRLSDGALLLRVLGIIAPSSRGGPRMLRGLDGPA
AWRVWNLNHLWGRLRDFYQEELQLLILSPPPDLQTLGFDPLSEEAVEQLEGVLRLLLGASVQCEHRELFIRHIQGLSLEVQSELAAA
IQEVTQPGAGVVLALSGPDPGELAPAELEMLSRSLMGTLSKLARERDLGAQRLAELLLEREPLCLRPEAPSRAPAEGPSHHLALQLA
NAKAQLRRLRQELEEKAELLLDSQAEVQGLEAEIRRLRQEAQALSGQAKRAELYREEAEALRERAGRLPRLQEELRRCRERLQAAEA

YKSQLEEERVLSGVLEASKALLEEQLEAARERCARLHETQRENLLLRTRLGEAHAELDSLRHQVDQLAEENVELELELQRSLEPPPG
SPGEAPLAGAAPSLQDEVREAEAGRLRTLERENRELRGLLQVLQGQPGGQHPLLEAPREDPVLPVLEEAPQTPVAFDHSPQGLVQKA
RDGGPQALDLAPPALDSVLEASAECPQAPDSDPQEAESPLQAAAMDPQASDWSPQESGSPVETQESPEKAGRRSSLQSPASVAPPQG
PGTKIQAPQLLGGETEGREAPQGELVPEAWGLRQEGPEHKPGPSEPSSVQLEEQEGPNQGLDLATGQAEAREHDQRLEGTVRDPAWQ
KPQQKSEGALEVQVWEGPIPGESLASGVAEQEALREEVAQLRRKAEALGDELEAQARKLEAQNTEAARLSKELAQARRAEAEAHREA
EAQAWEQARLREAVEAAGQELESASQEREALVEALAAAGRERRQWEREGSRLRAQSEAAEERMQVLESEGRQHLEEAERERREKEAL
QAELEKAVVRGKELGDRLEHLQRELEQAALERQEFLREKESQHQRYQGLEQRLEAELQAAATSKEEALMELKTRALQLEEELFQLRQ
GPAGLGPKKRAEPQLVETQNVRLIEVERSNAMLVAEKAALQGQLQHLEGQLGSLQGRAQELLLQSQRAQEHSSRLQAEKSVLEIQGQ
ELHRKLEVLEEEVRAARQSQEETRGQQQALLRDHKALAQLQRRQEAELEGLLVRHRDLKANMRALELAHRELQGRHEQLQAQRASVE
AQEVALLAERERLMQDGHRQRGLEEELRRLQSEHDRAQMLLAELSRERGELQGERGELRGRLARLELERAQLEMQSQQLRESNQQLD
LSACRLTTQCELLTQLRSAQEEENRQLLAEVQALSRENRELLERSLESRDHLHREQREYLDQLNALRREKQKLVEKIMDQYRVLEPV
PLPRTKKGSWLADKVKRLMRPRREGGPPGGLRLGADGAGSTESLGGPPETELPEGREADGTGSPSPAPMRRAQSSLCLRDETLAGGQ
RRKLSSRFPVGRSSESFSPGDTPRQRFRQRHPGPLGAPVSHSKGPGVGWENSAETLQEHETDANREGPEVQEPEKRPLTPSLSQ
>sp|Q9BSE5|SPEB_HUMAN
MLRLLASGCARGPGPGVGARPAAGLFHPGRRQSRQASDAPRNQPPSPEFVARPVGVCSMMRLPVQTSPEGLDAAFIGVPLDTGTSNR
PGARFGPRRIREESVMLGTVNPSTGALPFQSLMVADLGDVNVNLYNLQDSCRRIQEAYEKIVAAGCIPLTLGGDHTITYPILQAMAK
KHGPVGLLHVDAHTDTTDKALGEKLYHGAPFRRCVDEGLLDCKRVVQIGIRGSSTTLDPYRYNRSQGFRVVLAEDCWMKSLVPLMGE
VRQQMGGKPIYISFDIDALDPAYAPGTGTPEIAGLTPSQALEIIRGCQGLNVMGCDLVEVSPPYDLSGNTALLAANLLFEMLCALPK
VTTV
>sp|Q3SY46|KR133_HUMAN
MSYNCCSRNFSSCSHGGYLHYPGSSCGSSYPSNLVYSTDLCSPSTCQLGSSLYRGCQETCWRPNSCQTLCVESSPCHTSCYYPRTHM
LCNSCLTMHVGSRGFGSNSCCSLSCGSRSCSSLGCGSNGFRYLNYRIHTSPSQSYRSRFCHPIYFPPRRWFHSSCYQPFCRSGFY
>sp|Q8TBB0|THAP6_HUMAN
MVKCCSAIGCASRCLPNSKLKGLTFHVFPTDENIKRKWVLAMKRLDVNAAGIWEPKKGDVLCSRHFKKTDFDRSAPNIKLKPGVIPS
IFDSPYHLQGKREKLHCRKNFTLKTVPATNYNHHLVGASSCIEEFQSQFIFEHSYSVMDSPKKLKHKLDHVIGELEDTKESLRNVLD
REKRFQKSLRKTIRELKDECLISQETANRLDTFCWDCCQESIEQDYIS
>sp|P07947|YES_HUMAN
MGCIKSKENKSPAIKYRPENTPEPVSTSVSHYGAEPTTVSPCPSSSAKGTAVNFSSLSMTPFGGSSGVTPFGGASSSFSVVPSSYPA
GLTGGVTIFVALYDYEARTTEDLSFKKGERFQIINNTEGDWWEARSIATGKNGYIPSNYVAPADSIQAEEWYFGKMGRKDAERLLLN
PGNQRGIFLVRESETTKGAYSLSIRDWDEIRGDNVKHYKIRKLDNGGYYITTRAQFDTLQKLVKHYTEHADGLCHKLTTVCPTVKPQ
TQGLAKDAWEIPRESLRLEVKLGQGCFGEVWMGTWNGTTKVAIKTLKPGTMMPEAFLQEAQIMKKLRHDKLVPLYAVVSEEPIYIVT
EFMSKGSLLDFLKEGDGKYLKLPQLVDMAAQIADGMAYIERMNYIHRDLRAANILVGENLVCKIADFGLARLIEDNEYTARQGAKFP
IKWTAPEAALYGRFTIKSDVWSFGILQTELVTKGRVPYPGMVNREVLEQVERGYRMPCPQGCPESLHELMNLCWKKDPDERPTFEYI
QSFLEDYFTATEPQYQPGENL
>sp|Q9NVX0|HAUS2_HUMAN
MAAANPWDPASAPNGAGLVLGHFIASGMVNQEMLNMSKKTVSCFVNFTRLQQITNIQAEIYQKNLEIELLKLEKDTADVVHPFFLAQ
KCHTLQSMNNHLEAVLKEKRSLRQRLLKPMCQENLPIEAVYHRYMVHLLELAVTFIERLETHLETIRNIPHLAANLKKMNQALAKMD
ILVTETEELAENILKWRKQQNEVSSCIPKILAEESYLYKHDIIMPPLPFTSKVHVQTINAK
>sp|Q6IPR1|ETFR1_HUMAN
MKMANSLRGEVLKLYKNLLYLGRDYPKGADYFKKRLKNIFLKNKDVKNPEKIKELIAQGEFVMKELEALYFLRKYRAMKQRYYSDTN
KTN
>sp|Q8TF64|GIPC3_HUMAN
MEGAAAREARGTETPRASAPPPAPSEPPAAPRARPRLVFRTQLAHGSPTGKIEGFTNVRELYAKIAEAFGIAPTEILFCTLNSHKVD
MQKLLGGQIGLEDFIFAHVRGETKEVEVTKTEDALGLTITDNGAGYAFIKRIKEGSIINRIEAVCVGDSIEAINDHSIVGCRHYEVA

KMLRELPKSQPFTLRLVQPKRAFDMIGQRSRSSKCPVEAKVTSGRETLRLRSGGAATVEEAPSEFEEEASRKVDDLLESYMGIRDPE
LASTMVETSKKTASAQEFARCLDSVLGEFAFPDEFVVEVWAAIGEAREACG
>sp|Q9Y5A6|ZSC21_HUMAN
MMTKVLGMAPVLGPRPPQEQVGPLMVKVEEKEEKGKYLPSLEMFRQRFRQFGYHDTPGPREALSQLRVLCCEWLRPEIHTKEQILEL
LVLEQFLTILPQELQAWVQEHCPESAEEAVTLLEDLERELDEPGHQVSTPPNEQKPVWEKISSSGTAKESPSSMQPQPLETSHKYES
WGPLYIQESGEEQEFAQDPRKVRDCRLSTQHEESADEQKGSEAEGLKGDIISVIIANKPEASLERQCVNLENEKGTKPPLQEAGSKK
GRESVPTKPTPGERRYICAECGKAFSNSSNLTKHRRTHTGEKPYVCTKCGKAFSHSSNLTLHYRTHLVDRPYDCKCGKAFGQSSDLL
KHQRMHTEEAPYQCKDCGKAFSGKGSLIRHYRIHTGEKPYQCNECGKSFSQHAGLSSHQRLHTGEKPYKCKECGKAFNHSSNFNKHH
RIHTGEKPYWCHHCGKTFCSKSNLSKHQRVHTGEGEAP
>sp|P04004|VTNC_HUMAN
MAPLRPLLILALLAWVALADQESCKGRCTEGFNVDKKCQCDELCSYYQSCCTDYTAECKPQVTRGDVFTMPEDEYTVYDDGEEKNNA
TVHEQVGGPSLTSDLQAQSKGNPEQTPVLKPEEEAPAPEVGASKPEGIDSRPETLHPGRPQPPAEEELCSGKPFDAFTDLKNGSLFA
FRGQYCYELDEKAVRPGYPKLIRDVWGIEGPIDAAFTRINCQGKTYLFKGSQYWRFEDGVLDPDYPRNISDGFDGIPDNVDAALALP
AHSYSGRERVYFFKGKQYWEYQFQHQPSQEECEGSSLSAVFEHFAMMQRDSWEDIFELLFWGRTSAGTRQPQFISRDWHGVPGQVDA
AMAGRIYISGMAPRPSLAKKQRFRHRNRKGYRSQRGHSRGRNQNSRRPSRATWLSLFSSEESNLGANNYDDYRMDWLVPATCEPIQS
VFFFSGDKYYRVNLRTRRVDTVDPPYPRSIAQYWLGCPAPGHL
>sp|Q96MN5|TEAN2_HUMAN
MDKFVIRTPRIQNSPQKKDSGGKVYKQATIESLKRVVVVEDIKRWKTMLELPDQTKENLVEALQELKKKIPSREVLKSTRIGHTVNK
MRKHSDSEVASLAREVYTEWKTFTEKHSNRPSIEVRSDPKTESLRKNAQKLLSEALELKMDHLLVENIERETFHLCSRLINGPYRRT
VRALVFTLKHRAEIRAQVKSGSLPVGTFVQTHKK
>sp|P59990|KR121_HUMAN
MCHTSCSSGCQPACCAPSPCQASCYIPVGCQSSVCVPVSFKPAVCVPVRCQSSVCVPVSCRPVVYAAPSCQSSGCCQPSCTSVLCRP
ISCSTPSCC
>sp|Q1A5X6|IQCJ_HUMAN
MRLEELKRLQNPLEQVNDGKYSFENHQLAMDAENNIEKYPLNLQPLESKVKIIQRAWREYLQRQEPLGKRSPSPPSVSSEKLSSSVS
MNTFSDSSTPFARAPVGKIHPYISWRLQSPGDKLPGGRKVILLYLDQLARPTGFIHTLKEPQIERLGFLTLQ
>sp|Q8N4E7|FTMT_HUMAN
MLSCFRLLSRHISPSLASLRPVRCCFALPLRWAPGRPLDPRQIAPRRPLAAAASSRDPTGPAAGPSRVRQNFHPDSEAAINRQINLE
LYASYVYLSMAYYFSRDDVALNNFSRYFLHQSREETEHAEKLMRLQNQRGGRIRLQDIKKPEQDDWESGLHAMECALLLEKNVNQSL
LELHALASDKGDPHLCDFLETYYLNEQVKSIKELGDHVHNLVKMGAPDAGLAEYLFDTHTLGNENKQN
>sp|Q9UII5|ZN107_HUMAN
MVAKPPVMSFHFAQDLWPEQNIKDSFQKVTLRRYGKCEYENLQLRKGCKHVDECTGHKGGHNTVNQCLTATPSKIFQCNKYVKVFDK
FSNSNRYKRRHTGNKHFKCKECSKSFCVLSQLTQHRRIHTRVNSYKCEECGKAFNWFSTLTKHKRIHTGEKPYKCEECGKAFNQSSQ
LTRHKIIHTEEKPNKCEECGKAFKQASHLTIHKIIHTGEKPYKYEECGKVFSQSSHLTTQKILHTGENLYKCKECGKAFNLFSNLTN
HKRIHAGEKPYKCKECGRAFNISSNLNKQEKIHTGGKLNKCEECDKAFNRSLKLTAHKKILMEEKPYKCEECGKVFNQFSTLTRHKI
IHTGEKPYKCKECGKAFNQSSNLTEHKKIHTAEKSYKCEECGKAFNQHSNLINHRKIYSGEKPYKCEECGKAFNRSSTLTRHKKIHT
GEKPYKCEECDRAFSQSSNLTEHKKIHTGEKPYKCEECGKAFNRFSTLTKHKRIHTGEKPYKCEECGKAFNQSYQLTRHKIVHTKEK
LNKCEEFGKAFKQSSHRTIHKIIHTGEKPYKCEEHGKVFNQSSNLTTQKIIHTGENLYKFEEHGKAFNLFSNITNHKIIYTGEKPHK
CEECGKAYNRFSNLTIHKRIHTGEKPYQCAECGKAFNCSSTLNRHKIIHTGEKPYKCKECGKAFNLSSTLTAHKKIHTGEKPYKCEE
CGKAFNQSSNLTTHKKIHTSEKPYKCEECGKSFNQFSSLNIHKIIHTGEKPYKCGDYGRAFNLSSNLTTHKKIHTGEKPYKCEYGKT
>sp|P51809|VAMP7_HUMAN
MAILFAVVARGTTILAKHAWCGGNFLEVTEQILAKIPSENNKLTYSHGNYLFHYICQDRIVYLCITDDDFERSRAFNFLNEIKKRFQ
TTYGSRAQTALPYAMNSEFSSVLAAQLKHHSENKGLDKVMETQAQVDELKGIMVRNIDLVAQRGERLELLIDKTENLVDSSVTFKTT
SRNLARAMCMKNLKLTIIIIIVSIVFIYIIVSPLCGGFTWPSCVKK

>sp|Q969Q6|P2R3C_HUMAN
MDWKEVLRRRLATPNTCPNKKKSEQELKDEEMDLFTKYYSEWKGGRKNTNEFYKTIPRFYYRLPAEDEVLLQKLREESRAVFLQRKS
RELLDNEELQNLWFLLDKHQTPPMIGEEAMINYENFLKVGEKAGAKCKQFFTAKVFAKLLHTDSYGRISIMQFFNYVMRKVWLHQTR
IGLSLYDVAGQGYLRESDLENYILELIPTLPQLDGLEKSFYSFYVCTAVRKFFFFLDPLRTGKIKIQDILACSFLDDLLELRDEELS
KESQETNWFSAPSALRVYGQYLNLDKDHNGMLSKEELSRYGTATMTNVFLDRVFQECLTYDGEMDYKTYLDFVLALENRKEPAALQY
IFKLLDIENKGYLNVFSLNYFFRAIQELMKIHGQDPVSFQDVKDEIFDMVKPKDPLKISLQDLINSNQGDTVTTILIDLNGFWTYEN
REALVANDSENSADLDDT
>sp|Q96N46|TTC14_HUMAN
MDRDLLRQSLNCHGSSLLSLLRSEQQDNPHFRSLLGSAAEPARGPPPQHPLQGRKEKRVDNIEIQKFISKKADLLFALSWKSDAPAT
SEINEDSEDHYAIMPPLEQFMEIPSMDRRELFFRDIERGDIVIGRISSIREFGFFMVLICLGSGIMRDIAHLEITALCPLRDVPSHS
NHGDPLSYYQTGDIIRAGIKDIDRYHEKLAVSLYSSSLPPHLSGIKLGVISSEELPLYYRRSVELNSNSLESYENVMQSSLGFVNPG
VVEFLLEKLGIDESNPPSLMRGLQSKNFSEDDFASALRKKQSASWALKCVKIGVDYFKVGRHVDAMNEYNKALEIDKQNVEALVARG
ALYATKGSLNKAIEDFELALENCPTHRNARKYLCQTLVERGGQLEEEEKFLNAESYYKKALALDETFKDAEDALQKLHKYMQKSLEL
REKQAEKEEKQKTKKIETSAEKLRKLLKEEKRLKKKRRKSTSSSSVSSADESVSSSSSSSSSGHKRHKKHKRNRSESSRSSRRHSSR
ASSNQIDQNRKDECYPVPANTSASFLNHKQEVEKLLGKQDRLQYEKTQIKEKDRCPLSSSSLEIPDDFGGRSEDPRDFYNSYKTQAG
SSKTEKPYKSERHFSSRRNSSDSFCRNSEDKIYGYRRFEKDIEGRKEHYRRWEPGSVRHSTSPASSEYSWKSVEKYKKYAHSGSRDF
SRHEQRYRLNTNQGEYEREDNYGEDIKTEVPEEDALSSKEHSESSVKKNLPQNLLNIFNQIAEFEKEKGNKSKN
>sp|P17032|ZN37A_HUMAN
MITSQGSVSFRDVTVGFTQEEWQHLDPAQRTLYRDVMLENYSHLVSVGYCIPKPEVILKLEKGEEPWILEEKFPSQSHLELINTSRN
YSIMKFNEFNKGGKCFCDEKHEIIHSEEEPSEYNKNGNSFWLNEDLIWHQKIKNWEQSFEYNECGKAFPENSLFLVHKRGYTGQKTC
KYTEHGKTCDMSFFITHQQTHPRENHYGNECGENIFEESILLEHQSVYPFSQKLNLTPIQRTHSINNIIEYNECGTFFSEKLVLHLQ
QRTHTGEKPYECHECGKTFTQKSAHTRHQRTHTGGKPYECHECGKTFYKNSDLIKHQRIHTGERPYGCHECGKSFSEKSTLTQHQRT
HTGEKPYECHECGKTFSFKSVLTVHQKTHTGEKPYECYACGKAFLRKSDLIKHQRIHTGEKPYECNECGKSFSEKSTLTKHLRTHTG
EKPYECIQCGKFFCYYSGFTEHLRRHTGEKPFGCNECGKTFRQKSALIVHQRTHIRQKPYGCNQCGKSFCVKSKLIAHHRTHTGEKP
YECNVCGKSFYVKSKLTVHQRIHLGRNPINVVNEGNYSG
>sp|P20073|ANXA7_HUMAN
MSYPGYPPTGYPPFPGYPPAGQESSFPPSGQYPYPSGFPPMGGGAYPQVPSSGYPGAGGYPAPGGYPAPGGYPGAPQPGGAPSYPGV
PPGQGFGVPPGGAGFSGYPQPPSQSYGGGPAQVPLPGGFPGGQMPSQYPGGQPTYPSQINTDSFSSYPVFSPVSLDYSSEPATVTQV
TQGTIRPAANFDAIRDAEILRKAMKGFGTDEQAIVDVVANRSNDQRQKIKAAFKTSYGKDLIKDLKSELSGNMEELILALFMPPTYY
DAWSLRKAMQGAGTQERVLIEILCTRTNQEIREIVRCYQSEFGRDLEKDIRSDTSGHFERLLVSMCQGNRDENQSINHQMAQEDAQR
LYQAGEGRLGTDESCFNMILATRSFPQLRATMEAYSRMANRDLLSSVSREFSGYVESGLKTILQCALNRPAFFAERLYYAMKGAGTD
DSTLVRIVVTRSEIDLVQIKQMFAQMYQKTLGTMIAGDTSGDYRRLLLAIVGQ
>sp|Q93038|TNR25_HUMAN
MEQRPRGCAAVAAALLLVLLGARAQGGTRSPRCDCAGDFHKKIGLFCCRGCPAGHYLKAPCTEPCGNSTCLVCPQDTFLAWENHHNS
ECARCQACDEQASQVALENCSAVADTRCGCKPGWFVECQVSQCVSSSPFYCQPCLDCGALHRHTRLLCSRRDTDCGTCLPGFYEHGD
GCVSCPTSTLGSCPERCAAVCGWRQMFWVQVLLAGLVVPLLLGATLTYTYRHCWPHKPLVTADEAGMEALTPPPATHLSPLDSAHTL
LAPPDSSEKICTVQLVGNSWTPGYPETQEALCPQVTWSWDQLPSRALGPAAAPTLSPESPAGSPAMMLQPGPQLYDVMDAVPARRWK
EFVRTLGLREAEIEAVEVEIGRFRDQQYEMLKRWRQQQPAGLGAVYAALERMGLDGCVEDLRSRLQRGP
>sp|P49773|HINT1_HUMAN
MADEIAKAQVARPGGDTIFGKIIRKEIPAKIIFEDDRCLAFHDISPQAPTHFLVIPKKHISQISVAEDDDESLLGHLMIVGKKCAAD
LGLNKGYRMVVNEGSDGGQSVYHVHLHVLGGRQMHWPPG
>sp|Q3KNT7|NSN5B_HUMAN
MATLLAWVGVSCCELAEEDFLAVSPLDPRYREVHYVLLDPSCSGSGMPSRQLEDPGAGTPSPVRLHALAGFQQRALCHALTFPSLQR
LVYSMCSLCQEENEDMVPDALQQNPGAFRLAPALPARPHRGLSTFPGAEHCLRASPKTTLSGGFFVAVIERVEMPT

>sp|P11055|MYH3_HUMAN
MSSDTEMEVFGIAAPFLRKSEKERIEAQNQPFDAKTYCFVVDSKEEYAKGKIKSSQDGKVTVETEDNRTLVVKPEDVYAMNPPKFDR
IEDMAMLTHLNEPAVLYNLKDRYTSWMIYTYSGLFCVTVNPYKWLPVYNPEVVEGYRGKKRQEAPPHIFSISDNAYQFMLTDRENQS
ILITGESGAGKTVNTKRVIQYFATIAATGDLAKKKDSKMKGTLEDQIISANPLLEAFGNAKTVRNDNSSRFGKFIRIHFGTTGKLAS
ADIETYLLEKSRVTFQLKAERSYHIFYQILSNKKPELIELLLITTNPYDYPFISQGEILVASIDDAEELLATDSAIDILGFTPEEKS
GLYKLTGAVMHYGNMKFKQKQREEQAEPDGTEVADKTAYLMGLNSSDLLKALCFPRVKVGNEYVTKGQTVDQVHHAVNALSKSVYEK
LFLWMVTRINQQLDTKLPRQHFIGVLDIAGFEIFEYNSLEQLCINFTNEKLQQFFNHHMFVLEQEEYKKEGIEWTFIDFGMDLAACI
ELIEKPMGIFSILEEECMFPKATDTSFKNKLYDQHLGKSNNFQKPKVVKGRAEAHFSLIHYAGTVDYSVSGWLEKNKDPLNETVVGL
YQKSSNRLLAHLYATFATADADSGKKKVAKKKGSSFQTVSALFRENLNKLMSNLRTTHPHFVRCIIPNETKTPGAMEHSLVLHQLRC
NGVLEGIRICRKGFPNRILYGDFKQRYRVLNASAIPEGQFIDSKKACEKLLASIDIDHTQYKFGHTKVFFKAGLLGTLEEMRDDRLA
KLITRTQAVCRGFLMRVEFQKMVQRRESIFCIQYNIRSFMNVKHWPWMKLFFKIKPLLKSAETEKEMATMKEEFQKTKDELAKSEAK
RKELEEKLVTLVQEKNDLQLQVQAESENLLDAEERCDQLIKAKFQLEAKIKEVTERAEDEEEINAELTAKKRKLEDECSELKKDIDD
LELTLAKVEKEKHATENKVKNLTEELSGLDETIAKLTREKKALQEAHQQALDDLQAEEDKVNSLNKTKSKLEQQVEDLESSLEQEKK
LRVDLERNKRKLEGDLKLAQESILDLENDKQQLDERLKKKDFEYCQLQSKVEDEQTLGLQFQKKIKELQARIEELEEEIEAERATRA
KTEKQRSDYARELEELSERLEEAGGVTSTQIELNKKREAEFLKLRRDLEEATLQHEAMVAALRKKHADSVAELGEQIDNLQRVKQKL
EKEKSEFKLEIDDLSSSMESVSKSKANLEKICRTLEDQLSEARGKNEEIQRSLSELTTQKSRLQTEAGELSRQLEEKESIVSQLSRS
KQAFTQQTEELKRQLEEENKAKNALAHALQSSRHDCDLLREQYEEEQEGKAELQRALSKANSEVAQWRTKYETDAIQRTEELEEAKK
KLAQRLQDSEEQVEAVNAKCASLEKTKQRLQGEVEDLMVDVERANSLAAALDKKQRNFDKVLAEWKTKCEESQAELEASLKESRSLS
TELFKLKNAYEEALDQLETVKRENKNLEQEIADLTEQIAENGKTIHELEKSRKQIELEKADIQLALEEAEAALEHEEAKILRIQLEL
TQVKSEIDRKIAEKDEEIEQLKRNYQRTVETMQSALDAEVRSRNEAIRLKKKMEGDLNEIEIQLSHANRQAAETLKHLRSVQGQLKD
TQLHLDDALRGQEDLKEQLAIVERRANLLQAEVEELRATLEQTERARKLAEQELLDSNERVQLLHTQNTSLIHTKKKLETDLMQLQS
EVEDASRDARNAEEKAKKAITDAAMMAEELKKEQDTSAHLERMKKNLEQTVKDLQHRLDEAEQLALKGGKKQIQKLETRIRELEFEL
EGEQKKNTESVKGLRKYERRVKELTYQSEEDRKNVLRLQDLVDKLQVKVKSYKRQAEEADEQANAHLTKFRKAQHELEEAEERADIA
ESQVNKLRAKTRDFTSSRMVVHESEE
>sp|Q8NHR9|PROF4_HUMAN
MSHLQSLLLDTLLGTKHVDSAALIKIQERSLCVASPGFNVTPSDVRTLVNGFAKNPLQARREGLYFKGKDYRCVRADEYSLYAKNEN
TGVVVVKTHLYLLVATYTEGMYPSICVEATESLGDYLRKKGS
>sp|O15400|STX7_HUMAN
MSYTPGVGGDPAQLAQRISSNIQKITQCSVEIQRTLNQLGTPQDSPELRQQLQQKQQYTNQLAKETDKYIKEFGSLPTTPSEQRQRK
IQKDRLVAEFTTSLTNFQKVQRQAAEREKEFVARVRASSRVSGSFPEDSSKERNLVSWESQTQPQVQVQDEEITEDDLRLIHERESS
IRQLEADIMDINEIFKDLGMMIHEQGDVIDSIEANVENAEVHVQQANQQLSRAADYQRKSRKTLCIIILILVIGVAIISLIIWGLNH
>sp|Q9H257|CARD9_HUMAN
MSDYENDDECWSVLEGFRVTLTSVIDPSRITPYLRQCKVLNPDDEEQVLSDPNLVIRKRKVGVLLDILQRTGHKGYVAFLESLELYY
PQLYKKVTGKEPARVFSMIIDASGESGLTQLLMTEVMKLQKKVQDLTALLSSKDDFIKELRVKDSLLRKHQERVQRLKEECEAGSRE
LKRCKEENYDLAMRLAHQSEEKGAALMRNRDLQLEIDQLKHSLMKAEDDCKVERKHTLKLRHAMEQRPSQELLWELQQEKALLQARV
QELEASVQEGKLDRSSPYIQVLEEDWRQALRDHQEQANTIFSLRKDLRQGEARRLRCMEEKEMFELQCLALRKDSKMYKDRIEAILL
QMEEVAIERDQAIATREELHAQHARGLQEKDALRKQVRELGEKADELQLQVFQCEAQLLAVEGRLRRQQLETLVLSSDLEDGSPRRS
QELSLPQDLEDTQLSDKGCLAGGGSPKQPFAALHQEQVLRNPHDAGLSSGEPPEKERRRLKESFENYRRKRALRKMQKGWRQGEEDR
ENTTGSDNTDTEGS
>sp|Q9NP79|VTA1_HUMAN
MAALAPLPPLPAQFKSIQHHLRTAQEHDKRDPVVAYYCRLYAMQTGMKIDSKTPECRKFLSKLMDQLEALKKQLGDNEAITQEIVGC
AHLENYALKMFLYADNEDRAGRFHKNMIKSFYTASLLIDVITVFGELTDENVKHRKYARWKATYIHNCLKNGETPQAGPVGIEEDND
IEENEDAGAASLPTQPTQPSSSSTYDPSNMPSGNYTGIQIPPGAHAPANTPAEVPHSTGVASNTIQPTPQTIPAIDPALFNTISQGD
VRLTPEDFARAQKYCKYAGSALQYEDVSTAVQNLQKALKLLTTGRE

>sp|Q9HAS3|S28A3_HUMAN
MELRSTAAPRAEGYSNVGFQNEENFLENENTSGNNSIRSRAVQSREHTNTKQDEEQVTVEQDSPRNREHMEDDDEEMQQKGCLERRY
DTVCGFCRKHKTTLRHIIWGILLAGYLVMVISACVLNFHRALPLFVITVAAIFFVVWDHLMAKYEHRIDEMLSPGRRLLNSHWFWLK
WVIWSSLVLAVIFWLAFDTAKLGQQQLVSFGGLIMYIVLLFLFSKYPTRVYWRPVLWGIGLQFLLGLLILRTDPGFIAFDWLGRQVQ
TFLEYTDAGASFVFGEKYKDHFFAFKVLPIVVFFSTVMSMLYYLGLMQWIIRKVGWIMLVTTGSSPIESVVASGNIFVGQTESPLLV
RPYLPYITKSELHAIMTAGFSTIAGSVLGAYISFGVPSSHLLTASVMSAPASLAAAKLFWPETEKPKITLKNAMKMESGDSGNLLEA
ATQGASSSISLVANIAVNLIAFLALLSFMNSALSWFGNMFDYPQLSFELICSYIFMPFSFMMGVEWQDSFMVARLIGYKTFFNEFVA
YEHLSKWIHLRKEGGPKFVNGVQQYISIRSEIIATYALCGFANIGSLGIVIGGLTSMAPSRKRDIASGAVRALIAGTVACFMTACIA
GILSSTPVDINCHHVLENAFNSTFPGNTTKVIACCQSLLSSTVAKGPGEVIPGGNHSLYSLKGCCTLLNPSTFNCNGISNTF
>sp|Q96LW2|SG494_HUMAN
MGAVSCRQGQHTQQGEHTRVAVPHKQGGNIRGPWARGWKSLWTGLGTIRSDLEELWELRGHHYLHQESLKPAPVLVEKPLPEWPVPQ
FINLFLPEFPIRPIRGQQQLKILGLVAKGSFGTVLKVLDCTQKAVFAVKVVPKVKVLQRDTVRQCKEEVSIQRQINHPFVHSLGDSW
QGKRHLFIMCSYCSTDLYSLWSAVGCFPEASIRLFAAELVLVLCYLHDLGIMHRDVKMENILLDERGHLKLTDFGLSRHVPQGAQAY
TICGTLQYMGERG
>sp|Q5HYW2|NHSL2_HUMAN
MESMGMVYSVPSSCNGPTESTFSTSWKGDAFTYMTPSATSQSNQVNENGKNPSCGNSWVSLNKVPPLVPKEAATLLVARDNPAGCSG
SAGYPERLIQQRHMPERPSKIGLLTSGTSRLETGPGGASRFRERSLSVPTDSGTTDVDYDEEQKANEACALPFASTSSEGSNSADNI
ASLSAQQEAQHRRQRSKSISLRKAKKKPSPPTRSVSLVKDEPGLLPEGGSALPKDQRPKSLCLSLEHQGHHSSHPDAQGHPAIPNHK
DPESTQFSHHWYLTDWKSGDTYQSLSSSSTATGTTVIECTQVQGSSESLASPSTSRATTPSQLSIEVEAREISSPGRPPGLMSPSSG
YSSQSETPTPTVSMSLTLGHLPPPSSSVRVRPVVPERKSSLPPTSPMEKFPKSRLSFDLPLTSSPNLDLSGMSISIRSKTKVSRHHS
ETNFGVKLAQKTNPNQPIMPMVTQSDLRSVRLRSVSKSEPEDDIESPEYAEEPRAEEVFTLPERKTKPPVAEKPPVARRPPSLVHKP
PSVPEEYALTSPTLAMPPRSSIQHARPLPQDSYTVVRKPKPSSFPDGRSPGESTAPSSLVFTPFASSSDAFFSGTQQPPQGSVEDEG
PKVRVLPERISLQSQEEAEKKKGKIPPPVPKKPSVLYLPLTSPTAQMEAYVAEPRLPLSPIITLEEDTKCPATGDDLQSLGQRVTST
PQADSEREASPLG
>sp|P26441|CNTF_HUMAN
MAFTEHSPLTPHRRDLCSRSIWLARKIRSDLTALTESYVKHQGLNKNINLDSADGMPVASTDQWSELTEAERLQENLQAYRTFHVLL
ARLLEDQQVHFTPTEGDFHQAIHTLLLQVAAFAYQIEELMILLEYKIPRNEADGMPINVGDGGLFEKKLWGLKVLQELSQWTVRSIH
DLRFISSHQTGIPARGSHYIANNKKM
>sp|Q96JL9|ZN333_HUMAN
MESVTFEDVAVEFIQEWALLDSARRSLCKYRMLDQCRTLASRGTPPCKPSCVSQLGQRAEPKATERGILRATGVAWESQLKPEELPS
MQDLLEEASSRDMQMGPGLFLRMQLVPSIEERETPLTREDRPALQEPPWSLGCTGLKAAMQIQRVVIPVPTLGHRNPWVARDSAVPA
RDPAWLQEDKVEEEAMAPGLPTACSQEPVTFADVAVVFTPEEWVFLDSTQRSLYRDVMLENYRNLASVADQLCKPNALSYLEERGEQ
WTTDRGVLSDTCAEPQCQPQEAIPSQDTFTEILSIDVKGEQPQPGEKLYKYNELEKPFNSIEPLFQYQRIHAGEASCECQEIRNSFF
QSAHLIVPEKIRSGDKSYACNKCEKSFRYSSDLIRHEKTHTAEKCFDCQECGQAFKYSSNLRRHMRTHTGEKPFECSQCGKTFTRNF
NLILHQRNHTGEKPYECKDCGKAFNQPSSLRSHVRTHTGEKPFECSQCGKAFREHSSLKTHLRTHTREKPYECNQCGKPFRTSTHLN
VHKRIHTGEKLYECATCGQVLSRLSTLKSHMRTHTGEKPYVCQECGRAFSEPSSLRKHARTHSGKKPYACQECGRAFGQSSHLIVHV
RTHSAGRPYQCNQCEKAFRHSSSLTVHKRTHVGRETIRNGSLPLSMSHPYCGPLAN
>sp|Q14587|ZN268_HUMAN
MATRVRTASIWVPPLQERNSSWDRIRKLQGQESILGQGTPGLQPLPGTPRQKQKSRRIEKVLEWLFISQEQPKITKSWGPLSFMDVF
VDFTWEEWQLLDPAQKCLYRSVMLENYSNLVSLGYQHTKPDIIFKLEQGEELCMVQAQVPNQTCPNTVWKIDDLMDWHQENKDKLGS
TAKSFECTTFGKLCLLSTKYLSRQKPHKCGTHGKSLKYIDFTSDYARNNPNGFQVHGKSFFHSKHEQTVIGIKYCESIESGKTVNKK
SQLMCQQMYMGEKPFGCSCCEKAFSSKSYLLVHQQTHAEEKPYGCNECGKDFSSKSYLIVHQRIHTGEKLHECSECRKTFSFHSQLV
IHQRIHTGENPYECCECGKVFSRKDQLVSHQKTHSGQKPYVCNECGKAFGLKSQLIIHERIHTGEKPYECNECQKAFNTKSNLMVHQ
RTHTGEKPYVCSDCGKAFTFKSQLIVHQGIHTGVKPYGCIQCGKGFSLKSQLIVHQRSHTGMKPYVCNECGKAFRSKSYLIIHTRTH

TGEKLHECNNCGKAFSFKSQLIIHQRIHTGENPYECHECGKAFSRKYQLISHQRTHAGEKPYECTDCGKAFGLKSQLIIHQRTHTGE
KPFECSECQKAFNTKSNLIVHQRTHTGEKPYSCNECGKAFTFKSQLIVHKGVHTGVKPYGCSQCAKTFSLKSQLIVHQRSHTGVKPY
GCSECGKAFRSKSYLIIHMRTHTGEKPHECRECGKSFSFNSQLIVHQRIHTGENPYECSECGKAFNRKDQLISHQRTHAGEKPYGCS
ECGKAFSSKSYLIIHMRTHSGEKPYECNECGKAFIWKSLLIVHERTHAGVNPYKCSQCEKSFSGKLRLLVHQRMHTREKPYECSECG
KAFIRNSQLIVHQRTHSGEKPYGCNECGKTFSQKSILSAHQRTHTGEKPCKCTECGKAFCWKSQLIMHQRTHVDDKH
>sp|Q6P2I3|FAH2B_HUMAN
MLVSGRRRLLTALLQAQKWPFQPSRDMRLVQFRAPHLVGPHLGLETGNGGGVINLNAFDPTLPKTMTQFLEQGEATLSVARRALAAQ
LPVLPWSEVTFLAPVTWPDKVVCVGMNYVDHCKEQNVPVPKEPIIFSKFASSIVGPYDEVVLPPQSQEVDWEVELAVVIGKKGKHIK
ATDAMAHVAGFTVAHDVSARDWLTRRNGKQWLLGKTFDTFCPLGPALVTKDSVADPHNLKICCRVNGEVVQSSNTNQMVFKTEDLIA
WVSQFVTFYPGDVILTGTPPGVGVFRKPPVFLKKGDEVQCEIEELGVIINKVV
>sp|Q8IUR6|CRERF_HUMAN
MPQPSVSGMDPPFGDAFRSHTFSEQTLMSTDLLANSSDPDFMYELDREMNYQQNPRDNFLSLEDCKDIENLESFTDVLDNEGALTSN
WEQWDTYCEDLTKYTKLTSCDIWGTKEVDYLGLDDFSSPYQDEEVISKTPTLAQLNSEDSQSVSDSLYYPDSLFSVKQNPLPSSFPG
KKITSRAAAPVCSSKTLQAEVPLSDCVQKASKPTSSTQIMVKTNMYHNEKVNFHVECKDYVKKAKVKINPVQQSRPLLSQIHTDAAK
ENTCYCGAVAKRQEKKGMEPLQGHATPALPFKETQELLLSPLPQEGPGSLAAGESSSLSASTSVSDSSQKKEEHNYSLFVSDNLGEQ
PTKCSPEEDEEDEEDVDDEDHDEGFGSEHELSENEEEEEEEEDYEDDKDDDISDTFSEPGYENDSVEDLKEVTSISSRKRGKRRYFW
EYSEQLTPSQQERMLRPSEWNRDTLPSNMYQKNGLHHGKYAVKKSRRTDVEDLTPNPKKLLQIGNELRKLNKVISDLTPVSELPLTA
RPRSRKEKNKLASRACRLKKKAQYEANKVKLWGLNTEYDNLLFVINSIKQEIVNRVQNPRDERGPNMGQKLEILIKDTLGLPVAGQT
SEFVNQVLEKTAEGNPTGGLVGLRIPTSKV
>sp|A8MXJ8|F90A5_HUMAN
MMARRDPTSWAKRLVRAQTLQKQRRAPVGPRAPPPDEEDPRLKCKNCGAFGHTARSTRCPMKCWKAALVPATLGKKEGKENLKPWKP
RVEANPGPLNKDKGEKEERPRQQDPQRKALLHMFSGKPPEKPLPNGKGSTEPSDYLRVASGPMPVHTTSKRPRVDPVLADGSATEMS
DRGSVLASLSPLRKASLSSSSSLGPKERQTGAAADMPQPAVRHQGREPLLVVKPTHSRPEGGCREVPQAASKTHGLLQAARPQAQDK
RPAVTSQPCPPAATHSLGLGSNLSFGPGAKRPAQAPIQACLNFPKKPRLGPFQIPESAIQGGELGAPENLQPPPAATELGPSTSPQM
GRRTPAQVPSVDRQPPHSRPCLPTAQACTMSHHPAASHDGAQPLRVLFRRLENGRWSSSLLAAPSFHSPEKPGAFLAQSPHVSEKSE
APCVRVPPSVLYEDLQVSSSSEDSDSDLE
>sp|P52824|DGKQ_HUMAN
MAAAAEPGARAWLGGGSPRPGSPACSPVLGSGGRARPGPGPGPGPERAGVRAPGPAAAPGHSFRKVTLTKPTFCHLCSDFIWGLAGF
LCDVCNFMSHEKCLKHVRIPCTSVAPSLVRVPVAHCFGPRGLHKRKFCAVCRKVLEAPALHCEVCELHLHPDCVPFACSDCRQCHQD
GHQDHDTHHHHWREGNLPSGARCEVCRKTCGSSDVLAGVRCEWCGVQAHSLCSAALAPECGFGRLRSLVLPPACVRLLPGGFSKTQS
FRIVEAAEPGEGGDGADGSAAVGPGRETQATPESGKQTLKIFDGDDAVRRSQFRLVTVSRLAGAEEVLEAALRAHHIPEDPGHLELC
RLPPSSQACDAWAGGKAGSAVISEEGRSPGSGEATPEAWVIRALPRAQEVLKIYPGWLKVGVAYVSVRVTPKSTARSVVLEVLPLLG
RQAESPESFQLVEVAMGCRHVQRTMLMDEQPLLDRLQDIRQMSVRQVSQTRFYVAESRDVAPHVSLFVGGLPPGLSPEEYSSLLHEA
GATKATVVSVSHIYSSQGAVVLDVACFAEAERLYMLLKDMAVRGRLLTALVLPDLLHAKLPPDSCPLLVFVNPKSGGLKGRDLLCSF
RKLLNPHQVFDLTNGGPLPGLHLFSQVPCFRVLVCGGDGTVGWVLGALEETRYRLACPEPSVAILPLGTGNDLGRVLRWGAGYSGED
PFSVLLSVDEADAVLMDRWTILLDAHEAGSAENDTADAEPPKIVQMSNYCGIGIDAELSLDFHQAREEEPGKFTSRLHNKGVYVRVG
LQKISHSRSLHKQIRLQVERQEVELPSIEGLIFINIPSWGSGADLWGSDSDTRFEKPRMDDGLLEVVGVTGVVHMGQVQGGLRSGIR
IAQGSYFRVTLLKATPVQVDGEPWVQAPGHMIISAAGPKVHMLRKAKQKPRRAGTTRDARADAAPAPESDPR
>sp|O75604|UBP2_HUMAN
MSQLSSTLKRYTESARYTDAHYAKSGYGAYTPSSYGANLAASLLEKEKLGFKPVPTSSFLTRPRTYGPSSLLDYDRGRPLLRPDITG
GGKRAESQTRGTERPLGSGLSGGSGFPYGVTNNCLSYLPINAYDQGVTLTQKLDSQSDLARDFSSLRTSDSYRIDPRNLGRSPMLAR
TRKELCTLQGLYQTASCPEYLVDYLENYGRKGSASQVPSQAPPSRVPEIISPTYRPIGRYTLWETGKGQAPGPSRSSSPGRDGMNSK
SAQGLAGLRNLGNTCFMNSILQCLSNTRELRDYCLQRLYMRDLHHGSNAHTALVEEFAKLIQTIWTSSPNDVVSPSEFKTQIQRYAP
RFVGYNQQDAQEFLRFLLDGLHNEVNRVTLRPKSNPENLDHLPDDEKGRQMWRKYLEREDSRIGDLFVGQLKSSLTCTDCGYCSTVF

DPFWDLSLPIAKRGYPEVTLMDCMRLFTKEDVLDGDEKPTCCRCRGRKRCIKKFSIQRFPKILVLHLKRFSESRIRTSKLTTFVNFP
LRDLDLREFASENTNHAVYNLYAVSNHSGTTMGGHYTAYCRSPGTGEWHTFNDSSVTPMSSSQVRTSDAYLLFYELASPPSRM
>sp|P54619|AAKG1_HUMAN
METVISSDSSPAVENEHPQETPESNNSVYTSFMKSHRCYDLIPTSSKLVVFDTSLQVKKAFFALVTNGVRAAPLWDSKKQSFVGMLT
ITDFINILHRYYKSALVQIYELEEHKIETWREVYLQDSFKPLVCISPNASLFDAVSSLIRNKIHRLPVIDPESGNTLYILTHKRILK
FLKLFITEFPKPEFMSKSLEELQIGTYANIAMVRTTTPVYVALGIFVQHRVSALPVVDEKGRVVDIYSKFDVINLAAEKTYNNLDVS
VTKALQHRSHYFEGVLKCYLHETLETIINRLVEAEVHRLVVVDENDVVKGIVSLSDILQALVLTGGEKKP
>sp|P83436|COG7_HUMAN
MDFSKFLADDFDVKEWINAAFRAGSKEAASGKADGHAATLVMKLQLFIQEVNHAVEETSHQALQNMPKVLRDVEALKQEASFLKEQM
ILVKEDIKKFEQDTSQSMQVLVEIDQVKSRMQLAAESLQEADKWSTLSADIEETFKTQDIAVISAKLTGMQNSLMMLVDTPDYSEKC
VHLEALKNRLEALASPQIVAAFTSQAVDQSKVFVKVFTEIDRMPQLLAYYYKCHKVQLLAAWQELCQSDLSLDRQLTGLYDALLGAW
HTQIQWATQVFQKPHEVVMVLLIQTLGALMPSLPSCLSNGVERAGPEQELTRLLEFYDATAHFAKGLEMALLPHLHEHNLVKVTELV
DAVYDPYKPYQLKYGDMEESNLLIQMSAVPLEHGEVIDCVQELSHSVNKLFGLASAAVDRCVRFTNGLGTCGLLSALKSLFAKYVSD
FTSTLQSIRKKCKLDHIPPNSLFQEDWTAFQNSIRIIATCGELLRHCGDFEQQLANRILSTAGKYLSDSCSPRSLAGFQESILTDKK
NSAKNPWQEYNYLQKDNPAEYASLMEILYTLKEKGSSNHNLLAAPRAALTRLNQQAHQLAFDSVFLRIKQQLLLISKMDSWNTAGIG
ETLTDELPAFSLTPLEYISNIGQYIMSLPLNLEPFVTQEDSALELALHAGKLPFPPEQGDELPELDNMADNWLGSIARATMQTYCDA
ILQIPELSPHSAKQLATDIDYLINVMDALGLQPSRTLQHIVTLLKTRPEDYRQVSKGLPRRLATTVATMRSVNY
>sp|Q86YC3|NRROS_HUMAN
MELLPLWLCLGFHFLTVGWRNRSGTATAASQGVCKLVGGAADCRGQSLASVPSSLPPHARMLTLDANPLKTLWNHSLQPYPLLESLS
LHSCHLERISRGAFQEQGHLRSLVLGDNCLSENYEETAAALHALPGLRRLDLSGNALTEDMAALMLQNLSSLRSVSLAGNTIMRLDD
SVFEGLERLRELDLQRNYIFEIEGGAFDGLAELRHLNLAFNNLPCIVDFGLTRLRVLNVSYNVLEWFLATGGEAAFELETLDLSHNQ
LLFFPLLPQYSKLRTLLLRDNNMGFYRDLYNTSSPREMVAQFLLVDGNVTNITTVSLWEEFSSSDLADLRFLDMSQNQFQYLPDGFL
RKMPSLSHLNLHQNCLMTLHIREHEPPGALTELDLSHNQLSELHLAPGLASCLGSLRLFNLSSNQLLGVPPGLFANARNITTLDMSH
NQISLCPLPAASDRVGPPSCVDFRNMASLRSLSLEGCGLGALPDCPFQGTSLTYLDLSSNWGVLNGSLAPLQDVAPMLQVLSLRNMG
LHSSFMALDFSGFGNLRDLDLSGNCLTTFPRFGGSLALETLDLRRNSLTALPQKAVSEQLSRGLRTIYLSQNPYDCCGVDGWGALQH
GQTVADWAMVTCNLSSKIIRVTELPGGVPRDCKWERLDLGLLYLVLILPSCLTLLVACTVIVLTFKKPLLQVIKSRCHWSSVY
>sp|Q86T96|RN180_HUMAN
MKRSKELITKNHSQEETSILRCWKCRKCIASSGCFMEYLENQVIKDKDDSVDAQNICHVWHMNVEALPEWISCLIQKAQWTVGKLNC
PFCGARLGGFNFVSTPKCSCGQLAAVHLSKSRTDYQPTQAGRLMRPSVKYLSHPRVQSGCDKEALLTGGGSENRNHRLLNMARNNND
PGRLTEALCLEVRPTYFEMKNEKLLSKASEPKYQLFVPQLVTGRCATRAFHRKSHSLDLNISEKLTLLPTLYEIHSKTTAYSRLNET
QPIDLSGLPLQSSKNSYSFQNPSSFDPSMLLQRFSVAPHETQTQRGGEFQCGLEAASVYSDHTNTNNLTFLMDLPSAGRSMPEASDQ
EEHLSPLDFLHSANFSLGSINQRLNKRERSKLKNLRRKQRRRERWLQKQGKYSGVGLLDHMTLNNEMSTDEDNEYAEEKDSYICAVC
LDVYFNPYMCYPCHHIFCEPCLRTLAKDNPSSTPCPLCRTIISRVFFQTELNNATKTFFTKEYLKIKQSFQKSNSAKWPLPSCRKAF
HLFGGFRRHAAPVTRRQFPHGAHRMDYLHFEDDSRGWWFDMDMVIIYIYSVNWVIGFIVFCFLCYFFFPF
>sp|P05000|IFNW1_HUMAN
MALLFPLLAALVMTSYSPVGSLGCDLPQNHGLLSRNTLVLLHQMRRISPFLCLKDRRDFRFPQEMVKGSQLQKAHVMSVLHEMLQQI
FSLFHTERSSAAWNMTLLDQLHTGLHQQLQHLETCLLQVVGEGESAGAISSPALTLRRYFQGIRVYLKEKKYSDCAWEVVRMEIMKS
LFLSTNMQERLRSKDRDLGSS
>sp|O14599|VCY2_HUMAN
MMTLVPRARTRAGQDHYSHPCPRFSQVLLTEGIMTYCLTKNLSDVNILHRLLKNGNVRNTLLQSKVGLLTYYVKLYPGEVTLLTRPS
IQMRLCCITGSVSRPRSQK
>sp|Q15415|RBY1F_HUMAN
MVEADHPGKLFIGGLNRETNEKMLKAVFGKHGPISEVLLIKDRTSKSRGFAFITFENPADAKNAAKDMNGTSLHGKAIKVEQAKKPS
FQSGGRRRPPASSRNRSPSGSLRSARGSSGGTRGWLPSHEGHLDDGGYTPDLKMSYSRGLIPVKRGPSSRSGGPPPKKSAPSAVARS

NSWMGSQGPMSQRRENYGVPPRRATISSWRNDRMSTRHDGYATNDGNHPSCQETRDYAPPSRGYAYRDNGHSNRDEHSSRGYRNHRS
SRETRDYAPPSRGHAYRDYGHSRRDESYSRGYRNHRSSRETREYAPPSRGHGYRDYGHSRRHESYSRGYRNHPSSRETRDYAPPHRD
YAYRDYGHSSWDEHSSRGYSYHDGYGEALGRDHSEHLSGSSYRDALQRYGTSHGAPPARGPRMSYGGSTCHAYSNTRDRYGRSWESY
SSCGDFHYCDREHVCRKDQRNPPSLGRVLPDPREAYGSSSYVASIVDGGESRSEKGDSSRY
>sp|Q93074|MED12_HUMAN
MAAFGILSYEHRPLKRPRLGPPDVYPQDPKQKEDELTALNVKQGFNNQPAVSGDEHGSAKNVSFNPAKISSNFSSIIAEKLRCNTLP
DTGRRKPQVNQKDNFWLVTARSQSAINTWFTDLAGTKPLTQLAKKVPIFSKKEEVFGYLAKYTVPVMRAAWLIKMTCAYYAAISETK
VKKRHVDPFMEWTQIITKYLWEQLQKMAEYYRPGPAGSGGCGSTIGPLPHDVEVAIRQWDYTEKLAMFMFQDGMLDRHEFLTWVLEC
FEKIRPGEDELLKLLLPLLLRYSGEFVQSAYLSRRLAYFCTRRLALQLDGVSSHSSHVISAQSTSTLPTTPAPQPPTSSTPSTPFSD
LLMCPQHRPLVFGLSCILQTILLCCPSALVWHYSLTDSRIKTGSPLDHLPIAPSNLPMPEGNSAFTQQVRAKLREIEQQIKERGQAV
EVRWSFDKCQEATAGFTIGRVLHTLEVLDSHSFERSDFSNSLDSLCNRIFGLGPSKDGHEISSDDDAVVSLLCEWAVSCKRSGRHRA
MVVAKLLEKRQAEIEAERCGESEAADEKGSIASGSLSAPSAPIFQDVLLQFLDTQAPMLTDPRSESERVEFFNLVLLFCELIRHDVF
SHNMYTCTLISRGDLAFGAPGPRPPSPFDDPADDPEHKEAEGSSSSKLEDPGLSESMDIDPSSSVLFEDMEKPDFSLFSPTMPCEGK
GSPSPEKPDVEKEVKPPPKEKIEGTLGVLYDQPRHVQYATHFPIPQEESCSHECNQRLVVLFGVGKQRDDARHAIKKITKDILKVLN
RKGTAETDQLAPIVPLNPGDLTFLGGEDGQKRRRNRPEAFPTAEDIFAKFQHLSHYDQHQVTAQVSRNVLEQITSFALGMSYHLPLV
QHVQFIFDLMEYSLSISGLIDFAIQLLNELSVVEAELLLKSSDLVGSYTTSLCLCIVAVLRHYHACLILNQDQMAQVFEGLCGVVKH
GMNRSDGSSAERCILAYLYDLYTSCSHLKNKFGELFSDFCSKVKNTIYCNVEPSESNMRWAPEFMIDTLENPAAHTFTYTGLGKSLS
ENPANRYSFVCNALMHVCVGHHDPDRVNDIAILCAELTGYCKSLSAEWLGVLKALCCSSNNGTCGFNDLLCNVDVSDLSFHDSLATF
VAILIARQCLLLEDLIRCAAIPSLLNAACSEQDSEPGARLTCRILLHLFKTPQLNPCQSDGNKPTVGIRSSCDRHLLAASQNRIVDG
AVFAVLKAVFVLGDAELKGSGFTVTGGTEELPEEEGGGGSGGRRQGGRNISVETASLDVYAKYVLRSICQQEWVGERCLKSLCEDSN
DLQDPVLSSAQAQRLMQLICYPHRLLDNEDGENPQRQRIKRILQNLDQWTMRQSSLELQLMIKQTPNNEMNSLLENIAKATIEVFQQ
SAETGSSSGSTASNMPSSSKTKPVLSSLERSGVWLVAPLIAKLPTSVQGHVLKAAGEELEKGQHLGSSSRKERDRQKQKSMSLLSQQ
PFLSLVLTCLKGQDEQREGLLTSLYSQVHQIVNNWRDDQYLDDCKPKQLMHEALKLRLNLVGGMFDTVQRSTQQTTEWAMLLLEIII
SGTVDMQSNNELFTTVLDMLSVLINGTLAADMSSISQGSMEENKRAYMNLAKKLQKELGERQSDSLEKVRQLLPLPKQTRDVITCEP
QGSLIDTKGNKIAGFDSIFKKEGLQVSTKQKISPWDLFEGLKPSAPLSWGWFGTVRVDRRVARGEEQQRLLLYHTHLRPRPRAYYLE
PLPLPPEDEEPPAPTLLEPEKKAPEPPKTDKPGAAPPSTEERKKKSTKGKKRSQPATKTEDYGMGPGRSGPYGVTVPPDLLHHPNPG
SITHLNYRQGSIGLYTQNQPLPAGGPRVDPYRPVRLPMQKLPTRPTYPGVLPTTMTGVMGLEPSSYKTSVYRQQQPAVPQGQRLRQQ
LQQSQGMLGQSSVHQMTPSSSYGLQTSQGYTPYVSHVGLQQHTGPAGTMVPPSYSSQPYQSTHPSTNPTLVDPTRHLQQRPSGYVHQ
QAPTYGHGLTSTQRFSHQTLQQTPMISTMTPMSAQGVQAGVRSTAILPEQQQQQQQQQQQQQQQQQQQQQQQQQQQYHIRQQQQQQIL
RQQQQQQQQQQQQQQQQQQQQQQQQQQQHQQQQQQQAAPPQPQPQSQPQFQRQGLQQTQQQQQTAALVRQLQQQLSNTQPQPSTNIFG
RY
>sp|P10155|RO60_HUMAN
MEESVNQMQPLNEKQIANSQDGYVWQVTDMNRLHRFLCFGSEGGTYYIKEQKLGLENAEALIRLIEDGRGCEVIQEIKSFSQEGRTT
KQEPMLFALAICSQCSDISTKQAAFKAVSEVCRIPTHLFTFIQFKKDLKESMKCGMWGRALRKAIADWYNEKGGMALALAVTKYKQR
NGWSHKDLLRLSHLKPSSEGLAIVTKYITKGWKEVHELYKEKALSVETEKLLKYLEAVEKVKRTRDELEVIHLIEEHRLVREHLLTN
HLKSKEVWKALLQEMPLTALLRNLGKMTANSVLEPGNSEVSLVCEKLCNEKLLKKARIHPFHILIALETYKTGHGLRGKLKWRPDEE
ILKALDAAFYKTFKTVEPTGKRFLLAVDVSASMNQRVLGSILNASTVAAAMCMVVTRTEKDSYVVAFSDEMVPCPVTTDMTLQQVLM
AMSQIPAGGTDCSLPMIWAQKTNTPADVFIVFTDNETFAGGVHPAIALREYRKKMDIPAKLIVCGMTSNGFTIADPDDRGMLDMCGF
DTGALDVIRNFTLDMI
>sp|Q13554|KCC2B_HUMAN
MATTVTCTRFTDEYQLYEDIGKGAFSVVRRCVKLCTGHEYAAKIINTKKLSARDHQKLEREARICRLLKHSNIVRLHDSISEEGFHY
LVFDLVTGGELFEDIVAREYYSEADASHCIQQILEAVLHCHQMGVVHRDLKPENLLLASKCKGAAVKLADFGLAIEVQGDQQAWFGF
AGTPGYLSPEVLRKEAYGKPVDIWACGVILYILLVGYPPFWDEDQHKLYQQIKAGAYDFPSPEWDTVTPEAKNLINQMLTINPAKRI
TAHEALKHPWVCQRSTVASMMHRQETVECLKKFNARRKLKGAILTTMLATRNFSVGRQTTAPATMSTAASGTTMGLVEQAKSLLNKK

ADGVKPQTNSTKNSAAATSPKGTLPPAALEPQTTVIHNPVDGIKESSDSANTTIEDEDAKAPRVPDILSSVRRGSGAPEAEGPLPCP
SPAPFSPLPAPSPRISDILNSVRRGSGTPEAEGPLSAGPPPCLSPALLGPLSSPSPRISDILNSVRRGSGTPEAEGPSPVGPPPCPS
PTIPGPLPTPSRKQEIIKTTEQLIEAVNNGDFEAYAKICDPGLTSFEPEALGNLVEGMDFHRFYFENLLAKNSKPIHTTILNPHVHV
IGEDAACIAYIRLTQYIDGQGRPRTSQSEETRVWHRRDGKWQNVHFHCSGAPVAPLQ
>sp|Q9Y312|AAR2_HUMAN
MAAVQMDPELAKRLFFEGATVVILNMPKGTEFGIDYNSWEVGPKFRGVKMIPPGIHFLHYSSVDKANPKEVGPRMGFFLSLHQRGLT
VLRWSTLREEVDLSPAPESEVEAMRANLQELDQFLGPYPYATLKKWISLTNFISEATVEKLQPENRQICAFSDVLPVLSMKHTKDRV
GQNLPRCGIECKSYQEGLARLPEMKPRAGTEIRFSELPTQMFPEGATPAEITKHSMDLSYALETVLNKQFPSSPQDVLGELQFAFVC
FLLGNVYEAFEHWKRLLNLLCRSEAAMMKHHTLYINLISILYHQLGEIPADFFVDIVSQDNFLTSTLQVFFSSACSIAVDATLRKKA
EKFQAHLTKKFRWDFAAEPEDCAPVVVELPEGIEMG
>sp|Q96DR4|STAR4_HUMAN
MEGLSDVASFATKLKNTLIQYHSIEEDKWRVAKKTKDVTVWRKPSEEFNGYLYKAQGVIDDLVYSIIDHIRPGPCRLDWDSLMTSLD
ILENFEENCCVMRYTTAGQLWNIISPREFVDFSYTVGYKEGLLSCGISLDWDEKRPEFVRGYNHPCGWFCVPLKDNPNQSLLTGYIQ
TDLRGMIPQSAVDTAMASTLTNFYGDLRKAL
>sp|P19544|WT1_HUMAN
MGSDVRDLNALLPAVPSLGGGGGCALPVSGAAQWAPVLDFAPPGASAYGSLGGPAPPPAPPPPPPPPPHSFIKQEPSWGGAEPHEEQ
CLSAFTVHFSGQFTGTAGACRYGPFGPPPPSQASSGQARMFPNAPYLPSCLESQPAIRNQGYSTVTFDGTPSYGHTPSHHAAQFPNH
SFKHEDPMGQQGSLGEQQYSVPPPVYGCHTPTDSCTGSQALLLRTPYSSDNLYQMTSQLECMTWNQMNLGATLKGVAAGSSSSVKWT
EGQSNHSTGYESDNHTTPILCGAQYRIHTHGVFRGIQDVRRVPGVAPTLVRSASETSEKRPFMCAYPGCNKRYFKLSHLQMHSRKHT
GEKPYQCDFKDCERRFSRSDQLKRHQRRHTGVKPFQCKTCQRKFSRSDHLKTHTRTHTGKTSEKPFSCRWPSCQKKFARSDELVRHH
NMHQRNMTKLQLAL
>sp|Q9BV81|EMC6_HUMAN
MAAVVAKREGPPFISEAAVRGNAAVLDYCRTSVSALSGATAGILGLTGLYGFIFYLLASVLLSLLLILKAGRRWNKYFKSRRPLFTG
GLIGGLFTYVLFWTFLYGMVHVY
>sp|Q8TBZ3|WDR20_HUMAN
MATEGGGKEMNEIKTQFTTREGLYKLLPHSEYSRPNRVPFNSQGSNPVRVSFVNLNDQSGNGDRLCFNVGRELYFYIYKGVRKAADL
SKPIDKRIYKGTQPTCHDFNHLTATAESVSLLVGFSAGQVQLIDPIKKETSKLFNEERLIDKSRVTCVKWVPGSESLFLVAHSSGNM
YLYNVEHTCGTTAPHYQLLKQGESFAVHTCKSKSTRNPLLKWTVGEGALNEFAFSPDGKFLACVSQDGFLRVFNFDSVELHGTMKSY
FGGLLCVCWSPDGKYIVTGGEDDLVTVWSFVDCRVIARGHGHKSWVSVVAFDPYTTSVEEGDPMEFSGSDEDFQDLLHFGRDRANST
QSRLSKRNSTDSRPVSVTYRFGSVGQDTQLCLWDLTEDILFPHQPLSRARTHTNVMNATSPPAGSNGNSVTTPGNSVPPPLPRSNSL
PHSAVSNAGSKSSVMDGAIASGVSKFATLSLHDRKERHHEKDHKRNHSMGHISSKSSDKLNLVTKTKTDPAKTLGTPLCPRMEDVPL
LEPLICKKIAHERLTVLIFLEDCIVTACQEGFICTWGRPGKVVSFNP
>sp|Q9HD87|CF050_HUMAN
MANTQLDHLHYTTEFTRNDLLIICKKFNLMLMDEDIISLLAIFIKMCLWLWKQFLKRGSKCSETSELLEKVKLQLAFTAYKYVDICF
PEQMAYSRYIRWYIH
>sp|Q9Y3D2|MSRB2_HUMAN
MARLLWLLRGLTLGTAPRRAVRGQAGGGGPGTGPGLGEAGSLATCELPLAKSEWQKKLTPEQFYVTREKGTEPPFSGIYLNNKEAGM
YHCVCCDSPLFSSEKKYCSGTGWPSFSEAHGTSGSDESHTGILRRLDTSLGSARTEVVCKQCEAHLGHVFPDGPGPNGQRFCINSVA
LKFKPRKH
>sp|P01130|LDLR_HUMAN
MGPWGWKLRWTVALLLAAAGTAVGDRCERNEFQCQDGKCISYKWVCDGSAECQDGSDESQETCLSVTCKSGDFSCGGRVNRCIPQFW
RCDGQVDCDNGSDEQGCPPKTCSQDEFRCHDGKCISRQFVCDSDRDCLDGSDEASCPVLTCGPASFQCNSSTCIPQLWACDNDPDCE
DGSDEWPQRCRGLYVFQGDSSPCSAFEFHCLSGECIHSSWRCDGGPDCKDKSDEENCAVATCRPDEFQCSDGNCIHGSRQCDREYDC
KDMSDEVGCVNVTLCEGPNKFKCHSGECITLDKVCNMARDCRDWSDEPIKECGTNECLDNNGGCSHVCNDLKIGYECLCPDGFQLVA

QRRCEDIDECQDPDTCSQLCVNLEGGYKCQCEEGFQLDPHTKACKAVGSIAYLFFTNRHEVRKMTLDRSEYTSLIPNLRNVVALDTE
VASNRIYWSDLSQRMICSTQLDRAHGVSSYDTVISRDIQAPDGLAVDWIHSNIYWTDSVLGTVSVADTKGVKRKTLFRENGSKPRAI
VVDPVHGFMYWTDWGTPAKIKKGGLNGVDIYSLVTENIQWPNGITLDLLSGRLYWVDSKLHSISSIDVNGGNRKTILEDEKRLAHPF
SLAVFEDKVFWTDIINEAIFSANRLTGSDVNLLAENLLSPEDMVLFHNLTQPRGVNWCERTTLSNGGCQYLCLPAPQINPHSPKFTC
ACPDGMLLARDMRSCLTEAEAAVATQETSTVRLKVSSTAVRTQHTTTRPVPDTSRLPGATPGLTTVEIVTMSHQALGDVAGRGNEKK
PSSVRALSIVLPIVLLVFLCLGVFLLWKNWRLKNINSINFDNPVYQKTTEDEVHICHNQDGYSYPSRQMVSLEDDVA
>sp|Q04118|PRB3_HUMAN
MLLILLSVALLALSSAQSLNEDVSQEESPSVISGKPEGRRPQGGNQPQRTPPPPGKPEGRPPQGGNQSQGPPPRPGKPEGPPPQGGN
QSQGPPPRPGKPEGQPPQGGNQSQGPPPRPGKPEGPPPQGGNQSQGPPPRPGKPEGPPPQGGNQSQGPPPHPGKPEGPPPQGGNQSQ
GPPPRPGKPEGPPPQGGNQSQGPPPRPGKPEGPPPQGGNQSQGPPPRPGKPEGSPSQGGNKPQGPPPHPGKPQGPPPQEGNKPQRPP
PPGRPQGPPPPGGNPQQPLPPPAGKPQGPPPPPQGGRPHRPPQGQPPQ
>sp|Q9UKL0|RCOR1_HUMAN
MVEKGPEVSGKRRGRNNAAASASAAAASAAASAACASPAATAASGAAASSASAAAASAAAAPNNGQNKSLAAAAPNGNSSSNSWEEG
SSGSSSDEEHGGGGMRVGPQYQAVVPDFDPAKLARRSQERDNLGMLVWSPNQNLSEAKLDEYIAIAKEKHGYNMEQALGMLFWHKHN
IEKSLADLPNFTPFPDEWTVEDKVLFEQAFSFHGKTFHRIQQMLPDKSIASLVKFYYSWKKTRTKTSVMDRHARKQKREREESEDEL
EEANGNNPIDIEVDQNKESKKEVPPTETVPQVKKEKHSTQAKNRAKRKPPKGMFLSQEDVEAVSANATAATTVLRQLDMELVSVKRQ
IQNIKQTNSALKEKLDGGIEPYRLPEVIQKCNARWTTEEQLLAVQAIRKYGRDFQAISDVIGNKSVVQVKNFFVNYRRRFNIDEVLQ
EWEAEHGKEETNGPSNQKPVKSPDNSIKMPEEEDEAPVLDVRYASAS
>sp|Q9UFV1|TBC29_HUMAN
MGHLDKEGLCTQGSSFSWLLRVLNDGISLGLTPCLWDMYLLEGEQMLMLITSIAFKVQRSLYEETNKETWGPATPRALKGTGRARPI
CESLHSSLQALTASESSRGPSLLQTPPRVPGQQALSRGDKGISVSLSLPSLPSRRGRCGRIIG
>sp|Q86VG3|CK074_HUMAN
MSAHMSGLEIMDEDQLIKDVLDKFLNCHEQTYDEEFLNTFTHLSQEDHVSKRGVFGTDSSENIFTSAKVTHKNEADDYHLRNKTIFL
RTSSQCLEEQVDNFLDLEDLDMDEEIKPQMSEDLLLLPGEVEQDVSTSIPSCIPFVAQPPTCEVKPKPSVKRMDKQTEEILGDEVQL
FSLDEEFDYDNVMLTSKFSPAEIENIKELCKQQKRKDTSPDLEKSCD
>sp|P22455|FGFR4_HUMAN
MRLLLALLGVLLSVPGPPVLSLEASEEVELEPCLAPSLEQQEQELTVALGQPVRLCCGRAERGGHWYKEGSRLAPAGRVRGWRGRLE
IASFLPEDAGRYLCLARGSMIVLQNLTLITGDSLTSSNDDEDPKSHRDPSNRHSYPQQAPYWTHPQRMEKKLHAVPAGNTVKFRCPA
AGNPTPTIRWLKDGQAFHGENRIGGIRLRHQHWSLVMESVVPSDRGTYTCLVENAVGSIRYNYLLDVLERSPHRPILQAGLPANTTA
VVGSDVELLCKVYSDAQPHIQWLKHIVINGSSFGADGFPYVQVLKTADINSSEVEVLYLRNVSAEDAGEYTCLAGNSIGLSYQSAWL
TVLPEEDPTWTAAAPEARYTDIILYASGSLALAVLLLLAGLYRGQALHGRHPRPPATVQKLSRFPLARQFSLESGSSGKSSSSLVRG
VRLSSSGPALLAGLVSLDLPLDPLWEFPRDRLVLGKPLGEGCFGQVVRAEAFGMDPARPDQASTVAVKMLKDNASDKDLADLVSEME
VMKLIGRHKNIINLLGVCTQEGPLYVIVECAAKGNLREFLRARRPPGPDLSPDGPRSSEGPLSFPVLVSCAYQVARGMQYLESRKCI
HRDLAARNVLVTEDNVMKIADFGLARGVHHIDYYKKTSNGRLPVKWMAPEALFDRVYTHQSDVWSFGILLWEIFTLGGSPYPGIPVE
ELFSLLREGHRMDRPPHCPPELYGLMRECWHAAPSQRPTFKQLVEALDKVLLAVSEEYLDLRLTFGPYSPSGGDASSTCSSSDSVFS
HDPLPLGSSSFPFGSGVQT
>sp|P62714|PP2AB_HUMAN
MDDKAFTKELDQWVEQLNECKQLNENQVRTLCEKAKEILTKESNVQEVRCPVTVCGDVHGQFHDLMELFRIGGKSPDTNYLFMGDYV
DRGYYSVETVTLLVALKVRYPERITILRGNHESRQITQVYGFYDECLRKYGNANVWKYFTDLFDYLPLTALVDGQIFCLHGGLSPSI
DTLDHIRALDRLQEVPHEGPMCDLLWSDPDDRGGWGISPRGAGYTFGQDISETFNHANGLTLVSRAHQLVMEGYNWCHDRNVVTIFS
APNYCYRCGNQAAIMELDDTLKYSFLQFDPAPRRGEPHVTRRTPDYFL
>sp|B7Z8K6|TRDC_HUMAN
SQPHTKPSVFVMKNGTNVACLVKEFYPKDIRINLVSSKKITEFDPAIVISPSGKYNAVKLGKYEDSNSVTCSVQHDNKTVHSTDFEV
KTDSTDHVKPKETENTKQPSKSCHKPKAIVHTEKVNMMSLTVLGLRMLFAKTVAVNFLLTAKLFFL

>sp|Q5TEZ5|CF163_HUMAN
MIRNSDYKNFVCCAVCNKIIPPAPFGKTFKRIHEYKPLKTRFYTHKDILDIGANILKKEEQFQEDILREHIAKAEAEVWAQANERQK
QAVEKALEEANDRHKIEIQILKEEHQKDLQEVTAKTKTEMYQNMDDEMKREHLAAEQRMVHRIQRIMMECHREKVEAVEKARAEERH
IAQEAIQAQKSKAVEEIVNTGVTVIKDEKTSVARLMREKEHEMSILYGIAQRQRQEEVQEVLQEAEKTHQATLGNMMDKLANTQGEL
LSIAKQLGIMTNWKDFLEEELQETRMAFQKYINYTFPKLSPGHADFILPERKKTPSNLVIKENKTTLD
>sp|Q9H0R6|GATA_HUMAN
MLGRSLREVSAALKQGQITPTELCQKCLSLIKKTKFLNAYITVSEEVALKQAEESEKRYKNGQSLGDLDGIPIAVKDNFSTSGIETT
CASNMLKGYIPPYNATVVQKLLDQGALLMGKTNLDEFAMGSGSTDGVFGPVKNPWSYSKQYREKRKQNPHSENEDSDWLITGGSSGG
SAAAVSAFTCYAALGSDTGGSTRNPAAHCGLVGFKPSYGLVSRHGLIPLVNSMDVPGILTRCVDDAAIVLGALAGPDPRDSTTVHEP
INKPFMLPSLADVSKLCIGIPKEYLVPELSSEVQSLWSKAADLFESEGAKVIEVSLPHTSYSIVCYHVLCTSEVASNMARFDGLQYG
HRCDIDVSTEAMYAATRREGFNDVVRGRILSGNFFLLKENYENYFVKAQKVRRLIANDFVNAFNSGVDVLLTPTTLSEAVPYLEFIK
EDNRTRSAQDDIFTQAVNMAGLPAVSIPVALSNQGLPIGLQFIGRAFCDQQLLTVAKWFEKQVQFPVIQLQELMDDCSAVLENEKLA
SVSLKQ
>sp|P22087|FBRL_HUMAN
MKPGFSPRGGGFGGRGGFGDRGGRGGRGGFGGGRGRGGGFRGRGRGGGGGGGGGGGGRGGGGFHSGGNRGRGRGGKRGNQSGKNVM
VEPHRHEGVFICRGKEDALVTKNLVPGESVYGEKRVSISEGDDKIEYRAWNPFRSKLAAAILGGVDQIHIKPGAKVLYLGAASGTTV
SHVSDIVGPDGLVYAVEFSHRSGRDLINLAKKRTNIIPVIEDARHPHKYRMLIAMVDVIFADVAQPDQTRIVALNAHTFLRNGGHFV
ISIKANCIDSTASAEAVFASEVKKMQQENMKPQEQLTLEPYERDHAVVVGVYRPPPKVKN
>sp|Q5T0F9|C2D1B_HUMAN
MMPGPRPRKGPQARGQGVAAAKQMGLFMEFGPEDMLLGMDEAEDDEDLEAELLALTGEAQTTGKKPAPKGQAPLPMAHIEKLAADCM
RDVEEEEEEEGLEEDAELLTELQEVLGVDEETEPLDGDEVADPGGSEEENGLEDTEPPVQTAVLTASAPAAQAGASQGLHALLEERI
HNYREAAASAKEAGEAAKARRCERGLKTLESQLASVRRGRKINEDEIPPPVALGKRPLAPQEPANRSPETDPPAPPALESDNPSQPE
TSLPGISAQPVSDLDPDPRALLSSRQREYKVAALSAKRAGELDRARELMRIGKRFGAVLEALEKGQPVDLSAMPPAPEDLKPQQASQ
APTAPSVIPPAVERVQPVMAPDVPATPVAPTESQTVLDALQQRLNKYREAGIQARSGGDERKARMHERIAKQYQDAIRAHRAGRKVN
FAELPVPPGFPPIPGLESTMGVEEDAVAATLAAAEKLASAEDSAPADKDEDEPPGHLQGEPPAQAPVAKKPARPTVPSSQRLPEPRA
SSSKESPSPSVREQLALLEARKLQYQRAALQAKRSQDLEQAKAYLRVAKWLEAQIIQARSGRPVDLSKVPSPLTDEEGDFILIHHED
LRLSQKAEEVYAQLQKMLLEQQEKCLLFSKQFMHQGNVAETTRFEKLAQDRKKQLEILQLAQAQGLDPPTHHFELKTFQTVRIFSEL
NSTEMHLIIVRGMNLPAPPGVTPDDLDAFVRFEFHYPNSDQAQKSKTAVVKNTNSPEFDQLFKLNINRNHRGFKRVIQSKGIKFEIF
HKGSFFRSDKLVGTAHLKLERLENECEIREIVEVLDGRKPTGGKLEVKVRLREPLSGQDVQMVTENWLVLEPRGL
>sp|Q8WXH6|RB40A_HUMAN
MSAPGSPDQAYDFLLKFLLVGDRDVGKSEILESLQDGAAESPYSHLGGIDYKTTTILLDGQRVKLKLWDTSGQGRFCTIFRSYSRGA
QGVILVYDIANRWSFEGMDRWIKKIEEHAPGVPKILVGNRLHLAFKRQVPREQAQAYAERLGVTFFEVSPLCNFNIIESFTELARIV
LLRHRMNWLGRPSKVLSLQDLCCRTIVSCTPVHLVDKLPLPSTLRSHLKSFSMAKGLNARMMRGLSYSLTTSSTHKSSLCKVEIVCP
PQSPPKNCTRNSCKIS
>sp|Q8NDI1|EHBP1_HUMAN
MASVWKRLQRVGKHASKFQFVASYQELMVECTKKWQPDKLVVVWTRRSRRKSSKAHSWQPGIKNPYRGVVVWPVPENIEITVTLFKD
PHAEEFEDKEWTFVIENESPSGRRKALATSSINMKQYASPMPTQTDVKLKFKPLSKKVVSAALQFSLSCIFLREGKATDEDMQSLAS
LMSMKQADIGNLDDFEEDNEDDDENRVNQEEKAAKITEIVNQLNALSSLDEDQDDCIKQANMRSAKSASSSEELINKLNFLDEAEKD
LATVNSNPFDDPDAAELNPFGDPDSEEPITETASPRKTEDSFYNNSYNPFKEVQTPQYLNPFDEPEAFVTIKDSPPQSTKRKNIRPV
DMSKYLYADSSKTEEEELDESNPFYEPKSTPPPNNLVNPVQELETERRVKRKAPAPPVLSPKTGVLNENTVSAGKDLSTSPKPSPIP
SPVLGRKPNASQSLLVWCKEVTKNYRGVKITNFTTSWRNGLSFCAILHHFRPDLIDYKSLNPQDIKENNKKAYDGFASIGISRLLEP
SDMVLLAIPDKLTVMTYLYQIRAHFSGQELNVVQIEENSSKSTYKVGNYETDTNSSVDQEKFYAELSDLKREPELQQPISGAVDFLS
QDDSVFVNDSGVGESESEHQTPDDHLSPSTASPYCRRTKSDTEPQKSQQSSGRTSGSDDPGICSNTDSTQAQVLLGKKRLLKAETLE
LSDLYVSDKKKDMSPPFICEETDEQKLQTLDIGSNLEKEKLENSRSLECRSDPESPIKKTSLSPTSKLGYSYSRDLDLAKKKHASLR

QTESDPDADRTTLNHADHSSKIVQHRLLSRQEELKERARVLLEQARRDAALKAGNKHNTNTATPFCNRQLSDQQDEERRRQLRERAR
QLIAEARSGVKMSELPSYGEMAAEKLKERSKASGDENDNIEIDTNEEIPEGFVVGGGDELTNLENDLDTPEQNSKLVDLKLKKLLEV
QPQVANSPSSAAQKAVTESSEQDMKSGTEDLRTERLQKTTERFRNPVVFSKDSTVRKTQLQSFSQYIENRPEMKRQRSIQEDTKKGN
EEKAAITETQRKPSEDEVLNKGFKDTSQYVVGELAALENEQKQIDTRAALVEKRLRYLMDTGRNTEEEEAMMQEWFMLVNKKNALIR
RMNQLSLLEKEHDLERRYELLNRELRAMLAIEDWQKTEAQKRREQLLLDELVALVNKRDALVRDLDAQEKQAEEEDEHLERTLEQNK
GKMAKKEEKCVLQ
>sp|Q9BPW4|APOL4_HUMAN
MEGAALLKIFVVCIWVQQNHPGWTVAGQFQEKKRFTEEVIEYFQKKVSPVHLKILLTSDEAWKRFVRVAELPREEADALYEALKNLT
PYVAIEDKDMQQKEQQFREWFLKEFPQIRWKIQESIERLRVIANEIEKVHRGCVIANVVSGSTGILSVIGVMLAPFTAGLSLSITAA
GVGLGIASATAGIASSIVENTYTRSAELTASRLTATSTDQLEALRDILRDITPNVLSFALDFDEATKMIANDVHTLRRSKATVGRPL
IAWRYVPINVVETLRTRGAPTRIVRKVARNLGKATSGVLVVLDVVNLVQDSLDLHKGAKSESAESLRQWAQELEENLNELTHIHQSL
KAG
>sp|B9ZVM9|TCP2L_HUMAN
MLEGQLEAREPKEGTHPEDPCPGAGAAMEKTPAAAEVPREDSNAGEMPSLQQQITSLHQELGRQQSLWADIHRKLQSHMDALRKQNR
ELREELRGLQRQQWEAGKKPAASPHAGRESHTLALEPAFGKISPLSADEETTPKYAGRKSQSATLLGQRWSSNHLAPPKPMSLKTER
INSGKTPPQEDREKSPPGRRQDRSPAPTGRPTPGAERREVSEDGKIMHPSSRSLQNSGGRKSPVQASQAATLQEQTAAAGVADRSSS
VLGSSEGGFLSRVQAEEFASSSPDSAERQNLPVNPPSSLEIAQAMDTKMKKEEVQEEKRHPNGKADDCRRSGFPSEFPGALHAAPSR
QDMGP
>sp|P68431|H31_HUMAN
MARTKQTARKSTGGKAPRKQLATKAARKSAPATGGVKKPHRYRPGTVALREIRRYQKSTELLIRKLPFQRLVREIAQDFKTDLRFQS
SAVMALQEACEAYLVGLFEDTNLCAIHAKRVTIMPKDIQLARRIRGERA
>sp|Q6ZN30|BNC2_HUMAN
MAHLGPTPPPHSLNYKSEDRLSEQDWPAYFKVPCCGVDTSQIESEEAEVDVRERETQRDREPKRARDLTLRDSCTDNSMQFGTRTTT
AEPGFMGTWQNADTNLLFRMSQQAIRCTLVNCTCECFQPGKINLRTCDQCKHGWVAHALDKLSTQHLYHPTQVEIVQSNVVFDISSL
MLYGTQAVPVRLKILLDRLFSVLKQEEVLHILHGLGWTLRDYVRGYILQDAAGKVLDRWAIMSREEEIITLQQFLRFGETKSIVELM
AIQEKEGQAVAVPSSKTDSDIRTFIESNNRTRSPSLLAHLENSNPSSIHHFENIPNSLAFLLPFQYINPVSAPLLGLPPNGLLLEQP
GLRLREPSLSTQNEYNESSESEVSPTPYKNDQTPNRNALTSITNVEPKTEPACVSPIQNSAPVSDLTKTEHPKSSFRIHRMRRMGSA
SRKGRVFCNACGKTFYDKGTLKIHYNAVHLKIKHRCTIEGCNMVFSSLRSRNRHSANPNPRLHMPMLRNNRDKDLIRATSGAATPVI
ASTKSNLALTSPGRPPMGFTTPPLDPVLQNPLPSQLVFSGLKTVQPVPPFYRSLLTPGEMVSPPTSLPTSPIIPTSGTIEQHPPPPS
EPVVPAVMMATHEPSADLAPKKKPRKSSMPVKIEKEIIDTADEFDDEDDDPNDGGAVVNDMSHDNHCHSQEEMSPGMSVKDFSKHNR
TRCISRTEIRRADSMTSEDQEPERDYENESESSEPKLGEESMEGDEHIHSEVSEKVLMNSERPDENHSEPSHQDVIKVKEEFTDPTY
DMFYMSQYGLYNGGGASMAALHESFTSSLNYGSPQKFSPEGDLCSSPDPKICYVCKKSFKSSYSVKLHYRNVHLKEMHVCTVAGCNA
AFPSRRSRDRHSANINLHRKLLTKELDDMGLDSSQPSLSKDLRDEFLVKIYGAQHPMGLDVREDASSPAGTEDSHLNGYGRGMAEDY
MVLDLSTTSSLQSSSSIHSSRESDAGSDEGILLDDIDGASDSGESAHKAEAPALPGSLGAEVSGSLMFSSLSGSNGGIMCNICHKMY
SNKGTLRVHYKTVHLREMHKCKVPGCNMMFSSVRSRNRHSQNPNLHKNIPFTSVD
>sp|Q8NFZ3|NLGNY_HUMAN
MLRPQGLLWLPLLFTSVCVMLNSNVLLWITALAIKFTLIDSQAQYPVVNTNYGKIQGLRTPLPSEILGPVEQYLGVPYASPPTGERR
FQPPESPSSWTGIRNATQFSAVCPQHLDERFLLHDMLPIWFTTSLDTLMTYVQDQNEDCLYLNIYVPMEDDIHEQNSKKPVMVYIHG
GSYMEGTGNMIDGSILASYGNVIVITINYRLGILGFLSTGDQAAKGNYGLLDQIQALRWIEENVGAFGGDPKRVTIFGSGAGASCVS
LLTLSHYSEGLFQKAIIQSGTALSSWAVNYQPAKYTRILADKVGCNMLDTTDMVECLKNKNYKELIQQTITPATYHIAFGPVIDGDV
IPDDPQILMEQGEFLNYDIMLGVNQGEGLKFVDGIVDNEDGVTPNDFDFSVSNFVDNLYGYPEGKDTLRETIKFMYTDWADKENPET
RRKTLVALFTDHQWVAPAVATADLHAQYGSPTYFYAFYHHCQSEMKPSWADSAHGDEVPYVFGIPMIGPTELFSCNFSKNDVMLSAV
VMTYWTNFAKTGDPNQPVPQDTKFIHTKPNRFEEVAWSKYNPKDQLYLHIGLKPRVRDHYRATKVAFWLELVPHLHNLNEIFQYVST
TTKVPPPDMTSFPYGTRRSPAKIWPTTKRPAITPANNPKHSKDPHKTGPEDTTVLIETKRDYSTELSVTIAVGASLLFLNILAFAAL

YYKKDKRRHETHRHPSPQRNTTNDITHIQNEEIMSLQMKQLEHDHECESLQAHDTLRLTCPPDYTLTLRRSPDDIPFMTPNTITMIP
NTLMGMQPLHTFKTFSGGQNSTNLPHGHSTTRV
>sp|Q9ULQ0|STRP2_HUMAN
MEDPAAPGTGGPPANGNGNGGGKGKQAAPKGREAFRSQRRESEGSVDCPTLEFEYGDADGHAAELSELYSYTENLEFTNNRRCFEED
FKTQVQGKEWLELEEDAQKAYIMGLLDRLEVVSRERRLKVARAVLYLAQGTFGECDSEVDVLHWSRYNCFLLYQMGTFSTFLELLHM
EIDNSQACSSALRKPAVSIADSTELRVLLSVMYLMVENIRLERETDPCGWRTARETFRTELSFSMHNEEPFALLLFSMVTKFCSGLA
PHFPIKKVLLLLWKVVMFTLGGFEHLQTLKVQKRAELGLPPLAEDSIQVVKSMRAASPPSYTLDLGESQLAPPPSKLRGRRGSRRQL
LTKQDSLDIYNERDLFKTEEPATEEEEESAGDGERTLDGELDLLEQDPLVPPPPSQAPLSAERVAFPKGLPWAPKVRQKDIEHFLEM
SRNKFIGFTLGQDTDTLVGLPRPIHESVKTLKQHKYISIADVQIKNEEELEKCPMSLGEEVVPETPCEILYQGMLYSLPQYMIALLK
ILLAAAPTSKAKTDSINILADVLPEEMPITVLQSMKLGIDVNRHKEIIVKSISTLLLLLLKHFKLNHIYQFEYVSQHLVFANCIPLI
LKFFNQNILSYITAKNSISVLDYPCCTIQDLPELTTESLEAGDNSQFCWRNLFSCINLLRLLNKLTKWKHSRTMMLVVFKSAPILKR
ALKVKQAMLQLYVLKLLKLQTKYLGRQWRKSNMKTMSAIYQKVRHRMNDDWAYGNDIDARPWDFQAEECTLRANIEAFNSRRYDRPQ
DSEFSPVDNCLQSVLGQRLDLPEDFHYSYELWLEREVFSQPICWEELLQNH
>sp|Q8NHC6|O14L1_HUMAN
MSPDTFLKGFAEFFLMGFSNSWDIQIVHAALFFLVYLAAVIGNLLIIILTTLDVHLQTPMYFFLRNLSFLDFCYISVTIPKSIVSSL
THDTSISFFGCALQAFFFMDLATTEVAILTVMSYDRYMAICRPLHYEVIINQGVCLRMMAMSWLSGVICGFMHVIATFSLPFCGRNR
IRQFFCNIPQLLSLLDPKVITIEIGVMVFGTSLVIISFVVITLSYMYIFSVIMRIPSKEGRSKTFSTCIPHLVVVTLFMISGSIAYV
KPISNSPPVLDVFLSAFYTVVPPTLNPVIYSLRNRDMKAALRRQCGP
>sp|O00628|PEX7_HUMAN
MSAVCGGAARMLRTPGRHGYAAEFSPYLPGRLACATAQHYGIAGCGTLLILDPDEAGLRLFRSFDWNDGLFDVTWSENNEHVLITCS
GDGSLQLWDTAKAAGPLQVYKEHAQEVYSVDWSQTRGEQLVVSGSWDQTVKLWDPTVGKSLCTFRGHESIIYSTIWSPHIPGCFASA
SGDQTLRIWDVKAAGVRIVIPAHQAEILSCDWCKYNENLLVTGAVDCSLRGWDLRNVRQPVFELLGHTYAIRRVKFSPFHASVLASC
SYDFTVRFWNFSKPDSLLETVEHHTEFTCGLDFSLQSPTQVADCSWDETIKIYDPACLTIPA
>sp|Q15435|PP1R7_HUMAN
MAAERGAGQQQSQEMMEVDRRVESEESGDEEGKKHSSGIVADLSEQSLKDGEERGEEDPEEEHELPVDMETINLDRDAEDVDLNHYR
IGKIEGFEVLKKVKTLCLRQNLIKCIENLEELQSLRELDLYDNQIKKIENLEALTELEILDISFNLLRNIEGVDKLTRLKKLFLVNN
KISKIENLSNLHQLQMLELGSNRIRAIENIDTLTNLESLFLGKNKITKLQNLDALTNLTVLSMQSNRLTKIEGLQNLVNLRELYLSH
NGIEVIEGLENNNKLTMLDIASNRIKKIENISHLTELQEFWMNDNLLESWSDLDELKGARSLETVYLERNPLQKDPQYRRKVMLALP
SVRQIDATFVRF
>sp|P22234|PUR6_HUMAN
MATAEVLNIGKKLYEGKTKEVYELLDSPGKVLLQSKDQITAGNAARKNHLEGKAAISNKITSCIFQLLQEAGIKTAFTRKCGETAFI
APQCEMIPIEWVCRRIATGSFLKRNPGVKEGYKFYPPKVELFFKDDANNDPQWSEEQLIAAKFCFAGLLIGQTEVDIMSHATQAIFE
ILEKSWLPQNCTLVDMKIEFGVDVTTKEIVLADVIDNDSWRLWPSGDRSQQKDKQSYRDLKEVTPEGLQMVKKNFEWVAERVELLLK
SESQCRVVVLMGSTSDLGHCEKIKKACGNFGIPCELRVTSAHKGPDETLRIKAEYEGDGIPTVFVAVAGRSNGLGPVMSGNTAYPVI
SCPPLTPDWGVQDVWSSLRLPSGLGCSTVLSPEGSAQFAAQIFGLSNHLVWSKLRASILNTWISLKQADKKIRECNL
>sp|Q9Y376|CAB39_HUMAN
MPFPFGKSHKSPADIVKNLKESMAVLEKQDISDKKAEKATEEVSKNLVAMKEILYGTNEKEPQTEAVAQLAQELYNSGLLSTLVADL
QLIDFEGKKDVAQIFNNILRRQIGTRTPTVEYICTQQNILFMLLKGYESPEIALNCGIMLRECIRHEPLAKIILWSEQFYDFFRYVE
MSTFDIASDAFATFKDLLTRHKLLSAEFLEQHYDRFFSEYEKLLHSENYVTKRQSLKLLGELLLDRHNFTIMTKYISKPENLKLMMN
LLRDKSRNIQFEAFHVFKVFVANPNKTQPILDILLKNQAKLIEFLSKFQNDRTEDEQFNDEKTYLVKQIRDLKRPAQQEA
>sp|Q5T1B1|CJ091_HUMAN
MWSFLPGAESVSMGPVPGVSSLGACWTHDQDSGRAEDRPQAPRITQYTWVLSFLFTEKPQTRSTSPISHQGQPQTTRALSLRQPQHP
SAPASGRPRPPHSSGPDLAEAAPVVDQASQAAGRASSGLGLWEQASVSQGFRNAAFEA
>sp|O60732|MAGC1_HUMAN

MGDKDMPTAGMPSLLQSSSESPQSCPEGEDSQSPLQIPQSSPESDDTLYPLQSPQSRSEGEDSSDPLQRPPEGKDSQSPLQIPQSSP
EGDDTQSPLQNSQSSPEGKDSLSPLEISQSPPEGEDVQSPLQNPASSFFSSALLSIFQSSPESTQSPFEGFPQSVLQIPVSAASSST
LVSIFQSSPESTQSPFEGFPQSPLQIPVSRSFSSTLLSIFQSSPERTQSTFEGFAQSPLQIPVSPSSSSTLLSLFQSFSERTQSTFE
GFAQSSLQIPVSPSFSSTLVSLFQSSPERTQSTFEGFPQSPLQIPVSSSSSSSTLLSLFQSSPERTHSTFEGFPQSLLQIPMTSSFSS
TLLSIFQSSPESAQSTFEGFPQSPLQIPGSPSFSSTLLSLFQSSPERTHSTFEGFPQSPLQIPMTSSFSSTLLSILQSSPESAQSAF
EGFPQSPLQIPVSSSFSYTLLSLFQSSPERTHSTFEGFPQSPLQIPVSSSSSSSTLLSLFQSSPECTQSTFEGFPQSPLQIPQSPPE
GENTHSPLQIVPSLPEWEDSLSPHYFPQSPPQGEDSLSPHYFPQSPPQGEDSLSPHYFPQSPQGEDSLSPHYFPQSPPQGEDSMSPL
YFPQSPLQGEEFQSSLQSPVSICSSSTPSSLPQSFPESSQSPPEGPVQSPLHSPQSPPEGMHSQSPLQSPESAPEGEDSLSPLQIPQ
SPLEGEDSLSSLHFPQSPPEWEDSLSPLHFPQFPPQGEDFQSSLQSPVSICSSSTSLSLPQSFPESPQSPPEGPAQSPLQRPVSSFF
SYTLASLLQSSHESPQSPPEGPAQSPLQSPVSSFPSSTSSSLSQSSPVSSFPSSTSSSLSKSSPESPLQSPVISFSSSTSLSPFSEE
SSSPVDEYTSSSDTLLESDSLTDSESLIESEPLFTYTLDEKVDELARFLLLKYQVKQPITKAEMLTNVISRYTGYFPVIFRKAREFI
EILFGISLREVDPDDSYVFVNTLDLTSEGCLSDEQGMSQNRLLILILSIIFIKGTYASEEVIWDVLSGIGVRAGREHFAFGEPRELL
TKVWVQEHYLEYREVPNSSPPRYEFLWGPRAHSEVIKRKVVEFLAMLKNTVPITFPSSYKDALKDVEERAQAIIDTTDDSTATESAS
SSVMSPSFSSE
>sp|O14581|OR7AH_HUMAN
MEPENDTGISEFVLLGLSEEPELQPFLFGLFLSMYLVTVLGNLLIILATISDSHLHTPMYFFLSNLSFADICFISTTIPKMLINIQT
QSRVITYAGCITQMCFFVLFGGLDSLLLAVMAYDRFVAICHPLHYTVIMNPRLCGLLVLASWMIAALNSLSQSLMVLWLSFCTDLEI
PHFFCELNQVIHLACSDTFLNDMGMYFAAGLLAGGPLVGILCSYSKIVSSIRAISSAQGKYKAFSTCASHLSVVSLFCCTGLGVYLT
SAATHNSHTSATASVMYTVATPMLNPFIYSLRNKDIKRALKMSFRGKQ
>sp|Q8N5Q1|F71E2_HUMAN
MIWLRNRRCLEPLQGTPKWVPVLGELQKTLQKGEYLPLRPLPMFESNFVQVTHQGGPVFVNHRTNRLAMGVAASLPGLVLPDILLIG
QPAEDRDCSGLVLTRMIPLDLVHLCVHDLSAWRLKLRLVSGRQYYLALDAPDNEVGFLFHCWVRLINLLQEPAPTWTPRTTRTAPLD
MPLAEAPASTWHLQDQPISRHAVRVAERNFPHKTVAAQRQRKAKALKRSFKSQAVGDSVPLIWSQLEHADVRKKPAEKKSHSDPRPD
RTHTQIRLPEKTSITTWTIFSIISSTANQTQSSPKACTSASDEATGQGHVVESPSHCVSADSPDGFFLGSCSSLDPCLWHQDTEDLM
DSGGSTLSSAASGLAPYPPAACLSTPYSSIPRGREKAGPMGSHQGPGPPPCQKAPSGLVTSCKAPFLVDQSQKLPAVPASSWKPPPG
LAPPQKAPAASAPPRKAPAVPAPSQKAPAVPAPSQKAPAIPAPSRKASAASASPRKASAVPAPPQKTPPPSQKAPSVPTIPQKAVSP
TAPKKKSLLLPAPSQKALPTSPTEYQMALSPPASRGKLPGDFDVLPTGIPGRAVLERSQSGGKPEPVVTVRTQETDVVEMTTQAKSP
ESPFTVTKKESKDILISQTKEVTLEAFRGQGKLEDWAHWAKLEERSPDLPGVRSKELEQRKRWVKAKELAVEGPSQEHSRPFSVEAL
TLTKLMITANSKEQPSKSALVSLPSWLLATPQASATSMMASVPSRPGQLSLLEGKPVVVREQPESHTWVKEGKRPWGEMKEQPWGEM
KEPPWDPKGPPKVPFRSKPTSASLKREGISQAPIPLTASPWEDLRPSPLSETLISKMEATARASQQPKRVSQEPMRMPAQHPLATVG
SSSEILLPMLLELETVRNTATKAEEIQEESGVLNLLPSLQHSQHSEWPDAGA
>sp|Q8IV33|K0825_HUMAN
MDWDDEYSHNSFDLHCLLNSFPGDLEFEQIFSDIDEKIEQNAASIKHCIKEIQSEINKQCPGVQLQTTTDCFEWLTNYNYSTSESSF
ISHGDLIKFFKTLQDLLKNEQNQEEMTLDLLWDLSCHSSVSFPSTLSGTSFHFLSRTSLHSVEDNSSMDVKSMWDDIRLHLRRFLVS
KLQSHNEINNSQQKILLKKQCLQQLLFLYPESEVIIKYQNIQNKLLANLLWNCFPSYNRDSNLDVIAHGYQSTMLKLYSVIKEDFNT
LCEILAPSSMVKFIKETYLDTVTEEMAKFLENFCELQFRENAVRVVKTSKSSSKHRGAVHALVTTECPQKGRNFSLPLDKVEFLSQL
IKSFMKLEKGVQELFDEILLSLKITRDTSGILEKSDREVVMEKPRANETNIPSEQSLPGKEATLLDFGWRSAFKEVSLPMAHCVVTA
IEGFSTKILQQEQNERSSAVSYAMNLVNVQQVWQDSHMFPEEEQPKKIGKFCSDIMEKLDTMLPLALACRDDSFQEIRANLVEACCK
VATAVLQRLQERAKEVPSKAPLKNLHTYLSTAVYVFQHFKRYDNLMKEMTKKPIFLVLVQRYQEFINTLQFQVTNYCVRVCATSILQ
DAESHHWDDYKAFYEGERCSFSIQMWHYFCWSLHYDLWTILPPKLAQEILVEVLEKSLSLLASRYARAHPSRKRTPQLRLDVTTILI
CTENMLWSVCTSVQKLLNPHQHTDDKIFKIHTHCNNLFTTLVILTSPLTELYKTFQHGLDESASDSLKSFFKQPLYWVSCISHFYPS
LLRTPSAGGLKAEGQLKLLLSQPRCNWNLLLETLLHHDGLLLRILLKSSKQVSDTENNLNQGPSLMEAIFKILYHCSFSPQTFANVF
VSYMEEEQLWDFLYNIPVSTCVEYELEVIRCLRLALTDAIKDTVQQIVSVMSSRRNCETNLNKHIVPDCLLESMPKEWNYSPKETNR
KESCKSFTRLTAQAVSIVISKLPTVIACLPPPVKYFFFLSERKMSKKFVELKKAGLLVWNLIVIICRIFEDGNTVELLTGASLDRWS

KEKLGLICMCLKSIMGDQTSIHNQMIQKVIQSIEQQKPNWIERQLLKARKLSTECAFMTIEKSTALQEGDVALELTEQKINTMVLDL
CHKPGGREYLRQIYHIMQLNEEYLKEQLFSMNSSEEKPLPIRPLKTTLRSIEDQPSAFNPFHVYKAFSENMLDQVSITKWNWNWAKL
LPNYLRLDKMTFSVLLKNRWEMKKDETLEEEEKAILEHLKQICTPQNSSASDNIEEQ
>sp|Q01167|FOXK2_HUMAN
MAAAAAALSGAGTPPAGGGAGGGGAGGGGSPPGGWAVARLEGREFEYLMKKRSVTIGRNSSQGSVDVSMGHSSFISRRHLEIFTPPG
GGGHGGAAPELPPAQPRPDAGGDFYLRCLGKNGVFVDGVFQRRGAPPLQLPRVCTFRFPSTNIKITFTALSSEKREKQEASESPVKA
VQPHISPLTINIPDTMAHLISPLPSPTGTISAANSCPSSPRGAGSSGYKVGRVMPSDLNLMADNSQPENEKEASGGDSPKDDSKPPY
SYAQLIVQAITMAPDKQLTLNGIYTHITKNYPYYRTADKGWQNSIRHNLSLNRYFIKVPRSQEEPGKGSFWRIDPASESKLIEQAFR
KRRPRGVPCFRTPLGPLSSRSAPASPNHAGVLSAHSSGAQTPESLSREGSPAPLEPEPGAAQPKLAVIQEARFAQSAPGSPLSSQPV
LITVQRQLPQAIKPVTYTVATPVTTSTSQPPVVQTVHVVHQIPAVSVTSVAGLAPANTYTVSGQAVVTPAAVLAPPKAEAQENGDHR
EVKVKVEPIPAIGHATLGTASRIIQTAQTTPVQTVTIVQQAPLGQHQLPIKTVTQNGTHVASVPTAVHGQVNNAAASPLHMLATHAS
ASASLPTKRHNGDQPEQPELKRIKTEDGEGIVIALSVDTPPAAVREKGVQN
>sp|Q9NVS2|RT18A_HUMAN
MAALKALVSGCGRLLRGLLAGPAATSWSRLPARGFREVVETQEGKTTIIEGRITATPKESPNPPNPSGQCPICRWNLKHKYNYDDVL
LLSQFIRPHGGMLPRKITGLCQEEHRKIEECVKMAHRAGLLPNHRPRLPEGVVPKSKPQLNRYLTRWAPGSVKPIYKKGPRWNRVRM
PVGSPLLRDNVCYSRTPWKLYH
>sp|Q96HA8|NTAQ1_HUMAN
MEGNGPAAVHYQPASPPRDACVYSSCYCEENIWKLCEYIKNHDQYPLEECYAVFISNERKMIPIWKQQARPGDGPVIWDYHVVLLHV
SSGGQNFIYDLDTVLPFPCLFDTYVEDAFKSDDDIHPQFRRKFRVIRADSYLKNFASDRSHMKDSSGNWREPPPPYPCIETGDSKMN
LNDFISMDPKVGWGAVYTLSEFTHRFGSKNC
>sp|P36956|SRBP1_HUMAN
MDEPPFSEAALEQALGEPCDLDAALLTDIEDMLQLINNQDSDFPGLFDPPYAGSGAGGTDPASPDTSSPGSLSPPPATLSSSLEAFL
SGPQAAPSPLSPPQPAPTPLKMYPSMPAFSPGPGIKEESVPLSILQTPTPQPLPGALLPQSFPAPAPPQFSSTPVLGYPSPPGGFST
GSPPGNTQQPLPGLPLASPPGVPPVSLHTQVQSVVPQQLLTVTAAPTAAPVTTTVTSQIQQVPVLLQPHFIKADSLLLTAMKTDGAT
VKAAGLSPLVSGTTVQTGPLPTLVSGGTILATVPLVVDAEKLPINRLAAGSKAPASAQSRGEKRTAHNAIEKRYRSSINDKIIELKD
LVVGTEAKLNKSAVLRKAIDYIRFLQHSNQKLKQENLSLRTAVHKSKSLKDLVSACGSGGNTDVLMEGVKTEVEDTLTPPPSDAGSP
FQSSPLSLGSRGSGSGGSGSDSEPDSPVFEDSKAKPEQRPSLHSRGMLDRSRLALCTLVFLCLSCNPLASLLGARGLPSPSDTTSVY
HSPGRNVLGTESRDGPGWAQWLLPPVVWLLNGLLVLVSLVLLFVYGEPVTRPHSGPAVYFWRHRKQADLDLARGDFAQAAQQLWLAL
RALGRPLPTSHLDLACSLLWNLIRHLLQRLWVGRWLAGRAGGLQQDCALRVDASASARDAALVYHKLHQLHTMGKHTGGHLTATNLA
LSALNLAECAGDAVSVATLAEIYVAAALRVKTSLPRALHFLTRFFLSSARQACLAQSGSVPPAMQWLCHPVGHRFFVDGDWSVLSTP
WESLYSLAGNPVDPLAQVTQLFREHLLERALNCVTQPNPSPGSADGDKEFSDALGYLQLLNSCSDAAGAPAYSFSISSSMATTTGVD
PVAKWWASLTAVVIHWLRRDEEAAERLCPLVEHLPRVLQESERPLPRAALHSFKAARALLGCAKAESGPASLTICEKASGYLQDSLA
TTPASSSIDKAVQLFLCDLLLVVRTSLWRQQQPPAPAPAAQGTSSRPQASALELRGFQRDLSSLRRLAQSFRPAMRRVFLHEATARL
MAGASPTRTHQLLDRSLRRRAGPGGKGGAVAELEPRPTRREHAEALLLASCYLPPGFLSAPGQRVGMLAEAARTLEKLGDRRLLHDC
QQMLMRLGGGTTVTSS
>sp|Q9P0M6|H2AW_HUMAN
MSGRSGKKKMSKLSRSARAGVIFPVGRLMRYLKKGTFKYRISVGAPVYMAAVIEYLAAEILELAGNAARDNKKARIAPRHILLAVAN
DEELNQLLKGVTIASGGVLPRIHPELLAKKRGTKGKSETILSPPPEKRGRKATSGKKGGKKSKAAKPRTSKKSKPKDSDKEGTSNST
SEDGPGDGFTILSSKSLVLGQKLSLTQSDISHIGSMRVEGIVHPTTAEIDLKEDIGKALEKAGGKEFLETVKELRKSQGPLEVAEAA
VSQSSGLAAKFVIHCHIPQWGSDKCEEQLEETIKNCLSAAEDKKLKSVAFPPFPSGRNCFPKQTAAQVTLKAISAHFDDSSASSLKN
VYFLLFDSESIGIYVQEMAKLDAK
>sp|Q15771|RAB30_HUMAN

MSMEDYDFLFKIVLIGNAGVGKTCLVRRFTQGLFPPGQGATIGVDFMIKTVEINGEKVKLQIWDTAGQERFRSITQSYYRSANALIL
TYDITCEESFRCLPEWLREIEQYASNKVITVLVGNKIDLAERREVSQQRAEEFSEAQDMYYLETSAKESDNVEKLFLDLACRLISEA
RQNTLVNNVSSPLPGEGKSISYLTCCNFN
>sp|Q7RTS6|OTOP2_HUMAN
MSEELAQGPKESPPAPRAGPREVWKKGGRLLSVLLAVNVLLLACTLISGGAFNKVAVYDTDVFALLTAMMLLATLWILFYLLRTVRC
PCAVPYRDAHAGPIWLRGGLVLFGICTLIMDVFKTGYYSSFFECQSAIKILHPLIQAVFVIIQTYFLWVSAKDCVHVHLDLTWCGLM
FTLTTNLAIWMAAVVDESVHQSHSYSSSHSNASHARLISDQHADNPVGGDSCLCSTAVCQIFQQGYFYLYPFNIEYSLFASTMLYVM
WKNVGRFLASTPGHSHTPTPVSLFRETFFAGPVLGLLLFVVGLAVFIIYEVQVSGDGSRTRQALVIYYSFNIVCLGLTTLVSLSGSI
IYRFDRRAMDHHKNPTRTLDVALLMGAALGQYAISYYSIVAVVAGTPQDLLAGLNLTHALLMIAQHTFQNMFIIESLHRGPPGAEPH
STHPKEPCQDLTFTNLDALHTLSACPPNPGLVSPSPSDQREAVAIVSTPRSQWRRQCLKDISLFLLLCNVILWIMPAFGARPHFSNT
VEVDFYGYSLWAVIVNICLPFGIFYRMHAVSSLLEVYVLS
>sp|Q5TCY1|TTBK1_HUMAN
MQCLAAALKDETNMSGGGEQADILPANYVVKDRWKVLKKIGGGGFGEIYEAMDLLTRENVALKVESAQQPKQVLKMEVAVLKKLQGK
DHVCRFIGCGRNEKFNYVVMQLQGRNLADLRRSQPRGTFTLSTTLRLGKQILESIEAIHSVGFLHRDIKPSNFAMGRLPSTYRKCYM
LDFGLARQYTNTTGDVRPPRNVAGFRGTVRYASVNAHKNREMGRHDDLWSLFYMLVEFAVGQLPWRKIKDKEQVGMIKEKYEHRMLL
KHMPSEFHLFLDHIASLDYFTKPDYQLIMSVFENSMKERGIAENEAFDWEKAGTDALLSTSTSTPPQQNTRQTAAMFGVVNVTPVPG
DLLRENTEDVLQGEHLSDQENAPPILPGRPSEGLGPSPHLVPHPGGPEAEVWEETDVNRNKLRINIGKSPCVEEEQSRGMGVPSSPV
RAPPDSPTTPVRSLRYRRVNSPESERLSTADGRVELPERRSRMDLPGSPSRQACSSQPAQMLSVDTGHADRQASGRMDVSASVEQEA
LSNAFRSVPLAEEEDFDSKEWVIIDKETELKDFPPGAEPSTSGTTDEEPEELRPLPEEGEERRRLGAEPTVRPRGRSMQALAEEDLQ
HLPPQPLPPQLSQGDGRSETSQPPTPGSPSHSPLHSGPRPRRRESDPTGPQRQVFSVAPPFEVNGLPRAVPLSLPYQDFKRDLSDYR
ERARLLNRVRRVGFSHMLLTTPQVPLAPVQPQANGKEEEEEEEEDEEEEEEDEEEEEEEEEEEEEEEEEEEEEEEEAAAAVALGEVLG
PRSGSSSEGSERSTDRSQEGAPSTLLADDQKESRGRASMADGDLEPEEGSKTLVLVSPGDMKKSPVTAELAPDPDLGTLAALTPQHE
RPQPTGSQLDVSEPGTLSSVLKSEPKPPGPGAGLGAGTVTTGVGGVAVTSSPFTKVERTFVHIAEKTHLNVMSSGGQALRSEEFSAG
GELGLELASDGGAVEEGARAPLENGLALSGLNGAEIEGSALSGAPRETPSEMATNSLPNGPALADGPAPVSPLEPSPEKVATISPRR
HAMPGSRPRSRIPVLLSEEDTGSEPSGSLSAKERWSKRARPQQDLARLVMEKRQGRLLLRLASGASSSSSEEQRRASETLSGTGSEE
DTPASEPAAALPRKSGRAAATRSRIPRPIGLRMPMPVAAQQPASRSHGAAPALDTAITSRLQLQTPPGSATAADLRPKQPPGRGLGP
GRAQAGARPPAPRSPRLPASTSAARNASASPRSQSLSRRESPSPSHQARPGVPPPRGVPPARAQPDGTPSPGGSKKGPRGKLQAQRA
TTKGRAGGAEGRAGAR
>sp|Q9NZV7|ZIM2_HUMAN
MYQPEDDNNSDVTSDDDMTRNRRESSPPHSVHSFSGDRDWDRRGRSRDMEPRDRWSHTRNPRSRMPPRDLSLPVVAKTSFEMDREDD
RDSRAYESRSQDAESYQNVVDLAEDRKPHNTIQDNMENYRKLLSLGFLAQDSVPAEKRNTEMLDNLPSAGSQFPDFKHLGTFLVFEE
LVTFEDVLVDFSPEELSSLSAAQRNLYREVMLENYRNLVSLGHQFSKPDIISRLEEEESYAMETDSRHTVICQGESHDDPLEPHQGN
QEKLLTPITMNDPKTLTPERSYGSDEFERSSNLSKQSKDPLGKDPQEGTAPGICTSPQSASQENKHNRCEFCKRTFSTQVALRRHER
IHTGKKPYECKQCAEAFYLMPHLNRHQKTHSGRKTSGCNEGRKPSVQCANLCERVRIHSQEDYFECFQCGKAFLQNVHLLQHLKAHE
AARVLPPGLSHSKTYLIRYQRKHDYVGERACQCCDCGRVFSRNSYLIQHYRTHTQERPYQCQLCGKCFGRPSYLTQHYQLHSQEKTV
ECDHC
>sp|E9PGG2|ANHX_HUMAN
MQSFLTLLKEHEDTCAPPAELVTLAGRLCRDFQDDLAQLQPLVTAILDSQLRLHLLDNADVALACARVLDQQEQQQAACRLLEGCQV
PGGSQELVQLWNDIHYRLVMRRLGVAALTPVQKFRCRKRNPPPPSLCPEGLKSRNFPREVREKLHNFAVGVNTNPSKAERENLALET
SLTPEQVYNWFANYRRRQRALPQHMKPAQQATAEDPGARERGPDLLQPSGNPRVDSGFVDRPQWSEEREEKGPPQSPQTTQGPWEPL
ALAPDFPADETVSKPLDVSGHPQSVQLEEGLGTSSGRTELRVGSFLVTQPPLQAPEFILTQSPPELAPAPSAFPGPVSAMELSQALP
SSQVQCSDSQASGDAFWGARMLLEFSGSSLG
>sp|P09629|HXB7_HUMAN

MSSLYYANTLFSKYPASSSVFATGAFPEQTSCAFASNPQRPGYGAGSGASFAASMQGLYPGGGGMAGQSAAGVYAAGYGLEPSSFNM
HCAPFEQNLSGVCPGDSAKAAGAKEQRDSDLAAESNFRIYPWMRSSGTDRKRGRQTYTRYQTLELEKEFHYNRYLTRRRRIEIAHTL
CLTERQIKIWFQNRRMKWKKENKTAGPGTTGQDRAEAEEEEEE
>sp|O43301|HS12A_HUMAN
MADKEAGGSDGPRETAPTSAYSSPARSLGDTGITPLSPSHIVNDTDSNVSEQQSFLVVVAVDFGTTSSGYAYSFTKEPECIHVMRRW
EGGDPGVSNQKTPTTILLTPERKFHSFGYAARDFYHDLDPNEAKQWLYLEKFKMKLHTTGDLTMDTDLTAANGKKVKALEIFAYALQ
YFKEQALKELSDQAGSEFENSDVRWVITVPAIWKQPAKQFMRQAAYQAGLASPENSEQLIIALEPEAASIYCRKLRLHQMIELSSKA
AVNGYSGSDTVGAGFTQAKEHIRRNRQSRTFLVENVIGEIWSELEEGDKYVVVDSGGGTVDLTVHQIRLPEGHLKELYKATGGPYGS
LGVDYEFEKLLYKIFGEDFIEQFKIKRPAAWVDLMIAFESRKRAAAPDRTNPLNITLPFSFIDYYKKFRGHSVEHALRKSNVDFVKW
SSQGMLRMSPDAMNALFKPTIDSIIEHLRDLFQKPEVSTVKFLFLVGGFAEAPLLQQAVQAAFGDQCRIIIPQDVGLTILKGAVLFG
LDPAVIKVRRSPLTYGVGVLNRYVEGKHPPEKLLVKDGTRWCTDVFDKFISADQSVALGELVKRSYTPAKPSQLVIVINIYSSEHDN
VSFITDPGVKKCGTLRLDLTGTSGTAVPARREIQTLMQFGDTEIKATAIDIATSKSVKVGIDFLNY
>sp|Q96F25|ALG14_HUMAN
MVCVLVLAAAAGAVAVFLILRIWVVLRSMDVTPRESLSILVVAGSGGHTTEILRLLGSLSNAYSPRHYVIADTDEMSANKINSFELD
RADRDPSNMYTKYYIHRIPRSREVQQSWPSTVFTTLHSMWLSFPLIHRVKPDLVLCNGPGTCVPICVSALLLGILGIKKVIIVYVES
ICRVETLSMSGKILFHLSDYFIVQWPALKEKYPKSVYLGRIV
>sp|Q15080|NCF4_HUMAN
MAVAQQLRAESDFEQLPDDVAISANIADIEEKRGFTSHFVFVIEVKTKGGSKYLIYRRYRQFHALQSKLEERFGPDSKSSALACTLP
TLPAKVYVGVKQEIAEMRIPALNAYMKSLLSLPVWVLMDEDVRIFFYQSPYDSEQVPQALRRLRPRTRKVKSVSPQGNSVDRMAAPR
AEALFDFTGNSKLELNFKAGDVIFLLSRINKDWLEGTVRGATGIFPLSFVKILKDFPEEDDPTNWLRCYYYEDTISTIKDIAVEEDL
SSTPLLKDLLELTRREFQREDIALNYRDAEGDLVRLLSDEDVALMVRQARGLPSQKRLFPWKLHITQKDNYRVYNTMP
>sp|Q9UBT6|POLK_HUMAN
MDSTKEKCDSYKDDLLLRMGLNDNKAGMEGLDKEKINKIIMEATKGSRFYGNELKKEKQVNQRIENMMQQKAQITSQQLRKAQLQVD
RFAMELEQSRNLSNTIVHIDMDAFYAAVEMRDNPELKDKPIAVGSMSMLSTSNYHARRFGVRAAMPGFIAKRLCPQLIIVPPNFDKY
RAVSKEVKEILADYDPNFMAMSLDEAYLNITKHLEERQNWPEDKRRYFIKMGSSVENDNPGKEVNKLSEHERSISPLLFEESPSDVQ
PPGDPFQVNFEEQNNPQILQNSVVFGTSAQEVVKEIRFRIEQKTTLTASAGIAPNTMLAKVCSDKNKPNGQYQILPNRQAVMDFIKD
LPIRKVSGIGKVTEKMLKALGIITCTELYQQRALLSLLFSETSWHYFLHISLGLGSTHLTRDGERKSMSVERTFSEINKAEEQYSLC
QELCSELAQDLQKERLKGRTVTIKLKNVNFEVKTRASTVSSVVSTAEEIFAIAKELLKTEIDADFPHPLRLRLMGVRISSFPNEEDR
KHQQRSIIGFLQAGNQALSATECTLEKTDKDKFVKPLEMSHKKSFFDKKRSERKWSHQDTFKCEAVNKQSFQTSQPFQVLKKKMNEN
LEISENSDDCQILTCPVCFRAQGCISLEALNKHVDECLDGPSISENFKMFSCSHVSATKVNKKENVPASSLCEKQDYEAHPKIKEIS
SVDCIALVDTIDNSSKAESIDALSNKHSKEECSSLPSKSFNIEHCHQNSSSTVSLENEDVGSFRQEYRQPYLCEVKTGQALVCPVCN
VEQKTSDLTLFNVHVDVCLNKSFIQELRKDKFNPVNQPKESSRSTGSSSGVQKAVTRTKRPGLMTKYSTSKKIKPNNPKHTLDIFFK
>sp|Q8NGH9|O52E4_HUMAN
MPSINDTHFYPPFFLLLGIPGLDTLHIWISFPFCIVYLIAIVGNMTILFVIKTEHSLHQPMFYFLAMLSMIDLGLSTSTIPKMLGIF
WFNLQEISFGGCLLQMFFIHMFTGMETVLLVVMAYDRFVAICNPLQYTMILTNKTISILASVVVGRNLVLVTPFVFLILRLPFCGHN
IVPHTYCEHRGLAGLACAPIKINIIYGLMVISYIIVDVILIASSYVLILRAVFRLPSQDVRLKAFNTCGSHVCVMLCFYTPAFFSFM
THRFGQNIPHYIHILLANLYVVVPPALNPVIYGVRTKQIREQIVKIFVQKE
>sp|Q9BQE3|TBA1C_HUMAN
MRECISIHVGQAGVQIGNACWELYCLEHGIQPDGQMPSDKTIGGGDDSFNTFFSETGAGKHVPRAVFVDLEPTVIDEVRTGTYRQLF
HPEQLITGKEDAANNYARGHYTIGKEIIDLVLDRIRKLADQCTGLQGFLVFHSFGGGTGSGFTSLLMERLSVDYGKKSKLEFSIYPA
PQVSTAVVEPYNSILTTHTTLEHSDCAFMVDNEAIYDICRRNLDIERPTYTNLNRLISQIVSSITASLRFDGALNVDLTEFQTNLVP
YPRIHFPLATYAPVISAEKAYHEQLTVAEITNACFEPANQMVKCDPRHGKYMACCLLYRGDVVPKDVNAAIATIKTKRTIQFVDWCP
TGFKVGINYQPPTVVPGGDLAKVQRAVCMLSNTTAVAEAWARLDHKFDLMYAKRAFVHWYVGEGMEEGEFSEAREDMAALEKDYEEV
GADSADGEDEGEEY

>sp|Q96MD7|CI085_HUMAN
MSSQKGNVARSRPQKHQNTFSFKNDKFDKSVQTKKINAKLHDGVCQRCKEVLEWRVKYSKYKPLSKPKKCVKCLQKTVKDSYHIMCR
PCACELEVCAKCGKKEDIVIPWSLPLLPRLECSGRILAHHNLRLPCSSDSPASASRVAGTTGAHHHAQLIFVFLVEMGFHYVGQAGL
ELLTS
>sp|Q6IA17|SIGIR_HUMAN
MPGVCDRAPDFLSPSEDQVLRPALGSSVALNCTAWVVSGPHCSLPSVQWLKDGLPLGIGGHYSLHEYSWVKANLSEVLVSSVLGVNV
TSTEVYGAFTCSIQNISFSSFTLQRAGPTSHVAAVLASLLVLLALLLAALLYVKCRLNVLLWYQDAYGEVEINDGKLYDAYVSYSDC
PEDRKFVNFILKPQLERRRGYKLFLDDRDLLPRAEPSADLLVNLSRCRRLIVVLSDAFLSRAWCSHSFREGLCRLLELTRRPIFITF
EGQRRDPAHPALRLLRQHRHLVTLLLWRPGSVTPSSDFWKEVQLALPRKVQYRPVEGDPQTQLQDDKDPMLILRGRVPEGRALDSEV
DPDPEGDLGVRGPVFGEPSAPPHTSGVSLGESRSSEVDVSDLGSRNYSARTDFYCLVSKDDM
>sp|Q9HAC8|UBTD1_HUMAN
MGNCVGRQRRERPAAPGHPRKRAGRNEPLKKERLKWKSDYPMTDGQLRSKRDEFWDTAPAFEGRKEIWDALKAAAYAAEANDHELAQ
AILDGASITLPHGTLCECYDELGNRYQLPIYCLSPPVNLLLEHTEEESLEPPEPPPSVRREFPLKVRLSTGKDVRLSASLPDTVGQL
KRQLHAQEGIEPSWQRWFFSGKLLTDRTRLQETKIQKDFVIQVIINQPPPPQD
>sp|Q6NUJ1|SAPL1_HUMAN
MLCALLLLPSLLGATRASPTSGPQECAKGSTVWCQDLQTAARCGAVGYCQGAVWNKPTAKSLPCDVCQDIAAAAGNGLNPDATESDI
LALVMKTCEWLPSQESSAGCKWMVDAHSSAILSMLRGAPDSAPAQVCTALSLCEPLQRHLATLRPLSKEDTFEAVAPFMANGPLTFH
PRQAPEGALCQDCVRQVSRLQEAVRSNLTLADLNIQEQCESLGPGLAVLCKNYLFQFFVPADQALRLLPPQELCRKGGFCEELGAPA
RLTQVVAMDGVPSLELGLPRKQSEMQMKAGVTCEVCMNVVQKLDHWLMSNSSELMITHALERVCSVMPASITKECIILVDTYSPSLV
QLVAKITPEKVCKFIRLCGNRRRARAVHDAYAIVPSPEWDAENQGSFCNGCKRLLTVSSHNLESKSTKRDILVAFKGGCSILPLPYM
IQCKHFVTQYEPVLIESLKDMMDPVAVCKKVGACHGPRTPLLGTDQCALGPSFWCRSQEAAKLCNAVQHCQKHVWKEMHLHAGEHA
>sp|A6NNM8|TTL13_HUMAN
MEPSTCRTMESEEDYVEEKESEKCVKEGVTNPSNSSQQALLKADYKALKNGVPSPIMATKIPKKVIAPVDTGDLEAGRRKRRRKRRS
LAINLTNCKYESVRRAAQMCGLKEVGEDEEWTLYWTDCAVSLERVMDMKRFQKINHFPGMTEICRKDLLARNLNRMYKLYPSEYNIF
PRTWCLPADYGDFQSYGRQRKARTYICKPDSGCQGRGIFITRNPREIKPGEHMICQQYISKPLLIDGFKFDMRVYVLITSCDPLRIF
TYEEGLARFATTPYMEPSHNNLDNVCMHLTNYAINKHNENFVRDGAVGSKRKLSTLNIWLQEHSYNPGELWGDIEDIIIKTIISAHS
VLRHNYRTCFPQYLNGGTCACFEILGFDILLDHKLKPWLLEVNHSPSFTTDSCLDQEVKDALLCDAMTLVNLRGCDKRKVMEEDKRR
VKERLFQCYRQPRESRKEKTESSHVAMLDQERYEDSHLGKYRRIYPGPDTEKYARFFKHNGSLFQETAASKAREECARQQLEEIRLK
QEQQETSGTKRQKARDQNQGESAGEKSRPRAGLQSLSTHLAYRNRNWEKELLPGQLDTMRPQEIVEEEELERMKALLQRETLIRSLG
IVEQLTRLQHPGPQGQKKLHESRDRLGSQELKSMSLVLLVLLRGAATEQGAPHFLHPVLPHESIPRILGALPSMNAAIPHVPRYHLQ
PKNFNWTGEPAAINSCSLSMKKAGRCYFSSARIRLTSQGQASRRLEAINRVLAGSVPPTLTPKQGYFLQPERVASDSWTECTLPSMV
NSEHRAAKVPLCPASAPMLQRSRALLNINQFR
>sp|P34932|HSP74_HUMAN
MSVVGIDLGFQSCYVAVARAGGIETIANEYSDRCTPACISFGPKNRSIGAAAKSQVISNAKNTVQGFKRFHGRAFSDPFVEAEKSNL
AYDIVQLPTGLTGIKVTYMEEERNFTTEQVTAMLLSKLKETAESVLKKPVVDCVVSVPCFYTDAERRSVMDATQIAGLNCLRLMNET
TAVALAYGIYKQDLPALEEKPRNVVFVDMGHSAYQVSVCAFNRGKLKVLATAFDTTLGGRKFDEVLVNHFCEEFGKKYKLDIKSKIR
ALLRLSQECEKLKKLMSANASDLPLSIECFMNDVDVSGTMNRGKFLEMCNDLLARVEPPLRSVLEQTKLKKEDIYAVEIVGGATRIP
AVKEKISKFFGKELSTTLNADEAVTRGCALQCAILSPAFKVREFSITDVVPYPISLRWNSPAEEGSSDCEVFSKNHAAPFSKVLTFY
RKEPFTLEAYYSSPQDLPYPDPAIAQFSVQKVTPQSDGSSSKVKVKVRVNVHGIFSVSSASLVEVHKSEENEEPMETDQNAKEEEKM
QVDQEEPHVEEQQQQTPAENKAESEEMETSQAGSKDKKMDQPPQAKKAKVKTSTVDLPIENQLLWQIDREMLNLYIENEGKMIMQDK
LEKERNDAKNAVEEYVYEMRDKLSGEYEKFVSEDDRNSFTLKLEDTENWLYEDGEDQPKQVYVDKLAELKNLGQPIKIRFQESEERP
KLFEELGKQIQQYMKIISSFKNKEDQYDHLDAADMTKVEKSTNEAMEWMNNKLNLQNKQSLTMDPVVKSKEIEAKIKELTSTCSPII
SKPKPKVEPPKEEQKNAEQNGPVDGQGDNPGPQAAEQGTDTAVPSDSDKKLPEMDID
>sp|Q53H12|AGK_HUMAN

MTVFFKTLRNHWKKTTAGLCLLTWGGHWLYGKHCDNLLRRAACQEAQVFGNQLIPPNAQVKKATVFLNPAACKGKARTLFEKNAAPI
LHLSGMDVTIVKTDYEGQAKKLLELMENTDVIIVAGGDGTLQEVVTGVLRRTDEATFSKIPIGFIPLGETSSLSHTLFAESGNKVQH
ITDATLAIVKGETVPLDVLQIKGEKEQPVFAMTGLRWGSFRDAGVKVSKYWYLGPLKIKAAHFFSTLKEWPQTHQASISYTGPTERP
PNEPEETPVQRPSLYRRILRRLASYWAQPQDALSQEVSPEVWKDVQLSTIELSITTRNNQLDPTSKEDFLNICIEPDTISKGDFITI
GSRKVRNPKLHVEGTECLQASQCTLLIPEGAGGSFSIDSEEYEAMPVEVKLLPRKLQFFCDPRKREQMLTSPTQ
>sp|Q8NGC5|OR6J1_HUMAN
MGNWTAAVTEFVLLGFSLSREVELLLLVLLLPTFLLTLLGNLLIISTVLSCSRLHTPMYFFLCNLSILDILFTSVISPKVLANLGSR
DKTISFAGCITQCYFYFFLGTVEFLLLTVMSYDRYATICCPLRYTTIMRPSVCIGTVVFSWVGGFLSVLFPTILISQLPFCGSNIIN
HFFCDSGPLLALACADTTAIELMDFMLSSMVILCCIVLVAYSYTYIILTIVRIPSASGRKKAFNTCASHLTIVIIPSGITVFIYVTP
SQKEYLEINKIPLVLSSVVTPFLNPFIYTLRNDTVQGVLRDVWVRVRGVFEKRMRAVLRSRLSSNKDHQGRACSSPPCVYSVKLQC
>sp|Q9H832|UBE2Z_HUMAN
MAESPTEEAATAGAGAAGPGASSVAGVVGVSGSGGGFGPPFLPDVWAAAAAAGGAGGPGSGLAPLPGLPPSAAAHGAALLSHWDPTL
SSDWDGERTAPQCLLRIKRDIMSIYKEPPPGMFVVPDTVDMTKIHALITGPFDTPYEGGFFLFVFRCPPDYPIHPPRVKLMTTGNNT
VRFNPNFYRNGKVCLSILGTWTGPAWSPAQSISSVLISIQSLMTENPYHNEPGFEQERHPGDSKNYNECIRHETIRVAVCDMMEGKC
PCPEPLRGVMEKSFLEYYDFYEVACKDRLHLQGQTMQDPFGEKRGHFDYQSLLMRLGLIRQKVLERLHNENAEMDSDSSSSGTETDL
HGSLRV
>sp|Q9H9J2|RM44_HUMAN
MASGLVRLLQQGHRCLLAPVAPKLVPPVRGVKKGFRAAFRFQKELERQRLLRCPPPPVRRSEKPNWDYHAEIQAFGHRLQENFSLDL
LKTAFVNSCYIKSEEAKRQQLGIEKEAVLLNLKSNQELSEQGTSFSQTCLTQFLEDEYPDMPTEGIKNLVDFLTGEEVVCHVARNLA
VEQLTLSEEFPVPPAVLQQTFFAVIGALLQSSGPERTALFIRDFLITQMTGKELFEMWKIINPMGLLVEELKKRNVSAPESRLTRQS
GGTTALPLYFVGLYCDKKLIAEGPGETVLVAEEEAARVALRKLYGFTENRRPWNYSKPKETLRAEKSITAS
>sp|Q86VF2|IGFN1_HUMAN
MAGKLRKSHIPGVSIWQLVEEIPEGCSTPDFEQKPVTSALPEGKNAVFRAVVCGEPRPEVRWQNSKGDLSDSSKYKISSSPGSKEHV
LQINKLTGEDTDLYRCTAVNAYGEAACSVRLTVIEVGFRKNRKRHREPQEDLRKELMDFRKLLKKRAPPAPKKKMDLEQIWQLLMTA
DRKDYEKICLKYGIVDYRGMLRRLQEMKKEQEDKMAQYINTISSLRHIRVTKDGNAKFDLELDLKDSQSKIYLYKDGEMIPYGFNNQ
TKHCLRRLGKRYEFQIQDLRPEDSGIYQVKVEDAVVFSTELEASAIPPRVVVPLAETHCEEQGDAVFECTLSSPCPSAAWHFRHRLL
HPSDKYEVYVSPDGLTHRLVVRGARFSDMGPYSLGTGLYTSSAWLVVEAGKDKDLQSTSADHKLQSRRSGKDGRLDIYGERRDATRS
STSRYKPGTGSFSKDAQGPMGHFSQGLADMEVQPGEAATLSCTLTSDLGPGTWFKDGVKLTTQDGVIFKQDGLVHSLFITHVQGTQA
GRYTFVAGDQQSEATLTVQDSPTIAPDVTEKLREPLVVKAGKPVIVKIPFQSHLPIQAAWRKDGAEVVGSSDREAQVDLGDGYTRLC
LPSAGRKDCGQYSVTLRSEGGSVQAELTLQVIDKPDPPQGPMEVQDCHRAGVCLRWRPPRDNGGRTVECYVVERRQAGRSTWLKVGE
APADSTTFTDAHVEPGRKYTFRVRAVTSEGAGEALESEEILVAPEALPKAPSAPAILSASSQGITLTWTAPRGPGSAHILGYLIERR
KKGSNTWTAVNDQPVPERRWTVADVRQGCQYEFRVTAVAPSGPGEPGPPSDAVFARDPMRPPGLVRNLQVTDRSNTSITLSWAGPDT
QEGDEAQGYVVELCSSDSLQWLPCHVGTVPVTTYTAKGLRPGEGYFVRVTAVNEGGQSQPSALDTLVQAMPVTVCPKFLVDSSTKDL
LTVKVGDTVRVPVSFEAMPMPEVTWLKDGLPLPKRSVTVTKDGLTQLLIPVAGLSDSGLYTVVLRTLQGKEVAHSFRIRVAACPQAP
GPIHLQENVPGTVTAEWEPSPDEAQDVPLHYAVFTRSSAHGPWHEAADRIHTNRFTLLGILPGHEYHFRVVAKNELGASKPSDTSQP
WCIPRQRDRFTVKAPCYREPDLSQKPRFLVGLRSHLLPQGCECCMSCAVQGSPRPHVTWFKNDRSLEGNPAVYSTDLLGVCSLTIPS
VSPKDSGEYKAVAENTLGQAVSTATLIVIEPST
>sp|H3BSY2|GOG8M_HUMAN
MAEETQHNKLAAAKKKLKEYWQKNSPRVPAGANRNRKTNGSIPQTATSGGCQPPGDSATGFHREGPTSSATLKDLESPCQERAVVLD
SRSVEISQLKNTIKSLKQQKKQVEHQLEEEKKANNKKQKAKRVLEVQLQTLNIQKEELNTDLYHMKRSLRYFEEKSKDLAVRLQHSL
QRKGELESVLSDVMATQKKKANQLSSPSKAGTEWKLEQSMREEALLKVQLTQLKESFQQVQLERDEYSEHLKGERARWQQRMRKMSQ
EICTLKKEKQQDMRRVEKLERSLSKLKNQMAEPLPPEPPAVPSEVELQHLRKELERVAGELQAQVKNNQRISLLNQRQEERIREQEE
RLRKQEERIQEQHKSLQQLAKPQSVFEEPNNENKSTLQLEQQVKELQEKLGEEHLEAASQQNQQLTAQLSLMALPGEGHGGEHLDSE
GEEAPQPMPSVPEDPESREAMSSFMDHLEEKADLSELVKKQELRFIQYWQERCHQKIHHLLSEPGGRAKDAALGGGHHQAGAQGGDE

GEAAGAAADGIAAYSNYNNGHRKFLAAAHNSADEPGPGAPAPQELGAADKHGDLCEVSLTSSAQGEAREDPLLDKPTAQPIVQDHQE
HPGLGSNCCVPFFCWAWLPRRRR
>sp|Q14106|TOB2_HUMAN
MQLEIKVALNFIISYLYNKLPRRRADLFGEELERLLKKKYEGHWYPEKPLKGSGFRCVHIGEMVDPVVELAAKRSGLAVEDVRANVP
EELSVWIDPFEVSYQIGEKGAVKVLYLDDSEGCGAPELDKEIKSSFNPDAQVFVPIGSQDSSLSNSPSPSFGQSPSPTFIPRSAQPI
TFTTASFAATKFGSTKMKKGGGAASGGGVASSGAGGQQPPQQPRMARSPTNSLLKHKSLSLSMHSLNFITANPAPQSQLSPNAKEFV
YNGGGSPSLFFDAADGQGSGTPGPFGGSGAGTCNSSSFDMAQVFGGGANSLFLEKTPFVEGLSYNLNTMQYPSQQFQPVVLAN
>sp|O00338|ST1C2_HUMAN
MALTSDLGKQIKLKEVEGTLLQPATVDNWSQIQSFEAKPDDLLICTYPKAGTTWIQEIVDMIEQNGDVEKCQRAIIQHRHPFIEWAR
PPQPSGVEKAKAMPSPRILKTHLSTQLLPPSFWENNCKFLYVARNAKDCMVSYYHFQRMNHMLPDPGTWEEYFETFINGKVVWGSWF
DHVKGWWEMKDRHQILFLFYEDIKRDPKHEIRKVMQFMGKKVDETVLDKIVQETSFEKMKENPMTNRSTVSKSILDQSISSFMRKGT
VGDWKNHFTVAQNERFDEIYRRKMEGTSINFCMEL
>sp|Q9NX45|SOLH2_HUMAN
MASSIICQEHCQISGQAKIDILLVGDVTVGYLADTVQKLFANIAEVTITISDTKEAAALLDDCIFNMVLLKVPSSLSAEELEAIKLI
RFGKKKNTHSLFVFIIPENFKGCISGHGMDIALTEPLTMEKMSNVVKYWTTCPSNTVKTENATGPEELGLPLQRSYSEHLGYFPTDL
FACSESLRNGNGLELNASLSEFEKNKKISLLHSSKEKLRRERIKYCCEQLRTLLPYVKGRKNDAASVLEATVDYVKYIREKISPAVM
AQITEALQSNMRFCKKQQTPIELSLPGTVMAQRENSVMSTYSPERGLQFLTNTCWNGCSTPDAESSLDEAVRVPSSSASENAIGDPY
KTHISSAALSLNSLHTVRYYSKVTPSYDATAVTNQNISIHLPSAMPPVSKLLPRHCTSGLGQTCTTHPNCLQQFWAY
>sp|P46663|BKRB1_HUMAN
MASSWPPLELQSSNQSQLFPQNATACDNAPEAWDLLHRVLPTFIISICFFGLLGNLFVLLVFLLPRRQLNVAEIYLANLAASDLVFV
LGLPFWAENIWNQFNWPFGALLCRVINGVIKANLFISIFLVVAISQDRYRVLVHPMASRRQQRRRQARVTCVLIWVVGGLLSIPTFL
LRSIQAVPDLNITACILLLPHEAWHFARIVELNILGFLLPLAAIVFFNYHILASLRTREEVSRTRCGGRKDSKTTALILTLVVAFLV
CWAPYHFFAFLEFLFQVQAVRGCFWEDFIDLGLQLANFFAFTNSSLNPVIYVFVGRLFRTKVWELYKQCTPKSLAPISSSHRKEIFQ
LFWRN
>sp|Q04864|REL_HUMAN
MASGAYNPYIEIIEQPRQRGMRFRYKCEGRSAGSIPGEHSTDNNRTYPSIQIMNYYGKGKVRITLVTKNDPYKPHPHDLVGKDCRDG
YYEAEFGQERRPLFFQNLGIRCVKKKEVKEAIITRIKAGINPFNVPEKQLNDIEDCDLNVVRLCFQVFLPDEHGNLTTALPPVVSNP
IYDNRAPNTAELRICRVNKNCGSVRGGDEIFLLCDKVQKDDIEVRFVLNDWEAKGIFSQADVHRQVAIVFKTPPYCKAITEPVTVKM
QLRRPSDQEVSESMDFRYLPDEKDTYGNKAKKQKTTLLFQKLCQDHVETGFRHVDQDGLELLTSGDPPTLASQSAGITVNFPERPRP
GLLGSIGEGRYFKKEPNLFSHDAVVREMPTGVSSQAESYYPSPGPISSGLSHHASMAPLPSSSWSSVAHPTPRSGNTNPLSSFSTRT
LPSNSQGIPPFLRIPVGNDLNASNACIYNNADDIVGMEASSMPSADLYGISDPNMLSNCSVNMMTTSSDSMGETDNPRLLSMNLENP
SCNSVLDPRDLRQLHQMSSSSMSAGANSNTTVFVSQSDAFEGSDFSCADNSMINESGPSNSTNPNSHGFVQDSQYSGIGSMQNEQLS
DSFPYEFFQV
>sp|P56696|KCNQ4_HUMAN
MAEAPPRRLGLGPPPGDAPRAELVALTAVQSEQGEAGGGGSPRRLGLLGSPLPPGAPLPGPGSGSGSACGQRSSAAHKRYRRLQNWV
YNVLERPRGWAFVYHVFIFLLVFSCLVLSVLSTIQEHQELANECLLILEFVMIVVFGLEYIVRVWSAGCCCRYRGWQGRFRFARKPF
CVIDFIVFVASVAVIAAGTQGNIFATSALRSMRFLQILRMVRMDRRGGTWKLLGSVVYAHSKELITAWYIGFLVLIFASFLVYLAEK
DANSDFSSYADSLWWGTITLTTIGYGDKTPHTWLGRVLAAGFALLGISFFALPAGILGSGFALKVQEQHRQKHFEKRRMPAANLIQA
AWRLYSTDMSRAYLTATWYYYDSILPSFRELALLFEHVQRARNGGLRPLEVRRAPVPDGAPSRYPPVATCHRPGSTSFCPGESSRMG
IKDRIRMGSSQRRTGPSKQHLAPPTMPTSPSSEQVGEATSPTKVQKSWSFNDRTRFRASLRLKPRTSAEDAPSEEVAEEKSYQCELT
VDDIMPAVKTVIRSIRILKFLVAKRKFKETLRPYDVKDVIEQYSAGHLDMLGRIKSLQTRVDQIVGRGPGDRKAREKGDKGPSDAEV
VDEISMMGRVVKVEKQVQSIEHKLDLLLGFYSRCLRSGTSASLGAVQVPLFDPDITSDYHSPVDHEDISVSAQTLSISRSVSTNMD
>sp|O95968|SG1D1_HUMAN

MRLSVCLLLLTLALCCYRANAVVCQALGSEITGFLLAGKPVFKFQLAKFKAPLEAVAAKMEVKKCVDTMAYEKRVLITKTLGKIAEK
CDR
>sp|Q9BZS9|YG041_HUMAN
MPYDQDSFSTLLGFLQASRKYSEFTLKCPICIYVPCQCFAVGFLKQSDQ
>sp|Q4VC05|BCL7A_HUMAN
MSGRSVRAETRSRAKDDIKRVMAAIEKVRKWEKKWVTVGDTSLRIYKWVPVTEPKVDDKNKNKKKGKDEKCGSEVTTPENSSSPGMM
DMHDDNSNQSSIADASPIKQENSSNSSPAPEPNSAVPSDGTEAKVDEAQADGKEHPGAEDASDEQNSQSSMEHSMNSSEKVDRQPSG
DSGLAAETSAISQDLEGVPPSKKMKLEASQQNSEEM
>sp|P43119|PI2R_HUMAN
MADSCRNLTYVRGSVGPATSTLMFVAGVVGNGLALGILSARRPARPSAFAVLVTGLAATDLLGTSFLSPAVFVAYARNSSLLGLARG
GPALCDAFAFAMTFFGLASMLILFAMAVERCLALSHPYLYAQLDGPRCARLALPAIYAFCVLFCALPLLGLGQHQQYCPGSWCFLRM
RWAQPGGAAFSLAYAGLVALLVAAIFLCNGSVTLSLCRMYRQQKRHQGSLGPRPRTGEDEVDHLILLALMTVVMAVCSLPLTIRCFT
QAVAPDSSSEMGDLLAFRFYAFNPILDPWVFILFRKAVFQRLKLWVCCLCLGPAHGDSQTPLSQLASGRRDPRAPSAPVGKEGSCVP
LSAWGEGQVEPLPPTQQSSGSAVGTSSKAEASVACSLC
>sp|Q9BU76|MMTA2_HUMAN
MFGSSRGGVRGGQDQFNWEDVKTDKQRENYLGNSLMAPVGRWQKGRDLTWYAKGRAPCAGPSREEELAAVREAEREALLAALGYKNV
KKQPTGLSKEDFAEVCKREGGDPEEKGVDRLLGLGSASGSVGRVAMSREDKEAAKLGLSVFTHHRVESGGPGTSAASARRKPRAEDQ
TESSCESHRKSKKEKKKKKKRKHKKEKKKKDKEHRRPAEATSSPTSPERPRHHHHDSDSNSPCCKRRKRGHSGDRRSPSRRWHDRGS
EA
>sp|Q9H7L2|KI3X1_HUMAN
MAPKLITVLCLGFCLNQKICPHAGAQDKFSLSAWPSPVVPLGGRVTLSCHSHLRFVIWTIFQTTGTRSHELHTGLSNNITISPVTPE
HAGTYRCVGIYKHASKWSAESNSLKIIVTGLFTKPSISAHPSSLVHAGARVSLRCHSELAFDEFILYKEGHIQHSQQLDQGMEAGIH
YVEAVFSMGPVTPAHAGAYRCCGCFSHSRYEWSAPSDPLDIVITGKYKKPSLSTQVDPMMRLGEKLTLFCSSEISFDQYHLFRHGVA
HGQWLSGGQRHREAFQANFSVGRATPVPGGTYRCYGSFNDSPYKPPVTRCNFTPQETLRVLLCHSQNPPLNLTHLALKDSPATCICS
LDSQ
>sp|Q6UX72|B3GN9_HUMAN
MRRRLRLRRDALLTLLLGASLGLLLYAQRDGAAPTASAPRGRGRAAPRPTPGPRAFQLPDAGAAPPAYEGDTPAPPTPTGPFDFARY
LRAKDQRRFPLLINQPHKCRGDGAPGGRPDLLIAVKSVAEDFERRQAVRQTWGAEGRVQGALVRRVFLLGVPRGAGSGGADEVGEGA
RTHWRALLRAESLAYADILLWAFDDTFFNLTLKEIHFLAWASAFCPDVRFVFKGDADVFVNVGNLLEFLAPRDPAQDLLAGDVIVHA
RPIRTRASKYYIPEAVYGLPAYPAYAGGGGFVLSGATLHRLAGACAQVELFPIDDVFLGMCLQRLRLTPEPHPAFRTFGIPQPSAAP
HLSTFDPCFYRELVVVHGLSAADIWLMWRLLHGPHGPACAHPQPVAAGPFQWDS
>sp|Q5FVE4|ACBG2_HUMAN
MTGTPKTQEGAKDLEVDMNKTEVTPRLWTTCRDGEVLLRLSKHGPGHETPMTIPEFFRESVNRFGTYPALASKNGKKWEILNFNQYY
EACRKAAKSLIKLGLERFHGVGILGFNSAEWFITAVGAILAGGLCVGIYATNSAEVCQYVITHAKVNILLVENDQQLQKILSIPQSS
LEPLKAIIQYRLPMKKNNNLYSWDDFMELGRSIPDTQLEQVIESQKANQCAVLIYTSGTTGIPKGVMLSHDNITWIAGAVTKDFKLT
DKHETVVSYLPLSHIAAQMMDIWVPIKIGALTYFAQADALKGTLVSTLKEVKPTVFIGVPQIWEKIHEMVKKNSAKSMGLKKKAFVW
ARNIGFKVNSKKMLGKYNTPVSYRMAKTLVFSKVKTSLGLDHCHSFISGTAPLNQETAEFFLSLDIPIGELYGLSESSGPHTISNQN
NYRLLSCGKILTGCKNMLFQQNKDGIGEICLWGRHIFMGYLESETETTEAIDDEGWLHSGDLGQLDGLGFLYVTGHIKEILITAGGE
NVPPIPVETLVKKKIPIISNAMLVGDKLKFLSMLLTLKCEMNQMSGEPLDKLNFEAINFCRGLGSQASTVTEIVKQQDPLVYKAIQQ
GINAVNQEAMNNAQRIEKWVILEKDFSIYGGELGPMMKLKRHFVAQKYKKQIDHMYH
>sp|Q16644|MAPK3_HUMAN
MDGETAEEQGGPVPPPVAPGGPGLGGAPGGRREPKKYAVTDDYQLSKQVLGLGVNGKVLECFHRRTGQKCALKLLYDSPKARQEVDH
HWQASGGPHIVCILDVYENMHHGKRCLLIIMECMEGGELFSRIQERGDQAFTEREAAEIMRDIGTAIQFLHSHNIAHRDVKPENLLY
TSKEKDAVLKLTDFGFAKETTQNALQTPCYTPYYVAPEVLGPEKYDKSCDMWSLGVIMYILLCGFPPFYSNTGQAISPGMKRRIRLG

QYGFPNPEWSEVSEDAKQLIRLLLKTDPTERLTITQFMNHPWINQSMVVPQTPLHTARVLQEDKDHWDEVKEEMTSALATMRVDYDQ
VKIKDLKTSNNRLLNKRRKKQAGSSSASQGCNNQ
>sp|Q96E39|RMXL1_HUMAN
MVEADRPGKLFIGGLNTETNEKALETVFGKYGRIVEVLLIKDRETNKSRGFAFVTFESPADAKDAARDMNGKSLDGKAIKVEQATKP
SFERGRHGPPPPPRSRGPPRGFGAGRGGSGGTRGPPSRGGHMDDGGYSMNFNMSSSRGPLPVKRGPPPRSGGPSPKRSAPSGLVRSS
SGMGGRAPLSRGRDSYGGPPRREPLPSRRDVYLSPRDDGYSTKDSYSSRDYPSSRDTRDYAPPPRDYTYRDYGHSSSRDDYPSRGYG
DRDGYGRDRDYSDHPSGGSYRDSYESYGNSRSAPLTRGPPPSYGGSSRYDDYSSSRDGYGGSRDSYSSSRSDLYSSCDRVGRQERGL
PPSVERGYPSSRDSYSSSSRGAPRGAGPGGSRSDRGGGRSRY
>sp|Q30KR1|DB109_HUMAN
MRLHLLLLILLLFSILLSPVRGGLGPAEGHCLNLFGVCRTDVCNIVEDQIGACRRRMKCCRAWWILMPIPTPLIMSDYQEPLKPNLK
>sp|Q15543|TAF13_HUMAN
MADEEEDPTFEEENEEIGGGAEGGQGKRKRLFSKELRCMMYGFGDDQNPYTESVDILEDLVIEFITEMTHKAMSIGRQGRVQVEDIV
FLIRKDPRKFARVKDLLTMNEELKRARKAFDEANYGS
>sp|P0CH98|F106C_HUMAN
MLPSTMFLVHLPLSTNRLHCLRNTSLESCLCSFVHLNHPLHISDPVILISLHEAVRFSFAFSFPRGTLSIAYCLMSSVSTSSEAIMS
TELLANYCHSSLHVCICISSFPNETGNHDSFPGAVVSISDQPTDQCKLAAKELPLRNLLECRFFDCMGEEDLINLGVIGTER
>sp|Q6UXN9|WDR82_HUMAN
MKLTDSVLRSFRVAKVFRENSDKINCFDFSPNGETVISSSDDDSIVLYDCQEGKPKRTLYSKKYGVDLIRYTHAANTVVYSSNKIDD
TIRYLSLHDNKYIRYFPGHSKRVVALSMSPVDDTFISGSLDKTIRLWDLRSPNCQGLMHLQGKPVCSFDPEGLIFAAGVNSEMVKLY
DLRSFDKGPFATFKMQYDRTCEWTGLKFSNDGKLILISTNGSFIRLIDAFKGVVMHTFGGYANSKAVTLEASFTPDSQFIMIGSEDG
KIHVWNGESGIKVAVLDGKHTGPITCLQFNPKFMTFASACSNMAFWLPTIDD
>sp|Q9BU23|LMF2_HUMAN
MAGSRLPRQLFLQGVAAVFMFAFASLYTQIPGLYGPEGILPARRTLRPQGKGRWQQLWETPTLLWEAPRLGLDTAQGLELLSLLGAL
VALGALLLSPLRHPVIYLLLWAAYLSACQVGQVFLYFQWDSLLLETGFLAVLVAPLRPASHRKEAPQGRQAGALPHEDLPFWLVRWL
LFRLMFASGVVKLTSRCPAWWGLTALTYHYETQCLPTPAAWFAHHLPVWLHKLSVVATFLIEIAVPPLFFAPIRRLRLAAFYSQVLL
QVLIIITGNYNFFNLMTLVLTTALLDDQHLAAEPGHGSRKKTATSWPKALLATLSLLLELAVYGLLAYGTVHYFGLEVDWQQRTIHS
RTTFTFHQFSQWLKTLTLPTVWLGVASLVWELLSALWRWTQVRGWLRKLSAVVQLSLVGTATVALFLISLVPYSYVEPGTHGRLWTG
AHRLFGAVEHLQLANSYGLFRRMTGLGGRPEVVLEGSYDGHHWTEIEFMYKPGNLSRPPPVVVPHQPRLDWQMWFAALGPHTHSPWF
TSLVLRLLQGKEPVIRLVQSQVARYPFHKQPPTYVRAQRYKYWFSQPGEQGQWWRRQWVEEFFPSVSLGDPTLETLLRQFGLQEKSP
PRTRSANSTLAQALHWTRSQLSPLEAPALLWGLLMAVGAVRFVQALLAPCSLRSSPLAPVSGEKRRPASQKDSGAASEQATAAPNPC
SSSSRTTRRKK
>sp|Q9UIX4|KCNG1_HUMAN
MTLLPGDNSDYDYSALSCTSDASFHPAFLPQRQAIKGAFYRRAQRLRPQDEPRQGCQPEDRRRRIIINVGGIKYSLPWTTLDEFPLT
RLGQLKACTNFDDILNVCDDYDVTCNEFFFDRNPGAFGTILTFLRAGKLRLLREMCALSFQEELLYWGIAEDHLDGCCKRRYLQKIE
EFAEMVEREEEDDALDSEGRDSEGPAEGEGRLGRCMRRLRDMVERPHSGLPGKVFACLSVLFVTVTAVNLSVSTLPSLREEEEQGHC
SQMCHNVFIVESVCVGWFSLEFLLRLIQAPSKFAFLRSPLTLIDLVAILPYYITLLVDGAAAGRRKPGAGNSYLDKVGLVLRVLRAL
RILYVMRLARHSLGLQTLGLTARRCTREFGLLLLFLCVAIALFAPLLYVIENEMADSPEFTSIPACYWWAVITMTTVGYGDMVPRST
PGQVVALSSILSGILLMAFPVTSIFHTFSRSYLELKQEQERVMFRRAQFLIKTKSQLSVSQDSDILFGSASSDTRDNN
>sp|A1A519|F170A_HUMAN
MKRRQKRKHLENEESQETAEKGGGMSKSQEDALQPGSTRVAKGWSQGVGEVTSTSEYCSCVSSSRKLIHSGIQRIHRDSPQPQSPLA
QVQERGETPPRSQHVSLSSYSSYKTCVSSLCVNKEERGMKIYYMQVQMNKGVAVSWETEETLESLEKQPRMEEVTLSEVVRVGTPPS
DVSTRNLLSDSEPSGEEKEHEERTESDSLPGSPTVEDTPRAKTPDWLVTMENGFRCMACCRVFTTMEALQEHVQFGIREGFSCHVFH
LTMAQLTGNMESESTQDEQEEENGNEKEEEEKPEAKEEEGQPTEEDLGLRRSWSQCPGCVFHSPKDRNS
>sp|P62266|RS23_HUMAN

MGKCRGLRTARKLRSHRRDQKWHDKQYKKAHLGTALKANPFGGASHAKGIVLEKVGVEAKQPNSAIRKCVRVQLIKNGKKITAFVPN
DGCLNFIEENDEVLVAGFGRKGHAVGDIPGVRFKVVKVANVSLLALYKGKKERPRS
>sp|Q8IU81|I2BP1_HUMAN
MASVQASRRQWCYLCDLPKMPWAMVWDFSEAVCRGCVNFEGADRIELLIDAARQLKRSHVLPEGRSPGPPALKHPATKDLAAAAAQG
PQLPPPQAQPQPSGTGGGVSGQDRYDRATSSGRLPLPSPALEYTLGSRLANGLGREEAVAEGARRALLGSMPGLMPPGLLAAAVSGL
GSRGLTLAPGLSPARPLFGSDFEKEKQQRNADCLAELNEAMRGRAEEWHGRPKAVREQLLALSACAPFNVRFKKDHGLVGRVFAFDA
TARPPGYEFELKLFTEYPCGSGNVYAGVLAVARQMFHDALREPGKALASSGFKYLEYERRHGSGEWRQLGELLTDGVRSFREPAPAE
ALPQQYPEPAPAALCGPPPRAPSRNLAPTPRRRKASPEPEGEAAGKMTTEEQQQRHWVAPGGPYSAETPGVPSPIAALKNVAEALGH
SPKDPGGGGGPVRAGGASPAASSTAQPPTQHRLVARNGEAEVSPTAGAEAVSGGGSGTGATPGAPLCCTLCRERLEDTHFVQCPSVP
GHKFCFPCSREFIKAQGPAGEVYCPSGDKCPLVGSSVPWAFMQGEIATILAGDIKVKKERDP
>sp|Q15041|AR6P1_HUMAN
MAEGDNRSTNLLAAETASLEEQLQGWGEVMLMADKVLRWERAWFPPAIMGVVSLVFLIIYYLDPSVLSGVSCFVMFLCLADYLVPIL
APRIFGSNKWTTEQQQRFHEICSNLVKTRRRAVGWWKRLFTLKEEKPKMYFMTMIVSLAAVAWVGQQVHNLLLTYLIVTSLLLLPGL
NQHGIILKYIGMAKREINKLLKQKEKKNE
>sp|P58658|EVA1C_HUMAN
MLLPGRARQPPTPQPVQHPGLRRQVEPPGQLLRLFYCTVLVCSKEISALTDFSGYLTKLLQNHTTYACDGDYLNLQCPRHSTISVQS
AFYGQDYQMCSSQKPASQREDSLTCVAATTFQKVLDECQNQRACHLLVNSRVFGPDLCPGSSKYLLVSFKCQPNELKNKTVCEDQEL
KLHCHESKFLNIYSATYGRRTQERDICSSKAERLPPFDCLSYSALQVLSRRCYGKQRCKIIVNNHHFGSPCLPGVKKYLTVTYACVP
KNILTAIDPAIANLKPSLKQKDGEYGINFDPSGSKVLRKDGILVSNSLAAFAYIRAHPERAALLFVSSVCIGLALTLCALVIRESCA
KDFRDLQLGREQLVPGSDKVEEDSEDEEEEEDPSESDFPGELSGFCRTSYPIYSSIEAAELAERIERREQIIQEIWMNSGLDTSLPR
NMGQFY
>sp|Q13127|REST_HUMAN
MATQVMGQSSGGGGLFTSSGNIGMALPNDMYDLHDLSKAELAAPQLIMLANVALTGEVNGSCCDYLVGEERQMAELMPVGDNNFSDS
EEGEGLEESADIKGEPHGLENMELRSLELSVVEPQPVFEASGAPDIYSSNKDLPPETPGAEDKGKSSKTKPFRCKPCQYEAESEEQF
VHHIRVHSAKKFFVEESAEKQAKARESGSSTAEEGDFSKGPIRCDRCGYNTNRYDHYTAHLKHHTRAGDNERVYKCIICTYTTVSEY
HWRKHLRNHFPRKVYTCGKCNYFSDRKNNYVQHVRTHTGERPYKCELCPYSSSQKTHLTRHMRTHSGEKPFKCDQCSYVASNQHEVT
RHARQVHNGPKPLNCPHCDYKTADRSNFKKHVELHVNPRQFNCPVCDYAASKKCNLQYHFKSKHPTCPNKTMDVSKVKLKKTKKREA
DLPDNITNEKTEIEQTKIKGDVAGKKNEKSVKAEKRDVSKEKKPSNNVSVIQVTTRTRKSVTEVKEMDVHTGSNSEKFSKTKKSKRK
LEVDSHSLHGPVNDEESSTKKKKKVESKSKNNSQEVPKGDSKVEENKKQNTCMKKSTKKKTLKNKSSKKSSKPPQKEPVEKGSAQMD
PPQMGPAPTEAVQKGPVQVEPPPPMEHAQMEGAQIRPAPDEPVQMEVVQEGPAQKELLPPVEPAQMVGAQIVLAHMELPPPMETAQT
EVAQMGPAPMEPAQMEVAQVESAPMQVVQKEPVQMELSPPMEVVQKEPVQIELSPPMEVVQKEPVKIELSPPIEVVQKEPVQMELSP
PMGVVQKEPAQREPPPPREPPLHMEPISKKPPLRKDKKEKSNMQSERARKEQVLIEVGLVPVKDSWLLKESVSTEDLSPPSPPLPKE
NLREEASGDQKLLNTGEGNKEAPLQKVGAEEADESLPGLAANINESTHISSSGQNLNTPEGETLNGKHQTDSIVCEMKMDTDQNTRE
NLTGINSTVEEPVSPMLPPSAVEEREAVSKTALASPPATMAANESQEIDEDEGIHSHEGSDLSDNMSEGSDDSGLHGARPVPQESSR
KNAKEALAVKAAKGDFVCIFCDRSFRKGKDYSKHLNRHLVNVYYLEEAAQGQE
>sp|Q6PIU2|NCEH1_HUMAN
MRSSCVLLTALVALAAYYVYIPLPGSVSDPWKLMLLDATFRGAQQVSNLIHYLGLSHHLLALNFIIVSFGKKSAWSSAQVKVTDTDF
DGVEVRVFEGPPKPEEPLKRSVVYIHGGGWALASAKIRYYDELCTAMAEELNAVIVSIEYRLVPKVYFPEQIHDVVRATKYFLKPEV
LQKYMVDPGRICISGDSAGGNLAAALGQQFTQDASLKNKLKLQALIYPVLQALDFNTPSYQQNVNTPILPRYVMVKYWVDYFKGNYD
FVQAMIVNNHTSLDVEEAAAVRARLNWTSLLPASFTKNYKPVVQTTGNARIVQELPQLLDARSAPLIADQAVLQLLPKTYILTCEHD
VLRDDGIMYAKRLESAGVEVTLDHFEDGFHGCMIFTSWPTNFSVGIRTRNSYIKWLDQNL
>sp|H0YL14|CI069_HUMAN
MPVMPIPRRVRSFHGPHTTCLHAACGPVRASHLARTKYNNFDVYIKTRWLYGFIRFLLYFSCSLFTAALWGALAALFCLQYLGVRVL
LRFQRKLSVLLLLLGRRRVDFRLVNELLVYGIHVTMLLVGGLGWCFMVFVDM

>sp|P63267|ACTH_HUMAN
MCEEETTALVCDNGSGLCKAGFAGDDAPRAVFPSIVGRPRHQGVMVGMGQKDSYVGDEAQSKRGILTLKYPIEHGIITNWDDMEKIW
HHSFYNELRVAPEEHPTLLTEAPLNPKANREKMTQIMFETFNVPAMYVAIQAVLSLYASGRTTGIVLDSGDGVTHNVPIYEGYALPH
AIMRLDLAGRDLTDYLMKILTERGYSFVTTAEREIVRDIKEKLCYVALDFENEMATAASSSSLEKSYELPDGQVITIGNERFRCPET
LFQPSFIGMESAGIHETTYNSIMKCDIDIRKDLYANNVLSGGTTMYPGIADRMQKEITALAPSTMKIKIIAPPERKYSVWIGGSILA
SLSTFQQMWISKPEYDEAGPSIVHRKCF
>sp|Q8IYU8|MICU2_HUMAN
MAAAAGSCARVAAWGGKLRRGLAVSRQAVRSPGPLAAAVAGAALAGAGAAWHHSRVSVAARDGSFTVSAQKNVEHGIIYIGKPSLRK
QRFMQFSSLEHEGEYYMTPRDFLFSVMFEQMERKTSVKKLTKKDIEDTLSGIQTAGCGSTFFRDLGDKGLISYTEYLFLLTILTKPH
SGFHVAFKMLDTDGNEMIEKREFFKLQKIISKQDDLMTVKTNETGYQEAIVKEPEINTTLQMRFFGKRGQRKLHYKEFRRFMENLQT
EIQEMEFLQFSKGLSFMRKEDFAEWLLFFTNTENKDIYWKNVREKLSAGESISLDEFKSFCHFTTHLEDFAIAMQMFSLAHRPVRLA
EFKRAVKVATGQELSNNILDTVFKIFDLDGDECLSHEEFLGVLKNRMHRGLWVPQHQSIQEYWKCVKKESIKGVKEVWKQAGKGLF
>sp|Q9UEW3|MARCO_HUMAN
MRNKKILKEDELLSETQQAAFHQIAMEPFEINVPKPKRRNGVNFSLAVVVIYLILLTAGAGLLVVQVLNLQARLRVLEMYFLNDTLA
AEDSPSFSLLQSAHPGEHLAQGASRLQVLQAQLTWVRVSHEHLLQRVDNFTQNPGMFRIKGEQGAPGLQGHKGAMGMPGAPGPPGPP
AEKGAKGAMGRDGATGPSGPQGPPGVKGEAGLQGPQGAPGKQGATGTPGPQGEKGSKGDGGLIGPKGETGTKGEKGDLGLPGSKGDR
GMKGDAGVMGPPGAQGSKGDFGRPGPPGLAGFPGAKGDQGQPGLQGVPGPPGAVGHPGAKGEPGSAGSPGRAGLPGSPGSPGATGLK
GSKGDTGLQGQQGRKGESGVPGPAGVKGEQGSPGLAGPKGAPGQAGQKGDQGVKGSSGEQGVKGEKGERGENSVSVRIVGSSNRGRA
EVYYSGTWGTICDDEWQNSDAIVFCRMLGYSKGRALYKVGAGTGQIWLDNVQCRGTESTLWSCTKNSWGHHDCSHEEDAGVECSV
>sp|Q9Y2Y1|RPC10_HUMAN
MLLFCPGCGNGLIVEEGQRCHRFSCNTCPYVHNITRKVTNRKYPKLKEVDDVLGGAAAWENVDSTAESCPKCEHPRAYFMQLQTRSA
DEPMTTFYKCCNAQCGHRWRD
>sp|Q9H1B7|I2BPL_HUMAN
MSAAQVSSSRRQSCYLCDLPRMPWAMIWDFSEPVCRGCVNYEGADRIEFVIETARQLKRAHGCFQDGRSPGPPPPVGVKTVALSAKE
AAAAAAAAAAAAAAAQQQQQQQQQQQQQQQQQQQQQQQQQLNHVDGSSKPAVLAAPSGLERYGLSAAAAAAAAAAAAVEQRSRFEYP
PPPVSLGSSSHTARLPNGLGGPNGFPKPTPEEGPPELNRQSPNSSSAAASVASRRGTHGGLVTGLPNPGGGGGPQLTVPPNLLPQTL
LNGPASAAVLPPPPPHALGSRGPPTPAPPGAPGGPACLGGTPGVSATSSSASSSTSSSVAEVGVGAGGKRPGSVSSTDQERELKEKQ
RNAEALAELSESLRNRAEEWASKPKMVRDTLLTLAGCTPYEVRFKKDHSLLGRVFAFDAVSKPGMDYELKLFIEYPTGSGNVYSSAS
GVAKQMYQDCMKDFGRGLSSGFKYLEYEKKHGSGDWRLLGDLLPEAVRFFKEGVPGADMLPQPYLDASCPMLPTALVSLSRAPSAPP
GTGALPPAAPSGRGAAASLRKRKASPEPPDSAEGALKLGEEQQRQQWMANQSEALKLTMSAGGFAAPGHAAGGPPPPPPPLGPHSNR
TTPPESAPQNGPSPMAALMSVADTLGTAHSPKDGSSVHSTTASARRNSSSPVSPASVPGQRRLASRNGDLNLQVAPPPPSAHPGMDQ
VHPQNIPDSPMANSGPLCCTICHERLEDTHFVQCPSVPSHKFCFPCSRESIKAQGATGEVYCPSGEKCPLVGSNVPWAFMQGEIATI
LAGDVKVKKERDP
>sp|E9PI22|P23D1_HUMAN
MYGYRRLRSPRDSQTEPQNDNEGETSLATTQMNPPKRRQVEQGPSTGAKKPSISGAPHLNSYQSLELPQNQQDSGTEELMIVLEQGT
EVRLSLEEVILILAPETVLQLTLENTVLVIVPEHVLRSEDGLQSPVQIQYIIPSVDDFSLEFHAQDGDISDMRRENVPFSPAEEGKA
APLYQQPLMIPQANHMAGISPSFLVTPLCIPRCRAAFPQCYPLPPTPSPVGRPRPADSSFSLHGMELLCTSSLRPMPPSPSPGPQVY
HRVHHRPPSRARRCLFRK
>sp|Q9BXU3|TX13A_HUMAN
MALRPEDPSSGFRHSNVVAFINEKMARHTKGPEFYLENISLSWEKVEDKLRAILEDSEVPSEVKEACTWGSLALGVRFAHRQAQLQR
HRVRWLHGFAKLHKSAAQALASDLKKLREQQETERKEAASRLRMAQTSLVEVQKERDKELVSPHEWEQGAGWPGLATAGGVCTEGAA
EEEEEAAVAAAGAAGGKGAEEEQRDVEVVAAPVEAMAPPVEAGAAPMETQFPHVEARAASMETTEKLERILLQLLGDADQEKYTYWG
QKEGDLRSVETATSYFSGTTNPWSRASSEPLPVQLPASYSYSYSSPFSSFSDIPTISPPQATVTAPVPPQLPSDWEAFDTSLWSDGG
PHRIDHQEHPRDRRYSEPHQQRPPVYRRPGDWDCPWCNAVNFSRRDTCFDCGKGIWLQKPH

>sp|Q6ZQW0|I2302_HUMAN
MLHFHYYDTSNKIMEPHRPNVKTAVPLSLESYHISEEYGFLLPDSLKELPDHYRPWMEIANKLPQLIDAHQLQAHVDKMPLLSCQFL
KGHREQRLAHLVLSFLTMGYVWQEGEAQPAEVLPRNLALPFVEVSRNLGLPPILVHSDLVLTNWTKKDPDGFLEIGNLETIISFPGG
ESLHGFILVTALVEKEAVPGIKALVQATNAILQPNQEALLQALQRLRLSIQDITKTLGQMHDYVDPDIFYAGIRIFLSGWKDNPAMP
AGLMYEGVSQEPLKYSGGSAAQSTVLHAFDEFLGIRHSKESGDFLYRMRDYMPPSHKAFIEDIHSAPSLRDYILSSGQDHLLTAYNQ
CVQALAELRSYHITMVTKYLITAAAKAKHGKPNHLPGPPQALKDRGTGGTAVMSFLKSVRDKTLESILHPRG
>sp|Q7RTT9|S29A4_HUMAN
MGSVGSQRLEEPSVAGTPDPGVVMSFTFDSHQLEEAAEAAQGQGLRARGVPAFTDTTLDEPVPDDRYHAIYFAMLLAGVGFLLPYNS
FITDVDYLHHKYPGTSIVFDMSLTYILVALAAVLLNNVLVERLTLHTRITAGYLLALGPLLFISICDVWLQLFSRDQAYAINLAAVG
TVAFGCTVQQSSFYGYTGMLPKRYTQGVMTGESTAGVMISLSRILTKLLLPDERASTLIFFLVSVALELLCFLLHLLVRRSRFVLFY
TTRPRDSHRGRPGLGRGYGYRVHHDVVAGDVHFEHPAPALAPNESPKDSPAHEVTGSGGAYMRFDVPRPRVQRSWPTFRALLLHRYV
VARVIWADMLSIAVTYFITLCLFPGLESEIRHCILGEWLPILIMAVFNLSDFVGKILAALPVDWRGTHLLACSCLRVVFIPLFILCV
YPSGMPALRHPAWPCIFSLLMGISNGYFGSVPMILAAGKVSPKQRELAGNTMTVSYMSGLTLGSAVAYCTYSLTRDAHGSCLHASTA
NGSILAGL
>sp|Q7Z3F1|GP155_HUMAN
MNSNLPAENLTIAVNMTKTLPTAVTHGFNSTNDPPSMSITRLFPALLECFGIVLCGYIAGRANVITSTQAKGLGNFVSRFALPALLF
KNMVVLNFSNVDWSFLYSILIAKASVFFIVCVLTLLVASPDSRFSKAGLFPIFATQSNDFALGYPIVEALYQTTYPEYLQYIYLVAP
ISLMMLNPIGFIFCEIQKWKDTQNASQNKIKIVGLGLLRVLQNPIVFMVFIGIAFNFILDRKVPVYVENFLDGLGNSFSGSALFYLG
LTMVGKIKRLKKSAFVVLILLITAKLLVLPLLCREMVELLDKGDSVVNHTSLSNYAFLYGVFPVAPGVAIFATQFNMEVEIITSGMV
ISTFVSAPIMYVSAWLLTFPTMDPKPLAYAIQNVSFDISIVSLISLIWSLAILLLSKKYKQLPHMLTTNLLIAQSIVCAGMMIWNFV
KEKNFVGQILVFVLLYSSLYSTYLWTGLLAISLFLLKKRERVQIPVGIIIISGWGIPALLVGVLLITGKHNGDSIDSAFFYGKEQMI
TTAVTLFCSILIAGISLMCMNQTAQAGSYEGFDQSQSHKVVEPGNTAFEESPAPVNEPELFTSSIPETSCCSCSMGNGELHCPSIEP
IANTSTSEPVIPSFEKNNHCVSRCNSQSCILAQEEEQYLQSGDQQLTRHVLLCLLLIIGLFANLSSCLWWLFNQEPGRLYVELQFFC
AVFNFGQGFISFGIFGLDKHLIILPFKRRLEFLWNNKDTAENRDSPVSEEIKMTCQQFIHYHRDLCIRNIVKERRCGAKTSAGTFCG
CDLVSWLIEVGLASDRGEAVIYGDRLVQGGVIQHITNEYEFRDEYLFYRFLQKSPEQSPPAINANTLQQERYKEIEHSSPPSHSPKT
>sp|Q06124|PTN11_HUMAN
MTSRRWFHPNITGVEAENLLLTRGVDGSFLARPSKSNPGDFTLSVRRNGAVTHIKIQNTGDYYDLYGGEKFATLAELVQYYMEHHGQ
LKEKNGDVIELKYPLNCADPTSERWFHGHLSGKEAEKLLTEKGKHGSFLVRESQSHPGDFVLSVRTGDDKGESNDGKSKVTHVMIRC
QELKYDVGGGERFDSLTDLVEHYKKNPMVETLGTVLQLKQPLNTTRINAAEIESRVRELSKLAETTDKVKQGFWEEFETLQQQECKL
LYSRKEGQRQENKNKNRYKNILPFDHTRVVLHDGDPNEPVSDYINANIIMPEFETKCNNSKPKKSYIATQGCLQNTVNDFWRMVFQE
NSRVIVMTTKEVERGKSKCVKYWPDEYALKEYGVMRVRNVKESAAHDYTLRELKLSKVGQALLQGNTERTVWQYHFRTWPDHGVPSD
PGGVLDFLEEVHHKQESIMDAGPVVVHCSAGIGRTGTFIVIDILIDIIREKGVDCDIDVPKTIQMVRSQRSGMVQTEAQYRFIYMAV
QHYIETLQRRIEEEQKSKRKGHEYTNIKYSLADQTSGDQSPLPPCTPTPPCAEMREDSARVYENVGLMQQQKSFR
>sp|Q8WXF3|REL3_HUMAN
MARYMLLLLLLAVWVLTGELWPGAEARAAPYGVRLCGREFIRAVIFTCGGSRWRRSDILAHEAMGDTFPDADADEDSLAGELDEAMGS
SEWLALTKSPQAFYRGRPSWQGTPGVLRGSRDVLAGLSSSCCKWGCSKSEISSLC
>sp|Q2KHT4|GSG1_HUMAN
MAKMELSKAFSGQRTLLSAILSMLSLSFSTTSLLSNYWFVGTQKVPKPLCEKGLAAKCFDMPVSLDGDTNTSTQEVVQYNWETGDDR
FSFRSFRSGMWLSCEETVEEPALLHPQSWKQFRALRSSGTAAAKGERCRSFIELTPPAKREILWLSLGTQITYIGLQFISFLLLLTD
LLLTGNPACGLKLSAFAAVSSVLSGLLGMVAHMMYSQVFQATVNLGPEDWRPHVWNYGWAFYMAWLSFTCCMASAVTTFNTYTRMVL
EFKCKHSKSFKENPNCLPHHHQCFPRRLSSAAPTVGPLTSYHQYHNQPIHSVSEGVDFYSELRNKGFQRGASQELKEAVRSSVEEEQ
C
>sp|Q9BZF2|OSBL7_HUMAN

MDFQERDPPFLPESAQSSKPSSAQQASELWEVVEEPRVRLGTEGVMPERQEGHLLKKRKWPLKGWHKRYFVLEDGILHYATTRQDIT
KGKLHGSIDVRLSVMSINKKAQRIDLDTEDNIYHLKIKSQDLFQSWVAQLRAHRLAHRLDMPRGSLPSTAHRKVPGAQLPTAATASA
LPGLGPREKVSSWLRDSDGLDRCSHELSECQGKLQELHRLLQSLESLHRIPSAPVIPTHQASVTTERPKKGKRTSRMWCTQSFAKDD
TIGRVGRLHGSVPNLSRYLESRDSSGTRGLPPTDYAHLQRSFWALAQKVHSSLSSVLAALTMERDQLRDMHQGSELSRMGVSEASTG
QRRLHSLSTSSDTTADSFSSLNPEEQEALYMKGRELTPQLSQTSILSLADSHTEFFDACEVLLSASSSENEGSEEEESCTSEITTSL
SEEMLDLRGAERCQKGGCVPGRPMGPPRRRCLPAASGPGADVSLWNILRNNIGKDLSKVSMPVQLNEPLNTLQRLCEELEYSSLLDQ
ASRIADPCERMVYIAAFAVSAYSSTYHRAGCKPFNPVLGETYECERPDRGFRFISEQVSHHPPISACHAESENFAFWQDMKWKNKFW
GKSLEIVPVGTVNVSLPRFGDHFEWNKVTSCIHNVLSGQRWIEHYGEVLIRNTQDSSCHCKITFCKAKYWSSNVHEVQGAVLSRSGR
VLHRLFGKWHEGLYRGPTPGGQCIWKPNSMPPDHERNFGFTQFALELNELTAELKRSLPSTDTRLRPDQRYLEEGNIQAAEAQKRRI
EQLQRDRRKVMEENNIVHQARFFRRQTDSSGKEWWVTNNTYWRLRAEPGYGNMDGAVLW
>sp|Q8N584|TT39C_HUMAN
MAGSEQQRPRRRDDGDSDAAAAAAAPLQDAELALAGINMLLNNGFRESDQLFKQYRNHSPLMSFGASFVSFLNAMMTFEEEKMQLAC
DDLKTTEKLCESEEAGVIETIKNKIKKNVDVRKSAPSMVDRLQRQIIIADCQVYLAVLSFVKQELSAYIKGGWILRKAWKIYNKCYL
DINALQELYQKKLTEESLTSDAANDNHIVAEGVSEESLNRLKGAVSFGYGLFHLCISMVPPNLLKIINLLGFPGDRLQGLSSLMYAS
ESKDMKAPLATLALLWYHTVVRPFFALDGSDNKAGLDEAKEILLKKEAAYPNSSLFMFFKGRIQRLECQINSALTSFHTALELAVDQ
REIQHVCLYEIGWCSMIELNFKDAFDSFERLKNESRWSQCYYAYLTAVCQGATGDVDGAQIVFKEVQKLFKRKNNQIEQFSVKKAER
FRKQTPTKALCVLASIEVLYLWKALPNCSFPNLQRMSQACHEVDDSSVVGLKYLLLGAIHKCLGNSEDAVQYFQRAVKDELCRQNNL
YVQPYACYELGCLLLDKPETVGRGRALLLQAKEDFSGYDFENRLHVRIHAALASLRELVPQ
>sp|Q5T750|XP32_HUMAN
MCDQQKQPQFPPSCVKGSGLGAGQGSNGASVKCPVPCQTQTVCVTGPAPCPTQTYVKYQVPCQTQTYVKCPAPCQRTYVKYPTPCQT
YVKCPAPCQTTYVKCPTPCQTYVKCPAPCQMTYIKSPAPCQTQTCYVQGASPCQSYYVQAPASGSTSQYCVTDPCSAPCSTSYCCLA
PRTFGVSPLRRWIQRPQNCNTGSSGCCENSGSSGCCGSGGCGCSCGCGSSGCCCLGIIPMRSRGPACCDHEDDCCC
>sp|Q96I24|FUBP3_HUMAN
MAELVQGQSAPVGMKAEGFVDALHRVRQIAAKIDSIPHLNNSTPLVDPSVYGYGVQKRPLDDGVGNQLGALVHQRTVITEEFKVPDK
MVGFIIGRGGEQISRIQAESGCKIQIASESSGIPERPCVLTGTPESIEQAKRLLGQIVDRCRNGPGFHNDIDSNSTIQEILIPASKV
GLVIGRGGETIKQLQERTGVKMVMIQDGPLPTGADKPLRITGDAFKVQQAREMVLEIIREKDQADFRGVRGDFNSRMGGGSIEVSVP
RFAVGIVIGRNGEMIKKIQNDAGVRIQFKPDDGISPERAAQVMGPPDRCQHAAHIISELILTAQERDGFGGLAAARGRGRGRGDWSV
GAPGGVQEITYTVPADKCGLVIGKGGENIKSINQQSGAHVELQRNPPPNSDPNLRRFTIRGVPQQIEVARQLIDEKVGGTNLGAPGA
FGQSPFSQPPAPPHQNTFPPRSSGCFPNMAAKVNGNPHSTPVSGPPAFLTQGWGSTYQAWQQPTQQVPSQQSQPQSSQPNYSKAWED
YYKKQSHAASAAPQASSPPDYTMAWAEYYRQQVAFYGQTLGQAQAHSQEQ
>sp|Q92823|NRCAM_HUMAN
MQLKIMPKKKRLSAGRVPLILFLCQMISALEVPLDPKLLEDLVQPPTITQQSPKDYIIDPRENIVIQCEAKGKPPPSFSWTRNGTHF
DIDKDPLVTMKPGTGTLIINIMSEGKAETYEGVYQCTARNERGAAVSNNIVVRPSRSPLWTKEKLEPITLQSGQSLVLPCRPPIGLP
PPIIFWMDNSFQRLPQSERVSQGLNGDLYFSNVLPEDTREDYICYARFNHTQTIQQKQPISVKVISVDELNDTIAANLSDTEFYGAK
SSRERPPTFLTPEGNASNKEELRGNVLSLECIAEGLPTPIIYWAKEDGMLPKNRTVYKNFEKTLQIIHVSEADSGNYQCIAKNALGA
IHHTISVRVKAAPYWITAPQNLVLSPGEDGTLICRANGNPKPRISWLTNGVPIEIAPDDPSRKIDGDTIIFSNVQERSSAVYQCNAS
NEYGYLLANAFVNVLAEPPRILTPANTLYQVIANRPALLDCAFFGSPLPTIEWFKGAKGSALHEDIYVLHENGTLEIPVAQKDSTGT
YTCVARNKLGMAKNEVHLEIKDPTWIVKQPEYAVVQRGSMVSFECKVKHDHTLSLTVLWLKDNRELPSDERFTVDKDHLVVADVSDD
DSGTYTCVANTTLDSVSASAVLSVVAPTPTPAPVYDVPNPPFDLELTDQLDKSVQLSWTPGDDNNSPITKFIIEYEDAMHKPGLWHH
QTEVSGTQTTAQLKLSPYVNYSFRVMAVNSIGKSLPSEASEQYLTKASEPDKNPTAVEGLGSEPDNLVITWKPLNGFESNGPGLQYK
VSWRQKDGDDEWTSVVVANVSKYIVSGTPTFVPYLIKVQALNDMGFAPEPAVVMGHSGEDLPMVAPGNVRVNVVNSTLAEVHWDPVP
LKSIRGHLQGYRIYYWKTQSSSKRNRRHIEKKILTFQGSKTHGMLPGLEPFSHYTLNVRVVNGKGEGPASPDRVFNTPEGVPSAPSS
LKIVNPTLDSLTLEWDPPSHPNGILTEYTLKYQPINSTHELGPLVDLKIPANKTRWTLKNLNFSTRYKFYFYAQTSAGSGSQITEEA
VTTVDEAGILPPDVGAGKVQAVNPRISNLTAAAAETYANISWEYEGPEHVNFYVEYGVAGSKEEWRKEIVNGSRSFFGLKGLMPGTA

YKVRVGAVGDSGFVSSEDVFETGPAMASRQVDIATQGWFIGLMCAVALLILILLIVCFIRRNKGGKYPVKEKEDAHADPEIQPMKED
DGTFGEYSDAEDHKPLKKGSRTPSDRTVKKEDSDDSLVDYGEGVNGQFNEDGSFIGQYSGKKEKEPAEGNESSEAPSPVNAMNSFV
>sp|Q8NEG5|ZSWM2_HUMAN
MLRRGYKASERRRHLSERLSWHQDQALSSSIYLLREMGPTGFLLREEEPEYMDFRVFLGNPHVCNCSTFPKGGELCKHICWVLLKKF
KLPRNHESALQLGLGEREISDLLRGIHRVQTPQPGTNDENEHVEEDGYIKQKEIDSEDICSICQELLLEKKLPVTFCRFGCGNSIHI
KCMKILANYQSTSNTSMLKCPLCRKEFAPLKLILEEFKNSSKLVAAAEKERLDKHLGIPCNNCKQFPIEGKCYKCTECIEYHLCQEC
FDSCCHLSHTFTFREKRNQKWRSLEKRADEVVKYIDTKNEIEEKMSHFQEKQGQVYTPKHIVRSLPLQLITKNSKLLAPGYQCLLCL
KAFHLGQHTRLLPCTHKFHRKCIDNWLFHKCNSCPIDGQVIYNPLTWKNSAVNGQAHQSVSNRDIIHLSKQKEPDLFIPGTGLVLKQ
NRLGILPSIPQCNFDELNTPQSPKDAYENTTIDNLCSIKLDNSNSKKLTYDYKISQHFPRYLQDLPTVSFGKIPSQTLLPPIVHKNI
VCPTAMESPCISGKFHTSLSRMTKGCKCNNHNLKKTPATKIREDNKRSTLLPEDFNLIVNWSTAKLSLSKRYSNCMGEITRKCSHLS
RQPVSHSVNTKSTELSLIIEGVQL
>sp|A8K7I4|CLCA1_HUMAN
MGPFKSSVFILILHLLEGALSNSLIQLNNNGYEGIVVAIDPNVPEDETLIQQIKDMVTQASLYLLEATGKRFYFKNVAILIPETWKT
KADYVRPKLETYKNADVLVAESTPPGNDEPYTEQMGNCGEKGERIHLTPDFIAGKKLAEYGPQGRAFVHEWAHLRWGVFDEYNNDEK
FYLSNGRIQAVRCSAGITGTNVVKKCQGGSCYTKRCTFNKVTGLYEKGCEFVLQSRQTEKASIMFAQHVDSIVEFCTEQNHNKEAPN
KQNQKCNLRSTWEVIRDSEDFKKTTPMTTQPPNPTFSLLQIGQRIVCLVLDKSGSMATGNRLNRLNQAGQLFLLQTVELGSWVGMVT
FDSAAHVQNELIQINSGSDRDTLAKRLPAAASGGTSICSGLRSAFTVIRKKYPTDGSEIVLLTDGEDNTISGCFNEVKQSGAIIHTV
ALGPSAAQELEELSKMTGGLQTYASDQVQNNGLIDAFGALSSGNGAVSQRSIQLESKGLTLQNSQWMNGTVIVDSTVGKDTLFLITW
TMQPPQILLWDPSGQKQGGFVVDKNTKMAYLQIPGIAKVGTWKYSLQASSQTLTLTVTSRASNATLPPITVTSKTNKDTSKFPSPLV
VYANIRQGASPILRASVTALIESVNGKTVTLELLDNGAGADATKDDGVYSRYFTTYDTNGRYSVKVRALGGVNAARRRVIPQQSGAL
YIPGWIENDEIQWNPPRPEINKDDVQHKQVCFSRTSSGGSFVASDVPNAPIPDLFPPGQITDLKAEIHGGSLINLTWTAPGDDYDHG
TAHKYIIRISTSILDLRDKFNESLQVNTTALIPKEANSEEVFLFKPENITFENGTDLFIAIQAVDKVDLKSEISNIARVSLFIPPQT
PPETPSPDETSAPCPNIHINSTIPGIHILKIMWKWIGELQLSIA
>sp|Q13133|NR1H3_HUMAN
MSLWLGAPVPDIPPDSAVELWKPGAQDASSQAQGGSSCILREEARMPHSAGGTAGVGLEAAEPTALLTRAEPPSEPTEIRPQKRKKG
PAPKMLGNELCSVCGDKASGFHYNVLSCEGCKGFFRRSVIKGAHYICHSGGHCPMDTYMRRKCQECRLRKCRQAGMREECVLSEEQI
RLKKLKRQEEEQAHATSLPPRASSPPQILPQLSPEQLGMIEKLVAAQQQCNRRSFSDRLRVTPWPMAPDPHSREARQQRFAHFTELA
IVSVQEIVDFAKQLPGFLQLSREDQIALLKTSAIEVMLLETSRRYNPGSESITFLKDFSYNREDFAKAGLQVEFINPIFEFSRAMNE
LQLNDAEFALLIAISIFSADRPNVQDQLQVERLQHTYVEALHAYVSIHHPHDRLMFPRMLMKLVSLRTLSSVHSEQVFALRLQDKKL
PPLLSEIWDVHE
>sp|Q6ZNQ3|LRC69_HUMAN
MTERLLIKALSGGKNTKIITLNGKKMTKMPSALGKLPGLKTLVLQNNLIPKVCPELCNLTQLTTLNLGNNLLEEVPEEMKYLTSLKN
LHLSGNRICRFAPGACDGLQNLILLNLNNNHLTQLPQEVSRLKSLTYMSINYNQLASIPRELCFLENLVELQLNYNQLICIPEEIKF
LKKLQKLLLARNNIGVLPEELCDLKKLRILDIAGNIIQIFPSGFQDLKLREFYCEGNPLFLQQPVISTQQENVWSLQEITSRFVMNQ
LAENNPFLMDDIERYPQVRSMISQGKTCAICGQYFITVWLECVRFVPPPKDWKISKNLKLVPLQVLICSYKCFTQRDPNLFGIAQV
>sp|Q6P2S7|TTC41_HUMAN
MSQKTNENVERYTQFLQKPQKPIQPYICSTLNDFQEERDFLANNIFPQLNELCNSWGTYFKAVDLSWSALKAPKSLPTHLFRQYSCL
RSQRLKLCLDYVNSCFPFFICMLGQTYGDFLPDYSHFMTSKVTRLSSLSKVENLYVAAKNGYPWVLENPSCSLTEFEIIQAAFLNES
QFQYFYFRTGTTLLKALDDEKKGERLPSSSSTNEEETLRIGKLKAKIISKGLPVRFYSDLHELGELVFKDWSVVIEKLHPATLMIEN
IDYKHSFERFYHEEFTEKCKQMCVISKESDRTFEILEKFALKDVELDFNNVAADSSLDSVPRFFRINPTPTYKSILLLSREHGCGKS
TLIANWVNYFKKKHPSMLLIPHFVGSTCESSYIMSVIHYFITELQYRNYGTQLETDILNEDSDGLVFSFLVEVFIASISLKPCILVL
DGIEELIGIYGISGQKVKDFSWLPHSLSPHCKFIMSTVSSSLSYKSLCARPDVRTVELISTGDEETKLNIFRKHLSIPMMDPFEQST
QALRKKPDLSPLKLTILANELKEYRINHNEFQCMKEYLEAVSVQELWELVLKRWIEDYSWTFQPKRANSDTVASGEGLDSWVADALC
LLCLSHGGLAEDELLQLLDMLGYRNHYKVTALHWAAFRNATKQWVQEKPNGLLYFWHQSLSAVEHKLLGVITPVESSPCSFQTPMNH

KKTHFHQVLIRYFQRQTSFWRVYQELPWHMKMSGCLRGLCGFLSSPTITDFISKIQSLGFWTRLHLIHFWNVLLEAGYDVSEAYLLS
VAKIKADQCHTMRKSGTLSVLQCRLIELTVLDKCRLMFFIGSFLKFMGKTNEAEELFLSVEDMLVQSQSMTDMLLKVQNAIGELYLE
TGMTQEGFQYFQKAWSSMLRLSLSDLEDSRDLVKQKVRVLDNLAKSASEEYLKENHILEYATEISNLLDNNPRDQATMKYIEGVLMF
VAGNTSLAKMKLRECLNIRKSLFGKKNMLVGEVMEFLADLLFFPQRDSKKSQRKQVLKYYKQVIKIKENAETLAKSSLLRKQLSISL
SDTLCKLAGHLLASDSCHHVMIEAVGYLYRSVDLRVIHLGSSHSSIHGIHGILHLLREIEWIRSRRYWPQGMSQQHSEGSRNGFSLW
EHLVKLNYHSAQSSNTVSSAMCMNADKLHRARRMDLAPQTISDKSKCAPGKGKKKPIICISAEEKIQRKTQNNAEIWNGSGKEASKK
KTDYSSNILSLGKMNGLIKLSRQRILLAKSESGEGEITTIYHHPLPWPVSTKNPGESEFISEKWLFHSPDYISISQKSFLQRRLHIE
TKLLKTSNDINKE
>sp|Q2V2M9|FHOD3_HUMAN
MATLACRVQFLDDTDPFNSTNFPEPSRPPLFTFREDLALGTQLAGVHRLLQAPHKLDDCTLQLSHNGAYLDLEATLAEQRDELEGFQ
DDAGRGKKHSIILRTQLSVRVHACIEKLYNSSGRDLRRALFSLKQIFQDDKDLVHEFVVAEGLTCLIKVGAEADQNYQNYILRALGQ
IMLYVDGMNGVINRNETIQWLYTLIGSKFRLVVKTALKLLLVFVEYSESNAPLLIQAVTAVDTKRGVKPWSNIMEILEEKDGVDTEL
LVYAMTLVNKTLSGLPDQDTFYDVVDCLEELGIAAVSQRHLNKKGTDLDLVEQLNIYEVALRHEDGDETTEPPPSGCRDRRRASVCS
SGGGEHRGLDRRRSRRHSVQSIKSTLSAPTSPCSQSAPSFKPNQVRDLREKYSNFGNNSYHSSRPSSGSSVPTTPTSSVSPPQEARL
ERSSPSGLLTSSFRQHQESLAAERERRRQEREERLQRIEREERNKFRYKYLEQLAAEEHEKELRSRSVSRGRADLSLDLTSPAAPAC
LAPLSHSPSSSDSQEALTVSASSPGTPHHPQASAGDPEPESEAEPEAEAGAGQVADEAGQDIASAHEGAETEVEQALEQEPEERASL
SEKERQNEGVNERDNCSASSVSSSSSTLEREEKEDKLSRDRTTGLWPAGVQDAGVNGQCGDILTNKRFMLDMLYAHNRKSPDDEEKG
DGEAGRTQQEAEAVASLATRISTLQANSQTQDESVRRVDVGCLDNRGSVKAFAEKFNSGDLGRGSISPDAEPNDKVPETAPVQPKTE
SDYIWDQLMANPRELRIQDMDFTDLGEEDDIDVLDVDLGHREAPGPPPPPPPTFLGLPPPPPPPLLDSIPPPPVPGNLLVPPPPVFN
APQGLGWSQVPRGQPTFTKKKKTIRLFWNEVRPFDWPCKNNRRCREFLWSKLEPIKVDTSRLEHLFESKSKELSVSKKTAADGKRQE
IIVLDSKRSNAINIGLTVLPPPRTIKIAILNFDEYALNKEGIEKILTMIPTDEEKQKIQEAQLANPEIPLGSAEQFLLTLSSISELS
ARLHLWAFKMDYETTEKEVAEPLLDLKEGIDQLENNKTLGFILSTLLAIGNFLNGTNAKAFELSYLEKVPEVKDTVHKQSLLHHVCT
MVVENFPDSSDLYSEIGAITRSAKVDFDQLQDNLCQMERRCKASWDHLKAIAKHEMKPVLKQRMSEFLKDCAERIIILKIVHRRIIN
RFHSFLLFMGHPPYAIREVNINKFCRIISEFALEYRTTRERVLQQKQKRANHRERNKTRGKMITDSGKFSGSSPAPPSQPQGLSYAE
DAAEHENMKAVLKTSSPSVEDATPALGVRTRSRASRGSTSSWTMGTDDSPNVTDDAADEIMDRIVKSATQVPSQRVVPRERKRSRAN
RKSLRRTLKSGLTPEEARALGLVGTSELQL
>sp|P08842|STS_HUMAN
MPLRKMKIPFLLLFFLWEAESHAASRPNIILVMADDLGIGDPGCYGNKTIRTPNIDRLASGGVKLTQHLAASPLCTPSRAAFMTGRY
PVRSGMASWSRTGVFLFTASSGGLPTDEITFAKLLKDQGYSTALIGKWHLGMSCHSKTDFCHHPLHHGFNYFYGISLTNLRDCKPGE
GSVFTTGFKRLVFLPLQIVGVTLLTLAALNCLGLLHVPLGVFFSLLFLAALILTLFLGFLHYFRPLNCFMMRNYEIIQQPMSYDNLT
QRLTVEAAQFIQRNTETPFLLVLSYLHVHTALFSSKDFAGKSQHGVYGDAVEEMDWSVGQILNLLDELRLANDTLIYFTSDQGAHVE
EVSSKGEIHGGSNGIYKGGKANNWEGGIRVPGILRWPRVIQAGQKIDEPTSNMDIFPTVAKLAGAPLPEDRIIDGRDLMPLLEGKSQ
RSDHEFLFHYCNAYLNAVRWHPQNSTSIWKAFFFTPNFNPVGSNGCFATHVCFCFGSYVTHHDPPLLFDISKDPRERNPLTPASEPR
FYEILKVMQEAADRHTQTLPEVPDQFSWNNFLWKPWLQLCCPSTGLSCQCDREKQDKRLSR
>sp|P01763|HV348_HUMAN
MELGLCWVFLVAILEGVQCEVQLVESGGGLVQPGGSLRLSCAASGFTFSSYEMNWVRQAPGKGLEWVSYISSSGSTIYYADSVKGRF
TISRDNAKNSLYLQMNSLRAEDTAVYYCAR
>sp|Q9NP84|TNR12_HUMAN
MARGSLRRLLRLLVLGLWLALLRSVAGEQAPGTAPCSRGSSWSADLDKCMDCASCRARPHSDFCLGCAAAPPAPFRLLWPILGGALS
LTFVLGLLSGFLVWRRCRRREKFTTPIEETGGEGCPAVALIQ
>sp|P0C604|OR4A8_HUMAN
MRQNNNITEFVLLGFSQYPDVQNALFVMFLLIYIVTMVGNLLIVVSIIASPFLGSPVYFFLACLSFIDAVYSTTISPVLIVDLLCDK
KTISFPACMGQLFIEHLFGDTDVFLLVVMAYDRYVATCKPLRYLTIMNRQVCILLLVVAVTGGFLHSVFQILVVYSLPFCGPNVIYH

FFCNIYPLLDLECTDTYFVGLAVVFNGGAICMVIFTLLLISYGVILNSLKTYSPEGRHKAPFICSSHFIMVILFFVPCIFLYVRPVS
NFPIDKFLTVFYSVITPKLNPFIYMLRNSEMRNAIENLLGYQSGKTGFRCSKLN
>sp|Q9Y2Y6|TMM98_HUMAN
METVVIVAIGVLATIFLASFAALVLVCRQRYCRPRDLLQRYDSKPIVDLIGAMETQSEPSELELDDVVITNPHIEAILENEDWIEDA
SGLMSHCIAILKICHTLTEKLVAMTMGSGAKMKTSASVSDIIVVAKRISPRVDDVVKSMYPPLDPKLLDARTTALLLSVSHLVLVTR
NACHLTGGLDWIDQSLSAAEEHLEVLREAALASEPDKGLPGPEGFLQEQSAI
>sp|Q6X9E4|FBW12_HUMAN
MEIRLPDLALKRIFSFLDLFGLLQVSQVNKHWNRIADSDYLWRSLSLQRWDCSNFTNQHLGTHTWKQFFLHQRRKELRLALAQPHNF
IYKVTKNIAFETELAYLSGNRLTVDEQEKSIICSVSPKQELCAWDVQEGTMIWSSPVQEFHFSNLVTLPQMHLAITMDRKKTIKVWN
CQDRDALAVLPMPQPCYCMEAYLTKDGPFLMVGDAAGDIYTFTLPGLRDVSKVTAFQYGIVLLHCSPDKKWVFACGTYSRTLPQVFL
TESLLRPSEGSVPLSTFLPHKLCASACWTPKVKNRITLMSQSSTGKKTEFITFDLTTKKTGGQTVIQAYEIASFQVAAHLKCPIWMG
ASDGYMIVFTSGPYLLLFSITGFLLQRFEDHQAAINNFWVDPCYVLTTSENSVHVYMWEEGGRHPYLRSCCHLENTWHDHTTDSCIS
SVMCDNASIVLRVRKVSDSSILVMYSLNT
>sp|A6NH00|OR2T8_HUMAN
MENGSYTSYFILLGLFNHTRAHQVLFMMVLSIVLTSLFGNSLMILLIHWDHRLHTPMYFLLSQLSLMDVMLVSTTVPKMAADYLTGS
KAISRAGCGAQIFFLPTLGGGECFLLAAMAYDRYAAVCHPLRYPTLMSWQLCLRMNLSCWLLGAADGLLQAVATLSFPYCGAHEIDH
FFCETPVLVRLACADTSVFENAMYICCVLMLLVPFSLILSSYGLILAAVLHMRSTEARKKAFATCSSHVAVVGLFYGAAIFTYMRPK
SHRSTNHDKVVSAFYTMFTPLLNPLIYSVKNSEVKGALTRCMGRCVALSRE
>sp|Q9BYC2|SCOT2_HUMAN
MAALRLLASVLGRGVPAGGSGLALSQGCARCFATSPRLRAKFYADPVEMVKDISDGATVMIGGFGLCGIPENLIAALLRTRVKDLQV
VSSNVGVEDFGLGLLLAARQVRRIVCSYVGENTLCESQYLAGELELELTPQGTLAERIRAGGAGVPAFYTPTGYGTLVQEGGAPIRY
TPDGHLALMSQPREVREFNGDHFLLERAIRADFALVKGWKADRAGNVVFRRSARNFNVPMCKAADVTAVEVEEIVEVGAFPPEDIHV
PNIYVDRVIKGQKYEKRIERLTILKEEDGDAGKEEDARTRIIRRAALEFEDGMYANLGIGIPLLASNFISPSMTVHLHSENGILGLG
PFPTEDEVDADLINAGKQTVTVLPGGCFFASDDSFAMIRGGHIQLTMLGAMQVSKYGDLANWMIPGKKVKGMGGAMDLVSSQKTRVV
VTMQHCTKDNTPKIMEKCTMPLTGKRCVDRIITEKAVFDVHRKKELTLRELWEGLTVDDIKKSTGCAFAVSPNLRPMQQVAP
>sp|Q13326|SGCG_HUMAN
MVREQYTTATEGICIERPENQYVYKIGIYGWRKRCLYLFVLLLLIILVVNLALTIWILKVMWFSPAGMGHLCVTKDGLRLEGESEFL
FPLYAKEIHSRVDSSLLLQSTQNVTVNARNSEGEVTGRLKVGPKMVEVQNQQFQINSNDGKPLFTVDEKEVVVGTDKLRVTGPEGAL
FEHSVETPLVRADPFQDLRLESPTRSLSMDAPRGVHIQAHAGKIEALSQMDILFHSSDGMLVLDAETVCLPKLVQGTWGPSGSSQSL
YEICVCPDGKLYLSVAGVSTTCQEHNHICL
>sp|Q9BQG2|NUD12_HUMAN
MSSVKRSLKQEIVTQFHCSAAEGDIAKLTGILSHSPSLLNETSENGWTALMYAARNGHPEIVQFLLEKGCDRSIVNKSRQTALDIAV
FWGYKHIANLLATAKGGKKPWFLTNEVEECENYFSKTLLDRKSEKRNNSDWLLAKESHPATVFILFSDLNPLVTLGGNKESFQQPEV
RLCQLNYTDIKDYLAQPEKITLIFLGVELEIKDKLLNYAGEVPREEEDGLVAWFALGIDPIAAEEFKQRHENCYFLHPPMPALLQLK
EKEAGVVAQARSVLAWHSRYKFCPTCGNATKIEEGGYKRLCLKEDCPSLNGVHNTSYPRVDPVVIMQVIHPDGTKCLLGRQKRFPPG
MFTCLAGFIEPGETIEDAVRREVEEESGVKVGHVQYVACQPWPMPSSLMIGCLALAVSTEIKVDKNEIEDARWFTREQVLDVLTKGK
QQAFFVPPSRAIAHQLIKHWIRINPNL
>sp|Q9BYG0|B3GN5_HUMAN
MRMLVSGRRVKKWQLIIQLFATCFLASLMFFWEPIDNHIVSHMKSYSYRYLINSYDFVNDTLSLKHTSAGPRYQYLINHKEKCQAQD
VLLLLFVKTAPENYDRRSGIRRTWGNENYVRSQLNANIKTLFALGTPNPLEGEELQRKLAWEDQRYNDIIQQDFVDSFYNLTLKLLM
QFSWANTYCPHAKFLMTADDDIFIHMPNLIEYLQSLEQIGVQDFWIGRVHRGAPPIRDKSSKYYVSYEMYQWPAYPDYTAGAAYVIS
GDVAAKVYEASQTLNSSLYIDDVFMGLCANKIGIVPQDHVFFSGEGKTPYHPCIYEKMMTSHGHLEDLQDLWKNATDPKVKTISKGF
FGQIYCRLMKIILLCKISYVDTYPCRAAFI
>sp|Q96AQ1|CC74A_HUMAN

MSGAGVAAGTRPPSSPTPGSRRRRQRPSVGVQSLRPQSPQLRQSDPQKRNLDLEKSLQFLQQQHSEMLAKLHEEIEHLKRENKDLHY
KLIMNQTSQKKDGPSGNHLSRASAPLGARWVCINGVWVEPGGPSPARLKEGSSRTHRPGGKRGRLAGGSADTVRSPADSLSMSSFQS
VKSISNSGKARPQPGSFNKQDSKADVSQKADLEEEPLLHNSKLDKVPGVQGQARKEKAEASNAGAACMGNSQHQGRQMGAGAHPPMI
LPLPLRKPTTLRQCEVLIRELWNTNLLQTQELRHLKSLLEGSQRPQAAPEEASFPRDQEATHFPKVSTKSLSKKCLSPPVAERAILP
ALKQTPKNNFAERQKRLQAMQKRRLHRSVL
>sp|O60502|OGA_HUMAN
MVQKESQATLEERESELSSNPAASAGASLEPPAAPAPGEDNPAGAGGAAVAGAAGGARRFLCGVVEGFYGRPWVMEQRKELFRRLQK
WELNTYLYAPKDDYKHRMFWREMYSVEEAEQLMTLISAAREYEIEFIYAISPGLDITFSNPKEVSTLKRKLDQVSQFGCRSFALLFD
DIDHNMCAADKEVFSSFAHAQVSITNEIYQYLGEPETFLFCPTEYCGTFCYPNVSQSPYLRTVGEKLLPGIEVLWTGPKVVSKEIPV
ESIEEVSKIIKRAPVIWDNIHANDYDQKRLFLGPYKGRSTELIPRLKGVLTNPNCEFEANYVAIHTLATWYKSNMNGVRKDVVMTDS
EDSTVSIQIKLENEGSDEDIETDVLYSPQMALKLALTEWLQEFGVPHQYSSRQVAHSGAKASVVDGTPLVAAPSLNATTVVTTVYQE
PIMSQGAALSGEPTTLTKEEEKKQPDEEPMDMVVEKQEETDHKNDNQILSEIVEAKMAEELKPMDTDKESIAESKSPEMSMQEDCIS
DIAPMQTDEQTNKEQFVPGPNEKPLYTAEPVTLEDLQLLADLFYLPYEHGPKGAQMLREFQWLRANSSVVSVNCKGKDSEKIEEWRS
RAAKFEEMCGLVMGMFTRLSNCANRTILYDMYSYVWDIKSIMSMVKSFVQWLGCRSHSSAQFLIGDQEPWAFRGGLAGEFQRLLPID
GANDLFFQPPPLTPTSKVYTIRPYFPKDEASVYKICREMYDDGVGLPFQSQPDLIGDKLVGGLLSLSLDYCFVLEDEDGICGYALGT
VDVTPFIKKCKISWIPFMQEKYTKPNGDKELSEAEKIMLSFHEEQEVLPETFLANFPSLIKMDIHKKVTDPSVAKSMMACLLSSLKA
NGSRGAFCEVRPDDKRILEFYSKLGCFEIAKMEGFPKDVVILGRSL
>sp|Q9BZ29|DOCK9_HUMAN
MSQPPLLPASAETRKFTRALSKPGTAAELRQSVSEVVRGSVLLAKPKLIEPLDYENVIVQKKTQILNDCLREMLLFPYDDFQTAILR
RQGRYICSTVPAKAEEEAQSLFVTECIKTYNSDWHLVNYKYEDYSGEFRQLPNKVVKLDKLPVHVYEVDEEVDKDEDAASLGSQKGG
ITKHGWLYKGNMNSAISVTMRSFKRRFFHLIQLGDGSYNLNFYKDEKISKEPKGSIFLDSCMGVVQNNKVRRFAFELKMQDKSSYLL
AADSEVEMEEWITILNKILQLNFEAAMQEKRNGDSHEDDEQSKLEGSGSGLDSYLPELAKSAREAEIKLKSESRVKLFYLDPDAQKL
DFSSAEPEVKSFEEKFGKRILVKCNDLSFNLQCCVAENEEGPTTNVEPFFVTLSLFDIKYNRKISADFHVDLNHFSVRQMLATTSPA
LMNGSGQSPSVLKGILHEAAMQYPKQGIFSVTCPHPDIFLVARIEKVLQGSITHCAEPYMKSSDSSKVAQKVLKNAKQACQRLGQYR
MPFAWAARTLFKDASGNLDKNARFSAIYRQDSNKLSNDDMLKLLADFRKPEKMAKLPVILGNLDITIDNVSSDFPNYVNSSYIPTKQ
FETCSKTPITFEVEEFVPCIPKHTQPYTIYTNHLYVYPKYLKYDSQKSFAKARNIAICIEFKDSDEEDSQPLKCIYGRPGGPVFTRS
AFAAVLHHHQNPEFYDEIKIELPTQLHEKHHLLLTFFHVSCDNSSKGSTKKRDVVETQVGYSWLPLLKDGRVVTSEQHIPVSANLPS
GYLGYQELGMGRHYGPEIKWVDGGKPLLKISTHLVSTVYTQDQHLHNFFQYCQKTESGAQALGNELVKYLKSLHAMEGHVMIAFLPT
ILNQLFRVLTRATQEEVAVNVTRVIIHVVAQCHEEGLESHLRSYVKYAYKAEPYVASEYKTVHEELTKSMTTILKPSADFLTSNKLL
KYSWFFFDVLIKSMAQHLIENSKVKLLRNQRFPASYHHAVETVVNMLMPHITQKFRDNPEASKNANHSLAVFIKRCFTFMDRGFVFK
QINNYISCFAPGDPKTLFEYKFEFLRVVCNHEHYIPLNLPMPFGKGRIQRYQDLQLDYSLTDEFCRNHFLVGLLLREVGTALQEFRE
VRLIAISVLKNLLIKHSFDDRYASRSHQARIATLYLPLFGLLIENVQRINVRDVSPFPVNAGMTVKDESLALPAVNPLVTPQKGSTL
DNSLHKDLLGAISGIASPYTTSTPNINSVRNADSRGSLISTDSGNSLPERNSEKSNSLDKHQQSSTLGNSVVRCDKLDQSEIKSLLM
CFLYILKSMSDDALFTYWNKASTSELMDFFTISEVCLHQFQYMGKRYIARTGMMHARLQQLGSLDNSLTFNHSYGHSDADVLHQSLL
EANIATEVCLTALDTLSLFTLAFKNQLLADHGHNPLMKKVFDVYLCFLQKHQSETALKNVFTALRSLIYKFPSTFYEGRADMCAALC
YEILKCCNSKLSSIRTEASQLLYFLMRNNFDYTGKKSFVRTHLQVIISVSQLIADVVGIGGTRFQQSLSIINNCANSDRLIKHTSFS
SDVKDLTKRIRTVLMATAQMKEHENDPEMLVDLQYSLAKSYASTPELRKTWLDSMARIHVKNGDLSEAAMCYVHVTALVAEYLTRKE
AVQWEPPLLPHSHSACLRRSRGGVFRQGCTAFRVITPNIDEEASMMEDVGMQDVHFNEDVLMELLEQCADGLWKAERYELIADIYKL
IIPIYEKRRDFERLAHLYDTLHRAYSKVTEVMHSGRRLLGTYFRVAFFGQAAQYQFTDSETDVEGFFEDEDGKEYIYKEPKLTPLSE
ISQRLLKLYSDKFGSENVKMIQDSGKVNPKDLDSKYAYIQVTHVIPFFDEKELQERKTEFERSHNIRRFMFEMPFTQTGKRQGGVEE
QCKRRTILTAIHCFPYVKKRIPVMYQHHTDLNPIEVAIDEMSKKVAELRQLCSSAEVDMIKLQLKLQGSVSVQVNAGPLAYARAFLD
DTNTKRYPDNKVKLLKEVFRQFVEACGQALAVNERLIKEDQLEYQEEMKANYREMAKELSEIMHEQLG
>sp|Q96LA9|MRGX4_HUMAN

MDPTVPVFGTKLTPINGREETPCYNQTLSFTVLTCIISLVGLTGNAVVLWLLGYRMRRNAVSIYILNLAAADFLFLSFQIIRLPLRL
INISHLIRKILVSVMTFPYFTGLSMLSAISTERCLSVLWPIWYRCRRPTHLSAVVCVLLWGLSLLFSMLEWRFCDFLFSGADSSWCE
TSDFIPVAWLIFLCVVLCVSSLVLLVRILCGSRKMPLTRLYVTILLTVLVFLLCGLPFGILGALIYRMHLNLEVLYCHVYLVCMSLS
SLNSSANPIIYFFVGSFRQRQNRQNLKLVLQRALQDKPEVDKGEGQLPEESLELSGSRLGP
>sp|Q99689|FEZ1_HUMAN
MEAPLVSLDEEFEDLRPSCSEDPEEKPQCFYGSSPHHLEDPSLSELENFSSEIISFKSMEDLVNEFDEKLNVCFRNYNAKTENLAPV
KNQLQIQEEEETLQDEEVWDALTDNYIPSLSEDWRDPNIEALNGNCSDTEIHEKEEEEFNEKSENDSGINEEPLLTADQVIEEIEEM
MQNSPDPEEEEEEVLEEEDGGETSSQADSVLLQEMQALTQTFNNNWSYEGLRHMSGSELTELLDQVEGAIRDFSEELVQQLARRDELE
FEKEVKNSFITVLIEVQNKQKEQRELMKKRRKEKGLSLQSSRIEKGNQMPLKRFSMEGISNILQSGIRQTFGSSGTDKQYLNTVIPY
EKKASPPSVEDLQMLTNILFAMKEDNEKVPTLLTDYILKVLCPT
>sp|P54098|DPOG1_HUMAN
MSRLLWRKVAGATVGPGPVPAPGRWVSSSVPASDPSDGQRRRQQQQQQQQQQQQPQQPQVLSSEGGQLRHNPLDIQMLSRGLHEQI
FGQGGEMPGEAAVRRSVEHLQKHGLWGQPAVPLPDVELRLPPLYGDNLDQHFRLLAQKQSLPYLEAANLLLQAQLPPKPPAWAWAEG
WTRYGPEGEAVPVAIPEERALVFDVEVCLAEGTCPTLAVAISPSAWYSWCSQRLVEERYSWTSQLSPADLIPLEVPTGASSPTQRDW
QEQLVVGHNVSFDRAHIREQYLIQGSRMRFLDTMSMHMAISGLSSFQRSLWIAAKQGKHKVQPPTKQGQKSQRKARRGPAISSWDWL
DISSVNSLAEVHRLYVGGPPLEKEPRELFVKGTMKDIRENFQDLMQYCAQDVWATHEVFQQQLPLFLERCPHPVTLAGMLEMGVSYL
PVNQNWERYLAEAQGTYEELQREMKKSLMDLANDACQLLSGERYKEDPWLWDLEWDLQEFKQKKAKKVKKEPATASKLPIEGAGAPG
DPMDQEDLGPCSEEEEFQQDVMARACLQKLKGTTELLPKRPQHLPGHPGWYRKLCPRLDDPAWTPGPSLLSLQMRVTPKLMALTWDG
FPLHYSERHGWGYLVPGRRDNLAKLPTGTTLESAGVVCPYRAIESLYRKHCLEQGKQQLMPQEAGLAEEFLLTDNSAIWQTVEELDY
LEVEAEAKMENLRAAVPGQPLALTARGGPKDTQPSYHHGNGPYNDVDIPGCWFFKLPHKDGNSCNVGSPFAKDFLPKMEDGTLQAGP
GGASGPRALEINKMISFWRNAHKRISSQMVVWLPRSALPRAVIRHPDYDEEGLYGAILPQVVTAGTITRRAVEPTWLTASNARPDRV
GSELKAMVQAPPGYTLVGADVDSQELWIAAVLGDAHFAGMHGCTAFGWMTLQGRKSRGTDLHSKTATTVGISREHAKIFNYGRIYGA
GQPFAERLLMQFNHRLTQQEAAEKAQQMYAATKGLRWYRLSDEGEWLVRELNLPVDRTEGGWISLQDLRKVQRETARKSQWKKWEVV
AERAWKGGTESEMFNKLESIATSDIPRTPVLGCCISRALEPSAVQEEFMTSRVNWVVQSSAVDYLHLMLVAMKWLFEEFAIDGRFCI
SIHDEVRYLVREEDRYRAALALQITNLLTRCMFAYKLGLNDLPQSVAFFSAVDIDRCLRKEVTMDCKTPSNPTGMERRYGIPQGEAL
DIYQIIELTKGSLEKRSQPGP
>sp|P15018|LIF_HUMAN
MKVLAAGVVPLLLVLHWKHGAGSPLPITPVNATCAIRHPCHNNLMNQIRSQLAQLNGSANALFILYYTAQGEPFPNNLDKLCGPNVT
DFPPFHANGTEKAKLVELYRIVVYLGTSLGNITRDQKILNPSALSLHSKLNATADILRGLLSNVLCRLCSKYHVGHVDVTYGPDTSG
KDVFQKKKLGCQLLGKYKQIIAVLAQAF
>sp|P48048|KCNJ1_HUMAN
MNASSRNVFDTLIRVLTESMFKHLRKWVVTRFFGHSRQRARLVSKDGRCNIEFGNVEAQSRFIFFVDIWTTVLDLKWRYKMTIFITA
FLGSWFFFGLLWYAVAYIHKDLPEFHPSANHTPCVENINGLTSAFLFSLETQVTIGYGFRCVTEQCATAIFLLIFQSILGVIINSFM
CGAILAKISRPKKRAKTITFSKNAVISKRGGKLCLLIRVANLRKSLLIGSHIYGKLLKTTVTPEGETIILDQININFVVDAGNENLF
FISPLTIYHVIDHNSPFFHMAAETLLQQDFELVVFLDGTVESTSATCQVRTSYVPEEVLWGYRFAPIVSKTKEGKYRVDFHNFSKTV
EVETPHCAMCLYNEKDVRARMKRGYDNPNFILSEVNETDDTKM
>sp|Q96NE9|FRMD6_HUMAN
MNKLNFHNNRVMQDRRSVCIFLPNDESLNIIINVKILCHQLLVQVCDLLRLKDCHLFGLSVIQNNEHVYMELSQKLYKYCPKEWKKE
ASKVRQYEVTWGIDQFGPPMIIHFRVQYYVENGRLISDRAARYYYYWHLRKQVLHSQCVLREEAYFLLAAFALQADLGNFKRNKHYG
KYFEPEAYFPSWVVSKRGKDYILKHIPNMHKDQFALTASEAHLKYIKEAVRLDDVAVHYYRLYKDKREIEASLTLGLTMRGIQIFQN
LDEEKQLLYDFPWTNVGKLVFVGKKFEILPDGLPSARKLIYYTGCPMRSRHLLQLLSNSHRLYMNLQPVLRHIRKLEENEEKKQYRE
SYISDNLDLDMDQLEKRSRASGSSAGSMKHKRLSRHSTASHSSSHTSGIEADTKPRDTGPEDSYSSSAIHRKLKTCSSMTSHGSSHT
SGVESGGKDRLEEDLQDDEIEMLVDDPRDLEQMNEESLEVSPDMCIYITEDMLMSRKLNGHSGLIVKEIGSSTSSSSETVVKLRGQS

TDSLPQTICRKPKTSTDRHSLSLDDIRLYQKDFLRIAGLCQDTAQSYTFGCGHELDEEGLYCNSCLAQQCINIQDAFPVKRTSKYFS
LDLTHDEVPEFVV
>sp|Q6P5R6|RL22L_HUMAN
MAPQKDRKPKRSTWRFNLDLTHPVEDGIFDSGNFEQFLREKVKVNGKTGNLGNVVHIERFKNKITVVSEKQFSKRYLKYLTKKYLKK
NNLRDWLRVVASDKETYELRYFQISQDEDESESED
>sp|Q96FG2|ELMD3_HUMAN
MNEKSCSFHSKEELRDGQGERLSAGYSPSYDKDKSVLAFRGIPISELKNHGILQALTTEAYEWEPRVVSTEVVRAQEEWEAVDTIQP
ETGSQASSEQPGQLISFSEALQHFQTVDLSPFKKRIQPTIRRTGLAALRHYLFGPPKLHQRLREERDLVLTIAQCGLDSQDPVHGRV
LQTIYKKLTGSKFDCALHGNHWEDLGFQGANPATDLRGAGFLALLHLLYLVMDSKTLPMAQEIFRLSRHHIQQFPFCLMSVNITHIA
IQALREECLSRECNRQQKVIPVVNSFYAATFLHLAHVWRTQRKTISDSGFVLKELEVLAKKSPRRLLKTLELYLARVSKGQASLLGA
QKCYGPEAPPFKDLTFTGESDLQSHSSEGVWLI
>sp|A6NFE3|EFC10_HUMAN
METSSRELQAAEYLEKHQIKEVVSYLTSALLFFRPEKPKEYLISLLERLRIAKVTGVAFPFFMDNSNIVAMFEMMDSSGRGTISFVQ
YKEALKTLGLCTEDEDLQDDGHKITLDKFKEEVNKRMKEI
>sp|B7Z1M9|C2D4D_HUMAN
MWLLEKAGYKVGAAEPAARWAPSGLFSKRRAPGPPTSACPNVLTPDRIPQFFIPPRLPDPGGAVPAARRHVAGRGLPATCSLPHLAG
REGWAFLPESPHTRRRESLFHGPPPAPAGGLPAAQSRLHVSAPDLRLCRAPDSDTASSPDSSPFGSPRPGLGRRRVSRPHSLSPEKA
SSADTSPHSPRRAGPPTPPLFHLDFLCCQLRPTRESVLRLGPRGGQLRLSTEYQAGPGRLRLRLVSAEGLPRPRSRPGSGGGGCCVV
LRLRPRVRPREQQSRVVKCSANPIFNEDFFFDGLGPPDLAARSLRAKVLDRGAGLRRDVLLGECETPLIALLPPLGGGLGPGSSLAP
THLSL
>sp|Q9UI09|NDUAC_HUMAN
MELVQVLKRGLQQITGHGGLRGYLRVFFRTNDAKVGTLVGEDKYGNKYYEDNKQFFGRHRWVVYTTEMNGKNTFWDVDGSMVPPEWH
RWLHSMTDDPPTTKPLTARKFIWTNHKFNVTGTPEQYVPYSTTRKKIQEWIPPSTPYK
>sp|Q7Z434|MAVS_HUMAN
MPFAEDKTYKYICRNFSNFCNVDVVEILPYLPCLTARDQDRLRATCTLSGNRDTLWHLFNTLQRRPGWVEYFIAALRGCELVDLADE
VASVYQSYQPRTSDRPPDPLEPPSLPAERPGPPTPAAAHSIPYNSCREKEPSYPMPVQETQAPESPGENSEQALQTLSPRAIPRNPD
GGPLESSSDLAALSPLTSSGHQEQDTELGSTHTAGATSSLTPSRGPVSPSVSFQPLARSTPRASRLPGPTGSVVSTGTSFSSSSPGL
ASAGAAEGKQGAESDQAEPIICSSGAEAPANSLPSKVPTTLMPVNTVALKVPANPASVSTVPSKLPTSSKPPGAVPSNALTNPAPSK
LPINSTRAGMVPSKVPTSMVLTKVSASTVPTDGSSRNEETPAAPTPAGATGGSSAWLDSSSENRGLGSELSKPGVLASQVDSPFSGC
FEDLAISASTSLGMGPCHGPEENEYKSEGTFGIHVAENPSIQLLEGNPGPPADPDGGPRPQADRKFQEREVPCHRPSPGALWLQVAV
TGVLVVTLLVVLYRRRLH
>sp|Q92766|RREB1_HUMAN
MTSSSPAGLEGSDLSSINTMMSAVMSVGKVTENGGSPQGIKSPSKPPGPNRIGRRNQETKEEKSSYNCPLCEKICTTQHQLTMHIRQ
HNTDTGGADHSCSICGKSLSSASSLDRHMLVHSGERPYKCTVCGQSFTTNGNMHRHMKIHEKDPNSATATAPPSPLKRRRLSSKRKL
SHDAESEREDPAPAKKMVEDGQSGDLEKKADEVFHCPVCFKEFVCKYGLETHMETHSDNPLRCDICCVTFRTHRGLLRHNALVHKQL
PRDAMGRPFIQNNPSIPAGFHDLGFTDFSCRKFPRISQAWCETNLRRCISEQHRFVCDTCDKAFPMLCSLALHKQTHVAADQGQEKP
QATPLPGDALDQKGFLALLGLQHTKDVRPAPAEEPLPDDNQAIQLQTLKCQLPQDPGCTNLLSLSPFEAASLGGSLTVLPATKDSIK
HLSLQPFQKGFIIQPDSSIVVKPISGESAIELADIQQILKMAASAPPQISLPPFSKAPAAPLQAIFKHMPPLKPKPLVTPRTVVATS
TPPPLINAQQASPGCISPSLPPPPLKLLKGSVEAASNAHLLQSKSGTQPHAATRLSLQQPRAELPGQPEMKTQLEQDSIIEALLPLS
MEAKIKQEITEGELKAFMTAPGGKKTPAMRKVLYPCRFCNQVFAFSGVLRAHVRSHLGISPYQCNICDYIAADKAALIRHLRTHSGE
RPYICKICHYPFTVKANCERHLRKKHLKATRKDIEKNIEYVSSSAAELVDAFCAPDTVCRLCGEDLKHYRALRIHMRTHCGRGLGGG
HKGRKPFECKECSAAFAAKRNCIHHILKQHLHVPEQDIESYVLAADGLGPAEAPAAEASGRGEDSGCAALGDCKPLTAFLEPQNGFL
HRGPTQPPPPHVSIKLEPASSFAVDFNEPLDFSQKGLALVQVKQENISFLSPSSLVPYDCSMEPIDLSIPKNFRKGDKDLATPSEAK
KPEEEAGSSEQPSPCPAPGPSLPVTLGPSGILESPMAPAPAATPEPPAQPLQGPVQLAVPIYSSALVSSPPLVGSSALLSGTALLRP

LRPKPPLLLPKPPVTEELPPLASIAQIISSVSSAPTLLKTKVADPGPASTGSNTTASDSLGGSVPKAATTATPAATTSPKESSEPPA
PASSPEAASPTEQGPAGTSKKRGRKRGMRSRPRANSGGVDLDSSGEFASIEKMLATTDTNKFSPFLQTAEDNTQDEVAGAPADHHGP
SDEEQGSPPEDKLLRAKRNSYTNCLQKITCPHCPRVFPWASSLQRHMLTHTDSQSDAETAAAAGEVLDLTSRDREQPSEGATELRQV
AGDAPVEQATAETASPVHREEHGRGESHEPEEEHGTEESTGDADGAEEDASSNQSLDLDFATKLMDFKLAEGDGEAGAGGAASQEQK
LACDTCGKSFKFLGTLSRHRKAHGRQEPKDEKGDGASTAEEGPQPAPEQEEKPPETPAEVVESAPGAGEAPAEKLAEETEGPSDGES
AAEKRSSEKSDDDKKPKTDSPKSVASKADKRKKVCSVCNKRFWSLQDLTRHMRSHTGERPYKCQTCERTFTLKHSLVRHQRIHQKAR
HAKHHGKDSDKEERGEEDSENESTHSGNNAVSENEAELAPNASNHMAVTRSRKEGLASATKDCSHREEKVTAGWPSEPGQGDLNPES
PAALGQDLLEPRSKRPAHPILATADGASQLVGME
>sp|Q6IAA8|LTOR1_HUMAN
MGCCYSSENEDSDQDREERKLLLDPSSPPTKALNGAEPNYHSLPSARTDEQALLSSILAKTASNIIDVSAADSQGMEQHEYMDRARQ
YSTRLAVLSSSLTHWKKLPPLPSLTSQPHQVLASEPIPFSDLQQVSRIAAYAYSALSQIRVDAKEELVVQFGIP
>sp|Q86X59|CQ082_HUMAN
MGRPLEGQPLRALDLYPEPAFLRSGKDPKSSPASSPSFAVLGPEVRSTGGQAGSRRRPSAPCSQDRAAAEGAPALLGGSPSSGSPGH
PPGSAFGVEAGCRALNVSEHARGGFALGLPFGLSGGAYLFLLLDGAGDPKPTPEAPISSADGRAWFPSESSWQLPQLPAGSTSGSEP
RARPGLGPRQLLTGPRDGAAGQGPGRGLTARLGREREIDCGPRQAGHGGTATDTGRAGSGARHRPPRDRGTPGLRTH
>sp|Q6IEV9|OR4CB_HUMAN
MQQNNSVPEFILLGLTQDPLRQKIVFVIFLIFYMGTVVGNMLIIVTIKSSRTLGSPMYFFLFYLSFADSCFSTSTAPRLIVDALSEK
KIITYNECMTQVFALHLFGCMEIFVLILMAVDRYVAICKPLRYPTIMSQQVCIILIVLAWIGSLIHSTAQIILALRLPFCGPYLIDH
YCCDLQPLLKLACMDTYMINLLLVSNSGAICSSSFMILIISYIVILHSLRNHSAKGKKKALSACTSHIIVVILFFGPCIFIYTRPPT
TFPMDKMVAVFYTIGTPFLNPLIYTLRNAEVKNAMRKLWHGKIISENKG
>sp|Q32P41|TRM5_HUMAN
MVLWILWRPFGFSGRFLKLESHSITESKSLIPVAWTSLTQMLLEAPGIFLLGQRKRFSTMPETETHERETELFSPPSDVRGMTKLDR
TAFKKTVNIPVLKVRKEIVSKLMRSLKRAALQRPGIRRVIEDPEDKESRLIMLDPYKIFTHDSFEKAELSVLEQLNVSPQISKYNLE
LTYEHFKSEEILRAVLPEGQDVTSGFSRIGHIAHLNLRDHQLSFKHLIGQVMIDKNPGITSAVNKINNIDNMYRNFQMEVLSGEQNM
MTKVRENNYTYEFDFSKVYWNPRLSTEHSRITELLKPGDVLFDVFAGVGPFAIPVAKKNCTVFANDLNPESHKWLLYNCKLNKVDQK
VKVFNLDGKDFLQGPVKEELMQLLGLSKERKPSVHVVMNLPAKAIEFLSAFKWLLDGQPCSSEFLPIVHCYSFSKDANPAEDVRQRA
GAVLGISLEACSSVHLVRNVAPNKEMLCITFQIPASVLYKNQTRNPENHEDPPLKRQRTAEAFSDEKTQIVSNT
>sp|Q9H7Z3|NRDE2_HUMAN
MALFPAFAGLSEAPDGGSSRKELDWLSNPSFCVGSITSLSQQTEAAPAHVSEGLPLTRSHLKSESSDESDTNKKLKQTSRKKKKEKK
KKRKHQHHKKTKRKHGPSSSSRSETDTDSEKDKPSRGVGGSKKESEEPNQGNNAAADTGHRFVWLEDIQAVTGETFRTDKKPDPANW
EYKSLYRGDIARYKRKGDSCLGINPKKQCISWEGTSTEKKHSRKQVERYFTKKSVGLMNIDGVAISSKTEPPSSEPISFIPVKDLED
AAPVTTWLNPLGIYDQSTTHWLQGQGPPEQESKQPDAQPDSESAALKAKVEEFNRRVRENPRDTQLWMAFVAFQDEVMKSPGLYAIE
EGEQEKRKRSLKLILEKKLAILERAIESNQSSVDLKLAKLKLCTEFWEPSTLVKEWQKLIFLHPNNTALWQKYLLFCQSQFSTFSIS
KIHSLYGKCLSTLSAVKDGSILSHPALPGTEEAMFALFLQQCHFLRQAGHSEKAISLFQAMVDFTFFKPDSVKDLPTKGQVEFFEPF
WDSGEPRAGEKGARGWKAWMHQQERGGWVVINPDEDDDEPEEDDQEIKDKTLPRWQIWLAAERSRDQRHWRPWRPDKTKKQTEEDCE
DPERQVLFDDIGQSLIRLSSHDLQFQLVEAFLQFLGVPSGFTPPASCLYLAMDENSIFDNGLYDEKPLTFFNPLFSGASCVGRMDRL
GYPRWTRGQNREGEEFIRNVFHLVMPLFSGKEKSQLCFSWLQYEIAKVIWCLHTKNKKRLKSQGKNCKKLAKNLLKEPENCNNFCLW
KQYAHLEWLLGNTEDARKVFDTALGMAGSRELKDSDLCELSLLYAELEVELSPEVRRAATARAVHILTKLTESSPYGPYTGQVLAVH
ILKARKAYEHALQDCLGDSCVSNPAPTDSCSRLISLAKCFMLFQYLTIGIDAAVQIYEQVFAKLNSSVFPEGSGEGDSASSQSWTSV
LEAITLMHTSLLRFHMKVSVYPLAPLREALSQALKLYPGNQVLWRSYVQIQNKSHSASKTRRFFDTITRSAKPLEPWLFAIEAEKLR
KRLVETVQRLDGREIHATIPETGLMHRIQALFENAMRSDSGSQCPLLWRMYLNFLVSLGNKERSKGVFYKALQNCPWAKVLYLDAVE
YFPDEMQEILDLMTEKELRVRLPLEELELLLED
>sp|Q8WVV4|POF1B_HUMAN

MSSSYWSETSSSSCGTQQLPEVLQCQPQHYHCYHQSSQAQQPPEKNVVYERVRTYSGPMNKVVQALDPFNSREVLSPLKTTSSYQNL
VWSDHSQELHSPTLKISTCAPSTLHITQNTEQELHSPTVKLTTYPQTTIRKYVVQNPEQEPLSQFLRGSHFFPGNNVIYEKTIRKVE
KLNTDQGCHPQAQCHHHIIQQPQVIHSAHWQQPDSSQQIQAITGNNPISTHIGNELCHSGSSQICEQVIIQDDGPEKLDPRYFGELL
ADLSRKNTDLYHCLLEHLQRIGGSKQDFESTDESEDIESLIPKGLSEFTKQQIRYILQMRGMSDKSLRLVLSTFSNIREELGHLQND
MTSLENDKMRLEKDLSFKDTQLKEYEELLASVRANNHQQQQGLQDSSSKCQALEENNLSLRHTLSDMEYRLKELEYCKRNLEQENQN
LRMQVSETCTGPMLQAKMDEIGNHYTEMVKNLRMEKDREICRLRSQLNQYHKDVSKREGSCSDFQFKLHELTSLLEEKDSLIKRQSE
ELSKLRQEIYSSHNQPSTGGRTTITTKKYRTQYPILGLLYDDYEYIPPGSETQTIVIEKTEDKYTCP
>sp|Q6TDU7|CASC1_HUMAN
MSGSKKKKVTKAERLKLLQEEEERRLKEEEEARLKYEKEEMERLEIQRIEKEKWHRLEAKDLERRNEELEELYLLERCFPEAEKLKQ
ETKLLSQWKHYIQCDGSPDPSVAQEMNTFISLWKEKTNETFEEVIEKSKVVLNLIEKLKFILLETPPCDLQDKNIIQYQESILQLQE
LLHLKFGVATEILLKQASTLADLDSGNMEKVIKDENVTLYVWANLKKNPRHRSVRFSETQIGFEIPRILATSDIAVRLLHTHYDHVS
ALHPVSTPSKEYTSAVTELVKDDVKNVEKAISKEVEEESKQQERGSHLIQEEEIKVEEEQGDIEVKMSSAEEESEAIKCEREMKVLS
ETVSAAQLLLVENSSEKPDFFEDNVVDLCQFTTLGGVYHLDILELPPQCKPVKGWMIVEILKEGLQKYTYPPETTEEFETENAFPPI
EVTLEVHENVIFFEDPVVVRWDAEGKHWRTDGISNVSYKPKERLVTFSLDTFGPVTLIQDAHINMPYQSWELRPLDVNKVLLTVTTV
FTEIQIQIKENLCMLSSIKLKDKKHISILEGTWMTPIPFIIALKEAGLNIFPTRHSHFYVIINNKVPLVEVKAYRQMALLSSAFAFG
WSKWNLLCNSTKVVFKVREHLTEACTENPNWALLMFSGDRAQRLKIKEESEAFSEALKEETEFHSTLYHMVKDFASEEAMEKVRSSN
CQFVNSVCHMLLSTRLLSYS
>sp|Q02833|RASF7_HUMAN
MLLGLAAMELKVWVDGIQRVVCGVSEQTTCQEVVIALAQAIGQTGRFVLVQRLREKERQLLPQECPVGAQATCGQFASDVQFVLRRT
GPSLAGRPSSDSCPPPERCLIRASLPVKPRAALGCEPRKTLTPEPAPSLSRPGPAAPVTPTPGCCTDLRGLELRVQRNAEELGHEAF
WEQELRREQAREREGQARLQALSAATAEHAARLQALDAQARALEAELQLAAEAPGPPSPMASATERLHQDLAVQERQSAEVQGSLAL
VSRALEAAERALQAQAQELEELNRELRQCNLQQFIQQTGAALPPPPRPDRGPPGTQGPLPPAREESLLGAPSESHAGAQPRPRGGPH
DAELLEVAAAPAPEWCPLAAQPQAL
>sp|Q9BVL2|NUP58_HUMAN
MSTGFSFGSGTLGSTTVAAGGTSTGGVFSFGTGASSNPSVGLNFGNLGSTSTPATTSAPSSGFGTGLFGSKPATGFTLGGTNTGIAT
TITTGLTLGTPATTSAATTGFSLGFNKPAASATPFALPITSTSASGLTLSSALTSTPAASTGFTLNNLGGTTATTTASTGLSLGGA
LAGLGGSLFQSTNTGTSGLGQNALGLTLGTTAATSTAGNEGLGGIDFSSSSDKKSDKTGTRPEDSKALKDENLPPVICQDVENLQKF
VKEQKQVQEEISRMSSKAMLKVQEDIKALKQLLSLAANGIQRNTLNIDKLKIETAQELKNAEIALRTQKTPPGLQHEYAAPADYFRI
LVQQFEVQLQQYRQQIEELENHLATQANNSHITPQDLSMAMQKIYQTFVALAAQLQSIHENVKVLKEQYLGYRKMFLGDAVDVFETR
RAEAKKWQNTPRVTTGPTPFSTMPNAAAVAMAATLTQQQQPATGPQPSLGVSFGTPFGSGIGTGLQSSGLGSSNLGGFGTSSGFGCS
TTGASTFGFGTTNKPSGSLSAGFGSSSTSGFNFSNPGITASAGLTFGVSNPASAGFGTGGQLLQLKKPPAGNKRGKR
>sp|Q92826|HXB13_HUMAN
MEPGNYATLDGAKDIEGLLGAGGGRNLVAHSPLTSHPAAPTLMPAVNYAPLDLPGSAEPPKQCHPCPGVPQGTSPAPVPYGYFGGGY
YSCRVSRSSLKPCAQAATLAAYPAETPTAGEEYPSRPTEFAFYPGYPGTYQPMASYLDVSVVQTLGAPGEPRHDSLLPVDSYQSWAL
AGGWNSQMCCQGEQNPPGPFWKAAFADSSGQHPPDACAFRRGRKKRIPYSKGQLRELEREYAANKFITKDKRRKISAATSLSERQIT
IWFQNRRVKEKKVLAKVKNSATP
>sp|Q8N423|LIRB2_HUMAN
MTPIVTVLICLGLSLGPRTHVQTGTIPKPTLWAEPDSVITQGSPVTLSCQGSLEAQEYRLYREKKSASWITRIRPELVKNGQFHIPS
ITWEHTGRYGCQYYSRARWSELSDPLVLVMTGAYPKPTLSAQPSPVVTSGGRVTLQCESQVAFGGFILCKEGEEEHPQCLNSQPHAR
GSSRAIFSVGPVSPNRRWSHRCYGYDLNSPYVWSSPSDLLELLVPGVSKKPSLSVQPGPVVAPGESLTLQCVSDVGYDRFVLYKEGE
RDLRQLPGRQPQAGLSQANFTLGPVSRSYGGQYRCYGAHNLSSECSAPSDPLDILITGQIRGTPFISVQPGPTVASGENVTLLCQSW
RQFHTFLLTKAGAADAPLRLRSIHEYPKYQAEFPMSPVTSAHAGTYRCYGSLNSDPYLLSHPSEPLELVVSGPSMGSSPPPTGPIST
PAGPEDQPLTPTGSDPQSGLGRHLGVVIGILVAVVLLLLLLLLLFLILRHRRQGKHWTSTQRKADFQHPAGAVGPEPTDRGLQWRSS
PAADAQEENLYAAVKDTQPEDGVEMDTRAAASEAPQDVTYAQLHSLTLRRKATEPPPSQEREPPAEPSIYATLAIH

>sp|Q12866|MERTK_HUMAN

MGPAPLPLLLGLFLPALWRRAITEAREEAKPYPLFPGPFPGSLQTDHTPLLSLPHASGYQPALMFSPTQPGRPHTGNVAIPQVTSVE
SKPLPPLAFKHTVGHIILSEHKGVKFNCSISVPNIYQDTTISWWKDGKELLGAHHAITQFYPDDEVTAIIASFSITSVQRSDNGSYI
CKMKINNEEIVSDPIYIEVQGLPHFTKQPESMNVTRNTAFNLTCQAVGPPEPVNIFWVQNSSRVNEQPEKSPSVLTVPGLTEMAVFS
CEAHNDKGLTVSKGVQINIKAIPSPPTEVSIRNSTAHSILISWVPGFDGYSPFRNCSIQVKEADPLSNGSVMIFNTSALPHLYQIKQ
LQALANYSIGVSCMNEIGWSAVSPWILASTTEGAPSVAPLNVTVFLNESSDNVDIRWMKPPTKQQDGELVGYRISHVWQSAGISKEL
LEEVGQNGSRARISVQVHNATCTVRIAAVTRGGVGPFSDPVKIFIPAHGWVDYAPSSTPAPGNADPVLIIFGCFCGFILIGLILYIS
LAIRKRVQETKFGNAFTEEDSELVVNYIAKKSFCRRAIELTLHSLGVSEELQNKLEDVVIDRNLLILGKILGEGEFGSVMEGNLKQE
DGTSLKVAVKTMKLDNSSQREIEEFLSEAACMKDFSHPNVIRLLGVCIEMSSQGIPKPMVILPFMKYGDLHTYLLYSRLETGPKHIP
LQTLLKFMVDIALGMEYLSNRNFLHRDLAARNCMLRDDMTVCVADFGLSKKIYSGDYYRQGRIAKMPVKWIAIESLADRVYTSKSDV
WAFGVTMWEIATRGMTPYPGVQNHEMYDYLLHGHRLKQPEDCLDELYEIMYSCWRTDPLDRPTFSVLRLQLEKLLESLPDVRNQADV
IYVNTQLLESSEGLAQGSTLAPLDLNIDPDSIIASCTPRAAISVVTAEVHDSKPHEGRYILNGGSEEWEDLTSAPSAAVTAEKNSVL
PGERLVRNGVSWSHSSMLPLGSSLPDELLFADDSSEGSEVLM

>sp|Q02153|GCYB1_HUMAN

MYGFVNHALELLVIRNYGPEVWEDIKKEAQLDEEGQFLVRIIYDDSKTYDLVAAASKVLNLNAGEILQMFGKMFFVFCQESGYDTIL
RVLGSNVREFLQNLDALHDHLATIYPGMRAPSFRCTDAEKGKGLILHYYSEREGLQDIVIGIIKTVAQQIHGTEIDMKVIQQRNEEC
DHTQFLIEEKESKEEDFYEDLDRFEENGTQESRISPYTFCKAFPFHIIFDRDLVVTQCGNAIYRVLPQLQPGNCSLLSVFSLVRPHI
DISFHGILSHINTVFVLRSKEGLLDVEKLECEDELTGTEISCLRLKGQMIYLPEADSILFLCSPSVMNLDDLTRRGLYLSDIPLHDA
TRDLVLLGEQFREEYKLTQELEILTDRLQLTLRALEDEKKKTDTLLYSVLPPSVANELRHKRPVPAKRYDNVTILFSGIVGFNAFCS
KHASGEGAMKIVNLLNDLYTRFDTLTDSRKNPFVYKVETVGDKYMTVSGLPEPCIHHARSICHLALDMMEIAGQVQVDGESVQITIG
IHTGEVVTGVIGQRMPRYCLFGNTVNLTSRTETTGEKGKINVSEYTYRCLMSPENSDPQFHLEHRGPVSMKGKKEPMQVWFLSRKNT
GTEETKQDDD

>sp|Q12797|ASPH_HUMAN

MAQRKNAKSSGNSSSSGSGSGSTSAGSSSPGARRETKHGGHKNGRKGGLSGTSFFTWFMVIALLGVWTSVAVVWFDLVDYEEVLGKL
GIYDADGDGDFDVDDAKVLLGLKERSTSEPAVPPEEAEPHTEPEEQVPVEAEPQNIEDEAKEQIQSLLHEMVHAEHVEGEDLQQEDG
PTGEPQQEDDEFLMATDVDDRFETLEPEVSHEETEHSYHVEETVSQDCNQDMEEMMSEQENPDSSEPVVEDERLHHDTDDVTYQVYE
EQAVYEPLENEGIEITEVTAPPEDNPVEDSQVIVEEVSIFPVEEQQEVPPETNRKTDDPEQKAKVKKKKPKLLNKFDKTIKAELDAA
EKLRKRGKIEEAVNAFKELVRKYPQSPRARYGKAQCEDDLAEKRRSNEVLRGAIETYQEVASLPDVPADLLKLSLKRRSDRQQFLGH
MRGSLLTLQRLVQLFPNDTSLKNDLGVGYLLIGDNDNAKKVYEEVLSVTPNDGFAKVHYGFILKAQNKIAESIPYLKEGIESGDPGT
DDGRFYFHLGDAMQRVGNKEAYKWYELGHKRGHFASVWQRSLYNVNGLKAQPWWTPKETGYTELVKSLERNWKLIRDEGLAVMDKAK
GLFLPEDENLREKGDWSQFTLWQQGRRNENACKGAPKTCTLLEKFPETTGCRRGQIKYSIMHPGTHVWPHTGPTNCRLRMHLGLVIP
KEGCKIRCANETKTWEEGKVLIFDDSFEHEVWQDASSFRLIFIVDVWHPELTPQQRRSLPAI

>sp|Q5HYC2|K2026_HUMAN

MSVPGTPGAMEPAGEEERPPPAAEGEDDEEEVAAAAQTSGPAHGRSASSLEDADDQEEEMEAMVIGGGCCKEQELTYELQQGYRILG
EFLQEKHRGLTAPFLQPLGGVATAEEEVAEGPRSGGRGGRAFPQQPGQGMCLLQMEEKFASGQYGGITEFVADFRLMLETCYRLHGV
DHWISKQGQKLEMMLEQKLALLSRHLREKTTIAVTSRGYYGLEDEKGTACTSTRRRSTPRSLAGLTSGVFESIMVQVLRQEEQLRAK
EEKRLREQERKEAEEEASQKEIEEWERKLLAQAAPTCMETMWEIPAIGHFLCLAQQILNLPEIVFYELERCLLMPQCNAFLSKIMTSL
LSPPHRRPTLHRRPTLPYRTWEAALRQKVQQWYTAVGQTENPDNCAEKLGLCPQFFKVLGEVNPLEEKPFHELPFYQKVWLLKGLCD
FVYETQKEVQDAVLGQPIHECREVILGYDYLENAYVHFPQFCGADVRIYKQRPFQAPEFPIPPIKIQRVPRIKLEKLKCDYVSTSNG
EHRCSRDSLPSSFKKEQENNFDPACCPAKMILDNHDISVEMGVKSNYEIRIRRPCEIKKTDCCKENLEKPRSPGEVTGFGEPLSPGE
IRFIENQEKYGEASRIKIEPSPLKENTLKSCQIHVNGSHSDHPEINCHKVVRDILLEQSLQSHKKLKLTKMRAKKKKKKKKLKDVL
NENLQRKREGLHSLAFKSYKPEIQNKLLIIKKKAKHKKHKSGKKSVSKKAITKKRKTVIKSPTVPEFQLICTNLDELRELITKIENE
LKDLENSRKKSGKWYHRRQAVKELHSTLIRLLNELLPWEPKLMKAFQRNRSRLKKDYDDFRRQPDHDTFNRELWTTDEGEGDLGKDS
PKGEISKSIDSTEPLDILEKDHFDSDDMKLSEIDFPMARSKLLKKELPSKDLPKTLLKTLKRQSKQTDYVDDSTKELSPRKKAKLST

NETTVENLESDVQIDCFSESKHTEPSFPESFASLDSVPVSTLQKGTKPIQALLAKNIGNKVTLTNQLPPSTGRNALAVEKPVLSPPE
ASPIKPALTCHTNTKGPLQMVYKMPCGQWLPIDLQNSSVKIQVQPMVDPKTGEKIMQQVLILPKNFVIQHKEGKAVAKEVPPLQQKG
TEQHCSSFPQTTNINSSLASVFVNSPGTVSTQLPNTAFNKTITPLSNISSARPQPLSPVTSVSNLLTPSVKTSQSEAGKAKNAVSAA
TFSLPSASPTISSTGQPLSSTTTLNGSTNPGSSFNCFAQQTADSSEAKQELKTVCIRDSQSILVRTRGGNTGVVKVQTNPDQNSPNT
VSSSSVFTFAPQLQAFLVPKSTTSSSAFSPVAGTTTTSSLSPFSQTPTSVSIPASFAPSMGKNLKLTLGHTTGSGDLGHVIDKTSHM
PSSPLKSSICSSTLLPSTTSSSVSVISISAANFGQNNANIIHTPTKQQQVDYITKSYPVTRSEATAATNGDVISGTPVQKLMLVSAP
SILSSGNGTAINMTPALTSTGVSAQKLVFINAPVPSGTSTPTLVAESLKQTLPPPLHKAYVKTPEQPQIVLIPSTVGTPIKINSSPA
VSQIKDVKIGLNIGQAIVNTSGTVPAIPSINILQNVTPKGEDKSSKGYILPLSTSGNSVPVSSNFVSQNITPVNESVVSSARAVNVL
SVTGANLSLGSFPVTSASASAGAQPPVLVSGNDTSSRIMPILSNRLCSSSLGNTVAISTVKTGHLASSVLISTTQPVVSPKCLTSAL
QIPVTVALPTPATTSPKIINTVPHSAAVPGATRSVSISKRQSRTSLQFHSPGISTTVPTNVNTNKPQTELSSLSTSPGKITNTSNFA
SLPNQQALVKTPSYSSAPGGTTIHTASAPSNVTSLVGSQFSEPCIQQKIVINTSTPLAPGTQIMINGTRFIVPPQGLGAGSHVLLIS
TNPKYGAPLVLNSGQGIQSTPIDNSAQKITLASNNSLSGQPLQHPLRSPTKFINSFGNASSIPTVHTSPQLINTTAKVPVPPPVPTV
SLTSVIKSPATLLAKTSLVSAICPSNPPLPSSTSVFHLDPPVKKLLVSPEGAILNTINTPASKVSSLSPSLSQIVVSASRSPASVFP
AFQSSGLEKPDRAAS
>sp|Q6PML9|ZNT9_HUMAN
MLPGLAAAAAHRCSWSSLCRLRLRCRAAACNPSDRQEWQNLVTFGSFSNMVPCSHPYIGTLSQVKLYSTNVQKEGQGSQTLRVEKVP
SFETAEGIGTELKAPLKQEPLQVRVKAVLKKREYGSKYTQNNFITGVRAINEFCLKSSDLEQLRKIRRRSPHEDTESFTVYLRSDVE
AKSLEVWGSPEALAREKKLRKEAEIEYRERLFRNQKILREYRDFLGNTKPRSRTASVFFKGPGKVVMVAICINGLNCFFKFLAWIYT
GSASMFSEAIHSLSDTCNQGLLALGISKSVQTPDPSHPYGFSNMRYISSLISGVGIFMMGAGLSWYHGVMGLLHPQPIESLLWAYCI
LAGSLVSEGATLLVAVNELRRNARAKGMSFYKYVMESRDPSTNVILLEDTAAVLGVIIAATCMGLTSITGNPLYDSLGSLGVGTLLG
MVSAFLIYTNTEALLGRSIQPEQVQRLTELLENDPSVRAIHDVKATDLGLGKVRFKAEVDFDGRVVTRSYLEKQDFDQMLQEIQEVK
TPEELETFMLKHGENIIDTLGAEVDRLEKELKKRNPEVRHVDLEIL
>sp|Q15835|RK_HUMAN
MDFGSLETVVANSAFIAARGSFDGSSSQPSRDKKYLAKLKLPPLSKCESLRDSLSLEFESVCLEQPIGKKLFQQFLQSAEKHLPALE
LWKDIEDYDTADNDLQPQKAQTILAQYLDPQAKLFCSFLDEGIVAKFKEGPVEIQDGLFQPLLQATLAHLGQAPFQEYLGSLYFLRF
LQWKWLEAQPMGEDWFLDFRVLGKGGFGEVSACQMKATGKLYACKKLNKKRLKKRKGYQGAMVEKKILMKVHSRFIVSLAYAFETKA
DLCLVMTIMNGGDIRYHIYNVNEENPGFPEPRALFYTAQIICGLEHLHQRRIVYRDLKPENVLLDNDGNVRISDLGLAVELLDGQSK
TKGYAGTPGFMAPELLQGEEYDFSVDYFALGVTLYEMIAARGPFRARGEKVENKELKHRIISEPVKYPDKFSQASKDFCEALLEKDP
EKRLGFRDETCDKLRAHPLFKDLNWRQLEAGMLMPPFIPDSKTVYAKDIQDVGAFSTVKGVAFDKTDTEFFQEFATGNCPIPWQEEM
IETGIFGELNVWRSDGQMPDDMKGISGGSSSSSKSGMCLVS
>sp|Q92889|XPF_HUMAN
MESGQPARRIAMAPLLEYERQLVLELLDTDGLVVCARGLGADRLLYHFLQLHCHPACLVLVLNTQPAEEEYFINQLKIEGVEHLPRR
VTNEITSNSRYEVYTQGGVIFATSRILVVDFLTDRIPSDLITGILVYRAHRIIESCQEAFILRLFRQKNKRGFIKAFTDNAVAFDTG
FCHVERVMRNLFVRKLYLWPRFHVAVNSFLEQHKPEVVEIHVSMTPTMLAIQTAILDILNACLKELKCHNPSLEVEDLSLENAIGKP
FDKTIRHYLDPLWHQLGAKTKSLVQDLKILRTLLQYLSQYDCVTFLNLLESLRATEKAFGQNSGWLFLDSSTSMFINARARVYHLPD
AKMSKKEKISEKMEIKEGEETKKELVLESNPKWEALTEVLKEIEAENKESEALGGPGQVLICASDDRTCSQLRDYITLGAEAFLLRL
YRKTFEKDSKAEEVWMKFRKEDSSKRIRKSHKRPKDPQNKERASTKERTLKKKKRKLTLTQMVGKPEELEEEGDVEEGYRREISSSP
ESCPEEIKHEEFDVNLSSDAAFGILKEPLTIIHPLLGCSDPYALTRVLHEVEPRYVVLYDAELTFVRQLEIYRASRPGKPLRVYFLI
YGGSTEEQRYLTALRKEKEAFEKLIREKASMVVPEEREGRDETNLDLVRGTASADVSTDTRKAGGQEQNGTQQSIVVDMREFRSELP
SLIHRRGIDIEPVTLEVGDYILTPEMCVERKSISDLIGSLNNGRLYSQCISMSRYYKRPVLLIEFDPSKPFSLTSRGALFQEISSND
ISSKLTLLTLHFPRLRILWCPSPHATAELFEELKQSKPQPDAATALAITADSETLPESEKYNPGPQDFLLKMPGVNAKNCRSLMHHV
KNIAELAALSQDELTSILGNAANAKQLYDFIHTSFAEVVSKGKGKK
>sp|O15342|VA0E1_HUMAN
MAYHGLTVPLIVMSVFWGFVGFLVPWFIPKGPNRGVIITMLVTCSVCCYLFWLIAILAQLNPLFGPQLKNETIWYLKYHWP

>sp|O43251|RFOX2_HUMAN
MQNEPLTPGYHGFPARDSQGNQEPTTTPDAMVQPFTTIPFPPPPQNGIPTEYGVPHTQDYAGQTGEHNLTLYGSTQAHGEQSSNSPS
TQNGSLTTEGGAQTDGQQSQTQSSENSESKSTPKRLHVSNIPFRFRDPDLRQMFGQFGKILDVEIIFNERGSKGFGFVTFENSADAD
RAREKLHGTVVEGRKIEVNNATARVMTNKKMVTPYANGWKLSPVVGAVYGPELYAASSFQADVSLGNDAAVPLSGRGGINTYIPLIS
LPLVPGFPYPTAATTAAAFRGAHLRGRGRTVYGAVRAVPPTAIPAYPGVVYQDGFYGADLYGGYAAYRYAQPATATAATAAAAAAAA
YSDGYGRVYTADPYHALAPAASYGVGAVASLYRGGYSRFAPY
>sp|Q6EMB2|TTLL5_HUMAN
MPIVMARDLEETASSSEDEEVISQEDHPCIMWTGGCRRIPVLVFHADAILTKDNNIRVIGERYHLSYKIVRTDSRLVRSILTAHGFH
EVHPSSTDYNLMWTGSHLKPFLLRTLSEAQKVNHFPRSYELTRKDRLYKNIIRMQHTHGFKAFHILPQTFLLPAEYAEFCNSYSKDR
GPWIVKPVASSRGRGVYLINNPNQISLEENILVSRYINNPLLIDDFKFDVRLYVLVTSYDPLVIYLYEEGLARFATVRYDQGAKNIR
NQFMHLTNYSVNKKSGDYVSCDDPEVEDYGNKWSMSAMLRYLKQEGRDTTALMAHVEDLIIKTIISAELAIATACKTFVPHRSSCFE
LYGFDVLIDSTLKPWLLEVNLSPSLACDAPLDLKIKASMISDMFTVVGFVCQDPAQRASTRPIYPTFESSRRNPFQKPQRCRPLSAS
DAEMKNLVGSAREKGPGKLGGSVLGLSMEEIKVLRRVKEENDRRGGFIRIFPTSETWEIYGSYLEHKTSMNYMLATRLFQDRMTADG
APELKIESLNSKAKLHAALYERKLLSLEVRKRRRRSSRLRAMRPKYPVITQPAEMNVKTETESEEEEEVALDNEDEEQEASQEESAG
FLRENQAKYTPSLTALVENTPKENSMKVREWNNKGGHCCKLETQELEPKFNLMQILQDNGNLSKMQARIAFSAYLQHVQIRLMKDSG
GQTFSASWAAKEDEQMELVVRFLKRASNNLQHSLRMVLPSRRLALLERRRILAHQLGDFIIVYNKETEQMAEKKSKKKVEEEEEDGV
NMENFQEFIRQASEAELEEVLTFYTQKNKSASVFLGTHSKISKNNNNYSDSGAKGDHPETIMEEVKIKPPKQQQTTEIHSDKLSRFT
TSAEKEAKLVYSNSSSGPTATLQKIPNTHLSSVTTSDLSPGPCHHSSLSQIPSAIPSMPHQPTILLNTVSASASPCLHPGAQNIPSP
TGLPRCRSGSHTIGPFSSFQSAAHIYSQKLSRPSSAKAGSCYLNKHHSGIAKTQKEGEDASLYSKRYNQSMVTAELQRLAEKQAARQ
YSPSSHINLLTQQVTNLNLATGIINRSSASAPPTLRPIISPSGPTWSTQSDPQAPENHSSSPGSRSLQTGGFAWEGEVENNVYSQAT
GVVPQHKYHPTAGSYQLQFALQQLEQQKLQSRQLLDQSRARHQAIFGSQTLPNSNLWTMNNGAGCRISSATASGQKPTTLPQKVVPP
PSSCASLVPKKPPPNHEQVLRRATSQKASKGSSAEGQLNGLQSSLNPAAFVPITSSTDPAHTKI
>sp|P30487|1B49_HUMAN
MRVTAPRTVLLLLSAALALTETWAGSHSMRYFHTAMSRPGRGEPRFITVGYVDDTLFVRFDSDATSPRKEPRAPWIEQEGPEYWDRE
TQISKTNTQTYRENLRIALRYYNQSEAGSHTWQRMYGCDLGPDGRLLRGYNQLAYDGKDYIALNEDLSSWTAADTAAQITQRKWEAA
REAEQLRAYLEGLCVEWLRRYLENGKETLQRADPPKTHVTHHPISDHEATLRCWALGFYPAEITLTWQRDGEDQTQDTELVETRPAG
DRTFQKWAAVVVPSGEEQRYTCHVQHEGLPKPLTLRWEPSSQSTIPIVGIVAGLAVLAVVVIGAVVATVMCRRKSSGGKGGSYSQAA
SSDSAQGSDVSLTA
>sp|Q96NU0|CNT3B_HUMAN
MASVAWAVLKVLLLLPTQTWSPVGAGNPPDCDSPLASALPRSSFSSSSELSSSHGPGFSRLNRRDGAGGWTPLVSNKYQWLQIDLGE
RMEVTAVATQGGYGSSDWVTSYLLMFSDGGRNWKQYRREESIWGFPGNTNADSVVHYRLQPPFEARFLRFLPLAWNPRGRIGMRIEV
YGCAYKSEVVYFDGQSALLYTLDKKPLKPIRDVISLKFKAMQSNGILLHREGQHGNHITLELIKGKLVFFLNSGNAKLPSTIAPVTL
TLGSLLDDQHWHSVLIELLDTQVNFTVDKHTHHFQAKGDSSNLDLNFEISFGGILSPGRSRAFTRKSFHGCLENLYYNGVDVTELAK
KHKPQILMMGNVSFSCPQPQTVPVTFLSSRSYLALPGNSGEDKVSVTFQFRTWNRAGHLLFGELQRGSGSFVLFLKDGKLKLSLFQA
GQSPRNVTAGAGLNDGQWHSVSFSAKWSHMNVVVDDDTAVQPLVAVLIDSGDTYYFGGCLGNSSGSGCKSPLGGFQGCLRLITIGDK
AVDPILVQQGALGSFRDLQIDSCGITDRCLPSYCEHGGECSQSWDTFSCDCLGTGYTGETCHSSLYEQSCEAHKHRGNPSGLYYIDA
DGSGPLGPFLVYCNMTADSAWTVVRHGGPDAVTLRGAPSGHPLSAVSFAYAAGAGQLRAAVNLAERCEQRLALRCGTARRPDSRDGT
PLSWWVGRTNETHTSWGGSLPDAQKCTCGLEGNCIDSQYYCNCDAGQNEWTSDTIVLSQKEHLPVTQIVMTDTGQPHSEADYTLGPL
LCRGDKSFWNSASFNTETSYLHFPAFHGELTADVCFFFKTTVSSGVFMENLGITDFIRIELRAPTEVTFSFDVGNGPCEVTVQSPTP
FNDNQWHHVRAERNVKGASLQVDQLPQKMQPAPADGHVRLQLNSQLFIGGTATRQRGFLGCIRSLQLNGVALDLEERATVTPGVEPG
CAGHCSTYGHLCRNGGRCREKRRGVTCDCAFSAYDGPFCSNEISAYFATGSSMTYHFQEHYTLSENSSSLVSSLYRDVTLTREMITL
SFRTTRSPSLLLYVSSFYEEYLSVILANNGSLQIRYKLDRHQNPDAFTFDFKNMADGQLHQVKINREEAVVMVEVNQSAKKQVILSS
GTEFNAVKSLILGKVLEAAGADPDTRRAATSGFTGCLSAVRFGRAAPLKAALRPSGPSRVTVRGHVAPMARCAAGAASGSPARELAP
RLAGGAGRSGPVDEGEPLVNADRRDSAVMGGVIAVVIFILLCITAIAIRIYQQRKLRKENESKVSKKEEC

>sp|Q8IWY9|CDAN1_HUMAN
MAAVLESLLREEVSVAAVVRWIARSTQGSEDNAGEAAALSSLRALRKEFVPFLLNFLREQSSRVLPQGPPTPAKTPGASAALPGRPG
GPPRGSRGARSQLFPPTEAQSTAAEAPLARRGGRRRGPGPARERGGRGLEEGVSGESLPGAGGRRLRGSGSPSRPSLTLSDPPNLSN
LEEFPPVGSVPPGPTGTKPSRRINPTPVSEERSLSKPKTCFTSPPISCVPSSQPSALDTSPWGLGLPPGCRSLQEEREMLRKERSKQ
LQQSPTPTCPTPELGSPLPSRTGSLTDEPADPARVSSRQRLELVALVYSSCIAENLVPNLFLELFFVFQLLTARRMVTAKDSDPELS
PAVLDSLESPLFQSIHDCVFFAVQVLECHFQVLSNLDKGTLKLLAENERLLCFSPALQGRLRAAYEGSVAKVSLVMPPSTQAVSFQP
ETDNRANFSSDRAFHTFKKQRDVFYEVLREWEDHHEEPGWDFEKGLGSRIRAMMGQLSAACSHSHFVRLFQKQLLQMCQSPGGAGGT
VLGEAPDVLSMLGADKLGRLWRLQERLMAPQSSGGPCPPPTFPGCQGFFRDFILSASSFQFNQHLMDSLSLKIQELNGLALPQHEPN
DEDGESDVDWQGERKQFAVVLLSLRLLAKFLGFVAFLPYRGPEPPPTGELQDSILALRSQVPPVLDVRTLLQRGLQARRAVLTVPWL
VEFLSFADHVVPLLEYYRDIFTLLLRLHRSLVLSQESEGKMCFLNKLLLLAVLGWLFQIPTVPEDLFFLEEGPSYAFEVDTVAPEHG
LDNAPVVDQQLLYTCCPYIGELRKLLASWVSGSSGRSGGFMRKITPTTTTSLGAQPSQTSQGLQAQLAQAFFHNQPPSLRRTVEFVA
ERIGSNCVKHIKATLVADLVRQAESLLQEQLVTQGEEGGDPAQLLEILCSQLCPHGAQALALGREFCQRKSPGAVRALLPEETPAAV
LSSAENIAVGLATEKACAWLSANITALIRREVKAAVSRTLRAQGPEPAARGERRGCSRACEHHAPLPSHLISEIKDVLSLAVGPRDP
DEGVSPEHLEQLLGQLGQTLRCRQFLCPPAEQHLAKCSVELASLLVADQIPILGPPAQYRLERGQARRLLHMLLSLWKEDFQGPVPL
QLLLSPRNVGLLADTRPREWDLLLFLLRELVEKGLMGRMEIEACLGSLHQAQWPGDFAEELATLSNLFLAEPHLPEPQLRACELVQP
NRGTVLAQS
>sp|P16860|ANFB_HUMAN
MDPQTAPSRALLLLLFLHLAFLGGRSHPLGSPGSASDLETSGLQEQRNHLQGKLSELQVEQTSLEPLQESPRPTGVWKSREVATEGI
RGHRKMVLYTLRAPRSPKMVQGSGCFGRKMDRISSSSGLGCKVLRRH
>sp|Q9H8S9|MOB1A_HUMAN
MSFLFSSRSSKTFKPKKNIPEGSHQYELLKHAEATLGSGNLRQAVMLPEGEDLNEWIAVNTVDFFNQINMLYGTITEFCTEASCPVM
SAGPRYEYHWADGTNIKKPIKCSAPKYIDYLMTWVQDQLDDETLFPSKIGVPFPKNFMSVAKTILKRLFRVYAHIYHQHFDSVMQLQ
EEAHLNTSFKHFIFFVQEFNLIDRRELAPLQELIEKLGSKDR
>sp|P60509|ERB1_HUMAN
MDPLHTIEKVPARRNIHDRGHQGHRMGDGTPGRPKISVQQMTRFSLIIFFLSAPFVVNASTSNVFLQWAHSYADGLQQGDPCWVCGS
LPVTNTMELPWWVSPLQGKDWVFFQSFIGDLKQWTGAQMTGVTRKNISEWPINKTLNEPGHDKPFSVNETRDKVIAFAIPLLDTKVF
VQTSRPQNTQYRNGFLQIWDGFIWLTATKGHLSQIAPLCWEQRNHSLDNWPNTTRVMGWIPPGQCRHTILLQQRDLFATDWSQQPGL
NWYAPNGTQWLCSPNLWPWLPSGWLGCCTLGIPWAQGRWVKTMEVYPYLPHVVNQGTRAIVHRNDHLPTIFMPSVGLGTVIQHIEAL
ANFTQRALNDSLQSISLMNAEVYYMHEDILQNRMALDILTAAEGGTCALIKTECCVYIPNNSRNISLALEDTCRQIQVISSSALSLH
DWIASQFSGRPSWWQKILIVLATLWSVGIALCCGLYFCRMFSQHIPQTHSIIFQQELPLSPPSQEHYQSQRDIFHSNAP
>sp|O75347|TBCA_HUMAN
MADPRVRQIKIKTGVVKRLVKEKVMYEKEAKQQEEKIEKMRAEDGENYDIKKQAEILQESRMMIPDCQRRLEAAYLDLQRILENEKD
LEEAEEYKEARLVLDSVKLEA
>sp|Q9BYE2|TMPSD_HUMAN
MERDSHGNASPARTPSAGASPAQASPAGTPPGRASPAQASPAQASPAGTPPGRASPAQASPAGTPPGRASPGRASPAQASPAQASPA
RASPALASLSRSSSGRSSSARSASVTTSPTRVYLVRATPVGAVPIRSSPARSAPATRATRESPGTSLPKFTWREGQKQLPLIGCVLL
LIALVVSLIILFQFWQGHTGIRYKEQRESCPKHAVRCDGVVDCKLKSDELGCVRFDWDKSLLKIYSGSSHQWLPICSSNWNDSYSEK
TCQQLGFESAHRTTEVAHRDFANSFSILRYNSTIQESLHRSECPSQRYISLQCSHCGLRAMTGRIVGGALASDSKWPWQVSLHFGTT
HICGGTLIDAQWVLTAAHCFFVTREKVLEGWKVYAGTSNLHQLPEAASIAEIIINSNYTDEEDDYDIALMRLSKPLTLSAHIHPACL
PMHGQTFSLNETCWITGFGKTRETDDKTSPFLREVQVNLIDFKKCNDYLVYDSYLTPRMMCAGDLRGGRDSCQGDSGGPLVCEQNNR
WYLAGVTSWGTGCGQRNKPGVYTKVTEVLPWIYSKMEVRSLQQDTAPSRLGTSSGGDPGGAPRV
>sp|P22760|AAAD_HUMAN
MGRKSLYLLIVGILIAYYIYTPLPDNVEEPWRMMWINAHLKTIQNLATFVELLGLHHFMDSFKVVGSFDEVPPTSDENVTVTETKFN
NILVRVYVPKRKSEALRRGLFYIHGGGWCVGSAALSGYDLLSRWTADRLDAVVVSTNYRLAPKYHFPIQFEDVYNALRWFLRKKVLA

KYGVNPERIGISGDSAGGNLAAAVTQQLLDDPDVKIKLKIQSLIYPALQPLDVDLPSYQENSNFLFLSKSLMVRFWSEYFTTDRSLE

KAMLSRQHVPVESSHLFKFVNWSSLLPERFIKGHVYNNPNYGSSELAKKYPGFLDVRAAPLLADDNKLRGLPLTYVITCQYDLLRDD

GLMYVTRLRNTGVQVTHNHVEDGFHGAFSFLGLKISHRLINQYIEWLKENL

>sp|P00750|TPA_HUMAN

MDAMKRGLCCVLLLCGAVFVSPSQEIHARFRRGARSYQVICRDEKTQMIYQQHQSWLRPVLRSNRVEYCWCNSGRAQCHSVPVKSCS

EPRCFNGGTCQQALYFSDFVCQCPEGFAGKCCEIDTRATCYEDQGISYRGTWSTAESGAECTNWNSSALAQKPYSGRRPDAIRLGLG

NHNYCRNPDRDSKPWCYVFKAGKYSSEFCSTPACSEGNSDCYFGNGSAYRGTHSLTESGASCLPWNSMILIGKVYTAQNPSAQALGL

GKHNYCRNPDGDAKPWCHVLKNRRLTWEYCDVPSCSTCGLRQYSQPQFRIKGGLFADIASHPWQAAIFAKHRRSPGERFLCGGILIS

SCWILSAAHCFQERFPPHHLTVILGRTYRVVPGEEEQKFEVEKYIVHKEFDDDTYDNDIALLQLKSDSSRCAQESSVVRTVCLPPAD

LQLPDWTECELSGYGKHEALSPFYSERLKEAHVRLYPSSRCTSQHLLNRTVTDNMLCAGDTRSGGPQANLHDACQGDSGGPLVCLND

GRMTLVGIISWGLGCGQKDVPGVYTKVTNYLDWIRDNMRP

>sp|Q9H3H1|MOD5_HUMAN

MASVAAARAVPVGSGLRGLQRTLPLVVILGATGTGKSTLALQLGQRLGGEIVSADSMQVYEGLDIITNKVSAQEQRICRHHMISFVD

PLVTNYTVVDFRNRATALIEDIFARDKIPIVVGGTNYYIESLLWKVLVNTKPQEMGTEKVIDRKVELEKEDGLVLHKRLSQVDPEMA

AKLHPHDKRKVARSLQVFEETGISHSEFLHRQHTEEGGGPLGGPLKFSNPCILWLHADQAVLDERLDKRVDDMLAAGLLEELRDFHR

RYNQKNVSENSQDYQHGIFQSIGFKEFHEYLITEGKCTLETSNQLLKKGIEALKQVTKRYARKQNRWVKNRFLSRPGPIVPPVYGLE

VSDVSKWEESVLEPALEIVQSFIQGHKPTATPIKMPYNEAENKRSYHLCDLCDRIIIGDREWAAHIKSKSHLNQLKKRRRLDSDAVN

TIESQSVSPDHNKEPKEKGSPGQNDQELKCSV

>sp|Q8NEV1|CSK23_HUMAN

MSGPVPSRARVYTDVNTHRPREYWDYESHVVEWGNQDDYQLVRKLGRGKYSEVFEAINITNNEKVVVKILKPVKKKKIKREIKILEN

LRGGPNIITLADIVKDPVSRTPALVFEHVNNTDFKQLYQTLTDYDIRFYMYEILKALDYCHSMGIMHRDVKPHNVMIDHEHRKLRLI

DWGLAEFYHPGQEYNVRVASRYFKGPELLVDYQMYDYSLDMWRLGCMLASMIFRKEPFFHGRDNYDQLVRIAKFLGTEDLYGYIDKY

NIELDPRFNDILGRHSRKRWERFVHSENQHLVSPEALDFLDKLLRYDHQSRLTAREAMEHPYFYTVVKDQARMGSSSMPGGSTPVSS

ANVMSGISSVPTPSPLGPLAGSPVIAAANPLGMPVPAATGAQQ

>sp|Q9H2S9|IKZF4_HUMAN

MHTPPALPRRFQGGGRVRTPGSHRQGKDNLERDPSGGCVPDFLPQAQDSNHFIMESLFCESSGDSSLEKEFLGAPVGPSVSTPNSQH

SSPSRSLSANSIKVEMYSDEESSRLLGPDERLLEKDDSVIVEDSLSEPLGYCDGSGPEPHSPGGIRLPNGKLKCDVCGMVCIGPNVL

MVHKRSHTGERPFHCNQCGASFTQKGNLLRHIKLHSGEKPFKCPFCNYACRRRDALTGHLRTHSVSSPTVGKPYKCNYCGRSYKQQS

TLEEHKERCHNYLQSLSTEAQALAGQPGDEIRDLEMVPDSMLHSSSERPTFIDRLANSLTKRKRSTPQKFVGEKQMRFSLSDLPYDV

NSGGYEKDVELVAHHSLEPGFGSSLAFVGAEHLRPLRLPPTNCISELTPVISSVYTQMQPLPGRLELPGSREAGEGPEDLADGGPLL

YRPRGPLTDPGASPSNGCQDSTDTESNHEDRVAGVVSLPQGPPPQPPPTIVVGRHSPAYAKEDPKPQEGLLRGTPGPSKEVLRVVGE

SGEPVKAFKCEHCRILFLDHVMFTIHMGCHGFRDPFECNICGYHSQDRYEFSSHIVRGEHKVG

>sp|Q6NZI2|PTRF_HUMAN

MEDPTLYIVERPLPGYPDAEAPEPSSAGAQAAEEPSGAGSEELIKSDQVNGVLVLSLLDKIIGAVDQIQLTQAQLEERQAEMEGAVQ

SIQGELSKLGKAHATTSNTVSKLLEKVRKVSVNVKTVRGSLERQAGQIKKLEVNEAELLRRRNFKVMIYQDEVKLPAKLSISKSLKE

SEALPEKEGEELGEGERPEEDAAALELSSDEAVEVEEVIEESRAERIKRSGLRRVDDFKKAFSKEKMEKTKVRTRENLEKTRLKTKE

NLEKTRHTLEKRMNKLGTRLVPAERREKLKTSRDKLRKSFTPDHVVYARSKTAVYKVPPFTFHVKKIREGQVEVLKATEMVEVGADD

DEGGAERGEAGDLRRGSSPDVHALLEITEESDAVLVDKSDSD

>sp|Q9P241|AT10D_HUMAN

MTEALQWARYHWRRLIRGATRDDDSGPYNYSSLLACGRKSSQTPKLSGRHRIVVPHIQPFKDEYEKFSGAYVNNRIRTTKYTLLNFV

PRNLFEQFHRAANLYFLFLVVLNWVPLVEAFQKEITMLPLVVVLTIIAIKDGLEDYRKYKIDKQINNLITKVYSRKEKKYIDRCWKD

VTVGDFIRLSCNEVIPADMVLLFSTDPDGICHIETSGLDGESNLKQRQVVRGYAEQDSEVDPEKFSSRIECESPNNDLSRFRGFLEH

SNKERVGLSKENLLLRGCTIRNTEAVVGIVVYAGHETKAMLNNSGPRYKRSKLERRANTDVLWCVMLLVIMCLTGAVGHGIWLSRYE

KMHFFNVPEPDGHIISPLLAGFYMFWTMIILLQVLIPISLYVSIEIVKLGQIYFIQSDVDFYNEKMDSIVQCRALNIAEDLGQIQYL

FSDKTGTLTENKMVFRRCSVAGFDYCHEENARRLESYQEAVSEDEDFIDTVSGSLSNMAKPRAPSCRTVHNGPLGNKPSNHLAGSSF
TLGSGEGASEVPHSRQAAFSSPIETDVVPDTRLLDKFSQITPRLFMPLDETIQNPPMETLYIIDFFIALAICNTVVVSAPNQPRQKI
RHPSLGGLPIKSLEEIKSLFQRWSVRRSSSPSLNSGKEPSSGVPNAFVSRLPLFSRMKPASPVEEEVSQVCESPQCSSSSACCTETE
KQHGDAGLLNGKAESLPGQPLACNLCYEAESPDEAALVYAARAYQCTLRSRTPEQVMVDFAALGPLTFQLLHILPFDSVRKRMSVVV
RHPLSNQVVVYTKGADSVIMELLSVASPDGASLEKQQMIVREKTQKHLDDYAKQGLRTLCIAKKVMSDTEYAEWLRNHFLAETSIDN
REELLLESAMRLENKLTLLGATGIEDRLQEGVPESIEALHKAGIKIWMLTGDKQETAVNIAYACKLLEPDDKLFILNTQSKDACGML
MSTILKELQKKTQALPEQVSLSEDLLQPPVPRDSGLRAGLIITGKTLEFALQESLQKQFLELTSWCQAVVCCRATPLQKSEVVKLVR
SHLQVMTLAIGDGANDVSMIQVADIGIGVSGQEGMQAVMASDFAVSQFKHLSKLLLVHGHWCYTRLSNMILYFFYKNVAYVNLLFWY
QFFCGFSGTSMTDYWVLIFFNLLFTSAPPVIYGVLEKDVSAETLMQLPELYRSGQKSEAYLPHTFWITLLDAFYQSLVCFFVPYFTY
QGSDTDIFAFGNPLNTAALFIVLLHLVIESKSLTWIHLLVIIGSILSYFLFAIVFGAMCVTCNPPSNPYWIMQEHMLDPVFYLVCIL
TTSIALLPRFVYRVLQGSLFPSPILRAKHFDRLTPEERTKALKKWRGAGKMNQVTSKYANQSAGKSGRRPMPGPSAVFAMKSASSCA
IEQGNLSLCETALDQGYSETKAFEMAGPSKGKES
>sp|Q96RY7|IF140_HUMAN
MALYYDHQIEAPDAAGSPSFISWHPVHPFLAVAYISTTSTGSVDIYLEQGECVPDTHVERPFRVASLCWHPTRLVLAVGWETGEVTV
FNKQDKEQHTMPLTHTADITVLRWSPSGNCLLSGDRLGVLLLWRLDQRGRVQGTPLLKHEYGKHLTHCIFRLPPPGEDLVQLAKAAV
SGDEKALDMFNWKKSSSGSLLKMGSHEGLLFFVSLMDGTVHYVDEKGKTTQVVSADSTIQMLFYMEKREALVVVTENLRLSLYTVPP
EGKAEEVMKVKLSGKTGRRADIALIEGSLLVMAVGEAALRFWDIERGENYILSPDEKFGFEKGENMNCVCYCKVKGLLAAGTDRGRV
AMWRKVPDFLGSPGAEGKDRWALQTPTELQGNITQIQWGSRKNLLAVNSVISVAILSERAMSSHFHQQVAAMQVSPSLLNVCFLSTG
VAHSLRTDMHISGVFATKDAVAVWNGRQVAIFELSGAAIRSAGTFLCETPVLAMHEENVYTVESNRVQVRTWQGTVKQLLLFSETEG
NPCFLDICGNFLVVGTDLAHFKSFDLSRREAKAHCSCRSLAELVPGVGGIASLRCSSSGSTISILPSKADNSPDSKICFYDVEMDTV
TVFDFKTGQIDRRETLSFNEQETNKSHLFVDEGLKNYVPVNHFWDQSEPRLFVCEAVQETPRSQPQSANGQPQDGRAGPAADVLILS
FFISEEHGFLLHESFPRPATSHSLLGMEVPYYYFTRKPEEADREDEVEPGCHHIPQMVSRRPLRDFVGLEDCDKATRDAMLHFSFFV
TIGDMDEAFKSIKLIKSEAVWENMARMCVKTQRLDVAKVCLGNMGHARGARALREAEQEPELEARVAVLATQLGMLEDAEQLYRKCK
RHDLLNKFYQAAGRWQEALQVAEHHDRVHLRSTYHRYAGHLEASADCSRALSYYEKSDTHRFEVPRMLSEDLPSLELYVNKMKDKTL
WRWWAQYLESQGEMDAALHYYELARDHFSLVRIHCFQGNVQKAAQIANETGNLAASYHLARQYESQEEVGQAVHFYTRAQAFKNAIR
LCKENGLDDQLMNLALLSSPEDMIEAARYYEEKGVQMDRAVMLYHKAGHFSKALELAFATQQFVALQLIAEDLDETSDPALLARCSD
FFIEHSQYERAVELLLAARKYQEALQLCLGQNMSITEEMAEKMTVAKDSSDLPEESRRELLEQIADCCMRQGSYHLATKKYTQAGNK
LKAMRALLKSGDTEKITFFASVSRQKEIYIMAANYLQSLDWRKEPEIMKNIIGFYTKGRALDLLAGFYDACAQVEIDEYQNYDKAHG
ALTEAYKCLAKAKAKSPLDQETRLAQLQSRMALVKRFIQARRTYTEDPKESIKQCELLLEEPDLDSTIRIGDVYGFLVEHYVRKEEY
QTAYRFLEEMRRRLPLANMSYYVSPQAVDAVHRGLGLPLPRTVPEQVRHNSMEDARELDEEVVEEADDDP
>sp|Q96H15|TIMD4_HUMAN
MSKEPLILWLMIEFWWLYLTPVTSETVVTEVLGHRVTLPCLYSSWSHNSNSMCWGKDQCPYSGCKEALIRTDGMRVTSRKSAKYRLQ
GTIPRGDVSLTILNPSESDSGVYCCRIEVPGWFNDVKINVRLNLQRASTTTHRTATTTTRRTTTTSPTTTRQMTTTPAALPTTVVTT
PDLTTGTPLQMTTIAVFTTANTCLSLTPSTLPEEATGLLTPEPSKEGPILTAESETVLPSDSWSSVESTSADTVLLTSKESKVWDLP
STSHVSMWKTSDSVSSPQPGASDTAVPEQNKTTKTGQMDGIPMSMKNEMPISQLLMIIAPSLGFVLFALFVAFLLRGKLMETYCSQK
HTRLDYIGDSKNVLNDVQHGREDEDGLFTL
>sp|P26012|ITB8_HUMAN
MCGSALAFFTAAFVCLQNDRRGPASFLWAAWVFSLVLGLGQGEDNRCASSNAASCARCLALGPECGWCVQEDFISGGSRSERCDIVS
NLISKGCSVDSIEYPSVHVIIPTENEINTQVTPGEVSIQLRPGAEANFMLKVHPLKKYPVDLYYLVDVSASMHNNIEKLNSVGNDLS
RKMAFFSRDFRLGFGSYVDKTVSPYISIHPERIHNQCSDYNLDCMPPHGYIHVLSLTENITEFEKAVHRQKISGNIDTPEGGFDAML
QAAVCESHIGWRKEAKRLLLVMTDQTSHLALDSKLAGIVVPNDGNCHLKNNVYVKSTTMEHPSLGQLSEKLIDNNINVIFAVQGKQF
HWYKDLLPLLPGTIAGEIESKAANLNNLVVEAYQKLISEVKVQVENQVQGIYFNITAICPDGSRKPGMEGCRNVTSNDEVLFNVTVT
MKKCDVTGGKNYAIIKPIGFNETAKIHIHRNCSCQCEDNRGPKGKCVDETFLDSKCFQCDENKCHFDEDQFSSESCKSHKDQPVCSG
RGVCVCGKCSCHKIKLGKVYGKYCEKDDFSCPYHHGNLCAGHGECEAGRCQCFSGWEGDRCQCPSAAAQHCVNSKGQVCSGRGTCVC

GRCECTDPRSIGRFCEHCPTCYTACKENWNCMQCLHPHNLSQAILDQCKTSCALMEQQHYVDQTSECFSSPSYLRIFFIIFIVTFLI
GLLKVLIIRQVILQWNSNKIKSSSDYRVSASKKDKLILQSVCTRAVTYRREKPEEIKMDISKLNAHETFRCNF
>sp|Q96M42|CU129_HUMAN
MDGGSLRASPAAMDGGALEPAQQLSSLEGWTGQDRLLIPRWREARSLSWMQSPDLESSKCLTQGCCYPQAWILACSAALLQALASSD
LQGSLDRVNYSRGLGPRVRLFVFPDTVGSTRKSGSTGNICSVMLNVATGCVRIEK
>sp|Q9NYA3|GOG6A_HUMAN
MWPQPYLPPHPMMLEESRQNKLAAAKKKLKEYQQRKSPGIPAGAKTKKKKTDSSPETTTSGGCHSPGDSQYQELAVALESSSVTISQ
LNENIESLKQQKKQVEHQLEEAKKTNNEIHKAQMERLETINILTLEKADLKTTLYHTKRAARHFEEESKDLAGRLQYSLQRIQELER
ALCAVSTQQQEEDRSSSCREAVLQRWLQQTIKERALLNAHVTQVTESLKQVQLERDEYAKHIKGERARWQERMWKMSVEARTLKEEK
KRDIHRIQELERSLSELKNQMAEPPSLAPPAVTSVVEQLQDEAKHLRQEVEGLEGKLQSQVENNQALSLLSKEQKQRLQEQEEMLRE
QEAQRVREQERLCEQNERLREQQKTLQEQGERLRKQEQRLRKQEERLRKEEERLQKQEKRLWDQEERLWKKEERLQKQEERLALSQN
HKLDKQLAEPQCSFEDLNNEKKSALQLEQQVKELQEKLDEEHLEAASHQNQQLETQLSLVALPGEGDGGQHLDSEEEEAPRPTPNIP
EDLESREATSSFMDLPKEKADGTEQVERRELGFVQPSGVTDGMRESFTVYESQGAVPNTRHQEMEDVIRLAQKEEEMKVKLLELQEL
VLPLVGNHEGHGKFLIAAQNPADEPTPGAPAPQELGAAGEQDVFYEVSLDNNVEPAPGAAREGSPHDNPTVQQIVQLSPVMQDT
>sp|Q6PRD1|GP179_HUMAN
MGTRGAVMPPPMWGLLGCCFVCAWALGGPRPIRSLPPLSSQVKPGSVPMQVPLEGAEAALAYLYSGDAQQLSQVNCSERYEARGAGA
MPGLPPSLQGAAGTLAQAANFLNMLLQANDIRESSVEEDVEWYQALVRSVAEGDPRVYRALLTFNPPPGASHLQLALQATRTGEETI
LQDLSGNWVQEENPPGDLDTPALKKRVLTNDLGSLGSPKWPQADGYVGDTQQVRLSPPFLECQEGRLRPGWLITLSATFYGLKPDLS
PEVRGQVQMDVDLQSVDINQCASGPGWYSNTHLCDLNSTQCVPLESQGFVLGRYLCRCRPGFYGASPSGGLEESDFQTTGQFGFPEG
RSGRLLQCLPCPEGCTSCMDATPCLVEEAAVLRAAVLACQACCMLAIFLSMLVSYRCRRNKRIWASGVVLLETVLFGFLLLYFPVFI
LYFKPSVFRCIALRWVRLLGFAIVYGTIILKLYRVLQLFLSRTAQRSALLSSGRLLRRLGLLLLPVLGFLAVWTVGALERGIQHAPL
VIRGHTPSGRHFYLCHHDRWDYIMVVAELLLLCWGSFLCYATRAVLSAFHEPRYMGIALHNELLLSAAFHTARFVLVPSLHPDWTLL
LFFFHTHSTVTTTLALIFIPKFWKLGAPPREEMVDEVCEDELDLQHSGSYLGSSIASAWSEHSLDPGDIRDELKKLYAQLEVHKTKE
MAANNPHLPKKRGSSCQGLGRSFMRYLAEFPEALARQHSRDSGSPGHGSLPGSSRRRLLSSSLQEPEGTPALHKSRSTYDQRREQDP
PLLDSLLRRKLAKKASRTESRESVEGPPALGFRSASAHNLTVGERLPRARPASLQKSLSVASSREKALLMASQAYLEETYRQAKERE
ERKKAKAAMASLVRRPSARRLERPRGAPLSAPPSPAKSSSVDSSHTSGRLHEEARRRLPHPPIRHQVSTPILALSGGLGEPRMLSPT
STLAPALLPALAPTPAPALAPVPVSPQSPNLLTYICPWENAELPAKQENVPQEGPSGPERGHHSPAPARARLWRALSVAVEKSRAGE
NEMDAEDAHHQREANDVDEDRPKIFPKSHSLKAPVQQGSMRSLGLAIKALTRSRSTYREKESVEESPEGQNSGTAGESMGAPSRSPR
LGRPKAVSKQAALIPSDDKESLQNQQNAHTSRMLQVCQREGSREQEDRGRRMTQGLGERKAERAGKTGLAMLRQVSRDKNIKQSKET
PVGWQELPKAGLQSLGSADHRVAEVCPWEVTESETRQPDSGNKAEICPWETSEGAPESRALRQDPGDSQKKRGEARGKSEPIDVVPM
MRKKPERLVREQEAVCPWESADRGGLSPGSAPQDPGRIRDKSEAGDSVEARKVEKPGWEAAGPEAHTPDITKAEPCPWEASEGGEDG
KPAQEAVKDLPQEKQKTRKATFWKEQKPGGDLESLCPWESTDFRGPSAVSIQAPGSSECSGSLGSGIAEVCLWEAGDAPAIQKAEIC
PWELDDNVMGQEMLSLGTGRESLQEKEKASRKGSFGEMGEQTVKAVQKLSQQQESVCPRESTVPGHSSPCLDNSSSKAGSQFLCNGG
SRATQVCPQEDLRPEAQEATPAKTEICPWEVNERTREEWTSAQVPRGGESQKDKEKMPGKSEIEDVTAWEKPEGQIQKQEAVGPWES
VDPGSFSPQPRPQDTERPQTLLQMSGSVGSKAADICPLDVEENLTAGKAEICPWEVGAGAGEERALGAEAIRKSPNDTGKVSADLGP
RERAVTAPEKPQKPTPEWEVACPWGSVGPGACSQHPGTLDADGPKAGFQELDHMGCRPGEVCPWEAQEAATSEKAKICPWEVSEGTT
GKGLDQKAGSESAEQREKALEKGRLTSLGEDVSKGMAKLCQQQETICIWENKDLRESPAQAPKISDLPSSMSSEVAEGHSLEATEKG
DLRQDPKTGSFPEHITQEKAPAADTEEFTTEDGEKTSHELQSVCPWETTAPADSVSHLDRQRPDQPKASSQRLVSTGGRAADVCPWD
VPDAGVYKSDSSAKAETCPWEVTERIPVKGVSRQDGKGDSQEEKGRAPEKSEPKGVPVQKKPEMADFRQQEAVCPWESQDGKGLSPQ
PAPDASDRSRGSSEAAGSVETRVAEVCLWEVVEAPSAKKAEICPWEAGGGAAEEGEQERESQGQGEMFLQKAGPGGTEEHFSKAAAK
PREQEAVCPGEGTGSGGLLPQSGALDPELKVSPKEAGSMGSRMAELCQWEITDPEGNKIKGTMADICPGEETGVPSEESGLLALTAT
RREFFPTAPEKPLCLLVHGPLDHFFPESKIPCPKVSRPASTFTLEGVRELQGPSGLEPRTSLAPEPSLQEAESQSSSLTEDSGQVAF
EAQYEEFTPPTVYPWDWE
>sp|P30447|1A23_HUMAN

MAVMAPRTLVLLLSGALALTQTWAGSHSMRYFSTSVSRPGRGEPRFIAVGYVDDTQFVRFDSDAASQRMEPRAPWIEQEGPEYWDEE
TGKVKAHSQTDRENLRIALRYYNQSEAGSHTLQMMFGCDVGSDGRFLRGYHQYAYDGKDYIALKEDLRSWTAADMAAQITQRKWEAA
RVAEQLRAYLEGTCVDGLRRYLENGKETLQRTDPPKTHMTHHPISDHEATLRCWALGFYPAEITLTWQRDGEDQTQDTELVETRPAG
DGTFQKWAAVVVPSGEEQRYTCHVQHEGLPKPLTLRWEPSSQPTVHIVGIIAGLVLLGAVITGAVVAAVMWRRNSSDRKGGSYSQAA
SSDSAQGSDVSLTACKV
>sp|Q14003|KCNC3_HUMAN
MLSSVCVSSFRGRQGASKQQPAPPPQPPESPPPPPLPPQQQQPAQPGPAASPAGPPAPRGPGDRRAEPCPGLPAAAMGRHGGGGGDS
GKIVINVGGVRHETYRSTLRTLPGTRLAGLTEPEAAARFDYDPGADEFFFDRHPGVFAYVLNYYRTGKLHCPADVCGPLFEEELGFW
GIDETDVEACCWMTYRQHRDAEEALDSFEAPDPAGAANAANAAGAHDGGLDDEAGAGGGGLDGAGGELKRLCFQDAGGGAGGPPGGA
GGAGGTWWRRWQPRVWALFEDPYSSRAARYVAFASLFFILISITTFCLETHEGFIHISNKTVTQASPIPGAPPENITNVEVETEPFL
TYVEGVCVVWFTFEFLMRITFCPDKVEFLKSSLNIIDCVAILPFYLEVGLSGLSSKAAKDVLGFLRVVRFVRILRIFKLTRHFVGLR
VLGHTLRASTNEFLLLIIFLALGVLIFATMIYYAERIGADPDDILGSNHTYFKNIPIGFWWAVVTMTTLGYGDMYPKTWSGMLVGAL
CALAGVLTIAMPVPVIVNNFGMYYSLAMAKQKLPKKKNKHIPRPPQPGSPNYCKPDPPPPPPPHPHHGSGGISPPPPITPPSMGVTV
AGAYPAGPHTHPGLLRGGAGGLGIMGLPPLPAPGEPCPLAQEEVIEINRADPRPNGDPAAAALAHEDCPAIDQPAMSPEDKSPITPG
SRGRYSRDRACFLLTDYAPSPDGSIRKATGAPPLPPQDWRKPGPPSFLPDLNANAAAWISP
>sp|Q9NR16|C163B_HUMAN
MMLPQNSWHIDFGRCCCHQNLFSAVVTCILLLNSCFLISSFNGTDLELRLVNGDGPCSGTVEVKFQGQWGTVCDDGWNTTASTVVCK
QLGCPFSFAMFRFGQAVTRHGKIWLDDVSCYGNESALWECQHREWGSHNCYHGEDVGVNCYGEANLGLRLVDGNNSCSGRVEVKFQE
RWGTICDDGWNLNTAAVVCRQLGCPSSFISSGVVNSPAVLRPIWLDDILCQGNELALWNCRHRGWGNHDCSHNEDVTLTCYDSSDLE
LRLVGGTNRCMGRVELKIQGRWGTVCHHKWNNAAADVVCKQLGCGTALHFAGLPHLQSGSDVVWLDGVSCSGNESFLWDCRHSGTVN
FDCLHQNDVSVICSDGADLELRLADGSNNCSGRVEVRIHEQWWTICDQNWKNEQALVVCKQLGCPFSVFGSRRAKPSNEARDIWINS
ISCTGNESALWDCTYDGKAKRTCFRRSDAGVICSDKADLDLRLVGAHSPCYGRLEVKYQGEWGTVCHDRWSTRNAAVVCKQLGCGKP
LHVFGMTYFKEASGPIWLDDVSCIGNESNIWDCEHSGWGKHNCVHREDVIVTCSGDATWGLRLVGGSNRCSGRLEVYFQGRWGTVCD
DGWNSKAAAVVCSQLDCPSSIIGMGLGNASTGYGKIWLDDVSCDGDESDLWSCRNSGWGNNDCSHSEDVGVICSDASDMELRLVGGS
SRCAGKVEVNVQGAVGILCANGWGMNIAEVVCRQLECGSAIRVSREPHFTERTLHILMSNSGCTGGEASLWDCIRWEWKQTACHLNM
EASLICSAHRQPRLVGADMPCSGRVEVKHADTWRSVCDSDFSLHAANVLCRELNCGDAISLSVGDHFGKGNGLTWAEKFQCEGSETH
LALCPIVQHPEDTCIHSREVGVVCSRYTDVRLVNGKSQCDGQVEINVLGHWGSLCDTHWDPEDARVLCRQLSCGTALSTTGGKYIGE
RSVRVWGHRFHCLGNESLLDNCQMTVLGAPPCIHGNTVSVICTGSLTQPLFPCLANVSDPYLSAVPEGSALICLEDKRLRLVDGDSR
CAGRVEIYHDGFWGTICDDGWDLSDAHVVCQKLGCGVAFNATVSAHFGEGSGPIWLDDLNCTGMESHLWQCPSRGWGQHDCRHKEDA
GVICSEFTALRLYSETETESCAGRLEVFYNGTWGSVGRRNITTAIAGIVCRQLGCGENGVVSLAPLSKTGSGFMWVDDIQCPKTHIS
IWQCLSAPWERRISSPAEETWITCEDRIRVRGGDTECSGRVEIWHAGSWGTVCDDSWDLAEAEVVCQQLGCGSALAALRDASFGQGT
GTIWLDDMRCKGNESFLWDCHAKPWGQSDCGHKEDAGVRCSGQSLKSLNASSGHLALILSSIFGLLLLVLFILFLTWCRVQKQKHLP
LRVSTRRRGSLEENLFHEMETCLKREDPHGTRTSDDTPNHGCEDASDTSLLGVLPASEATK
>sp|Q9UHA4|LTOR3_HUMAN
MADDLKRFLYKKLPSVEGLHAIVVSDRDGVPVIKVANDNAPEHALRPGFLSTFALATDQGSKLGLSKNKSIICYYNTYQVVQFNRLP
LVVSFIASSSANTGLIVSLEKELAPLFEELRQVVEVS
>sp|P23759|PAX7_HUMAN
MAALPGTVPRMMRPAPGQNYPRTGFPLEVSTPLGQGRVNQLGGVFINGRPLPNHIRHKIVEMAHHGIRPCVISRQLRVSHGCVSKIL
CRYQETGSIRPGAIGGSKPRQVATPDVEKKIEEYKRENPGMFSWEIRDRLLKDGHCDRSTVPSGLVSSISRVLRIKFGKKEEEDEAD
KKEDDGEKKAKHSIDGILGDKGNRLDEGSDVESEPDLPLKRKQRRSRTTFTAEQLEELEKAFERTHYPDIYTREELAQRTKLTEARV
QVWFSNRRARWRKQAGANQLAAFNHLLPGGFPPTGMPTLPPYQLPDSTYPTTTISQDGGSTVHRPQPLPPSTMHQGGLAAAAAAADT
SSAYGARHSFSSYSDSFMNPAAPSNHMNPVSNGLSPQVMSILGNPSAVPPQPQADFSISPLHGGLDSATSISASCSQRADSIKPGDS
LPTSQAYCPPTYSTTGYSVDPVAGYQYGQYGQTAVDYLAKNVSLSTQRRMKLGEHSAVLGLLPVETGQAY
>sp|Q7Z460|CLAP1_HUMAN

MEPRMESCLAQVLQKDVGKRLQVGQELIDYFSDKQKSADLEHDQTMLDKLVDGLATSWVNSSNYKVVLLGMDILSALVTRLQDRFKA
QIGTVLPSLIDRLGDAKDSVREQDQTLLLKIMDQAANPQYVWDRMLGGFKHKNFRTREGICLCLIATLNASGAQTLTLSKIVPHICN
LLGDPNSQVRDAAINSLVEIYRHVGERVRADLSKKGLPQSRLNVIFTKFDEVQKSGNMIQSANDKNFDDEDSVDGNRPSSASSTSSK
APPSSRRNVGMGTTRRLGSSTLGSKSSAAKEGAGAVDEEDFIKAFDDVPVVQIYSSRDLEESINKIREILSDDKHDWEQRVNALKKI
RSLLLAGAAEYDNFFQHLRLLDGAFKLSAKDLRSQVVREACITLGHLSSVLGNKFDHGAEAIMPTIFNLIPNSAKIMATSGVVAVRL
IIRHTHIPRLIPVITSNCTSKSVAVRRRCFEFLDLLLQEWQTHSLERHISVLAETIKKGIHDADSEARIEARKCYWGFHSHFSREAE
HLYHTLESSYQKALQSHLKNSDSIVSLPQSDRSSSSSQESLNRPLSAKRSPTGSTTSRASTVSTKSVSTTGSLQRSRSDIDVNAAAS
AKSKVSSSSGTTPFSSAAALPPGSYASLGRIRTRRQSSGSATNVASTPDNRGRSRAKVVSQSQRSRSANPAGAGSRSSSPGKLLGSG
YGGLTGGSSRGPPVTPSSEKRSKIPRSQGCSRETSPNRIGLARSSRIPRPSMSQGCSRDTSRESSRDTSPARGFPPLDRFGLGQPGR
IPGSVNAMRVLSTSTDLEAAVADALKKPVRRRYEPYGMYSDDDANSDASSVCSERSYGSRNGGIPHYLRQTEDVAEVLNHCASSNWS
ERKEGLLGLQNLLKSQRTLSRVELKRLCEIFTRMFADPHSKRVFSMFLETLVDFIIIHKDDLQDWLFVLLTQLLKKMGADLLGSVQA
KVQKALDVTRDSFPFDQQFNILMRFIVDQTQTPNLKVKVAILKYIESLARQMDPTDFVNSSETRLAVSRIITWTTEPKSSDVRKAAQ
IVLISLFELNTPEFTMLLGALPKTFQDGATKLLHNHLKNSSNTSVGSPSNTIGRTPSRHTSSRTSPLTSPTNCSHGGLSPSRLWGWS
ADGLAKHPPPFSQPNSIPTAPSHKALRRSYSPSMLDYDTENLNSEEIYSSLRGVTEAIEKFSFRSQEDLNEPIKRDGKKECDIVSRD
GGAASPATEGRGGSEVEGGRTALDNKTSLLNTQPPRAFPGPRARDYNPYPYSDAINTYDKTALKEAVFDDDMEQLRDVPIDHSDLVA
DLLKELSNHNERVEERKGALLELLKITREDSLGVWEEHFKTILLLLLETLGDKDHSIRALALRVLREILRNQPARFKNYAELTIMKT
LEAHKDSHKEVVRAAEEAASTLASSIHPEQCIKVLCPIIQTADYPINLAAIKMQTKVVERIAKESLLQLLVDIIPGLLQGYDNTESS
VRKASVFCLVAIYSVIGEDLKPHLAQLTGSKMKLLNLYIKRAQTTNSNSSSSSDVSTHS
>sp|Q9NPU4|CN132_HUMAN
MDLSFMAAQLPMMGGAFMDSPNEDFSTEYSLFNSSANVHAAANGQGQPEDPPRSSNDAVLLWIAIIATLGNIVVVGVVYAFTF
>sp|A6NHQ4|CQ096_HUMAN
METLCPAPRLAVPASPRGSPCSPTPRKPCRGTQEFSPLCLRALAFCALAKPRASSLGPGPGELAARSPVLRGPQAPLRPGGWAPDGL
KHLWAPTGRPGVPNTAAGEDADVAACPRRGEEEEGGGGFPHFGVRSCAPPGRCPAPPHPRESTTSFASAPPRPAPGLEPQRGPAASP
PQEPSSRPPSPPAGLSTEPAGPGTAPRPFLPGQPAEVDGNPPPAAPEAPAASPSTASPAPAAPGDLRQEHFDRLIRRSKLWCYAKGF
ALDTPSLRRGPERPPAKGPARGAAKKRRLPAPPPRTAQPRRPAPTLPTTSTFSLLNCFPCPPALVVGEDGDLKPASSLRLQGDSKPP
PAHPLWRWQMGGPAVPEPPGLKFWGINMDES
>sp|O60336|MABP1_HUMAN
MAVEGSTITSRIKNLLRSPSIKLRRSKAGNRREDLSSKVTLEKVLGITVSGGRGLACDPRSGLVAYPAGCVVVLFNPRKHKQHHILN
SSRKTITALAFSPDGKYLVTGESGHMPAVRVWDVAEHSQVAELQEHKYGVACVAFSPSAKYIVSVGYQHDMIVNVWAWKKNIVVASN
KVSSRVTAVSFSEDCSYFVTAGNRHIKFWYLDDSKTSKVNATVPLLGRSGLLGELRNNLFTDVACGRGKKADSTFCITSSGLLCEFS
DRRLLDKWVELRNIDSFTTTVAHCISVSQDYIFCGCADGTVRLFNPSNLHFLSTLPRPHALGTDIASVTEASRLFSGVANARYPDTI
ALTFDPTNQWLSCVYNDHSIYVWDVRDPKKVGKVYSALYHSSCVWSVEVYPEVKDSNQACLPPSSFITCSSDNTIRLWNTESSGVHG
STLHRNILSSDLIKIIYVDGNTQALLDTELPGGDKADASLLDPRVGIRSVCVSPNGQHLASGDRMGTLRVHELQSLSEMLKVEAHDS
EILCLEYSKPDTGLKLLASASRDRLIHVLDAGREYSLQQTLDEHSSSITAVKFAASDGQVRMISCGADKSIYFRTAQKSGDGVQFTR
THHVVRKTTLYDMDVEPSWKYTAIGCQDRNIRIFNISSGKQKKLFKGSQGEDGTLIKVQTDPSGIYIATSCSDKNLSIFDFSSGECV
ATMFGHSEIVTGMKFSNDCKHLISVSGDSCIFVWRLSSEMTISMRQRLAELRQRQRGGKQQGPSSPQRASGPNRHQAPSMLSPGPAL
SSDSDKEGEDEGTEEELPALPVLAKSTKKALASVPSPALPRSLSHWEMSRAQESVGFLDPAPAANPGPRRRGRWVQPGVELSVRSML
DLRQLETLAPSLQDPSQDSLAIIPSGPRKHGQEALETSLTSQNEKPPRPQASQPCSYPHIIRLLSQEEGVFAQDLEPAPIEDGIVYP
EPSDNPTMDTSEFQVQAPARGTLGRVYPGSRSSEKHSPDSACSVDYSSSCLSSPEHPTEDSESTEPLSVDGISSDLEEPAEGDEEEE
EEEGGMGPYGLQEGSPQTPDQEQFLKQHFETLASGAAPGAPVQVPERSESRSISSRFLLQVQTRPLREPSPSSSSLALMSRPAQVPQ
ASGEQPRGNGANPPGAPPEVEPSSGNPSPQQAASVLLPRCRLNPDSSWAPKRVATASPFSGLQKAQSVHSLVPQERHEASLQAPSPG
ALLSREIEAQDGLGSLPPADGRPSRPHSYQNPTTSSMAKISRSISVGENLGLVAEPQAHAPIRVSPLSKLALPSRAHLVLDIPKPLP
DRPTLAAFSPVTKGRAPGEAEKPGFPVGLGKAHSTTERWACLGEGTTPKPRTECQAHPGPSSPCAQQLPVSSLFQGPENLQPPPPEK

TPNPMECTKPGAALSQDSEPAVSLEQCEQLVAELRGSVRQAVRLYHSVAGCKMPSAEQSRIAQLLRDTFSSVRQELEAVAGAVLSSP
GSSPGAVGAEQTQALLEQYSELLLRAVERRMERKL
>sp|O15303|GRM6_HUMAN
MARPRRAREPLLVALLPLAWLAQAGLARAAGSVRLAGGLTLGGLFPVHARGAAGRACGQLKKEQGVHRLEAMLYALDRVNADPELLP
GVRLGARLLDTCSRDTYALEQALSFVQALIRGRGDGDEVGVRCPGGVPPLRPAPPERVVAVVGASASSVSIMVANVLRLFAIPQISY
ASTAPELSDSTRYDFFSRVVPPDSYQAQAMVDIVRALGWNYVSTLASEGNYGESGVEAFVQISREAGGVCIAQSIKIPREPKPGEFS
KVIRRLMETPNARGIIIFANEDDIRRVLEAARQANLTGHFLWVGSDSWGAKTSPILSLEDVAVGAITILPKRASIDGFDQYFMTRSL
ENNRRNIWFAEFWEENFNCKLTSSGTQSDDSTRKCTGEERIGRDSTYEQEGKVQFVIDAVYAIAHALHSMHQALCPGHTGLCPAMEP
TDGRMLLQYIRAVRFNGSAGTPVMFNENGDAPGRYDIFQYQATNGSASSGGYQAVGQWAETLRLDVEALQWSGDPHEVPSSLCSLPC
GPGERKKMVKGVPCCWHCEACDGYRFQVDEFTCEACPGDMRPTPNHTGCRPTPVVRLSWSSPWAAPPLLLAVLGIVATTTVVATFVR
YNNTPIVRASGRELSYVLLTGIFLIYAITFLMVAEPGAAVCAARRLFLGLGTTLSYSALLTKTNRIYRIFEQGKRSVTPPPFISPTS
QLVITFSLTSLQVVGMIAWLGARPPHSVIDYEEQRTVDPEQARGVLKCDMSDLSLIGCLGYSLLLMVTCTVYAIKARGVPETFNEAK
PIGFTMYTTCIIWLAFVPIFFGTAQSAEKIYIQTTTLTVSLSLSASVSLGMLYVPKTYVILFHPEQNVQKRKRSLKATSTVAAPPKG
EDAEAHK
>sp|Q96M43|NBPF4_HUMAN
MVVSADPLSSERAEMNILEINQELRSQLAESNQQFRDLKEKFLITQATAYSLANQLKKYKCEEYKDIIDSVLRDELQSMEKLAEKLR
QAEELRQYKALVHSQAKELTQLREKLREGRDASRWLNKHLKTLLTPDDPDKSQGQDLREQLAEGHRLAEHLVHKLSPENDEDEDEDE
DDKDEEVEKVQESPAPREVQKTEEKEVPQDSLEECAVTCSNSHNPSNSNQPHRSTKITFKEHEVDSALVVESEHPHDEEEEALNIPP
ENQNDHEEEEGKAPVPPRHHDKSNSYRHREVSFLALDEQKVCSAQDVARDYSNPKWDETSLGFLEKQSDLEEVKGQETVAPRLSRGP
LRVDKHEIPQESLDGCCLTPSILPDLTPSYHPYWSTLYSFEDKQVSLALVDKIKKDQEEIEDQSPPCPRLSQELPEVKEQEVPEDSV
NEVYLTPSVHHDVSDCHQPYSSTLSSLEDQLACSALDVASPTEAACPQGTWSGDLSHHQSEVQVSQAQLEPSTLVPSCLRLQLDQGF
HCGNGLAQRGLSSTTCSFSANADSGNQWPFQELVLEPSLGMKNPPQLEDDALEGSASNTQGRQVTGRIRASLVLILKTIRRRLPFSK
WRLAFRFAGPHAESAEIPNTAGRTQRMAG
>sp|Q9NRR2|TRYG1_HUMAN
MALGACGLLLLLAVPGVSLRTLQPGCGRPQVSDAGGRIVGGHAAPAGAWPWQASLRLRRMHVCGGSLLSPQWVLTAAHCFSGSLNSS
DYQVHLGELEITLSPHFSTVRQIILHSSPSGQPGTSGDIALVELSVPVTLSSRILPVCLPEASDDFCPGIRCWVTGWGYTREGEPLP
PPYSLREVKVSVVDTETCRRDYPGPGGSILQPDMLCARGPGDACQDDSGGPLVCQVNGAWVQAGTVSWGEGCGRPNRPGVYTRVPAY
VNWIRRHITASGGSESGYPRLPLLAGLFLPGLFLLLVSCVLLAKCLLHPSADGTPFPAPD
>sp|Q9H4M9|EHD1_HUMAN
MFSWVSKDARRKKEPELFQTVAEGLRQLYAQKLLPLEEHYRFHEFHSPALEDADFDNKPMVLLVGQYSTGKTTFIRHLIEQDFPGMR
IGPEPTTDSFIAVMHGPTEGVVPGNALVVDPRRPFRKLNAFGNAFLNRFMCAQLPNPVLDSISIIDTPGILSGEKQRISRGYDFAAV
LEWFAERVDRIILLFDAHKLDISDEFSEVIKALKNHEDKIRVVLNKADQIETQQLMRVYGALMWSLGKIINTPEVVRVYIGSFWSHP
LLIPDNRKLFEAEEQDLFKDIQSLPRNAALRKLNDLIKRARLAKVHAYIISSLKKEMPNVFGKESKKKELVNNLGEIYQKIEREHQI
SPGDFPSLRKMQELLQTQDFSKFQALKPKLLDTVDDMLANDIARLMVMVRQEESLMPSQVVKGGAFDGTMNGPFGHGYGEGAGEGID
DVEWVVGKDKPTYDEIFYTLSPVNGKITGANAKKEMVKSKLPNTVLGKIWKLADVDKDGLLDDEEFALANHLIKVKLEGHELPADLP
PHLVPPSKRRHE
>sp|Q93084|AT2A3_HUMAN
MEAAHLLPAADVLRHFSVTAEGGLSPAQVTGARERYGPNELPSEEGKSLWELVLEQFEDLLVRILLLAALVSFVLAWFEEGEETTTA
FVEPLVIMLILVANAIVGVWQERNAESAIEALKEYEPEMGKVIRSDRKGVQRIRARDIVPGDIVEVAVGDKVPADLRLIEIKSTTLR
VDQSILTGESVSVTKHTEAIPDPRAVNQDKKNMLFSGTNITSGKAVGVAVATGLHTELGKIRSQMAAVEPERTPLQRKLDEFGRQLS
HAISVICVAVWVINIGHFADPAHGGSWLRGAVYYFKIAVALAVAAIPEGLPAVITTCLALGTRRMARKNAIVRSLPSVETLGCTSVI
CSDKTGTLTTNQMSVCRMFVVAEADAGSCLLHEFTISGTTYTPEGEVRQGDQPVRCGQFDGLVELATICALCNDSALDYNEAKGVYE
KVGEATETALTCLVEKMNVFDTDLQALSRVERAGACNTVIKQLMRKEFTLEFSRDRKSMSVYCTPTRPHPTGQGSKMFVKGAPESVI
ERCSSVRVGSRTAPLTPTSREQILAKIRDWGSGSDTLRCLALATRDAPPRKEDMELDDCSKFVQYETDLTFVGCVGMLDPPRPEVAA

CITRCYQAGIRVVMITGDNKGTAVAICRRLGIFGDTEDVAGKAYTGREFDDLSPEQQRQACRTARCFARVEPAHKSRIVENLQSFNE
ITAMTGDGVNDAPALKKAEIGIAMGSGTAVAKSAAEMVLSDDNFASIVAAVEEGRAIYSNMKQFIRYLISSNVGEVVCIFLTAILGL
PEALIPVQLLWVNLVTDGLPATALGFNPPDLDIMEKLPRSPREALISGWLFFRYLAIGVYVGLATVAAATWWFVYDAEGPHINFYQL
RNFLKCSEDNPLFAGIDCEVFESRFPTTMALSVLVTIEMCNALNSVSENQSLLRMPPWMNPWLLVAVAMSMALHFLILLVPPLPLIF
QVTPLSGRQWVVVLQISLPVILLDEALKYLSRNHMHACLYPGLLRTVSQAWSRQPLTTSWTPDHTGRNEPEVSAGNRVESPVCTSD
>sp|Q6IEU7|OR5MA_HUMAN
MLSPNHTIVTEFILLGLTDDPVLEKILFGVFLAIYLITLAGNLCMILLIRTNSQLQTPMYFFLGHLSFVDICYSSNVTPNMLHNFLS
EQKTISYAGCFTQCLLFIALVITEFYFLASMALDRYVAICSPLHYSSRMSKNICISLVTVPYMYGFLNGLSQTLLTFHLSFCGSLEI
NHFYCADPPLIMLACSDTRVKKMAMFVVAGFTLSSSLFIILLSYLFIFAAIFRIRSAEGRHKAFSTCASHLTIVTLFYGTLFCMYVR
PPSEKSVEESKIIAVFYTFLSPMLNPLIYSLRNRDVILAIQQMIRGKSFCKIAV
>sp|O75167|PHAR2_HUMAN
MDNAVDGLDKASIANSDGPTAGSQTPPFKRKGKLSTIGKIFKPWKWRKKKTSDKFRETSAVLERKISTRQSREELIRRGVLKELPDQ
DGDVTVNFENSNGHMIPIGEESTREENVVKSEEGNGSVSEKTPPLEEQAEDKKENTENHSETPAAPALPPSAPPKPRPKPKPKKSPV
PPKGATAGASHKGDEVPPIKKNTKAPGKQAPVPPPKPASRNTTREAAGSSHSKKTTGSKASASPSTSSTSSRPKASKETVSSKAGTV
GTTKGKRKTDKQPITSHLSSDTTTSGTSDLKGEPAETRVESFKLEQTVPGAEEQNTGKFKSMVPPPPVAPAPSPLAPPLPLEDQCIT
ASDTPVVLVSVGADLPVSALDPSQLLWAEEPTNRTTLYSGTGLSVNRENAKCFTTKEELGKTVPQLLTPGLMGESSESFSASEDEGH
REYQANDSDSDGPILYTDDEDEDEDEDGSGESALASKIRRRDTLAIKLGNRPSKKELEDKNILQRTSEEERQEIRQQIGTKLVRRLS
QRPTTEELEQRNILKQKNEEEEQEAKMELKRRLSRKLSLRPTVAELQARRILRFNEYVEVTDSPDYDRRADKPWARLTPADKAAIRK
ELNEFKSTEMEVHEESRQFTRFHRP
>sp|Q9BYP8|KR171_HUMAN
MGCCPGDCFTCCTQEQNCCEECCCQPGCCGCCGSCCGCGGSGCGGSGCGGSCCGSSCCGSGCGGCGGCGGGCCGSSCCGSSCCG
SGCCGPVCCQPTPICDTK
>sp|A6NGS2|ERIC4_HUMAN
MELWRQLNQAGLVPPGLGPPPQALREVSPVEIPGQTLRTAGADTGGACDSLLWIREELGNLRRVDVQLLGQLCSLGLEMGALREELV
TILEEEEESSKEEEEDQEPQRKQEEEHLEACPAPHPPDFEMMI
>sp|Q02548|PAX5_HUMAN
MDLEKNYPTPRTSRTGHGGVNQLGGVFVNGRPLPDVVRQRIVELAHQGVRPCDISRQLRVSHGCVSKILGRYYETGSIKPGVIGGSK
PKVATPKVVEKIAEYKRQNPTMFAWEIRDRLLAERVCDNDTVPSVSSINRIIRTKVQQPPNQPVPASSHSIVSTGSVTQVSSVSTDS
AGSSYSISGILGITSPSADTNKRKRDEGIQESPVPNGHSLPGRDFLRKQMRGDLFTQQQLEVLDRVFERQHYSDIFTTTEPIKPEQT
TEYSAMASLAGGLDDMKANLASPTPADIGSSVPGPQSYPIVTGRDLASTTLPGYPPHVPPAGQGSYSAPTLTGMVPGSEFSGSPYSH
PQYSSYNDSWRFPNPGLLGSPYYYSAAARGAAPPAAATAYDRH
>sp|O94812|BAIP3_HUMAN
MRPRGAAFAAGPPGDLHLGTAIGFAGAIWRSRSPAMSTLLDIKSSVLRQVQVCPSFRRRTEQDPGSASADPQEPATGAWKPGDGVEF
FAHMRLMLKKGEGRQGLPCLEVPLRSGSPAPPEPVDPSLGLRALAPEEVEMLYEEALYTVLYRAGTMGPDQVDDEEALLSYLQQVFG
TSLEEHTEAIERVRKAKAPTYALKVSVMRAKNLLAKDPNGFSDPYCMLGILPASDATREPRAQKEQRFGFRKGSKRGGPLPAKCIQV
TEVKSSTLNPVWKEHFLFEIEDVSTDQLHLDIWDHDDDVSLVEACRKLNEVIGLKGMGRYFKQIVKSARANGTAGPTEDHTDDFLGC
LNIPVREVPVAGVDRWFKLEPRSSASRVQGHCHLVLKLITTQRDTAMSQRGRSGFLSHLLLLSHLLRLEHSAEEPNSSSWRGELSTP
AATILCLHGAQSNLSPLQLAVLHWQVSSRHHQTCTLDYSYLLGLLEDMQAHWEEAPSLPQEQEESLADSLSAFSEFGLQLLRQLRDY
FPATNSTAVHRLELLLKCLGKLQLFQPSFEICPFESELNMDIAAALKRGNREWYDRILNDKSPREQPGPQRLPGLVVLADAVYDDLQ
FCYSVYASLFHSILNVDVFTLTFRQLERLVAEEAWVLTEELSPKMTLEVASGLFELYLTLADLQRFWDSIPGRDSRSLALAGIHAPF
LPAVKLWFQVLRDQAKWRLQGAVDMDTLEPVDASSRHSSSAATAGLCLSHIQELWVRLAWPDPAQAQGLGTQLGQDVCEATLFYTEL
LRKKVDTQPGAAGEAVSEALCVVLNNVELVRKAAGQALKGLAWPEGATGPEGVLPRPLLSCTQALDDDLQREAHTVTAHLTSKMVGD
IRKYVQHISLSPDSIQNDEAVAPLMKYLDEKLALLNASLVKGNLSRVLEALWELLLQAILQALGANRDVSADFYSRFHFTLEALVSF
FHAEGQGLPLESLRDGSYKRLKEELRLHKCSTRECIEQFYLDKLKQRTLEQNRFGRLSVRCHYEAAEQRLAVEVLHAADLLPLDANG

LSDPFVIVELGPPHLFPLVRSQRTQVKTRTLHPVYDELFYFSVPAEACRRRAACVLFTVMDHDWLSTNDFAGEAALGLGGVTGVARP
QVGGGARAGQPVTLHLCRPRAQVRSALRRLEGRTSKEAQEFVKKLKELEKCMEADP
>sp|Q14207|NPAT_HUMAN
MLLPSDVARLVLGYLQQENLISTCQTFILESSDLKEYAEHCTDEGFIPACLLSLFGKNLTTILNEYVAMKTKETSNNVPAIMSSLWK
KLDHTLSQIRSMQSSPRFAGSQRARTRTGIAEIKRQRKLASQTAPASAELLTLPYLSGQFTTPPSTGTQVTRPSGQISDPSRSYFVV
VNHSQSQDTVTTGEALNVIPGAQEKKAHASLMSPGRRKSESQRKSTTLSGPHSTIRNFQDPNAFAVEKQMVIENAREKILSNKSLQE
KLAENINKFLTSDNNIAQVPKQTDNNPTEPETSIDEFLGLPSEIHMSEEAIQDILEQTESDPAFQALFDLFDYGKTKNNKNISQSIS
SQPMESNPSIVLADETNLAVKGSFETEESDGQSGQPAFCTSYQNDDPLNALKNSNNHDVLRQEDQENFSQISTSIQKKAFKTAVPTE
QKCDIDITFESVPNLNDFNQRGNSNAECNPHCAELYTNQMSTETEMAIGIEKNSLSSNVPSESQLQPDQPDIPITSFVSLGCEANNE
NLILSGKSSQLLSQDTSLTGKPSKKSQFCENSNDTVKLKINFHGSKSSDSSEVHKSKIEINVLEPVMSQLSNCQDNSCLQSEILPVS
VESSHLNVSGQVEIHLGDSLSSTKQPSNDSASVELNHTENEAQASKSENSQEPSSSVKEENTIFLSLGGNANCEKVALTPPEGTPVE
NSHSLPPESVCSSVGDSHPESQNTDDKPSSNNSAEIDASNIVSLKVIISDDPFVSSDTELTSAVSSINGENLPTIILSSPTKSPTKN
AELVKCLSSEETVGAVVYAEVGDSASMEQSLLTFKSEDSAVNNTQNEDGIAFSANVTPCVSKDGGYIQLMPATSTAFGNSNNILIAT
CVTDPTALGTSVSQSNVVVLPGNSAPMTAQPLPPQLQTPPRSNSVFAVNQAVSPNFSQGSAIIIASPVQPVLQGMVGMIPVSVVGQN
GNNFSTPPRQVLHMPLTAPVCNRSIPQFPVPPKSQKAQGLRNKPCIGKQVNNLVDSSGHSVGCHAQKTEVSDKSIATDLGKKSEETT
VPFPEESIVPAAKPCHRRVLCFDSTTAPVANTQGPNHKMVSQNKERNAVSFPNLDSPNVSSTLKPPSNNAIKREKEKPPLPKILSKS
ESAISRHTTIRETQSEKKVSPTEIVLESFHKATANKENELCSDVERQKNPENSKLSIGQQNGGLRSEKSIASLQEMTKKQGTSSNNK
NVLSVGTAVKDLKQEQTKSASSLITTEMLQDIQRHSSVSRLADSSDLPVPRTPGSGAGEKHKEEPIDIIKAPSSRRFSEDSSTSKVM
VPPVTPDLPACSPASETGSENSVNMAAHTLMILSRAAISRTTSATPLKDNTQQFRASSRSTTKKRKIEELDERERNSRPSSKNLTNS
SIPMKKKKIKKKKLPSSFPAGMDVDKFLLSLHYDE
>sp|Q9NUP1|BL1S4_HUMAN
MEGSFSDGGALPEGLAEEAEPQGAAWSGDSGTVSQSHSSASGPWEDEGAEDGAPGRDLPLLRRAAAGYAACLLPGAGARPEVEALDA
SLEDLLTRVDEFVGMLDMLRGDSSHVVSEGVPRIHAKAAEMRRIYSRIDRLEAFVRMVGGRVARMEEQVTKAEAELGTFPRAFKKLL
HTMNVPSLFSKSAPSRPQQAGYEAPVLFRTEDYFPCCSERPQL
>sp|Q14493|SLBP_HUMAN
MACRPRSPPRHQSRCDGDASPPSPARWSLGRKRRADGRRWRPEDAEEAEHRGAERRPESFTTPEGPKPRSRCSDWASAVEEDEMRTR
VNKEMARYKRKLLINDFGRERKSSSGSSDSKESMSTVPADFETDESVLMRRQKQINYGKNTIAYDRYIKEVPRHLRQPGIHPKTPNK
FKKYSRRSWDQQIKLWKVALHFWDPPAEEGCDLQEIHPVDLESAESSSEPQTSSQDDFDVYSGTPTKVRHMDSQVEDEFDLEACLTE
PLRDFSAMS
>sp|Q92870|APBB2_HUMAN
MSEVLPADSGVDTLAVFMASSGTTDVTNRNSPATPPNTLNLRSSHNELLNAEIKHTETKNSTPPKCRKKYALTNIQAAMGLSDPAAQ
PLLGNGSANIKLVKNGENQLRKAAEQGQQDPNKNLSPTAVINITSEKLEGKEPHPQDSSSCEILPSQPRRTKSFLNYYADLETSARE
LEQNRGNHHGTAEEKSQPVQGQASTIIGNGDLLLQKPNRPQSSPEDGQVATVSSSPETKKDHPKTGAKTDCALHRIQNLAPSDEESS
WTTLSQDSASPSSPDETDIWSDHSFQTDPDLPPGWKRVSDIAGTYYWHIPTGTTQWERPVSIPADLQGSRKGSLSSVTPSPTPENEK
QPWSDFAVLNGGKINSDIWKDLHAATVNPDPSLKEFEGATLRYASLKLRNAPHPDDDDSCSINSDPEAKCFAVRSLGWVEMAEEDLA
PGKSSVAVNNCIRQLSYCKNDIRDTVGIWGEGKDMYLILENDMLSLVDPMDRSVLHSQPIVSIRVWGVGRDNGRDFAYVARDKDTRI
LKCHVFRCDTPAKAIATSLHEICSKIMAERKNAKALACSSLQERANVNLDVPLQVDFPTPKTELVQKFHVQYLGMLPVDKPVGMDIL
NSAIENLMTSSNKEDWLSVNMNVADATVTVISEKNEEEVLVECRVRFLSFMGVGKDVHTFAFIMDTGNQRFECHVFWCEPNAGNVSE
AVQAACMLRYQKCLVARPPSQKVRPPPPPADSVTRRVTTNVKRGVLSLIDTLKQKRPVTEMP
>sp|P57087|JAM2_HUMAN
MARRSRHRLLLLLLLRYLVVALGYHKAYGFSAPKDQQVVTAVEYQEAILACKTPKKTVSSRLEWKKLGRSVSFVYYQQTLQGDFKNRA
EMIDFNIRIKNVTRSDAGKYRCEVSAPSEQGQNLEEDTVTLEVLVAPAVPSCEVPSSALSGTVVELRCQDKEGNPAPEYTWFKDGIR
LLENPRLGSQSTNSSYTMNTKTGTLQFNTVSKLDTGEYSCEARNSVGYRRCPGKRMQVDDLNISGIIAAVVVVALVISVCGLGVCYA
QRKGYFSKETSFQKSNSSSKATTMSENDFKHTKSFII

>sp|O75528|TADA3_HUMAN
MSELKDCPLQFHDFKSVDHLKVCPRYTAVLARSEDDGIGIEELDTLQLELETLLSSASRRLRVLEAETQILTDWQDKKGDRRFLKLG
RDHELGAPPKHGKPKKQKLEGKAGHGPGPGPGRPKSKNLQPKIQEYEFTDDPIDVPRIPKNDAPNRFWASVEPYCADITSEEVRTLE
ELLKPPEDEAEHYKIPPLGKHYSQRWAQEDLLEEQKDGARAAAVADKKKGLMGPLTELDTKDVDALLKKSEAQHEQPEDGCPFGALT
QRLLQALVEENIISPMEDSPIPDMSGKESGADGASTSPRNQNKPFSVPHTKSLESRIKEELIAQGLLESEDRPAEDSEDEVLAELRK
RQAELKALSAHNRTKKHDLLRLAKEEVSRQELRQRVRMADNEVMDAFRKIMAARQKKRTPTKKEKDQAWKTLKERESILKLLDG
>sp|Q9NQ90|ANO2_HUMAN
MATPGPRDIPLLPGSPRRLSPQAGSRGGQGPKHGQQCLKMPGPRAPGLQGGSNRDPGQPCGGESTRSSSVINNYLDANEPVSLEARL
SRMHFHDSQRKVDYVLAYHYRKRGVHLAQGFPGHSLAIVSNGETGKEPHAGGPGDIELGPLDALEEERKEQREEFEHNLMEAGLELE
KDLENKSQGSIFVRIHAPWQVLAREAEFLKIKVPTKKEMYEIKAGGSIAKKFSAALQKLSSHLQPRVPEHSNNKMKNLSYPFSREKM
YLYNIQEKDTFFDNATRSRIVHEILKRTACSRANNTMGINSLIANNIYEAAYPLHDGEYDSPEDDMNDRKLLYQEWARYGVFYKFQP
IDLIRKYFGEKIGLYFAWLGLYTSFLIPSSVIGVIVFLYGCATIEEDIPSREMCDQQNAFTMCPLCDKSCDYWNLSSACGTAQASHL
FDNPATVFFSIFMALWATMFLENWKRLQMRLGYFWDLTGIEEEEERAQEHSRPEYETKVREKMLKESNQSAVQKLETNTTECGDEDD
EDKLTWKDRFPGYLMNFASILFMIALTFSIVFGVIVYRITTAAALSLNKATRSNVRVTVTATAVIINLVVILILDEIYGAVAKWLTK
IEVPKTEQTFEERLILKAFLLKFVNAYSPIFYVAFFKGRFVGRPGSYVYVFDGYRMEECAPGGCLMELCIQLSIIMLGKQLIQNNIF
EIGVPKLKKLFRKLKDETEAGETDSAHSKHPEQWDLDYSLEPYTGLTPEYMEMIIQFGFVTLFVASFPLAPVFALLNNVIEVRLDAK
KFVTELRRPDAVRTKDIGIWFDILSGIGKFSVISNAFVIAITSDFIPRLVYQYSYSHNGTLHGFVNHTLSFFNVSQLKEGTQPENSQ
FDQEVQFCRFKDYREPPWAPNPYEFSKQYWFILSARLAFVIIFQNLVMFLSVLVDWMIPDIPTDISDQIKKEKSLLVDFFLKEEHEK
LKLMDEPALRSPGGGDRSRSRAASSAPSGQSQLGSMMSSGSQHTNV
>sp|A6NMN3|F170B_HUMAN
MKCYFTDHRGEQSPTDGTTLSLTSPESTEESVEVFWPGTIQREGSSPRPGPAIPREEGLYFAARDRGMRDWSSSPSSESSEYQSYSQ
YQSCCSCMCDEDNAAPQSVCAFYTHVQTVRGVAVAWETEAGFEPVTRKPRIHEAQFIKRQRWNGSSFEMASNTDMRWDLEACKSNCS
PEPEDIDLLECCLQELREPPDWLVTTNYGVRCVACCRVLPSLDALLEHAQHGIREGFSCQIFFEEMLERRRAQGQAHDQQLEEEQSP
SDNSECSRPQGEVLSAQQQEKQ
>sp|A6NIV6|LRIQ4_HUMAN
MSKDIKSVEHSPKIHQRNDPQHVNDRTFFIDASNQSLTAIPLEIFTFTELEEVHLENNQIEEIPQEIQRLKNIRVLYLDKNNLRSLC
PALGLLSSLESLDLSYNPIFSSSLVVVSFLHALRELRLYQTDLKEIPVVIFKNLHHLELLGLTGNHLKCLPKEIVNQTKLREIYLKR
NQFEVFPQELCVLYTLEIIDLDENKIGAIPEEIGHLTGLQKFYMASNNLPVLPASLCQCSQLSVLDLSHNLLHSIPKSFAELRKMTE
IGLSGNRLEKVPRLICRWTSLHLLYLGNTGLHRLRGSFRCLVNLRFLDLSQNHLHHCPLQICALKNLEVLGLDDNKIGQLPSELGSL
SKLKILGLTGNEFLSFPEEVLSLASLEKLYIGQDQGFKLTYVPEHIRKLQSLKELYIENNHLEYLPVSLGSMPNLEVLDCRHNLLKQ
LPDAICQAQALKELRLEDNLLTHLPENLDSLVNLKVLTLMDNPMEEPPKEVCAEGNEAIWKYLKENRNRNIMATKIQAWWRGTMVQR
GFGKFGELLKPQKKGKTSPKDKKGKKDVKGKPGKGKKK
>sp|Q14264|ENR1_HUMAN
MLGMNMLLITLFLLLPLSMLKGEPWEGCLHCTHTTWSGNIMTKTLLYHTYYECAGTCLGTCTHNQTTYSVCDPGRGQPYVCYDPKSS
PGTWFEIHVGSKEGDLLNQTKVFPSGKDVVSLYFDVCQIVSMGSLFPVIFSSMEYYSSCHKNRYAHPACSTDSPVTTCWDCTTWSTN
QQSLGPIMLTKIPLEPDCKTSTCNSVNLTILEPDQPIWTTGLKAPLGARVSGEEIGPGAYVVLYIIKKTRTRSTQQFRVFESFYEHV
NQKLPEPPPLASNLFAQLAENIASSLHVASCYVCGGMNMGDQWPWEARELMPQDNFTLTASSLEPAPSSQSIWFLKTSIIGKFCIAR
WGKAFTDPVGELTCLGQQYYNETLGKTLWRGKSNNSESPHPSPFSRFPSLNHSWYQLEAPNTWQAPSGLYWICGPQAYRQLPAKWSG
ACVLGTIRPSFFLMPLKQGEALGYPIYDETKRKSKRGITIGDWKDNEWPPERIIQYYGPATWAEDGMWGYRTPVYMLNRIIRLQAVL
EIITNETAGALNLLAQQATKMRNVIYQNRLALDYLLAQEEGVCGKFNLTNCCLELDDEGKVIKEITAKIQKLAHIPVQTWKG
>sp|P17023|ZNF19_HUMAN
MAAMPLKAQYQEMVTFEDVAVHFTKTEWTGLSPAQRALYRSVMLENFGNLTALGYPVPKPALISLLERGDMAWGLEAQDDPPAERTK
NVCKDVETNIDSESTLIQGISEERDGMMSHGQLKSVPQRTDFPETRNVEKHQDIPTVKNIQGKVPRIPCARKPFICEECGKSFSYFS
YYARHQRIHTGEKPFECSECGKAFNGNSSLIRHQRIHTGERPYQCEECGRAFNDNANLIRHQRIHSGDRPYYCTECGNSFTSSSEFV

IHQRIHTGEKPYECNECGKAFVGNSPLLRHQKIHTGEKPYECNECGKSFGRTSHLSQHQRIHTGEKPYSCKVCGQAFNFHTKLTRHQ
RIHSEEKPFDCVDCGKAFSAQEQLKRHLRIHTQESSYVCDECGKALTSKRNLHQHQRIHTGEKPYECSKYEKAFGTSSQLGHLEHVY
SGEKPVLDICRFGLPEFFTPFYW
>sp|P63220|RS21_HUMAN
MQNDAGEFVDLYVPRKCSASNRIIGAKDHASIQMNVAEVDKVTGRFNGQFKTYAICGAIRRMGESDDSILRLAKADGIVSKNF
>sp|Q14781|CBX2_HUMAN
MEELSSVGEQVFAAECILSKRLRKGKLEYLVKWRGWSSKHNSWEPEENILDPRLLLAFQKKEHEKEVQNRKRGKRPRGRPRKLTAMS
SCSRRSKLKEPDAPSKSKSSSSSSSSTSSSSSSDEEDDSDLDAKRGPRGRETHPVPQKKAQILVAKPELKDPIRKKRGRKPLPPEQK
ATRRPVSLAKVLKTARKDLGAPASKLPPPLSAPVAGLAALKAHAKEACGGPSAMATPENLASLMKGMASSPGRGGISWQSSIVHYMN
RMTQSQAQAASRLALKAQATNKCGLGLDLKVRTQKGELGMSPPGSKIPKAPSGGAVEQKVGNTGGPPHTHGASRVPAGCPGPQPAPT
QELSLQVLDLQSVKNGMPGVGLLARHATATKGVPATNPAPGKGTGSGLIGASGATMPTDTSKSEKLASRAVAPPTPASKRDCVKGSA
TPSGQESRTAPGEARKAATLPEMSAGEESSSSDSDPDSASPPSTGQNPSVSVQTSQDWKPTRSLIEHVFVTDVTANLITVTVKESPT
SVGFFNLRHY
>sp|Q14739|LBR_HUMAN
MPSRKFADGEVVRGRWPGSSLYYEVEILSHDSTSQLYTVKYKDGTELELKENDIKPLTSFRQRKGGSTSSSPSRRRGSRSRSRSRSP
GRPPKSARRSASASHQADIKEARREVEVKLTPLILKPFGNSISRYNGEPEHIERNDAPHKNTQEKFSLSQESSYIATQYSLRPRREE
VKLKEIDSKEEKYVAKELAVRTFEVTPIRAKDLEFGGVPGVFLIMFGLPVFLFLLLLMCKQKDPSLLNFPPPLPALYELWETRVFGV
YLLWFLIQVLFYLLPIGKVVEGTPLIDGRRLKYRLNGFYAFILTSAVIGTSLFQGVEFHYVYSHFLQFALAATVFCVVLSVYLYMRS
LKAPRNDLSPASSGNAVYDFFIGRELNPRIGTFDLKYFCELRPGLIGWVVINLVMLLAEMKIQDRAVPSLAMILVNSFQLLYVVDAL
WNEEALLTTMDIIHDGFGFMLAFGDLVWVPFIYSFQAFYLVSHPNEVSWPMASLIIVLKLCGYVIFRGANSQKNAFRKNPSDPKLAH
LKTIHTSTGKNLLVSGWWGFVRHPNYLGDLIMALAWSLPCGFNHILPYFYIIYFTMLLVHREARDEYHCKKKYGVAWEKYCQRVPYR
IFPYIY
>sp|Q5VUY2|ADCL4_HUMAN
MAVPWLVLLLALPIFFLGVFVWAVFEHFLTTDIPATLQHPAKLRFLHCIFLYLVTLGNIFEKLGICSMPKFIRFLHDSVRIKKDPEL
VVTDLRFGTIPVRLFQPKAASSRPRRGIIFYHGGATVFGSLDCYHGLCNYLARETESVLLMIGYRKLPDHHSPALFQDCMNASIHFL
KALETYGVDPSRVVVCGESVGGAAVAAITQALVGRSDLPRIRAQVLIYPVVQAFCLQLPSFQQNQNVPLLSRKFMVTSLCNYLAIDL
SWRDAILNGTCVPPDVWRKYEKWLSPDNIPKKFKNRGYQPWSPGPFNEAAYLEAKHMLDVENSPLIADDEVIAQLPEAFLVSCENDI
LRDDSLLYKKRLEDQGVRVTWYHLYDGFHGSIIFFDKKALSFPCSLKIVNAVVSYIKGI
>sp|Q9H845|ACAD9_HUMAN
MSGCGLFLRTTAAARACRGLVVSTANRRLLRTSPPVRAFAKELFLGKIKKKEVFPFPEVSQDELNEINQFLGPVEKFFTEEVDSRKI
DQEGKIPDETLEKLKSLGLFGLQVPEEYGGLGFSNTMYSRLGEIISMDGSITVTLAAHQAIGLKGIILAGTEEQKAKYLPKLASGEH
IAAFCLTEPASGSDAASIRSRATLSEDKKHYILNGSKVWITNGGLANIFTVFAKTEVVDSDGSVKDKITAFIVERDFGGVTNGKPED
KLGIRGSNTCEVHFENTKIPVENILGEVGDGFKVAMNILNSGRFSMGSVVAGLLKRLIEMTAEYACTRKQFNKRLSEFGLIQEKFAL
MAQKAYVMESMTYLTAGMLDQPGFPDCSIEAAMVKVFSSEAAWQCVSEALQILGGLGYTRDYPYERILRDTRILLIFEGTNEILRMY
IALTGLQHAGRILTTRIHELKQAKVSTVMDTVGRRLRDSLGRTVDLGLTGNHGVVHPSLADSANKFEENTYCFGRTVETLLLRFGKT
IMEEQLVLKRVANILINLYGMTAVLSRASRSIRIGLRNHDHEVLLANTFCVEAYLQNLFSLSQLDKYAPENLDEQIKKVSQQILEKR
AYICAHPLDRTC
>sp|Q9UEE9|CFDP1_HUMAN
MEEFDSEDFSTSEEDEDYVPSGGEYSEDDVNELVKEDEVDGEEQTQKTQGKKRKAQSIPARKRRQGGLSLEEEEEEDANSESEGSSS
EEEDDAAEQEKGIGSEDARKKKEDELWASFLNDVGPKSKVPPSTQVKKGEETEETSSSKLLVKAEELEKPKETEKVKITKVFDFAGE
EVRVTKEVDATSKEAKSFFKQNEKEKPQANVPSALPSLPAGSGLKRSSGMSSLLGKIGAKKQKMSTLEKSKLDWESFKEEEGIGEEL
AIHNRGKEGYIERKAFLDRVDHRQFEIERDLRLSKMKP
>sp|Q9BX10|GTPB2_HUMAN

MDSRVSELFGGCCRPGGGPAVGGTLKARGAGSSSGCGGPKGKKKNGRNRGGKANNPPYLPPEAEDGNIEYKLKLVNPSQYRFEHLVT
QMKWRLQEGRGEAVYQIGVEDNGLLVGLAEEEMRASLKTLHRMAEKVGADITVLREREVDYDSDMPRKITEVLVRKVPDNQQFLDLR
VAVLGNVDSGKSTLLGVLTQGELDNGRGRARLNLFRHLHEIQSGRTSSISFEILGFNSKGEVVNYSDSRTAEEICESSSKMITFIDL
AGHHKYLHTTIFGLTSYCPDCALLLVSANTGIAGTTREHLGLALALKVPFFIVVSKIDLCAKTTVERTVRQLERVLKQPGCHKVPML
VTSEDDAVTAAQQFAQSPNVTPIFTLSSVSGESLDLLKVFLNILPPLTNSKEQEELMQQLTEFQVDEIYTVPEVGTVVGGTLSSGIC
REGDQLVVGPTDDGCFLELRVCSIQRNRSACRVLRAGQAATLALGDFDRALLRKGMVMVSPEMNPTICSVFEAEIVLLFHATTFRRG
FQVTVHVGNVRQTAVVEKIHAKDKLRTGEKAVVRFRFLKHPEYLKVGAKLLFREGVTKGIGHVTDVQAITAGEAQANMGF
>sp|Q6ZN19|ZN841_HUMAN
MLEGHESYDTENFYFREIRKNLQEVDFQWKDGEINYKEGPMTHKNNLTGQRVRHSQGDVENKHMENQLILRFQSGLGELQKFQTAEK
IYGCNQIERTVNNCFLASPLQRIFPGVQTNISRKYGNDFLQLSLPTQDEKTHIREKPYIGNECGKAFRVSSSLINHQMIHTTEKPYR
CNESGKAFHRGSLLTVHQIVHTRGKPYQCDVCGRIFRQNSDLVNHRRSHTGDKPYICNECGKSFSKSSHLAVHQRIHTGEKPYKCNR
CGKCFSQSSSLATHQTVHTGDKPYKCNECGKTFKRNSSLTAHHIIHAGKKPYTCDVCGKVFYQNSQLVRHQIIHTGETPYKCNECGK
VFFQRSRLAGHRRIHTGEKPYKCNECGKVFSQHSHLAVHQRVHTGEKPYKCNECGKAFNWGSLLTVHQRIHTGEKPYKCNVCGKVFN
YGGYLSVHMRCHTGEKPLHCNKCGMVFTYYSCLARHQRMHTGEKPYKCNVCGKVFIDSGNLSIHRRSHTGEKPFQCNECGKVFSYYS
CLARHRKIHTGEKPYKCNDCGKAYTQRSSLTKHLVIHTGENPYHCNEFGEAFIQSSKLARYHRNPTGEKPHKCSECGRTFSHKTSLV
YHQRRHTGEMPYKCIECGKVFNSTTTLARHRRIHTGEKPYKCNECGKVFRYRSGLARHWSIHTGEKPYKCNECGKAFRVRSILLNHQ
MMHTGEKPYKCNECGKAFIERSNLVYHQRNHTGEKPYKCMECGKAFGRRSCLTKHQRIHSSEKPYKCNECGKSYISRSGLTKHQIKH
AGENLTTKLNVERPLDVVLTSGIPK
>sp|Q8IYE1|CCD13_HUMAN
MAADESSQNTLRLQFKAMQEMQHKRLQKQMEKKREKELSLKSRADDQEEPLEVSDGLSLLHAGEPNSKNSFEKRVLEDEIEHLRNEL
RETVDENGRLYKLLKERDFEIKHLKKKIEEDRFAFTGTAGVAGDVVATKIVELSKKNRLLMAESEGAKTRVKQLTNRIQELERELQT
ALTRLSAKGATDAGAKPPRAQMGDRALLETPEVKALQDRLVATNLKMSDLRNQIQSVKQELRMAQKVLAREVGEDINVQQLLSSPGT
WRGRAQQILVLQSKVQELEKQLGQARSQSAGTASDELSVYPDPRKLSAQEKNLLRIRSLEREKQEGLEKLASERDVLQRELEELKKK
FEGMRSRNKLLSSEMKTLKSQMGTLVEKGRHDDELIDALMDQLKQLQEILGSLSLQEEKTRVSQHHLDQQLNSEAQRSNSLVAQLQA
MVAEREAKVRQLEMEIGQLNVHYLRNKGVGEGSSGREVSPAYTQFLEDPGLTKSPASAGDHVGRLGSSRSVTSLGHTLVESALTRPS
LPSPHRTSPRFSDSPEQKGWQAQVSEIKALWQAAEVERDRLTEFVTVLQKRVEESNSKLLESERKLQEERHRTVVLEQHLEKIRLEP
GKASASQRAAPRTKTGLPTSNNRHNPTGSEKKDPSFAQLSDVPVESQMEELTTRLAIQVEENEMLKAALGSALRGKEEDFRMYHEIL
GQVKSVFLQALRQQKTGKQ
>sp|P60014|KR10A_HUMAN
MAASTMSICSSACTDSWRVVDCPESCCEPCCCAPAPSLTLVCTPVSCVSSPCCQTACEPSACQSGYTSSCTTPCYQQSSCQPDCCTS
SPCQQACCVPVCCVPVCCVPVCNKPVCFVPTCSESSPSCCQQSSCQPTCCTSSPCQQACCVPVCSKSVCYVPVCSGASTSCCQQSSC
QPACCTASCCRPSSSVSLLCHPVCKSTCCVPVPSCGASASSCQPSCCRTASCVSLLCRPVCSRPACYSLCSGQKSSC
>sp|Q96PZ0|PUS7_HUMAN
MEMTEMTGVSLKRGALVVEDNDSGVPVEETKKQKLSECSLTKGQDGLQNDFLSISEDVPRPPDTVSTGKGGKNSEAQLEDEEEEEED
GLSEECEEEESESFADMMKHGLTEADVGITKFVSSHQGFSGILKERYSDFVVHEIGKDGRISHLNDLSIPVDEEDPSEDIFTVLTAE
EKQRLEELQLFKNKETSVAIEVIEDTKEKRTIIHQAIKSLFPGLETKTEDREGKKYIVAYHAAGKKALANPRKHSWPKSRGSYCHFV
LYKENKDTMDAINVLSKYLRVKPNIFSYMGTKDKRAITVQEIAVLKITAQRLAHLNKCLMNFKLGNFSYQKNPLKLGELQGNHFTVV
LRNITGTDDQVQQAMNSLKEIGFINYYGMQRFGTTAVPTYQVGRAILQNSWTEVMDLILKPRSGAEKGYLVKCREEWAKTKDPTAAL
RKLPVKRCVEGQLLRGLSKYGMKNIVSAFGIIPRNNRLMYIHSYQSYVWNNMVSKRIEDYGLKPVPGDLVLKGATATYIEEDDVNNY
SIHDVVMPLPGFDVIYPKHKIQEAYREMLTADNLDIDNMRHKIRDYSLSGAYRKIIIRPQNVSWEVVAYDDPKIPLFNTDVDNLEGK
TPPVFASEGKYRALKMDFSLPPSTYATMAIREVLKMDTSIKNQTQLNTTWLR
>sp|O76075|DFFB_HUMAN
MLQKPKSVKLRALRSPRKFGVAGRSCQEVLRKGCLRFQLPERGSRLCLYEDGTELTEDYFPSVPDNAELVLLTLGQAWQGYVSDIRR
FLSAFHEPQVGLIQAAQQLLCDEQAPQRQRLLADLLHNVSQNIAAETRAEDPPWFEGLESRFQSKSGYLRYSCESRIRSYLREVSSY

PSTVGAEAQEEFLRVLGSMCQRLRSMQYNGSYFDRGAKGGSRLCTPEGWFSCQGPFDMDSCLSRHSINPYSNRESRILFSTWNLDHI
IEKKRTIIPTLVEAIKEQDGREVDWEYFYGLLFTSENLKLVHIVCHKKTTHKLNCDPSRIYKPQTRLKRKQPVRKRQ
>sp|Q70J99|UN13D_HUMAN
MATLLSHPQQRPPFLRQAIKIRRRRVRDLQDPPPQMAPEIQPPSHHFSPEQRALLYEDALYTVLHRLGHPEPNHVTEASELLRYLQE
AFHVEPEEHQQTLQRVRELEKPIFCLKATVKQAKGILGKDVSGFSDPYCLLGIEQGVGVPGGSPGSRHRQKAVVRHTIPEEETHRTQ
VITQTLNPVWDETFILEFEDITNASFHLDMWDLDTVESVRQKLGELTDLHGLRRIFKEARKDKGQDDFLGNVVLRLQDLRCREDQWY
PLEPRTETYPDRGQCHLQFQLIHKRRATSASRSQPSYTVHLHLLQQLVSHEVTQHEAGSTSWDGSLSPQAATVLFLHATQKDLSDFH
QSMAQWLAYSRLYQSLEFPSSCLLHPITSIEYQWIQGRLKAEQQEELAASFSSLLTYGLSLIRRFRSVFPLSVSDSPARLQSLLRVL
VQMCKMKAFGELCPNTAPLPQLVTEALQTGTTEWFHLKQQHHQPMVQGIPEAGKALLGLVQDVIGDLHQCQRTWDKIFHNTLKIHLF
SMAFRELQWLVAKRVQDHTTVVGDVVSPEMGESLFQLYISLKELCQLRMSSSERDGVLALDNFHRWFQPAIPSWLQKTYNEALARVQ
RAVQMDELVPLGELTKHSTSAVDLSTCFAQISHTARQLDWPDPEEAFMITVKFVEDTCRLALVYCSLIKARARELSSGQKDQGQAAN
MLCVVVNDMEQLRLVIGKLPAQLAWEALEQRVGAVLEQGQLQNTLHAQLQSALAGLGHEIRTGVRTLAEQLEVGIAKHIQKLVGVRE
SVLPEDAILPLMKFLEVELCYMNTNLVQENFSSLLTLLWTHTLTVLVEAAASQRSSSLASNRLKIALQNLEICFHAEGCGLPPKALH
TATFQALQRDLELQAASSRELIRKYFCSRIQQQAETTSEELGAVTVKASYRASEQKLRVELLSASSLLPLDSNGSSDPFVQLTLEPR
HEFPELAARETQKHKKDLHPLFDETFEFLVPAEPCRKAGACLLLTVLDYDTLGADDLEGEAFLPLREVPGLSGSEEPGEVPQTRLPL
TYPAPNGDPILQLLEGRKGDREAQVFVRLRRHRAKQASQHALRPAP
>sp|Q9BT30|ALKB7_HUMAN
MAGTGLLALRTLPGPSWVRGSGPSVLSRLQDAAVVRPGFLSTAEEETLSRELEPELRRRRYEYDHWDAAIHGFRETEKSRWSEASRA
ILQRVQAAAFGPGQTLLSSVHVLDLEARGYIKPHVDSIKFCGATIAGLSLLSPSVMRLVHTQEPGEWLELLLEPGSLYILRGSARYD
FSHEILRDEESFFGERRIPRGRRISVICRSLPEGMGPGESGQPPPAC
>sp|Q9UQQ1|NALDL_HUMAN
MQWTKVLGLGLGAAALLGLGIILGHFAIPKKANSLAPQDLDLEILETVMGQLDAHRIRENLRELSREPHLASSPRDEDLVQLLLQRW
KDPESGLDSAEASTYEVLLSFPSQEQPNVVDIVGPTGGIIHSCHRTEENVTGEQGGPDVVQPYAAYAPSGTPQGLLVYANRGAEEDF
KELQTQGIKLEGTIALTRYGGVGRGAKAVNAAKHGVAGVLVYTDPADINDGLSSPDETFPNSWYLPPSGVERGSYYEYFGDPLTPYL
PAVPSSFRVDLANVSGFPPIPTQPIGFQDARDLLCNLNGTLAPATWQGALGCHYRLGPGFRPDGDFPADSQVNVSVYNRLELRNSSN
VLGIIRGAVEPDRYVLYGNHRDSWVHGAVDPSSGTAVLLELSRVLGTLLKKGTWRPRRSIVFASWGAEEFGLIGSTEFTEEFFNKLQ
ERTVAYINVDISVFANATLRVQGTPPVQSVVFSATKEIRSPGPGDLSIYDNWIRYFNRSSPVYGLVPSLGSLGAGSDYAPFVHFLGI
SSMDIAYTYDRSKTSARIYPTYHTAFDTFDYVDKFLDPGFSSHQAVARTAGSVILRLSDSFFLPLKVSDYSETLRSFLQAAQQDLGA
LLEQHSISLGPLVTAVEKFEAEAAALGQRISTLQKGSPDPLQVRMLNDQLMLLERTFLNPRAFPEERYYSHVLWAPRTGSVVTFPGL
SNACSRARDTASGSEAWAEVQRQLSIVVTALEGAAATLRPVADL
>sp|A6NDH6|O5H15_HUMAN
MEEENATLLTEFVLTGFLYQPQWKIPLFLAFLVIYLITIMGNLGLIAVIWKDPHLHIPMYLLLGNLAFVDAWISSTVTPKMLNNFLA
KSKMISLSECKIQFFSIAIGVTTECFLLATMAYDRYVAICKPLLYPAIMTNGLCIRLLILSYIAGILHALIHEGFLFRLTFCNSNIV
HHIYCDTIPLSKISCTDSSINFLMVFIFSGSIQVFSIVTILISYTFVLFTVLEKKSDKGVRKAFSTCGAHLFSVCLYYGPLLLMYVG
PASPQADGQNMVEPLFYTVIIPLLNPIIYSLRNKQVIVSFIKMLKRNVKVSY
>sp|P0C879|YJ018_HUMAN
MYNPWQVGASLAPARAGPRPFPTPRARPPDCSGGHAPSGFPALGLRTAQRPRPFSSSPRSASGRLRGPRPVARGPAHSSSPTGLPAY
LPPAAALDSQTSAPTVSPVTRPRVVARGKTPRVSLAGLETLSSLLHQQQLFD
>sp|O00303|EIF3F_HUMAN
MATPAVPVSAPPATPTPVPAAAPASVPAPTPAPAAAPVPAAAPASSSDPAAAAAATAAPGQTPASAQAPAQTPAPALPGPALPGPFP
GGRVVRLHPVILASIVDSYERRNEGAARVIGTLLGTVDKHSVEVTNCFSVPHNESEDEVAVDMEFAKNMYELHKKVSPNELILGWYA
TGHDITEHSVLIHEYYSREAPNPIHLTVDTSLQNGRMSIKAYVSTLMGVPGRTMGVMFTPLTVKYAYYDTERIGVDLIMKTCFSPNR
VIGLSSDLQQVGGASARIQDALSTVLQYAEDVLSGKVSADNTVGRFLMSLVNQVPKIVPDDFETMLNSNINDLLMVTYLANLTQSQI
ALNEKLVNL

>sp|P25789|PSA4_HUMAN
MSRRYDSRTTIFSPEGRLYQVEYAMEAIGHAGTCLGILANDGVLLAAERRNIHKLLDEVFFSEKIYKLNEDMACSVAGITSDANVLT
NELRLIAQRYLLQYQEPIPCEQLVTALCDIKQAYTQFGGKRPFGVSLLYIGWDKHYGFQLYQSDPSGNYGGWKATCIGNNSAAAVSM
LKQDYKEGEMTLKSALALAIKVLNKTMDVSKLSAEKVEIATLTRENGKTVIRVLKQKEVEQLIKKHEEEEAKAEREKKEKEQKEKDK
>sp|O75747|P3C2G_HUMAN
MAYSWQTDPNPNESHEKQYEHQEFLFVNQPHSSSQVSLGFDQIVDEISGKIPHYESEIDENTFFVPTAPKWDSTGHSLNEAHQISLN
EFTSKSRELSWHQVSKAPAIGFSPSVLPKPQNTNKECSWGSPIGKHHGADDSRFSILAPSFTSLDKINLEKELENENHNYHIGFESS
IPPTNSSFSSDFMPKEENKRSGHVNIVEPSLMLLKGSLQPGMWESTWQKNIESIGCSIQLVEVPQSSNTSLASFCNKVKKIRERYHA
ADVNFNSGKIWSTTTAFPYQLFSKTKFNIHIFIDNSTQPLHFMPCANYLVKDLIAEILHFCTNDQLLPKDHILSVCGSEEFLQNDHC
LGSHKMFQKDKSVIQLHLQKSREAPGKLSRKHEEDHSQFYLNQLLEFMHIWKVSRQCLLTLIRKYDFHLKYLLKTQENVYNIIEEVK
KICSVLGCVETKQITDAVNELSLILQRKGENFYQSSETSAKGLIEKVTTELSTSIYQLINVYCNSFYADFQPVNVPRCTSYLNPGLP
SHLSFTVYAAHNIPETWVHRINFPLEIKSLPRESMLTVKLFGIACATNNANLLAWTCLPLFPKEKSILGSMLFSMTLQSEPPVEMIT
PGVWDVSQPSPVTLQIDFPATGWEYMKPDSEENRSNLEEPLKECIKHIARLSQKQTPLLLSEEKKRYLWFYRFYCNNENCSLPLVLG
SAPGWDERTVSEMHTILRRWTFSQPLEALGLLTSSFPDQEIRKVAVQQLDNLLNDELLEYLPQLVQAVKFEWNLESPLVQLLLHRSL
QSIQVAHRLYWLLKNAENEAYFKSWYQKLLAALQFCAGKALNDEFSKEQKLIKILGDIGERVKSASDHQRQEVLKKEIGRLEEFFQD
VNTCHLPLNPALCIKGIDHDACSYFTSNALPLKITFINANPMGKNISIIFKAGDDLRQDMLVLQLIQVMDNIWLQEGLDMQMIIYRC
LSTGKDQGLVQMVPDAVTLAKIHRHSGLIGPLKENTIKKWFSQHNHLKADYEKALRNFFYSCAGWCVVTFILGVCDRHNDNIMLTKS
GHMFHIDFGKFLGHAQTFGGIKRDRAPFIFTSEMEYFITEGGKNPQHFQDFVELCCRAYNIIRKHSQLLLNLLEMMLYAGLPELSGI
QDLKYVYNNLRPQDTDLEATSHFTKKIKESLECFPVKLNNLIHTLAQMSAISPAKSTSQTFPQESCLLSTTRSIERATILGFSKKSS
NLYLIQVTHSNNETSLTEKSFEQFSKLHSQLQKQFASLTLPEFPHWWHLPFTNSDHRRFRDLNHYMEQILNVSHEVTNSDCVLSFFL
SEAVQQTVEESSPVYLGEKFPDKKPKVQLVISYEDVKLTILVKHMKNIHLPDGSAPSAHVEFYLLPYPSEVRRRKTKSVPKCTDPTY
NEIVVYDEVTELQGHVLMLIVKSKTVFVGAINIRLCSVPLDKEKWYPLGNSII
>sp|O15160|RPAC1_HUMAN
MAASQAVEEMRSRVVLGEFGVRNVHTTDFPGNYSGYDDAWDQDRFEKNFRVDVVHMDENSLEFDMVGIDAAIANAFRRILLAEVPTM
AVEKVLVYNNTSIVQDEILAHRLGLIPIHADPRLFEYRNQGDEEGTEIDTLQFRLQVRCTRNPHAAKDSSDPNELYVNHKVYTRHMT
WIPLGNQADLFPEGTIRPVHDDILIAQLRPGQEIDLLMHCVKGIGKDHAKFSPVATASYRLLPDITLLEPVEGEAAEELSRCFSPGV
IEVQEVQGKKVARVANPRLDTFSREIFRNEKLKKVVRLARVRDHYIFSVESTGVLPPDVLVSEAIKVLMGKCRRFLDELDAVQMD
>sp|P57682|KLF3_HUMAN
MLMFDPVPVKQEAMDPVSVSYPSNYMESMKPNKYGVIYSTPLPEKFFQTPEGLSHGIQMEPVDLTVNKRSSPPSAGNSPSSLKFPSS
HRRASPGLSMPSSSPPIKKYSPPSPGVQPFGVPLSMPPVMAAALSRHGIRSPGILPVIQPVVVQPVPFMYTSHLQQPLMVSLSEEME
NSSSSMQVPVIESYEKPISQKKIKIEPGIEPQRTDYYPEEMSPPLMNSVSPPQALLQENHPSVIVQPGKRPLPVESPDTQRKRRIHR
CDYDGCNKVYTKSSHLKAHRRTHTGEKPYKCTWEGCTWKFARSDELTRHFRKHTGIKPFQCPDCDRSFSRSDHLALHRKRHMLV
>sp|Q3KQU3|MA7D1_HUMAN
MESGPRAELGAGAPPAVVARTPPEPRPSPEGDPSPPPPPMSALVPDTPPDTPPAMKNATSSKQLPLEPESPSGQVGPRPAPPQEESP
SSEAKSRGPTPPAMGPRDARPPRRSSQPSPTAVPASDSPPTKQEVKKAGERHKLAKERREERAKYLAAKKAVWLEKEEKAKALREKQ
LQERRRRLEEQRLKAEQRRAALEERQRQKLEKNKERYEAAIQRSVKKTWAEIRQQRWSWAGALHHSSPGHKTSGSRCSVSAVNLPKH
VDSIINKRLSKSSATLWNSPSRNRSLQLSAWESSIVDRLMTPTLSFLARSRSAVTLPRNGRDQGRGCDPGRGPTWGRAGASLARGPQ
PDRTHPSAAVPVCPRSASASPLTPCSVTRSVHRCAPAGERGERRKPNAGGSPAPVRRRPEASPVQKKEKKDKERENEKEKSALARER
SLKKRQSLPASPRARLSASTASELSPKSKARPSSPSTSWHRPASPCPSPGPGHTLPPKPPSPRGTTASPKGRVRRKEEAKESPSAAG
PEDKSQSKRRASNEKESAAPASPAPSPAPSPTPAPPQKEQPPAETPTDAAVLTSPPAPAPPVTPSKPMAGTTDREEATRLLAEKRRQ
AREQREREEQERRLQAERDKRMREEQLAREAEARAEREAEARRREEQEAREKAQAEQEEQERLQKQKEEAEARSREEAERQRLEREK
HFQQQEQERQERRKRLEEIMKRTRKSEVSETKQKQDSKEANANGSSPEPVKAVEARSPGLQKEAVQKEEPIPQEPQWSLPSKELPAS
LVNGLQPLPAHQENGFSTNGPSGDKSLSRTPETLLPFAEAEAFLKKAVVQSPQVTEVL
>sp|Q6UWP2|DHR11_HUMAN

MARPGMERWRDRLALVTGASGGIGAAVARALVQQGLKVVGCARTVGNIEELAAECKSAGYPGTLIPYRCDLSNEEDILSMFSAIRSQ
HSGVDICINNAGLARPDTLLSGSTSGWKDMFNVNVLALSICTREAYQSMKERNVDDGHIININSMSGHRVLPLSVTHFYSATKYAVT
ALTEGLRQELREAQTHIRATCISPGVVETQFAFKLHDKDPEKAAATYEQMKCLKPEDVAEAVIYVLSTPAHIQIGDIQMRPTEQVT
>sp|Q9BTE1|DCTN5_HUMAN
MELGELLYNKSEYIETASGNKVSRQSVLCGSQNIVLNGKTIVMNDCIIRGDLANVRVGRHCVVKSRSVIRPPFKKFSKGVAFFPLHI
GDHVFIEEDCVVNAAQIGSYVHVGKNCVIGRRCVLKDCCKILDNTVLPPETVVPPFTVFSGCPGLFSGELPECTQELMIDVTKSYYQ
KFLPLTQV
>sp|Q75VX8|GARE2_HUMAN
MEKLAAGLAGLRWSMGAFPLDLIVSRCRLPTLACLGPGEYAEGVSERDILLIHSCRQWTTVTAHTLEEGHYVIGPKIDIPLQYPGKF
KLLEQARDVREPVRYFSSVEEVASVFPDRIFVMEAITFSVKVVSGEFSEDSEVYNFTLHAGDELTLMGQAEILCAKTTKERSRFTTL
LRKLGRAGALAGVGGGGPASAGAAGGTGGGGARPVKGKMPCLICMNHRTNESLSLPFQCQGRFSTRSPLELQMQEGEHTVRAIIERV
RLPVNVLVPSRPPRNPYDLHPVREGHCYKLVSIISKTVVLGLALRREGPAPLHFLLLTDTPRFALPQGLLAGDPRVERLVRDSASYC
RERFDPDEYSTAVREAPAELAEDCASPRRARLCLPAPRAPGLARAPGPLAPAPAGEGDQEYVSPDWAAAPEPAAPPAEIPYEELWAH
QGPEGLVRPPPGLDLISFGAAGPPRREPEAPPPPVPPKSEAVKEECRLLNAPPVPPRGGNGSGRLSSSPPVPPRFPKLQPVHSPSSS
LSYYSSGLQDGAGSRSGSGSPSPDTYSLYCYPCTWGDCKVGESSSRPAPGPLPSTTQPSQASRALTEPLSGRAASLLGADTPVKTYH
SCPPLFKPSHPQKRFAPFGALNPFSGPAYPSGPSAALSSGPRTTSGPVATSGPAYSPGPASPGGQAYSAAPPSSCAPSSSSSSEWQEP
VLEPFDPFELGQGSSPEPELLRSQEPRAVGTPGPGPRLSPLGPSKAFEPEGLVLHQVPTPLSPAALQGPEAGGALFLTQGRLEGPPA
SPRDGATGFGVRDASSWQPPADLSALSLEEVSRSLRFIGLSEDVVSFFARERIDGSIFVQLSEDILADDFHLTKLQVKKIMQFIKGW
RPKI
>sp|Q9GZS9|CHST5_HUMAN
MGMRARVPKVAHSTRRPPAARMWLPRFSSKTVTVLLLAQTTCLLLFIISRPGPSSPAGGEDRVHVLVLSSWRSGSSFLGQLFSQHPD
VFYLMEPAWHVWTTLSQGSAATLHMAVRDLMRSIFLCDMDVFDAYMPQSRNLSAFFNWATSRALCSPPACSAFPRGTISKQDVCKTL
CTRQPFSLAREACRSYSHVVLKEVRFFNLQVLYPLLSDPALNLRIVHLVRDPRAVLRSREAAGPILARDNGIVLGTNGKWVEADPHL
RLIREVCRSHVRIAEAATLKPPPFLRGRYRLVRFEDLAREPLAEIRALYAFTGLTLTPQLEAWIHNITHGSGIGKPIEAFHTSSRNA
RNVSQAWRHALPFTKILRVQEVCAGALQLLGYRPVYSADQQRDLTLDLVLPRGPDHFSWASPD
>sp|Q9HCE5|MET14_HUMAN
MDSRLQEIRERQKLRRQLLAQQLGAESADSIGAVLNSKDEQREIAETRETCRASYDTSAPNAKRKYLDEGETDEDKMEEYKDELEMQ
QDEENLPYEEEIYKDSSTFLKGTQSLNPHNDYCQHFVDTGHRPQNFIRDVGLADRFEEYPKLRELIRLKDELIAKSNTPPMYLQADI
EAFDIRELTPKFDVILLEPPLEEYYRETGITANEKCWTWDDIMKLEIDEIAAPRSFIFLWCGSGEGLDLGRVCLRKWGYRRCEDICW
IKTNKNNPGKTKTLDPKAVFQRTKEHCLMGIKGTVKRSTDGDFIHANVDIDLIITEEPEIGNIEKPVEIFHIIEHFCLGRRRLHLFG
RDSTIRPGWLTVGPTLTNSNYNAETYASYFSAPNSYLTGCTEEIERLRPKSPPPKSKSDRGGGAPRGGGRGGTSAGRGRERNRSNFR
GERGGFRGGRGGAHRGGFPPR
>sp|P11047|LAMC1_HUMAN
MRGSHRAAPALRPRGRLWPVLAVLAAAAAAGCAQAAMDECTDEGGRPQRCMPEFVNAAFNVTVVATNTCGTPPEEYCVQTGVTGVTK
SCHLCDAGQPHLQHGAAFLTDYNNQADTTWWQSQTMLAGVQYPSSINLTLHLGKAFDITYVRLKFHTSRPESFAIYKRTREDGPWIP
YQYYSGSCENTYSKANRGFIRTGGDEQQALCTDEFSDISPLTGGNVAFSTLEGRPSAYNFDNSPVLQEWVTATDIRVTLNRLNTFGD
EVFNDPKVLKSYYYAISDFAVGGRCKCNGHASECMKNEFDKLVCNCKHNTYGVDCEKCLPFFNDRPWRRATAESASECLPCDCNGRS
QECYFDPELYRSTGHGGHCTNCQDNTDGAHCERCRENFFRLGNNEACSSCHCSPVGSLSTQCDSYGRCSCKPGVMGDKCDRCQPGFH
SLTEAGCRPCSCDPSGSIDECNIETGRCVCKDNVEGFNCERCKPGFFNLESSNPRGCTPCFCFGHSSVCTNAVGYSVYSISSTFQID
EDGWRAEQRDGSEASLEWSSERQDIAVISDSYFPRYFIAPAKFLGKQVLSYGQNLSFSFRVDRRDTRLSAEDLVLEGAGLRVSVPLI
AQGNSYPSETTVKYVFRLHEATDYPWRPALTPFEFQKLLNNLTSIKIRGTYSERSAGYLDDVTLASARPGPGVPATWVESCTCPVGY
GGQFCEMCLSGYRRETPNLGPYSPCVLCACNGHSETCDPETGVCNCRDNTAGPHCEKCSDGYYGDSTAGTSSDCQPCPCPGGSSCAV
VPKTKEVVCTNCPTGTTGKRCELCDDGYFGDPLGRNGPVRLCRLCQCSDNIDPNAVGNCNRLTGECLKCIYNTAGFYCDRCKDGFFG
NPLAPNPADKCKACNCNLYGTMKQQSSCNPVTGQCECLPHVTGQDCGACDPGFYNLQSGQGCERCDCHALGSTNGQCDIRTGQCECQ

PGITGQHCERCEVNHFGFGPEGCKPCDCHPEGSLSLQCKDDGRCECREGFVGNRCDQCEENYFYNRSWPGCQECPACYRLVKDKVAD
HRVKLQELESLIANLGTGDEMVTDQAFEDRLKEAEREVMDLLREAQDVKDVDQNLMDRLQRVNNTLSSQISRLQNIRNTIEETGNLA
EQARAHVENTERLIEIASRELEKAKVAAANVSVTQPESTGDPNNMTLLAEEARKLAERHKQEADDIVRVAKTANDTSTEAYNLLLRT
LAGENQTAFEIEELNRKYEQAKNISQDLEKQAARVHEEAKRAGDKAVEIYASVAQLSPLDSETLENEANNIKMEAENLEQLIDQKLK
DYEDLREDMRGKELEVKNLLEKGKTEQQTADQLLARADAAKALAEEAAKKGRDTLQEANDILNNLKDFDRRVNDNKTAAEEALRKIP
AINQTITEANEKTREAQQALGSAAADATEAKNKAHEAERIASAVQKNATSTKAEAERTFAEVTDLDNEVNNMLKQLQEAEKELKRKQ
DDADQDMMMAGMASQAAQEAEINARKAKNSVTSLLSIINDLLEQLGQLDTVDLNKLNEIEGTLNKAKDEMKVSDLDRKVSDLENEAK
KQEAAIMDYNRDIEEIMKDIRNLEDIRKTLPSGCFNTPSIEKP
>sp|P0CAP1|MYZAP_HUMAN
MLRSTSTVTLLSGGAARTPGAPSRRANVCRLRLTVPPESPVPEQCEKKIERKEQLLDLSNGEPTRKLPQGVVYGVVRRSDQNQQKEM
VVYGWSTSQLKEEMNYIKDVRATLEKVRKRMYGDYDEMRQKIRQLTQELSVSHAQQEYLENHIQTQSSALDRFNAMNSALASDSIGL
QKTLVDVTLENSNIKDQIRNLQQTYEASMDKLREKQRQLEVAQVENQLLKMKVESSQEANAEVMREMTKKLYSQYEEKLQEEQRKHS
AEKEALLEETNSFLKAIEEANKKMQAAEISLEEKDQRIGELDRLIERMEKERHQLQLQLLEHETEMSGELTDSDKERYQQLEEASAS
LRERIRHLDDMVHCQQKKVKQMVEEIESLKKKLQQKQLLILQLLEKISFLEGENNELQSRLDYLTETQAKTEVETREIGVGCDLLPS
QTGRTREIVMPSRNYTPYTRVLELTMKKTLT
>sp|Q9GZU5|NYX_HUMAN
MKGRGMLVLLLHAVVLGLPSAWAVGACARACPAACACSTVERGCSVRCDRAGLLRVPAELPCEAVSIDLDRNGLRFLGERAFGTLPS
LRRLSLRHNNLSFITPGAFKGLPRLAELRLAHNGDLRYLHARTFAALSRLRRLDLAACRLFSVPERLLAELPALRELAAFDNLFRRV
PGALRGLANLTHAHLERGRIEAVASSSLQGLRRLRSLSLQANRVRAVHAGAFGDCGVLEHLLLNDNLLAELPADAFRGLRRLRTLNL
GGNALDRVARAWFADLAELELLYLDRNSIAFVEEGAFQNLSGLLALHLNGNRLTVLAWVAFQPGFFLGRLFLFRNPWCCDCRLEWLR
DWMEGSGRVTDVPCASPGSVAGLDLSQVTFGRSSDGLCVDPEELNLTTSSPGPSPEPAATTVSRFSSLLSKLLAPRVPVEEAANTTG
GLANASLSDSLSSRGVGGAGRQPWFLLASCLLPSVAQHVVFGLQMD
>sp|P19338|NUCL_HUMAN
MVKLAKAGKNQGDPKKMAPPPKEVEEDSEDEEMSEDEEDDSSGEEVVIPQKKGKKAAATSAKKVVVSPTKKVAVATPAKKAAVTPGK
KAAATPAKKTVTPAKAVTTPGKKGATPGKALVATPGKKGAAIPAKGAKNGKNAKKEDSDEEEDDDSEEDEEDDEDEDEDEDEIEPAA
MKAAAAAPASEDEDDEDDEDDEDDDDDEEDDSEEEAMETTPAKGKKAAKVVPVKAKNVAEDEDEEEDDEDEDDDDDEDDEDDDDEDD
EEEEEEEEEEPVKEAPGKRKKEMAKQKAAPEAKKQKVEGTEPTTAFNLFVGNLNFNKSAPELKTGISDVFAKNDLAVVDVRIGMTRK
FGYVDFESAEDLEKALELTGLKVFGNEIKLEKPKGKDSKKERDARTLLAKNLPYKVTQDELKEVFEDAAEIRLVSKDGKSKGIAYIE
FKTEADAEKTFEEKQGTEIDGRSISLYYTGEKGQNQDYRGGKNSTWSGESKTLVLSNLSYSATEETLQEVFEKATFIKVPQNQNGKS
KGYAFIEFASFEDAKEALNSCNKREIEGRAIRLELQGPRGSPNARSQPSKTLFVKGLSEDTTEETLKESFDGSVRARIVTDRETGSS
KGFGFVDFNSEEDAKAAKEAMEDGEIDGNKVTLDWAKPKGEGGFGGRGGGRGGFGGRGGGRGGRGGFGGRGRGGFGGRGGFRGGRGG
GGDHKPQGKKTKFE
>sp|Q9HCJ0|TNR6C_HUMAN
MATGSAQGNFTGHTKKTNGNNGTNGALVQSPSNQSALGAGGANSNGSAARVWGVATGSSSGLAHCSVSGGDGKMDTMIGDGRSQNCW
GASNSNAGINLNLNPNANPAAWPVLGHEGTVATGNPSSICSPVSAIGQNMGNQNGNPTGTLGAWGNLLPQESTEPQTSTSQNVSFSA
QPQNLNTDGPNNTNPMNSSPNPINAMQTNGLPNWGMAVGMGAIIPPHLQGLPGANGSSVSQVSGGSAEGISNSVWGLSPGNPATGNS
NSGFSQGNGDTVNSALSAKQNGSSSAVQKEGSGGNAWDSGPPAGPGILAWGRGSGNNGVGNIHSGAWGHPSRSTSNGVNGEWGKPPN
QHSNSDINGKGSTGWESPSVTSQNPTVQPGGEHMNSWAKAASSGTTASEGSSDGSGNHNEGSTGREGTGEGRRRDKGIIDQGHIQLP
RNDLDPRVLSNTGWGQTPVKQNTAWEFEESPRSERKNDNGTEAWGCAATQASNSGGKNDGSIMNSTNTSSVSGWVNAPPAAVPANTG
WGDSNNKAPSGPGVWGDSISSTAVSTAAAAKSGHAWSGAANQEDKSPTWGEPPKPKSQHWGDGQRSNPAWSAGGGDWADSSSVLGHL
GDGKKNGSGWDADSNRSGSGWNDTTRSGNSGWGNSTNTKANPGTNWGETLKPGPQQNWASKPQDNNVSNWGGAASVKQTGTGWIGGP
VPVKQKDSSEATGWEEPSPPSIRRKMEIDDGTSAWGDPSNYNNKTVNMWDRNNPVIQSSTTTNTTTTTTTTTSNTTHRVETPPPHQA
GTQLNRSPLLGPGRKVSSGWGEMPNVHSKTENSWGEPSSPSTLVDNGTAAWGKPPSSGSGWGDHPAEPPVAFGRAGAPVAASALCKP
ASKSMQEGWGSGGDEMNLSTSQWEDEEGDVWNNAASQESTSSCSSWGNAPKKGLQKGMKTSGKQDEAWIMSRLIKQLTDMGFPREPA

EEALKSNNMNLDQAMSALLEKKVDVDKRGLGVTDHNGMAAKPLGCRPPISKESSVDRPTFLDKDGGLVEEPTPSPFLPSPSLKLPLS
HSALPSQALGGIASGLGMQNLNSSRQIPSGNLGMFGNSGAAQARTMQQPPQPPVQPLNSSQPSLRAQVPQFLSPQVQAQLLQFAAKN
IGLNPALLTSPINPQHMTMLNQLYQLQLAYQRLQIQQQMLQAQRNVSGSMRQQEQQVARTITNLQQQIQQHQRQLAQALLVKQPPPP
PPPPHLSLHPSAGKSAMDSFPSHPQTPGLPDLQTKEQQSSPNTFAPYPLAGLNPNMNVNSMDMTGGLSVKDPSQSQSRLPQWTHPNS
MDNLPSAASPLEQNPSKHGAIPGGLSIGPPGKSSIDDSYGRYDLIQNSESPASPPVAVPHSWSRAKSDSDKISNGSSINWPPEFHPG
VPWKGLQNIDPENDPDVTPGSVPTGPTINTTIQDVNRYLLKSGGKLSDIKSTWSSGPTSHTQASLSHELWKVPRNSTAPTRPPPGLT
NPKPSSTWGASPLGWTSSYSSGSAWSTDTSGRTSSWLVLRNLTPQIDGSTLRTLCLQHGPLITFHLNLTQGNAVVRYSSKEEAAKAQ
KSLHMCVLGNTTILAEFAGEEEVNRFLAQGQALPPTSSWQSSSASSQPRLSAAGSSHGLVRSDAGHWNAPCLGGKGSSELLWGGVPQ
YSSSLWGPPSADDSRVIGSPTPLTTLLPGDLLSGESL
>sp|Q7RTT4|SSX8_HUMAN
MNGDDAFAKRPRDDDKASEKRSKAFNDIATYFSKKEWEKMKYSEKISYVYMKRNYEAMTKLGFNVTLPPFMCNKQATDFQGNYFDND
RNRRIQVERPQMTFGRLQRIIPKIMPKKPAEEGNDSKGVSEASGPQNDGKQLAPGKANTSEKINKRSGPKRGRHAWTHRLRERNQLV
IYEEIRDPEEDDE
>sp|Q53HI1|UNC50_HUMAN
MLPSTSVNSLVQGNGVLNSRDAARHTAGAKRYKYLRRLFRFRQMDFEFAAWQMLYLFTSPQRVYRNFHYRKQTKDQWARDDPAFLVL
LSIWLCVSTIGFGFVLDMGFFETIKLLLWVVLIDCVGVGLLIATLMWFISNKYLVKRQSRDYDVEWGYAFDVHLNAFYPLLVILHFI
QLFFINHVILTDTFIGYLVGNTLWLVAVGYYIYVTFLGYSALPFLKNTVILLYPFAPLILLYGLSLALGWNFTHTLCSFYKYRVK
>sp|Q8TCQ1|MARH1_HUMAN
MLGWCEAIARNPHRIPNNTRTPEISGDLADASQTSTLNEKSPGRSASRSSNISKASSPTTGTAPRSQSRLSVCPSTQDICRICHCEG
DEESPLITPCRCTGTLRFVHQSCLHQWIKSSDTRCCELCKYDFIMETKLKPLRKWEKLQMTTSERRKIFCSVTFHVIAITCVVWSLY
VLIDRTAEEIKQGNDNGVLEWPFWTKLVVVAIGFTGGLVFMYVQCKVYVQLWRRLKAYNRVIFVQNCPDTAKKLEKNFSCNVNTDIK
DAVVVPVPQTGANSLPSAEGGPPEVVSV
>sp|Q7Z5L4|SPT19_HUMAN
MIITTWIVYILARKGVGLPFLPITSSDIDVVESEAVSVLHHWLKKTEEEASRGIKEKLSINHPSQGVREKMSTDSPPTHGQDIHVTR
DVVKHHLSKSDLLANQSQEVLEERTRIQFIRWSHTRIFQVPSEMTEDIMRDRIEQVRRSISRLTDVSAQDFSMRPSSSDC
>sp|Q8IXQ6|PARP9_HUMAN
MDFSMVAGAAAYNEKSGRITSLSLLFQKVFAQIFPQWRKGNTEECLPYKCSETGALGENYSWQIPINHNDFKILKNNERQLCEVLQN
KFGCISTLVSPVQEGNSKSLQVFRKMLTPRIELSVWKDDLTTHAVDAVVNAANEDLLHGGGLALALVKAGGFEIQEESKQFVARYGK
VSAGEIAVTGAGRLPCKQIIHAVGPRWMEWDKQGCTGKLQRAIVSILNYVIYKNTHIKTVAIPALSSGIFQFPLNLCTKTIVETIRV
SLQGKPMMSNLKEIHLVSNEDPTVAAFKAASEFILGKSELGQETTPSFNAMVVNNLTLQIVQGHIEWQTADVIVNSVNPHDITVGPV
AKSILQQAGVEMKSEFLATKAKQFQRSQLVLVTKGFNLFCKYIYHVLWHSEFPKPQILKHAMKECLEKCIEQNITSISFPALGTGNM
EIKKETAAEILFDEVLTFAKDHVKHQLTVKFVIFPTDLEIYKAFSSEMAKRSKMLSLNNYSVPQSTREEKRENGLEARSPAINLMGF
NVEEMYEAHAWIQRILSLQNHHIIENNHILYLGRKEHDILSQLQKTSSVSITEIISPGRTELEIEGARADLIEVVMNIEDMLCKVQE
EMARKKERGLWRSLGQWTIQQQKTQDEMKENIIFLKCPVPPTQELLDQKKQFEKCGLQVLKVEKIDNEVLMAAFQRKKKMMEEKLHR
QPVSHRLFQQVPYQFCNVVCRVGFQRMYSTPCDPKYGAGIYFTKNLKNLAEKAKKISAADKLIYVFEAEVLTGFFCQGHPLNIVPPP
LSPGAIDGHDSVVDNVSSPETFVIFSGMQAIPQYLWTCTQEYVQSQDYSSGPMRPFAQHPWRGFASGSPVD
>sp|Q8N490|PNKD_HUMAN
MAAVVAATALKGRGARNARVLRGILAGATANKASHNRTRALQSHSSPEGKEEPEPLSPELEYIPRKRGKNPMKAVGLAWYSLYTRTW
LGYLFYRQQLRRARNRYPKGHSKTQPRLFNGVKVLPIPVLSDNYSYLIIDTQAQLAVAVDPSDPRAVQASIEKEGVTLVAILCTHKH
WDHSGGNRDLSRRHRDCRVYGSPQDGIPYLTHPLCHQDVVSVGRLQIRALATPGHTQGHLVYLLDGEPYKGPSCLFSGDLLFLSGCG
RTFEGNAETMLSSLDTVLGLGDDTLLWPGHEYAEENLGFAGVVEPENLARERKMQWVQRQRLERKGTCPSTLGEERSYNPFLRTHCL
ALQEALGPGPGPTGDDDYSRAQLLEELRRLKDMHKSK
>sp|P23381|SYWC_HUMAN

MPNSEPASLLELFNSIATQGELVRSLKAGNASKDEIDSAVKMLVSLKMSYKAAAGEDYKADCPPGNPAPTSNHGPDATEAEEDFVDP
WTVQTSSAKGIDYDKLIVRFGSSKIDKELINRIERATGQRPHHFLRRGIFFSHRDMNQVLDAYENKKPFYLYTGRGPSSEAMHVGHL
IPFIFTKWLQDVFNVPLVIQMTDDEKYLWKDLTLDQAYSYAVENAKDIIACGFDINKTFIFSDLDYMGMSSGFYKNVVKIQKHVTFN
QVKGIFGFTDSDCIGKISFPAIQAAPSFSNSFPQIFRDRTDIQCLIPCAIDQDPYFRMTRDVAPRIGYPKPALLHSTFFPALQGAQT
KMSASDPNSSIFLTDTAKQIKTKVNKHAFSGGRDTIEEHRQFGGNCDVDVSFMYLTFFLEDDDKLEQIRKDYTSGAMLTGELKKALI
EVLQPLIAEHQARRKEVTDEIVKEFMTPRKLSFDFQ
>sp|P11474|ERR1_HUMAN
MSSQVVGIEPLYIKAEPASPDSPKGSSETETEPPVALAPGPAPTRCLPGHKEEEDGEGAGPGEQGGGKLVLSSLPKRLCLVCGDVAS
GYHYGVASCEACKAFFKRTIQGSIEYSCPASNECEITKRRRKACQACRFTKCLRVGMLKEGVRLDRVRGGRQKYKRRPEVDPLPFPG
PFPAGPLAVAGGPRKTAAPVNALVSHLLVVEPEKLYAMPDPAGPDGHLPAVATLCDLFDREIVVTISWAKSIPGFSSLSLSDQMSVL
QSVWMEVLVLGVAQRSLPLQDELAFAEDLVLDEEGARAAGLGELGAALLQLVRRLQALRLEREEYVLLKALALANSDSVHIEDAEAV
EQLREALHEALLEYEAGRAGPGGGAERRRAGRLLLTLPLLRQTAGKVLAHFYGVKLEGKVPMHKLFLEMLEAMMD
>sp|Q8TCT0|CERK1_HUMAN
MGATGAAEPLQSVLWVKQQRCAVSLEPARALLRWWRSPGPGAGAPGADACSVPVSEIIAVEETDVHGKHQGSGKWQKMEKPYAFTVH
CVKRARRHRWKWAQVTFWCPEEQLCHLWLQTLREMLEKLTSRPKHLLVFINPFGGKGQGKRIYERKVAPLFTLASITTDIIVTEHAN
QAKETLYEINIDKYDGIVCVGGDGMFSEVLHGLIGRTQRSAGVDQNHPRAVLVPSSLRIGIIPAGSTDCVCYSTVGTSDAETSALHI
VVGDSLAMDVSSVHHNSTLLRYSVSLLGYGFYGDIIKDSEKKRWLGLARYDFSGLKTFLSHHCYEGTVSFLPAQHTVGSPRDRKPCR
AGCFVCRQSKQQLEEEQKKALYGLEAAEDVEEWQVVCGKFLAINATNMSCACRRSPRGLSPAAHLGDGSSDLILIRKCSRFNFLRFL
IRHTNQQDQFDFTFVEVYRVKKFQFTSKHMEDEDSDLKEGGKKRFGHICSSHPSCCCTVSNSSWNCDGEVLHSPAIEVRVHCQLVRL
FARGIEENPKPDSHS
>sp|Q3MIV0|KR221_HUMAN
MSFDNNYHGGQGYAKGGLGCSYGCGLSGYGYACYCPWCYERSWFSGCF
>sp|Q9UFF9|CNOT8_HUMAN
MPAALVENSQVICEVWASNLEEEMRKIREIVLSYSYIAMDTEFPGVVVRPIGEFRSSIDYQYQLLRCNVDLLKIIQLGLTFTNEKGE
YPSGINTWQFNFKFNLTEDMYSQDSIDLLANSGLQFQKHEEEGIDTLHFAELLMTSGVVLCDNVKWLSFHSGYDFGYMVKLLTDSRL
PEEEHEFFHILNLFFPSIYDVKYLMKSCKNLKGGLQEVADQLDLQRIGRQHQAGSDSLLTGMAFFRMKELFFEDSIDDAKYCGRLYG
LGTGVAQKQNEDVDSAQEKMSILAIINNMQQ
>sp|Q96KS9|F167A_HUMAN
MSVPQIHVEEVGAEEGAGAAAPPDDHLRSLKALTEKLRLETRRPSYLEWQARLEEHTWPFPRPAAEPQASLEEGERGGQEPLLPLRE
AGQHPPSARSASQGARPLSTGKLEGFQSIDEAIAWLRKELTEMRLQDQQLARQLMRLRGDINKLKIEHTCRLHRRMLNDATYELEER
DELADLFCDSPLASSFSLSTPLKLIGVTKMNINSRRFSLC
>sp|Q14966|ZN638_HUMAN
MSRPRFNPRGDFPLQRPRAPNPSGMRPPGPFMRPGSMGLPRFYPAGRARGIPHRFAGHESYQNMGPQRMNVQVTQHRTDPRLTKEKL
DFHEAQQKKGKPHGSRWDDEPHISASVAVKQSSVTQVTEQSPKVQSRYTKESASSILASFGLSNEDLEELSRYPDEQLTPENMPLIL
RDIRMRKMGRRLPNLPSQSRNKETLGSEAVSSNVIDYGHASKYGYTEDPLEVRIYDPEIPTDEVENEFQSQQNISASVPNPNVICNS
MFPVEDVFRQMDFPGESSNNRSFFSVESGTKMSGLHISGGQSVLEPIKSVNQSINQTVSQTMSQSLIPPSMNQQPFSSELISSVSQQ
ERIPHEPVINSSNVHVGSRGSKKNYQSQADIPIRSPFGIVKASWLPKFSHADAQKMKRLPTPSMMNDYYAASPRIFPHLCSLCNVEC
SHLKDWIQHQNTSTHIESCRQLRQQYPDWNPEILPSRRNEGNRKENETPRRRSHSPSPRRSRRSSSSHRFRRSRSPMHYMYRPRSRS
PRICHRFISRYRSRSRSRSPYRIRNPFRGSPKCFRSVSPERMSRRSVRSSDRKKALEDVVQRSGHGTEFNKQKHLEAADKGHSPAQK
PKTSSGTKPSVKPTSATKSDSNLGGHSIRCKSKNLEDDTLSECKQVSDKAVSLQRKLRKEQSLHYGSVLLITELPEDGCTEEDVRKL
FQPFGKVNDVLIVPYRKEAYLEMEFKEAITAIMKYIETTPLTIKGKSVKICVPGKKKAQNKEVKKKTLESKKVSASTLKRDADASKA
VEIVTSTSAAKTGQAKASVAKVNKSTGKSASSVKSVVTVAVKGNKASIKTAKSGGKKSLEAKKTGNVKNKDSNKPVTIPENSEIKTS
IEVKATENCAKEAISDAALEATENEPLNKETEEMCVMLVSNLPNKGYSVEEVYDLAKPFGGLKDILILSSHKKAYIEINRKAAESMV
KFYTCFPVLMDGNQLSISMAPENMNIKDEEAIFITLVKENDPEANIDTIYDRFVHLDNLPEDGLQCVLCVGLQFGKVDHHVFISNRN

KAILQLDSPESAQSMYSFLKQNPQNIGDHMLTCSLSPKIDLPEVQIEHDPELEKESPGLKNSPIDESEVQTATDSPSVKPNELEEES
TPSIQTETLVQQEEPCEEEAEKATCDSDFAVETLELETQGEEVKEEIPLVASASVSIEQFTENAEECALNQQMFNSDLEKKGAEIIN
PKTALLPSDSVFAEERNLKGILEESPSEAEDFISGITQTMVEAVAEVEKNETVSEILPSTCIVTLVPGIPTGDEKTVDKKNISEKKG
NMDEKEEKEFNTKETRMDLQIGTEKAEKNEGRMDAEKVEKMAAMKEKPAENTLFKAYPNKGVGQANKPDETSKTSILAVSDVSSSKP
SIKAVIVSSPKAKATVSKTENQKSFPKSVPRDQINAEKKLSAKEFGLLKPTSARSGLAESSSKFKPTQSSLTRGGSGRISALQGKLS
KLDYRDITKQSQETEARPSIMKRDDSNNKTLAEQNTKNPKSTTGRSSKSKEEPLFPFNLDEFVTVDEVIEEVNPSQAKQNPLKGKRK
ETLKNVPFSELNLKKKKGKTSTPRGVEGELSFVTLDEIGEEEDAAAHLAQALVTVDEVIDEEELNMEEMVKNSNSLFTLDELIDQDD
CISHSEPKDVTVLSVAEEQDLLKQERLVTVDEIGEVEELPLNESADITFATLNTKGNEGDTVRDSIGFISSQVPEDPSTLVTVDEIQ
DDSSDLHLVTLDEVTEEDEDSLADFNNLKEELNFVTVDEVGEEEDGDNDLKVELAQSKNDHPTDKKGNRKKRAVDTKKTKLESLSQV
GPVNENVMEEDLKTMIERHLTAKTPTKRVRIGKTLPSEKAVVTEPAKGEEAFQMSEVDEESGLKDSEPERKRKKTEDSSSGKSVASD
VPEELDFLVPKAGFFCPICSLFYSGEKAMTNHCKSTRHKQNTEKFMAKQRKEKEQNEAEERSSR
>sp|Q32ZL2|PLPR5_HUMAN
MPLLPAALTSSMLYFQMVIMAGTVMLAYYFEYTDTFTVNVQGFFCHDSAYRKPYPGPEDSSAVPPVLLYSLAAGVPVLVIIVGETAV
FCLQLATRDFENQEKTILTGDCCYINPLVRRTVRFLGIYTFGLFATDIFVNAGQVVTGNLAPHFLALCKPNYTALGCQQYTQFISGE
EACTGNPDLIMRARKTFPSKEAALSVYAAMYLTMYITNTIKAKGTRLAKPVLCLGLMCLAFLTGLNRVAEYRNHWSDVIAGFLVGIS
IAVFLVVCVVNNFKGRQAENEHIHMDNLAQMPMISIPRVESPLEKVTSVQNHITAFAEVT
>sp|O75949|F155B_HUMAN
MFRGAWMWPGKDAAALTICCCCCCWAPRPSDKPCADSERAQRWRLSLASLLFFTVLLADHLWLCAGARPRARELSSAMRPPWGAGRE
RQPVPPRAVLPLPPPPPGEPSAPPGTCGPRYSNLTKAAPAAGSRPVCGGVPEPTGLDAACTKLQSLQRLFEPTTPAPPLRPPDSLSR
APAEFPSAKKNLLKGHFRNFTLSFCDTYTVWDLLLGMDRPDSLDCSLDTLMGDLLAVVASPGSGAWEACSNCIEAYQRLDRHAQEKY
DEFDLVLHKYLQAEEYSIRSCTKGCKAVYKAWLCSEYFSVTQQECQRWVPCKQYCLEVQTRCPFILPDNEEMVYGGLPGFICTGLLD
TSPKRLETKCCDVQWVSCEAKKKKKFKESEAPKTHQQQFHHSYFHHYHQQYHHYHPHHDPPGRVSNKPALLPVSGGSRLSPSRIRLC
VLVLMLLHTVVSFSSNQGGGGLGLETLPALEEGLTREE
>sp|Q9NYT6|ZN226_HUMAN
MNMFKEAVTFKDVAVAFTEEELGLLGPAQRKLYRDVMVENFRNLLSVGHPPFKQDVSPIERNEQLWIMTTATRRQGNLGEKNQSKLI
TVQDRESEEELSCWQIWQQIANDLTRCQDSMINNSQCHKQGDFPYQVGTELSIQISEDENYIVNKADGPNNTGNPEFPILRTQDSWR
KTFLTESQRLNRDQQISIKNKLCQCKKGVDPIGWISHHDGHRVHKSEKSYRPNDYEKDNMKILTFDHNSMIHTGQKSYQCNECKKPF
SDLSSFDLHQQLQSGEKSLTCVERGKGFCYSPVLPVHQKVHVGEKLKCDECGKEFSQGAHLQTHQKVHVIEKPYKCKQCGKGFSRRS
ALNVHCKVHTAEKPYNCEECGRAFSQASHLQDHQRLHTGEKPFKCDACGKSFSRNSHLQSHQRVHTGEKPYKCEECGKGFICSSNLY
IHQRVHTGEKPYKCEECGKGFSRPSSLQAHQGVHTGEKSYICTVCGKGFTLSSNLQAHQRVHTGEKPYKCNECGKSFRRNSHYQVHL
VVHTGEKPYKCEICGKGFSQSSYLQIHQKAHSIEKPFKCEECGQGFNQSSRLQIHQLIHTGEKPYKCEECGKGFSRRADLKIHCRIH
TGEKPYNCEECGKVFRQASNLLAHQRVHSGEKPFKCEECGKSFGRSAHLQAHQKVHTGDKPYKCDECGKGFKWSLNLDMHQRVHTGE
KPYKCGECGKYFSQASSLQLHQSVHTGEKPYKCDVCGKVFSRSSQLQSHQRVHTGEKPYKCEICGKSFSWRSNLTVHHRIHVGDKSY
KSNRGGKNIRESTQEKKSIK
>sp|Q9P0S2|COX16_HUMAN
MFAPAVMRAFRKNKTLGYGVPMLLLIVGGSFGLREFSQIRYDAVKSKMDPELEKKLKENKISLESEYEKIKDSKFDDWKNIRGPRPW
EDPDLLQGRNPESLKTKTT
>sp|Q9UBC3|DNM3B_HUMAN
MKGDTRHLNGEEDAGGREDSILVNGACSDQSSDSPPILEAIRTPEIRGRRSSSRLSKREVSSLLSYTQDLTGDGDGEDGDGSDTPVM
PKLFRETRTRSESPAVRTRNNNSVSSRERHRPSPRSTRGRQGRNHVDESPVEFPATRSLRRRATASAGTPWPSPPSSYLTIDLTDDT
EDTHGTPQSSSTPYARLAQDSQQGGMESPQVEADSGDGDSSEYQDGKEFGIGDLVWGKIKGFSWWPAMVVSWKATSKRQAMSGMRWV
QWFGDGKFSEVSADKLVALGLFSQHFNLATFNKLVSYRKAMYHALEKARVRAGKTFPSSPGDSLEDQLKPMLEWAHGGFKPTGIEGL
KPNNTQPVVNKSKVRRAGSRKLESRKYENKTRRRTADDSATSDYCPAPKRLKTNCYNNGKDRGDEDQSREQMASDVANNKSSLEDGC
LSCGRKNPVSFHPLFEGGLCQTCRDRFLELFYMYDDDGYQSYCTVCCEGRELLLCSNTSCCRCFCVECLEVLVGTGTAAEAKLQEPW

SCYMCLPQRCHGVLRRRKDWNVRLQAFFTSDTGLEYEAPKLYPAIPAARRRPIRVLSLFDGIATGYLVLKELGIKVGKYVASEVCEE
SIAVGTVKHEGNIKYVNDVRNITKKNIEEWGPFDLVIGGSPCNDLSNVNPARKGLYEGTGRLFFEFYHLLNYSRPKEGDDRPFFWMF
ENVVAMKVGDKRDISRFLECNPVMIDAIKVSAAHRARYFWGNLPGMNRPVIASKNDKLELQDCLEYNRIAKLKKVQTITTKSNSIKQ
GKNQLFPVVMNGKEDVLWCTELERIFGFPVHYTDVSNMGRGARQKLLGRSWSVPVIRHLFAPLKDYFACE
>sp|P61962|DCAF7_HUMAN
MSLHGKRKEIYKYEAPWTVYAMNWSVRPDKRFRLALGSFVEEYNNKVQLVGLDEESSEFICRNTFDHPYPTTKLMWIPDTKGVYPDL
LATSGDYLRVWRVGETETRLECLLNNNKNSDFCAPLTSFDWNEVDPYLLGTSSIDTTCTIWGLETGQVLGRVNLVSGHVKTQLIAHD
KEVYDIAFSRAGGGRDMFASVGADGSVRMFDLRHLEHSTIIYEDPQHHPLLRLCWNKQDPNYLATMAMDGMEVVILDVRVPCTPVAR
LNNHRACVNGIAWAPHSSCHICTAADDHQALIWDIQQMPRAIEDPILAYTAEGEINNVQWASTQPDWIAICYNNCLEILRV
>sp|Q969G5|PRDBP_HUMAN
MRESALERGPVPEAPAGGPVHAVTVVTLLEKLASMLETLRERQGGLARRQGGLAGSVRRIQSGLGALSRSHDTTSNTLAQLLAKAER
VSSHANAAQERAVRRAAQVQRLEANHGLLVARGKLHVLLFKEEGEVPASAFQKAPEPLGPADQSELGPEQLEAEVGESSDEEPVESR
AQRLRRTGLQKVQSLRRALSGRKGPAAPPPTPVKPPRLGPGRSAEAQPEAQPALEPTLEPEPPQDTEEDPGRPGAAEEALLQMESVA
>sp|O95405|ZFYV9_HUMAN
MENYFQAEAYNLDKVLDEFEQNEDETVSSTLLDTKWNKILDPPSHRLSFNPTLASVNESAVSNESQPQLKVFSLAHSAPLTTEEEDH
CANGQDCNLNPEIATMWIDENAVAEDQLIKRNYSWDDQCSAVEVGEKKCGNLACLPDEKNVLVVAVMHNCDKRTLQNDLQDCNNYNS
QSLMDAFSCSLDNENRQTDQFSFSINESTEKDMNSEKQMDPLNRPKTEGRSVNHLCPTSSDSLASVCSPSQLKDDGSIGRDPSMSAI
TSLTVDSVISSQGTDGCPAVKKQENYIPDEDLTGKISSPRTDLGSPNSFSHMSEGILMKKEPAEESTTEESLRSGLPLLLKPDMPNG
SGRNNDCERCSDCLVPNEVRADENEGYEHEETLGTTEFLNMTEHFSESQDMTNWKLTKLNEMNDSQVNEEKEKFLQISQPEDTNGDS
GGQCVGLADAGLDLKGTCISESEECDFSTVIDTPAANYLSNGCDSYGMQDPGVSFVPKTLPSKEDSVTEEKEIEESKSECYSNIYEQ
RGNEATEGSGLLLNSTGDLMKKNYLHNFCSQVPSVLGQSSPKVVASLPSISVPFGGARPKQPSNLKLQIPKPLSDHLQNDFPANSGN
NTKNKNDILGKAKLGENSATNVCSPSLGNISNVDTNGEHLESYEAEISTRPCLALAPDSPDNDLRAGQFGISARKPFTTLGEVAPVW
VPDSQAPNCMKCEARFTFTKRRHHCRACGKVFCASCCSLKCKLLYMDRKEARVCVICHSVLMNAQAWENMMSASSQSPNPNNPAEYC
STIPPLQQAQASGALSSPPPTVMVPVGVLKHPGAEVAQPREQRRVWFADGILPNGEVADAAKLTMNGTSSAGTLAVSHDPVKPVTTS
PLPAETDICLFSGSITQVGSPVGSAMNLIPEDGLPPILISTGVKGDYAVEEKPSQISVMQQLEDGGPDPLVFVLNANLLSMVKIVNY
VNRKCWCFTTKGMHAVGQSEIVILLQCLPDEKCLPKDIFNHFVQLYRDALAGNVVSNLGHSFFSQSFLGSKEHGGFLYVTSTYQSLQ
DLVLPTPPYLFGILIQKWETPWAKVFPIRLMLRLGAEYRLYPCPLFSVRFRKPLFGETGHTIMNLLADFRNYQYTLPVVQGLVVDME
VRKTSIKIPSNRYNEMMKAMNKSNEHVLAGGACFNEKADSHLVCVQNDDGNYQTQAISIHNQPRKVTGASFFVFSGALKSSSGYLAK
SSIVEDGVMVQITAENMDSLRQALREMKDFTITCGKADAEEPQEHIHIQWVDDDKNVSKGVVSPIDGKSMETITNVKIFHGSEYKAN
GKVIRWTEVFFLENDDQHNCLSDPADHSRLTEHVAKAFCLALCPHLKLLKEDGMTKLGLRVTLDSDQVGYQAGSNGQPLPSQYMNDL
DSALVPVIHGGACQLSEGPVVMELIFYILENIV
>sp|Q8N4W9|ZN808_HUMAN
MLREEAAQKRKGKESGMALPQGRLTFRDVAIEFSLAEWKFLNPAQRALYREVMLENYRNLEAVDISSKHMMKEVLSTGQGNREVIHT
GTLQRHQSYHIGDFCFQEIEKEIHNIEFQCQEDERNGHEAPTTKIKKLTGSTDQHDHRHAGNKPIKDQLGSSFYSHLPELHIFQIKG
EIANQLEKSTSDASSVSTSQRISCRPQIHISNNYGNNPLNSSLLPQKQEVHMREKSFPCNESGKAFNCSSLLRKHQIPHLGDKQYKC
DVCGKLFNHKQYLACHRRCHTGEKPYKCKECGKSFSYKSSLTCHHRLHTGVKPYKCNECGKVFRQNSALVIHKAIHTGEKPYKCNEC
GKAFNQQSHLSRHQRLHTGVKPYKCKICEKAFACHSYLANHTRIHSGEKTYKCNECGKAFNHQSSLARHHILHTGEKPYKCEECDKV
FSQKSTLERHKRIHTGEKPYKCKVCDTAFTCNSQLARHRRIHTGEKTYKCNECRKTFSRRSSLLCHRRLHSGEKPYKCNQCGNTFRH
RASLVYHRRLHTLEKSYKCTVCNKVFMRNSVLAVHTRIHTAKKPYKCNECGKAFNQQSHLSRHRRLHTGEKPYKCEACDKVFGQKSA
LESHKRIHTGEKPYRCQVCDTAFTWNSQLARHTRIHTGEKTYKCNECGKTFSYKSSLVWHRRLHGGEKSYKCKVCDKAFVCRSYVAK
HTRIHSGMKPYKCNECSKTFSNRSSLVCHRRIHSGEKPYKCSECSKTFSQKATLLCHRRLHSGEKPYKCNDCGNTFRHWSSLVYHRR
LHTGEKSYKCTVCDKAFVRNSYLARHIRIHTAEKPYKCNECGKAFNEQSHLSRHHRIHTGEKPYKCEACDKVFSRKSHLKRHRIIHT
GEKPYKCNECGKAFSDRSTLIHHQAIHGIGKFD
>sp|O75575|RPC9_HUMAN

MEVKDANSALLSNYEVFQLLTDLKEQRKESGKNKHSSGQQNLNTITYETLKYISKTPCRHQSPEIVREFLTALKSHKLTKAEKLQLL
NHRPVTAVEIQLMVEESEERLTEEQIEALLHTVTSILPAEPEAEQKKNTNSNVAMDEEDPA
>sp|Q2M1Z3|RHG31_HUMAN
MKNKGAKQKLKRKGAASAFGCDLTEYLESSGQDVPYVLKSCAEFIETHGIVDGIYRLSGVTSNIQRLRQEFGSDQCPDLTREVYLQD
IHCVGSLCKLYFRELPNPLLTYELYEKFTEAVSHCPEEGQLARIQNVIQELPPSHYRTLEYLIRHLAHIASFSSKTNMHARNLALVW
APNLLRSKEIEATGCNGDAAFLAVRVQQVVIEFILNHVDQIFNNGAPGSLENDENRPIMKSLTLPALSLPMKLVSLEEAQARSLATN
HPARKERRENSLPEIVPPMGTLFHTVLELPDNKRKLSSKSKKWKSIFNLGRSGSDSKSKLSRNGSVFVRGQRLSVEKATIRPAKSMD
SLCSVPVEGKETKGNFNRTVTTGGFFIPATKMHSTGTGSSCDLTKQEGEWGQEGMPPGAEGGFDVSSDRSHLQGAQARPPPEQLKVF
RPVEDPESEQTAPKMLGMFYTSNDSPSKSVFTSSLFQMEPSPRNQRKALNISEPFAVSVPLRVSAVISTNSTPCRTPPKELQSLSSL
EEFSFHGSESGGWPEEEKPLGAETSAASVPKKAGLEDAKAVPEAPGTVECSKGLSQEPGAHLEEKKTPESSLSSQHLNELEKRPNPE
KVVEEGREAGEMESSTLQESPRARAEAVLLHEMDEDDLANALIWPEIQQELKIIESEEELSSLPPPALKTSPIQPILESSLGPFIPS
EPPGSLPCGSFPAPVSTPLEVWTRDPANQSTQGASTAASREKPEPEQGLHPDLASLAPLEIVPFEKASPQATVEVGGPGNLSPPLPP
APPPPTPLEESTPVLLSKGGPEREDSSRKLRTDLYIDQLKSQDSPEISSLCQGEEATPRHSDKQNSKNAASEGKGCGFPSPTREVEI
VSQEEEDVTHSVQEPSDCDEDDTVTDIAQHGLEMVEPWEEPQWVTSPLHSPTLKDAHKAQVQGLQGHQLEKRLSHRPSLRQSHSLDS
KPTVKSQWTLEVPSSSSCANLETERNSDPLQPQAPRREITGWDEKALRSFREFSGLKGAEAPPNQKGPSGVQPNPAETSPISLAEGK
ELGTHLGHSSPQIRQGGVPGPESSKESSPSVQDSTSPGEHPAKLQLKSTECGPPKGKNRPSSLNLDPAIPIADLFWFENVASFSSPG
MQVSEPGDPKVTWMTSSYCKADPWRVYSQDPQDLDIVAHALTGRRNSAPVSVSAVRTSFMVKMCQARAVPVIPPKIQYTQIPQPLPS
QSSGENGVQPLERSQEGPSSTSGTTQKPAKDDSPSSLESSKEEKPKQDPGAIKSSPVDATAPCMCEGPTLSPEPGSSNLLSTQDAVV
QCRKRMSETEPSGDNLLSSKLERPSGGSKPFHRSRPGRPQSLILFSPPFPIMDHLPPSSTVTDSKVLLSPIRSPTQTVSPGLLCGEL
AENTWVTPEGVTLRNKMTIPKNGQRLETSTSCFYQPQRRSVILDGRSGRQIE
>sp|Q9H310|RHBG_HUMAN
MAGSPSRAAGRRLQLPLLCLFLQGATAVLFAVFVRYNHKTDAALWHRSNHSNADNEFYFRYPSFQDVHAMVFVGFGFLMVFLQRYGF
SSVGFTFLLAAFALQWSTLVQGFLHSFHGGHIHVGVESMINADFCAGAVLISFGAVLGKTGPTQLLLMALLEVVLFGINEFVLLHLL
GVRDAGGSMTIHTFGAYFGLVLSRVLYRPQLEKSKHRQGSVYHSDLFAMIGTIFLWIFWPSFNAALTALGAGQHRTALNTYYSLAAS
TLGTFALSALVGEDGRLDMVHIQNAALAGGVVVGTSSEMMLTPFGALAAGFLAGTVSTLGYKFFTPILESKFKVQDTCGVHNLHGMP
GVLGALLGVLVAGLATHEAYGDGLESVFPLIAEGQRSATSQAMHQLFGLFVTLMFASVGGGLGGLLLKLPFLDSPPRLPALRGPSSL
AGAWRA
>sp|P61574|RE113_HUMAN
MNPSEMQRKAPPRRRRHRNRAPLTHKMNKMVTSEEQMKLPSTKKAEPPTWAQLKKLTQLATKYLENTKVTQTPESMLLAALMIVSMV
SYGVPNSSEETATIENGP
>sp|A6NHG4|DDTL_HUMAN
MPFLELDTNLPANRVPAGLEKRLCAAAASILGKPADRVNVTVRPGLAMALSGSTEPCAQLSISSIGVVGTAEDNRSHSAHFFEFLTK
ELALGQDRFPTVLSTSPAAHGGPRCPGEIIEGKKSCLNEEALFIYFI
>sp|Q9P2X3|IMPCT_HUMAN
MAEGDAGSDQRQNEEIEAMAAIYGEEWCVIDDCAKIFCIRISDDIDDPKWTLCLQVMLPNEYPGTAPPIYQLNAPWLKGQERADLSN
SLEEIYIQNIGESILYLWVEKIRDVLIQKSQMTEPGPDVKKKTEEEDVECEDDLILACQPESSLKALDFDISETRTEVEVEELPPID
HGIPITDRRSTFQAHLAPVVCPKQVKMVLSKLYENKKIASATHNIYAYRIYCEDKQTFLQDCEDDGETAAGGRLLHLMEILNVKNVM
VVVSRWYGGILLGPDRFKHINNCARNILVEKNYTNSPEESSKALGKNKKVRKDKKRNEH
>sp|O00155|GPR25_HUMAN
MAPTEPWSPSPGSAPWDYSGLDGLEELELCPAGDLPYGYVYIPALYLAAFAVGLLGNAFVVWLLAGRRGPRRLVDTFVLHLAAADLG
FVLTLPLWAAAAALGGRWPFGDGLCKLSSFALAGTRCAGALLLAGMSVDRYLAVVKLLEARPLRTPRCALASCCGVWAVALLAGLPS
LVYRGLQPLPGGQDSQCGEEPSHAFQGLSLLLLLLLTFVLPLVVTLFCYCRISRRLRRPPHVGRARRNSLRIIFAIESTFVGSWLPFS
ALRAVFHLARLGALPLPCPLLLALRWGLTIATCLAFVNSCANPLIYLLLDRSFRARALDGACGRTGRLARRISSASSLSRDDSSVFR
CRAQAANTASASW

>sp|Q96DT7|ZBT10_HUMAN
MSFSEMNRRTLAFRGGGLVTASGGGSTNNNAGGEASAWPPQPQPRQPPPPAPPALQPPNGRGADEEVELEGLEPQDLEASAGPAAGA
AEEAKELLLPQDAGGPTSLGGGAGGPLLAERNRRTLAFRGGGGGGLGNNGSSRGRPETSVWPLRHFNGRGPATVDLELDALEGKELM
QDGASLSDSTEDEEEGASLGDGSGAEGGSCSSSRRSGGDGGDEVEGSGVGAGEGETVQHFPLARPKSLMQKLQCSFQTSWLKDFPWL
RYSKDTGLMSCGWCQKTPADGGSVDLPPVGHDELSRGTRNYKKTLLLRHHVSTEHKLHEANAQESEIPSEEGYCDFNSRPNENSYCY
QLLRQLNEQRKKGILCDVSIVVSGKIFKAHKNILVAGSRFFKTLYCFSNKESPNQNNTTHLDIAAVQGFSVILDFLYSGNLVLTSQN
AIEVMTVASYLQMSEVVQTCRNFIKDALNISIKSEAPESVVVDYNNRKPVNRDGLSSSRDQKIASFWATRNLTNLASNVKIENDGCN
VDEGQIENYQMNDSSWVQDGSPEMAENESEGQTKVFIWNNMGSQGIQETGKTRRKNQTTKRFIYNIPPNNETNLEDCSVMQPPVAYP
EENTLLIKEEPDLDGALLSGPDGDRNVNANLLAEAGTSQDGGDAGTSHDFKYGLMPGPSNDFKYGLIPGTSNDFKYGLIPGASNDFK
YGLLPESWPKQETWENGESSLIMNKLKCPHCSYVAKYRRTLKRHLLIHTGVRSFSCDICGKLFTRREHVKRHSLVHKKDKKYKCMVC
KKIFMLAASVGIRHGSRRYGVCVDCADKSQPGGQEGVDQGQDTEFPRDEEYEENEVGEADEELVDDGEDQNDPSRWDESGEVCMSLD
D
>sp|Q9H4E5|RHOJ_HUMAN
MNCKEGTDSSCGCRGNDEKKMLKCVVVGDGAVGKTCLLMSYANDAFPEEYVPTVFDHYAVTVTVGGKQHLLGLYDTAGQEDYNQLRP
LSYPNTDVFLICFSVVNPASYHNVQEEWVPELKDCMPHVPYVLIGTQIDLRDDPKTLARLLYMKEKPLTYEHGVKLAKAIGAQCYLE
CSALTQKGLKAVFDEAILTIFHPKKKKKRCSEGHSCCSII
>sp|Q9NUB4|CT141_HUMAN
MTRLCLPRPEAREDPIPVPPRGLGAGEGSGSPVRPPVSTWGPSWAQLLDSVLWLGALGLTIQAVFSTTGPALLLLLVSFLTFDLLHR
PAGHTLPQRKLLTRGQSQGAGEGPGQQEALLLQMGTVSGQLSLQDALLLLLMGLGPLLRACGMPLTLLGLAFCLHPWA
>sp|Q629K1|TRIQK_HUMAN
MGRKDAATIKLPVDQYRKQIGKQDYKKTKPILRATKLKAEAKKTAIGIKEVGLVLAAILALLLAFYAFFYLRLTTDVDPDLDQDED
>sp|Q5VTA0|PRA17_HUMAN
MSLQSPSRLLELAGQSLLRNQFLTIFILDELPREVFPLMFMEASSMRHFEALKLMVQAWPFLRLPLGSLMKTPHLETLQAVLKGLDT
LLAQKLRPRRWKLQVLDLRDVDGNFWTIWSGARALSCSPEAMSKRQTVEDYPRTGEHQPLKVFIDLCQKESTLDECLSYLCRWIHYR
RGLVHLCCNKVQNYSMPTSSFRNLLKRVYPDSIQELEIKRKCSLNKTGKFAPYLSQMSNLRKLFLAFGYDDELYVSGQQQFVPDLDC
PFLCLYYPQMLYIRKISNIKEHLEHLLRCLKNPLGTFIFCHAYLADQDMECLSQYPSLSQLKELHLIHILMWTTNLEPLGALLEKVA
ATLEILTLKDCQIQDSQLRVLLPALSRCSQLTTFYFRGNETSTNALKDLLCHTGGLSKLGLELYPAPLECLDNRGHVNWEILAPIRA
ELMCTLREVRQPKRIFFGPIPCPSCGSWPSEKVDFHLCS
>sp|Q8N271|PROM2_HUMAN
MKHTLALLAPLLGLGLGLALSQLAAGATDCKFLGPAEHLTFTPAARARWLAPRVRAPGLLDSLYGTVRRFLSVVQLNPFPSELVKAL
LNELASVKVNEVVRYEAGYVVCAVIAGLYLLLVPTAGLCFCCCRCHRRCGGRVKTEHKALACERAALMVFLLLTTLLLLIGVVCAFV
TNQRTHEQMGPSIEAMPETLLSLWGLVSDVPQELQAVAQQFSLPQEQVSEELDGVGVSIGSAIHTQLRSSVYPLLAAVGSLGQVLQV
SVHHLQTLNATVVELQAGQQDLEPAIREHRDRLLELLQEARCQGDCAGALSWARTLELGADFSQVPSVDHVLHQLKGVPEANFSSMV
QEENSTFNALPALAAMQTSSVVQELKKAVAQQPEGVRTLAEGFPGLEAASRWAQALQEVEESSRPYLQEVQRYETYRWIVGCVLCSV
VLFVVLCNLLGLNLGIWGLSARDDPSHPEAKGEAGARFLMAGVGLSFLFAAPLILLVFATFLVGGNVQTLVCQSWENGELFEFADTP
GNLPPSMNLSQLLGLRKNISIHQAYQQCKEGAALWTVLQLNDSYDLEEHLDINQYTNKLRQELQSLKVDTQSLDLLSSAARRDLEAL
QSSGLQRIHYPDFLVQIQRPVVKTSMEQLAQELQGLAQAQDNSVLGQRLQEEAQGLRNLHQEKVVPQQSLVAKLNLSVRALESSAPN
LQLETSDVLANVTYLKGELPAWAARILRNVSECFLAREMGYFSQYVAWVREEVTQRIATCQPLSGALDNSRVILCDMMADPWNAFWF
CLAWCTFFLIPSIIFAVKTSKYFRPIRKRLSSTSSEETQLFHIPRVTSLKL
>sp|Q5VX52|SPAT1_HUMAN
MSLNPSRPSSSELVELHVFYVPEGSWNYKLNTISTEVVNKFISAGFLRVSPQLTLRALRERLGEFLGEDAIAEKFLFLKCIGNNLAV
VKEKQESELKLKSFAPPYALQPELYLLPVMDHLGNVYSPSTVILDERQTNNGVNEADGTIHRPISVTLFKEELGRDPSLLENTLKEL
PNKNQEEAGGKATAEKSQIAKNQIGNSELPGSLEDSNNDCFGTKKSQCLWENEDDTAISRRQDNQTAEKEYITLPDHPSLPCQPVLS
SGITDISLLQTEREKIIKQMKQVKEERRYLERNREELVKTVEKLFEQSKLKRYHAYNGWKKKYLETKKVTASMEEVLTKLREDLELY

YKKLLMQLEAREIKMRPKNLANITDSKNYLIIQITEVQHAIDQLKRKLDTDKMKLIVEVKMRKQAVSDLRTLKTELAQKKKIIHLYN
LN
>sp|Q9H936|GHC1_HUMAN
MADKQISLPAKLINGGIAGLIGVTCVFPIDLAKTRLQNQQNGQRVYTSMSDCLIKTVRSEGYFGMYRGAAVNLTLVTPEKAIKLAAN
DFFRHQLSKDGQKLTLLKEMLAGCGAGTCQVIVTTPMEMLKIQLQDAGRIAAQRKILAAQGQLSAQGGAQPSVEAPAAPRPTATQLT
RDLLRSRGIAGLYKGLGATLLRDVPFSVVYFPLFANLNQLGRPASEEKSPFYVSFLAGCVAGSAAAVAVNPCDVVKTRLQSLQRGVN
EDTYSGILDCARKILRHEGPSAFLKGAYCRALVIAPLFGIAQVVYFLGIAESLLGLLQDPQA
>sp|Q8NGJ2|O52H1_HUMAN
MPSASAMIIFNLSSYNPGPFILVGIPGLEQFHVWIGIPFCIIYIVAVVGNCILLYLIVVEHSLHEPMFFFLSMLAMTDLILSTAGVP
KALSIFWLGAREITFPGCLTQMFFLHYNFVLDSAILMAMAFDHYVAICSPLRYTTILTPKTIIKSAMGISFRSFCIILPDVFLLTCL
PFCRTRIIPHTYCEHIGVAQLACADISINFWYGFCVPIMTVISDVILIAVSYAHILCAVFGLPSQDACQKALGTCGSHVCVILMFYT
PAFFSILAHRFGHNVSRTFHIMFANLYIVIPPALNPMVYGVKTKQIRDKVILLFSKGTG
>sp|Q9Y5S2|MRCKB_HUMAN
MSAKVRLKKLEQLLLDGPWRNESALSVETLLDVLVCLYTECSHSALRRDKYVAEFLEWAKPFTQLVKEMQLHREDFEIIKVIGRGAF
GEVAVVKMKNTERIYAMKILNKWEMLKRAETACFREERDVLVNGDCQWITALHYAFQDENHLYLVMDYYVGGDLLTLLSKFEDKLPE
DMARFYIGEMVLAIDSIHQLHYVHRDIKPDNVLLDVNGHIRLADFGSCLKMNDDGTVQSSVAVGTPDYISPEILQAMEDGMGKYGPE
CDWWSLGVCMYEMLYGETPFYAESLVETYGKIMNHEERFQFPSHVTDVSEEAKDLIQRLICSRERRLGQNGIEDFKKHAFFEGLNWE
NIRNLEAPYIPDVSSPSDTSNFDVDDDVLRNTEILPPGSHTGFSGLHLPFIGFTFTTESCFSDRGSLKSIMQSNTLTKDEDVQRDLE
HSLQMEAYERRIRRLEQEKLELSRKLQESTQTVQSLHGSSRALSNSNRDKEIKKLNEEIERLKNKIADSNRLERQLEDTVALRQERE
DSTQRLRGLEKQHRVVRQEKEELHKQLVEASERLKSQAKELKDAHQQRKLALQEFSELNERMAELRAQKQKVSRQLRDKEEEMEVAT
QKVDAMRQEMRRAEKLRKELEAQLDDAVAEASKERKLREHSENFCKQMESELEALKVKQGGRGAGATLEHQQEISKIKSELEKKVLF
YEEELVRREASHVLEVKNVKKEVHDSESHQLALQKEILMLKDKLEKSKRERHNEMEEAVGTIKDKYERERAMLFDENKKLTAENEKL
CSFVDKLTAQNRQLEDELQDLAAKKESVAHWEAQIAEIIQWVSDEKDARGYLQALASKMTEELEALRSSSLGSRTLDPLWKVRRSQK
LDMSARLELQSALEAEIRAKQLVQEELRKVKDANLTLESKLKDSEAKNRELLEEMEILKKKMEEKFRADTGLKLPDFQDSIFEYFNT
APLAHDLTFRTSSASEQETQAPKPEASPSMSVAASEQQEDMARPPQRPSAVPLPTTQALALAGPKPKAHQFSIKSFSSPTQCSHCTS
LMVGLIRQGYACEVCSFACHVSCKDGAPQVCPIPPEQSKRPLGVDVQRGIGTAYKGHVKVPKPTGVKKGWQRAYAVVCDCKLFLYDL
PEGKSTQPGVIASQVLDLRDDEFSVSSVLASDVIHATRRDIPCIFRVTASLLGAPSKTSSLLILTENENEKRKWVGILEGLQSILHK
NRLRNQVVHVPLEAYDSSLPLIKAILTAAIVDADRIAVGLEEGLYVIEVTRDVIVRAADCKKVHQIELAPREKIVILLCGRNHHVHL
YPWSSLDGAEGSFDIKLPETKGCQLMATATLKRNSGTCLFVAVKRLILCYEIQRTKPFHRKFNEIVAPGSVQCLAVLRDRLCVGYPS
GFCLLSIQGDGQPLNLVNPNDPSLAFLSQQSFDALCAVELESEEYLLCFSHMGLYVDPQGRRARAQELMWPAAPVACSCSPTHVTVY
SEYGVDVFDVRTMEWVQTIGLRRIRPLNSEGTLNLLNCEPPRLIYFKSKFSGAVLNVPDTSDNSKKQMLRTRSKRRFVFKVPEEERL
QQRREMLRDPELRSKMISNPTNFNHVAHMGPGDGMQVLMDLPLSAVPPSQEERPGPAPTNLARQPPSRNKPYISWPSSGGSEPSVTV
PLRSMSDPDQDFDKEPDSDSTKHSTPSNSSNPSGPPSPNSPHRSQLPLEGLEQPACDT
>sp|O43772|MCAT_HUMAN
MADQPKPISPLKNLLAGGFGGVCLVFVGHPLDTVKVRLQTQPPSLPGQPPMYSGTFDCFRKTLFREGITGLYRGMAAPIIGVTPMFA
VCFFGFGLGKKLQQKHPEDVLSYPQLFAAGMLSGVFTTGIMTPGERIKCLLQIQASSGESKYTGTLDCAKKLYQEFGIRGIYKGTVL
TLMRDVPASGMYFMTYEWLKNIFTPEGKRVSELSAPRILVAGGIAGIFNWAVAIPPDVLKSRFQTAPPGKYPNGFRDVLRELIRDEG
VTSLYKGFNAVMIRAFPANAACFLGFEVAMKFLNWATPNL
>sp|Q9NTX5|ECHD1_HUMAN
MALKQEMAKSLLKTASLSGRTKLLHQTGLSLYSTSHGFYEEEVKKTLQQFPGGSIDLQKEDNGIGILTLNNPSRMNAFSGVMMLQLL
EKVIELENWTEGKGLIVRGAKNTFSSGSDLNAVKSLGTPEDGMAVCMFMQNTLTRFMRLPLISVALVQGWALGGGAEFTTACDFRLM
TPESKIRFVHKEMGIIPSWGGTTRLVEIIGSRQALKVLSGALKLDSKNALNIGMVEEVLQSSDETKSLEEAQEWLKQFIQGPPEVIR
ALKKSVCSGRELYLEEALQNERDLLGTVWGGPANLEAIAKKGKFNK
>sp|Q9C009|FOXQ1_HUMAN

MKLEVFVPRAAHGDKQGSDLEGAGGSDAPSPLSAAGDDSLGSDGDCAANSPAAGGGARDTQGDGEQSAGGGPGAEEAIPAAAAAAVV
AEGAEAGAAGPGAGGAGSGEGARSKPYTRRPKPPYSYIALIAMAIRDSAGGRLTLAEINEYLMGKFPFFRGSYTGWRNSVRHNLSLN
DCFVKVLRDPSRPWGKDNYWMLNPNSEYTFADGVFRRRRKRLSHRAPVPAPGLRPEEAPGLPAAPPPAPAAPASPRMRSPARQEERA
SPAGKFSSSFAIDSILRKPFRSRRLRDTAPGTTLQWGAAPCPPLPAFPALLPAAPCRALLPLCAYGAGEPARLGAREAEVPPTAPPL
LLAPLPAAAPAKPLRGPAAGGAHLYCPLRLPAALQAASVRRPGPHLPYPVETLLA
>sp|Q9UK08|GBG8_HUMAN
MSNNMAKIAEARKTVEQLKLEVNIDRMKVSQAAAELLAFCETHAKDDPLVTPVPAAENPFRDKRLFCVLL
>sp|Q8NGC3|O10G2_HUMAN
MGKTKNTSLDAVVTDFILLGLSHPPNLRSLLFLVFFIIYILTQLGNLLILLTMWADPKLCARPMYILLGVLSFLDMWLSSVTVPRLI
LDFTPSIKAIPFGGCVAQLYFFHFLGSTQCFLYTLMAYDRYLAICQPLHYPVLMNGRLCTVLVAGAWVAGSMHGSIQATLTFRLPYC
GPNQVDYFICDIRAVLRLACADTTVNELVTFVDVRVVAASCFMLILLSYANIVHAILKIRTADGRRRAFSTCGSHLIVVTVYYVPCI
FIYLRAGSKDPLDGAAAVFYTVVTPLLNPLIYTLRNQEVKSALKRITAG
>sp|Q96FX2|DPH3_HUMAN
MAVFHDEVEIEDFQYDEDSETYFYPCPCGDNFSITKEDLENGEDVATCPSCSLIIKVIYDKDQFVCGETVPAPSANKELVKC
>sp|P35998|PRS7_HUMAN
MPDYLGADQRKTKEDEKDDKPIRALDEGDIALLKTYGQSTYSRQIKQVEDDIQQLLKKINELTGIKESDTGLAPPALWDLAADKQTL
QSEQPLQVARCTKIINADSEDPKYIINVKQFAKFVVDLSDQVAPTDIEEGMRVGVDRNKYQIHIPLPPKIDPTVTMMQVEEKPDVTY
SDVGGCKEQIEKLREVVETPLLHPERFVNLGIEPPKGVLLFGPPGTGKTLCARAVANRTDACFIRVIGSELVQKYVGEGARMVRELF
EMARTKKACLIFFDEIDAIGGARFDDGAGGDNEVQRTMLELINQLDGFDPRGNIKVLMATNRPDTLDPALMRPGRLDRKIEFSLPDL
EGRTHIFKIHARSMSVERDIRFELLARLCPNSTGAEIRSVCTEAGMFAIRARRKIATEKDFLEAVNKVIKSYAKFSATPRYMTYN
>sp|Q9Y3E7|CHMP3_HUMAN
MGLFGKTQEKPPKELVNEWSLKIRKEMRVVDRQIRDIQREEEKVKRSVKDAAKKGQKDVCIVLAKEMIRSRKAVSKLYASKAHMNSV
LMGMKNQLAVLRVAGSLQKSTEVMKAMQSLVKIPEIQATMRELSKEMMKAGIIEEMLEDTFESMDDQEEMEEEAEMEIDRILFEITA
GALGKAPSKVTDALPEPEPPGAMAASEDEEEEEEALEAMQSRLATLRS
>sp|Q13637|RAB32_HUMAN
MAGGGAGDPGLGAAAAPAPETREHLFKVLVIGELGVGKTSIIKRYVHQLFSQHYRATIGVDFALKVLNWDSRTLVRLQLWDIAGQER
FGNMTRVYYKEAVGAFVVFDISRSSTFEAVLKWKSDLDSKVHLPNGSPIPAVLLANKCDQNKDSSQSPSQVDQFCKEHGFAGWFETS
AKDNINIEEAARFLVEKILVNHQSFPNEENDVDKIKLDQETLRAENKSQCC
>sp|Q14554|PDIA5_HUMAN
MARAGPAWLLLAIWVVLPSWLSSAKVSSLIERISDPKDLKKLLRTRNNVLVLYSKSEVAAENHLRLLSTVAQAVKGQGTICWVDCGD
AESRKLCKKMKVDLSPKDKKVELFHYQDGAFHTEYNRAVTFKSIVAFLKDPKGPPLWEEDPGAKDVVHLDSEKDFRRLLKKEEKPLL
IMFYAPWCSMCKRMMPHFQKAATQLRGHAVLAGMNVYSSEFENIKEEYSVRGFPTICYFEKGRFLFQYDNYGSTAEDIVEWLKNPQP
PQPQVPETPWADEGGSVYHLTDEDFDQFVKEHSSVLVMFHAPWCGHCKKMKPEFEKAAEALHGEADSSGVLAAVDATVNKALAERFH
ISEFPTLKYFKNGEKYAVPVLRTKKKFLEWMQNPEAPPPPEPTWEEQQTSVLHLVGDNFRETLKKKKHTLVMFYAPWCPHCKKVIPH
FTATADAFKDDRKIACAAVDCVKDKNQDLCQQEAVKGYPTFHYYHYGKFAEKYDSDRTELGFTNYIRALREGDHERLGKKKEEL
>sp|O43719|HTSF1_HUMAN
MSGTNLDGNDEFDEQLRMQELYGDGKDGDTQTDAGGEPDSLGQQPTDTPYEWDLDKKAWFPKITEDFIATYQANYGFSNDGASSSTA
NVEDVHARTAEEPPQEKAPEPTDARKKGEKRKAESGWFHVEEDRNTNVYVSGLPPDITVDEFIQLMSKFGIIMRDPQTEEFKVKLYK
DNQGNLKGDGLCCYLKRESVELALKLLDEDEIRGYKLHVEVAKFQLKGEYDASKKKKKCKDYKKKLSMQQKQLDWRPERRAGPSRMR
HERVVIIKNMFHPMDFEDDPLVLNEIREDLRVECSKFGQIRKLLLFDRHPDGVASVSFRDPEEADYCIQTLDGRWFGGRQITAQAWD
GTTDYQVEETSREREERLRGWEAFLNAPEANRGLRRSDSVSASERAGPSRARHFSEHPSTSKMNAQETATGMAFEEPIDEKKFEKTE
DGGEFEEGASENNAKESSPEKEAEEGCPEKESEEGCPKRGFEGSCSQKESEEGNPVRGSEEDSPKKESKKKTLKNDCEENGLAKESE
DDLNKESEEEVGPTKESEEDDSEKESDEDCSEKQSEDGSEREFEENGLEKDLDEEGSEKELHENVLDKELEENDSENSEFEDDGSEK

VLDEEGSEREFDEDSDEKEEEEDTYEKVFDDESDEKEDEEYADEKGLEAADKKAEEGDADEKLFEESDDKEDEDADGKEVEDADEKL
FEDDDSNEKLFDEEEDSSEKLFDDSDERGTLGGFGSVEEGPLSTGSSFILSSDDDDDDI
>sp|P49755|TMEDA_HUMAN
MSGLSGPPARRGPFPLALLLLFLLGPRLVLAISFHLPINSRKCLREEIHKDLLVTGAYEISDQSGGAGGLRSHLKITDSAGHILYSK
EDATKGKFAFTTEDYDMFEVCFESKGTGRIPDQLVILDMKHGVEAKNYEEIAKVEKLKPLEVELRRLEDLSESIVNDFAYMKKREEE
MRDTNESTNTRVLYFSIFSMFCLIGLATWQVFYLRRFFKAKKLIE
>sp|A6NE52|WDR97_HUMAN
MEAEVWEAEGYNLVLDSDLYDADGYDVPDPGLLTEKNELTFTEPSQVLPFLTSSQQWQSLTPRARARRLWLLLRTSLHEVVEKEKRA
ELRAARLTHGLEPLRRLEVAAGLRSVAQDPVGGRFVVLDGAGRLHLHKEDGWAQETLLAPVRLTGLVTVLGPLGAVGRFVGWGPAGL
AILRPNLSLLWLSEQGVGRAPGWAPTCCLPVPDLRLLLVAEMNSSLALWQFRSGGRRLVLRGSALHPPPSPTGRLMRLAVAPVPPHH
VLRCFAAYGSAVLTFDLHAWTLVDVRRDLHKTTISDLAYCEEVEAMVTASRDSTVKVWEADWQIRMVFVGHTGPVTAMTVLPNTTLV
LSASQDGTLRTWDLQAAAQVGEVALGFWGQDKLSRRVGRLLAPVRPGWPVLSLCASSMQLWRVRELYSPLAQLPAKVLHVQVAPALP
APAHQSLPTRLVCACADGSVYLLSAATGRIVSSLLLEPEDCAAAVAYCLPREALWLLTRAGHLVRANAARCPMSVLHRVCPPPPPAP
QPCCLHLYSHLTDLEGAFSSWEIVRQHWGELRCSSVACAWKNKNRYLPVVGHTDGTLSVLEWLSSKTVFQTEAHSPGPVVAIASTWN
SIVSSGGDLTVKMWRVFPYAEESLSLLRTFSCCYPAVALCALGRRVTAGFEDPDSATYGLVQFGLGDSPRLDHRPQDDPTDHITGLC
CCPTLKLYACSSLDCTVRIWTAENRLLRLLQLNGAPQALAFCSNSGDLVLALGSRLCLVSHRLYLPTSYLVKKMCRKAPDVVDDPPL
PLMSQESLTSAQLQRLTNLHGAASLSEALSLIHRRRATSQHLVPKEDLDAIVARDRDLQQLRLGLVVPAAQPPPSWQQRQEGFDNYL
RLIYGSGLLGMQSGRGSQQWSAGTLRVERETRDVCAVPQAAHCLARAEVSTAAQTVPTALSPQDLGALGQHFSQSPRVTVPIPPTHR
RVHSKASQLLARSSLSHYLGISLDLQLQLEQLRGRTTMALDLPSSHLQCRIPLLPKRWDKEPLSSLRGFFPATVQPHKHCLRPICFP
GYVPNSAVLQQMWLNAEPGASQDALWLWRPRPSQTQWQRKLLQWMGEKPGEEGEEDKKEEEEEKEDEELDWALASLSPHSNQQLDSW
ELEDQSAVDWTQEPRRRSCKVARTHPHPWHRHGSLLLDEHYGHLPKFLHFFIYQTWFKKLFPIFSLQAYPEAGTIEGLASLLVALLE
KTTWVDRVHILQVLLRLLPNMSSDLQGQLQGLLVHLLNLDQPPSLQDQTQKKFVILALQLLLACSLESRDVVLELMSYFLYSPVHCR
PELKKLLHGLGLQDPEGFLFKEMMTWVQGPDLDSKAGLRTCCHQKLEDMIQELQETPSQTSVVSGAPTRASVIPSGTSWSASGIFGR
LSQVSEVPLMVVSPAEPHSLAPELQAQRMLAPKRSWGTPQLRLRVLSETLKSFCLEPEARLHPAGPAQLPGEPPPLEETDWSHSQLL
DLGPIDALNFFCEQLRAQQRSSLQEKAAHPHPPEPYTVAPVPDMVVPPPREHWYHPILRLQEAKPQRSARSAMRLRGPMRSRLCAGR
TLDGPIRTLKLPLPRVEPQPFPLDWPMPPRPLPPRLLQPALQRYFLPADADPDTYS
>sp|P50993|AT1A2_HUMAN
MGRGAGREYSPAATTAENGGGKKKQKEKELDELKKEVAMDDHKLSLDELGRKYQVDLSKGLTNQRAQDVLARDGPNALTPPPTTPEW
VKFCRQLFGGFSILLWIGAILCFLAYGIQAAMEDEPSNDNLYLGVVLAAVVIVTGCFSYYQEAKSSKIMDSFKNMVPQQALVIREGE
KMQINAEEVVVGDLVEVKGGDRVPADLRIISSHGCKVDNSSLTGESEPQTRSPEFTHENPLETRNICFFSTNCVEGTARGIVIATGD
RTVMGRIATLASGLEVGRTPIAMEIEHFIQLITGVAVFLGVSFFVLSLILGYSWLEAVIFLIGIIVANVPEGLLATVTVCLTLTAKR
MARKNCLVKNLEAVETLGSTSTICSDKTGTLTQNRMTVAHMWFDNQIHEADTTEDQSGATFDKRSPTWTALSRIAGLCNRAVFKAGQ
ENISVSKRDTAGDASESALLKCIELSCGSVRKMRDRNPKVAEIPFNSTNKYQLSIHEREDSPQSHVLVMKGAPERILDRCSTILVQG
KEIPLDKEMQDAFQNAYMELGGLGERVLGFCQLNLPSGKFPRGFKFDTDELNFPTEKLCFVGLMSMIDPPRAAVPDAVGKCRSAGIK
VIMVTGDHPITAKAIAKGVGIISEGNETVEDIAARLNIPMSQVNPREAKACVVHGSDLKDMTSEQLDEILKNHTEIVFARTSPQQKL
IIVEGCQRQGAIVAVTGDGVNDSPALKKADIGIAMGISGSDVSKQAADMILLDDNFASIVTGVEEGRLIFDNLKKSIAYTLTSNIPE
ITPFLLFIIANIPLPLGTVTILCIDLGTDMVPAISLAYEAAESDIMKRQPRNSQTDKLVNERLISMAYGQIGMIQALGGFFTYFVIL
AENGFLPSRLLGIRLDWDDRTMNDLEDSYGQEWTYEQRKVVEFTCHTAFFASIVVVQWADLIICKTRRNSVFQQGMKNKILIFGLLE
ETALAAFLSYCPGMGVALRMYPLKVTWWFCAFPYSLLIFIYDEVRKLILRRYPGGWVEKETYY
>sp|Q92482|AQP3_HUMAN
MGRQKELVSRCGEMLHIRYRLLRQALAECLGTLILVMFGCGSVAQVVLSRGTHGGFLTINLAFGFAVTLGILIAGQVSGAHLNPAVT
FAMCFLAREPWIKLPIYTLAQTLGAFLGAGIVFGLYYDAIWHFADNQLFVSGPNGTAGIFATYPSGHLDMINGFFDQFIGTASLIVC
VLAIVDPYNNPVPRGLEAFTVGLVVLVIGTSMGFNSGYAVNPARDFGPRLFTALAGWGSAVFTTGQHWWWVPIVSPLLGSIAGVFVY
QLMIGCHLEQPPPSNEEENVKLAHVKHKEQI

>sp|P62306|RUXF_HUMAN
MSLPLNPKPFLNGLTGKPVMVKLKWGMEYKGYLVSVDGYMNMQLANTEEYIDGALSGHLGEVLIRCNNVLYIRGVEEEEEDGEMRE
>sp|P58173|OR2B6_HUMAN
MNWVNDSIIQEFILLGFSDRPWLEFPLLVVFLISYTVTIFGNLTIILVSRLDTKLHTPMYFFLTNLSLLDLCYTTCTVPQMLVNLCS
IRKVISYRGCVAQLFIFLALGATEYLLLAVMSFDRFVAICRPLHYSVIMHQRLCLQLAAASWVTGFSNSVWLSTLTLQLPLCDPYVI
DHFLCEVPALLKLSCVETTANEAELFLVSELFHLIPLTLILISYAFIVRAVLRIQSAEGRQKAFGTCGSHLIVVSLFYSTAVSVYLQ
PPSPSSKDQGKMVSLFYGIIAPMLNPLIYTLRNKEVKEGFKRLVARVFLIKK
>sp|A8MW99|MEI4L_HUMAN
MDVQKWYLRTSKLALALAIIRSKPADKSSREYTEHLAMLLSEEQSKWRSKVEILEAEVMQLRQKLLVSRLCSGSFKSGYVSSQLEAQ
EPKSSESTLTSMEDSGCDLSNEQRTESSDLSQHFVESCTPTHFPPLPLVKRPCAILQNPLSSHMQFLQYLLELKNLTESGNLKRDLT
HFEKDSSTVSDSVFQLLDGLITFYRNPKLPFSRFWTEAVGTLASLISDYNLSSHILKKCSKKLEEFEKTLLHAILGNNHINQFQVQH
YVSQSLVTLGNCSLLRKSIISLLLSEVNGFADDLGAINQEQASYDVSRYENIFYLFWVLEQLLQKETEEGNTSSIGHDDQEIKKFLQ
KHDETIFQLSDAFPLFTFYLWRVGILLSSAQIETLRK
>sp|Q8IVC4|ZN584_HUMAN
MAGEAEAQLDPSLQGLVMFEDVTVYFSREEWGLLNVTQKGLYRDVMLENFALVSSLGLAPSRSPVFTQLEDDEQSWVPSWVDVTPVS
RAEARRGFGLDGLCRVEDERAHPEHLKSYRVIQHQDTHSEGKPRRHTEHGAAFPPGSSCGQQQEVHVAEKLFKCSDCGKVFLKAFAL
LDHLITHSEERPFRCPTGRSAFKKSAHINPRKIHTGETAHVCNECGKAFSYPSKLRKHQKVHTGIKPFKCSDCGKTFNRKDALVLHQ
RIHTGERPYECSKCGKTFSVLSTLIRHRKVHIGERPYECTECGKFFKYNNSFILHQRVHTGERPFECKQCGKGYVTRSGLYQHWKVH
TGERPYECSLCGKTFTTRSYRNRHQQFHTEERSYECTECGKAFKHSSTLLQHKKVHTPERRQEDRAHGKVVSC
>sp|Q9BSG0|PADC1_HUMAN
MVPGAAGWCCLVLWLPACVAAHGFRIHDYLYFQVLSPGDIRYIFTATPAKDFGGIFHTRYEQIHLVPAEPPEACGELSNGFFIQDQI
ALVERGGCSFLSKTRVVQEHGGRAVIISDNAVDNDSFYVEMIQDSTQRTADIPALFLLGRDGYMIRRSLEQHGLPWAIISIPVNVTS
IPTFELLQPPWTFW
>sp|Q9HAV5|TNR27_HUMAN
MDCQENEYWDQWGRCVTCQRCGPGQELSKDCGYGEGGDAYCTACPPRRYKSSWGHHRCQSCITCAVINRVQKVNCTATSNAVCGDCL
PRFYRKTRIGGLQDQECIPCTKQTPTSEVQCAFQLSLVEADTPTVPPQEATLVALVSSLLVVFTLAFLGLFFLYCKQFFNRHCQRGG
LLQFEADKTAKEESLFPVPPSKETSAESQVSENIFQTQPLNPILEDDCSSTSGFPTQESFTMASCTSESHSHWVHSPIECTELDLQK
FSSSASYTGAETLGGNTVESTGDRLELNVPFEVPSP
>sp|Q9BXR0|TGT_HUMAN
MAGAATQASLESAPRIMRLVAECSRSRARAGELWLPHGTVATPVFMPVGTQATMKGITTEQLDALGCRICLGNTYHLGLRPGPELIQ
KANGLHGFMNWPHNLLTDSGGFQMVSLVSLSEVTEEGVRFRSPYDGNETLLSPEKSVQIQNALGSDIIMQLDDVVSSTVTGPRVEEA
MYRSIRWLDRCIAAHQRPDKQNLFAIIQGGLDADLRATCLEEMTKRDVPGFAIGGLSGGESKSQFWRMVALSTSRLPKDKPRYLMGV
GYATDLVVCVALGCDMFDCVFPTRTARFGSALVPTGNLQLRKKVFEKDFGPIDPECTCPTCQKHSRAFLHALLHSDNTAALHHLTVH
NIAYQLQLMSAVRTSIVEKRFPDFVRDFMGAMYGDPTLCPTWATDALASVGITLG
>sp|Q9BQE9|BCL7B_HUMAN
MSGRSVRAETRSRAKDDIKKVMAAIEKVRKWEKKWVTVGDTSLRIFKWVPVTDSKEKEKSKSNSSAAREPNGFPSDASANSSLLLEF
QDENSNQSSVSDVYQLKVDSSTNSSPSPQQSESLSPAHTSDFRTDDSQPPTLGQEILEEPSLPSSEVADEPPTLTKEEPVPLETQVV
EEEEDSGAPPLKRFCVDQPTVPQTASES
>sp|Q9BS91|S35A5_HUMAN
MEKQCCSHPVICSLSTMYTFLLGAIFIALSSSRILLVKYSANEENKYDYLPTTVNVCSELVKLVFCVLVSFCVIKKDHQSRNLKYAS
WKEFSDFMKWSIPAFLYFLDNLIVFYVLSYLQPAMAVIFSNFSIITTALLFRIVLKRRLNWIQWASLLTLFLSIVALTAGTKTLQHN
LAGRGFHHDAFFSPSNSCLLFRSECPRKDNCTAKEWTFPEAKWNTTARVFSHIRLGMGHVLIIVQCFISSMANIYNEKILKEGNQLT
ESIFIQNSKLYFFGILFNGLTLGLQRSNRDQIKNCGFFYGHSAFSVALIFVTAFQGLSVAFILKFLDNMFHVLMAQVTTVIITTVSV
LVFDFRPSLEFFLEAPSVLLSIFIYNASKPQVPEYAPRQERIRDLSGNLWERSSGDGEELERLTKPKSDESDEDTF

>sp|Q86WW8|COA5_HUMAN
MPKYYEDKPQGGACAGLKEDLGACLLQSDCVVQEGKSPRQCLKEGYCNSLKYAFFECKRSVLDNRARFRGRKGY
>sp|Q92615|LAR4B_HUMAN
MTSDQDAKVVAEPQTQRVQEGKDSAHLMNGPISQTTSQTSSIPPLSQVPATKVSELNPNAEVWGAPVLHLEASSAADGVSAAWEEVA
GHHADRGPQGSDANGDGDQGHENAALPDPQESDPADMNALALGPSEYDSLPENSETGGNESQPDSQEDPREVLKKTLEFCLSRENLA
SDMYLISQMDSDQYVPITTVANLDHIKKLSTDVDLIVEVLRSLPLVQVDEKGEKVRPNQNRCIVILREISESTPVEEVEALFKGDNL
PKFINCEFAYNDNWFITFETEADAQQAYKYLREEVKTFQGKPIKARIKAKAIAINTFLPKNGFRPLDVSLYAQQRYATSFYFPPMYS
PQQQFPLYSLITPQTWSATHSYLDPPLVTPFPNTGFINGFTSPAFKPAASPLTSLRQYPPRSRNPSKSHLRHAIPSAERGPGLLESP
SIFNFTADRLINGVRSPQTRQAGQTRTRIQNPSAYAKREAGPGRVEPGSLESSPGLGRGRKNSFGYRKKREEKFTSSQTQSPTPPKP
PSPSFELGLSSFPPLPGAAGNLKTEDLFENRLSSLIIGPSKERTLSADASVNTLPVVVSREPSVPASCAVSATYERSPSPAHLPDDP
KVAEKQRETHSVDRLPSALTATACKSVQVNGAATELRKPSYAEICQRTSKEPPSSPLQPQKEQKPNTVGCGKEEKKLAEPAERYREP
PALKSTPGAPRDQRRPAGGRPSPSAMGKRLSREQSTPPKSPQ
>sp|Q86Y01|DTX1_HUMAN
MSRPGHGGLMPVNGLGFPPQNVARVVVWEWLNEHSRWRPYTATVCHHIENVLKEDARGSVVLGQVDAQLVPYIIDLQSMHQFRQDTG
TMRPVRRNFYDPSSAPGKGIVWEWENDGGAWTAYDMDICITIQNAYEKQHPWLDLSSLGFCYLIYFNSMSQMNRQTRRRRRLRRRLD
LAYPLTVGSIPKSQSWPVGASSGQPCSCQQCLLVNSTRAASNAILASQRRKAPPAPPLPPPPPPGGPPGALAVRPSATFTGAALWAA
PAAGPAEPAPPPGAPPRSPGAPGGARTPGQNNLNRPGPQRTTSVSARASIPPGVPALPVKNLNGTGPVHPALAGMTGILLCAAGLPV
CLTRAPKPILHPPPVSKSDVKPVPGVPGVCRKTKKKHLKKSKNPEDVVRRYMQKVKNPPDEDCTICMERLVTASGYEGVLRHKGVRP
ELVGRLGRCGHMYHLLCLVAMYSNGNKDGSLQCPTCKAIYGEKTGTQPPGKMEFHLIPHSLPGFPDTQTIRIVYDIPTGIQGPEHPN
PGKKFTARGFPRHCYLPNNEKGRKVLRLLITAWERRLIFTIGTSNTTGESDTVVWNEIHHKTEFGSNLTGHGYPDASYLDNVLAELT
AQGVSEAAAKA
>sp|P26006|ITA3_HUMAN
MGPGPSRAPRAPRLMLCALALMVAAGGCVVSAFNLDTRFLVVKEAGNPGSLFGYSVALHRQTERQQRYLLLAGAPRELAVPDGYTNR
TGAVYLCPLTAHKDDCERMNITVKNDPGHHIIEDMWLGVTVASQGPAGRVLVCAHRYTQVLWSGSEDQRRMVGKCYVRGNDLELDSS
DDWQTYHNEMCNSNTDYLETGMCQLGTSGGFTQNTVYFGAPGAYNWKGNSYMIQRKEWDLSEYSYKDPEDQGNLYIGYTMQVGSFIL
HPKNITIVTGAPRHRHMGAVFLLSQEAGGDLRRRQVLEGSQVGAYFGSAIALADLNNDGWQDLLVGAPYYFERKEEVGGAIYVFMNQ
AGTSFPAHPSLLLHGPSGSAFGLSVASIGDINQDGFQDIAVGAPFEGLGKVYIYHSSSKGLLRQPQQVIHGEKLGLPGLATFGYSLS
GQMDVDENFYPDLLVGSLSDHIVLLRARPVINIVHKTLVPRPAVLDPALCTATSCVQVELCFAYNQSAGNPNYRRNITLAYTLEADR
DRRPPRLRFAGSESAVFHGFFSMPEMRCQKLELLLMDNLRDKLRPIIISMNYSLPLRMPDRPRLGLRSLDAYPILNQAQALENHTEV
QFQKECGPDNKCESNLQMRAAFVSEQQQKLSRLQYSRDVRKLLLSINVTNTRTSERSGEDAHEALLTLVVPPALLLSSVRPPGACQA
NETIFCELGNPFKRNQRMELLIAFEVIGVTLHTRDLQVQLQLSTSSHQDNLWPMILTLLVDYTLQTSLSMVNHRLQSFFGGTVMGES
GMKTVEDVGSPLKYEFQVGPMGEGLVGLGTLVLGLEWPYEVSNGKWLLYPTEITVHGNGSWPCRPPGDLINPLNLTLSDPGDRPSSP
QRRRRQLDPGGGQGPPPVTLAAAKKAKSETVLTCATGRAHCVWLECPIPDAPVVTNVTVKARVWNSTFIEDYRDFDRVRVNGWATLF
LRTSIPTINMENKTTWFSVDIDSELVEELPAEIELWLVLVAVGAGLLLLGLIILLLWKCGFFKRARTRALYEAKRQKAEMKSQPSET
ERLTDDY
>sp|O14545|TRAD1_HUMAN
MAEFLDDQETRLCDNCKKEIPVFNFTIHEIHCQRNIGMCPTCKEPFPKSDMETHMAAEHCQVTCKCNKKLEKRLLKKHEETECPLRL
AVCQHCDLELSILKLKEHEDYCGARTELCGNCGRNVLVKDLKTHPEVCGREGEEKRNEVAIPPNAYDESWGQDGIWIASQLLRQIEA
LDPPMRLPRRPLRAFESDVFHNRTTNQRNITAQVSIQNNLFEEQERQERNRGQQPPKEGGEESANLDFMLALSLQNEGQASSVAEQD
FWRAVCEADQSHGGPRSLSDIKGAADEIMLPCEFCEELYPEELLIDHQTSCNPSRALPSLNTGSSSPRGVEEPDVIFQNFLQQAASN
QLDSLMGLSNSHPVEESIIIPCEFCGVQLEEEVLFHHQDQCDQRPATATNHVTEGIPRLDSQPQETSPELPRRRVRHQGDLSSGYLD
DTKQETANGPTSCLPPSRPINNMTATYNQLSRSTSGPRPGCQPSSPCVPKLSNSDSQDIQGRNRDSQNGAIAPGHVSVIRPPQNLYP
ENIVPSFSPGPSGRYGASGRSEGGRNSRVTPAAANYRSRTAKAKPSKQQGAGDAEEEEEE
>sp|Q9Y512|SAM50_HUMAN

MGTVHARSLEPLPSSGPDFGGLGEEAEFVEVEPEAKQEILENKDVVVQHVHFDGLGRTKDDIIICEIGDVFKAKNLIEVMRKSHEAR
EKLLRLGIFRQVDVLIDTCQGDDALPNGLDVTFEVTELRRLTGSYNTMVGNNEGSMVLGLKLPNLLGRAEKVTFQFSYGTKETSYGL
SFFKPRPGNFERNFSVNLYKVTGQFPWSSLRETDRGMSAEYSFPIWKTSHTVKWEGVWRELGCLSRTASFAVRKESGHSLKSSLSHA
MVIDSRNSSILPRRGALLKVNQELAGYTGGDVSFIKEDFELQLNKQLIFDSVFSASFWGGMLVPIGDKPSSIADRFYLGGPTSIRGF
SMHSIGPQSEGDYLGGEAYWAGGLHLYTPLPFRPGQGGFGELFRTHFFLNAGNLCNLNYGEGPKAHIRKLAECIRWSYGAGIVLRLG
NIARLELNYCVPMGVQTGDRICDGVQFGAGIRFL
>sp|O60831|PRAF2_HUMAN
MSEVRLPPLRALDDFVLGSARLAAPDPCDPQRWCHRVINNLLYYQTNYLLCFGIGLALAGYVRPLHTLLSALVVAVALGVLVWAAET
RAAVRRCRRSHPAACLAAVLAVGLLVLWVAGGACTFLFSIAGPVLLILVHASLRLRNLKNKIENKIESIGLKRTPMGLLLEALGQEQ
EAGS
>sp|P0DJD9|PEPA5_HUMAN
MKWLLLLGLVALSECIMYKVPLIRKKSLRRTLSERGLLKDFLKKHNLNPARKYFPQWEAPTLVDEQPLENYLDMEYFGTIGIGTPAQ
DFTVVFDTGSSNLWVPSVYCSSLACTNHNRFNPEDSSTYQSTSETVSITYGTGSMTGILGYDTVQVGGISDTNQIFGLSETEPGSFL
YYAPFDGILGLAYPSISSSGATPVFDNIWNQGLVSQDLFSVYLSADDKSGSVVIFGGIDSSYYTGSLNWVPVTVEGYWQITVDSITM
NGETIACAEGCQAIVDTGTSLLTGPTSPIANIQSDIGASENSDGDMVVSCSAISSLPDIVFTINGVQYPVPPSAYILQSEGSCISGF
QGMNVPTESGELWILGDVFIRQYFTVFDRANNQVGLAPVA
>sp|Q5VTQ0|TT39B_HUMAN
MDAVLACRLRGRGNRVAALRPRPRPGGSAGPSPFALLCAGLSPEPRAGVGSEFPAWFLGGSSQRRNMALLGSRAELEADEDVFEDAL
ETISISSHSDMATSSLHFASCDTQQAPRQRGASTVSSSSSTKVDLKSGLEECAVALNLFLSNKFTDALELLRPWAKESMYHALGYST
IVVLQAVLTFEQQDIQNGISAMKDALQTCQKYRKKYTVVESFSSLLSRGSLEQLSEEEMHAEICYAECLLQKAALTFVQDENMINFI
KGGLKIRTSYQIYKECLSILHEIQKNKLQQEFFYEFEGGVKLGSGAFNLMLSLLPARIIRLLEFIGFSGNRELGLLQLREGASGRSM
RSALCCLTILAFHTYISLILGTGEVNVAEAERLLAPFLQQFPNGSLVLFYHARIELLKGNLEEAQEVFQKCISVQEEWKQFHHLCYW
ELMWINVFQQNWMQAYYYSDLLCKESKWSKATYVFLKAAILSMLPEEDVVATNENVVTLFRQVDSLKQRIAGKSIPTEKFAVRKARR
YSASLPAPVKLILPALEMMYVWNGFSIVSKRKDLSENLLVTVEKAEAALQSQNFNSFSVDDECLVKLLKGCCLKNLQRPLQAELCYN
HVVESEKLLKYDHYLVPFTLFELASLYKSQGEIDKAIKFLETARNNYKDYSLESRLHFRIQAALHLWRKPSSD
>sp|Q9C0E2|XPO4_HUMAN
MMAAALGPPEVIAQLENAAKVLMAPPSMVNNEQRQHAEHIFLSFRKSKSPFAVCKHILETSKVDYVLFQAATAIMEAVVREWILLEK
GSIESLRTFLLTYVLQRPNLQKYVREQILLAVAVIVKRGSLDKSIDCKSIFHEVSQLISSGNPTVQTLACSILTALLSEFSSSSKTS
NIGLSMEFHGNCKRVFQEEDLRQIFMLTVEVLQEFSRRENLNAQMSSVFQRYLALANQVLSWNFLPPNLGRHYIAMFESSQNVLLKP
TESWRETLLDSRVMELFFTVHRKIREDSDMAQDSLQCLAQLASLHGPIFPDEGSQVDYLAHFIEGLLNTINGIEIEDSEAVGISSII
SNLITVFPRNVLTAIPSELFSSFVNCLTHLTCSFGRSAALEEVLDKDDMVYMEAYDKLLESWLTLVQDDKHFHKGFFTQHAVQVFNS
YIQCHLAAPDGTRNLTANGVASREEEEISELQEDDRDQFSDQLASVGMLGRIAAEHCIPLLTSLLEERVTRLHGQLQRHQQQLLASP
GSSTVDNKMLDDLYEDIHWLILVTGYLLADDTQGETPLIPPEIMEYSIKHSSEVDINTTLQILGSPGEKASSIPGYNRTDSVIRLLS
AILRVSEVESRAIRADLTHLLSPQMGKDIVWFLKRWAKTYLLVDEKLYDQISLPFSTAFGADTEGSQWIIGYLLQKVISNLSVWSSE
QDLANDTVQLLVTLVERRERANLVIQCENWWNLAKQFASRSPPLNFLSSPVQRTLMKALVLGGFAHMDTETKQQYWTEVLQPLQQRF
LRVINQENFQQMCQQEEVKQEITATLEALCGIAEATQIDNVAILFNFLMDFLTNCIGLMEVYKNTPETVNLIIEVFVEVAHKQICYL
GESKAMNLYEACLTLLQVYSKNNLGRQRIDVTAEEEQYQDLLLIMELLTNLLSKEFIDFSDTDEVFRGHEPGQAANRSVSAADVVLY
GVNLILPLMSQDLLKFPTLCNQYYKLITFICEIFPEKIPQLPEDLFKSLMYSLELGMTSMSSEVCQLCLEALTPLAEQCAKAQETDS
PLFLATRHFLKLVFDMLVLQKHNTEMTTAAGEAFYTLVCLHQAEYSELVETLLSSQQDPVIYQRLADAFNKLTASSTPPTLDRKQKM
AFLKSLEEFMANVGGLLCVK
>sp|O94911|ABCA8_HUMAN
MRKRKISVCQQTWALLCKNFLKKWRMKRESLMEWLNSLLLLLCLYIYPHSHQVNDFSSLLTMDLGRVDTFNESRFSVVYTPVTNTTQ
QIMNKVASTPFLAGKEVLGLPDEESIKEFTANYPEEIVRVTFTNTYSYHLKFLLGHGMPAKKEHKDHTAHCYETNEDVYCEVSVFWK
EGFVALQAAINAAIIEITTNHSVMEELMSVTGKNMKMHSFIGQSGVITDLYLFSCIISFSSFIYYASVNVTRERKRMKALMTMMGLR

DSAFWLSWGLLYAGFIFIMALFLALVIRSTQFIILSGFMVVFSLFLLYGLSLVALAFLMSILVKKSFLTGLVVFLLTVFWGCLGFTS
LYRHLPASLEWILSLLSPFAFMLGMAQLLHLDYDLNSNAFPHPSDGSNLIVATNFMLAFDTCLYLALAIYFEKILPNEYGHRRPPLF
FLKSSFWSQTQKTDHVALEDEMDADPSFHDSFEQAPPEFQGKEAIRIRNVTKEYKGKPDKIEALKDLVFDIYEGQITAILGHSGAGK
STLLNILSGLSVPTKGSVTIYNNKLSEMADLENLSKLTGVCPQSNVQFDFLTVRENLRLFAKIKGILPQEVDKEIFLLDEPTAGLDP
FSRHQVWNLLKERKTDRVILFSTQFMDEADILADRKVFLSQGKLKCAGSSLFLKKKWGIGYHLSLQLNEICVEENITSLVKQHIPDA
KLSAKSEGKLIYTLPLERTNKFPELYKDLDSYPDLGIENYGVSMTTLNEVFLKLEGKSTINESDIAILGEVQAEKADDTERLVEMEQ
VLSSLNKMRKTIGGVALWRQQICAIARVRLLKLKHERKALLALLLILMAGFCPLLVEYTMVKIYQNSYTWELSPHLYFLAPGQQPHD
PLTQLLIINKTGASIDDFIQSVEHQNIALEVDAFGTRNGTDDPSYNGAITVCCNEKNYSFSLACNAKRLNCFPVLMDIVSNGLLGMV
KPSVHIRTERSTFLENGQDNPIGFLAYIMFWLVLTSSCPPYIAMSSIDDYKNRARSQLRISGLSPSAYWFGQALVDVSLYFLVFVFI
YLMSYISNFEDMLLTIIHIIQIPCAVGYSFSLIFMTYVISFIFRKGRKNSGIWSFCFYVVTVFSVAGFAFSIFESDIPFIFTFLIPP
ATMIGCLFLSSHLLFSSLFSEERMDVQPFLVFLIPFLHFIIFLFTLRCLEWKFGKKSMRKDPFFRISPRSSDVCQNPEEPEGEDEDV
QMERVRTANALNSTNFDEKPVIIASCLRKEYAGKRKGCFSKRKNKIATRNVSFCVRKGEVLGLLGHNGAGKSTSIKVITGDTKPTAG
QVLLKGSGGGDALEFLGYCPQENALWPNLTVRQHLEVYAAVKGLRKGDAEVAITRLVDALKLQDQLKSPVKTLSEGIKRKLCFVLSI
LGNPSVVLLDEPSTGMDPEGQQQMWQAIRATFRNTERGALLTTHYMAEAEAVCDRVAIMVSGRLRCIGSIQHLKSKFGKDYLLEMKV
KNLAQVEPLHAEILRLFPQAARQERYSSLMVYKLPVEDVQPLAQAFFKLEKVKQSFDLEEYSLSQSTLEQVFLELSKEQELGDFEED
FDPSVKWKLLPQEEP
>sp|A8MW92|P20L1_HUMAN
MSKKPPNRPGITFEIGARLEALDYLQKWYPSRIEKIDYEEGKMLVHFERWSHRYDEWIYWDSNRLRPLERPALRKEGLKDEEDFFDF
KAGEEVLARWTDCRYYPAKIEAINKEGTFTVQFYDGVIRCLKRMHIKAMPEDAKGQVKSQHPLSWCCPIDPAGSCNQSMGSEDWIAL
VKAAAAAAAKNKTGSKPRTSANSNKDKDKDERKWFKVPSKKEETSTCIATPDVEKKEDLPTSSETFGLHVENVPKMVFPQPESTLSN
KRKNNQGNSFQAKRARLNKITGLLASKAVGVDGAEKKEDYNETAPMLEQAISPKPQSQKKNEADISSSANTQKPALLSSTLSSGKAR
SKKCKHESGDSSGCIKPPKSPLSPELIQVEDLTLVSQLSSSVINKTSPPQPVNPPRPFKHSERRRRSQRLATLPMPDDSVEKVSSPS
PATDGKVFSISSQNQQESSVPEVPDVAHLPLEKLGPCLPLDLSRGSEVTAPVASDSSYRNECPRAEKEDTQMLPNPSSKAIADGRGA
PAAAGISKTEKKVKLEDKSSTAFGKRKEKDKERREKRDKDHYRPKQKKKKKKKKSKQHDYSDYEDSSLEFLERCSSPLTRSSGSSL
ASRSMFTEKTTTYQYPRAILSVDLSGENLSDVDFLDDSSTESLLLSGDEYNQDFDSTNFEESQDEDDALNEIVRCICEMDEENGFMI
QCEECLCWQHSVCMGLLEESIPEQYICYICRDPPGQRWSAKYRYDKEWLNNGRMCGLSFFKENYSHLNAKKIVSTHHLLADVYGVTE
VLHGLQLKIGILKNKHHPDLHLWACSGKRKDQDQIIAGVEKKIAQDTVNREEKKYVQNHKEPPRLPLKMEGTYITSEHSYQKPQSFG
QDCKSLADPGSSDDDDVSSLEEEQEFHMRSKNSLQYSAKEHGMPEKNPAEGNTVFVYNDKKGTEDPGDSHLQWQLNLLTHIENVQNE
VTSRMDLIEKEVDVLESWLDFTGELEPPDPLARLPQLKRHIKQLLIDMGKVQQIATLCSV
>sp|Q8N9S9|SNX31_HUMAN
MKMHFCIPVSQQRSDALGGRYVLYSVHLDGFLFCRVRYSQLHGWNEQLRRVFGNCLPPFPPKYYLAMTTAMADERRDQLEQYLQNVT
MDPNVLRSDVFVEFLKLAQLNTFDIATKKAYLDIFLPNEQSIRIEIITSDTAERVLEVVSHKIGLCRELLGYFGLFLIRFGKEGKLS
VVKKLADFELPYVSLGSSEVENCKVGLRKWYMAPSLDSVLMDCRVAVDLLYMQAIQDIEKGWAKPTQAQRQKLEAFQKEDSQTKFLE
LAREVRHYGYLQLDPCTCDYPESGSGAVLSVGNNEISCCITLPDSQTQDIVFQMSRVKCWQVTFLGTLLDTDGPQRTLNQNLELRFQ
YSEDSCWQWFVIYTKQAFLLSSCLKKMISEKMVKLAAENTEMQIEVPEQSKSKKYHIQQSQQKDYSSFLSRKSKIKIAKDDCVFGNI
KEEDL
>sp|Q8N335|GPD1L_HUMAN
MAAAPLKVCIVGSGNWGSAVAKIIGNNVKKLQKFASTVKMWVFEETVNGRKLTDIINNDHENVKYLPGHKLPENVVAMSNLSEAVQD
ADLLVFVIPHQFIHRICDEITGRVPKKALGITLIKGIDEGPEGLKLISDIIREKMGIDISVLMGANIANEVAAEKFCETTIGSKVME
NGLLFKELLQTPNFRITVVDDADTVELCGALKNIVAVGAGFCDGLRCGDNTKAAVIRLGLMEMIAFARIFCKGQVSTATFLESCGVA
DLITTCYGGRNRRVAEAFARTGKTIEELEKEMLNGQKLQGPQTSAEVYRILKQKGLLDKFPLFTAVYQICYESRPVQEMLSCLQSHP
EHT
>sp|O15528|CP27B_HUMAN

MTQTLKYASRVFHRVRWAPELGASLGYREYHSARRSLADIPGPSTPSFLAELFCKGGLSRLHELQVQGAAHFGPVWLASFGTVRTVY
VAAPALVEELLRQEGPRPERCSFSPWTEHRRCRQRACGLLTAEGEEWQRLRSLLAPLLLRPQAAARYAGTLNNVVCDLVRRLRRQRG
RGTGPPALVRDVAGEFYKFGLEGIAAVLLGSRLGCLEAQVPPDTETFIRAVGSVFVSTLLTMAMPHWLRHLVPGPWGRLCRDWDQMF
AFAQRHVERREAEAAMRNGGQPEKDLESGAHLTHFLFREELPAQSILGNVTELLLAGVDTVSNTLSWALYELSRHPEVQTALHSEIT
AALSPGSSAYPSATVLSQLPLLKAVVKEVLRLYPVVPGNSRVPDKDIHVGDYIIPKNTLVTLCHYATSRDPAQFPEPNSFRPARWLG
EGPTPHPFASLPFGFGKRSCMGRRLAELELQMALAQILTHFEVQPEPGAAPVRPKTRTVLVPERSINLQFLDR
>sp|Q9HD26|GOPC_HUMAN
MSAGGPCPAAAGGGPGGASCSVGAPGGVSMFRWLEVLEKEFDKAFVDVDLLLGEIDPDQADITYEGRQKMTSLSSCFAQLCHKAQSV
SQINHKLEAQLVDLKSELTETQAEKVVLEKEVHDQLLQLHSIQLQLHAKTGQSADSGTIKAKLSGPSVEELERELEANKKEKMKEAQ
LEAEVKLLRKENEALRRHIAVLQAEVYGARLAAKYLDKELAGRVQQIQLLGRDMKGPAHDKLWNQLEAEIHLHRHKTVIRACRGRND
LKRPMQAPPGHDQDSLKKSQGVGPIRKVLLLKEDHEGLGISITGGKEHGVPILISEIHPGQPADRCGGLHVGDAILAVNGVNLRDTK
HKEAVTILSQQRGEIEFEVVYVAPEVDSDDENVEYEDESGHRYRLYLDELEGGGNPGASCKDTSGEIKVLQGFNKKAVTDTHENGDL
GTASETPLDDGASKLDDLHTLYHKKSY
>sp|Q8IVW1|ARL17_HUMAN
MGNIFEKLFKSLLGKKKMRILILSLDTAGKTTILYKLKLGETVPAVPTVGFCVETVEYKNNTFAVWDVGSHFKIRPLWQHFFQNTKG
ARSPGSTHQGSLASGVLPIKCSHVEFGMWKGGRSHPFLPHSSRCAGSGGQLDSILPHQSPAWGPWGCKDLSSGFPSFLTSSILWKSA
VVK
>sp|Q5UAW9|GP157_HUMAN
MQPSPPPTELVPSERAVVLLSCALSALGSGLLVATHALWPDLRSRARRLLLFLSLADLLSAASYFYGVLQNFAGPSWDCVLQGALST
FANTSSFFWTVAIALYLYLSIVRAARGPRTDRLLWAFHVVSWGVPLVITVAAVALKKIGYDASDVSVGWCWIDLEAKDHVLWMLLTG
KLWEMLAYVLLPLLYLLVRKHINRAHTALSEYRPILSQEHRLLRHSSMADKKLVLIPLIFIGLRVWSTVRFVLTLCGSPAVQTPVLV
VLHGIGNTFQGGANCIMFVLCTRAVRTRLFSLCCCCCSSQPPTKSPAGTPKAPAPSKPGESQESQGTPGELPST
>sp|O15374|MOT5_HUMAN
MLKREGKVQPYTKTLDGGWGWMIVIHFFLVNVFVMGMTKTFAIFFVVFQEEFEGTSEQIGWIGSIMSSLRFCAGPLVAIICDILGEK
TTSILGAFVVTGGYLISSWATSIPFLCVTMGLLPGLGSAFLYQVAAVVTTKYFKKRLALSTAIARSGMGLTFLLAPFTKFLIDLYDW
TGALILFGAIALNLVPSSMLLRPIHIKSENNSGIKDKGSSLSAHGPEAHATETHCHETEESTIKDSTTQKAGLPSKNLTVSQNQSEE
FYNGPNRNRLLLKSDEESDKVISWSCKQLFDISLFRNPFFYIFTWSFLLSQLAYFIPTFHLVARAKTLGIDIMDASYLVSVAGILET
VSQIISGWVADQNWIKKYHYHKSYLILCGITNLLAPLATTFPLLMTYTICFAIFAGGYLALILPVLVDLCRNSTVNRFLGLASFFAG
MAVLSGPPIAGWLYDYTQTYNGSFYFSGICYLLSSVSFFFVPLAERWKNSLT
>sp|P13928|ANXA8_HUMAN
MAWWKSWIEQEGVTVKSSSHFNPDPDAETLYKAMKGIGTNEQAIIDVLTKRSNTQRQQIAKSFKAQFGKDLTETLKSELSGKFERLI
VALMYPPYRYEAKELHDAMKGLGTKEGVIIEILASRTKNQLREIMKAYEEDYGSSLEEDIQADTSGYLERILVCLLQGSRDDVSSFV
DPGLALQDAQDLYAAGEKIRGTDEMKFITILCTRSATHLLRVFEEYEKIANKSIEDSIKSETHGSLEEAMLTVVKCTQNLHSYFAER
LYYAMKGAGTRDGTLIRNIVSRSEIDLNLIKCHFKKMYGKTLSSMIMEDTSGDYKNALLSLVGSDP
>sp|O15243|OBRG_HUMAN
MAGVKALVALSFSGAIGLTFLMLGCALEDYGVYWPLFVLIFHAISPIPHFIAKRVTYDSDATSSACRELAYFFTTGIVVSAFGFPVI
LARVAVIKWGACGLVLAGNAVIFLTIQGFFLIFGRGDDFSWEQW
>sp|Q9BZI7|REN3B_HUMAN
MKEEKEHRPKEKRVTLLTPAGATGSGGGTSGDSSKGEDKQDRNKEKKEALSKVVIRRLPPTLTKEQLQEHLQPMPEHDYFEFFSNDT
SLYPHMYARAYINFKNQEDIILFRDRFDGYVFLDNKGQEYPAIVEFAPFQKAAKKKTKKRDTKVGTIDDDPEYRKFLESYATDNEKM
TSTPETLLEEIEAKNRELIAKKTTPLLSFLKNKQRMREEKREERRRREIERKRQREEERRKWKEEEKRKRKDIEKLKKIDRIPERDK
LKDEPKIKVHRFLLQAVNQKNLLKKPEKGDEKELDKREKAKKLDKENLSDERASGQSCTLPKRSDSELKDEKPKRPEDESGRDYRER
EREYERDQERILRERERLKRQEEERRRQKERYEKEKTFKRKEEEMKKEKDTLRDKGKKAESTESIGSSEKTEKKEEVVKRDRIRNKD
RPAMQLYQPGARSRNRLCPPDDSTKSGDSAAERKQESGISHRKEGGEE

>sp|P01880|IGHD_HUMAN
APTKAPDVFPIISGCRHPKDNSPVVLACLITGYHPTSVTVTWYMGTQSQPQRTFPEIQRRDSYYMTSSQLSTPLQQWRQGEYKCVVQ
HTASKSKKEIFRWPESPKAQASSVPTAQPQAEGSLAKATTAPATTRNTGRGGEEKKKEKEKEEQEERETKTPECPSHTQPLGVYLLT
PAVQDLWLRDKATFTCFVVGSDLKDAHLTWEVAGKVPTGGVEEGLLERHSNGSQSQHSRLTLPRSLWNAGTSVTCTLNHPSLPPQRL
MALREPAAQAPVKLSLNLLASSDPPEAASWLLCEVSGFSPPNILLMWLEDQREVNTSGFAPARPPPQPGSTTFWAWSVLRVPAPPSP
QPATYTCVVSHEDSRTLLNASRSLEVSYVTDHGPMK
>sp|O43813|LANC1_HUMAN
MAQRAFPNPYADYNKSLAEGYFDAAGRLTPEFSQRLTNKIRELLQQMERGLKSADPRDGTGYTGWAGIAVLYLHLYDVFGDPAYLQL
AHGYVKQSLNCLTKRSITFLCGDAGPLAVAAVLYHKMNNEKQAEDCITRLIHLNKIDPHAPNEMLYGRIGYIYALLFVNKNFGVEKI
PQSHIQQICETILTSGENLARKRNFTAKSPLMYEWYQEYYVGAAHGLAGIYYYLMQPSLQVSQGKLHSLVKPSVDYVCQLKFPSGNY
PPCIGDNRDLLVHWCHGAPGVIYMLIQAYKVFREEKYLCDAYQCADVIWQYGLLKKGYGLCHGSAGNAYAFLTLYNLTQDMKYLYRA
CKFAEWCLEYGEHGCRTPDTPFSLFEGMAGTIYFLADLLVPTKARFPAFEL
>sp|Q8N3F8|MILK1_HUMAN
MAGPRGALLAWCRRQCEGYRGVEIRDLSSSFRDGLAFCAILHRHRPDLLDFDSLSKDNVFENNRLAFEVAEKELGIPALLDPNDMVS
MSVPDCLSIMTYVSQYYNHFCSPGQAGVSPPRKGLAPCSPPSVAPTPVEPEDVAQGEELSSGSLSEQGTGQTPSSTCAACQQHVHLV
QRYLADGRLYHRHCFRCRRCSSTLLPGAYENGPEEGTFVCAEHCARLGPGTRSGTRPGPFSQPKQQHQQQLAEDAKDVPGGGPSSSA
PAGAEADGPKASPEARPQIPTKPRVPGKLQELASPPAGRPTPAPRKASESTTPAPPTPRPRSSLQQENLVEQAGSSSLVNGRLHELP
VPKPRGTPKPSEGTPAPRKDPPWITLVQAEPKKKPAPLPPSSSPGPPSQDSRQVENGGTEEVAQPSPTASLESKPYNPFEEEEEDKE
EEAPAAPSLATSPALGHPESTPKSLHPWYGITPTSSPKTKKRPAPRAPSASPLALHASRLSHSEPPSATPSPALSVESLSSESASQT
AGAELLEPPAVPKSSSEPAVHAPGTPGNPVSLSTNSSLASSGELVEPRVEQMPQASPGLAPRTRGSSGPQPAKPCSGATPTPLLLVG
DRSPVPSPGSSSPQLQVKSSCKENPFNRKPSPAASPATKKATKGSKPVRPPAPGHGFPLIKRKVQADQYIPEEDIHGEMDTIERRLD
ALEHRGVLLEEKLRGGLNEGREDDMLVDWFKLIHEKHLLVRRESELIYVFKQQNLEQRQADVEYELRCLLNKPEKDWTEEDRAREKV
LMQELVTLIEQRNAIINCLDEDRQREEEEDKMLEAMIKKKEFQREAEPEGKKKGKFKTMKMLKLLGNKRDAKSKSPRDKS
>sp|Q96J02|ITCH_HUMAN
MSDSGSQLGSMGSLTMKSQLQITVISAKLKENKKNWFGPSPYVEVTVDGQSKKTEKCNNTNSPKWKQPLTVIVTPVSKLHFRVWSHQ
TLKSDVLLGTAALDIYETLKSNNMKLEEVVVTLQLGGDKEPTETIGDLSICLDGLQLESEVVTNGETTCSENGVSLCLPRLECNSAI
SAHCNLCLPGLSDSPISASRVAGFTGASQNDDGSRSKDETRVSTNGSDDPEDAGAGENRRVSGNNSPSLSNGGFKPSRPPRPSRPPP
PTPRRPASVNGSPSATSESDGSSTGSLPPTNTNTNTSEGATSGLIIPLTISGGSGPRPLNPVTQAPLPPGWEQRVDQHGRVYYVDHV
EKRTTWDRPEPLPPGWERRVDNMGRIYYVDHFTRTTTWQRPTLESVRNYEQWQLQRSQLQGAMQQFNQRFIYGNQDLFATSQSKEFD
PLGPLPPGWEKRTDSNGRVYFVNHNTRITQWEDPRSQGQLNEKPLPEGWEMRFTVDGIPYFVDHNRRTTTYIDPRTGKSALDNGPQI
AYVRDFKAKVQYFRFWCQQLAMPQHIKITVTRKTLFEDSFQQIMSFSPQDLRRRLWVIFPGEEGLDYGGVAREWFFLLSHEVLNPMY
CLFEYAGKDNYCLQINPASYINPDHLKYFRFIGRFIAMALFHGKFIDTGFSLPFYKRILNKPVGLKDLESIDPEFYNSLIWVKENNI
EECDLEMYFSVDKEILGEIKSHDLKPNGGNILVTEENKEEYIRMVAEWRLSRGVEEQTQAFFEGFNEILPQQYLQYFDAKELEVLLC
GMQEIDLNDWQRHAIYRHYARTSKQIMWFWQFVKEIDNEKRMRLLQFVTGTCRLPVGGFADLMGSNGPQKFCIEKVGKENWLPRSHT
CFNRLDLPPYKSYEQLKEKLLFAIEETEGFGQE
>sp|Q6IE37|OVOS1_HUMAN
MHVHVCVCLCVCIYTSSCVCACVHMCMRDALLAEGRGGGLAAADDFLYLECCKCFSQESQIAMVCQERSQNETYEVKMNNDTEACRA
TLNLEERRSVAIRSRENVVFVQTDKPTYKPGQKDVNGIAQFFLDTYTFTYPNITLKDPQNNRIFQRQNVTSFRNITQLSFQLISEPM
FGDYWIVVKRNSRETVTHQFAVKRYVLPKFEVTVNAPQTVTISDDEFQVDVCAYNFGQPVQGETQIRVCREYFSSSNCEKNENEICE
QFIAQVQTNLDIFTLLCSSFLTVMQISEKTSVFITQLLGTVNFENMDTFYRRGISYFGQLKFSDPNNVPMVNKLLQLELNDEFIGNY
TTDENGEAQFSIDTSDIFDPEFNLKVRHQRTEECYLPSWLTPQYLDAHFLVSRFYSRTNSFLKIVPEPKQLECNQQKVVTVHYSLNS
EAYEDDSNVKFFYLNGNFSFPISISADLAPAAVLFVYTLHPSGEIVADSVRFQVDKCFKHKVNIKFSNEQGLPGSNASLCLQAAPVL
FCALRAVDRNVLLLKSEQQLSAESVSSLYNMVPSIEPYGYFYHGLNLDDGKEDPCIPQRDMFYNGLYYTPVSNYGDGDIYNIVRVRS
LRILENIIQTVRTNFPETWMWDLVSVSSSGSANLSFLIPDTITQWEASGFCVNGDVGFGISSTTTLEVSQPFFIEIASPFSVVQNEQ

FDLIVNVFSYRNTCVEVSYIWECLPGKVNITVVAESKQSSACPNEGMEQQKLNWKDTVVQSFLVEFLFLGDILGLALQNLVVLQMPY
GSGEQNAALLASDTYVLDYLKSTEQLTEEVQSKAFFLSILGYQRQLSFKNSDGSYSVFWQQSQKGSIWLSALTFKTLERMKKYVFID
ENVQKQTLIWLSSQQKTSGCFKNDGQLFNHALRNALFCLEAALDSGVTNGYNHAILAYAFALAGKEKQVESLLQTLDQSAPKLSKRY
YWERERKPKTEEFPSFIPWAPSAQTEKSCYVLLAVISRKIPDLTYASKIVQWLAQRMNSHGGFSSNQVINVGLILIAARGEEGLFSK
DQNTVTFSSEGSSEIFQVNGHNRLLVQRSEVTQAPGEYTVDVEGHGCTFIQIFRYTGIRNKSSMVVIDVKMLSGFTPTMSSIEEVNN
RSLIFQHKDSYIEYKRADSFPFSVEQSNLVFNIQPAPAMVYDYYEKGRQATAMP
>sp|Q9Y2M2|SSUH2_HUMAN
MPSPVGLLRALPLPWPQFLACTLRRLAGPRESTGPSQKPPPLCSVPCRVPAMTEEVAREALLSFVDSKCCYSSTVAGDLVIQELKRQ
TLCRYRLETFSESRISEWTFQPFTNHSVDGPQRGASPRLWDIKVQGPPMFQEDTRKFQVPHSSLVKECHKCHGRGRYKCSGCHGAGT
VRCPSCCGAKRKAKQSRRCQLCAGSGRRRCSTCSGRGNKTCATCKGEKKLLHFIQLVIMWKNSLFEFVSEHRLNCPRELLAKAKGEN
LFKDENSVVYPIVDFPLRDISLASQRGIAEHSAALASRARVLQQRQTIELIPLTEVHYWYQGKTYVYYIYGTDHQVYAVDYPERYCC
GCTIV
>sp|Q8NHW3|MAFA_HUMAN
MAAELAMGAELPSSPLAIEYVNDFDLMKFEVKKEPPEAERFCHRLPPGSLSSTPLSTPCSSVPSSPSFCAPSPGTGGGGGAGGGGGS
SQAGGAPGPPSGGPGAVGGTSGKPALEDLYWMSGYQHHLNPEALNLTPEDAVEALIGSGHHGAHHGAHHPAAAAAYEAFRGPGFAGG
GGADDMGAGHHHGAHHAAHHHHAAHHHHHHHHHHGGAGHGGGAGHHVRLEERFSDDQLVSMSVRELNRQLRGFSKEEVIRLKQKRRT
LKNRGYAQSCRFKRVQQRHILESEKCQLQSQVEQLKLEVGRLAKERDLYKEKYEKLAGRGGPGSAGGAGFPREPSPPQAGPGGAKGT
ADFFL
>sp|Q01968|OCRL_HUMAN
MEPPLPVGAQPLATVEGMEMKGPLREPCALTLAQRNGQYELIIQLHEKEQHVQDIIPINSHFRCVQEAEETLLIDIASNSGCKIRVQ
GDWIRERRFEIPDEEHCLKFLSAVLAAQKAQSQLLVPEQKDSSSWYQKLDTKDKPSVFSGLLGFEDNFSSMNLDKKINSQNQPTGIH
REPPPPPFSVNKMLPREKEASNKEQPKVTNTMRKLFVPNTQSGQREGLIKHILAKREKEYVNIQTFRFFVGTWNVNGQSPDSGLEPW
LNCDPNPPDIYCIGFQELDLSTEAFFYFESVKEQEWSMAVERGLHSKAKYKKVQLVRLVGMMLLIFARKDQCRYIRDIATETVGTGI
MGKMGNKGGVAVRFVFHNTTFCIVNSHLAAHVEDFERRNQDYKDICARMSFVVPNQTLPQLNIMKHEVVIWLGDLNYRLCMPDANEV
KSLINKKDLQRLLKFDQLNIQRTQKKAFVDFNEGEIKFIPTYKYDSKTDRWDSSGKCRVPAWCDRILWRGTNVNQLNYRSHMELKTS
DHKPVSALFHIGVKVVDERRYRKVFEDSVRIMDRMENDFLPSLELSRREFVFENVKFRQLQKEKFQISNNGQVPCHFSFIPKLNDSQ
YCKPWLRAEPFEGYLEPNETVDISLDVYVSKDSVTILNSGEDKIEDILVLHLDRGKDYFLTISGNYLPSCFGTSLEALCRMKRPIRE
VPVTKLIDLEEDSFLEKEKSLLQMVPLDEGASERPLQVPKEIWLLVDHLFKYACHQEDLFQTPGMQEELQQIIDCLDTSIPETIPGS
NHSVAEALLIFLEALPEPVICYELYQRCLDSAYDPRICRQVISQLPRCHRNVFRYLMAFLRELLKFSEYNSVNANMIATLFTSLLLR
PPPNLMARQTPSDRQRAIQFLLGFLLGSEED
>sp|P48454|PP2BC_HUMAN
MSGRRFHLSTTDRVIKAVPFPPTQRLTFKEVFENGKPKVDVLKNHLVKEGRLEEEVALKIINDGAAILRQEKTMIEVDAPITVCGDI
HGQFFDLMKLFEVGGSPSNTRYLFLGDYVDRGYFSIECVLYLWSLKINHPKTLFLLRGNHECRHLTDYFTFKQECRIKYSEQVYDAC
METFDCLPLAALLNQQFLCVHGGMSPEITSLDDIRKLDRFTEPPAFGPVCDLLWSDPSEDYGNEKTLEHYTHNTVRGCSYFYSYPAV
CEFLQNNNLLSIIRAHEAQDAGYRMYRKSQATGFPSLITIFSAPNYLDVYNNKAAVLKYENNVMNIRQFNCSPHPYWLPNFMDVFTW
SLPFVGEKVTEMLVNVLNICSDDELISDDEAEGSTTVRKEIIRNKIRAIGKMARVFSILRQESESVLTLKGLTPTGTLPLGVLSGGK
QTIETATVEAVEAREAIRGFSLQHKIRSFEEARGLDRINERMPPRKDSIHAGGPMKSVTSAHSHAAHRSDQGKKAHS
>sp|Q13410|BT1A1_HUMAN
MAVFPSSGLPRCLLTLILLQLPKLDSAPFDVIGPPEPILAVVGEDAELPCRLSPNASAEHLELRWFRKKVSPAVLVHRDGREQEAEQ
MPEYRGRATLVQDGIAKGRVALRIRGVRVSDDGEYTCFFREDGSYEEALVHLKVAALGSDPHISMQVQENGEICLECTSVGWYPEPQ
VQWRTSKGEKFPSTSESRNPDEEGLFTVAASVIIRDTSAKNVSCYIQNLLLGQEKKVEISIPASSLPRLTPWIVAVAVILMVLGLLT
IGSIFFTWRLYNERPRERRNEFSSKERLLEELKWKKATLHAVDVTLDPDTAHPHLFLYEDSKSVRLEDSRQKLPEKTERFDSWPCVL
GRETFTSGRHYWEVEVGDRTDWAIGVCRENVMKKGFDPMTPENGFWAVELYGNGYWALTPLRTPLPLAGPPRRVGIFLDYESGDISF

YNMNDGSDIYTFSNVTFSGPLRPFFCLWSSGKKPLTICPIADGPERVTVIANAQDLSKEIPLSPMGEDSAPRDADTLHSKLIPTQPS
QGAP
>sp|Q6UWE3|COLL2_HUMAN
MAAALALVAGVLSGAVLPLWSALPQYKKKITDRCFHHSECYSGCCLMDLDSGGAFCAPRARITMICLPQTKGATNIICPCRMGLTCI
SKDLMCSRRCHMI
>sp|A6NDN8|UBIML_HUMAN
MRGRRRAWRGAWRGGGAADLSLLCPQVAYVRARELHTLEVTGLETVAQSKAHVASLEGLIPEDKVVLLAGSPLQNEATLGQCGVEAL
TTLEVVGRRLGVHNV
>sp|P61578|REC16_HUMAN
MNPSEMQRKAPPRRRRHRNRAPSSHKMNKMMMSEEQMKLPSTNKAEPLTWAQLNKLTQLATKCLENTKMTQTPESMLLAALMIVSTV
SAGVPNSSEETVTIENGP
>sp|Q9Y5H2|PCDGB_HUMAN
MANRLQRGDRSRLLLLLCIFLGTLRGFRARQIRYSVPEETEKGSFVGNISKDLGLEPRELAKRGVRIVSRGKTQLFAVNPRSGSLIT
AGRIDREELCETVSSCFLNMELLVEDTLKIYGVEVEIIDINDNAPSFQEDEVEIKVSEHAIPGARFALPNARDPDVGVNSLQSYQLS
PNNYFSLQLRGRTDGAKNPELVLEGSLDREKEAAHLLLLTALDGGDPIRKGAVPIRVVVLDVNDHIPMFTQSVYRVSVPENISSGTR
VLMVNATDPDEGINGEVMYSFRNMESKASEIFQLDSQTGEVQVRGSLDFEKYRFYEMEIQGQDGGGLFTTTTMLITVVDVNDNAPEI
TITSSINSILENSPPGTVIALLNVQDQDSGENGQVSCFIPNHLPFKLEKTYGNYYKLITSRVLDRELVQSYNITLTATDQGSPPLSA
ETHVWLNVADDNDNPPVFPHSSYSAYIPENNPRGASIFSVTALDPDSKQNALVTYSLTDDTVQGVPLSSYVSINSNTGVLYALQSFD
YEQFRDLELRVIARDSGDPPLSSNVSLSLFVLDQNDNAPEILYPALPTDGSTGVELAPRSAEPGYLVTKVVAVDKDSGQNAWLSYRL
LKASEPGLFAVGEHTGEVRTARALLDRDALKQSLVVAVQDHGQPPLSATVTLTVAVADSIPEVLADLGSLESLANSETSDLSLYLVV
AVAAVSCIFLVFVIVLLALRLWRWHKSRLLQASEGGLAGMPTSHFVGVDGVQAFLQTYSHEVSLIADSQKSHLIFPQPNYGDTLISQ
ESCEKSEPLLIAEDSAIILGKCDPTSNQQAPPNTDWRFSQAQRPGTSGSQNGDDTGTWPNNQFDTEMLQAMILASASEAADGSSTLG
GGAGTMGLSARYGPQFTLQHVPDYRQNVYIPGSNATLTNAAGKRDGKAPAGGNGNKKKSGKKEKK
>sp|A8MWA4|Z705E_HUMAN
MHSLKKVTFEDVAIDFTQEEWAMMDTSKRKLYRDVMLENISHLVSLVTGYQISKSYIILQLEQGKELWQEGREFLQDQNPDRESALK
KTHMISMHPIIRKDAPTSMTMENSLILEDPFECNDSGEDCTHSSTIIQCLLTHSGKKPYVSKQCGKSLSNLLSPKPHKQIHTKGKSY
QCNLCEKAYTNCFHLRRPKMTHTGERPYTCHLCRKAFTQCSHLRRHEKTHTGERPYKCHQCGKAFIQSFNLRRHERTHLGEKWYECD
NSGKAFSQSSGFRGNKIIHTGEKPHACLLCGKAFSLSSDLR
>sp|Q9P2T1|GMPR2_HUMAN
MPHIDNDVKLDFKDVLLRPKRSTLKSRSEVDLTRSFSFRNSKQTYSGVPIIAANMDTVGTFEMAKVLCKFSLFTAVHKHYSLVQWQE
FAGQNPDCLEHLAASSGTGSSDFEQLEQILEAIPQVKYICLDVANGYSEHFVEFVKDVRKRFPQHTIMAGNVVTGEMVEELILSGAD
IIKVGIGPGSVCTTRKKTGVGYPQLSAVMECADAAHGLKGHIISDGGCSCPGDVAKAFGAGADFVMLGGMLAGHSESGGELIERDGK
KYKLFYGMSSEMAMKKYAGGVAEYRASEGKTVEVPFKGDVEHTIRDILGGIRSTCTYVGAAKLKELSRRTTFIRVTQQVNPIFSEAC
>sp|P09564|CD7_HUMAN
MAGPPRLLLLPLLLALARGLPGALAAQEVQQSPHCTTVPVGASVNITCSTSGGLRGIYLRQLGPQPQDIIYYEDGVVPTTDRRFRGR
IDFSGSQDNLTITMHRLQLSDTGTYTCQAITEVNVYGSGTLVLVTEEQSQGWHRCSDAPPRASALPAPPTGSALPDPQTASALPDPP
AASALPAALAVISFLLGLGLGVACVLARTQIKKLCSWRDKNSAACVVYEDMSHSRCNTLSSPNQYQ
>sp|O95471|CLD7_HUMAN
MANSGLQLLGFSMALLGWVGLVACTAIPQWQMSSYAGDNIITAQAMYKGLWMDCVTQSTGMMSCKMYDSVLALSAALQATRALMVVS
LVLGFLAMFVATMGMKCTRCGGDDKVKKARIAMGGGIIFIVAGLAALVACSWYGHQIVTDFYNPLIPTNIKYEFGPAIFIGWAGSAL
VILGGALLSCSCPGNESKAGYRVPRSYPKSNSSKEYV
>sp|Q6PKH6|DR4L2_HUMAN

MARLLGLCAWARKSVRLASSRMTRRDPLTNKVALVTASTDGIGFAIARRLAQDRAHVVVSSRKQQNVDQAVATLQGEGLSVTGTVCH
VGKAEDRERLVAMAVKLHGGIDILVSNAAVNPFFGSLMDVTEEVWDKTLDINVKAPALMTKAVVPEMEKRGGGSVVIVSSIAAFSPS
PGFSPYNVSKTALLGLNNTLAIELAPRNIRVNCLHLDLSRLASAGCSGWTRKKRKA
>sp|Q9NUQ7|UFSP2_HUMAN
MVISESMDILFRIRGGLDLAFQLATPNEIFLKKALKHVLSDLSTKLSSNALVFRICHSSVYIWPSSDINTIPGELTDASACKNILRF
IQFEPEEDIKRKFMRKKDKKLSDMHQIVNIDLMLEMSTSLAAVTPIIERESGGHHYVNMTLPVDAVISVAPEETWGKVRKLLVDAIH
NQLTDMEKCILKYMKGTSIVVPEPLHFLLPGKKNLVTISYPSGIPDGQLQAYRKELHDLFNLPHDRPYFKRSNAYHFPDEPYKDGYI
RNPHTYLNPPNMETGMIYVVQGIYGYHHYMQDRIDDNGWGCAYRSLQTICSWFKHQGYTERSIPTHREIQQALVDAGDKPATFVGSR
QWIGSIEVQLVLNQLIGITSKILFVSQGSEIASQGRELANHFQSEGTPVMIGGGVLAHTILGVAWNEITGQIKFLILDPHYTGAEDL
QVILEKGWCGWKGPDFWNKDAYYNLCLPQRPNMI
>sp|Q96T92|INSM2_HUMAN
MPRGFLVKRTKRTGGLYRVRLAERVFPLLGPQGAPPFLEEAPSASLPGAERATPPTREEPGKGLTAEAAREQSGSPCRAAGVSPGTG
GREGAEWRAGGREGPGPSPSPSPSPAKPAGAELRRAFLERCLSSPVSAESFPGGAAAVAAFSCSVAPAAAPTPGEQFLLPLRAPFPE
PALQPDPAPLSAALQSLKRAAGGERRGKAPTDCASGPAAAGIKKPKAMRKLSFADEVTTSPVLGLKIKEEEPGAPSRGLGGSRTPLG
EFICQLCKEQYADPFALAQHRCSRIVRVEYRCPECDKVFSCPANLASHRRWHKPRPAAANAATVSSADGKPPSSSSSSSRDSGAIAS
FLAEGKENSRIERTADQHPQARDSSGADQHPDSAPRQGLQVLTHPEPPLPQGPYTEGVLGRRVPVPGSTSGGRGSEIFVCPYCHKKF
RRQAYLRKHLSTHEAGSARALAPGFGSERGAPLAFACPLCGAHFPTADIREKHRLWHAVREELLLPALAGAPPETSGPSGPSDGSAQ
QIFSCKHCPSTFFSSPGLTRHINKCHPSESRQVLLLQMPLRPGC
>sp|O15047|SET1A_HUMAN
MDQEGGGDGQKAPSFQWRNYKLIVDPALDPALRRPSQKVYRYDGVHFSVNDSKYIPVEDLQDPRCHVRSKNRDFSLPVPKFKLDEFY
IGQIPLKEVTFARLNDNVRETFLKDMCRKYGEVEEVEILLHPRTRKHLGLARVLFTSTRGAKETVKNLHLTSVMGNIIHAQLDIKGQ
QRMKYYELIVNGSYTPQTVPTGGKALSEKFQGSGAATETAESRRRSSSDTAAYPAGTTAVGTPGNGTPCSQDTSFSSSRQDTPSSFG
QFTPQSSQGTPYTSRGSTPYSQDSAYSSSTTSTSFKPRRSENSYQDAFSRRHFSASSASTTASTAIAATTAATASSSASSSSLSSSS
SSSSSSSSSQFRSSDANYPAYYESWNRYQRHTSYPPRRATREEPPGAPFAENTAERFPPSYTSYLPPEPSRPTDQDYRPPASEAPPP
EPPEPGGGGGGGGPSPEREEVRTSPRPASPARSGSPAPETTNESVPFAQHSSLDSRIEMLLKEQRSKFSFLASDTEEEEENSSMVLG
ARDTGSEVPSGSGHGPCTPPPAPANFEDVAPTGSGEPGATRESPKANGQNQASPCSSGDDMEISDDDRGGSPPPAPTPPQQPPPPPP
PPPPPPPYLASLPLGYPPHQPAYLLPPRPDGPPPPEYPPPPPPPPHIYDFVNSLELMDRLGAQWGGMPMSFQMQTQMLTRLHQLRQG
KGLIAASAGPPGGAFGEAFLPFPPPQEAAYGLPYALYAQGQEGRGAYSREAYHLPMPMAAEPLPSSSVSGEEARLPPREEAELAEGK
TLPTAGTVGRVLAMLVQEMKSIMQRDLNRKMVENVAFGAFDQWWESKEEKAKPFQNAAKQQAKEEDKEKTKLKEPGLLSLVDWAKSG
GTTGIEAFAFGSGLRGALRLPSFKVKRKEPSEISEASEEKRPRPSTPAEEDEDDPEQEKEAGEPGRPGTKPPKRDEERGKTQGKHRK
SFALDSEGEEASQESSSEKDEEDDEEDEEDEDREEAVDTTKKETEVSDGEDEESDSSSKCSLYADSDGENDSTSDSESSSSSSSSSS
SSSSSSSSSSSSSSSESSSEDEEEEERPAALPSASPPPREVPVPTPAPVEVPVPERVAGSPVTPLPEQEASPARPAGPTEESPPSAPL
RPPEPPAGPPAPAPRPDERPSSPIPLLPPPKKRRKTVSFSAIEVVPAPEPPPATPPQAKFPGPASRKAPRGVERTIRNLPLDHASLV
KSWPEEVSRGGRSRAGGRGRLTEEEEAEPGTEVDLAVLADLALTPARRGLPALPAVEDSEATETSDEAERPRPLLSHILLEHNYALA
VKPTPPAPALRPPEPVPAPAALFSSPADEVLEAPEVVVAEAEEPKPQQLQQQREEGEEEGEEEGEEEEEESSDSSSSSDGEGALRRR
SLRSHARRRRPPPPPPPPPPRAYEPRSEFEQMTILYDIWNSGLDSEDMSYLRLTYERLLQQTSGADWLNDTHWVHHTITNLTTPKRK
RRPQDGPREHQTGSARSEGYYPISKKEKDKYLDVCPVSARQLEGVDTQGTNRVLSERRSEQRRLLSAIGTSAIMDSDLLKLNQLKFR
KKKLRFGRSRIHEWGLFAMEPIAADEMVIEYVGQNIRQMVADMREKRYVQEGIGSSYLFRVDHDTIIDATKCGNLARFINHCCTPNC
YAKVITIESQKKIVIYSKQPIGVDEEITYDYKFPLEDNKIPCLCGTESCRGSLN
>sp|P16112|PGCA_HUMAN
MTTLLWVFVTLRVITAAVTVETSDHDNSLSVSIPQPSPLRVLLGTSLTIPCYFIDPMHPVTTAPSTAPLAPRIKWSRVSKEKEVVLL
VATEGRVRVNSAYQDKVSLPNYPAIPSDATLEVQSLRSNDSGVYRCEVMHGIEDSEATLEVVVKGIVFHYRAISTRYTLDFDRAQRA
CLQNSAIIATPEQLQAAYEDGFHQCDAGWLADQTVRYPIHTPREGCYGDKDEFPGVRTYGIRDTNETYDVYCFAEEMEGEVFYATSP
EKFTFQEAANECRRLGARLATTGHVYLAWQAGMDMCSAGWLADRSVRYPISKARPNCGGNLLGVRTVYVHANQTGYPDPSSRYDAIC

YTGEDFVDIPENFFGVGGEEDITVQTVTWPDMELPLPRNITEGEARGSVILTVKPIFEVSPSPLEPEEPFTFAPEIGATAFAEVENE
TGEATRPWGFPTPGLGPATAFTSEDLVVQVTAVPGQPHLPGGVVFHYRPGPTRYSLTFEEAQQACPGTGAVIASPEQLQAAYEAGYE
QCDAGWLRDQTVRYPIVSPRTPCVGDKDSSPGVRTYGVRPSTETYDVYCFVDRLEGEVFFATRLEQFTFQEALEFCESHNATATTGQ
LYAAWSRGLDKCYAGWLADGSLRYPIVTPRPACGGDKPGVRTVYLYPNQTGLPDPLSRHHAFCFRGISAVPSPGEEEGGTPTSPSGV
EEWIVTQVVPGVAAVPVEEETTAVPSGETTAILEFTTEPENQTEWEPAYTPVGTSPLPGILPTWPPTGAETEESTEGPSATEVPSAS
EEPSPSEVPFPSEEPSPSEEPFPSVRPFPSVELFPSEEPFPSKEPSPSEEPSASEEPYTPSPPEPSWTELPSSGEESGAPDVSGDFT
GSGDVSGHLDFSGQLSGDRASGLPSGDLDSSGLTSTVGSGLTVESGLPSGDEERIEWPSTPTVGELPSGAEILEGSASGVGDLSGLP
SGEVLETSASGVGDLSGLPSGEVLETTAPGVEDISGLPSGEVLETTAPGVEDISGLPSGEVLETTAPGVEDISGLPSGEVLETTAPG
VEDISGLPSGEVLETTAPGVEDISGLPSGEVLETAAPGVEDISGLPSGEVLETAAPGVEDISGLPSGEVLETAAPGVEDISGLPSGE
VLETAAPGVEDISGLPSGEVLETAAPGVEDISGLPSGEVLETAAPGVEDISGLPSGEVLETAAPGVEDISGLPSGEVLETAAPGVED
ISGLPSGEVLETAAPGVEDISGLPSGEVLETAAPGVEDISGLPSGEVLETAAPGVEDISGLPSGEVLETTAPGVEEISGLPSGEVLE
TTAPGVDEISGLPSGEVLETTAPGVEEISGLPSGEVLETSTSAVGDLSGLPSGGEVLEISVSGVEDISGLPSGEVVETSASGIEDVS
ELPSGEGLETSASGVEDLSRLPSGEEVLEISASGFGDLSGVPSGGEGLETSASEVGTDLSGLPSGREGLETSASGAEDLSGLPSGKE
DLVGSASGDLDLGKLPSGTLGSGQAPETSGLPSGFSGEYSGVDLGSGPPSGLPDFSGLPSGFPTVSLVDSTLVEVVTASTASELEGR
GTIGISGAGEISGLPSSELDISGRASGLPSGTELSGQASGSPDVSGEIPGLFGVSGQPSGFPDTSGETSGVTELSGLSSGQPGVSGE
ASGVLYGTSQPFGITDLSGETSGVPDLSGQPSGLPGFSGATSGVPDLVSGTTSGSGESSGITFVDTSLVEVAPTTFKEEEGLGSVEL
SGLPSGEADLSGKSGMVDVSGQFSGTVDSSGFTSQTPEFSGLPSGIAEVSGESSRAEIGSSLPSGAYYGSGTPSSFPTVSLVDRTLV
ESVTQAPTAQEAGEGPSGILELSGAHSGAPDMSGEHSGFLDLSGLQSGLIEPSGEPPGTPYFSGDFASTTNVSGESSVAMGTSGEAS
GLPEVTLITSEFVEGVTEPTISQELGQRPPVTHTPQLFESSGKVSTAGDISGATPVLPGSGVEVSSVPESSSETSAYPEAGFGASAA
PEASREDSGSPDLSETTSAFHEANLERSSGLGVSGSTLTFQEGEASAAPEVSGESTTTSDVGTEAPGLPSATPTASGDRTEISGDLS
GHTSQLGVVISTSIPESEWTQQTQRPAETHLEIESSSLLYSGEETHTVETATSPTDASIPASPEWKRESESTAAAPARSCAEEPCGA
GTCKETEGHVICLCPPGYTGEHCNIDQEVCEEGWNKYQGHCYRHFPDRETWVDAERRCREQQSHLSSIVTPEEQEFVNNNAQDYQWI
GLNDRTIEGDFRWSDGHPMQFENWRPNQPDNFFAAGEDCVVMIWHEKGEWNDVPCNYHLPFTCKKGTVACGEPPVVEHARTFGQKKD
RYEINSLVRYQCTEGFVQRHMPTIRCQPSGHWEEPRITCTDATTYKRRLQKRSSRHPRRSRPSTAH
>sp|O60911|CATL2_HUMAN
MNLSLVLAAFCLGIASAVPKFDQNLDTKWYQWKATHRRLYGANEEGWRRAVWEKNMKMIELHNGEYSQGKHGFTMAMNAFGDMTNEE
FRQMMGCFRNQKFRKGKVFREPLFLDLPKSVDWRKKGYVTPVKNQKQCGSCWAFSATGALEGQMFRKTGKLVSLSEQNLVDCSRPQG
NQGCNGGFMARAFQYVKENGGLDSEESYPYVAVDEICKYRPENSVANDTGFTVVAPGKEKALMKAVATVGPISVAMDAGHSSFQFYK
SGIYFEPDCSSKNLDHGVLVVGYGFEGANSNNSKYWLVKNSWGPEWGSNGYVKIAKDKNNHCGIATAASYPNV
>sp|Q01831|XPC_HUMAN
MARKRAAGGEPRGRELRSQKSKAKSKARREEEEEDAFEDEKPPKKSLLSKVSQGKRKRGCSHPGGSADGPAKKKVAKVTVKSENLKV
IKDEALSDGDDLRDFPSDLKKAHHLKRGATMNEDSNEEEEESENDWEEVEELSEPVLGDVRESTAFSRSLLPVKPVEIEIETPEQAK
TRERSEKIKLEFETYLRRAMKRFNKGVHEDTHKVHLLCLLANGFYRNNICSQPDLHAIGLSIIPARFTRVLPRDVDTYYLSNLVKWF
IGTFTVNAELSASEQDNLQTTLERRFAIYSARDDEELVHIFLLILRALQLLTRLVLSLQPIPLKSATAKGKKPSKERLTADPGGSSE
TSSQVLENHTKPKTSKGTKQEETFAKGTCRPSAKGKRNKGGRKKRSKPSSSEEDEGPGDKQEKATQRRPHGRERRVASRVSYKEESG
SDEAGSGSDFELSSGEASDPSDEDSEPGPPKQRKAPAPQRTKAGSKSASRTHRGSHRKDPSLPAASSSSSSSKRGKKMCSDGEKAEK
RSIAGIDQWLEVFCEQEEKWVCVDCVHGVVGQPLTCYKYATKPMTYVVGIDSDGWVRDVTQRYDPVWMTVTRKCRVDAEWWAETLRP
YQSPFMDREKKEDLEFQAKHMDQPLPTAIGLYKNHPLYALKRHLLKYEAIYPETAAILGYCRGEAVYSRDCVHTLHSRDTWLKKARV
VRLGEVPYKMVKGFSNRARKARLAEPQLREENDLGLFGYWQTEEYQPPVAVDGKVPRNEFGNVYLFLPSMMPIGCVQLNLPNLHRVA
RKLDIDCVQAITGFDFHGGYSHPVTDGYIVCEEFKDVLLTAWENEQAVIERKEKEKKEKRALGNWKLLAKGLLIRERLKRRYGPKSE
AAAPHTDAGGGLSSDEEEGTSSQAEAARILAASWPQNREDEEKQKLKGGPKKTKREKKAAASHLFPFEQL
>sp|Q9BZ72|PITM2_HUMAN
MIIKEYRIPLPMTVEEYRIAQLYMIQKKSRNETYGEGSGVEILENRPYTDGPGGSGQYTHKVYHVGMHIPSWFRSILPKAALRVVEE
SWNAYPYTRTRFTCPFVEKFSIDIETFYKTDAGENPDVFNLSPVEKNQLTIDFIDIVKDPVPHNEYKTEEDPKLFQSTKTQRGPLSE

NWIEEYKKQVFPIMCAYKLCKVEFRYWGMQSKIERFIHDTGLRRVMVRAHRQAWCWQDEWYGLSMENIRELEKEAQLMLSRKMAQFN
EDGEEATELVKHEAVSDQTSGEPPEPSSSNGEPLVGRGLKKQWSTSSKSSRSSKRGASPSRHSISEWRMQSIARDSDESSDDEFFDA
HEDLSDTEEMFPKDITKWSSNDLMDKIESPEPEDTQDGLYRQGAPEFRVASSVEQLNIIEDEVSQPLAAPPSKIHVLLLVLHGGTIL
DTGAGDPSSKKGDANTIANVFDTVMRVHYPSALGRLAIRLVPCPPVCSDAFALVSNLSPYSHDEGCLSSSQDHIPLAALPLLATSSP
QYQEAVATVIQRANLAYGDFIKSQEGMTFNGQVCLIGDCVGGILAFDALCYSNQPVSESQSSSRRGSVVSMQDNDLLSPGILMNAAH
CCGGGGGGGGGGGSSGGGGSSGGSSLESSRHLSRSNVDIPRSNGTEDPKRQLPRKRSDSSTYELDTIQQHQAFLSSLHASVLRTEPC
SRHSSSSTMLDGTGALGRFDFEITDLFLFGCPLGLVLALRKTVIPALDVFQLRPACQQVYNLFHPADPSASRLEPLLERRFHALPPF
SVPRYQRYPLGDGCSTLLADVLQTHNAAFQEHGAPSSPGTAPASRGFRRASEISIASQVSGMAESYTASSIAQKAPDALSHTPSVRR
LSLLALPAPSPTTPGPHPPARKASPGLERAPGLPELDIGEVAAKWWGQKRIDYALYCPDALTAFPTVALPHLFHASYWESTDVVSFL
LRQVMRHDNSSILELDGKEVSVFTPSKPREKWQRKRTHVKLRNVTANHRINDALANEDGPQVLTGRFMYGPLDMVTLTGEKVDVHIM
TQPPSGEWLYLDTLVTNNSGRVSYTIPESHRLGVGVYPIKMVVRGDHTFADSYITVLPKGTEFVVFSIDGSFAASVSIMGSDPKVRA
GAVDVVRHWQDLGYLIIYVTGRPDMQKQRVVAWLAQHNFPHGVVSFCDGLVHDPLRHKANFLKLLISELHLRVHAAYGSTKDVAVYS
AISLSPMQIYIVGRPTKKLQQQCQFITDGYAAHLAQLKYSHRARPARNTATRMALRKGSFGLPGQGDFLRSRNHLLRTISAQPSGPS
HRHERTQSQADGEQRGQRSMSVAAGCWGRAMTGRLEPGAAAGPK
>sp|Q9Y462|ZN711_HUMAN
MDSGGGSLGLHTPDSRMAHTMIMQDFVAGMAGTAHIDGDHIVVSVPEAVLVSDVVTDDGITLDHGLAAEVVHGPDIITETDVVTEGV
IVPEAVLEADVAIEEDLEEDDGDHILTSELITETVRVPEQVFVADLVTGPNGHLEHVVQDCVSGVDSPTMVSEEVLVTNSDTETVIQ
AAGGVPGSTVTIKTEDDDDDDVKSTSEDYLMISLDDVGEKLEHMGNTPLKIGSDGSQEDAKEDGFGSEVIKVYIFKAEAEDDVEIGG
TEIVTESEYTSGHSVAGVLDQSRMQREKMVYMAVKDSSQEEDDIRDERRVSRRYEDCQASGNTLDSALESRSSTAAQYLQICDGINT
NKVLKQKAKKRRRGETRQWQTAVIIGPDGQPLTVYPCHICTKKFKSRGFLKRHMKNHPDHLMRKKYQCTDCDFTTNKKVSFHNHLES
HKLINKVDKTHEFTEYTRRYREASPLSSNKLILRDKEPKMHKCKYCDYETAEQGLLNRHLLAVHSKNFPHVCVECGKGFRHPSELKK
HMRTHTGEKPYQCQYCIFRCADQSNLKTHIKSKHGNNLPYKCEHCPQAFGDERELQRHLDLFQGHKTHQCPHCDHKSTNSSDLKRHI
ISVHTKDFPHKCEVCDKGFHRPSELKKHSDIHKGRKIHQCRHCDFKTSDPFILSGHILSVHTKDQPLKCKRCKRGFRQQNELKKHMK
THTGRKIYQCEYCEYSTTDASGFKRHVISIHTKDYPHRCEFCKKGFRRPSEKNQHIMRHHKEALM
>sp|O95157|NXPH3_HUMAN
MQLTRCCFVFLVQGSLYLVICGQDDGPPGSEDPERDDHEGQPRPRVPRKRGHISPKSRPMANSTLLGLLAPPGEAWGILGQPPNRPN
HSPPPSAKVKKIFGWGDFYSNIKTVALNLLVTGKIVDHGNGTFSVHFQHNATGQGNISISLVPPSKAVEFHQEQQIFIEAKASKIFN
CRMEWEKVERGRRTSLCTHDPAKICSRDHAQSSATWSCSQPFKVVCVYIAFYSTDYRLVQKVCPDYNYHSDTPYYPSG
>sp|Q8IYA2|C144C_HUMAN
MVSWGGEKRGGAEGSPKPAVYATRKTGSVRSQEDQWYLGYPGDQWSSGFSYSWWKNSVGSESKHGEGALDQPQHDVRLEDLGELHRA
ARSGDVPGVEHVLVPGDTGVDKRDRKKSIQQLVPEYKEKQTPESLPQNNNPDWHPTNLTLSDETCQRSKNLKVDDKCPSVSPSMPEN
QSATKELGQMNLTEREKMDTGVKTSQEPEMAKDCDREDIPIYPVLPHVQKSEEMRIEQGKLEWKNQLKLVINELKQRFGEIYEKYKI
PACPEEEPLLDNSTRGTDVKDIPFNLTNNIPGCEEEDASEISVSVVFETFPEQKEPSLKNIIHSYYHPYSGSQEHVCQSSSKLHLHE
NKLDCDNDNKPGIGHIFSTDKNFHNDASTKKARNPEVVTVEMKEDQEFDLQMTKNMNQNSDSGSTNNYKSLKPKLENLSSLPPDSDR
TSEVYLHEELQQDMQKFKNEVNTLEEEFLALKKENVQLHKEVEEEMEKHRSNSTELSGTLTDGTTVGNDDDGLNQQIPRKENGEHDR
LALKQENEEKRNADMLYNKDSEQLRIKEEECGKVVETKQQLKWNLRRLVKELRTVVQERNDAQKQLSEEQDARILQDQILTSKQKEL
EMAQKKRNPEISHRHQKEKDLFHENCMLQEEIALLRLEIDTIKNQNKQKEKKYFEDIEVVKEKNDNLQKIIKRNEETLTETILQYSG
QLNNLTAENKMLNSELENGKENQERLEIEMESYRCRLAAAVHDCDQSQTARDLKLDFQRTRQEWVRLHDKMKVDMSGLQAKNEILSE
KLSNAESKINSLQIQLHNTRDALGRESLILERVQRDLSQTQCQKKETEQMYQSKLKKYIAKQESVEERLSQLQSENMLLRQQLDDVH
KKANSQEKTISTIQDQFHSAAKNLQAESEKQILSLQEKNKELMDEYNHLKERMDQCEKEKAGRKIDLTEAQETVPSRCLHLDAENEV
LQLQQTLFSMKAIQKQCETLQKNKKQLKQEVVNLKSYMERNMLERGEAEWHKLLIEERARKEIEEKLNEAILTLQKQAAVSHEQLAQ
LREDNTTSIKTQMELTVIDLESEISRIKTSQADFNKTKLERYKELYLEEVKVRESLSNELSRTNEMIAEVSTQLTVEKEQTRSRSLF
TAYATRPVLESPCVGNLNDSEGLNRKHIPRKKRSALKDMESYLLKMQQKLQNDLTAEVAGSSQTGLHRIPQCSSFSSSSLHLLLCSI
CQPFFLILQLLLNMNLDPI

>sp|P35626|ARBK2_HUMAN
MADLEAVLADVSYLMAMEKSKATPAARASKRIVLPEPSIRSVMQKYLAERNEITFDKIFNQKIGFLLFKDFCLNEINEAVPQVKFYE
EIKEYEKLDNEEDRLCRSRQIYDAYIMKELLSCSHPFSKQAVEHVQSHLSKKQVTSTLFQPYIEEICESLRGDIFQKFMESDKFTRF
CQWKNVELNIHLTMNEFSVHRIIGRGGFGEVYGCRKADTGKMYAMKCLDKKRIKMKQGETLALNERIMLSLVSTGDCPFIVCMTYAF
HTPDKLCFILDLMNGGDLHYHLSQHGVFSEKEMRFYATEIILGLEHMHNRFVVYRDLKPANILLDEHGHARISDLGLACDFSKKKPH
ASVGTHGYMAPEVLQKGTAYDSSADWFSLGCMLFKLLRGHSPFRQHKTKDKHEIDRMTLTVNVELPDTFSPELKSLLEGLLQRDVSK
RLGCHGGGSQEVKEHSFFKGVDWQHVYLQKYPPPLIPPRGEVNAADAFDIGSFDEEDTKGIKLLDCDQELYKNFPLVISERWQQEVT
ETVYEAVNADTDKIEARKRAKNKQLGHEEDYALGKDCIMHGYMLKLGNPFLTQWQRRYFYLFPNRLEWRGEGESRQNLLTMEQILSV
EETQIKDKKCILFRIKGGKQFVLQCESDPEFVQWKKELNETFKEAQRLLRRAPKFLNKPRSGTVELPKPSLCHRNSNGL
>sp|P51161|FABP6_HUMAN
MAFTGKFEMESEKNYDEFMKLLGISSDVIEKARNFKIVTEVQQDGQDFTWSQHYSGGHTMTNKFTVGKESNIQTMGGKTFKATVQME
GGKLVVNFPNYHQTSEIVGDKLVEVSTIGGVTYERVSKRLA
>sp|Q9Y6X8|ZHX2_HUMAN
MASKRKSTTPCMVRTSQVVEQDVPEEVDRAKEKGIGTPQPDVAKDSWAAELENSSKENEVIEVKSMGESQSKKLQGGYECKYCPYST
QNLNEFTEHVDMQHPNVILNPLYVCAECNFTTKKYDSLSDHNSKFHPGEANFKLKLIKRNNQTVLEQSIETTNHVVSITTSGPGTGD
SDSGISVSKTPIMKPGKPKADAKKVPKKPEEITPENHVEGTARLVTDTAEILSRLGGVELLQDTLGHVMPSVQLPPNINLVPKVPVP
LNTTKYNSALDTNATMINSFNKFPYPTQAELSWLTAASKHPEEHIRIWFATQRLKHGISWSPEEVEEARKKMFNGTIQSVPPTITVL
PAQLAPTKVTQPILQTALPCQILGQTSLVLTQVTSGSTTVSCSPITLAVAGVTNHGQKRPLVTPQAAPEPKRPHIAQVPEPPPKVAN
PPLTPASDRKKTKEQIAHLKASFLQSQFPDDAEVYRLIEVTGLARSEIKKWFSDHRYRCQRGIVHITSESLAKDQLAIAASRHGRTY
HAYPDFAPQKFKEKTQGQVKILEDSFLKSSFPTQAELDRLRVETKLSRREIDSWFSERRKLRDSMEQAVLDSMGSGKKGQDVGAPNG
ALSRLDQLSGAQLTSSLPSPSPAIAKSQEQVHLLRSTFARTQWPTPQEYDQLAAKTGLVRTEIVRWFKENRCLLKTGTVKWMEQYQH
QPMADDHGYDAVARKATKPMAESPKNGGDVVPQYYKDPKKLCEEDLEKLVTRVKVGSEPAKDCLPAKPSEATSDRSEGSSRDGQGSD
ENEESSVVDYVEVTVGEEDAISDRSDSWSQAAAEGVSELAESDSDCVPAEAGQA
>sp|Q9P1Z3|HCN3_HUMAN
MEAEQRPAAGASEGATPGLEAVPPVAPPPATAASGPIPKSGPEPKRRHLGTLLQPTVNKFSLRVFGSHKAVEIEQERVKSAGAWIIH
PYSDFRFYWDLIMLLLMVGNLIVLPVGITFFKEENSPPWIVFNVLSDTFFLLDLVLNFRTGIVVEEGAEILLAPRAIRTRYLRTWFL
VDLISSIPVDYIFLVVELEPRLDAEVYKTARALRIVRFTKILSLLRLLRLSRLIRYIHQWEEIFHMTYDLASAVVRIFNLIGMMLLL
CHWDGCLQFLVPMLQDFPPDCWVSINHMVNHSWGRQYSHALFKAMSHMLCIGYGQQAPVGMPDVWLTMLSMIVGATCYAMFIGHATA
LIQSLDSSRRQYQEKYKQVEQYMSFHKLPADTRQRIHEYYEHRYQGKMFDEESILGELSEPLREEIINFTCRGLVAHMPLFAHADPS
FVTAVLTKLRFEVFQPGDLVVREGSVGRKMYFIQHGLLSVLARGARDTRLTDGSYFGEICLLTRGRRTASVRADTYCRLYSLSVDHF
NAVLEEFPMMRRAFETVAMDRLLRIGKKNSILQRKRSEPSPGSSGGIMEQHLVQHDRDMARGVRGRAPSTGAQLSGKPVLWEPLVHA
PLQAAAVTSNVAIALTHQRGPLPLSPDSPATLLARSAWRSAGSPASPLVPVRAGPWASTSRLPAPPARTLHASLSRAGRSQVSLLGP
PPGGGGRRLGPRGRPLSASQPSLPQRATGDGSPGRKGSGSERLPPSGLLAKPPRTAQPPRPPVPEPATPRGLQLSANM
>sp|P22695|QCR2_HUMAN
MKLLTRAGSFSRFYSLKVAPKVKATAAPAGAPPQPQDLEFTKLPNGLVIASLENYSPVSRIGLFIKAGSRYEDFSNLGTTHLLRLTS
SLTTKGASSFKITRGIEAVGGKLSVTATRENMAYTVECLRGDVDILMEFLLNVTTAPEFRRWEVADLQPQLKIDKAVAFQNPQTHVI
ENLHAAAYRNALANPLYCPDYRIGKVTSEELHYFVQNHFTSARMALIGLGVSHPVLKQVAEQFLNMRGGLGLSGAKANYRGGEIREQ
NGDSLVHAAFVAESAVAGSAEANAFSVLQHVLGAGPHVKRGSNTTSHLHQAVAKATQQPFDVSAFNASYSDSGLFGIYTISQATAAG
DVIKAAYNQVKTIAQGNLSNTDVQAAKNKLKAGYLMSVESSECFLEEVGSQALVAGSYMPPSTVLQQIDSVANADIINAAKKFVSGQ
KSMAASGNLGHTPFVDEL
>sp|Q8N6G5|CGAT2_HUMAN
MPRRGLILHTRTHWLLLGLALLCSLVLFMYLLECAPQTDGNASLPGVVGENYGKEYYQALLQEQEEHYQTRATSLKRQIAQLKQELQ
EMSEKMRSLQERRNVGANGIGYQSNKEQAPSDLLEFLHSQIDKAEVSIGAKLPSEYGVIPFESFTLMKVFQLEMGLTRHPEEKPVRK
DKRDELVEVIEAGLEVINNPDEDDEQEDEEGPLGEKLIFNENDFVEGYYRTERDKGTQYELFFKKADLTEYRHVTLFRPFGPLMKVK

SEMIDITRSIINIIVPLAERTEAFVQFMQNFRDVCIHQDKKIHLTVVYFGKEGLSKVKSILESVTSESNFHNYTLVSLNEEFNRGRG
LNVGARAWDKGEVLMFFCDVDIYFSAEFLNSCRLNAEPGKKVFYPVVFSLYNPAIVYANQEVPPPVEQQLVHKKDSGFWRDFGFGMT
CQYRSDFLTIGGFDMEVKGWGGEDVHLYRKYLHGDLIVIRTPVPGLFHLWHEKRCADELTPEQYRMCIQSKAMNEASHSHLGMLVFR
EEIETHLHKQAYRTNSEAVG
>sp|Q9HBQ8|GGA2B_HUMAN
MDSEEEEEVPQPMPSIPEDLESQKAMVAFFNSAVASAEEEQARLCGQLKECTASAWLICWPRPRRNLRQQPQPQELGVIPCVGRPTR
PCRGPWRSCGRVHRTVPEPEGSAEGGGVHQQAGPGQGRGEGEAAGAGVACGRLQQVA
>sp|Q8NBU5|ATAD1_HUMAN
MVHAEAFSRPLSRNEVVGLIFRLTIFGAVTYFTIKWMVDAIDPTRKQKVEAQKQAEKLMKQIGVKNVKLSEYEMSIAAHLVDPLNMH
VTWSDIAGLDDVITDLKDTVILPIKKKHLFENSRLLQPPKGVLLYGPPGCGKTLIAKATAKEAGCRFINLQPSTLTDKWYGESQKLA
AAVFSLAIKLQPSIIFIDEIDSFLRNRSSSDHEATAMMKAQFMSLWDGLDTDHSCQVIVMGATNRPQDLDSAIMRRMPTRFHINQPA
LKQREAILKLILKNENVDRHVDLLEVAQETDGFSGSDLKEMCRDAALLCVREYVNSTSEESHDEDEIRPVQQQDLHRAIEKMKKSKD
AAFQNVLTHVCLD
>sp|Q6PKG0|LARP1_HUMAN
MATQVEPLLPGGATLLQAEEHGGLVRKKPPPAPEGKGEPGPNDVRGGEPDGSARRPRPPCAKPHKEGTGQQERESPRPLQLPGAEGP
AISDGEEGGGEPGAGGGAAGAAGAGRRDFVEAPPPKVNPWTKNALPPVLTTVNGQSPPEHSAPAKVVRAAVPKQRKGSKVGDFGDAI
NWPTPGEIAHKSVQPQSHKPQPTRKLPPKKDMKEQEKGEGSDSKESPKTKSDESGEEKNGDEDCQRGGQKKKGNKHKWVPLQIDMKP
EVPREKLASRPTRPPEPRHIPANRGEIKGSESATYVPVAPPTPAWQPEIKPEPAWHDQDETSSVKSDGAGGARASFRGRGRGRGRGR
GRGRGGTRTHFDYQFGYRKFDGVEGPRTPKYMNNITYYFDNVSSTELYSVDQELLKDYIKRQIEYYFSVDNLERDFFLRRKMDADGF
LPITLIASFHRVQALTTDISLIFAALKDSKVVEIVDEKVRRREEPEKWPLPPIVDYSQTDFSQLLNCPEFVPRQHYQKETESAPGSP
RAVTPVPTKTEEVSNLKTLPKGLSASLPDLDSENWIEVKKRPRPSPARPKKSEESRFSHLTSLPQQLPSQQLMSKDQDEQEELDFLF
DEEMEQMDGRKNTFTAWSDEESDYEIDDRDVNKILIVTQTPHYMRRHPGGDRTGNHTSRAKMSAELAKVINDGLFYYEQDLWAEKFE
PEYSQIKQEVENFKKVNMISREQFDTLTPEPPVDPNQEVPPGPPRFQQVPTDALANKLFGAPEPSTIARSLPTTVPESPNYRNTRTP
RTPRTPQLKDSSQTSRFYPVVKEGRTLDAKMPRKRKTRHSSNPPLESHVGWVMDSREHRPRTASISSSPSEGTPTVGSYGCTPQSLP
KFQHPSHELLKENGFTQHVYHKYRRRCLNERKRLGIGQSQEMNTLFRFWSFFLRDHFNKKMYEEFKQLALEDAKEGYRYGLECLFRY
YSYGLEKKFRLDIFKDFQEETVKDYEAGQLYGLEKFWAFLKYSKAKNLDIDPKLQEYLGKFRRLEDFRVDPPMGEEGNHKRHSVVAG
GGGGEGRKRCPSQSSSRPAAMISQPPTPPTGQPVREDAKWTSQHSNTQTLGK
>sp|Q9Y296|TPPC4_HUMAN
MAIFSVYVVNKAGGLIYQLDSYAPRAEAEKTFSYPLDLLLKLHDERVLVAFGQRDGIRVGHAVLAINGMDVNGRYTADGKEVLEYLG
NPANYPVSIRFGRPRLTSNEKLMLASMFHSLFAIGSQLSPEQGSSGIEMLETDTFKLHCYQTLTGIKFVVLADPRQAGIDSLLRKIY
EIYSDFALKNPFYSLEMPIRCELFDQNLKLALEVAEKAGTFGPGS
>sp|Q6ZN03|CU136_HUMAN
MALLITPAGVATVNRHSTIPSDTHTSREKPRFHKPCRNDLESLLSEGRLDTSVQTPCPQHPHTQLSCEPQPLEHSSCLSTCLAGCFL
PVPSSPHTHPLLPGSRWLPPPLALLMGTLSPGLAVKPSWVPRFPLLARQSPATSVGMPLSAATQPGSVGRLHFPKLRSSSPFSGHSD
ENKATGQGRENRDQPQRPSHLCECPEAAKQSATNGVAETNRSVFPLGSEARSLSLRRQESQPHSGSSRRESVSCSPSFWCCWQPLAF
LTCGCAAPISVPGVTRPSPRPCCVSPPLVRLQSLGLGPTQI
>sp|Q9NRP7|STK36_HUMAN
MEKYHVLEMIGEGSFGRVYKGRRKYSAQVVALKFIPKLGRSEKELRNLQREIEIMRGLRHPNIVHMLDSFETDKEVVVVTDYAEGEL
FQILEDDGKLPEDQVQAIAAQLVSALYYLHSHRILHRDMKPQNILLAKGGGIKLCDFGFARAMSTNTMVLTSIKGTPLYMSPELVEE
RPYDHTADLWSVGCILYELAVGTPPFYATSIFQLVSLILKDPVRWPSTISPCFKNFLQGLLTKDPRQRLSWPDLLYHPFIAGHVTII
TEPAGPDLGTPFTSRLPPELQVLKDEQAHRLAPKGNQSRILTQAYKRMAEEAMQKKHQNTGPALEQEDKTSKVAPGTAPLPRLGATP
QESSLLAGILASELKSSWAKSGTGEVPSAPRENRTTPDCERAFPEERPEVLGQRSTDVVDLENEEPDSDNEWQHLLETTEPVPIQLK
APLTLLCNPDFCQRIQSQLHEAGGQILKGILEGASHILPAFRVLSSLLSSCSDSVALYSFCREAGLPGLLLSLLRHSQESNSLQQQS
WYGTFLQDLMAVIQAYFACTFNLERSQTSDSLQVFQEAANLFLDLLGKLLAQPDDSEQTLRRDSLMCFTVLCEAMDGNSRAISKAFY

SSLLTTQQVVLDGLLHGLTVPQLPVHTPQGAPQVSQPLREQSEDIPGAISSALAAICTAPVGLPDCWDAKEQVCWHLANQLTEDSSQ
LRPSLISGLQHPILCLHLLKVLYSCCLVSEGLCRLLGQEPLALESLFMLIQGKVKVVDWEESTEVTLYFLSLLVFRLQNLPCGMEKL
GSDVATLFTHSHVVSLVSAAACLLGQLGQQGVTFDLQPMEWMAAATHALSAPAEVRLTPPGSCGFYDGLLILLLQLLTEQGKASLIR
DMSSSEMWTVLWHRFSMVLRLPEEASAQEGELSLSSPPSPEPDWTLISPQGMAALLSLAMATFTQEPQLCLSCLSQHGSILMSILKH
LLCPSFLNQLRQAPHGSEFLPVVVLSVCQLLCFPFALDMDADLLIGVLADLRDSEVAAHLLQVCCYHLPLMQVELPISLLTRLALMD
PTSLNQFVNTVSASPRTIVSFLSVALLSDQPLLTSDLLSLLAHTARVLSPSHLSFIQELLAGSDESYRPLRSLLGHPENSVRAHTYR
LLGHLLQHSMALRGALQSQSGLLSLLLLGLGDKDPVVRCSASFAVGNAAYQAGPLGPALAAAVPSMTQLLGDPQAGIRRNVASALGN
LGPEGLGEELLQCEVPQRLLEMACGDPQPNVKEAALIALRSLQQEPGIHQVLVSLGASEKLSLLSLGNQSLPHSSPRPASAKHCRKL
IHLLRPAHSM
>sp|O75475|PSIP1_HUMAN
MTRDFKPGDLIFAKMKGYPHWPARVDEVPDGAVKPPTNKLPIFFFGTHETAFLGPKDIFPYSENKEKYGKPNKRKGFNEGLWEIDNN
PKVKFSSQQAATKQSNASSDVEVEEKETSVSKEDTDHEEKASNEDVTKAVDITTPKAARRGRKRKAEKQVETEEAGVVTTATASVNL
KVSPKRGRPAATEVKIPKPRGRPKMVKQPCPSESDIITEEDKSKKKGQEEKQPKKQPKKDEEGQKEEDKPRKEPDKKEGKKEVESKR
KNLAKTGVTSTSDSEEEGDDQEGEKKRKGGRNFQTAHRRNMLKGQHEKEAADRKRKQEEQMETEQQNKDEGKKPEVKKVEKKRETSM
DSRLQRIHAEIKNSLKIDNLDVNRCIEALDELASLQVTMQQAQKHTEMITTLKKIRRFKVSQVIMEKSTMLYNKFKNMFLVGEGDSV
ITQVLNKSLAEQRQHEEANKTKDQGKKGPNKKLEKEQTGSKTLNGGSDAQDGNQPQHNGESNEDSKDNHEASTKKKPSSEERETEIS
LKDSTLDN
>sp|Q9Y3Q8|T22D4_HUMAN
MSGGKKKSSFQITSVTTDYEGPGSPGASDPPTPQPPTGPPPRLPNGEPSPDPGGKGTPRNGSPPPGAPSSRFRVVKLPHGLGEPYRR
GRWTCVDVYERDLEPHSFGGLLEGIRGASGGAGGRSLDSRLELASLGLGAPTPPSGLSQGPTSWLRPPPTSPGPQARSFTGGLGQLV
VPSKAKAEKPPLSASSPQQRPPEPETGESAGTSRAATPLPSLRVEAEAGGSGARTPPLSRRKAVDMRLRMELGAPEEMGQVPPLDSR
PSSPALYFTHDASLVHKSPDPFGAVAAQKFSLAHSMLAISGHLDSDDDSGSGSLVGIDNKIEQAMDLVKSHLMFAVREEVEVLKEQI
RELAERNAALEQENGLLRALASPEQLAQLPSSGVPRLGPPAPNGPSV
>sp|Q9UI38|TSP50_HUMAN
MGRWCQTVARGQRPRTSAPSRAGALLLLLLLLRSAGCWGAGEAPGALSTADPADQSVQCVPKATCPSSRPRLLWQTPTTQTLPSTTM
ETQFPVSEGKVDPYRSCGFSYEQDPTLRDPEAVARRWPWMVSVRANGTHICAGTIIASQWVLTVAHCLIWRDVIYSVRVGSPWIDQM
TQTASDVPVLQVIMHSRYRAQRFWSWVGQANDIGLLKLKQELKYSNYVRPICLPGTDYVLKDHSRCTVTGWGLSKADGMWPQFRTIQ
EKEVIILNNKECDNFYHNFTKIPTLVQIIKSQMMCAEDTHREKFCYELTGEPLVCSMEGTWYLVGLVSWGAGCQKSEAPPIYLQVSS
YQHWIWDCLNGQALALPAPSRTLLLALPLPLSLLAAL
>sp|O95219|SNX4_HUMAN
MEQAPPDPERQLQPAPLEPLGSPDAGLGAAVGKEAEGAGEESSGVDTMTHNNFWLKKIEISVSEAEKRTGRNAMNMQETYTAYLIET
RSVEHTDGQSVLTDSLWRRYSEFELLRSYLLVYYPHIVVPPLPEKRAEFVWHKLSADNMDPDFVERRRIGLENFLLRIASHPILCRD
KIFYLFLTQEGNWKETVNETGFQLKADSRLKALNATFRVKNPDKRFTDLKHYSDELQSVISHLLRVRARVADRLYGVYKVHGNYGRV
FSEWSAIEKEMGDGLQSAGHHMDVYASSIDDILEDEEHYADQLKEYLFYAEALRAVCRKHELMQYDLEMAAQDLASKKQQCEELVTG
TVRTFSLKGMTTKLFGQETPEQREARIKVLEEQINEGEQQLKSKNLEGREFVKNAWADIERFKEQKNRDLKEALISYAVMQISMCKK
GIQVWTNAKECFSKM
>sp|Q96A54|ADR1_HUMAN
MSSHKGSVVAQGNGAPASNREADTVELAELGPLLEEKGKRVIANPPKAEEEQTCPVPQEEEEEVRVLTLPLQAHHAMEKMEEFVYKV
WEGRWRVIPYDVLPDWLKDNDYLLHGHRPPMPSFRACFKSIFRIHTETGNIWTHLLGFVLFLFLGILTMLRPNMYFMAPLQEKVVFG
MFFLGAVLCLSFSWLFHTVYCHSEKVSRTFSKLDYSGIALLIMGSFVPWLYYSFYCSPQPRLIYLSIVCVLGISAIIVAQWDRFATP
KHRQTRAGVFLGLGLSGVVPTMHFTIAEGFVKATTVGQMGWFFLMAVMYITGAGLYAARIPERFFPGKFDIWFQSHQIFHVLVVAAA
FVHFYGVSNLQEFRYGLEGGCTDDTLL
>sp|P25786|PSA1_HUMAN

MFRNQYDNDVTVWSPQGRIHQIEYAMEAVKQGSATVGLKSKTHAVLVALKRAQSELAAHQKKILHVDNHIGISIAGLTADARLLCNF
MRQECLDSRFVFDRPLPVSRLVSLIGSKTQIPTQRYGRRPYGVGLLIAGYDDMGPHIFQTCPSANYFDCRAMSIGARSQSARTYLER
HMSEFMECNLNELVKHGLRALRETLPAEQDLTTKNVSIGIVGKDLEFTIYDDDDVSPFLEGLEERPQRKAQPAQPADEPAEKADEPM
EH
>sp|O75367|H2AY_HUMAN
MSSRGGKKKSTKTSRSAKAGVIFPVGRMLRYIKKGHPKYRIGVGAPVYMAAVLEYLTAEILELAGNAARDNKKGRVTPRHILLAVAN
DEELNQLLKGVTIASGGVLPNIHPELLAKKRGSKGKLEAIITPPPAKKAKSPSQKKPVSKKAGGKKGARKSKKKQGEVSKAASADST
TEGTPADGFTVLSTKSLFLGQKLNLIHSEISNLAGFEVEAIINPTNADIDLKDDLGNTLEKKGGKEFVEAVLELRKKNGPLEVAGAA
VSAGHGLPAKFVIHCNSPVWGADKCEELLEKTVKNCLALADDKKLKSIAFPSIGSGRNGFPKQTAAQLILKAISSYFVSTMSSSIKT
VYFVLFDSESIGIYVQEMAKLDAN
>sp|Q8N4A0|GALT4_HUMAN
MAVRWTWAGKSCLLLAFLTVAYIFVELLVSTFHASAGAGRARELGSRRLSDLQKNTEDLSRPLYKKPPADSRALGEWGKASKLQLNE
DELKQQEELIERYAINIYLSDRISLHRHIEDKRMYECKSQKFNYRTLPTTSVIIAFYNEAWSTLLRTIHSVLETSPAVLLKEIILVD
DLSDRVYLKTQLETYISNLDRVRLIRTNKREGLVRARLIGATFATGDVLTFLDCHCECNSGWLEPLLERIGRDETAVVCPVIDTIDW
NTFEFYMQIGEPMIGGFDWRLTFQWHSVPKQERDRRISRIDPIRSPTMAGGLFAVSKKYFQYLGTYDTGMEVWGGENLELSFRVWQC
GGKLEIHPCSHVGHVFPKRAPYARPNFLQNTARAAEVWMDEYKEHFYNRNPPARKEAYGDISERKLLRERLRCKSFDWYLKNVFPNL
HVPEDRPGWHGAIRSRGISSECLDYNSPDNNPTGANLSLFGCHGQGGNQFFEYTSNKEIRFNSVTELCAEVPEQKNYVGMQNCPKDG
FPVPANIIWHFKEDGTIFHPHSGLCLSAYRTPEGRPDVQMRTCDALDKNQIWSFEK
>sp|Q96BU1|S1PBP_HUMAN
MMCSRVPSEQSSGTSLLPKDGAPFSWDSLDEDGLDDSLLELSEGEEDDGDVNYTEEEIDALLKEDDPSYEQSSGEDDGGHVEKGERG
SQILLDTPREKNSSYSLGPVAETPDLFKLPQLSTSSGHGPAHTKPLNRRSVLEKNLIKVTVAPFNPTVCDALLDKDETDSSKDTEKL
SSLGEEMREDGLSPNESKLCTESEGISPNNSAWNGPQLSSSNNNFQQTVSDKNMPDSENPTSVFSRISDHSETPNMELSCRNGGSHK
SSCEMRSLVVSTSSNKQDVLNKDSGKMKGHERRLGKVIPVLQTKTRTNVPTFSQSNLEQQKQLYLRSVIAHIEDPEDTNQGISGELC
ALMDQVHHMQHSKWQHPSDLTTRNYARRQKHLQRYSLTQWVDRNMRSHHRFQRLPDFSYS
>sp|A6NFN3|RFOX3_HUMAN
MAQPYPPAQYPPPPQNGIPAEYAPPPPHPTQDYSGQTPVPTEHGMTLYTPAQTHPEQPGSEASTQPIAGTQTVPQTDEAAQTDSQPL
HPSDPTEKQQPKRLHVSNIPFRFRDPDLRQMFGQFGKILDVEIIFNERGSKGFGFVTFETSSDADRAREKLNGTIVEGRKIEVNNAT
ARVMTNKKTGNPYTNGWKLNPVVGAVYGPEFYAVTGFPYPTTGTAVAYRGAHLRGRGRAVYNTFRAAPPPPPIPTYGAVVYQDGFYG
AEIYGGYAAYRYAQPAAAAAAYSDSYGRVYAAADPYHHTIGPAATYSIGTM
>sp|Q99742|NPAS1_HUMAN
MAAPYPGSGGGSEVKCVGGRGASVPWDFLPGLMVKAPSGPCLQAQRKEKSRNAARSRRGKENLEFFELAKLLPLPGAISSQLDKASI
VRLSVTYLRLRRFAALGAPPWGLRAAGPPAGLAPGRRGPAALVSEVFEQHLGGHILQSLDGFVFALNQEGKFLYISETVSIYLGLSQ
VEMTGSSVFDYIHPGDHSEVLEQLGLRTPTPGPPTPPSVSSSSSSSSSLADTPEIEASLTKVPPSSLVQERSFFVRMKSTLTKRGLH
VKASGYKVIHVTGRLRAHALGLVALGHTLPPAPLAELPLHGHMIVFRLSLGLTILACESRVSDHMDLGPSELVGRSCYQFVHGQDAT
RIRQSHVDLLDKGQVMTGYYRWLQRAGGFVWLQSVATVAGSGKSPGEHHVLWVSHVLSQAEGGQTPLDAFQLPASVACEEASSPGPE
PTEPEPPTEGKQAAPAENEAPQTQGKRIKVEPGPRETKGSEDSGDEDPSSHPATPRPEFTSVIRAGVLKQDPVRPWGLAPPGDPPPT
LLHAGFLPPVVRGLCTPGTIRYGPAELGLVYPHLQRLGPGPALPEAFYPPLGLPYPGPAGTRLPRKGD
>sp|Q6UUV7|CRTC3_HUMAN
MAASPGSGSANPRKFSEKIALHTQRQAEETRAFEQLMTDLTLSRVQFQKLQQLRLTQYHGGSLPNVSQLRSSASEFQPSFHQADNVR
GTRHHGLVERPSRNRFHPLHRRSGDKPGRQFDGSAFGANYSSQPLDESWPRQQPPWKDEKHPGFRLTSALNRTNSDSALHTSALSTK
PQDPYGGGGQSAWPAPYMGFCDGENNGHGEVASFPGPLKEENLLNVPKPLPKQLWETKEIQSLSGRPRSCDVGGGNAFPHNGQNLGL
SPFLGTLNTGGSLPDLTNLHYSTPLPASLDTTDHHFGSMSVGNSVNNIPAAMTHLGIRSSSGLQSSRSNPSIQATLNKTVLSSSLNN
HPQTSVPNASALHPSLRLFSLSNPSLSTTNLSGPSRRRQPPVSPLTLSPGPEAHQGFSRQLSSTSPLAPYPTSQMVSSDRSQLSFLP
TEAQAQVSPPPPYPAPQELTQPLLQQPRAPEAPAQQPQAASSLPQSDFQLLPAQGSSLTNFFPDVGFDQQSMRPGPAFPQQVPLVQQ

GSRELQDSFHLRPSPYSNCGSLPNTILPEDSSTSLFKDLNSALAGLPEVSLNVDTPFPLEEELQIEPLSLDGLNMLSDSSMGLLDPS
VEETFRADRL
>sp|Q6QEF8|CORO6_HUMAN
MSRRVVRQSKFRHVFGQAAKADQAYEDIRVSKVTWDSSFCAVNPKFLAIIVEAGGGGAFIVLPLAKTGRVDKNYPLVTGHTAPVLDI
DWCPHNDNVIASASDDTTIMVWQIPDYTPMRNITEPIITLEGHSKRVGILSWHPTARNVLLSAGGDNVIIIWNVGTGEVLLSLDDMH
PDVIHSVCWNSNGSLLATTCKDKTLRIIDPRKGQVVAEQARPHEGARPLRAVFTADGKLLSTGFSRMSERQLALWDPNNFEEPVALQ
EMDTSNGVLLPFYDPDSSIVYLCGKGDSSIRYFEITDEPPFVHYLNTFSSKEPQRGMGFMPKRGLDVSKCEIARFYKLHERKCEPII
MTVPRKSDLFQDDLYPDTPGPEPALEADEWLSGQDAEPVLISLRDGYVPPKHRELRVTKRNILDVRPPSGPRRSQSASDAPLSQQHT
LETLLEEIKALRERVQAQEQRITALENMLCELVDGTD
>sp|Q9H3M0|KCNF1_HUMAN
MDGSGERSLPEPGSQSSAASDDIEIVVNVGGVRQVLYGDLLSQYPETRLAELINCLAGGYDTIFSLCDDYDPGKREFYFDRDPDAFK
CVIEVYYFGEVHMKKGICPICFKNEMDFWKVDLKFLDDCCKSHLSEKREELEEIARRVQLILDDLGVDAAEGRWRRCQKCVWKFLEK
PESSCPARVVAVLSFLLILVSSVVMCMGTIPELQVLDAEGNRVEHPTLENVETACIGWFTLEYLLRLFSSPNKLHFALSFMNIVDVL
AILPFYVSLTLTHLGARMMELTNVQQAVQALRIMRIARIFKLARHSSGLQTLTYALKRSFKELGLLLMYLAVGIFVFSALGYTMEQS
HPETLFKSIPQSFWWAIITMTTVGYGDIYPKTTLGKLNAAISFLCGVIAIALPIHPIINNFVRYYNKQRVLETAAKHELELMELNSS
SGGEGKTGGSRSDLDNLPPEPAGKEAPSCSSRLKLSHSDTFIPLLTEEKHHRTRLQSCK
>sp|Q9Y2W6|TDRKH_HUMAN
MSTERTSWTSLSTIQKIALGLGIPASATVAYILYRRYRESREERLTFVGEDDIEIEMRVPQEAVKLIIGRQGANIKQLRKQTGARID
VDTEDVGDERVLLISGFPVQVCKAKAAIHQILTENTPVSEQLSVPQRSVGRIIGRGGETIRSICKASGAKITCDKESEGTLLLSRLI
KISGTQKEVAAAKHLILEKVSEDEELRKRIAHSAETRVPRKQPISVRREDMTEPGGAGEPALWKNTSSSMEPTAPLVTPPPKGGGDM
AVVVSKEGSWEKPSDDSFQKSEAQAIPEMPMFEIPSPDFSFHADEYLEVYVSASEHPNHFWIQIVGSRSLQLDKLVNEMTQHYENSV
PEDLTVHVGDIVAAPLPTNGSWYRARVLGTLENGNLDLYFVDFGDNGDCPLKDLRALRSDFLSLPFQAIECSLARIAPSGDQWEEEA
LDEFDRLTHCADWKPLVAKISSYVQTGISTWPKIYLYDTSNGKKLDIGLELVHKGYAIELPEDIEENRAVPDMLKDMATETDASLST
LLTETKKSSGEITHTLSCLSLSEAASMSGDDNLEDDYLL
>sp|Q9H015|S22A4_HUMAN
MRDYDEVIAFLGEWGPFQRLIFFLLSASIIPNGFNGMSVVFLAGTPEHRCRVPDAANLSSAWRNNSVPLRLRDGREVPHSCSRYRLA
TIANFSALGLEPGRDVDLGQLEQESCLDGWEFSQDVYLSTVVTEWNLVCEDNWKVPLTTSLFFVGVLLGSFVSGQLSDRFGRKNVLF
ATMAVQTGFSFLQIFSISWEMFTVLFVIVGMGQISNYVVAFILGTEILGKSVRIIFSTLGVCTFFAVGYMLLPLFAYFIRDWRMLLL
ALTVPGVLCVPLWWFIPESPRWLISQRRFREAEDIIQKAAKMNNIAVPAVIFDSVEELNPLKQQKAFILDLFRTRNIAIMTIMSLLL
WMLTSVGYFALSLDAPNLHGDAYLNCFLSALIEIPAYITAWLLLRTLPRRYIIAAVLFWGGGVLLFIQLVPVDYYFLSIGLVMLGKF
GITSAFSMLYVFTAELYPTLVRNMAVGVTSTASRVGSIIAPYFVYLGAYNRMLPYIVMGSLTVLIGILTLFFPESLGMTLPETLEQM
QKVKWFRSGKKTRDSMETEENPKVLITAF
>sp|O75443|TECTA_HUMAN
MNYSSFLRIWVSFIFALVQHQAQPRELMYPFWQNDTKTPKVDDGSSSEIKLAIPVFFFGVPYRTVYVNNNGVVSFNVLVSQFTPESF
PLTDGRAFVAPFWADVHNGIRGEIYYRETMEPAILKRATKDIRKYFKDMATFSATWVFIVTWEEVTFYGGSSTTPVNTFQAVLVSDG
SYTFTLFNYYEINWTTGTASGGDPLTGLGGVMAQAGFNGGNLTNFFSLPGSRTPEIVNIQETTNVNVPGRWAFKVDGKEIDPANGCT
SRGQFLRRGEVFWDDLNCTVKCRCLDFNNEIYCQEASCSPYEVCEPKGKFFYCSAVETSTCVVFGEPHYHTFDGFLFHFQGSCAYLL
ARQCLQTSSLPFFSVEAKNEHRRGSAVSWVKELSVEVNGYKILIPKGSYGRVKVNDLVTSLPVTLDLGTVKIYQSGISTAVETDFGL
LVTFDGQHYASISVPGSYINSTCGLCGNYNKNPLDDFLRPDGRPAMSVLDLGESWRVYHADWKCDSGCVDNCTQCDAATEALYFGSD
YCGFLNKTDGPLWECGTVVDPTAFVHSCVYDLCSVRDNGTLLCQAIQAYALVCQALGIPIGDWRTQTGCVSTVQCPSFSHYSVCTSS
CPDTCSDLTASRNCATPCTEGCECNQGFVLSTSQCVPLHKCGCDFDGHYYTMGEFFWATANCTVQCLCEEGGDVYCFNKTCGSGEVC
AVEDGYQGCFPKRETVCLLSQNQVLHTFDGASYAFPSEFSYTLLKTCPERPEYLEIDINKKKPDAGPAWLRGLRILVADQEVKIGGI
GASEVKLNGQEVELPFFHPSGKLEIYRNKNSTTVESKGVVTVQYSDIGLLYIRLSTTYFNCTGGLCGFYNANASDEFCLPNGKCTDN
LAVFLESWTTFEEICNGECGDLLKACNNDSELLKFYRSRSRCGIINDPSNSSFLECHGVVNVTAYYRTCLFRLCQSGGNESELCDSV

ARYASACKNADVEVGPWRTYDFCPLECPENSHFEECITCTETCETLTLGPICVDSCSEGCQCDEGYALLGSQCVTRSECGCNFEGHQ
LATNETFWVDLDCQIFCYCSGTDNRVHCETIPCKDDEYCMEEGGLYYCQARTDASCIVSGYGHYLTFDGFPFDFQTSCPLILCTTGS
RPSSDSFPKFVVTAKNEDRDPSLALWVKQVDVTVFGYSIVIHRAYKHTVLVNSERLYLPLKLGQGKINIFSFGFHVVVETDFGLKVV
YDWKTFLSITVPRSMQNSTYGLCGRYNGNPDDDLEMPMGLLASSVNEFGQSWVKRDTFCQVGCGDRCPSCAKVEGFSKVQQLCSLIP
NQNAAFSKCHSKVNPTFFYKNCLFDSCIDGGAVQTACSWLQNYASTCQTQGITVTGWRNYTSCTVTCPPNSHYESCVSVCQPRCAAI
RLKSDCSHYCVEGCHCDAGYVLNGKSCILPHSCGCYSDGKYYEPKQLFWNSDCTRRCRCFRRNVIQCDPRQCKSDEECALRNGVRGC
FSTKTSYCLAAGGGVFRTFDGAFLRFPANCAFVLSTICQKLPDISFQLIINFDKWSAPNLTIISPVYFYINEEQILINDRNTVKVNG
TQVNVPFITGLATKIYSSEGFLVIDTSPDIQIYYNGFNVIKISISERLQNKVCGLCGNFNGDLTDDYVTLRGKPVVSSVVLAQSWKT
NGMQKRPLAPSCNELQFSQYAAMCDNVHIQKMQGDGYCLKLTDMKGFFQPCYGLLDPLPFYESCYLDGCYSHKKFQLCGSLAAYGEA
CRSFGILSTEWIEKENCSGVVEDPCVGADCPNRTCELGNGRELCGCIEPPPYGNNSHDIIDAEVTCKAAQMEVSISKCKLFQLGFER
EGVRINDRQCTGIEGEDFISFQINNTKGNCGNIVQSNGTHIMYKNTLWIESANNTGNIITRDRTINVEFSCAYELDIKISLDSVVKP
MLSVINLTVPTQEGSFITKMALYKNASYKHPYRQGEVVLTTRDVLYVGVFVVGADATHLILTLNKCYATPTRDSNDKLRYFIIEGGC
QNLKDNTIGIEENAVSLTCRFHVTVFKFIGDYDEVHLHCAVSLCDSEKYSCKITCPHNSRIATDYTKEPKEQIISVGPIRRKRLDWC
EDNGGCEQICTSRVDGPLCSCVTGTLQEDGKSCRASNSSMELQVWTLLLIMIQISLWHFVYKSGTTS
>sp|Q8N130|NPT2C_HUMAN
MPSSLPGSQVPHPTLDAVDLVEKTLRNEGTSSSAPVLEEGDTDPWTLPQLKDTSQPWKELRVAGRLRRVAGSVLKACGLLGSLYFFI
CSLDVLSSAFQLLGSKVAGDIFKDNVVLSNPVAGLVIGVLVTALVQSSSTSSSIVVSMVAAKLLTVRVSVPIIMGVNVGTSITSTLV
SMAQSGDRDEFQRAFSGSAVHGIFNWLTVLVLLPLESATALLERLSELALGAASLTPRAQAPDILKVLTKPLTHLIVQLDSDMIMSS
ATGNATNSSLIKHWCGTTGQPTQENSSCGAFGPCTEKNSTAPADRLPCRHLFAGTELTDLAVGCILLAGSLLVLCGCLVLIVKLLNS
VLRGRVAQVVRTVINADFPFPLGWLGGYLAVLAGAGLTFALQSSSVFTAAVVPLMGVGVISLDRAYPLLLGSNIGTTTTALLAALAS
PADRMLSALQVALIHFFFNLAGILLWYLVPALRLPIPLARHFGVVTARYRWVAGVYLLLGFLLLPLAAFGLSLAGGMELAAVGGPLV
GLVLLVILVTVLQRRRPAWLPVRLRSWAWLPVWLHSLEPWDRLVTRCCPCNVCSPPKATTKEAYCYENPEILASQQL
>sp|Q8TDU6|GPBAR_HUMAN
MTPNSTGEVPSPIPKGALGLSLALASLIITANLLLALGIAWDRRLRSPPAGCFFLSLLLAGLLTGLALPTLPGLWNQSRRGYWSCLL
VYLAPNFSFLSLLANLLLVHGERYMAVLRPLQPPGSIRLALLLTWAGPLLFASLPALGWNHWTPGANCSSQAIFPAPYLYLEVYGLL
LPAVGAAAFLSVRVLATAHRQLQDICRLERAVCRDEPSALARALTWRQARAQAGAMLLFGLCWGPYVATLLLSVLAYEQRPPLGPGT
LLSLLSLGSASAAAVPVAMGLGDQRYTAPWRAAAQRCLQGLWGRASRDSPGPSIAYHPSSQSSVDLDLN
>sp|Q6UX41|BTNL8_HUMAN
MALMLSLVLSLLKLGSGQWQVFGPDKPVQALVGEDAAFSCFLSPKTNAEAMEVRFFRGQFSSVVHLYRDGKDQPFMQMPQYQGRTKL
VKDSIAEGRISLRLENITVLDAGLYGCRISSQSYYQKAIWELQVSALGSVPLISITGYVDRDIQLLCQSSGWFPRPTAKWKGPQGQD
LSTDSRTNRDMHGLFDVEISLTVQENAGSISCSMRHAHLSREVESRVQIGDTFFEPISWHLATKVLGILCCGLFFGIVGLKIFFSKF
QWKIQAELDWRRKHGQAELRDARKHAVEVTLDPETAHPKLCVSDLKTVTHRKAPQEVPHSEKRFTRKSVVASQSFQAGKHYWEVDGG
HNKRWRVGVCRDDVDRRKEYVTLSPDHGYWVLRLNGEHLYFTLNPRFISVFPRTPPTKIGVFLDYECGTISFFNINDQSLIYTLTCR
FEGLLRPYIEYPSYNEQNGTPIVICPVTQESEKEASWQRASAIPETSNSESSSQATTPFLPRGEM
>sp|Q9P244|LRFN1_HUMAN
MAPGPFSSALLSPPPAALPFLLLLWAGASRGQPCPGRCICQNVAPTLTMLCAKTGLLFVPPAIDRRVVELRLTDNFIAAVRRRDFAN
MTSLVHLTLSRNTIGQVAAGAFADLRALRALHLDSNRLAEVRGDQLRGLGNLRHLILGNNQIRRVESAAFDAFLSTVEDLDLSYNNL
EALPWEAVGQMVNLNTLTLDHNLIDHIAEGTFVQLHKLVRLDMTSNRLHKLPPDGLFLRSQGTGPKPPTPLTVSFGGNPLHCNCELL
WLRRLTREDDLETCATPEHLTDRYFWSIPEEEFLCEPPLITRQAGGRALVVEGQAVSLRCRAVGDPEPVVHWVAPDGRLLGNSSRTR
VRGDGTLDVTITTLRDSGTFTCIASNAAGEATAPVEVCVVPLPLMAPPPAAPPPLTEPGSSDIATPGRPGANDSAAERRLVAAELTS
NSVLIRWPAQRPVPGIRMYQVQYNSSVDDSLVYRMIPSTSQTFLVNDLAAGRAYDLCVLAVYDDGATALPATRVVGCVQFTTAGDPA
PCRPLRAHFLGGTMIIAIGGVIVASVLVFIVLLMIRYKVYGDGDSRRVKGSRSLPRVSHVCSQTNGAGTGAAQAPALPAQDHYEALR
EVESQAAPAVAVEAKAMEAETASAEPEVVLGRSLGGSATSLCLLPSEETSGEESRAAVGPRRSRSGALEPPTSAPPTLALVPGGAAA
RPRPQQRYSFDGDYGALFQSHSYPRRARRTKRHRSTPHLDGAGGGAAGEDGDLGLGSARACLAFTSTEWMLESTV

>sp|Q96AB6|NTAN1_HUMAN
MPLLVEGRRVRLPQSAGDLVRAHPPLEERARLLRGQSVQQVGPQGLLYVQQRELAVTSPKDGSISILGSDDATTCHIVVLRHTGNGA
TCLTHCDGTDTKAEVPLIMNSIKSFSDHAQCGRLEVHLVGGFSDDRQLSQKLTHQLLSEFDRQEDDIHLVTLCVTELNDREENENHF
PVIYGIAVNIKTAEIYRASFQDRGPEEQLRAARTLAGGPMISIYDAETEQLRIGPYSWTPFPHVDFWLHQDDKQILENLSTSPLAEP
PHFVEHIRSTLMFLKKHPSPAHTLFSGNKALLYKKNEDGLWEKISSPGS
>sp|Q96H22|CENPN_HUMAN
MDETVAEFIKRTILKIPMNELTTILKAWDFLSENQLQTVNFRQRKESVVQHLIHLCEEKRASISDAALLDIIYMQFHQHQKVWEVFQ
MSKGPGEDVDLFDMKQFKNSFKKILQRALKNVTVSFRETEENAVWIRIAWGTQYTKPNQYKPTYVVYYSQTPYAFTSSSMLRRNTPL
LGQALTIASKHHQIVKMDLRSRYLDSLKAIVFKQYNQTFETHNSTTPLQERSLGLDINMDSRIIHENIVEKERVQRITQETFGDYPQ
PQLEFAQYKLETKFKSGLNGSILAEREEPLRCLIKFSSPHLLEALKSLAPAGIADAPLSPLLTCIPNKRMNYFKIRDK
>sp|P27105|STOM_HUMAN
MAEKRHTRDSEAQRLPDSFKDSPSKGLGPCGWILVAFSFLFTVITFPISIWMCIKIIKEYERAIIFRLGRILQGGAKGPGLFFILPC
TDSFIKVDMRTISFDIPPQEILTKDSVTISVDGVVYYRVQNATLAVANITNADSATRLLAQTTLRNVLGTKNLSQILSDREEIAHNM
QSTLDDATDAWGIKVERVEIKDVKLPVQLQRAMAAEAEASREARAKVIAAEGEMNASRALKEASMVITESPAALQLRYLQTLTTIAA
EKNSTIVFPLPIDMLQGIIGAKHSHLG
>sp|Q9H063|MAF1_HUMAN
MKLLENSSFEAINSQLTVETGDAHIIGRIESYSCKMAGDDKHMFKQFCQEGQPHVLEALSPPQTSGLSPSRLSKSQGGEEEGPLSDK
CSRKTLFYLIATLNESFRPDYDFSTARSHEFSREPSLSWVVNAVNCSLFSAVREDFKDLKPQLWNAVDEEICLAECDIYSYNPDLDS
DPFGEDGSLWSFNYFFYNKRLKRIVFFSCRSISGSTYTPSEAGNELDMELGEEEVEEESRSGGSGAEETSTMEEDRVPVICI
>sp|P59020|DSCR9_HUMAN
MGRICPVNSRARRLRARPGRPSGDSLPYHQLQGGAPRLWSPDPGRPAAYRRAHVCDVTAPRWGSTSRQGEGAVLQRMLGRRAPPSWS
RDHAYSRRGWENAALFLNRKRKQEGTENTSICCRPESALACGGNLSPQFLKKVIQIQTQELW
>sp|P53990|IST1_HUMAN
MLGSGFKAERLRVNLRLVINRLKLLEKKKTELAQKARKEIADYLAAGKDERARIRVEHIIREDYLVEAMEILELYCDLLLARFGLIQ
SMKELDSGLAESVSTLIWAAPRLQSEVAELKIVADQLCAKYSKEYGKLCRTNQIGTVNDRLMHKLSVEAPPKILVERYLIEIAKNYN
VPYEPDSVVMAEAPPGVETDLIDVGFTDDVKKGGPGRGGSGGFTAPVGGPDGTVPMPMPMPMPSANTPFSYPLPKGPSDFNGLPMGT
YQAFPNIHPPQIPATPPSYESVDDINADKNISSAQIVGPGPKPEASAKLPSRPADNYDNFVLPELPSVPDTLPTASAGASTSASEDI
DFDDLSRRFEELKKKT
>sp|Q8N966|ZDH22_HUMAN
MLALRLLNVVAPAYFLCISLVTFVLQLFLFLPSMREDPAAARLFSPALLHGALFLFLSANALGNYVLVIQNSPDDLGACQGASARKT
PCPSPSTHFCRVCARVTLRHDHHCFFTGNCIGSRNMRNFVLFCLYTSLACLYSMVAGVAYISAVLSISFAHPLAFLTLLPTSISQFF
SGAVLGSEMFVILMLYLWFAIGLACAGFCCHQLLLILRGQTRHQVRKGVAVRARPWRKNLQEVFGKRWLLGLLVPMFNVGSESSKQQ
DK
>sp|P51685|CCR8_HUMAN
MDYTLDLSVTTVTDYYYPDIFSSPCDAELIQTNGKLLLAVFYCLLFVFSLLGNSLVILVLVVCKKLRSITDVYLLNLALSDLLFVFS
FPFQTYYLLDQWVFGTVMCKVVSGFYYIGFYSSMFFITLMSVDRYLAVVHAVYALKVRTIRMGTTLCLAVWLTAIMATIPLLVFYQV
ASEDGVLQCYSFYNQQTLKWKIFTNFKMNILGLLIPFTIFMFCYIKILHQLKRCQNHNKTKAIRLVLIVVIASLLFWVPFNVVLFLT
SLHSMHILDGCSISQQLTYATHVTEIISFTHCCVNPVIYAFVGEKFKKHLSEIFQKSCSQIFNYLGRQMPRESCEKSSSCQQHSSRS
SSVDYIL
>sp|Q96JA3|PKHA8_HUMAN
MEGVLYKWTNYLSGWQPRWFLLCGGILSYYDSPEDAWKGCKGSIQMAVCEIQVHSVDNTRMDLIIPGEQYFYLKARSVAERQRWLVA
LGSAKACLTDSRTQKEKEFAENTENLKTKMSELRLYCDLLVQQVDKTKEVTTTGVSNSEEGIDVGTLLKSTCNTFLKTLEECMQIAN
AAFTSELLYRTPPGSPQLAMLKSSKMKHPIIPIHNSLERQMELSTCENGSLNMEINGEEEILMKNKNSLYLKSAEIDCSISSEENTD
DNITVQGEIRKEDGMENLKNHDNNLTQSGSDSSCSPECLWEEGKEVIPTFFSTMNTSFSDIELLEDSGIPTEAFLASCYAVVPVLDK

LGPTVFAPVKMDLVGNIKKVNQKYITNKEEFTTLQKIVLHEVEADVAQVRNSATEALLWLKRGLKFLKGFLTEVKNGEKDIQTALNN
AYGKTLRQHHGWVVRGVFALALRAAPSYEDFVAALTVKEGDHQKEAFSIGMQRDLSLYLPAMEKQLAILDTLYEVHGLESDEVV
>sp|Q9Y6R1|S4A4_HUMAN
MEDEAVLDRGASFLKHVCDEEEVEGHHTIYIGVHVPKSYRRRRRHKRKTGHKEKKEKERISENYSDKSDIENADESSSSILKPLISP
AAERIRFILGEEDDSPAPPQLFTELDELLAVDGQEMEWKETARWIKFEEKVEQGGERWSKPHVATLSLHSLFELRTCMEKGSIMLDR
EASSLPQLVEMIVDHQIETGLLKPELKDKVTYTLLRKHRHQTKKSNLRSLADIGKTVSSASRMFTNPDNGSPAMTHRNLTSSSLNDI
SDKPEKDQLKNKFMKKLPRDAEASNVLVGEVDFLDTPFIAFVRLQQAVMLGALTEVPVPTRFLFILLGPKGKAKSYHEIGRAIATLM
SDEVFHDIAYKAKDRHDLIAGIDEFLDEVIVLPPGEWDPAIRIEPPKSLPSSDKRKNMYSGGENVQMNGDTPHDGGHGGGGHGDCEE
LQRTGRFCGGLIKDIKRKAPFFASDFYDALNIQALSAILFIYLATVTNAITFGGLLGDATDNMQGVLESFLGTAVSGAIFCLFAGQP
LTILSSTGPVLVFERLLFNFSKDNNFDYLEFRLWIGLWSAFLCLILVATDASFLVQYFTRFTEEGFSSLISFIFIYDAFKKMIKLAD
YYPINSNFKVGYNTLFSCTCVPPDPANISISNDTTLAPEYLPTMSSTDMYHNTTFDWAFLSKKECSKYGGNLVGNNCNFVPDITLMS
FILFLGTYTSSMALKKFKTSPYFPTTARKLISDFAIILSILIFCVIDALVGVDTPKLIVPSEFKPTSPNRGWFVPPFGENPWWVCLA
AAIPALLVTILIFMDQQITAVIVNRKEHKLKKGAGYHLDLFWVAILMVICSLMALPWYVAATVISIAHIDSLKMETETSAPGEQPKF
LGVREQRVTGTLVFILTGLSVFMAPILKFIPMPVLYGVFLYMGVASLNGVQFMDRLKLLLMPLKHQPDFIYLRHVPLRRVHLFTFLQ
VLCLALLWILKSTVAAIIFPVMILALVAVRKGMDYLFSQHDLSFLDDVIPEKDKKKKEDEKKKKKKKGSLDSDNDDSDCPYSEKVPS
IKIPMDIMEQQPFLSDSKPSDRERSPTFLERHTSC
>sp|Q8NDW4|ZN248_HUMAN
MNKSQEQVSFKDVCVDFTQEEWYLLDPAQKILYRDVILENYSNLVSVGYCITKPEVIFKIEQGEEPWILEKGFPSQCHPERKWKVDD
VLESSQENEDDHFWELLFHNNKTVSVENGDRGSKTFNLGTDPVSLRNYPYKICDSCEMNLKNISGLIISKKNCSRKKPDEFNVCEKL
LLDIRHEKIPIGEKSYKYDQKRNAINYHQDLSQPSFGQSFEYSKNGQGFHDEAAFFTNKRSQIGETVCKYNECGRTFIESLKLNISQ
RPHLEMEPYGCSICGKSFCMNLRFGHQRALTKDNPYEYNEYGEIFCDNSAFIIHQGAYTRKILREYKVSDKTWEKSALLKHQIVHMG
GKSYDYNENGSNFSKKSHLTQLRRAHTGEKTFECGECGKTFWEKSNLTQHQRTHTGEKPYECTECGKAFCQKPHLTNHQRTHTGEKP
YECKQCGKTFCVKSNLTEHQRTHTGEKPYECNACGKSFCHRSALTVHQRTHTGEKPFICNECGKSFCVKSNLIVHQRTHTGEKPYKC
NECGKTFCEKSALTKHQRTHTGEKPYECNACGKTFSQRSVLTKHQRIHTRVKALSTS
>sp|Q13571|LAPM5_HUMAN
MDPRLSTVRQTCCCFNVRIATTALAIYHVIMSVLLFIEHSVEVAHGKASCKLSQMGYLRIADLISSFLLITMLFIISLSLLIGVVKN
REKYLLPFLSLQIMDYLLCLLTLLGSYIELPAYLKLASRSRASSSKFPLMTLQLLDFCLSILTLCSSYMEVPTYLNFKSMNHMNYLP
SQEDMPHNQFIKMMIIFSIAFITVLIFKVYMFKCVWRCYRLIKCMNSVEEKRNSKMLQKVVLPSYEEALSLPSKTPEGGPAPPPYSE
V
>sp|O95182|NDUA7_HUMAN
MASATRLIQRLRNWASGHDLQGKLQLRYQEISKRTQPPPKLPVGPSHKLSNNYYCTRDGRRESVPPSIIMSSQKALVSGKPAESSAV
AATEKKAVTPAPPIKRWELSSDQPYL
>sp|Q6PIF2|SYCE2_HUMAN
MERQGVDVPHVKCKDQEPQPLGESKEHPRWEENCEEEAGGGPASASCQLTVLEGKSGLYFSSLDSSIDILQKRAQELIENINKSRQK
DHALMTNFRNSLKTKVSDLTEKLEERIYQIYNDHNKIIQEKLQEFTQKMAKISHLETELKQVCHSVETVYKDLCLQPEQSLRLRWGP
DHSRGKSPPRPGNSQPPDVFVSSVAETTSQATASEVQTNRDGEC
>sp|Q9H0L4|CSTFT_HUMAN
MSSLAVRDPAMDRSLRSVFVGNIPYEATEEQLKDIFSEVGSVVSFRLVYDRETGKPKGYGFCEYQDQETALSAMRNLNGREFSGRAL
RVDNAASEKNKEELKSLGPAAPIIDSPYGDPIDPEDAPESITRAVASLPPEQMFELMKQMKLCVQNSHQEARNMLLQNPQLAYALLQ
AQVVMRIMDPEIALKILHRKIHVTPLIPGKSQSVSVSGPGPGPGPGLCPGPNVLLNQQNPPAPQPQHLARRPVKDIPPLMQTPIQGG
IPAPGPIPAAVPGAGPGSLTPGGAMQPQLGMPGVGPVPLERGQVQMSDPRAPIPRGPVTPGGLPPRGLLGDAPNDPRGGTLLSVTGE
VEPRGYLGPPHQGPPMHHASGHDTRGPSSHEMRGGPLGDPRLLIGEPRGPMIDQRGLPMDGRGGRDSRAMETRAMETEVLETRVMER
RGMETCAMETRGMEARGMDARGLEMRGPVPSSRGPMTGGIQGPGPINIGAGGPPQGPRQVPGISGVGNPGAGMQGTGIQGTGMQGAG

IQGGGMQGAGIQGVSIQGGGIQGGGIQGASKQGGSQPSSFSPGQSQVTPQDQEKAALIMQVLQLTADQIAMLPPEQRQSILILKEQI
QKSTGAS
>sp|Q8NBT2|SPC24_HUMAN
MAAFRDIEEVSQGLLSLLGANRAEAQQRRLLGRHEQVVERLLETQDGAEKQLREILTMEKEVAQSLLNAKEQVHQGGVELQQLEAGL
QEAGEEDTRLKASLLYLTRELEELKEIEADLERQEKEVDEDTTVTIPSAVYVAQLYHQVSKIEWDYECEPGMVKGIHHGPSVAQPIH
LDSTQLSRKFISDYLWSLVDTEW
>sp|P34810|CD68_HUMAN
MRLAVLFSGALLGLLAAQGTGNDCPHKKSATLLPSFTVTPTVTESTGTTSHRTTKSHKTTTHRTTTTGTTSHGPTTATHNPTTTSHG
NVTVHPTSNSTATSQGPSTATHSPATTSHGNATVHPTSNSTATSPGFTSSAHPEPPPPSPSPSPTSKETIGDYTWTNGSQPCVHLQA
QIQIRVMYTTQGGGEAWGISVLNPNKTKVQGSCEGAHPHLLLSFPYGHLSFGFMQDLQQKVVYLSYMAVEYNVSFPHAAQWTFSAQN
ASLRDLQAPLGQSFSCSNSSIILSPAVHLDLLSLRLQAAQLPHTGVFGQSFSCPSDRSILLPLIIGLILLGLLALVLIAFCIIRRRP
SAYQAL
>sp|Q8NGQ3|OR1S2_HUMAN
MKTLCSFLQISRNMHQENQTTITEFILLGLSNQAEHQNLLFVLFLSMYVVTVVGNGLIIVAISLDIYLHTPMYLFLAYLSFADISSI
SNSVPKMLVNIQTNSQSISYESCITQMYFSIVFVVTDNLLLGTMAFDHFVAICHPLNYTTFMRARFGTLLTVISWFLSNIIALTHTL
LLIQLLFCDHNTLPHFFCDLAPLLKLSCSDTMINELVLFIVGLSVIIFPFVLIFFSYVCIIRAVLGVSSTQGKWKAFSTCGSHLTIA
LLFYGTTVGVYFFPSSTHPEDTDKIGAVLFTVVTPMMNPFIYSLRNKDMKGALRKLINRKISSL
>sp|Q8IZ57|NRSN1_HUMAN
MSSCSNVCGSRQAQAAAEGGYQRYGVRSYLHQFYEDCTASIWEYEDDFQIQRSPNRWSSVFWKVGLISGTVFVILGLTVLAVGFLVP
PKIEAFGEADFVVVDTHAVQFNSALDMYKLAGAVLFCIGGTSMAGCLLMSVFVKSYSKEEKFLQQKFKERIADIKAHTQPVTKAPGP
GETKIPVTLSRVQNVQPLLAT
>sp|P40337|VHL_HUMAN
MPRRAENWDEAEVGAEEAGVEEYGPEEDGGEESGAEESGPEESGPEELGAEEEMEAGRPRPVLRSVNSREPSQVIFCNRSPRVVLPV
WLNFDGEPQPYPTLPPGTGRRIHSYRGHLWLFRDAGTHDGLLVNQTELFVPSLNVDGQPIFANITLPVYTLKERCLQVVRSLVKPEN
YRRLDIVRSLYEDLEDHPNVQKDLERLTQERIAHQRMGD
>sp|Q6NVV7|CDPF1_HUMAN
MASHVECRPLGVFECELCTLTAPYSYVGQKPPNTQSMVLLEESYVMKDPFTSDKDRFLVLGSCCSLCSRLVCVGPECSLFYSKRFCL
PCVRENINAFPQEIRQDLEKRKAPSKRTPSQPGSRT
>sp|Q9BYG4|PAR6G_HUMAN
MNRSFHKSQTLRFYDCSAVEVKSKFGAEFRRFSLDRHKPGKFEDFYKLVVHTHHISNSDVTIGYADVHGDLLPINNDDNFCKAVSSA
NPLLRVFIQKREEAERGSLGAGSLCRRRRALGALRDEGPRRRAHLDIGLPRDFRPVSSIIDVDLVPETHRRVRLHRHGCEKPLGFYI
RDGASVRVTPHGLEKVPGIFISRMVPGGLAESTGLLAVNDEVLEVNGIEVAGKTLDQVTDMMIANSHNLIVTVKPANQRNNVVRGGR
ALGSSGPPSDGTAGFVGPPAPRVLQNFHPDEAESDEDNDVVIEGTLEPARPPQTPGAPAGSLSRVNGAGLAQRLQRDLALDGGLQRL
LSSLRADPRHSLALPPGGVEEHGPAVTL
>sp|Q8WTS1|ABHD5_HUMAN
MAAEEEEVDSADTGERSGWLTGWLPTWCPTSISHLKEAEEKMLKCVPCTYKKEPVRISNGNKIWTLKFSHNISNKTPLVLLHGFGGG
LGLWALNFGDLCTNRPVYAFDLLGFGRSSRPRFDSDAEEVENQFVESIEEWRCALGLDKMILLGHNLGGFLAAAYSLKYPSRVNHLI
LVEPWGFPERPDLADQDRPIPVWIRALGAALTPFNPLAGLRIAGPFGLSLVQRLRPDFKRKYSSMFEDDTVTEYIYHCNVQTPSGET
AFKNMTIPYGWAKRPMLQRIGKMHPDIPVSVIFGARSCIDGNSGTSIQSLRPHSYVKTIAILGAGHYVYADQPEEFNQKVKEICDTV
D
>sp|Q9P2Y5|UVRAG_HUMAN
MSASASVGGPVPQPPPGPAAALPPGSAARALHVELPSQQRRLRHLRNIAARNIVNRNGHQLLDTYFTLHLCSTEKIYKEFYRSEVIK
NSLNPTWRSLDFGIMPDRLDTSVSCFVVKIWGGKENIYQLLIEWKVCLDGLKYLGQQIHARNQNEIIFGLNDGYYGAPFEHKGYSNA
QKTILLQVDQNCVRNSYDVFSLLRLHRAQCAIKQTQVTVQKIGKEIEEKLRLTSTSNELKKKSECLQLKILVLQNELERQKKALGRE

VALLHKQQIALQDKGSAFSAEHLKLQLQKESLNELRKECTAKRELFLKTNAQLTIRCRQLLSELSYIYPIDLNEHKDYFVCGVKLPN
SEDFQAKDDGSIAVALGYTAHLVSMISFFLQVPLRYPIIHKGSRSTIKDNINDKLTEKEREFPLYPKGGEKLQFDYGVYLLNKNIAQ
LRYQHGLGTPDLRQTLPNLKNFMEHGLMVRCDRHHTSSAIPVPKRQSSIFGGADVGFSGGIPSPDKGHRKRASSENERLQYKTPPPS
YNSALAQPVTTVPSMGETERKITSLSSSLDTSLDFSKENKKKGEDLVGSLNGGHANVHPSQEQGEALSGHRATVNGTLLPSEQAGSA
SVQLPGEFHPVSEAELCCTVEQAEEIIGLEATGFASGDQLEAFNCIPVDSAVAVECDEQVLGEFEEFSRRIYALNENVSSFRRPRRS
SDK
>sp|O43610|SPY3_HUMAN
MDAAVTDDFQQILPIEQLRSTHASNDYVERPPAPCKQALSSPSLIVQTHKSDWSLATMPTSLPRSLSQCHQLQPLPQHLSQSSIASS
MSHSTTASDQRLLASITPSPSGQSIIRTQPGAGVHPKADGALKGEAEQSAGHPSEHLFICEECGRCKCVPCTAARPLPSCWLCNQRC
LCSAESLLDYGTCLCCVKGLFYHCSTDDEDNCADEPCSCGPSSCFVRWAAMSLISLFLPCLCCYLPTRGCLHLCQQGYDSLRRPGCR
CKRHTNTVCRKISSGSAPFPKAQEKSV
>sp|A8K8P3|SFI1_HUMAN
MKNLLTEKCISSHNFHQKVIKQRMEKKVDSRYFKDGAVKKPYSAKTLSNKKSSASFGIRRELPSTSHLVQYRGTHTCTRQGRLRELR
IRCVARKFLYLWIRMTFGRVFPSKARFYYEQRLLRKVFEEWKEEWWVFQHEWKLCVRADCHYRYYLYNLMFQTWKTYVRQQQEMRNK
YIRAEVHDAKQKMRQAWKSWLIYVVVRRTKLQMQTTALEFRQRIILRVWWSTWRQRLGQVRVSRALHASALKHRALSLQVQAWSQWR
EQLLYVQKEKQKVVSAVKHHQHWQKRRFLKAWLEYLQVRRVKRQQNEMAERFHHVTVLQIYFCDWQQAWERRESLYAHHAQVEKLAR
KMALRRAFTHWKHYMLLCAEEAAQFEMAEEHHRHSQLYFCFRALKDNVTHAHLQQIRRNLAHQQHGVTLLHRFWNLWRSQIEQKKER
ELLPLLHAAWDHYRIALLCKCIELWLQYTQKRRYKQLLQARADGHFQQRALPAAFHTWNRLWRWRHQENVLSARATRFHRETLEKQV
FSLWRQKMFQHRENRLAERMAILHAERQLLYRSWFMWHQQAAARHQEQEWQTVACAHHRHGRLKKAFCLWRESAQGLRTERTGRVRA
AEFHMAQLLRWAWSQWRECLALRGAERQKLMRADLHHQHSVLHRALQAWVTYQGRVRSILREVAARESQHNRQLLRGALRRWKENTM
ARVDEAKKTFQASTHYRRTICSKVLVQWREAVSVQMYYRQQEDCAIWEAQKVLDRGCLRTWFQRWWDCSRRSAQQRLQLERAVQHHH
RQLLLEGLARWKTHHLQCVRKRLLHRQSTQLLAQRLSRTCFRQWRQQLAARRQEQRATVRALWFWAFSLQAKVWATWLAFVLERRRK
KARLQWALQAYQGQLLQEGATRLLRFAASMKASRQQLQAQQQVQAAHSLHRAVRRCATLWKQKVLGRGGKPQPLAAIAPSRKVTFEG
PLLNRIAAGAGDGTLETKRPQASRPLGALGRLAAEEPHALELNTAHSARKQPRRPHFLLEPAQSQRPQKPQEHGLGMAQPAAPSLTR
PFLAEAPTALVPHSPLPGALSSAPGPKQPPTASTGPELLLLPLSSFMPCGAAAPARVSAQRATPRDKPPVPSSLASVPDPHLLLPGD
FSATRAGPGLSTAGSLDLEAELEEIQQQLLHYQTTKQNLWSCRRQASSLRRWLELNREEPGPEDQEVEQQVQKELEQVEMQIQLLAE
ELQAQRQPIGACVARIQALRQALC
>sp|Q96S86|HPLN3_HUMAN
MGLLLLVPLLLLPGSYGLPFYNGFYYSNSANDQNLGNGHGKDLLNGVKLVVETPEETLFTYQGASVILPCRYRYEPALVSPRRVRVK
WWKLSENGAPEKDVLVAIGLRHRSFGDYQGRVHLRQDKEHDVSLEIQDLRLEDYGRYRCEVIDGLEDESGLVELELRGVVFPYQSPN
GRYQFNFHEGQQVCAEQAAVVASFEQLFRAWEEGLDWCNAGWLQDATVQYPIMLPRQPCGGPGLAPGVRSYGPRHRRLHRYDVFCFA
TALKGRVYYLEHPEKLTLTEAREACQEDDATIAKVGQLFAAWKFHGLDRCDAGWLADGSVRYPVVHPHPNCGPPEPGVRSFGFPDPQ
SRLYGVYCYRQH
>sp|Q5VXM1|CDCP2_HUMAN
MLAEWGACLLLAVALLGPGLQAQAMEGVKCGGVLSAPSGNFSSPNFPRLYPYNTECSWLIVVAEGSSVLLTFHAFDLEYHDTCSFDF
LEIYNGASPDKGNLLGRFCGKVPPPPFTSSWHVMSVIFHSDKHVASHGFSAGYQKDVCGGVLTGLSGVLTSPEYPNNYPNSMECHWV
IRAAGPAHVKLVFVDFQVEGNEECTYDYVAVLGGPGPTRGHHYCGSTRPPTLVSLGHELQVVFKSDFNIGGRGFKAYYFSGECQEVY
MAMRGNFSSPQYPSSYPNNIRCHWTIRLPPGYQVKVFFLDLDLEEPNSLTKTCDFDHLAAFDGASEEAPLLGNWCGHHLPPPVTSSH
NQLLLLLHTDRSTTRRGFSVAYIGGQLGCGSGSTEGEGEALQPQSLQSPSSIPPVCPAPPMNGLLQLLLHWLHPCPLSGPLRLDGTA
PACFHYCRASFPSF
>sp|Q6ZMT4|KDM7A_HUMAN
MAGAAAAVAAGAAAGAAAAAVSVAAPGRASAPPPPPPVYCVCRQPYDVNRFMIECDICKDWFHGSCVGVEEHHAVDIDLYHCPNCAV
LHGSSLMKKRRNWHRHDYTEIDDGSKPVQAGTRTFIKELRSRVFPSADEIIIKMHGSQLTQRYLEKHGFDVPIMVPKLDDLGLRLPS
PTFSVMDVERYVGGDKVIDVIDVARQADSKMTLHNYVVKYFMNPNRPKVLNVISLEFSDTKMSELVEVPDIAKKLSWVENYWPDDSVF

PKPFVQKYCLMGVQDSYTDFHIDFGGTSVWYHVLWGEKIFYLIKPTDENLARYESWSSSVTQSEVFFGDKVDKCYKCVVKQGHTLFV
PTGWIHAVLTSQDCMAFGGNFLHNLNIGMQLRCYEMEKRLKTPDLFKFPFFEAICWFVAKNLLETLKELREDGFQPQTYLVQGVKAL
HTALKLWMKKELVSEHAFEIPDNVRPGHLIKELSKVIRAIEEENGKPVKSQGIPIVCPVSRSSNEATSPYHSRRKMRKLRDHNVRTP
SNLDILELHTREVLKRLEMCPWEEDILSSKLNGKFNKHLQPSSTVPEWRAKDNDLRLLLTNGRIIKDERQPFADQSLYTADSENEED
KRRTKKAKMKIEESSGVEGVEHEESQKPLNGFFTRVKSELRSRSSGYSDISESEDSGPECTALKSIFTTEESESSGDEKKQEITSNF
KEESNVMRNFLQKSQKPSRSEIPIKRECPTSTSTEEEAIQGMLSMAGLHYSTCLQRQIQSTDCSGERNSLQDPSSCHGSNHEVRQLY
RYDKPVECGYHVKTEDPDLRTSSWIKQFDTSRFHPQDLSRSQKCIRKEGSSEISQRVQSRNYVDSSGSSLQNGKYMQNSNLTSGACQ
ISNGSLSPERPVGETSFSVPLHPTKRPASNPPPISNQATKGKRPKKGMATAKQRLGKILKLNRNGHARFFV
>sp|P51690|ARSE_HUMAN
MLHLHHSCLCFRSWLPAMLAVLLSLAPSASSDISASRPNILLLMADDLGIGDIGCYGNNTMRTPNIDRLAEDGVKLTQHISAASLCT
PSRAAFLTGRYPVRSGMVSSIGYRVLQWTGASGGLPTNETTFAKILKEKGYATGLIGKWHLGLNCESASDHCHHPLHHGFDHFYGMP
FSLMGDCARWELSEKRVNLEQKLNFLFQVLALVALTLVAGKLTHLIPVSWMPVIWSALSAVLLLASSYFVGALIVHADCFLMRNHTI
TEQPMCFQRTTPLILQEVASFLKRNKHGPFLLFVSFLHVHIPLITMENFLGKSLHGLYGDNVEEMDWMVGRILDTLDVEGLSNSTLI
YFTSDHGGSLENQLGNTQYGGWNGIYKGGKGMGGWEGGIRVPGIFRWPGVLPAGRVIGEPTSLMDVFPTVVRLAGGEVPQDRVIDGQ
DLLPLLLGTAQHSDHEFLMHYCERFLHAARWHQRDRGTMWKVHFVTPVFQPEGAGACYGRKVCPCFGEKVVHHDPPLLFDLSRDPSE
THILTPASEPVFYQVMERVQQAVWEHQRTLSPVPLQLDRLGNIWRPWLQPCCGPFPLCWCLREDDPQ
>sp|Q8WXD2|SCG3_HUMAN
MGFLGTGTWILVLVLPIQAFPKPGGSQDKSLHNRELSAERPLNEQIAEAEEDKIKKTYPPENKPGQSNYSFVDNLNLLKAITEKEKI
EKERQSIRSSPLDNKLNVEDVDSTKNRKLIDDYDSTKSGLDHKFQDDPDGLHQLDGTPLTAEDIVHKIAARIYEENDRAVFDKIVSK
LLNLGLITESQAHTLEDEVAEVLQKLISKEANNYEEDPNKPTSWTENQAGKIPEKVTPMAAIQDGLAKGENDETVSNTLTLTNGLER
RTKTYSEDNFEELQYFPNFYALLKSIDSEKEAKEKETLITIMKTLIDFVKMMVKYGTISPEEGVSYLENLDEMIALQTKNKLEKNAT
DNISKLFPAPSEKSHEETDSTKEEAAKMEKEYGSLKDSTKDDNSNPGGKTDEPKGKTEAYLEAIRKNIEWLKKHDKKGNKEDYDLSK
MRDFINKQADAYVEKGILDKEEAEAIKRIYSSL
>sp|P10253|LYAG_HUMAN
MGVRHPPCSHRLLAVCALVSLATAALLGHILLHDFLLVPRELSGSSPVLEETHPAHQQGASRPGPRDAQAHPGRPRAVPTQCDVPPN
SRFDCAPDKAITQEQCEARGCCYIPAKQGLQGAQMGQPWCFFPPSYPSYKLENLSSSEMGYTATLTRTTPTFFPKDILTLRLDVMME
TENRLHFTIKDPANRRYEVPLETPHVHSRAPSPLYSVEFSEEPFGVIVRRQLDGRVLLNTTVAPLFFADQFLQLSTSLPSQYITGLA
EHLSPLMLSTSWTRITLWNRDLAPTPGANLYGSHPFYLALEDGGSAHGVFLLNSNAMDVVLQPSPALSWRSTGGILDVYIFLGPEPK
SVVQQYLDVVGYPFMPPYWGLGFHLCRWGYSSTAITRQVVENMTRAHFPLDVQWNDLDYMDSRRDFTFNKDGFRDFPAMVQELHQGG
RRYMMIVDPAISSSGPAGSYRPYDEGLRRGVFITNETGQPLIGKVWPGSTAFPDFTNPTALAWWEDMVAEFHDQVPFDGMWIDMNEP
SNFIRGSEDGCPNNELENPPYVPGVVGGTLQAATICASSHQFLSTHYNLHNLYGLTEAIASHRALVKARGTRPFVISRSTFAGHGRY
AGHWTGDVWSSWEQLASSVPEILQFNLLGVPLVGADVCGFLGNTSEELCVRWTQLGAFYPFMRNHNSLLSLPQEPYSFSEPAQQAMR
KALTLRYALLPHLYTLFHQAHVAGETVARPLFLEFPKDSSTWTVDHQLLWGEALLITPVLQAGKAEVTGYFPLGTWYDLQTVPVEAL
GSLPPPPAAPREPAIHSEGQWVTLPAPLDTINVHLRAGYIIPLQGPGLTTTESRQQPMALAVALTKGGEARGELFWDDGESLEVLER
GAYTQVIFLARNNTIVNELVRVTSEGAGLQLQKVTVLGVATAPQQVLSNGVPVSNFTYSPDTKVLDICVSLLMGEQFLVSWC
>sp|O15155|BET1_HUMAN
MRRAGLGEGVPPGNYGNYGYANSGYSACEEENERLTESLRSKVTAIKSLSIEIGHEVKTQNKLLAEMDSQFDSTTGFLGKTMGKLKI
LSRGSQTKLLCYMMLFSLFVFFIIYWIIKLR
>sp|Q06413|MEF2C_HUMAN
MGRKKIQITRIMDERNRQVTFTKRKFGLMKKAYELSVLCDCEIALIIFNSTNKLFQYASTDMDKVLLKYTEYNEPHESRTNSDIVET
LRKKGLNGCDSPDPDADDSVGHSPESEDKYRKINEDIDLMISRQRLCAVPPPNFEMPVSIPVSSHNSLVYSNPVSSLGNPNLLPLAH
PSLQRNSMSPGVTHRPPSAGNTGGLMGGDLTSGAGTSAGNGYGNPRNSPGLLVSPGNLNKNMQAKSPPPMNLGMNNRKPDLRVLIPP
GSKNTMPSVSEDVDLLLNQRINNSQSAQSLATPVVSVATPTLPGQGMGGYPSAISTTYGTEYSLSSADLSSLSGFNTASALHLGSVT

GWQQQHLHNMPPSALSQLGACTSTHLSQSSNLSLPSTQSLNIKSEPVSPPRDRTTTPSRYPQHTRHEAGRSPVDSLSSCSSSYDGSD
REDHRNEFHSPIGLTRPSPDERESPSVKRMRLSEGWAT
>sp|Q0D2K5|EGFEM_HUMAN
MDELRWYHITVCLDHIFGHNCSLSCKDCMNGGKCQEGKSECSCPAGCRVILCNENCLEGAYGAGCTSECQCVEENTLECSAKNGSCT
CKSGYQGNRCQKDGLWGPEGWFSSAPCENGGQCNKKTGNCDCTPDYTRKSCTILRCISLTNLALSRRSSPMKYQQNVSSHREVRQRQ
QCSSDRPFKKLLCKFSFKIGM
>sp|Q9BU02|THTPA_HUMAN
MAQGLIEVERKFLPGPGTEERLQELGGTLEYRVTFRDTYYDTPELSLMQADHWLRRREDSGWELKCPGAAGVLGPHTEYKELTAEPT
IVAQLCKVLRADGLGAGDVAAVLGPLGLQEVASFVTKRSAWKLVLLGADEEEPQLRVDLDTADFGYAVGEVEALVHEEAEVPTALEK
IHRLSSMLGVPAQETAPAKLIVYLQRFRPQDYQRLLEVNSSRERPQETEDPDHCLG
>sp|Q99523|SORT_HUMAN
MERPWGAADGLSRWPHGLGLLLLLQLLPPSTLSQDRLDAPPPPAAPLPRWSGPIGVSWGLRAAAAGGAFPRGGRWRRSAPGEDEECG
RVRDFVAKLANNTHQHVFDDLRGSVSLSWVGDSTGVILVLTTFHVPLVIMTFGQSKLYRSEDYGKNFKDITDLINNTFIRTEFGMAI
GPENSGKVVLTAEVSGGSRGGRIFRSSDFAKNFVQTDLPFHPLTQMMYSPQNSDYLLALSTENGLWVSKNFGGKWEEIHKAVCLAKW
GSDNTIFFTTYANGSCKADLGALELWRTSDLGKSFKTIGVKIYSFGLGGRFLFASVMADKDTTRRIHVSTDQGDTWSMAQLPSVGQE
QFYSILAANDDMVFMHVDEPGDTGFGTIFTSDDRGIVYSKSLDRHLYTTTGGETDFTNVTSLRGVYITSVLSEDNSIQTMITFDQGG
RWTHLRKPENSECDATAKNKNECSLHIHASYSISQKLNVPMAPLSEPNAVGIVIAHGSVGDAISVMVPDVYISDDGGYSWTKMLEGP
HYYTILDSGGIIVAIEHSSRPINVIKFSTDEGQCWQTYTFTRDPIYFTGLASEPGARSMNISIWGFTESFLTSQWVSYTIDFKDILE
RNCEEKDYTIWLAHSTDPEDYEDGCILGYKEQFLRLRKSSVCQNGRDYVVTKQPSICLCSLEDFLCDFGYYRPENDSKCVEQPELKG
HDLEFCLYGREEHLTTNGYRKIPGDKCQGGVNPVREVKDLKKKCTSNFLSPEKQNSKSNSVPIILAIVGLMLVTVVAGVLIVKKYVC
GGRFLVHRYSVLQQHAEANGVDGVDALDTASHTNKSGYHDDSDEDLLE
>sp|P0C6P0|BCL8_HUMAN
MSCCLSSRVHITRPVLEQFLSFAKYLDGLSHGVPLLKQLCDHILFINPAIWIHTPAKVQLSLYTYLSAEFIGTATIYTTICRIGTVI
KDNAHLKILLLGY
>sp|Q15029|U5S1_HUMAN
MDTDLYDEFGNYIGPELDSDEDDDELGRETKDLDEMDDDDDDDDVGDHDDDHPGMEVVLHEDKKYYPTAEEVYGPEVETIVQEEDTQ
PLTEPIIKPVKTKKFTLMEQTLPVTVYEMDFLADLMDNSELIRNVTLCGHLHHGKTCFVDCLIEQTHPEIRKRYDQDLCYTDILFTE
QERGVGIKSTPVTVVLPDTKGKSYLFNIMDTPGHVNFSDEVTAGLRISDGVVLFIDAAEGVMLNTERLIKHAVQERLAVTVCINKID
RLILELKLPPTDAYYKLRHIVDEVNGLISMYSTDENLILSPLLGNVCFSSSQYSICFTLGSFAKIYADTFGDINYQEFAKRLWGDIY
FNPKTRKFTKKAPTSSSQRSFVEFILEPLYKILAQVVGDVDTSLPRTLDELGIHLTKEELKLNIRPLLRLVCKKFFGEFTGFVDMCV
QHIPSPKVGAKPKIEHTYTGGVDSDLGEAMSDCDPDGPLMCHTTKMYSTDDGVQFHAFGRVLSGTIHAGQPVKVLGENYTLEDEEDS
QICTVGRLWISVARYHIEVNRVPAGNWVLIEGVDQPIVKTATITEPRGNEEAQIFRPLKFNTTSVIKIAVEPVNPSELPKMLDGLRK
VNKSYPSLTTKVEESGEHVILGTGELYLDCVMHDLRKMYSEIDIKVADPVVTFCETVVETSSLKCFAETPNKKNKITMIAEPLEKGL
AEDIENEVVQITWNRKKLGEFFQTKYDWDLLAARSIWAFGPDATGPNILVDDTLPSEVDKALLGSVKDSIVQGFQWGTREGPLCDEL
IRNVKFKILDAVVAQEPLHRGGGQIIPTARRVVYSAFLMATPRLMEPYYFVEVQAPADCVSAVYTVLARRRGHVTQDAPIPGSPLYT
IKAFIPAIDSFGFETDLRTHTQGQAFSLSVFHHWQIVPGDPLDKSIVIRPLEPQPAPHLAREFMIKTRRRKGLSEDVSISKFFDDPM
LLELAKQDVVLNYPM
>sp|Q8NGG2|OR5T2_HUMAN
MSYSIYKSTVNIPLSHGVVHSFCHNMNCNFMHIFKFVLDFNMKNVTEVTLFVLKGFTDNLELQTIFFFLFLAIYLFTLMGNLGLILV
VIRDSQLHKPMYYFLSMLSSVDACYSSVITPNMLVDFTTKNKVISFLGCVAQVFLACSFGTTECFLLAAMAYDRYVAIYNPLLYSVS
MSPRVYMPLINASYVAGILHATIHTVATFSLSFCGANEIRRVFCDIPPLLAISYSDTHTNQLLLFYFVGSIELVTILIVLISYGLIL
LAILKMYSAEGRRKVFSTCGAHLTGVSIYYGTILFMYVRPSSSYASDHDMIVSIFYTIVIPLLNPVIYSLRNKDVKDSMKKMFGKNQ
VINKVYFHTKK
>sp|Q53T59|H1BP3_HUMAN

MQSPAVLVTSRRLQNAHTGLDLTVPQHQEVRGKMMSGHVEYQILVVTRLAAFKSAKHRPEDVVQFLVSKKYSEIEEFYQKLSSRYAA
ASLPPLPRKVLFVGESDIRERRAVFNEILRCVSKDAELAGSPELLEFLGTRSPGAAGLTSRDSSVLDGTDSQTGNDEEAFDFFEEQD
QVAEEGPPVQSLKGEDAEESLEEEEALDPLGIMRSKKPKKHPKVAVKAKPSPRLTIFDEEVDPDEGLFGPGRKLSPQDPSEDVSSVD
PLKLFDDPDLGGAIPLGDSLLLPAACESGGPTPSLSHRDASKELFRVEEDLDQILNLGAEPKPKPQLKPKPPVAAKPVIPRKPAVPP
KAGPAEAVAGQQKPQEQIQAMDEMDILQYIQDHDTPAQAAPSLF
>sp|Q9HC44|GPBL1_HUMAN
MAQHDFVPAWLNFSTPQSAKSPTATFEKHGEHLPRGEGRFGVSRRRHNSSDGFFNNGPLRTAGDSWHQPSLFRHDSVDSGVSKGAYA
GITGNPSGWHSSSRGHDGMSQRSGGGTGNHRHWNGSFHSRKGCAFQEKPPMEIREEKKEDKVEKLQFEEEDFPSLNPEAGKQHQPCR
PIGTPSGVWENPPSAKQPSKMLVIKKVSKEDPAAAFSAAFTSPGSHHANGNKLSSVVPSVYKNLVPKPVPPPSKPNAWKANRMEHKS
GSLSSSRESAFTSPISVTKPVVLASGAALSSPKESPSSTTPPIEISSSRLTKLTRRTTDRKSEFLKTLKDDRNGDFSENRDCDKLED
LEDNSTPEPKENGEEGCHQNGLALPVVEEGEVLSHSLEAEHRLLKAMGWQEYPENDENCLPLTEDELKEFHMKTEQLRRNGFGKNGF
LQSRSSSLFSPWRSTCKAEFEDSDTETSSSETSDDDAWK
>sp|Q99719|SEPT5_HUMAN
MSTGLRYKSKLATPEDKQDIDKQYVGFATLPNQVHRKSVKKGFDFTLMVAGESGLGKSTLVHSLFLTDLYKDRKLLSAEERISQTVE
ILKHTVDIEEKGVKLKLTIVDTPGFGDAVNNTECWKPITDYVDQQFEQYFRDESGLNRKNIQDNRVHCCLYFISPFGHGLRPVDVGF
MKALHEKVNIVPLIAKADCLVPSEIRKLKERIREEIDKFGIHVYQFPECDSDEDEDFKQQDRELKESAPFAVIGSNTVVEAKGQRVR
GRLYPWGIVEVENQAHCDFVKLRNMLIRTHMHDLKDVTCDVHYENYRAHCIQQMTSKLTQDSRMESPIPILPLPTPDAETEKLIRMK
DEELRRMQEMLQRMKQQMQDQ
>sp|P11171|41_HUMAN
MTTEKSLVTEAENSQHQQKEEGEEAINSGQQEPQQEESCQTAAEGDNWCEQKLKASNGDTPTHEDLTKNKERTSESRGLSRLFSSFL
KRPKSQVSEEEGKEVESDKEKGEGGQKEIEFGTSLDEEIILKAPIAAPEPELKTDPSLDLHSLSSAETQPAQEELREDPDFEIKEGE
GLEECSKIEVKEESPQSKAETELKASQKPIRKHRNMHCKVSLLDDTVYECVVEKHAKGQDLLKRVCEHLNLLEEDYFGLAIWDNATS
KTWLDSAKEIKKQVRGVPWNFTFNVKFYPPDPAQLTEDITRYYLCLQLRQDIVAGRLPCSFATLALLGSYTIQSELGDYDPELHGVD
YVSDFKLAPNQTKELEEKVMELHKSYRSMTPAQADLEFLENAKKLSMYGVDLHKAKDLEGVDIILGVCSSGLLVYKDKLRINRFPWP
KVLKISYKRSSFFIKIRPGEQEQYESTIGFKLPSYRAAKKLWKVCVEHHTFFRLTSTDTIPKSKFLALGSKFRYSGRTQAQTRQASA
LIDRPAPHFERTASKRASRSLDGAAAVDSADRSPRPTSAPAITQGQVAEGGVLDASAKKTVVPKAQKETVKAEVKKEDEPPEQAEPE
PTEAWKVEKTHIEVTVPTSNGDQTQKLAEKTEDLIRMRKKKRERLDGENIYIRHSNLMLEDLDKSQEEIKKHHASISELKKNFMESV
PEPRPSEWDKRLSTHSPFRTLNINGQIPTGEGPPLVKTQTVTISDNANAVKSEIPTKDVPIVHTETKTITYEAAQTDDNSGDLDPGV
LLTAQTITSETPSSTTTTQITKTVKGGISETRIEKRIVITGDADIDHDQVLVQAIKEAKEQHPDMSVTKVVVHQETEIADE
>sp|P49238|CX3C1_HUMAN
MDQFPESVTENFEYDDLAEACYIGDIVVFGTVFLSIFYSVIFAIGLVGNLLVVFALTNSKKPKSVTDIYLLNLALSDLLFVATLPFW
THYLINEKGLHNAMCKFTTAFFFIGFFGSIFFITVISIDRYLAIVLAANSMNNRTVQHGVTISLGVWAAAILVAAPQFMFTKQKENE
CLGDYPEVLQEIWPVLRNVETNFLGFLLPLLIMSYCYFRIIQTLFSCKNHKKAKAIKLILLVVIVFFLFWTPYNVMIFLETLKLYDF
FPSCDMRKDLRLALSVTETVAFSHCCLNPLIYAFAGEKFRRYLYHLYGKCLAVLCGRSVHVDFSSSESQRSRHGSVLSSNFTYHTSD
GDALLLL
>sp|P17858|PFKAL_HUMAN
MAAVDLEKLRASGAGKAIGVLTSGGDAQGMNAAVRAVTRMGIYVGAKVFLIYEGYEGLVEGGENIKQANWLSVSNIIQLGGTIIGSA
RCKAFTTREGRRAAAYNLVQHGITNLCVIGGDGSLTGANIFRSEWGSLLEELVAEGKISETTARTYSHLNIAGLVGSIDNDFCGTDM
TIGTDSALHRIMEVIDAITTTAQSHQRTFVLEVMGRHCGYLALVSALASGADWLFIPEAPPEDGWENFMCERLGETRSRGSRLNIII
IAEGAIDRNGKPISSSYVKDLVVQRLGFDTRVTVLGHVQRGGTPSAFDRILSSKMGMEAVMALLEATPDTPACVVTLSGNQSVRLPL
MECVQMTKEVQKAMDDKRFDEATQLRGGSFENNWNIYKLLAHQKPPKEKSNFSLAILNVGAPAAGMNAAVRSAVRTGISHGHTVYVV
HDGFEGLAKGQVQEVGWHDVAGWLGRGGSMLGTKRTLPKGQLESIVENIRIYGIHALLVVGGFEAYEGVLQLVEARGRYEELCIVMC
VIPATISNNVPGTDFSLGSDTAVNAAMESCDRIKQSASGTKRRVFIVETMGGYCGYLATVTGIAVGADAAYVFEDPFNIHDLKVNVE

HMTEKMKTDIQRGLVLRNEKCHDYYTTEFLYNLYSSEGKGVFDCRTNVLGHLQQGGAPTPFDRNYGTKLGVKAMLWLSEKLREVYRK
GRVFANAPDSACVIGLKKKAVAFSPVTELKKDTDFEHRMPREQWWLSLRLMLKMLAQYRISMAAYVSGELEHVTRRTLSMDKGF
>sp|Q5VUJ9|EFCB2_HUMAN
MLGPGQVRLRPRVWRDKAGGRVADGASGLPPARGSWRETGTGRALGASSPPRPAQGSSSPGIQSGPSSRPGSPRGAEQAGTPRPRLS
LGISQATGSAARWRTRRTGKGLGYNSDEIRPRTLLIEHLMEGGRRDHHTMTVLWGTQEIIVAEFHKKIKEAFEVFDHESNNTVDVRE
IGTIIRSLGCCPTEGELHDLIAEVEEEEPTGYIRFEKFLPVMTEILLERKYRPIPEDVLLRAFEVLDSAKRGFLTKDELIKYMTEED
GVSLRRPG
>sp|Q9H0W7|THAP2_HUMAN
MPTNCAAAGCATTYNKHINISFHRFPLDPKRRKEWVRLVRRKNFVPGKHTFLCSKHFEASCFDLTGQTRRLKMDAVPTIFDFCTHIK
SMKLKSRNLLKKNNSCSPAGPSNLKSNISSQQVLLEHSYAFRNPMEAKKRIIKLEKEIASLRRKMKTCLQKERRATRRWIKATCLVK
NLEANSVLPKGTSEHMLPTALSSLPLEDFKILEQDQQDKTLLSLNLKQTKSTFI
>sp|P30486|1B48_HUMAN
MLVMAPRTVLLLLSAALALTETWAGSHSMRYFYTSVSRPGRGEPRFISVGYVDDTQFVRFDSDAASPREEPRAPWIEQEGPEYWDRE
TQISKTNTQTYRESLRNLRGYYNQSEAGSHTLQSMYGCDVGPDGRLLRGHNQYAYDGKDYIALNEDLRSWTAADTAAQISQRKLEAA
RVAEQLRAYLEGECVEWLRRYLENGKDKLERADPPKTHVTHHPISDHEATLRCWALGFYPAEITLTWQRDGEDQTQDTELVETRPAG
DRTFQKWTAVVVPSGEEQRYTCHVQHEGLPKPLTLRWEPSSQSTVPIVGIVAGLAVLAVVVIGAVVAAVMCRRKSSGGKGGSYSQAA
CSDSAQGSDVSLTA
>sp|Q9UNZ2|NSF1C_HUMAN
MAAERQEALREFVAVTGAEEDRARFFLESAGWDLQIALASFYEDGGDEDIVTISQATPSSVSRGTAPSDNRVTSFRDLIHDQDEDEE
EEEGQRFYAGGSERSGQQIVGPPRKKSPNELVDDLFKGAKEHGAVAVERVTKSPGETSKPRPFAGGGYRLGAAPEEESAYVAGEKRQ
HSSQDVHVVLKLWKSGFSLDNGELRSYQDPSNAQFLESIRRGEVPAELRRLAHGGQVNLDMEDHRDEDFVKPKGAFKAFTGEGQKLG
STAPQVLSTSSPAQQAENEAKASSSILIDESEPTTNIQIRLADGGRLVQKFNHSHRISDIRLFIVDARPAMAATSFILMTTFPNKEL
ADESQTLKEANLLNAVIVQRLT
>sp|Q8IW93|ARHGJ_HUMAN
MDCGPPATLQPHLTGPPGTAHHPVAVCQQESLSFAELPALKPPSPVCLDLFPVAPEELRAPGSRWSLGTPAPLQGLLWPLSPGGSDT
EITSGGMRPSRAGSWPHCPGAQPPALEGPWSPRHTQPQRRASHGSEKKSAWRKMRVYQREEVPGCPEAHAVFLEPGQVVQEQALSTE
EPRVELSGSTRVSLEGPERRRFSASELMTRLHSSLRLGRNSAARALISGSGTGAAREGKASGMEARSVEMSGDRVSRPAPGDSREGD
WSEPRLDTQEEPPLGSRSTNERRQSRFLLNSVLYQEYSDVASARELRRQQREEEGPGDEAEGAEEGPGPPRANLSPSSSFRAQRSAR
GSTFSLWQDIPDVRGSGVLATLSLRDCKLQEAKFELITSEASYIHSLSVAVGHFLGSAELSECLGAQDKQWLFSKLPEVKSTSERFL
QDLEQRLEADVLRFSVCDVVLDHCPAFRRVYLPYVTNQAYQERTYQRLLLENPRFPGILARLEESPVCQRLPLTSFLILPFQRITRL
KMLVENILKRTAQGSEDEDMATKAFNALKELVQECNASVQSMKRTEELIHLSKKIHFEGKIFPLISQARWLVRHGELVELAPLPAAP
PAKLKLSSKAVYLHLFNDCLLLSRRKELGKFAVFVHAKMAELQVRDLSLKLQGIPGHVFLLQLLHGQHMKHQFLLRARTESEKQRWI
SALCPSSPQEDKEVISEGEDCPQVQCVRTYKALHPDELTLEKTDILSVRTWTSDGWLEGVRLADGEKGWVPQAYVEEISSLSARLRN
LRENKRVTSATSKLGEAPV
>sp|Q96L08|SUSD3_HUMAN
MRWAAATLRGKARPRGRAGVTTPAPGNRTGTCAKLRLPPQATFQVLRGNGASVGTVLMFRCPSNHQMVGSGLLTCTWKGSIAEWSSG
SPVCKLVPPHETFGFKVAVIASIVSCAIILLMSMAFLTCCLLKCVKKSKRRRSNRSAQLWSQLKDEDLETVQAAYLGLKHFNKPVSG
PSQAHDNHSFTTDHGESTSKLASVTRSVDKDPGIPRALSLSGSSSSPQAQVMVHMANPRQPLPASGLATGMPQQPAAYALG
>sp|Q8NC67|NETO2_HUMAN
MALERLCSVLKVLLITVLVVEGIAVAQKTQDGQNIGIKHIPATQCGIWVRTSNGGHFASPNYPDSYPPNKECIYILEAAPRQRIELT
FDEHYYIEPSFECRFDHLEVRDGPFGFSPLIDRYCGVKSPPLIRSTGRFMWIKFSSDEELEGLGFRAKYSFIPDPDFTYLGGILNPI
PDCQFELSGADGIVRSSQVEQEEKTKPGQAVDCIWTIKATPKAKIYLRFLDYQMEHSNECKRNFVAVYDGSSSIENLKAKFCSTVAN
DVMLKTGIGVIRMWADEGSRLSRFRMLFTSFVEPPCTSSTFFCHSNMCINNSLVCNGVQNCAYPWDENHCKEKKKAGVFEQITKTHG
TIIGITSGIVLVLLIISILVQVKQPRKKVMACKTAFNKTGFQEVFDPPHYELFSLRDKEISADLADLSEELDNYQKMRRSSTASRCI

HDHHCGSQASSVKQSRTNLSSMELPFRNDFAQPQPMKTFNSTFKKSSYTFKQGHECPEQALEDRVMEEIPCEIYVRGREDSAQASIS
IDF
>sp|P05164|PERM_HUMAN
MGVPFFSSLRCMVDLGPCWAGGLTAEMKLLLALAGLLAILATPQPSEGAAPAVLGEVDTSLVLSSMEEAKQLVDKAYKERRESIKQR
LRSGSASPMELLSYFKQPVAATRTAVRAADYLHVALDLLERKLRSLWRRPFNVTDVLTPAQLNVLSKSSGCAYQDVGVTCPEQDKYR
TITGMCNNRRSPTLGASNRAFVRWLPAEYEDGFSLPYGWTPGVKRNGFPVALARAVSNEIVRFPTDQLTPDQERSLMFMQWGQLLDH
DLDFTPEPAARASFVTGVNCETSCVQQPPCFPLKIPPNDPRIKNQADCIPFFRSCPACPGSNITIRNQINALTSFVDASMVYGSEEP
LARNLRNMSNQLGLLAVNQRFQDNGRALLPFDNLHDDPCLLTNRSARIPCFLAGDTRSSEMPELTSMHTLLLREHNRLATELKSLNP
RWDGERLYQEARKIVGAMVQIITYRDYLPLVLGPTAMRKYLPTYRSYNDSVDPRIANVFTNAFRYGHTLIQPFMFRLDNRYQPMEPN
PRVPLSRVFFASWRVVLEGGIDPILRGLMATPAKLNRQNQIAVDEIRERLFEQVMRIGLDLPALNMQRSRDHGLPGYNAWRRFCGLP
QPETVGQLGTVLRNLKLARKLMEQYGTPNNIDIWMGGVSEPLKRKGRVGPLLACIIGTQFRKLRDGDRFWWENEGVFSMQQRQALAQ
ISLPRIICDNTGITTVSKNNIFMSNSYPRDFVNCSTLPALNLASWREAS
>sp|P24071|FCAR_HUMAN
MDPKQTTLLCLVLCLGQRIQAQEGDFPMPFISAKSSPVIPLDGSVKIQCQAIREAYLTQLMIIKNSTYREIGRRLKFWNETDPEFVI
DHMDANKAGRYQCQYRIGHYRFRYSDTLELVVTGLYGKPFLSADRGLVLMPGENISLTCSSAHIPFDRFSLAKEGELSLPQHQSGEH
PANFSLGPVDLNVSGIYRCYGWYNRSPYLWSFPSNALELVVTDSIHQDYTTQNLIRMAVAGLVLVALLAILVENWHSHTALNKEASA
DVAEPSWSQQMCQPGLTFARTPSVCK
>sp|P23526|SAHH_HUMAN
MSDKLPYKVADIGLAAWGRKALDIAENEMPGLMRMRERYSASKPLKGARIAGCLHMTVETAVLIETLVTLGAEVQWSSCNIFSTQDH
AAAAIAKAGIPVYAWKGETDEEYLWCIEQTLYFKDGPLNMILDDGGDLTNLIHTKYPQLLPGIRGISEETTTGVHNLYKMMANGILK
VPAINVNDSVTKSKFDNLYGCRESLIDGIKRATDVMIAGKVAVVAGYGDVGKGCAQALRGFGARVIITEIDPINALQAAMEGYEVTT
MDEACQEGNIFVTTTGCIDIILGRHFEQMKDDAIVCNIGHFDVEIDVKWLNENAVEKVNIKPQVDRYRLKNGRRIILLAEGRLVNLG
CAMGHPSFVMSNSFTNQVMAQIELWTHPDKYPVGVHFLPKKLDEAVAEAHLGKLNVKLTKLTEKQAQYLGMSCDGPFKPDHYRY
>sp|P86434|AAS1_HUMAN
MEQDWQPGEEVTPGPEPCSKGQAPLYPIVHVTELKHTDPNFPSNSNAVGTSSGWNRIGTGCSHTWDWRFSCTQQALLPLLGAWEWSI
DTEAGGGRREQSQKPCSNGGPAAAGEGRVLPSPCFPWSTCQAAIHKVCRWQGCTRPALLAPSLATLKEHSYP
>sp|Q7Z7K2|ZN467_HUMAN
MRETLEALSSLGFSVGGQPEMAPQSEPREGSHNAQEQMSSSREERALGVCSGHEAPTPEEGAHTEQAEAPCRGQACSAQKAQPVGTCP
GEEWMIRKVKVEDEDQEAEEEVEWPQHLSLLPSPFPAPDLGHLAAAYKLEPGAPGALSGLALSGWGPMPEKPYGCGECERRFRDQLT
LRLHQRLHRGEGPCACPDCGRSFTQRAHMLLHQRSHRGERPFPCSECDKRFSKKAHLTRHLRTHTGERPYPCAECGKRFSQKIHLGS
HQKTHTGERPFPCTECEKRFRKKTHLIRHQRIHTGERPYQCAQCARSFTHKQHLVRHQRVHQTAGPARPSPDSSASPHSTAPSPTPS
FPGPKPFACSDCGLSFGWKKNLATHQCLHRSEGRPFGCDECALGATVDAPAAKPLASAPGGPGCGPGSDPVVPQRAPSGERSFFCPD
CGRGFSHGQHLARHPRVHTGERPFACTQCDRRFGSRPNLVAHSRAHSGARPFACAQCGRRFSRKSHLGRHQAVHTGSRPHACAVCAR
SFSSKTNLVRHQAIHTGSRPFSCPQCGKSFSRKTHLVRHQLIHGEAAHAAPDAALAAPAWSAPPEVAPPPLFF
>sp|P23409|MYF6_HUMAN
MMMDLFETGSYFFYLDGENVTLQPLEVAEGSPLYPGSDGTLSPCQDQMPPEAGSDSSGEEHVLAPPGLQPPHCPGQCLIWACKTCKR
KSAPTDRRKAATLRERRRLKKINEAFEALKRRTVANPNQRLPKVEILRSAISYIERLQDLLHRLDQQEKMQELGVDPFSYRPKQENL
EGADFLRTCSSQWPSVSDHSRGLVITAKEGGASIDSSASSSLRCLSSIVDSISSEERKLPCVEEVVEK
>sp|Q96HS1|PGAM5_HUMAN
MAFRQALQLAACGLAGGSAAVLFSAVAVGKPRAGGDAEPRPAEPPAWAGGARPGPGVWDPNWDRREPLSLINVRKRNVESGEEELAS
KLDHYKAKATRHIFLIRHSQYHVDGSLEKDRTLTPLGREQAELTGLRLASLGLKFNKIVHSSMTRAIETTDIISRHLPGVCKVSTDL
LREGAPIEPDPPVSHWKPEAVQYYEDGARIEAAFRNYIHRADARQEEDSYEIFICHANVIRYIVCRALQFPPEGWLRLSLNNGSITH
LVIRPNGRVALRTLGDTGFMPPDKITRS
>sp|Q8NGC8|O11H7_HUMAN

MNNSQISTVTQFVLLGFPGPWKIQIIFFSMILLVYIFTLTGNMAIICAVRWDHRLHTPMYVLLANFSFLEIWYVTCTVPNMLVNFFS
KTKTISFSGCFTQFHFFFSLGTTECFFLCVMAYDRYLAICHPLHYPSIMTGQLCGILVSLCWLIGFLGHSISIFFIFQLPFCGPNII
DHFLCDVDPLMALSSAPTHIIGHVFHSVSSLFINLTMVYILGSYTLVLRTVLQVPSSAGWQKAISTCGSHLVVVSLFYGAIMLMYVS
PTPGNSVAMHKLITLIYSVVTPVLNPLIYSLRNKDMKYALHHVFCGMRIIQRS
>sp|Q99612|KLF6_HUMAN
MDVLPMCSIFQELQIVHETGYFSALPSLEEYWQQTCLELERYLQSEPCYVSASEIKFDSQEDLWTKIILAREKKEESELKISSSPPE
DTLISPSFCYNLETNSLNSDVSSESSDSSEELSPTAKFTSDPIGEVLVSSGKLSSSVTSTPPSSPELSREPSQLWGCVPGELPSPGK
VRSGTSGKPGDKGNGDASPDGRRRVHRCHFNGCRKVYTKSSHLKAHQRTHTGEKPYRCSWEGCEWRFARSDELTRHFRKHTGAKPFK
CSHCDRCFSRSDHLALHMKRHL
>sp|Q8NCR0|B3GL2_HUMAN
MRNWLVLLCPCVLGAALHLWLRLRSPPPACASGAGPADQLALFPQWKSTHYDVVVGVLSARNNHELRNVIRSTWMRHLLQHPTLSQR
VLVKFIIGAHGCEVPVEDREDPYSCKLLNITNPVLNQEIEAFSLSEDTSSGLPEDRVVSVSFRVLYPIVITSLGVFYDANDVGFQRN
ITVKLYQAEQEEALFIARFSPPSCGVQVNKLWYKPVEQFILPESFEGTIVWESQDLHGLVSRNLHKVTVNDGGGVLRVITAGEGALP
HEFLEGVEGVAGGFIYTIQEGDALLHNLHSRPQRLIDHIRNLHEEDALLKEESSIYDDIVFVDVVDTYRNVPAKLLNFYRWTVETTS
FNLLLKTDDDCYIDLEAVFNRIVQKNLDGPNFWWGNFRLNWAVDRTGKWQELEYPSPAYPAFACGSGYVISKDIVKWLASNSGRLKT
YQGEDVSMGIWMAAIGPKRYQDSLWLCEKTCETGMLSSPQYSPWELTELWKLKERCGDPCRCQAR
>sp|Q9BXT2|CCG6_HUMAN
MMWSNFFLQEENRRRGAAGRRRAHGQGRSGLTPEREGKVKLALLLAAVGATLAVLSVGTEFWVELNTYKANGSAVCEAAHLGLWKAC
TKRLWQADVPVDRDTCGPAELPGEANCTYFKFFTTGENARIFQRTTKKEVNLAAAVIAVLGLAVMALGCLCIIMVLSKGAEFLLRVG
AVCFGLSGLLLLVSLEVFRHSVRALLQRVSPEPPPAPRLTYEYSWSLGCGVGAGLILLLGAGCFLLLTLPSWPWGSLCPKRGHRAT
>sp|O95813|CER1_HUMAN
MHLLLFQLLVLLPLGKTTRHQDGRQNQSSLSPVLLPRNQRELPTGNHEEAEEKPDLFVAVPHLVATSPAGEGQRQREKMLSRFGRFW
KKPEREMHPSRDSDSEPFPPGTQSLIQPIDGMKMEKSPLREEAKKFWHHFMFRKTPASQGVILPIKSHEVHWETCRTVPFSQTITHE
GCEKVVVQNNLCFGKCGSVHFPGAAQHSHTSCSHCLPAKFTTMHLPLNCTELSSVIKVVMLVEECQCKVKTEHEDGHILHAGSQDSF
IPGVSA
>sp|P39019|RS19_HUMAN
MPGVTVKDVNQQEFVRALAAFLKKSGKLKVPEWVDTVKLAKHKELAPYDENWFYTRAASTARHLYLRGGAGVGSMTKIYGGRQRNGV
MPSHFSRGSKSVARRVLQALEGLKMVEKDQDGGRKLTPQGQRDLDRIAGQVAAANKKH
>sp|Q8TE23|TS1R2_HUMAN
MGPRAKTISSLFFLLWVLAEPAENSDFYLPGDYLLGGLFSLHANMKGIVHLNFLQVPMCKEYEVKVIGYNLMQAMRFAVEEINNDSS
LLPGVLLGYEIVDVCYISNNVQPVLYFLAHEDNLLPIQEDYSNYISRVVAVIGPDNSESVMTVANFLSLFLLPQITYSAISDELRDK
VRFPALLRTTPSADHHIEAMVQLMLHFRWNWIIVLVSSDTYGRDNGQLLGERVARRDICIAFQETLPTLQPNQNMTSEERQRLVTIV
DKLQQSTARVVVVFSPDLTLYHFFNEVLRQNFTGAVWIASESWAIDPVLHNLTELRHLGTFLGITIQSVPIPGFSEFREWGPQAGPP
PLSRTSQSYTCNQECDNCLNATLSFNTILRLSGERVVYSVYSAVYAVAHALHSLLGCDKSTCTKRVVYPWQLLEEIWKVNFTLLDHQ
IFFDPQGDVALHLEIVQWQWDRSQNPFQSVASYYPLQRQLKNIQDISWHTINNTIPMSMCSKRCQSGQKKKPVGIHVCCFECIDCLP
GTFLNHTEDEYECQACPNNEWSYQSETSCFKRQLVFLEWHEAPTIAVALLAALGFLSTLAILVIFWRHFQTPIVRSAGGPMCFLMLT
LLLVAYMVVPVYVGPPKVSTCLCRQALFPLCFTICISCIAVRSFQIVCAFKMASRFPRAYSYWVRYQGPYVSMAFITVLKMVIVVIG
MLATGLSPTTRTDPDDPKITIVSCNPNYRNSLLFNTSLDLLLSVVGFSFAYMGKELPTNYNEAKFITLSMTFYFTSSVSLCTFMSAY
SGVLVTIVDLLVTVLNLLAISLGYFGPKCYMILFYPERNTPAYFNSMIQGYTMRRD
>sp|Q9UHA7|IL36A_HUMAN
MEKALKIDTPQQGSIQDINHRVWVLQDQTLIAVPRKDRMSPVTIALISCRHVETLEKDRGNPIYLGLNGLNLCLMCAKVGDQPTLQL
KEKDIMDLYNQPEPVKSFLFYHSQSGRNSTFESVAFPGWFIAVSSEGGCPLILTQELGKANTTDFGLTMLF
>sp|P10323|ACRO_HUMAN

MVEMLPTAILLVLAVSVVAKDNATCDGPCGLRFRQNPQGGVRIVGGKAAQHGAWPWMVSLQIFTYNSHRYHTCGGSLLNSRWVLTAA
HCFVGKNNVHDWRLVFGAKEITYGNNKPVKAPLQERYVEKIIIHEKYNSATEGNDIALVEITPPISCGRFIGPGCLPHFKAGLPRGS
QSCWVAGWGYIEEKAPRPSSILMEARVDLIDLDLCNSTQWYNGRVQPTNVCAGYPVGKIDTCQGDSGGPLMCKDSKESAYVVVGITS
WGVGCARAKRPGIYTATWPYLNWIASKIGSNALRMIQSATPPPPTTRPPPIRPPFSHPISAHLPWYFQPPPRPLPPRPPAAQPRPPP
SPPPPPPPPASPLPPPPPPPPPTPSSTTKLPQGLSFAKRLQQLIEVLKGKTYSDGKNHYDMETTELPELTSTS
>sp|Q8NBR6|FA63B_HUMAN
MESSPESLQPLEHGVAAGPASGTGSSQEGLQETRLAAGDGPGVWAAETSGGNGLGAAAARRSLPDSASPAGSPEVPGPCSSSAGLDL
KDSGLESPAAAEAPLRGQYKVTASPETAVAGVGHELGTAGDAGARPDLAGTCQAELTAAGSEEPSSAGGLSSSCSDPSPPGESPSLD
SLESFSNLHSFPSSCEFNSEEGAENRVPEEEEGAAVLPGAVPLCKEEEGEETAQVLAASKERFPGQSVYHIKWIQWKEENTPIITQN
ENGPCPLLAILNVLLLAWKVKLPPMMEIITAEQLMEYLGDYMLDAKPKEISEIQRLNYEQNMSDAMAILHKLQTGLDVNVRFTGVRV
FEYTPECIVFDLLDIPLYHGWLVDPQIDDIVKAVGNCSYNQLVEKIISCKQSDNSELVSEGFVAEQFLNNTATQLTYHGLCELTSTV
QEGELCVFFRNNHFSTMTKYKGQLYLLVTDQGFLTEEKVVWESLHNVDGDGNFCDSEFHLRPPSDPETVYKGQQDQIDQDYLMALSL
QQEQQSQEINWEQIPEGISDLELAKKLQEEEDRRASQYYQEQEQAAAAAAAASTQAQQGQPAQASPSSGRQSGNSERKRKEPREKDK
EKEKEKNSCVIL
>sp|Q8N7X2|STPG3_HUMAN
MMNSDQKAVKFLANFYINGGKHWTHGHLRQTQPEPTQPKASVLLLGPEPGMAWDETQPPKMKEIPVGLRLQTGTPQESLPTYTQTLR
ELLLEQRPLITADLEVPSPTRYQVPSPSVRESSPHPHYSIGCKHQGREGGGRRAWQTLWFQSESPFTQKADFDQEQKWPSPAHYQLL
SRPAFPAFSFRGCHSASKTPEGHTHLGLPGARGLGLRVQPQSLLQASLQAPGKRCPGPNTYNILPGSRLQSPRSPAFSMSRSPAFTS
WLSTWLPAFSFGSPNPWPSRLPRGGLQLTLPFGAWRGHPGCTQTQAPRHRPLLHALEPAGHNLLGPATEWNHGLPRGFPRPPLGLGA
PAWPSDPLGTPMHPSGWPTLLWAVDKAGPAWIPHLPISPLH
>sp|Q9UHV7|MED13_HUMAN
MSASFVPNGASLEDCHCNLFCLADLTGIKWKKYVWQGPTSAPILFPVTEEDPILSSFSRCLKADVLGVWRRDQRPGRRELWIFWWGE
DPSFADLIHHDLSEEEDGVWENGLSYECRTLLFKAVHNLLERCLMNRNFVRIGKWFVKPYEKDEKPINKSEHLSCSFTFFLHGDSNV
CTSVEINQHQPVYLLSEEHITLAQQSNSPFQVILCPFGLNGTLTGQAFKMSDSATKKLIGEWKQFYPISCCLKEMSEEKQEDMDWED
DSLAAVEVLVAGVRMIYPACFVLVPQSDIPTPSPVGSTHCSSSCLGVHQVPASTRDPAMSSVTLTPPTSPEEVQTVDPQSVQKWVKF
SSVSDGFNSDSTSHHGGKIPRKLANHVVDRVWQECNMNRAQNKRKYSASSGGLCEEATAAKVASWDFVEATQRTNCSCLRHKNLKSR
NAGQQGQAPSLGQQQQILPKHKTNEKQEKSEKPQKRPLTPFHHRVSVSDDVGMDADSASQRLVISAPDSQVRFSNIRTNDVAKTPQM
HGTEMANSPQPPPLSPHPCDVVDEGVTKTPSTPQSQHFYQMPTPDPLVPSKPMEDRIDSLSQSFPPQYQEAVEPTVYVGTAVNLEED
EANIAWKYYKFPKKKDVEFLPPQLPSDKFKDDPVGPFGQESVTSVTELMVQCKKPLKVSDELVQQYQIKNQCLSAIASDAEQEPKID
PYAFVEGDEEFLFPDKKDRQNSEREAGKKHKVEDGTSSVTVLSHEEDAMSLFSPSIKQDAPRPTSHARPPSTSLIYDSDLAVSYTDL
DNLFNSDEDELTPGSKKSANGSDDKASCKESKTGNLDPLSCISTADLHKMYPTPPSLEQHIMGFSPMNMNNKEYGSMDTTPGGTVLE
GNSSSIGAQFKIEVDEGFCSPKPSEIKDFSYVYKPENCQILVGCSMFAPLKTLPSQYLPPIKLPEECIYRQSWTVGKLELLSSGPSM
PFIKEGDGSNMDQEYGTAYTPQTHTSFGMPPSSAPPSNSGAGILPSPSTPRFPTPRTPRTPRTPRGAGGPASAQGSVKYENSDLYSP
ASTPSTCRPLNSVEPATVPSIPEAHSLYVNLILSESVMNLFKDCNFDSCCICVCNMNIKGADVGVYIPDPTQEAQYRCTCGFSAVMN
RKFGNNSGLFLEDELDIIGRNTDCGKEAEKRFEALRATSAEHVNGGLKESEKLSDDLILLLQDQCTNLFSPFGAADQDPFPKSGVIS
NWVRVEERDCCNDCYLALEHGRQFMDNMSGGKVDEALVKSSCLHPWSKRNDVSMQCSQDILRMLLSLQPVLQDAIQKKRTVRPWGVQ
GPLTWQQFHKMAGRGSYGTDESPEPLPIPTFLLGYDYDYLVLSPFALPYWERLMLEPYGSQRDIAYVVLCPENEALLNGAKSFFRDL
TAIYESCRLGQHRPVSRLLTDGIMRVGSTASKKLSEKLVAEWFSQAADGNNEAFSKLKLYAQVCRYDLGPYLASLPLDSSLLSQPNL
VAPTSQSLITPPQMTNTGNANTPSATLASAASSTMTVTSGVAISTSVATANSTLTTASTSSSSSSNLNSGVSSNKLPSFPPFGSMNS
NAAGSMSTQANTVQSGQLGGQQTSALQTAGISGESSSLPTQPHPDVSESTMDRDKVGIPTDGDSHAVTYPPAIVVYIIDPFTYENTD
ESTNSSSVWTLGLLRCFLEMVQTLPPHIKSTVSVQIIPCQYLLQPVKHEDREIYPQHLKSLAFSAFTQCRRPLPTSTNVKTLTGFGP
GLAMETALRSPDRPECIRLYAPPFILAPVKDKQTELGETFGEAGQKYNVLFVGYCLSHDQRWILASCTDLYGELLETCIINIDVPNR
ARRKKSSARKFGLQKLWEWCLGLVQMSSLPWRVVIGRLGRIGHGELKDWSCLLSRRNLQSLSKRLKDMCRMCGISAADSPSILSACL
VAMEPQGSFVIMPDSVSTGSVFGRSTTLNMQTSQLNTPQDTSCTHILVFPTSASVQVASATYTTENLDLAFNPNNDGADGMGIFDLL

DTGDDLDPDIINILPASPTGSPVHSPGSHYPHGGDAGKGQSTDRLLSTEPHEEVPNILQQPLALGYFVSTAKAGPLPDWFWSACPQA
QYQCPLFLKASLHLHVPSVQSDELLHSKHSHPLDSNQTSDVLRFVLEQYNALSWLTCDPATQDRRSCLPIHFVVLNQLYNFIMNML
>sp|Q15061|WDR43_HUMAN
MAAGGGGSCDPLAPAGVPCAFSPHSQAYFALASTDGHLRVWETANNRLHQEYVPSAHLSGTCTCLAWAPARLQAKESPQRKKRKSEA
VGMSNQTDLLALGTAVGSILLYSTVKGELHSKLISGGHDNRVNCIQWHQDSGCLYSCSDDKHIVEWNVQTCKVKCKWKGDNSSVSSL
CISPDGKMLLSAGRTIKLWVLETKEVYRHFTGHATPVSSLMFTTIRPPNESQPFDGITGLYFLSGAVHDRLLNVWQVRSENKEKSAV
MSFTVTDEPVYIDLTLSENKEEPVKLAVVCRDGQVHLFEHILNGYCKKPLTSNCTIQIATPGKGKKSTPKPIPILAAGFCSDKMSLL
LVYGSWFQPTIERVALNSREPHMCLVRDISNCWAPKVETAITKVRTPVMNSEAKVLVPGIPGHHAAIKPAPPQTEQVESKRKSGGNE
VSIEERLGAMDIDTHKKGKEDLQTNSFPVLLTQGLESNDFEMLNKVLQTRNVNLIKKTVLRMPLHTIIPLLQELTKRLQGHPNSAVL
MVQWLKCVLTVHASYLSTLPDLVPQLGTLYQLMESRVKTFQKLSHLHGKLILLITQVTASEKTKGATSPGQKAKLVYEEESSEEESD
DEIADKDSEDNWDEDEEESESEKDEDVEEEDEDAEGKDEENGEDRDTASEKELNGDSDLDPENESEEE
>sp|P48163|MAOX_HUMAN
MEPEAPRRRHTHQRGYLLTRNPHLNKDLAFTLEERQQLNIHGLLPPSFNSQEIQVLRVVKNFEHLNSDFDRYLLLMDLQDRNEKLFY
RVLTSDIEKFMPIVYTPTVGLACQQYSLVFRKPRGLFITIHDRGHIASVLNAWPEDVIKAIVVTDGERILGLGDLGCNGMGIPVGKL
ALYTACGGMNPQECLPVILDVGTENEELLKDPLYIGLRQRRVRGSEYDDFLDEFMEAVSSKYGMNCLIQFEDFANVNAFRLLNKYRN
QYCTFNDDIQGTASVAVAGLLAALRITKNKLSDQTILFQGAGEAALGIAHLIVMALEKEGLPKEKAIKKIWLVDSKGLIVKGRASLT
QEKEKFAHEHEEMKNLEAIVQEIKPTALIGVAAIGGAFSEQILKDMAAFNERPIIFALSNPTSKAECSAEQCYKITKGRAIFASGSP
FDPVTLPNGQTLYPGQGNNSYVFPGVALGVVACGLRQITDNIFLTTAEVIAQQVSDKHLEEGRLYPPLNTIRDVSLKIAEKIVKDAY
QEKTATVYPEPQNKEAFVRSQMYSTDYDQILPDCYSWPEEVQKIQTKVDQ
>sp|Q6ZV70|LANC3_HUMAN
MDTKRCFANRFDDYQGSLLAGQCEEAVAPLVTATIERILQELPPLGGGAEARGATAGASACQGGLYGGVAGVAYMLYHVSQSPLFAT
ARERYLRSAKRLIDACARAEEWGEPDADTRAAFLLGGAGVYAVATLVYHALGRSDYVQPLGKFRALCAVCAPVSFLECGSDELFVGR
AGYLCAALVLKQKLAQEVLTPAQIKSICQAILDSGKQYAIKKRKPFPLMYSYYGTEYLGAAHGLSSILQMLLSYHEHLKPSDRELVW
QSVDFLMEQEQNCNWPPELGETIERENELVHWCHGAPGIAYLFAKAYLVSKKPQYLDTCIRCGELTWQKGLLKKGPGICHGVAGSAY
VFLLLYRLTGNSKYIYRAQRFAQFLFTEEFKAGSRVLESIYSLYEGFSGTVCFLIDLLQPNQAEFPLFSVFV
>sp|A6NKT7|RGPD3_HUMAN
MSCSKAYGERYVASVQGSAPSPRKKSTRGFYFAKLYYEAKEYDLAKKYICTYINVREMDPRAHRFLGLLYELEENTEKAVECYRRSV
ELNPTQKDLVLKIAELLCKNDVTDGRAEYWVERAAKLFPGSPAIYKLKEQLLDCEGEDGWNKLFDLIQSELYVRPDDVHVNIRLVEL
YRSTKRLKDAVARCHEAERNIALRSSLEWNSCVVQTLKEYLESLQCLESDKSDWRATNTDLLLAYANLMLLTLSTRDVQESRELLES
FDSALQSAKSSLGGNDELSATFLEMKGHFYMHAGSLLLKMGQHGNNVQWRALSELAALCYLIAFQVPRPKIKLIKGEAGQNLLEMMA
CDRLSQSGHMLLNLSRGKQDFLKVVVETFANKSGQSALYDALFSSQSPKDTSFLGSDDIGNIDVQEPELEDLARYDVGAIRAHNGSL
QHLTWLGLQWNSLPALPGIRKWLKQLFHHLPQETSRLETNAPESICILDLEVFLLGVVYTSHLQLKEKCNSHHSSYQPLCLPLPVCK
QLCTERQKSWWDAVCTLIHRKAVPGNSAKLRLLVQHEINTLRAQEKHGLQPALLVHWAKCLQKMGSGLNSFYDQREYIGRSVHYWKK
VLPLLKIIKKKNSIPEPIDPLFKHFHSVDIQASEIVEYEEDAHVTFAILDAVNGNIEDAMTAFESIKSVVSYWNLALIFHRKAEDIA
NDALSPEEQEECKNYLRKTRGYLIKILDDSDSNLSVVKKLPVPLESVKEMLKSVMQELENYSEGGPLYKNGSLRNADSEIKHSTPSP
TKYSLSPSKSYKYSPKTPPRWAEDQNSLLKMIRQEVKAIKEEMQELKLNSSKSASHHRWPTENYGPDSVPDGYQGSQTFHGAPLTVA
TTGPSVYYSQSPAYNSQYLLRPAANVTPTKGSSNTEFKSTKEGFSIPVSADGFKFGISEPGNQEKKSEKPLENDTGLQAQDISGRKK
GRGVIFGQTSSTFTFADVAKSTSGEGFQFGKKDLNFKGFSGAGEKLFSSQYGKMANKANTSGDFEKDDDAYKTEDSDDIHFEPVVQM
PEKVELVTGEEGEKVLYSQGVKLFRFDAEVSQWKERGLGNLKILKNEVNGKVRMLMQREQVLKVCANHWITTTMNLKPLSGSDRAWM
WSASDFSDGDAKLERLAAKFKTPELAEEFKQKFEECQRLLLDIPLQTPHKLVDTGRAAKLIQRAEEMKSGLKDFKTFLTNDQTKVAE
EENKGSGTGAAGASDTTIKPNAENTGPTLEWDNYDLREDALDDSVSSSSVHASPLASSPVRKNLFHFDESTTGSNFSFKSALSLSKS
PAKLNQSGTSVGTDEESDVTQEEERDGQYFEPVVPLPDLVEVSSGEENEQVVFSHRAEFYRYDKDVGQWKERGIGDIKILQNYDNKH
VRILMRRDQVLKLCANHRITPDMSLQNMKGTERVWVWTACDFADGERKVEHLAVRFKLQDVADSFKKIFDEAKTAQEKDSLITPHVS
RSSTPRESPCGKIAVAVLEETTRERTDVIQGDDVADAASEVEVSSTSETTTKAVVSPPKFVFGSESVKRIFSSEKSKPFAFGNSSAT

GSLFGFSFNAPLKSNNSETSSVAQSGSESKVEPKKCELSKNSDIEQSSDSKVKNLSASFPTEESSINYTFKTPEKEPPLWYAEFTKE
ELVQKLSSTTKSADHLNGLLREIEATNAVLMEQIKLLKSEIRRLERNQEQEVSAANVEHLKNVLLQFIFLKPGSERERLLPVINTML
QLSLEEKGKLAAVAQGEE
>sp|P34910|EVI2B_HUMAN
MDPKYFILILFCGHLNNTFFSKTETITTEKQSQPTLFTSSMSQVLANSQNTTGNPLGQPTQFSDTFSGQSISPAKVTAGQPTPAVYT
SSEKPEAHTSAGQPLAYNTKQPTPIANTSSQQAVFTSARQLPSARTSTTQPPKSFVYTFTQQSSSVQIPSRKQITVHNPSTQPTSTV
KNSPRSTPGFILDTTSNKQTPQKNNYNSIAAILIGVLLTSMLVAIIIIVLWKCLRKPVLNDQNWAGRSPFADGETPDICMDNIRENE
ISTKRTSIISLTPWKPSKSTLLADDLEIKLFESSENIEDSNNPKTEKIKDQVNGTSEDSADGSTVGTAVSSSDDADLPPPPPLLDLE
GQESNQSDKPTMTIVSPLPNDSTSLPPSLDCLNQDCGDHKSEIIQSFPPLDSLNLPLPPVDFMKNQEDSNLEIQCQEFSIPPNSDQD
LNESLPPPPAELL
>sp|Q9H5K3|SG196_HUMAN
MEKQPQNSRRGLAPREVPPAVGLLLIMALMNTLLYLCLDHFFIAPRQSTVDPTHCPYGHFRIGQMKNCSPWLSCEELRTEVRQLKRV
GEGAVKRVFLSEWKEHKVALSQLTSLEMKDDFLHGLQMLKSLQGTHVVTLLGYCEDDNTMLTEYHPLGSLSNLEETLNLSKYQNVNT
WQHRLELAMDYVSIINYLHHSPVGTRVMCDSNDLPKTLSQYLLTSNFSILANDLDALPLVNHSSGMLVKCGHRELHGDFVAPEQLWP
YGEDVPFHDDLMPSYDEKIDIWKIPDISSFLLGHIEGSDMVRFHLFDIHKACKSQTPSERPTAQDVLETYQKVLDTLRDAMMSQARE
ML
>sp|Q00653|NFKB2_HUMAN
MESCYNPGLDGIIEYDDFKLNSSIVEPKEPAPETADGPYLVIVEQPKQRGFRFRYGCEGPSHGGLPGASSEKGRKTYPTVKICNYEG
PAKIEVDLVTHSDPPRAHAHSLVGKQCSELGICAVSVGPKDMTAQFNNLGVLHVTKKNMMGTMIQKLQRQRLRSRPQGLTEAEQREL
EQEAKELKKVMDLSIVRLRFSAFLRASDGSFSLPLKPVISQPIHDSKSPGASNLKISRMDKTAGSVRGGDEVYLLCDKVQKDDIEVR
FYEDDENGWQAFGDFSPTDVHKQYAIVFRTPPYHKMKIERPVTVFLQLKRKRGGDVSDSKQFTYYPLVEDKEEVQRKRRKALPTFSQ
PFGGGSHMGGGSGGAAGGYGGAGGGGSLGFFPSSLAYSPYQSGAGPMGCYPGGGGGAQMAATVPSRDSGEEAAEPSAPSRTPQCEPQ
APEMLQRAREYNARLFGLAQRSARALLDYGVTADARALLAGQRHLLTAQDENGDTPLHLAIIHGQTSVIEQIVYVIHHAQDLGVVNL
TNHLHQTPLHLAVITGQTSVVSFLLRVGADPALLDRHGDSAMHLALRAGAGAPELLRALLQSGAPAVPQLLHMPDFEGLYPVHLAVR
ARSPECLDLLVDSGAEVEATERQGGRTALHLATEMEELGLVTHLVTKLRANVNARTFAGNTPLHLAAGLGYPTLTRLLLKAGADIHA
ENEEPLCPLPSPPTSDSDSDSEGPEKDTRSSFRGHTPLDLTCSTKVKTLLLNAAQNTMEPPLTPPSPAGPGLSLGDTALQNLEQLLD
GPEAQGSWAELAERLGLRSLVDTYRQTTSPSGSLLRSYELAGGDLAGLLEALSDMGLEEGVRLLRGPETRDKLPSTAEVKEDSAYGS
QSVEQEAEKLGPPPEPPGGLCHGHPQPQVH
>sp|Q9UBS5|GABR1_HUMAN
MLLLLLLLAPLFLRPPGAGGAQTPNATSEGCQIIHPPWEGGIRYRGLTRDQVKAINFLPVDYEIEYVCRGEREVVGPKVRKCLANGSW
TDMDTPSRCVRICSKSYLTLENGKVFLTGGDLPALDGARVDFRCDPDFHLVGSSRSICSQGQWSTPKPHCQVNRTPHSERRAVYIGA
LFPMSGGWPGGQACQPAVEMALEDVNSRRDILPDYELKLIHHDSKCDPGQATKYLYELLYNDPIKIILMPGCSSVSTLVAEAARMWN
LIVLSYGSSSPALSNRQRFPTFFRTHPSATLHNPTRVKLFEKWGWKKIATIQQTTEVFTSTLDDLEERVKEAGIEITFRQSFFSDPA
VPVKNLKRQDARIIVGLFYETEARKVFCEVYKERLFGKKYVWFLIGWYADNWFKIYDPSINCTVDEMTEAVEGHITTEIVMLNPANT
RSISNMTSQEFVEKLTKRLKRHPEETGGFQEAPLAYDAIWALALALNKTSGGGGRSGVRLEDFNYNNQTITDQIYRAMNSSSFEGVS
GHVVFDASGSRMAWTLIEQLQGGSYKKIGYYDSTKDDLSWSKTDKWIGGSPPADQTLVIKTFRFLSQKLFISVSVLSSLGIVLAVVC
LSFNIYNSHVRYIQNSQPNLNNLTAVGCSLALAAVFPLGLDGYHIGRNQFPFVCQARLWLLGLGFSLGYGSMFTKIWWVHTVFTKKE
EKKEWRKTLEPWKLYATVGLLVGMDVLTLAIWQIVDPLHRTIETFAKEEPKEDIDVSILPQLEHCSSRKMNTWLGIFYGYKGLLLLL
GIFLAYETKSVSTEKINDHRAVGMAIYNVAVLCLITAPVTMILSSQQDAAFAFASLAIVFSSYITLVVLFVPKMRRLITRGEWQSEA
QDTMKTGSSTNNNEEEKSRLLEKENRELEKIIAEKEERVSELRHQLQSRQQLRSRRHPPTPPEPSGGLPRGPPEPPDRLSCDGSRVH
LLYK
>sp|Q9H4Z3|PCIF1_HUMAN
MANENHGSPREEASLLSHSPGTSNQSQPCSPKPIRLVQDLPEELVHAGWEKCWSRRENRPYYFNRFTNQSLWEMPVLGQHDVISDPL
GLNATPLPQDSSLVETPPAENKPRKRQLSEEQPSGNGVKKPKIEIPVTPTGQSVPSSPSIPGTPTLKMWGTSPEDKQQAALLRPTEV

YWDLDIQTNAVIKHRGPSEVLPPHPEVELLRSQLILKLRQHYRELCQQREGIEPPRESFNRWMLERKVVDKGSDPLLPSNCEPVVSP
SMFREIMNDIPIRLSRIKFREEAKRLLFKYAEAARRLIESRSASPDSRKVVKWNVEDTFSWLRKDHSASKEDYMDRLEHLRRQCGPH
VSAAAKDSVEGICSKIYHISLEYVKRIREKHLAILKENNISEEVEAPEVEPRLVYCYPVRLAVSAPPMPSVEMHMENNVVCIRYKGE
MVKVSRNYFSKLWLLYRYSCIDDSAFERFLPRVWCLLRRYQMMFGVGLYEGTGLQGSLPVHVFEALHRLFGVSFECFASPLNCYFRQ
YCSAFPDTDGYFGSRGPCLDFAPLSGSFEANPPFCEELMDAMVSHFERLLESSPEPLSFIVFIPEWREPPTPALTRMEQSRFKRHQL
ILPAFEHEYRSGSQHICKKEEMHYKAVHNTAVLFLQNDPGFAKWAPTPERLQELSAAYRQSGRSHSSGSSSSSSSEAKDRDSGREQG
PSREPHPT
>sp|Q9BVS5|TR61B_HUMAN
MLMAWCRGPVLLCLRQGLGTNSFLHGLGQEPFEGARSLCCRSSPRDLRDGEREHEAAQRKAPGAESCPSLPLSISDIGTGCLSSLEN
LRLPTLREESSPRELEDSSGDQGRCGPTHQGSEDPSMLSQAQSATEVEERHVSPSCSTSRERPFQAGELILAETGEGETKFKKLFRL
NNFGLLNSNWGAVPFGKIVGKFPGQILRSSFGKQYMLRRPALEDYVVLMKRGTAITFPKDINMILSMMDINPGDTVLEAGSGSGGMS
LFLSKAVGSQGRVISFEVRKDHHDLAKKNYKHWRDSWKLSHVEEWPDNVDFIHKDISGATEDIKSLTFDAVALDMLNPHVTLPVFYP
HLKHGGVCAVYVVNITQVIELLDGIRTCELALSCEKISEVIVRDWLVCLAKQKNGILAQKVESKINTDVQLDSQEKIGVKGELFQED
DHEESHSDFPYGSFPYVARPVHWQPGHTAFLVKLRKVKPQLN
>sp|Q00526|CDK3_HUMAN
MDMFQKVEKIGEGTYGVVYKAKNRETGQLVALKKIRLDLEMEGVPSTAIREISLLKELKHPNIVRLLDVVHNERKLYLVFEFLSQDL
KKYMDSTPGSELPLHLIKSYLFQLLQGVSFCHSHRVIHRDLKPQNLLINELGAIKLADFGLARAFGVPLRTYTHEVVTLWYRAPEIL
LGSKFYTTAVDIWSIGCIFAEMVTRKALFPGDSEIDQLFRIFRMLGTPSEDTWPGVTQLPDYKGSFPKWTRKGLEEIVPNLEPEGRD
LLMQLLQYDPSQRITAKTALAHPYFSSPEPSPAARQYVLQRFRH
>sp|Q9UK97|FBX9_HUMAN
MPDIIWVFPPQAEAEEDCHSDTVRADDDEENESPAETDLQAQLQMFRAQWMFELAPGVSSSNLENRPCRAARGSLQKTSADTKGKQE
QAKEEKARELFLKAVEEEQNGALYEAIKFYRRAMQLVPDIEFKITYTRSPDGDGVGNSYIEDNDDDSKMADLLSYFQQQLTFQESVL
KLCQPELESSQIHISVLPMEVLMYIFRWVVSSDLDLRSLEQLSLVCRGFYICARDPEIWRLACLKVWGRSCIKLVPYTSWREMFLER
PRVRFDGVYISKTTYIRQGEQSLDGFYRAWHQVEYYRYIRFFPDGHVMMLTTPEEPQSIVPRLRTRNTRTDAILLGHYRLSQDTDNQ
TKVFAVITKKKEEKPLDYKYRYFRRVPVQEADQSFHVGLQLCSSGHQRFNKLIWIHHSCHITYKSTGETAVSAFEIDKMYTPLFFAR
VRSYTAFSERPL
>sp|O95707|RPP29_HUMAN
MKSVIYHALSQKEANDSDVQPSGAQRAEAFVRAFLKRSTPRMSPQAREDQLQRKAVVLEYFTRHKRKEKKKKAKGLSARQRRELRLF
DIKPEQQRYSLFLPLHELWKQYIRDLCSGLKPDTQPQMIQAKLLKADLHGAIISVTKSKCPSYVGITGILLQETKHIFKIITKEDRL
KVIPKLNCVFTVETDGFISYIYGSKFQLRSSERSAKKFKAKGTIDL
>sp|Q8IYS8|BD1L2_HUMAN
MADGGGGGSGGAGPASTRASGGGGPINPASLPPGDPQLIAIIVGQLKSRGLFDSFRRDCKADVDTKPAYQNLSQKADNFVSTHLDKQ
EWNPPANDNQLHDGLRQSVVQSGRSEAGVDRISSQVVDPKLNHIFRPQIEQIIHEFLVAQKEAAVPALPPEPEGQDPPAPSQDTS
>sp|Q7Z7L1|SLN11_HUMAN
MEANQCPLVVEPSYPDLVINVGEVTLGEENRKKLQKIQRDQEKERVMRAACALLNSGGGVIRMAKKVEHPVEMGLDLEQSLRELIQS
SDLQAFFETKQQGRCFYIFVKSWSSGPFPEDRSVKPRLCSLSSSLYRRSETSVRSMDSREAFCFLKTKRKPKILEEGPFHKIHKGVY
QELPNSDPADPNSDPADLIFQKDYLEYGEILPFPESQLVEFKQFSTKHFQEYVKRTIPEYVPAFANTGGGYLFIGVDDKSREVLGCA
KENVDPDSLRRKIEQAIYKLPCVHFCQPQRPITFTLKIVNVLKRGELYGYACMIRVNPFCCAVFSEAPNSWIVEDKYVCSLTTEKWV
GMMTDTDPDLLQLSEDFECQLSLSSGPPLSRPVYSKKGLEHKKELQQLLFSVPPGYLRYTPESLWRDLISEHRGLEELINKQMQPFF
RGILIFSRSWAVDLNLQEKPGVICDALLIAQNSTPILYTILREQDAEGQDYCTRTAFTLKQKLVNMGGYTGKVCVRAKVLCLSPESS
AEALEAAVSPMDYPASYSLAGTQHMEALLQSLVIVLLGFRSLLSDQLGCEVLNLLTAQQYEIFSRSLRKNRELFVHGLPGSGKTIMA
MKIMEKIRNVFHCEAHRILYVCENQPLRNFISDRNICRAETRKTFLRENFEHIQHIVIDEAQNFRTEDGDWYGKAKSITRRAKGGPG
ILWIFLDYFQTSHLDCSGLPPLSDQYPREELTRIVRNADPIAKYLQKEMQVIRSNPSFNIPTGCLEVFPEAEWSQGVQGTLRIKKYL

TVEQIMTCVADTCRRFFDRGYSPKDVAVLVSTAKEVEHYKYELLKAMRKKRVVQLSDACDMLGDHIVLDSVRRFSGLERSIVFGIHP
RTADPAILPNVLICLASRAKQHLYIFPWGGH
>sp|P0CG23|ZN853_HUMAN
MLHQPTPGNRGLTARMEVGPATETFVLELQCLEDGGPGPDTLSGGSGGSESQEEEEPQERNSSPQRPAVSAPVGASEIAEETRPGQR
ELQLQQLEQQPEPQQQPQHEQLQQPQPHLELQQQPQQDGQQQLSQLQQEKHQSVHHQELKPELQLMHQQQQLQPQQVQEQQRLQQQQ
EQLQTQQAQEQQVLQQQEQLQQQVQEQQLLQQQQEQLQQQQLLQQQEQLQQQQFQQQQEQLQQQQQLLLLQQQGQLQQQLLQQQQAQ
LQQQLLEQQQAQLQQQLLLQQQEQLQQQQQQQLLQQQQEQLQQQQLQPPPLEPEEEEEVELELMPVDLGSEQELEQQRQELERQQEL
ERQQEQRQLQLKLQEELQQLEQQLEQQQQQLEQQEVQLELTPVELGAQQQEVQLELTPVQPELQLELVPAAGGGGAAVPGAPAAVVV
APPGYVVVQELMVLPAVAAPAVVAIPGPAGSAALTPARQRRRRRARDRPTICGECGKGFSRSTDLVRHQATHTGERPHRCGECGKGF
SQHSNLVTHQRIHTGEKPYACSYCAKRFSESSALVQHQRTHTGERPYACGDCGKRFSVSSNLLRHRRTHSGERPYVCEDCGERFRHK
VQIRRHERQLHGAGRSRGLGLLRASRPAALGGPARAEQAATATAPADKAL
>sp|Q9Y3M8|STA13_HUMAN
MFSQVPRTPASGCYYLNSMTPEGQEMYLRFDQTTRRSPYRMSRILARHQLVTKIQQEIEAKEACDWLRAAGFPQYAQLYEDSQFPIN
IVAVKNDHDFLEKDLVEPLCRRLNTLNKCASMKLDVNFQRKKGDDSDEEDLCISNKWTFQRTSRRWSRVDDLYTLLPRGDRNGSPGG
TGMRNTTSSESVLTDLSEPEVCSIHSESSGGSDSRSQPGQCCTDNPVMLDAPLVSSSLPQPPRDVLNHPFHPKNEKPTRARAKSFLK
RMETLRGKGAHGRHKGSGRTGGLVISGPMLQQEPESFKAMQCIQIPNGDLQNSPPPACRKGLPCSGKSSGESSPSEHSSSGVSTPCL
KERKCHEANKRGGMYLEDLDVLAGTALPDAGDQSRMHEFHSQENLVVHIPKDHKPGTFPKALSIESLSPTDSSNGVNWRTGSISLGR
EQVPGAREPRLMASCHRASRVSIYDNVPGSHLYASTGDLLDLEKDDLFPHLDDILQHVNGLQEVVDDWSKDVLPELQTHDTLVGEPG
LSTFPSPNQITLDFEGNSVSEGRTTPSDVERDVTSLNESEPPGVRDRRDSGVGASLTRPNRRLRWNSFQLSHQPRPAPASPHISSQT
ASQLSLLQRFSLLRLTAIMEKHSMSNKHGWTWSVPKFMKRMKVPDYKDKAVFGVPLIVHVQRTGQPLPQSIQQALRYLRSNCLDQVG
LFRKSGVKSRIHALRQMNENFPENVNYEDQSAYDVADMVKQFFRDLPEPLFTNKLSETFLHIYQYVSKEQRLQAVQAAILLLADENR
EVLQTLLCFLNDVVNLVEENQMTPMNLAVCLAPSLFHLNLLKKESSPRVIQKKYATGKPDQKDLNENLAAAQGLAHMIMECDRLFEV
PHELVAQSRNSYVEAEIHVPTLEELGTQLEESGATFHTYLNHLIQGLQKEAKEKFKGWVTCSSTDNTDLAFKKVGDGNPLKLWKASV
EVEAPPSVVLNRVLRERHLWDEDFVQWKVVETLDRQTEIYQYVLNSMAPHPSRDFVVLRTWKTDLPKGMCTLVSLSVEHEEAQLLGG
VRAVVMDSQYLIEPCGSGKSRLTHICRIDLKGHSPEWYSKGFGHLCAAEVARIRNSFQPLIAEGPETKI
>sp|Q9UKA1|FBXL5_HUMAN
MAPFPEEVDVFTAPHWRMKQLVGLYCDKLSKTNFSNNNDFRALLQSLYATFKEFKMHEQIENEYIIGLLQQRSQTIYNVHSDNKLSE
MLSLFEKGLKNVKNEYEQLNYAKQLKERLEAFTRDFLPHMKEEEEVFQPMLMEYFTYEELKDIKKKVIAQHCSQKDTAELLRGLSLW
NHAEERQKFFKYSVDEKSDKEAEVSEHSTGITHLPPEVMLSIFSYLNPQELCRCSQVSMKWSQLTKTGSLWKHLYPVHWARGDWYSG
PATELDTEPDDEWVKNRKDESRAFHEWDEDADIDESEESAEESIAISIAQMEKRLLHGLIHNVLPYVGTSVKTLVLAYSSAVSSKMV
RQILELCPNLEHLDLTQTDISDSAFDSWSWLGCCQSLRHLDLSGCEKITDVALEKISRALGILTSHQSGFLKTSTSKITSTAWKNKD
ITMQSTKQYACLHDLTNKGIGEEIDNEHPWTKPVSSENFTSPYVWMLDAEDLADIEDTVEWRHRNVESLCVMETASNFSCSTSGCFS
KDIVGLRTSVCWQQHCASPAFAYCGHSFCCTGTALRTMSSLPESSAMCRKAARTRLPRGKDLIYFGSEKSDQETGRVLLFLSLSGCY
QITDHGLRVLTLGGGLPYLEHLNLSGCLTITGAGLQDLVSACPSLNDEYFYYCDNINGPHADTASGCQNLQCGFRACCRSGE
>sp|Q15629|TRAM1_HUMAN
MAIRKKSTKSPPVLSHEFVLQNHADIVSCVAMVFLLGLMFEITAKASIIFVTLQYNVTLPATEEQATESVSLYYYGIKDLATVFFYM
LVAIIIHAVIQEYMLDKINRRMHFSKTKHSKFNESGQLSAFYLFACVWGTFILISENYISDPTILWRAYPHNLMTFQMKFFYISQLA
YWLHAFPELYFQKTKKEDIPRQLVYIGLYLFHIAGAYLLNLNHLGLVLLVLHYFVEFLFHISRLFYFSNEKYQKGFSLWAVLFVLGR
LLTLILSVLTVGFGLARAENQKLDFSTGNFNVLAVRIAVLASICVTQAFMMWKFINFQLRRWREHSAFQAPAVKKKPTVTKGRSSKK
GTENGVNGTLTSNVADSPRNKKEKSS
>sp|Q92765|SFRP3_HUMAN
MVCGSPGGMLLLRAGLLALAALCLLRVPGARAAACEPVRIPLCKSLPWNMTKMPNHLHHSTQANAILAIEQFEGLLGTHCSPDLLFF
LCAMYAPICTIDFQHEPIKPCKSVCERARQGCEPILIKYRHSWPENLACEELPVYDRGVCISPEAIVTADGADFPMDSSNGNCRGAS

SERCKCKPIRATQKTYFRNNYNYVIRAKVKEIKTKCHDVTAVVEVKEILKSSLVNIPRDTVNLYTSSGCLCPPLNVNEEYIIMGYED
EERSRLLLVEGSIAEKWKDRLGKKVKRWDMKLRHLGLSKSDSSNSDSTQSQKSGRNSNPRQARN
>sp|Q8TAC2|JOS2_HUMAN
MSQAPGAQPSPPTVYHERQRLELCAVHALNNVLQQQLFSQEAADEICKRLAPDSRLNPHRSLLGTGNYDVNVIMAALQGLGLAAVWW
DRRRPLSQLALPQVLGLILNLPSPVSLGLLSLPLRRRHWVALRQVDGVYYNLDSKLRAPEALGDEDGVRAFLAAALAQGLCEVLLVV
TKEVEEKGSWLRTD
>sp|Q8IZS7|CLCL1_HUMAN
MVSNFFHVIQVFEKSATLISKTEHIGFVIYSWRKSTTHLGSRRKFAISIYLSEVSLQKYDCPFSGTSFVVFSLFLICAMAGDVVYAD
IKTVRTSPLELAFPLQRSVSFNFSTVHKSCPAKDWKVHKGKCYWIAETKKSWNKSQNDCAINNSYLMVIQDITAMVRFNI
>sp|P36507|MP2K2_HUMAN
MLARRKPVLPALTINPTIAEGPSPTSEGASEANLVDLQKKLEELELDEQQKKRLEAFLTQKAKVGELKDDDFERISELGAGNGGVVT
KVQHRPSGLIMARKLIHLEIKPAIRNQIIRELQVLHECNSPYIVGFYGAFYSDGEISICMEHMDGGSLDQVLKEAKRIPEEILGKVS
IAVLRGLAYLREKHQIMHRDVKPSNILVNSRGEIKLCDFGVSGQLIDSMANSFVGTRSYMAPERLQGTHYSVQSDIWSMGLSLVELA
VGRYPIPPPDAKELEAIFGRPVVDGEEGEPHSISPRPRPPGRPVSGHGMDSRPAMAIFELLDYIVNEPPPKLPNGVFTPDFQEFVNK
CLIKNPAERADLKMLTNHTFIKRSEVEEVDFAGWLCKTLRLNQPGTPTRTAV
>sp|P98073|ENTK_HUMAN
MGSKRGISSRHHSLSSYEIMFAALFAILVVLCAGLIAVSCLTIKESQRGAALGQSHEARATFKITSGVTYNPNLQDKLSVDFKVLAF
DLQQMIDEIFLSSNLKNEYKNSRVLQFENGSIIVVFDLFFAQWVSDENVKEELIQGLEANKSSQLVTFHIDLNSVDILDKLTTTSHL
ATPGNVSIECLPGSSPCTDALTCIKADLFCDGEVNCPDGSDEDNKMCATVCDGRFLLTGSSGSFQATHYPKPSETSVVCQWIIRVNQ
GLSIKLSFDDFNTYYTDILDIYEGVGSSKILRASIWETNPGTIRIFSNQVTATFLIESDESDYVGFNATYTAFNSSELNNYEKINCN
FEDGFCFWVQDLNDDNEWERIQGSTFSPFTGPNFDHTFGNASGFYISTPTGPGGRQERVGLLSLPLDPTLEPACLSFWYHMYGENVH
KLSINISNDQNMEKTVFQKEGNYGDNWNYGQVTLNETVKFKVAFNAFKNKILSDIALDDISLTYGICNGSLYPEPTLVPTPPPELPT
DCGGPFELWEPNTTFSSTNFPNSYPNLAFCVWILNAQKGKNIQLHFQEFDLENINDVVEIRDGEEADSLLLAVYTGPGPVKDVFSTT
NRMTVLLITNDVLARGGFKANFTTGYHLGIPEPCKADHFQCKNGECVPLVNLCDGHLHCEDGSDEADCVRFFNGTTNNNGLVRFRIQ
SIWHTACAENWTTQISNDVCQLLGLGSGNSSKPIFPTDGGPFVKLNTAPDGHLILTPSQQCLQDSLIRLQCNHKSCGKKLAAQDITP
KIVGGSNAKEGAWPWVVGLYYGGRLLCGASLVSSDWLVSAAHCVYGRNLEPSKWTAILGLHMKSNLTSPQTVPRLIDEIVINPHYNR
RRKDNDIAMMHLEFKVNYTDYIQPICLPEENQVFPPGRNCSIAGWGTVVYQGTTANILQEADVPLLSNERCQQQMPEYNITENMICA
GYEEGGIDSCQGDSGGPLMCQENNRWFLAGVTSFGYKCALPNRPGVYARVSRFTEWIQSFLH
>sp|P0C628|O5AC1_HUMAN
MAEENKILVTHFVLTGLTDHPGLQAPLFLVFLVIYLITLVGNLGLMALIWKDPHLHTPIYLFLGSLAFADACTSSSVTSKMLINFLS
KNHMLSMAKCATQFYFFGSNATTECFLLVVMAYDRYVAICNPLLYPVVMSNSLCTQFIGISYFIGFLHSAIHVGLLFRLTFCRSNII
HYFYCEILQLFKISCTNPTVNILLIFIFSAFIQVFTFMTLIVSYSYILSAILKKKSEKGRSKAFSTCSAHLLSVSLFYGTLFFMYVS
SRSGSAADQAKMYSLFYTIIIPLLNPFIYSLRNKEVIDALRRIMKK
>sp|Q7M4L6|SHF_HUMAN
MQQEGGPVRSAPCRTGTLEGSRQGSPGHRKRASPKGSLSSAQPHSWMLTPSPLNSHCAHREPISSSPQPVANGPKQKKKSNWRSTTR
LRIIRLRDRLEPRPLAILEDYADPFDVQETGEGSAGASGAPEKVPENDGYMEPYEAQKMMAEIRGSKETATQPLPLYDTPYEPEEDG
ATAEGEGAPWPRESRLPEDDERPPEEYDQPWEWKKERISKAFAVDIKVIKDLPWPPPVGQLDSSPSLPDGDRDISGPASPLPEPSLE
DSSAQFEGPEKSCLSPGREEKGRLPPRLSAGNPKSAKPLSMEPSSPLGEWTDPALPLENQVWYHGAISRTDAENLLRLCKEASYLVR
NSETSKNDFSLSLKSSQGFMHMKLSRTKEHKYVLGQNSPPFSSVPEIVHHYASRKLPIKGAEHMSLLYPVAIRTL
>sp|Q86SS6|SYT9_HUMAN
MPGARDALCHQALQLLAELCARGALEHDSCQDFIYHLRDRARPRLRDPDISVSLLTLVVTACGLALFGVSLFVSWKLCWVPWRERGL
PSGSKDNNQEPLNYMDTETNEQENSEDFLDPPTPCPDSSMKISHTSPDIPLSTQTGIQENCAHGVRVQRQVTEPTSSARHNSIRRQL
NLSNPDFNIQQLQKQEQLTGIGRIKPELYKQRSLDNDDGRRSNSKACGKLNFILKYDCDLEQLIVKIHKAVNLPAKDFSGTSDPYVK
IYLLPDRKTKHQTKVHRKTLNPVFDEVFLFPVPYNDLEARKLHFSVYDFDRFSRHDLIGQVVVDHFLDLADFPRECILWKDIEYVTN

DNVDLGELMFSLCYLPTAGRLTITIIKARNLKAMDITGASDPYVKVSLMCDGRRLKKRKTSTKRNTLNPVYNEAIVFDVPPENIDQI
HLSIAVMDYDRVGHNEIIGVCQVGNEAERLGRDHWSEMLSYPRKPIAHWHSLVEKR
>sp|Q5U5X0|LYRM7_HUMAN
MGRAVKVLQLFKTLHRTRQQVFKNDARALEAARIKINEEFKNNKSETSSKKIEELMKIGSDVELLLRTSVIQGIHTDHNTLKLVPRK
DLLVENVPYCDAPTQKQ
>sp|Q9P218|COKA1_HUMAN
MSSGDPAHLGLCLWLWLGATLGREQVQASGLLRLAVLPEDRLQMKWRESEGSGLGYLVQVKPMAGDSEQEVILTTKTPKATVGGLSP
SKGYTLQIFELTGSGRFLLARREFVIEDLKSSSLDRSSQRPLGSGAPEPTPSHTGSPDPEQASEPQVAFTPSQDPRTPAGPQFRCLP
PVPADMVFLVDGSWSIGHSHFQQVKDFLASVIAPFEIGPDKVQVGLTQYSGDAQTEWDLNSLSTKEQVLAAVRRLRYKGGNTFTGLA
LTHVLGQNLQPAAGLRPEAAKVVILVTDGKSQDDVHTAARVLKDLGVNVFAVGVKNADEAELRLLASPPRDITVHSVLDFLQLGALA
GLLSRLICQRLQGGSPRQGPAAAPALDTLPAPTSLVLSQVTSSSIRLSWTPAPRHPLKYLIVWRASRGGTPREVVVEGPAASTELHN
LASRTEYLVSVFPIYEGGVGEGLRGLVTTAPLPPPRALTLAAVTPRTVHLTWQPSAGATHYLVRCSPASPKGEEEEREVQVGRPEVL
LDGLEPGRDYEVSVQSLRGPEGSEARGIRARTPTLAPPRHLGFSDVSHDAARVFWEGAPRPVRLVRVTYVSSEGGHSGQTEAPGNAT
SATLGPLSSSTTYTVRVTCLYPGGGSSTLTGRVTTKKAPSPSQLSMTELPGDAVQLAWVAAAPSGVLVYQITWTPLGEGKAHEISVP
GNLGTAVLPGLGRHTEYDVTILAYYRDGARSDPVSLRYTPSTVSRSPPSNLALASETPDSLQVSWTPPLGRVLHYWLTYAPASGLGP
EKSVSVPGARSHVTLPDLQAATKYRVLVSAIYAAGRSEAVSATGQTACPALRPDGSLPGFDLMVAFSLVEKAYASIRGVAMEPSAFG
GTPTFTLFKDAQLTRRVSDVYPAPLPPEHTIVFLVRLLPETPREAFALWQMTAEDFQPLLGVLLDAGKKSLTYFHRDPRAALQEATF
DPQEVRKIFFGSFHKVHVAVGRSKVRLYVDCRKVAERPLGEMGSPPAAGFVTLGRLAKARGPRSSSAAFQLQMLQIVCSDTWADEDR
CCELPASRDGETCPAFVSACSCSSETPGPPGPQGPPGLPGRNGTPGEQGFPGPRGPPGVKGEKGDHGLPGLQGHPGHQGIPGRVGLQ
GPKGMRGLEGTAGLPGPPGPRGFQGMAGARGTSGERGPPGTVGPTGLPGPKGERGEKGEPQSLATLYQLVSQASHVSKFDSFHENTR
PPMPILEQKLEPGTEPLGSPGTRSKALVPGEWGRGGRHLEGRGEPGAVGQMGSPGQQGASTQGLWE
>sp|Q5JUX0|SPIN3_HUMAN
MKTPFGKAAAGQRSRTGAGHGSVSVTMIKRKAAHKKHRSRPTSQPRGNIVGCRIQHGWKDGDEPLTQWKGTVLDQVPVNPSLYLIKY
DGFDCVYGLELHRDERVSSLEVLPNRVASSRISDTHLAEIMVGKAVEHIFETEEGSKNEWRGMVLAQAPVMNTWFYITYEKDPVLYM
YQLLDDYKDGDLRILQDSNDSPLAEREPGEVIDSLVGKQVEYAKDDGSKRTGMVIHQVEAKPSVYFIKFDDDFHIYVYDLVKTS
>sp|O95445|APOM_HUMAN
MFHQIWAALLYFYGIILNSIYQCPEHSQLTTLGVDGKEFPEVHLGQWYFIAGAAPTKEELATFDPVDNIVFNMAAGSAPMQLHLRAT
IRMKDGLCVPRKWIYHLTEGSTDLRTEGRPDMKTELFSSSCPGGIMLNETGQGYQRFLLYNRSPHPPEKCVEEFKSLTSCLDSKAFL
LTPRNQEACELSNN
>sp|Q7L2E3|DHX30_HUMAN
MFSLDSFRKDRAQHRQRQCKLPPPRLPPMCVNPTPGGTISRASRDLLKEFPQPKNLLNSVIGRALGISHAKDKLVYVHTNGPKKKKV
TLHIKWPKSVEVEGYGSKKIDAERQAAAAACQLFKGWGLLGPRNELFDAAKYRVLADRFGSPADSWWRPEPTMPPTSWRQLNPESIR
PGGPGGLSRSLGREEEEDEEEELEEGTIDVTDFLSMTQQDSHAPLRDSRGSSFEMTDDDSAIRALTQFPLPKNLLAKVIQIATSSST
AKNLMQFHTVGTKTKLSTLTLLWPCPMTFVAKGRRKAEAENKAAALACKKLKSLGLVDRNNEPLTHAMYNLASLRELGETQRRPCTI
QVPEPILRKIETFLNHYPVESSWIAPELRLQSDDILPLGKDSGPLSDPITGKPYVPLLEAEEVRLSQSLLELWRRRGPVWQEAPQLP
VDPHRDTILNAIEQHPVVVISGDTGCGKTTRIPQLLLERYVTEGRGARCNVIITQPRRISAVSVAQRVSHELGPSLRRNVGFQVRLE
SKPPSRGGALLFCTVGILLRKLQSNPSLEGVSHVIVDEVHERDVNTDFLLILLKGLQRLNPALRLVLMSATGDNERFSRYFGGCPVI
KVPGFMYPVKEHYLEDILAKLGKHQYLHRHRHHESEDECALDLDLVTDLVLHIDARGEPGGILCFLPGWQEIKGVQQRLQEALGMHE
SKYLILPVHSNIPMMDQKAIFQQPPVGVRKIVLATNIAETSITINDIVHVVDSGLHKEERYDLKTKVSCLETVWVSRANVIQRRGRA
GRCQSGFAYHLFPRSRLEKMVPFQVPEILRTPLENLVLQAKIHMPEKTAVEFLSKAVDSPNIKAVDEAVILLQEIGVLDQREYLTTL
GQRLAHISTDPRLAKAIVLAAIFRCLHPLLVVVSCLTRDPFSSSLQNRAEVDKVKALLSHDSGSDHLAFVRAVAGWEEVLRWQDRSS
RENYLEENLLYAPSLRFIHGLIKQFSENIYEAFLVGKPSDCTLASAQCNEYSEEEELVKGVLMAGLYPNLIQVRQGKVTRQGKFKPN
SVTYRTKSGNILLHKSTINREATRLRSRWLTYFMAVKSNGSVFVRDSSQVHPLAVLLLTDGDVHIRDDGRRATISLSDSDLLRLEGD
SRTVRLLKELRRALGRMVERSLRSELAALPPSVQEEHGQLLALLAELLRGPCGSFDVRKTADD

>sp|Q9NZW4|DSPP_HUMAN
MKIITYFCIWAVAWAIPVPQSKPLERHVEKSMNLHLLARSNVSVQDELNASGTIKESGVLVHEGDRGRQENTQDGHKGEGNGSKWAE
VGGKSFSTYSTLANEEGNIEGWNGDTGKAETYGHDGIHGKEENITANGIQGQVSIIDNAGATNRSNTNGNTDKNTQNGDVGDAGHNE
DVAVVQEDGPQVAGSNNSTDNEDEIIENSCRNEGNTSEITPQINSKRNGTKEAEVTPGTGEDAGLDNSDGSPSGNGADEDEDEGSGD
DEDEEAGNGKDSSNNSKGQEGQDHGKEDDHDSSIGQNSDSKEYYDPEGKEDPHNEVDGDKTSKSEENSAGIPEDNGSQRIEDTQKLN
HRESKRVENRITKESETHAVGKSQDKGIEIKGPSSGNRNITKEVGKGNEGKEDKGQHGMILGKGNVKTQGEVVNIEGPGQKSEPGNK
VGHSNTGSDSNSDGYDSYDFDDKSMQGDDPNSSDESNGNDDANSESDNNSSSRGDASYNSDESKDNGNGSDSKGAEDDDSDSTSDTN
NSDSNGNGNNGNDDNDKSDSGKGKSDSSDSDSSDSSNSSDSSDSSDSDSSDSNSSSDSDSSDSDSSDSSDSDSSDSSNSSDSSDSSD
SSDSSDSSDSSDSKSDSSKSESDSSDSDSKSDSSDSNSSDSSDNSDSSDSSNSSNSSDSSDSSDSSDSSSSSDSSNSSDSSDSSDSS
NSSESSDSSDSSDSDSSDSSDSSNSNSSDSDSSNSSDSSDSSNSSDSSDSSDSSNSSDSSDSSDSSNSSDSSDSSDSSDSSDSSNSS
DSNDSSNSSDSSDSSNSSDSSNSSDSSDSSDSSDSDSSNSSDSSNSSDSSDSSNSSDSSDSSDSSDGSDSDSSNRSDSSNSSDSSDS
SDSSNSSDSSDSSDSNESSNSSDSSDSSNSSDSDSSDSSNSSDSSDSSNSSDSSESSNSSDNSNSSDSSNSSDSSDSSDSSNSSDSS
NSSDSSNSSDSSDSNSSDSSDSSNSSDSSDSSDSSDSSDSSDSSNSSDSSDSSDSSDSSNSSDSSNSSDSSNSSDSSDSSDSSDSSD
SSDSSDSSDSSNSSDSSDSSDSSDSSDSSDSSDSSDSSESSDSSDSSNSSDSSDSSDSSDSSDSSDSSDSSDSSDSSNSSDSSDSSD
SSDSSDSSNSSDSSDSSESSDSSDSSDSSDSSDSSDSSDSSDSSDSSNSSDSSDSSDSSDSSDSSDSSDSSDSSDSSDSSDSSDSSD
SSDSSDSSDSSDSNESSDSSDSSDSSDSSNSSDSSDSSDSSDSTSDSNDESDSQSKSGNGNNNGSDSDSDSEGSDSNHSTSDD
>sp|Q8NGP4|OR5M3_HUMAN
MLNFTDVTEFILLGLTSRREWQVLFFIIFLVVYIITMVGNIGMMVLIKVSPQLNNPMYFFLSHLSFVDVWFSSNVTPKMLENLLSDK
KTITYAGCLVQCFFFIALVHVEIFILAAMAFDRYMAIGNPLLYGSKMSRVVCIRLITFPYIYGFLTSLAATLWTYGLYFCGKIEINH
FYCADPPLIKMACAGTFVKEYTMIILAGINFTYSLTVIIISYLFILIAILRMRSAEGRQKAFSTCGSHLTAVIIFYGTLIFMYLRRP
TEESVEQGKMVAVFYTTVIPMLNPMIYSLRNKDVKKAMMKVISRSC
>sp|Q96DU9|PABP5_HUMAN
MGSGEPNPAGKKKKYLKAALYVGDLDPDVTEDMLYKKFRPAGPLRFTRICRDPVTRSPLGYGYVNFRFPADAEWALNTMNFDLINGK
PFRLMWSQPDDRLRKSGVGNIFIKNLDKSIDNRALFYLFSAFGNILSCKVVCDDNGSKGYAYVHFDSLAAANRAIWHMNGVRLNNRQ
VYVGRFKFPEERAAEVRTRDRATFTNVFVKNIGDDIDDEKLKELFCEYGPTESVKVIRDASGKSKGFGFVRYETHEAAQKAVLDLHG
KSIDGKVLYVGRAQKKIERLAELRRRFERLRLKEKSRPPGVPIYIKNLDETINDEKLKEEFSSFGSISRAKVMMEVGQGKGFGVVCF
SSFEEATKAVDEMNGRIVGSKPLHVTLGQARRRC
>sp|Q9UI56|YYY4_HUMAN
MQSRDTVNVKFTPRSFWGSRPKHTGGRSEMRAHVPQAPWARQAPPVLPLWTVVNDHPHEKPVSRPQNT
>sp|P53611|PGTB2_HUMAN
MGTPQKDVIIKSDAPDTLLLEKHADYIASYGSKKDDYEYCMSEYLRMSGIYWGLTVMDLMGQLHRMNREEILAFIKSCQHECGGISA
SIGHDPHLLYTLSAVQILTLYDSINVIDVNKVVEYVKGLQKEDGSFAGDIWGEIDTRFSFCAVATLALLGKLDAINVEKAIEFVLSC
MNFDGGFGCRPGSESHAGQIYCCTGFLAITSQLHQVNSDLLGWWLCERQLPSGGLNGRPEKLPDVCYSWWVLASLKIIGRLHWIDRE
KLRNFILACQDEETGGFADRPGDMVDPFHTLFGIAGLSLLGEEQIKPVNPVFCMPEEVLQRVNVQPELVS
>sp|Q53RE8|ANR39_HUMAN
MATPRPCADGPCCSHPSAVLGVQQTLEEMDFERGIWSAALNGDLGRVKHLIQKAEDPSQPDSAGYTALHYASRNGHYAVCQFLLESG
AKCDAQTHGGATALHRASYCGHTEIARLLLSHGSNPRVVDDDGMTSLHKAAERGHGDICSLLLQHSPALKAIRDRKARLACDLLPCN
SDLRDLLSS
>sp|P30520|PURA2_HUMAN
MAFAETYPAASSLPNGDCGRPRARPGGNRVTVVLGAQWGDEGKGKVVDLLAQDADIVCRCQGGNNAGHTVVVDSVEYDFHLLPSGII
NPNVTAFIGNGVVIHLPGLFEEAEKNVQKGKGLEGWEKRLIISDRAHIVFDFHQAADGIQEQQRQEQAGKNLGTTKKGIGPVYSSKA
ARSGLRMCDLVSDFDGFSERFKVLANQYKSIYPTLEIDIEGELQKLKGYMEKIKPMVRDGVYFLYEALHGPPKKILVEGANAALLDI
DFGTYPFVTSSNCTVGGVCTGLGMPPQNVGEVYGVVKAYTTRVGIGAFPTEQDNEIGELLQTRGREFGVTTGRKRRCGWLDLVLLKY

AHMINGFTALALTKLDILDMFTEIKVGVAYKLDGEIIPHIPANQEVLNKVEVQYKTLPGWNTDISNARAFKELPVNAQNYVRFIEDE
LQIPVKWIGVGKSRESMIQLF
>sp|Q9NZI6|TF2L1_HUMAN
MLFWHTQPEHYNQHNSGSYLRDVLALPIFKQEEPQLSPENEARLPPLQYVLCAATSPAVKLHEETLTYLNQGQSYEIRLLENRKLGD
FQDLNTKYVKSIIRVVFHDRRLQYTEHQQLEGWRWSRPGDRILDIDIPLSVGILDPRASPTQLNAVEFLWDPAKRASAFIQVHCIST
EFTPRKHGGEKGVPFRVQIDTFKQNENGEYTEHLHSASCQIKVFKPKGADRKQKTDREKMEKRTAQEKEKYQPSYETTILTECSPWP
DVAYQVNSAPSPSYNGSPNSFGLGEGNASPTHPVEALPVGSDHLLPSASIQDAQQWLHRNRFSQFCRLFASFSGADLLKMSRDDLVQ
ICGPADGIRLFNAIKGRNVRPKMTIYVCQELEQNRVPLQQKRDGSGDSNLSVYHAIFLEELTTLELIEKIANLYSISPQHIHRVYRQ
GPTGIHVVVSNEMVQNFQDESCFVLSTIKAESNDGYHIILKCGL
>sp|Q9Y5F8|PCDGJ_HUMAN
MGGSCAQRRRAGPRQVLFPLLLPLFYPTLCEPIRYSIPEELAKGSVVGNLAKDLGLSVLDVSARELRVSAEKLHFSVDAQSGDLLVK
DRIDREQICKERRRCELQLEAVVENPLNIFHVIVVIEDVNDHAPQFRKDEINLEISESVSLGMGTILESAEDPDISMNSLSKYQLSP
NEYFSLVEKDNPDGGKYPELVLQKTLDRETQSAHHLVLTALDGGDPPRSGTAQIRILVIDANDNPPVFSQDVYRVSLREDVPPGTSI
LRVKATDQDEGINSEITYSFFGVADKAQHVFSLDYTTGNILTQQPLDFEEVERYTINIEAKDRGSLSTRCKVIVEVVDENDNSPEII
ITSLSDQIMEDSPPGVVVALFKTRDQDSGENGEVRCSLSRGVPFKIHSSSNNYYKLVTDEALDREQTPEYNVTIAATDRGKPPLSSS
KTITLHITDVNDNAPVFGQSAYLVHVPENNQPGASIAQVSASDPDFGLNGRVSYSLIASDLESRTLSSYVSVSAQSGVVFAQRAFDH
EQLRTFELTLQARDQGSPALSANVSLRVLVGDRNDNAPRVLYPALGPDGSALFDTVPRAAQPGYLVTKVVAVDADSGHNAWLSYHVV
QASEPGLFSLGLRTGEVRMVRALGDKDSVRQRLLVAVRDGGQPPLSATATLHLVFADSLQEVLPDFSDHPTPSDSQAEMQFYLVVAL
ALISVLFLLAVILAIALRLRQSFSPTAGDCFESVLCSKSGPVGPPNYSEGTLPYAYNFCVPGDQMNPEFNFFTSVDHCPATQDNLNK
DSMLLASILTPSVEADKKILKQQAPPNTDWRFSQAQRPGTSGSQNGDDTGTWPNNQFDTEMLQAMILASASEAADGSSTLGGGAGTM
GLSARYGPQFTLQHVPDYRQNVYIPGSNATLTNAAGKRDGKAPAGGNGNKKKSGKKEKK
>sp|P39060|COIA1_HUMAN
MAPYPCGCHILLLLLFCCLAAARANLLNLNWLWFNNEDTSHAATTIPEPQGPLPVQPTADTTTHVTPRNGSTEPATAPGSPEPPSELL
EDGQDTPTSAESPDAPEENIAGVGAEILNVAKGIRSFVQLWNDTVPTESLARAETLVLETPVGPLALAGPSSTPQENGTTLWPSRGI
PSSPGAHTTEAGTLPAPTPSPPSLGRPWAPLTGPSVPPPSSGRASLSSLLGGAPPWGSLQDPDSQGLSPAAAAPSQQLQRPDVRLRT
PLLHPLVMGSLGKHAAPSAFSSGLPGALSQVAVTTLTRDSGAWVSHVANSVGPGLANNSALLGADPEAPAGRCLPLPPSLPVCGHLG
ISRFWLPNHLHHESGEQVRAGARAWGGLLQTHCHPFLAWFFCLLLVPPCGSVPPPAPPPCCQFCEALQDACWSRLGGGRLPVACASL
PTQEDGYCVLIGPAAERISEEVGLLQLLGDPPPQQVTQTDDPDVGLAYVFGPDANSGQVARYHFPSLFFRDFSLLFHIRPATEGPGV
LFAITDSAQAMVLLGVKLSGVQDGHQDISLLYTEPGAGQTHTAASFRLPAFVGQWTHLALSVAGGFVALYVDCEEFQRMPLARSSRG
LELEPGAGLFVAQAGGADPDKFQGVIAELKVRRDPQVSPMHCLDEEGDDSDGASGDSGSGLGDARELLREETGAALKPRLPAPPPVT
TPPLAGGSSTEDSRSEEVEEQTTVASLGAQTLPGSDSVSTWDGSVRTPGGRVKEGGLKGQKGEPGVPGPPGRAGPPGSPCLPGPPGL
PCPVSPLGPAGPALQTVPGPQGPPGPPGRDGTPGRDGEPGDPGEDGKPGDTGPQGFPGTPGDVGPKGDKGDPGVGERGPPGPQGPPG
PPGPSFRHDKLTFIDMEGSGFGGDLEALRGPRGFPGPPGPPGVPGLPGEPGRFGVNSSDVPGPAGLPGVPGREGPPGFPGLPGPPGP
PGREGPPGRTGQKGSLGEAGAPGHKGSKGAPGPAGARGESGLAGAPGPAGPPGPPGPPGPPGPGLPAGFDDMEGSGGPFWSTARSAD
GPQGPPGLPGLKGDPGVPGLPGAKGEVGADGVPGFPGLPGREGIAGPQGPKGDRGSRGEKGDPGKDGVGQPGLPGPPGPPGPVVYVS
EQDGSVLSVPGPEGRPGFAGFPGPAGPKGNLGSKGERGSPGPKGEKGEPGSIFSPDGGALGPAQKGAKGEPGFRGPPGPYGRPGYKG
EIGFPGRPGRPGMNGLKGEKGEPGDASLGFGMRGMPGPPGPPGPPGPPGTPVYDSNVFAESSRPGPPGLPGNQGPPGPKGAKGEVGP
PGPPGQFPFDFLQLEAEMKGEKGDRGDAGQKGERGEPGGGGFFGSSLPGPPGPPGPPGPRGYPGIPGPKGESIRGQPGPPGPQGPPG
IGYEGRQGPPGPPGPPGPPSFPGPHRQTISVPGPPGPPGPPGPPGTMGASSGVRLWATRQAMLGQVHEVPEGWLIFVAEQEELYVRV
QNGFRKVQLEARTPLPRGTDNEVAALQPPVVQLHDSNPYPRREHPHPTARPWRADDILASPPRLPEPQPYPGAPHHSSYVHLRPARP
TSPPAHSHRDFQPVLHLVALNSPLSGGMRGIRGADFQCFQQARAVGLAGTFRAFLSSRLQDLYSIVRRADRAAVPIVNLKDELLFPS
WEALFSGSEGPLKPGARIFSFDGKDVLRHPTWPQKSVWHGSDPNGRRLTESYCETWRTEAPSATGQASSLLGGRLLGQSAASCHHAY
IVLCIENSFMTASK
>sp|O60353|FZD6_HUMAN

MEMFTFLLTCIFLPLLRGHSLFTCEPITVPRCMKMAYNMTFFPNLMGHYDQSIAAVEMEHFLPLANLECSPNIETFLCKAFVPTCIE
QIHVVPPCRKLCEKVYSDCKKLIDTFGIRWPEELECDRLQYCDETVPVTFDPHTEFLGPQKKTEQVQRDIGFWCPRHLKTSGGQGYK
FLGIDQCAPPCPNMYFKSDELEFAKSFIGTVSIFCLCATLFTFLTFLIDVRRFRYPERPIIYYSVCYSIVSLMYFIGFLLGDSTACN
KADEKLELGDTVVLGSQNKACTVLFMLLYFFTMAGTVWWVILTITWFLAAGRKWSCEAIEQKAVWFHAVAWGTPGFLTVMLLAMNKV
EGDNISGVCFVGLYDLDASRYFVLLPLCLCVFVGLSLLLAGIISLNHVRQVIQHDGRNQEKLKKFMIRIGVFSGLYLVPLVTLLGCY
VYEQVNRITWEITWVSDHCRQYHIPCPYQAKAKARPELALFMIKYLMTLIVGISAVFWVGSKKTCTEWAGFFKRNRKRDPISESRRV
LQESCEFFLKHNSKVKHKKKHYKPSSHKLKVISKSMGTSTGATANHGTSAVAITSHDYLGQETLTEIQTSPETSMREVKADGASTPR
LREQDCGEPASPAASISRLSGEQVDGKGQAGSVSESARSEGRISPKSDITDTGLAQSNNLQVPSSSEPSSLKGSTSLLVHPVSGVRK
EQGGGCHSDT
>sp|Q9P2M4|TBC14_HUMAN
MTDGKLSTSTNGVAFMGILDGRPGNPLQNLQHVNLKAPRLLSAPEYGPKLKLRALEDRHSLQSVDSGIPTLEIGNPEPVPCSAVHVR
RKQSDSDLIPERAFQSACALPSCAPPAPSSTEREQSVRKSSTFPRTGYDSVKLYSPTSKALTRSDDVSVCSVSSLGTELSTTLSVSN
EDILDLVVTSSSSAIVTLENDDDPQFTNVTLSSIKETRGLHQQDCVHEAEEGSKLKILGPFSNFFARNLLARKQSARLDKHNDLGWK
LFGKAPLRENAQKDSKRIQKEYEDKAGRPSKPPSPKQNVRKNLDFEPLSTTALILEDRPANLPAKPAEEAQKHRQQYEEMVVQAKKR
ELKEAQRRKKQLEERCRVEESIGNAVLTWNNEILPNWETMWCSRKVRDLWWQGIPPSVRGKVWSLAIGNELNITHELFDICLARAKE
RWRSLSTGGSEVENEDAGFSAADREASLELIKLDISRTFPNLCIFQQGGPYHDMLHSILGAYTCYRPDVGYVQGMSFIAAVLILNLD
TADAFIAFSNLLNKPCQMAFFRVDHGLMLTYFAAFEVFFEENLPKLFAHFKKNNLTPDIYLIDWIFTLYSKSLPLDLACRIWDVFCR
DGEEFLFRTALGILKLFEDILTKMDFIHMAQFLTRLPEDLPAEELFASIATIQMQSRNKKWAQVLTALQKDSREMEKGSPSLRH
>sp|P57105|SYJ2B_HUMAN
MNGRVDYLVTEEEINLTRGPSGLGFNIVGGTDQQYVSNDSGIYVSRIKENGAAALDGRLQEGDKILSVNGQDLKNLLHQDAVDLFRN
AGYAVSLRVQHRLQVQNGPIGHRGEGDPSGIPIFMVLVPVFALTMVAAWAFMRYRQQL
>sp|Q8N8Y5|ZFP41_HUMAN
MEKPAGRKKKTPTPREEADVQKSALREEKVSGDRKPPERPTVPRKPRTEPCLSPEDEEHVFDAFDASFKDDFEGVPVFIPFQRKKPY
ECSECGRIFKHKTDHIRHQRVHTGEKPFKCAQCGKAFRHSSDVTKHQRTHTGEKPFKCGECGKAFNCGSNLLKHQKTHTGEKPYECT
HCGKAFAYSSCLIRHQKRHPRKKP
>sp|P23921|RIR1_HUMAN
MHVIKRDGRQERVMFDKITSRIQKLCYGLNMDFVDPAQITMKVIQGLYSGVTTVELDTLAAETAATLTTKHPDYAILAARIAVSNLH
KETKKVFSDVMEDLYNYINPHNGKHSPMVAKSTLDIVLANKDRLNSAIIYDRDFSYNYFGFKTLERSYLLKINGKVAERPQHMLMRV
SVGIHKEDIDAAIETYNLLSERWFTHASPTLFNAGTNRPQLSSCFLLSMKDDSIEGIYDTLKQCALISKSAGGIGVAVSCIRATGSY
IAGTNGNSNGLVPMLRVYNNTARYVDQGGNKRPGAFAIYLEPWHLDIFEFLDLKKNTGKEEQRARDLFFALWIPDLFMKRVETNQDW
SLMCPNECPGLDEVWGEEFEKLYASYEKQGRVRKVVKAQQLWYAIIESQTETGTPYMLYKDSCNRKSNQQNLGTIKCSNLCTEIVEY
TSKDEVAVCNLASLALNMYVTSEHTYDFKKLAEVTKVVVRNLNKIIDINYYPVPEACLSNKRHRPIGIGVQGLADAFILMRYPFESA
EAQLLNKQIFETIYYGALEASCDLAKEQGPYETYEGSPVSKGILQYDMWNVTPTDLWDWKVLKEKIAKYGIRNSLLIAPMPTASTAQ
ILGNNESIEPYTSNIYTRRVLSGEFQIVNPHLLKDLTERGLWHEEMKNQIIACNGSIQSIPEIPDDLKQLYKTVWEISQKTVLKMAA
ERGAFIDQSQSLNIHIAEPNYGKLTSMHFYGWKQGLKTGMYYLRTRPAANPIQFTLNKEKLKDKEKVSKEEEEKERNTAAMVCSLEN
RDECLMCGS
>sp|P32248|CCR7_HUMAN
MDLGKPMKSVLVVALLVIFQVCLCQDEVTDDYIGDNTTVDYTLFESLCSKKDVRNFKAWFLPIMYSIICFVGLLGNGLVVLTYIYFK
RLKTMTDTYLLNLAVADILFLLTLPFWAYSAAKSWVFGVHFCKLIFAIYKMSFFSGMLLLLCISIDRYVAIVQAVSAHRHRARVLLI
SKLSCVGIWILATVLSIPELLYSDLQRSSSEQAMRCSLITEHVEAFITIQVAQMVIGFLVPLLAMSFCYLVIIRTLLQARNFERNKA
IKVIIAVVVVFIVFQLPYNGVVLAQTVANFNITSSTCELSKQLNIAYDVTYSLACVRCCVNPFLYAFIGVKFRNDLFKLFKDLGCLS
QEQLRQWSSCRHIRRSSMSVEAETTTTFSP
>sp|Q8N414|PGBD5_HUMAN

MPDYRTRALFVQSTLGDSAGPELQLLSIVPGRDLQPSDSFTGPTRKMPPSASAVDFFQLFVPDNVLKNMVVQTNMYAKKFQERFGSD
GAWVEVTLTEMKAFLGYMISTSISHCESVLSIWSGGFYSNRSLALVMSQARFEKILKYFHVVAFRSSQTTHGLYKVQPFLDSLQNSF
DSAFRPSQTQVLHEPLIDEDPVFIATCTERELRKRKKRKFSLWVRQCSSTGFIIQIYVHLKEGGGPDGLDALKNKPQLHSMVARSLC
RNAAGKNYIIFTGPSITSLTLFEEFEKQGIYCCGLLRARKSDCTGLPLSMLTNPATPPARGQYQIKMKGNMSLICWYNKGHFRFLTN
AYSPVQQGVIIKRKSGEIPCPLAVEAFAAHLSYICRYDDKYSKYFISHKPNKTWQQVFWFAISIAINNAYILYKMSDAYHVKRYSRA
QFGERLVRELLGLEDASPTH
>sp|O75366|AVIL_HUMAN
MPLTSAFRAVDNDPGIIVWRIEKMELALVPVSAHGNFYEGDCYVILSTRRVASLLSQDIHFWIGKDSSQDEQSCAAIYTTQLDDYLG
GSPVQHREVQYHESDTFRGYFKQGIIYKQGGVASGMKHVETNTYDVKRLLHVKGKRNIRATEVEMSWDSFNRGDVFLLDLGKVIIQW
NGPESNSGERLKAMLLAKDIRDRERGGRAKIGVIEGDKEAASPELMKVLQDTLGRRSIIKPTVPDEIIDQKQKSTIMLYHISDSAGQ
LAVTEVATRPLVQDLLNHDDCYILDQSGTKIYVWKGKGATKAEKQAAMSKALGFIKMKSYPSSTNVETVNDGAESAMFKQLFQKWSV
KDQTMGLGKTFSIGKIAKVFQDKFDVTLLHTKPEVAAQERMVDDGNGKVEVWRIENLELVPVEYQWYGFFYGGDCYLVLYTYEVNGK
PHHILYIWQGRHASQDELAASAYQAVEVDRQFDGAAVQVRVRMGTEPRHFMAIFKGKLVIFEGGTSRKGNAEPDPPVRLFQIHGNDK
SNTKAVEVPAFASSLNSNDVFLLRTQAEHYLWYGKGSSGDERAMAKELASLLCDGSENTVAEGQEPAEFWDLLGGKTPYANDKRLQQ
EILDVQSRLFECSNKTGQFVVTEITDFTQDDLNPTDVMLLDTWDQVFLWIGAEANATEKESALATAQQYLHTHPSGRDPDTPILIIK
QGFEPPIFTGWFLAWDPNIWSAGKTYEQLKEELGDAAAIMRITADMKNATLSLNSNDSEPKYYPIAVLLKNQNQELPEDVNPAKKEN
YLSEQDFVSVFGITRGQFAALPGWKQLQMKKEKGLF
>sp|P11441|UBL4A_HUMAN
MQLTVKALQGRECSLQVPEDELVSTLKQLVSEKLNVPVRQQRLLFKGKALADGKRLSDYSIGPNSKLNLVVKPLEKVLLEEGEAQRL
ADSPPPQVWQLISKVLARHFSAADASRVLEQLQRDYERSLSRLTLDDIERLASRFLHPEVTETMEKGFSK
>sp|Q7Z449|CP2U1_HUMAN
MSSPGPSQPPAEDPPWPARLLRAPLGLLRLDPSGGALLLCGLVALLGWSWLRRRRARGIPPGPTPWPLVGNFGHVLLPPFLRRRSWL
SSRTRAAGIDPSVIGPQVLLAHLARVYGSIFSFFIGHYLVVVLSDFHSVREALVQQAEVFSDRPRVPLISIVTKEKGVVFAHYGPVW
RQQRKFSHSTLRHFGLGKLSLEPKIIEEFKYVKAEMQKHGEDPFCPFSIISNAVSNIICSLCFGQRFDYTNSEFKKMLGFMSRGLEI
CLNSQVLLVNICPWLYYLPFGPFKELRQIEKDITSFLKKIIKDHQESLDRENPQDFIDMYLLHMEEERKNNSNSSFDEEYLFYIIGD
LFIAGTDTTTNSLLWCLLYMSLNPDVQEKVHEEIERVIGANRAPSLTDKAQMPYTEATIMEVQRLTVVVPLAIPHMTSENTVLQGYT
IPKGTLILPNLWSVHRDPAIWEKPEDFYPNRFLDDQGQLIKKETFIPFGIGKRVCMGEQLAKMELFLMFVSLMQSFAFALPEDSKKP
LLTGRFGLTLAPHPFNITISRR
>sp|Q92783|STAM1_HUMAN
MPLFATNPFDQDVEKATSEMNTAEDWGLILDICDKVGQSRTGPKDCLRSIMRRVNHKDPHVAMQALTLLGACVSNCGKIFHLEVCSR
DFASEVSNVLNKGHPKVCEKLKALMVEWTDEFKNDPQLSLISAMIKNLKEQGVTFPAIGSQAAEQAKASPALVAKDPGTVANKKEEE
DLAKAIELSLKEQRQQSTTLSTLYPSTSSLLTNHQHEGRKVRAIYDFEAAEDNELTFKAGEIITVLDDSDPNWWKGETHQGIGLFPS
NFVTADLTAEPEMIKTEKKTVQFSDDVQVETIEPEPEPAFIDEDKMDQLLQMLQSTDPSDDQPDLPELLHLEAMCHQMGPLIDEKLE
DIDRKHSELSELNVKVMEALSLYTKLMNEDPMYSMYAKLQNQPYYMQSSGVSGSQVYAGPPPSGAYLVAGNAQMSHLQSYSLPPEQL
SSLSQAVVPPSANPALPSQQTQAAYPNTMVSSVQGNTYPSQAPVYSPPPAATAAAATADVTLYQNAGPNMPQVPNYNLTSSTLPQPG
GSQQPPQPQQPYSQKALL
>sp|Q6PIW4|FIGL1_HUMAN
MQTSSSRSVHLSEWQKNYFAITSGICTGPKADAYRAQILRIQYAWANSEISQVCATKLFKKYAEKYSAIIDSDNVESGLNNYAENIL
TLAGSQQTDSDKWQSGLSINNVFKMSSVQKMMQAGKKFKDSLLEPALASVVIHKEATVFDLPKFSVCGSSQESDSLPNSAHDRDRTQ
DFPESNRLKLLQNAQPPMVTNTARTCPTFSAPVGESATAKFHVTPLFGNVKKENHSSAKENIGLNVFLSNQSCFPAACENPQRKSFY
GSGTIDALSNPILNKACSKTEDNGPKEDSSLPTFKTAKEQLWVDQQKKYHQPQRASGSSYGGVKKSLGASRSRGILGKFVPPIPKQD
GGEQNGGMQCKPYGAGPTEPAHPVDERLKNLEPKMIELIMNEIMDHGPPVNWEDIAGVEFAKATIKEIVVWPMLRPDIFTGLRGPPK
GILLFGPPGTGKTLIGKCIASQSGATFFSISASSLTSKWVGEGEKMVRALFAVARCQQPAVIFIDEIDSLLSQRGDGEHESSRRIKT

EFLVQLDGATTSSEDRILVVGATNRPQEIDEAARRRLVKRLYIPLPEASARKQIVINLMSKEQCCLSEEEIEQIVQQSDAFSGADMT
QLCREASLGPIRSLQTADIATITPDQVRPIAYIDFENAFRTVRPSVSPKDLELYENWNKTFGCGK
>sp|O60676|CST8_HUMAN
MPRCRWLSLILLTIPLALVARKDPKKNETGVLRKLKPVNASNANVKQCLWFAMQEYNKESEDKYVFLVVKTLQAQLQVTNLLEYLID
VEIARSDCRKPLSTNEICAIQENSKLKRKLSCSFLVGALPWNGEFTVMEKKCEDA
>sp|Q9BSJ1|TRI51_HUMAN
MNSGILQVFQRALTCPICMNYFLDPVTIDCGHSFCRPCLYLNWQDTAVLAQCSECKKTTRQRNLNTDICLKNMAFIARKASLRQFLS
SEEQICGMHRETKKMFCEVDKSLLCLPCSNSQEHRNHIHCPIEWAAEERREELLKKMQSLWEKACENLRNLNMETTRTRCWKDYVSL
RIEAIRAEYQKMPAFLHEEEQHHLERLRKEGEDIFQQLNESKARMEHSRELLRGMYEDLKQMCHKADVELLQAFGDILHRYESLLLQ
VSEPVNPELSAGPITGLLDSLSGFRVDFTLQPERANSHIFLCGDLRSMNVGCDPQDDPDITGKSECFLVWGAQAFTSGKYYWEVHMG
DSWNWAFGVCNNYWKEKRQNDKIDGEEGLFLLGCVKEDTHCSLFTTSPLVVQYVPRPTSTVGLFLDCEGRTVSFVDVDQSSLIYTIP
NCSFSPPLRPIFCCSHF
>sp|O43566|RGS14_HUMAN
MPGKPKHLGVPNGRMVLAVSDGELSSTTGPQGQGEGRGSSLSIHSLPSGPSSPFPTEEQPVASWALSFERLLQDPLGLAYFTEFLKK
EFSAENVTFWKACERFQQIPASDTQQLAQEARNIYQEFLSSQALSPVNIDRQAWLGEEVLAEPRPDMFRAQQLQIFNLMKFDSYARF
VKSPLYRECLLAEAEGRPLREPGSSRLGSPDATRKKPKLKPGKSLPLGVEELGQLPPVEGPGGRPLRKSFRRELGGTANAALRRESQ
GSLNSSASLDLGFLAFVSSKSESHRKSLGSTEGESESRPGKYCCVYLPDGTASLALARPGLTIRDMLAGICEKRGLSLPDIKVYLVG
NEQALVLDQDCTVLADQEVRLENRITFELELTALERVVRISAKPTKRLQEALQPILEKHGLSPLEVVLHRPGEKQPLDLGKLVSSVA
AQRLVLDTLPGVKISKARDKSPCRSQGCPPRTQDKATHPPPASPSSLVKVPSSATGKRQTCDIEGLVELLNRVQSSGAHDQRGLLRK
EDLVLPEFLQLPAQGPSSEETPPQTKSAAQPIGGSLNSTTDSAL
>sp|Q8WXK4|ASB12_HUMAN
MNLMDITKIFSLLQPDKEEEDTDTEEKQALNQAVYDNDSYTLDQLLRQERYKRFINSRSGWGVPGTPLRLAASYGHLSCLQVLLAHG
ADVDSLDVKAQTPLFTAVSHGHLDCVRVLLEAGASPGGSIYNNCSPVLTAARDGAVAILQELLDHGAEANVKAKLPVWASNIASCSG
PLYLAAVYGHLDCFRLLLLHGADPDYNCTDQGLLARVPRPRTLLEICLHHNCEPEYIQLLIDFGANIYLPSLSLDLTSQDDKGIALL
LQARATPRSLLSQVRLVVRRALCQAGQPQAINQLDIPPMLISYLKHQL
>sp|Q86YV0|RASL3_HUMAN
MDPPSPSRTSQTQPTATSPLTSYRWHTGGGGEKAAGGFRWGRFAGWGRALSHQEPMVSTQPAPRSIFRRVLSAPPKESRTSRLRLSK
ALWGRHKNPPPEPDPEPEQEAPELEPEPELEPPTPQIPEAPTPNVPVWDIGGFTLLDGKLVLLGGEEEGPRRPRVGSASSEGSIHVA
MGNFRDPDRMPGKTEPETAGPNQVHNVRGLLKRLKEKKKARLEPRDGPPSALGSRESLATLSELDLGAERDVRIWPLHPSLLGEPHC
FQVTWTGGSRCFSCRSAAERDRWIEDLRRQFQPTQDNVEREETWLSVWVHEAKGLPRAAAGAPGVRAELWLDGALLARTAPRAGPGQ
LFWAERFHFEALPPARRLSLRLRGLGPGSAVLGRVALALEELDAPRAPAAGLERWFPLLGAPAGAALRARIRARRLRVLPSERYKEL
AEFLTFHYARLCGALEPALPAQAKEELAAAMVRVLRATGRAQALVTDLGTAELARCGGREALLFRENTLATKAIDEYMKLVAQDYLQ
ETLGQVVRRLCASTEDCEVDPSKCPASELPEHQARLRNSCEEVFETIIHSYDWFPAELGIVFSSWREACKERGSEVLGPRLVCASLF
LRLLCPAILAPSLFGLAPDHPAPGPARTLTLIAKVIQNLANRAPFGEKEAYMGFMNSFLEEHGPAMQCFLDQVAMVDVDAAPSGYQG
SGDLALQLAVLHAQLCTIFAELDQTTRDTLEPLPTILRAIEEGQPVLVSVPMRLPLPPAQVHSSLSAGEKPGFLAPRDLPKHTPLIS
KSQSLRSVRRSESWARPRPDEERPLRRPRPVQRTQSVPVRRPARRRQSAGPWPRPKGSLSMGPAPRARPWTRDSASLPRKPSVPWQR
QMDQPQDRNQALGTHRPVNKLAELQCEVAALREEQKVLSRLVESLSTQIRALTEQQEQLRGQLQDLDSRLRAGSSEFDSEHNLTSNE
GHSLKNLEHRLNEMERTQAQLRDAVQSLQLSPRTRGSWSQPQPLKAPCLNGDTT
>sp|Q6N043|Z280D_HUMAN
MGDNPFQPKSNSKMAELFMECEEEELEPWQKKVKEVEDDDDDEPIFVGEISSSKPAISNILNRVNPSSYSRGLKNGALSRGITAAFK
PTSQHYTNPTSNPVPASPINFHPESRSSDSSVIVQPFSKPGYITNSSRVVSNKSSELLFDLTQDTGLSHYQGGPTLSMAGMSESSFL
SKRPSTSEVNNVNPKKPKPSESVSGANSSAVLPSVKSPSVTSSQAMLAKGTNTSSNQSKNGTPFPRACPKCNIHFNLLDPLKNHMKY
CCPDMINNFLGLAKTEFSSTVNKNTTIDSEKGKLIMLVNDFYYGKHEGDVQEEQKTHTTFKCFSCLKILKNNIRFMNHMKHHLELEK
QSSESWENHTTCQHCYRQFPTPFQLQCHIESTHTPHEFSTICKICELSFETEHVLLQHMKDNHKPGEMPYVCQVCNYRSSSFSDVET

HFRTSHENTKNLLCPFCLKVIKIATPYMHHYMKHQKKGIHRCTKCRLQFLTCKEKMDHKTQHHRTFIKPKQLEGLPPGTKVTIRASV
GPLQSGASPTPSISASASTLQLSPPRTKNITAKNPAKSNTSKPNTVKSNASKPNTSKPNGSKSKYKPKISNMQKKQSTLASSNKKSK
VNTALRNLRYRRGIHKCIECCSEIKDFANHFPTYVHCSFCRYNTSCSKAYVNHMMSFHSNRPSKRFCIFKKHSENLRGITLVCLNCD
FLSDVSGLDNMATHLSQHKTHTCQVVMQKVSVCIPTSEHLSELKKEAPAKEQEPVSKEIARPNMAERETETSNSESKQDKAASSKEK
NGCNANSFEGSSTTKSEESITVSDKENETCLADQETGSKNIVSCDSNIGADKVEKKKQIQHVCQEMELKMCQSSENIILSDQIKDHN
SSEARFSSKNIKDLRLASDNVSIDQFLRKRHEPESVSSDVSEQGSIHLEPLTPSEVLEYEATEILQKGSGDPSAKTDEVVSDQTDDI
PGGNNPSTTEATVDLEDEKERS
>sp|O95025|SEM3D_HUMAN
MNANKDERLKARSQDFHLFPALMMLSMTMLFLPVTGTLKQNIPRLKLTYKDLLLSNSCIPFLGSSEGLDFQTLLLDEERGRLLLGAK
DHIFLLSLVDLNKNFKKIYWPAAKERVELCKLAGKDANTECANFIRVLQPYNKTHIYVCGTGAFHPICGYIDLGVYKEDIIFKLDTH
NLESGRLKCPFDPQQPFASVMTDEYLYSGTASDFLGKDTAFTRSLGPTHDHHYIRTDISEHYWLNGAKFIGTFFIPDTYNPDDDKIY
FFFRESSQEGSTSDKTILSRVGRVCKNDVGGQRSLINKWTTFLKARLICSIPGSDGADTYFDELQDIYLLPTRDERNPVVYGVFTTT
SSIFKGSAVCVYSMADIRAVFNGPYAHKESADHRWVQYDGRIPYPRPGTCPSKTYDPLIKSTRDFPDDVISFIKRHSVMYKSVYPVA
GGPTFKRINVDYRLTQIVVDHVIAEDGQYDVMFLGTDIGTVLKVVSISKEKWNMEEVVLEELQIFKHSSIILNMELSLKQQQLYIGS
RDGLVQLSLHRCDTYGKACADCCLARDPYCAWDGNACSRYAPTSKRRARRQDVKYGDPITQCWDIEDSISHETADEKVIFGIEFNST
FLECIPKSQQATIKWYIQRSGDEHREELKPDERIIKTEYGLLIRSLQKKDSGMYYCKAQEHTFIHTIVKLTLNVIENEQMENTQRAE
HEEGKVKDLLAESRLRYKDYIQILSSPNFSLDQYCEQMWHREKRRQRNKGGPKWKHMQEMKKKRNRRHHRDLDELPRAVAT
>sp|Q8NA75|DC4L2_HUMAN
MESKRPRLLEEADKQKKTVRVGLNAPSMLRKNQLGFLRFANYCRIARELRVSCMQRKKVQIHSWDPSSLASDRFNRILANTNTDQLF
TVNQVEAGGSKYGIITMRGLTTPELRVYPHKTLYVPNRKVNSMCWASLNHLDSHLLLCFVGLADTPSCAVLLPASLFIGSFPGMRRP
GMLCSFQIPDAWSCAWSLSIHAYHSFSTGLSQQVLLTNVVTGHQQSFGTSSDVLAQQFAIMTPLLFNGCRSGEIFGIDLRCGNQGSG
WKAICLSHDSAVTSLQILQDGQFLVSSDMTGTIKLWDLRATKCVTQYEGHVNNSAYLPVHVNEEEGVVAAVGQDCYTRIWSLRHGHL
LTTIPSPYPASENDIPSVAFSSRLGGFRGAPGLLMAVREDLYCFSYG
>sp|Q86SM5|MRGRG_HUMAN
MFGLFGLWRTFDSVVFYLTLIVGLGGPVGNGLVLWNLGFRIKKGPFSIYLLHLAAADFLFLSCRVGFSVAQAALGAQDTLYFVLTFL
WFAVGLWLLAAFSVERCLSDLFPACYQGCRPRHASAVLCALVWTPTLPAVPLPANACGLLRNSACPLVCPRYHVASVTWFLVLARVA
WTAGVVLFVWVTCCSTRPRPRLYGIVLGALLLLFFCGLPSVFYWSLQPLLNFLLPVFSPLATLLACVNSSSKPLIYSGLGRQPGKRE
PLRSVLRRALGEGAELGARGQSLPMGLL
>sp|O43172|PRP4_HUMAN
MASSRASSTQATKTKAPDDLVAPVVKKPHIYYGSLEEKERERLAKGESGILGKDGLKAGIEAGNINITSGEVFEIEEHISERQAEVL
AEFERRKRARQINVSTDDSEVKACLRALGEPITLFGEGPAERRERLRNILSVVGTDALKKTKKDDEKSKKSKEEYQQTWYHEGPNSL
KVARLWIANYSLPRAMKRLEEARLHKEIPETTRTSQMQELHKSLRSLNNFCSQIGDDRPISYCHFSPNSKMLATACWSGLCKLWSVP
DCNLLHTLRGHNTNVGAIVFHPKSTVSLDPKDVNLASCAADGSVKLWSLDSDEPVADIEGHTVRVARVMWHPSGRFLGTTCYDRSWR
LWDLEAQEEILHQEGHSMGVYDIAFHQDGSLAGTGGLDAFGRVWDLRTGRCIMFLEGHLKEIYGINFSPNGYHIATGSGDNTCKVWD
LRQRRCVYTIPAHQNLVTGVKFEPIHGNFLLTGAYDNTAKIWTHPGWSPLKTLAGHEGKVMGLDISSDGQLIATCSYDRTFKLWMAE
>sp|P05060|SCG1_HUMAN
MQPTLLLSLLGAVGLAAVNSMPVDNRNHNEGMVTRCIIEVLSNALSKSSAPPITPECRQVLKTSRKDVKDKETTENENTKFEVRLLR
DPADASEAHESSSRGEAGAPGEEDIQGPTKADTEKWAEGGGHSRERADEPQWSLYPSDSQVSEEVKTRHSEKSQREDEEEEEGENYQ
KGERGEDSSEEKHLEEPGETQNAFLNERKQASAIKKEELVARSETHAAGHSQEKTHSREKSSQESGEETGSQENHPQESKGQPRSQE
ESEEGEEDATSEVDKRRTRPRHHHGRSRPDRSSQGGSLPSEEKGHPQEESEESNVSMASLGEKRDHHSTHYRASEEEPEYGEEIKGY
PGVQAPEDLEWERYRGRGSEEYRAPRPQSEESWDEEDKRNYPSLELDKMAHGYGEESEEERGLEPGKGRHHRGRGGEPRAYFMSDTR
EEKRFLGEGHHRVQENQMDKARRHPQGAWKELDRNYLNYGEEGAPGKWQQQGDLQDTKENREEARFQDKQYSSHHTAEKRKRLGELF
NPYYDPLQWKSSHFERRDNMNDNFLEGEEENELTLNEKNFFPEYNYDWWEKKPFSEDVNWGYEKRNLARVPKLDLKRQYDRVAQLDQ
LLHYRKKSAEFPDFYDSEEPVSTHQEAENEKDRADQTVLTEDEKKELENLAAMDLELQKIAEKFSQRG

>sp|Q96F44|TRI11_HUMAN
MAAPDLSTNLQEEATCAICLDYFTDPVMTDCGHNFCRECIRRCWGQPEGPYACPECRELSPQRNLRPNRPLAKMAEMARRLHPPSPV
PQGVCPAHREPLAAFCGDELRLLCAACERSGEHWAHRVRPLQDAAEDLKAKLEKSLEHLRKQMQDALLFQAQADETCVLWQKMVESQ
RQNVLGEFERLRRLLAEEEQQLLQRLEEEELEVLPRLREGAAHLGQQSAHLAELIAELEGRCQLPALGLLQDIKDALRRVQDVKLQP
PEVVPMELRTVCRVPGLVETLRRFRGDVTLDPDTANPELILSEDRRSVQRGDLRQALPDSPERFDPGPCVLGQERFTSGRHYWEVEV
GDRTSWALGVCRENVNRKEKGELSAGNGFWILVFLGSYYNSSERALAPLRDPPRRVGIFLDYEAGHLSFYSATDGSLLFIFPEIPFS
GTLRPLFSPLSSSPTPMTICRPKGGSGDTLAPQ
>sp|P11233|RALA_HUMAN
MAANKPKGQNSLALHKVIMVGSGGVGKSALTLQFMYDEFVEDYEPTKADSYRKKVVLDGEEVQIDILDTAGQEDYAAIRDNYFRSGE
GFLCVFSITEMESFAATADFREQILRVKEDENVPFLLVGNKSDLEDKRQVSVEEAKNRAEQWNVNYVETSAKTRANVDKVFFDLMRE
IRARKMEDSKEKNGKKKRKSLAKRIRERCCIL
>sp|Q9Y264|ANGP4_HUMAN
MLSQLAMLQGSLLLVVATMSVAQQTRQEADRGCETLVVQHGHCSYTFLLPKSEPCPPGPEVSRDSNTLQRESLANPLHLGKLPTQQV
KQLEQALQNNTQWLKKLERAIKTILRSKLEQVQQQMAQNQTAPMLELGTSLLNQTTAQIRKLTDMEAQLLNQTSRMDAQMPETFLST
NKLENQLLLQRQKLQQLQGQNSALEKRLQALETKQQEELASILSKKAKLLNTLSRQSAALTNIERGLRGVRHNSSLLQDQQHSLRQL
LVLLRHLVQERANASAPAFIMAGEQVFQDCAEIQRSGASASGVYTIQVSNATKPRKVFCDLQSSGGRWTLIQRRENGTVNFQRNWKD
YKQGFGDPAGEHWLGNEVVHQLTRRAAYSLRVELQDWEGHEAYAQYEHFHLGSENQLYRLSVVGYSGSAGRQSSLVLQNTSFSTLDS
DNDHCLCKCAQVMSGGWWFDACGLSNLNGVYYHAPDNKYKMDGIRWHYFKGPSYSLRASRMMIRPLDI
>sp|P22314|UBA1_HUMAN
MSSSPLSKKRRVSGPDPKPGSNCSPAQSVLSEVPSVPTNGMAKNGSEADIDEGLYSRQLYVLGHEAMKRLQTSSVLVSGLRGLGVEI
AKNIILGGVKAVTLHDQGTAQWADLSSQFYLREEDIGKNRAEVSQPRLAELNSYVPVTAYTGPLVEDFLSGFQVVVLTNTPLEDQLR
VGEFCHNRGIKLVVADTRGLFGQLFCDFGEEMILTDSNGEQPLSAMVSMVTKDNPGVVTCLDEARHGFESGDFVSFSEVQGMVELNG
NQPMEIKVLGPYTFSICDTSNFSDYIRGGIVSQVKVPKKISFKSLVASLAEPDFVVTDFAKFSRPAQLHIGFQALHQFCAQHGRPPR
PRNEEDAAELVALAQAVNARALPAVQQNNLDEDLIRKLAYVAAGDLAPINAFIGGLAAQEVMKACSGKFMPIMQWLYFDALECLPED
KEVLTEDKCLQRQNRYDGQVAVFGSDLQEKLGKQKYFLVGAGAIGCELLKNFAMIGLGCGEGGEIIVTDMDTIEKSNLNRQFLFRPW
DVTKLKSDTAAAAVRQMNPHIRVTSHQNRVGPDTERIYDDDFFQNLDGVANALDNVDARMYMDRRCVYYRKPLLESGTLGTKGNVQV
VIPFLTESYSSSQDPPEKSIPICTLKNFPNAIEHTLQWARDEFEGLFKQPAENVNQYLTDPKFVERTLRLAGTQPLEVLEAVQRSLV
LQRPQTWADCVTWACHHWHTQYSNNIRQLLHNFPPDQLTSSGAPFWSGPKRCPHPLTFDVNNPLHLDYVMAAANLFAQTYGLTGSQD
RAAVATFLQSVQVPEFTPKSGVKIHVSDQELQSANASVDDSRLEELKATLPSPDKLPGFKMYPIDFEKDDDSNFHMDFIVAASNLRA
ENYDIPSADRHKSKLIAGKIIPAIATTTAAVVGLVCLELYKVVQGHRQLDSYKNGFLNLALPFFGFSEPLAAPRHQYYNQEWTLWDR
FEVQGLQPNGEEMTLKQFLDYFKTEHKLEITMLSQGVSMLYSFFMPAAKLKERLDQPMTEIVSRVSKRKLGRHVRALVLELCCNDES
GEDVEVPYVRYTIR
>sp|Q6NUK1|SCMC1_HUMAN
MLRWLRDFVLPTAACQDAEQPTRYETLFQALDRNGDGVVDIGELQEGLRNLGIPLGQDAEEKIFTTGDVNKDGKLDFEEFMKYLKDH
EKKMKLAFKSLDKNNDGKIEASEIVQSLQTLGLTISEQQAELILQSIDVDGTMTVDWNEWRDYFLFNPVTDIEEIIRFWKHSTGIDI
GDSLTIPDEFTEDEKKSGQWWRQLLAGGIAGAVSRTSTAPLDRLKIMMQVHGSKSDKMNIFGGFRQMVKEGGIRSLWRGNGTNVIKI
APETAVKFWAYEQYKKLLTEEGQKIGTFERFISGSMAGATAQTFIYPMEVMKTRLAVGKTGQYSGIYDCAKKILKHEGLGAFYKGYV
PNLLGIIPYAGIDLAVYELLKSYWLDNFAKDSVNPGVMVLLGCGALSSTCGQLASYPLALVRTRMQAQAMLEGSPQLNMVGLFRRII
SKEGIPGLYRGITPNFMKVLPAVGISYVVYENMKQTLGVTQK
>sp|Q5H9E4|S2553_HUMAN
MGEQNHSPGKELQHRTRAEAPGKKSWHSQAYALGAVSNFMSTFLTFPIYKVVFRQQIHAMAVSEAVRQLWHEGPQYFYRGIYPPLLS
KTLQGTLLFGTYDSLLCFLSPVGPHTLGHRWAAGLMSGVVEAVALSPFERVQNVLQDGRKQARFPSTFSILKEFNSYGLWGRLSLGY
YRGFWPVLARNSLGSALYFSFKDPIQDGLAEQGLPHWVPALVSGSVNGTITCLVLYPLIVLVANMQSHIGWQNMPSLWASAQDVWNT
RGRKLLLIYRGGSLVILRSSVTWGLTTAIHDFLQRKSHSRKELKTD

>sp|Q13219|PAPP1_HUMAN
MRLWSWVLHLGLLSAALGCGLAERPRRARRDPRAGRPPRPAAGPATCATRAARGRRASPPPPPPPGGAWEAVRVPRRRQQREARGAT
EEPSPPSRALYFSGRGEQLRLRADLELPRDAFTLQVWLRAEGGQRSPAVITGLYDKCSYISRDRGWVVGIHTISDQDNKDPRYFFSL
KTDRARQVTTINAHRSYLPGQWVYLAATYDGQFMKLYVNGAQVATSGEQVGGIFSPLTQKCKVLMLGGSALNHNYRGYIEHFSLWKV
ARTQREILSDMETHGAHTALPQLLLQENWDNVKHAWSPMKDGSSPKVEFSNAHGFLLDTSLEPPLCGQTLCDNTEVIASYNQLSSFR
QPKVVRYRVVNLYEDDHKNPTVTREQVDFQHHQLAEAFKQYNISWELDVLEVSNSSLRRRLILANCDISKIGDENCDPECNHTLTGH
DGGDCRHLRHPAFVKKQHNGVCDMDCNYERFNFDGGECCDPEITNVTQTCFDPDSPHRAYLDVNELKNILKLDGSTHLNIFFAKSSE
EELAGVATWPWDKEALMHLGGIVLNPSFYGMPGHTHTMIHEIGHSLGLYHVFRGISEIQSCSDPCMETEPSFETGDLCNDTNPAPKH
KSCGDPGPGNDTCGFHSFFNTPYNNFMSYADDDCTDSFTPNQVARMHCYLDLVYQGWQPSRKPAPVALAPQVLGHTTDSVTLEWFPP
IDGHFFERELGSACHLCLEGRILVQYASNASSPMPCSPSGHWSPREAEGHPDVEQPCKSSVRTWSPNSAVNPHTVPPACPEPQGCYL
ELEFLYPLVPESLTIWVTFVSTDWDSSGAVNDIKLLAVSGKNISLGPQNVFCDVPLTIRLWDVGEEVYGIQIYTLDEHLEIDAAMLT
STADTPLCLQCKPLKYKVVRDPPLQMDVASILHLNRKFVDMDLNLGSVYQYWVITISGTEESEPSPAVTYIHGSGYCGDGIIQKDQG
EQCDDMNKINGDGCSLFCRQEVSFNCIDEPSRCYFHDGDGVCEEFEQKTSIKDCGVYTPQGFLDQWASNASVSHQDQQCPGWVIIGQ
PAASQVCRTKVIDLSEGISQHAWYPCTISYPYSQLAQTTFWLRAYFSQPMVAAAVIVHLVTDGTYYGDQKQETISVQLLDTKDQSHD
LGLHVLSCRNNPLIIPVVHDLSQPFYHSQAVRVSFSSPLVAISGVALRSFDNFDPVTLSSCQRGETYSPAEQSCVHFACEKTDCPEL
AVENASLNCSSSDRYHGAQCTVSCRTGYVLQIRRDDELIKSQTGPSVTVTCTEGKWNKQVACEPVDCSIPDHHQVYAASFSCPEGTT
FGSQCSFQCRHPAQLKGNNSLLTCMEDGLWSFPEALCELMCLAPPPVPNADLQTARCRENKHKVGSFCKYKCKPGYHVPGSSRKSKK
RAFKTQCTQDGSWQEGACVPVTCDPPPPKFHGLYQCTNGFQFNSECRIKCEDSDASQGLGSNVIHCRKDGTWNGSFHVCQEMQGQCS
VPNELNSNLKLQCPDGYAIGSECATSCLDHNSESIILPMNVTVRDIPHWLNPTRVERVVCTAGLKWYPHPALIHCVKGCEPFMGDNY
CDAINNRAFCNYDGGDCCTSTVKTKKVTPFPMSCDLQGDCACRDPQAQEHSRKDLRGYSHG
>sp|P30613|KPYR_HUMAN
MSIQENISSLQLRSWVSKSQRDLAKSILIGAPGGPAGYLRRASVAQLTQELGTAFFQQQQLPAAMADTFLEHLCLLDIDSEPVAARS
TSIIATIGPASRSVERLKEMIKAGMNIARLNFSHGSHEYHAESIANVREAVESFAGSPLSYRPVAIALDTKGPEIRTGILQGGPESE
VELVKGSQVLVTVDPAFRTRGNANTVWVDYPNIVRVVPVGGRIYIDDGLISLVVQKIGPEGLVTQVENGGVLGSRKGVNLPGAQVDL
PGLSEQDVRDLRFGVEHGVDIVFASFVRKASDVAAVRAALGPEGHGIKIISKIENHEGVKRFDEILEVSDGIMVARGDLGIEIPAEK
VFLAQKMMIGRCNLAGKPVVCATQMLESMITKPRPTRAETSDVANAVLDGADCIMLSGETAKGNFPVEAVKMQHAIAREAEAAVYHR
QLFEELRRAAPLSRDPTEVTAIGAVEAAFKCCAAAIIVLTTTGRSAQLLSRYRPRAAVIAVTRSAQAARQVHLCRGVFPLLYREPPE
AIWADDVDRRVQFGIESGKLRGFLRVGDLVIVVTGWRPGSGYTNIMRVLSIS
>sp|P09661|RU2A_HUMAN
MVKLTAELIEQAAQYTNAVRDRELDLRGYKIPVIENLGATLDQFDAIDFSDNEIRKLDGFPLLRRLKTLLVNNNRICRIGEGLDQAL
PCLTELILTNNSLVELGDLDPLASLKSLTYLSILRNPVTNKKHYRLYVIYKVPQVRVLDFQKVKLKERQEAEKMFKGKRGAQLAKDI
ARRSKTFNPGAGLPTDKKKGGPSPGDVEAIKNAIANASTLAEVERLKGLLQSGQIPGRERRSGPTDDGEEEMEEDTVTNGS
>sp|Q6IE38|ISK14_HUMAN
MAKSFPVFSLLSFILIHLVLSSVSGPRHWWPPRGIIKVKCPYEKVNLSWYNGTVNPCPGLYQPICGTNFITYDNPCILCVESLKSHG
RIRFYHDGKC
>sp|Q8IY57|YAF2_HUMAN
MGDKKSPTRPKRQPKPSSDEGYWDCSVCTFRNSAEAFKCMMCDVRKGTSTRKPRPVSQLVAQQVTQQFVPPTQSKKEKKDKVEKEKS
EKETTSKKNSHKKTRPRLKNVDRSSAQHLEVTVGDLTVIITDFKEKTKSPPASSAASADQHSQSGSSSDNTERGMSRSSSPRGEASS
LNGESH
>sp|P55196|AFAD_HUMAN
MSAGGRDEERRKLADIIHHWNANRLDLFEISQPTEDLEFHGVMRFYFQDKAAGNFATKCIRVSSTATTQDVIETLAEKFRPDMRMLS
SPKYSLYEVHVSGERRLDIDEKPLVVQLNWNKDDREGRFVLKNENDAIPPKKAQSNGPEKQEKEGVIQNFKRTLSKKEKKEKKKREK
EALRQASDKDDRPFQGEDVENSRLAAEVYKDMPETSFTRTISNPEVVMKRRRQQKLEKRMQEFRSSDGRPDSGGTLRIYADSLKPNI
PYKTILLSTTDPADFAVAEALEKYGLEKENPKDYCIARVMLPPGAQHSDEKGAKEIILDDDECPLQIFREWPSDKGILVFQLKRRPP

DHIPKKTKKHLEGKTPKGKERADGSGYGSTLPPEKLPYLVELSPGRRNHFAYYNYHTYEDGSDSRDKPKLYRLQLSVTEVGTEKLDD
NSIQLFGPGIQPHHCDLTNMDGVVTVTPRSMDAETYVEGQRISETTMLQSGMKVQFGASHVFKFVDPSQDHALAKRSVDGGLMVKGP
RHKPGIVQETTFDLGGDIHSGTALPTSKSTTRLDSDRVSSASSTAERGMVKPMIRVEQQPDYRRQESRTQDASGPELILPASIEFRE
SSEDSFLSAIINYTNSSTVHFKLSPTYVLYMACRYVLSNQYRPDISPTERTHKVIAVVNKMVSMMEGVIQKQKNIAGALAFWMANAS
ELLNFIKQDRDLSRITLDAQDVLAHLVQMAFKYLVHCLQSELNNYMPAFLDDPEENSLQRPKIDDVLHTLTGAMSLLRRCRVNAALT
IQLFSQLFHFINMWLFNRLVTDPDSGLCSHYWGAIIRQQLGHIEAWAEKQGLELAADCHLSRIVQATTLLTMDKYAPDDIPNINSTC
FKLNSLQLQALLQNYHCAPDEPFIPTDLIENVVTVAENTADELARSDGREVQLEEDPDLQLPFLLPEDGYSCDVVRNIPNGLQEFLD
PLCQRGFCRLIPHTRSPGTWTIYFEGADYESHLLRENTELAQPLRKEPEIITVTLKKQNGMGLSIVAAKGAGQDKLGIYVKSVVKGG
AADVDGRLAAGDQLLSVDGRSLVGLSQERAAELMTRTSSVVTLEVAKQGAIYHGLATLLNQPSPMMQRISDRRGSGKPRPKSEGFEL
YNNSTQNGSPESPQLPWAEYSEPKKLPGDDRLMKNRADHRSSPNVANQPPSPGGKSAYASGTTAKITSVSTGNLCTEEQTPPPRPEA
YPIPTQTYTREYFTFPASKSQDRMAPPQNQWPNYEEKPHMHTDSNHSSIAIQRVTRSQEELREDKAYQLERHRIEAAMDRKSDSDMW
INQSSSLDSSTSSQEHLNHSSKSVTPASTLTKSGPGRWKTPAAIPATPVAVSQPIRTDLPPPPPPPPVHYAGDFDGMSMDLPLPPPP
SANQIGLPSAQVAAAERRKREEHQRWYEKEKARLEEERERKRREQERKLGQMRTQSLNPAPFSPLTAQQMKPEKPSTLQRPQETVIR
ELQPQQQPRTIERRDLQYITVSKEELSSGDSLSPDPWKRDAKEKLEKQQQMHIVDMLSKEIQELQSKPDRSAEESDRLRKLMLEWQF
QKRLQESKQKDEDDEEEEDDDVDTMLIMQRLEAERRARLQDEERRRQQQLEEMRKREAEDRARQEEERRRQEEERTKRDAEEKRRQE
EGYYSRLEAERRRQHDEAARRLLEPEAPGLCRPPLPRDYEPPSPSPAPGAPPPPPQRNASYLKTQVLSPDSLFTAKFVAYNEEEEEE
DCSLAGPNSYPGSTGAAVGAHDACRDAKEKRSKSQDADSPGSSGAPENLTFKERQRLFSQGQDVSNKVKASRKLTELENELNTK
>sp|Q6ZU69|F205A_HUMAN
MLSPTFVLWEVGYPLYIYGSIFIVIVIIWQVKRSHHELSSEPKRSCCRCHQKVRQRARDAASTARRRSREEAEKPQKLLSIIKSQGW
LPLERSVRRILCADPCCQICNSVALEIQQLLVGENNQISLTLSGPLQGSSCLEMLSTSSMSLDQSLEFHSWHTRELSLSSVTPTLSQ
LTDQKSLTQSAAQSTYADGIQDYWADHLQLGQEFQVPDVLRGPNTIASSRIEKPRAPLNQEEMTQSNPSLVQGNQGQHHLNSQVSLL
SLNPETLNRMHPMALHMVLPAHLPFLSPEVLRLLEVHVKKWMHFQRWGLPRRVEESLRQLMPNPPLYYQPGNDQPVSFNLKNTPQVS
LHRFETISLQTWCSCVAGQPIQTFWVSEWSTMNPEQRHHCQQTPNPMALALPSPALKALSGPHPQSGGQDNDSGSDLQQKYSQLFCG
LPSLHSESLVATFMGSQGLPKIENVPKPPLKDPFLFNELSFPQLLPKTSPQSAPPSSPLSPNWMSPSDHQRAQINVPFLTLAEYEAL
EWHLLQRQLQLQWGWPAALQRSQHTQCLMQHEPCGKAQSPETTTASQTGKSISVLTRELLFFPEHARKLLEFHIQKQLIRHRWGLPQ
KIQQSIQLLLTSTDQQTVSSSSTALANVSIPQPVALEANGACDVLSPIAAPVSIPRPHLLTQVKAILQSHIDSKCGQIHQGKIPACV
HRSWDCRISGVLAVAPFPCIPESQFLELQTASDPDLHHKVMPWMPTALDQQQQALPGTVTEHPKLLRVLSVEAIEKLETTLRHKHLA
FLSGLPALYYVALPRALAPAVTSQSVITEMEPSPVEIPAEPLIQMVSFEEQCISLGPCPQGNNESCTDVAKEFQPAVPVKGTMETLP
LESQTHPTSPHSLQTHILTKLNFHLRKKVLEIQWGIPIRARKSREQTVAAPENISTQKSLESLNHQGETLLQELPIPPDTLPAPNPE
GVHLKEQLANDLKAVQQNQKQSNSKAVPQGSAHSVSKISQPSGDMTEAHMPCVQVEANVNKPSLEEPCGPEPQSPSKSKDPAHVPML
AGNREDPEETKAARDHREGDAGFGRSSTREERRPAEDQRPAGMLPNKTPRGSWRWSRSFHLADPCQHSPQHHPQLKLPQLPPRVPGE
KESEKDLQDSQTKLTVILEPATIPENAQTVLPQASQGQPFLSQPTQAKPLQGQTLQGQVLHGLVMPVHAQKKPSLTESSFRNKIKCF
LQHINPKTKGKGHEDSMFSAAAKVAKTRKENVAKSLAPAKSPVGRSKTEKPTGCSKAQSRPAQKLVGPAFLDGPQSLDDKLRLHSRQ
PGSASALGYPRHCPRHCPREACANKPGHPT
>sp|Q9BU70|TRMO_HUMAN
MRGLEESGPRPTATPCGCVKPALETGNLLTEPVGYLESCFSAKNGTPRQPSICSYSRACLRIRKRIFNNPEHSLMGLEQFSHVWILF
VFHKNGHLSCKAKVQPPRLNGAKTGVFSTRSPHRPNAIGLTLAKLEKVEGGAIYLSGIDMIHGTPVLDIKPYIAEYDSPQNVMEPLA
DFNLQNNQHTPNTVSQSDSKTDSCDQRQLSGCDEPQPHHSTKRKPKCPEDRTSEENYLTHSDTARIQQAFPMHREIAVDFGLESRRD
QSSSVAEEQIGPYCPEKSFSEKGTDKKLERVEGAAVLQGSRAETQPMAPHCPAGRADGAPRSVVPAWVTEAPVATLEVRFTPHAEMD
LGQLSSQDVGQASFKYFQSAEEAKRAIEAVLSADPRSVYRRKLCQDRLFYFTVDIAHVTCWFGDGFAEVLRIKPASEPVHMTGPVGS
LVSLGS
>sp|I3L3R5|CCER2_HUMAN
MPPRGPASELLLLRLLLLGAATAAPLAPRPSKEELTRCLAEVVTEVLTVGQVQRGPCTALLHKELCGTEPHGCASTEEKGLLLGDFK
KQEAGKMRSSQEVRDEEEEEVAERTHKSEVQEQAIRMQGHRQLHQEEDEEEEKEERKRGPMETFEDLWQRHLENGGDLQKRVAEKAS

DKETAQFQAEEKGVRVLGGDRSLWQGAERGGGERREDLPHHHHHHHQPEAEPRQEKEEASEREEKEVEQLEHLRDELKKVTETLGEQ
LRREG
>sp|O75362|ZN217_HUMAN
MQSKVTGNMPTQSLLMYMDGPEVIGSSLGSPMEMEDALSMKGTAVVPFRATQEKNVIQIEGYMPLDCMFCSQTFTHSEDLNKHVLMQ
HRPTLCEPAVLRVEAEYLSPLDKSQVRTEPPKEKNCKENEFSCEVCGQTFRVAFDVEIHMRTHKDSFTYGCNMCGRRFKEPWFLKNH
MRTHNGKSGARSKLQQGLESSPATINEVVQVHAAESISSPYKICMVCGFLFPNKESLIEHRKVHTKKTAFGTSSAQTDSPQGGMPSS
REDFLQLFNLRPKSHPETGKKPVRCIPQLDPFTTFQAWQLATKGKVAICQEVKESGQEGSTDNDDSSSEKELGETNKGSCAGLSQEK
EKCKHSHGEAPSVDADPKLPSSKEKPTHCSECGKAFRTYHQLVLHSRVHKKDRRAGAESPTMSVDGRQPGTCSPDLAAPLDENGAVD
RGEGGSEDGSEDGLPEGIHLDKNDDGGKIKHLTSSRECSYCGKFFRSNYYLNIHLRTHTGEKPYKCEFCEYAAAQKTSLRYHLERHH
KEKQTDVAAEVKNDGKNQDTEDALLTADSAQTKNLKRFFDGAKDVTGSPPAKQLKEMPSVFQNVLGSAVLSPAHKDTQDFHKNAADD
SADKVNKNPTPAYLDLLKKRSAVETQANNLICRTKADVTPPPDGSTTHNLEVSPKEKQTETAADCRYRPSVDCHEKPLNLSVGALHN
CPAISLSKSLIPSITCPFCTFKTFYPEVLMMHQRLEHKYNPDVHKNCRNKSLLRSRRTGCPPALLGKDVPPLSSFCKPKPKSAFPAQ
SKSLPSAKGKQSPPGPGKAPLTSGIDSSTLAPSNLKSHRPQQNVGVQGAATRQQQSEMFPKTSVSPAPDKTKRPETKLKPLPVAPSQ
PTLGSSNINGSIDYPAKNDSPWAPPGRDYFCNRSASNTAAEFGEPLPKRLKSSVVALDVDQPGANYRRGYDLPKYHMVRGITSLLPQ
DCVYPSQALPPKPRFLSSSEVDSPNVLTVQKPYGGSGPLYTCVPAGSPASSSTLEGKRPVSYQHLSNSMAQKRNYENFIGNAHYRPN
DKKT
>sp|O43261|LEU1_HUMAN
MRPCIWIHVHLKPPCRLVELLPFSSALQGLSHLSLGTTLPVILPERNEEQNLQELSHNADKYQMGDCCKEEIDDSIFY
>sp|Q6ZSB3|CB046_HUMAN
MVQREKARDNFEGGCLAELIGSPRDWKCFLAVPDPLLGVQHWLHLWRPQTKDGNSLHRHGDQAWGKHRRQNSLKSPALSGHSIDYHF
YPRLRCGMLIGPDKQAVASGLEVLVTSSTKILGQLFPDAAHFLEEASEFKAE
>sp|Q8N2Q7|NLGN1_HUMAN
MALPRCTWPNYVWRAVMACLVHRGLGAPLTLCMLGCLLQAGHVLSQKLDDVDPLVATNFGKIRGIKKELNNEILGPVIQFLGVPYAA
PPTGERRFQPPEPPSPWSDIRNATQFAPVCPQNIIDGRLPEVMLPVWFTNNLDVVSSYVQDQSEDCLYLNIYVPTEDGPLTKKRDEA
TLNPPDTDIRDSGGPKPVMVYIHGGSYMEGTGNLYDGSVLASYGNVIVITVNYRLGVLGFLSTGDQAAKGNYGLLDLIQALRWTSEN
IGFFGGDPLRITVFGSGAGGSCVNLLTLSHYSEGNRWSNSTKGLFQRAIAQSGTALSSWAVSFQPAKYARMLATKVGCNVSDTVELV
ECLQKKPYKELVDQDIQPARYHIAFGPVIDGDVIPDDPQILMEQGEFLNYDIMLGVNQGEGLKFVENIVDSDDGISASDFDFAVSNF
VDNLYGYPEGKDVLRETIKFMYTDWADRHNPETRRKTLLALFTDHQWVAPAVATADLHSNFGSPTYFYAFYHHCQTDQVPAWADAAH
GDEVPYVLGIPMIGPTELFPCNFSKNDVMLSAVVMTYWTNFAKTGDPNQPVPQDTKFIHTKPNRFEEVAWTRYSQKDQLYLHIGLKP
RVKEHYRANKVNLWLELVPHLHNLNDISQYTSTTTKVPSTDITFRPTRKNSVPVTSAFPTAKQDDPKQQPSPFSVDQRDYSTELSVT
IAVGASLLFLNILAFAALYYKKDKRRHDVHRRCSPQRTTTNDLTHAQEEEIMSLQMKHTDLDHECESIHPHEVVLRTACPPDYTLAM
RRSPDDVPLMTPNTITMIPNTIPGIQPLHTFNTFTGGQNNTLPHPHPHPHSHSTTRV
>sp|O60637|TSN3_HUMAN
MGQCGITSSKTVLVFLNLIFWGAAGILCYVGAYVFITYDDYDHFFEDVYTLIPAVVIIAVGALLFIIGLIGCCATIRESRCGLATFV
IILLLVFVTEVVVVVLGYVYRAKVENEVDRSIQKVYKTYNGTNPDAASRAIDYVQRQLHCCGIHNYSDWENTDWFKETKNQSVPLSC
CRETASNCNGSLAHPSDLYAEGCEALVVKKLQEIMMHVIWAALAFAAIQLLGMLCACIVLCRRSRDPAYELLITGGTYA
>sp|Q8WV16|DCAF4_HUMAN
MNKSRWQSRRRHGRRSHQQNPWFRLRDSEDRSDSRAAQPAHDSGHGDDESPSTSSGTAGTSSVPELPGFYFDPEKKRYFRLLPGHNN
CNPLTKESIRQKEMESKRLRLLQEEDRRKKIARMGFNASSMLRKSQLGFLNVTNYCHLAHELRLSCMERKKVQIRSMDPSALASDRF
NLILADTNSDRLFTVNDVKVGGSKYGIINLQSLKTPTLKVFMHENLYFTNRKVNSVCWASLNHLDSHILLCLMGLAETPGCATLLPA
SLFVNSHPGIDRPGMLCSFRIPGAWSCAWSLNIQANNCFSTGLSRRVLLTNVVTGHRQSFGTNSDVLAQQFALMAPLLFNGCRSGEI
FAIDLRCGNQGKGWKATRLFHDSAVTSVRILQDEQYLMASDMAGKIKLWDLRTTKCVRQYEGHVNEYAYLPLHVHEEEGILVAVGQD
CYTRIWSLHDARLLRTIPSPYPASKADIPSVAFSSRLGGSRGAPGLLMAVGQDLYCYSYS
>sp|Q8TCG5|CPT1C_HUMAN

MAEAHQAVGFRPSLTSDGAEVELSAPVLQEIYLSGLRSWKRHLSRFWNDFLTGVFPASPLSWLFLFSAIQLAWFLQLDPSLGLMEKI
KELLPDWGGQHHGLRGVLAAALFASCLWGALIFTLHVALRLLLSYHGWLLEPHGAMSSPTKTWLALVRIFSGRHPMLFSYQRSLPRQ
PVPSVQDTVRKYLESVRPILSDEDFDWTAVLAQEFLRLQASLLQWYLRLKSWWASNYVSDWWEEFVYLRSRNPLMVNSNYYMMDFLY
VTPTPLQAARAGNAVHALLLYRHRLNRQEIPPTLLMGMRPLCSAQYEKIFNTTRIPGVQKDYIRHLHDSQHVAVFHRGRFFRMGTHS
RNSLLSPRALEQQFQRILDDPSPACPHEEHLAALTAAPRGTWAQVRTSLKTQAAEALEAVEGAAFFVSLDAEPAGLTREDPAASLDA
YAHALLAGRGHDRWFDKSFTLIVFSNGKLGLSVEHSWADCPISGHMWEFTLATECFQLGYSTDGHCKGHPDPTLPQPQRLQWDLPDQ
IHSSISLALRGAKILSENVDCHVVPFSLFGKSFIRRCHLSSDSFIQIALQLAHFRDRGQFCLTYESAMTRLFLEGRTETVRSCTREA
CNFVRAMEDKEKTDPQCLALFRVAVDKHQALLKAAMSGQGVDRHLFALYIVSRFLHLQSPFLTQVHSEQWQLSTSQIPVQQMHLFDV
HNYPDYVSSGGGFGPADDHGYGVSYIFMGDGMITFHISSKKSSTKTDSHRLGQHIEDALLDVASLFQAGQHFKRRFRGSGKENSRHR
CGFLSRQTGASKASMTSTDF
>sp|Q9Y2S0|RPAC2_HUMAN
MEEDQELERKISGLKTSMAEGERKTALEMVQAAGTDRHCVTFVLHEEDHTLGNSLRYMIMKNPEVEFCGYTTTHPSESKINLRIQTR
GTLPAVEPFQRGLNELMNVCQHVLDKFEASIKDYKDQKASRNESTF
>sp|Q8N9V3|WSDU1_HUMAN
MVKLIHTLADHGDDVNCCAFSFSLLATCSLDKTIRLYSLRDFTELPHSPLKFHTYAVHCCCFSPSGHILASCSTDGTTVLWNTENGQ
MLAVMEQPSGSPVRVCQFSPDSTCLASGAADGTVVLWNAQSYKLYRCGSVKDGSLAACAFSPNGSFFVTGSSCGDLTVWDDKMRCPH
SEKAHDLGITCCDFSSQPVSDGEQGLQFFRLASCGQDCQVKIWIVSFTHILGFELKYKSTLSGHCAPVLACAFSHDGQMLVSGSVDK
SVIVYDTNTENILHTLTQHTRYVTTCAFAPNTLLLATGSMDKTVNIWQFDLETLCQARRTEHQLKQFTEDWSEEDVSTWLCAQDLKD
LVGIFKMNNIDGKELLNLTKESLADDLKIESLGLRSKVLRKIEELRTKVKSLSSGIPDEFICPITRELMKDPVIASDGYSYEKEAME
NWISKKKRTSPMTNLVLPSAVLTPNRTLKMAINRWLETHQK
>sp|Q15063|POSTN_HUMAN
MIPFLPMFSLLLLLIVNPINANNHYDKILAHSRIRGRDQGPNVCALQQILGTKKKYFSTCKNWYKKSICGQKTTVLYECCPGYMRME
GMKGCPAVLPIDHVYGTLGIVGATTTQRYSDASKLREEIEGKGSFTYFAPSNEAWDNLDSDIRRGLESNVNVELLNALHSHMINKRM
LTKDLKNGMIIPSMYNNLGLFINHYPNGVVTVNCARIIHGNQIATNGVVHVIDRVLTQIGTSIQDFIEAEDDLSSFRAAAITSDILE
ALGRDGHFTLFAPTNEAFEKLPRGVLERIMGDKVASEALMKYHILNTLQCSESIMGGAVFETLEGNTIEIGCDGDSITVNGIKMVNK
KDIVTNNGVIHLIDQVLIPDSAKQVIELAGKQQTTFTDLVAQLGLASALRPDGEYTLLAPVNNAFSDDTLSMDQRLLKLILQNHILK
VKVGLNELYNGQILETIGGKQLRVFVYRTAVCIENSCMEKGSKQGRNGAIHIFREIIKPAEKSLHEKLKQDKRFSTFLSLLEAADLK
ELLTQPGDWTLFVPTNDAFKGMTSEEKEILIRDKNALQNIILYHLTPGVFIGKGFEPGVTNILKTTQGSKIFLKEVNDTLLVNELKS
KESDIMTTNGVIHVVDKLLYPADTPVGNDQLLEILNKLIKYIQIKFVRGSTFKEIPVTVYTTKIITKVVEPKIKVIEGSLQPIIKTE
GPTLTKVKIEGEPEFRLIKEGETITEVIHGEPIIKKYTKIIDGVPVEITEKETREERIITGPEIKYTRISTGGGETEETLKKLLQEE
VTKVTKFIEGGDGHLFEDEEIKRLLQGDTPVRKLQANKKVQGSRRRLREGRSQ
>sp|Q8TCZ2|C99L2_HUMAN
MVAWRSAFLVCLAFSLATLVQRGSGDFDDFNLEDAVKETSSVKQPWDHTTTTTTNRPGTTRAPAKPPGSGLDLADALDDQDDGRRKP
GIGGRERWNHVTTTTKRPVTTRAPANTLGNDFDLADALDDRNDRDDGRRKPIAGGGGFSDKDLEDIVGGGEYKPDKGKGDGRYGSND
DPGSGMVAEPGTIAGVASALAMALIGAVSSYISYQQKKFCFSIQQGLNADYVKGENLEAVVCEEPQVKYSTLHTQSAEPPPPPEPAR
I
>sp|Q5T2S8|ARMC4_HUMAN
MGVALRKLTQWTAAGHGTGILEITPLNEAILKEIIVFVESFIYKHPQEAKFVFVEPLEWNTSLAPSAFESGYVVSETTVKSEEVDKN
GQPLLFLSVPQIKIRSFGQLSRLLLIAKTGKLKEAQACVEANRDPIVKILGSDYNTMKENSIALNILGKITRDDDPESEIKMKIAML
LKQLDLHLLNHSLKHISLEISLSPMTVKKDIELLKRFSGKGNQTVLESIEYTSDYEFSNGCRAPPWRQIRGEICYVLVKPHDGETLC
ITCSAGGVFLNGGKTDDEGDVNYERKGSIYKNLVTFLREKSPKFSENMSKLGISFSEDQQKEKDQLGKAPKKEEAAALRKDISGSDK
RSLEKNQINFWRNQMTKRWEPSLNWKTTVNYKGKGSAKEIQEDKHTGKLEKPRPSVSHGRAQLLRKSAEKIEETVSDSSSESEEDEE
PPDHRQEASADLPSEYWQIQKLVKYLKGGNQTATVIALCSMRDFSLAQETCQLAIRDVGGLEVLINLLETDEVKCKIGSLKILKEIS
HNPQIRQNIVDLGGLPIMVNILDSPHKSLKCLAAETIANVAKFKRARRVVRQHGGITKLVALLDCAHDSTKPAQSSLYEARDVEVAR

CGALALWSCSKSHTNKEAIRKAGGIPLLARLLKTSHENMLIPVVGTLQECASEENYRAAIKAERIIENLVKNLNSENEQLQEHCAMA
IYQCAEDKETRDLVRLHGGLKPLASLLNNTDNKERLAAVTGAIWKCSISKENVTKFREYKAIETLVGLLTDQPEEVLVNVVGALGEC
CQERENRVIVRKCGGIQPLVNLLVGINQALLVNVTKAVGACAVEPESMMIIDRLDGVRLLWSLLKNPHPDVKASAAWALCPCIKNAK
DAGEMVRSFVGGLELIVNLLKSDNKEVLASVCAAITNIAKDQENLAVITDHGVVPLLSKLANTNNNKLRHHLAEAISRCCMWGRNRV
AFGEHKAVAPLVRYLKSNDTNVHRATAQALYQLSEDADNCITMHENGAVKLLLDMVGSPDQDLQEAAAGCISNIRRLALATEKARYT
>sp|Q8N9B4|ANR42_HUMAN
MPGVANSGPSTSSRETANPCSRKKVHFGSIHDAVRAGDVKQLSEIVCLHWLLWHGADITHVTTRGWTASHIAAIRGQDACVQALIMN
GANLTAQDDRGCTPLHLAATHGHSFTLQIMLRSGVDPSVTDKREWRPVHYAAFHGRLGCLQLLVKWGCSIEDVDYNGNLPVHLAAME
GHLHCFKFLVSRMSSATQVLKAFNDNGENVLDLAQRFFKQNILQFIQGAEYEGKDLEDQETLAFPGHVAAFKGDLGMLKKLVEDGVI
NINERADNGSTPMHKAAGQGHIECLQWLIKMGADSNITNKAGERPSDVAKRFAHLAAVKLLEELQKYDIDDENEIDENDVKYFIRHG
VEGSTDAKDDLCLSDLDKTDARRPSKNCRASWSMNDYVEKN
>sp|P35226|BMI1_HUMAN
MHRTTRIKITELNPHLMCVLCGGYFIDATTIIECLHSFCKTCIVRYLETSKYCPICDVQVHKTRPLLNIRSDKTLQDIVYKLVPGLF
KNEMKRRRDFYAAHPSADAANGSNEDRGEVADEDKRIITDDEIISLSIEFFDQNRLDRKVNKDKEKSKEEVNDKRYLRCPAAMTVMH
LRKFLRSKMDIPNTFQIDVMYEEEPLKDYYTLMDIAYIYTWRRNGPLPLKYRVRPTCKRMKISHQRDGLTNAGELESDSGSDKANSP
AGGIPSTSSCLPSPSTPVQSPHPQFPHISSTMNGTSNSPSGNHQSSFANRPRKSSVNGSSATSSG
>sp|A8K979|ERI2_HUMAN
MATKRLARQLGLIRRKSIAPANGNLGRSKSKQLFDYLIVIDFESTCWNDGKHHHSQEIIEFPAVLLNTSTGQIDSEFQAYVQPQEHP
ILSEFCMELTGIKQAQVDEGVPLKICLSQFCKWIHKIQQQKNIIFATGISEPSASEVKLCAFVTWSDWDLGVCLEYECKRKQLLKPV
FLNSWIDLRATYKLFYRRKPKGLSGALQEVGIEFSGREHSGLDDSRNTALLAWKMIRDGCVMKITRSLNKVPTKKNFSILARNLNTI
QVEEMSACNISIQGPSIYNKEPKNIINPHEKVQMKSICANSPIKAQQDQLQVKNNIKASLHNVKSSLPLFNTKSSTSVGQLQSPTLN
SPIYMQKQGKNEHLAFNTKSKASTVGSELVLVSTTVPTVHHVSDLEMSSTLDCLPVLADWEDVVLLPASQPEENVDCTVPISDSDLE
ISFNSGERLMVLKELEMSSHENFGDIEETPQKSETSKSIVYKSPHTTIYNVKEAKDPGSDISAFKLPEHKSSTFNRVNANMSHPLVL
GKHPLLSGGTKRNPCSPQAFPPAKKQPFTIHEEKPTSSDCSPVRSSSWRRLPSILTSTVNLQEPWKSGKMTPPLCKCGRRSKRLVVS
NNGPNHGKVFYCCPIGKYQENRKCCGYFKWEQTLQKERANSMVPSHSTGGLTFSSPETSHICDRNLSISTKNSLRLRPSMRN
>sp|P26927|HGFL_HUMAN
MGWLPLLLLLTQCLGVPGQRSPLNDFQVLRGTELQHLLHAVVPGPWQEDVADAEECAGRCGPLMDCRAFHYNVSSHGCQLLPWTQHS
PHTRLRRSGRCDLFQKKDYVRTCIMNNGVGYRGTMATTVGGLPCQAWSHKFPNDHKYTPTLRNGLEENFCRNPDGDPGGPWCYTTDP
AVRFQSCGIKSCREAACVWCNGEEYRGAVDRTESGRECQRWDLQHPHQHPFEPGKFLDQGLDDNYCRNPDGSERPWCYTTDPQIERE
FCDLPRCGSEAQPRQEATTVSCFRGKGEGYRGTANTTTAGVPCQRWDAQIPHQHRFTPEKYACKDLRENFCRNPDGSEAPWCFTLRP
GMRAAFCYQIRRCTDDVRPQDCYHGAGEQYRGTVSKTRKGVQCQRWSAETPHKPQFTFTSEPHAQLEENFCRNPDGDSHGPWCYTMD
PRTPFDYCALRRCADDQPPSILDPPDQVQFEKCGKRVDRLDQRRSKLRVVGGHPGNSPWTVSLRNRQGQHFCGGSLVKEQWILTARQ
CFSSCHMPLTGYEVWLGTLFQNPQHGEPSLQRVPVAKMVCGPSGSQLVLLKLERSVTLNQRVALICLPPEWYVVPPGTKCEIAGWGE
TKGTGNDTVLNVALLNVISNQECNIKHRGRVRESEMCTEGLLAPVGACEGDYGGPLACFTHNCWVLEGIIIPNRVCARSRWPAVFTR
VSVFVDWIHKVMRLG
>sp|P14555|PA2GA_HUMAN
MKTLLLLAVIMIFGLLQAHGNLVNFHRMIKLTTGKEAALSYGFYGCHCGVGGRGSPKDATDRCCVTHDCCYKRLEKRGCGTKFLSYK
FSNSGSRITCAKQDSCRSQLCECDKAAATCFARNKTTYNKKYQYYSNKHCRGSTPRC
>sp|Q86VI3|IQGA3_HUMAN
MERRAAGPGWAAYERLTAEEMDEQRRQNVAYQYLCRLEEAKRWMEACLKEELPSPVELEESLRNGVLLAKLGHCFAPSVVPLKKIYD
VEQLRYQATGLHFRHTDNINFWLSAIAHIGLPSTFFPETTDIYDKKNMPRVVYCIHALSLFLFRLGLAPQIHDLYGKVKFTAEELSN
MASELAKYGLQLPAFSKIGGILANELSVDEAAVHAAVLAINEAVERGVVEDTLAALQNPSALLENLREPLAAVYQEMLAQAKMEKAA
NARNHDDRESQDIYDHYLTQAEIQGNINHVNVHGALEVVDDALERQSPEALLKALQDPALALRGVRRDFADWYLEQLNSDREQKAQE
LGLVELLEKEEVQAGVAAANTKGDQEQAMLHAVQRINKAIRRRVAADTVKELMCPEAQLPPVYPVASSMYQLELAVLQQQQGELGQE

ELFVAVEMLSAVVLINRALEARDASGFWSSLVNPATGLAEVEGENAQRYFDALLKLRQERGMGEDFLSWNDLQATVSQVNAQTQEET
DRVLAVSLINEALDKGSPEKTLSALLLPAAGLDDVSLPVAPRYHLLLVAAKRQKAQVTGDPGAVLWLEEIRQGVVRANQDTNTAQRM
ALGVAAINQAIKEGKAAQTERVLRNPAVALRGVVPDCANGYQRALESAMAKKQRPADTAFWVQHDMKDGTAYYFHLQTFQGIWEQPP
GCPLNTSHLTREEIQSAVTKVTAAYDRQQLWKANVGFVIQLQARLRGFLVRQKFAEHSHFLRTWLPAVIKIQAHWRGYRQRKIYLEW
LQYFKANLDAIIKIQAWARMWAARRQYLRRLHYFQKNVNSIVKIQAFFRARKAQDDYRILVHAPHPPLSVVRRFAHLLNQSQQDFLA
EAELLKLQEEVVRKIRSNQQLEQDLNIMDIKIGLLVKNRITLQEVVSHCKKLTKRNKEQLSDMMVLDKQKGLKSLSKEKRQKLEAYQ
HLFYLLQTQPIYLAKLIFQMPQNKTTKFMEAVIFSLYNYASSRREAYLLLQLFKTALQEEIKSKVEQPQDVVTGNPTVVRLVVRFYR
NGRGQSALQEILGKVIQDVLEDKVLSVHTDPVHLYKNWINQTEAQTGQRSHLPYDVTPEQALSHPEVQRRLDIALRNLLAMTDKFLL
AITSSVDQIPYGMRYVAKVLKATLAEKFPDATDSEVYKVVGNLLYYRFLNPAVVAPDAFDIVAMAAGGALAAPQRHALGAVAQLLQH
AAAGKAFSGQSQHLRVLNDYLEETHLKFRKFIHRACQVPEPEERFAVDEYSDMVAVAKPMVYITVGELVNTHRLLLEHQDCIAPDHQ
DPLHELLEDLGELPTIPDLIGESIAADGHTDLSKLEVSLTLTNKFEGLEADADDSNTRSLLLSTKQLLADIIQFHPGDTLKEILSLS
ASREQEAAHKQLMSRRQACTAQTPEPLRRHRSLTAHSLLPLAEKQRRVLRNLRRLEALGLVSARNGYQGLVDELAKDIRNQHRHRHR
RKAELVKLQATLQGLSTKTTFYEEQGDYYSQYIRACLDHLAPDSKSSGKGKKQPSLHYTAAQLLEKGVLVEIEDLPASHFRNVIFDI
TPGDEAGKFEVNAKFLGVDMERFQLHYQDLLQLQYEGVAVMKLFNKAKVNVNLLIFLLNKKFLRK
>sp|Q9HBU9|POPD2_HUMAN
MSANSSRVGQLLLQGSACIRWKQDVEGAVYHLANCLLLLGFMGGSGVYGCFYLFGFLSAGYLCCVLWGWFSACGLDIVLWSFLLAVV
CLLQLAHLVYRLREDTLPEEFDLLYKTLCLPLQVPLQTYKEIVHCCEEQVLTLATEQTYAVEGETPINRLSLLLSGRVRVSQDGQFL
HYIFPYQFMDSPEWESLQPSEEGVFQVTLTAETSCSYISWPRKSLHLLLTKERYISCLFSALLGYDISEKLYTLNDKLFAKFGLRFD
IRLPSLYHVLGPTAADAGPESEKGDEEVCEPAVSPPQATPTSLQQTPPCSTPPATTNFPAPPTRARLSRPDSGILASRIPLQSYSQV
ISRGQAPLAPTHTPEL
>sp|Q5THK1|PR14L_HUMAN
MLSSGVETQPVPLDSSMSAVVQELYSELPVSVSRELHADPEPSVIPDVKPGASSSLLSQNRALPLELQRTHVESCCEETYETLDHGS
EPGRCGLVDSTAGGSVASGILDRAKRSESMEPKVFRDPGGQAGIIREPSEGAKEDPHQHSTAAEEKTSPSQEDLLMQSSKELSHVDL
PEDFLRSKEGNVQITAETLLKSAEVQGMKVNGTKTDNNEGHKNGNVSKDLSAGCGEFQEVDKIMTSDEVSETSTLVTPEPLTFVDPV
LTEATPKEKECEELKSCPWLSLPGNSAISNVDNGKEELCKPNLVCEADDNHQQLHGHHNEQPSSTHDSPTATSPLKENSEVSCFTSD
LSGPESRTISLENCGFEGGGLLKRSAEKTDSSYFYRGDDQGKNLASREENEERLLIPRSERGGPFLFNAREPEKEISGRCSGEKEPV
VSPKENIHNNCIQDSLHTGNSSSLMPNSFTEATEVMLNKNDLKITVHVQGNLTNPEDHKETFTNMSHPGGHSEESSFSSLMQIEEAG
QTTPVEPNILSKSFYTKDCNSLVSIQRNLEGNTQLNEASCNDFLFERKSIVSLMPEDQISPVSEVLKPKQGTALLLPSPEFDYRPES
EKVIQTSHDDIPLLDEQSIACEMNELSCTNELVVNKVESECVLNQQVSLNSQEHANLPTDSLLHLNKEMPLATGRDAHQSHHPPLEG
RADVIADIQTIPIQTKIKDISPPGNQTCGASSNCPTLNIKPVSLERKKEMADSGTKALHSRLRSNKREAAGFPQVVSVIECHSVQSQ
DISSCHRVRKNVSQENMCSASAAFKSSKISLQVDNSLITKYENAFQHRDHCCQGTGHSVEKSSCKVSYTSQERELDGKETNGSLPGD
KIRNKMVAGLLNSGISNKTIHTSSSIKLSEEGLEGKEQDVSKETVFCKYNISDHAIQELNQTVNIPGPEKVLDQSPTVMFSSFKNVK
SVETLDQKADEVLDCQSNQNRPDECKSEGQSAKEMLSSDQRETVTEPHGEVNHNQKDLLVSSGSNNSLPCGSPKKCNLKGAFVKMSG
CDESTEGMVDIVYTDCSNKLAEGVLDVKASNLLDCGARQEKLAFQEDSRSTLSRRELDAAHTGTTGQDSDFPVTAASTVDFLKIKKS
CEENVCRSLKDCEMEKCPDSCAHEMESVADHEPNKRILGRVNLSLNDSHYGQQDKGTSLRETQEMTEGSRLEPNSEFGKESTFGISS
KESMSCHDESSVSLRSLKSIEIMPSQENSETNVNSEETDLKNLCKPKDGEMLCENVKDCTVLPEMKEIVSRDWSNSSDRDSVCTCVE
KNACKACHPHENSSDRHLPLTVKTDIKVKGEETEEHQRGRLGYLTVGEQSEELVTRETGDGDPVSNISQTHFKCRGILNHAEKQQSP
EVLDYMLQKEEKYIRQQKAHTISQQCISSSLLLDDAQNQNQPKADKDESTMINEITLAKLAKDSIVAQTQKLEDQKEERLHHPLRKD
TESCTSPCLLGAPRKAQDPSSAGCDQIHGAFAKKGVLPLKKQPHRTCKKVSYQEQIIVGRKIGKIRSSAFLKSSSNPIPTKAHRLLS
LCTLSAPTRLEPETAPTKSLVSHIPKQMSTPCHPLRSLNFRKTTKESALLNKLSILASKLAPAMKTQKLRYRRCSSELLPMAKSYKR
LRYKRLLDGFSSSTEQLNPYLAASGWDKRPNSKPMALYSLESIKMTFIDLSNKMPSLLFGSEIFPVSFHVKSSSSDCTTESSRTFPE
HCAPARLALGEALQCPSQPPKWTFSFFLSHGCPGMATFREDTGVHSQTHTQAPPQPPAPLQDYGGTAIVQTRADCSVLGLHTLLALC
SPGCYRIWTKKRSFSSHMPTMQRLFMTQFTQGLKGLRSPASIADKVFCSLPYSVGRVLSIWSQHGPSVCSFEISSLHSPHCKRQPSL
GTTSSHTMLPYVPLPGMEATYNTSGSQTRLEPPFPALVPKSCLVAESAVSKLLLSASEFQVRGLDELDGVKAACPCPQSSPPEQKEA

EPEKRPKKVSQIRIRKTIPRPDPNLTPMGLPRPKRLKKKEFSLEEIYTNKNYKSPPANRCLETIFEEPKERNGTLISISQQKRKRVL
EFQDFTVPRKRRARGKVKVAGSFTRAQKAAVQSRELDALLIQKLMELETFFAKEEEQEQSSGC
>sp|Q92886|NGN1_HUMAN
MPARLETCISDLDCASSSGSDLSGFLTDEEDCARLQQAASASGPPAPARRGAPNISRASEVPGAQDDEQERRRRRGRTRVRSEALLH
SLRRSRRVKANDRERNRMHNLNAALDALRSVLPSFPDDTKLTKIETLRFAYNYIWALAETLRLADQGLPGGGARERLLPPQCVPCLP
GPPSPASDAESWGSGAAAASPLSDPSSPAASEDFTYRPGDPVFSFPSLPKDLLHTTPCFIPYH
>sp|P47710|CASA1_HUMAN
MRLLILTCLVAVALARPKLPLRYPERLQNPSESSEPIPLESREEYMNGMNRQRNILREKQTDEIKDTRNESTQNCVVAEPEKMESSI
SSSSEEMSLSKCAEQFCRLNEYNQLQLQAAHAQEQIRRMNENSHVQVPFQQLNQLAAYPYAVWYYPQIMQYVPFPPFSDISNPTAHE
NYEKNNVMLQW
>sp|Q6Q4G3|AMPQ_HUMAN
MGPPSSSGFYVSRAVALLLAGLVAALLLALAVLAALYGHCERVPPSELPGLRDLEAESSPPLRQKPTPTPKPSSARELAVTTTPSNW
RPPGPWDQLRLPPWLVPLHYDLELWPQLRPDELPAGSLPFTGRVNITVRCTVATSRLLLHSLFQDCERAEVRGPLSPGTGNATVGRV
PVDDVWFALDTEYMVLELSEPLKPGSSYELQLSFSGLVKEDLREGLFLNVYTDQGERRALLASQLEPTFARYVFPCFDEPALKATFN
ITMIHHPSYVALSNMPKLGQSEKEDVNGSKWTVTTFSTTPHMPTYLVAFVICDYDHVNRTERGKEIRIWARKDAIANGSADFALNIT
GPIFSFLEDLFNISYSLPKTDIIALPSFDNHAMENWGLMIFDESGLLLEPKDQLTEKKTLISYVVSHEIGHQWFGNLVTMNWWNNIW
LNEGFASYFEFEVINYFNPKLPRNEIFFSNILHNILREDHALVTRAVAMKVENFKTSEIQELFDIFTYSKGASMARMLSCFLNEHLF
VSALKSYLKTFSYSNAEQDDLWRHFQMAIDDQSTVILPATIKNIMDSWTHQSGFPVITLNVSTGVMKQEPFYLENIKNRTLLTSNDT
WIVPILWIKNGTTQPLVWLDQSSKVFPEMQVSDSDHDWVILNLNMTGYYRVNYDKLGWKKLNQQLEKDPKAIPVIHRLQLIDDAFSL
SKNNYIEIETALELTKYLAEEDEIIVWHTVLVNLVTRDLVSEVNIYDIYSLLKRYLLKRLNLIWNIYSTIIRENVLALQDDYLALIS
LEKLFVTACWLGLEDCLQLSKELFAKWVDHPENEIPYPIKDVVLCYGIALGSDKEWDILLNTYTNTTNKEEKIQLAYAMSCSKDPWI
LNRYMEYAISTSPFTSNETNIIEVVASSEVGRYVAKDFLVNNWQAVSKRYGTQSLINLIYTIGRTVTTDLQIVELQQFFSNMLEEHQ
RIRVHANLQTIKNENLKNKKLSARIAAWLRRNT
>sp|Q9GZV3|SC5A7_HUMAN
MAFHVEGLIAIIVFYLLILLVGIWAAWRTKNSGSAEERSEAIIVGGRDIGLLVGGFTMTATWVGGGYINGTAEAVYVPGYGLAWAQA
PIGYSLSLILGGLFFAKPMRSKGYVTMLDPFQQIYGKRMGGLLFIPALMGEMFWAAAIFSALGATISVIIDVDMHISVIISALIATL
YTLVGGLYSVAYTDVVQLFCIFVGLWISVPFALSHPAVADIGFTAVHAKYQKPWLGTVDSSEVYSWLDSFLLLMLGGIPWQAYFQRV
LSSSSATYAQVLSFLAAFGCLVMAIPAILIGAIGASTDWNQTAYGLPDPKTTEEADMILPIVLQYLCPVYISFFGLGAVSAAVMSSA
DSSILSASSMFARNIYQLSFRQNASDKEIVWVMRITVFVFGASATAMALLTKTVYGLWYLSSDLVYIVIFPQLLCVLFVKGTNTYGA
VAGYVSGLFLRITGGEPYLYLQPLIFYPGYYPDDNGIYNQKFPFKTLAMVTSFLTNICISYLAKYLFESGTLPPKLDVFDAVVARHS
EENMDKTILVKNENIKLDELALVKPRQSMTLSSTFTNKEAFLDVDSSPEGSGTEDNLQ
>sp|Q96RS6|NUDC1_HUMAN
MEVAANCSLRVKRPLLDPRFEGYKLSLEPLPCYQLELDAAVAEVKLRDDQYTLEHMHAFGMYNYLHCDSWYQDSVYYIDTLGRIMNL
TVMLDTALGKPREVFRLPTDLTACDNRLCASIHFSSSTWVTLSDGTGRLYVIGTGERGNSASEKWEIMFNEELGDPFIIIHSISLLN
AEEHSIATLLLRIEKEELDMKGSGFYVSLEWVTISKKNQDNKKYEIIKRDILRGKSVPHYAAIEPDGNGLMIVSYKSLTFVQAGQDL
EENMDEDISEKIKEPLYYWQQTEDDLTVTIRLPEDSTKEDIQIQFLPDHINIVLKDHQFLEGKLYSSIDHESSTWIIKESNSLEISL
IKKNEGLTWPELVIGDKQGELIRDSAQCAAIAERLMHLTSEELNPNPDKEKPPCNAQELEECDIFFEESSSLCRFDGNTLKTTHVVN
LGSNQYLFSVIVDPKEMPCFCLRHDVDALLWQPHSSKQDDMWEHIATFNALGYVQASKRDKKFFACAPNYSYAALCECLRRVFIYRQ
PAPMSTVLYNRKEGRQVGQVAKQQVASLETNDPILGFQATNERLFVLTTKNLFLIKVNTEN
>sp|Q6P3W7|SCYL2_HUMAN
MESMLNKLKSTVTKVTADVTSAVMGNPVTREFDVGRHIASGGNGLAWKIFNGTKKSTKQEVAVFVFDKKLIDKYQKFEKDQIIDSLK
RGVQQLTRLRHPRLLTVQHPLEESRDCLAFCTEPVFASLANVLGNWENLPSPISPDIKDYKLYDVETKYGLLQVSEGLSFLHSSVKM
VHGNITPENIILNKSGAWKIMGFDFCVSSTNPSEQEPKFPCKEWDPNLPSLCLPNPEYLAPEYILSVSCETASDMYSLGTVMYAVFN
KGKPIFEVNKQDIYKSFSRQLDQLSRLGSSSLTNIPEEVREHVKLLLNVTPTVRPDADQMTKIPFFDDVGAVTLQYFDTLFQRDNLQ

KSQFFKGLPKVLPKLPKRVIVQRILPCLTSEFVNPDMVPFVLPNVLLIAEECTKEEYVKLILPELGPVFKQQEPIQILLIFLQKMDL
LLTKTPPDEIKNSVLPMVYRALEAPSIQIQELCLNIIPTFANLIDYPSMKNALIPRIKNACLQTSSLAVRVNSLVCLGKILEYLDKW
FVLDDILPFLQQIPSKEPAVLMGILGIYKCTFTHKKLGITKEQLAGKVLPHLIPLSIENNLNLNQFNSFISVIKEMLNRLESEHKTK
LEQLHIMQEQQKSLDIGNQMNVSEEMKVTNIGNQQIDKVFNNIGADLLTGSESENKEDGLQNKHKRASLTLEEKQKLAKEQEQAQKL
KSQQPLKPQVHTPVATVKQTKDLTDTLMDNMSSLTSLSVSTPKSSASSTFTSVPSMGIGMMFSTPTDNTKRNLTNGLNANMGFQTSG
FNMPVNTNQNFYSSPSTVGVTKMTLGTPPTLPNFNALSVPPAGAKQTQQRPTDMSALNNLFGPQKPKVSMNQLSQQKPNQWLNQFVP
PQGSPTMGSSVMGTQMNVIGQSAFGMQGNPFFNPQNFAQPPTTMTNSSSASNDLKDLFG
>sp|Q16854|DGUOK_HUMAN
MAAGRLFLSRLRAPFSSMAKSPLEGVSSSRGLHAGRGPRRLSIEGNIAVGKSTFVKLLTKTYPEWHVATEPVATWQNIQAAGTQKAC
TAQSLGNLLDMMYREPARWSYTFQTFSFLSRLKVQLEPFPEKLLQARKPVQIFERSVYSDRYIFAKNLFENGSLSDIEWHIYQDWHS
FLLWEFASRITLHGFIYLQASPQVCLKRLYQRAREEEKGIELAYLEQLHGQHEAWLIHKTTKLHFEALMNIPVLVLDVNDDFSEEVT
KQEDLMREVNTFVKNL
>sp|O95166|GBRAP_HUMAN
MKFVYKEEHPFEKRRSEGEKIRKKYPDRVPVIVEKAPKARIGDLDKKKYLVPSDLTVGQFYFLIRKRIHLRAEDALFFFVNNVIPPT
SATMGQLYQEHHEEDFFLYIAYSDESVYGL
>sp|Q14802|FXYD3_HUMAN
MQKVTLGLLVFLAGFPVLDANDLEDKNSPFYYDWHSLQVGGLICAGVLCAMGIIIVMSAKCKCKFGQKSGHHPGETPPLITPGSAQS
>sp|P32243|OTX2_HUMAN
MMSYLKQPPYAVNGLSLTTSGMDLLHPSVGYPATPRKQRRERTTFTRAQLDVLEALFAKTRYPDIFMREEVALKINLPESRVQVWFK
NRRAKCRQQQQQQQNGGQNKVRPAKKKTSPAREVSSESGTSGQFTPPSSTSVPTIASSSAPVSIWSPASISPLSDPLSTSSSCMQRS
YPMTYTQASGYSQGYAGSTSYFGGMDCGSYLTPMHHQLPGPGATLSPMGTNAVTSHLNQSPASLSTQGYGASSLGFNSTTDCLDYKD
QTASWKLNFNADCLDYKDQTSSWKFQVL
>sp|Q8NG92|O13H1_HUMAN
MAMDNVTAVFQFLLIGISNYPQWRDTFFTLVLIIYLSTLLGNGFMIFLIHFDPNLHTPIYFFLSNLSFLDLCYGTASMPQALVHCFS
THPYLSYPRCLAQTSVSLALATAECLLLAAMAYDRVVAISNPLRYSVVMNGPVCVCLVATSWGTSLVLTAMLILSLRLHFCGANVIN
HFACEILSLIKLTCSDTSLNEFMILITSIFTLLLPFGFVLLSYIRIAMAIIRIRSLQGRLKAFTTCGSHLTVVTIFYGSAISMYMKT
QSKSYPDQDKFISVFYGALTPMLNPLIYSLRKKDVKRAIRKVMLKRT
>sp|Q8NAJ2|CI106_HUMAN
MAYVALSDKPHLSGEVGEEACSSWNPPLFSPRPLPRLWPLPGTPFLPIRALPFSASSSGKSSLVPSPSSSAHSGFRTPCLGPDCLLC
TQGCELHEGRNHMAVHSCVARAWPGDPQEVRHLNPLLCDPGSQVEPSWPWHPGLEQAAASWVGNHVSPAHRQALRGHSLGSALRALM
PGRHCPLCVPCKRGCDLRGGRGKHGPRPCCPLLRKFPVLPVHPWPFPCAVWDSGWSCR
>sp|P62081|RS7_HUMAN
MFSSSAKIVKPNGEKPDEFESGISQALLELEMNSDLKAQLRELNITAAKEIEVGGGRKAIIIFVPVPQLKSFQKIQVRLVRELEKKF
SGKHVVFIAQRRILPKPTRKSRTKNKQKRPRSRTLTAVHDAILEDLVFPSEIVGKRIRVKLDGSRLIKVHLDKAQQNNVEHKVETFS
GVYKKLTGKDVNFEFPEFQL
>sp|O00421|CCRL2_HUMAN
MANYTLAPEDEYDVLIEGELESDEAEQCDKYDAQALSAQLVPSLCSAVFVIGVLDNLLVVLILVKYKGLKRVENIYLLNLAVSNLCF
LLTLPFWAHAGGDPMCKILIGLYFVGLYSETFFNCLLTVQRYLVFLHKGNFFSARRRVPCGIITSVLAWVTAILATLPEFVVYKPQM
EDQKYKCAFSRTPFLPADETFWKHFLTLKMNISVLVLPLFIFTFLYVQMRKTLRFREQRYSLFKLVFAIMVVFLLMWAPYNIAFFLS
TFKEHFSLSDCKSSYNLDKSVHITKLIATTHCCINPLLYAFLDGTFSKYLCRCFHLRSNTPLQPRGQSAQGTSREEPDHSTEV
>sp|P61088|UBE2N_HUMAN
MAGLPRRIIKETQRLLAEPVPGIKAEPDESNARYFHVVIAGPQDSPFEGGTFKLELFLPEEYPMAAPKVRFMTKIYHPNVDKLGRIC
LDILKDKWSPALQIRTVLLSIQALLSAPNPDDPLANDVAEQWKTNEAQAIETARAWTRLYAMNNI
>sp|Q99583|MNT_HUMAN

MSIETLLEAARFLEWQAQQQQRAREEQERLRLEQEREQEQKKANSLARLAHTLPVEEPRMEAPPLPLSPPAPPPAPPPPLATPAPLT
VIPIPVVTNSPQPLPPPPPLPAAAQPLPLAPRQPALVGAPGLSIKEPAPLPSRPQVPTPAPLLPDSKATIPPNGSPKPLQPLPTPVL
TIAPHPGVQPQLAPQQPPPPTLGTLKLAPAEEVKSSEQKKRPGGIGTREVHNKLEKNRRAHLKECFETLKRNIPNVDDKKTSNLSVL
RTALRYIQSLKRKEKEYEHEMERLAREKIATQQRLAELKHELSQWMDVLEIDRVLRQTGQPEDDQASTSTASEGEDNIDEDMEEDRA
GLGPPKLSHRPQPELLKSTLPPPSTTPAPLPPHPHPHPHSVALPPAHLPVQQQQPQQKTPLPAPPPPPAAPAQTLVPAPAHLVATAG
GGSTVIAHTATTHASVIQTVNHVLQGPGGKHIAHIAPSAPSPAVQLAPATPPIGHITVHPATLNHVAHLGSQLPLYPQPVAVSHIAH
TLSHQQVNGTAGLGPPATVMAKPAVGAQVVHHPQLVGQTVLNPVTMVTMPSFPVSTLKLA
>sp|Q15661|TRYB1_HUMAN
MLNLLLLALPVLASRAYAAPAPGQALQRVGIVGGQEAPRSKWPWQVSLRVHGPYWMHFCGGSLIHPQWVLTAAHCVGPDVKDLAALR
VQLREQHLYYQDQLLPVSRIIVHPQFYTAQIGADIALLELEEPVNVSSHVHTVTLPPASETFPPGMPCWVTGWGDVDNDERLPPPFP
LKQVKVPIMENHICDAKYHLGAYTGDDVRIVRDDMLCAGNTRRDSCQGDSGGPLVCKVNGTWLQAGVVSWGEGCAQPNRPGIYTRVT
YYLDWIHHYVPKKP
>sp|P42127|ASIP_HUMAN
MDVTRLLLATLLVFLCFFTANSHLPPEEKLRDDRSLRSNSSVNLLDVPSVSIVALNKKSKQIGRKAAEKKRSSKKEASMKKVVRPRT
PLSAPCVATRNSCKPPAPACCDPCASCQCRFFRSACSCRVLSLNC
>sp|O75817|POP7_HUMAN
MAENREPRGAVEAELDPVEYTLRKRLPSRLPRRPNDIYVNMKTDFKAQLARCQKLLDGGARGQNACSEIYIHGLGLAINRAINIALQ
LQAGSFGSLQVAANTSTVELVDELEPETDTREPLTRIRNNSAIHIRVFRVTPK
>sp|Q2NKX8|ERC6L_HUMAN
MEASRRFPEAEALSPEQAAHYLRYVKEAKEATKNGDLEEAFKLFNLAKDIFPNEKVLSRIQKIQEALEELAEQGDDEFTDVCNSGLL
LYRELHNQLFEHQKEGIAFLYSLYRDGRKGGILADDMGLGKTVQIIAFLSGMFDASLVNHVLLIMPTNLINTWVKEFIKWTPGMRVK
TFHGPSKDERTRNLNRIQQRNGVIITTYQMLINNWQQLSSFRGQEFVWDYVILDEAHKIKTSSTKSAICARAIPASNRLLLTGTPIQ
NNLQELWSLFDFACQGSLLGTLKTFKMEYENPITRAREKDATPGEKALGFKISENLMAIIKPYFLRRTKEDVQKKKSSNPEARLNEK
NPDVDAICEMPSLSRKNDLIIWIRLVPLQEEIYRKFVSLDHIKELLMETRSPLAELGVLKKLCDHPRLLSARACCLLNLGTFSAQDG
NEGEDSPDVDHIDQVTDDTLMEESGKMIFLMDLLKRLRDEGHQTLVFSQSRQILNIIERLLKNRHFKTLRIDGTVTHLLEREKRINL
FQQNKDYSVFLLTTQVGGVGLTLTAATRVVIFDPSWNPATDAQAVDRVYRIGQKENVVVYRLITCGTVEEKIYRRQVFKDSLIRQTT
GEKKNPFRYFSKQELRELFTIEDLQNSVTQLQLQSLHAAQRKSDIKLDEHIAYLQSLGIAGISDHDLMYTCDLSVKEELDVVEESHY
IQQRVQKAQFLVEFESQNKEFLMEQQRTRNEGAWLREPVFPSSTKKKCPKLNKPQPQPSPLLSTHHTQEEDISSKMASVVIDDLPKE
GEKQDLSSIKVNVTTLQDGKGTGSADSIATLPKGFGSVEELCTNSSLGMEKSFATKNEAVQKETLQEGPKQEALQEDPLESFNYVLS
KSTKADIGPNLDQLKDDEILRHCNPWPIISITNESQNAESNVSIIEIADDLSASHSALQDAQASEAKLEEEPSASSPQYACDFNLFL
EDSADNRQNFSSQSLEHVEKENSLCGSAPNSRAGFVHSKTCLSWEFSEKDDEPEEVVVKAKIRSKARRIVSDGEDEDDSFKDTSSIN
PFNTSLFQFSSVKQFDASTPKNDISPPGRFFSSQIPSSVNKSMNSRRSLASRRSLINMVLDHVEDMEERLDDSSEAKGPEDYPEEGV
EESSGEASKYTEEDPSGETLSSENKSSWLMTSKPSALAQETSLGAPEPLSGEQLVGSPQDKAAEATNDYETLVKRGKELKECGKIQE
ALNCLVKALDIKSADPEVMLLTLSLYKQLNNN
>sp|Q9NQI0|DDX4_HUMAN
MGDEDWEAEINPHMSSYVPIFEKDRYSGENGDNFNRTPASSSEMDDGPSRRDHFMKSGFASGRNFGNRDAGECNKRDNTSTMGGFGV
GKSFGNRGFSNSRFEDGDSSGFWRESSNDCEDNPTRNRGFSKRGGYRDGNNSEASGPYRRGGRGSFRGCRGGFGLGSPNNDLDPDEC
MQRTGGLFGSRRPVLSGTGNGDTSQSRSGSGSERGGYKGLNEEVITGSGKNSWKSEAEGGESSDTQGPKVTYIPPPPPEDEDSIFAH
YQTGINFDKYDTILVEVSGHDAPPAILTFEEANLCQTLNNNIAKAGYTKLTPVQKYSIPIILAGRDLMACAQTGSGKTAAFLLPILA
HMMHDGITASRFKELQEPECIIVAPTRELVNQIYLEARKFSFGTCVRAVVIYGGTQLGHSIRQIVQGCNILCATPGRLMDIIGKEKI
GLKQIKYLVLDEADRMLDMGFGPEMKKLISCPGMPSKEQRQTLMFSATFPEEIQRLAAEFLKSNYLFVAVGQVGGACRDVQQTVLQV
GQFSKREKLVEILRNIGDERTMVFVETKKKADFIATFLCQEKISTTSIHGDREQREREQALGDFRFGKCPVLVATSVAARGLDIENV
QHVINFDLPSTIDEYVHRIGRTGRCGNTGRAISFFDLESDNHLAQPLVKVLTDAQQDVPAWLEEIAFSTYIPGFSGSTRGNVFASVD
TRKGKSTLNTAGFSSSQAPNPVDDESWD

>sp|Q86WJ1|CHD1L_HUMAN
MERAGATSRGGQAPGFLLRLHTEGRAEAARVQEQDLRQWGLTGIHLRSYQLEGVNWLAQRFHCQNGCILGDEMGLGKTCQTIALFIY
LAGRLNDEGPFLILCPLSVLSNWKEEMQRFAPGLSCVTYAGDKEERACLQQDLKQESRFHVLLTTYEICLKDASFLKSFPWSVLVVD
EAHRLKNQSSLLHKTLSEFSVVFSLLLTGTPIQNSLQELYSLLSFVEPDLFSKEEVGDFIQRYQDIEKESESASELHKLLQPFLLRR
VKAEVATELPKKTEVVIYHGMSALQKKYYKAILMKDLDAFENETAKKVKLQNILSQLRKCVDHPYLFDGVEPEPFEVGDHLTEASGK
LHLLDKLLAFLYSGGHRVLLFSQMTQMLDILQDYMDYRGYSYERVDGSVRGEERHLAIKNFGQQPIFVFLLSTRAGGVGMNLTAADT
VIFVDSDFNPQNDLQAAARAHRIGQNKSVKVIRLIGRDTVEEIVYRKAASKLQLTNMIIEGGHFTLGAQKPAADADLQLSEILKFGL
DKLLASEGSTMDEIDLESILGETKDGQWVSDALPAAEGGSRDQEEGKNHMYLFEGKDYSKEPSKEDRKSFEQLVNLQKTLLEKASQE
GRSLRNKGSVLIPGLVEGSTKRKRVLSPEELEDRQKKRQEAAAKRRRLIEEKKRQKEEAEHKKKMAWWESNNYQSFCLPSEESEPED
LENGEESSAELDYQDPDATSLKYVSGDVTHPQAGAEDALIVHCVDDSGHWGRGGLFTALEKRSAEPRKIYELAGKMKDLSLGGVLLF
PVDDKESRNKGQDLLALIVAQHRDRSNVLSGIKMAALEEGLKKIFLAAKKKKASVHLPRIGHATKGFNWYGTERLIRKHLAARGIPT
YIYYFPRSKSAVLHAQSSSSSSRQLVP
>sp|A6NC62|RAKDN_HUMAN
MLTPRKAFRTCSKESGLAETESCGQTHTWPRALAVLMGLWWPRDQKAGEEDLRFRERRPGLQATATGSGEHGAFPVHSQGVWASTHW
QGTAVCPLQTPPPDAFIRNNKVLS
>sp|Q9HCE6|ARGAL_HUMAN
MASSNPPPQPAIGDQLVPGVPGPSSEAEDDPGEAFEFDDSDDEEDTSAALGVPSLAPERDTDPPLIHLDSIPVTDPDPAAAPPGTGV
PAWVSNGDAADAAFSGARHSSWKRKSSRRIDRFTFPALEEDVIYDDVPCESPDAHQPGAERNLLYEDAHRAGAPRQAEDLGWSSSEF
ESYSEDSGEEAKPEVEVEPAKHRVSFQPKLSPDLTRLKERYARTKRDILALRVGGRDMQELKHKYDCKMTQLMKAAKSGTKDGLEKT
RMAVMRKVSFLHRKDVLGDSEEEDMGLLEVSVSDIKPPAPELGPMPEGLSPQQVVRRHILGSIVQSEGSYVESLKRILQDYRNPLME
MEPKALSARKCQVVFFRVKEILHCHSMFQIALSSRVAEWDSTEKIGDLFVASFSKSMVLDVYSDYVNNFTSAMSIIKKACLTKPAFL
EFLKRRQVCSPDRVTLYGLMVKPIQRFPQFILLLQDMLKNTPRGHPDRLSLQLALTELETLAEKLNEQKRLADQVAEIQQLTKSVSD
RSSLNKLLTSGQRQLLLCETLTETVYGDRGQLIKSKERRVFLLNDMLVCANINFKPANHRGQLEISSLVPLGPKYVVKWNTALPQVQ
VVEVGQDGGTYDKDNVLIQHSGAKKASASGGQAQNKVYLGPPRLFQELQDLQKDLAVVEQITLLISTLHGTYQNLNMTVAQDWCLALQ
RLMRVKEEEIHSANKCRLRLLLPGKPDKSGRPISFMVVFITPNPLSKISWVNRLHLAKIGLREENQPGWLCPDEDKKSKAPFWCPIL
ACCIPAFSSRALSLQLGALVHSPVNCPLLGFSAVSTSLPQGYLWVGGGQEGAGGQVEIFSLNRPSPRTVKSFPLAAPVLCMEYIPEL
EEEAESRDESPTVADPSATVHPTICLGLQDGSILLYSSVDTGTQCLVSCRSPGLQPVLCLRHSPFHLLAGLQDGTLAAYPRTSGGVL
WDLESPPVCLTVGPGPVRTLLSLEDAVWASCGPWVTVLEATTLQPQQSFEAHQDEAVSVTHMVKAGSGVWMAFSSGTSIRLFHTETL
EHLQEINIATRTTFLLPGQKHLCVTSLLICQGLLWVGTDQGVIVLLPVPRLEGIPKITGKGMVSLNGHCGPVAFLAVATSILAPDIL
RSDQEEAEGPRAEEDKPDGQAHEPMPDSHVGRELTRKKGILLQYRLRSTAHLPGPLLSMREPAPADGAALEHSEEDGSIYEMADDPD
IWVRSRPCARDAHRKEICSVAIISGGQGYRNFGSALGSSGRQAPCGETDSTLLIWQVPLML
>sp|Q6P2C8|MED27_HUMAN
MADVINVSVNLEAFSQAISAIQALRSSVSRVFDCLKDGMRNKETLEGREKAFIAHFQDNLHSVNRDLNELERLSNLVGKPSENHPLH
NSGLLSLDPVQDKTPLYSQLLQAYKWSNKLQYHAGLASGLLNQQSLKRSANQMGVSAKRRPKAQPTTLVLPPQYVDDVISRIDRMFP
EMSIHLSRPNGTSAMLLVTLGKVLKVIVVMRSLFIDRTIVKGYNENVYTEDGKLDIWSKSNYQVFQKVTDHATTALLHYQLPQMPDV
VVRSFMTWLRSYIKLFQAPCQRCGKFLQDGLPPTWRDFRTLEAFHDTCRQ
>sp|Q9UQC9|CLCA2_HUMAN
MTQRSIAGPICNLKFVTLLVALSSELPFLGAGVQLQDNGYNGLLIAINPQVPENQNLISNIKEMITEASFYLFNATKRRVFFRNIKI
LIPATWKANNNSKIKQESYEKANVIVTDWYGAHGDDPYTLQYRGCGKEGKYIHFTPNFLLNDNLTAGYGSRGRVFVHEWAHLRWGVF
DEYNNDKPFYINGQNQIKVTRCSSDITGIFVCEKGPCPQENCIISKLFKEGCTFIYNSTQNATASIMFMQSLSSVVEFCNASTHNQE
APNLQNQMCSLRSAWDVITDSADFHHSFPMNGTELPPPPTFSLVQAGDKVVCLVLDVSSKMAEADRLLQLQQAAEFYLMQIVEIHTF
VGIASFDSKGEIRAQLHQINSNDDRKLLVSYLPTTVSAKTDISICSGLKKGFEVVEKLNGKAYGSVMILVTSGDDKLLGNCLPTVLS
SGSTIHSIALGSSAAPNLEELSRLTGGLKFFVPDISNSNSMIDAFSRISSGTGDIFQQHIQLESTGENVKPHHQLKNTVTVDNTVGN
DTMFLVTWQASGPPEIILFDPDGRKYYTNNFITNLTFRTASLWIPGTAKPGHWTYTLNNTHHSLQALKVTVTSRASNSAVPPATVEA

FVERDSLHFPHPVMIYANVKQGFYPILNATVTATVEPETGDPVTLRLLDDGAGADVIKNDGIYSRYFFSFAANGRYSLKVHVNHSPS
ISTPAHSIPGSHAMYVPGYTANGNIQMNAPRKSVGRNEEERKWGFSRVSSGGSFSVLGVPAGPHPDVFPPCKIIDLEAVKVEEELTL
SWTAPGEDFDQGQATSYEIRMSKSLQNIQDDFNNAILVNTSKRNPQQAGIREIFTFSPQISTNGPEHQPNGETHESHRIYVAIRAMD
RNSLQSAVSNIAQAPLFIPPNSDPVPARDYLILKGVLTAMGLIGIICLIIVVTHHTLSRKKRADKKENGTKLL
>sp|P52907|CAZA1_HUMAN
MADFDDRVSDEEKVRIAAKFITHAPPGEFNEVFNDVRLLLNNDNLLREGAAHAFAQYNMDQFTPVKIEGYEDQVLITEHGDLGNSRF
LDPRNKISFKFDHLRKEASDPQPEEADGGLKSWRESCDSALRAYVKDHYSNGFCTVYAKTIDGQQTIIACIESHQFQPKNFWNGRWR
SEWKFTITPPTAQVVGVLKIQVHYYEDGNVQLVSHKDVQDSLTVSNEAQTAKEFIKIIENAENEYQTAISENYQTMSDTTFKALRRQ
LPVTRTKIDWNKILSYKIGKEMQNA
>sp|Q9NUJ3|T11L1_HUMAN
MSENLDKSNVNEAGKSKSNDSEEGLEDAVEGADEALQKAIKSDSSSPQRVQRPHSSPPRFVTVEELLETARGVTNMALAHEIVVNGD
FQIKPVELPENSLKKRVKEIVHKAFWDCLSVQLSEDPPAYDHAIKLVGEIKETLLSFLLPGHTRLRNQITEVLDLDLIKQEAENGAL
DISKLAEFIIGMMGTLCAPARDEEVKKLKDIKEIVPLFREIFSVLDLMKVDMANFAISSIRPHLMQQSVEYERKKFQEILERQPNSL
DFVTQWLEEASEDLMTQKYKHALPVGGMAAGSGDMPRLSPVAVQNYAYLKLLKWDHLQRPFPETVLMDQSRFHELQLQLEQLTILGA
VLLVTFSMAAPGISSQADFAEKLKMIVKILLTDMHLPSFHLKDVLTTIGEKVCLEVSSCLSLCGSSPFTTDKETVLKGQIQAVASPD
DPIRRIMESRILTFLETYLASGHQKPLPTVPGGLSPVQRELEEVAIKFARLVNYNKMVFCPYYDAILSKILVRS
>sp|Q9UBW7|ZMYM2_HUMAN
MDTSSVGGLELTDQTPVLLGSTAMATSLTNVGNSFSGPANPLVSRSNKFQNSSVEDDDDVVFIEPVQPPPPSVPVVADQRTITFTSS
KNEELQGNDSKITPSSKELASQKGSVSETIVIDDEEDMETNQGQEKNSSNFIERRPPETKNRTNDVDFSTSSFSRSKVNAGMGNSGI
TTEPDSEIQIANVTTLETGVSSVNDGQLENTDGRDMNLMITHVTSLQNTNLGDVSNGLQSSNFGVNIQTYTPSLTSQTKTGVGPFNP
GRMNVAGDVFQNGESATHHNPDSWISQSASFPRNQKQPGVDSLSPVASLPKQIFQPSVQQQPTKPVKVTCANCKKPLQKGQTAYQRK
GSAHLFCSTTCLSSFSHKPAPKKLCVMCKKDITTMKGTIVAQVDSSESFQEFCSTSCLSLYEDKQNPTKGALNKSRCTICGKLTEIR
HEVSFKNMTHKLCSDHCFNRYRMANGLIMNCCEQCGEYLPSKGAGNNVLVIDGQQKRFCCQSCVSEYKQVGSHPSFLKEVRDHMQDS
FLMQPEKYGKLTTCTGCRTQCRFFDMTQCIGPNGYMEPYCSTACMNSHKTKYAKSQSLGIICHFCKRNSLPQYQATMPDGKLYNFCN
SSCVAKFQALSMQSSPNGQFVAPSDIQLKCNYCKNSFCSKPEILEWENKVHQFCSKTCSDDYKKLHCIVTYCEYCQEEKTLHETVNF
SGVKRPFCSEGCKLLYKQDFARRLGLRCVTCNYCSQLCKKGATKELDGVVRDFCSEDCCKKFQDWYYKAARCDCCKSQGTLKERVQW
RGEMKHFCDQHCLLRFYCQQNEPNMTTQKGPENLHYDQGCQTSRTKMTGSAPPPSPTPNKEMKNKAVLCKPLTMTKATYCKPHMQTK
SCQTDDTWRTEYVPVPIPVPVYIPVPMHMYSQNIPVPTTVPVPVPVPVFLPAPLDSSEKIPAAIEELKSKVSSDALDTELLTMTDMM
SEDEGKTETTNINSVIIETDIIGSDLLKNSDPETQSSMPDVPYEPDLDIEIDFPRAAEELDMENEFLLPPVFGEEYEEQPRPRSKKK
GAKRKAVSGYQSHDDSSDNSECSFPFKYTYGVNAWKHWVKTRQLDEDLLVLDELKSSKSVKLKEDLLSHTTAELNYGLAHFVNEIRR
PNGENYAPDSIYYLCLGIQEYLCGSNRKDNIFIDPGYQTFEQELNKILRSWQPSILPDGSIFSRVEEDYLWRIKQLGSHSPVALLNT
LFYFNTKYFGLKTVEQHLRLSFGTVFRHWKKNPLTMENKACLRYQVSSLCGTDNEDKITTGKRKHEDDEPVFEQIENTANPSRCPVK
MFECYLSKSPQNLNQRMDVFYLQPECSSSTDSPVWYTSTSLDRNTLENMLVRVLLVKDIYDKDNYELDEDTD
>sp|Q96NU1|SAM11_HUMAN
MSKGILQVHPPICDCPGCRISSPVNRGRLADKRTVALPAARNLKKERTPSFSASDGDSDGSGPTCGRRPGLKQEDGPHIRIMKRRVH
THWDVNISFREASCSQDGNLPTLISSVHRSRHLVMPEHQSRCEFQRGSLEIGLRPAGDLLGKRLGRSPRISSDCFSEKRARSESPQE
ALLLPRELGPSMAPEDHYRRLVSALSEASTFEDPQRLYHLGLPSHGEDPPWHDPPHHLPSHDLLRVRQEVAAAALRGPSGLEAHLPS
STAGQRRKQGLAQHREGAAPAAAPSFSERELPQPPPLLSPQNAPHVALGPHLRPPFLGVPSALCQTPGYGFLPPAQAEMFAWQQELL
RKQNLARLELPADLLRQKELESARPQLLAPETALRPNDGAEELQRRGALLVLNHGAAPLLALPPQGPPGSGPPTPSRDSARRAPRKG
GPGPASARPSESKEMTGARLWAQDGSEDEPPKDSDGEDPETAAVGCRGPTPGQAPAGGAGAEGKGLFPGSTLPLGFPYAVSPYFHTG
AVGGLSMDGEEAPAPEDVTKWTVDDVCSFVGGLSGCGEYTRVFREQGIDGETLPLLTEEHLLTNMGLKLGPALKIRAQVARRLGRVF
YVASFPVALPLQPPTLRAPERELGTGEQPLSPTTATSPYGGGHALAGQTSPKQENGTLALLPGAPDPSQPLC
>sp|Q6ZQN5|FOXI2_HUMAN

MATYCDDLGPSSAPPGQAQATAHPPGYEPGDLGAVGGGPLLWVNAPALSPKSYASGPGPAPPYAAPSYGAPGPLLGAPGGLAGADLA
WLSLSGQQELLRLVRPPYSYSALIAMAIQSAPLRKLTLSQIYQYVAGNFPFYKRSKAGWQNSIRHNLSLNDCFKKVPRDEDDPGKGN
YWTLDPNCEKMFDNGNFRRKRKRRAEASAAVRSGARSVGGAEAPALEPPSAACLDLQASPSPSAPEAATCFSGFASAMSALAGGLGT
FPGGLAGDFSFGRRPPTVATHAPQTLNPSPGFAPGHQTAAAGFRLSHLLYSREGTEV
>sp|P31946|1433B_HUMAN
MTMDKSELVQKAKLAEQAERYDDMAAAMKAVTEQGHELSNEERNLLSVAYKNVVGARRSSWRVISSIEQKTERNEKKQQMGKEYREK
IEAELQDICNDVLELLDKYLIPNATQPESKVFYLKMKGDYFRYLSEVASGDNKQTTVSNSQQAYQEAFEISKKEMQPTHPIRLGLAL
NFSVFYYEILNSPEKACSLAKTAFDEAIAELDTLNEESYKDSTLIMQLLRDNLTLWTSENQGDEGDAGEGEN
>sp|Q8NEM7|SP20H_HUMAN
MQQALELALDRAEYVIESARQRPPKRKYLSSGRKSVFQKLYDLYIEECEKEPEVKKLRRNVNLLEKLVMQETLSCLVVNLYPGNEGY
SLMLRGKNGSDSETIRLPYEEGELLEYLDAEELPPILVDLLEKSQVNIFHCGCVIAEIRDYRQSSNMKSPGYQSRHILLRPTMQTLI
CDVHSITSDNHKWTQEDKLLLESQLILATAEPLCLDPSIAVTCTANRLLYNKQKMNTRPMKRCFKRYSRSSLNRQQDLSHCPPPPQL
RLLDFLQKRKERKAGQHYDLKISKAGNCVDMWKRSPCNLAIPSEVDVEKYAKVEKSIKSDDSQPTVWPAHDVKDDYVFECEAGTQYQ
KTKLTILQSLGDPLYYGKIQPCKADEESDSQMSPSHSSTDDHSNWFIIGSKTDAERVVNQYQELVQNEAKCPVKMSHSSSGSASLSQ
VSPGKETDQTETVSVQSSVLGKGVKHRPPPIKLPSSSGNSSSGNYFTPQQTSSFLKSPTPPPSSKPSSIPRKSSVDLNQVSMLSPAA
LSPASSSQRTTATQVMANSAGLNFINVVGSVCGAQALMSGSNPMLGCNTGAITPAGINLSGLLPSGGLLPNALPSAMQAASQAGVPF
GLKNTSSLRPLNLLQLPGGSLIFNTLQQQQQQLSQFTPQQPQQPTTCSPQQPGEQGSEQGSTSQEQALSAQQAAVINLTGVGSFMQS
QAAVLSQLGSAENRPEQSLPQQRFQLSSAFQQQQQQIQQLRFLQHQMAMAAAAAQTAQLHHHRHTGSQSKSKMKRGTPTTPKF
>sp|Q12912|LRMP_HUMAN
MESTPFSGVANQIHTLCERPTYGEVKDGALDVKRQHKCPGPTSGPSPGTNLSGCIRMNDDPSMEENGVERVCPESLLQSREYSSLPL
PRHTSSTDGTITSSDPGLEILNMASCDLDRNSLCKKEEDTRSASPTIEAQGTSPAHDNIAFQDSTSKDKTILNLEAKEEPETIEEHK
KEHASGDSVVSPLPVTTVKSVNLRQSENTSANEKEVEAEFLRLSLGFKCDWFTLEKRVKLEERSRDLAEENLKKEITNCLKLLESLT
PLCEDDNQAQEIIKKLEKSIKFLSQCAARVASRAEMLGAINQESRVSKAVEVMIQHVENLKRMYAKEHAELEELKQVLLQNERSFNP
LEDDDDCQIKKRSASLNSKPSSLRRVTIASLPRNIGNAGMVAGMENNDRFSRRSSSWRILGSKQSEHRPSLPRFISTYSWADAEEEK
CELKTKDDSEPSGEETVERTRKPSLSEKKNNPSKWDVSSVYDTIASWATNLKSSIRKANKALWLSIAFIVLFAALMSFLTGQLFQKS
VDAAPTQQEDSWTSLEHILWPFTRLRHNGPPPV
>sp|Q9BZF1|OSBL8_HUMAN
MEGGLADGEPDRTSLLGDSKDVLGPSTVVANSDESQLLTPGKMSQRQGKEAYPTPTKDLHQPSLSPASPHSQGFERGKEDISQNKDE
SSLSMSKSKSESKLYNGSEKDSSTSSKLTKKESLKVQKKNYREEKKRATKELLSTITDPSVIVMADWLKIRGTLKSWTKLWCVLKPG
VLLIYKTQKNGQWVGTVLLNACEIIERPSKKDGFCFKLFHPLEQSIWAVKGPKGEAVGSITQPLPSSYLIIRATSESDGRCWMDALE
LALKCSSLLKRTMIREGKEHDLSVSSDSTHVTFYGLLRANNLHSGDNFQLNDSEIERQHFKDQDMYSDKSDKENDQEHDESDNEVMG
KSEESDTDTSERQDDSYIEPEPVEPLKETTYTEQSHEELGEAGEASQTETVSEENKSLIWTLLKQVRPGMDLSKVVLPTFILEPRSF
LDKLSDYYYHADFLSEAALEENPYFRLKKVVKWYLSGFYKKPKGLKKPYNPILGETFRCLWIHPRTNSKTFYIAEQVSHHPPISAFY
VSNRKDGFCLSGSILAKSKFYGNSLSAILEGEARLTFLNRGEDYVMTMPYAHCKGILYGTMTLELGGTVNITCQKTGYSAILEFKLK
PFLGSSDCVNQISGKLKLGKEVLATLEGHWDSEVFITDKKTDNSEVFWNPTPDIKQWRLIRHTVKFEEQGDFESEKLWQRVTRAINA
KDQTEATQEKYVLEEAQRQAARDRKTKNEEWSCKLFELDPLTGEWHYKFADTRPWDPLNDMIQFEKDGVIQTKVKHRTPMVSVPKMK
HKPTRQQKKVAKGYSSPEPDIQDSSGSEAQSVKPSTRRKKGIELGDIQSSIESIKQTQEEIKRNIMALRNHLVSSTPATDYFLQQKD
YFIIFLLILLQVIINFMFK
>sp|Q8NBZ0|IN80E_HUMAN
MNGPADGEVDYKKKYRNLKRKLKFLIYEHECFQEELRKAQRKLLKVSRDKSFLLDRLLQYENVDEDSSDSDATASSDNSETEGTPKL
SDTPAPKRKRSPPLGGAPSPSSLSLPPSTGFPLQASGVPSPYLSSLASSRYPPFPSDYLALQLPEPSPLRPKREKRPRLPRKLKMAV
GPPDCPVGGPLTFPGRGSGAGVGTTLTPLPPPKMPPPTILSTVPRQMFSDAGSGDDALDGDDDLVIDIPE
>sp|O60479|DLX3_HUMAN

MSGSFDRKLSSILTDISSSLSCHAGSKDSPTLPESSVTDLGYYSAPQHDYYSGQPYGQTVNPYTYHHQFNLNGLAGTGAYSPKSEYT
YGASYRQYGAYREQPLPAQDPVSVKEEPEAEVRMVNGKPKKVRKPRTIYSSYQLAALQRRFQKAQYLALPERAELAAQLGLTQTQVK
IWFQNRRSKFKKLYKNGEVPLEHSPNNSDSMACNSPPSPALWDTSSHSTPAPARSQLPPPLPYSASPSYLDDPTNSWYHAQNLSGPH
LQQQPPQPATLHHASPGPPPNPGAVY
>sp|Q8NBB2|STAS1_HUMAN
MPRPASESWSPIQSRIPAYKAVSHLRLLPTANSPSGSNQPTNPNRAQHPEGPQSREGATSPVLASLEPPTPTDLPSARARPRVPAES
GPGWLHKARRPTRGILRDVLRHRGVPAQPRPAPGAHQLSSPSS
>sp|Q9H1J7|WNT5B_HUMAN
MPSLLLLFTAALLSSWAQLLTDANSWWSLALNPVQRPEMFIIGAQPVCSQLPGLSPGQRKLCQLYQEHMAYIGEGAKTGIKECQHQF
RQRRWNCSTADNASVFGRVMQIGSRETAFTHAVSAAGVVNAISRACREGELSTCGCSRTARPKDLPRDWLWGGCGDNVEYGYRFAKE
FVDAREREKNFAKGSEEQGRVLMNLQNNEAGRRAVYKMADVACKCHGVSGSCSLKTCWLQLAEFRKVGDRLKEKYDSAAAMRVTRKG
RLELVNSRFTQPTPEDLVYVDPSPDYCLRNESTGSLGTQGRLCNKTSEGMDGCELMCCGRGYNQFKSVQVERCHCKFHWCCFVRCKK
CTEIVDQYICK
>sp|P51679|CCR4_HUMAN
MNPTDIADTTLDESIYSNYYLYESIPKPCTKEGIKAFGELFLPPLYSLVFVFGLLGNSVVVLVLFKYKRLRSMTDVYLLNLAISDLL
FVFSLPFWGYYAADQWVFGLGLCKMISWMYLVGFYSGIFFVMLMSIDRYLAIVHAVFSLRARTLTYGVITSLATWSVAVFASLPGFL
FSTCYTERNHTYCKTKYSLNSTTWKVLSSLEINILGLVIPLGIMLFCYSMIIRTLQHCKNEKKNKAVKMIFAVVVLFLGFWTPYNIV
LFLETLVELEVLQDCTFERYLDYAIQATETLAFVHCCLNPIIYFFLGEKFRKYILQLFKTCRGLFVLCQYCGLLQIYSADTPSSSYT
QSTMDHDLHDAL
>sp|Q9NR46|SHLB2_HUMAN
MDFNMKKLASDAGIFFTRAVQFTEEKFGQAEKTELDAHFENLLARADSTKNWTEKILRQTEVLLQPNPSARVEEFLYEKLDRKVPSR
VTNGELLAQYMADAASELGPTTPYGKTLIKVAEAEKQLGAAERDFIHTASISFLTPLRNFLEGDWKTISKERRLLQNRRLDLDACKA
RLKKAKAAEAKATTVPDFQETRPRNYILSASASALWNDEVDKAEQELRVAQTEFDRQAEVTRLLLEGISSTHVNHLRCLHEFVKSQT
TYYAQCYRHMLDLQKQLGRFPGTFVGTTEPASPPLSSTSPTTAAATMPVVPSVASLAPPGEASLCLEEVAPPASGTRKARVLYDYEA
ADSSELALLADELITVYSLPGMDPDWLIGERGNKKGKVPVTYLELLS
>sp|Q96H35|RBM18_HUMAN
MEAETKTLPLENASILSEGSLQEGHRLWIGNLDPKITEYHLLKLLQKFGKVKQFDFLFHKSGALEGQPRGYCFVNFETKQEAEQAIQ
CLNGKLALSKKLVVRWAHAQVKRYDHNKNDKILPISLEPSSSTEPTQSNLSVTAKIKAIEAKLKMMAENPDAEYPAAPVYSYFKPPD
KKRTTPYSRTAWKSRR
>sp|Q6P5X5|CV039_HUMAN
MCRCSLVLLSVDHEVPFSSFFIGWRTEGRAWRAGRPDMADGSGWQPPRPCEAYRAEWKLCRSARHFLHHYYVHGERPACEQWQRDLA
SCRDWEERRNAEAQQSLCESERARVRAARKHILVWAPRQSPPPDWHLPLPQEKDE
>sp|Q9Y2T7|YBOX2_HUMAN
MSEVEAAAGATAVPAATVPATAAGVVAVVVPVPAGEPQKGGGAGGGGGAASGPAAGTPSAPGSRTPGNPATAVSGTPAPPARSQADK
PVLAIQVLGTVKWFNVRNGYGFINRNDTKEDVFVHQTAIKRNNPRKFLRSVGDGETVEFDVVEGEKGAEATNVTGPGGVPVKGSRYA
PNRRKSRRFIPRPPSVAPPPMVAEIPSAGTGPGSKGERAEDSGQRPRRWCPPPFFYRRRFVRGPRPPNQQQPIEGTDRVEPKETAPL
EGHQQQGDERVPPPRFRPRYRRPFRPRPRQQPTTEGGDGETKPSQGPADGSRPEPQRPRNRPYFQRRRQQAPGPQQAPGPRQPAAPE
TSAPVNSGDPTTTILE
>sp|P16083|NQO2_HUMAN
MAGKKVLIVYAHQEPKSFNGSLKNVAVDELSRQGCTVTVSDLYAMNLEPRATDKDITGTLSNPEVFNYGVETHEAYKQRSLASDITD
EQKKVREADLVIFQFPLYWFSVPAILKGWMDRVLCQGFAFDIPGFYDSGLLQGKLALLSVTTGGTAEMYTKTGVNGDSRYFLWPLQH
GTLHFCGFKVLAPQISFAPEIASEEERKGMVAAWSQRLQTIWKEEPIPCTAHWHFGQ
>sp|Q9ULL0|K1210_HUMAN

MRAGWTPRGFSAFHASLLPGRHPYLAHLGPRDRGARIGSRAYSQGCCSCLWLTYKGKKEGSTKGELGPAAVTDLEIPSYSRGFLPCT
PRFPTTWCRGPGCFCGTAVIAGNLGDLARIVGPSHHASQLLLLQEQDSGNHPTMAESLSEISDSLDVLEAGDEGKKKCKFKALKSFF
VKKKEKEAEDTQEEEMLELSLSSSNINISSLQPVRENQPTKARAKSSMGSKALSHDSIFMLGPEPERSASKMFPSMDPQRGRPQQRS
HISRTLPKPRSKVPGVVSGAMSGAVLQNVPTSAVWVAGPKITENPPSRRRRLSIIPPVIQPEIISKNLVEISLDDESPKNPQKKALP
HKSLTATQSFSELSSGPDCSQSLTAFATLASTSSTQLPIGFSTPATTQGCLDSSAARHKMTLNPRKQKKNLQVIIRGLPVWFSHFQG
ILEGSLQCVTQTLETPNLDEPLPVEPKEEEPNLPLVSEEEKSITKPKEINEKKLGMDSADSSSQKQNNKTEMYDKKTTDQAPNTDAS
RSQGYPMSAAYGRRWRRKGASVSGLSGCEFKGRSLKQSSEGYGLGDRAGSSPTNKTARNVPFSHLSLEKDNMEQPTTSQPETTTPQG
LLSDKDDMGRRNAGIDFGSRKASAAQPIPENMDNSMVSDPQPYHEDAASGAEKTEARASLSLMVESLSTTQEEAILSVAAEAQVFMN
PSHIQLEDQEAFSFDLQKAQSKMESAQDVQTICKEKPSGNVHQTFTASVLGMTSTTAKGDVYAKTLPPRSLFQSSRKPDAEEVSSDS
ENIPEEGDGSEELAHGHSSQSLGKFEDEQEVFSESKSFVEDLSSSEEELDLRCLSQALEEPEDAEVFTESSSYVEKYNTSDDCSSSE
EDLPLRHPAQALGKPKNQQEVSSASNNTPEEQNDFMQQLPSRCPSQPIMNPTVQQQVPTSSVGTSIKQSDSVEPIPPRHPFQPWVNP
KVEQEVSSSPKSMAVEESISMKPLPPKLLCQPLMNPKVQQNMFSGSEDIAVERVISVEPLLPRYSPQSLTDPQIRQISESTAVEEGT
YVEPLPPRCLSQPSERPKFLDSMSTSAEWSSPVAPTPSKYTSPPWVTPKFEELYQLSAHPESTTVEEDISKEQLLPRHLSQLTVGNK
VQQLSSNFERAAIEADISGSPLPPQYATQFLKRSKVQEMTSRLEKMAVEGTSNKSPIPRRPTQSFVKFMAQQIFSESSALKRGSDVA
PLPPNLPSKSLSKPEVKHQVFSDSGSANPKGGISSKMLPMKHPLQSLGRPEDPQKVFSYSERAPGKCSSFKEQLSPRQLSQALRKPE
YEQKVSPVSASSPKEWRNSKKQLPPKHSSQASDRSKFQPQMSSKGPVNVPVKQSSGEKHLPSSSPFQQQVHSSSVNAAARRSVFESN
SDNWFLGRDEAFAIKTKKFSQGSKNPIKSIPAPATKPGKFTIAPVRQTSTSGGIYSKKEDLESGDGNNNQHANLSNQDDVEKLFGVR
LKRAPPSQKYKSEKQDNFTQLASVPSGPISSSVGRGHKIRSTSQGLLDAAGNLTKISYVADKQQSRPKSESMAKKQPACKTPGKPAG
QQSDYAVSEPVWITMAKQKQKSFKAHISVKELKTKSNAGADAETKEPKYEGAGSANENQPKKMFTSSVHKQEKTAQMKPPKPTKSVG
FEAQKILQVPAMEKETKRSSTLPAKFQNPVEPIEPVWFSLARKKAKAWSHMAEITQ
>sp|Q7RTY9|PRS41_HUMAN
MGARGALLLALLLARAGLGKPGELGALQAGPGAARRPGGGGREEACGHREIHALVAGGVESARGRWPWQASLRLRRRHRCGGSLLSR
RWVLSAAHCFQKHYYPSEWTVQLGELTSRPTPWNLRAYSSRYKVQDIIVNPDALGVLRNDIALLRLASSVTYNAYIQPICIESSTFN
FVHRPDCWVTGWGLISPSGTPLPPPYNLREAQVTILNNTRCNYLFEQPSSRSMIWDSMFCAGAEDGSVDTCKGDSGGPLVCDKDGLW
YQVGIVSWGMDCGQPNRPGVYTNISVYFHWIRRVMSHSTPRPNPSQLLLLLALLWAP
>sp|Q9UPQ0|LIMC1_HUMAN
MACPALGLEALQPLQPEPPPEPAFSEAQKWIEQVTGRSFGDKDFRTGLENGILLCELLNAIKPGLVKKINRLPTPIAGLDNIILFLR
GCKELGLKESQLFDPSDLQDTSNRVTVKSLDYSRKLKNVLVTIYWLGKAANSCTSYSGTTLNLKEFEGLLAQMRKDTDDIESPKRSI
RDSGYIDCWDSERSDSLSPPRHGRDDSFDSLDSFGSRSRQTPSPDVVLRGSSDGRGSDSESDLPHRKLPDVKKDDMSARRTSHGEPK
SAVPFNQYLPNKSNQTAYVPAPLRKKKAEREEYRKSWSTATSPLGGERPFRYGPRTPVSDDAESTSMFDMRCEEEAAVQPHSRARQE
QLQLINNQLREEDDKWQDDLARWKSRRRSVSQDLIKKEEERKKMEKLLAGEDGTSERRKSIKTYREIVQEKERRERELHEAYKNARS
QEEAEGILQQYIERFTISEAVLERLEMPKILERSHSTEPNLSSFLNDPNPMKYLRQQSLPPPKFTATVETTIARASVLDTSMSAGSG
SPSKTVTPKAVPMLTPKPYSQPKNSQDVLKTFKVDGKVSVNGETVHREEEKERECPTVAPAHSLTKSQMFEGVARVHGSPLELKQDN
GSIEINIKKPNSVPQELAATTEKTEPNSQEDKNDGGKSRKGNIELASSEPQHFTTTVTRCSPTVAFVEFPSSPQLKNDVSEEKDQKK
PENEMSGKVELVLSQKVVKPKSPEPEATLTFPFLDKMPEANQLHLPNLNSQVDSPSSEKSPVMTPQFKFWAWDPEEERRRQEKWQQE
QERLLQERYQKEQDKLKEEWEKAQKEVEEEERRYYEEERKIIEDTVVPFTVSSSSADQLSTSSSMTEGSGTMNKIDLGNCQDEKQDR
RWKKSFQGDDSDLLLKTRESDRLEEKGSLTEGALAHSGNPVSKGVHEDHQLDTEAGAPHCGTNPQLAQDPSQNQQTSNPTHSSEDVK
PKTLPLDKSINHQIESPSERRKKSPREHFQAGPFSPCSPTPPGQSPNRSISGKKLCSSCGLPLGKGAAMIIETLNLYFHIQCFRCGI
CKGQLGDAVSGTDVRIRNGLLNCNDCYMRSRSAGQPTTL
>sp|Q8NDF8|PAPD5_HUMAN
MYRSGERLLGSHALPAEQRDFLPLETTNNNNNHHQPGAWARRAGSSASSPPSASSSPHPSAAVPAADPADSASGSSNKRKRDNKASG
GRAAGGGRADGGGVVYSGTPWKRRNYNQGVVGLHEEISDFYEYMSPRPEEEKMRMEVVNRIESVIKELWPSADVQIFGSFKTGLYLP
TSDIDLVVFGKWENLPLWTLEEALRKHKVADEDSVKVLDKATVPIIKLTDSFTEVKVDISFNVQNGVRAADLIKDFTKKYPVLPYLV
LVLKQFLLQRDLNEVFTGGIGSYSLFLMAVSFLQLHPREDACIPNTNYGVLLIEFFELYGRHFNYLKTGIRIKDGGSYVAKDEVQKN

MLDGYRPSMLYIEDPLQPGNDVGRSSYGAMQVKQAFDYAYVVLSHAVSPIAKYYPNNETESILGRIIRVTDEVATYRDWISKQWGLK
NRPEPSCNGPVSSSSATQSSSSDVDSDATPCKTPKQLLCRPSTGNRVGSQDVSLESSQAVGKMQSTQTTNTSNSTNKSQHGSARLFR
SSSKGFQGTTQTSHGSLMTNKQHQGKSNNQYYHGKKRKHKRDAPLSDLCR
>sp|Q5TKA1|LIN9_HUMAN
MAELDQLPDESSSAKALVSLKEGSLSNTWNEKYSSLQKTPVWKGRNTSSAVEMPFRNSKRSRLFSDEDDRQINTRSPKRNQRVAMVP
QKFTATMSTPDKKASQKIGFRLRNLLKLPKAHKWCIYEWFYSNIDKPLFEGDNDFCVCLKESFPNLKTRKLTRVEWGKIRRLMGKPR
RCSSAFFEEERSALKQKRQKIRLLQQRKVADVSQFKDLPDEIPLPLVIGTKVTARLRGVHDGLFTGQIDAVDTLNATYRVTFDRTGL
GTHTIPDYEVLSNEPHETMPIAAFGQKQRPSRFFMTPPRLHYTPPLQSPIIDNDPLLGQSPWRSKISGSDTETLGGFPVEFLIQVTR
LSKILMIKKEHIKKLREMNTEAEKLKSYSMPISIEFQRRYATIVLELEQLNKDLNKVLHKVQQYCYELAPDQGLQPADQPTDMRRRC
EEEAQEIVRHANSSTGQPCVENENLTDLISRLTAILLQIKCLAEGGDLNSFEFKSLTDSLNDIKSTIDASNISCFQNNVEIHVAHIQ
SGLSQMGNLHAFAANNTNRD
>sp|P16050|LOX15_HUMAN
MGLYRIRVSTGASLYAGSNNQVQLWLVGQHGEAALGKRLWPARGKETELKVEVPEYLGPLLFVKLRKRHLLKDDAWFCNWISVQGPG
AGDEVRFPCYRWVEGNGVLSLPEGTGRTVGEDPQGLFQKHREEELEERRKLYRWGNWKDGLILNMAGAKLYDLPVDERFLEDKRVDF
EVSLAKGLADLAIKDSLNVLTCWKDLDDFNRIFWCGQSKLAERVRDSWKEDALFGYQFLNGANPVVLRRSAHLPARLVFPPGMEELQ
AQLEKELEGGTLFEADFSLLDGIKANVILCSQQHLAAPLVMLKLQPDGKLLPMVIQLQLPRTGSPPPPLFLPTDPPMAWLLAKCWVR
SSDFQLHELQSHLLRGHLMAEVIVVATMRCLPSIHPIFKLIIPHLRYTLEINVRARTGLVSDMGIFDQIMSTGGGGHVQLLKQAGAF
LTYSSFCPPDDLADRGLLGVKSSFYAQDALRLWEIIYRYVEGIVSLHYKTDVAVKDDPELQTWCREITEIGLQGAQDRGFPVSLQAR
DQVCHFVTMCIFTCTGQHASVHLGQLDWYSWVPNAPCTMRLPPPTTKDATLETVMATLPNFHQASLQMSITWQLGRRQPVMVAVGQH
EEEYFSGPEPKAVLKKFREELAALDKEIEIRNAKLDMPYEYLRPSVVENSVAI
>sp|Q9H0T7|RAB17_HUMAN
MAQAHRTPQPRAAPSQPRVFKLVLLGSGSVGKSSLALRYVKNDFKSILPTVGCAFFTKVVDVGATSLKLEIWDTAGQEKYHSVCHLY
FRGANAALLVYDITRKDSFLKAQQWLKDLEEELHPGEVLVMLVGNKTDLSQEREVTFQEGKEFADSQKLLFMETSAKLNHQVSEVFN
TVAQELLQRSDEEGQALRGDAAVALNKGPARQAKCCAH
>sp|Q8IVG9|HUNIN_HUMAN
MAPRGFSCLLLLTSEIDLPVKRRA
>sp|Q5H9F3|BCORL_HUMAN
MISTAPLYSGVHNWTSSDRIRMCGINEERRAPLSDEESTTGDCQHFGSQEFCVSSSFSKVELTAVGSGSNARGADPDGSATEKLGHK
SEDKPDDPQPKMDYAGNVAEAEGFLVPLSSPGDGLKLPASDSAEASNSRADCSWTPLNTQMSKQVDCSPAGVKALDSRQGVGEKNTF
ILATLGTGVPVEGTLPLVTTNFSPLPAPICPPAPGSASVPHSVPDAFQVPLSVPAPVPHSGLVPVQVATSVPAPSPPLAPVPALAPA
PPSVPTLISDSNPLSVSASVLVPVPASAPPSGPVPLSAPAPAPLSVPVSAPPLALIQAPVPPSAPTLVLAPVPTPVLAPMPASTPPA
APAPPSVPMPTPTPSSGPPSTPTLIPAFAPTPVPAPTPAPIFTPAPTPMPAATPAAIPTSAPIPASFSLSRVCFPAAQAPAMQKVPL
SFQPGTVLTPSQPLVYIPPPSCGQPLSVATLPTTLGVSSTLTLPVLPSYLQDRCLPGVLASPELRSYPYAFSVARPLTSDSKLVSLE
VNRLPCTSPSGSTTTQPAPDGVPGPLADTSLVTASAKVLPTPQPLLPAPSGSSAPPHPAKMPSGTEQQTEGTSVTFSPLKSPPQLER
EMASPPECSEMPLDLSSKSNRQKLPLPNQRKTPPMPVLTPVHTSSKALLSTVLSRSQRTTQAAGGNVTSCLGSTSSPFVIFPEIVRN
GDPSTWVKNSTALISTIPGTYVGVANPVPASLLLNKDPNLGLNRDPRHLPKQEPISIIDQGEPKGTGATCGKKGSQAGAEGQPSTVK
RYTPARIAPGLPGCQTKELSLWKPTGPANIYPRCSVNGKPTSTQVLPVGWSPYHQASLLSIGISSAGQLTPSQGAPIRPTSVVSEFS
GVPSLSSSEAVHGLPEGQPRPGGSFVPEQDPVTKNKTCRIAAKPYEEQVNPVLLTLSPQTGTLALSVQPSGGDIRMNQGPEESESHL
CSDSTPKMEGPQGACGLKLAGDTKPKNQVLATYMSHELVLATPQNLPKMPELPLLPHDSHPKELILDVVPSSRRGSSTERPQLGSQV
DLGRVKMEKVDGDVVFNLATCFRADGLPVAPQRGQAEVRAKAGQARVKQESVGVFACKNKWQPDDVTESLPPKKMKCGKEKDSEEQQ
LQPQAKAVVRSSHRPKCRKLPSDPQESTKKSPRGASDSGKEHNGVRGKHKHRKPTKPESQSPGKRADSHEEGSLEKKAKSSFRDFIP
VVLSTRTRSQSGSICSSFAGMADSDMGSQEVFPTEEEEEVTPTPAKRRKVRKTQRDTQYRSHHAQDKSLLSQGRRHLWRAREMPWRT
EAARQMWDTNEEEEEEEEEGLLKRKKRRRQKSRKYQTGEYLTEQEDEQRRKGRADLKARKQKTSSSQSLEHRLRNRNLLLPNKVQGI
SDSPNGFLPNNLEEPACLENSEKPSGKRKCKTKHMATVSEEAKDVVLYCLQKDSEDVNHRDNAGYTALHEACSRGWTDILNILLEHG

ANVNCSAQDGTRPVHDAVVNDNLETIWLLLSYGADPTLATYSGQTAMKLASSDTMKRFLSDHLSDLQGRAEGDPGVSWDFYSSSVLE
EKDGFACDLLHNPPGSSDQEGDDPMEEDDFMFELSDKPLLPCYNLQVSVSRGPCNWFLFSDVLKRLKLSSRIFQARFPHFEITTMPK
AEFYRQVASSQLLTPAERPGGLDDRSPPGSSETVELVRYEPDLLRLLGSEVEFQSCNS
>sp|Q86U10|LPP60_HUMAN
MARAVGPERRLLAVYTGGTIGMRSELGVLVPGTGLAAILRTLPMFHDEEHARARGLSEDTLVLPPASRNQRILYTVLECQPLFDSSD
MTIAEWVCLAQTIKRHYEQYHGFVVIHGTDTMAFAASMLSFMLENLQKTVILTGAQVPIHALWSDGRENLLGALLMAGQYVIPEVCL
FFQNQLFRGNRATKVDARRFAAFCSPNLLPLATVGADITINRELVRKVDGKAGLVVHSSMEQDVGLLRLYPGIPAALVRAFLQPPLK
GVVMETFGSGNGPTKPDLLQELRVATERGLVIVNCTHCLQGAVTTDYAAGMAMAGAGVISGFDMTSEAALAKLSYVLGQPGLSLDVR
KELLTKDLRGEMTPPSVEERRPSLQGNTLGGGVSWLLSLSGSQEADALRNALVPSLACAAAHAGDVEALQALVELGSDLGLVDFNGQ
TPLHAAARGGHTEAVTMLLQRGVDVNTRDTDGFSPLLLAVRGRHPGVIGLLREAGASLSTQELEEAGTELCRLAYRADLEGLQVWWQ
AGADLGQPGYDGHSALHVAEAAGNLAVVAFLQSLEGAVGAQAPCPEVLPGV
>sp|Q8NG48|LINES_HUMAN
MKVFCEVLEELYKKVLLGATLENDSHDYIFYLNPAVSDQDCSTATSLEWANTCGIQGRHQPISVGVAPIAVAPVCLKTNSQMSGSRE
VMLLQLTVIKVMTTRILSVKTEFHAKEQYRDVIKILLESAKVDSKLICMFQNSDKLLSHMAAQCLALLLYFQLREKITLSNSWIAFC
QKNLSEYSESNKAIYCLWTLTAIIKEIFKDSCSQKTEILKQFLTHFDTIFEVFYNSLFSQHFENCRDTSKIVNILMCFLDLLELLIA
SRIHLKLHFTCQRILFLKPSCMLEVITWPIQAFVKRKVIIFLKKCLLCKVGEDLCRGSVPALMPPDHHVAVDMLALANAVLQAVNSG
LLKTLSVYEKHSFFGGDEVQPECELITSPDHVILRAASLVIMKSLEIKFQNYSSASEVKVDLQRFMSELLTFLKPHLQPSLQLHNPC
KWLSRVFIEQDDDMLEAAKASLGIYLTLTRGCEATESLTQGKEMWDHHTHENGYNPHCIFLFFLKNIGFDSTVLLDFLISSETCFLE
YFVRYLKLLQKDWDNFFTICNNFDATESKYDISICGCVPSLVQDQSSNQTIPHRLTAPHSHRDVCARHSWASDAPSEPLKAVMSKGA
HTMCASSLSSPRASQSLVDYDSSDDSDVESTEQCLANSKQTSLHQQATKEIQDAAGTSRDKKEFSLEPPSRPLVLKEFDTAFSFDCE
VAPNDVVSEVGIFYRIVKCFQELQDAICRLQKKNLFPYNPTALLKLLKYIEVISNKTMNTL
>sp|Q8WXF7|ATLA1_HUMAN
MAKNRRDRNSWGGFSEKTYEWSSEEEEPVKKAGPVQVLIVKDDHSFELDETALNRILLSEAVRDKEVVAVSVAGAFRKGKSFLMDFM
LRYMYNQESVDWVGDYNEPLTGFSWRGGSERETTGIQIWSEIFLINKPDGKKVAVLLMDTQGTFDSQSTLRDSATVFALSTMISSIQ
VYNLSQNVQEDDLQHLQLFTEYGRLAMEETFLKPFQSLIFLVRDWSFPYEFSYGADGGAKFLEKRLKVSGNQHEELQNVRKHIHSCF
TNISCFLLPHPGLKVATNPNFDGKLKEIDDEFIKNLKILIPWLLSPESLDIKEINGNKITCRGLVEYFKAYIKIYQGEELPHPKSML
QATAEANNLAAVATAKDTYNKKMEEICGGDKPFLAPNDLQTKHLQLKEESVKLFRGVKKMGGEEFSRRYLQQLESEIDELYIQYIKH
NDSKNIFHAARTPATLFVVIFITYVIAGVTGFIGLDIIASLCNMIMGLTLITLCTWAYIRYSGEYRELGAVIDQVAAALWDQGSTNE
ALYKLYSAAATHRHLYHQAFPTPKSESTEQSEKKKM
>sp|Q8WVZ7|RN133_HUMAN
MHLLKVGTWRNNTASSWLMKFSVLWLVSQNCCRASVVWMAYMNISFHVGNHVLSELGETGVFGRSSTLKRVAGVIVPPEGKIQNACN
PNTIFSRSKYSETWLALIERGGCTFTQKIKVATEKGASGVIIYNVPGTGNQVFPMFHQAFEDVVVVMIGNLKGTEIFHLIKKGVLIT
AVVEVGRKHIIWMNHYLVSFVIVTTATLAYFIFYHIHRLCLARIQNRRWQRLTTDLQNTFGQLQLRVVKEGDEEINPNGDSCVICFE
RYKPNDIVRILTCKHFFHKNCIDPWILPHGTCPICKCDILKVLGIQVVVENGTEPLQVLMSNELPETLSPSEEETNNEVSPAGTSDK
VIHVEENPTSQNNDIQPHSVVEDVHPSP
>sp|Q9Y536|PAL4A_HUMAN
MVNSVVFFDITVDGKPLGRISIKLFADKILKTAENFRALSTGEKGFRYKGSCFHRIIPGFMCQGGDFTRHNGTGDKSIYGEKFDDEN
LIRKHTGSGILSMANAGPNTNGSQFFICAAKTEWLDGKHVAFGKVKERVNIVEAMEHFGYRNSKTSKKITIADCGQF
>sp|Q96MN2|NALP4_HUMAN
MAASFFSDFGLMWYLEELKKEEFRKFKEHLKQMTLQLELKQIPWTEVKKASREELANLLIKHYEEQQAWNITLRIFQKMDRKDLCMK
VMRERTGYTKTYQAHAKQKFSRLWSSKSVTEIHLYFEEEVKQEECDHLDRLFAPKEAGKQPRTVIIQGPQGIGKTTLLMKLMMAWSD
NKIFRDRFLYTFYFCCRELRELPPTSLADLISREWPDPAAPITEIVSQPERLLFVIDSFEELQGGLNEPDSDLCGDLMEKRPVQVLL
SSLLRKKMLPEASLLIAIKPVCPKELRDQVTISEIYQPRGFNESDRLVYFCCFFKDPKRAMEAFNLVRESEQLFSICQIPLLCWILC
TSLKQEMQKGKDLALTCQSTTSVYSSFVFNLFTPEGAEGPTPQTQHQLKALCSLAAEGMWTDTFEFCEDDLRRNGVVDADIPALLGT

KILLKYGERESSYVFLHVCIQEFCAALFYLLKSHLDHPHPAVRCVQELLVANFEKARRAHWIFLGCFLTGLLNKKEQEKLDAFFGFQ
LSQEIKQQIHQCLKSLGERGNPQGQVDSLAIFYCLFEMQDPAFVKQAVNLLQEANFHIIDNVDLVVSAYCLKYCSSLRKLCFSVQNV
FKKEDEHSSTSDYSLICWHHICSVLTTSGHLRELQVQDSTLSESTFVTWCNQLRHPSCRLQKLGINNVSFSGQSVLLFEVLFYQPDL
KYLSFTLTKLSRDDIRSLCDALNYPAGNVKELALVNCHLSPIDCEVLAGLLTNNKKLTYLNVSCNQLDTGVPLLCEALCSPDTVLVY
LMLAFCHLSEQCCEYISEMLLRNKSVRYLDLSANVLKDEGLKTLCEALKHPDCCLDSLCLVKCFITAAGCEDLASALISNQNLKILQ
IGCNEIGDVGVQLLCRALTHTDCRLEILGLEECGLTSTCCKDLASVLTCSKTLQQLNLTLNTLDHTGVVVLCEALRHPECALQVLGL
RKTDFDEETQALLTAEEERNPNLTITDDCDTITRVEI
>sp|Q9P2R7|SUCB1_HUMAN
MAASMFYGRLVAVATLRNHRPRTAQRAAAQVLGSSGLFNNHGLQVQQQQQRNLSLHEYMSMELLQEAGVSVPKGYVAKSPDEAYAIA
KKLGSKDVVIKAQVLAGGRGKGTFESGLKGGVKIVFSPEEAKAVSSQMIGKKLFTKQTGEKGRICNQVLVCERKYPRREYYFAITME
RSFQGPVLIGSSHGGVNIEDVAAESPEAIIKEPIDIEEGIKKEQALQLAQKMGFPPNIVESAAENMVKLYSLFLKYDATMIEINPMV
EDSDGAVLCMDAKINFDSNSAYRQKKIFDLQDWTQEDERDKDAAKANLNYIGLDGNIGCLVNGAGLAMATMDIIKLHGGTPANFLDV
GGGATVHQVTEAFKLITSDKKVLAILVNIFGGIMRCDVIAQGIVMAVKDLEIKIPVVVRLQGTRVDDAKALIADSGLKILACDDLDE
AARMVVKLSEIVTLAKQAHVDVKFQLPI
>sp|Q14974|IMB1_HUMAN
MELITILEKTVSPDRLELEAAQKFLERAAVENLPTFLVELSRVLANPGNSQVARVAAGLQIKNSLTSKDPDIKAQYQQRWLAIDANA
RREVKNYVLQTLGTETYRPSSASQCVAGIACAEIPVNQWPELIPQLVANVTNPNSTEHMKESTLEAIGYICQDIDPEQLQDKSNEIL
TAIIQGMRKEEPSNNVKLAATNALLNSLEFTKANFDKESERHFIMQVVCEATQCPDTRVRVAALQNLVKIMSLYYQYMETYMGPALF
AITIEAMKSDIDEVALQGIEFWSNVCDEEMDLAIEASEAAEQGRPPEHTSKFYAKGALQYLVPILTQTLTKQDENDDDDDWNPCKAA
GVCLMLLATCCEDDIVPHVLPFIKEHIKNPDWRYRDAAVMAFGCILEGPEPSQLKPLVIQAMPTLIELMKDPSVVVRDTAAWTVGRI
CELLPEAAINDVYLAPLLQCLIEGLSAEPRVASNVCWAFSSLAEAAYEAADVADDQEEPATYCLSSSFELIVQKLLETTDRPDGHQN
NLRSSAYESLMEIVKNSAKDCYPAVQKTTLVIMERLQQVLQMESHIQSTSDRIQFNDLQSLLCATLQNVLRKVQHQDALQISDVVMA
SLLRMFQSTAGSGGVQEDALMAVSTLVEVLGGEFLKYMEAFKPFLGIGLKNYAEYQVCLAAVGLVGDLCRALQSNIIPFCDEVMQLL
LENLGNENVHRSVKPQILSVFGDIALAIGGEFKKYLEVVLNTLQQASQAQVDKSDYDMVDYLNELRESCLEAYTGIVQGLKGDQENV
HPDVMLVQPRVEFILSFIDHIAGDEDHTDGVVACAAGLIGDLCTAFGKDVLKLVEARPMIHELLTEGRRSKTNKAKTLATWATKELR
KLKNQA
>sp|Q5VZ46|K1614_HUMAN
MEGTEAAAAKPAGGSPQGPKTGSGTASPVEGTSAVEWSGPEPQLDNGHPPRPWPCPQENRTSSLMAPQPPRVWGVQLQGPSVLESKV
RALKEKMTVAKQGVSPCSASQEWSSPKKPQCRRGKAGRAGTPSEGSFLPGAVVAPRTQNLPDGQLDGSINEEQPARDGGPRLPRPPA
PGREYCNRGSPWPPEAEWTLPDHDRGPLLGPSSLQQSPIHGVTPGRPGGPGHCNKIIHIPSPRTGRSYPFPDGVVTEADLDSTSLTS
EEVFVPRTALLGERWRAGDLEALGAGSSVLSLSDRVERNRLLLQEMLNVSGQSPRKVGTPAWTPSWDTAAPERPVGDVDWASGTSLQ
DSGQNRTVGPNPEPVLSPRHEEATHLLQRARMKARTRPLRASHDIVPTITQGSRDGHRSPARDPRTTPACRDSLQNGHTSDSSSGES
SGGHRPRRGPSPSHVRFEDESAREAEFRHLERLQQRQRQVLSTVLQAADQGPLRSKPDLADYINGAPRLRDAGQGTFHRLVGSLDRR
GHPAPPAPGSERRCQACGSCIDDPRPAQGKAPPVPRTLQELQAACGMERVLGGLSSPLRLLPAEPRLHMEWIRETHIGDTVCPAEVD
SALDSTDNSDNCRTDSEEAGTSQAGWACGRTQGSSPRLRLRGSRPRGHRWSKKAEAELPWGLQAQQHLPRADDVEVENEVKEGRGHT
PEGTLFLREDAKPPDLELKRVSLGPQWQPGPGLGSHQPHPLDSRTPCRTAYATTAPMTPESSGPGGQAQVTESHESLEIVSPSSLQQ
SHAEPSAPHQAWQPTASLCPEGWAPTPPPSRKTTSPVSHRKAALAGLLRLGDQTEPVGIPRPPSRSAVLRTCELPPSQTQPSRPQVR
HPLLALSTNNCNNSAPRGLQEPYGGAVHEGRVERGPCSREPEPPLENSRDGGPQGFLGSADVATINSTGITLSLSSEESESSKESEG
SLQRTGSGSGGHVLSRASAGAGTGPGSPSAAPLDQNKKRSSSIASTLGLKKLFSALGQSSRPKLGKSRSYSVEQLQPAPPGLTSQSR
APSLQSLHPVSPSHQRRKAASFQNLHSLLSSKGNRSSLYLVAGPGDHSAAGRPAKTSPRRALSVEDVGAPSLARTVGRLVEVFPDGT
SQLQLQRSPGGTFGFCVASGNGRPDSGMPSPLPQPHGWGGLSKQGRAFWLWSEAFLVFG
>sp|O60503|ADCY9_HUMAN
MASPPHQQLLHHHSTEVSCDSSGDSNSVRVKINPKQLSSNSHPKHCKYSISSSCSSSGDSGGVPRRVGGGGRLRRQKKLPQLFERAS
SRWWDPKFDSVNLEEACLERCFPQTQRRFRYALFYIGFACLLWSIYFAVHMRSRLIVMVAPALCFLLVCVGFFLFTFTKLYARHYAW

TSLALTLLVFALTLAAQFQVLTPVSGRGDSSNLTATARPTDTCLSQVGSFSMCIEVLFLLYTVMHLPLYLSLCLGVAYSVLFETFGY
HFRDEACFPSPGAGALHWELLSRGLLHGCIHAIGVHLFVMSQVRSRSTFLKVGQSIMHGKDLEVEKALKERMIHSVMPRIIADDLMK
QGDEESENSVKRHATSSPKNRKKKSSIQKAPIAFRPFKMQQIEEVSILFADIVGFTKMSANKSAHALVGLLNDLFGRFDRLCEETKC
EKISTLGDCYYCVAGCPEPRADHAYCCIEMGLGMIKAIEQFCQEKKEMVNMRVGVHTGTVLCGILGMRRFKFDVWSNDVNLANLMEQ
LGVAGKVHISEATAKYLDDRYEMEDGKVIERLGQSVVADQLKGLKTYLISGQRAKESRCSCAEALLSGFEVIDGSQVSSGPRGQGTA
SSGNVSDLAQTVKTFDNLKTCPSCGITFAPKSEAGAEGGAPQNGCQDEHKNSTKASGGPNPKTQNGLLSPPQEEKLTNSQTSLCEIL
QEKGRWAGVSLDQSALLPLRFKNIREKTDAHFVDVIKEDSLMKDYFFKPPINQFSLNFLDQELERSYRTSYQEEVIKNSPVKTFASP
TFSSLLDVFLSTTVFLTLSTTCFLKYEAATVPPPPAALAVFSAALLLEVLSLAVSIRMVFFLEDVMACTKRLLEWIAGWLPRHCIGA
ILVSLPALAVYSHVTSEYETNIHFPVFTGSAALIAVVHYCNFCQLSSWMRSSLATVVGAGPLLLLYVSLCPDSSVLTSPLDAVQNFS
SERNPCNSSVPRDLRRPASLIGQEVVLVFFLLLLLVWFLNREFEVSYRLHYHGDVEADLHRTKIQSMRDQADWLLRNIIPYHVAEQL
KVSQTYSKNHDSGGVIFASIVNFSEFYEENYEGGKECYRVLNELIGDFDELLSKPDYSSIEKIKTIGATYMAASGLNTAQAQDGSHP
QEHLQILFEFAKEMMRVVDDFNNNMLWFNFKLRVGFNHGPLTAGVIGTTKLLYDIWGDTVNIASRMDTTGVECRIQVSEESYRVLSK
MGYDFDYRGTVNVKGKGQMKTYLYPKCTDHRVIPQHQLSISPDIRVQVDGSIGRSPTDEIANLVPSVQYVDKTSLGSDSSTQAKDAH
LSPKRPWKEPVKAEERGRFGKAIEKDDCDETGIEEANELTKLNVSKSV
>sp|Q8N6N7|ACBD7_HUMAN
MALQADFDRAAEDVRKLKARPDDGELKELYGLYKQAIVGDINIACPGMLDLKGKAKWEAWNLKKGLSTEDATSAYISKAKELIEKYG
I
>sp|Q99501|GA2L1_HUMAN
MADPVAGIAGSAAKSVRPFRSSEAYVEAMKEDLAEWLNALYGLGLPGGGDGFLTGLATGTTLCQHANAVTEAARALAAARPARGVAF
QAHSVVPGSFMARDNVATFIGWCRVELGVPEVLMFETEDLVLRKNEKSVVLCLLEVARRGARLGLLAPRLVQFEQEIERELRAAPPA
PNAPAAGEDTTETAPAPGTPARGPRMTPSDLRNLDELVREILGRCTCPDQFPMIKVSEGKYRVGDSSLLIFVRVLRSHVMVRVGGGW
DTLEHYLDKHDPCRCSSTAHRPPQPRVCTFSPQRVSPTTSPRPASPVPGSERRGSRPEMTPVSLRSTKEGPETPPRPRDQLPPHPRS
RRYSGDSDSSASSAQSGPLGTRSDDTGTGPRRERPSRRLTTGTPASPRRPPALRSQSRDRLDRGRPRGAPGGRGAQLSVPSPARRAR
SQSREEQAVLLVRRDRDGQHSWVPRGRGSGGSGRSTPQTPRARSPAAPRLSRVSSPSPELGTTPASIFRTPLQLDPQQEQQLFRRLE
EEFLANARALEAVASVTPTGPVPDPARAPDPPAPDSAYCSSSSSSSSLSVLGGKCGQPGDSGRTANGLPGPRSQALSSSSDEGSPCP
GMGGPLDAPGSPLACTEPSRTWARGRMDTQPDRKPSRIPTPRGPRRPSGPAELGTWHALHSVTPRAEPDSWM
>sp|O15294|OGT1_HUMAN
MASSVGNVADSTEPTKRMLSFQGLAELAHREYQAGDFEAAERHCMQLWRQEPDNTGVLLLLSSIHFQCRRLDRSAHFSTLAIKQNPL
LAEAYSNLGNVYKERGQLQEAIEHYRHALRLKPDFIDGYINLAAALVAAGDMEGAVQAYVSALQYNPDLYCVRSDLGNLLKALGRLE
EAKACYLKAIETQPNFAVAWSNLGCVFNAQGEIWLAIHHFEKAVTLDPNFLDAYINLGNVLKEARIFDRAVAAYLRALSLSPNHAVV
HGNLACVYYEQGLIDLAIDTYRRAIELQPHFPDAYCNLANALKEKGSVAEAEDCYNTALRLCPTHADSLNNLANIKREQGNIEEAVR
LYRKALEVFPEFAAAHSNLASVLQQQGKLQEALMHYKEAIRISPTFADAYSNMGNTLKEMQDVQGALQCYTRAIQINPAFADAHSNL
ASIHKDSGNIPEAIASYRTALKLKPDFPDAYCNLAHCLQIVCDWTDYDERMKKLVSIVADQLEKNRLPSVHPHHSMLYPLSHGFRKA
IAERHGNLCLDKINVLHKPPYEHPKDLKLSDGRLRVGYVSSDFGNHPTSHLMQSIPGMHNPDKFEVFCYALSPDDGTNFRVKVMAEA
NHFIDLSQIPCNGKAADRIHQDGIHILVNMNGYTKGARNELFALRPAPIQAMWLGYPGTSGALFMDYIITDQETSPAEVAEQYSEKL
AYMPHTFFIGDHANMFPHLKKKAVIDFKSNGHIYDNRIVLNGIDLKAFLDSLPDVKIVKMKCPDGGDNADSSNTALNMPVIPMNTIA
EAVIEMINRGQIQITINGFSISNGLATTQINNKAATGEEVPRTIIVTTRSQYGLPEDAIVYCNFNQLYKIDPSTLQMWANILKRVPN
SVLWLLRFPAVGEPNIQQYAQNMGLPQNRIIFSPVAPKEEHVRRGQLADVCLDTPLCNGHTTGMDVLWAGTPMVTMPGETLASRVAA
SQLTCLGCLELIAKNRQEYEDIAVKLGTDLEYLKKVRGKVWKQRISSPLFNTKQYTMELERLYLQMWEHYAAGNKPDHMIKPVEVTE
SA
>sp|Q5VVQ6|OTU1_HUMAN
MFGPAKGRHFGVHPAPGFPGGVSQQAAGTKAGPAGAWPVGSRTDTMWRLRCKAKDGTHVLQGLSSRTRVRELQGQIAAITGIAPGGQ
RILVGYPPECLDLSNGDTILEDLPIQSGDMLIIEEDQTRPRSSPAFTKRGASSYVRETLPVLTRTVVPADNSCLFTSVYYVVEGGVL

NPACAPEMRRLIAQIVASDPDFYSEAILGKTNQEYCDWIKRDDTWGGAIEISILSKFYQCEICVVDTQTVRIDRFGEDAGYTKRVLL
IYDGIHYDPLQRNFPDPDTPPLTIFSSNDDIVLVQALELADEARRRRQFTDVNRFTLRCMVCQKGLTGQAEAREHAKETGHTNFGEV
>sp|Q9BZV2|S19A3_HUMAN
MDCYRTSLSSSWIYPTVILCLFGFFSMMRPSEPFLIPYLSGPDKNLTSAEITNEIFPVWTYSYLVLLLPVFVLTDYVRYKPVIILQG
ISFIITWLLLLFGQGVKTMQVVEFFYGMVTAAEVAYYAYIYSVVSPEHYQRVSGYCRSVTLAAYTAGSVLAQLLVSLANMSYFYLNV
ISLASVSVAFLFSLFLPMPKKSMFFHAKPSREIKKSSSVNPVLEETHEGEAPGCEEQKPTSEILSTSGKLNKGQLNSLKPSNVTVDV
FVQWFQDLKECYSSKRLFYWSLWWAFATAGFNQVLNYVQILWDYKAPSQDSSIYNGAVEAIATFGGAVAAFAVGYVKVNWDLLGELA
LVVFSVVNAGSLFLMHYTANIWACYAGYLIFKSSYMLLITIAVFQIAVNLNVERYALVFGINTFIALVIQTIMTVIVVDQRGLNLPV
SIQFLVYGSYFAVIAGIFLMRSMYITYSTKSQKDVQSPAPSENPDVSHPEEESNIIMSTKL
>sp|A6NMD2|GOG8J_HUMAN
MAEETQHNKLAAAKKKLKEYWQKNSPRVPAGANRNRKTNGSIPEKATSGGCQPPRDSATGFHREGPTSSATLKDLESPCQERAVVLD
SRSVEISQLKNTIKSLKQQKKQVEHQLEEEKKANNKKQKAKRVLEVQIQTLNIQKEELNTDLYHMKRSLRYFEEKSKDLAVRLQHSL
QRKGELESVLSNVMATQKKKANQLSSRSKARTEWKLEQSMREEALLKVQLTQFKESFQQVQLERDEYSEHLKGERARWQQRMRKMSQ
EICTLKKEKQQDMRRVEKLERSLSKLKNQMAEPLPPEPPAVPSEVELQHLRKELERVAGELQAQVKNNQRISLLNQRQEERIREQEE
RLRKQEERIQEQHKSLQQLAKPQSVFKEPNNENKNALQLEQQVKELQEKLGEEHLEAASQQNQQLTAQLSLMALPGEGHGGEHLDSE
GEEAPRPMPSVPEDPESREAMSSFMDHLEEKADLSELVKKKELCFIHHWRERCHQKTHHLLSEPGGRAKDAALGGGHHQAGAQGGDE
GEAAGAAADGIAAYSNYNNGHRKFLAAAHNSADEPGPGAPAPQELGAADKHGHLCEVSLTSSAQGEAREDPLLDKPTAQPIVQDHQE
HPGLGSNCCVPFLCWAWLPRRRR
>sp|Q03692|COAA1_HUMAN
MLPQIPFLLLVSLNLVHGVFYAERYQMPTGIKGPLPNTKTQFFIPYTIKSKGIAVRGEQGTPGPPGPAGPRGHPGPSGPPGKPGYGS
PGLQGEPGLPGPPGPSAVGKPGVPGLPGKPGERGPYGPKGDVGPAGLPGPRGPPGPPGIPGPAGISVPGKPGQQGPTGAPGPRGFPG
EKGAPGVPGMNGQKGEMGYGAPGRPGERGLPGPQGPTGPSGPPGVGKRGENGVPGQPGIKGDRGFPGEMGPIGPPGPQGPPGERGPE
GIGKPGAAGAPGQPGIPGTKGLPGAPGIAGPPGPPGFGKPGLPGLKGERGPAGLPGGPGAKGEQGPAGLPGKPGLTGPPGNMGPQGP
KGIPGSHGLPGPKGETGPAGPAGYPGAKGERGSPGSDGKPGYPGKPGLDGPKGNPGLPGPKGDPGVGGPPGLPGPVGPAGAKGMPGH
NGEAGPRGAPGIPGTRGPIGPPGIPGFPGSKGDPGSPGPPGPAGIATKGLNGPTGPPGPPGPRGHSGEPGLPGPPGPPGPPGQAVMP
EGFIKAGQRPSLSGTPLVSANQGVTGMPVSAFTVILSKAYPAIGTPIPFDKILYNRQQHYDPRTGIFTCQIPGIYYFSYHVHVKGTH
VWVGLYKNGTPVMYTYDEYTKGYLDQASGSAIIDLTENDQVWLQLPNAESNGLYSSEYVHSSFSGFLVAPM
>sp|Q9Y2J4|AMOL2_HUMAN
MRTLEDSSGTVLHRLIQEQLRYGNLTETRTLLAIQQQALRGGAGTGGTGSPQASLEILAPEDSQVLQQATRQEPQGQEHQGGENHLA
ENTLYRLCPQPSKGEELPTYEEAKAHSQYYAAQQAGTRPHAGDRDPRGAPGGSRRQDEALRELRHGHVRSLSERLLQLSLERNGARA
PSHMSSSHSFPQLARNQQGPPLRGPPAEGPESRGPPPQYPHVVLAHETTTAVTDPRYRARGSPHFQHAEVRILQAQVPPVFLQQQQQ
YQYLQQSQEHPPPPHPAALGHGPLSSLSPPAVEGPVSAQASSATSGSAHLAQMEAVLRENARLQRDNERLQRELESSAEKAGRIEKL
ESEIQRLSEAHESLTRASSKREALEKTMRNKMDSEMRRLQDFNRDLRERLESANRRLASKTQEAQAGSQDMVAKLLAQSYEQQQEQE
KLEREMALLRGAIEDQRRRAELLEQALGNAQGRAARAEEELRKKQAYVEKVERLQQALGQLQAACEKREQLELRLRTRLEQELKALR
AQQRQAGAPGGSSGSGGSPELSALRLSEQLREKEEQILALEADMTKWEQKYLEERAMRQFAMDAAATAAAQRDTTLIRHSPQPSPSS
SFNEGLLTGGHRHQEMESRLKVLHAQILEKDAVIKVLQQRSRRDPGKAIQGSLRPAKSVPSVFAAAAAGTQGWQGLSSSERQTADAP
ARLTTDRAPTEEPVVTAPPAAHAKHGSRDGSTQTEGPPDSTSTCLPPEPDSLLGCSSSQRAASLDSVATSRVQDLSDMVEILI
>sp|Q7Z6B0|CCD91_HUMAN
MDDDDFGGFEAAETFDGGSGETQTTSPAIPWAAFPAVSGVHLSPSSPEIVLDRDHSSSIGCLSSDAIISSPENTHAANSIVSQTIPK
AQIQQSTHTHLDISLFPLGLTDEKSNGTIALVDDSEDPGANVSNIQLQQKISSLEIKLKVSEEEKQRIKQDVESLMEKHNVLEKGFL
KEKEQEAISFQDRYKELQEKHKQELEDMRKAGHEALSIIVDEYKALLQSSVKQQVEAIEKQYISAIEKQAHKCEELLNAQHQRLLEM
LDTEKELLKEKIKEALIQQSQEQKEILEKCLEEERQRNKEALVSAAKLEKEAVKDAVLKVVEEERKNLEKAHAEERELWKTEHAKDQ
EKVSQEIQKAIQEQRKISQETVKAAIIEEQKRSEKAVEEAVKRTRDELIEYIKEQKRLDQVIRQRSLSSLELFLSCAQKQLSALIAT
EPVDIE

>sp|Q6P5X7|TMM71_HUMAN
MYRISQLMSTPVASSSRLEREYAGELSPTCIFPSFTCDSLDGYHSFECGSIDPLTGSHYTCRRSPRLLTNGYYIWTEDSFLCDKDGN
ITLNPSQTSVMYKENLVRIFRKKKRICHSFSSLFNLSTSKSWLHGSIFGDINSSPSEDNWLKGTRRLDTDHCNGNADDLDCSSLTDD
WESGKMNAESVITSSSSHIISQPPGGNSHSLSLQSQLTASERFQENSSDHSETRLLQEVFFQAILLAVCLIISACARWFMGEILASV
FTCSLMITVAYVKSLFLSLASYFKTTACARFVKI
>sp|Q96KR6|F210B_HUMAN
MAGLLALLGPAGRVGARVRPRATWLLGATAPCAPPPLALALLPPRLDARLLRTARGDCRGHQDPSQATGTTGSSVSCTEEKKQSKSQ
QLKKIFQEYGTVGVSLHIGISLISLGIFYMVVSSGVDMPAILLKLGFKESLVQSKMAAGTSTFVVAYAIHKLFAPVRISITLVSVPL
IVRYFRKVGFFKPPAAKP
>sp|Q8N6V9|TEX9_HUMAN
MAGRSLCLTRSSVPGTPFPPPVQQPSTPGPDLLALEEEYKRLNAELQAKTADVVQQAKEIIRDRQEVRSRPVSTQMKSCDDEDDYSL
RGLLPSEGIVHLHSETKPKTKNIDPVNKVQNKLHSANKGRKTNSSVKLKYSDVQTADDVAIPEDFSDFSLAKTISKIEGQLEEEGLP
EYIDDIFSGVSNDIGTEAQIRFLKAKLHVMQEELDNVVCECNKKEDEIQNLKSQVKNFEEDFMRQQRTINMQQSQVEKYKTLFEEAN
KKYDGLQQQLSSVERELENKRRLQKQAASSQSATEVRLNRALEEAEKYKLELSKLRQNNKDIANEEHKKIEVLKSENKKLEKQKGEL
MIGFKKQLKLIDVLKRQKMHIEAAKMLSFTEEEFMKALEWGNS
>sp|Q8NFD5|ARI1B_HUMAN
MAHNAGAAAAAGTHSAKSGGSEAALKEGGSAAALSSSSSSSAAAAAASSSSSSGPGSAMETGLLPNHKLKTVGEAPAAPPHQQHHHH
HHAHHHHHHAHHLHHHHALQQQLNQFQQQQQQQQQQQQQQQQQQHPISNNNSLGGAGGGAPQPGPDMEQPQHGGAKDSAAGGQADPP
GPPLLSKPGDEDDAPPKMGEPAGGRYEHPGLGALGTQQPPVAVPGGGGGPAAVPEFNNYYGSAAPASGGPGGRAGPCFDQHGGQQSP
GMGMMHSASAAAAGAPGSMDPLQNSHEGYPNSQCNHYPGYSRPGAGGGGGGGGGGGGGSGGGGGGGGAGAGGAGAGAVAAAAAAAAA
AAGGGGGGGYGGSSAGYGVLSSPRQQGGGMMMGPGGGGAASLSKAAAGSAAGGFQRFAGQNQHPSGATPTLNQLLTSPSPMMRSYGG
SYPEYSSPSAPPPPPPSQPQSQAAAAGAAAGGQQAAAGMGLGKDMGAQYAAASPAWAAAQQRSHPAMSPGTPGPTMGRSQGSPMDPMV
MKRPQLYGMGSNPHSQPQQSSPYPGGSYGPPGPQRYPIGIQGRTPGAMAGMQYPQQQMPPQYGQQGVSGYCQQGQQPYYSQQPQPPH
LPPQAQYLPSQSQQRYQPQQDMSQEGYGTRSQPPLAPGKPNHEDLNLIQQERPSSLPDLSGSIDDLPTGTEATLSSAVSASGSTSSQ
GDQSNPAQSPFSPHASPHLSSIPGGPSPSPVGSPVGSNQSRSGPISPASIPGSQMPPQPPGSQSESSSHPALSQSPMPQERGFMAGT
QRNPQMAQYGPQQTGPSMSPHPSPGGQMHAGISSFQQSNSSGTYGPQMSQYGPQGNYSRPPAYSGVPSASYSGPGPGMGISANNQMH
GQGPSQPCGAVPLGRMPSAGMQNRPFPGNMSSMTPSSPGMSQQGGPGMGPPMPTVNRKAQEAAAAVMQAAANSAQSRQGSFPGMNQS
GLMASSSPYSQPMNNSSSLMNTQAPPYSMAPAMVNSSAASVGLADMMSPGESKLPLPLKADGKEEGTPQPESKSKKSSSSTTTGEKI
TKVYELGNEPERKLWVDRYLTFMEERGSPVSSLPAVGKKPLDLFRLYVCVKEIGGLAQVNKNKKWRELATNLNVGTSSSAASSLKKQ
YIQYLFAFECKIERGEEPPPEVFSTGDTKKQPKLQPPSPANSGSLQGPQTPQSTGSNSMAEVPGDLKPPTPASTPHGQMTPMQGGRS
STISVHDPFSDVSDSSFPKRNSMTPNAPYQQGMSMPDVMGRMPYEPNKDPFGGMRKVPGSSEPFMTQGQMPNSSMQDMYNQSPSGAM
SNLGMGQRQQFPYGASYDRRHEPYGQQYPGQGPPSGQPPYGGHQPGLYPQQPNYKRHMDGMYGPPAKRHEGDMYNMQYSSQQQEMYN
QYGGSYSGPDRRPIQGQYPYPYSRERMQGPGQIQTHGIPPQMMGGPLQSSSSEGPQQNMWAARNDMPYPYQNRQGPGGPTQAPPYPG
MNRTDDMMVPDQRINHESQWPSHVSQRQPYMSSSASMQPITRPPQPSYQTPPSLPNHISRAPSPASFQRSLENRMSPSKSPFLPSMK
MQKVMPTVPTSQVTGPPPQPPPIRREITFPPGSVEASQPVLKQRRKITSKDIVTPEAWRVMMSLKSGLLAESTWALDTINILLYDDS
TVATFNLSQLSGFLELLVEYFRKCLIDIFGILMEYEVGDPSQKALDHNAARKDDSQSLADDSGKEEEDAECIDDDEEDEEDEEEDSE
KTESDEKSSIALTAPDAAADPKEKPKQASKFDKLPIKIVKKNNLFVVDRSDKLGRVQEFNSGLLHWQLGGGDTTEHIQTHFESKMEI
PPRRRPPPPLSSAGRKKEQEGKGDSEEQQEKSIIATIDDVLSARPGALPEDANPGPQTESSKFPFGIQQAKSHRNIKLLEDEPRSRD
ETPLCTIAHWQDSLAKRCICVSNIVRSLSFVPGNDAEMSKHPGLVLILGKLILLHHEHPERKRAPQTYEKEEDEDKGVACSKDEWWW
DCLEVLRDNTLVTLANISGQLDLSAYTESICLPILDGLLHWMVCPSAEAQDPFPTVGPNSVLSPQRLVLETLCKLSIQDNNVDLILA
TPPFSRQEKFYATLVRYVGDRKNPVCREMSMALLSNLAQGDALAARAIAVQKGSIGNLISFLEDGVTMAQYQQSQHNLMHMQPPPLE
PPSVDMMCRAAKALLAMARVDENRSEFLLHEGRLLDISISAVLNSLVASVICDVLFQIGQL
>sp|P58182|O12D2_HUMAN

MLNTTSVTEFLLLGVTDIQELQPFLFVVFLTIYFISVTGNGAVLMIVISDPRLHSLMYFFLGNLSYLDICYSTVTLPKMLQNFLSTH
KAISFLGCISQLHFFHSLGSTESMLFAVMAFDLSVAICKPLRYTVIMNPQLCTQMAITIWVIGFFHALLHSVMTSRLNFCGSNRIHH
FLCDIKPLLKLACGNTELNQWLLSTVTGTIAMGPFFLTLLSYFYIITYLFFKTRSCSMLCKALSTCASHFMVVILFYAPVLFTYIHP
ALESFMDQDRIVAIMYTVVTPVLNPLIYTLRNKEVKGALGRVIRRL
>sp|P08571|CD14_HUMAN
MERASCLLLLLLPLVHVSATTPEPCELDDEDFRCVCNFSEPQPDWSEAFQCVSAVEVEIHAGGLNLEPFLKRVDADADPRQYADTVK
ALRVRRLTVGAAQVPAQLLVGALRVLAYSRLKELTLEDLKITGTMPPLPLEATGLALSSLRLRNVSWATGRSWLAELQQWLKPGLKV
LSIAQAHSPAFSCEQVRAFPALTSLDLSDNPGLGERGLMAALCPHKFPAIQNLALRNTGMETPTGVCAALAAAGVQPHSLDLSHNSL
RATVNPSAPRCMWSSALNSLNLSFAGLEQVPKGLPAKLRVLDLSCNRLNRAPQPDELPEVDNLTLDGNPFLVPGTALPHEGSMNSGV
VPACARSTLSVGVSGTLVLLQGARGFA
>sp|Q9BQ39|DDX50_HUMAN
MPGKLLWGDIMELEAPLEESESQKKERQKSDRRKSRHHYDSDEKSETRENGVTDDLDAPKAKKSKMKEKLNGDTEEGFNRLSDEFSK
SHKSRRKDLPNGDIDEYEKKSKRVSSLDTSTHKSSDNKLEETLTREQKEGAFSNFPISEETIKLLKGRGVTYLFPIQVKTFGPVYEG
KDLIAQARTGTGKTFSFAIPLIERLQRNQETIKKSRSPKVLVLAPTRELANQVAKDFKDITRKLSVACFYGGTSYQSQINHIRNGID
ILVGTPGRIKDHLQSGRLDLSKLRHVVLDEVDQMLDLGFAEQVEDIIHESYKTDSEDNPQTLLFSATCPQWVYKVAKKYMKSRYEQV
DLVGKMTQKAATTVEHLAIQCHWSQRPAVIGDVLQVYSGSEGRAIIFCETKKNVTEMAMNPHIKQNAQCLHGDIAQSQREITLKGFR
EGSFKVLVATNVAARGLDIPEVDLVIQSSPPQDVESYIHRSGRTGRAGRTGICICFYQPRERGQLRYVEQKAGITFKRVGVPSTMDL
VKSKSMDAIRSLASVSYAAVDFFRPSAQRLIEEKGAVDALAAALAHISGASSFEPRSLITSDKGFVTMTLESLEEIQDVSCAWKELN
RKLSSNAVSQITRMCLLKGNMGVCFDVPTTESERLQAEWHDSDWILSVPAKLPEIEEYYDGNTSSNSRQRSGWSSGRSGRSGRSGGR
SGGRSGRQSRQGSRSGSRQDGRRRSGNRNRSRSGGHKRSFD
>sp|Q9UHE8|STEA1_HUMAN
MESRKDITNQEELWKMKPRRNLEEDDYLHKDTGETSMLKRPVLLHLHQTAHADEFDCPSELQHTQELFPQWHLPIKIAAIIASLTFL
YTLLREVIHPLATSHQQYFYKIPILVINKVLPMVSITLLALVYLPGVIAAIVQLHNGTKYKKFPHWLDKWMLTRKQFGLLSFFFAVL
HAIYSLSYPMRRSYRYKLLNWAYQQVQQNKEDAWIEHDVWRMEIYVSLGIVGLAILALLAVTSIPSVSDSLTWREFHYIQSKLGIVS
LLLGTIHALIFAWNKWIDIKQFVWYTPPTFMIAVFLPIVVLIFKSILFLPCLRKKILKIRHGWEDVTKINKTEICSQL
>sp|Q9NRD1|FBX6_HUMAN
MDAPHSKAALDSINELPENILLELFTHVPARQLLLNCRLVCSLWRDLIDLMTLWKRKCLREGFITKDWDQPVADWKIFYFLRSLHRN
LLRNPCAEEDMFAWQIDFNGGDRWKVESLPGAHGTDFPDPKVKKYFVTSYEMCLKSQLVDLVAEGYWEELLDTFRPDIVVKDWFAAR
ADCGCTYQLKVQLASADYFVLASFEPPPVTIQQWNNATWTEVSYTFSDYPRGVRYILFQHGGRDTQYWAGWYGPRVTNSSIVVSPKM
TRNQASSEAQPGQKHGQEEAAQSPYRAVVQIF
>sp|Q30KP9|DB135_HUMAN
MATRSVLLALVVLNLLFYVPPGRSGPNVYIQKIFASCWRLQGTCRPKCLKNEQYRILCDTIHLCCVNPKYLPILTGK
>sp|P0DJ07|PT100_HUMAN
MGVKLEIFRMIIYLTFPVAMFWVSNQAEWFEDDVIQRKRELWPPEKLQEIEEFKERLRKRREEKLLRDAQQNS
>sp|Q9UKD2|MRT4_HUMAN
MPKSKRDKKVSLTKTAKKGLELKQNLIEELRKCVDTYKYLFIFSVANMRNSKLKDIRNAWKHSRMFFGKNKVMMVALGRSPSDEYKD
NLHQVSKRLRGEVGLLFTNRTKEEVNEWFTKYTEMDYARAGNKAAFTVSLDPGPLEQFPHSMEPQLRQLGLPTALKRGVVTLLSDYE
VCKEGDVLTPEQARVLKLFGYEMAEFKVTIKYMWDSQSGRFQQMGDDLPESASESTEESDSEDDD
>sp|P42338|PK3CB_HUMAN
MCFSFIMPPAMADILDIWAVDSQIASDGSIPVDFLLPTGIYIQLEVPREATISYIKQMLWKQVHNYPMFNLLMDIDSYMFACVNQTA
VYEELEDETRRLCDVRPFLPVLKLVTRSCDPGEKLDSKIGVLIGKGLHEFDSLKDPEVNEFRRKMRKFSEEKILSLVGLSWMDWLKQ
TYPPEHEPSIPENLEDKLYGGKLIVAVHFENCQDVFSFQVSPNMNPIKVNELAIQKRLTIHGKEDEVSPYDYVLQVSGRVEYVFGDH
PLIQFQYIRNCVMNRALPHFILVECCKIKKMYEQEMIAIEAAINRNSSNLPLPLPPKKTRIISHVWENNNPFQIVLVKGNKLNTEET
VKVHVRAGLFHGTELLCKTIVSSEVSGKNDHIWNEPLEFDINICDLPRMARLCFAVYAVLDKVKTKKSTKTINPSKYQTIRKAGKVH

YPVAWVNTMVFDFKGQLRTGDIILHSWSSFPDELEEMLNPMGTVQTNPYTENATALHVKFPENKKQPYYYPPFDKIIEKAAEIASSD
SANVSSRGGKKFLPVLKEILDRDPLSQLCENEMDLIWTLRQDCREIFPQSLPKLLLSIKWNKLEDVAQLQALLQIWPKLPPREALEL
LDFNYPDQYVREYAVGCLRQMSDEELSQYLLQLVQVLKYEPFLDCALSRFLLERALGNRRIGQFLFWHLRSEVHIPAVSVQFGVILE
AYCRGSVGHMKVLSKQVEALNKLKTLNSLIKLNAVKLNRAKGKEAMHTCLKQSAYREALSDLQSPLNPCVILSELYVEKCKYMDSKM
KPLWLVYNNKVFGEDSVGVIFKNGDDLRQDMLTLQMLRLMDLLWKEAGLDLRMLPYGCLATGDRSGLIEVVSTSETIADIQLNSSNV
AAAAAFNKDALLNWLKEYNSGDDLDRAIEEFTLSCAGYCVASYVLGIGDRHSDNIMVKKTGQLFHIDFGHILGNFKSKFGIKRERVP
FILTYDFIHVIQQGKTGNTEKFGRFRQCCEDAYLILRRHGNLFITLFALMLTAGLPELTSVKDIQYLKDSLALGKSEEEALKQFKQK
FDEALRESWTTKVNWMAHTVRKDYRS
>sp|Q9BPU9|B9D2_HUMAN
MAEVHVIGQIIGASGFSESSLFCKWGIHTGAAWKLLSGVREGQTQVDTPQIGDMAYWSHPIDLHFATKGLQGWPRLHFQVWSQDSFG
RCQLAGYGFCHVPSSPGTHQLACPTWRPLGSWREQLARAFVGGGPQLLHGDTIYSGADRYRLHTAAGGTVHLEIGLLLRNFDRYGVE
C
>sp|Q5DT21|ISK9_HUMAN
MRATAIVLLLALTLATMFSIECAKQTKQMVDCSHYKKLPPGQQRFCHHMYDPICGSDGKTYKNDCFFCSKVKKTDGTLKFVHFGKC
>sp|Q9H9Q4|NHEJ1_HUMAN
MEELEQGLLMQPWAWLQLAENSLLAKVFITKQGYALLVSDLQQVWHEQVDTSVVSQRAKELNKRLTAPPAAFLCHLDNLLRPLLKDA
AHPSEATFSCDCVADALILRVRSELSGLPFYWNFHCMLASPSLVSQHLIRPLMGMSLALQCQVRELATLLHMKDLEIQDYQESGATL
IRDRLKTEPFEENSFLEQFMIEKLPEACSIGDGKPFVMNLQDLYMAVTTQEVQVGQKHQGAGDPHTSNSASLQGIDSQCVNQPEQLV
SSAPTLSAPEKESTGTSGPLQRPQLSKVKRKKPRGLFS
>sp|Q8WUD6|CHPT1_HUMAN
MAAGAGAGSAPRWLRALSEPLSAAQLRRLEEHRYSAAGVSLLEPPLQLYWTWLLQWIPLWMAPNSITLLGLAVNVVTTLVLISYCPT
ATEEAPYWTYLLCALGLFIYQSLDAIDGKQARRTNSCSPLGELFDHGCDSLSTVFMAVGASIAARLGTYPDWFFFCSFIGMFVFYCA
HWQTYVSGMLRFGKVDVTEIQIALVIVFVLSAFGGATMWDYTIPILEIKLKILPVLGFLGGVIFSCSNYFHVILHGGVGKNGSTIAG
TSVLSPGLHIGLIIILAIMIYKKSATDVFEKHPCLYILMFGCVFAKVSQKLVVAHMTKSELYLQDTVFLGPGLLFLDQYFNNFIDEY
VVLWMAMVISSFDMVIYFSALCLQISRHLHLNIFKTACHQAPEQVQVLSSKSHQNNMD
>sp|Q9H4B4|PLK3_HUMAN
MEPAAGFLSPRPFQRAAAAPAPPAGPGPPPSALRGPELEMLAGLPTSDPGRLITDPRSGRTYLKGRLLGKGGFARCYEATDTETGSA
YAVKVIPQSRVAKPHQREKILNEIELHRDLQHRHIVRFSHHFEDADNIYIFLELCSRKSLAHIWKARHTLLEPEVRYYLRQILSGLK
YLHQRGILHRDLKLGNFFITENMELKVGDFGLAARLEPPEQRKKTICGTPNYVAPEVLLRQGHGPEADVWSLGCVMYTLLCGSPPFE
TADLKETYRCIKQVHYTLPASLSLPARQLLAAILRASPRDRPSIDQILRHDFFTKGYTPDRLPISSCVTVPDLTPPNPARSLFAKVT
KSLFGRKKKSKNHAQERDEVSGLVSGLMRTSVGHQDARPEAPAASGPAPVSLVETAPEDSSPRGTLASSGDGFEEGLTVATVVESAL
CALRNCIAFMPPAEQNPAPLAQPEPLVWVSKWVDYSNKFGFGYQLSSRRVAVLFNDGTHMALSANRKTVHYNPTSTKHFSFSVGAVP
RALQPQLGILRYFASYMEQHLMKGGDLPSVEEVEVPAPPLLLQWVKTDQALLMLFSDGTVQVNFYGDHTKLILSGWEPLLVTFVARN
RSACTYLASHLRQLGCSPDLRQRLRYALRLLRDRSPA
>sp|O60496|DOK2_HUMAN
MGDGAVKQGFLYLQQQQTFGKKWRRFGASLYGGSDCALARLELQEGPEKPRRCEAARKVIRLSDCLRVAEAGGEASSPRDTSAFFLE
TKERLYLLAAPAAERGDWVQAICLLAFPGQRKELSGPEGKQSRPCMEENELYSSAVTVGPHKEFAVTMRPTEASERCHLRGSYTLRA
GESALELWGGPEPGTQLYDWPYRFLRRFGRDKVTFSFEAGRRCVSGEGNFEFETRQGNEIFLALEEAISAQKNAAPATPQPQPATIP
ASLPRPDSPYSRPHDSLPPPSPTTPVPAPRPRGQEGEYAVPFDAVARSLGKNFRGILAVPPQLLADPLYDSIEETLPPRPDHIYDEP
EGVAALSLYDSPQEPRGEAWRRQATADRDPAGLQHVQPAGQDFSASGWQPGTEYDNVVLKKGPK
>sp|Q9NYI0|PSD3_HUMAN
MEGRSAAAETFVWVNNASAHSQSVAKAKYEFLFGRSEGKAPDTSDHGGSTLLPPNVTNEFPEYGTMEEGGEGLRASLEFDGEALPCH
PQEQQGVQPLTGCHSGLDSVTEGPKDVREAPSQSHLKEQSLQPIDSLISALKATEARIISGTLQATKVLDQDAVSSFSVQQVEKELD
TASRKTQRVNKTLPAGQKNLPEIPLSAEVTTEESFYLSIQKDLTALLTGDTQAEISQIMNNGRKGAVCVQEPSCPLASLGSSAVTCH

SAGSVGFLKEQRSALGREHPGGCDRSSSMGRPGRVKHVEFQGVEILWTGGDKRETQHPIDFETSLQRTASPDSKESSKVPRHLISSA
GLCNSSSLTENVWDESWKAPSERPGTSSGTFSPVRLDESGEDEVFLQENKQHLEKTPKPERDRERISEQEEHVKGEDEDILGPGYTE
DSTDVYSSQFETILDNTSLYYSAESLETLYSEPDSYFSFEMPLTPMIQQRIKEGGQFLERTSGGGHQDILSVSADGGIVMGYSSGVT
NGLNDASDSIYTKGTPEIAFWGSNAGVKTTRLEAHSEMGSTEILEKETPENLSNGTSSNVEAAKRLAKRLYQLDRFKRSDVAKHLGK
NNEFSKLVAEEYLKFFDFTGMTLDQSLRYFFKAFSLVGETQERERVLIHFSNRYFYCNPDTIASQDGVHCLTCAIMLLNTDLHGHVN
IGKKMTCQEFIANLQGVNEGVDFSKDLLKALYNSIKNEKLEWAVDDEEKKKSPSESTEEKANGTHPKTISRIGSTTNPFLDIPHDPN
AAVYKSGFLARKIHADMDGKKTPRGKRGWKTFYAVLKGTVLYLQKDEYKPEKALSEEDLKNAVSVHHALASKATDYEKKPNVFKLKT
ADWRVLLFQTQSPEEMQGWINKINCVAAVFSAPPFPAAIGSQKKFSRPLLPATTTKLSQEEQLKSHESKLKQITTELAEHRSYPPDK
KVKAKDVDEYKLKDHYLEFEKTRYEMYVSILKEGGKELLSNDESEAAGLKKSHSSPSLNPDTSPITAKVKRNVSERKDHRPETPSIK
QKVT
>sp|Q8N1F1|C1AS1_HUMAN
MFPGSLSRGRRAAVEMAWLPGSCARVAFAAGAAARYWTAWQGSAGPNPAAVAEAHGSLFCGRATSARAWSLRRPGPGSPAHSGGVQT
RENWIAYPLQSAEDGVATRLQIREESASCLAAEYWSQEPAMRF
>sp|Q92692|NECT2_HUMAN
MARAAALLPSRSPPTPLLWPLLLLLLLETGAQDVRVQVLPEVRGQLGGTVELPCHLLPPVPGLYISLVTWQRPDAPANHQNVAAFHP
KMGPSFPSPKPGSERLSFVSAKQSTGQDTEAELQDATLALHGLTVEDEGNYTCEFATFPKGSVRGMTWLRVIAKPKNQAEAQKVTFS
QDPTTVALCISKEGRPPARISWLSSLDWEAKETQVSGTLAGTVTVTSRFTLVPSGRADGVTVTCKVEHESFEEPALIPVTLSVRYPP
EVSISGYDDNWYLGRTDATLSCDVRSNPEPTGYDWSTTSGTFPTSAVAQGSQLVIHAVDSLFNTTFVCTVTNAVGMGRAEQVIFVRE
TPNTAGAGATGGIIGGIIAAIIATAVAATGILICRQQRKEQTLQGAEEDEDLEGPPSYKPPTPKAKLEAQEMPSQLFTLGASEHSPL
KTPYFDAGASCTEQEMPRYHELPTLEERSGPLHPGATSLGSPIPVPPGPPAVEDVSLDLEDEEGEEEEEYLDKINPIYDALSYSSPS
DSYQGKGFVMSRAMYV
>sp|Q5T440|CAF17_HUMAN
MATAALLRGATPGRGGPVWRWRLRAAPRCRLAHSSCSPGGDPTAGAAWACFRLDGRTLLRVRGPDAAPFLLGLLTNELPLPSPAAAG
APPAARAGYAHFLNVQGRTLYDVILYGLQEHSEVSGFLLECDSSVQGALQKHLALYRIRRKVTVEPHPELRVWAVLPSSPEACGAAS
LQERAGAAAILIRDPRTARMGWRLLTQDEGPALVPGGRLGDLWDYHQHRYLQGVPEGVRDLPPGVALPLESNLAFMNGVSFTKGCYI
GQELTARTHHMGVIRKRLFPVRFLDPLPTSGITPGATVLTASGQTVGKFRAGQGNVGLALLWSEKIKGPLHIRASEGAQVALAASVP
DWWPTVSK
>sp|Q92609|TBCD5_HUMAN
MYHSLSETRHPLQPEEQEVGIDPLSSYSNKSGGDSNKNGRRTSSTLDSEGTFNSYRKEWEELFVNNNYLATIRQKGINGQLRSSRFR
SICWKLFLCVLPQDKSQWISRIEELRAWYSNIKEIHITNPRKVVGQQDLMINNPLSQDEGSLWNKFFQDKELRSMIEQDVKRTFPEM
QFFQQENVRKILTDVLFCYARENEQLLYKQGMHELLAPIVFVLHCDHQAFLHASESAQPSEEMKTVLNPEYLEHDAYAVFSQLMETA
EPWFSTFEHDGQKGKETLMTPIPFARPQDLGPTIAIVTKVNQIQDHLLKKHDIELYMHLNRLEIAPQIYGLRWVRLLFGREFPLQDL
LVVWDALFADGLSLGLVDYIFVAMLLYIRDALISSNYQTCLGLLMHYPFIGDVHSLILKALFLRDPKRNPRPVTYQFHPNLDYYKAR
GADLMNKSRTNAKGAPLNINKVSNSLINFGRKLISPAMAPGSAGGPVPGGNSSSSSSVVIPTRTSAEAPSHHLQQQQQQQRLMKSES
MPVQLNKGLSSKNISSSPSVESLPGGREFTGSPPSSATKKDSFFSNISRSRSHSKTMGRKESEEELEAQISFLQGQLNDLDAMCKYC
AKVMDTHLVNIQDVILQENLEKEDQILVSLAGLKQIKDILKGSLRFNQSQLEAEENEQITIADNHYCSSGQGQGRGQGQSVQMSGAI
KQASSETPGCTDRGNSDDFILISKDDDGSSARGSFSGQAQPLRTLRSTSGKSQAPVCSPLVFSDPLMGPASASSSNPSSSPDDDSSK
DSGFTIVSPLDI
>sp|P04437|TVA2_HUMAN
MAMLLGASVLILWLQPDWVNSQQKNDDQQVKQNSPSLSVQEGRISILNCDYTNSMFDYFLWYKKYPAEGPTFLISISSIKDKNEDGR
FTVFLNKSAKHLSLHIVPSQPGDSAVYFCAAKGAGTASKLTFGTGTRLQVTL
>sp|Q96LA6|FCRL1_HUMAN
MLPRLLLLICAPLCEPAELFLIASPSHPTEGSPVTLTCKMPFLQSSDAQFQFCFFRDTRALGPGWSSSPKLQIAAMWKEDTGSYWCE
AQTMASKVLRSRRSQINVHRVPVADVSLETQPPGGQVMEGDRLVLICSVAMGTGDITFLWYKGAVGLNLQSKTQRSLTAEYEIPSVR

ESDAEQYYCVAENGYGPSPSGLVSITVRIPVSRPILMLRAPRAQAAVEDVLELHCEALRGSPPILYWFYHEDITLGSRSAPSGGGAS
FNLSLTEEHSGNYSCEANNGLGAQRSEAVTLNFTVPTGARSNHLTSGVIEGLLSTLGPATVALLFCYGLKRKIGRRSARDPLRSLPS
PLPQEFTYLNSPTPGQLQPIYENVNVVSGDEVYSLAYYNQPEQESVAAETLGTHMEDKVSLDIYSRLRKANITDVDYEDAM
>sp|P42768|WASP_HUMAN
MSGGPMGGRPGGRGAPAVQQNIPSTLLQDHENQRLFEMLGRKCLTLATAVVQLYLALPPGAEHWTKEHCGAVCFVKDNPQKSYFIRL
YGLQAGRLLWEQELYSQLVYSTPTPFFHTFAGDDCQAGLNFADEDEAQAFRALVQEKIQKRNQRQSGDRRQLPPPPTPANEERRGGL
PPLPLHPGGDQGGPPVGPLSLGLATVDIQNPDITSSRYRGLPAPGPSPADKKRSGKKKISKADIGAPSGFKHVSHVGWDPQNGFDVN
NLDPDLRSLFSRAGISEAQLTDAETSKLIYDFIEDQGGLEAVRQEMRRQEPLPPPPPPSRGGNQLPRPPIVGGNKGRSGPLPPVPLG
IAPPPPTPRGPPPPGRGGPPPPPPPATGRSGPLPPPPPGAGGPPMPPPPPPPPPPPPSSGNGPAPPPLPPALVPAGGLAPGGGRGALL
DQIRQGIQLNKTPGAPESSALQPPPQSSEGLVGALMHVMQKRSRAIHSSDEGEDQAGDEDEDDEWDD
>sp|Q6ZU65|UBN2_HUMAN
MAEPRRVAFISLSPVRRREAEYPGPEREPEYPREPPRLEPQPYREPARAEPPAPREPAPRSDAQPPSREKPLPQREVSRAEPPMSLQ
REPPRPEPPPPFPPLPLQPPPPRESASRAEQPPRPPRETVRLELVLKDPTDESCVEFSYPELLLCGEQRKKLIHTEDPFNDEHQERQ
EVEMLAKKFEMKYGGKPRKHRKDRLQDLIDIGFGYDETDPFIDNSEAYDELVPASLTTKYGGFYINTGTLQFRQASDTEEDDITDNQ
KHKPPKVPKIKEDDIEMKKRKRKEEGEKEKKPRKKVPKQLGVVALNSHKSEKKKKRYKDSLSLAAMIRKFQKEKDALKKESNPKVPV
TLSTPSLNKPPCAAAALGNDVPDLNLSSGDPDLPIFVSTNEHELFQEAENALEMLDDFDFDRLLDAASDGSPLSESGGENGTTTQPT
YTSQVMPKVVPTLPEGLPVLLEKRIEDLRVAAKLFDEEGRKKFFTQDMNNILLDIELQLQELGPVIRSGVYSHLEAFVPCNKETLVK
RLKKLHLNVQDDRLREPLQKLKLAVSNVMPEQLFKYQEDCQARSQAKCAKLQTDEEREKNGSEEDDDEKPGKRVIGPRKKFHWDDTI
RTLLCNLVEIKLGCYELEPNKSQSAEDYLKSFMETEVKPLWPKGWMQARMLFKESRSVHNHLTSAPAKKKVIPAPKPKVKEVMVKTL
PLHSFPTMLKECSPKKDQKTPTSLVASVSGPPTSSSTAAIAAASSSSAPAQETICLDDSLDEDLSFHSPSLDLVSEALAVINNGNKG
PPVGSRISMPTTKPRPGLREEKLASIMSKLPLATPKKLDSTQTTHSSSLIAGHTGPVPKKPQDLAHTGISSGLIAGSSIQNPKVSLE
PLPARLLQQGLQRSSQIHTSSSSQTHVSSSSQAQIAASSHALGTSEAQDASSLTQVTKVHQHSAVQQNYVSPLQATISKSQTNPVVK
LSNNPQLSCSSSLIKTSDKPLMYRLPLSTPSPGNGSQGSHPLVSRTVPSTTTSSNYLAKAMVSQISTQGFKSPFSMAASPKLAASPK
PATSPKPLPSPKPSASPKPSLSAKPSVSTKLISKSNPTPKPTVSPSSSSPNALVAQGSHSSTNSPVHKQPSGMNISRQSPTLNLLPS
SRTSGLPPTKNLQAPSKLTNSSSTGTVGKNSLSGIAMNVPASRGSNLNSSGANRTSLSGGTGSGTQGATKPLSTPHRPSTASGSSVV
TASVQSTAGASLLANASPLTLMTSPLSVTNQNVTPFGMLGGLVPVTMPFQFPLEIFGFGTDTAGVTTTSGSTSAAFHHSLTQNLLKG
LQPGGAQHAATLSHSPLPAHLQQAFHDGGQSKGDTKLPRKSQ
>sp|Q9H7M6|ZSWM4_HUMAN
MEPPAAKRSRGCPAGPEERDAGAGAARGRGRPEALLDLSAKRVAESWAFEQVEERFSRVPEPVQKRIVFWSFPRSEREICMYSSLGY
PPPEGEHDARVPFTRGLHLLQSGAVDRVLQVGFHLSGNIREPGSPGEPERLYHVSISFDRCKITSVSCGCDNRDLFYCAHVVALSLY
RIRHAHQVELRLPISETLSQMNRDQLQKFVQYLISAHHTEVLPTAQRLADEILLLGSEINLVNGAPDPTAGAGIEDANCWHLDEEQI
QEQVKQLLSNGGYYGASQQLRSMFSKVREMLRMRDSNGARMLILMTEQFLQDTRLALWRQQGAGMTDKCRQLWDELGALWVCVVLSP
HCKPEERAGWLQLLSRWDKLDVCPLEEGNYSFDGPSLQPTMAPAPELLQKGSTCITNTEGWVGHPLDPIGCLCRALLEACRLEEETL
TLYPDSGPEKRKVAYQHVPVPGSPGESYLVLALEVALLGLGQQRALPEGLYAQDKVVRNEEQLLALLEEVELDERLVQVLRKQAGLL
LEGGPFSGFGEVLFRESVPMHTCARYLFTALLPHDPDLAYRLALRAMRLPILETAFPAGEPHPSPLDSIMSNRFPRWFILGHLETRQ
CELASTMLTAAKGDPKWLHTVLGSIQQNIHSPALLFKLAQDACKTATPVSAPPDTTLLGIALELGLQVMRMTLNVMTWRRREMVRWL
VSCATEIGPQALMNIMQNWYSLFTPVEAATIVAVTGTTHATLLRLQLDTSRREELWACARTLALQCAMKDPQNCALPALTLCEKNHS
AFEAAYQIVLDAAAGGLGHAHLFTVARYMEHRGLPLRAYKLATLALAQLSIAFNQDSHPAVNDVLWACSLSHSLGRHELSAIVPLII
RSIHCAPMLSDILRRWTLSAPGLGPLGARRAAKPLGADRAPLCQLLDAAVTAYITTSHSRLTHISPRHYGDFIEFLGKARETFLLAP
DGHLQFSQFLENLKQTYKGKKKLMLLVRERFG
>sp|Q86YN1|DOPP1_HUMAN
MAADGQCSLPASWRPVTLTHVEYPAGDLSGHLLAYLSLSPVFVIVGFVTLIIFKRELHTISFLGGLALNEGVNWLIKNVIQEPRPCG
GPHTAVGTKYGMPSSHSQFMWFFSVYSFLFLYLRMHQTNNARFLDLLWRHVLSLGLLAVAFLVSYSRVYLLYHTWSQVLYGGIAGGL
MAIAWFIFTQEVLTPLFPRIAAWPVSEFFLIRDTSLIPNVLWFEYTVTRAEARNRQRKLGTKLQ

>sp|P62987|RL40_HUMAN
MQIFVKTLTGKTITLEVEPSDTIENVKAKIQDKEGIPPDQQRLIFAGKQLEDGRTLSDYNIQKESTLHLVLRLRGGIIEPSLRQLAQ
KYNCDKMICRKCYARLHPRAVNCRKKKCGHTNNLRPKKKVK
>sp|Q9BTC0|DIDO1_HUMAN
MDDKGDPSNEEAPKAIKPTSKEFRKTWGFRRTTIAKREGAGDAEADPLEPPPPQQQLGLSLRRSGRQPKRTERVEQFLTIARRRGRR
SMPVSLEDSGEPTSCPATDAETASEGSVESASETRSGPQSASTAVKERPASSEKVKGGDDHDDTSDSDSDGLTLKELQNRLRRKREQ
EPTERPLKGIQSRLRKKRREEGPAETVGSEASDTVEGVLPSKQEPENDQGVVSQAGKDDRESKLEGKAAQDIKDEEPGDLGRPKPEC
EGYDPNALYCICRQPHNNRFMICCDRCEEWFHGDCVGISEARGRLLERNGEDYICPNCTILQVQDETHSETADQQEAKWRPGDADGT
DCTSIGTIEQKSSEDQGIKGRIEKAANPSGKKKLKIFQPVIEAPGASKCIGPGCCHVAQPDSVYCSNDCILKHAAATMKFLSSGKEQ
KPKPKEKMKMKPEKPSLPKCGAQAGIKISSVHKRPAPEKKETTVKKAVVVPARSEALGKEAACESSTPSWASDHNYNAVKPEKTAAP
SPSLLYKSTKEDRRSEEKAAAMAASKKTAPPGSAVGKQPAPRNLVPKKSSFANVAAATPAIKKPPSGFKGTIPKRPWLSATPSSGAS
AARQAGPAPAAATAASKKFPGSAALVGAVRKPVVPSVPMASPAPGRLGAMSAAPSQPNSQIRQNIRRSLKEILWKRVNDSDDLIMTE
NEVGKIALHIEKEMFNLFQVTDNRYKSKYRSIMFNLKDPKNQGLFHRVLREEISLAKLVRLKPEELVSKELSTWKERPARSVMESRT
KLHNESKKTAPRQEAIPDLEDSPPVSDSEEQQESARAVPEKSTAPLLDVFSSMLKDTTSQHRAHLFDLNCKICTGQVPSAEDEPAPK
KQKLSASVKKEDLKSKHDSSAPDPAPDSADEVMPEAVPEVASEPGLESASHPNVDRTYFPGPPGDGHPEPSPLEDLSPCPASCGSGV
VTTVTVSGRDPRTAPSSSCTAVASAASRPDSTHMVEARQDVPKPVLTSVMVPKSILAKPSSSPDPRYLSVPPSPNISTSESRSPPEG
DTTLFLSRLSTIWKGFINMQSVAKFVTKAYPVSGCFDYLSEDLPDTIHIGGRIAPKTVWDYVGKLKSSVSKELCLIRFHPATEEEEV
AYISLYSYFSSRGRFGVVANNNRHVKDLYLIPLSAQDPVPSKLLPFEGPGLESPRPNIILGLVICQKIKRPANSGELDKMDEKRTRL
QPEEADVPAYPKVATVPQSEKKPSKYPLCSADAAVSTTPPGSPPPPPPLPEPPVLKVLSSLKPAAPSPATAATTAAAASTAASSTAS
SASKTASPLEHILQTLFGKKKSFDPSAREPPGSTAGLPQEPKTTAEDGVPAPPLLDPIVQQFGQFSKDKALEEEEDDRPYDPEEEYD
PERAFDTQLVERGRRHEVERAPEAAAAEREEVAYDPEDETILEEAKVTVDDLPNRMCADVRRNSVERPAEPVAGAATPSLVEQQKML
EELNKQIEEQKRQLEEQEEALRQQRAAVGVSMAHFSVSDALMSPPPKSSLPKAELFQQEQQSADKPASLPPASQASNHRDPRQARRL
ATETGEGEGEPLSRLSARGAQGALPERDASRGGLVGQAPMPVPEEKEPASSPWASGEKPPAGSEQDGWKAEPGEGTRPATVGDSSAR
PARRVLLPTPPCGALQPGFPLQHDGERDPFTCPGFASQDKALGSAQYEDPRNLHSAGRSSSPAGETEGDREPQARPGEGTAPLPPPG
QKVGGSQPPFQGQREPGPHALGMSGLHGPNFPGPRGPAPPFPEENIASNDGPRGPPPARFGAQKGPIPSLFSGQHGPPPYGDSRGPS
PSYLGGPRGVAPSQFEERKDPHGEKREFQDAPYNEVTGAPAQFEGTEQAPFLGSRGGAPFQFGGQRRPLLSQLKGPRGGPPPSQFGG
QRGPPPGHFVGPRGPHPSQFETARGPHPNQFEGPRGQAPNFMPGPRGIQPQQFEDQRVHSPPRFTNQRAPAPLQFGGLRGSAPFSEK
NEQTPSRFHFQGQAPQVMKPGPRPLLELPSHPPQHRKDRWEEAGPPSALSSSAPGQGPEADGQWASADFREGKGHEYRNQTFEGRQR
ERFDVGPKEKPLEEPDAQGRASEDRRRERERGRNWSRERDWDRPREWDRHRDKDSSRDWDRNRERSANRDREREADRGKEWDRSRER
SRNRERERDRRRDRDRSRSRERDRDKARDRERGRDRKDRSKSKESARDPKPEASRASDAGTASQA
>sp|Q8IZY5|BLID_HUMAN
MVTLLPIEGQEIHFFEILESECVLYTGWIERASGSSIYPEAKARLPLEALLGSNKEPMLPKETVLSLKRYNLGSSAMKRNVPGHVLQ
RPSYLTRIQVTLLCNSSAEAL
>sp|Q14761|PTCA_HUMAN
MALPCTLGLGMLLALPGALGSGGSAEDSVGSSSVTVVLLLLLLLLLATGLALAWRRLSRDSGGYYHPARLGAALWGRTRRLLWASPP
GRWLQARAELGSTDNDLERQEDEQDTDYDHVADGGLQADPGEGEQQCGEASSPEQVPVRAEEARDSDTEGDLVLGSPGPASAGGSAE
ALLSDLHAFAGSAAWDDSARAAGGQGLHVTAL
>sp|O95969|SG1D2_HUMAN
MKLSVCLLLVTLALCCYQANAEFCPALVSELLDFFFISEPLFKLSLAKFDAPPEAVAAKLGVKRCTDQMSLQKRSLIAEVLVKILKK
CSV
>sp|Q92572|AP3S1_HUMAN
MIKAILIFNNHGKPRLSKFYQPYSEDTQQQIIRETFHLVSKRDENVCNFLEGGLLIGGSDNKLIYRHYATLYFVFCVDSSESELGIL
DLIQVFVETLDKCFENVCELDLIFHVDKVHNILAEMVMGGMVLETNMNEIVTQIDAQNKLEKSEAGLAGAPARAVSAVKNMNLPEIP
RNINIGDISIKVPNLPSFK

>sp|Q6ZN54|DEFI8_HUMAN
MAILSLRAPGPWQAMQVWADRTLLTPHTGVTSQVLGVAAAVMTPLPGGHAAGRTREARWDAMEYDEKLARFRQAHLNPFNKQSGPRQ
HEQGPGEEVPDVTPEEALPELPPGEPEFRCPERVMDLGLSEDHFSRPVGLFLASDVQQLRQAIEECKQVILELPEQSEKQKDAVVRL
IHLRLKLQELKDPNEDEPNIRVLLEHRFYKEKSKSVKQTCDKCNTIIWGLIQTWYTCTGCYYRCHSKCLNLISKPCVSSKVSHQAEY
ELNICPETGLDSQDYRCAECRAPISLRGVPSEARQCDYTGQYYCSHCHWNDLAVIPARVVHNWDFEPRKVSRCSMRYLALMVSRPVL
RLREINPLLFSYVEELVEIRKLRQDILLMKPYFITCREAMEARLLLQLQDRQHFVENDEMYSVQDLLDVHAGRLGCSLTEIHTLFAK
HIKLDCERCQAKGFVCELCREGDVLFPFDSHTSVCADCSAVFHRDCYYDNSTTCPKCARLSLRKQSLFQEPGPDVEA
>sp|O94822|LTN1_HUMAN
MGGKNKQRTKGNLRPSNSGRAAELLAKEQGTVPGFIGFGTSQSDLGYVPAIQGAEEIDSLVDSDFRMVLRKLSKKDVTTKLKAMQEF
GTMCTERDTETVKGVLPYWPRIFCKISLDHDRRVREATQQAFEKLILKVKKQLAPYLKSLMGYWLMAQCDTYTPAAFAAKDAFEAAF
PPSKQPEAIAFCKDEITSVLQDHLIKETPDTLSDPQTVPEEEREAKFYRVVTCSLLALKRLLCLLPDNELDSLEEKFKSLLSQNKFW
KYGKHSVPQIRSAYFELVSALCQRIPQLMKEEASKVSPSVLLSIDDSDPIVCPALWEAVLYTLTTIEDCWLHVNAKKSVFPKLSTVI
REGGRGLATVIYPYLLPFISKLPQSITNPKLDFFKNFLTSLVAGLSTERTKTSSLESSAVISAFFECLRFIMQQNLGEEEIEQMLVN
DQLIPFIDAVLKDPGLQHGQLFNHLAETLSSWEAKADTEKDEKTAHNLENVLIHFWERLSEICVAKISEPEADVESVLGVSNLLQVL
QKPKSSLKSSKKKNGKVRFADEILESNKENEKCVSSEGEKIEGWELTTEPSLTHNSSGLLSPLRKKPLEDLVCKLADISINYVNERK
SEQHLRFLSTLLDSFSSSRVFKMLLGDEKQSIVQAKPLEIAKLVQKNPAVQFLYQKLIGWLNEDQRKDFGFLVDILYSALRCCDNDM
ERKKVLDDLTKVDLKWNSLLKIIEKACPSSDKHALVTPWLKGDILGEKLVNLADCLCNEDLESRVSSESHFSERWTLLSLVLSQHVK
NDYLIGDVYVERIIVRLHETLFKTKKLSEAESSDSSVSFICDVAYNYFSSAKGCLLMPSSEDLLLTLFQLCAQSKEKTHLPDFLICK
LKNTWLSGVNLLVHQTDSSYKESTFLHLSALWLKNQVQASSLDINSLQVLLSAVDDLLNTLLESEDSYLMGVYIGSVMPNDSEWEKM
RQSLPMQWLHRPLLEGRLSLNYECFKTDFKEQDIKTLPSHLCTSALLSKMVLIALRKETVLENNELEKIIAELLYSLQWCEELDNPP
IFLIGFCEILQKMNITYDNLRVLGNTSGLLQLLFNRSREHGTLWSLIIAKLILSRSISSDEVKPHYKRKESFFPLTEGNLHTIQSLC
PFLSKEEKKEFSAQCIPALLGWTKKDLCSTNGGFGHLAIFNSCLQTKSIDDGELLHGILKIIISWKKEHEDIFLFSCNLSEASPEVL
GVNIEIIRFLSLFLKYCSSPLAESEWDFIMCSMLAWLETTSENQALYSIPLVQLFACVSCDLACDLSAFFDSTTLDTIGNLPVNLIS
EWKEFFSQGIHSLLLPILVTVTGENKDVSETSFQNAMLKPMCETLTYISKEQLLSHKLPARLVADQKTNLPEYLQTLLNTLAPLLLF
RARPVQIAVYHMLYKLMPELPQYDQDNLKSYGDEEEEPALSPPAALMSLLSIQEDLLENVLGCIPVGQIVTIKPLSEDFCYVLGYLL
TWKLILTFFKAASSQLRALYSMYLRKTKSLNKLLYHLFRLMPENPTYAETAVEVPNKDPKTFFTEELQLSIRETTMLPYHIPHLACS
VYHMTLKDLPAMVRLWWNSSEKRVFNIVDRFTSKYVSSVLSFQEISSVQTSTQLFNGMTVKARATTREVMATYTIEDIVIELIIQLP
SNYPLGSIIVESGKRVGVAVQQWRNWMLQLSTYLTHQNGSIMEGLALWKNNVDKRFEGVEDCMICFSVIHGFNYSLPKKACRTCKKK
FHSACLYKWFTSSNKSTCPLCRETFF
>sp|P22079|PERL_HUMAN
MRVLLHLPALLASLILLQAAASTTRAQTTRTSAISDTVSQAKVQVNKAFLDSRTRLKTAMSSETPTSRQLSEYLKHAKGRTRTAIRN
GQVWEESLKRLRQKASLTNVTDPSLDLTSLSLEVGCGAPAPVVRCDPCSPYRTITGDCNNRRKPALGAANRALARWLPAEYEDGLSL
PFGWTPGKTRNGFPLPLAREVSNKIVGYLNEEGVLDQNRSLLFMQWGQIVDHDLDFAPDTELGSSEYSKAQCDEYCIQGDNCFPIMF
PPNDPKAGTQGKCMPFFRAGFVCPTPPYKSLAREQINALTSFLDASFVYSSEPSLASRLRNLSSPLGLMAVNQEVSDHGLPYLPYDS
KKPSPCEFINTTARVPCFLAGDSRASEHILLATSHTLFLREHNRLARELKRLNPQWDGEKLYQEARKILGAFVQIITFRDYLPILLG
DHMQKWIPPYQGYSESVDPRISNVFTFAFRFGHLEVPSSMFRLDENYQPWGPEPELPLHTLFFNTWRMVKDGGIDPLVRGLLAKKSK
LMKQNKMMTGELRNKLFQPTHRIHGFDLAAINTQRCRDHGQPGYNSWRAFCDLSQPQTLEELNTVLKSKMLAKKLLGLYGTPDNIDI
WIGAIAEPLVERGRVGPLLACLLGKQFQQIRDGDRFWWENPGVFTNEQKDSLQKMSFSRLVCDNTRITKVPRDPFWANSYPYDFVDC
SAIDKLDLSPWASVKN
>sp|Q96B45|BORC7_HUMAN
MATGTPESQARFGQSVKGLLTEKVTTCGTDVIALTKQVLKGSRSSELLGQAARNMVLQEDAILHSEDSLRKMAIITTHLQYQQEAIQ
KNVEQSSDLQDQLNHLLK
>sp|O60667|FAIM3_HUMAN

MDFWLWPLYFLPVSGALRILPEVKVEGELGGSVTIKCPLPEMHVRIYLCREMAGSGTCGTVVSTTNFIKAEYKGRVTLKQYPRKNLF
LVEVTQLTESDSGVYACGAGMNTDRGKTQKVTLNVHSEYEPSWEEQPMPETPKWFHLPYLFQMPAYASSSKFVTRVTTPAQRGKVPP
VHHSSPTTQITHRPRVSRASSVAGDKPRTFLPSTTASKISALEGLLKPQTPSYNHHTRLHRQRALDYGSQSGREGQGFHILIPTILG
LFLLALLGLVVKRAVERRKALSRRARRLAVRMRALESSQRPRGSPRPRSQNNIYSACPRRARGADAAGTGEAPVPGPGAPLPPAPLQ
VSESPWLHAPSLKTSCEYVSLYHQPAAMMEDSDSDDYINVPA
>sp|Q6NXP0|EFC12_HUMAN
MDDDYEAYHSLFLSLLGLCPSKTPINENAPVFDPEPVIAHCFKQFQQKDFRLPQTRRRIIMVPRKEDQTPLNPASQPQAPPKPIPSF
KVLEARDIQEQPEDRKTWLSQRSKLRQELESFGDVKRWLENKPSITPSEAKVLHMIHEEQSAQPNASQATTRTTRKKAPRLSRLSRQ
MVPQLQLPEPPALSVMYSYLHSRKIKILEIFHKVGQGENQRITREEFIAAVKAVGVPLKNQEVEDIVIYLSSLGKHNTITMDILANT
YKQWSMAQQRSSLATAREHYILAKHRDSLKGPLKKQEVDSAPQLPKVDLLTVPAVDTQMETRPMTLEEMEEVGKRYRERQRQHKLTI
PSIQYTEQCHLVRCGNRHFDEHCLPSTIHGDMRELIDSARRHNFLVYLQCWKLCKSYGLPLTEDILMKALLYPGDKIIFQMDKVCPI
RQPGGYYSDWKVFSPNLALLRSQGPGKSKRTDKKTPKKSKKMRFKEFEEFTRKLKVKRSSGLQQTHPNSFWPGHLLDKLQLYLPTVA
TDRSLALFSCVQHQPHVYPATYHPDHWWPLRNKNYMTHAHYDAAKVYYIN
>sp|Q7Z392|TPC11_HUMAN
MSPTQWDFPVELCCRPMAFVTLTGLDVVYNAVHRAVWDAFCANRRADRVPISFKVLPGDHEYPKCRPKRTSYEWYIPKGILKTGWMN
KHLNLVPALVVVFYELDWDEPQWKEKQSECATRVEIVRQSLQGRNTKVAVVLIQKKTPLPPGEDVIASERAAALCNACELSGKSLFV
LPHTDHLVGYIIRLENAFYEHAQTYYYTEIRRVKSHKEFLNKTTHQLLFVRHQFKIAFFSELKQDTQNALKNYRTAYNLVHELRAHE
TNILEIKTMAGFINYKICRLCFQHNTPLDAIAQFRKHIDLCKKKIGSAELSFEHDAWMSKQFQAFGDLFDEAIKLGLTAIQTQNPGF
YYQQAAYYAQERKQLAKTLCNHEASVMYPNPDPLETQTGVLDFYGQRSWRQGILSFDLSDPEKEKVGILAIQLKERNVVHSEIIITL
LSNAVAQFKKYKCPRMKSHLMVQMGEEYYYAKDYTKALKLLDYVMCDYRSEGWWTLLTSVLTTALKCSYLMAQLKDYITYSLELLGR
ASTLKDDQKSRIEKNLINVLMNESPDPEPDCDILAVKTAQKLWADRISLAGSNIFTIGVQDFVPFVQCKAKFHAPSFHVDVPVQFDI
YLKADCPHPIRFSKLCVSFNNQEYNQFCVIEEASKANEVLENLTQGKMCLVPGKTRKLLFKFVAKTEDVGKKIEITSVDLALGNETG
RCVVLNWQGGGGDAASSQEALQAARSFKRRPKLPDNEVHWDSIIIQASTMIISRVPNISVHLLHEPPALTNEMYCLVVTVQSHEKTQ
IRDVKLTAGLKPGQDANLTQKTHVTLHGTELCDESYPALLTDIPVGDLHPGEQLEKMLYVRCGTVGSRMFLVYVSYLINTTVEEKEI
VCKCHKDETVTIETVFPFDVAVKFVSTKFEHLERVYADIPFLLMTDLLSASPWALTIVSSELQLAPSMTTVDQLESQVDNVILQTGE
SASECFCLQCPSLGNIEGGVATGHYIISWKRTSAMENIPIITTVITLPHVIVENIPLHVNADLPSFGRVRESLPVKYHLQNKTDLVQ
DVEISVEPSDAFMFSGLKQIRLRILPGTEQEMLYNFYPLMAGYQQLPSLNINLLRFPNFTNQLLRRFIPTSIFVKPQGRLMDDTSIA
AA
>sp|Q9Y2W7|CSEN_HUMAN
MQPAKEVTKASDGSLLGDLGHTPLSKKEGIKWQRPRLSRQALMRCCLVKWILSSTAPQGSDSSDSELELSTVRHQPEGLDQLQAQTK
FTKKELQSLYRGFKNECPTGLVDEDTFKLIYAQFFPQGDATTYAHFLFNAFDADGNGAIHFEDFVVGLSILLRGTVHEKLKWAFNLY
DINKDGYITKEEMLAIMKSIYDMMGRHTYPILREDAPAEHVERFFEKMDRNQDGVVTIEEFLEACQKDENIMSSMQLFENVI
>sp|Q02779|M3K10_HUMAN
MEEEEGAVAKEWGTTPAGPVWTAVFDYEAAGDEELTLRRGDRVQVLSQDCAVSGDEGWWTGQLPSGRVGVFPSNYVAPGAPAAPAGL
QLPQEIPFHELQLEEIIGVGGFGKVYRALWRGEEVAVKAARLDPEKDPAVTAEQVCQEARLFGALQHPNIIALRGACLNPPHLCLVM
EYARGGALSRVLAGRRVPPHVLVNWAVQVARGMNYLHNDAPVPIIHRDLKSINILILEAIENHNLADTVLKITDFGLAREWHKTTKM
SAAGTYAWMAPEVIRLSLFSKSSDVWSFGVLLWELLTGEVPYREIDALAVAYGVAMNKLTLPIPSTCPEPFARLLEECWDPDPHGRP
DFGSILKRLEVIEQSALFQMPLESFHSLQEDWKLEIQHMFDDLRTKEKELRSREEELLRAAQEQRFQEEQLRRREQELAEREMDIVE
RELHLLMCQLSQEKPRVRKRKGNFKRSRLLKLREGGSHISLPSGFEHKITVQASPTLDKRKGSDGASPPASPSIIPRLRAIRLTPVD
CGGSSSGSSSGGSGTWSRGGPPKKEELVGGKKKGRTWGPSSTLQKERVGGEERLKGLGEGSKQWSSSAPNLGKSPKHTPIAPGFASL
NEMEEFAEAEDGGSSVPPSPYSTPSYLSVPLPAEPSPGARAPWEPTPSAPPARWGHGARRRCDLALLGCATLLGAVGLGADVAEARA
ADGEEQRRWLDGLFFPRAGRFPRGLSPPARPHGRREDVGPGLGLAPSATLVSLSSVSDCNSTRSLLRSDSDEAAPAAPSPPPSPPAP
TPTPSPSTNPLVDLELESFKKDPRQSLTPTHVTAACAVSRGHRRTPSDGALGQRGPPEPAGHGPGPRDLLDFPRLPDPQALFPARRR
PPEFPGRPTTLTFAPRPRPAASRPRLDPWKLVSFGRTLTISPPSRPDTPESPGPPSVQPTLLDMDMEGQNQDSTVPLCGAHGSH

>sp|Q9NUK0|MBNL3_HUMAN
MTAVNVALIRDTKWLTLEVCREFQRGTCSRADADCKFAHPPRVCHVENGRVVACFDSLKGRCTRENCKYLHPPPHLKTQLEINGRNN
LIQQKTAAAMFAQQMQLMLQNAQMSSLGSFPMTPSIPANPPMAFNPYIPHPGMGLVPAELVPNTPVLIPGNPPLAMPGAVGPKLMRS
DKLEVCREFQRGNCTRGENDCRYAHPTDASMIEASDNTVTICMDYIKGRCSREKCKYFHPPAHLQARLKAAHHQMNHSAASAMALQP
GTLQLIPKRSALEKPNGATPVFNPTVFHCQQALTNLQLPQPAFIPAGPILCMAPASNIVPMMHGATPTTVSAATTPATSVPFAAPTT
GNQLKF
>sp|P78345|RPP38_HUMAN
MAAAPQAPGRGSLRKTRPLVVKTSLNNPYIIRWSALESEDMHFILQTLEDRLKAIGLQKIEDKKKKNKTPFLKKESREKCSIAVDIS
ENLKEKKTDAKQQVSGWTPAHVRKQLAIGVNEVTRALERRELLLVLVCKSVKPAMITSHLIQLSLSRSVPACQVPRLSERIAPVIGL
KCVLALAFKKNTTDFVDEVRAIIPRVPSLSVPWLQDRIEDSGENLETEPLESQDRELLDTSFEDLSKPKRKLADGRQASVTLQPLKI
KKLIPNPNKIRKPPKSKKATPK
>sp|P0C7P2|RFL3S_HUMAN
MQTDTSNLSARSCRFCVMSPLRTLLRSSEMRRKLLAVSASKVISTVKRKTSCSASGQKPTPCLSSTSKAQISPDFSFFNSVSSSKIK
TFHEETSLFQIFIGMLCGNT
>sp|O75128|COBL_HUMAN
MDAPRASAAKPPTGRKMKARAPPPPGKAATLHVHSDQKPPHDGALGSQQNLVRMKEALRASTMDVTVVLPSGLEKRSVLNGSHAMMD
LLVELCLQNHLNPSHHALEIRSSETQQPLSFKPNTLIGTLNVHTVFLKEKVPEEKVKPGPPKVPEKSVRLVVNYLRTQKAVVRVSPE
VPLQNILPVICAKCEVSPEHVVLLRDNIAGEELELSKSLNELGIKELYAWDNRRETFRKSSLGNDETDKEKKKFLGFFKVNKRSNSK
GCLTTPNSPSMHSRSLTLGPSLSLGSISGVSVKSEMKKRRAPPPPGSGPPVQDKASEKVSLGSQIDLQKKKRRAPAPPPPQPPPPSP
LIPNRTEDKEENRKSTMVSLPLGSGSHCSPDGAPQVLSEAEETVSVGSCFASEDTTEDSGVMSSPSDIVSLDSQQDSMKYKDKWATD
QEDCSDQDLAGTPDLGPQKSPLWEKNGSENSHLRTEKAVTASNDEEDLLIAGEFRKTLAELDEDLEEMEDSYETDTSSLTSSIHGAS
NHCPQDAMIPHGDTDAIPVTFIGEVSDDPVDSGLFSNRNNNAGSFDSEGVASRRDSLAPLQAEHSQPHEKAREEVPALHPASHDVGK
GIRVALSNISKDGNLMETAPRVTSFASNLHTDNLNAKVKDKVYGCADGERTQATERVNSQPVNEKDSNDKNAALAPTSWHQRGQNPG
KSYRLKHGLTTYKIIPPKSEMRCYDRDVSLSTGAIKIDELGNLVSPHATGIRIISLSSSVPEAESQPIGKVREFWRCNSVEKHLGRP
SESSARGPPSTPVPTQTQNPESRLQADPKPISPQQKSAHHEGRNPLGEGRNQPPTMGMGHVRVPAAHTTEVTFLKPQRRTSSQYVAS
AIAKRIGAPKVHADVVRPHGYAEKGYAGKAPVLAAPPVTVKDDRTSSPHSETQGWKDGAQWPCVTPPNNHGEDLAVGAPPRGEVIGP
HRKLSTQDRPAAIHRSSCFSLVQSSQRDRVSVGQSCGFSGKQSTSSQEASSASEPRRAPDGTDPPPPHTSDTQACSRELVNGSVRAP
GHGEPSHPPGGSGTESHILLEREEKPSVFSTDGNETDSIWPPSIFGPKKKFKPVVQRPVPKDTSLHSALMEAIHSAGGKDRLRKTAE
HTGEGRPAKLSYTEAEGERSALLAAIRGHSGTCSLRKVASSASEELQSFRDAALSAQGSESPLLEDLGLLSPPAIPPPPPPPSQALS
APRTASRFSTGTLSNTADARQALMDAIRSGTGAARLRKVPLLV
>sp|O14657|TOR1B_HUMAN
MLRAGWLRGAAALALLLAARVVAAFEPITVGLAIGAASAITGYLSYNDIYCRFAECCREERPLNASALKLDLEEKLFGQHLATEVIF
KALTGFRNNKNPKKPLTLSLHGWAGTGKNFVSQIVAENLHPKGLKSNFVHLFVSTLHFPHEQKIKLYQDQLQKWIRGNVSACANSVF
IFDEMDKLHPGIIDAIKPFLDYYEQVDGVSYRKAIFIFLSNAGGDLITKTALDFWRAGRKREDIQLKDLEPVLSVGVFNNKHSGLWH
SGLIDKNLIDYFIPFLPLEYRHVKMCVRAEMRARGSAIDEDIVTRVAEEMTFFPRDEKIYSDKGCKTVQSRLDFH
>sp|Q9UPT6|JIP3_HUMAN
MMEIQMDEGGGVVVYQDDYCSGSVMSERVSGLAGSIYREFERLIHCYDEEVVKELMPLVVNVLENLDSVLSENQEHEVELELLREDN
EQLLTQYEREKALRRQAEEKFIEFEDALEQEKKELQIQVEHYEFQTRQLELKAKNYADQISRLEERESEMKKEYNALHQRHTEMIQT
YVEHIERSKMQQVGGNSQTESSLPGRRKERPTSLNVFPLADGTVRAQIGGKLVPAGDHWHLSDLGQLQSSSSYQCPQDEMSESGQSS
AAATPSTTGTKSNTPTSSVPSAAVTPLNESLQPLGDYGVGSKNSKRAREKRDSRNMEVQVTQEMRNVSIGMGSSDEWSDVQDIIDST
PELDMCPETRLDRTGSSPTQGIVNKAFGINTDSLYHELSTAGSEVIGDVDEGADLLGEFSVRDDFFGMGKEVGNLLLENSQLLETKN
ALNVVKNDLIAKVDQLSGEQEVLRGELEAAKQAKVKLENRIKELEEELKRVKSEAIIARREPKEEAEDVSSYLCTESDKIPMAQRRR
FTRVEMARVLMERNQYKERLMELQEAVRWTEMIRASREHPSVQEKKKSTIWQFFSRLFSSSSSPPPAKRPYPSVNIHYKSPTTAGFS
QRRNHAMCPISAGSRPLEFFPDDDCTSSARREQKREQYRQVREHVRNDDGRLQACGWSLPAKYKQLSPNGGQEDTRMKNVPVPVYCR

PLVEKDPTMKLWCAAGVNLSGWRPNEDDAGNGVKPAPGRDPLTCDREGDGEPKSAHTSPEKKKAKELPEMDATSSRVWILTSTLTTS
KVVIIDANQPGTVVDQFTVCNAHVLCISSIPAASDSDYPPGEMFLDSDVNPEDPGADGVLAGITLVGCATRCNVPRSNCSSRGDTPV
LDKGQGEVATIANGKVNPSQSTEEATEATEVPDPGPSEPETATLRPGPLTEHVFTDPAPTPSSGPQPGSENGPEPDSSSTRPEPEPS
GDPTGAGSSAAPTMWLGAQNGWLYVHSAVANWKKCLHSIKLKDSVLSLVHVKGRVLVALADGTLAIFHRGEDGQWDLSNYHLMDLGH
PHHSIRCMAVVYDRVWCGYKNKVHVIQPKTMQIEKSFDAHPRRESQVRQLAWIGDGVWVSIRLDSTLRLYHAHTHQHLQDVDIEPYV
SKMLGTGKLGFSFVRITALLVAGSRLWVGTGNGVVISIPLTETVVLHRGQLLGLRANKTSPTSGEGARPGGIIHVYGDDSSDRAASS
FIPYCSMAQAQLCFHGHRDAVKFFVSVPGNVLATLNGSVLDSPAEGPGPAAPASEVEGQKLRNVLVLSGGEGYIDFRIGDGEDDETE
EGAGDMSQVKPVLSKAERSHIIVWQVSYTPE
>sp|Q8NEB7|ACRBP_HUMAN
MRKPAAGFLPSLLKVLLLPLAPAAAQDSTQASTPGSPLSPTEYERFFALLTPTWKAETTCRLRATHGCRNPTLVQLDQYENHGLVPD
GAVCSNLPYASWFESFCQFTHYRCSNHVYYAKRVLCSQPVSILSPNTLKEIEASAEVSPTTMTSPISPHFTVTERQTFQPWPERLSN
NVEELLQSSLSLGGQEQAPEHKQEQGVEHRQEPTQEHKQEEGQKQEEQEEEQEEEGKQEEGQGTKEGREAVSQLQTDSEPKFHSESL
SSNPSSFAPRVREVESTPMIMENIQELIRSAQEIDEMNEIYDENSYWRNQNPGSLLQLPHTEALLVLCYSIVENTCIITPTAKAWKY
MEEEILGFGKSVCDSLGRRHMSTCALCDFCSLKLEQCHSEASLQRQQCDTSHKTPFVSPLLASQSLSIGNQVGSPESGRFYGLDLYG
GLHMDFWCARLATKGCEDVRVSGWLQTEFLSFQDGDFPTKICDTDYIQYPNYCSFKSQQCLMRNRNRKVSRMRCLQNETYSALSPGK
SEDVVLRWSQEFSTLTLGQFG
>sp|P29120|NEC1_HUMAN
MERRAWSLQCTAFVLFCAWCALNSAKAKRQFVNEWAAEIPGGPEAASAIAEELGYDLLGQIGSLENHYLFKHKNHPRRSRRSAFHIT
KRLSDDDRVIWAEQQYEKERSKRSALRDSALNLFNDPMWNQQWYLQDTRMTAALPKLDLHVIPVWQKGITGKGVVITVLDDGLEWNH
TDIYANYDPEASYDFNDNDHDPFPRYDPTNENKHGTRCAGEIAMQANNHKCGVGVAYNSKVGGIRMLDGIVTDAIEASSIGFNPGHV
DIYSASWGPNDDGKTVEGPGRLAQKAFEYGVKQGRQGKGSIFVWASGNGGRQGDNCDCDGYTDSIYTISISSASQQGLSPWYAEKCS
STLATSYSSGDYTDQRITSADLHNDCTETHTGTSASAPLAAGIFALALEANPNLTWRDMQHLVVWTSEYDPLANNPGWKKNGAGLMV
NSRFGFGLLNAKALVDLADPRTWRSVPEKKECVVKDNDFEPRALKANGEVIIEIPTRACEGQENAIKSLEHVQFEATIEYSRRGDLH
VTLTSAAGTSTVLLAERERDTSPNGFKNWDFMSVHTWGENPIGTWTLRITDMSGRIQNEGRIVNWKLILHGTSSQPEHMKQPRVYTS
YNTVQNDRRGVEKMVDPGEEQPTQENPKENTLVSKSPSSSSVGGRRDELEEGAPSQAMLRLLQSAFSKNSPPKQSPKKSPSAKLNIP
YENFYEALEKLNKPSQLKDSEDSLYNDYVDVFYNTKPYKHRDDRLLQALVDILNEEN
>sp|P00441|SODC_HUMAN
MATKAVCVLKGDGPVQGIINFEQKESNGPVKVWGSIKGLTEGLHGFHVHEFGDNTAGCTSAGPHFNPLSRKHGGPKDEERHVGDLGN
VTADKDGVADVSIEDSVISLSGDHCIIGRTLVVHEKADDLGKGGNEESTKTGNAGSRLACGVIGIAQ
>sp|O95125|ZN202_HUMAN
MATAVEPEDQDLWEEEGILMVKLEDDFTCRPESVLQRDDPVLETSHQNFRRFRYQEAASPREALIRLRELCHQWLRPERRTKEQILE
LLVLEQFLTVLPGELQSWVRGQRPESGEEAVTLVEGLQKQPRRPRRWVTVHVHGQEVLSEETVHLGVEPESPNELQDPVQSSTPEQS
PEETTQSPDLGAPAEQRPHQEEELQTLQESEVPVPEDPDLPAERSSGDSEMVALLTALSQGLVTFKDVAVCFSQDQWSDLDPTQKEF
YGEYVLEEDCGIVVSLSFPIPRPDEISQVREEEPWVPDIQEPQETQEPEILSFTYTGDRSKDEEECLEQEDLSLEDIHRPVLGEPEI
HQTPDWEIVFEDNPGRLNERRFGTNISQVNSFVNLRETTPVHPLLGRHHDCSVCGKSFTCNSHLVRHLRTHTGEKPYKCMECGKSYT
RSSHLARHQKVHKMNAPYKYPLNRKNLEETSPVTQAERTPSVEKPYRCDDCGKHFRWTSDLVRHQRTHTGEKPFFCTICGKSFSQKS
VLTTHQRIHLGGKPYLCGECGEDFSEHRRYLAHRKTHAAEELYLCSECGRCFTHSAAFAKHLRGHASVRPCRCNECGKSFSRRDHLV
RHQRTHTGEKPFTCPTCGKSFSRGYHLIRHQRTHSEKTS
>sp|Q86V85|GP180_HUMAN
MGGLRLLAVALTCCWWPQGSQGKTLRGSFSSTAAQDAQGQRIGHFEFHGDHALLCVRINNIAVAVGKEAKLYLFQAQEWLKLQQSSH
GYSCSEKLSKAQLTMTMNQTEHNLTVSQIPSPQTWHVFYADKYTCQDDKENSQVEDIPFEMVLLNPDAEGNPFDHFSAGESGLHEFF
FLLVLVYFVIACIYAQSLWQAIKKGGPMHMILKVLTTALLLQAGSALANYIHFSSYSKDGIGVPFMGSLAEFFDIASQIQMLYLLLS
LCMGWTIVRMKKSQSRPLQWDSTPASTGIAVFIVMTQSVLLLWEQFEDISHHSYHSHHNLAGILLIVLRICLALSLGCGLYQIITVE

RSTLKREFYITFAKGCILWFLCHPVLACISVIFSDYQRDKVITIGVILCQSVSMVILYRLFLSHSLYWEVSSLSSVTLPLTISSGHK
SRPHF
>sp|Q5T0J3|CA220_HUMAN
MIPVRGLWYCYLQVKKVRLREAERLGPKSQCPAECGAASWISWVLQHQQLLASEWATKDGRGTWGSKPSCTVPALSATSQLLQLGRL
NSDLQFVRQRAKKSVSSPSLLQFWCCLSRLSFHALNQKINSTNINIT
>sp|Q9NU02|ANKE1_HUMAN
MALADKRLENLQIYKVLQCVRNKDKKQIEKLTKLGYPELINYTEPINGLSALHLASVSNDIDMVSFLLDLGAHPDVQDRMGCTPTMR
AAELGHELSMEILAKAKADMTIVDNEGKGVLFYCILPTKRHYRCALIALEHGADVNNSTYEGKPIFLRACEDAHDVKDVCLTFLEKG
ANPNAINSSTGRTALMEASREGVVEIVRGILERGGEVNAFDNDRHHAAHFAAKGGFFDILKLLFAYNGDVGLISINGNTPLHYAAMG
GFADCCKYIAQRGCDLKWKNLDHKTPRAVAKEGGFKAASKEIRRAERIANKLARPGAKNPNPLWALRLHDWSVEREAFLREAFAVLD
RGDGSISKNDFVMVLEERQDYASSEQLAAIAHLHEKTRGGGVNINEFFKGTRYLNKSFVLGSYGPKKKEKGMGKKGKKGKFVLPLPI
CVIPEYAFPRRQDGGPPYYMIETYKNVTDSSRFNRDHPPEHPIQDDSVWYIDDSEKVFSNINIITKAGDLASLKKAFESGIPVDMKD
NYYKTPLMTACASGNIDVVKFLLEKGANVNATDNFLWTPLHFACHAGQQDIVELLVESGALIDAASINNSTPLNRAIESCRLDTVKY
LLDIGAKFQLENRKGHSAMDVAKAYADYRIIDLIKEKLDNLPKPAENQKLKGKTPPILKTEGPEIKKEEELLSSIYGVPTTSEGKKV
QKGNVVHLNSLITSGYTKKVDITFIPRRIWSPEATTAELIRKRELRRERFTHEVDFDDFMMPFQKNITEKARALEAALKT
>sp|Q96GX1|TECT2_HUMAN
MGFQPPAALLLRLFLLQGILRLLWGDLAFIPPFIRMSGPAVSASLVGDTEGVTVSLAVLQDEAGILPIPTCGVLNNETEDWSVTVIP
GAKVLEVTVRWKRGLDWCSSNETDSFSESPCILQTLLVSASHNSSCSAHLLIQVEIYANSSLTHNASENVTVIPNQVYQPLGPCPCN
LTAGACDVRCCCDQECSSNLTTLFRRSCFTGVFGGDVNPPFDQLCSAGTTTRGVPDWFPFLCVQSPLANTPFLGYFYHGAVSPKQDS
SFEVYVDTDAKDFADFGYKQGDPIMTVKKAYFTIPQVSLAGQCMQNAPVAFLHNFDVKCVTNLELYQERDGIINAKIKNVALGGIVT
PKVIYEEATDLDKFITNTETPLNNGSTPRIVNVEEHYIFKWNNNTISEINVKIFRAEINAHQKGIMTQRFVVKFLSYNSGNEEELSG
NPGYQLGKPVRALNINRMNNVTTLHLWQSAGRGLCTSATFKPILFGENVLSGCLLEVGINENCTQLRENAVERLDSLIQATHVAMRG
NSDYADLSDGWLEIIRVDAPDPGADPLASSVNGMCLDIPAHLSIRILISDAGAVEGITQQEILGVETRFSSVNWQYQCGLTCEHKAD
LLPISASVQFIKIPAQLPHPLTRFQINYTEYDCNRNEVCWPQLLYPWTQYYQGELHSQCVAKGLLLLLFLTLALFLSNPWTRICKAY
S
>sp|A6NKW6|F159B_HUMAN
MSEASRLCSGYYSLNQSFVEPFQCPRRGEGAALQYCCGFADLKYCCSEPGSYFPYKHSYMWSLSIGALIGLGIAALVLLAFVISVCV
LCYLFLYTKPQRLDTGLKLQHLEASSTQEGKSNGKTKALNSNAASNATNETYYEADDIIQEKTMDATQIHIAY
>sp|Q9C0G0|ZN407_HUMAN
MMDSENKPENDEDEKINKEAQDLTKLSSHNEDGGPVSDVIASFPENSMGKRGFSESSNSDSVVIGEDRNKHASKRRKLDEAEPLKSG
KQGICRLETSESSVTEGGIALDETGKETFLSDCTVGGTCLPNALSPSCNFSTIDVVSLKTDTEKTSAQEMVSLDLERESPFPPKEIS
VSCTIGNVDTVLKCSICGHLFSSCSDLEKHAESHMQQPKEHTCCHCSHKAESSSALHMHIKQAHGPQKVFSCDLCGFQCSEENLLNA
HYLGKTHLRRQNLAARGGFVQILTKQPFFPKKSRTMATKNVHSKPRTSKSIAKNSDSKGLRNVGSTFKDFRGSISKQSGSSSELLVEM
MPSRNTLSQEVEIVEEHVTSLGLAQNPENQSRKLDTLVTSEGLLEKLESTKNTLQAAHGNSVTSRPRPERNILVLGNSFRRRSSTFT
LKGGQAKKRFNLLGIKRGTSETQRMYMKHLRTQMKTHDAESVLKHLEACSSVQRVCVTTSETQEAEQGQGSARPPDSGLHSLTVKPAS
GSQTLCACTDCGQVATNRTDLEIHVKRCHAREMKFYCRTCDFSSMSRRDLDEHLHSNQHQQTASVLSCQCCSFISLDEINLRDHMKE
KHNMHFLCTPCNLFFLSEKDVEEHKATEKHINSLVQPKTLQSSNSDLVLQTLPLSTLESENAKESMDDSGKASQEEPLKSRVSHGNE
VRHSSKPQFQCKKCFYKTRSSTVLTRHIKLRHGQDYHFLCKACNLYSLSKEGMEKHIKRSKHLENAKKNNIGLSFEECIERVCIGAN
DKKEEFDVSGNGRIEGHIGVQLQEHSYLEKGMLASEELSQSGGSTKDDELASTTTPKRGRPKGNISRTCSHCGLLASSITNLTVHIR
RKHSHQYSYLCKVCKYYTVTKGDMERHCATKKHKGRVEIEASGKHSSDIIVGPEGGSLEAGKKNAGSAVTMSDEHANKPAESPTSVL
EKPDRGNSIEAEVENVFHSLDGEVNSHLLDKKEQISSEPEDFAQPGDVYSQRDVTGTGENKCLHCEFSAHSSASLELHVKRKHTKEF
EFYCMACDYYAVTRREMTRHAATEKHKMKRQSYLNSANVEAGSADMSKNIIMPEEEHQQNSEEFQIISGQPSDTLKSRNAADCSILN
ENTNLDMSKVLCAADSVEVETEEESNFNEDHSFCETFQQAPVKDKVRKPEEMMSLTMSSNYGSPSRFQNENSGSSALNCETAKKNHE
ISNDAGELRVHCEGEGGNAGDGGGVVPHRHLCPVTLDGERSAESPVLVVTRITREQGNLESGGQNRVARGHGLEDLKGVQEDPVLGN

KEILMNSQHETEFILEEDGPASDSTVESSDVYETIISIDDKGQAMYSFGRFDSSIIRIKNPEDGELIDQSEEGLIATGVRISELPLK
DCAQGVKKKKSEGSSIGESTRIRCDDCGFLADGLSGLNVHIAMKHPTKEKHFHCLLCGKSFYTESNLHQHLASAGHMRNEQASVEEL
PEGGATFKCVKCTEPFDSEQNLFLHIKGQHEELLREVNKYIVEDTEQINREREENQGNVCKYCGKMCRSSNSMAFLAHIRTHTGSKP
FKCKICHFATAQLGDARNHVKRHLGMREYKCHVCGVAFVMKKHLNTHLLGKHGVGTPKERKFTCHLCDRSFTEKWALNNHMKLHTGE
KPFKCTWPTCHYSFLTASAMKDHYRTHTGEKSFLCDLCGFAGGTRHALTKHRRQHTGEKPFKCDECNFASTTQSHLTRHKRVHTGEK
PYRCPWCDYRSNCAENIRKHILHTGKHEGVKMYNCPKCDYGTNVPVEFRNHLKEQHPDIENPDLAYLHAGIVSKSYECRLKGQGATF
VETDSPFTAAALAEEPLVKEKPLRSSRRPAPPPEQVQQVIIFQGYDGEFALDPSVEETAAATLQTLAMAGQVARVVHITEDGQVIAT
SQSGAHVGSVVPGPILPEQLADGATQVVVVGGSMEGHGMDESLSPGGAVIQQVTKQEILNLSEAGVAPPEASSALDALLCAVTELGE
VEGRAGLEEQGRPGAKDVLIQLPGQEVSHVAADPEAPEIQMFPQAQESPAAVEVLTQVVHPSAAMASQERAQVAFKKMVQGVLQFAV
CDTAAAGQLVKDGVTQVVVSEEGAVHMVAGEGAQIIMQEAQGEHMDLVESDGEISQIIVTEELVQAMVQESSGGFSEGTTHYILTEL
PPGVQDEPGLYSHTVLETADSQELLQAGATLGTEAGAPSRAEQLASVVIYTQEGSSAAAAIQSQRESSELQEA
>sp|Q9UJ72|ANX10_HUMAN
MFCGDYVQGTIFPAPNFNPIMDAQMLGGALQGFDCDKDMLINILTQRCNAQRMMIAEAYQSMYGRDLIGDMREQLSDHFKDVMAGLM
YPPPLYDAHELWHAMKGVGTDENCLIEILASRTNGEIFQMREAYCLQYSNNLQEDIYSETSGHFRDTLMNLVQGTREEGYTDPAMAA
QDAMVLWEACQQKTGEHKTMLQMILCNKSYQQLRLVFQEFQNISGQDMVDAINECYDGYFQELLVAIVLCVRDKPAYFAYRLYSAIH
DFGFHNKTVIRILIARSEIDLLTIRKRYKERYGKSLFHDIRNFASGHYKKALLAICAGDAEDY
>sp|B7Z368|CJ142_HUMAN
MRMYSSDAHERPPSPSLGTTPPHPLPPTGSPRPRQDSAAGNSEEREPRGLRRASGVGSSCKRPTVCMGRQQGLPFCTVCGYRCSSPE
RTRGRCAVGKVRVAGGGGAPGGGAGMRCCGCRERNINKELELF
>sp|Q19AV6|ZSWM7_HUMAN
MAVVLPAVVEELLSEMAAAVQESARIPDEYLLSLKFLFGSSATQALDLVDRQSITLISSPSGRRVYQVLGSSSKTYTCLASCHYCSC
PAFAFSVLRKSDSILCKHLLAVYLSQVMRTCQQLSVSDKQLTDILLMEKKQEA
>sp|Q9P0K8|FOXJ2_HUMAN
MASDLESSLTSIDWLPQLTLRATIEKLGSASQAGPPGSSRKCSPGSPTDPNATLSKDEAAVHQDGKPRYSYATLITYAINSSPAKKM
TLSEIYRWICDNFPYYKNAGIGWKNSIRHNLSLNKCFRKVPRPRDDPGKGSYWTIDTCPDISRKRRHPPDDDLSQDSPEQEASKSPR
GGVAGSGEASLPPEGNPQMSLQSPTSIASYSQGTGSVDGGAVAAGASGRESAEGPPPLYNTNHDFKFSYSEINFQDLSWSFRNLYKS
MLEKSSSSSQHGFSSLLGDIPPSNNYYMYQQQQPPPPQQQQQQQPPQPPPQQSQPQQQQAPAQGPSAVGGAPPLHTPSTDGCTPPG
GKQAGAEGYGPPPVMAMHPPPLQHGGYHPHQHHPHSHPAQQPPPPQPQAQGQAPINNTGFAFPSDWCSNIDSLKESFKMVNRLNWSS
IEQSQFSELMESLRQAEQKNWTLDQHHIANLCDSLNHFLTQTGHVPPQGGTHRPPAPARIADSCALTSGKQESAMSQVNSYGHPQAP
HLYPGPSPMYPIPTQDSAGYNRPAHHMVPRPSVPPPGANEEIPDDFDWDLIT
>sp|Q9H329|E41LB_HUMAN
MLRFLRRTFGRRSMQRYARGAAGRGAAGLGDERDGGPRGGPAAAASSSALPAAPGGSVFPAGGGPLLTGGAAVHISAAGAAKATLYC
RVFLLDGTEVSVDLPKHAKGQDLFDQIVYHLDLVETDYFGLQFLDSAQVAHWLDHAKPIKKQMKIGPAYALHFRVKYYSSEPNNLRE
EFTRYLFVLQLRHDILSGKLKCPYETAVELAALCLQAELGECELPEHTPELVSEFRFIPNQTEAMEFDIFQRWKECRGKSPAQAELS
YLNKAKWLEMYGVDMHVVRGRDGCEYSLGLTPTGILIFEGANKIGLFFWPKITKMDFKKSKLTLVVVEDDDQGREQEHTFVFRLDSA
RTCKHLWKCAVEHHAFFRLRTPGNSKSNRSDFIRLGSRFRFSGRTEYQATHGSRLRRTSTFERKPSKRYPSRRHSTFKASNPVIAAQ
LCSKTNPEVHNYQPQYHPNIHPSQPRWHPHSPNVSYPLPSPVLSSSDRLPFGIEENGGTPFLTAASGRHHHQHQHQHQHQHHSNYSL
SLTLENKEGPLRSPNSSSKSLTKLSPGTPALFSEAAAHLKKLELETVKAAGPWPPLHININKAEEKKVSEKTLQTPLLPSPVADHVK
CNILKAQLENASRVNIQGGKEESPFVNINKKSSLQDASVRSPIPIRVETAQPAVEKPEIKPPRVRKLTRQYSFDEDDLPPDLAEAVG
VTTSTTTNTTTAATQVSVPLPSPKVQNVSSPHKSEGKGLLSPGAKSPSDRGGAFTLEPGDLLMDFTEATPLAEPASNPHCAHSRCSP
PLSLPMKEETTGVCMYPPIKTRLIKTFPVDTMNPFPDTFTTGPQFTADFRDSKLQCCPGPTSPLIPAATLRPLTETVSTVQTIYTTR
KPVSLAASAETLRQELEREKMMKRLLMTEL
>sp|Q53HC0|CCD92_HUMAN

MTSPHFSSYDEGPLDVSMAATNLENQLHSAQKNLLFLQREHASTLKGLHSEIRRLQQHCTDLTYELTVKSSEQTGDGTSKSSELKKR
CEELEAQLKVKENENAELLKELEQKNAMITVLENTIKEREKKYLEELKAKSHKLTLLSSELEQRASTIAYLTSQLHAAKKKLMSSSG
TSDASPSGSPVLASYKPAPPKDKLPETPRRRMKKSLSAPLHPEFEEVYRFGAESRKLLLREPVDAMPDPTPFLLARESAEVHLIKER
PLVIPPIASDRSGEQHSPAREKPHKAHVGVAHRIHHATPPQAQPEVKTLAVDQVNGGKVVRKHSGTDRTV
>sp|Q9HB29|ILRL2_HUMAN
MWSLLLCGLSIALPLSVTADGCKDIFMKNEILSASQPFAFNCTFPPITSGEVSVTWYKNSSKIPVSKIIQSRIHQDETWILFLPMEW
GDSGVYQCVIKGRDSCHRIHVNLTVFEKHWCDTSIGGLPNLSDEYKQILHLGKDDSLTCHLHFPKSCVLGPIKWYKDCNEIKGERFT
VLETRLLVSNVSAEDRGNYACQAILTHSGKQYEVLNGITVSITERAGYGGSVPKIIYPKNHSIEVQLGTTLIVDCNVTDTKDNTNLR
CWRVNNTLVDDYYDESKRIREGVETHVSFREHNLYTVNITFLEVKMEDYGLPFMCHAGVSTAYIILQLPAPDFRAYLIGGLIALVAV
AVSVVYIYNIFKIDIVLWYRSAFHSTETIVDGKLYDAYVLYPKPHKESQRHAVDALVLNILPEVLERQCGYKLFIFGRDEFPGQAVA
NVIDENVKLCRRLIVIVVPESLGFGLLKNLSEEQIAVYSALIQDGMKVILIELEKIEDYTVMPESIQYIKQKHGAIRWHGDFTEQSQ
CMKTKFWKTVRYHMPPRRCRPFPPVQLLQHTPCYRTAGPELGSRRKKCTLTTG
>sp|O60290|ZN862_HUMAN
MEPRESGKAPVTFDDITVYLLQEEWVLLSQQQKELCGSNKLVAPLGPTVANPELFRKFGRGPEPWLGSVQGQRSLLEHHPGKKQMGY
MGEMEVQGPTRESGQSLPPQKKAYLSHLSTGSGHIEGDWAGRNRKLLKPRSIQKSWFVQFPWLIMNEEQTALFCSACREYPSIRDKR
SRLIEGYTGPFKVETLKYHAKSKAHMFCVNALAARDPIWAARFRSIRDPPGDVLASPEPLFTADCPIFYPPGPLGGFDSMAELLPSS
RAELEDPGGDGAIPAMYLDCISDLRQKEITDGIHSSSDINILYNDAVESCIQDPSAEGLSEEVPVVFEELPVVFEDVAVYFTREEWG
MLDKRQKELYRDVMRMNYELLASLGPAAAKPDLISKLERRAAPWIKDPNGPKWGKGRPPGNKKMVAVREADTQASAADSALLPGSPV
EARASCCSSSICEEGDGPRRIKRTYRPRSIQRSWFGQFPWLVIDPKETKLFCSACIERPNLHDKSSRLVRGYTGPFKVETLKYHEVS
KAHRLCVNTVEIKEDTPHTALVPEISSDLMANMEHFFNAAYSIAYHSRPLNDFEKILQLLQSTGTVILGKYRNRTACTQFIKYISET
LKREILEDVRNSPCVSVLLDSSTDASEQACVGIYIRYFKQMEVKESYITLAPLYSETADGYFETIVSALDELDIPFRKPGWVVGLGT
DGSAMLSCRGGLVEKFQEVIPQLLPVHCVAHRLHLAVVDACGSIDLVKKCDRHIRTVFKFYQSSNKRLNELQEGAAPLEQEIIRLKD
LNAVRWVASRRRTLHALLVSWPALARHLQRVAEAGGQIGHRAKGMLKLMRGFHFVKFCHFLLDFLSIYRPLSEVCQKEIVLITEVNA
TLGRAYVALESLRHQAGPKEEEFNASFKDGRLHGICLDKLEVAEQRFQADRERTVLTGIEYLQQRFDADRPPQLKNMEVFDTMAWPS
GIELASFGNDDILNLARYFECSLPTGYSEEALLEEWLGLKTIAQHLPFSMLCKNALAQHCRFPLLSKLMAVVVCVPISTSCCERGFK
AMNRIRTDERTKLSNEVLNMLMMTAVNGVAVTEYDPQPAIQHWYLTSSGRRFSHVYTCAQVPARSPASARLRKEEMGALYVEEPRTQ
KPPILPSREAAEVLKDCIMEPPERLLYPHTSQEAPGMS
>sp|Q9NQ89|CL004_HUMAN
MKKNRERFCNREREFVYKFKVGSQCLELRVPLKFPVQENASHLHGRLMLLHSLPCFIEKDLKEALTQFIEEESLSDYDRDAEASLAA
VKSGEVDLHQLASTWAKAYAETTLEHARPEEPSWDEDFADVYHDLIHSPASETLLNLEHNYFVSISELIGERDVELKKLRERQGIEM
EKVMQELGKSLTDQDVNSLAAQHFESQQDLENKWSNELKQSTAIQKQEYQEWVIKLHQDLKNPNNSSLSEEIKVQPSQFRESVEAIG
RIYEEQRKLEESFTIHLGAQLKTMHNLRLLRADMLDFCKHKRNHRSGVKLHRLQTALSLYSTSLCGLVLLVDNRINSYSGIKRDFAT
VCQECTDFHFPRIEEQLEVVQQVVLYARTQRRSKLKESLDSGNQNGGNDDKTKNAERNYLNVLPGEFYITRHSNLSEIHVAFHLCVD
DHVKSGNITARDPAIMGLRNILKVCCTHDITTISIPLLLVHDMSEEMTIPWCLRRAELVFKCVKGFMMEMASWDGGISRTVQFLVPQ
SISEEMFYQLSNMLPQIFRVSSTLTLTSKH
>sp|P29466|CASP1_HUMAN
MADKVLKEKRKLFIRSMGEGTINGLLDELLQTRVLNKEEMEKVKRENATVMDKTRALIDSVIPKGAQACQICITYICEEDSYLAGTL
GLSADQTSGNYLNMQDSQGVLSSFPAPQAVQDNPAMPTSSGSEGNVKLCSLEEAQRIWKQKSAEIYPIMDKSSRTRLALIICNEEFD
SIPRRTGAEVDITGMTMLLQNLGYSVDVKKNLTASDMTTELEAFAHRPEHKTSDSTFLVFMSHGIREGICGKKHSEQVPDILQLNAI
FNMLNTKNCPSLKDKPKVIIIQACRGDSPGVVWFKDSVGVSGNLSLPTTEEFEDDAIKKAHIEKDFIAFCSSTPDNVSWRHPTMGSV
FIGRLIEHMQEYACSCDVEEIFRKVRFSFEQPDGRAQMPTTERVTLTRCFYLFPGH
>sp|Q01668|CAC1D_HUMAN
MMMMMMMKKMQHQRQQQADHANEANYARGTRLPLSGEGPTSQPNSSKQTVLSWQAAIDAARQAKAAQTMSTSAPPPVGSLSQRKRQQ
YAKSKKQGNSSNSRPARALFCLSLNNPIRRACISIVEWKPFDIFILLAIFANCVALAIYIPFPEDDSNSTNHNLEKVEYAFLIIFTV

ETFLKIIAYGLLLHPNAYVRNGWNLLDFVIVIVGLFSVILEQLTKETEGGNHSSGKSGGFDVKALRAFRVLRPLRLVSGVPSLQVVL
NSIIKAMVPLLHIALLVLFVIIIYAIIGLELFIGKMHKTCFFADSDIVAEEDPAPCAFSGNGRQCTANGTECRSGWVGPNGGITNFD
NFAFAMLTVFQCITMEGWTDVLYWMNDAMGFELPWVYFVSLVIFGSFFVLNLVLGVLSGEFSKEREKAKARGDFQKLREKQQLEEDL
KGYLDWITQAEDIDPENEEEGGEEGKRNTSMPTSETESVNTENVSGEGENRGCCGSLCQAISKSKLSRRWRRWNRFNRRRCRAAVKS
VTFYWLVIVLVFLNTLTISSEHYNQPDWLTQIQDIANKVLLALFTCEMLVKMYSLGLQAYFVSLFNRFDCFVVCGGITETILVELEI
MSPLGISVFRCVRLLRIFKVTRHWTSLSNLVASLLNSMKSIASLLLLLFLFIIIFSLLGMQLFGGKFNFDETQTKRSTFDNFPQALL
TVFQILTGEDWNAVMYDGIMAYGGPSSSGMIVCIYFIILFICGNYILLNVFLAIAVDNLADAESLNTAQKEEAEEKERKKIARKESL
ENKKNNKPEVNQIANSDNKVTIDDYREEDEDKDPYPPCDVPVGEEEEEEEEDEPEVPAGPRPRRISELNMKEKIAPIPEGSAFFILS
KTNPIRVGCHKLINHHIFTNLILVFIMLSSAALAAEDPIRSHSFRNTILGYFDYAFTAIFTVEILLKMTTFGAFLHKGAFCRNYFNL
LDMLVVGVSLVSFGIQSSAISVVKILRVLRVLRPLRAINRAKGLKHVVQCVFVAIRTIGNIMIVTTLLQFMFACIGVQLFKGKFYRC
TDEAKSNPEECRGLFILYKDGDVDSPVVRERIWQNSDFNFDNVLSAMMALFTVSTFEGWPALLYKAIDSNGENIGPIYNHRVEISIF
FIIYIIIVAFFMMNIFVGFVIVTFQEQGEKEYKNCELDKNQRQCVEYALKARPLRRYIPKNPYQYKFWYVVNSSPFEYMMFVLIMLN
TLCLAMQHYEQSKMFNDAMDILNMVFTGVFTVEMVLKVIAFKPKGYFSDAWNTFDSLIVIGSIIDVALSEADPTESENVPVPTATPG
NSEESNRISITFFRLFRVMRLVKLLSRGEGIRTLLWTFIKSFQALPYVALLIAMLFFIYAVIGMQMFGKVAMRDNNQINRNNNFQTF
PQAVLLLFRCATGEAWQEIMLACLPGKLCDPESDYNPGEEYTCGSNFAIVYFISFYMLCAFLIINLFVAVIMDNFDYLTRDWSILGP
HHLDEFKRIWSEYDPEAKGRIKHLDVVTLLRRIQPPLGFGKLCPHRVACKRLVAMNMPLNSDGTVMFNATLFALVRTALKIKTEGNL
EQANEELRAVIKKIWKKTSMKLLDQVVPPAGDDEVTVGKFYATFLIQDYFRKFKKRKEQGLVGKYPAKNTTIALQAGLRTLHDIGPE
IRRAISCDLQDDEPEETKREEEDDVFKRNGALLGNHVNHVNSDRRDSLQQTNTTHRPLHVQRPSIPPASDTEKPLFPPAGNSVCHNH
HNHNSIGKQVPTSTNANLNNANMSKAAHGKRPSIGNLEHVSENGHHSSHKHDREPQRRSSVKRTRYYETYIRSDSGDEQLPTICRED
PEIHGYFRDPHCLGEQEYFSSEECYEDDSSPTWSRQNYGYYSRYPGRNIDSERPRGYHHPQGFLEDDDSPVCYDSRRSPRRRLLPPT
PASHRRSSFNFECLRRQSSQEEVPSSPIFPHRTALPLHLMQQQIMAVAGLDSSKAQKYSPSHSTRSWATPPATPPYRDWTPCYTPLI
QVEQSEALDQVNGSLPSLHRSSWYTDEPDISYRTFTPASLTVPSSFRNKNSDKQRSADSLVEAVLISEGLGRYARDPKFVSATKHEI
ADACDLTIDEMESAASTLLNGNVRPRANGDVGPLSHRQDYELQDFGPGYSDEEPDPGRDEEDLADEMICITTL
>sp|Q58WW2|DCAF6_HUMAN
MSRGGSYPHLLWDVRKRSLGLEDPSRLRSRYLGRREFIQRLKLEATLNVHDGCVNTICWNDTGEYILSGSDDTKLVISNPYSRKVLT
TIRSGHRANIFSAKFLPCTNDKQIVSCSGDGVIFYTNVEQDAETNRQCQFTCHYGTTYEIMTVPNDPYTFLSCGEDGTVRWFDTRIK
TSCTKEDCKDDILINCRRAATSVAICPPIPYYLAVGCSDSSVRIYDRRMLGTRATGNYAGRGTTGMVARFIPSHLNNKSCRVTSLCY
SEDGQEILVSYSSDYIYLFDPKDDTARELKTPSAEERREELRQPPVKRLRLRGDWSDTGPRARPESERERDGEQSPNVSLMQRMSDM
LSRWFEEASEVAQSNRGRGRSRPRGGTSQSDISTLPTVPSSPDLEVSETAMEVDTPAEQFLQPSTSSTMSAQAHSTSSPTESPHSTP
LLSSPDSEQRQSVEASGHHTHHQSDNNNEKLSPKPGTGEPVLSLHYSTEGTTTSTIKLNFTDEWSSIASSSRGIGSHCKSEGQEESF
VPQSSVQPPEGDSETKAPEESSEDVTKYQEGVSAENPVENHINITQSDKFTAKPLDSNSGERNDLNLDRSCGVPEESASSEKAKEPE
TSDQTSTESATNENNTNPEPQFQTEATGPSAHEETSTRDSALQDTDDSDDDPVLIPGARYRAGPGDRRSAVARIQEFFRRRKERKEM
EELDTLNIRRPLVKMVYKGHRNSRTMIKEANFWGANFVMSGSDCGHIFIWDRHTAEHLMLLEADNHVVNCLQPHPFDPILASSGIDY
DIKIWSPLEESRIFNRKLADEVITRNELMLEETRNTITVPASFMLRMLASLNHIRADRLEGDRSEGSGQENENEDEE
>sp|P0C0L4|CO4A_HUMAN
MRLLWGLIWASSFFTLSLQKPRLLLFSPSVVHLGVPLSVGVQLQDVPRGQVVKGSVFLRNPSRNNVPCSPKVDFTLSSERDFALLSL
QVPLKDAKSCGLHQLLRGPEVQLVAHSPWLKDSLSRTTNIQGINLLFSSRRGHLFLQTDQPIYNPGQRVRYRVFALDQKMRPSTDTI
TVMVENSHGLRVRKKEVYMPSSIFQDDFVIPDISEPGTWKISARFSDGLESNSSTQFEVKKYVLPNFEVKITPGKPYILTVPGHLDE
MQLDIQARYIYGKPVQGVAYVRFGLLDEDGKKTFFRGLESQTKLVNGQSHISLSKAEFQDALEKLNMGITDLQGLRLYVAAAIIESP
GGEMEEAELTSWYFVSSPFSLDLSKTKRHLVPGAPFLLQALVREMSGSPASGIPVKVSATVSSPGSVPEVQDIQQNTDGSGQVSIPI
IIPQTISELQLSVSAGSPHPAIARLTVAAPPSGGPGFLSIERPDSRPPRVGDTLNLNLRAVGSGATFSHYYYMILSRGQIVFMNREP
KRTLTSVSVFVDHHLAPSFYFVAFYYHGDHPVANSLRVDVQAGACEGKLELSVDGAKQYRNGESVKLHLETDSLALVALGALDTALY
AAGSKSHKPLNMGKVFEAMNSYDLGCGPGGGDSALQVFQAAGLAFSDGDQWTLSRKRLSCPKEKTTRKKRNVNFQKAINEKLGQYAS
PTAKRCCQDGVTRLPMMRSCEQRAARVQQPDCREPFLSCCQFAESLRKKSRDKGQAGLQRALEILQEEDLIDEDDIPVRSFFPENWL

WRVETVDRFQILTLWLPDSLTTWEIHGLSLSKTKGLCVATPVQLRVFREFHLHLRLPMSVRRFEQLELRPVLYNYLDKNLTVSVHVS
PVEGLCLAGGGGLAQQVLVPAGSARPVAFSVVPTAAAAVSLKVVARGSFEFPVGDAVSKVLQIEKEGAIHREELVYELNPLDHRGRT
LEIPGNSDPNMIPDGDFNSYVRVTASDPLDTLGSEGALSPGGVASLLRLPRGCGEQTMIYLAPTLAASRYLDKTEQWSTLPPETKDH
AVDLIQKGYMRIQQFRKADGSYAAWLSRDSSTWLTAFVLKVLSLAQEQVGGSPEKLQETSNWLLSQQQADGSFQDPCPVLDRSMQGG
LVGNDETVALTAFVTIALHHGLAVFQDEGAEPLKQRVEASISKANSFLGEKASAGLLGAHAAAITAYALTLTKAPVDLLGVAHNNLM
AMAQETGDNLYWGSVTGSQSNAVSPTPAPRNPSDPMPQAPALWIETTAYALLHLLLHEGKAEMADQASAWLTRQGSFQGGFRSTQDT
VIALDALSAYWIASHTTEERGLNVTLSSTGRNGFKSHALQLNNRQIRGLEEELQFSLGSKINVKVGGNSKGTLKVLRTYNVLDMKNT
TCQDLQIEVTVKGHVEYTMEANEDYEDYEYDELPAKDDPDAPLQPVTPLQLFEGRRNRRRREAPKVVEEQESRVHYTVCIWRNGKVG
LSGMAIADVTLLSGFHALRADLEKLTSLSDRYVSHFETEGPHVLLYFDSVPTSRECVGFEAVQEVPVGLVQPASATLYDYYNPERRC
SVFYGAPSKSRLLATLCSAEVCQCAEGKCPRQRRALERGLQDEDGYRMKFACYYPRVEYGFQVKVLREDSRAAFRLFETKITQVLHF
TKDVKAAANQMRNFLVRASCRLRLEPGKEYLIMGLDGATYDLEGHPQYLLDSNSWIEEMPSERLCRSTRQRAACAQLNDFLQEYGTQ
GCQV
>sp|P52948|NUP98_HUMAN
MFNKSFGTPFGGGTGGFGTTSTFGQNTGFGTTSGGAFGTSAFGSSNNTGGLFGNSQTKPGGLFGTSSFSQPATSTSTGFGFGTSTGT
ANTLFGTASTGTSLFSSQNNAFAQNKPTGFGNFGTSTSSGGLFGTTNTTSNPFGSTSGSLFGPSSFTAAPTGTTIKFNPPTGTDTMV
KAGVSTNISTKHQCITAMKEYESKSLEELRLEDYQANRKGPQNQVGAGTTTGLFGSSPATSSATGLFSSSTTNSGFAYGQNKTAFGT
STTGFGTNPGGLFGQQNQQTTSLFSKPFGQATTTQNTGFSFGNTSTIGQPSTNTMGLFGVTQASQPGGLFGTATNTSTGTAFGTGTG
LFGQTNTGFGAVGSTLFGNNKLTTFGSSTTSAPSFGTTSGGLFGNKPTLTLGTNTNTSNFGFGTNTSGNSIFGSKPAPGTLGTGLGA
GFGTALGAGQASLFGNNQPKIGGPLGTGAFGAPGFNTTTATLGFGAPQAPVALTDPNASAAQQAVLQQHINSLTYSPFGDSPLFRNP
MSDPKKKEERLKPTNPAAQKALTTPTHYKLTPRPATRVRPKALQTTGTAKSHLFDGLDDDEPSLANGAFMPKKSIKKLVLKNLNNSN
LFSPVNRDSENLASPSEYPENGERFSFLSKPVDENHQQDGDEDSLVSHFYTNPIAKPIPQTPESAGNKHSNSNSVDDTIVALNMRAA
LRNGLEGSSEETSFHDESLQDDREEIENNSYHMHPAGIILTKVGYYTIPSMDDLAKITNEKGECIVSDFTIGRKGYGSIYFEGDVNL
TNLNLDDIVHIRRKEVVVYLDDNQKPPVGEGLNRKAEVTLDGVWPTDKTSRCLIKSPDRLADINYEGRLEAVSRKQGAQFKEYRPET
GSWVFKVSHFSKYGLQDSDEEEEEHPSKTSTKKLKTAPLPPASQTTPLQMALNGKPAPPPQSQSPEVEQLGRVVELDSDMVDITQEP
VLDTMLEESMPEDQEPVSASTHIASSLGINPHVLQIMKASLLTDEEDVDMALDQRFSRLPSKADTSQEICSPRLPISASHSSKTRSL
VGGLLQSKFTSGAFLSPSVSVQECRTPRAASLMNIPSTSSWSVPPPLTSVFTMPSPAPEVPLKTVGTRRQLGLVPREKSVTYGKGKL
LMDMALFMGRSFRVGWGPNWTLANSGEQLNGSHELENHQIADSMEFGFLPNPVAVKPLTESPFKVHLEKLSLRQRKPDEDMKLYQTP
LELKLKHSTVHVDELCPLIVPNLGVAVIHDYADWVKEASGDLPEAQIVKHWSLTWTLCEALWGHLKELDSQLNEPREYIQILERRRA
FSRWLSCTATPQIEEEVSLTQKNSPVEAVFSYLTGKRISEACSLAQQSGDHRLALLLSQFVGSQSVRELLTMQLVDWHQLQADSFIQ
DERLRIFALLAGKPVWQLSEKKQINVCSQLDWKRSLAIHLWYLLPPTASISRALSMYEEAFQNTSDSDRYACSPLPSYLEGSGCVIA
EEQNSQTPLRDVCFHLLKLYSDRHYDLNQLLEPRSITADPLDYRLSWHLWEVLRALNYTHLSAQCEGVLQASYAGQLESEGLWEWAI
FVLLHIDNSGIREKAVRELLTRHCQLLETPESWAKETFLTQKLRVPAKWIHEAKAVRAHMESDKHLEALCLFKAEHWNRCHKLIIRH
LASDAIINENYDYLKGFLEDLAPPERSSLIQDWETSGLVYLDYIRVIEMLRHIQQVDCSGNDLEQLHIKVTSLCSRIEQIQCYSAKD
RLAQSDMAKRVANLLRVVLSLHHPPDRTSDSTPDPQRVPLRLLAPHIGRLPMPEDYAMDELRSLTQSYLRELAVGSL
>sp|Q9BUA6|MYL10_HUMAN
MLLRLVSNSWPQVILPPRPPKVLGLQAPRRARKRAEGTASSNVFSMFDQSQIQEFKESLALSPRLERNGMISAHCNLCLTGSSNSPA
SASQAFTIMDQNRDGFIDKEDLRDTFAALGRINVKNEELEAMVKEAPGPINFTVFLTMFGEKLKGTDPEETILHAFKVFDTEGKGFV
KADVIKEKLMTQADRFSEEEVKQMFAAFPPDVCGNLDYRNLCYVITHGEEKD
>sp|Q13003|GRIK3_HUMAN
MTAPWRRLRSLVWEYWAGLLVCAFWIPDSRGMPHVIRIGGIFEYADGPNAQVMNAEEHAFRFSANIINRNRTLLPNTTLTYDIQRIH
FHDSFEATKKACDQLALGVVAIFGPSQGSCTNAVQSICNALEVPHIQLRWKHHPLDNKDTFYVNLYPDYASLSHAILDLVQYLKWRS
ATVVYDDSTGLIRLQELIMAPSRYNIRLKIRQLPIDSDDSRPLLKEMKRGREFRIIFDCSHTMAAQILKQAMAMGMMTEYYHFIFTT
LDLYALDLEPYRYSGVNLTGFRILNVDNPHVSAIVEKWSMERLQAAPRSESGLLDGVMMTDAALLYDAVHIVSVCYQRAPQMTVNSL
QCHRHKAWRFGGRFMNFIKEAQWEGLTGRIVFNKTSGLRTDFDLDIISLKEDGLEKVGVWSPADGLNITEVAKGRGPNVTDSLTNRS

LIVTTVLEEPFVMFRKSDRTLYGNDRFEGYCIDLLKELAHILGFSYEIRLVEDGKYGAQDDKGQWNGMVKELIDHKADLAVAPLTIT
HVREKAIDFSKPFMTLGVSILYRKPNGTNPSVFSFLNPLSPDIWMYVLLAYLGVSCVLFVIARFSPYEWYDAHPCNPGSEVVENNFT
LLNSFWFGMGSLMQQGSELMPKALSTRIIGGIWWFFTLIIISSYTANLAAFLTVERMESPIDSADDLAKQTKIEYGAVKDGATMTFF
KKSKISTFEKMWAFMSSKPSALVKNNEEGIQRALTADYALLMESTTIEYVTQRNCNLTQIGGLIDSKGYGIGTPMGSPYRDKITIAI
LQLQEEDKLHIMKEKWWRGSGCPEEENKEASALGIQKIGGIFIVLAAGLVLSVLVAVGEFVYKLRKTAEREQRSFCSTVADEIRFSL
TCQRRVKHKPQPPMMVKTDAVINMHTFNDRRLPGKDSMACSTSLAPVFP
>sp|Q5VWN6|F208B_HUMAN
MAPPAHKSILERSENVLMSPWKGKLIVQDRMLCDIALWSTYGAMIPTQLPQELDFKYVMKVSSLKKRLPEAAFRKQNYLEEKVCFQD
LCFNLYEVELSNRQGENIDKLTECIKNKQLAIIKCLEDRGFFILLTSSALLSEPDFGGKQMGLHGLHLFRSPLSTGVKDLKVEDDIS
MKVIPILSTLNCALLETKKSLPEERIHPNTLVKRHFQELYKADRSPSLSVAPQDRMKDPTFLGKLPSGFDLIPPAEKCPSESLTQLN
SYFSDPSAYILEVSTALDLLAEHPQSPCVSDGICDAGFSLVMTPDPEFLVSEAEVRKETETKKDSEEMLKAKKRVFPLSPASNLRVQ
PKRKASMPHMVQSKKVNLCRPFPKRTASRADNSSDSPTTLKLVKGQFPQKRKRGAEVLTAQFVQKTKLDRKNQEAPISKDVPVPTNA
KRARKQEKSPVKTVPRAKPPVKKSPQKQRVNIVKGNENPRNRKQLQPVKGETASKLQSEISRGCQEDGISINSVQPENTTAAHNDLP
ENSIVNYDSQALNMLADLALSSATSSTPVSEARNLHCSSELPQNDVLLSKENSLRGTSDHEYHRGVKTQKGELLPNPSSDRKSNSGS
DLTVSQDEESLVPCSQAPAKAQSALTEEMLESSDASQSSSVSVEHSYALLLTEHSKKHLQEREILSPLFPRNGTKSPEAATPVGKVM
PFRHQPGLLLQQKPPDDPVVKPKDRPPSARVKKSSCSRIVLSCDDSVKITFKCETEYAFSLDSKYTNNPLEKTVVRALHGPWNTDLP
DNVEEVKLLLHMWVALFYSNQNKIIRSSRKVVEHSNPAKYVSINSTLESCELREIEESLGLEKCSADSLLETNEISRAHAAEVSFRD
PNCLLPFIKTPLTQGLELCVQNEQKKTFARECDPDTQEDQNFICSYNNEVTGEEAKQESLETSNLVLSGIGSTQTNGPSVPSEEEIV
QPLDSTRVASYSGTVTQATFTRTYDGPGSQPVICQSSVYGTLENKVDILDAAVQTKTGTLQDLIQHGSPINNECHPSLERKDDNMGC
AVINPEPITLTFEKNAHVPIQTEGVNTADERTTFKKELIKQVSPAASLRHPVSTSENARTQGLRDIPSLVVAGQKGTKYLCASSVGG
ETLDKAVCSLQKETPLPVSLPSDKTMVMEALSLAKSSSHLSPSEEVRCTQDFLSQTQSLLGLSSEGLLELTQVEVDSSSASTTLGRQ
CSLNCISSGCHTSGDSLELRKNHKNGPNTENMNLEAFDSVFIKQTSLSVSREVSLELSEEDSDIDLALTISPPTSPREEMPAGEIEQ
FEEAPFSNLELQDVAEEIGEPEEVALTESREVSSADNVSVYPSVSEEPVENKERKGDNLQPVTLILSKENCTLEIAEEINVTSDFPF
DSVIEEVSPASSPEPPVPVKETRPYQAVTPCILKLHGTQCEKSNQISQCESEDLGITEKENVFVGPTHPVGQDNFTQVQQMQVSAEM
PLILTDHPGRTGRPTLPGKVTEEIVSSEHDEGLSFSGKVQCYGRELNQPASAAKCTGDFSPSPEKLVKSGNPLQPVSIENRNLDLKH
LVLESSEPPFGPRNVIENKSLSDTLVSTTAPSGIVNVSVKQQTSPKSSQNHLFPGDLKTDEGIYLQVKSLTAASVDGAYSTQGCMCS
VVPTLCSSSDNATLTHYVRPINAEPVFQAQEIPAGRMASLLKNGEPEAELHKETTGPGTAGPQSNTTSSLKGERKAIHTLQDVSTCE
TKELLNVGVSSLCAGPYQNTADTKENLSKEPLASFVSESFDTSVCGIATEHVEIENSGEGLRAEAGSETLGRDGEVGVNSDMHYELS
GDSDLDLLGDCRNPRLDLEDSYTLRGSYTRKKDVPTDGYESSLNFHNNNQEDWGCSSWVPGMETSLPPGHWTAAVKKEEKCVPPYVQ
IRDLHGILRTYANFSITKELKDTMRTSHGLRRHPSFSANCGLPSSWTSTWQVADDLTQNTLDLEYLRFAHKLKQTIKNGDSQHSASS
ANVFPKESPTQISIGAFPSTKISEAPFLHPAPRSRSPLLVTVVESDPRPQGQPRRGYTASSLDSSSSWRERCSHNRDLRNSQRNHTV
SFHLNKLKYNSTVKESRNDISLILNEYAEFNKVMKNSNQFIFQDKELNDVSGEATAQEMYLPFPGRSASYEDIIIDVCTNLHVKLRS
VVKEACKSTFLFYLVETEDKSFFVRTKNLLRKGGHTEIEPQHFCQAFHRENDTLIIIIRNEDISSHLHQIPSLLKLKHFPSVIFAGV
DSPGDVLDHTYQELFRAGGFVISDDKILEAVTLVQLKEIIKILEKLNGNGRWKWLLHYRENKKLKEDERVDSTAHKKNIMLKSFQSA
NIIELLHYHQCDSRSSTKAEILKCLLNLQIQHIDARFAVLLTDKPTIPREVFENSGILVTDVNNFIENIEKIAAPFRSSYW
>sp|P34998|CRFR1_HUMAN
MGGHPQLRLVKALLLLGLNPVSASLQDQHCESLSLASNISGLQCNASVDLIGTCWPRSPAGQLVVRPCPAFFYGVRYNTTNNGYREC
LANGSWAARVNYSECQEILNEEKKSKVHYHVAVIINYLGHCISLVALLVAFVLFLRLRPGCTHWGDQADGALEVGAPWSGAPFQVRR
SIRCLRNIIHWNLISAFILRNATWFVVQLTMSPEVHQSNVGWCRLVTAAYNYFHVTNFFWMFGEGCYLHTAIVLTYSTDRLRKWMFI
CIGWGVPFPIIVAWAIGKLYYDNEKCWFGKRPGVYTDYIYQGPMILVLLINFIFLFNIVRILMTKLRASTTSETIQYRKAVKATLVL
LPLLGITYMLFFVNPGEDEVSRVVFIYFNSFLESFQGFFVSVFYCFLNSEVRSAIRKRWHRWQDKHSIRARVARAMSIPTSPTRVSF
HSIKQSTAV
>sp|Q8TEH3|DEN1A_HUMAN

MGSRIKQNPETTFEVYVEVAYPRTGGTLSDPEVQRQFPEDYSDQEVLQTLTKFCFPFYVDSLTVSQVGQNFTFVLTDIDSKQRFGFC
RLSSGAKSCFCILSYLPWFEVFYKLLNILADYTTKRQENQWNELLETLHKLPIPDPGVSVHLSVHSYFTVPDTRELPSIPENRNLTE
YFVAVDVNNMLHLYASMLYERRILIICSKLSTLTACIHGSAAMLYPMYWQHVYIPVLPPHLLDYCCAPMPYLIGIHLSLMEKVRNMA
LDDVVILNVDTNTLETPFDDLQSLPNDVISSLKNRLKKVSTTTGDGVARAFLKAQAAFFGSYRNALKIEPEEPITFCEEAFVSHYRS
GAMRQFLQNATQLQLFKQFIDGRLDLLNSGEGFSDVFEEEINMGEYAGSDKLYHQWLSTVRKGSGAILNTVKTKANPAMKTVYKFAK
DHAKMGIKEVKNRLKQKDIAENGCAPTPEEQLPKTAPSPLVEAKDPKLREDRRPITVHFGQVRPPRPHVVKRPKSNIAVEGRRTSVP
SPEQPQPYRTLRESDSAEGDEAESPEQQVRKSTGPVPAPPDRAASIDLLEDVFSNLDMEAALQPLGQAKSLEDLRAPKDLREQPGTF
DYQRLDLGGSERSRGVTVALKLTHPYNKLWSLGQDDMAIPSKPPAASPEKPSALLGNSLALPRRPQNRDSILNPSDKEEVPTPTLGS
ITIPRPQGRKTPELGIVPPPPIPRPAKLQAAGAALGDVSERLQTDRDRRAALSPGLLPGVVPQGPTELLQPLSPGPGAAGTSSDALL
ALLDPLSTAWSGSTLPSRPATPNVATPFTPQFSFPPAGTPTPFPQPPLNPFVPSMPAAPPTLPLVSTPAGPFGAPPASLGPAFASGL
LLSSAGFCAPHRSQPNLSALSMPNLFGQMPMGTHTSPLQPLGPPAVAPSRIRTLPLARSSARAAETKQGLALRPGDPPLLPPRPPQG
LEPTLQPSAPQQARDPFEDLLQKTKQDVSPSPALAPAPDSVEQLRKQWETFE
>sp|O95273|CCDB1_HUMAN
MASATAPAAAVPTLASPLEQLRHLAEELRLLLPRVRVGEAQETTEEFNREMFWRRLNEAAVTVSREATTLTIVFSQLPLPSPQETQK
FCEQVHAAIKAFIAVYYLLPKDQGITLRKLVRGATLDIVDGMAQLMEVLSVTPTQSPENNDLISYNSVWVACQQMPQIPRDNKAAAL
LMLTKNVDFVKDAHEEMEQAVEECDPYSGLLNDTEENNSDNHNHEDDVLGFPSNQDLYWSEDDQELIIPCLALVRASKACLKKIRML
VAENGKKDQVAQLDDIVDISDEISPSVDDLALSIYPPMCHLTVRINSAKLVSVLKKALEITKASHVTPQPEDSWIPLLINAIDHCMN
RIKELTQSELEL
>sp|Q9UBM7|DHCR7_HUMAN
MAAKSQPNIPKAKSLDGVTNDRTASQGQWGRAWEVDWFSLASVIFLLLFAPFIVYYFIMACDQYSCALTGPVVDIVTGHARLSDIWA
KTPPITRKAAQLYTLWVTFQVLLYTSLPDFCHKFLPGYVGGIQEGAVTPAGVVNKYQINGLQAWLLTHLLWFANAHLLSWFSPTIIF
DNWIPLLWCANILGYAVSTFAMVKGYFFPTSARDCKFTGNFFYNYMMGIEFNPRIGKWFDFKLFFNGRPGIVAWTLINLSFAAKQRE
LHSHVTNAMVLVNVLQAIYVIDFFWNETWYLKTIDICHDHFGWYLGWGDCVWLPYLYTLQGLYLVYHPVQLSTPHAVGVLLLGLVGY
YIFRVANHQKDLFRRTDGRCLIWGRKPKVIECSYTSADGQRHHSKLLVSGFWGVARHFNYVGDLMGSLAYCLACGGGHLLPYFYIIY
MAILLTHRCLRDEHRCASKYGRDWERYTAAVPYRLLPGIF
>sp|Q96ER9|CCD51_HUMAN
MMGRSPGFAMQHIVGVPHVLVRRGLLGRDLFMTRTLCSPGPSQPGEKRPEEVALGLHHRLPALGRALGHSIQQRATSTAKTWWDRYE
EFVGLNEVREAQGKVTEAEKVFMVARGLVREAREDLEVHQAKLKEVRDRLDRVSREDSQYLELATLEHRMLQEEKRLRTAYLRAEDS
EREKFSLFSAAVRESHEKERTRAERTKNWSLIGSVLGALIGVAGSTYVNRVRLQELKALLLEAQKGPVSLQEAIREQASSYSRQQRD
LHNLMVDLRGLVHAAGPGQDSGSQAGSPPTRDRDVDVLSAALKEQLSHSRQVHSCLEGLREQLDGLEKTCSQMAGVVQLVKSAAHPG
LVEPADGAMPSFLLEQGSMILALSDTEQRLEAQVNRNTIYSTLVTCVTFVATLPVLYMLFKAS
>sp|Q13258|PD2R_HUMAN
MKSPFYRCQNTTSVEKGNSAVMGGVLFSTGLLGNLLALGLLARSGLGWCSRRPLRPLPSVFYMLVCGLTVTDLLGKCLLSPVVLAAY
AQNRSLRVLAPALDNSLCQAFAFFMSFFGLSSTLQLLAMALECWLSLGHPFFYRRHITLRLGALVAPVVSAFSLAFCALPFMGFGKF
VQYCPGTWCFIQMVHEEGSLSVLGYSVLYSSLMALLVLATVLCNLGAMRNLYAMHRRLQRHPRSCTRDCAEPRADGREASPQPLEEL
DHLLLLALMTVLFTMCSLPVIYRAYYGAFKDVKEKNRTSEEAEDLRALRFLSVISIVDPWIFIIFRSPVFRIFFHKIFIRPLRYRSR
CSNSTNMESSL
>sp|Q68DE3|USF3_HUMAN
MPEMTENETPTKKQHRKKNRETHNAVERHRKKKINAGINRIGELIPCSPALKQSKNMILDQAFKYITELKRQNDELLLNGGNNEQAE
EIKKLRKQLEEIQKENGRYIELLKANDICLYDDPTIHWKGNLKNSKVSVVIPSDQVQKKIIVYSNGNQPGGNSQGTAVQGITFNVSH
NLQKQTANVVPVQRTCNLVTPVSISGVYPSENKPWHQTTVPALATNQPVPLCLPAAISAQSILELPTSESESNVLGATSGSLIAVSI
ESEPHQHHSLHTCLNDQNSSENKNGQENPKVLKKMTPCVTNIPHSSSATATKVHHGNKSCLSIQDFRGDFQNTFVVSVTTTVCSQPP
RTAGDSSPMSISKSADLTSTATVVASSAPGVGKATIPISTLSGNPLDNGWTLSCSLPSSSVSTSDLKNINSLTRISSAGNTQTTWTT
LQLAGNTIQPLSQTPSSAVTPVLNESGTSPTTSNHSRYVATDINLNNSFPADGQPVEQVVVTLPSCPSLPMQPLIAQPQVKSQPPKN

ILPLNSAMQVIQMAQPVGSAVNSAPTNQNVIILQPPSTTPCPTVMRAEVSNQTVGQQIVIIQAANQNPLPLLPAPPPGSVRLPINGA
NTVIGSNNSVQNVPTPQTFGGKHLVHILPRPSSLSASNSTQTFSVTMSNQQPQTISLNGQLFALQPVMSSSGTTNQTPMQIIQPTTS
EDPNTNVALNTFGALASLNQSISQMAGQSCVQLSISQPANSQTAANSQTTTANCVSLTTTAAPPVTTDSSATLASTYNLVSTSSMNT
VACLPNMKSKRLNKKPGGRKHLAANKSACPLNSVRDVSKLDCPNTEGSAEPPCNDGLLESFPAVLPSVSVSQANSVSVSASHSLGVL
SSESLIPESVSKSKSAEKSSPPSQESVTSEHFAMAAAKSKDSTPNLQQETSQDKPPSSLALSDAAKPCASANVLIPSPSDPHILVSQ
VPGLSSTTSTTSTDCVSEVEIIAEPCRVEQDSSDTMQTTGLLKGQGLTTLLSDLAKKKNPQKSSLSDQMDHPDFSSENPKIVDSSVN
LHPKQELLLMNNDDRDPPQHHSCLPDQEVINGSLINGRQADSPMSTSSGSSRSFSVASMLPETTREDVTSNATTNTCDSCTFVEQTD
IVALAARAIFDQENLEKGRVGLQADIREVASKPSEASLLEGDPPFKSQIPKESGTGQAEATPNEFNSQGSIEATMERPLEKPSCSLG
IKTSNASLQDSTSQPPSITSLSVNNLIHQSSISHPLASCAGLSPTSEQTTVPATVNLTVSSSSYGSQPPGPSLMTEYSQEQLNTMTS
TIPNSQIQEPLLKPSHESRKDSAKRAVQDDLLLSSAKRQKHCQPAPLRLESMSLMSRTPDTISDQTQMMVSQIPPNSSNSVVPVSNP
AHGDGLTRLFPPSNNFVTPALRQTEVQCGSQPSVAEQQQTQASQHLQALQQHVPAQGVSHLHSNHLYIKQQQQQQQQQQQQQQQQQA
GQLRERHHLYQMQHHVPHAESSVHSQPHNVHQQRTLQQEVQMQKKRNLVQGTQTSQLSLQPKHHGTDQSRSKTGQPHPHHQQMQQQM
QQHFGSSQTEKSCENPSTSRNHHNHPQNHLNQDIMHQQQDVGSRQQGSGVSSEHVSGHNPMQRLLTSRGLEQQMVSQPSIVTRSSDM
TCTPHRPERNRVSSYSAEALIGKTSSNSEQRMGISIQGSRVSDQLEMRSYLDVPRNKSLAIHNMQGRVDHTVASDIRLSDCQTFKPS
GASQQPQSNFEVQSSRNNEIGNPVSSLRSMQSQAFRISQNTGPPPIDRQKRLSYPPVQSIPTGNGIPSRDSENTCHQSFMQSLLAPH
LSDQVIGSQRSLSEHQRNTQCGPSSAIEYNCPPTHENVHIRRESESQNRESCDMSLGAINTRNSTLNIPFSSSSSSGDIQGRNTSPN
VSVQKSNPMRITESHATKGHMNPPVTTNMHGVARPALPHPSVSHGNGDQGPAVRQANSSVPQRSRHPLQDSSGSKIRQPERNRSGNQ
RQSTVFDPSLPHLPLSTGGSMILGRQQPATEKRGSIVRFMPDSPQVPNDNSGPDQHTLSQNFGFSFIPEGGMNPPINANASFIPQVT
QPSATRTPALIPVDPQNTLPSFYPPYSPAHPTLSNDISIPYFPNQMFSNPSTEKVNSGSLNNRFGSILSPPRPVGFAQPSFPLLPDM
PPMHMTNSHLSNFNMTSLFPEIATVLPDGSAMSPLLTIANSSASDSSKQSSNRPAHNISHILGHDCSSAV
>sp|Q9NTI2|AT8A2_HUMAN
MSRATSVGDQLEAPARTIYLNQPHLNKFRDNQISTAKYSVLTFLPRFLYEQIRRAANAFFLFIALLQQIPDVSPTGRYTTLVPLIII
LTIAGIKEIVEDFKRHKADNAVNKKKTIVLRNGMWHTIMWKEVAVGDIVKVVNGQYLPADVVLLSSSEPQAMCYVETANLDGETNLK
IRQGLSHTADMQTREVLMKLSGTIECEGPNRHLYDFTGNLNLDGKSLVALGPDQILLRGTQLRNTQWVFGIVVYTGHDTKLMQNSTK
APLKRSNVEKVTNVQILVLFGILLVMALVSSAGALYWNRSHGEKNWYIKKMDTTSDNFGYNLLTFIILYNNLIPISLLVTLEVVKYT
QALFINWDTDMYYIGNDTPAMARTSNLNEELGQVKYLFSDKTGTLTCNIMNFKKCSIAGVTYGHFPELAREPSSDDFCRMPPPCSDS
CDFDDPRLLKNIEDRHPTAPCIQEFLTLLAVCHTVVPEKDGDNIIYQASSPDEAALVKGAKKLGFVFTARTPFSVIIEAMGQEQTFG
ILNVLEFSSDRKRMSVIVRTPSGRLRLYCKGADNVIFERLSKDSKYMEETLCHLEYFATEGLRTLCVAYADLSENEYEEWLKVYQEA
STILKDRAQRLEECYEIIEKNLLLLGATAIEDRLQAGVPETIATLLKAEIKIWVLTGDKQETAINIGYSCRLVSQNMALILLKEDSL
DATRAAITQHCTDLGNLLGKENDVALIIDGHTLKYALSFEVRRSFLDLALSCKAVICCRVSPLQKSEIVDVVKKRVKAITLAIGDGA
NDVGMIQTAHVGVGISGNEGMQATNNSDYAIAQFSYLEKLLLVHGAWSYNRVTKCILYCFYKNVVLYIIELWFAFVNGFSGQILFER
WCIGLYNVIFTALPPFTLGIFERSCTQESMLRFPQLYKITQNGEGFNTKVFWGHCINALVHSLILFWFPMKALEHDTVLTSGHATDY
LFVGNIVYTYVVVTVCLKAGLETTAWTKFSHLAVWGSMLTWLVFFGIYSTIWPTIPIAPDMRGQATMVLSSAHFWLGLFLVPTACLI
EDVAWRAAKHTCKKTLLEEVQELETKSRVLGKAVLRDSNGKRLNERDRLIKRLGRKTPPTLFRGSSLQQGVPHGYAFSQEEHGAVSQ
EEVIRAYDTTKKKSRKK
>sp|Q6UXZ4|UNC5D_HUMAN
MGRAAATAGGGGGGARRWLPWLGLCFWAAGTAAARGTDNGEALPESIPSAPGTLPHFIEEPDDAYIIKSNPIALRCKARPAMQIFFKC
NGEWVHQNEHVSEETLDESSGLKVREVFINVTRQQVEDFHGPEDYWCQCVAWSHLGTSKSRKASVRIAYLRKNFEQDPQGREVPIEG
MIVLHCRPPEGVPAAEVEWLKNEEPIDSEQDENIDTRADHNLIIRQARLSDSGNYTCMAANIVAKRRSLSATVVVYVNGGWSSWTEW
SACNVRCGRGWQKRSRTCTNPAPLNGGAFCEGMSVQKITCTSLCPVDGSWEVWSEWSVCSPECEHLRIRECTAPPPRNGGKFCEGLS
QESENCTDGLCILDKKPLHEIKPQSIENASDIALYSGLGAAVVAVAVLVIGVTLYRRSQSDYGVDVIDSSALTGGFQTFNFKTVRQG
NSLLLNSAMQPDLTVSRTYSGPICLQDPLDKELMTESSLFNPLSDIKVKVQSSFMVSLGVSERAEYHGKNHSRTFPHGNNHSFSTMH
PRNKMPYIQNLSSLPTRTELRTTGVFGHLGGRLVMPNTGVSLLIPHGAIPEENSWEIYMSINQGEPSLQSDGSEVLLSPEVTCGPPD
MIVTTPFALTIPHCADVSSEHWNIHLKKRTQQGKWEEVMSVEDESTSCYCLLDPFACHVLLDSFGTYALTGEPITDCAVKQLKVAVF

GCMSCNSLDYNLRVYCVDNTPCAFQEVVSDERHQGGQLLEEPKLLHFKGNTFSLQISVLDIPPFLWRIKPFTACQEVPFSRVWCSNR
QPLHCAFSLERYTPTTTQLSCKICIRQLKGHEQILQVQTSILESERETITFFAQEDSTFPAQTGPKAFKIPYSIRQRICATFDTPNA
KGKDWQMLAQKNSINRNLSYFATQSSPSAVILNLWEARHQHDGDLDSLACALEEIGRTHTKLSNISESQLDEADFNYSRQNGL
>sp|Q6P3R8|NEK5_HUMAN
MDKYDVIKAIGQGAFGKAYLAKGKSDSKHCVIKEINFEKMPIQEKEASKKEVILLEKMKHPNIVAFFNSFQENGRLFIVMEYCDGGD
LMKRINRQRGVLFSEDQILGWFVQISLGLKHIHDRKILHRDIKAQNIFLSKNGMVAKLGDFGIARVLNNSMELARTCIGTPYYLSPE
ICQNKPYNNKTDIWSLGCVLYELCTLKHPFEGNNLQQLVLKICQAHFAPISPGFSRELHSLISQLFQVSPRDRPSINSILKRPFLEN
LIPKYLTPEVIQEEFSHMLICRAGAPASRHAGKVVQKCKIQKVRFQGKCPPRSRISVPIKRNAILHRNEWRPPAGAQKARSIKMIER
PKIAAVCGHYDYYYAQLDMLRRRAHKPSYHPIPQENTGVEDYGQETRHGPSPSQWPAEYLQRKFEAQQYKLKVEKQLGLRPSSAEPN
YNQRQELRSNGEEPRFQELPFRKNEMKEQEYWKQLEEIRQQYHNDMKEIRKKMGREPEENSKISHKTYLVKKSNLPVHQDASEGEAP
VQMEFRSCCPGWSAMARSWLTATSASQDIEKDLKQMRLQNTKESKNPEQKYKAKKGVKFEINLDKCISDENILQEEEAMDIPNETLT
FEDGMKFKEYECVKEHGDYTDKAFEKLHCPEAGFSTQTVAAVGNRRQWDGGAPQTLLQMMAVADITSTCPTGPDSESVLSVSRQEGK
TKDPYSPVLILM
>sp|Q16473|TENXA_HUMAN
MEDKNGAPHGAPHSDSPLGFSHAAPEEDTPAPELAPEAPEPPEEPRLGVLTVTDTTPDSMRLSWSVAQGPFDSFVVQYEDTNGQPQA
LLVDGDQSKILISGLEPSTPYRFLLYGLHEGKRLGPLSAEGTTGLAPAGQTSEESRPRLSQLSVTDVTTSSLRLNWEAPPGAFDSFL
LRFGVPSPSTLEPHPRPLLQRELMVPGTRHSAVLRDLRSGTLYSLTLYGLRGPHKADSIQGTARTLSPVLESPRDLQFSEIRETSAK
VNWMPPPSRADSFKVSYQLADGGEPQSVQVDGRARTQKLQFLTVPHSCVH
>sp|P51636|CAV2_HUMAN
MGLETEKADVQLFMDDDSYSHHSGLEYADPEKFADSDQDRDPHRLNSHLKLGFEDVIAEPVTTHSFDKVWICSHALFEISKYVMYKF
LTVFLAIPLAFIAGILFATLSCLHIWILMPFVKTCLMVLPSVQTIWKSVTDVIIAPLCTSVGRCFSSVSLQLSQD
>sp|Q8TE67|ES8L3_HUMAN
MSRPSSRAIYLHRKEYSQNLTSEPTLLQHRVEHLMTCKQGSQRVQGPEDALQKLFEMDAQGRVWSQDLILQVRDGWLQLLDIETKEE
LDSYRLDSIQAMNVALNTCSYNSILSITVQEPGLPGTSTLLFQCQEVGAERLKTSLQKALEEELEQRPRLGGLQPGQDRWRGPAMER
PLPMEQARYLEPGIPPEQPHQRTLEHSLPPSPRPLPRHTSAREPSAFTLPPPRRSSSPEDPERDEEVLNHVLRDIELFMGKLEKAQA
KTSRKKKFGKKNKDQGGLTQAQYIDCFQKIKHSFNLLGRLATWLKETSAPELVHILFKSLNFILARCPEAGLAAQVISPLLTPKAIN
LLQSCLSPPESNLWMGLGPAWTTSRADWTGDEPLPYQPTFSDDWQLPEPSSQAPLGYQDPVSLRRGSHRLGSTSHFPQEKTHNHDPQ
PGDPNSRPSSPKPAQPALKMQVLYEFEARNPRELTVVQGEKLEVLDHSKRWWLVKNEAGRSGYIPSNILEPLQPGTPGTQGQSPSRV
PMLRLSSRPEEVTDWLQAENFSTATVRTLGSLTGSQLLRIRPGELQMLCPQEAPRILSRLEAVRRMLGISP
>sp|P47893|OR3A2_HUMAN
MSLQKLMEPEAGTNRTAVAEFILLGLVQTEEMQPVVFVLLLFAYLVTTGGNLSILAAVLVEPKLHAPMYFFLGNLSVLDVGCITVTV
PAMLGRLLSHKSTISYDACLSQLFFFHLLAGMDCFLLTAMAYDRLLAICQPLTYSTRMSQTVQRMLVAASLACAFTNALTHTVAMST
LNFCGPNEVNHFYCDLPQLFQLSCSSTQLNELLLFAVGFIMAGTPLVLIITAYSHVAAAVLRIRSVEGRKKAFSTCGSHLTVVCLFF
GRGIFNYMRLGSEEASDKDKGVGVFNTVINPMLNPLIYSLRNPDVQGALWQIFLGRRSLT
>sp|Q59EK9|RUN3A_HUMAN
MEASFVQTTMALGLSSKKASSRNVAVERKNLITVCRFSVKTLLEKYTAEPIDDSSEEFVNFAAILEQILSHRFKACAPAGPVSWFSS
DGQRGFWDYIRLACSKVPNNCVSSIENMENISTARAKGRAWIRVALMEKRMSEYITTALRDTRTTRRFYDSGAIMLRDEATILTGML
IGLSAIDFSFCLKGEVLDGKTPVVIDYTPYLKFTQSYDYLTDEEERHSAESSTSEDNSPEHPYLPLVTDEDSWYSKWHKMEQKFRIV
YAQKGYLEELVRLRESQLKDLEAENRRLQLQLEEAAAQNQREKRELEGVILELQEQLTGLIPSDHAPLAQGSKELTTPLVNQWPSLG
TLNGAEGASNSKLYRRHSFMSTEPLSAEASLSSDSQRLGEGTRDEEPWGPIGKDPTPSMLGLCGSLASIPSCKSLASFKSNECLVSD
SPEGSPALSPS
>sp|Q9NVJ2|ARL8B_HUMAN

MLALISRLLDWFRSLFWKEEMELTLVGLQYSGKTTFVNVIASGQFSEDMIPTVGFNMRKVTKGNVTIKIWDIGGQPRFRSMWERYCR
GVNAIVYMIDAADREKIEASRNELHNLLDKPQLQGIPVLVLGNKRDLPNALDEKQLIEKMNLSAIQDREICCYSISCKEKDNIDITL
QWLIQHSKSRRS
>sp|A4UGR9|XIRP2_HUMAN
MSPESGHSRIFEATAGPNKPESGFAEDSAARGEGVSDLHEVVSLKERMARYQAAVSRGDCRSFSANMMEESEMCAVPGGLAKVKKQF
EDEITSSRNTFAQYQYQHQNRSEQEAIHSSQVGTSRSSQEMARNEQEGSKVQKIDVHGTEMVSHLEKHTEEVNQASQFHQYVQETVI
DTPEDEEIPKVSTKLLKEQFEKSAQEKILYSDKEMTTPAKQIKTESEYEETFKPSSVVSTSSTSCVSTSQRKETSTTRYSDHSVTSS
TLAQINATSSGMTEEFPPPPPDVLQTSVDVTAFSQSPELPSPPRRLPVPKDVYSKQRNLYELNRLYKHIHPELRKNLEKDYISEVSE
IVSSQMNSGSSVSADVQQARYVFENTNDSSQKDLNSEREYLEWDEILKGEVQSIRWIFENQPLDSINNGSPDEGDISRGIADQEIIA
GGDVKYTTWMFETQPIDTLGAYSSDTVENAEKIPELARGDVCTARWMFETRPLDSMNKMHQSQEESAVTISKDITGGDVKTVRYMFE
TQHLDQLGQLHSVDEVHLLQLRSELKEIKGNVKRSIKCFETQPLYVIRDGSGQMLEIKTVHREDVEKGDVRTARWMFETQPLDTINK
DITEIKVVRGISMEENVKGGVSKAKWLFETQPLEKIKESEEVIIEKEKIIGTDVSRKCWMFETQPLDILKEVPDADSLQREEIIGGD
VQTTKHLFETLPIEALKDSPDIGKLQKITASEEEKGDVRHQKWIFETQPLEDIRKDKKEYTRTVKLEEVDRGDVKNYTHIFESNNLI
KFDASHKIEVEGVTRGAVELNKSLFETTPLYAIQDPLGKYHQVKTVQQEEIVRGDVRSCRWLFETRPIDQFDESIHKFQIIRGISAQ
EIQTGNVKSAKWLFETQPLDSIKYFSDVEETESKTEQTRDIVKGDVKTCKWLFETQPMESLYEKVSLMTSSEEIHKGDVKTCTWLFE
TQPLDTIKDDSETAVKLQTVKQEEIQGGDVRTACFLFETENLDSIQGEEVKEIKPVEMDIQAGDVSSMRYKFENQSLDSISSSSEEV
LKKIKTLKTEDIQKGNVLNCRWLFENQPIDKIKESQEGDECVKTVTDIQGGDVRKGCFIFETFSLDEIKEESDYISTKKTITEEVIQ
GDVKSYRMLFETQPLYAIQDREGSYHEVTTVKKEEVIHGDVRGTRWLFETKPLDSINKSETVYVIKSVTQEDIQKGDVSSVRYRFET
QPLDQISEESHNIMPSIDHIQGGNVKTSRQFFESENFDKNNYIRTVSVNEIQKGNVKTSTWLFETHTMDELRGEGLEYENIKTVTQE
DVQKGDVKQAVWLFENRTFDSIMEAHKGITKMTKEEIPPSDVKTTTWLFETTPLHEFNETRVEKIEIIGKSIKETLEDLYSQKVIQA
PGIIIEADEIGDVRMAKYKLMNQASPEIQKEEIIRADLRNIMVNLLSKRDCTEREILISEEEKGNVNLTKTQLLNRSTEFHAEKEEI
VKGDVQQAIKNLFSEERSVKKGILIQEDEKGDINMTIYCLLHENDGDTIEREEVIGGDVKRTIHNLLSSTSNNKISERAKIDASERG
NVQFFTTCIEAGALDYLKQLHTESNETLTAKKQEGEKEIIGGDVEGTKLLLKKRQSLVERTVSETDIIPGDVHNTVKVFMTEPQSTF
GKIPKEEIIKGDLTSTLNSLSQAVNQKTVTKTEEIIKGNMLATLKSLKESSHRWKESKQPDAIPGDIEKAIECLEKATNTKTEILKK
ELLKDDLETSLRSLKEAQRSFKEVHKEGVIKKDAKAVMAGSSGEQKTDIHQVAVQRNKNSLLQPKPGPFEPAAKWQGGADTLSQTMG
KSCHGNLVEERTEVNLPKAPKGTVKIVIDREQNNDALEKSLRRLSNSHHKSNVLESGDKTGVWTDTTGEQHLRDEYMSRQLTSTVSV
KNNLTTKESDRAVRELKKDDVFNSIQSAGKTVGKQQTYELRNDHQKMEGFHIKSPKKTKNIKILTDTQSSKPSPTQHPVSMPVGGTY
DLSGDFQKQTLLKQETKYSNKDIKKKNINLQPMWQLLPVEQDTSNVTEMKVSEKSHNTFKATNKKRETDVHLKSQDFLMKTNTSTGL
KMAMERSLNPINFNPENNVKESECPLPPPSPPPPPPSNASSEIEFPLPPPPPLMMFPEKNGFLPSLSTEKIKAEFESFPGLPLPPPP
VDEKSERESSSMFLPPPPPPTPSQKPAHLLSSSAPEKHSGDFMQQYSQKEASNSQNSQAKIITGKTGVLPPPTLPKPKLPKHIKDNK
NDFSPKVELATSLSDMECKITTSKDQKKVMVMTSSEHTETKQNVISKSLDERKQLSIDSANCLSHTVPGTSAPRKKQIAPLIKSHSF
PESSGQQNPKPYMRKFKTPLMIAEEKYRQQKEEIEKQKQESSYYNIVKTQSQNQHITEVEKEMPLQKTNEEVSLSGIDSECTVVQPS
PGSQSNARILGVCSDNQLSTTSPETVAAKRLHHVLAASEDKDKMKKEVLQSSRDIMQSKSACEIKQSHQECSTQQTQQKKYLEQLHL
PQSKPISPNFKVKTIKLPTLDHTLNETDHSYESHKQQSEIDVQTFTKKQYLKTKKTEASTECSHKQSLAERHYQLPKKEKRVTVQLP
TESIQKNQEDKLKMVPRKQREFSGSDRGKLPGSEEKNQGPSMIGRKEERLITERKHEHLKNKSAPKVVKQKVIDAHLDSQTQNFQQT
QIQTAESKAEHKKLPQPYNSLQEEKCLEVKGIQEKQVFSNTKDSKQEITQNKSFFSSVKESQRDDGKGALNIVEFLRKREELQQILS
RVKQFEAEPNKSGLKTFQTLLNTIPGWLISEDKREYAVHIAMENNLEKVKEEITHIKTQAEDMLVSYENIIQTAMMSSKTGKPGNKP
TSLDETSSKVSNVHVSNNKNSEQKENKIAKEKTVQHQVAAHHEATVRSHVKTHQEIKLDDSNIPPPSLKTRPPSPTFITIESTARRT
ENPTKNELSQSPKKDSYVEPPPRRPMSQKSEIHRANTSPSPPRSRSEQLVRLKDTTAKLSKGAIPCPAATPVPIVEKRSEIIMSPAT
LRRQIKIETRGRDSPPTITIPVNINHAASGSFRESVDAQEEIRKVEKRATYVHKDGLNSTDHMVPDTESYDAVEIIRKVAVPPRLSE
HTQRYEAANRTVQMAENFVNDPENEINRWFREFEHGPVSEAKSNRRVYAKGETNHNIQQESRTFCKEEFGLTSLGNTSFTDFSCKHP
RELREKIPVKQPRICSETRSLSEHFSGMDAFESQIVESKMKTSSSHSSEAGKSGCDFKHAPPTYEDVIAGHILDISDSPKEVRKNFQ
KTWQESGRVFKGLGYATADASATEMRTTFQEESAFISEAAAPRQGNMYTLSKDSLSNGVPSGRQAEFS
>sp|A8MU76|YP034_HUMAN

MRLCLSPQNTGTPQRVLPPVVWSPPSRKKPALSARNSTMFGHLRPLRIPRLRGKFNLQLPSFDEQVIPARLPKTEMRAEEPKEATEV
KDQVETQGQEDNKRGPCSNGEAASTSRPLETQGNLTSSWYNPRTLEGNVHLKSLTENNQTDKAQVHAVSFYSKGHGVASSHSPAGGI
LPFGKPDPLPTVLPAPVPGCSLWPEKGALKVLGKDHLPSSPGLLMVGEDMQPKDPAALGSSRSSPPRAAGHSSRKRKLSGPLLQLQL
TPPLQLRWDRDEGPPPAKLPCLSPEALLVGQASQREGRLQQGNMHKNMRVLSRTSKFRRLRELLRRRKERRQGRRGGPRL
>sp|Q15181|IPYR_HUMAN
MSGFSTEERAAPFSLEYRVFLKNEKGQYISPFHDIPIYADKDVFHMVVEVPRWSNAKMEIATKDPLNPIKQDVKKGKLRYVANLFPY
KGYIWNYGAIPQTWEDPGHNDKHTGCCGDNDPIDVCEIGSKVCARGEIIGVKVLGILAMIDEGETDWKVIAINVDDPDAANYNDIND
VKRLKPGYLEATVDWFRRYKVPDGKPENEFAFNAEFKDKDFAIDIIKSTHDHWKALVTKKTNGKGISCMNTTLSESPFKCDPDAARA
IVDALPPPCESACTVPTDVDKWFHHQKN
>sp|Q14954|KI2S1_HUMAN
MSLTVVSMACVGFFLLQGAWPHEGVHRKPSLLAHPGRLVKSEETVILQCWSDVMFEHFLLHREGMFNDTLRLIGEHHDGVSKANFSI
SRMRQDLAGTYRCYGSVTHSPYQLSAPSDPLDIVIIGLYEKPSLSAQPGPTVLAGENVTLSCSSRSSYDMYHLSREGEAHERRLPAG
TKVNGTFQANFPLGPATHGGTYRCFGSFRDSPYEWSKSSDPLLVSVTGNPSNSWPSPTEPSSETGNPRHLHVLIGTSVVKIPFTILL
FFLLHRWCSDKKNAAVMDQEPAGNRTVNSEDSDEQDHQEVSYA
>sp|P04180|LCAT_HUMAN
MGPPGSPWQWVTLLLGLLLPPAAPFWLLNVLFPPHTTPKAELSNHTRPVILVPGCLGNQLEAKLDKPDVVNWMCYRKTEDFFTIWLD
LNMFLPLGVDCWIDNTRVVYNRSSGLVSNAPGVQIRVPGFGKTYSVEYLDSSKLAGYLHTLVQNLVNNGYVRDETVRAAPYDWRLEP
GQQEEYYRKLAGLVEEMHAAYGKPVFLIGHSLGCLHLLYFLLRQPQAWKDRFIDGFISLGAPWGGSIKPMLVLASGDNQGIPIMSSI
KLKEEQRITTTSPWMFPSRMAWPEDHVFISTPSFNYTGRDFQRFFADLHFEEGWYMWLQSRDLLAGLPAPGVEVYCLYGVGLPTPRT
YIYDHGFPYTDPVGVLYEDGDDTVATRSTELCGLWQGRQPQPVHLLPLHGIQHLNMVFSNLTLEHINAILLGAYRQGPPASPTASPE
PPPPE
>sp|Q8NH05|OR4Q3_HUMAN
MKKEQDSNVTEFVLLGLSSSWELQLFLFLLFLFFYIAIVLGNLLIVVTVQAHAHLLQSPMYYFLGHLSFIDLCLSCVTVPKMLGDFL
QQGKSISFSGCLAQIYFLHFLGASEMFLLTVMAYDRYVAICNPLRYLTVMNPQLCLWLVLACWCGGFIHSIMQVILVIQLPFCGPNE
LDNFYCDVPQVIKLACMDTYVVEVLVIANSGLLSLVCFLVLLFSYAIILITLRTHFCQGQNKVFSTCASHLTVVSLIFVPCVFIYLR
PFCSFSVDKIFSLFYTVITPMLNPLIYTLRNTDMKTAMKKLRIKPCGIPLPC
>sp|Q1AE95|T183B_HUMAN
MARGPGPLGRPRPDTVAMPKRGKRLKFRAHDACSGRVTVADYADSDLAVVRSGRVKKAVANAVRQEVKSLCGLEASQVPAEEALSGA
GEPYDIIDSSDEMDAQEENIHERTVSRKKKSKRHKEELDGAGGEEYPMDIWLLLASYIRPEDIVNFSLICKNAWTVTCTAAFWTRLY
RRHYTLDASLPLRLRPESMEKLHCLRACVIRSLYHMYEPFAARISKNPAIPESTPSTLKNSKCLLFWCRKIVGNRQEPMWEFNFKFK
KQSPRLKSKCTGGLQPPVQYEDVHTNPDQDCCLLQVTTLNFIFIPIVMGMIFTLFTINVSTDMRHHRVRLVFQDSPVHGGRKLRSEQ
GVQVILDPVHSVRLFDWWHPQYPFSLRA
>sp|Q6B9Z1|IGFL4_HUMAN
MVPRISAAIFIFELLGSNSEGVTDLRLWLCQPAPRCGEWTYNPLEQCCDDGVILDLNQTRLCGSSCTFWPCFQHCCLESLGSQNQTV
VRFKVPGMKPDCKSSPITRICAQEYHPKSPVSRSDLI
>sp|Q5JV73|FRPD3_HUMAN
METLDSQRVQDRLLAAPGCSSPSGQQELFSSHVMQEESANDMECEQLPAEILRQVTVHRDPIYGFGFVAGSERPVVVRSVRPGGPSE
NKLLAGDQIVAINEEDVSEAPRERLIELIRSAKEFIVLTVLHTHQSPKSAFISAAKKAKLRSNPVKVRFSEQVAVGETDAKMMKKEA
LLLIPNVLKVFLENGQIKSFTFDGRTTVKDVMLTLQDRLSLRFIEHFALVLEYAGPEQNHKFLLLQDKQPLAYVVQRTHYHGMKCLF
RISFFPKDPVELLRRDPAAFEYLYIQSRNDVIRERFGMDPKPEMLLGLAALHIYITVSATRPSQKISLKNVEKEWGLEPFLPPSLLQ
VIKEKNLRKSLSQQLKAHQTHPSCGTKGSAIQAKLQYLRILNELPTFTGVLFNTVGLDEKQSATTLLVGPRHGISHVIDLKTNLTTV
LSEFSKISKIQLFRENQGVARVETSIMDAKPLVLLMEWPEATNFACLIAGYCRLLLDSRKMVFSRPASQPLPPPMIKADYMHSAHRP
VTGGHLGKKESSYVGSVGTSPRKSSRCTPPPADSELVSFCYLHMREQRKEQESRTDVNENLIFFEETRPRTKSDPTSKSSGQGYEVV
PDDFDAASLDHEPCASRARSYTLDNSLGAEALNFYCDSCKAKLQEQLGPRKGGKPGSSRDNIVDLMSLPPPGSEEEEEEEDETTSLL

PAIAAPPPGFRDNSSDEDDPKRRAVQSQEQGRHLRGLLYDEIPVTLIDSVQTRTVRDHAQELDDALVSTLQALEALAASEDGPHPPP
PQTAGLIVLATITPESSLDSGHETNSSELTDMSEMMSAMKQHQNTTYFLAQHLNKDSLLARKDLPFRIQSCAAQAVLTAPYSLGRPD
PNPSLQPIATGQSPGPPGARRKLPQSEGQVQGERTYSLAVHPALSPQLSEQKNLSLLSPVPEDKGPGHTRAGLEMSLRAATSSLSEE
QVSELRDNLPKEVRLSPKLILDPKSSVTPAIISAALQQVVHNKSLVTAGGALGNPPSRGERRLEASMGRPEVSMMSSSASKNLKFKI
SPSAPETSWNSQHQLGAEVSSSPRAPTGSRADSLHLSQQEDSLPVQNFPPKSYLLRTSRESVGKQATGEVAGKGGPVGGKPTLQKQG
TISSQGEKAQLESTPKRSKLEETSLVPRATYPMALQSPSCQSRSHSPSCQPHGHSPSSQSRGQSPSCQPRGQSPLRSQAASRQVSTM
PSRKLETTLNGAHSTSEGPAKPKSSRGPFRLRNLFSATFPTRQKKETDERQAQLQKVKQYELEFLEELLKPPSQGELPGTEYLQPPA
PGRCSCQLRSSPVQQGPGMSREQRRSCDCKRICRGGRPQATQTPVPSLRGRERDRVLPSQRQPEAGPGVSLSSPINVQRIRSTSLES
RECRSDPESGVSCLTTCASGGECLGAPNYRKLMRRYSISELDQGDRASLTSDVYPHPPLGMLPREAKEVEASLPIALGPKSRSLESP
TLGDPSYVQVAPETKGPRQMAVFSLPEEVYRKPAELDEDSESSKCCSIRYCFYYRKCDMADDASDGKDELSYSIPMKILPGMKLDEQ
VVPVVSRTLQVLDAATCSSSSPEASRTQEIDLRVSTFEGSLAKINALRAHAYGLPDGFLAARLDTNELLTVLRQCVASPEARAPKPY
VSQISEYKLELALKFKELRASCRRVANVDKSPTHMLAAITGSFQVLSSLIETFVRLVFIVRSEAQRQELLAKVEEVVRNYTFLLRAA
EESTARNLNQQQQQQQQQQQQQQQQQQQQQQQQQQQVAAAAGAATEHPPGSPTSATVMSTFTHSLKTLIK
>sp|A8MTJ6|FOXI3_HUMAN
MALYCGDNFGVYSQPGLPPPAATAAAPGAPPAARAPYGLADYAAPPAAAANPYLWLNGPGVGGPPSAAAAAAAAYLGAPPPPPPPGA
AAGPFLQPPPAAGTFGCSQRPFAQPAPAAPASPAAPAGPGELGWLSMASREDLMKMVRPPYSYSALIAMAIQSAPERKLTLSHIYQF
VADSFPFYQRSKAGWQNSIRHNLSLNDCFKKVPRDEDDPGKGNYWTLDPNCEKMFDNGNFRRKRKRRSEASNGSTVAAGTSKSEEGL
SSGLGSGVGGKPEEESPSTLLRPSHSPEPPEGTKSTASSPGGPMLTSTPCLNTFFSSLSSLSVSSSVSTQRALPGSRHLGIQGAQLP
SSGVFSPTSISEASADTLQLSNSTSNSTGQRSSYYSPFPASTSGGQSSPFSSPFHNFSMVNSLIYPREGSEV
>sp|Q9H8W5|TRI45_HUMAN
MSENRKPLLGFVSKLTSGTALGNSGKTHCPLCLGLFKAPRLLPCLHTVCTTCLEQLEPFSVVDIRGGDSDTSSEGSIFQELKPRSLQ
SQIGILCPVCDAQVDLPMGGVKALTIDHLAVNDVMLESLRGEGQGLVCDLCNDREVEKRCQTCKANLCHFCCQAHRRQKKTTYHTMV
DLKDLKGYSRIGKPILCPVHPAEELRLFCEFCDRPVCQDCVVGEHREHPCDFTSNVIHKHGDSVWELLKGTQPHVEALEEALAQIHI
INSALQKRVEAVAADVRTFSEGYIKAIEEHRDKLLKQLEDIRAQKENSLQLQKAQLEQLLADMRTGVEFTEHLLTSGSDLEILITKR
VVVERLRKLNKVQYSTRPGVNDKIRFCPQEKAGQCRGYEIYGTINTKEVDPAKCVLQGEDLHRAREKQTASFTLLCKDAAGEIMGRG
GDNVQVAVVPKDKKDSPVRTMVQDNKDGTYYISYTPKEPGVYTVWVCIKEQHVQGSPFTVMVRRKHRPHSGVFHCCTFCSSGGQKTA
RCACGGTMPGGYLGCGHGHKGHPGHPHWSCCGKFNEKSECTWTGGQSAPRSLLRTVAL
>sp|Q9P243|ZFAT_HUMAN
METRAAENTAIFMCKCCNLFSPNQSELLSHVSEKHMEEGVNVDEIIIPLRPLSTPEPPNSSKTGDEFLVMKRKRGRPKGSTKKSSTE
EELAENIVSPTEDSPLAPEEGNSLPPSSLECSKCCRKFSNTRQLRKHICIIVLNLGEEEGEAGNESDLELEKKCKEDDREKASKRPR
SQKTEKVQKISGKEARQLSGAKKPIISVVLTAHEAIPGATKIVPVEAGPPETGATNSETTSADLVPRRGYQEYAIQQTPYEQPMKSS
RLGPTQLKIFTCEYCNKVFKFKHSLQAHLRIHTNEKPYKCPQCSYASAIKANLNVHLRKHTGEKFACDYCSFTCLSKGHLKVHIERV
HKKIKQHCRFCKKKYSDVKNLIKHIRDAHDPQDKKVKEALDELCLMTREGKRQLLYDCHICERKFKNELDRDRHMLVHGDKWPFACE
LCGHGATKYQALELHVRKHPFVYVCAVCRKKFVSSIRLRTHIKEVHGAAQEALVFTSSINQSFCLLEPGGDIQQEALGDQLQLVEEE
FALQGVNALKEEACPGDTQLEEGRKEPEAPGEMPAPAVHLASPQAESTALPPCELETTVVSSSDLHSQEVVSDDFLLKNDTSSAEAH
AAPEKPPDMQHRSSVQTQGEVITLLLSKAQSAGSDQESHGAQSPLGEGQNMAVLSAGDPDPSRCLRSNPAEASDLLPPVAGGGDTIT
HQPDSCKAAPEHRSGITAFMKVLNSLQKKQMNTSLCERIRKVYGDLECEYCGKLFWYQVHFDMHVRTHTREHLYYCSQCHYSSITKN
CLKRHVIQKHSNILLKCPTDGCDYSTPDKYKLQAHLKVHTALDKRSYSCPVCEKSFSEDRLIKSHIKTNHPEVSMSTISEVLGRRVQ
LKGLIGKRAMKCPYCDFYFMKNGSDLQRHIWAHEGVKPFKCSLCEYATRSKSNLKAHMNRHSTEKTHLCDMCGKKFKSKGTLKSHKL
LHTADGKQFKCTVCDYTAAQKPQLLRHMEQHVSFKPFRCAHCHYSCNISGSLKRHYNRKHPNEEYANVGTGELAAEVLIQQGGLKCP
VCSFVYGTKWEFNRHLKNKHGLKVVEIDGDPKWETATEAPEEPSTQYLHITEAEEDVQGTQAAVAALQDLRYTSESGDRLDPTAVNI
LQQIIELGAETHDATALASVVAMAPGTVTVVKQVTEEEPSSNHTVMIQETVQQASVELAEQHHLVVSSDDVEGIETVTVYTQGGEAS
EFIVYVQEAMQPVEEQAVEQPAQEL
>sp|P07919|QCR6_HUMAN

MGLEDEQKMLTESGDPEEEEEEEELVDPLTTVREQCEQLEKCVKARERLELCDERVSSRSHTEEDCTEELFDFLHARDHCVAHKLF
NNLK
>sp|O75901|RASF9_HUMAN
MAPFGRNLLKTRHKNRSPTKDMDSEEKEIVVWVCQEEKLVCGLTKRTTSADVIQALLEEHEATFGEKRFLLGKPSDYCIIEKWRGSE
RVLPPLTRILKLWKAWGDEQPNMQFVLVKADAFLPVPLWRTAEAKLVQNTEKLWELSPANYMKTLPPDKQKRIVRKTFRKLAKIKQD
TVSHDRDNMETLVHLIISQDHTIHQQVKRMKELDLEIEKCEAKFHLDRVENDGENYVQDAYLMPSFSEVEQNLDLQYEENQTLEDLS
ESDGIEQLEERLKYYRILIDKLSAEIEKEVKSVCIDINEDAEGEAASELESSNLESVKCDLEKSMKAGLKIHSHLSGIQKEIKYSDS
LLQMKAKEYELLAKEFNSLHISNKDGCQLKENRAKESEVPSSNGEIPPFTQRVFSNYTNDTDSDTGISSNHSQDSETTVGDVVLLST
>sp|Q16678|CP1B1_HUMAN
MGTSLSPNDPWPLNPLSIQQTTLLLLLSVLATVHVGQRLLRQRRRQLRSAPPGPFAWPLIGNAAAVGQAAHLSFARLARRYGDVFQI
RLGSCPIVVLNGERAIHQALVQQGSAFADRPAFASFRVVSGGRSMAFGHYSEHWKVQRRAAHSMMRNFFTRQPRSRQVLEGHVLSEA
RELVALLVRGSADGAFLDPRPLTVVAVANVMSAVCFGCRYSHDDPEFRELLSHNEEFGRTVGAGSLVDVMPWLQYFPNPVRTVFREF
EQLNRNFSNFILDKFLRHCESLRPGAAPRDMMDAFILSAEKKAAGDSHGGGARLDLENVPATITDIFGASQDTLSTALQWLLLLFTR
YPDVQTRVQAELDQVVGRDRLPCMGDQPNLPYVLAFLYEAMRFSSFVPVTIPHATTANTSVLGYHIPKDTVVFVNQWSVNHDPLKWP
NPENFDPARFLDKDGLINKDLTSRVMIFSVGKRRCIGEELSKMQLFLFISILAHQCDFRANPNEPAKMNFSYGLTIKPKSFKVNVTL
RESMELLDSAVQNLQAKETCQ
>sp|O00469|PLOD2_HUMAN
MGGCTVKPQLLLLALVLHPWNPCLGADSEKPSSIPTDKLLVITVATKESDGFHRFMQSAKYFNYTVKVLGQGEEWRGGDGINSIGGG
QKVRLMKEVMEHYADQDDLVVMFTECFDVIFAGGPEEVLKKFQKANHKVVFAADGILWPDKRLADKYPVVHIGKRYLNSGGFIGYAP
YVNRIVQQWNLQDNDDDQLFYTKVYIDPLKREAINITLDHKCKIFQTLNGAVDEVVLKFENGKARAKNTFYETLPVAINGNGPTKIL
LNYFGNYVPNSWTQDNGCTLCEFDTVDLSAVDVHPNVSIGVFIEQPTPFLPRFLDILLTLDYPKEALKLFIHNKEVYHEKDIKVFFD
KAKHEIKTIKIVGPEENLSQAEARNMGMDFCRQDEKCDYYFSVDADVVLTNPRTLKILIEQNRKIIAPLVTRHGKLWSNFWGALSPD
GYYARSEDYVDIVQGNRVGVWNVPYMANVYLIKGKTLRSEMNERNYFVRDKLDPDMALCRNAREMGVFMYISNRHEFGRLLSTANYN
TSHYNNDLWQIFENPVDWKEKYINRDYSKIFTENIVEQPCPDVFWFPIFSEKACDELVEEMEHYGKWSGGKHHDSRISGGYENVPTD
DIHMKQVDLENVWLHFIREFIAPVTLKVFAGYYTKGFALLNFVVKYSPERQRSLRPHHDASTFTINIALNNVGEDFQGGGCKFLRYN
CSIESPRKGWSFMHPGRLTHLHEGLPVKNGTRYIAVSFIDP
>sp|P00505|AATM_HUMAN
MALLHSGRVLPGIAAAFHPGLAAAASARASSWWTHVEMGPPDPILGVTEAFKRDTNSKKMNLGVGAYRDDNGKPYVLPSVRKAEAQI
AAKNLDKEYLPIGGLAEFCKASAELALGENSEVLKSGRFVTVQTISGTGALRIGASFLQRFFKFSRDVFLPKPTWGNHTPIFRDAGM
QLQGYRYYDPKTCGFDFTGAVEDISKIPEQSVLLLHACAHNPTGVDPRPEQWKEIATVVKKRNLFAFFDMAYQGFASGDGDKDAWAV
RHFIEQGINVCLCQSYAKNMGLYGERVGAFTMVCKDADEAKRVESQLKILIRPMYSNPPLNGARIAAAILNTPDLRKQWLQEVKVMA
DRIIGMRTQLVSNLKKEGSTHNWQHITDQIGMFCFTGLKPEQVERLIKEFSIYMTKDGRISVAGVTSSNVGYLAHAIHQVTK
>sp|Q9NXC2|GFOD1_HUMAN
MLPGVGVFGTSLTARVIIPLLKDEGFAVKALWGRTQEEAEELAKEMSVPFYTSRIDEVLLHQDVDLVCINLPPPLTRQIAVKTLGIG
KNVICDRTATPLDAFRMTSAAHYYPKLMSIMGNVLRFLPAFVRMKQLIEEGYVGEPLVCEVQVHGGSLLGKKYNWSCDDLMGGGGLH
SVGTYIIDLLTFLTGQKAVKVHGLLKTFVKQTDHIKGIRQITSDDFCTFQMVLEGGVCCTVTLNFNVPGEFKQDVTVVGSAGRLLAV
GTDLYGQRNSAPEQELLVQDATPVSNSLLPEKAFSDIPSPYLRGTIKMMQAVRQAFQDQDDRRTWDGRPLTMAATFDDCLYALCVVD
TIKRSSQTGEWQNIAIMTEEPELSPAYLISEAMRRSRMSLYC
>sp|Q16670|ZSC26_HUMAN
MATALVSAHSLAPLNLKKEGLRVVREDHYSTWEQGFKLQGNSKGLGQEPLCKQFRQLRYEETTGPREALSRLRELCQQWLQPETHTK
EQILELLVLEQFLIILPKELQARVQEHHPESREDVVVVLEDLQLDLGETGQQDPDQPKKQKILVEEMAPLKGVQEQQVRHECEVTKP
EKEKGEETRIENGKLIVVTDSCGRVESSGKISEPMEAHNEGSNLERHQAKPKEKIEYKCSEREQRFIQHLDLIEHASTHTGKKLCES
DVCQSSSLTGHKKVLSREKGHQCHECGKAFQRSSHLVRHQKIHLGEKPYQCNECGKVFSQNAGLLEHLRIHTGEKPYLCIHCGKNFR

RSSHLNRHQRIHSQEEPCECKECGKTFSQALLLTHHQRIHSHSKSHQCNECGKAFSLTSDLIRHHRIHTGEKPFKCNICQKAFRLNS
HLAQHVRIHNEEKPYQCSECGEAFRQRSGLFQHQRYHHKDKLA
>sp|E9PJI5|NPIA7_HUMAN
MFCCLGYEWLSGGCTTWHSAWVINTLADHRHRGTDFGGSPWLLIITVFLRSYKFAISLCTSYLCVSFLKTIFPSQNGHDGSTDVQQR
ARRSNRRRQEGIKIVLEDIFTLWRQVETKVRAKIRKMKVTTKVNRHDKINGKRKTAKEHLRKLSMKEREHREEERQVSEAEENGKLD
MKEIHTYMEMFQRAQALRRRAEDYYRCKITPSARKPLCNRVRMAAVEHRHSSGLPYWPYLTAETLKNRMGHQPPPPTQQHSIIDNSL
SLKTPSECVLYPLPPSADDNLKTPPECLLTPLPPSALPSADDNLKTPAECLLYPLPPSADDNLKTPPECLLTPLPPSAPPSADDNLK
TPPECVCSLPFHPQRMIISRN
>sp|P59541|T2R30_HUMAN
MITFLPIIFSILIVVIFVIGNFANGFIALVNSIEWVKRQKISFVDQILTALAVSRVGLLWVLLLHWYATQLNPAFYSVEVRITAYNV
WAVTNHFSSWLATSLSMFYLLRIANFSNLIFLRIKRRVKSVVLVILLGPLLFLVCHLFVINMDETVWTKEYEGNVTWKIKLRSAMYH
SNMTLTMLANFVPLTLTLISFLLLICSLCKHLKKMQLHGKGSQDPSTKVHIKALQTVTSFLLLCAIYFLSMIISVCNFGRLEKQPVF
MFCQAIIFSYPSTHPFILILGNKKLKQIFLSVLRHVRYWVKDRSLRLHRFTRGALCVF
>sp|Q99747|SNAG_HUMAN
MAAQKINEGLEHLAKAEKYLKTGFLKWKPDYDSAASEYGKAAVAFKNAKQFEQAKDACLREAVAHENNRALFHAAKAYEQAGMMLKE
MQKLPEAVQLIEKASMMYLENGTPDTAAMALERAGKLIENVDPEKAVQLYQQTANVFENEERLRQAVELLGKASRLLVRGRRFDEAA
LSIQKEKNIYKEIENYPTCYKKTIAQVLVHLHRNDYVAAERCVRESYSIPGFNGSEDCAALEQLLEGYDQQDQDQVSDVCNSPLFKY
MDNDYAKLGLSLVVPGGGIKKKSPATPQAKPDGVTATAADEEEDEYSGGLC
>sp|P48729|KC1A_HUMAN
MASSSGSKAEFIVGGKYKLVRKIGSGSFGDIYLAINITNGEEVAVKLESQKARHPQLLYESKLYKILQGGVGIPHIRWYGQEKDYNV
LVMDLLGPSLEDLFNFCSRRFTMKTVLMLADQMISRIEYVHTKNFIHRDIKPDNFLMGIGRHCNKLFLIDFGLAKKYRDNRTRQHIP
YREDKNLTGTARYASINAHLGIEQSRRDDMESLGYVLMYFNRTSLPWQGLKAATKKQKYEKISEKKMSTPVEVLCKGFPAEFAMYLN
YCRGLRFEEAPDYMYLRQLFRILFRTLNHQYDYTFDWTMLKQKAAQQAASSSGQGQQAQTPTGKQTDKTKSNMKGF
>sp|Q8N5R6|CCD33_HUMAN
MAFRGPEPWVSASLLRQRLKAEEKTLDLEFEVLSVGFNEAGRYALRLSAENPLQVGSGAGVQLQVNDGDPFPACSAITDVIEQQEPG
QSLTLTRSKFIFTLPKGFCKNDGQHDAQLHVEALRLDEPLGRAAQRVGEAIFPIYPRPDQPRMNPKAQDHEDLYRYCGNLALLRAST
DPTARHCGSLAYSVAFHVHRGPQPPVSDSPPRAGQPELMSPEEPLIASQSTEPEIGHLSPSKKETIMVTLHGATNLPACKDGSEPWP
YVVVKSTSEEKNNQSSKAVTSVTSEPTRAPIWGDTVNVEIQAEDAGQEDVILKVVDNRKKQELLSYKIPIKYLRVFHPYHFELVKPT
ESGKADEATAKTQLYATVVRKSSFIPRYIGCNHMALEIFLRGVNEPLANNPNPIVVIARVVPNYKEFKVSQANRDLASVGLPITPLS
FPIPSMMNFDVPRVSQNGCPQLSKPGGPPEQPLWNQSFLFQGRDGATSFSEDTALVLEYYSSTSMKGSQPWTLNQPLGISVLPLKSR
LYQKMLTGKGLDGLHVERLPIMDTSLKTINDEAPTVALSFQLLSSERPENFLTPNNSKALPTLDPKILDKKLRTIQESWSKDTVSST
MDLSTSTPREAEEEPLVPEMSHDTEMNNYRRAMQKMAEDILSLRRQASILEGENRILRSRLAQQEEEEGQGKASEAQNTVSMKQKLL
LSELDMKKLRDRVQHLQNELIRKNDREKELLLLYQAQQPQAALLKQYQGKLQKMKALEETVRHQEKVIEKMERVLEDRLQDRSKPPP
LNRQQGKPYTGFPMLSASGLPLGSMGENLPVELYSVLLAENAKLRTELDKNRHQQAPIILQQQALPDLLSGTSDKFNLLAKLEHAQS
RILSLESQLEDSARRWGREKQDLATRLQEQEKGFRHPSNSIIIEQPSALTHSMDLKQPSELEPLLPSSDSKLNKPLSPQKETANSQQ
T
>sp|P05496|AT5G1_HUMAN
MQTAGALFISPALIRCCTRGLIRPVSASFLNSPVNSSKQPSYSNFPLQVARREFQTSVVSRDIDTAAKFIGAGAATVGVAGSGAGIG
TVFGSLIIGYARNPSLKQQLFSYAILGFALSEAMGLFCLMVAFLILFAM
>sp|A6NKC9|SH2D7_HUMAN
MEDSLKQLSLGRDPEGAGDSQALAELQELALKWFMETQAPFILQNGALPPWFHGFITRKQTEQLLRDKALGSFLIRLSDRATGYILS
YRGSDRCRHFVINQLRNRRYIISGDTQSHSTLAELVHHYQEAQLEPFKEMLTAACPRPEDNDLYDAITRGLHQTIVDPENPPATAFL
TVVPDKAASPRSSPKPQVSFLHAQKSLDVSPRNLSQEESMEAPIRVSPLPEKSSSLLEESFGGPSDIIYADLRRMNQARLGLGTEGS
GRHGPVPAGSQAYSPGREAQRRLSDGEQNRPDGLGPVLSGVSPDQGPTESPTSWGCSDAMGSLGATWRQEFPKLSQEAQPCSQGSSA

DIYEFIGTEGLLQEARDTPDQEGSTYEQIPACWGGPARAPHPGASPTYSPWVHGYKRISGTPELSEPGNTYEQIPATKSKETGRTHK
PDKLRRLFFTYRKHKF
>sp|Q9UL19|HRSL4_HUMAN
MASPHQEPKPGDLIEIFRLGYEHWALYIGDGYVIHLAPPSEYPGAGSSSVFSVLSNSAEVKRERLEDVVGGCCYRVNNSLDHEYQPR
PVEVIISSAKEMVGQKMKYSIVSRNCEHFVTQLRYGKSRCKQVEKAKVEVGVATALGILVVAGCSFAIRRYQKKATA
>sp|Q9NYT0|PLEK2_HUMAN
MEDGVLKEGFLVKRGHIVHNWKARWFILRQNTLVYYKLEGGRRVTPPKGRILLDGCTITCPCLEYENRPLLIKLKTQTSTEYFLEAC
SREERDAWAFEITGAIHAGQPGKVQQLHSLRNSFKLPPHISLHRIVDKMHDSNTGIRSSPNMEQGSTYKKTFLGSSLVDWLISNSFT
ASRLEAVTLASMLMEENFLRPVGVRSMGAIRSGDLAEQFLDDSTALYTFAESYKKKISPKEEISLSTVELSGTVVKQGYLAKQGHKR
KNWKVRRFVLRKDPAFLHYYDPSKEENRPVGGFSLRGSLVSALEDNGVPTGVKGNVQGNLFKVITKDDTHYYIQASSKAERAEWIEA
IKKLT
>sp|Q9Y2W1|TR150_HUMAN
MSKTNKSKSGSRSSRSRSASRSRSRSFSKSRSRSRSLSRSRKRRLSSRSRSRSYSPAHNRERNHPRVYQNRDFRGHNRGYRRPYYFR
GRNRGFYPWGQYNRGGYGNYRSNWQNYRQAYSPRRGRSRSRSPKRRSPSPRSRSHSRNSDKSSSDRSRRSSSSRSSSNHSRVESSKR
KSAKEKKSSSKDSRPSQAAGDNQGDEAKEQTFSGGTSQDTKASESSKPWPDATYGTGSASRASAVSELSPRERSPALKSPLQSVVVR
RRSPRPSPVPKPSPPLSSTSQMGSTLPSGAGYQSGTHQGQFDHGSGSLSPSKKSPVGKSPPSTGSTYGSSQKEESAASGGAAYTKRY
LEEQKTENGKDKEQKQTNTDKEKIKEKGSFSDTGLGDGKMKSDSFAPKTDSEKPFRGSQSPKRYKLRDDFEKKMADFHKEEMDDQDK
DKAKGRKESEFDDEPKFMSKVIGANKNQEEEKSGKWEGLVYAPPGKEKQRKTEELEEESFPERSKKEDRGKRSEGGHRGFVPEKNFR
VTAYKAVQEKSSSPPPRKTSESRDKLGAKGDFPTGKSSFSITREAQVNVRMDSFDEDLARPSGLLAQERKLCRDLVHSNKKEQEFRS
IFQHIQSAQSQRSPSELFAQHIVTIVHHVKEHHFGSSGMTLHERFTKYLKRGTEQEAAKNKKSPEIHRRIDISPSTFRKHGLAHDEM
KSPREPGYKAEGKYKDDPVDLRLDIERRKKHKERDLKRGKSRESVDSRDSSHSRERSAEKTEKTHKGSKKQKKHRRARDRSRSSSSS
SQSSHSYKAEEYTEETEEREESTTGFDKSRLGTKDFVGPSERGGGRARGTFQFRARGRGWGRGNYSGNNNNNSNNDFQKRNREEEWD
PEYTPKSKKYYLHDDREGEGSDKWVSRGRGRGAFPRGRGRFMFRKSSTSPKWAHDKFSGEEGEIEDDESGTENREEKDNIQPTTE
>sp|P49356|FNTB_HUMAN
MASPSSFTYYCPPSSSPVWSEPLYSLRPEHARERLQDDSVETVTSIEQAKVEEKIQEVFSSYKFNHLVPRLVLQREKHFHYLKRGLR
QLTDAYECLDASRPWLCYWILHSLELLDEPIPQIVATDVCQFLELCQSPEGGFGGGPGQYPHLAPTYAAVNALCIIGTEEAYDIINR
EKLLQYLYSLKQPDGSFLMHVGGEVDVRSAYCAASVASLTNIITPDLFEGTAEWIARCQNWEGGIGGVPGMEAHGGYTFCGLAALVI
LKRERSLNLKSLLQWVTSRQMRFEGGFQGRCNKLVDGCYSFWQAGLLPLLHRALHAQGDPALSMSHWMFHQQALQEYILMCCQCPAG
GLLDKPGKSRDFYHTCYCLSGLSIAQHFGSGAMLHDVVLGVPENALQPTHPVYNIGPDKVIQATTYFLQKPVPGFEELKDETSAEPA
TD
>sp|P52951|GBX2_HUMAN
MSAAFPPSLMMMQRPLGSSTAFSIDSLIGSPPQPSPGHFVYTGYPMFMPYRPVVLPPPPPPPPALPQAALQPALPPAHPHHQIPSLP
TGFCSSLAQGMALTSTLMATLPGGFSASPQHQEAAAARKFAPQPLPGGGNFDKAEALQADAEDGKGFLAKEGSLLAFSAAETVQASL
VGAVRGQGKDESKVEDDPKGKEESFSLESDVDYSSDDNLTGQAAHKEEDPGHALEETPPSSGAAGSTTSTGKNRRRRTAFTSEQLLE
LEKEFHCKKYLSLTERSQIAHALKLSEVQVKIWFQNRRAKWKRVKAGNANSKTGEPSRNPKIVVPIPVHVSRFAIRSQHQQLEQARP
>sp|Q9UFP1|F198A_HUMAN
MASWLRRKLRGKRRPVIAFCLLMILSAMAVTRFPPQRPSAGPDPGPMEPQGVTGAPATHIRQALSSSRRQRARNMGFWRSRALPRNS
ILVCAEEQGHRARVDRSRESPGGDLRHPGRVRRDITLSGHPRLSTQHVVLLREDEVGDPGTKDLGHPQHGSPIQETQSEVVTLVSPL
PGSDMAALPAWRATSGLTLWPHTAEGRDLLGAENRALTGGQQAEDPTLASGAHQWPGSVEKLQGSVWCDAETLLSSSRTGGQAPPWL
TDHDVQMLRLLAQGEVVDKARVPAHGQVLQVGFSTEAALQDLSSPRLSQLCSQGLCGLIKRPGDLPEVLSFHVDRVLGLRRSLPAVA
RRFHSPLLPYRYTDGGARPVIWWAPDVQHLSDPDEDQNSLALGWLQYQALLAHSCNWPGQAPCPGIHHTEWARLALFDFLLQVHDRL
DRYCCGFEPEPSDPCVEERLREKCQNPAELRLVHILVRSSDPSHLVYIDNAGNLQHPEDKLNFRLLEGIDGFPESAVKVLASGCLQN
MLLKSLQMDPVFWESQGGAQGLKQVLQTLEQRGQVLLGHIQKHNLTLFRDEDP
>sp|Q6P387|CP046_HUMAN

MDLCQKNETDLENAENNEIQFTEETEPTYTCPDGKSEKNHVYCLLDVSDITLEQDEKAKEFIIGTGWEEAVQGWGRTSPAACIWPRK
IPKKARVGEGACSDCLVCVNLSHWSLQTKPPTEGGPEKDQSSPSQTQAAPQGPSTASRAISDICFPTYFRAEKKSLQIKEFIWCNKD
WAIPGTNRGKASGNPSGGAHRGLSIPGPLTSRALLVLPPLKASLSNALDVLGKKSKNSFLQSEEKVLDVEKDGCVAYAYGLKTADGK
GEKRASELAKHPMVNDTPSSPSPAAQISLLTDPEQRCLHWSLLSEKNLACPPDPSNVRYLAALQLLQKRGVQSYKSKFKAKEPRSPV
ITRKHVLPKAKQENRPQMLETKVFPRPVLPSLTVSRVIIPVSTHRIL
>sp|Q9Y365|PCTL_HUMAN
MEKLAASTEPQGPRPVLGRESVQVPDDQDFRSFRSECEAEVGWNLTYSRAGVSVWVQAVEMDRTLHKIKCRMECCDVPAETLYDVLH
DIEYRKKWDSNVIETFDIARLTVNADVGYYSWRCPKPLKNRDVITLRSWLPMGADYIIMNYSVKHPKYPPRKDLVRAVSIQTGYLIQ
STGPKSCVITYLAQVDPKGSLPKWVVNKSSQFLAPKAMKKMYKACLKYPEWKQKHLPHFKPWLHPEQSPLPSLALSELSVQHADSLE
NIDESAVAESREERMGGAGGEGSDDDTSLT
>sp|Q9NYJ7|DLL3_HUMAN
MVSPRMSGLLSQTVILALIFLPQTRPAGVFELQIHSFGPGPGPGAPRSPCSARLPCRLFFRVCLKPGLSEEAAESPCALGAALSARG
PVYTEQPGAPAPDLPLPDGLLQVPFRDAWPGTFSFIIETWREELGDQIGGPAWSLLARVAGRRRLAAGGPWARDIQRAGAWELRFSY
RARCEPPAVGTACTRLCRPRSAPSRCGPGLRPCAPLEDECEAPLVCRAGCSPEHGFCEQPGECRCLEGWTGPLCTVPVSTSSCLSPR
GPSSATTGCLVPGPGPCDGNPCANGGSCSETPRSFECTCPRGFYGLRCEVSGVTCADGPCFNGGLCVGGADPDSAYICHCPPGFQGS
NCEKRVDRCSLQPCRNGGLCLDLGHALRCRCRAGFAGPRCEHDLDDCAGRACANGGTCVEGGGAHRCSCALGFGGRDCRERADPCAA
RPCAHGGRCYAHFSGLVCACAPGYMGARCEFPVHPDGASALPAAPPGLRPGDPQRYLLPPALGLLVAAGVAGAALLLVHVRRRGHSQ
DAGSRLLAGTPEPSVHALPDALNNLRTQEGSGDGPSSSVDWNRPEDVDPQGIYVISAPSIYAREVATPLFPPLHTGRAGQRQHLLFP
YPSSILSVK
>sp|D6RA61|U17LM_HUMAN
MEDDSLYLGGEWQFNHFSKLTSSRPDAAFAEIQRTSLPEKSPLSCETRVDLCDDLAPVARQLAPREKLPLSSRRPAAVGAGLQNMGN
TCYVNASLQCLTYTPPLANYMLSREHSQTCHRHKGCMLCTMQAHITRALHNPGHVIQPSQALAAGFHRGKQEDAHEFLMFTVDAMKK
ACLPGHKQVDHHSKDTTLIHQIFGGYWRSQIKCLHCHGISDTFDPYLDIALDIQAAQSVQQALEQLVKPEELNGENAYHCGVCLQRA
PASKTLTLHTSAKVLILVLKRFSDVTGNKIAKNVQYPECLDMQPYMSQQNTGPLVYVLYAVLVHAGWSCHNGHYFSYVKAQEGQWYK
MDDAEVTASSITSVLSQQAYVLFYIQKSEWERHSESVSRGREPRALGAEDTDRRATQGELKRDHPCLQAPELDEHLVERATQESTLD
HWKFLQEQNKTKPEFNVRKVEGTLPPDVLVIHQSKYKCGMKNHHPEQQSSLLKLSSTTPTHQESMNTGTLASLRGRARRSKGKNKHS
KRALLVCQ
>sp|Q8WWC4|MAIP1_HUMAN
MALAARLLPQFLHSRSLPCGAVRLRTPAVAEVRLPSATLCYFCRCRLGLGAALFPRSARALAASALPAQGSRWPVLSSPGLPAAFAS
FPACPQRSYSTEEKPQQHQKTKMIVLGFSNPINWVRTRIKAFLIWAYFDKEFSITEFSEGAKQAFAHVSKLLSQCKFDLLEELVAKE
VLHALKEKVTSLPDNHKNALAANIDEIVFTSTGDISIYYDEKGRKFVNILMCFWYLTSANIPSETLRGASVFQVKLGNQNVETKQLL
SASYEFQREFTQGVKPDWTIARIEHSKLLE
>sp|P28070|PSB4_HUMAN
MEAFLGSRSGLWAGGPAPGQFYRIPSTPDSFMDPASALYRGPITRTQNPMVTGTSVLGVKFEGGVVIAADMLGSYGSLARFRNISRI
MRVNNSTMLGASGDYADFQYLKQVLGQMVIDEELLGDGHSYSPRAIHSWLTRAMYSRRSKMNPLWNTMVIGGYADGESFLGYVDMLG
VAYEAPSLATGYGAYLAQPLLREVLEKQPVLSQTEARDLVERCMRVLYYRDARSYNRFQIATVTEKGVEIEGPLSTETNWDIAHMIS
GFE
>sp|Q99683|M3K5_HUMAN
MSTEADEGITFSVPPFAPSGFCTIPEGGICRRGGAAAVGEGEEHQLPPPPPGSFWNVESAAAPGIGCPAATSSSSATRGRGSSVGGG
SRRTTVAYVINEASQGQLVVAESEALQSLREACETVGATLETLHFGKLDFGETTVLDRFYNADIAVVEMSDAFRQPSLFYHLGVRES
FSMANNIILYCDTNSDSLQSLKEIICQKNTMCTGNYTFVPYMITPHNKVYCCDSSFMKGLTELMQPNFELLLGPICLPLVDRFIQLL
KVAQASSSQYFRESILNDIRKARNLYTGKELAAELARIRQRVDNIEVLTADIVINLLLSYRDIQDYDSIVKLVETLEKLPTFDLASH
HHVKFHYAFALNRRNLPGDRAKALDIMIPMVQSEGQVASDMYCLVGRIYKDMFLDSNFTDTESRDHGASWFKKAFESEPTLQSGINY
AVLLLAAGHQFESSFELRKVGVKLSSLLGKKGNLEKLQSYWEVGFFLGASVLANDHMRVIQASEKLFKLKTPAWYLKSIVETILIYK

HFVKLTTEQPVAKQELVDFWMDFLVEATKTDVTVVRFPVLILEPTKIYQPSYLSINNEVEEKTISIWHVLPDDKKGIHEWNFSASSV
RGVSISKFEERCCFLYVLHNSDDFQIYFCTELHCKKFFEMVNTITEEKGRSTEEGDCESDLLEYDYEYDENGDRVVLGKGTYGIVYA
GRDLSNQVRIAIKEIPERDSRYSQPLHEEIALHKHLKHKNIVQYLGSFSENGFIKIFMEQVPGGSLSALLRSKWGPLKDNEQTIGFY
TKQILEGLKYLHDNQIVHRDIKGDNVLINTYSGVLKISDFGTSKRLAGINPCTETFTGTLQYMAPEIIDKGPRGYGKAADIWSLGCT
IIEMATGKPPFYELGEPQAAMFKVGMFKVHPEIPESMSAEAKAFILKCFEPDPDKRACANDLLVDEFLKVSSKKKKTQPKLSALSAG
SNEYLRSISLPVPVLVEDTSSSSEYGSVSPDTELKVDPFSFKTRAKSCGERDVKGIRTLFLGIPDENFEDHSAPPSPEEKDSGFFML
RKDSERRATLHRILTEDQDKIVRNLMESLAQGAEEPKLKWEHITTLIASLREFVRSTDRKIIATTLSKLKLELDFDSHGISQVQVVL
FGFQDAVNKVLRNHNIKPHWMFALDSIIRKAVQTAITILVPELRPHFSLASESDTADQEDLDVEDDHEEQPSNQTVRRPQAVIEDAV
ATSGVSTLSSTVSHDSQSAHRSLNVQLGRMKIETNRLLEELVRKEKELQALLHRAIEEKDQEIKHLKLKSQPIEIPELPVFHLNSSG
TNTEDSELTDWLRVNGADEDTISRFLAEDYTLLDVLYYVTRDDLKCLRLRGGMLCTLWKAIIDFRNKQT
>sp|Q6ZRX8|YL004_HUMAN
MSPTTGPQPNPRAWECHHTTGPQPNPRAWECHRMKGPQPNPRAWECHLRRVHNLTPEPGNVTIRGVHILTPEPGNVTIRGVHNLTPE
PGNVTERGVHNLTPEPGNVTERGVHNLIPEPGNVTVKRGPQPNPRAWECHRTRGPQPNPRAWECHRTRGPQPNPRAWECHR
>sp|Q8N239|KLH34_HUMAN
MSYFLSYCKAHGGALLTGYQALRAEGFLCDVTLETEGSEFPAHRSLLACSSDYFRALFKSHTQESRARVIHLHVPSAAGLQRLLDFI
YTAWLSLSMDTVEDTLEAASYLQVTEALGLCGRYLERQLAPENCCFAANVAARFGLAHTLDAAERCIVSHLQELLARGAGPAGLLEL
NPTSLRAVLGAPDVARVPEARLLGLALAWLRQEPTTERLAHCTELLERVRFGLVPADVLRRVYSGSGLVLPARVKGLIIQALNYHTT
PSRQPLMQGEQTSIRSPQTRILLVGGRRAREVVIEEVAAPQRAARGQVAAPEPEEEEEELEEEEEEEEWELTQNVVAFDVYNHRWRS
LTQLPTPLLGHSVCTAGNFLFVLGGESPSGSASSPLADDSRVVTAQVHRYDPRFHAWTEVPAMREARAHFWCGAVGERLLAVGGLGA
GGEVLASVEMYDLRRDRWTAAGALPRALHGHAGAVGDRGVVYISGGKAGRGEGGASSLRDLYVLGPEEQVWSKKAPMGTARFGHHMA
VLRGAVFAFLGRYEPFSEIERYDPGADQWTRLRPLPYDRFCYGLAVVEETALLLGGLKWRDSRQVPTRNVVGYDLDLDRWEDIGCAL
PWAWSGLRCAVLQLAEGGDDEREGEVGEALDLVLG
>sp|Q9GZP7|VN1R1_HUMAN
MVGDTLKLLSPLMTRYFFLLFYSTDSSDLNENQHPLDFDEMAFGKVKSGISFLIQTGVGILGNSFLLCFYNLILFTGHKLRPTDLIL
SQLALANSMVLFFKGIPQTMAAFGLKYLLNDTGCKFVFYYHRVGTRVSLSTICLLNGFQAIKLNPSICRWMEIKIRSPRFIDFCCLL
CWAPHVLMNASVLLLVNGPLNSKNSSAKNNYGYCSYKASKRFSSLHAVLYFSPDFMSLGFMVWASGSMVFFLYRHKQQVQHNHSNRL
SCRPSQEARATHTIMVLVSSFFVFYSVHSFLTIWTTVVANPGQWIVTNSVLVASCFPARSPFVLIMSDTHISQFCFACRTRKTLFPN
LVVMP
>sp|P14921|ETS1_HUMAN
MKAAVDLKPTLTIIKTEKVDLELFPSPDMECADVPLLTPSSKEMMSQALKATFSGFTKEQQRLGIPKDPRQWTETHVRDWVMWAVNE
FSLKGVDFQKFCMNGAALCALGKDCFLELAPDFVGDILWEHLEILQKEDVKPYQVNGVNPAYPESRYTSDYFISYGIEHAQCVPPSE
FSEPSFITESYQTLHPISSEELLSLKYENDYPSVILRDPLQTDTLQNDYFAIKQEVVTPDNMCMGRTSRGKLGGQDSFESIESYDSC
DRLTQSWSSQSSFNSLQRVPSYDSFDSEDYPAALPNHKPKGTFKDYVRDRADLNKDKPVIPAAALAGYTGSGPIQLWQFLLELLTDK
SCQSFISWTGDGWEFKLSDPDEVARRWGKRKNKPKMNYEKLSRGLRYYYDKNIIHKTAGKRYVYRFVCDLQSLLGYTPEELHAMLDV
KPDADE
>sp|A6NGB0|T191C_HUMAN
MCRATLGLPLPPIVIQPARRSLPPIVTPASRRLGPRGGRHLGSVSTAMAATQELLLQLQKDNRDGRQRKQELEKLMRGLEAESESLN
QRLQDLSERERSLLRRRSQAAQPLQGEAREAARERAERVRRRLEEAERHKEYLEQHSRQLQEQWEELSSQLFYYGGELQSQKSTEQQ
LAAQLVTLQNELELAETKCALQEEKLQQDALQTAEAWAIFQEQTVVLQVRPHSDAKVPPASPPPDLGRCDGQLRGVQYSTESLMEEM
ARADRETRLFGGPRALAIRRCVLGALQVLLTLPLLFLGLSLLWTVLLDPGAVSAWLWSLTSETTLRRLRYTLSPLLELRANGLLPT
>sp|Q9GZP8|IMUP_HUMAN
MEFDLGAALEPTSQKPGVGAGHGGDPKLSPHKVQGRSEAGAGPGPKQGHHSSSDSSSSSSDSDTDVKSHAAGSKQHESIPGKAKKPK
VKKKEKGKKEKGKKKEAPH
>sp|Q9H3U7|SMOC2_HUMAN

MLLPQLCWLPLLAGLLPPVPAQKFSALTFLRVDQDKDKDCSLDCAGSPQKPLCASDGRTFLSRCEFQRAKCKDPQLEIAYRGNCKDV
SRCVAERKYTQEQARKEFQQVFIPECNDDGTYSQVQCHSYTGYCWCVTPNGRPISGTAVAHKTPRCPGSVNEKLPQREGTGKTDDAA
APALETQPQGDEEDIASRYPTLWTEQVKSRQNKTNKNSVSSCDQEHQSALEEAKQPKNDNVVIPECAHGGLYKPVQCHPSTGYCWCV
LVDTGRPIPGTSTRYEQPKCDNTARAHPAKARDLYKGRQLQGCPGAKKHEFLTSVLDALSTDMVHAASDPSSSSGRLSEPDPSHTLE
ERVVHWYFKLLDKNSSGDIGKKEIKPFKRFLRKKSKPKKCVKKFVEYCDVNNDKSISVQELMGCLGVAKEDGKADTKKRHTPRGHAE
STSNRQPRKQG
>sp|Q68BL8|OLM2B_HUMAN
MAKPRLLVLYFALIVVPAWVSSIVLTGTSEPPDAQTVAPAEDETLQNEADNQENVLSQLLGDYDKVKAMSEGSDCQCKCVVRPLGRD
ACQRINAGASRKEDFYTVETITSGSSCKCACVAPPSALNPCEGDFRLQKLREADSQDLKLSTIIDMLEGAFYGLDLLKLHSVTTKLV
GRVDKLEEEVSKNLTKENEQIKEDMEEIRTEMNKRGKENCSENILDSMPDIRSALQRDAAAAYAHPEYEERFLQEETVSQQINSIEL
LQTRPLALPEVVKSQRPLQRQVHLRGRPASQPTVIRGITYYKAKVSEEENDIEEQQDEFFSGDNGVDLLIEDQLLRHNGLMTSVTRR
PAATRQGHSTAVTSDLNARTAPWSSALPQPSTSDPSIANHASVGPTLQTTSVSPDPTRESVLQPSPQVPATTVAHTATQQPAAPAPP
AVSPREALMEAMHTVPVPPTTVRTDSLGKDAPAGWGTTPASPTLSPEEEDDIRNVIGRCKDTLSTITGPTTQNTYGRNEGAWMKDPL
AKDERIYVTNYYYGNTLVEFRNLENFKQGRWSNSYKLPYSWIGTGHVVYNGAFYYNRAFTRNIIKYDLKQRYVAAWAMLHDVAYEEA
TPWRWQGHSDVDFAVDENGLWLIYPALDDEGFSQEVIVLSKLNAADLSTQKETTWRTGLRRNFYGNCFVICGVLYAVDSYNQRNANI
SYAFDTHTNTQIVPRLLFENEYSYTTQIDYNPKDRLLYAWDNGHQVTYHVIFAY
>sp|Q9Y4P1|ATG4B_HUMAN
MDAATLTYDTLRFAEFEDFPETSEPVWILGRKYSIFTEKDEILSDVASRLWFTYRKNFPAIGGTGPTSDTGWGCMLRCGQMIFAQAL
VCRHLGRDWRWTQRKRQPDSYFSVLNAFIDRKDSYYSIHQIAQMGVGEGKSIGQWYGPNTVAQVLKKLAVFDTWSSLAVHIAMDNTV
VMEEIRRLCRTSVPCAGATAFPADSDRHCNGFPAGAEVTNRPSPWRPLVLLIPLRLGLTDINEAYVETLKHCFMMPQSLGVIGGKPN
SAHYFIGYVGEELIYLDPHTTQPAVEPTDGCFIPDESFHCQHPPCRMSIAELDPSIAVGFFCKTEDDFNDWCQQVKKLSLLGGALPM
FELVELQPSHLACPDVLNLSLDSSDVERLERFFDSEDEDFEILSL
>sp|P24592|IBP6_HUMAN
MTPHRLLPPLLLLLALLLAASPGGALARCPGCGQGVQAGCPGGCVEEEDGGSPAEGCAEAEGCLRREGQECGVYTPNCAPGLQCHPP
KDDEAPLRALLLGRGRCLPARAPAVAEENPKESKPQAGTARPQDVNRRDQQRNPGTSTTPSQPNSAGVQDTEMGPCRRHLDSVLQQL
QTEVYRGAQTLYVPNCDHRGFYRKRQCRSSQGQRRGPCWCVDRMGKSLPGSPDGNGSSSCPTGSSG
>sp|Q9BZR6|RTN4R_HUMAN
MKRASAGGSRLLAWVLWLQAWQVAAPCPGACVCYNEPKVTTSCPQQGLQAVPVGIPAASQRIFLHGNRISHVPAASFRACRNLTILW
LHSNVLARIDAAAFTGLALLEQLDLSDNAQLRSVDPATFHGLGRLHTLHLDRCGLQELGPGLFRGLAALQYLYLQDNALQALPDDTF
RDLGNLTHLFLHGNRISSVPERAFRGLHSLDRLLLHQNRVAHVHPHAFRDLGRLMTLYLFANNLSALPTEALAPLRALQYLRLNDNP
WVCDCRARPLWAWLQKFRGSSSEVPCSLPQRLAGRDLKRLAANDLQGCAVATGPYHPIWTGRATDEEPLGLPKCCQPDAADKASVLE
PGRPASAGNALKGRVPPGDSPPGNGSGPRHINDSPFGTLPGSAEPPLTAVRPEGSEPPGFPTSGPRRRPGCSRKNRTRSHCRLGQAG
SGGGGTGDSEGSGALPSLTCSLTPLGLALVLWTVLGPC
>sp|Q9Y6N9|USH1C_HUMAN
MDRKVAREFRHKVDFLIENDAEKDYLYDVLRMYHQTMDVAVLVGDLKLVINEPSRLPLFDAIRPLIPLKHQVEYDQLTPRRSRKLKE
VRLDRLHPEGLGLSVRGGLEFGCGLFISHLIKGGQADSVGLQVGDEIVRINGYSISSCTHEEVINLIRTKKTVSIKVRHIGLIPVKS
SPDEPLTWQYVDQFVSESGGVRGSLGSPGNRENKEKKVFISLVGSRGLGCSISSGPIQKPGIFISHVKPGSLSAEVGLEIGDQIVEV
NGVDFSNLDHKEAVNVLKSSRSLTISIVAAAGRELFMTDRERLAEARQRELQRQELLMQKRLAMESNKILQEQQEMERQRRKEIAQK
AAEENERYRKEMEQIVEEEEKFKKQWEEDWGSKEQLLLPKTITAEVHPVPLRKPKYDQGVEPELEPADDLDGGTEEQGEQDFRKYEE
GFDPYSMFTPEQIMGKDVRLLRIKKEGSLDLALEGGVDSPIGKVVVSAVYERGAAERHGGIVKGDEIMAINGKIVTDYTLAEAEAAL
QKAWNQGGDWIDLVVAVCPPKEYDDELTFF
>sp|Q9ULH1|ASAP1_HUMAN
MRSSASRLSSFSSRDSLWNRMPDQISVSEFIAETTEDYNSPTTSSFTTRLHNCRNTVTLLEEALDQDRTALQKVKKSVKAIYNSGQD
HVQNEENYAQVLDKFGSNFLSRDNPDLGTAFVKFSTLTKELSTLLKNLLQGLSHNVIFTLDSLLKGDLKGVKGDLKKPFDKAWKDYE

TKFTKIEKEKREHAKQHGMIRTEITGAEIAEEMEKERRLFQLQMCEYLIKVNEIKTKKGVDLLQNLIKYYHAQCNFFQDGLKTADKL
KQYIEKLAADLYNIKQTQDEEKKQLTALRDLIKSSLQLDQKEDSQSRQGGYSMHQLQGNKEYGSEKKGYLLKKSDGIRKVWQRRKCS
VKNGILTISHATSNRQPAKLNLLTCQVKPNAEDKKSFDLISHNRTYHFQAEDEQDYVAWISVLTNSKEEALTMAFRGEQSAGENSLE
DLTKAIIEDVQRLPGNDICCDCGSSEPTWLSTNLGILTCIECSGIHREMGVHISRIQSLELDKLGTSELLLAKNVGNNSFNDIMEAN
LPSPSPKPTPSSDMTVRKEYITAKYVDHRFSRKTCSTSSAKLNELLEAIKSRDLLALIQVYAEGVELMEPLLEPGQELGETALHLAV
RTADQTSLHLVDFLVQNCGNLDKQTALGNTVLHYCSMYSKPECLKLLLRSKPTVDIVNQAGETALDIAKRLKATQCEDLLSQAKSGK
FNPHVHVEYEWNLRQEEIDESDDDLDDKPSPIKKERSPRPQSFCHSSSISPQDKLALPGFSTPRDKQRLSYGAFTNQIFVSTSTDSP
TSPTTEAPPLPPRNAGKGPTGPPSTLPLSTQTSSGSSTLSKKRPPPPPPGHKRTLSDPPSPLPHGPPNKGAVPWGNDGGPSSSSKTT
NKFEGLSQQSSTSSAKTALGPRVLPKLPQKVALRKTDHLSLDKATIPPEIFQKSSQLAELPQKPPPGDLPPKPTELAPKPQIGDLPP
KPGELPPKPQLGDLPPKPQLSDLPPKPQMKDLPPKPQLGDLLAKSQTGDVSPKAQQPSEVTLKSHPLDLSPNVQSRDAIQKQASEDS
NDLTPTLPETPVPLPRKINTGKNKVRRVKTIYDCQADNDDELTFIEGEVIIVTGEEDQEWWIGHIEGQPERKGVFPVSFVHILSD
>sp|Q8N5S3|CB073_HUMAN
MEEKEDKHQQHKIEDAAITYVSENEEIKHEEKPGKSIHHSKSHVGRGRIYYAKFINTNARTYNEPFPYIDPKKGPEIQGDWWSHGKA
LEPVFLPPYDSKSTQRSDFQKPSCPLVLPVKHSKMQKPSCGIVPLASPGTSAELQNNFIEYISFIHQYDARKTPNEPLQGKRHGAFV
QREIKPGSRPTVPKGAEVLLNTPGSRSSEQSKKTEKGNSAESRMISPGLCQQNSQELLEPKTHLSETDVRQAAKACPSTPESREKTS
GATQTTVGDALFTRHKPLNPPIKKSE
>sp|Q02809|PLOD1_HUMAN
MRPLLLLALLGWLLLAEAKGDAKPEDNLLVLTVATKETEGFRRFKRSAQFFNYKIQALGLGEDWNVEKGTSAGGGQKVRLLKKALEK
HADKEDLVILFADSYDVLFASGPRELLKKFRQARSQVVFSAEELIYPDRRLETKYPVVSDGKRFLGSGGFIGYAPNLSKLVAEWEGQ
DSDSDQLFYTKIFLDPEKREQINITLDHRCRIFQNLDGALDEVVLKFEMGHVRARNLAYDTLPVLIHGNGPTKLQLNYLGNYIPRFW
TFETGCTVCDEGLRSLKGIGDEALPTVLVGVFIEQPTPFVSLFFQRLLRLHYPQKHMRLFIHNHEQHHKAQVEEFLAQHGSEYQSVK
LVGPEVRMANADARNMGADLCRQDRSCTYYFSVDADVALTEPNSLRLLIQQNKNVIAPLMTRHGRLWSNFWGALSADGYYARSEDYV
DIVQGRRVGVWNVPYISNIYLIKGSALRGELQSSDLFHHSKLDPDMAFCANIRQQDVFMFLTNRHTLGHLLSLDSYRTTHLHNDLWE
VFSNPEDWKEKYIHQNYTKALAGKLVETPCPDVYWFPIFTEVACDELVEEMEHFGQWSLGNNKDNRIQGGYENVPTIDIHMNQIGFE
REWHKFLLEYIAPMTEKLYPGYYTRAQFDLAFVVRYKPDEQPSLMPHHDASTFTINIALNRVGVDYEGGGCRFLRYNCSIRAPRKGW
TLMHPGRLTHYHEGLPTTRGTRYIAVSFVDP
>sp|Q6PI48|SYDM_HUMAN
MYFPSWLSQLYRGLSRPIRRTTQPIWGSLYRSLLQSSQRRIPEFSSFVVRTNTCGELRSSHLGQEVTLCGWIQYRRQNTFLVLRDFD
GLVQVIIPQDESAASVKKILCEAPVESVVQVSGTVISRPAGQENPKMPTGEIEIKVKTAELLNACKKLPFEIKNFVKKTEALRLQYR
YLDLRSFQMQYNLRLRSQMVMKMREYLCNLHGFVDIETPTLFKRTPGGAKEFLVPSREPGKFYSLPQSPQQFKQLLMVGGLDRYFQV
ARCYRDEGSRPDRQPEFTQIDIEMSFVDQTGIQSLIEGLLQYSWPNDKDPVVVPFPTMTFAEVLATYGTDKPDTRFGMKIIDISDVF
RNTEIGFLQDALSKPHGTVKAICIPEGAKYLKRKDIESIRNFAADHFNQEILPVFLNANRNWNSPVANFIMESQRLELIRLMETQEE
DVVLLTAGEHNKACSLLGKLRLECADLLETRGVVLRDPTLFSFLWVVDFPLFLPKEENPRELESAHHPFTAPHPSDIHLLYTEPKKA
RSQHYDLVLNGNEIGGGSIRIHNAELQRYILATLLKEDVKMLSHLLQALDYGAPPHGGIALGLDRLICLVTGSPSIRDVIAFPKSFR
GHDLMSNTPDSVPPEELKPYHIRVSKPTDSKAERAH
>sp|O95136|S1PR2_HUMAN
MGSLYSEYLNPNKVQEHYNYTKETLETQETTSRQVASAFIVILCCAIVVENLLVLIAVARNSKFHSAMYLFLGNLAASDLLAGVAFV
ANTLLSGSVTLRLTPVQWFAREGSAFITLSASVFSLLAIAIERHVAIAKVKLYGSDKSCRMLLLIGASWLISLVLGGLPILGWNCLG
HLEACSTVLPLYAKHYVLCVVTIFSIILLAIVALYVRIYCVVRSSHADMAAPQTLALLKTVTIVLGVFIVCWLPAFSILLLDYACPV
HSCPILYKAHYFFAVSTLNSLLNPVIYTWRSRDLRREVLRPLQCWRPGVGVQGRRRGGTPGHHLLPLRSSSSLERGMHMPTSPTFLE
GNTVV
>sp|Q1HG43|DOXA1_HUMAN
MATLGHTFPFYAGPKPTFPMDTTLASIIMIFLTALATFIVILPGIRGKTRLFWLLRVVTSLFIGAAILAVNFSSEWSVGQVSTNTSY
KAFSSEWISADIGLQVGLGGVNITLTGTPVQQLNETINYNEEFTWRLGENYAEEYAKALEKGLPDPVLYLAEKFTPRSPCGLYRQYR

LAGHYTSAMLWVAFLCWLLANVMLSMPVLVYGGYMLLATGIFQLLALLFFSMATSLTSPCPLHLGASVLHTHHGPAFWITLTTGLLC
VLLGLAMAVAHRMQPHRLKAFFNQSVDEDPMLEWSPEEGGLLSPRYRSMADSPKSQDIPLSEASSTKAYCKEAHPKDPDCAL
>sp|P0CI01|SPDE6_HUMAN
MDRTETRFRKRGQITGKITTSRQPHPQNEQSPQRSTSGYPLQEVVDDEMLGPSAPGVDPSPPCRSLGWKRKREWSDESEEEPEKELA
PEPEETWVVEMLCGLKMKLKQQRVSSILPEHHKDFNSQLAPGVDPSPPHRSFCWKRKMEWWDESEESLEEEPRKVLAPEPEEIWVAE
MLCGLKMKLKRRRVSLVLPEHHEAFNRLLEDPVIKRFLAWDKDLRVSDKYLLAMVIAYFSRAGFPSWQYQRIHFFLALYLANDMEED
DEDSKQNIFHFLYRKNRSRIPLLRKRWFQLGHSMNPRARKNRSRIPLLRKRRFQLYRSTNPRARKNRSRIPLLRKRRFQLYRSMNSR
ARKNRSQIVLFQKRRFHFFCSMSCRAWVSPEELEEIQAYDPEHWVWARDRAHLS
>sp|P51530|DNA2_HUMAN
MEQLNELELLMEKSFWEEAELPAELFQKKVVASFPRTVLSTGMDNRYLVLAVNTVQNKEGNCEKRLVITASQSLENKELCILRNDWC
SVPVEPGDIIHLEGDCTSDTWIIDKDFGYLILYPDMLISGTSIASSIRCMRRAVLSETFRSSDPATRQMLIGTVLHEVFQKAINNSF
APEKLQELAFQTIQEIRHLKEMYRLNLSQDEIKQEVEDYLPSFCKWAGDFMHKNTSTDFPQMQLSLPSDNSKDNSTCNIEVVKPMDI
EESIWSPRFGLKGKIDVTVGVKIHRGYKTKYKIMPLELKTGKESNSIEHRSQVVLYTLLSQERRADPEAGLLLYLKTGQMYPVPANH
LDKRELLKLRNQMAFSLFHRISKSATRQKTQLASLPQIIEEEKTCKYCSQIGNCALYSRAVEQQMDCSSVPIVMLPKIEEETQHLKQ
THLEYFSLWCLMLTLESQSKDNKKNHQNIWLMPASEMEKSGSCIGNLIRMEHVKIVCDGQYLHNFQCKHGAIPVTNLMAGDRVIVSG
EERSLFALSRGYVKEINMTTVTCLLDRNLSVLPESTLFRLDQEEKNCDIDTPLGNLSKLMENTFVSKKLRDLIIDFREPQFISYLSS
VLPHDAKDTVACILKGLNKPQRQAMKKVLLSKDYTLIVGMPGTGKTTTICTLVRILYACGFSVLLTSYTHSAVDNILLKLAKFKIGF
LRLGQIQKVHPAIQQFTEQEICRSKSIKSLALLEELYNSQLIVATTCMGINHPIFSRKIFDFCIVDEASQISQPICLGPLFFSRRFV
LVGDHQQLPPLVLNREARALGMSESLFKRLEQNKSAVVQLTVQYRMNSKIMSLSNKLTYEGKLECGSDKVANAVINLRHFKDVKLEL
EFYADYSDNPWLMGVFEPNNPVCFLNTDKVPAPEQVEKGGVSNVTEAKLIVFLTSIFVKAGCSPSDIGIIAPYRQQLKIINDLLARS
IGMVEVNTVDKYQGRDKSIVLVSFVRSNKDGTVGELLKDWRRLNVAITRAKHKLILLGCVPSLNCYPPLEKLLNHLNSEKLIIDLPS
REHESLCHILGDFQRE
>sp|Q9NRW7|VPS45_HUMAN
MNVVFAVKQYISKMIEDSGPGMKVLLMDKETTGIVSMVYTQSEILQKEVYLFERIDSQNREIMKHLKAICFLRPTKENVDYIIQELR
RPKYTIYFIYFSNVISKSDVKSLAEADEQEVVAEVQEFYGDYIAVNPHLFSLNILGCCQGRNWDPAQLSRTTQGLTALLLSLKKCPM
IRYQLSSEAAKRLAECVKQVITKEYELFEFRRTEVPPLLLILDRCDDAITPLLNQWTYQAMVHELLGINNNRIDLSRVPGISKDLRE
VVLSAENDEFYANNMYLNFAEIGSNIKNLMEDFQKKKPKEQQKLESIADMKAFVENYPQFKKMSGTVSKHVTVVGELSRLVSERNLL
EVSEVEQELACQNDHSSALQNIKRLLQNPKVTEFDAARLVMLYALHYERHSSNSLPGLMMDLRNKGVSEKYRKLVSAVVEYGGKRVR
GSDLFSPKDAVAITKQFLKGLKGVENVYTQHQPFLHETLDHLIKGRLKENLYPYLGPSTLRDRPQDIIVFVIGGATYEEALTVYNLN
RTTPGVRIVLGGTTVHNTKSFLEEVLASGLHSRSKESSQVTSRSASRR
>sp|Q14116|IL18_HUMAN
MAAEPVEDNCINFVAMKFIDNTLYFIAEDDENLESDYFGKLESKLSVIRNLNDQVLFIDQGNRPLFEDMTDSDCRDNAPRTIFIISM
YKDSQPRGMAVTISVKCEKISTLSCENKIISFKEMNPPDNIKDTKSDIIFFQRSVPGHDNKMQFESSSYEGYFLACEKERDLFKLIL
KKEDELGDRSIMFTVQNED
>sp|Q8WXU2|DYXC1_HUMAN
MPLQVSDYSWQQTKTAVFLSLPLKGVCVRDTDVFCTENYLKVNFPPFLFEAFLYAPIDDESSKAKIGNDTIVFTLYKKEAAMWETLS
VTGVDKEMMQRIREKSILQAQERAKEATEAKAAAKREDQKYALSVMMKIEEEERKKIEDMKENERIKATKALEAWKEYQRKAEEQKK
IQREEKLCQKEKQIKEERKKIKYKSLTRNLASRNLAPKGRNSENIFTEKLKEDSIPAPRSVGSIKINFTPRVFPTALRESQVAEEEE
WLHKQAEARRAMNTDIAELCDLKEEEKNPEWLKDKGNKLFATENYLAAINAYNLAIRLNNKMPLLYLNRAACHLKLKNLHKAIEDSS
KALELLMPPVTDNANARMKAHVRRGTAFCQLELYVEGLQDYEAALKIDPSNKIVQIDAEKIRNVIQGTELKS
>sp|P50458|LHX2_HUMAN
MLFHSLSGPEVHGVIDEMDRRAKSEAPAISSAIDRGDTETTMPSISSDRAALCAGCGGKISDRYYLLAVDKQWHMRCLKCCECKLNL
ESELTCFSKDGSIYCKEDYYRRFSVQRCARCHLGISASEMVMRARDLVYHLNCFTCTTCNKMLTTGDHFGMKDSLVYCRLHFEALLQ
GEYPAHFNHADVAAAAAAAAAAAKSAGLGAAGANPLGLPYYNGVGTVQKGRPRKRKSPGPGADLAAYNAALSCNENDAEHLDRDQPYP

SSQKTKRMRTSFKHHQLRTMKSYFAINHNPDAKDLKQLAQKTGLTKRVLQVWFQNARAKFRRNLLRQENTGVDKSTDAALQTGTPSG
PASELSNASLSPSSTPTTLTDLTSPTLPTVTSVLTSVPGNLEGHEPHSPSQTTLTNLF
>sp|Q9NS87|KIF15_HUMAN
MAPGCKTELRSVTNGQSNQPSNEGDAIKVFVRIRPPAERSGSADGEQNLCLSVLSSTSLRLHSNPEPKTFTFDHVADVDTTQESVFA
TVAKSIVESCMSGYNGTIFAYGQTGSGKTFTMMGPSESDNFSHNLRGVIPRSFEYLFSLIDREKEKAGAGKSFLCKCSFIEIYNEQI
YDLLDSASAGLYLREHIKKGVFVVGAVEQVVTSAAEAYQVLSGGWRNRRVASTSMNRESSRSHAVFTITIESMEKSNEIVNIRTSLL
NLVDLAGSERQKDTHAEGMRLKEAGNINRSLSCLGQVITALVDVGNGKQRHVCYRDSKLTFLLRDSLGGNAKTAIIANVHPGSRCFG
ETLSTLNFAQRAKLIKNKAVVNEDTQGNVSQLQAEVKRLKEQLAELASGQTPPESFLTRDKKKTNYMEYFQEAMLFFKKSEQEKKSL
IEKVTQLEDLTLKKEKFIQSNKMIVKFREDQIIRLEKLHKESRGGFLPEEQDRLLSELRNEIQTLREQIEHHPRVAKYAMENHSLRE
ENRRLRLLEPVKRAQEMDAQTIAKLEKAFSEISGMEKSDKNQQGFSPKAQKEPCLFANTEKLKAQLLQIQTELNNSKQEYEEFKELT
RKRQLELESELQSLQKANLNLENLLEATKACKRQEVSQLNKIHAETLKIITTPTKAYQLHSRPVPKLSPEMGSFGSLYTQNSSILDN
DILNEPVPPEMNEQAFEAISEELRTVQEQMSALQAKLDEEEHKNLKLQQHVDKLEHHSTQMQELFSSERIDWTKQQEELLSQLNVLE
KQLQETQTKNDFLKSEVHDLRVVLHSADKELSSVKLEYSSFKTNQEKEFNKLSERHMHVQLQLDNLRLENEKLLESKACLQDSYDNL
QEIMKFEIDQLSRNLQNFKKENETLKSDLNNLMELLEAEKERNNKLSLQFEEDKENSSKEILKVLEAVRQEKQKETAKCEQQMAKVQ
KLEESLLATEKVISSLEKSRDSDKKVVADLMNQIQELRTSVCEKTETIDTLKQELKDINCKYNSALVDREESRVLIKKQEVDILDLK
ETLRLRILSEDIERDMLCEDLAHATEQLNMLTEASKKHSGLLQSAQEELTKKEALIQELQHKLNQKKEEVEQKKNEYNFKMRQLEHV
MDSAAEDPQSPKTPPHFQTHLAKLLETQEQEIEDGRASKTSLEHLVTKLNEDREVKNAEILRMKEQLREMENLRLESQQLIEKNWLL
QGQLDDIKRQKENSDQNHPDNQQLKNEQEESIKERLAKSKIVEEMLKMKADLEEVQSALYNKEMECLRMTDEVERTQTLESKAFQEK
EQLRSKLEEMYEERERTSQEMEMLRKQVECLAEENGKLVGHQNLHQKIQYVVRLKKENVRLAEETEKLRAENVFLKEKKRSES
>sp|Q9NQX5|NPDC1_HUMAN
MATPLPPPSPRHLRLLRLLLSGLVLGAALRGAAAGHPDVAACPGSLDCALKRRARCPPGAHACGPCLQPFQEDQQGLCVPRMRRPPG
GGRPQPRLEDEIDFLAQELARKESGHSTPPLPKDRQRLPEPATLGFSARGQGLELGLPSTPGTPTPTPHTSLGSPVSSDPVHMSPLE
PRGGQGDGLALVLILAFCVAGAAALSVASLCWCRLQREIRLTQKADYATAKAPGSPAAPRISPGDQRLAQSAEMYHYQHQRQQMLCL
ERHKEPPKELDTASSDEENEDGDFTVYECPGLAPTGEMEVRNPLFDHAALSAPLPAPSSPPALP
>sp|Q8N9Q2|SR1IP_HUMAN
MAVPGCNKDSVRAGCKKCGYPGHLTFECRNFLRVDPKRDIVLDVSSTSSEDSDEENEELNKLQALQEKRINEEEEKKKEKSKEKIKL
KKKRKRSYSSSSTEEDTSKQKKQKYQKKEKKKEKKSKSKKGKHHKKEKKKRKKEKHSSTPNSSEFSRK
>sp|Q6NSW5|FA45B_HUMAN
MAAAELADTQLMLGVGLIEKDTNGEVLWVWCYPSTTATLRNLLLRKCCLTDENKLLHPFVFGQYRRTWFYITIIEVPDSSILKKVTH
FSIVLTAKDFNPEKYAAFTRILCRMYLKHGSPVKMMESYIAVLTKGICQSEENGSFLSKDFDARKAYPAGSIKDIVSQFGMETVILH
TALMLKKRIVVYHPKIEAVQEFTRTLPALVWHRQDWTILHSYVHLNADELEALQMCTGYVAGFVDLEVSNRPDLYDVFVNLAESEIT
IAPLAKEAMAMGKLHKEMGQLIVQSAEDPEKSESQVIQDIALKTREIFTNLAPFSEVSADGEKRVLNLEALKQKRFPPATENFLYHL
AAAEQMLKI
>sp|P10243|MYBA_HUMAN
MAKRSRSEDEDDDLQYADHDYEVPQQKGLKKLWNRVKWTRDEDDKLKKLVEQHGTDDWTLIASHLQNRSDFQCQHRWQKVLNPELIK
GPWTKEEDQRVIELVQKYGPKRWSLIAKHLKGRIGKQCRERWHNHLNPEVKKSSWTEEEDRIIYEAHKRLGNRWAEIAKLLPGRTDN
SIKNHWNSTMRRKVEQEGYLQDGIKSERSSSKLQHKPCAAMDHMQTQNQFYIPVQIPGYQYVSPEGNCIEHVQPTSAFIQQPFIDED
PDKEKKIKELEMLLMSAENEVRRKRIPSQPGSFSSWSGSFLMDDNMSNTLNSLDEHTSEFYSMDENQPVSAQQNSPTKFLAVEANAV
LSSLQTIPEFAETLELIESDPVAWSDVTSFDISDAAASPIKSTPVKLMRIQHNEGAMECQFNVSLVLEGKKNTCNGGNSEAVPLTSP
NIAKFSTPPAILRKKRKMRVGHSPGSELRDGSLNDGGNMALKHTPLKTLPFSPSQFFNTCPGNEQLNIENPSFTSTPICGQKALITT
PLHKETTPKDQKENVGFRTPTIRRSILGTTPRTPTPFKNALAAQEKKYGPLKIVSQPLAFLEEDIREVLKEETGTDLFLKEEDEPAY
KSCKQENTASGKKVRKSLVLDNWEKEESGTQLLTEDISDMQSENRFTTSLLMIPLLEIHDNRCNLIPEKQDINSTNKTYTLTKKKPN
PNTSKVVKLEKNLQSNCEWETVVYGKTEDQLIMTEQARRYLSTYTATSSTSRALIL
>sp|Q4ZG55|GREB1_HUMAN

MGNSYAGQLKTTRFEEVLHNSIEASLRSNNLVPRPIFSQLYLEAEQQLAALEGGSRVDNEEEEEEGEGGLETNGPPNPFQLHPLPEG
CCTTDGFCQAGKDLRLVSISNEPMDVPAGFLLVGVKSPSLPDHLLVCAVDKRFLPDDNGHNALLGFSGNCVGCGKKGFCYFTEFSNH
INLKLTTQPKKQKHLKYYLVRNAQGTLTKGPLICWKGSEFRSRQIPASTCSSSLFPALESTAAFPSEPVPGTNPSILMGAQQAGPAS
DHPSLNAAMGPAVFNGKDSPKCQQLAKNNLLALPRPSALGILSNSGPPKKRHKGWSPESPSAPDGGCPQGGGNRAKYESAGMSCVPQ
VGLVGPASVTFPVVASGEPVSVPDNLLKICKAKPVIFKGHGNFPYLCGNLNDVVVSPLLYTCYQNSQSVSRAYEQYGASAIQPISEE
MQLLLTVYYLVQLAADQVPLMEDLEQIFLRSWRESHLTEIRQYQQAPPQPFPPAPSAAAPVTSAQLPWLASLAASSCNDSVHVIECA
YSLAEGLSEMFRLLVEGKLAKTNYVVIICACRSAAIDSCIAVTGKYQARILSESLLTPAEYQKEVNYELVTGKVDSLGAFFSTLCPE
GDIDILLDKFHQENQGHISSSLAASSVTKAASLDVSGTPVCTSYNLEPHSIRPFQLAVAQKLLSHVCSIADSSTQNLDLGSFEKVDF
LICIPPSEVTYQQTLLHVWHSGVLLELGLKKEHMTKQRVEQYVLKLDTEAQTKFKAFLQNSFQNPHTLFVLIHDHAHWDLVSSTVHN
LYSQSDPSVGLVDRLLNCREVKEAPNIVTLHVTSFPYALQTQHTLISPYNEIHWPASCSNGVDLYHENKKYFGLSEFIESTLSGHSL
PLLRYDSSFEAMVTALGKRFPRLHSAVIRTFVLVQHYAAALMAVSGLPQMKNYTSVETLEITQNLLNSPKQCPCGHGLMVLLRVPCS
PLAVVAYERLAHVRARLALEEHFEIILGSPSSGVTVGKHFVKQLRMWQKIEDVEWRPQTYLELEGLPCILIFSGMDPHGESLPRSLR
YCDLRLINSSCLVRTALEQELGLAAYFVSNEVPLEKGARNEALESDAEKLSSTDNEDEELGTEGSTSEKRSPMKRERSRSHDSASSS
LSSKASGSALGGESSAQPTALPQGEHARSPQPRGPAEEGRAPGEKQRPRASQGPPSAISRHSPGPTPQPDCSLRTGQRSVQVSVTSS
CSQLSSSSGSSSSSVAPAAGTWVLQASQCSLTKACRQPPIVFLPKLVYDMVVSTDSSGLPKAASLLPSPSVMWASSFRPLLSKTMTS
TEQSLYYRQWTVPRPSHMDYGNRAEGRVDGFHPRRLLLSGPPQIGKTGAYLQFLSVLSRMLVRLTEVDVYDEEEININLREESDWHY
LQLSDPWPDLELFKKLPFDYIIHDPKYEDASLICSHYQGIKSEDRGMSRKPEDLYVRRQTARMRLSKYAAYNTYHHCEQCHQYMGFH
PRYQLYESTLHAFAFSYSMLGEEIQLHFIIPKSKEHHFVFSQPGGQLESMRLPLVTDKSHEYIKSPTFTPTTGRHEHGLFNLYHAMD
GASHLHVLVVKEYEMAIYKKYWPNHIMLVLPSIFNSAGVGAAHFLIKELSYHNLELERNRQEELGIKPQDIWPFIVISDDSCVMWNV
VDVNSAGERSREFSWSERNVSLKHIMQHIEAAPDIMHYALLGLRKWSSKTRASEVQEPFSRCHVHNFIILNVDLTQNVQYNQNRFLC
DDVDFNLRVHSAGLLLCRFNRFSVMKKQIVVGGHRSFHITSKVSDNSAAVVPAQYICAPDSKHTFLAAPAQLLLEKFLQHHSHLFFP
LSLKNHDHPVLSVDCYLNLGSQISVCYVSSRPHSLNISCSDLLFSGLLLYLCDSFVGASFLKKFHFLKGATLCVICQDRSSLRQTVV
RLELEDEWQFRLRDEFQTANAREDRPLFFLTGRHI
>sp|P17643|TYRP1_HUMAN
MSAPKLLSLGCIFFPLLLFQQARAQFPRQCATVEALRSGMCCPDLSPVSGPGTDRCGSSSGRGRCEAVTADSRPHSPQYPHDGRDDR
EVWPLRFFNRTCHCNGNFSGHNCGTCRPGWRGAACDQRVLIVRRNLLDLSKEEKNHFVRALDMAKRTTHPLFVIATRRSEEILGPDG
NTPQFENISIYNYFVWTHYYSVKKTFLGVGQESFGEVDFSHEGPAFLTWHRYHLLRLEKDMQEMLQEPSFSLPYWNFATGKNVCDIC
TDDLMGSRSNFDSTLISPNSVFSQWRVVCDSLEDYDTLGTLCNSTEDGPIRRNPAGNVARPMVQRLPEPQDVAQCLEVGLFDTPPFY
SNSTNSFRNTVEGYSDPTGKYDPAVRSLHNLAHLFLNGTGGQTHLSPNDPIFVLLHTFTDAVFDEWLRRYNADISTFPLENAPIGHN
RQYNMVPFWPPVTNTEMFVTAPDNLGYTYEIQWPSREFSVPEIIAIAVVGALLLVALIFGTASYLIRARRSMDEANQPLLTDQYQCY
AEEYEKLQNPNQSVV
>sp|Q684P5|RPGP2_HUMAN
MFGRKRSVSFGGFGWIDKTMLASLKVKKQELANSSDATLPDRPLSPPLTAPPTMKSSEFFEMLEKMQGIKLEEQKPGPQKNKDDYIP
YPSIDEVVEKGGPYPQVILPQFGGYWIEDPENVGTPTSLGSSICEEEEEDNLSPNTFGYKLECKGEARAYRRHFLGKDHLNFYCTGS
SLGNLILSVKCEEAEGIEYLRVILRSKLKTVHERIPLAGLSKLPSVPQIAKAFCDDAVGLRFNPVLYPKASQMIVSYDEHEVNNTFK
FGVIYQKARQTLEEELFGNNEESPAFKEFLDLLGDTITLQDFKGFRGGLDVTHGQTGVESVYTTFRDREIMFHVSTKLPFTDGDAQQ
LQRKRHIGNDIVAIIFQEENTPFVPDMIASNFLHAYIVVQVETPGTETPSYKVSVTAREDVPTFGPPLPSPPVFQKGPEFREFLLTK
LTNAENACCKSDKFAKLEDRTRAALLDNLHDELHAHTQAMLGLGPEEDKFENGGHGGFLESFKRAIRVRSHSMETMVGGQKKSHSGG
IPGSLSGGISHNSMEVTKTTFSPPVVAATVKNQSRSPIKRRSGLFPRLHTGSEGQGDSRARCDSTSSTPKTPDGGHSSQEIKSETSS
NPSSPEICPNKEKPFMKLKENGRAISRSSSSTSSVSSTAGEGEAMEEGDSGGSQPSTTSPFKQEVFVYSPSPSSESPSLGAAATPII
MSRSPTDAKSRNSPRSNLKFRFDKLSHASSGAGH
>sp|Q92734|TFG_HUMAN
MNGQLDLSGKLIIKAQLGEDIRRIPIHNEDITYDELVLMMQRVFRGKLLSNDEVTIKYKDEDGDLITIFDSSDLSFAIQCSRILKLT
LFVNGQPRPLESSQVKYLRRELIELRNKVNRLLDSLEPPGEPGPSTNIPENDTVDGREEKSASDSSGKQSTQVMAASMSAFDPLKNQ

DEINKNVMSAFGLTDDQVSGPPSAPAEDRSGTPDSIASSSSAAHPPGVQPQQPPYTGAQTQAGQIEGQMYQQYQQQAGYGAQQPQAP
PQQPQQYGIQYSASYSQQTGPQQPQQFQGYGQQPTSQAPAPAFSGQPQQLPAQPPQQYQASNYPAQTYTAQTSQPTNYTVAPASQPG
MAPSQPGAYQPRPGFTSLPGSTMTPPPSGPNPYARNRPPFGQGYTQPGPGYR
>sp|O15121|DEGS1_HUMAN
MGSRVSREDFEWVYTDQPHADRRREILAKYPEIKSLMKPDPNLIWIIIMMVLTQLGAFYIVKDLDWKWVIFGAYAFGSCINHSMTLA
IHEIAHNAAFGNCKAMWNRWFGMFANLPIGIPYSISFKRYHMDHHRYLGADGVDVDIPTDFEGWFFCTAFRKFIWVILQPLFYAFRP
LFINPKPITYLEVINTVAQVTFDILIYYFLGIKSLVYMLAASLLGLGLHPISGHFIAEHYMFLKGHETYSYYGPLNLLTFNVGYHNE
HHDFPNIPGKSLPLVRKIAAEYYDNLPHYNSWIKVLYDFVMDDTISPYSRMKRHQKGEMVLE
>sp|P57679|EVC_HUMAN
MARGGAACKSDARLLLGRDALRPAPALLAPAVLLGAALGLGLGLWLGCRAGRQRTRHQKDDTQNLLKNLESNAQTPSETGSPSRRRK
REVQMSKDKEAVDECEPPSNSNITAFALKAKVIYPINQKFRPLADGSSNPSLHENLKQAVLPHQPVEASPSSSLGSLSQGEKDDCSS
SSSVHSATSDDRFLSRTFLRVNAFPEVLACESVDVDLCIYSLHLKDLLHLDTALRQEKHMMFIQIFKMCLLDLLPKKKSDDELYQKI
LSKQEKDLEELEKGLQVKLSNTEMSGAGDSEYITLADVEKKEREYSEQLIDNMEAFWKQMANIQHFLVDQFKCSSSKARQLMMTLTE
RMIAAEGLLCDSQELQALDALERTMGRAHMAKVIEFLKLQVQEETRCRLAAISHGLELLAGEGKLSGRQKEELLTQQHKAFWQEAER
FSREFVQRGKDLVTASLAHQVEGTAKLTLAQEEEQRSFLAEAQPTADPEKFLEAFHEVLERQRLMQCDLEEEENVRATEAVVALCQE
LYFSTVDTFQKFVDALFLQTLPGMTGLPPEECDYLRQEVQENAAWQLGKSNRFRRQQWKLFQELLEQDQQVWMEECALSSVLQTHLR
EDHEGTIRGVLGRLGGLTEESTRCVLQGHDLLLRSALRRLALRGNALATLTQMRLSGKKHLLQELREQRALEQGSSQCLDEHQWQLL
RALEARVLEEASRLEEEAQQTRLQLQQRLLAEAQEVGQLLQQHMECAIGQALLVHARNAATKSRAKDRDDFKRTLMEAAVESVYVTS
AGVSRLVQAYYQQIGRIMEDHEERKLQHLKTLQGERMENYKLRKKQELSNPSSGSRTAGGAHETSQAVHQRMLSQQKRFLAQFPVHQ
QMRLHAQQQQAGVMDLLEAQLETQLQEAEQNFISELAALARVPLAESKLLPAKRGLLEKPLRTKRKKPLPQERGDLGVPNNEDLASG
DQTSGSLSSKRLSQQESEAGDSGNSKKMLKRRSNL
>sp|Q6UWJ1|TMCO3_HUMAN
MKVLGRSFFWVLFPVLPWAVQAVEHEEVAQRVIKLHRGRGVAAMQSRQWVRDSCRKLSGLLRQKNAVLNKLKTAIGAVEKDVGLSDE
EKLFQVHTFEIFQKELNESENSVFQAVYGLQRALQGDYKDVVNMKESSRQRLEALREAAIKEETEYMELLAAEKHQVEALKNMQHQN
QSLSMLDEILEDVRKAADRLEEEIEEHAFDDNKSVKGVNFEAVLRVEEEEANSKQNITKREVEDDLGLSMLIDSQNNQYILTKPRDS
TIPRADHHFIKDIVTIGMLSLPCGWLCTAIGLPTMFGYIICGVLLGPSGLNSIKSIVQVETLGEFGVFFTLFLVGLEFSPEKLRKVW
KISLQGPCYMTLLMIAFGLLWGHLLRIKPTQSVFISTCLSLSSTPLVSRFLMGSARGDKEGDIDYSTVLLGMLVTQDVQLGLFMAVM
PTLIQAGASASSSIVVEVLRILVLIGQILFSLAAVFLLCLVIKKYLIGPYYRKLHMESKGNKEILILGISAFIFLMLTVTELLDVSM
ELGCFLAGALVSSQGPVVTEEIATSIEPIRDFLAIVFFASIGLHVFPTFVAYELTVLVFLTLSVVVMKFLLAALVLSLILPRSSQYI
KWIVSAGLAQVSEFSFVLGSRARRAGVISREVYLLILSVTTLSLLLAPVLWRAAITRCVPRPERRSSL
>sp|Q9H972|CN093_HUMAN
MSFSATILFSPPSGSEARCCCCACKSETNGGNTGSQGGNPPPSTPITVTGHGLAVQSSEQLLHVIYQRVDKAVGLAEAALGLARANN
ELLKRLQEEVGDLRQGKVSIPDEDGESRAHSSPPEEPGPLKESPGEAFKALSAVEEECDSVGSGVQVVIEELRQLGAASVGPGPLGF
PATQRDMRLPGCTLAASEAAPLLNPLVDDYVASEGAVQRVLVPAYAKQLSPATQLAIQRATPETGPENGTKLPPPRPEDMLNAAAAL
DSALEESGPGSTGELRHSLGLTVSPCRTRGSGQKNSRRKRDLVLSKLVHNVHNHITNDKRFNGSESIKSSWNISVVKFLLEKLKQEL
VTSPHNYTDKELKGACVAYFLTKRREYRNSLNPFKGLKEKEEKKLRSRRYRLFANRSSIMRHFGPEDQRLWNDVTEELMSDEEDSLN
EPGVWVARPPRFRAQRLTELCYHLDANSKHGTKANRVYGPPSDRLPSAEAQLLPPELYNPNFQEEEDEGGDENAPGSPSFDQPHKTC
CPDLNSFIEIKVEKDE
>sp|Q96I51|RCC1L_HUMAN
MALVALVAGARLGRRLSGPGLGRGHWTAARRSRSRREAAEAEAEVPVVQYVGERAARADRVFVWGFSFSGALGVPSFVVPSSGPGPR
AGARPRRRIQPVPYRLELDQKISSAACGYGFTLLSSKTADVTKVWGMGLNKDSQLGFHRSRKDKTRGYEYVLEPSPVSLPLDRPQET
RVLQVSCGRAHSLVLTDREGVFSMGNNSYGQCGRKVVENEIYSESHRVHRMQDFDGQVVQVACGQDHSLFLTDKGEVYSCGWGADGQ
TGLGHYNITSSPTKLGGDLAGVNVIQVATYGDCCLAVSADGGLFGWGNSEYLQLASVTDSTQVNVPRCLHFSGVGKVRQAACGGTGC

AVLNGEGHVFVWGYGILGKGPNLVESAVPEMIPPTLFGLTEFNPEIQVSRIRCGLSHFAALTNKGELFVWGKNIRGCLGIGRLEDQY
FPWRVTMPGEPVDVACGVDHMVTLAKSFI
>sp|Q9P2M1|LR2BP_HUMAN
MKLTSEKLPKNPFYASVSQYAAKNQKFFQWKKEKTDYTHANLVDKALQLLKERILKGDTLAYFLRGQLYFEEGWYEEALEQFEEIKE
KDHQATYQLGVMYYDGLGTTLDAEKGVDYMKKILDSPCPKARHLKFAAAYNLGRAYYEGKGVKRSNEEAERLWLIAADNGNPKASVK
AQSMLGLYYSTKEPKELEKAFYWHSEACGNGNLESQGALGLMYLYGQGIRQDTEAALQCLREAAERGNVYAQGNLVEYYYKMKFFTK
CVAFSKRIADYDEVHDIPMIAQVTDCLPEFIGRGMAMASFYHARCLQLGLGITRDETTAKHYYSKACRLNPALADELHSLLIRQRI
>sp|Q01538|MYT1_HUMAN
MSLENEDKRARTRSKALRGPPETTAADLSCPTPGCTGSGHVRGKYSRHRSLQSCPLAKKRKLEGAEAEHLVSKRKSHPLKLALDEGY
GVDSDGSEDTEVKDASVSDESEGTLEGAEAETSGQDEIHRPETAEGRSPVKSHFGSNPIGSATASSKGSYSSYQGIIATSLLNLGQI
AEETLVEEDLGQAAKPGPGIVHLLQEAAEGAASEEGEKGLFIQPEDAEEVVEVTTERSQDLCPQSLEDAASEESSKQKGILSHEEED
EEEEEEEEEEEDEEEEEEEEEEEEEEEEEEEEEEEEEEEEEEEEEEAAPDVIFQEDTSHTSAQKAPELRGPESPSPKPEYSVIVEVRS
DDDKDEDTHSRKSTVTDESEMQDMMTRGNLGLLEQAIALKAEQVRTVCEPGCPPAEQSQLGLGEPGKAAKPLDTVRKSYYSKDPSRA
EKREIKCPTPGCDGTGHVTGLYPHHRSLSGCPHKDRIPPEILAMHENVLKCPTPGCTGQGHVNSNRNTHRSLSGCPIAAAEKLAKSH
EKQQPQTGDPSKSSSNSDRILRPMCFVKQLEVPPYGSYRPNVAPATPRANLAKELEKFSKVTFDYASFDAQVFGKRMLAPKIQTSET
SPKAFQCFDYSQDAEAAHMAATAILNLSTRCWEMPENLSTKPQDLPSKSVDIEVDENGTLDLSMHKHRKRENAFPSSSSCSSSPGVK
SPDASQRHSSTSAPSSSMTSPQSSQASRQDEWDRPLDYTKPSRLREEEPEESEPAAHSFASSEADDQEVSEENFEERKYPGEVTLTN
FKLKFLSKDIKKELLTCPTPGCDGSGHITGNYASHRSLSGCPLADKSLRNLMAAHSADLKCPTPGCDGSGHITGNYASHRSLSGCPR
AKKSGVKVAPTKDDKEDPELMKCPVPGCVGLGHISGKYASHRSASGCPLAARRQKEGSLNGSSFSWKSLKNEGPTCPTPGCDGSGHA
NGSFLTHRSLSGCPRATFAGKKGKLSGDEVLSPKFKTSDVLENDEEIKQLNQEIRDLNESNSEMEAAMVQLQSQISSMEKNLKNIEE
ENKLIEEQNEALFLELSGLSQALIQSLANIRLPHMEPICEQNFDAYVSTLTDMYSNQDPENKDLLESIKQAVRGIQV
>sp|A8MZA4|GG6L6_HUMAN
MLMWPQPHLPTHPHLPTHPHLPTHPHLPTHPHLPTHPMMSKETRQSKLAEAKEQLTDHHPQTNPSVGTAASDTKKKKINNGTNPETT
TSGGCHSPEDEQKASHQHQEALRRELEAQVHTIRILTCQKTELQMALYYSQHAVKQLEGEARDLISRLHDSWKFAGELEQALSAVAT
QKKKADRYIEELTKERDALSLELYRNTITDEELKEKNAELQEKLQLVESEKSEIQLNVKELKRKLERAKLLLPQQQLQAEADHLGKE
LQSVSAKLQAQVEENELWNRLNQQQEEKMWRQEEKIQEWEEKIQEQEEKIREQEEKIREQEEKMRRQEEMMWEKEEKMRRQEEMMWE
KEEKMRRQEEMMWEKEEKIRELEEKMHEQEKIREQEEKRQEEEKIREQEKRQEQEAKMWRQEEKIREQEEKIREQEKKMWRQEEKIH
EQEKIREEEKRQEQEEMWRQEEKIREQEEIWRQKEKMHEQEEKIRKQEEKVWRQEEKIREQEEKIREQEEKMWRQEEKIREQEEMWR
EEEKMHEQEKIWEEEKRQEQEDKMWRQEEKIREQEEKVWRQEEKIREQEEKRQEQEEKMWKQEEKIREQEEKIREQEEKIREQEEKI
REQEEMTQEQEEKMGEQEEKMCEQEEKMQEQEETMWRQEEKIREQEKKIREQEEKIREQEEMMQEQEEKMWEQEEKMCEQEEKMQEQ
EEKMRRQEEKMWEQEVRLRQQEEKMQEH
>sp|O14983|AT2A1_HUMAN
MEAAHAKTTEECLAYFGVSETTGLTPDQVKRNLEKYGLNELPAEEGKTLWELVIEQFEDLLVRILLLAACISFVLAWFEEGEETITA
FVEPFVILLILIANAIVGVWQERNAENAIEALKEYEPEMGKVYRADRKSVQRIKARDIVPGDIVEVAVGDKVPADIRILAIKSTTLR
VDQSILTGESVSVIKHTEPVPDPRAVNQDKKNMLFSGTNIAAGKALGIVATTGVGTEIGKIRDQMAATEQDKTPLQQKLDEFGEQLS
KVISLICVAVWLINIGHFNDPVHGGSWFRGAIYYFKIAVALAVAAIPEGLPAVITTCLALGTRRMAKKNAIVRSLPSVETLGCTSVI
CSDKTGTLTTNQMSVCKMFIIDKVDGDICLLNEFSITGSTYAPEGEVLKNDKPVRPGQYDGLVELATICALCNDSSLDFNEAKGVYE
KVGEATETALTTLVEKMNVFNTDVRSLSKVERANACNSVIRQLMKKEFTLEFSRDRKSMSVYCSPAKSSRAAVGNKMFVKGAPEGVI
DRCNYVRVGTTRVPLTGPVKEKIMAVIKEWGTGRDTLRCLALATRDTPPKREEMVLDDSARFLEYETDLTFVGVVGMLDPPRKEVTG
SIQLCRDAGIRVIMITGDNKGTAIAICRRIGIFGENEEVADRAYTGREFDDLPLAEQREACRRACCFARVEPSHKSKIVEYLQSYDE
ITAMTGDGVNDAPALKKAEIGIAMGSGTAVAKTASEMVLADDNFSTIVAAVEEGRAIYNNMKQFIRYLISSNVGEVVCIFLTAALGL
PEALIPVQLLWVNLVTDGLPATALGFNPPDLDIMDRPPRSPKEPLISGWLFFRYMAIGGYVGAATVGAAAWWFLYAEDGPHVNYSQL
THFMQCTEDNTHFEGIDCEVFEAPEPMTMALSVLVTIEMCNALNSLSENQSLLRMPPWVNIWLLGSICLSMSLHFLILYVDPLPMIF
KLRALDLTQWLMVLKISLPVIGLDEILKFVARNYLEDPEDERRK

>sp|P30475|1B39_HUMAN
MLVMAPRTVLLLLSAALALTETWAGSHSMRYFYTSVSRPGRGEPRFISVGYVDDTQFVRFDSDAASPREEPRAPWIEQEGPEYWDRN
TQICKTNTQTDRESLRNLRGYYNQSEAGSHTLQRMYGCDVGPDGRLLRGHNQFAYDGKDYIALNEDLSSWTAADTAAQITQRKWEAA
RVAEQLRTYLEGTCVEWLRRYLENGKETLQRADPPKTHVTHHPISDHEATLRCWALGFYPAEITLTWQRDGEDQTQDTELVETRPAG
DRTFQKWAAVVVPSGEEQRYTCHVQHEGLPKPLTLRWEPSSQSTVPIVGIVAGLAVLAVVVIGAVVAAVMCRRKSSGGKGGSYSQAA
SSDSAQGSDVSLTA
>sp|Q9NP61|ARFG3_HUMAN
MGDPSKQDILTIFKRLRSVPTNKVCFDCGAKNPSWASITYGVFLCIDCSGSHRSLGVHLSFIRSTELDSNWSWFQLRCMQVGGNASA
SSFFHQHGCSTNDTNAKYNSRAAQLYREKIKSLASQATRKHGTDLWLDSCVVPPLSPPPKEEDFFASHVSPEVSDTAWASAIAEPSS
LTSRPVETTLENNEGGQEQGPSVEGLNVPTKATLEVSSIIKKKPNQAKKGLGAKKGSLGAQKLANTCFNEIEKQAQAADKMKEQEDL
AKVVSKEESIVSSLRLAYKDLEIQMKKDEKMNISGKKNVDSDRLGMGFGNCRSVISHSVTSDMQTIEQESPIMAKPRKKYNDDSDDS
YFTSSSSYFDEPVELRSSSFSSWDDSSDSYWKKETSKDTETVLKTTGYSDRPTARRKPDYEPVENTDEAQKKFGNVKAISSDMYFGR
QSQADYETRARLERLSASSSISSADLFEEPRKQPAGNYSLSSVLPNAPDMAQFKQGVRSVAGKLSVFANGVVTSIQDRYGS
>sp|Q5JTD7|LRC73_HUMAN
MLPSSIQISGEPLSGAEVRDICRGLRDNAVRLLSLRGCRLCDRDFGRICRALAGATSLAQLNLNLGVVSSPSRIKQLAEALRTNRSI
QSLFLHGSPLTDAGLALLNPALALHPALVALDLGDCMLGDEAINLICGLLPPDGAKSGLKELTLSANPGITPKGWSRLAIAVAHSSQ
VRVLNLDYNPLGDHVAGMLAVAVASSRTLEVLDLEGTGLTNQSAQTLLDMVENYPTALRSLVLAENSISPELQQQICDLLSEGEEEE
EVAGGAGDTQEWERGREPAAHQRGSSSWMCPSDPSSQMVLMTSGLGDSLLAETEM
>sp|O95677|EYA4_HUMAN
MEDSQDLNEQSVKKTCTESDVSQSQNSRSMEMQDLASPHTLVGGGDTPGSSKLEKSNLSSTSVTTNGTGGENMTVLNTADWLLSCNT
PSSATMSLLAVKTEPLNSSETTATTGDGALDTFTGSVITSSGYSPRSAHQYSPQLYPSKPYPHILSTPAAQTMSAYAGQTQYSGMQQ
PAVYTAYSQTGQPYSLPTYDLGVMLPAIKTESGLSQTQSPLQSGCLSYSPGFSTPQPGQTPYSYQMPGSSFAPSSTIYANNSVSNST
NFSGSQQDYPSYTAFGQNQYAQYYSASTYGAYMTSNNTADGTPSSTSTYQLQESLPGLTNQPGEFDTMQSPSTPIKDLDERTCRSSG
SKSRGRGRKNNPSPPPDSDLERVFVWDLDETIIVFHSLLTGSYAQKYGKDPPMAVTLGLRMEEMIFNLADTHLFFNDLEECDQVHID
DVSSDDNGQDLSTYSFATDGFHAAASSANLCLPTGVRGGVDWMRKLAFRYRRVKELYNTYKNNVGGLLGPAKRDAWLQLRAEIEGLT
DSWLTNALKSLSIISTRSNCINVLVTTTQLIPALAKVLLYSLGGAFPIENIYSATKIGKESCFERIMQRFGRKVVYVVIGDGVEEEQ
AAKKHNMPFWRISSHSDLLALHQALELEYL
>sp|P09912|IFI6_HUMAN
MRQKAVSLFLCYLLLFTCSGVEAGKKKCSESSDSGSGFWKALTFMAVGGGLAVAGLPALGFTGAGIAANSVAASLMSWSAILNGGGV
PAGGLVATLQSLGAGGSSVVIGNIGALMGYATHKYLDSEEDEE
>sp|Q9NWW0|HPIP_HUMAN
MILQQPLQRGPQGGAQRLPRAALGVTWGLDASSPLRGAVPMSTKRRLEEEQEPLRKQFLSEENMATHFSQLSLHNDHPYCSPPMTFS
PALPPLRSPCSELLLWRYPGSLIPEALRLLRLGDTPSPPYPATPAGDIMEL
>sp|A6NNA5|DRGX_HUMAN
MFYFHCPPQLEGTATFGNHSSGDFDDGFLRRKQRRNRTTFTLQQLEALEAVFAQTHYPDVFTREELAMKINLTEARVQVWFQNRRAK
WRKTERGASDQEPGAKEPMAEVTPPPVRNINSPPPGDQARSKKEALEAQQSLGRTVGPAGPFFPSCLPGTLLNTATYAQALSHVASL
KGGPLCSCCVPDPMGLSFLPTYGCQSNRTASVATLRMKAREHSEAVLQSANLLPSTSSSPGPVAKPAPPDGSQEKTSPTKEQSEAEK
SV
>sp|Q9H4I2|ZHX3_HUMAN
MASKRKSTTPCMIPVKTVVLQDASMEAQPAETLPEGPQQDLPPEASAASSEAAQNPSSTDGSTLANGHRSTLDGYLYSCKYCDFRSH
DMTQFVGHMNSEHTDFNKDPTFVCSGCSFLAKTPEGLSLHNATCHSGEASFVWNVAKPDNHVVVEQSIPESTSTPDLAGEPSAEGAD
GQAEIIITKTPIMKIMKGKAEAKKIHTLKENVPSQPVGEALPKLSTGEMEVREGDHSFINGAVPVSQASASSAKNPHAANGPLIGTV
PVLPAGIAQFLSLQQQPPVHAQHHVHQPLPTAKALPKVMIPLSSIPTYNAAMDSNSFLKNSFHKFPYPTKAELCYLTVVTKYPEEQL
KIWFTAQRLKQGISWSPEEIEDARKKMFNTVIQSVPQPTITVLNTPLVASAGNVQHLIQAALPGHVVGQPEGTGGGLLVTQPLMANG

LQATSSPLPLTVTSVPKQPGVAPINTVCSNTTSAVKVVNAAQSLLTACPSITSQAFLDASIYKNKKSHEQLSALKGSFCRNQFPGQS
EVEHLTKVTGLSTREVRKWFSDRRYHCRNLKGSRAMIPGDHSSIIIDSVPEVSFSPSSKVPEVTCIPTTATLATHPSAKRQSWHQTP
DFTPTKYKERAPEQLRALESSFAQNPLPLDEELDRLRSETKMTRREIDSWFSERRKKVNAEETKKAEENASQEEEEAAEDEGGEEDL
ASELRVSGENGSLEMPSSHILAERKVSPIKINLKNLRVTEANGRNEIPGLGACDPEDDESNKLAEQLPGKVSCKKTAQQRHLLRQLF
VQTQWPSNQDYDSIMAQTGLPRPEVVRWFGDSRYALKNGQLKWYEDYKRGNFPPGLLVIAPGNRELLQDYYMTHKMLYEEDLQNLCD
KTQMSSQQVKQWFAEKMGEETRAVADTGSEDQGPGTGELTAVHKGMGDTYSEVSENSESWEPRVPEASSEPFDTSSPQAGRQLETD
>sp|O14863|ZNT4_HUMAN
MAGSGAWKRLKSMLRKDDAPLFLNDTSAFDFSDEAGDEGLSRFNKLRVVVADDGSEAPERPVNGAHPTLQADDDSLLDQDLPLTNSQ
LSLKVDSCDNCSKQREILKQRKVKARLTIAAVLYLLFMIGELVGGYIANSLAIMTDALHMLTDLSAIILTLLALWLSSKSPTKRFTF
GFHRLEVLSAMISVLLVYILMGFLLYEAVQRTIHMNYEINGDIMLITAAVGVAVNVIMGFLLNQSGHRHSHSHSLPSNSPTRGSGCE
RNHGQDSLAVRAAFVHALGDLVQSVGVLIAAYIIRFKPEYKIADPICTYVFSLLVAFTTFRIIWDTVVIILEGVPSHLNVDYIKEAL
MKIEDVYSVEDLNIWSLTSGKSTAIVHIQLIPGSSSKWEEVQSKANHLLLNTFGMYRCTIQLQSYRQEVDRTCANCQSSSP
>sp|Q16769|QPCT_HUMAN
MAGGRHRRVVGTLHLLLLVAALPWASRGVSPSASAWPEEKNYHQPAILNSSALRQIAEGTSISEMWQNDLQPLLIERYPGSPGSYAA
RQHIMQRIQRLQADWVLEIDTFLSQTPYGYRSFSNIISTLNPTAKRHLVLACHYDSKYFSHWNNRVFVGATDSAVPCAMMLELARAL
DKKLLSLKTVSDSKPDLSLQLIFFDGEEAFLHWSPQDSLYGSRHLAAKMASTPHPPGARGTSQLHGMDLLVLLDLIGAPNPTFPNFF
PNSARWFERLQAIEHELHELGLLKDHSLEGRYFQNYSYGGVIQDDHIPFLRRGVPVLHLIPSPFPEVWHTMDDNEENLDESTIDNLN
KILQVFVLEYLHL
>sp|Q9NR23|GDF3_HUMAN
MLRFLPDLAFSFLLILALGQAVQFQEYVFLQFLGLDKAPSPQKFQPVPYILKKIFQDREAAATTGVSRDLCYVKELGVRGNVLRFLP
DQGFFLYPKKISQASSCLQKLLYFNLSAIKEREQLTLAQLGLDLGPNSYYNLGPELELALFLVQEPHVWGQTTPKPGKMFVLRSVPW
PQGAVHFNLLDVAKDWNDNPRKNFGLFLEILVKEDRDSGVNFQPEDTCARLRCSLHASLLVVTLNPDQCHPSRKRRAAIPVPKLSCK
NLCHRHQLFINFRDLGWHKWIIAPKGFMANYCHGECPFSLTISLNSSNYAFMQALMHAVDPEIPQAVCIPTKLSPISMLYQDNNDNV
ILRHYEDMVVDECGCG
>sp|Q9NYV9|T2R13_HUMAN
MESALPSIFTLVIIAEFIIGNLSNGFIVLINCIDWVSKRELSSVDKLLIILAISRIGLIWEILVSWFLALHYLAIFVSGTGLRIMIF
SWIVSNHFNLWLATIFSIFYLLKIASFSSPAFLYLKWRVNKVILMILLGTLVFLFLNLIQINMHIKDWLDRYERNTTWNFSMSDFET
FSVSVKFTMTMFSLTPFTVAFISFLLLIFSLQKHLQKMQLNYKGHRDPRTKVHTNALKIVISFLLFYASFFLCVLISWISELYQNTV
IYMLCETIGVFSPSSHSFLLILGNAKLRQAFLLVAAKVWAKR
>sp|Q5JT25|RAB41_HUMAN
MSAFGHDEAWMEAGGFGLEAAERTEYQSLCKSKLLFLGEQSVGKTSIISRFMYNSFGCACQATVGIDFLSKTMYLEDQIVQLQLWDT
AGQERFHSLIPSYIRDSTIAVVVYDITNINSFKETDKWVEHVRAERGDDVVIMLLGNKIDLDNKRQVTAEQGEEKSRNLNVMFIETS
AKTGYNVKKLFRRVASALLSTRTSPPPKEGTVEIELESFEESGNRSYC
>sp|Q494R0|FBAS1_HUMAN
MAEPGGRGDYRKDGRLPSLSRSPLSTTLGTSPACGLEIPPTSGARPDGSCSLPAPVYHLKSRQWKGMGRGYRQRWRLQGRGDCDMGC
VVQASGLFPAEMRERTTKKMATSPLDLCAGACWEM
>sp|Q8WVJ2|NUDC2_HUMAN
MSAPFEERSGVVPCGTPWGQWYQTLEEVFIEVQVPPGTRAQDIQCGLQSRHVALSVGGREILKGKLFDSTIADEGTWTLEDRKMVRI
VLTKTKRDAANCWTSLLESEYAADPWVQDQMQRKLTLERFQKENPGFDFSGAEISGNYTKGGPDFSNLEK
>sp|Q9GZK7|O11A1_HUMAN
MEIVSTGNETITEFVLLGFYDIPELHFLFFIVFTAVYVFIIIGNMLIIVAVVSSQRLHKPMYIFLANLSFLDILYTSAVMPKMLEGF
LQEATISVAGCLLQFFIFGSLATAECLLLAVMAYDRYLAICYPLHYPLLMGPRRYMGLVVTTWLSGFVVDGLVVALVAQLRFCGPNH
IDQFYCDFMLFVGLACSDPRVAQVTTLILSVFCLTIPFGLILTSYARIVVAVLRVPAGASRRRAFSTCSSHLAVVTTFYGTLMIFYV
APSAVHSQLLSKVFSLLYTVVTPLFNPVIYTMRNKEVHQALRKILCIKQTETLD

>sp|Q9NUG6|PDRG1_HUMAN
MLSPEAERVLRYLVEVEELAEEVLADKRQIVDLDTKRNQNREGLRALQKDLSLSEDVMVCFGNMFIKMPHPETKEMIEKDQDHLDKE
IEKLRKQLKVKVNRLFEAQGKPELKGFNLNPLNQDELKALKVILKG
>sp|Q6IQ22|RAB12_HUMAN
MDPGAALQRRAGGGGGLGAGSPALSGGQGRRRKQPPRPADFKLQVIIIGSRGVGKTSLMERFTDDTFCEACKSTVGVDFKIKTVELR
GKKIRLQIWDTAGQERFNSITSAYYRSAKGIILVYDITKKETFDDLPKWMKMIDKYASEDAELLLVGNKLDCETDREITRQQGEKFA
QQITGMRFCEASAKDNFNVDEIFLKLVDDILKKMPLDILRNELSNSILSLQPEPEIPPELPPPRPHVRCC
>sp|O95447|LCA5L_HUMAN
MSLADLTKTNIDEHFFGVALENNRRSAACKRSPGTGDFSRNSNASNKSVDYSRSQCSCGSLSSQYDYSEDFLCDCSEKAINRNYLKQ
PVVKEKEKKKYNVSKISQSKGQKEISVEKKHTWNASLFNSQIHMIAQRRDAMAHRILSARLHKIKGLKNELADMHHKLEAILTENQF
LKQLQLRHLKAIGKYENSQNNLPQIMAKHQNEVKNLRQLLRKSQEKERTLSRKLRETDSQLLKTKDILQALQKLSEDKNLAEREELT
HKLSIITTKMDANDKKIQSLEKQLRLNCRAFSRQLAIETRKTLAAQTATKTLQVEVKHLQQKLKEKDRELEIKNIYSHRILKNLHDT
EDYPKVSSTKSVQADRKILPFTSMRHQGTQKSDVPPLTTKGKKATGNIDHKEKSTEINHEIPHCVNKLPKQEDSKRKYEDLSGEEKH
LEVQILLENTGRQKDKKEDQEKKNIFVKEEQELPPKIIEVIHPERESNQEDVLVREKFKRSMQRNGVDDTLGKGTAPYTKGPLRQRR
HYSFTEATENLHHGLPASGGPANAGNMRYSHSTGKHLSNREEMELEHSDSGYEPSFGKSSRIKVKDTTFRDKKSSLMEELFGSGYVL
KTDQSSPGVAKGSEEPLQSKESHPLPPSQASTSHAFGDSKVTVVNSIKPSSPTEGKRKIII
>sp|P24723|KPCL_HUMAN
MSSGTMKFNGYLRVRIGEAVGLQPTRWSLRHSLFKKGHQLLDPYLTVSVDQVRVGQTSTKQKTNKPTYNEEFCANVTDGGHLELAVF
HETPLGYDHFVANCTLQFQELLRTTGASDTFEGWVDLEPEGKVFVVITLTGSFTEATLQRDRIFKHFTRKRQRAMRRRVHQINGHKF
MATYLRQPTYCSHCREFIWGVFGKQGYQCQVCTCVVHKRCHHLIVTACTCQNNINKVDSKIAEQRFGINIPHKFSIHNYKVPTFCDH
CGSLLWGIMRQGLQCKICKMNVHIRCQANVAPNCGVNAVELAKTLAGMGLQPGNISPTSKLVSRSTLRRQGKESSKEGNGIGVNSSN
RLGIDNFEFIRVLGKGSFGKVMLARVKETGDLYAVKVLKKDVILQDDDVECTMTEKRILSLARNHPFLTQLFCCFQTPDRLFFVMEF
VNGGDLMFHIQKSRRFDEARARFYAAEIISALMFLHDKGIIYRDLKLDNVLLDHEGHCKLADFGMCKEGICNGVTTATFCGTPDYIA
PEILQEMLYGPAVDWWAMGVLLYEMLCGHAPFEAENEDDLFEAILNDEVVYPTWLHEDATGILKSFMTKNPTMRLGSLTQGGEHAIL
RHPFFKEIDWAQLNHRQIEPPFRPRIKSREDVSNFDPDFIKEEPVLTPIDEGHLPMINQDEFRNFSYVSPELQP
>sp|Q9UJ37|SIA7B_HUMAN
MGLPRGSFFWLLLLLTAACSGLLFALYFSAVQRYPGPAAGARDTTSFEAFFQSKASNSWTGKGGQACRHLLHLAIQRHPHFRGLFNLS
IPVLLWGDLFTPALWDRLSQHKAPYGWRGLSHQVIASTLSLLNGSESAKLFAPPRDTPPKCIRCAVVGNGGILNGSRQGPNIDAHDY
VFRLNGAVIKGFERDVGTKTSFYGFTVNTMKNSLVSYWNLGFTSVPQGQDLQYIFIPSDIRDYVMLRSAILGVPVPEGLDKGDRPHA
YFGPEASASKFKLLHPDFISYLTERFLKSKLINTHFGDLYMPSTGALMLLTALHTCDQVSAYGFITSNYWKFSDHYFERKMKPLIFY
ANHDLSLEAALWRDLHKAGILQLYQR
>sp|Q15833|STXB2_HUMAN
MAPSGLKAVVGEKILSGVIRSVKKDGEWKVLIMDHPSMRILSSCCKMSDILAEGITIVEDINKRREPIPSLEAIYLLSPTEKSVQAL
IKDFQGTPTFTYKAAHIFFTDTCPEPLFSELGRSRLAKVVKTLKEIHLAFLPYEAQVFSLDAPHSTYNLYCPFRAEERTRQLEVLAQ
QIATLCATLQEYPAIRYRKGPEDTAQLAHAVLAKLNAFKADTPSLGEGPEKTRSQLLIMDRAADPVSPLLHELTFQAMAYDLLDIEQ
DTYRYETTGLSEAREKAVLLDEDDDLWVELRHMHIADVSKKVTELLRTFCESKRLTTDKANIKDLSQILKKMPQYQKELNKYSTHLH
LADDCMKHFKGSVEKLCSVEQDLAMGSDAEGEKIKDSMKLIVPVLLDAAVPAYDKIRVLLLYILLRNGVSEENLAKLIQHANVQAHS
SLIRNLEQLGGTVTNPGGSGTSSRLEPRERMEPTYQLSRWTPVIKDVMEDAVEDRLDRNLWPFVSDPAPTASSQAAVSARFGHWHKN
KAGIEARAGPRLIVYVMGGVAMSEMRAAYEVTRATEGKWEVLIGSSHILTPTRFLDDLKALDKKLEDIALP
>sp|P33764|S10A3_HUMAN
MARPLEQAVAAIVCTFQEYAGRCGDKYKLCQAELKELLQKELATWTPTEFRECDYNKFMSVLDTNKDCEVDFVEYVRSLACLCLYCH
EYFKDCPSEPPCSQ
>sp|P04062|GLCM_HUMAN

MEFSSPSREECPKPLSRVSIMAGSLTGLLLLQAVSWASGARPCIPKSFGYSSVVCVCNATYCDSFDPPTFPALGTFSRYESTRSGRR
MELSMGPIQANHTGTGLLLTLQPEQKFQKVKGFGGAMTDAAALNILALSPPAQNLLLKSYFSEEGIGYNIIRVPMASCDFSIRTYTY
ADTPDDFQLHNFSLPEEDTKLKIPLIHRALQLAQRPVSLLASPWTSPTWLKTNGAVNGKGSLKGQPGDIYHQTWARYFVKFLDAYAE
HKLQFWAVTAENEPSAGLLSGYPFQCLGFTPEHQRDFIARDLGPTLANSTHHNVRLLMLDDQRLLLPHWAKVVLTDPEAAKYVHGIA
VHWYLDFLAPAKATLGETHRLFPNTMLFASEACVGSKFWEQSVRLGSWDRGMQYSHSIITNLLYHVVGWTDWNLALNPEGGPNWVRN
FVDSPIIVDITKDTFYKQPMFYHLGHFSKFIPEGSQRVGLVASQKNDLDAVALMHPDGSAVVVVLNRSSKDVPLTIKDPAVGFLETI
SPGYSIHTYLWRRQ
>sp|P47804|RGR_HUMAN
MAETSALPTGFGELEVLAVGMVLLVEALSGLSLNTLTIFSFCKTPELRTPCHLLVLSLALADSGISLNALVAATSSLLRRWPYGSDG
CQAHGFQGFVTALASICSSAAIAWGRYHHYCTRSQLAWNSAVSLVLFVWLSSAFWAALPLLGWGHYDYEPLGTCCTLDYSKGDRNFT
SFLFTMSFFNFAMPLFITITSYSLMEQKLGKSGHLQVNTTLPARTLLLGWGPYAILYLYAVIADVTSISPKLQMVPALIAKMVPTIN
AINYALGNEMVCRGIWQCLSPQKREKDRTK
>sp|O60294|TYW4_HUMAN
MGPRSRERRAGAVQNTNDSSALSKRSLAARGYVQDPFAALLVPGAARRAPLIHRGYYVRARAVRHCVRAFLEQIGAPQAALRAQILS
LGAGFDSLYFRLKTAGRLARAAVWEVDFPDVARRKAERIGETPELCALTGPFERGEPASALCFESADYCILGLDLRQLQRVEEALGA
AGLDAASPTLLLAEAVLTYLEPESAAALIAWAAQRFPNALFVVYEQMRPQDAFGQFMLQHFRQLNSPLHGLERFPDVEAQRRRFLQA
GWTACGAVDMNEFYHCFLPAEERRRVENIEPFDEFEEWHLKCAHYFILAASRGDTLSHTLVFPSSEAFPRVNPASPSGVFPASVVSS
EGQVPNLKRYGHASVFLSPDVILSAGGFGEQEGRHCRVSQFHLLSRDCDSEWKGSQIGSCGTGVQWDGRLYHTMTRLSESRVLVLGG
RLSPVSPALGVLQLHFFKSEDNNTEDLKVTITKAGRKDDSTLCCWRHSTTEVSCQNQEYLFVYGGRSVVEPVLSDWHFLHVGTMAWV
RIPVEGEVPEARHSHSACTWQGGALIAGGLGASEEPLNSVLFLRPISCGFLWESVDIQPPITPRYSHTAHVLNGKLLLVGGIWIHSS
SFPGVTVINLTTGLSSEYQIDTTYVPWPLMLHNHTSILLPEEQQLLLLGGGGNCFSFGTYFNPHTVTLDLSSLSAGQ
>sp|Q52MB2|CC184_HUMAN
MEDGLLEIMTKDGGDMPAPLEVSTVPAVGDVISGEYNGGMKELMEHLKAQLQALFEDVRAMRGALDEQASHIQVLSDDVCANQRAIV
SMCQIMTTAPRQGGLGVVGGKGSFQSDPQEPETPSPGIGDSGLLGRDPEDEEEEEEEEKEMPSPATPSSHCERPESPCAGLLGGDGPL
VEPLDMPDITLLQLEGEASL
>sp|O94933|SLIK3_HUMAN
MKPSIAEMLHRGRMLWIILLSTIALGWTTPIPLIEDSEEIDEPCFDPCYCEVKESLFHIHCDSKGFTNISQITEFWSRPFKLYLQRN
SMRKLYTNSFLHLNNAVSINLGNNALQDIQTGAFNGLKILKRLYLHENKLDVFRNDTFLGLESLEYLQADYNVIKRIESGAFRNLSK
LRVLILNDNLIPMLPTNLFKAVSLTHLDLRGNRLKVLFYRGMLDHIGRSLMELQLEENPWNCTCEIVQLKSWLERIPYTALVGDITC
ETPFHFHGKDLREIRKTELCPLLSDSEVEASLGIPHSSSSKENAWPTKPSSMLSSVHFTASSVEYKSSNKQPKPTKQPRTPRPPSTS
QALYPGPNQPPIAPYQTRPPIPIICPTGCTCNLHINDLGLTVNCKERGFNNISELLPRPLNAKKLYLSSNLIQKIYRSDFWNFSSLD
LLHLGNNRISYVQDGAFINLPNLKSLFLNGNDIEKLTPGMFRGLQSLHYLYFEFNVIREIQPAAFSLMPNLKLLFLNNNLLRTLPTD
AFAGTSLARLNLRKNYFLYLPVAGVLEHLNAIVQIDLNENPWDCTCDLVPFKQWIETISSVSVVGDVLCRSPENLTHRDVRTIELEV
LCPEMLHVAPAGESPAQPGDSHLIGAPTSASPYEFSPPGGPVPLSVLILSLLVLFFSAVFVAAGLFAYVLRRRRKKLPFRSKRQEGV
DLTGIQMQCHRLFEDGGGGGGGSGGGGRPTLSSPEKAPPVGHVYEYIPHPVTQMCNNPIYKPREEEEVAVSSAQEAGSAERGGPGTQ
PPGMGEALLGSEQFAETPKENHSNYRTLLEKEKEWALAVSSSQLNTIVTVNHHHPHHPAVGGVSGVVGGTGGDLAGFRHHEKNGGVV
LFPPGGGCGSGSMLLDRERPQPAPCTVGFVDCLYGTVPKLKELHVHPPGMQYPDLQQDARLKETLLFSAGKGFTDHQTQKSDYLELR
AKLQTKPDYLEVLEKTTYRF
>sp|P06702|S10A9_HUMAN
MTCKMSQLERNIETIINTFHQYSVKLGHPDTLNQGEFKELVRKDLQNFLKKENKNEKVIEHIMEDLDTNADKQLSFEEFIMLMARLT
WASHEKMHEGDEGPGHHHKPGLGEGTP
>sp|Q6P575|BGP11_HUMAN
MTAAETGRGKPRLGGGSGLGGSPAAVVWLHVGATGRDAVSPREPVVAAQAAGRPLKLPRRLLRQPTPGLRGAESGPTVDMPVPSSFN
DIGQGWRLRHFVSWLWYEREVTLLERWIQDLCTRVVLRIGSAHFYAIVWVNGVDTVEHEGGYLPFEADISSLFQVEPLPSHLCITIA

INNTLTPQPCHQGPSVHDRHLQVGTILPPLHAPTFPPHPVVFLPGTGYPKGYFVQNTDFDFFSYAGLLWSLLLYTTPPTYIDDVTVT
TGVKRDSGEGFW
>sp|Q9Y467|SALL2_HUMAN
MSRRKQRKPQQLISDCEGPSASENGDASEEDHPQVCAKCCAQFTDPTEFLAHQNACSTDPPVMVIIGGQENPNNSSASSEPRPEGHN
NPQVMDTEHSNPPDSGSSVPTDPTWGPERRGEESPGHFLVAATGTAAGGGGGLILASPKLGATPLPPESTPAPPPPPPPPPPPGVGS
GHLNIPLILEELRVLQQRQIHQMQMTEQICRQVLLLGSLGQTVGAPASPSELPGTGTASSTKPLLPLFSPIKPVQTSKTLASSSSSS
SSSSGAETPKQAFFHLYHPLGSQHPFSAGGVGRSHKPTPAPSPALPGSTDQLIASPHLAFPSTTGLLAAQCLGAARGLEATASPGLL
KPKNGSGELSYGEVMGPLEKPGGRHKCRFCAKVFGSDSALQIHLRSHTGERPYKCNVCGNRFTTRGNLKVHFHRHREKYPHVQMNPH
PVPEHLDYVITSSGLPYGMSVPPEKAEEEAATPGGGVERKPLVASTTALSATESLTLLSTSAGTATAPGLPAFNKFVLMKAVEPKNK
ADENTPPGSEGSAISGVAESSTATRMQLSKLVTSLPSWALLTNHFKSTGSFPFPYVLEPLGASPSETSKLQQLVEKIDRQGAVAVTS
AASGAPTTSAPAPSSSASSGPNQCVICLRVLSCPRALRLHYGQHGGERPFKCKVCGRAFSTRGNLRAHFVGHKASPAARAQNSCPIC
QKKFTNAVTLQQHVRMHLGGQIPNGGTALPEGGGAAQENGSEQSTVSGAGSFPQQQSQQPSPEEELSEEEEEEDEEEEEDVTDEDSL
AGRGSESGGEKAISVRGDSEEASGAEEEVGTVAAAATAGKEMDSNEKTTQQSSLPPPPPPDSLDQPQPMEQGSSGVLGGKEEGGKPE
RSSSPASALTPEGEATSVTLVEELSLQEAMRKEPGESSSRKACEVCGQAFPSQAALEEHQKTHPKEGPLFTCVFCRQGFLERATLKK
HMLLAHHQVQPFAPHGPQNIAALSLVPGCSPSITSTGLSPFPRKDDPTIP
>sp|Q8TAE7|KCNG3_HUMAN
MTFGRSGAASVVLNVGGARYSLSRELLKDFPLRRVSRLHGCRSERDVLEVCDDYDRERNEYFFDRHSEAFGFILLYVRGHGKLRFAP
RMCELSFYNEMIYWGLEGAHLEYCCQRRLDDRMSDTYTFYSADEPGVLGRDEARPGGAEAAPSRRWLERMRRTFEEPTSSLAAQILA
SVSVVFVIVSMVVLCASTLPDWRNAAADNRSLDDRSRYSAGPGREPSGIIEAICIGWFTAECIVRFIVSKNKCEFVKRPLNIIDLLA
ITPYYISVLMTVFTGENSQLQRAGVTLRVLRMMRIFWVIKLARHFIGLQTLGLTLKRCYREMVMLLVFICVAMAIFSALSQLLEHGL
DLETSNKDFTSIPAACWWVIISMTTVGYGDMYPITVPGRILGGVCVVSGIVLLALPITFIYHSFVQCYHELKFRSARYSRSLSTEFL
N
>sp|Q13217|DNJC3_HUMAN
MVAPGSVTSRLGSVFPFLLVLVDLQYEGAECGVNADVEKHLELGKKLLAAGQLADALSQFHAAVDGDPDNYIAYYRRATVFLAMGKS
KAALPDLTKVIQLKMDFTAARLQRGHLLLKQGKLDEAEDDFKKVLKSNPSENEEKEAQSQLIKSDEMQRLRSQALNAFGSGDYTAAI
AFLDKILEVCVWDAELRELRAECFIKEGEPRKAISDLKAASKLKNDNTEAFYKISTLYYQLGDHELSLSEVRECLKLDQDHKRCFAH
YKQVKKLNKLIESAEELIRDGRYTDATSKYESVMKTEPSIAEYTVRSKERICHCFSKDEKPVEAIRVCSEVLQMEPDNVNALKDRAE
AYLIEEMYDEAIQDYETAQEHNENDQQIREGLEKAQRLLKQSQKRDYYKILGVKRNAKKQEIIKAYRKLALQWHPDNFQNEEEKKKA
EKKFIDIAAAKEVLSDPEMRKKFDDGEDPLDAESQQGGGGNPFHRSWNSWQGFNPFSSGGPFRFKFHFN
>sp|Q9UHF0|TKNK_HUMAN
MRIMLLFTAILAFSLAQSFGAVCKEPQEEVVPGGGRSKRDPDLYQLLQRLFKSHSSLEGLLKALSQASTDPKESTSPEKRDMHDFFV
GLMGKRSVQPDSPTDVNQENVPSFGILKYPPRAE
>sp|Q96TA1|NIBL1_HUMAN
MGDVLSTHLDDARRQHIAEKTGKILTEFLQFYEDQYGVALFNSMRHEIEGTGLPQAQLLWRKVPLDERIVFSGNLFQHQEDSKKWRN
RFSLVPHNYGLVLYENKAAYERQVPPRAVINSAGYKILTSVDQYLELIGNSLPGTTAKSGSAPILKCPTQFPLILWHPYARHYYFCM
MTEAEQDKWQAVLQDCIRHCNNGIPEDSKVEGPAFTDAIRMYRQSKELYGTWEMLCGNEVQILSNLVMEELGPELKAELGPRLKGKP
QERQRQWIQISDAVYHMVYEQAKARFEEVLSKVQQVQPAMQAVIRTDMDQIITSKEHLASKIRAFILPKAEVCVRNHVQPYIPSILE
ALMVPTSQGFTEVRDVFFKEVTDMNLNVINEGGIDKLGEYMEKLSRLAYHPLKMQSCYEKMESLRLDGLQQRFDVSSTSVFKQRAQI
HMREQMDNAVYTFETLLHQELGKGPTKEELCKSIQRVLERVLKKYDYDSSSVRKRFFREALLQISIPFLLKKLAPTCKSELPRFQEL
IFEDFARFILVENTYEEVVLQTVMKDILQAVKEAAVQRKHNLYRDSMVMHNSDPNLHLLAEGAPIDWGEEYSNSGGGGSPSPSTPES
ATLSEKRRRAKQVVSVVQDEEVGLPFEASPESPPPASPDGVTEIRGLLAQGLRPESPPPAGPLLNGAPAGESPQPKAAPEASSPPAS
PLQHLLPGKAVDLGPPKPSDQETGEQVSSPSSHPALHTTTEDSAGVQTEF
>sp|Q96LR5|UB2E2_HUMAN

MSTEAQRVDDSPSTSGGSSDGDQRESVQQEPEREQVQPKKKEGKISSKTAAKLSTSAKRIQKELAEITLDPPPNCSAGPKGDNIYEW
RSTILGPPGSVYEGGVFFLDITFSPDYPFKPPKVTFRTRIYHCNINSQGVICLDILKDNWSPALTISKVLLSICSLLTDCNPADPLV
GSIATQYMTNRAEHDRMARQWTKRYAT
>sp|A2RUS2|DEND3_HUMAN
MRSLRKKREKPRPEQWKGLPGPPRAPEPEDVAVPGGVDLLTLPQLCFPGGVCVATEPKEDCVHFLVLTDVCGNRTYGVVAQYYRPLH
DEYCFYNGKTHRECPGCFVPFAVCVVSRFPYYNSLKDCLSCLLALLKPCKDFEVDSHIKDFAAKLSLIPSPPPGPLHLVFNMKSLQI
VLPARADPESPILDLDLHLPLLCFRPEKVLQILTCILTEQRIVFFSSDWALLTLVTECFMAYLYPLQWQHPFVPILSDQMLDFVMAP
TSFLMGCHLDHFEEVSKEADGLVLINIDHGSITYSKSTDDNVDIPDVPLLAAQTFIQRVQSLQLHHELHAAHLLSSTDLKEGRAHRR
SWQQKLNCQIQQTTLQLLVSIFRDVKNHLNYEHRVFNSEEFLKTRAPGDHQFYKQVLDTYMFHSFLKARLNRRMDAFAQMDLDTQSE
EDRINGMLLSPRRPTVEKRASRKSSHLHVTHRRMVVSMPNLQDIAMPELAPRNSSLRLTDTAGCRGSSAVLNVTPKSPYTFKIPEIH
FPLESKCVQAYHAHFVSMLSEAMCFLAPDNSLLLARYLYLRGLVYLMQGQLLNALLDFQNLYKTDIRIFPTDLVKRTVESMSAPEWE
GAEQAPELMRLISEILDKPHEASKLDDHVKKFKLPKKHMQLGDFMKRVQESGIVKDASIIHRLFEALTVGQEKQIDPETFKDFYNCW
KETEAEAQEVSLPWLVMEHLDKNECVCKLSSSVKTNLGVGKIAMTQKRLFLLTEGRPGYLEISTFRNIEEVRRTTTTFLLRRIPTLK
IRVASKKEVFEANLKTECDLWHLMVKEMWAGKKLADDHKDPHYVQQALTNVLLMDAVVGTLQSPGAIYAASKLSYFDKMSNEMPMTL
PETTLETLKHKINPSAGEAFPQAVDVLLYTPGHLDPAEKVEDAHPKLWCALSEGKVTVFNASSWTIHQHSFKVGTAKVNCMVMADQN
QVWVGSEDSVIYIINVHSMSCNKQLTAHCSSVTDLIVQDGQEAPSNVYSCSMDGMVLVWNVSTLQVTSRFQLPRGGLTSIRLHGGRL
WCCTGNSIMVMKMNGSLHQELKIEENFKDTSTSFLAFQLLPEEEQLWAACAGRSEVYIWSLKDLAQPPQRVPLEDCSEINCMIRVKK
QVWVGSRGLGQGTPKGKIYVIDAERKTVEKELVAHMDTVRTLCSAEDRYVLSGSGREEGKVAIWKGE
>sp|O94985|CSTN1_HUMAN
MLRRPAPALAPAARLLLAGLLCGGGVWAARVNKHKPWLEPTYHGIVTENDNTVLLDPPLIALDKDAPLRFAESFEVTVTKEGEICGF
KIHGQNVPFDAVVVDKSTGEGVIRSKEKLDCELQKDYSFTIQAYDCGKGPDGTNVKKSHKATVHIQVNDVNEYAPVFKEKSYKATVI
EGKQYDSILRVEAVDADCSPQFSQICSYEIITPDVPFTVDKDGYIKNTEKLNYGKEHQYKLTVTAYDCGKKRATEDVLVKISIKPTC
TPGWQGWNNRIEYEPGTGALAVFPNIHLETCDEPVASVQATVELETSHIGKGCDRDTYSEKSLHRLCGAAAGTAELLPSPSGSLNWT
MGLPTDNGHDSDQVFEFNGTQAVRIPDGVVSVSPKEPFTISVWMRHGPFGRKKETILCSSDKTDMNRHHYSLYVHGCRLIFLFRQDP
SEEKKYRPAEFHWKLNQVCDEEWHHYVLNVEFPSVTLYVDGTSHEPFSVTEDYPLHPSKIETQLVVGACWQEFSGVENDNETEPVTV
ASAGGDLHMTQFFRGNLAGLTLRSGKLADKKVIDCLYTCKEGLDLQVLEDSGRGVQIQAHPSQLVLTLEGEDLGELDKAMQHISYLN
SRQFPTPGIRRLKITSTIKCFNEATCISVPPVDGYVMVLQPEEPKISLSGVHHFARAASEFESSEGVFLFPELRIISTITREVEPEG
DGAEDPTVQESLVSEEIVHDLDTCEVTVEGEELNHEQESLEVDMARLQQKGIEVSSSELGMTFTGVDTMASYEEVLHLLRYRNWHAR
SLLDRKFKLICSELNGRYISNEFKVEVNVIHTANPMEHANHMAAQPQFVHPEHRSFVDLSGHNLANPHPFAVVPSTATVVIVVCVSF
LVFMIILGVFRIRAAHRRTMRDQDTGKENEMDWDDSALTITVNPMETYEDQHSSEEEEEEEEEEESEDGEEEDDITSAESESSEEEE
GEQGDPQNATRQQQLEWDDSTLSY
>sp|Q12792|TWF1_HUMAN
MSHQTGIQASEDVKEIFARARNGKYRLLKISIENEQLVIGSYSQPSDSWDKDYDSFVLPLLEDKQPCYILFRLDSQNAQGYEWIFIA
WSPDHSHVRQKMLYAATRATLKKEFGGGHIKDEVFGTVKEDVSLHGYKKYLLSQSSPAPLTAAEEELRQIKINEVQTDVGVDTKHQT
LQGVAFPISREAFQALEKLNNRQLNYVQLEIDIKNEIIILANTTNTELKDLPKRIPKDSARYHFFLYKHSHEGDYLESIVFIYSMPG
YTCSIRERMLYSSCKSRLLEIVERQLQMDVIRKIEIDNGDELTADFLYEEVHPKQHAHKQSFAKPKGPAGKRGIRRLIRGPAETEAT
TD
>sp|Q8IXK2|GLT12_HUMAN
MWGRTARRRCPRELRRGREALLVLLALLALAGLGSVLRAQRGAGAGAAEPGPPRTPRPGRREPVMPRPPVPANALGARGEAVRLQLQ
GEELRLQEESVRLHQINIYLSDRISLHRRLPERWNPLCKEKKYDYDNLPRTSVIIAFYNEAWSTLLRTVYSVLETSPDILLEEVILV
DDYSDREHLKERLANELSGLPKVRLIRANKREGLVRARLLGASAARGDVLTFLDCHCECHEGWLEPLLQRIHEEESAVVCPVIDVID
WNTFEYLGNSGEPQIGGFDWRLVFTWHTVPERERIRMQSPVDVIRSPTMAGGLFAVSKKYFEYLGSYDTGMEVWGGENLEFSFRIWQ
CGGVLETHPCSHVGHVFPKQAPYSRNKALANSVRAAEVWMDEFKELYYHRNPRARLEPFGDVTERKQLRDKLQCKDFKWFLETVYPE

LHVPEDRPGFFGMLQNKGLTDYCFDYNPPDENQIVGHQVILYLCHGMGQNQFFEYTSQKEIRYNTHQPEGCIAVEAGMDTLIMHLCE
ETAPENQKFILQEDGSLFHEQSKKCVQAARKESSDSFVPLLRDCTNSDHQKWFFKERML
>sp|P78312|F193A_HUMAN
MKVRLLRQLSAAAKVKAPSGLQGPPQAHQFISLLLEEYGALCQAARSISTFLGTLENEHLKKFQVTWELHNKHLFENLVFSEPLLQS
NLPALVSQIRLGTTTHDTCSEDTYSTLLQRYQRSEEELRRVAEEWLECQKRIDAYVDEQMTMKTKQRMLTEDWELFKQRRFIEEQLT
NKKAVTGENNFTDTMRHMLSSRLSMPDCPNCNYRRRCACDDCSLSHILTCGIMDPPVTDDIHIHQLPLQVDPAPDYLAERSPPSVSS
ASSGSGSSSPITIQQHPRLILTDSGSAPTFCSDDEDVAPLSAKFADIYPLSNYDDTEVVANMNGIHSELNGGGENMALKDESPQISS
TSSSSSEADDEEADGESSGEPPGAPKEDGVLGSRSPRTEESKADSPPPSYPTQQAEQAPNTCECHVCKQEASGLTPSAMTAGALPPG
HQFLSPEKPTHPALHLYPHIHGHVPLHTVPHLPRPLIHPTLYATPPFTHSKALPPAPVQNHTNKHQVFNASLQDHIYPSCFGNTPEW
NSSKFISLWGSEVMNDKNWNPGTFLPDTISGSEILGPTLSETRPEALPPPSSNETPAVSDSKEKKNAAKKKCLYNFQDAFMEANKVV
MATSSATSSVSCTATTVQSSNSQFRVSSKRPPSVGDVFHGISKEDHRHSAPAAPRNSPTGLAPLPALSPAALSPAALSPASTPHLAN
LAAPSFPKTATTTPGFVDTRKSFCPAPLPPATDGSISAPPSVCSDPDCEGHRCENGVYDPQQDDGDESADEDSCSEHSSSTSTSTNQ
KEGKYCDCCYCEFFGHGGPPAAPTSRNYAEMREKLRLRLTKRKEEQPKKMDQISERESVVDHRRVEDLLQFINSSETKPVSSTRAAK
RARHKQRKLEEKARLEAEARAREHLHLQEEQRRREEEEDEEEEEDRFKEEFQRLQELQKLRAVKKKKKERPSKDCPKLDMLTRNFQA
ATESVPNSGNIHNGSLEQTEEPETSSHSPSRHMNHSEPRPGLGADGDAADPVDTRDSKFLLPKEVNGKQHEPLSFFFDIMQHHKEGN
GKQKLRQTSKASSEPARRPTEPPKATEGQSKPRAQTESKAKVVDLMSITEQKREERKVNSNNNNKKQLNHIKDEKSNPTPMEPTSPG
EHQQNSKLVLAESPQPKGKNKKNKKKKGDRVNNSIDGVSLLLPSLGYNGAILAHCNLRLPGSSDCAASASQVVGITDDVFLPKDIDL
DSVDMDETEREVEYFKRFCLDSARQTRQRLSINWSNFSLKKATFAAH
>sp|O60884|DNJA2_HUMAN
MANVADTKLYDILGVPPGASENELKKAYRKLAKEYHPDKNPNAGDKFKEISFAYEVLSNPEKRELYDRYGEQGLREGSGGGGGMDDI
FSHIFGGGLFGFMGNQSRSRNGRRRGEDMMHPLKVSLEDLYNGKTTKLQLSKNVLCSACSGQGGKSGAVQKCSACRGRGVRIMIRQL
APGMVQQMQSVCSDCNGEGEVINEKDRCKKCEGKKVIKEVKILEVHVDKGMKHGQRITFTGEADQAPGVEPGDIVLLLQEKEHEVFQ
RDGNDLHMTYKIGLVEALCGFQFTFKHLDGRQIVVKYPPGKVIEPGCVRVVRGEGMPQYRNPFEKGDLYIKFDVQFPENNWINPDKL
SELEDLLPSRPEVPNIIGETEEVELQEFDSTRGSGGGQRREAYNDSSDEESSSHHGPGVQCAHQ
>sp|Q9P1Y5|CAMP3_HUMAN
MVEAAPPGPGPLRRTFLVPEIKSLDQYDFSRAKAAASLAWVLRAAFGGAEHVPPELWEPFYTDQYAQEHVKPPVTRLLLSAELYCRA
WRQALPQLETPPNPSALLALLARRGTVPALPERPVREADLRHQPILMGAHLAVIDALMAAFAFEWTKTLPGPLALTSLEHKLLFWVD
TTVRRLQEKTEQEAAQRASPAAPADGAAPAQPSIRYRKDRVVARRAPCFPTVTSLQDLASGAALAATIHCYCPQLLRLEEVCLKDPM
SVADSLYNLQLVQDFCASRLPRGCPLSLEDLLYVPPPLKVNLVVMLAELFMCFEVLKPDFVQVKDLPDGHAASPRGTEASPPQNNSG
SSSPVFTFRHPLLSSGGPQSPLRGSTGSLKSSPSMSHMEALGKAWNRQLSRPLSQAVSFSTPFGLDSDVDVVMGDPVLLRSVSSDSL
GPPRPAPARTPTQPPPEPGDLPTIEEALQIIHSAEPRLLPDGAADGSFYLHSPEGPSKPSLASPYLPEGTSKPLSDRPTKAPVYMPH
PETPSKPSPCLVGEASKPPAPSEGSPKAVASSPAATNSEVKMTSFAERKKQLVKAEAEAGAGSPTSTPAPPEALSSEMSELSARLEE
KRRAIEAQKRRIEAIFAKHRQRLGKSAFLQVQPREASGEAEAEAEEADSGPVPGGERPAGEGQGEPTSRPKAVTFSPDLGPVPHEGL
GEYNRAVSKLSAALSSLQRDMQRLTDQQQRLLAPPEAPGSAPPPAAWVIPGPTTGPKAASPSPARRVPATRRSPGPGPSQSPRSPKH
TRPAELRLAPLTRVLTPPHDVDSLPHLRKFSPSQVPVQTRSSILLAEETPPEEPAARPGLIEIPLGSLADPAAEDEGDGSPAGAEDS
LEEEASSEGEPRVGLGFFYKDEDKPEDEMAQKRASLLERQQRRAEEARRRKQWQEVEKEQRREEAARLAQEEAPGPAPLVSAVPMAT
PAPAARAPAEEEVGPRKGDFTRQEYERRAQLKLMDDLDKVLRPRAAGSGGPGRGGRRATRPRSGCCDDSALARSPARGLLGSRLSKI
YSQSTLSLSTVANEAHNNLGVKRPTSRAPSPSGLMSPSRLPGSRERDWENGSNASSPASVPEYTGPRLYKEPSAKSNKFIIHNALSH
CCLAGKVNEPQKNRILEEIEKSKANHFLILFRDSSCQFRALYTLSGETEELSRLAGYGPRTVTPAMVEGIYKYNSDRKRFTQIPAKT
MSMSVDAFTIQGHLWQGKKPTTPKKGGGTPK
>sp|P02753|RET4_HUMAN
MKWVWALLLLAALGSGRAERDCRVSSFRVKENFDKARFSGTWYAMAKKDPEGLFLQDNIVAEFSVDETGQMSATAKGRVRLLNNWDV
CADMVGTFTDTEDPAKFKMKYWGVASFLQKGNDDHWIVDTDYDTYAVQYSCRLLNLDGTCADSYSFVFSRDPNGLPPEAQKIVRQRQ
EELCLARQYRLIVHNGYCDGRSERNLL

>sp|Q9UHL3|F153A_HUMAN
MVDKDTERDIEMKRQLRRLRELHLYSTWKKYQEAMKTSLGVPQCERDEGSLGKPLCPPEILSETLPGSVKKRVCFPSEDHLEEFIAE
HLPEASNQSLLTVAHADAGTQTNGDLEDLEEHGPGQTVSEEATEVHTMEGDPDTLAEFLIRDVLQELSSYNGEEEDPEEVKTSLGVP
QRGDLEDLEEHVPGQTVSEEATGVHMMQVDPATLAKSDLEDLEEHVPEQTVSEEATGVHMMQVDPATLAKQLEDSTITGSHQQMSAS
PSSAPAEEATEKTKVEEEVKTRKPKKKTRKPSKKSRWNVLKCWDIFNIF
>sp|Q2M2H8|MGAL_HUMAN
MARKLSVLEVLLIIFCLIVVTIDILLLLLVLEETSDTSFTPECPEIPQSERIDCTPDQEVTEDICRWQYKCCWSPVADANVPRCFFP
WNWGYEASNGHTNTSTGFTAQLKRLPSPSLFGNDVATTLFTAEYQTSNRFHFKITDFNNIRYEVSHENINLVDGIADASNLSYYVEV
TDKPFSIKIMRTSNRRVLLDTSIGPLQFAQQYLQLSFRLPSANVYGLGEHVHQQYRHNMTWKTWPIFTRDATPTEGMINLYGAHTFF
LCLEDARGSSFGVFLMNSNAMEVTLQPAPAITYRTIGGILDFYVFLGNTPEQVVQEYLELVGRPFFPPYWSLGFQLSRRDYGGINKL
KEVVSRNRLAEIPYDVQYSDIDYMDGKKDFTVDEVAYSGLPDFVKELHDNGQKYLIIMNPGISKNSNYEPYNNGSLKRVWILGSNGF
AVGEGYPGPTVFPDYTNPVCTEWWTDQVAKFHDHLEFDGVWIEMNEVSSLLQASNNQCESNNLNFPPFLPRVLDHLLFARTLCMDTE
FHGGLHYDIHSLYGHSMARTTNLALETIFMNNRSFILSRSTFAGSGKFAAHWLGDNAATWDDLRWSIPTILEFNLFGIPMVGANICG
YNNNVTEELCRRWMQLGAFYPLPRNHNGPGFRDQDPAAFGVDSLLLKSSRHYLNIRYTLLPYLYTLFYHAHTRGETVARPLVHEFYQ
DSATWDVHEQFLWGPGLLITPVLYEGVDEVKAYIPDATWYDYETGVAISWRKQLVNMLLPGDKIGLHLRGGYIFPTQKPNTTTEASR
RNSLGLIIALDYKREAKGELYWDDGVSKDAVTEKKYILYDFSVTSNHLQAKIINNNYMDTDNLMFTDITILGMDKQPANFIVLLNNV
ATSSPSVVYNASTKVVTITDLQGLVLGQEFSIRWNLPVSDLEKFNCYPDDPTASEESCRQRGCLWEDTSTPGVPTCYYDTIPNYVAS
DIQYLNTSITADLSLPMAPESAAAAASDSLSAKISFLHLKVIYHTATMLQVKIYDPTNKRYEVPVPLNTPPQPVGDPENRLYDVRIQ
NNPFGIQIQRKNSSTVIWDSQLPGFIFNDMFLSISTRLPSQYIYGFGETEHTTFRRNMNWNTWGMFAHDEPPAYKKNSYGVHPYYMA
LEEDGSAHGVLLLNSNAMDVTLQPTPALTYRTTGGILDFYIVLGPTPELVTQQYTELIGRPAMIPYWALGFHLSRYGYQNDAEISSL
YDAMVAAQIPYDVQHVDIDYMNRKLDFTLSANFQNLSLLIEQMKKNGMRFILILDPAISGNETQYLPFIRGQENNVFIKWPDTNDIV
WGKVWPDLPNVIVDGSLDHETQVKLYRAYVAFPDFFRNSTAAWWKKEIEELYANPREPEKSLKFDGLWIDMNEPSNFVDGSVRGCSN
EMLNNPPYMPYLESRDKGLSSKTLCMESQQILPDSSPVEHYNVHNLYGWSQTRPTYEAVQEVTGQRGVIITRSTFPSSGRWGGHRLG
NNTAAWDQLGKSIIGMMEFSLFGIPYTGADICGFFGDAEYEMCVRWMQLGAFYPFSRNHNNIGTRRQDPVAWNSTFEMLSRKVLETR
YTLLPYLYTLMHKAHVEGSTVVRPLLHEFTDDRTTWDIDRQFMLGPAILISPVLETSTFEISAYFPRARWYDYSTGTSSTSTGQRKI
LKAPLDHINLHVRGGYILPWQEPAMNTHSSRQNFMGLIVALDDNGTAEGQVFWDDGQSIDTYENGNYFLANFIAAQNILQIQTIHNK
YLSDSNPLKVGYIRIWGVNTYVTQVSFTYDNRQFMETNFKSEPYNQILTIQLTDKTINLEKLTEVTWIDGGPVLPTPTKTSTIPMSS
HPSPSTTNATSSETITSSASANTTTGTTDTVPITTTSFPSTTSVTTNTTVPDTTSPFPTSTTNASTNATVPITTTPFPTSTIGVTTN
ATVPNTTAPFPTNASTASTNATVPITTTCFATSTIGVTTNATVPDTTAPFPTNTTTASTNATIPITTTPFATSTISVTTSTTVPDTT
APFPTSTTSASTNATPVPITTTLFATSTIGVTTGTTVPDTTAPFPTSTTSTSTSATVPITTTPSPTNTADANTSNTVPNTTMPSPTS
STTVSTIATVPISVTPSLTSTADATISTTVLIATTSSLTGTTDVSTSTTINNISTPVQTNTTNASTSTNVANITATSHTSTDDTVPN
NTVPVTAIPSLANTGVDTTSNSFSIMTTSFSESTNAMNTTVIMATTSPTSTDVASTNNDASMTNFLLATMSAGNITSNSISITTTSF
GNSVPFVTTPSPSTDATTTSNNTNPGMTTYYQTSPTIPTHTLTSIPSSITSILSMFPTSNTFTTDKITNFTTPTNANTIIFNTLDTK
STMVIDATVTTTSTKDNTMSPDTTVTSIDKFTTHITQFATPHSATTTTLALSHTSLAPTNLSNLGTMDITDADNSSSVTGNTTHISV
SNLTTASVTITATGLDSQTPHMVINSVATYLPITATSATTDTTNITKYALNTTTPDSTVHTSATAPTYIANAINATQVP
>sp|Q70EL2|UBP45_HUMAN
MRVKDPTKALPEKAKRSKRPTVPHDEDSSDDIAVGLTCQHVSHAISVNHVKRAIAENLWSVCSECLKERRFYDGQLVLTSDIWLCLK
CGFQGCGKNSESQHSLKHFKSSRTEPHCIIINLSTWIIWCYECDEKLSTHCNKKVLAQIVDFLQKHASKTQTSAFSRIMKLCEEKCE
TDEIQKGGKCRNLSVRGITNLGNTCFFNAVMQNLAQTYTLTDLMNEIKESSTKLKIFPSSDSQLDPLVVELSRPGPLTSALFLFLHS
MKETEKGPLSPKVLFNQLCQKAPRFKDFQQQDSQELLHYLLDAVRTEETKRIQASILKAFNNPTTKTADDETRKKVKAYGKEGVKMN
FIDRIFIGELTSTVMCEECANISTVKDPFIDISLPIIEERVSKPLLWGRMNKYRSLRETDHDRYSGNVTIENIHQPRAAKKHSSSKD
KSQLIHDRKCIRKLSSGETVTYQKNENLEMNGDSLMFASLMNSESRLNESPTDDSEKEASHSESNVDADSEPSESESASKQTGLFRS
SSGSGVQPDGPLYPLSAGKLLYTKETDSGDKEMAEAISELRLSSTVTGDQDFDRENQPLNISNNLCFLEGKHLRSYSPQNAFQTLSQ
SYITTSKECSIQSCLYQFTSMELLMGNNKLLCENCTKNKQKYQEETSFAEKKVEGVYTNARKQLLISAVPAVLILHLKRFHQAGLSL

RKVNRHVDFPLMLDLAPFCSATCKNASVGDKVLYGLYGIVEHSGSMREGHYTAYVKVRTPSRKLSEHNTKKKNVPGLKAADNESAGQ
WVHVSDTYLQVVPESRALSAQAYLLFYERVL
>sp|Q8N612|F16A2_HUMAN
MERMNWLSRLASRGPGHRIPQGANLQTPVMADPETCLMVFKNHWSQVVRILERQGPRAAPGGADDLSAVRNHTYQMLTLLAEDRAVP
SAPTGPGPLLEFALHEDLLTRVLTWQLQWDELGDGVEERRAEQLKLFEMLVSEARQPLLRHGPVREALLTLLDACGRPVPSSPALDE
GLVLLLSQLCVCVAQEPSLLEFFLQPPPEPGAAPRLLLFSRLVPFVHREGTLGQQARDALLLLMALSAGSPTVGRYIADHSYFCPVL
ATGLSALYSSLPRKIEVPGDDWHCLRREDWLGVPALALFMSSLEFCNAVIQVAHPLVQKQLVDYIHNGFLVPVMGPALHKTSVEEMI
ASTAYLELFLRSISEPALLRTFLRFLLLHRHDTHTILDTLVARIGSNSRLCMVSLSLFRTLLNLSCEDVLLQLVLRYLVPCNHVMLS
QKPAVRDVDLYGRAADKFLSLIPRCCRHHAPSPPRPEHASWARGPGSPSVDSSSVTTVPRPSTPSRLALFLRQQSLGGSESPGPAPC
SPGLSASPASSPGRRPTPAEEPGELEDNYLEYLREARRGVDRCVRACRTWSAPYDGERPSPEPSPFGSRTKKRSLLPEEDRNNVGEG
EEEELGRRGRAGGAGEGPGHLPPPQLNGVPGSWPEGAKKVRLVPKEGAGELLEGISEGMAGLEGFGQELRELEVALSNGGTGSESPL
EPPLPLEEEEAYESFTCPPEPPGPFLSSPLRTLNQLPSQPFTGPFMAVLFAKLENMLQNSVYVNFLLTGLVAQLACHPQPLLRSFLL
NTNMVFQPSVKSLLQVLGSVKNKIENFAASQEDFPALLSKAKKYLIARGKLDWAEGPAAGPAPRRSDPLVKSRRPSLGELLLRHAHS
PTRARQAAQLVLQPGRDGAGLGLSGGSPGASTPVLLTRGGAPERQGEALRVKNAVYCAVIFPEFLKELAAISQAHAVTSPFLLETSE
EGSGPLISGCGPLNP
>sp|Q06732|ZN33B_HUMAN
MNKVDQKFQGSVSFKDVTVGFTQEEWQHLDPSQRALYRDVMLENYSNLVSVGYCAHKPEVIFRLEQGEEPWRLEEEFPSQSFPEVWT
ADHLKERSQENQSKHLWEVVFINNEMLTKEQGNVIGIPFNMDVSSFPSRKMFCQYDSRGMSFNTVSELVISKINYLGKKSDEFNACG
KLLLNIKHDETHTREKNEVLKNRNTLSHRENTLQHEKIQTLDHNFEYSICQETLLEKAVFNTRKRENAEENNCDYNEFGRTFCDSSS
LLFHQIPPSKDSHYEFSDCEKFLCVKSTLSKHDGVPVKHYDCGESGNNFRRKLCLSQLQKGDKGEKHFECNECGKAFWEKSHLTRHQ
RVHTGEKHFQCNQCGKTFWEKSNLTKHQRSHTGEKPFECNECGKAFSHKSALTLHQRTHTGEKPYQCNACGKTFYQKSDLTKHQRTH
TGQKPYECYECGKSFCMNSHLTVHQRTHTGEKPFECLECGKSFCQKSHLTQHQRTHIGDKPYECNACGKTFYHKSVLTRHQIIHTGL
KPYECYECGKTFCLKSDLTIHQRTHTGEKPFACPECGKFFSHKSTLSQHYRTHTGEKPYECHECGKIFYNKSYLTKHNRTHTGEKPY
ECNECGKTFCQKSQLTQHQRIHIGEKPYECNECGKAFCHKSALIVHQRTHTQEKPYKCNECGKSFCVKSGLILHERKHTGEKPYECN
ECGKSFSHKSSLTVHHRAHTGEKSCQCNECGKIFYRKSDLAKHQRSHTGEKPYECNTCRKTFSQKSNLIVHQRTHIGEKPYE
>sp|Q96KR4|LMLN_HUMAN
MVTTLGPKMAAEWGGGVGYSGSGPGRSRWRWSGSVWVRSVLLLLGGLRASATSTPVSLGSSPPCRHHVPSDTEVINKVHLKANHVVK
RDVDEHLRIKTVYDKSVEELLPEKKNLVKNKLFPQAISYLEKTFQVRRPAGTILLSRQCATNQYLRKENDPHRYCTGECAAHTKCGP
VIVPEEHLQQCRVYRGGKWPHGAVGVPDQEGISDADFVLYVGALATERCSHENIISYAAYCQQEANMDRPIAGYANLCPNMISTQPQ
EFVGMLSTVKHEVIHALGFSAGLFAFYHDKDGNPLTSRFADGLPPFNYSLGLYQWSDKVVRKVERLWDVRDNKIVRHTVYLLVTPRV
VEEARKHFDCPVLEGMELENQGGVGTELNHWEKRLLENEAMTGSHTQNRVLSRITLALMEDTGRQMLSPYCDTLRSNPLQLTCRQDQ
RAVAVCNLQKFPKPLPQEYQYFDELSGIPAEDLPYYGGSVEIADYCPFSQEFSWHLSGEYQRSSDCRILENQPEIFKNYGAEKYGPH
SVCLIQKSAFVMEKCERKLSYPDWGSGCYQVSCSPQGLKVWVQDTSYLCSRAGQVLPVSIQMNGWIHDGNLLCPSCWDFCELCPPET
DPPATNLTRALPLDLCSCSSSLVVTLWLLLGNLFPLLAGFLLCIWH
>sp|P52815|RM12_HUMAN
MLPAAARPLWGPCLGLRAAAFRLARRQVPCVCAVRHMRSSGHQRCEALAGAPLDNAPKEYPPKIQQLVQDIASLTLLEISDLNELLK
KTLKIQDVGLVPMGGVMSGAVPAAAAQEAVEEDIPIAKERTHFTVRLTEAKPVDKVKLIKEIKNYIQGINLVQAKKLVESLPQEIKA
NVAKAEAEKIKAALEAVGGTVVLE
>sp|Q99445|GML_HUMAN
MLLFALLLAMELPLVAASATMRAQWTYSLRCHDCAVINDFNCPNIRVCPYHIRRCMTISIRINSRELLVYKNCTNNCTFVYAAEQPP
EAPGKIFKTNSFYWVCCCNSMVCNAGGPTNLERDMLPDEVTEEELPEGTVRLGVSKLLLSFASIIVSNILP
>sp|Q92947|GCDH_HUMAN
MALRGVSVRLLSRGPGLHVLRTWVSSAAQTEKGGRTQSQLAKSSRPEFDWQDPLVLEEQLTTDEILIRDTFRTYCQERLMPRILLAN
RNEVFHREIISEMGELGVLGPTIKGYGCAGVSSVAYGLLARELERVDSGYRSAMSVQSSLVMHPIYAYGSEEQRQKYLPQLAKGELL

GCFGLTEPNSGSDPSSMETRAHYNSSNKSYTLNGTKTWITNSPMADLFVVWARCEDGCIRGFLLEKGMRGLSAPRIQGKFSLRASAT
GMIIMDGVEVPEENVLPGASSLGGPFGCLNNARYGIAWGVLGASEFCLHTARQYALDRMQFGVPLARNQLIQKKLADMLTEITLGLH
ACLQLGRLKDQDKAAPEMVSLLKRNNCGKALDIARQARDMLGGNGISDEYHVIRHAMNLEAVNTYEGTHDIHALILGRAITGIQAFT
ASK
>sp|Q06889|EGR3_HUMAN
MTGKLAEKLPVTMSSLLNQLPDNLYPEEIPSALNLFSGSSDSVVHYNQMATENVMDIGLTNEKPNPELSYSGSFQPAPGNKTVTYLG
KFAFDSPSNWCQDNIISLMSAGILGVPPASGALSTQTSTASMVQPPQGDVEAMYPALPPYSNCGDLYSEPVSFHDPQGNPGLAYSPQ
DYQSAKPALDSNLFPMIPDYNLYHHPNDMGSIPEHKPFQGMDPIRVNPPPITPLETIKAFKDKQIHPGFGSLPQPPLTLKPIRPRKY
PNRPSKTPLHERPHACPAEGCDRRFSRSDELTRHLRIHTGHKPFQCRICMRSFSRSDHLTTHIRTHTGEKPFACEFCGRKFARSDER
KRHAKIHLKQKEKKAEKGGAPSASSAPPVSLAPVVTTCA
>sp|P51587|BRCA2_HUMAN
MPIGSKERPTFFEIFKTRCNKADLGPISLNWFEELSSEAPPYNSEPAEESEHKNNNYEPNLFKTPQRKPSYNQLASTPIIFKEQGLT
LPLYQSPVKELDKFKLDLGRNVPNSRHKSLRTVKTKMDQADDVSCPLLNSCLSESPVVLQCTHVTPQRDKSVVCGSLFHTPKFVKGR
QTPKHISESLGAEVDPDMSWSSSLATPPTLSSTVLIVRNEEASETVFPHDTTANVKSYFSNHDESLKKNDRFIASVTDSENTNQREA
ASHGFGKTSGNSFKVNSCKDHIGKSMPNVLEDEVYETVVDTSEEDSFSLCFSKCRTKNLQKVRTSKTRKKIFHEANADECEKSKNQV
KEKYSFVSEVEPNDTDPLDSNVANQKPFESGSDKISKEVVPSLACEWSQLTLSGLNGAQMEKIPLLHISSCDQNISEKDLLDTENKR
KKDFLTSENSLPRISSLPKSEKPLNEETVVNKRDEEQHLESHTDCILAVKQAISGTSPVASSFQGIKKSIFRIRESPKETFNASFSG
HMTDPNFKKETEASESGLEIHTVCSQKEDSLCPNLIDNGSWPATTTQNSVALKNAGLISTLKKKTNKFIYAIHDETSYKGKKIPKDQ
KSELINCSAQFEANAFEAPLTFANADSGLLHSSVKRSCSQNDSEEPTLSLTSSFGTILRKCSRNETCSNNTVISQDLDYKEAKCNKE
KLQLFITPEADSLSCLQEGQCENDPKSKKVSDIKEEVLAAACHPVQHSKVEYSDTDFQSQKSLLYDHENASTLILTPTSKDVLSNLV
MISRGKESYKMSDKLKGNNYESDVELTKNIPMEKNQDVCALNENYKNVELLPPEKYMRVASPSRKVQFNQNTNLRVIQKNQEETTSI
SKITVNPDSEELFSDNENNFVFQVANERNNLALGNTKELHETDLTCVNEPIFKNSTMVLYGDTGDKQATQVSIKKDLVYVLAEENKN
SVKQHIKMTLGQDLKSDISLNIDKIPEKNNDYMNKWAGLLGPISNHSFGGSFRTASNKEIKLSEHNIKKSKMFFKDIEEQYPTSLAC
VEIVNTLALDNQKKLSKPQSINTVSAHLQSSVVVSDCKNSHITPQMLFSKQDFNSNHNLTPSQKAEITELSTILEESGSQFEFTQFR
KPSYILQKSTFEVPENQMTILKTTSEECRDADLHVIMNAPSIGQVDSSKQFEGTVEIKRKFAGLLKNDCNKSASGYLTDENEVGFRG
FYSAHGTKLNVSTEALQKAVKLFSDIENISEETSAEVHPISLSSSKCHDSVVSMFKIENHNDKTVSEKNNKCQLILQNNIEMTTGTF
VEEITENYKRNTENEDNKYTAASRNSHNLEFDGSDSSKNDTVCIHKDETDLLFTDQHNICLKLSGQFMKEGNTQIKEDLSDLTFLEV
AKAQEACHGNTSNKEQLTATKTEQNIKDFETSDTFFQTASGKNISVAKESFNKIVNFFDQKPEELHNFSLNSELHSDIRKNKMDILS
YEETDIVKHKILKESVPVGTGNQLVTFQGQPERDEKIKEPTLLGFHTASGKKVKIAKESLDKVKNLFDEKEQGTSEITSFSHQWAKT
LKYREACKDLELACETIEITAAPKCKEMQNSLNNDKNLVSIETVVPPKLLSDNLCRQTENLKTSKSIFLKVKVHENVEKETAKSPAT
CYTNQSPYSVIENSALAFYTSCSRKTSVSQTSLLEAKKWLREGIFDGQPERINTADYVGNYLYENNSNSTIAENDKNHLSEKQDTYL
SNSSMSNSYSYHSDEVYNDSGYLSKNKLDSGIEPVLKNVEDQKNTSFSKVISNVKDANAYPQTVNEDICVEELVTSSSPCKNKNAAI
KLSISNSNNFEVGPPAFRIASGKIVCVSHETIKKVKDIFTDSFSKVIKENNENKSKICQTKIMAGCYEALDDSEDILHNSLDNDECS
THSHKVFADIQSEEILQHNQNMSGLEKVSKISPCDVSLETSDICKCSIGKLHKSVSSANTCGIFSTASGKSVQVSDASLQNARQVFS
EIEDSTKQVFSKVLFKSNEHSDQLTREENTAIRTPEHLISQKGFSYNVVNSSAFSGFSTASGKQVSILESSLHKVKGVLEEFDLIRT
EHSLHYSPTSRQNVSKILPRVDKRNPEHCVNSEMEKTCSKEFKLSNNLNVEGGSSENNHSIKVSPYLSQFQQDKQQLVLGTKVSLVE
NIHVLGKEQASPKNVKMEIGKTETFSDVPVKTNIEVCSTYSKDSENYFETEAVEIAKAFMEDDELTDSKLPSHATHSLFTCPENEEM
VLSNSRIGKRRGEPLILVGEPSIKRNLLNEFDRIIENQEKSLKASKSTPDGTIKDRRLFMHHVSLEPITCVPFRTTKERQEIQNPNF
TAPGQEFLSKSHLYEHLTLEKSSSNLAVSGHPFYQVSATRNEKMRHLITTGRPTKVFVPPFKTKSHFHRVEQCVRNINLEENRQKQN
IDGHGSDDSKNKINDNEIHQFNKNNSNQAAAVTFTKCEEEPLDLITSLQNARDIQDMRIKKKQRQRVFPQPGSLYLAKTSTLPRISL
KAAVGGQVPSACSHKQLYTYGVSKHCIKINSKNAESFQFHTEDYFGKESLWTGKGIQLADGGWLIPSNDGKAGKEEFYRALCDTPGV
DPKLISRIWVYNHYRWIIWKLAAMECAFPKEFANRCLSPERVLLQLKYRYDTEIDRSRRSAIKKIMERDDTAAKTLVLCVSDIISLS
ANISETSSNKTSSADTQKVAIIELTDGWYAVKAQLDPPLLAVLKNGRLTVGQKIILHGAELVGSPDACTPLEAPESLMLKISANSTR
PARWYTKLGFFPDPRPFPLPLSSLFSDGGNVGCVDVIIQRAYPIQWMEKTSSGLYIFRNEREEEKEAAKYVEAQQKRLEALFTKIQE

EFEEHEENTTKPYLPSRALTRQQVRALQDGAELYEAVKNAADPAYLEGYFSEEQLRALNNHRQMLNDKKQAQIQLEIRKAMESAEQK
EQGLSRDVTTVWKLRIVSYSKKEKDSVILSIWRPSSDLYSLLTEGKRYRIYHLATSKSKSKSERANIQLAATKKTQYQQLPVSDEIL
FQIYQPREPLHFSKFLDPDFQPSCSEVDLIGFVVSVVKKTGLAPFVYLSDECYNLLAIKFWIDLNEDIIKPHMLIAASNLQWRPESK
SGLLTLFAGDFSVFSASPKEGHFQETFNKMKNTVENIDILCNEAENKLMHILHANDPKWSTPTKDCTSGPYTAQIIPGTGNKLLMSS
PNCEIYYQSPLSLCMAKRKSVSTPVSAQMTSKSCKGEKEIDDQKNCKKRRALDFLSRLPLPPPVSPICTFVSPAAQKAFQPPRSCGT
KYETPIKKKELNSPQMTPFKKFNEISLLESNSIADEELALINTQALLSGSTGEKQFISVSESTRTAPTSSEDYLRLKRRCTTSLIKE
QESSQASTEECEKNKQDTITTKKYI
>sp|Q16772|GSTA3_HUMAN
MAGKPKLHYFNGRGRMEPIRWLLAAAGVEFEEKFIGSAEDLGKLRNDGSLMFQQVPMVEIDGMKLVQTRAILNYIASKYNLYGKDIK
ERALIDMYTEGMADLNEMILLLPLCRPEEKDAKIALIKEKTKSRYFPAFEKVLQSHGQDYLVGNKLSRADISLVELLYYVEELDSSL
ISNFPLLKALKTRISNLPTVKKFLQPGSPRKPPADAKALEEARKIFRF
>sp|Q15784|NDF2_HUMAN
MLTRLFSEPGLLSDVPKFASWGDGEDDEPRSDKGDAPPPPPPAPGPGAPGPARAAKPVPLRGEEGTEATLAEVKEEGELGGEEEEEE
EEEEGLDEAEGERPKKRGPKKRKMTKARLERSKLRRQKANARERNRMHDLNAALDNLRKVVPCYSKTQKLSKIETLRLAKNYIWALS
EILRSGKRPDLVSYVQTLCKGLSQPTTNLVAGCLQLNSRNFLTEQGADGAGRFHGSGGPFAMHPYPYPCSRLAGAQCQAAGGLGGGA
AHALRTHGYCAAYETLYAAAGGGGASPDYNSSEYEGPLSPPLCLNGNFSLKQDSSPDHEKSYHYSMHYSALPGSRPTGHGLVFGSSA
VRGGVHSENLLSYDMHLHHDRGPMYEELNAFFHN
>sp|O14776|TCRG1_HUMAN
MAERGGDGGESERFNPGELRMAQQQALRFRGPAPPPNAVMRGPPPLMRPPPPFGMMRGPPPPPRPPFGRPPFDPNMPPMPPPGGIPP
PMGPPHLQRPPFMPPPMSSMPPPPGMMFPPGMPPVTAPGTPALPPTEEIWVENKTPDGKVYYYNARTRESAWTKPDGVKVIQQSELT
PMLAAQAQVQAQAQAQAQAQAQAQAQAQAQAQAQAQAQAQAQAQAQAQAQAQAQAQAQAQAQAQVQAQVQAQVQAQAVGASTP
TTSSPAPAVSTSTSSSTPSSTTSTTTTATSVAQTVSTPTTQDQTPSSAVSVATPTVSVSTPAPTATPVQTVPQPHPQTLPPAVPHSV
PQPTTAIPAFPPVMVPPFRVPLPGMPIPLPGVAMMQIVSCPYVKTVATTKTGVLPGMAPPIVPMIHPQVAIAASPATLAGATAVSEW
TEYKTADGKTYYYNNRTLESTWEKPQELKEKEKLEEKIKEPIKEPSEEPLPMETEEEDPKEEPIKEIKEEPKEEEMTEEEKAAQKAK
PVATAPIPGTPWCVVWTGDERVFFYNPTTRLSMWDRPDDLIGRADVDKIIQEPPHKKGMEELKKLRHPTPTMLSIQKWQFSMSAIKE
EQELMEEINEDEPVKAKKRKRDDNKDIDSEKEAAMEAEIKAARERAIVPLEARMKQFKDMLLERGVSAFSTWEKELHKIVFDPRYLL
LNPKERKQVFDQYVKTRAEEERREKKNKIMQAKEDFKKMMEEAKFNPRATFSEFAAKHAKDSRFKAIEKMKDREALFNEFVAAARKK
EKEDSKTRGEKIKSDFFELLSNHHLDSQSRWSKVKDKVESDPRYKAVDSSSMREDLFKQYIEKIAKNLDSEKEKELERQARIEASLR
EREREVQKARSEQTKEIDREREQHKREEAIQNFKALLSDMVRSSDVSWSDTRRTLRKDHRWESGSLLEREEKEKLFNEHIEALTKKK
REHFRQLLDETSAITLTSTWKEVKKIIKEDPRCIKFSSSDRKKQREFEEYIRDKYITAKADFRTLLKETKFITYRSKKLIQESDQHL
KDVEKILQNDKRYLVLDCVPEERRKLIVAYVDDLDRRGPPPPPTASEPTRRSTK
>sp|O60361|NDK8_HUMAN
MQCGLVGKIIKRFEQKGFRLVAMKFLPASEEHLKQHYIDLKDRPFFPGLVKYMNSGPVVAMVWEGLNVVKTGRVMLGETNPADSKPG
TIRGDFCIQVGRNIIHGSDSVKSAEKEISLRFKPEELVDYKSCAHDWVYE
>sp|Q9NXF1|TEX10_HUMAN
MTKKRKRQHDFQKVKLKVGKKKPKLQNATPTNFKTKTIHLPEQLKEDGTLPTNNRKLNIKDLLSQMHHYNAGVKQSALLGLKDLLSQ
YPFIIDAHLSNILSEVTAVFTDKDANVRLAAVQLLQFLAPKIRAEQISPFFPLVSAHLSSAMTHITEGIQEDSLKVLDILLEQYPAL
ITGRSSILLKNFVELISHQQLSKGLINRDRSQSWILSVNPNRRLTSQQWRLKVLVRLSKFLQALADGSSRLRESEGLQEQKENPHAT
SNSIFINWKEHANDQQHIQVYENGGSQPNVSSQFRLRYLVGGLSGVDEGLSSTENLKGFIEIIIPLLIECWVEAVPPQLATPVGNGI
EREPLQVMQQVLNIISLLWKLSKQQDETHKLESWLRKNYLIDFKHHFMSRFPYVLKEITKHKRKEPNKSIKHCTVLSNNIDHLLLNL
TLSDIMVSLANASTLQKDCSWIEMIRKFVTETLEDGSRLNSKQLNRLLGVSWRLMQIQPNREDTETLIKAVYTLYQQRGLILPVRTL
LLKFFSKIYQTEELRSCRFRYRSKVLSRWLAGLPLQLAHLGSRNPELSTQLIDIIHTAAARANKELLKSLQATALRIYDPQEGAVVV
LPADSQQRLVQLVYFLPSLPADLLSRLSRCCIMGRLSSSLAAMLIGILHMRSSFSGWKYSAKDWLMSDVDYFSFLFSTLTGFSKEEL
TWLQSLRGVPHVIQTQLSPVLLYLTDLDQFLHHWDVTEAVFHSLLVIPARSQNFDILQSAISKHLVGLTVIPDSTAGCVFGVICKLL

DHTCVVSETLLPFLASCCYSLLYFLLTIEKGEAEHLRKRDKLWGVCVSILALLPRVLRLMLQSLRVNRVGPEELPVVGQLLRLLLQH
APLRTHMLTNAILVQQIIKNITTLKSGSVQEQWLTDLHYCFNVYITGHPQGPSALATVY
>sp|P0DMV2|CT459_HUMAN
MTDKTEKVAVDPETVFKRPRECDSPSYQKRQRMALLARKQGAGDSLIAGSAMSKEKKLMTGHAIPPSQLDSQIDDFTGFSKDRMMQK
PGSNAPVGGNVTSSFSGDDLECRETAFSPKSQQEINADIKRQLVKELRCVGQKYEKIFEMLEGVQGPTAVRKRFFESIIKEAARCMR
RDFVKHLKKKLKRMI
>sp|Q9H0C3|TM117_HUMAN
MGKDFRYYFQHPWSRMIVAYLVIFFNFLIFAEDPVSHSQTEANVIVVGNCFSFVTNKYPRGVGWRILKVLLWLLAILTGLIAGKFLF
HQRLFGQLLRLKMFREDHGSWMTMFFSTILFLFIFSHIYNTILLMDGNMGAYIITDYMGIRNESFMKLAAVGTWMGDFVTAWMVTDM
MLQDKPYPDWGKSARAFWKKGNVRITLFWTVLFTLTSVVVLVITTDWISWDKLNRGFLPSDEVSRAFLASFILVFDLLIVMQDWEFP
HFMGDVDVNLPGLHTPHMQFKIPFFQKIFKEEYRIHITGKWFNYGIIFLVLILDLNMWKNQIFYKPHEYGQYIGPGQKIYTVKDSES
LKDLNRTKLSWEWRSNHTNPRTNKTYVEGDMFLHSRFIGASLDVKCLAFVPSLIAFVWFGFFIWFFGRFLKNEPRMENQDKTYTRMK
RKSPSEHSKDMGITRENTQASVEDPLNDPSLVCIRSDFNEIVYKSSHLTSENLSSQLNESTSATEADQDPTTSKSTPTN
>sp|Q5T1A1|DCST2_HUMAN
MPKVMKDVVHPLGGEEPSMARAVVRSVGGFTLGLSLATAYGLLELLVEGHSPWGCLVGTLTLAAFLSLGMGFSRQVRATVLLLLPQA
FSRQGRTLLLVAAFGLVLQGPCANTLRNFTRASEAVACGAELALNQTAEVLQRAKQPLVSALNKIKAIARKTKEVADRVRKFFRSIM
DGVKHIARALRNVWQWLLHIGDVCNSELGNPYLKCARVFDDAKDSCMMVIPQAYHLCYVLMPFKLALCGLASLVQVFCVIPKYIQPF
LRQTIGTPVIQLLNRVRQEFEFNMTATHHFSVDLNASRSLSQVAMDLHEAVSMKLHRVREALALMGFTTPLLLVLLYLQALFYRYCY
LNWDHYDNIYITSRFLRMEAVRSTAGLPTVLPLSAHEARRYIPPGSIFLSQWEKFFYILETFNLIRHLLLVLFLVFLDYAVFWVLDL
ARHQLQGEIVARSPVLVSLTVEGTGYAGNIYRDLVSAFDVLQQGNISILSRRCLLRPSEPDSTGYIVIGVMYGLCFFITLFGSYVSR
LRRVICASYYPSREQERISYLYNVLLSRRTNLLAALHRSVRRRAADQGHRSAFLVLASRCPCLGPFVSHFWLHQAYCLGCGQPQDEG
DMENTVSCSTPGCQGLYCLTCFRLLDNTCSVCASPLSYQGDLDLELDSSDEEGPQLWLAAAQRKDPEQAWLLQQQLQEVLGRSLSME
STSESSDLDEEKGPQQRKHGQQPLPEAHQPVSILTSPEPHRPPETSSATKGAPTPASEPSVPLSPPSLPDPSHPPPK
>sp|Q8IUI8|CRLF3_HUMAN
MRGAMELEPELLLQEARENVEAAQSYRRELGHRLEGLREARRQIKESASQTRDVLKQHFNDLKGTLGKLLDERLVTLLQEVDTIEQE
TIKPLDDCQKLIEHGVNTAEDLVREGEIAMLGGVGEENEKLWSFTKKASHIQLDSLPEVPLLVDVPCLSAQLDDSILNIVKDHIFKH
GTVASRPPVQIEELIEKPGGIIVRWCKVDDDFTAQDYRLQFRKCTSNHFEDVYVGSETEFIVLHIDPNVDYQFRVCARGDGRQEWSP
WSVPQIGHSTLVPHEWTAGFEGYSLSSRRNIALRNDSESSGVLYSRAPTYFCGQTLTFRVETVGQPDRRDSIGVCAEKQDGYDSLQR
DQAVCISTNGAVFVNGKEMTNQLPAVTSGSTVTFDIEAVTLGTTSNNEGGHFKLRVTISSNNREVVFDWLLDQSCGSLYFGCSFFYP
GWKVLVF
>sp|Q86X02|CDR2L_HUMAN
MRRAAGMEDFSAEEEESWYDQQDLEQDLHLAAELGKTLLERNKELEGSLQQMYSTNEEQVQEIEYLTKQLDTLRHVNEQHAKVYEQL
DLTARDLELTNHRLVLESKAAQQKIHGLTETIERLQAQVEELQAQVEQLRGLEQLRVLREKRERRRTIHTFPCLKELCTSPRCKDAF
RLHSSSLELGPRPLEQENERLQTLVGALRSQVSQERQRKERAEREYTAVLQEYSELERQLCEMEACRLRVQELEAELLELQQMKQAK
TYLLGPDDHLAEALLAPLTQAPEADDPQPGRGDDLGAQDGVSSPAASPGHVVRKSCSDTALNAIVAKDPASRHAGNLTLHANSVRKR
GMSILREVDEQYHALLEKYEELLSKCRQHGAGVRHAGVQTSRPISRDSSWRDLRGGEEGQGEVKAGEKSLSQHVEAVDKRLEQSQPE
YKALFKEIFSRIQKTKADINATKVKTHSSK
>sp|Q9BUP3|HTAI2_HUMAN
MAETEALSKLREDFRMQNKSVFILGASGETGRVLLKEILEQGLFSKVTLIGRRKLTFDEEAYKNVNQEVVDFEKLDDYASAFQGHDV
GFCCLGTTRGKAGAEGFVRVDRDYVLKSAELAKAGGCKHFNLLSSKGADKSSNFLYLQVKGEVEAKVEELKFDRYSVFRPGVLLCDR
QESRPGEWLVRKFFGSLPDSWASGHSVPVVTVVRAMLNNVVRPRDKQMELLENKAIHDLGKAHGSLKP
>sp|Q2VIR3|IF2GL_HUMAN
MAGGEAGVTLGQPHLSRQDLTTLDVTKLTPLSHEVISRQATINIGTIGHVAHGKSTVVKAISGVHTVRFKNELERNITIKLGYANAK
IYQLDDPSCPRPECYRSCGSSMPDEFPTDIPGTKGNFRLVRHVSFVDCPGHDILMATMLNGAAVMDAALLLIAGNESCPQPQTSEHL

AAIEIMKLKHILILQNKIDLVKERQAKEQYEQILAFVQGTVAEGAPIIPISAQLKYNIEVVCEYIVKKIPVPPRDFTSEPRLIVIRS
FDVNKPGCEVDDLKGGVAGGSILKGVLKVGQETEVRPGIVSKDSEGKLMCKSIFSKIVSLFAEHNDLQYAAPGGLIGVGTKIDPTLC
RADRMVGQILGAVGALPEIFTELEISYFLLRRLLGVRTEGDKKAAKVQKLSKNEVLMVNIGSLSTGGRVSAVKADLGKIVLTNPVCT
EVGEKIALSRRVEKHWRLIGWGQIRRGVTIKPTVDDD
>sp|Q9HCN8|SDF2L_HUMAN
MWSAGRGGAAWPVLLGLLLALLVPGGGAAKTGAELVTCGSVLKLLNTHHRVRLHSHDIKYGSGSGQQSVTGVEASDDANSYWRIRGG
SEGGCPRGSPVRCGQAVRLTHVLTGKNLHTHHFPSPLSNNQEVSAFGEDGEGDDLDLWTVRCSGQHWEREAAVRFQHVGTSVFLSVT
GEQYGSPIRGQHEVHGMPSANTHNTWKAMEGIFIKPSVEPSAGHDEL
>sp|Q8NGL0|OR5L2_HUMAN
MGKENCTTVAEFILLGLSDVPELRVCLFLLFLLIYGVTLLANLGMTALIQVSSRLHTPVYFFLSHLSFVDFCYSSIIVPKMLANIFN
KDKAISFLGCMVQFYLFCTCGVTEVFLLAVMAYDRFVAICNPLLYMVTMSQKLRVELTSCCYFCGTVCSLIHSSLALRILFYRSNVI
NHFFCDLPPLLSLACSDVTVNETLLFLVATLNESVTIMIILTSYLLILTTILKIHSAESRHKAFSTCASHLTAITVSHGTILYIYCR
PSSGNSGDVDKVATVFYTVVIPMLNPLIYSLRNKDVNKALRKVMGSKIHS
>sp|O43889|CREB3_HUMAN
MELELDAGDQDLLAFLLEESGDLGTAPDEAVRAPLDWALPLSEVPSDWEVDDLLCSLLSPPASLNILSSSNPCLVHHDHTYSLPRET
VSMDLGECEISLTGRTGFMGLAIHTFPFAESESCRKEGTQMTPQHMEELAEQEIARLVLTDEEKSLLEKEGLILPETLPLTKTEEQI
LKRVRRKIRNKRSAQESRRKKKVYVGGLESRVLKYTAQNMELQNKVQLLEEQNLSLLDQLRKLQAMVIEISNKTSSSSTCILVLLVS
FCLLLVPAMYSSDTRGSLPAEHGVLSRQLRALPSEDPYQLELPALQSEVPKDSTHQWLDGSDCVLQAPGNTSCLLHYMPQAPSAEPP
LEWPFPDLFSEPLCRGPILPLQANLTRKGGWLPTGSPSVILQDRYSG
>sp|P06132|DCUP_HUMAN
MEANGLGPQGFPELKNDTFLRAAWGEETDYTPVWCMRQAGRYLPEFRETRAAQDFFSTCRSPEACCELTLQPLRRFPLDAAIIFSDI
LVVPQALGMEVTMVPGKGPSFPEPLREEQDLERLRDPEVVASELGYVFQAITLTRQRLAGRVPLIGFAGAPWTLMTYMVEGGGSSTM
AQAKRWLYQRPQASHQLLRILTDALVPYLVGQVVAGAQALQLFESHAGHLGPQLFNKFALPYIRDVAKQVKARLREAGLAPVPMIIF
AKDGHFALEELAQAGYEVVGLDWTVAPKKARECVGKTVTLQGNLDPCALYASEEEIGQLVKQMLDDFGPHRYIANLGHGLYPDMDPE
HVGAFVDAVHKHSRLLRQN
>sp|P16234|PGFRA_HUMAN
MGTSHPAFLVLGCLLTGLSLILCQLSLPSILPNENEKVVQLNSSFSLRCFGESEVSWQYPMSEEESSDVEIRNEENNSGLFVTVLEV
SSASAAHTGLYTCYYNHTQTEENELEGRHIYIYVPDPDVAFVPLGMTDYLVIVEDDDSAIIPCRTTDPETPVTLHNSEGVVPASYDS
RQGFNGTFTVGPYICEATVKGKKFQTIPFNVYALKATSELDLEMEALKTVYKSGETIVVTCAVFNNEVVDLQWTYPGEVKGKGITML
EEIKVPSIKLVYTLTVPEATVKDSGDYECAARQATREVKEMKKVTISVHEKGFIEIKPTFSQLEAVNLHEVKHFVVEVRAYPPPRIS
WLKNNLTLIENLTEITTDVEKIQEIRYRSKLKLIRAKEEDSGHYTIVAQNEDAVKSYTFELLTQVPSSILDLVDDHHGSTGGQTVRC
TAEGTPLPDIEWMICKDIKKCNNETSWTILANNVSNIITEIHSRDRSTVEGRVTFAKVEETIAVRCLAKNLLGAENRELKLVAPTLR
SELTVAAAVLVLLVIVIISLIVLVVIWKQKPRYEIRWRVIESISPDGHEYIYVDPMQLPYDSRWEFPRDGLVLGRVLGSGAFGKVVE
GTAYGLSRSQPVMKVAVKMLKPTARSSEKQALMSELKIMTHLGPHLNIVNLLGACTKSGPIYIITEYCFYGDLVNYLHKNRDSFLSH
HPEKPKKELDIFGLNPADESTRSYVILSFENNGDYMDMKQADTTQYVPMLERKEVSKYSDIQRSLYDRPASYKKKSMLDSEVKNLLS
DDNSEGLTLLDLLSFTYQVARGMEFLASKNCVHRDLAARNVLLAQGKIVKICDFGLARDIMHDSNYVSKGSTFLPVKWMAPESIFDN
LYTTLSDVWSYGILLWEIFSLGGTPYPGMMVDSTFYNKIKSGYRMAKPDHATSEVYEIMVKCWNSEPEKRPSFYHLSEIVENLLPGQ
YKKSYEKIHLDFLKSDHPAVARMRVDSDNAYIGVTYKNEEDKLKDWEGGLDEQRLSADSGYIIPLPDIDPVPEEEDLGKRNRHSSQT
SEESAIETGSSSSTFIKREDETIEDIDMMDDIGIDSSDLVEDSFL
>sp|O15079|SNPH_HUMAN
MAMSLPGSRRTSAGSRRRTSPPVSVRDAYGTSSLSSSSNSGSYKGSDSSPTPRRSMKYTLCSDNHGIKPPTPEQYLTPLQQKEVCIR
HLKARLKDTQDRLQDRDTEIDDLKTQLSRMQEDWIEEECHRVEAQLALKEARKEIKQLKQVIDTVKNNLIDKDKGLQKYFVDINIQN
KKLETLLHSMEVAQNGMAKEDGTGESAGGSPARSLTRSSTYTKLSDPAVCGDRQPGDPSSGSAEDGADSGFAAADDTLSRTDALEAS
SLLSSGVDCGTEETSLHSSFGLGPRFPASNTYEKLLCGMEAGVQASCMQERAIQTDFVQYQPDLDTILEKVTQAQVCGTDPESGDRC

PELDAHPSGPRDPNSAVVVTVGDELEAPEPITRGPTPQRPGANPNPGQSVSVVCPMEEEEEAAVAEKEPKSYWSRHYIVDLLAVVVP
AVPTVAWLCRSQRRQGQPIYNISSLLRGCCTVALHSIRRISCRSLSQPSPSPAGGGSQL
>sp|Q13882|PTK6_HUMAN
MVSRDQAHLGPKYVGLWDFKSRTDEELSFRAGDVFHVARKEEQWWWATLLDEAGGAVAQGYVPHNYLAERETVESEPWFFGCISRSE
AVRRLQAEGNATGAFLIRVSEKPSADYVLSVRDTQAVRHYKIWRRAGGRLHLNEAVSFLSLPELVNYHRAQSLSHGLRLAAPCRKHE
PEPLPHWDDWERPREEFTLCRKLGSGYFGEVFEGLWKDRVQVAIKVISRDNLLHQQMLQSEIQAMKKLRHKHILALYAVVSVGDPVY
IITELMAKGSLLELLRDSDEKVLPVSELLDIAWQVAEGMCYLESQNYIHRDLAARNILVGENTLCKVGDFGLARLIKEDVYLSHDHN
IPYKWTAPEALSRGHYSTKSDVWSFGILLHEMFSRGQVPYPGMSNHEAFLRVDAGYRMPCPLECPPSVHKLMLTCWCRDPEQRPCFK
ALRERLSSFTSYENPT
>sp|Q4ZJI4|SL9B1_HUMAN
MHTTESKNEHLEDENFQTSTTPQSLIDPNNTAQEETKTVLSDTEEIKPQTKKETYISCPLRGVLNVIITNGVILFVIWCMTWSILGS
EALPGGNLFGLFIIFYSAIIGGKILQLIRIPLVPPLPPLLGMLLAGFTIRNVPFINEHVHVPNTWSSILRSIALTIILIRAGLGLDP
QALRHLKVVCFRLAVGPCLMEASAAAVFSHFIMKFPWQWAFLLGFVLGAVSPAVVVPYMMVLQENGYGVEEGIPTLLMAASSMDDIL
AITGFNTCLSIVFSSGGILNNAIASIRNVCISLLAGIVLGFFVRYFPSEDQKKLTLKRGFLVLTMCVSAVLGSQRIGLHGSGGLCTL
VLSFIAGTKWSQEKMKVQKIITTVWDIFQPLLFGLVGAEVSVSSLESNIVGISVATLSLALCVRILTTYLLMCFAGFSFKEKIFIAL
AWMPKATVQAVLGPLALETARVSAPHLEPYAKDVMTVAFLAILITAPNGALLMGILGPKMLTRHYDPSKIKLQLSTLEHH
>sp|O00570|SOX1_HUMAN
MYSMMMETDLHSPGGAQAPTNLSGPAGAGGGGGGGGGGGGGGGGAKANQDRVKRPMNAFMVWSRGQRRKMAQENPKMHNSEISKRLGA
EWKVMSEAEKRPFIDEAKRLRALHMKEHPDYKYRPRRKTKTLLKKDKYSLAGGLLAAGAGGGGAAVAMGVGVGVGAAAVGQRLESPG
GAAGGGYAHVNGWANGAYPGSVAAAAAAAAMMQEAQLAYGQHPGAGGAHPHAHPAHPHPHHPHAHPHNPQPMHRYDMGALQYSPISN
SQGYMSASPSGYGGLPYGAAAAAAAAAGGAHQNSAVAAAAAAAAASSGALGALGSLVKSEPSGSPPAPAHSRAPCPGDLREMISMYL
PAGEGGDPAAAAAAAAQSRLHSLPQHYQGAGAGVNGTVPLTHI
>sp|Q92835|SHIP1_HUMAN
MVPCWNHGNITRSKAEELLSRTGKDGSFLVRASESISRAYALCVLYRNCVYTYRILPNEDDKFTVQASEGVSMRFFTKLDQLIEFYK
KENMGLVTHLQYPVPLEEEDTGDDPEEDTVESVVSPPELPPRNIPLTASSCEAKEVPFSNENPRATETSRPSLSETLFQRLQSMDTS
GLPEEHLKAIQDYLSTQLAQDSEFVKTGSSSLPHLKKLTTLLCKELYGEVIRTLPSLESLQRLFDQQLSPGLRPRPQVPGEANPINM
VSKLSQLTSLLSSIEDKVKALLHEGPESPHRPSLIPPVTFEVKAESLGIPQKMQLKVDVESGKLIIKKSKDGSEDKFYSHKKILQLI
KSQKFLNKLVILVETEKEKILRKEYVFADSKKREGFCQLLQQMKNKHSEQPEPDMITIFIGTWNMGNAPPPKKITSWFLSKGQGKTR
DDSADYIPHDIYVIGTQEDPLSEKEWLEILKHSLQEITSVTFKTVAIHTLWNIRIVVLAKPEHENRISHICTDNVKTGIANTLGNKG
AVGVSFMFNGTSLGFVNSHLTSGSEKKLRRNQNYMNILRFLALGDKKLSPFNITHRFTHLFWFGDLNYRVDLPTWEAETIIQKIKQQ
QYADLLSHDQLLTERREQKVFLHFEEEEITFAPTYRFERLTRDKYAYTKQKATGMKYNLPSWCDRVLWKSYPLVHVVCQSYGSTSDI
MTSDHSPVFATFEAGVTSQFVSKNGPGTVDSQGQIEFLRCYATLKTKSQTKFYLEFHSSCLESFVKSQEGENEEGSEGELVVKFGET
LPKLKPIISDPEYLLDQHILISIKSSDSDESYGEGCIALRLEATETQLPIYTPLTHHGELTGHFQGEIKLQTSQGKTREKLYDFVKT
ERDESSGPKTLKSLTSHDPMKQWEVTSRAPPCSGSSITEIINPNYMGVGPFGPPMPLHVKQTLSPDQQPTAWSYDQPPKDSPLGPCR
GESPPTPPGQPPISPKKFLPSTANRGLPPRTQESRPSDLGKNAGDTLPQEDLPLTKPEMFENPLYGSLSSFPKPAPRKDQESPKMPR
KEPPPCPEPGILSPSIVLTKAQEADRGEGPGKQVPAPRLRSFTCSSSAEGRAAGGDKSQGKPKTPVSSQAPVPAKRPIKPSRSEINQ
QTPPTPTPRPPLPVKSPAVLHLQHSKGRDYRDNTELPHHGKHRPEEGPPGPLGRTAMQ
>sp|O75351|VPS4B_HUMAN
MSSTSPNLQKAIDLASKAAQEDKAGNYEEALQLYQHAVQYFLHVVKYEAQGDKAKQSIRAKCTEYLDRAEKLKEYLKNKEKKAQKPV
KEGQPSPADEKGNDSDGEGESDDPEKKKLQNQLQGAIVIERPNVKWSDVAGLEGAKEALKEAVILPIKFPHLFTGKRTPWRGILLFG
PPGTGKSYLAKAVATEANNSTFFSISSSDLVSKWLGESEKLVKNLFQLARENKPSIIFIDEIDSLCGSRSENESEAARRIKTEFLVQ
MQGVGVDNDGILVLGATNIPWVLDSAIRRRFEKRIYIPLPEPHARAAMFKLHLGTTQNSLTEADFRELGRKTDGYSGADISIIVRDA
LMQPVRKVQSATHFKKVRGPSRADPNHLVDDLLTPCSPGDPGAIEMTWMDVPGDKLLEPVVSMSDMLRSLSNTKPTVNEHDLLKLKK
FTEDFGQEG

>sp|Q9Y2L1|RRP44_HUMAN
MLKSKTFLKKTRAGGVMKIVREHYLRDDIGCGAPGCAACGGAHEGPALEPQPQDPASSVCPQPHYLLPDTNVLLHQIDVLEDPAIRN
VIVLQTVLQEVRNRSAPVYKRIRDVTNNQEKHFYTFTNEHHRETYVEQEQGENANDRNDRAIRVAAKWYNEHLKKMSADNQLQVIFI
TNDRRNKEKAIEEGIPAFTCEEYVKSLTANPELIDRLACLSEEGNEIESGKIIFSEHLPLSKLQQGIKSGTYLQGTFRASRENYLEA
TVWIHGDNEENKEIILQGLKHLNRAVHEDIVAVELLPKSQWVAPSSVVLHDEGQNEEDVEKEEETERMLKTAVSEKMLKPTGRVVGI
IKRNWRPYCGMLSKSDIKESRRHLFTPADKRIPRIRIETRQASTLEGRRIIVAIDGWPRNSRYPNGHFVRNLGDVGEKETETEVLLL
EHDVPHQPFSQAVLSFLPKMPWSITEKDMKNREDLRHLCICSVDPPGCTDIDDALHCRELENGNLEVGVHIADVSHFIRPGNALDQE
SARRGTTVYLCEKRIDMVPELLSSNLCSLKCDVDRLAFSCIWEMNHNAEILKTKFTKSVINSKASLTYAEAQLRIDSANMNDDITTS
LRGLNKLAKILKKRRIEKGALTLSSPEVRFHMDSETHDPIDLQTKELRETNSMVEEFMLLANISVAKKIHEEFSEHALLRKHPAPPP
SNYEILVKAARSRNLEIKTDTAKSLAESLDQAESPTFPYLNTLLRILATRCMMQAVYFCSGMDNDFHHYGLASPIYTHFTSPIRRYA
DVIVHRLLAVAIGADCTYPELTDKHKLADICKNLNFRHKMAQYAQRASVAFHTQLFFKSKGIVSEEAYILFVRKNAIVVLIPKYGLE
GTVFFEEKDKPNPQLIYDDEIPSLKIEDTVFHVFDKVKVKIMLDSSNLQHQKIRMSLVEPQIPGISIPTDTSNMDLNGPKKKKMKLG
K
>sp|Q8N1E6|FXL14_HUMAN
METHISCLFPELLAMIFGYLDVRDKGRAAQVCTAWRDAAYHKSVWRGVEAKLHLRRANPSLFPSLQARGIRRVQILSLRRSLSYVIQ
GMANIESLNLSGCYNLTDNGLGHAFVQEIGSLRALNLSLCKQITDSSLGRIAQYLKGLEVLELGGCSNITNTGLLLIAWGLQRLKSL
NLRSCRHLSDVGIGHLAGMTRSAAEGCLGLEQLTLQDCQKLTDLSLKHISRGLTGLRLLNLSFCGGISDAGLLHLSHMGSLRSLNLR
SCDNISDTGIMHLAMGSLRLSGLDVSFCDKVGDQSLAYIAQGLDGLKSLSLCSCHISDDGINRMVRQMHGLRTLNIGQCVRITDKGL
ELIAEHLSQLTGIDLYGCTRITKRGLERITQLPCLKVLNLGLWQMTDSEKEARGDFSPLFTVRTRGSSRR
>sp|Q8NEK5|ZN548_HUMAN
MNLTEGRVVFEDVAIYFSQEEWGHLDEAQRLLYRDVMLENLALLSSLGSWHGAEDEEAPSQQGFSVGVSEVTASKPCLSSQKVHPSE
TCGPPLKDILCLVEHNGIHPEQHIYICEAELFQHPKQQIGENLSRGDDWIPSFGKNHRVHMAEEIFTCMEGWKDLPATSCLLQHQGP
QSEWKPYRDTEDREAFQTGQNDYKCSECGKTFTCSYSFVEHQKIHTGERSYECNKCGKFFKYSANFMKHQTVHTSERTYECRECGKS
FMYNYRLMRHKRVHTGERPYECNTCGKFFRYSSTFVRHQRVHTGERPYECRECGKFFMDSSTLIKHQRVHTGERPYKCNDCGKFFRY
ISTLIRHQRIHTGERPYECSVCGELFRYNSSLVKHWRNHTGERPYKCSECGKSFRYHCRLIRHQRVHTGERPYECSECGKFFRYNSN
LIKHWRNHTGERPYECRECGKAFSHKHILVEHQKIHSGERPYECSECQKAFIRKSHLVHHQKIHSEERLVCSMNVGNSLAKTPTSLN
IRDFTMEKVYH
>sp|Q96S59|RANB9_HUMAN
MSGQPPPPPPPQQQQQQQQLSPPPPAALAPVSGVVLPAPPAVSAGSSPAGSPGGGAGGEGLGAAAAALLLHPPPPPPPATAAPPPPPP
PPPPPASAAAPASGPPAPPGLAAGPGPAGGAPTPALVAGSSAAAPFPHGDSALNEQEKELQRRLKRLYPAVDEQETPLPRSWSPKDK
FSYIGLSQNNLRVHYKGHGKTPKDAASVRATHPIPAACGIYYFEVKIVSKGRDGYMGIGLSAQGVNMNRLPGWDKHSYGYHGDDGHS
FCSSGTGQPYGPTFTTGDVIGCCVNLINNTCFYTKNGHSLGIAFTDLPPNLYPTVGLQTPGEVVDANFGQHPFVFDIEDYMREWRTK
IQAQIDRFPIGDREGEWQTMIQKMVSSYLVHHGYCATAEAFARSTDQTVLEELASIKNRQRIQKLVLAGRMGEAIETTQQLYPSLLE
RNPNLLFTLKVRQFIEMVNGTDSEVRCLGGRSPKSQDSYPVSPRPFSSPSMSPSHGMNIHNLASGKGSTAHFSGFESCSNGVISNKA
HQSYCHSNKHQSSNLNVPELNSINMSRSQQVNNFTSNDVDMETDHYSNGVGETSSNGFLNGSSKHDHEMEDCDTEMEVDSSQLRRQL
CGGSQAAIERMIHFGRELQAMSEQLRRDCGKNTANKKMLKDAFSLLAYSDPWNSPVGNQLDPIQREPVCSALNSAILETHNLPKQPP
LALAMGQATQCLGLMARSGIGSCAFATVEDYLH
>sp|P49915|GUAA_HUMAN
MALCNGDSKLENAGGDLKDGHHHYEGAVVILDAGAQYGKVIDRRVRELFVQSEIFPLETPAFAIKEQGFRAIIISGGPNSVYAEDAP
WFDPAIFTIGKPVLGICYGMQMMNKVFGGTVHKKSVREDGVFNISVDNTCSLFRGLQKEEVVLLTHGDSVDKVADGFKVVARSGNIV
AGIANESKKLYGAQFHPEVGLTENGKVILKNFLYDIAGCSGTFTVQNRELECIREIKERVGTSKVLVLLSGGVDSTVCTALLNRALN
QEQVIAVHIDNGFMRKRESQSVEEALKKLGIQVKVINAAHSFYNGTTTLPISDEDRTPRKRISKTLNMTTSPEEKRKIIGDTFVKIA
NEVIGEMNLKPEEVFLAQGTLRPDLIESASLVASGKAELIKTHHNDTELIRKLREEGKVIEPLKDFHKDEVRILGRELGLPEELVSR
HPFPGPGLAIRVICAEEPYICKDFPETNNILKIVADFSASVKKPHTLLQRVKACTTEEDQEKLMQITSLHSLNAFLLPIKTVGVQGD

CRSYSYVCGISSKDEPDWESLIFLARLIPRMCHNVNRVVYIFGPPVKEPPTDVTPTFLTTGVLSTLRQADFEAHNILRESGYAGKIS
QMPVILTPLHFDRDPLQKQPSCQRSVVIRTFITSDFMTGIPATPGNEIPVEVVLKMVTEIKKIPGISRIMYDLTSKPPGTTEWE
>sp|P06731|CEAM5_HUMAN
MESPSAPPHRWCIPWQRLLLTASLLTFWNPPTTAKLTIESTPFNVAEGKEVLLLVHNLPQHLFGYSWYKGERVDGNRQIIGYVIGTQ
QATPGPAYSGREIIYPNASLLIQNIIQNDTGFYTLHVIKSDLVNEEATGQFRVYPELPKPSISSNNSKPVEDKDAVAFTCEPETQDA
TYLWWVNNQSLPVSPRLQLSNGNRTLTLFNVTRNDTASYKCETQNPVSARRSDSVILNVLYGPDAPTISPLNTSYRSGENLNLSCHA
ASNPPAQYSWFVNGTFQQSTQELFIPNITVNNSGSYTCQAHNSDTGLNRTTVTTITVYAEPPKPFITSNNSNPVEDEDAVALTCEPE
IQNTTYLWWVNNQSLPVSPRLQLSNDNRTLTLLSVTRNDVGPYECGIQNKLSVDHSDPVILNVLYGPDDPTISPSYTYYRPGVNLSL
SCHAASNPPAQYSWLIDGNIQQHTQELFISNITEKNSGLYTCQANNSASGHSRTTVKTITVSAELPKPSISSNNSKPVEDKDAVAFT
CEPEAQNTTYLWWVNGQSLPVSPRLQLSNGNRTLTLFNVTRNDARAYVCGIQNSVSANRSDPVTLDVLYGPDTPIISPPDSSYLSGA
NLNLSCHSASNPSPQYSWRINGIPQQHTQVLFIAKITPNNNGTYACFVSNLATGRNNSIVKSITVSASGTSPGLSAGATVGIMIGVL
VGVALI
>sp|Q96M95|CCD42_HUMAN
MSLGIMEEEDLAEYFRLQYGERLLQMLQKLPNVEGASESPSIWLLEKKKETEIMHQTMVQKKKMFQRRMETLNLRWEELGVKEAQLK
AHIQKSEQFIQENDQKRIRAMKKANKERELKCQHMQELTKRKQEMVALRLEHQRLSAKLKDYYIFNKYLEKVVENSEFEEIHEVIAR
YKTLVSMRHDLMQSAQEGQEKIERAKARLARYMEEKDDEILQQNNELARLQMRFDRARSNVIFWESRWAHIQNTAAKKTLLLGTIKM
ATLNLFQIVSKHLKEVTEVALEDTHKQLDMIQQFIQDRSDIWAEVKKKEQQRVRI
>sp|P54886|P5CS_HUMAN
MLSQVYRCGFQPFNQHLLPWVKCTTVFRSHCIQPSVIRHVRSWSNIPFITVPLSRTHGKSFAHRSELKHAKRIVVKLGSAVVTRGDE
CGLALGRLASIVEQVSVLQNQGREMMLVTSGAVAFGKQRLRHEILLSQSVRQALHSGQNQLKEMAIPVLEARACAAAGQSGLMALYE
AMFTQYSICAAQILVTNLDFHDEQKRRNLNGTLHELLRMNIVPIVNTNDAVVPPAEPNSDLQGVNVISVKDNDSLAARLAVEMKTDL
LIVLSDVEGLFDSPPGSDDAKLIDIFYPGDQQSVTFGTKSRVGMGGMEAKVKAALWALQGGTSVVIANGTHPKVSGHVITDIVEGKK
VGTFFSEVKPAGPTVEQQGEMARSGGRMLATLEPEQRAEIIHHLADLLTDQRDEILLANKKDLEEAEGRLAAPLLKRLSLSTSKLNS
LAIGLRQIAASSQDSVGRVLRRTRIAKNLELEQVTVPIGVLLVIFESRPDCLPQVAALAIASGNGLLLKGGKEAAHSNRILHLLTQE
ALSIHGVKEAVQLVNTREEVEDLCRLDKMIDLIIPRGSSQLVRDIQKAAKGIPVMGHSEGICHMYVDSEASVDKVTRLVRDSKCEYP
AACNALETLLIHRDLLRTPLFDQIIDMLRVEQVKIHAGPKFASYLTFSPSEVKSLRTEYGDLELCIEVVDNVQDAIDHIHKYGSSHT
DVIVTEDENTAEFFLQHVDSACVFWNASTRFSDGYRFGLGAEVGISTSRIHARGPVGLEGLLTTKWLLRGKDHVVSDFSEHGSLKYL
HENLPIPQRNTN
>sp|P58876|H2B1D_HUMAN
MPEPTKSAPAPKKGSKKAVTKAQKKDGKKRKRSRKESYSVYVYKVLKQVHPDTGISSKAMGIMNSFVNDIFERIAGEASRLAHYNKR
STITSREIQTAVRLLLPGELAKHAVSEGTKAVTKYTSSK
>sp|Q13046|PSG7_HUMAN
MGPLSAPPCTQHITWKGLLLTASLLNFWNPPTTAQVTIEAQPPKVSEGKDVLLLVHNLPQNLTGHIWYKGQIRDLYHYVTSYIVDGQ
IIKYGPAYSGRETVYSNASLLIQNVTQEDTGSYTLHIIKRGDGTGGVTGRFTFTLYLETPKPSISSSNFNPREATEAVILTCDPETP
DASYLWWMNGQSLPMTHSLQLSETNRTLYLFGVTNYTAGPYECEIRNPVSASRSDPVTLNLLPKLPKPYITINNLNPRENKDVSTFT
CEPKSENYTYIWWLNGQSLPVSPRVKRRIENRILILPSVTRNETGPYQCEIRDRYGGIRSDPVTLNVLYGPDLPRIYPSFTYYHSGQ
NLYLSCFADSNPPAQYSWTINGKFQLSGQKLSIPQITTKHSGLYACSVRNSATGKESSKSVTVRVSDWTLP
>sp|P07199|CENPB_HUMAN
MGPKRRQLTFREKSRIIQEVEENPDLRKGEIARRFNIPPSTLSTILKNKRAILASERKYGVASTCRKTNKLSPYDKLEGLLIAWFQQ
IRAAGLPVKGIILKEKALRIAEELGMDDFTASNGWLDRFRRRHGVVSCSGVARARARNAAPRTPAAPASPAAVPSEGSGGSTTGWRA
REEQPPSVAEGYASQDVFSATETSLWYDFLPDQAAGLCGGDGRPRQATQRLSVLLCANADGSEKLPPLVAGKSAKPRAGQAGLPCDY
TANSKGGVTTQALAKYLKALDTRMAAESRRVLLLAGRLAAQSLDTSGLRHVQLAFFPPGTVHPLERGVVQQVKGHYRQAMLLKAMAA
LEGQDPSGLQLGLTEALHFVAAAWQAVEPSDIAACFREAGFGGGPNATITTSLKSEGEEEEEEEEEEEEEEGEGEEEEEEGEEEEEE

GGEGEELGEEEEVEEEGDVDSDEEEEEDEESSSEGLEAEDWAQGVVEAGGSFGAYGAQEEAQCPTLHFLEGGEDSDSDSEEEDDEEE
DDEDEDDDDDEEDGDEVPVPSFGEAMAYFAMVKRYLTSFPIDDRVQSHILHLEHDLVHVTRKNHARQAGVRGLGHQS
>sp|Q9BY44|EIF2A_HUMAN
MAPSTPLLTVRGSEGLYMVNGPPHFTESTVFPRESGKNCKVCIFSKDGTLFAWGNGEKVNIISVTNKGLLHSFDLLKAVCLEFSPKN
TVLATWQPYTTSKDGTAGIPNLQLYDVKTGTCLKSFIQKKMQNWCPSWSEDETLCARNVNNEVHFFENNNFNTIANKLHLQKINDFV
LSPGPQPYKVAVYVPGSKGAPSFVRLYQYPNFAGPHAALANKSFFKADKVTMLWNKKATAVLVIASTDVDKTGASYYGEQTLHYIAT
NGESAVVQLPKNGPIYDVVWNSSSTEFCAVYGFMPAKATIFNLKCDPVFDFGTGPRNAAYYSPHGHILVLAGFGNLRGQMEVWDVKN
YKLISKPVASDSTYFAWCPDGEHILTATCAPRLRVNNGYKIWHYTGSILHKYDVPSNAELWQVSWQPFLDGIFPAKTITYQAVPSEV
PNEEPKVATAYRPPALRNKPITNSKLHEEEPPQNMKPQSGNDKPLSKTALKNQRKHEAKKAAKQEARSDKSPDLAPTPAPQSTPRNT
VSQSISGDPEIDKKIKNLKKKLKAIEQLKEQAATGKQLEKNQLEKIQKETALLQELEDLELGI
>sp|A6NGG8|CB071_HUMAN
MGCTPSHSDLVNSVAKSGIQFLKKPKAIRPGCQGGSERGSIPLLVKNSTCYDAGEGLAEEQPSPRRNQTTAKGLCQLMGDPASGKRK
DMEGLIPGTKTSSSQLNKSQSHMAKDIPFKTQGSHGSQGADFSGDESEESSTQDTSKWKRTAKCHTSSTQSHCYQTIHPAHEPEGKV
DFPEPLVKAHQQAYTYLHSSLSKYEAILCIIHQATQTRELLQPMVSFLLLCFEEISQLLGEISKDGEVLLQEVREDLAWPLKKREPQ
EQPNLLQQLLQYTVSKLQVLNGTVASLTGSFLEGSSSYLHSTATHLENKLSTKRNVDERLLRALRQLESLASGCGDPGVQGLPLCSE
DSGIGADNESVQSVDKLGKQTSWDLAPEPEEWKSVTSPHTEARQSGHTWQQSPFCLGSGRPQDCLLSGAPMAKVQPRAQDEARSPCL
SSTSPENITSPPLKLGTSTPCDSFGIGVSVEPHLSKTSRPMDASSLSDSEDSSPEEEEEDKMSSMSLCAWQEKTPHSRPQSSPADRE
SPFQARTRRLRSLQAQEMILKMKESISERIKFVPVPCGHQDWSEEEEGRTVVPPRPSTVSGSRRAPERQTRSQSESCLQSHVEDPTF
QELRRVQRDLSQKLEAFYALGAKGQGQSQEQILQPRAAAVWPNGTCRVSPSNTTSRLKASLTKNFSILPSQDKSILQKCNPHPEDEQ
GKAGKLPNAIPSGEVSEAAKATDWNVRGCPTRTSVKKLIETFSPTESLRMLGDSKDAGASPCLRNCIMPPRFPKYTGLAPLYPKPQI
SPASGRESLKMGIGWKPLAPIFPPLPKAEAAKSEELSCEMEGNLEHLPPPPMEVLMDKSFASLESPESSKSTENSPKETQEPGPGEA
GPTRRTWASPKLRASVSPLDLLPSKSTASLTKPHSTGPGSGRSSCQPRKPALDLSSPPATSQSPEVKGGTWSQAEKATSLYRQPRKA
IAWHHSGPPSGQNRTSESSLARPRQSRERSPPVGRKASPTRTHWVPQADKRRRSLPSSYRPAQPSPSAVQTPPSPPVSPRVLSPPTT
KRRTSPPHQPKLPNPPPESAPAQCKVPSPPTQHPEASPPFSIPSPSPPMSPSQEHKETRDSEDSQAVIAKVSGNTHSIFCPATSSLF
EAKPPLSTAHPLTPPSLPPEAGGPLGNPAECWKNSSGPWLRADSQRRAALCALNPLPFLRRTASDRQPGGRPQPPTLDPTSTSYESQ
LGQNSSSEESPKKDTEPGSSPCSPELQGGTRRASPPEFCVLGHGLQPEPRTGHIQDKSQPEAQPQQEEVS
>sp|P40189|IL6RB_HUMAN
MLTLQTWLVQALFIFLTTESTGELLDPCGYISPESPVVQLHSNFTAVCVLKEKCMDYFHVNANYIVWKTNHFTIPKEQYTIINRTAS
SVTFTDIASLNIQLTCNILTFGQLEQNVYGITIISGLPPEKPKNLSCIVNEGKKMRCEWDGGRETHLETNFTLKSEWATHKFADCKA
KRDTPTSCTVDYSTVYFVNIEVWVEAENALGKVTSDHINFDPVYKVKPNPPHNLSVINSEELSSILKLTWTNPSIKSVIILKYNIQY
RTKDASTWSQIPPEDTASTRSSFTVQDLKPFTEYVFRIRCMKEDGKGYWSDWSEEASGITYEDRPSKAPSFWYKIDPSHTQGYRTVQ
LVWKTLPPFEANGKILDYEVTLTRWKSHLQNYTVNATKLTVNLTNDRYLATLTVRNLVGKSDAAVLTIPACDFQATHPVMDLKAFPK
DNMLWVEWTTPRESVKKYILEWCVLSDKAPCITDWQQEDGTVHRTYLRGNLAESKCYLITVTPVYADGPGSPESIKAYLKQAPPSKG
PTVRTKKVGKNEAVLEWDQLPVDVQNGFIRNYTIFYRTIIGNETAVNVDSSHTEYTLSSLTSDTLYMVRMAAYTDEGGKDGPEFTFT
TPKFAQGEIEAIVVPVCLAFLLTTLLGVLFCFNKRDLIKKHIWPNVPDPSKSHIAQWSPHTPPRHNFNSKDQMYSDGNFTDVSVVEI
EANDKKPFPEDLKSLDLFKKEKINTEGHSSGIGGSSCMSSSRPSISSSDENESSQNTSSTVQYSTVVHSGYRHQVPSVQVFSRSEST
QPLLDSEERPEDLQLVDHVDGGDGILPRQQYFKQNCSQHESSPDISHFERSKQVSSVNEEDFVRLKQQISDHISQSCGSGQMKMFQE
VSAADAFGPGTEGQVERFETVGMEAATDEGMPKSYLPQTVRQGGYMPQ
>sp|Q9P2D8|UNC79_HUMAN
MSTKAEQFASKIRYLQEYHNRVLHNIYPVPSGTDIANTLKYFSQTLLSILSRTGKKENQDASNLTVPMTMCLFPVPFPLTPSLRPQV
SSINPTVTRSLLYSVLRDAPSERGPQSRDAQLSDYPSLDYQGLYVTLVTLLDLVPLLQHGQHDLGQSIFYTTTCLLPFLNDDILSTL
PYTMISTLATFPPFLHKDIIEYLSTSFLPMAILGSSRREGVPAHVNLSASSMLMIAMQYTSNPVYHCQLLECLMKYKQEVWKDLLYV
IAYGPSQVKPPAVQMLFHYWPNLKPPGAISEYRGLQYTAWNPIHCQHIECHNAINKPAVKMCIDPSLSVALGDKPPPLYLCEECSER
IAGDHSEWLIDVLLPQAEISAICQKKNCSSHVRRAVVTCFSAGCCGRHGNRPVRYCKRCHSNHHSNEVGAAAETHLYQTSPPPINTR

ECGAEELVCAVEAVISLLKEAEFHAEQREHELNRRRQLGLSSSHHSLDNADFDNKDDDKHDQRLLSQFGIWFLVSLCTPSENTPTES
LARLVAMVFQWFHSTAYMMDDEVGSLVEKLKPQFVTKWLKTVCDVRFDVMVMCLLPKPMEFARVGGYWDKSCSTVTQLKEGLNRILC
LIPYNVINQSVWECIMPEWLEAIRTEVPDNQLKEFREVLSKMFDIELCPLPFSMEEMFGFISCRFTGYPSSVQEQALLWLHVLSELD
IMVPLQLLISMFSDGVNSVKELANQRKSRVSELAGNLASRRVSVASDPGRRVQHNMLSPFHSPFQSPFRSPLRSPFRSPFKNFGHPG
GRTIDFDCEDDEMNLNCFILMFDLLLKQMELQDDGITMGLEHSLSKDIISIINNVFQAPWGGSHTCQKDEKAIECNLCQSSILCYQL
ACELLERLAPKEESRLVEPTDSLEDSLLSSRPEFIIGPEGEEEENPASKHGENPGNCTEPVEHAAVKNDTERKFCYQQLPVTLRLIY
TIFQEMAKFEEPDILFNMLNCLKILCLHGECLYIARKDHPQFLAYIQDHMLIASLWRVVKSEFSQLSSLAVPLLLHALSLPHGADIF
WTIINGNFNSKDWKMRFEAVEKVAVICRFLDIHSVTKNHLLKYSLAHAFCCFLTAVEDVNPAVATRAGLLLDTIKRPALQGLCLCLD
FQFDTVVKDRPTILSKLLLLHFLKQDIPALSWEFFVNRFETLSLEAQLHLDCNKEFPFPTTITAVRTNVANLSDAALWKIKRARFAR
NRQKSVRSLRDSVKGPVESKRALSLPETLTSKIRQQSPENDNTIKDLLPEDAGIDHQTVHQLITVLMKFMAKDESSAESDISSAKAF
NTVKRHLYVLLGYDQQEGCFMIAPQKMRLSTCFNAFIAGIAQVMDYNINLGKHLLPLVVQVLKYCSCPQLRHYFQQPPRCSLWSLKP
HIRQMWLKALLVILYKYPYRDCDISKILLHLIHITVNTLNAQYHSCKPHATAGPLYSDNSNISRYSEKEKGEIELAEYRETGALQDS
LLHCVREESIPKKKLRSFKQKSLDIGNADSLLFTLDEHRRKSCIDRCDIEKPPTQAAYIAQRPNDPGRSRQNSATRPDNSEIPENPA
MEGFPDARRPVIPEVRLNCMETFEVKVDSPVKPAPKEDLDLIDLSSDSTSGPEKHSILSTSDSDSLVFEPLPPLRIVESDEEEETMN
QGDDGPSGKNAASSPSVPSHPSVLSLSTAPLVQVSVEDCSKDFSSKDSGNNQSAGNTDSALITLEDPMDAEGSSKPEELPEFSCGSP
LTLKQKRDLLQKSFALPEMSLDDHPDPGTEGEKPGELMPSSGAKTVLLKVPEDAENPTESEKPDTSAESDTEQNPERKVEEDGAEES
EFKIQIVPRQRKQRKIAVSAIQREYLDISFNILDKLGEQKDPDPSTKGLSTLEMPRESSSAPTLDAGVPETSSHSSISTQYRQMKRG
SLGVLTMSQLMKRQLEHQSSAPHNISNWDTEQIQPGKRQCNVPTCLNPDLEGQPLRMRGATKSSLLSAPSIVSMFVPAPEEFTDEQP
TVMTDKCHDCGAILEEYDEETLGLAIVVLSTFIHLSPDLAAPLLLDIMQSVGRLASSTTFSNQAESMMVPGNAAGVAKQFLRCIFHQ
LAPNGIFPQLFQSTIKDGTFLRTLASSLMDFNELSSIAALSQLLEGLNNKKNLPAGGAMIRCLENIATFMEALPMDSPSSLWTTISN
QFQTFFAKLPCVLPLKCSLDSSLRIMICLLKIPSTNATRSLLEPFSKLLSFVIQNAVFTLAYLVELCGLCYRAFTKERDKFYLSRSV
VLELLQALKLKSPLPDTNLLLLVQFICADAGTKLAESTILSKQMIASVPGCGTAAMECVRQYINEVLDFMADMHTLTKLKSHMKTCS
QPLHEDTFGGHLKVGLAQIAAMDISRGNHRDNKAVIRYLPWLYHPPSAMQQGPKEFIECVSHIRLLSWLLLGSLTHNAVCPNASSPC
LPIPLDAGSHVADHLIVILIGFPEQSKTSVLHMCSLFHAFIFAQLWTVYCEQSAVATNLQNQNEFSFTAILTALEFWSRVTPSILQL
MAHNKVMVEMVCLHVISLMEALQECNSTIFVKLIPMWLPMIQSNIKHLSAGLQLRLQAIQNHVNHHSLRTLPGSGQSSAGLAALRKW
LQCTQFKMAQVEIQSSEAASQFYPL
>sp|Q9BW66|CINP_HUMAN
MEAKTLGTVTPRKPVLSVSARKIKDNAADWHNLILKWETLNDAGFTTANNIANLKISLLNKDKIELDSSSPASKENEEKVCLEYNEE
LEKLCEELQATLDGLTKIQVKMEKLSSTTKGICELENYHYGEESKRPPLFHTWPTTHFYEVSHKLLEMYRKELLLKRTVAKELAHTG
DPDLTLSYLSMWLHQPYVESDSRLHLESMLLETGHRAL
>sp|Q86W33|TPRA1_HUMAN
MDTLEEVTWANGSTALPPPLAPNISVPHRCLLLLYEDIGTSRVRYWDLLLLIPNVLFLIFLLWKLPSARAKIRITSSPIFITFYILV
FVVALVGIARAVVSMTVSTSNAATVADKILWEITRFFLLAIELSVIILGLAFGHLESKSSIKRVLAITTVLSLAYSVTQGTLEILYP
DAHLSAEDFNIYGHGGRQFWLVSSCFFFLVYSLVVILPKTPLKERISLPSRRSFYVYAGILALLNLLQGLGSVLLCFDIIEGLCCVD
ATTFLYFSFFAPLIYVAFLRGFFGSEPKILFSYKCQVDETEEPDVHLPQPYAVARREGLEAAGAAGASAASYSSTQFDSAGGVAYLD
DIASMPCHTGSINSTDSERWKAINA
>sp|O60759|CYTIP_HUMAN
MSLQRLLQHSSNGNLADFCAGPAYSSYSTLTGSLTMDDNRRIQMLADTVATLPRGRKQLALTRSSSLSDFSWSQRKLVTVEKQDNET
FGFEIQSYRPQNQNACSSEMFTLICKIQEDSPAHCAGLQAGDVLANINGVSTEGFTYKQVVDLIRSSGNLLTIETLNGTMILKRTEL
EAKLQVLKQTLKQKWVEYRSLQLQEHRLLHGDAANCPSLENMDLDELSLFGPLPGPGPALVDRNRLSSESSCKSWLSSMTMDSEDGY
QTCVSEDSSRGAFSRQTSTDDECFIPKEGDDFLRRSSSRRNRSISNTSSGSMSPLWEGNLSSMFGTLPRKSRKGSVRKQLLKFIPGL
HRAVEEEESRF
>sp|P28676|GRAN_HUMAN

MAYPGYGGGFGNFSIQVPGMQMGQPVPETGPAILLDGYSGPAYSDTYSSAGDSVYTYFSAVAGQDGEVDAEELQRCLTQSGINGTYS
PFSLETCRIMIAMLDRDHTGKMGFNAFKELWAALNAWKENFMTVDQDGSGTVEHHELRQAIGLMGYRLSPQTLTTIVKRYSKNGRIF
FDDYVACCVKLRALTDFFRKRDHLQQGSANFIYDDFLQGTMAI
>sp|O94892|ZN432_HUMAN
MINAQELLTLEDVTVEFTWEEWQLLGPFQKDLYRDVMLEIYSNLLSMGYQVSKPDALSKLERGEEPWTMEDERHSRICPENNEVDDH
LQDHLENQRMLKSVEQYHEHNAFGNTASQTKSLCLFRENHDTFELYIKTLKSNLSLVNQNKSCEINNSTKFSGDGKSFLHGNYEELY
SAAKFSVSTKANSTKSQVSKHQRTHEIEKNHVCSECGKAFVKKSQLTDHERVHTGEKPYGCTLCAKVFSRKSRLNEHQRIHKREKSF
ICSECGKVFTMKSRLIEHQRTHTGEKPYICNECGKGFPGKRNLIVHQRNHTGEKSYICSECGKGFTGKSMLIIHQRTHTGEKPYICS
ECGKGFTTKHYVIIHQRNHTGEKPYICNECGKGFTMKSRMIEHQRTHTGEKPYICSECGKGFPRKSNLIVHQRNHTVEKSYLCSECG
KGFTVKSMLIIHQRTHTGEKPYTCSECGKGFPLKSRLIVHQRTHTGEKPYRCSECGKGFIVNSGLMLHQRTHTGEKPYICNECGKGF
AFKSNLVVHQRTHTGEKPFMCSECGKGFTMKRYLIVHQQIHTEEKSCICSECGRGFAKETELALHKQVHTGEKPYGCNECGKGFTMK
SRLIVHQRTHTGEKPFVCSECRKAFSSKRNLIVHQRTHNGNKP
>sp|Q01726|MSHR_HUMAN
MAVQGSQRRLLGSLNSTPTAIPQLGLAANQTGARCLEVSISDGLFLSLGLVSLVENALVVATIAKNRNLHSPMYCFICCLALSDLLV
SGSNVLETAVILLLEAGALVARAAVLQQLDNVIDVITCSSMLSSLCFLGAIAVDRYISIFYALRYHSIVTLPRARRAVAAIWVASVV
FSTLFIAYYDHVAVLLCLVVFFLAMLVLMAVLYVHMLARACQHAQGIARLHKRQRPVHQGFGLKGAVTLTILLGIFFLCWGPFFLHL
TLIVLCPEHPTCGCIFKNFNLFLALIICNAIIDPLIYAFHSQELRRTLKEVLTCSW
>sp|Q9Y5A7|NUB1_HUMAN
MAQKKYLQAKLTQFLREDRIQLWKPPYTDENKKVGLALKDLAKQYSDRLECCENEVEKVIEEIRCKAIERGTGNDNYRTTGIATIEV
FLPPRLKKDRKNLLETRLHITGRELRSKIAETFGLQENYIKIVINKKQLQLGKTLEEQGVAHNVKAMVLELKQSEEDARKNFQLEEE
EQNEAKLKEKQIQRTKRGLEILAKRAAETVVDPEMTPYLDIANQTGRSIRIPPSERKALMLAMGYHEKGRAFLKRKEYGIALPCLLD
ADKYFCECCRELLDTVDNYAVLQLDIVWCYFRLEQLECLDDAEKKLNLAQKCFKNCYGENHQRLVHIKGNCGKEKVLFLRLYLLQGI
RNYHSGNDVEAYEYLNKARQLFKELYIDPSKVDNLLQLGFTAQEARLGLRACDGNVDHAATHITNRREELAQIRKEEKEKKRRRLEN
IRFLKGMGYSTHAAQQVLHAASGNLDEALKILLSNPQMWWLNDSNPETDNRQESPSQENIDRLVYMGFDALVAEAALRVFRGNVQLA
AQTLAHNGGSLPPELPLSPEDSLSPPATSPSDSAGTSSASTDEDMETEAVNEILEDIPEHEEDYLDSTLEDEEIIIAEYLSYVENRK
SATKKN
>sp|Q9UMS4|PRP19_HUMAN
MSLICSISNEVPEHPCVSPVSNHVYERRLIEKYIAENGTDPINNQPLSEEQLIDIKVAHPIRPKPPSATSIPAILKALQDEWDAVML
HSFTLRQQLQTTRQELSHALYQHDAACRVIARLTKEVTAAREALATLKPQAGLIVPQAVPSSQPSVVGAGEPMDLGELVGMTPEIIQ
KLQDKATVLTTERKKRGKTVPEELVKPEELSKYRQVASHVGLHSASIPGILALDLCPSDTNKILTGGADKNVVVFDKSSEQILATLK
GHTKKVTSVVFHPSQDLVFSASPDATIRIWSVPNASCVQVVRAHESAVTGLSLHATGDYLLSSSDDQYWAFSDIQTGRVLTKVTDET
SGCSLTCAQFHPDGLIFGTGTMDSQIKIWDLKERTNVANFPGHSGPITSIAFSENGYYLATAADDSSVKLWDLRKLKNFKTLQLDNN
FEVKSLIFDQSGTYLALGGTDVQIYICKQWTEILHFTEHSGLTTGVAFGHHAKFIASTGMDRSLKFYSL
>sp|Q6GV28|TM225_HUMAN
MVHVSNRSIQGMNILFSSWAVVLMVMGITLDKWVELISEDERAKMNHSPWMMCCPALWPEDDLKVVRIMMTSSLGLSFLLNLILGMK
FTYLIPQNKYIQLFTTILSFFSGISLLWALILYHNKLKQGQSMHFSNYRITWIMYTAYLNVFFLSVCGVLSLLECKLSTSSCTCLNI
HKSDNECKESENSIEDISLPECTAMPRSIVRAHTVNSLNKKVQTRHVTWAL
>sp|P35321|SPR1A_HUMAN
MNSQQQKQPCTPPPQPQQQQVKQPCQPPPQEPCIPKTKEPCHPKVPEPCHPKVPEPCQPKVPEPCQPKVPEPCPSTVTPAPAQQKTK
QK
>sp|Q14353|GAMT_HUMAN
MSAPSATPIFAPGENCSPAWGAAPAAYDAADTHLRILGKPVMERWETPYMHALAAAASSKGGRVLEVGFGMAIAASKVQEAPIDEHW
IIECNDGVFQRLRDWAPRQTHKVIPLKGLWEDVAPTLPDGHFDGILYDTYPLSEETWHTHQFNFIKNHAFRLLKPGGVLTYCNLTSW
GELMKSKYSDITIMFEETQVPALLEAGFRRENIRTEVMALVPPADCRYYAFPQMITPLVTKG

>sp|P31271|HXA13_HUMAN
MTASVLLHPRWIEPTVMFLYDNGGGLVADELNKNMEGAAAAAAAAAAAAAAGAGGGGFPHPAAAAAGGNFSVAAAAAAAAAAAANQC
RNLMAHPAPLAPGAASAYSSAPGEAPPSAAAAAAAAAAAAAAAAAASSSGGPGPAGPAGAEAAKQCSPCSAAAQSSSGPAALPYGYF
GSGYYPCARMGPHPNAIKSCAQPASAAAAAAFADKYMDTAGPAAEEFSSRAKEFAFYHQGYAAGPYHHHQPMPGYLDMPVVPGLGGP
GESRHEPLGLPMESYQPWALPNGWNGQMYCPKEQAQPPHLWKSTLPDVVSHPSDASSYRRGRKKRVPYTKVQLKELEREYATNKFIT
KDKRRRISATTNLSERQVTIWFQNRRVKEKKVINKLKTTS
>sp|P51513|NOVA1_HUMAN
MMAAAPIQQNGTHTGVPIDLDPPDSRKRPLEAPPEAGSTKRTNTGEDGQYFLKVLIPSYAAGSIIGKGGQTIVQLQKETGATIKLSK
LSKSKDFYPGTTERVCLIQGTVEALNAVHGFIAEKIREMPQNVAKTEPVSILQPQTTVNPDRIKQTLPSSPTTTKSSPSDPMTTSRA
NQVKIIVPNSTAGLIIGKGGATVKAVMEQSGAWVQLSQKPDGINLQERVVTVSGEPEQNRKAVELIIQKIQEDPQSGSCLNISYANV
TGPVANSNPTGSPYANTAEVLPTAAAAAGLLGHANLAGVAAFPAVLSGFTGNDLVAITSALNTLASYGYNLNTLGLGLSQAAATGAL
AAAAASANPAAAAANLLATYASEASASGSTAGGTAGTFALGSLAAATAATNGYFGAASPLAASAILGTEKSTDGSKDVVEIAVPENL
VGAILGKGGKTLVEYQELTGARIQISKKGEFVPGTRNRKVTITGTPAATQAAQYLITQRITYEQGVRAANPQKVG
>sp|P01714|LV319_HUMAN
MAWTPLWLTLLTLCIGSVVSSELTQDPAVSVALGQTVRITCQGDSLRSYYASWYQQKPGQAPVLVIYGKNNRPSGIPDRFSGSSSGN
TASLTITGAQAEDEADYYCNSRDSS
>sp|A8MXZ3|KRA91_HUMAN
MTHCCSPCCQPTCCRTTCCRTTCWKPTTVTTCSSTPCCQPSCCVPSCCQPCCHPTCCQNTCCRTTCCQPTCVASCCQPSCCSTPCCQ
PTCCGSSCCGQTSCGSSCCQPICGSSCCQPCCHPTCYQTICFRTTCCQPTCCQPTCCRNTSCQPTCCGSSCCQPCCHPTCCQTICRS
TCCQPSCVTRCCSTPCCQPTCGGSSCCSQTCNESSYCLPCCRPTCCQTTCYRTTCCRPSCCCSPCCVSSCCQPSCC
>sp|P48169|GBRA4_HUMAN
MVSAKKVPAIALSAGVSFALLRFLCLAVCLNESPGQNQKEEKLCTENFTRILDSLLDGYDNRLRPGFGGPVTEVKTDIYVTSFGPVS
DVEMEYTMDVFFRQTWIDKRLKYDGPIEILRLNNMMVTKVWTPDTFFRNGKKSVSHNMTAPNKLFRIMRNGTILYTMRLTISAECPM
RLVDFPMDGHACPLKFGSYAYPKSEMIYTWTKGPEKSVEVPKESSSLVQYDLIGQTVSSETIKSITGEYIVMTVYFHLRRKMGYFMI
QTYIPCIMTVILSQVSFWINKESVPARTVFGITTVLTMTTLSISARHSLPKVSYATAMDWFIAVCFAFVFSALIEFAAVNYFTNIQM
EKAKRKTSKPPQEVPAAPVQREKHPEAPLQNTNANLNMRKRTNALVHSESDVGNRTEVGNHSSKSSTVVQESSKGTPRSYLASSPNP
FSRANAAETISAARALPSASPTSIRTGYMPRKASVGSASTRHVFGSRLQRIKTTVNTIGATGKLSATPPPSAPPPSGSGTSKIDKYA
RILFPVTFGAFNMVYWVVYLSKDTMEKSESLM
>sp|Q96HY6|DDRGK_HUMAN
MVAPVWYLVAAALLVGFILFLTRSRGRAASAGQEPLHNEELAGAGRVAQPGPLEPEEPRAGGRPRRRRDLGSRLQAQRRAQRVAWAE
ADENEEEAVILAQEEEGVEKPAETHLSGKIGAKKLRKLEEKQARKAQREAEEAEREERKRLESQREAEWKKEEERLRLEEEQKEEEE
RKAREEQAQREHEEYLKLKEAFVVEEEGVGETMTEEQSQSFLTEFINYIKQSKVVLLEDLASQVGLRTQDTINRIQDLLAEGTITGV
IDDRGKFIYITPEELAAVANFIRQRGRVSIAELAQASNSLIAWGRESPAQAPA
>sp|P25098|ARBK1_HUMAN
MADLEAVLADVSYLMAMEKSKATPAARASKKILLPEPSIRSVMQKYLEDRGEVTFEKIFSQKLGYLLFRDFCLNHLEEARPLVEFYE
EIKKYEKLETEEERVARSREIFDSYIMKELLACSHPFSKSATEHVQGHLGKKQVPPDLFQPYIEEICQNLRGDVFQKFIESDKFTRF
CQWKNVELNIHLTMNDFSVHRIIGRGGFGEVYGCRKADTGKMYAMKCLDKKRIKMKQGETLALNERIMLSLVSTGDCPFIVCMSYAF
HTPDKLSFILDLMNGGDLHYHLSQHGVFSEADMRFYAAEIILGLEHMHNRFVVYRDLKPANILLDEHGHVRISDLGLACDFSKKKPH
ASVGTHGYMAPEVLQKGVAYDSSADWFSLGCMLFKLLRGHSPFRQHKTKDKHEIDRMTLTMAVELPDSFSPELRSLLEGLLQRDVNR
RLGCLGRGAQEVKESPFFRSLDWQMVFLQKYPPPLIPPRGEVNAADAFDIGSFDEEDTKGIKLLDSDQELYRNFPLTISERWQQEVA
ETVFDTINAETDRLEARKKAKNKQLGHEEDYALGKDCIMHGYMSKMGNPFLTQWQRRYFYLFPNRLEWRGEGEAPQSLLTMEEIQSV
EETQIKERKCLLLKIRGGKQFILQCDSDPELVQWKKELRDAYREAQQLVQRVPKMKNKPRSPVVELSKVPLVQRGSANGL
>sp|Q5M8T2|S35D3_HUMAN

MRQLCRGRVLGISVAIAHGVFSGSLNILLKFLISRYQFSFLTLVQCLTSSTAALSLELLRRLGLIAVPPFGLSLARSFAGVAVLSTL
QSSLTLWSLRGLSLPMYVVFKRCLPLVTMLIGVLVLKNGAPSPGVLAAVLITTCGAALAGAGDLTGDPIGYVTGVLAVLVHAAYLVL
IQKASADTEHGPLTAQYVIAVSATPLLVICSFASTDSIHAWTFPGWKDPAMVCIFVACILIGCAMNFTTLHCTYINSAVTTSFVGVV
KSIATITVGMVAFSDVEPTSLFIAGVVVNTLGSIIYCVAKFMETRKQSNYEDLEAQPRGEEAQLSGDQLPFVMEELPGEGGNGRSEG
GEAAGGPAQESRQEVRGSPRGVPLVAGSSEEGSRRSLKDAYLEVWRLVRGTRYMKKDYLIENEELPSP
>sp|Q9H2H8|PPIL3_HUMAN
MSVTLHTDVGDIKIEVFCERTPKTCENFLALCASNYYNGCIFHRNIKGFMVQTGDPTGTGRGGNSIWGKKFEDEYSEYLKHNVRGVV
SMANNGPNTNGSQFFITYGKQPHLDMKYTVFGKVIDGLETLDELEKLPVNEKTYRPLNDVHIKDITIHANPFAQ
>sp|P47881|OR3A1_HUMAN
MQPESGANGTVIAEFILLGLLEAPGLQPVVFVLFLFAYLVTVRGNLSILAAVLVEPKLHTPMYFFLGNLSVLDVGCISVTVPSMLSR
LLSRKRAVPCGACLTQLFFFHLFVGVDCFLLTAMAYDRFLAICRPLTYSTRMSQTVQRMLVAASWACAFTNALTHTVAMSTLNFCGP
NVINHFYCDLPQLFQLSCSSTQLNELLLFAVGFIMAGTPMALIVISYIHVAAAVLRIRSVEGRKKAFSTCGSHLTVVAIFYGSGIFN
YMRLGSTKLSDKDKAVGIFNTVINPMLNPIIYSFRNPDVQSAIWRMLTGRRSLA
>sp|Q8IYS4|CP071_HUMAN
MASNDKGMAPSLGSPWASQMGPWDAILKAVKDQLPSLDSDSPLSDYGEEELFIFQRNQTSLIPDLSEELAEDPADGDKSRAWVAAAE
ESLPEPVLVPAELATEPGCRQNTRTKDASSQEGRDPGRPFESSGEVSALLGMAEEPPRWLEGDLGSLSFNTKGSQGPPWDPQAEATL
SCHEGDPKAEPLSTASQESVNRRALRQERRKMIETDILQKVTRDACGPTSSDKGGVKEAPCHAAESAPRSKMPLVEPPEGPPVLSLQ
QLEAWDLDDILQSLAGQEDNQGNRAPGTVWWAADHRQVQDRMVPSAHNRLMEQLALLCTTQSKASACARKVPADTPQDTKEADSGSR
CASRKQGSQAGPGPQLAQGMRLNAESPTIFIDLRQMELPDHLSPESSSHSSSDSEEEEEEEMAALGDAEGASPSSLGLRTCTGKSQL
LQQLRAFQKGTAQPELPASKGPAGGRAQAPEDTAGSRTGRKQHMKLCAKGQSAQARLPRGRPRALGDVPEPGAAREALMPPLEQL
>sp|O00305|CACB4_HUMAN
MSSSSYAKNGTADGPHSPTSQVARGTTTRRSRLKRSDGSTTSTSFILRQGSADSYTSRPSDSDVSLEEDREAIRQEREQQAAIQLER
AKSKPVAFAVKTNVSYCGALDEDVPVPSTAISFDAKDFLHIKEKYNNDWWIGRLVKEGCEIGFIPSPLRLENIRIQQEQKRGRFHGG
KSSGNSSSSLGEMVSGTFRATPTSTAKQKQKVTEHIPPYDVVPSMRPVVLVGPSLKGYEVTDMMQKALFDFLKHRFDGRISITRVTA
DISLAKRSVLNNPSKRAIIERSNTRSSLAEVQSEIERIFELARSLQLVVLDADTINHPAQLIKTSLAPIIVHVKVSSPKVLQRLIKS
RGKSQSKHLNVQLVAADKLAQCPPEMFDVILDENQLEDACEHLGEYLEAYWRATHTTSSTPMTPLLGRNLGSTALSPYPTAISGLQS
QRMRHSNHSTENSPIERRSLMTSDENYHNERARKSRNRLSSSSQHSRDHYPLVEEDYPDSYQDTYKPHRNRGSPGGYSHDSRHRL
>sp|P20151|KLK2_HUMAN
MWDLVLSIALSVGCTGAVPLIQSRIVGGWECEKHSQPWQVAVYSHGWAHCGGVLVHPQWVLTAAHCLKKNSQVWLGRHNLFEPEDTG
QRVPVSHSFPHPLYNMSLLKHQSLRPDEDSSHDLMLLRLSEPAKITDVVKVLGLPTQEPALGTTCYASGWGSIEPEEFLRPRSLQCV
SLHLLSNDMCARAYSEKVTEFMLCAGLWTGGKDTCGGDSGGPLVCNGVLQGITSWGPEPCALPEKPAVYTKVVHYRKWIKDTIAANP
>sp|Q9UGF6|OR5V1_HUMAN
MERKNQTAITEFIILGFSNLNELQFLLFTIFFLTYFCTLGGNILIILTTVTDPHLHTPMYYFLGNLAFIDICYTTSNVPQMMVHLLS
KKKSISYVGCVVQLFAFVFFVGSECLLLAAMAYDRYIAICNPLRYSVILSKVLCNQLAASCWAAGFLNSVVHTVLTFCLPFCGNNQI
NYFFCDIPPLLILSCGNTSVNELALLSTGVFIGWTPFLCIVLSYICIISTILRIQSSEGRRKAFSTCASHLAIVFLFYGSAIFTYVR
PISTYSLKKDRLVSVLYSVVTPMLNPIIYTLRNKDIKEAVKTIGSKWQPPISSLDSKLTY
>sp|P14923|PLAK_HUMAN
MEVMNLMEQPIKVTEWQQTYTYDSGIHSGANTCVPSVSSKGIMEEDEACGRQYTLKKTTTYTQGVPPSQGDLEYQMSTTARAKRVRE
AMCPGVSGEDSSLLLATQVEGQATNLQRLAEPSQLLKSAIVHLINYQDDAELATRALPELTKLLNDEDPVVVTKAAMIVNQLSKKEA
SRRALMGSPQLVAAVVRTMQNTSDLDTARCTTSILHNLSHHREGLLAIFKSGGIPALVRMLSSPVESVLFYAITTLHNLLLYQEGAK
MAVRLADGLQKMVPLLNKNNPKFLAITTDCLQLLAYGNQESKLIILANGGPQALVQIMRNYSYEKLLWTTSRVLKVLSVCPSNKPAI
VEAGGMQALGKHLTSNSPRLVQNCLWTLRNLSDVATKQEGLESVLKILVNQLSVDDVNVLTCATGTLSNLTCNNSKNKTLVTQNSGV
EALIHAILRAGDKDDITEPAVCALRHLTSRHPEAEMAQNSVRLNYGIPAIVKLLNQPNQWPLVKATIGLIRNLALCPANHAPLQEAA
VIPRLVQLLVKAHQDAQRHVAAGTQQPYTDGVRMEEIVEGCTGALHILARDPMNRMEIFRLNTIPLFVQLLYSSVENIQRVAAGVLC

ELAQDKEAADAIDAEGASAPLMELLHSRNEGTATYAAAVLFRISEDKNPDYRKRVSVELTNSLFKHDPAAWEAAQSMIPINEPYGDD
MDATYRPMYSSDVPLDPLEMHMDMDGDYPIDTYSDGLRPPYPTADHMLA
>sp|O15014|ZN609_HUMAN
MSLSSGASGGKGVDANPVETYDSGDEWDIGVGNLIIDLDADLEKDQQKLEMSGSKEVGIPAPNAVATLPDNIKFVTPVPGPQGKEGK
SKSKRSKSGKDTSKPTPGTSLFTPSEGAASKKEVQGRSGDGANAGGLVAAIAPKGSEKAAKASRSVAGSKKEKENSSSKSKKERSEG
VGTCSEKDPGVLQPVPLGGRGGQYDGSAGVDTGAVEPLGSIAIEPGAALNPLGTKPEPEEGENECRLLKKVKSEKMESPVSTPAVLP
IHLLVPVVNNDISSPCEQIMVRTRSVGVNTCDVALATEPECLGPCEPGTSVNLEGIVWQETEDGMLVVNVTWRNKTYVGTLLDCTRH
DWAPPRFCDSPTSDLEMRNGRGRGKRMRPNSNTPVNETATASDSKGTSNSSKTRAGANSKGRRGSQNSSEHRPPASSTSEDVKASPS
SANKRKNKPLSDMELNSSSEDSKGSKRVRTNSMGSATGPLPGTKVEPTVLDRNCPSPVLIDCPHPNCNKKYKHINGLKYHQAHAHTD
DDSKPEADGDSEYGEEPILHADLGSCNGASVSQKGSLSPARSATPKVRLVEPHSPSPSSKFSTKGLCKKKLSGEGDTDLGALSNDGS
DDGPSVMDETSNDAFDSLERKCMEKEKCKKPSSLKPEKIPSKSLKSARPIAPAIPPQQIYTFQTATFTAASPGSSSGLTATVAQAMP
NSPQLKPIQPKPTVMGEPFTVNPALTPAKDKKKKDKKKKESSKELESPLTPGKVCRAEEGKSPFRESSGDGMKMEGLLNGSSDPHQS
RLASIKAEADKIYSFTDNAPSPSIGGSSRLENTTPTQPLTPLHVVTQNGAEASSVKTNSPAYSDISDAGEDGEGKVDSVKSKDAEQL
VKEGAKKTLFPPQPQSKDSPYYQGFESYYSPSYAQSSPGALNPSSQAGVESQALKTKRDEEPESIEGKVKNDICEEKKPELSSSSQQ
PSVIQQRPNMYMQSLYYNQYAYVPPYGYSDQSYHTHLLSTNTAYRQQYEEQQKRQSLEQQQRGVDKKAEMGLKEREAALKEEWKQKP
SIPPTLTKAPSLTDLVKSGPGKAKEPGADPAKSVIIPKLDDSSKLPGQAPEGLKVKLSDASHLSKEASEAKTGAECGRQAEMDPILW
YRQEAEPRMWTYVVYPAKYSDIKSEDERWKEERDRKLKEERSRSKDSVPKEDGKESTSSDCKLPTSEESRLGSKEPRPSVHVPVSSPL
TQHQSYIPYMHGYSYSQSYDPNHPSYRSMPAVMMQNYPGSYLPSSYSFSPYGSKVSGGEDADKARASPSVTCKSSSESKALDILQQH
ASHYKSKSPTISDKTSQERDRGGCGVVGGGGSCSSVGGASGGERSVDRPRTSPSQRLMSTHHHHHHLGYSLLPAQYNLPYAAGLSST
AIVASQQGSTPSLYPPPRR
>sp|O15119|TBX3_HUMAN
MSLSMRDPVIPGTSMAYHPFLPHRAPDFAMSAVLGHQPPFFPALTLPPNGAAALSLPGALAKPIMDQLVGAAETGIPFSSLGPQAHL
RPLKTMEPEEEVEDDPKVHLEAKELWDQFHKRGTEMVITKSGRRMFPPFKVRCSGLDKKAKYILLMDIIAADDCRYKFHNSRWMVAG
KADPEMPKRMYIHPDSPATGEQWMSKVVTFHKLKLTNNISDKHGFTLAFPSDHATWQGNYSFGTQTILNSMHKYQPRFHIVRANDIL
KLPYSTFRTYLFPETEFIAVTAYQNDKITQLKIDNNPFAKGFRDTGNGRREKRKQLTLQSMRVFDERHKKENGTSDESSSEQAAFNC
FAQASSPAASTVGTSNLKDLCPSEGESDAEAESKEEHGPEACDAAKISTTTSEEPCRDKGSPAVKAHLFAAERPRDSGRLDKASPDS
RHSPATISSSTRGLGAEERRSPVREGTAPAKVEEARALPGKEAFAPLTVQTDAAAAHLAQGPLPGLGFAPGLAGQQFFNGHPLFLHP
SQFAMGGAFSSMAAAGMGPLLATVSGASTGVSGLDSTAMASAAAAQGLSGASAATLPFHLQQHVLASQGLAMSPFGSLFPYPYTYMA
AAAAASSAAASSSVHRHPFLNLNTMRPRLRYSPYSIPVPVPDGSSLLTTALPSMAAAAGPLDGKVAALAASPASVAVDSGSELNSRS
STLSSSSMSLSPKLCAEKEAATSELQSIQRLVSGLEAKPDRSRSASP
>sp|P16403|H12_HUMAN
MSETAPAAPAAAPPAEKAPVKKKAAKKAGGTPRKASGPPVSELITKAVAASKERSGVSLAALKKALAAAGYDVEKNNSRIKLGLKSL
VSKGTLVQTKGTGASGSFKLNKKAASGEAKPKVKKAGGTKPKKPVGAAKKPKKAAGGATPKKSAKKTPKKAKKPAAATVTKKVAKSP
KKAKVAKPKKAAKSAAKAVKPKAAKPKVVKPKKAAPKKK
>sp|Q8WW52|F151A_HUMAN
MVCREQLSKNQVKWVFAGITCVSVVVIAAIVLAITLRRPGCELEACSPDADMLDYLLSLGQISRRDALEVTWYHAANSKKAMTAALN
SNITVLEADVNVEGLGTANETGVPIMAHPPTIYSDNTLEQWLDAVLGSSQKGIKLDFKNIKAVGPSLDLLRQLTEEGKVRRPIWINA
DILKGPNMLISTEVNATQFLALVQEKYPKATLSPGWTTFYMSTSPNRTYTQAMVEKMHELVGGVPQRVTFPVRSSMVRAAWPHFSWL
LSQSERYSLTLWQAASDPMSVEDLLYVRDNTAVHQVYYDIFEPLLSQFKQLALNATRKPMYYTGGSLIPLLQLPGDDGLNVEWLVPD
VQGSGKTATMTLPDTEGMILLNTGLEGTVAENPVPIVHTPSGNILTLESCLQQLATHPGHWGIHLQIAEPAALRPSLALLARLSSLG
LLHWPVWVGAKISHGSFSVPGHVAGRELLTAVAEVFPHVTVAPGWPEEVLGSGYREQLLTDMLELCQGLWQPVSFQMQAMLLGHSTA
GAIGRLLASSPRATVTVEHNPAGGDYASVRTALLAARAVDRTRVYYRLPQGYHKDLLAHVGRN
>sp|P17097|ZNF7_HUMAN

MEVVTFGDVAVHFSREEWQCLDPGQRALYREVMLENHSSVAGLAGFLVFKPELISRLEQGEEPWVLDLQGAEGTEAPRTSKTDSTIR
TENEQACEDMDILKSESYGTVVRISPQDFPQNPGFGDVSDSEVWLDSHLGSPGLKVTGFTFQNNCLNEETVVPKTFTKDAPQGCKEL
GSSGLDCQPLESQGESAEGMSQRCEECGKGIRATSDIALHWEINTQKISRCQECQKKLSDCLQGKHTNNCHGEKPYECAECGKVFRL
CSQLNQHQRIHTGEKPFKCTECGKAFRLSSKLIQHQRIHTGEKPYRCEECGKAFGQSSSLIHHQRIHTGERPYGCRECGKAFSQQSQ
LVRHQRTHTGERPYPCKECGKAFSQSSTLAQHQRMHTGEKAQILKASDSPSLVAHQRIHAVEKPFKCDECGKAFRWISRLSQHQLIH
TGEKPYKCNKCTKAFGCSSRLIRHQRTHTGEKPFKCDECGKGFVQGSHLIQHQRIHTGEKPYVCNDCGKAFSQSSSLIYHQRIHKGE
KPYECLQCGKAFSMSTQLTIHQRVHTGERPYKCNECGKAFSQNSTLFQHQIIHAGVKPYECSECGKAFSRSSYLIEHQRIHTRAQWF
YEYGNALEGSTFVSRKKVNTIKKLHQCEDCEKIFRWRSHLIIHQRIHTGEKPYKCNDCGKAFNRSSRLTQHQKIHMG
>sp|O43747|AP1G1_HUMAN
MPAPIRLRELIRTIRTARTQAEEREMIQKECAAIRSSFREEDNTYRCRNVAKLLYMHMLGYPAHFGQLECLKLIASQKFTDKRIGYL
GAMLLLDERQDVHLLMTNCIKNDLNHSTQFVQGLALCTLGCMGSSEMCRDLAGEVEKLLKTSNSYLRKKAALCAVHVIRKVPELMEM
FLPATKNLLNEKNHGVLHTSVVLLTEMCERSPDMLAHFRKLVPQLVRILKNLIMSGYSPEHDVSGISDPFLQVRILRLLRILGRNDD
DSSEAMNDILAQVATNTETSKNVGNAILYETVLTIMDIKSESGLRVLAINILGRFLLNNDKNIRYVALTSLLKTVQTDHNAVQRHRS
TIVDCLKDLDVSIKRRAMELSFALVNGNNIRGMMKELLYFLDSCEPEFKADCASGIFLAAEKYAPSKRWHIDTIMRVLTTAGSYVRD
DAVPNLIQLITNSVEMHAYTVQRLYKAILGDYSQQPLVQVAAWCIGEYGDLLVSGQCEEEEPIQVTEDEVLDILESVLISNMSTSVT
RGYALTAIMKLSTRFTCTVNRIKKVVSIYGSSIDVELQQRAVEYNALFKKYDHMRSALLERMPVMEKVTTNGPTEIVQTNGETEPAP
LETKPPPSGPQPTSQANDLLDLLGGNDITPVIPTAPTSKPSSAGGELLDLLGDINLTGAPAAAPAPASVPQISQPPFLLDGLSSQPL
FNDIAAGIPSITAYSKNGLKIEFTFERSNTNPSVTVITIQASNSTELDMTDFVFQAAVPKTFQLQLLSPSSSIVPAFNTGTITQVIK
VLNPQKQQLRMRIKLTYNHKGSAMQDLAEVNNFPPQSWQ
>sp|Q495B1|AKD1A_HUMAN
MQEELAWETDGLLPLERQLHEAARQNNVGRMQELIGRRVNTRARNHVGRVALHWAAGAGHEQAVRLLLEHEAAVDEEDAVGALTEAR
LCFGMNALLLSAWFGHLRILQILVNSGAKIHCESKDGLTLLHCAAQKGHVPVLAFIMEDLEDVALDHVDKLGRTAFHRAAEHGQLDA
LDFLVGSGCDHNVKDKEGNTALHLAAGRGHMAVLQRLVDIGLDLEEQNAEGLTALHSAAGGSHPDCVQLLLRAGSTVNALTQKNLSC
LHYAALSGSEDVSRVLIHAGGCANVVDHQGASPLHLAVRHNFPALVRLLINSDSDVNAVDNRQQTPLHLAAEHAWQDIADMLLIAGV
DLNLRDKQGKTALAVAVRSNHVSLVDMIIKADRFYRWEKDHPSDPSGKSLSFKQDHRQETQQLRSVLWRLASRYLQPREWKKLAYSW
EFTEAHVDAIEQQWTGTRSYQEHGHRMLLIWLHGVATAGENPSKALFEGLVAIGRRDLAGWSTMARSQLTATSASRVQMILVPQPPE
>sp|Q9Y446|PKP3_HUMAN
MQDGNFLLSALQPEAGVCSLALPSDLQLDRRGAEGPEAERLRAARVQEQVRARLLQLGQQPRHNGAAEPEPEAETARGTSRGQYHTL
QAGFSSRSQGLSGDKTSGFRPIAKPAYSPASWSSRSAVDLSCSRRLSSAHNGGSAFGAAGYGGAQPTPPMPTRPVSFHERGGVGSRA
DYDTLSLRSLRLGPGGLDDRYSLVSEQLEPAATSTYRAFAYERQASSSSSRAGGLDWPEATEVSPSRTIRAPAVRTLQRFQSSHRSR
GVGGAVPGAVLEPVARAPSVRSLSLSLADSGHLPDVHGFNSYGSHRTLQRLSSGFDDIDLPSAVKYLMASDPNLQVLGAAYIQHKCY
SDAAAKKQARSLQAVPRLVKLFNHANQEVQRHATGAMRNLIYDNADNKLALVEENGIFELLRTLREQDDELRKNVTGILWNLSSSDH
LKDRLARDTLEQLTDLVLSPLSGAGGPPLIQQNASEAEIFYNATGFLRNLSSASQATRQKMRECHGLVDALVTSINHALDAGKCEDK
SVENAVCVLRNLSYRLYDEMPPSALQRLEGRGRRDLAGAPPGEVVGCFTPQSRRLRELPLAADALTFAEVSKDPKGLEWLWSPQIVG
LYNRLLQRCELNRHTTEAAAGALQNITAGDRRWAGVLSRLALEQERILNPLLDRVRTADHHQLRSLTGLIRNLSRNARNKDEMSTKV
VSHLIEKLPGSVGEKSPPAEVLVNIIAVLNNLVVASPIAARDLLYFDGLRKLIFIKKKRDSPDSEKSSRAASSLLANLWQYNKLHRD
FRAKGYRKEDFLGP
>sp|Q9Y6E2|BZW2_HUMAN
MNKHQKPVLTGQRFKTRKRDEKEKFEPTVFRDTLVQGLNEAGDDLEAVAKFLDSTGSRLDYRRYADTLFDILVAGSMLAPGGTRIDD
GDKTKMTNHCVFSANEDHETIRNYAQVFNKLIRRYKYLEKAFEDEMKKLLLFLKAFSETEQTKLAMLSGILLGNGTLPATILTSLFT
DSLVKEGIAASFAVKLFKAWMAEKDANSVTSSLRKANLDKRLLELFPVNRQSVDHFAKYFTDAGLKELSDFLRVQQSLGTRKELQKE
LQERLSQECPIKEVVLYVKEEMKRNDLPETAVIGLLWTCIMNAVEWNKKEELVAEQALKHLKQYAPLLAVFSSQGQSELILLQKVQE
YCYDNIHFMKAFQKIVVLFYKADVLSEEAILKWYKEAHVAKGKSVFLDQMKKFVEWLQNAEEESESEGEEN
>sp|P0DN80|OR5H8_HUMAN

MDDENATLLTEFVLTGLTYQSEWKIPLFLAFLVIYLITIMANLGLIAVIWKDSHLHIPMYLFLGSLAFVDAWLSSSVTPKMLISFLA
KSMIISVSECKIQFFSFGISGTTECFLLATMAYDRYVAICKPLLYPVIMTNGLCIWLLVLSFIGGFLHALIHEGILFRLTFCNSNII
HHFYCDIIPLLKISCTDPSINFLMLFILSGSIQVFTILTVLVSYTFVLFTILKKKAKDIRKAFSTCGAHLLSVSLYYGPLLFMYVHP
ASPQADDQDMVESLFYTVIIPFLNPIIYSLRNKQVIDSLTKTLKGNV
>sp|Q13454|TUSC3_HUMAN
MGARGAPSRRRQAGRRLRYLPTGSFPFLLLLLLLCIQLGGGQKKKENLLAEKVEQLMEWSSRRSIFRMNGDKFRKFIKAPPRNYSMI
VMFTALQPQRQCSVCRQANEEYQILANSWRYSSAFCNKLFFSMVDYDEGTDVFQQLNMNSAPTFMHFPPKGRPKRADTFDLQRIGFA
AEQLAKWIADRTDVHIRVFRPPNYSGTIALALLVSLVGGLLYLRRNNLEFIYNKTGWAMVSLCIVFAMTSGQMWNHIRGPPYAHKNP
HNGQVSYIHGSSQAQFVAESHIILVLNAAITMGMVLLNEAATSKGDVGKRRIICLVGLGLVVFFFSFLLSIFRSKYHGYPYSDLDFE
>sp|Q8NCS7|CTL5_HUMAN
MNDTEKPADTPSEEEDFGDPRTYDPDFKGPVANRSCTDVLCCMIFLLCIIGYIVLGLVAWVHGDPRRAAYPTDSQGHFCGQKGTPNE
NKTILFYFNLLRCTSPSVLLNLQCPTTQICVSKCPEKFLTYVEMQLLYTKDKSYWEDYRQFCKTTAKPVKSLTQLLLDDDCPTAIFP
SKPFLQRCFPDFSTKNGTLTIGSKMMFQDGNGGTRSVVELGIAANGINKLLDAKSLGLKVFEDYARTWYWILIGLTIAMVLSWIFLI
LLRFIAGCLFWVFMIGVIGIIGYGIWHCYQQYTNLQERPSSVLTIYDIGIQTNISMYFELQQTWFTFMIILCIIEVIVILMLIFLRN
RIRVAIILLKEGSKAIGYVPSTLVYPALTFILLSICICYWVVTAVFLATSGVPVYKVIAPGGHCIHENQTCDPEIFNTTEIAKACPG
ALCNFAFYGGKSLYHQYIPTFHVYNLFVFLWLINFVIALGQCALAGAFATYYWAMKKPDDIPRYPLFTAFGRAIRYHTGSLAFGSLI
IALIQMFKIVLEYLDHRLKRTQNTLSKFLQCCLRCCFWCLENAIKFLNRNAYIMIAIYGRNFCRSAKDAFNLLMRNVLKVAVTDEVT
YFVLFLGKLLVAGSIGVLAFLFFTQRLPVIAQGPASLNYYWVPLLTVIFGSYLIAHGFFSVYAMCVETIFICFLEDLERNDGSTARP
YFVTPNLHGILIKKQLVPQKQKE
>sp|Q8N127|O5AS1_HUMAN
MLESNYTMPTEFLFVGFTDYLPLRVTLFLVFLLVYTLTMVGNILLIILVNINSSLQIPMYYFLSNLSFLDISCSTAITPKMLANFLA
SRKSISPYGCALQMFFFASFADAECLILAAMAYDRYAAICNPLLYTTLMSRRVCVCFIVLAYFSGSTTSLVHVCLTFRLSFCGSNIV
NHFFCDIPPLLALSCTDTQINQLLLFALCSFIQTSTFVVIFISYFCILITVLSIKSSGGRSKTFSTCASHLIAVTLFYGALLFMYLQ
PTTSYSLDTDKVVAVFYTVVFPMFNPIIYSFRNKDVKNALKKLLERIGYSNEWYLNRLRIVNI
>sp|P35670|ATP7B_HUMAN
MPEQERQITAREGASRKILSKLSLPTRAWEPAMKKSFAFDNVGYEGGLDGLGPSSQVATSTVRILGMTCQSCVKSIEDRISNLKGII
SMKVSLEQGSATVKYVPSVVCLQQVCHQIGDMGFEASIAEGKAASWPSRSLPAQEAVVKLRVEGMTCQSCVSSIEGKVRKLQGVVRV
KVSLSNQEAVITYQPYLIQPEDLRDHVNDMGFEAAIKSKVAPLSLGPIDIERLQSTNPKRPLSSANQNFNNSETLGHQGSHVVTLQL
RIDGMHCKSCVLNIEENIGQLLGVQSIQVSLENKTAQVKYDPSCTSPVALQRAIEALPPGNFKVSLPDGAEGSGTDHRSSSSHSPGS
PPRNQVQGTCSTTLIAIAGMTCASCVHSIEGMISQLEGVQQISVSLAEGTATVLYNPSVISPEELRAAIEDMGFEASVVSESCSTNP
LGNHSAGNSMVQTTDGTPTSVQEVAPHTGRLPANHAPDILAKSPQSTRAVAPQKCFLQIKGMTCASCVSNIERNLQKEAGVLSVLVA
LMAGKAEIKYDPEVIQPLEIAQFIQDLGFEAAVMEDYAGSDGNIELTITGMTCASCVHNIESKLTRTNGITYASVALATSKALVKFD
PEIIGPRDIIKIIEEIGFHASLAQRNPNAHHLDHKMEIKQWKKSFLCSLVFGIPVMALMIYMLIPSNEPHQSMVLDHNIIPGLSILN
LIFFILCTFVQLLGGWYFYVQAYKSLRHRSANMDVLIVLATSIAYVYSLVILVVAVAEKAERSPVTFFDTPPMLFVFIALGRWLEHL
AKSKTSEALAKLMSLQATEATVVTLGEDNLIIREEQVPMELVQRGDIVKVVPGGKFPVDGKVLEGNTMADESLITGEAMPVTKKPGS
TVIAGSINAHGSVLIKATHVGNDTTLAQIVKLVEEAQMSKAPIQQLADRFSGYFVPFIIIMSTLTLVVWIVIGFIDFGVVQRYFPNP
NKHISQTEVIIRFAFQTSITVLCIACPCSLGLATPTAVMVGTGVAAQNGILIKGGKPLEMAHKIKTVMFDKTGTITHGVPRVMRVLL
LGDVATLPLRKVLAVVGTAEASSEHPLGVAVTKYCKEELGTETLGYCTDFQAVPGCGIGCKVSNVEGILAHSERPLSAPASHLNEAG
SLPAEKDAVPQTFSVLIGNREWLRRNGLTISSDVSDAMTDHEMKGQTAILVAIDGVLCGMIAIADAVKQEAALAVHTLQSMGVDVVL
ITGDNRKTARAIATQVGINKVFAEVLPSHKVAKVQELQNKGKKVAMVGDGVNDSPALAQADMGVAIGTGTDVAIEAADVVLIRNDLL
DVVASIHLSKRTVRRIRINLVLALIYNLVGIPIAAGVFMPIGIVLQPWMGSAAMAASSVSVVLSSLQLKCYKKPDLERYEAQAHGHM
KPLTASQVSVHIGMDDRWRDSPRATPWDQVSYVSQVSLSSLTSDKPSRHSAAADDDGDKWSLLLNGRDEEQYI
>sp|O15234|CASC3_HUMAN

MADRRRQRASQDTEDEESGASGSDSGGSPLRGGGSCSGSAGGGGSGSLPSQRGGRTGALHLRRVESGGAKSAEESECESEDGIEGDA
VLSDYESAEDSEGEEGEYSEEENSKVELKSEANDAVNSSTKEEKGEEKPDTKSTVTGERQSGDGQESTEPVENKVGKKGPKHLDDDE
DRKNPAYIPRKGLFFEHDLRGQTQEEEVRPKGRQRKLWKDEGRWEHDKFREDEQAPKSRQELIALYGYDIRSAHNPDDIKPRRIRKP
RYGSPPQRDPNWNGERLNKSHRHQGLGGTLPPRTFINRNAAGTGRMSAPRNYSRSGGFKEGRAGFRPVEAGGQHGGRSGETVKHEIS
YRSRRLEQTSVRDPSPEADAPVLGSPEKEEAASEPPAAAPDAAPPPPDRPIEKKSYSRARRTRTKVGDAVKLAEEVPPPPEGLIPAP
PVPETTPTPPTKTGTWEAPVDSSTSGLEQDVAQLNIAEQNWSPGQPSFLQPRELRGMPNHIHMGAGPPPQFNRMEEMGVQGGRAKRY
SSQRQRPVPEPPAPPVHISIMEGHYYDPLQFQGPIYTHGDSPAPLPPQGMLVQPGMNLPHPGLHPHQTPAPLPNPGLYPPPVSMSPG
QPPPQQLLAPTYFSAPGVMNFGNPSYPYAPGALPPPPPPHLYPNTQAPSQVYGGVTYYNPAQQQVQPKPSPPRRTPQPVTIKPPPPE
VVSRGSS
>sp|Q15070|OXA1L_HUMAN
MAMGLMCGRRELLRLLQSGRRVHSVAGPSQWLGKPLTTRLLFPVAPCCCRPHYLFLAASGPRSLSTSAISFAEVQVQAPPVVAATPS
PTAVPEVASGETADVVQTAAEQSFAELGLGSYTPVGLIQNLLEFMHVDLGLPWWGAIAACTVFARCLIFPLIVTGQREAARIHNHLP
EIQKFSSRIREAKLAGDHIEYYKASSEMALYQKKHGIKLYKPLILPVTQAPIFISFFIALREMANLPVPSLQTGGLWWFQDLTVSDP
IYILPLAVTATMWAVLELGAETGVQSSDLQWMRNVIRMMPLITLPITMHFPTAVFMYWLSSNLFSLVQVSCLRIPAVRTVLKIPQRV
VHDLDKLPPREGFLESFKKGWKNAEMTRQLREREQRMRNQLELAARGPLRQTFTHNPLLQPGKDNPPNIPSSSSKPKSKYPWHDTLG
>sp|Q96PG8|BBC3B_HUMAN
MKFGMGSAQACPCQVPRAASTTWVPCQICGPRERHGPRTPGGQLPGARRGPGPRRPAPLPARPPGALGSVLRPLRARPGCRPRRPHP
AARCLPLRPHRPTRRHRRPGGFPLAWGSPQPAPRPAPGRSSALALAGGAAPGVARAQRPGGSGGRSHPGGPGSPRGGGTVGPGDRGP
AAADGGRPQRTVRAAETRGAAAAPPLTLEGPVQSHHGTPALTQGPQSPRDGAQLGACTRPVDVRDSGGRPLPPPDTLASAGDFLCTM
>sp|Q9UHW9|S12A6_HUMAN
MHPPETTTKMASVRFMVTPTKIDDIPGLSDTSPDLSSRSSSRVRFSSRESVPETSRSEPMSEMSGATTSLATVALDPPSDRTSHPQD
VIEDLSQNSITGEHSQLLDDGHKKARNAYLNNSNYEEGDEYFDKNLALFEEEMDTRPKVSSLLNRMANYTNLTQGAKEHEEAENITE
GKKKPTKTPQMGTFMGVYLPCLQNIFGVILFLRLTWVVGTAGVLQAFAIVLICCCCTMLTAISMSAIATNGVVPAGGSYFMISRALG
PEFGGAVGLCFYLGTTFAAAMYILGAIEIFLVYIVPRAAIFHSDDALKESAAMLNNMRVYGTAFLVLMVLVVFIGVRYVNKFASLFL
ACVIVSILAIYAGAIKSSFAPPHFPVCMLGNRTLSSRHIDVCSKTKEINNMTVPSKLWGFFCNSSQFFNATCDEYFVHNNVTSIQGI
PGLASGIITENLWSNYLPKGEIIEKPSAKSSDVLGSLNHEYVLVDITTSFTLLVGIFFPSVTGIMAGSNRSGDLKDAQKSIPIGTIL
AILTTSFVYLSNVVLFGACIEGVVLRDKFGDAVKGNLVVGTLSWPSPWVIVIGSFFSTCGAGLQSLTGAPRLLQAIAKDNIIPFLRV
FGHSKANGEPTWALLLTAAIAELGILIASLDLVAPILSMFFLMCYLFVNLACALQTLLRTPNWRPRFRYYHWALSFMGMSICLALMF
ISSWYYAIVAMVIAGMIYKYIEYQGAEKEWGDGIRGLSLSAARFALLRLEEGPPHTKNWRPQLLVLLKLDEDLHVKHPRLLTFASQL
KAGKGLTIVGSVIVGNFLENYGEALAAEQTIKHLMEAEKVKGFCQLVVAAKLREGISHLIQSCGLGGMKHNTVVMGWPNGWRQSEDA
RAWKTFIGTVRVTTAAHLALLVAKNISFFPSNVEQFSEGNIDVWWIVHDGGMLMLLPFLLKQHKVWRKCSIRIFTVAQLEDNSIQMK
KDLATFLYHLRIEAEVEVVEMHDSDISAYTYERTLMMEQRSQMLRHMRLSKTERDREAQLVKDRNSMLRLTSIGSDEDEETETYQEK
VHMTWTKDKYMASRGQKAKSMEGFQDLLNMRPDQSNVRRMHTAVKLNEVIVNKSHEAKLVLLNMPGPPRNPEGDENYMEFLEVLTEG
LERVLLVRGGGSEVITIYS
>sp|Q8N183|MIMIT_HUMAN
MGWSQDLFRALWRSLSREVKEHVGTDQFGNKYYYIPQYKNWRGQTIREKRIVEAANKKEVDYEAGDIPTEWEAWIRRTRKTPPTMEE
ILKNEKHREEIKIKSQDFYEKEKLLSKETSEELLPPPVQTQIKGHASAPYFGKEEPSVAPSSTGKTFQPGSWMPRDGKSHNQ
>sp|O15205|UBD_HUMAN
MAPNASCLCVHVRSEEWDLMTFDANPYDSVKKIKEHVRSKTKVPVQDQVLLLGSKILKPRRSLSSYGIDKEKTIHLTLKVVKPSDEE
LPLFLVESGDEAKRHLLQVRRSSSVAQVKAMIETKTGIIPETQIVTCNGKRLEDGKMMADYGIRKGNLLFLACYCIGG
>sp|Q8N960|CE120_HUMAN
MVSKSDQLLIVVSILEGRHFPKRPKHMLVVEAKFDGEQLATDPVDHTDQPEFATELAWEIDRKALHQHRLQRTPIKLQCFALDPVTS
AKETIGYIVLDLRTAQETKQAPKWYQLLSNKYTKFKSEIQISIALETDTKPPVDSFKAKGAPPRDGKVPAILAGLDPRDIVAVLNEE
GGYHQIGPAEYCTDSFIMSVTIAFATQLEQLIPCTMKLPERQPEFFFYYSLLGNDVTNEPFNDLINPNFEPERASVRIRSSVEILRV

YLALQSKLQIHLCCGDQSLGSTEIPLTGLLKKGSTEINQHPVTVEGAFTLDPPNRAKQKLAPIPVELAPTVGVSVALQREGIDSQSL
IELKTQNEHEPEHSKKKVLTPIKEKTLTGPKSPTVSPVPSHNQSPPTKDDATESEVESLQYDKDTKPNPKASSSVPASLAQLVTTSN
ASEVASGQKIAVPATSHHFCFSIDLRSIHALEIGFPINCILRYSYPFFGSAAPIMTNPPVEVRKNMEVFLPQSYCAFDFATMPHQLQ
DTFLRIPLLVELWHKDKMSKDLLLGIARIQLSNILSSEKTRFLGSNGEQCWRQTYSESVPVIAAQGSNNRIADLSYTVTLEDYGLVK
MREIFISDSSQGVSAVQQKPSSLPPAPCPSEIQTEPRETLEYKAALELEMWKEMQEDIFENQLKQKELAHMQALAEEWKKRDRERES
LVKKKVAEYTILEGKLQKTLIDLEKREQQLASVESELQREKKELQSERQRNLQELQDSIRRAKEDCIHQVELERLKIKQLEEDKHRL
QQQLNDAENKYKILEKEFQQFKDQQNNKPEIRLQSEINLLTLEKVELERKLESATKSKLHYKQQWGRALKELARLKQREQESQMARL
KKQQEELEQMRLRYLAAEEKDTVKTERQELLDIRNELNRLRQQEQKQYQDSTEIASGKKDGPHGSVLEEGLDDYLTRLIEERDTLMR
TGVYNHEDRIISELDRQIREILAKSNASN
>sp|P56945|BCAR1_HUMAN
MNHLNVLAKALYDNVAESPDELSFRKGDIMTVLEQDTQGLDGWWLCSLHGRQGIVPGNRLKILVGMYDKKPAGPGPGPPATPAQPQP
GLHAPAPPASQYTPMLPNTYQPQPDSVYLVPTPSKAQQGLYQVPGPSPQFQSPPAKQTSTFSKQTPHHPFPSPATDLYQVPPGPGGP
AQDIYQVPPSAGMGHDIYQVPPSMDTRSWEGTKPPAKVVVPTRVGQGYVYEAAQPEQDEYDIPRHLLAPGPQDIYDVPPVRGLLPSQ
YGQEVYDTPPMAVKGPNGRDPLLEVYDVPPSVEKGLPPSNHHAVYDVPPSVSKDVPDGPLLREETYDVPPAFAKAKPFDPARTPLVL
AAPPPDSPPAEDVYDVPPPAPDLYDVPPGLRRPGPGTLYDVPRERVLPPEVADGGVVDSGVYAVPPPAEREAPAEGKRLSASSTGST
RSSQSASSLEVAGPGREPLELEVAVEALARLQQGVSATVAHLLDLAGSAGATGSWRSPSEPQEPLVQDLQAAVAAVQSAVHELLEFA
RSAVGNAAHTSDRALHAKLSRQLQKMEDVHQTLVAHGQALDAGRGGSGATLEDLDRLVACSRAVPEDAKQLASFLHGNASLLFRRTK
ATAPGPEGGGTLHPNPTDKTSSIQSRPLPSPPKFTSQDSPDGQYENSEGGWMEDYDYVHLQGKEEFEKTQKELLEKGSITRQGKSQL
ELQQLKQFERLEQEVSRPIDHDLANWTPAQPLAPGRTGGLGPSDRQLLLFYLEQCEANLTTLTNAVDAFFTAVATNQPPKIFVAHSK
FVILSAHKLVFIGDTLSRQAKAADVRSQVTHYSNLLCDLLRGIVATTKAAALQYPSPSAAQDMVERVKELGHSTQQFRRVLGQLAAA
>sp|A8MVA2|KRA96_HUMAN
MTHCCSPGCQPTCCRTTCCRTTCWQPTIVTTCSSTPCCQPSCCVSSCCQPYCHPTCCQNTCCRTTCCQPTCVTSCCQPSCCSTPCYQ
PICCGSSCCGQTSCGSSCGQSSSCAPVYCRRTCYHPTTVCLPGCLNQSCGSSCCQPCYCPACCVSSCCQHSCC
>sp|P52741|ZN134_HUMAN
MTLVTAGGAWTGPGCWHEVKDEESSSEQSISIAVSHVNTSKAGLPAQTALPCDICGPILKDILHLDEHQGTHHGLKLHTCGACGRQF
WFSANLHQYQKCYSIEQPLRRDKSEASIVKNCTVSKEPHPSEKPFTCKEEQKNFQATLGGCQQKAIHSKRKTHRSTESGDAFHGEQM
HYKCSECGKAFSRKDTLVQHQRIHSGEKPYECSECGKAFSRKATLVQHQRIHTGERPYECSECGKTFSRKDNLTQHKRIHTGEMPYK
CNECGKYFSHHSNLIVHQRVHNGARPYKCSDCGKVFRHKSTLVQHESIHTGENPYDCSDCGKSFGHKYTLIKHQRIHTESKPFECIE
CGKFFSRSSDYIAHQRVHTGERPFVCSKCGKDFIRTSHLVRHQRVHTGERPYECSECGKAYSLSSHLNRHQKVHTAGRL
>sp|Q9UBQ5|EIF3K_HUMAN
MAMFEQMRANVGKLLKGIDRYNPENLATLERYVETQAKENAYDLEANLAVLKLYQFNPAFFQTTVTAQILLKALTNLPHTDFTLCKC
MIDQAHQEERPIRQILYLGDLLETCHFQAFWQALDENMDLLEGITGFEDSVRKFICHVVGITYQHIDRWLLAEMLGDLSDSQLKVWM
SKYGWSADESGQIFICSQEESIKPKNIVEKIDFDSVSSIMASSQ
>sp|P08758|ANXA5_HUMAN
MAQVLRGTVTDFPGFDERADAETLRKAMKGLGTDEESILTLLTSRSNAQRQEISAAFKTLFGRDLLDDLKSELTGKFEKLIVALMKP
SRLYDAYELKHALKGAGTNEKVLTEIIASRTPEELRAIKQVYEEEYGSSLEDDVVGDTSGYYQRMLVVLLQANRDPDAGIDEAQVEQ
DAQALFQAGELKWGTDEEKFITIFGTRSVSHLRKVFDKYMTISGFQIEETIDRETSGNLEQLLLAVVKSIRSIPAYLAETLYYAMKG
AGTDDHTLIRVMVSRSEIDLFNIRKEFRKNFATSLYSMIKGDTSGDYKKALLLLCGEDD
>sp|Q7RTX1|TS1R1_HUMAN
MLLCTARLVGLQLLISCCWAFACHSTESSPDFTLPGDYLLAGLFPLHSGCLQVRHRPEVTLCDRSCSFNEHGYHLFQAMRLGVEEIN
NSTALLPNITLGYQLYDVCSDSANVYATLRVLSLPGQHHIELQGDLLHYSPTVLAVIGPDSTNRAATTAALLSPFLVPMISYAASSE
TLSVKRQYPSFLRTIPNDKYQVETMVLLLQKFGWTWISLVGSSDDYGQLGVQALENQATGQGICIAFKDIMPFSAQVGDERMQCLMR
HLAQAGATVVVVFSSRQLARVFFESVVLTNLTGKVWVASEAWALSRHITGVPGIQRIGMVLGVAIQKRAVPGLKAFEEAYARADKKA
PRPCHKGSWCSSNQLCRECQAFMAHTMPKLKAFSMSSAYNAYRAVYAVAHGLHQLLGCASGACSRGRVYPWQLLEQIHKVHFLLHKD

TVAFNDNRDPLSSYNIIAWDWNGPKWTFTVLGSSTWSPVQLNINETKIQWHGKDNQVPKSVCSSDCLEGHQRVVTGFHHCCFECVPC
GAGTFLNKSDLYRCQPCGKEEWAPEGSQTCFPRTVVFLALREHTSWVLLAANTLLLLLLLGTAGLFAWHLDTPVVRSAGGRLCFLML
GSLAAGSGSLYGFFGEPTRPACLLRQALFALGFTIFLSCLTVRSFQLIIIFKFSTKVPTFYHAWVQNHGAGLFVMISSAAQLLICLT
WLVVWTPLPAREYQRFPHLVMLECTETNSLGFILAFLYNGLLSISAFACSYLGKDLPENYNEAKCVTFSLLFNFVSWIAFFTTASVY
DGKYLPAANMMAGLSSLSSGFGGYFLPKCYVILCRPDLNSTEHFQASIQDYTRRCGST
>sp|O75439|MPPB_HUMAN
MAAAAARVVLSSAARRRLWGFSESLLIRGAAGRSLYFGENRLRSTQAATQVVLNVPETRVTCLESGLRVASEDSGLSTCTVGLWIDA
GSRYENEKNNGTAHFLEHMAFKGTKKRSQLDLELEIENMGAHLNAYTSREQTVYYAKAFSKDLPRAVEILADIIQNSTLGEAEIERE
RGVILREMQEVETNLQEVVFDYLHATAYQNTALGRTILGPTENIKSISRKDLVDYITTHYKGPRIVLAAAGGVSHDELLDLAKFHFG
DSLCTHKGEIPALPPCKFTGSEIRVRDDKMPLAHLAIAVEAVGWAHPDTICLMVANTLIGNWDRSFGGGMNLSSKLAQLTCHGNLCH
SFQSFNTSYTDTGLWGLYMVCESSTVADMLHVVQKEWMRLCTSVTESEVARARNLLKTNMLLQLDGSTPICEDIGRQMLCYNRRIPI
PELEARIDAVNAETIREVCTKYIYNRSPAIAAVGPIKQLPDFKQIRSNMCWLRD
>sp|Q96BD0|SO4A1_HUMAN
MPLHQLGDKPLTFPSPNSAMENGLDHTPPSRRASPGTPLSPGSLRSAAHSPLDTSKQPLCQLWAEKHGARGTHEVRYVSAGQSVACG
WWAFAPPCLQVLNTPKGILFFLCAAAFLQGMTVNGFINTVITSLERRYDLHSYQSGLIASSYDIAACLCLTFVSYFGGSGHKPRWLG
WGVLLMGTGSLVFALPHFTAGRYEVELDAGVRTCPANPGAVCADSTSGLSRYQLVFMLGQFLHGVGATPLYTLGVTYLDENVKSSCS
PVYIAIFYTAAILGPAAGYLIGGALLNIYTEMGRRTELTTESPLWVGAWWVGFLGSGAAAFFTAVPILGYPRQLPGSQRYAVMRAAE
MHQLKDSSRGEASNPDFGKTIRDLPLSIWLLLKNPTFILLCLAGATEATLITGMSTFSPKFLESQFSLSASEAATLFGYLVVPAGGG
GTFLGGFFVNKLRLRGSAVIKFCLFCTVVSLLGILVFSLHCPSVPMAGVTASYGGSLLPEGHLNLTAPCNAACSCQPEHYSPVCGSD
GLMYFSLCHAGCPAATETNVDGQKVYRDCSCIPQNLSSGFGHATAGKCTSTCQRKPLLLVFIFVVIFFTFLSSIPALTATLRCVRDP
QRSFALGIQWIVVRILGGIPGPIAFGWVIDKACLLWQDQCGQQGSCLVYQNSAMSRYILIMGLLYKVLGVLFFAIACFLYKPLSESS
DGLETCLPSQSSAPDSATDSQLQSSV
>sp|A1L168|CT202_HUMAN
MYKSKIPRAQNQVSVKVTPKNTEMKIAEEPSPSLGQTLEWLRKELSEMQIQDQSLLLTLRHLHSVLEELRADSAHWEDARSSGGTSP
IRARAGSEGRGCQPVCSRGLAQLLRGEDSRRSSLP
>sp|Q9UBU6|FA8A1_HUMAN
MAEGPEEARGHPPGQDDGGGDHEPVPSLRGPPTTAVPCPRDDPQAEPQAPGRPTAPGLAAAAAADKLEPPRELRKRGEAASGSGAEL
QEQAGCEAPEAAAPRERPARLSAREYSRQVHEWLWQSYCGYLTWHSGLAAFPAYCSPQPSPQSFPSGGAAVPQAAAPPPPQLGYYNP
FYFLSPGAAGPDPRTAAGISTPAPVAGLGPRAPHVQASVRATPVTRVGSAAPSRSPSETGRQAGREYVIPSLAHRFMAEMVDFFILF
FIKATIVLSIMHLSGIKDISKFAMHYIIEEIDEDTSMEDLQKMMVVALIYRLLVCFYEIICIWGAGGATPGKFLLGLRVVTCDTSVL
IAPSRVLVIPSSNVSITTSTIRALIKNFSIASFFPAFITLLFFQHNRTAYDIVAGTIVVKRNGVR
>sp|Q5PSV4|BRM1L_HUMAN
MPVHSRGDKKETNHHDEMEVDYAENEGSSSEDEDTESSSVSEDGDSSEMDDEDCERRRMECLDEMSNLEKQFTDLKDQLYKERLSQV
DAKLQEVIAGKAPEYLEPLATLQENMQIRTKVAGIYRELCLESVKNKYECEIQASRQHCESEKLLLYDTVQSELEEKIRRLEEDRHS
IDITSELWNDELQSRKKRKDPFSPDKKKPVVVSGPYIVYMLQDLDILEDWTTIRKAMATLGPHRVKTEPPVKLEKHLHSARSEEGRL
YYDGEWYIRGQTICIDKKDECPTSAVITTINHDEVWFKRPDGSKSKLYISQLQKGKYSIKHS
>sp|Q9H3S1|SEM4A_HUMAN
MALPALGLDPWSLLGLFLFQLLQLLLPTTTAGGGGQGPMPRVRYYAGDERRALSFFHQKGLQDFDTLLLSGDGNTLYVGAREAILAL
DIQDPGVPRLKNMIPWPASDRKKSECAFKKKSNETQCFNFIRVLVSYNVTHLYTCGTFAFSPACTFIELQDSYLLPISEDKVMEGKG
QSPFDPAHKHTAVLVDGMLYSGTMNNFLGSEPILMRTLGSQPVLKTDNFLRWLHHDASFVAAIPSTQVVYFFFEETASEFDFFERLH
TSRVARVCKNDVGGEKLLQKKWTTFLKAQLLCTQPGQLPFNVIRHAVLLPADSPTAPHIYAVFTSQWQVGGTRSSAVCAFSLLDIER
VFKGKYKELNKETSRWTTYRGPETNPRPGSCSVGPSSDKALTFMKDHFLMDEQVVGTPLLVKSGVEYTRLAVETAQGLDGHSHLVMY
LGTTTGSLHKAVVSGDSSAHLVEEIQLFPDPEPVRNLQLAPTQGAVFVGFSGGVWRVPRANCSVYESCVDCVLARDPHCAWDPESRT
CCLLSAPNLNSWKQDMERGNPEWACASGPMSRSLRPQSRPQIIKEVLAVPNSILELPCPHLSALASYYWSHGPAAVPEASSTVYNGS

LLLIVQDGVGGLYQCWATENGFSYPVISYWVDSQDQTLALDPELAGIPREHVKVPLTRVSGGAALAAQQSYWPHFVTVTVLFALVLS
GALIILVASPLRALRARGKVQGCETLRPGEKAPLSREQHLQSPKECRTSASDVDADNNCLGTEVA
>sp|Q8N6L0|KASH5_HUMAN
MDLPEGPVGGPTAEMYLRERPEEARLGMPVSLEEQILNSTFEACDPQRTGTVAVAQVLAYLEAVTGQGPQDARLQTLANSLDPNGEG
PKATVDLDTFLVVMRDWIAACQLHGGLELEEETAFQGALTSRQLPSGCPEAEEPANLESFGGEDPRPELQATADLLSSLEDLELSNR
RLVGENAKLQRSMETAEEGSARLGEEILALRKQLHSTQQALQFAKAMDEELEDLKTLARSLEEQNRSLLAQARQAEKEQQHLVAEME
TLQEENGKLLAERDGVKKRSQELAMEKDTLKRQLFECEHLICQRDTILSERTRDVESLAQTLEEYRVTTQELRLEISRLEEQLSQTY
EGPDELPEGAQLRRVGWTELLPPSLGLEIEAIRQKQEVATADLSNPLCGVWQWEEVIHETSEETEFPSEAPAGGQRNFQGEPAHPEE
GRKEPSMWLTRREEEEDAESQVTADLPVPLGAPRPGDIPENPPERPARRELQQALVPVMKKLVPVRRRAWGQLCLPPQRLRVTRHPL
IPAPVLGLLLLLLLSVLLLGPSPPPTWPHLQLCYLQPPPV
>sp|Q9UJA2|CRLS1_HUMAN
MLALRVARGSWGALRGAAWAPGTRPSKRRACWALLPPVPCCLGCLAERWRLRPAALGLRLPGIGQRNHCSGAGKAAPRPAAGAGAAA
EAPGGQWGPASTPSLYENPWTIPNMLSMTRIGLAPVLGYLIIEEDFNIALGVFALAGLTDLLDGFIARNWANQRSALGSALDPLADK
ILISILYVSLTYADLIPVPLTYMIISRDVMLIAAVFYVRYRTLPTPRTLAKYFNPCYATARLKPTFISKVNTAVQLILVAASLAAPV
FNYADSIYLQILWCFTAFTTAASAYSYYHYGRKTVQVIKD
>sp|Q3B7T1|EDRF1_HUMAN
MGDAKEAGAEGPPAGAAARGGLSLLSQGESEESSAQGSALFLGGNEVKSRAVVKYSSAPPRTAFARLEEKTDLKLPPANWLRESAKL
GPAGTTILGNSKKSKPFSSFGMAYDFIDSVGNDVDVVSDSENIKKLLKIPYSKSHVSMAVHRIGRTLLLDELDIQELFMRSSQTGDW
TWLKEFYQRLIDQKWQRKKKSKEHWYQKAILSKFLYYSINGDGAAQPVSSTAEQQESSSSDQTNDSEGASWPAPFEMPSSVSEDPSA
SSQGSEPLEPSYIVGHVASAPKEQNLITLFNDGEHSQGLKNDFVRNILWTFEDIHMLVGSNMPIFGGGRYPAVSLRLRDNNKPINVL
TGIDYWLDNLICNVPELVMCFHVNGIVQKYEMIKTEEIPNLENSNFSTKVIKDIAQNILSFLKSNCTKEGHTYWLFKASGSDIVKLY
DLTTLCEETEDKYQNPFTMPVAILLYKVACNMMMKKNQNKKHYGTIRTLLLNCLKLLDKSRHPQIIASANYMLSELFQLDEPKKEEN
SESPLNENSDESYSEEEEEMPDSDENGSYSTSSDPSDDSKAVAIIKSVGELSVPEKYKSIHQIRPSCAFPVCHDTEERCRLVLSYVL
EGLKSVDSSIKKESDLPAADPSTPIPLKYEDESSRGGPEGLEKQMALFLDKMGSLQKGNYSSQSGMIPGSWQHKMKLQLILKSSKAY
YVLSDAAMSLQKYGRALRYIKLALQSHDTYCCLCTNMLSEVLLFLSQYLTLCGDIQLMLAQNANNRAAHLEEFHYQTKEDQEILHSL
HRESSCQGFAWATDLSTDLESQLSVSCKCYEAANEILQFSDLKSQNPEHYVQVLKRMGNIRNEIGVFYMNQAAALQSERLVSKSVSA
AEQQLWKKSFSCFEKGIHNFESIEDATNAALLLCNTGRLMRICAQAHCGAGDELKREFSPEEGLYYNKAIDYYLKALRSLGTRDIHP
AVWDSVNWELSTTYFTMATLQQDYAPLSRKAQEQIEKEVSEAMMKSLKYCDVDSVSARQPLCQYRAATIHHRLASMYHSCLRNQVGD
EHLRKQHRVLADLHYSKAAKLFQLLKDAPCELLRVQLERVAFAEFQMTSQNSNVGKLKTLSGALDIMVRTEHAFQLIQKELIEEFGQ
PKSGDAAAAADASPSLNREEVMKLLSIFESRLSFLLLQSIKLLSSTKKKTSNNIEDDTILKTNKHIYSQLLRATANKTATLLERINV
IVHLLGQLAAGSAASSNAVQ
>sp|Q15743|OGR1_HUMAN
MGNITADNSSMSCTIDHTIHQTLAPVVYVTVLVVGFPANCLSLYFGYLQIKARNELGVYLCNLTVADLFYICSLPFWLQYVLQHDNW
SHGDLSCQVCGILLYENIYISVGFLCCISVDRYLAVAHPFRFHQFRTLKAAVGVSVVIWAKELLTSIYFLMHEEVIEDENQHRVCFE
HYPIQAWQRAINYYRFLVGFLFPICLLLASYQGILRAVRRSHGTQKSRKDQIQRLVLSTVVIFLACFLPYHVLLLVRSVWEASCDFA
KGVFNAYHFSLLLTSFNCVADPVLYCFVSETTHRDLARLRGACLAFLTCSRTGRAREAYPLGAPEASGKSGAQGEEPELLTKLHPAF
QTPNSPGSGGFPTGRLA
>sp|O00482|NR5A2_HUMAN
MSSNSDTGDLQESLKHGLTPIGAGLPDRHGSPIPARGRLVMLPKVETEALGLARSHGEQGQMPENMQVSQFKMVNYSYDEDLEELCP
VCGDKVSGYHYGLLTCESCKGFFKRTVQNNKRYTCIENQNCQIDKTQRKRCPYCRFQKCLSVGMKLEAVRADRMRGGRNKFGPMYKR
DRALKQQKKALIRANGLKLEAMSQVIQAMPSDLTISSAIQNIHSASKGLPLNHAALPPTDYDRSPFVTSPISMTMPPHGSLQGYQTY
GHFPSRAIKSEYPDPYTSSPESIMGYSYMDSYQTSSPASIPHLILELLKCEPDEPQVQAKIMAYLQQEQANRSKHEKLSTFGLMCKM
ADQTLFSIVEWARSSIFFRELKVDDQMKLLQNCWSELLILDHIYRQVVHGKEGSIFLVTGQQVDYSIIASQAGATLNNLMSHAQELV

AKLRSLQFDQREFVCLKFLVLFSLDVKNLENFQLVEGVQEQVNAALLDYTMCNYPQQTEKFGQLLLRLPEIRAISMQAEEYLYYKHL
NGDVPYNNLLIEMLHAKRA
>sp|Q5TAH2|SL9C2_HUMAN
MSSYFWAQNESNRPDLLCGQPADYLVEEKHFTTLVCFIVVLGGLLKMCLKNCEVIVLTILSLSGFVIGHMAYNSVEVHQIVYPLLRT
SSFSLYSYFSPLIIFMVALDVEFYTLKKMFWQVLLTGLISFSTASIIIGYVVIKFNKDSWDLQSCLLFSITLGIIDPLRSVNSLKTI
GISKIYIDLIRGESLIICSIASIFFGNFRGNRIHFSIFRDLHVGIELSYDILGSIIFGYWCAKIIQCILADVFSNMLTNIILCFSMV
YMTFYIVEFLGMSGTLALAAVGLNLDSLTFKPKIELVITKFLRIFSSVYEHLIYAFFGIVIGCGELSHYEFHTIPFIFILFTTVNLV
RLLTILLVSPILMHSNYEYNWRWGVVITWSGIKGVFNLLWAPDVYNLAERKVEVPQMFILYVQVISLLTMGINSYVMTQSARKLDLC
VLSLPRQMILQNATQHIQEIVQNTITLFKTEKILTNVNWTLVEDKTRIEYIPFSHVSHNDMKTESTTDEALMEEARLHVAAIQMSSF
EKQRNNGILEIEAARILIGAAKCYYSIQGKFMSIYDVSTYMRTRSWLIKFKNVLTFLEYCIEKIHFIPPESNTFLTFIFHIVFSEEF
EYTGQIINLIYIYPMIIHLWPMARGLNVSALISINYYFMFLYVLESTLKIIILKRKYFQQCWNTLEFFILVIGIIDIFCVYFVKLRP
DNLALIQLTVIMGYLRIIRFLPLFKIIVPILIRIADVQIKKRLSLMYSITKGYIKSQEDAKLLIKQIAVCESIYQKLCEILETNKQD
AVKELVLMEHEGRDVVIALKTKQAIRNVIAKALKNLTFLCSRGIIDKHEVIEINKVLLKKLKALNNFPKAIPPPTPDIYLHNIIWLE
GKDVLIDFFKERAKLACFDSGDTICKGGEMPQGIYLIISGMAILHSLSPTFGIESNQRCDRGSRDMFTEFCTTGDIIGELSCLLKRE
IEYTVICETSLQACFISLEDLYEGFDAFWPSLEYKIWLKLALSTAYQYFESSLIDEDLRFQNCVMFNQAYVETLSSYSDMIIDNMTM
KFVIIVYGSVIDTKTEEPYFAPCIIPTTCEQVQGTSDLSKLLIIQASELTQRNSNTNVMASVNTVFEQPGKNINGRQKMS
>sp|P17405|ASM_HUMAN
MPRYGASLRQSCPRSGREQGQDGTAGAPGLLWMGLVLALALALALALSDSRVLWAPAEAHPLSPQGHPARLHRIVPRLRDVFGWGNL
TCPICKGLFTAINLGLKKEPNVARVGSVAIKLCNLLKIAPPAVCQSIVHLFEDDMVEVWRRSVLSPSEACGLLLGSTCGHWDIFSSW
NISLPTVPKPPPKPPSPPAPGAPVSRILFLTDLHWDHDYLEGTDPDCADPLCCRRGSGLPPASRPGAGYWGEYSKCDLPLRTLESLL
SGLGPAGPFDMVYWTGDIPAHDVWHQTRQDQLRALTTVTALVRKFLGPVPVYPAVGNHESTPVNSFPPPFIEGNHSSRWLYEAMAKA
WEPWLPAEALRTLRIGGFYALSPYPGLRLISLNMNFCSRENFWLLINSTDPAGQLQWLVGELQAAEDRGDKVHIIGHIPPGHCLKSW
SWNYYRIVARYENTLAAQFFGHTHVDEFEVFYDEETLSRPLAVAFLAPSATTYIGLNPGYRVYQIDGNYSGSSHVVLDHETYILNLT
QANIPGAIPHWQLLYRARETYGLPNTLPTAWHNLVYRMRGDMQLFQTFWFLYHKGHPPSEPCGTPCRLATLCAQLSARADSPALCRH
LMPDGSLPEAQSLWPRPLFC
>sp|Q6IBS0|TWF2_HUMAN
MAHQTGIHATEELKEFFAKARAGSVRLIKVVIEDEQLVLGASQEPVGRWDQDYDRAVLPLLDAQQPCYLLYRLDSQNAQGFEWLFLA
WSPDNSPVRLKMLYAATRATVKKEFGGGHIKDELFGTVKDDLSFAGYQKHLSSCAAPAPLTSAERELQQIRINEVKTEISVESKHQT
LQGLAFPLQPEAQRALQQLKQKMVNYIQMKLDLERETIELVHTEPTDVAQLPSRVPRDAARYHFFLYKHTHEGDPLESVVFIYSMPG
YKCSIKERMLYSSCKSRLLDSVEQDFHLEIAKKIEIGDGAELTAEFLYDEVHPKQHAFKQAFAKPKGPGGKRGHKRLIRGPGENGDD
S
>sp|Q9NXJ5|PGPI_HUMAN
MEQPRKAVVVTGFGPFGEHTVNASWIAVQELEKLGLGDSVDLHVYEIPVEYQTVQRLIPALWEKHSPQLVVHVGVSGMATTVTLEKC
GHNKGYKGLDNCRFCPGSQCCVEDGPESIDSIIDMDAVCKRVTTLGLDVSVTISQDAGRYLCDFTYYTSLYQSHGRSAFVHVPPLGK
PYNADQLGRALRAIIEEMLDLLEQSEGKINYCHKH
>sp|Q14257|RCN2_HUMAN
MRLGPRTAALGLLLLCAAAAGAGKAEELHYPLGERRSDYDREALLGVQEDVDEYVKLGHEEQQKRLQAIIKKIDLDSDGFLTESELS
SWIQMSFKHYAMQEAKQQFVEYDKNSDDTVTWDEYNIQMYDRVIDFDENTALDDAEEESFRKLHLKDKKRFEKANQDSGPGLSLEEF
IAFEHPEEVDYMTEFVIQEALEEHDKNGDGFVSLEEFLGDYRWDPTANEDPEWILVEKDRFVNDYDKDNDGRLDPQELLPWVVPNNQ
GIAQEEALHLIDEMDLNGDKKLSEEEILENPDLFLTSEATDYGRQLHDDYFYHDEL
>sp|Q9UQC2|GAB2_HUMAN
MSGGGDVVCTGWLRKSPPEKKLRRYAWKKRWFILRSGRMSGDPDVLEYYKNDHSKKPLRIINLNFCEQVDAGLTFNKKELQDSFVFD
IKTSERTFYLVAETEEDMNKWVQSICQICGFNQAEESTDSLRNVSSAGHGPRSSPAELSSSSQHLLRERKSSAPSHSSQPTLFTFEP
PVSNHMQPTLSTSAPQEYLYLHQCISRRAENARSASFSQGTRASFLMRSDTAVQKLAQGNGHCVNGISGQVHGFYSLPKPSRHNTEF

RDSTYDLPRSLASHGHTKGSLTGSETDNEDVYTFKTPSNTLCREFGDLLVDNMDVPATPLSAYQIPRTFTLDKNHNAMTVATPGDSA
IAPPPRPPKPSQAETPRWGSPQQRPPISENSRSVAATIPRRNTLPAMDNSRLHRASSCETYEYPQRGGESAGRSAESMSDGVGSFLP
GKMIVGRSDSTNSEDNYVPMNPGSSTLLAMERAGDNSQSVYIPMSPGAHHFDSLGYPSTTLPVHRGPSRGSEIQPPPVNRNLKPDRK
AKPTPLDLRNNTVIDELPFKSPITKSWSRANHTFNSSSSQYCRPISTQSITSTDSGDSEENYVPMQNPVSASPVPSGTNSPAPKKST
GSVDYLALDFQPSSPSPHRKPSTSSVTSDEKVDYVQVDKEKTQALQNTMQEWTDVRQSSEPSKGAKL
>sp|Q9Y3E0|GOT1B_HUMAN
MISLTDTQKIGMGLTGFGVFFLFFGMILFFDKALLAIGNVLFVAGLAFVIGLERTFRFFFQKHKMKATGFFLGGVFVVLIGWPLIGM
IFEIYGFFLLFRGFFPVVVGFIRRVPVLGSLLNLPGIRSFVDKVGESNNMV
>sp|Q5VTE0|EF1A3_HUMAN
MGKEKTHINIVVIGHVDSGKSTTTGHLIYKCGGIDKRTIEKFEKEAAEMGKGSFKYAWVLDKLKAERERGITIDISLWKFETSKYYV
TIIDAPGHRDFIKNMITGTSQADCAVLIVAAGVGEFEAGISKNGQTREHALLAYTLGVKQLIVGVNKMDSTEPPYSQKRYEEIVKEV
STYIKKIGYNPDTVAFVPISGWNGDNMLEPSANMPWFKGWKVTRKDGNASGTTLLEALDCILPPTRPTDKPLRLPLQDVYKIGGIGT
VPVGRVETGVLKPGMVVTFAPVNVTTEVKSVEMHHEALSEALPGDNVGFKVKNVSVKDVRRGNVAGDSKNDPPMEAAGFTAQVIILN
HPGQISAGYAPVLDCHMAHIACKFAELKEKIDRRSGKKLEDGPKFLKSGDAAIVDMVPGKPMCVESFSDYPPLGRFAVRDMRQTVAV
GVIKAVDKKAAGAGKVTKSAQKAQKAK
>sp|P54136|SYRC_HUMAN
MDVLVSECSARLLQQEEEIKSLTAEIDRLKNCGCLGASPNLEQLQEENLKLKYRLNILRKSLQAERNKPTKNMINIISRLQEVFGHA
IKAAYPDLENPPLLVTPSQQAKFGDYQCNSAMGISQMLKTKEQKVNPREIAENITKHLPDNECIEKVEIAGPGFINVHLRKDFVSEQ
LTSLLVNGVQLPALGENKKVIVDFSSPNIAKEMHVGHLRSTIIGESISRLFEFAGYDVLRLNHVGDWGTQFGMLIAHLQDKFPDYLT
VSPPIGDLQVFYKESKKRFDTEEEFKKRAYQCVVLLQGKNPDITKAWKLICDVSRQELNKIYDALDVSLIERGESFYQDRMNDIVKE
FEDRGFVQVDDGRKIVFVPGCSIPLTIVKSDGGYTYDTSDLAAIKQRLFEEKADMIIYVVDNGQSVHFQTIFAAAQMIGWYDPKVTR
VFHAGFGVVLGEDKKKFKTRSGETVRLMDLLGEGLKRSMDKLKEKERDKVLTAEELNAAQTSVAYGCIKYADLSHNRLNDYIFSFDK
MLDDRGNTAAYLLYAFTRIRSIARLANIDEEMLQKAARETKILLDHEKEWKLGRCILRFPEILQKILDDLFLHTLCDYIYELATAFT
EFYDSCYCVEKDRQTGKILKVNMWRMLLCEAVAAVMAKGFDILGIKPVQRM
>sp|P63129|VPK24_HUMAN
WASQVSENRPVCKAIIQGKQFEGLVDTGADVSIIALNQWPKNWPKQKAVTGLVGIGTASEVYQSTEILHCLGPDNQESTVQPMITSI
PLNLWGRDLLQQWGAEITMPAPLYSPTSQKIMTKMGYIPGKGLGKNEDGIKIPFEAKINQKREGIGYPF
>sp|Q5TGY1|TMCO4_HUMAN
MAMWNRPCQRLPQQPLVAEPTAEGEPHLPTGRELTEANRFAYAALCGISLSQLFPEPEHSSFCTEFMAGLVQWLELSEAVLPTMTAF
ASGLGGEGADVFVQILLKDPILKDDPTVITQDLLSFSLKDGHYDARARVLVCHMTSLLQVPLEELDVLEEMFLESLKEIKEEESEMA
EASRKKKENRRKWKRYLLIGLATVGGGTVIGVTGGLAAPLVAAGAATIIGSAGAAALGSAAGIAIMTSLFGAAGAGLTGYKMKKRVG
AIEEFTFLPLTEGRQLHITIAVTGWLASGKYRTFSAPWAALAHSREQYCLAWEAKYLMELGNALETILSGLANMVAQEALKYTVLSG
IVAALTWPASLLSVANVIDNPWGVCLHRSAEVGKHLAHILLSRQQGRRPVTLIGFSLGARVIYFCLQEMAQEKDCQGIIEDVILLGA
PVEGEAKHWEPFRKVVSGRIINGYCRGDWLLSFVYRTSSVQLRVAGLQPVLLQDRRVENVDLTSVVSGHLDYAKQMDAILKAVGIRT
KPGWDEKGLLLAPGCLPSEEPRQAAAAASSGETPHQVGQTQGPISGDTSKLAMSTDPSQAQVPVGLDQSEGASLPAAASPERPPICS
HGMDPNPLGCPDCACKTQGPSTGLD
>sp|O95551|TYDP2_HUMAN
MELGSCLEGGREAAEEEGEPEVKKRRLLCVEFASVASCDAAVAQCFLAENDWEMERALNSYFEPPVEESALERRPETISEPKTYVDL
TNEETTDSTTSKISPSEDTQQENGSMFSLITWNIDGLDLNNLSERARGVCSYLALYSPDVIFLQEVIPPYYSYLKKRSSNYEIITGH
EEGYFTAIMLKKSRVKLKSQEIIPFPSTKMMRNLLCVHVNVSGNELCLMTSHLESTRGHAAERMNQLKMVLKKMQEAPESATVIFAG
DTNLRDREVTRCGGLPNNIVDVWEFLGKPKHCQYTWDTQMNSNLGITAACKLRFDRIFFRAAAEEGHIIPRSLDLLGLEKLDCGRFP
SDHWGLLCNLDIIL
>sp|A0AVI2|FR1L5_HUMAN

MLRLVVQSAKIDPPLAPLPRPCMSIDFRDIKKRTRVVEGNDPVWNETLIWHLWNRPLENDSFLQVTLQDMGSQKKERFIGLATVLLK
PLLKQPSEVLFVKDLTLLNHSMKPTDCTVTLQVAHMSNQDIEKTGAEDHLGITAREAASQKLMVPGSTAHRALSSKPQHFQVRVKVF
EARQLMGNNIKPVVKVSIAGQQHQTRIKMGNNPFFNEIFFQNFHEVPAKFFDETILIQTDIGFIYHSPGHTLLRKWLGLCQPNNPGS
GVTGYLKVTIYALGVGDQALIDQKLLYGTDDTDIQIFKSAVVPINMAYLQLFIYCAEDLHLKKHQSVNPQLEVELIGEKLRTHMQTQ
TDNPIWNQILTFRIQLPCLSSYIKFRVLDCRKKDCPDEIGTASLSLNQISSTGEEIEGKQSLEPTSYTPRVYSGFLPCFGPSFLTLH
GGKKAPFRIQEEGACIPDSVRDGLAYRGRVFLELITQIKSYQDSTIKDLSHEVTRIEKHQNRQKYGLCVIFLSCTMMPNFKELIHFE
VSIGHYGNKMDLNYKPLVSSTPYSPVIYDGNIYHYVPWYNTKPVVAVTSNWEDVSFRMNCLNLLHFTRDRLKANLDTLKSTRNPKDP
ALLYQWEKLLRELAEDCKRPLPCMTYQPKATSLDRKRWQLRSLLLQELAQKAKQAKPKDMVATAEDWLYRLNTVLPEPQMGLPDVMI
WLVAKEQRVAYAQVPAHSVLFSPAGALHSGRLCGKIQTLFLQYPEGEGQKDVLPAHLRVCMWLGNVTDSKDLQLLRQGDTAVYAEMY
ENQAKYKDQWGQQGLYHCPNFSDVMGNKTLPMTDFQPPLGWHWQDSWTVEPQRRLLLDIDINKSQVLEEVYENQGRDTRGAWGPAAI
PNTDVNGQPMEARENVKCPQGWHFKKDWVVELNHAVDSWEYGVGIPPSGLPQVWSPVEKTYHSCRRRRWARVRFRNHGELSHEQETL
SFLQLGLAKGEEEGWEYDTFGSKFHLNPQPQSRFRRRCWRRRLAPNKDKGIAPIFLLEGSLACEGGSVREEGDWAWARGNRSSTQEP
QGRLSWVQAMDLKYHAGKEEDSKTWPWGLDRQFRDPQRQDTRPPNLPFIYCTFNKPHYYQLFCYIYQARNLVSNQILTFQGPFIRVV
FLNHSQCTQTLRSSAGPTWAQTLIFQHLLLYENPQDTKESPPLVVLELWQRDFWGKESLWGRSVWPPMVWLDLQDRILPPMRWHPLV
KELGKEEGEILASCELILQTEKLGEKQLPILSVPWKNGAYTLPKSIQPTIKRMAIEILAWGLRNMKKASSPQLLVEFGEESLRTEPI
RDFQTNPNFPESESVLVLTVLMPTEEAYALPLVVKVVDNWAFGQQTVTGQANIDFLQPYFCDPWAQDYMHPKLPTLSEKKHQDFLGY
LYRKFWFKSSKAEDEYEHEVDWWSKLFWATDEHKSLKYKYKDYHTLKVYECELEAVPAFQGLQDFCQTFKLYQEQPKLDSPVVGEFK
GLFRIYPFPENPEAPKPPLQFLVWPEREDFPQPCLVRVYMVRAINLQPQDYNGLCDPYVILKLGKTELGNRDMYQPNTLDPIFGMMF
ELTCNIPLEKDLEIQLYDFDLFSPDDKIGTTVIDLENRLLSGFGAHCGLSKSYCQSGPFRWRDQMPPSYLLERYAKRKGLPPPLFSP
EEDAVFYNGKKFKLQSFEPKTPTVHGLGPKKERLALYLLHTQGLVPEHVETRTLYSHSQPGIDQGKVQMWVDIFPKKLGPPGPQVNI
NPRKPKRKASEHSGHRYELRCIIWKTANVDLVDDNLSREKTSDIYIKGWLYGLEKDMQKTDIHYHSLTGEADFNWRFIFTMDYLAAE
RTCVQSQKDYIWSLDATSMKFPARLIIQVWDNDIFSPDDFLGVLELDLSDMPLPARHAKQCSIRMMDADPKWPYFIQYKHFSLFKKK
TVTGWWPCQVLDGGKWRLSGKVKMSLEILSEKEALIKPAGRGQSEPNQYPTLHPPLRTNTSFTWLRSPVQNFCYIFWKRYRFKLIAF
MVISIIALMLFNFIYSAPHYLAMSWIKPQLQLYPPIKIFNIINSLNTSNASSSILPTQDPNLKPTIDHEWKLHPGPTNHLSDIFPEL
PAPGD
>sp|Q9NX78|TM260_HUMAN
MSPHGDGRGQAQGRAVRVGLRRSGGIRGGVAVFAAVAAVFTFTLPPSVPGGDSGELITAAHELGVAHPPGYPLFTLVAKLAITLFPF
GSIAYRVNLLCGLFGAVAASLLFFTVFRLSGSSAGGILAAGVFSFSRLTWQWSIAAEVFSLNNLFVGLLMALTVHFEEAATAKERSK
VAKIGAFCCGLSLCNQHTIILYVLCIIPWILFQLLKKKELSLGSLLKLSLYFSAGLLPYVHLPISSYLNHARWTWGDQTTLQGFLTH
FLREEYGTFSLAKSEIGSSMSEILLSQVTNMRTELSFNIQALAVCANICLATKDRQNPSLVWLFTGMFCIYSLFFAWRANLDISKPL
FMGVVERFWMQSNAVVAVLAGIGLAAVVSETNRVLNSNGLQCLEWLSATLFVVVYQIYSNYSVCDQRTNYVIDKFAKNLLTSMPHDAI
ILLRGDLPGNSLRYMHYCEGLRPDISLVDQEMMTYEWYLPKMAKHLPGVNFPGNRWNPVEGILPSGMVTFNLYHFLEVNKQKETFVC
IGIHEGDPTWKKNYSLWPWGSCDKLVPLEIVFNPEEWIKLTKSIYNWTEEYGRFDPSSWESVANEEMWQARMKTPFFIFNLAETAHM
PSKVKAQLYAQAYDLYKEIVYLQKEHPVNWHKNYAIACERMLRLQARDADPEVLLSETIRHFRLYSQKAPNDPQQADILGALKHLRK
ELQSLRNRKNV
>sp|Q8TBR4|ST3L4_HUMAN
MSGWIATSKTRMQDFWSLLTFSSDLVDVKDSGDYPLTAPGLSWKKFQGSFCEFVGTLVCRCQYILLHDDFPMDNLISLLTGFSDSQV
CAFCHTSTLAAMKLMTSLVRVALQLSLHEDINQRQYEAERNKGPGQRAPERLESLLEKHKELH
>sp|Q6NSJ5|LRC8E_HUMAN
MIPVAEFKQFTEQQPAFKVLKPWWDVLAEYLTVAMLMIGVFGCTLQVTQDKIICLPNHELQENLSEAPCQQLLPRGIPEQIGALQEV
KGLKNNLDLQQYSFINQLCYETALHWYAKYFPYLVVIHTLIFMVCTSFWFKFPGTSSKIEHFISILGKCFDSPWTTRALSEVSGENQ
KGPAATERAAATIVAMAGTGPGKAGEGEKEKVLAEPEKVVTEPPVVTLLDKKEGEQAKALFEKVKKFRMHVEEGDILYTMYIRQTVL
KVCKFLAILVYNLVYVEKISFLVACRVETSEVTGYASFCCNHTKAHLFSKLAFCYISFVCIYGLTCIYTLYWLFHRPLKEYSFRSVR
EETGMGDIPDVKNDFAFMLHLIDQYDSLYSKRFAVFLSEVSESRLKQLNLNHEWTPEKLRQKLQRNAAGRLELALCMLPGLPDTVFE

LSEVESLRLEAICDITFPPGLSQLVHLQELSLLHSPARLPFSLQVFLRDHLKVMRVKCEELREVPLWVFGLRGLEELHLEGLFPQEL
ARAATLESLRELKQLKVLSLRSNAGKVPASVTDVAGHLQRLSLHNDGARLVALNSLKKLAALRELELVACGLERIPHAVFSLGALQE
LDLKDNHLRSIEEILSFQHCRKLVTLRLWHNQIAYVPEHVRKLRSLEQLYLSYNKLETLPSQLGLCSGLRLLDVSHNGLHSLPPEVG
LLQNLQHLALSYNALEALPEELFFCRKLRTLLLGDNQLSQLSPHVGALRALSRLELKGNRLEALPEELGNCGGLKKAGLLVEDTLYQ
GLPAEVRDKMEEE
>sp|Q9UHG2|PCSK1_HUMAN
MAGSPLLWGPRAGGVGLLVLLLLGLFRPPPALCARPVKEPRGLSAASPPLAETGAPRRFRRSVPRGEAAGAVQELARALAHLLEAER
QERARAEAQEAEDQQARVLAQLLRVWGAPRNSDPALGLDDDPDAPAAQLARALLRARLDPAALAAQLVPAPVPAAALRPRPPVYDDG
PAGPDAEEAGDETPDVDPELLRYLLGRILAGSADSEGVAAPRRLRRAADHDVGSELPPEGVLGALLRVKRLETPAPQVPARRLLPP
>sp|Q8NFW9|MYRIP_HUMAN
MGRKLDLSGLTDDETEHVLQVVQRDFNLRKKEEERLSELKQKLDEEGSKCSILSKHQQFVEHCCMRCCSPFTFLVNTKRQCGDCKFN
VCKSCCSYQKHEKAWVCCVCQQARLLRAQSLEWFYNNVKSRFKRFGSAKVLKNLYRKHRLESGACFDILGGSLFESNLENEGSISGS
DSTFYRQSEGHSVMDTLAVALRVAEEAIEEAISKAEAYGDSLDKQNEASYLRDHKEELTEELATTILQKIIRKQKSKSEQQVEEEPG
WPHPQSCSTKVADEGTSASPGGYRAPAALWRSQSAFSITGEEALKTPPVEAPSRQPRDQGQHPRAESALPSWKSVDRLDETNLAPVL
QSPDGNWVALKDGAPPPTRLLAKPKSGTFQALEVASSVASAYDEMGSDSEEDFDWSEALSKLCPRSRALPRNPQPQPTQAQSSDQGP
IAASPSSALSPNPEAMCSDSETSSAGSSREVGHQARLSWLQRKAPRNPAAEKMRLHGELDVNFNPQLASRETSDSSEPEEAPHTTDR
RARRWRRARLGSEEPSKEPSSPSAQLRDLDTHQVSDDLSETDISNEARDPQTLTDTTEEKRRNRLYELAMKMSEKETSSGEDQESEP
KTESENQKESLSSEDNSQSVQEELKKKFSAVSLCNISTEVLKVINATEELIAGSTGPWESPQVPPDRQKGMFPRGTDQVRLDEQLTS
LEENVYLAAGTVYGLETQLTELEDAARCIHSGTDETHLADLEDQVATAAAQVHHAELQISDIESRISALTIAGLNIAPCVRFTRRRD
QKQRTQVQTIDTSRQQRRKLPAPPVKAEKIETSSVTTIKTFNHNFILQGSSTNRTKERKGTTKDLMEPALESAVMY
>sp|P20936|RASA1_HUMAN
MMAAEAGSEEGGPVTAGAGGGGAAAGSSAYPAVCRVKIPAALPVAAAPYPGLVETGVAGTLGGGAALGSEFLGAGSVAGALGGAGLT
GGGTAAGVAGAAAGVAGAAVAGPSGDMALTKLPTSLLAETLGPGGGFPPLPPPPYLPPLGAGLGTVDEGDSLDGPEYEEEEVAIPLT
APPTNQWYHGKLDRTIAEERLRQAGKSGSYLIRESDRRPGSFVLSFLSQMNVVNHFRIIAMCGDYYIGGRRFSSLSDLIGYYSHVSC
LLKGEKLLYPVAPPEPVEDRRRVRAILPYTKVPDTDEISFLKGDMFIVHNELEDGWMWVTNLRTDEQGLIVEDLVEEVGREEDPHEG
KIWFHGKISKQEAYNLLMTVGQVCSFLVRPSDNTPGDYSLYFRTNENIQRFKICPTPNNQFMMGGRYYNSIGDIIDHYRKEQIVEGY
YLKEPVPMQDQEQVLNDTVDGKEIYNTIRRKTKDAFYKNIVKKGYLLKKGKGKRWKNLYFILEGSDAQLIYFESEKRATKPKGLIDL
SVCSVYVVHDSLFGRPNCFQIVVQHFSEEHYIFYFAGETPEQAEDWMKGLQAFCNLRKSSPGTSNKRLRQVSSLVLHIEEAHKLPVK
HFTNPYCNIYLNSVQVAKTHAREGQNPVWSEEFVFDDLPPDINRFEITLSNKTKKSKDPDILFMRCQLSRLQKGHATDEWFLLSSHI
PLKGIEPGSLRVRARYSMEKIMPEEEYSEFKELILQKELHVVYALSHVCGQDRTLLASILLRIFLHEKLESLLLCTLNDREISMEDE
ATTLFRATTLASTLMEQYMKATATQFVHHALKDSILKIMESKQSCELSPSKLEKNEDVNTNLTHLLNILSELVEKIFMASEILPPTL
RYIYGCLQKSVQHKWPTNTTMRTRVVSGFVFLRLICPAILNPRMFNIISDSPSPIAARTLILVAKSVQNLANLVEFGAKEPYMEGVN
PFIKSNKHRMIMFLDELGNVPELPDTTEHSRTDLSRDLAALHEICVAHSDELRTLSNERGAQQHVLKKLLAITELLQQKQNQYTKTN
DVR
>sp|O60431|OR1I1_HUMAN
MEPEKQTEISEFFLQGLSEKPEHQTLLFTMFLSTYLVTIIGNALIILAIITDSHLHTPMYFFLFNLSLVDTLLSSTTVPKMLANIQA
QSRAIPFVGCLTQMYAFHLFGTMDSFLLAVMAIDRFVAIVHPQRYLVLMCSPVCGLLLGASWMITNLQSLIHTCLMAQLTFCAGSEI
SHFFCDLMPLLKLSGSDTHTNELVIFAFGIVVGTSPFSCILLSYIRIFWTVFKIPSTRGKWKAFSTCGLHLTVVSLSYGTIFAVYLQ
PTSPSSSQKDKAAALMCGVFIPMLNPFIYSIRNKDMKAALGKLIGKVAVPCPRPEQLLDVYHVPGSLLAARDTEMHPIPYPGGVQSL
AGNRDME
>sp|Q6NUK4|REEP3_HUMAN
MVSWMISRAVVLVFGMLYPAYYSYKAVKTKNVKEYVRWMMYWIVFALYTVIETVADQTVAWFPLYYELKIAFVIWLLSPYTKGASLI
YRKFLHPLLSSKEREIDDYIVQAKERGYETMVNFGRQGLNLAATAAVTAAVKSQGAITERLRSFSMHDLTTIQGDEPVGQRPYQPLP
EAKKKSKPAPSESAGYGIPLKDGDEKTDEEAEGPYSDNEMLTHKGLRRSQSMKSVKTTKGRKEVRYGSLKYKVKKRPQVYF

>sp|Q9Y2Y4|ZBT32_HUMAN
MSLPPIRLPSPYGSDRLVQLAARLRPALCDTLITVGSQEFPAHSLVLAGVSQQLGRRGQWALGEGISPSTFAQLLNFVYGESVELQP
GELRPLQEAARALGVQSLEEACWRARGDRAKKPDPGLKKHQEEPEKPSRNPERELGDPGEKQKPEQVSRTGGREQEMLHKHSPPRGR
PEMAGATQEAQQEQTRSKEKRLQAPVGQRGADGKHGVLTWLRENPGGSEESLRKLPGPLPPAGSLQTSVTPRPSWAEAPWLVGGQPA
LWSILLMPPRYGIPFYHSTPTTGAWQEVWREQRIPLSLNAPKGLWSQNQLASSSPTPGSLPQGPAQLSPGEMEESDQGHTGALATCA
GHEDKAGCPPRPHPPPAPPARSRPYACSVCGKRFSLKHQMETHYRVHTGEKPFSCSLCPQRSRDFSAMTKHLRTHGAAPYRCSLCGA
GCPSLASMQAHMRGHSPSQLPPGWTIRSTFLYSSSRPSRPSTSPCCPSSSTT
>sp|Q8N187|CARTF_HUMAN
MEQSNDSLRVNHNDGEESKTSAQVFEHLICMDSRDSSFGQNDSPTVLPITTREANNSLISQNIPGPLTQTQTLSAEQFHLVDQNGQA
IQYELQSLGESNAQMMIVASPTENGQVLRVIPPTQTGMAQVIIPQGQLVDVNSPRDVPEEKPSNRNLPTVRVDTLADNTSNYILHPQ
TSFPLPKKSVTGMLEEPLLGPLQPLSSNTPIWACRLRSCEKIGDSYRGYCVSETELESVLTFHKQQTQSVWGTRQSPSPAKPATRLM
WKSQYVPYDGIPFVNAGSRAVVMECQYGPRRKGFQLKKVSEQESRSCQLYKATCPARIYIKKVQKFPEYRVPTDPKIDKKIIRMEQE
KAFNMLKKNLVDAGGVLRWYVQLPTQQAHQYHELETPCLTLSPSPFPVSSLEEEETAVRDENCALPSRLHPQVAHKIQELVSQGIEQ
VYAVRKQLRKFVERELFKPDEVPERHNLSFFPTVNDIKNHIHEVQKSLRNGDTVYNSEIIPATLQWTTDSGNILKETMTVTFAEGNS
PGESITTKVETNQTRGSLSPEPTHLLSSLSSFQPKIFTQLQGLQLQPRYTSPDESPAVVSVNNQPSSSPSGLLDTIGSAVMNNNSLL
LGQSHSLQRDTCLTQNNSTASTMGNLPEPDQNLVAMDELVEVGDVEDTGNLEGTVHRILLGDVQTIPIQIIDNHSALIEENPESTIS
VSQVKQEPKEPALSMEAKKTVDYKKLSAT
>sp|P51693|APLP1_HUMAN
MGPASPAARGLSRRPGQPPLPLLLPLLLLLLRAQPAIGSLAGGSPGAAEAPGSAQVAGLCGRLTLHRDLRTGRWEPDPQRSRRCLRD
PQRVLEYCRQMYPELQIARVEQATQAIPMERWCGGSRSGSCAHPHHQVVPFRCLPGEFVSEALLVPEGCRFLHQERMDQCESSTRRH
QEAQEACSSQGLILHGSGMLLPCGSDRFRGVEYVCCPPPGTPDPSGTAVGDPSTRSWPPGSRVEGAEDEEEEESFPQPVDDYFVEPP
QAEEEEETVPPPSSHTLAVVGKVTPTPRPTDGVDIYFGMPGEISEHEGFLRAKMDLEERRMRQINEVMREWAMADNQSKNLPKADRQ
ALNEHFQSILQTLEEQVSGERQRLVETHATRVIALINDQRRAALEGFLAALQADPPQAERVLLALRRYLRAEQKEQRHTLRHYQHVA
AVDPEKAQQMRFQVHTHLQVIEERVNQSLGLLDQNPHLAQELRPQIQELLHSEHLGPSELEAPAPGGSSEDKGGLQPPDSKDDTPMT
LPKGSTEQDAASPEKEKMNPLEQYERKVNASVPRGFPFHSSEIQRDELAPAGTGVSREAVSGLLIMGAGGGSLIVLSMLLLRRKKPY
GAISHGVVEVDPMLTLEEQQLRELQRHGYENPTYRFLEERP
>sp|Q96PF1|TGM7_HUMAN
MDQVATLRLESVDLQSSRNNKEHHTQEMGVKRLTVRRGQPFYLRLSFSRPFQSQNDHITFVAETGPKPSELLGTRATFFLTRVQPGN
VWSASDFTIDSNSLQVSLFTPANAVIGHYTLKIEISQGQGHSVTYPLGTFILLFNPWSPEDDVYLPSEILLQEYIMRDYGFVYKGHE
RFITSWPWNYGQFEEDIIDICFEILNKSLYHLKNPAKDCSQRNDVVYVCRVVSAMINSNDDNGVLQGNWGEDYSKGVSPLEWKGSVA
ILQQWSARGGQPVKYGQCWVFASVMCTVMRCLGVPTRVVSNFRSAHNVDRNLTIDTYYDRNAEMLSTQKRDKIWNFHVWNECWMIRK
DLPPGYNGWQVLDPTPQQTSSGLFCCGPASVKAIREGDVHLAYDTPFVYAEVNADEVIWLLGDGQAQEILAHNTSSIGKEISTKMVG
SDQRQSITSSYKYPEGSPEERAVFMKASRKMLGPQRASLPFLDLLESGGLRDQPAQLQLHLARIPEWGQDLQLLLRIQRVPDSTHPR
GPIGLVVRFCAQALLHGGGTQKPFWRHTVRMNLDFGKETQWPLLLPYSNYRNKLTDEKLIRVSGIAEVEETGRSMLVLKDICLEPPH
LSIEVSERAEVGKALRVHVTLTNTLMVALSSCTMVLEGSGLINGQIAKDLGTLVAGHTLQIQLDLYPTKAGPRQLQVLISSNEVKEI
KGYKDIFVTVAGAP
>sp|Q6V9R5|ZN562_HUMAN
MSAFDMSHGFFPREPICPFEEKTKIGTMVEDHRSNSYQDSVTFDDVAVEFTPEEWALLDTTQKYLYRDVMLENYMNLASVDFFFCLT
SEWEIQPRTKRSSLQQGFLKNQIFTGIQMQTRSYSGWKLCENCGEVFSEQFCLKTHMRAQNGGNTFEGNCYGKDSISVHKEASIGQE
LSKFNPCGKVFTLTPGLAVHLEILNGRQPYKCKECGKGFKYFASLDNHMGIHIGEKLCEFQECERAITTSSHLKQCVAVHTGKKSEK
TKNCGKSFTNFSQLSAHAKTHKGEKSFECKECGRSFRNSSSFNVHIQIHTGIKPHKCTECGKAFTRSTHLTQHVRTHTGIKPYECKE
CGQAFTQYTGLAIHIRNHTGEKPYQCKECGKAFNRSSTLTQHRRIHTGEKPYECVECGKTFITSSHRSKHLKTHSGER
>sp|Q92759|TF2H4_HUMAN

MESTPSRGLNRVHLQCRNLQEFLGGLSPGVLDRLYGHPATCLAVFRELPSLAKNWVMRMLFLEQPLPQAAVALWVKKEFSKAQEEST
GLLSGLRIWHTQLLPGGLQGLILNPIFRQNLRIALLGGGKAWSDDTSQLGPDKHARDVPSLDKYAEERWEVVLHFMVGSPSAAVSQD
LAQLLSQAGLMKSTEPGEPPCITSAGFQFLLLDTPAQLWYFMLQYLQTAQSRGMDLVEILSFLFQLSFSTLGKDYSVEGMSDSLLNF
LQHLREFGLVFQRKRKSRRYYPTRLAINLSSGVSGAGGTVHQPGFIVVETNYRLYAYTESELQIALIALFSEMLYRFPNMVVAQVTR
ESVQQAIASGITAQQIIHFLRTRAHPVMLKQTPVLPPTITDQIRLWELERDRLRFTEGVLYNQFLSQVDFELLLAHARELGVLVFEN
SAKRLMVVTPAGHSDVKRFWKRQKHSS
>sp|P30456|1A43_HUMAN
MAVMAPRTLVLLLSGALALTQTWAGSHSMRYFYTSVSRPGRGEPRFIAVGYVDDTQFVRFDSDAASQRMEPRAPWIEQEGPEYWDLQ
TRNVKAHSQTDRANLGTLRGYYNQSEDGSHTIQRMYGCDVGPDGRFLRGYQQDAYDGKDYIALNEDLRSWTAADMAAQITQRKWETA
HEAEQWRAYLEGRCVEWLRRYLENGKETLQRTDAPKTHMTHHAVSDHEATLRCWALSFYPAEITLTWQRDGEDQTQDTELVETRPAG
DGTFQKWASVVVPSGQEQRYTCHVQHEGLPKPLTLRWEPSSQPTIPIVGIIAGLVLFGAVIAGAVVAAVMWRRKSSDRKGGSYSQAA
SSDSAQGSDMSLTACKV
>sp|Q14137|BOP1_HUMAN
MAGSRGAGRTAAPSVRPEKRRSEPELEPEPEPEPPLLCTSPLSHSTGSDSGVSDSEESVFSGLEDSGSDSSEDDDEGDEEGEDGALD
DEGHSGIKKTTEEQVQASTPCPRTEMASARIGDEYAEDSSDEEDIRNTVGNVPLEWYDDFPHVGYDLDGRRIYKPLRTRDELDQFLD
KMDDPDYWRTVQDPMTGRDLRLTDEQVALVRRLQSGQFGDVGFNPYEPAVDFFSGDVMIHPVTNRPADKRSFIPSLVEKEKVSRMVH
AIKMGWIQPRRPRDPTPSFYDLWAQEDPNAVLGRHKMHVPAPKLALPGHAESYNPPPEYLLSEEERLAWEQQEPGERKLSFLPRKFP
SLRAVPAYGRFIQERFERCLDLYLCPRQRKMRVNVDPEDLIPKLPRPRDLQPFPTCQALVYRGHSDLVRCLSVSPGGQWLVSGSDDG
SLRLWEVATARCVRTVPVGGVVKSVAWNPSPAVCLVAAAVEDSVLLLNPALGDRLVAGSTDQLLSAFVPPEEPPLQPARWLEASEEE
RQVGLRLRICHGKPVTQVTWHGRGDYLAVVLATQGHTQVLIHQLSRRRSQSPFRRSHGQVQRVAFHPARPFLLVASQRSVRLYHLLR
QELTKKLMPNCKWVSSLAVHPAGDNVICGSYDSKLVWFDLDLSTKPYRMLRHHKKALRAVAFHPRYPLFASGSDDGSVIVCHGMVYN
DLLQNPLLVPVKVLKGHVLTRDLGVLDVIFHPTQPWVFSSGADGTVRLFT
>sp|Q8NH93|OR1L3_HUMAN
MGMSNLTRLSEFILLGLSSRSEDQRPLFALFLIIYLVTLMGNLLIILAIHSDPRLQNPMYFFLSILSFADICYTTVIVPKMLVNFLS
EKKTISYAECLAQMYFFLVFGNIDSYLLAAMAINRCVAICNPFHYVTVMNRRCCVLLLAFPITFSYFHSLLHVLLVNRLTFCTSNVI
HHFFCDVNPVLKLSCSSTFVNEIVAMTEGLASVMAPFVCIIISYLRILIAVLKIPSAAGKHKAFSTCSSHLTVVILFYGSISYVVLQ
PLSSYTVKDRIATINYTVLTSVLNPFIYSLRNKDMKRGLQKLINKIKSQMSRFSTKTNKICGP
>sp|Q9BZP3|CR002_HUMAN
MVRHPYSVQTQLSTEAKAIWRSMQQQETNLLANLTTNDARDNSKDFQNSKVGAAATSRDEGCNCPIIGEIVISCYWLFEIPPLISE
>sp|P0CF74|LAC6_HUMAN
GQPKAAPSVTLFPPSSEELQANKATLVCLISDFYPGAVKVAWKADGSPVNTGVETTTPSKQSNNKYAASSYLSLTPEQWKSHRSYSC
QVTHEGSTVEKTVAPAECS
>sp|Q96KN1|FA84B_HUMAN
MGNQVEKLTHLSYKEVPTADPTGVDRDDGPRIGVSYIFSNDDEDVEPQPPPQGPDGGGLPDGGDGPPPPQPQPYDPRLHEVECSVFY
RDECIYQKSFAPGSAALSTYTPENLLNKCKPGDLVEFVSQAQYPHWAVYVGNFQVVHLHRLEVINSFLTDASQGRRGRVVNDLYRYK
PLSSSAVVRNALAHVGAKERELSWRNSESFAAWCRYGKREFKIGGELRIGKQPYRLQIQLSAQRSHTLEFQSLEDLIMEKRRNDQIG
RAAVLQELATHLHPAEPEEGDSNVARTTPPPGRPPAPSSEEEDGEAVAH
>sp|Q8IV56|PRR15_HUMAN
MADSGDAGSSGPWWKSLTNSRKKSKEAAVGVPPPAQPAPGEPTPPAPPSPDWTSSSRENQHPNLLGGAGEPPKPDKLYGDKSGSSRR
NLKISRSGRFKEKRKVRATLLPEAGRSPEEAGFPGDPHEDKQ
>sp|O15211|RGL2_HUMAN
MLPRPLRLLLDTSPPGGVVLSSFRSRDPEEGGGPGGLVVGGGQEEEEEEEEEAPVSVWDEEEDGAVFTVTSRQYRPLDPLVPMPPPR
SSRRLRAGTLEALVRHLLDTRTSGTDVSFMSAFLATHRAFTSTPALLGLMADRLEALESHPTDELERTTEVAISVLSTWLASHPEDF
GSEAKGQLDRLESFLLQTGYAAGKGVGGGSADLIRNLRSRVDPQAPDLPKPLALPGDPPADPTDVLVFLADHLAEQLTLLDAELFLN

LIPSQCLGGLWGHRDRPGHSHLCPSVRATVTQFNKVAGAVVSSVLGATSTGEGPGEVTIRPLRPPQRARLLEKWIRVAEECRLLRNF
SSVYAVVSALQSSPIHRLRAAWGEATRDSLRVFSSLCQIFSEEDNYSQSRELLVQEVKLQSPLEPHSKKAPRSGSRGGGVVPYLGTF
LKDLVMLDAASKDELENGYINFDKRRKEFAVLSELRRLQNECRGYNLQPDHDIQRWLQGLRPLTEAQSHRVSCEVEPPGSSDPPAPR
VLRPTLVISQWTEVLGSVGVPTPLVSCDRPSTGGDEAPTTPAPLLTRLAQHMKWPSVSSLDSALESSPSLHSPADPSHLSPPASSPR
PSRGHRRSASCGSPLSGGAEEASGGTGYGGEGSGPGASDCRIIRVQMELGEDGSVYKSILVTSQDKAPSVISRVLKKNNRDSAVASE
YELVQLLPGERELTIPASANVFYAMDGASHDFLLRQRRRSSTATPGVTSGPSASGTPPSEGGGGSFPRIKATGRKIARALF
>sp|P0DN77|OPSG2_HUMAN
MAQQWSLQRLAGRHPQDSYEDSTQSSIFTYTNSNSTRGPFEGPNYHIAPRWVYHLTSVWMIFVVIASVFTNGLVLAATMKFKKLRHP
LNWILVNLAVADLAETVIASTISVVNQVYGYFVLGHPMCVLEGYTVSLCGITGLWSLAIISWERWMVVCKPFGNVRFDAKLAIVGIA
FSWIWAAVWTAPPIFGWSRYWPHGLKTSCGPDVFSGSSYPGVQSYMIVLMVTCCITPLSIIVLCYLQVWLAIRAVAKQQKESESTQK
AEKEVTRMVVVMVLAFCFCWGPYAFFACFAAANPGYPFHPLMAALPAFFAKSATIYNPVIYVFMNRQFRNCILQLFGKKVDDGSELS
SASKTEVSSVSSVSPA
>sp|Q9UQ53|MGT4B_HUMAN
MRLRNGTFLTLLLFCLCAFLSLSWYAALSGQKGDVVDVYQREFLALRDRLHAAEQESLKRSKELNLVLDEIKRAVSERQALRDGDGN
RTWGRLTEDPRLKPWNGSHRHVLHLPTVFHHLPHLLAKESSLQPAVRVGQGRTGVSVVMGIPSVRREVHSYLTDTLHSLISELSPQE
KEDSVIVVLIAETDSQYTSAVTENIKALFPTEIHSGLLEVISPSPHFYPDFSRLRESFGDPKERVRWRTKQNLDYCFLMMYAQSKGI
YYVQLEDDIVAKPNYLSTMKNFALQQPSEDWMILEFSQLGFIGKMFKSLDLSLIVEFILMFYRDKPIDWLLDHILWVKVCNPEKDAK
HCDRQKANLRIRFKPSLFQHVGTHSSLAGKIQKLKDKDFGKQALRKEHVNPPAEVSTSLKTYQHFTLEKAYLREDFFWAFTPAAGDF
IRFRFFQPLRLERFFFRSGNIEHPEDKLFNTSVEVLPFDNPQSDKEALQEGRTATLRYPRSPDGYLQIGSFYKGVAEGEVDPAFGPL
EALRLSIQTDSPVWVILSEIFLKKAD
>sp|Q8IX01|SUGP2_HUMAN
MAARRITQETFDAVLQEKAKRYHMDASGEAVSETLQFKAQDLLRAVPRSRAEMYDDVHSDGRYSLSGSVAHSRDAGREGLRSDVFPG
PSFRSSNPSISDDSYFRKECGRDLEFSHSDSRDQVIGHRKLGHFRSQDWKFALRGSWEQDFGHPVSQESSWSQEYSFGPSAVLGDFG
SSRLIEKECLEKESRDYDVDHPGEADSVLRGGSQVQARGRALNIVDQEGSLLGKGETQGLLTAKGGVGKLVTLRNVSTKKIPTVNRI
TPKTQGTNQIQKNTPSPDVTLGTNPGTEDIQFPIQKIPLGLDLKNLRLPRRKMSFDIIDKSDVFSRFGIEIIKWAGFHTIKDDIKFS
QLFQTLFELETETCAKMLASFKCSLKPEHRDFCFFTIKFLKHSALKTPRVDNEFLNMLLDKGAVKTKNCFFEIIKPFDKYIMRLQDR
LLKSVTPLLMACNAYELSVKMKTLSNPLDLALALETTNSLCRKSLALLGQTFSLASSFRQEKILEAVGLQDIAPSPAAFPNFEDSTL
FGREYIDHLKAWLVSSGCPLQVKKAEPEPMREEEKMIPPTKPEIQAKAPSSLSDAVPQRADHRVVGTIDQLVKRVIEGSLSPKERTL
LKEDPAYWFLSDENSLEYKYYKLKLAEMQRMSENLRGADQKPTSADCAVRAMLYSRAVRNLKKKLLPWQRRGLLRAQGLRGWKARRA
TTGTQTLLSSGTRLKHHGRQAPGLSQAKPSLPDRNDAAKDCPPDPVGPSPQDPSLEASGPSPKPAGVDISEAPQTSSPCPSADIDMK
TMETAEKLARFVAQVGPEIEQFSIENSTDNPDLWFLHDQNSSAFKFYRKKVFELCPSICFTSSPHNLHTGGGDTTGSQESPVDLMEG
EAEFEDEPPPREAELESPEVMPEEEDEDDEDGGEEAPAPGGAGKSEGSTPADGLPGEAAEDDLAGAPALSQASSGTCFPRKRISSKS
LKVGMIPAPKRVCLIQEPKVHEPVRIAYDRPRGRPMSKKKKPKDLDFAQQKLTDKNLGFQMLQKMGWKEGHGLGSLGKGIREPVSVG
TPSEGEGLGADGQEHKEDTFDVFRQRMMQMYRHKRANK
>sp|Q9H4Y5|GSTO2_HUMAN
MSGDATRTLGKGSQPPGPVPEGLIRIYSMRFCPYSHRTRLVLKAKDIRHEVVNINLRNKPEWYYTKHPFGHIPVLETSQCQLIYESV
IACEYLDDAYPGRKLFPYDPYERARQKMLLELFCKVPHLTKECLVALRCGRECTNLKAALRQEFSNLEEILEYQNTTFFGGTCISMI
DYLLWPWFERLDVYGILDCVSHTPALRLWISAMKWDPTVCALLMDKSIFQGFLNLYFQNNPNAFDFGLC
>sp|Q8NGF8|OR4B1_HUMAN
MASTSNVTELIFTGLFQDPAVQSVCFVVFLPVYLATVVGNGLIVLTVSISKSLDSPMYFFLSCLSLVEISYSSTIAPKFIIDLLAKI
KTISLEGCLTQIFFFHFFGVAEILLIVVMAYDCYVAICKPLHYMNIISRQLCHLLVAGSWLGGFCHSIIQILVIIQLPFCGPNVIDH
YFCDLQPLFKLACTDTFMEGVIVLANSGLFSVFSFLILVSSYIVILVNLRNHSAEGRHKALSTCASHITVVILFFGPAIFLYMRPSS
TFTEDKLVAVFYTVITPMLNPIIYTLRNAEVKIAIRRLWSKKENPGRE
>sp|Q5RHP9|ERIC3_HUMAN

MSHSHPAGLLAAYNSLMDKHLAGYFNNTRIRRHLLRSGLITRSGRILSEKEYKLNMMKRDHQKYIRECLAQAIFHKVLDMERYHQLE
IKKKLETLARKERIQRFKGEHTRRSVENNMPILSPHPPVGPKSNRGHSVLVDEGHSSPLALTAPRPYTAPGNMQPPIRLQPLPSNPA
VETVPKVTSRSRSKTSLLENEALFPIGGKKAVMKFRNSIGNSQRMNSYQLPNINSYMMPIPPPLPPTGKITRENRSETWRRRRFRPT
TAPNGLEPLLTKDSRRIHKTSLHSNAAITMIYLGKNVHLSSDNPDFRDEIKVYQQHCGGENLCVYKGKLLEKETFQFISKRHHGFPF
SLTFFLNGMQVNRLSSCCEYKHRKGSRLGGKRGYFGFVCVERSSPCYKCIIAMGLDKKPSLPKSRKEKSTEKGEELKKAEGKVRKER
EYVIPKRNEIKENKTSVSAKFSAQEIKTGLKEVVTAVEEMTSKGKPGQEVLEDDQENTLKYEYEEDFEVDEEKQGEKSNEEGQADVQ
MNGIPQSPLDDKKDNLDPEKESETSSQKAPDARDNVKDENDGCSESELEEDKQDMKTASSTSSRSHPYSSDSEDESAVGDREAHTDS
STDESARRSSSQELSENDKPRKSHLPIEESLEIEIEDQEITKADVETKPMPIDESFENVLKEGTEKGTQEIAEGLSEKSGKHVSAEE
KEKDKSKLWEESTAQVKDKKAGLPGLEEGGKDSLPLAYVLALGAPTMNFMVDETAAINSNKESQQLVQKTYTLEKKEAMEEDEAPQH
RDADIVQGKGEAALWGEAGAVHEAPLRAWKPTAEQPELAEEFTEKREIPPGIERGAEGAAEAEGVRRLGEGGSDPIGQAAAKDAVGL
SKDEAPEKQALMLTVLETDKAASEGEQGLEKAVLANEAAALNLEHLHEVAALREAATSEEGEAEGGVAVSDVGESEEEASIDLEDTG
PMEDTASKREDGSEEAILGGEEPAKERKEVMRTETRLSPFTGEAEASRMQVSEGSPEEGSLAKEAFLCKEDVEGEEMVTEAEANRED
DRKEILPKELDLARERRKAERPKTSLRKTDSEREEVTRANALKDEDAFKEEQKLKAEEGETETEVRAEEETKAPPNEMGSDAENEAP
VEASELSDNPGLLGEDSLKETVVPIFEATPGFEKSLENITALRKEGGGERLSEARDTEHKDREELSSRENRALKEGHRQDGEGALAA
PEAEPAGKVQAPEGLIPATGQAEELAAKDHDSCAGLEGRAEGQGGVDVVLRTQEAVAEEDPIMAEKFREEAVDEDPEEEEDKECTLE
TEAMQDRNSEGDGDMEGEGNTQKNEGMGGGRVVAVEVLHGGGETAETAAEEREVLAGSETAEEKTIANKASSFSDVAEEETWHQQDE
LVGKTAAAGKVVVEELARSGEEVPAAEEMTVTYTTEAGVGTPGALERKTSGLGQEQEEGSEGQEAATGSGDGRQETGAAEKFRLGLS
REGERELSPESLQAMATLPVKPDFTETREKQQHMVQGESETADVSPNNVQV
>sp|Q16512|PKN1_HUMAN
MASDAVQSEPRSWSLLEQLGLAGADLAAPGVQQQLELERERLRREIRKELKLKEGAENLRRATTDLGRSLGPVELLLRGSSRRLDLL
HQQLQELHAHVVLPDPAATHDGPQSPGAGGPTCSATNLSRVAGLEKQLAIELKVKQGAENMIQTYSNGSTKDRKLLLTAQQMLQDSK
TKIDIIRMQLRRALQAGQLENQAAPDDTQGSPDLGAVELRIEELRHHFRVEHAVAEGAKNVLRLLSAAKAPDRKAVSEAQEKLTESN
QKLGLLREALERRLGELPADHPKGRLLREELAAASSAAFSTRLAGPFPATHYSTLCKPAPLTGTLEVRVVGCRDLPETIPWNPTPSM
GGPGTPDSRPPFLSRPARGLYSRSGSLSGRSSLKAEAENTSEVSTVLKLDNTVVGQTSWKPCGPNAWDQSFTLELERARELELAVFW
RDQRGLCALKFLKLEDFLDNERHEVQLDMEPQGCLVAEVTFRNPVIERIPRLRRQKKIFSKQQGKAFQRARQMNIDVATWVRLLRRL
IPNATGTGTFSPGASPGSEARTTGDISVEKLNLGTDSDSSPQKSSRDPPSSPSSLSSPIQESTAPELPSETQETPGPALCSPLRKSP
LTLEDFKFLAVLGRGHFGKVLLSEFRPSGELFAIKALKKGDIVARDEVESLMCEKRILAAVTSAGHPFLVNLFGCFQTPEHVCFVME
YSAGGDLMLHIHSDVFSEPRAIFYSACVVLGLQFLHEHKIVYRDLKLDNLLLDTEGYVKIADFGLCKEGMGYGDRTSTFCGTPEFLA
PEVLTDTSYTRAVDWWGLGVLLYEMLVGESPFPGDDEEEVFDSIVNDEVRYPRFLSAEAIGIMRRLLRRNPERRLGSSERDAEDVKK
QPFFRTLGWEALLARRLPPPFVPTLSGRTDVSNFDEEFTGEAPTLSPPRDARPLTAAEQAAFLDFDFVAGGC
>sp|A8MV57|MPTX_HUMAN
MGMYLLHIGNAAVTFNGPTPCPRSPYASTHVNVSWESASGIATLWANGKLVGRKGVWKGYSVGEEAKIILGQEQDSFGGHFDENQSF
VGVIWDVFLWDHVLPPKEMCDSCYSGSLLNRHTLTYEDNGYVVTKPKVWA
>sp|Q86YN6|PRGC2_HUMAN
MAGNDCGALLDEELSSFFLNYLADTQGGGSGEEQLYADFPELDLSQLDASDFDSATCFGELQWCPENSETEPNQYSPDDSELFQIDS
ENEALLAELTKTLDDIPEDDVGLAAFPALDGGDALSCTSASPAPSSAPPSPAPEKPSAPAPEVDELSLLQKLLLATSYPTSSSDTQK
EGTAWRQAGLRSKSQRPCVKADSTQDKKAPMMQSQSRSCTELHKHLTSAQCCLQDRGLQPPCLQSPRLPAKEDKEPGEDCPSPQPAP
ASPRDSLALGRADPGAPVSQEDMQAMVQLIRYMHTYCLPQRKLPPQTPEPLPKACSNPSQQVRSRPWSRHHSKASWAEFSILRELLA
QDVLCDVSKPYRLATPVYASLTPRSRPRPPKDSQASPGRPSSVEEVRIAASPKSTGPRPSLRPLRLEVKREVRRPARLQQQEEEDEE
EEEEEEEEEKEEEEEEWGRKRPGRGLPWTKLGRKLESSVCPVRRSRRLNPELGPWLTFADEPLVPSEPQGALPSLCLAPKAYDVEREL
GSPTDEDSGQDQQLLRGPQIPALESPCESGCGDMDEDPSCPQLPPRDSPRCLMLALSQSDPTFGKKSFEQTLTVELCGTAGLTPPTT
PPYKPTEEDPFKPDIKHSLGKEIALSLPSPEGLSLKATPGAAHKLPKKHPERSELLSHLRHATAQPASQAGQKRPFSCSFGDHDYCQ
VLRPEGVLQRKVLRSWEPSGVHLEDWPQQGAPWAEAQAPGREEDRSCDAGAPPKDSTLLRDHEIRASLTKHFGLLETALEEEDLASC
KSPEYDTVFEDSSSSSGESSFLPEEEEEEGEEEEEDDEEEDSGVSPTCSDHCPYQSPPSKANRQLCSRSRSSSGSSPCHSWSPATRR

NFRCESRGPCSDRTPSIRHARKRREKAIGEGRVVYIQNLSSDMSSRELKRRFEVFGEIEECEVLTRNRRGEKYGFITYRCSEHAALS
LTKGAALRKRNEPSFQLSYGGLRHFCWPRYTDYDSNSEEALPASGKSKYEAMDFDSLLKEAQQSLH
>sp|O96011|PX11B_HUMAN
MDAWVRFSAQSQARERLCRAAQYACSLLGHALQRHGASPELQKQIRQLESHLSLGRKLLRLGNSADALESAKRAVHLSDVVLRFCIT
VSHLNRALYFACDNVLWAGKSGLAPRVDQEKWAQRSFRYYLFSLIMNLSRDAYEIRLLMEQESSACSRRLKGSGGGVPGGSETGGLG
GPGTPGGGLPQLALKLRLQVLLLARVLRGHPPLLLDVVRNACDLFIPLDKLGLWRCGPGIVGLCGLVSSILSILTLIYPWLRLKP
>sp|Q9UBT7|CTNL1_HUMAN
MAASPGPAGVGGAGAVYGSGSSGFALDSGLEIKTRSVEQTLLPLVSQITTLINHKDNTKKSDKTLQAIQRVGQAVNLAVGRFVKVGE
AIANENWDLKEEINIACIEAKQAGETIAALTDITNLNHLESDGQITIFTDKTGVIKAARLLLSSVTKVLLLADRVVIKQIITSRNKV
LATMERLEKVNSFQEFVQIFSQFGNEMVEFAHLSGDRQNDLKDEKKKAKMAAARAVLEKCTMMLLTASKTCLRHPNCESAHKNKEGV
FDRMKVALDKVIEIVTDCKPNGETDISSISIFTGIKEFKMNIEALRENLYFQSKENLSVTLEVILERMEDFTDSAYTSHEHRERILE
LSTQARMELQQLISVWIQAQSKKTKSIAEELELSILKISHSLNELKKELHSTATQLAADLLKYHADHVVLKALKLTGVEGNLEALAE
YACKLSEQKEQLVETCRLLRHISGTEPLEITCIHAEETFQVTGQQIISAAETLTLHPSSKIAKENLDVFCEAWESQISDMSTLLREI
NDVFEGRRGEKYGYLSLPKPMKNNANLKSLKPDKPDSEEQAKIAKLGLKLGLLTSDADCEIEKWEDQENEIVQYGRNMSSMAYSLYL
FTRGEGPLKTSQDLIHQLEVFAAEGLKLTSSVQAFSKQLKDDDKLMLLLEINKLIPLCHQLQTVTKTSLQNKVFLKVDKCITKTRSM
MALLVQLLSLCYKLLKKLQMENNGWVSVTNKDTMDSKT
>sp|P0CG42|F157B_HUMAN
MGPLFTTIPGAHSGPMRPLPKKHVEPMAVRQLLLGNSTMIRHTCPMSVPLSRQVKEVAAQKPSEDIYKNRQRRQQQQQQQQQQQQLD
LLFHQRIQISLWPRKQKRRKTEQHSHPFVKKAFRFSAGPGCRRPSSNKMLRSMGGGQRPMGLGSEFFRLLHDLHLLAFATKRIWIHR
RGEATARPRAPEHPAPPATAVRGRDAASQNLKRRPGSGTDGLRLQGAEPSRLLRTYAGGAVIPTGTPERAQPPPPQDPLGRRRWLSR
NTWGPWPGTTQPPSPQLLRNDWGSCGFMVPEAARGKVFQDSQEGAHIRRETVSKSVCAEPWRHQRARDPAPTNFPLKCQKQRGASTS
SGQHGGRVNLVFFIDSPTVIAVPDLQCPTKYSGILY
>sp|P82650|RT22_HUMAN
MAPLGTTVLLWSLLRSSPGVERVCFRARIQPWHGGLLQPLPCSFEMGLPRRRFSSEAAESGSPETKKPTFMDEEVQSILTKMTGLNL
QKTFKPAIQELKPPTYKLMTQAQLEEATRQAVEAAKVRLKMPPVLEERVPINDVLAEDKILEGTETTKYVFTDISYSIPHRERFIVV
REPSGTLRKASWEERDRMIQVYFPKEGRKILTPIIFKEENLRTMYSQDRHVDVLNLCFAQFEPDSTEYIKVHHKTYEDIDKRGKYDL
LRSTRYFGGMVWYFVNNKKIDGLLIDQIQRDLIDDATNLVQLYHVLHPDGQSAQGAKDQAAEGINLIKVFAKTEAQKGAYIELTLQT
YQEALSRHSAAS
>sp|Q12798|CETN1_HUMAN
MASGFKKPSAASTGQKRKVAPKPELTEDQKQEVREAFDLFDVDGSGTIDAKELKVAMRALGFEPRKEEMKKMISEVDREGTGKISFN
DFLAVMTQKMSEKDTKEEILKAFRLFDDDETGKISFKNLKRVANELGENLTDEELQEMIDEADRDGDGEVNEEEFLRIMKKTSLY
>sp|Q96N22|ZN681_HUMAN
MEPLKFRDVAIEFSLEEWQCLDTIQQNLYRNVMLENYRNLVFLGIVVSKPDLITCLEQEKEPWTRKRHRMVAEPPVICSHFAQDFSP
EQNIKDSFQKVTPRRYGKCEHENLQLSKSVDECKVQKGGYNGLNQCLPTTQSKIFQCDKYMKIFHKFSNLNGHKVRHTRKKPFKYKE
FGKSFCIFSNLTQHKIICTRVNFYKCEDCGKAFNGSSIFTKHKRIHIGEKSYICEECGKACNQFTNLTTHKIIYTRDKLYKREECSK
AFNLSSHITTHTIIHTGENPYKREECDKAFNQSLTLTTHKIIHTREKLNEYKECGKAFNQSSHLTRHKIIHTGEKPYKCEECGKAFN
QSSHLTRHKIIHTGEKPYRCEECGKAFRQSSHLTTHKIIHTGEKPYKCEECGKAFNKSSHLTRHKSIHTGEKPYQCEKCGKASNQSS
NLTEHKNIHTEEKPYKCEECGKAFNQFSNLTTHKRIHTGEKPYKCEECGKAFNQSSILTTHKRIHTGEKSYKCEECGKAFYRSSKLT
EHKKIHTGEKPYTCEECGKAFNHSSHLATHKVIHTGEKPYQCEECGKAFNQSSHLTRHKRIHTGEKPYQCEKCGKAFNQSSNLTGHK
KIHTGEKLYKPKRCNSDFENTSKFSKHKRNYAGEKS
>sp|Q5T7V8|GORAB_HUMAN
MSWAAVLAVAAARFGHFWGCRWPGPMAQGWAGFSEEELRRLKQTKDPFEPQRRLPAKKSRQQLQREKALVEQSQKLGLQDGSTSLLP
EQLLSAPKQRVNVQKPPFSSPTLPSHFTLTSPVGDGQPQGIESQPKELGLENSHDGHNNVEILPPKPDCKLEKKKVELQEKSRWEVL
QQEQRLMEEKNKRKKALLAKAIAERSKRTQAETMKLKRIQKELQALDDMVSADIGILRNRIDQASLDYSYARKRFDRAEAEYIAAKL

DIQRKTEIKEQLTEHLCTIIQQNELRKAKKLEELMQQLDVEADEETLELEVEVERLLHEQEVESRRPVVRLERPFQPAEESVTLEFA
KENRKCQEQAVSPKVDDQCGNSSSIPFLSPNCPNQEGNDISAALAT
>sp|Q9BZZ5|API5_HUMAN
MPTVEELYRNYGILADATEQVGQHKDAYQVILDGVKGGTKEKRLAAQFIPKFFKHFPELADSAINAQLDLCEDEDVSIRRQAIKELP
QFATGENLPRVADILTQLLQTDDSAEFNLVNNALLSIFKMDAKGTLGGLFSQILQGEDIVRERAIKFLSTKLKTLPDEVLTKEVEEL
ILTESKKVLEDVTGEEFVLFMKILSGLKSLQTVSGRQQLVELVAEQADLEQTFNPSDPDCVDRLLQCTRQAVPLFSKNVHSTRFVTY
FCEQVLPNLGTLTTPVEGLDIQLEVLKLLAEMSSFCGDMEKLETNLRKLFDKLLEYMPLPPEEAENGENAGNEEPKLQFSYVECLLY
SFHQLGRKLPDFLTAKLNAEKLKDFKIRLQYFARGLQVYIRQLRLALQGKTGEALKTEENKIKVVALKITNNINVLIKDLFHIPPSY
KSTVTLSWKPVQKVEIGQKRASEDTTSGSPPKKSSAGPKRDARQIYNPPSGKYSSNLGNFNYEQRGAFRGSRGGRGWGTRGNRSRGR
LY
>sp|A8MVW0|F1712_HUMAN
MPPASGPSVLARLLPLLGLLLGSASRAPGKSPPEPPSPQEILIKVQVYVSGELVPLARASVDVFGNRTLLAAGTTDSEGVATLPLSY
RLGTWVLVTAARPGFLTNSVPWRVDKLPLYASVSLYLLPERPATLILYEDLVHILLGSPGARSQPLVQFQRRAARLPVSSTYSQLWA
SLTPASTQQEMRAFPAFLGTEASSSGNGSWLELMPLTAVSVHLLTGNGTEVPLSGPIHLSLPVPSETRALTVGTSIPAWRFDPKSGL
WVRNGTGVIRKEGRQLYWTFVSPQLGYWVAAMASPTAGLVTITSGIQDIGTYHTIFLLTILAALALLVLILLCLLIYYCRRRCLKPR
QQHRKLQLSGPSDGNKRDQATSMSQLHLICGGPLEPAPSGDPEAPPPGPLHSAFSSSRDLASSRDDFFRTKPRSASRPAAEPSGARG
GESAGLKGARSAEGPGGLEPGLEEHRRGPSGAAAFLHEPPSPPPPFDHYLGHKGAAEGKTPDFLLSQSVDQLARPPSLGQAGQLIFC
GSIDHLKDNVYRNVMPTLVIPAHYVRLGGEAGAAGVGDEPAPPEGTAPGPARAFPQPDPQRPQMPGHSGPGGEGGGGGGEGWGAGRA
APVSGSVTIPVLFNESTMAQLNGELQALTEKKLLELGVKPHPRAWFVSLDGRSNSQVRHSYIDLQAGGGARSTDASLDSGVDVHEAR
PARRRPAREERERAPPAAPPPPPAPPRLALSEDTEPSSSESRTGLCSPEDNSLTPLLDEVAAPEGRAATVPRGRGRSRGDSSRSSAS
ELRRDSLTSPEDELGAEVGDEAGDKKSPWQRREERPLMVFNVK
>sp|A6QL64|AN36A_HUMAN
MEDGKRERWPTLMERLCSDGFAFPQYPIKPYHLKRIHRAVLHGNLEKLKYLLLTYYDANKRDRKERTALHLACATGQPEMVHLLVSR
RCELNLCDREDRTPLIKAVQLRQEACATLLLQNGANPNITDFFGRTALHYAVYNEDTSMIEKLLSHGTNIEECSKCEYQPLLFAVSR
RKVKMVEFLLKKKANVNAIDYLGRSALIHAVTLGEKDIVILLLQHNIDVLSRDAFRKIAGDYAIEAKNRVIFDLIYEYERKRYEDLP
INSNPVSSQKQPALKATSGKEDSISNIATEIKDGQKSGTVSSQKQPALKDTSDKDDSVSNTATEIKDEQKSGTVLPAVEQCLNRSLY
RPDAVAQPVTENEFSLESEIISKLYIPKRKIISPRSIKDVLPPVEEAVDRCLYLLDRFAQPVTKDKFALESENISEPYFTNRRTISQ
QSAENLDAACGIDKTENGNMFEDQNVDKEGKALPATGQKANVSPEQPPLFTHTVKDRDHISTRFLGGMDSLTSSEESSERPPLSTLT
LKEADPSSKAAMRRKDSPPPGKVSSQKQPAEKATSDDKDSVSNIATEIKEGPISGTVSSQKQPAEKATSDEKDSVSNIATEIKKGQQ
SGTVSPQKQSAWKVIFKKKVSLLNIATRIMGGGKSGTVSSQKQPASKATSDKTDSALNIATEIKDGLQCGTVSSQKQPALKATTDEE
DSVSNIATEIKDGEKSGTVSSQKQPALKATTDEEDSVSNIATEIKDGEKSGTVSSQKQPALKATTDEKDSVSNIATEIKDGEKSGTV
SSQKPPALTATSDEEGSVLSIARENKDGEKSRTVSSRKKPALKATSDEKDSFSNITRGKKDGEISRKVSSQKPPTLKGTSDEEDSVL
GIARENKDGEKSRTVSSEKPPGLKASSAEKDSVLNIARGKKDGEKTKRVSSRKKPSLEATSDEKDSFSNITREKKDGEISRKVSSQK
PPALKGTSDEEDSVLGIARENKDGEKSRTVSSEKPPGLKATSDEKDSVLNIARGKKDGEKTRTVSSQKPPTLKATSDEEDSVLSIAR
ENKDGEKSRTVSSEKPSGLKATSAEKDSVLNIARGKKYGEKTKRVSSRKKPALKATSDEKDSVLYIAREKKDGEKSRTVSSPKQPAL
KAICDKEDSVPNMATEKKDEQISGTVSCQKQPALKATSDKKDSVSNIPTEIKDGQQSGTVSSQKQPAWKATSVKKDSVSNIATEIKD
GQIRGTGILEYTFNVMFDQIEEKFTSLNKSAGVSPQKQSAQKVIFKKKVSLLNIATRITGGWKSGTEYPENLPTLKATIENKNSVLN
TATKMKDVQTSTPAEQDLEMASEGEQKRLEEYENNQPQVKNQIHSRDDLDDIIQSSQTVSEDGDSLCCNCKNVILLIDQHEMKCKDC
VHLLKIKNTFCLWKRLIKLKDNHCEQLRVKIRKLKNKASVLQKRISEKEEIKSQLKHEILELEKELCSLRFAIQQEKKKRRNVEEVH
QKVREKLRITEEQYRIEADVTKPIKPALKSAEVELKTGGNNSNQVSETDEKEDLLHENRLMQDEIARLRLEKDTIKNQNLEKKYLKD
FEIVKRKHEDLQKALKRNGETLAKTIACYSGQLAALTDENTTLRSKLEKQRESRQRLETEMQSYHCRLNAARCDHDQSHSSKRDQEL
AFQGTVDKCRHLQENLNSHVLILSLQLSKAESKSRVLKTELHYTGEALKEKALVFEHVQSELKQKQSQMKDIEKMYKSGYNTMEKCI
EKQERFCQLKKQNMLLQQQLDDARNKADNQEKAILNIQARCDARVQNLQAECRKHRLLLEEDNKMLVNELNHSKEKECQYEKEKAER

EVAVRQLQQKRDDVLNKGSATKALLDASSRHCTYLENGMQDSRKKLDQMRSQFQEIQDQLTATIRCTKEMEGDTQKLEVEHVMMRKI
IKKQDDQIERLEKILQHSSLMLQVFES
>sp|Q5T7B8|KIF24_HUMAN
MASWLYECLCEAELAQYYSHFTALGLQKIDELAKITMKDYSKLGVHDMNDRKRLFQLIKIIKIMQEEDKAVSIPERHLQTSSLRIKS
QELRSGPRRQLNFDSPADNKDRNASNDGFEMCSLSDFSANEQKSTYLKVLEHMLPDDSQYHTKTGILNATAGDSYVQTEISTSLFSP
NYLSAILGDCDIPIIQRISHVSGYNYGIPHSCIRQNTSEKQNPWTEMEKIRVCVRKRPLGMREVRRGEINIITVEDKETLLVHEKKE
AVDLTQYILQHVFYFDEVFGEACTNQDVYMKTTHPLIQHIFNGGNATCFAYGQTGAGKTYTMIGTHENPGLYALAAKDIFRQLEVSQ
PRKHLFVWISFYEIYCGQLYDLLNRRKRLFAREDSKHMVQIVGLQELQVDSVELLLEVILKGSKERSTGATGVNADSSRSHAVIQIQ
IKDSAKRTFGRISFIDLAGSERAADARDSDRQTKMEGAEINQSLLALKECIRALDQEHTHTPFRQSKLTQVLKDSFIGNAKTCMIAN
ISPSHVATEHTLNTLRYADRVKELKKGIKCCTSVTSRNRTSGNSSPKRIQSSPGALSEDKCSPKKVKLGFQQSLTVAAPGSTRGKVH
PLTSHPPNIPFTSAPKVSGKRGGSRGSPSQEWVIHASPVKGTVRSGHVAKKKPEESAPLCSEKNRMGNKTVLGWESRASGPGEGLVR
GKLSTKCKKVQTVQPVQKQLVSRVELSFGNAHHRAEYSQDSQRGTPARPASEAWTNIPPHQKEREEHLRFYHQQFQQPPLLQQKLKY
QPLKRSLRQYRPPEGQLTNETPPLFHSYSENHDGAQVEELDDSDFSEDSFSHISSQRATKQRNTLENSEDSFFLHQTWGQGPEKQVA
ERQQSLFSSPRTGDKKDLTKSWVDSRDPINHRRAALDHSCSPSKGPVDWSRENSTSSGPSPRDSLAEKPYCSQVDFIYRQERGGGSS
FDLRKDASQSEVSGENEGNLPSPEEDGFTISLSHVAVPGSPDQRDTVTTPLREVSADGPIQVTSTVKNGHAVPGEDPRGQLGTHAEY
ASGLMSPLTMSLLENPDNEGSPPSEQLVQDGATHSLVAESTGGPVVSHTVPSGDQEAALPVSSATRHLWLSSSPPDNKPGGDLPALS
PSPIRQHPADKLPSREADLGEACQSRETVLFSHEHMGSEQYDADAEETGLDGSWGFPGKPFTTIHMGVPHSGPTLTPRTGSSDVADQ
LWAQERKHPTRLGWQEFGLSTDPIKLPCNSENVTWLKPRPISRCLARPSSPLVPSCSPKTAGTLRQPTLEQAQQVVIRAHQEQLDEM
AELGFKEETLMSQLASNDFEDFVTQLDEIMVLKSKCIQSLRSQLQLYLTCHGPTAAPEGTVPS
>sp|P0CG31|Z286B_HUMAN
METDLAEMPEKGVLSSQDSPHFQEKSTEEGEVAALRLTARSQAAAAAAAPGSRSLRGVHVPPPLHPAPAREEIKSTCSLKACFSLSL
TLTYYRTAFLLSTENEGNLHFQCPSDVETRPQSKDSTSVQDFSKAESCKVAIIDRLTRNSVYDSNLEAALECENWLEKQQGNQERHL
REMFTHMNSLSEETDHEHDVYWKSFNQKSVLITEDRVPKGSYAFHTLEKSLKQKSNLMKKQRTYKEKKPHKCNDCGELFTCHSVHIQ
HQRVHTGEKPYTCNECGKSFSHRANLTKHQRTHTRILFECRECKKTFTESSSLATHQRIHVGERPYECNECGKGFNRSTHLVQHQLI
HTGVRPYECNECDKAFIHSSALIKHQRTHTGEKPYKCQECGKAFSHCSSLTKHQRVHTGEKPYECSECGKTFSQSTHLVQHQRIHTG
EKPYECSECGKTFSQSSNFAKHQRIHIGKKPYKCSECGKAFIHSSALIQHQRTHTGEKPFRCNECGKSFKCSSSLIRHQRVHTEEQP
>sp|P04000|OPSR_HUMAN
MAQQWSLQRLAGRHPQDSYEDSTQSSIFTYTNSNSTRGPFEGPNYHIAPRWVYHLTSVWMIFVVTASVFTNGLVLAATMKFKKLRHP
LNWILVNLAVADLAETVIASTISIVNQVSGYFVLGHPMCVLEGYTVSLCGITGLWSLAIISWERWLVVCKPFGNVRFDAKLAIVGIA
FSWIWSAVWTAPPIFGWSRYWPHGLKTSCGPDVFSGSSYPGVQSYMIVLMVTCCIIPLAIIMLCYLQVWLAIRAVAKQQKESESTQK
AEKEVTRMVVVMIFAYCVCWGPYTFFACFAAANPGYAFHPLMAALPAYFAKSATIYNPVIYVFMNRQFRNCILQLFGKKVDDGSELS
SASKTEVSSVSSVSPA
>sp|P60510|PP4C_HUMAN
MAEISDLDRQIEQLRRCELIKESEVKALCAKAREILVEESNVQRVDSPVTVCGDIHGQFYDLKELFRVGGDVPETNYLFMGDFVDRG
FYSVETFLLLLALKVRYPDRITLIRGNHESRQITQVYGFYDECLRKYGSVTVWRYCTEIFDYLSLSAIIDGKIFCVHGGLSPSIQTL
DQIRTIDRKQEVPHDGPMCDLLWSDPEDTTGWGVSPRGAGYLFGSDVVAQFNAANDIDMICRAHQLVMEGYKWHFNETVLTVWSAPN
YCYRCGNVAAILELDEHLQKDFIIFEAAPQETRGIPSKKPVADYFL
>sp|O43296|ZN264_HUMAN
MAAAVLTDRAQVSVTFDDVAVTFTKEEWGQLDLAQRTLYQEVMLENCGLLVSLGCPVPKAELICHLEHGQEPWTRKEDLSQDTCPGD
KGKPKTTEPTTCEPALSEGISLQGQVTQGNSVDSQLGQAEDQDGLSEMQEGHFRPGIDPQEKSPGKMSPECDGLGTADGVCSRIGQE
QVSPGDRVRSHNSCESGKDPMIQEEENNFKCSECGKVFNKKHLLAGHEKIHSGVKPYECTECGKTFIKSTHLLQHHMIHTGERPYEC
MECGKAFNRKSYLTQHQRIHSGEKPYKCNECGKAFTHRSNFVLHNRRHTGEKSFVCTECGQVFRHRPGFLRHYVVHSGENPYECLEC
GKVFKHRSYLMWHQQTHTGEKPYECSECGKVFLESAALIHHYVIHTGEKPFECLECGKAFNHRSYLKRHQRIHTGEKPFVCSECGKA
FTHCSTFILHKRAHTGEKPFECKECGKAFSNRKDLIRHFSIHTGEKPYECVECGKAFTRMSGLTRHKRIHSGEKPYECVECGKSFCW

STNLIRHAIIHTGEKPYKCSECGKAFSRSSSLTQHQRMHTGKNPISVTDVGRPFTSGQTSVTLRELLLGKDFLNVTTEANILPEETS
SSASDQPYQRETPQVSSL
>sp|Q9ULJ1|ODF2L_HUMAN
MEKAVNDGSHSEELFCHLKTISEKEDLPRCTSESHLSCLKQDILNEKTELEATLKEAELVTHSVELLLPLFKDTIEKINFENANLSA
LNLKISEQKEILIKELDTFKSVKLALEHLLRKRDYKQTGDNLSSMLLENLTDNESENTNLKKKVFEKEAHIQELSCLFQSEKANTLK
ANRFSQSVKVVHERLQIQIHKREAENDKLKEYVKSLETKIAKWNLQSRMNKNEAIVMKEASRQKTVALKKASKVYKQRLDHFTGAIE
KLTSQIRDQEAKLSETISASNAWKSHYEKIVIEKTELEVQIETMKKQIINLLEDLKKMEDHGKNSCEEILRKVHSIEYENETLNLEN
TKLKLRFPCRITESKNMNILIVLDMLCYISSEKTTLAALKDEVVSVENELSELQEVEKKQKTLIEMYKTQVQKLQEAAEIVKSRCEN
LLHKNNQITKTKNKNVEKMRGQMESHLKELERVCDSLTAAERRLHECQESLQCCKGKCADQEHTIRELQGQVDGNHNLLTKLSLEEE
NCLIQLKCENLQQKLEQMDAENKELEKKLANQEECLKHSNLKFKEKSAEYTALARQLEAALEEGRQKVAEEIEKMSSRESALQIKIL
DLETELRKKNEEQNQLVCKMNSDPETP
>sp|Q4ZIN3|MBRL_HUMAN
MSEHVEPAAPGPGPNGGGGGPAPARGPRTPNLNPNPLINVRDRLFHALFFKMAVTYSRLFPPAFRRLFEFFVLLKALFVLFVLAYIH
IVFSRSPINCLEHVRDKWPREGILRVEVRHNSSRAPVFLQFCDSGGRGSFPGLAVEPGSNLDMEDEEEEELTMEMFGNSSIKFELDI
EPKVFKPPSSTEALNDSQEFPFPETPTKVWPQDEYIVEYSLEYGFLRLSQATRQRLSIPVMVVTLDPTRDQCFGDRFSRLLLDEFLG
YDDILMSSVKGLAENEENKGFLRNVVSGEHYRFVSMWMARTSYLAAFAIMVIFTLSVSMLLRYSHHQIFVFIVDLLQMLEMNMAIAF
PAAPLLTVILALVGMEAIMSEFFNDTTTAFYIILIVWLADQYDAICCHTSTSKRHWLRFFYLYHFAFYAYHYRFNGQYSSLALVTSW
LFIQHSMIYFFHHYELPAILQQVRIQEMLLQAPPLGPGTPTALPDDMNNNSGAPATAPDSAGQPPALGPVSPGASGSPGPVAAAPSS
LVAAAASVAAAAGGDLGWMAETAAIITDASFLSGLSASLLERRPASPLGPAGGLPHAPQDSVPPSDSAASDTTPLGAAVGGPSPASM
APTEAPSEVGS
>sp|Q14D04|MELT_HUMAN
MHQLFRLVLGQKDLSRAGDLFSLDDSEIEDSLTEALEQIKIISSSSDYQTNNNDQAVVEICITRITTAIRETESIEKHAKALVGLWD
SCLEHNLRPFGKDEDTPHAKIASDIMSCILQNYNRPPVMALAIPIAVKFLHRGNKELCRNMSNYLSLAAITKADLLADHTEVIVKSI
LQGNTMLLRVLPAVYEKQPQPINRHLTELLALMSQLEQPEQYHLLRLLHVAAKKKQLEVVQKCIPFLIGHLKDSTHNDIILNILIEI
AVYEPVALNSFLPMLKEIGERFPYLTGQMARIYGAVGHVDEERARSCLTYLVSQLANMEHSFHHILLLEIKSITDTFSSILGPQSRD
IFRMSNSFTAIAKLLTRQLENTKAGSGRRKISTEIEFPEKLEETKLIVTENEDHEKLQVKIQAFEDKINAGSNTPGSIRRYSLGQVS
KEERKNIRFNRSKSLAFHTMLTKGVGSDDGEDENRGDIPASISLSEIDPLGQGNDKLPFKTDTERSQLGESSVSYPNIIHIDSENLS
ETVKENSQEETPETTASPIEYQDKLYLHLKKNLSKVKAYAMEIGKKIPVPDQCTIEDTVRSCVAKLFFTCSLKGHYCLYSKSSFILI
SQEPQPWIQIMFLFQQSLFPEPLSIQSHSVQFLRALWEKTQAGGAHSFETAMMESTFPQQKDLDQVQLHLEEVRFFDVFGFSETAGA
WQCFMCNNPEKATVVNQDGQPLIEGKLKEKQVRWKFIKRWKTRYFTLAGNQLLFQKGKSKDDPDDCPIELSKVQSVKAVAKKRRDRS
LPRAFEIFTDNKTYVFKAKDEKNAEEWLQCINVAVAQAKERESREVTTYL
>sp|P49137|MAPK2_HUMAN
MLSNSQGQSPPVPFPAPAPPPQPPTPALPHPPAQPPPPPPQQFPQFHVKSGLQIKKNAIIDDYKVTSQVLGLGINGKVLQIFNKRTQ
EKFALKMLQDCPKARREVELHWRASQCPHIVRIVDVYENLYAGRKCLLIVMECLDGGELFSRIQDRGDQAFTEREASEIMKSIGEAI
QYLHSINIAHRDVKPENLLYTSKRPNAILKLTDFGFAKETTSHNSLTTPCYTPYYVAPEVLGPEKYDKSCDMWSLGVIMYILLCGYP
PFYSNHGLAISPGMKTRIRMGQYEFPNPEWSEVSEEVKMLIRNLLKTEPTQRMTITEFMNHPWIMQSTKVPQTPLHTSRVLKEDKER
WEDVKEEMTSALATMRVDYEQIKIKKIEDASNPLLLKRRKKARALEAAALAH
>sp|P17927|CR1_HUMAN
MGASSPRSPEPVGPPAPGLPFCCGGSLLAVVVLLALPVAWGQCNAPEWLPFARPTNLTDEFEFPIGTYLNYECRPGYSGRPFSIICL
KNSVWTGAKDRCRRKSCRNPPDPVNGMVHVIKGIQFGSQIKYSCTKGYRLIGSSSATCIISGDTVIWDNETPICDRIPCGLPPTITN
GDFISTNRENFHYGSVVTYRCNPGSGGRKVFELVGEPSIYCTSNDDQVGIWSGPAPQCIIPNKCTPPNVENGILVSDNRSLFSLNEV
VEFRCQPGFVMKGPRRVKCQALNKWEPELPSCSRVCQPPPDVLHAERTQRDKDNFSPGQEVFYSCEPGYDLRGAASMRCTPQGDWSP
AAPTCEVKSCDDFMGQLLNGRVLFPVNLQLGAKVDFVCDEGFQLKGSSASYCVLAGMESLWNSSVPVCEQIFCPSPPVIPNGRHTGK
PLEVFPFGKTVNYTCDPHPDRGTSFDLIGESTIRCTSDPQGNGVWSSPAPRCGILGHCQAPDHFLFAKLKTQTNASDFPIGTSLKYE

CRPEYYGRPFSITCLDNLVWSSPKDVCKRKSCKTPPDPVNGMVHVITDIQVGSRINYSCTTGHRLIGHSSAECILSGNAAHWSTKPP
ICQRIPCGLPPTIANGDFISTNRENFHYGSVVTYRCNPGSGGRKVFELVGEPSIYCTSNDDQVGIWSGPAPQCIIPNKCTPPNVENG
ILVSDNRSLFSLNEVVEFRCQPGFVMKGPRRVKCQALNKWEPELPSCSRVCQPPPDVLHAERTQRDKDNFSPGQEVFYSCEPGYDLR
GAASMRCTPQGDWSPAAPTCEVKSCDDFMGQLLNGRVLFPVNLQLGAKVDFVCDEGFQLKGSSASYCVLAGMESLWNSSVPVCEQIF
CPSPPVIPNGRHTGKPLEVFPFGKAVNYTCDPHPDRGTSFDLIGESTIRCTSDPQGNGVWSSPAPRCGILGHCQAPDHFLFAKLKTQ
TNASDFPIGTSLKYECRPEYYGRPFSITCLDNLVWSSPKDVCKRKSCKTPPDPVNGMVHVITDIQVGSRINYSCTTGHRLIGHSSAE
CILSGNTAHWSTKPPICQRIPCGLPPTIANGDFISTNRENFHYGSVVTYRCNLGSRGRKVFELVGEPSIYCTSNDDQVGIWSGPAPQ
CIIPNKCTPPNVENGILVSDNRSLFSLNEVVEFRCQPGFVMKGPRRVKCQALNKWEPELPSCSRVCQPPPEILHGEHTPSHQDNFSP
GQEVFYSCEPGYDLRGAASLHCTPQGDWSPEAPRCAVKSCDDFLGQLPHGRVLFPLNLQLGAKVSFVCDEGFRLKGSSVSHCVLVGM
RSLWNNSVPVCEHIFCPNPPAILNGRHTGTPSGDIPYGKEISYTCDPHPDRGMTFNLIGESTIRCTSDPHGNGVWSSPAPRCELSVR
AGHCKTPEQFPFASPTIPINDFEFPVGTSLNYECRPGYFGKMFSISCLENLVWSSVEDNCRRKSCGPPPEPFNGMVHINTDTQFGST
VNYSCNEGFRLIGSPSTTCLVSGNNVTWDKKAPICEIISCEPPPTISNGDFYSNNRTSFHNGTVVTYQCHTGPDGEQLFELVGERSI
YCTSKDDQVGVWSSPPPRCISTNKCTAPEVENAIRVPGNRSFFSLTEIIRFRCQPGFVMVGSHTVQCQTNGRWGPKLPHCSRVCQPP
PEILHGEHTLSHQDNFSPGQEVFYSCEPSYDLRGAASLHCTPQGDWSPEAPRCTVKSCDDFLGQLPHGRVLLPLNLQLGAKVSFVCD
EGFRLKGRSASHCVLAGMKALWNSSVPVCEQIFCPNPPAILNGRHTGTPFGDIPYGKEISYACDTHPDRGMTFNLIGESSIRCTSDP
QGNGVWSSPAPRCELSVPAACPHPPKIQNGHYIGGHVSLYLPGMTISYICDPGYLLVGKGFIFCTDQGIWSQLDHYCKEVNCSFPLF
MNGISKELEMKKVYHYGDYVTLKCEDGYTLEGSPWSQCQADDRWDPPLAKCTSRTHDALIVGTLSGTIFFILLIIFLSWIILKHRKG
NNAHENPKEVAIHLHSQGGSSVHPRTLQTNEENSRVLP
>sp|O15305|PMM2_HUMAN
MAAPGPALCLFDVDGTLTAPRQKITKEMDDFLQKLRQKIKIGVVGGSDFEKVQEQLGNDVVEKYDYVFPENGLVAYKDGKLLCRQNI
QSHLGEALIQDLINYCLSYIAKIKLPKKRGTFIEFRNGMLNVSPIGRSCSQEERIEFYELDKKENIRQKFVADLRKEFAGKGLTFSI
GGQISFDVFPDGWDKRYCLRHVENDGYKTIYFFGDKTMPGGNDHEIFTDPRTMGYSVTAPEDTRRICELLFS
>sp|Q9BUR4|WAP53_HUMAN
MKTLETQPLAPDCCPSDQDPAPAHPSPHASPMNKNADSELMPPPPERGDPPRLSPDPVAGSAVSQELREGDPVSLSTPLETEFGSPS
ELSPRIEEQELSENTSLPAEEANGSLSEEEANGPELGSGKAMEDTSGEPAAEDEGDTAWNYSFSQLPRFLSGSWSEFSTQPENFLKG
CKWAPDGSCILTNSADNILRIYNLPPELYHEGEQVEYAEMVPVLRMVEGDTIYDYCWYSLMSSAQPDTSYVASSSRENPIHIWDAFT
GELRASFRAYNHLDELTAAHSLCFSPDGSQLFCGFNRTVRVFSTARPGRDCEVRATFAKKQGQSGIISCIAFSPAQPLYACGSYGRS
LGLYAWDDGSPLALLGGHQGGITHLCFHPDGNRFFSGARKDAELLCWDLRQSGYPLWSLGREVTTNQRIYFDLDPTGQFLVSGSTSG
AVSVWDTDGPGNDGKPEPVLSFLPQKDCTNGVSLHPSLPLLATASGQRVFPEPTESGDEGEELGLPLLSTRHVHLECRLQLWWCGGA
PDSSIPDDHQGEKGQGGTEGGVGELI
>sp|Q86Y78|LYPD6_HUMAN
MEPGPALAWLLLLSLLADCLKAAQSRDFTVKDIIYLHPSTTPYPGGFKCFTCEKAADNYECNRWAPDIYCPRETRYCYTQHTMEVTG
NSISVTKRCVPLEECLSTGCRDSEHEGHKVCTSCCEGNICNLPLPRNETDATFATTSPINQTNGHPRCMSVIVSCLWLWLGLML
>sp|P23527|H2B1O_HUMAN
MPDPAKSAPAPKKGSKKAVTKAQKKDGKKRKRSRKESYSIYVYKVLKQVHPDTGISSKAMGIMNSFVNDIFERIAGEASRLAHYNKR
STITSREIQTAVRLLLPGELAKHAVSEGTKAVTKYTSSK
>sp|A4D263|CG072_HUMAN
MDVEIQDTPGKISISKRSILSGTVENIDYPHYCDLLRKMNMPFVKGLENRHNYGRFEKKCNPAFLKFHPYPPSVLPDYHLHDPYPPP
YGPHYPLFPLRDDVTLGDSCSGFMSPGGDADLNPGIGRTIPTLVDFSDVKPQHRVPRPDTGFQTTIKRQKILSEELQQNRRWNSREV
PDISIRARLGGWTSPLKVTPLQPHHEGRSLSHIFTFDEEATCTDEGEPLVQTNKKCNAKDSFYKSSTQKAYEDVPWDKMLPPKLVPE
ETTLEKTADPISQCFTLKRYKGVPAITQMVGELWDRFQTRSFLAPVKPINFVSSSSRSKYIPLYTGHVQSTNADDVDNPLGDIASLA
KQRYSKPLYTNTSRAANIPGYTGKVHFTATHPANSNIPSTTPSPDSELHRVFQKEMAVDLFRHQAPLSRLVTTVRPYNPFNKKDKET
IDY
>sp|A6NL05|F74A7_HUMAN

MWRELRGCPGGDVETVQRLSRRRRGKSSEAVPEKTWRAQRMSQTRESSEAVPEKTWREFRGCPGEDVERAQRLRDCPGEDMETAQRL
SARRRAESSEAVPEKTWRELKGCPQEDVERVQRLSLLLHLAVFLWIIIAINFSNSGVKSQSSTYLPSGKILK
>sp|Q17R55|F187B_HUMAN
MPPMLWLLLHFAAPALGFYFSISCPSGKQCQQALLSGNDILLYCNSSGAHWYYLFTQGKKGRLTSLTNISNMEIMPEGSLLIKDPLP
SQTGLYHCWNKNGRQVVQYEIDFQDVTTLHITHKDLGQRPLQNETLHLGSKQLIFTWWEPWQDCNRCEEPGECKRLGYRYIEEPLEE
AMPCWLYLGEVLVWSSRLRPELQVEACHVQCTNNTQLRVDYVIFDNFRLDEKTEFVWLDCPLGSMYRPVNWRANDTPLTWESQLSGQ
DFTTFLDPSTGGRQLQVFQPAVYKCFVQQELVAQFKPAASLETLEAQWRENDAQWREARKALRGRADSVLKGLKLVLLVVTVLALLG
ALLKCIHPSPGRRSTQVLVVK
>sp|Q9BXT6|M10L1_HUMAN
MLSLAAKLVAFFWRTADTPREEAGQLEPELAEGDTKLKTVRGVVTRYCSDYGMIDDMIYFSSDAVTSRVLLNVGQEVIAVVEENKVS
NGLKAIRVEAVSDKWEDDSRNHGSPSDCGPRVLIGCVTSLVEGAGCISQTTYFSLESVCEGFEPCKGDWVEAEYRIRPGTWSSEATS
VKPLRYKRVDKVCISSLCGRNGVLEESIFFTLDSLKLPDGYTPRRGDVVNAVVVESSQSCYVWRALCMTLVKRRDAAPVHEATHFYG
TILLKNKGDIEVTQVTHFGTLKEGRSKTMVIWIENKGDIPQNLVSCKLAGWDKSKQFRFQMLDKDQMCPVVSFVSVPEKENSSDENI
NSLNSHTKNKTSQMSESSLVNNRGISPGDCTCKGENGEKDNILSRKQMTEPEPGGLVPPGGKTFIVVICDGKNPGRCKELLLLCFSD
FLIGRYLEVNVISGEESLIAAREPFSWKKLKSSQALTSAKTTVVVTAQKRNSRRQLPSFLPQYPIPDRLRKCVEQKIDILTFQPLLA
ELLNMSNYKEKFSTLLWLEEIYAEMELKEYNMSGIILRRNGDLLVLEVPGLAEGRPSLYAGDKLILKTQEYNGHAIEYISYVTEIHE
EDVTLKINPEFEQAYNFEPMDVEFTYNRTTSRRCHFALEHVIHLGVKVLFPEEIILQSPQVTGNWNHAQDTKSSGQSTSKKNRKTMT
DQAEHGTEERRVGDKDLPVLAPFTAEMSDWVDEIQTPKARKMEFFNPVLNENQKLAVKRILSGDCRPLPYILFGPPGTGKTVTIIEA
VLQVHFALPDSRILVCAPSNSAADLVCLRLHESKVLQPATMVRVNATCRFEEIVIDAVKPYCRDGEDIWKASRFRIIITTCSSSGLF
YQIGVRVGHFTHVFVDEAGQASEPECLIPLGLMSDISGQIVLAGDPMQLGPVIKSRLAMAYGLNVSFLERLMSRPAYQRDENAFGAC
GAHNPLLVTKLVKNYRSHEALLMLPSRLFYHRELEVCADPTVVTSLLGWEKLPKKGFPLIFHGVRGSEAREGKSPSWFNPAEAVQVL
RYCCLLAHSISSQVSASDIGVITPYRKQVEKIRILLRNVDLMDIKVGSVEEFQGQEYLVIIISTVRSNEDRFEDDRYFLGFLSNSKR
FNVAITRPKALLIVLGNPHVLVRDPCFGALLEYSITNGVYMGCDLPPALQSLQNCGEGVADPSYPVVPESTGPEKHQEPS
>sp|Q32MK0|MYLK3_HUMAN
MSGTSKESLGHGGLPGLGKTCLTTMDTKLNMLNEKVDQLLHFQEDVTEKLQSMCRDMGHLERGLHRLEASRAPGPGGADGVPHIDTQ
AGWPEVLELVRAMQQDAAQHGARLEALFRMVAAVDRAIALVGATFQKSKVADFLMQGRVPWRRGSPGDSPEENKERVEEEGGKPKHV
LSTSGVQSDAREPGEESQKADVLEGTAERLPPIRASGLGADPAQAVVSPGQGDGVPGPAQAFPGHLPLPTKVEAKAPETPSENLRTG
LELAPAPGRVNVVSPSLEVAPGAGQGASSSRPDPEPLEEGTRLTPGPGPQCPGPPGLPAQARATHSGGETPPRISIHIQEMDTPGEM
LMTGRGSLGPTLTTEAPAAAQPGKQGPPGTGRCLQAPGTEPGEQTPEGARELSPLQESSSPGGVKAEEEQRAGAEPGTRPSLARSDD
NDHEVGALGLQQGKSPGAGNPEPEQDCAARAPVRAEAVRRMPPGAEAGSVVLDDSPAPPAPFEHRVVSVKETSISAGYEVCQHEVLG
GGRFGQVHRCTEKSTGLPLAAKIIKVKSAKDREDVKNEINIMNQLSHVNLIQLYDAFESKHSCTLVMEYVDGGELFDRITDEKYHLT
ELDVVLFTRQICEGVHYLHQHYILHLDLKPENILCVNQTGHQIKIIDFGLARRYKPREKLKVNFGTPEFLAPEVVNYEFVSFPTDMW
SVGVITYMLLSGLSPFLGETDAETMNFIVNCSWDFDADTFEGLSEEAKDFVSRLLVKEKSCRMSATQCLKHEWLNNLPAKASRSKTR
LKSQLLLQKYIAQRKWKKHFYVVTAANRLRKFPTSP
>sp|O15482|TEX28_HUMAN
MVLKAEHTRSPSATLPSNVPSCRSLSSSEDGPSGPSSLADGGLAHNLQDSVRHRILYLSEQLRVEKASRDGNTVSYLKLVSKADRHQ
VPHIQQAFEKVNQRASATIAQIEHRLHQCHQQLQELEEGCRPEGLLLMAESDPANCEPPSEKALLSEPPEPGGEDGPVNLPHASRPF
ILESRFQSLQQGTCLETEDVAQQQNLLLQKVKAELEEAKRFHISLQESYHSLKERSLTDLQLLLESLQEEKCRQALMEEQVNGRLQG
QLNEIYNLKHNLACSEERMAYLSYERAKEIWEITETFKSRISKLEMLQQVTQLEAAEHLQSRPPQMLFKFLSPRLSLATVLLVFVST
LCACPSSLISSRLCTCTMLMLIGLGVLAWQRWRAIPATDWQEWVPSRCRLYSKDSGPPADGP
>sp|Q156A1|ATX8_HUMAN
MQQQQQQQQQQQQQQQQQQQQQQQQQQQQQQQQQQQQQQQQQQQQQQQQQQQQQQQQQQQQQQQQQQQQQQQQQQQQQQQ
>sp|P49792|RBP2_HUMAN

MRRSKADVERYIASVQGSTPSPRQKSMKGFYFAKLYYEAKEYDLAKKYICTYINVQERDPKAHRFLGLLYELEENTDKAVECYRRSV
ELNPTQKDLVLKIAELLCKNDVTDGRAKYWLERAAKLFPGSPAIYKLKEQLLDCEGEDGWNKLFDLIQSELYVRPDDVHVNIRLVEV
YRSTKRLKDAVAHCHEAERNIALRSSLEWNSCVVQTLKEYLESLQCLESDKSDWRATNTDLLLAYANLMLLTLSTRDVQESRELLQS
FDSALQSVKSLGGNDELSATFLEMKGHFYMHAGSLLLKMGQHSSNVQWRALSELAALCYLIAFQVPRPKIKLIKGEAGQNLLEMMAC
DRLSQSGHMLLNLSRGKQDFLKEIVETFANKSGQSALYDALFSSQSPKDTSFLGSDDIGNIDVREPELEDLTRYDVGAIRAHNGSLQ
HLTWLGLQWNSLPALPGIRKWLKQLFHHLPHETSRLETNAPESICILDLEVFLLGVVYTSHLQLKEKCNSHHSSYQPLCLPLPVCKQ
LCTERQKSWWDAVCTLIHRKAVPGNVAKLRLLVQHEINTLRAQEKHGLQPALLVHWAECLQKTGSGLNSFYDQREYIGRSVHYWKKV
LPLLKIIKKKNSIPEPIDPLFKHFHSVDIQASEIVEYEEDAHITFAILDAVNGNIEDAVTAFESIKSVVSYWNLALIFHRKAEDIEN
DALSPEEQEECKNYLRKTRDYLIKIIDDSDSNLSVVKKLPVPLESVKEMLNSVMQELEDYSEGGPLYKNGSLRNADSEIKHSTPSPT
RYSLSPSKSYKYSPKTPPRWAEDQNSLLKMICQQVEAIKKEMQELKLNSSNSASPHRWPTENYGPDSVPDGYQGSQTFHGAPLTVAT
TGPSVYYSQSPAYNSQYLLRPAANVTPTKGPVYGMNRLPPQQHIYAYPQQMHTPPVQSSSACMFSQEMYGPPALRFESPATGILSPR
GDDYFNYNVQQTSTNPPLPEPGYFTKPPIAAHASRSAESKTIEFGKTNFVQPMPGEGLRPSLPTQAHTTQPTPFKFNSNFKSNDGDF
TFSSPQVVTQPPPAAYSNSESLLGLLTSDKPLQGDGYSGAKPIPGGQTIGPRNTFNFGSKNVSGISFTENMGSSQQKNSGFRRSDDM
FTFHGPGKSVFGTPTLETANKNHETDGGSAHGDDDDDGPHFEPVVPLPDKIEVKTGEEDEEEFFCNRAKLFRFDVESKEWKERGIGN
VKILRHKTSGKIRLLMRREQVLKICANHYISPDMKLTPNAGSDRSFVWHALDYADELPKPEQLAIRFKTPEEAALFKCKFEEAQSIL
KAPGTNVAMASNQAVRIVKEPTSHDNKDICKSDAGNLNFEFQVAKKEGSWWHCNSCSLKNASTAKKCVSCQNLNPSNKELVGPPLAE
TVFTPKTSPENVQDRFALVTPKKEGHWDCSICLVRNEPTVSRCIACQNTKSANKSGSSFVHQASFKFGQGDLPKPINSDFRSVFSTK
EGQWDCSACLVQNEGSSTKCAACQNPRKQSLPATSIPTPASFKFGTSETSKTLKSGFEDMFAKKEGQWDCSSCLVRNEANATRCVAC
QNPDKPSPSTSVPAPASFKFGTSETSKAPKSGFEGMFTKKEGQWDCSVCLVRNEASATKCIACQNPGKQNQTTSAVSTPASSETSKA
PKSGFEGMFTKKEGQWDCSVCLVRNEASATKCIACQNPGKQNQTTSAVSTPASSETSKAPKSGFEGMFTKKEGQWDCSVCLVRNEAS
ATKCIACQCPSKQNQTTAISTPASSEISKAPKSGFEGMFIRKGQWDCSVCCVQNESSSLKCVACDASKPTHKPIAEAPSAFTLGSEM
KLHDSSGSQVGTGFKSNFSEKASKFGNTEQGFKFGHVDQENSPSFMFQGSSNTEFKSTKEGFSIPVSADGFKFGISEPGNQEKKSEK
PLENGTGFQAQDISGQKNGRGVIFGQTSSTFTFADLAKSTSGEGFQFGKKDPNFKGFSGAGEKLFSSQYGKMANKANTSGDFEKDDD
AYKTEDSDDIHFEPVVQMPEKVELVTGEEDEKVLYSQRVKLFRFDAEVSQWKERGLGNLKILKNEVNGKLRMLMRREQVLKVCANHW
ITTTMNLKPLSGSDRAWMWLASDFSDGDAKLEQLAAKFKTPELAEEFKQKFEECQRLLLDIPLQTPHKLVDTGRAAKLIQRAEEMKS
GLKDFKTFLTNDQTKVTEEENKGSGTGAAGASDTTIKPNPENTGPTLEWDNYDLREDALDDSVSSSSVHASPLASSPVRKNLFRFGE
STTGFNFSFKSALSPSKSPAKLNQSGTSVGTDEESDVTQEEERDGQYFEPVVPLPDLVEVSSGEENEQVVFSHRAKLYRYDKDVGQW
KERGIGDIKILQNYDNKQVRIVMRRDQVLKLCANHRITPDMTLQNMKGTERVWLWTACDFADGERKVEHLAVRFKLQDVADSFKKIF
DEAKTAQEKDSLITPHVSRSSTPRESPCGKIAVAVLEETTRERTDVIQGDDVADATSEVEVSSTSETTPKAVVSPPKFVFGSESVKS
IFSSEKSKPFAFGNSSATGSLFGFSFNAPLKSNNSETSSVAQSGSESKVEPKKCELSKNSDIEQSSDSKVKNLFASFPTEESSINYT
FKTPEKAKEKKKPEDSPSDDDVLIVYELTPTAEQKALATKLKLPPTFFCYKNRPDYVSEEEEDDEDFETAVKKLNGKLYLDGSEKCR
PLEENTADNEKECIIVWEKKPTVEEKAKADTLKLPPTFFCGVCSDTDEDNGNGEDFQSELQKVQEAQKSQTEEITSTTDSVYTGGTE
VMVPSFCKSEEPDSITKSISSPSVSSETMDKPVDLSTRKEIDTDSTSQGESKIVSFGFGSSTGLSFADLASSNSGDFAFGSKDKNFQ
WANTGAAVFGTQSVGTQSAGKVGEDEDGSDEEVVHNEDIHFEPIVSLPEVEVKSGEEDEEILFKERAKLYRWDRDVSQWKERGVGDI
KILWHTMKNYYRILMRRDQVFKVCANHVITKTMELKPLNVSNNALVWTASDYADGEAKVEQLAVRFKTKEVADCFKKTFEECQQNLM
KLQKGHVSLAAELSKETNPVVFFDVCADGEPLGRITMELFSNIVPRTAENFRALCTGEKGFGFKNSIFHRVIPDFVCQGGDITKHDG
TGGQSIYGDKFEDENFDVKHTGPGLLSMANQGQNTNNSQFVITLKKAEHLDFKHVVFGFVKDGMDTVKKIESFGSPKGSVCRRITIT
ECGQI
>sp|Q8WU67|ABHD3_HUMAN
MQRLAMDLRMLSRELSLYLEHQVRVGFFGSGVGLSLILGFSVAYAFYYLSSIAKKPQLVTGGESFSRFLQDHCPVVTETYYPTVWCW
EGRGQTLLRPFITSKPPVQYRNELIKTADGGQISLDWFDNDNSTCYMDASTRPTILLLPGLTGTSKESYILHMIHLSEELGYRCVVF
NNRGVAGENLLTPRTYCCANTEDLETVIHHVHSLYPSAPFLAAGVSMGGMLLLNYLGKIGSKTPLMAAATFSVGWNTFACSESLEKP
LNWLLFNYYLTTCLQSSVNKHRHMFVKQVDMDHVMKAKSIREFDKRFTSVMFGYQTIDDYYTDASPSPRLKSVGIPVLCLNSVDDVF
SPSHAIPIETAKQNPNVALVLTSYGGHIGFLEGIWPRQSTYMDRVFKQFVQAMVEHGHELS

>sp|P25391|LAMA1_HUMAN
MRGGVLLVLLLCVAAQCRQRGLFPAILNLASNAHISTNATCGEKGPEMFCKLVEHVPGRPVRNPQCRICDGNSANPRERHPISHAID
GTNNWWQSPSIQNGREYHWVTITLDLRQVFQVAYVIIKAANAPRPGNWILERSLDGTTFSPWQYYAVSDSECLSRYNITPRRGPPTY
RADDEVICTSYYSRLVPLEHGEIHTSLINGRPSADDLSPKLLEFTSARYIRLRLQRIRTLNADLMTLSHREPKELDPIVTRRYYYSI
KDISVGGMCICYGHASSCPWDETTKKLQCQCEHNTCGESCNRCCPGYHQQPWRPGTVSSGNTCEACNCHNKAKDCYYDESVAKQKKS
LNTAGQFRGGGVCINCLQNTMGINCETCIDGYYRPHKVSPYEDEPCRPCNCDPVGSLSSVCIKDDLHSDLHNGKQPGQCPCKEGYTG
EKCDRCQLGYKDYPTCVSCGCNPVGSASDEPCTGPCVCKENVEGKACDRCKPGFYNLKEKNPRGCSECFCFGVSDVCSSLSWPVGQV
NSMSGWLVTDLISPRKIPSQQDALGGRHQVSINNTAVMQRLAPKYYWAAPEAYLGNKLTAFGGFLKYTVSYDIPVETVDSNLMSHAD
VIIKGNGLTLSTQAEGLSLQPYEEYLNVVRLVPENFQDFHSKRQIDRDQLMTVLANVTHLLIRANYNSAKMALYRLESVSLDIASSN
AIDLVVAADVEHCECPQGYTGTSCESCLSGYYRVDGILFGGICQPCECHGHAAECNVHGVCIACAHNTTGVHCEQCLPGFYGEPSRG
TPGDCQPCACPLTIASNNFSPTCHLNDGDEVVCDWCAPGYSGAWCERCADGYYGNPTVPGESCVPCDCSGNVDPSEAGHCDSVTGEC
LKCLGNTDGAHCERCADGFYGDAVTAKNCRACECHVKGSHSAVCHLETGLCDCKPNVTGQQCDQCLHGYYGLDSGHGCRPCNCSVAG
SVSDGCTDEGQCHCVPGVAGKRCDRCAHGFYAYQDGSCTPCDCPHTQNTCDPETGECVCPPHTQGVKCEECEDGHWGYDAEVGCQAC
NCSLVGSTHHRCDVVTGHCQCKSKFGGRACDQCSLGYRDFPDCVPCDCDLRGTSGDACNLEQGLCGCVEETGACPCKENVFGPQCNE
CREGTFALRADNPLGCSPCFCSGLSHLCSELEDYVRTPVTLGSDQPLLRVVSQSNLRGTTEGVYYQAPDFLLDAATVRQHIRAEPFY
WRLPQQFQGDQLMAYGGKLKYSVAFYSLDGVGTSNFEPQVLIKGGRIRKQVIYMDAPAPENGVRQEQEVAMRENFWKYFNSVSEKPV
TREDFMSVLSDIEYILIKASYGQGLQQSRISDISMEVGRKAEKLHPEEEVASLLENCVCPPGTVGFSCQDCAPGYHRGKLPAGSDRG
PRPLVAPCVPCSCNNHSDTCDPNTGKCLNCGDNTAGDHCDVCTSGYYGKVTGSASDCALCACPHSPPASFSPTCVLEGDHDFRCDAC
LLGYEGKHCERCSSSYYGNPQTPGGSCQKCDCNPHGSVHGDCDRTSGQCVCRLGASGLRCDECEPRHILMETDCVSCDDECVGVLLN
DLDEIGDAVLSLNLTGIIPVPYGILSNLENTTKYLQESLLKENMQKDLGKIKLEGVAEETDNLQKKLTRMLASTQKVNRATERIFKE
SQDLAIAIERLQMSITEIMEKTTLNQTLDEDFLLPNSTLQNMQQNGTSLLEIMQIRDFTQLHQNATLELKAAEDLLSQIQENYQKPL
EELEVLKEAASHVLSKHNNELKAAEALVREAEAKMQESNHLLLMVNANLREFSDKKLHVQEEQNLTSELIVQGRGLIDAAAAQTDAV
QDALEHLEDHQDKLLLWSAKIRHHIDDLVMHMSQRNAVDLVYRAEDHAAEFQRLADVLYSGLENIRNVSLNATSAAYVHYNIQSLIE
ESEELARDAHRTVTETSLLSESLVSNGKAAVQRSSRFLKEGNNLSRKLPGIALELSELRNKTNRFQENAVEITRQTNESLLILRAIP
KGIRDKGAKTKELATSASQSAVSTLRDVAGLSQELLNTSASLSRVNTTLRETHQLLQDSTMATLLAGRKVKDVEIQANLLFDRLKPL
KMLEENLSRNLSEIKLLISQARKQAASIKVAVSADRDCIRAYQPQISSTNYNTLTLNVKTQEPDNLLFYLGSSTASDFLAVEMRRGR
VAFLWDLGSGSTRLEFPDFPIDDNRWHSIHVARFGNIGSLSVKEMSSNQKSPTKTSKSPGTANVLDVNNSTLMFVGGLGGQIKKSPA
VKVTHFKGCLGEAFLNGKSIGLWNYIEREGKCRGCFGSSQNEDPSFHFDGSGYSVVEKSLPATVTQIIMLFNTFSPNGLLLYLGSYG
TKDFLSIELFRGRVKVMTDLGSGPITLLTDRRYNNGTWYKIAFQRNRKQGVLAVIDAYNTSNKETKQGETPGASSDLNRLDKDPIYV
GGLPRSRVVRRGVTTKSFVGCIKNLEISRSTFDLLRNSYGVRKGCLLEPIRSVSFLKGGYIELPPKSLSPESEWLVTFATTNSSGII
LAALGGDVEKRGDREEAHVPFFSVMLIGGNIEVHVNPGDGTGLRKALLHAPTGTCSDGQAHSISLVRNRRIITVQLDENNPVEMKLG
TLVESRTINVSNLYVGGIPEGEGTSLLTMRRSFHGCIKNLIFNLELLDFNSAVGHEQVDLDTCWLSERPKLAPDAEDSKLLPEPRAF
PEQCVVDAALEYVPGAHQFGLTQNSHFILPFNQSAVRKKLSVELSIRTFASSGLIYYMAHQNQADYAVLQLHGGRLHFMFDLGKGRT
KVSHPALLSDGKWHTVKTDYVKRKGFITVDGRESPMVTVVGDGTMLDVEGLFYLGGLPSQYQARKIGNITHSIPACIGDVTVNSKQL
DKDSPVSAFTVNRCYAVAQEGTYFDGSGYAALVKEGYKVQSDVNITLEFRTSSQNGVLLGISTAKVDAIGLELVDGKVLFHVNNGAG
RITAAYEPKTATVLCDGKWHTLQANKSKHRITLIVDGNAVGAESPHTQSTSVDTNNPIYVGGYPAGVKQKCLRSQTSFRGCLRKLAL
IKSPQVQSFDFSRAFELHGVFLHSCPGTES
>sp|Q6P2H3|CEP85_HUMAN
MAMQEKYPTEGISHVTSPSSDVIQKGSSLGTEWQTPVISEPFRSRFSRCSSVADSGDTAIGTSCSDIAEDFCSSSGSPPFQPIKSHV
TIPTAHVMPSTLGTSPAKPNSTPVGPSSSKLPLSGLAESVGMTRNGDLGAMKHSPGLSRDLMYFSGATGENGIEQSWFPAVGHERQE
EARKFDIPSMESTLNQSAMMETLYSDPHHRVRFHNPRTSTSKELYRVLPEAKKAPGSGAVFERNGPHSNSSGVLPLGLQPAPGLSKP
LPSQVWQPSPDTWHPREQSCELSTCRQQLELIRLQMEQMQLQNGAICHHPAAFGPSLPILEPAQWISILNSNEHLLKEKELLIDKQR
KHISQLEQKVRESELQVHSALLGRPAPFGDVCLLRLQELQRENTFLRAQFAQKTEALSREKIDLEKKLSASEVEVQLIRESLKVALQ
KHSEEVKKQEERVKGRDKHINNLKKKCQKESEQNREKQQRIETLERYLADLPTLEDHQKQSQQLKDSELKSTELQEKVTELESLLEE

TQAICREKEIQLESLRQREAEFSSAGHSLQDKQSVEETSGEGPEVEMESWQKRYDSLQKIVEKQQQKMDQLRSQVQSLEQEVAQEEG
TSQALREEAQRRDSALQQLRTAVKELSVQNQDLIEKNLTLQEHLRQAQPGSPPSPDTAQLALELHQELASCLQDLQAVCSIVTQRAQ
GHDPNLSLLLGIHSAQHPETQLDLQKPDVIKRKLEEVQQLRRDIEDLRTTMSDRYAQDMGENCVTQ
>sp|Q96PZ2|F111A_HUMAN
MSCKKQRSRKHSVNEKCNMKIEHYFSPVSKEQQNNCSTSLMRMESRGDPRATTNTQAQRFHSPKKNPEDQTMPQNRTIYVTLKVNHR
RNQDMKLKLTHSENSSLYMALNTLQAVRKEIETHQGQEMLVRGTEGIKEYINLGMPLSCFPEGGQVVITFSQSKSKQKEDNHIFGRQ
DKASTECVKFYIHAIGIGKCKRRIVKCGKLHKKGRKLCVYAFKGETIKDALCKDGRFLSFLENDDWKLIENNDTILESTQPVDELEG
RYFQVEVEKRMVPSAAASQNPESEKRNTCVLREQIVAQYPSLKRESEKIIENFKKKMKVKNGETLFELHRTTFGKVTKNSSSIKVVK
LLVRLSDSVGYLFWDSATTGYATCFVFKGLFILTCRHVIDSIVGDGIEPSKWATIIGQCVRVTFGYEELKDKETNYFFVEPWFEIHN
EELDYAVLKLKENGQQVPMELYNGITPVPLSGLIHIIGHPYGEKKQIDACAVIPQGQRAKKCQERVQSKKAESPEYVHMYTQRSFQK
IVHNPDVITYDTEFFFGASGSPVFDSKGSLVAMHAAGFAYTYQNETRSIIEFGSTMESILLDIKQRHKPWYEEVFVNQQDVEMMSDE
DL
>sp|P08700|IL3_HUMAN
MSRLPVLLLLQLLVRPGLQAPMTQTTPLKTSWVNCSNMIDEIITHLKQPPLPLLDFNNLNGEDQDILMENNLRRPNLEAFNRAVKSL
QNASAIESILKNLLPCLPLATAAPTRHPIHIKDGDWNEFRRKLTFYLKTLENAQAQQTTLSLAIF
>sp|Q4VC31|CCD58_HUMAN
MAAPSGGVNCEEFAEFQELLKVMRTIDDRIVHELNTTVPTASFAGKIDASQTCKQLYESLMAAHASRDRVIKNCIAQTSAVVKNLRE
EREKNLDDLTLLKQLRKEQTKLKWMQSELNVEEVVNDRSWKVFNERCRIHFKPPKNE
>sp|O95139|NDUB6_HUMAN
MTGYTPDEKLRLQQLRELRRRWLKDQELSPREPVLPPQKMGPMEKFWNKFLENKSPWRKMVHGVYKKSIFVFTHVLVPVWIIHYYMK
YHVSEKPYGIVEKKSRIFPGDTILETGEVIPPMKEFPDQHH
>sp|P19388|RPAB1_HUMAN
MDDEEETYRLWKIRKTIMQLCHDRGYLVTQDELDQTLEEFKAQSGDKPSEGRPRRTDLTVLVAHNDDPTDQMFVFFPEEPKVGIKTI
KVYCQRMQEENITRALIVVQQGMTPSAKQSLVDMAPKYILEQFLQQELLINITEHELVPEHVVMTKEEVTELLARYKLRENQLPRIQ
AGDPVARYFGIKRGQVVKIIRPSETAGRYITYRLVQ
>sp|Q8N9I9|DTX3_HUMAN
MSFVLSRMAACGGTCKNKVTVSKPVWDFLSKETPARLARLREEHRVSILIDGETSDIYVLQLSPQGPPPAPPNGLYLARKALKGLLK
EAEKELKKAQRQGELMGCLALGGGGEHPEMHRAGPPPLRAAPLLPPGARGLPPPPPPLPPPLPPRLREEAEEQESTCPICLGEIQNA
KTLEKCRHSFCEGCITRALQVKKACPMCGRFYGQLVGNQPQNGRMLVSKDATLLLPSYEKYGTIVIQYVFPPGVQGAEHPNPGVRYP
GTTRVAYLPDCPEGNKVLTLFRKAFDQRLTFTIGTSMTTGRPNVITWNDIHHKTSCTGGPQLFGYPDPTYLTRVQEELRAKGITDD
>sp|P62820|RAB1A_HUMAN
MSSMNPEYDYLFKLLLIGDSGVGKSCLLLRFADDTYTESYISTIGVDFKIRTIELDGKTIKLQIWDTAGQERFRTITSSYYRGAHGI
IVVYDVTDQESFNNVKQWLQEIDRYASENVNKLLVGNKCDLTTKKVVDYTTAKEFADSLGIPFLETSAKNATNVEQSFMTMAAEIKK
RMGPGATAGGAEKSNVKIQSTPVKQSGGGCC
>sp|Q15788|NCOA1_HUMAN
MSGLGDSSSDPANPDSHKRKGSPCDTLASSTEKRRREQENKYLEELAELLSANISDIDSLSVKPDKCKILKKTVDQIQLMKRMEQEK
STTDDDVQKSDISSSSQGVIEKESLGPLLLEALDGFFFVVNCEGRIVFVSENVTSYLGYNQEELMNTSVYSILHVGDHAEFVKNLLP
KSLVNGVPWPQEATRRNSHTFNCRMLIHPPDEPGTENQEACQRYEVMQCFTVSQPKSIQEDGEDFQSCLICIARRLPRPPAITGVES
FMTKQDTTGKIISIDTSSLRAAGRTGWEDLVRKCIYAFFQPQGREPSYARQLFQEVMTRGTASSPSYRFILNDGTMLSAHTKCKLCY
PQSPDMQPFIMGIHIIDREHSGLSPQDDTNSGMSIPRVNPSVNPSISPAHGVARSSTLPPSNSNMVSTRINRQQSSDLHSSSHSNSS
NSQGSFGCSPGSQIVANVALNQGQASSQSSNPSLNLNNSPMEGTGISLAQFMSPRRQVTSGLATRPRMPNNSFPPNISTLSSPVGMT
SSACNNNNRSYSNIPVTSLQGMNEGPNNSVGFSASSPVLRQMSSQNSPSRLNIQPAKAESKDNKEIASILNEMIQSDNSSSDGKPLD
SGLLHNNDRLSDGDSKYSQTSHKLVQLLTTTAEQQLRHADIDTSCKDVLSCTGTSNSASANSSGGSCPSSHSSLTERHKILHRLLQE
GSPSDITTLSVEPDKKDSASTSVSVTGQVQGNSSIKLELDASKKKESKDHQLLRYLLDKDEKDLRSTPNLSLDDVKVKVEKKEQMDP

CNTNPTPMTKPTPEEIKLEAQSQFTADLDQFDQLLPTLEKAAQLPGLCETDRMDGAVTSVTIKSEILPASLQSATARPTSRLNRLPE
LELEAIDNQFGQPGTGDQIPWTNNTVTAINQSKSEDQCISSQLDELLCPPTTVEGRNDEKALLEQLVSFLSGKDETELAELDRALGI
DKLVQGGGLDVLSERFPPQQATPPLIMEERPNLYSQPYSSPSPTANLPSPFQGMVRQKPSLGTMPVQVTPPRGAFSPGMGMQPRQTL
NRPPAAPNQLRLQLQQRLQGQQQLIHQNRQAILNQFAATAPVGINMRSGMQQQITPQPPLNAQMLAQRQRELYSQQHRQRQLIQQQR
AMLMRQQSFGNNLPPSSGLPVQMGNPRLPQGAPQQFPYPPNYGTNPGTPPASTSPFSQLAANPEASLANRNSMVSRGMTGNIGGQFG
TGINPQMQQNVFQYPGAGMVPQGEANFAPSLSPGSSMVPMPIPPPQSSLLQQTPPASGYQSPDMKAWQQGAIGNNNVFSQAVQNQPT
PAQPGVYNNMSITVSMAGGNTNVQNMNPMMAQMQMSSLQMPGMNTVCPEQINDPALRHTGLYCNQLSSTDLLKTEADGTQQVQQVQV
FADVQCTVNLVGGDPYLNQPGPLGTQKPTSGPQTPQAQQKSLLQQLLTE
>sp|Q96GM8|TOE1_HUMAN
MAADSDDGAVSAPAASDGGVSKSTTSGEELVVQVPVVDVQSNNFKEMWPSLLLAIKTANFVAVDTELSGLGDRKSLLNQCIEERYKA
VCHAARTRSILSLGLACFKRQPDKGEHSYLAQVFNLTLLCMEEYVIEPKSVQFLIQHGFNFNQQYAQGIPYHKGNDKGDESQSQSVR
TLFLELIRARRPLVLHNGLIDLVFLYQNFYAHLPESLGTFTADLCEMFPAGIYDTKYAAEFHARFVASYLEYAFRKCERENGKQRAA
GSPHLTLEFCNYPSSMRDHIDYRCCLPPATHRPHPTSICDNFSAYGWCPLGPQCPQSHDIDLIIDTDEAAAEDKRRRRRRREKRKRA
LLNLPGTQTSGEAKDGPPKKQVCGDSIKPEETEQEVAADETRNLPHSKQGNKNDLEMGIKAARPEIADRATSEVPGSQASPNPVPGD
GLHRAGFDAFMTGYVMAYVEVSQGPQPCSSGPWLPECHNKVYLSGKAVPLTVAKSQFSRSSKAHNQKMKLTWGSS
>sp|A6NEQ2|F181B_HUMAN
MAVQAALLSTHPFVPFGFGGSPDGLGGAFGALDKGCCFEDDETGAPAGALLSGAEGGDVREATRDLLSFIDSASSNIKLALDKPGKS
KRKVNHRKYLQKQIKRCSGLMGAAPPGPPSPSAADTPAKRPLAAPSAPTVAAPAHGKAAPRREASQAAAAASLQSRSLAALFDSLRH
VPGGAEPAGGEVAAPAAGLGGAGTGGAGGDVAGPAGATAIPGARKVPLRARNLPPSFFTEPSRAGGGGCGPSGPDVSLGDLEKGAEA
VEFFELLGPDYGAGTEAAVLLAAEPLDVFPAGASVLRGPPELEPGLFEPPPAVVGNLLYPEPWSVPGCSPTKKSPLTAPRGGLTLNE
PLSPLYPAAADSPGGEDGRGHLASFAPFFPDCALPPPPPPHQVSYDYSAGYSRTAYSSLWRSDGVWEGAPGEEGAHRD
>sp|P30493|1B55_HUMAN
MRVTAPRTLLLLLWGALALTETWAGSHSMRYFYTAMSRPGRGEPRFIAVGYVDDTQFVRFDSDAASPREEPRAPWIEQEGPEYWDRN
TQIYKAQAQTDRESLRNLRGYYNQSEAGSHTWQTMYGCDLGPDGRLLRGHNQLAYDGKDYIALNEDLSSWTAADTAAQITQRKWEAA
REAEQLRAYLEGTCVEWLRRYLENGKETLQRADPPKTHVTHHPISDHEATLRCWALGFYPAEITLTWQRDGEDQTQDTELVETRPAG
DRTFQKWAAVVVPSGEEQRYTCHVQHEGLPKPLTLRWEPSSQSTIPIVGIVAGLAVLAVVVIGAVVATVMCRRKSSGGKGGSYSQAA
SSDSAQGSDVSLTA
>sp|Q8N8E1|MAAS1_HUMAN
MALSMSLSSDILSGAPTAGRGGGCSAALSPRGRGSKGLGTRAPGPRGDGQQPPLGTGGDEDPGAGSASAGGSRLAAAAAAEAAAPGD
RSLCWAPRGRLLASSPAGEAGSWGRARRGGPRPGAPCKGLAGPPLRPGLARS
>sp|Q9UKF2|ADA30_HUMAN
MRSVQIFLSQCRLLLLLVPTMLLKSLGEDVIFHPEGEFDSYEVTIPEKLSFRGEVQGVVSPVSYLLQLKGKKHVLHLWPKRLLLPRH
LRVFSFTEHGELLEDHPYIPKDCNYMGSVKESLDSKATISTCMGGLRGVFNIDAKHYQIEPLKASPSFEHVVYLLKKEQFGNQVCGL
SDDEIEWQMAPYENKARLRDFPGSYKHPKYLELILLFDQSRYRFVNNNLSQVIHDAILLTGIMDTYFQDVRMRIHLKALEVWTDFNK
IRVGYPELAEVLGRFVIYKKSVLNARLSSDWAHLYLQRKYNDALAWSFGKVCSLEYAGSVSTLLDTNILAPATWSAHELGHAVGMSH
DEQYCQCRGRLNCIMGSGRTGFSNCSYISFFKHISSGATCLNNIPGLGYVLKRCGNKIVEDNEECDCGSTEECQKDRCCQSNCKLQP
GANCSIGLCCHDCRFRPSGYVCRQEGNECDLAEYCDGNSSSCPNDVYKQDGTPCKYEGRCFRKGCRSRYMQCQSIFGPDAMEAPSEC
YDAVNLIGDQFGNCEITGIRNFKKCESANSICGRLQCINVETIPDLPEHTTIISTHLQAENLMCWGTGYHLSMKPMGIPDLGMINDG
TSCGEGRVCFKKNCVNSSVLQFDCLPEKCNTRGVCNNRKNCHCMYGWAPPFCEEVGYGGSIDSGPPGLLRGAIPSSIWVVSIIMFRL
ILLILSVVFVFFRQVIGNHLKPKQEKMPLSKAKTEQEESKTKTVQEESKTKTGQEESEAKTGQEESKAKTGQEESKANIESKRPKAK
SVKKQKK
>sp|Q5TZJ5|S31A1_HUMAN
MENLPFPLKLLSASSLNAPSSTPWVLDIFLTLVFALGFFFLLLPYLSYFRCDDPPSPSPGKRKCPVGRRRRPRGRMKNHSLRAGREC
PRGLQETSDLLSQLQSLLGPHLDKGDFGQLSGPDPPGEVGERAPDGASQSSHEPMEDAAPILSPLASPDPQAKHPQDLASTPSPGPM

TTSVSSLSASQPPEPSLPLEHPSPEPPALFPHPPHTPDPLACSPPPPKGFTAPPLRDSTLITPSHCDSVALPLGTVPQSLSPHEDLV
ASVPAISGLGGSNSHVSASSRWQETARTSCAFNSSVQQDHLSRHPPETYQMEAGSLFLLSSDGQNAVGIQVTETAKVNIWEEKENVG
SFTDRMTPEKHLNSLRNLAKSLDAEQDTTNPKPFWNMGENSKQLPGPQKLSDPRLWQESFWKNYSQLFWGLPSLHSESLVANAWVTD
RSYTLQSPPFLFNEMSNVCPIQRETTMSPLLFQAQPPSHLGPECQPFISSTPQFRPTPMAQAEAQAHLQSSFPVLSPAFPSLIKNTG
VACPASQNKVQALSLPETQHPEWPLLRRQLEGRLALPSRVQKSQDVFSVSTPNLPQESLTSILPENFPVSPELRRQLEQHIKKWIIQ
HWGNLGRIQESLDLMQLRDESPGTSQAKGKPSPWQSSMSTGESSKEAQKVKFQLERDPCPHLGQILGETPQNLSRDMKSFPRKVLGV
TSEESERNLRKPLRSDSGSDLLRCTERTHIENILKAHMGRNLGQTNEGLIPVRVRRSWLAVNQALPVSNTHVKTSNLAAPKSGKACV
NTAQVLSFLEPCTQQGLGAHIVRFWAKHRWGLPLRVLKPIQCFKLEKVSSLSLTQLAGPSSATCESGAGSEVEVDMFLRKPPMASLR
KQVLTKASDHMPESLLASSPAWKQFQRAPRGIPSWNDHGPLKPPPAGQEGRWPSKPLTYSLTGSTQQSRSLGAQSSKAGETREAVPQ
CRVPLETCMLANLQATSEDMHGFEAPGTSKSSLHPRVSVSQDPRKLCLMEEVVNEFEPGMATKSETQPQVCAAVVLLPDGQASVVPH
ASENLVSQVPQGHLQSMPAGNMRASQELHDLMAARRSKLVHEEPRNPNCQGSCKNQRPMFPPIHKSEKSRKPNLEKHEERLEGLRTP
QLTPVRKTEDTHQDEGVQLLPSKKQPPSVSHFGGNIKQFFQWIFSKKKSKPAPVTAESQKTVKNRSCVYSSSAEAQGLMTAVGQMLD
EKMSLCHARHASKVNQHKQKFQAPVCGFPCNHRHLFYSEHGRILSYAASSQQATLKSQGCPNRDRQIRNQQPLKSVRCNNEQWGLRH
PQILHPKKAVSPVSPLQHWPKTSGASSHHHHCPRHCLLWEGI
>sp|Q9UL40|ZN346_HUMAN
MEYPAPATVQAADGGAAGPYSSSELLEGQEPDGVRFDRERARRLWEAVSGAQPVGREEVEHMIQKNQCLFTNTQCKVCCALLISESQ
KLAHYQSKKHANKVKRYLAIHGMETLKGETKKLDSDQKSSRSKDKNQCCPICNMTFSSPVVAQSHYLGKTHAKNLKLKQQSTKVEAL
HQNREMIDPDKFCSLCHATFNDPVMAQQHYVGKKHRKQETKLKLMARYGRLADPAVTDFPAGKGYPCKTCKIVLNSIEQYQAHVSGF
KHKNQSPKTVASSLGQIPMQRQPIQKDSTTLED
>sp|Q6XYQ8|SYT10_HUMAN
MSFHKEDGVNSLCQKALHIVTELCFAGQVEWEKCSGIFPRDRGSQGGSSTDISVSLLAVVVSFCGLALLVVSLFVFWKLCWPCWKSK
PVTSNITTLPQSISSAPTEVFETEEKKEIKENEKPAVKAIEPAIKISHTSPDIPAEVQTALKEHLIKHARVQRQITEPTSSTRHSSF
RRHLPRQMQVSSVDFSMGTEPVLQRGETTTSIGRIKPELYKQKSVDSEGNQNEDVKICGKLNFTLQYDYENELLVVKIIKALDLPAK
DFTGTSDPYVKMYLLPDRKKKFQTRVHRKTLNPLFDETFQFPVAYDQLSNRKLHFSVYDFDRFSRHDMIGEVILDNLFEVSDLSREA
TVWKDIHCATTESIDLGEIMFSLCYLPTAGRMTLTVIKCRNLKAMDITGSSDPYVKVSLMCEGRRLKKRKTTTKKNTLNPVYNEAII
FDIPPENVDQVSLSIAVMDYDRVGHNEVIGVCRTGLDAEGLGRDHWNEMLAYHRKPITHWHPLLELPGRATSFDSQGSCPSPKPPST
P
>sp|Q0IIN9|ZNFS1_HUMAN
MLRQSCSFPVTSLPALGGVCGREGAGAEVPPAACGCEGRDPDTERSCGRSSTGGCSPCSGPGPSSPRTSRGALSPSLGRLFPHLQVV
IKLRIQLAPAVHLALPTCSLLTSSPPLEGCCHRLNEEAEVQRGFRPIAVELEFENQPLGAETRLRNGRRAGVKRSEGRGQVRPGQVR
STGPEGGLTRMERKAARLQWDSGSIKMSENEQLWEEP
>sp|Q16659|MK06_HUMAN
MAEKFESLMNIHGFDLGSRYMDLKPLGCGGNGLVFSAVDNDCDKRVAIKKIVLTDPQSVKHALREIKIIRRLDHDNIVKVFEILGPS
GSQLTDDVGSLTELNSVYIVQEYMETDLANVLEQGPLLEEHARLFMYQLLRGLKYIHSANVLHRDLKPANLFINTEDLVLKIGDFGL
ARIMDPHYSHKGHLSEGLVTKWYRSPRLLLSPNNYTKAIDMWAAGCIFAEMLTGKTLFAGAHELEQMQLILESIPVVHEEDRQELLS
VIPVYIRNDMTEPHKPLTQLLPGISREALDFLEQILTFSPMDRLTAEEALSHPYMSIYSFPMDEPISSHPFHIEDEVDDILLMDETH
SHIYNWERYHDCQFSEHDWPVHNNFDIDEVQLDPRALSDVTDEEEVQVDPRKYLDGDREKYLEDPAFDTNYSTEPCWQYSDHHENKY
CDLECSHTCNYKTRSSSYLDNLVWRESEVNHYYEPKLIIDLSNWKEQSKEKSDKKGKSKCERNGLVKAQIALEEASQQLAGKEREKN
QGFDFDSFIAGTIQLSSQHEPTDVVDKLNDLNSSVSQLELKSLISKSVSQEKQEKGMANLAQLEALYQSSWDSQFVSGGEDCFFINQ
FCEVRKDEQVEKENTYTSYLDKFFSRKEDTEMLETEPVEDGKLGERGHEEGFLNNSGEFLFNKQLESIGIPQFHSPVGSPLKSIQAT
LTPSAMKSSPQIPHQTYSSILKHLN
>sp|Q9HCM4|E41L5_HUMAN
MLSFFRRTLGRRSMRKHAEKERLREAQRAATHIPAAGDSKSIITCRVSLLDGTDVSVDLPKKAKGQELFDQIMYHLDLIESDYFGLR
FMDSAQVAHWLDGTKSIKKQVKIGSPYCLHLRVKFYSSEPNNLREELTRYLFVLQLKQDILSGKLDCPFDTAVQLAAYNLQAELGDY

DLAEHSPELVSEFRFVPIQTEEMELAIFEKWKEYRGQTPAQAETNYLNKAKWLEMYGVDMHVVKARDGNDYSLGLTPTGVLVFEGDT
KIGLFFWPKITRLDFKKNKLTLVVVEDDDQGKEQEHTFVFRLDHPKACKHLWKCAVEHHAFFRLRGPVQKSSHRSGFIRLGSRFRYS
GKTEYQTTKTNKARRSTSFERRPSKRYSRRTLQMKACATKPEELSVHNNVSTQSNGSQQAWGMRSALPVSPSISSAPVPVEIENLPQ
SPGTDQHDRKCIPLNIDLLNSPDLLEATIGDVIGASDTMETSQALNDVNVATRLPGLGEPEVEYETLKDTSEKLKQLEMENSPLLSP
RSNIDVNINSQEEVVKLTEKCLNNVIESPGLNVMRVPPDFKSNILKAQVEAVHKVTKEDSLLSHKNANVQDAATNSAVLNENNVPLP
KESLETLMLITPADSGSVLKEATDELDALLASLTENLIDHTVAPQVSSTSMITPRWIVPQSGAMSNGLAGCEMLLTGKEGHGNKDGI
SLISPPAPFLVDAVTSSGPILAEEAVLKQKCLLTTEL
>sp|B7ZBB8|PP13G_HUMAN
MEPIGARLSLEAPGPAPFREAPPAEELPAPVVPCVQGGGDGGASETPSPDAQLGDRPLSPKEEAAPQEQEELLECRRRCRARSFSL
PADPILQAAKFLQQQQQQAVALGGEGAEDAQLGPGGCCAKCKKRVQFADTLGLSLASVKHFSEAEEPQVPPAVLSRLRSFPMRAEDL
EQLGGLLAAAAVAAPLSAPPSRLRPLFQLPGPSAAAERLQRQRVCLERVQCSTASGAEVKGSGRVLSCPGPRAVTVRYTFTEWRSFL
DVPAELQPEPLEPQQPEAPSGASEPGSGDAKKEPGAECFHFSLCLPPGLQPEDEEDADERGVAVHFAVCYRCAQGEYWDNNAGANYT
LRYARPADAL
>sp|P40126|TYRP2_HUMAN
MSPLWWGFLLSCLGCKILPGAQGQFPRVCMTVDSLVNKECCPRLGAESANVCGSQQGRGQCTEVRADTRPWSGPYILRNQDDRELWP
RKFFHRTCKCTGNFAGYNCGDCKFGWTGPNCERKKPPVIRQNIHSLSPQEREQFLGALDLAKKRVHPDYVITTQHWLGLLGPNGTQP
QFANCSVYDFFVWLHYYSVRDTLLGPGRPYRAIDFSHQGPAFVTWHRYHLLCLERDLQRLIGNESFALPYWNFATGRNECDVCTDQL
FGAARPDDPTLISRNSRFSSWETVCDSLDDYNHLVTLCNGTYEGLLRRNQMGRNSMKLPTLKDIRDCLSLQKFDNPPFFQNSTFSFR
NALEGFDKADGTLDSQVMSLHNLVHSFLNGTNALPHSAANDPIFVVLHSFTDAIFDEWMKRFNPPADAWPQELAPIGHNRMYNMVPF
FPPVTNEELFLTSDQLGYSYAIDLPVSVEETPGWPTTLLVVMGTLVALVGLFVLLAFLQYRRLRKGYTPLMETHLSSKRYTEEA
>sp|Q9NPP4|NLRC4_HUMAN
MNFIKDNSRALIQRMGMTVIKQITDDLFVWNVLNREEVNIICCEKVEQDAARGIIHMILKKGSESCNLFLKSLKEWNYPLFQDLNGQ
SLFHQTSEGDLDDLAQDLKDLYHTPSFLNFYPLGEDIDIIFNLKSTFTEPVLWRKDQHHHRVEQLTLNGLLQALQSPCIIEGESGKG
KSTLLQRIAMLWGSGKCKALTKFKFVFFLRLSRAQGGLFETLCDQLLDIPGTIRKQTFMAMLLKLRQRVLFLLDGYNEFKPQNCPEI
EALIKENHRFKNMVIVTTTTECLRHIRQFGALTAEVGDMTEDSAQALIREVLIKELAEGLLLQIQKSRCLRNLMKTPLFVVITCAIQ
MGESEFHSHTQTTLFHTFYDLLIQKNKHKHKGVAASDFIRSLDHCGDLALEGVFSHKFDFELQDVSSVNEDVLLTTGLLCKYTAQRF
KPKYKFFHKSFQEYTAGRRLSSLLTSHEPEEVTKGNGYLQKMVSISDITSTYSSLLRYTCGSSVEATRAVMKHLAAVYQHGCLLGLS
IAKRPLWRQESLQSVKNTTEQEILKAININSFVECGIHLYQESTSKSALSQEFEAFFQGKSLYINSGNIPDYLFDFFEHLPNCASAL
DFIKLDFYGGAMASWEKAAEDTGGIHMEEAPETYIPSRAVSLFFNWKQEFRTLEVTLRDFSKLNKQDIRYLGKIFSSATSLRLQIKR
CAGVAGSLSLVLSTCKNIYSLMVEASPLTIEDERHITSVTNLKTLSIHDLQNQRLPGGLTDSLGNLKNLTKLIMDNIKMNEEDAIKL
AEGLKNLKKMCLFHLTHLSDIGEGMDYIVKSLSSEPCDLEEIQLVSCCLSANAVKILAQNLHNLVKLSILDLSENYLEKDGNEALHE
LIDRMNVLEQLTALMLPWGCDVQGSLSSLLKHLEEVPQLVKLGLKNWRLTDTEIRILGAFFGKNPLKNFQQLNLAGNRVSSDGWLAF
MGVFENLKQLVFFDFSTKEFLPDPALVRKLSQVLSKLTFLQEARLVGWQFDDDDLSVITGAFKLVTA
>sp|Q99426|TBCB_HUMAN
MEVTGVSAPTVTVFISSSLNTFRSEKRYSRSLTIAEFKCKLELLVGSPASCMELELYGVDDKFYSKLDQEDALLGSYPVDDGCRIHV
IDHSGARLGEYEDVSRVEKYTISQEAYDQRQDTVRSFLKRSKLGRYNEEERAQQEAEAAQRLAEEKAQASSIPVGSRCEVRAAGQSP
RRGTVMYVGLTDFKPGYWIGVRYDEPLGKNDGSVNGKRYFECQAKYGAFVKPAVVTVGDFPEEDYGLDEI
>sp|P22105|TENX_HUMAN
MMPAQYALTSSLVLLVLLSTARAGPFSSRSNVTLPAPRPPPQPGGHTVGAGVGSPSSQLYEHTVEGGEKQVVFTHRINLPPSTGCGC
PPGTEPPVLASEVQALRVRLEILEELVKGLKEQCTGGCCPASAQAGTGQTDVRTLCSLHGVFDLSRCTCSCEPGWGGPTCSDPTDAE
IPPSSPPSASGSCPDDCNDQGRCVRGRCVCFPGYTGPSCGWPSCPGDCQGRGRCVQGVCVCRAGFSGPDCSQRSCPRGCSQRGRCEG
GRCVCDPGYTGDDCGMRSCPRGCSQRGRCENGRCVCNPGYTGEDCGVRSCPRGCSQRGRCKDGRCVCDPGYTGEDCGTRSCPWDCGE
GGRCVDGRCVCWPGYTGEDCSTRTCPRDCRGRGRCEDGECICDTGYSGDDCGVRSCPGDCNQRGRCEDGRCVCWPGYTGTDCGSRAC
PRDCRGRGRCENGVCVCNAGYSGEDCGVRSCPGDCRGRGRCESGRCMCWPGYTGRDCGTRACPGDCRGRGRCVDGRCVCNPGFTGED

CGSRRCPGDCRGHGLCEDGVCVCDAGYSGEDCSTRSCPGGCRGRGQCLDGRCVCEDGYSGEDCGVRQCPNDCSQHGVCQDGVCICWE
GYVSEDCSIRTCPSNCHGRGRCEEGRCLCDPGYTGPTCATRMCPADCRGRGRCVQGVCLCHVGYGGEDCGQEEPPASACPGGCGPRE
LCRAGQCVCVEGFRGPDCAIQTCPGDCRGRGECHDGSCVCKDGYAGEDCGEEVPTIEGMRMHLLEETTVRTEWTPAPGPVDAYEIQF
IPTTEGASPPFTARVPSSASAYDQRGLAPGQEYQVTVRALRGTSWGLPASKTITTMIDGPQDLRVVAVTPTTLELGWLRPQAEVDRF
VVSYVSAGNQRVRLEVPPEADGTLLTDLMPGVEYVVTVTAERGRAVSYPASVRANTGSSPLGLLGTTDEPPPSGPSTTQGAQAPLLQ
QRPQELGELRVLGRDETGRLRVVWTAQPDTFAYFQLRMRVPEGPGAHEEVLPGDVRQALVPPPPPGTPYELSLHGVPPGGKPSDPII
YQGIMDKDEEKPGKSSGPPRLGELTVTDRTSDSLLLRWTVPEGEFDSFVIQYKDRDGQPQVVPVEGPQRSAVITSLDPGRKYKFVLY
GFVGKKRHGPLVAEAKILPQSDPSPGTPPHLGNLWVTDPTPDSLHLSWTVPEGQFDTFMVQYRDRDGRPQVVPVEGPERSFVVSSLD
PDHKYRFTLFGIANKKRYGPLTADGTTAPERKEEPPRPEFLEQPLLGELTVTGVTPDSLRLSWTVAQGPFDSFMVQYKDAQGQPQAV
PVAGDENEVTVPGLDPDRKYKMNLYGLRGRQRVGPESVVAKTAPQEDVDETPSPTELGTEAPESPEEPLLGELTVTGSSPDSLSLFW
TVPQGSFDSFTVQYKDRDGRPRAVRVGGKESEVTVGGLEPGHKYKMHLYGLHEGQRVGPVSAVGVTAPQQEETPPATESPLEPRLGE
LTVTDVTPNSVGLSWTVPEGQFDSFIVQYKDKDGQPQVVPVAADQREVTVYNLEPERKYKMNMYGLHDGQRMGPLSVVIVTAPLPPA
PATEASKPPLEPRLGELTVTDITPDSVGLSWTVPEGEFDSFVVQYKDRDGQPQVVPVAADQREVTIPDLEPSRKYKFLLFGIQDGKR
RSPVSVEAKTVARGDASPGAPPRLGELWVTDPTPDSLRLSWTVPEGQFDSFVVQFKDKDGPQVVPVEGHERSVTVTPLDAGRKYRFL
LYGLLGKKRHGPLTADGTTEARSAMDDTGTKRPPKPRLGEELQVTTVTQNSVGLSWTVPEGQFDSFVVQYKDRDGQPQVVPVEGSLR
EVSVPGLDPAHRYKLLLYGLHHGKRVGPISAVAITAGREETETETTAPTPPAPEPHLGELTVEEATSHTLHLSWMVTEGEFDSFEIQ
YTDRDGQLQMVRIGGDRNDITLSGLESDHRYLVTLYGFSDGKHVGPVHVEALTVPEEEKPSEPPTATPEPPIKPRLGELTVTDATPD
SLSLSWTVPEGQFDHFLVQYRNGDGQPKAVRVPGHEEGVTISGLEPDHKYKMNLYGFHGGQRMGPVSVVGVTAAEEETPSPTEPSME
APEPAEEPLLGELTVTGSSPDSLSLSWTVPQGRFDSFTVQYKDRDGRPQVVRVGGEESEVTVGGLEPGRKYKMHLYGLHEGRRVGPV
SAVGVTAPEEESPDAPLAKLRLGQMTVRDITSDSLSLSWTVPEGQFDHFLVQFKNGDGQPKAVRVPGHEDGVTISGLEPDHKYKMNL
YGFHGGQRVGPVSAVGLTAPGKDEEMAPASTEPPTPEPPIKPRLEELTVTDATPDSLSLSWTVPEGQFDHFLVQYKNGDGQPKATRV
PGHEDRVTISGLEPDNKYKMNLYGFHGGQRVGPVSAIGVTAAEEETPSPTEPSMEAPEPPEEPLLGELTVTGSSPDSLSLSWTVPQG
RFDSFTVQYKDRDGRPQVVRVGGEESEVTVGGLEPGRKYKMHLYGLHEGRRVGPVSTVGVTAPQEDVDETPSPTEPGTEAPGPPEEP
LLGELTVTGSSPDSLSLSWTVPQGRFDSFTVQYKDRDGRPQAVRVGGQESKVTVRGLEPGRKYKMHLYGLHEGRRLGPVSAVGVTED
EAETTQAVPTMTPEPPIKPRLGELTMTDATPDSLSLSWTVPEGQFDHFLVQYRNGDGQPKAVRVPGHEDGVTISGLEPDHKYKMNLY
GFHGGQRVGPISVIGVTAAEEETPSPTELSTEAPEPPEEPLLGELTVTGSSPDSLSLSWTIPQGHFDSFTVQYKDRDGRPQVMRVRG
EESEVTVGGLEPGRKYKMHLYGLHEGRRVGPVSTVGVTEDEAETTQAVPTTTPEPPNKPRLGELTVTDATPDSLSLSWMVPEGQFDH
FLVQYRNGDGQPKVVRVPGHEDGVTISGLEPDHKYKMNLYGFHGGQRVGPISVIGVTAAEEETPAPTEPSTEAPEPPEEPLLGELTV
TGSSPDSLSLSWTIPQGRFDSFTVQYKDRDGRPQVVRVRGEESEVTVGGLEPGCKYKMHLYGLHEGQRVGPVSAVGVTAPKDEAETT
QAVPTMTPEPPIKPRLGELTVTDATPDSLSLSWMVPEGQFDHFLVQYRNGDGQPKAVRVPGHEDGVTISGLEPDHKYKMNLYGFHGG
QRVGPVSAIGVTEEETPSPTEPSTEAPEAPEEPLLGELTVTGSSPDSLSLSWTVPQGRFDSFTVQYKDRDGQPQVVRVRGEESEVTV
GGLEPGRKYKMHLYGLHEGQRVGPVSTVGITAPLPTPLPVEPRLGELAVAAVTSDSVGLSWTVAQGPFDSFLVQYRDAQGQPQAVPV
SGDLRAVAVSGLDPARKYKFLLFGLQNGKRHGPVPVEARTAPDTKPSPRLGELTVTDATPDSVGLSWTVPEGEFDSFVVQYKDKDGR
LQVVPVAANQREVTVQGLEPSRKYRFLLYGLSGRKRLGPISADSTTAPLEKELPPHLGELTVAEETSSSLRLSWTVAQGPFDSFVVQ
YRDTDGQPRAVPVAADQRTVTVEDLEPGKKYKFLLYGLLGGKRLGPVSALGMTAPEEDTPAPELAPEAPEPPEEPRLGVLTVTDTTP
DSMRLSWSVAQGPFDSFVVQYEDTNGQPQALLVDGDQSKILISGLEPSTPYRFLLYGLHEGKRLGPLSAEGTTGLAPAGQTSEESRP
RLSQLSVTDVTTSSLRLNWEAPPGAFDSFLLRFGVPSPSTLEPHPRPLLQRELMVPGTRHSAVLRDLRSGTLYSLTLYGLRGPHKAD
SIQGTARTLSPVLESPRDLQFSEIRETSAKVNWMPPPSRADSFKVSYQLADGGEPQSVQVDGQARTQKLQGLIPGARYEVTVVSVRG
FEESEPLTGFLTTVPDGPTQLRALNLTEGFAVLHWKPPQNPVDTYDVQVTAPGAPPLQAETPGSAVDYPLHDLVLHTNYTATVRGLR
GPNLTSPASITFTTGLEAPRDLEAKEVTPRTALLTWTEPPVRPAGYLLSFHTPGGQNQEILLPGGITSHQLLGLFPSTSYNARLQAM
WGQSLLPPVSTSFTTGGLRIPFPRDCGEEMQNGAGASRTSTIFLNGNRERPLNVFCDMETDGGGWLVFQRRMDGQTDFWRDWEDYAH
GFGNISGEFWLGNEALHSLTQAGDYSMRVDLRAGDEAVFAQYDSFHVDSAAEYYRLHLEGYHGTAGDSMSYHSGSVFSARDRDPNSL
LISCAVSYRGAWWYRNCHYANLNGLYGSTVDHQGVSWYHWKGFEFSVPFTEMKLRPRNFRSPAGGG
>sp|Q14190|SIM2_HUMAN

MKEKSKNAAKTRREKENGEFYELAKLLPLPSAITSQLDKASIIRLTTSYLKMRAVFPEGLGDAWGQPSRAGPLDGVAKELGSHLLQT
LDGFVFVVASDGKIMYISETASVHLGLSQVELTGNSIYEYIHPSDHDEMTAVLTAHQPLHHHLLQEYEIERSFFLRMKCVLAKRNAG
LTCSGYKVIHCSGYLKIRQYMLDMSLYDSCYQIVGLVAVGQSLPPSAITEIKLYSNMFMFRASLDLKLIFLDSRVTEVTGYEPQDLI
EKTLYHHVHGCDVFHLRYAHHLLLVKGQVTTKYYRLLSKRGGWVWVQSYATVVHNSRSSRPHCIVSVNYVLTEIEYKELQLSLEQVS
TAKSQDSWRTALSTSQETRKLVKPKNTKMKTKLRTNPYPPQQYSSFQMDKLECGQLGNWRASPPASAAAPPELQPHSESSDLLYTPS
YSLPFSYHYGHFPLDSHVFSSKKPMLPAKFGQPQGSPCEVARFFLSTLPASGECQWHYANPLVPSSSSPAKNPPEPPANTARHSLVP
SYEAPAAAVRRFGEDTAPPSFPSCGHYREEPALGPAKAARQAARDGARLALARAAPECCAPPTPEAPGAPAQLPFVLLNYHRVLARR
GPLGGAAPAASGLACAPGGPEAATGALRLRHPSPAATSPPGAPLPHYLGASVIITNGR
>sp|A8MZG2|CP090_HUMAN
MEALVYAVSQAQGRPGHPDAPPNIYEGGLGSPQPQCPSAQGSKPKNFRLRHLRGLGLYLESHPPPTGQCESHWLGRLMAGGCLPQPE
GTAWALDLPQGTLGPRNSLCSALLEARLPRDSLGSSASSSSMDPDKGALPQPSPSRLRPKRSWGTWEEAMCPLCKRTRSGALERP
>sp|Q9P1W8|SIRPG_HUMAN
MPVPASWPHPPGPFLLLTLLLGLTEVAGEEELQMIQPEKLLLVTVGKTATLHCTVTSLLPVGPVLWFRGVGPGRELIYNQKEGHFPR
VTTVSDLTKRNNMDFSIRISSITPADVGTYYCVKFRKGSPENVEFKSGPGTEMALGAKPSAPVVLGPAARTTPEHTVSFTCESHGFS
PRDITLKWFKNGNELSDFQTNVDPTGQSVAYSIRSTARVVLDPWDVRSQVICEVAHVTLQGDPLRGTANLSEAIRVPPTLEVTQQPM
RVGNQVNVTCQVRKFYPQSLQLTWSENGNVCQRETASTLTENKDGTYNWTSWFLVNISDQRDDVVLTCQVKHDGQLAVSKRLALEVT
VHQKDQSSDATPGPASSLTALLLIAVLLGPIYVPWKQKT
>sp|Q86UW7|CAPS2_HUMAN
MLDPSSSEEESDEGLEEESRDVLVAAGSSQRAPPAPTREGRRDAPGRAGGGGAARSVSPSPSVLSEGRDEPQRQLDDEQERRIRLQL
YVFVVRCIAYPFNAKQPTDMARRQQKLNKQQLQLLKERFQAFLNGETQIVADEAFCNAVRSYYEVFLKSDRVARMVQSGGCSANDFR
EVFKKNIEKRVRSLPEIDGLSKETVLSSWIAKYDAIYRGEEDLCKQPNRMALSAVSELILSKEQLYEMFQQILGIKKLEHQLLYNAC
QLDNADEQAAQIRRELDGRLQLADKMAKERKFPKFIAKDMENMYIEELRSSVNLLMANLESLPVSKGGPEFKLQKLKRSQNSAFLDI
GDENEIQLSKSDVVLSFTLEIVIMEVQGLKSVAPNRIVYCTMEVEGEKLQTDQAEASRPQWGTQGDFTTTHPRPVVKVKLFTESTGV
LALEDKELGRVILYPTSNSSKSAELHRMVVPKNSQDSDLKIKLAVRMDKPAHMKHSGYLYALGQKVWKRWKKRYFVLVQVSQYTFAM
CSYREKKSEPQELMQLEGYTVDYTDPHPGLQGGCMFFNAVKEGDTVIFASDDEQDRILWVQAMYRATGQSYKPVPAIQTQKLNPKGG
TLHADAQLSGKDADRFQKHGMDEFISANPCKLDHAFLFRILQRQTLDHRLNDSYSCLGWFSPGQVFVLDEYCARYGVRGCHRHLCYL
AELMEHSENGAVIDPTLLHYSFAFCASHVHGNRPDGIGTVSVEEKERFEEIKERLSSLLENQISHFRYCFPFGRPEGALKATLSLLE
RVLMKDIATPIPAEEVKKVVRKCLEKAALINYTRLTEYAKIEETMNQASPARKLEEILHLAELCIEVLQQNEEHHAEGREAFAWWPD
LLAEHAEKFWALFTVDMDTALEAQPQDSWDSFPLFQLLNNFLRNDTLLCNGKFHKHLQEIFVPLVVRYVDLMESSIAQSIHRGFEQE
TWQPVKNIANSLPNVALPKVPSLPLNLPQIPNISTASWMPSLYESTNGSATSEDLFWKLDALQMFVFDLHWPEQEFAHHLEQRLKLM
ASDMLEACVKRTRTAFELKLQKASKTTDLRIPASVCTMFNVLVDAKKQSTKLCALDGGQEQQYHSKIDDLIDNSVKEIISLLVSKFV
SVLEGVLSKLSRYDEGTFFSSILSFTVKAAAKYVDVPKPGMDLADTYIMFVRQNQDILREKVNEEMYIEKLFDQWYSSSMKVICVWL
TDRLDLQLHIYQLKTLIKIVKKTYRDFRLQGVLEGTLNSKTYDTVHRRLTVEEATASVSEGGGLQGITMKDSDEEEEG
>sp|Q9NYV4|CDK12_HUMAN
MPNSERHGGKKDGSGGASGTLQPSSGGGSSNSRERHRLVSKHKRHKSKHSKDMGLVTPEAASLGTVIKPLVEYDDISSDSDTFSDDM
AFKLDRRENDERRGSDRSDRLHKHRHHQHRRSRDLLKAKQTEKEKSQEVSSKSGSMKDRISGSSKRSNEETDDYGKAQVAKSSSKES
RSSKLHKEKTRKERELKSGHKDRSKSHRKRETPKSYKTVDSPKRRSRSPHRKWSDSSKQDDSPSGASYGQDYDLSPSRSHTSSNYDS
YKKSPGSTSRRQSVSPPYKEPSAYQSSTRSPSPYSRRQRSVSPYSRRRSSSYERSGSYSGRSPSPYGRRRSSSPFLSKRSLSRSPLP
SRKSMKSRSRSPAYSRHSSSHSKKKRSSSRSRHSSISPVRLPLNSSLGAELSRKKKERAAAAAAAKMDGKESKGSPVFLPRKENSSV
EAKDSGLESKKLPRSVKLEKSAPDTELVNVTHLNTEVKNSSDTGKVKLDENSEKHLVKDLKAQGTRDSKPIALKEEIVTPKETETSE
KETPPPLPTIASPPPPLPTTTPPPQTPPLPPLPPIPALPQQPPLPPSQPAFSQVPASSTSTLPPSTHSKTSAVSSQANSQPPVQVSV
KTQVSVTAAIPHLKTSTLPPLPLPPLLPGDDDMDSPKETLPSKPVKKEKEQRTRHLLTDLPLPPELPGGDLSPPDSPEPKAITPPQQ
PYKKRPKICCPRYGERRQTESDWGKRCVDKFDIIGIIGEGTYGQVYKAKDKDTGELVALKKVRLDNEKEGFPITAIREIKILRQLIH
RSVVNMKEIVTDKQDALDFKKDKGAFYLVFEYMDHDLMGLLESGLVHFSEDHIKSFMKQLMEGLEYCHKKNFLHRDIKCSNILLNNS

GQIKLADFGLARLYNSEESRPYTNKVITLWYRPPELLLGEERYTPAIDVWSCGCILGELFTKKPIFQANLELAQLELISRLCGSPCP
AVWPDVIKLPYFNTMKPKKQYRRRLREEFSFIPSAALDLLDHMLTLDPSKRCTAEQTLQSDFLKDVELSKMAPPDLPHWQDCHELWS
KKRRRQRQSGVVVEEPPPSKTSRKETTSGTSTEPVKNSSPAPPQPAPGKVESGAGDAIGLADITQQLNQSELAVLLNLLQSQTDLSI
PQMAQLLNIHSNPEMQQQLEALNQSISALTEATSQQQDSETMAPEESLKEAPSAPVILPSAEQTTLEASSTPADMQNILAVLLSQLM
KTQEPAGSLEENNSDKNSGPQGPRRTPTMPQEEAAACPPHILPPEKRPPEPPGPPPPPPPPPLVEGDLSSAPQELNPAVTAALLQLL
SQPEAEPPGHLPHEHQALRPMEYSTRPRPNRTYGNTDGPETGFSAIDTDERNSGPALTESLVQTLVKNRTFSGSLSHLGESSSYQGT
GSVQFPGDQDLRFARVPLALHPVVGQPFLKAEGSSNSVVHAETKLQNYGELGPGTTGASSSGAGLHWGGPTQSSAYGKLYRGPTRVP
PRGGRGRGVPY
>sp|Q96N19|G137A_HUMAN
MESNLSGLVPAAGLVPALPPAVTLGLTAAYTTLYALLFFSVYAQLWLVLLYGHKRLSYQTVFLALCLLWAALRTTLFSFYFRDTPRA
NRLGPLPFWLLYCCPVCLQFFTLTLMNLYFAQVVFKAKVKRRPEMSRGLLAVRGAFVGASLLFLLVNVLCAVLSHRRRAQPWALLLV
RVLVSDSLFVICALSLAACLCLVARRAPSTSIYLEAKGTSVCQAAAMGGAMVLLYASRACYNLTALALAPQSRLDTFDYDWYNVSDQ
ADLVNDLGNKGYLVFGLILFVWELLPTTLLVGFFRVHRPPQDLSTSHILNGQVFASRSYFFDRAGHCEDEGCSWEHSRGESTRCQDQ
AATTTVSTPPHRRDPPPSPTEYPGPSPPHPRPLCQVCLPLLAQDPGGRGYPLLWPAPCCSCHSELVPSP
>sp|Q9NVR2|INT10_HUMAN
MSAQGDCEFLVQRARELVPQDLWAAKAWLITARSLYPADFNIQYEMYTIERNAERTATAGRLLYDMFVNFPDQPVVWREISIITSAL
RNDSQDKQTQFLRSLFETLPGRVQCEMLLKVTEQCFNTLERSEMLLLLLRRFPETVVQHGVGLGEALLEAETIEEQESPVNCFRKLF
VCDVLPLIINNHDVRLPANLLYKYLNKAAEFYINYVTRSTQIENQHQGAQDTSDLMSPSKRSSQKYIIEGLTEKSSQIVDPWERLFK
ILNVVGMRCEWQMDKGRRSYGDILHRMKDLCRYMNNFDSEAHAKYKNQVVYSTMLVFFKNAFQYVNSIQPSLFQGPNAPSQVPLVLL
EDVSNVYGDVEIDRNKHIHKKRKLAEGREKTMSSDDEDCSAKGRNRHIVVNKAELANSTEVLESFKLARESWELLYSLEFLDKEFTR
ICLAWKTDTWLWLRIFLTDMIIYQGQYKKAIASLHHLAALQGSISQPQITGQGTLEHQRALIQLATCHFALGEYRMTCEKVLDLMCY
MVLPIQDGGKSQEEPSKVKPKFRKGSDLKLLPCTSKAIMPYCLHLMLACFKLRAFTDNRDDMALGHVIVLLQQEWPRGENLFLKAVN
KICQQGNFQYENFFNYVTNIDMLEEFAYLRTQEGGKIHLELLPNQGMLIKHHTVTRGITKGVKEDFRLAMERQVSRCGENLMVVLHR
FCINEKILLLQTLT
>sp|O75489|NDUS3_HUMAN
MAAAAVARLWWRGILGASALTRGTGRPSVLLLPVRRESAGADTRPTVRPRNDVAHKQLSAFGEYVAEILPKYVQQVQVSCFNELEVC
IHPDGVIPVLTFLRDHTNAQFKSLVDLTAVDVPTRQNRFEIVYNLLSLRFNSRIRVKTYTDELTPIESAVSVFKAANWYEREIWDMF
GVFFANHPDLRRILTDYGFEGHPFRKDFPLSGYVELRYDDEVKRVVAEPVELAQEFRKFDLNSPWEAFPVYRQPPESLKLEAGDKKP
DAK
>sp|Q9Y6B7|AP4B1_HUMAN
MPYLGSEDVVKELKKALCNPHIQADRLRYRNVIQRVIRYMTQGLDMSGVFMEMVKASATVDIVQKKLVYLYMCTYAPLKPDLALLAI
NTLCKDCSDPNPMVRGLALRSMCSLRMPGVQEYIQQPILNGLRDKASYVRRVAVLGCAKMHNLHGDSEVDGALVNELYSLLRDQDPI
VVVNCLRSLEEILKQEGGVVINKPIAHHLLNRMSKLDQWGQAEVLNFLLRYQPRSEEELFDILNLLDSFLKSSSPGVVMGATKLFLI
LAKMFPHVQTDVLVRVKGPLLAACSSESRELCFVALCHVRQILHSLPGHFSSHYKKFFCSYSEPHYIKLQKVEVLCELVNDENVQQV
LEELRGYCTDVSADFAQAAIFAIGGIARTYTDQCVQILTELLGLRQEHITTVVVQTFRDLVWLCPQCTEAVCQALPGCEENIQDSEG
KQALIWLLGVHGERIPNAPYVLEDFVENVKSETFPAVKMELLTALLRLFLSRPAECQDMLGRLLYYCIEEEKDMAVRDRGLFYYRLL
LVGIDEVKRILCSPKSDPTLGLLEDPAERPVNSWASDFNTLVPVYGKAHWATISKCQGAERCDPELPKTSSFAASGPLIPEENKERV
QELPDSGALMLVPNRQLTADYFEKTWLSLKVAHQQVLPWRGEFHPDTLQMALQVVNIQTIAMSRAGSRPWKAYLSAQDDTGCLFLTE
LLLEPGNSEMQISVKQNEARTETLNSFISVLETVIGTIEEIKS
>sp|Q96H12|MSD3_HUMAN
MQNNEIIKPAKYFSELEKSILLALVEKYKYVLECKKSDARTIALKQRTWQALAHEYNSQPSVSLRDFKQLKKCWENIKARTKKIMAH
ERREKVKRSVSPLLSTHVLGKEKIASMLPEQLYFLQSPPEEEPEYHPDASAQESFAVSNRELCDDEKEFIHFPVCEGTSQPEPSCSA
VRITANKNYRSKTSQEGALKKMHEEEHHQQMSILQLQLIQMNEVHVAKIQQIERECEMAEEEHRIKMEVLNKKKMYWERKLQTFTKE
WPVSSFNRPFPNSP

>sp|Q8TAP6|CEP76_HUMAN
MSLPPEKASELKQLIHQQLSKMDVHGRIREILAETIREELAPDQQHLSTEDLIKALRRRGIIDDVMKELNFVTDSVEQELPSSPKQP
ICFDRQSTLKKTNIDPTRRYLYLQVLGGKAFLEHLQEPEPLPGQVCSTFTLCLHYRNQRFRSKPVPCACEPDFHDGFLLEVHRESLG
DGTRMADSTTMLSISDPIHMVLIKTDIFGETTLVASYFLEWRSVLGSENGVTSLTVELMGVGTESKVSVGILNIKLEMYPPLNQTLS
QEVVNTQLALERQKTAEKERLFLVYAKQWWREYLQIRPSHNSRLVKIFAQDENGINRPVCSYVKPLRAGRLLDTPRQAARFVNVLGY
ERAPVIGGGGKQEQWCTLLAFLCRNKGDCEDHANLLCSLLLGYGLEAFVCVGTKAKGVPHAWVMTCGTDGAITFWESLTGHRYIHKP
TNPDEPPVAEQPKPLYPYRTIGCVFNHQMFLGNCQPSDAVETCVFDLNDESKWKPMSEEAIKSVCAPGATTSLPPFPPLCASTIDAS
VTSNEIEMQLRLLVSEHRKDLGLTTVWEDQLSYLLSPALASYEFERTTSISAGNEEFQDAIRRAVPDGHTFKGFPIHFVYRNARRAF
ATCLRSPFCEEIICCRGDQVRLAVRVRVFTYPESACAVWIMFACKYRSVL
>sp|Q0VAA5|PLCX2_HUMAN
MLAVRKARRKLRMGTICSPNPSGTKTSSEVCNADWMASLPPHLHNLPLSNLAIPGSHDSFSYWVDEKSPVGPDQTQAIKRLARISLV
KKLMKKWSVTQNLTFREQLEAGIRYFDLRVSSKPGDADQEIYFIHGLFGIKVWDGLMEIDSFLTQHPQEIIFLDFNHFYAMDETHHK
CLVLRIQEAFGNKLCPACSVESLTLRTLWEKNCQVLIFYHCPFYKQYPFLWPGKKIPAPWANTTSVRKLILFLETTLSERASRGSFH
VSQAILTPRVKTIARGLVGGLKNTLVHSNRWNSHGPSLLSQERS
>sp|P60371|KR106_HUMAN
MAASTMSVCSSDLSYGSRVCLPGSCDSCSDSWQVDDCPESCCEPPCCAPAPCLSLVCTPVSRVSSPCCPVTCEPSPCQSGCTSSCTP
SCCQQSSCQLACCASSPCQQACCVPVCCKTVCCKPVCCVSVCCGDSSCCQQSSCQSACCTSSPCQQACCVPVCCKPVCSGISSSCCQ
QSSCVSCVSSPCCQAVCEPSPCQSGCTSSCTPSCCQQSSCQPTCCTSSPCQQACCVPVCCVPVCCVPTCSEDSSSCCQQSSCQPACC
TSSPCQHACCVPVCSGASTSCCQQSSCQPACCTASCCRPSSSVSLLCHPVCKSTCCVPVPSCGASASSCQPSCCRTASCVSLLCRPM
CSRPACYSLCSGQKSSC
>sp|Q8N972|ZN709_HUMAN
MDSVVFEDVAVNFTQEEWALLGPSQKKLYRDVMQETFVNLASIGENWEEKNIEDHKNQGRKLRSHMVERLCERKEGSQFGETISQTP
NPKPNKKTFTRVKPYECSVCGKDYMCHSSLNRHMRSHTEHRSYEYHKYGEKSYECKECGKRFSFRSSFRIHERTHTGEKPYKCKQCG
KAFSWPSSFQIHERTHTGEKPYECKECGKAFIYHTTFRGHMRMHTGEKPYKCKECGKTFSHPSSFRNHERTHSGEKPYECKQCGKAF
RYYQTFQIHERTHTGEKPYQCKQCGKALSCPTSFRSHERIHTGEKPYKCKKCGKAFSFPSSFRKHERIHTGEKPYDCKECGKAFISL
PSYRRHMIMHTGNGPYKCKECGKAFDCPSSFQIHERTHTGEKPYECKQCGKAFSCSSSFRMHERTHTGEKPHECKQCGKAFSCSSSV
RIHERTHTGEKPYECKQCGKAFSCSSSFRMHERIHTGEKPYECKQCGKAFSFSSSFRMHERTHTGEKPYECKQCGKAFSCSSSFRMH
ERTHTGEKPYECKQCGKAFSCSSSIRIHERTHTGEKPYECKQCGKAFSCSSSVRMHERTHTGVKPYECKQCDKAFSCSRSFRIHERT
HTGEKPYACQQCGKAFKCSRSFRIHERVHSGE
>sp|Q86YT6|MIB1_HUMAN
MSNSRNNRVMVEGVGARVVRGPDWKWGKQDGGEGHVGTVRSFESPEEVVVVWDNGTAANYRCSGAYDLRILDSAPTGIKHDGTMCDT
CRQQPIIGIRWKCAECTNYDLCTVCYHGDKHHLRHRFYRITTPGSERVLLESRRKSKKITARGIFAGARVVRGVDWQWEDQDGGNGR
RGKVTEIQDWSASSPHSAAYVLWDNGAKNLYRVGFEGMSDLKCVQDAKGGSFYRDHCPVLGEQNGNRNPGGLQIGDLVNIDLDLEIV
QSLQHGHGGWTDGMFETLTTTGTVCGIDEDHDIVVQYPSGNRWTFNPAVLTKANIVRSGDAAQGAEGGTSQFQVGDLVQVCYDLERI
KLLQRGHGEWAEAMLPTLGKVGRVQQIYSDSDLKVEVCGTSWTYNPAAVSKVASAGSAISNASGERLSQLLKKLFETQESGDLNEEL
VKAAANGDVAKVEDLLKRPDVDVNGQCAGHTAMQAASQNGHVDILKLLLKQNVDVEAEDKDGDRAVHHAAFGDEGAVIEVLHRGSAD
LNARNKRRQTPLHIAVNKGHLQVVKTLLDFGCHPSLQDSEGDTPLHDAISKKRDDILAVLLEAGADVTITNNNGFNALHHAALRGNP
SAMRVLLSKLPRPWIVDEKKDDGYTALHLAALNNHVEVAELLVHQGNANLDIQNVNQQTALHLAVERQHTQIVRLLVRAGAKLDIQD
KDGDTPLHEALRHHTLSQLRQLQDMQDVGKVDAAWEPSKNTLIMGLGTQGAEKKSAASIACFLAANGADLSIRNKKGQSPLDLCPDP
NLCKALAKCHKEKVSGQVGSRSPSMISNDSETLEECMVCSDMKRDTLFGPCGHIATCSLCSPRVKKCLICKEQVQSRTKIEECVVCS
DKKAAVLFQPCGHMCACENCANLMKKCVQCRAVVERRVPFIMCCGGKSSEDATDDISSGNIPVLQKDKDNTNVNADVQKLQQQLQDI
KEQTMCPVCLDRLKNMIFLCGHGTCQLCGDRMSECPICRKAIERRILLY
>sp|P35790|CHKA_HUMAN

MKTKFCTGGEAEPSPLGLLLSCGSGSAAPAPGVGQQRDAASDLESKQLGGQQPPLALPPPPPLPLPLPLPQPPPPQPPADEQPEPRT
RRRAYLWCKEFLPGAWRGLREDEFHISVIRGGLSNMLFQCSLPDTTATLGDEPRKVLLRLYGAILQMRSCNKEGSEQAQKENEFQGA
EAMVLESVMFAILAERSLGPKLYGIFPQGRLEQFIPSRRLDTEELSLPDISAEIAEKMATFHGMKMPFNKEPKWLFGTMEKYLKEVL
RIKFTEESRIKKLHKLLSYNLPLELENLRSLLESTPSPVVFCHNDCQEGNILLLEGRENSEKQKLMLIDFEYSSYNYRGFDIGNHFC
EWMYDYSYEKYPFFRANIRKYPTKKQQLHFISSYLPAFQNDFENLSTEEKSIIKEEMLLEVNRFALASHFLWGLWSIVQAKISSIEF
GYMDYAQARFDAYFHQKRKLGV
>sp|Q5T6L9|EMARD_HUMAN
MEVLIGDPITTCLSPSVYDIICNLGFQLRENCDINSIVTQNGEVCWKTITDCVSYTESEQGLDYWGSVRLLGPVCEAVHSHFLSLTK
GQFEIRYAPWFQWTSFPELFPEIFDALESLQSPAISLSLMKLTSCLERALGDVFLLIGKECPFLLRDLLSSEELAQVFSQSVMNVLK
VFVGSPCGLNLRNVLWHGFASPEEIPPKYCSMMILLTAGLGQLLKSYLQNTKLTLAHRSFISLTNLEDLIVFPDVTYEVLSVLEEVM
MKSAFILKIMLPYWEVALVKFKSHRFADCAILLLTQLETGLRNVFATLNRCPKRLLTAESTALYTTFDQILAKHLNDGKINQLPLFL
GEPAMEFLWDFLNHQEGPRIRDHLSHGEINLHEFSKETTNQLLAFSLVLLLRFVDDCLLSVFKEKSAVELLISLAEGYSSRCHPVFQ
LKKQVLSCEESIRVWALLPFPEELTRQAVRLEDNSETNACHSLITKMTDELYHHMPENRCVLKDLDRLPTETWPQLLRELCSTPVPT
LFCPRIVLEVLVVLRSISEQCRRVSSQVTVASELRHRQWVERTLRSRQRQNYLRMWSSIRLLSPVLSLILLLIALELVNIHAVCGKN
AHEYQQYLKFVKSILQYTENLVAYTSYEKNKWNETINLTHTALLKMWTFSEKKQMLIHLAKKSTSKVLL
>sp|Q9HCU4|CELR2_HUMAN
MRSPATGVPLPTPPPPLLLLLLLLLPPPLLGDQVGPCRSLGSRGRGSSGACAPMGWLCPSSASNLWLYTSRCRDAGTELTGHLVPHH
DGLRVWCPESEAHIPLPPAPEGCPWSCRLLGIGGHLSPQGKLTLPEEHPCLKAPRLRCQSCKLAQAPGLRAGERSPEESLGGRRKRN
VNTAPQFQPPSYQATVPENQPAGTPVASLRAIDPDEGEAGRLEYTMDALFDSRSNQFFSLDPVTGAVTTAEELDRETKSTHVFRVTA
QDHGMPRRSALATLTILVTDTNDHDPVFEQQEYKESLRENLEVGYEVLTVRATDGDAPPNANILYRLLEGSGGSPSEVFEIDPRSGV
IRTRGPVDREEVESYQLTVEASDQGRDPGPRSTTAAVFLSVEDDNDNAPQFSEKRYVVQVREDVTPGAPVLRVTASDRDKGSNAVVH
YSIMSGNARGQFYLDAQTGALDVVSPLDYETTKEYTLRVRAQDGGRPPLSNVSGLVTVQVLDINDNAPIFVSTPFQATVLESVPLGY
LVLHVQAIDADAGDNARLEYRLAGVGHDFPFTINNGTGWISVAAELDREEVDFYSFGVEARDHGTPALTASASVSVTVLDVNDNNPT
FTQPEYTVRLNEDAAVGTSVVTVSAVDRDAHSVITYQITSGNTRNRFSITSQSGGGLVSLALPLDYKLERQYVLAVTASDGTRQDTA
QIVVNVTDANTHRPVFQSSHYTVNVNEDRPAGTTVVLISATDEDTGENARITYFMEDSIPQFRIDADTGAVTTQAELDYEDQVSYTL
AITARDNGIPQKSDTTYLEILVNDVNDNAPQFLRDSYQGSVYEDVPPFTSVLQISATDRDSGLNGRVFYTFQGGDDGDGDFIVESTS
GIVRTLRRLDRENVAQYVLRAYAVDKGMPPARTPMEVTVTVLDVNDNPPVFEQDEFDVFVEENSPIGLAVARVTATDPDEGTNAQIM
YQIVEGNIPEVFQLDIFSGELTALVDLDYEDRPEYVLVIQATSAPLVSRATVHVRLLDRNDNPPVLGNFEILFNNYVTNRSSSFPGG
AIGRVPAHDPDISDSLTYSFERGNELSLVLLNASTGELKLSRALDNNRPLEAIMSVLVSDGVHSVTAQCALRVTIITDEMLTHSITL
RLEDMSPERFLSPLLGLFIQAVAATLATPPDHVVVFNVQRDTDAPGGHILNVSLSVGQPPGPGGGPPFLPSEDLQERLYLNRSLLTA
ISAQRVLPFDDNICLREPCENYMRCVSVLRFDSSAPFIASSSVLFRPIHPVGGLRCRCPPGFTGDYCETEVDLCYSRPCGPHGRCRS
REGGYTCLCRDGYTGEHCEVSARSGRCTPGVCKNGGTCVNLLVGGFKCDCPSGDFEKPYCQVTTRSFPAHSFITFRGLRQRFHFTLA
LSFATKERDGLLLYNGRFNEKHDFVALEVIQEQVQLTFSAGESTTTVSPFVPGGVSDGQWHTVQLKYYNKPLLGQTGLPQGPSEQKV
AVVTVDGCDTGVALRFGSVLGNYSCAAQGTQGGSKKSLDLTGPLLLGGVPDLPESFPVRMRQFVGCMRNLQVDSRHIDMADFIANNG
TVPGCPAKKNVCDSNTCHNGGTCVNQWDAFSCECPLGFGGKSCAQEMANPQHFLGSSLVAWHGLSLPISQPWYLSLMFRTRQADGVL
LQAITRGRSTITLQLREGHVMLSVEGTGLQASSLRLEPGRANDGDWHHAQLALGASGGPGHAILSFDYGQQRAEGNLGPRLHGLHLS
NITVGGIPGPAGGVARGFRGCLQGVRVSDTPEGVNSLDPSHGESINVEQGCSLPDPCDSNPCPANSYCSNDWDSYSCSCDPGYYGDN
CTNVCDLNPCEHQSVCTRKPSAPHGYTCECPPNYLGPYCETRIDQPCPRGWWGHPTCGPCNCDVSKGFDPDCNKTSGECHCKENHYR
PPGSPTCLLCDCYPTGSLSRVCDPEDGQCPCKPGVIGRQCDRCDNPFAEVTTNGCEVNYDSCPRAIEAGIWWPRTRFGLPAAAPCPK
GSFGTAVRHCDEHRGWLPPNLFNCTSITFSELKGFAERLQRNESGLDSGRSQQLALLLRNATQHTAGYFGSDVKVAYQLATRLLAHE
STQRGFGLSATQDVHFTENLLRVGSALLDTANKRHWELIQQTEGGTAWLLQHYEAYASALAQNMRHTYLSPFTIVTPNIVISVVRLD
KGNFAGAKLPRYEALRGEQPPDLETTVILPESVFRETPPVVRPAGPGEAQEPEELARRQRRHPELSQGEAVASVIIYRTLAGLLPHN
YDPDKRSLRVPKRPIINTPVVSISVHDDEELLPRALDKPVTVQFRLLETEERTKPICVFWNHSILVSGTGGWSARGCEVVFRNESHV
SCQCNHMTSFAVLMDVSRRENGEILPLKTLTYVALGVTLAALLLTFFFLTLLRILRSNQHGIRRNLTAALGLAQLVFLLGINQADLP

FACTVIAILLHFLYLCTFSWALLEALHLYRALTEVRDVNTGPMRFYYMLGWGVPAFITGLAVGLDPEGYGNPDFCWLSIYDTLIWSF
AGPVAFAVSMSVFLYILAARASCAAQRQGFEKKGPVSGLQPSFAVLLLLSATWLLALLSVNSDTLLFHYLFATCNCIQGPFIFLSYV
VLSKEVRKALKLACSRKPSPDPALTTKSTLTSSYNCPSPYADGRLYQPYGDSAGSLHSTSRSGKSQPSYIPFLLREESALNPGQGPP
GLGDPGSLFLEGQDQQHDPDTDSDSDLSLEDDQSGSYASTHSSDSEEEEEEEEEEAAFPGEQGWDSLLGPGAERLPLHSTPKDGGPG
PGKAPWPGDFGTTAKESSGNGAPEERLRENGDALSREGSLGPLPGSSAQPHKGILKKKCLPTISEKSSLLRLPLEQCTGSSRGSSAS
EGSRGGPPPRPPPRQSLQEQLNGVMPIAMSIKAGTVDEDSSGSEFLFFNFLH
>sp|Q9BWT6|MND1_HUMAN
MSKKKGLSAEEKRTRMMEIFSETKDVFQLKDLEKIAPKEKGITAMSVKEVLQSLVDDGMVDCERIGTSNYYWAFPSKALHARKHKLE
VLESQLSEGSQKHASLQKSIEKAKIGRCETEERTRLAKELSSLRDQREQLKAEVEKYKDCDPQVVEEIRQANKVAKEAANRWTDNIF
AIKSWAKRKFGFEENKIDRTFGIPEDFDYID
>sp|Q8N5G2|MACOI_HUMAN
MKRRNADCSKLRRPLKRNRITEGIYGSTFLYLKFLVVWALVLLADFVLEFRFEYLWPFWLFIRSVYDSFRYQGLAFSVFFVCVAFTS
NIICLLFIPIQWLFFAASTYVWVQYVWHTERGVCLPTVSLWILFVYIEAAIRFKDLKNFHVDLCRPFAAHCIGYPVVTLGFGFKSYV
SYKMRLRKQKEVQKENEFYMQLLQQALPPEQQMLQKQEKEAEEAAKGLPDMDSSILIHHNGGIPANKKLSTTLPEIEYREKGKEKDK
DAKKHNLGINNNNILQPVDSKIQEIEYMENHINSKRLNNDLVGSTENLLKEDSCTASSKNYKNASGVVNSSPRSHSATNGSIPSSSS
KNEKKQKCTSKSPSTHKDLMENCIPNNQLSKPDALVRLEQDIKKLKADLQASRQVEQELRSQISSLSSTERGIRSEMGQLRQENELL
QNKLHNAVQMKQKDKQNISQLEKKLKAEQEARSFVEKQLMEEKKRKKLEEATAARAVAFAAASRGECTETLRNRIRELEAEGKKLTM
DMKVKEDQIRELELKVQELRKYKENEKDTEVLMSALSAMQDKTQHLENSLSAETRIKLDLFSALGDAKRQLEIAQGQILQKDQEIKD
LKQKIAEVMAVMPSITYSAATSPLSPVSPHYSSKFVETSPSGLDPNASVYQPLKK
>sp|Q5VU97|CAHD1_HUMAN
MARQPEEEETAVARARRPPLWLLCLVACWLLGAGAEADFSILDEAQVLASQMRRLAAEELGVVTMQRIFNSFVYTEKISNGESEVQQ
LAKKIREKFNRYLDVVNRNKQVVEASYTAHLTSPLTAIQDCCTIPPSMMEFDGNFNTNVSRTISCDRLSTTVNSRAFNPGRDLNSVL
ADNLKSNPGIKWQYFSSEEGIFTVFPAHKFRCKGSYEHRSRPIYVSTVRPQSKHIVVILDHGASVTDTQLQIAKDAAQVILSAIDEH
DKISVLTVADTVRTCSLDQCYKTFLSPATSETKRKMSTFVSSVKSSDSPTQHAVGFQKAFQLIRSTNNNTKFQANTDMVIIYLSAGI
TSKDSSEEDKKATLQVINEENSFLNNSVMILTYALMNDGVTGLKELAFLRDLAEQNSGKYGVPDRMALPVIKGSMMVLNQLSNLETT
VGRFYTNLPNRMIDEAVFSLPFSDEMGDGLIMTVSKPCYFGNLLLGIVGVDVNLAYILEDVTYYQDSLASYTFLIDDKGYTLMHPSL
TRPYLLSEPPLHTDIIHYENIPKFELVRQNILSLPLGSQIIAVPVNSSLSWHINKLRETGKEAYNVSYAWKMVQDTSFILCIVVIQP
EIPVKQLKNLNTVPSSKLLYHRLDLLGQPSACLHFKQLATLESPTIMLSAGSFSSPYEHLSQPETKRMVEHYTAYLSDNTRLIANPG
LKFSVRNEVMATSHVTDEWMTQMEMSSLNTYIVRRYIATPNGVLRIYPGSLMDKAFDPTRRQWYLHAVANPGLISLTGPYLDVGGAG
YVVTISHTIHSSSTQLSSGHTVAVMGIDFTLRYFYKVLMDLLPVCNQDGGNKIRCFIMEDRGYLVAHPTLIDPKGHAPVEQQHITHK
EPLVANDILNHPNFVKKNLCNSFSDRTVQRFYKFNTSLAGDLTNLVHGSHCSKYRLARIPGTNAFVGIVNETCDSLAFCACSMVDRL
CLNCHRMEQNECECPCECPLEVNECTGNLTNAENRNPSCEVHQEPVTYTAIDPGLQDALHQCVNSRCSQRLESGDCFGVLDCEWCMV
DSDGKTHLDKPYCAPQKECFGGIVGAKSPYVDDMGAIGDEVITLNMIKSAPVGPVAGGIMGCIMVLVLAVYAYRHQIHRRSHQHMSP
LAAQEMSVRMSNLENDRDERDDDSHEDRGIISNTRFIAAVIERHAHSPERRRRYWGRSGTESDHGYSTMSPQEDSENPPCNNDPLSA
GVDVGNHDEDLDLDTPPQTAALLSHKFHHYRSHHPTLHHSHHLQAAVTVHTVDAEC
>sp|Q7Z2X7|PAGE2_HUMAN
MSELLRARSQSSERGNDQESSQPVGSVIVQEPTEEKRQEEEPPTDNQGIAPSGEIENQAVPAFQGPDMEAFQQELALLKIEDEPGDG
PDVREGIMPTFDLTKVLEAGDAQP
>sp|P08861|CEL3B_HUMAN
MMLRLLSSLLLVAVASGYGPPSSRPSSRVVNGEDAVPYSWPWQVSLQYEKSGSFYHTCGGSLIAPDWVVTAGHCISSSRTYQVVLGE
YDRAVKEGPEQVIPINSGDLFVHPLWNRSCVACGNDIALIKLSRSAQLGDAVQLASLPPAGDILPNETPCYITGWGRLYTNGPLPDK
LQEALLPVVDYEHCSRWNWWGSSVKKTMVCAGGDIRSGCNGDSGGPLNCPTEDGGWQVHGVTSFVSAFGCNTRRKPTVFTRVSAFID
WIEETIASH
>sp|P14653|HXB1_HUMAN

MDYNRMNSFLEYPLCNRGPSAYSAHSAPTSFPPSSAQAVDSYASEGRYGGGLSSPAFQQNSGYPAQQPPSTLGVPFPSSAPSGYAPA
ACSPSYGPSQYYPLGQSEGDGGYFHPSSYGAQLGGLSDGYGAGGAGPGPYPPQHPPYGNEQTASFAPAYADLLSEDKETPCPSEPNT
PTARTFDWMKVKRNPPKTAKVSEPGLGSPSGLRTNFTTRQLTELEKEFHFNKYLSRARRVEIAATLELNETQVKIWFQNRRMKQKKR
EREEGRVPPAPPGCPKEAAGDASDQSTCTSPEASPSSVTS
>sp|A0PJY2|FEZF1_HUMAN
MDSSCHNATTKMLATAPARGNMMSTSKPLAFSIERIMARTPEPKALPVPHFLQGALPKGEPKHSLHLNSSIPCMIPFVPVAYDTSPK
AGVTGSEPRKASLEAPAAPAAVPSAPAFSCSDLLNCALSLKGDLARDALPLQQYKLVRPRVVNHSSFHAMGALCYLNRGDGPCHPAA
GVNIHPVASYFLSSPLHPQPKTYLAERNKLVVPAVEKYPSGVAFKDLSQAQLQHYMKESAQLLSEKIAFKTSDFSRGSPNAKPKVFT
CEVCGKVFNAHYNLTRHMPVHTGARPFVCKVCGKGFRQASTLCRHKIIHTQEKPHKCNQCGKAFNRSSTLNTHTRIHAGYKPFVCEF
CGKGFHQKGNYKNHKLTHSGEKQFKCNICNKAFHQVYNLTFHMHTHNDKKPFTCPTCGKGFCRNFDLKKHVRKLHDSSLGLARTPAG
EPGTEPPPPLPQQPPMTLPPLQPPLPTPGPLQPGLHQGHQ
>sp|Q7Z4V0|ZN438_HUMAN
MQNSVSVPPKDEGESNIPSGTIQSRKGLQNKSQFRTIAPKIVPKVLTSRMLPCHSPSRSDQVNLGPSINSKLLGMSTQNYALMQVAG
QEGTFSLVALPHVASAQPIQKPRMSLPENLKLPIPRYQPPRNSKASRKKPILIFPKSGCSKAPAQTQMCPQMSPSPPHHPELLYKPS
PFEEVPSLEQAPASISTAALTNGSDHGDLRPPVTNTHGSLNPPATPASSTPEEPAKQDLTALSGKAHFVSKITSSKPSAVASEKFKE
QVDLAKTMTNLSPTILGNAVQLISSVPKGKLPIPPYSRMKTMEVYKIKSDANIAGFSLPGPKADCDKIPSTTEGFNAATKVASRLPV
PQVSQQSACESAFCPPTKLDLNHKTKLNSGAAKRKGRKRKVPDEILAFQGKRRKYIINKCRDGKERVKNDPQEFRDQKLGTLKKYRS
IMPKPIMVIPTLASLASPTTLQSQMLGGLGQDVLLNNSLTPKYLGCKQDNSSSPKPSSVFRNGFSGIKKPWHRCHVCNHHFQFKQHL
RDHMNTHTNRRPYSCRICRKSYVRPGSLSTHMKLHHGENRLKKLMCCEFCAKVFGHIRVYFGHLKEVHRVVISTEPAPSELQPGDIP
KNRDMSVRGMEGSLERENKSNLEEDFLLNQADEVKLQIKCGRCQITAQSFAEIKFHLLDVHGEEIEGRLQEGTFPGSKGTQEELVQH
ASPDWKRHPERGKPEKVHSSSEESHACPRLKRQLHLHQNGVEMLMENEGPQSGTNKPRETCQGPECPGLHTFLLWSHSGFNCLLCAE
MLGRKEDLLHHWKHQHNCEDPSKLWAILNTVSNQGVIELSSEAEK
>sp|Q8NG94|O11H1_HUMAN
MCPLTLQVTGLMNVSEPNSSFAFVNEFILQGFSCEWTIQIFLFSLFTTTYALTITGNGAIAFVLWCDRRLHTPMYMFLGNFSFLEIW
YVSSTVPKMLVNFLSEKKNISFAGCFLQFYFFFSLGTSECLLLTVMAFDQYLAICRPLLYPNIMTGHLYAKLVILCWVCGFLWFLIP
IVLISQMPFCGPNIIDHVVCDPGPRFALDCVSAPRIQLFCYTLSSLVIFGNFLFIIGSYTLVLKAMLGMPSSTGRHKAFSTCGSHLA
VVSLCYSSLMVMYVSPGLGHSTGMQKIETLFYAMVTPLFNPLIYSLQNKEIKAALRKVLGSSNII
>sp|Q96Q15|SMG1_HUMAN
MSRRAPGSRLSSGGGGGGTKYPRSWNDWQPRTDSASADPDNLKYSSSRDRGGSSSYGLQPSNSAVVSRQRHDDTRVHADIQNDEKGG
YSVNGGSGENTYGRKSLGQELRVNNVTSPEFTSVQHGSRALATKDMRKSQERSMSYSDESRLSNLLRRITREDDRDRRLATVKQLKE
FIQQPENKLVLVKQLDNILAAVHDVLNESSKLLQELRQEGACCLGLLCASLSYEAEKIFKWIFSKFSSSAKDEVKLLYLCATYKALE
TVGEKKAFSSVMQLVMTSLQSILENVDTPELLCKCVKCILLVARCYPHIFSTNFRDTVDILVGWHIDHTQKPSLTQQVSGWLQSLEP
FWVADLAFSTTLLGQFLEDMEAYAEDLSHVASGESVDEDVPPPSVSLPKLAALLRVFSTVVRSIGERFSPIRGPPITEAYVTDVLYR
VMRCVTAANQVFFSEAVLTAANECVGVLLGSLDPSMTIHCDMVITYGLDQLENCQTCGTDYIISVLNLLTLIVEQINTKLPSSFVEK
LFIPSSKLLFLRYHKEKEVVAVAHAVYQAVLSLKNIPVLETAYKLILGEMTCALNNLLHSLQLPEACSEIKHEAFKNHVFNVDNAKF
VVIFDLSALTTIGNAKNSLIGMWALSPTVFALLSKNLMIVHSDLAVHFPAIQYAVLYTLYSHCTRHDHFISSSLSSSSPSLFDGAVI
STVTTATKKHFSIILNLLGILLKKDNLNQDTRKLLMTWALEAAVLMKKSETYAPLFSLPSFHKFCKGLLANTLVEDVNICLQACSSL
HALSSSLPDDLLQRCVDVCRVQLVHSGTRIRQAFGKLLKSIPLDVVLSNNNHTEIQEISLALRSHMSKAPSNTFHPQDFSDVISFIL
YGNSHRTGKDNWLERLFYSCQRLDKRDQSTIPRNLLKTDAVLWQWAIWEAAQFTVLSKLRTPLGRAQDTFQTIEGIIRSLAAHTLNP
DQDVSQWTTADNDEGHGNNQLRLVLLLQYLENLEKLMYNAYEGCANALTSPPKVIRTFFYTNRQTCQDWLTRIRLSIMRVGLLAGQP
AVTVRHGFDLLTEMKTTSLSQGNELEVTIMMVVEALCELHCPEAIQGIAVWSSSIVGKNLLWINSVAQQAEGRFEKASVEYQEHLCA
MTGVDCCISSFDKSVLTLANAGRNSASPKHSLNGESRKTVLSKPTDSSPEVINYLGNKACECYISIADWAAVQEWQNAIHDLKKSTS
STSLNLKADFNYIKSLSSFESGKFVECTEQLELLPGENINLLAGGSKEKIDMKKLLPNMLSPDPRELQKSIEVQLLRSSVCLATALN
PIEQDQKWQSITENVVKYLKQTSRIAIGPLRLSTLTVSQSLPVLSTLQLYCSSALENTVSNRLSTEDCLIPLFSEALRSCKQHDVRP

WMQALRYTMYQNQLLEKIKEQTVPIRSHLMELGLTAAKFARKRGNVSLATRLLAQCSEVQLGKTTTAQDLVQHFKKLSTQGQVDEKW
GPELDIEKTKLLYTAGQSTHAMEMLSSCAISFCKSVKAEYAVAKSILTLAKWIQAEWKEISGQLKQVYRAQHQQNFTGLSTLSKNIL
TLIELPSVNTMEEEYPRIESESTVHIGVGEPDFILGQLYHLSSVQAPEVAKSWAALASWAYRWGRKVVDNASQGEGVRLLPREKSEV
QNLLPDTITEEEKERIYGILGQAVCRPAGIQDEDITLQITESEDNEEDDMVDVIWRQLISSCPWLSELDESATEGVIKVWRKVVDRI
FSLYKLSCSAYFTFLKLNAGQIPLDEDDPRLHLSHRVEQSTDDMIVMATLRLLRLLVKHAGELRQYLEHGLETTPTAPWRGIIPQLF
SRLNHPEVYVRQSICNLLCRVAQDSPHLILYPAIVGTISLSSESQASGNKFSTAIPTLLGNIQGEELLVSECEGGSPPASQDSNKDE
PKSGLNEDQAMMQDCYSKIVDKLSSANPTMVLQVQMLVAELRRVTVLWDELWLGVLLQQHMYVLRRIQQLEDEVKRVQNNNTLRKEE
KIAIMREKHTALMKPIVFALEHVRSITAAPAETPHEKWFQDNYGDAIENALEKLKTPLNPAKPGSSWIPFKEIMLSLQQRAQKRASY
ILRLEEISPWLAAMTNTEIALPGEVSARDTVTIHSVGGTITILPTKTKPKKLLFLGSDGKSYPYLFKGLEDLHLDERIMQFLSIVNT
MFATINRQETPRFHARHYSVTPLGTRSGLIQWVDGATPLFGLYKRWQQREAALQAQKAQDSYQTPQNPGIVPRPSELYYSKIGPALK
TVGLSLDVSRRDWPLHVMKAVLEELMEATPPNLLAKELWSSCTTPDEWWRVTQSYARSTAVMSMVGYIIGLGDRHLDNVLIDMTTGE
VVHIDYNVCFEKGKSLRVPEKVPFRMTQNIETALGVTGVEGVFRLSCEQVLHIMRRGRETLLTLLEAFVYDPLVDWTAGGEAGFAGA
VYGGGGQQAESKQSKREMEREITRSLFSSRVAEIKVNWFKNRDEMLVVLPKLDGSLDEYLSLQEQLTDVEKLQGKLLEEIEFLEGAE
GVDHPSHTLQHRYSEHTQLQTQQRAVQEAIQVKLNEFEQWITHYQAAFNNLEATQLASLLQEISTQMDLGPPSYVPATAFLQNAGQA
HLISQCEQLEGEVGALLQQRRSVLRGCLEQLHHYATVALQYPKAIFQKHRIEQWKTWMEELICNTTVERCQELYRKYEMQYAPQPPP
TVCQFITATEMTLQRYAADINSRLIRQVERLKQEAVTVPVCEDQLKEIERCIKVFLHENGEEGSLSLASVIISALCTLTRRNLMMEG
AASSAGEQLVDLTSRDGAWFLEELCSMSGNVTCLVQLLKQCHLVPQDLDIPNPMEASETVHLANGVYTSLQELNSNFRQIIFPEALR
CLMKGEYTLESMLHELDGLIEQTTDGVPLQTLVESLQAYLRNAAMGLEEETHAHYIDVARLLHAQYGELIQPRNGSVDETPKMSAGQ
MLLVAFDGMFAQVETAFSLLVEKLNKMEIPIAWRKIDIIREARSTQVNFFDDDNHRQVLEEIFFLKRLQTIKEFFRLCGTFSKTLSG
SSSLEDQNTVNGPVQIVNVKTLFRNSCFSEDQMAKPIKAFTADFVRQLLIGLPNQALGLTLCSFISALGVDIIAQVEAKDFGAESKV
SVDDLCKKAVEHNIQIGKFSQLVMNRATVLASSYDTAWKKHDLVRRLETSISSCKTSLQRVQLHIAMFQWQHEDLLINRPQAMSVTP
PPRSAILTSMKKKLHTLSQIETSIATVQEKLAALESSIEQRLKWAGGANPALAPVLQDFEATIAERRNLVLKESQRASQVTFLCSNI
IHFESLRTRTAEALNLDAALFELIKRCQQMCSFASQFNSSVSELELRLLQRVDTGLEHPIGSSEWLLSAHKQLTQDMSTQRAIQTEK
EQQIETVCETIQNLVDNIKTVLTGHNRQLGDVKHLLKAMAKDEEAALADGEDVPYENSVRQFLGEYKSWQDNIQTVLFTLVQAMGQV
RSQEHVEMLQEITPTLKELKTQSQSIYNNLVSFASPLVTDATNECSSPTSSATYQPSFAAAVRSNTGQKTQPDVMSQNARKLIQKNL
ATSADTPPSTVPGTGKSVACSPKKAVRDPKTGKAVQERNSYAVSVWKRVKAKLEGRDVDPNRRMSVAEQVDYVIKEATNLDNLAQLY
EGWTAWV
>sp|Q13336|UT1_HUMAN
MEDSPTMVRVDSPTMVRGENQVSPCQGRRCFPKALGYVTGDMKELANQLKDKPVVLQFIDWILRGISQVVFVNNPVSGILILVGLLV
QNPWWALTGWLGTVVSTLMALLLSQDRSLIASGLYGYNATLVGVLMAVFSDKGDYFWWLLLPVCAMSMTCPIFSSALNSMLSKWDLP
VFTLPFNMALSMYLSATGHYNPFFPAKLVIPITTAPNISWSDLSALELLKSIPVGVGQIYGCDNPWTGGIFLGAILLSSPLMCLHAA
IGSLLGIAAGLSLSAPFEDIYFGLWGFNSSLACIAMGGMFMALTWQTHLLALGCALFTAYLGVGMANFMAEVGLPACTWPFCLATLL
FLIMTTKNSNIYKMPLSKVTYPEENRIFYLQAKKRMVESPL
>sp|P43652|AFAM_HUMAN
MKLLKLTGFIFFLFFLTESLTLPTQPRDIENFNSTQKFIEDNIEYITIIAFAQYVQEATFEEMEKLVKDMVEYKDRCMADKTLPECS
KLPNNVLQEKICAMEGLPQKHNFSHCCSKVDAQRRLCFFYNKKSDVGFLPPFPTLDPEEKCQAYESNRESLLNHFLYEVARRNPFVF
APTLLTVAVHFEEVAKSCCEEQNKVNCLQTRAIPVTQYLKAFSSYQKHVCGALLKFGTKVVHFIYIAILSQKFPKIEFKELISLVED
VSSNYDGCCEGDVVQCIRDTSKVMNHICSKQDSISSKIKECCEKKIPERGQCIINSNKDDRPKDLSLREGKFTDSENVCQERDADPD
TFFAKFTFEYSRRHPDLSIPELLRIVQIYKDLLRNCCNTENPPGCYRYAEDKFNETTEKSLKMVQQECKHFQNLGKDGLKYHYLIRL
TKIAPQLSTEELVSLGEKMVTAFTTCCTLSEEFACVDNLADLVFGELCGVNENRTINPAVDHCCKTNFAFRRPCFESLKADKTYVPP
PFSQDLFTFHADMCQSQNEELQRKTDRFLVNLVKLKHELTDEELQSLFTNFANVVDKCCKAESPEVCFNEESPKIGN
>sp|Q9BZQ4|NMNA2_HUMAN
MTETTKTHVILLACGSFNPITKGHIQMFERARDYLHKTGRFIVIGGIVSPVHDSYGKQGLVSSRHRLIMCQLAVQNSDWIRVDPWEC
YQDTWQTTCSVLEHHRDLMKRVTGCILSNVNTPSMTPVIGQPQNETPQPIYQNSNVATKPTAAKILGKVGESLSRICCVRPPVERFT

FVDENANLGTVMRYEEIELRILLLCGSDLLESFCIPGLWNEADMEVIVGDFGIVVVPRDAADTDRIMNHSSILRKYKNNIMVVKDDI
NHPMSVVSSTKSRLALQHGDGHVVDYLSQPVIDYILKSQLYINASG
>sp|Q9Y698|CCG2_HUMAN
MGLFDRGVQMLLTTVGAFAAFSLMTIAVGTDYWLYSRGVCKTKSVSENETSKKNEEVMTHSGLWRTCCLEGNFKGLCKQIDHFPEDA
DYEADTAEYFLRAVRASSIFPILSVILLFMGGLCIAASEFYKTRHNIILSAGIFFVSAGLSNIIGIIVYISANAGDPSKSDSKKNSY
SYGWSFYFGALSFIIAEMVGVLAVHMFIDRHKQLRATARATDYLQASAITRIPSYRYRYQRRSRSSSRSTEPSHSRDASPVGIKGFN
TLPSTEISMYTLSRDPLKAATTPTATYNSDRDNSFLQVHNCIQKENKDSLHSNTANRRTTPV
>sp|Q13023|AKAP6_HUMAN
MLTMSVTLSPLRSQDLDPMATDASPMAINMTPTVEQGEGEEAMKDMDSDQQYEKPPPLHTGADWKIVLHLPEIETWLRMTSERVRDL
TYSVQQDSDSKHVDVHLVQLKDICEDISDHVEQIHALLETEFSLKLLSYSVNVIVDIHAVQLLWHQLRVSVLVLRERILQGLQDANG
NYTRQTDILQAFSEETKEGRLDSLTEVDDSGQLTIKCSQNYLSLDCGITAFELSDYSPSEDLLSGLGDMTSSQVKTKPFDSWSYSEM
EKEFPELIRSVGLLTVAADSISTNGSEAVTEEVSQVSLSVDDKGGCEEDNASAVEEQPGLTLGVSSSSGEALTNAAQPSSETVQQES
SSSSHHDAKNQQPVPCENATPKRTIRDCFNYNEDSPTQPTLPKRGLFLKEETFKNDLKGNGGKRQMVDLKPEMSRSTPSLVDPPDRS
KLCLVLQSSYPNSPSAASQSYECLHKVGNGNLENTVKFHIKEISSSLGRLNDCYKEKSRLKKPHKTSEEVPPCRTPKRGTGSGKQAK
NTKSSAVPNGELSYTSKAIEGPQTNSASTSSLEPCNQRSWNAKLQLQSETSSSPAFTQSSESSVGSDNIMSPVPLLSKHKSKKGQAS
SPSHVTRNGEVVEAWYGSDEYLALPSHLKQTEVLALKLENLTKLLPQKPRGETIQNIDDWELSEMNSDSEIYPTYHVKKKHTRLGRV
SPSSSSDIASSLGESIESGPLSDILSDEESSMPLAGMKKYADEKSERASSSEKNESHSATKSALIQKLMQDIQHQDNYEAIWEKIEG
FVNKLDEFIQWLNEAMETTENWTPPKAEMDDLKLYLETHLSFKLNVDSHCALKEAVEEEGHQLLELIASHKAGLKDMLRMIASQWKE
LQRQIKRQHSWILRALDTIKAEILATDVSVEDEEGTGSPKAEVQLCYLEAQRDAVEQMSLKLYSEQYTSSSKRKEEFADMSKVHSVG
SNGLLDFDSEYQELWDWLIDMESLVMDSHDLMMSEEQQQHLYKRYSVEMSIRHLKKTELLSKVEALKKGGVLLPNDLLEKVDSINEK
WELLGKTLGEKIQDTMAGHSGSSPRDLLSPESGSLVRQLEVRIKELKGWLRDTELFIFNSCLRQEKEGTMNTEKQLQYFKSLCREIK
QRRRGVASILRLCQHLLDDRETCNLNADHQPMQLIIVNLERRWEAIVMQAVQWQTRLQKKMGKESETLNVIDPGLMDLNGMSEDALE
WDEMDISNKLISLNEESNDLDQELQPVIPSLKLGETSNEDPGYDEEADNHGGSQYASNITAPSSPHIYQVYSLHNVELYEDNHMPFL
KNNPKVTGMTQPNVLTKSLSKDSSFSSTKSLPDLLGGSNLVKPCACHGGDMSQNSGSESGIVSEGDTETTTNSEMCLLNAVDGSPSN
LETEHLDPQMGDAVNVLKQKFTDEGESIKLPNSSQSSISPVGCVNGKVGDLNSITKHTPDCLGEELQGKHDVFTFYDYSYLQGSKLK
LPMIMKQSQSEKAHVEDPLLRGFYFDKKSCKSKHQTTELQPDVPPHERILASASHEMDRISYKSGNIEKTFTGMQNAKQLSLLSHSS
SIESLSPGGDLFGLGIFKNGSDSLQRSTSLESWLTSYKSNEDLFSCHSSGDISVSSGSVGELSKRTLDLLNRLENIQSPSEQKIKRS
VSDITLQSSSQKMSFTGQMSLDIASSINEDSAASLTELSSSDELSLCSEDIVLHKNKIPESNASFRKRLTRSVADESDVNVSMIVNV
SCTSACTDDEDDSDLLSSSTLTLTEEELCIKDEDDDSSIATDDEIYEDCTLMSGLDYIKNELQTWIRPKLSLTRDKKRCNVSDEMKG
SKDISSSEMTNPSDTLNIETLLNGSVKRVSENNGNGKNSSHTHELGTKRENKKTIFKVNKDPYVADMENGNIEGIPERQKGKPNVTS
KVSENLGSHGKEISESEHCKCKALMDSLDDSNTAGKEFVSQDVRHLPKKCPNHHHFENQSTASTPTEKSFSELALETRFNNRQDSDA
LKSSDDAPSMAGKSAGCCLALEQNGTEENASISNISCCNCEPDVFHQKDAEDCSVHNFVKEIIDMASTALKSKSQPENEVAAPTSLT
QIKEKVLEHSHRPIQLRKGDFYSYLSLSSHDSDCGEVTNYIEEKSSTPLPLDTTDSGLDDKEDIECFFEACVEGDSDGEEPCFSSAP
PNESAVPSEAAMPLQATACSSEFSDSSLSADDADTVALSSPSSQERAEVGKEVNGLPQTSSGCAENLEFTPSKLDSEKESSGKPGES
GMPEEHNAASAKSKVQDLSLKANQPTDKAALHPSPKTLTCEENLLNLHEKRHRNMHR
>sp|Q9H1Y0|ATG5_HUMAN
MTDDKDVLRDVWFGRIPTCFTLYQDEITEREAEPYYLLLPRVSYLTLVTDKVKKHFQKVMRQEDISEIWFEYEGTPLKWHYPIGLLF
DLLASSSALPWNITVHFKSFPEKDLLHCPSKDAIEAHFMSCMKEADALKHKSQVINEMQKKDHKQLWMGLQNDRFDQFWAINRKLME
YPAEENGFRYIPFRIYQTTTERPFIQKLFRPVAADGQLHTLGDLLKEVCPSAIDPEDGEKKNQVMIHGIEPMLETPLQWLSEHLSYP
DNFLHISIIPQPTD
>sp|P06401|PRGR_HUMAN
MTELKAKGPRAPHVAGGPPSPEVGSPLLCRPAAGPFPGSQTSDTLPEVSAIPISLDGLLFPRPCQGQDPSDEKTQDQQSLSDVEGAY
SRAEATRGAGGSSSSPPEKDSGLLDSVLDTLLAPSGPGQSQPSPPACEVTSSWCLFGPELPEDPPAAPATQRVLSPLMSRSGCKVGD
SSGTAAAHKVLPRGLSPARQLLLPASESPHWSGAPVKPSPQAAAVEVEEEDGSESEESAGPLLKGKPRALGGAAAGGGAAAVPPGAA

AGGVALVPKEDSRFSAPRVALVEQDAPMAPGRSPLATTVMDFIHVPILPLNHALLAARTRQLLEDESYDGGAGAASAFAPPRSSPCA
SSTPVAVGDFPDCAYPPDAEPKDDAYPLYSDFQPPALKIKEEEEGAEASARSPRSYLVAGANPAAFPDFPLGPPPPLPPRATPSRPG
EAAVTAAPASASVSSASSSGSTLECILYKAEGAPPQQGPFAPPPCKAPGASGCLLPRDGLPSTSASAAAAGAAPALYPALGLNGLPQ
LGYQAAVLKEGLPQVYPPYLNYLRPDSEASQSPQYSFESLPQKICLICGDEASGCHYGVLTCGSCKVFFKRAMEGQHNYLCAGRNDC
IVDKIRRKNCPACRLRKCCQAGMVLGGRKFKKFNKVRVVRALDAVALPQPVGVPNESQALSQRFTFSPGQDIQLIPPLINLLMSIEP
DVIYAGHDNTKPDTSSSLLTSLNQLGERQLLSVVKWSKSLPGFRNLHIDDQITLIQYSWMSLMVFGLGWRSYKHVSGQMLYFAPDLI
LNEQRMKESSFYSLCLTMWQIPQEFVKLQVSQEEFLCMKVLLLLNTIPLEGLRSQTQFEEMRSSYIRELIKAIGLRQKGVVSSSQRF
YQLTKLLDNLHDLVKQLHLYCLNTFIQSRALSVEFPEMMSEVIAAQLPKILAGMVKPLLFHKK
>sp|Q86VW2|ARHGP_HUMAN
MRGGHKGGRCACPRVIRKVLAKCGCCFARGGRESYSIAGSEGSISASAASGLAAPSGPSSGLSSGPCSPGPPGPVSGLRRWLDHSKH
CLSVETEADSGQAGPYENWMLEPALATGEELPELTLLTTLLEGPGDKTQPPEEETLSQAPESEEEQKKKALERSMYVLSELVETEKM
YVDDLGQIVEGYMATMAAQGVPESLRGRDRIVFGNIQQIYEWHRDYFLQELQRCLKDPDWLAQLFIKHERRLHMYVVYCQNKPKSEH
VVSEFGDSYFEELRQQLGHRLQLNDLLIKPVQRIMKYQLLLKDFLKYYNRAGMDTADLEQAVEVMCFVPKRCNDMMTLGRLRGFEGK
LTAQGKLLGQDTFWVTEPEAGGLLSSRGRERRVFLFEQIIIFSEALGGGVRGGTQPGYVYKNSIKVSCLGLEGNLQGDPCRFALTSR
GPEGGIQRYVLQAADPAISQAWIKHVAQILESQRDFLNALQSPIEYQRRESQTNSLGRPRGPGVGSPGRIQLGDQAQGSTHTPINGS
LPSLLLSPKGEVARALLPLDKQALGDIPQAPHDSPPVSPTPKTPPCQARLAKLDEDEL
>sp|P31270|HXA11_HUMAN
MDFDERGPCSSNMYLPSCTYYVSGPDFSSLPSFLPQTPSSRPMTYSYSSNLPQVQPVREVTFREYAIEPATKWHPRGNLAHCYSAEE
LVHRDCLQAPSAAGVPGDVLAKSSANVYHHPTPAVSSNFYSTVGRNGVLPQAFDQFFETAYGTPENLASSDYPGDKSAEKGPPAATA
TSAAAAAAATGAPATSSSDSGGGGGCRETAAAAEEKERRRRPESSSSPESSSGHTEDKAGGSSGQRTRKKRCPYTKYQIRELEREFF
FSVYINKEKRLQLSRMLNLTDRQVKIWFQNRRMKEKKINRDRLQYYSANPLL
>sp|Q8TB52|FBX30_HUMAN
MEEELQHSHCVNCVSRRCMTRPEPGISCDLIGCPLVCGAVFHSCKADEHRLLCPFERVPCLNSDFGCPFTMARNKVAEHLEMCPASV
VCCTMEWNRWPVSYADRKSYENLSRDVDEVAQLDMALALQDQRMLLESLKVATMMSKATDKVSKPREQISVKSSVPEIPHANGLVSV
DEESYGALYQATVETTRSLAAALDILNTATRDIGMLNTSVPNDMDEQQNARESLEDQNLKDQDHLYEEEIGAVGGIDYNDTNQNAQS
EQNGSSDLLCDLNTSSYDTSALCNGFPLENICTQVIDQNQNLHGDSKQSNLTNGDCVASSDGTSKPSSSLAVAAQLREIIPSSALPN
GTVQHILMPDDEGEGELCWKKVDLGDVKNVDVLSFSHAPSFNFLSNSCWSKPKEDKAVDTSDLEVAEDPMGLQGIDLITAALLFCLG
DSPGGRGISDSRMADIYHIDVGTQTFSLPSAILATSTMVGEIASASACDHANPQLSNPSPFQTLGLDLVLECVARYQPKQRSMFTFV
CGQLFRRKEFSSHFKNVHGDIHAGLNGWMEQRCPLAYYGCTYSQRRFCPSIQGAKIIHDRHLRSFGVQPCVSTVLVEPARNCVLGLH
NDHLSSLPFEVLQHIAGFLDGFSLCQLSCVSKLMRDVCGSLLQSRGMVILQWGKRKYPEGNSSWQIKEKVWRFSTAFCSVNEWKFAD
ILSMADHLKKCSYNVVEKREEAIPLPCMCVTRELTKEGRSLRSVLKPVL
>sp|P51553|IDH3G_HUMAN
MALKVATVAGSAAKAVLGPALLCRPWEVLGAHEVPSRNIFSEQTIPPSAKYGGRHTVTMIPGDGIGPELMLHVKSVFRHACVPVDFE
EVHVSSNADEEDIRNAIMAIRRNRVALKGNIETNHNLPPSHKSRNNILRTSLDLYANVIHCKSLPGVVTRHKDIDILIVRENTEGEY
SSLEHESVAGVVESLKIITKAKSLRIAEYAFKLAQESGRKKVTAVHKANIMKLGDGLFLQCCREVAARYPQITFENMIVDNTTMQLV
SRPQQFDVMVMPNLYGNIVNNVCAGLVGGPGLVAGANYGHVYAVFETATRNTGKSIANKNIANPTATLLASCMMLDHLKLHSYATSI
RKAVLASMDNENMHTPDIGGQGTTSEAIQDVIRHIRVINGRAVEA
>sp|Q9H008|LHPP_HUMAN
MAPWGKRLAGVRGVLLDISGVLYDSGAGGGTAIAGSVEAVARLKRSRLKVRFCTNESQKSRAELVGQLQRLGFDISEQEVTAPAPAA
CQILKEQGLRPYLLIHDGVRSEFDQIDTSNPNCVVIADAGESFSYQNMNNAFQVLMELEKPVLISLGKGRYYKETSGLMLDVGPYMK
ALEYACGIKAEVVGKPSPEFFKSALQAIGVEAHQAVMIGDDIVGDVGGAQRCGMRALQVRTGKFRPSDEHHPEVKADGYVDNLAEAV
DLLLQHADK
>sp|Q15052|ARHG6_HUMAN

MNPEEQIVTWLISLGVLESPKKTICDPEEFLKSSLKNGVVLCKLINRLMPGSVEKFCLDPQTEADCINNINDFLKGCATLQVEIFDP
DDLYSGVNFSKVLSTLLAVNKATEDQLSERPCGRSSSLSAANTSQTNPQGAVSSTVSGLQRQSKTVEMTENGSHQLIVKARFNFKQT
NEDELSVCKGDIIYVTRVEEGGWWEGTLNGRTGWFPSNYVREIKSSERPLSPKAVKGFETAPLTKNYYTVVLQNILDTEKEYAKELQ
SLLVTYLRPLQSNNNLSTVEVTSLLGNFEEVCTFQQTLCQALEECSKFPENQHKVGGCLLSLMPHFKSMYLAYCANHPSAVNVLTQH
SDELEQFMENQGASSPGILILTTNLSKPFMRLEKYVTLLQELERHMEDTHPDHQDILKAIVAFKTLMGQCQDLRKRKQLELQILSEP
IQAWEGEDIKNLGNVIFMSQVMVQYGACEEKEERYLMLFSNVLIMLSASPRMSGFIYQGKIPIAGTVVTRLDEIEGNDCTFEITGNT
VERIVVHCNNNQDFQEWLEQLNRLIRGPASCSSLSKTSSSSCSAHSSFSSTGQPRGPLEPPQIIKPWSLSCLRPAPPLRPSAALGYK
ERMSYILKESSKSPKTMKKFLHKRKTERKPSEEEYVIRKSTAALEEDAQILKVIEAYCTSANFQQGHGSSTRKDSIPQVLLPEEEKL
IIEETRSNGQTIMEEKSLVDTVYALKDEVRELKQENKRMKQCLEEELKSRRDLEKLVRRLLKQTDECIRGESSSKTSILP
>sp|P04629|NTRK1_HUMAN
MLRGGRRGQLGWHSWAAGPGSLLAWLILASAGAAPCPDACCPHGSSGLRCTRDGALDSLHHLPGAENLTELYIENQQHLQHLELRDL
RGLGELRNLTIVKSGLRFVAPDAFHFTPRLSRLNLSFNALESLSWKTVQGLSLQELVLSGNPLHCSCALRWLQRWEEEGLGGVPEQK
LQCHGQGPLAHMPNASCGVPTLKVQVPNASVDVGDDVLLRCQVEGRGLEQAGWILTELEQSATVMKSGGLPSLGLTLANVTSDLNRK
NVTCWAENDVGRAEVSVQVNVSFPASVQLHTAVEMHHWCIPFSVDGQPAPSLRWLFNGSVLNETSFIFTEFLEPAANETVRHGCLRL
NQPTHVNNGNYTLLAANPFGQASASIMAAFMDNPFEFNPEDPIPVSFSPVDTNSTSGDPVEKKDETPFGVSVAVGLAVFACLFLSTL
LLVLNKCGRRNKFGINRPAVLAPEDGLAMSLHFMTLGGSSLSPTEGKGSGLQGHIIENPQYFSDACVHHIKRRDIVLKWELGEGAFG
KVFLAECHNLLPEQDKMLVAVKALKEASESARQDFQREAELLTMLQHQHIVRFFGVCTEGRPLLMVFEYMRHGDLNRFLRSHGPDAK
LLAGGEDVAPGPLGLGQLLAVASQVAAGMVYLAGLHFVHRDLATRNCLVGQGLVVKIGDFGMSRDIYSTDYYRVGGRTMLPIRWMPP
ESILYRKFTTESDVWSFGVVLWEIFTYGKQPWYQLSNTEAIDCITQGRELERPRACPPEVYAIMRGCWQREPQQRHSIKDVHARLQA
LAQAPPVYLDVLG
>sp|P47972|NPTX2_HUMAN
MLALLAASVALAVAAGAQDSPAPGSRFVCTALPPEAVHAGCPLPAMPMQGGAQSPEEELRAAVLQLRETVVQQKETLGAQREAIREL
TGKLARCEGLAGGKARGAGATGKDTMGDLPRDPGHVVEQLSRSLQTLKDRLESLEHQLRANVSNAGLPGDFREVLQQRLGELERQLL
RKVAELEDEKSLLHNETSAHRQKTESTLNALLQRVTELERGNSAFKSPDAFKVSLPLRTNYLYGKIKKTLPELYAFTICLWLRSSAS
PGIGTPFSYAVPGQANEIVLIEWGNNPIELLINDKVAQLPLFVSDGKWHHICVTWTTRDGMWEAFQDGEKLGTGENLAPWHPIKPGG
VLILGQEQDTVGGRFDATQAFVGELSQFNIWDRVLRAQEIVNIANCSTNMPGNIIPWVDNNVDVFGGASKWPVETCEERLLDL
>sp|Q9Y385|UB2J1_HUMAN
METRYNLKSPAVKRLMKEAAELKDPTDHYHAQPLEDNLFEWHFTVRGPPDSDFDGGVYHGRIVLPPEYPMKPPSIILLTANGRFEVG
KKICLSISGHHPETWQPSWSIRTALLAIIGFMPTKGEGAIGSLDYTPEERRALAKKSQDFCCEGCGSAMKDVLLPLKSGSDSSQADQ
EAKELARQISFKAEVNSSGKTISESDLNHSFSLTDLQDDIPTTFQGATASTSYGLQNSSAASFHQPTQPVAKNTSMSPRQRRAQQQS
QRRLSTSPDVIQGHQPRDNHTDHGGSAVLIVILTLALAALIFRRIYLANEYIFDFEL
>sp|A6NKX1|F223B_HUMAN
MYVQEHRLWGLMDKPHSAPCLMSLSLSFLICNKGRNAIRVQQSTDERMDAMLLRQCPTQGTRKNHESNSSLHHVPNWIFHSTIIPPN
KGSKRCLRKVDWLLPRAGGVGGKRGVTADGDRVSF
>sp|Q15382|RHEB_HUMAN
MPQSKSRKIAILGYRSVGKSSLTIQFVEGQFVDSYDPTIENTFTKLITVNGQEYHLQLVDTAGQDEYSIFPQTYSIDINGYILVYSV
TSIKSFEVIKVIHGKLLDMVGKVQIPIMLVGNKKDLHMERVISYEEGKALAESWNAAFLESSAKENQTAVDVFRRIILEAEKMDGAA
SQGKSSCSVM
>sp|Q49AH0|CDNF_HUMAN
MWCASPVAVVAFCAGLLVSHPVLTQGQEAGGRPGADCEVCKEFLNRFYKSLIDRGVNFSLDTIEKELISFCLDTKGKENRLCYYLGA
TKDAATKILSEVTRPMSVHMPAMKICEKLKKLDSQICELKYEKTLDLASVDLRKMRVAELKQILHSWGEECRACAEKTDYVNLIQEL
APKYAATHPKTEL
>sp|O60493|SNX3_HUMAN

MAETVADTRRLITKPQNLNDAYGPPSNFLEIDVSNPQTVGVGRGRFTTYEIRVKTNLPIFKLKESTVRRRYSDFEWLRSELERESKV
VVPPLPGKAFLRQLPFRGDDGIFDDNFIEERKQGLEQFINKVAGHPLAQNERCLHMFLQDEIIDKSYTPSKIRHA
>sp|P19099|C11B2_HUMAN
MALRAKAEVCVAAPWLSLQRARALGTRAARAPRTVLPFEAMPQHPGNRWLRLLQIWREQGYEHLHLEMHQTFQELGPIFRYNLGGPR
MVCVMLPEDVEKLQQVDSLHPCRMILEPWVAYRQHRGHKCGVFLLNGPEWRFNRLRLNPDVLSPKAVQRFLPMVDAVARDFSQALKK
KVLQNARGSLTLDVQPSIFHYTIEASNLALFGERLGLVGHSPSSASLNFLHALEVMFKSTVQLMFMPRSLSRWISPKVWKEHFEAWD
CIFQYGDNCIQKIYQELAFNRPQHYTGIVAELLLKAELSLEAIKANSMELTAGSVDTTAFPLLMTLFELARNPDVQQILRQESLAAA
ASISEHPQKATTELPLLRAALKETLRLYPVGLFLERVVSSDLVLQNYHIPAGTLVQVFLYSLGRNAALFPRPERYNPQRWLDIRGSG
RNFHHVPFGFGMRQCLGRRLAEAEMLLLLHHVLKHFLVETLTQEDIKMVYSFILRPGTSPLLTFRAIN
>sp|Q6PL45|BRID5_HUMAN
MEPASCCAERPKPGPTGVKTKPSCGGWRAVSLLLLLLLLLVLAAVGVVAGGLLGSAQGPPKPRLQTLRMTLPSPHMPRPNQTILVDVA
RNAATITVTPPQSNHSWAVLFDGQSGCICYRPEEHQVCFLRLMEDSDRETLRLLVDTSKVQEAWVPSQDTHHTQELLAVQGSLEVDP
AQAGALVQRLCMRTPIYWARRAEGESGPLWGKARPSGWFEELGAEPLEIHGTLATGPRRQRLIYLCIDICFPSNICVSVCFYYLPD
>sp|Q6ZU35|K1211_HUMAN
MGTRAFSHDSIFIPDGGAESEQTVQAMSQDNILGKVKTLQQQLGKNIKFGQRSPNAIPMNKANSGEASLEEDLFLTSPMEIVTQQDI
VLSDAENKSSDTPSSLSPLNLPGAGSEMEEKVAPVKPSRPKRHFSSAGTIESVNLDAIPLAIARLDNSAAKHKLAVKPKKQRVSKKH
RRLAQDPQHEQGGLESRPCLDQNGHPGEDKPTWHEEEPNPLDSEEERRRQEDYWRELEAKCKRQKAEAAEKRRLEEQRLQALERRLW
EENRRQELLEEEGEGQEPPLEAERAPREEQQRSLEAPGWEDAERREREERERLEAEEERRRLQAQAQAEERRRLEEDARLEERRRQE
EEEGRCAEELKRQEEEEAEGWEELEQQEAEVQGPPEALEETGEGRRGAEEEDLGEEEEEGQAHLEDWRGQLSELLNDFEERLEDQER
LKPEGQREHSEEPGICEEQNPEAERRREQQGRSGDFQGADRPGPEEKREEGDTEPLLKQEGPVEAAQPPVERKEAAALEQGRKVEEL
RWQEVDERQTMPRPYTFQVSSGGKQILFPKVNLSPVTPAKDTGLTAAPQEPKAPKASPVQHALPSSLSVPHTAILVTGAQLCGPAVN
LSQIKDTACKSLLGLEEKKHAEAPAGENPPRGPGDARAGSGKAKPRQESPSSASALAEWASIRSRILKNAESDPRSSERDQLRPGDE
STPRGRCDSRGNQRKTPPVNAKFSIMPAWQKFSDGGTETSKQSTEAESIRKRPMLGPSEETAPQPPPAGVRELGKGPEKSEMHREPA
DTTEGCKFAKDLPSFLVPSLPYPPQKVVAHTEFTTSSDSETANGIAKPDPVMPGGEEKASPFGIKLRRTNYSLRFNCDQQAEQKKKK
RHSSTGDSADAGPPAAGSARGEKEMEGVALKHGPSLPQERKQAPSTRRDSAEPSSSRSVPVAHPGPPPASSQTPAPEHDKAANKMPL
AQKPALAPKPTSQTPPASPLSKLSRPYLVELLSRRAGRPDPEPSEPSKEDQESSDRRPPSPPGPEERKGQKRDEEEEATERKPASPP
LPATQQEKPSQTPEAGRKEKPMLQSRHSLDGSKLTEKVETAQPLWITLALQKQKGFREQQATREERKQAREAKQAEKLSKENVSVSV
QPGSSSVSRAGSLHKSTALPEEKRPETAVSRLERREQLKKANTLPTSVTVEISDSAPPAPLVKEVTKRFSTPDAAPVSTEPAWLALA
KRKAKAWSDCPQIIK
>sp|Q9BQI9|NRIP2_HUMAN
MLFIFPLSLPWRPSCWKESCSTGQRQAGRSREDSVTPPPSSPWPTPPAGAMSTKQEARRDEGEARTRGQEAQLRDRAHLSQQRRLKQ
ATQFLHKDSADLLPLDSLKRLGTSKDLQPRSVIQRRLVEGNPNWLQGEPPRMQDLIHGQESRRKTSRTEIPALLVNCKCQDQLLRVA
VDTGTQYNRISAGCLSRLGLEKRVLKASAGDLAPGPPTQVEQLELQLGQETVVCSAQVVDAESPEFCLGLQTLLSLKCCIDLEHGVL
RLKAPFSELPFLPLYQEPGQ
>sp|Q9P1G2|RBAS1_HUMAN
MAQDFSQHPQTGIIRRHRFYSPKPLSTTPRGQSFLLSRQAKVATWDPMLSPDFQPKSAFKLTWTAQPPCLSVTPSQGQTFHPNSPEG
ELPPDLTENTCPSQA
>sp|Q8NDH6|ICA1L_HUMAN
MDSFGQPRPEDNQSVVRRMQKKYWKTKQVFIKATGKKEDEHLVASDAELDAKLEVFHSVQETCTELLKIIEKYQLRLNVISEEENEL
GLFLKFQAERDATQAGKMMDATGKALCSSAKQRLALCTPLSRLKQEVATFSQRAVSDTLMTINRMEQARTEYRGALLWMKDVSQELD
PDTLKQMEKFRKVQMQVRNSKASFDKLKMDVCQKVDLLGASRCNMLSHSLTTYQRTLLGFWKKTARMMSQIHEACIGFHPYDFVALK
QLQDTPSKISEDNKDEQIGGFLTEQLNKLVLSDEEASFESEQANKDHNEKHSQMREFGAPQFSNSENVAKDLPVDSLEGEDFEKEFS
FLNNLLSSGSSSTSEFTQECQTAFGSPSASLTSQEPSMGSEPLAHSSRFLPSQLFDLGFHVAGAFNNWVSQEESELCLSHTDNQPVP
SQSPKKLTRSPNNGNQDMSAWFNLFADLDPLSNPDAIGHSDDELLNA

>sp|A6NJZ3|O6C65_HUMAN
MPNMTSIREFILLGFTDNPELQVVIFFFMLITYLLSVSGNMIIIMLTLSNIHLKTPMYFFLRNFSFLEISFTTVFIPRFLINIATGD
TTISYNASMAQVFFLILLGSTEFFLLAVMSYDRYVAICKPLHYTTIMSNKVCNWLVISSWLAGFLIIFPPVIMGLQLDFCDSSTIDH
FICDSSPMLLIACTDTQFLELMAFLLAVFTLMVTLALVVLSYTLILKTILKIPSAQQRKKAFSTCSSHMIVVSVSYGSCIFMCVKTS
AKEGMALSKGVAVLNTSVAPMLNPFIYTLRNQQVKQALREFTKKILSLNKQ
>sp|Q6ZT83|YR005_HUMAN
MGALNSISLETNKSSFSMSWTPSVEHSSGTARTLRSCKLALVGFSSMSEFLFRASKTQPVTLFVPNVNSALCDQPVVRGVGIHEQRK
RNISSLISVFNHWLPPLQLHLGRALICRFNSRGNFTCLMTAVK
>sp|Q8WTV0|SCRB1_HUMAN
MGCSAKARWAAGALGVAGLLCAVLGAVMIVMVPSLIKQQVLKNVRIDPSSLSFNMWKEIPIPFYLSVYFFDVMNPSEILKGEKPQVR
ERGPYVYREFRHKSNITFNNNDTVSFLEYRTFQFQPSKSHGSESDYIVMPNILVLGAAVMMENKPMTLKLIMTLAFTTLGERAFMNR
TVGEIMWGYKDPLVNLINKYFPGMFPFKDKFGLFAELNNSDSGLFTVFTGVQNISRIHLVDKWNGLSKVDFWHSDQCNMINGTSGQM
WPPFMTPESSLEFYSPEACRSMKLMYKESGVFEGIPTYRFVAPKTLFANGSIYPPNEGFCPCLESGIQNVSTCRFSAPLFLSHPHFL
NADPVLAEAVTGLHPNQEAHSLFLDIHPVTGIPMNCSVKLQLSLYMKSVAGIGQTGKIEPVVLPLLWFAESGAMEGETLHTFYTQLV
LMPKVMHYAQYVLLALGCVLLLVPVICQIRSQVGAGQRAARADSHSLACWGKGASDRTLWPTAAWSPPPAAVLRLCRSGSGHCWGLR
STLASFACRVATTLPVLEGLGPSLGGGTGS
>sp|Q8IZR5|CKLF4_HUMAN
MRSGEELDGFEGEASSTSMISGASSPYQPTTEPVSQRRGLAGLRCDPDYLRGALGRLKVAQVILALIAFICIETIMACSPCEGLYFF
EFVSCSAFVVTGVLLIMFSLNLHMRIPQINWNLTDLVNTGLSAFLFFIASIVLAALNHRAGAEIAAVIFGFLATAAYAVNTFLAVQK
WRVSVRQQSTNDYIRARTESRDVDSRPEIQRLDTFSYSTNVTVRKKSPTNLLSLNHWQLA
>sp|Q05048|CSTF1_HUMAN
MYRTKVGLKDRQQLYKLIISQLLYDGYISIANGLINEIKPQSVCAPSEQLLHLIKLGMENDDTAVQYAIGRSDTVAPGTGIDLEFDA
DVQTMSPEASEYETCYVTSHKGPCRVATYSRDGQLIATGSADASIKILDTERMLAKSAMPIEVMMNETAQQNMENHPVIRTLYDHVD
EVTCLAFHPTEQILASGSRDYTLKLFDYSKPSAKRAFKYIQEAEMLRSISFHPSGDFILVGTQHPTLRLYDINTFQCFVSCNPQDQH
TDAICSVNYNSSANMYVTGSKDGCIKLWDGVSNRCITTFEKAHDGAEVCSAIFSKNSKYILSSGKDSVAKLWEISTGRTLVRYTGAG
LSGRQVHRTQAVFNHTEDYVLLPDERTISLCCWDSRTAERRNLLSLGHNNIVRCIVHSPTNPGFMTCSDDFRARFWYRRSTTD
>sp|Q9NVR7|TBCC1_HUMAN
MDQSRVLLWVKAEPFIVGALQVPPPSKFSLHYLRKISTYVQIRATEGAYPRLYWSTWRHIACGKLQLAKDLAWLYFEIFDSLSMKTP
EERLEWSEVLSNCMSEEEVEKQRNQLSVDTLQFLLFLYIQQLNKVSLRTSLIGEEWPSPRNKSQSPDLTEKSNCHNKNWNDYSHQAF
VYDHLSDLLELLLDPKQLTASFHSTHSSLVSREAVVALSFLIEGTISRARKIYPLHELALWQPLHADSGFSKISKTFSFYKLETWLR
SCLTGNPFGTSACLKSGKKLAWAHQVEGTTKRAKIACNTHVAPRMHRLVVMSQVYKQTLAKSSDTLAGAHVKIHRCNESFIYLLSPL
RSVTIEKCRNSIFVLGPVGTTLHLHSCDNVKVIAVCHRLSISSTTGCIFHVLTPTRPLILSGNQTVTFAPFHTHYPMLEDHMARTGL
ATVPNYWDNPMVVCRENSDTRVFQLLPPCEFYVFIIPFEMEGDTTEIPGGLPSVYQKALGQREQKIQIWQKTVKEAHLTKDQRKQFQ
VLVENKFYEWLINTGHRQQLDSLVPPAAGSKQAAG
>sp|P55851|UCP2_HUMAN
MVGFKATDVPPTATVKFLGAGTAACIADLITFPLDTAKVRLQIQGESQGPVRATASAQYRGVMGTILTMVRTEGPRSLYNGLVAGLQ
RQMSFASVRIGLYDSVKQFYTKGSEHASIGSRLLAGSTTGALAVAVAQPTDVVKVRFQAQARAGGGRRYQSTVNAYKTIAREEGFRG
LWKGTSPNVARNAIVNCAELVTYDLIKDALLKANLMTDDLPCHFTSAFGAGFCTTVIASPVDVVKTRYMNSALGQYSSAGHCALTML
QKEGPRAFYKGFMPSFLRLGSWNVVMFVTYEQLKRALMAACTSREAPF
>sp|O75061|AUXI_HUMAN
MKDSENKGASSPDMEPSYGGGLFDMVKGGAGRLFSNLKDNLKDTLKDTSSRVIQSVTSYTKGDLDFTYVTSRIIVMSFPLDNVDIGF
RNQVDDIRSFLDSRHLDHYTVYNLSPKSYRTAKFHSRVSECSWPIRQAPSLHNLFAVCRNMYNWLLQNPKNVCVVHCLDGRAASSIL
VGAMFIFCNLYSTPGPAIRLLYAKRPGIGLSPSHRRYLGYMCDLLADKPYRPHFKPLTIKSITVSPIPFFNKQRNGCRPYCDVLIGE
TKIYSTCTDFERMKEYRVQDGKIFIPLNITVQGDVVVSMYHLRSTIGSRLQAKVTNTQIFQLQFHTGFIPLDTTVLKFTKPELDACD

VPEKYPQLFQVTLDVELQPHDKVIDLTPPWEHYCTKDVNPSILFSSHQEHQDTLALGGQAPIDIPPDNPRHYGQSGFFASLCWQDQK
SEKSFCEEDHAALVNQESEQSDDELLTLSSPHGNANGDKPHGVKKPSKKQQEPAAPPPPEDVDLLGLEGSAMSNSFSPPAAPPTNSE
LLSDLFGGGGAAGPTQAGQSGVEDVFHPSGPASTQSTPRRSATSTSASPTLRVGEGATFDPFGAPSKPSGQDLLGSFLNTSSASSDP
FLQPTRSPSPTVHASSTPAVNIQPDVSGGWDWHAKPGGFGMGSKSAATSPTGSSHGTPTHQSKPQTLDPFADLGTLGSSSFASKPTT
PTGLGGGFPPLSSPQKASPQPMGGGWQQGGAYNWQQPQPKPQPSMPHSSPQNRPNYNVSFSAMPGGQNERGKGSSNLEGKQKAADFE
DLLSGQGFNAHKDKKGPRTIAEMRKEEMAKEMDPEKLKILEWIEGKERNIRALLSTMHTVLWAGETKWKPVGMADLVTPEQVKKVYR
KAVLVVHPDKATGQPYEQYAKMIFMELNDAWSEFENQGQKPLY
>sp|P52954|LBX1_HUMAN
MTSKEDGKAAPGEERRRSPLDHLPPPANSNKPLTPFSIEDILNKPSVRRSYSLCGAAHLLAAADKHAQGGLPLAGRALLSQTSPLCA
LEELASKTFKGLEVSVLQAAEGRDGMTIFGQRQTPKKRRKSRTAFTNHQIYELEKRFLYQKYLSPADRDQIAQQLGLTNAQVITWFQ
NRRAKLKRDLEEMKADVESAKKLGPSGQMDIVALAELEQNSEATAGGGGGCGRAKSRPGSPVLPPGAPKAPGAGALQLSPASPLTDQ
PASSQDCSEDEEDEEIDVDD
>sp|Q8IVF7|FMNL3_HUMAN
MGNLESAEGVPGEPPSVPLLLPPGKMPMPEPCELEERFALVLSSMNLPPDKARLLRQYDNEKKWDLICDQERFQVKNPPHTYIQKLQ
SFLDPSVTRKKFRRRVQESTKVLRELEISLRTNHIGWVREFLNDENKGLDVLVDYLSFAQCSVMFDFEGLESGDDGAFDKLRSWSRS
IEDLQPPSALSAPFTNSLARSARQSVLRYSTLPGRRALKNSRLVSQKDDVHVCILCLRAIMNYQYGFNLVMSHPHAVNEIALSLNNK
NPRTKALVLELLAAVCLVRGGHEIILAAFDNFKEVCKELHRFEKLMEYFRNEDSNIDFMVACMQFINIVVHSVEDMNFRVHLQYEFT
KLGLEEFLQKSRHTESEKLQVQIQAYLDNVFDVGGLLEDAETKNVALEKVEELEEHVSHLTEKLLDLENENMMRVAELEKQLLQREK
ELESIKETYENTSHQVHTLRRLIKEKEEAFQRRCHLEPNVRGLESVDSEALARVGPAELSEGMPPSDLDLLAPAPPPEEVLPLPPPP
APPLPPPPPPLPDKCPPAPPLPGAAPSVVLTVGLSAIRIKKPIKTKFRLPVFNWTALKPNQISGTVFSELDDEKILEDLDLDKFEEL
FKTKAQGPALDLICSKNKTAQKAASKVTLLEANRAKNLAITLRKAGRSAEEICRAIHTFDLQTLPVDFVECLMRFLPTEAEVKLLRQ
YERERQPLEELAAEDRFMLLFSKVERLTQRMAGMAFLGNFQDNLQMLTPQLNAIIAASASVKSSQKLKQMLEIILALGNYMNSSKRG
AVYGFKLQSLDLLLDTKSTDRKMTLLHFIALTVKEKYPDLANFWHELHFVEKAAAVSLENVLLDVKELGRGMELIRRECSIHDNSVL
RNFLSTNEGKLDKLQRDAKTAEEAYNAVVRYFGESPKTTPPSVFFPVFVRFIRSYKEAEQENEARKKQEEVMREKQLAQEAKKLDAK
TPSQRNKWQQQELIAELRRRQAKEHRPVYEGKDGTIEDIITVLKSVPFTARTAKRGSRFFCDAAHHDESNC
>sp|A6NJJ6|CS067_HUMAN
MATEQWFEGSLPLDPGETPPPDALEPGTPPCGDPSRSTPPGRPGNPSEPDPEDAEGRLAEARASTSSPKPLVPRPGPAPPRLSLDTL
FSPITQQLRYLLKKADDFQSYLLYSRDQVQKEQLAKAMPTFLQMCEPYFLYLEAAARSIPPIYGPLQELVRKGLLEISQQLTLRLEQ
LVLMYASFGFVDLEEMNPLSISCFFCGRFSISLSHEVSIFRYCAPTAYTASRFPRYLYKKMRWHLEATPEAPGRGQDSLVDYYFLCY
RDTWEDTGQSPANSCPQIQKLWSIGRWVPLGPAEDDLYSWILCPQPLGDYQQLLTIGFEEPTPTLATDLLVQILTGQAGQARPPSAA
GPAGWAAQGS
>sp|Q8WWU5|TCP11_HUMAN
MPDVKESVPPKYPGDSEGRSCKPETSGPPQEDKSGSEDPPPFLSVTGLTETVNEVSKLSNKIGMNCDYYMEEKVLPPSSLEGKVKET
VHNAFWDHLKEQLSATPPDFSCALELLKEIKEILLSLLLPRQNRLRIEIEEALDMDLLKQEAEHGALKVLYLSKYVLNMMALLCAPV
RDEAVQKLENITDPVWLLRGIFQVLGRMKMDMVNYTIQSLQPHLQEHSIQYERAKFQELLNKQPSLLNHTTKWLTQAAGDLTMSPPT
CPDTSDSSSVAGPSPNEAANNPEPLSPTMVLCQGFLNLLLWDLENEEFPETLLMDRTRLQELKSQLHQLTVMASVLLVASSFSGSVL
FGSPQFVDKLKRITKSLLEDFHSRPEEAILTVSEQVSQEIHQSLKNMGLVALSSDNTASLMGQLQNIAKKENCVCSVIDQRIHLFLK
CCLVLGVQRSLLDLPGGLTLIEAELAELGQKFVNLTHHNQQVFGPYYTEILKTLISPAQALETKVESV
>sp|O00592|PODXL_HUMAN
MRCALALSALLLLLSTPPLLPSSPSPSPSPSQNATQTTTDSSNKTAPTPASSVTIMATDTAQQSTVPTSKANEILASVKATTLGVSS
DSPGTTTLAQQVSGPVNTTVARGGGSGNPTTTIESPKSTKSADTTTVATSTATAKPNTTSSQNGAEDTTNSGGKSSHSVTTDLTSTK
AEHLTTPHPTSPLSPRQPTSTHPVATPTSSGHDHLMKISSSSSTVAIPGYTFTSPGMTTTLLETVFHHVSQAGLELLTSGDLPTLAS
QSAGITASSVISQRTQQTSSQMPASSTAPSSQETVQPTSPATALRTPTLPETMSSSPTAASTTHRYPKTPSPTVAHESNWAKCEDLE
TQTQSEKQLVLNLTGNTLCAGGASDEKLISLICRAVKATFNPAQDKCGIRLASVPGSQTVVVKEITIHTKLPAKDVYERLKDKWDEL

KEAGVSDMKLGDQGPPEEAEDRFSMPLIITIVCMASFLLLVAALYGCCHQRLSQRKDQQRLTEELQTVENGYHDNPTLEVMETSSEM
QEKKVVSLNGELGDSWIVPLDNLTKDDLDEEEDTHL
>sp|Q9BV47|DUS26_HUMAN
MCPGNWLWASMTFMARFSRSSSRSPVRTRGTLEEMPTVQHPFLNVFELERLLYTGKTACNHADEVWPGLYLGDQDMANNRRELRRLG
ITHVLNASHSRWRGTPEAYEGLGIRYLGVEAHDSPAFDMSIHFQTAADFIHRALSQPGGKILVHCAVGVSRSATLVLAYLMLYHHLT
LVEAIKKVKDHRGIIPNRGFLRQLLALDRRLRQGLEA
>sp|Q16134|ETFD_HUMAN
MLVPLAKLSCLAYQCFHALKIKKNYLPLCATRWSSTSTVPRITTHYTIYPRDKDKRWEGVNMERFAEEADVVIVGAGPAGLSAAVRL
KQLAVAHEKDIRVCLVEKAAQIGAHTLSGACLDPGAFKELFPDWKEKGAPLNTPVTEDRFGILTEKYRIPVPILPGLPMNNHGNYIV
RLGHLVSWMGEQAEALGVEVYPGYAAAEVLFHDDGSVKGIATNDVGIQKDGAPKATFERGLELHAKVTIFAEGCHGHLAKQLYKKFD
LRANCEPQTYGIGLKELWVIDEKNWKPGRVDHTVGWPLDRHTYGGSFLYHLNEGEPLVALGLVVGLDYQNPYLSPFREFQRWKHHPS
IRPTLEGGKRIAYGARALNEGGFQSIPKLTFPGGLLIGCSPGFMNVPKIKGTHTAMKSGILAAESIFNQLTSENLQSKTIGLHVTEY
EDNLKNSWVWKELYSVRNIRPSCHGVLGVYGGMIYTGIFYWILRGMEPWTLKHKGSDFERLKPAKDCTPIEYPKPDGQISFDLLSSV
ALSGTNHEHDQPAHLTLRDDSIPVNRNLSIYDGPEQRFCPAGVYEFVPVEQGDGFRLQINAQNCVHCKTCDIKDPSQNINWVVPEGG
GGPAYNGM
>sp|Q9NP97|DLRB1_HUMAN
MAEVEETLKRLQSQKGVQGIIVVNTEGIPIKSTMDNPTTTQYASLMHSFILKARSTVRDIDPQNDLTFLRIRSKKNEIMVAPDKDYF
LIVIQNPTE
>sp|Q9P203|BTBD7_HUMAN
MGANASNYPHSCSPRVGGNSQAQQTFIGTSSYSQQGYGCESKLYSLDHGHEKPQDKKKRTSGLATLKKKFIKRRKSNRSADHAKQMR
ELLSGWDVRDVNALVEEYEGTSALKELSLQASLARPEARTLQKDMADLYEYKYCTDVDLIFQETCFPVHRAILAARCPFFKTLLSSS
PEYGAEIIMDINTAGIDMPMFSALLHYLYTGEFGMEDSRFQNVDILVQLSEEFGTPNSLDVDMRGLFDYMCYYDVVLSFSSDSELVE
AFGGNQNCLDEELKAHKAVISARSPFFRNLLQRRIRTGEEITDRTLRTPTRIILDESIIPKKYATVILHCMYTDVVDLSVLHCSPSV
GSLSEVQALVAGKPNMTRAEEAMELYHIALFLEFNMLAQGCEDIIAESISLDTLIAILKWSSHPYGSKWVHRQALHFLCEEFSQVMT
SDVFYELSKDHLLTAIQSDYLQASEQDILKYLIKWGEHQLMKRIADREPNLLSGTAHSVNKRGVKRRDLDMEELREILSSLLPFVRI
EHILPINSEVLSDAMKRGLISTPPSDMLPTTEGGKSNAWLRQKNAGIYVRPRLFSPYVEEAKSVLDEMMVEQTDLVRLRMVRMSNVP
DTLYMVNNAVPQCCHMISHQQISSNQSSPPSVVANEIPVPRLLIMKDMVRRLQELRHTEQVQRAYALNCGEGATVSYEIQIRVLREF
GLADAAAELLQNPHKFFPDERFGDESPLLTMRQPGRCRVNSTPPAETMFTDLDSFVAFHPPLPPPPPPYHPPATPIHNQLKAGWKQR
PPSQHPSRSFSYPCNHSLFHSRTAPKAGPPPVYLPSVKAAPPDCTSTAGLGRQTVAAAAATTTSTATAAAAAASEKQVRTQPVLNDL
MPDIAVGVSTLSLKDRRLPELAVDTELSQSVSEAGPGPPQHLSCIPQRHTHTSRKKHTLEQKTDTRENPQEYPDFYDFSNAACRPST
PALSRRTPSPSQGGYFGPDLYSHNKASPSGLKSAYLPGQTSPKKQEEARREYPLSPDGHLHRQKNEPIHLDVVEQPPQRSDFPLAAP
ENASTGPAHVRGRTAVETDLTFGLTPNRPSLSACSSEAPEERSGRRLADSESLGHGAQRNTDLEREDSISRGRRSPSKPDFLYKKSA
L
>sp|Q6P9F5|TRI40_HUMAN
MIPLQKDNQEEGVCPICQESLKEAVSTNCGHLFCRVCLTQHVEKASASGVFCCPLCRKPCSEEVLGTGYICPNHQKRVCRFCEESRL
LLCVECLVSPEHMSHHELTIENALSHYKERLNRRSRKLRKDIAELQRLKAQQEKKLQALQFQVDHGNHRLEAGPESQHQTREQLGAL
PQQWLGQLEHMPAEAARILDISRAVTQLRSLVIDLERTAKELDTNTLKNAGDLLNRSAPQKLEVIYPQLEKGVSELLLQPPQKL
>sp|Q96KQ7|EHMT2_HUMAN
MAAAAGAAAAAAAEGEAPAEMGALLLEKETRGATERVHGSLGDTPRSEETLPKATPDSLEPAGPSSPASVTVTVGDEGADTPVGATP
LIGDESENLEGDGDLRGGRILLGHATKSFPSSPSKGGSCPSRAKMSMTGAGKSPPSVQSLAMRLLSMPGAQGAAAAGSEPPPATTSP
EGQPKVHRARKTMSKPGNGQPPVPEKRPPEIQHFRMSDDVHSLGKVTSDLAKRRKLNSGGGLSEELGSARRSGEVTLTKGDPGSLEE
WETVVGDDFSLYYDSYSVDERVDSDSKSEVEALTEQLSEEEEEEEEEEEEEEEEEEEEEEEEEDEESGNQSDRSGSSGRRKAKKKWRK
DSPWVKPSRKRRKREPPRAKEPRGVNGVGSSGPSEYMEVPLGSLELPSEGTLSPNHAGVSNDTSSLETERGFEELPLCSCRMEAPKI
DRISERAGHKCMATESVDGELSGCNAAILKRETMRPSSRVALMVLCETHRARMVKHHCCPGCGYFCTAGTFLECHPDFRVAHRFHKA

CVSQLNGMVFCPHCGEDASEAQEVTIPRGDGVTPPAGTAAPAPPPLSQDVPGRADTSQPSARMRGHGEPRRPPCDPLADTIDSSGPS
LTLPNGGCLSAVGLPLGPGREALEKALVIQESERRKKLRFHPRQLYLSVKQGELQKVILMLLDNLDPNFQSDQQSKRTPLHAAAQKG
SVEICHVLLQAGANINAVDKQQRTPLMEAVVNNHLEVARYMVQRGGCVYSKEEDGSTCLHHAAKIGNLEMVSLLLSTGQVDVNAQDS
GGWTPIIWAAEHKHIEVIRMLLTRGADVTLTDNEENICLHWASFTGSAAIAEVLLNARCDLHAVNYHGDTPLHIAARESYHDCVLLF
LSRGANPELRNKEGDTAWDLTPERSDVWFALQLNRKLRLGVGNRAIRTEKIICRDVARGYENVPIPCVNGVDGEPCPEDYKYISENC
ETSTMNIDRNITHLQHCTCVDDCSSSNCLCGQLSIRCWYDKDGRLLQEFNKIEPPLIFECNQACSCWRNCKNRVVQSGIKVRLQLYR
TAKMGWGVRALQTIPQGTFICEYVGELISDAEADVREDDSYLFDLDNKDGEVYCIDARYYGNISRFINHLCDPNIIPVRVFMLHQDL
RFPRIAFFSSRDIRTGEELGFDYGDRFWDIKSKYFTCQCGSEKCKHSAEAIALEQSRLARLDPHPELLPELGSLPPVNT
>sp|Q8NFH4|NUP37_HUMAN
MKQDASRNAAYTVDCEDYVHVVEFNPFENGDSGNLIAYGGNNYVVIGTCTFQEEEADVEGIQYKTLRTFHHGVRVDGIAWSPETRLD
SLPPVIKFCTSAADMKIRLFTSDLQDKNEYKVLEGHTDFINGLVFDPKEGQEIASVSDDHTCRIWNLEGVQTAHFVLHSPGMSVCWH
PEETFKLMVAEKNGTIRFYDLLAQQAILSLESEQVPLMSAHWCLKNTFKVGAVAGNDWLIWDITRSSYPQNKRPVHMDRACLFRWST
ISENLFATTGYPGKMASQFQIHHLGHPQPILMGSVAVGSGLSWHRTLPLCVIGGDHKLLFWVTEV
>sp|Q9UEG4|ZN629_HUMAN
MEPETALWGPDLQGPEQSPNDAHRGAESENEEESPRQESSGEEIIMGDPAQSPESKDSTEMSLERSSQDPSVPQNPPTPLGHSNPLD
HQIPLDPPAPEVVPTPSDWTKACEASWQWGALTTWNSPPVVPANEPSLRELVQGRPAGAEKPYICNECGKSFSQWSKLLRHQRIHTG
ERPNTCSECGKSFTQSSHLVQHQRTHTGEKPYKCPDCGKCFSWSSNLVQHQRTHTGEKPYKCTECEKAFTQSTNLIKHQRSHTGEKP
YKCGECRRAFYRSSDLIQHQATHTGEKPYKCPECGKRFGQNHNLLKHQKIHAGEKPYRCTECGKSFIQSSELTQHQRTHTGEKPYEC
LECGKSFGHSSTLIKHQRTHLREDPFKCPVCGKTFTLSATLLRHQRTHTGERPYKCPECGKSFSVSSNLINHQRIHRGERPYICADC
GKSFIMSSTLIRHQRIHTGEKPYKCSDCGKSFIRSSHLIQHRRTHTGEKPYKCPECGKSFSQSSNLITHVRTHMDENLFVCSDCGKA
FLEAHELEQHRVIHERGKTPARRAQGDSLLGLGDPSLLTPPPGAKPHKCLVCGKGFNDEGIFMQHQRIHIGENPYKNADGLIAHAAP
KPPQLRSPRLPFRGNSYPGAAEGRAEAPGQPLKPPEGQEGFSQRRGLLSSKTYICSHCGESFLDRSVLLQHQLTHGNEKPFLFPDYR
IGLGEGAGPSPFLSGKPFKCPECKQSFGLSSELLLHQKVHAGGKSSQKSPELGKSSSVLLEHLRSPLGARPYRCSDCRASFLDRVAL
TRHQETHTQEKPPNPEDPPPEAVTLSTDQEGEGETPTPTESSSHGEGQNPKTLVEEKPYLCPECGAGFTEVAALLLHRSCHPGVSL
>sp|P09326|CD48_HUMAN
MCSRGWDSCLALELLLLPLSLLVTSIQGHLVHMTVVSGSNVTLNISESLPENYKQLTWFYTFDQKIVEWDSRKSKYFESKFKGRVRL
DPQSGALYISKVQKEDNSTYIMRVLKKTGNEQEWKIKLQVLDPVPKPVIKIEKIEDMDDNCYLKLSCVIPGESVNYTWYGDKRPFPK
ELQNSVLETTLMPHNYSRCYTCQVSNSVSSKNGTVCLSPPCTLARSFGVEWIASWLVVTVPTILGLLLT
>sp|P13501|CCL5_HUMAN
MKVSAAALAVILIATALCAPASASPYSSDTTPCCFAYIARPLPRAHIKEYFYTSGKCSNPAVVFVTRKNRQVCANPEKKWVREYINS
LEMS
>sp|Q96M11|HYLS1_HUMAN
MEELLPDGQIWANMDPEERMLAAATAFTHICAGQGEGDVRREAQSIQYDPYSKASVAPGKRPALPVQLQYPHVESNVPSETVSEASQ
RLRKPVMKRKVLRRKPDGEVLVTDESIISESESGTENDQDLWDLRQRLMNVQFQEDKESSFDVSQKFNLPHEYQGISQDQLICSLQR
EGMGSPAYEQDLIVASRPKSFILPKLDQLSRNRGKTDRVARYFEYKRDWDSIRLPGEDHRKELRWGVREQMLCRAEPQSKPQHIYVP
NNYLVPTEKKRSALRWGVRCDLANGVIPRKLPFPLSPS
>sp|Q12851|M4K2_HUMAN
MALLRDVSLQDPRDRFELLQRVGAGTYGDVYKARDTVTSELAAVKIVKLDPGDDISSLQQEITILRECRHPNVVAYIGSYLRNDRLW
ICMEFCGGGSLQEIYHATGPLEERQIAYVCREALKGLHHLHSQGKIHRDIKGANLLLTLQGDVKLADFGVSGELTASVAKRRSFIGT
PYWMAPEVAAVERKGGYNELCDVWALGITAIELGELQPPLFHLHPMRALMLMSKSSFQPPKLRDKTRWTQNFHHFLKLALTKNPKKR
PTAEKLLQHPFTTQQLPRALLTQLLDKASDPHLGTPSPEDCELETYDMFPDTIHSRGQHGPAERTPSEIQFHQVKFGAPRRKETDPL
NEPWEEEWTLLGKEELSGSLLQSVQEALEERSLTIRSASEFQELDSPDDTMGTIKRAPFLGPLPTDPPAEEPLSSPPGTLPPPPSGP
NSSPLLPTAWATMKQREDPERSSCHGLPPTPKVHMGACFSKVFNGCPLRIHAAVTWIHPVTRDQFLVVGAEEGIYTLNLHELHEDTL
EKLISHRCSWLYCVNNVLLSLSGKSTHIWAHDLPGLFEQRRLQQQVPLSIPTNRLTQRIIPRRFALSTKIPDTKGCLQCRVVRNPYT

GATFLLAALPTSLLLLQWYEPLQKFLLLKNFSSPLPSPAGMLEPLVLDGKELPQVCVGAEGPEGPGCRVLFHVLPLEAGLTPDILIP
PEGIPGSAQQVIQVDRDTILVSFERCVRIVNMQGEPTATLAPELTFDFPIETVVCLQDSVLAFWSHGMQGRSLDTNEVTQEITDETR
IFRVLGAHRDIILESIPTDNPEAHSNLYILTGHQSTY
>sp|Q9HC77|CENPJ_HUMAN
MFLMPTSSELNSGQNFLTQWMTNPSRAGVILNRGFPILEADKEKRAAVDISTSFPIKGTHFSDSFSFINEEDSLLEEQKLESNNPYK
PQSDKSETHTAFPCIKKGPQVAACHSAPGHQEENKNDFIPDLASEFKEGAYKDPLFKKLEQLKEVQQKKQEQLKRQQLEQLQRLMEE
QEKLLTMVSGQCTLPGLSLLPDDQSQKHRSPGNTTTGERATCCFPSYVYPDPTQEETYPSNILSHEQSNFCRTAHGDFVLTSKRASP
NLFSEAQYQEAPVEKNNLKEENRNHPTGESILCWEKVTEQIQEANDKNLQKHDDSSEVANIEERPIKAAIGERKQTFEDYLEEQIQL
EEQELKQKQLKEAEGPLPIKAKPKQPFLKRGEGLARFTNAKSKFQKGKESKLVTNQSTSEDQPLFKMDRQQLQRKTALKNKELCADN
PILKKDSKARTKSGSVTLSQKPKMLKCSNRKSLSPSGLKIQTGKKCDGQFRDQIKFENKVTSNNKENVTECPKPCDTGCTGWNKTQG
KDRLPLSTGPASRLAAKSPIRETMKESESSLDVSLQKKLETWEREKEKENLELDEFLFLEQAADEISFSSNSSFVLKILERDQQICK
GHRMSSTPVKAVPQKTNPADPISHCNRSEDLDHTAREKESECEVAPKQLHSLSSADELREQPCKIRKAVQKSTSENQTEWNARDDEG
VPNSDSSTDSEEQLDVTIKPSTEDRERGISSREDSPQVCDDKGPFKDTRTQEDKRRDVDLDLSDKDYSSDESIMESIKHKVSEPSRS
SSLSLSKMDFDDERTWTDLEENLCNHDVVLGNESTYGTPQTCYPNNEIGILDKTIKRKIAPVKRGEDLSKSRRSRSPPTSELMMKFF
PSLKPKPKSDSHLGNELKLNISQDQPPGDNARSQVLREKIIELETEIEKFKAENASLAKLRIERESALEKLRKEIADFEQQKAKELA
RIEEFKKEEMRKLQKERKVFEKYTTAARTFPDKKEREEIQTLKQQIADLREDLKRKETKWSSTHSRLRSQIQMLVRENTDLREEIKV
MERFRLDAWKRAEAIESSLEVEKKDKLANTSVRFQNSQISSGTQVEKYKKNYLPMQGNPPRRSKSAPPRDLGNLDKGQAASPREPLE
PLNFPDPEYKEEEDQDIQGEISHPDGKVEKVYKNGCRVILFPNGTRKEVSADGKTITVTFFNGDVKQVMPDQRVIYYYAAAQTTHT
TYPEGLEVLHFSSGQIEKHYPDGRKEITFPDQTVKNLFPDGQEESIFPDGTIVRVQRDGNKLIEFNNGQRELHTAQFKRREYPDGTV
KTVYANGHQETKYRSGRIRVKDKEGNVLMDTEL
>sp|Q9BSH4|TACO1_HUMAN
MSAWAAASLSRAAARCLLARGPGVRAAPPRDPRPSHPEPRGCGAAPGRTLHFTAAVPAGHNKWSKVRHIKGPKDVERSRIFSKLCLN
IRLAVKEGGPNPEHNSNLANILEVCRSKHMPKSTIETALKMEKSKDTYLLYEGRGPGGSSLLIEALSNSSHKCQADIRHILNKNGGV
MAVGARHSFDKKGVIVVEVEDREKKAVNLERALEMAIEAGAEDVKETEDEEERNVFKFICDASSLHQVRKKLDSLGLCSVSCALEFI
PNSKVQLAEPDLEQAAHLIQALSNHEDVIHVYDNIE
>sp|Q8IYF1|ELOA2_HUMAN
MAAGSTTLHAVEKLQVRLATKTEPKKLEKYLQKLSALPMTADILAETGIRKTVKRLRKHQHVGDFARDLAARWKKLVLVDRNTRPGP
QDPEESASRQRFGEALQDQEKAWGFPENATAPRSPSHSPEHRRTARRTPPGQQRPHPRSHSREPRAERKCPRIAPADSGRYRASPTR
TAPLRMPEGPEPAAPGKQPGRGHTHAAQGGPLLCPGCQGQPQGKAVVSHSKGHKSSRQEKRPLCAQGDWHSPTLIREKSCGACLREE
TPRMPSWASARDRQPSDFKTDKEGGQAGSGGQRVPALEEAPDSHQKRPQHSHSNKKRPSLDGRDPGNGTHGLSPEEKEQLSNDRETQE
GKPPTAHLDRTSVSSLSEVEEVDMAEEFEQPTLSCEKYLTYDQLRKQKKKTGKSATTALGDKQRKANESKGTRESWDSAKKLPPVQE
SQSERLQAAGADSAGPKTVPSHVFSELWDLSEAWMQANYDPLSDSDSMTSQAKPEALSSPKFREEAAFPGRRVNAKMPVYSGSRPAC
QLQVPTLRQQCAQVLRNNPDALSDVGEVPYWVLEPVLEGWRPDQLYRRKKDNHALVRETDELRRNHCFQDFKEEKPQENKTWREQYL
RLPDAPEQRLRVMTTNIRSARGNNPNGREAKMICFKSVAKTPYDTSRRQEKSAGDADPENGEIKPASKPAGSSHTPSSQSSSGGGRD
SSSSILRWLPEKRANPCLSSSNEHAAPAAKTRKQAAKKVAPLMAKAIRDYKRRFSRR
>sp|P13945|ADRB3_HUMAN
MAPWPHENSSLAPWPDLPTLAPNTANTSGLPGVPWEAALAGALLALAVLATVGGNLLVIVAIAWTPRLQTMTNVFVTSLAAADLVMG
LLVVPPAATLALTGHWPLGATGCELWTSVDVLCVTASIETLCALAVDRYLAVTNPLRYGALVTKRCARTAVVLVWVVSAAVSFAPIM
SQWWRVGADAEAQRCHSNPRCCAFASNMPYVLLSSSVSFYLPLLVMLFVYARVFVVATRQLRLLRGELGRFPPEESPPAPSRSLAPA
PVGTCAPPEGVPACGRRPARLLPLREHRALCTLGLIMGTFTLCWLPFFLANVLRALGGPSLVPGPAFLALNWLGYANSAFNPLIYCR
SPDFRSAFRRLLCRCGRRLPPEPCAAARPALFPSGVPAARSSPAQPRLCQRLDGASWGVS
>sp|Q9Y5B0|CTDP1_HUMAN
MEVPAAGRVPAEGAPTAAVAEVRCPGPAPLRLLEWRVAAGAAVRIGSVLAVFEAAASAQSSGASQSRVASGGCVRPARPERRLRSER
AGVVRELCAQPGQVVAPGAVLVRLEGCSHPVVMKGLCAECGQDLTQLQSKNGKQQVPLSTATVSMVHSVPELMVSSEQAEQLGREDQ

QRLHRNRKLVLMVDLDQTLIHTTEQHCQQMSNKGIFHFQLGRGEPMLHTRLRPHCKDFLEKIAKLYELHVFTFGSRLYAHTIAGFLD
PEKKLFSHRILSRDECIDPFSKTGNLRNLFPCGDSMVCIIDDREDVWKFAPNLITVKKYVYFQGTGDMNAPPGSRESQTRKKVNHSR
GTEVSEPSPPVRDPEGVTQAPGVEPSNGLEKPARELNGSEAATPRDSPRPGKPDERDIWPPAQAPTSSQELAGAPEPQGSCAQGGRV
APGQRPAQGATGTDLDFDLSSDSESSSESEGTKSSSSASDGESEGKRGRQKPKAAPEGAGALAQGSSLEPGRPAAPSLPGEAEPGAH
APDKEPELGGQEEGERDGLCGLGNGCADRKEAETESQNSELSGVTAGESLDQSMEEEEEEDTDEDDHLIYLEEILVRVHTDYYAKYD
RYLNKEIEEAPDIRKIVPELKSKVLADVAIIFSGLHPTNFPIEKTREHYHATALGAKILTRLVLSPDAPDRATHLIAARAGTEKVLQ
AQECGHLHVVNPDWLWSCLERWDKVEEQLFPLRDDHTKAQRENSPAAFPDREGVPPTALFHPMPVLPKAQPGPEVRIYDSNTGKLIR
TGARGPPAPSSSLPIRQEPSSFRAVPPPQPQMFGEELPDAQDGEQPGPSRRKRQPSMSETMPLYTLCKEDLESMDKEVDDILGEGSD
DSDSEKRRPEEQEEEPQPRKPGTRRERTLGAPASSERSAAGGRGPRGHKRKLNEEDAASESSRESSNEDEGSSSEADEMAKALEAEL
NDLM
>sp|P63151|2ABA_HUMAN
MAGAGGGNDIQWCFSQVKGAVDDDVAEADIISTVEFNHSGELLATGDKGGRVVIFQQEQENKIQSHSRGEYNVYSTFQSHEPEFDYL
KSLEIEEKINKIRWLPQKNAAQFLLSTNDKTIKLWKISERDKRPEGYNLKEEDGRYRDPTTVTTLRVPVFRPMDLMVEASPRRIFAN
AHTYHINSISINSDYETYLSADDLRINLWHLEITDRSFNIVDIKPANMEELTEVITAAEFHPNSCNTFVYSSSKGTIRLCDMRASAL
CDRHSKLFEEPEDPSNRSFFSEIISSISDVKFSHSGRYMMTRDYLSVKIWDLNMENRPVETYQVHEYLRSKLCSLYENDCIFDKFEC
CWNGSDSVVMTGSYNNFFRMFDRNTKRDITLEASRENNKPRTVLKPRKVCASGKRKKDEISVDSLDFNKKILHTAWHPKENIIAVAT
TNNLYIFQDKVN
>sp|Q9BRS2|RIOK1_HUMAN
MDYRRLLMSRVVPGQFDDADSSDSENRDLKTVKEKDDILFEDLQDNVNENGEGEIEDEEEEGYDDDDDDWDWDEGVGKLAKGYVWNG
GSNPQANRQTSDSSSAKMSTPADKVLRKFENKINLDKLNVTDSVINKVTEKSRQKEADMYRIKDKADRATVEQVLDPRTRMILFKML
TRGIITEINGCISTGKEANVYHASTANGESRAIKIYKTSILVFKDRDKYVSGEFRFRHGYCKGNPRKMVKTWAEKEMRNLIRLNTAE
IPCPEPIMLRSHVLVMSFIGKDDMPAPLLKNVQLSESKARELYLQVIQYMRRMYQDARLVHADLSEFNMLYHGGGVYIIDVSQSVEH
DHPHALEFLRKDCANVNDFFMRHSVAVMTVRELFEFVTDPSITHENMDAYLSKAMEIASQRTKEERSSQDHVDEEVFKRAYIPRTLN
EVKNYERDMDIIMKLKEEDMAMNAQQDNILYQTVTGLKKDLSGVQKVPALLENQVEERTCSDSEDIGSSECSDTDSEEQGDHARPKK
HTTDPDIDKKERKKMVKEAQREKRKNKIPKHVKKRKEKTAKTKKGK
>sp|Q6P087|RUSD3_HUMAN
MRAVLAREMDGRRVLGRFWSGWRRGLGVRPVPEDAGFGTEARHQRQPRGSCQRSGPLGDQPFAGLLPKNLSREELVDALRAAVVDRK
GPLVTLNKPQGLPVTGKPGELTLFSVLPELSQSLGLREQELQVVRASGKESSGLVLLSSCPQTASRLQKYFTHARRAQRPTATYCAV
TDGIPAASEGKIQAALKLEHIDGVNLTVPVKAPSRKDILEGVKKTLSHFRVVATGSGCALVQLQPLTVFSSQLQVHMVLQLCPVLGD
HMYSARVGTVLGQRFLLPAENNKPQRQVLDEALLRRLHLTPSQAAQLPLHLHLHRLLLPGTRARDTPVELLAPLPPYFSRTLQCLGL
RLQ
>sp|Q8TCJ2|STT3B_HUMAN
MAEPSAPESKHKSSLNSSPWSGLMALGNSRHGHHGPGAQCAHKAAGGAAPPKPAPAGLSGGLSQPAGWQSLLSFTILFLAWLAGFSS
RLFAVIRFESIIHEFDPWFNYRSTHHLASHGFYEFLNWFDERAWYPLGRIVGGTVYPGLMITAGLIHWILNTLNITVHIRDVCVFLA
PTFSGLTSISTFLLTRELWNQGAGLLAACFIAIVPGYISRSVAGSFDNEGIAIFALQFTYYLWVKSVKTGSVFWTMCCCLSYFYMVS
AWGGYVFIINLIPLHVFVLLLMQRYSKRVYIAYSTFYIVGLILSMQIPFVGFQPIRTSEHMAAAGVFALLQAYAFLQYLRDRLTKQE
FQTLFFLGVSLAAGAVFLSVIYLTYTGYIAPWSGRFYSLWDTGYAKIHIPIIASVSEHQPTTWVSFFFDLHILVCTFPAGLWFCIKN
INDERVFVALYAISAVYFAGVMVRLMLTLTPVVCMLSAIAFSNVFEHYLGDDMKRENPPVEDSSDEDDKRNQGNLYDKAGKVRKHAT
EQEKTEEGLGPNIKSIVTMLMLMLLMMFAVHCTWVTSNAYSSPSVVLASYNHDGTRNILDDFREAYFWLRQNTDEHARVMSWWDYGY
QIAGMANRTTLVDNNTWNNSHIALVGKAMSSNETAAYKIMRTLDVDYVLVIFGGVIGYSGDDINKFLWMVRIAEGEHPKDIRESDYF
TPQGEFRVDKAGSPTLLNCLMYKMSYYRFGEMQLDFRTPPGFDRTRNAEIGNKDIKFKHLEEAFTSEHWLVRIYKVKAPDNRETLDH
KPRVTNIFPKQKYLSKKTTKRKRGYIKNKLVFKKGKKISKKTV
>sp|P30533|AMRP_HUMAN

MAPRRVRSFLRGLPALLLLLLFLGPWPAASHGGKYSREKNQPKPSPKRESGEEFRMEKLNQLWEKAQRLHLPPVRLAELHADLKIQE
RDELAWKKLKLDGLDEDGEKEARLIRNLNVILAKYGLDGKKDARQVTSNSLSGTQEDGLDDPRLEKLWHKAKTSGKFSGEELDKLWR
EFLHHKEKVHEYNVLLETLSRTEEIHENVISPSDLSDIKGSVLHSRHTELKEKLRSINQGLDRLRRVSHQGYSTEAEFEEPRVIDLW
DLAQSANLTDKELEAFREELKHFEAKIEKHNHYQKQLEIAHEKLRHAESVGDGERVSRSREKHALLEGRTKELGYTVKKHLQDLSGR
ISRARHNEL
>sp|P0C2S0|CTXN2_HUMAN
MSSTYCGNSSAKMSVNEVSAFSLTLEQKTGFAFVGILCIFLGLLIIRCFKILLDPYSSMPSSTWEDEVEEFDKGTFEYALA
>sp|Q9H165|BC11A_HUMAN
MSRRKQGKPQHLSKREFSPEPLEAILTDDEPDHGPLGAPEGDHDLLTCGQCQMNFPLGDILIFIEHKRKQCNGSLCLEKAVDKPPSP
SPIEMKKASNPVEVGIQVTPEDDDCLSTSSRGICPKQEHIADKLLHWRGLSSPRSAHGALIPTPGMSAEYAPQGICKDEPSSYTCTT
CKQPFTSAWFLLQHAQNTHGLRIYLESEHGSPLTPRVGIPSGLGAECPSQPPLHGIHIADNNPFNLLRIPGSVSREASGLAEGRFPP
TPPLFSPPPRHHLDPHRIERLGAEEMALATHHPSAFDRVLRLNPMAMEPPAMDFSRRLRELAGNTSSPPLSPGRPSPMQRLLQPFQP
GSKPPFLATPPLPPLQSAPPPSQPPVKSKSCEFCGKTFKFQSNLVVHRRSHTGEKPYKCNLCDHACTQASKLKRHMKTHMHKSSPMT
VKSDDGLSTASSPEPGTSDLVGSASSALKSVVAKFKSENDPNLIPENGDEEEEEDDEEEEEEEEEEEEEELTESERVDYGFGLSLEAA
RHHENSSRGAVVGVGDESRALPDVMQGMVLSSMQHFSEAFHQVLGEKHKRGHLAEAEGHRDTCDEDSVAGESDRIDDGTVNGRGCSP
GESASGGLSKKLLLGSPSSLSPFSKRIKLEKEFDLPPAAMPNTENVYSQWLAGYAASRQLKDPFLSFGDSRQSPFASSSEHSSENGS
LRFSTPPGELDGGISGRSGTGSGGSTPHISGPGPGRPSSKEGRRSDTCEYCGKVFKNCSNLTVHRRSHTGERPYKCELCNYACAQSS
KLTRHMKTHGQVGKDVYKCEICKMPFSVYSTLEKHMKKWHSDRVLNNDIKTE
>sp|Q8IWA5|CTL2_HUMAN
MGDERPHYYGKHGTPQKYDPTFKGPIYNRGCTDIICCVFLLLAIVGYVAVGIIAWTHGDPRKVIYPTDSRGEFCGQKGTKNENKPYL
FYFNIVKCASPLVLLEFQCPTPQICVEKCPDRYLTYLNARSSRDFEYYKQFCVPGFKNNKGVAEVLQDGDCPAVLIPSKPLARRCFP
AIHAYKGVLMVGNETTYEDGHGSRKNITDLVEGAKKANGVLEARQLAMRIFEDYTVSWYWIIIGLVIAMAMSLLFIILLRFLAGIMV
WVMIIMVILVLGYGIFHCYMEYSRLRGEAGSDVSLVDLGFQTDFRVYLHLRQTWLAFMIILSILEVIIILLLIFLRKRILIAIALIK
EASRAVGYVMCSLLYPLVTFFLLCLCIAYWASTAVFLSTSNEAVYKIFDDSPCPFTAKTCNPETFPSSNESRQCPNARCQFAFYGGE
SGYHRALLGLQIFNAFMFFWLANFVLALGQVTLAGAFASYYWALRKPDDLPAFPLFSAFGRALRYHTGSLAFGALILAIVQIIRVIL
EYLDQRLKAAENKFAKCLMTCLKCCFWCLEKFIKFLNRNAYIMIAIYGTNFCTSARNAFFLLMRNIIRVAVLDKVTDFLFLLGKLLI
VGSVGILAFFFFTHRIRIVQDTAPPLNYYWVPILTVIVGSYLIAHGFFSVYGMCVDTLFLCFLEDLERNDGSAERPYFMSSTLKKLL
NKTNKKAAES
>sp|Q9UJ78|ZMYM5_HUMAN
MEKCSVGGLELTEQTPALLGNMAMATSLMDIGDSFGHPACPLVSRSRNSPVEDDDDDDDVVFIESIQPPSISAPAIADQRNFIFASS
KNEKPQGNYSVIPPSSRDLASQKGNISETIVIDDEEDIETNGGAEKKSSCFIEWGLPGTKNKTNDLDFSTSSLSRSKTKTGVRPFNP
GRMNVAGDLFQNGEFATHHSPDSWISQSASFPSNQKQPGVDSLSPVALLRKQNFQPTAQQQLTKPAKITCANCKKPLQKGQTAYQRK
GSAHLFCSTTCLSSFSHKRTQNTRSIICKKDASTKKANVILPVESSKSFQEFYSTSCLSPCENNWNLKKGVFNKSRCTICSKLAEIR
HEVSVNNVTHKLCSNHCFNKYRLANGLIMNCCEHCGEYMPSKSTGNNILVIGGQQKRFCCQSCINEYKQMMETKSKKLTASENRKRN
AFREENEKQLYGSSNTLLKKIEGIPEKKEKTSQLQLSVECGTDTLLIQENVNLPPSSTSTIADTFQEQLEEKNFEDSIVPVVLSADP
GTWPRILNIKQRDTLVENVPPQVRNFNFPKDNTGRKFSETYYTRILPNGEKTTRSWLLYSTSKDSVFCLYCKLFGEGKNQLKNENGC
KDWQHLSHILSKHEESEMHVNNSVKYSKLKSDLKKNKAIDAAEHRLYENEKNDGVLLLYT
>sp|Q86XI6|PPR3B_HUMAN
MMAVDIEYRYNCMAPSLRQERFAFKISPKPSKPLRPCIQLSSKNEASGMVAPAVQEKKVKKRVSFADNQGLALTMVKVFSEFDDPLD
MPFNITELLDNIVSLTTAESESFVLDFSQPSADYLDFRNRLQADHVCLENCVLKDKAIAGTVKVQNLAFEKTVKIRMTFDTWKSYTD
FPCQYVKDTYAGSDRDTFSFDISLPEKIQSYERMEFAVYYECNGQTYWDSNRGKNYRIIRAELKSTQGMTKPHSGPDLGISFDQFGS
PRCSYGLFPEWPSYLGYEKLGPYY
>sp|Q9UKA2|FBXL4_HUMAN

MSPVFPMLTVLTMFYYICLRRRARTATRGEMMNTHRAIESNSQTSPLNAEVVQYAKEVVDFSSHYGSENSMSYTMWNLAGVPNVFPS
SGDFTQTAVFRTYGTWWDQCPSASLPFKRTPPNFQSQDYVELTFEQQVYPTAVHVLETYHPGAVIRILACSANPYSPNPPAEVRWEI
LWSERPTKVNASQARQFKPCIKQINFPTNLIRLEVNSSLLEYYTELDAVVLHGVKDKPVLSLKTSLIDMNDIEDDAYAEKDGCGMDS
LNKKFSSAVLGEGPNNGYFDKLPYELIQLILNHLTLPDLCRLAQTCKLLSQHCCDPLQYIHLNLQPYWAKLDDTSLEFLQSRCTLVQ
WLNLSWTGNRGFISVAGFSRFLKVCGSELVRLELSCSHFLNETCLEVISEMCPNLQALNLSSCDKLPPQAFNHIAKLCSLKRLVLYR
TKVEQTALLSILNFCSELQHLSLGSCVMIEDYDVIASMIGAKCKKLRTLDLWRCKNITENGIAELASGCPLLEELDLGWCPTLQSST
GCFTRLAHQLPNLQKLFLTANRSVCDTDIDELACNCTRLQQLDILGTRMVSPASLRKLLESCKDLSLLDVSFCSQIDNRAVLELNAS
FPKVFIKKSFTQ
>sp|P01210|PENK_HUMAN
MARFLTLCTWLLLLGPGLLATVRAECSQDCATCSYRLVRPADINFLACVMECEGKLPSLKIWETCKELLQLSKPELPQDGTSTLREN
SKPEESHLLAKRYGGFMKRYGGFMKKMDELYPMEPEEEANGSEILAKRYGGFMKKDAEEDDSLANSSDLLKELLETGDNRERSHHQD
GSDNEEEVSKRYGGFMRGLKRSPQLEDEAKELQKRYGGFMRRVGRPEWWMDYQKRYGGFLKRFAEALPSDEEGESYSKEVPEMEKRY
GGFMRF
>sp|P53365|ARFP2_HUMAN
MTDGILGKAATMEIPIHGNGEARQLPEDDGLEQDLQQVMVSGPNLNETSIVSGGYGGSGDGLIPTGSGRHPSHSTTPSGPGDEVARG
IAGEKFDIVKKWGINTYKCTKQLLSERFGRGSRTVDLELELQIELLRETKRKYESVLQLGRALTAHLYSLLQTQHALGDAFADLSQK
SPELQEEFGYNAETQKLLCKNGETLLGAVNFFVSSINTLVTKTMEDTLMTVKQYEAARLEYDAYRTDLEELSLGPRDAGTRGRLESA
QATFQAHRDKYEKLRGDVAIKLKFLEENKIKVMHKQLLLFHNAVSAYFAGNQKQLEQTLQQFNIKLRPPGAEKPSWLEEQ
>sp|Q09328|MGT5A_HUMAN
MALFTPWKLSSQKLGFFLVTFGFIWGMMLLHFTIQQRTQPESSSMLREQILDLSKRYIKALAEENRNVVDGPYAGVMTAYDLKKTLA
VLLDNILQRIGKLESKVDNLVVNGTGTNSTNSTTAVPSLVALEKINVADIINGAQEKCVLPPMDGYPHCEGKIKWMKDMWRSDPCYA
DYGVDGSTCSFFIYLSEVENWCPHLPWRAKNPYEEADHNSLAEIRTDFNILYSMMKKHEEFRWMRLRIRRMADAWIQAIKSLAEKQN
LEKRKRKKVLVHLGLLTKESGFKIAETAFSGGPLGELVQWSDLITSLYLLGHDIRISASLAELKEIMKKVVGNRSGCPTVGDRIVEL
IYIDIVGLAQFKKTLGPSWVHYQCMLRVLDSFGTEPEFNHANYAQSKGHKTPWGKWNLNPQQFYTMFPHTPDNSFLGFVVEQHLNSS
DIHHINEIKRQNQSLVYGKVDSFWKNKKIYLDIIHTYMEVHATVYGSSTKNIPSYVKNHGILSGRDLQFLLRETKLFVGLGFPYEGP
APLEAIANGCAFLNPKFNPPKSSKNTDFFIGKPTLRELTSQHPYAEVFIGRPHVWTVDLNNQEEVEDAVKAILNQKIEPYMPYEFTC
EGMLQRINAFIEKQDFCHGQVMWPPLSALQVKLAEPGQSCKQVCQESQLICEPSFFQHLNKDKDMLKYKVTCQSSELAKDILVPSFD
PKNKHCVFQGDLLLFSCAGAHPRHQRVCPCRDFIKGQVALCKDCL
>sp|P61599|NAA20_HUMAN
MTTLRAFTCDDLFRFNNINLDPLTETYGIPFYLQYLAHWPEYFIVAEAPGGELMGYIMGKAEGSVAREEWHGHVTALSVAPEFRRLG
LAAKLMELLEEISERKGGFFVDLFVRVSNQVAVNMYKQLGYSVYRTVIEYYSASNGEPDEDAYDMRKALSRDTEKKSIIPLPHPVRP
EDIE
>sp|Q8NFZ8|CADM4_HUMAN
MGRARRFQWPLLLLWAAAAGPGAGQEVQTENVTVAEGGVAEITCRLHQYDGSIVVIQNPARQTLFFNGTRALKDERFQLEEFSPRRV
RIRLSDARLEDEGGYFCQLYTEDTHHQIATLTVLVAPENPVVEVREQAVEGGEVELSCLVPRSRPAATLRWYRDRKELKGVSSSQEN
GKVWSVASTVRFRVDRKDDGGIIICEAQNQALPSGHSKQTQYVLDVQYSPTARIHASQAVVREGDTLVLTCAVTGNPRPNQIRWNRG
NESLPERAEAVGETLTLPGLVSADNGTYTCEASNKHGHARALYVLVVYDPGAVVEAQTSVPYAIVGGILALLVFLIICVLVGMVWCS
VRQKGSYLTHEASGLDEQGEAREAFLNGSDGHKRKEEFFI
>sp|P32926|DSG3_HUMAN
MMGLFPRTTGALAIFVVVILVHGELRIETKGQYDEEEMTMQQAKRRQKREWVKFAKPCREGEDNSKRNPIAKITSDYQATQKITYRI
SGVGIDQPPFGIFVVDKNTGDINITAIVDREETPSFLITCRALNAQGLDVEKPLILTVKILDINDNPPVFSQQIFMGEIEENSASNS
LVMILNATDADEPNHLNSKIAFKIVSQEPAGTPMFLLSRNTGEVRTLTNSLDREQASSYRLVVSGADKDGEGLSTQCECNIKVKDVN
DNFPMFRDSQYSARIEENILSSELLRFQVTDLDEEYTDNWLAVYFFTSGNEGNWFEIQTDPRTNEGILKVVKALDYEQLQSVKLSIA
VKNKAEFHQSVISRYRVQSTPVTIQVINVREGIAFRPASKTFTVQKGISSKKLVDYILGTYQAIDEDTNKAASNVKYVMGRNDGGYL

MIDSKTAEIKFVKNMNRDSTFIVNKTITAEVLAIDEYTGKTSTGTVYVRVPDFNDNCPTAVLEKDAVCSSSPSVVVSARTLNNRYTG
PYTFALEDQPVKLPAVWSITTLNATSALLRAQEQIPPGVYHISLVLTDSQNNRCEMPRSLTLEVCQCDNRGICGTSYPTTSPGTRYG
RPHSGRLGPAAIGLLLLGLLLLLLAPLLLLTCDCGAGSTGGVTGGFIPVPDGSEGTIHQWGIEGAHPEDKEITNICVPPVTANGADF
MESSEVCTNTYARGTAVEGTSGMEMTTKLGAATESGGAAGFATGTVSGAASGFGAATGVGICSSGQSGTMRTRHSTGGTNKDYADGA
ISMNFLDSYFSQKAFACAEEDDGQEANDCLLIYDNEGADATGSPVGSVGCCSFIADDLDDSFLDSLGPKFKKLAEISLGVDGEGKEV
QPPSKDSGYGIESCGHPIEVQQTGFVKCQTLSGSQGASALSTSGSVQPAVSIPDPLQHGNYLVTETYSASGSLVQPSTAGFDPLLTQ
NVIVTERVICPISSVPGNLAGPTQLRGSHTMLCTEDPCSRLI
>sp|Q8N8P7|CK044_HUMAN
MVLLCLFLASLAATPRAGVTGAWPTSGLSILAQLQPSCELRWTQLSRSSWRDLVMTTSLWSLAYFQHFSQETTTQEQLDEMEEIPRE
SQCGRQDFHALSCPTASPRDRVLLCCPGWRAMVPS
>sp|P00797|RENI_HUMAN
MDGWRRMPRWGLLLLLWGSCTFGLPTDTTTFKRIFLKRMPSIRESLKERGVDMARLGPEWSQPMKRLTLGNTTSSVILTNYMDTQYY
GEIGIGTPPQTFKVVFDTGSSNVWVPSSKCSRLYTACVYHKLFDASDSSSYKHNGTELTLRYSTGTVSGFLSQDIITVGGITVTQMF
GEVTEMPALPFMLAEFDGVVGMGFIEQAIGRVTPIFDNIISQGVLKEDVFSFYYNRDSENSQSLGGQIVLGGSDPQHYEGNFHYINL
IKTGVWQIQMKGVSVGSSTLLCEDGCLALVDTGASYISGSTSSIEKLMEALGAKKRLFDYVVKCNEGPTLPDISFHLGGKEYTLTSA
DYVFQESYSSKKLCTLAIHAMDIPPPTGPTWALGATFIRKFYTEFDRRNNRIGFALAR
>sp|Q96MU8|KREM1_HUMAN
MAPPAARLALLSAAALTLAARPAPSPGLGPECFTANGADYRGTQNWTALQGGKPCLFWNETFQHPYNTLKYPNGEGGLGEHNYCRNP
DGDVSPWCYVAEHEDGVYWKYCEIPACQMPGNLGCYKDHGNPPPLTGTSKTSNKLTIQTCISFCRSQRFKFAGMESGYACFCGNNPD
YWKYGEAASTECNSVCFGDHTQPCGGDGRIILFDTLVGACGGNYSAMSSVVYSPDFPDTYATGRVCYWTIRVPGASHIHFSFPLFDI
RDSADMVELLDGYTHRVLARFHGRSRPPLSFNVSLDFVILYFFSDRINQAQGFAVLYQAVKEELPQERPAVNQTVAEVITEQANLSV
SAARSSKVLYVITTSPSHPPQTVPGSNSWAPPMGAGSHRVEGWTVYGLATLLILTVTAIVAKILLHVTFKSHRVPASGDLRDCHQPG
TSGEIWSIFYKPSTSISIFKKKLKGQSQQDDRNPLVSD
>sp|O75477|ERLN1_HUMAN
MTQARVLVAAVVGLVAVLLYASIHKIEEGHLAVYYRGGALLTSPSGPGYHIMLPFITTFRSVQTTLQTDEVKNVPCGTSGGVMIYID
RIEVVNMLAPYAVFDIVRNYTADYDKTLIFNKIHHELNQFCSAHTLQEVYIELFDQIDENLKQALQKDLNLMAPGLTIQAVRVTKPK
IPEAIRRNFELMEAEKTKLLIAAQKQKVVEKEAETERKKAVIEAEKIAQVAKIRFQQKVMEKETEKRISEIEDAAFLAREKAKADAE
YYAAHKYATSNKHKLTPEYLELKKYQAIASNSKIYFGSNIPNMFVDSSCALKYSDIRTGRESSLPSKEALEPSGENVIQNKESTG
>sp|Q8NFJ8|BHE22_HUMAN
MERGMHLGAAAAGEDDLFLHKSLSASTSKRLEAAFRSTPPGMDLSLAPPPRERPASSSSSPLGCFEPADPEGAGLLLPPPGGGGGGS
AGSGGGGGGGVGVPGLLVGSAGVGGDPSLSSLPAGAALCLKYGESASRGSVAESSGGEQSPDDDSDGRCELVLRAGVADPRASPGAG
GGGAKAAEGCSNAHLHGGASVPPGGLGGGGGGGSSSGSSGGGGGSGSGSGGSSSSSSSSSKKSKEQKALRLNINARERRRMHDLNDA
LDELRAVIPYAHSPSVRKLSKIATLLLAKNYILMQAQALEEMRRLVAYLNQGQAISAASLPSSAAAAAAAAALHPALGAYEQAAGYP
FSAGLPPAASCPEKCALFNSVSSSLCKQCTEKP
>sp|Q96S82|UBL7_HUMAN
MSLSDWHLAVKLADQPLTPKSILRLPETELGEYSLGGYSISFLKQLIAGKLQESVPDPELIDLIYCGRKLKDDQTLDFYGIQPGSTV
HVLRKSWPEPDQKPEPVDKVAAMREFRVLHTALHSSSSYREAVFKMLSNKESLDQIIVATPGLSSDPIALGVLQDKDLFSVFADPNM
LDTLVPAHPALVNAIVLVLHSVAGSAPMPGTDSSSRSMPSSSYRDMPGGFLFEGLSDDEDDFHPNTRSTPSSSTPSSRPASLGYSGA
AGPRPITQSELATALALASTPESSSHTPTPGTQGHSSGTSPMSSGVQSGTPITNDLFSQALQHALQASGQPSLQSQWQPQLQQLRDM
GIQDDELSLRALQATGGDIQAALELIFAGGAP
>sp|Q14532|K1H2_HUMAN
MTSSCCVTNNLQASLKSCPRPASVCSSGVNCRPELCLGYVCQPMACLPSVCLPTTFRPASCLSKTYLSSSCQAASGISGSMGPGSWY
SEGAFNGNEKETMQFLNDRLASYLTRVRQLEQENAELESRIQEASHSQVLTMTPDYQSHFRTIEELQQKILCTKAENARMVVNIDNA
KLAADDFRAKYEAELAMRQLVEADINGLRRILDDLTLCKADLEAQVESLKEELMCLKKNHEEEVGSLRCQLGDRLNIEVDAAPPVDL

TRVLEEMRCQYEAMVEANRRDVEEWFNMQMEELNQQVATSSEQLQNYQSDIIDLRRTVNTLEIELQAQHSLRDSLENTLTESEARYS
SQLAQMQCMITNVEAQLAEIRADLERQNQEYQVLLDVRARLEGEINTYRSLLENEDCKLPCNPCSTPSCTTCVPSPCVPRTVCVPRT
VGMPCSPCPQGRY
>sp|P43116|PE2R2_HUMAN
MGNASNDSQSEDCETRQWLPPGESPAISSVMFSAGVLGNLIALALLARRWRGDVGCSAGRRSSLSLFHVLVTELVFTDLLGTCLISP
VVLASYARNQTLVALAPESRACTYFAFAMTFFSLATMLMLFAMALERYLSIGHPYFYQRRVSRSGGLAVLPVIYAVSLLFCSLPLLD
YGQYVQYCPGTWCFIRHGRTAYLQLYATLLLLLIVSVLACNFSVILNLIRMHRRSRRSRCGPSLGSGRGGPGARRRGERVSMAEETD
HLILLAIMTITFAVCSLPFTIFAYMNETSSRKEKWDLQALRFLSINSIIDPWVFAILRPPVLRLMRSVLCCRISLRTQDATQTSCST
QSDASKQADL
>sp|Q16048|MCHL1_HUMAN
MLSQKPKKKHNFLNHGLSLNLVIKPYLALEGSVAFPAENGVQDTESTQEKRETGDEENSAKFPVGRRDFDTLSCMLGRVYQSCWQV
>sp|F7VJQ1|APRIO_HUMAN
MEHWGQPIPGAGQPWRQPLPTSGRWWLGAASWWWLGAASWWWLGAAPWWWLGTASWWWLGSRRWHPQSVEQAE
>sp|Q7Z3E5|ARMC9_HUMAN
MGDILAHESELLGLVKEYLDFAEFEDTLKTFSKECKIKGKPLCKTVGGSFRDSKSLTIQKDLVAAFDNGDQKVFFDLWEEHISSSIR
DGDSFAQKLEFYLHIHFAIYLLKYSVGRPDKEELDEKISYFKTYLETKGAALSQTTEFLPFYALPFVPNPMVHPSFKELFQDSWTPE
LKLKLIKFLALISKASNTPKLLTIYKENGQSNKEILQQLHQQLVEAERRSVTYLKRYNKIQADYHNLIGVTAELVDSLEATVSGKMI
TPEYLQSVCVRLFSNQMRQSLAHSVDFTRPGTASTMLRASLAPVKLKDVPLLPSLDYEKLKKDLILGSDRLKAFLLQALRWRLTTSH
PGEQRETVLQAYISNDLLDCYSHNQRSVLQLLHSTSDVVRQYMARLINAFASLAEGRLYLAQNTKVLQMLEGRLKEEDKDIITRENV
LGALQKFSLRRPLQTAMIQDGLIFWLVDVLKDPDCLSDYTLEYSVALLMNLCLRSTGKNMCAKVAGLVLKVLSDLLGHENHEIQPYV
NGALYSILSVPSIREEARAMGMEDILRCFIKEGNAEMIRQIEFIIKQLNSEELPDGVLESDDDEDEDDEEDHDIMEADLDKDELIQP
QLGELSGEKLLTTEYLGIMTNTGKTRRKGLANVQWSGDEPLQRPVTPGGHRNGYPVVEDQHTPPQTAQHARNGHPQALPAAHEAVYR
EGKPSTPESCVSSSSAIIAKPGEWLPRGRQEEPRPAPTTPRQPREAPQDPGNGVTTRECASAFTCKPRAPCTPEMLDWNPPKAKASV
LAPLFSSCGPQQASRPGSTASSTRGLPSSQSHRK
>sp|P59923|ZN445_HUMAN
MPPGRWHAAYPAQAQSSRERGRLQTVKKEEEDESYTPVQAARPQTLNRPGQELFRQLFRQLRYHESSGPLETLSRLRELCRWWLRPD
VLSKAQILELLVLEQFLSILPGELRVWVQLHNPESGEEAVALLEELQRDLDGTSWRDPGPAQSPDVHWMGTGALRSAQIWSLASPLR
SSSALGDHLEPPYEIEARDFLAGQSDTPAAQMPALFPREGCPGDQVTPTRSLTAQLQETMTFKDVEVTFSQDEWGWLDSAQRNLYRD
VMLENYRNMASLVGPFTKPALISWLEAREPWGLNMQAAQPKGNPVAAPTGDDLQSKTNKFILNQEPLEEAETLAVSSGCPATSVSEG
IGLRESFQQKSRQKDQCENPIQVRVKKEETNFSHRTGKDSEVSGSNSLDLKHVTYLRVSGRKESLKHGCGKHFRMSSHHYDYKKYGK
GLRHMIGGFSLHQRIHSGLKGNKKDVCGKDFSLSSHHQRGQSLHTVGVSFKCSDCGRTFSHSSHLAYHQRLHTQEKAFKCRVCGKAF
RWSSNCARHEKIHTGVKPYKCDLCEKAFRRLSAYRLHRETHAKKKFLELNQYRAALTYSSGFDHHLGDQSGEKLFDCSQCRKSFHCK
SYVLEHQRIHTQEKPYKCTKCRKTFRWRSNFTRHMRLHEEEKFYKQDECREGFRQSPDCSQPQGAPAVEKTFLCQQCGKTFTRKKTL
VDHQRIHTGEKPYQCSDCGKDFAYRSAFIVHKKKHAMKRKPEGGPSFSQDTVFQVPQSSHSKEEPYKCSQCGKAFRNHSFLLIHQRV
HTGEKPYKCRECGKAFRWSSNLYRHQRIHSLQKQYDCHESEKTPNVEPKILTGEKRFWCQECGKTFTRKRTLLDHKGIHSGEKRYKC
NLCGKSYDRNYRLVNHQRIHSTERPFKCQWCGKEFIGRHTLSSHQRKHTRAAQAERSPPARSSSQDTKLRLQKLKPSEEMPLEDCKE
ACSQSSRLTGLQDISIGKKCHKCSICGKTFNKSSQLISHKRFHTRERPFKCSKCGKTFRWSSNLARHMKNHIRD
>sp|Q6XXX2|CU024_HUMAN
MQTTWQPGCSYPTSWLSSQESFSKMRTGWRGAIPLRWRNRARNREKPHSPRAVSSPATHSLPPSNPCRLTPTLSSARPREGSCPSKC
SCPGGNWSNTALSAELMWAEGRFSGGCLPVYMRQNINPGCQEQWEGEERSRWL
>sp|Q5TFQ8|SIRBL_HUMAN
MPVPASWPHLPSPFLLMTLLLGRLTGVAGEEELQVIQPDKSISVAAGESATLHCTVTSLIPVGPIQWFRGAGPGRELIYNQKEGHFP
RVTTVSDLTKRNNMDFSIRISNITPADAGTYYCVKFRKGSPDHVEFKSGAGTELSVRAKPSAPVVSGPAARATPQHTVSFTCESHGF
SPRDITLKWFKNGNELSDFQTNVDPAGDSVSYSIHSTAKVVLTREDVHSQVICEVAHVTLQGDPLRGTANLSETIRVPPTLEVTQQP

VRAENQVNVTCQVRKFYPQRLQLTWLENGNVSRTETASTLTENKDGTYNWMSWLLVNVSAHRDDVKLTCQVEHDGQPAVSKSHDLKV
SAHPKEQGSNTAPGPALASAAPLLIAFLLGPKVLLVVGVSVIYVYWKQKA
>sp|Q96GL9|F163A_HUMAN
MTAGTVVITGGILATVILLCIIAVLCYCRLQYYCCKKSGTEVADEEEEREHDLPTHPRGPTCNACSSQALDGRGSLAPLTSEPCSQP
CGVAASHCTTCSPYSSPFYIRTADMVPNGGGGERLSFAPTYYKEGGPPSLKLAAPQSYPVTWPGSGREAFTNPRAISTDV
>sp|Q9HCM9|TRI39_HUMAN
MAETSLLEAGASAASTAAALENLQVEASCSVCLEYLKEPVIIECGHNFCKACITRWWEDLERDFPCPVCRKTSRYRSLRPNRQLGSM
VEIAKQLQAVKRKIRDESLCPQHHEALSLFCYEDQEAVCLICAISHTHRAHTVVPLDDATQEYKEKLQKCLEPLEQKLQEITRCKSS
EEKKPGELKRLVESRRQQILREFEELHRRLDEEQQVLLSRLEEEEQDILQRLRENAAHLGDKRRDLAHLAAEVEGKCLQSGFEMLKD
VKSTLEKNIPRKFGGSLSTICPRDHKALLGLVKEINRCEKVKTMEVTSVSIELEKNFSNFPRQYFALRKILKQLIADVTLDPETAHP
NLVLSEDRKSVKFVETRLRDLPDTPRRFTFYPCVLATEGFTSGRHYWEVEVGDKTHWAVGVCRDSVSRKGELTPLPETGYWRVRLWN
GDKYAATTTPFTPLHIKVKPKRVGIFLDYEAGTLSFYNVTDRSHIYTFTDTFTEKLWPLFYPGIRAGRKNAAPLTIRPPTDWE
>sp|Q8TC56|FA71B_HUMAN
MSNESCLPYYTAHSYSSMSAFKTSMGDLQRQLYNRGEYNIFKYAPMFESNFIQINKKGEVIDVHNRVRMVTVGIVCTSPILPLPDVM
VLAQPTKICEQHVRWGRFAKGRGRRPVKTLELTRLLPLKFVKISIHDHEKQQLRLKLATGRTFYLQLCPSSDTREDLFCYWEKLVYL
LRPPVESYCSTPTLLSGDAPPEDNKSLVAAELHREGDQSETGLYKPCDVSAATSSAYAGGEGIQHASHGTASAASPSTSTPGAAEGG
AARTAGGMAVAGTATGPRTDVAIAGAAMSPATGAMSIATTKSAGPGQVTTALAGAAIKNPGENESSKSMAGAANISSEGISLALVGA
ASTSLEGTSTSMAGAASLSQDSSLSAAFAGSITTSKCAAERTEGPAVGPLISTLQSEGYMSERDGSQKVSQPSAEVWNENKERREKK
DRHPSRKSSHHRKAGESHRRRAGDKNQKASSHRSASGHKNTRDDKKEKGYSNVRGKRHGSSRKSSTHSSTKKESRTTQELGKNQSAS
STGALQKKASKISSFLRSLRATPGSKTRVTSHDREVDIVAKMVEKQNIEAKVEKAQGGQELEMISGTMTSEKTEMIVFETKSI
>sp|Q6P1K2|PMF1_HUMAN
MAEASSANLGSGCEEKRHEGSSSESVPPGTTISRVKLLDTMVDTFLQKLVAAGSYQRFTDCYKCFYQLQPAMTQQIYDKFIAQLQTS
IREEISDIKEEGNLEAVLNALDKIVEEGKVRKEPAWRPSGIPEKDLHSVMAPYFLQQRDTLRRHVQKQEAENQQLADAVLAGRRQVE
ELQLQVQAQQQAWQALHREQRELVAVLREPE
>sp|Q8IWU9|TPH2_HUMAN
MQPAMMMFSSKYWARRGFSLDSAVPEEHQLLGSSTLNKPNSGKNDDKGNKGSSKREAATESGKTAVVFSLKNEVGGLVKALRLFQEK
RVNMVHIESRKSRRRSSEVEIFVDCECGKTEFNELIQLLKFQTTIVTLNPPENIWTEEEELEDVPWFPRKISELDKCSHRVLMYGSE
LDADHPGFKDNVYRQRRKYFVDVAMGYKYGQPIPRVEYTEEETKTWGVVFRELSKLYPTHACREYLKNFPLLTKYCGYREDNVPQLE
DVSMFLKERSGFTVRPVAGYLSPRDFLAGLAYRVFHCTQYIRHGSDPLYTPEPDTCHELLGHVPLLADPKFAQFSQEIGLASLGASD
EDVQKLATCYFFTIEFGLCKQEGQLRAYGAGLLSSIGELKHALSDKACVKAFDPKTTCLQECLITTFQEAYFVSESFEEAKEKMRDF
AKSITRPFSVYFNPYTQSIEILKDTRSIENVVQDLRSDLNTVCDALNKMNQYLGI
>sp|Q92914|FGF11_HUMAN
MAALASSLIRQKREVREPGGSRPVSAQRRVCPRGTKSLCQKQLLILLSKVRLCGGRPARPDRGPEPQLKGIVTKLFCRQGFYLQANP
DGSIQGTPEDTSSFTHFNLIPVGLRVVTIQSAKLGHYMAMNAEGLLYSSPHFTAECRFKECVFENYYVLYASALYRQRRSGRAWYLG
LDKEGQVMKGNRVKKTKAAAHFLPKLLEVAMYQEPSLHSVPEASPSSPPAP
>sp|P16444|DPEP1_HUMAN
MWSGWWLWPLVAVCTADFFRDEAERIMRDSPVIDGHNDLPWQLLDMFNNRLQDERANLTTLAGTHTNIPKLRAGFVGGQFWSVYTPC
DTQNKDAVRRTLEQMDVVHRMCRMYPETFLYVTSSAGIRQAFREGKVASLIGVEGGHSIDSSLGVLRALYQLGMRYLTLTHSCNTPW
ADNWLVDTGDSEPQSQGLSPFGQRVVKELNRLGVLIDLAHVSVATMKATLQLSRAPVIFSHSSAYSVCASRRNVPDDVLRLVKQTDS
LVMVNFYNNYISCTNKANLSQVADHLDHIKEVAGARAVGFGGDFDGVPRVPEGLEDVSKYPDLIAELLRRNWTEAEVKGALADNLLR
VFEAVEQASNLTQAPEEEPIPLDQLGGSCRTHYGYSSGASSLHRHWGLLLASLAPLVLCLSLL
>sp|Q9Y5L5|LENEP_HUMAN
MQPRTQPLAQTLPFFLGGAPRDTGLRVPVIKMGTGWEGFQRTLKEVAYILLCCWCIKELLD
>sp|Q86VX2|COMD7_HUMAN

MGRLHCTEDPVPEAVGGDMQQLNQLGAQQFSALTEVLFHFLTEPKEVERFLAQLSEFATTNQISLGSLRSIVKSLLLVPNGALKKSL
TAKQVQADFITLGLSEEKATYFSEKWKQNAPTLARWAIGQTLMINQLIDMEWKFGVTSGSSELEKVGSIFLQLKLVVKKGNQTENVY
IELTLPQFYSFLHEMERVRTSMECFC
>sp|A4GXA9|EME2_HUMAN
MARVGPGRAGVSCQGRGRGRGGSGQRRPPTWEISDSDAEDSAGSEAAARARDPAGERRAAAEALRLLRPEQVLKRLAVCVDTAILED
AGADVLMEALEALGCECRIEPQRPARSLRWTRASPDPCPRSLPPEVWAAGEQELLLLLEPEEFLQGVATLTQISGPTHWVPWISPET
TARPHLAVIGLDAYLWSRQHVSRGTQQPESPKVAGAEVAVSWPEVEEALVLLQLWANLDVLLVASWQELSRHVCAVTKALAQYPLKQ
YRESQAFSFCTAGRWAAGEPVARDGAGLQAAWRRQIRQFSRVSPAVADAVVTAFPSPRLLQQALEACSTERERMGLLADLPVPPSEG
GRPRRVGPDLSRRICLFLTTANPDLLLDLGS
>sp|Q9NQ87|HEYL_HUMAN
MKRPKEPSGSDGESDGPIDVGQEGQLSQMARPLSTPSSSQMQARKKHRGIIEKRRRDRINSSLSELRRLVPTAFEKQGSSKLEKAEV
LQMTVDHLKMLHATGGTGFFDARALAVDFRSIGFRECLTEVIRYLGVLEGPSSRADPVRIRLLSHLNSYAAEMEPSPTPTGPLAFPA
WPWSFFHSCPGLPALSNQLAILGRVPSPVLPGVSSPAYPIPALRTAPLRRATGIILPARRNVLPSRGASSTRRARPLERPATPVPVA
PSSRAARSSHIAPLLQSSSPTPPGPTGSAAYVAVPTPNSSSPGPAGRPAGAMLYHSWVSEITEIGAF
>sp|Q9NPI7|KRCC1_HUMAN
MKHSKKTYDSFQDELEDYIKVQKARGLEPKTCFRKMKGDYLETCGYKGEVNSRPTYRMFDQRLPSETIQTYPRSCNIPQTVENRLPQ
WLPAHDSRLRLDSLSYCQFTRDCFSEKPVPLNFNQQEYICGSHGVEHRVYKHFSSDNSTSTHQASHKQIHQKRKRHPEEGREKSEEE
RSKHKRKKSCEEIDLDKHKSIQRKKTEVEIETVHVSTEKLKNRKEKKSRDVVSKKEERKRTKKKKEQGQERTEEEMLWDQSILGF
>sp|Q96MH2|HEXI2_HUMAN
MMATPNQTACNAESPVALEEAKTSGAPGSPQTPPERHDSGGSLPLTPRMESHSEDEDLAGAVGGLGWNSRSPRTQSPGGCSAEAVLA
RKKHRRRPSKRKRHWRPYLELSWAEKQQRDERQSQRASRVREEMFAKGQPVAPYNTTQFLMNDRDPEEPNLDVPHGISHPGSSGESE
AGDSDGRGRAHGEFQRKDFSETYERFHTESLQGRSKQELVRDYLELEKRLSQAEEETRRLQQLQACTGQQSCRQVEELAAEVQRLRT
ENQRLRQENQMWNREGCRCDEEPGT
>sp|Q9HAW0|BRF2_HUMAN
MPGRGRCPDCGSTELVEDSHYSQSQLVCSDCGCVVTEGVLTTTFSDEGNLREVTYSRSTGENEQVSRSQQRGLRRVRDLCRVLQLPP
TFEDTAVAYYQQAYRHSGIRAARLQKKEVLVGCCVLITCRQHNWPLTMGAICTLLYADLDVFSSTYMQIVKLLGLDVPSLCLAELVK
TYCSSFKLFQASPSVPAKYVEDKEKMLSRTMQLVELANETWLVTGRHPLPVITAATFLAWQSLQPADRLSCSLARFCKLANVDLPYP
ASSRLQELLAVLLRMAEQLAWLRVLRLDKRSVVKHIGDLLQHRQSLVRSAFRDGTAEVETREKEPPGWGQGQGEGEVGNNSLGLPQG
KRPASPALLLPPCMLKSPKRICPVPPVSTVTGDENISDSEIEQYLRTPQEVRDFQRAQAARQAATSVPNPP
>sp|Q6IFH4|OR6B2_HUMAN
MSGENVTKVSTFILVGLPTAPGLQYLLFLLFLLTYLFVLVENLAIILIVWSSTSLHRPMYYFLSSMSFLEIWYVSDITPKMLEGFLL
QQKRISFVGCMTQLYFFSSLVCTECVLLASMAYDRYVAICHPLRYHVLVTPGLCLQLVGFSFVSGFTISMIKVCFISSVTFCGSNVL
NHFFCDISPILKLACTDFSTAELVDFILAFIILVFPLLATILSYWHITLAVLRIPSATGCWRAFSTCASHLTVVTVFYTALLFMYVR
PQAIDSQSSNKLISAVYTVVTPIINPLIYCLRNKEFKDALKKALGLGQTSH
>sp|Q8IYT8|ULK2_HUMAN
MEVVGDFEYSKRDLVGHGAFAVVFRGRHRQKTDWEVAIKSINKKNLSKSQILLGKEIKILKELQHENIVALYDVQELPNSVFLVMEY
CNGGDLADYLQAKGTLSEDTIRVFLHQIAAAMRILHSKGIIHRDLKPQNILLSYANRRKSSVSGIRIKIADFGFARYLHSNMMAATL
CGSPMYMAPEVIMSQHYDAKADLWSIGTVIYQCLVGKPPFQANSPQDLRMFYEKNRSLMPSIPRETSPYLANLLLGLLQRNQKDRMD
FEAFFSHPFLEQGPVKKSCPVPVPMYSGSVSGSSCGSSPSCRFASPPSLPDMQHIQEENLSSPPLGPPNYLQVSKDSASTSSKNSSC
DTDDFVLVPHNISSDHSCDMPVGTAGRRASNEFLVCGGQCQPTVSPHSETAPIPVPTQIRNYQRIEQNLTSTASSGTNVHGSPRSAV
VRRSNTSPMGFLRPGSCSPVPADTAQTVGRRLSTGSSRPYSPSPLVGTIPEQFSQCCCGHPQGHDSRSRNSSGSPVPQAQSPQSLLS
GARLQSAPTLTDIYQNKQKLRKQHSDPVCPSHTGAGYSYSPQPSRPGSLGTSPTKHLGSSPRSSDWFFKTPLPTIIGSPTKTTAPFK
IPKTQASSNLLALVTRHGPAEEQSKDGNEPRECAHCLLVQGSERQRAEQQSKAVFGRSVSTGKLSDQQGKTPICRHQGSTDSLNTER
PMDIAPAGACGGVLAPPAGTAASSKAVLFTVGSPPHSAAAPTCTHMFLRTRTTSVGPSNSGGSLCAMSGRVCVGSPPGPGFGSSPPG

AEAAPSLRYVPYGASPPSLEGLITFEAPELPEETLMEREHTDTLRHLNVMLMFTECVLDLTAMRGGNPELCTSAVSLYQIQESVVVD
QISQLSKDWGRVEQLVLYMKAAQLLAASLHLAKAQIKSGKLSPSTAVKQVVKNLNERYKFCITMCKKLTEKLNRFFSDKQRFIDEIN
SVTAEKLIYNCAVEMVQSAALDEMFQQTEDIVYRYHKAALLLEGLSRILQDPADIENVHKYKCSIERRLSALCHSTATV
>sp|P35813|PPM1A_HUMAN
MGAFLDKPKMEKHNAQGQGNGLRYGLSSMQGWRVEMEDAHTAVIGLPSGLESWSFFAVYDGHAGSQVAKYCCEHLLDHITNNQDFKG
SAGAPSVENVKNGIRTGFLEIDEHMRVMSEKKHGADRSGSTAVGVLISPQHTYFINCGDSRGLLCRNRKVHFFTQDHKPSNPLEKER
IQNAGGSVMIQRVNGSLAVSRALGDFDYKCVHGKGPTEQLVSPEPEVHDIERSEEDDQFIILACDGIWDVMGNEELCDFVRSRLEVT
DDLEKVCNEVVDTCLYKGSRDNMSVILICFPNAPKVSPEAVKKEAELDKYLECRVEEIIKKQGEGVPDLVHVMRTLASENIPSLPPG
GELASKRNVIEAVYNRLNPYKNDDTDSTSTDDMW
>sp|O75908|SOAT2_HUMAN
MEPGGARLRLQRTEGLGGERERQPCGDGNTETHRAPDLVQWTRHMEAVKAQLLEQAQGQLRELLDRAMREAIQSYPSQDKPLPPPPP
GSLSRTQEPSLGKQKVFIIRKSLLDELMEVQHFRTIYHMFIAGLCVFIISTLAIDFIDEGRLLLEFDLLIFSFGQLPLALVTWVPMF
LSTLLAPYQALRLWARGTWTQATGLGCALLAAHAVVLCALPVHVAVEHQLPPASRCVLVFEQVRFLMKSYSFLREAVPGTLRARRGE
GIQAPSFSSYLYFLFCPTLIYRETYPRTPYVRWNYVAKNFAQALGCVLYACFILGRLCVPVFANMSREPFSTRALVLSILHATLPGI
FMLLLIFFAFLHCWLNAFAEMLRFGDRMFYRDWWNSTSFSNYYRTWNVVVHDWLYSYVYQDGLRLLGARARGVAMLGVFLVSAVAHE
YIFCFVLGFFYPVMLILFLVIGGMLNFMMHDQRTGPAWNVLMWTMLFLGQGIQVSLYCQEWYARRHCPLPQATFWGLVTPRSWSCHT
>sp|O95867|LY66C_HUMAN
MKALMLLTLSVLLCWVSADIRCHSCYKVPVLGCVDRQSCRLEPGQQCLTTHAYLGKMWVFSNLRCGTPEEPCQEAFNQTNRKLGLTY
NTTCCNKDNCNSAGPRPTPALGLVFLTSLAGLGLWLLH
>sp|Q9NYM9|BET1L_HUMAN
MADWARAQSPGAVEEILDRENKRMADSLASKVTRLKSLALDIDRDAEDQNRYLDGMDSDFTSMTSLLTGSVKRFSTMARSGQDNRKL
LCGMAVGLIVAFFILSYFLSRART
>sp|P48546|GIPR_HUMAN
MTTSPILQLLLRLSLCGLLLQRAETGSKGQTAGELYQRWERYRRECQETLAAAEPPSGLACNGSFDMYVCWDYAAPNATARASCPWY
LPWHHHVAAGFVLRQCGSDGQWGLWRDHTQCENPEKNEAFLDQRLILERLQVMYTVGYSLSLATLLLALLILSLFRRLHCTRNYIHI
NLFTSFMLRAAAILSRDRLLPRPGPYLGDQALALWNQALAACRTAQIVTQYCVGANYTWLLVEGVYLHSLLVLVGGSEEGHFRYYLL
LGWGAPALFVIPWVIVRYLYENTQCWERNEVKAIWWIIRTPILMTILINFLIFIRILGILLSKLRTRQMRCRDYRLRLARSTLTLVP
LLGVHEVVFAPVTEEQARGALRFAKLGFEIFLSSFQGFLVSVLYCFINKEVQSEIRRGWHHCRLRRSLGEEQRQLPERAFRALPSGS
GPGEVPTSRGLSSGTLPGPGNEASRELESYC
>sp|Q6P1L8|RM14_HUMAN
MAFFTGLWGPFTCVSRVLSHHCFSTTGSLSAIQKMTRVRVVDNSALGNSPYHRAPRCIHVYKKNGVGKVGDQILLAIKGQKKKALIV
GHCMPGPRMTPRFDSNNVVLIEDNGNPVGTRIKTPIPTSLRKREGEYSKVLAIAQNFV
>sp|P29274|AA2AR_HUMAN
MPIMGSSVYITVELAIAVLAILGNVLVCWAVWLNSNLQNVTNYFVVSLAAADIAVGVLAIPFAITISTGFCAACHGCLFIACFVLVL
TQSSIFSLLAIAIDRYIAIRIPLRYNGLVTGTRAKGIIAICWVLSFAIGLTPMLGWNNCGQPKEGKNHSQGCGEGQVACLFEDVVPM
NYMVYFNFFACVLVPLLLMLGVYLRIFLAARRQLKQMESQPLPGERARSTLQKEVHAAKSLAIIVGLFALCWLPLHIINCFTFFCPD
CSHAPLWLMYLAIVLSHTNSVVNPFIYAYRIREFRQTFRKIIRSHVLRQQEPFKAAGTSARVLAAHGSDGEQVSLRLNGHPPGVWAN
GSAPHPERRPNGYALGLVSGGSAQESQGNTGLPDVELLSHELKGVCPEPPGLDDPLAQDGAGVS
>sp|Q8NFZ5|TNIP2_HUMAN
MSRDPGSGGWEEAPRAAAALCTLYHEAGGQRLRRLQDQLAARDALIARLRARLAALEGDAAPSLVDALLEQVARFREQLRRQEGGAAE
AQMRQEIERLTERLEEKEREMQQLLSQPQHEREKEVVLLRRSMAEGERARAASDVLCRSLANETHQLRRTLTATAHMCQHLAKCLDE
RQHAQRNVGERSPDQSEHTDGHTSVQSVIEKLQEENRLLKQKVTHVEDLNAKWQRYNASRDEYVRGLHAQLRGLQIPHEPELMRKEI
SRLNRQLEEKINDCAEVKQELAASRTARDAALERVQMLEQQILAYKDDFMSERADRERAQSRIQELEEKVASLLHQVSWRQDSREPD
AGRIHAGSKTAKYLAADALELMVPGGWRPGTGSQQPEPPAEGGHPGAAQRGQGDLQCPHCLQCFSDEQGEELLRHVAECCQ

>sp|Q149M9|NWD1_HUMAN
MQRGKPCRALPTLKCQTFCQRHGLMFEVVDLRWGIRNIEATDHLTTELCLEEVDRCWKTSIGPAFVALIGDQYGPCLIPSRIDEKEW
EVLRDHLTARPSDLELVARYFQRDENAFPPTYVLQAPGTGEACEPEEATLTSVLRSGAQEARRLGLITQEQWQHYHRSVIEWEIERS
LLSSEDREQGATVFLREIQDLHKHILEDCALRMVDRLADGCLDADAQNLLSSLKSHITDMHPGVLKTHRLPWSRDLVNPKNKTHACY
LKELGEQFVVRANHQVLTRLRELDTAGQELAWLYQEIRHHLWQSSEVIQTFCGRQELLARLGQQLRHDDSKQHTPLVLFGPPGIGKT
ALMCKLAEQMPRLLGHKTVTVLRLLGTSQMSSDARGLLKSICFQVCLAYGLPLPPAQVLDAHTRVVQFFHTLLHTVSCRNFESLVLL
LDAMDDLDSVRHARRVPWLPLNCPPRVHLILSACSGALGVLDTLQRVLLDPEAYWEVKPLSGNQGQQMIQLLLAAARRTLSPVHTDL
LWASLPECGNPGRLRLAFEEARKWASFTVPVPLATTAEEATHQLCTRLEQTHGQLLVAHVLGYIVSSRHGLSEAELKDVLSLDDEVL
QDVYRDWTPPSKELLRFPPLLWVRLRRDLGYYLARRPVDGFTLLAIAHRQLVEVVRERYLSGSERAKRHGVLADFFSGTWSQGTKKL
ITLPLVGKPLNLDRKVAPQPLWFSHTVANLRKLKELPYHLLHSGRLEELKQEVLGSMSWISCRGISGGIEDLLDDFDLCAPHLDSPE
VGLVREALQLCRPAVELRGMERSLLYTELLARLHFFATSHPALVGQLCQQAQSWFQLCAHPVLVPLGGFLQPPGGPLRATLSGCHKG
ITAMAWGVEEKLLVIGTQDGIMAVWDMEEQHVIHMLTGHTGEVRCVKIFAKGTLANSASKDYTLHLWNLLSGQEKFTIWDGGSKNPA
EPQIWNLHVDEAHKVVYSASGSKINAWNLETAEPVFHILGDASDPWMCMAVLASQATLLTVSRDGVVSLWSSATGKLQGKQHMSSIK
EETPTCAVSVQKQGKLVTGFSNGSISLVSSKGDRLLEKLPDAVRFLVVSEDESLLAAGFGRSVRIFLADSRGFRRFMAMDLEHEDMV
ETAVFGTENNLIITGSLDALIQVWSLSEQGTLLDILEGVGAPVSLLARGGALVASASPQSSSFKVWDLSDAHRSRVPAPFLDRTGLT
AVSHNGSYVYFPKIGDKNKVTIWDLAEGEEQDSLDTSSEIRCLEVAEQRKLLFTGLVSGVVLVFPLNSRQDVICIPPPEARKAINCM
SLSKCEDRLAIAYDNIVLVLDITSGDPCPVIDGPRYTFYTQLPETLSSVAILTDYRVVYSMTNGDLFLYECATSKAFPLETHRSRVA
CVEVSHKEQLVVSGSEDALLCLWDLQARKWKFEMSYTSSYCRGVQCACFSKDDKYVYVGLKDRSILVWSVLDGTLLTVQFVHAVVNR
IIPTTSGFIAPTRHGYLIRENFQCLSAKASPQDPLKNFKKAMWMVKSRQREELVAAAGAPQDLESESAQGNETKSNKCSQVCLIV
>sp|Q9UQV4|LAMP3_HUMAN
MPRQLSAAAALFASLAVILHDGSQMRAKAFPETRDYSQPTAAATVQDIKKPVQQPAKQAPHQTLAARFMDGHITFQTAATVKIPTTT
PATTKNTATTSPITYTLVTTQATPNNSHTAPPVTEVTVGPSLAPYSLPPTITPPAHTTGTSSSTVSHTTGNTTQPSNQTTLPATLSI
ALHKSTTGQKPVQPTHAPGTTAAAHNTTRTAAPASTVPGPTLAPQPSSVKTGIYQVLNGSRLCIKAEMGIQLIVQDKESVFSPRRYF
NIDPNATQASGNCGTRKSNLLLNFQGGFVNLTFTKDEESYYISEVGAYLTVSDPETIYQGIKHAVVMFQTAVGHSFKCVSEQSLQLS
AHLQVKTTDVQLQAFDFEDDHFGNVDECSSDYTIVLPVIGAIVVGLCLMGMGVYKIRLRCQSSGYQRI
>sp|Q9NY72|SCN3B_HUMAN
MPAFNRLFPLASLVLIYWVSVCFPVCVEVPSETEAVQGNPMKLRCISCMKREEVEATTVVEWFYRPEGGKDFLIYEYRNGHQEVESP
FQGRLQWNGSKDLQDVSITVLNVTLNDSGLYTCNVSREFEFEAHRPFVKTTRLIPLRVTEEAGEDFTSVVSEIMMYILLVFLTLWLL
IEMIYCYRKVSKAEEAAQENASDYLAIPSENKENSAVPVEE
>sp|Q8WV41|SNX33_HUMAN
MALKGRALYDFHSENKEEISIQQDEDLVIFSETSLDGWLQGQNSRGETGLFPASYVEIVRSGISTNHADYSSSPAGSPGAQVSLYNS
PSVASPARSGGGSGFLSNQGSFEEDDDDDWDDWDDGCTVVEEPRAGGLGTNGHPPLNLSYPGAYPSQHMAFRPKPPLERQDSLASAK
RGSVVGRNLNRFSCFVRSGVEAFILGDVPMMAKIAETYSIEMGPRGPQWKANPHPFACSVEDPTKQTKFKGIKSYISYKLTPTHAAS
PVYRRYKHFDWLYNRLLHKFTVISVPHLPEKQATGRFEEDFIEKRKRRLILWMDHMTSHPVLSQYEGFQHFLSCLDDKQWKMGKRRA
EKDEMVGASFLLTFQIPTEHQDLQDVEDRVDTFKAFSKKMDDSVLQLSTVASELVRKHVGGFRKEFQKLGSAFQAISHSFQMDPPFC
SEALNSAISHTGRTYEAIGEMFAEQPKNDLFQMLDTLSLYQGLLSNFPDIIHLQKGAFAKVKESQRMSDEGRMVQDEADGIRRRCRV
VGFALQAEMNHFHQRRELDFKHMMQNYLRQQILFYQRVGQQLEKTLRMYDNL
>sp|Q9P206|K1522_HUMAN
MVVFVGRRLPALLGLFKKKGSAKAENDKHLSVGPGQGPGSAVDEHQDNVFFPSGRPPHLEELHTQAQEGLRSLQHQEKQKLNKGGWD
HGDTQSIQSSRTGPDEDNISFCSQTTSYVAESSTAEDALSIRSEMIQRKGSTFRPHDSFPKSGKSGRRRRERRSTVLGLPQHVQKEL
GLRNEREAPGTPRAPGARDAVRIPTVDGRPRGTSGMGARVSLQALEAEAEAGAETEAMLQRHIDRVYRDDTFVGRSTGTRAPPLTRP
MSLAVPGLTGGAGPAEPLSPAMSISPQATYLSKLIPHAVLPPTVDVVALGRCSLRTLSRCSLHSASPASVRSLGRFSSVSSPQPRSR
HPSSSSDTWSHSQSSDTIVSDGSTLSSKGGSEGQPESSTASNSVVPPPQGGSGRGSPSGGSTAEASDTLSIRSSGQLSGRSVSLRKL
KRPPPPPRRTHSLHQRGLAVPDGPLGLPPKPERKQQPQLPRPPTTGGSEGAGAAPCPPNPANSWVPGLSPGGSRRPPRSPERTLSPS

SGYSSQSGTPTLPPKGLAGPPASPGKAQPPKPERVTSLRSPGASVSSSLTSLCSSSSDPAPSDRSGPQILTPLGDRFVIPPHPKVPA
PFSPPPSKPRSPNPAAPALAAPAVVPGPVSTTDASPQSPPTPQTTLTPLQESPVISKDQSPPPSPPPSYHPPPPPTKKPEVVVEAPS
ASETAEEPLQDPNWPPPPPPAPEEQDLSMADFPPPEEAFFSVASPEPAGPSGSPELVSSPAASSSSATALQIQPPGSPDPPPAPPAP
APASSAPGHVAKLPQKEPVGCSKGGGPPREDVGAPLVTPSLLQMVRLRSVGAPGGAPTPALGPSAPQKPLRRALSGRASPVPAPSSG
LHAAVRLKACSLAASEGLSSAQPNGPPEAEPRPPQSPASTASFIFSKGSRKLQLERPVSPETQADLQRNLVAELRSISEQRPPQAPK
KSPKAPPPVARKPSVGVPPPASPSYPRAEPLTAPPTNGLPHTQDRTKRELAENGGVLQLVGPEEKMGLPGSDSQKELA
>sp|Q8NGK3|O52K2_HUMAN
MSASNITLTHPTAFLLVGIPGLEHLHIWISIPFCLAYTLALLGNCTLLLIIQADAALHEPMYLFLAMLAAIDLVLSSSALPKMLAIF
WFRDREINFFACLAQMFFLHSFSIMESAVLLAMAFDRYVAICKPLHYTKVLTGSLITKIGMAAVARAVTLMTPLPFLLRCFHYCRGP
VIAHCYCEHMAVVRLACGDTSFNNIYGIAVAMFIVVLDLLLVILSYIFILQAVLLLASQEARYKAFGTCVSHIGAILAFYTTVVISS
VMHRVARHAAPHVHILLANFYLLFPPMVNPIIYGVKTKQIRESILGVFPRKDM
>sp|Q8IWZ8|SUGP1_HUMAN
MSLKMDNRDVAGKANRWFGVAPPKSGKMNMNILHQEELIAQKKREIEAKMEQKAKQNQVASPQPPHPGEITNAHNSSCISNKFANDG
SFLQQFLKLQKAQTSTDAPTSAPSAPPSTPTPSAGKRSLLISRRTGLGLASLPGPVKSYSHAKQLPVAHRPSVFQSPDEDEEEDYEQ
WLEIKVSPPEGAETRKVIEKLARFVAEGGPELEKVAMEDYKDNPAFAFLHDKNSREFLYYRKKVAEIRKEAQKSQAASQKVSPPEDE
EVKNLAEKLARFIADGGPEVETIALQNNRENQAFSFLYEPNSQGYKYYRQKLEEFRKAKASSTGSFTAPDPGLKRKSPPEALSGSLP
PATTCPASSTPAPTIIPAPAAPGKPASAATVKRKRKSRWGPEEDKVELPPAELVQRDVDASPSPLSVQDLKGLGYEKGKPVGLVGVT
ELSDAQKKQLKEQQEMQQMYDMIMQHKRAMQDMQLLWEKAVQQHQHGYDSDEEVDSELGTWEHQLRRMEMDKTREWAEQLTKMGRGK
HFIGDFLPPDELEKFMETFKALKEGREPDYSEYKEFKLTVENIGYQMLMKMGWKEGEGLGSEGQGIKNPVNKGTTTVDGAGFGIDRP
AELSKEDDEYEAFRKRMMLAYRFRPNPLNNPRRPYY
>sp|P04083|ANXA1_HUMAN
MAMVSEFLKQAWFIENEEQEYVQTVKSSKGGPGSAVSPYPTFNPSSDVAALHKAIMVKGVDEATIIDILTKRNNAQRQQIKAAYLQE
TGKPLDETLKKALTGHLEEVVLALLKTPAQFDADELRAAMKGLGTDEDTLIEILASRTNKEIRDINRVYREELKRDLAKDITSDTSG
DFRNALLSLAKGDRSEDFGVNEDLADSDARALYEAGERRKGTDVNVFNTILTTRSYPQLRRVFQKYTKYSKHDMNKVLDLELKGDIE
KCLTAIVKCATSKPAFFAEKLHQAMKGVGTRHKALIRIMVSRSEIDMNDIKAFYQKMYGISLCQAILDETKGDYEKILVALCGGN
>sp|Q96S94|CCNL2_HUMAN
MAAAAAAAGAAGSAAPAAAAGAPGSGGAPSGSQGVLIGDRLYSGVLITLENCLLPDDKLRFTPSMSSGLDTDTETDLRVVGCELIQA
AGILLRLPQVAMATGQVLFQRFFYTKSFVKHSMEHVSMACVHLASKIEEAPRRIRDVINVFHRLRQLRDKKKPVPLLLDQDYVNLKN
QIIKAERRVLKELGFCVHVKHPHKIIVMYLQVLECERNQHLVQTSWNYMNDSLRTDVFVRFQPESIACACIYLAARTLEIPLPNRPH
WFLLFGATEEEIQEICLKILQLYARKKVDLTHLEGEVEKRKHAIEEAKAQARGLLPGGTQVLDGTSGFSPAPKLVESPKEGKGSKPS
PLSVKNTKRRLEGAKKAKADSPVNGLPKGRESRSRSRSREQSYSRSPSRSASPKRRKSDSGSTSGGSKSQSRSRSRSDSPPRQAPRS
APYKGSEIRGSRKSKDCKYPQKPHKSRSRSSSRSRSRSRERADNPGKYKKKSHYYRDQRRERSRSYERTGRRYERDHPGHSRHRR
>sp|Q9NX94|WBP1L_HUMAN
MPFLLGLRQDKEACVGTNNQSYICDTGHCCGQSQCCNYYYELWWFWLVWTIIIILSCCCVCHHRRAKHRLQAQQRQHEINLIAYREA
HNYSALPFYFRFLPNYLLPPYEEVVNRPPTPPPPYSAFQLQQQQLLPPQCGPAGGSPPGIDPTRGSQGAQSSPLSEPSRSSTRPPSI
ADPDPSDLPVDRAATKAPGMEPSGSVAGLGELDPGAFLDKDAECREELLKDDSSEHGAPDSKEKTPGRHRRFTGDSGIEVCVCNRGH
HDDDLKEFNTLIDDALDGPLDFCDSCHVRPPGDEEEGLCQSSEEQAREPGHPHLPRPPACLLLNTINEQDSPNSQSSSSPS
>sp|P50135|HNMT_HUMAN
MASSMRSLFSDHGKYVESFRRFLNHSTEHQCMQEFMDKKLPGIIGRIGDTKSEIKILSIGGGAGEIDLQILSKVQAQYPGVCINNEV
VEPSAEQIAKYKELVAKTSNLENVKFAWHKETSSEYQSRMLEKKELQKWDFIHMIQMLYYVKDIPATLKFFHSLLGTNAKMLIIVVS
GSSGWDKLWKKYGSRFPQDDLCQYITSDDLTQMLDNLGLKYECYDLLSTMDISDCFIDGNENGDLLWDFLTETCNFNATAPPDLRAE
LGKDLQEPEFSAKKEGKVLFNNTLSFIVIEA
>sp|P47870|GBRB2_HUMAN

MWRVRKRGYFGIWSFPLIIAAVCAQSVNDPSNMSLVKETVDRLLKGYDIRLRPDFGGPPVAVGMNIDIASIDMVSEVNMDYTLTMYF
QQAWRDKRLSYNVIPLNLTLDNRVADQLWVPDTYFLNDKKSFVHGVTVKNRMIRLHPDGTVLYGLRITTTAACMMDLRRYPLDEQNC
TLEIESYGYTTDDIEFYWRGDDNAVTGVTKIELPQFSIVDYKLITKKVVFSTGSYPRLSLSFKLKRNIGYFILQTYMPSILITILSW
VSFWINYDASAARVALGITTVLTMTTINTHLRETLPKIPYVKAIDMYLMGCFVFVFMALLEYALVNYIFFGRGPQRQKKAAEKAASA
NNEKMRLDVNKIFYKDIKQNGTQYRSLWDPTGNLSPTRRTTNYDFSLYTMDPHENILLSTLEIKNEMATSEAVMGLGDPRSTMLAYD
ASSIQYRKAGLPRHSFGRNALERHVAQKKSRLRRRASQLKITIPDLTDVNAIDRWSRIFFPVVFSFFNIVYWLYYVN
>sp|Q8NG50|RDM1_HUMAN
MAELVPFAVPIESDKTLLVWELSSGPTAEALHHSLFTAFSQFGLLYSVRVFPNAAVAHPGFYAVIKFYSARAAHRAQKACDRKQLFQ
KSPVKVRLGTRHKAVQHQALALNSSKCQELANYYFGFNGCSKRIIKLQELSDLEERENEDSMVPLPKQSLKFFCALEVVLPSCDCRS
PGIGLVEEPMDKVEEGPLSFLMKRKTAQKLAIQKALSDAFQKLLIVVLESGKIAVEYRPSEDIVGVRCEEELHGLIQVPCSPWKQYG
QEEEGYLSDFSLEEEEFRLPELD
>sp|P11362|FGFR1_HUMAN
MWSWKCLLFWAVLVTATLCTARPSPTLPEQAQPWGAPVEVESFLVHPGDLLQLRCRLRDDVQSINWLRDGVQLAESNRTRITGEEVE
VQDSVPADSGLYACVTSSPSGSDTTYFSVNVSDALPSSEDDDDDDDSSSEEKETDNTKPNRMPVAPYWTSPEKMEKKLHAVPAAKTV
KFKCPSSGTPNPTLRWLKNGKEFKPDHRIGGYKVRYATWSIIMDSVVPSDKGNYTCIVENEYGSINHTYQLDVVERSPHRPILQAGL
PANKTVALGSNVEFMCKVYSDPQPHIQWLKHIEVNGSKIGPDNLPYVQILKTAGVNTTDKEMEVLHLRNVSFEDAGEYTCLAGNSIG
LSHHSAWLTVLEALEERPAVMTSPLYLEIIIYCTGAFLISCMVGSVIVYKMKSGTKKSDFHSQMAVHKLAKSIPLRRQVTVSADSSA
SMNSGVLLVRPSRLSSSGTPMLAGVSEYELPEDPRWELPRDRLVLGKPLGEGCFGQVVLAEAIGLDKDKPNRVTKVAVKMLKSDATE
KDLSDLISEMEMMKMIGKHKNIINLLGACTQDGPLYVIVEYASKGNLREYLQARRPPGLEYCYNPSHNPEEQLSSKDLVSCAYQVAR
GMEYLASKKCIHRDLAARNVLVTEDNVMKIADFGLARDIHHIDYYKKTTNGRLPVKWMAPEALFDRIYTHQSDVWSFGVLLWEIFTL
GGSPYPGVPVEELFKLLKEGHRMDKPSNCTNELYMMMRDCWHAVPSQRPTFKQLVEDLDRIVALTSNQEYLDLSMPLDQYSPSFPDT
RSSTCSSGEDSVFSHEPLPEEPCLPRHPAQLANGGLKRR
>sp|Q502X0|MORN2_HUMAN
MNGFGRLEHFSGAVYEGQFKDNMFHGLGTYTFPNGAKYTGNFNENRVEGEGEYTDIQGLEWSGNFHFTAAPDLKLKLHM
>sp|Q4KN68|CT062_HUMAN
MAYYFHFYGELPSQESPHPGVYSPHPQGGWQPTADSYREWHNEDLNPRMSHWTYAQELVSYKKTSFIVWRHRISPNGVTSRKPYCHF
HGGQWPGPTPPSCLSSAFASGSFSHFEDSPYILLHVVLQMCWLRAQRLNQRDLGLNPTSAIEHQLSDLDCVPLLLWASDSPSE
>sp|Q99418|CYH2_HUMAN
MEDGVYEPPDLTPEERMELENIRRRKQELLVEIQRLREELSEAMSEVEGLEANEGSKTLQRNRKMAMGRKKFNMDPKKGIQFLVENE
LLQNTPEEIARFLYKGEGLNKTAIGDYLGEREELNLAVLHAFVDLHEFTDLNLVQALRQFLWSFRLPGEAQKIDRMMEAFAQRYCLC
NPGVFQSTDTCYVLSFAVIMLNTSLHNPNVRDKPGLERFVAMNRGINEGGDLPEELLRNLYDSIRNEPFKIPEDDGNDLTHTFFNPD
REGWLLKLGGGRVKTWKRRWFILTDNCLYYFEYTTDKEPRGIIPLENLSIREVDDPRKPNCFELYIPNNKGQLIKACKTEADGRVVE
GNHMVYRISAPTQEEKDEWIKSIQAAVSVDPFYEMLAARKKRISVKKKQEQP
>sp|Q9NWW9|HRSL2_HUMAN
MALARPRPRLGDLIEISRFGYAHWAIYVGDGYVVHLAPASEIAGAGAASVLSALTNKAIVKKELLSVVAGGDNYRVNNKHDDRYTPL
PSNKIVKRAEELVGQELPYSLTSDNCEHFVNHLRYGVSRSDQVTGAVTTVGVAAGLLAAASLVGILLARSKRERQ
>sp|Q9UK22|FBX2_HUMAN
MDGDGDPESVGQPEEASPEEQPEEASAEEERPEDQQEEEAAAAAAYLDELPEPLLLRVLAALPAAELVQACRLVCLRWKELVDGAPL
WLLKCQQEGLVPEGGVEEERDHWQQFYFLSKRRRNLLRNPCGEEDLEGWCDVEHGGDGWRVEELPGDSGVEFTHDESVKKYFASSFE
WCRKAQVIDLQAEGYWEELLDTTQPAIVVKDWYSGRSDAGCLYELTVKLLSEHENVLAEFSSGQVAVPQDSDGGGWMEISHTFTDYG
PGVRFVRFEHGGQDSVYWKGWFGARVTNSSVWVEP
>sp|O75054|IGSF3_HUMAN
MKCFFPVLSCLAVLGVVSAQRQVTVQEGPLYRTEGSHITIWCNVSGYQGPSEQNFQWSIYLPSSPEREVQIVSTMDSSFPYAIYTQR
VRGGKIFIERVQGNSTLLHITDLQARDAGEYECHTPSTDKQYFGSYSAKMNLVVIPDSLQTTAMPQTLHRVEQDPLELTCEVASETI

QHSHLSVAWLRQKVGEKPVEVISLSRDFMLHSSSEYAQRQSLGEVRLDKLGRTTFRLTIFHLQPSDQGEFYCEAAEWIQDPDGSWYA
MTRKRSEGAVVNVQPTDKEFTVRLETEKRLHTVGEPVEFRCILEAQNVPDRYFAVSWAFNSSLIATMGPNAVPVLNSEFAHREARGQ
LKVAKESDSVFVLKIYHLRQEDSGKYNCRVTEREKTVTGEFIDKESKRPKNIPIIVLPLKSSISVEVASNASVILEGEDLRFSCSVR
TAGRPQGRFSVIWQLVDRQNRRSNIMWLDRDGTVQPGSSYWERSSFGGVQMEQVQPNSFSLGIFNSRKEDEGQYECHVTEWVRAVDG
EWQIVGERRASTPISITALEMGFAVTAISRTPGVTYSDSFDLQCIIKPHYPAWVPVSVTWRFQPVGTVEFHDLVTFTRDGGVQWGDR
SSSFRTRTAIEKAESSNNVRLSISRASDTEAGKYQCVAELWRKNYNNTWTRLAERTSNLLEIRVLQPVTKLQVSKSKRTLTLVENKP
IQLNCSVKSQTSQNSHFAVLWYVHKPSDADGKLILKTTHNSAFEYGTYAEEEGLRARLQFERHVSGGLFSLTVQRAEVSDSGSYYCH
VEEWLLSPNYAWYKLAEEVSGRTEVTVKQPDSRLRLSQAQGNLSVLETRQVQLECVVLNRTSITSQLMVEWFVWKPNHPERETVARL
SRDATFHYGEQAAKNNLKGRLHLESPSPGVYRLFIQNVAVQDSGTYSCHVEEWLPSPSGMWYKRAEDTAGQTALTVMRPDASLQVDT
VVPNATVSEKAAFQLDCSIVSRSSQDSRFAVAWYSLRTKAGGKRSSPGLEEQEEEREEEEEEDDDDDDDPTERTALLSVGPDAVFGP
EGSPWEGRLRFQRLSPVLYRLTVLQASPQDTGNYSCHVEEWLPSPQKEWYRLTEEESAPIGIRVLDTSPTLQSIICSNDALFYFVFF
YPFPIFGILIITILLVRFKSRNSSKNSDGKNGVPLLWIKEPHLNYSPTCLEPPVLSIHPGAID
>sp|Q96LT9|RBM40_HUMAN
MAAPEQPLAISRGCTSSSSLSPPRGDRTLLVRHLPAELTAEEKEDLLKYFGAQSVRVLSDKGRLKHTAFATFPNEKAAIKALTRLHQ
LKLLGHTLVVEFAKEQDRVHSPCPTSGSEKKKRSDDPVEDDKEKKELGYLTVENGIAPNHGLTFPLNSCLKYMYPPPSSTILANIVN
ALASVPKFYVQVLHLMNKMNLPTPFGPITARPPMYEDYMPLHAPLPPTSPQPPEEPPLPDEDEELSSEESEYESTDDEDRQRMNKLM
ELANLQPKRPKTIKQRHVRKKRKIKDMLNTPLCPSHSSLHPVLLPSDVFDQPQPVGNKRIEFHISTDMPAAFKKDLEKEQNCEEKNH
DLPATEVDASNIGFGKIFPKPNLDITEEIKEDSDEMPSECISRRELEKGRISREEMETLSVFRSYEPGEPNCRIYVKNLAKHVQEKD
LKYIFGRYVDFSSETQRIMFDIRLMKEGRMKGQAFIGLPNEKAAAKALKEANGYVLFGKPMVVQFARSARPKQDPKEGKRKC
>sp|P43243|MATR3_HUMAN
MSKSFQQSSLSRDSQGHGRDLSAAGIGLLAAATQSLSMPASLGRMNQGTARLASLMNLGMSSSLNQQGAHSALSSASTSSHNLQSIF
NIGSRGPLPLSSQHRGDADQASNILASFGLSARDLDELSRYPEDKITPENLPQILLQLKRRRTEEGPTLSYGRDGRSATREPPYRVP
RDDWEEKRHFRRDSFDDRGPSLNPVLDYDHGSRSQESGYYDRMDYEDDRLRDGERCRDDSFFGETSHNYHKFDSEYERMGRGPGPLQ
ERSLFEKKRGAPPSSNIEDFHGLLPKGYPHLCSICDLPVHSNKEWSQHINGASHSRRCQLLLEIYPEWNPDNDTGHTMGDPFMLQQS
TNPAPGILGPPPPSFHLGGPAVGPRGNLGAGNGNLQGPRHMQKGRVETSRVVHIMDFQRGKNLRYQLLQLVEPFGVISNHLILNKIN
EAFIEMATTEDAQAAVDYYTTTPALVFGKPVRVHLSQKYKRIKKPEGKPDQKFDQKQELGRVIHLSNLPHSGYSDSAVLKLAEPYGK
IKNYILMRMKSQAFIEMETREDAMAMVDHCLKKALWFQGRCVKVDLSEKYKKLVLRIPNRGIDLLKKDKSRKRSYSPDGKESPSDKK
SKTDGSQKTESSTEGKEQEEKSGEDGEKDTKDDQTEQEPNMLLESEDELLVDEEEAAALLESGSSVGDETDLANLGDVASDGKKEPS
DKAVKKDGSASAAAKKKLKKVDKIEELDQENEAALENGIKNEENTEPGAESSENADDPNKDTSENADGQSDENKDDYTIPDEYRIGP
YQPNVPVGIDYVIPKTGFYCKLCSLFYTNEEVAKNTHCSSLPHYQKLKKFLNKLAEERRQKKET
>sp|O14904|WNT9A_HUMAN
MLDGSPLARWLAAAFGLTLLLAALRPSAAYFGLTGSEPLTILPLTLEPEAAAQAHYKACDRLKLERKQRRMCRRDPGVAETLVEAVS
MSALECQFQFRFERWNCTLEGRYRASLLKRGFKETAFLYAISSAGLTHALAKACSAGRMERCTCDEAPDLENREAWQWGGCGDNLKY
SSKFVKEFLGRRSSKDLRARVDFHNNLVGVKVIKAGVETTCKCHGVSGSCTVRTCWRQLAPFHEVGKHLKHKYETALKVGSTTNEAA
GEAGAISPPRGRASGAGGSDPLPRTPELVHLDDSPSFCLAGRFSPGTAGRRCHREKNCESICCGRGHNTQSRVVTRPCQCQVRWCCY
VECRQCTQREEVYTCKG
>sp|O75223|GGCT_HUMAN
MANSGCKDVTGPDEESFLYFAYGSNLLTERIHLRNPSAAFFCVARLQDFKLDFGNSQGKTSQTWHGGIATIFQSPGDEVWGVVWKMN
KSNLNSLDEQEGVKSGMYVVIEVKVATQEGKEITCRSYLMTNYESAPPSPQYKKIICMGAKENGLPLEYQEKLKAIEPNDYTGKVSE
EIEDIIKKGETQTL
>sp|Q8NBK3|SUMF1_HUMAN
MAAPALGLVCGRCPELGLVLLLLLLSLLCGAAGSQEAGTGAGAGSLAGSCGCGTPQRPGAHGSSAAAHRYSREANAPGPVPGERQLA
HSKMVPIPAGVFTMGTDDPQIKQDGEAPARRVTIDAFYMDAYEVSNTEFEKFVNSTGYLTEAEKFGDSFVFEGMLSEQVKTNIQQAV
AAAPWWLPVKGANWRHPEGPDSTILHRPDHPVLHVSWNDAVAYCTWAGKRLPTEAEWEYSCRGGLHNRLFPWGNKLQPKGQHYANIW

QGEFPVTNTGEDGFQGTAPVDAFPPNGYGLYNIVGNAWEWTSDWWTVHHSVEETLNPKGPPSGKDRVKKGGSYMCHRSYCYRYRCAA
RSQNTPDSSASNLGFRCAADRLPTMD
>sp|Q8J025|APCD1_HUMAN
MSWPRRLLLRYLFPALLLHGLGEGSALLHPDSRSHPRSLEKSAWRAFKESQCHHMLKHLHNGARITVQMPPTIEGHWVSTGCEVRSG
PEFITRSYRFYHNNTFKAYQFYYGSNRCTNPTYTLIIRGKIRLRQASWIIRGGTEADYQLHNVQVICHTEAVAEKLGQQVNRTCPGF
LADGGPWVQDVAYDLWREENGCECTKAVNFAMHELQLIRVEKQYLHHNLDHLVEELFLGDIHTDATQRMFYRPSSYQPPLQNAKNHD
HACIACRIIYRSDEHHPPILPPKADLTIGLHGEWVSQRCEVRPEVLFLTRHFIFHDNNNTWEGHYYHYSDPVCKHPTFSIYARGRYS
RGVLSSRVMGGTEFVFKVNHMKVTPMDAATASLLNVFNGNECGAEGSWQVGIQQDVTHTNGCVALGIKLPHTEYEIFKMEQDARGRY
LLFNGQRPSDGSSPDRPEKRATSYQMPLVQCASSSPRAEDLAEDSGSSLYGRAPGRHTWSLLLAALACLVPLLHWNIRR
>sp|Q9NR64|KLHL1_HUMAN
MSGSGRKDFDVKHILRLRWKLFSHPSPSTGGPAGGGCLQQDGSGSFEHWGPSQSRLLKSQERSGVSTFWKKPSSSSSSSSPSSSSS
SFNPLNGTLLPVATRLQQGAPGQGTQQPARTLFYVESLEEEVVPGMDFPGPHEKGLVLQELKVEPDNSSQATGEGCGHRLSSTGHSM
TPQSDLDSSSSEEFYQAVHHAEQTFRKMESYLKQQQLCDVILIVGNRKIPAHRLVLSSVSDYFAAMFTSDVCEAKQEEIKMEGIDPN
ALWDLVQFAYTGCLELKEDTIENLLAAACLLQLPQVVEVCCHFLMKLLHPSNCLGIRAFADAQGCIELMKVAHSYTMENIMEVIRNQ
EFLLLPAEELHKLLASDDVNVPDEETIFHALMMWVKYDMQSRCNDLSMLLAFIRLPLLPPQILADLENHALFKNDLECQKLILEAMK
YHLLPERRTLMQSPRTKPRKSTVGTLYAVGGMDNNKGATTIEKYDLRTNLWIQAGMMNGRRLQFGVAVIDDKLFVIGGRDGLKTLNT
VECYNPKTKTWTVLPPMSTHRHGLGVTVLEGPIYAVGGHDGWSYLNTVERWDPQSQQWTFVASMSIARSTVGVAALNGKLYSVGGRD
GSSCLSSMEYYDPHTNKWNMCAPMCKRRGGVGVATCDGFLYAVGGHDAPASNHCSRLLDYVERYDPKTDTWTMVAPLSMPRDAVGVC
LLGDRLYAVGGYDGQTYLNTMESYDPQTNEWTQMASLNIGRAGACVVVIKQP
>sp|Q92968|PEX13_HUMAN
MASQPPPPPKPWETRRIPGAGPGPGPGPTFQSADLGPTLMTRPGQPALTRVPPPILPRPSQQTGSSSVNTFRPAYSSFSSGYGAYGN
SFYGGYSPYSYGYNGLGYNRLRVDDLPPSRFVQQAEESSRGAFQSIESIVHAFASVSMMMDATFSAVYNSFRAVLDVANHFSRLKIH
FTKVFSAFALVRTIRYLYRRLQRMLGLRRGSENEDLWAESEGTVACLGAEDRAATSAKSWPIFLFFAVILGGPYLIWKLLSTHSDEV
TDSINWASGEDDHVVARAEYDFAAVSEEEISFRAGDMLNLALKEQQPKVRGWLLASLDGQTTGLIPANYVKILGKRKGRKTVESSKV
SKQQQSFTNPTLTKGATVADSLDEQEAAFESVFVETNKVPVAPDSIGKDGEKQDL
>sp|P26373|RL13_HUMAN
MAPSRNGMVLKPHFHKDWQRRVATWFNQPARKIRRRKARQAKARRIAPRPASGPIRPIVRCPTVRYHTKVRAGRGFSLEELRVAGIH
KKVARTIGISVDPRRRNKSTESLQANVQRLKEYRSKLILFPRKPSAPKKGDSSAEELKLATQLTGPVMPVRNVYKKEKARVITEEEK
NFKAFASLRMARANARLFGIRAKRAKEAAEQDVEKKK
>sp|O95926|SYF2_HUMAN
MAAIAASEVLVDSAEEGSLAAAAELAAQKREQRLRKFRELHLMRNEARKLNHQEVVEEDKRLKLPANWEAKKARLEWELKEEEKKKE
CAARGEDYEKVKLLEISAEDAERWERKKKRKNPDLGFSDYAAAQLRQYHRLTKQIKPDMETYERLREKHGEEFFPTSNSLLHGTHVP
STEEIDRMVIDLEKQIEKRDKYSRRRPYNDDADIDYINERNAKFNKKAERFYGKYTAEIKQNLERGTAV
>sp|Q8WW33|GTSF1_HUMAN
MEETYTDSLDPEKLLQCPYDKNHQIRACRFPYHLIKCRKNHPDVASKLATCPFNARHQVPRAEISHHISSCDDRSCIEQDVVNQTRS
LRQETLAESTWQCPPCDEDWDKDLWEQTSTPFVWGTTHYSDNNSPASNIVTEHKNNLASGMRVPKSLPYVLPWKNNGNAQ
>sp|P51817|PRKX_HUMAN
MEAPGLAQAAAAESDSRKVAEETPDGAPALCPSPEALSPEPPVYSLQDFDTLATVGTGTFGRVHLVKEKTAKHFFALKVMSIPDVIR
LKQEQHVHNEKSVLKEVSHPFLIRLFWTWHDERFLYMLMEYVPGGELFSYLRNRGRFSSTTGLFYSAEIICAIEYLHSKEIVYRDLK
PENILLDRDGHIKLTDFGFAKKLVDRTWTLCGTPEYLAPEVIQSKGHGRAVDWWALGILIFEMLSGFPPFFDDNPFGIYQKILAGKI
DFPRHLDFHVKDLIKKLLVVDRTRRLGNMKNGANDVKHHRWFRSVDWEAVPQRKLKPPIVPKIAGDGDTSNFETYPENDWDTAAPVP
QKDLEIFKNF
>sp|Q9HAW8|UD110_HUMAN

MARAGWTSPVPLCVCLLLTCGFAEAGKLLVVPMDGSHWFTMQSVVEKLILRGHEVVVVMPEVSWQLERSLNCTVKTYSTSYTLEDQN
REFMVFAHAQWKAQAQSIFSLLMSSSSGFLDLFFSHCRSLFNDRKLVEYLKESSFDAVFLDPFDTCGLIVAKYFSLPSVVFTRGIFC
HHLEEGAQCPAPLSYVPNDLLGFSDAMTFKERVWNHIVHLEDHLFCQYLFRNALEIASEILQTPVTAYDLYSHTSIWLLRTDFVLDY
PKPVMPNMIFIGGINCHQGKPLPMEFEAYINASGEHGIVVFSLGSMVSEIPEKKAMAIADALGKIPQTVLWRYTGTRPSNLANNTIL
VKWLPQNDLLGHPMTRAFITHAGSHGVYESICNGVPMVMMPLFGDQMDNAKRMETKGAGVTLNVLEMTSEDLENALKAVINDKSYKE
NIMRLSSLHKDRPVEPLDLAVFWVEFVMRHKGAPHLRPAAHDLTWYQYHSLDVIGFLLAVVLTVAFITFKCCAYGYRKCLGKKGRVK
KAHKSKTH
>sp|Q6P1W5|CA094_HUMAN
MRGGGGCVLALGGQRGFQKERRRMASGNGLPSSSALVAKGPCALGPFPRYIWIHQDTPQDSLDKTCHEIWKRVQGLPEASQPWTSME
QLSVPVVGTLRGNELSFQEEALELSSGKDEISLLVEQEFLSLTKEHSILVEESSGELEVPGSSPEGTRELAPCILAPPLVAGSNERP
RASIIVGDKLLKQKVAMPVISSRQDCDSATSTVTDILCAAEVKSSKGTEDRGRILGDSNLQVSKLLSQFPLKSTETSKVPDNKNVLD
KTRVTKDFLQDNLFSGPGPKEPTGLSPFLLLPPRPPPARPDKLPELPAQKRQLPVFAKICSKPKADPAVERHHLMEWSPGTKEPKKG
QGSLFLSQWPQSQKDACGEEGCCDAVGTASLTLPPKKPTCPAEKNLLYEFLGATKNPSGQPRLRNKVEVDGPELKFNAPVTVADKNN
PKYTGNVFTPHFPTAMTSATLNQPLWLNLNYPPPPVFTNHSTFLQYQGLYPQQAARMPYQQALHPQLGCYSQQVMPYNPQQMGQQIF
RSSYTPLLSYIPFVQPNYPYPQRTPPKMSANPRDPPLMAGDGPQYLFPQGYGFGSTSGGPLMHSPYFSSSGNGINF
>sp|Q8WW38|FOG2_HUMAN
MSRRKQSKPRQIKRPLEDAIEDEEEECPSEETDIISKGDFPLEESFSTEFGPENLSCEEVEYFCNKGDDEGIQETAESDGDTQSEKP
GQPGVETDDWDGPGELEVFQKDGERKIQSRQQLPVGTTWGPFPGKMDLNNNSLKTKAQVPMVLTAGPKWLLDVTWQGVEDNKNNCIV
YSKGGQLWCTTTKAISEGEELIAFVVDFDSRLQAASQMTLTEGMYPARLLDSIQLLPQQAAMASILPTAIVNKDIFPCKSCGIWYRS
ERNLQAHLMYYCSGRQREAAPVSEENEDSAHQISSLCPFPQCTKSFSNARALEMHLNSHSGVKMEEFLPPGASLKCTVCSYTADSVI
NFHQHLFSHLTQAAFRCNHCHFGFQTQRELLQHQELHVPSGKLPRESDMEHSPSATEDSLQPATDLLTRSELPQSQKAMQTKDASSD
TELDKCEKKTQLFLTNQRPEIQPTTNKQSFSYTKIKSEPSSPRLASSPVQPNIGPSFPVGPFLSQFSFPQDITMVPQASEILAKMSE
LVHRRLRHGSSSYPPVIYSPLMPKGATCFECNITFNNLDNYLVHKKHYCSSRWQQMAKSPEFPSVSEKMPEALSPNTGQTSINLLNP
AAHSADPENPLLQTSCINSSTVLDLIGPNGKGHDKDFSTQTKKLSTSSNNDDKINGKPVDVKNPSVPLVDGESDPNKTTCEACNITF
SRHETYMVHKQYYCATRHDPPLKRSASNKVPAMQRTMRTRKRRKMYEMCLPEQEQRPPLVQQRFLDVANLNNPCTSTQEPTEGLGEC
YHPRCDIFPGIVSKHLETSLTINKCVPVSKCDTTHSSVSCLEMDVPIDLSKKCLSQSERTTTSPKRLLDYHECTVCKISFNKVENYL
AHKQNFCPVTAHQRNDLGQLDGKVFPNPESERNSPDVSYERSIIKCEKNGNLKQPSPNGNLFSSHLATLQGLKVFSEAAQLIATKEE
NRHLFLPQCLYPGAIKKAKGADQLSPYYGIKPSDYISGSLVIHNTDIEQSRNAENESPKGQASSNGCAALKKDSLPLLPKNRGMVIV
NGGLKQDERPAANPQQENISQNPQHEDDHKSPSWISENPLAANENVSPGIPSAEEQLSSIAKGVNGSSQAPTSGKYCRLCDIQFNNL
SNFITHKKFYCSSHAAEHVK
>sp|Q14151|SAFB2_HUMAN
MAETLPGSGDSGPGTASLGPGVAETGTRRLSELRVIDLRAELKKRNLDTGGNKSVLMERLKKAVKEEGQDPDEIGIELEATSKKSAK
RCVKGLKMEEEGTEDNGLEDDSRDGQEDMEASLENLQNMGMMDMSVLDETEVANSSAPDFGEDGTDGLLDSFCDSKEYVAAQLRQLP
AQPPEHAVDGEGFKNTLETSSLNFKVTPDIEESLLEPENEKILDILGETCKSEPVKEESSELEQPFAQDTSSVGPDRKLAEEEDLFD
SAHPEEGDLDLASESTAHAQSSKADSLLAVVKREPAEQPGDGERTDCEPVGLEPAVEQSSAASELAEASSEELAEAPTEAPSPEARD
SKEDGRKFDFDACNEVPPAPKESSTSEGADQKMSSFKEEKDIKPIIKDEKGRVGSGSGRNLWVSGLSSTTRATDLKNLFSKYGKVVG
AKVVTNARSPGARCYGFVTMSTSDEATKCISHLHRTELHGRMISVEKAKNEPAGKKLSDRKECEVKKEKLSSVDRHHSVEIKIEKTV
IKKEEKIEKKEEKKPEDIKKEEKDQDELKPGPTNRSRVTKSGSRGMERTVVMDKSKGEPVISVKTTSRSKERSSKSQDRKSESKEKR
DILSFDKIKEQREREREQRQREREIRETERRREREQREREQRLEAFHERKEKARLQRERLQLECQRQRLERERMERERLERERMRVER
ERRKEQERIHREREELRRQQEQLRYEQERRPGRRPYDLDRRDDAYWPEGKRVAMEDRYRADFPRPDHRFHDFDHRDRGQYQDHAIDR
REGSRPMMGDHRDGQHYGDDRHGHGGPPERHGRDSRDGWGGYGSDKRLSEGRGLPPPPRGGRDWGEHNQRLEEHQARAWQGAMDAGA
ASREHARWQGGERGLSGPSGPGHMASRGGVAGRGGFAQGGHSQGHVVPGGGLEGGGVASQDRGSRVPHPHPHPPPYPHFTRRY
>sp|Q8IYX3|CC116_HUMAN

MARCRHHSGYLADDEASHSMCSARVQLPKKPLVPEMRPACKPGRVPHPPSTCGSSALQGQRRNKRHPQPFGHFLDFLTESQVLDSLE
TVVEKATERMAAMKTEAGVPLVEVQDPVEVPSGGRRAHARPSLSTVHRHRVRPTLCTGHPNNYPSSSSSMSNCHSSLMAGCLGSHSR
DSDLGAQGSLPPVRDKLLLEKNLKRLLQLEREGKGLSQSCSQRDSLLWDSLGSQTSFQWTQEQPLSWFSGLLGSSSGVPEASEPRPG
EQEPIFRKREFNKEIKSLLSQLESLDLPGYCPLREPHRTLNFLADHRLFPALQSVVSQAVDKLRGAHCRDGRPLFPTSLEPTSDLPP
LGSEPAKPTNGGQPYASPRPTVSSPKMLQRKRKDRGGSPSMSSAQVATRFKLKSPCSSSRFTKKKPLPSISSKSSMSHFSNRLYEEL
ADFLTQQAASLVIRKYEFEKDLSKQLGFFSFPITHVLRDLSLGLKKVKGSRIHLSSETHRSCLLRKLEESKRAAAVSLEA
>sp|Q9P253|VPS18_HUMAN
MASILDEYENSLSRSAVLQPGCPSVGIPHSGYVNAQLEKEVPIFTKQRIDFTPSERITSLVVSSNQLCMSLGKDTLLRIDLGKANEP
NHVELGRKDDAKVHKMFLDHTGSHLLIALSSTEVLYVNRNGQKVRPLARWKGQLVESVGWNKALGTESSTGPILVGTAQGHIFEAEL
SASEGGLFGPAPDLYFRPLYVLNEEGGPAPVCSLEAERGPDGRSFVIATTRQRLFQFIGRAAEGAEAQGFSGLFAAYTDHPPPFREF
PSNLGYSELAFYTPKLRSAPRAFAWMMGDGVLYGALDCGRPDSLLSEERVWEYPEGVGPGASPPLAIVLTQFHFLLLLADRVEAVCT
LTGQVVLRDHFLEKFGPLKHMVKDSSTGQLWAYTERAVFRYHVQREARDVWRTYLDMNRFDLAKEYCRERPDCLDTVLAREADFCFR
QRRYLESARCYALTQSYFEEIALKFLEARQEEALAEFLQRKLASLKPAERTQATLLTTWLTELYLSRLGALQGDPEALTLYRETKEC
FRTFLSSPRHKEWLFASRASIHELLASHGDTEHMVYFAVIMQDYERVVAYHCQHEAYEEALAVLARHRDPQLFYKFSPILIRHIPRQ
LVDAWIEMGSRLDARQLIPALVNYSQGGEVQQVSQAIRYMEFCVNVLGETEQAIHNYLLSLYARGRPDSLLAYLEQAGASPHRVHYD
LKYALRLCAEHGHHRACVHVYKVLELYEEAVDLALQVDVDLAKQCADLPEEDEELRKKLWLKIARHVVQEEEDVQTAMACLASCPLL
KIEDVLPFFPDFVTIDHFKEAICSSLKAYNHHIQELQREMEEATASAQRIRRDLQELRGRYGTVEPQDKCATCDFPLLNRPFYLFLC
GHMFHADCLLQAVRPGLPAYKQARLEELQRKLGAAPPPAKGSARAKEAEGGAATAGPSREQLKADLDELVAAECVYCGELMIRSIDR
PFIDPQRYEEEQLSWL
>sp|Q9NZU1|FLRT1_HUMAN
MDLRDWLFLCYGLIAFLTEVIDSTTCPSVCRCDNGFIYCNDRGLTSIPADIPDDATTLYLQNNQINNAGIPQDLKTKVNVQVIYLYE
NDLDEFPINLPRSLRELHLQDNNVRTIARDSLARIPLLEKLHLDDNSVSTVSIEEDAFADSKQLKLLFLSRNHLSSIPSGLPHTLEE
LRLDDNRISTIPLHAFKGLNSLRRLVLDGNLLANQRIADDTFSRLQNLTELSLVRNSLAAPPLNLPSAHLQKLYLQDNAISHIPYNT
LAKMRELERLDLSNNNLTTLPRGLFDDLGNLAQLLLRNNPWFCGCNLMWLRDWVKARAAVVNVRGLMCQGPEKVRGMAIKDITSEMD
ECFETGPQGGVANAAAKTTASNHASATTPQGSLFTLKAKRPGLRLPDSNIDYPMATGDGAKTLAIHVKALTADSIRITWKATLPASS
FRLSWLRLGHSPAVGSITETLVQGDKTEYLLTALEPKSTYIICMVTMETSNAYVADETPVCAKAETADSYGPTTTLNQEQNAGPMAS
LPLAGIIGGAVALVFLFLVLGAICWYVHQAGELLTRERAYNRGSRKKDDYMESGTKKDNSILEIRGPGLQMLPINPYRAKEEYVVHT
IFPSNGSSLCKATHTIGYGTTRGYRDGGIPDIDYSYT
>sp|Q07283|TRHY_HUMAN
MSPLLRSICDITEIFNQYVSHDCDGAALTKKDLKNLLEREFGAVLRRPHDPKTVDLILELLDLDSNGRVDFNEFLLFIFKVAQACYY
ALGQATGLDEEKRARCDGKESLLQDRRQEEDQRRFEPRDRQLEEEPGQRRRQKRQEQERELAEGEEQSEKQERLEQRDRQRRDEELW
RQRQEWQEREERRAEEEQLQSCKGHETEEFPDEEQLRRRELLELRRKGREEKQQQRRERQDRVFQEEEEKEWRKRETVLRKEEEKLQ
EEEPQRQRELQEEEEQLRKLERQELRRERQEEEQQQQRLRREQQLRRKQEEERREQQEERREQQERREQQEERREQQLRREQEERRE
QQLRREQEEERREQQLRREQEEERREQQLRREQQLRREQQLRREQQLRREQQLRREQQLRREQQLRREQQLRREQQLRREQEEERHE
QKHEQERREQRLKREQEERRDWLKREEETERHEQERRKQQLKRDQEEERRERWLKLEEEERREQQERREQQLRREQEERREQRLKRQ
EEEERLQQRLRSEQQLRREQEERREQLLKREEEKRLEQERREQRLKREQEERRDQLLKREEERRQQRLKREQEERLEQRLKREEVER
LEQEERREQRLKREEPEEERRQQLLKSEEQEERRQQQLRREQQERREQRLKREEEEERLEQRLKREHEEERREQELAEEEQEQARER
IKSRIPKWQWQLESEADARQSKVYSRPRKQEGQRRRQEQEEKRRRRESELQWQEEERAHRQQQEEEQRRDFTWQWQAEEKSERGRQR
LSARPPLREQRERQLRAEERQQREQRFLPEEEEKEQRRRQRREREKELQFLEEEEQLQRRERAQQLQEEEDGLQEDQERRRSQEQRR
DQKWRWQLEEERKRRRHTLYAKPALQEQLRKEQQLLQEEEEELQREEREKRRRQEQERQYREEEQLQQEEEQLLREEREKRRRQERE
RQYRKDKKLQQKEEQLLGEEPEKRRRQEREKKYREEEELQQEEEQLLREEREKRRRQEWERQYRKKDELQQEEEQLLREEREKRRLQ
ERERQYREEEELQQEEEQLLGEERETRRRQELERQYRKEEELQQEEEQLLREEPEKRRRQERERQCREEEELQQEEEQLLREEREKR
RRQELERQYREEEEVQQEEEQLLREEPEKRRRQELERQYREEEELQQEEEQLLREEQEKRRQERERQYREEEELQRQKRKQRYRDED
QRSDLKWQWEPEKENAVRDNKVYCKGRENEQFRQLEDSQLRDRQSQQDLQHLLGEQQERDREQERRRWQQRDRHFPEEEQLEREEQK

EAKRRDRKSQEEKQLLREEREEKRRRQETDRKFREEEQLLQEREEQPLRRQERDRKFREEELRHQEQGRKFLEEEQRLRRQERERKF
LKEEQQLRCQEREQQLRQDRDRKFREEEQQLSRQERDRKFREEEQQVRRQERERKFLEEEQQLRQERHRKFREEEQLLQEREEQQLH
RQERDRKFLEEEQQLRRQERDRKFREQELRSQEPERKFLEEEQQLHRQQRQRKFLQEEQQLRRQERGQQRRQDRDRKFREEEQLRQE
REEQQLSRQERDRKFRLEEQKVRRQEQERKFMEDEQQLRRQEGQQQLRQERDRKFREDEQLLQEREEQQLHRQERDRKFLEEEPQLR
RQEREQQLRHDRDRKFREEEQLLQEGEEQQLRRQERDRKFREEEQQLRRQERERKFLQEEQQLRRQELERKFREEEQLRQETEQEQL
RRQERYRKILEEEQLRPEREEQQLRRQERDRKFREEEQLRQEREEQQLRSQESDRKFREEEQLRQEREEQQLRPQQRDGKYRWEEEQ
LQLEEQEQRLRQERDRQYRAEEQFATQEKSRREEQELWQEEEQKRRQERERKLREEHIRRQQKEEQRHRQVGEIKSQEGKGHGRLLE
PGTHQFASVPVRSSPLYEYIQEQRSQYRP
>sp|Q53EP0|FND3B_HUMAN
MYVTMMMTDQIPLELPPLLNGEVAMMPHLVNGDAAQQVILVQVNPGETFTIRAEDGTLQCIQGPAEVPMMSPNGSIPPIHVPPGYIS
QVIEDSTGVRRVVVTPQSPECYPPSYPSAMSPTHHLPPYLTHHPHFIHNSHTAYYPPVTGPGDMPPQFFPQHHLPHTIYGEQEIIPF
YGMSTYITREDQYSKPPHKKLKDRQIDRQNRLNSPPSSIYKSSCTTVYNGYGKGHSGGSGGGGSGSGPGIKKTERRARSSPKSNDSD
LQEYELEVKRVQDILSGIEKPQVSNIQARAVVLSWAPPVGLSCGPHSGLSFPYSYEVALSDKGRDGKYKIIYSGEELECNLKDLRPA
TDYHVRVYAMYNSVKGSCSEPVSFTTHSCAPECPFPPKLAHRSKSSLTLQWKAPIDNGSKITNYLLEWDEGKRNSGFRQCFFGSQKH
CKLTKLCPAMGYTFRLAARNDIGTSGYSQEVVCYTLGNIPQMPSAPRLVRAGITWVTLQWSKPEGCSPEEVITYTLEIQEDENDNLF
HPKYTGEDLTCTVKNLKRSTQYKFRLTASNTEGKSCPSEVLVCTTSPDRPGPPTRPLVKGPVTSHGFSVKWDPPKDNGGSEILKYLL
EITDGNSEANQWEVAYSGSATEYTFTHLKPGTLYKLRACCISTGGHSQCSESLPVRTLSIAPGQCRPPRVLGRPKHKEVHLEWDVPA
SESGCEVSEYSVEMTEPEDVASEVYHGPELECTVGNLLPGTVYRFRVRALNDGGYGPYSDVSEITTAAGPPGQCKAPCISCTPDGCV
LVGWESPDSSGADISEYRLEWGEDEESLELIYHGTDTRFEIRDLLPAAQYCCRLQAFNQAGAGPYSELVLCQTPASAPDPVSTLCVL
EEEPLDAYPDSPSACLVLNWEEPCNNGSEILAYTIDLGDTSITVGNTTMHVMKDLLPETTYRIRIQAINEIGAGPFSQFIKAKTRPL
PPLPPRLECAAAGPQSLKLKWGDSNSKTHAAEDIVYTLQLEDRNKRFISIYRGPSHTYKVQRLTEFTCYSFRIQAASEAGEGPFSET
YTFSTTKSVPPTIKAPRVTQLEGNSCEILWETVPSMKGDPVNYILQVLVGRESEYKQVYKGEEATFQISGLQTNTDYRFRVCACRRC
LDTSQELSGAFSPSAAFVLQRSEVMLTGDMGSLDDPKMKSMMPTDEQFAAIIVLGFATLSILFAFILQYFLMK
>sp|Q9NX70|MED29_HUMAN
MAASQQQASAASSAAGVSGPSSAGGPGPQQQPQPPAQLVGPAQSGLLQQQQQDFDPVQRYKMLIPQLKESLQTLMKVAAQNLIQNTN
IDNGQKSSDGPIQRFDKCLEEFYALCDQLELCLRLAHECLSQSCDSAKHSPTLVPTATKPDAVQPDSLPYPQYLAVIKAQISCAKDI
HTALLDCANKVTGKTPAPPAGPGGTL
>sp|Q9UK28|TM59L_HUMAN
MAAVALMPPPLLLLLLLASPPAASAPSARDPFAPQLGDTQNCQLRCRDRDLGPQPSQAGLEGASESPYDRAVLISACERGCRLFSIC
RFVARSSKPNATQTECEAACVEAYVKEAEQQACSHGCWSQPAEPEPEQKRKVLEAPSGALSLLDLFSTLCNDLVNSAQGFVSSTWTY
YLQTDNGKVVVFQTQPIVESLGFQGGRLQRVEVTWRGSHPEALEVHVDPVGPLDKVRKAKIRVKTSSKAKVESEEPQDNDFLSCMSR
RSGLPRWILACCLFLSVLVMLWLSCSTLVTAPGQHLKFQPLTLEQHKGFMMEPDWPLYPPPSHACEDSLPPYKLKLDLTKL
>sp|P34896|GLYC_HUMAN
MTMPVNGAHKDADLWSSHDKMLAQPLKDSDVEVYNIIKKESNRQRVGLELIASENFASRAVLEALGSCLNNKYSEGYPGQRYYGGTE
FIDELETLCQKRALQAYKLDPQCWGVNVQPYSGSPANFAVYTALVEPHGRIMGLDLPDGGHLTHGFMTDKKKISATSIFFESMPYKV
NPDTGYINYDQLEENARLFHPKLIIAGTSCYSRNLEYARLRKIADENGAYLMADMAHISGLVAAGVVPSPFEHCHVVTTTTHKTLRG
CRAGMIFYRKGVKSVDPKTGKEILYNLESLINSAVFPGLQGGPHNHAIAGVAVALKQAMTLEFKVYQHQVVANCRALSEALTELGYK
IVTGGSDNHLILVDLRSKGTDGGRAEKVLEACSIACNKNTCPGDRSALRPSGLRLGTPALTSRGLLEKDFQKVAHFIHRGIELTLQI
QSDTGVRATLKEFKERLAGDKYQAAVQALREEVESFASLFPLPGLPDF
>sp|Q9NXS3|KLH28_HUMAN
MDHTSPTYMLANLTHLHSEQLLQGLNLLRQHHELCDIILRVGDVKIHAHKVVLASVSPYFKAMFTGNLSEKENSEVEFQCIDETALQ
AIVEYAYTGTVFISQDTVESLLPAANLLQIKLVLKECCAFLESQLDPGNCIGISRFAETYGCRDLYLAATKYICQNFEAVCQTEEFF
ELTHADLDEIVSNDCLNVATEETVFYALESWIKYDVQERQKYLAQLLNSVRLPLLSVKFLTRLYEANHLIRDDRTCKHLLNEALKYH
FMPEHRLSHQTVLMTRPRCAPKVLCAVGGKSGLFACLDSVEMYFPQNDSWIGLAPLNIPRYEFGICVLDQKVYVIGGIATNVRPGVT

IRKHENSVECWNPDTNTWTSLERMNESRSTLGVVVLAGELYALGGYDGQSYLQSVEKYIPKIRKWQPVAPMTTTRSCFAAAVLDGMI
YAIGGYGPAHMNSVERYDPSKDSWEMVASMADKRIHFGVGVMLGFIFVVGGHNGVSHLSSIERYDPHQNQWTVCRPMKEPRTGVGAA
VIDNYLYVVGGHSGSSYLNTVQKYDPISDTWLDSAGMIYCRCNFGLTAL
>sp|O75882|ATRN_HUMAN
MVAAAAATEARLRRRTAATAALAGRSGGPHWDWDVTRAGRPGLGAGLRLPRLLSPPLRPRLLLLLLLLSPPLLLLLLPCEAEAAAAA
AAVSGSAAAEAKECDRPCVNGGRCNPGTGQCVCPAGWVGEQCQHCGGRFRLTGSSGFVTDGPGNYKYKTKCTWLIEGQPNRIMRLRF
NHFATECSWDHLYVYDGDSIYAPLVAAFSGLIVPERDGNETVPEVVATSGYALLHFFSDAAYNLTGFNITYSFDMCPNNCSGRGECK
ISNSSDTVECECSENWKGEACDIPHCTDNCGFPHRGICNSSDVRGCSCFSDWQGPGCSVPVPANQSFWTREEYSNLKLPRASHKAVV
NGNIMWVVGGYMFNHSDYNMVLAYDLASREWLPLNRSVNNVVVRYGHSLALYKDKIYMYGGKIDSTGNVTNELRVFHIHNESWVLLT
PKAKEQYAVVGHSAHIVTLKNGRVVMLVIFGHCPLYGYISNVQEYDLDKNTWSILHTQGALVQGGYGHSSVYDHRTRALYVHGGYKA
FSANKYRLADDLYRYDVDTQMWTILKDSRFFRYLHTAVIVSGTMLVFGGNTHNDTSMSHGAKCFSSDFMAYDIACDRWSVLPRPDLH
HDVNRFGHSAVLHNSTMYVFGGFNSLLLSDILVFTSEQCDAHRSEAACLAAGPGIRCVWNTGSSQCISWALATDEQEEKLKSECFSK
RTLDHDRCDQHTDCYSCTANTNDCHWCNDHCVPRNHSCSEGQISIFRYENCPKDNPMYYCNKKTSCRSCALDQNCQWEPRNQECIAL
PENICGIGWHLVGNSCLKITTAKENYDNAKLFCRNHNALLASLTTQKKVEFVLKQLRIMQSSQSMSKLTLTPWVGLRKINVSYWCWE
DMSPFTNSLLQWMPSEPSDAGFCGILSEPSTRGLKAATCINPLNGSVCERPANHSAKQCRTPCALRTACGDCTSGSSECMWCSNMKQ
CVDSNAYVASFPFGQCMEWYTMSTCPPENCSGYCTCSHCLEQPGCGWCTDPSNTGKGKCIEGSYKGPVKMPSQAPTGNFYPQPLLNS
SMCLEDSRYNWSFIHCPACQCNGHSKCINQSICEKCENLTTGKHCETCISGFYGDPTNGGKCQPCKCNGHASLCNTNTGKCFCTTKG
VKGDECQLCEVENRYQGNPLRGTCYYTLLIDYQFTFSLSQEDDRYYTAINFVATPDEQNRDLDMFINASKNFNLNITWAASFSAGTQ
AGEEMPVVSKTNIKEYKDSFSNEKFDFRNHPNITFFVYVSNFTWPIKIQIAFSQHSNFMDLVQFFVTFFSCFLSLLLVAAVVWKIKQ
SCWASRRREQLLREMQQMASRPFASVNVALETDEEPPDLIGGSIKTVPKPIALEPCFGNKAAVLSVFVRLPRGLGGIPPPGQSGLAV
ASALVDISQQMPIVYKEKSGAVRNRKQQPPAQPGTCI
>sp|P11177|ODPB_HUMAN
MAAVSGLVRRPLREVSGLLKRRFHWTAPAALQVTVRDAINQGMDEELERDEKVFLLGEEVAQYDGAYKVSRGLWKKYGDKRIIDTPI
SEMGFAGIAVGAAMAGLRPICEFMTFNFSMQAIDQVINSAAKTYYMSGGLQPVPIVFRGPNGASAGVAAQHSQCFAAWYGHCPGLKV
VSPWNSEDAKGLIKSAIRDNNPVVVLENELMYGVPFEFPPEAQSKDFLIPIGKAKIERQGTHITVVSHSRPVGHCLEAAAVLSKEGV
ECEVINMRTIRPMDMETIEASVMKTNHLVTVEGGWPQFGVGAEICARIMEGPAFNFLDAPAVRVTGADVPMPYAKILEDNSIPQVKD
IIFAIKKTLNI
>sp|Q7Z398|ZN550_HUMAN
MAETKDAAQMLVTFKDVAVTFTREEWRQLDLAQRTLYREVMLETCGLLVSLGHRVPKPELVHLLEHGQELWIVKRGLSHATCAGDRA
QVHTREPTTYPPVLSERAFLRGSLTLESSTSSDSRLGRARDEEGLLEMQKGKVTPETDLHKETHLGKVSLEGEGLGTDDGLHSRALQ
EWLSADVLHECDSQQPGKDALIHAGTNPYKCKQCGKGFNRKWYLVRHQRVHTGMKPYECNACGKAFSQSSTLIRHYLIHTGEKPYKC
LECGKAFKRRSYLMQHHPIHTGEKPYECSQCRKAFTHRSTFIRHNRTHTGEKPFECKECEKAFSNRAHLIQHYIIHTGEKPYDCMAC
GKAFRCSSELIQHQRIHTGEKPYECTQCGKAFHRSTYLIQHSVIHTGEMPYKCIECGKAFKRRSHLLQHQRVHT
>sp|O75330|HMMR_HUMAN
MSFPKAPLKRFNDPSGCAPSPGAYDVKTLEVLKGPVSFQKSQRFKQQKESKQNLNVDKDTTLPASARKVKSSESKESQKNDKDLKIL
EKEIRVLLQERGAQDRRIQDLETELEKMEARLNAALREKTSLSANNATLEKQLIELTRTNELLKSKFSENGNQKNLRILSLELMKLR
NKRETKMRGMMAKQEGMEMKLQVTQRSLEESQGKIAQLEGKLVSIEKEKIDEKSETEKLLEYIEEISCASDQVEKYKLDIAQLEENL
KEKNDEILSLKQSLEENIVILSKQVEDLNVKCQLLEKEKEDHVNRNREHNENLNAEMQNLKQKFILEQQEREKLQQKELQIDSLLQQ
EKELSSSLHQKLCSFQEEMVKEKNLFEEELKQTLDELDKLQQKEEQAERLVKQLEEEAKSRAEELKLLEEKLKGKEAELEKSSAAHT
QATLLLQEKYDSMVQSLEDVTAQFESYKALTASEIEDLKLENSSLQEKAAKAGKNAEDVQHQILATESSNQEYVRMLLDLQTKSALK
ETEIKEITVSFLQKITDLQNQLKQQEEDFRKQLEDEEGRKAEKENTTAELTEEINKWRLLYEELYNKTKPFQLQLDAFEVEKQALLN
EHGAAQEQLNKIRDSYAKLLGHQNLKQKIKHVVKLKDENSQLKSEVSKLRCQLAKKKQSETKLQEELNKVLGIKHFDPSKAFHHESK
ENFALKTPLKEGNTNCYRAPMECQESWK
>sp|Q13950|RUNX2_HUMAN

MASNSLFSTVTPCQQNFFWDPSTSRRFSPPSSSLQPGKMSDVSPVVAAQQQQQQQQQQQQQQQQQQQQQQQEAAAAAAAAAAAAAAA
AAVPRLRPPHDNRTMVEIIADHPAELVRTDSPNFLCSVLPSHWRCNKTLPVAFKVVALGEVPDGTVVTVMAGNDENYSAELRNASAV
MKNQVARFNDLRFVGRSGRGKSFTLTITVFTNPPQVATYHRAIKVTVDGPREPRRHRQKLDDSKPSLFSDRLSDLGRIPHPSMRVGV
PPQNPRPSLNSAPSPFNPQGQSQITDPRQAQSSPPWSYDQSYPSYLSQMTSPSIHSTTPLSSTRGTGLPAITDVPRRISDDDTATSD
FCLWPSTLSKKSQAGASELGPFSDPRQFPSISSLTESRFSNPRMHYPATFTYTPPVTSGMSLGMSATTHYHTYLPPPYPGSSQSQSG
PFQTSSTPYLYYGTSSGSYQFPMVPGGDRSPSRMLPPCTTTSNGSTLLNPNLPNQNDGVDADGSHSSSPTVLNSSGRMDESVWRPY
>sp|O60488|ACSL4_HUMAN
MKLKLNVLTIILLPVHLLITIYSALIFIPWYFLTNAKKKNAMAKRIKAKPTSDKPGSPYRSVTHFDSLAVIDIPGADTLDKLFDHAV
SKFGKKDSLGTREILSEENEMQPNGKVFKKLILGNYKWMNYLEVNRRVNNFGSGLTALGLKPKNTIAIFCETRAEWMIAAQTCFKYN
FPLVTLYATLGKEAVVHGLNESEASYLITSVELLESKLKTALLDISCVKHIIYVDNKAINKAEYPEGFEIHSMQSVEELGSNPENLG
IPPSRPTPSDMAIVMYTSGSTGRPKGVMMHHSNLIAGMTGQCERIPGLGPKDTYIGYLPLAHVLELTAEISCFTYGCRIGYSSPLTL
SDQSSKIKKGSKGDCTVLKPTLMAAVPEIMDRIYKNVMSKVQEMNYIQKTLFKIGYDYKLEQIKKGYDAPLCNLLLFKKVKALLGGN
VRMMLSGGAPLSPQTHRFMNVCFCCPIGQGYGLTESCGAGTVTEVTDYTTGRVGAPLICCEIKLKDWQEGGYTINDKPNPRGEIVIG
GQNISMGYFKNEEKTAEDYSVDENGQRWFCTGDIGEFHPDGCLQIIDRKKDLVKLQAGEYVSLGKVEAALKNCPLIDNICAFAKSDQ
SYVISFVVPNQKRLTLLAQQKGVEGTWVDICNNPAMEAEILKEIREAANAMKLERFEIPIKVRLSPEPWTPETGLVTDAFKLKRKEL
RNHYLKDIERMYGGK
>sp|Q8NHZ7|MB3L2_HUMAN
MGEPAFTSFPSLPVLGKLKRNMMPWALQKKREIHMAKAHRRRAARSALPMRLTSCIFRRPVTRIRSHPDNQVRRRKGDEHLEKPQQL
CAYRRLQALQPCSSQGEGSSPLHLESVLSILAPGTAGESLDRAGAERVRSPLEPTPGRFPAVAGGPTPGMGCQLPPPLSGQLVTPAD
IRRQARRVKKARERLAKALQADRLARRAEMLTGG
>sp|O15394|NCAM2_HUMAN
MSLLLSFYLLGLLVSSGQALLQVTISLSKVELSVGESKFFTCTAIGEPESIDWYNPQGEKIISTQRVVVQKEGVRSRLTIYNANIED
AGIYRCQATDAKGQTQEATVVLEIYQKLTFREVVSPQEFKQGEDAEVVCRVSSSPAPAVSWLYHNEEVTTISDNRFAMLANNNLQIL
NINKSDEGIYRCEGRVEARGEIDFRDIIVIVNVPPAISMPQKSFNATAERGEEMTFSCRASGSPEPAISWFRNGKLIEENEKYILKG
SNTELTVRNIINSDGGPYVCRATNKAGEDEKQAFLQVFVQPHIIQLKNETTYENGQVTLVCDAEGEPIPEITWKRAVDGFTFTEGDK
SLDGRIEVKGQHGSSSLHIKDVKLSDSGRYDCEAASRIGGHQKSMYLDIEYAPKFISNQTIYYSWEGNPINISCDVKSNPPASIHWR
RDKLVLPAKNTTNLKTYSTGRKMILEIAPTSDNDFGRYNCTATNHIGTRFQEYILALADVPSSPYGVKIIELSQTTAKVSFNKPDSH
GGVPIHHYQVDVKEVASEIWKIVRSHGVQTMVVLNNLEPNTTYEIRVAAVNGKGQGDYSKIEIFQTLPVREPSPPSIHGQPSSGKSF
KLSITKQDDGGAPILEYIVKYRSKDKEDQWLEKKVQGNKDHIILEHLQWTMGYEVQITAANRLGYSEPTVYEFSMPPKPNIIKDTLF
NGLGLGAVIGLGVAALLLILVVTDVSCFFIRQCGLLMCITRRMCGKKSGSSGKSKELEEGKAAYLKDGSKEPIVEMRTEDERVTNHE
DGSPVNEPNETTPLTEPEKLPLKEEDGKEALNPETIEIKVSNDIIQSKEDDSKA
>sp|Q6NV75|GP153_HUMAN
MSDERRLPGSAVGWLVCGGLSLLANAWGILSVGAKQKKWKPLEFLLCTLAATHMLNVAVPIATYSVVQLRRQRPDFEWNEGLCKVFV
STFYTLTLATCFSVTSLSYHRMWMVCWPVNYRLSNAKKQAVHTVMGIWMVSFILSALPAVGWHDTSERFYTHGCRFIVAEIGLGFGV
CFLLLVGGSVAMGVICTAIALFQTLAVQVGRQADRRAFTVPTIVVEDAQGKRRSSIDGSEPAKTSLQTTGLVTTIVFIYDCLMGFPV
LVVSFSSLRADASAPWMALCVLWCSVAQALLLPVFLWACDRYRADLKAVREKCMALMANDEESDDETSLEGGISPDLVLERSLDYGY
GGDFVALDRMAKYEISALEGGLPQLYPLRPLQEDKMQYLQVPPTRRFSHDDADVWAAVPLPAFLPRWGSGEDLAALAHLVLPAGPER
RRASLLAFAEDAPPSRARRRSAESLLSLRPSALDSGPRGARDSPPGSPRRRPGPGPRSASASLLPDAFALTAFECEPQALRRPPGPF
PAAPAAPDGADPGEAPTPPSSAQRSPGPRPSAHSHAGSLRPGLSASWGEPGGLRAAGGGGSTSSFLSSPSESSGYATLHSDSLGSAS
>sp|Q9UQ05|KCNH4_HUMAN
MPVMKGLLAPQNTFLDTIATRFDGTHSNFLLANAQGTRGFPIVYCSDGFCELTGYGRTEVMQKTCSCRFLYGPETSEPALQRLHKAL
EGHQEHRAEICFYRKDGSAFWCLLDMMPIKNEMGEVVLFLFSFKDITQSGSPGLGPQGGRGDSNHENSLGRRGATWKFRSARRRSRT
VLHRLTGHFGRRGQGGMKANNNVFEPKPSVPEYKVASVGGSRCLLLHYSVSKAIWDGLILLATFYVAVTVPYNVCFSGDDDTPITSR
HTLVSDIAVEMLFILDIILNFRTTYVSQSGQVISAPRSIGLHYLATWFFIDLIAALPFDLLYIFNITVTSLVHLLKTVRLLRLLRLL

QKLERYSQCSAVVLTLLMSVFALLAHWMACIWYVIGRREMEANDPLLWDIGWLHELGKRLEVPYVNGSVGGPSRRSAYIAALYFTLS
SLTSVGFGNVCANTDAEKIFSICTMLIGALMHAVVFGNVTAIIQRMYSRRSLYHSRMKDLKDFIRVHRLPRPLKQRMLEYFQTTWAV
NSGIDANELLRDFPDELRADIAMHLNREILQLPLFGAASRGCLRALSLHIKTSFCAPGEYLLRRGDALQAHYYVCSGSLEVLRDNMV
LAILGKGDLIGADIPEPGQEPGLGADPNFVLKTSADVKALTYCGLQQLSSRGLAEVLRLYPEYGAAFRAGLPRDLTFNLRQGSDTSG
LSRFSRSPRLSQPRSESLGSSSDKTLPSITEAESGAEPGGGPRPRRPLLLPNLSPARPRGSLVSLLGEELPPFSALVSSPSLSPSLS
PALAGQGHSASPHGPPRCSAAWKPPQLLIPPLGTFGPPDLSPRIVDGIEDSGSTAEAPSFRFSRRPELPRPRSQAPPTGTRPSPELA
SEAEEVKEKVCRLNQEISRLNQEVSQLSRELRHIMGLLQARLGPPGHPAGSAWTPDPPCPQLRPPCLSPCASRPPPSLQDTTLAEVH
CPASVGTMETGTALLDLRPSILPPYPSEPDPLGPSPVPEASPPTPSLLRHSFQSRSDTFH
>sp|Q68G74|LHX8_HUMAN
MQILSRCQGLMSEECGRTTALAAGRTRKGAGEEGLVSPEGAGDEDSCSSSAPLSPSSSPRSMASGSGCPPGKCVCNSCGLEIVDKYL
LKVNDLCWHVRCLSCSVCRTSLGRHTSCYIKDKDIFCKLDYFRRYGTRCSRCGRHIHSTDWVRRAKGNVYHLACFACFSCKRQLSTG
EEFALVEEKVLCRVHYDCMLDNLKREVENGNGISVEGALLTEQDVNHPKPAKRARTSFTADQLQVMQAQFAQDNNPDAQTLQKLAER
TGLSRRVIQVWFQNCRARHKKHVSPNHSSSTPVTAVPPSRLSPPMLEEMAYSAYVPQDGTMLTALHSYMDAHSPTTLGLQPLLPHSM
TQLPISHT
>sp|A6NHS1|YK042_HUMAN
MVLLAGTRPQGGEARCMIPPPPSPLLGAQVEEDRTEFKEFQDFSSLPDTRSVASDDSLYPFQDEEEHGVEGVESVPEEGILEAWGSC
GRWCGVG
>sp|Q9NS67|GPR27_HUMAN
MANASEPGGSGGGEAAALGLKLATLSLLLCVSLAGNVLFALLIVRERSLHRAPYYLLLDLCLADGLRALACLPAVMLAARRAAAAAG
[truncated: 928,873 more chars]
