# Supplementary material for: The Amino Acid Composition of Quadruplex Binding Proteins Reveals a Shared Motif and Predicts New Potential Quadruplex Interactors
Source: Molecules. 2018 Sep 13;23(9):2341. doi: 10.3390/molecules23092341 (PMC6225207; doi:10.3390/molecules23092341)
Supplement: Supplementary file 1 [file molecules-23-02341-s001.zip › molecules-355581-supplementary/Supplementary material 3D.pdf]

>ENSG00000011478|ENST00000012049
MRSGGRGRPRLRLGERGLMEPLLPPKRRLLPRVRLLPLLLALAVGSAFYTIWSGWHRRTE
ELPLGRELRVPLIGSLPEARLRRVVGQLDPQRLWSTYLRPLLVVRTPGSPGNLQVRKFLE
ATLRSLTAGWHVELDPFTASTPLGPVDFGNVVATLDPRAARHLTLACHYDSKLFPPGSTP
FVGATDSAVPCALLLELAQALDLELSRAKKQAAPVTLQLLFLDGEEALKEWGPKDSLYGS
RHLAQLMESIPHSPGPTRIQAIELFMLLDLLGAPNPTFYSHFPRTVRWFHRLRSIEKRLH
RLNLLQSHPQEVMYFQPGEPFGSVEDDHIPFLRRGVPVLHLISTPFPAVWHTPADTEVNL
HPPTVHNLCRILAVFLAEYLGL*
>ENSG00000008118|ENST00000009105
MGRKEEDDCSSWKKQTTNIRKTFIFMEVLGSGAFSEVFLVKQRLTGKLFALKCIKKSPAF
RDSSLENEIAVLKKIKHENIVTLEDIYESTTHYYLVMQLVSGGELFDRILERGVYTEKDA
SLVIQQVLSAVKYLHENGIVHRDLKPENLLYLTPEENSKIMITDFGLSKMEQNGIMSTAC
GTPGYVAPEVLAQKPYSKAVDCWSIGVITYILLCGYPPFYEETESKLFEKIKEGYYEFES
PFWDDISESAKDFICHLLEKDPNERYTCEKALSHPWIDGNTALHRDIYPSVSLQIQKNFA
KSKWRQAFNAAAVVHHMRKLHMNLHSPGVRPEVENRPPETQASETSRPSSPEITITEAPV
LDHSVALPALTQLPCQHGRRPTAPGGRSLNCLVNGSLHISSSLVPMHQGSLAAGPCGCCS
SCLNIGSKGKSSYCSEPTLLKKANKKQNFKSEVMVPVKASGSSHCRAGQTGVCLIM*
>ENSG00000003056|ENST00000000412
MFPFYSCWRTGLLLLLLAVAVRESWQTEEKTCDLVGEKGKESEKELALVKRLKPLFNKSF
ESTVGQGSDTYIYIFRVCREAGNHTSGAGLVQINKSNGKETVVGRLNETHIFNGSNWIML
IYKGGDEYDNHCGKEQRRAVVMISCNRHTLADNFNPVSEERGKVQDCFYLFEMDSSLACS
PEISHLSVGSILLVTFASLVAVYVVGGFLYQRLVVGAKGMEQFPHLAFWQDLGNLVADGC
DFVCRSKPRNVPAAYRGVGDDQLGEESEERDDHLLPM*
>ENSG00000047932|ENST00000052569
MSAGGPCPAAAGGGPGGASCSVGAPGGVSMFRWLEVLEKEFDKAFVDVDLLLGEIDPDQA
DITYEGRQKMTSLSSCFAQLCHKAQSVSQINHKLEAQLVDLKSELTETQAEKVVLEKEVH
DQLLQLHSIQLQLHAKTGQSADSGTIKAKLERELEANKKEKMKEAQLEAEVKLLRKENEA
LRRHIAVLQAEVYGARLAAKYLDKELAGRVQQIQLLGRDMKGPAHDKLWNQLEAEIHLHR
HKTVIRACRGRNDLKRPMQAPPGHDQDSLKKSQGVGPIRKVLLLKEDHEGLGISITGGKE
HGVPILISEIHPGQPADRCGGLHVGDAILAVNGVNLRDTKHKEAVTILSQQRGEIEFEVV
YVAPEVDSDDENVEYEDESGHRYRLYLDELEGGGNPGASCKDTSGEIKVLQGFNKKAVTD
THENGDLGTASETPLDDGASKLDDLHTLYHKKSY*
>ENSG00000005486|ENST00000006777
MAASGPGCRSWCLCPEVPSATFFTALLSLLVSGPRLFLLQQPLAPSGLTLKSEALRNWQV
YRLVTYIFVYENPISLLCGAIIIWRFAGNFERTVGTVRHCFFTVIFAIFSAIIFLSFEAV
SSLSKLGEVEDARGFTPVAFAMLGVTTVRSRMRRALVFGMVVPSVLVPWLLLGASWLIPQ
TSFLSNVCGLSIGLAYGLTYCYSIDLSERVALKLDQTFPFSLMRRISVFKYVSGSSAERR
AAQSRKLNPVPGSYPTQSCHPHLSPSHPVSQTQHASGQKLASWPSCTPGHMPTLPPYQPA
SGLCYVQNHFGPNPTSSSVYPASAGTSLGIQPPTPVNSPGTVYSGALGTPGAAGSKESSR
VPMP*
>ENSG00000015532|ENST00000017003
MVASARVQKLVRRYKLAIATALAILLLQGLVVWSFSGLEEDEAGEKGRQRKPRPLDPGEG
SKDTDSSAGRRGSTGRRHGRWRGRAESPGVPVAKVVRAVTSRQRASRRVPPAPPPEAPGR
QNLSGAAAGEALVGAAGFPPHGDTGSVEGAPQPTDNGFTPKCEIVGKDALSALARASTKQ

CQQEIANVVCLHQAGSLMPKAVPRHCQLTGKMSPGIQWDESQAQQPMDGPPVRIAYMLVV
HGRAIRQLKRLLKAVYHEQHFFYIHVDKRSDYLHREVVELAQGYDNVRVTPWRMVTIWGG
ASLLRMYLRSMRDLLEVPGWAWDFFINLSATDYPTRTNEELVAFLSKNRDKNFLKSHGRD
NSRFIKKQGLDRLFHECDSHMWRLGERQIPAGIVVDGGSDWFVLTRSFVEYVVYTDDPLV
AQLRQFYTYTLLPAESFFHTVLENSLACETLVDNNLRVTNWNRKLGCKCQYKHIVDWCGC
SPNDFKPQDFLRLQQVSRPTFFARKFESTVNQEVLEILDFHLYGSYPPGTPALKAYWENT
YDAADGPSGLSDVMLTAYTAFARLSLHHAATAAPPMGTPLCRFEPRGLPSSVHLYFYDDH
FQGYLVTQAVQPSAQGPAETLEMWLMPQGSLKLLGRSDQASRLQSLEVGTDWDPKERLFR
NFGGLLGPLDEPVAVQRWARGPNLTATVVWIDPTYVVATSYDITVDTETEVTQYKPPLSR
PLRPGPWTVRLLQFWEPLGETRFLVLPLTFNRKLPLRKDDASWLHAGPPHNEYMEQSFQG
LSSILNLPQPELAEEAAQRHTQLTGPALEAWTDRELSSFWSVAGLCAIGPSPCPSLEPCR
LTSWSSLSPDPKSELGPVKADGRLR*
>ENSG00000033100|ENST00000035307
MRLSSLLALLRPALPLILGLSLGCSLSLLRVSWIQGEGEDPCVEAVGERGGPQNPDSRAR
LDQSDEDFKPRIVPYYRDPNKPYKKVLRTRYIQTELGSRERLLVAVLTSRATLSTLAVAV
NRTVAHHFPRLLYFTGQRGARAPAGMQVVSHGDERPAWLMSETLRHLHTHFGADYDWFFI
MQDDTYVQAPRLAALAGHLSINQDLYLGRAEEFIGAGEQARYCHGGFGYLLSRSLLLRLR
PHLDGCRGDILSARPDEWLGRCLIDSLGVGCVSQHQGQQYRSFELAKNRDPEKEGSSAFL
SAFAVHPVSEGTLMYRLHKRFSALELERAYSEIEQLQAQIRNLTVLTPEGEAGLSWPVGL
PAPFTPHSRFEVLGWDYFTEQHTFSCADGAPKCPLQGASRADVGDALETALEQLNRRYQP
RLRFQKQRLLNGYRRFDPARGMEYTLDLLLECVTQRGHRRALARRVSLLRPLSRVEILPM
PYVTEATRVQLVLPLLVAEAAAAPAFLEAFAANVLEPREHALLTLLLVYGPREGGRGAPD
PFLGVKAAAAELERRYPGTRLAWLAVRAEAPSQVRLMDVVSKKHPVDTLFFLTTVWTRPG
PEVLNRCRMNAISGWQAFFPVHFQEFNPALSPQRSPPGPPGAGPDPPSPPGADPSRGAPI
GGRFDRQASAEGCFYNADYLAARARLAGELAGQEEEEALEGLEVMDVFLRFSGLHLFRAV
EPGLVQKFSLRDCSPRLSEELYHRCRLSNLEGLGGRAQLAMALFEQEQANST*
>ENSG00000001626|ENST00000003084
MQRSPLEKASVVSKLFFSWTRPILRKGYRQRLELSDIYQIPSVDSADNLSEKLEREWDRE
LASKKNPKLINALRRCFFWRFMFYGIFLYLGEVTKAVQPLLLGRIIASYDPDNKEERSIA
IYLGIGLCLLFIVRTLLLHPAIFGLHHIGMQMRIAMFSLIYKKTLKLSSRVLDKISIGQL
VSLLSNNLNKFDEGLALAHFVWIAPLQVALLMGLIWELLQASAFCGLGFLIVLALFQAGL
GRMMMKYRDQRAGKISERLVITSEMIENIQSVKAYCWEEAMEKMIENLRQTELKLTRKAA
YVRYFNSSAFFFSGFFVVFLSVLPYALIKGIILRKIFTTISFCIVLRMAVTRQFPWAVQT
WYDSLGAINKIQDFLQKQEYKTLEYNLTTTEVVMENVTAFWEEGFGELFEKAKQNNNNRK
TSNGDDSLFFSNFSLLGTPVLKDINFKIERGQLLAVAGSTGAGKTSLLMVIMGELEPSEG
KIKHSGRISFCSQFSWIMPGTIKENIIFGVSYDEYRYRSVIKACQLEEDISKFAEKDNIV
LGEGGITLSGGQRARISLARAVYKDADLYLLDSPFGYLDVLTEKEIFESCVCKLMANKTR
ILVTSKMEHLKKADKILILHEGSSYFYGTFSELQNLQPDFSSKLMGCDSFDQFSAERRNS
ILTETLHRFSLEGDAPVSWTETKKQSFKQTGEFGEKRKNSILNPINSIRKFSIVQKTPLQ
MNGIEEDSDEPLERRLSLVPDSEQGEAILPRISVISTGPTLQARRRQSVLNLMTHSVNQG
QNIHRKTTASTRKVSLAPQANLTELDIYSRRLSQETGLEISEEINEEDLKECFFDDMESI
PAVTTWNTYLRYITVHKSLIFVLIWCLVIFLAEVAASLVVLWLLGNTPLQDKGNSTHSRN
NSYAVIITSTSSYYVFYIYVGVADTLLAMGFFRGLPLVHTLITVSKILHHKMLHSVLQAP
MSTLNTLKAGGILNRFSKDIAILDDLLPLTIFDFIQLLLIVIGAIAVVAVLQPYIFVATV

PVIVAFIMLRAYFLQTSQQLKQLESEGRSPIFTHLVTSLKGLWTLRAFGRQPYFETLFHK
ALNLHTANWFLYLSTLRWFQMRIEMIFVIFFIAVTFISILTTGEGEGRVGIILTLAMNIM
STLQWAVNSSIDVDSLMRSVSRVFKFIDMPTEGKPTKSTKPYKNGQLSKVMIIENSHVKK
DDIWPSGGQMTVKDLTAKYTEGGNAILENISFSISPGQRVGLLGRTGSGKSTLLSAFLRL
LNTEGEIQIDGVSWDSITLQQWRKAFGVIPQKVFIFSGTFRKNLDPYEQWSDQEIWKVAD
EVGLRSVIEQFPGKLDFVLVDGGCVLSHGHKQLMCLARSVLSKAKILLLDEPSAHLDPVT
YQIIRRTLKQAFADCTVILCEHRIEAMLECQQFLVIEENKVRQYDSIQKLLNERSLFRQA
ISPSDRVKLFPHRNSSKCKSKPQIAALKEETEEEVQDTRL*
>ENSG00000034713|ENST00000037243
MKWMFKEDHSLEHRCVESAKIRAKYPDRVPVIVEKVSGSQIVDIDKRKYLVPSDITVAQF
MWIIRKRIQLPSEKAIFLFVDKTVPQSSLTMGQLYEKEKDEDGFLYVAYSGENTFGF*
>ENSG00000019582|ENST00000009530
MHRRRSRSCREDQKPVMDDQRDLISNNEQLPMLGRRPGAPESKCSRGALYTGFSILVTLL
LAGQATTAYFLYQQQGRLDKLTVTSQNLQLENLRMKLPKPPKPVSKMRMATPLLMQALPM
GALPQGPMQNATKYGNMTEDHVMHLLQNADPLKVYPPLKGSFPENLRHLKNTMETIDWKV
FESWMHHWLLFEMSRHSLEQKPTDAPPKVLTKCQEEVSHIPAVHPGSFRPKCDENGNYLP
LQCYGSIGYCWCVFPNGTEVPNTRSRGHHNCSESLELEDPSSGLGVTKQDLGPVPM*
>ENSG00000007255|ENST00000006275
MADTVLFEFLHTEMVAELWAHDPDPGPGVSAGLRGEEAGATKGQKMSLSVLEGMGFRVGQ
ALGERLPRETLAFREELDVLKFLCKDLWVAVFQKQMDSLRTNHQGTYVLQDNSFPLLLPM
ASGLQYLEEAPKFLAFTCGLLRGALYTLGIESVVTASVAALPVCKFQVVIPKS*
>ENSG00000002587|ENST00000002596
MAALLLGAVLLVAQPQLVPSRPAELGQQELLRKAGTLQDDVRDGVAPNGSAQQLPQTIII
GVRKGGTRALLEMLSLHPDVAAAENEVHFFDWEEHYSHGLGWYLSQMPFSWPHQLTVEKT
PAYFTSPKVPERVYSMNPSIRLLLILRDPSERVLSDYTQVFYNHMQKHKPYPSIEEFLVR
DGRLNVDYKALNRSLYHVHMQNWLRFFPLRHIHIVDGDRLIRDPFPEIQKVERFLKLSPQ
INASNFYFNKTKGFYCLRDSGRDRCLHESKGRAHPQVDPKLLNKLHEYFHEPNKKFFELV
GRTFDWH*
>ENSG00000027847|ENST00000029410
MFPSRRKAAQLPWEDGRSGLLSGGLPRKCSVFHLFVACLSLGFFSLLWLQLSCSGDVARA
VRGQGQETSGPPRACPPEPPPEHWEEDASWGPHRLAVLVPFRERFEELLVFVPHMRRFLS
RKKIRHHIYVLNQVDHFRFNRAALINVGFLESSNSTDYIAMHDVDLLPLNEELDYGFPEA
GPFHVASPELHPLYHYKTYVGGILLLSKQHYRLCNGMSNRFWGWGREDDEFYRRIKGAGL
QLFRPSGITTGYKTFRHLHDPAWRKRDQKRIAAQKQEQFKVDREGGLNTVKYHVASRTAL
SVGGAPCTVLNIMLDCDKTATPWCTFS*
>ENSG00000006118|ENST00000005286
MCARMAGRTTAAPRGPYGPWLCLLVALALDVVRVDCGQAPLDPVYLPAALELLDAPEHFR
VQQVGHYPPANSSLSSRSETFLLLQPWPRAQPLLRASYPPFATQQVVPPRVTEPHQRPVP
WDVRAVSVEAAVTPAEPYARVLFHLKGQDWPPGSGSLPCARLHATHPAGTAHQACRFQPS
LGACVVELELPSHWFSQASTTRAELAYTLEPAAEGPGGCGSGEENDPGEQALPVGGVELR
PADPPQYQEVPLDEAVTLRVPDMPVRPGQLFSATLLLRHNFTASLLTLRIKVKKGLHVTA
ARPAQPTLWTAKLDRFKGSRHHTTLITCHRAGLTEPDSSSPLELSEFLWVDFVVENSTGG
GVAVTRPVTWQLEYPGGQAPEAEKDKMVWEILVSERDIRALIPLAKAEELVNTAPLTGVPQ
HVPVRLVTVDGGGALVEVTEHVGCESANTQVLQVSEACDAVFVAGKESRGARGVRVDFWW

RRLRASLRLTVWAPLLPLRIELTDTTLEQVRGWRVPGPAEGPAEPAAEASDEAERRARGC
HLQYQRAGVRFLAPFAAHPLDGGRRLTHLLGPDWLLDVSHLVAPHARVLDSRVASLEGGR
VVVGREPGVTSIEVRSPLSDSILGEQALAVTDDKVSVLELRVQPVMGISLTLSRGTAHPG
EVTATCWAQSALPAPKQEVALSLWLSFSDHTVAPAELYDRRDLGLSVSAEEPGAILPAEE
QGAQLGVVVSGAGAEGLPLHVALHPPEPCRRGRHRVPLASGTAWLGLPPASTPAPALPSS
PAWSPPATEATMGGKRQVAGSVGGNTGVRGKFERAEEEARKEETEAREEEEEEEEEMVPA
PQHVTELELGMYALLGVFCVAIFIFLVNGVVFVLRYQRKEPPDSATDPTSPQPHNWVWLG
TDQEELSRQLDRQSPGPPKGEGSCPCESGGGGEAPTLAPGPPGGTTSSSSTLARKEAGGR
RKRVEFVTFAPAPPAQSPEEPVGAPAVQSILVAGEEDIRWVCEDMGLKDPEELRNYMERI
RGSS*
>ENSG00000011465|ENST00000052754
MKATIILLLLAQVSWAGPFQQRGLFDFMLEDEASGIGPEVPDDRDFEPSLGPVCPFRCQC
HLRVVQCSDLGLDKVPKDLPPDTTLLDLQNNKITEIKDGDFKNLKNLHALILVNNKISKV
SPGAFTPLVKLERLYLSKNQLKELPEKMPKTLQELRAHENEITKVRKVTFNGLNQMIVIE
LGTNPLKSSGIENGAFQGMKKLSYIRIADTNITSIPQGLPPSLTELHLDGNKISRVDAAS
LKGLNNLAKLGLSFNSISAVDNGSLANTPHLRELHLDNNKLTRVPGGLAEHKYIQVVYLH
NNNISVVGSSDFCPPGHNTKKASYSGVSLFSNPVQYWEIQPSTFRCVYVRSAIQLGNYK*
>ENSG00000090487|ENST00000204566
MGEIKVSPDYNWFRGTVPLKKIIVDDDDSKIWSLYDAGPRSIRCPLIFLPPVSGTADVFF
RQILALTGWGYRVIALQYPVYWDHLEFCDGFRKLLDHLQLDKVHLFGASLGGFLAQKFAE
YTHKSPRVHSLILCNSFSDTSIFNQTWTANSFWLMPAFMLKKIVLGNFSSGPVDPMMADA
IDFMVDRLESLGQSELASRLTLNCQNSYVEPHKIRDIPVTIMDVFDQSALSTEAKEEMYK
LYPNARRAHLKTGGNFPYLCRSAEVNLYVQIHLLQFHGTKYAAIDPSMVSAEELEVQKGS
LGISQEEQ*
>ENSG00000049245|ENST00000054666
MSTGPTAATGSNRRLQQTQNQVDEVVDIMRVNVDKVLERDQKLSELDDRADALQAGASQF
ETSAAKLKRKYWWKNCKMWAIGITVLVIFIIIIIVWVVSS*
>ENSG00000089063|ENST00000202834
MMPSRTNLATGIPSSKVKYSRLSSTDDGYIDLQFKKTPPKIPYKAIALATVLFLIGAFLI
IIGSLLLSGYISKGGADRAVPVLIIGILVFLPGFYHLRIAYYASKGYRGYSYDDIPDFDD
*
>ENSG00000073849|ENST00000169298
MIHTNLKKKFSCCVLVFLLFAVICVWKEKKKGSYYDSFKLQTKEFQVLKSLGKLAMGSDS
QSVSSSSTQDPHRGRQTLGSLRGLAKAKPEASFQVWNKDSSSKNLIPRLQKIWKNYLSMN
KYKVSYKGPGPGIKFSAEALRCHLRDHVNVSMVEVTDFPFNTSEWEGYLPKESIRTKAGP
WGRCAVVSSAGSLKSSQLGREIDDHDAVLRFNGAPTANFQQDVGTKTTIRLMNSQLVTTE
KRFLKDSLYNEGILIVWDPSVYHSDIPKWYQNPDYNFFNNYKTYRKLHPNQPFYILKPQM
PWELWDILQEISPEEIQPNPPSSGMLGIIIMMTLCDQVDIYEFLPSKRKTDVCYYYQKFF
DSACTMGAYHPLLYEKNLVKHLNQGTDEDIYLLGKATLPGFRTIHC*
>ENSG00000067182|ENST00000162749
MGLSTVPDLLLPLVLLELLVGIYPSGVIGLVPHLGDREKRDSVCPQGKYIHPQNNSICCT
KCHKGTYLYNDCPGPGQDTDCRECESGSFTASENHLRHCLSCSKCRKEMGQVEISSCTVD
RDTVCGCRKNQYRHYWSENLFQCFNCSLCLNGTVHLSCQEKQNTVCTCHAGFFLRENECV
SCSNCKKSLECTKLCLPQIENVKGTEDSGTTVLLPLVIFFGLCLLSLLFIGLMYRYQRWK

SKLYSIVCGKSTPEKEGELEGTTTKPLAPNPSFSPTPGFTPTLGFSPVPSSTFTSSSTYT
PGDCPNFAAPRREVAPPYQGADPILATALASDPIPNPLQKWEDSAHKPQSLDTDDPATLY
AVVENVPPLRWKEFVRRLGLSDHEIDRLELQNGRCLREAQYSMLATWRRRTPRREATLEL
LGRVLRDMDLLGCLEDIEEALCGPAALPPAPSLLR*
>ENSG00000154252|ENST00000192314
MMSMLGGLQRYFRVILLLLLALTLLLLAGFLHSDLELDTPLFGGQAEGPPVTNIMFLKTH
KTASSTVLNILYRFAETHNLSVALPAGSRVHLGYPWLFLARYVEGVGSQQRFNIMCNHLR
FNLPQVQKVMPNDTFYFSILRNPVFQLESSFIYYKTYAPAFRGAPSLDAFLASPRTFYND
SRHLRNVYAKNNMWFDFGFDPNAQCEEGYVRARIAEVERRFRLVLIAEHLDESLVLLRRR
LRWALDDVVAFRLNSRSARSVARLSPETRERARSWCALDWRLYEHFNRTLWAQLRAELGP
RRLRGEVERLRARRRELASLCLQDGGALKNHTQIRDPRLRPYQSGKADILGYNLRPGLDN
QTLGVCQRLVMPELQYMARLYALQFPEKPLKNIPFLGA*
>ENSG00000066455|ENST00000163416
MSWFVDLAGKAEDLLNRVDQGAATALSRKDNASNIYSKNTDYTELHQQNTDLIYQTGPKS
TYISSAADNIRNQKATILAGTANVKVGSRTPVEASHPVENASVPRPSSHFVRRKKSEPDD
ELLFDFLNSSQKEPTGRVEIRKEKGKTPVFQSSQTSSVSSVNPSVTTIKTIEENSFGSQT
HEAASNSDSSHEGQEESSKENVSSNAACPDHTPTPNDDGKSHELSNLRLENQLLRNEVQS
LNQEMASLLQRSKETQEELNKARARVEKWNADHSKSDRMTRGLRAQVDDLTEAVAAKDSQ
LAVLKVRLQEADQLLSTRTEALEALQSEKSRIMQDQSEGNSLQNQALQTFQERLHEADAT
LKREQESYKQMQSEFAARLNKVEMERQNLAEAITLAERKYSDEKKRVDELQQQVKLYKLN
LESSKQELIDYKQKATRILQSKEKLINSLKEGSGFEGLDSSTASSMELEELRHEKEMQRE
EIQKLMGQIHQLRSELQDMEAQQVNEAESAREQLQDLHDQIAGQKASKQELETELERLKQ
EFHYIEEDLYRTKNTLQSRIKDRDEEIQKLRNQLTNKTLSNSSQSELENRLHQLTETLIQ
KQTMLESLSTEKNSLVFQLERLEQQMNSASGSSSNGSSINMSGIDNGEGTRLRNVPVLFN
DTETNLAGMYGKVRKAASSIDQFSIRLGIFLRRYPIARVFVIIYMALLHLWVMIVLLTYT
PEMHHDQPYGK*
>ENSG00000058799|ENST00000072644
MAAVDDLQFEEFGNAATSLTANPDATTVNIEDPGETPKHQPGSPRGSGREEDDELLGNDD
SDKTELLAGQKKSSPFWTFEYYQTFFDVDTYQVFDRIKGSLLPIPGKNFVRLYIRSNPDL
YGPFWICATLVFAIAISGNLSNFLIHLGEKTYHYVPEFRKVSIAATIIYAYAWLVPLALW
GFLMWRNSKVMNIVSYSFLEIVCVYGYSLFIYIPTAILWIIPQKAVRWILVMIALGISGS
LLAMTFWPAVREDNRRVALATIVTIVLLHMLLSVGCLAYFFDAPEMDHLPTTTATPNQTV
AAAKSS*
>ENSG00000070526|ENST00000156626
MRSCLWRCRHLSQGVQWSLLLAVLVFFLFALPSFIKEPQTKPSRHQRTENIKERSLQSLA
KPKSQAPTRARRTTIYAEPVPENNALNTQTQPKAHTTGDRGKEANQAPPEEQDKVPHTAQ
RAAWKSPEKEKTMVNTLSPRGQDAGMASGRTEAQSWKSQDTKTTQGNGGQTRKLTASRTV
SEKHQGKAATTAKTLIPKSQHRMLAPTGAVSTRTRQKGVTTAVIPPKEKKPQATPPPAPF
QSPTTQRNQRLKAANFKSEPRWDFEEKYSFEIGGLQTTCPDSVKIKASKSLWLQKLFLPN
LTLFLDSRHFNQSEWDRLEHFAPPFGFMELNYSLVQKVVTRFPPVPQQQLLLASLPAGSL
RCITCAVVGNGGILNNSHMGQEIDSHDYVFRLSGALIKGYEQDVGTRTSFYGFTAFSLTQ
SLLILGNRGFKNVPLGKDVRYLHFLEGTRDYEWLEALLMNQTVMSKNLFWFRHRPQEAFR
EALHMDRYLLLHPDFLRYMKNRFLRSKTLDGAHWRIYRPTTGALLLLTALQLCDQVSAYG
FITEGHERFSDHYYDTSWKRLIFYINHDFKLEREVWKRLHDEGIIRLYQRPGPGTAKAKN

*
>ENSG00000060566|ENST00000078445
MNTDLAAGKMASAACSMDPIDSFELLDLLFDRQDGILRHVELGEGWGHVKDQQVLPNPDS
DDFLSSILGSGDSLPSSPLWSPEGSDSGISEDLPSDPQDTPPRSGPATSPAGCHPAQPGK
GPCLSYHPGNSCSTTTPGPVIQVPEASVTIDLEMWSPGGRICAEKPADPVDLSPRCNLTV
KDLLLSGSSGDLQQHHLGASYLLRPGAGHCQELVLTEDEKKLLAKEGITLPTQLPLTKYE
ERVLKKIRRKIRNKQSAQESRKKKKEYIDGLETRMSACTAQNQELQRKVLHLEKQNLSLL
EQLKKLQAIVVQSTSKSAQTGTCVAVLLLSFALIILPSISPFGPNKTESPGDFAPVRVFS
RTLHNDAASRVAADAVPGSEAPGPRPEADTTREESPGSPGADWGFQDTANLTNSTEELDN
ATLVLRNATEGLGQVALLDWVAPGPSTGSGRAGLEAAGDEL*
>ENSG00000090581|ENST00000204679
MAAGLARLLLLLGLSAGGPAPAGAAKMKVVEEPNAFGVNNPFLPQASRLQAKRDPSPVSG
PVHLFRLSGKCFSLVESTYKYEFCPFHNVTQHEQTFRWNAYSGILGIWHEWEIANNTFTG
MWMRDGDACRSRSRQSKVELACGKSNRLAHVSEPSTCVYALTFETPLVCHPHALLVYPTL
PEALQRQWDQVEQDLADELITPQGHEKLLRTLFEDAGYLKTPEENEPTQLEGGPDSLGFE
TLENCRKAHKELSKEIKRLKGLLTQHGIPYTRPTETSNLEHLGHETPRAKSPEQLRGDPG
LRGSL*
>ENSG00000099953|ENST00000215743
MAPAAWLRSAAARALLPPMLLLLLQPPPLLARALPPDAHHLHAERRGPQPWHAALPSSPA
PAPATQEAPRPASSLRPPRCGVPDPSDGLSARNRQKRFVLSGGRWEKTDLTYRILRFPWQ
LVQEQVRQTMAEALKVWSDVTPLTFTEVHEGRADIMIDFARYWHGDDLPFDGPGGILAHA
FFPKTHREGDVHFDYDETWTIGDDQGTDLLQVAAHEFGHVLGLQHTTAAKALMSAFYTFR
YPLSLSPDDCRGVQHLYGQPWPTVTSRTPALGPQAGIDTNEIAPLEPDAPPDACEASFDA
VSTIRGELFFFKAGFVWRLRGGQLQPGYPALASRHWQGLPSPVDAAFEDAQGHIWFFQGA
QYWVYDGEKPVLGPAPLTELGLVRFPVHAALVWGPEKNKIYFFRGRDYWRFHPSTRRVDS
PVPRRATDWRGVPSEIDAAFQDADGYAYFLRGRLYWKFDPVKVKALEGFPRLVGPDFFGC
AEPANTFL*
>ENSG00000102109|ENST00000218230
MAGSPLLWGPRAGGVGLLVLLLLGLFRPPPALCARPVKEPRGLSAASPPLAETGAPRRFR
RSVPRGEAAGAVQELARALAHLLEAERQERARAEAQEAEDQQARVLAQLLRVWGAPRNSD
PALGLDDDPDAPAAQLARALLRARLDPAALAAQLVPAPVPAAALRPRPPVYDDGPAGPDA
EEAGDETPDVDPELLRYLLGRILAGSADSEGVAAPRRLRRAADHDVGSELPPEGVLGALL
RVKRLETPAPQVPARRLLPP*
>ENSG00000101695|ENST00000217740
MGSVLSTDSGKSAPASATARALERRRDPELPVTSFDCAVCLEVLHQPVRTRCGHVFCRSC
IATSLKNNKWTCPYCRAYLPSEGVPATDVAKRMKSEYKNCAECDTLVCLSEMRAHIRTCQ
KYIDKYGPLQELEETAARCVCPFCQRELYEDSLLDHCITHHRSERRPVFCPLCRLIPDEN
PSSFSGSLIRHLQVSHTLFYDDFIDFNIIEEALIRRVLDRSLLEYVNHSNTT*
>ENSG00000099940|ENST00000215730
MSAYPKSYNPFDDDGEDEGARPAPWRDARDLPDGPDAPADRQQYLRQEVLRRAEATAAST
SRSLALMYESEKVGVASSEELARQRGVLERTEKMVDKMDQDLKISQKHINSIKSVFGGLV
NYFKSKPVETPPEQNGTLTSQPNNRLKEAISTSKEQEAKYQASHPNLRKLDDTDPVPRGA
GSAMSTDAYPKNPHLRAYHQKIDSNLDELSMGLGRLKDIALGMQTEIEEQDDILDRLTTK
VDKLDVNIKSTERKVRQL*

>ENSG00000090615|ENST00000204726
MDGASAEQDGLQEDRSHSGPSSLPEAPLKPPGPLVPPDQQDKVQCAEVNRASTEGESPDG
PGQGGLCQNGPTPPFPDPPSSLDPTTSPVGPDASPGVAGFHDNLRKSQGTSAEGSVRKEA
LQSLRLSLPMQETQLCSTDSPLPLEKEEQVRLQARKWLEEQLKQYRVKRQQERSSQPATK
TRLFSTLDPELMLNPENLPRASTLAMTKEYSFLRTSVPRGPKVGSLGLPAHPREKKTSKS
SKIRSLADYRTEDSNAGNSGGNVPAPDSTKGSLKQNRSSAASVVSEISLSPDTDDRLENT
SLAGDSVSEVDGNDSDSSSYSSASTRGTYGILSKTVGTQDTPYMVNGQEIPADTLGQFPS
IKDVLQAAAAEHQDQGQEVNGEVRSRRDSICSSVSLESSAAETQEEMLQVLKEKMRLEGQ
LEALSLEASQALKEKAELQAQLAALSTKLQAQVECSHSSQQRQDSLSSEVDTLKQSCWDL
ERAMTDLQNMLEAKNASLASSNNDLQVAEEQYQRLMAKVEDMQRSMLSKDNTVHDLRQQM
TALQSQLQQVQLERTTLTSKLKASQAEISSLQSVRQWYQQQLALAQEARVRLQGEMAHIQ
VGQMTQAGLLEHLKLENVSLSQQLTETQHRSMKEKGRIAAQLQGIEADMLDQEAAFMQIQ
EAKTMVEEDLQRRLEEFEGERERLQRMADSAASLEQQLEQVKLTLLQRDQQLEALQQEHL
DLMKQLTLTQEALQSREQSLDALQTHYDELQARLGELQGEAASREDTICLLQNEKIILEA
ALQAAKSGKEELDRGARRLEEGTEETSETLEKLREELAIKSGQVEHLQQETAALKKQMQK
IKEQFLQQKVMVEAYRRDATSKDQLISELKATRKRLDSELKELRQELMQVHGEKRTAEAE
LSRLHREVAQVRQHMADLEGHLQSAQKERDEMETHLQSLQFDKEQMVAVTEANEALKKQI
EELQQEARKAITEQKQKMRRLGSDLTSAQKEMKTKHKAYENAVGILSRRLQEALAAKEAA
DAELGQLRAQGGSSDSSLALHERIQALEAELQAVSHSKTLLEKELQEVIALTSQELEESR
EKVLELEDELQESRGFRKKIKRLEESNKKLALELEHEKGKLTGLGQSNAALREHNSILET
ALAKREADLVQLNLQVQAVLQRKEEEDRQMKHLVQALQASLEKEKEKVNSLKEQVAAAKV
EAGHNRRHFKAASLELSEVKKELQAKEHLVQKLQAEADDLQIREGKHSQEIAQFQAELAE
ARAQLQLLQKQLDEQLSKQPVGNQEMENLKWEVDQKEREIQSLKQQLDLTEQQGRKELEG
LQQLLQNVKSELEMAQEDLSMTQKDKFMLQAKVSELKNNMKTLLQQNQQLKLDLRRGAAK
TRKEPKGEASSSNPATPIKIPDCPVPASLLEELLRPPPAVSKEPLKNLNSCLQQLKQEMD
SLQRQMEEHALTVHESLSSWTPLEPATASPVPPGGHAGPRGDPQRHSQSRASKEGPGE*
>ENSG00000100522|ENST00000216410
MKPDETPMFDPSLLKEVDWSQNTATFSPAISPTHPGEGLVLRPLCTADLNRGFFKVLGQL
TETGVVSPEQFMKSFEHMKKSGDYYVTVVEDVTLGQIVATATLIIEHKFIHSCAKRGRVE
DVVVSDECRGKQLGKLLLSTLTLLSKKLNCYKITLECLPQNVGFYKKFGYTVSEENYMCR
RFLK*
>ENSG00000100528|ENST00000216416
MAFTFAAFCYMLALLLTAALIFFAIWHIIAFDELKTDYKNPIDQCNTLNPLVLPEYLIHA
FFCVMFLCAAEWLTLGLNMPLLAYHIWRYMSRPVMSGPGLYDPTTIMNADILAYCQKEGW
CKLAFYLLAFFYYLYGMIYVLVSS*
>ENSG00000100478|ENST00000216366
MIKFFLMVNKQGQTRLSKYYEHVDINKRTLLETEVIKSCLSRSNEQCSFIEYKDFKLIYR
QYAALFIVVGVNDTENEMAIYEFIHNFVEVLDEYFSRVSELDVSFFNTVFHSTWQMHSGP
YQEPIDELPKICSALEPQQTCFSPDSSSFKGAASTTPIY*
>ENSG00000101160|ENST00000217131
MARRGPGWRPLLLLVLLAGAAQGGLYFRRGQTCYRPLRGDGLAPLGRSTYPRPHEYLSPA
DLPKSWDWRNVDGVNYASITRNQHIPQYCGSCWAHASTSAMADRINIKRKGAWPSTLLSV
QNVIDCGNAGSCEGGNDLSVWDYAHQHGIPDETCNNYQAKDQECDKFNQCGTCNEFKECH
AIRNYTLWRVGDYGSLSGREKMMAEIYANGPISCGIMATERLANYTGGIYAEYQDTTYIN

HVVSVAGWGISDGTEYWIVRNSWGEPWGERGWLRIVTSTYKDGKGARYNLAIEEHCTFGD
PIV*
>ENSG00000088833|ENST00000216879
MAAERQEALREFVAVTGAEEDRARFFLESAGWDLQIALASFYEDGGDEDIVTISQATPSS
VSRGTAPSDNRVTSFRDLIHDQDEDEEEEEGQRFYAGGSERSGQQIVGPPRKKSPNELVD
DLFKGAKEHGAVAVERVTKSPGETSKPRPFAGGGYRLGAAPEEESAYVAGEKRQHSSQDV
HVVLKLWKSGFSLDNGELRSYQDPSNAQFLESIRRGEVPAELRRLAHGGQVNLDMEDHRD
EDFVKPKGAFKAFTGEGQKLGSTAPQVLSTSSPAQQAENEAKASSSILIDESEPTTNIQI
RLADGGRLVQKFNHSHRISDIRLFIVDARPAMAATSFILMTTFPNKELADESQTLKEANL
LNAVIVQRLT*
>ENSG00000102471|ENST00000218652
MARRRSQRVCASGPSMLNSARGAPELLRGTATNAEVSAAAAGATGSEELPPGDRGCRNGG
GRGPAATTSSTGVAVGAEHGEDSLSRKPDPEPGRMDHHQPGTGRYQVLLNEEDNSESSAI
EQPPTSNPAPQIVQAASSAPALETDSSPPPYSSITVEVPTTSDTEVYGEFYPVPPPYSVA
TSLPTYDEAEKAKAAAMAAAAAETSQRIQEEECPPRDDFSDADQLRVGNDGIFMLAFFMA
FIFNWLGFCLSFCITNTIAGRYGAICGFGLSLIKWILIVRFSDYFTGYFNGQYWLWWIFL
VLGLLLFFRGFVNYLKVRNMSESMAAAHRTRYFFLL*
>ENSG00000100749|ENST00000216639
MPRVKAAQAGRQSSAKRHLAEQFAVGEIITDMAKKEWKVGLPIGQGGFGCIYLADMNSSE
SVGSDAPCVVKVEPSDNGPLFTELKFYQRAAKPEQIQKWIRTRKLKYLGVPKYWGSGLHD
KNGKSYRFMIMDRFGSDLQKIYEANAKRFSRKTVLQLSLRILDILEYIHEHEYVHGDIKA
SNLLLNYKNPDQVYLVDYGLAYRYCPEGVHKEYKEDPKRCHDGTIEFTSIDAHNGVAPSR
RGDLEILGYCMIQWLTGHLPWEDNLKDPKYVRDSKIRYRENIASLMDKCFPEKNKPGEIA
KYMETVKLLDYTEKPLYENLRDILLQGLKAIGSKDDGKLDLSVVENGGLKAKTITKKRKK
EIEESKEPGVEDTEWSNTQTEEAIQTRSRTRKRVQK*
>ENSG00000100211|ENST00000216029
MPFFGNTFSPKKTPPRKSASLSNLHSLDRSTREVELGLEYGSPTMNLAGQSLKFENGQWI
AETGVSGGVDRREVQRLRRRNQQLEEENNLLRLKVDILLDMLSESTAESHLMEKELDELR
ISRKRK*
>ENSG00000076321|ENST00000209884
MEGKPMRRCTNIRPGETGMDVTSRCTLGDPNKLPEGVPQPARMPYISDKHPRQTLEVINL
LRKHRELCDVVLVVGAKKIYAHRVILSACSPYFRAMFTGELAESRQTEVVIRDIDERAME
LLIDFAYTSQITVEEGNVQTLLPAACLLQLAEIQEACCEFLKRQLDPSNCLGIRAFADTH
SCRELLRIADKFTQHNFQEVMESEEFMLLPANQLIDIISSDELNVRSEEQVFNAVMAWVK
YSIQERRPQLPQVLQHVRLPLLSPKFLVGTVGSDPLIKSDEECRDLVDEAKNYLLLPQER
PLMQGPRTRPRKPIRCGEVLFAVGGWCSGDAISSVERYDPQTNEWRMVASMSKRRCGVGV
SVLDDLLYAVGGHDGSSYLNSVERYDPKTNQWSSDVAPTSTCRTSVGVAVLGGFLYAVGG
QDGVSCLNIVERYDPKENKWTRVASMSTRRLGVAVAVLGGFLYAVGGSDGTSPLNTVERY
NPQENRWHTIAPMGTRRKHLGCAVYQDMIYAVGGRDDTTELSSAERYNPRTNQWSPVVAM
TSRRSGVGLAVVNGQLMAVGGFDGTTYLKTIEVFDPDANTWRLYGGMNYRRLGGGVGVIK
MTHCESHIW*
>ENSG00000101916|ENST00000218032
MENMFLQSSMLTCIFLLISGSCELCAEENFSRSYPCDEKKQNDSVIAECSNRRLQEVPQT
VGKYVTELDLSDNFITHITNESFQGLQNLTKINLNHNPNVQHQNGNPGIQSNGLNITDGA

FLNLKNLRELLLEDNQLPQIPSGLPESLTELSLIQNNIYNITKEGISRLINLKNLYLAWN
CYFNKVCEKTNIEDGVFETLTNLELLSLSFNSLSHVPPKLPSSLRKLFLSNTQIKYISEE
DFKGLINLTLLDLSGNCPRCFNAPFPCVPCDGGASINIDRFAFQNLTQLRYLNLSSTSLR
KINAAWFKNMPHLKVLDLEFNYLVGEIASGAFLTMLPRLEILDLSFNYIKGSYPQHINIS
RNFSKLLSLRALHLRGYVFQELREDDFQPLMQLPNLSTINLGINFIKQIDFKLFQNFSNL
EIIYLSENRISPLVKDTRQSYANSSSFQRHIRKRRSTDFEFDPHSNFYHFTRPLIKPQCA
AYGKALDLSLNSIFFIGPNQFENLPDIACLNLSANSNAQVLSGTEFSAIPHVKYLDLTNN
RLDFDNASALTELSDLEVLDLSYNSHYFRIAGVTHHLEFIQNFTNLKVLNLSHNNIYTLT
DKYNLESKSLVELVFSGNRLDILWNDDDNRYISIFKGLKNLTRLDLSLNRLKHIPNEAFL
NLPASLTELHINDNMLKFFNWTLLQQFPRLELLDLRGNKLLFLTDSLSDFTSSLRTLLLS
HNRISHLPSGFLSEVSSLKHLDLSSNLLKTINKSALETKTTTKLSMLELHGNPFECTCDI
GDFRRWMDEHLNVKIPRLVDVICASPGDQRGKSIVSLELTTCVSDVTAVILFFFTFFITT
MVMLAALAHHLFYWDVWFIYNVCLAKVKGYRSLSTSQTFYDAYISYDTKDASVTDWVINE
LRYHLEESRDKNVLLCLEERDWDPGLAIIDNLMQSINQSKKTVFVLTKKYAKSWNFKTAF
YLALQRLMDENMDVIIFILLEPVLQHSQYLRLRQRICKSSILQWPDNPKAEGLFWQTLRN
VVLTENDSRYNNMYVDSIKQY*
>ENSG00000105329|ENST00000221930
MPPSGLRLLPLLLPLLWLLVLTPGRPAAGLSTCKTIDMELVKRKRIEAIRGQILSKLRLA
SPPSQGEVPPGPLPEAVLALYNSTRDRVAGESAEPEPEPEADYYAKEVTRVLMVETHNEI
YDKFKQSTHSIYMFFNTSELREAVPEPVLLSRAELRLLRLKLKVEQHVELYQKYSNNSWR
YLSNRLLAPSDSPEWLSFDVTGVVRQWLSRGGEIEGFRLSAHCSCDSRDNTLQVDINGFT
TGRRGDLATIHGMNRPFLLLMATPLERAQHLQSSRHRRALDTNYCFSSTEKNCCVRQLYI
DFRKDLGWKWIHEPKGYHANFCLGPCPYIWSLDTQYSKVLALYNQHNPGASAAPCCVPQA
LEPLPIVYYVGRKPKVEQLSNMIVRSCKCS*
>ENSG00000103502|ENST00000219789
MPDENIFLFVPNLIGYARIVFAIISFYFMPCCPLTASSFYLLSGLLDAFDGHAARALNQG
TRFGAMLDMLTDRCSTMCLLVNLALLYPGATLFFQISMSLDVASHWLHLHSSVVRGSESH
KMIDLSGNPVLRIYYTSRPALFTLCAGNELFYCLLYLFHFSEGPLVGSVGLFRMGLWVTA
PIALLKSLISVIHLITAARNMAALDAADRAKKK*
>ENSG00000103056|ENST00000219334
MVLYTTPFPNSCLSALHCVSWALIFPCYWLVDRLAASFIPTTYEKRQRADDPCCLQLLCT
ALFTPIYLALLVASLPFAFLGFLFWSPLQSARRPYIYSRLEDKGLAGGAALLSEWKGTGP
GKSFCFATANVCLLPDSLARVNNLFNTQARAKEIGQRIRNGAARPQIKIYIDSPTNTSIS
AASFSSLVSPQGGDGVARAVPGSIKRTASVEYKGDGGRHPGDEAANGPASGDPVDSSSPE
DACIVRIGGEEGGRPPEADDPVPGGQARNGAGGGPRGQTPNHNQQDGDSGSLGSPSASRE
SLVKGRAGPDTSASGEPGANSKLLYKASVVKKAAARRRRHPDEAFDHEVSAFFPANLDFL
CLQEVFDKRAATKLKEQLHGYFEYILYDVGVYGCQGCCSFKCLNSGLLFASRYPIMDVAY
HCYPNKCNDDALASKGALFLKVQVGSTPQDQRIVGYIACTHLHAPQEDSAIRCGQLDLLQ
DWLADFRKSTSSSSAANPEELVAFDVVCGDFNFDNCSSDDKLEQQHSLFTHYRDPCRLGP
GEEKPWAIGTLLDTNGLYDEDVCTPDNLQKVLESEEGRREYLAFPTSKSSGQKGRKELLK
GNGRRIDYMLHAEEGLCPDWKAEVEEFSFITQLSGLTDHLPVAMRLMVSSGEEEA*
>ENSG00000167964|ENST00000210187
MSRKKTPKSKGASTPAASTLPTANGARPARSGTALSGPDAPPNGPLQPGRPSLGGGVDFY
DVAFKVMLVGDSGVGKTCLLVRFKDGAFLAGTFISTVGIDFRNKVLDVDGVKVKLQMWDT

AGQERFRSVTHAYYRDAHALLLLYDVTNKASFDNIQAWLTEIHEYAQHDVALMLLGNKVD
SAHERVVKREDGEKLAKEYGLPFMETSAKTGLNVDLAFTAIAKELKQRSMKAPSEPRFRL
HDYVKREGRGASCCRP*
>ENSG00000100312|ENST00000216139
MVEMLPTAILLVLAVSVVAKDNATCDGPCGLRFRQNPQGGVRIVGGKAAQHGAWPWMVSL
QIFTYNSHRYHTCGGSLLNSRWVLTAAHCFVGKNNVHDWRLVFGAKEITYGNNKPVKAPL
QERYVEKIIIHEKYNSATEGNDIALVEITPPISCGRFIGPGCLPHFKAGLPRGSQSCWVA
GWGYIEEKAPRPSSILMEARVDLIDLDLCNSTQWYNGRVQPTNVCAGYPVGKIDTCQGDS
GGPLMCKDSKESAYVVVGITSWGVGCARAKRPGIYTATWPYLNWIASKIGSNALRMIQSA
TPPPPTTRPPPIRPPFSHPISAHLPWYFQPPPRPLPPRPPAAQPRPPPSPPPPPPPPASP
LPPPPPPPPPTPSSTTKLPQGLSFAKRLQQLIEVLKGKTYSDGKNHYDMETTELPELTST
S*
>ENSG00000090863|ENST00000205061
MAACGRVRRMFRLSAALHLLLLFAAGAEKLPGQGVHSQGQGPGANFVSFVGQAGGGGPAG
QQLPQLPQSSQLQQQQQQQQQQQPQPPQPPFPAGGPPARRGGAGAGGGWKLAEEESCRE
DVTRVCPKHTWSNNLAVLECLQDVREPENEISSDCNHLLWNYKLNLTTDPKFESVAREVC
KSTITEIKECADEPVGKGYMVSCLVDHRGNITEYQCHQYITKMTAIIFSDYRLICGFMDD
CKNDINILKCGSIRLGEKDAHSQGEVVSCLEKGLVKEAEEREPKIQVSELCKKAILRVAE
LSSDDFHLDRHLYFACRDDRERFCENTQAGEGRVYKCLFNHKFEESMSEKCREALTTRQK
LIAQDYKVSYSLAKSCKSDLKKYRCNVENLPRSREARLSYLLMCLESAVHRGRQVSSECQ
GEMLDYRRMLMEDFSLSPEIILSCRGEIEHHCSGLHRKGRTLHCLMKVVRGEKGNLGMNC
QQALQTLIQETDPGADYRIDRALNEACESVIQTACKHIRSGDPMILSCLMEHLYTEKMVE
DCEHRLLELQYFISRDWKLDPVLYRKCQGDASRLCHTHGWNETSEFMPQGAVFSCLYRHA
YRTEEQGRRLSRECRAEVQRILHQRAMDVKLDPALQDKCLIDLGKWCSEKTETGQELECL
QDHLDDLVVECRDIVGNLTELESEDIQIEALLMRACEPIIQNFCHDVADNQIDSGDLMEC
LIQNKHQKDMNEKCAIGVTHFQLVQMKDFRFSYKFKMACKEDVLKLCPNIKKKVDVVICL
STTVRNDTLQEAKEHRVSLKCRRQLRVEELEMTEDIRLEPDLYEACKSDIKNFCSAVQYG
NAQIIECLKENKKQLSTRCHQKVFKLQETEMMDPELDYTLMRVCKQMIKRFCPEADSKTM
LQCLKQNKNSELMDPKCKQMITKRQITQNTDYRLNPMLRKACKADIPKFCHGILTKAKDD
SELEGQVISCLKLRYADQRLSSDCEDQIRIIIQESALDYRLDPQLQLHCSDEISSLCAEE
AAAQEQTGQVEECLKVNLLKIKTELCKKEVLNMLKESKADIFVDPVLHTACALDIKHHCA
AITPGRGRQMSCLMEALEDKRVRLQPECKKRLNDRIEMWSYAAKVAPADGFSDLAMQVMT
SPSKNYILSVISGSICILFLIGLMCGRITKRVTRELKDRLQYRSETMAYKGLVWSQDVTG
SPA*
>ENSG00000104826|ENST00000221421
MEMLQGLLLLLLLSMGGAWASREPLRPWCHPINAILAVEKEGCPVCITVNTTICAGYCPT
MMRVLQAVLPPLPQVVCTYRDVRFESIRLPGCPRGVDPVVSFPVALSCRCGPCRRSTSDC
GGPKDHPLTCDHPQLSGLLFL*
>ENSG00000104490|ENST00000220931
MGKQNSKLRPEVMQDLLESTDFTEHEIQEWYKGFLRDCPSGHLSMEEFKKIYGNFFPYGD
ASKFAEHVFRTFDANGDGTIDFREFIIALSVTSRGKLEQKLKWAFSMYDLDGNGYISKAE
MLEIVQAIYKMVSSVMKMPEDESTPEKRTEKIFRQMDTNRDGKLSLEEFIRGAKSDPSIV
RLLQCDPSSAGQF*
>ENSG00000101981|ENST00000218099

MQRVNMIMAESPGLITICLLGYLLSAECTVFLDHENANKILNRPKRYNSGKLEEFVQGNL
ERECMEEKCSFEEAREVFENTERTTEFWKQYVDGDQCESNPCLNGGSCKDDINSYECWCP
FGFEGKNCELDVTCNIKNGRCEQFCKNSADNKVVCSCTEGYRLAENQKSCEPAVPFPCGR
VSVSQTSKLTRAETVFPDVDYVNSTEAETILDNITQSTQSFNDFTRVVGGEDAKPGQFPW
QVVLNGKVDAFCGGSIVNEKWIVTAAHCVETGVKITVVAGEHNIEETEHTEQKRNVIRII
PHHNYNAAINKYNHDIALLELDEPLVLNSYVTPICIADKEYTNIFLKFGSGYVSGWGRVF
HKGRSALVLQYLRVPLVDRATCLRSTKFTIYNNMFCAGFHEGGRDSCQGDSGGPHVTEVE
GTSFLTGIISWGEECAMKGKYGIYTKVSRYVNWIKEKTKLT*
>ENSG00000100314|ENST00000216144
MPFHPVTAALMYRGIYTVPNLLSEQRPVDIPEDELEEIREAFKVFDRDGNGFISKQELGT
AMRSLGYMPNEVELEVIIQRLDMDGDGQVDFEEFVTLLGPKLSTSGIPEKFHGTDFDTVF
WKCDMQKLTVDELKRLLYDTFCEHLSMKDIENIIMTEEESHLGTAEECPVDVETCSNQQI
RQTCVRKSLICAFAIAFIISVMLIAANQVLRSGMK*
>ENSG00000100196|ENST00000216014
MNVFRILGDLSHLLAMILLLGKIWRSKCCKGISGKSQILFALVFTTRYLDLFTNFISIYN
TVMKVVFLLCAYVTVYMIYGKFRKTFDSENDTFRLEFLLVPVIGLSFLENYSFTLLEILW
TFSIYLESVAILPQLFMISKTGEAETITTHYLFFLGLYRALYLANWIRRYQTENFYDQIA
VVSGVVQTIFYCDFFYLYVTKVLKGKKLSLPMPI*
>ENSG00000111666|ENST00000229266
MAAGAGAGSAPRWLRALSEPLSAAQLRRLEEHRYSAAGVSLLEPPLQLYWTWLLQWIPLW
MAPNSITLLGLAVNVVTTLVLISYCPTATEEAPYWTYLLCALGLFIYQSLDAIDGKQARR
TNSCSPLGELFDHGCDSLSTVFMAVGASIAARLGTYPDWFFFCSFIGMFVFYCAHWQTYV
SGMLRFGKVDVTEIQIALVIVFVLSAFGGATMWDYTIPILEIKLKILPVLGFLGGVIFSC
SNYFHVILHGGVGKNGSTIAGTSVLSPGLHIGLIIILAIMIYKKSATDVFEKHPCLYILM
FGCVFAKVSQKLVVAHMTKSELYLQDTVFLGPGLLFLDQYFNNFIDEYVVLWMAMVISSF
DMVIYFSALCLQISRHLHLNIFKTACHQAPEQVQVLSSKSHQNNMD*
>ENSG00000116209|ENST00000234831
MAAPKGSLWVRTQLGLPPLLLLTMALAGGSGTASAEAFDSVLGDTASCHRACQLTYPLHT
YPKEEELYACQRGCRLFSICQFVDDGIDLNRTKLECESACTEAYSQSDEQYACHLGCQNQ
LPFAELRQEQLMSLMPKMHLLFPLTLVRSFWSDMMDSAQSFITSSWTFYLQADDGKIVIF
QSKPEIQYAPHLEQEPTNLRESSLSKMSYLQMRNSQAHRNFLEDGESDGFLRCLSLNSGW
ILTTTLVLSVMVLLWICCATVATAVEQYVPSEKLSIYGDLEFMNEQKLNRYPASSLVVVR
SKTEDHEEAGPLPTKVNLAHSEI*
>ENSG00000002549|ENST00000226299
MFLLPLPAAGRVVVRRLAVRRFGSRSLSTADMTKGLVLGIYSKEKEDDVPQFTSAGENFD
KLLAGKLRETLNISGPPLKAGKTRTFYGLHQDFPSVVLVGLGKKAAGIDEQENWHEGKEN
IRAAVAAGCRQIQDLELSSVEVDPCGDAQAAAEGAVLGLYEYDDLKQKKKMAVSAKLYGS
GDQEAWQKGVLFASGQNLARQLMETPANEMTPTRFAEIIEKNLKSASSKTEVHIRPKSWI
EEQAMGSFLSVAKGSDEPPVFLEIHYKGSPNANEPPLVFVGKGITFDSGGISIKASANMD
LMRADMGGAATICSAIVSAAKLNLPINIIGLAPLCENMPSGKANKPGDVVRAKNGKTIQV
DNTDAEGRLILADALCYAHTFNPKVILNAATLTGAMDVALGSGATGVFTNSSWLWNKLFE
ASIETGDRVWRMPLFEHYTRQVVDCQLADVNNIGKYRSAGACTAAAFLKEFVTHPKWAHL
DIAGVMTNKDEVPYLRKGMTGRPTRTLIEFLLRFSQDNA*
>ENSG00000109072|ENST00000226218

MAPLRPLLILALLAWVALADQESCKGRCTEGFNVDKKCQCDELCSYYQSCCTDYTAECKP
QVTRGDVFTMPEDEYTVYDDGEEKNNATVHEQVGGPSLTSDLQAQSKGNPEQTPVLKPEE
EAPAPEVGASKPEGIDSRPETLHPGRPQPPAEEELCSGKPFDAFTDLKNGSLFAFRGQYC
YELDEKAVRPGYPKLIRDVWGIEGPIDAAFTRINCQGKTYLFKGSQYWRFEDGVLDPDYP
RNISDGFDGIPDNVDAALALPAHSYSGRERVYFFKGKQYWEYQFQHQPSQEECEGSSLSA
VFEHFAMMQRDSWEDIFELLFWGRTSAGTRQPQFISRDWHGVPGQVDAAMAGRIYISGMA
PRPSLAKKQRFRHRNRKGYRSQRGHSRGRNQNSRRPSRATWLSLFSSEESNLGANNYDDY
RMDWLVPATCEPIQSVFFFSGDKYYRVNLRTRRVDTVDPPYPRSIAQYWLGCPAPGHL*
>ENSG00000114354|ENST00000240851
MNGQLDLSGKLIIKAQLGEDIRRIPIHNEDITYDELVLMMQRVFRGKLLSNDEVTIKYKD
EDGDLITIFDSSDLSFAIQCSRILKLTLFVNGQPRPLESSQVKYLRRELIELRNKVNRLL
DSLEPPGEPGPSTNIPENDTVDGREEKSASDSSGKQSTQVMAASMSAFDPLKNQDEINKN
VMSAFGLTDDQVSGPPSAPAEDRSGTPDSIASSSSAAHPPGVQPQQPPYTGAQTQAGQIE
GQMYQQYQQQAGYGAQQPQAPPQQPQQYGIQYSASYSQQTGPQQPQQFQGYGQQPTSQAP
APAFSGQPQQLPAQPPQQYQASNYPAQTYTAQTSQPTNYTVAPASQPGMAPSQPGAYQPR
PGFTSLPGSTMTPPPSGPNPYARNRPPFGQGYTQPGPGYR*
>ENSG00000105971|ENST00000222693
MGLETEKADVQLFMDDDSYSHHSGLEYADPEKFADSDQDRDPHRLNSHLKLGFEDVIAEP
VTTHSFDKVWICSHALFEISKYVMYKFLTVFLAIPLAFIAGILFATLSCLHIWILMPFVK
TCLMVLPSVQTIWKSVTDVIIAPLCTSVGRCFSSVSLQLSQD*
>ENSG00000205155|ENST00000222266
MNLERVSNEEKLNLCRKYYLGGFAFLPFLWLVNIFWFFREAFLVPAYTEQSQIKGYVWRS
AVGFLFWVIVLTSWITIFQIYRPRWGALGDYLSFTIPLGTP*
>ENSG00000109861|ENST00000227266
MGAGPSLLLAALLLLLSGDGAVRCDTPANCTYLDLLGTWVFQVGSSGSQRDVNCSVMGPQ
EKKVVVYLQKLDTAYDDLGNSGHFTIIYNQGFEIVLNDYKWFAFFKYKEEGSKVTTYCNE
TMTGWVHDVLGRNWACFTGKKVGTASENVYYVNIAHLKNSQEKYSNRLYKYDHNFVKAINA
IQKSWTATTYMEYETLTLGDMIRRSGGHSRKIPRPKPAPLTAEIQQKILHLPTSWDWRNV
HGINFVSPVRNQASCGSCYSFASMGMLEARIRILTNNSQTPILSPQEVVSCSQYAQGCEG
GFPYLIAGKYAQDFGLVEEACFPYTGTDSPCKMKEDCFRYYSSEYHYVGGFYGGCNEALM
KLELVHHGPMAVAFEVYDDFLHYKKGIYHHTGLRDPFNPFELTNHAVLLVGYGTDSASGM
DYWIVKNSWGTGWGENGYFRIRRGTDECAIESIAVAATPIPKL*
>ENSG00000108587|ENST00000225724
MAAGTSSYWEDLRKQARQLENELDLKLVSFSKLCTSYSHSSTRDGRRDRYSSDTTPLLNG
SSQDRMFETMAIEIEQLLARLTGVNDKMAEYTNSAGVPSLNAALMHTLQRHRDILQDYTH
EFHKTKANFMAIRERENLMGSVRKDIESYKSGSGVNNRRTELFLKEHDHLRNSDRLIEET
ISIAMATKENMTSQRGMLKSIHSKMNTLANRFPAVNSLIQRINLRKRRDSLILGGVIGIC
TILLLLYAFH*
>ENSG00000106397|ENST00000223127
MTSSGPGPRFLLLLPLLLPPAASASDRPRGRDPVNPEKLLVITVATAETEGYLRFLRSAE
FFNYTVRTLGLGEEWRGGDVARTVGGGQKVRWLKKEMEKYADREDMIIMFVDSYDVILAG
SPTELLKKFVQSGSRLLFSAESFCWPEWGLAEQYPEVGTGKRFLNSGGFIGFATTIHQIV
RQWKYKDDDDDQLFYTRLYLDPGLREKLSLNLDHKSRIFQNLNGALDEVVLKFDRNRVRI
RNVAYDTLPIVVHGNGPTKLQLNYLGNYVPNGWTPEGGCGFCNQDRRTLPGGQPPPRVFL

AVFVEQPTPFLPRFLQRLLLLDYPPDRVTLFLHNNEVFHEPHIADSWPQLQDHFSAVKLV
GPEEALSPGEARDMAMDLCRQDPECEFYFSLDADAVLTNLQTLRILIEENRKVIAPMLSR
HGKLWSNFWGALSPDEYYARSEDYVELVQRKRVGVWNVPYISQAYVIRGDTLRMELPQRD
VFSGSDTDPDMAFCKSFRDKGIFLHLSNQHEFGRLLATSRYDTEHLHPDLWQIFDNPVDW
KEQYIHENYSRALEGEGIVEQPCPDVYWFPLLSEQMCDELVAEMEHYGQWSGGRHEDSRL
AGGYENVPTVDIHMKQVGYEDQWLQLLRTYVGPMTESLFPGYHTKARAVMNFVVRYRPDE
QPSLRPHHDSSTFTLNVALNHKGLDYEGGGCRFLRYDCVISSPRKGWALLHPGRLTHYHE
GLPTTWGTRYIMVSFVDP*
>ENSG00000117280|ENST00000235932
MGSRDHLFKVLVVGDAAVGKTSLVQRYSQDSFSKHYKSTVGVDFALKVLQWSDYEIVRLQ
LWDIAGQERFTSMTRLYYRDASACVIMFDVTNATTFSNSQRWKQDLDSKLTLPNGEPVPC
LLLANKCDLSPWAVSRDQIDRFSKENGFTGWTETSVKENKNINEAMRVLIEKMMRNSTED
IMSLSTQGDYINLQTKSSSWSCC*
>ENSG00000121073|ENST00000240333
MRPLPPVGDVRLELSPPPPLLPVPVVSGSPVGSSGRLMASSSSLVPDRLRLPLCFLGVFV
CYFYYGILQEKITRGKYGEGAKQETFTFALTLVFIQCVINAVFAKILIQFFDTARVDRTR
SWLYAACSISYLGAMVSSNSALQFVNYPTQVLGKSCKPIPVMLLGVTLLKKKYPLAKYLC
VLLIVAGVALFMYKPKKVVGIEEHTVGYGELLLLLSLTLDGLTGVSQDHMRAHYQTGSNH
MMLNINLWSTLLLGMGILFTGELWEFLSFAERYPAIIYNILLFGLTSALGQSFIFMTVVY
FGPLTCSIITTTRKFFTILASVILFANPISPMQWVGTVLVFLGLGLDAKFGKGAKKTSH*
>ENSG00000118972|ENST00000237837
MLGARLRLWVCALCSVCSMSVLRAYPNASPLLGSSWGGLIHLYTATARNSYHLQIHKNGH
VDGAPHQTIYSALMIRSEDAGFVVITGVMSRRYLCMDFRGNIFGSHYFDPENCRFQHQTL
ENGYDVYHSPQYHFLVSLGRAKRAFLPGMNPPPYSQFLSRRNEIPLIHFNTPIPRRHTRS
AEDDSERDPLNVLKPRARMTPAPASCSQELPSAEDNSPMASDPLGVVRGGRVNTHAGGTG
PEGCRPFAKFI*
>ENSG00000115718|ENST00000234071
MWQLTSLLLFVATWGISGTPAPLDSVFSSSERAHQVLRIRKRANSFLEELRHSSLERECI
EEICDFEEAKEIFQNVDDTLAFWSKHVDGDQCLVLPLEHPCASLCCGHGTCIDGIGSFSC
DCRSGWEGRFCQREVSFLNCSLDNGGCTHYCLEEVGWRRCSCAPGYKLGDDLLQCHPAVK
FPCGRPWKRMEKKRSHLKRDTEDQEDQVDPRLIDGKMTRRGDSPWQVVLLDSKKKLACGA
VLIHPSWVLTAAHCMDESKKLLVRLGEYDLRRWEKWELDLDIKEVFVHPNYSKSTTDNDI
ALLHLAQPATLSQTIVPICLPDSGLAERELNQAGQETLVTGWGYHSSREKEAKRNRTFVL
NFIKIPVVPHNECSEVMSNMVSENMLCAGILGDRQDACEGDSGGPMVASFHGTWFLVGLV
SWGEGCGLLHNYGVYTKVSRYLDWIHGHIRDKEAPQKSWAP*
>ENSG00000110328|ENST00000227756
MVCTRKTKTLVSTCVILSGMTNIICLLYVGWVTNYIASVYVRGQEPAPDKKLEEDKGDTL
KIIERLDHLENVIKQHIQEAPAKPEEAEAEPFTDSSLFAHWGQELSPEGRRVALKQFQYY
GYNAYLSDRLPLDRPLPDLRPSGCRNLSFPDSLPEVSIVFIFVNEALSVLLRSIHSAMER
TPPHLLKEIILVDDNSSNEELKEKLTEYVDKVNSQKPGFIKVVRHSKQEGLIRSRVSGWR
AATAPVVALFDAHVEFNVGWAEPVLTRIKENRKRIISPSFDNIKYDNFEIEEYPLAAQGF
DWELWCRYLNPPKAWWKLENSTAPIRSPALIGCFIVDRQYFQEIGLLDEGMEVYGGENVE
LGIRVWQCGGSVEVLPCSRIAHIERAHKPYTEDLTAHVRRNALRVAEVWMDEFKSHVYMA
WNIPQEDSGIDIGDITARKALRKQLQCKTFRWYLVSVYPEMRMYSDIIAYGVLQNSLKTD

LCLDQGPDTENVPIMYICHGMTPQNVYYTSSQQIHVGILSPTVDDDDNRCLVDVNSRPRL
IECSYAKAKRMKLHWQFSQGGPIQNRKSKRCLELQENSDLEFGFQLVLQKCSGQHWSITN
VLRSLAS*
>ENSG00000122705|ENST00000242285
MAELDPFGAPAGAPGGPALGNGVAGAGEEDPAAAFLAQQESEIAGIENDEAFAILDGGAP
GPQPHGEPPGGPDAVDGVMNGEYYQESNGPTDSYAAISQVDRLQSEPESIRKWREEQMER
LEALDANSRKQEAEWKEKAIKELEEWYARQDEQLQKTKANNRVADEAFYKQPFADVIGYV
TNINHPCYSLEQAAEEAFVNDIDESSPGTEWERVARLCDFNPKSSKQAKDVSRMRSVLIS
LKQAPLVH*
>ENSG00000106392|ENST00000223122
MASKSWLNFLTFLCGSAIGFLLCSQLFSILLGEKVDTQPNVLHNDPHARHSDDNGQNHLE
GQMNFNADSSQHKDENTDIAENLYQKVRILCWVMTGPQNLEKKAKHVKATWAQRCNKVLF
MSSEENKDFPAVGLKTKEGRDQLYWKTIKAFQYVHEHYLEDADWFLKADDDTYVILDNLR
WLLSKYDPEEPIYFGRRFKPYVKQGYMSGGAGYVLSKEALKRFVDAFKTDKCTHSSSIED
LALGRCMEIMNVEAGDSRDTIGKETFHPFVPEHHLIKGYLPRTFWYWNYNYYPPVEGPGC
CSDLAVSFHYVDSTTMYELEYLVYHLRPYGYLYRYQPTLPERILKEISQANKNEDTKVKL
GNP*
>ENSG00000115806|ENST00000234160
MGSSQSVEIPGGGTEGYHVLRVQENSPGHRAGLEPFFDFIVSINGSRLNKDNDTLKDLLK
ANVEKPVKMLIYSSKTLELRETSVTPSNLWGGQGLLGVSIRFCSFDGANENVWHVLEVES
NSPAALAGLRPHSDYIIGADTVMNESEDLFSLIETHEAKPLKLYVYNTDTDNCREVIITP
NSAWGGEGSLGCGIGYGYLHRIPTRPFEEGKKISLPGQMAGTPITPLKDGFTEVQLSSVN
PPSLSPPGTTGIEQSLTGLSISSTPPAVSSVLSTGVPTVPLLPPQVNQSLTSVPPMNPAT
TLPGLMPLPAGLPNLPNLNLNLPAPHIMPGVGLPELVNPGLPPLPSMPPRNLPGIAPLPL
PSEFLPSFPLVPESSSAASSGELLSSLPPTSNAPSDPATTTAKADAASSLTVDVTPPTAK
APTTVEDRVGDSTPVSEKPVSAAVDANASESP*
>ENSG00000117533|ENST00000236192
MPPKFKRHLNDDDVTGSVKSERRNLLEDDSDEEEDFFLRGPSGPRFGPRNDKIKHVQNQV
DEVIDVMQENITKVIERGERLDELQDKSESLSDNATAFSNRSKQLRRQMWWRGCKIKAIM
ALVAAILLLVIIILIVMKYRT*
>ENSG00000175106|ENST00000225576
MLQQDSNDDTEDVSLFDAEEETTNRPRKAKIRHPVASFFHLFFRVSAIIVCLLCELLSSS
FITCMVTIILLLSCDFWAVKNVTGRLMVGLRWWNHIDEDGKSHWVFESRKESSQENKTVS
EAESRIFWLGLIACSVLWVIFAFSALFSFTVKWLRRSRHIAQTGLKVLGSRDPPASAFQS
AGITGVSRCPGHPSRKFHQVDINSFTRITDRALYWKPAPRLSSPPLRAAPGNCQQMAPAR
LFLSLRLWAWRGGGESPNSRGTGEPGPKFHLASGMH*
>ENSG00000112309|ENST00000230053
MKSALFTRFFILLPWILIVIIMLDVDTRRPVPPLTPRPYFSPYAVGRGGARLPLRRGGPA
HGTQKRNQSRPQPQPEPQLPTIYAITPTYSRPVQKAELTRLANTFRQVAQLHWILVEDAA
ARSELVSRFLARAGLPSTHLHVPTPRRYKRPGLPRATEQRNAGLAWLRQRHQHQRAQPGV
LFFADDDNTYSLELFQEMRTTRKVSVWPVGLVGGRRYERPLVENGKVVGWYTGWRADRPF
AIDMAGFAVSLQVILSNPKAVFKRRGSQPGMQESDFLKQITTVEELEPKANNCTKVLVWH
TRTEKVNLANEPKYHLDTVKIEV*
>ENSG00000119844|ENST00000238855

MEPDIIRMYSSSPPPLDNGAEDDDDDEFGEFGGFSEVSPSGVGFVDFDTPDYTRPKEEFV
PSNHFMPIHEFSENVDSLTSFKSIKNGNDKDITAELSAPVKGQSDVLLSTTSKEIISSEM
LATSIDGMERPGNLNKVVEQRQNVGTLESFSPGDFRTNMNVVHQNKQLESCNGEKPPCLE
ILTNGFAVLETVNPQGTDDLDNVADSKGRKPLSTHSTEYNLDSVPSPAEEFADFATFSKK
ERIQLEEIECAVLNDREALTIRENNKINRVNELNSVKEVALGRSLDNKGDTDGEDQVCVS
EISIVTNRGFSVEKQGLPTLQQDEFLQSGVQSKAWSLVDSADNSEAIRREQCKTEEKLDL
LTSKCAHLCMDSVKTSDDEVGSPKEESRKFTNFQSPNIDPTEENDLDDSLSVKNGDSSND
FVTCNDINEDDFGDFGDFGSASGSTPPFVTGTQDSMSDATFEESSEHFPHFSEPGDDFGE
FGDINAVSCQEETILTKSDLKQTSDNLSEECQLARKSSGTGTEPVAKLKNGQEGEIGHFD
SVPNIQDDCNGFQDSDDFADFSSAGPSQVVDWNAFEDEQKDSCSWAAFGDQQATESHHRK
EAWQSHRTDENIDTPGTPKTHSVPSATSKGAVASGHLQESATSVQTALLNRLERIFEACF
PSILVPDAEEEVTSLKHLLETSTLPIKTREALPESGELLDVWTELQDIHDAHGLRYQWGG
SHSNKKLLSSLGIDTRNILFTGNKKQPVIVPMYAAGLGMLEPTKEPLKPLSAAEKIASIG
QTATMSPDMNTCTSDQFQESLPPVQFDWSSSGLTNPLDASGGSTLLNLDFFGPVDDSSSS
SSTTIPGVDPELYELTTSKLEISTSSLKVTDAFARLMSTVEKTSTSTRKPKREEHLSEEA
IKVIAGLPDLTFMHAKVLMFPATLTPSTSSQEKADG*
>ENSG00000111711|ENST00000229314
MISLTDTQKIGMGLTGFGVFFLFFGMILFFDKALLAIGNVLFVAGLAFVIGLERTFRFFF
QKHKMKATGFFLGGVFVVLIGWPLIGMIFEIYGFFLLFRGFFPVVVGFIRRVPVLGSLLN
LPGIRSFVDKVGESNNMV*
>ENSG00000122218|ENST00000241704
MLTKFETKSARVKGLSFHPKRPWILTSLHNGVIQLWDYRMCTLIDKFDEHDGPVRGIDFH
KQQPLFVSGGDDYKIKVWNYKLRRCLFTLLGHLDYIRTTFFHHEYPWILSASDDQTIRVW
NWQSRTCVCVLTGHNHYVMCAQFHPTEDLVVSASLDQTVRVWDISGLRKKNLSPGAVESD
VRGITGVDLFGTTDAVVKHVLEGHDRGVNWAAFHPTMPLIVSGADDRQVKIWRMNESKAW
EVDTCRGHYNNVSCAVFHPRQELILSNSEDKSIRVWDMSKRTGVQTFRRDHDRFWVLAAH
PNLNLFAAGHDGGMIVFKLERERPAYAVHGNMLHYVKDRFLRQLDFNSSKDVAVMQLRSG
SKFPVFNMSYNPAENAVLLCTRASNLENSTYDLYTIPKDADSQNPDAPEGKRSSGLTAVW
VARNRFAVLDRMHSLLIKNLKNEITKKVQVPNCDEIFYAGTGNLLLRDADSITLFDVQQK
RTLASVKISKVKYVIWSADMSHVALLAKHAIVICNRKLDALCNIHENIRVKSGAWDESGV
FIYTTSNHIKYAVTTGDHGIIRTLDLPIYVTRVKGNNVYCLDRECRPRVLTIDPTEFKFK
LALINRKYDEVLHMVRNAKLVGQSIIAYLQKKGYPEVALHFVKDEKTRFSLALECGNIEI
ALEAAKALDDKNCWEKLGEVALLQGNHQIVEMCYQRTKNFDKLSFLYLITGNLEKLRKMM
KIAEIRKDMSGHYQNALYLGDVSERVRILKNCGQKSLAYLTAATHGLDEEAESLKETFDP
EKETIPDIDPNAKLLQPPAPIMPLDTNWPLLTVSKGFFEGTIASKGKGGALAADIDIDTV
GTEGWGEDAELQLDEDGFVEATEGLGDDALGKGQEEGGGWDVEEDLELPPELDISPGAAG
GAEDGFFVPPTKGTSPTQIWCNNSQLPVDHILAGSFETAMRLLHDQVGVIQFGPYKQLFL
QTYARGRTTYQALPCLPSMYGYPNRNWKDAGLKNGVPAVGLKLNDLIQRLQLCYQLTTVG
KFEEAVEKFRSILLSVPLLVVDNKQEIAEAQQLITICREYIVGLSVETERKKLPKETLEQ
QKRICEMAAYFTHSNLQPVHMILVLRTALNLFFKLKNFKTAATFARRLLELGPKPEVAQQ
TRKILSACEKNPTDAYQLNYDMHNPFDICAASYRPIYRGKPVEKCPLSGACYSPEFKGQI
CRVTTVTEIGKDVIGLRISPLQFR*
>ENSG00000112367|ENST00000230124
MPTAAAPIISSVQKLVLYETRARYFLVGSNNAETKYRVLKIDRTEPKDLVIIDDRHVYTQ

QEVRELLGRLDLGNRTKMGQKGSSGLFRAVSAFGVVGFVRFLEGYYIVLITKRRKMADIG
GHAIYKVEDTNMIYIPNDSVRVTHPDEARYLRIFQNVDLSSNFYFSYSYDLSHSLQYNLT
VLRMPLEMLKSEMTQNRQESFDIFEDEGLITQGGSGVFGICSEPYMKYVWNGELLDIIKS
TVHRDWLLYIIHGFCGQSKLLIYGRPVYVTLIARRSSKFAGTRFLKRGANCEGDVANEVE
TEQILCDASVMSFTAGSYSSYVQVRGSVPLYWSQDISTMMPKPPITLDQADPFAHVAALH
FDQMFQRFGSPIIILNLVKEREKRKHERILSEELVAAVTYLNQFLPPEHTIVYIPWDMAK
YTKSKLCNVLDRLNVIAESVVKKTGFFVNRPDSYCSILRPDEKWNELGGCVIPTGRLQTG
ILRTNCVDCLDRTNTAQFMVGKCALAYQLYSLGLIDKPNLQFDTDAVRLFEELYEDHGDT
LSLQYGGSQLVHRVKTYRKIAPWTQHSKDIMQTLSRYYSNAFSDADRQDSINLFLGVFHP
TEGKPHLWELPTDFYLHHKNTMRLLPTRRSYTYWWTPEVIKHLPLPYDEVICAVNLKKLI
VKKFHKYEEEIDIHNEFFRPYELSSFDDTFCLAMTSSARDFMPKTVGIDPSPFTVRKPDE
TGKSVLGNKSNREEAVLQRKTAASAPPPPSEEAVSSSSEDDSGTDREEEGSVSQRSTPVK
MTDAGDSAKVTENVVQPMKELYGINLSDGLSEEDFSIYSRFVQLGQSQHKQDKNSQQPCS
RCSDGVIKLTPISAFSQDNIYEVQPPRVDRKSTEIFQAHIQASQGIMQPLGKEDSSMYRE
YIRNRYL*
>ENSG00000105538|ENST00000222145
MLSGERKEGGSPRFGKLHLPVGLWINSPRKQLAKLGRRWPSAASVKSSSSDTGSRSSEPL
PPPPPHVELRRVGAVKAAGGASGSRAKRISQLFRGSGTGTTGSSGAGGPGTPGGAQRWAS
EKKLPELAAGVAPEPPLATRATAPPGVLKIFGAGLASGANYKSVLATARSTARELVAEAL
ERYGLAGSPGGGPGESSCVDAFALCDALGRPAAAGVGSGEWRAEHLRVLGDSERPLLVQE
LWRARPGWARRFELRGREEARRLEQEAFGAADSEGTGAPSWRPQKNRSRAASGGAALASP
GPGTGSGAPAGSGGKERSENLSLRRSVSELSLQGRRRRQQERRQQALSMAPGAADAQIGT
ADPGDFDQLTQCLIQAPSNRPYFLLLQGYQDAQDFVVYVMTREQHVFGRGGNSSGRGGSP
APYVDTFLNAPDILPRHCTVRAGPEHPAMVRPSRGAPVTHNGCLLLREAELHPGDLLGLG
EHFLFMYKDPRTGGSGPARPPWLPARPGATPPGPGWAFSCRLCGRGLQERGEALAAYLDG
REPVLRFRPREEEALLGEIVRAAAAGSGDLPPLGPATLLALCVQHSARELELGHLPRLLG
RLARLIKEAVWEKIKEIGDRQPENHPEGVPEVPLTPEAVSVELRPLMLWMANTTELLSFV
QEKVLEMEKEADQEDPQLCNDLELCDEAMALLDEVIMCTFQQSVYYLTKTLYSTLPALLD
SNPFTAGAELPGPGAELGAMPPGLRPTLGVFQAALELTSQCELHPDLVSQTFGYLFFFSN
ASLLNSLMERGQGRPFYQWSRAVQIRTNLDLVLDWLQGAGLGDIATEFFRKLSMAVNLLC
VPRTSLLKASWSSLRTDHPTLTPAQLHHLLSHYQLGPGRGPPAAWDPPPAEREAVDTGDI
FESFSSHPPLILPLGSSRLRLTGPVTDDALHRELRRLRRLLWDLEQQELPANYRHGPPVA
TSP*
>ENSG00000106636|ENST00000223369
MKLYSLSVLYKGEAKVVLLKAAYDVSSFSFFQRSSVQEFMTFTSQLIVERSSKGTRASVK
EQDYLCHVYVRNDSLAGVVIADNEYPSRVAFTLLEKVLDEFSKQVDRIDWPVGSPATIHY
PALDGHLSRYQNPREADPMTKVQAELDETKIILHNTMESLLERGEKLDDLVSKSEVLGTQ
SKAFYKTARKQNSCCAIM*
>ENSG00000108379|ENST00000225512
MEPHLLGLLLGLLLGGTRVLAGYPIWWSLALGQQYTSLGSQPLLCGSIPGLVPKQLRFCR
NYIEIMPSVAEGVKLGIQECQHQFRGRRWNCTTIDDSLAIFGPVLDKATRESAFVHAIAS
AGVAFAVTRSCAEGTSTICGCDSHHKGPPGEGWKWGGCSEDADFGVLVSREFADARENRP
DARSAMNKHNNEAGRTTILDHMHLKCKCHGLSGSCEVKTCWWAQPDFRAIGDFLKDKYDS
ASEMVVEKHRESRGWVETLRAKYSLFKPPTERDLVYYENSPNFCEPNPETGSFGTRDRTC

NVTSHGIDGCDLLCCGRGHNTRTEKRKEKCHCIFHWCCYVSCQECIRIYDVHTCK*
>ENSG00000070731|ENST00000225276
MGLPRGSFFWLLLLLTAACSGLLFALYFSAVQRYPGPAAGARDTTSFEAFFQSKASNSWT
GKGQACRHLLHLAIQRHPHFRGLFNLSIPVLLWGDLFTPALWDRLSQHKAPYGWRGLSHQ
VIASTLSLLNGSESAKLFAPPRDTPPKCIRCAVVGNGGILNGSRQGPNIDAHDYVFRLNG
AVIKGFERDVGTKTSFYGFTVNTMKNSLVSYWNLGFTSVPQGQDLQYIFIPSDIRDYVML
RSAILGVPVPEGLDKGDRPHAYFGPEASASKFKLLHPDFISYLTERFLKSKLINTHFGDL
YMPSTGALMLLTALHTCDQVSAYGFITSNYWKFSDHYFERKMKPLIFYANHDLSLEAALW
RDLHKAGILQLYQR*
>ENSG00000119844|ENST00000238856
MEPDIIRMYSSSPPPLDNGAEDDDDDEFGEFGGFSEVSPSGVGFVDFDTPDYTRPKEEFV
PSNHFMPIHEFSENVDSLTSFKSIKNGNDKDITAELSAPVKGQSDVLLSTTSKEIISSEM
LATSIDGMERPGNLNKVVEQRQNVGTLESFSPGDFRTNMNVVHQNKQLESCNGEKPPCLE
ILTNGFAVLETVNPQGTDDLDNVADSKGRKPLSTHSTEYNLDSVPSPAEEFADFATFSKK
ERIQLEEIECAVLNDREALTIRENNKINRVNELNSVKEVALGRSLDNKGDTDGEDQVCVS
EISIVTNRGFSVEKQGLPTLQQDEFLQSGVQSKAWSLVDSADNSEAIRREQCKTEEKLDL
LTSKCAHLCMDSVKTSDDEVGSPKEESRKFTNFQSPNIDPTEENDLDDSLSVKNGDSSND
FVTCNDINEDDFGDFGDFGSASGSTPPFVTGTQDSMSDATFEESSEHFPHFSEPGDDFGE
FGDINAVSCQEETILTKSDLKQTSDNLSEECQLARKSSGTGTEPVAKLKNGQEGEIGHFD
SVPNIQDDCNGFQDSDDFADFSSAGPSQVVDWNAFEDEQKDSCSWAAFGDQQATESHHRK
EAWQSHRTDENIDTPGTPKTHSVPSATSKGAVASGHLQESATSVQTALLNRLERIFEACF
PSILVPDAEEEVTSLKHLLETSTLPIKTREALPESGELLDVWTELQDIHDAHGLRYQWGG
SHSNKKLLSSLGIDTRNILFTGNKKQPVIVPMYAAGLGMLEPTKEPLKPLSAAEKIASIG
QTATMSPDMNTCTSDQFQESLPPVQFDWSSSGLTNPLDGVDPELYELTTSKLEISTSSLK
VTDAFARLMSTVEKTSTSTSRKPKREEHLSEEAIKVIAGLPDLTFMHAKVLMFPATLTPS
TSSQEKADG*
>ENSG00000206301|ENST00000241802
MILNKALLLGALALTAVMSPCGGEDIVADHVASYGVNFYQSHGPSGQYTHEFDGDEEFYV
DLETKETVWQLPMFSKFISFDPQSALRNMAVGKHTLEFMMRQSNSTAATNEVPEVTVFSK
FPVTLGQPNTLICLVDNIFPPVVNITWLSNGHSVTEGVSETSFLSKSDHSFFKISYLTFL
PSADEIYDCKVEHWGLDEPLLKHWEPEIPAPMSELTETLVCALGLSVGLMGIVVGTVFII
QGLRSVDASRHQGLL*
>ENSG00000110080|ENST00000227495
MVSKSRWKLLAMLALVLVVMVWYSISREDSFYFPIPEKKEPCLQGEAESKASKLFGNYSR
DQPIFLRLEDYFWVKTPSAYELPYGTKGSEDLLLRVLAITSSSIPKNIQSLRCRRCVVVG
NGHRLRNSSLGDAINKYDVVIRLNNAPVAGYEGDVGSKTTMRLFYPESAHFDPKVENNPD
TLLVLVAFKAMDFHWIETILSDKKRVRKGFWKQPPLIWDVNPKQIRILNPFFMEIAADKL
LSLPMQQPRKIKQKPTTGLLAITLALHLCDLVHIAGFGYPDAYNKKQTIHYYEQITLKSM
AGSGHNVSQEALAIKRMLEMGAIKNLTSF*
>ENSG00000113262|ENST00000231188
MARPRRAREPLLVALLPLAWLAQAGLARAAGSVRLAGGLTLGGLFPVHARGAAGRACGQL
KKEQGVHRLEAMLYALDRVNADPELLPGVRLGARLLDTCSRDTYALEQALSFVQALIRGR
GDGDEVGVRCPGGVPPLRPAPPERVVAVVGASASSVSIMVANVLRLFAIPQISYASTAPE
LSDSTRYDFFSRVVPPDSYQAQAMVDIVRALGWNYVSTLASEGNYGESGVEAFVQISREA

GGVCIAQSIKIPREPKPGEFSKVIRRLMETPNARGIIIFANEDDIRRVLEAARQANLTGH
FLWVGSDSWGAKTSPILSLEDVAVGAITILPKRASIDGFDQYFMTRSLENNRRNIWFAEF
WEENFNCKLTSSGTQSDDSTRKCTGEERIGRDSTYEQEGKVQFVIDAVYAIAHALHSMHQ
ALCPGHTGLCPAMEPTDGRMLLQYIRAVRFNGSAGTPVMFNENGDAPGRYDIFQYQATNG
SASSGGYQAVGQWAETLRLDVEALQWSGDPHEVPSSLCSLPCGPGERKKMVKGVPCCWHC
EACDGYRFQVDEFTCEACPGDMRPTPNHTGCRPTPVVRLSWSSPWAAPPLLLAVLGIVAT
TTVVATFVRYNNTPIVRASGRELSYVLLTGIFLIYAITFLMVAEPGAAVCAARRLFLGLG
TTLSYSALLTKTNRIYRIFEQGKRSVTPPPFISPTSQLVITFSLTSLQVVGMIAWLGARP
PHSVIDYEEQRTVDPEQARGVLKCDMSDLSLIGCLGYSLLLMVTCTVYAIKARGVPETFN
EAKPIGFTMYTTCIIWLAFVPIFFGTAQSAEKIYIQTTTLTVSLSLSASVSLGMLYVPKT
YVILFHPEQNVQKRKRSLKATSTVAAPPKGEDAEAHK*
>ENSG00000106003|ENST00000222725
MLKRCGRRLLLALAGALLACLLVLTADPPPPPLPAERGRRALRSLAGPAGAAPAPGLGAA
AAAPGALVRDVHSLSEYFSLLTRARRDAGPPPGAAPRPADGHPRPLAEPLAPRDVFIAVK
TTKKFHRARLDLLLETWISRHKEMTFIFTDGEDEALARHTGNVVITNCSAAHSRQALSCK
MAVEYDRFIESGRKWFCHVDDDNYVNLRALLRLLASYPHTRDVYVGKPSLDRPIQAMERV
SENKVRPVHFWFATGGAGFCISRGLALKMSPWASGGHFMNTAERIRLPDDCTIGYIVEAL
LGVPLIRSGLFHSHLENLQQVPTSELHEQVTLSYGMFENKRNAVHVKGPFSVEADPSRFR
SIHCHLYPDTPWCPRTAIF*
>ENSG00000110057|ENST00000227471
MEAEPPLYPMAGAAGPQGDEDLLGVPDGPEAPLDELVGAYPNYNEEEEERRYYRRKRLGV
LKNVLAASAGGMLTYGVYLGLLQMQLILHYDETYREVKYGNMGLPDIDSKMLMGINVTPI
AALLYTPVLIRFFGTKWMMFLAVGIYALFVSTNYWERYYTLVPSAVALGMAIVPLWASMG
NYITRMAQKYHEYSHYKEQDGQGMKQRPPRGSHAPYLLVFQAIFYSFFHLSFACAQLPMI
YFLNHYLYDLNHTLYNVQSCGTNSHGILSGFNKTVLRTLPRSGNLIVVESVLMAVAFLAM
LLVLGLCGAAYRPTEEIDLRSVGWGNIFQLPFKHVRDYRLRHLVPFFIYSGFEVLFACTG
IALGYGVCSVGLERLAYLLVAYSLGASAASLLGLLGLWLPRPVPLVAGAGVHLLLTFILF
FWAPVPRVLQHSWILYVAAALWGVGSALNKTGLSTLLGILYEDKERQDFIFTIYHWWQAV
AIFTVYLGSSLHMKAKLAVLLVTLVAAAVSYLRMEQKLRRGVAPRQPRIPRPQHKVRGYR
YLEEDNSDESDAEGEHGDGAEEEAPPAGPRPGPEPAGLGRRPCPYEQAQGGDGPEEQ*
>ENSG00000115596|ENST00000233948
MLPPLPSRLGLLLLLLLCPAHVGGLWWAVGSPLVMDPTSICRKARRLAGRQAELCQAEPE
VVAELARGARLGVRECQFQFRFRRWNCSSHSKAFGRILQQDIRETAFVFAITAAGASHAV
TQACSMGELLQCGCQAPRGRAPPRPSGLPGTPGPPGPAGSPEGSAAWEWGGCGDDVDFGD
EKSRLFMDARHKRGRGDIRALVQLHNNEAGRLAVRSHTRTECKCHGLSGSCALRTCWQKL
PPFREVGARLLERFHGASRVMGTNDGKALLPAVRTLKPPGRADLLYAADSPDFCAPNRRT
GSPGTRGRACNSSAPDLSGCDLLCCGRGHRQESVQLEENCLCRFHWCCVVQCHRCRVRKE
LSLCL*
>ENSG00000105829|ENST00000222547
MRRAGLGEGVPPGNYGNYGYANSGYSACEEENERLTESLRSKVTAIKSLSIEIGHEVKTQ
NKLLAEMDSQFDSTTGFLGKTMGKLKILSRGSQTKLLCYMMLFSLFVFFIIYWIIKLR*
>ENSG00000116095|ENST00000234453
MEGVLYKWTNYLTGWQPRWFVLDNGILSYYDSQDDVCKGSKGSIKMAVCEIKVHSADNTR
MELIIPGEQHFYMKAVNAAERQRWLVALGSSKACLTDTRTKKEKEISETSESLKTKMSEL

RLYCDLLMQQVHTIQEFVHHDENHSSPSAENMNEASSLLSATCNTFITTLEECVKIANAK
FKPEMFQLHHPDPLVSPVSPSPVQMMKRSVSHPGSCSSERSSHSIKEPVSTLHRLSQRRR
RTYSDTDSCSDIPLEDPDRPVHCSKNTLNGDLASATIPEESRLMAKKQSESEDTLPSFSS
*
>ENSG00000083097|ENST00000237163
MNTEELELLSDSKYRNYVAAIDKALKNFEYSSEWADLISALGKLNKVLQNNAKYQVVPKK
LTIGKRLAQCLHPALPGGVHRKALETYEIIFKIIGPKRLAKDLFLYSSGLFPLLANAAMS
VKPTLLSLYEIYYLPLGKTLKPGLQGLLTGILPGLEEGSEYYERTNMLLEKVAAAVDQSA
FYSALWGSLLTSPAVRLPGITYVLAHLNRKLSMEDQLYIIGSDIELMVEAVSTSVQDSSV
LVQRSTLDLILFCFPFHMSQATRPDMIRILSAALHVVLRRDMSLNRRLYAWLLGPRSTRH
SNPEEHATYYFTTFSKELLVQAMVGILQVNGFGEENTLMQDLKPFRILISLLDKPELGPV
ILEDVLIEVFRTLYSQCKAELDLQTEPPFSKDHAQLSSKLRENKKTAELIKTANLLFNSF
EPYYMWDYVARWFEECCRRTLHVRLQIGPGDSNDSSELQLTNFCLLVDFLLDIVSLPTRS
MRVLCQETYIEIQTEHLPQLLLRMISALTSHLQTLHLSELTDSLRLCSKILSKVQPPLLS
ASTGGVLQFPSGQNNSVKEWEDKKVSSVSHENPTEVFEDGENPPSSRSSESGFTEFIQYQ
ADRTDDIDRELSEGQGAAAIPIGSTSSETETASTVGSEETIIQTPSVVTQGTATRSRKTA
QKTAMQCCLEYVQQFLTRLINLYIIQNNSFSQSLATEHQGDLGREQGETSKWDRNSQGDV
KEKNISKQKTSKEYLSAFLAACQLFLECSSFPVYIAEGNHTSELRSEKLETDCEHVQPPQ
WLQTLMNACSQASDFSVQSVAISLVMDLVGLTQSVAMVTGENINSVEPAQPLSPNQGRVA
VVIRPPLTQGNLRYIAEKTEFFKHVALTLWDQLGDGTPQHHQKSVELFYQLHNLVPSSSI
CEDVISQQLTHKDKKIRMEAHAKFAVLWHLTRDLHINKSSSFVRSFDRSLFIMLDSLNSL
DGSTSSVGQAWLNQVLQRHDIARVLEPLLLLLLHPKTQRVSVQRVQAERYWNKSPCYPGE
ESDKHFMQNFACSNVSQVQLITSKGNGEKPLTMDEIENFSLTVNPLSDRLSLLSTSSETI
PMVVSDFDLPDQQIEILQSSDSGCSQSSAGDNLSYEVDPETVNAQEDSQMPKESSPDDDV
QQVVFDLICKVVSGLEVESASVTSQLEIEAMPPKCSDIDPDEETIKIEDDSIQQSQNALL
SNESSQFLSVSAEGGHECVANGISRNSSSPCISGTTHTLHDSSVASIETKSRQRSHSSIQ
FSFKEKLSEKVSEKETIVKESGKQPGAKPKVKLARKKDDDKKKSSNEKLKQTSVFFSDGL
DLENWYSCGEGDISEIESDMGSPGSRKSPNFNIHPLYQHVLLYLQLYDSSRTLYAFSAIK
AILKTNPIAFVNAISTTSVNNAYTPQLSLLQNLLARHRISVMGKDFYSHIPVDSNHNFRS
SMYIEILISLCLYYMRSHYPTHVKVTAQDLIGNRNMQMMSIEILTLLFTELAKVIESSAK
GFPSFISDMLSKCKVQKVILHCLLSSIFSAQKWHSEKMAGKNLVAVEEGFSEDSLINFSE
DEFDNGSTLQSQLLKVLQRLIVLEHRVMTIPEENETGFDFVVSDLEHISPHQPMTSLQYL
HAQPITCQGMFLCAVIRALHQHCACKMHPQWIGLITSTLPYMGKVLQRVVVSVTLQLCRN
LDNLIQQYKYETGLSDSRPLWMASIIPPDMILTLLEGITAIIHYCLLDPTTQYHQLLVSV
DQKHLFEARSGILSILHMIMSSVTLLWSILHQADSSEKMTIAASASLTTINLGATKNLRQ
QILELLGPISMNHGVHFMAAIAFVWNERRQNKTTTRTKVIPAASEEQLLLVELVRSISVM
RAETVIQTVKEVLKQPPAIAKDKKHLSLEVCMLQFFYAYIQRIPVPNLVDSWASLLILLK
DSIQLSLPAPGQFLILGVLNEFIMKNPSLENKKDQRDLQDVTHKIVDAIGAIAGSSLEQT
TWLRRNLEVKPSPKIMVDGTNLESDVEDMLSPAMETANITPSVYSVHALTLLSEVLAHLL
DMVFYSDEKERVIPLLVNIMHYVVPYLRNHSAHNAPSYRACVQLLSSLSGYQYTRRAWKK
EAFDLFMDPSFFQMDASCVNHWRAIMDNLMTHDKTTFRDLMTRVAVAQSSSLNLFANRDV
ELEQRAMLLKRLAFAIFSSEIDQYQKYLPDIQERLVESLRLPQVPTLHSQVFLFFRVLLL
RMSPQHLTSLWPTMITELVQVFLLMEQELTADEDISRTSGPSVAGLETTYTGGNGFSTSY
NSQRWLNLYLSACKFLDLALALPSENLPQFQMYRWAFIPEASDDSGLEVRRQGIHQREFK

PYVVRLAKLLRKRAKDKEEDFKTVILEGLEMAKHQKNPEEDNSGRTLGWEPGHLLLTICT
VRSMEQLLPFFNVLSQVFNSKVTSRCGGHSGSPILYSNAFPNKDMKLENHKPCSSKARQK
IEEMVEKDFLEGMIKT*
>ENSG00000108433|ENST00000225567
MDPLFQQTHKQVHEIQSCMGRLETADKQSVHIVENEIQASIDQIFSRLERLEILSSKEPP
NKRQNARLRVDQLKYDVQHLQTALRNFQHRRHAREQQERQREELLSRTFTTNDSDTTIPM
DESLQFNSSLQKVHNGMDDLILDGHNILDGLRTQRLTLKGTQKKILDIANMLGLSNTVMR
LIEKRAFQDKYFMIGTQGSCQTAHFGGRSAGSS*
>ENSG00000115540|ENST00000233892
MDSTLAVQQYIQQNIRADCSNIDKILEPPEGQDEGVWKYEHLRQFCLELNGLAVKLQSEC
HPDTCTQMTATEQWIFLCAAHKTPKECPAIDYTRHTLDGAACLLNSNKYFPSRVSIKESS
VAKLGSVCRRIYRIFSHAYFHHRQIFDEYENETFLCHRFTKFVMKYNLMSKDNLIVPILE
EEVQNSVSGESEA*
>ENSG00000119729|ENST00000238738
MAHGPGALMLKCVVVGDGAVGKTCLLMSYANDAFPEEYVPTVFDHYAVSVTVGGKQYLLG
LYDTAGQEDYDRLRPLSYPMTDVFLICFSVVNPASFQNVKEEWVPELKEYAPNVPFLLIG
TQIDLRDDPKTLARLNDMKEKPICVEQGQKLAKEIGACCYVECSALTQKGLKTVFDEAII
AILTPKKHTVKKRIGSRCINCCLIT*
>ENSG00000106692|ENST00000223528
MSRINKNVVLALLTLTSSAFLLFQLYYYKHYLSTKNGAGLSKSKGSRIGFDSTQWRAVKK
FIMLTSNQNVPVFLIDPLILELINKNFEQVKNTSHGSTSQCKFFCVPRDFTAFALQYHLW
KNEEGWFRIAENMGFQCLKIESKDPRLDGIDSLSGTEIPLHYICKLATHAIHLVVFHERS
GNYLWHGHLRLKEHIDRKFVPFRKLQFGRYPGAFDRPELQQVTVDGLEVLIPKDPMHFVE
EVPHSRFIECRYKEARAFFQQYLDDNTVEAVAFRKSAKELLQLAAKTLNKLGVPFWLSSG
TCLGWYRQCNIIPYSKDVDLGIFIQDYKSDIILAFQDAGLPLKHKFGKVEDSLELSFQGK
DDVKLDVFFFYEETDHMWNGGTQAKTGKKFKYLFPKFTLCWTEFVDMKVHVPCETLEYIE
ANYGKTWKIPVKTWDWKRSPPNVQPNGIWPISEWDEVIQLY*
>ENSG00000113595|ENST00000231524
MATLVVNKLGAGVDSGRQGSRGTAVVKVLECGVCEDVFSLQGDKVPRLLLCGHTVCHDCL
TRLPLHGRAIRCPFDRQVTDLGDSGVWGLKKNFALLELLERLQNGPIGQYGAAEESIGIS
GESIIRCDEDEAHLASVYCTVCATHLCSECSQVTHSTKTLAKHRRVPLADKPHEKTMCSQ
HQVHAIEFVCLEEGCQTSPLMCCVCKEYGKHQGHKHSVLEPEANQIRASILDMAHCIRTF
TEEISDYSRKLVGIVQHIEGGEQIVEDGIGMAHTEHVPGTAENARSCIRAYFYDLHETLC
RQEEMALSVVDAHVREKLIWLRQQQEDMTILLSEVSAACLHCEKTLQQDDCRVVLAKQEI
TRLLETLQKQQQQFTEVADHIQLDASIPVTFTKDNRVHIGPKMEIRVVTLGLDGAGKTTI
LFKLKQDEFMQPIPTIGFNVETVEYKNLKFTIWDVGGKHKLRPLWKHYYLNTQAVVFVVD
SSHRDRISEAHSELAKLLTEKELRDALLLIFANKQDVAGALSVEEITELLSLHKLCCGRS
WYIQGCDARSGMGLYEGLDWLSRQLVAAGVLDVA*
>ENSG00000118017|ENST00000236709
MRKELQLSLSVTLLLVCGFLYQFTLKSSCLFCLPSFKSHQGLEALLSHRRGIVFLETSER
MEPPHLVSCSVESAAKIYPEWPVVFFMKGLTDSTPMPSNSTYPAFSFLSAIDNVFLFPLD
MKRLLEDTPLFSWYNQINASAERNWLHISSDASRLAIIWKYGGIYMDTDVISIRPIPEEN
FLAAQASRYSSNGIFGFLPHHPFLWECMENFVEHYNSAIWGNQGPELMTRMLRVWCKLED
FQEVSDLRCLNISFLHPQRFYPISYREWRRYYEVWDTEPSFNVSYALHLWNHMNQEGRAV

IRGSNTLVENLYRKHCPRTYRDLIKGPEGSVTGELGPGNK*
>ENSG00000113532|ENST00000231461
MRSIRKRWTICTISLLLIFYKTKEIARTEEHQETQLIGDGELSLSRSLVNSSDKIIRKAG
SSIFQHNVEGWKINSSLVLEIRKNILRFLDAERDVSVVKSSFKPGDVIHYVLDRRRTLNI
SHDLHSLLPEVSPMKNRRFKTCAVVGNSGILLDSECGKEIDSHNFVIRCNLAPVVEFAAD
VGTKSDFITMNPSVVQRAFGGFRNESDREKFVHRLSMLNDSVLWIPAFMVKGGEKHVEWV
NALILKNKLKVRTAYPSLRLIHAVRGYWLTNKVPIKRPSTGLLMYTLATRFCDEIHLYGF
WPFPKDLNGKAVKYHYYDDLKYRYFSNASPHRMPLEFKTLNVLHNRGALKLTTGKCVKQ*
>ENSG00000132639|ENST00000254976
MAEDADMRNELEEMQRRADQLADESLESTRRMLQLVEESKDAGIRTLVMLDEQGEQLERI
EEGMDQINKDMKEAEKNLTDLGKFCGLCVCPCNKLKSSDAYKKAWGNNQDGVVASQPARV
VDEREQMAISGGFIRRVTNDARENEMDENLEQVSGIIGNLRHMALDMGNEIDTQNRQIDR
IMEKADSNKTRIDEANQRATKMLGSG*
>ENSG00000107036|ENST00000251879
MYFLSGWPKRLLCPLGSPAEAPFHVQSDPQRAFFAVLAAARLSIWYSRPSVLIVTYKEPA
KSSTQFGSYKQAEWRPDSTMIAVSTANGYILFFHITSTRGDKYLYEPVYPKGSPQMKGTP
HFKEEQCAPALNLEMRKILDLQAPIMSLQSVLEDLLVATSDGLLHLIHWEGMTNGRKAIN
LCTVPFSVDLQSSRVGSFLGFTDVHIRDMEYCATLDGFAVVFNDGKVGFITPVSSRFTAE
QLHGVWPQDVVDGTCVAVNNKYRLMAFGCVSGSVQVYTIDNSTGAMLLSHKLELTAKQYP
DIWNKTGAVKLMRWSPDNSVVIVTWEYGGLSLWSVFGAQLICTLGGDFAYRSDGTKKDPL
KINSMSWGAEGYHLWVISGFGSQNTEIESDLRSVVKQPSILLFQFIKSVLTVNPCMSNQE
QVLLQGEDRLYLNCGEASQTQNPRSSSTHSEHKPSREKSPFADGGLESQGLSTLLGHRHW
HVVQISSTYLESNWPIRFSAIDKLGQNIAVVGKFGFAHYSLLTKKWKLFGNITQEQNMIV
TGGLAWWNDFMVLACYNINDRQEELRVYLRTSNLDNAFAHVTKAQAETLLLSVFQDMVIV
FRADCSICLYSIERKSDGPNTTAGIQVLQEVSMSRYIPHPFLVVSVTLTSVSTENGITLK
MPQQARGAESIMLNLAGQLIMMQRDRSGPQIREKDSNPNNQRKLLPFCPPVVLAQSVENV
WTTCRANKQKRHLLEALWLSCGGAGMKVWLPLFPRDHRKPHSFLSQRIMLPFHINIYPLA
VLFEDALVLGAVNDTLLYDSLYTRNNAREQLEVLFPFCVVERTSQIYLHHILRQLLVRNL
GEQALLLAQSCATLPYFPHVLELMLHEVLEEEATSREPIPDPLLPTVAKFITEFPLFLQT
VVHCARKTEYALWNYLFAAVGNPKDLFEECLMAQDLDTAASYLIILQNMEVPAVSRQHAT
LLFNTALEQGKWDLCRHMIRFLKAIGSGESETPPSTPTAQEPSSSGGFEFFRNRSISLSQ
SAENVPASKFSLQKTLSMPSGPSGKRWSKDSDCAENMYIDMMLWRHARRLLEDVRLKDLG
CFAAQLGFELISWLCKERTRAARVDNFVIALKRLHKDFLWPLPIIPASSISSPFKNGKYR
TGNVDFMSLVQGELYFTPCIYTFCY*
>ENSG00000136710|ENST00000259229
MAALDLRAELDSLVLQLLGDLEELEGKRTVLNARVEEGWLSLAKARYAMGAKSVGPLQYA
SHMEPQVCLHASEAQEGLQKFKVVRAGVHAPEEVGPREAGLRRRKGPTKTPEPESSEAPQ
DPLNWFGILVPHSLRQAQASFRDGLQLAADIASLQNRIDWGRSQLRGLQEKLKQLEPGAA
*
>ENSG00000137642|ENST00000260197
MATRSSRRESRLPFLFTLVALLPPGALCEVWTQRLHGGSAPLPQDRGFLVVQGDPRELRL
WARGDARGASRADEKPLRRKRSAALQPEPIKVYGQVSLNDSHNQMVVHWAGEKSNVIVAL
ARDSLALARPKSSDVYVSYDYGKSFKKISDKLNFGLGNRSEAVIAQFYHSPADNKRYIFA
DAYAQYLWITFDFCNTLQGFSIPFRAADLLLHSKASNLLLGFDRSHPNKQLWKSDDFGQT

WIMIQEHVKSFSWGIDPYDKPNTIYIERHEPSGYSTVFRSTDFFQSRENQEVILEEVRDF
QLRDKYMFATKVVHLLGSEQQSSVQLWVSFGRKPMRAAQFVTRHPINEYYIADASEDQVF
VCVSHSNNRTNLYISEAEGLKFSLSLENVLYYSPGGAGSDTLVRYFANEPFADFHRVEGL
QGVYIATLINGSMNEENMRSVITFDKGGTWEFLQAPAFTGYGEKINCELSQGCSLHLAQR
LSQLLNLQLRRMPILSKESAPGLIIATGSVGKNLASKTNVYISSSAGARWREALPGPHYY
TWGDHGGIITAIAQGMETNELKYSTNEGETWKTFIFSEKPVFVYGLLTEPGEKSTVFTIF
GSNKENVHSWLILQVNATDALGVPCTENDYKLWSPSDERGNECLLGHKTVFKRRTPHATC
FNGEDFDRPVVVSNCSCTREDYECDFGFKMSEDLSLEVCVPDPEFSGKSYSPPVPCPVGS
TYRRTRGYRKISGDTCSGGDVEARLEGELVPCPLAEENEFILYAVRKSIYRYDLASGATE
QLPLTGLRAAVALDFDYEHNCLYWSDLALDVIQRLCLNGSTGQEVIINSGLETVEALAFE
PLSQLLYWVDAGFKKIEVANPDGDFRLTIVNSSVLDRPRALVLVPQEGVMFWTDWGDLKP
GIYRSNMDGSAAYHLVSEDVKWPNGISVDDQWIYWTDAYLECIERITFSGQQRSVILDNL
PHPYAIAVFKNEIYWDDWSQLSIFRASKYSGSQMEILANQLTGLMDMKIFYKGKNTGSNA
CVPRPCSLLCLPKANNSRSCRCPEDVSSSVLPSGDLMCDCPQGYQLKNNTCVKQENTCLR
NQYRCSNGNCINSIWWCDFDNDCGDMSDERNCPTTICDLDTQFRCQESGTCIPLSYKCDL
EDDCGDNSDESHCEMHQCRSDEYNCSSGMCIRSSWVCDGDNDCRDWSDEANCTAIYHTCE
ASNFQCRNGHCIPQRWACDGDTDCQDGSDEDPVNCEKKCNGFRCPNGTCIPSSKHCDGLR
DCSDGSDEQHCEPLCTHFMDFVCKNRQQCLFHSMVCDGIIQCRDGSDEDAAFAGCSQDPE
FHKVCDEFGFQCQNGVCISLIWKCDGMDDCGDYSDEANCENPTEAPNCSRYFQFRCENGH
CIPNRWKCDRENDCGDWSDEKDCGDSHILPFSTPGPSTCLPNYYRCSSGTCVMDTWVCDG
YRDCADGSDEEACPLLANVTAASTPTQLGRCDRFEFECHQPKTCIPNWKRCDGHQDCQDG
RDEANCPTHSTLTCMSREFQCEDGEACIVLSERCDGFLDCSDESDEKACSDELTVYKVQN
LQWTADFSGDVTLTWMRPKKMPSASCVYNVYYRVVGESIWKTLETHSNKTNTVLKVLKPD
TTYQVKVQVQCLSKAHNTNDFVTLRTPEGLPDAPRNLQLSLPREAEGVIVGHWAPPIHTH
GLIREYIVEYSRSGSKMWASQRAASNFTEIKNLLVNTLYTVRVAAVTSRGIGNWSDSKSI
TTIKGKVIPPPDIHIDSYGENYLSFTLTMESDIKVNGYVVNLFWAFDTHKQERRTLNFRG
SILSHKVGNLTAHTSYEISAWAKTDLGDSPLAFEHVMTRGVRPPAPSLKAKAINQTAVEC
TWTGPRNVVYGIFYATSFLDLYRNPKSLTTSLHNKTVIVSKDEQYLFLVRVVVPYQGPSS
DYVVVKMIPDSRLPPRHLHVVHTGKTSVVIKWESPYDSPDQDLLYAVAVKDLIRKTDRSY
KVKSRNSTVEYTLNKLEPGGKYHIIVQLGNMSKDSSIKITTVSLSAPDALKIITENDHVL
LFWKSLALKEKHFNESRGYEIHMFDSAMNITAYLGNTTDNFFKISNLKMGHNYTFTVQAR
CLFGNQICGEPAILLYDELGSGADASATQAARSTDVAAVVVPILFLILLSLGVGFAILYT
KHRRLQSSFTAFANSHYSSRLGSAIFSSGDDLGEDDEDAPMITGFSDDVPMVIA*
>ENSG00000115884|ENST00000254351
MRRAALWLWLCALALSLQPALPQIVATNLPPEDQDGSGDDSDNFSGSGAGALQDITLSQQ
TPSTWKDTQLLTAIPTSPEPTGLEATAASTSTLPAGEGPKEGEAVVLPEVEPGLTAREQE
ATPRPRETTQLPTTHLASTTTATTAQEPATSHPHRDMQPGHHETSTPAGPSQADLHTPHT
EDGGPSATERAAEDGASSQLPAAEGSGEQDFTFETSGENTAVVAVEPDRRNQSPVDQGAT
GASQGLLDRKEVLGGVIAGGLVGLIFAVCLVGFMLYRMKKKDEGSYSLEEPKQANGGAYQ
KPTKQEEFYA*
>ENSG00000125967|ENST00000246190
MACAGLLTVCLLRPPAPQPQPQTPRHPQLAPDPGPAGHTLFQDVFRRADKNDDGKLSFEE
FQNYFADGVLSLGELQELFSGIDGHLTDNLETEKLCDYFSEHLGVYRPVLAALESLNRAV
LAAMDATKLEYERASKVDQFVTRFLLRETVSQLQALQSSLEGASDTLEAQAHGWRSDAES

VEAQSRLCGSRRAGRRALRSVSRSSTWSPGSSDTGRSSEAEMQWRLQVNRLQELIDQLEC
KVRAVGPGPHKGGPSWYPPEPGPCWRPGPHSVPSQAPRLEPLREEDLAKGPDLHILMAQR
QVQVAEEGLQDFHRALRCYVDFTGAQSHCLHVSAQKMLDGASFTLYEFWQDEASWRRHQQ
SPGSKAFQRILIDHLRAPDTLTTVFFPASWWIMNNN*
>ENSG00000111728|ENST00000261197
MSPCGRARRQTSRGAMAVLAWKFPRTRLPMGASALCVVVLCWLYIFPVYRLPNEKEIVQG
VLQQGTAWRRNQTAARAFRKQMEDCCDPAHLFAMTKMNSPMGKSMWYDGEFLYSFTIDNS
TYSLFPQMQSPSFVK*
>ENSG00000132254|ENST00000254584
MTDGILGKAATMEIPIHGNGEARQLPEDDGLEQDLQQVMVSGPNLNETSIVSGGYGGSGD
GLIPTGSGRHPSHSTTPSGPGDEVARGIAGEKFDIVKKWGINTYKCTKQLLSERFGRGSR
TVDLELELQIELLRETKRKYESVLQLGRALTAHLYSLLQTQHALGDAFADLSQKSPELQE
EFGYNAETQKLLCKNGETLLGAVNFFVSSINTLVTKTMEDTLMTVKQYEAARLEYDAYRT
DLEELSLGPRDAGTRGRLESAQATFQAHRDKYEKLRGDVAIKLKFLEENKIKVMHKQLLL
FHNAVSAYFAGNQKQLEQTLQQFNIKLRPPGAEKPSWLEEQ*
>ENSG00000128274|ENST00000249005
MSKPPDLLLRLLRGAPRQRVCTLFIIGFKFTFFVSIMIYWHVVGEPKEKGQLYNLPAEIP
CPTLTPPTPPSHGPTPGNIFFLETSDRTNPNFLFMCSVESAARTHPESHVLVLMKGLPGG
NASLPRHLGISLLSCFPNVQMLPLDLRELFRDTPLADWYAAVQGRWEPYLLPVLSDASRI
ALMWKFGGIYLDTDFIVLKNLRNLTNVLGTQSRYVLNGAFLAFERRHEFMALCMRDFVDH
YNGWIWGHQGPQLLTRVFKKWCSIRSLAESRACRGVTTLPPEAFYPIPWQDWKKYFEDIN
PEELPRLLSATYAVHVWNKKSQGTRFEATSRALLAQLHARYCPTTHEAMKMYL*
>ENSG00000137177|ENST00000259711
MSDTKVKVAVRVRPMNRRELELNTKCVVEMEGNQTVLHPPPSNTKQGERKPPKVFAFDYC
FWSMDESNTTKYAGQEVVFKCLGEGILEKAFQGYNACIFAYGQTGSGKSFSMMGHAEQLG
LIPRLCCALFKRISLEQNESQTFKVEVSYMEIYNEKVRDLLDPKGSRQSLKVREHKVLGP
YVDGLSQLAVTSFEDIESLMSEGNKSRTVAATNMNEESSRSHAVFNIIITQTLYDLQSGN
SGEKVSKVSLVDLAGSERVSKTGAAGERLKEGSNINKSLTTLGLVISSLADQAAGKGKSK
FVPYRDSVLTWLLKDNLGGNSQTSMIATISPAADNYEETLSTLRYADRAKRIVNHAVVNE
DPNAKVIRELREEVEKLREQLSQAEAMKAPELKEKLEESEKLIKELTVTWEEKLRKTEEI
AQERQRQLESMGISLEMSGIKVGDDKCYLVNLNADPALNELLVYYLKDHTRVGADTSQDI
QLFGIGIQPQHCEIDIASDGDVTLTPKENARSCVNGTLVCSTTQLWHGDRILWGNNHFFR
INLPKRKRRDWLKDFEKETGPPEHDLDAASEASSEPDYNYEFAQMEVIMKTLNSNDPVQN
VVQVLEKQYLEEKRSALEEQRLMYERELEQLRQQLSPDRQPQSSGPDRLAYSSQTAQQKV
TQWAEERDELFRQSLAKLREQLVKANTLVREANFLAEEMSKLTDYQVTLQIPAANLSANR
KRGAIVSEPAIQVRRKGKSTQVWTIEKLENKLIDMRDLYQEWKEKVPEAKRLYGKRGDPF
YEAQENHNLIGVANVFLECLFCDVKLQYAVPIISQQGEVAGRLHVEVMRVTGAVPERVVE
DDSSENSSESGSLEVVDSSGEIIHRVKKLTCRVKIKEATGLPLNLSNFVFCQYTFWDQCE
STVAAPVVDPEVPSPQSKDAQYTVTFSHCKDYVVNVTEEFLEFISDGALAIEVWGHRCAG
NGSSIWEVDSLHAKTRTLHDRWNEVTRRIEMWISILELNELGEYAAVELHQAKDVNTGGI
FQLRQGHSRRVQVTVKPVQHSGTLPLMVEAILSVSIGCVTARSTKLQRGLDSYQRDDEDG
DDMDSYQEEDLNCVRERWSDALIKRREYLDEQIKKVSNKTEKTEDDVEREAQLVEQWVGL
TEERNAVLVPAPGSGIPGAPADWIPPPGMETHIPVLFLDLNADDLSANEQLVGPHASGVN
SILPKEHGSQFFYLPIIKHSDDEVSATASWDSSVHDSVHLNRVTPQNERIYLIVKTTVQL

SHPAAMELVLRKRIAANIYNKQSFTQSLKRRISLKNIFYSCGVTYEIVSNIPKATEEIED
RETLALLAARSENEGTSDGETYIEKYTRGVLQVENILSLERLRQAVTVKEALSTKARHIR
RSLSTPNVHNVSSSRPDLSGFDEDDKGWPENQLDMSDYSSSYQDVACYGTLPRDSPRRNK
EGCTSETPHALTVSPFKAFSPQPPKFFKPLMPVKEEHKKRIALEARPLLSQESMPPPQAH
NPGCIVPSGSNGSSMPVEHNSKREKKIDSEEEENELEAINRKLISSQPYVPVEFADFSVY
NASLENREWFSSKVDLSNSRVLEKEVSRSPTTSSITSGYFSHSASNATLSDMVVPSSDSS
DQLAIQTKDADSTEHSTPSLVHDFRPSSNKELTEVEKGLVKDKIIVVPLKENSALAKGSP
SSQSIPEKNSKSLCRTGSCSELDACPSKISQPARGFCPREVTVEHTTNILEDHSFTEFMG
VSEGKDFDGLTDSSAGELSSRRSLPNKTGGKTVSDGLHHPSQLHSKLENDQVIIPEAAFW
VLCCQ*
>ENSG00000132872|ENST00000255224
MAPITTSREEFDEIPTVVGIFSAFGLVFTVSLFAWICCQRKSSKSNKTPPYKFVHVLKGV
DIYPENLNSKKKFGADDKNEVKNKPAVPKNSLHLDLEKRDLNGNFPKTNLKPGSPSDLEN
ATPKLFLEGEKESVSPESLKSSTSLTSEEKQEKLGTLFFSLEYNFERKAFVVNIKEARGL
PAMDEQSMTSDPYIKMTILPEKKHKVKTRVLRKTLDPAFDETFTFYGIPYTQIQELALHF
TILSFDRFSRDDIIGEVLIPLSGIELSEGKMLMNREIIKRNVRKSSGRGELLISLCYQST
TNTLTVVVLKARHLPKSDVSGLSDPYVKVNLYHAKKRISKKKTHVKKCTPNAVFNELFVF
DIPCEGLEDISVEFLVLDSERGSRNEVIGQLVLGAAAEGTGGEHWKEICDYPRRQIAKWH
VLCDG*
>ENSG00000123989|ENST00000243776
MRASLLLSVLRPAGPVAVGISLGFTLSLLSVTWVEEPCGPGPPQPGDSELPPRGNTNAAR
RPNSVQPGAEREKPGAGEGAGENWEPRVLPYHPAQPGQAAKKAVRTRYISTELGIRQRLL
VAVLTSQTTLPTLGVAVNRTLGHRLERVVFLTGARGRRAPPGMAVVTLGEERPIGHLHLA
LRHLLEQHGDDFDWFFLVPDTTYTEAHGLARLTGHLSLASAAHLYLGRPQDFIGGEPTPG
RYCHGGFGVLLSRMLLQQLRPHLEGCRNDIVSARPDEWLGRCILDATGVGCTGDHEGVHY
SHLELSPGEPVQEGDPHFRSALTAHPVRDPVHMYQLHKAFARAELERTYQEIQELQWEIQ
NTSHLAVDGDQAAAWPVGIPAPSRPASRFEVLRWDYFTEQHAFSCADGSPRCPLRGADRA
DVADVLGTALEELNRRYHPALRLQKQQLVNGYRRFDPARGMEYTLDLQLEALTPQGGRRP
LTRRVQLLRPLSRVEILPVPYVTEASRLTVLLPLAAAERDLAPGFLEAFATAALEPGDAA
AALTLLLLYEPRQAQRVAHADVFAPVKAHVAELERRFPGARVPWLSVQTAAPSPLRLMDL
LSKKHPLDTLFLLAGPDTVLTPDFLNRCRMHAISGWQAFFPMHFQAFHPAVAPPQGPGPP
ELGRDTGRFDRQAASEACFYNSDYVAARGRLAAASEQEEELLESLDVYELFLHFSSLHVL
RAVEPALLQRYRAQTCSARLSEDLYHRCLQSVLEGLGSRTQLAMLLFEQEQGNST*
>ENSG00000136933|ENST00000259460
MKQLPVLEPGDKPRKATWYTLTVPGDSPCARVGHSCSYLPPVGNAKRGKVFIVGGANPNR
SFSDVHTMDLETRTWTTPEVTSPPPSPRTFHTSSAAIGNQLYVFGGGERGAQPVQDTKLH
VFDANTLTWSQPETLGNPPSPRHGHVMVAAGTKLFIHGGLAGDRFYDDLHCIDISDMKWQ
KLNPTGAAPAGCAAHSAVAMGKHVYIFGGMTPAGALDTMYQYHTEEQHWTLLKFDTLLPP
GRLDHSMCIIPWPVTCASEKEDSNSLTLNHEAEKEDSADKVMSHSGDSHEESQTATLLCL
VFGGMNTEGEIYDDCIVTVVD*
>ENSG00000128519|ENST00000249284
MIPIQLTVFFMIIYVLESLTIIVQSSLIVAVLGREWLQVRRLMPVDMILISLGISRFCLQ
WASMLNNFCSYFNLNYVLCNLTITWEFFNILTFWLNSLLTVFYCIKVSSFTHHIFLWLRW
RILRLFPWILLGSLMITCVTIIPSAIGNYIQIQLLTMEHLPRNSTVTDKLENFHQYQFQA

HTVALVIPFILFLASTIFLMASLTKQIQHHSTGHCNPSMKARFTALRSLAVLFIVFTSYF
LTILITIIGTLFDKRCWLWVWEAFVYAFILMHSTSLMLSSPTLKRILKGKC*
>ENSG00000133067|ENST00000255432
MGRPRLTLVCQVSIIISARDLSMNNLTELQPGLFHHLRFLEELRLSGNHLSHIPGQAFSG
LYSLKILMLQNNQLGGIPAEALWELPSLQSLRLDANLISLVPERSFEGLSSLRHLWLDDN
ALTEIPVRALNNLPALQAMTLALNRISHIPDYAFQNLTSLVVLHLHNNRIQHLGTHSFEG
LHNLETLDLNYNKLQEFPVAIRTLGRLQELGFHNNNIKAIPEKAFMGNPLLQTIHFYDNP
IQFVGRSAFQYLPKLHTLSLNGAMDIQEFPDLKGTTSLEILTLTRAGIRLLPSGMCQQLP
RLRVLELSHNQIEELPSLHRCQKLEEIGLQHNRIWEIGADTFSQLSSLQALDLSWNAIRS
IHPEAFSTLHSLVKLDLTDNQLTTLPLAGLGGLMHLKLKGNLALSQAFSKDSFPKLRILE
VPYAYQCCPYGMCASFFKASGQWEAEDLHLDDEESSKRPLGLLARQAENHYDQDLDELQL
EMEDSKPHPSVQCSPTPGPFKPCEYLFESWGIRLAVWAIVLLSVLCNGLVLLTVFAGGPV
PLPPVKFVVGAIAGANTLTGISCGLLASVDALTFGQFSEYGARWETGLGCRATGFLAVLG
SEASVLLLTLAAVQCSVSVSCVRAYGKSPSLGSVRAGVLGCLALAGLAAALPLASVGEYG
ASPLCLPYAPPEGQPAALGFTVALVMMNSFCFLVVAGAYIKLYCDLPRGDFEAVWDCAMV
RHVAWLIFADGLLYCPVAFLSFASMLGLFPVTPEAVKSVLLVVLPLPACLNPLLYLLFNP
HFRDDLRRLRPRAGDSGPLAYAAAGELEKSSCDSTQALVAFSDVDLILEASEAGRPPGLE
TYGFPSVTLISCQQPGAPRLEGSHCVEPEGNHFGNPQPSMDGELLLRAEGSTPAGGGLSG
GGGFQPSGLAFASHV*
>ENSG00000138459|ENST00000261034
MEKQCCSHPVICSLSTMYTFLLGAIFIALSSSRILLVKYSANEENKYDYLPTTVNVCSEL
VKLVFCVLVSFCVIKKDHQSRNLKYASWKEFSDFMKWSIPAFLYFLDNLIVFYVLSYLQP
AMAVIFSNFSIITTALLFRIVLKRRLNWIQWASLLTLFLSIVALTAGTKTLQHNLAGRGF
HHDAFFSPSNSCLLFRSPPWNFSWKPHQSFSLYLFIMPASLKFRNTHLGKKGSEI*
>ENSG00000132824|ENST00000255175
MGAVLGVFSLASWVPCLCSGASCLLCSCCPNSKNSTVTRLIYAFILLLSTVVSYIMQRKE
METYLKKIPGFCEGGFKIHEADINADKDCDVLVGYKAVYRISFAMAIFFFVFSLLMFKVK
TSKDLRAAVHNGFWFFKIAALIGIMVGSFYIPGGYFSSVWFVVGMIGAALFILIQLVLLV
DFAHSWNESWVNRMEEGNPRLWYAALLSFTSAFYILSIICVGLLYTYYTKPDGCTENKFF
ISINLILCVVASIISIHPKIQEHQPRSGLLQSSLITLYTMYLTWSAMSNEPDRSCNPNLM
SFITRITAPTLAPGNSTAVVPTPTPPSKSGSLLDSDNFIGLFVFVLCLLYSSIRTSTNSQ
VDKLTLSGSDSVILGDTTTSGASDEEDGQPRRAVDNEKEGVQYSYSLFHLMLCLASLYIM
MTLTSWYSPDAKFQSMTSKWPAVWVKISSSWVCLLLYVWTLVAPLVLTSRDFS*
>ENSG00000135823|ENST00000258301
MSMEDPFFVVKGEVQKAVNTAQGLFQRWTELLQDPSTATREEIDWTTNELRNNLRSIEWD
LEDLDETISIVEANPRKFNLDATELSIRKAFITSTRQVVRDMKDQMSTSSVQALAERKNR
QALLGDSGSQNWSTGTTDKYGRLDRELQRANSHFIEEQQAQQQLIVEQQDEQLELVSGSI
GVLKNMSQRIGGELEEQAVMLEDFSHELESTQSRLDNVMKKLAKVSHMTSDRRQWCAIAI
LFAVLLVVLILFLVL*
>ENSG00000129354|ENST00000250244
MSASAVFILDVKGKPLISRNYKGDVAMSKIEHFMPLLVQREEEGALAPLLSHGQVHFLWI
KHSNLYLVATTSKNANASLVYSFLYKTIEVFCEYFKELEEESIRDNFVIVYELLDELMDF
GFPQTTDSKILQEYITQQSNKLETGKSRVPPTVTNAVSWRSEGIKYKKNEVFIDVIESVN
LLVNANGSVLLSEIVGTIKLKVFLSGMPELRLGLNDRVLFELTGRSKNKSVELEDVKFHQ

CVRLSRFDNDRTISFIPPDGDFELMSYRLSTQVKPLIWIESVIEKFSHSRVEIMVKAKGQ
FKKQSVANGVEISVPVPSDADSPRFKTSVGSAKYVPERNVVIWSIKSFPGGKEYLMRAHF
GLPSVEKEEVEGRPPIGVKFEIPYFTVSGIQVRYMKIIEKSGYQALPWVRYITQSGDYQL
RTS*
>ENSG00000130035|ENST00000252318
MMFWRKLPKALFIGLTLAIAVNLLLVFSSKGTLQNLFTGGLHRELPLHLNKRYGAVIKRL
SHLEVELQDLKESMKLALRQQENVNSTLKRAKDEVRPLLKAMETKVNETKKHKTQMKLFP
HSQLFRQWGEDLSEAQQKAAQDLFRKFGYNAYLSNQLPLNRTIPDTRDYRCLRKTYPSQL
PSLSVILIFVNEALSIIQRAITSIINRTPSRLLKEIILVDDFSSNGELKVHLDEKIKLYN
QKYPGLLKIIRHPERKGLAQARNTGWEAATADVVAILDAHIEVNVGWAEPILARIQEDRT
VIVSPVFDNIRFDTFKLDKYELAVDGFNWELWCRYDALPQAWIDLHDVTAPVKSPSIMGI
LAANRHFLGEIGSLDGGMLIYGGENVELSLRVWQCGGKVEILPCSRIAHLERHHKPYALD
LTAALKRNALRVAEIWMDEHKHMVYLAWNIPLQNSGIDFGDVSSRMALREKLKCKTFDWY
LKNVYPLLKPLHTIVGYGRMKNLLDENVCLDQGPVPGNTPIMYYCHEFSSQNVYYHLTGE
LYVGQLIAEASASDRCLTDPGKAEKPTLEPCSKAAKNRLHIYWDFKPGGAVINRDTKRCL
EMKKDLLGSHVLVLQTCSTQVWEIQHTVRDWGQTNSQ*
>ENSG00000123191|ENST00000242839
MPEQERQITAREGASRKILSKLSLPTRAWEPAMKKSFAFDNVGYEGGLDGLGPSSQVATS
TVRILGMTCQSCVKSIEDRISNLKGIISMKVSLEQGSATVKYVPSVVCLQQVCHQIGDMG
FEASIAEGKAASWPSRSLPAQEAVVKLRVEGMTCQSCVSSIEGKVRKLQGVVRVKVSLSN
QEAVITYQPYLIQPEDLRDHVNDMGFEAAIKSKVAPLSLGPIDIERLQSTNPKRPLSSAN
QNFNNSETLGHQGSHVVTLQLRIDGMHCKSCVLNIEENIGQLLGVQSIQVSLENKTAQVK
YDPSCTSPVALQRAIEALPPGNFKVSLPDGAEGSGTDHRSSSSHSPGSPPRNQVQGTCST
TLIAIAGMTCASCVHSIEGMISQLEGVQQISVSLAEGTATVLYNPSVISPEELRAAIEDM
GFEASVVSESCSTNPLGNHSAGNSMVQTTDGTPTSVQEVAPHTGRLPANHAPDILAKSPQ
STRAVAPQKCFLQIKGMTCASCVSNIERNLQKEAGVLSVLVALMAGKAEIKYDPEVIQPL
EIAQFIQDLGFEAAVMEDYAGSDGNIELTITGMTCASCVHNIESKLTRTNGITYASVALA
TSKALVKFDPEIIGPRDIIKIIEEIGFHASLAQRNPNAHHLDHKMEIKQWKKSFLCSLVF
GIPVMALMIYMLIPSNEPHQSMVLDHNIIPGLSILNLIFFILCTFVQLLGGWYFYVQAYK
SLRHRSANMDVLIVLATSIAYVYSLVILVVAVAEKAERSPVTFFDTPPMLFVFIALGRWL
EHLAKSKTSEALAKLMSLQATEATVVTLGEDNLIIREEQVPMELVQRGDIVKVVPGGKFP
VDGKVLEGNTMADESLITGEAMPVTKKPGSTVIAGSINAHGSVLIKATHVGNDTTLAQIV
KLVEEAQMSKAPIQQLADRFSGYFVPFIIIMSTLTLVVWIVIGFIDFGVVQRYFPNPNKH
ISQTEVIIRFAFQTSITVLCIACPCSLGLATPTAVMVGTGVAAQNGILIKGGKPLEMAHK
IKTVMFDKTGTITHGVPRVMRVLLLGDVATLPLRKVLAVVGTAEASSEHPLGVAVTKYCK
EELGTETLGYCTDFQAVPGCGIGCKVSNVEGILAHSERPLSAPASHLNEAGSLPAEKDAV
PQTFSVLIGNREWLRRNGLTISSDVSDAMTDHEMKGQTAILVAIDGVLCGMIAIADAVKQ
EAALAVHTLQSMGVDVVLITGDNRKTARAIATQVGINKVFAEVLPSHKVAKVQELQNKGK
KVAMVGDGVNDSPALAQADMGVAIGTGTDVAIEAADVVLIRNDLLDVVASIHLSKRTVRR
IRINLVLALIYNLVGIPIAAGVFMPIGIVLQPWMGSAAMAASSVSVVLSSLQLKCYKKPD
LERYEAQAHGHMKPLTASQVSVHIGMDDRWRDSPRATPWDQVSYVSQVSLSSLTSDKPSR
HSAAADDDGDKWSLLLNGRDEEQYI*
>ENSG00000136720|ENST00000259241
MRRRRAGGRTMVERASKFVLVVAGSVCFMLILYQYAGPGLSLGAPGGRAPPDDLDLFPTP

DPHYEKKYYFPVRELERSLRFDMKGDDVIVFLHIQKTGGTTFGRHLVQNVRLEVPCDCRP
GQKKCTCYRPNRRETWLFSRFSTGWSCGLHADWTELTNCVPGVLDRRDSAALRTPRKFYY
ITLLRDPVSRYLSEWRHVQRGATWKTSLHMCDGRTPTPEELPPCYEGTDWSGCTLQEFMD
CPYNLANNRQVRMLADLSLVGCYNLSFIPEGKRAQLLLESAKKNLRGMAFFGLTEFQRKT
QYLFERTFNLKFIRPFMQYNSTRAGGVEVDEDTIRRIEELNDLDMQLYDYAKDLFQQRYQ
YKRQLERREQRLRSREERLLHRAKEALPREDADEPGRVPTEDYMSHIIEKW*
>ENSG00000134109|ENST00000256497
MQWRALVLGLVLLRLGLHGVLWLVFGLGPSMGFYQRFPLSFGFQRLRSPDGPASPTSGPV
GRPGGVSGPSWLQPPGTGAAQSPRKAPRRPGPGMCGPANWGYVLGGRGRGPDEYEKRYSG
AFPPQLRAQMRDLARGMFVFGYDNYMAHAFPQDELNPIHCRGRGPDRGDPSNLNINDVLG
NYSLTLVDALDTLAIMGNSSEFQKAVKLVINTVSFDKDSTVQVFEATIRVLGSLLSAHRI
ITDSKQPFGDMTIKDYDNELLYMAHDLAVRLLPAFENTKTGIPYPRVNLKTGVPPDTNNE
TCTAGAGSLLVEFGILSRLLGDSTFEWVARRAVKALWNLRSNDTGLLGNVVNIQTGHWVG
KQSGLGAGLDSFYEYLLKSYILFGEKEDLEMFNAAYQSIQNYLRRGREACNEGEGDPPLY
VNVNMFSGQLMNTWIDSLQAFFPGLQVLIGDVEDAICLHAFYYAIWKRYGALPERYNWQL
QAPDVLFYPLRPELVESTYLLYQATKNPFYLHVGMDILQSLEKYTKVKCGYATLHHVIDK
STEDRMESFFLSETCKYLYLLFDEDNPVHKSGTRYMFTTEGHIVSVDEHLRELPWKEFFS
EEGGQDQGGKSVHRPKPHELKVINSSSNCNRVPDERRYSLPLKSIYMRQIDQMVGLI*
>ENSG00000134049|ENST00000256433
MAFTLYSLLQAALLCVNAIAVLHEERFLKNIGWGTDQGIGGFGEEPGIKSQLMNLIRSVR
TVMRVPLIIVNSIAIVLLLLFG*
>ENSG00000134594|ENST00000257017
MAQPILGHGSLQPASAAGLASLELDSSLDQYVQIRIFKIIVIGDSNVGKTCLTFRFCGGT
FPDKTEATIGVDFREKTVEIEGEKIKVQVWDTAGQERFRKSMVEHYYRNVHAVVFVYDVT
KMTSFTNLKMWIQECNGHAVPPLVPKVLVGNKCDLREQIQVPSNLALKFADAHNMLLFET
SAKDPKESQNVESIFMCLACRLKAQKSLLYRDAERQQGKVQKLEFPQEANSKTSCPC*
>ENSG00000106086|ENST00000258679
MEGVLYKWTNYLSGWQPRWFLLCGGILSYYDSPEDAWKGCKGSIQMAVCEIQVHSVDNTR
MDLIIPGEQYFYLKARSVAERQRWLVALGSAKACLTDSRTQKEKEFAENTENLKTKMSEL
RLYCDLLVQQVDKTKEVTTTGVSNSEEGIDVGTLLKSTCNTFLKTLEECMQIANAAFTSE
LLYRTPPGSPQLAMLKSSKMKHPIIPIHNSLERQMELSTCENGSLNMEINGEEEILMKNK
NSLYLKSAEIDCSISSEENTDDNITVQGEIRKEDGMENLKNHDNNLTQSGSDSSCSPECL
WEEGKEVIPTFFSTMNTSFSDIELLEDSGIPTEAFLASCYAVVPVLDKLGPTVFAPVKMD
LVGNIKKVNQKYITNKEEFTTLQKIVLHEVEADVAQVRNSATEALLWLKRGLKFLKGFLT
EVKNGEKDIQTALRNPTENT*
>ENSG00000130958|ENST00000253270
MTAGGQAEAEGAGGEPGAARLPSRVARLLSALFYGTCSFLIVLVNKALLTTYGFPSPIFL
GIGQMAATIMILYVSKLNKIIHFPDFDKKIPVKLFPLPLLYVGNHISGLSSTSKLSLPMF
TVLRKFTIPLTLLLETIILGKQYSLNIILSVFAIILGAFIAAGSDLAFNLEGYIFVFLND
IFTAANGVYTKQKMDPKELGKYGVLFYNACFMIIPTLIISVSTGDLQQATEFNQWKNVVF
ILQFLLSCFLGFLLMYSTVLCSYYNSALTTAVVGAIKNVSVAYIGILIGGDYIFSLLNFV
GLNICMAGGLRYSFLTLSSQLKPKPVGEENICLDLKS*
>ENSG00000136750|ENST00000259271
MASPGSGFWSFGSEDGSGDSENPGTARAWCQVAQKFTGGIGNKLCALLYGDAEKPAESGG

SQPPRAAARKAACACDQKPCSCSKVDVNYAFLHATDLLPACDGERPTLAFLQDVMNILLQ
YVVKSFDRSTKVIDFHYPNELLQEYNWELADQPQNLEEILMHCQTTLKYAIKTGHPRYFN
QLSTGLDMVGLAADWLTSTANTNMFTYEIAPVFVLLEYVTLKKMREIIGWPGGSGDGIFS
PGGAISNMYAMMIARFKMFPEVKEKGMAALPRLIAFTSEHSHFSLKKGAAALGIGTDSVI
LIKCDERGKMIPSDLERRILEAKQKGFVPFLVSATAGTTVYGAFDPLLAVADICKKYKIW
MHVDAAWGGGLLMSRKHKWKLSGVERANSVTWNPHKMMGVPLQCSALLVREEGLMQNCNQ
MHASYLFQQDKHYDLSYDTGDKALQCGRHVDVFKLWLMWRAKGTTGFEAHVDKCLELAEY
LYNIIKNREGYEMVFDGKPQHTNVCFWYIPPSLRTLEDNEERMSRLSKVAPVIKARMMEY
GTTMVSYQPLGDKVNFFRMVISNPAATHQDIDFLIEEIERLGQDL*
>ENSG00000123892|ENST00000243662
MQAPHKEHLYKLLVIGDLGVGKTSIIKRYVHQNFSSHYRATIGVDFALKVLHWDPETVVR
LQLWDIAGQERFGNMTRVYYREAMGAFIVFDVTRPATFEAVAKWKNDLDSKLSLPNGKPV
SVVLLANKCDQGKDVLMNNGLKMDQFCKEHGFVGWFETSAKENINIDEASRCLVKHILAN
ECDLMESIEPDVVKPHLTSTKVASCSGCAKS*
>ENSG00000137845|ENST00000260408
MVLLRVLILLLLSWAAGMGGQYGNPLNKYIRHYEGLSYNVDSLHQKHQRAKRAVSHEDQFL
RLDFHAHGRHFNLRMKRDTSLFSDEFKVETSNKVLDYDTSHIYTGHIYGEEGSFSHGSVI
DGRFEGFIQTRGGTFYVEPAERYIKDRTLPFHSVIYHEDDINYPHKYGPQGGCADHSVFE
RMRKYQMTGVEEVTQIPQEEHAANGPELLRKKRTTSAEKNTCQLYIQTDHLFFKYYGTRE
AVIAQISSHVKAIDTIYQTTDFSGIRNISFMVKRIRINTTADEKDPTNPFRFPNIGVEKF
LELNSEQNHDDYCLAYVFTDRDFDDGVLGLAWVGAPSGSSGGICEKSKLYSDGKKKSLNT
GIITVQNYGSHVPPKVSHITFAHEVGHNFGSPHDSGTECTPGESKNLGQKENGNYIMYAR
ATSGDKLNNNKFSLCSIRNISQVLEKKRNNCFVESGQPICGNGMVEQGEECDCGYSDQCK
DECCFDANQPEGRKCKLKPGKQCSPSQGPCCTAQCAFKSKSEKCRDDSDCAREGICNGFT
ALCPASDPKPNFTDCNRHTQVCINGQCAGSICEKYGLEECTCASSDGKDDKELCHVCCMK
KMDPSTCASTGSVQWSRHFSGRTITLQPGSPCNDFRGYCDVFMRCRLVDADGPLARLKKA
IFSPELYENIAEWIVAHWWAVLLMGIALIMLMAGFIKICSVHTPSSNPKLPPPKPLPGTL
KRRRPPQPIQQPQRQRPRESYQMGHMRR*
>ENSG00000100055|ENST00000248901
MDLCHPEPAELSSGETEELQRIKWHRKQLLEDIQKLKDEIADVFAQIDCFESAEESRMAQ
KEKELCIGRKKFNMDPAKGIQYFIEHKLLTPDVQDIARFLYKGEGLNKTAIGTYLGERDP
INLQVLQAFVDCHEFANLNLVQALRQFLWSFRLPGEAQKIDRMMEAFATRYCLCNPGVFQ
STDTCYVLSFSIIMLNTSLHNPNVRDRPPFERFVSMNRGINNGSDLPEDQLRNLFDSIKS
EPFSIPEDDGNDLTHTFFNPDREGWLLKLGGRVKTWKRRWFILTDNCLYYFEFTTDKEPR
GIIPLENLSVQKVDDPKKPFCLELYNPSCRGQKIKACKTDGDGRVVEGKHESYRISATSA
EERDQWIESIRASITRVPFYDLVSTRKKKIASKQ*
>ENSG00000134243|ENST00000256637
MERPWGAADGLSRWPHGLGLLLLLQLLPPSTLSQDRLDAPPPPAAPLPRWSGPIGVSWGL
RAAAAGGAFPRGGRWRRSAPGEDEECGRVRDFVAKLANNTHQHVFDDLRGSVSLSWVGDS
TGVILVLTTFHVPLVIMTFGQSKLYRSEDYGKNFKDITDLINNTFIRTEFGMAIGPENSG
KVVLTAEVSGGSRGGRIFRSSDFAKNFVQTDLPFHPLTQMMYSPQNSDYLLALSTENGLW
VSKNFGGKWEEIHKAVCLAKWGSDNTIFFTTYANGSCKADLGALELWRTSDLGKSFKTIG
VKIYSFGLGGRFLFASVMADKDTTRRIHVSTDQGDTWSMAQLPSVGQEQFYSILAANDDM
VFMHVDEPGDTGFGTIFTSDDRGIVYSKSLDRHLYTTTGGETDFTNVTSLRGVYITSVLS

EDNSIQTMITFDQGGRWTHLRKPENSECDATAKNKNECSLHIHASYSISQKLNVPMAPLS
EPNAVGIVIAHGSVGDAISVMVPDVYISDDGGYSWTKMLEGPHYYTILDSGGIIVAIEHS
SRPINVIKFSTDEGQCWQTYTFTRDPIYFTGLASEPGARSMNISIWGFTESFLTSQWVSY
TIDFKDILERNCEEKDYTIWLAHSTDPEDYEDGCILGYKEQFLRLRKSSVCQNGRDYVVT
KQPSICLCSLEDFLCDFGYYRPENDSKCVEQPELKGHDLEFCLYGREEHLTTNGYRKIPG
DKCQGGVNPVREVKDLKKKCTSNFLSPEKQNSKSNSVPIILAIVGLMLVTVVAGVLIVKK
YVCGGRFLVHRYSVLQQHAEANGVDGVDALDTASHTNKSGYHDDSDEDLLE*
>ENSG00000074695|ENST00000251047
MAGSRQRGLRARVRPLFCALLLSLGRFVRGDGVGGDPAVALPHRRFEYKYSFKGPHLVQS
DGTVPFWAHAGNAIPSSDQIRVAPSLKSQRGSVWTKTKAAFENWEVEVTFRVTGRGRIGA
DGLAIWYAENQGLEGPVFGSADLWNGVGIFFDSFDNDGKKNNPAIVIIGNNGQIHYDHQN
DGASQALASCQRDFRNKPYPVRAKITYYQNTLTVMINNGFTPDKNDYEFCAKVENMIIPA
QGHFGISAATGGLADDHDVLSFLTFQLTEPGKEPPTPDKEISEKEKEKYQEEFEHFQQEL
DKKKEEFQKGHPDLQGQPAEEIFESVGDRELRQVFEGQNRIHLEIKQLNRQLDMILDEQR
RYVSSLTEEISKRGAGMPGQHGQITQQELDTVVKTQHEILRQVNEMKNSMSETVRLVSGM
QHPGSAGGVYETTQHFIDIKEHLHIVKRDIDNLVQRNMPSNEKPKCPELPPFPSCLSTVH
FIIFVVVQTVLFIGYIMYRSQQEAAAKKFF*
>ENSG00000070729|ENST00000251102
MLGWVQRVLPQPPGTPRKTKMQEEEEVEPEPEMEAEVEPEPNPEEAETESESMPPEESFK
EEEVAVADPSPQETKEAALTSTISLRAQGAEISEMNSPSRRVLTWLMKGVEKVIPQPVHS
ITEDPAQILGHGSTGDTGCTDEPNEALEAQDTRPGLRLLLWLEQNLERVLPQPPKSSEVW
RDEPAVATGAASDPAPPGRPQEMGPKLQARETPSLPTPIPLQPKEEPKEAPAPEPQPGSQ
AQTSSLPPTRDPARLVAWVLHRLEMALPQPVLHGKIGEQEPDSPGICDVQTISILPGGQV
EPDLVLEEVEPPWEDAHQDVSTSPQGTEVVPAYEEENKAVEKMPRELSRIEEEKEDEEEE
EEEEEEEEEEVTEVLLDSCVVSQVGVGQSEEDGTRPQSTSDQKLWEEVGEEAKKEAEEK
AKEEAEEVAEEEAEKEPQDWAETKEEPEAEAEAASSGVPATKQHPEVQVEDTDADSCPLM
AEENPPSTVLPPPSPAKSDTLIVPSSASGTHRKKLPSEDDEAEELKALSPAESPVVAWSD
PTTPKDTDGQDRAASTASTNSAIINDRLQELVKLFKERTEKVKEKLIDPDVTSDEESPKP
SPAKKAPEPAPDTKPAEAEPVEEEHYCDMLCCKFKHRPWKKYQFPQSIDPLTNLMYVLWL
FFVVMAWNWNCWLIPVRWAFPYQTPDNIHHWLLMDYLCDLIYFLDITVFQTRLQFVRGGD
IITDKKDMRNNYLKSRRFKMDLLSLLPLDFLYLKVGVNPLLRLPRCLKYMAFFEFNSRLE
SILSKAYVYRVIRTTAYLLYSLHLNSCLYYWASAYQGLGSTHWVYDGVGNSYIRCYYFAV
KTLITIGGLPDPKTLFEIVFQLLNYFTGVFAFSVMIGQMRDVVGAATAGQTYYRSCMDST
VKYMNFYKIPKSVQNRVKTWYEYTWHSQGMLDESELMVQLPDKMRLDLAIDVNYNIVSKV
ALFQGCDRQMIFDMLKRLRSVVYLPNDYVCKKGEIGREMYIIQAGQVQVLGGPDGKSVLV
TLKAGSVFGEISLLAVGGGNRRTANVVAHGFTNLFILDKKDLNEILVHYPESQKLLRKKA
RRMLRSNNKPKEEKSVLILPPRAGTPKLFNAALAMTGKMGGKGAKGGKLAHLRARLKELA
ALEAAAKQQELVEQAKSSQDVKGEEGSAAPDQHTHPKEAATDPPAPRTPPEPPGSPPSSP
PPASLGRPEGEEEGPAEPEEHSVRICMSPGPEPGEQILSVKMPEEREEKAE*
>ENSG00000102100|ENST00000247138
MAAVGAGGSTAAPGPGAVSAGALEPGTASAAHRRLKYISLAVLVVQNASLILSIRYARTL
PGDRFFATTAVVMAEVLKGLTCLLLLFAQKRGNVKHLVLFLHEAVLVQYVDTLKLAVPSL
IYTLQNNLQYVAISNLPAATFQVTYQLKILTTALFSVLMLNRSLSRLQWASLLLLFTGVA
IVQAQQAGGGGPRPLDQNPGAGLAAVVASCLSSGFAGVYFEKILKGSSGSVWLRNLQLGL

FGTALGLVGLWWAEGTAVATRGFFFGYTPAVWGVVLNQAFGGLLVAVVVKYADNILKGFA
TSLSIVLSTVASIRLFGFHVDPLFALGAGLVIGAVYLYSLPRGAAKAIASASASASGPCV
HQQPPGQPPPPQLSSHRGDLITEPFLPKLLTKVKGS*
>ENSG00000130733|ENST00000253031
MASADELTFHEFEEATNLLADTPDAATTSRSDQLTPQGHVAVAVGSGGSYGAEDEVEEES
DKAALLQEQQQQQQPGFWTFSYYQSFFDVDTSQVLDRIKGSLLPRPGHNFVRHHLRNRPD
LYGPFWICATLAFVLAVTGNLTLVLAQRRDPSIHYSPQFHKVTVAGISIYCYAWLVPLAL
WGFLRWRKGVQERMGPYTFLETVCIYGYSLFVFIPMVVLWLIPVPWLQWLFGALALGLSA
AGLVFTLWPVVREDTRLVATVLLSVVVLLHALLAMGCKLYFFQSLPPENVAPPPQITSLP
SNIALSPTLPQSLAPS*
>ENSG00000100365|ENST00000248899
MAVAQQLRAESDFEQLPDDVAISANIADIEEKRGFTSHFVFVIEVKTKGGSKYLIYRRYR
QFHALQSKLEERFGPDSKSSALACTLPTLPAKVYVGVKQEIAEMRIPALNAYMKSLLSLP
VWVLMDEDVRIFFYQSPYDSEQVPQALRRLRPRTRKVKSVSPQGNSVDRMAAPRAEALFD
FTGNSKLELNFKAGDVIFLLSRINKDWLEGTVRGATGIFPLSFVKILKDFPEEDDPTNWL
RCYYYEDTISTIKDIAVEEDLSSTPLLKDLLELTRREFQREDIALNYRDAEGDLVRLLSD
EDVALMVRQARGLPSQKRLFPWKLHITQKDNYRVYNTMP*
>ENSG00000125975|ENST00000246199
MLSGPHPSPTFRPNPCPWPCLHSLWMEISPTQLCFLSPGPSPQSPSCCFQGMNSGSELGK
LWRKLFKGIPRLSVSHFDFYCGTCVLLGRPQIPQGSSLGNDIDQYPVVFRNASDQGSWMQ
LEMLLRKLSDLVWTSDALSDKILEDGLVP*
>ENSG00000129083|ENST00000249923
MTAAENVCYTLINVPMDSEPPSEISLKNDLEKGDVKSKTEALKKVIIMILNGEKLPGLLM
TIIRFVLPLQDHTIKKLLLVFWEIVPKTTPDGRLLHEMILVCDAYRKDLQHPNEFIRGST
LRFLCKLKEAELLEPLMPAIRACLEHRHSYVRRNAVLAIYTIYRNFEHLIPDAPELIHDF
LVNEKDASCKRNAFMMLIHADQDRALDYLSTCIDQVQTFGDILQLVIVELIYKVCHANPS
ERARFIRCIYNLLQSSSPAVKYEAAGTLVTLSSAPTAIKAAAQCYIDLIIKESDNNVKLI
VLDRLIELKEHPAHERVLQDLVMDILRVLSTPDLEVRKKTLQLALDLVSSRNVEELVIVL
KKEVIKTNNVSEHEDTDKYRQLLVRTLHSCSVRFPDMAANVIPVLMEFLSDNNEAAAADV
LEFVREAIQRFDNLRMLIVEKMLEVFHAIKSVKIYRGALWILGEYCSTKEDIQSVMTEIR
RSLGEIPIVESEIKKEAGELKPEEEITVGPVQKLVTEMGTYATQSALSSSRPTKKEEDRP
PLRGFLLDGDFFVAASLATTLTKIALRYVALVQEKKKQNSFVAEAMLLMATILHLGKSSL
PKKPITDDDVDRISLCLKVLSECSPLMNDIFNKECRQSLSHMLSAKLEEEKLSQKKESEK
RNVTVQPDDPISFMQLTAKNEMNCKEDQFQLSLLAAMGNTQRKEAADPLASKLNKVTQLT
GFSDPVYAEAYVHVNQYDIVLDVLVVNQTSDTLQNCTLELATLGDLKLVEKPSPLTLAPH
DFANIKANVKVASTENGIIFGNIVYDVSGAASDRNCVVLSDIHIDIMDYIQPATCTDAEF
RQMWAEFEWENKVTVNTNMVDLNDYLQHILKSTNMKCLTPEKALSGYCGFMAANLYARSI
FGEDALANVSIEKPIHQGPDAAVTGHIRIRAKSQGMALSLGDKINLSQKKTSI*
>ENSG00000125966|ENST00000246186
MPRSRGGRAAPGPPPPPPPPGQAPRWSRWRVPGRLLLLLLPALCCLPGAARAAAAAAGAG
NRAAVAVAVARADEAEAPFAGQNWLKSYGYLLPYDSRASALHSAKALQSAVSTMQQFYGI
PVTGVLDQTTIEWMKKPRCGVPDHPHLSRRRRNKRYALTGQKWRQKHITYSIHNYTPKVG
ELDTRKAIRQAFDVWQKVTPLTFEEVPYHEIKSDRKEADIMIFFASGFHGDSSPFDGEGG
FLAHAYFPGPGIGGDTHFDSDEPWTLGNANHDGNDLFLVAVHELGHALGLEHSSDPSAIM

APFYQYMETHNFKLPQDDLQGIQKIYGPPAEPLEPTRPLPTLPVRRIHSPSERKHERQPR
PPRPPLGDRPSTPGTKPNICDGNFNTVALFRGEMFVFKDRWFWRLRNNRVQEGYPMQIEQ
FWKGLPARIDAAYERADGRFVFFKGDKYWVFKEVTVEPGYPHSLGELGSCLPREGIDTAL
RWEPVGKTYFFKGERYWRYSEERRATDPGYPKPITVWKGIPQAPQGAFISKEGYYTYFYK
GRDYWKFDNQKLSVEPGYPRNILRDWMGCNQKEVERRKERRLPQDDVDIMVTINDVPGSV
NAVAVVIPCILSLCILVLVYTIFQFKNKTGPQPVTYYKRPVQEWV*
>ENSG00000136542|ENST00000259056
MNRIRKFFRGSGRVLAFIFVASVIWLLFDMAALRLSFSEINTRVIKEDIVRRERIGFRVQ
PDQGKIFYSSIKEMKPPLRGHGKGAWGKENVRKTEESVLKVEVDLDQTQRERKMQNALGR
GKVVPLWHPAHLQTLPVTPNKQKTDGRGTKPEASSHQGTPKQTTAQGAPKTSFIAAKGTQ
VVKISVHMGRVSLKQEPRKSHSPSSDTSKLAAERDLNVTISLSTDRPKQRSQAVANERAH
PASTAVPKSGEAMALNKTKTQSKEVNANKHKANTSLPFPKFTVNSNRLRKQSINETPLGS
LSKDDGARGAHGKKLNFSESHLVIITKEEEQKADPKEVSNSKTKTIFPKVLGKSQSKHIS
RNRSEMSSSSLAPHRVPLSQTNHALTGGLEPAKINITAKAPSTEYNQSHIKALLPEDSGT
HQVLRIDVTLSPRDPKAPGQFGRPVVVPHGKEKEAERRWKEGNFNVYLSDLIPVDRAIED
TRPAGCAEQLVHNNLPTTSVIMCFVDEVWSTLLRSVHSVINRSPPHLIKEILLVDDFSTK
DYLKDNLDKYMSQFPKVRILRLKERHGLIRARLAGAQNATGDVLTFLDSHVECNVGWLEP
LLERVYLSRKKVACPVIEVINDKDMSYMTVDNFQRGIFVWPMNFGWRTIPPDVIAKNRIK
ETDTIRCPVMAGGLFSIDKSYFFELGTYDPGLDVWGGENMELSFKVWMCGGEIEIIPCSR
VGHIFRNDNPYSFPKDRMKTVERNLVRVAEVWLDEYKELFYGHGDHLIDQGLDVGNLTQQ
RELRKKLKCKSFKWYLENVFPDLRAPIVRASGVLINVALGKCISIENTTVILEDCDGSKE
LQQFNYTWLRLIKCGEWCIAPIPDKGAVRLHPCDNRNKGLKWLHKSTSVFHPELVNHIVF
ENNQQLLCLEGNFSQKILKVAACDPVKPYQKWKFEKYYEA*
>ENSG00000138834|ENST00000250894
MMEIQMDEGGGVVVYQDDYCSGSVMSERVSGLAGSIYREFERLIHCYDEEVVKELMPLVV
NVLENLDSVLSENQEHEVELELLREDNEQLLTQYEREKALRRQAEEKFIEFEDALEQEKK
ELQIQVEHYEFQTRQLELKAKNYADQISRLEERESEMKKEYNALHQRHTEMIQTYVEHIE
RSKMQQVGGNSQTESSLPGRRKERPTSLNVFPLADGTVRAQIGGKLVPAGDHWHLSDLGQ
LQSSSSYQCPQDEMSESGQSSAAATPSTTGTKSNTPTSSVPSAAVTPLNESLQPLGDYGV
GSKNSKRAREKRDSRNMEVQVTQEMRNVSIGMGSSDEWSDVQDIIDSTPELDMCPETRLD
RTGSSPTQGIVNKAFGINTDSLYHELSTAGSEVIGDVDEGADLLGEFSVRDDFFGMGKEV
GNLLLENSQLLETKNALNVVKNDLIAKVDQLSGEQEVLRGELEAAKQAKVKLENRIKELE
EELKRVKSEAIIARREPKEEAEDVSSYLCTESDKIPMAQRRRFTRVEMARVLMERNQYKE
RLMELQEAVRWTEMIRASREHPSVQEKKKSTIWQFFSRLFSSSSSPPPAKRPYPSVNIHY
KSPTTAGFSQRRNHAMCPISAGSRPLEFFPDDDCTSSARREQKREQYRQVREHVRNDDGR
LQACGWSLPAKYKQLSPNGGQEDTRMKNVPVPVYCRPLVEKDPTMKLWCAAGVNLSGWRP
NEDDAGNGVKPAPGRDPLTCDREGDGEPKSAHTSPEKKKAKELPEMDATSSRVWILTSTL
TTSKVVIIDANQPGTVVDQFTVCNAHVLCISSIPAASDSDYPPGEMFLDSDVNPEDPGAD
GVLAGITLVGCATRCNVPRSNCSSRGDTPVLDKGQGEVATIANGKVNPSQSTEEATEATE
VPDPGPSEPETATLRPGPLTEHVFTDPAPTPSSGPQPGSENGPEPDSSSTRPEPEPSGDP
TGAGSSAAPTMWLGAQNGWLYVHSAVANWKKCLHSIKLKDSVLSLVHVKGRVLVALADGT
LAIFHRGEDGQWDLSNYHLMDLGHPHHSIRCMAVVYDRVWCGYKNKVHVIQPKTMQIEKS
FDAHPRRESQVRQLAWIGDGVWVSIRLDSTLRLYHAHTHQHLQDVDIEPYVSKMLGTGKL
GFSFVRITALLVAGSRLWVGTGNGVVISIPLTETVVLHRGQLLGLRANKTSPTSGEGARP

GGIIHVYGDDSSDRAASSFIPYCSMAQAQLCFHGHRDAVKFFVSVPGNVLATLNGSVLDS
PAEGPGPAAPASEVEGQKLRNVLVLSGGEGYIDFRIGDGEDDETEEGAGDMSQVKPVLSK
AERSHIIVWQVSYTPE*
>ENSG00000137573|ENST00000260128
MKYSCCALVLAVLGTELLGSLCSTVRSPRFRGRIQQERKNIRPNIILVLTDDQDVELGSL
QVMNKTRKIMEHGGATFINAFVTTPMCCPSRSSMLTGKYVHNHNVYTNNENCSSPSWQAM
HEPRTFAVYLNNTGYRTAFFGKYLNEYNGSYIPPGWREWLGLIKNSRFYNYTVCRNGIKE
KHGFDYAKDYFTDLITNESINYFKMSKRMYPHRPVMMVISHAAPHGPEDSAPQFSKLYPN
ASQHITPSYNYAPNMDKHWIMQYTGPMLPIHMEFTNILQRKRLQTLMSVDDSVERLYNML
VETGELENTYIIYTADHGYHIGQFGLVKGKSMPYDFDIRVPFFIRGPSVEPGSIVPQIVL
NIDLAPTILDIAGLDTPPDVDGKSVLKLLDPEKPGNRFRTNKKAKIWRDTFLVERGKFLR
KKEESSKNIQQSNHLPKYERVKELCQQARYQTACEQPGQKWQCIEDTSGKLRIHKCKGPS
DLLTVRQSTRNLYARGFHDKDKECSCRESGYRASRSQRKSQRQFLRNQGTPKYKPRFVHT
RQTRSLSVEFEGEIYDINLEEEEELQVLQPRNIAKRHDEGHKGPRDLQASSGGNRGRMLA
DSSNAVGPPTTVRVTHKCFILPNDSIHCERELYQSARAWKDHKAYIDKEIEALQDKIKNL
REVRGHLKRRKPEECSCSKQSYYNKEKGVKKQEKLKSHLHPFKEAAQEVDSKLQLFKENN
RRRKKERKEKRRQRKGEECSLPGLTCFTHDNNHWQTAPFWNLGSFCACTSSNNNTYWCLR
TVNETHNFLFCEFATGFLEYFDMNTDPYQLTNTVHTVERGILNQLHVQLMELRSCQGYKQ
CNPRPKNLDVGNKDGGSYDLHRGQLWDGWEG*
>ENSG00000124091|ENST00000243913
MFVQLLRAIYVPQNVYCIHVDEKAPMKYKTAVQTLVNCFENVFISSKTEKVAYAGFTRLQ
ADINCMKVLVHSKFQWNYVINLCGQDFPIKTNREIIHYIRSKWSDKNITPGVIQPLHIKS
KTSQSHLEFVPKGSIYAPPNNRFKDKPPHNLTIYFGSAYYVLTRKFVEFILTDIHAKDML
QWSKDIRSPEQHYWVTLNRLKDAPGATPNAGWEGNVRAIKRKSEEGNVHDGCKGRYVEDI
CVYGPGDLPWLIQSPSLFANKFEPSTDPLVVTCLERRHRLQVLRQAEVPIEPHWHFQQQS
HFNMRLNR*
>ENSG00000130287|ENST00000252575
MGAPFVWALGLLMLQMLLFVAGEQGTQDITDASERGLHMQKLGSGSVQAALAELVALPCL
FTLQPRPSAARDAPRIKWTKVRTASGQRQDLPILVAKDNVVRVAKSWQGRVSLPSYPRRR
ANATLLLGPLRASDSGLYRCQVVRGIEDEQDLVPLEVTGVVFHYRSARDRYALTFAEAQE
ACRLSSAIIAAPRHLQAAFEDGFDNCDAGWLSDRTVRYPITQSRPGCYGDRSSLPGVRSY
GRRNPQELYDVYCFARELGGEVFYVGPARRLTLAGARAQCRRQGAALASVGQLHLAWHEG
LDQCDPGWLADGSVRYPIQTPRRRCGGPAPGVRTVYRFANRTGFPSPAERFDAYCFRAHH
PTSQHGDLETPSSGDEGEILSAEGPPVRELEPTLEEEEVVTPDFQEPLVSSGEEETLILE
EKQESQQTLSPTPGDPMLASWPTGEVWLSTVAPSPSDMGAGTAASSHTEVAPTDPMPRRR
GRFKGLNGRYFQQQEPEPGLQGGMEASAQPPTSEAAVNQMEPPLAMAVTEMLGSGQSRSP
WADLTNEVDMPGAGSAGGKSSPEPWLWPPTMVPPSISGHSRAPVLELEKAEGPSARPATP
DLFWSPLEATVSAPSPAPWEAFPVATSPDLPMMAMLRGPKEWMLPHPTPISTEANRVEAH
GEATATAPPSPAAETKVYSLPLSLTPTGQGGEAMPTTPESPRADFRETGETSPAQVNKAE
HSSSSPWPSVNRNVAVGFVPTETATEPTGLRGIPGSESGVFDTAESPTSGLQATVDEVQD
PWPSVYSKGLDASSPSAPLGSPGVFLVPKVTPNLEPWVATDEGPTVNPMDSTVTPAPSDA
SGIWEPGSQVFEEAESTTLSPQVALDTSIVTPLTTLEQGDKVGVPAMSTLGSSSSQPHPE
PEDQVETQGTSGASVPPHQSSPLGKPAVPPGTPTAASVGESASVSSGEPTVPWDPSSTLL
PVTLGIEDFELEVLAGSPGVESFWEEVASGEEPALPGTPMNAGAEEVHSDPCENNPCLHG

GTCNANGTMYGCSCDQGFAGENCEIDIDDCLCSPCENGGTCIDEVNGFVCLCLPSYGGSF
CEKDTEGCDRGWHKFQGHCYRYFAHRRAWEDAEKDCRRRSGHLTSVHSPEEHSFINSFGH
ENTWIGLNDRIVERDFQWTDNTGLQFENWRENQPDNFFAGGEDCVVMVAHESGRWNDVPC
NYNLPYVCKKGTVLCGPPPAVENASLIGARKAKYNVHATVRYQCNEGFAQHHVATIRCRS
NGKWDRPQIVCTKPRRSHRMRRHHHHHQHHHQHHHHKSRKERRKHKKHPTEDWEKDEGNF
C*
>ENSG00000262246|ENST00000251166
MNRFRVSKFRHTEARPPRRESWISDIRAGTAPSCRNHIKSSCSLIAFNSDRPGVLGIVPL
QGQGEDKRRVAHLGCHSDLVTDLDFSPFDDFLLATGSADRTVKLWRLPGPGQALPSAPGV
VLGPEDLPVEVLQFHPTSDGILVSAAGTTVKVWDAAKQQPLTELAAHGDLVQSAVWSRDG
ALVGTACKDKQLRIFDPRTKPRASQSTQAHENSRDSRLAWMGTWEHLVSTGFNQMREREV
KLWDTRFFSSALASLTLDTSLGCLVPLLDPDSGLLVLAGKGERQLYCYEVVPQQPALSPV
TQCVLESVLRGAALVPRQALAVMSCEVLRVLQLSDTAIVPIGYHVPRKAVEFHEDLFPDT
AGCVPATDPHSWWAGDNQQVQKVSLNPACRPHPSFTSCLVPPAEPLPDTAQPAVMETPVG
DADASEGFSSPPSSLTSPSTPSSLGPSLSSTSGIGTSPSLRSLQSLLGPSSKFRHAQGTV
LHRDSHITNLKGLNLTTPGESDGFCANKLRVAVPLLSSGGQVAVLELRKPGRLPDTALPT
LQNGAAVTDLAWDPFDPHRLAVAGEDARIRLWRVPAEGLEEVLTTPETVLTGHTEKICSL
RFHPLAANVLASSSYDLTVRIWDLQAGADRLKLQGHQDQIFSLAWSPDGQQLATVCKDGR
VRVYRPRSGPEPLQEGPGPKGGRGARIVWVCDGRCLLVSGFDSQSERQLLLYEAEALAGG
PLAVLGLDVAPSTLLPSYDPDTGLVLLTGKGDTRVFLYELLPESPFFLECNSFTSPDPHK
GLVLLPKTECDVREVELMRCLRLRQSSLEPVAFRLPRVRKEFFQDDVFPDTAVIWEPVLS
AEAWLQGANGQPWLLSLQPPDMSPVSQAPREAPARRAPSSAQYLEEKSDQQKKEELLNAM
VAKLGNREDPLPQDSFEGVDEDEWD*
>ENSG00000254647|ENST00000250971
MALWMRLLPLLALLALWGPDPAAAFVNQHLCGSHLVEALYLVCGERGFFYTPKTRREAED
LQVGQVELGGGPGAGSLQPLALEGSLQKRGIVEQCCTSICSLYQLENYCN*
>ENSG00000136240|ENST00000258739
MNIFRLTGDLSHLAAIVILLLKIWKTRSCAGISGKSQLLFALVFTTRYLDLFTSFISLYN
TSMKVIYLACSYATVYLIYLKFKATYDGNHDTFRVEFLVVPVGGLSFLVNHDFSPLEILW
TFSIYLESVAILPQLFMISKTGEAETITTHYLFFLGLYRALYLVNWIWRFYFEGFFDLIA
VVAGVVQTILYCDFFYLYITKVLKGKKLSLPA*
>ENSG00000204642|ENST00000259951
MAPRSLLLLLSGALALTDTWAGSHSLRYFSTAVSRPGRGEPRYIAVEYVDDTQFLRFDSD
AAIPRMEPREPWVEQEGPQYWEWTTGYAKANAQTDRVALRNLLRRYNQSEAGSHTLQGMN
GCDMGPDGRLLRGYHQHAYDGKDYISLNEDLRSWTAADTVAQITQRFYEAEEYAEEFRTY
LEGECLELLRRYLENGKETLQRADPPKAHVAHHPISDHEATLRCWALGFYPAEITLTWQR
DGEEQTQDTELVETRPAGDGTFQKWAAVVVPPGEEQRYTCHVQHEGLPQPLILRWEQSPQ
PTIPIVGIVAGLVVLGAVVTGAVVAAVMWRKKSSDRNRGSYSQAAAYSVVSGNLMITWWS
SLFLLGVLFQGYLGCLRSHSVLGRRKVGDMWILFFLWLWTSFNTAFLALQSLRFGFGFRR
GRSFLLRSWHHLMKRVQIKIFD*
>ENSG00000118600|ENST00000261234
MRLTRKRLCSFLIALYCLFSLYAAYHVFFGRRRQAPAGSPRGLRKGAAPARERRGREQST
LESEEWNPWEGDEKNEQQHRFKTSLQILDKSTKGKTDLSVQIWGKAAIGLYLWEHIFEGL
LDPSDVTAQWREGKSIVGRTQYSFITGPAVIPGYFSVDVNNVVLILNGREKAKIFYATQW

LLYAQNLVQIQKLQHLAVVLLGNEHCDNEWINPFLKRNGGFVELLFIIYDSPWINDVDVF
QWPLGVATYRNFPVVEASWSMLHDERPYLCNFLGTIYENSSRQALMNILKKDGNDKLCWV
SAREHWQPQETNESLKNYQDALLQSDLTLCPVGVNTECYRIYEACSYGSIPVVEDVMTAG
NCGNTSVHHGAPLQLLKSMGAPFIFIKNWKELPAVLEKEKTIILQEKIERRKMLLQWYQH
FKTELKMKFTNILESSFLMNNKS*
>ENSG00000138073|ENST00000260643
MGRRRAPELYRAPFPLYALQVDPSTGLLIAAGGGGAAKTGIKNGVHFLQLELINGRLSAS
LLHSHDTETRATMNLALAGDILAAGQDAHCQLLRFQAHQQQGNKAEKAGSKEQGPRQRKG
AAPAEKKCGAETQHEGLELRVENLQAVQTDFSSDPLQKVVCFNHDNTLLATGGTDGYVRV
WKVPSLEKVLEFKAHEGEIEDLALGPDGKLVTVGRDLKASVWQKDQLVTQLHWQENGPTF
SSTPYRYQACRFGQVPDQPAGLRLFTVQIPHKRLRQPPPCYLTAWDGSNFLPLRTKSCGH
EVVSCLDVSESGTFLGLGTVTGSVAIYIAFSLQCLYYVREAHGIVVTDVAFLPEKGRGPE
LLGSHETALFSVAVDSRCQLHLLPSRRSVPVWLLLLLCVGLIIVTILLLQSAFPGFL*
>ENSG00000134262|ENST00000256658
MPYLGSEDVVKELKKALCNPHIQADRLRYRNVIQRVIRYMTQGLDMSGVFMEMVKASATV
DIVQKKLVYLYMCTYAPLKPDLALLAINTLCKDCSDPNPMVRGLALRSMCSLRMPGVQEY
IQQPILNGLRDKASYVRRVAVLGCAKMHNLHGDSEVDGALVNELYSLLRDQDPIVVVNCL
RSLEEILKQEGGVVINKPIAHHLLNRMSKLDQWGQAEVLNFLLRYQPRSEEELFDILNLL
DSFLKSSSPGVVMGATKLFLILAKMFPHVQTDVLVRVKGPLLAACSSESRELCFVALCHV
RQILHSLPGHFSSHYKKFFCSYSEPHYIKLQKVEVLCELVNDENVQQVLEELRGYCTDVS
ADFAQAAIFAIGGIARTYTDQCVQILTELLGLRQEHITTVVVQTFRDLVWLCPQCTEAVC
QALPGCEENIQDSEGKQALIWLLGVHGERIPNAPYVLEDFVENVKSETFPAVKMELLTAL
LRLFLSRPAECQDMLGRLLYYCIEEEKDMAVRDRGLFYYRLLLVGIDEVKRILCSPKSDP
TLGLLEDPAERPVNSWASDFNTLVPVYGKAHWATISKCQGAERCDPELPKTSSFAASGPL
IPEENKERVQELPDSGALMLVPNRQLTADYFEKTWLSLKVAHQQVLPWRGEFHPDTLQMA
LQVVNIQTIAMSRAGSRPWKAYLSAQDDTGCLFLTELLLEPGNSEMQISVKQNEARTETL
NSFISVLETVIGTIEEIKS*
>ENSG00000132958|ENST00000255310
MNESPQTNEFKGTTEEAPAKESPHTSEFKGAALVSPISKRIFGVFLVLLDVTLLLADLIF
TDSKLYIPLEYRSISLAIGLFFLMDVLLRVFVEGWTHLVRLLRLIILIRIFHLLHQKRQL
EKLMRRLVSENKRRYTRDGFDLDLTYVTERIIAMSFPSSGRQSFYRNPIEEVVRFLDKKH
RNHYRVYNLCSERAYDPKHFHNRVSRIMIDDHNVPTLHEMVVFTKEVNEWMAQDLENIVA
IHCKGGKGRTGTMVCALLIASEIFLTAEESLYYFGERRTNKTHSNKFQGVETPSQNRYVG
YFAQVKHLYNWNLPPRRILFIKRFIIYSIRGDVCDLKVQVVMEKKVVFSSTSLGNCSILH
DIETDKILINVYDGPPLYDDVKVQFFSSNLPKYYDNCPFFFWFNTSFIQNNRLCLPRNEL
DNPHKQKAWKIYPPEFAVEILFGEK*
>ENSG00000135241|ENST00000257694
MSINLTVDIYIYLLSNARSVCGKQRSKQLYFLFSPKHYWRISHISLQRGFHTNIIRCKWT
KSEAHSCSKHCYSPSNHGLHIGILKLSTSAPKGLTKVNICMSRIKSTLNSVSKAVFGNQN
EMISRLAQFKPSSQILRKVSDSGWLKQKNIKQAIKSLKKYSDKSAEKSPFPEEKSHIIDK
EEDIGKRSLFHYTSSITTKFGDSFYFLSNHINSYFKRKEKMSQQKENEHFRDKSELEDKK
VEEGKLRSPDPGILAYKPGSESVHTVDKPTSPSAIPDVLQVSTKQSIANFLSRPTEGVQA
LVGGYIGGLVPKLKYDSKSQSEEQEEPAKTDQAVSKDRNAEEKKRLSLQREKIIARVSID
NRTRALVQALRRTTDPKLCITRVEELTFHLLEFPEGKGVAVKERIIPYLLRLRQIKDETL

QAAVREILALIGYVDPVKGRGIRILSIDGGGTRGVVALQTLRKLVELTQKPVHQLFDYIC
GVSTGAILAFMLGLFHMPLDECEELYRKLGSDVFSQNVIVGTVKMSWSHAFYDSQTWENI
LKDRMGSALMIETARNPTCPKVAAVSTIVNRGITPKAFVFRNYGHFPGINSHYLGGCQYK
MWQAIRASSAAPGYFAEYALGNDLHQDGGLLLNNPSALAMHECKCLWPDVPLECIVSLGT
GRYESDVRNTVTYTSLKTKLSNVINSATDTEEVHIMLDGLLPPDTYFRFNPVMCENIPLD
ESRNEKLDQLQLEGLKYIERNEQKMKKVAKILSQEKTTLQKINDWIKLKTDMYEGLPFFS
KL*
>ENSG00000137502|ENST00000260056
MSMEDYDFLFKIVLIGNAGVGKTCLVRRFTQGLFPPGQGATIGVDFMIKTVEINGEKVKL
QIWDTAGQERFRSITQSYYRSANALILTYDITCEESFRCLPEWLREIEQYASNKVITVLV
GNKIDLAERREVSQQRAEEFSEAQDMYYLETSAKESDNVEKLFLDLACRLISEARQNTLV
NNVSSPLPGEGKSISYLTCCNFN*
>ENSG00000136213|ENST00000258711
MTKARLFRLWLVLGSVFMILLIIVYWDSAGAAHFYLHTSFSRPHTGPPLPTPGPDRDREL
TADSDVDEFLDKFLSAGVKQSDLPRKETEQPPAPGSMEESVRGYDWSPRDARRSPDQGRQ
QAERRSVLRGFCANSSLAFPTKERAFDDIPNSELSHLIVDDRHGAIYCYVPKVACTNWKR
VMIVLSGSLLHRGAPYRDPLRIPREHVHNASAHLTFNKFWRRYGKLSRHLMKVKLKKYTK
FLFVRDPFVRLISAFRSKFELENEEFYRKFAVPMLRLYANHTSLPASAREAFRAGLKVSF
ANFIQYLLDPHTEKLAPFNEHWRQVYRLCHPCQIDYDFVGKLETLDEDAAQLLQLLQVDR
QLRFPPSYRNRTASSWEEDWFAKIPLAWRQQLYKLYEADFVLFGYPKPENLLRD*
>ENSG00000134250|ENST00000256646
MPALRPALLWALLALWLCCAAPAHALQCRDGYEPCVNEGMCVTYHNGTGYCKCPEGFLGE
YCQHRDPCEKNRCQNGGTCVAQAMLGKATCRCASGFTGEDCQYSTSHPCFVSRPCLNGGT
CHMLSRDTYECTCQVGFTGKECQWTDACLSHPCANGSTCTTVANQFSCKCLTGFTGQKCE
TDVNECDIPGHCQHGGTCLNLPGSYQCQCPQGFTGQYCDSLYVPCAPSPCVNGGTCRQTG
DFTFECNCLPGFEGSTCERNIDDCPNHRCQNGGVCVDGVNTYNCRCPPQWTGQFCTEDVD
ECLLQPNACQNGGTCANRNGGYGCVCVNGWSGDDCSENIDDCAFASCTPGSTCIDRVASF
SCMCPEGKAGLLCHLDDACISNPCHKGALCDTNPLNGQYICTCPQGYKGADCTEDVDECA
MANSNPCEHAGKCVNTDGAFHCECLKGYAGPRCEMDINECHSDPCQNDATCLDKIGGFTC
LCMPGFKGVHCELEINECQSNPCVNNGQCVDKVNRFQCLCPPGFTGPVCQIDIDDCSSTP
CLNGAKCIDHPNGYECQCATGFTGVLCEENIDNCDPDPCHHGQCQDGIDSYTCICNPGYM
GAICSDQIDECYSSPCLNDGRCIDLVNGYQCNCQPGTSGVNCEINFDDCASNPCIHGICM
DGINRYSCVCSPGFTGQRCNIDIDECASNPCRKGATCINGVNGFRCICPEGPHHPSCYSQ
VNECLSNPCIHGNCTGGLSGYKCLCDAGWVGINCEVDKNECLSNPCQNGGTCDNLVNGYR
CTCKKGFKGYNCQVNIDECASNPCLNQGTCFDDISGYTCHCVLPYTGKNCQTVLAPCSPN
PCENAAVCKESPNFESYTCLCAPGWQGQRCTIDIDECISKPCMNHGLCHNTQGSYMCECP
PGFSGMDCEEDIDDCLANPCQNGGSCMDGVNTFSCLCLPGFTGDKCQTDMNECLSEPCKN
GGTCSDYVNSYTCKCQAGFDGVHCENNINECTESSCFNGGTCVDGINSFSCLCPVGFTGS
FCLHEINECSSHPCLNEGTCVDGLGTYRCSCPLGYTGKNCQTLVNLCSRSPCKNKGTCVQ
KKAESQCLCPSGWAGAYCDVPNVSCDIAASRRGVLVEHLCQHSGVCINAGNTHYCQCPLG
YTGSYCEEQLDECASNPCQHGATCSDFIGGYRCECVPGYQGVNCEYEVDECQNQPCQNGG
TCIDLVNHFKCSCPPGTRGLLCEENIDDCARGPHCLNGGQCMDRIGGYSCRCLPGFAGER
CEGDINECLSNPCSSEGSLDCIQLTNDYLCVCRSAFTGRHCETFVDVCPQMPCLNGGTCA
VASNMPDGFICRCPPGFSGARCQSSCGQVKCRKGEQCVHTASGPRCFCPSPRDCESGCAS

SPCQHGGSCHPQRQPPYYSCQCAPPFSGSRCELYTAPPSTPPATCLSQYCADKARDGVCD
EACNSHACQWDGGDCSLTMENPWANCSSPLPCWDYINNQCDELCNTVECLFDNFECQGNS
KTCKYDKYCADHFKDNHCDQGCNSEECGWDGLDCAADQPENLAEGTLVIVVLMPPEQLLQ
DARSFLRALGTLLHTNLRIKRDSQGELMVYPYYGEKSAAMKKQRMTRRSLPGEQEQEVAG
SKVFLEIDNRQCVQDSDHCFKNTDAAAALLASHAIQGTLSYPLVSVVSESLTPERTQLLY
LLAVAVVIILFIILLGVIMAKRKRKHGSLWLPEGFTLRRDASNHKRREPVGQDAVGLKNL
SVQVSEANLIGTGTSEHWVDDEGPQPKKVKAEDEALLSEEDDPIDRRPWTQQHLEAADIR
RTPSLALTPPQAEQEVDVLDVNVRGPDGCTPLMLASLRGGSSDLSDEDEDAEDSSANIIT
DLVYQGASLQAQTDRTGEMALHLAARYSRADAAKRLLDAGADANAQDNMGRCPLHAAVAA
DAQGVFQILIRNRVTDLDARMNDGTTPLILAARLAVEGMVAELINCQADVNAVDDHGKSA
LHWAAAVNNVEATLLLLKNGANRDMQDNKEETPLFLAAREGSYEAAKILLDHFANRDITD
HMDRLPRDVARDRMHHDIVRLLDEYNVTPSPPGTVLTSALSPVICGPNRSFLSLKHTPMG
KKSRRPSAKSTMPTSLPNLAKEAKDAKGSRRKKSLSEKVQLSESSVTLSPVDSLESPHTY
VSDTTSSPMITSPGILQASPNPMLATAAPPAPVHAQHALSFSNLHEMQPLAHGASTVLPS
VSQLLSHHHIVSPGSGSAGSLSRLHPVPVPADWMNRMEVNETQYNEMFGMVLAPAEGTHP
GIAPQSRPPEGKHITTPREPLPPIVTFQLIPKGSIAQPAGAPQPQSTCPPAVAGPLPTMY
QIPEMARLPSVAFPTAMMPQQDGQVAQTILPAYHPFPASVGKYPTPPSQHSYASSNAAER
TPSHSGHLQGEHPYLTPSPESPDQWSSSSPHSASDWSDVTTSPTPGGAGGGQRGPGTHMS
EPPHNNMQVYA*
>ENSG00000135631|ENST00000258098
MALVRGAEPAAGPSRWLPTHVQVTVLRARGLRGKSSGAGSTSDAYTVIQVGREKYSTSVV
EKTHGCPEWREECSFELPPGALDGLLRAQEADAGPAPWAASSAAACELVLTTMHRSLIGV
DKFLGQATVALDEVFGAGRAQHTQWYKLHSKPGKKEKERGEIEVTIQFTRNNLSASMFDL
SMKDKPRSPFSKIRDKMKGKKKYDLESASAILPSSAIEDPDLGSLGKMGKAKGFFLRNKL
RKSSLTQSNTSLGSDSTLSSASGSLAYQGPGAELLTRSPSRSSWLSTEGGRDSAQSPKLF
THKRTYSDEANQMRVAPPRALLDLQGHLDAASRSSLCVNGSHIYNEEPQGPVRHRSSISG
SLPSSGSLQAVSSRFSEEGPRSTDDTWPRGSRSNSSSEAVLGQEELSAQAKVLAPGASHP
GEEEGARLPEGKPVQVATPIVASSEAVAEKEGARKEERKPRMGLFHHHHQGLSRSELGRR
SSLGEKGGPILGASPHHSSSGEEKAKSSWFGLREAKDPTQKPSPHPVKPLSAAPVEGSPD
RKQSRSSLSIALSSGLEKLKTVTSGSIQPVTQAPQAGQMVDTKRLKDSAVLDQSAKYYHL
THDELISLLLQRERELSQRDEHVQELESYIDRLLVRIMETSPTLLQIPPGPPK*
>ENSG00000138175|ENST00000260746
MGLLSILRKLKSAPDQEVRILLLGLDNAGKTTLLKQLASEDISHITPTQGFNIKSVQSQG
FKLNVWDIGGQRKIRPYWKNYFENTDILIYVIDSADRKRFEETGQELAELLEEEKLSCVP
VLIFANKQDLLTAAPASEIAEGLNLHTIRDRVWQIQSCSALTGEGVQDGMNWVCKNVNAK
KK*
>ENSG00000131873|ENST00000254190
MAARGRRAWLSVLLGLVLGFVLASRLVLPRASELKRAGPRRRASPEGCRSGQAAASQAGG
ARGDARGAQLWPPGSDPDGGPRDRNFLFVGVMTAQKYLQTRAVAAYRTWSKTIPGKVQFF
SSEGSDTSVPIPVVPLRGVDDSYPPQKKSFMMLKYMHDHYLDKYEWFMRADDDVYIKGDR
LENFLRSLNSSEPLFLGQTGLGTTEEMGKLALEPGENFCMGGPGVIMSREVLRRMVPHIG
KCLREMYTTHEDVEVGRCVRRFAGVQCVWSYEMQQLFYENYEQNKKGYIRDLHNSKIHQA
ITLHPNKNPPYQYRLHSYMLSRKISELRHRTIQLHREIVLMSKYSNTEIHKEDLQLGIPP
SFMRFQPRQREEILEWEFLTGKYLYSAVDGQPPRRGMDSAQREALDDIVMQVMEMINANA

KTRGRIIDFKEIQYGYRRVNPMYGAEYILDLLLLYKKHKGKKMTVPVRRHAYLQQTFSKI
QFVEHEELDAQELAKRINQESGSLSFLSNSLKKLVPFQLPGSKSEHKEPKDKKINILIPL
SGRFDMFVRFMGNFEKTCLIPNQNVKLVVLLFNSDSNPDKAKQVELMRDYRIKYPKADMQ
ILPVSGEFSRALALEVGSSQFNNESLLFFCDVDLVFTTEFLQRCRANTVLGQQIYFPIIF
SQYDPKIVYSGKVPSDNHFAFTQKTGFWRNYGFGITCIYKGDLVRVGGFDVSIQGWGLED
VDLFNKVVQAGLKTFRSQEVGVVHVHHPVFCDPNLDPKQYKMCLGSKASTYGSTQQLAEM
WLEKNDPSYSKSSNNNGSVRTA*
>ENSG00000241973|ENST00000255882
MAAAPARGGGGGGGGGGGCSGSGSSASRGFYFNTVLSLARSLAVQRPASLEKVQKLLCMC
PVDFHGIFQLDERRRDAVIALGIFLIESDLQHKDCVVPYLLRLLKGLPKVYWVEESTARK
GRGALPVAESFSFCLVTLLSDVAYRDPSLRDEILEVLLQVLHVLLGMCQALEIQDKEYLC
KYAIPCLIGISRAFGRYSNMEESLLSKLFPKIPPHSLRVLEELEGVRRRSFNDFRSILPS
NLLTVCQEGTLKRKTSSVSSISQVSPERGMPPPSSPGGSAFHYFEASCLPDGTALEPEYY
FSTISSSFSVSPLFNGVTYKEFNIPLEMLRELLNLVKKIVEEAVLKSLDAIVASVMEANP
SADLYYTSFSDPLYLTMFKMLRDTLYYMKDLPTSFVKEIHDFVLEQFNTSQGELQKILHD
ADRIHNELSPLKLRCQANAACVDLMVWAVKDEQGAENLCIKLSEKLQSKTSSKVIIAHLP
LLICCLQGLGRLCERFPVVVHSVTPSLRDFLVIPSPVLVKLYKYHSQYHTVAGNDIKISV
TNEHSESTLNVMSGKKSQPSMYEQLRDIAIDNICRCLKAGLTVDPVIVEAFLASLSNRLY
ISQESDKDAHLIPDHTIRALGHIAVALRDTPKVMEPILQILQQKFCQPPSPLDVLIIDQL
GCLVITGNQYIYQEVWNLFQQISVKASSVVYSATKDYKDHGYRHCSLAVINALANIAANI
QDEHLVDELLMNLLELFVQLGLEGKRASERASEKGPALKASSSAGNLGVLIPVIAVLTRR
LPPIKEAKPRLQKLFRDFWLYSVLMGFAVEGSGLWPEEWYEGVCEIATKSPLLTFPSKEP
LRSVLQYNSAMKNDTVTPAELSELRSTIINLLDPPPEVSALINKLDFAMSTYLLSVYRLE
YMRVLRSTDPDRFQVMFCYFEDKAIQKDKSGMMQCVIAVADKVFDAFLNMMADKAKTKEN
EEELERHAQFLLVNFNHIHKRIRRVADKYLSGLVDKFPHLLWSGTVLKTMLDILQTLSLS
LSADIHKDQPYYDIPDAPYRITVPDTYEARESIVKDFAARCGMILQEAMKWAPTVTKSHL
QEYLNKHQNWVSGLSQHTGLAMATESILHFAGYNKQNTTLGATQLSERPACVKKDYSNFM
ASLNLRNRYAGEVYGMIRFSGTTGQMSDLNKMMVQDLHSALDRSHPQHYTQAMFKLTAML
ISSKDCDPQLLHHLCWGPLRMFNEHGMETALACWEWLLAGKDGVEVPFMREMAGAWHMTV
EQKFGLFSAEIKEADPLAASEASQPKPCPPEVTPHYIWIDFLVQRFEIAKYCSSDQVEIF
SSLLQRSMSLNIGGAKGSMNRHVAAIGPRFKLLTLGLSLLHADVVPNATIRNVLREKIYS
TAFDYFSCPPKFPTQGEKRLREDISIMIKFWTAMFSDKKYLTASQLVPPDNQDTRSNLDI
TVGSRQQATQGWINTYPLSSGMSTISKKSGMSKKTNRGSQLHKYYMKRRTLLLSLLATEI
ERLITWYNPLSAPELELDQAGENSVANWRSKYISLSEKQWKDNVNLAWSISPYLAVQLPA
RFKNTEAIGNEVTRLVRLDPGAVSDVPEAIKFLVTWHTIDADAPELSHVLCWAPTDPPTG
LSYFSSMYPPHPLTAQYGVKVLRSFPPDAILFYIPQIVQALRYDKMGYVREYILWAASKS
QLLAHQFIWNMKTNIYLDEEGHQKDPDIGDLLDQLVEEITGSLSGPAKDFYQREFDFFNK
ITNVSAIIKPYPKGDERKKACLSALSEVKVQPGCYLPSNPEAIVLDIDYKSGTPMQSAAK
APYLAKFKVKRCGVSELEKEGLRCRSDSEDECSTQEADGQKISWQAAIFKVGDDCRQDML
ALQIIDLFKNIFQLVGLDLFVFPYRVVATAPGCGVIECIPDCTSRDQLGRQTDFGMYDYF
TRQYGDESTLAFQQARYNFIRSMAAYSLLLFLLQIKDRHNGNIMLDKKGHIIHIDFGFMF
ESSPGGNLGWEPDIKLTDEMVMIMGGKMEATPFKWFMEMCVRGYLAVRPYMDAVVSLVTL
MLDTGLPCFRGQTIKLLKHRFSPNMTEREAANFIMKVIQSCFLSNRSRTYDMIQYYQNDI
PY*

>ENSG00000131507|ENST00000253814
MALALAALAAVEPACGSRYQQLQNEEESGEPEQAAGDAPPPYSSISAESAAYFDYKDESG
FPKPPSYNVATTLPSYDEAERTKAEATIPLVPGRDEDFVGRDDFDDADQLRIGNDGIFML
TFFMAFLFNWIGFFLSFCLTTSAAGRYGAISGFGLSLIKWILIVRFSTYFPGYFDGQYWL
WWVFLVLGFLLFLRGFINYAKVRKMPETFSNLPRTRVLFIY*
>ENSG00000204070|ENST00000243918
MAGQFRSYVWDPLLILSQIVLMQTVYYGSLGLWLALVDGLVRSSPSLDQMFDAEILGFST
PPGRLSMMSFILNALTCALGLLYFIRRGKQCLDFTVTVHFFHLLGCWFYSSRFPSALTWW
LVQAVCIALMAVIGEYLCMRTELKEIPLNSAPKSNV*
>ENSG00000186088|ENST00000257626
MALRLVADFDLGKDVLPWLRAQRAVSEASGAGSGGADVLENDYESLHVLNVERNGNIIYT
YKDDKGNVVFGLYDCQTRQNELLYTFEKDLQVFSCSVNSERTLLAASLVQSTKEGKRNEL
QPGSKCLTLLVEIHPVNNVKVLKAVDSYIWVQFLYPHIESHPLPENHLLLISEEKYIEQF
RIHVAQEDGNRVVIKNSGHLPRDRIAEDFVWAQWDMSEQRLYYIDLKKSRSILKCIQFYA
DESYNLMFEVPLDISLSNSGFKLVNFGCDYHQYRDKFSKHLTLCVFTNHTGSLCVCYSPK
CASWGQITYSVFYIHKGHSKTFTTSLENVGSHMTKGITFLNLDYYVAVYLPGHFFHLLNV
QHPDLICHNLFLTGNNEMIDMLPHCPLQSLSGSLVLDCCSGKLYRALLSQSSLLQLLQNT
CLDCEKMAALHCALYCGQGAQFLEAQIIQWISENVSACHSFDLIQEFIIASSYWSVYSET
SNMDKLLPHSSVLTWNTEIPGITLVTEDIALPLMKVLSFKGYWEKLNSNLEYVKYAKPHF
HYNNSVVRREWHNLISEEKTGKRRSAAYVRNILDNAVKVISNLEARNLGPRLTPLLQEED
SHQRLLMGLMVSELKDHFLRHLQGVEKKKIEQMVLDYISKLLDLICHIVETNWRKHNLHS
WVLHFNSRGSAAEFAVFHIMTRILEATNSLFLPLPPGFHTLHTILGVQCLPLHNLLHCID
SGVLLLTETAVIRLMKDLDNTEKNEKLKFSIIVRLPPLIGQKICRLWDHPMSSNIISRNH
VTRLLQNYKKQPRNSMINKSSFSVEFLPLNYFIEILTDIESSNQALYPFEGHDNVDAEFV
EEAALKHTAMLLGL*
>ENSG00000130383|ENST00000252675
MDPLGPAKPQWLWRRCLAGLLFQLLVAVCFFSYLRVSRDDATGSPRPGLMAVEPVTGAPN
GSRCQDSMATPAHPTLLILLWTWPFNTPVALPRCSEMVPGAADCNITADSSVYPQADAVI
VHHWDIMYNPSANLPPPTRPQGQRWIWFSMESPSNCRHLEALDGYFNLTMSYRSDSDIFT
PYGWLEPWSGQPAHPPLNLSAKTELVAWAVSNWKPDSARVRYYQSLQAHLKVDVYGRSHK
PLPKGTMMETLSRYKFYLAFENSLHPDYITEKLWRNALEAWAVPVVLGPSRSNYERFLPP
DAFIHVDDFQSPKDLARYLQELDKDHARYLSYFHWRETLRPRSFSWALAFCKACWKLQQE
SRYQTVRSIAAWFT*
>ENSG00000134287|ENST00000256682
MGNIFGNLLKSLIGKKEMRILMVGLDAAGKTTILYKLKLGEIVTTIPTIGFNVETVEYKN
ISFTVWDVGGQDKIRPLWRHYFQNTQGLIFVVDSNDRERVNEAREELMRMLAEDELRDAV
LLVFANKQDLPNAMNAAEITDKLGLHSLRHRNWYIQATCATSGDGLYEGLDWLANQLKNK
K*
>ENSG00000148985|ENST00000278243
MYQVPLPLDRDGTLVRLRFTMVALVTVCCPLVAFLFCILWSLLFHFKETTATHCGATPCR
MFSAASQPLDPDGTLFRLRFTAMVWWAITFPVFGFFFCIIWSLVFHFEYTVATDCGVPNY
LPSVSSAIGGEVPQRYVWRFCIGLHSAPRFLVAFAYWNHYLSCTSPCSCYRPLCRLNFGL
NVVENLALLVLTYVSSSEDFTIHENAFIVFIASSLGHMLLTCILWRLTKKHTVSQEDRKS
YSWKQRLFIINFISFFSALAVYFRHNMYCEAGVYTIFAILEYTVVLTNMAFHMTAWWDFG

NKELLITSQPEEKRF*
>ENSG00000141252|ENST00000291074
MMEEEELEFVEELEAVLQLTPEVQLAIEQVFPSQDPLDRADFNAVEYINTLFPTEQSLAN
IDEVVNKIRLKIRRLDDNIRTVVRGQTNVGQDGRQVKEITRDIKQLDHAKRHLTTSITTL
NHLHMLAGGVDSLEAMTRRRQYGEVANLLQGVMNVLEHFHKYMGIPQIRQLSERVKAAQT
ELGQQILADFEEAFPSQGTKRPGGPSNVLRDACLVANILDPRIKQEIIKKFIKQHLSEYL
VLFQENQDVAWLDKIDRRYAWIKRQLVDYEEKYGRMFPREWCMAERIAVEFCHVTRAELA
KIMRTRAKEIEVKLLLFAIQRTTNFEGFLAKRFSGCTLTDGTLKKLESPPPSTNPFLEDE
PTPEMEELATEKGDLDQPKKPKAPDNPFHGIVSKCFEPHLYVYIESQDKNLGELIDRFVA
DFKAQGPPKPNTDEGGAVLPSCADLFVYYKKCMVQCSQLSTGEPMIALTTIFQKYLREYA
WKILSGNLPKTTTSSGGLTISSLLKEKEGSEVAKFTLEELCLICNILSTAEYCLATTQQL
EEKLKEKVDVSLIERINLTGEMDTFSTVISSSIQLLVQDLDAACDPALTAMSKMQWQNVE
HVGDQSPYVTSVILHIKQNVPIIRDNLASTRKYFTQFCVKFANSFIPKFITHLFKCKPIS
MVGAEQVRWT*
>ENSG00000086598|ENST00000262225
MVTLAELLVLLAALLATVSGYFVSIDAHAEECFFERVTSGTKMGLIFEVAEGGFLDIDVE
ITGPDNKGIYKGDRESSGKYTFAAHMDGTYKFCFSNRMSTMTPKIVMFTIDIGEAPKGQD
METEAHQNKLEEMINELAVAMTAVKHEQEYMEVRERIHRAINDNTNSRVVLWSFFEALVL
VAMTLGQIYYLKRFFEVRRVV*
>ENSG00000152291|ENST00000282120
MRFVVALVLLNVAAAGAVPLLATESVKQEEAGVRPSAGNVSTHPSLSQRPGGSTKSHPEP
QTPKDSPSKSSAEAQTPEDTPNKSGAEAKTQKDSSNKSGAEAKTQKGSTSKSGSEAQTTK
DSTSKSHPELQTPKDSTGKSGAEAQTPEDSPNRSGAEAKTQKDSPSKSGSEAQTTKDVPN
KSGADGQTPKDGSSKSGAEDQTPKDVPNKSGAEKQTPKDGSNKSGAEEQGPIDGPSKSGA
EEQTSKDSPNKVVPEQPSRKDHSKPISNPSDNKELPKADTNQLADKGKLSPHAFKTESGE
ETDLISPPQEEVKSSEPTEDVEPKEAEDDDTGPEEGSPPKEEKEKMSGSASSENREGTLS
DSTGSEKDDLYPNGSGNGSAESSHFFAYLVTAAILVAVLYIAHHNKRKIIAFVLEGKRSK
VTRRPKASDYQRLDQKYVLILNVFPAPPKRSFFPVLTEWYIPLEKDERHQWIVLLSFQL*
>ENSG00000106809|ENST00000262551
MKTLQSTLLLLLLVPLIKPAPPTQQDSRIIYDYGTDNFEESIFSQDYEDKYLDGKNIKEK
ETVIIPNEKSLQLQKDEAITPLPPKKENDEMPTCLLCVCLSGSVYCEEVDIDAVPPLPKE
SAYLYARFNKIKKLTAKDFADIPNLRRLDFTGNLIEDIEDGTFSKLSLLEELSLAENQLL
KLPVLPPKLTLFNAKYNKIKSRGIKANAFKKLNNLTFLYLDHNALESVPLNLPESLRVIH
LQFNNIASITDDTFCKANDTSYIRDRIEEIRLEGNPIVLGKHPNSFICLKRLPIGSYF*
>ENSG00000144583|ENST00000273067
MLMPLCGLLWWWWCCCSGWYCYGLCAPAPQMLRHQGLLKCRCRMLFNDLKVFLLRRPPQA
PLPMHGDPQPPGLAANNTLPALGAGGWAGWRGPREVVGREPPPVPPPPPLPPSSVEDDWG
GPATEPPASLLSSASSDDFCKEKTEDRYSLGSSLDSGMRTPLCRICFQGPEQGELLSPCR
CDGSVKCTHQPCLIKWISERGCWSCELCYYKYHVIAISTKNPLQWQAISLTVIEKVQVAA
AILGSLFLIASISWLIWSTFSPSARWQRQDLLFQICYGMYGFMDVVCIGLIIHEGPSVYR
IFKRWQAVNQQWKVLNYDKTKDLEDQKAGGRTNPRTSSSTQANIPSSEEETAGTPAPEQG
PAQAAGHPSGPLSHHHCAYTILHILSHLRPHEQRSPPGSSRELVMRVTTV*
>ENSG00000154342|ENST00000284523
MAPLGYFLLLCSLKQALGSYPIWWSLAVGPQYSSLGSQPILCASIPGLVPKQLRFCRNYV

EIMPSVAEGIKIGIQECQHQFRGRRWNCTTVHDSLAIFGPVLDKATRESAFVHAIASAGV
AFAVTRSCAEGTAAICGCSSRHQGSPGKGWKWGGCSEDIEFGGMVSREFADARENRPDAR
SAMNRHNNEAGRQAIASHMHLKCKCHGLSGSCEVKTCWWSQPDFRAIGDFLKDKYDSASE
MVVEKHRESRGWVETLRPRYTYFKVPTERDLVYYEASPNFCEPNPETGSFGTRDRTCNVS
SHGIDGCDLLCCGRGHNARAERRREKCRCVFHWCCYVSCQECTRVYDVHTCK*
>ENSG00000162552|ENST00000290167
MSPRSCLRSLRLLVFAVFSAAASNWLYLAKLSSVGSISEEETCEKLKGLIQRQVQMCKRN
LEVMDSVRRGAQLAIEECQYQFRNRRWNCSTLDSLPVFGKVVTQGTREAAFVYAISSAGV
AFAVTRACSSGELEKCGCDRTVHGVSPQGFQWSGCSDNIAYGVAFSQSFVDVRERSKGAS
SSRALMNLHNNEAGRKAILTHMRVECKCHGVSGSCEVKTCWRAVPPFRQVGHALKEKFDG
ATEVEPRRVGSSRALVPRNAQFKPHTDEDLVYLEPSPDFCEQDMRSGVLGTRGRTCNKTS
KAIDGCELLCCGRGFHTAQVELAERCSCKFHWCCFVKCRQCQRLVELHTCR*
>ENSG00000126062|ENST00000266025
MQRALPGARQHLGAILASASVVVKALCAAVLFLYLLSFAVDTGCLAVTPGYLFPPNFWIW
TLATHGLMEQHVWDVAISLTTVVVAGRLLEPLWGALELLIFFSVVNVSVGLLGAFAYLLT
YMASFNLVYLFTVRIHGALGFLGGVLVALKQTMGDCVVLRVPQVRVSVMPMLLLALLLLL
RLATLLQSPALASYGFGLLSSWVYLRFYQRHSRGRGDMADHFAFATFFPEILQPVVGLLA
NLVHSLLVKVKICQKTVKRYDVGAPSSITISLPGTDPQDAERRRQLALKALNERLKRVED
QSIWPSMDDDEEESGAKVDSPLPSDKAPTPPGKGAAPESSLITFEAAPPTL*
>ENSG00000007384|ENST00000262316
MSEARRDSTSSLQRKKPPWLKLDIPSAVPLTAEEPSFLQPLRRQAFLRSVSMPAETAHIS
SPHHELRRPVLQRQTSITQTIRRGTADWFGVSKDSDSTQKWQRKSIRHCSQRYGKLKPQV
LRELDLPSQDNVSLTSTETPPPLYVGPCQLGMQKIIDPLARGRAFRVADDTAEGLSAPHT
PVTPGAASLCSFSSSRSGFHRLPRRRKRESVAKMSFRAAAALMKGRSVRDGTFRRAQRRS
FTPASFLEEDTTDFPDELDTSFFAREGILHEELSTYPDEVFESPSEAALKDWEKAPEQAD
LTGGALDRSELERSHLMLPLERGWRKQKEGAAAPQPKVRLRQEVVSTAGPRRGQRIAVPV
RKLFAREKRPYGLGMVGRLTNRTYRKRIDSFVKRQIEDMDDHRPFFTYWLTFVHSLVTIL
AVCIYGIAPVGFSQHETVDSVLRNRGVYENVKYVQQENFWIGPSSEALIHLGAKFSPCMR
QDPQVHSFIRSAREREKHSACCVRNDRSGCVQTSEEECSSTLAVWVKWPIHPSAPELAGH
KRQFGSVCHQDPRVCDEPSSEDPHEWPEDITKWPICTKNSAGNHTNHPHMDCVITGRPCC
IGTKGRCEITSREYCDFMRGYFHEEATLCSQVHCMDDVCGLLPFLNPEVPDQFYRLWLSL
FLHAGILHCLVSICFQMTVLRDLEKLAGWHRIAIIYLLSGVTGNLASAIFLPYRAEVGPA
GSQFGILACLFVELFQSWQILARPWRAFFKLLAVVLFLFTFGLLPWIDNFAHISGFISGL
FLSFAFLPYISFGKFDLYRKRCQIIIFQVVFLGLLAGLVVLFYVYPVRCEWCEFLTCIPF
TDKFCEKYELDAQLH*
>ENSG00000104388|ENST00000262646
MAYAYLFKYIIIGDTGVGKSCLLLQFTDKRFQPVHDLTIGVEFGARMITIDGKQIKLQIW
DTAGQESFRSITRSYYRGAAGALLVYDITRRDTFNHLTTWLEDARQHSNSNMVIMLIGNK
SDLESRREVKKEEGEAFAREHGLIFMETSAKTASNVEEAFINTAKEIYEKIQEGVFDINN
EANGIKIGPQHAATNATHAGNQGGQQAGGGCC*
>ENSG00000156232|ENST00000286760
MEDEQPDSLEGWVPVREGLFAEPERHRLRFLVAWNGAEGKFAVTCHDRTAQQRRLREGAR
LGPEPEPKPEAAVSPSSWAGLLSAAGLRGAHRQLAALWPPLERCFPRLPPELDVGGGGAW
GLGLGLWALLWPTRAGPGEAALQELCGQLERYLGAAADGCGGATVRDALFPAEGGAADCE

SPREFRERALRARWVEADARLRQVIQGHGKANTMVALMNVYQEEDEAYQELVTVATMFFQ
YLLQPFRAMREVATLCKLDILKSLDEDDLGPRRVVALEKEAEEWTRRAEEAVVSIQDITV
NYFKETVKALAGMQKEMEQDAKRFGQAAWATAIPRLEKLQLMLARETLQLMRAKELCLNH
KRAEIQGKMEDLPEQEKNTNVVDELEIQFYEIQLELYEVKFEILKNEEILLTTQLDSLKR
LIKEKQDEVVYYDPCENPEELKVIDCVVGLQDDKNLEVKELRRQCQQLESKRGRICAKRA
SLRSRKDQCKENHRFRLQQAEESIRYSRQHHSIQMKRDKIKEEEQKKKEWINQERQKTLQ
RLRSFKDKRLAQSVRNTSGSEPVAPNLPSDLSQQMCLPASHAVSVIHPSSRKTRGVPLSE
AGNVKSPKCQNCHGNIPVQVFVPVGDQTHSKSSEELSLPPPPPPPPPPPPPPPPPPPPLR
ALSSSSQAATHQNLGFRAPVKDDQPRPLVCESPAERPRDSLESFSCPGSMDEVLASLRHG
RAPLRKVEVPAVRPPHASINEHILAAIRQGVKLKKVHPDLGPNPSSKPTSNRRTSDLERS
IKAALQRIKRVSADSEEDSDEQDPGQWDG*
>ENSG00000141367|ENST00000269122
MAQILPIRFQEHLQLQNLGINPANIGFSTLTMESDKFICIREKVGEQAQVVIIDMNDPSN
PIRRPISADSAIMNPASKVIALKAGKTLQIFNIEMKSKMKAHTMTDDVTFWKWISLNTVA
LVTDNAVYHWSMEGESQPVKMFDRHSSLAGCQIINYRTDAKQKWLLLTGISAQQNRVVGA
MQLYSVDRKVSQPIEGHAASFAQFKMEGNAEESTLFCFAVRGQAGGKLHIIEVGTPPTGN
QPFPKKAVDVFFPPEAQNDFPVAMQISEKHDVVFLITKYGYIHLYDLETGTCIYMNRISG
ETIFVTAPHEATAGIIGVNRKGQVLSVCVEEENIIPYITNVLQNPDLALRMAVRNNLAGA
EELFARKFNALFAQGNYSEAAKVAANAPKGILRTPDTIRRFQSVPAQPGQTSPLLQYFGI
LLDQGQLNKYESLELCRPVLQQGRKQLLEKWLKEDKLECSEELGDLVKSVDPTLALSVYL
RANVPNKVIQCFAETGQVQKIVLYAKKVGYTPDWIFLLRNVMRISPDQGQQFAQMLVQDE
EPLADITQIVDVFMEYNLIQQCTAFLLDALKNNRPSEGPLQTRLLEMNLMHAPQVADAIL
GNQMFTHYDRAHIAQLCEKAGLLQRALEHFTDLYDIKRAVVHTHLLNPEWLVNYFGSLSV
EDSLECLRAMLSANIRQNLQICVQVASKYHEQLSTQSLIELFESFKSFEGLFYFLGSIVN
FSQDPDVHFKYIQAACKTGQIKEVERICRESNCYDPERVKNFLKEAKLTDQLPLIIVCDR
FDFVHDLVLYLYRNNLQKYIEIYVQKVNPSRLPVVIGGLLDVDCSEDVIKNLILVVRGQF
STDELVAEVEKRNRLKLLLPWLEARIHEGCEEPATHNALAKIYIDSNNNPERFLRENPYY
DSRVVGKYCEKRDPHLACVAYERGQCDLELINVCNENSLFKSLSRYLVRRKDPELWGSVL
LESNPYRRPLIDQVVQTALSETQDPEEVSVTVKAFMTADLPNELIELLEKIVLDNSVFSE
HRNLQNLLILTAIKADRTRVMEYINRLDNYDAPDIANIAISNELFEEAFAIFRKFDVNTS
AVQVLIEHIGNLDRAYEFAERCNEPAVWSQLAKAQLQKGMVKEAIDSYIKADDPSSYMEV
VQAANTSGNWEELVKYLQMARKKARESYVETELIFALAKTNRLAELEEFINGPNNAHIQQ
VGDRCYDEKMYDAAKLLYNNVSNFGRLASTLVHLGEYQAAVDGARKANSTRTWKEVCFAC
VDGKEFRLAQMCGLHIVVHADELEELINYYQDRGYFEELITMLEAALGLERAHMGMFTEL
AILYSKFKPQKMREHLELFWSRVNIPKVLRAAEQAHLWAELVFLYDKYEEYDNAIITMMN
HPTDAWKEGQFKDIITKVANVELYYRAIQFYLEFKPLLLNDLLMVLSPRLDHTRAVNYFS
KVKQLPLVKPYLRSVQNHNNKSVNESLNNLFITEEDYQALRTSIDAYDNFDNISLAQRLE
KHELIEFRRIAAYLFKGNNRWKQSVELCKKDSLYKDAMQYASESKDTELAEELLQWFLQE
EKRECFGACLFTCYDLLRPDVVLETAWRHNIMDFAMPYFIQVMKEYLTKVDKLDASESLR
KEEEQATETQPIVYGQPQLMLTAGPSVAVPPQAPFGYGYTAPPYGQPQPGFGYSM*
>ENSG00000148396|ENST00000290037
MQPPPQTVPSGMAGPPPAGNPRSVFWASSPYRRRANNNAAVAPTTCPLQPVTDPFAFSRQ
ALQSTPLGSSSKSSPPVLQGPAPAGFSQHPGLLVPHTHARDSSQGPCEPLPGPLTQPRAH
ASPFSGALTPSAPPGPEMNRSAEVGPSSEPEVQTLPYLPHYIPGVDPETSHGGHPHGNMP

GLDRPLSRQNPHDGVVTPAASPSLPQPGLQMPGQWGPVQGGPQPSGQHRSPCPEGPVPSG
VPCATSVPHFPTPSILHQGPGHEQHSPLVAPPAALPSDGRDEVSHLQSGSHLANNSDPES
TFRQNPRIVNHWASPELRQNPGVKNEHRPASALVNPLARGDSPENRTHHPLGAGAGSGCA
PLEADSGASGALAMFFQGGETENEENLSSEKAGLSGQADFDDFCSSPGLGRPPAPTHVGA
GSLCQALLPGPSNEAAGDVWGDTASTGVPDASGSQYENVENLEFVQNQEVLPSEPLNLDP
SSPSDQFRYGPLPGPAVPRHGAVCHTGAPDATLHTVHPDSVSSSYSSRSHGRLSGSARPQ
ELVGTFIQQEVGKPEDEASGSFFKQIDSSPVGGETDETTVSQNYRGSVSQPSTPSPPKPT
GIFQTSANSSFEPVKSHLVGVKPFEADRANVVGEVRETCVRQKQCRPAAALPDASPGNLE
QPPDNMETLCAPQVCPLPLNSTTEAVHMLPHAGAPPLDTVYPAPEKRPSARTQGPVKCES
PATTLWAQSELPDFGGNVLLAPAAPALYVCAKPQPPVVQPPEEAMSGQQSRNPSSAAPVQ
SRGGIGASENLENPPKMGEEEALQSQASSGYASLLSSPPTESLQNPPVLIAQPDHSYNLA
QPINFSVSLSNSHEKNQSWREALVGDRPAVSSWALGGDSGENTSLSGIPTSSVLSLSLPS
SVAQSNFPQGSGASEMVSNQPANLLVQPPSQPVPENLVPESQKDRKAGSALPGFANSPAG
STSVVLVPPAHGTLVPDGNKANHSSHQEDTYGALDFTLSRTLENPVNVYNPSHSDSLASQ
QSVASHPRQSGPGAPNLDRFYQQVTKDAQGQPGLERAQQELVPPQQQASPPQLPKAMFSE
LSNPESLPAQGQAQNSAQSPASLVLVDAGQQLPPRPPQSSSVSLVSSGSGQAAVPSEQPW
PQPVPALAPGPPPQDLAAYYYYRPLYDAYQPQYSLPYPPEPGAASLYYQDVYSLYEPRYR
PYDGAASAYAQNYRYPEPERPSSRASHSSERPPPRQGYPEGYYSSKSGWSSQSDYYASYY
SSQYDYGDPGHWDRYHYSARVRDPRTYDRRYWCDAEYDAYRREHSAFGDRPEKRDNNWRY
DPRFTGSFDDDPDPHRDPYGEEVDRRSVHSEHSARSLHSAHSLASRRSSLSSHSHQSQIY
RSHNVAAGSYEAPLPPGSFHGDFAYGTYRSNFSSGPGFPEYGYPADTVWPAMEQVSSRPT
SPEKFSVPHVCARFGPGGQLIKVIPNLPSEGQPALVEVHSMEALLQHTSEQEEMRAFPGP
LAKDDTHKVDVINFAQNKAMKCLQNENLIDKESASLLWNFIVLLCRQNGTVVGTDIAELL
LRDHRTVWLPGKSPNEANLIDFTNEAVEQVEEEESGEAQLSFLTGGPAAAASSLERETER
FRELLLYGRKKDALESAMKNGLWGHALLLASKMDSRTHARVMTRFANSLPINDPLQTVYQ
LMSGRMPAASTCCGDEKWGDWRPHLAMVLSNLNNNMDVESRTMATMGDTLASRGLLDAAH
FCYLMAQAGFGVYTKKTTKLVLIGSNHSLPFLKFATNEAIQRTEAYEYAQSLGAETCPLP
SFQVFKFIYSCRLAEMGLATQAFHYCEAIAKSILTQPHLYSPVLISQLVQMASQLRLFDP
QLKEKPEEESLAAPTWLVHLQQVERQIKEGAGVWHQDGALPQQCPGTPSSEMEQLDRPGL
SQPGALGIANPLLAVPAPSPEHSSPSVRLLPSAPQTLPDGPLASPARVPMFPVPLPPGPL
EPGPGCVTPGPALGFLEPSGPGLPPGVPPLQERRHLLQEARSPDPGIVPQEAPVGNSLSE
LSEENFDGKFANLTPSRTVPDSEAPPGWDRADSGPTQPPLSLSPAPETKRPGQAAKKETK
EPKKGESWFFRWLPGKKKTEAYLPDDKNKSIVWDEKKNQWVNLNEPEEEKKAPPPPPTSM
PKTVQAAPPALPGPPGAPVNMYSRRAAGTRARYVDVLNPSGTQRSEPALAPADFVAPLAP
LPIPSNLFVPTPVSSVRPQGRSGRNDGLLALSSPDAEEPQLPDGTGREGPAAARGLANPE
PAPEPKAPGDLPAAGGPPSGAMPFYNPAQLAQACATSGSSRLGRIGQRKHLVLN*
>ENSG00000151693|ENST00000281419
MPDQISVSEFVAETHEDYKAPTASSFTTRTAQCRNTVAAIEEALDVDRMVLYKMKKSVKA
INSSGLAHVENEEQYTQALEKFGGNCVCRDDPDLGSAFLKFSVFTKELTALFKNLIQNMN
NIISFPLDSLLKGDLKGVKGDLKKPFDKAWKDYETKITKIEKEKKEHAKLHGMIRTEISG
AEIAEEMEKERRFFQLQMCEYLLKVNEIKIKKGVDLLQNLIKYFHAQCNFFQDGLKAVES
LKPSIETLSTDLHTIKQAQDEERRQLIQLRDILKSALQVEQKEDSQIRQSTAYSLHQPQG
NKEHGTERNGSLYKKSDGIRKVWQKRKCSVKNGFLTISHGTANRPPAKLNLLTCQVKTNP
EEKKCFDLISHDRTYHFQAEDEQECQIWMSVLQNSKEEALNNAFKGDDNTGENNIVQELT

KEIISEVQRMTGNDVCCDCGAPDPTWLSTNLGILTCIECSGIHRELGVHYSRMQSLTLDV
LGTSELLLAKNIGNAGFNEIMECCLPAEDSVKPNPGSDMNARKDYITAKYIERRYARKKH
ADNAAKLHSLCEAVKTRDIFGLLQAYADGVDLTEKIPLANGHEPDETALHLAVRSVDRTS
LHIVDFLVQNSGNLDKQTGKGSTALHYCCLTDNAECLKLLLRGKASIEIANESGETPLDI
AKRLKHEHCEELLTQALSGRFNSHVHVEYEWRLLHEDLDESDDDMDEKLQPSPNRREDRP
ISFYQLGSNQLQSNAVSLARDAANLAKEKQRAFMPSILQNETYGALLSGSPPPAQPAAPS
TTSAPPLPPRNVGKVQTASSANTLWKTNSVSVDGGSRQRSSSDPPAVHPPLPPLRVTSTN
PLTPTPPPPVAKTPSVMEALSQPSKPAPPGISQIRPPPLPPQPPSRLPQKKPAPGADKST
PLTNKGQPRGPVDLSATEALGPLSNAMVLQPPAPMPRKSQATKLKPKRVKALYNCVADNP
DELTFSEGDVIIVDGEEDQEWWIGHIDGDPGRKGAFPVSFVHFIAD*
>ENSG00000139330|ENST00000266719
MAGTICFIMWVLFITDTVWSRSVRQVYEVHDSDDWTIHDFECPMECFCPPSFPTALYCEN
RGLKEIPAIPSRIWYLYLQNNLIETIPEKPFENATQLRWINLNKNKITNYGIEKGALSQL
KKLLFLFLEDNELEEVPSPLPRSLEQLQLARNKVSRIPQGTFSNLENLTLLDLQNNKLVD
NAFQRDTFKGLKNLMQLNMAKNALRNMPPRLPANTMQLFLDNNSIEGIPENYFNVIPKVA
FLRLNHNKLSDEGLPSRGFDVSSILDLQLSHNQLTKVPRISAHLQHLHLDHNKIKSVNVS
VICPSPSMLPAERDSFSYGPHLRYLRLDGNEIKPPIPMALMTCFRLLQAVII*
>ENSG00000147642|ENST00000276646
MGPLRESKKEHRVQHHDKEISRSRIPRLILRPHMPQQQHKVSPASESPFSEEESREFNPS
SSGRSARTVSSNSFCSDDTGCPSSQSVSPVKTPSDAGNSPIGFCPGSDEGFTRKKCTIGM
VGEGSIQSSRYKKESKSGLVKPGSEADFSSSSSTGSISAPEVHMSTAGSKRSSSSRNRGP
HGRSNGASSHKPGSSPSSPREKDLLSMLCRNQLSPVNIHPSYAPSSPSSSNSGSYKGSDC
SPIMRRSGRYMSCGENHGVRPPNPEQYLTPLQQKEVTVRHLKTKLKESERRLHERESEIV
ELKSQLARMREDWIEEECHRVEAQLALKEARKEIKQLKQVIETMRSSLADKDKGIQKYFV
DINIQNKKLESLLQSMEMAHSGSLRDELCLDFPCDSPEKSLTLNPPLDTMADGLSLEEQV
TGEGADRELLVGDSIANSTDLFDEIVTATTTESGDLELVHSTPGANVLELLPIVMGQEEG
SVVVERAVQTDVVPYSPAISELIQSVLQKLQDPCPSSLASPDESEPDSMESFPESLSALV
VDLTPRNPNSAILLSPVETPYANVDAEVHANRLMRELDFAACVEERLDGVIPLARGGVVR
QYWSSSFLVDLLAVAAPVVPTVLWAFSTQRGGTDPVYNIGALLRGCCVVALHSLRRTAFR
IKT*
>ENSG00000123240|ENST00000263036
MSHQPLSCLTEKEDSPSESTGNGPPHLAHPNLDTFTPEELLQQMKELLTENHQLKEAMKL
NNQAMKGRFEELSAWTEKQKEERQFFEIQSKEAKERLMALSHENEKLKEELGKLKGKSER
SSEDPTDDSRLPRAEAEQEKDQLRTQVVRLQAEKADLLGIVSELQLKLNSSGSSEDSFVE
IRMAEGEAEGSVKEIKHSPGPTRTVSTGTALSKYRSRSADGAKNYFEHEELTVSQLLLCL
REGNQKVERLEVALKEAKERVSDFEKKTSNRSEIETQTEGSTEKENDEEKGPETVGSEVE
ALNLQVTSLFKELQEAHTKLSEAELMKKRLQEKCQALERKNSAIPSELNEKQELVYTNKK
LELQVESMLSEIKMEQAKTEDEKSKLTVLQMTHNKLLQEHNNALKTIEELTRKESEKVDR
AVLKELSEKLELAEKALASKQLQMDEMKQTIAKQEEDLETMTILRAQMEVYCSDFHAERA
AREKIHEEKEQLALQLAVLLKENDAFEDGGRQSLMEMQSRHGARTSDSDQQAYLVQRGAE
DRDWRQQRNIPIHSCPKCGEVLPDIDTLQIHVMDCII*
>ENSG00000036257|ENST00000264414
MSNLSKGTGSRKDTKMRIRAFPMTMDEKYVNSIWDLLKNAIQEIQRKNNSGLSFEELYRN
AYTMVLHKHGEKLYTGLREVVTEHLINKVREDVLNSLNNNFLQTLNQAWNDHQTAMVMIR

DILMYMDRVYVQQNNVENVYNLGLIIFRDQVVRYGCIRDHLRQTLLDMIARERKGEVVDR
GAIRNACQMLMILGLEGRSVYEEDFEAPFLEMSAEFFQMESQKFLAENSASVYIKKVEAR
INEEIERVMHCLDKSTEEPIVKVVERELISKHMKTIVEMENSGLVHMLKNGKTEDLGCMY
KLFSRVPNGLKTMCECMSSYLREQGKALVSEEGEGKNPVDYIQGLLDLKSRFDRFLLESF
NNDRLFKQTIAGDFEYFLNLNSRSPEYLSLFIDDKLKKGVKGLTEQEVETILDKAMVLFR
FMQEKDVFERYYKQHLARRLLTNKSVSDDSEKNMISKLKTECGCQFTSKLEGMFRDMSIS
NTTMDEFRQHLQATGVSLGGVDLTVRVLTTGYWPTQSATPKCNIPPAPRHAFEIFRRFYL
AKHSGRQLTLQHHMGSADLNATFYGPVKKEDGSEVGVGGAQVTGSNTRKHILQVSTFQMT
ILMLFNNREKYTFEEIQQETDIPERELVRALQSLACGKPTQRVLTKEPKSKEIENGHIFT
VNDQFTSKLHRVKIQTVAAKQGESDPERKETRQKVDDDRKHEIEAAIVRIMKSRKKMQHN
VLVAEVTQQLKARFLPSPVVIKKRIEGLIEREYLARTPEDRKVYTYVA*
>ENSG00000103723|ENST00000261722
MSAAPAYSEDKGGSAGPGEPEYGHDPASGGIFSSDYKRHDDLKEMLDTNKDSLKLEAMKR
IVAMIARGKNASDLFPAVVKNVACKNIEVKKLVYVYLVRYAEEQQDLALLSISTFQRGLK
DPNQLIRASALRVLSSIRVPIIVPIMMLAIKEAASDMSPYVRKTAAHAIPKLYSLDSDQK
DQLIEVIEKLLADKTTLVAGSVVMAFEEVCPERIDLIHKNYRKLCNLLIDVEEWGQVVII
SMLTRYARTQFLSPTQNESLLEENAEKAFYGSEEDEAKGAGSEETAAAAAPSRKPYVMDP
DHRLLLRNTKPLLQSRSAAVVMAVAQLYFHLAPKAEVGVIAKALVRLLRSHSEVQYVVLQ
NVATMSIKRRGMFEPYLKSFYIRSTDPTQIKILKLEVLTNLANETNIPTVLREFQTYIRS
MDKDFVAATIQAIGRCATNIGRVRDTCLNGLVQLLSNRDELVVAESVVVIKKLLQMQPAQ
HGEIIKHLAKLTDNIQVPMARASILWLIGEYCEHVPRIAPDVLRKMAKSFTAEEDIVKLQ
VINLAAKLYLTNSKQTKLLTQYVLSLAKYDQNYDIRDRARFTRQLIVPSEQGGALSRHAK
KLFLAPKPAPVLESSFKDRDHFQLGSLSHLLNAKATGYQELPDWPEEAPDPSVRNVEVPE
WTKCSNREKRKEKEKPFYSDSEGESGPTESADSDPESESESDSKSSSESGSGESSSESDN
EDQDEDEEKGRGSESEQSEEDGKRKTKKKVPERKGEASSSDEGSDSSSSSSESEMTSESE
EEQLEPASWSRKTPPSSKSAPATKEISLLDLEDFTPPSVQPVSPPAIVSTSLAADLEGLT
LTDSTLVPSLLSPVSGVGRQELLHRVAGEGLAVDYTFSRQPFSGDPHMVSVHIHFSNSSD
TPIKGLHVGTPKLPAGISIQEFPEIESLAPGESATAVMGINFCDSTQAANFQLCTQTRQF
YVSIQPPVGELMAPVFMSENEFKKEQGKLMGMNEITEKLMLPDTCRSDHIVVQKVTATAN
LGRVPCGTSDEYRFAGRTLTGGSLVLLTLDARPAGAAQLTVNSEKMVIGTMLVKDVIQAL
TQ*
>ENSG00000145416|ENST00000274056
MLGWCEAIARNPHRIPNNTRTPEISGDLADASQTSTLNEKSPGRSASRSSNISKASSPTT
GTAPRSQSRLSVCPSTQDICRICHCEGDEESPLITPCRCTGTLRFVHQSCLHQWIKSSDT
RCCELCKYDFIMETKLKPLRKWEKLQMTTSERRKIFCSVTFHVIAITCVVWSLYVLIDRT
AEEIKQGNDNGVLEWPFWTKLVVVAIGFTGGLVFMYVQCKVYVQLWRRLKAYNRVIFVQN
CPDTAKKLEKNFSCNVNTDIKDAVVVPVPQTGANSLPSAEGGPPEVVSV*
>ENSG00000070540|ENST00000262139
MEAEAADAPPGGVESALSCFSFNQDCTSLATGTKAGYKLFSLSSVEQLDQVHGSNEIPDV
YIVERLFSSSLVVVVSHTKPRQMNVYHFKKGTEICNYSYSSNILSIRLNRQRLLVCLEES
IYIHNIKDMKLLKTLLDIPANPTGLCALSINHSNSYLAYPGSLTSGEIVLYDGNSLKTVC
TIAAHEGTLAAITFNASGSKLASASEKGTVIRVFSVPDGQKLYEFRRGMKRYVTISSLVF
SMDSQFLCASSNTETVHIFKLEQVTNSRPEEPSTWSGYMGKMFMAATNYLPTQVSDMMHQ
DRAFATARLNFSGQRNICTLSTIQKLPRLLVASSSGHLYMYNLDPQDGGECVLIKTHSLL

GSGTTEENKENDLRPSLPQSYAATVARPSASSASTVPGYSEDGGALRGEVIPEHEFATGP
VCLDDENEFPPIILCRGNQKGKTKQS*
>ENSG00000147509|ENST00000276500
MRTADGGEPAGASSPAGRVDGGLQMGSERMEMRKRQMPAAQDTPGAAPGQPGAGSRGSNA
CCFCWCCCCSCSCLTVRNQEDQRPTIASHELRADLPTWEESPAPTLEEVNAWAQSFDKLM
VTPAGRNAFREFLRTEFSEENMLFWMACEELKKEANKNIIEEKARIIYEDYISILSPKEV
SLDSRVREVINRNMVEPSQHIFDDAQLQIYTLMHRDSYPRFMNSAVYKDLLQSLSEKSIE
A*
>ENSG00000249915|ENST00000264933
MAAYSYRPGPGAGPGPAAGAALPDQSFLWNVFQRVDKDRSGVISDTELQQALSNGTWTPF
NPVTVRSIISMFDRENKAGVNFSEFTGVWKYITDWQNVFRTYDRDNSGMIDKNELKQALS
GFGYRLSDQFHDILIRKFDRQGRGQIAFDDFIQGCIVLQRLTDIFRRYDTDQDGWIQVSY
EQYLSMVFSIV*
>ENSG00000139292|ENST00000266674
MDTSRLGVLLSLPVLLQLATGGSSPRSGVLLRGCPTHCHCEPDGRMLLRVDCSDLGLSEL
PSNLSVFTSYLDLSMNNISQLLPNPLPSLRFLEELRLAGNALTYIPKGAFTGLYSLKVLM
LQNNQLRHVPTEALQNLRSLQSLRLDANHISYVPPSCFSGLHSLRHLWLDDNALTEIPVQ
AFRSLSALQAMTLALNKIHHIPDYAFGNLSSLVVLHLHNNRIHSLGKKCFDGLHSLETLD
LNYNNLDEFPTAIRTLSNLKELGFHSNNIRSIPEKAFVGNPSLITIHFYDNPIQFVGRSA
FQHLPELRTLTLNGASQITEFPDLTGTANLESLTLTGAQISSLPQTVCNQLPNLQVLDLS
YNLLEDLPSFSVCQKLQKIDLRHNEIYEIKVDTFQQLLSLRSLNLAWNKIAIIHPNAFST
LPSLIKLDLSSNLLSSFPITGLHGLTHLKLTGNHALQSLISSENFPELKVIEMPYAYQCC
AFGVCENAYKISNQWNKGDNSSMDDLHKKDAGMFQAQDERDLEDFLLDFEEDLKALHSVQ
CSPSPGPFKPCEHLLDGWLIRIGVWTIAVLALTCNALVTSTVFRSPLYISPIKLLIGVIA
AVNMLTGVSSAVLAGVDAFTFGSFARHGAWWENGVGCHVIGFLSIFASESSVFLLTLAAL
ERGFSVKYSAKFETKAPFSSLKVIILLCALLALTMAAVPLLGGSKYGASPLCLPLPFGEP
STMGYMVALILLNSLCFLMMTIAYTKLYCNLDKGDLENIWDCSMVKHIALLLFTNCILNC
PVAFLSFSSLINLTFISPEVIKFILLVVVPLPACLNPLLYILFNPHFKEDLVSLRKQTYV
WTRSKHPSLMSINSDDVEKQSCDSTQALVTFTSSSITYDLPPSSVPSPAYPVTESCHLSS
VAFVPCL*
>ENSG00000147437|ENST00000276414
MKPIQKLLAGLILLTWCVEGCSSQHWSYGLRPGGKRDAENLIDSFQEIVKEVGQLAETQR
FECTTHQPRSPLRDLKGALESLIEEETGQKKI*
>ENSG00000038210|ENST00000264864
MEDPSEPDRLASADGGSPEEEEDGEREPLLPRIAWAHPRRGAPGSAVRLLDAAGEEGEAG
DEELPLPPGDVGVSRSSSAELDRSRPAVSVTIGTSEMNAFLDDPEFADIMLRAEQAIEVG
IFPERISQGSSGSYFVKDPKRKIIGVFKPKSEEPYGQLNPKWTKYVHKVCCPCCFGRGCL
IPNQGYLSEAGAYLVDNKLHLSIVPKTKVVWLVSETFNYNAIDRAKSRGKKYALEKVPKV
GRKFHRIGLPPKIGSFQLFVEGYKEAEYWLRKFEADPLPENIRKQFQSQFERLVILDYII
RNTDRGNDNWLVRYEKQKCEKEIDHKESKWIDDEEFLIKIAAIDNGLAFPFKHPDEWRAY
PFHWAWLPQAKVPFSEEIRNLILPYISDMNFVQDLCEDLYELFKTDKGFDKATFESQMSV
MRGQILNLTQALRDGKSPFQLVQIPCVIVERSQGGSQGRIVHLSNSFTQTVNCRKPFFSS
W*
>ENSG00000140557|ENST00000268164

MQLQFRSWMLAALTLLVVFLIFADISEIEEEIGNSGGRGTIRSAVNSLHSKSNRAEVVIN
GSSSPAVVDRSNESIKHNIQPASSKWRHNQTLSLRIRKQILKFLDAEKDISVLKGTLKPG
DIIHYIFDRDSTMNVSQNLYELLPRTSPLKNKHFGTCAIVGNSGVLLNSGCGQEIDAHSF
VIRCNLAPVQEYARDVGLKTDLVTMNPSVIQRAFEDLVNATWREKLLQRLHSLNGSILWI
PAFMARGGKERVEWVNELILKHHVNVRTAYPSLRLLHAVRGYWLTNKVHIKRPTTGLLMY
TLATRFCKQIYLYGFWPFPLDQNQNPVKYHYYDSLKYGYTSQASPHTMPLEFKALKSLHE
QGALKLTVGQCDGAT*
>ENSG00000116729|ENST00000262348
MAGAIIENMSTKKLCIVGGILLVFQIIAFLVGGLIAPGPTTAVSYMSVKCVDARKNHHKT
KWFVPWGPNHCDKIRDIEEAIPREIEANDIVFSVHIPLPHMEMSPWFQFMLFILQLDIAF
KLNNQIRENAEVSMDVSLAYRDDAFAEWTEMAHERVPRKLKCTFTSPKTPEHEGRYYECD
VLPFMEIGSVAHKFYLLNIRLPVNEKKKINVGIGEIKDIRLVGIHQNGGFTKVWFAMKTF
LTPSIFIIMVWYWRRITMMSRPPVLLEKVIFALGISMTFINIPVEWFSIGFDWTWMLLFG
DIRQGIFYAMLLSFWIIFCGEHMMDQHERNHIAGYWKQVGPIAVGSFCLFIFDMCERGVQ
LTNPFYSIWTTDIGTELAMAFIIVAGICLCLYFLFLCFMVFQVFRNISGKQSSLPAMSKV
RRLHYEGLIFRFKFLMLITLACAAMTVIFFIVSQVTEGHWKWGGVTVQVNSAFFTGIYGM
WNLYVFALMFLYAPSHKNYGEDQSNGDLGVHSGEELQLTTTITHVDGPTEIYKLTRKEAQ
E*
>ENSG00000081014|ENST00000261842
MSDIVEKTLTALPGLFLQNQPGGGPAAAKASFSSRLGSLVRGITALTSKHEEEKLIQQEL
SSLKATVSAPTTTLKMMKECMVRLIYCEMLGYDASFGYIHAIKLAQQGNLLEKRVGYLAV
SLFLHESHELLLLLVNTVVKDLQSTNLVEVCMALTVVSQIFPCEMIPAVLPLIEDKLQHS
KEIVRRKAVLALYKFHLIAPNQVQHIHIKFRKALCDRDVGVMAASLHIYLRMIKENSSGY
KDLTGSFVTILKQVVGGKLPVEFNYHSVPAPWLQIQLLRILGLLGKDDQRTSELMYDVLD
ESLRRAELNHNVTYAILFECVHTVYSIYPKSELLEKAAKCIGKFVLSPKINLKYLGLKAL
TYVIQQDPTLALQHQMTIIECLDHPDPIIKRETLELLYRITNAQNITVIVQKMLEYLHQS
KEEYVIVNLVGKIAELAEKYAPDNAWFIQTMNAVFSVGGDVMHPDIPNNFLRLLAEGFDD
ETEDQQLRLYAVQSYLTLLDMENVFYPQRFLQVMSWVLGEYSYLLDKETPEEVIAKLYKL
LMNDSVSSETKAWLIAAVTKLTSQAHSSNTVERLIHEFTISLDTCMRQHAFELKHLHENV
ELMKSLLPVDRSCEDLVVDASLSFLDGFVAEGLSQGAAPYKPPHQRQEEKLSQEKVLNFE
PYGLSFSSSGFTGRQSPAGISLGSDVSGNSAETGLKETNSLKLEGIKKLWGKEGYLPKKE
SKTGDESGALPVPQESIMENVDQAITKKDQSQVLTQSKEEKEKQLLASSLFVGLGSESTI
NLLGKADTVSHKFRRKSKVKEAKSGETTSTHNMTCSSFSSLSNVAYEDDYYSNTLHDTGD
KELKKFSLTSELLDSESLTELPLVEKFSYCSLSTPSLFANNNMEIFHPPQSTAASVAKES
SLASSFLEETTEYIHSNAMEVCNNETISVSSYKIWKDDCLLMVWSVTNKSGLELKSADLE
IFPAENFKVTEQPGCCLPVMEAESTKSFQYSVQIEKPFTEGNLTGFISYHMMDTHSAQLE
FSVNLSLLDFIRPLKISSDDFGKLWLSFANDVKQNVKMSESQAALPSALKTLQQKLRLHI
IEIIGNEGLLACQLLPSIPCLLHCRVHADVLALWFRSSCSTLPDYLLYQCQKVMEGS*
>ENSG00000148396|ENST00000277537
PAAAASSLERETERFRELLLYGRKKDALESAMKNGLWGHALLLASKMDSRTHARVMTRFA
NSLPINDPLQTVYQLMSGRMPAASTCCGDEKWGDWRPHLAMVLSNLNNNMDVESRTMATM
GDTLASRGLLDAAHFCYLMAQAGFGVYTKKTTKLVLIGSNHSLPFLKFATNEAIQRTEAY
EYAQSLGAETCPLPSFQVFKFIYSCRLAEMGLATQAFHYCEAIAKSILTQPHLYSPVLIS
QLVQMASQLRLFDPQLKEKPEEESLAAPTWLVHLQQVERQIKEGAGVWHQDGALPQQCPG

TPSSEMEQLDRPGLSQPGALGIANPLLAVPAPSPEHSSPSVRLLPSAPQTLPDGPLASPA
RVPMFPVPLPPGPLEPGPGCVTPGPALGFLEPSGPGLPPGVPPLQERRHLLQEARSPDPG
IVPQEAPVGNSLSELSEENFDGKFANLTPSRTVPDSEAPPGWDRADSGPTQPPLSLSPAP
ETKRPGQAAKKETKEPKKGESWFFRWLPGKKKTEAYLPDDKNKSIVWDEKKNQWVNLNEP
EEEKKAPPPPPTSMPKTVQAAPPALPGPPGAPVNMYSRRAAGTRARYVDVLNPSGTQRSE
PALAPADFVAPLAPLPIPSNLFVPTPDAEEPQLPDGTGREGPAAARGLANPEPAPEPKVL
SSAASLPGSELPSSRPEGSQGGELSRCSSMSSLSREVSQHFNQAPGDLPAAGGPPSGAMP
FYNPAQLAQACATSGSSRLGRIGQRKHLVLN*
>ENSG00000138604|ENST00000261858
MRCLAARVNYKTLIIICALFTLVTVLLWNKCSSDKAIQFPRRSSSGFRVDGFEKRAAASE
SNNYMNHVAKQQSEEAFPQEQQKAPPVVGGFNSNVGSKVLGLKYEEIDCLINDEHTIKGR
REGNEVFLPFTWVEKYFDVYGKVVQYDGYDRFEFSHSYSKVYAQRAPYHPDGVFMSFEGY
NVEVRDRVKCISGVEGVPLSTQWGPQGYFYPIQIAQYGLSHYSKNLTEKPPHIEVYETAE
DRDKNKPNDWTVPKGCFMANVADKSRFTNVKQFIAPETSEGVSLQLGNTKDFIISFDLKF
LTNGSVSVVLETTEKNQLFTIHYVSNAQLIAFKERDIYYGIGPRTSWSTVTRDLVTDLRK
GVGLSNTKAVKPTKIMPKKVVRLIAKGKGFLDNITISTTAHMAAFFAASDWLVRNQDEKG
GWPIMVTRKLGEGFKSLEPGWYSAMAQGQAISTLVRAYLLTKDHIFLNSALRATAPYKFL
SEQHGVKAVFMNKHDWYEEYPTTPSSFVLNGFMYSLIGLYDLKETAGEKLGKEARSLYER
GMESLKAMLPLYDTGSGTIYDLRHFMLGIAPNLARWDYHTTHINQLQLLSTIDESPVFKE
FVKRWKSYLKGSRAKHN*
>ENSG00000115652|ENST00000283148
MVSKALLRLVSAVNRRRMKLLLGIALLAYVASVWGNFVNMSFLLNRSIQENGELKIESKI
EEMVEPLREKIRDLEKSFTQKYPPVKFLSEKDRKRILITGGAGFVGSHLTDKLMMDGHEV
TVVDNFFTGRKRNVEHWIGHENFELINHDVVEPLYIEVDQIYHLASPASPPNYMYNPIKT
LKTNTIGTLNMLGLAKRVGARLLLASTSEVYGDPEVHPQSEDYWGHVNPIGPRACYDEGK
RVAETMCYAYMKQEGVEVRVARIFNTFGPRMHMNDGRVVSNFILQALQGEPLTVYGSGSQ
TRAFQYVSDLVNGLVALMNSNVSSPVNLGNPEEHTILEFAQLIKNLVGSGSEIQFLSEAQ
DDPQKRKPDIKKAKLMLGWEPVVPLEEGLNKAIHYFRKELEYQANNQYIPKPKPARIKKG
RTRHS*
>ENSG00000110048|ENST00000263847
MAATELRGVVGPGPAAIAALGGGGAGPPVVGGGGGRGDAGPGSGAASGTVVAAAAGGPGP
GAGGVAAAGPAPAPPTGGSGGSGAGGSGSAREGWLFKWTNYIKGYQRRWFVLSNGLLSYY
RSKAEMRHTCRGTINLATANITVEDSCNFIISNGGAQTYHLKASSEVERQRWVTALELAK
AKAVKMLAESDESGDEESVSQTDKTELQNTLRTLSSKVEDLSTCNDLIAKHGTALQRSLS
ELESLKLPAESNEKIKQVNERATLFRITSNAMINACRDFLMLAQTHSKKWQKSLQYERDQ
RIRLEETLEQLAKQHNHLERAFRGATVLPANTPGNVGSGKDQCCSGKGDMSDEDDENEFF
DAPEIITMPENLGHKRTGSNISGASSDISLDEQYKHQLEETKKEKRTRIPYKPNYSLNLW
SIMKNCIGKELSKIPMPVNFNEPLSMLQRLTEDLEYHELLDRAAKCENSLEQLCYVAAFT
VSSYSTTVFRTSKPFNPLLGETFELDRLEENGYRSLCEQVSHHPPAAAHHAESKNGWTLR
QEIKITSKFRGKYLSIMPLGTIHCIFHATGHHYTWKKVTTTVHNIIVGKLWIDQSGEIDI
VNHKTGDKCNLKFVPYSYFSRDVARKVTGEVTDPSGKVHFALLGTWDEKMECFKVQPVIG
ENGGDARQRGHEAEESRVMLWKRNPLPKNAENMYYFSELALTLNAWESGTAPTDSRLRPD
QRLMENGRWDEANAEKQRLEEKQRLSRKKREAEAMKATEDGTPYDPYKALWFERKKDPVT
KELTHIYRGEYWECKEKQDWSSCPDIF*

>ENSG00000077498|ENST00000263321
MLLAVLYCLLWSFQTSAGHFPRACVSSKNLMEKECCPPWSGDRSPCGQLSGRGSCQNILL
SNAPLGPQFPFTGVDDRESWPSVFYNRTCQCSGNFMGFNCGNCKFGFWGPNCTERRLLVR
RNIFDLSAPEKDKFFAYLTLAKHTISSDYVIPIGTYGQMKNGSTPMFNDINIYDLFVWMH
YYVSMDALLGGSEIWRDIDFAHEAPAFLPWHRLFLLRWEQEIQKLTGDENFTIPYWDWRD
AEKCDICTDEYMGGQHPTNPNLLSPASFFSSWQIVCSRLEEYNSHQSLCNGTPEGPLRRN
PGNHDKSRTPRLPSSADVEFCLSLTQYESGSMDKAANFSFRNTLEGFASPLTGIADASQS
SMHNALHIYMNGTMSQVQGSANDPIFLLHHAFVDSIFEQWLRRHRPLQEVYPEANAPIGH
NRESYMVPFIPLYRNGDFFISSKDLGYDYSYLQDSDPDSFQDYIKSYLEQASRIWSWLLG
AAMVGAVLTALLAGLVSLLCRHKRKQLPEEKQPLLMEKEDYHSLYQSHL*
>ENSG00000153976|ENST00000284110
MAPPGPASALSTSAEPLSRSIFRKFLLMLCSLLTSLYVFYCLAERCQTLSGPVVGLSGGG
EEAGAPGGGVLAGGPRELAVWPAAAQRKRLLQLPQWRRRRPPAPRDDGEEAAWEEESPGL
SGGPGGSGAGSTVAEAPPGTLALLLDEGSKQLPQAIIIGVKKGGTRALLEFLRVHPDVRA
VGAEPHFFDRSYDKGLAWYRDLMPRTLDGQITMEKTPSYFVTREAPARISAMSKDTKLIV
VVRDPVTRAISDYTQTLSKRPDIPTFESLTFKNRTAGLIDTSWSAIQIGIYAKHLEHWLR
HFPIRQMLFVSGERLISDPAGELGRVQDFLGLKRIITDKHFYFNKTKGFPCLKKAEGSSR
PHCLGKTKGRTHPEIDREVVRRLREFYRPFNLKFYQMTGHDFGWDG*
>ENSG00000143457|ENST00000271732
MTTLTHRARRTEISKNSEKKMESEEDSNWEKSPDNEDSGDSKDIRLTLMEEVLLLGLKDK
EGYTSFWNDCISSGLRGGILIELAMRGRIYLEPPTMRKKRLLDRKVLLKSDSPTGDVLLD
ETLKHIKATEPTETVQTWIELLTGETWNPFKLQYQLRNVRERIAKNLVEKGILTTEKQNF
LLFDMTTHPVTNTTEKQRLVKKLQDSVLERWVNDPQRMDKRTLALLVLAHSSDVLENVFS
SLTDDKYDVAMNRAKDLVELDPEVEGTKPSATEMIWAVLAAFNKS*
>ENSG00000064225|ENST00000265261
MRTSAEYFRLALSKLQSCDLFDEFDKMNNGPVLGHEEEVGRRTTFRLFYPESVFSDPIHN
DPNTTVILTAFKPHDLRWLLELLMGDKINTNGFWKKPALNLIYKPYQIRILDPFIIRTAA
YELLHFPKVFPKNQKPKHPTTGIIAITLAFYICHEVHLAGFKYNFSDLKSPLHYYGNATM
SLMNKNAYHNVTAEQLFLKDIIEKNLVINLTQD*
>ENSG00000124225|ENST00000265626
MMVMVVVITCLLSHYKLSARSFISRHSQGRRREDALSSEGCLWPSESTVSGNGIPEPQVY
APPRPTDRLAVPPFAQRERFHRFQPTYPYLQHEIDLPPTISLSDGEEPPPYQGPCTLQLR
DPEQQLELNRESVRAPPNRTIFDSDLMDSARLGGPCPPSSNSGISATCYGSGGRMEGPPP
TYSEVIGHYPGSSFQHQQSSGPPSLLEGTRLHHTHIAPLESAAIWSKEKDKQKGHPL*
>ENSG00000152683|ENST00000282587
MGTIHLFRKPQRSFFGKLLREFRLVAADRRSWKILLFGVINLICTGFLLMWCSSTNSIAL
TAYTYLTIFDLFSLMTCLISYWVTLRKPSPVYSFGFERLEVLAVFASTVLAQLGALFILK
ESAERFLEQPEIHTGRLLVGTFVALCFNLFTMLSIRNKPFAYVSEAASTSWLQEHVADLS
RSLCGIIPGLSSIFLPRMNPFVLIDLAGAFALCITYMLIEINNYFAVDTASAIAIALMTF
GTMYPMSVYSGKVLLQTTPPHVIGQLDKLIREVSTLDGVLEVRNEHFWTLGFGSLAGSVH
VRIRRDANEQMVLAHVTNRLYTLVSTLTVQIFKDDWIRPALLSGPVAANVLNFSDHHVIP
MPLLKGTDDLNPVTSTPAKPSSPPPEFSFNTPGKNVNPVILLNTQTRPYGFGLNHGHTPY
SSMLNQGLGVPGIGATQGLRTGFTNIPSRYGTNNRIGQPRP*
>ENSG00000124302|ENST00000262622

MTLRPGTMRLACMFSSILLFGAAGLLLFISLQDPTELAPQQVPGIKFNIRPRQPHHDLPP
GGSQDGDLKEPTERVTRDLSSGAPRGRNLPAPDQPQPPLQRGTRLRLRQRRRRLLIKKMP
AAATIPANSSDAPFIRPGPGTLDGRWVSLHRSQQERKRVMQEACAKYRASSSRRAVTPRH
VSRIFVEDRHRVLYCEVPKAGCSNWKRVLMVLAGLASSTADIQHNTVHYGSALKRLDTFD
RQGILHRLSTYTKMLFVREPFERLVSAFRDKFEHPNSYYHPVFGKAILARYRANASREAL
RTGSGVRFPEFVQYLLDVHRPVGMDIHWDHVSRLCSPCLIDYDFVGKFESMEDDANFFLS
LIRAPRNLTFPRFKDRHSQEARTTARIAHQYFAQLSALQRQRTYDFYYMDYLMFNYSKPF
ADLY*
>ENSG00000112893|ENST00000261483
MKLSRQFTVFGSAIFCVVIFSLYLMLDRGHLDYPRNPRREGSFPQGQLSMLQEKIDHLER
LLAENNEIISNIRDSVINLSESVEDGPKSSQSNFSQGAGSHLLPSQLSLSVDTADCLFAS
QSGSHNSDVQMLDVYSLISFDNPDGGVWKQGFDITYESNEWDTEPLQVFVVPHSHNDPGW
LKTFNDYFRDKTQYIFNNMVLKLKEDSRRKFIWSEISYLSKWWDIIDIQKKDAVKSLIEN
GQLEIVTGGWVMPDEATPHYFALIDQLIEGHQWLENNIGVKPRSGWAIDPFGHSPTMAYL
LNRAGLSHMLIQRVHYAVKKHFALHKTLEFFWRQNWDLGSVTDILCHMMPFYSYDIPHTC
GPDPKICCQFDFKRLPGGRFGCPWGVPPETIHPGNVQSRARMLLDQYRKKSKLFRTKVLL
APLGDDFRYCEYTEWDLQFKNYQQLFDYMNSQSKFKVKIQFGTLSDFFDALDKADETQRD
KGQSMFPVLSGDFFTYADRDDHYWSGYFTSRPFYKRMDRIMESHLRAAEILYYFALRQAH
KYKINKFLSSSLYTALTEARRNLGLFQHHDAITGTAKDWVVVDYGTRLFHSLMVLEKIIG
NSAFLLILKDKLTYDSYSPDTFLEMDLKQKSQDSLPQKNIIRLSAEPRYLVVYNPLEQDR
ISLVSVYVSSPTVQVFSASGKPVEVQVSAVWDTANTISETAYEISFRAHIPPLGLKVYKI
LESASSNSHLADYVLYKNKVEDSGIFTIKNMINTEEGITLENSFVLLRFDQTGLMKQMMT
KEDGKHHEVNVQFSWYGTTIKRDKSGAYLFLPDGNAKPYVYTTPPFVRVTHGRIYSEVTC
FFDHVTHRVRLYHIQGIEGQSVEVSNIVDIRKVYNREIAMKISSDIKSQNRFYTDLNGYQ
IQPRMTLSKLPLQANVYPMTTMAYIQDAKHRLTLLSAQSLGVSSLNSGQIEVIMDRRLMQ
DDNRGLEQGIQDNKITANLFRILLEKRSAVNTEEEKKSVSYPSLLSHITSSLMNHPVIPM
ANKFSSPTLELQGEFSPLQSSLPCDIHLVNLRTIQSKVGNGHSNEAALILHRKGFDCRFS
SKGTGLFCSTTQGKILVQKLLNKFIVESLTPSSLSLMHSPPGTQNISEINLSPMEISTFR
IQLR*
>ENSG00000155511|ENST00000285900
MQHIFAFFCTGFLGAVVGANFPNNIQIGGLFPNQQSQEHAAFRFALSQLTEPPKLLPQID
IVNISDSFEMTYRFCSQFSKGVYAIFGFYERRTVNMLTSFCGALHVCFITPSFPVDTSNQ
FVLQLRPELQDALISIIDHYKWQKFVYIYDADRGLSVLQKVLDTAAEKNWQVTAVNILTT
TEEGYRMLFQDLEKKKERLVVVDCESERLNAILGQIIKLEKNGIGYHYILANLGFMDIDL
NKFKESGANVTGFQLVNYTDTIPAKIMQQWKNSDARDHTRVDWKRPKYTSALTYDGVKVM
AEAFQSLRRQRIDISRRGNAGDCLANPAVPWGQGIDIQRALQQVRFEGLTGNVQFNEKGR
RTNYTLHVIEMKHDGIRKIGYWNEDDKFVPAATDAQAGGDNSSVQNRTYIVTTILEDPYV
MLKKNANQFEGNDRYEGYCVELAAEIAKHVGYSYRLEIVSDGKYGARDPDTKAWNGMVGE
LVYGRADVAVAPLTITLVREEVIDFSKPFMSLGISIMIKKPQKSKPGVFSFLDPLAYEIW
MCIVFAYIGVSVVLFLVSRFSPYEWHSEEFEEGRDQTTSDQSNEFGIFNSLWFSLGAFMQ
QGCDISPRSLSGRIVGGVWWFFTLIIISSYTANLAAFLTVERMVSPIESAEDLAKQTEIA
YGTLEAGSTKEFFRRSKIAVFEKMWTYMKSAEPSVFVRTTEEGMIRVRKSKGKYAYLLES
TMNEYIEQRKPCDTMKVGGNLDSKGYGIATPKGSALRNPVNLAVLKLNEQGLLDKLKNKW
WYDKGECGSGGGDSKDKTSALSLSNVAGVFYILIGGLGLAMLVALIEFCYKSRSESKRMK

GFCLIPQQSINEAIRTSTLPRNSGAGASSGGSGENGRVVSHDFPKSMQSIPCMSHSSGMP
LGATGL*
>ENSG00000104331|ENST00000262644
MAPMGIRLSPLGVAVFCLLGLGVLYHLYSGFLAGRFSLFGLGGEPGGGAAGPAAAADGGT
VDLREMLAVSVLAAVRGGDEVRRVRESNVLHEKSKGKTREGAEDKMTSGDVLSNRKMFYL
LKTAFPSVQINTEEHVDAADQEVILWDHKIPEDILKEVTTPKEVPAESVTVWIDPLDATQ
EYTEDLRKYVTTMVCVAVNGKPMLGVIHKPFSEYTAWAMVDGGSNVKARSSYNEKTPRIV
VSRSHSGMVKQVALQTFGNQTTIIPAGGAGYKVLALLDVPDKSQEKADLYIHVTYIKKWD
ICAGNAILKALGGHMTTLSGEEISYTGSDGIEGGLLASIRMNHQALVRKLPDLEKTGHK*
>ENSG00000141429|ENST00000269195
MRKFAYCKVVLATSLIWVLLDMFLLLYFSECNKCDEKKERGLPAGDVLEPVQKPHEGPGE
MGKPVVIPKEDQEKMKEMFKINQFNLMASEMIALNRSLPDVRLEGCKTKVYPDNLPTTSV
VIVFHNEAWSTLLRTVHSVINRSPRHMIEEIVLVDDASERDFLKRPLESYVKKLKVPVHV
IRMEQRSGLIRARLKGAAVSKGQVITFLDAHCECTVGWLEPLLARIKHDRRTVVCPIIDV
ISDDTFEYMAGSDMTYGGFNWKLNFRWYPVPQREMDRRKGDRTLPVRTPTMAGGLFSIDR
DYFQEIGTYDAGMDIWGGENLEISFRIWQCGGTLEIVTCSHVGHVFRKATPYTFPGGTGQ
IINKNNRRLAEVWMDEFKNFFYIISPGVTKVDYGDISSRVGLRHKLQCKPFSWYLENIYP
DSQIPRHYFSLGEIRNVETNQCLDNMARKENEKVGIFNCHGMGGNQVFSYTANKEIRTDD
LCLDVSKLNGPVTMLKCHHLKGNQLWEYDPVKLTLQHVNSNQCLDKATEEDSQVPSIRDC
NGSRSQQWLLRNVTLPEIF*
>ENSG00000159723|ENST00000290953
MLTAAVLSCALLLALPATRGAQMGLAPMEGIRRPDQALLPELPGLGLRAPLKKTTAEQAE
EDLLQEAQALAEVLDLQDREPRSSRRCVRLHESCLGQQVPCCDPCATCYCRFFNAFCYCR
KLGTAMNPCSRT*
>ENSG00000114251|ENST00000264634
MKKSIGILSPGVALGMAGSAMSSKFFLVALAIFFSFAQVVIEANSWWSLGMNNPVQMSEV
YIIGAQPLCSQLAGLSQGQKKLCHLYQDHMQYIGEGAKTGIKECQYQFRHRRWNCSTVDN
TSVFGRVMQIGSRETAFTYAVSAAGVVNAMSRACREGELSTCGCSRAARPKDLPRDWLWG
GCGDNIDYGYRFAKEFVDARERERIHAKGSYESARILMNLHNNEAGRRTVYNLADVACKC
HGVSGSCSLKTCWLQLADFRKVGDALKEKYDSAAAMRLNSRGKLVQVNSRFNSPTTQDLV
YIDPSPDYCVRNESTGSLGTQGRLCNKTSEGMDGCELMCCGRGYDQFKTVQTERCHCKFH
WCCYVKCKKCTEIVDQFVCK*
>ENSG00000153214|ENST00000283206
MVAACRSVAGLLPRRRRCFPARAPLLRVALCLLCWTPAAVRAVPELGLWLETVNDKSGPL
IFRKTMFNSTDIKLSVKSFHCSGPVKFTIVWHLKYHTCHNEHSNLEELFQKHKLSVDEDF
CHYLKNDNCWTTKNENLDCNSDSQVFPSLNNKELINIRNVSNQERSMDVVARTQKDGFHI
FIVSIKTENTDASWNLNVSLSMIGPHGYISASDWPLMIFYMVMCIVYILYGILWLTWSAC
YWKDILRIQFWIAAVIFLGMLEKAVFYSEYQNISNTGLSTQGLLIFAELISAIKRTLARL
LVIIVSLGYGIVKPRLGTVMHRVIGLGLLYLIFAAVEGVMRVIGGSNHLAVVLDDIILAV
IDSIFVWFIFISLAQTMKTLRLRKNTVKFSLYRHFKNTLIFAVLASIVFMGWTTKTFRIA
KCQSDWMERWVDDAFWSFLFSLILIVIMFLWRPSANNQRYAFMPLIDDSDDEIEEFMVTS
ENLTEGIKLRASKSVSNGTAKPATSENFDEDLKWVEENIPSSFTDVALPVLVDSDEEIMT
RSEMAEKMFSSEKIM*
>ENSG00000109586|ENST00000265000

MRLKIGFILRSLLVVGSFLGLVVLWSSLTPRPDDPSPLSRMREDRDVNDPMPNRGGNGLA
PGEDRFKPVVPWPHVEGVEVDLESIRRINKAKNEQEHHAGGDSQKDIMQRQYLTFKPQTF
TYHDPVLRPGILGNFEPKEPEPPGVVGGPGEKAKPLVLGPEFKQAIQASIKEFGFNMVAS
DMISLDRSVNDLRQEECKYWHYDENLLTSSVVIVFHNEGWSTLMRTVHSVIKRTPRKYLA
EIVLIDDFSNKEHLKEKLDEYIKLWNGLVKVFRNERREGLIQARSIGAQKAKLGQVLIYL
DAHCEVAVNWYAPLVAPISKDRTICTVPLIDVINGNTYEIIPQGGGDEDGYARGAWDWSM
LWKRVPLTPQEKRLRKTKTEPYRSPAMAGGLFAIEREFFFELGLYDPGLQIWGGENFEIS
YKIWQCGGKLLFVPCSRVGHIYRLEGWQGNPPPIYVGSSPTLKNYVRVVEVWWDEYKDYF
YASRPESQALPYGDISELKKFREDHNCKSFKWFMEEIAYDITSHYPLPPKNVDWGEIRGF
ETAYCIDSMGKTNGGFVELGPCHRMGGNQLFRINEANQLMQYDQCLTKGADGSKVMITHC
NLNEFKEWQYFKNLHRFTHIPSGKCLDRSEVLHQVFISNCDSSKTTQKWEMNNIHSV*
>ENSG00000080371|ENST00000261263
MAAAGGGGGGAAAAGRAYSFKVVLLGEGCVGKTSLVLRYCENKFNDKHITTLQASFLTKK
LNIGGKRVNLAIWDTAGQERFHALGPIYYRDSNGAILVYDITDEDSFQKVKNWVKELRKM
LGNEICLCIVGNKIDLEKERHVSIQEAESYAESVGAKHYHTSAKQNKGIEELFLDLCKRM
IETAQVDERAKGNGSSQPGTARRGVQIIDDEPQAQTSGGGCCSSG*
>ENSG00000143952|ENST00000272322
MASSHSSSPVPQGSSSDVFFKIEVDPSKHIRPVPSLPDVCPKEPTGDSHSLYVAPSLVTD
QHRWTVYHSKVNLPAALNDPRLAKRESDFFTKTWGLDFVDTEVIPSFYLPQISKEHFTVY
QQEISQREKIHERCKNICPPKDTFERTLLHTHDKSRTDLEQVPKIFMKPDFALDDSLTFN
SVLPWSHFNTAGGKGNRDAASSKLLQEKLSHYLDIVEVNIAHQISLRSEAFFHAMTSQHE
LQDYLRKTSQAVKMLRDKIAQIDKVMCEGSLHILRLALTRNNCVKVYNKLKLMATVHQTQ
PTVQVLLSTSEFVGALDLIATTQEVLQQELQGIHSFRHLGSQLCELEKLIDKMMIAEFST
YSHSDLNRPLEDDCQVLEEERLISLVFGLLKQRKLNFLEIYGEKMVITAKNIIKQCVINK
VSQTEEIDTDVVVKLADQMRMLNFPQWFDLLKDIFSKFTIFLQRVKATLNIIHSVVLSVL
DKNQRTRELEEISQQKNAAKDNSLDTEVAYLIHEGMFISDAFGEGELTPIAVDTTSQRNA
SPNSEPCSSDSVSEPECTTDSSSSKEHTSSSAIPGGVDIMVSEDMKLTDSELGKLANNIQ
ELLYSASDICHDRAVKFLMSRAKDGFLEKLNSMEFITLSRLMETFILDTEQICGRKSTSL
LGALQSQAIKFVNRFHEERKTKLSLLLDNERWKQADVPAEFQDLVDSLSDGKIALPEKKS
GATEERKPAEVLIVEGQQYAVVGTVLLLIRIILEYCQCVDNIPSVTTDMLTRLSDLLKYF
NSRSCQLVLGAGALQVVGLKTITTKNLALSSRCLQLIVHYIPVIRAHFEARLPPKQYSML
RHFDHITKDYHDHIAEISAKLVAIMDSLFDKLLSKYEVKAPVPSACFRNICKQMTKMHEA
IFDLLPEEQTQMLFLRINASYKLHLKKQLSHLNVINDGGPQNGLVTADVAFYTGNLQALK
GLKDLDLNMAEIWEQKR*
>ENSG00000139329|ENST00000266718
MSLSAFTLFLALIGGTSGQYYDYDFPLSIYGQSSPNCAPECNCPESYPSAMYCDELKLKS
VPMVPPGIKYLYLRNNQIDHIDEKAFENVTDLQWLILDHNLLENSKIKGRVFSKLKQLKK
LHINHNNLTESVGPLPKSLEDLQLTHNKITKLGSFEGLVNLTFIHLQHNRLKEDAVSAAF
KGLKSLEYLDLSFNQIARLPSGLPVSLLTLYLDNNKISNIPDEYFKRFNALQYLRLSHNE
LADSGIPGNSFNVSSLVELDLSYNKLKNIPTVNENLENYYLEVNQLEKFDIKSFCKILGP
LSYSKIKHLRLDGNRISETSLPPDMYECLRVANEVTLN*
>ENSG00000105669|ENST00000262812
MAPPAPGPASGGSGEVDELFDVKNAFYIGSYQQCINEAQRVKLSSPERDVERDVFLYRAY
LAQRKFGVVLDEIKPSSAPELQAVRMFADYLAHESRRDSIVAELDREMSRSVDVTNTTFL

LMAASIYLHDQNPDAALRALHQGDSLECTAMTVQILLKLDRLDLARKELKRMQDLDEDAT
LTQLATAWVSLATGGEKLQDAYYIFQEMADKCSPTLLLLNGQAACHMAQGRWEAAEGLLQ
EALDKDSGYPETLVNLIVLSQHLGKPPEVTNRYLSQLKDAHRSHPFIKEYQAKENDFDRL
VLQYAPSA*
>ENSG00000139192|ENST00000266556
MGTQEGWCLLLCLALSGAAETKPHPAEGQWRAVDVVLDCFLAKDGAHRGALASSEDRARA
SLVLKQVPVLDDGSLEDFTDFQGGTLAQDDPPIIFEASVDLVQIPQAEALLHADCSGKEV
TCEISRYFLQMTETTVKTAAWFMANMQVSGGGPSISLVMKTPRVTKNEALWHPTLNLPLS
PQGTVRTAVEFQVMTQTQSLSFLLGSSASLDCGFSMAPGLDLISVEWRLQHKGRGQLVYS
WTAGQGQAVRKGATLEPAQLGMARDASLTLPGLTIQDEGTYICQITTSLYRAQQIIQLNI
QASPKVRLSLANEALLPTLICDIAGYYPLDVVVTWTREELGGSPAQVSGASFSSLRQSVA
GTYSISSSLTAEPGSAGATYTCQVTHISLEEPLGASTQVVPPERRTALGVIFASSLFLLA
LMFLGLQRRQAPTGLGLLQAERWETTSCADTQSSHLHEDRTARVSQPS*
>ENSG00000113595|ENST00000274327
MATLVVNKLGAGVDSGRQGSRGTAVVKVLECGVCEDVFSLQGDKVPRLLLCGHTVCHDCL
TRLPLHGRAIRCPFDRQVTDLGDSGVWGLKKNFALLELLERLQNGPIGQYGAAEESIGIS
GESIIRCDEDEAHLASVYCTVCATHLCSECSQVTHSTKTLAKHRRVPLADKPHEKTMCSQ
HQVHAIEFVCLEEGCQTSPLMCCVCKEYGKHQGHKHSVLEPEANQIRASILDMAHCIRTF
TEEISDYSRKLVGIVQHIEGGEQIVEDGIGMAHTEHVPGTAENARSCIRAYFYDLHETLC
RQEEMALSVVDAHVREKLIWLRQQQEDMTILLSEVSAACLHCEKTLQQDDCRVVLAKQEI
TRLLETLQKQQQQFTEVADHIQLDASIPVTFTKDNRVHIGPKMEIRVVTLGLDGAGKTTI
LFKLKQDEFMQPIPTIGFNVETVEYKNLKFTIWDVGGKHKLRPLWKHYYLNTQAVVFVVD
SSHRDRISEAHSELAKLLTEKELRDALLLIFANKQDVAGALSVEEITELLSLHKLCCGRS
CFSDNM*
>ENSG00000064270|ENST00000262429
MVEGRVSEFLKKLGFSGGGRQYQALEKDEEEALIDEQSELKAIEKEKKVTALPPKEACKC
QKEDLARAFCVDLHTGLSEFSVTQRRLAHGWNEFVADNSEPVWKKYLDQFKNPLILLLLG
SALVSVLTKEYEDAVSIATAVLVVVTVAFIQEYRSEKSLEELTKLVPPECNCLREGKLQH
LLARELVPGDVVSLSIGDRIPADIRLTEVTDLLVDESSFTGEAEPCSKTDSPLTGGGDLT
TLSNIVFMGTLVQYGRGQGVVIGTGESSQFGEVFKMMQAEETPKTPLQKSMDRLGKQLTL
FSFGIIGLIMLIGWSQGKQLLSMFTIGVSLAVAAIPEGLPIVVMVTLVLGVLRMAKKRVI
VKKLPIVETLGCCSVLCSDKTGTLTANEMTVTQLVTSDGLRAEVSGVGYDGQGTVCLLPS
KEVIKEFSNVSVGKLVEAGCVANNAVIRKNAVMGQPTEGALMALAMKMDLSDIKNSYIRK
KEIPFSSEQKWMAVKCSLKTEDQEDIYFMKGALEEVIRYCTMYNNGGIPLPLTPQQRSFC
LQEEKRMGSLGLRVLALASGPELGRLTFLGLVGIIDPPRVGVKEAVQVLSESGVSVKMIT
GDALETALAIGRNIGLCNGKLQAMSGEEVDSVEKGELADRVGKVSVFFRTSPKHKLKIIK
ALQESGAIVAMTGDGVNDAVALKSADIGIAMGQTGTDVSKEAANMILVDDDFSAIMNAVE
EGKGIFYNIKNFVRFQLSTSISALSLITLSTVFNLPSPLNAMQILWINIIMDGPPAQSLG
VEPVDKDAFRQPPRSVRDTILSRALILKILMSAAIIISGTLFIFWKEMPEDRASTPRTTT
MTFTCFVFFDLFNALTCRSQTKLIFEIGFLRNHMFLYSVLGSILGQLAVIYIPPLQRVFQ
TENLGALDLLFLTGLASSVFILSELLKLCEKYCCSPKRVQMHPEDV*
>ENSG00000100228|ENST00000263116
MVIAGASWMLGRAAASPTQTPPTTSTIRVARRSRVALVAMVIAAAGSGGPGRAEPQLSQP
SLDCGRMRSSLTPLGPPVSRDRVIASFPKWYTPEACLQLREHFHGQVSAACQRRNTGTVG

LKLSKVVVVGDLYVGKTSLIHRFCKNVFDRDYKATIGVDFEIERFEIAGIPYSLQIWDTA
GQEKFKCIASAYYRGAQVIITAFDLTDVQTLEHTRQWLEDALRENEAGSCFIFLVGTKKD
LLSGAACEQAEADAVHLAREMQAEYWSVSAKTGENVKAFFSRVAALAFEQSVLQDLERQS
SARLQVGNGDLIQMEGSPPETQESKRPSSLGCC*
>ENSG00000003147|ENST00000265577
MSGHKCYPWDLQDRYAQDKSVVNKMQQKYWETKQAFIKATGKKEDEHVVASDADLDAKLE
LFHSIQRTCLDLSKAIVLYQKRICFLSQEENELGKFLRSQGFQDKTRAGKMMQATGKALC
FSSQQRLALRNPLCRFHQEVETFRHRAISDTWLTVNRMEQCRTEYRGALLWMKDVSQELD
PDLYKQMEKFRKVQTQVRLAKKNFDKLKMDVCQKVDLLGASRCNLLSHMLATYQTTLLHF
WEKTSHTMAAIHESFKGYQPYEFTTLKSLQDPMKKLVEKEEKKKINQQESTDAAVQEPSQ
LISLEEENQRKESSSFKTEDGKSILSALDKGSTHTACSGPIDELLDMKSEEGACLGPVAG
TPEPEGADKDDLLLLSEIFNASSLEEGEFSKEWAAVFGDGQVKEPVPTMALGEPDPKAQT
GSGFLPSQLLDQNMKDLQASLQEPAKAASDLTAWFSLFADLDPLSNPDAVGKTDKEHELL
NA*
>ENSG00000087253|ENST00000262134
MSRCAQAAEVAATVPGAGVGNVGLRPPMVPRQASFFPPPVPNPFVQQTQIGSARRVQIVL
LGIILLPIRVLLVALILLLAWPFAAISTVCCPEKLTHPITGWRRKITQTALKFLGRAMFF
SMGFIVAVKGKIASPLEAPVFVAAPHSTFFDGIACVVAGLPSMVSRNENAQVPLIGRLLR
AVQPVLVSRVDPDSRKNTINEIIKRTTSGGEWPQILVFPEGTCTNRSCLITFKPGAFIPG
VPVQPVLLRYPNKLDTVTWTWQGYTFIQLCMLTFCQLFTKVEVEFMPVQVPNDEEKNDPV
LFANKVRNLMAEALGIPVTDHTYEDCRLMISAGQLTLPMEAGLVEFTKISRKLKLDWDGV
RKHLDEYASIASSSKGGRIGIEEFAKYLKLPVSDVLRQLFALFDRNHDGSIDFREYVIGL
AVLCNPSNTEEIIQVAFKLFDVDEDGYITEEEFSTILQASLGVPDLDVSGLFKEIAQGDS
ISYEEFKSFALKHPEYAKIFTTYLDLQTCHVFSLPKEVQTTPSTASNKVSPEKHEESTSD
KKDD*
>ENSG00000101310|ENST00000262544
MATYLEFIQQNEERDGVRFSWNVWPSSRLEATRMVVPLACLLTPLKERPDLPPVQYEPVL
CSRPTCKAVLNPLCQVDYRAKLWACNFCFQRNQFPPAYGGISEVNQPAELMPQFSTIEYV
IQRGAQSPLIFLYVVDTCLEEDDLQALKESLQMSLSLLPPDALVGLITFGRMVQVHELSC
EGISKSYVFRGTKDLTAKQIQDMLGLTKPAMPMQQARPAQPQEHPFASSRFLQPVHKIDM
NLTDLLGELQRDPWPVTQGKRPLRSTGVALSIAVGLLEGTFPNTGARIMLFTGGPPTQGP
GMVVGDELKIPIRSWHDIEKDNARFMKKATKHYEMLANRTAANGHCIDIYACALDQTGLL
EMKCCANLTGGYMVMGDSFNTSLFKQTFQRIFTKDFNGDFRMAFGATLDVKTSRELKIAG
AIGPCVSLNVKGPCVSENELGVGGTSQWKICGLDPTSTLGIYFEVVNQHNTPIPQGGRGA
IQFVTHYQHSSTQRRIRVTTIARNWADVQSQLRHIEAAFDQEAAAVLMARLGVFRAESEE
GPDVLRWLDRQLIRLCQKFGQYNKEDPTSFRLSDSFSLYPQFMFHLRRSPFLQVFNNSPD
ESSYYRHHFARQDLTQSLIMIQPILYSYSFHGPPEPVLLDSSSILADRILLMDTFFQIVI
YLGETIAQWRKAGYQDMPEYENFKHLLQAPLDDAQEILQARFPMPRYINTEHGGSQARFL
LSKVNPSQTHNNLYAWGQETGAPILTDDVSLQVFMDHLKKLAVSSAC*
>ENSG00000106615|ENST00000262187
MPQSKSRKIAILGYRSVGKSSLTIQFVEGQFVDSYDPTIENTFTKLITVNGQEYHLQLVD
TAGQDEYSIFPQTYSIDINGYILVYSVTSIKSFEVIKVIHGKLLDMVGKVQIPIMLVGNK
KDLHMERVISYEEGKALAESWNAAFLESSAKENQTAVDVFRRIILEAEKMDGAASQGKSS
CSVM*

>ENSG00000146648|ENST00000275493
MRPSGTAGAALLALLAALCPASRALEEKKVCQGTSNKLTQLGTFEDHFLSLQRMFNNCEV
VLGNLEITYVQRNYDLSFLKTIQEVAGYVLIALNTVERIPLENLQIIRGNMYYENSYALA
VLSNYDANKTGLKELPMRNLQEILHGAVRFSNNPALCNVESIQWRDIVSSDFLSNMSMDF
QNHLGSCQKCDPSCPNGSCWGAGEENCQKLTKIICAQQCSGRCRGKSPSDCCHNQCAAGC
TGPRESDCLVCRKFRDEATCKDTCPPLMLYNPTTYQMDVNPEGKYSFGATCVKKCPRNYV
VTDHGSCVRACGADSYEMEEDGVRKCKKCEGPCRKVCNGIGIGEFKDSLSINATNIKHFK
NCTSISGDLHILPVAFRGDSFTHTPPLDPQELDILKTVKEITGFLLIQAWPENRTDLHAF
ENLEIIRGRTKQHGQFSLAVVSLNITSLGLRSLKEISDGDVIISGNKNLCYANTINWKKL
FGTSGQKTKIISNRGENSCKATGQVCHALCSPEGCWGPEPRDCVSCRNVSRGRECVDKCN
LLEGEPREFVENSECIQCHPECLPQAMNITCTGRGPDNCIQCAHYIDGPHCVKTCPAGVM
GENNTLVWKYADAGHVCHLCHPNCTYGCTGPGLEGCPTNGPKIPSIATGMVGALLLLLVV
ALGIGLFMRRRHIVRKRTLRRLLQERELVEPLTPSGEAPNQALLRILKETEFKKIKVLGS
GAFGTVYKGLWIPEGEKVKIPVAIKELREATSPKANKEILDEAYVMASVDNPHVCRLLGI
CLTSTVQLITQLMPFGCLLDYVREHKDNIGSQYLLNWCVQIAKGMNYLEDRRLVHRDLAA
RNVLVKTPQHVKITDFGLAKLLGAEEKEYHAEGGKVPIKWMALESILHRIYTHQSDVWSY
GVTVWELMTFGSKPYDGIPASEISSILEKGERLPQPPICTIDVYMIMVKCWMIDADSRPK
FRELIIEFSKMARDPQRYLVIQGDERMHLPSPTDSNFYRALMDEEDMDDVVDADEYLIPQ
QGFFSSPSTSRTPLLSSLSATSNNSTVACIDRNGLQSCPIKEDSFLQRYSSDPTGALTED
SIDDTFLPVPEYINQSVPKRPAGSVQNPVYHNQPLNPAPSRDPHYQDPHSTAVGNPEYLN
TVQPTCVNSTFDSPAHWAQKGSHQISLDNPDYQQDFFPKEAKPNGIFKGSTAENAEYLRV
APQSSEFIGA*
>ENSG00000071073|ENST00000264968
MRLRNGTVATALAFITSFLTLSWYTTWQNGKEKLIAYQREFLALKERLRIAEHRISQRSS
ELNTIVQQFKRVGAETNGSKDALNKFSDNTLKLLKELTSKKSLQVPSIYYHLPHLLKNEG
SLQPAVQIGNGRTGVSIVMGIPTVKREVKSYLIETLHSLIDNLYPEEKLDCVIVVFIGET
DIDYVHGVVANLEKEFSKEISSGLVEVISPPESYYPDLTNLKETFGDSKERVRWRTKQNL
DYCFLMMYAQEKGIYYIQLEDDIIVKQNYFNTIKNFALQLSSEEWMILEFSQLGFIGKMF
QAPDLTLIVEFIFMFYKEKPIDWLLDHILWVKVCNPEKDAKHCDRQKANLRIRFRPSLFQ
HVGLHSSLSGKIQKLTDKDYMKPLLLKIHVNPPAEVSTSLKVYQGHTLEKTYMGEDFFWA
ITPIAGDYILFKFDKPVNVESYLFHSGNQEHPGDILLNTTVEVLPFKSEGLEISKETKDK
RLEDGYFRIGKFENGVAEGMVDPSLNPISAFRLSVIQNSAVWAILNEIHIKKATN*
>ENSG00000139044|ENST00000266383
MGSPRAARPPLLLRPVKLLRRRFRLLLALAVVSVGLWTLYLELVASAQVGGNPLNRRYGS
WRELAKALASRNIPAVDPHLQFYHPQRLSLEDHDIDQGVSSNSSYLKWNKPVPWLSEFRG
RANLHVFEDWCGSSIQQLRRNLHFPLYPHIRTTLRKLAVSPKWTNYGLRIFGYLHPFTDG
KIQFAIAADDNAEFWLSLDDQVSGLQLLASVGKTGKEWTAPGEFGKFRSQISKPVSLSAS
HRYYFEVLHKQNEEGTDHVEVAWRRNDPGAKFTIIDSLSLSLFTNETFLQMDEVGHIPQT
AASHVDSSNALPRDEQPPADMLRPDPRDTLYRVPLIPKSHLRHVLPDCPYKPSYLVDGLP
LQRYQGLRFVHLSFVYPNDYTRLSHMETHNKCFYQENAYYQDRFSFQEYIKIDQPEKQGL
EQPGFEENLLEESQYGEVAEETPASNNQNARMLEGRQTPASTLEQDATDYRLRSLRKLLA
QPREGLLAPFSKRNSTASFPGRTSHIPVQQPEKRKQKPSPEPSQDSPHSDKWPPGHPVKN
LPQMRGPRPRPAGDSPRKTQWLNQVESYIAEQRRGDRMRPQAPGRGWHGEEEVVAAAGQE
GQVEGEEEGEEEEEEEDMSEVFEYVPVFDPVVNWDQTFSARNLDFQALRTDWIDLSCNTS

GNLLLPEQEALEVTRVFLKKLNQRSRGRYQLQRIVNVEKRQDQLRGGRYLLELELLEQGQ
RVVRLSEYVSARGWQGIDPAGGEEVEARNLQGLVWDPHNRRRQVLNTRAQEPKLCWPQGF
SWSHRAVVHFVVPVKNQARWVQQFIKDMENLFQVTGDPHFNIVITDYSSEDMDVEMALKR
SKLRSYQYVKLSGNFERSAGLQAGIDLVKDPHSIIFLCDLHIHFPAGVIDAIRKHCVEGK
MAFAPMVMRLHCGATPQWPEGYWEVNGFGLLGIYKSDLDRIGGMNTKEFRDRWGGEDWEL
LDRILQAGLDVERLSLRNFFHHFHSKRGMWSRRQMKTL*
>ENSG00000072310|ENST00000261646
MDEPPFSEAALEQALGEPCDLDAALLTDIEDMLQLINNQDSDFPGLFDPPYAGSGAGGTD
PASPDTSSPGSLSPPPATLSSSLEAFLSGPQAAPSPLSPPQPAPTPLKMYPSMPAFSPGP
GIKEESVPLSILQTPTPQPLPGALLPQSFPAPAPPQFSSTPVLGYPSPPGGFSTGSPPGN
TQQPLPGLPLASPPGVPPVSLHTQVQSVVPQQLLTVTAAPTAAPVTTTVTSQIQQVPVLL
QPHFIKADSLLLTAMKTDGATVKAAGLSPLVSGTTVQTGPLPTLVSGGTILATVPLVVDA
EKLPINRLAAGSKAPASAQSRGEKRTAHNAIEKRYRSSINDKIIELKDLVVGTEAKLNKS
AVLRKAIDYIRFLQHSNQKLKQENLSLRTAVHKSKSLKDLVSACGSGGNTDVLMEGVKTE
VEDTLTPPPSDAGSPFQSSPLSLGSRGSGSGGSGSDSEPDSPVFEDSKAKPEQRPSLHSR
GMLDRSRLALCTLVFLCLSCNPLASLLGARGLPSPSDTTSVYHSPGRNVLGTESRDGPGW
AQWLLPPVVWLLNGLLVLVSLVLLFVYGEPVTRPHSGPAVYFWRHRKQADLDLARGDFAQ
AAQQLWLALRALGRPLPTSHLDLACSLLWNLIRHLLQRLWVGRWLAGRAGGLQQDCALRV
DASASARDAALVYHKLHQLHTMGKHTGGHLTATNLALSALNLAECAGDAVSVATLAEIYV
AAALRVKTSLPRALHFLTRFFLSSARQACLAQSGSVPPAMQWLCHPVGHRFFVDGDWSVL
STPWESLYSLAGNPVDPLAQVTQLFREHLLERALNCVTQPNPSPGSADGDKEFSDALGYL
QLLNSCSDAAGAPAYSFSISSSMATTTGVDPVAKWWASLTAVVIHWLRRDEEAAERLCPL
VEHLPRVLQESERPLPRAALHSFKAARALLGCAKAESGPASLTICEKASGYLQDSLATTP
ASSSIDKAVQLFLCDLLLVVRTSLWRQQQPPAPAPAAQGTSSRPQASALELRGFQRDLSS
LRRLAQSFRPAMRRVFLHEATARLMAGASPTRTHQLLDRSLRRRAGPGGKGGAVAELEPR
PTRREHAEALLLASCYLPPGFLSAPGQRVGMLAEAARTLEKLGDRRLLHDCQQMLMRLGG
GTTVTSS*
>ENSG00000156103|ENST00000286614
MILLTFSTGRRLDFVHHSGVFFLQTLLWILCATVCGTEQYFNVEVWLQKYGYLPPTDPRM
SVLRSAETMQSALAAMQQFYGINMTGKVDRNTIDWMKKPRCGVPDQTRGSSKFHIRRKRY
ALTGQKWQHKHITYSIKNVTPKVGDPETRKAIRRAFDVWQNVTPLTFEEVPYSELENGKR
DVDITIIFASGFHGDSSPFDGEGGFLAHAYFPGPGIGGDTHFDSDEPWTLGNPNHDGNDL
FLVAVHELGHALGLEHSNDPTAIMAPFYQYMETDNFKLPNDDLQGIQKIYGPPDKIPPPT
RPLPTVPPHRSIPPADPRKNDRPKPPRPPTGRPSYPGAKPNICDGNFNTLAILRREMFVF
KDQWFWRVRNNRVMDGYPMQITYFWRGLPPSIDAVYENSDGNFVFFKGNKYWVFKDTTLQ
PGYPHDLITLGSGIPPHGIDSAIWWEDVGKTYFFKGDRYWRYSEEMKTMDPGYPKPITVW
KGIPESPQGAFVHKENGFTYFYKGKEYWKFNNQILKVEPGYPRSILKDFMGCDGPTDRVK
EGHSPPDDVDIVIKLDNTASTVKAIAIVIPCILALCLLVLVYTVFQFKRKGTPRHILYCK
RSMQEWV*
>ENSG00000156966|ENST00000287590
MSLWKKTVYRSLCLALALLVAVTVFQRSLTPGQFLQEPPPPTLEPQKAQKPNGQLVNPNN
FWKNPKDVAAPTPMASQGPQAWDVTTTNCSANINLTHQPWFQVLEPQFRQFLFYRHCRYF
PMLLNHPEKCRGDVYLLVVVKSVITQHDRREAIRQTWGRERQSAGGGRGAVRTLFLLGTA
SKQEERTHYQQLLAYEDRLYGDILQWGFLDTFFNLTLKEIHFLKWLDIYCPHVPFIFKGD

DDVFVNPTNLLEFLADRQPQENLFVGDVLQHARPIRRKDNKYYIPGALYGKASYPPYAGG
GGFLMAGSLARRLHHACDTLELYPIDDVFLGMCLEVLGVQPTAHEGFKTFGISRNRNSRM
NKEPCFFRAMLVVHKLLPPELLAMWGLVHSNLTCSRKLQVL*
>ENSG00000154917|ENST00000285208
MSAGGDFGNPLRKFKLVFLGEQSVGKTSLITRFMYDSFDNTYQATIGIDFLSKTMYLEDR
TVRLQLWDTAGQERFRSLIPSYIRDSTVAVVVYDITNLNSFQQTSKWIDDVRTERGSDVI
IMLVGNKTDLADKRQITIEEGEQRAKELSVMFIETSAKTGYNVKQLFRRVASALPGMENV
QEKSKEGMIDIKLDKPQEPPASEGGCSC*
>ENSG00000038427|ENST00000265077
MFINIKSILWMCSTLIVTHALHKVKVGKSPPVRGSLSGKVSLPCHFSTMPTLPPSYNTSE
FLRIKWSKIEVDKNGKDLKETTVLVAQNGNIKIGQDYKGRVSVPTHPEAVGDASLTVVKL
LASDAGLYRCDVMYGIEDTQDTVSLTVDGVVFHYRAATSRYTLNFEAAQKACLDVGAVIA
TPEQLFAAYEDGFEQCDAGWLADQTVRYPIRAPRVGCYGDKMGKAGVRTYGFRSPQETYD
VYCYVDHLDGDVFHLTVPSKFTFEEAAKECENQDARLATVGELQAAWRNGFDQCDYGWLS
DASVRHPVTVARAQCGGGLLGVRTLYRFENQTGFPPPDSRFDAYCFKPKEATTIDLSILA
ETASPSLSKEPQMVSDRTTPIIPLVDELPVIPTEFPPVGNIVSFEQKATVQPQAITDSLA
TKLPTPTGSTKKPWDMDDYSPSASGPLGKLDISEIKEEVLQSTTGVSHYATDSWDGVVED
KQTQESVTQIEQIEVGPLVTSMEILKHIPSKEFPVTETPLVTARMILESKTEKKMVSTVS
ELVTTGHYGFTLGEEDDEDRTLTVGSDESTLIFDQIPEVITVSKTSEDTIHTHLEDLESV
SASTTVSPLIMPDNNGSSMDDWEERQTSGRITEEFLGKYLSTTPFPSQHRTEIELFPYSG
DKILVEGISTVIYPSLQTEMTHRRERTETLIPEMRTDTYTDEIQEEITKSPFMGKTEEEV
FSGMKLSTSLSEPIHVTESSVEMTKSFDFPTLITKLSAEPTEVRDMEEDFTATPGTTKYD
ENITTVLLAHGTLSVEAATVSKWSWDEDNTTSKPLESTEPSASSKLPPALLTTVGMNGKD
KDIPSFTEDGADEFTLIPDSTQKQLEEVTDEDIAAHGKFTIRFQPTTSTGIAEKSTLRDS
TTEEKVPPITSTEGQVYATMEGSALGEVEDVDLSKPVSTVPQFAHTSEVEGLAFVSYSST
QEPTTYVDSSHTIPLSVIPKTDWGVLVPSVPSEDEVLGEPSQDILVIDQTRLEATISPET
MRTTKITEGTTQEEFPWKEQTAEKPVPALSSTAWTPKEAVTPLDEQEGDGSAYTVSEDEL
LTGSERVPVLETTPVGKIDHSVSYPPGAVTEHKVKTDEVVTLTPRIGPKVSLSPGPEQKY
ETEGSSTTGFTSSLSPFSTHITQLMEETTTEKTSLEDIDLGSGLFEKPKATELIEFSTIK
VTVPSDITTAFSSVDRLHTTSAFKPSSAITKKPPLIDREPGEETTSDMVIIGESTSHVPP
TTLEDIVAKETETDIDREYFTTSSPPATQPTRPPTVEDKEAFGPQALSTPQPPASTKFHP
DINVYIIEVRENKTGRMSDLSVIGHPIDSESKEDEPCSEETDPVHDLMAEILPEFPDIIE
IDLYHSEENEEEEEECANATDVTTTPSVQYINGKHLVTTVPKDPEAAEARRGQFESVAPS
QNFSDSSESDTHPFVIAKTELSTAVQPNESTETTESLEVTWKPETYPETSEHFSGGEPDV
FPTVPFHEEFESGTAKKGAESVTERDTEVGHQAHEHTEPVSLFPEESSGEIAIDQESQKI
AFARATEVTFGEEVEKSTSVTYTPTIVPSSASAYVSEEEAVTLIGNPWPDDLLSTKESWV
EATPRQVVELSGSSSIPITEGSGEAEEDEDTMFTMVTDLSQRNTTDTLITLDTSRIITES
FFEVPATTIYPVSEQPSAKVVPTKFVSETDTSEWISSTTVEEKKRKEEEGTTGTASTFEV
YSSTQRSDQLILPFELESPNVATSSDSGTRKSFMSLTTPTQSEREMTDSTPVFTETNTLE
NLGAQTTEHSSIHQPGVQEGLTTLPRSPASVFMEQGSGEAAADPETTTVSSFSLNVEYAI
QAEKEVAGTLSPHVETTFSTEPTGLVLSTVMDRVVAENITQTSREIVISERLGEPNYGAE
IRGFSTGFPLEEDFSGDFREYSTVSHPIAKEETVMMEGSGDAAFRDTQTSPSTVPTSVHI
SHISDSEGPSSTMVSTSAFPWEEFTSSAEGSGEQLVTVSSSVVPVLPSAVQKFSGTASSI
IDEGLGEVGTVNEIDRRSTILPTAEVEGTKAPVEKEEVKVSGTVSTNFPQTIEPAKLWSR

QEVNPVRQEIESETTSEEQIQEEKSFESPQNSPATEQTIFDSQTFTETELKTTDYSVLTT
KKTYSDDKEMKEEDTSLVNMSTPDPDANGLESYTTLPEATEKSHFFLATALVTESIPAEH
VVTDSPIKKEESTKHFPKGMRPTIQESDTELLFSGLGSGEEVLPTLPTESVNFTEVEQIN
NTLYPHTSQVESTSSDKIEDFNRMENVAKEVGPLVSQTDIFEGSGSVTSTTLIEILSDTG
AEGPTVAPLPFSTDIGHPQNQTVRWAEEIQTSRPQTITEQDSNKNSSTAEINETTTSSTD
FLARAYGFEMAKEFVTSAPKPSDLYYEPSGEGSGEVDIVDSFHTSATTQATRQESSTTFV
SDGSLEKHPEVPSAKAVTADGFPTVSVMLPLHSEQNKSSPDPTSTLSNTVSYERSTDGSF
QDRFREFEDSTLKPNRKKPTENIIIDLDKEDKDLILTITESTILEILPELTSDKNTIIDI
DHTKPVYEDILGMQTDIDTEVPSEPHDSNDESNDDSTQVQEIYEAAVNLSLTEETFEGSA
DVLASYTQATHDESMTYEDRSQLDHMGFHFTTGIPAPSTETELDVLLPTATSLPIPRKSA
TVIPEIEGIKAEAKALDDMFESSTLSDGQAIADQSEIIPTLGQFERTQEEYEDKKHAGPS
FQPEFSSGAEEALVDHTPYLSIATTHLMDQSVTEVPDVMEGSNPPYYTDTTLAVSTFAKL
SSQTPSSPLTIYSGSEASGHTEIPQPSALPGIDVGSSVMSPQDSFKEIHVNIEATFKPSS
EEYLHITEPPSLSPDTKLEPSEDDGKPELLEEMEASPTELIAVEGTEILQDFQNKTDGQV
SGEAIKMFPTIKTPEAGTVITTADEIELEGATQWPHSTSASATYGVEAGVVPWLSPQTSE
RPTLSSSPEINPETQAALIRGQDSTIAASEQQVAARILDSNDQATVNPVEFNTEVATPPF
SLLETSNETDFLIGINEESVEGTAIYLPGPDRCKMNPCLNGGTCYPTETSYVCTCVPGYS
GDQCELDFDECHSNPCRNGATCVDGFNTFRCLCLPSYVGALCEQDTETCDYGWHKFQGQC
YKYFAHRRTWDAAERECRLQGAHLTSILSHEEQMFVNRVGHDYQWIGLNDKMFEHDFRWT
DGSTLQYENWRPNQPDSFFSAGEDCVVIIWHENGQWNDVPCNYHLTYTCKKGTVACGQPP
VVENAKTFGKMKPRYEINSLIRYHCKDGFIQRHLPTIRCLGNGRWAIPKITCMNPSAYQR
TYSMKYFKNSSSAKDNSINTSKHDHRWSRRWQESRR*
>ENSG00000138802|ENST00000265175
MSAPAGSSHPAASARIPPKFGGAAVSGAAAPAGPGAGPAPHQQNGPAQNQMQVPSGYGLH
HQNYIAPSGHYSQGPGKMTSLPLDTQCGDYYSALYTVPTQNVTPNTVNQQPGAQQLYSRG
PPAPHIVGSTLGSFQGAASSASHLHTSASQPYSSFVNHYNSPAMYSASSSVASQGFPSTC
GHYAMSTVSNAAYPSVSYPSLPAGDTYGQMFTSQNAPTVRPVKDNSFSGQNTAISHPSPL
PPLPSQQHHQQQSLSGYSTLTWSSPGLPSTQDNLIRNHTGSLAVANNNPTITVADSLSCP
VMQNVQPPKSSPVVSTVLSGSSGSSSTRTPPTANHPVEPVTSVTQPSELLQQKGVQYGEY
VNNQASSAPTPLSSTSDDEEEEEEDEEAGVDSSSTTSSASPMPNSYDALEGGSYPDMLSS
SASSPAPDPAPEPDPASAPAPASAPAPVVPQPSKMAKPFGYGYPTLQPGYQNATAPLISG
VQPSNPVYSGFQQYPQQYPGVNQLSSSIGGLSLQSSPQPESLRPVNLTQERNILPMTPVW
APVPNLNADLKKLNCSPDSFRCTLTNIPQTQALLNKAKLPLGLLLHPFRDLTQLPVITSN
TIVRCRSCRTYINPFVSFIDQRRWKCNLCYRVNDVPEEFMYNPLTRSYGEPHKRPEVQNS
TVEFIASSDYMLRPPQPAVYLFVLDVSHNAVEAGYLTILCQSLLENLDKLPGDSRTRIGF
MTFDSTIHFYNLQEGLSQPQMLIVSDIDDVFLPTPDSLLVNLYESKELIKDLLNALPNMF
TNTRETHSALGPALQAAFKLMSPTGGRVSVFQTQLPSLGAGLLQSREDPNQRSSTKVVQH
LGPATDFYKKLALDCSGQQTAVDLFLLSSQYSDLASLACMSKYSAGCIYYYPSFHYTHNP
SQAEKLQKDLKRYLTRKIGFEAVMRIRCTKGLSMHTFHGNFFVRSTDLLSLANINPDAGF
AVQLSIEESLTDTSLVCFQTALLYTSSKGERRIRVHTLCLPVVSSLADVYAGVDVQAAIC
LLANMAVDRSVSSSLSDARDALVNAVVDSLSAYGSTVSNLQHSALMAPSSLKLFPLYVLA
LLKQKAFRTGTSTRLDDRVYAMCQIKSQPLVHLMKMIHPNLYRIDRLTDEGAVHVNDRIV
PQPPLQKLSAEKLTREGAFLMDCGSVFYIWVGKGCDNNFIEDVLGYTNFASIPQKMTHLP
ELDTLSSERARSFITWLRDSRPLSPILHIVKDESPAKAEFFQHLIEDRTEAAFSYYEFLL

HVQQQICK*
>ENSG00000105402|ENST00000263354
MDNSGKEAEAMALLAEAERKVKNSQSFFSGLFGGSSKIEEACEIYARAANMFKMAKNWSA
AGNAFCQAAQLHLQLQSKHDAATCFVDAGNAFKKADPQEAINCLMRAIEIYTDMGRFTIA
AKHHISIAEIYETELVDIEKAIAHYEQSADYYKGEESNSSANKCLLKVAGYAALLEQYQK
AIDIYEQVGTNAMDSPLLKYSAKDYFFKAALCHFCIDMLNAKLAVQKYEELFPAFSDSRE
CKLMKKLLEAHEEQNVDSYTESVKEYDSISRLDQWLTTMLLRIKKTIQGDEEDLR*
>ENSG00000070614|ENST00000261797
MPALACLRRLCRHVSPQAVLFLLFIFCLFSVFISAYYLYGWKRGLEPSADAPEPDCGDPP
PVAPSRLLPLKPVQAATPSRTDPLVLVFVESLYSQLGQEVVAILESSRFKYRTEIAPGKG
DMPTLTDKGRGRFALIIYENILKYVNLDAWNRELLDKYCVAYGVGIIGFFKANENSLLSA
QLKGFPLFLHSNLGLKDCSINPKSPLLYVTRPSEVEKGVLPGEDWTVFQSNHSTYEPVLL
AKTRSSESIPHLGADAGLHAALHATVVQDLGLHDGIQRVLFGNNLNFWLHKLVFVDAVAF
LTGKRLSLPLDRYILVDIDDIFVGKEGTRMKVEDVKALFDTQNELRAHIPNFTFNLGYSG
KFFHTGTNAEDAGDDLLLSYVKEFWWFPHMWSHMQPHLFHNQSVLAEQMALNKKFAVEHG
IPTDMGYAVAPHHSGVYPVHVQLYEAWKQVWSIRVTSTEEYPHLKPARYRRGFIHNGIMV
LPRQTCGLFTHTIFYNEYPGGSSELDKIINGGELFLTVLLNPISIFMTHLSNYGNDRLGL
YTFKHLVRFLHSWTNLRLQTLPPVQLAQKYFQIFSEEKDPLWQDPCEDKRHKDIWSKEKT
CDRFPKLLIIGPQKTGTTALYLFLGMHPDLSSNYPSSETFEEIQFFNGHNYHKGIDWYME
FFPIPSNTTSDFYFEKSANYFDSEVAPRRAAALLPKAKVLTILINPADRAYSWYQHQRAH
DDPVALKYTFHEVITAGSDASSKLRALQNRCLVPGWYATHIERWLSAYHANQILVLDGKL
LRTEPAKVMDMVQKFLGVTNTIDYHKTLAFDPKKGFWCQLLEGGKTKCLGKSKGRKYPEM
DLDSRAFLKDYYRDHNIELSKLLYKMGQTLPTWLREDLQNTR*
>ENSG00000140564|ENST00000268171
MELRPWLLWVVAATGTLVLLAADAQGQKVFTNTWAVRIPGGPAVANSVARKHGFLNLGQI
FGDYYHFWHRGVTKRSLSPHRPRHSRLQREPQVQWLEQQVAKRRTKRDVYQEPTDPKFPQ
QWYLSGVTQRDLNVKAAWAQGYTGHGIVVSILDDGIEKNHPDLAGNYDPGASFDVNDQDP
DPQPRYTQMNDNRHGTRCAGEVAAVANNGVCGVGVAYNARIGGVRMLDGEVTDAVEARSL
GLNPNHIHIYSASWGPEDDGKTVDGPARLAEEAFFRGVSQGRGGLGSIFVWASGNGGREH
DSCNCDGYTNSIYTLSISSATQFGNVPWYSEACSSTLATTYSSGNQNEKQIVTTDLRQKC
TESHTGTSASAPLAAGIIALTLEANKNLTWRDMQHLVVQTSKPAHLNANDWATNGVGRKV
SHSYGYGLLDAGAMVALAQNWTTVAPQRKCIIDILTEPKDIGKRLEVRKTVTACLGEPNH
ITRLEHAQARLTLSYNRRGDLAIHLVSPMGTRSTLLAARPHDYSADGFNDWAFMTTHSWD
EDPSGEWVLEIENTSEANNYGTLTKFTLVLYGTAPEGLPVPPESSGCKTLTSSQACVVCE
EGFSLHQKSCVQHCPPGFAPQVLDTHYSTENDVETIRASVCAPCHASCATCQGPALTDCL
SCPSHASLDPVEQTCSRQSQSSRESPPQQQPPRLPPEVEAGQRLRAGLLPSHLPEVVAGL
SCAFIVLVFVTVFLVLQLRSGFSFRGVKVYTMDRGLISYKGLPPEAWQEECPSDSEEDEG
RGERTAFIKDQSAL*
>ENSG00000124406|ENST00000264449
MPTMRRTVSEIRSRAEGYEKTDDVSEKTSLADQEEVRTIFINQPQLTKFCNNHVSTAKYN
IITFLPRFLYSQFRRAANSFFLFIALLQQIPDVSPTGRYTTLVPLLFILAVAAIKEIIED
IKRHKADNAVNKKQTQVLRNGAWEIVHWEKVNVGDIVIIKGKEYIPADTVLLSSSEPQAM
CYIETSNLDGETNLKIRQGLPATSDIKDVDSLMRISGRIECESPNRHLYDFVGNIRLDGH
GTVPLGADQILLRGAQLRNTQWVHGIVVYTGHDTKLMQNSTSPPLKLSNVERITNVQILI

LFCILIAMSLVCSVGSAIWNRRHSGKDWYLNLNYGGASNFGLNFLTFIILFNNLIPISLL
VTLEVVKFTQAYFINWDLDMHYEPTDTAAMARTSNLNEELGQVKYIFSDKTGTLTCNVMQ
FKKCTIAGVAYGQNSQFGDEKTFSDSSLLENLQNNHPTAPIICEFLTMMAVCHTAVPERE
GDKIIYQAASPDEGALVRAAKQLNFVFTGRTPDSVIIDSLGQEERYELLNVLEFTSARKR
MSVIVRTPSGKLRLYCKGADTVIYDRLAETSKYKEITLKHLEQFATEGLRTLCFAVAEIS
ESDFQEWRAVYQRASTSVQNRLLKLEESYELIEKNLQLLGATAIEDKLQDQVPETIETLM
KADIKIWILTGDKQETAINIGHSCKLLKKNMGMIVINEGSLDGTRETLSRHCTTLGDALR
KENDFALIIDGKTLKYALTFGVRQYFLDLALSCKAVICCRVSPLQKSEVVEMVKKQVKVV
TLAIGDGANDVSMIQTAHVGVGISGNEGLQAANSSDYSIAQFKYLKNLLMIHGAWNYNRV
SKCILYCFYKNIVLYIIEIWFAFVNGFSGQILFERWCIGLYNVMFTAMPPLTLGIFERSC
RKENMLKYPELYKTSQNALDFNTKVFWVHCLNGLFHSVILFWFPLKALQYGTAFGNGKTS
DYLLLGNFVYTFVVITVCLKAGLETSYWTWFSHIAIWGSIALWVVFFGIYSSLWPAIPMA
PDMSGEAAMLFSSGVFWMGLLFIPVASLLLDVVYKVIKRTAFKTLVDEVQELEAKSQDPG
AVVLGKSLTERAQLLKNVFKKNHVNLYRSESLQQNLLHGYAFSQDENGIVSQSEVIRAYD
TTKQRPDEW*
>ENSG00000129993|ENST00000268679
MPASRLRDRAASSASGSTCGSMSQTHPVLESGLLASAGCSAPRGPRKGGPAPVDRKAKAS
AMPDSPAEVKTQPRSTPPSMPPPPPAASQGATRPPSFTPHTHREDGPATLPHGRFHGCLK
WSMVCLLMNGSSHSPTAINGAPCTPNGFSNGPATSSTASLSTQHLPPACGARQLSKLKRF
LTTLQQFGSDISPEIGERVRTLVLGLVNSTLTIEEFHSKLQEATNFPLRPFVIPFLKANL
PLLQRELLHCARLAKQTPAQYLAQHEQLLLDASASSPIDSSELLLEVNENGKRRTPDRTK
ENGSDRDPLHPEHLSKRPCTLNPAQRYSPSNGPPQPTPPPHYRLEDIAMAHHFRDAYRHP
DPRELRERHRPLVVPGSRQEEVIDHKLTEREWAEEWKHLNNLLNCIMDMVEKTRRSLTVL
RRCQEADREELNHWARRYSDAEDTKKGPAPAAARPRSSSAGPEGPQLDVPREFLPRTLTG
YVPEDIWRKAEEAVNEVKRQAMSELQKAVSDAERKAHELITTERAKMERALAEAKRQASE
DALTVINQQEDSSESCWNCGRKASETCSGCNAARYCGSFCQHRDWEKHHHVCGQSLQGPT
AVVADPVPGPPEAAHSLGPSLPVGAASPSEAGSAGPSRPGSPSPPGPLDTVPR*
>ENSG00000103489|ENST00000261381
MVAAPCARRLARRSHSALLAALTVLLLQTLVVVWNFSSLDSGAGERRGGAAVGGGEQPPPA
PAPRRERRDLPAEPAAARGGGGGGGGGGGGRGPQARARGGGPGEPRGQQPASRGALPARA
LDPHPSPLITLETQDGYFSHRPKEKVRTDSNNENSVPKDFENVDNSNFAPRTQKQKHQPE
LAKKPPSRQKELLKRKLEQQEKGKGHTFPGKGPGEVLPPGDRAAANSSHGKDVSRPPHAR
KTGGSSPETKYDQPPKCDISGKEAISALSRAKSKHCRQEIGETYCRHKLGLLMPEKVTRF
CPLEGKANKNVQWDEDSVEYMPANPVRIAFVLVVHGRASRQLQRMFKAIYHKDHFYYIHV
DKRSNYLHRQVLQVSRQYSNVRVTPWRMATIWGGASLLSTYLQSMRDLLEMTDWPWDFFI
NLSAADYPIRTNDQLVAFLSRYRDMNFLKSHGRDNARFIRKQGLDRLFLECDAHMWRLGD
RRIPEGIAVDGGSDWFLLNRRFVEYVTFSTDDLVTKMKQFYSYTLLPAESFFHTVLENSP
HCDTMVDNNLRITNWNRKLGCKCQYKHIVDWCGCSPNDFKPQDFHRFQQTARPTFFARKF
EAVVNQEIIGQLDYYLYGNYPAGTPGLRSYWENVYDEPDGIHSLSDVTLTLYHSFARLGL
RRAETSLHTDGENSCRYYPMGHPASVHLYFLADRFQGFLIKHHATNLAVSKLETLETWVM
PKKVFKIASPPSDFGRLQFSEVGTDWDAKERLFRNFGGLLGPMDEPVGMQKWGKGPNVTV
TVIWVDPVNVIAATYDILIESTAEFTHYKPPLNLPLRPGVWTVKILHHWVPVAETKFLVA
PLTFSNRQPIKPEEALKLHNGPLRNAYMEQSFQSLNPVLSLPINPAQVEQARRNAASTGT
ALEGWLDSLVGGMWTAMDICATGPTACPVMQTCSQTAWSSFSPDPKSELGAVKPDGRLR*

>ENSG00000149541|ENST00000265471
MKLKLKNVFLAYFLVSIAGLLYALVQLGQPCDCLPPLRAAAEQLRQKDLRISQLQAELRR
PPPAPAQPPEPEALPTIYVVTPTYARLVQKAELVRLSQTLSLVPRLHWLLVEDAEGPTPL
VSGLLAASGLLFTHLVVLTPKAQRLREGEPGWVHPRGVEQRNKALDWLRGRGGAVGGEKD
PPPPGTQGVVYFADDDNTYSRELFEEMRWTRGVSVWPVGLVGGLRFEGPQVQDGRVVGFH
TAWEPSRPFPVDMAGFAVALPLLLDKPNAQFDSTAPRGHLESSLLSHLVDPKDLEPRAAN
CTRVLVWHTRTEKPKMKQEEQLQRQGRGSDPAIEV*
>ENSG00000008710|ENST00000262304
MPPAAPARLALALGLGLWLGALAGGPGRGCGPCEPPCLCGPAPGAACRVNCSGRGLRTLG
PALRIPADATALDVSHNLLRALDVGLLANLSALAELDISNNKISTLEEGIFANLFNLSEI
NLSGNPFECDCGLAWLPRWAEEQQVRVVQPEAATCAGPGSLAGQPLLGIPLLDSGCGEEY
VACLPDNSSGTVAAVSFSAAHEGLLQPEACSAFCFSTGQGLAALSEQGWCLCGAAQPSSA
SFACLSLCSGPPPPPAPTCRGPTLLQHVFPASPGATLVGPHGPLASGQLAAFHIAAPLPV
TATRWDFGDGSAEVDAAGPAASHRYVLPGRYHVTAVLALGAGSALLGTDVQVEAAPAALE
LVCPSSVQSDESLDLSIQNRGGSGLEAAYSIVALGEEPARAVHPLCPSDTEIFPGNGHCY
RLVVEKAAWLQAQEQCQAWAGAALAMVDSPAVQRFLVSRVTRSLDVWIGFSTVQGVEVGP
APQGEAFSLESCQNWLPGEPHPATAEHCVRLGPTGWCNTDLCSAPHSYVCELQPGGPVQD
AENLLVGAPSGDLQGPLTPLAQQDGLSAPHEPVEVMVFPGLRLSREAFLTTAEFGTQELR
RPAQLRLQVYRLLSTAGTPENGSEPESRSPDNRTQLAPACMPGGRWCPGANICLPLDASC
HPQACANGCTSGPGLPGAPYALWREFLFSVPAGPPAQYSVTLHGQDVLMLPGDLVGLQHD
AGPGALLHCSPAPGHPGPRAPYLSANASSWLPHLPAQLEGTWACPACALRLLAATEQLTV
LLGLRPNPGLRLPGRYEVRAEVGNGVSRHNLSCSFDVVSPVAGLRVIYPAPRDGRLYVPT
NGSALVLQVDSGANATATARWPGGSVSARFENVCPALVATFVPGCPWETNDTLFSVVALP
WLSEGEHVVDVVVENSASRANLSLRVTAEEPICGLRATPSPEARVLQGVLVRYSPVVEAG
SDMVFRWTINDKQSLTFQNVVFNVIYQSAAVFKLSLTASNHVSNVTVNYNVTVERMNRMQ
GLQVSTVPAVLSPNATLALTAGVLVDSAVEVAFLWTFGDGEQALHQFQPPYNESFPVPDP
SVAQVLVEHNVMHTYAAPGEYLLTVLASNAFENLTQQVPVSVRASLPSVAVGVSDGVLVA
GRPVTFYPHPLPSPGGVLYTWDFGDGSPVLTQSQPAANHTYASRGTYHVRLEVNNTVSGA
AAQADVRVFEELRGLSVDMSLAVEQGAPVVVSAAVQTGDNITWTFDMGDGTVLSGPEATV
EHVYLRAQNCTVTVGAASPAGHLARSLHVLVFVLEVLRVEPAACIPTQPDARLTAYVTGN
PAHYLFDWTFGDGSSNTTVRGCPTVTHNFTRSGTFPLALVLSSRVNRAHYFTSICVEPEV
GNVTLQPERQFVQLGDEAWLVACAWPPFPYRYTWDFGTEEAAPTRARGPEVTFIYRDPGS
YLVTVTASNNISAANDSALVEVQEPVLVTSIKVNGSLGLELQQPYLFSAVGRGRPASYLW
DLGDGGWLEGPEVTHAYNSTGDFTVRVAGWNEVSRSEAWLNVTVKRRVRGLVVNASRTVV
PLNGSVSFSTSLEAGSDVRYSWVLCDRCTPIPGGPTISYTFRSVGTFNIIVTAENEVGSA
QDSIFVYVLQLIEGLQVVGGGRYFPTNHTVQLQAVVRDGTNVSYSWTAWRDRGPALAGSG
KGFSLTVLEAGTYHVQLRATNMLGSAWADCTMDFVEPVGWLMVAASPNPAAVNTSVTLSA
ELAGGSGVVYTWSLEEGLSWETSEPFTTHSFPTPGLHLVTMTAGNPLGSANATVEVDVQV
PVSGLSIRASEPGGSFVAAGSSVPFWGQLATGTNVSWCWAVPGGSSKRGPHVTMVFPDAG
TFSIRLNASNAVSWVSATYNLTAEEPIVGLVLWASSKVVAPGQLVHFQILLAAGSAVTFR
LQVGGANPEVLPGPRFSHSFPRVGDHVVSVRGKNHVSWAQAQVRIVVLEAVSGLQVPNCC
EPGIATGTERNFTARVQRGSRVAYAWYFSLQKVQGDSLVILSGRDVTYTPVAAGLLEIQV
RAFNALGSENRTLVLEVQDAVQYVALQSGPCFTNRSAQFEAATSPSPRRVAYHWDFGDGS
PGQDTDEPRAEHSYLRPGDYRVQVNASNLVSFFVAQATVTVQVLACREPEVDVVLPLQVL

MRRSQRNYLEAHVDLRDCVTYQTEYRWEVYRTASCQRPGRPARVALPGVDVSRPRLVLPR
LALPVGHYCFVFVVSFGDTPLTQSIQANVTVAPERLVPIIEGGSYRVWSDTRDLVLDGSE
SYDPNLEDGDQTPLSFHWACVASTQREAGGCALNFGPRGSSTVTIPRERLAAGVEYTFSL
TVWKAGRKEEATNQTVLIRSGRVPIVSLECVSCKAQAVYEVSRSSYVYLEGRCLNCSSGS
KRGRWAARTFSNKTLVLDETTTSTGSAGMRLVLRRGVLRDGEGYTFTLTVLGRSGEEEGC
ASIRLSPNRPPLGGSCRLFPLGAVHALTTKVHFECTGWHDAEDAGAPLVYALLLRRCRQG
HCEEFCVYKGSLSSYGAVLPPGFRPHFEVGLAVVVQDQLGAAVVALNRSLAITLPEPNGS
ATGLTVWLHGLTASVLPGLLRQADPQHVIEYSLALVTVLNEYERALDVAAEPKHERQHRA
QIRKNITETLVSLRVHTVDDIQQIAAALAQCMGPSRELVCRSCLKQTLHKLEAMMLILQA
ETTAGTVTPTAIGDSILNITGDLIHLASSDVRAPQPSELGAESPSRMVASQAYNLTSALM
RILMRSRVLNEEPLTLAGEEIVAQGKRSDPRSLLCYGGAPGPGCHFSIPEAFSGALANLS
DVVQLIFLVDSNPFPFGYISNYTVSTKVASMAFQTQAGAQIPIERLASERAITVKVPNNS
DWAARGHRSSANSANSVVVQPQASVGAVVTLDSSNPAAGLHLQLNYTLLDGHYLSEEPEP
YLAVYLHSEPRPNEHNCSASRRIRPESLQGADHRPYTFFISPGSRDPAGSYHLNLSSHFR
WSALQVSVGLYTSLCQYFSEEDMVWRTEGLLPLEETSPRQAVCLTRHLTAFGASLFVPPS
HVRFVFPEPTADVNYIVMLTCAVCLVTYMVMAAILHKLDQLDASRGRAIPFCGQRGRFKY
EILVKTGWGRGSGTTAHVGIMLYGVDSRSGHRHLDGDRAFHRNSLDIFRIATPHSLGSVW
KIRVWHDNKGLSPAWFLQHVIVRDLQTARSAFFLVNDWLSVETEANGGLVEKEVLAASDA
ALLRFRRLLVAELQRGFFDKHIWLSIWDRPPRSRFTRIQRATCCVLLICLFLGANAVWYG
AVGDSAYSTGHVSRLSPLSVDTVAVGLVSSVVVYPVYLAILFLFRMSRSKVAGSPSPTPA
GQQVLDIDSCLDSSVLDSSFLTFSGLHAEQAFVGQMKSDLFLDDSKSLVCWPSGEGTLSW
PDLLSDPSIVGSNLRQLARGQAGHGLGPEEDGFSLASPYSPAKSFSASDEDLIQQVLAEG
VSSPAPTQDTHMETDLLSSLSSTPGEKTETLALQRLGELGPPSPGLNWEQPQAARLSRTG
LVEGLRKRLLPAWCASLAHGLSLLLVAVAVAVSGWVGASFPPGVSVAWLLSSSASFLASF
LGWEPLKVLLEALYFSLVAKRLHPDEDDTLVESPAVTPVSARVPRVRPPHGFALFLAKEE
ARKVKRLHGMLRSLLVYMLFLLVTLLASYGDASCHGHAYRLQSAIKQELHSRAFLAITRS
EELWPWMAHVLLPYVHGNQSSPELGPPRLRQVRLQEALYPDPPGPRVHTCSAAGGFSTSD
YDVGWESPHNGSGTWAYSAPDLLGAWSWGSCAVYDSGGYVQELGLSLEESRDRLRFLQLH
NWLDNRSRAVFLELTRYSPAVGLHAAVTLRLEFPAAGRALAALSVRPFALRRLSAGLSLP
LLTSVCLLLFAVHFAVAEARTWHREGRWRVLRLGAWARWLLVALTAATALVRLAQLGAAD
RQWTRFVRGRPRRFTSFDQVAQLSSAARGLAASLLFLLLVKAAQQLRFVRQWSVFGKTLC
RALPELLGVTLGLVVLGVAYAQLAILLVSSCVDSLWSVAQALLVLCPGTGLSTLCPAESW
HLSPLLCVGLWALRLWGALRLGAVILRWRYHALRGELYRPAWEPQDYEMVELFLRRLRLW
MGLSKVKEFRHKVRFEGMEPLPSRSSRGSKVSPDVPPPSAGSDASHPSTSSSQLDGLSVS
LGRLGTRCEPEPSRLQAVFEALLTQFDRLNQATEDVYQLEQQLHSLQGRRSSRAPAGSSR
GPSPGLRPALPSRLARASRGVDLATGPSRTPLRAKNKVHPSST*
>ENSG00000124333|ENST00000286448
MAILFAVVARGTTILAKHAWCGGNFLEVTEQILAKIPSENNKLTYSHGNYLFHYICQDRI
VYLCITDDDFERSRAFNFLNEIKKRFQTTYGSRAQTALPYAMNSEFSSVLAAQLKHHSEN
KGLDKVMETQAQVDELKGIMVRNIDLVAQRGERLELLIDKTENLVDSSVTFKTTSRNLAR
AMCMKNLKLTIIIIIVSIVFIYIIVSPLCGGFTWPSCVKK*
>ENSG00000113384|ENST00000265070
MTSLTQRSSGLVQRRTEASRNAADKERAAGGGAGSSEDDAQSRRDEQDDDDKGDSKETRL
TLMEEVLLLGLKDREGYTSFWNDCISSGLRGCMLIELALRGRLQLEACGMRRKSLLTRKV

ICKSDAPTGDVLLDEALKHVKETQPPETVQNWIELLSGETWNPLKLHYQLRNVRERLAKN
LVEKGVLTTEKQNFLLFDMTTHPLTNNNIKQRLIKKVQEAVLDKWVNDPHRMDRRLLALI
YLAHASDVLENAFAPLLDEQYDLATKRVRQLLDLDPEVECLKANTNEVLWAVVAAFTK*
>ENSG00000084733|ENST00000264710
MAKKTYDLLFKLLLIGDSGVGKTCVLFRFSDDAFNTTFISTIGIDFKIKTVELQGKKIKL
QIWDTAGQERFHTITTSYYRGAMGIMLVYDITNGKSFENISKWLRNIDEHANEDVERMLL
GNKCDMDDKRVVPKGKGEQIAREHGIRFFETSAKANINIEKAFLTLAEDILRKTPVKEPN
SENVDISSGGGVTGWKSKCC*
>ENSG00000143786|ENST00000272133
MAFTFAAFCYMLSLVLCAALIFFAIWHIIAFDELRTDFKSPIDQCNPVHARERLRNIERI
CFLLRKLVLPEYSIHSLFCIMFLCAQEWLTLGLNVPLLFYHFWRYFHCPADSSELAYDPP
VVMNADTLSYCQKEAWCKLAFYLLSFFYYLYCMIYTLVSS*
>ENSG00000148400|ENST00000277541
MPPLLAPLLCLALLPALAARGPRCSQPGETCLNGGKCEAANGTEACVCGGAFVGPRCQDP
NPCLSTPCKNAGTCHVVDRRGVADYACSCALGFSGPLCLTPLDNACLTNPCRNGGTCDLL
TLTEYKCRCPPGWSGKSCQQADPCASNPCANGGQCLPFEASYICHCPPSFHGPTCRQDVN
ECGQKPGLCRHGGTCHNEVGSYRCVCRATHTGPNCERPYVPCSPSPCQNGGTCRPTGDVT
HECACLPGFTGQNCEENIDDCPGNNCKNGGACVDGVNTYNCRCPPEWTGQYCTEDVDECQ
LMPNACQNGGTCHNTHGGYNCVCVNGWTGEDCSENIDDCASAACFHGATCHDRVASFYCE
CPHGRTGLLCHLNDACISNPCNEGSNCDTNPVNGKAICTCPSGYTGPACSQDVDECSLGA
NPCEHAGKCINTLGSFECQCLQGYTGPRCEIDVNECVSNPCQNDATCLDQIGEFQCICMP
GYEGVHCEVNTDECASSPCLHNGRCLDKINEFQCECPTGFTGHLCQYDVDECASTPCKNG
AKCLDGPNTYTCVCTEGYTGTHCEVDIDECDPDPCHYGSCKDGVATFTCLCRPGYTGHHC
ETNINECSSQPCRHGGTCQDRDNAYLCFCLKGTTGPNCEINLDDCASSPCDSGTCLDKID
GYECACEPGYTGSMCNINIDECAGNPCHNGGTCEDGINGFTCRCPEGYHDPTCLSEVNEC
NSNPCVHGACRDSLNGYKCDCDPGWSGTNCDINNNECESNPCVNGGTCKDMTSGYVCTCR
EGFSGPNCQTNINECASNPCLNQGTCIDDVAGYKCNCLLPYTGATCEVVLAPCAPSPCRN
GGECRQSEDYESFSCVCPTGWQGQTCEVDINECVLSPCRHGASCQNTHGGYRCHCQAGYS
GRNCETDIDDCRPNPCHNGGSCTDGINTAFCDCLPGFRGTFCEEDINECASDPCRNGANC
TDCVDSYTCTCPAGFSGIHCENNTPDCTESSCFNGGTCVDGINSFTCLCPPGFTGSYCQH
DVNECDSQPCLHGGTCQDGCGSYRCTCPQGYTGPNCQNLVHWCDSSPCKNGGKCWQTHTQ
YRCECPSGWTGLYCDVPSVSCEVAAQRQGVDVARLCQHGGLCVDAGNTHHCRCQAGYTGS
YCEDLVDECSPSPCQNGATCTDYLGGYSCKCVAGYHGVNCSEEIDECLSHPCQNGGTCLD
LPNTYKCSCPRGTQGVHCEINVDDCNPPVDPVSRSPKCFNNGTCVDQVGGYSCTCPPGFV
GERCEGDVNECLSNPCDARGTQNCVQRVNDFHCECRAGHTGRRCESVINGCKGKPCKNGG
TCAVASNTARGFICKCPAGFEGATCENDARTCGSLRCLNGGTCISGPRSPTCLCLGPFTG
PECQFPASSPCLGGNPCYNQGTCEPTSESPFYRCLCPAKFNGLLCHILDYSFGGGAGRDI
PPPLIEEACELPECQEDAGNKVCSLQCNNHACGWDGGDCSLNFNDPWKNCTQSLQCWKYF
SDGHCDSQCNSAGCLFDGFDCQRAEGQCNPLYDQYCKDHFSDGHCDQGCNSAECEWDGLD
CAEHVPERLAAGTLVVVVLMPPEQLRNSSFHFLRELSRVLHTNVVFKRDAHGQQMIFPYY
GREEELRKHPIKRAAEGWAAPDALLGQVKASLLPGGSEGGRRRRELDPMDVRGSIVYLEI
DNRQCVQASSQCFQSATDVAAFLGALASLGSLNIPYKIEAVQSETVEPPPPAQLHFMYVA
AAAFVLLFFVGCGVLLSRKRRRQHGQLWFPEGFKVSEASKKKRREPLGEDSVGLKPLKNA
SDGALMDDNQNEWGDEDLETKKFRFEEPVVLPDLDDQTDHRQWTQQHLDAADLRMSAMAP

TPPQGEVDADCMDVNVRGPDGFTPLMIASCSGGGLETGNSEEEEDAPAVISDFIYQGASL
HNQTDRTGETALHLAARYSRSDAAKRLLEASADANIQDNMGRTPLHAAVSADAQGVFQIL
IRNRATDLDARMHDGTTPLILAARLAVEGMLEDLINSHADVNAVDDLGKSALHWAAAVNN
VDAAVVLLKNGANKDMQNNREETPLFLAAREGSYETAKVLLDHFANRDITDHMDRLPRDI
AQERMHHDIVRLLDEYNLVRSPQLHGAPLGGTPTLSPPLCSPNGYLGSLKPGVQGKKVRK
PSSKGLACGSKEAKDLKARRKKSQDGKGCLLDSSGMLSPVDSLESPHGYLSDVASPPLLP
SPFQQSPSVPLNHLPGMPDTHLGIGHLNVAAKPEMAALGGGGRLAFETGPPRLSHLPVAS
GTSTVLGSSSGGALNFTVGGSTSLNGQCEWLSRLQSGMVPNQYNPLRGSVAPGPLSTQAP
SLQHGMVGPLHSSLAASALSQMMSYQGLPSTRLATQPHLVQTQQVQPQNLQMQQQNLQPA
NIQQQQSLQPPPPPPQPHLGVSSAASGHLGRSFLSGEPSQADVQPLGPSSLAVHTILPQE
SPALPTSLPSSLVPPVTAAQFLTPPSQHSYSSPVDNTPSHQLQVPEHPFLTPSPESPDQW
SSSSPHSNVSDWSEGVSSPPTSMQSQIARIPEAFK*
>ENSG00000066777|ENST00000262215
MYEGKKTKNMFLTRALEKILADKEVKKAHHSQLRKACEVALEEIKAETEKQSPPHGEAKA
GSSTLPPVKSKTNFIEADKYFLPFELACQSKCPRIVSTSLDCLQKLIAYGHLTGNAPDST
TPGKKLIDRIIETICGCFQGPQTDEGVQLQIIKALLTAVTSQHIEIHEGTVLQAVRTCYN
IYLASKNLINQTTAKATLTQMLNVIFARMENQALQEAKQMEKERHRQHHHLLQSPVSHHE
PESPQLRYLPPQTVDHISQEHEGDLDLHTNDVDKSLQDDTEPENGSDISSAENEQTEADQ
ATAAETLSKNEVLYDGENHDCEEKPQDIVQNIVEEMVNIVVGDMGEGTTINASADGNIGT
IEDGSDSENIQANGIPGTPISVAYTPSLPDDRLSVSSNDTQESGNSSGPSPGAKFSHILQ
KDAFLVFRSLCKLSMKPLSDGPPDPKSHELRSKILSLQLLLSILQNAGPIFRTNEMFINA
IKQYLCVALSKNGVSSVPEVFELSLSIFLTLLSNFKTHLKMQIEVFFKEIFLYILETSTS
SFDHKWMVIQTLTRICADAQSVVDIYVNYDCDLNAANIFERLVNDLSKIAQGRGSQELGM
SNVQELSLRKKGLECLVSILKCMVEWSKDQYVNPNSQTTLGQEKPSEQEMSEIKHPETIN
RYGSLNSLESTSSSGIGSYSTQMSGTDNPEQFEVLKQQKEIIEQGIDLFNKKPKRGIQYL
QEQGMLGTTPEDIAQFLHQEERLDSTQVGEFLGDNDKFNKEVMYAYVDQHDFSGKDFVSA
LRMFLEGFRLPGEAQKIDRLMEKFAARYLECNQGQTLFASADTAYVLAYSIIMLTTDLHS
PQVKNKMTKEQYIKMNRGINDSKDLPEEYLSAIYNEIAGKKISMKETKELTIPTKSSKQN
VASEKQRRLLYNLEMEQMAKTAKALMEAVSHVQAPFTSATHLEHVRPMFKLAWTPFLAAF
SVGLQDCDDTEVASLCLEGIRCAIRIACIFSIQLERDAYVQALARFTLLTVSSGITEMKQ
KNIDTIKTLITVAHTDGNYLGNSWHEILKCISQLELAQLIGTGVKPRYISGTVRGREGSL
TGTKDQAPDEFVGLGLVGGNVDWKQIASIQESIGETSSQSVVVAVDRIFTGSTRLDGNAI
VDFVRWLCAVSMDELLSTTHPRMFSLQKIVEISYYNMGRIRLQWSRIWEVIGDHFNKVGC
NPNEDVAIFAVDSLRQLSMKFLEKGELANFRFQKDFLRPFEHIMKRNRSPTIRDMVVRCI
AQMVNSQAANIRSGWKNIFSVFHLAASDQDESIVELAFQTTGHIVTLVFEKHFPATIDSF
QDAVKCLSEFACNAAFPDTSMEAIRLIRHCAKYVSDRPQAFKEYTSDDMNVAPEDRVWVR
GWFPILFELSCIINRCKLDVRTRGLTVMFEIMKTYGHTYEKHWWQDLFRIVFRIFDNMKL
PEQQTEKAEWMTTTCNHALYAICDVFTQYLEVLSDVLLDDIFAQLYWCVQQDNEQLARSG
TNCLENVVILNGEKFTLEIWDKTCNCTLDIFKTTIPHALLTWRPNSGETAPPPPSPVSEK
PLDTISQKSVDIHDSIQPRSVDNRPQAPLVSASAVNEEVSKIKSTAKFPEQKLFAALLIK
CVVQLELIQTIDNIVFFPATSKKEDAENLAAAQRDAVDFDVRVDTQDQGMYRFLTSQQLF
KLLDCLLESHRFAKAFNSNNEQRTALWKAGFKGKSKPNLLKQETSSLACGLRILFRMYMD
ESRVSAWEEVQQRLLNVCSEALSYFLTLTSESHREAWTNLLLLFLTKVLKISDNRFKAHA
SFYYPLLCEIMQFDLIPELRAVLRRFFLRIGVVFQISQPPEQELGINKQ*

>ENSG00000147119|ENST00000276055
MKGRRRRRREYCKFALLLVLYTLVLLLVPSVLDGGRDGDKGAEHCPGLQRSLGVWSLEAA
AAGEREQGAEARAAEEGGANQSPRFPSNLSGAVGEAVSREKQHIYVHATWRTGSSFLGEL
FNQHPDVFYLYEPMWHLWQALYPGDAESLQGALRDMLRSLFRCDFSVLRLYAPPGDPAAR
APDTANLTTAALFRWRTNKVICSPPLCPGAPRARAEVGLVEDTACERSCPPVAIRALEAE
CRKYPVVVIKDVRLLDLGVLVPLLRDPGLNLKVVQLFRDPRAVHNSRLKSRQGLLRESIQ
VLRTRQRGDRFHRVLLAHGVGARPGGQSRALPAAPRADFFLTGALEVICEAWLRDLLFAR
GAPAWLRRRYLRLRYEDLVRQPRAQLRRLLRFSGLRALAALDAFALNMTRGAAYGADRPF
HLSARDAREAVHAWRERLSREQVRQVEAACAPAMRLLAYPRSGEEGDAEQPREGETPLEM
DADGAT*
>ENSG00000138600|ENST00000261854
MGPQRRLSPAGAALLWGFLLQLTAAQEAILHASGNGTTKDYCMLYNPYWTALPSTLENAT
SISLMNLTSTPLCNLSDIPPVGIKSKAVVVPWGSCHFLEKARIAQKGGAEAMLVVNNSVL
FPPSGNRSEFPDVKILIAFISYKDFRDMNQTLGDNITVKMYSPSWPNFDYTMVVIFVIAV
FTVALGGYWSGLVELENLKAVTTEDREMRKKKEEYLTFSPLTVVIFVVICCVMMVLLYFF
YKWLVYVMIAIFCIASAMSLYNCLAALIHKIPYGQCTIACRGKNMEVRLIFLSGLCIAVA
VVWAVFRNEDRWAWILQDILGIAFCLNLIKTLKLPNFKSCVILLGLLLLYDVFFVFITPF
ITKNGESIMVELAAGPFGNNEKLPVVIRVPKLIYFSVMSVCLMPVSILGFGDIIVPGLLI
AYCRRFDVQTGSSYIYYVSSTVAYAIGMILTFVVLVLMKKGQPALLYLVPCTLITASVVA
WRRKEMKKFWKGNSYQMMDHLDCATNEENPVISGEQIVQQ*
>ENSG00000151746|ENST00000281474
MAAEEVLQTVDHYKTEIERLTKELTETTHEKIQAAEYGLVVLEEKLTLKQQYDELEAEYD
SLKQELEQLKEAFGQSFSIHRKVAEDGETREETLLQESASKEAYYLGKILEMQNELKQSR
AVVTNVQAENERLTAVVQDLKENNEMVELQRIRMKDEIREYKFREARLLQDYTELEEENI
TLQKLVSTLKQNQVEYEGLKHEIKRFEEETVLLNSQLEDAIRLKEIAEHQLEEALETLKN
EREQKNNLRKELSQYISLNDNHISISVDGLKFAEDGSEPNNDDKMNGHIHGPLVKLNGDY
RTPTLRKGESLNPVSDLFSELNISEIQKLKQQLMQVEREKAILLANLQESQTQLEHTKGA
LTEQHERVHRLTEHVNAMRGLQSSKELKAELDGEKGRDSGEEAHDYEVDINGLEILECKY
RVAVTEVIDLKAEIKALKEKYNKSVENYTDEKAKYESKIQMYDEQVTSLEKTTKESGEKM
AHMEKELQKMTSIANENHSTLNTAQDELVTFSEELAQLYHHVCLCNNETPNRVMLDYYRQ
SRVTRSGSLKGPDDPRGLLSPRLARRGVSSPVETRTSSEPVAKESTEASKEPSPTKTPTI
SPVITAPPSSPVLDTSDIRKEPMNIYNLNAIIRDQIKHLQKAVDRSLQLSRQRAAARELA
PMIDKDKEALMEEILKLKSLLSTKREQIATLRAVLKANKQTAEVALANLKNKYENEKAMV
TETMTKLRNELKALKEDAATFSSLRAMFATRCDEYVTQLDEMQRQLAAAEDEKKTLNTLL
RMAIQQKLALTQRLEDLEFDHEQSRRSKGKLGKSKIGSPKVSGEASVTVPTIDTYLLHSQ
GPQTPNIRVSSGTQRKRQFSPSLCDQSRPRTSGASYLQNLLRVPPDPTSTESFLLKGPPS
MSEFIQGHRLSKEKRLTVAPPDCQQPAASVPPQCSQLAGRQDCPTVSPDTALPEEQPHSS
SQCAPLHCLSKPPHP*
>ENSG00000115526|ENST00000264249
MHHQWLLLAACFWVIFMFMVASKFITLTFKDPDVYSAKQEFLFLTTMPEVRKLPEEKHIP
EELKPTGKELPDSQLVQPLVYMERLELIRNVCRDDALKNLSHTPVSKFVLDRIFVCDKHK
ILFCQTPKVGNTQWKKVLIVLNGAFSSIEEIPENVVHDHEKNGLPRLSSFSDAEIQKRLK
TYFKFFIVRDPFERLISAFKDKFVHNPRFEPWYRHEIAPGIIRKYRRNRTETRGIQFEDF
VRYLGDPNHRWLDLQFGDHIIHWVTYVELCAPCEIMYSVIGHHETLEDDAPYILKEAGID

HLVSYPTIPPGITVYNRTKVEHYFLGISKRDIRRLYARFEGDFKLFGYQKPDFLLN*
>ENSG00000114988|ENST00000264963
MAATLGPLGSWQQWRRCLSARDGSRMLLLLLLLGSGQGPQQVGAGQTFEYLKREHSLSKP
YQGVGTGSSSLWNLMGNAMVMTQYIRLTPDMQSKQGALWNRVPCFLRDWELQVHFKIHGQ
GKKNLHGDGLAIWYTKDRMQPGPVFGNMDKFVGLGVFVDTYPNEEKQQERVFPYISAMVN
NGSLSYDHERDGRPTELGGCTAIVRNLHYDTFLVIRYVKRHLTIMMDIDGKHEWRDCIEV
PGVRLPRGYYFGTSSITGDLSDNHDVISLKLFELTVERTPEEEKLHRDVFLPSVDNMKLP
EMTAPLPPLSGLALFLIVFFSLVFSVFAIVIGIILYNKWQEQSRKRFY*
>ENSG00000110075|ENST00000265636
MFWKFDLHSSSHIDTLLEREDVTLKELMDEEDVLQECKAQNRKLIEFLLKAECLEDLVSF
IIEEPPQDMDEKIRYKYPNISCELLTSDVSQMNDRLGEDESLLMKLYSFLLNDSPLNPLL
ASFFSKVLSILISRKPEQIVDFLKKKHDFVDLIIKHIGTSAIMDLLLRLLTCIEPPQPRQ
DVLNWLNEEKIIQRLVEIVHPSQEEDRHSNASQSLCEIVRLSRDQMLQIQNSTEPDPLLA
TLEKQEIIEQLLSNIFHKEKNESAIVSAIQILLTLLETRRPTFEGHIEICPPGMSHSACS
VNKSVLEAIRGRLGSFHELLLEPPKNMFFKYTWNNFLHTQVEICIALILASPFENTENAT
ITDQDSTGDNLLLKHLFQKCQLIERILEAWEMNEKKQAEGGRRHGYMGHLTRIANCIVHS
TDKGPNSALVQQLIKDLPDEVRERWETFCTSSLGETNKRNTVDLAFSDYQMQQMTSNFID
QFGFNDEKFADQDDIGNVSFDRVSDINFTLNTNESGNIALFEACCKERIQQFDDGGSDEE
DIWEEKHIAFTPESQRRSSSGSTDSEESTDSEEEDGAKQDLFEPSSANTEDKMEVDLSEP
PNWSANFDVPMETTHGAPLDSVGSDVWSTEEPMPTKETGWASFSEFTSSLSTKDSLRSNS
PVEMETSTEPMDPLTPSAAALAVQPEAAGSVAMEASSDGEEDAESTDKVTETVMNGGMKE
TLSLTVDAKTETAVFKSEEGKLSTSQDAACKDAEECPETAEAKCAAPRPPSSSPEQRTGQ
PSAPGDTSVNGPV*
>ENSG00000157782|ENST00000288616
MGNCVKYPLRNLSRKMCQEEQTSYMVVQTSEEGLAADAELPGPLLMLAQNCAVMHNLLGP
ACIFLRKGFAENRQPDRSLRPEEIEELREAFREFDKDKDGYINCRDLGNCMRTMGYMPTE
MELIELSQQINMNLGGHVDFDDFVELMGPKLLAETADMIGVKELRDAFREFDTNGDGEIS
TSELREAMRKLLGHQVGHRDIEEIIRDVDLNGDGRVDFEEFVRMMSR*
>ENSG00000146352|ENST00000275162
MTHLQAGLSPETLEKARLELNENPDTLHQDIQEVRDMVITRPDIGFLRTDDAFILRFLRA
RKFHHFEAFRLLAQYFEYRQQNLDMFKSFKATDPGIKQALKDGFPGGLANLDHYGRKILV
LFAANWDQSRYTLVDILRAILLSLEAMIEDPELQVNGFVLIIDWSNFTFKQASKLTPSML
RLAIEGLQDSFPARFGGIHFVNQPWYIHALYTVIRPFLKEKTRKRIFLHGNNLNSLHQLI
HPEILPSEFGGMLPPYDMGTWARTLLDHEYDDDSEYNVDSYSMPVKEVEKELSPKSMKRS
QSVVDPTVLKRMDKNEEENMQPLLSLD*
>ENSG00000154764|ENST00000285018
MNRKARRCLGHLFLSLGMVYLRIGGFSSVVALGASIICNKIPGLAPRQRAICQSRPDAII
VIGEGSQMGLDECQFQFRNGRWNCSALGERTVFGKELKVGSREAAFTYAIIAAGVAHAIT
AACTQGNLSDCGCDKEKQGQYHRDEGWKWGGCSADIRYGIGFAKVFVDAREIKQNARTLM
NLHNNEAGRKILEENMKLECKCHGVSGSCTTKTCWTTLPQFRELGYVLKDKYNEAVHVEP
VRASRNKRPTFLKIKKPLSYRKPMDTDLVYIEKSPNYCEEDPVTGSVGTQGRACNKTAPQ
ASGCDLMCCGRGYNTHQYARVWQCNCKFHWCCYVKCNTCSERTEMYTCK*
>ENSG00000152127|ENST00000281923
MALFTPWKLSSQKLGFFLVTFGFIWGMMLLHFTIQQRTQPESSSMLREQILDLSKRYIKA

LAEENRNVVDGPYAGVMTAYDLKKTLAVLLDNILQRIGKLESKVDNLVVNGTGTNSTNST
TAVPSLVALEKINVADIINGAQEKCVLPPMDGYPHCEGKIKWMKDMWRSDPCYADYGVDG
STCSFFIYLSEVENWCPHLPWRAKNPYEEADHNSLAEIRTDFNILYSMMKKHEEFRWMRL
RIRRMADAWIQAIKSLAEKQNLEKRKRKKVLVHLGLLTKESGFKIAETAFSGGPLGELVQ
WSDLITSLYLLGHDIRISASLAELKEIMKKVVGNRSGCPTVGDRIVELIYIDIVGLAQFK
KTLGPSWVHYQCMLRVLDSFGTEPEFNHANYAQSKGHKTPWGKWNLNPQQFYTMFPHTPD
NSFLGFVVEQHLNSSDIHHINEIKRQNQSLVYGKVDSFWKNKKIYLDIIHTYMEVHATVY
GSSTKNIPSYVKNHGILSGRDLQFLLRETKLFVGLGFPYEGPAPLEAIANGCAFLNPKFN
PPKSSKNTDFFIGKPTLRELTSQHPYAEVFIGRPHVWTVDLNNQEEVEDAVKAILNQKIE
PYMPYEFTCEGMLQRINAFIEKQDFCHGQVMWPPLSALQVKLAEPGQSCKQVCQESQLIC
EPSFFQHLNKDKDMLKYKVTCQSSELAKDILVPSFDPKNKHCVFQGDLLLFSCAGAHPRH
QRVCPCRDFIKGQVALCKDCL*
>ENSG00000153395|ENST00000283415
MRLRGCGPRAAPASSAGASDARLLAPPGRNPFVHELRLSALQKAQVALMTLTLFPVRLLV
AAAMMLLAWPLALVASLGSAEKEPEQPPALWRKVVDFLLKAIMRTMWFAGGFHRVAVKGR
QALPTEAAILTLAPHSSYFDAIPVTMTMSSIVMKAESRDIPIWGTLIQYIRPVFVSRSDQ
DSRRKTVEEIKRRAQSNGKWPQIMIFPEGTCTNRTCLITFKPGAFIPGAPVQPVVLRYPN
KLDTITWTWQGPGALEILWLTLCQFHNQVEIEFLPVYSPSEEEKRNPALYASNVRRVMAE
ALGVSVTDYTFEDCQLALAEGQLRLPADTCLLEFARLVRGLGLKPEKLEKDLDRYSERAR
MKGGEKIGIAEFAASLEVPVSDLLEDMFSLFDESGSGEVDLRECVVALSVVCRPARTLDT
IQLAFKMYGAQEDGSVGEGDLSCILKTALGVAELTVTDLFRAIDQEEKGKITFADFHRFA
EMYPAFAEEYLYPDQTHFESCAETSPAPIPNGFCADFSPENSDAGRKPVRKKLD*
>ENSG00000147127|ENST00000276066
MSAFGHDEAWMEAGGFGLEAAERTEYQSLCKSKLLFLGEQSGKTSIISRFMYNSFGCACQ
ATVGIDFLSKTMYLEDQIVQLQLWDTAGQERFHSLIPSYIRDSTIAVVVYDITNINSFKE
TDKWVEHVRAERGDDVVIMLLGNKIDLDNKRQVTAEQGEEKSRNLNVMFIETSAKTGYNV
KKLFRRVASALLSTRTSPPPKEGTVEIELESFEESGNRSYC*
>ENSG00000157064|ENST00000287713
MTETTKTHVILLACGSFNPITKGHIQMFERARDYLHKTGRFIVIGGIVSPVHDSYGKQGL
VSSRHRLIMCQLAVQNSDWIRVDPWECYQDTWQTTCSVLEHHRDLMKRVTGCILSNVNTP
SMTPVIGQPQNETPQPIYQNSNVATKPTAAKILGKVGESLSRICCVRPPVERFTFVDENA
NLGTVMRYEEIELRILLLCGSDLLESFCIPGLWNEADMEVIVGDFGIVVVPRDAADTDRI
MNHSSILRKYKNNIMVVKDDINHPMSVVSSTKSRLALQHGDGHVVDYLSQPVIDYILKSQ
LYINASG*
>ENSG00000095139|ENST00000264028
MVLLAAAVCTKAGKAIVSRQFVEMTRTRIEGLLAAFPKLMNTGKQHTFVETESVRYVYQP
MEKLYMVLITTKNSNILEDLETLRLFSRVIPEYCRALEENEISEHCFDLIFAFDEIVALG
YRENVNLAQIRTFTEMDSHEEKVFRAVRETQEREAKAEMRRKAKELQQARRDAERQGKKA
PGFGGFGSSAVSGGSTAAMITETIIETDKPKVAPAPARPSGPSKALKLGAKGKEVDNFVD
KLKSEGETIMSSSMGKRTSEATKMHAPPINMESVHMKIEEKITLTCGRDGGLQNMELHGM
IMLRISDDKYGRIRLHVENEDKKGVQLQTHPNVDKKLFTAESLIGLKNPEKSFPVNSDVG
VLKWRLQTTEESFIPLTINCWPSESGNGCDVNIEYELQEDNLELNDVVITIPLPSGVGAP
VIGEIDGEYRHDSRRNTLEWCLPVIDAKNKSGSLEFSIAGQPNDFFPVQVSFVSKKNYCN
IQVTKVTQVDGNSPVRFSTETTFLVDKYEIL*

>ENSG00000064687|ENST00000263094
MAFWTQLMLLLWKNFMYRRRQPVQLLVELLWPLFLFFILVAVRHSHPPLEHHECHFPNKP
LPSAGTVPWLQGLICNVNNTCFPQLTPGEEPGRLSNFNDSLVSRLLADARTVLGGASAHR
TLAGLGKLIATLRAARSTAQPQPTKQSPLEPPMLDVAELLTSLLRTESLGLALGQAQEPL
HSLLEAAEDLAQELLALRSLVELRALLQRPRGTSGPLELLSEALCSVRGPSSTVGPSLNW
YEASDLMELVGQEPESALPDSSLSPACSELIGALDSHPLSRLLWRRLKPLILGKLLFAPD
TPFTRKLMAQVNRTFEELTLLRDVREVWEMLGPRIFTFMNDSSNVAMLQRLLQMQDEGRR
QPRPGGRDHMEALRSFLDPGSGGYSWQDAHADVGHLVGTLGRVTECLSLDKLEAAPSEAA
LVSRALQLLAEHRFWAGVVFLGPEDSSDPTEHPTPDLGPGHVRIKIRMDIDVVTRTNKIR
DRFWDPGPAADPLTDLRYVWGGFVYLQDLVERAAVRVLSGANPRAGLYLQQMPYPCYVDD
VFLRVLSRSLPLFLTLAWIYSVTLTVKAVVREKETRLRDTMRAMGLSRAVLWLGWFLSCL
GPFLLSAALLVLVLKLGDILPYSHPGVVFLFLAAFAVATVTQSFLLSAFFSRANLAAACG
GLAYFSLYLPYVLCVAWRDRLPAGGRVAASLLSPVAFGFGCESLALLEEQGEGAQWHNVG
TRPTADVFSLAQVSGLLLLDAALYGLATWYLEAVCPGQYGIPEPWNFPFRRSYWCGPRPP
KSPAPCPTPLDPKVLVEEAPPGLSPGVSVRSLEKRFPGSPQPALRGLSLDFYQGHITAFL
GHNGAGKTTTLSILSGLFPPSGGSAFILGHDVRSSMAAIRPHLGVCPQYNVLFDMLTVDE
HVWFYGRLKGLSAAVVGPEQDRLLQDVGLVSKQSVQTRHLSGGMQRKLSVAIAFVGGSQV
VILDEPTAGVDPASRRGIWELLLKYREGRTLILSTHHLDEAELLGDRVAVVAGGRLCCCG
SPLFLRRHLGSGYYLTLVKARLPLTTNEKADTDMEGSVDTRQEKKNGSQGSRVGTPQLLA
LVQHWVPGARLVEELPHELVLVLPYTGAHDGSFATLFRELDTRLAELRLTGYGISDTSLE
EIFLKVVEECAADTDMEDGSCGQHLCTGIAGLDVTLRLKMPPQETALENGEPAGSAPETD
QGSGPDAVGRVQGWALTRQQLQALLLKRFLLARRSRRGLFAQIVLPALFVGLALVFSLIV
PPFGHYPALRLSPTMYGAQVSFFSEDAPGDPGRARLLEALLQEAGLEEPPVQHSSHRFSA
PEVPAEVAKVLASGNWTPESPSPACQCSRPGARRLLPDCPAAAGGPPPPQAVTGSGEVVQ
NLTGRNLSDFLVKTYPRLVRQGLKTKKWVNEVRYGGFSLGGRDPGLPSGQELGRSVEELW
ALLSPLPGGALDRVLKNLTAWAHSLDAQDSLKIWFNNKGWHSMVAFVNRASNAILRAHLP
PGPARHAHSITTLNHPLNLTKEQLSEGALMASSVDVLVSICVVFAMSFVPASFTLVLIEE
RVTRAKHLQLMGGLSPTLYWLGNFLWDMCNYLVPACIVVLIFLAFQQRAYVAPANLPALL
LLLLLYGWSITPLMYPASFFFSVPSTAYVVLTCINLFIGINGSMATFVLELFSDQKLQEV
SRILKQVFLIFPHFCLGRGLIDMVRNQAMADAFERLGDRQFQSPLRWEVVGKNLLAMVIQ
GPLFLLFTLLLQHRSQLLPQPRVRSLPLLGEEDEDVARERERVVQGATQGDVLVLRNLTK
VYRGQRMPAVDRLCLGIPPGECFGLLGVNGAGKTSTFRMVTGDTLASRGEAVLAGHSVAR
EPSAAHLSMGYCPQSDAIFELLTGREHLELLARLRGVPEAQVAQTAGSGLARLGLSWYAD
RPAGTYSGGNKRKLATALALVGDPAVVFLDEPTTGMDPSARRFLWNSLLAVVREGRSVML
TSHSMEECEALCSRLAIMVNGRFRCLGSPQHLKGRFAAGHTLTLRVPAARSQPAAAFVAA
EFPGAELREAHGGRLRFQLPPGGRCALARVFGELAVHGAEHGVEDFSVSQTMLEEVFLYF
SKDQGKDEDTEEQKEAGVGVDPAPGLQHPKRVSQFLDDPSTAETVL*
>ENSG00000159128|ENST00000290219
MRPTLLWSLLLLLGVFAAAAAAPPDPLSQLPAPQHPKIRLYNAEQVLSWEPVALSNSTRP
VVYQVQFKYTDSKWFTADIMSIGVNCTQITATECDFTAASPSAGFPMDFNVTLRLRAELG
ALHSAWVTMPWFQHYRNVTVGPPENIEVTPGEGSLIIRFSSPFDIADTSTAFFCYYVHYW
EKGGIQQVKGPFRSNSISLDNLKPSRVYCLQVQAQLLWNKSNIFRVGHLSNISCYETMAD
ASTELQQVILISVGTFSLLSVLAGACFFLVLKYRGLIKYWFHTPPSIPLQIEEYLKDPTQ
PILEALDKDSSPKDDVWDSVSIISFPEKEQEDVLQTL*

>ENSG00000103043|ENST00000261776
MNPEKDFAPLTPNIVRALNDKLYEKRKVAALEIEKLVREFVAQNNTVQIKHVIQTLSQEF
ALSQHPHSRKGGLIGLAACSIALGKDSGLYLKELIEPVLTCFNDADSRLRYYACEALYNI
VKVARGAVLPHFNVLFDGLSKLAADPDPNVKSGSELLDRLLKDIVTESNKFDLVSFIPLL
RERIYSNNQYARQFIISWILVLESVPDINLLDYLPEILDGLFQILGDNGKEIRKMCEVVL
GEFLKEIKKNPSSVKFAEMANILVIHCQTTDDLIQLTAMCWMREFIQLAGRVMLPYSSGI
LTAVLPCLAYDDRKKSIKEVANVCNQSLMKLVTPEDDELDELRPGQRQAEPTPDDALPKQ
EGTASGGPDGSCDSSFSSGISVFTAASTERAPVTLHLDGIVQVLNCHLSDTAIGMMTRIA
VLKWLYHLYIKTPRKMFRHTDSLFPILLQTLSDESDEVILKDLEVLAEIASSPAGQTDDP
GPLDGPDLQASHSELQVPTPGRAGLLNTSGTKGLECSPSTPTMNSYFYKFMINLLKRFSS
ERKLLEVRGPFIIRQLCLLLNAENIFHSMADILLREEDLKFASTMVHALNTILLTSTELF
QLRNQLKDLKTLESQNLFCCLYRSWCHNPVTTVSLCFLTQNYRHAYDLIQKFGDLEVTVD
FLAEVDKLVQLIECPIFTYLRLQLLDVKNNPYLIKALYGLLMLLPQSSAFQLLSHRLQCV
PNPELLQTEDSLKAAPKSQKADSPSIDYAELLQHFEKVQNKHLEVRHQRSGRGDHLDRRV
VL*
>ENSG00000122254|ENST00000261374
MAYRVLGRAGPPQPRRARRLLFAFTLSLSCTYLCYSFLCCCDDLGRSRLLGAPRCLRGPS
AGGQKLLQKSRPCDPSGPTPSEPSAPSAPAAAVPAPRLSGSNHSGSPKLGTKRLPQALIV
GVKKGGTRAVLEFIRVHPDVRALGTEPHFFDRNYGRGLDWYRSLMPRTLESQITLEKTPS
YFVTQEAPRRIFNMSRDTKLIVVVRNPVTRAISDYTQTLSKKPDIPTFEGLSFRNRTLGL
VDVSWNAIRIGMYVLHLESWLQYFPLAQIHFVSGERLITDPAGEMGRVQDFLGIKRFITD
KHFYFNKTKGFPCLKKTESSLLPRCLGKSKGRTHVQIDPEVIDQLREFYRPYNIKFYETV
GQDFRWE*
>ENSG00000120805|ENST00000261636
MGGFFSSIFSSLFGTREMRILILGLDGAGKTTILYRLQVGEVVTTIPTIGFNVETVTYKN
LKFQVWDLGGQTSIRPYWRCYYSNTDAVIYVVDSCDRDRIGISKSELVAMLEEEELRKAI
LVVFANKQDMEQAMTSSEMANSLGLPALKDRKWQIFKTSATKGTGLDEAMEWLVETLKSR
Q*
>ENSG00000124333|ENST00000262640
MAILFAVVARGTTILAKHAWCGGNFLEVTEQILAKIPSENNKLTYSHGNYLFHYICQDRI
VYLCITDDDFERSRAFNFLNEIKKRFQTTYGSRAQTALPYAMNSEFSSVLAAQLKHHSEN
KGLDKVMETQAQVDELKGIMVRNIVCHLQNYQQKSCSSHVYEEPQAHYYHHHRINCVHLY
HCFTSLWWIYMAKLCEEIGKKKLPLTKDMREQGVKSNPCDSSLSHTDRWYLPVSSTLFSL
FKILFHASRFIFVLSTSLFL*
>ENSG00000114646|ENST00000264723
MGRAGGGGPGRGPPPLLLFLGAALVLASGAVPAREAGSAVEAEELVKGSPAWEPPANDTR
EEAGPPAAGEDEASWTAPGGELAGPEEVLQESAAVTGTAWLEADSPGLGGVTAEAGSGDA
QALPATLQAPHEVLGQSIMPPAIPEATEASGPPSPTPGDKLSPASELPKESPLEVWLNLG
GSTPDPQGPELTYPFQGTLEPQPASDIIDIDYFEGLDGEGRGADLGSFPGSPGTSENHPD
TEGETPSWSLLDLYDDFTPFDESDFYPTTSFYDDLDEEEEEEEDDKDAVGGGDLEDENEL
LVPTGKPGLGPGTGQPTSRWHAVPPQHTLGSVPGSSIALRPRPGEPGRDLASSENGTECR
SGFVRHNGSCRSVCDLFPSYCHNGGQCYLVENIGAFCRCNTQDYIWHKGMRCESIITDFQ
VMCVAVGSAALVLLLLFMMTVFFAKKLYLLKTENTKLRRTNKFRTPSELHNDNFSLSTIA
EGSHPNDDPSAPHKIQEVLKSCLKEEESFNIQNSMSPKLEGGKGDQADLDVNCLQNNLT*

>ENSG00000149926|ENST00000279389
MASTAGYIVSTSCKHIIDDQHWLSSAYTQFAVPYFIYDIYAMFLCHWHKHQVKGHGGDDG
AARAPGSTWAIARGYLHKEFLMVLHHAAMVLVCFPLSVVWRQGKGDFFLGCMLMAEVSTP
FVCLGKILIQYKQQHTLLHKVNGALMLLSFLCCRVLLFPYLYWAYGRHAGLPLLAVPLAI
PAHVNLGAALLLAPQLYWFFLICRGACRLFWPRSRPPPACQAQD*
>ENSG00000139182|ENST00000266546
MTLLLLPLLLASLLASCSCNKANKHKPWIEAEYQGIVMENDNTVLLNPPLFALDKDAPLR
YAGEICGFRLHGSGVPFEAVILDKATGEGLIRAKEPVDCEAQKEHTFTIQAYDCGEGPDG
ANTKKSHKATVHVRVNDVNEFAPVFVERLYRAAVTEGKLYDRILRVEAIDGDCSPQYSQI
CYYEILTPNTPFLIDNDGNIENTEKLQYSGERLYKFTVTAYDCGKKRAADDAEVEIQVKP
TCKPSWQGWNKRIEYAPGAGSLALFPGIRLETCDEPLWNIQATIELQTSHVAKGCDRDNY
SERALRKLCGAATGEVDLLPMPGPNANWTAGLSVHYSQDSSLIYWFNGTQAVQVPLGGPS
GLGSGPQDSLSDHFTLSFWMKHGVTPNKGKKEEETIVCNTVQNEDGFSHYSLTVHGCRIA
FLYWPLLESARPVKFLWKLEQVCDDEWHHYALNLEFPTVTLYTDGISFDPALIHDNGLIH
PPRREPALMIGACWTEEKNKEKEKGDNSTDTTQGDPLSIHHYFHGYLAGFSVRSGRLESR
EVIECLYACREGLDYRDFESLGKGMKVHVNPSQSLLTLEGDDVETFNHALQHVAYMNTLR
FATPGVRPLRLTTAVKCFSEESCVSIPEVEGYVVVLQPDAPQILLSGTAHFARPAVDFEG
TNGVPLFPDLQITCSISHQVEAKKDESWQGTVTDTRMSDEIVHNLDGCEISLVGDDLDPE
RESLLLDTTSLQQRGLELTNTSAYLTIAGVESITVYEEILRQARYRLRHGAALYTRKFRL
SCSEMNGRYSSNEFIVEVNVLHSMNRVAHPSHVLSSQQFLHRGHQPPPEMAGHSLASSHR
NSMIPSAATLIIVVCVGFLVLMVVLGLVRIHSLHRRVSGAGGPPGASSDPKDPDLFWDDS
ALTIIVNPMESYQNRQSCVTGAVGGQQEDEDSSDSEVADSPSSDERRIIETPPHRY*
>ENSG00000149823|ENST00000279281
MAAAAAAGPSPGSGPGDSPEGPEGEAPERRRKAHGMLKLYYGLSEGEAAGRPAGPDPLDP
TDLNGAHFDPEVYLDKLRRECPLAQLMDSETDMVRQIRALDSDMQTLVYENYNKFISATD
TIRKMKNDFRKMEDEMDRLATNMAVITDFSARISATLQDRHERITKLAGVHALLRKLQFL
FELPSRLTKCVELGAYGQAVRYQGRAQAVLQQYQHLPSFRAIQDDCQVITARLAQQLRQR
FREGGSGAPEQAECVELLLALGEPAEELCEEFLAHARGRLEKELRNLEAELGPSPPAPDV
LEFTDHGGSGFVGGLCQVAAAYQELFAAQGPAGAEKLAAFARQLGSRYFALVERRLAQEQ
GGGDNSLLVRALDRFHRRLRAPGALLAAAGLADAATEIVERVARERLGHHLQGLRAAFLG
CLTDVRQALAAPRVAGKEGPGLAELLANVASSILSHIKASLAAVHLFTAKEVSFSNKPYF
RGEFCSQGVREGLIVGFVHSMCQTAQSFCDSPGEKGGATPPALLLLLSRLCLDYETATIS
YILTLTDEQFLVQDQFPVTPVSTLCAEARETARRLLTHYVKVQGLVISQMLRKSVETRDW
LSTLEPRNVRAVMKRVVEDTTAIDVQVGLLYEEGVRKAQSSDSSKRTFSVYSSSRQQGRY
APSYTPSAPMDTNLLSNIQKLFSERIDVFSPVEFNKVSVLTGIIKISLKTLLECVRLRTF
GRFGLQQVQVDCHFLQLYLWRFVADEELVHLLLDEVVASAALRCPDPVPMEPSVVEVICE
RG*
>ENSG00000140497|ENST00000268099
MSAFDTNPFADPVDVNPFQDPSVTQLTNAPQGGLAEFNPFSETNAATTVPVTQLPGSSQP
AVLQPSVEPTQPTPQAVVSAAQAGLLRQQEELDRKAAELERKERELQNTVANLHVRQNNW
PPLPSWCPVKPCFYQDFSTEIPADYQRICKMLYYLWMLHSVTLFLNLLACLAWFSGNSSK
GVDFGLSILWFLIFTPCAFLCWYRPIYKAFRSDNSFSFFVFFFVFFCQIGIYIIQLVGIP
GLGDSGWIAALSTLDNHSLAISVIMMVVAGFFTLCAVLSVFLLQRVHSLYRRTGASFQQA
QEEFSQGIFSSRTFHRAASSAAQGAFQGN*

>ENSG00000138768|ENST00000264904
MNFLRGVMGGQSAGPQHTEAETIQKLCDRVASSTLLDDRRNAVRALKSLSKKYRLEVGIQ
AMEHLIHVLQTDRSDSEIIGYALDTLYNIISNEEEEEVDDVEEENSTRQSEDLGSQFTEI
FIKQQENVTLLLSLLEEFDFHVRWPGVKLLTSLLKQLGPQVQQIILVSPMGVSRLMDLLA
DSREVIRNDGVLLLQALTRSNGAIQKIVAFENAFERLLDIISEEGNSDGGIVVEDCLILL
QNLLKNNNSNQNFFKEGSYIQRMKPWFEVGDENSGWSAQKVTNLHLMLQLVRVLVSPTNP
PGATSSCQKAMFQCGLLQQLCTILMATGVPADILTETINTVSEVIRGCQVNQDYFASVNA
PSNPPRPAIVVLLMSMVNERQPFVLRCAVLYCFQCFLYKNQKGQGEIVSTLLPSTIDATG
NSVSAGQLLCGGLFSTDSLSNWCAAVALAHALQENATQKEQLLRVQLATSIGNPPVSLLQ
QCTNILSQGDKIDRRGSKIQTRVGLLMLLCTWLSNCPIAVTHFLHNSANVPFLTGQIAEN
LGEEEQLVQGLCALLLGISIYFNDNSLESYMKEKLKQLIEKRIGKENFIEKLGFISKHEL
YSRASQKPQPNFPSPEYMIFDHEFTKLVKELEGVITKAIYKSSEEDKKEEEVKKTLEQHD
NIVTHYKNMIREQDLQLEELRQQVSTLKCQNEQLQTAVTQQVSQIQQHKDQYNLLKIQLG
KDNQHQGSYSEGAQMNGIQPEEIGRLREEIEELKRNQELLQSQLTEKDSMIENMKSSQTS
GTNEQSSAIVSARDSEQVAELKQELATLKSQLNSQSVEITKLQTEKQELLQKTEAFAKSV
EVQGETETIIATKTTDVEGRLSALLQETKELKNEIKALSEERTAIKEQLDSSNSTIAILQ
TEKDKLELEITDSKKEQDDLLVLLADQDQKILSLKNKLKDLGHPVEEEDELESGDQEDED
DESEDPGKDLDHI*
>ENSG00000158104|ENST00000289004
MTTYSDKGAKPERGRFLHFHSVTFWVGNAKQATSFYCSKMGFEPLAYRGLETGSREVVSH
VIKQGKIVFVLSSALNPWNKEMGDHLVKHGDGVKDIAFEVEDCDYIVQKARERGAKIMRE
PWVEQDKFGKVKFAVLQTYGDTTHTLVEKMNYIGQFLPGYEAPAFMDPLLPKLPKCSLEM
IDHIVGNQPDQEMVSASEWYLKNLQFHRFWSVDDTQVHTEYSSLRSIVVANYEESIKMPI
NEPAPGKKKSQIQEYVDYNGGAGVQHIALKTEDIITAIRHLRERGLEFLSVPSTYYKQLR
EKLKTAKIKVKENIDALEELKILVDYDEKGYLLQIFTKPVQDRPTLFLEVIQRHNHQGFG
AGNFNSLFKAFEEEQNLRGNLTNMETNGVVPGM*
>ENSG00000160216|ENST00000291572
MGLLAFLKTQFVLHLLVGFVFVVSGLVINFVQLCTLALWPVSKQLYRRLNCRLAYSLWSQ
LVMLLEWWSCTECTLFTDQATVERFGKEHAVIILNHNFEIDFLCGWTMCERFGVLGSSKV
LAKKELLYVPLIGWTWYFLEIVFCKRKWEEDRDTVVEGLRRLSDYPEYMWFLLYCEGTRF
TETKHRVSMEVAAAKGLPVLKYHLLPRTKGFTTAVKCLRGTVAAVYDVTLNFRGNKNPSL
LGILYGKKYEADMCVRRFPLEDIPLDEKEAAQWLHKLYQEKDALQEIYNQKGMFPGEQFK
PARRPWTLLNFLSWATILLSPLFSFVLGVFASGSPLLILTFLGFVGAASFGVRRLIGVTE
IEKGSSYGNQEFKKKE*
>ENSG00000115020|ENST00000264380
MATDDKTSPTLDSANDLPRSPTSPSHLTHFKPLTPDQDEPPFKSAYSSFVNLFRFNKERA
EGGQGEQQPLSGSWTSPQLPSRTQSVRSPTPYKKQLNEELQRRSSALDTRRKAEPTFGGH
DPRTAVQLRSLSTVLKRLKEIMEGKSQDSDLKQYWMPDSQCKECYDCSEKFTTFRRRHHC
RLCGQIFCSRCCNQEIPGKFMGYTGDLRACTYCRKIALSYAHSTDSNSIGEDLNALSDSA
CSVSVLDPSEPRTPVGSRKASRNIFLEDDLAWQSLIHPDSSNTPLSTRLVSVQEDAGKSP
ARNRSASITNLSLDRSGSPMVPSYETSVSPQANRTYVRTETTEDERKILLDSVQLKDLWK
KICHHSSGMEFQDHRYWLRTHPNCIVGKELVNWLIRNGHIATRAQAIAIGQAMVDGRWLD
CVSHHDQLFRDEYALYRPLQSTEFSETPSPDSDSVNSVEGHSEPSWFKDIKFDDSDTEQI
AEEGDDNLANSASPSKRTSVSSFQSTVDSDSAASISLNVELDNVNFHIKKPSKYPHVPPH

PADQKEYLISDTGGQQLSISDAFIKESLFNRRVEEKSKELPFTPLGWHHNNLELLREENG
EKQAMERLLSANHNHMMALLQQLLHSDSLSSSWRDIIVSLVCQVVQTVRPDVKNQDDDMD
IRQFVHIKKIPGGKKFDSVVVNGFVCTKNIAHKKMSSCIKNPKILLLKCSIEYLYREETK
FTCIDPIVLQEREFLKNYVQRIVDVRPTLVLVEKTVSRIAQDMLLEHGITLVINVKSQVL
ERISRMTQGDLVMSMDQLLTKPHLGTCHKFYMQIFQLPNEQTKTLMFFEGCPQHLGCTIK
LRGGSDYELARVKEILIFMICVAYHSQLEISFLMDEFAMPPTLMQNPSFHSLIEGRGHEG
AVQEQYGGGSIPWDPDIPPESLPCDDSSLLELRIVFEKGEQENKNLPQAVASVKHQEHST
TACPAGLPCAFFAPVPESLLPLPVDDQQDALGSEQPETLQQTVVLQDPKSQIRAFRDPLQ
DDTGLYVTEEVTSSEDKRKTYSLAFKQELKDVILCISPVITFREPFLLTEKGMRCSTRDY
FAEQVYWSPLLNKEFKEMENRRKKQLLRDLSGLQGMNGSIQAKSIQVLPSHELVSTRIAE
HLGDSQSLGRMLADYRARGGRIQPKNSDPFAHSKDASSTSSGQSGSKNEGDEERGLILSD
AVWSTKVDCLNPINHQRLCVLFSSSSAQSSNAPSACVSPWIVTMEFYGKNDLTLGIFLER
YCFRPSYQCPSMFCDTPMVHHIRRFVHGQGCVQIILKELDSPVPGYQHTILTYSWCRICK
QVTPVVALSNESWSMSFAKYLELRFYGHQYTRRANAEPCGHSIHHDYHQYFSYNQMVASF
SYSPIRLLEVCVPLPKIFIKRQAPLKVSLLQDLKDFFQKVSQVYVAIDERLASLKTDTFS
KTREEKMEDIFAQKEMEEGEFKNWIEKMQARLMSSSVDTPQQLQSVFESLIAKKQSLCEV
LQAWNNRLQDLFQQEKGRKRPSVPPSPGRLRQGEESKISAMDASPRNISPGLQNGEKEDR
FLTTLSSQSSTSSTHLQLPTPPEVMSEQSVGGPPELDTASSSEDVFDGHLLGSTDSQVKE
KSTMKAIFANLLPGNSYNPIPFPFDPDKHYLMYEHERVPIAVCEKEPSSIIAFALSCKEY
RNALEELSKATQWNSAEEGLPTNSTSDSRPKSSSPIRLPEMSGGQTNRTTETEPQPTKKA
SGMLSFFRGTAGKSPDLSSQKRETLRGADSAYYQVGQTGKEGTENQGVEPQDEVDGGDTQ
KKQLINPHVELQFSDANAKFYCRLYYAGEFHKMREVILDSSEEDFIRSLSHSSPWQARGG
KSGAAFYATEDDRFILKQMPRLEVQSFLDFAPHYFNYITNAVQQKRPTALAKILGVYRIG
YKNSQNNTEKKLDLLVMENLFYGRKMAQVFDLKGSLRNRNVKTDTGKESCDVVLLDENLL
KMVRDNPLYIRSHSKAVLRTSIHSDSHFLSSHLIIDYSLLVGRDDTSNELVVGIIDYIRT
FTWDKKLEMVVKSTGILGGQGKMPTVVSPELYRTRFCEAMDKYFLMVPDHWTGLGLNC*
>ENSG00000063660|ENST00000264039
MELRARGWWLLCAAAALVACARGDPASKSRSCGEVRQIYGAKGFSLSDVPQAEISGEHLR
ICPQGYTCCTSEMEENLANRSHAELETALRDSSRVLQAMLATQLRSFDDHFQHLLNDSER
TLQATFPGAFGELYTQNARAFRDLYSELRLYYRGANLHLEETLAEFWARLLERLFKQLHP
QLLLPDDYLDCLGKQAEALRPFGEAPRELRLRATRAFVAARSFVQGLGVASDVVRKVAQV
PLGPECSRAVMKLVYCAHCLGVPGARPCPDYCRNVLKGCLANQADLDAEWRNLLDSMVLI
TDKFWGTSGVESVIGSVHTWLAEAINALQDNRDTLTAKVIQGCGNPKVNPQGPGPEEKRR
RGKLAPRERPPSGTLEKLVSEAKAQLRDVQDFWISLPGTLCSEKMALSTASDDRCWNGMA
RGRYLPEVMGDGLANQINNPEVEVDITKPDMTIRQQIMQLKIMTNRLRSAYNGNDVDFQD
ASDDGSGSGSGDGCLDDLCSRKVSRKSSSSRTPLTHALPGLSEQEGQKTSAASCPQPPTF
LLPLLLFLALTVARPRWR*
>ENSG00000139266|ENST00000266643
MLKSRLRMFLNELKLLVLTGGGRPRAEPQPRGGRGGGCGWAPFAGCSTRDGDGDEEEYYG
SEPRARGLAGDKEPRAGPLPPPAPPLPPPGALDALSLSSSLDSGLRTPQCRICFQGPEQG
ELLSPCRCDGSVRCTHQPCLIRWISERGSWSCELCYFKYQVLAISTKNPLQWQAISLTVI
EKVQIAAIVLGSLFLVASISWLIWSSLSPSAKWQRQDLLFQICYGMYGFMDVVCIGLIIH
EGSSVYRIFKRWQAVNQQWKVLNYDKTKDIGGDAGGGTAGKSGPRNSRTGPTSGATSRPP
AAQRMRTLLPQRCGYTILHLLGQLRPPDARSSSHSGREVVMRVTTV*

>ENSG00000111481|ENST00000262061
MEALILEPSLYTVKAILILDNDGDRLFAKYYDDTYPSVKEQKAFEKNIFNKTHRTDSEIA
LLEGLTVVYKSSIDLYFYVIGSSYENELMLMAVLNCLFDSLSQMLRKNVEKRALLENMEG
LFLAVDEIVDGGVILESDPQQVVHRVALRGEDVPLTEQTVSQVLQSAKEQIKWSLLR*
>ENSG00000126091|ENST00000262915
MGLLVFVRNLLLALCLFLVLGFLYYSAWKLHLLQWEEDSSKYSHSSSPQEKPVADSVVLS
FDSAGQTLGSEYDRLGFLLNLDSKLSPRTLCTVVFGLDCILESPGEPKKLLMPASHPLEI
LKSLSEDTAFALGFLKLPRPAELATKYANFSEGACKPGYASALMTAIFPRFSKPAPMFLD
DSFRKWARIREFVPPFGIKGQDNLIKAILSVTKEYRLTPALDSLRCRRCIIVGNGGVLAN
KSLGSRIDDYDIVVRLNSAPVKGFEKDVGSKTTLRITYPEGAMQRPEQYERDSLFVLAGF
KWQDFKWLKYIVYKERVSASDGFWKSVATRVPKEPPEIRILNPYFIQEAAFTLIGLPFNN
GLMGRGNIPTLGSVAVTMALHGCDEVAVAGFGYDMSTPNAPLHYYETVRMAAIKESWTHN
IQREKEFLRKLVKARVITDLSSGI*
>ENSG00000100815|ENST00000267622
MSSWLGGLGSGLGQSLGQVGGSLASLTGQISNFTKDMLMEGTEEVEAELPDSRTKEIEAI
HAILRSENERLKKLCTDLEEKHEASEIQIKQQSTSYRNQLQQKEVEISHLKARQIALQDQ
LLKLQSAAQSVPSGAGVPATTASSSFAYGISHHPSAFHDDDMDFGDIISSQQEINRLSNE
VSRLESEVGHWRHIAQTSKAQGTDNSDQSEICKLQNIIKELKQNRSQEIDDHQHEMSVLQ
NAHQQKLTEISRRHREELSDYEERIEELENLLQQGGSGVIETDLSKIYEMQKTIQVLQIE
KVESTKKMEQLEDKIKDINKKLSSAENDRDILRREQEQLNVEKRQIMEECENLKLECSKL
QPSAVKQSDTMTEKERILAQSASVEEVFRLQQALSDAENEIMRLSSLNQDNSLAEDNLKL
KMRIEVLEKEKSLLSQEKEELQMSLLKLNNEYEVIKSTATRDISLDSELHDLRLNLEAKE
QELNQSISEKETLIAEIEELDRQNQEATKHMILIKDQLSKQQNEGDSIISKLKQDLNDEK
KRVHQLEDDKMDITKELDVQKEKLIQSEVALNDLHLTKQKLEDKVENLVDQLNKSQESNV
SIQKENLELKEHIRQNEEELSRIRNELMQSLNQDSNSNFKDTLLKEREAEVRNLKQNLSE
LEQLNENLKKVAFDVKMENEKLVLACEDVRHQLEECLAGNNQLSLEKNTIVETLKMEKGE
IEAELCWAKKRLLEEANKYEKTIEELSNARNLNTSALQLEHEHLIKLNQKKDMEIAELKK
NIEQMDTDHKETKDVLSSSLEEQKQLTQLINKKEIFIEKLKERSSKLQEELDKYSQALRK
NEILRQTIEEKDRSLGSMKEENNHLQEELERLREEQSRTAPVADPKTLDSVTELASEVSQ
LNTIKEHLEEEIKHHQKIIEDQNQSKMQLLQSLQEQKKEMDEFRYQHEQMNATHTQLFLE
KDEEIKSLQKTIEQIKTQLHEERQDIQTDNSDIFQETKVQSLNIENGSEKHDLSKAETER
LVKGIKERELEIKLLNEKNISLTKQIDQLSKDEVGKLTQIIQQKDLEIQALHARISSTSH
TQDVVYLQQQLQAYAMEREKVFAVLNEKTRENSHLKTEYHKMMDIVAAKEAALIKLQDEN
KKLSTRFESSGQDMFRETIQNLSRIIREKDIEIDALSQKCQTLLAVLQTSSTGNEAGGVN
SNQFEELLQERDKLKQQVKKMEEWKQQVMTTVQNMQHESAQLQEELHQLQAQVLVDSDNN
SKLQVDYTGLIQSYEQNETKLKNFGQELAQVQHSIGQLCNTKDLLLGKLDIISPQLSSAS
LLTPQSAECLRASKSEVLSESSELLQQELEELRKSLQEKDATIRTLQENNHRLSDSIAAT
SELERKEHEQTDSEIKQLKEKQDVLQKLLKEKDLLIKAKSDQLLSSNENFTNKVNENELL
RQAVTNLKERILILEMDIGKLKGENEKIVETYRGKETEYQALQETNMKFSMMLREKEFEC
HSMKEKALAFEQLLKEKEQGKTGELNQLLNAVKSMQEKTVVFQQERDQVMLALKQKQMEN
TALQNEVQRLRDKEFRSNQELERLRNHLLESEDSYTREALAAEDREAKLRKKVTVLEEKL
VSSSNAMENASHQASVQVESLQEQLNVVSKQRDETALQLSVSQEQVKQYALSLANLQMVL
EHFQQEEKAMYSAELEKQKQLIAEWKKNAENLEGKVISLQECLDEANAALDSASRLTEQL
DVKEEQIEELKRQNELRQEMLDDVQKKLMSLANSSEGKVDKVLMRNLFIGHFHTPKNQRH

EVLRLMGSILGVRREEMEQLFHDDQGGVTRWMTGWLGGGSKSVPNTPLRPNQQSVVNSSF
SELFVKFLETESHPSIPPPKLSVHDMKPLDSPGRRKRDTNAPESFKDTAESRSGRRTDVN
PFLAPRSAAVPLINPAGLGPGGPGHLLLKPISDVLPTFTPLPALPDNSAGVVLKDLLKQ*
>ENSG00000154146|ENST00000284292
MDCCTENACSKPDDDILDIPLDDPGANAAAAKIQASFRGHMARKKIKSGERGRKGPGPGG
PGGAGVARGGAGGGPSGD*
>ENSG00000143578|ENST00000271889
MDLGIPDLLDAWLEPPEDIFSTGSVLELGLHCPPPEVPVTRLQEQGLQGWKSGGDRGCGL
QESEPEDFLKLFIDPNEVYCSEASPGSDSGISEDPCHPDSPPAPRATSSPMLYEVVYEAG
ALERMQGETGPNVGLISIQLDQWSPAFMVPDSCMVSELPFDAHAHILPRAGTVAPVPCTT
LLPCQTLFLTDEEKRLLGQEGVSLPSHLPLTKAEERVLKKVRRKIRNKQSAQDSRRRKKE
YIDGLESRVAACSAQNQELQKKVQELERHNISLVAQLRQLQTLIAQTSNKAAQTSTCVLI
LLFSLALIILPSFSPFQSRPEAGSEDYQPHGVTSRNILTHKDVTENLETQVVESRLREPP
GAKDANGSTRTLLEKMGGKPRPSGRIRSVLHADEM*
>ENSG00000116199|ENST00000263733
MKLKQRVVLLAILLVIFIFTKVFLIDNLDTSAANREDQRAFHRMMTGLRVELAPKLDHTL
QSPWEIAAQWVVPREVYPEETPELGAVMHAMATKKIIKADVGYKGTQLKALLILEGGQKV
VFKPKRYSRDHVVEGEPYAGYDRHNAEVAAFHLDRILGFHRAPLVVGRFVNLRTEIKPVA
TEQLLSTFLTVGNNTCFYGKCYYCRETEPACADGDIMEGSVTLWLPDVWPLQKHRHPWGR
TYREGKLARWEYDESYCDAVKKTSPYDSGPRLLDIIDTAVFDYLIGNADRHHYESFQDDE
GASMLILLDNAKSFGNPSLDERSILAPLYQCCIIRVSTWNRLNYLKNGVLKSALKSAMAH
DPISPVLSDPHLDAVDQRLLSVLATVKQCTDQFGMDTVLVEDRMPLSHL*
>ENSG00000142186|ENST00000270176
MWFFARDPVRDFPFELIPEPPEGGLPGPWALHRGRKKATGSPVSIFVYDVKPGAEEQTQV
AKAAFKRFKTLRHPNILAYIDGLETEKCLHVVTEAVTPLGIYLKARVEAGGLKELEISWG
LHQIVKALSFLVNDCSLIHNNVCMAAVFVDRAGEWKLGGLDYMYSAQGNGGGPPRKGIPE
LEQYDPPELADSSGRVVREKWSADMWRLGCLIWEVFNGPLPRAAALRNPGKIPKTLVPHY
CELVGANPKVRPNPARFLQNCRAPGGFMSNRFVETNLFLEEIQIKEPAEKQKFFQELSKS
LDAFPEDFCRHKVLPQLLTAFEFGNAGAVVLTPLFKVGKFLSAEEYQQKIIPVVVKMFSS
TDRAMRIRLLQQMEQFIQYLDEPTVNTQIFPHVVHGFLDTNPAIREQTVKSMLLLAPKLN
EANLNVELMKHFARLQAKDEQGPIRCNTTVCLGKIGSYLSASTRHRVLTSAFSRATRDPF
APSRVAGVLGFAATHNLYSMNDCAQKILPVLCGLTVDPEKSVRDQAFKAIRSFLSKLESV
SEDPTQLEEVEKDVHAASSPGMGGAAASWAGWAVTGVSSLTSKLIRSHPTTAPTETNIPQ
RPTPEGVPAPAPTPVPATPTTSGHWETQEEDKDTAEDSSTADRWDDEDWGSLEQEAESVL
AQQDDWSTGGQVSRASQVSNSDHKSSKSPESDWSSWEAEGSWEQGWQEPSSQEPPPDGTR
LASEYNWGGPESSDKGDPFATLSARPSTQPRPDSWGEDNWEGLETDSRQVKAELARKKRE
ERRREMEAKRAERKVAKGPMKLGARKLD*
>ENSG00000041353|ENST00000262094
MTDGDYDYLIKLLALGDSGVGKTTFLYRYTDNKFNPKFITTVGIDFREKRVVYNAQGPNG
SSGKAFKVHLQLWDTAGQERFRSLTTAFFRDAMGFLLMFDLTSQQSFLNVRNWMSQLQAN
AYCENPDIVLIGNKADLPDQREVNERQARELADKYGIPYFETSAATGQNVEKAVETLLDL
IMKRMEQCVEKTQIPDTVNGGNSGNLDGEKPPEKKCIC*
>ENSG00000157214|ENST00000287908
MESISMMGSPKSLSETFLPNGINGIKDARKVTVGVIGSGDFAKSLTIRLIRCGYHVVIGS

RNPKFASEFFPHVVDVTHHEDALTKTNIIFVAIHREHYTSLWDLRHLLVGKILIDVSNNM
RINQYPESNAEYLASLFPDSLIVKGFNVVSAWALQLGPKDASRQVYICSNNIQARQQVIE
LARQLNFIPIDLGSLSSAREIENLPLRLFTLWRGPVVVAISLATFFFLYSFVRDVIHPYA
RNQQSDFYKIPIEIVNKTLPIVAITLLSLVYLAGLLAAAYQLYYGTKYRRFPPWLETWLQ
CRKQLGLLSFFFAMVHVAYSLCLPMRRSERYLFLNMAYQQVHANIENSWNEEEVWRIEMY
ISFGIMSLGLLSLLAVTSIPSVSNALNWREFSFIQSTLGYVALLISTFHVLIYGWKRAFE
EEYYRFYTPPNFVLALVLPSIVILGKIILFLPCISRKLKRIKKGWEKSQFLEEGMGGTIP
HVSPERVTVM*
>ENSG00000150961|ENST00000280551
MSQQGYVATPPYSQPQPGIGLSPPHYGHYGDPSHTASPTGMMKPAGPLGATATRGMLPPG
PPPPGPHQFGQNGAHATGHPPQRFPGPPPVNNVASSHAPYQPSAQSSYPGPISTSSVTQL
GSQLSAMQINSYGSGMAPPSQGPPGPLSATSLQTPPRPPQPSILQPGSQVLPPPPTTLNG
PGASPLPLPMYRPDGLSGPPPPNAQYQPPPLPGQTLGAGYPPQQANSGPQMAGAQLSYPG
GFPGGPAQMAGPPQPQKKLDPDSIPSPIQVIENDRASRGGQVYATNTRGQIPPLVTTDCM
IQDQGNASPRFIRCTTYCFPCTSDMAKQAQIPLAAVIKPFATIPSNESPLYLVNHGESGP
VRCNRCKAYMCPFMQFIEGGRRYQCGFCNCVNDVPPFYFQHLDHIGRRLDHYEKPELSLG
SYEYVATLDYCRKSKPPNPPAFIFMIDVSYSNIKNGLVKLICEELKTMLEKIPKEEQEET
SAIRVGFITYNKVLHFFNVKSNLAQPQMMVVTDVGEVFVPLLDGFLVNYQESQSVIHNLL
DQIPDMFADSNENETVFAPVIQAGMEALKAADCPGKLFIFHSSLPTAEAPGKLKNRDDKK
LVNTDKEKILFQPQTNVYDSLAKDCVAHGCSVTLFLFPSQYVDVASLGLVPQLTGGTLYK
YNNFQMHLDRQQFLNDLRNDIEKKIGFDAIMRVRTSTGFRATDFFGGILMNNTTDVEMAA
IDCDKAVTVEFKHDDKLSEDSGALIQCAVLYTTISGQRRLRIHNLGLNCSSQLADLYKSC
ETDALINFFAKSAFKAVLHQPLKVIREILVNQTAHMLACYRKNCASPSAASQLILPDSMK
VLPVYMNCLLKNCVLLSRPEISTDERAYQRQLVMTMGVADSQLFFYPQLLPIHTLDVKST
MLPAAVRCSESRLSEEGIFLLANGLHMFLWLGVSSPPELIQGIFNVPSFAHINTDMTLLP
EVGNPYSQQLRMIMGIIQQKRPYSMKLTIVKQREQPEMVFRQFLVEDKGLYGGSSYVDFL
CCVHKEICQLLN*
>ENSG00000144043|ENST00000272438
MWFMYLLSWLSLFIQVAFITLAVAAGLYYLAELIEEYTVATSRIIKYMIWFSTAVLIGLY
VFERFPTSMIGVGLFTNLVYFGLLQTFPFIMLTSPNFILSCGLVVVNHYLAFQFFAEEYY
PFSEVLAYFTFCLWIIPFAFFVSLSAGENVLPSTMQPGDDVVSNYFTKGKRGKRLGILVV
FSFIKEAILPSRQKIY*
>ENSG00000109220|ENST00000263921
MADFDEIYEEEEDEERALEEQLLKYSPDPVVVRGSGHVTVFGLSNKFESEFPSSLTGKVA
PEEFKASINRVNSCLKKNLPVNVRWLLCGCLCCCCTLGCSMWPVICLSKRTRRSIEKLLE
WENNRLYHKLCLHWRLSKRKCETNNMMEYVILIEFLPKTPIFRPD*
>ENSG00000087460|ENST00000265620
MGCLGNSKTEDQRNEEKAQREANKKIEKQLQKDKQVYRATHRLLLLGAGESGKSTIVKQM
RILHVNGFNGDEKATKVQDIKNNLKEAIETIVAAMSNLVPPVELANPENQFRVDYILSVM
NVPDFDFPPEFYEHAKALWEDEGVRACYERSNEYQLIDCAQYFLDKIDVIKQADYVPSDQ
DLLRCRVLTSGIFETKFQVDKVNFHMFDVGGQRDERRKWIQCFNDVTAIIFVVASSSYNM
VIREDNQTNRLQEALNLFKSIWNNRWLRTISVILFLNKQDLLAEKVLAGKSKIEDYFPEF
ARYTTPEDATPEPGEDPRVTRAKYFIRDEFLRISTASGDGRHYCYPHFTCAVDTENIRRV
FNDCRDIIQRMHLRQYELL*

>ENSG00000144063|ENST00000272462
MASPDPPATSYAPSDVPSGVALFLTIPFAFFLPELIFGFLVWTMVAATHIVYPLLQGWVM
YVSLTSFLISLMFLLSYLFGFYKRFESWRVLDSLYHGTTGILYMSAAVLQVHATIVSEKL
LDPRIYYINSAASFFAFIATLLYILHAFSIYYH*
>ENSG00000105696|ENST00000262817
MAAVALMPPPLLLLLLLASPPAASAPSARDPFAPQLGDTQNCQLRCRDRDLGPQPSQAGL
EGASESPYDRAVLISACERGCRLFSICRFVARSSKPNATQTECEAACVEAYVKEAEQQAC
SHGCWSQPAEPEPEQKRKVLEAPSGALSLLDLFSTLCNDLVNSAQGFVSSTWTYYLQTDN
GKVVVFQTQPIVESLGFQGGRLQRVEVTWRGSHPEALEVHVDPVGPLDKVRKAKIRVKTS
SKAKVESEEPQDNDFLSCMSRRSGLPRWILACCLFLSVLVMLWLSCSTLVTAPGQHLKFQ
PLTLEQHKGFMMEPDWPLYPPPSHACEDSLPPYKLKLDLTKL*
>ENSG00000138653|ENST00000264363
MNLIVKLRRSFRTLIVLLATFCLVSIVISAYFLYSGYKQEMTLIETTAEAECTDIKILPY
RSMELKTVKPIDTSKTDPTVLLFVESQYSQLGQDIIAILESSRFQYHMVIAPGKGDIPPL
TDNGKGKYTLVIYENILKYVSMDSWNRELLEKYCVEYSVSIIGFHKANENSLPSTQLKGF
PLNLFNNLALKDCFVNPQSPLLHITKAPKVEKGPLPGEDWTIFQYNHSTYQPVLLTELQT
EKSLSSLSSKTLFATVIQDLGLHDGIQRVLFGNNLNFWLHKLIFIDAISFLSGKRLTLSL
DRYILVDIDDIFVGKEGTRMNVKDVKALLETQNLLRTQVANFTFNLGFSGKFYHTGTEEE
DEGDDLLLRSVDEFWWFPHMWSHMQPHLFHNESSLVEQMILNKEFALEHGIPINMGYAVA
PHHSGVYPVHIQLYAAWKKVWGIQVTSTEEYPHLKPARYRKGFIHNSIMVLPRQTCGLFT
HTIFYKEYPGGPQELDKSIRGGELFLTILLNPISIFMTHLSNYGNDRLGLYTFVNLVNFV
QSWTNLKLQTLPPVQLAHQYFELFPEQKDPLWQNPCDDKRHKDIWSREKTCDHLPKFLVI
GPQKTGTTALYLFLLMHPSIISNLPSPKTFEEVQFFNGNNYHKGIDWYMDFFPTPSNTTS
DFLFEKSANYFHSEEAPRRAASLVPKAKIITILIDPSDRAYSWYQHQRSHEDPAALRFNF
YEVISTGHWAPSDLKTLQRRCLVPGWYAVHIERWLTYFATSQLLIIDGQQLRSDPATVMD
EVQKFLGVTPRYNYSEALTFDPQKGFWCQLLEGGKTKCLGKSKGRKYPPMDPESRTFLSN
YYRDHNVELSKLLHRLGQPLPSWLRQELQKVR*
>ENSG00000138760|ENST00000264896
MGRCCFYTAGTLSLLLLVTSVTLLVARVFQKAVDQSIEKKIVLRNGTEAFDSWEKPPLPV
YTQFYFFNVTNPEEILRGETPRVEEVGPYTYRELRNKANIQFGDNGTTISAVSNKAYVFE
RDQSVGDPKIDLIRTLNIPVLTVIEWSQVHFLREIIEAMLKAYQQKLFVTHTVDELLWGY
KDEILSLIHVFRPDISPYFGLFYEKNGTNDGDYVFLTGEDSYLNFTKIVEWNGKTSLDWW
ITDKCNMINGTDGDSFHPLITKDEVLYVFPSDFCRSVYITFSDYESVQGLPAFRYKVPAE
ILANTSDNAGFCIPEGNCLGSGVLNVSICKNGAPIIMSFPHFYQADERFVSAIEGMHPNQ
EDHETFVDINPLTGIILKAAKRFQINIYVKKLDDFVETGDIRTMVFPVMYLNESVHIDKE
TASRLKSMINTTLIITNIPYIIMALGVFFGLVFTWLACKGQGSMDEGTADERAPLIRT*
>ENSG00000152661|ENST00000282561
MGDWSALGKLLDKVQAYSTAGGKVWLSVLFIFRILLLGTAVESAWGDEQSAFRCNTQQPG
CENVCYDKSFPISHVRFWVLQIIFVSVPTLLYLAHVFYVMRKEEKLNKKEEELKVAQTDG
VNVDMHLKQIEIKKFKYGIEEHGKVKMRGGLLRTYIISILFKSIFEVAFLLIQWYIYGFS
LSAVYTCKRDPCPHQVDCFLSRPTEKTIFIIFMLVVSLVSLALNIIELFYVFFKGVKDRV
KGKSDPYHATSGALSPAKDCGSQKYAYFNGCSSPTAPLSPMSPPGYKLVTGDRNNSSCRN
YNKQASEQNWANYSAEQNRMGQAGSTISNSHAQPFDFPDDNQNSKKLAAGHELQPLAIVD
QRPSSRASSRASSRPRPDDLEI*

>ENSG00000143761|ENST00000272102
MGNIFANLFKGLFGKKEMRILMVGLDAAGKTTILYKLKLGEIVTTIPTIGFNVETVEYKN
ISFTVWDVGGQDKIRPLWRHYFQNTQGLIFVVDSNDRERVNEAREELMRMLAEDELRDAV
LLVFANKQDLPNAMNAAEITDKLGLHSLRHRNWYIQATCATSGDGLYEGLDWLSNQLRNQ
K*
>ENSG00000072958|ENST00000291439
MSASAVYVLDLKGKVLICRNYRGDVDMSEVEHFMPILMEKEEEGMLSPILAHGGVRFMWI
KHNNLYLVATSKKNACVSLVFSFLYKVVQVFSEYFKELEEESIRDNFVIIYELLDELMDF
GYPQTTDSKILQEYITQEGHKLETGAPRPPATVTNAVSWRSEGIKYRKNEVFLDVIESVN
LLVSANGNVLRSEIVGSIKMRVFLSGMPELRLGLNDKVLFDNTGRGKSKSVELEDVKFHQ
CVRLSRFENDRTISFIPPDGEFELMSYRLNTHVKPLIWIESVIEKHSHSRIEYMIKAKSQ
FKRRSTANNVEIHIPVPNDADSPKFKTTVGSVKWVPENSEIVWSIKSFPGGKEYLMRAHF
GLPSVEAEDKEGKPPISVKFEIPYFTTSGIQVRYLKIIEKSGYQALPWVRYITQNGDYQL
RTQ*
>ENSG00000103769|ENST00000261890
MGTRDDEYDYLFKVVLIGDSGVGKSNLLSRFTRNEFNLESKSTIGVEFATRSIQVDGKTI
KAQIWDTAGQERYRAITSAYYRGAVGALLVYDIAKHLTYENVERWLKELRDHADSNIVIM
LVGNKSDLRHLRAVPTDEARAFAEKNGLSFIETSALDSTNVEAAFQTILTEIYRIVSQKQ
MSDRRENDMSPSNNVVPIHVPPTTENKPKVQCCQNI*
>ENSG00000039068|ENST00000261769
MGPWSRSLSALLLLLQVSSWLCQEPEPCHPGFDAESYTFTVPRRHLERGRVLGRVNFEDC
TGRQRTAYFSLDTRFKVGTDGVITVKRPLRFHNPQIHFLVYAWDSTYRKFSTKVTLNTVG
HHHRPPPHQASVSGIQAELLTFPNSSPGLRRQKRDWVIPPISCPENEKGPFPKNLVQIKS
NKDKEGKVFYSITGQGADTPPVGVFIIERETGWLKVTEPLDRERIATYTLFSHAVSSNGN
AVEDPMEILITVTDQNDNKPEFTQEVFKGSVMEGALPGTSVMEVTATDADDDVNTYNAAI
AYTILSQDPELPDKNMFTINRNTGVISVVTTGLDRESFPTYTLVVQAADLQGEGLSTTAT
AVITVTDTNDNPPIFNPTTYKGQVPENEANVVITTLKVTDADAPNTPAWEAVYTILNDDG
GQFVVTTNPVNNDGILKTAKGLDFEAKQQYILHVAVTNVVPFEVSLTTSTATVTVDVLDV
NEAPIFVPPEKRVEVSEDFGVGQEITSYTAQEPDTFMEQKITYRIWRDTANWLEINPDTG
AISTRAELDREDFEHVKNSTYTALIIATDNGSPVATGTGTLLLILSDVNDNAPIPEPRTI
FFCERNPKPQVINIIDADLPPNTSPFTAELTHGASANWTIQYNDPTQESIILKPKMALEV
GDYKINLKLMDNQNKDQVTTLEVSVCDCEGAAGVCRKAQPVEAGLQIPAILGILGGILAL
LILILLLLLFLRRRAVVKEPLLPPEDDTRDNVYYYDEEGGGEEDQDFDLSQLHRGLDARP
EVTRNDVAPTLMSVPRYLPRPANPDEIGNFIDENLKAADTDPTAPPYDSLLVFDYEGSGS
EAASLSSLNSSESDKDQDYDYLNEWGNRFKKLADMYGGGEDD*
>ENSG00000078808|ENST00000263741
MVWPWVAMASRWGPLIGLAPCCLWLLGAVLLMDASARPANHSSTRERVANREENEILPPD
HLNGVKLEMDGHLNRGFHQEVFLGKDLGGFDEDAEPRRSRRKLMVIFSKVDVNTDRKISA
KEMQRWIMEKTAEHFQEAMEESKTHFRAVDPDGDGHVSWDEYKVKFLASKGHSEKEVADA
IRLNEELKVDEETQEVLENLKDRWYQADSPPADLLLTEEEFLSFLHPEHSRGMLRFMVKE
IVRDLDQDGDKQLSVPEFISLPVGTVENQQGQDIDDNWVKDRKKEFEELIDSNHDGIVTA
EELENVPTLPLQPIGTLNSHFVRLAAELGGGKATLTCAPLARRATWTP*
>ENSG00000127564|ENST00000262300
MLERPPALAMPMPTEGTPPPLSGTPIPVPAYFRHAEPGFSLKRPRGLSRSLPPPPPAKGS

IPISRLFPPRTPGWHQLQPRRVSFRGEASETLQSPGYDPSRPESFFQQSFQRLSRLGHGS
YGEVFKVRSKEDGRLYAVKRSMSPFRGPKDRARKLAEVGSHEKVGQHPCCVRLEQAWEEG
GILYLQTELCGPSLQQHCEAWGASLPEAQVWGYLRDTLLALAHLHSQGLVHLDVKPANIF
LGPRGRCKLGDFGLLVELGTAGAGEVQEGDPRYMAPELLQGSYGTAADVFSLGLTILEVA
CNMELPHGGEGWQQLRQGYLPPEFTAGLSSELRSVLVMMLEPDPKLRATAEALLALPVLR
QPRAWGVLWCMAAEALSRGWALWQALLALLCWLWHGLAHPASWLQPLGPPATPPGSPPCS
LLLDSSLSSNWDDDSLGPSLSPEAVLARTVGSTSTPRSRCTPRDALDLSDINSEPPRGSF
PSFEPRNLLSLFEDTLDPT*
>ENSG00000074181|ENST00000263388
MGPGARGRRRRRRPMSPPPPPPPVRALPLLLLLAGPGAAAPPCLDGSPCANGGRCTQLPS
REAACLCPPGWVGERCQLEDPCHSGPCAGRGVCQSSVVAGTARFSCRCPRGFRGPDCSLP
DPCLSSPCAHGARCSVGPDGRFLCSCPPGYQGRSCRSDVDECRVGEPCRHGGTCLNTPGS
FRCQCPAGYTGPLCENPAVPCAPSPCRNGGTCRQSGDLTYDCACLPGFEGQNCEVNVDDC
PGHRCLNGGTCVDGVNTYNCQCPPEWTGQFCTEDVDECQLQPNACHNGGTCFNTLGGHSC
VCVNGWTGESCSQNIDDCATAVCFHGATCHDRVASFYCACPMGKTGLLCHLDDACVSNPC
HEDAICDTNPVNGRAICTCPPGFTGGACDQDVDECSIGANPCEHLGRCVNTQGSFLCQCG
RGYTGPRCETDVNECLSGPCRNQATCLDRIGQFTCICMAGFTGTYCEVDIDECQSSPCVN
GGVCKDRVNGFSCTCPSGFSGSTCQLDVDECASTPCRNGAKCVDQPDGYECRCAEGFEGT
LCDRNVDDCSPDPCHHGRCVDGIASFSCACAPGYTGTRCESQVDECRSQPCRHGGKCLDL
VDKYLCRCPSGTTGVNCEVNIDDCASNPCTFGVCRDGINRYDCVCQPGFTGPLCNVEINE
CASSPCGEGGSCVDGENGFRCLCPPGSLPPLCLPPSHPCAHEPCSHGICYDAPGGFRCVC
EPGWSGPRCSQSLARDACESQPCRAGGTCSSDGMGFHCTCPPGVQGRQCELLSPCTPNPC
EHGGRCESAPGQLPVCSCPQGWQGPRCQQDVDECAGPAPCGPHGICTNLAGSFSCTCHGG
YTGPSCDQDINDCDPNPCLNGGSCQDGVGSFSCSCLPGFAGPRCARDVDECLSNPCGPGT
CTDHVASFTCTCPPGYGGFHCEQDLPDCSPSSCFNGGTCVDGVNSFSCLCRPGYTGAHCQ
HEADPCLSRPCLHGGVCSAAHPGFRCTCLESFTGPQCQTLVDWCSRQPCQNGGRCVQTGA
YCLCPPGWSGRLCDIRSLPCREAAAQIGVRLEQLCQAGGQCVDEDSSHYCVCPEGRTGSH
CEQEVDPCLAQPCQHGGTCRGYMGGYMCECLPGYNGDNCEDDVDECASQPCQHGGSCIDL
VARYLCSCPPGTLGVLCEINEDDCGPGPPLDSGPRCLHNGTCVDLVGGFRCTCPPGYTGL
RCEADINECRSGACHAAHTRDCLQDPGGGFRCLCHAGFSGPRCQTVLSPCESQPCQHGGQ
CRPSPGPGGGLTFTCHCAQPFWGPRCERVARSCRELQCPVGVPCQQTPRGPRCACPPGLS
GPSCRSFPGSPPGASNASCAAAPCLHGGSCRPAPLAPFFRCACAQGWTGPRCEAPAAAPE
VSEEPRCPRAACQAKRGDQRCDRECNSPGCGWDGGDCSLSVGDPWRQCEALQCWRLFNNS
RCDPACSSPACLYDNFDCHAGGRERTCNPVYEKYCADHFADGRCDQGCNTEECGWDGLDC
ASEVPALLARGVLVLTVLLPPEELLRSSADFLQRLSAILRTSLRFRLDAHGQAMVFPYHR
PSPGSEPRARRELAPEVIGSVVMLEIDNRLCLQSPENDHCFPDAQSAADYLGALSAVERL
DFPYPLRDVRGEPLEPPEPSVPLLPLLVAGAVLLLVILVLGVMVARRKREHSTLWFPEGF
SLHKDVASGHKGRREPVGQDALGMKNMAKGESLMGEVATDWMDTECPEAKRLKVEEPGMG
AEEAVDCRQWTQHHLVAADIRVAPAMALTPPQGDADADGMDVNVRGPDGFTPLMLASFCG
GALEPMPTEEDEADDTSASIISDLICQGAQLGARTDRTGETALHLAARYARADAAKRLLD
AGADTNAQDHSGRTPLHTAVTADAQGVFQILIRNRSTDLDARMADGSTALILAARLAVEG
MVEELIASHADVNAVDELGKSALHWAAAVNNVEATLALLKNGANKDMQDSKEETPLFLAA
REGSYEAAKLLLDHFANREITDHLDRLPRDVAQERLHQDIVRLLDQPSGPRSPPGPHGLG
PLLCPPGAFLPGLKAAQSGSKKSRRPPGKAGLGPQGPRGRGKKLTLACPGPLADSSVTLS

PVDSLDSPRPFGGPPASPGGFPLEGPYAAATATAVSLAQLGGPGRAGLGRQPPGGCVLSL
GLLNPVAVPLDWARLPPPAPPGPSFLLPLAPGPQLLNPGTPVSPQERPPPYLAVPGHGEE
YPAAGAHSSPPKARFLRVPSEHPYLTPSPESPEHWASPSPPSLSDWSESTPSPATATGAM
ATTTGALPAQPLPLSVPSSLAQAQTQLGPQPEVTPKRQVLA*
>ENSG00000111249|ENST00000261726
MAANVGSMFQYWKRFDLRRLQKELNSVASELSARQEESEHSHKHLIELRREFKKNVPEEI
REMVAPVLKSFQAEVVALSKRSQEAEAAFLSVYKQLIEAPDPVPVFEAARSLDDRLQPPS
FDPSGQPRRDLHTSWKRNPELLSPKEQREGTSPAGPTLTEGSRLPGIPGKALLTETLLQR
NEAEKQKGLQEVQITLAARLGEAEEKIKVLHSALKATQAELLELRRKYDEEAASKADEVG
LIMTNLEKANQRAEAAQREVESLREQLASVNSSIRLACCSPQGPSGDKVNFTLCSGPRLE
AALASKDREILRLLKDVQHLQSSLQELEEASANQIADLERQLTAKSEAIEKLEEKLQAQS
DYEEIKTELSILKAMKLASSTCSLPQGMAKPEDSLLIAKEAFFPTQKFLLEKPSLLASPE
EDPSEDDSIKDSLGTEQSYPSPQQLPPPPGPEDPLSPSPGQPLLGPSLGPDGTRTFSLSP
FPSLASGERLMMPPAAFKGEAGGLLVFPPAFYGAKPPTAPATPAPGPEPLGGPEPADGGG
GGAAGPGAEEEQLDTAEIAFQVKEQLLKHNIGQRVFGHYVLGLSQGSVSEILARPKPWRK
LTVKGKEPFIKMKQFLSDEQNVLALRTIQVRQRGSITPRIRTPETGSDDAIKSILEQAKK
EIESQKGGEPKTSVAPLSIANGTTPASTSEDAIKSILEQARREMQAQQQALLEMEVAPRG
RSVPPSPPERPSLATASQNGAPALVKQEEGSGGPAQAPLPVLSPAAFVQSIIRKVKSEIG
DAGYFDHHWASDRGLLSRPYASVSPSLSSSSSSGYSGQPNGRAWPRGDEAPVPPEDEAAA
GAEDEPPRTGELKAEGATAEAGARLPYYPAYVPRTLKPTVPPLTPEQYELYMYREVDTLE
LTRQVKEKLAKNGICQRIFGEKVLGLSQGSVSDMLSRPKPWSKLTQKGREPFIRMQLWLS
DQLGQAVGQQPGASQASPTEPRSSPSPPPSPTEPEKSSQEPLSLSLESSKENQQPEGRSS
SSLSGKMYSGSQAPGGIQEIVAMSPELDTYSITKRVKEVLTDNNLGQRLFGESILGLTQG
SVSDLLSRPKPWHKLSLKGREPFVRMQLWLNDPHNVEKLRDMKKLEKKAYLKRRYGLIST
GSDSESPATRSECPSPCLQPQDLSLLQIKKPRVVLAPEEKEALRKAYQLEPYPSQQTIEL
LSFQLNLKTNTVINWFHNYRSRMRREMLVEGTQDEPDLDPSGGPGILPPGHSHPDPTPQS
PDSETEDQKPTVKELELQEGPEENSTPLTTQDKAQVRIKQEQMEEDAEEEAGSQPQDSGE
LDKGQGPPKEEHPDPPGNDGLPKVAPGPLLPGGSTPDCPSLHPQQESEAGERLHPDPLSF
KSASESSRCSLEVSLNSPSAASSPGLMMSVSPVPSSSAPISPSPPGAPPAKVPSASPTAD
MAGALHPSAKVNPNLQRRHEKMANLNNIIYRVERAANREEALEWEF*
>ENSG00000148429|ENST00000277575
MRTEAVVNSQGQTDYLTKDSDQDVALKLAQERAEIVAKYDRGREGAEIEPWEDADYLVYK
VTDRFGFLHEEELPDHNVAVERQKHLEIERTTKWLKMLKGWEKYKNTEKFHRRIYKGIPL
QLRGEVWALLLEIPKMKEETRDLYSKLKHRARGCSPDIRQIDLDVNRTFRDHIMFRDRYG
VKQQSLFHVLAAYSIYNTEVGYCQGMSQITALLLMYMNEEDAFWALVKLFSGPKHAMHGF
FVQGFPKLLRFQEHHEKILNKFLSKLKQHLDSQEIYTSFYTMKWFFQCFLDRTPFTLNLR
IWDIYIFEGERVLTAMSYTILKLHKKHLMKLSMEELVEFFQETLAKDFFFEDDFVIEQLQ
ISMTELKRAKLDLPEPGKEDEYPKKPLGQLPPELQSWGVHHLSNGQRSVGRPSPLASGRR
ESGAPHRRHEHSPHPQSRTGTPERAQPPRRKSVEEESKKLKDEADFQRKLPSGPQDSSRQ
YNHAAANQNSNATSNIRKEFVPKWNKPSDVSATERTAKYTMEGKGRAAHPALAVTVPGPA
EVRVSNVRPKMKALDAEDGKRGSTASQYDNVPGPELDSGASVEEALERAYSQSPRHALYP
PSPRKHAEPSSSPSKVSNKFTFKVQPPSHARYPSQLDGEARGLAHPPSYSNPPVYHGNSP
KHFPTANSSFASPQFSPGTQLNPSRRPHGSTLSVSASPEKSYSRPSPLVLPSSRIEVLPV
DTGAGGYSGNSGSPKNGKLIIPPVDYLPDNRTWSEVSYTYRPETQGQSWTRDASRGNLPK

YSAFQLAPFQDHGLPAVSVDSPVRYKASPAAEDASPSGYPYSGPPPPAYHYRNRDGLSIQ
ESVLL*
>ENSG00000115657|ENST00000265316
MVTVGNYCEAEGPVGPAWMQDGLSPCFFFTLVPSTRMALGTLALVLALPCRRRERPAGAD
SLSWGAGPRISPYVLQLLLATLQAALPLAGLAGRVGTARGAPLPSYLLLASVLESLAGAC
GLWLLVVERSQARQRLAMGIWIKFRHSPGLLLLWTVAFAAENLALVSWNSPQWWWARADL
GQQVQFSLWVLRYVVSGGLFVLGLWAPGLRPQSYTLQVHEEDQDVERSQVRSAAQQSTWR
DFGRKLRLLSGYLWPRGSPALQLVVLICLGLMGLERALNVLVPIFYRNIVNLLTEKAPWN
SLAWTVTSYVFLKFLQGGGTGSTGFVSNLRTFLWIRVQQFTSRRVELLIFSHLHELSLRW
HLGRRTGEVLRIADRGTSSVTGLLSYLVFNVIPTLADIIIGIIYFSMFFNAWFGLIVFLC
MSLYLTLTIVVTEWRTKFRRAMNTQENATRARAVDSLLNFETVKYYNAESYEVERYREAI
IKYQGLEWKSSASLVLLNQTQNLVIGLGLLAGSLLCAYFVTEQKLQVGDYVLFGTYIIQL
YMPLNWFGTYYRMIQTNFIDMENMFDLLKEETEVKDLPGAGPLRFQKGRIEFENVHFSYA
DGRETLQDVSFTVMPGQTLALVGPSGAGKSTILRLLFRFYDISSGCIRIDGQDISQVTQA
SLRSHIGVVPQDTVLFNDTIADNIRYGRVTAGNDEVEAAAQAAGIHDAIMAFPEGYRTQV
GERGLKLSGGEKQRVAIARTILKAPGIILLDEATSALDTSNERAIQASLAKVCANRTTIV
VAHRLSTVVNADQILVIKDGCIVERGRHEALLSRGGVYADMWQLQQGQEETSEDTKPQTM
ER*
>ENSG00000114650|ENST00000265565
MTLTERLREKISRAFYNHGLLCASYPIPIILFTGFCILACCYPLLKLPLPGTGPVEFTTP
VKDYSPPPVDSDRKQGEPTEQPEWYVGAPVAYVQQIFVKSSVFPWHKNLLAVDVFRSPLS
RAFQLVEEIRNHVLRDSSGIRSLEELCLQVTDLLPGLRKLRNLLPEHGCLLLSPGNFWQN
DWERFHADPDIIGTIHQHEPKTLQTSATLKDLLFGVPGKYSGVSLYTRKRMVSYTITLVF
QHYHAKFLGSLRARLMLLHPSPNCSLRAESLVHVHFKEEIGVAELIPLVTTYIILFAYIY
FSTRKIDMVKSKWGLALAAVVTVLSSLLMSVGLCTLFGLTPTLNGGEIFPYLVVVIGLEN
VLVLTKSVVSTPVDLEVKLRIAQGLSSESWSIMKNMATELGIILIGYFTLVPAIQEFCLF
AVVGLVSDFFLQMLFFTTVLSIDIRRMELADLNKRLPPEACLPSAKPVGQPTRYERQLAV
RPSTPHTITLQPSSFRNLRLPKRLRVVYFLARTRLAQRLIMAGTVVWIGILVYTDPAGLR
NYLAAQVTEQSPLGEGALAPMPVPSGMLPPSHPDPAFSIFPPDAPKLPENQTSPGESPER
GGPAEVVHDSPVPEVTWGPEDEELWRKLSFRHWPTLFSYYNITLAKRYISLLPVIPVTLR
LNPREALEGRHPQDGRSAWPPPGPIPAGHWEAGPKGPGGVQAHGDVTLYKVAALGLATGI
VLVLLLLCLYRVLCPRNYGQLGGGPGRRRRGELPCDDYGYAPPETEIVPLVLRGHLMDIE
CLASDGMLLVSCCLAGHVCVWDAQTGDCLTRIPRPGRQRRDSGVGSGLEAQESWERLSDG
GKAGPEEPGDSPPLRHRPRGPPPPSLFGDQPDLTCLIDTNFSAQPRSSQPTQPEPRHRAV
CGRSRDSPGYDFSCLVQRVYQEEGLAAVCTPALRPPSPGPVLSQAPEDEGGSPEKGSPSL
AWAPSAEGSIWSLELQGNLIVVGRSSGRLEVWDAIEGVLCCSSEEVSSGITALVFLDKRI
VAARLNGSLDFFSLETHTALSPLQFRGTPGRGSSPASPVYSSSDTVACHLTHTVPCAHQK
PITALKAAAGRLVTGSQDHTLRVFRLEDSCCLFTLQGHSGAITTVYIDQTMVLASGGQDG
AICLWDVLTGSRVSHVFAHRGDVTSLTCTTSCVISSGLDDLISIWDRSTGIKFYSIQQDL
GCGASLGVISDNLLVTGGQGCVSFWDLNYGDLLQTVYLGKNSEAQPARQILVLDNAAIVC
NFGSELSLVYVPSVLEKLD*
>ENSG00000155893|ENST00000286353
MLFRNRFLLLLALAALLAFVSLSLQFFHLIPVSTPKNGMSSKSRKRIMPDPVTEPPVTDP
VYEALLYCNIPSVAERSMEGHAPHHFKLVSVHVFIRHGDRYPLYVIPKTKRPEIDCTLVA

NRKPYHPKLEAFISHMSKGSGASFESPLNSLPLYPNHPLCEMGELTQTGVVQHLQNGQLL
RDIYLKKHKLLPNDWSADQLYLETTGKSRTLQSGLALLYGFLPDFDWKKIYFRHQPSALF
CSGSCYCPVRNQYLEKEQRRQYLLRLKNSQLEKTYGEMAKIVDVPTKQLRAANPIDSMLC
HFCHNVSFPCTRNGCVDMEHFKVIKTHQIEDERERREKKLYFGYSLLGAHPILNQTIGRM
QRATEGRKEELFALYSAHDVTLSPVLSALGLSEARFPRFAARLIFELWQDREKPSEHSVR
ILYNGVDVTFHTSFCQDHHKRSPKPMCPLENLVRFVKRDMFVALGGSGTNYYDACHREGF
*
>ENSG00000242247|ENST00000263245
MGDPSKQDILTIFKRLRSVPTNKVCFDCGAKNPSWASITYGVFLCIDCSGSHRSLGVHLS
FIRSTELDSNWSWFQLRCMQVGGNASASSFFHQHGCSTNDTNAKYNSRAAQLYREKIKSL
ASQATRKHGTDLWLDSCVVPPLSPPPKEEDFFASHVSPEVSDTAWASAIAEPSSLTSRPV
ETTLENNEGGQEQGPSVEGLNVPTKATLEVSSIIKKKPNQAKKGLGAKKGSLGAQKLANT
CFNEIEKQAQAADKMKEQEDLAKVVSKEESIVSSLRLAYKDLEIQMKKDEKMNISGKKNV
DSDRLGMGFGNCRSVISHSVTSDMQTIEQESPIMAKPRKKYNDDSDDSYFTSSSSYFDEP
VELRSSSFSSWDDSSDSYWKKETSKDTETVLKTTGYSDRPTARRKPDYEPVENTDEAQKK
FGNVKAISSDMYFGRQSQADYETRARLERLSASSSISSADLFEEPRKQPAGNYSLSSVLP
NAPDMAQFKQGVRSVAGKLSVFANGVVTSIQDRYGS*
>ENSG00000111846|ENST00000265012
MNFWRYCFFAFTLLSVVIFVRFYSSQLSPPKSYEKLNSSSERYFRKTACNHALEKMPVFL
WENILPSPLRSVPCKDYLTQNHYITSPLSEEEAAFPLAYVMVIHKDFDTFERLFRAIYMP
QNVYCVHVDEKAPAEYKESVRQLLSCFQNAFIASKTESVVYAGISRLQADLNCLKDLVAS
EVPWKYVINTCGQDFPLKTNREIVQHLKGFKGKNITPGVLPPDHAIKRTKYVHQEHTDKG
GFFVKNTNILKTSPPHQLTIYFGTAYVALTRDFVDFVLRDQRAIDLLQWSKDTYSPDEHF
WVTLNRVSGVPGSMPNASWTGNLRAIKWSDMEDRHGGCHGHYVHGICIYGNGDLKWLVNS
PSLFANKFELNTYPLTVECLELRHRERTLNQSETAIQPSWYF*
>ENSG00000156413|ENST00000286955
MDPLGPAKPQWSWRCCLTTLLFQLLMAVCFFSYLRVSQDDPTVYPNGSRFPDSTGTPAHS
IPLILLWTWPFNKPIALPRCSEMVPGTADCNITADRKVYPQADAVIVHHREVMYNPSAQL
PRSPRRQGQRWIWFSMESPSHCWQLKAMDGYFNLTMSYRSDSDIFTPYGWLEPWSGQPAH
PPLNLSAKTELVAWAVSNWGPNSARVRYYQSLQAHLKVDVYGRSHKPLPQGTMMETLSRY
KFYLAFENSLHPDYITEKLWRNALEAWAVPVVLGPSRSNYERFLPPDAFIHVDDFQSPKD
LARYLQELDKDHARYLSYFRWRETLRPRSFSWALAFCKACWKLQEESRYQTRGIAAWFT*
>ENSG00000115963|ENST00000263895
MKERRASQKLSSKSIMDPNQNVKCKIVVVGDSQCGKTALLHVFAKDCFPENYVPTVFENY
TASFEIDTQRIELSLWDTSGSPYYDNVRPLSYPDSDAVLICFDISRPETLDSVLKKWKGE
IQEFCPNTKMLLVGCKSDLRTDVSTLVELSNHRQTPVSYDQGANMAKQIGAATYIECSAL
QSENSVRDIFHVATLACVNKTNKNVKRNKSQRATKRISHMPSRPELSAVATDLRKDKAKS
CTVM*
>ENSG00000085382|ENST00000262903
MERAMEQLNRLTRSLRRARTVELPEDNETAVYTLMPMVMADQHRSVSELLSNSKFDVNYA
FGRVKRSLLHIAANCGSVECLVLLLKKGANPNYQDISGCTPLHLAARNGQKKCMSKLLEY
SADVNICNNEGLTAIHWLAVNGRTELLHDLVQHVSDVDVEDAMGQTALHVACQNGHKTTV
QCLLDSGADINRPNVSGATPLYFACSHGQRDTAQILLLRGAKYLPDKNGVTPLDLCVQGG
YGETCEVLIQYHPRLFQTIIQMTQNEDLRENMLRQVLEHLSQQSESQYLKILTSLAEVAT

TNGHKLLSLSSNYDAQMKSLLRIVRMFCHVFRIGPSSPSNGIDMGYNGNKTPRSQVFKPL
ELLWHSLDEWLVLIATELMKNKRDSTEITSILLKQKGQDQDAASIPPFEPPGPGSYENLS
TGTRESKPDALAGRQEASADCQDVISMTANRLSAVIQAFYMCCSCQMPPGMTSPRFIEFV
CKHDEVLKCFVNRNPKIIFDHFHFLLECPELMSRFMHIIKAQPFKDRCEWFYEHLHSGQP
DSDMVHRPVNENDILLVHRDSIFRSSCEVVSKANCAKLKQGIAVRFHGEEGMGQGVVREW
FDILSNEIVNPDYALFTQSADGTTFQPNSNSYVNPDHLNYFRFAGQILGLALNHRQLVNI
YFTRSFYKHILGIPVNYQDVASIDPEYAKNLQWILDNDISDLGLELTFSVETDVFGAMEE
VPLKPGGGSILVTQNNKAEYVQLVTELRMTRAIQPQINAFLQGFHMFIPPSLIQLFDEYE
LELLLSGMPEIDVSDWIKNTEYTSGYEREDPVIQWFWEVVEDITQEERVLLLQFVTGSSR
VPHGGFANIMGGSGLQNFTIAAVPYTPNLLPTSSTCINMLKLPEYPSKEILKDRLLVALH
CGSYGYTMA*
>ENSG00000153339|ENST00000283351
MAQCVQSVQELIPDSFVPCVAALCSDEAERLTRLNHLSFAELLKPFSRLTSEVHMRDPNN
QLHVIKNLKIAVSNIVTQPPQPGAIRKLLNDVVSGSQPAEGLVANVITAGDYDLNISATT
PWFESYRETFLQSMPALDHEFLNHYLACMLVASSSEAEPVEQFSKLSQEQHRIQHNSDYS
YPKWFIPNTLKYYVLLHDVSAGDEQRAESIYEEMKQKYGTQGCYLLKINSRTSNRASDEQ
IPDPWSQYLQKNSIQNQESYEDGPCTITSNKNSDNNLLSLDGLDNEVKDGLPNNFRAHPL
QLEQSSDPSNSIDGPDHLRSASSLHETKKGNTGIIHGACLTLTDHDRIRQFIQEFTFRGL
LPHIEKTIRQLNDQLISRKGLSRSLFSATKKWFSGSKVPEKSINDLKNTSGLLYPPEAPE
LQIRKMADLCFLVQHYDLAYSCYHTAKKDFLNDQAMLYAAGALEMAAVSAFLQPGAPRPY
PAHYMDTAIQTYRDICKNMVLAERCVLLSAELLKSQSKYSEAAALLIRLTSEDSDLRSAL
LLEQAAHCFINMKSPMVRKYAFHMILAGHRFSKAGQKKHALRCYCQAMQVYKGKGWSLAE
DHINFTIGRQSYTLRQLDNAVSAFRHILINESKQSAAQQGAFLREYLYVYKNVSQLSPDG
PLPQLPLPYINSSATRVFFGHDRRPADGEKQAATHVSLDQEYDSESSQQWRELEEQVVSV
VNKGVIPSNFHPTQYCLNSYSDNSRFPLAVVEEPITVEVAFRNPLKVLLLLTDLSLLWKF
HPKDFSGKDNEEVKQLVTSEPEMIGAEVISEFLINGEESKVARLKLFPHHIGELHILGVV
YNLGTIQGSMTVDGIGALPGCHTGKYSLSMSVRGKQDLEIQGPRLNNTKEEKTSVKYGPD
RRLDPIITEEMPLLEVFFIHFPTGLLCGEIRKAYVEFVNVSKCPLTGLKVVSKRPEFFTF
GGNTAVLTPLSPSASENCSAYKTVVTDATSVCTALISSASSVDFGIGTGSQPEVIPVPLP
DTVLLPGASVQLPMWLRGPDEEGVHEINFLFYYESVKKQPKIRHRILRHTAIICTSRSLN
VRATVCRSNSLENEEGRGGNMLVFVDVENTNTSEAGVKEFHIVQVSSSSKHWKLQKSVNL
SENKDTKLASREKGKFCFKAIRCEKEEAATQSSEKYTFADIIFGNEQIISSASPCADFFY
RSLSSELKKPQAHLPVHTEKQSTEDAVRLIQKCSEVDLNIVILWKAYVVEDSKQLILEGQ
HHVILRTIGKEAFSYPQKQEPPEMELLKFFRPENITVSSRPSVEQLSSLIKTSLHYPESF
NHPFHQKSLCLVPVTLLLSNCSKADVDVIVDLRHKTTSPEALEIHGSFTWLGQTQYKLQL
KSQEIHSLQLKACFVHTGVYNLGTPRVFAKLSDQVTVFETSQQNSMPALIIISNV*
>ENSG00000153130|ENST00000338517
MDGSRKEEEEDSTFTNISLADDIDHSSRILYPRPKSLLPKMMNADMDAVDAENQVELEEK
TRLINQVLELQHTLEDLSARVDAVKEENLKLKSENQVLGQYIENLMSASSVFQTTDTKSK
RK*
>ENSG00000065000|ENST00000345016
MALKMVKGSIDRMFDKNLQDLVRGIRNHKEDEAKYISQCIDEIKQELKQDNIAVKANAVC
KLTYLQMLGYDISWAAFNIIEVMSASKFTFKRIGYLAASQSFHEGTDVIMLTTNQIRKDL
SSPSQYDTGVALTGLSCFVTPDLARDLANDIMTLMSHTKPYIRKKAVLIMYKVFLKYPES

LRPAFPRLKEKLEDPDPGVQSAAVNVICELARRNPKNYLSLAPLFFKLMTSSTNNWVLIK
IIKLFGALTPLEPRLGKKLIEPLTNLIHSTSAMSLLYECVNTVIAVLISLSSGMPNHSAS
IQLCVQKLRILIEDSDQNLKYLGLLAMSKILKTHPKSVQSHKDLILQCLDDKDESIRLRA
LDLLYGMVSKKNLMEIVKKLMTHVDKAEGTTYRDELLTKIIDICSQSNYQYITNFEWYIS
ILVELTRLEGTRHGHLIAAQMLDVAIRVKAIRKFAVSQMSALLDSAHLLASSTQRNGICE
VLYAAAWICGEFSEHLQEPHHTLEAMLRPRVTTLPGHIQAVYVQNVVKLYASILQQKEQA
GEAEGAQAVTQLMVDRLPQFVQSADLEVQERASCILQLVKHIQKLQAKDVPVAEEVSALF
AGELNPVAPKAQKKVPVPEGLDLDAWINEPLSDSESEDERPRAVFHEEEQRRPKHRPSEA
DEEELARRREARKQEQANNPFYIKSSPSPQKRYQDTPGVEHIPVVQIDLSVPLKVPGLPM
SDQYVKLEEERRHRQKLEKDKRRKKRKEKEKKGKRRHSSLPTESDEDIAPAQQVDIVTEE
MPENALPSDEDDKDPNDPYRALDIDLDKPLADSEKLPIQKHRNTETSKSPEKDVPMVEKK
SKKPKKKEKKHKEKERDKEKKKEKEKKKSPKPKKKKHRKEKEERTKGKKKSKKQPPGSEE
AAGEPVQNGAPEEEQLPPESSYSLLAENSYVKMTCDIRGSLQEDSQVTVAIVLENRSSSI
LKGMELSVLDSLNARMARPQGSSVHDGVPVPFQLPPGVSNEAQYVFTIQSIVMAQKLKGT
LSFIAKNDEGATHEKLDFRLHFSCSSYLITTPCYSDAFAKLLESGDLSMSSIKVDGIRMS
FQNLLAKICFHHHFSVVERVDSCASMYSRSIQGHHVCLLVKKGENSVSVDGKCSDSTLLS
NLLEEMKATLAKC*
>ENSG00000109956|ENST00000312527
MPKRRDILAIVLIVLPWTLLITVWHQSTLAPLLAVHKDEGSDPRRETPPGADPREYCTSD
RDIVEVVRTEYVYTRPPPWSDTLPTIHVVTPTYSRPVQKAELTRMANTLLHVPNLHWLVV
EDAPRRTPLTARLLRDTGLNYTHLHVETPRNYKLRGDARDPRIPRGTMQRNLALRWLRET
FPRNSSQPGVVYFADDDNTYSLELFEEMRSTRRVSVWPVAFVGGLRYEAPRVNGAGKVVG
WKTVFDPHRPFAIDMAGFAVNLRLILQRSQAYFKLRGVKGGYQESSLLRELVTLNDLEPK
AANCTKILVWHTRTEKPVLVNEGKKGFTDPSVEI*
>ENSG00000147408|ENST00000332246
MMMVRRGLLAWISRVVVLLVLLCCAISVLYMLACTPKGDEEQLALPRANSPTGKEGYQAV
LQEWEEQHRNYVSSLKRQIAQLKEELQERSEQLRNGQYQASDAAGLGLDRSPPEKTQADL
LAFLHSQVDKAEVNAGVKLATEYAAVPFDSFTLQKVYQLETGLTRHPEEKPVRKDKRDEL
VEAIESALETLNSPAENSPNHRPYTASDFIEGIYRTERDKGTLYELTFKGDHKHEFKRLI
LFRPFGPIMKVKNEKLNMANTLINVIVPLAKRVDKFRQFMQNFREMCIEQDGRVHLTVVY
FGKEEINEVKGILENTSKAANFRNFTFIQLNGEFSRGKGLDVGARFWKGSNVLLFFCDVD
IYFTSEFLNTCRLNTQPGKKVFYPVLFSQYNPGIIYGHHDAVPPLEQQLVIKKETGFWRD
FGFGMTCQYRSDFINIGGFDLDIKGWGGEDVHLYRKYLHSNLIVVRTPVRGLFHLWHEKR
CMDELTPEQYKMCMQSKAMNEASHGQLGMLVFRHEIEAHLRKQKQKTSSKKT*
>ENSG00000100626|ENST00000337827
MRKIRANAIAILTVAWILGTFYYLWQDNRAHAASSGGRGAQRAGRRSEQLREDRTIPLIV
TGTPSKGFDEKAYLSAKQLKAGEDPYRQHAFNQLESDKLSPDRPIRDTRHYSCPSVSYSS
DLPATSVIITFHNEARSTLLRTVKSVLNRTPANLIQEIILVDDFSSDPEDCLLLTRIPKV
KCLRNDRREGLIRSRVRGADVAAATVLTFLDSHCEVNTEWLPPMLQRVKEDHTRVVSPII
DVISLDNFAYLAASADLRGGFDWSLHFKWEQIPLEQKMTRTDPTRPIRTPVIAGGIFVID
KSWFNHLGKYDAQMDIWGGENFELSFRVWMCGGSLEIVPCSRVGHVFRKRHPYNFPEGNA
LTYIRNTKRTAEVWMDEYKQYYYEARPSAIGKAFGSVATRIEQRKKMNCKSFRWYLENVY
PELTVPVKEALPGIIKQGVNCLESQGQNTAGDFLLGMGICRGSAKNPQPAQAWLFSDHLI
QQQGKCLAATSTLMSSPGSPVILQMCNPREGKQKWRRKGSFIQHSVSGLCLETKPAQLVT

SKCQADAQAQQWQLLPHT*
>ENSG00000220205|ENST00000316509
MSATAATAPPAAPAGEGGPPAPPPNLTSNRRLQQTQAQVDEVVDIMRVNVDKVLERDQKL
SELDDRADALQAGASQFETSAAKLKRKYWWKNLKMMIILGVICAIILIIIIVYFST*
>ENSG00000179913|ENST00000318683
MKYLRHRRPNATLILAIGAFTLLLFSLLVSPPTCKVQEQPPAIPEALAWPTPPTRPAPAP
CHANTSMVTHPDFATQPQHVQNFLLYRHCRHFPLLQDVPPSKCAQPVFLLLVIKSSPSNY
VRRELLRRTWGRERKVRGLQLRLLFLVGTASNPHEARKVNRLLELEAQTHGDILQWDFHD
SFFNLTLKQVLFLQWQETRCANASFVLNGDDDVFAHTDNMVFYLQDHDPGRHLFVGQLIQ
NVGPIRAFWSKYYVPEVVTQNERYPPYCGGGGFLLSRFTAAALRRAAHVLDIFPIDDVFL
GMCLELEGLKPASHSGIRTSGVRAPSQRLSSFDPCFYRDLLLVHRFLPYEMLLMWDALNQ
PNLTCGNQTQIY*
>ENSG00000198108|ENST00000305031
MAVRSRRPWMSVALGLVLGFTAASWLIAPRVAELSERKRRGSSLCSYYGRSAAGPRAGAQ
QPLPQPQSRPRQEQSPPPARQDLQGPPLPEAAPGITSFRSSPWQQPPPLQQRRRGREPEG
ATGLPGAPAAEGEPEEEDGGAAGQRRDGRPGSSHNGSGDGGAAAPSARPRDFLYVGVMTA
QKYLGSRALAAQRTWARFIPGRVEFFSSQQPPNAGQPPPPLPVIALPGVDDSYPPQKKSF
MMIKYMHDHYLDKYEWFMRADDDVYIKGDKLEEFLRSLNSSKPLYLGQTGLGNIEELGKL
GLEPGENFCMGGPGMIFSREVLRRMVPHIGECLREMYTTHEDVEVGRCVRRFGGTQCVWS
YEMQQLFHENYEHNRKGYIQDLHNSKIHAAITLHPNKRPAYQYRLHNYMLSRKISELRYR
TIQLHRESALMSKLSNTEVSKEDQQLGVIPSFNHFQPRERNEVIEWEFLTGKLLYSAAEN
QPPRQSLSSILRTALDDTVLQVMEMINENAKSRGRLIDFKEIQYGYRRVNPMHGVEYILD
LLLLYKRHKGRKLTVPVRRHAYLQQLFSKPFFRETEELDVNSLVESINSETQSFSFISNS
LKILSSFQGAKEMGGHNEKKVHILVPLIGRYDIFLRFMENFENMCLIPKQNVKLVIILFS
RDSGQDSSKHIELIKGYQNKYPKAEMTLIPMKGEFSRGLGLEMASAQFDNDTLLLFCDVD
LIFREDFLQRCRDNTIQGQQVYYPIIFSQYDPKVTNGGNPPTDDYFIFSKKTGFWRDYGY
GITCIYKSDLLGAGGFDTSIQGWGLEDVDLYNKVILSGLRPFRSQEVGVVHIFHPVHCDP
NLDPKQYKMCLGSKASTFASTMQLAELWLEKHLGVRYNRTLS*
>ENSG00000188064|ENST00000339464
MHRNFRKWIFYVFLCFGVLYVKLGALSSVVALGANIICNKIPGLAPRQRAICQSRPDAII
VIGEGAQMGINECQYQFRFGRWNCSALGEKTVFGQELRVGSREAAFTYAITAAGVAHAVT
AACSQGNLSNCGCDREKQGYYNQAEGWKWGGCSADVRYGIDFSRRFVDAREIKKNARRLM
NLHNNEAGRKVLEDRMQLECKCHGVSGSCTTKTCWTTLPKFREVGHLLKEKYNAAVQVEV
VRASRLRQPTFLRIKQLRSYQKPMETDLVYIEKSPNYCEEDAATGSVGTQGRLCNRTSPG
ADGCDTMCCGRGYNTHQYTKVWQCNCKFHWCCFVKCNTCSERTEVFTCK*
>ENSG00000140943|ENST00000343411
MKLVNIWLLLLVVLLCGKKHLGDRLEKKSFEKAPCPGCSHLTLKVEFSSTVVEYEYIVAF
NGYFTAKARNSFISSALKSSEVDNWRIIPRNNPSSDYPSDFEVIQIKEKQKAGLLTLEDH
PNIKRVTPQRKVFRSLKYAESDPTVPCNETRWSQKWQSSRPLRRASLSLGSGFWHATGRH
SSRRLLRAIPRQVAQTLQADVLWQMGYTGANVRVAVFDTGLSEKHPHFKNVKERTNWTNE
RTLDDGLGHGTFVAGVIASMRECQGFAPDAELHIFRVFTNNQVSYTSWFLDAFNYAILKK
IDVLNLSIGGPDFMDHPFVDKVWELTANNVIMVSAIGNDGPLYGTLNNPADQMDVIGVGG
IDFEDNIARFSSRGMTTWELPGGYGRMKPDIVTYGAGVRGSGVKGGCRALSGTSVASPVV
AGAVTLLVSTVQKRELVNPASMKQALIASARRLPGVNMFEQGHGKLDLLRAYQILNSYKP

QASLSPSYIDLTECPYMWPYCSQPIYYGGMPTVVNVTILNGMGVTGRIVDKPDWQPYLPQ
NGDNIEVAFSYSSVLWPWSGYLAISISVTKKAASWEGIAQGHVMITVASPAETESKNGAE
QTSTVKLPIKVKIIPTPPRSKRVLWDQYHNLRYPPGYFPRDNLRMKNDPLDWNGDHIHTN
FRDMYQHLRSMGYFVEVLGAPFTCFDASQYGTLLMVDSEEEYFPEEIAKLRRDVDNGLSL
VIFSDWYNTSVMRKVKFYDENTRQWWMPDTGGANIPALNELLSVWNMGFSDGLYEGEFTL
ANHDMYYASGCSIAKFPEDGVVITQTFKDQGLEVLKQETAVVENVPILGLYQIPAEGGGR
IVLYGDSNCLDDSHRQKDCFWLLDALLQYTSYGVTPPSLSHSGNRQRPPSGAGSVTPERM
EGNHLHRYSKVLEAHLGDPKPRPLPACPRLSWAKPQPLNETAPSNLWKHQKLLSIDLDKV
VLPNFRSNRPQVRPLSPGESGAWDIPGGIMPGRYNQEVGQTIPVFAFLGAMVVLAFFVVQ
INKAKSRPKRRKPRVKRPQLMQQVHPPKTPSV*
>ENSG00000107863|ENST00000320481
MATRRTGLSEGDGDKLKACEVSKNKDGKEQSETVSLSEDETFSWPGPKTVTLKRTSQGFG
FTLRHFIVYPPESAIQFSYKDEENGNRGGKQRNRLEPMDTIFVKQVKEGGPAFEAGLCTG
DRIIKVNGESVIGKTYSQVIALIQNSDTTLELSVMPKDEDILQVAYSQDAYLKGNEAYSG
NARNIPEPPPICYPWLPSAPSAMAQPVEISPPDSSLSKQQTSTPVLTQPGRAYRMEIQVP
PSPTDVAKSNTAVCVCNESVRTVIVPSEKVVDLLSNRNNHTGPSHRTEEVRYGVSEQTSL
KTVSRTTSPPLSIPTTHLIHQPAGSRSLEPSGILLKSGNYSGHSDGISSSRSQAVEAPSV
SVNHYSPNSHQHIDWKNYKTYKEYIDNRRLHIGCRTIQERLDSLRAASQSTTDYNQVVPN
RTTLQGRRRSTSHDRVPQSVQIRQRSVSQERLEDSVLMKYCPRSASQGALTSPSVSFSNH
RTRSWDYIEGQDETLENVNSGTPIPDSNGEKKQTYKWSGFTEQDDRRGICERPRQQEIHK
SFRGSNFTVAPSVVNSDNRRMSGRGVGSVSHWRKGIPSIMRKTFEKKPTATGTFGVRLDD
CPPAHTNRYIPLIVDICCKLVEERGLEYTGIYRVPGNNAAISSMQEELNKGMADIDIQDD
KWRDLNVISSLLKSFFRKLPEPLFTNDKYADFIEANRKEDPLDRLKTLKRLIHDLPEHHY
ETLKFLSAHLKTVAENSEKNKMEPRNLAIVFGPTLVRTSEDNMTHMVTHMPDQYKIVETL
IQHHDWFFTEEGAEEPLTTVQEESTVDSQPVPNIDHLLTNIGRTGVSPGDVSDSATSDST
KSKGSWGSGKDQYSRELLVSSIFAAASRKRKKPKEKAQPSSSEDELDNVFFKKENVEQCH
NDTKEESKKESETLGRKQKIIIAKENSTRKDPSTTKDEKISLGKESTPSEEPSPPHNSKH
NKSPTLSCRFAILKESPRSLLAQKSSHLEETGSDSGTLLSTSSQASLARFSMKKSTSPET
KHSEFLANVSTITSDYSTTSSATYLTSLDSSRLSPEVQSVAESKGDEADDERSELISEGR
PVETDSESEFPVFPTALTSERLFRGKLQEVTKSSRRNSEGSELSCTEGSLTSSLDSRRQL
FSSHKLIECDTLSRKKSARFKSDSGSLGDAKNEKEAPSLTKVFDVMKKGKSTGSLLTPTR
GESEKQEPTWKTKIADRLKLRPRAPADDMFGVGNHKVNAETAKRKSIRRRHTLGGHRDAT
EISVLNFWKVHEQSGERESELSAVNRLKPKCSAQDLSISDWLARERLRTSTSDLSRGEIG
DPQTENPSTREIATTDTPLSLHCNTGSSSSTLASTNRPLLSIPPQSPDQINGESFQNVSK
NASSAANAQPHKLSETPGSKAEFHPCL*
>ENSG00000157227|ENST00000311852
MSPAPRPPRCLLLPLLTLGTALASLGSAQSSSFSPEAWLQQYGYLPPGDLRTHTQRSPQS
LSAAIAAMQKFYGLQVTGKADADTMKAMRRPRCGVPDKFGAEIKANVRRKRYAIQGLKWQ
HNEITFCIQNYTPKVGEYATYEAIRKAFRVWESATPLRFREVPYAYIREGHEKQADIMIF
FAEGFHGDSTPFDGEGGFLAHAYFPGPNIGGDTHFDSAEPWTVRNEDLNGNDIFLVAVHE
LGHALGLEHSSDPSAIMAPFYQWMDTENFVLPDDDRRGIQQLYGGESGFPTKMPPQPRTT
SRPSVPDKPKNPTYGPNICDGNFDTVAMLRGEMFVFKERWFWRVRNNQVMDGYPMPIGQF
WRGLPASINTAYERKDGKFVFFKGDKHWVFDEASLEPGYPKHIKELGRGLPTDKIDAALF
WMPNGKTYFFRGNKYYRFNEELRAVDSEYPKNIKVWEGIPESPRGSFMGSDEVFTYFYKG

NKYWKFNNQKLKVEPGYPKSALRDWMGCPSGGRPDEGTEEETEVIIIEVDEEGGGAVSAA
AVVLPVLLLLLVLAVGLAVFFFRRHGTPRRLLYCQRSLLDKV*
>ENSG00000125084|ENST00000293549
MGLWALLPGWVSATLLLALAALPAALAANSSGRWWGIVNVASSTNLLTDSKSLQLVLEPS
LQLLSRKQRRLIRQNPGILHSVSGGLQSAVRECKWQFRNRRWNCPTAPGPHLFGKIVNRG
CRETAFIFAITSAGVTHSVARSCSEGSIESCTCDYRRRGPGGPDWHWGGCSDNIDFGRLF
GREFVDSGEKGRDLRFLMNLHNNEAGRTTVFSEMRQECKCHGMSGSCTVRTCWMRLPTLR
AVGDVLRDRFDGASRVLYGNRGSNRASRAELLRLEPEDPAHKPPSPHDLVYFEKSPNFCT
YSGRLGTAGTAGRACNSSSPALDGCELLCCGRGHRTRTQRVTERCNCTFHWCCHVSCRNC
THTRVLHECL*
>ENSG00000182287|ENST00000329235
MQFMLLFSRQGKLRLQKWYVPLSDKEKKKITRELVQTVLARKPKMCSFLEWRDLKIVYKR
YASLYFCCAIEDQDNELITLEIIHRYVELLDKYFGSVCELDIIFNFEKAYFILDEFLLGG
EVQETSKKNVLKAIEQADLLQEEAETPRSVLEEIGLT*
>ENSG00000169105|ENST00000306243
MFPRPLTPLAAPNGAEPLGRALRRAPLGRARAGLGGPPLLLPSMLMFAVIVASSGLLLMI
ERGILAEMKPLPLHPPGREGTAWRGKAPKPGGLSLRAGDADLQVRQDVRNRTLRAVCGQP
GMPRDPWDLPVGQRRTLLRHILVSDRYRFLYCYVPKVACSNWKRVMKVLAGVLDSVDVRL
KMDHRSDLVFLADLRPEEIRYRLQHYFKFLFVREPLERLLSAYRNKFGEIREYQQRYGAE
IVRRYRAGAGPSPAGDDVTFPEFLRYLVDEDPERMNEHWMPVYHLCQPCAVHYDFVGSYE
RLEADANQVLEWVRAPPHVRFPARQAWYRPASPESLHYHLCSAPRALLQDVLPKYILDFS
LFAYPLPNVTKEACQQ*
>ENSG00000101294|ENST00000340852
MDSALSDPHNGSAEAGGPTNSTTRPPSTPEGIALAYGSLLLMALLPIFFGALRSVRCARG
KNASDMPETITSRDAARFPIIASCTLLGLYLFFKIFSQEYINLLLSMYFFVLGILALSHT
ISPFMNKFFPASFPNRQYQLLFTQGSGENKEEIINYEFDTKDLVCLGLSSIVGVWYLLRK
HWIANNLFGLAFSLNGVELLHLNNVSTGCILLGGLFIYDVFWVFGTNVMVTVAKSFEAPI
KLVFPQDLLEKGLEANNFAMLGLGDVVIPGIFIALLLRFDISLKKNTHTYFYTSFAAYIF
GLGLTIFIMHIFKHAQPALLYLVPACIGFPVLVALAKGEVTEMFSYEESNPKDPAAVTES
KEGTEASASKGLEKKEK*
>ENSG00000166405|ENST00000335425
MAYSTVQRVALASGLVLALSLLLPKAFLSRGKRQEPPPTPEGYPEETYPIYDLSDCIKRR
QETILVDYPDPKELSAEEIAERMGMIEEEESDHLGWESLPTDPRAQEDNSVTSCDPKPET
CSCCFHEDEDPAVLAENAGFSADSYPEQEETTKEEWSQDFKDEGLGISTDKAYTGSMLRK
RNPQGLE*
>ENSG00000114650|ENST00000320017
MTLTERLREKISRAFYNHGLLCASYPIPIILFTGFCILACCYPLLKLPLPGTGPVEFTTP
VKDYSPPPVDSDRKQGEPTEQPEWRDFPLPCGGYWVRECVGAHQVCGLNPGRPGGEAADR
PRPKQRELVHHEEHGHGAGHHPHRLLHPSARHPGVLSLCCRGAGV*
>ENSG00000167302|ENST00000300714
MAAAPPLRDRLSFLHRLPILLKGTSDDDVPCPGYLFEEIAKISHESPGSSQCLLEYLLSR
LHSSSGHGKLKVLKILLYLCSHGSSFFLLILKRNSAFIQEAAAFAGPPDPLHGNSLYQKV
RAAAQDLGSTLFSDTVLPLAPSQPLGTPPATGMGSQARPHSTLQGFGYSKEHGRTAVRHQ
PGQAGGGWDELDSGPSSQNSSQNSDLSRVSDSGSHSGSDSHSGASREPGDLAERVEVVAL

SDCQQELSLVRTVTRGPRAFLSREEAQHFIKACGLLNCEAVLQLLTCHLRGTSECTQLRA
LCAIASLGSSDLLPQEHILLRTRPWLQELSMGSPGPVTNKATKILRHFEASCGQLSPARG
TSAEPGPTAALPGPSDLLTDAVPLPGSQVFLQPLSSTPVSSRSPAPSSGMPSSPVPTPPP
DASPIPAPGDPSEAEARLAESRRWRPERIPGGTDSPKRGPSSCAWSRDSLFAGMELVACP
RLVGAGAAAGESCPDAPRAPQTSSQRTAAKEPPGSEPSAFAFLNA*
>ENSG00000157782|ENST00000316803
MGGGDGAAFKRPGDGARLQRVLGLGSRREPRSLPAGGPAPRRTAPPPPGHASAGPAAMSS
HIAKSESKTSLLKAAAAAASGGSRAPRHGPARDPGLPSRRLPGSCPATPQSSGDPSSRRP
LCRPAPREEGARGSQRVLPQAHCRPREALPAAASRPSPSSPLPPARGRDGEERGLSPALG
LRGSLRARGRGDSVPAAASEADPFLHRLRPMLSSAFGQDRSLRPEEIEELREAFREFDKD
KDGYINCRDLGNCMRTMGYMPTEMELIELSQQINMNLGGHVDFDDFVELMGPKLLAETAD
MIGVKELRDAFREFDTNGDGEISTSELREAMRKLLGHQVGHRDIEEIIRDVDLNGDGRVD
FEEFVRMMSR*
>ENSG00000135454|ENST00000341156
MWLGRRALCALVLLLACASLGLLYASTRDAPGLRLPLAPWAPPQSPRRPELPDLAPEPRY
AHIPVRIKEQVVGLLAWNNCSCESSGGGLPLPFQKQVRAIDLTKAFDPAELRAASATREQ
EFQAFLSRSQSPADQLLIAPANSPLQYPLQGVEVQPLRSILVPGLSLQAASGQEVYQVNL
TASLGTWDVAGEVTGVTLTGEGQADLTLVSPGLDQLNRQLQLVTYSSRSYQTNTADTVRF
STEGHEAAFTIRIRHPPNPRLYPPGSLPQGAQYNISALVTIATKTFLRYDRLRALITSIR
RFYPTVTVVIADDSDKPERVSGPYVEHYLMPFGKGWFAGRNLAVSQVTTKYVLWVDDDFV
FTARTRLERLVDVLERTPLDLVGGAVREISGFATTYRQLLSVEPGAPGLGNCLRQRRGFH
HELVGFPGCVVTDGVVNFFLARTDKVREVGFDPRLSRVAHLEFFLDGLGSLRVGSCSDVV
VDHASKLKLPWTSRDAGAETYARYRYPGSLDESQMAKHRLLFFKHRLQCMTSQ*
>ENSG00000161013|ENST00000292591
MRLRNGTFLTLLLFCLCAFLSLSWYAALSGQKGDVVDVYQREFLALRDRLHAAEQESLKR
SKELNLVLDEIKRAVSERQALRDGDGNRTWGRLTEDPRLKPWNGSHRHVLHLPTVFHHLP
HLLAKESSLQPAVRVGQGRTGVSVVMGIPSVRREVHSYLTDTLHSLISELSPQEKEDSVI
VVLIAETDSQYTSAVTENIKALFPTEIHSGLLEVISPSPHFYPDFSRLRESFGDPKERVR
WRTKQNLDYCFLMMYAQSKGIYYVQLEDDIVAKPNYLSTMKNFALQQPSEDWMILEFSQL
GFIGKMFKSLDLSLIVEFILMFYRDKPIDWLLDHILWVKVCNPEKDAKHCDRQKANLRIR
FKPSLFQHVGTHSSLAGKIQKLKDKDFGKQALRKEHVNPPAEVSTSLKTYQHFTLEKAYL
REDFFWAFTPAAGDFIRFRFFQPLRLERFFFRSGNIEHPEDKLFNTSVEVLPFDNPQSDK
EALQEGRTATLRYPRSPDGYLQIGSFYKGVAEGEVDPAFGPLEALRLSIQTDSPVWVILS
EIFLKKAD*
>ENSG00000166448|ENST00000345589
MAQAVWSRLGRILWLACLLPWAPAGVAAEFLVGDLVVTQNTSLPWPSSYLTKTVLKVSFL
LHDPSNFLKTALFLYSWDFGDGTQMVTEDSVVYYNYSIIGTFTVKLKVVAEWEEVEPDAT
RAVKQKTGDFSASLKLQETLRGIQVLGPTLIQTFQKMTVTLNFLGSPPLTVCWRLKPECL
PLEEGECHPVSVASTAYNLTHTFRDPGDYCFSIRAENIISKTHQYHKIQVWPSRIQPAVF
AFPCATLITVMLAFIMYMTLRNATQQKDMVENPEPPSGVRCCCQMCCGPFLLETPSEYLE
IVRENHGLLPPLYKSVKTYTV*
>ENSG00000101916|ENST00000311912
MKESSLQNSSCSLGKETKKENMFLQSSMLTCIFLLISGSCELCAEENFSRSYPCDEKKQN
DSVIAECSNRRLQEVPQTVGKYVTELDLSDNFITHITNESFQGLQNLTKINLNHNPNVQH

QNGNPGIQSNGLNITDGAFLNLKNLRELLLEDNQLPQIPSGLPESLTELSLIQNNIYNIT
KEGISRLINLKNLYLAWNCYFNKVCEKTNIEDGVFETLTNLELLSLSFNSLSHVPPKLPS
SLRKLFLSNTQIKYISEEDFKGLINLTLLDLSGNCPRCFNAPFPCVPCDGGASINIDRFA
FQNLTQLRYLNLSSTSLRKINAAWFKNMPHLKVLDLEFNYLVGEIASGAFLTMLPRLEIL
DLSFNYIKGSYPQHINISRNFSKLLSLRALHLRGYVFQELREDDFQPLMQLPNLSTINLG
INFIKQIDFKLFQNFSNLEIIYLSENRISPLVKDTRQSYANSSSFQRHIRKRRSTDFEFD
PHSNFYHFTRPLIKPQCAAYGKALDLSLNSIFFIGPNQFENLPDIACLNLSANSNAQVLS
GTEFSAIPHVKYLDLTNNRLDFDNASALTELSDLEVLDLSYNSHYFRIAGVTHHLEFIQN
FTNLKVLNLSHNNIYTLTDKYNLESKSLVELVFSGNRLDILWNDDDNRYISIFKGLKNLT
RLDLSLNRLKHIPNEAFLNLPASLTELHINDNMLKFFNWTLLQQFPRLELLDLRGNKLLF
LTDSLSDFTSSLRTLLLSHNRISHLPSGFLSEVSSLKHLDLSSNLLKTINKSALETKTTT
KLSMLELHGNPFECTCDIGDFRRWMDEHLNVKIPRLVDVICASPGDQRGKSIVSLELTTC
VSDVTAVILFFFTFFITTMVMLAALAHHLFYWDVWFIYNVCLAKVKGYRSLSTSQTFYDA
YISYDTKDASVTDWVINELRYHLEESRDKNVLLCLEERDWDPGLAIIDNLMQSINQSKKT
VFVLTKKYAKSWNFKTAFYLALQRLMDENMDVIIFILLEPVLQHSQYLRLRQRICKSSIL
QWPDNPKAEGLFWQTLRNVVLTENDSRYNNMYVDSIKQY*
>ENSG00000106003|ENST00000338732
MTPGRCCLAADIQVETFIFTDGEDEALARHTGNVVITNCSAAHSRQALSCKMAVEYDRFI
ESGRKWFCHVDDDNYVNLRALLRLLASYPHTRDVYVGKPSLDRPIQAMERVSENKVRPVH
FWFATGGAGFCISRGLALKMSPWASGGHFMNTAERIRLPDDCTIGYIVEALLGVPLIRSG
LFHSHLENLQQVPTSELHEQVTLSYGMFENKRNAVHVKGPFSVEADPSRFRSIHCHLYPD
TPWCPRTAIF*
>ENSG00000100023|ENST00000335025
MGKRQHQKDKMYITCAEYTHFYGGKKPDLPQTNFRRLPFDHCSLSLQPFVYPVCTPDGIV
FDLLNIVPWLKKYGTNPSNGEKLDGRSLIKLNFSKNSEGKYHCPVLFTVFTNNTHIVAVR
TTGNVYAYEAVEQLNIKAKNFRDLLTDEPFSRQDIITLQDPTNLDKFNVSNFYHVKNNMK
IIDPDEEKAKQDPSYYLKNTNAETRETLQELYKEFKGDEILAATMKAPEKKKVDKLNAAH
YSTGKVSASFTSTAMVPETTHEAAAIDEDVLRYQFVKKKGYVRLHTNKGDLNLELHCDLT
PKTCENFIRLCKKHYYDGTIFHRSIRNFVIQGGDPTGTGTGGESYWGKPFKDEFRPNLSH
TGRGILSMANSGPNSNRSQFFITFRSCAYLDKKHTIFGRVVGGFDVLTAMENVESDPKTD
RPKEEIRIDATTVFVDPYEEADAQIAQERKTQLKVAPETKVKSSQPQAGSQGPQTFRQGV
GKYINPAATKRAAEEEPSTSATVPMSKKKPSRGFGDFSSW*
>ENSG00000131899|ENST00000316843
MMKFRFRRQGADPQREKLKQELFAFNKTVEHGFPNQPSALAFDPELRIMAIGTRSGAVKI
YGAPGVEFTGLHRDAATVTQMHFLTGQGRLLSLLDDSSLHLWEIVHHNGCAHLEEALSFQ
LPSRPGFDGASAPLSLTRVTVVLLVAASDIAALGTEGSSVFFLDVTTLTLLEGQTLAPGE
VLRSVPDDYRCGKALGPVESLQGHLRDPTKILIGYSRGLLVIWNQASQCVDHIFLGNQQL
ESLCWGRDSSTVVSSHSDGSYAVWSVDAGSFPTLQPTVATTPYGPFPCKAINKILWRNCE
SGGHFIIFSGGMPRASYGDRHCVSVLRAETLVTLDFTSRIIDFFTVHSTRPEDEFDDPQA
LAVLLEEELVVLDLQTPGWPAVPAPYLAPLHSSAITCSAHVASVPAKLWARIVSAGEQQS
PQPVSSALSWPITGGRNLAQEPSQRGLLLTGHEDGTVRFWDASGVALRPLYKLSTAGLFQ
TDCEHADSLAQAAEDDWPPFRKVGCFDPYSDDPRLGVQKVALCKYTAQMVVAGTAGQVLV
LELSDVPVEQAVSVAIIDLLQDREGFTWKGHERLSPRTGPLPWPAGFQPRVLVQCLPPAA
VTAVTLHTEWSLVAFGTSHGFGLFDYQRKSPVLARCTLHPNDSLAMEGPLSRVKSLKKSL

RQSFRRIRKSRVSGKKRAANASSKLQEANAQLAEQACPHDVEMTPVQRRIEPRSADDSLS
GVVRCLYFADTFLRDGAHHGPTMWAGTNSGSVFAYALEVPAAAVGGEKRPEQAVEAVLGK
EVQLMHRAPVVAIAVLDGRGRPLPEPYEASRDLAQAPDMQGGHAVLIASEEQFKVFTLPK
VSAKTKFKLTAHEGCRVRKVALATFASVACEDYAETCLACLTNLGDVHVFSVPGLRPQVH
YSCIRKEDISGIASCVFTRHGQGFYLISPSEFERFSLSARNITEPLCSLDINWPRDATQA
SYRIRESPKLSQANGTPSILLAPQSLDGSPDPAHSMGPDTPEPPEAALSPMSIDSATSAD
TTLDTTGDVTVEDVKDFLGSSEESEKNLRNLAEDEAHACAILIK*
>ENSG00000170091|ENST00000303177
MVKLNSNPSEKGTKPPSVEDGFQTVPLITPLEVNHLQLPAPEKVIVKTRTEYQPEQKNKG
KFRVPKIAEFTVTILVSLALAFLACIVFLVVYKAFTYDHSCPEGFVYKHKRCIPASLDAY
YSSQDPNSRSRFYTVISHYSVAKQSTARAIGPWLSAAAVIHEPKPPKTQGH*
>ENSG00000176597|ENST00000326505
MRMLVSGRRVKKWQLIIQLFATCFLASLMFFWEPIDNHIVSHMKSYSYRYLINSYDFVND
TLSLKHTSAGPRYQYLINHKEKCQAQDVLLLLFVKTAPENYDRRSGIRRTWGNENYVRSQ
LNANIKTLFALGTPNPLEGEELQRKLAWEDQRYNDIIQQDFVDSFYNLTLKLLMQFSWAN
TYCPHAKFLMTADDDIFIHMPNLIEYLQSLEQIGVQDFWIGRVHRGAPPIRDKSSKYYVS
YEMYQWPAYPDYTAGAAYVISGDVAAKVYEASQTLNSSLYIDDVFMGLCANKIGIVPQDH
VFFSGEGKTPYHPCIYEKMMTSHGHLEDLQDLWKNATDPKVKTISKGFFGQIYCRLMKII
LLCKISYVDTYPCRAAFI*
>ENSG00000173402|ENST00000308775
MRMSVGLSLLLPLSGRTFLLLLSVVMAQSHWPSEPSEAVRDWENQLEASMHSVLSDLHEA
VPTVVGIPDGTAVVGRSFRVTIPTDLIASSGDIIKVSAAGKEALPSWLHWDSQSHTLEGL
PLDTDKGVHYISVSATRLGANGSHIPQTSSVFSIEVYPEDHSELQSVRTASPDPGEVVSS
ACAADEPVTVLTVILDADLTKMTPKQRIDLLHRMRSFSEVELHNMKLVPVVNNRLFDMSA
FMAGPGNAKKVVENGALLSWKLGCSLNQNSVPDIHGVEAPAREGAMSAQLGYPVVGWHIA
NKKPPLPKRVRRQIHATPTPVTAIGPPTTAIQEPPSRIVPTPTSPAIAPPTETMAPPVRD
PVPGKPTVTIRTRGAIIQTPTLGPIQPTRVSEAGTTVPGQIRPTMTIPGYVEPTAVATPP
TTTTKKPRVSTPKPATPSTDSTTTTTRRPTKKPRTPRPVPRVTTKVSITRLETASPPTRI
RTTTSGVPRGGEPNQRPELKNHIDRVDAWVGTYFEVKIPSDTFYDHEDTTTDKLKLTLKL
REQQLVGEKSWVQFNSNSQLMYGLPDSSHVGKHEYFMHATDKGGLSAVDAFEIHVHRRPQ
GDRAPARFKAKFVGDPALVLNDIHKKIALVKKLAFAFGDRNCSTITLQNITRGSIVVEWT
NNTLPLEPCPKEQIAGLSRRIAEDDGKPRPAFSNALEPDFKATSITVTGSGSCRHLQFIP
VVPPRRVPSEAPPTEVPDRDPEKSSEDDVYLHTVIPAVVVAAILLIAGIIAMICYRKKRK
GKLTLEDQATFIKKGVPIIFADELDDSKPPPSSSMPLILQEEKAPLPPPEYPNQSVPETT
PLNQDTMGEYTPLRDEDPNAPPYQPPPPFTAPMEGKGSRPKNMTPYRSPPPYVPP*
>ENSG00000145113|ENST00000349607
MKGARWRRVPWVSLSCLCLCLLPHVVPGVSLFPYGAGAGDLEFVRRTVDFTSPLFKPATG
FPLGSSLRDSLYFTDNGQIIFPESDYQIFSYPNPLPTGFTGRDPVALVAPFWDDADFSTG
RGTTFYQEYETFYGEHSLLVQQAESWIRKMTNNGGYKARWALKVTWVNAHAYPAQWTLGS
NTYQAILSTDGSRSYALFLYQSGGMQWDVAQRSGNPVLMGFSSGDGYFENSPLMSQPVWE
RYRPDRFLNSNSGLQGLQFYRLHREERPNYRLECLQWLKSQPRWPSWGWNQVSCPCSWQQ
GRRDLRFQPVSIGRWGLGSRQLCSFTSWRGGVCCSYGPWGEFREGWHVQRPWQLAQELEP
QSWCCRWNDKPYLCALYQQRRPHVGCATYRPPQPAWMFGDPHITTLDGVSYTFNGLGDFL
LVGAQDGNSSFLLQGRTAQTGSAQATNFIAFAAQYRSSSLGPVTVQWLLEPHDAIRVLLD

NQTVTFQPDHEDGGGQETFNATGVLLSRNGSEVSASFDGWATVSVIALSNILHASASLPP
EYQNRTEGLLGVWNNNPEDDFRMPNGSTIPPGSPEEMLFHFGMTWQINGTGLLGKRNDQL
PSNFTPVFYSQLQKNSSWAEHLISNCDGDSSCIYDTLALRNASIGLHTREVSKNYEQANA
TLNQYPPSINGGRVIEAYKGQTTLIQYTSNAEDANFTLRDSCTDLELFENGTLLWTPKSL
EPFTLEILARSAKIGLASALQPRTVVCHCNAESQCLYNQTSRVGNSSLEVAGCKCDGGTF
GRYCEGSEDACEEPCFPSVHCVPGKGCEACPPNLTGDGRHCAALGSSFLCQNQSCPVNYC
YNQGHCYISQTLGCQPMCTCPPAFTDSRCFLAGNNFSPTVNLELPLRVIQLLLSEEENAS
MAEVNASVAYRLGTLDMRAFLRNSQVERIDSAAPASGSPIQHWMVISEFQYRPRGPVIDF
LNNQLLAAVVEAFLYHVPRRSEEPRNDVVFQPISGEDVRDVTALNVSTLKAYFRCDGYKG
YDLVYSPQSGFTCVSPCSRGYCDHGGQCQHLPSGPRCSCVSFSIYTAWGEHCEHLSMKLD
AFFGIFFGALGGLLLLGVGTFVVLRFWGCSGARFSYFLNSAEALP*
>ENSG00000114745|ENST00000319283
MGLGVSAEQPAGGAEGFHLHGVQENSPAQQAGLEPYFDFIITIGHSRLNKENDTLKALLK
ANVEKPVKLEVFNMKTMRVREVEVVPSNMWGGQGLLGASVRFCSFRRASEQVWHVLDVEP
SSPAALAGLRPYTDYVVGSDQILQESEDFFTLIESHEGKPLKLMVYNSKSDSCREVTVTP
NAAWGGEGSLGCGIGYGYLHRIPTQPPSYHKKPPGTPPPSALPLGAPPPDALPPGPTPED
SPSLETGSRQSDYMEALLQAPGSSMEDPLPGPGSPSHSAPDPDGLPHFMETPLQPPPPVQ
RVMDPGFLDVSGISLLDNSNASVWPSLPSSTELTTTAVSTSGPEDICSSSSSHERGGEAT
WSGSEFEVSFLDSPGAQAQADHLPQLTLPDSLTSAASPEDGLSAELLEAQAEEEPASTEG
LDTGTEAEGLDSQAQISTTE*
>ENSG00000115020|ENST00000308862
MATDDKTSPTLDSANDLPRSPTSPSHLTHFKPLTPDQDEPPFKSAYSSFVNLFRFNKERA
EGGQGEQQPLSGSWTSPQLPSRTQSVRSPTPYKKQLNEELQRRSSALDNSLQHPQENTGD
LRACTYCRKIALSYAHSTDSNSIGEDLNALSDSACSVSVLDPSEPRTPVGSRKASRNIFL
EDDLAWQSLIHPDSSNTPLSTRLVSVQEDAGKSPARNRSASITNLSLDRSGSPMVPSYET
SVSPQANRTYVRTETTEDERKILLDSVQLKDLWKKICHHSSGMEFQDHRYWLRTHPNCIV
GKELVNWLIRNGHIATRAQAIAIGQAMVDGRWLDCVSHHDQLFRDEYALYRPLQSTEFSE
TPSPDSDSVNSVEGHSEPSWFKDIKFDDSDTEQIAEEGDDNLANSASPSKRTSVSSFQST
VDSDSAASISLNVELDNVNFHIKKPSKYPHVPPHPADQKGRR*
>ENSG00000165905|ENST00000325468
MLPRGRPRALGAAALLLLLLLLGFLLFGGDLGCERREPGGRAGAPGCFPGPLMPRVPPDG
RLRRAAALDGDPGAGPGDHNRSDCGPQPPPPPKCELLHVAIVCAGHNSSRDVITLVKSML
FYRKNPLHLHLVTDAVARNILETLFHTWMVPAVRVSFYHADQLKPQVSWIPNKHYSGLYG
LMKLVLPSALPAELARVIVLDTDVTFASDISELWALFAHFSDTQAIGLVENQSDWYLGNL
WKNHRPWPALGRGFNTGVILLRLDRLRQAGWEQMWRLTARRELLSLPATSLADQDIFNAV
IKEHPGLVQRLPCVWNVQLSDHTLAERCYSEASDLKVIHWNSPKKLRVKNKHVEFFRNFY
LTFLEYDGNLLRRELFVCPSQPPPGAEQLQQALAQLDEEDPCFEFRQQQLTVHRVHVTFL
PHEPPPPRPHDVTLVAQLSMDRLQMLEALCRHWPGPMSLALYLTDAEAQQFLHFVEASPV
LAARQDVAYHVVYREGPLYPVNQLRNVALAQALTPYVFLSDIDFLPAYSLYDYLRASIEQ
LGLGSRRKAALVVPAFETLRYRFSFPHSKVELLALLDAGTLYTFRYHEWPRGHAPTDYAR
WREAQAPYRVQWAANYEPYVVVPRDCPRYDPRFVGFGWNKVAHIVELDAQEYELLVLPEA
FTIHLPHAPSLDISRFRSSPTYRDCLQALKDEFHQDLSRHHGAAALKYLPALQQPQSPAR
G*
>ENSG00000175416|ENST00000345807

MADDFGFFSSSESGAPEAAEEDPAAAFLAQQESEIAGIENDEGFGAPAGSHAAPAQPGPT
SGAGSEDMGTTVNGDVFQEANGPADGYAAIAQADRLTQEPESIRKWREEQRKRLQELDAA
SKVTEQEWREKAKKDLEEWNQRQSEQVEKNKINNRASEEAFVKESKEETPGTEWEKVAQL
CDFNPKSSKQCKDVSRLRSVLMSLKQTPLSR*
>ENSG00000141551|ENST00000314028
MELRVGNRYRLGRKIGSGSFGDIYLGTDIAAGEEVAIKLECVKTKHPQLHIESKIYKMMQ
GGVGIPTIRWCGAEGDYNVMVMELLGPSLEDLFNFCSRKFSLKTVLLLADQMISRIEYIH
SKNFIHRDVKPDNFLMGLGKKGNLVYIIDFGLAKKYRDARTHQHIPYRENKNLTGTARYA
SINTHLGIEQSRRDDLESLGYVLMYFNLGSLPWQGLKAATKRQKYERISEKKMSTPIEVL
CKGYPSEFATYLNFCRSLRFDDKPDYSYLRQLFRNLFHRQGFSYDYVFDWNMLKFGASRA
ADDAERERRDREERLRHSRNPATRGLPSTASGRLRGTQEVAPPTPLTPTSHTANTSPRPV
SGMERERKVSMRLHRGAPVNISSSDLTGRQDTSRMSTSQIPGRVASSGLQSVVHR*
>ENSG00000183166|ENST00000329008
MPFHHVTAGLLYKGNYLNRSLSAGSDSEQLANISVEELDEIREAFRVLDRDGNGFISKQE
LGMAMRSLGYMPSEVELAIIMQRLDMDGDGQVDFDEFMTILGPKLVSSEGRDGFLGNTID
SIFWQFDMQRITLEELKHILYHAFRDHLTMKDIENIIINEEESLNETSGNCQTEFEGVHS
QKQNRQTCVRKSLICAFAMAFIISVMLIAANQILRSGME*
>ENSG00000215717|ENST00000338272
MTNVYSLDGILVFGLLFVCTCAYFKKVPRLKTWLLSEKKGVWGVFYKAAVIGTRLHAAVA
IACVVMAFYVLFIK*
>ENSG00000103365|ENST00000309859
MAATAVAAAVAGTESAQGPPGPAASLELWLNKATDPSMSEQDWSAIQNFCEQVNTDPNGP
THAPWLLAHKIQSPQEKEALYALTVLEMCMNHCGEKFHSEVAKFRFLNELIKVLSPKYLG
SWATGKVKGRVIEILFSWTVWFPEDIKIRDAYQMLKKQGIIKQDPKLPVDKILPPPSPWP
KSSIFDADEEKSKLLTRLLKSNHPEDLQAANRLIKNLVKEEQEKSEKVSKRVSAVEEVRS
HVKVLQEMLSMYRRPGQAPPDQEALQVVYERCEKLRPTLFRLASDTTDDDDALAEILQAN
DLLTQGVLLYKQVMEGRVTFGNRVTSSLGDIPVSRVFQNPAGCMKTCPLIDLEVDNGPAQ
MGTVVPSLLHQDLAALGISDAPVTGMVSGQNCCEEKRNPSSSTLPGGGVQNPSADRNLLD
LLSAQPAPCPLNYVSQKSVPKEVPPGTKSSPGWSWEAGPLAPSPSSQNTPLAQVFVPLES
VKPSSLPPLIVYDRNGFRILLHFSQTGAPGHPEVQVLLLTMMSTAPQPVWDIMFQVAVPK
SMRVKLQPASSSKLPAFSPLMPPAVISQMLLLDNPHKEPIRLRYKLTFNQGGQPFSEVGE
VKDFPDLAVLGAA*
>ENSG00000171310|ENST00000303694
MKPALLEVMRMNRICRMVLATCLGSFILVIFYFQSMLHPVMRRNPFGVDICCRKGSRSPL
QELYNPIQLELSNTAVLHQMRRDQVTDTCRANSATSRKRRVLTPNDLKHLVVDEDHELIY
CYVPKVACTNWKRLMMVLTGRGKYSDPMEIPANEAHVSANLKTLNQYSIPEINHRLKSYM
KFLFVREPFERLVSAYRNKFTQKYNISFHKRYGTKIIKRQRKNATQEALRKGDDVKFEEF
VAYLIDPHTQREEPFNEHWQTVYSLCHPCHIHYDLVGKYETLEEDSNYVLQLAGVGSYLK
FPTYAKSTRTTDEMTTEFFQNISSEHQTQLYEVYKLDFLMFNYSVPSYLKLE*
>ENSG00000169902|ENST00000304842
MVGKLKQNLLLACLVISSVTVFYLGQHAMECHHRIEERSQPVKLESTRTTVRTGLDLKAN
KTFAYHKDMPLIFIGGVPRSGTTLMRAMLDAHPDIRCGEETRVIPRILALKQMWSRSSKE
KIRLDEAGVTDEVLDSAMQAFLLEIIVKHGEPAPYLCNKDPFALKSLTYLSRLFPNAKFL
LMVRDGRASVHSMISRKVTIAGFDLNSYRDCLTKWNRAIETMYNQCMEVGYKKCMLVHYE

QLVLHPERWMRTLLKFLQIPWNHSVLHHEEMIGKAGGVSLSKVERSTDQVIKPVNVGALS
KWVGKIPPDVLQDMAVIAPMLAKLGYDPYANPPNYGKPDPKIIENTRRVYKGEFQLPDFL
KEKPQTEQVE*
>ENSG00000153786|ENST00000344861
MQPSGHRLRDVEHHPLLAENDNYDSSSSSSSEADVADRVWFIRDGCGMICAVMTWLLVAY
ADFVVTFVMLLPSKDFWYSVVNGVIFNCLAVLALSSHLRTMLTDPEKSSDCRPSACTVKT
GLDPTLVGICGEGTESVQSLLLGAVPKGNATKEYMESLQLKPGEVIYKCPKCCCIKPERA
HHCSICKRCIRKMDHHCPWVNNCVGEKNQRFFVLFTMYIALSSVHALILCGFQFISCVRG
QWTECSDFSPPITVILLIFLCLEGLLFFTFTAVMFGTQIHSICNDETEIERLKSEKPTWE
RRLRWEGMKSVFGGPPSLLWMNPFVGFRFRRLPTRPRKGGPEFSV*
>ENSG00000182601|ENST00000331351
MARWPAPPPPPPPPPPLAAPPPPGASAKGPPARKLLFMCTLSLSVTYLCYSLLGGSGSLQ
FPLALQESPGAAAEPPPSPPPPSLLPTPVRLGAPSQPPAPPPLDNASHGEPPEPPEQPAA
PGTDGWGLPSGGGGAQDAWLRTPLAPSEMITAQSALPEREAQESSTTDEDLAGRRAANGS
SERGGAVSTPDYGEKKLPQALIIGVKKGGTRALLEAIRVHPDVRAVGVEPHFFDRNYEKG
LEWYRNVMPKTLDGQITMEKTPSYFVTNEAPKRIHSMAKDIKLIVVVRNPVTRAISDYTQ
TLSKKPEIPTFEVLAFKNRTLGLIDASWSAIRIGIYALHLENWLQYFPLSQILFVSGERL
IVDPAGEMAKVQDFLGLKRVVTEKHFYFNKTKGFPCLKKPEDSSAPRCLGKSKGRTHPRI
DPDVIHRLRKFYKPFNLMFYQMTGQDFQWEQEEGDK*
>ENSG00000006715|ENST00000310301
MAEAEEQETGSLEESTDESEEEESEEEPKLKYERLSNGVTEILQKDAASCMTVHDKFLAL
GTHYGKVYLLDVQGNITQKFDVSPVKINQISLDESGEHMGVCSEDGKVQVFGLYSGEEFH
ETFDCPIKIIAVHPHFVRSSCKQFVTGGKKLLLFERSWMNRWKSAVLHEGEGNIRSVKWR
GHLIAWANNMGVKIFDIISKQRITNVPRDDISLRPDMYPCSLCWKDNVTLIIGWGTSVKV
CSVKERHASEMRDLPSRYVEIVSQFETEFYISGLAPLCDQLVVLSYVKEISEKTEREYCA
RPRLDIIQPLSETCEEISSDALTVRGFQENECRDYHLEYSEGESLFYIVSPRDVVVAKER
DQDDHIDWLLEKKKYEEALMAAEISQKNIKRHKILDIGLAYINHLVERGDYDIAARKCQK
ILGKNAALWEYEVYKFKEIGQLKAISPYLPRGDPVLKPLIYEMILHEFLESDYEGFATLI
REWPGDLYNNSVIVQAVRDHLKKDSQNKTLLKTLAELYTYDKNYGNALEIYLTLRHKDVF
QLIHKHNLFSSIKDKIVLLMDFDSEKAVDMLLDNEDKISIKKVVEELEDRPELQHVYLHK
LFKRDHHKGQRYHEKQISLYAEYDRPNLLPFLRDSTHCPLEKALEICQQRNFVEETVYLL
SRMGNSRSALKMIMEELHDVDKAIEFAKEQDDGELWEDLILYSIDKPPFITGLLNNIGTH
VDPILLIHRIKEGMEIPNLRDSLVKILQDYNLQILLREGCKKILVADSLSLLKKMHRTQM
KGVLVDEENICESCLSPILPSDAAKPFSVVVFHCRHMFHKECLPMPSMNSAAQFCNICSA
KNRGPGSAILEMKK*
>ENSG00000158089|ENST00000324589
MRRLTRRLVLPVFGVLWITVLLFFWVTKRKLEVPTGPEVQTPKVWSLFFKVAGMSPWAPQ
VPVSPTPPYQRGHLPTGGHLAVCHFPCLLQEAQFHLQTQVFLQVRCTLLVYCTDLPPTSI
IITFHNEARSTLLRTIRSVLNRTPTHLIREIILVDDFSNDPDDCKQLIKLPKVKCLRNNE
RQGLVRSRIRGADIAQGTTLTFLDSHCEVNRDWLQPLLHRVKEDYTRVVCPVIDIINLDT
FTYIESASELRGGFDWSLHFQWEQLSPEQKARRLDPTEPIRTPIIAGGLFVIDKAWFDYL
GKYDMDMDIWGGENFEISFRVWMCGGSLEIVPCSRVGHVFRKKHPYVFPDGNANTYIKNT
KRTAEVWMDEYKQYYYAARPFALERPFGNVESRLDLRKNLRCQSFKWYLENIYPELSIPK
ESSIQKGNIRQRQKCLESQRQNNQETPNLKLSPCAKVKGEDAKSQVWAFTYTQQILQEEL

CLSVITLFPGAPVVLVLCKNGDDRQQWTKTGSHIEHIASHLCLDTDMFGDGTENGKEIVV
NPCESSLMSQHWDMVSS*
>ENSG00000065923|ENST00000328306
MEPGDAARPGSGRATGAPPPRLLLLPLLLGWGLRVAAAASASSSGAAAEDSSAMEELATE
KEAEESHRQDSVSLLTFILLLTLTILTIWLFKHRRVRFLHETGLAMIYGLIVGVILRYGT
PATSGRDKSLSCTQEDRAFSTLLVNVSGKFFEYTLKGEISPGKINSVEQNDMLRKVTFDP
EVFFNILLPPIIFHAGYSLKKRHFFRNLGSILAYAFLGTAVSCFIIGNLMYGVVKLMKIM
GQLSDKFYYTDCLFFGAIISATDPVTVLAIFNELHADVDLYALLFGESVLNDAVAIVLSS
SIVAYQPAGLNTHAFDAAAFFKSVGIFLGIFSGSFTMGAVTGVNANVTKFTKLHCFPLLE
TALFFLMSWSTFLLAEACGFTGVVAVLFCGITQAHYTYNNLSVESRSRTKQLFEVLHFLA
ENFIFSYMGLALFTFQKHVFSPIFIIGAFVAIFLGRAAHIYPLSFFLNLGRRHKIGWNFQ
HMMMFSGLRGAMAFALAIRDTASYARQMMFTTTLLIVFFTVWIIGGGTTPMLSWLNIRVG
VEEPSEEDQNEHHWQYFRVGVDPDQDPPPNNDSFQVLQGDGPDSARGNRTKQESAWIFRL
WYSFDHNYLKPILTHSGPPLTTTLPAWCGLLARCLTSPQVYDNQEPLREEDSDFILTEGD
LTLTYGDSTVTANGSSSSHTASTSLEGSRRTKSSSEEVLERDLGMGDQKVSSRGTRLVFP
LEDNA*
>ENSG00000177257|ENST00000318157
MRVLYLLFSFLFIFLMPLPGVFGGIGDPVTCLKSGAICHPVFCPRRYKQIGTCGLPGTKC
CKKP*
>ENSG00000138674|ENST00000311785
MKLKEVDRTAMQAWSPAQNHPIYLATGTSAQQLDATFSTNASLEIFELDLSDPSLDMKSC
ATFSSSHRYHKLIWGPYKMDSKGDVSGVLIAGGENGNIILYDPSKIIAGDKEVVIAQNDK
HTGPVRALDVNIFQTNLVASGANESEIYIWDLNNFATPMTPGAKTQPPEDISCIAWNRQV
QHILASASPSGRATVWDLRKNEPIIKVSDHSNRMHCSGLAWHPDVATQMVLASEDDRLPV
IQMWDLRFASSPLRVLENHARGILAIAWSMADPELLLSCGKDAKILCSNPNTGEVLYELP
TNTQWCFDIQWCPRNPAVLSAASFDGRISVYSIMGGSTDGLRQKQVDKLSSSFGNLDPFG
TGQPLPPLQIPQQTAQHSIVLPLKKPPKWIRRPVGASFSFGGKLVTFENVRMPSHQGAEQ
QQQQHHVFISQVVTEKEFLSRSDQLQQAVQSQGFINYCQKKIDASQTEFEKNVWSFLKVN
FEDDSRGKYLELLGYRKEDLGKKIALALNKVDGANVALKDSDQVAQSDGEESPAAEEQLL
GEHIKEEKEESEFLPSSGGTFNISVSGDIDGLITQALLTGNFESAVDLCLHDNRMADAII
LAIAGGQELLARTQKKYFAKSQSKITRLITAVVMKNWKEIVESCDLKNWREALAAVLTYA
KPDEFSALCDLLGTRLENEGDSLLQTQACLCYICAGNVEKLVACWTKAQDGSHPLSLQDL
IEKVVILRKAVQLTQAMDTSTVGVLLAAKMSQYANLLAAQGSIAAALAFLPDNTNQPNIM
QLRDRLCRAQGEPVAGHESPKIPYEKQQLPKGRPGPVAGHHQMPRVQTQQYYPHGENPPP
PGFIMHGNVNPNAAGQLPTSPGHMHTQVPPYPQPQRPQNGWNDPPALNRVPKKKKMPENF
MPPVPITSPIMNPLGDPQSQMLQQQPSAPVPLSSQSSFPQPHLPGGQPFHGVQQPLGQTG
MPPSFSKPNIEGAPGAPIGNTFQHVQSLPTKKITKKPIPDEHLILKTTFEDLIQRCLSSA
TDPQTKRKLDDASKRLEFLYDKLREQTLSPTITSGLHNIARSIETRNYSEGLTMHTHIVS
TSNFSETSAFMPVLKVVLTQANKLGV*
>ENSG00000160613|ENST00000320934
MPKGRQKVPHLDAPLGLPTCLWLELAGLFLLVPWVMGLAGTGGPDGQGTGGPSWAVHLES
LEGDGEEETLEQQADALAQAAGLVNAGRIGELQGHYLFVQPAGHRPALEVEAIRQQVEAV
LAGHEAVRWHSEQRLLRRAKRSVHFNDPKYPQQWHLNNRRSPGRDINVTGVWERNVTGRG
VTVVVVDDGVEHTIQDIAPNYSPEGSYDLNSNDPDPMPHPDVENGNHHGTRCAGEIAAVP

NNSFCAVGVAYGSRIAGIRVLDGPLTDSMEAVAFNKHYQINDIYSCSWGPDDDGKTVDGP
HQLGKAALQHGVIAGRQGFGSIFVVASGNGGQHNDNCNYDGYANSIYTVTIGAVDEEGRM
PFYAEECASMLAVTFSGGDKMLRSIVTTDWDLQKGTGCTEGHTGTSAAAPLAAGMIALML
QVRPCLTWRDVQHIIVFTATRYEDRRAEWVTNEAGFSHSHQHGFGLLNAWRLVNAAKIWT
SVPYLASYVSPVLKENKAIPQSPRSLEVLWNVSRMDLEMSGLKTLEHVAVTVSITHPRRG
SLELKLFCPSGMMSLIGAPRSMDSDPNGFNDWTFSTVRCWGERARGTYRLVIRDVGDESF
QVGILRQWQLTLYGSVWSAVDIRDRQRLLESAMSGKYLHDDFALPCPPGLKIPEEDGYTI
TPNTLKTLVLVGCFTVFWTVYYMLEVYLSQRNVASNQVCRSGPCHWPHRSRKAKEEGTEL
ESVPLCSSKDPDEVETESRGPPTTSDLLAPDLLEQGDWSLSQNKSALDCPHQHLDVPHGK
EEQIC*
>ENSG00000133318|ENST00000339997
MAEPSAATQSHSISSSSFGAEPSAPGGGGSPGACPALGTKSCSSSCAEGLSSLCSDEPSS
EIMTSSFLSSSEIHNTGLTILHGEKSHVLGSQPILAKEGKDHLDLLDMKKMEKPQGTSNN
VSDSSVSLAAGVHCDRPSIPASFPEHPAFLSKKIGQVEEQIDKETKNPNGVSSREAKTAL
DADDRFTLLTAQKPPTEYSKVEGIYTYSLSPSKVSGDDVIEKDSPESPFEVIIDKAAFDK
EFKDSYKESTDDFGSWSVHTDKESSEDISETNDKLFPLRNKEAGRYPMSALLSRQFSHTN
AALEEVSRCVNDMHNFTNEILTWDLVPQVKQQTDKSSDCITKTTGLDMSEYNSEIPVVNL
KTSTHQKTPVCSIDGSTPITKSTGDWAEASLQQENAITGKPVPDSLNSTKEFSIKGVQGN
MQKQDDTLAELPGSPPEKCDSLGSGVATVKVVLPDDHLKDEMDWQSSALGEITEADSSGE
SDDTVIEDITADTSFENNKIQAEKPVSIPSAVVKTGEREIKEIPSCEREEKTSKNFEELV
SDSELHQDQPDILGRSPASEAACSKVPDTNVSLEDVSEVAPEKPITTENPKLPSTVSPNV
FNETEFSLNVTTSAYLESLHGKNVKHIDDSSPEDLIAAFTETRDKGIVDSERNAFKAISE
KMTDFKTTPPVEVLHENESGGSEIKDIGSKYSEQSKETNGSEPLGVFPTQGTPVASLDLE
QEQLTIKALKELGERQVEKSTSAQRDAELPSEEVLKQTFTFAPESWPQRSYDILERNVKN
GSDLGISQKPITIRETTRVDAVSSLSKTELVKKHVLARLLTDFSVHDLIFWRDVKKTGFV
FGTTLIMLLSLAAFSVISVVSYLILALLSVTISFRIYKSVIQAVQKSEEGHPFKAYLDVD
ITLSSEAFHNYMNAAMVHINRALKLIIRLFLVEDLVDSLKLAVFMWLMTYVGAVFNGITL
LILAELLIFSVPIVYEKYKTQIDHYVGIARDQTKSIVEKIQAKLPGIAKKKAE*
>ENSG00000182400|ENST00000330149
MADEALFLLLHNEMVSGVYKSAEQGEVENGRCITKLENMGFRVGQGLIERFTKDTARFKD
ELDIMKFICKDFWTTVFKKQIDNLRTNHQGIYVLQDNKFRLLTQMSAGKQYLEHASKYLA
FTCGLIRGGLSNLGIKSIVTAEVSSMPACKFQVMIQKL*
>ENSG00000172007|ENST00000305626
MAEEMESSLEASFSSSGAVSGASGFLPPARSRIFKIIVIGDSNVGKTCLTYRFCAGRFPD
RTEATIGVDFRERAVEIDGERIKIQLWDTAGQERFRKSMVQHYYRNVHAVVFVYDMTNMA
SFHSLPSWIEECKQHLLANDIPRILVGNKCDLRSAIQVPTDLAQKFADTHSMPLFETSAK
NPNDNDHVEAIFMTLAHKLKSHKPLMLSQPPDNGIILKPEPKPAMTCWC*
>ENSG00000180210|ENST00000311907
MAHVRGLQLPGCLALAALCSLVHSQHVFLAPQQARSLLQRVRRANTFLEEVRKGNLEREC
VEETCSYEEAFEALESSTATDVFWAKYTACETARTPRDKLAACLEGNCAEGLGTNYRGHV
NITRSGIECQLWRSRYPHKPEINSTTHPGADLQENFCRNPDSSTTGPWCYTTDPTVRRQE
CSIPVCGQDQVTVAMTPRSEGSSVNLSPPLEQCVPDRGQQYQGRLAVTTHGLPCLAWASA
QAKALSKHQDFNSAVQLVENFCRNPDGDEEGVWCYVAGKPGDFGYCDLNYCEEAVEEETG
DGLDEDSDRAIEGRTATSEYQTFFNPRTFGSGEADCGLRPLFEKKSLEDKTERELLESYI

DGRIVEGSDAEIGMSPWQVMLFRKSPQELLCGASLISDRWVLTAAHCLLYPPWDKNFTEN
DLLVRIGKHSRTRYERNIEKISMLEKIYIHPRYNWRENLDRDIALMKLKKPVAFSDYIHP
VCLPDRETAASLLQAGYKGRVTGWGNLKETWTANVGKGQPSVLQVVNLPIVERPVCKDST
RIRITDNMFCAGYKPDEGKRGDACEGDSGGPFVMKSPFNNRWYQMGIVSWGEGCDRDGKY
GFYTHVFRLKKWIQKVIDQFGE*
>ENSG00000107554|ENST00000324109
MEAGSVVRAIFDFCPSVSEELPLFVGDIIEVLAVVDEFWLLGKKEDVTGQFPSSFVEIVT
IPSLKEGERLFVCICEFTSQELDNLPLHRGDLVILDGIPTAGWLQGRSCWGARGFFPSSC
VRELCLSSQSRQWHSQSALFQIPEYSMGQARALMGLSAQLDEELDFREGDVITIIGVPEP
GWFEGELEGRRGIFPEGFVELLGPLRTVDESVSSGNQDDCIVNGEVDTPVGEEEIGPDED
EEEPGTYGVALYRFQALEPNELDFEVGDKIRILATLEDGWLEGSLKGRTGIFPYRFVKLC
PDTRVEETMALPQEGSLARIPETSLDCLENTLGVEEQRHETSDHEAEEPDCIISEAPTSP
LGHLTSEYDTDRNSYQDEDTAGGPPRSPGVEWEMPLATDSPTSDPTEVVNGISSQPQVPF
HPNLQKSQYYSTVGGSHPHSEQYPDLLPLEARTRDYASLPPKRMYSQLKTLQKPVLPLYR
GSSVSASRVVKPRQSSPQLHNLASYTKKHHTSSVYSISERLEMKPGPQAQGLVMEAATHS
QGDGSTDLDSKLTQQLIEFEKSLAGPGTEPDKILRHFSIMDFNSEKDIVRGSSKLITEQE
LPERRKALRPPPPRPCTPVSTSPHLLVDQNLKPAPPLVVRPSRPAPLPPSAQQRTNAVSP
KLLSRHRPTCETLEKEGPGHMGRSLDQTSPCPLVLVRIEEMERDLDMYSRAQEELNLMLE
EKQDESSRAETLEDLKFCESNIESLNMELQQLREMTLLSSQSSSLVAPSGSVSAENPEQR
MLEKRAKVIEELLQTERDYIRDLEMCIERIMVPMQQAQVPNIDFEGLFGNMQMVIKVSKQ
LLAALEISDAVGPVFLGHRDELEGTYKIYCQNHDEAIALLEIYEKDEKIQKHLQDSLADL
KSLYNEWGCTNYINLGSFLIKPVQRVMRYPLLLMELLNSTPESHPDKVPLTNAVLAVKEI
NVNINEYKRRKDLVLKYRKGDEDSLMEKISKLNIHSIIKKSNRVSSHLKHLTGFAPQIKD
EVFEETEKNFRMQERLIKSFIRDLSLYLQHIRESACVKVVAAVSMWDVCMERGHRDLEQF
ERVHRYISDQLFTNFKERTERLVISPLNQLLSMFTGPHKLVQKRFDKLLDFYNCTERAEK
LKDKKTLEELQSARNNYEALNAQLLDELPKFHQYAQGLFTNCVHGYAEAHCDFVHQALEQ
LKPLLSLLKVAGREGNLIAIFHEEHSRVLQQLQVFTFFPESLPATKKPFERKTIDRQSAR
KPLLGLPSYMLQSEELRASLLARYPPEKLFQAERNFNAAQDLDVSLLEGDLVGVIKKKDP
MGSQNRWLIDNGVTKGFVYSSFLKPYNPRRSHSDASVGSHSSTESEHGSSSPRFPRQNSG
STLTFNPSSMAVSFTSGSCQKQPQDASPPPKECDQGTLSASLNPSNSESSPSRCPSDPDS
TSQPRSGDSADVARDVKQPTATPRSYRNFRHPEIVGYSVPGRNGQSQDLVKGCARTAQAP
EDRSTEPDGSEAEGNQVYFAVYTFKARNPNELSVSANQKLKILEFKDVTGNTEWWLAEVN
GKKGYVPSNYIRKTEYT*
>ENSG00000175416|ENST00000310418
MADDFGFFSSSESGAPEAAEEDPAAAFLAQQESEIAGIENDEGFGAPAGSHAAPAQPGPT
SGAGSEDMGTTVNGDVFQEANGPADGYAAIAQADRLTQEPESIRKWREEQRKRLQELDAA
SKVTEQEWREKAKKDLEEWNQRQSEQVEKNKINNRIADKAFYQQPDADIIGYVASEEAFV
KESKEETPGTEWEKVAQLCDFNPKSSKQCKDVSRLRSVLMSLKQTPLSR*
>ENSG00000244045|ENST00000292114
MASSLLAGERLVRALGPGGELEPERLPRKLRAELEAALGKKHKGGDSSSGPQRLVSFRLI
RDLHQHLRERDSKLYLHELLEGSEIYLPEVVKPPRNPELVARLEKIKIQLANEEYKRITR
NVTCQDTRHGGTLSDLGKQVRSLKALVITIFNFIVTVVAAFVCTYLGSQYIFTEMASRVL
AALIVASVVGLAELYVMVRAMEGELGEL*
>ENSG00000131446|ENST00000333055

MLKKQSAGLVLWGAILFVAWNALLLLFFWTRPAPGRPPSVSALDGDPASLTREVIRLAQD
AEVELERQRGLLQQIGDALSSQRGRVPTAAPPAQPRVPVTPAPAVIPILVIACDRSTVRR
CLDKLLHYRPSAELFPIIVSQDCGHEETAQAIASYGSAVTHIRQPDLSSIAVPPDHRKFQ
GYYKIARHYRWALGQVFRQFRFPAAVVVEDDLEVAPDFFEYFRATYPLLKADPSLWCVSA
WNDNGKEQMVDASRPELLYRTDFFPGLGWLLLAELWAELEPKWPKAFWDDWMRRPEQRQG
RACIRPEISRTMTFGRKGVSHGQFFDQHLKFIKLNQQFVHFTQLDLSYLQREAYDRDFLA
RVYGAPQLQVEKVRTNDRKELGEVRVQYTGRDSFKAFAKALGVMDDLKSGVPRAGYRGIV
TFQFRGRRVHLAPPLTWEGYDPSWN*
>ENSG00000172728|ENST00000327671
MVRIQRRKLLASCLCVTATVFLLVTLQVMVELGKFERKEFKSSSLQDGHTKMEEAPTHLN
SFLKKEGLTFNRKRKWELDSYPIMLWWSPLTGETGRLGQCGADACFFTINRTYLHHHMTK
AFLFYGTDFNIDSLPLPRKAHHDWAVFHEESPKNNYKLFHKPVITLFNYTATFSRHSHLP
LTTQYLESIEVLKSLRYLVPLQSKNKLRKRLAPLVYVQSDCDPPSDRDSYVRELMTYIEV
DSYGECLRNKDLPQQLKNPASMDADGFYRIIAQYKFILAFENAVCDDYITEKFWRPLKLG
VVPVYYGSPSITDWLPSNKSAILVSEFSHPRELASYIRRLDSDDRLYEAYVEWKLKGEIS
NQRLLTALRERKWGVQDVNQDNYIDAFECMVCTKVWANIRLQEKGLPPKRWEAEDTHLSC
PEPTVFAFSPLRTPPLSSLREMWISSFEQSKKEAQALRWLVDRNQNFSSQEFWGLVFKD*
>ENSG00000176542|ENST00000316407
MPEMTENETPTKKQHRKKNRETHNAVERHRKKKINAGINRIGELIPCSPALKQSKNMILD
QAFKYITELKRQNDELLLNGGNNEQAEEIKKLRKQLEEIQKENGRYIELLKANDICLYDD
PTIHWKGNLKNSKVSVVIPSDQVQKKIIVYSNGNQPGGNSQGTAVQGITFNVSHNLQKQT
ANVVPVQRTCNLVTPVSISGVYPSENKPWHQTTVPALATNQPVPLCLPAAISAQSILELP
TSESESNVLGATSGSLIAVSIESEPHQHHSLHTCLNDQNSSENKNGQENPKVLKKMTPCV
TNIPHSSSATATKVHHGNKSCLSIQDFRGDFQNTFVVSVTTTVCSQPPRTAGDSSPMSIS
KSADLTSTATVVASSAPGVGKATIPISTLSGNPLDNGWTLSCSLPSSSVSTSDLKNINSL
TRISSAGNTQTTWTTLQLAGNTIQPLSQTPSSAVTPVLNESGTSPTTSNHSRYVATDINL
NNSFPADGQPVEQVVVTLPSCPSLPMQPLIAQPQVKSQPPKNILPLNSAMQVIQMAQPVG
SAVNSAPTNQNVIILQPPSTTPCPTVMRAEVSNQTVGQQIVIIQAANQNPLPLLPAPPPG
SVRLPINGANTVIGSNNSVQNVPTPQTFGGKHLVHILPRPSSLSASNSTQTFSVTMSNQQ
PQTISLNGQLFALQPVMSSSGTTNQTPMQIIQPTTSEDPNTNVALNTFGALASLNQSISQ
MAGQSCVQLSISQPANSQTAANSQTTTANCVSLTTTAAPPVTTDSSATLASTYNLVSTSS
MNTVACLPNMKSKRLNKKPGGRKHLAANKSACPLNSVRDVSKLDCPNTEGSAEPPCNDGL
LESFPAVLPSVSVSQANSVSVSASHSLGVLSSESLIPESVSKSKSAEKSSPPSQESVTSE
HFAMAAAKSKDSTPNLQQETSQDKPPSSLALSDAAKPCASANVLIPSPSDPHILVSQVPG
LSSTTSTTSTDCVSEVEIIAEPCRVEQDSSDTMQTTGLLKGQGLTTLLSDLAKKKNPQKS
SLSDQMDHPDFSSENPKIVDSSVNLHPKQELLLMNNDDRDPPQHHSCLPDQEVINGSLIN
GRQADSPMSTSSGSSRSFSVASMLPETTREDVTSNATTNTCDSCTFVEQTDIVALAARAI
FDQENLEKGRVGLQADIREVASKPSEASLLEGDPPFKSQIPKESGTGQAEATPNEFNSQG
SIEATMERPLEKPSCSLGIKTSNASLQDSTSQPPSITSLSVNNLIHQSSISHPLASCAGL
SPTSEQTTVPATVNLTVSSSSYGSQPPGPSLMTEYSQEQLNTMTSTIPNSQIQEPLLKPS
HESRKDSAKRAVQDDLLLSSAKRQKHCQPAPLRLESMSLMSRTPDTISDQTQMMVSQIPP
NSSNSVVPVSNPAHGDGLTRLFPPSNNFVTPALRQTEVQCGSQPSVAEQQQTQASQHLQA
LQQHVPAQGVSHLHSNHLYIKQQQQQQQQQQQQQQQQAGQLRERHHLYQMQHHVPHAES
SVHSQPHNVHQQRTLQQEVQMQKKRNLVQGTQTSQLSLQPKHHGTDQSRSKTGQPHPHHQ

QMQQQMQQHFGSSQTEKSCENPSTSRNHHNHPQNHLNQDIMHQQQDVGSRQQGSGVSSEH
VSGHNPMQRLLTSRGLEQQMVSQPSIVTRSSDMTCTPHRPERNRVSSYSAEALIGKTSSN
SEQRMGISIQGSRVSDQLEMRSYLDVPRNKSLAIHNMQGRVDHTVASDIRLSDCQTFKPS
GASQQPQSNFEVQSSRNNEIGNPVSSLRSMQSQAFRISQNTGPPPIDRQKRLSYPPVQSI
PTGNGIPSRDSENTCHQSFMQSLLAPHLSDQVIGSQRSLSEHQRNTQCGPSSAIEYNCPP
THENVHIRRESESQNRESCDMSLGAINTRNSTLNIPFSSSSSSGDIQGRNTSPNVSVQKS
NPMRITESHATKGHMNPPVTTNMHGVARPALPHPSVSHGNGDQGPAVRQANSSVPQRSRH
PLQDSSGSKIRQPERNRSGNQRQSTVFDPSLPHLPLSTGGSMILGRQQPATEKRGSIVRF
MPDSPQVPNDNSGPDQHTLSQNFGFSFIPEGGMNPPINANASFIPQVTQPSATRTPALIP
VDPQNTLPSFYPPYSPAHPTLSNDISIPYFPNQMFSNPSTEKVNSGSLNNRFGSILSPPR
PVGFAQPSFPLLPDMPPMHMTNSHLSNFNMTSLFPEIATALPDGSAMSPLLTIANSSASD
SSKQSSNRPAHNISHILGHDCSSAV*
>ENSG00000170962|ENST00000302251
MHRLIFVYTLICANFCSCRDTSATPQSASIKALRNANLRRDDLYRRDETIQVKGNGYVQS
PRFPNSYPRNLLLTWRLHSQENTRIQLVFDNQFGLEEAENDICRYDFVEVEDISETSTII
RGRWCGHKEVPPRIKSRTNQIKITFKSDDYFVAKPGFKIYYSLLEDFQPAAASETNWESV
TSSISGVSYNSPSVTDPTLIADALDKKIAEFDTVEDLLKYFNPESWQEDLENMYLDTPRY
RGRSYHDRKSKVDLDRLNDDAKRYSCTPRNYSVNIREELKLANVVFFPRCLLVQRCGGNC
GCGTVNWRSCTCNSGKTVKKYHEVLQFEPGHIKRRGRAKTMALVDIQLDHHERCDCICSS
RPPR*
>ENSG00000100478|ENST00000334725
MIKFFLMVNKQGQTRLSKYYEHVDINKRTLLETEVIKSCLSRSNEQCSFIEYKDFKLIYR
QYAALFIVVGVNDTENEMAIYEFIHNFVEVLDEYFSRVSELDVSFFNTVFHSTWQMHSGP
YQTRSCSVTQAGVQRCDHGSLHPGSPGLK*
>ENSG00000175040|ENST00000309575
MSRSPQRALPPGALPRLLQAAPAAAPRALLPQWPRRPGRRWPASPLGMKVFRRKALVLCA
GYALLLVLTMLNLLDYKWHKEPLQQCNPDGPLGAAAGAAGGSWGRPGPPPAGPPRAHARL
DLRTPYRPPAAAVGAAPAAAAGMAGVAAPPGNGTRGTGGVGDKRQLVYVFTTWRSGSSFF
GELFNQNPEVFFLYEPVWHVWQKLYPGDAVSLQGAARDMLSALYRCDLSVFQLYSPAGSG
GRNLTTLGIFGAATNKVVCSSPLCPAYRKEVVGLVDDRVCKKCPPQRLARFEEECRKYRT
LVIKGVRVFDVAVLAPLLRDPALDLKVIHLVRDPRAVASSRIRSRHGLIRESLQVVRSRD
PRAHRMPFLEAAGHKLGAKKEGVGGPADYHALGAMEVICNSMAKTLQTALQPPDWLQGHY
LVVRYEDLVGDPVKTLRRVYDFVGLLVSPEMEQFALNMTSGSGSSSKPFVVSARNATQAA
NAWRTALTFQQIKQVEEFCYQPMAVLGYERVNSPEEVKDLSKTLLRKPRL*
>ENSG00000128641|ENST00000339514
MAKMEVKTSLLDNMIGVGDMVLLEPLNEETFINNLKKRFDHSEIYTYIGSVVISVNPYRS
LPIYSPEKVEEYRNRNFYELSPHIFALSDEAYRSLRDQDKDQCILITGESGAGKTEASKL
VMSYVAAVCGKGAEVNQVKEQLLQSNPVLEAFGNAKTVRNDNSSRFGKYMDIEFDFKGDP
LGGVISNYLLEKSRVVKQPRGERNFHVFYQLLSGASEELLNKLKLERDFSRYNYLSLDSA
KVNGVDDAANFRTVRNAMQIVGFMDHEAESVLAVVAAVLKLGNIEFKPESRVNGLDESKI
KDKNELKEICELTGIDQSVLERAFSFRTVEAKQEKVSTTLNVAQAYYARDALAKNLYSRL
FSWLVNRINESIKAQTKVRKKVMGVLDIYGFEIFEDNSFEQFIINYCNEKLQQIFIELTL
KEEQEEYIREDIEWTHIDYFNNAIICDLIENNTNGILAMLDEECLRPGTVTDETFLEKLN
QVCATHQHFESRMSKCSRFLNDTSLPHSCFRIQHYAGKVLYQVEGFVDKNNDLLYRDLSQ

AMWKASHALIKSLFPEGNPAKINLKRPPTAGSQFKASVATLMKNLQTKNPNYIRCIKPND
KKAAHIFNEALVCHQIRYLGLLENVRVRRAGYAFRQAYEPCLERYKMLCKQTWPHWKGPA
RSGVEVLFNELEIPVEEYSFGRSKIFIRNPRTLFKLEDLRKQRLEDLATLIQKIYRGWKC
RTHFLLMKKSQIVIAAWYRRYAQQKRYQQTKSSALVIQSYIRGWKARKILRELKHQKRCK
EAVTTIAAYWHGTQVRREYRKFFRANAGKKIYEFTLQRIVQKYFLEMKNKMPSLSPIDKN
WPSRPYLFLDSTHKELKRIFHLWRCKKYRDQFTDQQKLIYEEKLEASELFKDKKALYPSS
VGQPFQGAYLEINKNPKYKKLKDAIEEKIIIAEVVNKINRANGKSTSRIFLLTNNNLLLA
DQKSGQIKSEVPLVDVTKVSMSSQNDGFFAVHLKEGSEAASKGDFLFSSDHLIEMATKLY
RTTLSQTKQKLNIEISDEFLVQFRQDKVCVKFIQGNQKNGSVPTCKRKNNRLLEVAVP*
>ENSG00000180957|ENST00000335272
MVLIKEFRVVLPCSVQEYQVGQLYSVAEASKNETGGGEGIEVLKNEPYEKDGEKGQYTHK
IYHLKSKVPAFVRMIAPEGSLVFHEKAWNAYPYCRTIVTNEYMKDDFFIKIETWHKPDLG
TLENVHGLDPNTWKTVEIVHIDIADRSQVEPADYKADEDPALFQSVKTKRGPLGPNWKKE
LANSPDCPQMCAYKLVTIKFKWWGLQSKVENFIQKQEKRIFTNFHRQLFCWIDKWIDLTM
EDIRRMEDETQKELETMRKRGSVRGTSAADV*
>ENSG00000196101|ENST00000307137
MVCLKLPGGSSLAALTVTLMVLSSRLAFAGDTRPRFLELLKSECHFFNGTERVRFLERHF
HNQEEYARFDSDVGEYRAVRELGRPDAEYWNSQKDLLEQKRGQVDNYCRHNYGVGESFTV
QRRVHPQVTVYPAKTQPLQHHNLLVCSVSGFYPGSIEVRWFRNGQEEKAGVVSTGLIQNG
DWTFQTLVMLETVPRSGEVYTCQVEHPSVTSPLTVEWSARSESAQSKMLSGVGGFVLGLL
FLGAGLFIYFRNQKGHSGLQPTGFLS*
>ENSG00000113719|ENST00000326654
MERWAMRVNELYVDDPDKDSGGKIDVSLNISLPNLHCELVGLDIQDEMGRHEVGHIDNSM
KIPLNNGAGCRFEGQFSINKVWKPCLSPFYLLPFPAVSPLPGNWLWRHSLDLTLTQPPAS
EGSCPAAWPFLLRIWMGVQAPWGFKPLMAGSGRSYSSLQ*
>ENSG00000017260|ENST00000328560
MKVARFQKIPNGENETMIPVLTSKKASELPVSEVASILQADLQNGLNKCEVSHRRAFHGW
NEFDISEDEPLWKKYISQFKNPLIMLLLASAVISVLMHQFDDAVSITVAILIVVTVAFVQ
EYRSEKSLEELSKLVPPECHCVREGKLEHTLARDLVPGDTVCLSVGDRVPADLRLFEAVD
LSIDESSLTGETTPCSKVTAPQPAATNGDLASRSNIAFMGTLVRCGKAKGVVIGTGENSE
FGEVFKMMQAEEAPKTPLQKSMDLLGKQLSFYSFGIIGIIMLVGWLLGKDILEMFTISVS
LAVAAIPEGLPIVVTVTLALGVMRMVKKRAIVKKLPIVETLGCCNVICSDKTGTLTKNEM
TVTHIFTSDGLHAEVTGVGYNQFGEVIVDGDVVHGFYNPAVSRIVEAGCVCNDAVIRNNT
LMGKPTEGALIALAMKMGLDGLQQDYIRKAEYPFSSEQKWMAVKCVHRTQQDRPEICFMK
GAYEQVIKYCTTYQSKGQTLTLTQQQRDVYQQEKARMGSAGLRVLALASGPELGQLTFLG
LVGIIDPPRTGVKEAVTTLIASGVSIKMITGDSQETAVAIASRLGLYSKTSQSVSGEEID
AMDVQQLSQIVPKVAVFYRASPRHKMKIIKSLQKNGSVVAMTGDGVNDAVALKAADIGVA
MGQTGTDVCKEAADMILVDDDFQTIMSAIEEGKGIYNNIKNFVRFQLSTSIAALTLISLA
TLMNFPNPLNAMQILWINIIMDGPPAQSLGVEPVDKDVIRKPPRNWKDSILTKNLILKIL
VSSIIIVCGTLFVFWRELRDNVITPRDTTMTFTCFVFFDMFNALSSRSQTKSVFEIGLCS
NRMFCYAVLGSIMGQLLVIYFPPLQKVFQTESLSILGLALGEEWTAAG*
>ENSG00000134909|ENST00000310343
METESESSTLGDDSVFWLESEVIIQVTDCEEEEREEKFRKMKSSVHSEEDDFVPELHRNV
HPRERPDWEETLSAMARGADVPEIPGDLTLKTCGSTASMKVKHVKKLPFTKGHFPKMAEC

AHFHYENVEFGSIQLSLSEEQNEVMKNGCESKELVYLVQIACQGKSWIVKRSYEDFRVLD
KHLHLCIYDRRFSQLSELPRSDTLKDSPESVTQMLMAYLSRLSAIAGNKINCGPALTWME
IDNKGNHLLVHEESSINTPAVGAAHVIKRYTARAPDELTLEVGDIVSVIDMPPKVLSTWW
RGKHGFQVGLFPGHCVELINQKVPQSVTNSVPKPVSKKHGKLITFLRTFMKSRPTKQKLK
QRGILKERVFGCDLGEHLLNSGFEVPQVLQSCTAFIERYGIVDGIYRLSGVASNIQRLRH
EFDSEHVPDLTKEPYVQDIHSVGSLCKLYFRELPNPLLTYQLYEKFSDAVSAATDEERLI
KIHDVIQQLPPPHYRTLEFLMRHLSLLADYCSITNMHAKNLAIVWAPNLLRSKQIESACF
SGTAAFMEVRIQSVVVEFILNHVDVLFSGRISMAMQEGAASLSRPKSLLVSSPSTKLLTL
EEAQARTQAQVNSPIVTENKYIEVGEGPAALQGKFHTIIEFPLERKRPQNKMKKSPVGSW
RSFFNLGKSSSVSKRKLQRNESEPSEMKAMALKGGRAEGTLRSAKSEESLTSLHAVDGDS
KLFRPRRPRSSSDALSASFNGEMLGNRCNSYDNLPHDNESEEEGGLLHIPALMSPHSAED
VDLSPPDIGVASLDFDPMSFQCSPPKAESECLESGASFLDSPGYSKDKPSANKKDAETGS
SQCQTPGSTASSEPVSPLQEKLSPFFTLDLSPTEDKSSKPSSFTEKVVYAFSPKIGRKLS
KSPSMSISEPISVTLPPRVSEVIGTVSNTTAQNASSSTWDKCVEERDATNRSPTQIVKMK
TNETVAQEAYESEVQPLDQVAAEEVELPGKEDQSVSSSQSKAVASGQTQTGAVTHDPPQD
SVPVSSVSLIPPPPPPKNVARMLALALAESAQQASTQSLKRPGTSQAGYTNYGDIAVATT
EDNLSSSYSAVALDKAYFQTDRPAEQFHLQNNAPGNCDHPLPETTATGDPTHSNTTESGE
QHHQVDLTGNQPHQAYLSGDPEKARITSVPLDSEKSDDHVSFPEDQSGKNSMPTVSFLDQ
DQSPPRFYSGDQPPSYLGASVDKLHHPLEFADKSPTPPNLPSDKIYPPSGSPEENTSTAT
MTYMTTTPATAQMSTKEASWDVAEQPTTADFAAATLQRTHRTNRPLPPPPSQRSAEQPPV
VGQVQAATNIGLNNSHKVQGVVPVPERPPEPRAMDDPASAFISDSGAAAAQCPMATAVQP
GLPEKVRDGARVPLLHLRAESVPAHPCGFPAPLPPTRMMESKMIAAIHSSSADATSSSNY
HSFVTASSTSVDDALPLPLPVPQPKHASQKTVYSSFARPDVTTEPFGPDNCLHFNMTPNC
QYRPQSVPPHHNKLEQHQVYGARSEPPASMGLRYNTYVAPGRNASGHHSKPCSRVEYVSS
LSSSVRNTCYPEDIPPYPTIRRVQSLHAPPSSMIRSVPISRTEVPPDDEPAYCPRPLYQY
KPYQSSQARSDYHVTQLQPYFENGRVHYRYSPYSSSSSSYYSPDGALCDVDAYGTVQLRP
LHRLPNRDFAFYNPRLQGKSLYSYAGLAPRPRANVTGYFSPNDHNVVSMPPAADVKHTYT
SWDLEDMEKYRMQSIRRESRARQKVKGPVMSQYDNMTPAVQDDLGGIYVIHLRSKSDPGK
TGLLSVAEGKESRHAAKAISPEGEDRFYRRHPEAEMDRAHHHGGHGSTQPEKPSLPQKQS
SLRSRKLPDMGCSLPEHRAHQEASHRQFCESKNGPPYPQGAGQLDYGSKGIPDTSEPVSY
HNSGVKYAASGQESLRLNHKEVRLSKEMERPWVRQPSAPEKHSRDCYKEEEHLTQSIVPP
PKPERSHSLKLHHTQNVERDPSVLYQYQPHGKRQSSVTVVSQYDNLEDYHSLPQHQRGVF
GGGGMGTYVPPGFPHPQSRTYATALGQGAFLPAELSLQHPETQIHAE*
>ENSG00000172551|ENST00000308796
MKFLAVLVLLGVSIFLVSAQNPTTAAPADTYPATGPADDEAPDAETTAAATTATTAAPTT
ATTAASTTARKDIPVLPKWVGDLPNGRVCP*
>ENSG00000109113|ENST00000301043
MNILAPVRRDRVLAELPQCLRKEAALHGHKDFHPRVTCACQEHRTGTVGFKISKVIVVGD
LSVGKTCLINRFCKDTFDKNYKATIGVDFEMERFEVLGIPFSLQLWDTAGQERFKCIAST
YYRGAQAIIIVFNLNDVASLEHTKQWLADALKENDPSSVLLFLVGSKKDLSTPAQYALME
KDALQVAQEMKAEYWAVSSLTGENVREFFFRVAALTFEANVLAELEKSGARRIGDVVRIN
SDDSNLYLTASKKKPTCCP*
>ENSG00000148334|ENST00000338961
MDPAARVVRALWPGGCALAWRLGGRPQPLLPTQSRAGFAGAAGGPSPVAAARKGSPRLLG

AAALALGGALGLYHTARWHLRAQDLHAERSAAQLSLSSRLQLTLYQYKTCPFCSKVRAFL
DFHALPYQVVEVNPVRRAEIKFSSYRKVPILVAQEGESSQQLNDSSVIISALKTYLVSGQ
PLEEIITYYPAMKAVNEQGKEVTEFGNKYWLMLNEKEAQQVYGGKEARTEEMKWRQWADD
WLVHLISPNVYRTPTEALASFDYIVREGKFGAVEGAVAKYMGAAAMYLISKRLKSRHRLQ
DNVREDLYEAADKWVAAVGKDRPFMGGQKPNLADLAVYGVLRVMEGLDAFDDLMQHTHIQ
PWYLRVERAITEASPAH*
>ENSG00000167645|ENST00000329420
MPGSASKRRIPVSQPGMADPHQLFDDTSSAQSRGYGAQRAPGGLSYPAASPTPHAAFLAD
PVSNMAMAYGSSLAAQGKELVDKNIDRFIPITKLKYYFAVDTMYVGRKLGLLFFPYLHQD
WEVQYQQDTPVAPRFDVNAPDLYIPAMAFITYVLVAGLALGTQDRFSPDLLGLQASSALA
WLTLEVLAILLSLYLVTVNTDLTTIDLVAFLGYKYVGMIGGVLMGLLFGKIGYYLVLGWC
CVAIFVFMIRTLRLKILADAAAEGVPVRGARNQLRMYLTMAVAAAQPMLMYWLTFHLVR*
>ENSG00000181029|ENST00000317378
MEARFTRGKSALLERALARPRTEVSLSAFALLFSELVQHCQSRVFSVAELQSRLAALGRQ
VGARVLDALVAREKGARRETKVLGALLFVKGAVWKALFGKEADKLEQANDDARTFYIIER
EPLINTYISVPKENSTLNCASFTAGIVEAVLTHSGFPAKVTAHWHKGTTLMIKFEEAVIA
RDRALEGR*
>ENSG00000184304|ENST00000331968
MSAPPVLRPPSPLLPVAAAAAAAAAALVPGSGPGPAPFLAPVAAPVGGISFHLQIGLSRE
PVLLLQDSSGDYSLAHVREMACSIVDQKFPECGFYGMYDKILLFRHDPTSENILQLVKAA
SDIQEGDLIEVVLSASATFEDFQIRPHALFVHSYRAPAFCDHCGEMLWGLVRQGLKCEGC
GLNYHKRCAFKIPNNCSGVRRRRLSNVSLTGVSTIRTSSAELSTSAPDEPLLQKSPSESF
IGREKRSNSQSYIGRPIHLDKILMSKVKVPHTFVIHSYTRPTVCQYCKKLLKGLFRQGLQ
CKDCRFNCHKRCAPKVPNNCLGEVTINGDLLSPGAESDVVMEEGSDDNDSERNSGLMDDM
EEAMVQDAEMAMAECQNDSGEMQDPDPDHEDANRTISPSTSNNIPLMRVVQSVKHTKRKS
STVMKEGWMVHYTSKDTLRKRHYWRLDSKCITLFQNDTGSRYYKEIPLSEILSLEPVKTS
ALIPNGANPHCFEITTANVVYYVGENVVNPSSPSPNNSVLTSGVGADVARMWEIAIQHAL
MPVIPKGSSVGTGTNLHRDISVSISVSNCQIQENVDISTVYQIFPDEVLGSGQFGIVYGG
KHRKTGRDVAIKIIDKLRFPTKQESQLRNEVAILQNLHHPGVVNLECMFETPERVFVVME
KLHGDMLEMILSSEKGRLPEHITKFLITQILVALRHLHFKNIVHCDLKPENVLLASADPF
PQVKLCDFGFARIIGEKSFRRSVVGTPAYLAPEVLRNKGYNRSLDMWSVGVIIYVSLSGT
FPFNEDEDIHDQIQNAAFMYPPNPWKEISHEAIDLINNLLQVKMRKRYSVDKTLSHPWLQ
DYQTWLDLRELECKIGERYITHESDDLRWEKYAGEQGLQYPTHLINPSASHSDTPETEET
EMKALGERVSIL*
>ENSG00000176797|ENST00000314357
MRIHYLLFALLFLFLVPVPGHGGIINTLQKYYCRVRGGRCAVLSCLPKEEQIGKCSTRGR
KCCRRKK*
>ENSG00000070831|ENST00000315554
MQTIKCVVVGDGAVGKTCLLISYTTNKFPSEYVPTVFDNYAVTVMIGGEPYTLGLFDTAG
QEDYDRLRPLSYPQTDVFLVCFSVVSPSSFENVKEKWVPEITHHCPKTPFLLVGTQIDLR
DDPSTIEKLAKNKQKPITPETAEKLARDLKAVKYVECSALTQRGLKNVFDEAILAALEPP
ETQPKRKCCIF*
>ENSG00000103978|ENST00000307216
MAAAAWLQVLPVILLLLGAHPSPLSFFSAGPATVAAADRSKWHIPIPSGKNYFSFGKILF

RNTTIFLKFDGEPCDLSLNITWYLKSADCYNEIYNFKAEEVELYLEKLKEKRGLSGKYQT
SSKLFQNCSELFKTQTFSGDFMHRLPLLGEKQEAKENGTNLTFIGDKTIQMPFLKKHFLD
C*
>ENSG00000147509|ENST00000297313
MPQLSQDNQECLQKHFSRPSIWTQFLPLFRAQRYNTDIHQITENEGDLRAVPDIKSFPPA
QLPDSPAAPKLFGLLSSPLSSLARFFSHLLRRPPPEAPRRRLDFSPLLPALPAARLSRGH
EELPGRLSLLLGAALALPGRPSGGRPLRPPHPVAKPREEDATAGQSSPMPQMGSERMEMR
KRQMPAAQDTPGAAPGQPGAGSRGSNACCFCWCCCCSCSCLTVRNQEDQRPTIASHELRA
DLPTWEESPAPTLEEVNAWAQSFDKLMVTPAGRNAFREFLRTEFSEENMLFWMACEELKK
EANKNIIEEKARIIYEDYISILSPKEVSLDSRVREVINRNMVEPSQHIFDDAQLQIYTLM
HRDSYPRFMNSAVYKDLLQSLSEKSIEA*
>ENSG00000204482|ENST00000339530
MLSRNDDICIYGGLGLGGLLLLAVVLLSACLCWLHRRAPSVLVPGPGLLRAGTPLCISAE
AASAQQ*
>ENSG00000117262|ENST00000313835
MSFLIDSSIMITSQILFFGFGWLFFMRQLFKDYEIRQYVVQVIFSVTFAFSCTMFELIIF
EILGVLNSSSRYFHWKMNLCVILLILVFMVPFYIGYFIVSNIRLLHKQRLLFSCLLWLTF
MYFFWKLGDPFPILSPKHGILSIEQLISRVGVIGVTLMALLSGFGAVNCPYTYMSYFLRN
VTDTDILALERRLLQTMDMIISKKKRMAMARRTMFQKGEVHNKPSGFWGMIKSVTTSASG
SENLTLIQQEVDALEELSRQLFLETADLYATKERIEYSKTFKGKYFNFLGYFFSIYCVWK
IFMATINIVFDRVGKTDPVTRGIEITVNYLGIQFDVKFWSQHISFILVGIIIVTSIRGLL
ITLTKFFYAISSSKSSNVIVLLLAQIMGMYFVSSVLLIRMSMPLEYRTIITEVLGELQFN
FYHRWFDVIFLVSALSSILFLYLAHKQAPEKQMAP*
>ENSG00000160408|ENST00000291839
MACSRPPSQCEPTSLPPGPPAGRRHLPLSRRRREMSSNKEQRSAVFVILFALITILILYS
SNSANEVFHYGSLRGRSRRPVNLKKWSITDGYVPILGNKTLPSRCHQCVIVSSSSHLLGT
KLGPEIERAECTIRMNDAPTTGYSADVGNKTTYRVVAHSSVFRVLRRPQEFVNRTPETVF
IFWGPPSKMQKPQGSLVRVIQRAGLVFPNMEAYAVSPGRMRQFDDLFRGETGKDREKSHS
WLSTGWFTMVIAVELCDHVHVYGMVPPNYCSQRPRLQRMPYHYYEPKGPDECVTYIQNEH
SRKGNHHRFITEKRVFSSWAQLYGITFSHPSWT*
>ENSG00000113615|ENST00000322887
MSQPGIPASGGAPASLQAQNGAALASGSPYTNGPVQNALLSSQESVSQGYNFQLPGSYPH
PIPAKTLNPVSGQSNYGGSQGSGQTLNRPPVASNPVTPSLHSGPAPRMPLPASQNPATTP
MPSSSFLPEANLPPPLNWQYNYPSTASQTNHCPRASSQPTVSGNTSLTTNHQYVSSGYPS
LQNSFIKSGPSVPPLVNPPLPTTFQPGAPHGPPPAGGPPPVRALTPLTSSYRDVPQPLFN
SAVNQEGITSNTNNGSMVVHSSYDEIEGGGLLATPQLTNKNPKMSRSVGYSYPSLPPGYQ
NTTPPGATGVPPSSLNYPSGPQAFTQTPLGANHLTTSMSGLSLQPEGLRVVNLLQERNML
PSTPLKPPVPNLHEDIQKLNCNPELFRCTLTSIPQTQALLNKAKLPLGLLLHPFKDLVQL
PVVTSSTIVRCRSCRTYINPFVSFLDQRRWKCNLCYRVNDVPEEFLYNPLTRVYGEPHRR
PEVQNATIEFMAPSEYMLRPPQPPVYLFVFDVSHNAVETGYLNSVCQSLLDNLDLLPGNT
RTKIGFITFDSTIHFYGLQESLSQPQMLIVSDIEDVFIPMPENLLVNLNESKESVIGVSS
EETLITCLEIAMR*
>ENSG00000185499|ENST00000342482
MTPGTQSPFFLLLLLTVLTATTAPKPATVVTGSGHASSTPGGEKETSATQRSSVPSSTEK

NASGAGVPGWGIALLVLVCVLVALAIVYLIALAVCQCRRKNYGQLDIFPARDTYHPMSEY
PTYHTHGRYVPPSSTDRSPYEKVSAGNGGSSLSYTNPAVAATSANL*
>ENSG00000160218|ENST00000291574
MDASEEPLPPVIYTMENKPIVTCAGDQNLFTSVYPTLSQQLPREPMEWRRSYGRAPKMIH
LESNFVQFKEELLPKEGNKALLTFPFLHIYWTECCDTEVYKATVKDDLTKWQNVLKAHSS
VDWLIVIVENDAKKKNKTNILPRTSIVDKIRNDFCNKQSDRCVVLSDPLKDSSRTQESWN
AFLTKLRTLLLMSFTKNLGKFEDDMRTLREKRTEPGWSFCEYFMVQEELAFVFEMLQQFE
DALVQYDELDALFSQYVVNFGAGDGANWLTFFCQPVKSWNGLILRKPIDMEKRESIQRRE
ATLLDLRSYLFSRQCTLLLFLQRPWEVAQRALELLHNCVQELKLLEVSVPPGALDCWVFL
SCLEVLQRIEGCCDRAQIDSNIAHTVGLWSYATEKLKSLGYLCGLVSEKGPNSEDLNRTV
DLLAGLGAERPETANTAQSPYKKLKEALSSVEAFEKHYLDLSHATIEMYTSIGRIRSAKF
VGKDLAEFYMRKKAPQKAEIYLQGALKNYLAEGWALPITHTRKQLAECQKHLGQIENYLQ
TSSLLASDHHLTEEERKHFCQEILDFASQPSDSPGHKIVLPMHSFAQLRDLHFDPSNAVV
HVGGVLCVEITMYSQMPVPVHVEQIVVNVHFSIEKNSYRKTAEWLTKHKTSNGIINFPPE
TAPFPVSQNSLPALELYEMFERSPSDNSLNTTGIICRNVHMLLRRQESSSSLEMPSGVAL
EEGAHVLRCSHVTLEPGANQITFRTQAKEPGTYTLRQLCASVGSVWFVLPHIYPIVQYDV
YSQEPQLHVEPLADSLLAGIPQRVKFTVTTGHYTIKNGDSLQLSNAEAMLILCQAESRAV
VYSNTREQSSEAALRIQSSDKVTSISLPVAPAYHVIEFELEVLSLPSAPALGGESDMLGM
AEPHRKHKDKQRTGRCMVTTDHKVSIDCPWSIYSTVIALTFSVPFRTTHSLLSSGTRKYV
QVCVQNLSELDFQLSDSYLVDTGDSTDLQLVPLNTQSQQPIYSKQSVFFVWELKWTEEPP
PSLHCRFSVGFSPASEEQLSISLKPYTYEFKVENFFTLYNVKAEIFPPSGMEYCRTGSLC
SLEVLITRLSDLLEVDKDEALTESDEHFSTKLMYEVVDNSSNWAVCGKSCGVISMPVAAR
ATHRVHMEVMPLFAGYLPLPDVRLFKYLPHHSAHSSQLDADSWIENDSLSVDKHGDDQPD
SSSLKSRGSVHSACSSEHKGLPMPRLQALPAGQVFNSSSGTQVLVIPSQDDHVLEVSVT*
>ENSG00000166747|ENST00000299980
MPAPIRLRELIRTIRTARTQAEEREMIQKECAAIRSSFREEDNTYRCRNVAKLLYMHMLG
YPAHFGQLECLKLIASQKFTDKRIGYLGAMLLLDERQDVHLLMTNCIKNDLNHSTQFVQG
LALCTLGCMGSSEMCRDLAGEVEKLLKTSNSYLRKKAALCAVHVIRKVPELMEMFLPATK
NLLNEKNHGVLHTSVVLLTEMCERSPDMLAHFRKLVPQLVRILKNLIMSGYSPEHDVSGI
SDPFLQVRILRLLRILGRNDDDSSEAMNDILAQVATNTETSKNVGNAILYETVLTIMDIK
SESGLRVLAINILGRFLLNNDKNIRYVALTSLLKTVQTDHNAVQRHRSTIVDCLKDLDVS
IKRRAMELSFALVNGNNIRGMMKELLYFLDSCEPEFKADCASGIFLAAEKYAPSKRWHID
TIMRVLTTAGSYVRDDAVPNLIQLITNSVEMHAYTVQRLYKAILGDYSQQPLVQVAAWCI
GEYGDLLVSGQCEEEEPIQVTEDEVLDILESVLISNMSTSVTRGYALTAIMKLSTRFTCT
VNRIKKVVSIYGSSIDVELQQRAVEYNALFKKYDHMRSALLERMPVMEKVTTNGPTEIVQ
TNGETEPAPLETKPPPSGPQPTSQANDLLDLLGGNDITPVIPTAPTSKPSSAGGELLDLL
GDINLTGAPAAAPAPASVPQISQPPFLLDGLSSQPLFNDIAAGIPSITAYSKNGLKIEFT
FERSNTNPSVTVITIQASNSTELDMTDFVFQAAVPKTFQLQLLSPSSSIVPAFNTGTITQ
VIKVLNPQKQQLRMRIKLTYNHKGSAMQDLAEVNNFPPQSWQ*
>ENSG00000089063|ENST00000342308
MQPWALPTVGELWVCGRPGAALRWSLVLSPRLEPSGVISAHCNLHLLASSDSSASASRLC
QRVMMPSRTNLATGIPSSKVKYSRLSSTDDGYIDLQFKKTPPKIPYKAIALATVLFLIGA
FLIIIGSLLLSGYISKGGADRAVPVLIIGILVFLPGFYHLRIAYYASKGYRGYSYDDIPD
FDD*

>ENSG00000157064|ENST00000294868
MEIQELEEIQACQGLWEVFVTLSERARDYLHKTGRFIVIGGIVSPVHDSYGKQGLVSSRH
RLIMCQLAVQNSDWIRVDPWECYQDTWQTTCSVLEHHRDLMKRVTGCILSNVNTPSMTPV
IGQPQNETPQPIYQNSNVATKPTAAKILGKVGESLSRICCVRPPVERFTFVDENANLGTV
MRYEEIELRILLLCGSDLLESFCIPGLWNEADMEVIVGDFGIVVVPRDAADTDRIMNHSS
ILRKYKNNIMVVKDDINHPMSVVSSTKSRLALQHGDGHVVDYLSQPVIDYILKSQLYINA
SG*
>ENSG00000175582|ENST00000336083
MSTGGDFGNPLRKFKLVFLGEQSVGKTSLITRFMYDSFDNTYQATIGIDFLSKTMYLEDR
TVRLQLWDTAGQERFRSLIPSYIRDSTVAVVVYDITNVNSFQQTTKWIDDVRTERGSDVI
IMLVGNKTDLADKRQVSIEEGERKAKELNVMFIETSAKAGYNVKQLFRRVAAALPGMEST
QDRSREDMIDIKLEKPQEQPVSEGGCSC*
>ENSG00000177191|ENST00000321702
MRCPKCLLCLSALLTLLGLKVYIEWTSESRLSKAYPSPRGTPPSPTPANPEPTLPANLST
RLGQTIPLPFAYWNQQQWRLGSLPSGDSTETGGCQAWGAAAATEIPDFASYPKDLRRFLL
SAACRSFPQWLPGGGGSQVSSCSDTDVPYLLLAVKSEPGRFAERQAVRETWGSPAPGIRL
LFLLGSPVGEAGPDLDSLVAWESRRYSDLLLWDFLDVPFNQTLKDLLLLAWLGRHCPTVS
FVLRAQDDAFVHTPALLAHLRALPPASARSLYLGEVFTQAMPLRKPGGPFYVPESFFEGG
YPAYASGGGYVIAGRLAPWLLRAAARVAPFPFEDVYTGLCIRALGLVPQAHPGFLTAWPA
DRTADHCAFRNLLLVRPLGPQASIRLWKQLQDPRLQC*
>ENSG00000164597|ENST00000347053
MGWVGGRRRDSASPPGRSRSAADDINPAPANMEGGGGSVAVAGLGARGSGAAAATVRELL
QDGCYSDFLNEDFDVKTYTSQSIHQAVIAEQLAKLAQGISQLDRELHLQVVARHEDLLAQ
ATGIESLEGVLQMMQTRIGALQGAVDRIKAKIVEPYNKIVARTAQLARLQVACDLLRRII
RILNLSKRLQGQLQGGSREITKAAQSLNELDYLSQGIDLSGIEVIENDLLFIARARLEVE
NQAKRLLEQGLETQNPTQVGTALQVFYNLGTLKDTITSVVDGYCATLEENINSALDIKVL
TQPSQSAVRGGPGRSTMPTPGNTAALRASFWTNMEKLMDHIYAVCGQVQHLQKVLAKKRD
PVSHICFIEEIVKDGQPEIFYTFWNSVTQALSSQFHMATNSSMFLKQAFEGEYPKLLRLY
NDLWKRLQQYSQHIQGNFNASGTTDLYVDLQHMEDDAQDIFIPKKPDYDPEKALKDSLQP
YEAAYLSKSLSRLFDPINLVFPPGGRNPPSSDELDGIIKTIASELNVAAVDTNLTLAVSK
NVAKTIQLYSVKSEQLLSTQGDASQVIGPLTEGQRRNVAVVNSLYKLHQSVTKAIHALME
NAVQPLLTSVGDAIEAIIITMHQEDFSGSLSSSGKPDVPCSLYMKELQGFIARVMSDYFK
HFECLDFVFDNTEAIAQRAVELFIRHASLIRPLGEGGKMRLAADFAQMELAVGPFCRRVS
DLGKSYRMLRSFRPLLFQASEHVASSPALGDVIPFSIIIQFLFTRAPAELKSPFQRAEWS
HTRFSQWLDDHPSEKDRLLLIRGALEAYVQSVRSREGKEFAPVYPIMVQLLQKAMSALQ*
>ENSG00000180398|ENST00000319466
MTMRSLLRTPFLCGLLWAFCAPGARAEEPAASFSQPGSMGLDKNTVHDQEHIMEHLEGVI
NKPEAEMSPQELQLHYFKMHDYDGNNLLDGLELSTAITHVHKEEGSEQAPLMSEDELINI
IDGVLRDDDKNNDGYIDYAEFAKSLQ*
>ENSG00000163812|ENST00000296127
MMLIPTHHFRNIERKPEYLQPEKCVPPPYPGPVGTMWFIRDGCGIACAIVTWFLVLYAEF
VVLFVMLIPSRDYVYSIINGIVFNLLAFLALASHCRAMLTDPGAVPKGNATKEFIESLQL
KPGQVVYKCPKCCSIKPDRAHHCSVCKRCIRKMDHHCPWVNNCVGENNQKYFVLFTMYIA
LISLHALIMVGFHFLHCFEEDWTTYGLNREEMAETGISLHEKMQPLNFSSTECSSFSPPT

TVILLILLCFEGLLFLIFTSVMFGTQVHSICTDETGIEQLKKEERRWAKKTKWMNMKAVF
GHPFSLGWASPFATPDQGKADPYQYVV*
>ENSG00000174684|ENST00000311181
MQMSYAIRCAFYQLLLAALMLVAMLQLLYLSLLSGLHGQEEQDQYFEFFPPSPRSVDQVK
AQLRTALASGGVLDASGDYRVYRGLLKTTMDPNDVILATHASVDNLLHLSGLLERWEGPL
SVSVFAATKEEAQLATVLAYALSSHCPDMRARVAMHLVCPSRYEAAVPDPREPGEFALLR
SCQEVFDKLARVAQPGINYALGTNVSYPNNLLRNLAREGANYALVIDVDMVPSEGLWRGL
REMLDQSNQWGGTALVVPAFEIRRARRMPMNKNELVQLYQVGEVRPFYYGLCTPCQAPTN
YSRWVNLPEESLLRPAYVVPWQDPWEPFYVAGGKVPTFDERFRQYGFNRISQACELHVAG
FDFEVLNEGFLVHKGFKEALKFHPQKEAENQHNKILYRQFKQELKAKYPNSPRRC*
>ENSG00000162885|ENST00000313984
MRNWLVLLCPCVLGAALHLWLRLRSPPPACASGAGPAGGVSLLLPRLECNGAVSAHPNLH
LPGSRDSPASASQVAGITDQLALFPQWKSTHYDVVVGVLSARNNHELRNVIRSTWMRHLL
QHPTLSQRVLVKFIIGAHGCEVPVEDREDPYSCKLLNITNPVLNQEIEAFSLSEDTSSGL
PEDRVVSVSFRVLYPIVITSLGVFYDANDVGFQRNITVKLYQAEQEEALFIARFSPPSCG
VQVNKLWYKPVEQFILPESFEGTIVWESQDLHGLVSRNLHKVTVNDGGGVLRVITAGEGA
LPHEFLEGVEGVAGGFIYTIQGKFAS*
>ENSG00000181027|ENST00000318584
MRLTRCQAALAAAITLNLLVLFYVSWLQHQPRNSRARGPRRASAAGPRVTVLVREFEAFD
NAVPELVDSFLQQDPAQPVVVAADTLPYPPLALPRIPNVRLALLQPALDRPAAASRPETY
VATEFVALVPDGARAEAPGLLERMVEALRAGSARLVAAPVATANPARCLALNVSLREWTA
RYGAAPAAPRCDALDGDAVVLLRARDLFNLSAPLARPVGTSLFLQTALRGWAVQLLDLTF
AAARQPPLATAHARWKAEREGRARRAALLRALGIRLVSWEGGRLEWFGCNKETTRCFGTV
VGDTPAYLYEERWTPPCCLRALRETARYVVGVLEAAGVRYWLEGGSLLGAARHGDIIPWD
YDVDLGIYLEDVGNCEQLRGAEAGSVVDERGFVWEKAVEGDFFRVQYSESNHLHVDLWPF
YPRNGVMTKDTWLDHRQDVEFPEHFLQPLVPLPFAGFVAQAPNNYRRFLELKFGPGVIEN
PQYPNPALLSLTGSG*
>ENSG00000239839|ENST00000327857
MRTLAILAAILLVALQAQAEPLQARADEVAAAPEQIAADIPEVVVSLAWDESLAPKHPGS
RKNMDCYCRIPACIAGERRYGTCIYQGRLWAFCC*
>ENSG00000167515|ENST00000301021
MAVCIAVIAKENYPLYIRSTPTENELKFHYMVHTSLDVVDEKISAMGKALVDQRELYLGL
LYPTEDYKVYGYVTNSKVKFVMVVDSSNTALRDNEIRSMFRKLHNSYTDVMCNPFYNPGD
RIQSSRAFDNMVTSMMIQVC*
>ENSG00000272617|ENST00000306875
MATAATIPSVATATAAALGEVEDEGLLASLFRDRFPEAQWRERPDVGRYLRELSGSGLER
LRREPERLAEERAQLLQQTRDLAFANYKTFIRGAECTERIHRLFGDVEASLGRLLDRLPS
FQQSCRNFVKEAEEISSNRRMNSLTLNRHTEILEILEIPQLMDTCVRNSYYEEALELAAY
VRRLERKYSSIPVIQGIVNEVRQSMQLMLSQLIQQLRTNIQLPACLRVIGYLRRMDVFTE
AELRVKFLQARDAWLRSILTAIPNDDPYFHITKTIEASRVHLFDIITQYRAIFSDEDPLL
PPAMGEHTVNESAIFHGWVLQKVSQFLQVLETDLYRGIGGHLDSLLGQCMYFGLSFSRVG
ADFRGQLAPVFQRVAISTFQKAIQETVEKFQEEMNSYMLISAPAILGTSNMPAAVPATQP
GTLQPPMVLLDFPPLACFLNNILVAFNDLRLCCPVALAQDVTGALEDALAKVTKIILAFH
RAEEAAFSSGEQELFVQFCTVFLEDLVPYLNRCLQVLFPPAQIAQTLGIPPTQLSKYGNL

GHVNIGAIQEPLAFILPKRETLFTLDDQALGPELTAPAPEPPAEEPRLEPAGPACPEGGR
AETQAEPPSVGP*
>ENSG00000169223|ENST00000303127
MAAEGWIWRWGWGRRCLGRPGLLGPGPGPTTPLFLLLLLGSVTADITDGNSEHLKREHSL
IKPYQGVGSSSMPLWDFQGSTMLTSQYVRLTPDERSKEGSIWNHQPCFLKDWEMHVHFKV
HGTGKKNLHGDGIALWYTRDRLVPGPVFGSKDNFHGLAIFLDTYPNDETTERVFPYISVM
VNNGSLSYDHSKDGRWTELAGCTADFRNRDHDTFLAVRYSRGRLTVMTDLEDKNEWKNCI
DITGVRLPTGYYFGASAGTGDLSDNHDIISMKLFQLMVEHTPDEESIDWTKIEPSVNFLK
SPKDNVDDPTGNFRSGPLTGWRVFLLLLCALLGIVVCAVVGAVVFQKRQERNKRFY*
>ENSG00000204642|ENST00000334668
MAPRSLLLLLSGALALTDTWAGSHSLRYFSTAVSRPGRGEPRYIAVEYVDDTQFLRFDSD
AAIPRMEPREPWVEQEGPQYWEWTTGYAKANAQTDRVALRNLLRRYNQSEAGSHTLQGMN
GCDMGPDGRLLRGYHQHAYDGKDYISLNEDLRSWTAADTVAQITQRFYEAEEYAEEFRTY
LEGECLELLRRYLENGKETLQRADPPKAHVAHHPISDHEATLRCWALGFYPAEITLTWQR
DGEEQTQDTELVETRPAGDGTFQKWAAVVVPPGEEQRYTCHVQHEGLPQPLILRWEQSPQ
PTIPIVGIVAGLVVLGAVVTGAVVAAVMWRKKSSDRNRGSYSQAAV*
>ENSG00000127954|ENST00000301959
MEKTCIDALPLTMNSSEKQETVCIFGTGDFGRSLGLKMLQCGYSVVFGSRNPQKTTLLPS
GAEVLSYSEAAKKSGIIIIAIHREHYDFLTELTEVLNGKILVDISNNLKINQYPESNAEY
LAHLVPGAHVVKAFNTISAWALQSGALDASRQAILKKENPFSTSSAWLSDSYVALGILGF
FLFVLLGITSLPSVSNAVNWREFRFVQSKLGYLTLILCTAHTLVYGGKRFLSPSNLRWYL
PAAYVLGLIIPCTVLVIKFVLIMPCVDNTLTRIRQGWERNSKH*
>ENSG00000120341|ENST00000308284
MELWAPQRLPQTRGKATAPSKDPDRGFRRDGHHRPVPHSWHNGERFHQWQDNRGSPQPQQ
EPRADHQQQPHYASRPGDWHQPVSGVDYYEGGYRNQLYSRPGYENSYQSYQSPTMREEYA
YGSYYYHGHPQWLQEERVPRQRSPYIWHEDYREQKYLDEHHYENQHSPFGTNSETHFQSN
SRNPCKDSPASNSGQEWPGELFPGSLLAEAQKNKPSLASESNLLQQRESGLSSSSYELSQ
YIRDAPERDDPPASAAWSPVQADVSSAGPKAPMKFYIPHVPVSFGPGGQLVHVGPSSPTD
GQAALVELHSMEVILNDSEEQEEMRSFSGPLIREDVHKVDIMTFCQQKAAQSCKSETLGS
RDSALLWQLLVLLCRQNGSMVGSDIAELLMQDCKKLEKYKRQPPVANLINLTDEDWPVLS
SGTPNLLTGEIPPSVETPAQIVEKFTRLLYYGRKKEALEWAMKNHLWGHALFLSSKMDPQ
TYSWVMSGFTSTLALNDPLQTLFQLMSGRIPQAATCCGEKQWGDWRPHLAVILSNQAGDP
ELYQRAIVAIGDTLAGKGLVEAAHFCYLMAHVPFGHYTVKTDHLVLLGSSHSQEFLKFAT
TEAIQRTEIFEYCQMLGRPKSFIPSFQVYKLLYASRLADYGLVSQALHYCEAIGAAVLSQ
GESSHPVLLVELIKLAEKLKLSDPLVLERRSGDRDLEPDWLAQLRRQLEQKVAGDIGDPH
PTRSDISGAGGTTTENTFYQDFSGCQGYSEAPGYRSALWLTPEQTCLLQPSPQQPFPLQP
GSYPAGGGAGQTGTPRPFYSVPETHLPGTGSSVAVTEATGGTVWEEMLQTHLGPGENTVS
QETSQPPDGQEVISKPQTPLAARPRSISESSASSAKEDEKESSDEADKNSPRNTAQRGKL
GDGKEHTKSSGFGWFSWFRSKPTKNASPAGDEDSSDSPDSEETPRASSPHQAGLGLSLTP
SPESPPLPDVSAFSRGRGGGEGRGSASSGGAAAGAGVGGLSGPESVSFELCSNPGVLLPP
PALKGAVPLYNPSQVPQLPTATSLNRPNRLAQRRYPTQPC*
>ENSG00000182492|ENST00000331595
MWPLWRLVSLLALSQALPFEQRGFWDFTLDDGPFMMNDEEASGADTSGVLDPDSVTPTYS
AMCPFGCHCHLRVVQCSDLGLKSVPKEISPDTTLLDLQNNDISELRKDDFKGLQHLYALV

LVNNKISKIHEKAFSPLRKLQKLYISKNHLVEIPPNLPSSLVELRIHDNRIRKVPKGVFS
GLRNMNCIEMGGNPLENSGFEPGAFDGLKLNYLRISEAKLTGIPKDLPETLNELHLDHNK
IQAIELEDLLRYSKLYRLGLGHNQIRMIENGSLSFLPTLRELHLDNNKLARVPSGLPDLK
LLQVVYLHSNNITKVGVNDFCPMGFGVKRAYYNGISLFNNPVPYWEVQPATFRCVTDRLA
IQFGNYKK*
>ENSG00000179562|ENST00000321407
MEKFGMNFGGGPSKKDLLETIETQKKQLLQYQARLKDVVRAYKSLLKEKEALEASIKVLS
VSHEADVGLAGVQLPGLTFPDSVDDRCSTHSEDSTGTATSLDTAASLTSTKGEFGVEDDR
PARGPPPPKSEEASWSESGVSSSSGDGPFAGGEVDKRLHQLKTQLATLTSSLATVTQEKS
RMEASYLADKKKMKQDLEDASNKAEEERARLEGELKGLQEQIAETKARLITQQHDRAQEQ
SDHALMLRELQKLLQEERTQRQDLELRLEETREALAGRAYAAEQMEGFELQTKQLTREVE
ELKSELQAIRDEKNQPDPRLQELQEEAARLKSHFQAQLQQEMRKTALAEDQLRQQSQVEE
QRVAALENQISEVSELLGTYEKAKQKDQLAIQKLKERILQLDLENKTLALAASSRSPLDS
HGEESSLDVNVLKDKMEKLKRLLQVAARKSQVTLDVEKLCDLEIMPSSEAADGEKATALY
YQQELKQLKEEFERYKMRAQVVLKSKNTKDGNLGKELEAAQEQLAELKEKYISLRLSCEE
LEHQHQQEADDWKQELARLQQLHRQELERCQLDFRDRTLKLEEELHKQRDRALAVLTEKD
LELEQLRSVALASGLPGRRSPVGGGGPGDPADTSSSDSLTQALQLAAANEPTFFLYAEQL
ARKEVEITSLRKQKHRLEVEVHQLQDRLLEEGERHREEVAALQSHIEKNIRDQSREGANL
EYLKNIIYRFLTLPDSLGRQQTLTAILTILHFSPEEKQVIMRLPTSASWWPSGKR*
>ENSG00000180767|ENST00000319340
MGRRCCRRRVLAAACLGAALLLLCAAPRSLRPAFGNRALGSSWLGGEKRSPLQKLYDLDQ
DPRSTLAKVHRQRRDLLNSACSRHSRRQRLLQPEDLRHVLVDDAHGLLYCYVPKVACTNW
KRVLLALSGQARGDPRAISAQEAHAPGRLPSLADFSPAEINRRLRAYLAFLFVREPFERL
ASAYRNKLARPYSAAFQRRYGARIVQRLRPRALPDARARGHDVRFAEFLAYLLDPRTRRE
EPFNEHWERAHALCHPCRLRYDVVGKFETLAEDAAFVLGLAGASDLSFPGPPRPRGAAAS
RDLAARLFRDISPFYQRRLFDLYKMDFLLFNYSAPSYLRLL*
>ENSG00000003147|ENST00000339809
MSGHKCSYPWDLQDRYAQDKSVVNKMQQKYWETKQAFIKATGKKEDEHVVASDADLDAKL
ELFHSIQRTCLDLSKAIVLYQKRICFLSQEENELGKFLRSQGFQDKTRAGKMMQATGKAL
CFSSQQRLALRNPLCRFHQEVETFRHRAISDTWLTVNRMEQCRTEYRGALLWMKDVSQEL
DPDLYKQMEKFRKVQTQVRLAKKNFDKLKMDVCQKVDLLGASRCNLLSHMLATYQLAWDQ
WQGPRNLKVLTKMTCCC*
>ENSG00000173273|ENST00000310430
MAASRRSQHHHHHHQQQLQPAPGASAPPPPPPPPLSPGLAPGTTPASPTASGLAPFASPR
HGLALPEGDGSRDPPDRPRSPDPVDGTSCCSTTSTICTVAAAPVVPAVSTSSAAGVAPNP
AGSGSNNSPSSSSSPTSSSSSSPSSPGSSLAESPEAAGVSSTAPLGPGAAGPGTGVPAVS
GALRELLEACRNGDVSRVKRLVDAANVNAKDMAGRKSSPLHFAAGFGRKDVVEHLLQMGA
NVHARDDGGLIPLHNACSFGHAEVVSLLLCQGADPNARDNWNYTPLHEAAIKGKIDVCIV
LLQHGADPNIRNTDGKSALDLADPSAKAVLTGEYKKDELLEAARSGNEEKLMALLTPLNV
NCHASDGRKSTPLHLAAGYNRVRIVQLLLQHGADVHAKDKGGLVPLHNACSYGHYEVTEL
LLKHGACVNAMDLWQFTPLHEAASKNRVEVCSLLLSHGADPTLVNCHGKSAVDMAPTPEL
RERLTYEFKGHSLLQAAREADLAKVKKTLALEIINFKQPQSHETALHCAVASLHPKRKQV
TELLLRKGANVNEKNKDFMTPLHVAAERAHNDVMEVLHKHGAKMNALDTLGQTALHRAAL
AGHLQTCRLLLSYGSDPSIISLQGFTAAQMGNEAVQQILSESTPIRTSDVDYRLLEASKA

GDLETVKQLCSSQNVNCRDLEGRHSTPLHFAAGYNRVSVVEYLLHHGADVHAKDKGGLVP
LHNACSYGHYEVAELLVRHGASVNVADLWKFTPLHEAAAKGKYEICKLLLKHGADPTKKN
RDGNTPLDLVKEGDTDIQDLLRGDAALLDAAKKGCLARVQKLCTPENINCRDTQGRNSTP
LHLAAGYNNLEVAEYLLEHGADVNAQDKGGLIPLHNAASYGHVDIAALLIKYNTCVNATD
KWAFTPLHEAAQKGRTQLCALLLAHGADPTMKNQEGQTPLDLATADDIRALLIDAMPPEA
LPTCFKPQATVVSASLISPASTPSCLSAASSIDNLTGPLAELAVGGASNAGDGAAGTERK
EGEVAGLDMNISQFLKSLGLEHLRDIFETEQITLDVLADMGHEELKEIGINAYGHRHKLI
KGVERLLGGQQGTNPYLTFHCVNQGTILLDLAPEDKEYQSVEEEMQSTIREHRDGGNAGG
IFNRYNVIRIQKVVNKKLRERFCHRQKEVSEENHNHHNERMLFHGSPFINAIIHKGFDER
HAYIGGMFGAGIYFAENSSKSNQYVYGIGGGTGCPTHKDRSCYICHRQMLFCRVTLGKSF
LQFSTMKMAHAPPGHHSVIGRPSVNGLAYAEYVIYRGEQAYPEYLITYQIMKPEAPSQTA
TAAEQKT*
>ENSG00000083097|ENST00000349129
MNTEELELLSDSKYRNYVAAIDKALKNFEYSSEWADLISALGKLNKVLQNNAKYQVVPKK
LTIGKRLAQCLHPALPGGVHRKALETYEIIFKIIGPKRLAKDLFLYSSGLFPLLANAAMS
VKPTLLSLYEIYYLPLGKTLKPGLQGLLTGILPGLEEGSEYYERTNMLLEKVAAAVDQSA
FYSALWGSLLTSPAVRLPGITYVLAHLNRKLSMEDQLYIIGSDIELMVEAVSTSVQDSSV
LVQRSTLDLILFCFPFHMSQATRPDMIRILSAALHVVLRRDMSLNRRLYAWLLGFDNNGA
IIGPRSTRHSNPEEHATYYFTTFSKELLVQAMVGILQVNGFGEENTLMQDLKPFRILISL
LDKPELGPVILEDVLIEVFRTLYSQCKAELDLQTEPPFSKDHAQLSSKLRENKKTAELIK
TANLLFNSFEPYYMWDYVARWFEECCRRTLHVRLQIGPGDSNDSSELQLTNFCLLVDFLL
DIVSLPTRSMRVLCQETYIEIQTEHLPQLLLRMISALTSHLQTLHLSELTDSLRLCSKIL
SKVQPPLLSASTGGVLQFPSGQNNSVKEWEDKKVSSVSHENPTEVFEDGENPPSSRSSES
GFTEFIQYQADRTDDIDRELSEGQGAAAIPIGSTSSETETASTVGSEETIIQTPSVVTQG
TATRSRKTAQKTAMQCCLEYVQQFLTRLINLYIIQNNSFSQSLATEHQGDLGREQGETSK
WDRNSQGDVKEKNISKQKTSKEYLSAFLAACQLFLECSSFPVYIAEGNHTSELRSEKLET
DCEHVQPPQWLQTLMNACSQASDFSVQSVAISLVMDLVGLTQSVAMVTGENINSVEPAQP
LSPNQGRVAVVIRPPLTQGNLRYIAEKTEFFKHVALTLWDQLGDGTPQHHQKSVELFYQL
HNLVPSSSICEDVISQQLTHKDKKIRMEAHAKFAVLWHLTRDLHINKSSSFVRSFDRSLF
IMLDSLNSLDGSTSSVGQAWLNQVLQRHDIARVLEPLLLLLLHPKTQRVSVQRVQAERYW
NKSPCYPGEESDKHFMQNFACSNVSQVQLITSKGNGEKPLTMDEIENFSLTVNPLSDRLS
LLSTSSETIPMVVSDFDLPDQQIEILQSSDSGCSQSSAGDNLSYEVDPETVNAQEDSQMP
KESSPDDDVQQVVFDLICKVVSGLEVESASVTSQLEIEAMPPKCSDIDPDEETIKIEDDS
IQQSQNALLSNESSQFLSVSAEGGHECVANGISRNSSSPCISGTTHTLHDSSVASIETKS
RQRSHSSIQFSFKEKLSEKVSEKETIVKESGKQPGAKPKVKLARKKDDDKKKSSNEKLKQ
TSVFFSDGLDLENWYSCGEGDISEIESDMGSPGSRKSPNFNIHPLYQHVLLYLQLYDSSR
TLYAFSAIKAILKTNPIAFVNAISTTSVNNAYTPQLSLLQNLLARHRISVMGKDFYSHIP
VDSNHNFRSSMYIEILISLCLYYMRSHYPTHVKVTAQDLIGNRNMQMMSIEILTLLFTEL
AKVIESSAKGFPSFISDMLSKCKVQKVILHCLLSSIFSAQKWHSEKMAGKNLVAVEEGFS
EDSLINFSEDEFDNGSTLQSQLLKVLQRLIVLEHRVMTIPEENETGFDFVVSDLEHISPH
QPMTSLQYLHAQPITCQGMFLCAVIRALHQHCACKMHPQWIGLITSTLPYMGKVLQRVVV
SVTLQLCRNLDNLIQQYKYETGLSDSRPLWMASIIPPDMILTLLEGITAIIHYCLLDPTT
QYHQLLVSVDQKHLFEARSGILSILHMIMSSVTLLWSILHQADSSEKMTIAASASLTTIN
LGATKNLRQQILELLGPISMNHGVHFMAAIAFVWNERRQNKTTTRTKVIPAASEEQLLLV

ELVRSISVMRAETVIQTVKEVLKQPPAIAKDKKHLSLEVCMLQFFYAYIQRIPVPNLVDS
WASLLILLKDSIQLSLPAPGQFLILGVLNEFIMKNPSLENKKDQRDLQDVTHKIVDAIGA
IAGSSLEQTTWLRRNLEVKPSPKIMVDGTNLESDVEDMLSPAMETANITPSVYSVHALTL
LSEVLAHLLDMVFYSDEKERVIPLLVNIMHYVVPYLRNHSAHNAPSYRACVQLLSSLSGY
QYTRRAWKKEAFDLFMDPSFFQMDASCVNHWRAIMDNLMTHDKTTFRDLMTRVAVAQSSS
LNLFANRDVELEQRAMLLKRLAFAIFSSEIDQYQKYLPDIQERLVESLRLPQVPTLHSQV
FLFFRVLLLRMSPQHLTSLWPTMITELVQVFLLMEQELTADEDISRTSGPSVAGLETTYT
GGNGFSTSYNSQRWLNLYLSACKFLDLALALPSENLPQFQMYRWAFIPEASDDSGLEVRR
QGIHQREFKPYVVRLAKLLRKRAKKNPEEDNSGRTLGWEPGHLLLTICTVRSMEQLLPFF
NVLSQVFNSKVTSRCGGHSGSPILYSNAFPNKDMKLENHKPCSSKARQKIEEMVEKDFLE
GMIKT*
>ENSG00000169439|ENST00000302190
MRRAWILLTLGLVACVSAESRAELTSDKDMYLDNSSIEEASGVYPIDDDDYASASGSGAD
EDVESPELTTSRPLPKILLTSAAPKVETTTLNIQNKIPAQTKSPEETDKEKVHLSDSERK
MDPAEEDTNVYTEKHSDSLFKRTEVLAAVIAGGVIGFLFAIFLILLLVYRMRKKDEGSYD
LGERKPSSAAYQKAPTKEFYA*
>ENSG00000257923|ENST00000292535
MLCVAGARLKRELDATATVLANRQDESEQSRKRLIEQSREFKKNTPEDLRKQVAPLLKSF
QGEIDALSKRSKEAEAAFLNVYKRLIDVPDPVPALDLGQQLQLKVQRLHDIETENQKLRE
TLEEYNKEFAEVKNQEVTIKALKEKIREYEQTLKNQAETIALEKEQKLQNDFAEKERKLQ
ETQMSTTSKLEEAEHKVQSLQTALEKTRTELFDLKTKYDEETTAKADEIEMIMTDLERAN
QRAEVAQREAETLREQLSSANHSLQLASQIQKAPDVEQAIEVLTRSSLEVELAAKEREIA
QLVEDVQRLQASLTKLRENSASQISQLEQQLSAKNSTLKQLEEKLKGQADYEEVKKELNI
LKSMEFAPSEGAGTQDAAKPLEVLLLEKNRSLQSENAALRISNSDLSGSARRKGKDQPES
RRPGSLPAPPPSQLPRNPGEQASNTNGTHQFSPAGLSQDFFSSSLASPSLPLASTGKFAL
NSLLQRQLMQSFYSKAMQEAGSTSMIFSTGPYSTNSISSQSPLQQSPDVNGMAPSPSQSE
SAGSVSEGEEMDTAEIARQVKEQLIKHNIGQRIFGHYVLGLSQGSVSEILARPKPWNKLT
VRGKEPFHKMKQFLSDEQNILALRSIQGRQRENPGQSLNRLFQEVPKRRNGSEGNITTRI
RASETGSDEAIKSILEQAKRELQVQKTAEPAQPSSASGSGNSDDAIRSILQQARREMEAQ
QAALDPALKQAPLSQSDITILTPKLLSTSPMPTVSSYPPLAISLKKPSAAPEAGASALPN
PPALKKEAQDAPGLDPQGAADCAQGVLRQVKNEVGRSGAWKDHWWSAVQPERRNAASSEE
AKAEETGGGKEKGSGGSGGGSQPRAERSQLQGPSSSEYWKEWPSAESPYSQSSELSLTGA
SRSETPQNSPLPSSPIVPMSKPTKPSVPPLTPEQYEVYMYQEVDTIELTRQVKEKLAKNG
ICQRIFGEKVLGLSQGSVSDMLSRPKPWSKLTQKGREPFIRMQLWLNGELGQGVLPVQGQ
QQGPVLHSVTSLQDPLQQGCVSSESTPKTSASCSPAPESPMSSSESVKSLTELVQQPCPP
IEASKDSKPPEPSDPPASDSQPTTPLPLSGHSALSIQELVAMSPELDTYGITKRVKEVLT
DNNLGQRLFGETILGLTQGSVSDLLARPKPWHKLSLKGREPFVRMQLWLNDPNNVEKLMD
MKRMEKKAYMKRRHSSVSDSQPCEPPSVGTEYSQGASPQPQHQLKKPRVVLAPEEKEALK
RAYQQKPYPSPKTIEDLATQLNLKTSTVINWFHNYRSRIRRELFIEEIQAGSQGQAGASD
SPSARSGRAAPSSEGDSCDGVEATEGPGSADTEEPKSQGEAEREEVPRPAEQTEPPPSGT
PGPDDARDDDHEGGPVEGPGPLPSPASATATAAPAAPEDAATSAAAAPGEGPAAPSSAPP
PSNSSSSSAPRRPSSLQSLFGLPEAAGARDSRDNPLRKKKAANLNSIIHRLEKAASREEP
IEWEF*
>ENSG00000184014|ENST00000328194

MSGGGGGGGSAPSRFADYFVICGLDTETGLEPDELSALCQYIQASKARDGASPFISSTTE
GENFEQTPLRRTFKSKVLARYPENVEWNPFDQDAVGMLCMPKGLAFKTQADPREPQFHAF
IITREDGSRTFGFALTFYEEVTSKQICSAMQTLYHMHNAEYDVLHAPPADDRDQSSMEDG
EDTPVTKLQRFNSYDISRDTLYVSKCICLITPMSFMKACRSVLEQLHQAVTSPQPPPLPL
ESYIYNVLYEVPLPPPGRSLKFSGVYGPIICQRPSTNELPLFDFPVKEVFELLGVENVFQ
LFTCALLEFQILLYSQHYQRLMTVAETITALMFPFQWQHVYVPILPASLLHFLDAPVPYL
MGLHSNGLDDRSKLELPQEANLCFVDIDNHFIELPEDLPQFPNKLEFVQEVSEILMAFGI
PPEGNLHCSESASKLKRLRASELVSDKRNGNIAGSPLHSYELLKENETIARLQALVKRTG
VSLEKLEVREDPSSNKDLKVQCDEEELRIYQLNIQIREVFANRFTQMFADYEVFVIQPSQ
DKESWFTNREQMQNFDKASFLSDQPEPYLPFLSRFLETQMFASFIDNKIMCHDDDDKDPV
LRVFDSRVDKIRLLNVRTPTLRTSMYQKCTTVDEAEKAIELRLAKIDHTAIHPHLLDMKI
GQGKYEPGFFPKLQSDVLSTGPASNKWTKRNAPAQWRRKDRQKQHTEHLRLDNDQREKYI
QEARTMGSTIRQPKLSNLSPSVIAQTNWKFVEGLLKECRNKTKRMLVEKMGREAVELGHG
EVNITGVEENTLIASLCDLLERIWSHGLQVKQGKSALWSHLLHYQDNRQRKLTSGSLSTS
GILLDSERRKSDASSLMPPLRISLIQDMRHIQNIGEIKTDVGKARAWVRLSMEKKLLSRH
LKQLLSDHELTKKLYKRYAFLRCDDEKEQFLYHLLSFNAVDYFCFTNVFTTILIPYHILI
VPSKKLGGSMFTANPWICISGELGETQIMQIPRNVLEMTFECQNLGKLTTVQIGHDNSGL
YAKWLVEYVMVRNEITGHTYKFPCGRWLGKGMDDGSLERILVGELLTSQPEVDERPCRTP
PLQQSPSVIRRLVTISPNNKPKLNTGQIQESIGEAVNGIVKHFHKPEKERGSLTLLLCGE
CGLVSALEQAFQHGFKSPRLFKNVFIWDFLEKAQTYYETLEKNEVVPEENWHTRARNFCR
FVTAINNTPRNIGKDGKFQMLVCLGARDHLLHHWIALLADCPITAHMYEDVALIKDHTLV
NSLIRVLQTLQEFNITLETSLVKGIDI*
>ENSG00000163472|ENST00000295694
MTEQETLALLEVKRSDSPEKSSPQALVPNGRQPEGEGGAESPGAESLRVGSSAGSPTAIE
GAEDGLDSTVSEAATLPWGTGPQPSAPFPDPPGWRDIEPEPPESEPLTKLEELPEDDANL
LPEKAARAFVPIDLQCIERQPQEDLIVRCEAGEGECRTFMPPRVTHPDPTERKWAEAVVR
PPGCSCGGCGSCGDREWLRAVASVGAALILFPCLLYGAYAFLPFDVPRLPTMSSRLIYTL
RCGVFATFPIVLGILVYGLSLLCFSALRPFGEPRREVEIHRRYVAQSVQLFILYFFNLAV
LSTYLPQDTLKLLPLLTGLFAVSRLIYWLTFAVGRSFRGFGYGLTFLPLLSMLMWNLYYM
FVVEPERMLTATESRLDYPDHARSASDYRPRPWG*
>ENSG00000182022|ENST00000346248
MRHCINCCIQLLPDGAHKQQVNCQGGPHHGHQACPTCKGENKILFRVDSKQMNLLAVLEV
RTEGNENWGGFLRFKKGKRCSLVFGLIIMTLVMASYILSGAHQELLISSPFHYGGFPSNP
SLMDSENPSDTKEHHHQSSVNNISYMKDYPSIKLIINSITTRIEFTTRQLPDLEDLKKQE
LHMFSVIPNKFLPNSKSPCWYEEFSGQNTTDPYLTNSYVLYSKRFRSTFDALRKAFWGHL
AHAHGKHFRLRCLPHFYIIGQPKCGTTDLYDRLRLHPEVKFSAIKEPHWWTRKRFGIVRL
RDGLRDRYPVEDYLDLFDLAAHQIHQGLQASSAKEQSKMNTIIIGEASASTMWDNNAWTF
FYDNSTDGEPPFLTQDFIHAFQPNARLIVMLRDPVERLYSDYLYFASSNKSADDFHEKVT
EALQLFENCMLDYSLRACVYNNTLNNAMPVRLQVGLYAVYLLDWLSVFDKQQFLILRLED
HASNVKYTMHKVFQFLNLGPLSEKQEALMTKSPASNARRPEDRNLGPMWPITQKILRDFY
RPFNARLAQVLADEAFAWKTT*
>ENSG00000148396|ENST00000313050
MQPPPQTVPSGMAGPPPAGNPRSVFWASSPYRRRANNNAAVAPTTCPLQPVTDPFAFSRQ
ALQSTPLGSSSKSSPPVLQGPAPAGFSQHPGLLVPHTHARDSSQGPCEPLPGPLTQPRAH

ASPFSGALTPSAPPGPEMNRSAEVGPSSEPEVQTLPYLPHYIPGVDPETSHGGHPHGNMP
GLDRPLSRQNPHDGVVTPAASPSLPQPGLQMPGQWGPVQGGPQPSGQHRSPCPEGPVPSG
VPCATSVPHFPTPSILHQGPGHEQHSPLVAPPAALPSDGRDEVSHLQSGSHLANNSDPES
TFRQNPRIVNHWASPELRQNPGVKNEHRPASALVNPLARGDSPENRTHHPLGAGAGSGCA
PLEADSGASGALAMFFQGGETENEENLSSEKAGLSGQADFDDFCSSPGLGRPPAPTHVGA
GSLCQALLPGPSNEAAGDVWGDTASTGVPDASGSQYENVENLEFVQNQEVLPSEPLNLDP
SSPSDQFRYGPLPGPAVPRHGAVCHTGAPDATLHTVHPDSVSSSYSSRSHGRLSGSARPQ
ELVGTFIQQEVGKPEDEASGSFFKQIDSSPVGGETDETTVSQNYRGSVSQPSTPSPPKPT
GIFQTSANSSFEPVKSHLVGVKPFEADRANVVGEVRETCVRQKQCRPAAALPDASPGNLE
QPPDNMETLCAPQVCPLPLNSTTEAVHMLPHAGAPPLDTVYPAPEKRPSARTQGPVKCES
PATTLWAQSELPDFGGNVLLAPAAPALYVCAKPQPPVVQPPEEAMSGQQSRNPSSAAPVQ
SRGGIGASENLENPPKMGEEEALQSQASSGYASLLSSPPTESLQNPPVLIAQPDHSYNLA
QPINFSVSLSNSHEKNQSWREALVGDRPAVSSWALGGDSGENTSLSGIPTSSVLSLSLPS
SVAQSNFPQGSGASEMVSNQPANLLVQPPSQPVPENLVPESQKDRKAGSALPGFANSPAG
STSVVLVPPAHGTLVPDGNKANHSSHQEDTYGALDFTLSRTLENPVNVYNPSHSDSLASQ
QSVASHPRQSGPGAPNLDRFYQQVTKDAQGQPGLERAQQELVPPQQQASPPQLPKAMFSE
LSNPESLPAQGQAQNSAQSPASLVLVDAGQQLPPRPPQSSSVSLVSSGSGQAAVPSEQPW
PQPVPALAPGPPPQDLAAYYYYRPLYDAYQPQYSLPYPPEPGAASLYYQDVYSLYEPRYR
PYDGAASAYAQNYRYPEPERPSSRASHSSERPPPRQGYPEGYYSSKSGWSSQSDYYASYY
SSQYDYGDPGHWDRYHYSARVRDPRTYDRRYWCDAEYDAYRREHSAFGDRPEKRDNNWRY
DPRFTGSFDDDPDPHRDPYGEEVDRRSVHSEHSARSLHSAHSLASRRSSLSSHSHQSQIY
RSHNVAAGSYEAPLPPGSFHGDFAYGTYRSNFSSGPGFPEYGYPADTVWPAMEQVSSRPT
SPEKFSVPHVCARFGPGGQLIKVIPNLPSEGQPALVEVHSMEALLQHTSEQEEMRAFPGP
LAKDDTHKVDVINFAQNKAMKCLQNENLIDKESASLLWNFIVLLCRQNGTVVGTDIAELL
LRDHRTVWLPGKSPNEANLIDFTNEAVEQVEEEESGEAQLSFLTGGPAAAASSLERETER
FRELLLYGRKKDALESAMKNGLWGHALLLASKMDSRTHARVMTRFANSLPINDPLQTVYQ
LMSGRMPAASTCCGDEKWGDWRPHLAMVLSNLNNNMDVESRTMATMGDTLASRGLLDAAH
FCYLMAQAGFGVYTKKTTKLVLIGSNHSLPFLKFATNEAIQRTEAYEYAQSLGAETCPLP
SFQVFKFIYSCRLAEMGLATQAFHYCEAIAKSILTQPHLYSPVLISQLVQMASQLRLFDP
QLKEKPEEESLAAPTWLVHLQQVERQIKEGAGVWHQDGALPQQCPGTPSSEMEQLDRPGL
SQPGALGIANPLLAVPAPSPEHSSPSVRLLPSAPQTLPDGPLASPARVPMFPVPLPPGPL
EPGPGCVTPGPALGFLEPSGPGLPPGVPPLQERRHLLQEARSPDPGIVPQEAPVGNSLSE
LSEENFDGKFANLTPSRTVPDSEAPPGWDRADSGPTQPPLSLSPAPETKRPGQAAKKETK
EPKKGESWFFRWLPGKKKTEAYLPDDKNKSIVWDEKKNQWVNLNEPEEEKKAPPPPPTSM
PKTVQAAPPALPGPPGAPVNMYSRRAAGTRARYVDVLNPSGTQRSEPALAPADFVAPLAP
LPIPSNLFVPTPDAEEPQLPDGTGREGPAAARGLANPEPAPEPKVLSSAASLPGSELPSS
RPEGSQGGELSRCSSMSSLSREVSQHFNQAPGDLPAAGGPPSGAMPFYNPAQLAQACATS
GSSRLGRIGQRKHLVLN*
>ENSG00000169446|ENST00000305963
MAPSLWKGLVGIGLFALAHAAFSAAQHRSYMRLTEKEDESLPIDIVLQTLLAFAVTCYGI
VHIAGEFKDMDATSELKNKTFDTLRNHPSFYVFNHRGRVLFRPSDTANSSNQDALSSNTS
LKLRKLESLRR*
>ENSG00000176383|ENST00000324189
MLPPQPSAAHQGRGGRSGLLPKGPAMLCRLCWLVSYSLAVLLLGCLLFLRKAAKPAGDPT

AHQPFWAPPTPRHSRCPPNHTVSSASLSLPSRHRLFLTYRHCRNFSILLEPSGCSKDTFL
LLAIKSQPGHVERRAAIRSTWGRVGGWARGRQLKLVFLLGVAGSAPPAQLLAYESREFDD
ILQWDFTEDFFNLTLKELHLQRWVVAACPQAHFMLKGDDDVFVHVPNVLEFLDGWDPAQD
LLVGDVIRQALPNRNTKVKYFIPPSMYRATHYPPYAGGGGYVMSRATVRRLQAIMEDAEL
FPIDDVFVGMCLRRLGLSPMHHAGFKTFGIRRPLDPLDPCLYRGLLLVHRLSPLEMWTMW
ALVTDEGLKCAAGPIPQR*
>ENSG00000177182|ENST00000325897
MGPVSLLPKYQKLNTWNGDLAKMTHLQAGLSPETIEKARLELNENPDVLHQDIQQVRDMI
ITRPDIGFLRTDDAFILRFLRARKFHQADAFRLLAQYFQYRQLNLDMFKNFKADDPGIKR
ALIDGFPGVLENRDHYGRKILLLFAANWDQSRNSFTDILRAILLSLEVLIEDPELQINGF
ILIIDWSNFSFKQASKLTPSILKLAIEGLQDSFPARFGGVHFVNQPWYIHALYTLIKPFL
KDKTRKRIFLHGNNLNSLHQLIHPEFLPSEFGGTLPPYDMGTWARTLLGPDYSDENDYTH
TSYNAMHVKHTSSNLERECSPKLMKRSQSVVEAGTLKHEEKGENENTQPLLALD*
>ENSG00000172270|ENST00000333511
MAAALFVLLGFALLGTHGASGAAGFVQAPLSQQRWVGGSVELHCEAVGSPVPEIQWWFEG
QGPNDTCSQLWDGARLDRVHIHATYHQHAASTISIDTLVEEDTGTYECRASNDPDRNHLT
RAPRVKWVRAQAVVLVLEPGTVFTTVEDLGSKILLTCSLNDSATEVTGHRWLKGGVVLKE
DALPGQKTEFKVDSDDQWGEYSCVFLPEPMGTANIQLHGPPRVKAVKSSEHINEGETAML
VCKSESVPPVTDWAWYKITDSEDKALMNGSESRFFVSSSQGRSELHIENLNMEADPGQYR
CNGTSSKGSDQAIITLRVRSHLAALWPFLGIVAEVLVLVTIIFIYEKRRKPEDVLDDDDA
GSAPLKSSGQHQNDKGKNVRQRNSS*
>ENSG00000171302|ENST00000302345
MPVQLSEHPEWNESMHSLRISVGGLPVLASMTKAADPRFRPRWKVILTFFVGAAILWLLC
SHRPAPGRPPTHNAHNWRLGQAPANWYNDTYPLSPPQRTPAGIRYRIAVIADLDTESRAQ
EENTWFSYLKKGYLTLSDSGDKVAVEWDKDHGVLESHLAEKGRGMELSDLIVFNGKLYSV
DDRTGVVYQIEGSKAVPWVILSDGDGTVEKGFKAEWLAVKDERLYVGGLGKEWTTTTGDV
VNENPEWVKVVGYKGSVDHENWVSNYNALRAAAGIQPPGYLIHESACWSDTLQRWFFLPR
RASQERYSEKDDERKGANLLLSASPDFGDIAVSHVGAVVPTHGFSSFKFIPNTDDQIIVA
LKSEEDSGRVASYIMAFTLDGRFLLPETKIGSVKYEGIEFI*
>ENSG00000176087|ENST00000323146
MSVEDGGMPGLGRPRQARWTLMLLLSTAMYGAHAPLLALCHVDGRVPFRPSSAVLLTELT
KLLLCAFSLLVGWQAWPQGPPPWRQAAPFALSALLYGANNNLVIYLQRYMDPSTYQVLSN
LKIGSTAVLYCLCLRHRLSVRQGLALLLLMAAGACYAAGGLQVPGNTLPSPPPAAAASPM
PLHITPLGLLLLILYCLISGLSSVYTELLMKRQRLPLALQNLFLYTFGVLLNLGLHAGGG
SGPGLLEGFSGWAALVVLSQALNGLLMSAVMKHGSSITRLFVVSCSLVVNAVLSAVLLRL
QLTAAFFLATLLIGLAMRLYYGSR*
>ENSG00000171365|ENST00000307367
MDFLEEPIPGVGTYDDFNTIDWVREKSRDRDRHREITNKSKESTWALIHSVSDAFSGWLL
MLLIGLLSGSLAGLIDISAHWMTDLKEGICTGGFWFNHEHCCWNSEHVTFEERDKCPEWN
SWSQLIISTDEGAFAYIVNYFMYVLWALLFAFLAVSLVKVFAPYACGSGIPEIKTILSGF
IIRGYLGKWTLVIKTITLVLAVSSGLSLGKEGPLVHVACCCGNILCHCFNKYRKNEAKRR
EVLSAAAAAGVSVAFGAPIGGVLFSLEEVSYYFPLKTLWRSFFAALVAAFTLRSINPFGN
SRLVLFYVEFHTPWHLFELVPFILLGIFGGLWGALFIRTNIAWCRKRKTTQLGKYPVIEV
LVVTAITAILAFPNEYTRMSTSELISELFNDCGLLDSSKLCDYENRFNTSKGGELPDRPA

GVGVYSAMWQLALTLILKIVITIFTFGMKIPSGLFIPSMAVGAIAGRLLGVGMEQLAYYH
QEWTVFNSWCSQGADCITPGLYAMVGAAACLGGVTRMTVSLVVIMFELTGGLEYIVPLMA
AAMTSKWVADALGREGIYDAHIRLNGYPFLEAKEEFAHKTLAMDVMKPRRNDPLLTVLTQ
DSMTVEDVETIISETTYSGFPVVVSRESQRLVGFVLRRDLIISIENARKKQDGVVSTSII
YFTEHSPPLPPYTPPTLKLRNILDLSPFTVTDLTPMEIVVDIFRKLGLRQCLVTHNGRLL
GIITKKDVLKHIAQMANQDPDSILFN*
>ENSG00000215252|ENST00000342314
MAEETGQSKLAAAKKKFKEYWQRNRPGVPAAAKRNTKANGSSPETAASGGCHSSEASSSA
SSSLHARQSPCQEQAAVLNSRSIKISRLNDTIKSLKQQKKQVEHQLEEEKKANNEKQKAE
RELEGQIQRLNTEKKKLNTDLYHMKHSLRYFEEESKDLAGRLQRSSQRIGELEWSLCAVA
ATQKKKPDGFSSRSKALLKRQLEQSIREQILLKGHVTQLKESLKEVQLERDQYAEQIKGE
RAQWQQRMRKMSQEVCTLKEEKKHDTHRVEELERSLSRLKNQMAEPLPPDAPAVSSEVEL
QDLRKELERVAGELQAQVENNQCISLLNRGQKERLREQEERLQEQQERLREREKRLQQLA
EPQSDLEELKHENKSALQLEQQVKELQEKLGQVMETLTSAEKEPEAAVPASGTGGESSGL
MDLLEEKADLREHVEKLELGFIQYRRERCHQKVHRLLTEPGDSAKDASPGGGHHQAGPGQ
GGEEGEAAGAAGDGVAACGSYSEGHGKFLAAARNPAAEPSPGAPAPQELGAADKHGDLCE
ASLTNSVEPAQGEAREGSSQDNPTAQPVLQLLGEMQDHQEHPGLGSNCCVPCFCWAWLPR
RRR*
>ENSG00000179331|ENST00000320578
METIWIYQFRLIVIGDSTVGKSCLLHRFTQGRFPGLRSPACDPTVGVDFFSRLLEIEPGK
RIKLQLWDTAGQERFRSITRSYYRNSVGGFLVFDITNRRSFEHVKDWLEEAKMYVQPFRI
VFLLVGHKCDLASQRQVTREEAEKLSADCGMKYIETSAKDATNVEESFTILTRDIYELIK
KGEICIQDGWEGVKSGFVPNTVHSSEEAVKPRKECFC*
>ENSG00000131386|ENST00000339732
MLLRKRYRHRPCRLQFLLLLLMLGCVLMMVAMLHPPHHTLHQTVTAQASKHSPEARYRLD
FGESQDWVLEAEDEGEEYSPLEGLPPFISLREDQLLVAVALPQARRNQSQGRRGGSYRLI
KQPRRQDKEAPKRDWGADEDGEVSEEEELTPFSLDPRGLQEALSARIPLQRALPEVRHPL
CLQQHPQDSLPTASVILCFHDEAWSTLLRTVHSILDTVPRAFLKEIILVDDLSQQGQLKS
ALSEYVARLEGVKLLRSNKRLGAIRARMLGATRATGDVLVFMDAHCECHPGWLEPLLSRI
AGDRSRVVSPVIDVIDWKTFQYYPSKDLQRGVLDWKLDFHWEPLPEHVRKALQSPISPIR
SPVVPGEVVAMDRHYFQNTGAYDSLMSLRGGENLELSFKAWLCGGSVEILPCSRVGHIYQ
NQDSHSPLDQEATLRNRVRIAETWLGSFKETFYKHSPEAFSLSKAEKPDCMERLQLQRRL
GCRTFHWFLANVYPELYPSEPRPSFSGKLHNTGLGLCADCQAEGDILGCPMVLAPCSDSR
QQQYLQHTSRKEIHFGSPQHLCFAVRQEQVILQNCTEEGLAIHQQHWDFQENGMIVHILS
GKCMEAVVQENNKDLYLRPCDGKARQQWRFDQINAVDER*
>ENSG00000146648|ENST00000342916
MRPSGTAGAALLALLAALCPASRALEEKKVCQGTSNKLTQLGTFEDHFLSLQRMFNNCEV
VLGNLEITYVQRNYDLSFLKTIQEVAGYVLIALNTVERIPLENLQIIRGNMYYENSYALA
VLSNYDANKTGLKELPMRNLQEILHGAVRFSNNPALCNVESIQWRDIVSSDFLSNMSMDF
QNHLGSCQKCDPSCPNGSCWGAGEENCQKLTKIICAQQCSGRCRGKSPSDCCHNQCAAGC
TGPRESDCLVCRKFRDEATCKDTCPPLMLYNPTTYQMDVNPEGKYSFGATCVKKCPRNYV
VTDHGSCVRACGADSYEMEEDGVRKCKKCEGPCRKVCNGIGIGEFKDSLSINATNIKHFK
NCTSISGDLHILPVAFRGDSFTHTPPLDPQELDILKTVKEITGFLLIQAWPENRTDLHAF
ENLEIIRGRTKQHGQFSLAVVSLNITSLGLRSLKEISDGDVIISGNKNLCYANTINWKKL

FGTSGQKTKIISNRGENSCKATGQVCHALCSPEGCWGPEPRDCVSCRNVSRGRECVDKCN
LLEGEPREFVENSECIQCHPECLPQAMNITCTGRGPDNCIQCAHYIDGPHCVKTCPAGVM
GENNTLVWKYADAGHVCHLCHPNCTYGS*
>ENSG00000185274|ENST00000333538
MASLRRVKVLLVLNLIAVAGFVLFLAKCRPIAVRSGDAFHEIRPRAEVANLSAHSASPIQ
DAVLKRLSLLEDIVYRQLNGLSKSLGLIEGYGGRGKGGLPATLSPAEEEKAKGPHEKYGY
NSYLSEKISLDRSIPDYRPTKCKELKYSKDLPQISIIFIFVNEALSVILRSVHSAVNHTP
THLLKEIILVDDNSDEEELKVPLEEYVHKRYPGLVKVVRNQKREGLIRARIEGWKVATGQ
VTGFFDAHVEFTAGWAEPVLSRIQENRKRVILPSIDNIKQDNFEVQRYENSAHGYSWELW
CMYISPPKDWWDAGDPSLPIRTPAMIGCSFVVNRKFFGEIGLLDPGMDVYGGENIELGIK
VWLCGGSMEVLPCSRVAHIERKKKPYNSNIGFYTKRNALRVAEVWMDDYKSHVYIAWNLP
LENPGIDIGDVSERRALRKSLKCKNFQWYLDHVYPEMRRYNNTVAYGELRNNKAKDVCLD
QGPLENHTAILYPCHGWGPQLARYTKEGFLHLGALGTTTLLPDTRCLVDNSKSRLPQLLD
CDKVKSSLYKRWNFIQNGAIMNKGTGRCLEVENRGLAGIDLILRSCTGQRWTIKNSIK*
>ENSG00000070831|ENST00000344548
MQTIKCVVVGDGAVGKTCLLISYTTNKFPSEYVPTVFDNYAVTVMIGGEPYTLGLFDTAG
QEDYDRLRPLSYPQTDVFLVCFSVVSPSSFENVKEKWVPEITHHCPKTPFLLVGTQIDLR
DDPSTIEKLAKNKQKPITPETAEKLARDLKAVKYVECSALTQKGLKNVFDEAILAALEPP
EPKKSRRCVLL*
>ENSG00000105971|ENST00000343213
MGLETEKADVQLFMDDDSYSHHSGLEYADPEKFADSDQDRDPHRLNSHLKDFNAFCKDLP
NGSAFSADNMEECDRCYHCSIVYERRTMLLFCQPATEPGLNTWTPGLEIGIL*
>ENSG00000164574|ENST00000297107
MRRKEKRLLQAVALVLAALVLLPNVGLWALYRERQPDGTPGGSGAAVAPAAGQGSHSRQK
KTFFLGDGQKLKDWHDKEAIRRDAQRVGNGEQGRPYPMTDAERVDQAYRENGFNIYVSDK
ISLNRSLPDIRHPNCNSKRYLETLPNTSIIIPFHNEGWSSLLRTVHSVLNRSPPELVAEI
VLVDDFSDREHLKKPLEDYMALFPSVRILRTKKREGLIRTRMLGASVATGDVITFLDSHC
EANVNWLPPLLDRIARNRKTIVCPMIDVIDHDDFRYETQAGDAMRGAFDWEMYYKRIPIP
PELQKADPSDPFESPVMAGGLFAVDRKWFWELGGYDPGLEIWGGEQYEISFKVWMCGGRM
EDIPCSRVGHIYRKYVPYKVPAGVSLARNLKRVAEVWMDEYAEYIYQRRPEYRHLSAGDV
AVQKKLRSSLNCKSFKWFMTKIAWDLPKFYPPVEPPAAAWGEIRNVGTGLCADTKHGALG
SPLRLEGCVRGRGEAAWNNMQVFTFTWREDIRPGDPQHTKKFCFDAISHTSPVTLYDCHS
MKGNQLWKYRKDKTLYHPVSGSCMDCSESDHRIFMNTCNPSSLTQQWLFEHTNSTVLEKF
NRN*
>ENSG00000132692|ENST00000329117
MAQLFLPLLAALVLAQAPAALADVLEGDSSEDRAFRVRIAGDAPLQGVLGGALTIPCHVH
YLRPPPSRRAVLGSPRVKWTFLSRGREAEVLVARGVRVKVNEAYRFRVALPAYPASLTDV
SLALSELRPNDSGIYRCEVQHGIDDSSDAVEVKVKGVVFLYREGSARYAFSFSGAQEACA
RIGAHIATPEQLYAAYLGGYEQCDAGWLSDQTVRYPIQTPREACYGDMDGFPGVRNYGVV
DPDDLYDVYCYAEDLNGELFLGDPPEKLTLEEARAYCQERGAEIATTGQLYAAWDGGLDH
CSPGWLADGSVRYPIVTPSQRCGGGLPGVKTLFLFPNQTGFPNKHSRFNVYCFRDSAQPS
AIPEASNPASNPASDGLEAIVTVTETLEELQLPQEATESESRGAIYSIPIMEDGGGGSST
PEDPAEAPRTLLEFETQSMVPPTGFSEEEGKALEEEEKYEDEEEKEEEEEEEEVEDEALW
AWPSELSSPGPEASLPTEPAAQEESLSQAPARAVLQPGASPLPDGESEASRPPRVHGPPT

ETLPTPRERNLASPSPSTLVEAREVGEATGGPELSGVPRGESEETGSSEGAPSLLPATRA
PEGTRELEAPSEDNSGRTAPAGTSVQAQPVLPTDSASRGGVAVVPASGDCVPSPCHNGGT
CLEEEEGVRCLCLPGYGGDLCDVGLRFCNPGWDAFQGACYKHFSTRRSWEEAETQCRMYG
AHLASISTPEEQDFINNRYREYQWIGLNDRTIEGDFLWSDGVPLLYENWNPGQPDSYFLS
GENCVVMVWHDQGQWSDVPCNYHLSYTCKMGLVSCGPPPELPLAQVFGRPRLRYEVDTVL
RYRCREGLAQRNLPLIRCQENGRWEAPQISCVPRRPARALHPEEDPEGRQGRLLGRWKAL
LIPPSSPMPGP*
>ENSG00000123191|ENST00000344297
MPEQERQITAREGASRKILSKLSLPTRAWEPAMKKSFAFDNVGYEGGLDGLGPSSQVATS
TVRILGMTCQSCVKSIEDRISNLKGIISMKVSLEQGSATVKYVPSVVCLQQVCHQIGDMG
FEASIAEGKAASWPSRSLPAQEAVVKLRVEGMTCQSCVSSIEGKVRKLQGVVRVKVSLSN
QEAVITYQPYLIQPEDLRDHVNDMGFEAAIKSKVAPLSLGPIDIERLQSTNPKRPLSSAN
QNFNNSETLGHQGSHVVTLQLRIDGMHCKSCVLNIEENIGQLLGVQSIQVSLENKTAQVK
YDPSCTSPVALQRAIEALPPGNFKVSLPDGAEGSGTDHRSSSSHSPGSPPRNQVQGTCST
TLIAIAGMTCASCVHSIEGMISQLEGVQQISVSLAEGTATVLYNPSVISPEELRAAIEDM
GFEASVVSESCSTNPLGNHSAGNSMVQTTDGTPTSVQEVAPHTGRLPANHAPDILAKSPQ
STRAVAPQKCFLQIKGMTCASCVSNIERNLQKEAGVLSVLVALMAGKAEIKYDPEVIQPL
EIAQFIQDLGFEAAVMEDYAGSDGNIELTITGMTCASCVHNIESKLTRTNGITYASVALA
TSKALVKFDPEIIGPRDIIKIIESKTSEALAKLMSLQATEATVVTLGEDNLIIREEQVPM
ELVQRGDIVKVVPGGKFPVDGKVLEGNTMADESLITGEAMPVTKKPGSTVIAGSINAHGS
VLIKATHVGNDTTLAQIVKLVEEAQMSKNPNKHISQTEVIIRFAFQTSITVLCIACPCSL
GLATPTAVMVGTGVAAQNGILIKGGKPLEMAHKIKTVMFDKTGTITHGVPRVMRVLLLGD
VATLPLRKVLAVVGTAEASSEHPLGVAVTKYCKEELGTETLGYCTDFQAVPGCGIGCKVS
NVEGILAHSERPLSAPASHLNEAGSLPAEKDAVPQTFSVLIGNREWLRRNGLTISSDVSD
AMTDHEMKGQTAILVAIDGVLCGMIAIADAVKQEAALAVHTLQSMGVDVVLITGDNRKTA
RAIATQVGINKVFAEVLPSHKVAKVQELQNKGKKVAMVGDGVNDSPALAQADMGVAIGTG
TDVAIEAADVVLIRNDLLDVVASIHLSKRTVRRIRINLVLALIYNLVGIPIAAGVFMPIG
IVLQPWMGSAAMAASSVSVVLSSLQLKCYKKPDLERYEAQAHGHMKPLTASQVSVHIGMD
DRWRDSPRATPWDQVSYVSQVSLSSLTSDKPSRHSAAADDDGDKWSLLLNGRDEEQYI*
>ENSG00000131446|ENST00000307826
MLKKQSAGLVLWGAILFVAWNALLLLFFWTRPAPGRPPSVSALDGDPASLTREVIRLAQD
AEVELERQRGLLQQIGDALSSQRGRVPTAAPPAQPRVPVTPAPAVIPILVIACDRSTVRR
CLDKLLHYRPSAELFPIIVSQDCGHEETAQAIASYGSAVTHIRQPDLSSIAVPPDHRKFQ
GYYKIARHYRWALGQVFRQFRFPAAVVVEDDLEVAPDFFEYFRATYPLLKADPSLWCVSA
WNDNGKEQMVDASRPELLYRTDFFPGLGWLLLAELWAELEPKWPKAFWDDWMRRPEQRQG
RACIRPEISRTMTFGRKGVSHGQFFDQHLKFIKLNQQFVHFTQLDLSYLQREAYDRDFLA
RVYGAPQLQVEKVRTNDRKELGEVRVQYTGRDSFKAFAKALGVMDDLKSGVPRAGYRGIV
TFQFRGRRVHLAPPLTWEGYDPSWN*
>ENSG00000182870|ENST00000328957
MAVARKIRTLLTVNILVFVGIVLFSVYCRLQGRSQELVRIVSGDRRVRSRHAKVGTLGDR
EAILQRLDHLEEVVYNQLNGLAKPIGLVEGPGGLGQGGLAATLRDDGQEAEGKYEEYGYN
AQLSDRISLDRSIPDYRPRKCRQMSYAQDLPQVSVVFIFVNEALSVILRSVHSVVNHTPS
QLLKEVILVDDNSDNVELKFNLDQYVNKRYPGLVKIVRNSRREGLIRARLQGWKAATAPV
VGFFDAHVEFNTGWAEPALSRIREDRRRIVLPAIDNIKYSTFEVQQYANAAHGYNWGLWC

MYIIPPQDWLDRGDESAPIRTPAMIGCSFVVDREYFGDIGLLDPGMEVYGGENVELGMRV
WQCGGSMEVLPCSRVAHIERTRKPYNNDIDYYAKRNALRAAEVWMDDFKSHVYMAWNIPM
SNPGVDFGDVSERLALRQRLKCRSFKWYLENVYPEMRVYNNTLTYGEVRNSKASAYCLDQ
GAEDGDRAILYPCHGMSSQLVRYSADGLLQLGPLGSTAFLPDSKCLVDDGTGRMPTLKKC
EDVARPTQRLWDFTQSGPIVSRATGRCLEVEMSKDANFGLRLVVQRCSGQKWMIRNWIKH
ARH*
>ENSG00000170043|ENST00000303731
MTVHNLYLFDRNGVCLHYSEWHRKKQAGIPKEEEYKLMYGMLFSIRSFVSKMSPLDMKDG
FLAFQTSRYKLHYYETPTGIKVVMNTDLGVGPIRDVLHHIYSALYVELVVKNPLCPLGQT
VQSELFRSRLDSYVRSLPFFSARAG*
>ENSG00000177674|ENST00000314340
MELPAVNLKVILLGHWLLTTWGCIVFSGSYAWANFTILALGVWAVAQRDSIDAISMFLGG
LLATIFLDIVHISIFYPRVSLTDTGRFGVGMAILSLLLKPLSCCFVYHMYRERGGELLVH
TGFLGSSQDRSAYQTIDSAEAPADPFAVPEGRSQDARGY*
>ENSG00000164597|ENST00000297135
MGWVGGRRRDSASPPGRSRSAADDINPAPANMEGGGGSVAVAGLGARGSGAAAATVRELL
QDGCYSDFLNEDFDVKTYTSQSIHQAVIAEQLAKLAQGISQLDRELHLQVVARHEDLLAQ
ATGIESLEGVLQMMQTRIGALQGAVDRIKAKIVEPYNKIVARTAQLARLQVACDLLRRII
RILNLSKRLQGQLQGGSREITKAAQSLNELDYLSQGIDLSGIEVIENDLLFIARARLEVE
NQAKRLLEQGLETQNPTQVGTALQVFYNLGTLKDTITSVVDGYCATLEENINSALDIKVL
TQPSQSAVRGGPGRSTMPTPGNTAALRASFWTNMEKLMDHIYAVCGQVQHLQKVLAKKRD
PVSHICFIEEIVKDGQPEIFYTFWNSVTQALSSQFHMATNSSMFLKQAFEGEYPKLLRLY
NDLWKRLQQYSQHIQGNFNASGTTDLYVDLQHMEDDAQDIFIPKKPDYDPEKALKDSLQP
YEAAYLSKSLSRLFDPINLVFPPGGRNPPSSDELDGIIKTIASELNVAAVDTNLTLAVSK
NVAKTIQLYSVKSEQLLSTQGDASQVIGPLTEGQRRNVAVVNSLYKLHQSVTKVVSSQSS
FPLAAEQTIISALKAIHALMENAVQPLLTSVGDAIEAIIITMHQEDFSGSLSSSGKPDVP
CSLYMKELQGFIARVMSDYFKHFECLDFVFDNTEAIAQRAVELFIRHASLIRPLGEGGKM
RLAADFAQMELAVGPFCRRVSDLGKSYRMLRSFRPLLFQASEHVASSPALGDVIPFSIII
QFLFTRAPAELKSPFQRAEWSHTRFSQWLDDHPSEKDRLLLIRGALEAYVQSVRSREGKE
FAPVYPIMVQLLQKAMSALQ*
>ENSG00000167645|ENST00000339413
MHPAGLAAAAAGTPRLRKWPSKRRIPVSQPGMADPHQLFDDTSSAQSRGYGAQRAPGGLS
YPAASPTPHAAFLADPVSNMAMAYGSSLAAQGGKELVDKNIDRFIPITKLKYYFAVDTMYV
GRKLGLLFFPYLHQDWEVQYQQDTPVAPRFDVNAPDLYIPAMAFITYVLVAGLALGTQDR
FSPDLLGLQASSALAWLTLEVLAILLSLYLVTVNTDLTTIDLVAFLGYKYVGMIGGVLMG
LLFGKIGYYLVLGWCCVAIFVFMIRTLRLKILADAAAEGVPVRGARNQLRMYLTMAVAAA
QPMLMYWLTFHLVR*
>ENSG00000156413|ENST00000318336
MDPLGPAKPQWSWRCCLTTLLFQLLMAVCFFSYLRVSQDDPTVYPNGSRFPDSTGTPAHS
IPLILLWTWPFNKPIALPRCSEMVPGTADCNITADRKVYPQADAVIVHHREVMYNPSAQL
PRSPRRQGQRWIWFSMESPSHCWQLKAMDGYFNLTMSYRSDSDIFTPYGWLEPWSGQPAH
PPLNLSAKTELVAWAVSNWGPNSARVRYYQSLQAHLKVDVYGRSHKPLPQGTMMETLSRY
KFYLAFENSLHPDYITEKLWRNALEAWAVPVVLGPSRSNYERFLPPDAFIHVDDFQSPKD
LARYLQELDKDHARYLSYFRWRETLRPRSFSWALAFCKACWKLQEESRYQTRGIAAWFT*

>ENSG00000161013|ENST00000337755
MSRVAGTRTDVNELLQRWTPRCVRWHTGGARRVALDRPLVTACLPPAGDVVDVYQREFLA
LRDRLHAAEQESLKRSKELNLVLDEIKRAVSERQALRDGDGNRTWGRLTEDPRLKPWNGS
HRHVLHLPTVFHHLPHLLAKESSLQPAVRVGQGRTGVSVVMGIPSVRREVHSYLTDTLHS
LISELSPQEKEDSVIVVLIAETDSQYTSAVTENIKALFPTEIHSGLLEVISPSPHFYPDF
SRLRESFGDPKERVRWRTKQNLDYCFLMMYAQSKGIYYVQLEDDIVAKPNYLSTMKNFAL
QQPSEDWMILEFSQLGFIGKMFKSLDLSLIVEFILMFYRDKPIDWLLDHILWVKVCNPEK
DAKHCDRQKANLRIRFKPSLFQHVGTHSSLAGKIQKLKDKDFGKQALRKEHVNPPAEVST
SLKTYQHFTLEKAYLREDFFWAFTPAAGDFIRFRFFQPLRLERFFFRSGNIEHPEDKLFN
TSVEVLPFDNPQSDKEALQEGRTATLRYPRSPDGYLQIGSFYKGVAEGEVDPAFGPLEAL
RLSIQTDSPVWVILSEIFLKKAD*
>ENSG00000142192|ENST00000346798
MLPGLALLLLAAWTARALEVPTDGNAGLLAEPQIAMFCGRLNMHMNVQNGKWDSDPSGTK
TCIDTKEGILQYCQEVYPELQITNVVEANQPVTIQNWCKRGRKQCKTHPHFVIPYRCLVG
EFVSDALLVPDKCKFLHQERMDVCETHLHWHTVAKETCSEKSTNLHDYGMLLPCGIDKFR
GVEFVCCPLAEESDNVDSADAEEDDSDVWWGGADTDYADGSEDKVVEVAEEEEVAEVEEE
EADDDEDDEDGDEVEEEAEEPYEEATERTTSIATTTTTTTESVEEVVREVCSEQAETGPC
RAMISRWYFDVTEGKCAPFFYGGCGGNRNNFDTEEYCMAVCGSAMSQSLLKTTQEPLARD
PVKLPTTAASTPDAVDKYLETPGDENEHAHFQKAKERLEAKHRERMSQVMREWEEAERQA
KNLPKADKKAVIQHFQEKVESLEQEAANERQQLVETHMARVEAMLNDRRRLALENYITAL
QAVPPRPRHVFNMLKKYVRAEQKDRQHTLKHFEHVRMVDPKKAAQIRSQVMTHLRVIYER
MNQSLSLLYNVPAVAEEIQDEVDELLQKEQNYSDDVLANMISEPRISYGNDALMPSLTET
KTTVELLPVNGEFSLDDLQPWHSFGADSVPANTENEVEPVDARPAADRGLTTRPGSGLTN
IKTEEISEVKMDAEFRHDSGYEVHHQKLVFFAEDVGSNKGAIIGLMVGGVVIATVIVITL
VMLKKKQYTSIHHGVVEVDAAVTPEERHLSKMQQNGYENPTYKFFEQMQN*
>ENSG00000162512|ENST00000339394
MKPGPPHRAGAAHGAGAGAGAAAGPGARGLLLPPLLLLLLAGRAAGAQRWRSENFERPVD
LEGSGDDDSFPDDELDDLYSGSGSGYFEQESGIETAMRFSPDVALAVSTTPAVLPTTNIQ
PVGTPFEELPSERPTLEPATSPLVVTEVPEEPSQRATTVSTTMATTAATSTGDPTVATVP
ATVATATPSTPAAPPFTATTAVIRTTGVRRLLPLPLTTVATARATTPEAPSPPTTAAVLD
TEAPTPRLVSTATSRPRALPRPATTQEPDIPERSTLPLGTTAPGPTEVAQTPTPETFLTT
IRDEPEVPVSGGPSGDFELPEEETTQPDTANEVVAVGGAAAKASSPPGTLPKGARPGPGL
LDNAIDSGSSAAQLPQKSILERKEVLVAVIVGGVVGALFAAFLVTLLIYRMKKKDEGSYT
LEEPKQASVTYQKPDKQEEFYA*
>ENSG00000114270|ENST00000328333
MTLRLLVAALCAGILAEAPRVRAQHRERVTCTRLYAADIVFLLDGSSSIGRSNFREVRSF
LEGLVLPFSGAASAQGVRFATVQYSDDPRTEFGLDALGSGGDVIRAIRELSYKGGNTRTG
AAILHVADHVFLPQLARPGVPKVCILITDGKSQDLVDTAAQRLKGQGVKLFAVGIKNADP
EELKRVASQPTSDFFFFVNDFSILRTLLPLVSRRVCTTAGGVPVTRPPDDSTSAPRDLVL
SEPSSQSLRVQWTAASGPVTGYKVQYTPLTGLGQPLPSERQEVNVPAGETSVRLRGLRPL
TEYQVTVIALYANSIGEAVSGTARTTALEGPELTIQNTTAHSLLVAWRSVPGATGYRVTW
RVLSGGPTQQQELGPGQGSVLLRDLEPGTDYEVTVSTLFGRSVGPATSLMARTDASVEQT
LRPVILGPTSILLSWNLVPEARGYRLEWRRETGLEPPQKVVLPSDVTRYQLDGLQPGTEY
RLTLYTLLEGHEVATPATVVPTGPELPVSPVTDLQATELPGQRVRVSWSPVPGATQYRII

VRSTQGVERTLVLPGSQTAFDLDDVQAGLSYTVRVSARVGPREGSASVLTVRREPETPLA
VPGLRVVVSDATRVRVAWGPVPGASGFRISWSTGSGPESSQTLPPDSTATDITGLQPGTT
YQVAVSVLRGREEGPAAVIVARTDPLGPVRTVHVTQASSSSVTITWTRVPGATGYRVSWH
SAHGPEKSQLVSGEATVAELDGLEPDTEYTVHVRAHVAGVDGPPASVVVRTAPEPVGRVS
RLQILNASSDVLRITWVGVTGATAYRLAWGRSEGGPMRHQILPGNTDSAEIRGLEGGVSY
SVRVTALVGDREGTPVSIVVTTPPEAPPALGTLHVVQRGEHSLRLRWEPVPRAQGFLLHW
QPEGGQEQSRVLGPELSSYHLDGLEPATQYRVRLSVLGPAGEGPSAEVTARTESPRVPSI
ELRVVDTSIDSVTLAWTPVSRASSYILSWRPLRGPGQEVPGSPQTLPGISSSQRVTGLEP
GVSYIFSLTPVLDGVRGPEASVTQTPVCPRGLADVVFLPHATQDNAHRAEATRRVLERLV
LALGPLGPQAVQVGLLSYSHRPSPLFPLNGSHDLGIILQRIRDMPYMDPSGNNLGTAVVT
AHRYMLAPDAPGRRQHVPGVMVLLVDEPLRGDIFSPIREAQASGLNVVMLGMAGADPEQL
RRLAPGMDSVQTFFAVDDGPSLDQAVSGLATALCQASFTTQPRPEPCPVYCPKGQKGEPG
EMGLRGQVGPPGDPGLPGRTGAPGPQGPPGSATAKGERGFPGADGRPGSPGRAGNPGTPG
APGLKGSPGLPGPRGDPGERGPRGPKGEPGAPGQVIGGEGPGLPGRKGDPGPSGPPGPRG
PLGDPGPRGPPGLPGTAMKGDKGDRGERGPPGPGEGGIAPGEPGLPGLPGSPGPQGPVGP
PGKKGEKGDSEDGAPGLPGQPGSPGEQGPRGPPGAIGPKGDRGFPGPLGEAGEKGERGPP
GPAGSRGLPGVAGRPGAKGPEGPPGPTGRQGEKGEPGRPGDPAVVGPAVAGPKGEKGDVG
PAGPRGATGVQGERGPPGLVLPGDPGPKGDPGDRGPIGLTGRAGPPGDSGPPGEKGDPGR
PGPPGPVGPRGRDGEVGEKGDEGPPGDPGLPGKAGERGLRGAPGVRGPVGEKGDQGDPGE
DGRNGSPGSSGPKGDRGEPGPPGPPGRLVDTGPGAREKGEPGDRGQEGPRGPKGDPGLPG
APGERGIEGFRGPPGPQGDPGVRGPAGEKGDRGPPGLDGRSGLDGKPGAAGPSGPNGAAG
KAGDPGRDGLPGLRGEQGLPGPSGPPGLPGKPGEDGKPGLNGKNGEPGDPGEDGRKGEKG
DSGASGREGRDGPKGERGAPGILGPQGPPGLPGPVGPPGQGFPGVPGGTGPKGDRGETGS
KGEQGLPGERGLRGEPGSVPNVDRLLETAGIKASALREIVETWDESSGSFLPVPERRRGP
KGDSGEQGPPGKEGPIGFPGERGLKGDRGDPGPQGPPGLALGERGPPGPSGLAGEPGKPG
IPGLPGRAGGVGEAGRPGERGERGEKGERGEQGRDGPPGLPGTPGPPGPPGPKVSVDEPG
PGLSGEQGPPGLKGAKGEPGSNGDQGPKGDRGVPGIKGDRGEPGPRGQDGNPGLPGERGM
AGPEGKPGLQGPRGPPGPVGGHGDPGPPGAPGLAGPAGPQGPSGLKGEPGETGPPGRGLT
GPTGAVGLPGPPGPSGLVGPQGSPGLPGQVGETGKPGAPGRDGASGKDGDRGSPGVPGSP
GLPGPVGPKGEPGPTGAPGQAVVGLPGAKGEKGAPGGLAGDLVGEPGAKGDRGLPGPRGE
KGEAGRAGEPGDPGEDGQKGAPGPKGFKGDPGVGVPGSPGPPGPPGVKGDLGLPGLPGAP
GVVGFPGQTGPRGEMGQPGPSGERGLAGPPGREGIPGPLGPPGPPGSVGPPGASGLKGDK
GDPGVGLPGPRGERGEPGIRGEDGRPGQEGPRGLTGPPGSRGERGEKGDVGSAGLKGDKG
DSAVILGPPGPRGAKGDMGERGPRGLDGDKGPRGDNGDPGDKGSKGEPGDKGSAGLPGLR
GLLGPQGQPGAAGIPGDPGSPGKDGVPGIRGEKGDVGFMGPRGLKGERGVKGACGLDGEK
GDKGEAGPPGRPGLAGHKGEMGEPGVPGQSGAPGKEGLIGPKGDRGFDGQPGPKGDQGEK
GERGTPGIGGFPGPSGNDGSAGPPGPPGSVGPRGPEGLQGQKGERGPPGERVVGAPGVPG
APGERGEQGRPGPAGPRGEKGEAALTEDDIRGFVRQEMSQHCACQGQFIASGSRPLPSYA
ADTAGSQLHAVPVLRVSHAEEEERVPPEDDEYSEYSEYSVEEYQDPEAPWDSDDPCSLPL
DEGSCTAYTLRWYHRAVTGSTEACHPFVYGGCGGNANRFGTREACERRCPPRVVQSQGTG
TAQD*
>ENSG00000128641|ENST00000304164
MAKMEVKTSLLDNMIGVGDMVLLEPLNEETFINNLKKRFDHSEIYTYIGSVVISVNPYRS
LPIYSPEKVEEYRNRNFYELSPHIFALSDEAYRSLRDQDKDQCILITGESGAGKTEASKL

VMSYVAAVCGKGAEVNQVKEQLLQSNPVLEAFGNAKTVRNDNSSRFGKYMDIEFDFKGDP
LGGVISNYLLEKSRVVKQPRGERNFHVFYQLLSGASEELLNKLKLERDFSRYNYLSLDSA
KVNGVDDAANFRTVRNAMQIVGFMDHEAESVLAVVAAVLKLGNIEFKPESRVNGLDESKI
KDKNELKEICELTGIDQSVLERAFSFRTVEAKQEKVSTTLNVAQAYYARDALAKNLYSRL
FSWLVNRINESIKAQTKVRKKVMGVLDIYGFEIFEDNSFEQFIINYCNEKLQQIFIELTL
KEEQEEYIREDIEWTHIDYFNNAIICDLIENNTNGILAMLDEECLRPGTVTDETFLEKLN
QVCATHQHFESRMSKCSRFLNDTSLPHSCFRIQHYAGKVLYQVEGFVDKNNDLLYRDLSQ
AMWKASHALIKSLFPEGNPAKINLKRPPTAGSQFKASVATLMKNLQTKNPNYIRCIKPND
KKAAHIFNEALVCHQIRYLGLLENVRVRRAGYAFRQAYEPCLERYKMLCKQTWPHWKGPA
RSGVEVLFNELEIPVEEYSFGRSKIFIRNPRTLFKLEDLRKQRLEDLATLIQKIYRGWKC
RTHFLLMKKSQIVIAAWYRRYAQQKRYQQTKSSALVIQSYIRGWKARKILRELKHQKRCK
EAVTTIAAYWHGTQARRELRRLKEEARNKHAIAVIWAYWLGSKARRELKRLKEEARRKHA
VAVIWAYWLGLKVRREYRKFFRANAGKKIYEFTLQRIVQKYFLEMKNKMPSLSPIDKNWP
SRPYLFLDSTHKELKRIFHLWRCKKYRDQFTDQQKLIYEEKLEASELFKDKKALYPSSVG
QPFQGAYLEINKNPKYKKLKDAIEEKIIIAEVVNKINRANGKSTSRIFLLTNNNLLLADQ
KSGQIKSEVPLVDVTKVSMSSQNDGFFAVHLKEGSEAASKGDFLFSSDHLIEMATKLYRT
TLSQTKQKLNIEISDEFLVQFRQDKVCVKFIQGNQKNGSVPTCKRKNNRLLEVAVP*
>ENSG00000038427|ENST00000342785
MFINIKSILWMCSTLIVTHALHKVKVGKSPPVRGSLSGKVSLPCHFSTMPTLPPSYNTSE
FLRIKWSKIEVDKNGKDLKETTVLVAQNGNIKIGQDYKGRVSVPTHPEAVGDASLTVVKL
LASDAGLYRCDVMYGIEDTQDTVSLTVDGVVFHYRAATSRYTLNFEAAQKACLDVGAVIA
TPEQLFAAYEDGFEQCDAGWLADQTVRYPIRAPRVGCYGDKMGKAGVRTYGFRSPQETYD
VYCYVDHLDGDVFHLTVPSKFTFEEAAKECENQDARLATVGELQAAWRNGFDQCDYGWLS
DASVRHPVTVARAQCGGGLLGVRTLYRFENQTGFPPPDSRFDAYCFKPKEATTIDLSILA
ETASPSLSKEPQMVSDRTTPIIPLVDELPVIPTEFPPVGNIVSFEQKATVQPQAITDSLA
TKLPTPTGSTKKPWDMDDYSPSASGPLGKLDISEIKEEVLQSTTGVSHYATDSWDGVVED
KQTQESVTQIEQIEVGPLVTSMEILKHIPSKEFPVTETPLVTARMILESKTEKKMVSTVS
ELVTTGHYGFTLGEEDDEDRTLTVGSDESTLIFDQIPEVITVSKTSEDTIHTHLEDLESV
SASTTVSPLIMPDNNGSSMDDWEERQTSGRITEEFLGKYLSTTPFPSQHRTEIELFPYSG
DKILVEGISTVIYPSLQTEMTHRRERTETLIPEMRTDTYTDEIQEEITKSPFMGKTEEEV
FSGMKLSTSLSEPIHVTESSVEMTKSFDFPTLITKLSAEPTEVRDMEEDFTATPGTTKYD
ENITTVLLAHGTLSVEAATVSKWSWDEDNTTSKPLESTEPSASSKLPPALLTTVGMNGKD
KDIPSFTEDGADEFTLIPDSTQKQLEEVTDEDIAAHGKFTIRFQPTTSTGIAEKSTLRDS
TTEEKVPPITSTEGQVYATMEGSALGEVEDVDLSKPVSTVPQFAHTSEVEGLAFVSYSST
QEPTTYVDSSHTIPLSVIPKTDWGVLVPSVPSEDEVLGEPSQDILVIDQTRLEATISPET
MRTTKITEGTTQEEFPWKEQTAEKPVPALSSTAWTPKEAVTPLDEQEGDGSAYTVSEDEL
LTGSERVPVLETTPVGKIDHSVSYPPGAVTEHKVKTDEVVTLTPRIGPKVSLSPGPEQKY
ETEGSSTTGFTSSLSPFSTHITQLMEETTTEKTSLEDIDLGSGLFEKPKATELIEFSTIK
VTVPSDITTAFSSVDRLHTTSAFKPSSAITKKPPLIDREPGEETTSDMVIIGESTSHVPP
TTLEDIVAKETETDIDREYFTTSSPPATQPTRPPTVEDKEAFGPQALSTPQPPASTKFHP
DINVYIIEVRENKTGPDRCKMNPCLNGGTCYPTETSYVCTCVPGYSGDQCELDFDECHSN
PCRNGATCVDGFNTFRCLCLPSYVGALCEQDTETCDYGWHKFQGQCYKYFAHRRTWDAAE
RECRLQGAHLTSILSHEEQMFVNRVGHDYQWIGLNDKMFEHDFRWTDGSTLQYENWRPNQ
PDSFFSAGEDCVVIIWHENGQWNDVPCNYHLTYTCKKGTVACGQPPVVENAKTFGKMKPR

YEINSLIRYHCKDGFIQRHLPTIRCLGNGRWAIPKITCMNPSAYQRTYSMKYFKNSSSAK
DNSINTSKHDHRWSRRWQESRR*
>ENSG00000142687|ENST00000325722
MEKRLGVKPNPASWILSGYYWQTSAKWLRSLYLFYTCFCFSVLWLSTDASESRCQQGKTQ
FGVGLRSGGENHLWLLEGTPSLQSCWAACCQDSACHVFWWLEGMCIQADCSRPQSCRAFR
THSSNSMLVFLKKFQTADDLGFLPEDDVPHLLGLGWNWASWRQSPPRAALRPAVSSSDQQ
SLIRKLQKRGSPSDVVTPIVTQHSKVNDSNELGGLTTSGSAEVHKAITISSPLTTDLTAE
LSGGPKNVSVQPEISEGLATTPSTQQVKSSEKTQIAVPQPVAPSYSYATPTPQASFQSTS
APYPVIKELVVSAGESVQITLPKNEVQLNAYVLQEPPKGETYTYDWQLITHPRDYSGEME
GKHSQILKLSKLTPGLYEFKVIVEGQNAHGEGYVNVTVKPEPRKNRPPIAIVSPQFQEIS
LPTTSTVIDGSQSTDDDKIVQYHWEELKGPLREEKISEDTAILKLSKLVPGNYTFSLTVV
DSDGATNSTTANLTVNKAVDYPPVANAGPNQVITLPQNSITLFGNQSTDDHGITSYEWSL
SPSSKGKVVEMQGVRTPTLQLSAMQEGDYTYQLTVTDTIGQQATAQVTVIVQPENNKPPQ
ADAGPDKELTLPVDSTTLDGSKSSDDQKIISYLWEKTQGPDGVQLENANSSVATVTGLQV
GTYVFTLTVKDERNLQSQSSVNVIVKEEINKPPIAKITGNVVITLPTSTAELDGSKSSDD
KGIVSYLWTRDEGSPAAGEVLNHSDHHPILFLSNLVEGTYTFHLKVTDAKGESDTDRTTV
EVKPDPRKNNLVEIILDINVSQLTERLKGMFIRQIGVLLGVLDSDIIVQKIQPYTEQSTK
MVFFVQNEPPHQIFKGHEVAAMLKSELRKQKADFLIFRALEVNTVTCQLNCSDHGHCDSF
TKRCICDPFWMENFIKVQLRDGDSNCEWSVLYVIIATFVIVVALGILSWTVICCCKRQKG
KPKRKSKYKILDATDQESLELKPTSRAGIKQKGLLLSSSLMHSESELDSDDAIFTWPDRE
KGKLLHGQNGSVPNGQTPLKARSPREEIL*
>ENSG00000168077|ENST00000337221
MKVRSAGGDGDALCVTEEDLAGDDEDMPTFPCTQKGRPGPRCSRCQKNLSLHTSVRILYL
FLALLLVAVAVLASLVFRKVDSLSEDISLTQSIYDKKLVLMQKNLQGLDPKALNNCSFCH
EAGQLGPEIRKLQEELEGIQKLLLAQEVQLDQTLQAQEVLSTTSRQISQEMGSCSFSIHQ
VNQSLGLFLAQVRGWQATTAGLDLSLKDLTQECYDVKAAVHQINFTVGQTSEWIHGIQRK
TDEETLTLQKIVTDWQNYTRLFSGLRTTSTKTGEAVKNIQATLGASSQRISQNSESMHDL
VLQVMGLQLQLDNISSFLDDHEENMHDLQYHTHYAQNRTVERFESLEGRMASHEIEIGTI
FTNINATDNHVHSMLKYLDDVRLSCTLGFHTHAEELYYLNKSVSIMLGTTDLLRERFSLL
SARLDLNVRNLSMIVEEMKAVDTQHGEILRNVTILRGHTLFSHNRI*
>ENSG00000111846|ENST00000316170
MPLSMRYLFIISVSSVIIFIVFSVFNFGGDPSFQRLNISDPLRLTQVCTSFINGKTRFLW
KNKLMIHEKSSCKEYLTQSHYITAPLSKEEADFPLAYIMVIHHHFDTFARLFRAIYMPQN
IYCVHVDEKATTEFKDAVEQLLSCFPNAFLASKMEPVVYGGISRLQADLNCIRDLSAFEV
SWKYVINTCGQDFPLKTNKEIVQYLKGFKGKNITPGVLPPAHAIGRTKYVHQEHLGKELS
YVIRTTALKPPPPHNLTIYFGSAYVALSREFANFVLHDPRAVDLLQWSKDTFSPDEHFWV
TLNRIPGVPGSMPNASWTGNLRAIKWSDMEDRHGGCHGHYVHGICIYGNGDLKWLVNSPS
LFANKFELNTYPLTVECLELRHRERTLNQSETAIQPSWYF*
>ENSG00000174951|ENST00000310160
MWLRSHRQLCLAFLLVCVLSVIFFLHIHQDSFPHGLGLSILCPDRRLVTPPVAIFCLPGT
AMGPNASSSCPQHPASLSGTWTVYPNGRFGNQMGQYATLLALAQLNGRRAFILPAMHAAL
APVFRITLPVLAPEVDSRTPWRELQLHDWMSEEYADLRDPFLKLSGFPCSWTFFHHLREQ
IRREFTLHDHLREEAQSVLGQLRLGRTGDRPRTFVGVHVRRGDYLQVMPQRWKGVVGDSA
YLRQAMDWFRARHEAPVFVVTSNGMEWCKENIDTSQGDVTFAGDGQEATPWKDFALLTQC

NHTIMTIGTFGFWAAYLAGGDTVYLANFTLPDSEFLKIFKPEAAFLPEWVGINADLSPLW
TLAKP*
>ENSG00000169876|ENST00000306151
MPRPGTMALCLLTLVLSLLPPQAAAEQDLSVNRAVWDGGGCISQGDVLNRQCQQLSQHVR
TGSAANTATGTTSTNVVEPRMYLSCSTNPEMTSIESSVTSDTPGVSSTRMTPTESRTTSE
STSDSTTLFPSSTEDTSSPTTPEGTDVPMSTPSEESISSTMAFVSTAPLPSFEAYTSLTY
KVDMSTPLTTSTQASSSPTTPESTTIPKSTNSEGSTPLTSMPASTMKVASSEAITLLTTP
VEISTPVTISAQASSSPTTAEGPSLSNSAPSGGSTPLTRMPLSVMLVVSSEASTLSTTPA
ATNIPVITSTEASSSPTTAEGTSIPTSTYTEGSTPLTSTPASTMPVATSEMSTLSITPVD
TSTLVTTSTEPSSLPTTAEATSMLTSTLSEGSTPLTNMPVSTILVASSEASTTSTIPVDS
KTFVTTASEASSSPTTAEDTSIATSTPSEGSTPLTSMPVSTTPVASSEASNLSTTPVDSK
TQVTTSTEASSSPPTAEVNSMPTSTPSEGSTPLTSMSVSTMPVASSEASTLSTTPVDTST
PVTTSSEASSSSTTPEGTSIPTSTPSEGSTPLTNMPVSTRLVVSSEASTTSTTPADSNTF
VTTSSEASSSSTTAEGTSMPTSTYSERGTTITSMSVSTTLVASSEASTLSTTPVDSNTPV
TTSTEATSSSTTAEGTSMPTSTYTEGSTPLTSMPVNTTLVASSEASTLSTTPVDTSTPVT
TSTEASSSPTTADGASMPTSTPSEGSTPLTSMPVSKTLLTSSEASTLSTTPLDTSTHITT
STEASCSPTTTEGTSMPISTPSEGSPLLTSIPVSITPVTSPEASTLSTTPVDSNSPVTTS
TEVSSSPTPAEGTSMPTSTYSEGRTPLTSMPVSTTLVATSAISTLSTTPVDTSTPVTNST
EARSSPTTSEGTSMPTSTPGEGSTPLTSMPDSTTPVVSSEARTLSATPVDTSTPVTTSTE
ATSSPTTAEGTSIPTSTPSEGTTPLTSTPVSHTLVANSEASTLSTTPVDSNTPLTTSTEA
SSPPPTAEGTSMPTSTPSEGSTPLTRMPVSTTMVASSETSTLSTTPADTSTPVTTYSQAS
SSSTTADGTSMPTSTYSEGSTPLTSVPVSTRLVVSSEASTLSTTPVDTSIPVTTSTEASS
SPTTAEGTSIPTSPPSEGTTPLASMPVSTTLVVSSEANTLSTTPVDSKTQVATSTEASSP
PPTAEVTSMPTSTPGERSTPLTSMPVRHTPVASSEASTLSTSPVDTSTPVTTSAETSSSP
TTAEGTSLPTSTTSEGSTLLTSIPVSTTLVTSPEASTLLTTPVDTKGPVVTSNEVSSSPT
PAEGTSMPTSTYSEGRTPLTSIPVNTTLVASSAISILSTTPVDNSTPVTTSTEACSSPTT
SEGTSMPNSNPSEGTTPLTSIPVSTTPVVSSEASTLSATPVDTSTPGTTSAEATSSPTTA
EGISIPTSTPSEGKTPLKSIPVSNTPVANSEASTLSTTPVDSNSPVVTSTAVSSSPTPAE
GTSIAISTPSEGSTALTSIPVSTTTVASSEINSLSTTPAVTSTPVTTYSQASSSPTTADG
TSMQTSTYSEGSTPLTSLPVSTMLVVSSEANTLSTTPIDSKTQVTASTEASSSTTAEGSS
MTISTPSEGSPLLTSIPVSTTPVASPEASTLSTTPVDSNSPVITSTEVSSSPTPAEGTSM
PTSTYTEGRTPLTSITVRTTPVASSAISTLSTTPVDNSTPVTTSTEARSSPTTSEGTSMP
NSTPSEGTTPLTSIPVSTTPVLSSEASTLSATPIDTSTPVTTSTEATSSPTTAEGTSIPT
STLSEGMTPLTSTPVSHTLVANSEASTLSTTPVDSNSPVVTSTAVSSSPTPAEGTSIATS
TPSEGSTALTSIPVSTTTVASSETNTLSTTPAVTSTPVTTYAQVSSSPTTADGSSMPTST
PREGRPPLTSIPVSTTTVASSEINTLSTTLADTRTPVTTYSQASSSPTTADGTSMPTPAY
SEGSTPLTSMPLSTTLVVSSEASTLSTTPVDTSTPATTSTEGSSSPTTAGGTSIQTSTPS
ERTTPLAGMPVSTTLVVSSEGNTLSTTPVDSKTQVTNSTEASSSATAEGSSMTISAPSEG
SPLLTSIPLSTTPVASPEASTLSTTPVDSNSPVITSTEVSSSPIPTEGTSMQTSTYSDRR
TPLTSMPVSTTVVASSAISTLSTTPVDTSTPVTNSTEARSSPTTSEGTSMPTSTPSEGST
PFTSMPVSTMPVVTSEASTLSATPVDTSTPVTTSTEATSSPTTAEGTSIPTSTLSEGTTP
LTSIPVSHTLVANSEVSTLSTTPVDSNTPFTTSTEASSPPPTAEGTSMPTSTSSEGNTPL
TRMPVSTTMVASFETSTLSTTPADTSTPVTTYSQAGSSPTTADDTSMPTSTYSEGSTPLT
SVPVSTMPVVSSEASTHSTTPVDTSTPVTTSTEASSSPTTAEGTSIPTSPPSEGTTPLAS

MPVSTTPVVSSEAGTLSTTPVDTSTPMTTSTEASSSPTTAEDIVVPISTASEGSTLLTSI
PVSTTPVASPEASTLSTTPVDSNSPVVTSTEISSSATSAEGTSMPTSTYSEGSTPLRSMP
VSTKPLASSEASTLSTTPVDTSIPVTTSTETSSSPTTAKDTSMPISTPSEVSTSLTSILV
STMPVASSEASTLSTTPVDTRTLVTTSTGTSSSPTTAEGSSMPTSTPGERSTPLTNILVS
TTLLANSEASTLSTTPVDTSTPVTTSAEASSSPTTAEGTSMRISTPSDGSTPLTSILVST
LPVASSEASTVSTTAVDTSIPVTTSTEASSSPTTAEVTSMPTSTPSETSTPLTSMPVNHT
PVASSEAGTLSTTPVDTSTPVTTSTKASSSPTTAEGIVVPISTASEGSTLLTSIPVSTTP
VASSEASTLSTTPVDTSIPVTTSTEGSSSPTTAEGTSMPISTPSEVSTPLTSILVSTVPV
AGSEASTLSTTPVDTRTPVTTSAEASSSPTTAEGTSMPISTPGERRTPLTSMSVSTMPVA
SSEASTLSRTPADTSTPVTTSTEASSSPTTAEGTGIPISTPSEGSTPLTSIPVSTTPVAI
PEASTLSTTPVDSNSPVVTSTEVSSSPTPAEGTSMPISTYSEGSTPLTGVPVSTTPVTSS
AISTLSTTPVDTSTPVTTSTEAHSSPTTSEGTSMPTSTPSEGSTPLTYMPVSTMLVVSSE
DSTLSATPVDTSTPVTTSTEATSSTTAEGTSIPTSTPSEGMTPLTSVPVSNTPVASSEAS
ILSTTPVDSNTPLTTSTEASSSPPTAEGTSMPTSTPSEGSTPLTSMPVSTTTVASSETST
LSTTPADTSTPVTTYSQASSSPPIADGTSMPTSTYSEGSTPLTNMSFSTTPVVSSEASTL
STTPVDTSTPVTTSTEASLSPTTAEGTSIPTSSPSEGTTPLASMPVSTTPVVSSEVNTLS
TTPVDSNTLVTTSTEASSSPTIAEGTSLPTSTTSEGSTPLSIMPLSTTPVASSEASTLST
TPVDTSTPVTTSSPTNSSPTTAEVTSMPTSTAGEGSTPLTNMPVSTTPVASSEASTLSTT
PVDSNTFVTSSSQASSSPATLQVTTMRMSTPSEGSSSLTTMLLSSTYVTSSEASTPSTPS
VDRSTPVTTSTQSNSTPTPPEVITLPMSTPSEVSTPLTIMPVSTTSVTISEAGTASTLPV
DTSTPVITSTQVSSSPVTPEGTTMPIWTPSEGSTPLTTMPVSTTRVTSSEGSTLSTPSVV
TSTPVTTSTEAISSSATLDSTTMSVSMPMEISTLGTTILVSTTPVTRFPESSTPSIPSVY
TSMSMTTASEGSSSPTTLEGTTTMPMSTTSERSTLLTTVLISPISVMSPSEASTLSTPPG
DTSTPLLTSTKAGSFSIPAEVTTIRISITSERSTPLTTLLVSTTLPTSFPGASIASTPPL
DTSTTFTPSTDTASTPTIPVATTISVSVITEGSTPGTTIFIPSTPVTSSTADVFPATTGA
VSTPVITSTELNTPSTSSSSTTTSFSTTKEFTTPAMTTAAPLTYVTMSTAPSTPRTTSRG
CTTSASTLSATSTPHTSTSVTTRPVTPSSESSRPSTITSHTIPPTFPPAHSSTPPTTSAS
STTVNPEAVTTMTTRTKPSTRTTSFPTVTTTAVPTNTTIKSNPTSTPTVPRTTTCFGDGC
QNTASRCKNGGTWDGLKCQCPNLYYGELCEEVVSSIDIGPPETISAQMELTVTVTSVKFT
EELKNHSSQEFQEFKQTFTEQMNIVYSGIPEYVGVNITKLRLGSVVVEHDVLLRTKYTPE
YKTVLDNATEVVKEKITKVTTQQIMINDICSDMMCFNTTGTQVQNITVTQYDPEEDCRKM
AKEYGDYFVVEYRDQKPYCISPCEPGFSVSKNCNLGKCQMSLSGPQCLCVTTETHWYSGE
TCNQGTQKSLVYGLVGAGVVLMLIILVALLMLVFRSKREVKRQKYRLSQLYKWQEEDSGP
APGTFQNIGFDICQDDDSIHLESIYSNFQPSLRHIDPETKIRIQRPQVMTTSF*
>ENSG00000082805|ENST00000347735
MYGSARSVGKVEPSSQSPGRSPRLPRSPRLGHRRTNSTGGSSGSSVGGGSGKTLSMENIQ
SLNAAYATSGPMYLSDHENVGSETPKSTMTLGRSGGRLPYGVRMTAMGSSPNIASSGVAS
DTIAFGEHHLPPVSMASTVPHSLRQARDNTIMDLQTQLKEVLRENDLLRKDVEVKESKLS
SSMNSIKTFWSPELKKERALRKDEASKITIWKEQYRVVQEENQHMQMTIQALQDELRIQR
DLNQLFQQDSSSRTGEPCVAELTEENFQRLHAEHERQAKELFLLRKTLEEMELRIETQKQ
TLNARDESIKKLLEMLQSKGLSAKATEEDHERTRRLAEAEMHVHHLESLLEQKEKENSML
REEMHRRFENAPDSAKTKALQTVIEMKDSKISSMERGLRDLEEEIQMLKSNGALSTEERE
EEMKQMEVYRSHSKFMKNKIGQVKQELSRKDTELLALQTKLETLTNQFSDSKQHIEVLKE
SLTAKEQRAAILQTEVDALRLRLEEKETMLNKKTKQIQDMAEEKGTQAGEIHDLKDMLDV

KERKVNVLQKKIENLQEQLRDKEKQMSSLKERVKSLQADTTNTDTALTTLEEALAEKERT
IERLKEQRDRDEREKQEEIDNYKKDLKDLKEKVSLLQGDLSEKEASLLDLKEHASSLASS
GLKKDSRLKTLEIALEQKKEECLKMESQLKKAHEAALEARASPEMSDRIQHLEREITRYK
DESSKAQAEVDRLLEILKEVENEKNDKDKKIAELESLTSRQVKDQNKKVANLKHKEQVEK
KKSAQMLEEARRREDNLNDSSQQLQVEELLMAMEKVKQELESMKAKLSSTQQSLAEKETH
LTNLRAERRKHLEEVLEMKQEALLAAISEKDANIALLELSSSKKKTQEEVAALKREKDRL
VQQLKQQTQNRMKLMADNYEDDHFKSSHSNQTNHKPSPDQDEEEGIWA*
>ENSG00000173230|ENST00000340645
MLSRLSGLANVVLHELSGDDDTDQNMRAPLDPELHQESDMEFNNTTQEDVQERLAYAEQL
VVELKDIIRQKDVQLQQKDEALQEERKAADNKIKKLKLHAKAKLTSLNKYIEEMKAQGGT
VLPTEPQSEEQLSKHDKSSTEEEMEIEKIKHKLQEKEELISTLQAQLTQAQAEQPAQSST
EMEEFVMMKQQLQEKEEFISTLQAQLSQTQAEQAAQQVVREKDARFETQVRLHEDELLQL
VTQADVETEMQQKLRVLQRKLEEHEESLVGRAQVVDLLQQELTAAEQRNQILSQQLQQME
AEHNTLRNTVETEREESKILLEKMELEVAERKLSFHNLQEEMHHLLEQFEQAGQAQAELE
SRYSALEQKHKAEMEEKTSHILSLQKTGQELQSACDALKDQNSKLLQDKNEQAVQSAQTI
QQLEDQLQQKSKEISQFLNRLPLQQHETASQTSFPDVYNEGTQAVTEENIASLQKRVVEL
ENEKGALLLSSIELEELKAENEKLSSQITLLEAQNRTGEADREVSEISIVDIANKRSSSA
EESGQDVLENTFSQKHKELSVLLLEMKEAQEEIAFLKLQLQGKRAEEADHEVLDQKEMKQ
MEGEGIAPIKMKVFLEDTGQDFPLMPNEESSLPAVEKEQASTEHQSRTSEEISLNDAGVE
LKSTKQDGDKSLSAVPDIGQCHQDELERLKSQILELELNFHKAQEIYEKNLDEKAKEISN
LNQLIEEFKKNADNNSSAFTALSEERDQLLSQVKELSMVTELRAQVKQLEMNLAEAERQR
RLDYESQTAHDNLLTEQIHSLSIEAKSKDVKIEVLQNELDDVQLQFSEQSTLIRSLQSQL
QNKESEVLEGAERVRHISSKVEELSQALSQKELEITKMDQLLLEKKRDVETLQQTIEEKD
QQVTEISFSMTEKMVQLNEEKFSLGVEIKTLKEQLNLLSRAEEAKKEQVEEDNEVSSGLK
QNYDEMSPAGQISKEELQHEFDLLKKENEQRKRKLQAALINRKELLQRVSRLEEELANLK
DESKKEIPLSETERGEVEEDKENKEYSEKCVTSKCQEIEIYLKQTISEKEVELQHIRKDL
EEKLAAEEQFQALVKQMNQTLQDKTNQIDLLQAEISENQAIIQKLITSNTDASDGDSVAL
VKETVVISPPCTGSSEHWKPELEEKILALEKEKEQLQKKLQEALTSRKAILKKAQEKERH
LREELKQQKDDYNRLQEQFDEQSKENENIGDQLRQLQIQVRESIDGKLPSTDQQESCSST
PGLEEPLFKATEQHHTQPVLESNLCPDWPSHSEDASALQGGTSVAQIKAQLKEIEAEKVE
LELKVSSTTSELTKKSEEVFQLQEQINKQGLEIESLKTVSHEAEVHAESLQQKLESSQLQ
IAGLEHLRELQPKLDELQKLISKKEEDVSYLSGQLSEKEAALTKIQTEIIEQEDLIKALH
TQLEMQAKEHDERIKQLQVELCEMKQKPEEIGEESRAKQQIQRKLQAALISRKEALKENK
SLQEELSLARGTIERLTKSLADVESQVSAQNKEKDTVLGRLALLQEERDKLITEMDRSLL
ENQSLSSSCESLKLALEGLTEDKEKLVKEIESLKSSKIAESTEWQEKHKELQKEYEILLQ
SYENVSNEAERIQHVVEAVRQEKQELYGKLRSTEANKKETEKQLQEAEQEMEEMKEKMRK
FAKSKQQKILELEEENDRLRAEVHPAGDTAKECMETLLSSNASMKEELERVKMEYETLSK
KFQSLMSEKDSLSEEVQDLKHQIEGNVSKQANLEATEKHDNQTNVTEEGTQSIPGETEEQ
DSLSMSTRPTCSESVPSAKSANPAVSKDFSSHDEINNYLQQIDQLKERIAGLEEEKQKNK
EFSQTLENEKNTLLSQISTKDGELKMLQEEVTKMNLLNQQIQEELSRVTKLKETAEEEKD
DLEERLMNQLAELNGSIGNYCQDVTDAQIKNELLESEMKNLKKCVSELEEEKQQLVKEKT
KVESEIRKEYLEKIQGAQKEPGNKSHAKELQELLKEKQQEVKQLQKDCIRYQEKISALER
TVKALEFVQTESQKDLEITKENLAQAVEHRKKAQAELASFKVLLDDTQSEAARVLADNLK
LKKELQSNKESVKSQMKQKDEDLERRLEQAEEKHLKEKKNMQEKLDALRREKVHLEETIG

EIQVTLNKKDKEVQQLQENLDSTVTQLAAFTKSMSSLQDDRDRVIDEAKKWERKFSDAIQ
SKEEEIRLKEDNCSVLKDQLRQMSIHMEELKINISRLEHDKQIWESKAQTEVQLQQKVCD
TLQGENKELLSQLEETRHLYHSSQNELAKLESELKSLKDQLTDLSNSLEKCKEQKGNLEG
IIRQQEADIQNSKFSYEQLETDLQASRELTSRLHEEINMKEQKIISLLSGKEEAIQVAIA
ELRQQHDKEIKELENLLSQEEEENIVLEEENKKAVDKTNQLMETLKTIKKENIQQKAQLD
SFVKSMSSLQNDRDRIVGDYQQLEERHLSIILEKDQLIQEAAAENNKLKEEIRGLRSHMD
DLNSENAKLDAELIQYREDLNQVITIKDSQQKQLLEVQLQQNKELENKYAKLEEKLKESE
EANEDLRRSFNALQEEKQDLSKEIESLKVSISQLTRQVTALQEEGTLGLYHAQLKVKEEE
VHRLSALFSSSQKRIAELEEELVCVQKEAAKKVGEIEDKLKKELKHLHHDAGIMRNETET
AEERVAELARDLVEMEQKLLMVTKENKGLTAQIQSFGRSMSSLQNSRDHANEELDELKRK
YDASLKELAQLKEQGLLNRERDALLSETAFSMNSTEENSLSHLEKLNQQLLSKDEQLLHL
SSQLEDSYNQVQSFSKAMASLQNERDHLWNELEKFRKSEEGKQRSAAQPSTSPAEVQSLK
KAMSSLQNDRDRLLKELKNLQQQYLQINQEITELHPLKAQLQEYQDKTKAFQIMQEELRQ
ENLSWQHELHQLRMEKSSWEIHERRMKEQYLMAISDKDQQLSHLQNLIRELRSSSSQTQP
LKVQYQRQASPETSASPDGSQNLVYETELLRTQLNDSLKEIHQKELRIQQLNSNFSQLLE
EKNTLSIQLCDTSQSLRENQQHYGDLLNHCAVLEKQVQELQAGPLNIDVAPGAPQEKNGV
HRKSDPEELREPQQSFSEAQQQLCNTRQEVNELRKLLEEERDQRVAAENALSVAEEQIRR
LEHSEWDSSRTPIIGSCGTQEQALLIDLTSNSCRRTRSGVGWKRVLRSLCHSRTRVPLLA
AIYFLMIHVLLILCFTGHL*
>ENSG00000177830|ENST00000323578
MRTLFNLLWLALACSPVHTTLSKSDAKKAASKTLLEKSQFSDKPVQDRGLVVTDLKAESV
VLEHRSYCSAKARDRHFAGDVLGYVTPWNSHGYDVTKVFGSKFTQISPVWLQLKRRGREM
FEVTGLHDVDQGWMRAVRKHAKGLHIVPRLLFEDWTYDDFRNVLDSEDEIEELSKTVVQV
AKNQHFDGFVVEVWNQLLSQKRVGLIHMLTHLAEALHQARLLALLVIPPAITPGTDQLGM
FTHKEFEQLAPVLDGFSLMTYDYSTAHQPGPNAPLSWVRACVQVLDPKSKWRSKILLGLN
FYGMDYATSKDAREPVVGARYIQTLKDHRPRMVWDSQASEHFFEYKKSRSGRHVVFYPTL
KSLQVRLELARELGVGVSIWELGQGLDYFYDLL*
>ENSG00000170348|ENST00000303575
MSGLSGPPARRGPFPLALLLLFLLGPRLVLAISFHLPINSRKCLREEIHKDLLVTGAYEI
SDQSGGAGGLRSHLKITDSAGHILYSKEDATKGKFAFTTEDYDMFEVCFESKGTGRIPDQ
LVILDMKHGVEAKNYEEIAKVEKLKPLEVELRRLEDLSESIVNDFAYMKKREEEMRDTNE
STNTRVLYFSIFSMFCLIGLATWQVFYLRRFFKAKKLIE*
>ENSG00000082996|ENST00000344229
MLLSIGMLMLSATQVVYTILTVQLFAFLNLLPVEADILAYNFENASQTFDDLPARFGYRLP
AEGLKGFLINSKPENACEPIVPPPVKDNSSGTFIVLIRRLDCNFDIKVLNAQRAGYKAAI
VHNVDSDDLISMGSNDIEVLKKIDIPSVFIGESSANSLKDEFTYEKGGHLILVPEFSLPL
EYYLIPFLIIVGICLILIVIFMITKFVQDRHRARRNRLRKDQLKKLPVHKFKKGDEYDVC
AICLDEYEDGDKLRILPCSHAYHCKCVDPWLTKTKKTCPVCKQKVVPSQGDSDSDTDSSQ
EENEVTEHTPLLRPLASVSAQSFGALSESRSHQNMTESSDYEEDDNEDTDSSDAENEINE
HDVVVQLQPNGERDYNIANTV*
>ENSG00000213420|ENST00000292377
MSALRPLLLLLLLPLCPGPGPGPGSEAKVTRSCAETRQVLGARGYSLNLIPPALISGEHLR
VCPQEYTCCSSETEQRLIRETEATFRGLVEDSGSFLVHTLAARHRKFDEFFLEMLSVAQH
SLTQLFSHSYGRLYAQHALIFNGLFSRLRDFYGESGEGLDDTLADFWAQLLERVFPLLHP

QYSFPPDYLLCLSRLASSTDGSLQPFGDSPRRLRLQITRTLVAARAFVQGLETGRNVVSE
ALKVPVSEGCSQALMRLIGCPLCRGVPSLMPCQGFCLNVVRGCLSSRGLEPDWGNYLDGL
LILADKLQGPFSFELTAESIGVKISEGLMYLQENSAKVSAQVFQECGPPDPVPARNRRAP
PPREEAGRLWSMVTEEERPTTAAGTNLHRLVWELRERLARMRGFWARLSLTVCGDSRMAA
DASLEAAPCWTGAGRGRYLPPVVGGSPAEQVNNPELKVDASGPDVPTRRRRLQLRAATAR
MKTAALGHDLDGQDADEDASGSGGGQQYADDWMAGAVAPPARPPRPPYPPRRDGSGGKGG
GGSARYNQGRSRSGGASIGFHTQTILILSLSALALLGPR*
>ENSG00000105974|ENST00000341049
MSGGKYVDSEGHLYTVPIREQGNIYKPNNKAMADELSEKQVYDAHTKEIDLVNRDPKHLN
DDVVKIDFEDVIAEPEGTHSFDGIWKASFTTFTVTKYWFYRLLSALFGIPMALIWGIYFA
ILSFLHIWAVVPCIKSFLIEIQCISRVYSIYVHTVCDPLFEAVGKIFSNVRINLQKEI*
>ENSG00000100934|ENST00000307712
MTTYLEFIQQNEERDGVRFSWNVWPSSRLEATRMVVPVAALFTPLKERPDLPPIQYEPVL
CSRTTCRAVLNPLCQVDYRAKLWACNFCYQRNQFPPSYAGISELNQPAELLPQFSSIEYV
VLRGPQMPLIFLYVVDTCMEDEDLQALKESMQMSLSLLPPTALVGLITFGRMVQVHELGC
EGISKSYVFRGTKDLSAKQLQEMLGLSKVPLTQATRGPQVQQPPPSNRFLQPVQKIDMNL
TDLLGELQRDPWPVPQGKRPLRSSGVALSIAVGLLECTFPNTGARIMMFIGGPATQGPGM
VVGDELKTPIRSWHDIDKDNAKYVKKGTKHFEALANRAATTGHVIDIYACALDQTGLLEM
KCCPNLTGGYMVMGDSFNTSLFKQTFQRVFTKDMHGQFKMGFGGTLEIKTSREIKISGAI
GPCVSLNSKGPCVSENEIGTGGTCQWKICGLSPTTTLAIYFEVVNQHNAPIPQGGRGAIQ
FVTQYQHSSGQRRIRVTTIARNWADAQTQIQNIAASFDQEAAAILMARLAIYRAETEEGP
DVLRWLDRQLIRLCQKFGEYHKDDPSSFRFSETFSLYPQFMFHLRRSSFLQVFNNSPDES
SYYRHHFMRQDLTQSLIMIQPILYAYSFSGPPEPVLLDSSSILADRILLMDTFFQILIYH
GETIAQWRKSGYQDMPEYENFRHLLQAPVDDAQEILHSRFPMPRYIDTEHGGSQARFLLS
KVNPSQTHNNMYAWGQESGAPILTDDVSLQVFMDHLKKLAVSSAA*
>ENSG00000124225|ENST00000341744
MHRLMGVNSTAAAAAGQPNVSCTCNCKRSLFQSMEITELEFVQIIIIVVVMMVMVVVITC
LLSHYKLSARSFISRHSQGRRREDALSSEGCLWPSESTVSGNGIPEPQVYAPPRPTDRLA
VPPFAQRERFHRFQPTYPYLQHEIDLPPTISLSDGEEPPPYQGPCTLQLRDPEQQLELNR
ESVRAPPNRTIFDSDLMDSARLGGPCPPSSNSGISATCYGSGGRMEGPPPTYSEVIGHYP
GSSFQHQQSSGPPSLLEGTRLHHTHIAPLESAAIWSKEKDKQKGHPL*
>ENSG00000138674|ENST00000348405
MKLKEVDRTAMQAWSPAQNHPIYLATGTSAQQLDATFSTNASLEIFELDLSDPSLDMKSC
ATFSSSHRYHKLIWGPYKMDSKGDVSGVLIAGGENGNIILYDPSKIIAGDKEVVIAQNDK
HTGPVRALDVNIFQTNLVASGANESEIYIWDLNNFATPMTPGAKTQPPEDISCIAWNRQV
QHILASASPSGRATVWDLRKNEPIIKVSDHSNRMHCSGLAWHPDVATQMVLASEDDRLPV
IQMWDLRFASSPLRVLENHARGILAIAWSMADPELLLSCGKDAKILCSNPNTGEVLYELP
TNTQWCFDIQWCPRNPAVLSAASFDGRISVYSIMGGSTDGLRQKQVDKLSSSFGNLDPFG
TGQPLPPLQIPQQTAQHSIVLPLKKPPKWIRRPVGASFSFGGKLVTFENVRMPSHQGAEQ
QQQQHHVFISQVVTEKEFLSRSDQLQQAVQSQGFINYCQKKIDASQTEFEKNVWSFLKVN
FEDDSRGKYLELLGYRKEDLGKKHIKEEKEESEFLPSSGGTFNISVSGDIDGLITQALLT
GNFESAVDLCLHDNRMADAIILAIAGGQELLARTQKKYFAKSQSKITRLITAVVMKNWKE
IVESCDLKNWREALAAVLTYAKPDEFSALCDLLGTRLENEGDSLLQTQACLCYICAGNVE
KLVACWTKAQDGSHPLSLQDLIEKVVILRKAVQLTQAMDTSTVGVLLAAKMSQYANLLAA

QGSIAAALAFLPDNTNQPNIMQLRDRLCRAQGEPVAGHESPKIPYEKQQLPKGRPGPVAG
HHQMPRVQTQQYYPHGENPPPPGFIMHGNVNPNAAGQLPTSPGHMHTQVPPYPQPQPYQP
AQPYPFGTGGSAMYRPQQPVAPPTSNAYPNTPYISSASSYTGQSQLYAAQHQASSPTSSP
ATSFPPPPSSGASFQHGGPGAPPSSSAYALPPGTTGTLPAASELPASQRTGPQNGWNDPP
ALNRVPKKKKMPENFMPPVPITSPIMNPLGDPQSQMLQQQPSAPVPLSSQSSFPQPHLPG
GQPFHGVQQPLGQTGMPPSFSKPNIEGAPGAPIGNTFQHVQSLPTKKITKKPIPDEHLIL
KTTFEDLIQRCLSSATDPQTKRKLDDASKRLEFLYDKLREQTLSPTITSGLHNIARSIET
RNYSEGLTMHTHIVSTSNFSETSAFMPVLKVVLTQANKLGV*
>ENSG00000164821|ENST00000297435
MRIIALLAAILLVALQVRAGPLQARGDEAPGQEQRGPEDQDISISFAWDKSSALQVSGST
RGMVCSCRLVFCRRTELRVGNCLIGGVSFTYCCTRVD*
>ENSG00000174903|ENST00000311481
MNPEYDYLFKLLLIGDSGVGKSCLLLRFADDTYTESYISTIGVDFKIRTIELDGKTIKLQ
IWDTAGQERFRTITSSYYRGAHGIIVVYDVTDQESYANVKQWLQEIDRYASENVNKLLVG
NKSDLTTKKVVDNTTAKEFADSLGIPFLETSAKNATNVEQAFMTMAAEIKKRMGPGAASG
GERPNLKIDSTPVKPAGGGCC*
>ENSG00000183778|ENST00000343118
MAFPKMRLMYICLLVLGALCLYFSMYSLNPFKEQSFVYKKDGNFLKLPDTDCRQTPPFLV
LLVTSSHKQLAERMAIRQTWGKERMVKGKQLKTFFLLGTTSSAAETKEVDQESQRHGDII
QKDFLDVYYNLTLKTMMGIEWVHRFCPQAAFVMKTDSDMFINVDYLTELLLKKNRTTRFF
TGFLKLNEFPIRQPFSKWFVSKSEYPWDRYPPFCSGTGYVFSGDVASQVYNVSKSVPYIK
LEDVFVGLCLERLNIRLEELHSQPTFFPGGLRFSVCLFRRIVACHFIKPRTLLDYWQALE
NSRGEDCPPV*
>ENSG00000176853|ENST00000334705
MNIDVEFHIRHNYPWNKLPANVRQSLGNSQREYEKQVVLYSIRNQLRYRNNLVKHVKKDE
RRYYEELLKYSRDHLMLYPYHLSDIMVKGLRITPFSYYTGIMEDIMNSEKSYDSLPNFTA
ADCLRLLGIGRNQYIDLMNQCRSSKKFFRRKTARDLLPIKPVEIAIEAWWVVQAGYITED
DIKICTLPEKCAVDKIIDSGPQLSGSLDYNVVHSLYNKGFIYLDVPISDDSCIAVPPLEG
FVMNRVQGDYFETLLYKIFVSIDEHTNVAELANVLEIDLSLVKNAVSMYCRLGFAHKKGQ
VINLDQLHSSWKNVPSVNRLKSTLDPQKMLLSWDGGESRSPVQEASSATDTDTNSQEDPA
DTASVSSLSLSTGHTKRIAFLFDSTLTAFLMMGNLSPNLKSHAVTMFEVGKLSDESLDSF
LIELEKVQSTGEGEAQRYFDHALTLRNTILFLRHNKDLVAQTAQPDQPNYGFPLDLLRCE
SLLGLDPATCSRVLNKNYTLLVSMAPLTNEIRPVSSCTPQHIGPAIPEVSSVWFKLYIYH
VTGQGPPSLLLSKGTRLRKLPDIFQSYDRLLITSWGHDPGVVPTSNVLTMLNDALTHSAV
LIQGHGLHGIGETVHVPFPFDETELQGEFTRVNMGVHKALQILRNRVDLQHLCGYVTMLN
ASSQLADRKLSDASDERGEPDLASGSDVNGSTESFEMVIEEATIDSATKQTSGATTEADW
VPLELCFGIPLFSSELNRKVCRKIAAHGLCRKESLQNLLHSSRKLSLQVLNFVHSFQEGA
SILDIHTEPSFSSLLSQSSCADMGVPLPAKNLIFKDGVLSEWSGRSPSSLLIANLHLQ*
>ENSG00000057593|ENST00000346342
MVSQALRLLCLLLGLQGCLAAVFVTQEEAHGVLHRRRRANAFLEELRPGSLERECKEEQC
SFEEAREIFKDAERTKLFWISYSDGDQCASSPCQNGGSCKDQLQSYICFCLPAFEGRNCE
THKDDQLICVNENGGCEQYCSDHTGTKRSCRCHEGYSLLADGVSCTPTVEYPCGKIPILE
KRNASKPQGRIVGGKVCPKGECPWQVLLLVNGAQLCGGTLINTIWVVSAAHCFDKIKNWR
NLIAVLGEHDLSEHDGDEQSRRVAQVIIPSTYVPGTTNHDIALLRLHQPVVLTDHVVPLC

LPERTFSERTLAFVRFSLVSGWGQLLDRGATALELMVLNVPRLMTQDCLQQSRKVGDSPN
ITEYMFCAGYSDGSKDSCKGDSGGPHATHYRGTWYLTGIVSWGQGCATVGHFGVYTRVSQ
YIEWLQKLMRSEPRPGVLLRAPFP*
>ENSG00000167645|ENST00000337679
MHPAGLAAAAAGTPRLPSKRRIPVSQPGMADPHQLFDDTSSAQSRGYGAQRAPGGLSYPA
ASPTPHAAFLADPVSNMAMAYGSSLAAQGKELVDKNIDRFIPITKLKYYFAVDTMYVGRK
LGLLFFPYLHQDWEVQYQQDTPVAPRFDVNAPDLYIPAMAFITYVLVAGLALGTQDRFSP
DLLGLQASSALAWLTLEVLAILLSLYLVTVNTDLTTIDLVAFLGYKYVGMIGGVLMGLLF
GKIGYYLVLGWCCVAIFVFMFPLLPGAVAHACNPSTLGGRGGRITRSGRCG*
>ENSG00000176986|ENST00000345254
MNVNQSVPPVPPFGQPQPIYPGYHQSSYGGQSGSTAPAIPYGAYNGPVPGYQQTPPQGMS
RAPPSSGAPPASTAQAPCGQAAYGQFGQGDVQNGPSSTVQMQRLPGSQPFGSPLAPVGNQ
PPVLQPYGPPPTSAQVATQLSGMQISGAVAPAPPSSGLGFGPPTSLASASGSFPNSGLYG
SYPQGQAPPLSQAQGHPGIQTPQRSAPSQASSFTPPASGGPRLPSMTGPLLPGQSFGGPS
VSQPNHVSSPPQALPPGTQMTGPLGPLPPMHSPQQPGYQPQQNGSFGPARGPQSNYGGPY
PAAPTFGSQPGPPQPLPPKRLDPDAIPSPIQVIEDDRNNRGTEPFVTGVRGQVPPLVTTN
FLVKDQGNASPRYIRCTSYNIPCTSDMAKQAQVPLAAVIKPLARLPPEEASPYVVDHGES
GPLRCNRCKAYMCPFMQFIEGGRRFQCCFCSCINDVPPQYFQHLDHTGKRVDAYDRPELS
LGSYEFLATVDYCKNNKFPSPPAFIFMIDVSYNAIRTGLVRLLCEELKSLLDFLPREGGA
EESAIRVGFVTYNKVLHFYNVKSSLAQPQMMVVSDVADMFVPLLDGFLVNVNESRAVITS
LLDQIPEMFADTRETETVFVPVIQAGMEALKAAECAGKLFLFHTSLPIAEAPGKLKNRDD
RKLINTDKEKTLFQPQTGAYQTLAKECVAQGCCVDLFLFPNQYVDVATLSVVPQLTGGSV
YKYASFQVENDQERFLSDLRRDVQKVVGFDAVMRVRTSTGIRAVDFFGAFYMSNTTDVEL
AGLDGDKTVTVEFKHDDRLNEESGALLQCALLYTSCAGQRRLRIHNLALNCCTQLADLYR
NCETDTLINYMAKFAYRGVLNSPVKAVRDTLITQCAQILACYRKNCASPSSAGQLILPEC
MKLLPVYLNCVLKSDVLQPGAEVTTDDRAYVRQLVTSMDVTETNVFFYPRLLPLTKSPVE
STTEPPAVRASEERLSNGDIYLLENGLNLFLWVGASVQQGVVQSLFSVSSFSQITSGLSV
LPVLDNPLSKKVRGLIDSLRAQRSRYMKLTVVKQEDKMEMLFKHFLVEDKSLSGGASYVD
FLCHMHKEIRQLLS*
>ENSG00000169255|ENST00000320474
MASALWTVLPSRMSLRSLKWSLLLLSLLSFFVMWYLSLPHYNVIERVNWMYFYEYEPIYR
QDFHFTLREHSNCSHQNPFLVILVTSHPSDVKARQAIRVTWGEKKSWWGYEVLTFFLLGQ
EAEKEDKMLALSLEDEHLLYGDIIRQDFLDTYNNLTLKTIMAFRWVTEFCPNAKYVMKTD
TDVFINTGNLVKYLLNLNHSEKFFTGYPLIDNYSYRGFYQKTHISYQEYPFKVFPPYCSG
LGYIMSRDLVPRIYEMMGHVKPIKFEDVYVGICLNLLKVNIHIPEDTNLFFLYRIHLDVC
QLRRVIAAHGFSSKEIITFWQVMLRNTTCHY*
>ENSG00000183862|ENST00000329903
MTEKTNGVKSSPANNHNHHAPPAIKANGKDDHRTSSRPHSAADDDTSSELQRLADVDAPQ
QGRSGFRRIVRLVGIIREWANKNFREEEPRPDSFLERFRGPELQTVTTQEGDGKGDKDGE
DKGTKKKFELFVLDPAGDWYYCWLFVIAMPVLYNWCLLVARACFSDLQKGYYLVWLVLDY
VSDVVYIADLFIRLRTGFLEQGLLVKDTKKLRDNYIHTLQFKLDVASIIPTDLIYFAVDI
HSPEVRFNRLLHFARMFEFFDRTETRTNYPNIFRISNLVLYILVIIHWNACIYYAISKSI
GFGVDTWVYPNITDPEYGYLAREYIYCLYWSTLTLTTIGETPPPVKDEEYLFVIFDFLIG
VLIFATIVGNVGSMISNMNATRAEFQAKIDAVKHYMQFRKVSKGMEAKVIRWFDYLWTNK

KTVDEREILKNLPAKLRAEIAINVHLSTLKKVRIFHDCEAGLLVELVLKLRPQVFSPGDY
ICRKGDIGKEMYIIKEGKLAVVADDGVTQYALLSAGSCFGEISILNIKGSKMGNRRTANI
RSLGYSDLFCLSKDDLMEAVTEYPDAKKVLEERGREILMKEGLLDENEVATSMEVDVQEK
LGQLETNMETLYTRFGRLLAEYTGAQQKLKQRITVLETKMKQNNEDDYLSDGMNSPELAA
ADEP*
>ENSG00000172780|ENST00000315150
MAGPGPGPGDPDEQYDFLFKLVLVGDASVGKTCVVQRFKTGAFSERQGSTIGVDFTMKTL
EIQGKRVKLQIWDTAGQERFRTITQSYYRSANGAILAYDITKRSSFLSVPHWIEDVRKYA
GSNIVQLLIGNKSDLSELREVSLAEAQSLAEHYDILCAIETSAKDSSNVEEAFLRVATEL
IMRHGGPLFSEKSPDHIQLNSKDIGEGWGCGC*
>ENSG00000164125|ENST00000296530
MTCPDKPGQLINWFICSLCVPRVRKLWSSRRPRTRRNLLLGTACAIYLGFLVSQVGRASL
QHGQAAEKGPHRSRDTAEPSFPEIPLDGTLAPPESQGNGSTLQPNVVYITLRSKRSKPAN
IRGTVKPKRRKKHAVASAAPGQEALVGPSLQPQEAAREADAVAPGYAQGANLVKIGERPW
RLVRGPGVRAGGPDFLQPSSRESNIRIYSESAPSWLSKDDIRRMRLLADSAVAGLRPVSS
RSGARLLVLEGGAPGAVLRCGPSPCGLLKQPLDMSEVFAFHLDRILGLNRTLPSVSRKAE
FIQDGRPCPIILWDASLSSASNDTHSSVKLTWGTYQQLLKQKCWQNGRVPKPESGCTEIH
HHEWSKMALFDFLLQIYNRLDTNCCGFRPRKEDACVQNGLRPKCDDQGSAALAHIIQRKH
DPRHLVFIDNKGFFDRSEDNLNFKLLEGIKEFPASAVSVLKSQHLRQKLLQSLFLDKVYW
ESQGGRQGIEKLIDVIEHRAKILITYINAHGVKVLPMNE*
>ENSG00000180549|ENST00000314412
MNNAGHGPTRRLRGLGVLAGVALLAALWLLWLLGSAPRGTPAPQPTITILVWHWPFTDQP
PELPSDTCTRYGIARCHLSANRSLLASADAVVFHHRELQTRRSHLPLAQRPRGQPWVWAS
MESPSHTHGLSHLRGIFNWVLSYRRDSDIFVPYGRLEPHWGPSPPLPAKSRVAAWVVSNF
QERQLRARLYRQLAPHLRVDVFGRANGRPLCASCLVPTVAQYRFYLSFENSQHRDYITEK
FWRNALVAGTVPVVLGPPRATYEAFVPADAFVHVDDFGSARELAAFLTGMNESRYQRFFA
WRDRLRVRLFTDWRERFCAICDRYPHLPRSQVYEDLEGWFQA*
>ENSG00000172575|ENST00000310803
MGTLGKAREAPRKPSHGCRAASKARLEAKPANSPFPSHPSLAHITQFRMMVSLGHLAKGA
SLDDLIDSCIQSFDADGNLCRSNQLLQVMLTMHRIVISSAELLQKVITLYKDALAKNSPG
LCLKICYFVRYWITEFWVMFKMDASLTDTMEEFQELVKAKGEELHCRLIDTTQINARDWS
RKLTQRIKSNTSKKRKVSLLFDHLEPEELSEHLTYLEFKSFRRISFSDYQNYLVNSCVKE
NPTMERSIALCNGISQWVQLMVLSRPTPQLRAEVFIKFIQVAQKLHQLQNFNTLMAVIGG
LCHSSISRLKETSSHVPHEINKVLGEMTELLSSSRNYDNYRRAYGECTDFKIPILGVHLK
DLISLYEAMPDYLEDGKVNVHKLLALYNHISELVQLQEVAPPLEANKDLVHLLTLSLDLY
YTEDEIYELSYAREPRNHRAPPLTPSKPPVVVDWASGVSPKPDPKTISKHVQRMVDSVFK
NYDHDQDGYISQEEFEKIAASFPFSFCVMDKDREGLISRDEITAYFMRASSIYSKLGLGF
PHNFQETTYLKPTFCDNCAGFLWGVIKQGYRCKDCGMNCHKQCKDLVVFECKKRAKNPVA
PTENNTSVGPVSNLCSLGAKDLLHAPEEGPFTFPNGEAVEHGEESKDRTIMLMGVSSQKI
SLRLKRAVAHKATQTESQPWIGSEGPSGPFVLSSPRKTAQDTLYVLPSPTSPCPSPVLVR
KRAFVKWENKDSLIKSKEELRHLRLPTYQELEQEINTLKADNDALKIQLKYAQKKIESLQ
LEKSNHVLAQMEQGDCS*
>ENSG00000115540|ENST00000323303
MVMAEGTAVLRRNRPGTKAQDFYNWPDESFDEMDSTLAVQQYIQQNIRADCSNIDKILEP

PEGQDEGVWKYEHLRQFCLELNGLAVKLQSECHPDTCTQMTATEQWIFLCAAHKTPKECP
AIDYTRHTLDGAACLLNSNKYFPSRVSIKESSVAKLGSVCRRIYRIFSHAYFHHRQIFDE
YENETFLCHRFTKFVMKYNLMSKDNLIVPILEEEVQNSVSGESEA*
>ENSG00000157350|ENST00000342907
MKCSLRVWFLSVAFLLVFIMSLLFTYSHHSMATLPYLDSGALDGTHRVKLVPGYAGLQRL
SKERLSGKSCACRRCMGDAGASDWFDSHFDGNISPVWTRENMDLPPDVQRWWMMLQPQFK
SHNTNEVLEKLFQIVPGENPYRFRDPHQCRRCAVVGNSGNLRGSGYGQDVDGHNFIMRMN
QAPTVGFEQDVGSRTTHHFMYPESAKNLPANVSFVLVPFKVLDLLWIASALSTGQIRFTY
APVKSFLRVDKEKVQIYNPAFFKYIHDRWTEHHGRYPSTGMLVLFFALHVCDEVNVYGFG
ADSRGNWHHYWENNRYAGEFRKTGVHDADFEAHIIDMLAKASKIEVYRGN*
>ENSG00000128242|ENST00000338911
MLPPQKKPWESMAKGLVLGALFTSFLLLVYSYAVPPLHAGLASTTPEAAASCSPPALEPE
AVIRANGSAGECQPRRNIVFLKTHKTASSTLLNILFRFGQKHRLKFAFPNGRNDFDYPTF
FARSLVQDYRPGACFNIICNHMRFHYDEVRGLVPTNAIFITVLRDPARLFESSFHYFGPV
VPLTWKLSAGDKLTEFLQDPDRYYDPNGFNAHYLRNLLFFDLGYDNSLDPSSPQVQEHIL
EVERRFHLVLLQEYFDESLVLLKDLLCWELEDVLYFKLNARRDSPVPRLSGELYGRATAW
NMLDSHLYRHFNASFWRKVEAFGRERMAREVAALRHANERMRTICIDGGHAVDAAAIQDE
AMQPWQPLGTKSILGYNLKKSIGQRHAQLCRRMLTPEIQYLMDLGANLWVTKLWKFIRDF
LRW*
>ENSG00000070729|ENST00000311183
MLGWVQRVLPQPPGTPRKTKMQEEEEVEPEPEMEAEVEPEPNPEEAETESESMPPEESFK
EEEVAVADPSPQETKEAALTSTISLRAQGAEISEMNSPSRRVLTWLMKGVEKVIPQPVHS
ITEDPAQILGHGSTGDTGCTDEPNEALEAQDTRPGLRLLLWLEQNLERVLPQPPKSSEVW
RDEPAVATGAASDPAPPGRPQEMGPKLQARETPSLPTPIPLQPKEEPKEAPAPEPQPGSQ
AQTSSLPPTRDPARLVAWVLHRLEMALPQPVLHGKIGEQEPDSPGICDVQTRVMGAGGL*
>ENSG00000175073|ENST00000310421
MSQPPPPPPPLPPPPPPPEAPQTPSSLASAAASGGLLKRRDRRILSGSCPDPKCQARLFF
PASGSVSIECTECGQRHEQQQLLGVEEVTDPDVVLHNLLRNALLGVTGAPKKNTELVKVM
GLSNYHCKLLSPILARYGMDKQTGRAKLLRDMNQGELFDCALLGDRAFLIEPEHVNTVGY
GKDRSGSLLYLHDTLEDIKRANKSQECLIPVHVDGDGHCLVHAVSRALVGRELFWHALRE
NLKQHFQQHLARYQALFHDFIDAAEWEDIINECDPLFVPPEGVPLGLRNIHIFGLANVLH
RPIILLDSLSGMRSSGDYSATFLPGLIPAEKCTGKDGHLNKPICIAWSSSGRNHYIPLVG
IKGAALPKLPMNLLPKAWGVPQDLIKKYIKLEEDGGCVIGGDRSLQDKYLLRLVAAMEEV
FMDKHGIHPSLVADVHQYFYRRTGVIGVQPEEVTAAAKKAVMDNRLHKCLLCGALSELHV
PPEWLAPGGKLYNLAKSTHGQLRTDKNYSFPLNNLVCSYDSVKDVLVPDYGMSNLTACNW
CHGTSVRKVRGDGSIVYLDGDRTNSRSTGGKCGCGFKHFWDGKEYDNLPEAFPITLEWGG
RVVRETVYWFQYESDSSLNSNVYDVAMKLVTKHFPGEFGSEILVQKVVHTILHQTAKKNP
DDYTPVNIDGAHAQRVGDVQGQESESQLPTKIILTGQKTKTLHKEELNMSKTERTIQQNI
TEQASVMQKRKTEKLKQEQKGQPRTVSPSTIRDGPSSAPATPTKAPYSPTTSKEKKIRIT
TNDGRQSMVTLKSSTTFFELQESIAREFNIPPYLQCIRYGFPPKELMPPQAGMEKEPVPL
QHGDRITIEILKSKAEGGQSAAAHSAHTVKQEDIAVTGKLSSKELQEQAEKEMYSLCLLA
TLMGEDVWSYAKGLPHMFQQGGVFYSIMKKTMGMADGKHCTFPHLPGKTFVYNASEDRLE
LCVDAAGHFPIGPDVEDLVKEAVSQVRAEATTRSRESSPSHGLLKLGSGGVVKKKSEQLH
NVTAFQGKGHSLGTASGNPHLDPRARETSVVRKHNTGTDFSNSSTKTEPSVFTASSSNSE

LIRIAPGVVTMRDGRQLDPDLVEAQRKKLQEMVSSIQASMDRHLRDQSTEQSPSDLPQRK
TEVVSSSAKSGSLQTGLPESFPLTGGTENLNTETTDGCVADALGAAFATRSKAQRGNSVE
ELEEMDSQDAEMTNTTEPMDHS*
>ENSG00000101236|ENST00000336095
MSSDFPHYNFRMPNIGFQNLPLNIYIVVFGTAIFVFILSLLFCCYLIRLRHQAHKEFYAY
KQVILKEKVKELNLHELCAVCLEDFKPRDELGICPCKHAFHRKCLIKWLEVRKVCPLCNM
PVLQLAQLHSKQDRGPPQGPLPGAENIV*
>ENSG00000175229|ENST00000312006
MPPILQRLQQATKMMSRRKILLLVLGCSTVSLLIHQGAQLSWYPKLFPLSCPPLRNSPPR
PKHMTVAFLKTHKTAGTTVQNILFRFAERHNLTVALPHPSCEHQFCYPRNFSAHFVHPAT
RPPHVLASHLRFDRAELERLMPPSTVYVTILREPAAMFESLFSYYNQYCPAFRRVPNASL
EAFLRAPEAYYRAGEHFAMFAHNTLAYDLGGDNERSPRDDAAYLAGLIRQVEEVFSLVMI
AEYFDESLVLLRRLLAWDLDDVLYAKLNARAASSRLAAIPAALARAARTWNALDAGLYDH
FNATFWRHVARAGRACVEREARELREARQRLLRRCFGDEPLLRPAAQIRTKQLQPWQPSR
KVDIMGYDLPGGGAGPATEACLKLAMPEVQYSNYLLRKQKRRGGARARPEPVLDNPPPRP
IRVLPRGPQGP*
>ENSG00000163235|ENST00000295400
MVPSAGQLALFALGIVLAACQALENSTSPLSADPPVAAAVVSHFNDCPDSHTQFCFHGTC
RFLVQEDKPACVCHSGYVGARCEHADLLAVVAASQKKQAITALVVVSIVALAVLIITCVL
IHCCQVRKHCEWCRALICRHEKPSALLKGRTACCHSETVV*
>ENSG00000148985|ENST00000300730
MARLGSTGGVSGRVVTQHRDSAPFLGAPRLGLRELGPIRGTAPEWHGRWNAAERSDKMYQ
VPLPLDRDGTLVRLRFTMVALVTVCCPLVAFLFCILWSLLFHFKETTATHCGVPNYLPSV
SSAIGGEVPQRYVWRFCIGLHSAPRFLVAFAYWNHYLSCTSPCSCYRPLCRLNFGLNVVE
NLALLVLTYVSSSEDFTIHENAFIVFIASSLGHMLLTCILWRLTKKHTDRKSYSWKQRLF
IINFISFFSALAVYFRHNMYCEAGVYTIFAILEYTVVLTNMAFHMTAWWDFGNKELLITS
QPEEKRF*
>ENSG00000038427|ENST00000343200
MFINIKSILWMCSTLIVTHALHKVKVGKSPPVRGSLSGKVSLPCHFSTMPTLPPSYNTSE
FLRIKWSKIEVDKNGKDLKETTVLVAQNGNIKIGQDYKGRVSVPTHPEAVGDASLTVVKL
LASDAGLYRCDVMYGIEDTQDTVSLTVDGVVFHYRAATSRYTLNFEAAQKACLDVGAVIA
TPEQLFAAYEDGFEQCDAGWLADQTVRYPIRAPRVGCYGDKMGKAGVRTYGFRSPQETYD
VYCYVDHLDGDVFHLTVPSKFTFEEAAKECENQDARLATVGELQAAWRNGFDQCDYGWLS
DASVRHPVTVARAQCGGGLLGVRTLYRFENQTGFPPPDSRFDAYCFKRRMSDLSVIGHPI
DSESKEDEPCSEETDPVHDLMAEILPEFPDIIEIDLYHSEENEEEEEECANATDVTTTPS
VQYINGKHLVTTVPKDPEAAEARRGQFESVAPSQNFSDSSESDTHPFVIAKTELSTAVQP
NESTETTESLEVTWKPETYPETSEHFSGGEPDVFPTVPFHEEFESGTAKKGAESVTERDT
EVGHQAHEHTEPVSLFPEESSGEIAIDQESQKIAFARATEVTFGEEVEKSTSVTYTPTIV
PSSASAYVSEEEAVTLIGNPWPDDLLSTKESWVEATPRQVVELSGSSSIPITEGSGEAEE
DEDTMFTMVTDLSQRNTTDTLITLDTSRIITESFFEVPATTIYPVSEQPSAKVVPTKFVS
ETDTSEWISSTTVEEKKRKEEEGTTGTASTFEVYSSTQRSDQLILPFELESPNVATSSDS
GTRKSFMSLTTPTQSEREMTDSTPVFTETNTLENLGAQTTEHSSIHQPGVQEGLTTLPRS
PASVFMEQGSGEAAADPETTTVSSFSLNVEYAIQAEKEVAGTLSPHVETTFSTEPTGLVL
STVMDRVVAENITQTSREIVISERLGEPNYGAEIRGFSTGFPLEEDFSGDFREYSTVSHP

IAKEETVMMEGSGDAAFRDTQTSPSTVPTSVHISHISDSEGPSSTMVSTSAFPWEEFTSS
AEGSGEQLVTVSSSVVPVLPSAVQKFSGTASSIIDEGLGEVGTVNEIDRRSTILPTAEVE
GTKAPVEKEEVKVSGTVSTNFPQTIEPAKLWSRQEVNPVRQEIESETTSEEQIQEEKSFE
SPQNSPATEQTIFDSQTFTETELKTTDYSVLTTKKTYSDDKEMKEEDTSLVNMSTPDPDA
NGLESYTTLPEATEKSHFFLATALVTESIPAEHVVTDSPIKKEESTKHFPKGMRPTIQES
DTELLFSGLGSGEEVLPTLPTESVNFTEVEQINNTLYPHTSQVESTSSDKIEDFNRMENV
AKEVGPLVSQTDIFEGSGSVTSTTLIEILSDTGAEGPTVAPLPFSTDIGHPQNQTVRWAE
EIQTSRPQTITEQDSNKNSSTAEINETTTSSTDFLARAYGFEMAKEFVTSAPKPSDLYYE
PSGEGSGEVDIVDSFHTSATTQATRQESSTTFVSDGSLEKHPEVPSAKAVTADGFPTVSV
MLPLHSEQNKSSPDPTSTLSNTVSYERSTDGSFQDRFREFEDSTLKPNRKKPTENIIIDL
DKEDKDLILTITESTILEILPELTSDKNTIIDIDHTKPVYEDILGMQTDIDTEVPSEPHD
SNDESNDDSTQVQEIYEAAVNLSLTEETFEGSADVLASYTQATHDESMTYEDRSQLDHMG
FHFTTGIPAPSTETELDVLLPTATSLPIPRKSATVIPEIEGIKAEAKALDDMFESSTLSD
GQAIADQSEIIPTLGQFERTQEEYEDKKHAGPSFQPEFSSGAEEALVDHTPYLSIATTHL
MDQSVTEVPDVMEGSNPPYYTDTTLAVSTFAKLSSQTPSSPLTIYSGSEASGHTEIPQPS
ALPGIDVGSSVMSPQDSFKEIHVNIEATFKPSSEEYLHITEPPSLSPDTKLEPSEDDGKP
ELLEEMEASPTELIAVEGTEILQDFQNKTDGQVSGEAIKMFPTIKTPEAGTVITTADEIE
LEGATQWPHSTSASATYGVEAGVVPWLSPQTSERPTLSSSPEINPETQAALIRGQDSTIA
ASEQQVAARILDSNDQATVNPVEFNTEVATPPFSLLETSNETDFLIGINEESVEGTAIYL
PGPDRCKMNPCLNGGTCYPTETSYVCTCVPGYSGDQCELDFDECHSNPCRNGATCVDGFN
TFRCLCLPSYVGALCEQDTETCDYGWHKFQGQCYKYFAHRRTWDAAERECRLQGAHLTSI
LSHEEQMFVNRVGHDYQWIGLNDKMFEHDFRWTDGSTLQYENWRPNQPDSFFSAGEDCVV
IIWHENGQWNDVPCNYHLTYTCKKGTVACGQPPVVENAKTFGKMKPRYEINSLIRYHCKD
GFIQRHLPTIRCLGNGRWAIPKITCMNPSAYQRTYSMKYFKNSSSAKDNSINTSKHDHRW
SRRWQESRR*
>ENSG00000160360|ENST00000291775
MDDQRCPLDDGQAGAAEATAAPTLEDRIAQPSMTASPQTEEFFDLIASSQSRRLDDQRAS
VGSLPGLRITHSNAGHLRGHGEPQEPGDDFFNMLIKYQSSRIDDQRCPPPDVLPRGPTMP
DEDFFSLIQRVQAKRMDEQRVDLAGGPEQGAGGPPEPQQQCQPGAS*
>ENSG00000164867|ENST00000297494
MGNLKSVAQEPGPPCGLGLGLGLGLCGKQGPATPAPEPSRAPASLLPPAPEHSPPSSPLT
QPPEGPKFPRVKNWEVGSITYDTLSAQAQQDGPCTPRRCLGSLVFPRKLQGRPSPGPPAP
EQLLSQARDFINQYYSSIKRSGSQAHEQRLQEVEAEVAATGTYQLRESELVFGAKQAWRN
APRCVGRIQWGKLQVFDARDCRSAQEMFTYICNHIKYATNRGNLRSAITVFPQRCPGRGD
FRIWNSQLVRYAGYRQQDGSVRGDPANVEITELCIQHGWTPGNGRFDVLPLLLQAPDDPP
ELFLLPPELVLEVPLEHPTLEWFAALGLRWYALPAVSNMLLEIGGLEFPAAPFSGWYMST
EIGTRNLCDPHRYNILEDVAVCMDLDTRTTSSLWKDKAAVEINVAVLHSYQLAKVTIVDH
HAATASFMKHLENEQKARGGCPADWAWIVPPISGSLTPVFHQEMVNYFLSPAFRYQPDPW
KGSAAKGTGITRKKTFKEVANAVKISASLMGTVMAKRVKATILYGSETGRAQSYAQQLGR
LFRKAFDPRVLCMDEYDVVSLEHETLVLVVTSTFGNGDPPENGESFAAALMEMSGPYNSS
PRPEQHKSYKIRFNSISCSDPLVSSWRRKRKESSNTDSAGALGTLRFCVFGLGSRAYPHF
CAFARAVDTRLEELGGERLLQLGQGDELCGQEEAFRGWAQAAFQAACETFCVGEDAKAAA
RDIFSPKRSWKRQRYRLSAQAEGLQLLPGLIHVHRRKMFQATIRSVENLQSSKSTRATIL
VRLDTGGQEGLQYQPGDHIGVCPPNRPGLVEALLSRVEDPPAPTEPVAVEQLEKGSPGGP

PPGWVRDPRLPPCTLRQALTFFLDITSPPSPQLLRLLSTLAEEPREQQELEALSQDPRRY
EEWKWFRCPTLLEVLEQFPSVALPAPLLLTQLPLLQPRYYSVSSAPSTHPGEIHLTVAVL
AYRTQDGLGPLHYGVCSTWLSQLKPGDPVPCFIRGAPSFRLPPDPSLPCILVGPGTGIAP
FRGFWQERLHDIESKGLQPTPMTLVFGCRCSQLDHLYRDEVQNAQQRGVFGRVLTAFSRE
PDNPKTYVQDILRTELAAEVHRVLCLERGHMFVCGDVTMATNVLQTVQRILATEGDMELD
EAGDVIGVLRDQQRYHEDIFGLTLRTQEVTSRIRTQSFSLQERQLRGAVPWAFDPPGSDT
NSP*
>ENSG00000213203|ENST00000307194
MGGRKMATDEENVYGLEENAQSRQESTRRLILVGRTGAGKSATGNSILGQRRFFSRLGAT
SVTRACTTGSRRWDKCHVEVVDTPDIFSSQVSKTDPGCEERGHCYLLSAPGPHALLLVTQ
LGRFTAQDQQAVRQVRDMFGEDVLKWMVIVFTRKEDLAGGSLHDYVSNTENRALRELVAE
CGGRVCAFDNRATGREQEAQVEQLLGMVEGLVLEHKGAHYSNEVYELAQVLRWAGPEERL
RRVAERVAARVQRRPWGAWLSARLWKWLKSPRSWRLGLALLLGGALLFWVLLHRRWSEAV
AEVGPD*
>ENSG00000184840|ENST00000332598
MAVELGVLLVRPRPGTGLGRVMRTLLLVLWLATRGSALYFHIGETEKKCFIEEIPDETMV
IGNYRTQLYDKQREEYQPATPGLGMFVEVKDPEDKVILARQYGSEGRFTFTSHTPGEHQI
CLHSNSTKFSLFAGGMLRVHLDIQVGEHANDYAEIAAKDKLSELQLRVRQLVEQVEQIQK
EQNYQRWREERFRQTSESTNQRVLWWSILQTLILVAIGVWQMRHLKSFFEAKKLV*
>ENSG00000175582|ENST00000310653
MSTGGDFGNPLRKFKLVFLGEQSVGKTSLITRFMYDSFDNTYQATIGIDFLSKTMYLEDR
TIRLQLWDTAGQERFRSLIPSYIRDSAAAVVVYDITNVNSFQQTTKWIDDVRTERGSDVI
IMLVGNKTDLADKRQVSIEEGERKAKELNVMFIETSAKAGYNVKQLFRRVAAALPGMEST
QDRSREDMIDIKLEKPQEQPVSEGGCSC*
>ENSG00000175115|ENST00000320580
MAERGGAGGGPGGAGGGSGQRGSGVAQSPQQPPPQQQQQQPPQQPTPPKLAQATSSSSST
SAAAASSSSSSTSTSMAVAVASGSAPPGGPGPGRTPAPVQMNLYATWEVDRSSSSCVPRL
FSLTLKKLVMLKEMDKDLNSVVIAVKLQGSKRILRSNEIVLPASGLVETELQLTFSLQYP
HFLKRDANKLQIMLQRRKRYKNRTILGYKTLAVGLINMAEVMQHPNEGALVLGLHSNVKD
VSVPVAEIKIYSLSSQPIDHEGIKSKLSDRSPDIDNYSEEEEESFSSEQEGSDDPLHGQD
LFYEDEDLRKVKKTRRKLTSTSAITRQPNIKQKFVALLKRFKVSDEVGFGLEHVSREQIR
EVEEDLDELYDSLEMYNPSDSGPEMEETESILSTPKPKLKPFFEGMSQSSSQTEIGSLNS
KGSLGKDTTSPMELAALEKIKSTWIKNQDDSLTETDTLEITDQDMFGDASTSLVVPEKVK
TPMKSSKTDLQGSASPSKVEGVHTPRQKRSTPLKERQLSKPLSERTNSSDSERSPDLGHS
TQIPRKVVYDQLNQILVSDAALPENVILVNTTDWQGQYVAELLQDQRKPVVCTCSTVEVQ
AVLSALLTRIQRYCNCNSSMPRPVKVAAVGGQSYLSSILRFFVKSLANKTSDWLGYMRFL
IIPLGSHPVAKYLGSVDSKYSSSFLDSGWRDLFSRSEPPVSEQLDVAGRVMQYVNGAATT
HQLPVAEAMLTCRHKFPDEDSYQKFIPFIGVVKVGLVEDSPSTAGDGDDSPVVSLTVPST
SPPSSSGLSRDATATPPSSPSMSSALAIVGSPNSPYGDVIGLQVDYWLGHPGERRREGDK
RDASSKNTLKSVFRSVQVSRLPHSGEAQLSGTMAMTVVTKEKNKKVPTIFLSKKPREKEV
DSKSQVIEGISRLICSAKQQQTMLRVSIDGVEWSDIKFFQLAAQWPTHVKHFPVGLFSGS
KAT*
>ENSG00000167080|ENST00000300404
MGSAGFSVGKFHVEVASRGRECVSGTPECGNRLGSAGFGALCLELRGADPAWGPFAAHGR

SRRQGSRFLWLLKILVIILVLGIVGFMFGSMFLQAVFSSPKPELPSPAPGVQKLKLLPEE
RLRNLFSYDGIWLFPKNQCKCEANKEQGGYNFQDAYGQSDLPAVKARRQAEFEHFQRREG
LPRPLPLLVQPNLPFGYPVHGVEVMPLHTVPIPGLQFEGPDAPVYEVTLTASLGTLNTLA
DVPDSVVQGRGQKQLIISTSDRKLLKFILQHVTYTSTGYQHQKVDIVSLESRSSVAKFPV
TIRHPVIPKLYDPGPERKLRNLVTIATKTFLRPHKLMIMLRSIREYYPDLTVIVADDSQK
PLEIKDNHVEYYTMPFGKGWFAGRNLAISQVTTKYVLWVDDDFLFNEETKIEVLVDVLEK
TELDVVGGSVLGNVFQFKLLLEQSENGACLHKRMGFFQPLDGFPSCVVTSGVVNFFLAHT
ERLQRVGFDPRLQRVAHSEFFIDGLGTLLVGSCPEVIIGHQSRSPVVDSELAALEKTYNT
YRSNTLTRVQFKLALHYFKNHLQCAA*
>ENSG00000101294|ENST00000335574
MDSALSDPHNGSAEAGGPTNSTTRPPSTPEGIALAYGSLLLMALLPIFFGALRSVRCARG
KNASDMPETITSRDAARFPIIASCTLLGLYLFFKIFSQEYINLLLSMYFFVLGILALSHT
ISPFMNKFFPASFPNRQYQLLFTQGSGENKEEIINYEFDTKDLVCLGLSSIVGVWYLLRK
HWIANNLFGLAFSLNGVELLHLNNVSTGCILLGGLFIYDVFWVFGTNVMVTVAKSFEAPI
KLVFPQDLLEKGLEANNFAMLGLGDVVIPGIFIALLLRFDISLKKNTHTYFYTSFAAYIF
GLGLTIFIMHIFKHAQPALLYLVPACIGFPVLVALAKGEVTEMFSYESSAEILPHTPRLT
HFPTVSGSPASLADSMQQKLAGPRRRRPQNPSAM*
>ENSG00000164100|ENST00000296499
MSFIMKLHRHFQRTVILLATFCMVSIIISAYYLYSGYKQENELSETASEVDCGDLQHLPY
QLMEVKAMKLFDASRTDPTVLVFVESQYSSLGQDIIMILESSRFQYHIEIAPGKGDLPVL
IDKMKGKYILIIYENILKYINMDSWNRSLLDKYCVEYGVGVIGFHKTSEKSVQSFQLKGF
PFSIYGNLAVKDCCINPHSPLIRVTKSSKLEKGSLPGTDWTVFQINHSAYQPVIFAKVKT
PENLSPSISKGAFYATIIHDLGLHDGIQRVLFGNNLNFWLHKLIFIDAISFLSGKRLTLS
LDRYILVDIDDIFVGKEGTRMNTNDVKALLDTQNLLRAQITNFTFNLGFSGKFYHTGTEE
EDEGDDCLLGSVDEFWWFPHMWSHMQPHLFHNESSLVEQMILNKKFALEHGIPTDMGYAV
APHHSGVYPVHVQLYEAWKKVWNIKITSTEEYPHLKPARYRRGFIHKNIMVLPRQTCGLF
THTIFYKEYPGGPKELDKSIQGGELFFTVVLNPISIFMTHLSNYGNDRLGLYTFVNLANF
VKSWTNLRLQTLPPVQLAHKYFELFPDQKDPLWQNPCDDKRHRDIWSKEKTCDRLPKFLV
IGPQKTGTTALYLFLVMHPSILSNSPSPKTFEEVQFFNRNNYHRGIDWYMDFFPVPSNVT
TDFLFEKSANYFHSEEAPKRAASLVPKAKIITILIDPSDRAYSWYQHQRSHEDPAALKFS
FYEVISAGPRAPSELRALQKRCLVPGWYASHIERWLVYFPPFQLLIIDGQQLRTDPATVM
DEVQKFLGVLPHYNYSEALTFDSHKGFWCQLLEEGKTKCLGKSKGRKYPPMDSDSRTFLS
SYYRDHNVELSKLLHKLGQPLPSWLRQELQKVR*
>ENSG00000141198|ENST00000348161
MCVQNCGPSFQSLIVKKEFVKENLVKLLNPRYNLPLDIQNRILNFIKTWSQGFPGGVDVS
EVKEVYLDLVKKGVQFPPSEAEAETARQETAQISSNPPTSVPTAPALSSVIAPKNSTVTL
VPEQIGKLHSELDMVKMNVRVMSAILMENTPGSENHEDIELLQKLYKTGREMQERIMDLL
VVVENEDVTVELIQVNEDLNNAILGYERFTRNQQRILEQNKNQKEATNTTSEPSAPSQDL
LDLSPSPRMPRATLGELNTMNNQLSGLNFSLPSSDVTNNLKPSLHPQMNLLALENTEIPP
FAQRTSQNLTSSHAYDNFLEHSNSVFLQPVSLQTIAAAPSNQSLPPLPSNHPAMTKSDLQ
PPNYYEVMEFDPLAPAVTTEAIYEEIDAHQHKGAQNDGD*
>ENSG00000185499|ENST00000337604
MTPGTQSPFFLLLLLTVLTVVTGSGHASSTPGGEKETSATQRSSVPSSTEKNALSTGVSF
FFLSFHISNLQFNSSLEDPSTDYYQELQRDISEMFLQIYKQGGFLGLSNIKFRPGSVVVQ

LTLAFREGTINVHDVETQFNQYKTEAASRYNLTISDVSVSDVPFPFSAQSGAGVPGWGIA
LLVLVCVLVALAIVYLIALAVCQCRRKNYGQLDIFPARDTYHPMSEYPTYHTHGRYVPPS
STDRSPYEKVSAGNGGSSLSYTNPAVAATSANL*
>ENSG00000167434|ENST00000300900
MRMLLALLALSAARPSASAESHWCYEVQAESSNYPCLVPVKWGGNCQKDRQSPINIVTTK
AKVDKKLGRFFFSGYDKKQTWTVQNNGHSVMMLLENKASISGGGLPAPYQAKQLHLHWSD
LPYKGSEHSLDGEHFAMEMHIVHEKEKGTSRNVKEAQDPEDEIAVLAFLVEAGTQVNEGF
QPLVEALSNIPKPEMSTTMAESSLLDLLPKEEKLRHYFRYLGSLTTPTCDEKVVWTVFRE
PIQLHREQILAFSQKLYYDKEQTVSMKDNVRPLQQLGQRTVIKSGAPGRPLPWALPALLG
PMLACLLAGFLR*
>ENSG00000158623|ENST00000330992
MIKKFDKKDEESGSGSNPFQHLEKSAVLQEARIFNETPINPRRCLHILTKILYLLNQGEH
FGTTEATEAFFAMTRLFQSNDQTLRRMCYLTIKEMATISEDVIIVTSSLTKDMTGKEDVY
RGPAIRALCRITDGTMLQAIERYMKQAIVDKVSSVSSSALVSSLHMMKISYDVVKRWINE
AQEAASSDNIMVQYHALGVLYHLRKNDRLAVSKMLNKFTKSGLKSQFAYCMLIRIASRLL
KETEDGHESPLFDFIESCLRNKHEMVIYEAASAIIHLPNCTARELAPAVSVLQLFCSSPK
PALRYAAVRTLNKVAMKHPSAVTACNLDLENLITDSNRSIATLAITTLLKTGSESSVDRL
MKQISSFVSEISDEFKVVVVQAISALCQKYPRKHSVMMTFLSNMLRDDGGFEYKRAIVDC
IISIVEENPESKEAGLAHLCEFIEDCEHTVLATKILHLLGKEGPRTPVPSKYIRFIFNRV
VLENEAVRAAAVSALAKFGAQNESLLPSILVLLQRCMMDTDDEVRDRATFYLNVLQQRQM
ALNATYIFNGLTVSVPGMEKALHQYTLEPSEKPFDMKSIPLAMAPVFEQKAEITLVATKP
EKLAPSRQDIFQEQLAAIPEFLNIGPLFKSSEPVQLTEAETEYFVRCIKHMFTNHIVFQF
DCTNTLNDQLLEKVTVQMEPSDSYEVLSCIPAPSLPYNQPGICYTLVRLPDDDPTAGTNP
QKGGDT*
>ENSG00000054793|ENST00000338821
MTDNIPLQPVRQKKRMDSRPRAGCCEWLRCCGGGEARPRTVWLGHPEKRDQRYPRNVINN
QKYNFFTFLPGVLFNQFKYFFNLYFLLLACSQFVPEMRLGALYTYWVPLGFVLAVTVIRE
AVEEIRCYVRDKEVNSQVYSRLTARGTVKVKSSNIQVGDLIIVEKNQRVPADMIFLRTSE
KNGSCFLRTDQLDGETDWKLRLPVACTQRLPTAADLLQIRSYVYAEEPNIDIHNFVGTFT
REDSDPPISESLSIENTLWAGTVVASGTVVGVVLYTGRELRSVMNTSNPRSKIGLFDLEV
NCLTKILFGALVVVSLVMVALQHFAGRWYLQIIRFLLLFSNIIPISLRVNLDMGKIVYSW
VIRRDSKIPGTVVRSSTIPEQLGRISYLLTDKTGTLTQNEMIFKRLHLGTVAYGLDSMDE
VQSHIFSIYTQQSQDPPAQKGPTLTTKVRRTMSSRVHEAVKAIALCHNVTPVYESNGVTD
QAEAEKQYEDSCRVYQASSPDEVALVQWTESVGLTLVGRDQSSMQLRTPGDQILNFTILQ
IFPFTYESKRMGIIVRDESTGEITFYMKGADVVMAGIVQYNDWLEEECGNMAREGLRVLV
VAKKSLAEEQYQDFEARYVQAKLSVHDRSLKVATVIESLEMEMELLCLTGVEDQLQADVR
PTLETLRNAGIKVWMLTGDKLETATCTAKNAHLVTRNQDIHVFRLVTNRGEAHLELNAFR
RKHDCALVISGDSLEVCLKYYEYEFMELACQCPAVVCCRCAPTQKAQIVRLLQERTGKLT
CAVGDGGNDVSMIQESDCGVGVEGKEGKQASLAADFSITQFKHLGRLLMVHGRNSYKRSA
ALSQFVIHRSLCISTMQAVFSSVFYFASVPLYQGFLIIGYSTIYTMFPVFSLVLDKDVKS
EVAMLYPELYKDLLKGRPLSYKTFLIWVLISIYQGSTIMYGALLLFESEFVHIVAISFTS
LILTELLMVALTIQTWHWLMTVAELLSLACYIASLVFLHEFIDVYFIATLSFLWKVSVIT
LVSCLPLYVLKYLRRRFSPPSYSKLTS*
>ENSG00000160216|ENST00000327505

MGLLAFLKTQFVLHLLVGFVFVVSGLVINFVQLCTLALWPVSKQLYRRLNCRLAYSLWSQ
LVMLLEWWSCTECTLFTDQATVERFGKEHAVIILNHNFEIDFLCGWTMCERFGVLGSSKV
LAKKELLYVPLIGWTWYFLEIVFCKRKWEEDRDTVVEGLRRLSDYPEYMWFLLYCEGTRF
TETKHRVSMEVAAAKGLPVLKYHLLPRTKGFTTAVKCLRGTVAAVYDVTLNFRGNKNPSL
LGILYGKKYEADMCVRRFPLEDIPLDEKEAAQWLHKLYQEKDALQEIYNQKGMFPGEQFK
PARRPWTLLNFLSWATILLSPLFSFVLGVFASGSPLLILTFLGFVGAASFGVRRLIGVTE
IEKGSSYGNQEFKKKE*
>ENSG00000167889|ENST00000301618
MITVNPDGKIMVRRCLVTLRPFRLFVLGIGFFTLCFLMTSLGGQFSARRLGDSPFTIRTE
VMGGPESRGVLRKMSDLLELMVKRMDALARLENSSELHRAGGDLHFPADRMPPGAGLMER
IQAIAQNVSDIAVKVDQILRHSLLLHSKVSEGRRDQCEAPSDPKFPDCSGKVEWMRARWT
SDPCYAFFGVDGTECSFLIYLSEVEWFCPPLPWRNQTAAQRAPKPLPKVQAVFRSNLSHL
LDLMGSGKESLIFMKKRTKRLTAQWALAAQRLAQKLGATQRDQKQILVHIGFLTEESGDV
FSPRVLKGGPLGEMVQWADILTALYVLGHGLRVTVSLKELQSNLGVPPGRGSCPLTMPLP
FDLIYTDYHGLQQMKRHMGLSFKKYRCRIRVIDTFGTEPAYNHEEYATLHGYRTNWGYWN
LNPKQFMTMFPHTPDNSFMGFVSEELNETEKRLIKGGKASNMAVVYGKEASIWKGKEKFL
GILNKYMEIHGTVYYESQRPPEVPAFVKNHGLLPQPEFQQLLRKAKLFIGFGFPYEGPAP
LEAIANGCIFLQSRFSPPHSSLNHEFFRGKPTSREVFSQHPYAENFIGKPHVWTVDYNNS
EEFEAAIKAIMRTQVDPYLPYEYTCEGMLERIHAYIQHQDFCRAPDPALPEAHAPQSPFV
LAPNATHLEWARNTSLAPGAWPPAHALRAWLAVPGRACTDTCLDHGLICEPSFFPFLNSQ
DAFLKLQVPCDSTESEMNHLYPAFAQPGQECYLQKEPLLFSCAGSNTKYRRLCPCRDFRK
GQVALCQGCL*
>ENSG00000169733|ENST00000310496
MSRARGALCRACLALAAALAALLLLPLPLPRAPAPARTPAPAPRAPPSRPAAPSLRPDDV
FIAVKTTRKNHGPRLRLLLRTWISRARQQTFIFTDGDDPELELQGGDRVINTNCSAVRTR
QALCCKMSVEYDKFIESGRKWFCHVDDDNYVNARSLLHLLSSFSPSQDVYLGRPSLDHPI
EATERVQGGRTVTTVKFWFATGGAGFCLSRGLALKMSPWASLGSFMSTAEQVRLPDDCTV
GYIVEGLLGARLLHSPLFHSHLENLQRLPPDTLLQQVTLSHGGPENPHNVVNVAGGFSLH
QDPTRFKSIHCLLYPDTDWCPRQKQGAPTSR*
>ENSG00000104915|ENST00000343587
MSLEDPFFVVRGEVQKAVNTARGLYQRWCELLQESAAVGREELDWTTNELRNGLRSIEWD
LEDLEETIGIVEANPGKPAAQKSPSDLLDASAVSATSRYIEEQQATQQLIMDEQDQQLEM
VSGSIQVLKHMSGRVGEELDEQGIMLDAFAQEMDHTQSRMDGVLRKLAKVSHMTSDRRQW
CAIAVLVGVLLLVLILLFSL*
>ENSG00000163558|ENST00000295797
MPTQRDSSTMSHTVAGGGSGDHSHQVRVKAYYRGDIMITHFEPSISFEGLCNEVRDMCSF
DNEQLFTMKWIDEEGDPCTVSSQLELEEAFRLYELNKDSELLIHVFPCVPERPGMPCPGE
DKSIYRRGARRWRKLYCANGHTFQAKRFNRRAHCAICTDRIWGLGRQGYKCINCKLLVHK
KCHKLVTIECGRHSLPQEPVMPMDQSSMHSDHAQTVIPYNPSSHESLDQVGEEKEAMNTR
ESGKASSSLGLQDFDLLRVIGRGSYAKVLLVRLKKTDRIYAMKVVKKELVNDDEDIDWVQ
TEKHVFEQASNHPFLVGLHSCFQTESRLFFVIEYVNGGDLMFHMQRQRKLPEEHARFYSA
EISLALNYLHERGIIYRDLKLDNVLLDSEGHIKLTDYGMCKEGLRPGDTTSTFCGTPNYI
APEILRGEDYGFSVDWWALGVLMFEMMAGRSPFDIVGSSDNPDQNTEDYLFQVILEKQIR
IPRSLSVKAASVLKSFLNKDPKERLGCHPQTGFADIQGHPFFRNVDWDMMEQKQVVPPFK

PNISGEFGLDNFDSQFTNEPVQLTPDDDDIVRKIDQSEFEGFEYINPLLMSAEECV*
>ENSG00000168067|ENST00000294066
MALLRDVSLQDPRDRFELLQRVGAGTYGDVYKARDTVTSELAAVKIVKLDPGDDISSLQQ
EITILRECRHPNVVAYIGSYLRNDRLWICMEFCGGGSLQEIYHATGPLEERQIAYVCREA
LKGLHHLHSQGKIHRDIKGANLLLTLQGDVKLADFGVSGELTASVAKRRSFIGTPYWMAP
EVAAVERKGGYNELCDVWALGITAIELGELQPPLFHLHPMRALMLMSKSSFQPPKLRDKT
RWTQNFHHFLKLALTKNPKKRPTAEKLLQHPFTTQQLPRALLTQLLDKASDPHLGTPSPE
DCELETYDMFPDTIHSRGQHGPAERTPSEIQFHQVKFGAPRRKETDPLNEPWEEEWTLLG
KEELSGSLLQSVQEALEERSLTIRSASEFQELDSPDDTMGTIKRAPFLGPLPTDPPAEEP
LSSPPGTLPPPPSGPNSSPLLPTAWATMKQREDPERSSCHGLPPTPKVHMGACFSKVFNG
CPLRIHAAVTWIHPVTRDQFLVVGAEEGIYTLNLHELHEDTLEKLISHRCSWLYCVNNVL
LSLSGKSTHIWAHDLPGLFEQRRLQQQVPLSIPTNRLTQRIIPRRFALSTKIPDTKGCLQ
CRVVRNPYTGATFLLAALPTSLLLLQWYEPLQKFLLLKNFSSPLPSPAGMLEPLVLDGKE
LPQVCVGAEGPEGPGCRVLFHVLPLEAGLTPDILIPPEGIPGSAQQVIQVDRDTILVSFE
RCVRIVNMQGEPTATLAPELTFDFPIETVVCLQDSVLAFWSHGMQGRSLDTNEVTQEITD
ETRIFRVLGAHRDIILESIPTDNPEAHSNLYILTGHQSTY*
>ENSG00000145730|ENST00000304400
MAGRVPSLLVLLVFPSSCLAFRSPLSVFKRFKETTRPFSNECLGTTRPVVPIDSSDFALD
IRMPGVTPKQSDTYFCMSMRIPVDEEAFVIDFKPRASMDTVHHMLLFGCNMPSSTGSYWF
CDEGTCTDKANILYAWARNAPPTRLPKGVGFRVGGETGSKYFVLQVHYGDISAFRDNNKD
CSGVSLHLTRLPQPLIAGMYLMMSVDTVIPAGEKVVNSDISCHYKNYPMHVFAYRVHTHH
LGKVVSGYRVRNGQWTLIGRQSPQLPQAFYPVGHPVDVSFGDLLAARCVFTGEGRTEATH
IGGTSSDEMCNLYIMYYMEAKHAVSFMTCTQNVAPDMFRTIPPEANIPIPVKSDMVMMHE
HHKETEYKDKIPLLQQPKREEEEVLDQGDFYSLLSKLLGEREDVVHVHKYNPTEKAESES
DLVAEIANVVQKKDLGRSDAREGAEHERGNAILVRDRIHKFHRLVSTLRPPESRVFSLQQ
PPPGEGTWEPEHTGDFHMEEALDWPGVYLLPGQVSGVALDPKNNLVIFHRGDHVWDGNSF
DSKFVYQQIGLGPIEEDTILVIDPNNAAVLQSSGKNLFYLPHGLSIDKDGNYWVTDVALH
QVFKLDPNNKEGPVLILGRSMQPGSDQNHFCQPTDVAVDPGTGAIYVSDGYCNSRIVQFS
PSGKFITQWGEESSGSSPLPGQFTVPHSLALVPLLGQLCVADRENGRIQCFKTDTKEFVR
EIKHSSFGRNVFAISYIPGLLFAVNGKPHFGDQEPVQGFVMNFSNGEIIDIFKPVRKHFD
MPHDIVASEDGTVYIGDAHTNTVWKFTLTEKLEHRSVKKAGIEVQEIKEAEAVVETKMEN
KPTSSELQKMQEKQKLIKEPGSGVPVVLITTLLVIPVVVLLAIAIFIRWKKSRAFGADSE
HKLETSSGRVLGRFRGKGSGGLNLGNFFASRKGYSRKGFDRLSTEGSDQEKEDDGSESEE
EYSAPLPALAPSSS*
>ENSG00000176928|ENST00000322348
MKIFKCYFKHTLQQKVFILFLTLWLLSLLKLLNVRRLFPQKDIYLVEYSLSTSPFVRNRY
THVKDEVRYEVNCSGIYEQEPLEIGKSLEIRRRDIIDLEDDDVVAMTSDCDIYQTLRGYA
QKLVSKEEKSFPIAYSLVVHKDAIMVERLIHAIYNQHNIYCIHYDRKAPDTFKVAMNNLA
KCFSNIFIASKLEAVEYAHISRLQADLNCLSDLLKSSIQWKYVINLCGQDFPLKSNFELV
SELKKLNGANMLETVKPPNSKLERFTYHHELRRVPYEYVKLPIRTNISKEAPPHNIQIFV
GSAYFVLSQAFVKYIFNNSIVQDFFAWSKDTYSPDEHFWATLIRVPGIPGEISRSAQDVS
DLQSKTRLVKWNYYEGFFYPSCTGSHLRSVCIYGAAELRWLIKDGHWFANKFDSKVDPIL
IKCLAEKLEEQQRDWITLPSEKLFMDRNLTTTS*
>ENSG00000206418|ENST00000329286

MDPGAALQRRAGGGGGLGAGSPALSGGQGRRRKQPPRPADFKLQVIIIGSRGVGKTSLME
RFTDDTFCEACKSTVGVDFKIKTVELRGKKIRLQIWDTAGQERFNSITSAYYRSAKGIIL
VYDITKKETFDDLPKWMKMIDKYASEDAELLLVGNKLDCETDREITRQQGEKFAQQITGM
RFCEASAKDNFNVDEIFLKLVDDILKKMPLDILRNELSNSILSLQPEPEIPPELPPPRPH
VRCC*
>ENSG00000178690|ENST00000321600
MVADIKGNEQIEKYSWREACDTGSSRMDRKHGKYILNVEHSENQPPITHPNDQEAHSSIC
WCLPSNDITSDVSPNLTGVCVNPGILAHSRCLQSESCNTQVKEYCRNDWSMWKVFLACLL
ACVIMTAIGVLIICLVNNKGSANSSIVIQLSTNDGECVTVKPGTPSPACPPTMTTTSTVP
ASTATESTTSTATAATTSTEPITVAPTDHL*
>ENSG00000276333|ENST00000293937
MAGSGGLGGGAGGGQGAGAGQGAALRASRAPMLLVALVLGAYCLCALPGRCPPAARAPAP
APAPSEPSSSVHRPGAPGLPLASGPGRRRFPQALIVGVKKGGTRALLEFLRLHPDVRALG
SEPHFFDRCYERGLAWYRSLMPRTLDGQITMEKTPSYFVTREAPRRIHAMSPDTKLIVVV
RNPVTRAISDYAQTLSKTPGLPSFRALAFRHGLGPVDTAWSAVRIGLYAQHLDHWLRYFP
LSHFLFVSGERLVSDPAGEVGRVQDFLGLKRVVTDKHFYFNATKGFPCLKKAQGGSRPRC
LGKSKGRPHPRVPQALVRRLQEFYRPFNRRFYQMTGQDFGWG*
>ENSG00000100228|ENST00000341989
MVIAGASWMLGRAAASPTQTPPTTSTIRVARRSRVALVAMVIAAAGSGGPGRAEPQLSQP
SLDCGRMRSSLTPLGPPVSRDRVIASFPKWYTPEACLQLREHFHGQVSAACQRRNTGTVG
FCKNVFDRDYKATIGVDFEIERFEIAGIPYSLQIWDTAGQEKFKCIASAYYRGAQVIITA
FDLTDVQTLEHTRQWLEDALRENEAGSCFIFLVGTKKDLLSGAACEQAEADAVHLAREMQ
AEYWSVSAKTGENVKAFFSRVAALAFEQSVLQDLERQSSARLQVGNGDLIQMEGSPPETQ
ESKRPSSLGCC*
>ENSG00000160282|ENST00000291670
MSQLVECVPNFSEGKNQEVIDAISGAITQTPGCVLLDVDAGPSTNRTVYTFVGPPECVVE
GALNAARVASRLIDMSRHQGEHPRMGALDVCPFIPVRGVSVDECVLCAQAFGQRLAEELD
VPVYLYGEAARMDSRRTLPAIRAGEYEALPKKLQQADWAPDFGPSSFVPSWGATATGARK
FLIAFNINLLGTKEQAHRIALNLREQGRGKDQPGRLKKVQGIGWYLDEKNLAQVSTNLLD
FEVTALHTVYEETCREAQELSLPVVGSQLVGLVPLKALLDAAAFYCEKENLFILEEEQRI
RLVVSRLGLDSLCPFSPKERIIEYLVPERGPERGLGSKSLRAFVGEVGARSAAPGGGSVA
AAAAAMGAALGSMVGLMTYGRRQFQSLDTTMRRLIPPFREASAKLTTLVDADAEAFTAYL
EAMRLPKNTPEEKDRRTAALQEGLRRAVSVPLTLAETVASLWPALQELARCGNLACRSDL
QVAAKALEMGVFGAYFNVLINLRDITDEAFKDQIHHRVSSLLQEAKTQAALVLDCLETRQ
E*
>ENSG00000169344|ENST00000302509
MGQPSLTWMLMVVVASWFITTAATDTSEARWCSECHSNATCTEDEAVTTCTCQEGFTGDG
LTCVDLDECAIPGAHNCSANSSCVNTPGSFSCVCPEGFRLSPGLGCTDVDECAEPGLSHC
HALATCVNVVGSYLCVCPAGYRGDGWHCECSPGSCGPGLDCVPEGDALVCADPCQAHRTL
DEYWRSTEYGEGYACDTDLRGWYRFVGQGGARMAETCVPVLRCNTAAPMWLNGTHPSSDE
GIVSRKACAHWSGHCCLWDASVQVKACAGGYYVYNLTAPPECHLAYCTDPSSVEGTCEEC
SIDEDCKSNNGRWHCQCKQDFNITDISLLEHRLECGANDMKVSLGKCQLKSLGFDKVFMY
LSDSRCSGFNDRDNRDWVSVVTPARDGPCGTVLTRNETHATYSNTLYLADEIIIRDLNIK
INFACSYPLDMKVSLKTALQPMVSALNIRVGGTGMFTVRMALFQTPSYTQPYQGSSVTLS

TEAFLYVGTMLDGGDLSRFALLMTNCYATPSSNATDPLKYFIIQDRCPHTRDSTIQVVEN
GESSQGRFSVQMFRFAGNYDLVYLHCEVYLCDTMNEKCKPTCSGTRFRSGSVIDQSRVLN
LGPITRKGVQATVSRAFSSLGLLKVWLPLLLSATLTLTFQ*
>ENSG00000171451|ENST00000310045
MPKGGAPPWIMALMFTGHLLFLALLMFAFSTFEESVSNYSEWAVFTDDIDQFKTQKVQDF
RPNQKLKKSMLHPSLYFDAGEIQAMRQKSRASHLHLFRAIRSAVTVMLSNPTYYLPPPKH
ADFAAKWNEIYGNNLPPLALYCLLCPEDKVAFEFVLEYMDRMVGYKDWLVENAPGDEVPI
GHSLTGFATAFDFLYNLLDNHRRQKYLEKIWVITEEMYEYSKVRSWGKQLLHNHQATNMI
ALLTGALVTGVDKGSKANIWKQAVVDVMEKTMFLLNHIVDGSLDEGVAYGSYTAKSVTQY
VFLAQRHFNINNLDNNWLKMHFWFYYATLLPGFQRTVGIADSNYNWFYGPESQLVFLDKF
ILKNGAGNWLAQQIRKHRPKDGPMVPSTAQRWSTLHTEYIWYDPQLTPQPPADYGTAKIH
TFPNWGVVTYGAGLPNTQTNTFVSFKSGKLGGRAVYDIVHFQPYSWIDGWRSFNPGHEHP
DQNSFTFAPNGQVFVSEALYGPKLSHLNNVLVFAPSPSSQCNKPWEGQLGECAQWLKWTG
EEVGDAAGEIITASQHGEMVFVSGEAVSAYSSAMRLKSVYRALLLLNSQTLLVVDHIERQ
EDSPINSVSAFFHNLDIDFKYIPYKFMNRYNGAMMDVWDAHYKMFWFDHHGNSPMASIQE
AEQAAEFKKRWTQFVNVTFQMEPTITRIAYVFYGPYINVSSCRFIDSSNPGLQISLNVNN
TEHVVSIVTDYHNLKTRFNYLGFGGFASVADQGQITRFGLGTQAIVKPVRHDRIIFPFGF
KFNIAVGLILCISLVILTFQWRFYLSFRKLMRWILILVIALWFIELLDVWSTCSQPICAK
WTRTEAEGSKKSLSSEGHHMDLPDVVITSLPGSGAEILKQLFFNSSDFLYIRVPTAYIDI
PETELEIDSFVDACEWKVSDIRSGHFRLLRGWLQSLVQDTKLHLQNIHLHEPNRGKLAQY
FAMNKDKKRKFKRRESLPEQRSQMKGAFDRDAEYIRALRRHLVYYPSARPVLSLSSGSWT
LKLHFFQEVLGASMRALYIVRDPRAWIYSMLYNSKPSLYSLKNVPEHLAKLFKIEGGKGK
CNLNSGYAFEYEPLRKELSKSKSNAVSLLSHLWLANTAAALRINTDLLPTSYQLVKFEDI
VHFPQKTTERIFAFLGIPLSPASLNQILFATSTNLFYLPYEGEISPTNTNVWKQNLPRDE
IKLIENICWTLMDRLGYPKFMD*
>ENSG00000100280|ENST00000317368
MTDSKYFTTTKKGEIFELKAELNSDKKEKKKEAVKKVIASMTVGKDVSALFPDVVNCMQT
DNLELKKLVYLYLMNYAKSQPDMAIMAVNTFVKDCEDPNPLIRALAVRTMGCIRVDKITE
YLCEPLRKCLKDEDPYVRKTAAVCVAKLHDINAQLVEDQGFLDTLKDLISDSNPMVVANA
VAALSEIAESHPSSNLLDLNPQSINKLLTALNECTEWGQIFILDCLANYMPKDDREAQSI
CERVTPRLSHANSAVVLSAVKVLMKFMEMLSKDLDYYGTLLKKLAPPLVTLLSAEPELQY
VALRNINLIVQKRPEILKHEMKVFFVKYNDPIYVKLEKLDIMIRLASQANIAQVLAELKE
YATEVDVDFVRKAVRAIGRCAIKVEQSAERCVSTLLDLIQTKVNYVVQEAIVVIKDIFRK
YPNKYESVIATLCENLDSLDEPEARAAMIWIVGEYAERIDNADELLESFLEGFHDESTQV
QLQLLTAIVKLFLKKPTETQELVQQVLSLATQDSDNPDLRDRGYIYWRLLSTDPVAAKEV
VLAEKPLISEETDLIEPTLLDELICYIGTLASVYHKPPSAFVEGGRGVVHKSLPPRTASS
ESAESPETAPTGAPPGEQPDVIPAQGDLLGDLLNLDLGPPVSGPPLATSSVQMGAVDLLG
GGLDSLIGGTNFVAPPTAAVPANLGAPIGSGLSDLFDLTSGVGTLSGSYVAPKAVGSISM
DLQLTNKALQVMTDFAIQFNRNSFGLAPAAPLQVHAPLSPNQTVEISLPLSTVGSVMKME
PLNNLQVAVKNNIDVFYFSTLYPLHILFVEDGKMDRQMFLATWKDIPNENEAQFQIRDCP
LNAEAASSKLQSSNIFTVAKRNVEGQDMLYQSLKLTNGIWVLAELRIQPGNPSCTLSLKC
RAPEVSQHVYQAYETILKN*
>ENSG00000174327|ENST00000308027
MARRTEPPDGGWGWVVVLSAFFQSALVFGVLRSFGVFFVEFVAAFEEQAARVSWIASIGI

AVQQFGSPVGSALSTKFGPRPVVMTGGILAALGMLLASFATSLTHLYLSIGLLSGSGWAL
TFAPTLACLSCYFSRRRSLATGLALTGVGLSSFTFAPFFQWLLSHYAWRGSLLLVSALSL
HLVACGALLRPPSLAEDPAVGGPRAQLTSLLHHGPFLRYTVALTLINTGYFIPYLHLVAH
LQDLDWDPLPAAFLLSVVAISDLVGRVVSGWLGDAVPGPVTRLLMLWTTLTGVSLALFPV
AQAPTALVALAVAYGFTSGALAPLAFSVLPELIGTRRIYCGLGLLQMIESIGGLLGPPLS
GYLRDVTGNYTASFVVAGAFLLSGSGILLTLPHFFCFSTTTSGPQDLVTEALDTKVPLPK
EGLEED*
>ENSG00000144730|ENST00000296318
MAPWLQLCSVFFTVNACLNGSQLAVAAGGSGRARGADTCGWRGVGPASRNSGLYNITFKY
DNCTTYLNPVGKHVIADAQNITISQYACHDQVAVTILWSPGALGIEFLKGFRVILEELKS
EGRQCQQLILKDPKQLNSSFKRTGMESQPFLNMKFETDYFVKVVPFPSIKNESNYHPFFF
RTRACDLLLQPDNLACKPFWKPRNLNISQHGSDMQVSFDHAPHNFGFRFFYLHYKLKHEG
PFKRKTCKQEQTTETTSCLLQNVSPGDYIIELVDDTNTTRKVMHYALKPVHSPWAGPIRA
VAITVPLVVISAFATLFTVMCRKKQQENIYSHLDEESSESSTYTAALPRERLRPRPKVFL
CYSSKDGQNHMNVVQCFAYFLQDFCGCEVALDLWEDFSLCREGQREWVIQKIHESQFIIV
VCSKGMKYFVDKKNYKHKGGGRGSGKGELFLVAVSAIAEKLRQAKQSSSAALSKFIAVYF
DYSCEGDVPGILDLSTKYRLMDNLPQLCSHLHSRDHGLQEPGQHTRQGSRRNYFRSKSGR
SLYVAICNMHQFIDEEPDWFEKQFVPFHPPPLRYREPVLEKFDSGLVLNDVMCKPGPESD
FCLKVEAAVLGATGPADSQHESQHGGLDQDGEARPALDGSAALQPLLHTVKAGSPSDMPR
DSGIYDSSVPSSELSLPLMEGLSTDQTETSSLTESVSSSSGLGEEEPPALPSKLLSSGSC
KADLGCRSYTDELHAVAPL*
>ENSG00000181789|ENST00000314797
MLKKFDKKDEESGGGSNPFQHLEKSAVLQEARVFNETPINPRKCAHILTKILYLINQGEH
LGTTEATEAFFAMTKLFQSNDPTLRRMCYLTIKEMSCIAEDVIIVTSSLTKDMTGKEDNY
RGPAVRALCQITDSTMLQAIERYMKQAIVDKVPSVSSSALVSSLHLLKCSFDVVKRWVNE
AQEAASSDNIMVQYHALGLLYHVRKNDRLAVNKMISKVTRHGLKSPFAYCMMIRVASKQL
EEEDGSRDSPLFDFIESCLRNKHEMVVYEAASAIVNLPGCSAKELAPAVSVLQLFCSSPK
AALRYAAVRTLNKVAMKHPSAVTACNLDLENLVTDSNRSIATLAITTLLKTGSESSIDRL
MKQISSFMSEISDEFKVVVVQAISALCQKYPRKHAVLMNFLFTMLREEGGFEYKRAIVDC
IISIIEENSESKETGLSHLCEFIEDCEFTVLATRILHLLGQEGPKTTNPSKYIRFIYNRV
VLEHEEVRAGAVSALAKFGAQNEEMLPSILVLLKRCVMDDDNEVRDRATFYLNVLEQKQK
ALNAGYILNGLTVSIPGLERALQQYTLEPSEKPFDLKSVPLATAPMAEQRTESTPITAVK
QPEKVAATRQEIFQEQLAAVPEFRGLGPLFKSSPEPVALTESETEYVIRCTKHTFTNHMV
FQFDCTNTLNDQTLENVTVQMEPTEAYEVLCYVPARSLPYNQPGTCYTLVALPKEDPTAV
ACTFSCMMKFTVKDCDPTTGETDDEGYEDEYVLEDLEVTVADHIQKVMKLNFEAAWDEVG
DEFEKEETFTLSTIKTLEEAVGNIVKFLGMHPCERSDKVPDNKNTHTLLLAGVFRGGHDI
LVRSRLLLLDTVTMQVTARSLEELPVDIILASVG*
>ENSG00000177096|ENST00000321753
MKLNERSVAHYALSDSPADHMGFLRTWGGPGTPPTPSGTGRRCWFVLKGNLLFSFESREG
RAPLSLVVLEGCTVELAEAPVPEEFAFAICFDAPGVRPHLLAAEGPAAQEAWVKVLSRAS
FGYMRLVVRELESQLQDARQSLALQRRSSWKSVASRCKPQAPNHRAAGLENGHCLSKDSS
PVGLVEEAGSRSAGWGLAEWELQGPASLLLGKGQSPVSPETSCFSTLHDWYGQEIVELRQ
CWQKRAQGSHSKCEEQDRP*
>ENSG00000111186|ENST00000310594

MPSLLLLFTAALLSSWAQLLTDANSWWSLALNPVQRPEMFIIGAQPVCSQLPGLSPGQRK
LCQLYQEHMAYIGEGAKTGIKECQHQFRQRRWNCSTADNASVFGRVMQIGSRETAFTHAV
SAAGVVNAISRACREGELSTCGCSRTARPKDLPRDWLWGGCGDNVEYGYRFAKEFVDARE
REKNFAKGSEEQGRVLMNLQNNEAGRRAVYKMADVACKCHGVSGSCSLKTCWLQLAEFRK
VGDRLKEKYDSAAAMRVTRKGRLELVNSRFTQPTPEDLVYVDPSPDYCLRNESTGSLGTQ
GRLCNKTSEGMDGCELMCCGRGYNQFKSVQVERCHCKFHWCCFVRCKKCTEIVDQYICK*
>ENSG00000158089|ENST00000349752
MRRLTRRLVLPVFGVLWITVLLFFWVTKRKLEVPTGPEVQTPKPSDADWDDLWDQFDERR
YLNAKKWRVGDDPYKLYAFNQRESERISSNRAIPDTRHLRCTLLVYCTDLPPTSIIITFH
NEARSTLLRTIRSVLNRTPTHLIREIILVDDFSNDPDDCKQLIKLPKVKCLRNNERQGLV
RSRIRGADIAQGTTLTFLDSHCEVNRDWLQPLLHRVKEDYTRVVCPVIDIINLDTFTYIE
SASELRGGFDWSLHFQWEQLSPEQKARRLDPTEPIRTPIIAGGLFVIDKAWFDYLGKYDM
DMDIWGGENFEISFRVWMCGGSLEIVPCSRVGHVFRKKHPYVFPDGNANTYIKNTKRTAE
VWMDEYKQYYYAARPFALERPFGNVESRLDLRKNLRCQSFKWYLENIYPELSIPKESSIQ
KGNIRQRQKCLESQRQNNQETPNLKLSPCAKVKGEDAKSQVWAFTYTQQILQEELCLSVI
TLFPGAPVVLVLCKNGDDRQQWTKTGSHIEHIASHLCLDTDMFGDGTENGKEIVVNPCES
SLMSQHWDMVSS*
>ENSG00000164816|ENST00000330590
MRTIAILAAILLVALQAQAESLQERADEATTQKQSGEDNQDLAISFAGNGLSALRTSGSQ
ARATCYCRTGRCATRESLSGVCEISGRLYRLCCR*
>ENSG00000188906|ENST00000298910
MASGSCQGCEEDEETLKKLIVRLNNVQEGKQIETLVQILEDLLVFTYSERASKLFQGKNI
HVPLLIVLDSYMRVASVQQVGWSLLCKLIEVCPGTMQSLMGPQDVGNDWEVLGVHQLILK
MLTVHNASVNLSVIGLKTLDLLLTSGKITLLILDEESDIFMLIFDAMHSFPANDEVQKLG
CKALHVLFERVSEEQLTEFVENKDYMILLSALTNFKDEEEIVLHVLHCLHSLAIPCNNVE
VLMSGNVRCYNIVVEAMKAFPMSERIQEVSCCLLHRLTLGNFFNILVLNEVHEFVVKAVQ
QYPENAALQISALSCLALLTETIFLNQDLEEKNENQENDDEGEEDKLFWLEACYKALTWH
RKNKHVQEAACWALNNLLMYQNSLHEKIGDEDGHFPAHREVMLSMLMHSSSKEVFQASAN
ALSTLLEQNVNFRKILLSKGIHLNVLELMQKHIHSPEVAESGCKMLNHLFEGSNTSLDIM
AAVVPKILTVMKRHETSLPVQLEALRAILHFIVPGMPEESREDTEFHHKLNMVKKQCFKN
DIHKLVLAALNRFIGNPGIQKCGLKVISSIVHFPDALEMLSLEGAMDSVLHTLQMYPDDQ
EIQCLGLSLIGYLITKKNVFIGTGHLLAKILVSSLYRFKDVAEIQTKGFQTILAILKLSA
SFSKLLVHHSFDLVIFHQMSSNIMEQKDQQFLNLCCKCFAKVAMDDYLKNVMLERACDQN
NSIMVECLLLLGADANQAKEGSSLICQVCEKESSPKLVELLLNSGSREQDVRKALTISIG
KGDSQIISLLLRRLALDVANNSICLGGFCIGKVEPSWLGPLFPDKTSNLRKQTNIASTLA
RMVIRYQMKSAVEEGTASGSDGNFSEDVLSKFDEWTFIPDSSMDSVFAQSDDLDSEGSEG
SFLVKKKSNSISVGEFYRDAVLQRCSPNLQRHSNSLGPIFDHEDLLKRKRKILSSDDSLR
SSKLQSHMRHSDSISSLASEREYITSLDLSANELRDIDALSQKCCISVHLEHLEKLELHQ
NALTSFPQQLCETLKSLTHLDLHSNKFTSFPSYLLKMSCIANLDVSRNDIGPSVVLDPTV
KCPTLKQFNLSYNQLSFVPENLTDVVEKLEQLILEGNKISGICSPLRLKELKILNLSKNH
ISSLSENFLEACPKVESFSARMNFLAAMPFLPPSMTILKLSQNKFSCIPEAILNLPHLRS
LDMSSNDIQYLPGPAHWKSLNLRELLFSHNQISILDLSEKAYLWSRVEKLHLSHNKLKEI
PPEIGCLENLTSLDVSYNLELRSFPNEMGKLSKIWDLPLDELHLNFDFKHIGCKAKDIIR
FLQQRLKKAVPYNRMKLMIVGNTGSGKTTLLQQLMKTKKSDLGMQSATVGIDVKDWPIQI

RDKRKRDLVLNVWDFAGREEFYSTHPHFMTQRALYLAVYDLSKGQAEVDAMKPWLFNIKA
RASSSPVILVGTHLDVSDEKQRKACMSKITKELLNKRGFPAIRDYHFVNATEESDALAKL
RKTIINESLNFKIRDQLVVGQLIPDCYVELEKIILSERKNVPIEFPVIDRKRLLQLVREN
QLQLDENELPHAVHFLNESGVLLHFQDPALQLSDLYFVEPKWLCKIMAQILTVKVEGCPK
HPKGIISRRDVEKFLSKKRKFPKNYMSQYFKLLEKFQIALPIGEEYLLVPSSLSDHRPVI
ELPHCENSEIIIRLYEMPYFPMGFWSRLINRLLEISPYMLSGRERALRPNRMYWRQGIYL
NWSPEAYCLVGSEVLDNHPESFLKITVPSCRKGCILLGQVVDHIDSLMEEWFPGLLEIDI
CGEGETLLKKWALYSFNDGEEHQKILLDDLMKKAEEGDLLVNPDQPRLTIPISQIAPDLI
LADLPRNIMLNNDELEFEQAPEFLLGDGSFGSVYRAAYEGEEVAVKIFNKHTSLRLLRQE
LVVLCHLHHPSLISLLAAGIRPRMLVMELASKGSLDRLLQQDKASLTRTLQHRIALHVAD
GLRYLHSAMIIYRDLKPHNVLLFTLYPNAAIIAKIADYGIAQYCCRMGIKTSEGTPGFRA
PEVARGNVIYNQQADVYSFGLLLYDILTTGGRIVEGLKFPNEFDELEIQGKLPDPVKEYG
CAPWPMVEKLIKQCLKENPQERPTSAQVFDILNSAELVCLTRRILLPKNVIVECMVATHH
NSRNASIWLGCGHTDRGQLSFLDLNTEGYTSEEVADSRILCLALVHLPVEKESWIVSGTQ
SGTLLVINTEDGKKRHTLEKMTDSVTCLYCNSFSKQSKQKNFLLVGTADGKLAIFEDKTV
KLKGAAPLKILNIGNVSTPLMCLSESTNSTERNVMWGGCGTKIFSFSNDFTIQKLIETRT
SQLFSYAAFSDSNIITVVVDTALYIAKQNSPVVEVWDKKTEKLCGLIDCVHFLREVMVKE
NKESKHKMSYSGRVKTLCLQKNTALWIGTGGGHILLLDLSTRRLIRVIYNFCNSVRVMMT
AQLGSLKNVMLVLGYNRKNTEGTQKQKEIQSCLTVWDINLPHEVQNLEKHIEVRKELAEK
MRRTSVE*
>ENSG00000187446|ENST00000334660
MGSRASTLLRDEELEEIKKETGFSHSQITRLYSRFTSLDKGENGTLSREDFQRIPELAIN
PLGDRIINAFFPEGEDQVNFRGFMRTLAHFRPIEDNEKSKDVNGPEPLNSRSNKLHFAFR
LYDLDKDEKISRDELLQVLRMMVGVNISDEQLGSIADRTIQEADQDGDSAISFTEFVKVL
EKVDVEQKMSIRFLH*
>ENSG00000188092|ENST00000314163
MSFLIDSSIMITSQILFFGFGWLFFMRQLFKDYEIRQYVVQVIFSVTFAFSCTMFELIIF
EILGVLNSSSRYFHWKMNLCVILLILVFMVPFYIGYFIVSNIRLLHKQRLLFSCLLWLTF
MYFFWKLGDPFPILSPKHGILSIEQLISRVGVIGVTLMALLSGFGAVNCPYTYMSYFLRN
VTDTDILALERRLLQTMDMIISKKKRMAMARRTMFQKGEVHNKPSGFWGMIKSVTTSASG
SENLTLIQQEVDALEELSRQLFLETADLYATKERIEYSKTFKGKYFNFLGYFFSIYCVWK
IFMATINIVFDRVGKTDPVTRGIEITVNYLGIQFDVKFWSQHISFILVGIIIVTSIRGLL
ITLTKFFYAISSSKSSNVIVLLLAQIMGMYFVSSVLLIRMSMPLEYRTIITEVLGELQFN
FYHRWFDVIFLVSALSSILFLYLAHKQAPEKQMAP*
>ENSG00000166405|ENST00000343202
MAYSTVQRVALASGLVLALSLLLPKAFLSRGKRQEPPPTPEGKLGRFPPMMHHHQAPSDG
QTPGARFQRSHLAEAFAKAKGSGGGAGGGGSGRGLMGQIIPIYGFGIFLYILYILFKLSK
GKTTAEDGKCYTAMPGNTHRKITSFELAQLQEKLKETEAAMEKLINRVGPNGERAQTVTS
DQEKRLLHQLREITRVMKEGKFIDRFSPEKEAEEAPYMEDWEGYPEETYPIYDLSDCIKR
RQETILVDYPDPKELSAEEIAERMGMIEEEESDHLGWESLPTDPRAQEDNSVTSCDPKPE
TCSCCFHEDEDPAVLAENAGFSADSYPEQEETTKEEWSQDFKDEGLGISTDKAYTGSMLR
KRNPQGLE*
>ENSG00000188467|ENST00000341459
MQTKGGQTWARRALLLGILWATAHLPLSGTSLPQRLPRATGNSTQCVISPSSEFPEGFFT

RQERRDGGIIIYFLIIVYMFMAISIVCDEYFLPSLEIISESLGLSQDVAGTTFMAAGSSA
PELVTAFLGVFITKGDIGISTILGSAIYNLLGICAACGLLSNTVSTLSCWPLFRDCAAYT
ISAAAVLGIIYDNQVYWYEGALLLLIYGLYVLVLCFDIKINQYIIKKCSPCCACLAKAME
RSEQQPLMGWEDEGQPFIRRQSRTDSGIFYEDSGYSQLSISLHGLSQVSEDPPSVFNMPE
ADLKRIFWVLSLPIITLLFLTTPDCRKKFWKNYFVITFFMSAIWISAFTYILVWMVTITG
ETLEIPDTVMGLTLLAAGTSIPDTIASVLVARKGKGDMAMSNIVGSNVFDMLCLGIPWFI
KTAFINGSAPAEVNSRGLTYITISLNISIIFLFLAVHFNGWKLDRKLGIVCLLSYLGLAT
LSVLYELGIIGNNKIRGCGG*
>ENSG00000184005|ENST00000328299
MACILKRKSVIAVSFIAAFLFLLVVRLVNEVNFPLLLNCFGQPGTKWIPFSYTYRRPLRT
HYGYINVKTQEPLQLDCDLCAIVSNSGQMVGQKVGNEIDRSSCIWRMNNAPTKGYEEDVG
RMTMIRVVSHTSVPLLLKNPDYFFKEANTTIYVIWGPFRNMRKDGNGIVYNMLKKTVGIY
PNAQIYVTTEKRMSYCDGVFKKETGKDRVQSGSYLSTGWFTFLLAMDACYGIHVYGMIND
TYCKTEGYRKVPYHYYEQGRDECDEYFLHEHAPYGGHRFITEKKVFAKWAKKHRIIFTHP
NWTLS*
>ENSG00000196735|ENST00000343139
MILNKALLLGALALTTVMSPCGGEDIVADHVASCGVNLYQFYGPSGQYTHEFDGDEQFYV
DLERKETAWRWPEFSKFGGFDPQGALRNMAVAKHNLNIMIKRYNSTAATNEVPEVTVFSK
SPVTLGQPNTLICLVDNIFPPVVNITWLSNGQSVTEGVSETSFLSKSDHSFFKISYLTFL
PSADEIYDCKVEHWGLDQPLLKHWEPEIPAPMSELTETVVCALGLSVGLMGIVVGTVFII
QGLRSVGASRHQGPL*
>ENSG00000170340|ENST00000301998
MSVGRRRIKLLGILMMANVFIYFIMEVSKSSSQEKNGKGEVIIPKEKFWKISTPPEAYWN
REQEKLNRQYNPILSMLTNQTGEAGRLSNISHLNYCEPDLRVTSVVTGFNNLPDRFKDFL
LYLRCRNYSLLIDQPDKCAKKPFLLLAIKSLTPHFARRQAIRESWGQESNAGNQTVVRVF
LLGQTPPEDNHPDLSDMLKFESEKHQDILMWNYRDTFFNLSLKEVLFLRWVSTSCPDTEF
VFKGDDDVFVNTHHILNYLNSLSKTKAKDLFIGDVIHNAGPHRDKKLKYYIPEVVYSGLY
PPYAGGGGFLYSGHLALRLYHITDQVHLYPIDDVYTGMCLQKLGLVPEKHKGFRTFDIEE
KNKNNICSYVDLMLVHSRKPQEMIDIWSQLQSAHLKC*
>ENSG00000117411|ENST00000309519
MAVEVQEQWPCLPAAGCPGPLGGPVAACGMSRLLGGTLERVCKAVLLLCLLHFLVAVILY
FDVYAQHLAFFSRFSARGPAHALHPAASSSSSSSNCSRPNATASSSGLPEVPSALPGPTA
PTLPPCPDSPPGLVGRLLIEFTSPMPLERVQRENPGVLMGGRYTPPDCTPAQTVAVIIPF
RHREHHLRYWLHYLHPILRRQRLRYGVYVINQHGEDTFNRAKLLNVGFLEALKEDAAYDC
FIFSDVDLVPMDDRNLYRCGDQPRHFAIAMDKFGFRLPYAGYFGGVSGLSKAQFLRINGF
PNEYWGWGGEDDDIFNRISLTGMKISRPDIRIGRYRMIKHDRDKHNEPNPQRFTKIQNTK
LTMKRDGIGSVRYQVLEVSRQPLFTNITVDIGRPPSWPPRG*
>ENSG00000149294|ENST00000316851
MLQTKDLIWTLFFLGTAVSLQVDIVPSQGEISVGESKFFLCQVAGDAKDKDISWFSPNGE
KLTPNQQRISVVWNDDSSSTLTIYNANIDDAGIYKCVVTGEDGSESEATVNVKIFQKLMF
KNAPTPQEFREGEDAVIVCDVVSSLPPTIIWKHKGRDVILKKDVRFIVLSNNYLQIRGIK
KTDEGTYRCEGRILARGEINFKDIQVIVNVPPTIQARQNIVNATANLGQSVTLVCDAEGF
PEPTMSWTKDGEQIEQEEDDEKYIFSDDSSQLTIKKVDKNDEAEYICIAENKAGEQDATI
HLKVFAKPKITYVENQTAMELEEQVTLTCEASGDPIPSITWRTSTRNISSEEKASWTRPE

KQETLDGHMVVRSHARVSSLTLKSIQYTDAGEYICTASNTIGQDSQSMYLEVQYAPKLQG
PVAVYTWEGNQVNITCEVFAYPSATISWFRDGQLLPSSNYSNIKIYNTPSASYLEVTPDS
ENDFGNYNCTAVNRIGQESLEFILVQADTPSSPSIDQVEPYSSTAQVQFDEPEATGGVPI
LKYKAEWRAVGEEVWHSKWYDAKEASMEGIVTIVGLKPETTYAVRLAALNGKGLGEISAA
SEFKTQPVQGEPSAPKLEGQMGEDGNSIKVNLIKQDDGGSPIRHYLVRYRALSSEWKPEI
RLPSGSDHVMLKSLDWNAEYEVYVVAENQQGKSKAAHFVFRTSAQPTAIPANGSPTSGLS
TGAIVGILIVIFVLLLVVVDITCYFLNKCGLFMCIAVNLCGKAGPGAKGKDMEEGKAAFS
KDESKEPIVEVRTEEERTPNHDGGKHTEPNETTPLTEPEKGPVEAKPECQETETKPAPAE
VKTVPNDATQTKENESKA*
>ENSG00000111817|ENST00000331677
MRTHTRGAPSVFFIYLLCFVSAYITDENPEVMIPFTNANYDSHPMLYFSRAEVAELQLRA
ASSHEHIAARLTEAVHTMLSSPLEYLPPWDPKDYSARWNEIFGNNLGALAMFCVLYPENI
EARDMAKDYMERMAAQPSWLVKDAPWDEVPLAHSLVGFATAYDFLYNYLSKTQQEKFLEV
IANASGYMYETSYRRGWGFQYLHNHQPTNCMALLTGSLVLMNQGYLQEAYLWTKQVLTIM
EKSLVLLREVTDGSLYEGVAYGSYTTRSLFQYMFLVQRHFNINHFGHPWLKQHFAFMYRT
ILPGFQRTVAIADSNYNWFYGPESQLVFLDKFVMRNGSGNWLADQIRRNRVVEGPGTPSK
GQRWCTLHTEFLWYDGSLKSVPPPDFGTPTLHYFEDWGVVTYGSALPAEINRSFLSFKSG
KLGGRAIYDIVHRNKYKDWIKGWRNFNAGHEHPDQNSFTFAPNGVPFITEALYGPKYTFF
NNVLMFSPAVSKSCFSPWVGQVTEDCSSKWSKYKHDLAASCQGRVVAAEEKNGVVFIRGE
GVGAYNPQLNLKNVQRNLILLHPQLLLLVDQIHLGEESPLETAASFFHNVDVPFEETVVD
GVHGAFIRQRDGLYKMYWMDDTGYSEKATFASVTYPRGYPYNGTNYVNVTMHLRSPITRA
AYLFIGPSIDVQSFTVHGDSQQLDVFIATSKHAYATYLWTGEATGQSAFAQVIADRHKIL
FDRNSAIKSSIVPEVKDYAAIVEQNLQHFKPVFQLLEKQILSRVRNTASFRKTAERLLRF
SDKRQTEEAIDRIFAISQQQQQQSKSKKNRRAGKRYKFVDAVPDIFAQIEVNEKKIRQKA
QILAQKELPIDEDEEMKDLLDFADVTYEKHKNGGLIKGRFGQARMVTTTHSRAPSLSASY
TRLFLILNIAIFFVMLAMQLTYFQRAQSLHGQRCLYAVLLIDSCILLWLYSSCSQSQC*
>ENSG00000144730|ENST00000320057
MESQPFLNMKFETDYFVKVVPFPSIKNESNYHPFFFRTRACDLLLQPDNLACKPFWKPRN
LNISQHGSDMQVSFDHAPHNFGFRFFYLHYKLKHEGPFKRKTCKQEQTTETTSCLLQNVS
PGDYIIELVDDTNTTRKVMHYALKPVHSPWAGPIRAVAITVPLVVISAFATLFTVMCRKK
QQENIYSHLDEESSESSTYTAALPRERLRPRPKVFLCYSSKDGQNHMNVVQCFAYFLQDF
CGCEVALDLWEDFSLCREGQREWVIQKIHESQFIIVVCSKGMKYFVDKKNYKHKGGGRGS
GKGELFLVAVSAIAEKLRQAKQSSSAALSKFIAVYFDYSCEGDVPGILDLSTKYRLMDNL
PQLCSHLHSRDHGLQEPGQHTRQGSRRNYFRSKSGRSLYVAICNMHQFIDEEPDWFEKQF
VPFHPPPLRYREPVLEKFDSGLVLNDVMCKPGPESDFCLKVEAAVLGATGPADSQHESQH
GGLDQDGEARPALDGSAALQPLLHTVKAGSPSDMPRDSGIYDSSVPSSELSLPLMEGLST
DQTETSSLTESVSSSSGLGEEEPPALPSKLLSSGSCKADLGCRSYTDELHAVAPL*
>ENSG00000173546|ENST00000308508
MQSGPRPPLPAPGLALALTLTMLARLASAASFFGENHLEVPVATALTDIDLQLQFSTSQP
EALLLLAAGPADHLLLQLYSGRLQVRLVLGQEELRLQTPAETLLSDSIPHTVVLTVVEGW
ATLSVDGFLNASSAVPGAPLEVPYGLFVGGTGTLGLPYLRGTSRPLRGCLHAATLNGRSL
LRPLTPDVHEGCAEEFSASDDVALGFSGPHSLAAFPAWGTQDEGTLEFTLTTQSRQAPLA
FQAGGRRGDFIYVDIFEGHLRAVVEKGQGTVLLHNSVPVADGQPHEVSVHINAHRLEISV
DQYPTHTSNRGVLSYLEPRGSLLLGGLDAEASRHLQEHRLGLTPEATNASLLGCMEDLSV

NGQRRGLREALLTRNMAAGCRLEEEEYEDDAYGHYEAFSTLAPEAWPAMELPEPCVPEPG
LPPVFANFTQLLTISPLVVAEGGTAWLEWRHVQPTLDLMEAELRKSQVLFSVTRGARHGE
LELDIPGAQARKMFTLLDVVNRKARFIHDGSEDTSDQLVLEVSVTARVPMPSCLRRGQTY
LLPIQVNPVNDPPHIIFPHGSLMVILEHTQKPLGPEVFQAYDPDSACEGLTFQVLGTSSG
LPVERRDQPGEPATEFSCRELEAGSLVYVHRGGPAQDLTFRVSDGLQASPPATLKVVAIR
PAIQIHRSTGLRLAQGSAMPILPANLSVETNAVGQDVSVLFRVTGALQFGELQKQGAGGV
EGAEWWATQAFHQRDVEQGRVRYLSTDPQHHAYDTVENLALEVQVGQEILSNLSFPVTIQ
RATVWMLRLEPLHTQNTQQETLTTAHLEATLEEAGPSPPTFHYEVVQAPRKGNLQLQGTR
LSDGQGFTQDDIQAGRVTYGATARASEAVEDTFRFRVTAPPYFSPLYTFPIHIGGDPDAP
VLTNVLLVVPEGGEGVLSADHLFVKSLNSASYLYEVMERPRHGRLAWRGTQDKTTMVTSF
TNEDLLRGRLVYQHDDSETTEDDIPFVATRQGESSGDMAWEEVRGVFRVAIQPVNDHAPV
QTISRIFHVARGGRRLLTTDDVAFSDADSGFADAQLVLTRKDLLFGSIVAVDEPTRPIYR
FTQEDLRKRRVLFVHSGADRGWIQLQVSDGQHQATALLEVQASEPYLRVANGSSLVVPQG
GQGTIDTAVLHLDTNLDIRSGDEVHYHVTAGPRWGQLVRAGQPATAFSQQDLLDGAVLYS
HNGSLSPRDTMAFSVEAGPVHTDATLQVTIALEGPLAPLKLVRHKKIYVFQGEAAEIRRD
QLEAAQEAVPPADIVFSVKSPPSAGYLVMVSRGALADEPPSLDPVQSFSQEAVDTGRVLY
LHSRPEAWSDAFSLDVASGLGAPLEGVLVELEVLPAAIPLEAQNFSVPEGGSLTLAPPLL
RVSGPYFPTLLGLSLQVLEPPQHGALQKEDGPQARTLSAFSWRMVEEQLIRYVHDGSETL
TDSFVLMANASEMDRQSHPVAFTVTVLPVNDQPPILTTNTGLQMWEGATAPIPAEALRST
DGDSGSEDLVYTIEQPSNGRVVLRGAPGTEVRSFTQAQLDGGLVLFSHRGTLDGGFRFRL
SDGEHTSPGHFFRVTAQKQVLLSLKGSQTLTVCPGSVQPLSSQTLRASSSAGTDPQLLLY
RVVRGPQLGRLFHAQQDSTGEALVNFTQAEVYAGNILYEHEMPPEPFWEAHDTLELQLSS
PPARDVAATLAVAVSFEAACPQRPSHLWKNKGLWVPEGQRARITVAALDASNLLASVPSP
QRSEHDVLFQVTQFPSRGQLLVSEEPLHAGQPHFLQSQLAAGQLVYAHGGGGTQQDGFHF
RAHLQGPAGASVAGPQTSEAFAITVRDVNERPPQPQASVPLRLTRGSRAPISRAQLSVVD
PDSAPGEIEYEVQRAPHNGFLSLVGGGLGPVTRFTQADVDSGRLAFVANGSSVAGIFQLS
MSDGASPPLPMSLAVDILPSAIEVQLRAPLEVPQALGRSSLSQQQLRVVSDREEPEAAYR
LIQGPQYGHLLVGGRPTSAFSQFQIDQGEVVFAFTNFSSSHDHFRVLALARGVNASAVVN
VTVRALLHVWAGGPWPQGATLRLDPTVLDAGELANRTGSVPRFRLLEGPRHGRVVRVPRA
RTEPGGSQLVEQFTQQDLEDGRLGLEVGRPEGRAPGPAGDSLTLELWAQGVPPAVASLDF
ATEPYNAARPYSVALLSVPEAARTEAGKPESSTPTGEPGPMASSPEPAVAKGGFLSFLEA
NMFSVIIPMCLVLLLLALILPLLFYLRKRNKTGKHDVQVLTAKPRNGLAGDTETFRKVEP
GQAIPLTAVPGQGPPPGGQPDPELLQFCRTPNPALKNGQYWV*
>ENSG00000128268|ENST00000341184
MKMRRYKLFLMFCMAGLCLISFLHFFKTLSYVTFPRELASLSPNLVSSFFWNNAPVTPQA
SPEPGGPDLLRTPLYSHSPLLQPLPPSKAAEELHRVDLVLPEDTTEYFVRTKAGGVCFKP
GTKMLERPPPGRPEEKPEGANGSSARRPPRYLLSARERTGGRGARRKWVECVCLPGWHGP
SCGVPTVVQYSNLPTKERLVPREVPRRVINAINVNHEFDLLDVRFHELGDVVDAFVVCES
NFTAYGEPRPLKFREMLTNGTFEYIRHKVLYVFLDHFPPGGRQDGWIADDYLRTFLTQDG
VSRLRNLRPDDVFIIDDADEIPARDGVLFLKLYDGWTEPFAFHMRKSLYGFFWKQPGTLE
VVSGCTVDMLQAVYGLDGIRLRRRQYYTMPNFRQYENRTGHILVQWSLGSPLHFAGWHCS
WCFTPEGIYFKLVSAQNGDFPRWGDYEDKRDLNYIRGLIRTGGWFDGTQQEYPPADPSEH
MYAPKYLLKNYDRFHYLLDNPYQEPRSTAAGGWRHRGPEGRPPARGKLDEAEV*
>ENSG00000145428|ENST00000347063

MAAGTAARKAAPVLEAPPQQEQLSHTKLSAEDTWNLQQERMYKMHRGHDSMHVEMILIFL
CVLVIAQIVLVQWRQRHGRSYNLVTLLQMWVVPLYFTIKLYWWRFLSMWGMFSVITSYIL
FRATRKPLSGRTPRLVYKWFLLIYKLSYAFGVVGYLAIMFTMCGFNLFFKIKARDSMDFG
IVSLFYGLYYGVMGRDFAEICSDYMASTIGFYSVSRLPTRSLSDNICAVCGQKIIVELDE
EGLIENTYQLSCNHVFHEFCIRGWCIVGKKQTCPYCKEKVDLKRMISNPWERTHFLYGQI
LDWLRYLVAWQPVVIGIVQGIIYSLGLE*
>ENSG00000167531|ENST00000301046
MRFFVPLFLVGILFPAILAKQFTKCELSQLLKDIDGYGGIALPELICTMFHTSGYDTQAI
VENNESTEYGLFQISNKLWCKSSQVPQSRNICDISCDKFLDDDITDDIMCAKKILDIKGI
DYWLAHKALCTEKLEQWLCEKL*
>ENSG00000135702|ENST00000336257
MGMRARVPKVAHSTRRPPAARMWLPRFSSKTVTVLLLAQTTCLLLFIISRPGPSSPAGGE
DRVHVLVLSSWRSGSSFLGQLFSQHPDVFYLMEPAWHVWTTLSQGSAATLHMAVRDLMRS
IFLCDMDVFDAYMPQSRNLSAFFNWATSRALCSPPACSAFPRGTISKQDVCKTLCTRQPF
SLAREACRSYSHVVLKEVRFFNLQVLYPLLSDPALNLRIVHLVRDPRAVLRSREAAGPIL
ARDNGIVLGTNGKWVEADPHLRLIREVCRSHVRIAEAATLKPPPFLRGRYRLVRFEDLAR
EPLAEIRALYAFTGLTLTPQLEAWIHNITHGSGIGKPIEAFHTSSRNARNVSQAWRHALP
FTKILRVQEVCAGALQLLGYRPVYSADQQRDLTLDLVLPRGPDHFSWASPD*
>ENSG00000157020|ENST00000337354
MGKMVSVINTVDTSHEDMIHDAQMDYYGTRLATCSSDRSVKIFDVRNGGQILIADLRGHE
GPVWQVAWAHPMYGNILASCSYDRKVIIWREENGTWEKSHEHAGHDSSVNSVCWAPHDYG
LILACGSSDGAISLLTYTGEGQWEVKKINNAHTIGCNAVSWAPAVVPGSLIDHPSGQKPN
YIKRFASGGCDNLIKLWKEEEDGQWKEEQKLEAHSDWVRDVAWAPSIGLPTSTIASCSQD
GRVFIWTCDDASSNTWSPKLLHKFNDVVWHVSWSITANILAVSGGDNKVTLWKESVDGQW
VCISDVNKGQGSVSASVTEGQQNEQ*
>ENSG00000135968|ENST00000309863
MEDLVQDGVASPATPGTGKSKLETLPKEDLIKFAKKQMMLIQKAKSRCTELEKEIEELRS
KPVTEGTGDIIKALTERLDALLLEKAETEQQCLSLKKENIKMKQEVEDSVTKMGDAHKEL
EQSHINYVKEIENLKNELMAVRSKYSEDKANLQKQLEEAMNTQLELSEQLKFQNNSEDNV
KKLQEEIEKIRPGFEEQILYLQKQLDATTDEKKETVTQLQNIIEANSQHYQKNINSLQEE
LLQLKAIHQEEVKELMCQIEASAKEHEAEINKLNELKENLVKQCEASEKNIQKKYECELE
NLRKATSNANQDNQICSILLQENTFVEQVVNEKVKHLEDTLKELESQHSILKDEVTYMNN
LKLKLEMDAQHIKDEFFHEREDLEFKINELLLAKEEQGCVIEKLKSELAGLNKQFCYTVE
QHNREVQSLKEQHQKEISELNETFLSDSEKEKLTLMFEIQGLKEQCENLQQEKQEAILNY
ESLREIMEILQTELGESAGKISQEFESMKQQQASDVHELQQKLRTAFTEKDALLETVNRL
QGENEKLLSQQELVPELENTIKNLQEKNGVYLLSLSQRDTMLKELEGKINSLTEEKDDFI
NKLKNSHEEMDNFHKKCEREERLILELGKKVEQTIQYNSELEQKVNELTGGLEETLKEKD
QNDQKLEKLMVQMKVLSEDKEVLSAEVKSLYEENNKLSSEKKQLSRDLEVFLSQKEDVIL
KEHITQLEKKLQLMVEEQDNLNKLLENEQVQKLFVKTQLYGFLKEMGSEVSEDSEEKDVV
NVLQAVGESLAKINEEKCNLAFQRDEKVLELEKEIKCLQEESVVQCEELKSLLRDYEQEK
VLLRKELEEIQSEKEALQSDLLEMKNANEKTRLENQNLLIQVEEVSQTCSKSEIHNEKEK
CFIKEHENLKPLLEQKELRDRRAELILLKDSLAKSPSVKNDPLSSVKELEEKIENLEKEC
KEKEEKINKIKLVAVKAKKELDSSRKETQTVKEELESLRSEKDQLSASMRDLIQGAESYK
NLLLEYEKQSEQLDVEKERANNFEHRIEDLTRQLRNSTLQCETINSDNEDLLARIETLQS

NAKLLEVQILEVQRAKAMVDKELEAEKLQKEQKIKEHATTVNELEELQVQLQKQKKQLQK
TMQELELVKKDAQQTTLMNMEIADYERLMKELNQKLTNKNNKIEDLEQEIKIQKQKQETL
QEEITSLQSSVQQYEEKNTKIKQLLVKTKKELADSKQAETDHLILQASLKGELEASQQQV
EVYKIQLAEITSEKHKIHEHLKTSAEQHQRTLSAYQQRVTALQEECRAAKAEQATVTSEF
ESYKVRVHNVLKQQKNKSMSQAETEGAKQEREHLEMLIDQLKIKLQDSQNNLQINVSELQ
TLQSEHDTLLERHNKMLQETVSKEAELREKLCSIQSENMMMKSEHTQTVSQLTSQNEVLR
NSFRDQVRHLQEEHRKTVETLQQQLSKMEAQLFQLKNEPTTRSPVSSQQSLKNLRERRNT
DLPLLDMHTVTREEGEGMETTDTESVSSASTYTQSLEQLLNSPETKLEPPLWHAEFTKEE
LVQKLSSTTKSADHLNGLLRETEATNAILMEQIKLLKSEIRRLERNQEREKSAANLEYLK
NVLLQFIFLKPGSERERLLPVINTMLQLSPEEKGKLAAVAQGEEENASRSSGWASYLHSW
SGLR*
>ENSG00000033170|ENST00000342677
MRPWTGSWRWIMLILFAWGTLLFYIGGHLVRDNDHPDHSSRELSKILAKLERLKQQNEDL
RRMAESLRIPEGPIDQGPAIGRVRVLEEQLVKAKEQIENYKKQTRNGLGKDHEILRRRIE
NGAKELWFFLQSELKKLKNLEGNELQRHADEFLLDLGHHERSIMTDLYYLSQTDGAGDWR
EKEAKDLTELVQRRITYLQNPKDCSKAKKLVCNINKGCGYGCQLHHVVYCFMIAYGTQRT
LILESQNWRYATGGWETVFRPVSETCTDRSGISTGHWSGTPIMNLLVITLFPGQLDCTID
TQKIHFVE*
>ENSG00000181830|ENST00000314134
MNRAPLKRSRILHMALTGASDPSAEAEANGEKPFLLRALQIALVVSLYWVTSISMVFLNK
YLLDSPSLRLDTPIFVTFYQCLVTTLLCKGLSALAACCPGAVDFPSLRLDLRVARSVLPL
SVVFIGMITFNNLCLKYVGVAFYNVGRSLTTVFNVLLSYLLLKQTTSFYALLTCGIIIGG
FWLGVDQEGAEGTLSWLGTVFGVLASLCVSLNAIYTTKVLPAVDGSIWRLTFYNNVNACI
LFLPLLLLLGELQALRDFAQLGSAHFWGMMTLGGLFGFAIGYVTGLQIKFTSPLTHNVSG
TAKACAQTVLAVLYYEETKSFLWWTSNMMVLGGSSAYTWVRGWEMKKTPEEPSPKDSEKS
AMGV*
>ENSG00000080815|ENST00000324501
MTELPAPLSYFQNAQMSEDNHLSNTVRSQNDNRERQEHNDRRSLGHPEPLSNGRPQGNSR
QVVEQDEEEDEELTLKYGAKHVIMLFVPVTLCMVVVVATIKSVSFYTRKDGQLIYTPFTE
DTETVGQRALHSILNAAIMISVIVVMTILLVVLYKYRCYKVIHAWLIISSLLLLFFFSFI
YLGEVFKTYNVAVDYITVALLIWNFGVVGMISIHWKGPLRLQQAYLIMISALMALVFIKY
LPEWTAWLILAVISVYDLVAVLCPKGPLRMLVETAQERNETLFPALIYSSTMVWLVNMAE
GDPEAQRRVSKNSKYNAESTERESQDTVAENDDGGFSEEWEAQRDSHLGPHRSTPESRAA
VQELSSSILAGEDPEERGVKLGLGDFIFYSVLVGKASATASGDWNTTIACFVAILIGLCL
TLLLLAIFKKALPALPISITFGLVFYFATDYLVQPFMDQLAFHQFYI*
>ENSG00000171155|ENST00000304661
MLSESSSFLKGVMLGSIFCALITMLGHIRIGHGNRMHHHEHHHLQAPNKEDILKISEDER
MELSKSFRVYCIILVKPKDVSLWAAVKETWTKHCDKAEFFSSENVKVFESINMDTNDMWL
MMRKAYKYAFDKYRDQYNWFFLARPTTFAIIENLKYFLLKKDPSQPFYLGHTIKSGDLEY
VGMEGGIVLSVESMKRLNSLLNIPEKCPEQGGMIWKISEDKQLAVCLKYAGVFAENAEDA
DGKDVFNTKSVGLSIKEAMTYHPNQVVEGCCSDMAVTFNGLTPNQMHVMMYGVYRLRAFG
HIFNDALVFLPPNGSDND*
>ENSG00000164850|ENST00000297469
MDVTSQARGVGLEMYPGTAQPAAPNTTSPELNLSHPLLGTALANGTGELSEHQQYVIGLF

LSCLYTIFLFPIGFVGNILILVVNISFREKMTIPDLYFINLAVADLILVADSLIEVFNLH
ERYYDIAVLCTFMSLFLQVNMYSSVFFLTWMSFDRYIALARAMRCSLFRTKHHARLSCGL
IWMASVSATLVPFTAVHLQHTDEACFCFADVREVQWLEVTLGFIVPFAIIGLCYSLIVRV
LVRAHRHRGLRPRRQKALRMILAVVLVFFVCWLPENVFISVHLLQRTQPGAAPCKQSFRH
AHPLTGHIVNLAAFSNSCLNPLIYSFLGETFRDKLRLYIEQKTNLPALNRFCHAALKAVI
PDSTEQSDVRFSSAV*
>ENSG00000109572|ENST00000347613
MESEQLFHRGYYRNSYNSITSASSDEELLDGAGVIMDFQTSEDDNLLDGDTAVGTHYTMT
NGGSINSSTHLLDLLDEPIPGVGTYDDFHTIDWVREKCKDRERHRRINSKKKESAWEMTK
SLYDAWSGWLVVTLTGLASGALAGLIDIAADWMTDLKEGICLSALWYNHEQCCWGSNETT
FEERDKCPQWKTWAELIIGQAEGPGSYIMNYIMYIFWALSFAFLAVSLVKVFAPYACGSG
IPEIKTILSGFIIRGYLGKWTLMIKTITLVLAVASGLSLGKEGPLVHVACCCGNIFSYLF
PKYSTNEAKKREVLSAASAAGVSVAFGAPIGGVLFSLEEVSYYFPLKTLWRSFFAALVAA
FVLRSINPFGNSRLVLFYVEYHTPWYLFELFPFILLGVFGGLWGAFFIRANIAWCRRRKS
TKFGKYPVLEVIIVAAITAVIAFPNPYTRLNTSELIKELFTDCGPLESSSLCDYRNDMNA
SKIVDDIPDRPAGIGVYSAIWQLCLALIFKIIMTVFTFGIKVPSGLFIPSMAIGAIAGRI
VGIAVEQLAYYHHDWFIFKEWCEVGADCITPGLYAMVGAAACLGGVTRMTVSLVVIVFEL
TGGLEYIVPLMAAVMTSKWVGDAFGREGIYEAHIRLNGYPFLDAKEEFTHTTLAADVMRP
RRNDPPLAVLTQDNMTVDDIENMINETSYNGFPVIMSKESQRLVGFALRRDLTIAIESAR
KKQEGIVGSSRVCFAQHTPSLPAESPRPLKLRSILDMSPFTVTDHTPMEIVVDIFRKLGL
RQCLVTHNGIVLGIITKKNILEHLEQLKQHVEPLAPPWHYNKKRYPPAYGPDGKPRPRFN
NVQLNLTDEEREETEEEVYLLNSTTL*
>ENSG00000111670|ENST00000299314
MLFKLLQRQTYTCLSHRYGLYVCFLGVVVTIVSAFQFGEVVLEWSRDQYHVLFDSYRDNI
AGKSFQNRLCLPMPIDVVYTWVNGTDLELLKELQQVREQMEEEQKAMREILGKNTTEPTK
KSEKQLECLLTHCIKVPMLVLDPALPANITLKDLPSLYPSFHSASDIFNVAKPKNPSTNV
SVVVFDSTKDVEDAHSGLLKGNSRQTVWRGYLTTDKEVPGLVLMQDLAFLSGFPPTFKET
NQLKTKLPENLSSKVKLLQLYSEASVALLKLNNPKDFQELNKQTKKNMTIDGKELTISPA
YLLWDLSAISQSKQDEDISASRFEDNEELRYSLRSIERHAPWVRNIFIVTNGQIPSWLNL
DNPRVTIVTHQDVFRNLSHLPTFSSPAIESHIHRIEGLSQKFIYLNDDVMFGKDVWPDDF
YSHSKGQKVYLTWPVPNCAEGCPGSWIKDGYCDKACNNSACDWDGGDCSGNSGGSRYIAG
GGGTGSIGVGQPWQFGGGINSVSYCNQGCANSWLADKFCDQACNVLSCGFDAGDCGQDHF
HELYKVILLPNQTHYIIPKGECLPYFSFAEVAKRGVEGAYSDNPIIRHASIANKWKTIHL
IMHSGMNATTIHFNLTFQNTNDEEFKMQITVEVDTREGPKLNSTAQKGYENLVSPITLLP
EAEILFEDIPKEKRFPKFKRHDVNSTRRAQEEVKIPLVNISLLPKDAQLSLNTLDLQLEH
GDITLKGYNLSKSALLRSFLMNSQHAKIKNQAIITDETNDSLVAPQEKQVHKSILPNSLG
VSERLQRLTFPAVSVKVNGHDQGQNPPLDLETTARFRVETHTQKTIGGNVTKEKPPSLIV
PLESQMTKEKKITGKEKENSRMEENAENHIGVTEVLLGRKLQHYTDSYLGFLPWEKKKYF
QDLLDEEESLKTQLAYFTDSKNTGRQLKDTFADSLRYVNKILNSKFGFTSRKVPAHMPHM
IDRIVMQELQDMFPEEFDKTSFHKVRHSEDMQFAFSYFYYLMSAVQPLNISQVFDEVDTD
QSGVLSDREIRTLATRIHELPLSLQDLTGLEHMLINCSKMLPADITQLNNIPPTQESYYD
PNLPPVTKSLVTNCKPVTDKIHKAYKDKNKYRFEIMGEEEIAFKMIRTNVSHVVGQLDDI
RKNPRKFVCLNDNIDHNHKDAQTVKAVLRDFYESMFPIPSQFELPREYRNRFLHMHELQE
WRAYRDKLKFWTHCVLATLIMFTIFSFFAEQLIALKRKIFPRRRIHKEASPNRIRV*

>ENSG00000133318|ENST00000341307
MAEPSAATQSHSISSSSFGAEPSAPGGGGSPGACPALGTKSCSSSCAVHDLIFWRDVKKT
GFVFGTTLIMLLSLAAFSVISVVSYLILALLSVTISFRIYKSVIQAVQKSEEGHPFKAYL
DVDITLSSEAFHNYMNAAMVHINRALKLIIRLFLVEDLVDSLKLAVFMWLMTYVGAVFNG
ITLLILAELLIFSVPIVYEKYKDPSKTPWNRQKKGRISTWKPEMQQLLKHHLIVITSLLV
L*
>ENSG00000181744|ENST00000315691
MWRLVPPKLGRLSRSLKLAALGSLLVLMVLHSPSLLASWQRNELTDRRFLQLNKCPACFG
TSWCRRFLNGQVVFEAWGRLRLLDFLNVKNVYFAQYGEPREGGRRRVVLKRLGSQRELAQ
LDQSICKRATGRPRCDLLQAMPRTEFARLNGDVRLLTPEAVEGWSDLVHCPSQRLLDRLV
RRYAETKDSGSFLLRNLKDSERMQLLLTLAFNPEPLVLQSFPSDEGWPFAKYLGACGRMV
AVNYVGEELWSYFNAPWEKRVDLAWQLMEIAEQLTNNDFEFALYLLDVSFDNFAVGPRDG
KVIIVDAENVLVADKRLIRQNKPENWDVWYESKFDDCDKEACLSFSKEILCARATVDHNY
YAVCQNLLSRHATWRGTSGGLLHDPPSEIAKDGRLEALLDECANPKKRYGRFQAAKELRE
YLAQLSNNVR*
>ENSG00000126091|ENST00000335430
MGLLVFVRNLLLALCLFLVLGFLYYSAWKLHLLQWEEDSSKYSHSSSPQEKPVAEYDRLG
FLLNLDSKLPAELATKYANFSEGACKPGYASALMTAIFPRFSKPAPMFLDDSFRKWARIR
EFVPPFGIKGQDNLIKAILSVTKEYRLTPALDSLRCRRCIIVGNGGVLANKSLGSRIDDY
DIVVR*
>ENSG00000182400|ENST00000347691
MADEALFLLLHNEMVSGVYKSAEQGEVENGRCITKLENMGFRVGQGLIERFTKDTARFKD
ELDIMKFICKDFWTTVFKKQIDNLRTNHQYLAFTCGLIRGGLSNLGIKSIVTAEVSSMPA
CKFQVMIQKL*
>ENSG00000165240|ENST00000341514
MDPSMGVNSVTISVEGMTCNSCVWTIEQQIGKVNGVHHIKVSLEEKNATIIYDPKLQTPK
TLQEAIDDMGFDAVIHNPDPLPVLTDTLFLTVTASLTLPWDHIQSTLLKTKGVTDIKIYP
QKRTVAVTIIPSIVNANQIKELVPELSLDTGTLEKKSGACEDHSMAQAGEVVLKMKVEGM
TCHSCTSTIEGKIGKLQGVQRIKVSLDNQEATIVYQPHLISVEEMKKQIEAMGFPAFVKK
QPKYLKLGAIDVERLKNTPVKSSEGSQQRSPSYTNDSTATFIIDGMHCKSCVSNIESTLS
ALQYVSSIVVSLENRSAIVKYNASSVTPESLRKAIEAVSPGLYRVSITSEVESTSNSPSS
SSLQKIPLNVVSQPLTQETVINIDGMTCNSCVQSIEGVISKKPGVKSIRVSLANSNGTVE
YDPLLTSPETLRGAIEDMGFDATLSDTNEPLVVIAQPSSEMPLLTSTNEFYTKGMTPVQD
KEEGKNSSKCYIQVTGMTCASCVANIERNLRREEGIYSILVALMAGKAEVRYNPAVIQPP
MIAEFIRELGFGATVIENADEGDGVLELVVRGMTCASCVHKIESSLTKHRGILYCSVALA
TNKAHIKYDPEIIGPRDIIHTIESLGFEASLVKKDRSASHLDHKREIRQWRRSFLVSLFF
CIPVMGLMIYMMVMDHHFATLHHNQNMSKEEMINLHSSMFLERQILPGLSVMNLLSFLLC
VPVQFFGGWYFYIQAYKALKHKTANMDVLIVLATTIAFAYSLIILLVAMYERAKVNPITF
FDTPPMLFVFIALGRWLEHIAKGKTSEALAKLISLQATEATIVTLDSDNILLSEEQVDVE
LVQRGDIIKVVPGGKFPVDGRVIEGHSMVDESLITGEAMPVAKKPGSTVIAGSINQNGSL
LICATHVGADTTLSQIVKLVEEAQTSKAPIQQFADKLSGYFVPFIVFVSIATLLVWIVIG
FLNFEIVETYFPGYNRSISRTETIIRFAFQASITVLCIACPCSLGLATPTAVMVGTGVGA
QNGILIKGGEPLEMAHKVKVVVFDKTGTITHGTPVVNQVKVLTESNRISHHKILAIVGTA
ESNSEHPLGTAITKYCKQELDTETLGTCIDFQVVPGCGISCKVTNIEGLLHKNNWNIEDN

NIKNASLVQIDASNEQSSTSSSMIIDAQISNALNAQQYKVLIGNREWMIRNGLVINNDVN
DFMTEHERKGRTAVLVAVDDELCGLIAIADTVKPEAELAIHILKSMGLEVVLMTGDNSKT
ARSIASQVGITKVFAEVLPSHKVAKVKQLQEEGKRVAMVGDGINDSPALAMANVGIAIGT
GTDVAIEAADVVLIRNDLLDVVASIDLSRKTVKRIRINFVFALIYNLVGIPIAAGVFMPI
GLVLQPWMGSAAMAASSVSVVLSSLFLKLYRKPTYESYELPARSQIGQKSPSEISVHVGI
DDTSRNSPKLGLLDRIVNYSRASINSLLSDKRSLNSVVTSEPDKHSLLVGDFREDDDTAL
*
>ENSG00000185010|ENST00000330287
MRIQDPGKVFFGNVDSSGIKHNIFNPPIIARYIRLHPTHYSIRSTLRMELMGCDLNSCSM
PLGMESKAISDAQITASSYFTNMFATWSPSKARLHLQGRSNAWRPQVNNPKEWLQVDFQK
TMKVTGVTTQGVKSLLTSMYVKEFLISSSQDGHQWTLFFQNGKVKVFQGNQDSFTPVVNS
LDPPLLTRYLRIHPQSWVHQIALRMEVLGCEAQDLY*
>ENSG00000166377|ENST00000307671
MADQIPLYPVRSAAAAAANRKRAAYYSAAGPRPGADRHSRYQLEDESAHLDEMPLMMSEE
GFENEESDYHTLPRARIMQRKRGLEWFVCDGWKFLCTSCCGWLINICRRKKELKARTVWL
GCPEKCEEKHPRNSIKNQKYNVFTFIPGVLYEQFKFFLNLYFLVISCSQFVPALKIGYLY
TYWAPLGFVLAVTMTREAIDEFRRFQRDKEVNSQLYSKLTVRGKVQVKSSDIQVGDLIIV
EKNQRIPSDMVFLRTSEKAGSCFIRTDQLDGETDWKLKVAVSCTQQLPALGDLFSISAYV
YAQKPQMDIHSFEGTFTREDSDPPIHESLSIENTLWASTIVASGTVIGVVIYTGKETRSV
MNTSNPKNKVGLLDLELNRLTKALFLALVALSIVMVTLQGFVGPWYRNLFRFLLLFSYII
PISLRVNLDMGKAVYGWMMMKDENIPGTVVRTSTIPEELGRLVYLLTDKTGTLTQNEMIF
KRLHLGTVSYGADTMDEIQSHVRDSYSQMQSQAGGNNTGSTPLRKAQSSAPKVRKSVSSR
IHEAVKAIVLCHNVTPVYESRAGVTEETEFAEADQDFSDENRTYQASSPDEVALVQWTES
VGLTLVSRDLTSMQLKTPSGQVLSFCILQLFPFTSESKRMGVIVRDESTAEITFYMKGAD
VAMSPIVQYNDWLEEECGNMAREGLRTLVVAKKALTEEQYQDFESRYTQAKLSMHDRSLK
VAAVVESLEREMELLCLTGVEDQLQADVRPTLEMLRNAGIKIWMLTGDKLETATCIAKSS
HLVSRTQDIHIFRQVTSRGEAHLELNAFRRKHDCALVISGDSLEVCLKYYEHEFVELACQ
CPAVVCCRCSPTQKARIVTLLQQHTGRRTCAIGDGGNDVSMIQAADCGIGIEGKEGKQAS
LAADFSITQFRHIGRLLMVHGRNSYKRSAALGQFVMHRGLIISTMQAVFSSVFYFASVPL
YQGFLMVGYATIYTMFPVFSLVLDQDVKPEMAMLYPELYKDLTKGRSLSFKTFLIWVLIS
IYQGGILMYGALVLFESEFVHVVAISFTALILTELLMVALTVRTWHWLMVVAEFLSLGCY
VSSLAFLNEYFDVAFITTVTFLWKVSAITVVSCLPLYVLKYLRRKLSPPSYCKLAS*
>ENSG00000110195|ENST00000312293
MAQRMTTQLLLLLVWVAVVGEAQTRIAWARTELLNVCMNAKHHKEKPGPEDKLHEQCRPW
RKNACCSTNTSQEAHKDVSYLYRFNWNHCGEMAPACKRHFIQDTCLYECSPNLGPWIQQV
DQSWRKERVLNVPLCKEDCEQWWEDCRTSYTCKSNWHKGWNWTSGFNKCAVGAACQPFHF
YFPTPTVLCNEIWTHSYKVSNYSRGSGRCIQMWFDPAQGNPNEEVARFYAAAMSGAGPWA
AWPFLLSLALMLLWLLS*
>ENSG00000188603|ENST00000333496
MGGCAGSRRRFSDSEGEETVPEPRLPLLDHQGAHWKNAVGFWLLGLCNNFSYVVMLSAAH
DILSHKRTSGNQSHAVLLADILPTLVIKLLAPLGLHLLPYSPRVLVSGICAAGSFVLVAF
SHSVGTSLCGVVFASISSGLGEVTFLSLTAFYPRAVISWWSSGTGGAGLLGALSYLGLTQ
AGLSPQQTLLSMLGIPALLLASYFLLLTSPEAQDPGGEEEAESAARQPLIRTEAPESKPG
SSSSLSLRERWTVFKGLLWYIVPLVVVYFAEYFINQGLFELLFFWNTSLSHAQQYRWYQM

LYQAGVFASRSSLRCCRIRFTWALALLQCLNLVFLLADVWFGFLPSIYLVFLIILYEGLL
GGAAYVNTFHNIALETSDEHREFAMAATCISDTLGISLSGLLALPLHDFLCQLS*
>ENSG00000174871|ENST00000311445
MAFTFAAFCYMLTLVLCASLIFFVIWHIIAFDELRTDFKNPIDQGNPARARERLKNIERI
CCLLRKLVVPEYSIHGLFCLMFLCAAEWVTLGLNIPLLFYHLWRYFHRPADGSEVMYDAV
SIMNADILNYCQKESWCKLAFYLLSFFYYLYSMVYTLVSF*
>ENSG00000127995|ENST00000297273
MAALAYNLGKREINHYFSVRSAKVLALVAVLLLAACHLASRRYRGNDSCEYLLSSGRFLG
EKVWQPHSCMMHKYKISEAKNCLVDKHIAFIGDSRIRQLFYSFVKIINPQFKEEGNKHEN
IPFEDKTASVKVDFLWHPEVNGSMKQCIKVWTEDSIAKPHVIVAGAATWSIKIHNGSSEA
LSQYKMNITSIAPLLEKLAKTSDVYWVLQDPVYEDLLSENRKMITNEKIDAYNEAAVSIL
NSSTRNSKSNVKMFSVSKLIAQETIMESLDGLHLPESSRETTAMILMNVYCNKILKPVDG
SCCQPRPPVTLIQKLAACFFTLSIIGYLIFYIIHRNAHRKNKPCTDLESGEEKKNIINTP
VSSLEILLQSFCKLGLIMAYFYMCDRANLFMKENKFYTHSSFFIPIIYILVLGVFYNENT
KETKVLNREQTDEWKGWMQLVILIYHISGASTFLPVYMHIRVLVAAYLFQTGYGHFSYFW
IKGDFGIYRVCQVLFRLNFLVVVLCIVMDRPYQFYYFVPLVTVWFMVIYVTLALWPQIIQ
KKANGNCFWHFGLLLKLGFLLLFICFLAYSQGAFEKIFSLWPLSKCFELKGNVYEWWFRW
RLDRYVVFHGMLFAFIYLALQKRQILSEGKGEPLFSNKISNFLLFISVVSFLTYSIWASS
CKNKAECNELHPSVSVVQILAFILIRNIPGYARSVYSSFFAWFGKISLELFICQYHIWLA
ADTRGILVLIPGNPMLNIIVSTFIFVCVAHEISQITNDLAQIIIPKDNSSLLKRLACIAA
FFCGLLILSSIQDKSKH*
>ENSG00000162961|ENST00000342166
MEPEQMLEGQTQVAENPHSEYGLTDNVERIVENEKINAEKSSKQKVDLQSLPTRAYLDQT
VVPILLQGLAVLAKERPPNPIEFLASYLLKNKAQFEDRN*
>ENSG00000154305|ENST00000344922
MAAAPGLLVWLLVLRLPWRVPGQLDPSTGRRFSEHKLCADDECSMLMYRGEALEDFTGPD
CRFVNFKKGDPVYVYYKLARGWPEVWAGSVGRTFGYFPKDLIQVVHEYTKEELQVPTDET
DFVCFDGGRDDFHNYNVEELLGFLELYNSAATDSEKAVEKTLQDMEKNPELSKEREPEPE
PVEANSEESDSVFSENTEDLQEQFTTQKHHSHANSQANHAQGEQASFESFEEMLQDKLKV
PESENNKTSNSSQVSNEQDKIDAYKLLKKEMTLDLKTKFGSTADALVSDDETTRLVTSLE
DDFDEELDTEYYAVGKEDEENQEDFDELPLLTFTDGEDMKTPAKSGVEKYPTDKEQNSNE
EDKVQLTVPPGIKNDDKNILTTWGDTIFSIVTGGEETRDTMDLESSSSEEEKEDDDDALV
PDSKQGKPQSATDYSDPDNVDDGLFIVDIPKTNNDKEVNAEHHIKGKGRGVQESKRGLVQ
DKTELEDENQEGMTVHSSVHSNNLNSMPAAEKGKDTLKSAYDDTENDLKGAAIHISKGML
HEEKPGEQILEGGSESESAQKAAGNQMNDRKIQQESLGSAPLMGDDHPNASRDSVEGDAL
VNGAKLHTLSVEHQREELKEELVLKTQNQPRFSSPDEIDLPRELEDEVPILGRNLPWQQE
RDVAATASKQMSEKIRLSEGEAKEDSLDEEFFHHKAMQGTEVGQTDQTDSTGGPAFLSKV
EEDDYPSEELLEDENAINAKRSKEKNPGNQGRQFDVNLQVPDRAVLGTIHPDPEIEESKQ
ETSMILDSEKTSETAAKGVNTGGREPNTMVEKERPLADKKAQRPFERSDFSDSIKIQTPE
LGEVFQNKDSDYLKNDNPEEHLKTSGLAGEPEGELSKEDHENTEKYMGTESQGSAAAEPE
DDSFHWTPHTSVEPGHSDKREDLLIISSFFKEQQSLQRFQKYFNVHELEALLQEMSSKLK
SAQQESLPYNMEKVLDKVFRASESQILSIAEKMLDTRVAENRDLGMNENNIFEEAAVLDD
IQDLIYFVRYKHSTAEETATLVMAPPLEEGLGGAMEEMQPLHEDNFSREKTAELNVQVPE
EPTHLDQRVIGDTHASEVSQKPNTEKDLDPGPVTTEDTPMDAIDANKQPETAAEEPASVT

PLENAILLIYSFMFYLTKSLVATLPDDVQPGPDFYGLPWKPVFITAFLGIASFAIFLWRT
VLVVKDRVYQVTEQQISEKLKTIMKENTELVQKLSNYEQKIKESKKHVQETRKQNMILSD
EAIKYKDKIKTLEKNQEILDDTAKNLRVMLESEREQNVKNQDLISENKKSIEKLKDVISM
NASEFSEVQIALNEAKLSEEKVKSECHRVQEENARLKKKKEQLQQEIEDWSKLHAELSEQ
IKSFEKSQKDLEVALTHKDDNINALTNCITQLNLLECESESEGQNKGGNDSDELANGEVG
GDRNEKMKNQIKQMMDVSRTQTAISVVEEDLKLLQLKLRASVSTKCNLEDQVKKLEDDRN
SLQAAKAGLEDECKTLRQKVEILNELYQQKEMALQKKLSQEEYERQEREHRLSAADEKAV
SAAEEVKTYKRRIEEMEDELQKTERSFKNQIATHEKKAHENWLKARAAERAIAEEKREAA
NLRHKLLELTQKMAMLQEEPVIVKPMPGKPNTQNPPRRGPLSQNGSFGPSPVSGGECSPP
LTVEPPVRPLSATLNRRDMPRSEFGSVDGPLPHPRWSAEASGKPSPSDPGSGTATMMNSS
SRGSSPTRVLDEGKVNMAPKGPPPFPGVPLMSTPMGGPVPPPIRYGPPPQLCGPFGPRPL
PPPFGPGMRPPLGLREFAPGVPPGRRDLPLHPRGFLPGHAPFRPLGSLGPREYFIPGTRL
PPPTHGPQEYPPPPAVRDLLPSGSRDEPPPASQSTSQDCSQALKQSP*
>ENSG00000177511|ENST00000324000
MRNCKMARVASVLGLVMLSVALLILSLISYVSLKKENIFTTPKYASPGAPRMYMFHAGFR
SQFALKFLDPSFVPITNSLTQELQEKPSKWKFNRTAFLHQRQEILQHVDVIKNFSLTKNS
VRIGQLMHYDYSSHKYVVFSISNNFRSLLPDVSPIMNKHYNICAVVGNSGILTGSQCGQEI
DKSDFVFRCNFAPTEAFQRDVGRKTNLTTFNPSILEKYYNNLLTIQDRNNFFLSLKKLDG
AILWIPAFFFHTSATVTRTLVDFFVEHRGQLKVQLAWPGNIMQHVNRYWKNKHLSPKRLS
TGILMYTLASAICEEIHLYGFWPFGFDPNTREDLPYHYYDKKGTKFTTKWQESHQLPAEF
QLLYRMHGEGLTKLTLSHCA*
>ENSG00000162236|ENST00000294179
MIPRKRYGSKNTDQGVYLGLSKTQVLSPATAGSSSSDIAPLPPPVTLVPPPPDTMSCRDR
TQEFLSACKSLQTRQNGIQTNKPALRAVRQRSEFTLMAKRIGKDLSNTFAKLEKLTILAK
RKSLFDDKAVEIEELTYIIKQDINSLNKQIAQLQDFVRAKGSQSGRHLQTHSNTIVVSLQ
SKLASMSNDFKSVLEVRTENLKQQRSRREQFSRAPVSALPLAPNHLGGGAVVLGAESHAS
KDVAIDMMDSRTSQQLQLIDEQDSYIQSRADTMQNIESTIVELGSIFQQLAHMVKEQEET
IQRIDENVLGAQLDVEAAHSEILKYFQSVTSNRWLMVKIFLILIVFFIIFVVFLA*
>ENSG00000180957|ENST00000320996
MVLIKEFRVVLPCSVQEYQVGQLYSVAEASKNETGGGEGIEVLKNEPYEKDGEKGQYTHK
IYHLKSKVPAFVRMIAPEGSLVFHEKAWNAYPYCRTIVTNEYMKDDFFIKIETWHKPDLG
TLENVHGLDPNTWKTVEIVHIDIADRSQVEPADYKADEDPALFQSVKTKRGPLGPNWKKE
LANSPDCPQMCAYKLVTIKFKWWGLQSKVENFIQKQEKRIFTNFHRQLFCWIDKWIDLTM
EDIRRMEDETQKELETLRNQGQVRGTSAASDE*
>ENSG00000175264|ENST00000308064
MQCSWKAVLLLALASIAIQYTAIRTFTAKSFHTCPGLAEAGLAERLCEESPTFAYNLSRK
THILILATTRSGSSFVGQLFNQHLDVFYLFEPLYHVQNTLIPRFTQGKSPADRRVMLGAS
RDLLRSLYDCDLYFLENYIKPPPVNHTTDRIFRRGASRVLCSRPVCDPPGPADLVLEEGD
CVRKCGLLNLTVAAEACRERSHVAIKTVRVPEVNDLRALVEDPRLNLKVIQLVRDPRGIL
ASRSETFRDTYRLWRLWYGTGRKPYNLDVTQLTTVCEDFSNSVSTGLMRPPWLKGKYMLV
RYEDLARNPMKKTEEIYGFLGIPLDSHVARWIQNNTRGDPTLGKHKYGTVRNSAATAEKW
RFRLSYDIVAFAQNACQQVLAQLGYKIAASEEELKNPSVSLVEERDFRPFS*
>ENSG00000126091|ENST00000347631
MGLLVFVRNLLLALCLFLVLGFLYYSAWKLHLLQWEEDSNSVVLSFDSAGQTLGSEYDRL

GFLLNLDSKLPAELATKYANFSEGACKPGYASALMTAIFPRFSKPAPMFLDDSFRKWARI
REFVPPFGIKGQDNLIKAILSVTKEYRLTPALDSLRCRRCIIVGNGGVLANKSLGSRIDD
YDIVVRLNSAPVKGFEKDVGSKTTLRITYPEGAMQRPEQYERDSLFVLAGFKWQDFKWLK
YIVYKERVSASDGFWKSVATRVPKEPPEIRILNPYFIQEAAFTLIGLPFNNGLMGRGNIP
TLGSVAVTMALHGCDEVAVAGFGYDMSTPNAPLHYYETVRMAAIKESWTHNIQREKEFLR
KLVKARVITDLSSGI*
>ENSG00000126091|ENST00000330208
MGLLVFVRNLLLALCLFLVLGFLYYSAWKLHLLQWEEDSKYDRLGFLLNLDSKLPAELAT
KYANFSEGACKPGYASALMTAIFPRPGRTISSERKSFCGSW*
>ENSG00000132376|ENST00000320345
MDVLSPLSFIKVSHVRMQGILLLVFAKYQHLPYIQILSTKSTPTGLFGYWGNKGGVNICL
KLYGYYVSIINCHLPPHISNNYQRLEHFDRILEMQNCEGRDIPNILDHDLIIWFGDMNFR
IEDFGLHFVRESIKNRCYGGLWEKDQLSIAKKHDPLLREFQEGRLLFPPTYKFDRNSNDY
DTSEKKRKPAWTDRILWRLKRQPCAGPDTPIPPASHFSLSLRGYSSHMTYGISDHKPVSG
TFDLELKPLVSAPLIVLMPEDLWTVENDMMVSYSSTSDFPSSPWDWIGLYKVGLRDVNDY
VSYAWVGDSKVSCSDNLNQVYIDISNIPTTEDEFLLCYYSNSLRSVVGISRPFQIPPGSL
REDPLGEAQPQI*
>ENSG00000169241|ENST00000303343
MEAGGFLDSLIYGACVVFTLGMFSAGLSDLRHMRMTRSVDNVQFLPFLTTEVNNLGWLSY
GALKGDGILIVVNTVGAALQTLYILAYLHYCPRKAKVIQTKSTQCLSYPLTIATLLTSAS
WCLYGFRLRDPYIMVSNFPGIVTSFIRFWLFWKYPQEQDRNYWLLQT*
>ENSG00000166448|ENST00000339375
MAQAVWSRLGRILWLACLLPWAPAGVAAGLYELNLTTDSPATTGAVVTISASLVAKDNGS
LALPADAHLYRFHWIHTPLVLTGKMEKGLSSTIRVVGHVPGEFPVSVWVTAADCWMCQPV
ARGFVVLPITEFLVGDLVVTQNTSLPWPSSYLTKTVLKVSFLLHDPSNFLKTALFLYSWD
FGDGTQMVTEDSVVYYNYSIIGTFTVKLKVVAEWEEVEPDATRAVKQKTGDFSASLKLQE
TLRGIQVLGPTLIQTFQKMTVTLNFLGSPPLTVCWRLKPECLPLEEGECHPVSVASTAYN
LTHTFRDPGDYCFSIRAENIISKTHQYHKIQVWPSRIQPAVFAFPCATLITVMLAFIMYM
TLRNATQQKDMVENPEPPSGVRCCCQMCCGPFLLETPSEYLEIVRENHGLLPPLYKSVKT
YTV*
>ENSG00000101638|ENST00000315087
MRYADPSANRDLLGSRTLLFIFICAFALVTLLQQILYGRNYIKRYFEFYEGPFEYNSTRC
LELRHEILEVKVLSMVKQSELFDRWKSLQMCKWAMNISEANQFKSTLSRCCNAPAFLFTT
QKNTPLGTKLKYEVDTSGIYHINQEIFRMFPKDMPYYRSQFKKCAVVGNGGILKNSRCGR
EINSADFVFRCNLPPISEKYTMDVGVKTDVVTVNPSIITERFHKLEKWRRPFYRVLQVYE
NASVLLPAFYNTRNTDVSIRVKYVLDDFESPQAVYYFHPQYLVNVSRYWLSLGVRAKRIS
TGLILVTAALELCEEVHLFGFWAFPMNPSGLYITHHYYDNVKPRPGFHAMPSEIFNFLHL
HSRGILRVHTGTCSCC*
>ENSG00000168282|ENST00000305386
MRFRIYKRKVLILTLVVAACGFVLWSSNGRQRKNEALAPPLLDAEPARGAGGRGGDHPSV
AVGIRRVSNVSAASLVPAVPQPEADNLTLRYRSLVYQLNFDQTLRNVDKAGTWAPRELVL
VVQVHNRPEYLRLLLDSLRKAQGIDNVLVIFSHDFWSTEINQLIAGVNFCPVLQVFFPFS
IQLYPNEFPGSDPRDCPRDLPKNAALKLGCINAEYPDSFGHYREAKFSQTKHHWWWKLHF
VWERVKILRDYAGLILFLEEDHYLAPDFYHVFKKMWKLKQQECPECDVLSLGTYSASRSF

YGMADKVDVKTWKSTEHNMGLALTRNAYQKLIECTDTFCTYDDYNWDWTLQYLTVSCLPK
FWKVLVPQIPRIFHAGDCGMHHKKTCRPSTQSAQIESLLNNNKQYMFPETLTISEKFTVV
AISPPRKNGGWGDIRDHELCKSYRRLQ*
>ENSG00000128294|ENST00000338754
MRLSVRRVLLAAGCALVLVLAVQLGQQVLECRAVLAGLRSPRGAMRPEQEELVMVGTNHV
EYRYGKAMPLIFVGGVPRSGTTLMRAMLDAHPEVRCGEETRIIPRVLAMRQAWSKSGREK
LRLDEAGVTDEVLDAAMQAFILEVIAKHGEPARVLCNKDPFTLKSSVYLSRLFPNSKFLL
MVRDGRASVHSMITRKVTIAGFDLSSYRDCLTKWNKAIEVMYAQCMEVGKEKCLPVYYEQ
LVLHPRRSLKLILDFLGIAWSDAVLHHEDLIGKPGGVSLSKIERSTDQVIKPVNLEALSK
WTGHIPGDVVRDMAQIAPMLAQLGYDPYANPPNYGNPDPFVINNTQRVLKGDYKTPANLK
GYFQVNQNSTSSHLGSS*
>ENSG00000257923|ENST00000292538
MAANVGSMFQYWKRFDLQQLQRELDATATVLANRQDESEQSRKRLIEQSREFKKNTPEDL
RKQVAPLLKSFQGEIDALSKRSKEAEAAFLNVYKRLIDVPDPVPALDLGQQLQLKVQRLH
DIETENQKLRETLEEYNKEFAEVKNQEVTIKALKEKIREYEQTLKNQAETIALEKEQKLQ
NDFAEKERKLQETQMSTTSKLEEAEHKVQSLQTALEKTRTELFDLKTKYDEETTAKADEI
EMIMTDLERANQRAEVAQREAETLREQLSSANHSLQLASQIQKAPDVEQAIEVLTRSSLE
VELAAKEREIAQLVEDVQRLQASLTKLRENSASQISQLEQQLSAKNSTLKQLEEKLKGQA
DYEEVKKELNILKSMEFAPSEGAGTQDAAKPLEVLLLEKNRSLQSENAALRISNSDLSGR
CAELQVRITEAVATATEQRELIARLEQDLSIIQSIQRPDAEGAAEHRLEKIPEPIKEATA
LFYGPAAPASGALPEGQVDSLLSIISSQRERFRARNQELEAENRLAQHTLQALQSELDSL
RADNIKLFEKIKFLQSYPGRGSGSDDTELRYSSQYEERLDPFSSFSKRERQRKYLSLSPW
DKATLSMGRLVLSNKMARTIGFFYTLFLHCLVFLVLYKLAWSESMERDCATFCAKKFADH
LHKFHENDNGAAAGDLWQ*
>ENSG00000078328|ENST00000311745
MLASQGVLLHPYGVPMIVPAAPYLPGLIQGNQEAAAAPDTMAQPYASAQFAPPQNGIPAE
YTAPHPHPAPEYTGQTTVPEHTLNLYPPAQTHSEQSPADTSAQTVSGTATQTDDAAPTDG
QPQTQPSENTENKSQPKRLHVSNIPFRFRDPDLRQMFGQFGKILDVEIIFNERGSKGFGF
VTFENSADADRAREKLHGTVVEGRKIEVNNATARVMTNKKTVNPYTNGWKLNPVVGAVYS
PEFYAGTVLLCQANQEGSSMYSAPSSLVYTSAMPGFPYPAATAAAAYRGAHLRGRGRTVY
NTFRAAAPPPPIPAYGGVVYQEPVYGNKLLQGGYAAYRYAQPTPATAAAYSDSYGRVYAA
DPYHHALAPAPTYGVGAMNAFAPLTDAKTRSHADDVGLVLSSLQASIYRGGYNRFAPY*
>ENSG00000172270|ENST00000346916
MKQSDASPQERVDSDDQWGEYSCVFLPEPMGTANIQLHGPPRVKAVKSSEHINEGETAML
VCKSESVPPVTDWAWYKITDSEDKALMNGSESRFFVSSSQGRSELHIENLNMEADPGQYR
CNGTSSKGSDQAIITLRVRSHLAALWPFLGIVAEVLVLVTIIFIYEKRRKPEDVLDDDDA
GSAPLKSSGQHQNDKGKNVRQRNSS*
>ENSG00000183087|ENST00000327773
MAPSLSPGPAALRRAPQLLLLLLAAECALAALLPAREATQFLRPRQRRAFQVFEEAKQGH
LERECVEELCSREEAREVFENDPETDYFYPRYLDCINKYGSPYTKNSGFATCVQNLPDQC
TPNPCDRKGTQACQDLMGNFFCLCKAGWGGRLCDKDVNECSQENGGCLQICHNKPGSFHC
SCHSGFELSSDGRTCQDIDECADSEACGEARCKNLPGSYSCLCDEGFAYSSQEKACRDVD
ECLQGRCEQVCVNSPGSYTCHCDGRGGLKLSQDMDTCEDILPCVPFSVAKSVKSLYLGRM
FSGTPVIRLRFKRLQPTRLVAEFDFRTFDPEGILLFAGGHQDSTWIVLALRAGRLELQLR

YNGVGRVTSSGPVINHGMWQTISVEELARNLVIKVNRDAVMKIAVAGDLFQPERGLYHLN
LTVGGIPFHEKDLVQPINPRLDGCMRSWNWLNGEDTTIQETVKVNTRMQCFSVTERGSFY
PGSGFAFYSLDYMRTPLDVGTESTWEVEVVAHIRPAADTGVLFALWAPDLRAVPLSVALV
DYHSTKKLKKQLVVLAVEHTALALMEIKVCDGQEHVVTVSLRDGEATLEVDGTRGQSEVS
AAQLQERLAVLERHLRSPVLTFAGGLPDVPVTSAPVTAFYRGCMTLEVNRRLLDLDEAAY
KHSDITAHSCPPVEPAAA*
>ENSG00000182272|ENST00000329962
MPRLPVKKIRKQMKLLLLLLLLSCAAWLTYVHLGLVRQGRALRQRLGYGRDGEKLTSETD
GRGVHAAPSTQRAEDSSESREEEQAPEGRDLDMLFPGGAGRLPLNFTHQTPPWREEYKGQ
VNLHVFEDWCGGAVGHLRRNLHFPLFPHTRTTVKKLAVSPKWKNYGLRIFGFIHPARDGD
VQFSVASDDNSEFWLSLDESPAAAQLVAFVGKTGSEWTAPGEFTKFSSQVSKPRRLMASR
RYYFELLHKQDDRGSDHVEVGWRAFLPGLKFEVISSAHISLYTDESALKMDHVAHVPQSP
ASHVGGRPPQEETSADMLRPDPRDTFFLTPRMESSSLENVLEPCAYAPTYVVKDFPIARY
QGLQFVYLSFVYPNDYTRLTHMETDNKCFYRESPLYLERFGFYKYMKMDKEEGDEDEEDE
VQRRAFLFLNPDDFLDDEDEGELLDSLEPTEAAPPRSGPQSPAPAAPAQPGATLAPPTPP
RPRDGGTPRHSRALSWAARAARPLPLFLGRAPPPRPAVEQPPPKVYVTRVRPGQRASPRA
PAPRAPWPPFPGVFLHPRPLPRVQLRAPPRPPRPHGRRTGGPQATQPRPPARAQATQGGR
EGQARTLGPAAPTVDSNLSSEARPVTSFLSLSQVSGPQLPGEGEEEEEGEDDGAPGDEAA
SEDSEEAAGPALGRWREDAIDWQRTFSVGAVDFELLRSDWNDLRCNVSGNLQLPEAEAVD
VTAQYMERLNARHGGRFALLRIVNVEKRRDSARGSRFLLELELQERGGGRLRLSEYVFLR
LPGARVGDADGESPEPAPAASVRPDGRPELCRPLRLAWRQDVMVHFIVPVKNQARWVAQF
LADMAALHARTGDSRFSVVLVDFESEDMDVERALRAARLPRYQYLRRTGNFERSAGLQAG
VDAVEDASSIVFLCDLHIHFPPNILDGIRKHCVEGRLAFAPVVMRLSCGSSPRDPHGYWE
VNGFGLFGIYKSDFDRVGGMNTEEFRDQWGGEDWELLDRVLQAGLEVERLRLRNFYHHYH
SKRGMWSVRSRKGSRTGAS*
>ENSG00000129993|ENST00000327483
MPDSPAEVKTQPRSTPPSMPPPPPAASQGATRPPSFTPHTLMNGSSHSPTAINGAPCTPN
GFSNGPATSSTASLSTQHLPPACGARQLSKLKRFLTTLQQFGSDISPEIGERVRTLVLGL
VNSTLTIEEFHSKLQEATNFPLRPFVIPFLKANLPLLQRELLHCARLAKQTPAQYLAQHE
QLLLDASASSPIDSSELLLEVNENGKRRTPDRTKENGSDRDPLHPEHLSKRPCTLNPAQR
YSPSNGPPQPTPPPHYRLEDIAMAHHFRDAYRHPDPRELRERHRPLVVPGSRQEEVIDHK
LTEREWAEEWKHLNNLLNCIMDMVEKTRRSLTVLRRCQEADREELNHWARRYSDAEDTKK
GPAPAAARPRSSSAGPEGPQLDVPREFLPRTLTGYVPEDIWRKAEEAVNEVKRQAMSELQ
KAVSDAERKAHELITTERAKMERALAEAKRQASEDALTVINQQEDSSESCWNCGRKASET
CSGCNAARYCGSFCQHRDWEKHHHVCGQSLQGPTAVVADPVPGPPEAAHSLGPSLPVGAA
SPSEAGSAGPSRPGSPSPPGPLDTVPR*
>ENSG00000196352|ENST00000314754
MTVARPSVPAALPLLGELPRLLLLVLLCLPAVWGDCGLPPDVPNAQPALEGRTSFPEDTV
ITYKCEESFVKIPGEKDSVICLKGSQWSDIEEFCNRSCEVPTRLNSASLKQPYITQNYFP
VGTVVEYECRPGYRREPSLSPKLTCLQNLKWSTAVEFCKKKSCPNPGEIRNGQIDVPGGI
LFGATISFSCNTGYKLFGSTSSFCLISGSSVQWSDPLPECREIYCPAPPQIDNGIIQGER
DHYGYRQSVTYACNKGFTMIGEHSIYCTVNNDEGEWSGPPPECRGKSLTSKVPPTVQKPT
TVNVPTTEVSPTSQKTTTKTTTPNAQATRSTPVSRTTKHFHETTPNKGSGTTSGTTRLLS
GSRPVTQAGMRWCDRSSLQSRTPGFKRSFHFSLPSSWYYRAHVFHVDRFAWDASNHGLAD

LAKEELRRKYTQVYRLFLVS*
>ENSG00000177243|ENST00000318124
MRIHYLLFALLFLFLVPVPGHGGIINTLQKYYCRVRGGRCAVLSCLPKEEQIGKCSTRGR
KCCRRKK*
>ENSG00000164342|ENST00000296795
MRQTLPCIYFWGGLLPFGMLCASSTTKCTVSHEVADCSHLKLTQVPDDLPTNITVLNLTH
NQLRRLPAANFTRYSQLTSLDVGFNTISKLEPELCQKLPMLKVLNLQHNELSQLSDKTFA
FCTNLTELHLMSNSIQKIKNNPFVKQKNLITLDLSHNGLSSTKLGTQVQLENLQELLLSN
NKIQALKSEELDIFANSSLKKLELSSNQIKEFSPGCFHAIGRLFGLFLNNVQLGPSLTEK
LCLELANTSIRNLSLSNSQLSTTSNTTFLGLKWTNLTMLDLSYNNLNVVGNDSFAWLPQL
EYFFLEYNNIQHLFSHSLHGLFNVRYLNLKRSFTKQSISLASLPKIDDFSFQWLKCLEHL
NMEDNDIPGIKSNMFTGLINLKYLSLSNSFTSLRTLTNETFVSLAHSPLHILNLTKNKIS
KIESDAFSWLGHLEVLDLGLNEIGQELTGQEWRGLENIFEIYLSYNKYLQLTRNSFALVP
SLQRLMLRRVALKNVDSSPSPFQPLRNLTILDLSNNNIANINDDMLEGLEKLEILDLQHN
NLARLWKHANPGGPIYFLKGLSHLHILNLESNGFDEIPVEVFKDLFELKIIDLGLNNLNT
LPASVFNNQVSLKSLNLQKNLITSVEKKVFGPAFRNLTELDMRFNPFDCTCESIAWFVNW
INETHTNIPELSSHYLCNTPPHYHGFPVRLFDTSSCKDSAPFELFFMINTSILLIFIFIV
LLIHFEGWRISFYWNVSVHRVLGFKEIDRQTEQFEYAAYIIHAYKDKDWVWEHFSSMEKE
DQSLKFCLEERDFEAGVFELEAIVNSIKRSRKIIFVITHHLLKDPLCKRFKVHHAVQQAI
EQNLDSIILVFLEEIPDYKLNHALCLRRGMFKSHCILNWPVQKERIGAFRHKLQVALGSK
NSVH*
>ENSG00000176720|ENST00000318407
MEVLRRSSVFAAEIMDAFDRSPTDKELVAQAKALGREYVHARLLRAGLSWSAPERAAPVP
GRLAEVCAVLLRLGDELEMIRPSVYRNVARQLHISLQSEPVVTDAFLAVAGHIFSAGITW
GKVVSLYAVAAGLAVDCVRQAQPAMVHALVDCLGEFVRKTLATWLRRRGGWTDVLKCVVS
TDPGLRSHWLVAALCSFGRFLKAAFFVLLPER*
>ENSG00000126091|ENST00000332628
XSAWKLHLLQWEEDSKYDRLGFLLNLDSKLFSKPAPMFLDDSFRKWARIREFVPPFGIKG
QDNLIKAILSVTKEYRLTPALDSLRCRRCIIVGNGGVLANKSLGSRIDDYDIVVRLNSAP
VKGFEKDVGSKTTLRITYPEGAMQRPEQYERDSLFVLAGFKWQDFKWLKYIVYKERVSAS
DGFWKSVATRVPKEPPEIRILNPYFIQEAAFTLIGLPFNNGLMGRGNIPTLGSVAVTMAL
HGCDEVAVAGFGYDMSTPNAPLHYYETVRMAAIKESWTHNIQREKEFLRKLVKARVITDL
SSGI*
>ENSG00000147509|ENST00000344277
MPQLSQDNQECLQKHFSRPSIWTQFLPLFRAQRYNTDIHQITENEGDLRAVPDIKQMGSE
RMEMRKRQMPAAQDTPGAAPGGQPGAGSRGSNACCFCWCCCCSCSCLTVRNQEDQRPTIAS
HELRADLPTWEESPAPTLEEVNAWAQSFDKLMVTPAGRNAFREFLRTEFSEENMLFWMAC
EELKKEANKNIIEEKARIIYEDYISILSPKEVSLDSRVREVINRNMVEPSQHIFDDAQLQ
IYTLMHRDSYPRFMNSAVYKDLLQSLSEKSIEA*
>ENSG00000160179|ENST00000347800
MIMRLPQPHGTNASSYSAEMTEPKSVCVSVDEVVSSNMEATETDLLNGHLKKVDNNLTEA
QRFSSLPRRAAVNIEFRDLSYSVPEGPWWRKKGYKTLLKGISGKFNSGELVAIMGPSGAG
KSTLMNILAGYRETGMKGAVLINGLPRDLRCFRKVSCYIMQDDMLLPHLTVQEAMMVSAH
LKLQEKDEGRREMVKEILTALGLLSCANTRTGSLSGGQRKRLAIALELVNNPPVMFFDEP

TSGLDSASCFQVVSLMKGLAQGGRSIICTIHQPSAKLFELFDQLYVLSQGQCVYRGKVCN
LVPYLRDLGLNCPTYHNPADFVMEVASGEYGDQNSRLVRAVREGMCDSDHKRDLGGDAEV
NPFLWHRPSEEDSSSMEGCHSFSASCLTQFCILFKRTFLSIMRDSVLTHLRITSHIGIGL
LIGLLYLGIGNEAKKVLSNSGFLFFSMLFLMFAALMPTVLTFPLEMGVFLREHLNYWYSL
KAYYLAKTMADVPFQIMFPVAYCSIVYWMTSQPSDAVRFVLFAALGTMTSLVAQSLGLLI
GAASTSLQVATFVGPVTAIPVLLFSGFFVSFDTIPTYLQWMSYISYVRYGFEGVILSIYG
LDREDLHCDIDETCHFQKSEAILRELDVENAKLYLDFIVLGIFFISLRLIAYFVLRYKIR
AER*
>ENSG00000114631|ENST00000342480
MGRLLRAARLPPLLSPLLLLLVGGAFLGACVAGSDEPGPEGLTSTSLLDLLLPTGLEPLD
SEEPSETMGLGAGLGAPGSGFPSEENEESRILQPPQYFWEEEEELNDSSLDLGPTADYVF
PDLTEKAGSIEDTSQAQELPNLPSPLPKMNLVEPPWHMPPREEEEEEEEEEEREKEEVEK
QEEEEEEELLPVNGSQEEAKPQVRDFSLTSSSQTPGATKSRHEDSGDQASSGVEVESSMG
PSLLLPSVTPTTVTPGDQDSTSQEAEATVLPAAGLGVEFEAPQEASEEATAGAAGLSGQH
EEVPALPSFPQTTAPSGAEHPDEDPLGSRTSASSPLAPGDMELTPSSATLGQEDLNQQLL
EGQAAEAQSRIPWDSTQVICKDWSNLAGKNYIILNMTENIDCEVFRQHRGPQLLALVEEV
LPRHGSGHHGAWHISLSKPSEKEQHLLMTLVGEQGVVPTQDVLSMLGDIRRSLEEIGIQN
YSTTSSCQARASQVRSDYGTLFVVLVVIGAICIIIIALGLLYNCWQRRLPKLKHVSHGEE
LRFVENGCHDNPTLDVASDSQSEMQEKHPSLNGGGALNGPGSWGALMGGKRDPEDSDVFE
EDTHL*
>ENSG00000206306|ENST00000328980
MVCLRLPGGSCMAVLTVTLMVLSSPLALAGDTRPRFLEYSTSECHFFNGTERVRYLDRYF
HNQEENVRFDSDVGEFRAVTELGRPDAEYWNSQKDLLEQKRGRVDNYCRHNYGVVESFTV
QRRVHPKVTVYPSKTQPLQHHNLLVCSVSGFYPGSIEVRWFRNGQEEKTGVVSTGLIHNG
DWTFQTLVMLETVPRSGEVYTCQVEHPSVTSPLTVEWRARSESAQSKMLSGVGGFVLGLL
FLGAGLFIYFRNQKGHSGLQPRGFLS*
>ENSG00000172461|ENST00000302103
MTSTSKGILRPFLIVCIILGCFMACLLIYIKPTNSWIFSPMESASSVLKMKNFFSTKTDY
FNETTILVWVWPFGQTFDLTSCQAMFNIQGCHLTTDRSLYNKSHAVLIHHRDISWDLTNL
PQQARPPFQKWIWMNLESPTHTPQKSGIEHLFNLTLTYRRDSDIQVPYGFLTVSTNPFVF
EVPSKEKLVCWVVSNWNPEHARVKYYNELSKSIEIHTYGQAFGEYVNDKNLIPTISTCKF
YLSFENSIHKDYITEKLYNAFLAGSVPVVLGPSRENYENYIPADSFIHVEDYNSPSELAK
YLKEVDKNNKLYLSYFNWRKDFTVNLPRFWESHACLACDHVKRHQEYKSVGNLEKWFWN*
>ENSG00000105438|ENST00000330720
MNLFRFLGDLSHLLAIILLLLKIWKSRSCAGISGKSQVLFAVVFTARYLDLFTNYISLYN
TCMKVVYIACSFTTVWLIYSKFKATYDGNHDTFRVEFLVVPTAILAFLVNHDFTPLEILW
TFSIYLESVAILPQLFMVSKTGEAETITSHYLFALGVYRTLYLFNWIWRYHFEGFFDLIA
IVAGLVQTVLYCDFFYLYITKVLKGKKLSLPA*
>ENSG00000249853|ENST00000312719
MLFKQQAWLRQKLLVLGSLAVGSLLYLVARVGSLDRLQPICPIEGRLGGARTQAEFPLRA
LQFKRGLLHEFRKGNASKEQVRLHDLVQQLPKAIIIGVRKGGTRALLEMLNLHPAVVKAS
QEIHFFDNDENYGKGIEWYRKKMPFSYPQQITIEKSPAYFITEEVPERIYKMNSSIKLLI
IVREPTTRAISDYTQVLEGKERKNKTYYKFEKLAIDPNTCEVNTKYKAVRTSIYTKHLER
WLKYFPIEQFHVVDGDRLITEPLPELQLVEKFLNLPPRISQYNLYFNATRGFYCLRFNII

FNKCLAGSKGRIHPEVDPSVITKLRKFFHPFNQKFYQITGRTLNWP*
>ENSG00000104490|ENST00000311028
MGKQNSKLRPEVMQDLLESTDFTEHEIQEWYKGFLRDCPSGHLSMEEFKKIYGNFFPYGD
ASKFAEHVFRTFDANGDGTIDFREFIIALSVTSRGKLEQKLKWAFSMYDLDGNGYISKAE
MLEIVQAIYKMVSSVMKMPEDESTPEKRTEKIFRQMDTNRDGKLSLEEFIRGAKSDPSIV
RLLQCDPSSAGQF*
>ENSG00000169116|ENST00000307428
MVYKTLFALCILTAGWRVQSLPTSAPLSVSLPTNIVPPTTIWTSSPQNTDADTASPSNGT
HNNSVLPVTASAPTSLLPKNISIESREEEITSPGSNWEGTNTDPSPSGFSSTSGGVHLTT
TLEEHSSGTPEAGVAATLSQSAAEPPTLISPQAPASSPSSLSTSPPEVFSASVTTNHSST
VTSTQPTGAPTAPESPTEESSSDHTPTSHATAEPVPQEKTPPTTVSGKVMCELIDMETTT
TFPRVIMQEVEHALSSGSIAAITVTVIAVVLLVFGVAAYLKIRHSSYGRLLDDHDYGSWG
NYNNPLYDDS*
>ENSG00000184432|ENST00000333188
MPLRLDIKRKLTARSDRVKSVDLHPTEPWMLASLYNGSVCVWNHETQTLVKTFEVCDLPV
RAAKFVARKNWVVTGADDMQIRVFNYNTLERVHMFEAHSDYIRCIAVHPTQPFILTSSDD
MLIKLWDWDKKWSCSQVFEGHTHYVMQIVINPKDNNQFASASLDRTIKVWQLGSSSPNFT
LEGHEKGVNCIDYYSGGDKPYLISGADDRLVKIWDYQNKTCVQTLEGHAQNVSCASFHPE
LPIIITGSEDGTVRIWHSSTYRLESTLNYGMERVWCVASLRGSNNVALGYDEGSIIVKLG
REEPAMSMDANGKIIWAKHSEVQQANLKAMGDAEIKDGERLPLAVKDMGSCEIYPQTIQH
NPNGRFVVVCGDGEYIIYTAMALRNKSFGSAQEFAWAHDSSEYAIRESNSIVKIFKNFKE
KKSFKPDFGAESIYGGFLLGVRSVNGLAFYDWDNTELIRRIEIQPKHIFWSDSGELVCIA
TEESFFILKYLSEKVLAAQETHEGVTEDGIEDAFEVLGEIQEIVKTGLWVGDCFIYTSSV
NRLNYYVGGEIVTIAHLDRTMYLLGYIPKDNRLYLGDKELNIISYSLLVSVLEYQTAVMR
RDFSMADKVLPTIPKEQRTRVAHFLEKQGFKQQALTVSTDPEHRFELALQLGELKIAYQL
AVEAESEQKWKQLAELAISKCQFGLAQECLHHAQDYGGLLLLATASGNANMVNKLAEGAE
RDGKNNVAFMSYFLQGKVDACLELLIRTGRLPEAAFLARTYLPSQVSRVVKLWRENLSKV
NQKAAESLADPTEYENLFPGLKEAFVVEEWVKETHADLWPAKQYPLVTPNEERNVMEEGK
DFQPSRSTAQQELDGKPASPTPVIVASHTANKEEKSLLELEVDLDNLELEDIDTTDINLD
EDILDD*
>ENSG00000181856|ENST00000317370
MPSGFQQIGSEDGEPPQQRVTGTLVLAVFSAVLGSLQFGYNIGVINAPQKVIEQSYNETW
LGRQGPEGPSSIPPGTLTTLWALSVAIFSVGGMISSFLIGIISQWLGRKRAMLVNNVLAV
LGGSLMGLANAAASYEMLILGRFLIGAYSGLTSGLVPMYVGEIAPTHLRGALGTLNQLAI
VIGILIAQVLGLESLLGTASLWPLLLGLTVLPALLQLVLLPFCPESPRYLYIIQNLEGPA
RKSLKRLTGWADVSGVLAELKDEKRKLERERPLSLLQLLGSRTHRQPLIIAVVLQLSQQL
SGINAVFYYSTSIFETAGVGQPAYATIGAGVVNTVFTLVSVLLVERAGRRTLHLLGLAGM
CGCAILMTVALLLLERVPAMSYVSIVAIFGFVAFFEIGPGPIPWFIVAELFSQGPRPAAM
AVAGFSNWTSNFIIGMGFQYVAEAMGPYVFLLFAVLLLGFFIFTFLRVPETRGRTFDQIS
AAFHRTPSLLEQEVKPSTELEYLGPDEND*
>ENSG00000140835|ENST00000338482
MLLPKKMKLLLFLVSQMAILALFFHMYSHNISSLSMKAQPERMHVLVLSSWRSGSSFVGQ
LFGQHPDVFYLMEPAWHVWMTFKQSTAWMLHMAVRDLIRAVFLCDMSVFDAYMEPGPRRQ
SSLFQWENSRALCSAPACDIIPQDEIIPRAHCRLLCSQQPFEVVEKACRSYSHVVLKEVR

FFNLQSLYPLLKDPSLNLHIVHLVRDPRAVFRSRERTKGDLMIDSRIVMGQHEQKLKKED
QPYYVMQVICQSQLEIYKTIQSLPKALQERYLLVRYEDLARAPVAQTSRMYEFVGLEFLP
HLQTWVHNITRGKGMGDHAFHTNARDALNVSQAWRWSLPYEKVSRLQKACGDAMNLLGYR
HVRSEQEQRNLLLDLLSTWTVPEQIH*
>ENSG00000154305|ENST00000340535
MDSVPATVPSIAATPGDPELVGPLSVLYAAFIAKLLELVATLPDDVQPGPDFYGLPWKPV
FITAFLGIASFAIFLWRTVLVVKDRVYQVTEQQISEKLKTIMKENTELVQKLSNYEQKIK
ESKKHVQETRKQNMILSDEAIKYKDKIKTLEKNQEILDDTAKNLRVMLESEREQNVKNQD
LISENKKSIEKLKDVISMNASEFSEVQIALNEAKLSEEKVKSECHRVQEENARLKKKKEQ
LQQEIEDWSKLHAELSEQIKSFEKSQKDLEVALTHKDDNINALTNCITQLNLLECESESE
GQNKGGNDSDELANGEVGGDRNEKMKNQIKQMMDVSRTQTAISVVEEDLKLLQLKLRASV
STKCNLEDQVKKLEDDRNSLQAAKAGLEDECKTLRQKVEILNELYQQKEMALQKKLSQEE
YERQEREHRLSAADEKAVSAAEEVKTYKRRIEEMEDELQKTERSFKNQIATHEKKAHENW
LKARAAERAIAEEKREAANLRHKLLELTQKMAMLQEEPVIVKPMPGKPNTQNPPRRGPLS
QNGSFGPSPVSGGECSPPLTVEPPVRPLSATLNRRDMPRSEFGSVDGPLPHPRWSAEASG
KPSPSDPGSGTATMMNSSSRGSSPTRVLDEGKVNMAPKGPPPFPGVPLMSTPMGGPVPPP
IRYGPPPQLCGPFGPRPLPPPFGPGMRPPLGLREFAPGVPPGRRDLPLHPRGFLPGHAPF
RPLGSLGPREYFIPGTRLPPPTHGPQEYPPPPAVRDLLPSGSRDEPPPASQSTSQDCSQA
LKQSP*
>ENSG00000167461|ENST00000300935
MAKTYDYLFKLLLIGDSGVGKTCVLFRFSEDAFNSTFISTIGIDFKIRTIELDGKRIKLQ
IWDTAGQERFRTITTAYYRGAMGIMLVYDITNEKSFDNIRNWIRNIEEHASADVEKMILG
NKCDVNDKRQVSKERGEKLALDYGIKFMETSAKANINVENAFFTLARDIKAKMDKKLEGN
SPQGSNQGVKITPDQQKRSSFFRCVLL*
>ENSG00000168818|ENST00000306200
MAVDITLLFRASVKTVKTRNKALGVAVGGGVDGSRDELFRRSPRPKGDFSSRAREVISHI
GKLRDFLLEHRKDYINAYSHTMSEYGRMTDTERDQIDQDAQIFMRTCSEAIQQLRTEAHK
EIHSQQVKEHRTAVLDFIEDYLKRVCKLYSEQRAIRVKRVVDKKRLSKLEPEPNTKTRES
TSSEKVSQSPSKDSEENPATEERPEKILAETQPELGTWGDGKGEDELSPEEIQMFEQENQ
RLIGEMNSLFDEVRQIEGRVVEISRLQEIFTEKVLQQEAEIDSIHQLVVGATENIKEGNE
DIREAIKNNAGFRVWILFFLVMCSFSLLFLDWYDS*
>ENSG00000188783|ENST00000343110
MRSPLCWLLPLLILASVAQGGQPTRRPRPGTGPGRRPRPRPRPTPSFPQPDEPAEPTDLPP
PLPPGPPSIFPDCPRECYCPPDFPSALYCDSRNLRKVPVIPPRIHYLYLQNNFITELPVE
SFQNATGLRWINLDNNRIRKIDQRVLEKLPGLVFLYMEKNQLEEVPSALPRNLEQLRLSQ
NHISRIPPGVFSKLENLLLLDLQHNRLSDGVFKPDTFHGLKNLMQLNLAHNILRKMPPRV
PTAIHQLYLDSNKIETIPNGYFKSFPNLAFIRLNYNKLTDRGLPKNSFNISNLLVLHLSH
NRISSVPAINNRLEHLYLNNNSIEKINGTQICPNDLVAFHDFSSDLENVPHLRYLRLDGN
YLKPPIPLDLMMCFRLLQSVVI*
>ENSG00000142920|ENST00000294517
MAGYLSESDFVMVEEGFSTRDLLKELTLGASQATTDEVAAFFVADLGAIVRKHFCFLKCL
PRVRPFYAVKCNSSPGVLKVLAQLGLGFSCANKAEMELVQHIGIPASKIICANPCKQIAQ
IKYAAKHGIQLLSFDNEMELAKVVKSHPSAKMVLCIATDDSHSLSCLSLKFGVSLKSCRH
LLENAKKHHVEVVGVSFHIGSGCPDPQAYAQSIADARLVFEMGTELGHKMHVLDLGGGFP

GTEGAKVRFEEIASVINSALDLYFPEGCGVDIFAELGRYYVTSAFTVAVSIIAKKEVLLD
QPGREEENGSTSKTIVYHLDEGVYGIFNSVLFDNICPTPILQKKPSTEQPLYSSSLWGPA
VDGCDCVAEGLWLPQLHVGDWLVFDNMGAYTVGMGSPFWGTQACHITYAMSRVAWEALRR
QLMAAEQEDDVEGVCKPLSCGWEITDTLCVGPVFTPASIM*
>ENSG00000101310|ENST00000336714
MATYLEFIQQNEERDGVRFSWNVWPSSRLEATRMVVPLACLLTPLKERPDLPPVQYEPVL
CSRPTCKAVLNPLCQVDYRAKLWACNFCFQRNQFPPAYGGISEVNQPAELMPQFSTIEYV
IQRGAQSPLIFLYVVDTCLEEDDLQALKESLQMSLSLLPPDALVGLITFGRMVQVHELSC
EGISKSYVFRGTKDLTAKQIQDMLGLTKPAMPMQQARPAQPQEHPFASSRFLQPVHKIDM
NLTDLLGELQRDPWPVTQGKRPLRSTGVALSIAVGLLEGTFPNTGARIMLFTGGPPTQGP
GMVVGDELKIPIRSWHDIEKDNARFMKKATKHYEMLANRTAANGHCIDIYACALDQTGLL
EMKCCANLTGGYMVMGDSFNTSLFKQTFQRIFTKDFNGDFRMAFGATLDVKTSRELKIAG
AIGPCVSLNVKGPCVSENELGVGGTSQWKICGLDPTSTLGIYFEVVNQHNTPIPQGGRGA
IQFVTHYQHSSTQRRIRVTTIARNWADVQSQLRHIEAAFDQEAAAVLMARLGVFRAESEE
GPDVLRWLDRQLIRLCQKFGQYNKEDPTSFRLSDSFSLYPQFMFHLRRSPFLQVFNNSPD
ESSYYRHHFARQDLTQSLIMIQPILYSYSFHGPPEPVLLDSSSILADRILLMDTFFQIVI
YLGETIAQWRKAGYQDMPEYENFKHLLQAPLDDAQEILQARFPMPRYINTEHGGSQARFL
LSKVNPSQTHNNLYAWGQETGAPILTDDVSLQVFMDHLKKLAVSSAC*
>ENSG00000189067|ENST00000339430
MSVPGPYQAATGPSSAPSAPPSYEETVAVNSYYPTPPAPMPGPTTGLVTGPDGKGMNPPS
YYTQPAPIPNNNPITVQTVYVQHPITFLDRPIQMCCPSCNKMIVSQLSYNAGALTWLSCG
SLCLLGCIAGCCFIPFCVDALQDVDHYCPNCRALLGTYKRL*
>ENSG00000164822|ENST00000297436
MRTLTILTAVLLVALQAKAEPLQAEDDPLQAKAYEADAQEQRGANDQDFAVSFAEDASSS
LRALGSTRAFTCHCRRSCYSTEYSYGTCTVMGINHRFCCL*
>ENSG00000204482|ENST00000303757
MLSRNDDICIYGGLGLGGLLLLAVVLLSACLCWLHRRGPGLLRAGTPLCISAEAASAQQ*
>ENSG00000180834|ENST00000318631
MAWPCISRLCCLARRWNQLDRSDVAVPLTLHGYSDLDSEEPGTGGAASRRGQPPAGARDS
GRDVPLTQYQRDFGLWTTPAGPKDPPPGRGPGAGGRRGKSSAQSSAPPAPGARGVYVLPI
GDADAAAAVTTSYRQEFQAWTGVKPSRSTKTKPARVITTHTSGWDSSPGAGFQVPEVRKK
FTPNPSAIFQASAPRILNV*
>ENSG00000101558|ENST00000340541
MASASGAMAKHEQILVLDPPTDLKFKGPFTDVVTTNLKLRNPSDRKVCFKVKTTAPRRYC
VRPNSGIIDPGSTVTVSVMLQPFDYDPNEKSKHKFMVQTIFAPPNTSDMEAVWKEAKPDE
LMDSKLRCVFEMPNENDKLGITPPGNAPTVTSMSSINNTVATPASYHTKDDPRGLSVLKQ
EKQKNDMEPSKAVPLNASKQDGPMPKPHSVSLNDTETRKLMEECKRLQGEMMKLSEENRH
LRDEGLRLRKVAHSDKPGSTSTASFRDNVTSPLPSLLVVIAAIFIGFFLGKFIL*
>ENSG00000166676|ENST00000299866
MKQALVDDTEDVSLDFGNEEELAFRKAKIRHPLATFFHLFFRVSAIVTYVSCDWFSKSFV
GCFVMVLLLLSLDFWSVKNVTGRLLVGLRWWNQIDEDGKSHWIFEARKVSPNSIAATEAE
ARIFWLGLIICPMIWIVFFFSTLFSLKLKWLALVVAGISLQAANLYGYILCKMGGNSDIG
KVTASFLSQTVFQTACPGDFQKPGLEGLEIHQH*
>ENSG00000166405|ENST00000309737

MAYSTVQRVALASGLVLALSLLLPKAFLSRGKRQEPPPTPEGKLGRFPPMMHHHQAPSDG
QTPGARFQRSHLAEAFAKAKGSGGGAGGGGSGRGLMGQIIPIYGFGIFLYILYILFKLSK
GKTTAEDGKCYTAMPGNTHRKITSFELAQLQEKLKETEAAMEKLINRVGPNGESRAQTVT
SDQEKRLLHQLREITRVMKEGKFIDRFSPEKEAEEAPYMEDWEGYPEETYPIYDLSDCIK
RRQETILVDYPDPKELSAEEIAERMGMIEEEESDHLGWESLPTDPRAQEDNSVTSCDPKP
ETCSCCFHEDEDPAVLAENAGFSADSYPEQEETTKEEWSQDFKDEGLGISTDKAYTGSML
RKRNPQGLE*
>ENSG00000142192|ENST00000348990
MLPGLALLLLAAWTARALEVPTDGNAGLLAEPQIAMFCGRLNMHMNVQNGKWDSDPSGTK
TCIDTKEGILQYCQEVYPELQITNVVEANQPVTIQNWCKRGRKQCKTHPHFVIPYRCLVG
EFVSDALLVPDKCKFLHQERMDVCETHLHWHTVAKETCSEKSTNLHDYGMLLPCGIDKFR
GVEFVCCPLAEESDNVDSADAEEDDSDVWWGGADTDYADGSEDKVVEVAEEEEVAEVEEE
EADDDEDDEDGDEVEEEAEEPYEEATERTTSIATTTTTTTESVEEVVRVPTTAASTPDAV
DKYLETPGDENEHAHFQKAKERLEAKHRERMSQVMREWEEAERQAKNLPKADKKAVIQHF
QEKVESLEQEAANERQQLVETHMARVEAMLNDRRRLALENYITALQAVPPRPRHVFNMLK
KYVRAEQKDRQHTLKHFEHVRMVDPKKAAQIRSQVMTHLRVIYERMNQSLSLLYNVPAVA
EEIQDEVDELLQKEQNYSDDVLANMISEPRISYGNDALMPSLTETKTTVELLPVNGEFSL
DDLQPWHSFGADSVPANTENEVEPVDARPAADRGLTTRPGSGLTNIKTEEISEVKMDAEF
RHDSGYEVHHQKLVFFAEDVGSNKGAIIGLMVGGVVIATVIVITLVMLKKKQYTSIHHGV
VEVDAAVTPEERHLSKMQQNGYENPTYKFFEQMQN*
>ENSG00000177692|ENST00000314399
MNTMYVMMAQILRSHLIKATVIPNRVKMLPYFGIIRNRMMSTHKSKKKIREYYRLLNVEE
GCSADEVRESFHKLAKQYHPDSGSNTADSATFIRIEKAYRKVLSHVIEQTNASQSKGEEE
EDVEKFKYKTPQHRHYLSFEGIGFGTPTQREKHYRQFRADRAAEQVMEYQKQKLQSQYFP
DSVIVKNIRQSKQQKITQAIERLVEDLIQESMAKGDFDNLSGKGKPLKKFSDCSYIDPMT
HNLNRILIDNGYQPEWILKQKEISDTIEQLREAILVSRKKLGNPMTPTEKKQWNHVCEQF
QENIRKLNKRINDFNLIVPILTRQKVHFDAQKEIVRAQKIYETLIKTKEVTDRNPNNLDQ
GEGEKTPEIKKGFLNWMNLWKFIKIRSF*
>ENSG00000122705|ENST00000345519
MAELDPFGAPAGAPGGPALGNGVAGAGEEDPAAAFLAQQESEIAGIENDEAFAILDGGAP
GPQPHGEPPGGPDAVDGVMNGEYYQESNGPTDSYAAISQVDRLQSEPESIRKWREEQMER
LEALDANSRKQEAEWKEKAIKELEEWYARQDEQLQKTKANNRAAEEAFVNDIDESSPGTE
WERVARLCDFNPKSSKQAKDVSRMRSVLISLKQAPLVH*
>ENSG00000185090|ENST00000329006
MITGSPQMTWCPPFWTPPISTASRYGSHGAFYRYKNSMGKSLPLFYIYDSYLTSPEAWAH
LLTPNGPHSIRNTPYDGVFIALLVEEGHTHDILAAGFDGMYTYFASNGFSFGSSHQNWKA
VKNFCDANNLMFIPSVGPGYIDTSIRPWNNHNTRNRVNGKYYETALQAALTVRPEIVSIT
SFNEWHEGTQIEKAIPKKTPTRLYLDYLPHQPSLYLELTRRWAEHFIKEKEQWLM*
>ENSG00000174567|ENST00000308302
MISITEWQKIGVGITGFGIFFILFGTLLYFDSVLLAFGNLLFLTGLSLIIGLRKTFWFFF
QRHKLKGTSFLLGGVVIVLLRWPLLGMFLETYGFFSLFKGFFPVAFGFLGNVCNIPFLGA
LFRRLQGTSSMV*
>ENSG00000166507|ENST00000309979
MLQLWKVVRPARQLELHRLILLLIAFSLGSMGFLAYYVSTSPKAKEPLPLPLGDCSSGGA

AGPGPARPPVPPRPPPRPPETARTEPVVLVFVESAYSQLGQEIVAILESSRFRYSTELAPG
RGDMPTLTDNTHGRYVLVIYENLLKYVNLDAWSRELLDRYCVEYGVGIIGFFRAHEHSLL
SAQLKGFPLFLHSNLGLRDYQVNPSAPLLHLTRPSRLEPGPLPGDDWTIFQSNHSTYEPV
LLASLRPAEPAVPGPVLRRARLPTVVQDLGLHDGIQRVLFGHGLSFWLHKLIFVDAVAYL
TGKRLCLDLDRYILVDIDDIFVGKEGTRMKVADVEALLTTQNKLRTLVPNFTFNLGFSGK
FYHTGTEEEDAGDDMLLKHRKEFWWFPHMWSHMQPHLFHNRSVLADQMRLNKQFALEHGI
PTDLGYAVAPHHSGVYPIHTQLYEAWKSVWGIQVTSTEEYPHLRPARYRRGFIHNGIMVL
PRQTCGLFTHTIFYNEYPGGSRELDRSIRGGELFLTVLLNPISIFMTHLSNYGNDRLGLY
TFESLVRFLQCWTRLRLQTLPPVPLAQKYFELFPQERSPLWQNPCDDKRHKDIWSKEKTC
DRLPKFLIVGPQKTGTTAIHFFLSLHPAVTSSFPSPSTFEEIQFFNSPNYHKGIDWYMDF
FPVPSNASTDFLFEKSATYFDSEVVPRRGAALLPRAKIITVLTNPADRAYSWYQHQRAHG
DPVALNYTFYQVISASSQTPLALRSLQNRCLVPGYYSTHLQRWLTYYPSGQLLIVDGQEL
RTNPAASMESIQKFLGITPFLNYTRTLRFDDDKGFWCQGLEGGKTRCLGRSKGRRYPDMD
TESRLFLTDFFRNHNLELSKLLSRLGQPVPSWLREELQHSSLG*
>ENSG00000166557|ENST00000299705
MGSTVPRSASVLLLLLLLRRAEQPCGAELTFELPDNAKQCFHEEVEQGVKFSLDYQVITG
GHYDVDCYVEDPQGNTIYRETKKQYDSFTYRAEVKGVYQFCFSNEFSTFSHKTVYFDFQV
GDEPPILPDMGNRVTALTQMESACVTIHEALKTVIDSQTHYRLREAQDRARAEDLNSRVS
YWSVGETIALFVVSFSQVLLLKSFFTEKRPISRAVHS*
>ENSG00000166685|ENST00000299886
MATAATSPALKRLDLRDPAALFETHGAEEIRGLERQVRAEIEHKKEELRQMVGERYRDLI
EAADTIGQMRRCAVGLVDAVKATDQYCARLRQAGSAAPRPPRAQQPQQPSQEKFYSMAAQ
IKLLLEIPEKIWSSMEASQCLHATQLYLLCCHLHSLLQLDSSSSRYSPVLSRFPILIRQV
AAASHFRSTILHESKMLLKCQGVSDQAVAEALCSIMLLEESSPRQALTDFLLARKATIQK
LLNQPHHGAGIKAQICSLVELLATTLKQAHALFYTLPEGLLPDPALPCGLLFSTLETITG
QHPAGKGTGVLQEEMKLCSWFKHLPASIVEFQPTLRTLAHPISQEYLKDTLQKWIHMCNE
DIKNGITNLLMYVKSMKGLAGIRDAMWELLTNESTNHSWDVLCRRLLEKPLLFWEDMMQQ
LFLDRLQTLTKEGFDSISSSSKELLVSALQELESSTSNSPSNKHIHFEYNMSLFLWSESP
NDLPSDAAWVSVANRGQFASSGLSMKAQAISPCVQNFCSALDSKLKVKLDDLLAYLPSDD
SSLPKDVSPTQAKSSAFDRYADAGTVQEMLRTQSVACIKHIVDCIRAELQSIEEGVQGQQ
DALNSAKLHSVLFMARLCQSLGELCPHLKQCILGKSESSEKPAREFRALRKQGKVKTQEI
IPTQAKWQEVKEVLLQQSVMGYQVWSSAVVKVLIHGFTQSLLLDDAGSVLATATSWDELE
IQEEAESGSSVTSKIRLPAQPSWYVQSFLFSLCQEINRVGGHALPKVTLQEMLKSCMVQV
VAAYEKLSEEKQIKKEGAFPVTQNRALQLLYDLRYLNIVLTAKGDEVKSGRSKPDSRIEK
VTDHLEALIDPFDLDVFTPHLNSNLHRLVQRTSVLFGLVTGTENQLAPRSSTFNSQEPHN
ILPLASSQIRFGLLPLSMTSTRKAKSTRNIETKAQVVPPARSTAGDPTVPGSLFRQLVSE
EDNTSAPSLFKLGWLSSMTK*
>ENSG00000145113|ENST00000346145
MKGARWRRVPWVSLSCLCLCLLPHVVPGMTTPSLKTDGGRRTATSPPPTTSQTIISTIPS
TAMHTRSTAAPIPILPERGVSLFPYGAGAGDLEFVRRTVDFTSPLFKPATGFPLGSSLRD
SLYFTDNGQIIFPESDYQIFSYPNPLPTGFTGRDPVALVAPFWDDADFSTGRGTTFYQEY
ETFYGEHSLLVQQAESWIRKMTNNGGYKARWALKVTWVNAHAYPAQWTLGSNTYQAILST
DGSRSYALFLYQSGGMQWDVAQRSGNPVLMGFSSGDGYFENSPLMSQPVWERYRPDRFLN
SNSGLQGLQFYRLHREERPNYRLECLQWLKSQPRWPSWGWNQVSCPCSWQQGRRDLRFQP

VSIGRWGLGSRQLCSFTSWRGGVCCSYGPWGEFREGWHVQRPWQLAQELEPQSWCCRWND
KPYLCALYQQRRPHVGCATYRPPQPAWMFGDPHITTLDGVSYTFNGLGDFLLVGAQDGNS
SFLLQGRTAQTGSAQATNFIAFAAQYRSSSLGPVTVQWLLEPHDAIRVLLDNQTVTFQPD
HEDGGGQETFNATGVLLSRNGSEVSASFDGWATVSVIALSNILHASASLPPEYQNRTEGL
LGVWNNNPEDDFRMPNGSTIPPGSPEEMLFHFGMTWQINGTGLLGKRNDQLPSNFTPVFY
SQLQKNSSWAEHLISNCDGDSSCIYDTLALRNASIGLHTREVSKNYEQANATLNQYPPSI
NGGRVIEAYKGQTTLIQYTSNAEDANFTLRDSCTDLELFENGTLLWTPKSLEPFTLEILA
RSAKIGLASALQPRTVVCHCNAESQCLYNQTSRVGNSSLEVAGCKCDGGTFGRYCEGSED
ACEEPCFPSVHCVPGKGCEACPPNLTGDGRHCAALGSSFLCQNQSCPVNYCYNQGHCYIS
QTLGCQPMCTCPPAFTDSRCFLAGNNFSPTVNLELPLRVIQLLLSEEENASMAEVNASVA
YRLGTLDMRAFLRNSQVERIDSAAPASGSPIQHWMVISEFQYRPRGPVIDFLNNQLLAAV
VEAFLYHVPRRSEEPRNDVVFQPISGEDVRDVTALNVSTLKAYFRCDGYKGYDLVYSPQS
GFTCVSPCSRGYCDHGGQCQHLPSGPRCSCVSFSIYTAWGEHCEHLSMKLDAFFGIFFGA
LGGLLLLGVGTFVVLRFWGCSGARFSYFLNSAEALP*
>ENSG00000104886|ENST00000326631
MRYNEKELQALSRQPAEMAAELGMRGPKKGSVLKRRLVKLVVNFLFYFRTDEAEPVGALL
LERCRVVREEPGTFSISFIEDPERKYHFECSSEEQCQEWMEALRRASYEFMRRSLIFYRN
EIRKVTGKDPLEQFGISEEARFQLSGLQA*
>ENSG00000185499|ENST00000343256
MTPGTQSPFFLLLLLTVLTVVTGSGHASSTPGGEKETSATQRSSVPSSTEKNAIPAPTTT
KSCRETFLKCFCRFINKGVFWASPILSSVSDVPFPFSAQSGAGVPGWGIALLVLVCVLVA
LAIVYLIALAVCQCRRKNYGQLDIFPARDTYHPMSEYPTYHTHGRYVPPSSTDRSPYEKV
SAGNGGSSLSYTNPAVAATSANL*
>ENSG00000158850|ENST00000319769
MLRRLLERPCTLALLVGSQLAVMMYLSLGGFRSLSALFGRDQGPTFDYSHPRDVYSNLSH
LPGAPGGPPAPQGLPYCPERSPLLVGPVSVSFSPVPSLAEIVERNPRVEPGGRYRPAGCE
PRSRTAIIVPHRAREHHLRLLLYHLHPFLQRQQLAYGIYVIHQAGNGTFNRAKLLNVGVR
EALRDEEWDCLFLHDVDLLPENDHNLYVCDPRGPRHVAVAMNKFGYSLPYPQYFGGVSAL
TPDQYLKMNGFPNEYWGWGGEDDDIATRVRLAGMKISRPPTSVGHYKMVKHRGDKGNEEN
PHRFDLLVRTQNSWTQDGMNSLTYQLLARELGPLYTNITADIGTDPRGPRAPSGPRYPPG
SSQAFRQEMLQRRPPARPGPLSTANHTALRGSH*
>ENSG00000165861|ENST00000318876
MSAQTSPAEKGLNPGLMCQESYACSGTDEAIFECDECCSLQCLRCEEELHRQERLRNHER
IRLKPGHVPYCDLCKGLSGHLPGVRQRAIVRCQTCKINLCLECQKRTHSGGNKRRHPVTV
YNVSNLQESLEAEEMDEETKRKKMTEKVVSFLLVDENEEIQVTNEEDFIRKLDCKPDQHL
KVVSIFGNTGDGKSHTLNHTFFYGREVFKTSPTQESCTVGVWAAYDPVHKVAVIDTEGLL
GATVNLSQRTRLLLKVLAISDLVIYRTHADRLHNDLFKFLGDASEAYLKHFTKELKATTA
RCGLDVPLSTLGPAVIIFHETVHTQLLGSDHPSEVPEKLIQDRFRKLGRFPEAFSSIHYK
GTRTYNPPTDFSGLRRALEQLLENNTTRSPRHPGVIFKALKALSDRFSGEIPDDQMAHSS
FFPDEYFTCSSLCLSCGVGCKKSMNHGKEGVPHEAKSRCRYSHQYDNRVYTCKACYERGE
EVSVVPKTSASTDSPWMGLAKYAWSGRQYWFGNQDPVDTVVRTEIVHVWPGTDGFLKDNN
NAAQRLLDGMNFMAQSVSELSLGPTKAVTSWLTDQIAPAYWRPNSQILSCNKCATSFKDN
DTKHHCRACGEGFCDSCSSKTRPVPERGWGPAPVRVCDNCYEARNVQLAVTEAQVDDEGG
TLIARKVGEAVQNTLGAVVTAIDIPLGLVKDAARPAYWVPDHEILHCHNCRKEFSIKLSK

HHCRACGQGFCDECSHDRRAVPSRGWDHPVRVCFNCNKKPGDL*
>ENSG00000162961|ENST00000295066
MEPEQMLEGQTQVAENPHSEYGLTDNVERIVENEKINAEKSSKQKVDLQSLPTRAYLDQT
VVPILLQGLAVLAKERPPNPIEFLASYLLKNKAQFEDRN*
>ENSG00000106367|ENST00000337619
MMRFMLLFSRQGKLRLQKWYLATSDKERKKMVRELMQVVLARKPKMCSFLEWRDLKVVYK
RYASLYFCCAIEGQDNELITLELIHRYVELLDKYFGSVCELDIIFNFEKAYFILDEFLMG
GDVQDTSKKSVLKAIEQADLLQEEDESPRSVLEEMGLA*
>ENSG00000103496|ENST00000313843
MRDRTHELRQGDDSSDEEDKERVALVVHPGTARLGSPDEEFFHKVRTIRQTIVKLGNKVQ
ELEKQQVTILATPLPEESMKQELQNLRDEIKQLGREIRLQLKAIEPQKEEADENYNSVNT
RMRKTQHGVLSQQFVELINKCNSMQSEYREKNVERIRRQLKITNAGMVSDEELEQMLDSG
QSEVFVSNILKDTQVTRQALNEISARHSEIQQLERSIRELHDIFTFLATEVEMQGEMINR
IEKNILSSADYVERGQEHVKTALENQKKARKKKVLIAICVSITVVLLAVIIGVTVVG*
>ENSG00000163914|ENST00000296271
MNGTEGPNFYVPFSNATGVVRSPFEYPQYYLAEPWQFSMLAAYMFLLIVLGFPINFLTLY
VTVQHKKLRTPLNYILLNLAVADLFMVLGGFTSTLYTSLHGYFVFGPTGCNLEGFFATLG
GEIALWSLVVLAIERYVVVCKPMSNFRFGENHAIMGVAFTWVMALACAAPPLAGWSRYIP
EGLQCSCGIDYYTLKPEVNNESFVIYMFVVHFTIPMIIIFFCYGQLVFTVKEAAAQQQES
ATTQKAEKEVTRMVIIMVIAFLICWVPYASVAFYIFTHQGSNFGPIFMTIPAFFAKSAAI
YNPVIYIMMNKQFRNCMLTTICCGKNPLGDDEASATVSKTETSQVAPA*
>ENSG00000213983|ENST00000308724
MVVPSLKLQDLIEEIRGAKTQAQEREVIQKECAHIRASFRDGDPVHRHRQLAKLLYVHML
GYPAHFGQMECLKLIASSRFTDKRVGYLGAMLLLDERHDAHLLITNSIKNDLSQGIQPVQ
GLALCTLSTMGSAEMCRDLAPEVEKLLLQPSPYVRKKAILTAVHMIRKVPELSSVFLPPC
AQLLHERHHGILLGTITLITELCERSPAALRHFRKVVPQLVHILRTLVTMGYSTEHSISG
VSDPFLQVQILRLLRILGRNHEESSETMNDLLAQVATNTDTSRNAGNAVLFETVLTIMDI
RSAAGLRVLAVNILGRFLLNSDRNIRYVALTSLLRLVQSDHSAVQRHRPTVVECLRETDA
SLSRRALELSLALVNSSNVRAMMQELQAFLESCPPDLRADCASGILLAAERFAPTKRWHI
DTILHVLTTAGTHVRDDAVANLTQLIGGAQELHAYSVRRLYNALAEDISQQPLVQVAAWC
IGEYGDLLLAGNCEEIEPLQVDEEEVLALLEKVLQSHMSLPATRGYALTALMKLSTRLCG
DNNRIRQVVSIYGSCLDVELQQRAVEYDTLFRKYDHMRAAILEKMPLVERDGPQADEEAK
ESKEAAQLSEAAPVPTEPQASQLLDLLDLLDGASGDVQHPPHLDPSPGGALVHLLDLPCV
PPPPAPIPDLKVFEREGVQLNLSFIRPPENPALLLITITATNFSEGDVTHFICQAAVPKS
LQLQLQAPSGNTVPARGGLPITQLFRILNPNKAPLRLKLRLTYDHFHQSVQEIFEVNNLP
VESWQ*
>ENSG00000182533|ENST00000343849
MMAEEHTDLEAQIVKDIHCKEIDLVNRDPKNINEDIVKVDFEDVIAEPVGTYSFDGVWKV
SYTTFTVSKYWCYRLLSTLLGVPLALLWGFLFACISFCHIWAVVPCIKSYLIEIQCISHI
YSLCIRTFCNPLFAALGQVCSSIKVVLRKEV*
>ENSG00000155511|ENST00000340592
MQHIFAFFCTGFLGAVVGANFPNNIQIGGLFPNQQSQEHAAFRFALSQLTEPPKLLPQID
IVNISDSFEMTYRFCSQFSKGVYAIFGFYERRTVNMLTSFCGALHVCFITPSFPVDTSNQ
FVLQLRPELQDALISIIDHYKWQKFVYIYDADRGLSVLQKVLDTAAEKNWQVTAVNILTT

TEEGYRMLFQDLEKKKERLVVVDCESERLNAILGQIIKLEKNGIGYHYILANLGFMDIDL
NKFKESGANVTGFQLVNYTDTIPAKIMQQWKNSDARDHTRVDWKRPKYTSALTYDGVKVM
AEAFQSLRRQRIDISRRGNAGDCLANPAVPWGQGIDIQRALQQVRFEGLTGNVQFNEKGR
RTNYTLHVIEMKHDGIRKIGYWNEDDKFVPAATDAQAGGDNSSVQNRTYIVTTILEDPYV
MLKKNANQFEGNDRYEGYCVELAAEIAKHVGYSYRLEIVSDGKYGARDPDTKAWNGMVGE
LVYGRADVAVAPLTITLVREEVIDFSKPFMSLGISIMIKKPQKSKPGVFSFLDPLAYEIW
MCIVFAYIGVSVVLFLVSRFSPYEWHSEEFEEGRDQTTSDQSNEFGIFNSLWFSLGAFMQ
QGCDISPRSLSGRIVGGVWWFFTLIIISSYTANLAAFLTVERMVSPIESAEDLAKQTEIA
YGTLEAGSTKEFFRRSKIAVFEKMWTYMKSAEPSVFVRTTEEGMIRVRKSKGKYAYLLES
TMNEYIEQRKPCDTMKVGGNLDSKGYGIATPKGSALRGPVNLAVLKLSEQGVLDKLKSKW
WYDKGECGSKDSGSKDKTSALSLSNVAGVFYILIGGLGLAMLVALIEFCYKSRSESKRMK
GFCLIPQQSINEAIRTSTLPRNSGAGASSGGSGENGRVVSHDFPKSMQSIPCMSHSSGMP
LGATGL*
>ENSG00000171928|ENST00000307767
MLQQDSNDDTEDVSLFDAEEETTNRPRKAKIRHPVASFFHLFFRVSAIIVYLLCGLLSSS
FITCMVTIILLLSCDFWAVKNVTGRLMVGLRWWNHIDEDGKSHWVFESRKESSQENKTVS
EAESRIFWLGLIACPVLWVIFAFSALFSFRVKWLAVVIMGVVLQGANLYGYIRCKVRSRK
HLTSMATSYFGKQFLRQNTGDDQTS*
>ENSG00000183196|ENST00000332272
MWLPRVSSTAVTALLLAQTFLLLFLVSRPGPSSPAGGEARVHVLVLSSWRSGSSFVGQLF
NQHPDVFYLMEPAWHVWTTLSQGSAATLHMAVRDLVRSVFLCDMDVFDAYLPWRRNLSDL
FQWAVSRALCSPPACSAFPRGAISSEAVCKPLCARQSFTLAREACRSYSHVVLKEVRFFN
LQVLYPLLSDPALNLRIVHLVRDPRAVLRSREQTAKALARDNGIVLGTNGTWVEADPGLR
VVREVCRSHVRIAEAATLKPPPFLRGRYRLVRFEDLAREPLAEIRALYAFTGLSLTPQLE
AWIHNITHGSGPGARREAFKTSSRNALNVSQAWRHALPFAKIRRVQELCAGALQLLGYRP
VYSEDEQRNLALDLVLPRGLNGFTWASSTASHPRN*
>ENSG00000103174|ENST00000312251
MATSTGRWLLLRLALFGFLWEASGGLDSGASRDDDLLLPYPRARARLPRDCTRVRAGNRE
HESWPPPPATPGAGGLAVRTFVSHFRDRAVAGHLTRAVEPLRTFSVLEPGGPGGCAARRR
ATVEETARAADCRVAQNGGFFRMNSGECLGNVVSDERRVSSSGGLQNAQFGIRRDGTLVT
GYLSEEEVLDTENPFVQLLSGVVWLIRNGSIYINESQATECDETQETGSFSKFVNVISAR
TAIGHDRKGQLVLFHADGQTEQRGINLWEMAEFLLKQDVVNAINLDGGGSATFVLNGTLA
SYPSDHCQDNMWRCPRQVSTVVCVHEPRCQPPDCHGHGTCVDGHCQCTGHFWRGPGCDEL
DCGPSNCSQHGLCTETGCRCDAGWTGSNCSEECPLGWHGPGCQRPCKCEHHCPCDPKTGN
CSVSRVKQCLQPPEATLRAGELSFFTRTAWLALTLALAFLLLISTAANLSLLLSRAERNR
RLHGDYAYHPLQEMNGEPLAAEKEQPGGAHNPFKD*
>ENSG00000118276|ENST00000306851
MSVLRRMMRVSNRSLLAFIFFFSLSSSCLYFIYVAPGIANTYLFMVQARGIMLRENVKTI
GHMIRLYTNKNSTLNGTDYPEGNNSSDYLVQTTTYLPENFTYSPYLPCPEKLPYMRGFLN
VNVSEVSFDEIHQLFSKDLDIEPGGHWRPKDCKPRWKVAVLIPFRNRHEHLPIFFLHLIP
MLQKQRLEFAFYVIEQTGTQPFNRAMLFNVGFKEAMKDSVWDCVIFHDVDHLPENDRNYY
GCGEMPRHFAAKLDKYMYILPYKEFFGGVSGLTVEQFRKINGFPNAFWGWGGEDDDLWNR
VHYAGYNVTRPEGDLGKYKSIPHHHRGEVQFLGRYKLLRYSKERQYIDGLNNLIYRPKIL
VDRLYTNISVNLMPELAPIEDY*

>ENSG00000036257|ENST00000344951
MSNLSKGTGSRKDTKMRIRAFPVREDVLNSLNNNFLQTLNQAWNDHQTAMVMIRDILMYM
DRVYVQQNNVENVYNLGLIIFRDQVVRYGCIRDHLRQTLLDMIARERKGEVVDRGAIRNA
CQMLMILGLEGRSVYEEDFEAPFLEMSAEFFQMESQKFLAENSASVYIKKVEARINEEIE
RVMHCLDKSTEEPIVKVVERELISKHMKTIVEMENSGLVHMLKNGKTEDLGCMYKLFSRV
PNGLKTMCECMSSYLREQGKALVSEEGEGKNPVDYIQGLLDLKSRFDRFLLESFNNDRLF
KQTIAGDFEYFLNLNSRSPEYLSLFIDDKLKKGVKGLTEQEVETILDKAMVLFRFMQEKD
VFERYYKQHLARRLLTNKSVSDDSEKNMISKLKTECGCQFTSKLEGMFRDMSISNTTMDE
FRQHLQATGVSLGGVDLTVRVLTTGYWPTQSATPKCNIPPAPRHAFEIFRRFYLAKHSGR
QLTLQHHMGSADLNATFYGPVKKEDGSEVGVGGAQVTGSNTRKHILQVSTFQMTILMLFN
NREKYTFEEIQQETDIPERELVRALQSLACGKPTQRVLTKEPKSKEIENGHIFTVNDQFT
SKLHRVKIQTVAAKQGESDPERKETRQKVDDDRKHEIEAAIVRIMKSRKKMQHNVLVAEV
TQQLKARFLPSPVVIKKRIEGLIEREYLARTPEDRKVYTYVA*
>ENSG00000036054|ENST00000344949
MAEGEDVPPLPTSSGDGWEKDLEEALEAGGCDLETLRNIIQGRPLPADLRAKVWKIALNV
AGKGDSLASWDGILDLPEQNTIHKDCLQFIDQLSVPEEKAAELLLDIESVITFYCKSRNI
KYSTSLSWIHLLKPLVHLQLPRSDLYNCFYAIMNKYIPRDCSQKGRPFHLFRLLIQYHEP
ELCSYLDTKKITPDSYALNWLGSLFACYCSTEVTQAIWDGYLQQADPFFIYFLMLIILVN
AKEVILTQESDSKEEVIKFLENTPSSLNIEDIEDLFSLAQYYCSKTPASFRKDNHHLFGS
TLLGIKDDDADLSQALCLAISVSEILQANQLQGEGVRFFVVDCRPAEQYNAGHLSTAFHL
DSDLMLQNPSEFAQSVKSLLEAQKQSIESGSIAGGEHLCFMGSGREEEDMYMNMVLAHFL
QKNKEYVSIASGGFMALQQHLADINVDGPENGYGHWIASTSGSRSSINSVDGESPNGSSD
RGMKSLVNKMTVALKTKSVNVREKVISFIENTSTPVDRHVSSSDRVGKPYRGVKPVFSIG
DEEEYDTDEIDSSSMSDDDRKEVVNIQTWINKPDVKHHFPCKEVKESGHMFPSHLLVTAT
HMYCLREIVSRKGLAYIQSRQALNSVVKITSKKKHPELITFKYGNSSASGIEILAIERYL
IPNAGDATKAIKQQIMKVLDALES*
>ENSG00000136840|ENST00000335791
MKAPGRLVLIILCSVVFSAVYILLCCWAGLPLCLATCLDHHFPTGSRPTVPGPLHFSGYS
SVPDGKPLVREPCRSCAVVSSSGQMLGSGLGAEIDSAECVFRMNQAPTVGFEADVGQRST
LRVVSHTSVPLLLRNYSHYFQKARDTLYMVWGQGRHMDRVLGGRTYRTLLQLTRMYPGLQ
VYTFTERMMAYCDQIFQDETGKNRRQSGSFLSTGWFTMILALELCEEIVVYGMVSDSYCR
EKSHPSVPYHYFEKGRLDECQMYLAHEQAPRSAHRFITEKAVFSRWAKKRPIVFAHPSWR
TE*
>ENSG00000147654|ENST00000337573
MAITQFRLFKFCTCLATVFSFLKRLICRSGRGRKLSGDQITLPTTVDYSSVPKQTDVEEW
TSWDEDAPTSVKIEGGNGNVATQQNSLEQLEPDYFKDMTPTIRKTQKIVIKKREPLNFGI
PDGSTGFSSRLAATQDLPFIHQSSELGDLDTWQENTNAWEEEEDAAWQAEEVLRQQKLAD
REKRAAEQQRKKMEKEAQRLMKKEQNKIGVKLS*
>ENSG00000100311|ENST00000331163
MNRCWALFLSLCCYLRLVSAEGDPIPEELYEMLSDHSIRSFDDLQRLLHGDPGEEDGAEL
DLNMTRSHSGGELESLARGRRSLGSLTIAEPAMIAECKTRTEVFEISRRLIDRTNANFLV
WPPCVEVQRCSGCCNNRNVQCRPTQVQLRPVQVRKIEIVRKKPIFKKATVTLEDHLACKC
ETVAAARPVTRSPGGSQEQRAKTPQTRVTIRTVRVRRPPKGKHRKFKHTHDKTALKETLG
A*

>ENSG00000171195|ENST00000304887
MKTLPLFVCICALSACFSFSEGRERDHELRHRRHHHQSPKSHFELPHYPGLLAHQKPFIR
KSYKCLHKRCRPKLPPSPNNPPKFPNPHQPPKHPDKNSSVVNPTLVATTQIPSVTFPSAS
TKITTLPNVTFLPQNATTISSRENVNTSSSVATLAPVNSPAPQDTTAAPPTPSATTPAPP
SSSAPPETTAAPPTPSATTQAPPSSSAPPETTAAPPTPPATTPAPPSSSAPPETTAAPPT
PSATTPAPLSSSAPPETTAVPPTPSATTLDPSSASAPPETTAAPPTPSATTPAPPSSPAP
QETTAAPITTPNSSPTTLAPDTSETSAAPTHQTTTSVTTQTTTTKQPTSAPGQNKISRFL
LYMKNLLNRIIDDMVEQ*
>ENSG00000164825|ENST00000297439
MRTSYLLLFTLCLLLSEMASGGNFLTGLGHRSDHYNCVSSGGQCLYSACPIFTKIQGTCY
RGKAKCCK*
>ENSG00000136238|ENST00000348035
MQAIKCVVVGDGAVGKTCLLISYTTNAFPGEYIPTVFDNYSANVMVDGKPVNLGLWDTAG
QEDYDRLRPLSYPQTDVFLICFSLVSPASFENVRAKWYPEVRHHCPNTPIILVGTKLDLR
DDKDTIEKLKEKKLTPITYPQGLAMAKEIGAVKYLECSALTQRGLKTVFDEAIRAVLCPP
PVKKRKRKCLLL*
>ENSG00000188175|ENST00000341723
MWLKVFTTFLSFATGACSGLKVTVPSHTVHGVRGQALYLPVHYGFHTPASDIQIIWLFER
PHTMPKYLLGSVNKSVVPDLEYQHKFTMMPPNASLLINPLQFPDEGNYIVKVNIQGNGTL
SASQKIQVTVDDPVTKPVVQIHPPSGAVEYVGNMTLTCHVEGGTRLAYQWLKNGRPVHTS
STYSFSPQNNTLHIAPVTKEDIGNYSCLVRNPVSEMESDIIMPIIYYGPYGLQVNSDKGL
KVGEVFTVDLGEAILFDCSADSHPPNTYSWIRRTDNTTYIIKHGPRLEVASEKVAQKTMD
YVCCAYNNITGRQDETHFTVIITSVGLEKLAQKGKSLSPLASITGISLFLIISMCLLFLW
KKYQPYKVIKQKLEGRPETEYRKAQTFSGHEDALDDFGIYEFVAFPDVSGVSRIPSRSVP
ASDCVSGQDLHSTVYEVIQHIPAQQQDHPE*
>ENSG00000124225|ENST00000347215
MAELEFVQIIIIVVVMMVMVVVITCLLSHYKLSARSFISRHSQGRRREDALSSEGCLWPS
ESTVSGNGIPEPQVYAPPRPTDRLAVPPFAQRERFHRFQPTYPYLQHEIDLPPTISLSDG
EEPPPYQGPCTLQLRDPEQQLELNRESVRAPPNRTIFDSDLMDSARLGGPCPPSSNSGIS
ATCYGSGGRMEGPPPTYSEVIGHYPGSSFQHQQSSGPPSLLEGTRLHHTHIAPLESAAIW
SKEKDKQKGHPL*
>ENSG00000163251|ENST00000295417
MARPDPSAPPSLLLLLLAQLVGRAAAASKAPVCQEITVPMCRGIGYNLTHMPNQFNHDTQ
DEAGLEVHQFWPLVEIQCSPDLRFFLCSMYTPICLPDYHKPLPPCRSVCERAKAGCSPLM
RQYGFAWPERMSCDRLPVLGRDAEVLCMDYNRSEATTAPPRPFPAKPTLPGPPGAPASGG
ECPAGGPFVCKCREPFVPILKESHPLYNKVRTGQVPNCAVPCYQPSFSADERTFATFWIG
LWSVLCFISTSTTVATFLIDMERFRYPERPIIFLSACYLCVSLGFLVRLVVGHASVACSR
EHNHIHYETTGPALCTIVFLLVYFFGMASSIWWVILSLTWFLAAGMKWGNEAIAGYAQYF
HLAAWLIPSVKSITALALSSVDGDPVAGICYVGNQNLNSLRGFVLGPLVLYLLVGTLFLL
AGFVSLFRIRSVIKQGGTKTDKLEKLMIRIGIFTLLYTVPASIVVACYLYEQHYRESWEA
ALTCACPGHDTGQPRAKPEYWVLMLKYFMCLVVGITSGVWIWSGKTVESWRRFTSRCCCR
PRRGHKSGGAMAAGDYPEASAALTGRTGPPGPAATYHKQVSLSHV*
>ENSG00000170296|ENST00000302386
MKFVYKEEHPFEKRRSEGEKIRKKYPDRVPVIVEKAPKARIGDLDKKKYLVPSDLTVGQF

YFLIRKRIHLRAEDALFFFVNNVIPPTSATMGQLYQEHHEEDFFLYIAYSDESVYGL*
>ENSG00000177076|ENST00000340967
MGAPHWWDQLQAGSSEVDWCEDNYTIVPAIAEFYNTISNVLFFILPPICMCLFRQYATCF
NSGIYLIWTLLVVVGIGSVYFHATLSFLGQMLDELAVLWVLMCALAMWFPRRYLPKIFRN
DRGRFKVVVSVLSAVTTCLAFVKPAINNISLMTLGVPCTALLIAELKRCDNMRVFKLGLF
SGLWWTLALFCWISDRAFCELLSSFNFPYLHCMWHILICLAAYLGCVCFAYFDAASEIPE
QGPVIKFWPNEKWAFIGVPYVSLLCANKKSSVKIT*
>ENSG00000146648|ENST00000344576
MRPSGTAGAALLALLAALCPASRALEEKKVCQGTSNKLTQLGTFEDHFLSLQRMFNNCEV
VLGNLEITYVQRNYDLSFLKTIQEVAGYVLIALNTVERIPLENLQIIRGNMYYENSYALA
VLSNYDANKTGLKELPMRNLQEILHGAVRFSNNPALCNVESIQWRDIVSSDFLSNMSMDF
QNHLGSCQKCDPSCPNGSCWGAGEENCQKLTKIICAQQCSGRCRGKSPSDCCHNQCAAGC
TGPRESDCLVCRKFRDEATCKDTCPPLMLYNPTTYQMDVNPEGKYSFGATCVKKCPRNYV
VTDHGSCVRACGADSYEMEEDGVRKCKKCEGPCRKVCNGIGIGEFKDSLSINATNIKHFK
NCTSISGDLHILPVAFRGDSFTHTPPLDPQELDILKTVKEITGFLLIQAWPENRTDLHAF
ENLEIIRGRTKQHGQFSLAVVSLNITSLGLRSLKEISDGDVIISGNKNLCYANTINWKKL
FGTSGQKTKIISNRGENSCKATGQVCHALCSPEGCWGPEPRDCVSCRNVSRGRECVDKCN
LLEGEPREFVENSECIQCHPECLPQAMNITCTGRGPDNCIQCAHYIDGPHCVKTCPAGVM
GENNTLVWKYADAGHVCHLCHPNCTYGPGNESLKAMLFCLFKLSSCNQSNDGSVSHQSGS
PAAQESCLGWIPSLLPSEFQLGWGGCSHLHAWPSASVIITASSCH*
>ENSG00000151693|ENST00000315273
MPDQISVSEFVAETHEDYKAPTASSFTTRTAQCRNTVAAIEEALDVDRMVLYKMKKSVKA
INSSGLAHVENEEQYTQALEKFGGNCVCRDDPDLGSAFLKFSVFTKELTALFKNLIQNMN
NIISFPLDSLLKGDLKGVKGDLKKPFDKAWKDYETKITKIEKEKKEHAKLHGMIRTEISG
AEIAEEMEKERRFFQLQMCEYLLKVNEIKIKKGVDLLQNLIKYFHAQCNFFQDGLKAVES
LKPSIETLSTDLHTIKQAQDEERRQLIQLRDILKSALQVEQKEDSQIRQSTAYSLHQPQG
NKEHGTERNGSLYKKSDGIRKVWQKRKCSVKNGFLTISHGTANRPPAKLNLLTCQVKTNP
EEKKCFDLISHDRTYHFQAEDEQECQIWMSVLQNSKEEALNNAFKGDDNTGENNIVQELT
KEIISEVQRMTGNDVCCDCGAPDPTWLSTNLGILTCIECSGIHRELGVHYSRMQSLTLDV
LGTSELLLAKNIGNAGFNEIMECCLPAEDSVKPNPGSDMNARKDYITAKYIERRYARKKH
ADNAAKLHSLCEAVKTRDIFGLLQAYADGVDLTEKIPLANGHEPDETALHLAVRSVDRTS
LHIVDFLVQNSGNLDKQTGKGSTALHYCCLTDNAECLKLLLRGKASIEIANESGETPLDI
AKRLKHEHCEELLTQALSGRFNSHVHVEYEWRLLHEDLDESDDDMDEKLQPSPNRREDRP
ISFYQLGSNQLQSNAVSLARDAANLAKEKQRAFMPSILQNETYGALLSGSPPPAQPAAPS
TTSAPPLPPRNVGKDPLTPTPPPPVAKTPSVMEALSQPSKPAPPGISQIRPPPLPPQPPS
RLPQKKPAPGADKSTPLTNKGQPRGPVDLSATEALGPLSNAMVLQPPAPMPRKSQATKLK
PKRVKALYNCVADNPDELTFSEGDVIIVDGEEDQEWWIGHIDGDPGRKGAFPVSFVHFIA
D*
>ENSG00000151348|ENST00000343631
MCASVKYNIRGPALIPRMKTKHRIYYITLFSIVLLGLIATGMFQFWPHSIESSNDWNVEK
RSIRDVPVVRLPADSPIPERGDLSCRMHTCFDVYRCGFNPKNKIKVYIYALKKYVDDFGV
SVSNTISREYNELLMAISDSDYYTDDINRACLFVPSIDVLNQNTLRIKETAQAMAQLSRW
DRGTNHLLFNMLPGGPPDYNTALDVPRDRALLAGGGFSTWTYRQGYDVSIPVYSPLSAEV
DLPEKGPGPRQYFLLSSQVGLHPEYREDLEALQVKHGESVLVLDKCTNLSEGVLSVRKRC

HKHQVFDYPQVLQEATFCVVLRGARLGQAVLSDVLQAGCVPVVIADSYILPFSEVLDWKR
ASVVVPEEKMSDVYSILQSIPQRQIEEMQRQARWFWEAYFQSIKAIALATLQIINDRIYP
YAAISYEEWNDPPAVKWGSVSNPLFLPLIPPQSQGFTAIVLTYDRVESLFRVITEVSKVP
SLSKLLVVWNNQNKNPPEDSLWPKIRVPLKVVRTAENKLSNRFFPYDEIETEAVLAIDDD
IIMLTSDELQFGYEVWREFPDRLVGYPGRLHLWDHEMNKWKYESEWTNEVSMVLTGAAFY
HKYFNYLYTYKMPGDIKNWVDAHMNCEDIAMNFLVANVTGKAVIKVTPRKKFKCPECTAI
DGLSLDQTHMVERSECINKFASVFGTMPLKVVEHRADPVLYKDDFPEKLKSFPNIGSL*
>ENSG00000115694|ENST00000316586
MAHLRGFANQHSRVDPEELFTKLDRIGKGSFGEVYKGIDNHTKEVVAIKIIDLEEAEDEI
EDIQQEITVLSQCDSPYITRYFGSYLKSTKLWIIMEYLGGGSALDLLKPGPLEETYIATI
LREILKGLDYLHSERKIHRDIKAANVLLSEQGDVKLADFGVAGQLTDTQIKRNTFVGTPF
WMAPEVIKQSAYDFKADIWSLGITAIELAKGEPPNSDLHPMRVLFLIPKNSPPTLEGQHS
KPFKEFVEACLNKDPRFRPTAKELLKHKFITRYTKKTSFLTELIDRYKRWKSEGHGEESS
SEDSDIDGEAEDGEQGPIWTFPPTIRPSPHSKLHKGTALHSSQKPAEPVKRQPRSQCLST
LVRPVFGELKEKHKQSGGSVGALEELENAFSLAEESCPGISDKLMVHLVERVQRFSHNRN
HLTSTR*
>ENSG00000100478|ENST00000313566
MIKFFLMVNKQGQTRLSKYYEHVDINKRTLLETEVIKSCLSRSNEQCSFIEYKDFKLIYR
QYAALFIVVGVNDTENEMAIYEFIHNFVEVLDEYFSRVSELDVSFFNTVFHSTWQMHSGP
YQTRSCSVTQAGVQRCDHGSLHPGSPGLK*
>ENSG00000171711|ENST00000302247
MRVLYLLFSFLFIFLMPLPGVFGGIGDPVTCLKSGAICHPVFCPRRYKQIGTCGLPGTKC
CKKP*
>ENSG00000171124|ENST00000303225
MDPLGAAKPQWPWRRCLAALLFQLLVAVCFFSYLRVSRDDATGSPRAPSGSSRQDTTPTR
PTLLILLRTWPFHIPVALSRCSEMVPGTADCHITADRKVYPQADMVIVHHWDIMSNPKSR
LPPSPRPQGQRWIWFNLEPPPNCQHLEALDRYFNLTMSYRSDSDIFTPYGWLEPWSGQPA
HPPLNLSAKTELVAWAVSNWKPDSARVRYYQSLQAHLKVDVYGRSHKPLPKGTMMETLSR
YKFYLAFENSLHPDYITEKLWRNALEAWAVPVVLGPSRSNYERFLPPDAFIHVDDFQSPK
DLARYLQELDKDHARYLSYFRWRETLRPRSFSWALDFCKACWKLQQESRYQTVRSIAAWF
T*
>ENSG00000161395|ENST00000300658
MAGLAARLVLLAGAAALASGSQGDREPVYRDCVLQCEEQNCSGGALNHFRSRQPIYMSLA
GWTCRDDCKYECMWVTVGLYLQEGHKVPQFHGKWPFSRFLFFQEPASAVASFLNGLASLV
MLCRYRTFVPASSPMYHTCVAFAWVSLNAWFWSTVFHTRDTDLTEKMDYFCASTVILHSI
YLCCVRTVGLQHPAVVSAFRALLLLMLTVHVSYLSLIRFDYGYNLVANVAIGLVNVVWWL
AWCLWNQRRLPHVRKCVVVVLLLQGLSLLELLDFPPLFWVLDAHAIWHISTIPVHVLFFS
FLEDDSLYLLKESEDKFKLD*
>ENSG00000145416|ENST00000339875
MTSSHVCCNFLNMWKKSKISTMYYLNQDAKLSNLFLQASSPTTGTAPRSQSRLSVCPSTQ
DICRICHCEGDEESPLITPCRCTGTLRFVHQSCLHQWIKSSDTRCCELCKYDFIMETKLK
PLRKWEKLQMTTSERRKIFCSVTFHVIAITCVVWSLYVLIDRTAEEIKQGNDNGVLEWPF
WTKLVVVAIGFTGGLVFMYVQCKVYVQLWRRLKAYNRVIFVQNCPDTAKKLEKNFSCNVN
TDIKDAVVVPVPQTGANSLPSAEGGPPEVVSV*

>ENSG00000166507|ENST00000299641
MLQLWKVVRPARQLELHRLILLLIAFSLGSMGFLAYYVSTSPKAKEPLPLPLGDCSSGGA
AGPGPARPPVPPRPPRPPETARTEPVVLVFVESAYSQLGQEIVAILESSRFRYSTELAPG
RGDMPTLTDNTHGRYVLVIYENLLKYVNLDAWSRELLDRYCVEYGVGIIGFFRAHEHSLL
SAQLKGFPLFLHSNLGLRDYQVNPSAPLLHLTRPSRLEPGPLPGDDWTIFQSNHSTYEPV
LLASLRPAEPAVPGPVLRRARLPTVVQDLGLHDGIQRVLFGHGLSFWLHKLIFVDAVAYL
TGKRLCLDLDRYILVDIDDIFVGKEGTRMKVADVEALLTTQNKLRTLVPNFTFNLGFSGK
FYHTGTEEEDAGDDMLLKHRKEFWWFPHMWSHMQPHLFHNRSVLADQMRLNKQFALEHGI
PTDLGYAVAPHHSGVYPIHTQLYEAWKSVWGIQVTSTEEYPHLRPARYRRGFIHNGIMVL
PRQTCGLFTHTIFYNEYPGGSRELDRSIRGGELFLTVLLNPISIFMTHLSNYGNDRLGLY
TFESLVRFLQCWTRLRLQTLPPVPLAQKYFELFPQERSPLWQNPCDDKRHKDIWSKEKTC
DRLPKFLIVGPQKTGTTAIHFFLSLHPAVTSSFPSPSTFEEIQFFNSPNYHKGIDWYMDF
FPVPSNASTDFLFEKSATYFDSEVVPRRGAALLPRAKIITVLTNPADRAYSWYQHQRAHG
DPVALNYTFYQVISASSQTPLALRSLQNRCLVPGYYSTHLQRWLTYYPSGQLLIVDGQEL
RTNPAASMESIQKFLGITPFLNYTRTLRFDDDKGFWCQGLEGGKTRCLGRSKGRRYPDMD
TESRLFLTDFFRNHNLELSKLLSRLGQPVPSWLREELQHSSLG*
>ENSG00000168434|ENST00000307149
MDFSKFLADDFDVKEWINAAFRAGSKEAASGKADGHAATLVMKLQLFIQEVNHAVEETSH
QALQNMPKVLRDVEALKQEASFLKEQMILVKEDIKKFEQDTSQSMQVLVEIDQVKSRMQL
AAESLQEADKWSTLSADIEETFKTQDIAVISAKLTGMQNSLMMLVDTPDYSEKCVHLEAL
KNRLEALASPQIVAAFTSQAVDQSKVFVKVFTEIDRMPQLLAYYYKCHKVQLLAAWQELC
QSDLSLDRQLTGLYDALLGAWHTQIQWATQVFQKPHEVVMVLLIQTLGALMPSLPSCLSN
GVERAGPEQELTRLLEFYDATAHFAKGLEMALLPHLHEHNLVKVTELVDAVYDPYKPYQL
KYGDMEESNLLIQMSAVPLEHGEVIDCVQELSHSVNKLFGLASAAVDRCVRFTNGLGTCG
LLSALKSLFAKYVSDFTSTLQSIRKKCKLDHIPPNSLFQEDWTAFQNSIRIIATCGELLR
HCGDFEQQLANRILSTAGKYLSDSCSPRSLAGFQESILTDKKNSAKNPWQEYNYLQKDNP
AEYASLMEILYTLKEKGSSNHNLLAAPRAALTRLNQQAHQLAFDSVFLRIKQQLLLISKM
DSWNTAGIGETLTDELPAFSLTPLEYISNIGQYIMSLPLNLEPFVTQEDSALELALHAGK
LPFPPEQGDELPELDNMADNWLGSIARATMQTYCDAILQIPELSPHSAKQLATDIDYLIN
VMDALGLQPSRTLQHIVTLLKTRPEDYRQVSKGLPRRLATTVATMRSVNY*
>ENSG00000176986|ENST00000339365
MNVNQSVPPVPPFGQPQPIYPGYHQSSYGGQSGSTAPAIPYGAYNGPVPGYQQTPPQGMS
RAPPSSGAPPASTAQAPCGQAAYGQFGQGDVQNGPSSTVQMQRLPGSQPFGSPLAPVGNQ
PPVLQPYGPPPTSAQVATQLSGMQISGAVAPAPPSSGLGFGPPTSLASASGSFPNSGLYG
SYPQGQAPPLSQAQGHPGIQTPQRSAPSQASSFTPPASGGPRLPSMTGPLLPGQSFGGPS
VSQPNHVSSPPQALPPGTQMTGPLGPLPPMHSPQQPGYQPQQNGSFGPARGPQSNYGGPY
PAAPTFGSQPGPPQPLPPKRLDPDAIPSPIQVIEDDRNNRGTEPFVTGVRGQVPPLVTTN
FLVKDQGNASPRYIRCTSYNIPCTSDMAKQAQVPLAAVIKPLARLPPEEASPYVVDHGES
GPLRCNRCKAYMCPFMQFIEGGRRFQCCFCSCINDVPPQYFQHLDHTGKRVDAYDRPELS
LGSYEFLATVDYCKNNKFPSPPAFIFMIDVSYNAIRTGLVRLLCEELKSLLDFLPREGGA
EESAIRVGFVTYNKVLHFYNVKSSLAQPQMMVVSDVADMFVPLLDGFLVNVNESRAVITS
LLDQIPEMFADTRETETVFVPVIQAGMEALKAAECAGKLFLFHTSLPIAEAPGKLKNRDD
RKLINTDKEKTLFQPQTGAYQTLAKECVAQGCCVDLFLFPNQYVDVATLSVVPQLTGGSV
YKYASFQVENDQERFLSDLRRDVQKVVGFDAVMRVRTSTGIRAVDFFGAFYMSNTTDVEL

AGLDGDKTVTVEFKHDDRLNEESGALLQCALLYTSCAGQRRLRIHNLALNCCTQLADLYR
NCETDTLINYMAKFAYRGVLNSPVKAVRDTLITQCAQILACYRKNCASPSSAGQLILPEC
MKLLPVYLNCVLKSDVLQPGAEVTTDDRAYVRQLVTSMDVTETNVFFYPRLLPLTKSPVE
STTEPPAVRASEERLSNGDIYLLENGLNLFLWVGASVQQGVVQSLFSVSSFSQITSGLSV
LPVLDNPLSKKVRGLIDSLRAQRSRYMKLTVVKQEDKMEMLFKHFLVEDKSLSGGASYVD
FLCHMHKEIRQLLS*
>ENSG00000174775|ENST00000311189
MTEYKLVVVGAGGVGKSALTIQLIQNHFVDEYDPTIEDSYRKQVVIDGETCLLDILDTAG
QEEYSAMRDQYMRTGEGFLCVFAINNTKSFEDIHQYREQIKRVKDSDDVPMVLVGNKCDL
AARTVESRQAQDLARSYGIPYIETSAKTRQGVEDAFYTLVREIRQHKLRKLNPPDESGPG
CMSCKCVLS*
>ENSG00000168077|ENST00000301904
MKVRSAGGDGDALCVTEEDLAGDDEDMPTFPCTQKGRPGPRCSRCQKNLSLHTSVRILYL
FLALLLVAVAVLASLVFRKVDSLSEDISLTQSIYDKKLVLMQKNLQGLDPKALNNCSFCH
EAGQLGPEIRKLQEELEGIQKLLLAQEVQLDQTLQAQEVLSTTSRQISQEMGSCSFSIHQ
VNQSLGLFLAQVRGWQATTAGLDLSLKDLTQECYDVKAAVHQINFTVGQTSEWIHGIQRK
TDEETLTLQKIVTDWQNYTRLFSGLRTTSTKTGEAVKNIQATLGASSQRISQNSESMHDL
VLQVMGLQLQLDNISSFLDDHEENMHDLQYHTHYAQNRTVERFESLEGRMASHEIEIGTI
FTNINATDNHVHSMLKYLDDVRLSCTLGFHTHAEELYYLNKSVSIMLGTTDLLRERFSLL
SARLDLNVRNLSMIVEEMKAVDTQHGEILRNVTILRGAPGPPGPRGFKGDMGVKGPVGGR
GPKGDPGSLGPLGPQGPQGQPGEAGPVGERGPVGPRGFPGLKGSKGSFGTGGPRGQPGPK
GDIGPPGPEGPPGSPGPSGPQGKPGIAGKTGSPGQRGAMGPKGEPGIQGPPGLPGPPGPP
GSQSFY*
>ENSG00000185294|ENST00000329196
MACLGFLLPVGFLLLISTVAGGKYGVAHVVSENWSKDYCILFSSDYITLPRDLHHAPLLP
LYDGTKAPWCPGEDSPHQAQLRSPSQRPLRQTTAMVMRGNCSFHTKGWLAQGQGAHGLLI
VSRVSDQQCSDTTLAPQDPRQPLADLTIPVAMLHYADMLDILSHTRGEAVVRVAMYAPPE
PIIDYNMLVIFILAVGTVAAGGYWAGLTEANRLQRRRARRGGGSGGHHQLQEAAAAEGAQ
KEDNEDIPVDFTPAMTGVVVTLSCSLMLLLYFFYDHFVYVTIGIFGLGAGIGLYSCLSPL
VCRLSLRQYQRPPHSLWASLPLPLLLLASLCATVIIFWVAYRNEDRWAWLLQDTLGISYC
LFVLHRVRLPTLKNCSSFLLALLAFDVFFVFVTPFFTKTGESIMAQVALGPAESSSHERL
PMVLKVPRLRVSALTLCSQPFSILGFGDIVVPGFLVAYCCRFDVQVCSRQIYFVACTVAY
AVGLLVTFMAMVLMQMGQPALLYLVSSTLLTSLAVAACRQELSLFWTGQGRAKMCGLGCA
PSAGSRQKQEGAADAHTASTLERGTSRGAGDLDSNPGEDTTEIVTISENEATNPEDRSDS
SEGWSDAHLDPNELPFIPPGASEELMPLMPMAMLIPLMPLMPPPSELGHVHAQAQAHETG
LPWAGLHKRKGLKVRKSMSTQAPL*
>ENSG00000186318|ENST00000313005
MAQALPWLLLWMGAGVLPAHGTQHGIRLPLRSGLGGAPLGLRLPRETDEEPEEPGRRGSF
VEMVDNLRGKSGQGYYVEMTVGSPPQTLNILVDTGSSNFAVGAAPHPFLHRYYQRQLSST
YRDLRKGVYVPYTQGKWEGELGTDLVSIPHGPNVTVRANIAAITESDKFFINGSNWEGIL
GLAYAEIARPDDSLEPFFDSLVKQTHVPNLFSLQLCGAGFPLNQSEVLASVGGSMIIGGI
DHSLYTGSLWYTPIRREWYYEVIIVRVEINGQDLKMDCKEYNYDKSIVDSGTTNLRLPKK
VFEAAVKSIKAASSTEKFPDGFWLGEQLVCWQAGTTPWNIFPVISLYLMGEVTNQSFRIT
ILPQQYLRPVEDVATSQDDCYKFAISQSSTGTVMGAVIMEGFYVVFDRARKRIGFAVSAC

HVHDEFRTAAVEGPFVTLDMEDCGYNIPQTDESTLMTIAYVMAAICALFMLPLCLMVCQW
RCLRCLRQQHDDFADDISLLK*
>ENSG00000170310|ENST00000306357
MAPDPWFSTYDSTCQIAQEIAEKIQQRNQYERKGEKAPKLTVTIRALLQNLKEKIALLKD
LLLRAVSTHQITQLEGDRRQNLLDDLVTRERLLLASFKNEGAEPDLIRSSLMSEEAKRGA
PNPWLFEEPEETRGLGFDEIRQQQQKIIQEQDAGLDALSSIISRQKQMGQEIGNELDEQN
EIIDDLANLVENTDEKLRNETRRVNMVDRKSASCGMIMVILLLLVAIVVVAVWPTN*
>ENSG00000003147|ENST00000317367
MDRYAQDKSVVNKMQQKYWETKQAFIKATGKKEDEHVVASDADLDAKLELFHSIQRTCLD
LSKAIVLYQKRICFLSQEENELGKFLRSQGFQDKTRAGKMMQATGKALCFSSQQRLALRN
PLCRFHQEVETFRHRAISDTWLTVNRMEQCRTEYRGALLWMKDVSQELDPDLYKQMEKFR
KVQTQVRLAKKNFDKLKMDVCQKVDLLGASRCNLLSHMLATYQTTLLHFWEKTSHTMAAI
HESFKGYQPYEFTTLK
>ENSG00000160179|ENST00000343687
MRISLPRAPERDGGVSASSLLDTVTNASSYSAEMTEPKSVCVSVDEVVSSNMEATETDLL
NGHLKKVDNNLTEAQRFSSLPRRAAVNIEFRDLSYSVPEGPWWRKKGYKTLLKGISGKFN
SGELVAIMGPSGAGKSTLMNILAGYRETGMKGAVLINGLPRDLRCFRKVSCYIMQDDMLL
PHLTVQEAMMVSAHLKLQEKDEGRREMVKEILTALGLLSCANTRTGSLSGGQRKRLAIAL
ELVNNPPVMFFDEPTSGLDSASCFQVVSLMKGLAQGGRSIICTIHQPSAKLFELFDQLYV
LSQGQCVYRGKVCNLVPYLRDLGLNCPTYHNPADFVMEVASGEYGDQNSRLVRAVREGMC
DSDHKRDLGGDAEVNPFLWHRPSEEDSSSMEGCHSFSASCLTQFCILFKRTFLSIMRDSV
LTHLRITSHIGIGLLIGLLYLGIGNEAKKVLSNSGFLFFSMLFLMFAALMPTVLTFPLEM
GVFLREHLNYWYSLKAYYLAKTMADVPFQIMFPVAYCSIVYWMTSQPSDAVRFVLFAALG
TMTSLVAQSLGLLIGAASTSLQVATFVGPVTAIPVLLFSGFFVSFDTIPTYLQWMSYISY
VRYGFEGVILSIYGLDREDLHCDIDETCHFQKSEAILRELDVENAKLYLDFIVLGIFFIS
LRLIAYFVLRYKIRAER*
>ENSG00000132824|ENST00000342374
MGAVLGVFSLASWVPCLCSGASCLLCSCCPNSKNSTVTRLIYAFILLLSTVVSYIMQRKE
METYLKKIPGFCEGGFKIHEADINADKDCDVLVGYKAVYRISFAMAIFFFVFSLLMFKVK
TSKDLRAAVHNGFWFFKIAALIGIMVGSFYIPGGYFSSVWFVVGMIGAALFILIQLVLLV
DFAHSWNESWVNRMEEGNPRLWYAALLSFTSAFYILSIICVGLLYTYYTKPDGCTENKFF
ISINLILCVVASIISIHPKIQEHQPRSGLLQSSLITLYTMYLTWSAMSNEPDRSCNPNLM
SFITRITAPTLAPGNSTAVVPTPTPPSKSGSLLDSDNFIGLFVFVLCLLYSSIRTSTNSQ
VDKLTLSGSDSVILGDTTTSGASDEEDGQPRRAVDNEKEGVQYSYSLFHLMLCLASLYIM
MTLTSWYSPDAKFQSMTSKWPAVWVKISSSWVCLLLYVWTLVAPLVLTSRDFS*
>ENSG00000154305|ENST00000344507
MAAAPGLLVWLLVLRLPWRVPGQLDPSTGRRFSEHKLCADDECSMLMYRGEALEDFTGPD
CRFVNFKKGDPVYVYYKLARGWPEVWAGSVGRTFGYFPKDLIQVVHEYTKEELQVPTDET
DFVCFDGGRDDFHNYNVEELLGFLELYNSAATDSEKAVEKTLQDMEKNPELSKEREPEPE
PVEANSEESDSVFSENTEDLQEQFTTQKHHSHANSQANHAQGEQASFESFEEMLQDKLKV
PESENNKTSNSSQVSNEQDKIDAYKLLKKEMTLDLKTKFGSTADALVSDDETTRLVTSLE
DDFDEELDTEYYAVGKEDEENQEDFDELPLLTFTDGEDMKTPAKSGVEKYPTDKEQNSNE
EDKVQLTVPPGIKNDDKNILTTWGDTIFSIVTGGEETRDTMDLESSSSEEEKEDDDDALV
PDSKQGKPQSATDYSDPDNVDDGLFIVDIPKTNNDKEVNAEHHIKGKGRGVQESKRGLVQ

DKTELEDENQEGFKTEPIKL*
>ENSG00000126231|ENST00000342783
MAGCVPLLQGLVLVLALHRVEPSATSLKERHGLHSDSACTGVQESLFLPASKANDVLVRW
KRAGSYLLEELFEGNLEKECYEEICVYEEAREVFENEVVTDEFWRRYKGGSPCISQPCLH
NGSCQDSIWGYTCTCSPGYEGSNCELAKNECHPERTDGCQHFCLPGQESYTCSCAQGYRL
GEDHKQCVPHDQCACGVLTSEKRAPDLQDLPWQVKLTNSEGKDFCGGVIIRENFVLTTAK
CSLLHRNITVKTYFNRTSQDPLMIKITHVHVHMRYDADAGENDLSLLELEWPIQCPGAGL
PVCTPEKDFAEHLLIPRTRGLLSGWARNGTDLGNSLTTRPVTLVEGEECGQVLNVTVTTR
TYCERSSVAAMHWMDGSVVTREHRGSWFLTGVLGSQPVGGQAHMVLVTKVSRYSLWFKQI
MN*
>ENSG00000165240|ENST00000343533
MDPSMGVNSVTISVEGMTCNSCVWTIEQQIGKVNGVHHIKVSLEEKNATIIYDPKLQTPK
TLQEAIDDMGFDAVIHNPDPLPVLTDTLFLTVTASLTLPWDHIQSTLLKTKGVTDIKIYP
QKRTVAVTIIPSIVNANQIKELVPELSLDTGTLEKKSGACEDHSMAQAGEVVLKMKVEGM
TCHSCTSTIEGKIGKLQGVQRIKVSLDNQEATIVYQPHLISVEEMKKQIEAMGFPAFVKK
QPKYLKLGAIDVERLKNTPVKSSEGSQQRSPSYTNDSTATFIIDGMHCKSCVSNIESTLS
ALQYVSSIVVSLENRSAIVKYNASSVTPESLRKAIEAVSPGLYRVSITSEVESTSNSPSS
SSLQKIPLNVVSQPLTQETVINIDGMTCNSCVQSIEGVISKKPGVKSIRVSLANSNGTVE
YDPLLTSPETLRGAIEDMGFDATLSDTNEPLVVIAQPSSEMPLLTSTNEFYTKGMTPVQD
KEEGKNSSKCYIQVTGMTCASCVANIERNLRREEGIYSILVALMAGKAEVRYNPAVIQPP
MIAEFIRELGFGATVIENADEGDGVLELVVRGMTCASCVHKIESSLTKHRGILYCSVALA
TNKAHIKYDPEIIGPRDIIHTIESLGFEASLVKKDRSASHLDHKREIRQWRRSFLVSLFF
CIPVMGLMIYMMVMDHHFATLHHNQNMSKEEMINLHSSMFLERQILPGLSVMNLLSFLLC
VPVQGKTSEALAKLISLQATEATIVTLDSDNILLSEEQVDVELVQRGDIIKVVPGGKFPV
DGRVIEGHSMVDESLITGEAMPVAKKPGSTVIAGSINQNGSLLICATHVGADTTLSQIVK
LVEEAQTSKAPIQQFADKLSGYFVPFIVFVSIATLLVWIVIGFLNFEIVETYFPGYNRSI
SRTETIIRFAFQASITVLCIACPCSLGLATPTAVMVGTGVGAQNGILIKGGEPLEMAHKV
KVVVFDKTGTITHGTPVVNQVKVLTESNRISHHKILAIVGTAESNSEHPLGTAITKYCKQ
ELDTETLGTCIDFQVVPGCGISCKVTNIEGLLHKNNWNIEDNNIKNASLVQIDASNEQSS
TSSSMIIDAQISNALNAQQYKVLIGNREWMIRNGLVINNDVNDFMTEHERKGRTAVLVAV
DDELCGLIAIADTVKPEAELAIHILKSMGLEVVLMTGDNSKTARSIASQVGITKVFAEVL
PSHKVAKVKQLQEEGKRVAMVGDGINDSPALAMANVGIAIGTGTDVAIEAADVVLIRNDL
LDVVASIDLSRKTVKRIRINFVFALIYNLVGIPIAAGVFMPIGLVLQPWMGSAAMAASSV
SVVLSSLFLKLYRKPTYESYELPARSQIGQKSPSEISVHVGIDDTSRNSPKLGLLDRIVN
YSRASINSLLSDKRSLNSVVTSEPDKHSLLVGDFREDDDTAL*
>ENSG00000054793|ENST00000311637
MDSRPRAGCCEWLRCCGGGEARPRTVWLGHPEKRDQRYPRNVINNQKYNFFTFLPGVLFN
QFKYFFNLYFLLLACSQFVPEMRLGALYTYWVPLGFVLAVTVIREAVEEIRCYVRDKEVN
SQVYSRLTARGTVVGVVLYTGRELRSVMNTSNPRSKIGLFDLEVNCLTKILFGALVVVSL
VMVALQHFAGRWYLQIIRFLLLFSNIIPISLRVNLDMGKIVYSWVIRRDSKIPGTVVRSS
TIPEQLGRISYLLTDKTGTLTQNEMIFKRLHLGTVAYGLDSMDEVQSHIFSIYTQQSQDP
PAQKGPTLTTKVRRTMSSRVHEAVKAIALCHNVTPVYESNGVTDQAEAEKQYEDSCRVYQ
ASSPDEVALVQWTESVGLTLVGRDQSSMQLRTPGDQILNFTILQIFPFTYESKRMGIIVR
DESTGEITFYMKGADVVMAGIVQYNDWLEEECGNMAREGLRVLVVAKKSLAEEQYQDFEA

RYVQAKLSVHDRSLKVATVIESLEMEMELLCLTGVEDQLQADVRPTLETLRNAGIKVWML
TGDKLETATCTAKNAHLVTRNQDIHVFRLVTNRGEAHLELNAFRRKHDCALVISGDSLEV
CLKYYEYEFMELACQCPAVVCCRCAPTQKAQIVRLLQERTGKLTCAVGDGGNDVSMIQES
DCGVGVEGKEGKQASLAADFSITQFKHLGRLLMVHGRNSYKRSAALSQFVIHRSLCISTM
QAVFSSVFYFASVPLYQGFLIIGYSTIYTMFPVFSLVLDKDVKSEVAMLYPELYKDLLKG
RPLSYKTFLIWVLISIYQGSTIMYGALLLFESEFVHIVAISFTSLILTELLMVALTIQTW
HWLMTVAELLSLACYIASLVFLHEFIDVYFIATLSFLWKVSVITLVSCLPLYVLKYLRRR
FSPPSYSKLTS*
>ENSG00000153786|ENST00000313732
MQPSGHRLRDVEHHPLLAENDNYDSSSSSSSEADVADRVWFIRDGCGMICAVMTWLLVAY
ADFVVTFVMLLPSKDFWYSVVNGVIFNCLAVLALSSHLRTMLTDPGAVPKGNATKEYMES
LQLKPGEVIYKCPKCCCIKPERAHHCSICKRCIRKMDHHCPWVNNCVGEKNQRFFVLFTM
YIALSSVHALILCGFQFISCVRGQWTECSDFSPPITVILLIFLCLEGLLFFTFTAVMFGT
QIHSICNDETEIERLKSEKPTWERRLRWEGMKSVFGGPPSLLWMNPFVGFRFRRLPTRPR
KGGPEFSV*
>ENSG00000117475|ENST00000329281
MTTKNLETKVTVTSSPIRGAGDGMETEEPPKSVEVTSGVQSRKHHSLQSPWKKAVPSESP
GVLQLGKMLTEKAMEVKAVRILVPKAAITHDIPNKNTKVKSLGHHKGEFLGQSEGVIEPN
KELSEVKNVLEKLKNSERRLLQDKEGLSNQLRVQTEVNRELKKLLVASVGDDLQYHFERL
AREKNQLILENEALGRNTAQLSEQLERMSIQCDVWRSKFLASRVMADELTNSRAALQRQN
RDAHGAIQDLLSEREQFRQEMIATQKLLEELLVSLQWGREQTYSPSVQPHSTAELALTNH
KLAKAVNSHLLGNVGINNQKKIPSTVEFCSTPAEKMAETVLRILDPVTCKESSPDNPFFE
SSPTTLLATKKNIGRFHPYTRYENITFNCCNHCRGELIAL*
>ENSG00000186635|ENST00000334211
MTKKEEPPPSRVPRAVRVASLLSEGEELSGDDQGDEEEDDHAYEGVPNGGWHTSSLSLSL
PSTIAAPHPMDGPPGGSTPVTPVIKAGWLDKNPPQGSYIYQKRWVRLDTDHLRYFDSNKD
AYSKRFISVACISHVAAIGDQKFEVITNNRTFAFRAESDVERKEWMQALQQAMAEQRARA
RLSSAYLLGVPGSEQPDRAGSLELRGFKNKLYVAVVGDKVQLYKNLEEYHLGIGITFIDM
SVGNVKEVDRRSFDLTTPYRIFSFSADSELEKEQWLEAMQGAIAEALSTSEVAERIWAAA
PNRFCADCGAPQPDWASINLCVVICKRCAGEHRGLGAGVSKVRSLKMDRKVWTETLIELF
LQLGNGAGNRFWAANVPPSEALQPSSSPSTRRCHLEAKYREGKYRRYHPLFGNQEELDKA
LCAAVTTTDLAETQALLGCGAGINCFSGDPEAPTPLALAEQAGQTLQMEFLRNNRTTEVP
RLDSMKPLEKHYSVVLPTVSHSGFLYKTASAGKLLQDRRAREEFSRRWCVLGDGVLSYFE
NERAVTPNGEIRASEIVCLAVPPPDTHGFEHTFEVYTEGERLYLFGLESAEQAHEWVKCI
AKAFVPPLAEDLLARDFERLGRLPYKAGLSLQRAQEGWFSLSGSELRAVFPEGPCEEPLQ
LRKLQELSIQGDSENQVLVLVERRRTLYIQGERRLDFMGWLGAIQKAAASMGDTLSEQQL
GDSDIPVIVYRCVDYITQCGLTSEGIYRKCGQTSKTQRLLESLRQDARSVHLKEGEQHVD
DVSSALKRFLRDLPDGLFTRAQRLTWLEASEIEDEEEKVSRYRELLVRLPPVNRATVKAL
ISHLYCVQCFSDTNQMNVHNLAIVFGPTLFQTDGQDYKAGRVVEDLINHYVVVFSVDEEE
LRKQREEITAIVKMRVAGTASGTQHAGDFICTVYLEEKKAETEQHIKVPASMTAEELTLE
ILDRRNVGIREKDYWTCFEVNEREEAERPLHFAEKVLPILHGLGTDSHLVVKKHQAMEAM
LLYLASRVGDTKHGMMKFREDRSLLGLGLPSGGFHDRYFILNSSCLRLYKEVRSQRPWSG
APETSHRPEKEWPIKSLKVYLGVKKKLRPPTCWGFTVVHETEKHEKQQWYLCCDTQMELR
EWFATFLFVQHDGLVWPSEPSRVSRAVPEVRLGSVSLIPLRGSENEMRRSVAAFTADPLS

LLRNV*
>ENSG00000163539|ENST00000313350
MRRLICKRICDYKSFDDEESVDGNRPSSAASAFKVPAPKTSGNPANSARKPGSAGGPKVG
GASKEGGAGAVDEDDFIKAFTDVPSIQIYSSRELEETLNKIREILSDDKHDWDQRANALK
KIRSLLVAGAAQYDCFFQHLRLLDGALKLSAKDLRSQVVREACITVAHLSTVLGNKFDHG
AEAIVPTLFNLVPNSAKVMATSGCAAIRFIIRHTHVPRLIPLITSNCTSKSVPVRRRSFE
FLDLLLQEWQTHSLERHAAVLVETIKKGIHDADAEARVEARKTYMGLRNHFPGEAETLYN
SLEPSYQKSLQTYLKSSGSVASLPQSDRSSSSSQESLNRPFSSKWSTANPSTVAGRVSAG
SSKASSLPGSLQRSRSDIDVNAAAGAKAHHAAGQSVRSGRLGAGALNAGSYASLECEAFW
RSGRTAKLYSV*
>ENSG00000110195|ENST00000393679
MAQRMTTQLLLLLVWVAVVGEAQTRIAWARTELLNVCMNAKHHKEKPGPEDKLHEQCRPW
RKNACCSTNTSQEAHKDVSYLYRFNWNHCGEMAPACKRHFIQDTCLYECSPNLGPWIQQV
DQSWRKERVLNVPLCKEDCEQWWEDCRTSYTCKSNWHKGWNWTSGFNKCAVGAACQPFHF
YFPTPTVLCNEIWTHSYKVSNYSRGSGRCIQMWFDPAQGNPNEEVARFYAAAMSGAGPWA
AWPFLLSLALMLLWLLS*
>ENSG00000117153|ENST00000367261
MGGIMAPKDIMTNTHAKSILNSMNSLRKSNTLCDVTLRVEQKDFPAHRIVLAACSDYFCA
MFTSELSEKGKPYVDIQGLTASTMEILLDFVYTETVHVTVENVQELLPAACLLQLKGVKQ
ACCEFLESQLDPSNCLGIRDFAETHNCVDLMQAAEVFSQKHFPEVVQHEEFILLSQGEVE
KLIKCDEIQVDSEEPVFEAVINWVKHAKKEREESLPNLLQYVRMPLLTPRYITDVIDAEP
FIRCSLQCRDLVDEAKKFHLRPELRSQMQGPRTRARLGANEVLLVVGGFGSQQSPIDVVE
KYDPKTQEWSFLPSITRKRRYVASVSLHDRIYVIGGYDGRSRLSSVECLDYTADEDGVWY
SVAPMNVRRGLAGATTLGDMIYVSGGFDGSRRHTSMERYDPNIDQWSMLGDMQTAREGAG
LVVASGVIYCLGGYDGLNILNSVEKYDPHTGHWTNVTPMATKRSGAGVALLNDHIYVVGG
FDGTAHLSSVEAYNIRTDSWTTVTSMTTPRCYVGATVLRGRLYAIAGYDGNSLLSSIECY
DPIIDSWEVVTSMGTQRCDAGVCVLREK*
>ENSG00000164414|ENST00000369552
MAAPRDNVTLLFKLYCLAVMTLMAAVYTIALRYTRTSDKELYFSTTAVCITEVIKLLLSV
GILAKETGSLGRFKASLRENVLGSPKELLKLSVPSLVYAVQNNMAFLALSNLDAAVYQVT
YQLKIPCTALCTVLMLNRTLSKLQWVSVFMLCAGVTLVQWKPAQATKVVVEQNPLLGFGA
IAIAVLCSGFAGVYFEKVLKSSDTSLWVRNIQMYLSGIIVTLAGVYLSDGAEIKEKGFFY
GYTYYVWFVIFLASVGGLYTSVVVKYTDNIMKGFSAAAAIVLSTIASVMLFGLQITLTFA
LGTLLVCVSIYLYGLPRQDTTSIQQGETASKERVIGV*
>ENSG00000138802|ENST00000399100
MSAPAGSSHPAASARIPPKFGGAAVSGAAAPAGPGAGPAPHQQNGPAQNQMQVPSGYGLH
HQNYIAPSGHYSQGPGKMTSLPLDTQCGDYYSALYTVPTQNVTPNTVNQQPGAQQLYSRG
PPAPHIVGSTLGSFQGAASSASHLHTSASQPYSSFVNHYNSPAMYSASSSVASQGFPSTC
GHYAMSTVSNAAYPSVSYPSLPAGDTYGQMFTSQNAPTVRPVKDNSFSGQNTAISHPSPL
PPLPSQQHHQQQSLSGYSTLTWSSPGLPSTQDNLIRNHTGSLAVANNNPTITVADSLSCP
VMQNVQPPKSSPVVSTVLSGSSGSSSTRTPPTANHPVEPVTSVTQPSELLQQKGVDSSST
TSSASPMPNSYDALEGGSYPDMLSSSASSPAPDPAPEPDPASAPAPASAPAPVVPQPSKM
AKPFGYGYPTLQPGYQNATAPLISGVQPSNPVYSGFQQYPQQYPGVNQLSSSIGGLSLQS
SPQPESLRPVNLTQERNILPMTPVWAPVPNLNADLKKLNCSPDSFRCTLTNIPQTQALLN

KAKLPLGLLLHPFRDLTQLPVITSNTIVRCRSCRTYINPFVSFIDQRRWKCNLCYRVNDV
PEEFMYNPLTRSYGEPHKRPEVQNSTVEFIASSDYMLRPPQPAVYLFVLDVSHNAVEAGY
LTILCQSLLENLDKLPGDSRTRIGFMTFDSTIHFYNLQEGLSQPQMLIVSDIDDVFLPTP
DSLLVNLYESKELIKDLLNALPNMFTNTRETHSALGPALQAAFKLMSPTGGRVSVFQTQL
PSLGAGLLQSREDPNQRSSTKVVQHLGPATDFYKKLALDCSGQQTAVDLFLLSSQYSDLA
SLACMSKYSAGCIYYYPSFHYTHNPSQAEKLQKDLKRYLTRKIGFEAVMRIRCTKGLSMH
TFHGNFFVRSTDLLSLANINPDAGFAVQLSIEESLTDTSLVCFQTALLYTSSKGERRIRV
HTLCLPVVSSLADVYAGVDVQAAICLLANMAVDRSVSSSLSDARDALVNAVVDSLSAYGS
TVSNLQHSALMAPSSLKLFPLYVLALLKQKAFRTGTSTRLDDRVYAMCQIKSQPLVHLMK
MIHPNLYRIDRLTDEGAVHVNDRIVPQPPLQKLSAEKLTREGAFLMDCGSVFYIWVGKGC
DNNFIEDVLGYTNFASIPQKMTHLPELDTLSSERARSFITWLRDSRPLSPILHIVKDESP
AKAEFFQHLIEDRTEAAFSYYEFLLHVQQQICK*
>ENSG00000100280|ENST00000357586
MTDSKYFTTTKKGEIFELKAELNSDKKEKKKEAVKKVIASMTVGKDVSALFPDVVNCMQT
DNLELKKLVYLYLMNYAKSQPDMAIMAVNTFVKDCEDPNPLIRALAVRTMGCIRVDKITE
YLCEPLRKCLKDEDPYVRKTAAVCVAKLHDINAQLVEDQGFLDTLKDLISDSNPMVVANA
VAALSEIAESHPSSNLLDLNPQSINKLLTALNECTEWGQIFILDCLANYMPKDDREAQSI
CERVTPRLSHANSAVVLSAVKVLMKFMEMLSKDLDYYGTLLKKLAPPLVTLLSAEPELQY
VALRNINLIVQKRPEILKHEMKVFFVKYNDPIYVKLEKLDIMIRLASQANIAQVLAELKE
YATEVDVDFVRKAVRAIGRCAIKVEQSAERCVSTLLDLIQTKVNYVVQEAIVVIKDIFRK
YPNKYESVIATLCENLDSLDEPEARAAMIWIVGEYAERIDNADELLESFLEGFHDESTQV
QLQLLTAIVKLFLKKPTETQELVQQVLSLATQDSDNPDLRDRGYIYWRLLSTDPVAAKEV
VLAEKPLISEETDLIEPTLLDELICYIGTLASVYHKPPSAFVEGGRGVVHKSLPPRTASS
ESAESPETAPTGAPPGEQPDVIPAQGDLLGDLLNLDLGPPVSGPPLATSSVQMGAVDLLG
GGLDSLMGDEPEGIGGTNFVAPPTAAVPANLGAPIGSGLSDLFDLTSGVGTLSGSYVAPK
AVWLPAMKAKGLEISGTFTRQVGSISMDLQLTNKALQVMTDFAIQFNRNSFGLAPAAPLQ
VHAPLSPNQTVEISLPLSTVGSVMKMEPLNNLQVAVKNNIDVFYFSTLYPLHILFVEDGK
MDRQMFLATWKDIPNENEAQFQIRDCPLNAEAASSKLQSSNIFTVAKRNVEGQDMLYQSL
KLTNGIWVLAELRIQPGNPSCTDLELSLKCRAPEVSQHVYQAYETILKN*
>ENSG00000137177|ENST00000378826
MSDTKVKVAVRVRPMNRRELELNTKCVVEMEGNQTVLHPPPSNTKQGERKPPKVFAFDYC
FWSMDESNTTKYAGQEVVFKCLGEGILEKAFQGYNACIFAYGQTGSGKSFSMMGHAEQLG
LIPRLCCALFKRISLEQNESQTFKVEVSYMEIYNEKVRDLLDPKGSRQSLKVREHKVLGP
YVDGLSQLAVTSFEDIESLMSEGNKSRTVAATNMNEESSRSHAVFNIIITQTLYDLQSGN
SGEKVSKVSLVDLAGSERVSKTGAAGERLKEGSNINKSLTTLGLVISSLADQAAGKGKSK
FVPYRDSVLTWLLKDNLGGNSQTSMIATISPAADNYEETLSTLRYADRAKRIVNHAVVNE
DPNAKVIRELREEVEKLREQLSQAEAMKAPELKEKLEESEKLIKELTVTWEEKLRKTEEI
AQERQRQLESMGISLEMSGIKVGDDKCYLVNLNADPALNELLVYYLKDHTRVGADTSQDI
QLFGIGIQPQHCEIDIASDGDVTLTPKENARSCVNGTLVCSTTQLWHGDRILWGNNHFFR
INLPKRKRRDWLKDFEKETGPPEHDLDAASEASSEPDYNYEFAQMEVIMKTLNSNDPVQN
VVQVLEKQYLEEKRSALEEQRLMYERELEQLRQQLSPDRQPQSSGPDRLAYSSQTAQQKV
TQWAEERDELFRQSLAKLREQLVKANTLVREANFLAEEMSKLTDYQVTLQIPAANLSANR
KRGAIVSEPAIQVRRKGKSTQVWTIEKLENKLIDMRDLYQEWKEKVPEAKRLYGKRGDPF
YEAQENHNLIGVANVFLECLFCDVKLQYAVPIISQQGEVAGRLHVEVMRVTGAVPERVVE

DDSSENSSESGSLEVVDSSGEIIHRVKKLTCRVKIKEATGLPLNLSNFVFCQYTFWDQCE
STVAAPVVDPEVPSPQSKDAQYTVTFSHCKDYVVNVTEEFLEFISDGALAIEVWGHRCAG
NGSSIWEVDSLHAKTRTLHDRWNEVTRRIEMWISILELNELGEYAAVELHQAKDVNTGGI
FQLRQGHSRRVQVTVKPVQHSGTLPLMVEAILSVSIGCVTARSTKLQRGLDSYQRDDEDG
DDMDSYQEEDLNCVRERWSDALIKRREYLDEQIKKVSNKTEKTEDDVEREAQLVEQWVGL
TEERNAVLVPAPGSGIPGAPADWIPPPGMETHIPVLFLDLNADDLSANEQLVGPHASGVN
SILPKEHGSQFFYLPIIKHSDDEVSATASWDSSVHDSVHLNRVTPQNERIYLIVKTTVQL
SHPAAMELVLRKRIAANIYNKQSFTQSLKRRISLKNIFYSCGVTYEIVSNIPKATEEIED
RETLALLAARSENEGTSDGETYIEKYTRGVLQVENILSLERLRQAVTVKEALSTKARHIR
RSLSTPNVHNVSSSRPDLSGFDEDDKGWPENQLDMSDYSSSYQDVACYGTLPRDSPRRNK
EGCTSETPHALTVSPFKAFSPQPPKFFKPLMPVKEEHKKRIALEARPLLSQEDSEEEENE
LEAINRKLISSQPYVPVEFADFSVYNASLENREWFSSKVDLSNSRVLEKEVSRSPTTSSI
TSGYFSHSASNATLSDMVVPSSDSSDQLAIQTKDADSTEHSTPSLVHDFRPSSNKELTEV
EKGLVKDKIIVVPLKENSALAKGSPSSQSIPEKNSKSLCRTGSCSELDACPSKISQPARG
FCPREVTVEHTTNILEDHSFTEFMGVSEGKDFDGLTDSSAGELSSRRSLPNKTGGKTVSD
GLHHPSQLHSKLENDQVIIPEAAFWVLCCQ*
>ENSG00000047932|ENST00000368498
MSAGGPCPAAAGGGPGGASCSVGAPGGVSMFRWLEVLEKEFDKAFVDVDLLLGEIDPDQA
DITYEGRQKMTSLSSCFAQLCHKAQSVSQINHKLEAQLVDLKSELTETQAEKVVLEKEVH
DQLLQLHSIQLQLHAKTGQSADSGTIKAKLSGPSVEELERELEANKKEKMKEAQLEAEVK
LLRKENEALRRHIAVLQAEVYGARLAAKYLDKELAGRVQQIQLLGRDMKGPAHDKLWNQL
EAEIHLHRHKTVIRACRGRNDLKRPMQAPPGHDQDSLKKSQGVGPIRKVLLLKEDHEGLG
ISITGGKEHGVPILISEIHPGQPADRCGGLHVGDAILAVNGVNLRDTKHKEAVTILSQQR
GEIEFEVVYVAPEVDSDDENVEYEDESGHRYRLYLDELEGGGNPGASCKDTSGEIKVLQG
FNKKAVTDTHENGDLGTASETPLDDGASKLDDLHTLYHKKSY*
>ENSG00000206433|ENST00000400250
MLSRNDDICIYGGLGLGGLLLLAVVLLSACLCWLHRRGPGLLRAGTPLCISAEAASAQQ*
>ENSG00000206433|ENST00000400257
MLSRNDEAASAQQ*
>ENSG00000054116|ENST00000373162
MGFNIGVRLIEDFLARSNVGRCHDFRETADVIAKVAFKMYLGITPSITNWSPAGDEFSLI
LENNPLVDFVELPDNHSSLIYSNLLCGVLRGALEMVQMAVEAKFVQDTLKGDGVTEIRMR
FIRRIEDNLPAGEE*
>ENSG00000102100|ENST00000376515
MKLCRDAHRRLKYISLAVLVVQNASLILSIRYARTLPGDRFFATTAVVMAEVLKGLTCLL
LLFAQKRGNVKHLVLFLHEAVLVQYVDTLKLAVPSLIYTLQNNLQYVAISNLPAATFQPS
PRCSQSHSLCLCLRLRALRSPAASRAATTTAAVFPPWRPHHGALSAKVSAGEVRAGSNGG
TQGRGTGVEGVGHLQDPSRHPPGPGSSGFGRWSFLPGH*
>ENSG00000073969|ENST00000398238
MAGRSMQAARCPTDELSLTNCAVVNEKDFQSGQHVIVRTSPNHRYTFTLKTHPSVVPGSI
AFSLPQRKWAGLSIGQEIEVSLYTFDKAKQCIGTMTIEIDFLQKKSIDSNPYDTDKMAAE
FIQQFNNQAFSVGQQLVFSFNEKLFGLLVKDIEAMDPSILKGEPATGKRQKIEVGLVVGN
SQVAFEKAENSSLNLIGKAKTKENRQSIINPDWNFEKMGIGGLDKEFSDIFRRAFASRVF
PPEIVEQMGCKHVKGILLYGPPGCGKTLLARQIGKMLNAREPKVVNGPEILNKYVGESEA

NIRKLFADAEEEQRRLGANSGLHIIIFDEIDAICKQRGSMAGSTGVHDTVVNQLLSKIDG
VEQLNNILVIGMTNRPDLIDEALLRPGRLEVKMEIGLPDEKGRLQILHIHTARMRGHQLL
SADVDIKELAVETKNFSGAELEGLVRAAQSTAMNRHIKASTKVEVDMEKAESLQVTRGDF
LASLENDIKPAFGTNQEDYASYIMNGIIKWGDPVTRVLDDGELLVQQTKNSDRTPLVSVL
LEGPPHSGKTALAAKIAEESNFPFIKICSPDKMIGFSETAKCQAMKKIFDDAYKSQLSCV
VVDDIERLLDYVPIGPRFSNLVLQALLVLLKKAPPQGRKLLIIGTTSRKDVLQEMEMLNA
FSTTIHVPNIATGEQLLEALELLGNFKDKERTTIAQQVKGKKVWIGIKKLLMLIEMSLQM
DPEYRVRKFLALLREEGASPLDFD*
>ENSG00000075651|ENST00000351298
MSLKNEPRVNTSALQKIAADMSNIIENLDTRELHFEGEEVDYDVSPSDPKIQEVYIPFSA
IYNTQGFKEPNIQTYLSGCPIKAQVLEVERFTSTTRVPSINLYTIELTHGEFKWQVKRKF
KHFQEFHRELLKYKAFIRIPIPTRRHTFRRQNVREEPREMPSLPRSSENMIREEQFLGRR
KQLEDYLTKILKMPMYRNYHATTEFLDISQLSFIHDLGPKGIEGMIMKRSGGHRIPGLNC
CGQGRACYRWSKRWLIVKDSFLLYMKPDSGAIAFVLLVDKEFKIKVGKKETETKYGIRID
NLSRTLILKCNSYRHARWWGGAIEEFIQKHGTNFLKDHRFGSYAAIQENALAKWYVNAKG
YFEDVANAMEEANEEIFITDWWLSPEIFLKRPVVEGNRWRLDCILKRKAQQGVRIFIMLY
KEVELALGINSEYTKRTLMRLHPNIKVMRHPDHVSSTVYLWAHHEKLVIIDQSVAFVGGI
DLAYGRWDDNEHRLTDVGSVKRVTSGPSLGSLPPAAMESMESLRLKDKNEPVQNLPIQKS
IDDVDSKLKGIGKPRKFSKFSLYKQLHRHHLHDADSISSIDSTSSYFNHYRSHHNLIHGL
KPHFKLFHPSSESEQGLTRPHADTGSIRSLQTGVGELHGETRFWHGKDYCNFVFKDWVQL
DKPFADFIDRYSTPRMPWHDIASAVHGKAARDVARHFIQRWNFTKIMKSKYRSLSYPFLL
PKSQTTAHELRYQVPGSVHANVQLLRSAADWSAGIKYHEESIHAAYVHVIENSRHYIYIE
NQFFISCADDKVVFNKIGDAIAQRILKAHRENQKYRVYVVIPLLPGFEGDISTGGGNALQ
AIMHFNYRTMCRGENSILGQLKAELGNQWINYISFCGLRTHAELEGNLVTELIYVHSKLL
IADDNTVIIGSANINDRSMLGKRDSEMAVIVQDTETVPSVMDGKEYQAGRFARGLRLQCF
RVVLGYLDDPSEDIQDPVSDKFFKEVWVSTAARNATIYDKVFRCLPNDEVHNLIQLRDFI
NKPVLAKEDPIRAEEELKKIRGFLVQFPFYFLSEESLLPSVGTKEAIVPMEVWT*
>ENSG00000157214|ENST00000394626
MESISMMGSPKSLSETFLPNGINGIKDARKVTVGVIGSGDFAKSLTIRLIRCGYHVVIGS
RNPKFASEFFPHVVDVTHHEDALTKTNIIFVAIHREHYTSLWDLRHLLVGKILIDVSNNM
RINQYPESNAEYLASLFPDSLIVKGFNVVSAWALQLGPKDASRQVYICSNNIQARQQVIE
LARQLNFIPIDLGSLSSAREIENLPLRLFTLWRGPVVVAISLATFFFLYSFVRDVIHPYA
RNQQSDFYKIPIEIVNKTLPIVAITLLSLVYLAGLLAAAYQLYYGTKYRRFPPWLETWLQ
CRKQLGLLSFFFAMVHVAYSLCLPMRRSERYLFLNMAYQQVHANIENSWNEEEVWRIEMY
ISFGIMSLGLLSLLAVTSIPSVSNALNWREFSFIQSTLGYVALLISTFHVLIYGWKRAFE
EEYYRFYTPPNFVLALVLPSIVILDLLQLCRYPD*
>ENSG00000197586|ENST00000360031
MKKGIRYETSRKTSYIFQPQHGPWQTRMRKISNHGSLRVAKVAYPLGLCVGVFIYVAYIK
WHRATATQAFFSITRAAPGARWGQQAHSPLGTAADGHEVFYGIMFDAGSTGTRVHVFQFT
RPPRETPTLTHETFKALKPGLSAYADDVEKSAQGIRELLDVAKQDIPFDFWKATPLVLKA
TAGLRLLPGEKAQKLLQKVKKVFKASPFLVGDDCVSIMNGTDEGVSAWITINFLTGSLKT
PGGSSVGMLDLGGGSTQIAFLPRVEGTLQASPPGYLTALRMFNRTYKLYSYSYLGLGLMS
ARLAILGGVEGQPAKDGKELVSPCLSPSFKGEWEHAEVTYRVSGQKAAASLHELCAARVS
EVLQNRVHRTEEVKHVDFYAFSYYYDLAAGVGLIDAEKGGSLVVGDFEIAAKYVCRTLET

QPQSSPFSCMDLTYVSLLLQEFGFPRSKVLKLTRKIDNVETSWALGAIFHYIDSLNRQKS
PAS*
>ENSG00000181027|ENST00000391909
MRLTRCQAALAAAITLNLLVLFYVSWLQHQPRNSRARGPRRASAAGPRVTVLVREFEAFD
NAVPELVDSFLQQDPAQPVVVAADTLPYPPLALPRIPNVRLALLQPALDRPAAASRPETY
VATEFVALVPDGARAEAPGLLERMVEALRAGSARLVAAPVATANPARCLALNVSLREWTA
RYGAAPAAPRCDALDGDAVVLLRARDLFNLSAPLARPVGTSLFLQTALRGWAVQLLDLTF
AAARQPPLATAHARWKAEREGRARRAALLRALGIRLVSWEGGRLEWFGCNKETTRCFGTV
VGDTPAYLYEERWTPPCCLRALRETARYVVGVLEAAGVRYWLEGGSLLGAARHGDIIPWD
YDVDLGIYLEDVGNCEQLRGAEAGSVVDERGFVWEKAVEGDFFRVQYSESNHLHVDLWPF
YPRNGVMTKDTWLDHRQDVEFPEHFLQPLVPLPFAGFVAQAPNNYRRFLELKFGPGVIEN
PQYPNPALLSLTGSG*
>ENSG00000136156|ENST00000378565
MVKVTFNSALAQKEAKKDEPKSGEEALIIPPDAVAVDCKDPDDVVPVGQRRAWCWCMCFG
LAFMLAGVILGGAYLYKYFALQPDDVYYCGIKYIKDDVILNEPSADAPAALYQTIEENIK
IFEEEEVEFISVPVPEFADSDPANIVHDFNKKLTAYLDLNLDKCYVIPLNTSIVMPPRNL
LELLINIKAGTYLPQSYLIHEHMVITDRIENIDHLGFFIYRLCHDKETYKLQRRETIKGI
QKREASNCFAIRHFENKFAVETLICS*
>ENSG00000173846|ENST00000372201
MEPAAGFLSPRPFQRAAAAPAPPAGPGPPPSALRGPELEMLAGLPTSDPGRLITDPRSGR
TYLKGRLLGKGGFARCYEATDTETGSAYAVKVIPQSRVAKPHQREKILNEIELHRDLQHR
HIVRFSHHFEDADNIYIFLELCSRKSLAHIWKARHTLLEPEVRYYLRQILSGLKYLHQRG
ILHRDLKLGNFFITENMELKVGDFGLAARLEPPEQRKKTICGTPNYVAPEVLLRQGHGPE
ADVWSLGCVMYTLLCGSPPFETADLKETYRCIKQVHYTLPASLSLPARQLLAAILRASPR
DRPSIDQILRHDFFTKGYTPDRLPISSCVTVPDLTPPNPARSLFAKVTKSLFGRKKKSKN
HAQERDEVSGLVSGLMRTSVGHQDARPEAPAASGPAPVSLVETAPEDSSPRGTLASSGDG
FEEGLTVATVVESALCALRNCIAFMPPAEQNPAPLAQPEPLVWVSKWVDYSNKFGFGYQL
SSRRVAVLFNDGTHMALSANRKTVHYNPTSTKHFSFSVGAVPRALQPQLGILRYFASYME
QHLMKGGDLPSVEEVEVPAPPLLLQWVKTDQALLMLFSDGTVQVNFYGDHTKLILSGWEP
LLVTFVARNRSACTYLASHLRQLGCSPDLRQRLRYALRLLRDRSPA*
>ENSG00000206505|ENST00000383605
MAVMAPRTLVLLLSGALALTQTWAGSHSMRYFYTSVSRPGRGEPRFIAVGYVDDTQFVRF
DSDAASQRMEPRAPWIEQEGPEYWDRNTRNVKAHSQTDRANLGTLRGYYNQSEDGSHTIQ
RMYGCDVGPDGRFLRGYQQDAYDGKDYIALNEDLRSWTAADMAAQITQRKWETAHEAEQW
RAYLEGRCVEWLRRYLENGKETLQRTDAPKTHMTHHAVSDHEATLRCWALSFYPAEITLT
WQRDGEDQTQDTELVETRPAGDGTFQKWASVVVPSGQEQRYTCHVQHEGLPKPLTLRWEP
SSQPTIPIVGIIAGLVLFGAVIAGAVVAAVMWRRKSSDRKGGSYSQAASSDSAQGSDMSL
TACKV*
>ENSG00000134909|ENST00000392657
MKSRPTKQKLKQRGILKERVFGCDLGEHLLNSGFEVPQVLQSCTAFIERYGIVDGIYRLS
GVASNIQRLRHEFDSEHVPDLTKEPYVQDIHSVGSLCKLYFRELPNPLLTYQLYEKFSDA
VSAATDEERLIKIHDVIQQLPPPHYRTLEFLMRHLSLLADYCSITNMHAKNLAIVWAPNL
LRSKQIESACFSGTAAFMEVRIQSVVVEFILNHVDVLFSGRISMAMQEGAASLSRPKSLL
VSSPSTKLLTLEEAQARTQAQVNSPIVTENKYIEVGEGPAALQGKFHTIIEFPLERKRPQ

NKMKKSPVGSWRSFFNLGKSSSVSKRKLQRNESEPSEMKAMALKGGRAEGTLRSAKSEES
LTSLHAVDGDSKLFRPRRPRSSSDALSASFNGEMLGNRCNSYDNLPHDNESEEEGGLLHI
PALMSPHSAEDVDLSPPDIGVASLDFDPMSFQCSPPKAESECLESGASFLDSPGYSKDKP
SANKKDAETGSSQCQTPGSTASSEPVSPLQEKLSPFFTLDLSPTEDKSSKPSSFTEKVVY
AFSPKIGRKLSKSPSMSISEPISVTLPPRVSEVIGTVSNTTAQNASSSTWDKCVEERDAT
NRSPTQIVKMKTNETVAQEAYESEVQPLDQVAAEEVELPGKEDQSVSSSQSKAVASGQTQ
TGAVTHDPPQDSVPVSSVSLIPPPPPPKNVARMLALALAESAQQASTQSLKRPGTSQAGY
TNYGDIAVATTEDNLSSSYSAVALDKAYFQTDRPAEQFHLQNNAPGNCDHPLPETTATGD
PTHSNTTESGEQHHQVDLTGNQPHQAYLSGDPEKARITSVPLDSEKSDDHVSFPEDQSGK
NSMPTVSFLDQDQSPPRFYSGDQPPSYLGASVDKLHHPLEFADKSPTPPNLPSDKIYPPS
GSPEENTSTATMTYMTTTPATAQMSTKEASWDVAEQPTTADFAAATLQRTHRTNRPLPPP
PSQRSAEQPPVVGQVQAATNIGLNNSHKVQGVVPVPERPPEPRAMDDPASAFISDSGAAA
AQCPMATAVQPGLPEKVRDGARVPLLHLRAESVPAHPCGFPAPLPPTRMMESKMIAAIHS
SSADATSSSNYHSFVTASSTSVDDALPLPLPVPQPKHASQKTVYSSFARPDVTTEPFGPD
NCLHFNMTPNCQYRPQSVPPHHNKLEQHQVYGARSEPPASMGLRYNTYVAPGRNASGHHS
KPCSRVEYVSSLSSSVRNTCYPEDIPPYPTIRRVQSLHAPPSSMIRSVPISRTEVPPDDE
PAYCPRPLYQYKPYQSSQARSDYHVTQLQPYFENGRVHYRYSPYSSSSSSYYSPDGALCD
VDAYGTVQLRPLHRLPNRDFAFYNPRLQGKSLYSYAGLAPRPRANVTGYFSPNDHNVVSM
PPAADVKHTYTSWDLEDMEKYRMQSIRRESRARQKVKGPVMSQYDNMTPAVQDDLGGIYV
IHLRSKSDPGKTGLLSVAEGKESRHAAKAISPEGEDRFYRRHPEAEMDRAHHHGGHGSTQ
PEKPSLPQKQSSLRSRKLPDMGCSLPEHRAHQEASHRQFCESKNGPPYPQGAGQLDYGSK
GIPDTSEPVSYHNSGVKYAASGQESLRLNHKEVRLSKEMERPWVRQPSAPEKHSRDCYKE
EEHLTQSIVPPPKPERSHSLKLHHTQNVERDPSVLYQYQPHGKRQSSVTVVSQYDNLEDY
HSLPQHQRGVFGGGGMGTYVPPGFPHPQSRTYATALGQGAFLPAELSLQHPETQIHAE*
>ENSG00000106003|ENST00000402045
MTPGRCCLAADIQVETFIFTDGEDEALARHTGNVVITNCSAAHSRQALSCKMAVEYDRFI
ESGRKWFCHVDDDNYVNLRALLRLLASYPHTRDVYVGKPSLDRPIQAMERVSENKVRPVH
FWFATGGAGFCISRGLALKMSPWASGGHFMNTAERIRLPDDCTIGYIVEALLGVPLIRSG
LFHSHLENLQQVPTSELHEQVTLSYGMFENKRNAVHVKGPFSVEADPSRFRSIHCHLYPD
TPWCPRTAIF*
>ENSG00000169359|ENST00000359479
MSPTISHKDSSRQRRPGNFSHSLDMKSGPLPPGGWDDSHLDSAGREGDREALLGDTGTGD
FLKAPQSFRAELSSILLLLFLYVLQGIPLGLAGSIPLILQSKNVSYTDQAFFSFVFWPFS
LKLLWAPLVDAVYVKNFGRRKSWLVPTQYILGLFMIYLSTQVDRLLGNTDDRTPDVIALT
VAFFLFEFLAATQDIAVDGWALTMLSRENVGYASTCNSVGQTAGYFLGNVLFLALESADF
CNKYLRFQPQPRGIVTLSDFLFFWGTVFLITTTLVALLKKENEVSVVKEETQGITDTYKL
LFAIIKMPAVLTFCLLILTAKIGFSAADAVTGLKLVEEGVPKEHLALLAVPMVPLQIILP
LIISKYTAGPQPLNTFYKAMPYRLLLGLEYALLVWWTPKVEHQGGFPIYYYIVVLLSYAL
HQVTVYSMYVSIMAFNAKVSDPLIGGTYMTLLNTVSNLGGNWPSTVALWLVDPLTVKECV
GASNQNCRTPDAVELCKKLGGSCVTALDGYYVESIICVFIGFGWWFFLGPKFKKLQDEGS
SSWKCKRNN*
>ENSG00000169241|ENST00000368401
MRGLHPWHVLRRPLGPQAHANDPECGQRPVPALSHHGSQRVVLLQTATLLGVLLLGYGYF
WLLVPNPEARLQQLGLFCSVFTISMYLSPLADLAKVIQTKSTQCLSYPLTIATLLTSASW

CLYGFRLRDPYIMVSNFPGIVTSFIRFWLFWKYPQEQDRNYWLLQT*
>ENSG00000177692|ENST00000402202
MNTMYVMMAQILRSHLIKATVIPNRVKMLPYFGIIRNRMMSTHKSKKKIREYYRLLNVEE
GCSADEVRESFHKLAKQYHPDSGSNTADSATFIRIEKAYRKVLSHVIEQTNASQSKGEEE
EDVEKFKYKTPQHRHYLSFEGIGFGTPTQREKHYRQFRADRAAEQVMEYQKQKLQSQYFP
DSVIVKNIRQSKQQKITQAIERLVEDLIQESMAKGDFDNLSGKGKPLKKFSDCSYIDPMT
HNLNRILIDNGYQPEWILKQKEISDTIEQLREAILVSRKKLGNPMTPTEKKQWNHVCEQF
QENIRKLNKRINDFNLIVPILTRQKVHFDAQKEIVRAQKIYETLIKTKEVTDRNPNNLDQ
GEGEKTPEIKKGFLNWMNLWKFIKIRSF*
>ENSG00000134602|ENST00000394335
MAHSPVAVQVPGMQGSKLWIIMEYLGGGSALDLLRAGPFDEFQIATMLKEILKGLDYLHS
EKKIHRDIKAANVLLSEQGDVKLADFGVAGQLTDTQIKRNTFVGTPFWMAPEVIQQSAYD
SKADIWSLGITAIELAKGEPPNSDMHPMRVLFLIPKNNPPTLVGDFTKSFKEFIDACLNK
DPSFRPTAKELLKHKFIVKNSKKTSYLTELIDRFKRWKAEGHSDDESDSEGSDSESTSRE
NNTHPEWSFTTVRKKPDPKKVQNGAEQDLVQTLSCLSMIITPAFAELKQQDENNASRNQA
IEELEKSIAVAEAACPGITDKMVKKLIEKFQKCSADESP*
>ENSG00000171155|ENST00000371313
MLSESSSFLKGVMLGSIFCALITMLGHIRIGHGNRMHHHEHHHLQAPNKEDILKISEDER
MELSKSFRVYCIILVKPKDVSLWAAVKETWTKHCDKAEFFSSENVKVFESINMDTNDMWL
MMRKAYKYAFDKYRDQYNWFFLARPTTFAIIENLKYFLLKKDPSQPFYLGHTIKSGDLEY
VGMEGGIVLSVESMKRLNSLLNIPEKCPEQGGMIWKISEDKQLAVCLKYAGVFAENAEDA
DGKDVFNTKSVGLSIKEAMTYHPNQVVEGCCSDMAVTFNGLTPNQMHVMMYGVYRLRAFG
HIFNDALVFLPPNGSDND*
>ENSG00000120215|ENST00000381471
MPREDAHFIYGYPKKGHGHSYTTAEEAAGIGILTVILGVLLLIGCWYCRRRNGYRALMDK
SLHVGTQCALTRRCPQEGFDHRDSKVSLQEKNCEPVVPNAPPAYEKLSAEQSPPPYSP*
>ENSG00000205060|ENST00000378509
MRPALAVGLVFAGCCSNVIFLELLARKHPGCGNIVTFAQFLFIAVEGFLFEADLGRKPPA
IPIRYYAIMVTMFFTVSVVNNYALNLNIAMPLHMIFRSGSLIANMILGIIILKKRYSIFK
YTSIALVSVGIFICTFMSAKQVTSQSSLSENDGFQAFVWWLLGIGALTFALLMSARMGIF
QETLYKRFGKHSKEALFYNHALPLPGFVFLASDIYDHAVLFNKSELYEIPVIGVTLPIMW
FYLLMNIITQYVCIRGVFILTTECASLTVTLVVTLRKFVSLIFSILYFQNPFTLWHWLGT
LFVFIGTLMYTEVWNNLGTTKSEPQKDSKKN*
>ENSG00000162630|ENST00000367434
MLQWRRRHCCFAKMTWNAKRSLFRTHLIGVLSLVFLFAMFLFFNHHDWLPGRAGFKENPV
TYTFRGFRSTKSETNHSSLRNIWKETVPQTLRPQTATNSNNTDLSPQGVTGLENTLSANG
SIYNEKGTGHPNSYHFKYIINEPEKCQEKSPFLILLIAAEPGQIEARRAIRQTWGNESLA
PGIQITRIFLLGLSIKLNGYLQRAILEESRQYHDIIQQEYLDTYYNLTIKTLMGMNWVAT
YCPHIPYVMKTDSDMFVNTEYLINKLLKPDLPPRHNYFTGYLMRGYAPNRNKDSKWYMPP
DLYPSERYPVFCSGTGYVFSGDLAEKIFKVSLGIRRLHLEDVYVGICLAKLRIDPVPPPN
EFVFNHWRVSYSSCKYSHLITSHQFQPSELIKYWNHLQQNKHNACANAAKEKAGRYRHRK
LH*
>ENSG00000171603|ENST00000377298
MLRRPAPALAPAARLLLAGLLCGGGVWAARVNKHKPWLEPTYHGIVTENDNTVLLDPPLI

ALDKDAPLRFAESFEVTVTKEGEICGFKIHGQNVPFDAVVVDKSTGEGVIRSKEKLDCEL
QKDYSFTIQAYDCGKGPDGTNVKKSHKATVHIQVNDVNEYAPVFKEKSYKATVIEGKQYD
SILRVEAVDADCSPQFSQICSYEIITPDVPFTVDKDGYIKNTEKLNYGKEHQYKLTVTAY
DCGKKRATEDVLVKISIKPTCTPGWQGWNNRIEYEPGTGALAVFPNIHLETCDEPVASVQ
ATVELETSHIGKGCDRDTYSEKSLHRLCGAAAGTAELLPSPSGSLNWTMGLPTDNGHDSD
QVFEFNGTQAVRIPDGVVSVSPKEPFTISVWMRHGPFGRKKETILCSSDKTDMNRHHYSL
YVHGCRLIFLFRQDPSEEKKYRPAEFHWKLNQVCDEEWHHYVLNVEFPSVTLYVDGTSHE
PFSVTEDYPLHPSKIETQLVVGACWQEFSGVENDNETEPVTVASAGGDLHMTQFFRGNLA
GLTLRSGKLADKKVIDCLYTCKEGLDLQVLEDSGRGVQIQAHPSQLVLTLEGEDLGELDK
AMQHISYLNSRQFPTPGIRRLKITSTIKCFNEATCISVPPVDGYVMVLQPEEPKISLSGV
HHFARAASEFESSEGVFLFPELRIISTITREVEPEGDGAEDPTVQESLVSEEIVHDLDTC
EVTVEGEELNHEQESLEVDMARLQQKGIEVSSSELGMTFTGVDTMASYEEVLHLLRYRNW
HARSLLDRKFKLICSELNGRYISNEFKVEVNVIHTANPMEHANHMAAQPQFVHPEHRSFV
DLSGHNLANPHPFAVVPSTATVVIVVCVSFLVFMIILGVFRIRAAHRRTMRDQDTGKENE
MDWDDSALTITVNPMETYEDQHSSEEEEEEEEEEEESEDGEEEDDITSAESESSEEEEGEQ
GDPQNATRQQQLEWDDSTLSY*
>ENSG00000198961|ENST00000361557
MSQYTEKEPAAMDQESGKAVWPKPAGGYQTITGRRYGRRHAYVSFKPCMTRHERSLGRAG
DDYEVLELDDVPKENSSGSSPLDQVDSSLPSEPIFEKSETEIPTCGSALNQTTESSQSFV
AVHHSEEGRDTLGSSTNLHNHSEGEYIPGACSASSVQNGIALVHTDSYDPDGKHGEDNDH
LQLSAEVVEGSRYQESLGNTVFELENREAEAYTGLSPPVPSFNCEVRDEFEELDSVPLVK
SSAGDTEFVHQNSQEIQRSSQDEMVSTKQQNNTSQERQTEHSPEDAACGPGHICSEQNTN
DREKNHGSSPEQVVRPKVRKLISSSQVDQETGFNRHEAKQRSVQRWREALEVEESGSDDL
LIKCEEYDGEHDCMFLDPPYSRVITQRETENNQMTSESGATAGRQEVDNTFWNGCGDYYQ
LYDKDEDSSECSDGEWSASLPHRFSGTEKDQSSSDESWETLPGKDENEPELQSDSSGPEE
ENQELSLQEGEQTSLEEGEIPWLQYNEVNESSSDEGNEPANEFAQPAFMLDGNNNLEDDS
SVSEDLDVDWSLFDGFADGLGVAEAISYVDPQFLTYMALEERLAQAMETALAHLESLAVD
VEVANPPASKESIDGLPETLVLEDHTAIGQEQCCPICCSEYIKDDIATELPCHHFFHKPC
VSIWLQKSGTCPVCRRHFPPAVIEASAAPSSEPDPDAPPSNDSIAEAP*
>ENSG00000148248|ENST00000371989
MGQNDLMGTAEDFADQFLRVTKQYLPHVARLCLISTFLEDGIRMWFQWSEQRDYIDTTWN
CGYLLASSFVFLNLLGQLTGCVLVLSRNFVQYACFGLFGIIALQTIAYSILWDLKFLMRN
LALGGGLLLLLAESRSEGKSMFAGVPTMRESSPKQYMQLGGRVLLVLMFMTLLHFDASFF
SIVQNIVGTALMILVAIGFKTKLAALTLVVWLFAINVYFNAFWTIPVYKPMHDFLKYDFF
QTMSVIGGLLLVVALGPGGVSMDEKKKEW*
>ENSG00000158488|ENST00000368160
MLLLFLLFEGLCCPGENTAAPQALQSYHLAAEEQLSFRMLQTSSFANHSWAHSEGSGWLG
DLQTHGWDTVLGTIRFLKPWSHGNFSKQELKNLQSLFQLYFHSFIQIVQASAGQFQLEYP
FEIQILAGCRMNAPQIFLNMAYQGSDFLSFQGISWEPSPGAGIRAQNICKVLNRYLDIKE
ILQSLLGHTCPRFLAGLMEAGESELKRKVKPEAWLSCGPSPGPGRLQLVCHVSGFYPKPV
WVMWMRGEQEQRGTQRGDVLPNADETWYLRATLDVAAGEAAGLSCRVKHSSLGGHDLIIH
WGGYSIFLILICLTVIVTLVILVVVDSRLKKQSPVFLMGANTQDTKNSRHQFCLAQVSWI
KNRVLKKWKTRLNQLW*
>ENSG00000186635|ENST00000359373

MAEAGDAALSVAEWLRALHLEQYTGLFEQHGLVWATECQGLSDTRLMDMGMLLPGHRRRI
LAGLLRAHTSPAPAPRPTPRPVPMKRHIFRSPPVPATPPEPLPTTTEDEGLPAAPPIPPR
RSCLPPTCFTTPSTAAPDPVLPPLPAKRHLAELSVPPVPPRTGPPRLLVSLPTKEEESLL
PSLSSPPQPQSEEPLSTLPQGPPQPPSPPPCPPEIPPKPVRLFPEFDDSDYDEVPEEGPG
APARVMTKKEEPPPSRVPRAVRVASLLSEGEELSGDDQGDEEEDDHAYEGVPNGGWHTSS
LSLSLPSTIAAPHPMDGPPGGSTPVTPVIKAGWLDKNPPQGSYIYQKRWVRLDTDHLRYF
DSNKDAYSKRFISVACISHVAAIGDQKFEVITNNRTFAFRAESDVERKEWMQALQQAMAE
QRARARLSSAYLLGVPGSEQPDRAGSLELRGFKNKLYVAVVGDKVQLYKNLEEYHLGIGI
TFIDMSVGNVKEVDRRSFDLTTPYRIFSFSADSELEKEQWLEAMQGAIAEALSTSEVAER
IWAAAPNRFCADCGAPQPDWASINLCVVICKRCAGEHRGLGAGVSKVRSLKMDRKVWTET
LIELFLQLGNGAGNRFWAANVPPSEALQPSSSPSTRRCHLEAKYREGKYRRYHPLFGNQE
ELDKALCAAVTTTDLAETQALLGCGAGINCFSGDPEAPTPLALAEQAGQTLQMEFLRNNR
TTEVPRLDSMKPLEKHYSVVLPTVSHSGFLYKTASAGKLLQDRRAREEFSRRWCVLGDGV
LSYFENERAVTPNGEIRASEIVCLAVPPPDTHGFEHTFEVYTEGERLYLFGLESAEQAHE
WVKCIAKAFVPPLAEDLLARDFERLGRLPYKAGLSLQRAQEGWFSLSGSELRAVFPEGPC
EEPLQLRKLQELSIQGDSENQVLVLVERRRTLYIQGERRLDFMGWLGAIQKAAASMGDTL
SEQQLGDSDIPVIVYRCVDYITQCGLTSEGIYRKCGQTSKTQRLLESLRQDARSVHLKEG
EQHVDDVSSALKRFLRDLPDGLFTRAQRLTWLEASEIEDEEEKVSRYRELLVRLPPVNRA
TVKALISHLYCVQCFSDTNQMNVHNLAIVFGPTLFQTDGQDYKAGRVVEDLINHYVVVFS
VDEEELRKQREEITAIVKMRVAGTASGTQHAGDFICTVYLEEKKAETEQHIKVPASMTAE
ELTLEILDRRNVGIREKDYWTCFEVNEREEAERPLHFAEKVLPILHGLGTDSHLVVKKHQ
AMEAMLLYLASRVGDTKHGMMKFREDRSLLGLGLPSGGFHDRYFILNSSCLRLYKEVRSH
RPEKEWPIKSLKVYLGVKKKLRPPTCWGFTVVHETEKHEKQQWYLCCDTQMELREWFATF
LFVQHDGLVWPSEPSRVSRAVPEVRLGSVSLIPLRGSENEMRRSVAAFTADPLSLLRNV*
>ENSG00000198734|ENST00000367797
MFPGCPRLWVLVVLGTSWVGWGSQGTEAAQLRQFYVAAQGISWSYRPEPTNSSLNLSVTS
FKKIVYREYEPYFKKEKPQSTISGLLGPTLYAEVGDIIKVHFKNKADKPLSIHPQGIRYS
KLSEGASYLDHTFPAEKMDDAVAPGREYTYEWSISEDSGPTHDDPPCLTHIYYSHENLIE
DFNSGLIGPLLICKKGTLTEGGTQKTFDKQIVLLFAVFDESKSWSQSSSLMYTVNGYVNG
TMPDITVCAHDHISWHLLGMSSGPELFSIHFNGQVLEQNHHKVSAITLVSATSTTANMTV
GPEGKWIISSLTPKHLQAGMQAYIDIKNCPKKTRNLKKITREQRRHMKRWEYFIAAEEVI
WDYAPVIPANMDKKYRSQHLDNFSNQIGKHYKKVMYTQYEDESFTKHTVNPNMKEDGILG
PIIRAQVRDTLKIVFKNMASRPYSIYPHGVTFSPYEDEVNSSFTSGRNNTMIRAVQPGET
YTYKWNILEFDEPTENDAQCLTRPYYSDVDIMRDIASGLIGLLLICKSRSLDRRGIQRAA
DIEQQAVFAVFDENKSWYLEDNINKFCENPDEVKRDDPKFYESNIMSTINGYVPESITTL
GFCFDDTVQWHFCSVGTQNEILTIHFTGHSFIYGKRHEDTLTLFPMRGESVTVTMDNVGT
WMLTSMNSSPRSKKLRLKFRDVKCIPDDDEDSYEIFEPPESTVMATRKMHDRLEPEDEES
DADYDYQNRLAAALGIRSFRNSSLNQEEEEFNLTALALENGTEFVSSNTDIIVGSNYSSP
SNISKFTVNNLAEPQKAPSHQQATTAGSPLRHLIGKNSVLNSSTAEHSSPYSEDPIEDPL
QPDVTGIRLLSLGAGEFKSQEHAKHKGPKVERDQAAKHRFSWMKLLAHKVGRHLSQDTGS
PSGMRPWEDLPSQDTGSPSRMRPWKDPPSDLLLLKQSNSSKILVGRWHLASEKGSYEIIQ
DTDEDTAVNNWLISPQNASRAWGESTPLANKPGKQSGHPKFPRVRHKSLQVRQDGGKSRL
KKSQFLIKTRKKKKEKHTHHAPLSPRTFHPLRSEAYNTFSERRLKHSLVLHKSNETSLPT
DLNQTLPSMDFGWIASLPDHNQNSSNDTGQASCPPGLYQTVPPEEHYQTFPIQDPDQMHS

TSDPSHRSSSPELSEMLEYDRSHKSFPTDISQMSPSSEHEVWQTVISPDLSQVTLSPELS
QTNLSPDLSHTTLSPELIQRNLSPALGQMPISPDLSHTTLSPDLSHTTLSLDLSQTNLSP
ELSQTNLSPALGQMPLSPDLSHTTLSLDFSQTNLSPELSHMTLSPELSQTNLSPALGQMP
ISPDLSHTTLSLDFSQTNLSPELSQTNLSPALGQMPLSPDPSHTTLSLDLSQTNLSPELS
QTNLSPDLSEMPLFADLSQIPLTPDLDQMTLSPDLGETDLSPNFGQMSLSPDLSQVTLSP
DISDTTLLPDLSQISPPPDLDQIFYPSESSQSLLLQEFNESFPYPDLGQMPSPSSPTLND
TFLSKEFNPLVIVGLSKDGTDYIEIIPKEEVQSSEDDYAEIDYVPYDDPYKTDVRTNINS
SRDPDNIAAWYLRSNNGNRRNYYIAAEEISWDYSEFVQRETDIEDSDDIPEDTTYKKVVF
RKYLDSTFTKRDPRGEYEEHLGILGPIIRAEVDDVIQVRFKNLASRPYSLHAHGLSYEKS
SEGKTYEDDSPEWFKEDNAVQPNSSYTYVWHATERSGPESPGSACRAWAYYSAVNPEKDI
HSGLIGPLLICQKGILHKDSNMPMDMREFVLLFMTFDEKKSWYYEKKSRSSWRLTSSEMK
KSHEFHAINGMIYSLPGLKMYEQEWVRLHLLNIGGSQDIHVVHFHGQTLLENGNKQHQLG
VWPLLPGSFKTLEMKASKPGWWLLNTEVGENQRAGMQTPFLIMDRDCRMPMGLSTGIISD
SQIKASEFLGYWEPRLARLNNGGSYNAWSVEKLAAEFASKPWIQVDMQKEVIITGIQTQG
AKHYLKSCYTTEFYVAYSSNQINWQIFKGNSTRNVMYFNGNSDASTIKENQFDPPIVARY
IRISPTRAYNRPTLRLELQGCEVNGCSTPLGMENGKIENKQITASSFKKSWWGDYWEPFR
ARLNAQGRVNAWQAKANNNKQWLEIDLLKIKKITAIITQGCKSLSSEMYVKSYTIHYSEQ
GVEWKPYRLKSSMVDKIFEGNTNTKGHVKNFFNPPIISRFIRVIPKTWNQSIALRLELFG
CDIY*
>ENSG00000125975|ENST00000374345
MKRWQIFVLWVFWVLILWLMTPYLDLTPESAPQEKRMYLVPQHCDCPWFSSGKCGCPSET
LNCSSCHHTADEWNWLDACSRKTMGYLMRTRESMTSDTVLWWLGMNSGSELGKLWRKLFK
GIPRLSVSHFDFYCGTCVLLGRPQIPQGSSLGNDIDQYPVVFRNASDQGSWMQLEMLLRK
LSDLVWTSDALSDKILEDGLVP*
>ENSG00000072310|ENST00000355815
MDEPPFSEAALEQALGEPCDLDAALLTDIEGEVGAGRGRANGLDAPRAGADRGAMDCTFE
DMLQLINNQDSDFPGLFDPPYAGSGAGGTDPASPDTSSPGSLSPPPATLSSSLEAFLSGP
QAAPSPLSPPQPAPTPLKMYPSMPAFSPGPGIKEESVPLSILQTPTPQPLPGALLPQSFP
APAPPQFSSTPVLGYPSPPGGFSTGSPPGNTQQPLPGLPLASPPGVPPVSLHTQVQSVVP
QQLLTVTAAPTAAPVTTTVTSQIQQVPVLLQPHFIKADSLLLTAMKTDGATVKAAGLSPL
VSGTTVQTGPLPTLVSGGTILATVPLVVDAEKLPINRLAAGSKAPASAQSRGEKRTAHNA
IEKRYRSSINDKIIELKDLVVGTEAKLNKSAVLRKAIDYIRFLQHSNQKLKQENLSLRTA
VHKSKSLKDLVSACGSGGNTDVLMEGVKTEVEDTLTPPPSDAGSPFQSSPLSLGSRGSGS
GGSGSDSEPDSPVFEDSKAKPEQRPSLHSRGMLDRSRLALCTLVFLCLSCNPLASLLGAR
GLPSPSDTTSVYHSPGRNVLGTESRDGPGWAQWLLPPVVWLLNGLLVLVSLVLLFVYGEP
VTRPHSGPAVYFWRHRKQADLDLARGDFAQAAQQLWLALRALGRPLPTSHLDLACSLLWN
LIRHLLQRLWVGRWLAGRAGGLQQDCALRVDASASARDAALVYHKLHQLHTMGKHTGGHL
TATNLALSALNLAECAGDAVSVATLAEIYVAAALRVKTSLPRALHFLTRFFLSSARQACL
AQSGSVPPAMQWLCHPVGHRFFVDGDWSVLSTPWESLYSLAGNPVDPLAQVTQLFREHLL
ERALNCVTQPNPSPGSADGDKEFSDALGYLQLLNSCSDAAGAPAYSFSISSSMATTTGVD
PVAKWWASLTAVVIHWLRRDEEAAERLCPLVEHLPRVLQESERPLPRAALHSFKAARALL
GCAKAESGPASLTICEKASGYLQDSLATTPASSSIDKAVQLFLCDLLLVVRTSLWRQQQP
PAPAPAAQGTSSRPQASALELRGFQRDLSSLRRLAQSFRPAMRRVFLHEATARLMAGASP
TRTHQLLDRSLRRRAGPGGKGGAVAELEPRPTRREHAEALLLASCYLPPGFLSAPGQRVG

MLAEAARTLEKLGDRRLLHDCQQMLMRLGGGTTVTSS*
>ENSG00000126218|ENST00000375559
MGRPLHLVLLSASLAGLLLLGESLFIRREQANNILARVTRANSFLEEMKKGHLERECMEE
TCSYEEAREVFEDSDKTNEFWNKYKDGDQCETSPCQNQGKCKDGLGEYTCTCLEGFEGKN
CELFTRKLCSLDNGDCDQFCHEEQNSVVCSCARGYTLADNGKACIPTGPYPCGKQTLERR
KRSVAQATSSSGEAPDSITWKPYDAADLDPTENPFDLLDFNQTQPERGDNNLTRIVGGQE
CKDGECPWQALLINEENEGFCGGTILSEFYILTAAHCLYQAKRFKVRVGDRNTEQEEGGE
AVHEVEVVIKHNRFTKETYDFDIAVLRLKTPITFRMNVAPACLPERDWAESTLMTQKTGI
VSGFGRTHEKGRQSTRLKMLEVPYVDRNSCKLSSSFIITQNMFCAGYDTKQEDACQGDSG
GPHVTRFKDTYFVTGIVSWGEGCARKGKYGIYTKVTAFLKWIDRSMKTRGLPKAKSHAPE
VITSSPLK*
>ENSG00000080815|ENST00000394157
MTELPAPLSYFQNAQMSEDNHLSNTVRSQNDNRERQEHNDRRSLGHPEPLSNGRPQGNSR
QVVEQDEEEDEELTLKYGAKHVIMLFVPVTLCMVVVVATIKSVSFYTRKDGQLIYTPFTE
DTETVGQRALHSILNAAIMISVIVVMTILLVVLYKYRCYKVSMRHRSLLSTLFFLWLGIL
VTVT*
>ENSG00000196562|ENST00000359930
MGPPSLVLCLLSATVFSLLGGSSAFLSHHRLKGRFQRDRRNIRPNIILVLTDDQDVELGS
MQVMNKTRRIMEQGGAHFINAFVTTPMCCPSRSSILTGKYVHNHNTYTNNENCSSPSWQA
QHESRTFAVYLNSTGYRTAFFGKYLNEYNGSYVPPGWKEWVGLLKNSRFYNYTLCRNGVK
EKHGSDYSKDYLTDLITNDSVSFFRTSKKMYPHRPVLMVISHAAPHGPEDSAPQYSRLFP
NASQHITPSYNYAPNPDKHWIMRYTGPMKPIHMEFTNMLQRKRLQTLMSVDDSMETIYNM
LVETGELDNTYIVYTADHGYHIGQFGLVKGKSMPYEFDIRVPFYVRGPNVEAGCLNPHIV
LNIDLAPTILDIAGLDIPADMDGKSILKLLDTERPVNRFHLKKKMRVWRDSFLVERGKLL
HKRDNDKVDAQEENFLPKYQRVKDLCQRAEYQTACEQLGQKWQCVEDATGKLKLHKCKGP
MRLGGSRALSNLVPKYYGQGSEACTCDSGDYKLSLAGRRKKLFKKKYKASYVRSRSIRSV
AIEVDGRVYHVGLGDAAQPRNLTKRHWPGAPEDQDDKDGGDFSGTGGLPDYSAANPIKVT
HRCYILENDTVQCDLDLYKSLQAWKDHKLHIDHEIETLQNKIKNLREVRGHLKKKRPEEC
DCHKISYHTQHKGRLKHRGSSLHPFRKGLQEKDKVWLLREQKRKKKLRKLLKRLQNNDTC
SMPGLTCFTHDNQHWQTAPFWTLGPFCACTSANNNTYWCMRTINETHNFLFCEFATGFLE
YFDLNTDPYQLMNAVNTLDRDVLNQLHVQLMELRSCKGYKQCNPRTRNMDLGLKDGGSYE
QYRQFQRRKWPEMKRPSSKSLGQLWEGWEG*
>ENSG00000148288|ENST00000372040
MHRRRLALGLGFCLLAGTSLSVLWVYLENWLPVSYVPYYLPCPEIFNMKLHYKREKPLQP
VVWSQYPQPKLLEHRPTQLLTLTPWLAPIVSEGTFNPELLQHIYQPLNLTIGVTVFAVGK
YTHFIQSFLESAEEFFMRGYRVHYYIFTDNPAAVPGVPLGPHRLLSSIPIQGHSHWEETS
MRRMETISQHIAKRAHREVDYLFCLDVDMVFRNPWGPETLGDLVAAIHPSYYAVPRQQFP
YERRRVSTAFVADSEGDFYYGGAVFGGQVARVYEFTRGCHMAILADKANGIMAAWREESH
LNRHFISNKPSKVLSPEYLWDDRKPQPPSLKLIRFSTLDKDISCLRS*
>ENSG00000206208|ENST00000383066
MKSLSLLLAVALGLATAVSAGPAVIECWFVEDASGKGLAKRPGALLLRQGPGEPPPRPDL
DPELYLSVHDPAGALQAAFRRYPRGAPAPHCEMSRFVPLPASAKWASGLTPAQNCPRALD
GAWLMVSISSPVLSLSSLLRPQPEPQQEPVLITMATVVLTVLTHTPAPRVRLGQDALLDL
SFAYMPPTSEAASSLAPGPPPFGLEWRRQHLGKGHLLLAATPGLNGQMPAAQEGAVAFAA

WDDDEPWGPWTGNGTFWLPTVQPFQEGTYLATIHLPYLQGQVTLELAVYKPPKVSLMPAT
LARAAPGEAPPELLCLVSHFYPSGGLEVEWELRGGPGGRSQKAEGQRWLSALRHHSDGSV
SLSGHLQPPPVTTEQHGARYACRIHHPSLPASGRSAEVTLEVAGLSGPSLEDSVGLFLSA
FLLLGLFKALGWAAVYLSTCKDSKKKAE*
>ENSG00000126091|ENST00000372362
MGLLVFVRNLLLALCLFLVLGFLYYSAWKLHLLQWEEDSNSVVLSFDSAGQTLGSEYDRL
GFLLNLDSKLPAELATKYANFSEGACKPGYASALMTAIFPRFSKPAPMFLDDSFRKWARI
REFVPPFGIKGQVLDAQYPARERVSAEAGESSRHH*
>ENSG00000196968|ENST00000372841
MAAGPIRVVLVLLGVLSVCAASGHGSVAEREAGGEAEWAEPWDGAVFRPPSALGAVGVTR
SSGTPRPGREEAGDLPVLLWWSPGLFPHFPGDSERIECARGACVASRNRRALRDSRTRAL
LFYGTDFRASAAPLPRLAHQSWALLHEESPLNNFLLSHGPGIRLFNLTSTFSRHSDYPLS
LQWLPGTAYLRRPVPPPMERAEWRRRGYAPLLYLQSHCDVPADRDRYVRELMRHIPVDSY
GKCLQNRELPTARLQDTATATTEDPELLAFLSRYKFHLALENAICNDYMTEKLWRPMHLG
AVPVYRGSPSVRDWMPNNHSVILIDDFESPQKLAEFIDFLDKNDEEYMKYLAYKQPGGIT
NQFLLDSLKHREWGVNDPLLPNYLNGFECFVCDYELARLDAEKAHAASPGDSPVFEPHIA
QPSHMDCPVPTPGFGNVEEIPENDSWKEMWLQDYWQGLDQGEALTAMIHNNETEQTKFWD
YLHEIFMKRQHL*
>ENSG00000153936|ENST00000370551
MGLLRIMMPPKLQLLAVVAFAVAMLFLENQIQKLEESRSKLERAIARHEVREIEQRHTMD
GPRQDATLDEEEDMVIIYNRVPKTASTSFTNIAYDLCAKNKYHVLHINTTKNNPVMSLQD
QVRFVKNITSWKEMKPGFYHGHVSYLDFAKFGVKKKPIYINVIRDPIERLVSYYYFLRFG
DDYRPGLRRRKQGDKKTFDECVAEGGSDCAPEKLWLQIPFFCGHSSECW*
>ENSG00000124222|ENST00000358029
MATRRLTDAFLLLRNNSIQNRQLLAEQVSSHITSSPLHSRSIAALADDRMALVSGISLDP
EAAIGVTKRPPPKWVDGVDEIQYDVGRIKQKMKELASLHDKHLNRPTLDDSSEEEHAIEI
TTQEITQLFHRCQRAVQALPSRARACSEQEGRLLGNVVASLAQALQELSTSFRHAQSGYL
KRMKNREERSQHFFDTSVPLMDDGDDNTLYHRGFTEDQLVLVEQNTLMVEEREREIRQIV
QSISDLNEIFRDLGAMIVEQGTVLDRIDYNVEQSCIKTEDGLKQLHKAEQYQKKNRKMLV
ILILFVIIIVLIVVLVGVKSR*
>ENSG00000204482|ENST00000376092
MLSRNDAPSVLVPGPGLLRAGTPLCISAEAASAQQ*
>ENSG00000117362|ENST00000369109
MGAAVFFGCTFVAFGPAFALFLITVAGDPLRVIILVAGAFFWLVSLLLASVVWFILVHVT
DRSDARLQYGLLIFGAAVSVLLQEVFRFAYYKLLKKADEGLASLSEDGRSPISIRQMAYV
SGLSFGIISGVFSVINILADALGPGVVGIHGDSPYYFLTSAFLTAAIILLHTFWGVVFFD
ACERRRYWALGLVVGSHLLTSGLTFLNPWYEASLLPIYAVTVSMGLWAFITAGGSLRSIQ
RSLLCRRQEDSRVMVYSALRIPPED*
>ENSG00000087460|ENST00000354359
MGCLGNSKTEDQRNEEKAQREANKKIEKQLQKDKQVYRATHRLLLLGAGESGKSTIVKQM
RILHVNGFNGEGGEEDPQAARSNSDGSEKATKVQDIKNNLKEAIETIVAAMSNLVPPVEL
ANPENQFRVDYILSVMNVPDFDFPPEFYEHAKALWEDEGVRACYERSNEYQLIDCAQYFL
DKIDVIKQADYVPSDQDLLRCRVLTSGIFETKFQVDKVNFHMFDVGGQRDERRKWIQCFN
DVTAIIFVVASSSYNMVIREDNQTNRLQEALNLFKSIWNNRWLRTISVILFLNKQDLLAE

KVLAGKSKIEDYFPEFARYTTPEDATPEPGEDPRVTRAKYFIRDEFLRISTASGDGRHYC
YPHFTCAVDTENIRRVFNDCRDIIQRMHLRQYELL*
>ENSG00000189067|ENST00000381810
MSVPGPYQAATGPSSAPSAPPSYEETVAVNSYYPTPPAPMPGPTTGLVTGPDGKGMNPPS
YYTQPAPIPNNNPITVQTVYVQHPITFLDRPIQMCCPSCNKMIVSQLSYNAGALTWLSCG
SLCLLGVHSGLLLHPLLRGCPAGRGPLLSQLQSSPGHLQAFVGLSQTWREPGAAGSPFHL
SSSFTPGGGSALVVSPLQGAHLHVFFWGEYVAKLTNLQTPEIAAWSRA*
>ENSG00000126091|ENST00000372368
MGLLVFVRNLLLALCLFLVLGFLYYSAWKLHLLQWEEDSNSVVLSFDSAGQTLGSEYDRL
GFLLNLDSKLSPRTLCTVVFGLDCILESPGEPKKLLMPASHPLEILKSLSEDTAFALGFL
KLPRPAELATKYANFSEGACKPGYASALMTAIFPRFSKPAPMFLDDSFRKWARIREFVPP
FGIKGQDNLIKAILSVTKEYRLTPALDSLRCRRCIIVGNGGVLANKSLGSRIDDYDIVVR
LNSAPVKGFEKDVGSKTTLRITYPEGAMQRPEQYERDSLFVLAGFKWQDFKWLKYIVYKE
RVSASDGFWKSVATRVPKEPPEIRILNPYFIQEAAFTLIGLPFNNGLMGRGSWTHNIQRE
KEFLRKLVKARVITDLSSGI*
>ENSG00000152056|ENST00000396654
MIHFILLFSRQGKLRLQKWYITLPDKERKKITREIVQIILSRGHRTSSFVDWKELKLVYK
RYASLYFCCAIENQDNELLTLEIVHRYVELLDKYFGNVCELDIIFNFEKAYFILDEFIIG
GEIQETSKKIAVKAIEDSDMLQETMEEYMNKPTF*
>ENSG00000151532|ENST00000393077
MSSDFEGYEQDFAVLTAEITSKIARVPRLPPDEKKQMVANVEKQLEEAKELLEQMDLEVR
EIPPQSRGMYSNRMRSYKQEMGKLETDFKRSRIAYSDEVRNELLGDDGNSSENQRAHLLD
NTERLERSSRRLEAGYQIAVETEQIGQEMLENLSHDREKIQRARERLRETDANLGKSSRI
LTGMLRRIIQNRILLVILGIIVVITILMAITFSVRRH*
>ENSG00000124198|ENST00000371917
MQESQTKSMFVSRALEKILADKEVKRPQHSQLRRACQVALDEIKAEIEKQRLGTAAPPKA
NFIEADKYFLPFELACQSKSPRVVSTSLDCLQKLIAYGHITGNAPDSGAPGKRLIDRIVE
TICSCFQGPQTDEGVQLQIIKALLTAVTSPHIEIHEGTILQTVRTCYNIYLASKNLINQT
TAKATLTQMLNVIFTRMENQVLQEARELEKPIQSKPQSPVIQAAAVSPKFVRLKHSQAQS
KPTTPEKTDLTNGEHARSDSGKVSTENGDAPRERGSSLSGTDDGAQEVVKDILEDVVTSA
IKEAAEKHGLTEPERVLGELECQECAIPPGVDENSQTNGIADDRQSLSSADNLESDAQGH
QVAARFSHVLQKDAFLVFRSLCKLSMKPLGEGPPDPKSHELRSKVVSLQLLLSVLQNAGP
VFRTHEMFINAIKQYLCVALSKNGVSSVPDVFELSLAIFLTLLSNFKMHLKMQIEVFFKE
IFLNILETSTSSFEHRWMVIQTLTRICADAQCVVDIYVNYDCDLNAANIFERLVNDLSKI
AQGRSGHELGMTPLQELSLRKKGLECLVSILKCMVEWSKDLYVNPNHQTSLGQERLTDQE
IGDGKGLDMARRCSVTSMESTVSSGTQTTVQDDPEQFEVIKQQKEIIEHGIELFNKKPKR
GIQFLQEQGMLGTSVEDIAQFLHQEERLDSTQVGDFLGDSARFNKEVMYAYVDQLDFCEK
EFVSALRTFLEGFRLPGEAQKIDRLMEKFAARYIECNQGQTLFASADTAYVLAYSIIMLT
TDLHSPQVKNKMTKEQYIKMNRGINDSKDLPEEYLSSIYEEIEGKKIAMKETKELTIATK
STKQNVASEKQRRLLYNLEMEQMAKTAKALMEAVSHAKAPFTSATHLDHVRPMFKLVWTP
LLAAYSIGLQNCDDTEVASLCLEGIRCAIRIACIFGMQLERDAYVQALARFSLLTASSSI
TEMKQKNIDTIKTLITVAHTDGNYLGNSWHEILKCISQLELAQLIGTGVKTRYLSGSGRE
REGSLKGHTLAGEEFMGLGLGNLVSGGVDKRQMASFQESVGETSSQSVVVAVDRIFTGST
RLDGNAIVDFVRWLCAVSMDELASPHHPRMFSLQKIVEISYYNMNRIRLQWSRIWHVIGD

HFNKVGCNPNEDVAIFAVDSLRQLSMKFLEKGELANFRFQKDFLRPFEHIMKKNRSPTIR
DMAIRCIAQMVNSQAANIRSGWKNIFAVFHQAASDHDGNIVELAFQTTCHIVTTIFQHHF
PAAIDSFQDAVKCLSEFACNAAFPDTSMEAIRLIRFCGKYVSERPRVLQEYTSDDMNVAP
GDRVWVRGWFPILFELSCIINRCKLDVRTRGLTVMFEIMKSYGHTFEKHWWQDLFRIVFR
IFDNMKLPEQLSEKSEWMTTTCNHALYAICDVFTQFYEALNEVLLSDVFAQLQWCVKQDN
EQLARSGTNCLENLVISNGEKFSPEVWDETCNCMLDIFKTTIPHVLLTWRPVGMEEDSSE
KHLDVDLDRQSLSSIDKNPSERGQSQLSNPTDDSWKGRPYANQKLFASLLIKCVVQLELI
QTIDNIVFYPATSKKEDAEHMVAAQQDTLDADIHIETEDQGMYKYMSSQHLFKLLDCLQE
SHSFSKAFNSNYEQRTVLWRAGFKGKSKPNLLKQETSSLACCLRILFRMYVDENRRDSWE
EIQQRLLTVCSEALAYFITVNSESHREAWTSLLLLLLTKTLKINDEKFKAHASMYYPYLC
EIMQFDLIPELRAVLRKFFLRIGVVYKIWIPEEPSQVPAALSPVW*
>ENSG00000119396|ENST00000373840
MATAPYNYSYIFKYIIIGDMGVGKSCLLHQFTEKKFMADCPHTIGVEFGTRIIEVSGQKI
KLQIWDTAGQERFRAVTRSYYRGAAGALMVYDITRRSTYNHLSSWLTDARNLTNPNTVII
LIGNKADLEAQRDVTYEEAKQFAEENGLLFLEASAKTGENVEDAFLEAAKKIYQNIQDGS
LDLNAAESGVQHKPSAPQGGRLTSEPQPQREGCGC*
>ENSG00000206291|ENST00000383224
MRPEDRMFHIRAVILRALSLAFLLSLRGAGAIKADHVSTYAAFVQTHRPTGEFMFEFDED
EMFYVDLDKKETVWHLEEFGQAFSFEAQGGLANIAILNNNLNTLIQRSNHTQATNDPPEV
TVFPKEPVELGQPNTLICHIDKFFPPVLNVTWLCNGELVTEGVAESLFLPRTDYSFHKFH
YLTFVPSAEDFYDCRVEHWGLDQPLLKHWEAQEPIQMPETTETVLCALGLVLGLVGIIVG
TVLIIKSLRSGHDPRAQGTL*
>ENSG00000033170|ENST00000360689
MRPWTGSWRWIMLILFAWGTLLFYIGGHLVRDNDHPDHSSRELSKILAKLERLKQQNEDL
RRMAESLRIPEGPIDQGPAIGRVRVLEEQLVKAKEQIENYKKQTRNGLGKDHEILRRRIE
NGAKELWFFLQSELKKLKNLEGNELQRHADEFLLDLGHHERSIMTDLYYLSQTDGAGDWR
EKEAKDLTELVQRRITYLQNPKDCSKAKKLVCNINKGCGYGCQLHHVVYCFMIAYGTQRT
LILESQNWRYATGGWETVFRPVSETCTDRSGISTGHWSGEVKDKNVQVVELPIVDSLHPR
PPYLPLAVPEDLADRLVRVHGDPAVWWVSQFVKYLIRPQPWLEKEIEEATKKLGFKHPVI
GVHVRRTDKVGTEAAFHPIEEYMVHVEEHFQLLARRMQVDKKRVYLATDDPSLLKEAKTK
YPNYEFISDNSISWSAGLHNRYTENSLRGVILDIHFLSQADFLVCTFSSQVCRVAYEIMQ
TLHPDASANFHSLDDIYYFGGQNAHNQIAIYAHQPRTADEIPMEPGDIIGVAGNHWDGYS
KGVNRKLGRTGLYPSYKVREKIETVKYPTYPEAEK*
>ENSG00000153130|ENST00000394203
MDGSRKEEEEDSTFTNISLADDIDHSSRILYPRPKSLLPKMMNADMDAVDAENQVELEEK
TRLINQVLELQHTLEDLSARVDAVKEENLKLKSENQVLGQYIENLMSASSVFQTTDTKSK
RK*
>ENSG00000101294|ENST00000398174
MDSALSDPHNGSAEAGGPTNSTTRPPSTPEGIALAYGSLLLMALLPIFFGALRSVRCARG
KNASDMPETITSRDAARFPIIASCTLLGLYLFFKIFSQEYINLLLSMYFFVLGILALSHT
ISPFMNKFFPASFPNRQYQLLFTQGSGENKEEIINYEFDTKDLVCLGLSSIVGVWYLLRK
HWIANNLFGLAFSLNGVELLHLNNVSTGCILLGGLFIYDVFWVFGTNVMVTVAKSFEAPI
KLVFPQDLLEKGLEANNFAMLGLGDVVIPGIFIALLLRFDISLKKNTHTYFYTSFAAYIF
GLGLTIFIMHIFKHAQPALLYLVPACIGFPVLVALAKGEVTEMFSYESSAEILPHTPRLT

HFPTVSGSPASLADSMQQKLAGPRRRRPQNPSAIYEESNPKDPAAVTESKEGTEASASKG
LEKKEK*
>ENSG00000011465|ENST00000393155
MKATIILLLLAQVSWAGPFQQRGLFDFMLEDEASGIGPEVPDDRDFEPSLGPVCPFRCQC
HLRVVQCSDLGLDKVPKDLPPDTTLLDLQNNKITEIKDGDFKNLKNLHALILVNNKISKV
SPGAFTPLVKLERLYLSKNQLKELPEKMPKTLQELRAHENEITKVRKVTFNGLNQMIVIE
LGTNPLKSSGIENGAFQGMKKLSYIRIADTNITSIPQGLPPSLTELHLDGNKISRVDAAS
LKGLNNLAKLGLSFNSISAVDNGSLANTPHLRELHLDNNKLTRVPGGLAEHKYIQVVYLH
NNNISVVGSSDFCPPGHNTKKASYSGVSLFSNPVQYWEIQPSTFRCVYVRSAIQLGNYK*
>ENSG00000126091|ENST00000372365
MGLLVFVRNLLLALCLFLVLGFLYYSAWKLHLLQWEEDSNSVVLSFDSAGQTLGSEYDRL
GFLLNLDSKLPAELATKYANFSEGACKPGYASALMTAIFPRFSKPAPMFLDDSFRKWARI
REFVPPFGIKGQDNLIKAILSVTKEYRLTPALDSLRCRRCIIVGNGGVLANKSLGSRIDD
YDIVVR*
>ENSG00000133318|ENST00000377819
MAEPSAATQSHSISSSSFGAEPSAPGGGGSPGACPALGTKSCSSSCADSFVSSSSSQPVS
LFSTSQEGLSSLCSDEPSSEIMTSSFLSSSEIHNTGLTILHGEKSHVLGSQPILAKEGKD
HLDLLDMKKMEKPQGTSNNVSDSSVSLAAGVHCDRPSIPASFPEHPAFLSKKIGQVEEQI
DKETKNPNGVSSREAKTALDADDRFTLLTAQKPPTEYSKVEGIYTYSLSPSKVSGDDVIE
KDSPESPFEVIIDKAAFDKEFKDSYKESTDDFGSWSVHTDKESSEDISETNDKLFPLRNK
EAGRYPMSALLSRQFSHTNAALEEVSRCVNDMHNFTNEILTWDLVPQVKQQTDKSSDCIT
KTTGLDMSEYNSEIPVVNLKTSTHQKTPVCSIDGSTPITKSTGDWAEASLQQENAITGKP
VPDSLNSTKEFSIKGVQGNMQKQDDTLAELPGSPPEKCDSLGSGVATVKVVLPDDHLKDE
MDWQSSALGEITEADSSGESDDTVIEDITADTSFENNKIQAEKPVSIPSAVVKTGEREIK
EIPSCEREEKTSKNFEELVSDSELHQDQPDILGRSPASEAACSKVPDTNVSLEDVSEVAP
EKPITTENPKLPSTVSPNVFNETEFSLNVTTSAYLESLHGKNVKHIDDSSPEDLIAAFTE
TRDKGIVDSERNAFKAISEKMTDFKTTPPVEVLHENESGGSEIKDIGSKYSEQSKETNGS
EPLGVFPTQGTPVASLDLEQEQLTIKALKELGERQVEKSTSAQRDAELPSEEVLKQTFTF
APESWPQRSYDILERNVKNGSDLGISQKPITIRETTRVDAVSSLSKTELVKKHVLARLLT
DFSVHDLIFWRDVKKTGFVFGTTLIMLLSLAAFSVISVVSYLILALLSVTISFRIYKSVI
QAVQKSEEGHPFKAYLDVDITLSSEAFHNYMNAAMVHINRALKLIIRLFLVEDLVDSLKL
AVFMWLMTYVGAVFNGITLLILAELLIFSVPIVYEKYKTQIDHYVGIARDQTKSIVEKIQ
AKLPGIAKKKAE*
>ENSG00000147533|ENST00000357743
MRPQQAPVSGKVFIQRDYSSGTRCQFQTKFPAELENRIDRQQFEETVRTLNNLYAEAEKL
GGQSYLEGCLACLTAYTIFLCMETHYEKVLKKVSKYIQEQNEKIYAPQGLLLTDPIERGL
RVIEITIYEDRGMSSGR*
>ENSG00000143393|ENST00000368872
MGDTVVEPAPLKPTSEPTSGPPGNNGGSLLSVITEGVGELSVIDPEVAQKACQEVLEKVK
LLHGGVAVSSRGTPLELVNGDGVDSEIRCLDDPPAQIREEEDEMGAAVASGTAKGARRRR
QNNSAKQSWLLRLFESKLFDISMAISYLYNSKEPGVQAYIGNRLFCFRNEDVDFYLPQLL
NMYIHMDEDVGDAIKPYIVHRCRQSINFSLQCALLLGAYSSDMHISTQRHSRGTKLRKLI
LSDELKPAHRKRELPSLSPAPDTGLSPSKRTHQRSKSDATASISLSSNLKRTASNPKVEN
EDEPVRLAPEREFIKSLMAIGKRLATLPTKEQKTQRLISELSLLNHKLPARVWLPTAGFD

HHVVRVPHTQAVVLNSKDKAPYLIYVEVLECENFDTTSVPARIPENRIRSTRSVENLPEC
GITHEQRAGSFSTVPNYDNDDEAWSVDDIGELQVELPEVHTNSCDNISQFSVDSITSQES
KEPVFIAAGDIRRRLSEQLAHTPTAFKRDPEDPSAVALKEPWQEKVRRIREGSPYGHLPN
WRLLSVIVKCGDDLRQELLAFQVLKQLQSIWEQERVPLWIKPYKILVISADSGMIEPVVN
AVSIHQVKKQSQLSLLDYFLQEHGSYTTEAFLSAQRNFVQSCAGYCLVCYLLQVKDRHNG
NILLDAEGHIIHIDFGFILSSSPRNLGFETSAFKLTTEFVDVMGGLDGDMFNYYKMLMLQ
GLIAARKHMDKVVQIVEIMQQGSQLPCFHGSSTIRNLKERFHMSMTEEQLQLLVEQMVDG
SMRSITTKLYDGFQYLTNGIM*
>ENSG00000196126|ENST00000360004
MVCLKLPGGSCMTALTVTLMVLSSPLALSGDTRPRFLWQPKRECHFFNGTERVRFLDRYF
YNQEESVRFDSDVGEFRAVTELGRPDAEYWNSQKDILEQARAAVDTYCRHNYGVVESFTV
QRRVQPKVTVYPSKTQPLQHHNLLVCSVSGFYPGSIEVRWFLNGQEEKAGMVSTGLIQNG
DWTFQTLVMLETVPRSGEVYTCQVEHPSVTSPLTVEWRARSESAQSKMLSGVGGFVLGLL
FLGAGLFIYFRNQKGHSGLQPTGFLS*
>ENSG00000123240|ENST00000378752
MSHQPLSCLTEKEDSPSESTGNGPPHLAHPNLDTFTPEELLQQMKELLTENHQLKEAMKL
NNQAMKGRFEELSAWTEKQKEERQFFEIQSKEAKERLMALSHENEKLKEELGKLKGKSER
SSEDPTDDSRLPRAEAEQEKDQLRTQVVRLQAEKADLLGIVSELQLKLNSSGSSEDSFVE
IRMAEGEAEGSVKEIKHSPGPTRTVSTGTSRSADGAKNYFEHEELTVSQLLLCLREGNQK
VERLEVALKEAKERVSDFEKKTSNRSEIETQTEGSTEKENDEEKGPETVGSEVEALNLQV
TSLFKELQEAHTKLSEAELMKKRLQEKCQALERKNSAIPSELNEKQELVYTNKKLELQVE
SMLSEIKMEQAKTEDEKSKLTVLQMTHNKLLQEHNNALKTIEELTRKESEKVDRAVLKEL
SEKLELAEKALASKQLQMDEMKQTIAKQEEDLETMTILRAQMEVYCSDFHAERAAREKIH
EEKEQLALQLAVLLKENDAFEDGGRQSLMEMQSRHGARTSDSDQQAYLVQRGAEDRDWRQ
QRNIPIHSCPKCGEVLPDIDTLQIHVMDCII*
>ENSG00000132254|ENST00000396777
MTDGILGKAATMEIPIHGNGEARQLPEDDGLEQDLQQVMVSGPNLNETSIVSGGYGGSGD
GLIPTGSGRHPSHSTTPSGPGDEVARGIAGEKFDIVKKWGINTYKCTKQLLSERFGRGSR
TVDLELELQIELLRETKRKYESVLQLGRALTAHLYSLLQTQHALGDAFADLSQKSPELQE
EFGYNAETQKLLCKNGETLLGAVNFFVSSINTLVTKTMEDTLMTVKQYEAARLEYDAYRT
DLEELSLGPRDAGTRGRLESAQATFQAHRDKYEKLRGDVAIKLKFLEENKIKVMHKQLLL
FHNAVSAYFAGNQKQLEQTLQQFNIKLRPPGAEKPSWLEEQ*
>ENSG00000160179|ENST00000361802
MACLMAAFSVGTAMNASSYSAEMTEPKSVCVSVDEVVSSNMEATETDLLNGHLKKVDNNL
TEAQRFSSLPRRAAVNIEFRDLSYSVPEGPWWRKKGYKTLLKGISGKFNSGELVAIMGPS
GAGKSTLMNILAGYRETGMKGAVLINGLPRDLRCFRKVSCYIMQDDMLLPHLTVQEAMMV
SAHLKLQEKDEGRREMVKEILTALGLLSCANTRTGSLSGGQRKRLAIALELVNNPPVMFF
DEPTSGLDSASCFQVVSLMKGLAQGGRSIICTIHQPSAKLFELFDQLYVLSQGQCVYRGK
VCNLVPYLRDLGLNCPTYHNPADFVMEVASGEYGDQNSRLVRAVREGMCDSDHKRDLGGD
AEVNPFLWHRPSEEVKQTKRLKGLRKDSSSMEGCHSFSASCLTQFCILFKRTFLSIMRDS
VLTHLRITSHIGIGLLIGLLYLGIGNEAKKVLSNSGFLFFSMLFLMFAALMPTVLTFPLE
MGVFLREHLNYWYSLKAYYLAKTMADVPFQIMFPVAYCSIVYWMTSQPSDAVRFVLFAAL
GTMTSLVAQSLGLLIGAASTSLQVATFVGPVTAIPVLLFSGFFVSFDTIPTYLQWMSYIS
YVRYGFEGVILSIYGLDREDLHCDIDETCHFQKSEAILRELDVENAKLYLDFIVLGIFFI

SLRLIAYFVLRYKIRAER*
>ENSG00000160408|ENST00000373142
MACSRPPSQCEPTSLPPGPPAGRRHLPLSRRRREMSSNKEQRSAVFVILFALITILILYS
SNSANEVFHYGSLRGRSRRPVNLKKWSITDGYVPILGNKTLPSRCHQCVIVSSSSHLLGT
KLGPEIERAECTIRMNDAPTTGYSADVGNKTTYRVVAHSSVFRVLRRPQEFVNRTPETVF
IFWGPPSKMQKPQGSLVRVIQRAGLVFPNMEAYAVSPGRMRQFDDLFRGETGKDREKSHS
WLSTGWFTMVIAVELCDHVHVYGMVPPNYCSGPASSACPTTTTSPRGRTNVSPTSRMSTV
ARATTTASSPRKGSSHRGPSCMASPSPTPPGPRPPSLWDLRRVRGEAASAQPLGQGPSSG
QSRLAGVSPSQSGP*
>ENSG00000111186|ENST00000397196
MPSLLLLFTAALLSSWAQLLTDANSWWSLALNPVQRPEMFIIGAQPVCSQLPGLSPGQRK
LCQLYQEHMAYIGEGAKTGIKECQHQFRQRRWNCSTADNASVFGRVMQIGSRETAFTHAV
SAAGVVNAISRACREGELSTCGCSRTARPKDLPRDWLWGGCGDNVEYGYRFAKEFVDARE
REKNFAKGSEEQGRVLMNLQNNEAGRRAVYKMADVACKCHGVSGSCSLKTCWLQLAEFRK
VGDRLKEKYDSAAAMRVTRKGRLELVNSRFTQPTPEDLVYVDPSPDYCLRNESTGSLGTQ
GRLCNKTSEGMDGCELMCCGRGYNQFKSVQVERCHCKFHWCCFVRCKKCTEIVDQYICK*
>ENSG00000206503|ENST00000396634
MAVMAPRTLLLLLSGALALTQTWAGSHSMRYFFTSVSRPGRGEPRFIAVGYVDDTQFVRF
DSDAASQRMEPRAPWIEQEGPEYWDQETRNVKAQSQTDRVDLGTLRGYYNQSEAGSHTIQ
IMYGCDVGSDGRFLRGYRQDAYDGKDYIALNEDLRSWTAADMAAQITKRKWEAAHEAEQL
RAYLDGTCVEWLRRYLENGKETLQRTDPPKTHMTHHPISDHEATLRCWALGFYPAEITLT
WQRDGEDQTQDTELVETRPAGDGTFQKWAAVVVPSGEEQRYTCHVQHEGLPKPLTLRWEL
SSQPTIPIVGIIAGLVLLGAVITGAVVAAVMWRRKSSDRKGGSYTQAASSDSAQGSDVSL
TACKV*
>ENSG00000188603|ENST00000360019
MGGCAGSRRRFSDSEGEETVPEPRLPLLDHQGAHWKNAVGFWLLGLCNNFSYVVMLSAAH
DILSHKRTSGNQSHAVLLADILPTLVIKLLAPLGLHLLPYSPRVLVSGICAAGSFVLVAF
SHSVGTSLCGVVFASISSGLGEVTFLSLTAFYPRAVISWWSSGTGGAGLLGALSYLGLTQ
AGLSPQQTLLSMLGIPALLLASYFLLLTSPEAQDPGGEEEAESAARQPLIRTEAPESKPG
SSSSLSLRERWTVFKGLLWYIVPLVVVYFAEYFINQGLFELLFFWNTSLSHAQQYRWYQM
LYQAGVFASRSSLRCCRIRFTWALALLQCLNLVFLLADVWFGFLPSIYLVFLIILYEGLL
GGAAYVNTFHNIALETSDEHREFAMAATCISDTLGISLSGLLALPLHDFLCQLS*
>ENSG00000116729|ENST00000354777
MAGAIIENMSTKKLCIVGGILLVFQIIAFLVGGLIGPTTAVSYMSVKCVDARKNHHKTKW
FVPWGPNHCDKIRDIEEAIPREIEANDIVFSVHIPLPHMEMSPWFQFMLFILQLDIAFKL
NNQIRENAEVSMDVSLAYRDDAFAEWTEMAHERVPRKLKCTFTSPKTPEHEGRYYECDVL
PFMEIGSVAHKFYLLNIRLPVNEKKKINVGIGEIKDIRLVGIHQNGGFTKVWFAMKTFLT
PSIFIIMVWYWRRITMMSRPPVLLEKVIFALGISMTFINIPVEWFSIGFDWTWMLLFGDI
RQGIFYAMLLSFWIIFCGEHMMDQHERNHIAGYWKQVGPIAVGSFCLFIFDMCERGVQLT
NPFYSIWTTDIGTELAMAFIIVAGICLCLYFLFLCFMVFQVFRNISGKQSSLPAMSKVRR
LHYEGLIFRFKFLMLITLACAAMTVIFFIVSQVTEGHWKWGGVTVQVNSAFFTGIYGMWN
LYVFALMFLYAPSHKNYGEDQSNGMQLPCKSREDCALFVSELYQELFSASKYSFINDNAA
SGI*
>ENSG00000132958|ENST00000400103

MNESPQTNEFKGTTEEAPAKESPHTSEFKGAALVSPISKSMLERLSKFEVEDAENVASYE
WTHLVRLLRLIILIRIFHLLHQKRQLEKLMRRLVSENKRRYTRDGFDLDLTYVTERIIAM
SFPSSGRQSFYRNPIEEVVRFLDKKHRNHYRVYNLCSERAYDPKHFHNRVSRIMIDDHNV
PTLHEMVVFTKEVNEWMAQDLENIVAIHCKGGKGRTGTMVCALLIASEIFLTAEESLYYF
GERRTNKTHSNKFQGVETPSQNRYVGYFAQVKHLYNWNLPPRRILFIKRFIIYSIRGDVC
DLKVQVVMEKKVVFSSTSLGNCSILHDIETDKILINVYDGPPLYDDVKVQFFSSNLPKYY
DNCPFFFWFNTSFIQNNRLCLPRNELDNPHKQKAWKIYPPEFAVEILFGEK*
>ENSG00000164850|ENST00000397088
MDVTSQARGVGLEMYPGTAQPAAPNTTSPELNLSHPLLGTALANGTGELSEHQQYVIGLF
LSCLYTIFLFPIGFVGNILILVVNISFREKMTIPDLYFINLAVADLILVADSLIEVFNLH
ERYYDIAVLCTFMSLFLQVNMYSSVFFLTWMSFDRYIALARAMRCSLFRTKHHARLSCGL
IWMASVSATLVPFTAVHLQHTDEACFCFADVREVQWLEVTLGFIVPFAIIGLCYSLIVRV
LVRAHRHRGLRPRRQKALRMILAVVLVFFVCWLPENVFISVHLLQRTQPGAAPCKQSFRH
AHPLTGHIVNLAAFSNSCLNPLIYSFLGETFRDKLRLYIEQKTNLPALNRFCHAALKAVI
PDSTEQSDVRFSSAV*
>ENSG00000111817|ENST00000359564
MRTHTRGAPSVFFIYLLCFVSAYITDENPEVMIPFTNANYDSHPMLYFSRAEVAELQLRA
ASSHEHIAARLTEAVHTMLSSPLEYLPPWDPKDYSARWNEIFGNNLGALAMFCVLYPENI
EARDMAKDYMERMAAQPSWLVKDAPWDEVPLAHSLVGFATAYDFLYNYLSKTQQEKFLEV
IANASGYMYETSYRRGWGFQYLHNHQPTNCMALLTGSLVLMNQGYLQEAYLWTKQVLTIM
EKSLVLLREVTDGSLYEGVAYGSYTTRSLFQYMFLVQRHFNINHFGHPWLKQHFAFMYRT
ILPGMMAA*
>ENSG00000221838|ENST00000359593
MISQFFILSSKGDPLIYKDFRGDSGGRDVAELFYRKLTGLPGDESPVVMHHHGRHFIHIR
HSGLYLVVTTSENVSPFSLLELLSRLATLLGDYCGSLGEGTISRNVALVYELLDEVLDYG
YVQTTSTEMLRNFIQTEAVVSKPFSLFDLSSVGLFGAETQQSKVAPSSAASRPVLSSRSD
QSQKNEVFLDVVERLSVLIASNGSLLKVDVQGEIRLKSFLPSGSEMRIGLTEEFCVGKSE
LRGYGPGIRVDEVSFHSSVNLDEFESHRILRLQPPQGELTVMRYQLSDDLPSPLPFRLFP
SVQWDRGSGRLQVYLKLRCDLLSKSQALNVRLHLPLPRGVVSLSQELSSPEQKAELAEGA
LRWDLPRVQGGSQLSGLFQMDVPGPPGPPSHGLSTSASPLGLGPASLSFELPRHTCSGLQ
VRFLRLAFRPCGNANPHKWVRHLSHSDAYVIRI*
>ENSG00000148488|ENST00000377602
MRPGGALLALLASLLLLLLLRLLWCPADAPGRARILVEESREATHGTPAALRTLRSPATA
VPRATNSTYLNEKSLQLTEKCKNLQYGIESFSNKTKGYSENDYLQIITDIQSCPWKRQAE
EYANFRAKLASCCDAVQNFVVSQNNTPVGTNMSYEVESKKEIPIKKNIFHMFPVSQPFVD
YPYNQCAVVGNGGILNKSLCGTEIDKSDFVFRCNLPPTTGDVSKDVGSKTNLVTINPSII
TLKYGNLKEKKALFLEDIATYGDAFFLLPAFSFRANTGTSFKVYYTLEESKARQKVLFFH
PKYLKDLALFWRTKGVTAYRLSTGLMITSVAVELCKNVKLYGFWPFSKTVEDIPVSHHYY
DNKLPKHGFHQMPKEYSQILQLHMKGILKLQFSKCEVA*
>ENSG00000196961|ENST00000354293
MPAVSKGDGMRGLAVFISDIRNCKSKEAEIKRINKELANIRSKFKGDKALDGYSKKKYVC
KLLFIFLLGHDIDFGHMEAVNLLSSNKYTEKQIGYLFISVLVNSNSELIRLINNAIKNDL
ASRNPTFMCLALHCIANVGSREMGEAFAADIPRILVAGDSMDSVKQSAALCLLRLYKASP
DLVPMGEWTARVVHLLNDQHMGVVTAAVSLITCLCKKNPDDFKTCVSLAVSRLSRIVSSA

STDLQDYTYYFVPAPWLSVKLLRLLQCYPPPEDAAVKGRLVECLETVLNKAQEPPKSKKV
QHSNAKNAILFETISLIIHYDSEPNLLVRACNQLGQFLQHRETNLRYLALESMCTLASSE
FSHEAVKTHIDTVINALKTERDVSVRQRAADLLYAMCDRSNAKQIVSEMLRYLETADYAI
REEIVLKVAILAEKYAVDYSWYVDTILNLIRIAGDYVSEEVWYRVLQIVTNRDDVQGYAA
KTVFEALQAPACHENMVKVGGYILGEFGNLIAGDPRSSPPVQFSLLHSKFHLCSVATRAL
LLSTYIKFINLFPETKATIQGVLRAGSQLRNADVELQQRAVEYLTLSSVASTDVLATVLE
EMPPFPERESSILAKLKRKKGPGAGSALDDGRRDPSSNDINGGMEPTPSTVSTPSPSADL
LGLRAAPPPAAPPASAGAGNLLVDVFDGPAAQPSLGPTPEEAFLSPGPEDIGPPIPEADE
LLNKFVCKNNGVLFENQLLQIGVKSEFRQNLGRMYLFYGNKTSVQFQNFSPTVVHPGDLQ
TQLAVQTKRVAAQVDGGAQVQQVLNIECLRDFLTPPLLSVRFRYGGAPQALTLKLPVTIN
KFFQPTEMAAQDFFQRWKQLSLPQQEAQKIFKANHPMDAEVTKAKLLGFGSALLDNVDPN
PENFVGAGIIQTKALQVGCLLRLEPNAQAQMYRLTLRTSKEPVSRHLCELLAQQF*
>ENSG00000196664|ENST00000380659
MVFPMWTLKRQILILFNIILISKLLGARWFPKTLPCDVTLDVPKNHVIVDCTDKHLTEIP
GGIPTNTTNLTLTINHIPDISPASFHRLDHLVEIDFRCNCVPIPLGSKNNMCIKRLQIKP
RSFSGLTYLKSLYLDGNQLLEIPQGLPPSLQLLSLEANNIFSIRKENLTELANIEILYLG
QNCYYRNPCYVSYSIEKDAFLNLTKLKVLSLKDNNVTAVPTVLPSTLTELYLYNNMIAKI
QEDDFNNLNQLQILDLSGNCPRCYNAPFPCAPCKNNSPLQIPVNAFDALTELKVLRLHSN
SLQHVPPRWFKNINKLQELDLSQNFLAKEIGDAKFLHFLPSLIQLDLSFNFELQVYRASM
NLSQAFSSLKSLKILRIRGYVFKELKSFNLSPLHNLQNLEVLDLGTNFIKIANLSMFKQF
KRLKVIDLSVNKISPSGDSSEVGFCSNARTSVESYEPQVLEQLHYFRYDKYARSCRFKNK
EASFMSVNESCYKYGQTLDLSKNSIFFVKSSDFQHLSFLKCLNLSGNLISQTLNGSEFQP
LAELRYLDFSNNRLDLLHSTAFEELHKLEVLDISSNSHYFQSEGITHMLNFTKNLKVLQK
LMMNDNDISSSTSRTMESESLRTLEFRGNHLDVLWREGDNRYLQLFKNLLKLEELDISKN
SLSFLPSGVFDGMPPNLKNLSLAKNGLKSFSWKKLQCLKNLETLDLSHNQLTTVPERLSN
CSRSLKNLILKNNQIRSLTKYFLQDAFQLRYLDLSSNKIQMIQKTSFPENVLNNLKMLLL
HHNRFLCTCDAVWFVWWVNHTEVTIPYLATDVTCVGPGAHKGQSVISLDLYTCELDLTNL
ILFSLSISVSLFLMVMMTASHLYFWDVWYIYHFCKAKIKGYQRLISPDCCYDAFIVYDTK
DPAVTEWVLAELVAKLEDPREKHFNLCLEERDWLPGQPVLENLSQSIQLSKKTVFVMTDK
YAKTENFKIAFYLSHQRLMDEKVDVIILIFLEKPFQKSKFLQLRKRLCGSSVLEWPTNPQ
AHPYFWQCLKNALATDNHVAYSQVFKETV*
>ENSG00000196968|ENST00000394790
MAAGPIRVVLVLLGVLSVCAASGHGSVAEREAGGEAEWAEPWDGAVFRPPSALGAVGVTR
SSGTPRPGREEAGDLPVLLWWSPGLFPHFPGDSERIECARGACVASRNRRALRDSRTRAL
LFYGTDFRASAAPLPRLAHQSWALLHEESPLNNFLLSHGPGIRLFNLTSTFSRHSDYPLS
LQWLPGTAYLRRPVPPPMERAEWRRRGYAPLLYLQSHCDVPADRDRYVRELMRHIPVDSY
GKCLQNRELPTARLQDTATATTEDPELLAFLSRYKFHLALENAICNDYMTEKLWRPMHLG
AVPVYRGSPSVRDWMPNNHSVILIDDFESPQKLAEFIDFLDKNDEEYMKYLAYKQPGGIT
NQFLLDSLKHREWGVNDPLLPNYLNGFECFVCDYELARLDAEKAHAASPGDSPVFEPHIA
QPSHMDCPVPTPGFGNVEEIPENDSSLVQVRDHCICLRFLWPHSEKGRTHNYAAVP*
>ENSG00000196547|ENST00000360468
MKLKKQVTVCGAAIFCVAVFSLYLMLDRVQHDPTRHQNGGNFPRSQISVLQNRIEQLEQL
LEENHEIISHIKDSVLELTANAEGPPAMLPYYTVNGSWVVPPEPRPSFFSISPQDCQFAL
GGRGQKPELQMLTVSEELPFDNVDGGVWRQGFDISYDPHDWDAEDLQVFVVPHSHNDPGW

IKTFDKYYTEQTQHILNSMVSKLQEDPRRRFLWAEVSFFAKWWDNINVQKRAAVRRLVGN
GQLEIATGGWVMPDEANSHYFALIDQLIEGHQWLERNLGATPRSGWAVDPFGYSSTMPYL
LRRANLTSMLIQRVHYAIKKHFAATHSLEFMWRQTWDSDSSTDIFCHMMPFYSYDVPHTC
GPDPKICCQFDFKRLPGGRINCPWKVPPRAITEANVAERAALLLDQYRKKSQLFRSNVLL
VPLGDDFRYDKPQEWDAQFFNYQRLFDFFNSRPNLHVQAQFGTLSDYFDALYKRTGVEPG
ARPPGFPVLSGDFFSYADREDHYWTGYYTSRPFYKSLDRVLEAHLRGAEVLYSLAAAHAR
RSGLAGRYPLSDFTLLTEARRTLGLFQHHDAITGTAKEAVVVDYGVRLLRSLVNLKQVII
HAAHYLVLGDKETYHFDPEAPFLQVDDTRLSHDALPERTVIQLDSSPRFVVLFNPLEQER
FSMVSLLVNSPRVRVLSEEGQPLAVQISAHWSSATEAVPDVYQVSVPVRLPALGLGVLQL
QLGLDGHRTLPSSVRIYLHGRQLSVSRHEAFPLRVIDSGTSDFALSNRYMQVWFSGLTGL
LKSIRRVDEEHEQQVDMQVLVYGTRTSKDKSGAYLFLPDGEAKPYVPKEPPVLRVTEGPF
FSEVVAYYEHIHQAVRLYNLPGVEGLSLDISSLVDIRDYVNKELALHIHTDIDSQGIFFT
DLNGFQVQPRRYLKKLPLQANFYPMPVMAYIQDAQKRLTLHTAQALGVSSLKDGQLEVIL
DRRLMQDDNRGLGQGLKDNKRTCNRFRLLLERRTVGSEVQDSHSTSYPSLLSHLTSMYLN
APALALPVARMQLPGPGLRSFHPLASSLPCDFHLLNLRTLQAEEDTLPSAETALILHRKG
FDCGLEAKNLGFNCTTSQGKVALGSLFHGLDVVFLQPTSLTLLYPLASPSNSTDVYLEPM
EIATFRLRLG*
>ENSG00000119414|ENST00000373547
MAPLDLDKYVEIARLCKYLPENDLKRLCDYVCDLLLEESNVQPVSTPVTVCGDIHGQFYD
LCELFRTGGQVPDTNYIFMGDFVDRGYYSLETFTYLLALKAKWPDRITLLRGNHESRQIT
QVYGFYDECQTKYGNANAWRYCTKVFDMLTVAALIDEQILCVHGGLSPDIKTLDQIRTIE
RNQEIPHKGAFCDLVWSDPEDVDTWAISPRGAGWLFGAKVTNEFVHINNLKLICRAHQLV
HEGYKFMFDEKLVTVWSAPNYCYRCGNIASIMVFKDVNTREPKLFRAVPDSERVIPPRTT
TPYFL*
>ENSG00000185499|ENST00000368389
MTPGTQSPFFLLLLLTVLTVVTGSGHASSTPGGEKETSATQRSSVPSSTEKNALSTGVSF
FFLSFHISNLQFNSSLEDPSTDYYQELQRDISEMAVCQCRRKNYGQLDIFPARDTYHPMS
EYPTYHTHGRYVPPSSTDRSPYEKVSAGNGGSSLSYTNPAVAATSANL*
>ENSG00000137497|ENST00000358965
MTLHATRGAALLSWVNSLHVADPVEAVLQLQDCSIFIKIIDRIHGTEEGQQILKQPVSER
LDFVCSFLQKNRKHPSSPECLVSAQKVLEGSELELAKMTMLLLYHSTMSSKSPRDWEQFE
YKIQAELAVILKFVLDHEDGLNLNEDLENFLQKAPVPSTCSSTFPEELSPPSHQAKREIR
FLELQKVASSSSGNNFLSGSPASPMGDILQTPQFQMRRLKKQLADERSNRDELELELAEN
RKLLTEKDAQIAMMQQRIDRLALLNEKQAASPLEPKELEELRDKNESLTMRLHETLKQCQ
DLKTEKSQMDRKINQLSEENGDLSFKLREFASHLQQLQDALNELTEEHSKATQEWLEKQA
QLEKELSAALQDKKCLEEKNEILQGKLSQLEEHLSQLQDNPPQEKGEVLGDVLQLETLKQ
EAATLAANNTQLQARVEMLETERGQQEAKLLAERGHFEEEKQQLSSLITDLQSSISNLSQ
AKEELEQASQAHGARLTAQVASLTSELTTLNATIQQQDQELAGLKQQAKEKQAQLAQTLQ
QQEQASQGLRHQVEQLSSSLKQKEQQLKEVAEKQEATRQDHAQQLATAAEEREASLRERD
AALKQLEALEKEKAAKLEILQQQLQVANEARDSAQTSVTQAQREKAELSRKVEELQACVE
TARQEQHEAQAQVAELELQLRSEQQKATEKERVAQEKDQLQEQLQALKESLKVTKGSLEE
EKRRAADALEEQQRCISELKAETRSLVEQHKRERKELEEERAGRKGLEARLQQLGEAHQA
ETEVLRRELAEAMAAQHTAESECEQLVKEVAAWRERYEDSQQEEAQYGAMFQEQLMTLKE
ECEKARQELQEAKEKVAGIESHSELQISRQQNELAELHANLARALQQVQEKEVRAQKLAD

DLSTLQEKMAATSKEVARLETLVRKAGEQQETASRELVKEPARAGDRQPEWLEEQQGRQF
CSTQAALQAMEREAEQMGNELERLRAALMESQGQQQEERGQQEREVARLTQERGRAQADL
ALEKAARAELEMRLQNALNEQRVEFATLQEALAHALTEKEGKDQELAKLRGLEAAQIKEL
EELRQTVKQLKEQLAKKEKEHASGSGAQSEAAGRTEPTGPKLEALRAEVSKLEQQCQKQQ
EQADSLERSLEAERASRAERDSALETLQGQLEEKAQELGHSQSALASAQRELAAFRTKVQ
DHSKAEDEWKAQVARGRQEAERKNSLISSLEEEVSILNRQVLEKEGESKELKRLVMAESE
KSQKLEERLRLLQAETASNSARAAERSSALREEVQSLREEAEKQRVASENLRQELTSQAE
RAEELGQELKAWQEKFFQKEQALSTLQLEHTSTQALVSELLPAKHLCQQLQAEQAAAEKR
HREELEQSKQAAGGLRAELLRAQRELGELIPLRQKVAEQERTAQQLRAEKASYAEQLSML
KKAHGLLAEENRGLGERANLGRQFLEVELDQAREKYVQELAAVRADAETRLAEVQREAQS
TARELEVMTAKYEGAKVKVLEERQRFQEERQKLTAQVEELSKKLADSDQASKVQQQKLKA
VQAQGGESQQEAQRLQAQLNELQAQLSQKEQAAEHYKLQMEKAKTHYDAKKQQNQELQEQ
LRSLEQLQKENKELRAEAERLGHELQQAGLKTKEAEQTCRHLTAQVRSLEAQVAHADQQL
RDLGKFQVATDALKSREPQAKPQLDLSIDSLDLSCEEGTPLSITSKLPRTQPDGTSVPGE
PASPISQRLPPKVESLESLYFTPIPARSQAPLESSLDSLGDVFLDSGRKTRSARRRTTQI
INITMTKKLDVEEPDSANSSFYSTRSAPASQASLRATSSTQSLARLGSPDYGNSALLSLP
GYRPTTRSSARRSQAGVSSGAPPGRNSFYMGTCQDEPEQLDDWNRIAELQQRNRVCPPHL
KTCYPLESRPSLSLGTITDEEMKTGDPQETLRRASMQPIQIAEGTGITTRQQRKRVSLEP
HQGPGTPESKKATSCFPRPMTPRDRHEGRKQSTTEAQKKAAPASTKQADRRQSMAFSILN
TPKKLGNSLLRRGASKKALSKASPNTRSGTRRSPRIATTTASAATAAAIGATPRAKGKAK
H*
>ENSG00000134470|ENST00000379977
MAPRRARGCRTLGLPALLLLLLLLRPPATRGITCPPPMSVEHADIWVKSYSLYSRERYICN
SGFKRKAGTSSLTECVLNKATNVAHWTTPSLKCIRDPALVHQRPAPPSTVTTAGVTPQPE
SLSPSGKEPAASSPSSNNTAATTAAIVPGSQLMPSKSPSTGTTEISSHESSHGTPSQTTA
KNWELTASASHQPPGVYPQGHSDTTVAISTSTVLLCGLSAVSLLACYLKSRQTPPLASVE
MEAMEALPVTWGTSSRDEDLENCSHHL*
>ENSG00000113719|ENST00000393784
MPFDFRRFDIYRKVPKDLTQPTYTGAIISICCCLFILFLFLSELTGFITTEVVNELYVDD
PDKDSGGKIDVSLNISLPNLHCELVGLDIQDEMGRHEVGHIDNSMKIPLNNGAGCRFEGQ
FSINKVPGNFHVSTHSATAQPQNPDMTHVIHKLSFGDTLQVQNIHGAFNALGGADRLTSN
PLASHDYILKIVPTVYEDKSGKQRYSYQYTVANKEYVAYSHTGRIIPAIWFRYDLSPITV
KYTERRQPLYRFITTICAIIGGTFTVAGILDSCIFTASEAWKKIQLGKMH*
>ENSG00000012174|ENST00000379484
MIPVSLVVVVVGGWTVVYLTDLVLKSSVYFKHSYEDWLENNGLSISPFHIRWQTAVFNRA
FYSWGRRKARMLYQWFNFGMVFGVIAMFSSFFLLGKTLMQTLAQMMADSPSSYSSSSSSS
SSSSSSSSSSSSSSSSSLHNEQVLQVVVPGINLPVNQLTYFFTAVLISGVVHEIGHGIAAI
REQVRFNGFGIFLFIIYPGAFVDLFTTHLQLISPVQQLRIFCAGIWHNFVLALLGILALV
LLPVILLPFYYTGVGVLITEVAEDSPAIGPRGLFVGDLVTHLQDCPVTNVQDWNECLDTI
AYEPQIGYCISASTLQQLSFPVRAYKRLDGSTECCNNHSLTDVCFSYRNNFNKRLHTCLP
ARKAVEATQVCRTNKDCKKSSSSSFCIIPSLETHTRLIKVKHPPQIDMLYVGHPLHLHYT
VSITSFIPRFNFLSIDLPVVVETFVKYLISLSGALAIVNAVPCFALDGQWILNSFLDATL
TSVIGDNDVKDLIGFFILLGGSVLLAANVTLGLWMVTAR*
>ENSG00000185499|ENST00000368393

MTPGTQSPFFLLLLLTVLTVVTGSGHASSTPGGEKETSATQRSSVPSSTEKNALSTGVSF
FFLSFHISNLQFNSSLEDPSTDYYQELQRDISEMFLQIYKQGGFLGLSNIKFRPGSVVVQ
LTLAFREGTINVHDVETQFNQYKTEAASRYNLTISDVSGCLSVPPKELRAAGHLSSPGYL
PSYERVPHLPHPWALCAP*
>ENSG00000106003|ENST00000359574
MLKRCGRRLLLALAGALLACLLVLTADPPPPPLPAERGRRALRSLAGPAGAAPAPGLGAA
AAAPGALVRDVHSLSEYFSLLTRARRDAGPPPGAAPRPADGHPRPLAEPLAPRDVFIAVK
TTKKFHRARLDLLLETWISRHKEMTFIFTDGEDEALARHTGNVVITNCSAAHSRQALSCK
MAVEYDRFIESGRKWFCHVDDDNYVNLRALLRLLASYPHTRDVYVGKPSLDRPIQAMERV
SENKVRPVHFWFATGGAGFCISRGLALKMSPWASGGHFMNTAERIRLPDDCTIGYIVEAL
LGVPLIRSGLFHSHLENLQQVPTSELHEQVTLSYGMFENKRNAVHVKGPFSVEADPSRWG
N*
>ENSG00000185010|ENST00000360256
MQIELSTCFFLCLLRFCFSATRRYYLGAVELSWDYMQSDLGELPVDARFPPRVPKSFPFN
TSVVYKKTLFVEFTDHLFNIAKPRPPWMGLLGPTIQAEVYDTVVITLKNMASHPVSLHAV
GVSYWKASEGAEYDDQTSQREKEDDKVFPGGSHTYVWQVLKENGPMASDPLCLTYSYLSH
VDLVKDLNSGLIGALLVCREGSLAKEKTQTLHKFILLFAVFDEGKSWHSETKNSLMQDRD
AASARAWPKMHTVNGYVNRSLPGLIGCHRKSVYWHVIGMGTTPEVHSIFLEGHTFLVRNH
RQASLEISPITFLTAQTLLMDLGQFLLFCHISSHQHDGMEAYVKVDSCPEEPQLRMKNNE
EAEDYDDDLTDSEMDVVRFDDDNSPSFIQIRSVAKKHPKTWVHYIAAEEEDWDYAPLVLA
PDDRSYKSQYLNNGPQRIGRKYKKVRFMAYTDETFKTREAIQHESGILGPLLYGEVGDTL
LIIFKNQASRPYNIYPHGITDVRPLYSRRLPKGVKHLKDFPILPGEIFKYKWTVTVEDGP
TKSDPRCLTRYYSSFVNMERDLASGLIGPLLICYKESVDQRGNQIMSDKRNVILFSVFDE
NRSWYLTENIQRFLPNPAGVQLEDPEFQASNIMHSINGYVFDSLQLSVCLHEVAYWYILS
IGAQTDFLSVFFSGYTFKHKMVYEDTLTLFPFSGETVFMSMENPGLWILGCHNSDFRNRG
MTALLKVSSCDKNTGDYYEDSYEDISAYLLSKNNAIEPRSFSQNSRHPSTRQKQFNATTI
PENDIEKTDPWFAHRTPMPKIQNVSSSDLLMLLRQSPTPHGLSLSDLQEAKYETFSDDPS
PGAIDSNNSLSEMTHFRPQLHHSGDMVFTPESGLQLRLNEKLGTTAATELKKLDFKVSST
SNNLISTIPSDNLAAGTDNTSSLGPPSMPVHYDSQLDTTLFGKKSSPLTESGGPLSLSEE
NNDSKLLESGLMNSQESSWGKNVSSTESGRLFKGKRAHGPALLTKDNALFKVSISLLKTN
KTSNNSATNRKTHIDGPSLLIENSPSVWQNILESDTEFKKVTPLIHDRMLMDKNATALRL
NHMSNKTTSSKNMEMVQQKKEGPIPPDAQNPDMSFFKMLFLPESARWIQRTHGKNSLNSG
QGPSPKQLVSLGPEKSVEGQNFLSEKNKVVVGKGEFTKDVGLKEMVFPSSRNLFLTNLDN
LHENNTHNQEKKIQEEIEKKETLIQENVVLPQIHTVTGTKNFMKNLFLLSTRQNVEGSYD
GAYAPVLQDFRSLNDSTNRTKKHTAHFSKKGEEENLEGLGNQTKQIVEKYACTTRISPNT
SQQNFVTQRSKRALKQFRLPLEETELEKRIIVDDTSTQWSKNMKHLTPSTLTQIDYNEKE
KGAITQSPLSDCLTRSHSIPQANRSPLPIAKVSSFPSIRPIYLTRVLFQDNSSHLPAASY
RKKDSGVQESSHFLQGAKKNNLSLAILTLEMTGDQREVGSLGTSATNSVTYKKVENTVLP
KPDLPKTSGKVELLPKVHIYQKDLFPTETSNGSPGHLDLVEGSLLQGTEGAIKWNEANRP
GKVPFLRVATESSAKTPSKLLDPLAWDNHYGTQIPKEEWKSQEKSPEKTAFKKKDTILSL
NACESNHAIAAINEGQNKPEIEVTWAKQGRTERLCSQNPPVLKRHQREITRTTLQSDQEE
IDYDDTISVEMKKEDFDIYDEDENQSPRSFQKKTRHYFIAAVERLWDYGMSSSPHVLRNR
AQSGSVPQFKKVVFQEFTDGSFTQPLYRGELNEHLGLLGPYIRAEVEDNIMVTFRNQASR
PYSFYSSLISYEEDQRQGAEPRKNFVKPNETKTYFWKVQHHMAPTKDEFDCKAWAYFSDV

DLEKDVHSGLIGPLLVCHTNTLNPAHGRQVTVQEFALFFTIFDETKSWYFTENMERNCRA
PCNIQMEDPTFKENYRFHAINGYIMDTLPGLVMAQDQRIRWYLLSMGSNENIHSIHFSGH
VFTVRKKEEYKMALYNLYPGVFETVEMLPSKAGIWRVECLIGEHLHAGMSTLFLVYSNKC
QTPLGMASGHIRDFQITASGQYGQWAPKLARLHYSGSINAWSTKEPFSWIKVDLLAPMII
HGIKTQGARQKFSSLYISQFIIMYSLDGKKWQTYRGNSTGTLMVFFGNVDSSGIKHNIFN
PPIIARYIRLHPTHYSIRSTLRMELMGCDLNSCSMPLGMESKAISDAQITASSYFTNMFA
TWSPSKARLHLQGRSNAWRPQVNNPKEWLQVDFQKTMKVTGVTTQGVKSLLTSMYVKEFL
ISSSQDGHQWTLFFQNGKVKVFQGNQDSFTPVVNSLDPPLLTRYLRIHPQSWVHQIALRM
EVLGCEAQDLY*
>ENSG00000158488|ENST00000368157
MLLLFLLFEGLCCPGENTAVKPEAWLSCGPSPGPGRLQLVCHVSGFYPKPVWVMWMRGGY
SIFLILICLTVIVTLVILVVVDSRLKKQSPVFLMGANTQDTKNSRHQFCLAQVSWIKNRV
LKKWKTRLNQLW*
>ENSG00000117362|ENST00000360244
MGAAVFFGCTFVAFGPAFALFLITVAGDPLRVIILVAGAFFWLVSLLLASVVWFILVHVT
DRSDARLQYGLLIFGAAVSVLLQEVFRFAYYKLLKKADEGLASLSEDGRSPISIRQMAYV
SGLSFGIISGVFSVINILADALGPGVVGIHGDSPYYFLTSAFLTAAIILLHTFWGVVFFD
ACERRRYWALGLVVGSHLLTSGLTFLNPWYEASLLPIYAVTVSMGLWAFITAGGSLRSIQ
RSLLCKD*
>ENSG00000137177|ENST00000378843
MSDTKVKVAVRVRPMNRRELELNTKCVVEMEGNQTVLHPPPSNTKQGERKPPKVFAFDYC
FWSMDESNTTKYAGQEVVFKCLGEGILEKAFQGYNACIFAYGQTGSGKSFSMMGHAEQLG
LIPRLCCALFKRISLEQNESQTFKVEVSYMEIYNEKVRDLLDPKGSRQSLKVREHKVLGP
YVDGLSQLAVTSFEDIESLMSEGNKSRTVAATNMNEESSRSHAVFNIIITQTLYDLQSGN
SGEKVSKVSLVDLAGSERVSKTGAAGERLKEGSNINKSLTTLGLVISSLADQAAGKGKSK
FVPYRDSVLTWLLKDNLGGNSQTSMIATISPAADNYEETLSTLRYADRAKRIVNHAVVNE
DPNAKVIRELREEVEKLREQLSQAEAMKAPELKEKLEESEKLIKELTVTWEEKLRKTEEI
AQERQRQLESMGISLEMSGIKVGDDKCYLVNLNADPALNELLVYYLKDHTRVGADTSQDI
QLFGIGIQPQHCEIDIASDGDVTLTPKENARSCVNGTLVCSTTQLWHGDRILWGNNHFFR
INLPKRKRRDWLKDFEKETGPPEHDLDAASEASSEPDYNYEFAQMEVIMKTLNSNDPVQN
VVQVLEKQYLEEKRSALEEQRLMYERELEQLRQQLSPDRQPQSSGPDRLAYSSQTAQQKV
TQWAEERDELFRQSLAKLREQLVKANTLVREANFLAEEMSKLTDYQVTLQIPAANLSANR
KRGAIVSEPAIQVRRKGKSTQVWTIEKLENKLIDMRDLYQEWKEKVPEAKRLYGKRGDPF
YEAQENHNLIGVANVFLECLFCDVKLQYAVPIISQQGEVAGRLHVEVMRVTGAVPERVVE
DDSSENSSESGSLEVVDSSGEIIHRVKKLTCRVKIKEATGLPLNLSNFVFCQYTFWDQCE
STVAAPVVDPEVPSPQSKDAQYTVTFSHCKDYVVNVTEEFLEFISDGALAIEVWGHRCAG
NGSSIWEVDSLHAKTRTLHDRWNEVTRRIEMWISILELNELGEYAAVELHQAKDVNTGGI
FQLRQGHSRRVQVTVKPVQHSGTLPLMVEAILSVSIGCVTARSTKLQRGLDSYQEEDLNC
VRERWSDALIKRREYLDEQIKKVSNKTEKTEDDVEREAQLVEQWVGLTEERNAVLVPAPG
SGIPGAPADWIPPPGMETHIPVLFLDLNADDLSANEQLVGPHASGVNSILPKEHGSQFFY
LPIIKHSDDEVSATASWDSSVHDSVHLNRVTPQNERIYLIVKTTVQLSHPAAMELVLRKR
IAANIYNKQSFTQSLKRRISLKNIFYSCGVTYEIVSNIPKATEEIEDRETLALLAARSEN
EGTSDGETYIEKYTRGVLQVENILSLERLRQAVTVKEALSTKARHIRRSLSTPNVHNVSS
SRPDLSGFDEDDKGWPENQLDMSDYSSSYQDVACYGTLPRDSPRRNKEGCTSETPHALTV

SPFKAFSPQPPKFFKPLMPVKEEHKKRIALEARPLLSQEDSEEEENELEAINRKLISSQP
YVPVEFADFSVYNASLENREWFSSKVDLSNSRVLEKEVSRSPTTSSITSGYFSHSASNAT
LSDMVVPSSDSSDQLAIQTKDADSTEHSTPSLVHDFRPSSNKELTEVEKGLVKDKIIVVP
LKENSALAKGSPSSQSIPEKNSKSLCRTGSCSELDACPSKISQPARGFCPREVTVEHTTN
ILEDHSFTEFMGVSEGKDFDGLTDSSAGELSSRRSLPNKTGGKTVSDGLHHPSQLHSKLE
NDQVIIPEAAFWVLCCQ*
>ENSG00000086062|ENST00000379731
MRLREPLLSGSAAMPGASLQRACRLLVAVCALHLGVTLVYYLAGRDLSRLPQLVGVSTPL
QGGSNSAAAIGQSSGELRTGGARPPPPLGASSQPRPGGDSSPVVDSGPGPASNLTSVPVP
HTTALSLPACPEESPLLVGPMLIEFNMPVDLELVAKQNPNVKMGGRYAPRDCVSPHKVAI
IIPFRNRQEHLKYWLYYLHPVLQRQQLDYGIYVINQAGDTIFNRAKLLNVGFQEALKDYD
YTCFVFSDVDLIPMNDHNAYRCFSQPRHISVAMDKFGFSLPYVQYFGGVSALSKQQFLTI
NGFPNNYWGWGGEDDDIFNRLVFRGMSISRPNAVVGRCRMIRHSRDKKNEPNPQRFDRIA
HTKETMLSDGLNSLTYQVLDVQRYPLYTQITVDIGTPS*
>ENSG00000206305|ENST00000399678
MILNKALMLGALALTTVMSPCGGEDIVADHVASYGVNLYQSYGPSGQYTHEFDGDEQFYV
DLGRKETVWCLPVLRQFRFDPQFALTNIAVLKHNLNSLIKRSNSTAATNEVPEVTVFSKS
PVTLGQPNILICLVDNIFPPVVNITWLSNGHSVTEGVSETSFLSKSDHSFFKISYLTLLP
SAEESYDCKVEHWGLDKPLLKHWEPEIPAPMSELTETVVCALGLSVGLVGIVVGTVFIIR
GLRSVGASRHQGPL*
>ENSG00000138193|ENST00000371380
MTSEEMTASVLIPVTQRKVVSAQSAADESSEKVSDINISKAHTVRRSGETSHTISQLNKL
KEEPSGSNLPKILSIAREKIVSDENSNEKCWEKIMPDSAKNLNINCNNILRNHQHGLPQR
QFYEMYNSVAEEDLCLETGIPSPLERKVFPGIQLELDRPSMGISPLGNQSVIIETGRAHP
DSRRAVFHFHYEVDRRMSDTFCTLSENLILDDCGNCVPLPGGEEKQKKNYVAYTCKLMEL
AKNCDNKNEQLQCDHCDTLNDKYFCFEGSCEKVDMVYSGDSFCRKDFTDSQAAKTFLSHF
EDFPDNCDDVEEDAFKSKKERSTLLVRRFCKNDREVKKSVYTGTRAIVRTLPSGHIGLTA
WSYIDQKRNGPLLPCGRVMEPPSTVEIRQDGSQRLSEAQWYPIYNAVRREETENTVGSLL
HFLTKLPASETAHGRISVGPCLKQCVRDTVCEYRATLQRTSISQYITGSLLEATTSLGAR
SGLLSTFGGSTGRMMLKERQPGPSVANSNALPSSSAGISKELIDLQPLIQFPEEVASILM
EQEQTIYRRVLPVDYLCFLTRDLGTPECQSSLPCLKASISASILTTQNGEHNALEDLVMR
FNEVSSWVTWLILTAGSMEEKREVFSYLVHVAKCCWNMGNYNAVMEFLAGLRSRKVLKMW
QFMDQSDIETMRSLKDAMAQHESSCEYRKVVTRALHIPGCKVVPFCGVFLKELCEVLDGA
SGLMKLCPRYNSQEETLEFVADYSGQDNFLQRVGQNGLKNSEKESTVNSIFQVIRSCNRS
LETDEEDSPSEGNSSRKSSLKDKSRWQFIIGDLLDSDNDIFEQSKEYDSHGSEDSQKAFD
HGTELIPWYVLSIQADVHQFLLQGATVIHYDQDTHLSARCFLQLQPDNSTLTWVKPTTAS
PASSKAKLGVLNNTAEPGKFPLLGNAGLSSLTEGVLDLFAVKAVYMGHPGIDIHTVCVQN
KLGSMFLSETGVTLLYGLQTTDNRLLHFVAPKHTAKMLFSGLLELTRAVRKMRKFPDQRQ
QWLRKQYVSLYQEDGRYEGPTLAHAVELFGGRRWSARNPSPGTSAKNAEKPNMQRNNTLG
ISTTKKKKKILMRGESGEVTDDEMATRKAKMHKECRSRSGSDPQDINEQEESEVNAIANP
PNPLPSRRAHSLTTAGSPNLAAGTSSPIRPVSSPVLSSSNKSPSSAWSSSSWHGRIKGGM
KGFQSFMVSDSNMSFVEFVELFKSFSVRSRKDLKDLFDVYAVPCNRSGSESAPLYTNLTI
DENTSDLQPDLDLLTRNVSDLGLFIKSKQQLSDNQRQISDAIAAASIVTNGTGIESTSLG
IFGVGILQLNDFLVNCQGEHCTYDEILSIIQKFEPSISMCHQGLMSFEGFARFLMDKENF

ASKNDESQENIKELQLPLSYYYIESSHNTYLTGHQLKGESSVELYSQVLLQGCRSVELDC
WDGDDGMPIIYHGHTLTTKIPFKEVVEAIDRSAFINSDLPIIISIENHCSLPQQRKMAEI
FKTVFGEKLVTKFLFETDFSDDPMLPSPDQLRKKVLLKNKKLKAHQTPVDILKQKAHQLA
SMQVQAYNGGNANPRPANNEEEEDEEDEYDYDYESLSDDNILEDRPENKSCNDKLQFEYN
EEIPKRIKKADNSACNKGKVYDMELGEEFYLDQNKKESRQIAPELSDLVIYCQAVKFPGL
STLNASGSSRGKERKSRKSIFGNNPGRMSPGETASFNKTSGKSSCEGIRQTWEESSSPLN
PTTSLSAIIRTPKCYHISSLNENAAKRLCRRYSQKLTQHTACQLLRTYPAATRIDSSNPN
PLMFWLHGIQLVALNYQTDDLPLHLNAAMFEANGGCGYVLKPPVLWDKNCPMYQKFSPLE
RDLDSMDPAVYSLTIVSGQNVCPSNSMGSPCIEVDVLGMPLDSCHFRTKPIHRNTLNPMW
NEQFLFHVHFEDLVFLRFAVVENNSSAVTAQRIIPLKALKRGYRHLQLRNLHNEVLEISS
LFINSRRMEENSSGNTMSASSMFNTEERKCLQTHRVTVHGVPGPEPFTVFTINGGTKAKQ
LLQQILTNEQDIKPVTTDYFLMEEKYFISKEKNECRKQPFQRAIGPEEEIMQILSSWFPE
EGYMGRIVLKTQQENLEEKNIVQDDKEVILSSEEESFFVQVHDVSPEQPRTVIKAPRVST
AQDVIQQTLCKAKYSYSILSNPNPSDYVLLEEVVKDTTNKKTTTPKSSQRVLLDQECVFQ
AQSKWKGAGKFILKLKEQVQASREDKKKGISFASELKKLTKSTKQPRGLTSPSQLLTSES
IQTKEEKPVGGLSSSDTMDYRQ*
>ENSG00000157214|ENST00000394629
MESISMMGSPKSLSETFLPNGINGIKDARKVTVGVIGSGDFAKSLTIRLIRCGYHVVIGS
RNPKFASEFFPHVVDVTHHEDALTKTNIIFVAIHREHYTSLWDLRHLLVGKILIDVSNNM
RINQYPESNAEYLASLFPDSLIVKGFNVVSAWALQLGPKDASRQVYICSNNIQARQQVIE
LARQLNFIPIDLGSLSSAREIENLPLRLFTLWRGPVVVAISLATFFFLYSFVRDVIHPYA
RNQQSDFYKIPIEIVNKTLPIVAITLLSLVYLAGLLAAAYQLYYGTKYRRFPPWLETWLQ
CRKQLGLLSFFFAMVHVAYSLCLPMRRSERYLFLNMAYQQVHANIENSWNEEEVWRIEMY
ISFGIMSLGLLSLLAVTSIPSVSNALNWREFSFIQSTLGYVALLISTFHVLIYGWKRAFE
EEYYRFYTPPNFVLALVLPSIVILDLLQLCRYPD*
>ENSG00000008118|ENST00000361322
MGRKEEDDCSSWKKQTTNIRKTFIFMEVLGSGAFSEVFLVKQRLTGKLFALKCIKKSPAF
RDSSLENEIAVLKKIKHENIVTLEDIYESTTHYYLVMQLVSGGELFDRILERGVYTEKDA
SLVIQQVLSAVKYLHENGIVHRDLKPENLLYLTPEENSKIMITDFGLSKMEQNGIMSTAC
GTPGYVAPEVLAQKPYSKAVDCWSIGVITYILLCGYPPFYEETESKLFEKIKEGYYEFES
PFWDDISESAKDFICHLLEKDPNERYTCEKALSHPWIDGNTALHRDIYPSVSLQIQKNFA
KSKWRQAFNAAAVVHHMRKLHMNLHSPGVRPEVENRPPETQASETSRPSSPEITITEAPV
LDHSVALPALTQLPCQHGRRPTAPGGRSLNCLVNGSLHISSSLVPMHQGSLAAGPCGCCS
SCLNIGSKGKSSYCSEPTLLKKANKKQNFKSEVMVPVKASGSSHCRAGQTGVCLIM*
>ENSG00000206493|ENST00000383597
MVDGTLLLLLSEALALTQTWAGSHSLKYFHTSVSRPGRGEPRFISVGYVDDTQFVRFDND
AASPRMVPRAPWMEQEGSEYWDRETRSARDTAQIFRVNLRTLRGYYNQSEAGSHTLQWMH
GCELGPDRRFLRGYEQFAYDGKDYLTLNEDLRSWTAVDTAAQISEQKSNDASEAEHQRAY
LEDTCVEWLHKYLEKGKETLLHLEPPKTHVTHHPISDHEATLRCWALGFYPAEITLTWQQ
DGEGHTQDTELVETRPAGDGTFQKWAAVVVPSGEEQRYTCHVQHEGLPEPVTLRWKPASQ
PTIPIVGIIAGLVLLGSVVSGAVVAAVIWRKKSSGGKGGSYSKAEWSDSAQGSESHSL*
>ENSG00000198162|ENST00000356554
MTTPALLPLSGRRIPPLNLGPPSFPHHRATLRLSEKFILLLILSAFITLCFGAFFFLPDS
SKHKRFDLGLEDVLIPHVDAGKGAKNPGVFLIHGPDEHRHREEEERLRNKIRADHEKALE

EAKEKLRKSREEIRAEIQTEKNKVVQEMKIKENKPLPPVPIPNLVGIRGGDPEDNDIREK
REKIKEMMKHAWDNYRTYGWGHNELRPIARKGHSPNIFGSSQMGATIVDALDTLYIMGLH
DEFLDGQRWIEDNLDFSVNSEVSVFEVNIRFIGGLLAAYYLSGEEIFKIKAVQLAEKLLP
AFNTPTGIPWAMVNLKSGVGRNWGWASAGSSILAEFGTLHMEFIHLSYLTGDLTYYKKVM
HIRKLLQKMDRPNGLYPNYLNPRTGRWGQYHTSVGGLGDSFYEYLLKAWLMSDKTDHEAR
KMYDDAIEAIEKHLIKKSRGGLTFIGEWKNGHLEKKMGHLACFAGGMFALGADGSRADKA
GHYLELGAEIARTCHESYDRTALKLGPESFKFDGAVEAVAVRQAEKYYILRPEVIETYWY
LWRFTHDPRYRQWGWEAALAIEKYCRVNGGFSGVKDVYSSTPTHDDVQQSFFLAETLKYL
YLLFSGDDLLPLDHWVFNTEAHPLPVLHLANTTLSGNPAVR*
>ENSG00000085063|ENST00000351554
MGIQGGSVLFGLLLVLAVFCHSGHSLQCYNCPNPTADCKTAVNCSSDFDACLITKAGLQV
YNKCWKFEHCNFNDVTTRLRENELTYYCCKKDLCNFNEQLENGGTSLSEKTVLLLVTPFL
AAAWSLHP*
>ENSG00000144674|ENST00000356847
MFKKLKQKISEEQQQLQQALAPAQASSNSSTPTRMRSRTSSFTEQLDEGTPNRENASTHA
SKSPDSVNGSEPSIPQSGDTQSFAQKLQLRVPSVESLFRSPIKESLFRSSSKESLVRTSS
RESLNRLDLDSSTASFDPPSDMDSEAEDLVGNSDSLNKEQLIQRLRRMERSLSSYRGKYS
ELVTAYQMLQREKKKLQGILSQSQDKSLRRIAELREELQMDQQAKKHLQEEFDASLEEKD
QYISVLQTQVSLLKQRLRNGPMNVDVLKPLPQLEPQAEVFTKEENPESDGEPVVEDGTSV
KTLETLQQRVKRQENLLKRCKETIQSHKEQCTLLTSEKEALQEQLDERLQELEKIKDLHM
AEKTKLITQLRDAKNLIEQLEQDKGMVIAETKRQMHETLEMKEEEIAQLRSRIKQMTTQG
EELREQKEKSERAAFEELEKALSTAQKTEEARRKLKAEMDEQIKTIEKTSEEERISLQQE
LSRVKQEVVDVMKKSSEEQIAKLQKLHEKELARKEQELTKKLQTREREFQEQMKVALEKS
QSEYLKISQEKEQQESLALEELELQKKAILTESENKLRDLQQEAETYRTRILELESSLEK
SLQENKNQSKDLAVHLEAEKNKHNKEITVMVEKHKTELESLKHQQDALWTEKLQVLKQQY
QTEMEKLREKCEQEKETLLKDKEIIFQAHIEEMNEKTLEKLDVKQTELESLSSELSEVLK
ARHKLEEELSVLKDQTDKMKQELEAKMDEQKNHHQQQVDSIIKEHEVSIQRTEKALKDQI
NQLELLLKERDKHLKEHQAHVENLEADIKRSEGELQQASAKLDVFQSYQSATHEQTKAYE
EQLAQLQQKLLDLETERILLTKQVAEVEAQKKDVCTELDAHKIQVQDLMQQLEKQNSEME
QKVKSLTQVYESKLEDGNKEQEQTKQILVEKENMILQMREGQKKEIEILTQKLSAKEDSI
HILNEEYETKFKNQEKKMEKVKQKAKEMQETLKKKLLDQEAKLKKELENTALELSQKEKQ
FNAKMLEMAQANSAGISDAVSRLETNQKEQIESLTEVHRRELNDVISIWEKKLNQQAEEL
QEIHEIQLQEKEQEVAELKQKILLFGCEKEEMNKEITWLKEEGVKQDTTLNELQEQLKQK
SAHVNSLAQDETKLKAHLEKLEVDLNKSLKENTFLQEQLVELKMLAEEDKRKVSELTSKL
KTTDEEFQSLKSSHEKSNKSLEDKSLEFKKLSEELAIQLDICCKKTEALLEAKTNELINI
SSSKTNAILSRISHCQHRTTKVKEALLIKTCTVSELEAQLRQLTEEQNTLNISFQQATHQ
LEEKENQIKSMKADIESLVTEKEALQKEGGNQQQAASEKESCITQLKKELSENINAVTLM
KEELKEKKVEISSLSKQLTDLNVQLQNSISLSEKEAAISSLRKQYDEEKCELLDQVQDLS
FKVDTLSKEKISALEQVDDWSNKFSEWKKKAQSRFTQHQNTVKELQIQLELKSKEAYEKD
EQINLLKEELDQQNKRFDCLKGEMEDDKSKMEKKESNLETELKSQTARIMELEDHITQKT
IEIESLNEVLKNYNQQKDIEHKELVQKLQHFQELGEEKDNRVKEAEEKILTLENQVYSMK
AELETKKKELEHVNLSVKSKEEELKALEDRLESESAAKLAELKRKAEQKIAAIKKQLLSQ
MEEKEEQYKKGTESHLSELNTKLQEREREVHILEEKLKSVESSQSETLIVPRSAKNVAAY
TEQEEADSQGCVQKTYEEKISVLQRNLTEKEKLLQRVGQEKEETVSSHFEMRCQYQERLI

KLEHAEAKQHEDQSMIGHLQEELEEKNKKYSLIVAQHVEKEGGKNNIQAKQNLENVFDDV
QKTLQEKELTCQILEQKIKELDSCLVRQKEVHRVEMEELTSKYEKLQALQQMDGRNKPTE
LLEENTEEKSKSHLVQPKLLSNMEAQHNDLEFKLAGAEREKQKLGKEIVRLQKDLRMLRK
EHQQELEILKKEYDQEREEKIKQEQEDLELKHNSTLKQLMREFNTQLAQKEQELEMTIKE
TINKAQEVEAELLESHQEETNQLLKKIAEKDDDLKRTAKRYEEILDAREEEMTAKVRDLQ
TQLEELQKKYQQKLEQEENPGNDNTQLAQKTTLISDSKLKEQEFREQIHNLEDRLKKYEK
NVYATTVGTPYKGGNLYHTDVSLFGEPTEFEYLRKVLFEYMMGRETKTMAKVITTVLKFP
DDQTQKILEREDARLMSWLRSSS*
>ENSG00000122126|ENST00000371113
MEPPLPVGAQPLATVEGMEMKGPLREPCALTLAQRNGQYELIIQLHEKEQHVQDIIPINS
HFRCVQEAEETLLIDIASNSGCKIRVQGDWIRERRFEIPDEEHCLKFLSAVLAAQKAQSQ
LLVPEQKDSSSWYQKLDTKDKPSVFSGLLGFEDNFSSMNLDKKINSQNQPTGIHREPPPP
PFSVNKMLPREKEASNKEQPKVTNTMRKLFVPNTQSGQREGLIKHILAKREKEYVNIQTF
RFFVGTWNVNGQSPDSGLEPWLNCDPNPPDIYCIGFQELDLSTEAFFYFESVKEQEWSMA
VERGLHSKAKYKKVQLVRLVGMMLLIFARKDQCRYIRDIATETVGTGIMGKMGNKGGVAV
RFVFHNTTFCIVNSHLAAHVEDFERRNQDYKDICARMSFVVPNQTLPQLNIMKHEVVIWL
GDLNYRLCMPDANEVKSLINKKDLQRLLKFDQLNIQRTQKKAFVDFNEGEIKFIPTYKYD
SKTDRWDSSGKCRVPAWCDRILWRGTNVNQLNYRSHMELKTSDHKPVSALFHIGVKVVDE
RRYRKVFEDSVRIMDRMENDFLPSLELSRREFVFENVKFRQLQKEKFQISNNGQVPCHFS
FIPKLNDSQYCKPWLRAEPFEGYLEPNETVDISLDVYVSKDSVTILNSGEDKIEDILVLH
LDRGKDYFLTISGNYLPSCFGTSLEALCRMKRPIREVPVTKLIDLEEDSFLEKEKSLLQM
VPLDEGASERPLQVPKEIWLLVDHLFKYACHQEDLFQTPGMQEELQQIIDCLDTSIPETI
PGSNHSVAEALLIFLEALPEPVICYELYQRCLDSAYDPRICRQVISQLPRCHRNVFRYLM
AFLRELLKFSEYNSVNANMIATLFTSLLLRPPPNLMARQTPSDRQRAIQFLLGFLLGSEE
D*
>ENSG00000213281|ENST00000369535
MTEYKLVVVGAGGVGKSALTIQLIQNHFVDEYDPTIEDSYRKQVVIDGETCLLDILDTAG
QEEYSAMRDQYMRTGEGFLCVFAINNSKSFADINLYREQIKRVKDSDDVPMVLVGNKCDL
PTRTVDTKQAHELAKSYGIPFIETSAKTRQGVEDAFYTLVREIRQYRMKKLNSSDDGTQG
CMGLPCVVM*
>ENSG00000172780|ENST00000393308
MAGPGPGPGDPDEQYDFLFKLVLVGDASVGKTCVVQRFKTGAFSERQGSTIGVDFTMKTL
EIQGKRVKLQIWDTAGQERFRTITQSYYRSANGAILAYDITKRSSFLSVPHWIEDVRKYA
GSNIVQLLIGNKSDLSELREVSLAEAQSLAEHYDILCAIETSAKDSSNVEEAFLRVATEL
IMRHGGPLFSEKSPDHIQLNSKDIGEGWGCGC*
>ENSG00000158488|ENST00000368155
MLLLFLLFEGLCCPGENTAAPQALQSYHLAAEEQLSFRMLQTSSFANHSWAHSEGSGWLG
DLQTHGWDTVLGTIRFLKPWSHGNFSKQELKNLQSLFQLYFHSFIQIVQASAGQFQLELK
PEAWLSCGPSPGPGRLQLVCHVSGFYPKPVWVMWMRGGYSIFLILICLTVIVTLVILVVV
DSRLKKQSPVFLMGANTQDTKNSRHQFCLAQVSWIKNRVLKKWKTRLNQLW*
>ENSG00000115020|ENST00000392202
MATDDKTSPTLDSANDLPRSPTSPSHLTHFKPLTPDQDEPPFKSAYSSFVNLFRFNKERA
EGGQGEQQPLSGSWTSPQLPSRTQSVRSPTPYKKQLNEELQRRSSALGDLRACTYCRKIA
LSYAHSTDSNSIGEDLNALSDSACSVSVLDPSEPRTPVGSRKASRNIFLEDDLAWQSLIH

PDSSNTPLSTRLVSVQEDAGKSPARNRSASITNLSLDRSGSPMVPSYETSVSPQANRTYV
RTETTEDERKILLDSVQLKDLWKKICHHSSGMEFQDHRYWLRTHPNCIVGKELVNWLIRN
GHIATRAQAIAIGQAMVDGRWLDCVSHHDQLFRDEYALYRPLQSTEFSETPSPDSDSVNS
VEGHSEPSWFKDIKFDDSDTEQIAEEGDDNLANSASPSKRTSVSSFQSTVDSDSAASISL
NVELDNVNFHIKKPSKYPHVPPHPADQKGRR*
>ENSG00000157214|ENST00000394632
MESISMMGSPKSLSETFLPNGINGIKDARKVTVGVIGSGDFAKSLTIRLIRCGYHVVIGS
RNPKFASEFFPHVVDVTHHEDALTKTNIIFVAIHREHYTSLWDLRHLLVGKILIDVSNNM
RINQYPESNAEYLASLFPDSLIVKGFNVVSAWALQLGPKDASRQVYICSNNIQARQQVIE
LARQLNFIPIDLGSLSSAREIENLPLRLFTLWRGPVVVAISLATFFFLYSFVRDVIHPYA
RNQQSDFYKIPIEIVNKTLPIVAITLLSLVYLAGLLAAAYQLYYGTKYRRFPPWLETWLQ
CRKQLGLLSFFFAMVHVAYSLCLPMRRSERYLFLNMAYQQVHANIENSWNEEEVWRIEMY
ISFGIMSLGLLSLLAVTSIPSVSNALNWREFSFIQIFCSFADTQTELELEFVFLLTLLL*
>ENSG00000206433|ENST00000400264
MLSRNDDICIYGGLGLGGLLLLAVVLLSACLCWLHRRVKRLERSWAQGSSEQELHYASLQ
RLPVPSSEGPDLRGRDKRGTKEDPRADYACIAENKPT*
>ENSG00000165661|ENST00000358701
MAAAGAAVARSPGIGAGPALRARRSPPPRAARLPRLLVLLAAAAVGPGAGGAARLYRAGE
DAVWVLDSGSVRGATANSSAAWLVQFYSSWCGHCIGYAPTWRALAGDVRDWASAIRVAAL
DCMEEKNQAVCHDYDIHFYPTFRYFKAFTKEFTTGENFKGPDRELRTVRQTMIDFLQNHT
EGSRPPACPRLDPIQPSDVLSLLDNRGSHYVAIVFESNSSYLGREVILDLIPYESIVVTR
ALDGDKAFLEKLGVSSVPSCYLIYPNGSHGLINVVKPLRAFFSSYLKSLPDVRKKSLPLP
EKPHKEENSEIVVWREFDKSKLYTVDLESGLHYLLRVELAAHKSLAGAELKTLKDFVTVL
AKLFPGRPPVKKLLEMLQEWLASLPLDRIPYNAVLDLVNNKMRISGIFLTNHIKWVGCQG
SRSELRGYPCSLWKLFHTLTVEASTHPDALVGTGFEDDPQAVLQTMRRYVHTFFGCKECG
EHFEEMAKESMDSVKTPDQAILWLWKKHNMVNGRLAGHLSEDPRFPKLQWPTPDLCPACH
EEIKGLASWDEGHVLTFLKQHYGRDNLLDTYSADQGDSSEGGTLARGEEEEKRLTPPEVS
HGDRDTQSVRPPGALGPRPALPESLHHSLDGKLQSLDGPGAHKEVGGAAPFLGVDFSSLD
MSLCVVLYVASSLFLMVMYFFFRVRSRRWKVKHHHPAV*
>ENSG00000198961|ENST00000361189
MSQYTEKEPAAMDQESGKAVWPKPAGGYQTITGRRYGRRHAYVSFKPCMTRHERSLGRAG
DDYEVLELDDVPKENSSGSSPLDQVDSSLPSEPIFEKSETEIPTCGSALNQTTESSQSFV
AVHHSEEGRDTLGSSTNLHNHSEGEYIPGACSASSVQNGIALVHTDSYDPDGKHGEDNDH
LQLSAEVVEGSRYQESLGNTVFELENREAEAYTGLSPPVPSFNCEVRDEFEELDSVPLVK
SSAGDTEFVHQNSQEIQRSSQDEMVSTKQQNNTSQERQTEHSPEDAACGPGHICSEQNTN
DREKNHGSSPEQVVRPKVRKLISSSQVDQETGFNRHEAKQRSVQRWREALEVEESGSDDL
LIKCEEYDGEHDCMFLDPPYSRVITQRETENNQMTSESGATAGRQEVDNTFWNGCGDYYQ
LYDKDEDSSECSDGEWSASLPHRFSGTEKDQSSSDESWETLPGKDENEPELQSDSSGPEE
ENQELSLQEGEQTSLEEGEIPWLQYNEVNESSSDEGNEPANEFAQPAFMLDGNNNLEDDS
SVSEDLDVDWSLFDGFADGLGVAEAISYVDPQFLTYMALEERLAQAMETALAHLESLAVD
VEVANPPASKESIDGLPETLVLEDHTAIGQEQCCPICCSEYIKDDIATELPCHHFFHKPC
VSIWLQKSGTCPVCRRHFPPAVIEASAAPSSEPDPDAPPSNDSIAEAP*
>ENSG00000181704|ENST00000374622
MAEAEESPGDPGTASPRPLMRDLKAVGKKFMHVLYPRKSNTLLRDWDLWGPLILCVTLAL

MLQRDSADSEKDGGPQFAEVFVIVWFGAVTITLNSKLLGGNISFFQSLCVLGYCILPLTV
AMLICRLVLLADPGPVNFMVRLFVVIVMFAWSIVASTAFLADSQPPNRRALAVYPVFLFY
FVISWMILTFTPQ*
>ENSG00000100211|ENST00000396811
MPFFGNTFSPKKTPPRKSASLSNLHSLDRSTREVELGLEYGSPTMNLAGQSLKFENGQWI
AETGVSGGVDRREVQRLRRRNQQLEEENNLLRLKVDILLDMLSESTAESHLMEKELDELR
ISRKRK*
>ENSG00000206305|ENST00000383251
MILNKALMLGALALTTVMSPCGGEDIVADHVASYGVNLYQSYGPSGQYTHEFDGDEQFYV
DLGRKETVWCLPVLRQFRFDPQFALTNIAVLKHNLNSLIKRSNSTAATNEVPEVTVFSKS
PVTLGQPNILICLVDNIFPPVVNITWLSNGHSVTEGVSETSFLSKSDHSFFKISYLTLLP
SAEESYDCKVEHWGLDKPLLKHWEPEIPAPMSELTETVVCALGLSVGLVGIVVGTVFIIR
GLRSVGASRHQGPL*
>ENSG00000152291|ENST00000398263
MRFVVALVLLNVAAAGAVPLLATESVKQEEAGVRPSAGNVSTHPSLSQRPGGSTKSHPEP
QTPKDSPSKSSAEAQTPEDTPNKSGAEAKTQKDSSNKSGAEAKTQKGSTSKSGSEAQTTK
DSTSKSHPELQTPKDSTGKSGAEAQTPEDSPNRSGAEAKTQKDSPSKSGSEAQTTKDVPN
KSGADGQTPKDGSSKSGAEDQTPKDVPNKSGAEKQTPKDGSNKSGAEEQGPIDGPSKSGA
EEQTSKDSPNKEEVKSSEPTEDVEPKEAEDDDTGPEEGSPPKEEKEKMSGSASSENREGT
LSDSTGSEKDDLYPNGSGNGSAESSHFFAYLVTAAILVAVLYIAHHNKRKIIAFVLEGKR
SKVTRRPKASDYQRLDQKS*
>ENSG00000117643|ENST00000374332
MLMRKVPGFVPASPWGLRLPQKFLFLLFLSGLVTLCFGALFLLPHSSRLKRLFLAPRTQQ
PGLEVVAEIAGHAPAREQEPPPNPAPAAPAPGEDDPSSWASPRRRKGGLRRTRPTGPREE
ATAARGNSIPASRPGDEGVPFRFDFNAFRSRLRHPVLGTRADESQEPQSQVRAQREKIKE
MMQFAWQSYKRYAMGKNELRPLTKDGYEGNMFGGLSGATVIDSLDTLYLMELKEEFQEAK
AWVGESFHLNVSGEASLFEVNIRYIGGLLSAFYLTGEEVFRIKAIRLGEKLLPAFNTPTG
IPKGVVSFKSGNWGWATAGSSSILAEFGSLHLEFLHLTELSGNQVFAEKVRNIRKVLRKI
EKPFGLYPNFLSPVSGNWVQHHVSVGGLGDSFYEYLIKSWLMSGKTDMEAKNMYYEALEA
IETYLLNVSPGGLTYIAEWRGGILDHKMGHLACFSGGMIALGAEDAKEEKRAHYRELAAQ
ITKTCHESYARSDTKLGPEAFWFNSGREAVATQLSESYYILRPEVVESYMYLWRQTHNPI
YREWGWEVVLALEKYCRTEAGFSGIQDVYSSTPNHDNKQQSFFLAETLKYLYLLFSEDDL
LSLEDWVFNTEAHPLPVNHSDSSGRAWGRH*
>ENSG00000142192|ENST00000358918
MLPGLALLLLAAWTARALEVPTDGNAGLLAEPQIAMFCGRLNMHMNVQNGKWDSDPSGTK
TCIDTKEGILQYCQEVYPELQITNVVEANQPVTIQNWCKRGRKQCKTHPHFVIPYRCLVG
EFVSDALLVPDKCKFLHQERMDVCETHLHWHTVAKETCSEKSTNLHDYGMLLPCGIDKFR
GVEFVCCPLAEESDNVDSADAEEDDSDVWWGGADTDYADGSEDKVVEVAEEEEVAEVEEE
EADDDEDDEDGDEVEEEAEEPYEEATERTTSIATTTTTTTESVEEVVREVCSEQAETGPC
RAMISRWYFDVTEGKCAPFFYGGCGGNRNNFDTEEYCMAVCGSAMSQSLLKTTQEPLARD
PVKLPTTAASTPDAVDKYLETPGDENEHAHFQKAKERLEAKHRERMSQVMREWEEAERQA
KNLPKADKKAVIQHFQEKVESLEQEAANERQQLVETHMARVEAMLNDRRRLALENYITAL
QAVPPRPRHVFNMLKKYVRAEQKDRQHTLKHFEHVRMVDPKKAAQIRSQVMTHLRVIYER
MNQSLSLLYNVPAVAEEIQDEVDELLQKEQNYSDDVLANMISEPRISYGNDALMPSLTET

KTTVELLPVNGEFSLDDLQPWHSFGADSVPANTENEGSGLTNIKTEEISEVKMDAEFRHD
SGYEVHHQKLVFFAEDVGSNKGAIIGLMVGGVVIATVIVITLVMLKKKQYTSIHHGVVEV
DAAVTPEERHLSKMQQNGYENPTYKFFEQMQN*
>ENSG00000169344|ENST00000396134
MGQPSLTWMLMVVVASWFITTAATDTSEARTKYNCPARWSWRTPQRGGDTEQGPDEDFTS
QGRWCSECHSNATCTEDEAVTTCTCQEGFTGDGLTCVDLDECAIPGAHNCSANSSCVNTP
GSFSCVCPEGFRLSPGLGCTDVDECAEPGLSHCHALATCVNVVGSYLCVCPAGYRGDGWH
CECSPGSCGPGLDCVPEGDALVCADPCQAHRTLDEYWRSTEYGEGYACDTDLRGWYRFVG
QGGARMAETCVPVLRCNTAAPMWLNGTHPSSDEGIVSRKACAHWSGHCCLWDASVQVKAC
AGGYYVYNLTAPPECHLAYCTDPSSVEGTCEECSIDEDCKSNNGRWHCQCKQDFNITDIS
LLEHRLECGANDMKVSLGKCQLKSLGFDKVFMYLSDSRCSGFNDRDNRDWVSVVTPARDG
PCGTVLTRNETHATYSNTLYLADEIIIRDLNIKINFACSYPLDMKVSLKTALQPMVSALN
IRVGGTGMFTVRMALFQTPSYTQPYQGSSVTLSTEAFLYVGTMLDGGDLSRFALLMTNCY
ATPSSNATDPLKYFIIQDRCPHTRDSTIQVVENGESSQGRFSVQMFRFAGNYDLVYLHCE
VYLCDTMNEKCKPTCSGTRFRSGSVIDQSRVLNLGPITRKGVQATVSRAFSSLGLLKVWL
PLLLSATLTLTFQ*
>ENSG00000103978|ENST00000389834
MAAAAWLQVLPVILLLLGAHPSPLSFFSAGPATVAAADRSKWHIPIPSGKNYFSFGKILF
RNTTIFLKFDGEPCDLSLNITWYLKSADCYNEIYNFKAEEVELYLEKLKEKRGLSGKYQT
SSKLFQNCSELFKTQTFSGDFMHRLPLLGEKQEAKENGTNLTFIGDKTAMHEPLQTWQDA
PYIFIVHIGISSSKESSKENSLSNLFTMTVEVKGPYEYLTLEDYPLMIFFMVMCIVYVLF
GVLWLAWSACYWRDLLRIQFWIGAVIFLGMLEKAVFYAEFQNIRYKGESVQGALILAELL
SAVKRSLARTLVIIVSLGYGIVKPRLGVTLHKVVVAGALYLLFSGMEGVLRVTGAQTDLA
SLAFIPLAFLDTALCWWIFISLTQTMKLLKLRRNIVKLSLYRHFTNTLILAVAASIVFII
WTTMKFRIVTCQSDWRELWVDDAIWRLLFSMILFVIMVLWRPSANNQRFAFSPLSEEEEE
DEQKEPMLKESFEGMKMRSTKQEPNGNSKVNKAQEDDLKWVEENVPSSVTDVALPALLDS
DEERMITHFERSKME*
>ENSG00000122863|ENST00000373115
MEKGLTLPQDCRDFVHSLKMRSKYALFLVFVVIVFVFIEKENKIISRVSDKLKQIPQALA
DANSTDPALILAENASLLSLSELDSAFSQLQSRLRNLSLQLGVEPAMEAAGEEEEEQRKE
EEPPRPAVAGPRRHVLLMATTRTGSSFVGEFFNQQGNIFYLFEPLWHIERTVSFEPGGAN
AAGSALVYRDVLKQLFLCDLYVLEHFITPLPEDHLTQFMFRRGSSRSLCEDPVCTPFVKK
VFEKYHCKNRRCGPLNVTLAAEACRRKEHMALKAVRIRQLEFLQPLAEDPRLDLRVIQLV
RDPRAVLASRMVAFAGKYKTWKKWLDDEGQDGLREEEVQRLRGNCESIRLSAELGLRQPA
WLRGRYMLVRYEDVARGPLQKAREMYRFAGIPLTPQVEDWIQKNTQAAHDGSGIYSTQKN
SSEQFEKWRFSMPFKLAQVVQAACGPAMRLFGYKLARDAAALTNRSVSLLEERGTFWVT*
>ENSG00000117226|ENST00000370481
MAPEIHMTGPMCLIENTNGELVANPEALKILSAITQPVVVVAIVGLYRTGKSYLMNKLAG
KNKGFSLGSTVKSHTKGIWMWCVPHPKKPEHTLVLLDTEGLGDVKKGDNQNDSWIFTLAV
LLSSTLVYNSMGTINQQAMDQLYYVTELTHRIRSKSSPDENENEDSADFVSFFPDFVWTL
RDFSLDLEADGQPLTPDEYLEYSLKLTQGTSQKDKNFNLPRLCIRKFFPKKKCFVFDLPI
HRRKLAQLEKLQDEELDPEFVQQVADFCSYIFSNSKTKTLSGGIKVNGPRLESLVLTYIN
AISRGDLPCMENAVLALAQIENSAAVQKAIAHYDQQMGQKVQLPAETLQELLDLHRVSER
EATEVYMKNSFKDVDHLFQKKLAAQLDKKRDDFCKQNQEASSDRCSALLQVIFSPLEEEV

KAGIYSKPGGYCLFIQKLQDLEKKYYEEPRKGIQAEEILQTYLKSKESVTDAILQTDQIL
TEKEKEIEVECVKAESAQASAKMVEEMQIKYQQMMEEKEKSYQEHVKQLTEKMERERAQL
LEEQEKTLTSKLQEQARVLKERCQGESTQLQNEIQKLQKTLKKKTKRYMSHKLKI*
>ENSG00000100311|ENST00000381551
MFIMGLGDPIPEELYEMLSDHSIRSFDDLQRLLHGDPGEEDGAELDLNMTRSHSGGELES
LARGRRSLGSLTIAEPAMIAECKTRTEVFEISRRLIDRTNANFLVWPPCVEVQRCSGCCN
NRNVQCRPTQVQLRPVQVRKIEIVRKKPIFKKATVTLEDHLACKCETVAAARPVTRSPGG
SQEQRAKTPQTRVTIRTVRVRRPPKGKHRKFKHTHDKTALKETLGA*
>ENSG00000157399|ENST00000381134
MLHLHHSCLCFRSWLPAMLAVLLSLAPSASSDISASRPNILLLMADDLGIGDIGCYGNNT
MRTPNIDRLAEDGVKLTQHISAASLCTPSRAAFLTGRYPVRSGMVSSIGYRVLQWTGASG
GLPTNETTFAKILKEKGYATGLIGKWHLGLNCESASDHCHHPLHHGFDHFYGMPFSLMGD
CARWELSEKRVNLEQKLNFLFQVLALVALTLVAGKLTHLIPVSWMPVIWSALSAVLLLAS
SYFVGALIVHADCFLMRNHTITEQPMCFQRTTPLILQEVASFLKRNKHGPFLLFVSFLHV
HIPLITMENFLGKSLHGLYGDNVEEMDWMVGRILDTLDVEGLSNSTLIYFTSDHGGSLEN
QLGNTQYGGWNGIYKGGKGMGGWEGGIRVPGIFRWPGVLPAGRVIGEPTSLMDVFPTVVR
LAGGEVPQDRVIDGQDLLPLLLGTAQHSDHEFLMHYCERFLHAARWHQRDRGTMWKVHFV
TPVFQPEGAGACYGRKVCPCFGEKVVHHDPPLLFDLSRDPSETHILTPASEPVFYQVMER
VQQAVWEHQRTLSPVPLQLDRLGNIWRPWLQPCCGPFPLCWCLREDDPQ*
>ENSG00000120215|ENST00000381476
MPREDAHFIYGYPKKGHGHSYTTAEEAAGIGILTVILGVLLLIGCWYCRRRNGYRALMDK
SLHVGTQCALTRRCPQEGFDHRDSKVSLQEKNCEPVVPNAPPAYEKLSAEQSPPPYSP*
>ENSG00000198502|ENST00000374975
MVCLKLPGGSYMAKLTVTLMVLSSPLALAGDTRPRFLQQDKYECHFFNGTERVRFLHRDI
YNQEEDLRFDSDVGEYRAVTELGRPDAEYWNSQKDFLEDRRAAVDTYCRHNYGVGESFTV
QRRVEPKVTVYPARTQTLQHHNLLVCSVNGFYPGSIEVRWFRNSQEEKAGVVSTGLIQNG
DWTFQTLVMLETVPRSGEVYTCQVEHPSVTSPLTVEWRAQSESAQSKMLSGVGGFVLGLL
FLGAGLFIYFKNQKGHSGLHPTGLVS*
>ENSG00000126091|ENST00000361400
MGLLVFVRNLLLALCLFLVLGFLYYSAWKLHLLQWEEDSKYDRLGFLLNLDSKLPAELAT
KYANFSEGACKPGYASALMTAIFPRFSKPAPMFLDDSFRKWARIREFVPPFGIKGQDNLI
KAILSVTKEYRLTPALDSLRCRRCIIVGNGGVLANKSLGSRIDDYDIVVRLNSAPVKGFE
KDVGSKTTLRITYPEGAMQRPEQYERDSLFVLAGFKWQDFKWLKYIVYKERVSASDGFWK
SVATRVPKEPPEIRILNPYFIQEAAFTLIGLPFNNGLMGRGNIPTLGSVAVTMALHGCDE
VAVAGFGYDMSTPNAPLHYYETVRMAAIKESWTHNIQREKEFLRKLVKARVITDLSSGI*
>ENSG00000206560|ENST00000399451
MAFLKLRDQPSLVQAIFNGDPDEVRALIFKKEDVNFQDNEKRTPLHAAAYLGDAEIIELL
ILSGARVNAKDSKWLTPLHRAVASCSEEAVQVLLKHSADVNARDKNWQTPLHIAAANKAV
KCAEALVPLLSNVNVSDRAGRTALHHAAFSGHGEMVKLLLSRGANINAFDKKDRRAIHWA
AYMGHIEVVKLLVSHGAEVTCKDKKSYTPLHAAASSGMISVVKYLLDLGVDMNEPNAYGN
TPLHVACYNGQDVVVNELIDCGAIVNQKNEKGFTPLHFAAASTHGALCLELLVGNGADVN
MKSKDGKTPLHMTALHGRFSRSQTIIQSGAVIDCEDKNGNTPLHIAARYGHELLINTLIT
SGADTAKRGIHGMFPLHLAALSGFSDCCRKLLSSGFDIDTPDDFGRTCLHAAAAGGNLEC
LNLLLNTGADFNKKDKFGRSPLHYAAANCNYQCLFALVGSGASVNDLDERGCTPLHYAAT

SDTDGKCLEYLLRNDANPGIRDKQGYNAVHYSAAYGHRLCLQLIASETPLDVLMETSGTD
MLSDSDNRATISPLHLAAYHGHHQALEVLVQSLLDLDVRNSSGRTPLDLAAFKGHVECVD
VLINQGASILVKDYILKRTPIHAAATNGHSECLRLLIGNAEPQNAVDIQDGNGQTPLMLS
VLNGHTDCVYSLLNKGANVDAKDKWGRTALHRGAVTGHEECVDALLQHGAKCLLRDSRGR
TPIHLSAACGHIGVLGALLQSAASMDANPATADNHGYTALHWACYNGHETCVELLLEQEV
FQKTEGNAFSPLHCAVINDNEGAAEMLIDTLGASIVNATDSKGRTPLHAAAFTDHVECLQ
LLLSHNAQVNSVDSTGKTPLMMAAENGQTNTVEMLVSSASAELTLQDNSKNTALHLACSK
GHETSALLILEKITDRNLINATNAALQTPLHVAARNGLTMVVQELLGKGASVLAVDENGY
TPALACAPNKDVADCLALILATMMPVSSSSPLSSLTFNAINRYTNTSKTVSFEALPIMRN
EPSSYCSFNNIGGEQEYLYTDVDELNDSDSETY*
>ENSG00000143578|ENST00000368600
MDLGIPDLLDAWLEPPEDIFSTGSVLELGLHCPPPEVPGLQESEPEDFLKLFIDPNEVYC
SEASPGSDSGISEDPCHPDSPPAPRATSSPMLYEVVYEAGALERMQGETGPNVGLISIQL
DQWSPAFMVPDSCMVSELPFDAHAHILPRAGTVAPVPCTTLLPCQTLFLTDEEKRLLGQE
GVSLPSHLPLTKAEERVLKKVRRKIRNKQSAQDSRRRKKEYIDGLESRVAACSAQNQELQ
KKVQELERHNISLVAQLRQLQTLIAQTSNKAAQTSTCVLILLFSLALIILPSFSPFQSRP
EAGSEDYQPHGVTSRNILTHKDVTENLETQVVESRLREPPGAKDANGSTRTLLEKMGGKP
RPSGRIRSVLHADEM*
>ENSG00000079805|ENST00000359692
MGNRGMEELIPLVNKLQDAFSSIGQSCHLDLPQIAVVGGQSAGKSSVLENFVGRDFLPRG
SGIVTRRPLILQLIFSKTEHAEFLHCKSKKFTDFDEVRQEIEAETDRVTGTNKGISPVPI
NLRVYSPHVLNLTLIDLPGITKVPVGDQPPDIEYQIKDMILQFISRESSLILAVTPANMD
LANSDALKLAKEVDPQGLRTIGVITKLDLMDEGTDARDVLENKLLPLRRGYIGVVNRSQK
DIEGKKDIRAALAAERKFFLSHPAYRHMADRMGTPHLQKTLNQQLTNHIRESLPALRSKL
QSQLLSLEKEVEEYKNFRPDDPTRKTKALLQMVQQFGVDFEKRIEGSGDQVDTLELSGGA
RINRIFHERFPFELVKMEFDEKDLRREISYAIKNIHGVRTGLFTPDLAFEAIVKKQVVKL
KEPCLKCVDLVIQELINTVRQCTSKLSSYPRLREETERIVTTYIREREGRTKDQILLLID
IEQSYINTNHEDFIGFANAQQRSTQLNKKRAIPNQVIRRGWLTINNISLMKGGSKEYWFV
LTAESLSWYKDEEEKEKKYMLPLDNLKIRDVEKGFMSNKHVFAIFNTEQRNVYKDLRQIE
LACDSQEDVDSWKASFLRAGVYPEKDQAENEDGAQENTFSMDPQLERQVETIRNLVDSYV
AIINKSIRDLMPKTIMHLMINNTKAFIHHELLAYLYSSADQSSLMEESADQAQRRDDMLR
MYHALKEALNIIGDISTSTVSTPVPPPVDDTWLQSASSHSPTPQRRPVSSIHPPGRPPAV
RGPTPGPPLIPVPVGAAASFSAPPIPSRPGPQSVFANSDLFPAPPQIPSRPVRIPPGIPP
GVPSRRPPAAPSRPTIIRPAEPSLLD*
>ENSG00000213853|ENST00000359543
MLVLLAFIIAFHITSAALLFIATVDNAWWVGDEFFADVWRICTNNTNCTVINDSFQEYST
LQAVQATMILSTILCCIAFFIFVLQLFRLKQGERFVLTSIIQLMSCLCVMIAASIYTDRR
EDIHDKNAKFYPVTREGSYGYSYILAWVAFACTFISGMMYLILRKRK*
>ENSG00000177951|ENST00000382762
MADWARAQSPGAVEEILDRENKRMADSLASKVTRLKSLALDIDRDAEDQNRYLDGMDSDF
TSMTSLLTGSVKRFSTMARSGQDNRKLLCGMAVGLIVAFFILSYFLSRART*
>ENSG00000155252|ENST00000370631
MDETSPLVSPERAQPPDYTFPSGSGAHFPQVPGGAVRVAAAAGSGPSPPGSPGHDRERQP
LLDRARGAAAQGQTQTVAAQAQALAAQAAAAAHAAQAHRERNEFPEDPEFEAVVRQAELA

IERCIFPERIYQGSSGSYFVKDPQGRIIAVFKPKNEEPYGHLNPKWTKWLQKLCCPCCFG
RDCLVLNQGYLSEAGASLVDQKLELNIVPRTKVVYLASETFNYSAIDRVKSRGKRLALEK
VPKVGQRFNRIGLPPKVGSFQLFVEGYKDADYWLRRFEAEPLPENTNRQLLLQFERLVVL
DYIIRNTDRGNDNWLIKYDCPMDSSSSRDTDWVVVKEPVIKVAAIDNGLAFPLKHPDSWR
AYPFYWAWLPQAKVPFSQEIKDLILPKISDPNFVKDLEEDLYELFKKDPGFDRGQFHKQI
AVMRGQILNLTQALKDNKSPLHLVQMPPVIVETARSHQRSSSESYTQSFQSRKPFFSWW*
>ENSG00000054116|ENST00000373163
MGFNIGVRLIEDFLARSNVGRCHDFRETADVIAKVAFKMYLGITPSITNWSPAGDEFSLI
LENNPLVDFVELPDNHSSLIYSNLLCGVLRGALEMVQMAVEAKFVQDTLKGDGVTEIRMR
FIRRIEDNLPAGEE*
>ENSG00000142197|ENST00000399151
MDPEEQELLNDYRYRSYSSVIEKALRNFESSSEWADLISSLGKLNKALQSNLRYSLLPRR
LLISKRLAQCLHPALPSGVHLKALETYEIIFKIVGTKWLAKDLFLYSCGLFPLLAHAAVS
VRPVLLTLYEKYFLPLQKLLLPSLQAFIVGLLPGLEEGSEISDRTDALLLRLSLVVGKEV
FYTALWGSVLASPSIRLPASVFVVGHINRDAPGREQKYMLGTNHQLTVKSLRASLLDSNV
LVQRNNLEIVLFFFPFYTCLDSNERAIPLLRSDIVRILSAATQTLLRRDMSLNRRLYAWL
LGSDIKGNTVVPESEISNSYEDQSSYFFEKYSKDLLVEGLAEILHQKFIDADVEERHHAY
LKPFRVLISLLDKPEIGPQVVGNLFLEVIRAFYSYCRDALGSDLKLSYTQSGNSLISAIK
ENRNASEIVKTVNLLITSLSTDFLWDYMTRCFEECFRPVKQRYSVRNSVSPPPTVSELCA
LLVFLLDVIPLELYSEVQTQYLPQVLGCLVQPLAEDMEALSLPELTHALKTCFKVLSKVQ
MPPSYLDTESTSGTSSPVKGENGKIILETKAVIPGDEDASFPPLKSEDSGIGLSASSPEL
SEHLRVPRVSLERDDVWKKGGSMQRTFLCIQELIANFASKNIFGVQLTASGEESKSEEPA
GKRDRDGTQSLAANDSSRKNSWEPKPITVPQFKQMLSDLFTARGSPFKTKSSESPSSSPS
SPARKNGGEWDVEKVVIDLGGSREERREAFAAACHLLLDCATFPVYLSEEETEQLCATLF
QLPGAGDSSFPSWLKSLMTICCCVTDCYLQNVAISTLLEVINHSQSLALVIEDKMKRYKS
SGHNPFFGKLQMVTVPPIAPGILKVIAEKTDFYQRVARVLWNQLNKETREHHVTCVELFY
RLHCLAPTANICEDIICHALLDPDKGTRLEALFRFSVIWHLTREIQGSRVTSHNRSFDRS
LFVVLDSLACTDGAIGAAAQGWLVRALSLGDVARILEPVLLLLLQPKTQRTSIHCLKQEN
SADDLHRWFNRKKTSFREACAVPEPQESGSEEHLPLSQFTTVDREAIWAEVEKEPEKYPL
RGELSEEELPYYVELPDRTAHGAPDSSEHTESADTSSCHTDSENTSSFSSPSHDLQELSN
EENCCAPIPMGGRAYPKRSALLAAFQSESFKAGAKLSLVRVDSDKTQASESFSSDEEADL
ELQALTTSRLLKQQRERQEAVEALFKHILLYLQPYDSRRVLYAFSVLEAVLKTNPKEFIE
AVSRTSMDTSSTAHLNLISNLLARHQEALIGQSFYGKLQTQVPNVCPHSLLLELLTYLCL
SFLRSYYPCYLKVSHRDILGNRDVQVKSVEVLIRIMMQLVSVAKSSEGKNVEFIHSLLQR
CKVQEFVLLSLSASMYTSQKRYGLATAHHGRALPEDSLFEESLINLGQDQIWSEHPLQIE
LLKLLQVLIVLEHHLGRAHEEAENQPDLSREWQRALNFQQAISALQYVQPHPLTSQGLLV
SAVVRGLQPAYGYGMHPAWVSLVTHSLPYFGKSLGWTVTPFVVQICKNLDDLVKQYESES
VKLSVSTTSKRENISPDYPLTLLEGLTTISHFCLLEQANQNKKTMAAGDPANLRNARNAI
LEELPRTVNTMALLWNVLRKEETQKRPVDLLGATKGSSSVYFKTTKTIRQKILDFLNPLT
AHLGVQLTAAVAAVWSRKKAQRHSKMKIIPTASASQLTLVDLVCALSTLQTDTLLHLVKE
VVKRPPQVKGGDEKSPLVDIPVLQFCYAFLQRLPVPALQENFSSLLGVLKESVQLNLAPP
GYFLLLSMLNDFVTRTPNLENKKDQKDLQEITQKILEAVGNIAGSSLEQTSWLSRNLEVK
AQPQASLEESDAEEDLYDAAAASAMVSSSAPSVYSVQALSLLAEVLASLLDMVYRSDEKE
KAVPLISRLLYYVFPYLRNHSAYNAPSFRAGAQLLSSLSGYAYTKRAWRKEVLELFLDPA

FFQMDTSCVHWKSIIDHLLTHEKTMFKDLMNMQSSSLKLFSSFEQKAMLLKRQAFAVFSG
ELDQYHLYLPLIQERLTDNLRVGQTSIVAAQMFLFFRVLLLRISPQHLTSLWPIMVSELI
QTFTQLEEDLKDEDESLRSTNKVNRTKVSVPDANGPSVGEIPQSELILYLSACKFLDTAL
SFPPDKMPLFQIYRWAFIPEVDTEGPAFLSDVEENHQECKPHTVRILELLKLKFGEISSS
DEITMKSEFPLLRQHSVSSIRQLMPFFMTLNGAFKTQRQLPADSPGTPFLDFPVTDSPRI
LKQLEECIEYDFLEHPEC*
>ENSG00000080815|ENST00000394164
MTELPAPLSYFQNAQMSEDNHLSNTNDNRERQEHNDRRSLGHPEPLSNGRPQGNSRQVVE
QDEEEDEELTLKYGAKHVIMLFVPVTLCMVVVVATIKSVSFYTRKDGQLIYTPFTEDTET
VGQRALHSILNAAIMISVIVVMTILLVVLYKYRCYKVIHAWLIISSLLLLFFFSFIYLGE
VFKTYNVAVDYITVALLIWNFGVVGMISIHWKGPLRLQQAYLIMISALMALVFIKYLPEW
TAWLILAVISVYDLVAVLCPKGPLRMLVETAQERNETLFPALIYSSTMVWLVNMAEGDPE
AQRRVSKNSKYNAESTERESQDTVAENDDGGFSEEWEAQRDSHLGPHRSTPESRAAVQEL
SSSILAGEDPEERGVKLGLGDFIFYSVLVGKASATASGDWNTTIACFVAILIGLCLTLLL
LAIFKKALPALPISITFGLVFYFATDYLVQPFMDQLAFHQFYI*
>ENSG00000147257|ENST00000394299
MAGTVRTACLVVAMLLSLDFPGQAQPPPPPPDATCHQVRSFFQRLQPGLKWVPETPVPGS
DLQVCLPKGPTCCSRKMEEKYQLTARLNMEQLLQSASMELKFLIIQNAAVFQEAFEIVVR
HAKNYTNAMFKNNYPSLTPQAFEFVGEFFTDVSLYILGSDINVDDMVNELFDSLFPVIYT
QLMNPGLPDSALDINECLRGARRDLKVFGNFPKLIMTQVSKSLQVTRIFLQALNLGIEVI
NTTDHLKFSKDCGRMLTRMWYCSYCQGLMMVKPCGGYCNVVMQGCMAGVVEIDKYWREYI
LSLEELVNGMYRIYDMENVLLGLFSTIHDSIQYVQKNAGKLTTTETEKKIWHFKYPIFFL
CIGLDLQIGKLCAHSQQRQYRSAYYPEDLFIDKKVLKVAHVEHEETLSSRRRELIQKLKS
FISFYSALPGYICSHSPVAENDTLCWNGQELVERYSQKAARNGMKNQFNLHELKMKGPEP
VVSQIIDKLKHINQLLRTMSMPKGRVLDKNLDEEGFESGDCGDDEDECIGGSGDGMIKVK
NQLRFLAELAYDLDVDDAPGNSQQATPKDNEISTFHNLGNVHSPLKLLTSMAISVVCFFF
LVH*
>ENSG00000137497|ENST00000393695
MTLHATRGAALLSWVNSLHVADPVEAVLQLQDCSIFIKIIDRIHGTEEGQQILKQPVSER
LDFVCSFLQKNRKHPSSPECLVSAQKVLEGSELELAKMTMLLLYHSTMSSKSPRDWEQFE
YKIQAELAVILKFVLDHEDGLNLNEDLENFLQKAPVPSTCSSTFPEELSPPSHQAKREIR
FLELQKVASSSSGNNFLSGSPASPMGDILQTPQFQMRRLKKQLADERSNRDELELELAEN
RKLLTEKDAQIAMMQQRIDRLALLNEKQAASPLEPKELEELRDKNESLTMRLHETLKQCQ
DLKTEKSQMDRKINQLSEENGDLSFKLREFASHLQQLQDALNELTEEHSKATQEWLEKQA
QLEKELSAALQDKKCLEEKNEILQGKLSQLEEHLSQLQDNPPQEKGEVLGDVLQLETLKQ
EAATLAANNTQLQARVEMLETERGQQEAKLLAERGHFEEEKQQLSSLITDLQSSISNLSQ
AKEELEQASQAHGARLTAQVASLTSELTTLNATIQQQDQELAGLKQQAKEKQAQLAQTLQ
QQEQASQGLRHQVEQLSSSLKQKEQQLKEVAEKQEATRQDHAQQLATAAEEREASLRERD
AALKQLEALEKEKAAKLEILQQQLQVANEARDSAQTSVTQAQREKAELSRKVEELQACVE
TARQEQHEAQAQVAELELQLRSEQQKATEKERVAQEKDQLQEQLQALKESLKVTKGSLEE
EKRRAADALEEQQRCISELKAETRSLVEQHKRERKELEEERAGRKGLEARLQQLGEAHQA
ETEVLRRELAEAMAAQHTAESECEQLVKEVAAWRERYEDSQQEEAQYGAMFQEQLMTLKE
ECEKARQELQEAKEKVAGIESHSELQISRQQNELAELHANLARALQQVQEKEVRAQKLAD
DLSTLQEKMAATSKEVARLETLVRKAGEQQETASRELVKEPARAGDRQPEWLEEQQGRQF

CSTQAALQAMEREAEQMGNELERLRAALMESQGQQQEERGQQEREVARLTQERGRAQADL
ALEKAARAELEMRLQNALNEQRVEFATLQEALAHALTEKEGKDQELAKLRGLEAAQIKEL
EELRQTVKQLKEQLAKKEKEHASGSGAQSEAAGRTEPTGPKLEALRAEVSKLEQQCQKQQ
EQADSLERSLEAERASRAERDSALETLQGQLEEKAQELGHSQSALASAQRELAAFRTKVQ
DHSKAEDEWKAQVARGRQEAERKNSLISSLEEEVSILNRQVLEKEGESKELKRLVMAESE
KSQKLEERLRLLQAETASNSARAAERSSALREEVQSLREEAEKQRVASENLRQELTSQAE
RAEELGQELKAWQEKFFQKEQALSTLQLEHTSTQALVSELLPAKHLCQQLQAEQAAAEKR
HREELEQSKQAAGGLRAELLRAQRELGELIPLRQKVAEQERTAQQLRAEKASYAEQLSML
KKAHGLLAEENRGLGERANLGRQFLEVELDQAREKYVQELAAVRADAETRLAEVQREAQS
TARELEVMTAKYEGAKVKVLEERQRFQEERQKLTAQVEQLEVFQREQTKQVEELSKKLAD
SDQASKVQQQKLKAVQAQGGESQQEAQRLQAQLNELQAQLSQKEQAAEHYKLQMEKAKTH
YDAKKQQNQELQEQLRSLEQLQKENKELRAEAERLGHELQQAGLKTKEAEQTCRHLTAQV
RSLEAQVAHADQQLRDLGKFQVATDALKSREPQAKPQLDLSIDSLDLSCEEGTPLSITSK
LPRTQPDGTSVPGEPASPISQRLPPKVESLESLYFTPIPARSQAPLESSLDSLGDVFLDS
GRKTRSARRRTTQIINITMTKKLDVEEPDSANSSFYSTRSAPASQASLRATSSTQSLARL
GSPDYGNSALLSLPGYRPTTRSSARRSQAGVSSGAPPGRNSFYMGTCQDEPEQLDDWNRI
AELQQRNRVCPPHLKTCYPLESRPSLSLGTITDEEMKTGDPQETLRRASMQPIQIAEGTG
ITTRQQRKRVSLEPHQGPGTPESKKATSCFPRPMTPRDRHEGRKQSTTEAQKKAAPASTK
QADRRQSMAFSILNTPKKLGNSLLRRGASKKALSKASPNTRSGTRRSPRIATTTASAATA
AAIGATPRAKGKAKH*
>ENSG00000100280|ENST00000402502
MTDSKYFTTTKKGEIFELKAELNSDKKEKKKEAVKKVIASMTVGKDVSALFPDVVNCMQT
DNLELKKLVYLYLMNYAKSQPDMAIMAVNTFVKDCEDPNPLIRALAVRTMGCIRVDKITE
YLCEPLRKCLKDEDPYVRKTAAVCVAKLHDINAQLVEDQGFLDTLKDLISDSNPMVVANA
VAALSEIAESHPSSNLLDLNPQSINKLLTALNECTEWGQIFILDCLANYMPKDDREAQSI
CERVTPRLSHANSAVVLSAVKVLMKFMEMLSKDLDYYGTLLKKLAPPLVTLLSAEPELQY
VALRNINLIVQKRPEILKHEMKVFFVKYNDPIYVKLEKLDIMIRLASQANIAQVLAELKE
YATEVDVDFVRKAVRAIGRCAIKVEQSAERCVSTLLDLIQTKVNYVVQEAIVVIKDIFRK
YPNKYESVIATLCENLDSLDEPEARAAMIWIVGEYAERIDNADELLESFLEGFHDESTQV
QLQLLTAIVKLFLKKPTETQELVQQVLSLATQDSDNPDLRDRGYIYWRLLSTDPVAAKEV
VLAEKPLISEETDLIEPTLLDELICYIGTLASVYHKPPSAFVEGGRGVVHKSLPPRTASS
ESAESPETAPTGAPPGEQPDVIPAQGDLLGDLLNLDLGPPVSGPPLATSSVQMGAVDLLG
GGLDSLIGGTNFVAPPTAAVPANLGAPIGSGLSDLFDLTSGVGTLSGSYVAPKAVWLPAM
KAKGLEISGTFTRQVGSISMDLQLTNKALQVMTDFAIQFNRNSFGLAPAAPLQVHAPLSP
NQTVEISLPLSTVGSVMKMEPLNNLQVAVKNNIDVFYFSTLYPLHILFVEDGKMDRQMFL
ATWKDIPNENEAQFQIRDCPLNAEAASSKLQSSNIFTVAKRNVEGQDMLYQSLKLTNGIW
VLAELRIQPGNPSCTLSLKCRAPEVSQHVYQAYETILKN*
>ENSG00000185499|ENST00000368398
MTPGTQSPFFLLLLLTVLTVVTGSGHASSTPGGEKETSATQRSSVPSSTEKNAIPAPTTT
KSCRETFLKWPGSVVVQLTLAFREGTINVHDVETQFNQYKTEAASRYNLTISDVSVSDVP
FPFSAQSGAGVPGWGIALLVLVCVLVALAIVYLIALAVCQCRRKNYGQLDIFPARDTYHP
MSEYPTYHTHGRYVPPSSTDRSPYEKVSAGNGGSSLSYTNPAVAATSANL*
>ENSG00000204482|ENST00000376093
MLSRNDDICIYGGLGLGGLLLLAVVLLSACLCWLHRRVKRLERSWHLLSWSQAQGSSEQE

LHYASLQRLPVPSSEGPDLRGRDKRGTKEDPRADYACIAENKPT*
>ENSG00000137177|ENST00000378814
MSDTKVKVAVRVRPMNRRELELNTKCVVEMEGNQTVLHPPPSNTKQGERKPPKVFAFDYC
FWSMDESNTTKYAGQEVVFKCLGEGILEKAFQGYNACIFAYGQTGSGKSFSMMGHAEQLG
LIPRLCCALFKRISLEQNESQTFKVEVSYMEIYNEKVRDLLDPKGSRQSLKVREHKVLGP
YVDGLSQLAVTSFEDIESLMSEGNKSRTVAATNMNEESSRSHAVFNIIITQTLYDLQSGN
SGEKVSKVSLVDLAGSERVSKTGAAGERLKEGSNINKSLTTLGLVISSLADQAAGKGKSK
FVPYRDSVLTWLLKDNLGGNSQTSMIATISPAADNYEETLSTLRYADRAKRIVNHAVVNE
DPNAKVIRELREEVEKLREQLSQAEAMKAPELKEKLEESEKLIKELTVTWEEKLRKTEEI
AQERQRQLESMGISLEMSGIKVGDDKCYLVNLNADPALNELLVYYLKDHTRVGADTSQDI
QLFGIGIQPQHCEIDIASDGDVTLTPKENARSCVNGTLVCSTTQLWHGDRILWGNNHFFR
INLPKRKRRDWLKDFEKETGPPEHDLDAASEASSEPDYNYEFAQMEVIMKTLNSNDPVQN
VVQVLEKQYLEEKRSALEEQRLMYERELEQLRQQLSPDRQPQSSGPDRLAYSSQTAQQKV
TQWAEERDELFRQSLAKLREQLVKANTLVREANFLAEEMSKLTDYQVTLQIPAANLSANR
KRGAIVSEPAIQVRRKGKSTQVWTIEKLENKLIDMRDLYQEWKEKVPEAKRLYGKRGDPF
YEAQENHNLIGVANVFLECLFCDVKLQYAVPIISQQGEVAGRLHVEVMRVTGAVPERVVE
DDSSENSSESGSLEVVDSSGEIIHRVKKLTCRVKIKEATGLPLNLSNFVFCQYTFWDQCE
STVAAPVVDPEVPSPQSKDAQYTVTFSHCKDYVVNVTEEFLEFISDGALAIEVWGHRCAG
NGSSIWEVDSLHAKTRTLHDRWNEVTRRIEMWISILELNELGEYAAVELHQAKDVNTGGI
FQLRQGHSRRVQVTVKPVQHSGTLPLMVEAILSVSIGCVTARSTKLQRGLDSYQEEDLNC
VRERWSDALIKRREYLDEQIKKVSNKTEKTEDDVEREAQLVEQWVGLTEERNAVLVPAPG
SGIPGAPADWIPPPGMETHIPVLFLDLNADDLSANEQLVGPHASGVNSILPKEHGSQFFY
LPIIKHSDDEVSATASWDSSVHDSVHLNRVTPQNERIYLIVKTTVQLSHPAAMELVLRKR
IAANIYNKQSFTQSLKRRISLKNIFYSCGVTYEIVSNIPKATEEIEDRETLALLAARSEN
EGTSDGETYIEKYTRGVLQVENILSLERLRQAVTVKEALSTKARHIRRSLSTPNVHNVSS
SRPDLSGFDEDDKGWPENQLDMSDYSSSYQDVACYGTLPRDSPRRNKEGCTSETPHALTV
SPFKAFSPQPPKFFKPLMPVKEEHKKRIALEARPLLSQEDSEEEENELEAINRKLISSQP
YVPVEFADFSVYNASLENREWFSSKVDLSNSRVLEKEVSRSPTTSSITSGYFSHSASNAT
LSDMVVPSSDSSDQLAIQTKDADSTEHSTPSLVHDFRPSSNKELTEVEKGLVKDKIIVVP
LKENSALAKGSPSSQSIPEKNSKSLCRTGSCSELDACPSKISQPARGFCPREVTVEHTTN
ILEDHSFTEFMGVSEGKDFDGLTDSSAGELSSRRSLPNKTGGKTVSDGLHHPSQLHSKLE
NDQGGTTSR*
>ENSG00000101981|ENST00000394090
MQRVNMIMAESPGLITICLLGYLLSAECTVFLDHENANKILNRPKRYNSGKLEEFVQGNL
ERECMEEKCSFEEAREVFENTERTTEFWKQYVDVTCNIKNGRCEQFCKNSADNKVVCSCT
EGYRLAENQKSCEPAVPFPCGRVSVSQTSKLTRAETVFPDVDYVNSTEAETILDNITQST
QSFNDFTRVVGGEDAKPGQFPWQVVLNGKVDAFCGGSIVNEKWIVTAAHCVETGVKITVV
AGEHNIEETEHTEQKRNVIRIIPHHNYNAAINKYNHDIALLELDEPLVLNSYVTPICIAD
KEYTNIFLKFGSGYVSGWGRVFHKGRSALVLQYLRVPLVDRATCLRSTKFTIYNNMFCAG
FHEGGRDSCQGDSGGPHVTEVEGTSFLTGIISWGEECAMKGKYGIYTKVSRYVNWIKEKT
KLT*
>ENSG00000184500|ENST00000394236
MRVLGGRCGALLACLLLVLPVSEANFLSKQQASQVLVRKRRANSLLEETKQGNLERECIE
ELCNKEEAREVFENDPETDYFYPKYLVCLRSFQTGLFTAARQSTNAYPDLRSCVNAIPDQ

CSPLPCNEDGYMSCKDGKASFTCTCKPGWQGEKCEFDINECKDPSNINGGCSQICDNTPG
SYHCSCKNGFVMLSNKKDCKDVDECSLKPSICGTAVCKNIPGDFECECPEGYRYNLKSKS
CEDIDECSENMCAQLCVNYPGGYTCYCDGKKGFKLAQDQKSCEVVSVCLPLNLDTKYELL
YLAEQFAGVVLYLKFRLPEISRFSAEFDFRTYDSEGVILYAESIDHSAWLLIALRGGKIE
VQLKNEHTSKITTGGDVINNGLWNMVSVEELEHSISIKIAKEAVMDINKPGPLFKPENGL
LETKVYFAGFPRKVESELIKPINPRLDGCIRSWNLMKQGASGIKEIIQEKQNKHCLVTVE
KGSYYPGSGIAQFHIDYNNVSSAEGWHVNVTLNIRPSTGTGVMLALVSGNNTVPFAVSLV
DSTSEKSQDILLSVENTVIYRIQALSLCSDQQSHLEFRVNRNNLELSTPLKIETISHEDL
QRQLAVLDKAMKAKVATYLGGLPDVPFSATPVNAFYNGCMEVNINGVQLDLDEAISKHND
IRAHSCPSVWKKTKNS*
>ENSG00000102100|ENST00000376521
MAAVGAGGSTAAPGPGAVSAGALEPGTASAAHRRLKYISLAVLVVQNASLILSIRYARTL
PGDRFFATTAVVMAEVLKGLTCLLLLFAQKRGNVKHLVLFLHEAVLVQYVDTLKLAVPSL
IYTLQNNLQYVAISNLPAATFQVTYQLKILTTALFSVLMLNRSLSRLQWASLLLLFTGVA
IVQAQQAGGGGPRPLDQNPGAGLAAVVASCLSSGFAGVYFEKILKGSSGSVWLRNLQLGL
FGTALGLVGLWWAEGTAVATRGFFFGYTPAVWGVVLNQAFGGLLVAVVVKYADNILKGFA
TSLSIVLSTVASIRLFGFHVDPLFALGAGLVIGAVYLYSLPRGAAKAIASASASASGPCV
HQQPPGQPPPPQLSSHRGDLITEPFLPKSVLVK*
>ENSG00000128242|ENST00000401975
MLPPQKKPWESMAKGLVLGALFTSFLLLVYSYAVPPLHAGLASTTPEAAASCSPPALEPE
AVIRANGSAGECQPRRNIVFLKTHKTASSTLLNILFRFGQKHRLKFAFPNGRNDFDYPTF
FARSLVQDYRPGACFNIICNHMRFHYDEVRGLVPTNAIFITVLRDPARLFESSFHYFGPV
VPLTWKLSAGDKLTEFLQDPDRYYDPNGFNAHYLRNLLFFDLGYDNSLDPSSPQVQEHIL
EVERRFHLVLLQEYFDESLVLLKDLLCWELEDVLYFKLNARRDSPVPRLSGELYGRATAW
NMLDSHLYRHFNASFWRKVEAFGRERMAREVAALRHANERMRTICIDGGHAVDAAAIQDE
AMQPWQPLGTKSILGYNLKKSIGQRHAQLCRRMLTPEIQYLMDLGANLWVTKLWKFIRDF
LRW*
>ENSG00000160408|ENST00000373146
MACSRPPSQCEPTSLPPGPPAGRRHLPLSRRRREMSSNKEQRSAVFVILFALITILILYS
SNSANEVFHYGSLRGRSRRPVNLKKWSITDGYVPILGNKTLPSRCHQCVIVSSSSHLLGT
KLGPEIERAECTIRMNDAPTTGYSADVGNKTTYRVVAHSSVFRVLRRPQEFVNRTPETVF
IFWGPPSKMQKPQGSLVRVIQRAGLVFPNMEAYAVSPGRMRQFDDLFRGETGKDREKSHS
WLSTGWFTMVIAVELCDHVHVYGMVPPNYCSQRPRLQRMPYHYYEPKGPDECVTYIQNEH
SRKGNHHRFITEKRVFSSWAQLYGITFSHPSWT*
>ENSG00000067900|ENST00000399799
MSTGDSFETRFEKMDNLLRDPKSEVNSDCLLDGLDALVYDLDFPALRKNKNIDNFLSRYK
DTINKIRDLRMKAEDYEVVKVIGRGAFGEVQLVRHKSTRKVYAMKLLSKFEMIKRSDSAF
FWEERDIMAFANSPWVVQLFYAFQDDRYLYMVMEYMPGGDLVNLMSNYDVPEKWARFYTA
EVVLALDAIHSMGFIHRDVKPDNMLLDKSGHLKLADFGTCMKMNKEGMVRCDTAVGTPDY
ISPEVLKSQGGDGYYGRECDWWSVGVFLYEMLVGDTPFYADSLVGTYSKIMNHKNSLTFP
DDNDISKEAKNLICAFLTDREVRLGRNGVEEIKRHLFFKNDQWAWETLRDTVAPVVPDLS
SDIDTSNFDDLEEDKGEEETFPIPKAFVGNQLPFVGFTYYSNRRYLSSANPNDNRTSSNA
DKSLQESLQKTIYKLEEQLHNEMQLKDEMEQKCRTSNIKLDKIMKELDEEGNQRRNLEST
VSQIEKEKMLLQHRINEYQRKAEQENEKRRNVENEVSTLKDQLEDLKKVSQNSQLANEKL

SQLQKQLEEANDLLRTESDTAVRLRKSHTEMSKSISQLESLNRELQERNRILENSKSQTD
KDYYQLQAILEAERRDRGHDSEMIGDLQARITSLQEEVKHLKHNLEKVEGERKEAQDMLN
HSEKEKNNLEIDLNYKLKSLQQRLEQEVNEHKVTKARLTDKHQSIEEAKSVAMCEMEKKL
KEEREAREKAENRVVQIEKQCSMLDVDLKQSQQKLEHLTGNKERMEDEVKNLTLQLEQES
NKRLLLQNELKTQAFEADNLKGLEKQMKQEINTLLEAKRLLEFELAQLTKQYRGNEGQMR
ELQDQLEAEQYFSTLYKTQVKELKEEIEEKNRENLKKIQELQNEKETLATQLDLAETKAE
SEQLARGLLEEQYFELTQESKKAASRNRQEITDKDHTVSRLEEANSMLTKDIEILRRENE
ELTEKMKKAEEEYKLEKEEEISNLKAAFEKNINTERTLKTQAVNKLAEIMNRKDFKIDRK
KANTQDLRKKEKENRKLQLELNQEREKFNQMVVKHQKELNDMQAQLVEECAHRNELQMQL
ASKESDIEQLRAKLLDLSDSTSVASFPSADETDGNLPESRIEGWLSVPNRGNIKRYGWKK
QYVVVSSKKILFYNDEQDKEQSNPSMVLDIDKLFHVRPVTQGDVYRAETEEIPKIFQILY
ANEGECRKDVEMEPVQQAEKTNFQNHKGHEFIPTLYHFPANCDACAKPLWHVFKPPPALE
CRRCHVKCHRDHLDKKEDLICPCKVSYDVTSARDMLLLACSQDEQKKWVTHLVKKIPKNP
PSGFVRASPRTLSTRSTANQSFRKVVKNTSGKTS*
>ENSG00000166747|ENST00000393512
MPAPIRLRELIRTIRTARTQAEEREMIQKECAAIRSSFREEDNTYRCRNVAKLLYMHMLG
YPAHFGQLECLKLIASQKFTDKRIGYLGAMLLLDERQDVHLLMTNCIKNDLNHSTQFVQG
LALCTLGCMGSSEMCRDLAGEVEKLLKTSNSYLRKKAALCAVHVIRKVPELMEMFLPATK
NLLNEKNHGVLHTSVVLLTEMCERSPDMLAHFRKNEKLVPQLVRILKNLIMSGYSPEHDV
SGISDPFLQVRILRLLRILGRNDDDSSEAMNDILAQVATNTETSKNVGNAILYETVLTIM
DIKSESGLRVLAINILGRFLLNNDKNIRYVALTSLLKTVQTDHNAVQRHRSTIVDCLKDL
DVSIKRRAMELSFALVNGNNIRGMMKELLYFLDSCEPEFKADCASGIFLAAEKYAPSKRW
HIDTIMRVLTTAGSYVRDDAVPNLIQLITNSVEMHAYTVQRLYKAILGDYSQQPLVQVAA
WCIGEYGDLLVSGQCEEEEPIQVTEDEVLDILESVLISNMSTSVTRGYALTAIMKLSTRF
TCTVNRIKKVVSIYGSSIDVELQQRAVEYNALFKKYDHMRSALLERMPVMEKVTTNGPTE
IVQTNGETEPAPLETKPPPSGPQPTSQANDLLDLLGGNDITPVIPTAPTSKPSSAGGELL
DLLGDINLTGAPAAAPAPASVPQISQPPFLLDGLSSQPLFNDIAAGIPSITAYSKNGLKI
EFTFERSNTNPSVTVITIQASNSTELDMTDFVFQAAVPKTFQLQLLSPSSSIVPAFNTGT
ITQVIKVLNPQKQQLRMRIKLTYNHKGSAMQDLAEVNNFPPQSWQ*
>ENSG00000197249|ENST00000402629
MPSSVSWGILLLAGLCCLVPVSLAEDPQGDAAQKTDTSHHDQDHPTFNKITPNLAEFAFS
LYRQLAHQSNSTNIFFSPVSIATAFAMLSLGTKADTHDEILEGLNFNLTEIPEAQIHEGF
QELLRTLNQPDSQLQLTTGNGLFLSEGLKLVDKFLEDVKKLYHSEAFTVNFGDTEEAKKQ
INDYVEKGTQGKIVDLVKELDRDTVFALVNYIFFKGKWERPFEVKDTEEEDFHVDQVTTV
KVPMMKRLGMFNIQHCKKLSSWVLLMKYLGNATAIFFLPDEGKLQHLENELTHDIITKFL
ENEDRRSASLHLPKLSITGTYDLKSVLGQLGITKVFSNGADLSGVTEEAPLKLSKVRSP*
>ENSG00000065000|ENST00000355272
MALKMVKGSIDRMFDKNLQDLVRGIRNHKEDEAKYISQCIDEIKQELKQDNIAVKANAVC
KLTYLQMLGYDISWAAFNIIEVMSASKFTFKRIGYLAASQSFHEGTDVIMLTTNQIRKDL
SSPSQYDTGVALTGLSCFVTPDLARDLANDIMTLMSHTKPYIRKKAVLIMYKVFLKYPES
LRPAFPRLKEKLEDPDPGVQSAAVNVICELARRNPKNYLSLAPLFFKLMTSSTNNWVLIK
IIKLFGALTPLEPRLGKKLIEPLTNLIHSTSAMSLLYECVNTVIAVLISLSSGMPNHSAS
IQLCVQKLRILIEDSDQNLKYLGLLAMSKILKTHPKSVQSHKDLILQCLDDKDESIRLRA
LDLLYGMVSKKNLMEIVKKLMTHVDKAEGTTYRDELLTKIIDICSQSNYQYITNFEWYIS

ILVELTRLEGTRHGHLIAAQMLDVAIRVKAIRKFAVSQMSALLDSAHLLASSTQRNGICE
VLYAAAWICGEFSEHLQEPHHTLEAMLRPRVTTLPGHIQAVYVQNVVKLYASILQQKEQA
GEAEGAQAVTQLMVDRLPQFVQSADLEVQERASCILQLVKHIQKLQAKDVPVAEEVSALF
AGELNPVAPKAQKKVPVPEGLDLDAWINEPLSDSESEDERPRAVFHEEEQRRPKHRPSEA
DEEELARRREARKQEQANNPFYIKSSPSPQKRYQDTPGVEHIPVVQIDLSVPLKVPGLPM
SDQYVKLEEERRHRQKLEKDKRRKKRKEKEKKGKRRHSSLPTESDEDIAPAQQVDIVTEE
MPENALPSDEDDKDPNDPYRALDIDLDKPLADSEKLPIQKHRNTETSKSPEKDVPMVEKK
SKKPKKKEKKHKEKERDKEKKKEKEKKAEDLDFWLSTTPPPAPAPAPAPVPSTGELSVNT
VTTPKDECEDAKTEAQGEEDDAEGQDQDKKSPKPKKKKHRKEKEERTKGKKKSKKQPPGS
EEAAGEPVQNGAPEEEQLPPESSYSLLAENSYVKMTCDIRGSLQEDSQVTVAIVLENRSS
SILKGMELSVLDSLNARMARPQGSSVHDGVPVPFQLPPGVSNEAQYVFTIQSIVMAQKLK
GTLSFIAKNDEGATHEKLDFRLHFSCSSYLITTPCYSDAFAKLLESGDLSMSSIKVDGIR
MSFQNLLAKICFHHHFSVVERVDSCASMYSRSIQGHHVCLLVKKGENSVSVDGKCSDSTL
LSNLLEEMKATLAKC*
>ENSG00000157837|ENST00000353487
MAEQTYSWAYSLVDSSQVSTFLISILLIVYGSFRSLNMDFENQDKEKDSNSSSGSFNGNS
TNNSIQTIDSTQALFLPIGASVSLLVMFFFFDSVQVVFTICTAVLATIAFAFLLLPMCQY
LTRPCSPQNKISFGCCGRFTAAELLSFSLSVMLVLIWVLTGHWLLMDALAMGLCVAMIAF
VRLPSLKVSCLLLSGLLIYDVFWVFFSAYIFNSNVMVKVATQPADNPLDVLSRKLHLGPN
VGRDVPRLSLPGKLVFPSSTGSHFSMLGIGDIVMPGLLLCFVLRYDNYKKQASGDSCGAP
GPANISGRMQKVSYFHCTLIGYFVGLLTATVASRIHRAAQPALLYLVPFTLLPLLTMAYL
KGDLRRMWSEPFHSKSSSSRFLEV*
>ENSG00000164414|ENST00000369557
MAAPRDNVTLLFKLYCLAVMTLMAAVYTIALRYTRTSDKELYFSTTAVCITEVIKLLLSV
GILAKETGSLGRFKASLRENVLGSPKELLKLSVPSLVYAVQNNMAFLALSNLDAAVYQVT
YQLKIPCTALCTVLMLNRTLSKLQWVSVFMLCAGVTLVQWKPAQATKVVFLQVLVASTLL
LWLSTQTTS*
>ENSG00000183778|ENST00000380618
MAFPKMRLMYICLLVLGALCLYFSMYSLNPFKEQSFVYKKDGNFLKLPDTDCRQTPPFLV
LLVTSSHKQLAERMAIRQTWGKERMVKGKQLKTFFLLGTTSSAAETKEVDQESQRHGDII
QKDFLDVYYNLTLKTMMGIEWVHRFCPQAAFVMKTDSDMFINVDYLTELLLKKNRTTRFF
TGFLKLNEFPIRQPFSKWFVSKSEYPWDRYPPFCSGTGYVFSGDVASQVYNVSKSVPYIK
LEDVFVGLCLERLNIRLEELHSQPTFFPGGLRFSVCLFRRIVACHFIKPRTLLDYWQALE
NSRGEDCPPV*
>ENSG00000101928|ENST00000370783
MHQQKRQPELVEGNLPVFVFPTELIFYADDQSTHKQVLTLYNPYEFALKFKVLCTTPNKY
VVVDAAGAVKPQCCVDIVIRHRDVRSCHYGVIDKFRLQVSEQSQRKALGRKEVVATLLPS
AKEQQKEEEEKRLKEHLTESLFFEQSFQPENRAVSSGPSLLTVFLGVVCIAALMLPTLGD
VESLVPLYLHLSVNQKLVAAYILGLITMAILRT*
>ENSG00000143553|ENST00000368685
MAGAGSAAVSGAGTPVAGPTGRDLFAEGLLEFLRPAVQQLDSHVHAVRESQVELREQIDN
LATELCRINEDQKVALDLDPYVKKLLNARRRVVLVNNILQNAQERLRRLNHSVAKETARR
RAMLDSGIYPPGSPGK*
>ENSG00000197249|ENST00000355814

MPSSVSWGILLLAGLCCLVPVSLAEDPQGDAAQKTDTSHHDQDHPTFNKITPNLAEFAFS
LYRQLAHQSNSTNIFFSPVSIATAFAMLSLGTKADTHDEILEGLNFNLTEIPEAQIHEGF
QELLRTLNQPDSQLQLTTGNGLFLSEGLKLVDKFLEDVKKLYHSEAFTVNFGDTEEAKKQ
INDYVEKGTQGKIVDLVKELDRDTVFALVNYIFFKGKWERPFEVKDTEEEDFHVDQVTTV
KVPMMKRLGMFNIQHCKKLSSWVLLMKYLGNATAIFFLPDEGKLQHLENELTHDIITKFL
ENEDRRSASLHLPKLSITGTYDLKSVLGQLGITKVFSNGADLSGVTEEAPLKLSKAVHKA
VLTIDEKGTEAAGAMFLEAIPMSIPPEVKFNKPFVFLMIEQNTKSPLFMGKVVNPTQK*
>ENSG00000160216|ENST00000398063
MGLLAFLKTQFVLHLLVGFVFVVSGLVINFVQLCTLALWPVSKQLYRRLNCRLAYSLWSQ
LVMLLEWWSCTECTLFTDQATVERFGKEHAVIILNHNFEIDFLCGWTMCERFGVLGSSKV
LAKKELLYVPLIGWTWYFLEIVFCKRKWEEDRDTVVEGLRRLSDYPEYMWFLLYCEGTRF
TETKHRVSMEVAAAKGLPVLKYHLLPRTKGFTTAVKCLRGTVAAVYDVTLNFRGNKNPSL
LGILYGKKYEADMCVRRFPLEDIPLDEKEAAQWLHKLYQEKDALQEIYNQKGMFPGEQFK
PARRPWTLLNFLSWATILLSPLFSFVLGVFASGSPLLILTFLGFVGAASFGVRRLIGVTE
IEKGSSYGNQEFKKKE*
>ENSG00000186635|ENST00000393605
MTLSGSRGQEEPPPSRVPRAVRVASLLSEGEELSGDDQGDEEEDDHAYEGVPNGGWHTSS
LSLSLPSTIAAPHPMDGPPGGSTPVTPVIKAGWLDKNPPQGSYIYQKRWVRLDTDHLRYF
DSNKDAYSKRFISVACISHVAAIGDQKFEVITNNRTFAFRAESDVERKEWMQALQQAMAE
QRARARLSSAYLLGVPGSEQPDRAGSLELRGFKNKLYVAVVGDKVQLYKNLEEYHLGIGI
TFIDMSVGNVKEVDRRSFDLTTPYRIFSFSADSELEKEQWLEAMQGAIAEALSTSEVAER
IWAAAPNRFCADCGAPQPDWASINLCVVICKRCAGEHRGLGAGVSKVRSLKMDRKVWTET
LIELFLQLGNGAGNRFWAANVPPSEALQPSSSPSTRRCHLEAKYREGKYRRYHPLFGNQE
ELDKALCAAVTTTDLAETQALLGCGAGINCFSGDPEAPTPLALAEQAGQTLQMEFLRNNR
TTEVPRLDSMKPLEKHYSVVLPTVSHSGFLYKTASAGKLLQDRRAREEFSRRWCVLGDGV
LSYFENERAVTPNGEIRASEIVCLAVPPPDTHGFEHTFEVYTEGERLYLFGLESAEQAHE
WVKCIAKAFVPPLAEDLLARDFERLGRLPYKAGLSLQRAQEGWFSLSGSELRAVFPEGPC
EEPLQLRKLQELSIQGDSENQVLVLVERRRTLYIQGERRLDFMGWLGAIQKAAASMGDTL
SEQQLGDSDIPVIVYRCVDYITQCGLTSEGIYRKCGQTSKTQRLLESLRQDARSVHLKEG
EQHVDDVSSALKRFLRDLPDGLFTRAQRLTWLEASEIEDEEEKVSRYRELLVRLPPVNRA
TVKALISHLYCVQCFSDTNQMNVHNLAIVFGPTLFQTDGQDYKAGRVVEDLINHYVVVFS
VDEEELRKQREEITAIVKMRVAGTASGTQHAGDFICTVYLEEKKAETEQHIKVPASMTAE
ELTLEILDRRNVGIREKDYWTCFEVNEREEAERPLHFAEKVLPILHGLGTDSHLVVKKHQ
AMEAMLLYLASRVGDTKHGMMKFREDRSLLGLGLPSGGFHDRYFILNSSCLRLYKEVRSQ
RPWSGAPETSHRPEKEWPIKSLKVYLGVKKKLRPPTCWGFTVVHETEKHEKQQWYLCCDT
QMELREWFATFLFVQHDGLVWPSEPSRVSRAVPEVRLGSVSLIPLRGSENEMRRSVAAFT
ADPLSLLRNV*
>ENSG00000099139|ENST00000376752
MGWGSRCCCPGRLDLLCVLALLGGCLLPVCRTRVYTNHWAVKIAGGFPEANRIASKYGFI
NIGQIGALKDYYHFYHSRTIKRSVISSRGTHSFISMEPKVEWIQQQVVKKRTKRDYDFSR
AQSTYFNDPKWPSMWYMHCSDNTHPCQSDMNIEGAWKRGYTGKNIVVTILDDGIERTHPD
LMQNYDALASCDVNGNDLDPMPRYDASNENKHGTRCAGEVAAAANNSHCTVGIAFNAKIG
GVRMLDGDVTDMVEAKSVSFNPQHVHIYSASWGPDDDGKTVDGPAPLTRQAFENGVRMGR
RGLGSVFVWASGNGGRSKDHCSCDGYTNSIYTISISSTAESGKKPWYLEECSSTLATTYS

SGESYDKKIITTDLRQRCTDNHTGTSASAPMAAGIIALALEANPFLTWRDVQHVIVRTSR
AGHLNANDWKTNAAGFKVSHLYGFGLMDAEAMVMEAEKWTTVPRQHVCVESTDRQIKTIR
PNSAVRSIYKASGCSDNPNRHVNYLEHVVVRITITHPRRGDLAIYLTSPSGTRSQLLANR
LFDHSMEGFKNWEFMTIHCWGERAAGDWVLEVYDTPSQLRNFKTPGKLKEWSLVLYGTSV
QPYSPTNEFPKVERFRYSRVEDPTDDYGTEDYAGPCDPECSEVGCDGPGPDHCNDCLHYY
YKLKNNTRICVSSCPPGHYHADKKRCRKCAPNCESCFGSHGDQCMSCKYGYFLNEETNSC
VTHCPDGSYQDTKKNLCRKCSENCKTCTEFHNCTECRDGLSLQGSRCSVSCEDGRYFNGQ
DCQPCHRFCATCAGAGADGCINCTEGYFMEDGRCVQSCSISYYFDHSSENGYKSCKKCDI
SCLTCNGPGFKNCTSCPSGYLLDLGMCQMGAICKDATEESWAEGGFCMLVKKNNLCQRKV
LQQLCCKTCTFQG*
>ENSG00000184831|ENST00000379226
MFKVIQRSVGPASLSLLTFKVYAAPKKDSPPKNSVKVDELSLYSVPEGQSKYVEEARSQL
EESISQLRHYCEPYTTWCQETYSQTKPKMQSLVQWGLDSYDYLQNAPPGFFPRLGVIGFA
GLIGLLLARGSKIKKLVYPPGFMGLAASLYYPQQAIVFAQVSGERLYDWGLRGYIVIEDL
WKENFQKPGNVKNSPGTK*
>ENSG00000172915|ENST00000379922
MFVNSNGYNLGVREDEVVVNDVDLPPWAKKPEDFVRINRMALESEFVSCQLHQWIDLIFG
YKQRGPEAVRALNVFHYLTYEGSVNLDSITDPVLREIPEAYFIRDPHTFLLTKDFIKAME
AQIQNFGQTPSQLLIEPHPPRSSAMHLCFLPQSPLMFKDQMQQDVIMVLKFPSNSPVTHV
AANTLPHLTIPAVVTVTCSRLFAVNRWHNTVGLRGAPGYSLDQAHHLPIEMDPLIANNSG
VNKRQITDLVDQSIQINAHCFVVTADNRYILICGFWDKSFRVYSTETGKLTQIVFGHWDV
VTCLARSESYIGGDCYIVSGSRDATLLLWYWSGRHHIIGDNPNSSDYPAPRAVLTGHDHE
VVCVSVCAELGLVISGAKEGPCLVHTITGDLLRALEGPENCLFPRLISVSSEGHCIIYYE
RGRFSNFSINGKLLAQMEINDSTRAILLSSDGQNLVTGGDNGVVEVWQACDFKQLYIYPG
CDAGIRAMDLSHDQRTLITGMASGSIVAFNIDFNRWHYEHQNRY*
>ENSG00000186635|ENST00000393609
MAEAGDAALSVAEWLRALHLEQYTGLFEQHGLVWATECQGLSDTRLMDMGMLLPGHRRRI
LAGLLRAHTSPAPAPRPTPRPVPMKRHIFRSPPVPATPPEPLPTTTEDEGLPAAPPIPPR
RSCLPPTCFTTPSTAAPDPVLPPLPAKRHLAELSVPPVPPRTGPPRLLVSLPTKEEESLL
PSLSSPPQPQSEEPLSTLPQGPPQPPSPPPCPPEIPPKPVRLFPEFDDSDYDEVPEEGPG
APARVMTKKEEPPPSRVPRAVRVASLLSEGEELSGDDQGDEEEDDHAYEGVPNGGWHTSS
LSLSLPSTIAAPHPMDGPPGGSTPVTPVIKAGWLDKNPPQGSYIYQKRWVRLDTDHLRYF
DSNKDAYSKRFISVACISHVAAIGDQKFEVITNNRTFAFRAESDVERKEWMQALQQAMAE
QRARARLSSAYLLGVPGSEQPDRAGSLELRGFKNKLYVAVVGDKVQLYKNLEEYHLGIGI
TFIDMSVGNVKEVDRRSFDLTTPYRIFSFSADSELEKEQWLEAMQGAIAEALSTSEVAER
IWAAAPNRFCADCGAPQPDWASINLCVVICKRCAGEHRGLGAGVSKVRSLKMDRKVWTET
LIELFLQLGNGAGNRFWAANVPPSEALQPSSSPSTRRCHLEAKYREGKYRRYHPLFGNQE
ELDKALCAAVTTTDLAETQALLGCGAGINCFSGDPEAPTPLALAEQAGQTLQMEFLRNNR
TTEVPRLDSMKPLEKHYSVVLPTVSHSGFLYKTASAGKLLQDRRAREEFSRRWCVLGDGV
LSYFENERAVTPNGEIRASEIVCLAVPPPDTHGFEHTFEVYTEGERLYLFGLESAEQAHE
WVKCIAKAFVPPLAEDLLARDFERLGRLPYKAGLSLQRAQEGWFSLSGSELRAVFPEGPC
EEPLQLRKLQELSIQGDSENQVLVLVERRRTLYIQGERRLDFMGWLGAIQKAAASMGDTL
SEQQLGDSDIPVIVYRCVDYITQCGLTSEGIYRKCGQTSKTQRLLESLRQDARSVHLKEG
EQHVDDVSSALKRFLRDLPDGLFTRAQRLTWLEASEIEDEEEKVSRYRELLVRLPPVNRA

TVKALISHLYCVQCFSDTNQMNVHNLAIVFGPTLFQTDGQDYKAGRVVEDLINHYVVVFS
VDEEELRKQREEITAIVKMRVAGTASGTQHAGDFICTVYLEEKKAETEQHIKVPASMTAE
ELTLEILDRRNVGIREKDYWTCFEVNEREEAERPLHFAEKVLPILHGLGTDSHLVVKKHQ
AMEAMLLYLASRVGDTKHGMMKFREDRSLLGLGLPSGGFHDRYFILNSSCLRLYKEVRSQ
RPWSGAPETSHRPEKEWPIKSLKVYLGVKKKLRPPTCWGFTVVHETEKHEKQQWYLCCDT
QMELREWFATFLFVQHDGLVWPSEPSRVSRAVPEVRLGSVSLIPLRGSENEMRRSVAAFT
ADPLSLLRNV*
>ENSG00000102100|ENST00000376512
MAAVGAGGSTAAPGPGAVSAGALEPGTASAAHRRLKYISLAVLVVQNASLILSIRYARTL
PGDRFFATTAVVMAEVLKGLTCLLLLFAQKRGNVKHLVLFLHEAVLVQYVDTLKLAVPSL
IYTLQNNLQYVAISNLPAATFQVSPKPSMAPEELTGLGTGVWE*
>ENSG00000204287|ENST00000395388
MAISGVPVLGFFIIAVLMSAQESWAIKEEHVIIQAEFYLNPDQSGEFMFDFDGDEIFHVD
MAKKETVWRLEEFGRFASFEAQGALANIAVDKANLEIMTKRSNYTPITNVPPEVTVLTNS
PVELREPNVLICFIDKFTPPVVNVTWLRNGKPVTTGVSETVFLPREDHLFRKFHYLPFLP
STEDVYDCRVEHWGLDEPLLKHWEFDAPSPLPETTENVVCALGLTVGLVGIIIGTIFIIK
GLRKSNAAERRGPL*
>ENSG00000157214|ENST00000394621
MESISMMGSPKSLSETFLPNGINGIKDARKVTVGVIGSGDFAKSLTIRLIRCGYHVVIGS
RNPKFASEFFPHVVDVTHHEDALTKTNIIFVAIHREHYTSLWDLRHLLVGKILIDVSNNM
RINQYPESNAEYLASLFPDSLIVKGFNVVSAWALQLGPKDASRQVYICSNNIQARQQVIE
LARQLNFIPIDLGSLSSAREIENLPLRLFTLWRGPVVVAISLATFFFLYSFVRDVIHPYA
RNQQSDFYKIPIEIVNKTLPIVAITLLSLVYLAGLLAAAYQLYYGTKYRRFPPWLETWLQ
CRKQLGLLSFFFAMVHVAYSLCLPMRRSERYLFLNMAYQQVHANIENSWNEEEVWRIEMY
ISFGIMSLGLLSLLAVTSIPSVSNALNWREFSFIQSTLGYVALLISTFHVLIYGWKRAFE
EEYYRFYTPPNFVLALVLPSIVILGKIILFLPCISRKLKRIKKGWEKSQFLEEGMGGTIP
HVSPERVTVM*
>ENSG00000139629|ENST00000356317
MRLLRRRHMPLRLAMVGCAFVLFLFLLHRDVSSREEATEKPWLKSLVSRKDHVLDLMLEA
MNNLRDSMPKLQIRAPEAQQTLFSINQSCLPGFYTPAELKPFWERPPQDPNAPGADGKAF
QKSKWTPLETQEKEEGYKKHCFNAFASDRISLQRSLGPDTRPPECVDQKFRRCPPLATTS
VIIVFHNEAWSTLLRTVYSVLHTTPAILLKEIILVDDASTEEHLKEKLEQYVKQLQVVRV
VRQEERKGLITARLLGASVAQAEVLTFLDAHCECFHGWLEPLLARIAEDKTVVVSPDIVT
IDLNTFEFAKPVQRGRVHSRGNFDWSLTFGWETLPPHEKQRRKDETYPIKSPTFAGGLFS
ISKSYFEHIGTYDNQMEIWGGENVEMSFRVWQCGGQLEIIPCSVVGHVFRTKSPHTFPKG
TSVIARNQVRLAEVWMDSYKKIFYRRNLQAAKMAQEKSFGDISERLQLREQLHCHNFSWY
LHNVYPEMFVPDLTPTFYGAIKNLGTNQCLDVGENNRGGKPLIMYSCHGLGGNQYFEYTT
QRDLRHNIAKQLCLHVSKGALGLGSCHFTGKNSQVPKDEEWELAQDQLIRNSGSGTCLTS
QDKKPAMAPCNPSDPHQLWLFV*
>ENSG00000158850|ENST00000367998
MLRRLLERPCTLALLVGSQLAVMMYLSLGGFRSLSALFGRDQGPTFDYSHPRDVYSNLSH
LPGAPGGPPAPQGLPYCPERSPLLVGPVSVSFSPVPSLAEIVERNPRVEPGGRYRPAGCE
PRSRTAIIVPHRAREHHLRLLLYHLHPFLQRQQLAYGIYVIHQAGNGTFNRAKLLNVGVR
EALRDEEWDCLFLHDVDLLPENDHNLYVCDPRGPRHVAVAMNKFGYSLPYPQYFGGVSAL

TPDQYLKMNGFPNEYWGWGGEDDDIATRVRLAGMKISRPPTSVGHYKMVKHRGDKGNEEN
PHRFDLLVRTQNSWTQDGMNSLTYQLLARELGPLYTNITADIGTDPRGPRAPSGPRYPPG
SSQAFRQEMLQRRPPARPGPLSTANHTALRGSH*
>ENSG00000126091|ENST00000351035
MGLLVFVRNLLLALCLFLVLGFLYYSAWKLHLLQWEEDSNSVVLSFDSAGQTLGSEYDRL
GFLLNLDSKLSPRTLCTVVFGLDCILESPGEPKKLLMPASHPLEILKSLSEDTAFALGFL
KLPRPAELATKYANFSEGACKPGYASALMTAIFPRFSKPAPMFLDDSFRKWARIREFVPP
FGIKGQDNLIKAILSVTKEYRLTPALDSLRCRRCIIVGNGGVLANKSLGSRIDDYDIVVR
LNSAPVKGFEKDVGSKTTLRITYPEGAMQRPEQYERDSLFVLAGFKWQDFKWLKYIVYKE
RVSASDGFWKSVATRVPKEPPEIRILNPYFIQEAAFTLIGLPFNNGLMGRGNIPTLGSVA
VTMALHGCDEVAVAGFGYDMSTPNAPLHYYETVRMAAIKESWTHNIQREKEFLRKLVKAR
VITDLSSGI*
>ENSG00000143393|ENST00000368874
MGDTVVEPAPLKPTSEPTSGPPGNNGGSLLSVITEGVGELSVIDPEVAQKACQEVLEKVK
LLHGGVAVSSRGTPLELVNGDGVDSEIRCLDDPPAQIREEEDEMGAAVASGTAKGARRRR
QNNSAKQSWLLRLFESKLFDISMAISYLYNSKEPGVQAYIGNRLFCFRNEDVDFYLPQLL
NMYIHMDEDVGDAIKPYIVHRCRQSINFSLQCALLLGAYSSDMHISTQRHSRGTKLRKLI
LSDELKPAHRKRELPSLSPAPDTGLSPSKRTHQRSKSDATASISLSSNLKRTASNPKVEN
EDEPVRLAPEREFIKSLMAIGKRLATLPTKEQKTQRLISELSLLNHKLPARVWLPTAGFD
HHVVRVPHTQAVVLNSKDKAPYLIYVEVLECENFDTTSVPARIPENRIRSTRSVENLPEC
GITHEQRAGSFSTVPNYDNDDEAWSVDDIGELQVELPEVHTNSCDNISQFSVDSITSQES
KEPVFIAAGDIRRRLSEQLAHTPTAFKRDPEDPSAVALKEPWQEKVRRIREGSPYGHLPN
WRLLSVIVKCGDDLRQELLAFQVLKQLQSIWEQERVPLWIKPYKILVISADSGMIEPVVN
AVSIHQVKKQSQLSLLDYFLQEHGSYTTEAFLSAQRNFVQSCAGYCLVCYLLQVKDRHNG
NILLDAEGHIIHIDFGFILSSSPRNLGFETSAFKLTTEFVDVMGGLDGDMFNYYKMLMLQ
GLIAARKHMDKVVQIVEIMQQGSQLPCFHGSSTIRNLKERFHMSMTEEQLQLLVEQMVDG
SMRSITTKLYDGFQYLTNGIM*
>ENSG00000109083|ENST00000357896
MAKDILGEAGLHFDELNKLRVLDPEVTQQTIELKEECKDFVDKIGQFQKIVGGLIELVDQ
LAKEAENEKMKSLAVSPRLECTGAISAHCKLCLSDSSDSPTSPSRVGGTTGHRCSELAQI
YSKAERSSTAATSSPNSRKENAARKVSG*
>ENSG00000082996|ENST00000392894
MLLSIGMLMLSATQVVYTILTVQLFAFLNLLPVEADILAYNFENASQTFDDLPARFGYRLP
AEGLKGFLINSKPENACEPIVPPPVKDNSSGTFIVLIRRLDCNFDIKVLNAQRAGYKAAI
VHNVDSDDLISMGSNDIEVLKKIDIPSVFIGESSANSLKDEFTYEKGGHLILVPEFSLPL
EYYLIPFLIIVGICLILIVIFMITKFVQDRHRARRNRLRKDQLKKLPVHKFKKGDEYDVC
AICLDEYEDGDKLRILPCSHAYHCKCVDPWLTKTKKTCPVCKQKVVPSQGDSDSDTDSSQ
EENEVTEHTPLLRPLASVSAQSFGALSESRSHQNMTESSDYEEDDNEDTDSSDAENEINE
HDVVVQLQPNGERDYNIANTV*
>ENSG00000133067|ENST00000367278
MPSPPGLRALWLCAALCASRRAGGAPQPGPGPTACPAPCHCQEDGIMLSADCSELGLSAV
PGDLDPLTAYLDLSMNNLTELQPGLFHHLRFLEELRLSGNHLSHIPGQAFSGLYSLKILM
LQNNQLGGIPAEALWELPSLQSLRLDANLISLVPERSFEGLSSLRHLWLDDNALTEIPVR
ALNNLPALQAMTLALNRISHIPDYAFQNLTSLVVLHLHNNRIQHLGTHSFEGLHNLETLD

LNYNKLQEFPVAIRTLGRLQELGFHNNNIKAIPEKAFMGNPLLQTIHFYDNPIQFVGRSA
FQYLPKLHTLSLNGAMDIQEFPDLKGTTSLEILTLTRAGIRLLPSGMCQQLPRLRVLELS
HNQIEELPSLHRCQKLEEIGLQHNRIWEIGADTFSQLSSLQALDLSWNAIRSIHPEAFST
LHSLVKLDLTDNQLTTLPLAGLGGLMHLKLKGNLALSQAFSKDSFPKLRILEVPYAYQCC
PYGMCASFFKASGQWEAEDLHLDDEESSKRPLGLLARQAENHYDQDLDELQLEMEDSKPH
PSVQCSPTPGPFKPCEYLFESWGIRLAVWAIVLLSVLCNGLVLLTVFAGGPVPLPPVKFV
VGAIAGANTLTGISCGLLASVDALTFGQFSEYGARWETGLGCRATGFLAVLGSEASVLLL
TLAAVQCSVSVSCVRAYGKSPSLGSVRAGVLGCLALAGLAAALPLASVGEYGASPLCLPY
APPEGQPAALGFTVALVMMNSFCFLVVAGAYIKLYCDLPRGDFEAVWDCAMVRHVAWLIF
ADGLLYCPVAFLSFASMLGLFPVTPEAVKSVLLVVLPLPACLNPLLYLLFNPHFRDDLRR
LRPRAGDSGPLAYAAAGELEKSSCDSTQALVAFSDVDLILEASEAGRPPGLETYGFPSVT
LISCQQPGAPRLEGSHCVEPEGNHFGNPQPSMDGELLLRAEGSTPAGGGLSGGGGFQPSG
LAFASHV*
>ENSG00000133318|ENST00000356000
MAEPSAATQSHSISSSSFGAEPSAPGGGGSPGACPALGTKSCSSSCADSFVSSSSSQPVS
LFSTSQVHDLIFWRDVKKTGFVFGTTLIMLLSLAAFSVISVVSYLILALLSVTISFRIYK
SVIQAVQKSEEGHPFKAYLDVDITLSSEAFHNYMNAAMVHINRALKLIIRLFLVEDLVDS
LKLAVFMWLMTYVGAVFNGITLLILAELLIFSVPIVYEKYKTQIDHYVGIARDQTKSIVE
KIQAKLPGIAKKKAE*
>ENSG00000095139|ENST00000392859
MIPEYCRALEENEISEHCFDLIFAFDEIVALGYRENVNLAQIRTFTEMDSHEEKVFRAVR
ETQEREAKAEMRRKAKELQQARRDAERQGKKAPGFGGFGSSAVSGGSTAAMITETIIETD
KPKVAPAPARPSGPSKALKLGAKGKEVDNFVDKLKSEGETIMSSSMGKRTSEATKMHAPP
INMESVHMKIEEKITLTCGRDGGLQNMELHGMIMLRISDDKYGRIRLHVENEDKKGVQLQ
THPNVDKKLFTAESLIGLKNPEKSFPVNSDVGVLKWRLQTTEESFIPLTINCWPSESGNG
CDVNIEYELQEDNLELNDVVITIPLPSGVGAPVIGEIDGEYRHDSRRNTLEWCLPVIDAK
NKSGSLEFSIAGQPNDFFPVQVSFVSKKNYCNIQVTKVTQVDGNSPVRFSTETTFLVDKY
EIL*
>ENSG00000109610|ENST00000382120
MLALLCSCLLLAAGASDAWTGEDSAEPNSDSAEWIRDMYAKVTEIWQEVMQRRDDDGALH
AACQVQPSATLDAAQPRVTGVVLFRQLAPRAKLDAFFALEGFPTEPNSSSRAIHVHQFGD
LSQGCESTGPHYNPLAVPHPQHPGDFGNFAVRDGSLWRYRAGLAASLAGPHSIVGRAVVV
HAGEDDLGRGGNQASVENGNAGRRLACCVVGVCGPGLWERQAREHSERKKRRRESECKAA
*
>ENSG00000121578|ENST00000359213
MGFNLTFHLSYKFRLLLLLTLCLTVVGWATSNYFVGAIQEIPKAKEFMANFHKTLILGKG
KTLTNEASTKKVELDNCPSVSPYLRGQSKLIFKPDLTLEEVQAENPKVSRGRYRPQECKA
LQRVAILVPHRNREKHLMYLLEHLHPFLQRQQLDYGIYVIHQAEGKKFNRAKLLNVGYLE
ALKEENWDCFIFHDVDLVPENDFNLYKCEEHPKHLVVGRNSTGYRLRYSGYFGGVTALSR
EQFFKVNGFSNNYWGWGGEDDDLRLRVELQRMKISRPLPEVGKYTMVFHTRDKGNEVNAE
RMKLLHQVSRVWRTDGLSSCSYKLVSVEHNPLYINITVDFWFGA*
>ENSG00000100365|ENST00000397147
MAVAQQLRAESDFEQLPDDVAISANIADIEEKRGFTSHFVFVIEVKTKGGSKYLIYRRYR
QFHALQSKLEERFGPDSKSSALACTLPTLPAKVYVGVKQEIAEMRIPALNAYMKSLLSLP

VWVLMDEDVRIFFYQSPYDSEQVPQALRRLRPRTRKVKSVSPQGNSVDRMAAPRAEALFD
FTGNSKLELNFKAGDVIFLLSRINKDWLEGTVRGATGIFPLSFVKILKDFPEEDDPTNWL
RCYYYEDTISTIKSVAWEGGACPAFLPSLRPLPLTSPSHGSLSHSKAPSGSQMSHNAVTS
HQRPGWPGQPHSPFPHPTPHFQPDASLLQPVTPLGTSRWRKISAALPY*
>ENSG00000163291|ENST00000395594
MHQKLLKSAHYIELGSYQYWPVLVPRGIRLYTYEQIPGSLKDNPYITDGYRAYLPSRLCI
KSLFILSNETVNIWSHLLGFFLFFTLGIYDMTSVLPSASASREDFVICSICLFCFQVCML
CSVGYHLFSCHRSEKTCRRWMALDYAGISIGILGCYVSGVFYAFYCNNYWRQVYLITVLA
MILAVFFAQIHPNYLTQQWQRLRSIIFCSVSGYGVIPTLHWVWLNGGIGAPIVQDFAPRV
IVMYMIALLAFLFYISKVPERYFPESLPR*
>ENSG00000148384|ENST00000371712
MPSKAENLRPSEPAPQPPEGRTLQGQLPGAPPAQRAGSPPDAPGSESPALACSTPATPSG
EDPPARAAPIAPRPPARPRLERALSLDDKGWRRRRFRGSQEDLEARNGTSPSRGSVQSEG
PGAPAHSCSPPCLSTSLQEIPKSRGVLSSERGSPSSGGNPLSGVASSSPNLPHRDAAVAG
SSPRLPSLLPPRPPPALSLDIASDSLRTANKVDSDLADYKLRAQPLLVRAHSSLGPGRPR
SPLACDDCSLRSAKSSFSLLAPIRSKDVRSRSYLEGSLLASGALLGADELARYFPDRNVA
LFVATWNMQGQKELPPSLDEFLLPAEADYAQDLYVIGVQEGCSDRREWETRLQETLGPHY
VLLSSAAHGVLYMSLFIRRDLIWFCSEVECSTVTTRIVSQIKTKGALGISFTFFGTSFLF
ITSHFTSGDGKVAERLLDYTRTVQALVLPRNVPDTNPYRSSAADVTTRFDEVFWFGDFNF
RLSGGRTVVDALLCQGLVVDVPALLQHDQLIREMRKGSIFKGFQEPDIHFLPSYKFDIGK
DTYDSTSKQRTPSYTDRVLYRSRHKGDICPVSYSSCPGIKTSDHRPVYGLFRVKVRPGRD
NIPLAAGKFDRELYLLGIKRRISKEIQRQQALQSQNSSTICSVS*
>ENSG00000075651|ENST00000356327
MSLKNEPRVNTSALQKIAADMSNIIENLDTRELHFEGEEVDYDVSPSDPKIQEVYIPFSA
IYNTQGFKEPNIQTYLSGCPIKAQVLEVERFTSTTRVPSINLYTIELTHGEFKWQVKRKF
KHFQEFHRELLKYKAFIRIPIPTRRHTFRRQNVREEPREMPSLPRSSENMIREEQFLGRR
KQLEDYLTKILKMPMYRNYHATTEFLDISQLSFIHDLGPKGIEGMIMKRSGGHRIPGLNC
CGQGRACYRWSKRWLIVKDSFLLYMKPDSGAIAFVLLVDKEFKIKVGKKETETKYGIRID
NLSRTLILKCNSYRHARWWGGAIEEFIQKHGTNFLKDHRFGSYAAIQENALAKWYVNAKG
YFEDVANAMEEANEEIFITDWWLSPEIFLKRPVVEGNRWRLDCILKRKAQQGVRIFIMLY
KEVELALGINSEYTKRTLMRLHPNIKVMRHPDHVSSTVYLWAHHEKLVIIDQSVAFVGGI
DLAYGRWDDNEHRLTDVGSVKRVTSGPSLGSLPPAAMESMESLRLKDKNEPVQNLPIQKS
IDDVDSKLKGIGKPRKFSKFSLYKQLHRHHLHDADSISSIDSTSNTGSIRSLQTGVGELH
GETRFWHGKDYCNFVFKDWVQLDKPFADFIDRYSTPRMPWHDIASAVHGKAARDVARHFI
QRWNFTKIMKSKYRSLSYPFLLPKSQTTAHELRYQVPGSVHANVQLLRSAADWSAGIKYH
EESIHAAYVHVIENSRHYIYIENQFFISCADDKVVFNKIGDAIAQRILKAHRENQKYRVY
VVIPLLPGFEGDISTGGGNALQAIMHFNYRTMCRGENSILGQLKAELGNQWINYISFCGL
RTHAELEGNLVTELIYVHSKLLIADDNTVIIGSANINDRSMLGKRDSEMAVIVQDTETVP
SVMDGKEYQAGRFARGLRLQCFRVVLGYLDDPSEDIQDPVSDKFFKEVWVSTAARNATIY
DKVFRCLPNDEVHNLIQLRDFINKPVLAKEDPIRAEEELKKIRGFLVQFPFYFLSEESLL
PSVGTKEAIVPMEVWT*
>ENSG00000171004|ENST00000370836
MALPACAVREFEPPRQPERGAPVRTTCPRRHSRVEAELAASRPGSVAASVRAGPPRGVSH
GFHTRPLLDKPRKASSSLAGAACAPLFALLSRGRRRRMHVLRRRWDLGSLCRALLTRGLA

ALGHSLKHVLGAIFSKIFGPMASVGNMDEKSNKLLLALVMLFLFAVIVLQYVCPGTECQL
LRLQAFSSPVPDPYRSEDESSARFVPRYNFTRGDLLRKVDFDIKGDDLIVFLHIQKTGGT
TFGRHLVRNIQLEQPCECRVGQKKCTCHRPGKRETWLFSRFSTGWSCGLHADWTELTSCV
PSVVDGKRDARLRPSRNFHYITILRDPVSRYLSEWRHVQRGATWKASLHVCDGRPPTSEE
LPSCYTGDDWSGCPLKEFMDCPYNLANNRQVRMLSDLTLVGCYNLSVMPEKQRNKVLLES
AKSNLKHMAFFGLTEFQRKTQYLFEKTFNMNFISPFTQYNTTRASSVEINEEIQKRIEGL
NFLDMELYSYAKDLFLQRYQFMRQKEHQEARRKRQEQRKFLKGRLLQTHFQSQGQGQSQN
PNQNQSQNPNPNANQNLTQNLMQNLTQSLSQKENRESPKQNSGKEQNDNTSNGTNDYIGS
VEKWR*
>ENSG00000011478|ENST00000366382
MRSGGRGRPRLRLGERGLMEPLLPPKRRLLPRVRLLPLLLALAVGSAFYTIWSGWHRRTE
ELPLGRELRVPLIGSLPEARLRRVVGQLDPQRLWSTYLRPLLVVRTPGSPGNLQVRKAAP
VTLQLLFLDGEEALKEWGPKDSLYGSRHLAQLMESIPHSPGPTRIQAIELFMLLDLLGAP
NPTFYSHFPRTVRWFHRLRSIEKRLHRLNLLQSHPQEVMYFQPGEPFGSVEDDHIPFLRR
GVPVLHLISTPFPAVWHTPADTEVNLHPPTVHNLCRILAVFLAEYLGL*
>ENSG00000064225|ENST00000394162
MRGYLVAIFLSAVFLYYVLHCILWGTNVYWVAPVEMKRRNKIQPCLSKPAFASLLRFHQF
HPFLCAADFRKIASLYGSDKFDLPYGMRTSAEYFRLALSKLQSCDLFDEFDNIPCKKCVV
VGNGGVLKNKTLGEKIDSYDVIIRMNNGPVLGHEEEVGRRTTFRLFYPESVFSDPIHNDP
NTTVILTAFKPHDLRWLLELLMGDKINTNGFWKKPALNLIYKPYQIRILDPFIIRTAAYE
LLHFPKVFPKNQKPKHPTTGIIAITLAFYICHEVHLAGFKYNFSDLKSPLHYYGNATMSL
MNKNAYHNVTAEQLFLKDIIEKNLVINLTQD*
>ENSG00000117620|ENST00000370153
MSSRSVLSPVVGTDAPDQHLELKKPQELKEMERLPLANEDKTMFANLKYVSLGILVFQTT
SLVLTMRYSRTLKEEGPRYLSSTAVVVAELLKIMACILLVYKDSKCSLRALNRVLHDEIL
NKPMETLKLAIPSGIYTLQNNLLYVALSNLDAATYQVTYQLKILTTALFSVSMLSKKLGV
YQWLSLVILMTGVAFVQWPSDSQLDSKELSAGSQFVGLMAVLTACFSSGFAGVYFEKILK
ETKQSVWIRNIQLGFFGSIFGLMGVYIYDGELVSKNGFFQGYNRLTWIVVVLQALGGLVI
AAVIKYADNILKGFATSLSIILSTLISYFWLQDFVPTSVFFLGAILVITATFLYGYDPKP
AGNPTKA*
>ENSG00000185499|ENST00000368392
MTPGTQSPFFLLLLLTVLTATTAPKPATVVTGSGHASSTPGGEKETSATQRSSVPSSTEK
NAFNSSLEDPSTDYYQELQRDISEMFLQIYKQGGFLGLSNIKFRPGSVVVQLTLAFREGT
INVHDVETQFNQYKTEAASRYNLTISDVSVSDVPFPFSAQSGAGVPGWGIALLVLVCVLV
ALAIVYLIALAVCQCRRKNYGQLDIFPARDTYHPMSEYPTYHTHGRYVPPSSTDRSPYEK
VSAGNGGSSLSYTNPAVAATSANL*
>ENSG00000138674|ENST00000355196
MKLKEVDRTAMQAWSPAQNHPIYLATGTSAQQLDATFSTNASLEIFELDLSDPSLDMKSC
ATFSSSHRYHKLIWGPYKMDSKGDVSGVLIAGGENGNIILYDPSKIIAGDKEVVIAQNDK
HTGPVRALDVNIFQTNLVASGANESEIYIWDLNNFATPMTPGAKTQPPEDISCIAWNRQV
QHILASASPSGRATVWDLRKNEPIIKVSDHSNRMHCSGLAWHPDVATQMVLASEDDRLPV
IQMWDLRFASSPLRVLENHARGILAIAWSMADPELLLSCGKDAKILCSNPNTGEVLYELP
TNTQWCFDIQWCPRNPAVLSAASFDGRISVYSIMGGSTDGLRQKQVDKLSSSFGNLDPFG
TGQPLPPLQIPQQTAQHSIVLPLKKPPKWIRRPVGASFSFGGKLVTFENVRMPSHQGAEQ

QQQQHHVFISQVVTEKEFLSRSDQLQQAVQSQGFINYCQKKIDASQTEFEKNVWSFLKVN
FEDDSRGKYLELLGYRKEDLGKKIALALNKVDGANVALKDSDQVAQSDGEESPAAEEQLL
GEHIKEEKEESEFLPSSGGTFNISVSGDIDGLITQALLTGNFESAVDLCLHDNRMADAII
LAIAGGQELLARTQKKYFAKSQSKITRLITAVVMKNWKEIVESCDLKNWREALAAVLTYA
KPDEFSALCDLLGTRLENEGDSLLQTQACLCYICAGNVEKLVACWTKAQDGSHPLSLQDL
IEKVVILRKAVQLTQAMDTSTVGVLLAAKMSQYANLLAAQGSIAAALAFLPDNTNQPNIM
QLRDRLCRAQGEPVAGHESPKIPYEKQQLPKGRPGPVAGHHQMPRVQTQQYYPHGENPPP
PGFIMHGNVNPNAAGQLPTSPGHMHTQVPPYPQPQPYQPAQPYPFGTGGSAMYRPQQPVA
PPTSNAYPNTPYISSASSYTGQSQLYAAQHQASSPTSSPATSFPPPPSSGASFQHGGPGA
PPSSSAYALPPGTTGTLPAASELPASQRTGPQNGWNDPPALNRVPKKKKMPENFMPPVPI
TSPIMNPLGDPQSQMLQQQPSAPVPLSSQSSFPQPHLPGGQPFHGVQQPLGQTGMPPSFS
KPNIEGAPGAPIGNTFQHVQSLPTKKITKKPIPDEHLILKTTFEDLIQRCLSSATDPQTK
RKLDDASKRLEFLYDKLREQTLSPTITSGLHNIARSIETRNYSEGLTMHTHIVSTSNFSE
TSAFMPVLKVVLTQANKLGV*
>ENSG00000172780|ENST00000393305
MAGPGPGPGDPDEQYDFLFKLVLVGDASVGKTCVVQRFKTGAFSERQGSTIGVDFTMKTL
EIQGKRVKLQIWDTAGQERFRTITQSYYRSANGAILAYDITKRSSFLSVPHWIEDVRKYA
GSNIVQLLIGNKSDLSELREVSLAEAQSLAEHYDILCAIETSAKDSSNVEEAFLRVATEL
IMRHGGPLFSEKSPDHIQLNSKDIGEGWGCGC*
>ENSG00000257923|ENST00000360264
MAANVGSMFQYWKRFDLQQLQRELDATATVLANRQDESEQSRKRLIEQSREFKKNTPEDL
RKQVAPLLKSFQGEIDALSKRSKEAEAAFLNVYKRLIDVPDPVPALDLGQQLQLKVQRLH
DIETENQKLRETLEEYNKEFAEVKNQEVTIKALKEKIREYEQTLKNQAETIALEKEQKLQ
NDFAEKERKLQETQMSTTSKLEEAEHKVQSLQTALEKTRTELFDLKTKYDEETTAKADEI
EMIMTDLERANQRAEVAQREAETLREQLSSANHSLQLASQIQKAPDVEQAIEVLTRSSLE
VELAAKEREIAQLVEDVQRLQASLTKLRENSASQISQLEQQLSAKNSTLKQLEEKLKGQA
DYEEVKKELNILKSMEFAPSEGAGTQDAAKPLEVLLLEKNRSLQSENAALRISNSDLSGS
ARRKGKDQPESRRPGSLPAPPPSQLPRNPGEQASNTNGTHQFSPAGLSQDFFSSSLASPS
LPLASTGKFALNSLLQRQLMQSFYSKAMQEAGSTSMIFSTGPYSTNSISSQSPLQQSPDV
NGMAPSPSQSESAGSVSEGEEMDTAEIARQVKEQLIKHNIGQRIFGHYVLGLSQGSVSEI
LARPKPWNKLTVRGKEPFHKMKQFLSDEQNILALRSIQGRQRENPGQSLNRLFQEVPKRR
NGSEGNITTRIRASETGSDEAIKSILEQAKRELQVQKTAEPAQPSSASGSGNSDDAIRSI
LQQARREMEAQQAALDPALKQAPLSQSDITILTPKLLSTSPMPTVSSYPPLAISLKKPSA
APEAGASALPNPPALKKEAQDAPGLDPQGAADCAQGVLRQVKNEVGRSGAWKDHWWSAVQ
PERRNAASSEEAKAEETGGGKEKGSGGSGGGSQPRAERSQLQGPSSSEYWKEWPSAESPY
SQSSELSLTGASRSETPQNSPLPSSPIVPMSKPTKPSVPPLTPEQYEVYMYQEVDTIELT
RQVKEKLAKNGICQRIFGEKVLGLSQGSVSDMLSRPKPWSKLTQKGREPFIRMQLWLNGE
LGQGVLPVQGQQQGPVLHSVTSLQDPLQQGCVSSESTPKTSASCSPAPESPMSSSESVKS
LTELVQQPCPPIEASKDSKPPEPSDPPASDSQPTTPLPLSGHSALSIQELVAMSPELDTY
GITKRVKEVLTDNNLGQRLFGETILGLTQGSVSDLLARPKPWHKLSLKGREPFVRMQLWL
NDPNNVEKLMDMKRMEKKAYMKRRHSSVSDSQPCEPPSVGTEYSQGASPQPQHQLKKPRV
VLAPEEKEALKRAYQQKPYPSPKTIEDLATQLNLKTSTVINWFHNYRSRIRRELFIEEIQ
AGSQGQAGASDSPSARSGRAAPSSEGDSCDGVEATEGPGSADTEEPKSQGEAEREEVPRP
AEQTEPPPSGTPGPDDARDDDHEGGPVEGPGPLPSPASATATAAPAAPEDAATSAAAAPG

EGPAAPSSAPPPSNSSSSSAPRRPSSLQSLFGLPEAAGARDSRDNPLRKKKAANLNSIIH
RLEKAASREEPIEWEF*
>ENSG00000085998|ENST00000371984
MDDWKPSPLIKPFGARKKRSWYLTWKYKLTNQRALRRFCQTGAVLFLLVTVIVNIKLILD
TRRAISEANEDPEPEQDYDEALGRLEPPRRRGSGPRRVLDVEVYSSRSKVYVAVDGTTVL
EDEAREQGRGIHVIVLNQATGHVMAKRVFDTYSPHEDEAMVLFLNMVAPGRVLICTVKDE
GSFHLKDTAKALLRSLGSQAGPALGWRDTWAFVGRKGGPVFGEKHSKSPALSSWGDPVLL
KTDVPLSSAEEAECHWADTELNRRRRRFCSKVEGYGSVCSCKDPTPIEFSPDPLPDNKVL
NVPVAVIAGNRPNYLYRMLRSLLSAQGVSPQMITVFIDGYYEEPMDVVALFGLRGIQHTP
ISIKNARVSQHYKASLTATFNLFPEAKFAVVLEEDLDIAVDFFSFLSQSIHLLEEDDSLY
CISAWNDQGYEHTAEDPALLYRVETMPGLGWVLRRSLYKEELEPKWPTPEKLWDWDMWMR
MPEQRRGRECIIPDVSRSYHFGIVGLNMNGYFHEAYFKKHKFNTVPGVQLRNVDSLKKEA
YEVEVHRLLSEAEVLDHSKNPCEDSFLPDTEGHTYVAFIRMEKDDDFTTWTQLAKCLHIW
DLDVRGNHRGLWRLFRKKNHFLMVGVPASPYSVKKPPSVTPIFLEPPPKEEGAPGAPEQT
*
>ENSG00000141367|ENST00000393043
MAQILPIRFQEHLQLQNLGINPANIGFSTLTMESDKFICIREKVGEQAQVVIIDMNDPSN
PIRRPISADSAIMNPASKVIALKAGKTLQIFNIEMKSKMKAHTMTDDVTFWKWISLNTVA
LVTDNAVYHWSMEGESQPVKMFDRHSSLAGCQIINYRTDAKQKWLLLTGISAQQNRVVGA
MQLYSVDRKVSQPIEGHAASFAQFKMEGNAEESTLFCFAVRGQAGGKLHIIEVGTPPTGN
QPFPKKAVDVFFPPEAQNDFPVAMQISEKHDVVFLITKYGYIHLYDLETGTCIYMNRISG
ETIFVTAPHEATAGIIGVNRKGQVLSVCVEEENIIPYITNVLQNPDLALRMAVRNNLAGA
EELFARKFNALFAQGNYSEAAKVAANAPKGILRTPDTIRRFQSVPAQPGQTSPLLQYFGI
LLDQGQLNKYESLELCRPVLQQGRKQLLEKWLKEDKLECSEELGDLVKSVDPTLALSVYL
RANVPNKVIQCFAETGQVQKIVLYAKKVGYTPDWIFLLRNVMRISPDQGQQFAQMLVQDE
EPLADITQIVDVFMEYNLIQQCTAFLLDALKNNRPSEGPLQTRLLEMNLMHAPQVADAIL
GNQMFTHYDRAHIAQLCEKAGLLQRALEHFTDLYDIKRAVVHTHLLNPEWLVNYFGSLSV
EDSLECLRAMLSANIRQNLQICVQVASKYHEQLSTQSLIELFESFKSFEGLFYFLGSIVN
FSQDPDVHFKYIQAACKTGQIKEVERICRESNCYDPERVKNFLKEAKLTDQLPLIIVCDR
FDFVHDLVLYLYRNNLQKYIEIYVQKVNPSRLPVVIGGLLDVDCSEDVIKNLILVVRGQF
STDELVAEVEKRNRLKLLLPWLEARIHEGCEEPATHNALAKIYIDSNNNPERFLRENPYY
DSRVVGKYCEKRDPHLACVAYERGQCDLELINVCNENSLFKSLSRYLVRRKDPELWGSVL
LESNPYRRPLIDQVVQTALSETQDPEEVSVTVKAFMTADLPNELIELLEKIVLDNSVFSE
HRNLQNLLILTAIKADRTRVMEYINRLDNYDAPDIANIAISNELFEEAFAIFRKFDVNTS
AVQVLIEHIGNLDRAYEFAERCNEPAVWSQLAKAQLQKGMVKEAIDSYIKADDPSSYMEV
VQAANTSGNWEELVKYLQMARKKARESYVETELIFALAKTNRLAELEEFINGPNNAHIQQ
VGDRCYDEKMYDAAKLLYNNVSNFGRLASTLVHLGEYQAAVDGARKANSTRTWKEVCFAC
VDGKEFRLAQMCGLHIVVHADELEELINYYQDRGYFEELITMLEAALGLERAHMGMFTEL
AILYSKFKPQKMREHLELFWSRVNIPKVLRAAEQAHLWAELVFLYDKYEEYDNAIITMMN
HPTDAWKEGQFKDIITKVANVELYYRAIQFYLEFKPLLLNDLLMVLSPRLDHTRAVNYFS
KVKQLPLVKPYLRSVQNHNNKSVNESLNNLFITEEDYQALRTSIDAYDNFDNISLAQRLE
KHELIEFRRIAAYLFKGNNRWKQSVELCKKDSLYKDAMQYASESKDTELAEELLQWFLQE
EKRECFGACLFTCYDLLRPDVVLETAWRHNIMDFAMPYFIQVMKEYLTKVDKLDASESLR
KEEEQATETQPIVYGNLSL*

>ENSG00000188603|ENST00000359984
MGGCAGSRRRFSDSEGEETVPEPRLPLLDHQGAHWKNAVGFWLLGLCNNFSYVVMLSAAH
DILSHKRTSGNQSHVDPGPTPIPHNSSSRFDCNSVSTAAVLLADILPTLVIKLLAPLGLH
LLPYSPRVLVSGICAAGSFVLVAFSHSVGTSLCGVVFASISSGLGEVTFLSLTAFYPRAV
ISWWSSGTGGAGLLGALSYLGLTQAGLSPQQTLLSMLGIPALLLASYFLLLTSPEAQDPG
GEEEAESAARQPLIRTEAPESKPGSSSSLSLRERWTVFKGLLWYIVPLVVVYFAEYFINQ
GLFELLFFWNTSLSHAQQYRWYQMLYQAGVFASRSSLRCCRIRFTWALALLQCLNLVFLL
ADVWFGFLPSIYLVFLIILYEGLLGGAAYVNTFHNIALETSDEHREFAMAATCISDTLGI
SLSGLLALPLHDFLCQLS*
>ENSG00000117475|ENST00000367807
MTTKNLETKVTVTSSPIRGAGDGMETEEPPKSVEVTSGVQSRKHHSLQSPWKKAVPSESP
GVLQLGKMLTEKAMEVKAVRILVPKAAITHDIPNKNTKVKSLGHHKGEFLGQSEGVIEPN
KELSEVKNVLEKLKNSERRLLQDKEGLSNQLRVQTEVRRALIDKMSTTLYVWTF*
>ENSG00000213983|ENST00000397120
MVVPSLKLQDLIEEIRGAKTQAQEREVIQKECAHIRASFRDGDPVHRHRQLAKLLYVHML
GYPAHFGQMECLKLIASSRFTDKRVGYLGAMLLLDERHDAHLLITNSIKNDLSQGIQPVQ
GLALCTLSTMGSAEMCRDLAPEVEKLLLQPSPYVRKKAILTAVHMIRKVPELSSVFLPPC
AQLLHERHHGILLGTITLITELCERSPAALRHFRKVVPQLVHILRTLVTMGYSTEHSISG
VSDPFLQVQILRLLRILGRNHEESSETMNDLLAQVATNTDTSRNAGNAVLFETVLTIMDI
RSAAGLRVLAVNILGRFLLNSDRNIRYVALTSLLRLVQSDHSAVQRHRPTVVECLRETDA
SLSRRALELSLALVNSSNVRAMMQELQAFLESCPPDLRADCASGILLAAERFAPTKRWHI
DTILHVLTTAGTHVRDDAVANLTQLIGGAQELHAYSVRRLYNALAEDISQQPLVQVAAWC
IGEYGDLLLAGNCEEIEPLQVDEEEVLALLEKVLQSHMSLPATRGYALTALMKLSTRLCG
DNNRIRQVVSIYGSCLDVELQQRAVEYDTLFRKYDHMRAAILEKMPLVERDGPQADEEAK
ESKEAAQLSEAAPVPTEPQASQLLDLLDLLDGASGDVQHPPHLDPSPGGALVHLLDLPCV
PPPPAPIPDLKVFEREGVQLNLSFIRPPENPALLLITITATNFSEGDVTHFICQAAVPKS
LQLQLQAPSGNTVPARGGLPITQLFRILNPNKAPLRLKLRLTYDHFHQSVQEIFEVNNLP
VESWQ*
>ENSG00000175265|ENST00000359187
MAEETGQSKLAAAKKKFKEYWQRNRPGVPAAAKRNTKANGSSPETAASGGCHSSEASSSA
SSSLHARQSPCQEQAAVLNSRSIKISRLNDTIKSLKQQKKQVEHQLEEEKKANNEKQKAE
RELEGQIQRLNTEKKKLNTDLYHMKHSLRYFEEESKDLAGRLQRSSQRIGELEWSLCAVA
ATQKKKPDGFSSRSKALLKRQLEQSIREQILLKGHVTQLKESLKEVQLERDQYAEQIKGE
RAQWQQRMRKMSQEVCTLKEEKKHDTHRVEELERSLSRLKNQMAEPLPPDAPAVSSEVEL
QDLRKELERVAGELQAQVENNQCISLLNRGQKERLREQEERLQEQQERLREREKRLQQLA
EPQSDLEELKHENKSALQLEQQVKELQEKLGQVMETLTSAEKEPEAAVPASGTGGESSGL
MDLLEEKADLREHVEKLELGFIQYRRERCHQKVHRLLTEPGDSAKDASPGGGHHQAGPGQ
GGEEGEAAGAAGDGVAACGSYSEGHGKFLAAARNPAAEPSPGAPAPQELGAADKHGDLCE
ASLTNSVEPAQGEAREGSSQDNPTAQPVVQLLGEMQDHQEHPGLGSNCCVPCFCWAWLPR
RRR*
>ENSG00000206433|ENST00000383480
MLSRNDVKRLERSWHLLSWSQAQGSSEQELHYASLQRLPVPSSEGPDLRGRDKRGTKEDP
RADYACIAENKPT*
>ENSG00000117411|ENST00000372324

MSRLLGGTLERVCKAVLLLCLLHFLVAVILYFDVYAQHLAFFSRFSARGPAHALHPAASS
SSSSSNCSRPNATASSSGLPEVPSALPGPTAPTLPPCPDSPPGLVGRLLIEFTSPMPLER
VQRENPGVLMGGRYTPPDCTPAQTVAVIIPFRHREHHLRYWLHYLHPILRRQRLRYGVYV
INQHGEDTFNRAKLLNVGFLEALKEDAAYDCFIFSDVDLVPMDDRNLYRCGDQPRHFAIA
MDKFGFRLPYAGYFGGVSGLSKAQFLRINGFPNEYWGWGGEDDDIFNRISLTGMKISRPD
IRIGRYRMIKHDRDKHNEPNPQRFTKIQNTKLTMKRDGIGSVRYQVLEVSRQPLFTNITV
DIGRPPSWPPRG*
>ENSG00000136152|ENST00000349995
MAEAALLLLPEAAAERDAREKLALWDRRPDTTAPLTDRQTDSVLELKAAAENLPVPAELP
IEDLCSLTSQSLPIELTSVVPESTEDILLKGFTSLGMEEERIETAQQFFSWFAKLQTQMD
QDEGTKYRQMRDYLSGFQEQCDAILNDVNSALQHLESLQKQYLFVSNKTGTLHEACEQLL
KEQSELVDLAENIQQKLSYFNELETINTKLNSPTLSVNSDGFIPMLAKLDDCITYISSHP
NFKDYPIYLLKFKQCLSKALHLMKTYTVNTLQTLTSQLLKRDPSSVPNADNAFTLFYVKF
RAAAPKVRTLIEQIELRSEKIPEYQQLLNDIHQCYLDQRELLLGPSIACTVAELTSQNNR
DHCALVRSGCAFMVHVCQDEHQLYNEFFTKPTSKLDELLEKLCVSLYDVFRPLIIHVIHL
ETLSELCGILKNEVLEDHVQNNAEQLGAFAAGVKQMLEDVQERLVYRTHIYIQTDITGYK
PAPGDLAYPDKLVMMEQIAQSLKDEQKKVPSEASFSDVHLEEGESNSLTKSGSTESLNPR
PQTTISPADLHGMWYPTVRRTLVCLSKLYRCIDRAVFQGLSQEALSACIQSLLGASESIS
KNKTQIDGQLFLIKHLLILREQIAPFHTEFTIKEISLDLKKTRDAAFKILNPMTVPRFFR
LNSNNALIEFLLEGTPEIREHYLDSKKDVDRHLKSACEQFIQQQTKLFVEQLEEFMTKVS
ALKTMASQGGPKYTLSQQPWAQPAKVNDLAATAYKTIKTKLPVTLRSMSLYLSNKDTEFI
LFKPVRNNIQQVFQKFHALLKEEFSPEDIQIIACPSMEQLSLLLLVSK*
>ENSG00000078328|ENST00000355637
MLASQGVLLHPYGVPMIVPAAPYLPGLIQGNQEAAAAPDTMAQPYASAQFAPPQNGIPAE
YTAPHPHPAPEYTGQTTVPEHTLNLYPPAQTHSEQSPADTSAQTVSGTATQTDDAAPTDG
QPQTQPSENTENKSQPKRLHVSNIPFRFRDPDLRQMFGQFGKILDVEIIFNERGSKGFGF
VTFENSADADRAREKLHGTVVEGRKIEVNNATARVMTNKKTVNPYTNGWKLNPVVGAVYS
PEFYAGTVLLCQANQEGSSMYSAPSSLVYTSAMPGFPYPAATAAAAYRGAHLRGRGRTVY
NTFRAAAPPPPIPAYGGVVYQEPVYGNKLLQGGYAAYRYAQPTPATAAAYSDRNQFVFVA
ADEISCNTSAVTDEFMLPTPTTTHLLQPPPTALVP*
>ENSG00000160179|ENST00000398449
MACLMAAFSVGTAMNASSYSAEMTEPKSVCVSVDEVVSSNMEATETDLLNGHLKKVDNNL
TEAQRFSSLPRRAAVNIEFRDLSYSVPEGPWWRKKGYKTLLKGISGKFNSGELVAIMGPS
GAGKSTLMNILAGYRETGMKGAVLINGLPRDLRCFRKVSCYIMQDDMLLPHLTVQEAMMV
SAHLKLQEKDEGRREMVKEILTALGLLSCANTRTGSLSGGQRKRLAIALELVNNPPVMFF
DEPTSGLDSASCFQVVSLMKGLAQGGRSIICTIHQPSAKLFELFDQLYVLSQGQCVYRGK
VCNLVPYLRDLGLNCPTYHNPADFVMEVASGEYGDQNSRLVRAVREGMCDSDHKRDLGGD
AEVNPFLWHRPSEEDSSSMEGCHSFSASCLTQFCILFKRTFLSIMRDSVLTHLRITSHIG
IGLLIGLLYLGIGNEAKKVLSNSGFLFFSMLFLMFAALMPTVLTFPLEMGVFLREHLNYW
YSLKAYYLAKTMADVPFQIMFPVAYCSIVYWMTSQPSDAVRFVLFAALGTMTSLVAQSLG
LLIGAASTSLQVATFVGPVTAIPVLLFSGFFVSFDTIPTYLQWMSYISYVRYGFEGVILS
IYGLDREDLHCDIDETCHFQKSEAILRELDVENAKLYLDFIVLGIFFISLRLIAYFVLRY
KIRAER*
>ENSG00000128242|ENST00000402369

MLPPQKKPWESMAKGLVLGALFTSFLLLVYSYAVPPLHAGLASTTPEAAASCSPPALEPE
AVIRANGSAGECQPRRNIVFLKTHKTASSTLLNILFRFGQKHRLKFAFPNGRNDFDYPTF
FARSLVQDYRPGACFNIICNHMRFHYDEVRGLVPTNAIFITVLRDPARLFESSFHYFGPV
VPLTWKLSAGDKLTEFLQDPDRYYDPNGFNAHYLRNLLFFDLGYDNSLDPSSPQVQEHIL
EVERRFHLVLLQEYFDESLVLLKDLLCWELEDVLYFKLNARRDSPVPRLSGELYGRATAW
NMLDSHLYRHFNASFWRKVEAFGRERMAREVAALRHANERMRTICIDGGHAVDAAAIQDE
AMQPWQPLGTKSILGYNLKKSIGQRHAQLCRRMLTPEIQYLMDLGANLWVTKLWKFIRDF
LRW*
>ENSG00000183196|ENST00000390664
MWLPRVSSTAVTALLLAQTFLLLFLVSRPGPSSPAGGEARVHVLVLSSWRSGSSFVGQLF
NQHPDVFYLMEPAWHVWTTLSQGSAATLHMAVRDLVRSVFLCDMDVFDAYLPWRRNLSDL
FQWAVSRALCSPPACSAFPRGAISSEAVCKPLCARQSFTLAREACRSYSHVVLKEVRFFN
LQVLYPLLSDPALNLRIVHLVRDPRAVLRSREQTAKALARDNGIVLGTNGTWVEADPGLR
VVREVCRSHVRIAEAATLKPPPFLRGRYRLVRFEDLAREPLAEIRALYAFTGLSLTPQLE
AWIHNITHGSGPGARREAFKTSSRNALNVSQAWRHALPFAKIRRVQELCAGALQLLGYRP
VYSEDEQRNLALDLVLPRGLNGFTWASSTASHPRN*
>ENSG00000125430|ENST00000360954
MGQRLSGGRSCLDVPGRLLPQPPPPPPPVRRKLALLFAMLCVWLYMFLYSCAGSCAAAPG
LLLLGSGSRAAHDPPALATAPDGTPPRLPFRAPPATPLASGKEMAEGAASPEEQSPEVPD
SPSPISSFFSGSGSKQLPQAIIIGVKKGGTRALLEFLRVHPDVRAVGAEPHFFDRSYDKG
LAWYRDLMPRTLDGQITMEKTPSYFVTREAPARISAMSKDTKLIVVVRDPVTRAISDYTQ
TLSKRPDIPTFESLTFKNRTAGLIDTSWSAIQIGIYAKHLEHWLRHFPIRQMLFVSGERL
ISDPAGELGRVQDFLGLKRIITDKHFYFNKTKGFPCLKKAEGSSRPHCLGKTKGRTHPEI
DREVVRRLREFYRPFNLKFYQMTGHDFGWD*
>ENSG00000101236|ENST00000358395
MSSDFPHYNFRMPNIGFQNLPLNIYIVVFGTAIFVFILSLLFCCYLIRLRHQAHKEFYAY
KQVILKEKVKELNLHELCAVCLEDFKPRDELGICPCKHAFHRKCLIKWLEVRKVCPLCNM
PVLQLAQLHSKQDRGPPQGPLPGAENIV*
>ENSG00000148154|ENST00000374279
MALLDLALEGMAVFGFVLFLVLWLMHFMAIIYTRLHLNKKATDKQPYSKLPGVSLLKPLK
GVDPNLINNLETFFELDYPKYEVLLCVQDHDDPAIDVCKKLLGKYPNVDARLFIGGKKVG
INPKINNLMPGYEVAKYDLIWICDSGIRVIPDTLTDMVNQMTEKVGLVHGLPYVADRQGF
AATLEQVYFGTSHPRYYISANVTGFKCVTGMSCLMRKDVLDQAGGLIAFAQYIAEDYFMA
KAIADRGWRFAMSTQVAMQNSGSYSISQFQSRMIRWTKLRINMLPATIICEPISECFVAS
LIIGWAAHHVFRWDIMVFFMCHCLAWFIFDYIQLRGVQGGTLCFSKLDYAVAWFIRESMT
IYIFLSALWDPTISWRTGRYRLRCGGTAEEILDV*
>ENSG00000242252|ENST00000368272
MRALTLLALLALAALCIAGQAGAKPSGAESSKGAAFVSKQEGSEVVKRPRRYLYQWLGAP
VPYPDPLEPRREVCELNPDCDELADHIGFQEAYRRFYGPV*
>ENSG00000206433|ENST00000383484
MLSRNDDICIYGGLGLGGLLLLAVVLLSACLCWLHRRAPSVLVPGPGLLRAGTPLCISAE
AASAQQ*
>ENSG00000065135|ENST00000369851
MGCTLSAEDKAAVERSKMIDRNLREDGEKAAKEVKLLLLGAGESGKSTIVKQMKIIHEDG

YSEDECKQYKVVVYSNTIQSIIAIIRAMGRLKIDFGEAARADDARQLFVLAGSAEEGVMT
PELAGVIKRLWRDGGVQACFSRSREYQLNDSASYYLNDLDRISQSNYIPTQQDVLRTRVK
TTGIVETHFTFKDLYFKMFDVGGQRSERKKWIHCFEGVTAIIFCVALSDYDLVLAEDEEM
NRMHESMKLFDSICNNKWFTETSIILFLNKKDLFEEKIKRSPLTICYPEYTGSNTYEEAA
AYIQCQFEDLNRRKDTKEIYTHFTCATDTKNVQFVFDAVTDVIIKNNLKECGLY*
>ENSG00000126091|ENST00000361392
MGLLVFVRNLLLALCLFLVLGFLYYSAWKLHLLQWEEDSSKYSHSSSPQEKPVADSVVLS
FDSAGQTLGSEYDRLGFLLNLDSKLPAELATKYANFSEGACKPGYASALMTAIFPRFSKP
APMFLDDSFRKWARIREFVPPFGIKGQDNLIKAILSVTKEYRLTPALDSLRCRRCIIVGN
GGVLANKSLGSRIDDYDIVVRLNSAPVKGFEKDVGSKTTLRITYPEGAMQRPEQYERDSL
FVLAGFKWQDFKWLKYIVYKERVSASDGFWKSVATRVPKEPPEIRILNPYFIQEAAFTLI
GLPFNNGLMGRGNIPTLGSVAVTMALHGCDEVAVAGFGYDMSTPNAPLHYYETVRMAAIK
ESWTHNIQREKEFLRKLVKARVITDLSSGI*
>ENSG00000196371|ENST00000358752
MRRLWGAARKPSGAGWEKEWAEAPQEAPGAWSGRLGPGRSGRKGRAVPGWASWPAHLALA
ARPARHLGGAGQGPRPLHSGTAPFHSRASGERQRRLEPQLQHESRCRSSTPADAWRAEAA
LPVRAMGAPWGSPTAAAGGRRGWRRGRGLPWTVCVLAAAGLTCTALITYACWGQLPPLPW
ASPTPSRPVGVLLWWEPFGGRDSAPRPPPDCRLRFNISGCRLLTDRASYGEAQAVLFHHR
DLVKGPPDWPPPWGIQAHTAEEVDLRVLDYEEAAAAAEALATSSPRPPGQRWVWMNFESP
SHSPGLRSLASNLFNWTLSYRADSDVFVPYGYLYPRSHPGDPPSGLAPPLSRKQGLVAWV
VSHWDERQARVRYYHQLSQHVTVDVFGRGGPGQPVPEIGLLHTVARYKFYLAFENSQHLD
YITEKLWRNALLAGAVPVVLGPDRANYERFVPRGAFIHVDDFPSASSLASYLLFLDRNPA
VYRRYFHWRRSYAVHITSFWDEPWCRVCQAVQRAGDRPKSIRNLASWFER*
>ENSG00000143393|ENST00000368873
MGDTVVEPAPLKPTSEPTSGPPGNNGGSLLSVITEGVGELSVIDPEVAQKACQEVLEKVK
LLHGGVAVSSRGTPLELVNGDGVDSEIRCLDDPPAQIREEEDEMGAAVASGTAKGARRRR
QNNSAKQSWLLRLFESKLFDISMAISYLYNSKEPGVQAYIGNRLFCFRNEDVDFYLPQLL
NMYIHMDEDVGDAIKPYIVHRCRQSINFSLQCALLLGAYSSDMHISTQRHSRGTKLRKLI
LSDELKPAHRKRELPSLSPAPDTGLSPSKRTHQRSKSDATASISLSSNLKRTASNPKVEN
EDEELSSSTESIDNSFSSPVRLAPEREFIKSLMAIGKRLATLPTKEQKTQRLISELSLLN
HKLPARVWLPTAGFDHHVVRVPHTQAVVLNSKDKAPYLIYVEVLECENFDTTSVPARIPE
NRIRSTRSVENLPECGITHEQRAGSFSTVPNYDNDDEAWSVDDIGELQVELPEVHTNSCD
NISQFSVDSITSQESKEPVFIAAGDIRRRLSEQLAHTPTAFKRDPEDPSAVALKEPWQEK
VRRIREGSPYGHLPNWRLLSVIVKCGDDLRQELLAFQVLKQLQSIWEQERVPLWIKPYKI
LVISADSGMIEPVVNAVSIHQVKKQSQLSLLDYFLQEHGSYTTEAFLSAQRNFVQSCAGY
CLVCYLLQVKDRHNGNILLDAEGHIIHIDFGFILSSSPRNLGFETSAFKLTTEFVDVMGG
LDGDMFNYYKMLMLQGLIAARKHMDKVVQIVEIMQQGSQLPCFHGSSTIRNLKERFHMSM
TEEQLQLLVEQMVDGSMRSITTKLYDGFQYLTNGIM*
>ENSG00000147257|ENST00000370818
MAGTVRTACLVVAMLLSLDFPGQAQPPPPPPDATCHQVRSFFQRLQPGLKWVPETPVPGS
DLQVCLPKGPTCCSRKMEEKYQLTARLNMEQLLQSASMELKFLIIQNAAVFQEAFEIVVR
HAKNYTNAMFKNNYPSLTPQAFEFVGEFFTDVSLYILGSDINVDDMVNELFDSLFPVIYT
QLMNPGLPDSALDINECLRGARRDLKVFGNFPKLIMTQVSKSLQVTRIFLQALNLGIEVI
NTTDHLKFSKDCGRMLTRMWYCSYCQGLMMVKPCGGYCNVVMQGCMAGVVEIDKYWREYI

LSLEELVNGMYRIYDMENVLLGLFSTIHDSIQYVQKNAGKLTTTIGKLCAHSQQRQYRSA
YYPEDLFIDKKVLKVAHVEHEETLSSRRRELIQKLKSFISFYSALPGYICSHSPVAENDT
LCWNGQELVERYSQKAARNGMKNQFNLHELKMKGPEPVVSQIIDKLKHINQLLRTMSMPK
GRVLDKNLDEEGFESGDCGDDEDECIGGSGDGMIKVKNQLRFLAELAYDLDVDDAPGNSQ
QATPKDNEISTFHNLGNVHSPLKLLTSMAISVVCFFFLVH*
>ENSG00000115525|ENST00000393805
MPSEYTYVKLRSDCSRPSLQWYTRAQSKMRRPSLLLKDILKCTLLVFGVWILYILKLNYT
TEECDMKKMHYVDPDHVKRAQKYAQQVLQKECRPKFAKTSMALLFEHRYSVDLLPFVQKA
PKDSEAESKYDPPFGFRKFSSKVQTLLELLPEHDLPEHLKAKTCRRCVVIGSGGILHGLE
LGHTLNQFDVVIRLNSAPVEGYSEHVGNKTTIRMTYPEGAPLSDLEYYSNDLFVAVLFKS
VDFNWLQAMVKKETLPFWVRLFFWKQVAEKIPLQPKHFRILNPVIIKETAFDILQYSEPQ
SRFWGRDKNVPTIGVIAVVLATHLCDEVSLAGFGYDLNQPRTPLHYFDSQCMAAMNFQTM
HNVTTETKFLLKLVKEGVVKDLSGGIDREF*
>ENSG00000187210|ENST00000376730
MLRTLLRRRLFSYPTKYYFMVLVLSLITFSVLRIHQKPEFVSVRHLELAGENPSSDINCT
KVLQGDVNEIQKVKLEILTVKFKKRPRWTPDDYINMTSDCSSFIKRRKYIVEPLSKEEAE
FPIAYSIVVHHKIEMLDRLLRAIYMPQNFYCIHVDTKSEDSYLAAVMGIASCFSNVFVAS
RLESVVYASWSRVQADLNCMKDLYAMSANWKYLINLCGMDFPIKTNLEIVRKLKLLMGEN
NLETERMPSHKEERWKKRYEVVNGKLTNTGTVKMLPPLETPLFSGSAYFVVSREYVGYVL
QNEKIQKLMEWAQDTYSPDEYLWATIQRIPEVPGSLPASHKYDLSDMQAVARFVKWQYFE
GDVSKGAPYPPCDGVHVRSVCIFGAGDLNWMLRKHHLFANKFDVDVDLFAIQCLDEHLRH
KALETLKH*
>ENSG00000176022|ENST00000379198
MKLLRRAWRRRAALGLGTLALCGAALLYLARCAAEPGDPRAMSGRSPPPPAPARAAAFLA
VLVASAPRAAERRSVIRSTWLARRGAPGDVWARFAVGTAGLGAEERRALEREQARHGDLL
LLPALRDAYENLTAKVLAMLAWLDEHVAFEFVLKADDDSFARLDALLAELRAREPARRRR
LYWGFFSGRGRVKPGGRWREAAWQLCDYYLPYALGGGYVLSADLVHYLRLSRDYLRAWHS
EDVSLGAWLAPVDVQREHDPRFDTEYRSRGCSNQYLVTHKQSLEDMLEKHATLAREGRLC
KREVQLRLSYVYDWSAPPSQCCQRREGIP*
>ENSG00000135775|ENST00000366668
MEKSRMNLPKGPDTLCFDKDEFMKEDFDVDHFVSDCRKRVQLEELRDDLELYYKLLKTAM
VELINKDYADFVNLSTNLVGMDKALNQLSVPLGQLREEVLSLRSSVSEGIRAVDERMSKQ
EDIRKKKMCVLRLIQVIRSVEKIEKILNSQSSKETSALEASSPLLTGQILERIATEFNQL
QFHAVQSKGMPLLDKVRPRIAGITAMLQQSLEGLLLEGLQTSDVDIIRHCLRTYATIDKT
RDAEALVGQVLVKPYIDEVIIEQFVESHPNGLQVMYNKLLEFVPHHCRLLREVTGGAISS
EKGNTVPGYDFLVNSVWPQIVQGLEEKLPSLFNPGNPDAFHEKYTISMDFVRRLERQCGS
QASVKRLRAHPAYHSFNKKWNLPVYFQIRFREIAGSLEAALTDVLEDAPAESPYCLLASH
RTWSSLRRCWSDEMFLPLLVHRLWRLTLQILARYSVFVNELSLRPISNESPKEIKKPLVT
GSKEPSITQGNTEDQGSGPSETKPVVSISRTQLVYVVADLDKLQEQLPELLEIIKPKLEM
IGFKNFSSISALEDSQSSFSACVPSLSSKIIQDLSDSCFGFLKSALEVPRLYRRTNKEVP
TTASSYVDSALKPLFQLQSGHKDKLKQAIIQQWLEGTLSESTHKYYETVSDVLNSVKKME
ESLKRLKQARKTTPANPVGPSGGMSDDDKIRLQLALDVEYLGEQIQKLGLQASDIKSFSA
LAELVAAAKDQATAEQP*
>ENSG00000158488|ENST00000368166

MLLLFLLFEGLCCPGENTAVKPEAWLSCGPSPGPGRLQLVCHVSGFYPKPVWVMWMRGEQ
EQRGTQRGDVLPNADETWYLRATLDVAAGEAAGLSCRVKHSSLGGHDLIIHWGGYSIFLI
LICLTVIVTLVILVVVDSRLKKQSPVFLMGANTQDTKNSRHQFCLAQVSWIKNRVLKKWK
TRLNQLW*
>ENSG00000113615|ENST00000398844
MSQPGIPASGGAPASLQAQNGAALASGSPYTNGPVQNALLSSQESVSQGYNFQLPGSYPH
PIPAKTLNPVSGQSNYGGSQGSGQTLNRPPVASNPVTPSLHSGPAPRMPLPASQNPATTP
MPSSSFLPEANLPPPLNWQYNYPSTASQTNHCPRASSQPTVSGNTSLTTNHQYVSSGYPS
LQNSFIKSGPSVPPLVNPPLPTTFQPGAPHGPPPAGGPPPVRALTPLTSSYRDVPQPLFN
SAVNQEGITSNTNNGSMVVHSSYDEIEGGGLLATPQLTNKNPKMSRSVGYSYPSLPPGYQ
NTTPPGATGVPPSSLNYPSGPQAFTQTPLGANHLTTSMSGLSLQPEGLRVVNLLQERNML
PSTPLKPPVPNLHEDIQKLNCNPELFRCTLTSIPQTQALLNKAKLPLGLLLHPFKDLVQL
PVVTSSTIVRCRSCRTYINPFVSFLDQRRWKCNLCYRVNDVPEEFLYNPLTRVYGEPHRR
PEVQNATIEFMAPSEYMLRPPQPPVYLFVFDVSHNAVETGYLNSVCQSLLDNLDLLPGNT
RTKIGFITFDSTIHFYGLQESLSQPQMLIVSDIEDVFIPMPENLLVNLNESKELVQDLLK
TLPQMFTKTLETQSALGPALQAAFKLMSPTGGRMSVFQTQLPTLGVGALKPREEPNHRSS
AKDIHMTPSTDFYKKLALDCSGQQVAVDLFLLSGQYSDLASLGCISRYSAGSVYYYPSYH
HQHNPVQVQKLQKELQRYLTRKIGFEAVMRIRCTKGLSIHTFHGNFFVRSTDLLSLPNVN
PDAGYAVQMSVEESLTDTQLVSFQSALLYTSSKGERRIRVHTLCLPVVSTLNDVFLGADV
QAISGLLANMAVDRSMTASLSDARDALVNAVIDSLSAYRSSVLSNQQPGLMVPFSLRLFP
LFVLALLKQKSFQTGTNARLDERIFAMCQVKNQPLVYLMLTTHPSLYRVDNLSDEGALNI
SDRTIPQPPILQLSVEKLSRDGAFLMDAGSVLMLWVGKNCTQNFLSQVLGVQNYASIPQP
MTDLPELDTPESARIIAFISWLREQRPFFPILYVIRDESPMKANFLQNMIEDRTESALSY
YEFLLHIQQQVNK*
>ENSG00000206047|ENST00000382692
MRTLAILAAILLVALQAQAEPLQARADEVAAAPEQIAADIPEVVVSLAWDESLAPKHPGS
RKNMACYCRIPACIAGERRYGTCIYQGRLWAFCC*
>ENSG00000111962|ENST00000367463
MKKKQQHPGGGADPWPHGAPMGGAPPGLGSWKRRVPLLPFLRFSLRDYGFCMATLLVFCL
GSLLYQLSGGPPRFLLDLRQYLGNSTYLDDHGPPPSKVLPFPSQVVYNRVGKCGSRTVVL
LLRILSEKHGFNLVTSDIHNKTRLTKNEQMELIKNISTAEQPYLFTRHVHFLNFSRFGGD
QPVYINIIRDPVNRFLSNYFFRRFGDWRGEQNHMIRTPSMRQEERYLDINECILENYPEC
SNPRLFYIIPYFCGQHPRCREPGEWALERAKLNVNENFLLVGILEELEDVLLLLERFLPH
YFKGVLSIYKDPEHRKLGNMTVTVKKTVPSPEAVQILYQRMRYEYEFYHYVKEQFHLLKR
KFGLKSHVSKPPLRPHFFIPTPLETEEPIDDEEQDDEKWLEDIYKR*
>ENSG00000076716|ENST00000370828
MARFGLPALLCTLAVLSAALLAAELKSKSCSEVRRLYVSKGFNKNDAPLHEINGDHLKIC
PQGSTCCSQEMEEKYSLQSKDDFKSVVSEQCNHLQAVFASRYKKFDEFFKELLENAEKSL
NDMFVKTYGHLYMQNSELFKDLFVELKRYYVVGNVNLEEMLNDFWARLLERMFRLVNSQY
HFTDEYLECVSKYTEQLKPFGDVPRKLKLQVTRAFVAARTFAQGLAVAGDVVSKVSVVNP
TAQCTHALLKMIYCSHCRGLVTVKPCYNYCSNIMRGCLANQGDLDFEWNNFIDAMLMVAE
RLEGPFNIESVMDPIDVKISDAIMNMQDNSVQVSQKVFQGCGPPKPLPAGRISRSISESA
FSARFRPHHPEERPTTAAGTSLDRLVTDVKEKLKQAKKFWSSLPSNVCNDERMAAGNGNE
DDCWNGKGKSRYLFAVTGNGLANQGNNPEVQVDTSKPDILILRQIMALRVMTSKMKNAYN

GNDVDFFDISDESSGEGSGSGCEYQQCPSEFDYNATDHAGKSANEKADSAGVRPGAQAYL
LTVFCILFLVMQREWR*
>ENSG00000133103|ENST00000356576
MAEGSGEVVAVSATGAANGLNNGAGGTSATTCNPLSRKLHKILETRLDNDKELGILLLSF
SWLLFEDSVRDSRRC*
>ENSG00000144057|ENST00000361686
MKPHLKQWRQRMLFGIFAWGLLFLLIFIYFTDSNPAEPVPSSLSFLETRRLLPVQGKQRA
IMGAAHEPSPPGGLDARQALPRAHPAGSFHAGPGDLQKWAQSQDGFEHKEFFSSQVGRKS
QSAFYPEDDDYFFAAGQPGWHSHTQGTLGFPSPGEPGPREGAFPAAQVQRRRVKKRHRRQ
RRSHVLEEGDDGDRLYSSMSRAFLYRLWKGNVSSKMLNPRLQKAMKDYLTANKHGVRFRG
KREAGLSRAQLLCQLRSRARVRTLDGTEAPFSALGWRRLVPAVPLSQLHPRGLRSCAVVM
SAGAILNSSLGEEIDSHDAVLRFNSAPTRGYEKDVGNKTTIRIINSQILTNPSHHFIDSS
LYKDVILVAWDPAPYSANLNLWYKKPDYNLFTPYIQHRQRNPNQPFYILHPKFIWQLWDI
IQENTKEKIQPNPPSSGFIGILIMMSMCREVHVYEYIPSVRQTELCHYHELYYDAACTLG
AYHPLLYEKLLVQRLNMGTQGDLHRKGKVVLPGFQAVHCPAPSPVIPHS*
>ENSG00000134470|ENST00000397250
MAPRRARGCRTLGLPALLLLLLLLRPPATREPAASSPSSNNTAATTAAIVPGSQLMPSKSP
STGTTEISSHESSHGTPSQTTAKNWELTASASHQPPGVYPQGHSDTTVAISTSTVLLCGL
SAVSLLACYLKSRQTPPLASVEMEAMEALPVTWGTSSRDEDLENCSHHL*
>ENSG00000068001|ENST00000395139
MRAGPGPTVTLALVLAVSWAMELKPTAPPIFTGRPFVVAWDVPTQDCGPRLKVPLDLNAF
DVQASPNEGFVNQNITIFYRDRLGLYPRFDSAGRSVHGGVPQNVSLWAHRKMLQKRVEHY
IRTQESAGLAVIDWEDWRPVWVRNWQDKDVYRRLSRQLVASRHPDWPPDRIVKQAQYEFE
FAAQQFMLETLRYVKAVRPRHLWGFYLFPDCYNHDYVQNWESYTGRCPDVEVARNDQLAW
LWAESTALFPSVYLDETLASSRHGRNFVSFRVQEALRVARTHHANHALPVYVFTRPTYSR
RLTGLSEMDLISTIGESAALGAAGVILWGDAGYTTSTETCQYLKDYLTRLLVPYVVNVSW
ATQYCSRAQCHGHGRCVRRNPSASTFLHLSTNSFRLVPGHAPGEPQLRPVGELSWADIDH
LQTHFRCQCYLGWSGEQCQWDHRQAAGGASEAWAGSHLTSLLALAALAFTWTL*
>ENSG00000126091|ENST00000372369
MGLLVFVRNLLLALCLFLVLGFLYYSAWKLHLLQWEEDSNSVVLSFDSAGQTLGSEYDRL
GFLLNLDSKLPAELATKYANFSEGACKPGYASALMTAIFPRFSKPAPMFLDDSFRKWARI
REFVPPFGIKGQDNLIKAILSVTKEYRLTPALDSLRCRRCIIVGNGGVLANKSLGSRIDD
YDIVVRLNSAPVKGFEKDVGSKTTLRITYPEGAMQRPEQYERDSLFVLAGFKWQDFKWLK
YIVYKERVEAAFTLIGLPFNNGLMGRGNIPTLGSVAVTMALHGCDEVAVAGFGYDMSTPN
APLHYYETVRMAAIKESWTHNIQREKEFLRKLVKARVITDLSSGI*
>ENSG00000101310|ENST00000377475
MATYLEFIQQNEERDGVRFSWNVWPSSRLEATRMVVPLACLLTPLKERPDLPPVQYEPVL
CSRPTCKAVLNPLCQVDYRAKLWACNFCFQRNQFPPAYGGISEVNQPAELMPQFSTIEYV
IQRGAQSPLIFLYVVDTCLEEDDLQALKESLQMSLSLLPPDALVGLITFGRMVQVHELSC
EGISKSYVFRGTKDLTAKQIQDMLGLTKPAMPMQQARPAQPQEHPFASSRFLQPVHKIDM
NLTDLLGELQRDPWPVTQGKRPLRSTGVALSIAVGLLEGTFPNTGARIMLFTGGPPTQGP
GMVVGDELKIPIRSWHDIEKDNARFMKKATKHYEMLANRTAANGHCIDIYACALDQTGLL
EMKCCANLTGGYMVMGDSFNTSLFKQTFQRIFTKDFNGDFRMAFGATLDVKTSRELKIAG
AIGPCVSLNVKGPCVSENELGVGGTSQWKICGLDPTSTLGIYFEVVNQHNTPIPQGGRGA

IQFVTHYQHSSTQRRIRVTTIARNWADVQSQLRHIEAAFDQEAAAVLMARLGVFRAESEE
GPDVLRWLDRQLIRLCQKFGQYNKEDPTSFRLSDSFSLYPQFMFHLRRSPFLQVFNNSPD
ESSYYRHHFARQDLTQSLIMIQPILYSYSFHGPPEPVLLDSSSILADRILLMDTFFQIVI
YLGETIAQWRKAGYQDMPEYENFKHLLQAPLDDAQEILQARFPMPRYINTEHGGSQARFL
LSKVNPSQTHNNLYAWGQETGAPILTDDVSLQVFMDHLKKLAVSSAC*
>ENSG00000158488|ENST00000368161
MLLLFLLFEGLCCPGENTAAPQALQSYHLAAEEQLSFRMLQTSSFANHSWAHSEGSGWLG
DLQTHGWDTVLGTIRFLKPWSHGNFSKQELKNLQSLFQLYFHSFIQIVQASAGQFQLEYP
FEIQILAGCRMNAPQIFLNMAYQGSDFLSFQGISWEPSPGAGIRAQNICKVLNRYLDIKE
ILQSLLGHTCPRFLAGLMEAGESELKRKVKPEAWLSCGPSPGPGRLQLVCHVSGFYPKPV
WVMWMRGEQEQRGTQRGDVLPNADETWWIFHLSHPDLFDCDSYPGHIGCS*
>ENSG00000204482|ENST00000376089
MLSRNDVKRLERSWAQGSSEQELHYASLQRLPVPSSEGPDLRGRDKRGTKEDPRADYACI
AENKPT*
>ENSG00000196459|ENST00000380579
MSGSFYFVIVGHHDNPVFEMEFLPAGKAESKDDHRHLNQFIAHAALDLVDENMWLSNNMY
LKTVDKFNEWFVSAFVTAGHMRFIMLHDIRQEDGIKNFFTDVYDLYIKFSMNPFYEPNSP
IRSSAFDRKVQFLGKKHLLS*
>ENSG00000123240|ENST00000378748
MSHQPLSCLTEKEDSPSESTGNGPPHLAHPNLDTFTPEELLQQMKELLTENHQLKEAMKL
NNQAMKGRFEELSAWTEKQKEERQFFEIQSKEAKERLMALSHENEKLKEELGKLKGKSER
SSEDPTDDSRLPRAEAEQEKDQLRTQVVRLQAEKADLLGIVSELQLKLNSSGSSEDSFVE
IRMAEGEAEGSVKEIKHSPGPTRTVSTGTALSKYRSRSADGAKNYFEHEELTVSQLLLCL
REGNQKVERLEVALKEAKERVSDFEKKTSNRSEIETQTEGSTEKENDEEKGPETVGSEVE
ALNLQVTSLFKELQEAHTKLSEAELMKKRLQEKCQALERKNSAIPSELNEKQELVYTNKK
LELQVESMLSEIKMEQAKTEDEKSKLTVLQMTHNKLLQEHNNALKTIEELTRKESEKVDR
AVLKELSEKLELAEKALASKQLQMDEMKQTIAKQEEDLETMTILRAQMEVYCSDFHAERA
AREKIHEEKEQLALQLAVLLKENDAFEDGGRQSLMEMQSRHGARTSDSDQQAYLVQRGAE
DRDWRQQRNIPIHSCPKCGEVLPDIDTLQIHVMDCII*
>ENSG00000124145|ENST00000372733
MAPARLFALLLFFVGGVAESIRETEVIDPQDLLEGRYFSGALPDDEDVVGPGQESDDFEL
SGSGDLDDLEDSMIGPEVVHPLVPLDNHIPERAGSGSQVPTEPKKLEENEVIPKRISPVE
ESEDVSNKVSMSSTVQGSNIFERTEVLAALIVGGIVGILFAVFLILLLMYRMKKKDEGSY
DLGKKPIYKKAPTNEFYA*
>ENSG00000117411|ENST00000356836
MSRLLGGTLERVCKAVLLLCLLHFLVAVILYFDVYAQHLAFFSRFSARGPAHALHPAASS
SSSSSNCSRPNATASSSGLPEVPSALPGPTAPTLPPCPDSPPGLVGRLLIEFTSPMPLER
VQRENPGVLMGGRYTPPDCTPAQTVAVIIPFRHREHHLRYWLHYLHPILRRQRLRYGVYV
INQHGEDTFNRAKLLNVGFLEALKEDAAYDCFIFSDVDLVPMDDRNLYRCGDQPRHFAIA
MDKFGFRLPYAGYFGGVSGLSKAQFLRINGFPNEYWGWGGEDDDIFNRISLTGMKISRPD
IRIGRYRMIKHDRDKHNEPNPQRFTKIQNTKLTMKRDGIGSVRYQVLEVSRQPLFTNITV
DIGRPPSWPPRG*
>ENSG00000174992|ENST00000400752
MLTVALLALLCASASGNAIQARSSSYSGEYGGGGGKRFSHSGNQLDGPITALRVRVNTYY

IVGLQVRYGKVWSDYVGGRNGDLEEIFLHPGESVIQVSGKYKWYLKKLVFVTDKGRYLSF
GKDSGTSFNAVPLHPNTVLRFISGRSGSLIDAIGLHWDVYPSSCSRC*
>ENSG00000197818|ENST00000361573
MGEKMAEEERFPNTTHEGFNVTLHTTLVVTTKLVLPTPGKPILPVQTGEQAQQEEQSSGM
TIFFSLLVLAICIILVHLLIRYRLHFLPESVAVVSLGILMGAVIKIIEFKKLANWKEEEM
FRPNMFFLLLLPPIIFESGYSLHKGNFFQNIGSITLFAVFGTAISAFVVGGGIYFLGQAD
VISKLNMTDSFAFGSLISAVDPVATIAIFNALHVDPVLNMLVFGESILNDAVSIVLTNTA
EGLTRKNMSDVSGWQTFLQALDYFLKMFFGSAALGTLTGLISALVLKHIDLRKTPSLEFG
MMIIFAYLPYGLAEGISLSGIMAILFSGIVMSHYTHHNLSPVTQILMQQTLRTVAFLCET
CVFAFLGLSIFSFPHKFEISFVIWCIVLVLFGRAVNIFPLSYLLNFFRDHKITPKMMFIM
WFSGLRGAIPYALSLHLDLEPMEKRQLIGTTTIVIVLFTILLLGGSTMPLIRLMDIEDAK
AHRRNKKDVNLSKTEKMGNTVESEHLSELTEEEYEAHYIRRQDLKGFVWLDAKYLNPFFT
RRLTQEDLHHGRIQMKTLTNKWYEEVRQGPSGSEDDEQELL*
>ENSG00000172318|ENST00000392690
MASKVSCLYVLTVVCWASALWYLSITRPTSSYTGSKPFSHLTVARKNFTFGNIRTRPINP
HSFEFLINEPNKCEKNIPFLVILISTTHKEFDARQAIRETWGDENNFKGIKIATLFLLGK
NADPVLNQMVEQESQIFHDIIVEDFIDSYHNLTLKTLMGMRWVATFCSKAKYVMKTDSDI
FVNMDNLIYKLLKPSTKPRRRYFTGYVINGGPIRDVRSKWYMPRDLYPDSNYPPFCSGTG
YIFSADVAELIYKTSLHTRLLHLEDVYVGLCLRKLGIHPFQNSGFNHWKMAYSLCRYRRV
ITVHQISPEEMHRIWNDMSSKKHLRC*
>ENSG00000162852|ENST00000366512
MDDSDTPTYYLQIEPQDGCHPGDSVERSVTCLPSASDENENQLDGDGHEHLTSSDSAMGK
PQVSEQDSLNNNESCTLSCEVAAGENLQNTLCEASRDEQAFLGKDKKIPGKRSPRSKKGT
AKKIPPGLFSGDIAPLMQEKVLSAVTYAVDDEEAAEVNANEQPEAPKLVLQSLFSLIRGE
VEQLDSRALPLCLHQIAESYFQEEDYEKAMKFIQLERLYHEQLLANLSAIQEQWETKWKT
VQPHTVTALRNSEKGFNGEDFERLTKICATHQDPLLSKHKIAAVEKSQERKCSTQLLVSE
DPKEGGATTKESESKTCLGTESSKESQHTVEPLGSSPCCHQMDVQTDSPSLSVTAGKDHM
EELLCSAEATLALHTQSSETAGSPSGPDSSEDACEDDSRLQLAQTEACQDVARIEGIAED
PKVFLSSKSKTEPLISPGCDRIPPALISEGKYSQAQRKELRLPLRDASEALPTDQLENNE
LNELQQPDLTDSDGKSPQAQADSDGSENVLCGNNQISDLGILLPEVCMAPEEKGDKDDQL
NKETEDYLNSLLEGCLKDTEDSLSYEDNQDDDSDLLQDLSPEEASYSLQENLPSDESCLS
LDDLAKRIEIAEVNQR*
>ENSG00000138674|ENST00000395310
MKLKEVDRTAMQAWSPAQNHPIYLATGTSAQQLDATFSTNASLEIFELDLSDPSLDMKSC
ATFSSSHRYHKLIWGPYKMDSKGDVSGVLIAGGENGNIILYDPSKIIAGDKEVVIAQNDK
HTGPVRALDVNIFQTNLVASGANESEIYIWDLNNFATPMTPGAKTQPPEDISCIAWNRQV
QHILASASPSGRATVWDLRKNEPIIKVSDHSNRMHCSGLAWHPDVATQMVLASEDDRLPV
IQMWDLRFASSPLRVLENHARGILAIAWSMADPELLLSCGKDAKILCSNPNTGEVLYELP
TNTQWCFDIQWCPRNPAVLSAASFDGRISVYSIMGGSTDGLRQKQVDKLSSSFGNLDPFG
TGQPLPPLQIPQQTAQHSIVLPLKKPPKWIRRPVGASFSFGGKLVTFENVRMPSHQGAEQ
QQQQHHVFISQVVTEKEFLSRSDQLQQAVQSQGFINYCQKKIDASQTEFEKNVWSFLKVN
FEDDSRGKYLELLGYRKEDLGKKIALALNKVDGANVALKDSDQVAQSDGEESPAAEEQLL
GEHIKEEKEESEFLPSSGGTFNISVSGDIDGLITQALLTGNFESAVDLCLHDNRMADAII
LAIAGGQELLARTQKKYFAKSQSKITRLITAVVMKNWKEIVESCDLKNWREALAAVLTYA

KPDEFSALCDLLGTRLENEGDSLLQTQACLCYICAGNVEKLVACWTKAQDGSHPLSLQDL
IEKVVILRKAVQLTQAMDTSTVGVLLAAKMSQYANLLAAQGSIAAALAFLPDNTNQPNIM
QLRDRLCRAQGEPVAGHESPKIPYEKQQLPKGRPGPVAGHHQMPRVQTQQYYPHGENPPP
PGFIMHGNVNPNAAGQLPTSPGHMHTQVPPYPQPQPYQPAQPYPFGTGGSAMYRPQQPVA
PPTSNAYPNTPYISSASSYTGQSQLYAAQHQASSPTSSPATSFPPPPSSGASFQHGGPGA
PPSSSAYALPPGTTGTLPAASELPASQRTGPQNGWNDPPALNRVPKKKKMPENFMPPVPI
TSPIMNPLGDPQSQMLQQQPSAPVPLSSQSSFPQPHLPGGQPFHGVQQPLGQTGMPPSFS
KPNIEGAPGAPIGNTFQHVQSLPTKKITKKPIPDEHLILKTTFEDLIQRCLSSATDPQTK
RKLDDASKRLEFLYDKLREQTLSPTITSGLHNIARSIETRNYSEGLTMHTHIVSTSNFSE
TSAFMPVLKVVLTQANKLGV*
>ENSG00000120942|ENST00000376810
MAASQVLGEKINILSGETVKAGDRDPLGNDCPEQDRLPQRSWRQKCASYVLALRPWSFSA
SLTPVALGSALAYRSHGVLDPRLLVGCAVAVLAVHGAGNLVNTYYDFSKGIDHKKSDDRT
LVDRILEPQDVVRFGVFLYTLGCVCAACLYYLSPLKLEHLALIYFGGLSGSFLYTGGIGF
KYVALGDLIILITFGPLAVMFAYAIQVGSLAIFPLVYAIPLALSTEAILHSNNTRDMESD
REAGIVTLAILIGPTFSYILYNTLLFLPYLVFSILATHCTISLALPLLTIPMAFSLERQF
RSQAFNKLPQRTAKLNLLLGLFYVFGIILAPAGSLPKI*
>ENSG00000177054|ENST00000399351
MVILLLQHGADPTLIDGEGFSSIHLAVLFQHMPIIAYLISKGQSVNMTDVNGQTPLMLSA
HKVIGPEPTGFLLKFNPSLNVVDKIHQNTPLHWAVAAGNVNAVDKLLEAGSSLDIQNVKG
ETPLDMALQNKNQLIIHMLKTEAKMRANQKFRLWRWLQKCELFLLLMLSVITMWAIGYIL
DFNSDSWLLKGCLLVTLFFLTSLFPRFLVGYKNLVYLPTAFLLSSVFWIFMTWFILFFPD
LAGAPFYFSFIFSIVAFLYFFYKTWATDPGFTKASEEEKKVNIITLAETGSLDFRTFCTS
CLIRKPLRSLHCHVCNCCVARYDQHCLWTGRCIGFGNHHYYIFFLFFLSMVCGWIIYGSF
IYLSSHCATTFKEDGLWTYLNQIVACSPWVLYILMLATFHFSWSTFLLLNQLFQIAFLGL
TSHERISLQKQSKHMKQTLSLRKTPYNLGFMQNLADFFQCGCFGLVKPCVVDWTSQYTMV
FHPAREKVLRSV*
>ENSG00000138193|ENST00000371375
MVSEGSAAGRDFAGMEEVRQLHVRFCKGIKIWHQAWFLCSLLGREPQEREAGCQLWLCTL
SAVLKVGWLFPLSEVPNFTLLKDGCGCWRLKEDQIYNAVRREETENTVGSLLHFLTKLPA
SETAHGRISVGPCLKQCVRDTVCEYRATLQRTSISQYITGSLLEATTSLGARSGLLSTFG
GSTGRMMLKERQPGPSVANSNALPSSSAGISKELIDLQPLIQFPEEVASILMEQEQTIYR
RVLPVDYLCFLTRDLGTPECQSSLPCLKASISASILTTQNGEHNALEDLVMRFNEVSSWV
TWLILTAGSMEEKREVFSYLVHVAKCCWNMGNYNAVMEFLAGLRSRKVLKMWQFMDQSDI
ETMRSLKDAMAQHESSCEYRKVVTRALHIPGCKVVPFCGVFLKELCEVLDGASGLMKLCP
RYNSQEETLEFVADYSGQDNFLQRVGQNGLKNSEKESTVNSIFQVIRSCNRSLETDEEDS
PSEGNSSRKSSLKDKSRWQFIIGDLLDSDNDIFEQSKEYDSHGSEDSQKAFDHGTELIPW
YVLSIQADVHQFLLQGATVIHYDQDTHLSARCFLQLQPDNSTLTWVKPTTASPASSKAKL
GVLNNTAEPGKFPLLGNAGLSSLTEGVLDLFAVKAVYMGHPGIDIHTVCVQNKLGSMFLS
ETGVTLLYGLQTTDNRLLHFVAPKHTAKMLFSGLLELTRAVRKMRKFPDQRQQWLRKQYV
SLYQEDGRYEGPTLAHAVELFGGRRWSARNPSPGTSAKNAEKPNMQRNNTLGISTTKKKK
KILMRGESGEVTDDEMATRKAKMHKECRSRSGSDPQDINEQEESEVNAIANPPNPLPSRR
AHSLTTAGSPNLAAGTSSPIRPVSSPVLSSSNKSPSSAWSSSSWHGRIKGGMKGFQSFMV
SDSNMSFVEFVELFKSFSVRSRKDLKDLFDVYAVPCNRSGSESAPLYTNLTIDENTSDLQ

PDLDLLTRNVSDLGLFIKSKQQLSDNQRQISDAIAAASIVTNGTGIESTSLGIFGVGILQ
LNDFLVNCQGEHCTYDEILSIIQKFEPSISMCHQGLMSFEGFARFLMDKENFASKNDESQ
ENIKELQLPLSYYYIESSHNTYLTGHQLKGESSVELYSQVLLQGCRSVELDCWDGDDGMP
IIYHGHTLTTKIPFKEVVEAIDRSAFINSDLPIIISIENHCSLPQQRKMAEIFKTVFGEK
LVTKFLFETDFSDDPMLPSPDQLRKKVLLKNKKLKAHQTPVDILKQKAHQLASMQVQAYN
GGNANPRPANNEEEEDEEDEYDYDYESLSDDNILEDRPENKSCNDKLQFEYNEEIPKRIK
KADNSACNKGKVYDMELGEEFYLDQNKKESRQIAPELSDLVIYCQAVKFPGLSTLNASGS
SRGKERKSRKSIFGNNPGRMSPGETASFNKTSGKSSCEGIRQTWEESSSPLNPTTSLSAI
IRTPKCYHISSLNENAAKRLCRRYSQKLTQHTACQLLRTYPAATRIDSSNPNPLMFWLHG
IQLVALNYQTDDLPLHLNAAMFEANGGCGYVLKPPVLWDKNCPMYQKFSPLERDLDSMDP
AVYSLTIVSGQNVCPSNSMGSPCIEVDVLGMPLDSCHFRTKPIHRNTLNPMWNEQFLFHV
HFEDLVFLRFAVVENNSSAVTAQRIIPLKALKRGYRHLQLRNLHNEVLEISSLFINSRRM
EENSSGNTMSASSMFNTEERKCLQTHRVTVHGVPGPEPFTVFTINGGTKAKQLLQQILTN
EQDIKPVTTDYFLMEEKYFISKEKNECRKQPFQRAIGPEEEIMQILSSWFPEEGYMGRIV
LKTQQENLEEKNIVQDDKEVILSSEEESFFVQVHDVSPEQPRTVIKAPRVSTAQDVIQQT
LCKAKYSYSILSNPNPSDYVLLEEVVKDTTNKKTTTPKSSQRVLLDQECVFQAQSKWKGA
GKFILKLKEQVQASREDKKKGISFASELKKLTKSTKQPRGLTSPSQLLTSESIQTKEEKP
VGGLSSSDTMDYRQ*

>ENSG00000206308|ENST00000383259
MAISGVPVLGFFIIAVLMSAQESWAIKEEHVIIQAEFYLNPDQSGEFMFDFDGDEIFHVD
MAKKETVWRLEEFGRFASFEAQGALANIAVDKANLEIMTKRSNYTPITNVPPEVTVLTNS
PVELREPNVLICFIDKFTPPVVNVTWLRNGKPVTTGVSETVFLPREDHLFRKFHYLPFLP
STEDVYDCRVEHWGLDEPLLKHWEFDAPSPLPETTENVVCALGLTVGLVGIIIGTIFIIK
GVRKSNAAERRGPL*

>ENSG00000100023|ENST00000398831
MGKRQHQKDKMYITCAEYTHFYGGKKPDLPQTNFRRLPFDHCSLSLQPFVYPVCTPDGIV
FDLLNIVPWLKKYGTNPSNGEKLDGRSLIKLNFSKNSEGKYHCPVLFTVFTNNTHIVAVR
TTGNVYAYEAVEQLNIKAKNFRDLLTDEPFSRQDIITLQDPTNLDKFNVSNFYHVKNNMK
IIDPDEEKAKQDPSYYLKNTNAETRETLQELYKEFKGDEILAATMKAPEKKKVDKLNAAH
YSTGKVSASFTSTAMVPETTHEAAAIDEDVLRYQFVKKKGYVRLHTNKGDLNLELHCDLT
PKTCENFIRLCKKHYYDGTIFHRSIRNFVIQGGDPTGTGTGGESYWGKPFKDEFRPNLSH
TGRGILSMANSGPNSNRSQFFITFRSCAYLDKKHTIFGRVVGGFDVLTAMENVESDPKTD
RPKEEIRIDATTVFVDPYEEADAQIAQERKTQLKVAPETKVKSSQPQAGSQGPQTFRQGV
GKYINPAATKRAAEEEPSTSATVPMSKKKPSRGFGDFSSW*

>ENSG00000103174|ENST00000381955
MATSTGRWLLLRLALFGFLWEASGGLDSGASRDDDLLLPYPRARARLPRDCTRVRAGNRE
HESWPPPPATPGAGGLAVRTFVSHFRDRAVAGHLTRAVEPLRTFSVLEPGGPGGCAARRR
ATVEETARAADCRVAQNGGFFRMNSGECLGNVVSDERRVSSSGGLQNAQFGIRRDGTLVT
GYLSEEEVLDTENPFVQLLSGVVWLIRNGSIYINESQATECDETQETGSFSKFVNVISAR
TAIGHDRKGQLVLFHADGQTEQRGINLWEMAEFLLKQDVVNAINLDGGGSATFVLNGTLA
SYPSDHCQDNMWRCPRQVSTVVCVHEPRCQPPDCHGHGTCVDGHCQCTGHFWRGPGCDEL
DCGPSNCSQHGLCTETGCRCDAGWTGSNCSEVKQCLQPPEATLRAGELSFFTRTAWLALT
LALAFLLLISTAANLSLLLSRAERNRRLHGDYAYHPLQEMNGEPLAAEKEQPGGAHNPFK
D*

>ENSG00000102531|ENST00000398316
MSGPAQVPMMSPNGSVPPIYVPPGYAPQVIEDNGVRRVVVVPQAPEFHPGSHTVLHRSPH
PPLPGFIPVPTMMPPPPRHMYSPVTGAGDMTTQYMPQYQSSQVYGDVDAHSTHGRSNFRD
ERSSKTYERLQKKLKDRQGTQKDKMSSPPSSPQKCPSPINEHNGLIKGQIAGGINTGSAK
IKSGKGKGGTQVDTEIEEKDEETKAFEALLSNIVKPVASDIQARTVVLTWSPPSSLINGE
TDESSVPELYGYEVLISSTGKDGKYKSVYVGEETNITLNDLKPAMDYHAKVQAEYNSIKG
TPSEAEIFTTLSCEPDIPNPPRIANRTKNSLTLQWKAPSDNGSKIQNFVLEWDEGKGNGE
FCQCYMGSQKQFKITKLSPAMGCKFRLSARNDYGTSGFSEEVLYYTSGCAPSMPASPVLT
KAGITWLSLQWSKPSGTPSDEGISYILEMEEETSGYGFKPKYDGEDLAYTVKNLRRSTKY
KFKVIAYNSEGKSNPSEVVEFTTCPDKPGIPVKPSVKGKIHSHSFKITWDPPKDNGGATI
NKYVVEMAEGSNGNKWEMIYSGATREHLCDRLNPGCFYRLRVYCISDGGQSAVSESLLVQ
TPAVPPGPCLPPRLQGRPKAKEIQLRWGPPLVDGGSPISCYSVEMSPIEKDEPREVYQGS
EVECTVSSLLPGKTYSFRLRAANKMGFGPFSEKCDITTAPGPPDQCKPPQVTCRSATCAQ
VNWEVPLSNGTDVTEYRLEWGGVEGSMQICYCGPGLSYEIKGLSPATTYYCRVQALSVVG
AGPFSEVVACVTPPSVPGIVTCLQEISDDEIENPHYSPSTCLAISWEKPCDHGSEILAYS
IDFGDKQSLTVGKVTSYIINNLQPDTTYRIRIQALNSLGAGPFSHMIKLKTKPLPPDPPR
LECVAFSHQNLKLKWGEGTPKTLSTDSIQYHLQMEDKNGRFVSLYRGPCHTYKVQRLNES
TSYKFCIQACNEAGEGPLSQEYIFTTPKSVPAALKAPKIEKVNDHICEITWECLQPMKGD
PVIYSLQVMLGKDSEFKQIYKGPDSSFRYSSLQLNCEYRFRVCAIRQCQDSLGHQDLVGP
YSTTVLFISQRTEPPASTNRDTVESTRTRRALSDEQCAAVILVLFAFFSILIAFIIQYFV
IK*
>ENSG00000156671|ENST00000372687
MAGPNQLCIRRWTTKHVAVWLKDEGFFEYVDILCNKHRLDGITLLTLTEYDLRSPPLEIK
VLGDIKRLMLSVRKLQKIHIDVLEEMGYNSDSPMGSMTPFISALQSTDWLCNGELSHDCD
GPITDLNSDQYQYMNGKNKHSVRRLDPEYWKTILSCIYVFIVFGFTSFIMVIVHERVPDM
QTYPPLPDIFLDSVPRIPWAFAMTEVCGMILCYIWLLVLLLHKHRSILLRRLCSLMGTVF
LLRCFTMFVTSLSVPGQHLQCTGKIYGSVWEKLHRAFAIWSGFGMTLTGVHTCGDYMFSG
HTVVLTMLNFFVTECKYLFSASMRIR*
>ENSG00000101310|ENST00000377465
MATYLEFIQQNEERDGVRFSWNVWPSSRLEATRMVVPLACLLTPLKERPDLPPVQYEPVL
CSRPTCKAVLNPLCQVDYRAKLWACNFCFQRNQFPPAYGGISEVNQPAELMPQFSTIEYV
IQRGAQSPLIFLYVVDTCLEEDDLQALKESLQMSLSLLPPDALVGLITFGRMVQVHELSC
EGISKSYVFRGTKDLTAKQIQDMLGLTKPAMPMQQARPAQPQEHPFASSRFLQPVHKIDM
NLTDLLGELQRDPWPVTQGKRPLRSTGVALSIAVGLLEGTFPNTGARIMLFTGGPPTQGP
GMVVGDELKIPIRSWHDIEKDNARFMKKATKHYEMLANRTAANGHCIDIYACALDQTGLL
EMKCCANLTGGYMVMGDSFNTSLFKQTFQRIFTKDFNGDFRMAFGATLDVKTSRELKIAG
AIGPCVSLNVKGPCVSENELGVGGTSQWKICGLDPTSTLGIYFEVVNQHNTPIPQGGRGA
IQFVTHYQHSSTQRRIRVTTIARNWADVQSQLRHIEAAFDQEAAAVLMARLGVFRAESEE
GPDVLRWLDRQLIRLCQKFGQYNKEDPTSFRLSDSFSLYPQFMFHLRRSPFLQVFNNSPD
ESSYYRHHFARQDLTQSLIMIQPILYSYSFHGPPEPVLLDSSSILADRILLMDTFFQIVI
YLGETIAQWRKAGYQDMPEYENFKHLLQAPLDDAQEILQARFPMPRYINTEHGGSQARFL
LSKVNPSQTHNNLYAWGQETGAPILTDDVSLQVFMDHLKKLAVSSAC*
>ENSG00000158488|ENST00000368163
MLLLFLLFEGLCCPGENTAAPQALQSYHLAAEEQLSFRMLQTSSFANHSWAHSEGSGWLG

DLQTHGWDTVLGTIRFLKPWSHGNFSKQELKNLQSLFQLYFHSFIQIVQASAGQFQLEYP
FEIQILAGCRMNAPQIFLNMAYQGSDFLSFQGISWEPSPGAGIRAQNICKVLNRYLDIKE
ILQSLLGHTCPRFLAGLMEAGESELKRKVKPEAWLSCGPSPGPGRLQLVCHVSGFYPKPV
WVMWMRGGYSIFLILICLTVIVTLVILVVVDSRLKKQSPVFLMGANTQDTKNSRHQFCLA
QVSWIKNRVLKKWKTRLNQLW*
>ENSG00000137522|ENST00000361756
MAAVVEVEVGGGAAGERELDEVDMSDLSPEEQWRVEHARMHAKHRGHEAMHAEMVLILIA
TLVVAQLLLVQWKQRHPRSYNMVTLFQMWVVPLYFTVKLHWWRFLVIWILFSAVTAFVTF
RATRKPLVQTTPRLVYKWFLLIYKISYATGIVGYMAVMFTLFGLNLLFKIKPEDAMDFGI
SLLFYGLYYGVLERDFAEMCADYMASTIGFYSESGMPTKHLSDSVCAVCGQQIFVDVSEE
GIIENTYRLSCNHVFHEFCIRGWCIVGKKQTCPYCKEKVDLKRMFSNPWERPHVMYGQLL
DWLRYLVAWQPVIIGVVQGINYILGLE*
>ENSG00000149926|ENST00000380495
MLTPMVAGGVVFPGLFLLSKNTLQRLPQLRWEEADAVIVSARLVSSVQAIMASTAGYIVS
TSCKHIIDDQHWLSSAYTQFAVPYFIYDIYAMFLCHWHKHQVKGHGGDDGAARAPGSTWA
IARGYLHKEFLMVLHHAAMVLVCFPLSVVWRQGKGDFFLGCMLMAEVSTPFVCLGKILIQ
YKQQHTLLHKVNGALMLLSFLCCRVLLFPYLYWAYGRHAGLPLLAVPLAIPAHVNLGAAL
LLAPQLYWFFLICRGACRLFWPRSRPPPACQAQD*
>ENSG00000160216|ENST00000398058
MGLLAFLKTQFVLHLLVGFVFVVSGLVINFVQLCTLALWPVSKQLYRRLNCRLAYSLWSQ
LVMLLEWWSCTECTLFTDQATVERFGKEHAVIILNHNFEIDFLCGWTMCERFGVLGSSKV
LAKKELLYVPLIGWTWYFLEIVFCKRKWEEDRDTVVEGLRRLSDYPEYMWFLLYCEGTRF
TETKHRVSMEVAAAKGLPVLKYHLLPRTKGFTTAVKCLRGTVAAVYDVTLNFRGNKNPSL
LGILYGKKYEADMCVRRFPLEDIPLDEKEAAQWLHKLYQEKDALQEIYNQKGMFPGEQFK
PARRPWTLLNFLSWATILLSPLFSFVLGVFASGSPLLILTFLGFVGAASFGVRRLIGVTE
IEKGSSYGNQEFKKKE*
>ENSG00000168067|ENST00000377350
MALLRDVSLQDPRDRFELLQRVGAGTYGDVYKARDTVTSELAAVKIVKLDPGDDISSLQQ
EITILRECRHPNVVAYIGSYLRNDRLWICMEFCGGGSLQEIYHATGPLEERQIAYVCREA
LKGLHHLHSQGKIHRDIKGANLLLTLQGDVKLADFGVSGELTASVAKRRSFIGTPYWMAP
EVAAVERKGGYNELCDVWALGITAIELGELQPPLFHLHPMRALMLMSKSSFQPPKLRDKT
RWTQNFHHFLKLALTKNPKKRPTAEKLLQHPFTTQQLPRALLTQLLDKASDPHLGTPSPE
DCELETYDMFPDTIHSRGQHGPAERTPSEIQFHQVKFGAPRRKETDPLNEPWEEEWTLLG
KEELSGSLLQSVQEALEERSLTIRSASEFQELDSPDDTMGTIKRAPFLGPLPTDPPAEEP
LSSPPGPNSSPLLPTAWATMKQREDPERSSCHGLPPTPKVHMGACFSKVFNGCPLRIHAA
VTWIHPVTRDQFLVVGAEEGIYTLNLHELHEDTLEKLISHRCSWLYCVNNVLLSLSGKST
HIWAHDLPGLFEQRRLQQQVPLSIPTNRLTQRIIPRRFALSTKIPDTKGCLQCRVVRNPY
TGATFLLAALPTSLLLLQWYEPLQKFLLLKNFSSPLPSPAGMLEPLVLDGKELPQVCVGA
EGPEGPGCRVLFHVLPLEAGLTPDILIPPEGIPGSAQQVIQVDRDTILVSFERCVRIVNM
QGEPTATLAPELTFDFPIETVVCLQDSVLAFWSHGMQGRSLDTNEVTQEITDETRIFRVL
GAHRDIILESIPTDNPEAHSNLYILTGHQSTY*
>ENSG00000115339|ENST00000392701
MAHLKRLVKLHIKRHYHKKFWKLGAVIFFFIIVLVLMQREVSVQYSKEESRMERNMKNKN
KMLDLMLEAVNNIKDAMPKMQIGAPVRQNIDAGERPCLQGYYTAAELKPVLDRPPQDSNA

PGASGKAFKTTNLSVEEQKEKERGEAKHCFNAFASDRISLHRDLGPDTRPPECIEQKFKR
CPPLPTTSVIIVFHNEAWSTLLRTVHSVLYSSPAILLKEIILVDDASVDEYLHDKLDEYV
KQFSIVKIVRQRERKGLITARLLGATVATAETLTFLDAHCECFYGWLEPLLARIAENYTA
VVSPDIASIDLNTFEFNKPSPYGSNHNRGNFDWSLSFGWESLPDHEKQRRKDETYPIKTP
TFAGGLFSISKEYFEYIGSYDEEMEIWGGENIEMSFRVWQCGGQLEIMPCSVVGHVFRSK
SPHSFPKGTQVIARNQVRLAEVWMDEYKEIFYRRNTDAAKIVKQKAFGDLSKRFEIKHRL
QCKNFTWYLNNIYPEVYVPDLNPVISGYIKSVGQPLCLDVGENNQGGKPLIMYTCHGLGG
NQYFEYSAQHEIRHNIQKELCLHAAQGLVQLKACTYKGHKTVVTGEQIWEIQKDQLLYNP
FLKMCLSANGEHPSLVSCNPSDPLQKWILSQND*
>ENSG00000198793|ENST00000361445
MLGTGPAAATTAATTSSNVSVLQQFASGLKSRNEETRAKAAKELQHYVTMELREMSQEES
TRFYDQLNHHIFELVSSSDANERKGGILAIASLIGVEGGNATRIGRFANYLRNLLPSNDP
VVMEMASKAIGRLAMAGDTFTAEYVEFEVKRALEWLGADRNEGRRHAAVLVLRELAISVP
TFFFQQVQPFFDNIFVAVWDPKQAIREGAVAALRACLILTTQREPKEMQKPQWYRHTFEE
AEKGFDETLAKEKGMNRDDRIHGALLILNELVRISSMEGERLREEMEEITQQQLVHDKYC
KDLMGFGTKPRHITPFTSFQAVQPQQSNALVGLLGYSSHQGLMGFGTSPSPAKSTLVESR
CCRDLMEEKFDQVCQWVLKCRNSKNSLIQMTILNLLPRLAAFRPSAFTDTQYLQDTMNHV
LSCVKKEKERTAAFQALGLLSVAVRSEFKVYLPRVLDIIRAALPPKDFAHKRQKAMQVDA
TVFTCISMLARAMGPGIQQDIKELLEPMLAVGLSPALTAVLYDLSRQIPQLKKDIQDGLL
KMLSLVLMHKPLRHPGMPKGLAHQLASPGLTTLPEASDVGSITLALRTLGSFEFEGHSLT
QFVRHCADHFLNSEHKEIRMEAARTCSRLLTPSIHLISGHAHVVSQTAVQVVADVLSKLL
VVGITDPDPDIRYCVLASLDERFDAHLAQAENLQALFVALNDQVFEIRELAICTVGRLSS
MNPAFVMPFLRKMLIQILTELEHSGIGRIKEQSARMLGHLVSNAPRLIRPYMEPILKALI
LKLKDPDPDPNPGVINNVLATIGELAQVSGLEMRKWVDELFIIIMDMLQDSSLLAKRQVA
LWTLGQLVASTGYVVEPYRKYPTLLEVLLNFLKTEQNQGTRREAIRVLGLLGALDPYKHK
VNIGMIDQSRDASAVSLSESKSSQDSSDYSTSEMLVNMGNLPLDEFYPAVSMVALMRIFR
DQSLSHHHTMVVQAITFIFKSLGLKCVQFLPQVMPTFLNVIRVCDGAIREFLFQQLGMLV
SFVKSHIRPYMDEIVTLMREFWVMNTSIQSTIILLIEQIVVALGGEFKLYLPQLIPHMLR
VFMHDNSPGRIVSIKLLAAIQLFGANLDDYLHLLLPPIVKLFDAPEAPLPSRKAALETVD
RLTESLDFTDYASRIIHPIVRTLDQSPELRSTAMDTLSSLVFQLGKKYQIFIPMVNKVLV
RHRINHQRYDVLICRIVKGYTLADEEEDPLIYQHRMLRSGQGDALASGPVETGPMKKLHV
STINLQKAWGAARRVSKDDWLEWLRRLSLELLKDSSSPSLRSCWALAQAYNPMARDLFNA
AFVSCWSELNEDQQDELIRSIELALTSQDIAEVTQTLLNLAEFMEHSDKGPLPLRDDNGI
VLLGERAAKCRAYAKALHYKELEFQKGPTPAILESLISINNKLQQPEAAAGVLEYAMKHF
GELEIQATWYEKLHEWEDALVAYDKKMDTNKDDPELMLGRMRCLEALGEWGQLHQQCCEK
WTLVNDETQAKMARMAAAAAWGLGQWDSMEEYTCMIPRDTHDGAFYRAVLALHQDLFSLA
QQCIDKARDLLDAELTAMAGESYSRAYGAMVSCHMLSELEEVIQYKLVPERREIIRQIWW
ERLQGCQRIVEDWQKILMVRSLVVSPHEDMRTWLKYASLCGKSGRLALAHKTLVLLLGVD
PSRQLDHPLPTVHPQVTYAYMKNMWKSARKIDAFQHMQHFVQTMQQQAQHAIATEDQQHK
QELHKLMARCFLKLGEWQLNLQGINESTIPKVLQYYSAATEHDRSWYKAWHAWAVMNFEA
VLHYKHQNQARDEKKKLRHASGANITNATTAATTAATATTTASTEGSNSESEAESTENSP
TPSPLQKKVTEDLSKTLLMYTVPAVQGFFRSISLSRGNNLQDTLRVLTLWFDYGHWPDVN
EALVEGVKAIQIDTWLQVIPQLIARIDTPRPLVGRLIHQLLTDIGRYHPQALIYPLTVAS
KSTTTARHNAANKILKNMCEHSNTLVQQAMMVSEELIRVAILWHEMWHEGLEEASRLYFG

ERNVKGMFEVLEPLHAMMERGPQTLKETSFNQAYGRDLMEAQEWCRKYMKSGNVKDLTQA
WDLYYHVFRRISKQLPQLTSLELQYVSPKLLMCRDLELAVPGTYDPNQPIIRIQSIAPSL
QVITSKQRPRKLTLMGSNGHEFVFLLKGHEDLRQDERVMQLFGLVNTLLANDPTSLRKNL
SIQRYAVIPLSTNSGLIGWVPHCDTLHALIRDYREKKKILLNIEHRIMLRMAPDYDHLTL
MQKVEVFEHAVNNTAGDDLAKLLWLKSPSSEVWFDRRTNYTRSLAVMSMVGYILGLGDRH
PSNLMLDRLSGKILHIDFGDCFEVAMTREKFPEKIPFRLTRMLTNAMEVTGLDGNYRITC
HTVMEVLREHKDSVMAVLEAFVYDPLLNWRLMDTNTKGNKRSRTRTDSYSAGQSVEILDG
VELGEPAHKKTGTTVPESIHSFIGDGLVKPEALNKKAIQIINRVRDKLTGRDFSHDDTLD
VPTQVELLIKQATSHENLCQCYIGWCPFW*
>ENSG00000105270|ENST00000360535
MTKTDPAPMAPPPRGEEEEEEEEEDEPVPEAPSPTQERRQKPVVHPSAPAPLPKDYAFTFF
DPNDPACQEILFDPQTTIPELFAIVRQWVPQVQHKIDVIGNEILRRGCHVNDRDGLTDMT
LLHYACKAGAHGVGDPAAAVRLSQQLLALGADVTLRSRWTNMNALHYAAYFDVPDLVRVL
LKGARPRVVNSTCSDFNHGSALHIAASSLCLGAAKCLLEHGANPALRNRKGQVPAEVVPD
PMDMSLDKAEAALVAKELRTLLEEAVPLSCALPKVTLPNYDNVPGNLMLSALGLRLGDRV
LLDGQKTGTLRFCGTTEFASGQWVGVELDEPEGKNDGSVGGVRYFICPPKQGLFASVSKI
SKAVDAPPSSVTSTPRTPRMDFSRVTGKGRREHKGKKKTPSSPSLGSLQQRDGAKAEVGD
QVLVAGQKQGIVRFYGKTDFAPGYWYGIELDQPTGKHDGSVFGVRYFTCPPRHGVFAPAS
RIQRIGGSTDSPGDSVGAKKVHQVTMTQPKRTFTTVRTPKDIASENSISRLLFCCWFPWM
LRAEMQS*
>ENSG00000211456|ENST00000389061
MATAAYEQLKLHITPEKFYVEACDDGADDVLTIDRVSTEVTLAVKKDVPPSAVTRPIFGI
LGTIHLVAGNYLIVITKKIKVGEFFSHVVWKATDFDVLSYKKTMLHLTDIQLQDNKTFLA
MLNHVLNVDGFYFSTTYDLTHTLQRLSNTSPEFQEMSLLERADQRFVWNGHLLRELSAQP
EVHRFALPVLHGFITMHSCSINGKYFDWILISRRSCFRAGVRYYVRGIDSEGHAANFVET
EQIVHYNGSKASFVQTRGSIPVFWSQRPNLKYKPLPQISKVANHMDGFQRHFDSQVIIYG
KQVIINLINQKGSEKPLEQTFATMVSSLGSGMMRYIAFDFHKECKNMRWDRLSILLDQVA
EMQDELSYFLVDSAGQVVANQEGVFRSNCMDCLDRTNVIQSLLARRSLQAQLQRLGVLHV
GQKLEEQDEFEKIYKNAWADNANACAKQYAGTGALKTDFTRTGKRTHLGLIMDGWNSMIR
YYKNNFSDGFRQDSIDLFLGNYSVDELESHSPLSVPRDWKFLALPIIMVVAFSMCIICLL
MAGDTWTETLAYVLFWGVASIGTFFIILYNGKDFVDAPRLVQKEKID*
>ENSG00000197249|ENST00000393088
MPSSVSWGILLLAGLCCLVPVSLAEDPQGDAAQKTDTSHHDQDHPTFNKITPNLAEFAFS
LYRQLAHQSNSTNIFFSPVSIATAFAMLSLGTKADTHDEILEGLNFNLTEIPEAQIHEGF
QELLRTLNQPDSQLQLTTGNGLFLSEGLKLVDKFLEDVKKLYHSEAFTVNFGDTEEAKKQ
INDYVEKGTQGKIVDLVKELDRDTVFALVNYIFFKGKWERPFEVKDTEEEDFHVDQVTTV
KVPMMKRLGMFNIQHCKKLSSWVLLMKYLGNATAIFFLPDEGKLQHLENELTHDIITKFL
ENEDRRSASLHLPKLSITGTYDLKSVLGQLGITKVFSNGADLSGVTEEAPLKLSKAVHKA
VLTIDEKGTEAAGAMFLEAIPMSIPPEVKFNKPFVFLMIEQNTKSPLFMGKVVNPTQK*
>ENSG00000105974|ENST00000393468
MADELSEKQVYDAHTKEIDLVNRDPKHLNDDVVKIDFEDVIAEPEGTHSFDGIWKASFTT
FTVTKYWFYRLLSALFGIPMALIWGIYFAILSFLHIWAVVPCIKSFLIEIQCISRVYSIY
VHTVCDPLFEAVGKIFSNVRINLQKEI*
>ENSG00000169255|ENST00000392779

MASALWTVLPSRMSLRSLKWSLLLLSLLSFFVMWYLSLPHYNVIERVNWMYFYEYEPIYR
QDFHFTLREHSNCSHQNPFLVILVTSHPSDVKARQAIRVTWGEKKSWWGYEVLTFFLLGQ
EAEKEDKMLALSLEDEHLLYGDIIRQDFLDTYNNLTLKTIMAFRWVTEFCPNAKYVMKTD
TDVFINTGNLVKYLLNLNHSEKFFTGYPLIDNYSYRGFYQKTHISYQEYPFKVFPPYCSG
LGYIMSRDLVPRIYEMMGHVKPIKFEDVYVGICLNLLKVNIHIPEDTNLFFLYRIHLDVC
QLRRVIAAHGFSSKEIITFWQVMLRNTTCHY*
>ENSG00000160408|ENST00000373141
MSSNKEQRSAVFVILFALITILILYSSNSANEVFHYGSLRGRSRRPVNLKKWSITDGYVP
ILGNKTLPSRCHQCVIVSSSSHLLGTKLGPEIERAECTIRMNDAPTTGYSADVGNKTTYR
VVAHSSVFRVLRRPQEFVNRTPETVFIFWGPPSKMQKPQGSLVRVIQRAGLVFPNMEAYA
VSPGRMRQFDDLFRGETGKDREKSHSWLSTGWFTMVIAVELCDHVHVYGMVPPNYCSQRP
RLQRMPYHYYEPKGPDECVTYIQNEHSRKGNHHRFITEKRVFSSWAQLYGITFSHPSWT*
>ENSG00000206433|ENST00000383486
MLSRNDDICIYGGLGLGGLLLLAVVLLSACLCWLHRRVKRLERSWHLLSWSQAQGSSEQE
LHYASLQRLPVPSSEGPDLRGRDKRGTKEDPRADYACIAENKPT*
>ENSG00000147127|ENST00000374473
MSAFGHDEAWMEAGGFGLEAAERTEYQSLCKSKLLFLGEQSVGKTSIISRFMYNSFGCAC
QATVGIDFLSKTMYLEDQIVQLQLWDTAGQERFHSLIPSYIRDSTIAVVVYDITNINSFK
ETDKWVEHVRAERGDDVVIMLLGNKIDLDNKRQVTAEQGEEKSRNLNVMFIETSAKTGYN
VKKLFRRVASALLSTRTSPPPKEGTVEIELESFEESGNRSYC*
>ENSG00000113595|ENST00000381018
MATLVVNKLGAGVDSGRQGSRGTAVVKVLECGVCEDVFSLQGDKVPRLLLCGHTVCHDCL
TRLPLHGRAIRCPFDRQVTDLGDSGVWGLKKNFALLELLERLQNGPIGQYGAAEESIGIS
GESIIRCDEDEAHLASVYCTVCATHLCSECSQVTHSTKTLAKHRRVPLADKPHEKTMCSQ
HQVHAIEFVCLEEGCQTSPLMCCVCKEYGKHQGHKHSVLEPEANQIRASILDMAHCIRTF
TEEISDYSRKLVGIVQHIEGGEQIVEDGIGMAHTEHVPGTAENARSCIRAYFYDLHETLC
RQEEMALSVVDAHVREKLIWLRQQQEDMTILLSEVSAACLHCEKTLQQDDCRVVLAKQEI
TRLLETLQKQQQQFTEVADHIQLDASIPVTFTKDNRVHIGPKMEIRVVTLGLDGAGKTTI
LFKLKQDEFMQPIPTIGFNVETVEYKNLKFTIWDVGGKHKLRPLWKHYYLNTQAVVFVVD
SSHRDRISEAHSELAKLLTEKELRDALLLIFANKQDVAGALSVEEITELLSLHKLCCGRS
WYIQGCDARSVFQIICDQYTGKEVVTEKG*
>ENSG00000117475|ENST00000367808
MTTKNLETKVTVTSSPIRGAGDGMETEEPPKSVEVTSGVQSRKHHSLQSPWKKAVPSESP
GVLQLGKMLTEKAMEVKAVRILVPKAAITHDIPNKNTKVKSLGHHKGEFLGQSEGVIEPN
KELSEVKNVLEKLKNSERRLLQDKEGLSNQLRVQTEVNRELKKLLVASVGDDLQYHFERL
AREKNQLILENEALGRNTAQLSEQLERMSIQCDVWRSKFLASRVMADELTNSRAALQRQN
RDAHGAIQDLLSEREQFRQEMIATQKLLEELLVSLQWGREQTYSPSVQPHSTAELALTNH
KLAKAVNSHLLGNVGINNQKKIPSTVEFCSTPAEKMAETVLRILDPVTCKESSPDNPFFE
SSPTTLLATKKNIGRFHPYTRYENITFNCCNHCRGELIAL*
>ENSG00000173947|ENST00000369737
MCFSRADAADNYPFGTCQQRKLFPHFHPPNLIGNKFVPLRGSPHRGPGCYFSDEMTPHAG
RYQKVSPQQEKHKQNFAPFNVLVPRFKNYPKDTYYPSPGAYNPEKKPPPKIAWPMKFGSP
DWAQVPCLQKRTLKAELSTDKDFRKHRNRVAYLSLYYN*
>ENSG00000147408|ENST00000397998

MMMVRRGLLAWISRVVVLLVLLCCAISVLYMLACTPKGDEEQLALPRANSPTGKEGYQAV
LQEWEEQHRNYVSSLKRQIAQLKEELQERSEQLRNGQYQASDAAGLGLDRSPPEKTQADL
LAFLHSQVDKAEVNAGVKLATEYAAVPFDSFTLQKVYQLETGLTRHPEEKPVRKDKRDEL
VEAIESALETLNSPAENSPNHRPYTASDFIEGIYRTERDKGTLYELTFKGDHKHEFKRLI
LFRPFGPIMKVKNEKLNMANTLINVIVPLAKRVDKFRQFMQNFRPADEVFRCVPLSP*
>ENSG00000172270|ENST00000353555
MAAALFVLLGFALLGTHGASGAAGTVFTTVEDLGSKILLTCSLNDSATEVTGHRWLKGGV
VLKEDALPGQKTEFKVDSDDQWGEYSCVFLPEPMGTANIQLHGPPRVKAVKSSEHINEGE
TAMLVCKSESVPPVTDWAWYKITDSEDKALMNGSESRFFVSSSQGRSELHIENLNMEADP
GQYRCNGTSSKGSDQAIITLRVRSHLAALWPFLGIVAEVLVLVTIIFIYEKRRKPEDVLD
DDDAGSAPLKSSGQHQNDKGKNVRQRNSS*
>ENSG00000162852|ENST00000366513
MDDSDTPTYYLQIEPQDGCHPGDSVERSVTCLPSASDENENQLDGDGHEHLTSSDSAMGK
PQVSEQDSLNNNESCTLSCEVAAGENLQNTLCEASRDEQAFLGKDKKIPGKRSPRSKKGT
AKKIPPGLFSGDIAPLMQEKVLSAVTYAVDDEEAAEVNANEQPEAPKLVLQSLFSLIRGE
VEQLDSRALPLCLHQIAESYFQEEDYEKAMKFIQLERLYHEQLLANLSAIQEQWETKWKT
VQPHTVTALRNSEKGFNGEDFERLTKICATHQDPLLSKHKIAAVEKSQERKCSTQLLVSE
DPKEGGATTKESESKTCLGTESSKESQHTVEPLGSSPCCHQMDVQTDSPSLSVTAGKDHM
EELLCSAEATLALHTQSSETAGSPSGPDSSEDACEDDSRLQLAQTEACQDVARIEGIAED
PKVFLSSKSKTEPLISPGCDRIPPALISEGKYSQAQRKELRLPLRDASEALPTDQLENNE
LNELQQPDLTDSDGKSPQAQADSDGSENVLCGNNQISDLGILLPEVCMAPEEKGDKDDQL
NKETEDYLNSLLEGCLKDTEDSLSYEDNQDDDSDLLQDLSPEEASYSLQENLPSDESCLS
LDDLAKRIEIAEVVPTEGLVSILKKRNDTVGDHPAQMQHKPSKRRVRFQEIDDSLDQDEV
GGGSCILLVLLCIATVFLSVGGTALYCTFGDMESPVCTDFADNMDFYYTKLLQGVAELKH
WIYLS*
>ENSG00000085382|ENST00000369125
MERAMEQLNRLTRSLRRARTVELPEDNETAVYTLMPMVMADQHRSVSELLSNSKFDVNYA
FGRVKRSLLHIAANCGSVECLVLLLKKGANPNYQDISGCTPLHLAARNGQKKCMSKLLEY
SADVNICNNEGLTAIHWLAVNGRTELLHDLVQHVSDVDVEDAMGQTALHVACQNGHKTTV
QCLLDSGADINRPNVSGATPLYFACSHGQRDTAQILLLRGAKYLPDKNGVTPLDLCVQGG
YGETCEVLIQYHPRLFQTIIQMTQNEDLRENMLRQVLEHLSQQSESQYLKILTSLAEVAT
TNGHKLLSLSSNYDAQMKSLLRIVRMFCHVFRIGPSSPSNGIDMGYNGNKTPRSQVFKPL
ELLWHSLDEWLVLIATELMKNKRDSTEITSILLKQKGQDQDAASIPPFEPPGPGSYENLS
TGTRESKPDALAGRQEASADCQDVISMTANRLSAVIQAFYMCCSCQMPPGMTSPRFIEFV
CKHDEVLKCFVNRNPKIIFDHFHFLLECPELMSRFMHIIKAQAEYVQLVTELRMTRAIQP
QINAFLQGFHMFIPPSLIQLFDEYELELLLSGMPEIDVSDWIKNTEYTSGYEREDPVIQW
FWEVVEDITQEERVLLLQFVTGSSRVPHGGFANIMGGSGLQNFTIAAVPYTPNLLPTSST
CINMLKLPEYPSKEILKDRLLVALHCGSYGYTMA*
>ENSG00000105974|ENST00000393470
MSGGKYVDSEGNIYKPNNKAMADELSEKQVYDAHTKEIDLVNRDPKHLNDDVVKIDFEDV
IAEPEGTHSFDGIWKASFTTFTVTKYWFYRLLSALFGIPMALIWGIYFAILSFLHIWAVV
PCIKSFLIEIQCISRVYSIYVHTVCDPLFEAVGKIFSNVRINLQKEI*
>ENSG00000115884|ENST00000381150
MRRAALWLWLCALALSLQPALPQIVATNLPPEDQDGSGDDSDNFSGSGAGALQDITLSQQ

TPSTWKDTQLLTAIPTSPEPTGLEATAASTSTLPAGEGPKEGEAVVLPEVEPGLTAREQE
ATPRPRETTQLPTTHLASTTTATTAQEPATSHPHRDMQPGHHETSTPAGPSQADLHTPHT
EDGGPSATERAAEDGASSQLPAAEGSGEQDFTFETSGENTAVVAVEPDRRNQSPVDQGAT
GASQGLLDRKEVLGGVIAGGLVGLIFAVCLVGFMLYRMKKKDEGSYSLEEPKQANGGAYQ
KPTKQEEFYA*
>ENSG00000107295|ENST00000380607
MSVAGLKKQFHKATQKVSEKVGGAEGTKLDDDFKEMERKVDVTSRAVMEIMTKTIEYLQP
NPASRAKLSMINTMSKIRGQEKGPGYPQAEALLAEAMLKFGRELGDDCNFGPALGEVGEA
MRELSEVKDSLDIEVKQNFIDPLQNLHDKDLREIQHHLKKLEGRRLDFDYKKKRQGKIPD
EELRQALEKFDESKEIAESSMFNLLEMDIEQVSQLSALVQAQLEYHKQAVQILQQVTVRL
EERIRQASSQPRREYQPKPRMSLEFPTGDSTQPNGGLSHTGTPKPSGVQMDQPCCRALYD
FEPENEGELGFKEGDIITLTNQIDENWYEGMLHGHSGFFPINYVEILVALPH*
>ENSG00000183778|ENST00000398714
MCLEMAFPKMRLMYICLLVLGALCLYFSMYSLNPFKEQSFVYKKDGNFLKLPDTDCRQTP
PFLVLLVTSSHKQLAERMAIRQTWGKERMVKGKQLKTFFLLGTTSSAAETKEVDQESQRH
GDIIQKDFLDVYYNLTLKTMMGIEWVHRFCPQAAFVMKTDSDMFINVDYLTELLLKKNRT
TRFFTGFLKLNEFPIRQPFSKWFVSKSEYPWDRYPPFCSGTGYVFSGDVASQVYNVSKSV
PYIKLEDVFVGLCLERLNIRLEELHSQPTFFPGGLRFSVCLFRRIVACHFIKPRTLLDYW
QALENSRGEDCPPV*
>ENSG00000157020|ENST00000397117
MIHDAQMDYYGTRLATCSSDRSVKIFDVRNGGQILIADLRGHEGPVWQVAWAHPMYGNIL
ASCSYDRKVIIWREENGTWEKSHEHAGHDSSVNSVCWAPHDYGLILACGSSDGAISLLTY
TGEGQWEVKKINNAHTIGCNAVSWAPAVVPGSLIDHPSGQKPNYIKRFASGGCDNLIKLW
KEEEDGQWKEEQKLEAHSDWVRDVAWAPSIGLPTSTIASCSQDGRVFIWTCDDASSNTWS
PKLLHKFNDVVWHVSWSITANILAVSGGDNKEELQMQPQIDHLP*
>ENSG00000198324|ENST00000361483
MAPGSPPGPAIATMKLNERSLAFYATCDAPVDNAGFLYKKGGRHAAYHRRWFVLRGNMLF
YFEDAASREPVGVIILEGCTVELVEAAEEFAFAVRFAGTRARTYVLAAESQDAMEGWVKA
LSRASFDYLRLVVRELEQQLAAVRGGGGMALPQPQPQSLPLPPSLPSALAPVPSLPSAPA
PVPALPLPRRPSALPPKENGCAVWSTEATFRPGPEPPPPPPRRRASAPHGPLDMAPFARL
HECYGQEIRALRGQWLSSRVQP*
>ENSG00000197093|ENST00000360039
MGPLSPARTLRLWGPRSLGVALGVFMTIGFALQLLGGPFQRRLPGLQLRQPSAPSLRPAL
PSCPPRQRLVFLKTHKSGSSSVLSLLHRYGDQHGLRFALPARYQFGYPKLFQASRVKGYR
PQGGGTQLPFHILCHHMRFNLKEVLQVMPSDSFFFSIVRDPAALARSAFSYYKSTSSAFR
KSPSLAAFLANPRGFYRPGARGDHYARNLLWFDFGLPFPPEKRAKRGNIHPPRDPNPPQL
QVLPSGAGPRAQTLNPNALIHPVSTVTDHRSQISSPASFDLGSSSFIQWGLAWLDSVFDL
VMVAEYFDESLVLLADALCWGLDDVVGFMHNAQAGHKQGLSTVSNSGLTAEDRQLTARAR
AWNNLDWALYVHFNRSLWARIEKYGQGRLQTAVAELRARREALAKHCLVGGEASDPKYIT
DRRFRPFQFGSAKVLGYILRSGLSPQDQEECERLATPELQYKDKLDAKQFPPTVSLPLKT
SRPLSP*
>ENSG00000198513|ENST00000358385
MAKNRRDRNSWGGFSEKTYEWSSEEEEPVKKAGPVQVLIVKDDHSFELDETALNRILLSE
AVRDKEVVAVSVAGAFRKGKSFLMDFMLRYMYNQESVDWVGDYNEPLTGFSWRGGSERET

TGIQIWSEIFLINKPDGKKVAVLLMDTQGTFDSQSTLRDSATVFALSTMISSIQVVYNLSQ
NVQEDDLQHLQLFTEYGRLAMEETFLKPFQSLIFLVRDWSFPYEFSYGADGGAKFLEKRL
KVSGNQHEELQNVRKHIHSCFTNISCFLLPHPGLKVATNPNFDGKLKEIDDEFIKNLKIL
IPWLLSPESLDIKEINGNKITCRGLVEYFKAYIKIYQGEELPHPKSMLQATAEANNLAAV
ATAKDTYNKKMEEICGGDKPFLAPNDLQTKHLQLKEESVKLFRGVKKMGGEEFSRRYLQQ
LESEIDELYIQYIKHNDSKNIFHAARTPATLFVVIFITYVIAGVTGFIGLDIIASLCNMI
MGLTLITLCTWAYIRYSGEYRELGAVIDQVAAALWDQGSTNEALYKLYSAAATHRHLYHQ
AFPTPKSESTEQSEKKKM*
>ENSG00000095139|ENST00000359415
MAECNLVAILISSIDNPLDKNLDNGGNSCLDFRPLNSFSQPQVLLAAAVCTKAGKAIVSR
QFVEMTRTRIEGLLAAFPKLMNTGKQHTFVETESVRYVYQPMEKLYMVLITTKNSNILED
LETLRLFSRVIPEYCRALEENEISEHCFDLIFAFDEIVALGYRENVNLAQIRTFTEMDSH
EEKVFRAVRETQEREAKAEMRRKAKELQQARRDAERQGKKAPGFGGFGSSAVSGGSTAAM
ITETIIETDKPKVAPAPARPSGPSKALKLGAKGKEVDNFVDKLKSEGETIMSSSMGKRTS
EATKMHAPPINMESVHMKIEEKITLTCGRDGGLQNMELHGMIMLRISDDKYGRIRLHVEN
EDKKGVQLQTHPNVDKKLFTAESLIGLKNPEKSFPVNSDVGVLKWRLQTTEESFIPLTIN
CWPSESGNGCDVNIEYELQEDNLELNDVVITIPLPSGVGAPVIGEIDGEYRHDSRRNTLE
WCLPVIDAKNKSGSLEFSIAGQPNDFFPVQVSFVSKKNYCNIQVTKVTQVDGNSPVRFST
ETTFLVDKYEIL*
>ENSG00000164414|ENST00000369556
MAAPRDNVTLLFKLYCLAVMTLMAAVYTIALRYTRTSDKELYFSTTAVCITEVIKLLLSV
GILAKETGSLGRFKASLRENVLGSPKELLKLSVPSLVYAVQNNMAFLALSNLDAAVYQVT
YQLKIPCTALCTVLMLNRTLSKLQWVSVFMLCAGVTLVQWKPAQATKVVVEQNPLLGFGA
IAIAVLCSGFAVLASVGGLYTSVVVKYTDNIMKGFSAAAAIVLSTIASVMLFGLQITLTF
ALGTLLVCVSIYLYGLPRQDTTSIQQGETASKERVIGV*
>ENSG00000126581|ENST00000361523
MEGSKTSNNSTMQVSFVCQRCSQPLKLDTSFKILDRVTIQELTAPLLTTAQAKPGETQEE
ETNSGEEPFIETPRQDGVSRRFIPPARMMSTESANSFTLIGEASDGGTMENLSRRLKVTG
DLFDIMSGQTDVDHPLCEECTDTLLDQLDTQLNVTENECQNYKRCLEILEQMNEDDSEQL
QMELKELALEEERLIQELEDVEKNRKIVAENLEKVQAEAERLDQEEAQYQREYSEFKRQQ
LELDDELKSVENQMRYAQTQLDKLKKTNVFNATFHIWHSGQFGTINNFRLGRLPSVPVEW
NEINAAWGQTVLLLHALANKMGLKFQRYRLVPYGNHSYLESLTDKSKELPLYCSGGLRFF
WDNKFDHAMVAFLDCVQQFKEEVEKGETRFCLPYRMDVEKGKIEDTGGSGGSYSIKTQFN
SEEQWTKALKFMLTNLKWGLAWVSSQFYNK*
>ENSG00000158488|ENST00000368156
MLLLFLLFEGLCCPGENTAAPQALQSYHLAAEEQLSFRMLQTSSFANHSWAHSEGSGWLG
DLQTHGWDTVLGTIRFLKPWSHGNFSKQELKNLQSLFQLYFHSFIQIVQASAGQFQLELK
PEAWLSCGPSPGPGRLQLVCHVSGFYPKPVWVMWMRGEQEQRGTQRGDVLPNADETWYLR
ATLDVAAGEAAGLSCRVKHSSLGGHDLIIHWGGYSIFLILICLTVIVTLVILVVVDSRLK
KQSPVFLMGANTQDTKNSRHQFCLAQVSWIKNRVLKKWKTRLNQLW*
>ENSG00000136631|ENST00000369130
MNVVFAVKQYISKMIEDSGPGMKVLLMDKETTGIVSMVYTQSEILQKEVYLFERIDSQNR
EIMKHLKAICFLRPTKENVDYIIQELRRPKYTIYFIYFSNVISKSDVKSLAEADEQEVVA
EVQEFYGDYIAVNPHLFSLNILGCCQGRNWDPAQLSRTTQGLTALLLSLKKCPMIRYQLS

SEAAKRLAECVKQVITKEYELFEFRRTEVPPLLLILDRCDDAITPLLNQWTYQAMVHELL
GINNNRIDLSRVPGISKDLREVVLSAENDEFYANNMYLNFAEIGSNIKNLMEDFQKKKPK
EQQKLESIADMKAFVENYPQFKKMSGTVSKHVTVVGELSRLVSERNLLEVSEVEQELACQ
NDHSSALQNIKRLLQNPKVTEFDAARLVMLYALHYERHSSNSLPGLMMDLRNKGVSEKYR
KLVSAVVEYGGKRVRGSDLFSPKDAVAITKQFLKGLKGVENVYTQHQPFLHETLDHLIKG
RLKENLYPYLGPSTLRDRPQDIIVFVIGGATYEEALTVYNLNRTTPGVRIVLGGTTVHNT
KSFLEEVLASGLHSRSKESSQVTSRSASRR*
>ENSG00000196735|ENST00000374949
MILNKALLLGALALTTVMSPCGGEDIVADHVASCGVNLYQFYGPSGQYTHEFDGDEQFYV
DLERKETAWRWPEFSKFGGFDPQGALRNMAVAKHNLNIMIKRYNSTAATNEVPEVTVFSK
SPVTLGQPNTLICLVDNIFPPVVNITWLSNGQSVTEGVSETSFLSKSDHSFFKISYLTFL
PSADEIYDCKVEHWGLDQPLLKHWEPEIPAPMSELTETVVCALGLSVGLMGIVVGTVFII
QGLRSVGASRHQGPL*
>ENSG00000127083|ENST00000375550
MGFLSPIYVIFFFFGVKVHCQYETYQWDEDYDQEPDDDYQTGFPFRQNVDYGVPFHQYTL
GCVSECFCPTNFPSSMYCDNRKLKTIPNIPMHIQQLYLQFNEIEAVTANSFINATHLKEI
NLSHNKIKSQKIDYGVFAKLPNLLQLHLEHNNLEEFPFPLPKSLERLLLGYNEISKLQTN
AMDGLVNLTMLDLCYNYLHDSLLKDKIFAKMEKLMQLNLCSNRLESMPPGLPSSLMYLSL
ENNSISSIPEKYFDKLPKLHTLRMSHNKLQDIPYNIFNLPNIVELSVGHNKLKQAFYIPR
NLEHLYLQNNEIEKMNLTVMCPSIDPLHYHHLTYIRVDQNKLKEPISSYIFFCFPHIHTI
YYGEQRSTNGQTIQLKTQVFRRFPDDDDESEDHDDPDNAHESPEQEGAEGHFDLHYYENQ
E*
>ENSG00000128641|ENST00000392318
MAKMEVKTSLLDNMIGVGDMVLLEPLNEETFINNLKKRFDHSEIYTYIGSVVISVNPYRS
LPIYSPEKVEEYRNRNFYELSPHIFALSDEAYRSLRDQDKDQCILITGESGAGKTEASKL
VMSYVAAVCGKGAEVNQVKEQLLQSNPVLEAFGNAKTVRNDNSSRFGKYMDIEFDFKGDP
LGGVISNYLLEKSRVVKQPRGERNFHVFYQLLSGASEELLNKLKLERDFSRYNYLSLDSA
KVNGVDDAANFRTVRNAMQIVGFMDHEAESVLAVVAAVLKLGNIEFKPESRVNGLDESKI
KDKNELKEICELTGIDQSVLERAFSFRTVEAKQEKVSTTLNVAQAYYARDALAKNLYSRL
FSWLVNRINESIKAQTKVRKKVMGVLDIYGFEIFEDNSFEQFIINYCNEKLQQIFIELTL
KEEQEEYIREDIEWTHIDYFNNAIICDLIENNTNGILAMLDEECLRPGTVTDETFLEKLN
QVCATHQHFESRMSKCSRFLNDTSLPHSCFRIQHYAGKVLYQVEGFVDKNNDLLYRDLSQ
AMWKASHALIKSLFPEGNPAKINLKRPPTAGSQFKASVATLMKNLQTKNPNYIRCIKPND
KKAAHIFNEALVCHQIRYLGLLENVRVRRAGYAFRQAYEPCLERYKMLCKQTWPHWKGPA
RSGVEVLFNELEIPVEEYSFGRSKIFIRNPRTLFKLEDLRKQRLEDLATLIQKIYRGWKC
RTHFLLMKKSQIVIAAWYRRYAQQKRYQQTKSSALVIQSYIRGWKARKILRELKHQKRCK
EAVTTIAAYWHGTQARRELRRLKEEARNKHAIAVIWAYWLGSKARRELKRLKEEARRKHA
VAVIWAYWLGLKVRREYRKFFRANAGKKIYEFTLQRIVQKYFLEMKNKMPSLSPIDKNWP
SRPYLFLDSTHKELKRIFHLWRCKKYRDQFTDQQKLIYEEKLEASELFKDKKALYPSSVG
QPFQGAYLEINKNPKYKKLKDAIEEKIIIAEVVNKINRANGKSTSRIFLLTNNNLLLADQ
KSGQIKSEVPLVDVTKVSMSSQNDGFFAVHLKEGSEAASKGDFLFSSDHLIEMATKLYRT
TLSQTKQKLNIEISDEFLVQFRQDKVCVKFIQGNQKNGSVPTCKRKNNRLLEVAVP*
>ENSG00000133110|ENST00000379743
MIPFLPMFSLLLLLIVNPINANNHYDKILAHSRIRGRDQGPNVCALQQILGTKKKYFSTC

KNWYKKSICGQKTTVLYECCPGYMRMEGMKGCPAVLPIDHVYGTLGIVGATTTQRYSDAS
KLREEIEGKGSFTYFAPSNEAWDNLDSDIRRGLESNVNVELLNALHSHMINKRMLTKDLK
NGMIIPSMYNNLGLFINHYPNGVVTVNCARIIHGNQIATNGVVHVIDRVLTQIGTSIQDF
IEAEDDLSSFRAAAITSDILEALGRDGHFTLFAPTNEAFEKLPRGVLERIMGDKVASEAL
MKYHILNTLQCSESIMGGAVFETLEGNTIEIGCDGDSITVNGIKMVNKKDIVTNNGVIHL
IDQVLIPDSAKQVIELAGKQQTTFTDLVAQLGLASALRPDGEYTLLAPVNNAFSDDTLSM
DQRLLKLILQNHILKVKVGLNELYNGQILETIGGKQLRVFVYRTAVCIENSCMEKGSKQG
RNGAIHIFREIIKPAEKSLHEKLKQDKRFSTFLSLLEAADLKELLTQPGDWTLFVPTNDA
FKGMTSEEKEILIRDKNALQNIILYHLTPGVFIGKGFEPGVTNILKTTQGSKIFLKEVND
TLLVNELKSKESDIMTTNGVIHVVDKLLYPADTPVGNDQLLEILNKLIKYIQIKFVRGST
FKEIPVTVYRPTLTKVKIEGEPEFRLIKEGETITEVIHGEPIIKKYTKIIDGVPVEITEK
ETREERIITGPEIKYTRISTGGGETEETLKKLLQEEVTKVTKFIEGGDGHLFEDEEIKRL
LQGDTPVRKLQANKKVQGSRRRLREGRSQ*
>ENSG00000127914|ENST00000356239
MEDEERQKKLEAGKAKLAQFRQRKAQSDGQSPSKKQKKKRKTSSSKHDVSAHHDLNIDQS
QCNEMYINSSQRVESTVIPESTIMRTLHSGEITSHEQGFSVELESEISTTADDCSSEVNG
CSFVMRTGKPTNLLREEEFGVDDSYSEQGAQDSPTHLEMMESELAGKQHEIEELNRELEE
MRVTYGTEGLQQLQEFEAAIKQRDGIITQLTANLQQARREKDETMREFLELTEQSQKLQI
QFQQLQASETLRNSTHSSTAADLLQAKQQILTHQQQLEEQDHLLEDYQKKKEDFTMQISF
LQEKIKVYEMEQDKKVENSNKEEIQEKETIIEELNTKIIEEEKKTLELKDKLTTADKLLG
ELQEQIVQKNQEIKNMKLELTNSKQKERQSSEEIKQLMGTVEELQKRNHKDSQFETDIVQ
RMEQETQRKLEQLRAELDEMYGQQIVQMKQELIRQHMAQMEEMKTRHKGEMENALRSYSN
ITVNEDQIKLMNVAINELNIKLQDTNSQKEKLKEELGLILEEKCALQRQLEDLVEELSFS
REQIQRARQTIAEQESKLNEAHKSLSTVEDLKAEIVSASESRKELELKHEAEVTNYKIKL
EMLEKEKNAVLDRMAESQEAELERLRTQLLFSHEEELSKLKEDLEIEHRINIEKLKDNLG
IHYKQQIDGLQNEMSQKIETMQFEKDNLITKQNQLILEISKLKDLQQSLVNSKSEEMTLQ
INELQKEIEILRQEEKEKGTLEQEVQELQLKTELLEKQMKEKENDLQEKFAQLEAENSIL
KDEKKTLEDMLKIHTPVSQEERLIFLDSIKSKSKDSVWEKEIEILIEENEDLKQQCIQLN
EEIEKQRNTFSFAEKNFEVNYQELQEEYACLLKVKDDLEDSKNKQELEYKSKLKALNEEL
HLQRINPTTVKMKSSVFDEDKTFVAETLEMGEVVEKDTTELMEKLEVTKREKLELSQRLS
DLSEQLKQKHGEISFLNEEVKSLKQEKEQVSLRCRELEIIINHNRAENVQSCDTQVSSLL
DGVVTMTSRGAEGSVSKVNKSFGEESKIMVEDKVSFENMTVGEESKQEQLILDHLPSVTK
ESSLRATQPSENDKLQKELNVLKSEQNDLRLQMEAQRICLSLVYSTHVDQVREYMENEKD
KALCSLKEELIFAQEEKIKELQKIHQLELQTMKTQETGDEGKPLHLLIGKLQKAVSEECS
YFLQTLCSVLGEYYTPALKCEVNAEDKENSGDYISENEDPELQDYRYEVQDFQENMHTLL
NKVTEEYNKLLVLQTRLSKIWGQQTDGMKLEFGEENLPKEETEFLSIHSQMTNLEDIDVN
HKSKLSSLQDLEKTKLEEQVQELESLISSLQQQLKETEQNYEAEIHCLQKRLQAVSESTV
PPSLPVDSVVITESDAQRTMYPGSCVKKNIDGTIEFSGEFGVKEETNIVKLLEKQYQEQL
EEEVAKVIVSMSIAFAQQTELSRISGGKENTASSKQAHAVCQQEQHYFNEMKLSQDQIGF
QTFETVDVKFKEEFKPLSKELGEHGKEILLSNSDPHDIPESKDCVLTISEEMFSKDKTFI
VRQSIHDEISVSSMDASRQLMLNEEQLEDMRQELVRQYQEHQQATELLRQAHMRQMERQR
EDQEQLQEEIKRLNRQLAQRSSIDNENLVSERERVLLEELEALKQLSLAGREKLCCELRN
SSTQTQNGNENQGEVEEQTFKEKELDRKPEDVPPEILSNERYALQKANNRLLKILLEVVK
TTAAVEETIGRHVLGILDRSSKSQSSASLIWRSEAEASVKSCVHEEHTRVTDESIPSYSG

SDMPRNDINMWSKVTEEGTELSQRLVRSGFAGTEIDPENEELMLNISSRLQAAVEKLLEA
ISETSSQLEHAKVTQTELMRESFRQKQEATESLKCQEELRERLHEESRAREQLAVELSKA
EGVIDGYADEKTLFERQIQEKTDIIDRLEQELLCASNRLQELEAEQQQIQEERELLSRQK
EAMKAEAGPVEQQLLQETEKLMKEKLEVQCQAEKVRDDLQKQVKALEIDVEEQVSRFIEL
EQEKNTELMDLRQQNQALEKQLEKMRKFLDEQAIDREHERDVFQQEIQKLEQQLKVVPRF
QPISEHQTREVEQLANHLKEKTDKCSELLLSKEQLQRDIQERNEEIEKLEFRVRELEQAL
LVSADTFQKVEDRKHFGAVEAKPELSLEVQLQAERDAIDRKEKEITNLEEQLEQFREELE
NKNEEVQQLHMQLEIQKKESTTRLQELEQENKLFKDDMEKLGLAIKESDAMSTQDQHVLF
GKFAQIIQEKEVEIDQLNEQVTKLQQQLKITTDNKVIEEKNELIRDLETQIECLMSDQEC
VKRNREEEIEQLNEVIEKLQQELANIGQKTSMNAHSLSEEADSLKHQLDVVIAEKLALEQ
QVETANEEMTFMKNVLKETNFKMNQLTQELFSLKRERESVEKIQSIPENSVNVAIDHLSK
DKPELEVVLTEDALKSLENQTYFKSFEENGKGSIINLETRLLQLESTVSAKDLELTQCYK
QIKDMQEQGQFETEMLQKKIVNLQKIVEEKVAAALVSQIQLEAVQEYAKFCQDNQTISSE
PERTNIQNLNQLREDELGSDISALTLRISELESQVVEMHTSLILEKEQVEIAEKNVLEKE
KKLLELQKLLEGNEKKQREKEKKRSPQDVEVLKTTTELFHSNEESGFFNELEALRAESVA
TKAELASYKEKAEKLQEELLVKETNMTSLQKDLSQVRDHLAEAKEKLSILEKEDETEVQE
SKKACMFEPLPIKLSKSIASQTDGTLKISSSNQTPQILVKNAGIQINLQSECSSEEVTEI
ISQFTEKIEKMQELHAAEILDMESRHISETETLKREHYVAVQLLKEECGTLKAVIQCLRS
KEGSSIPELAHSDAYQTREICSSDSGSDWGQGIYLTHSQGFDIASEGRGEESESATDSFP
KKIKGLLRAVHNEGMQVLSLTESPYSDGEDHSIQQVSEPWLEERKAYINTISSLKDLITK
MQLQREAEVYDSSQSHESFSDWRGELLLALQQVFLEERSVLLAAFRTELTALGTTDAVGL
LNCLEQRIQEQGVEYQAAMECLQKADRRSLLSEIQALHAQMNGRKITLKREQESEKPSQE
LLEYNIQQKQSQMLEMQVELSSMKDRATELQEQLSSEKMVVAELKSELAQTKLELETTLK
AQHKHLKELEAFRLEVKDKTDEVHLLNDTLASEQKKSRELQWALEKEKAKLGRSEERDKE
ELEDLKFSLESQKQRNLQLNLLLEQQKQLLNESQQKIESQRMLYDAQLSEEQGRNLELQV
LLESEKVRIREMSSTLDRERELHAQLQSSDGTGQSRPPLPSEDLLKELQKQLEEKHSRIV
ELLNETEKYKLDSLQTRQQMEKDRQVHRKTLQTEQEANTEGQKKMHELQSKVEDLQRQLE
EKRQQVYKLDLEGQRLQGIMQEFQKQELEREEKRESRRILYQNLNEPTTWSLTSDRTRNW
VLQQKIEGETKESNYAKLIEMNGGGTGCNHELEMIRQKLQCVASKLQVLPQKASERLQFE
TADDEDFIWVQENIDEIILQLQKLTGQQGEEPSLVSPSTSCGSLTERLLRQNAELTGHIS
QLTEEKNDLRNMVMKLEEQIRWYRQTGAGRDNSSRFSLNGGANIEAIIASEKEVWNREKL
TLQKSLKRAEAEVYKLKAELRNDSLLQTLSPDSEHVTLKRIYGKYLRAESFRKALIYQKK
YLLLLLGGFQECEDATLALLARMGGQPAFTDLEVITNRPKGFTRFRSAVRVSIAISRMKF
LVRRWHRVTGSVSININRDGFGLNQGAEKTDSFYHSSGGLELYGEPRHTTYRSRSDLDYI
RSPLPFQNRYPGTPADFNPGSLACSQLQNYDPDRALTDYITRLEALQRRLGTIQSGSTTQ
FHAGMRR*
>ENSG00000172469|ENST00000358812
MAKFRRRTCIILALFILFIFSLMMGLKMLRPNTATFGAPFGLDLLPELHQRTIHLGKNFD
FQKSDRINSETNTKNLKSVEITMKPSKASELNLDELPPLNNYLHVFYYSWYGNPQFDGKY
IHWNHPVLEHWDPRIAKNYPQGRHNPPDDIGSSFYPELGSYSSRDPSVIETHMRQMRSAS
IGVLALSWYPPDVNDENGEPTDNLVPTILDKAHKYNLKVTFHIEPYSNRDDQNMYKNVKY
IIDKYGNHPAFYRYKTKTGNALPMFYVYDSYITKPEKWANLLTTSGSRSIRNSPYDGLFI
ALLVEEKHKYDILQSGFDGIYTYFATNGFTYGSSHQNWASLKLFCDKYNLIFIPSVGPGY
IDTSIRPWNTQNTRNRINGKYYEIGLSAALQTRPSLISITSFNEWHEGTQIEKAVPKRTS

NTVYLDYRPHKPGLYLELTRKWSEKYSKERATYALDRQLPVS*
>ENSG00000082996|ENST00000361785
MLILMTSLAWDPTTVSTVEVLKKIDIPSVFIGESSANSLKDEFTYEKGGHLILVPEFSLP
LEYYLIPFLIIVGICLILIVIFMITKFVQDRHRARRNRLRKDQLKKLPVHKFKKGDEYDV
CAICLDEYEDGDKLRILPCSHAYHCKCVDPWLTKTKKTCPVCKQKVVPSQGDSDSDTDSS
QEENEVTEHTPLLRPLASVSAQSFGALSESRSHQNMTESSDYEEDDNEDTDSSDAENEIN
EHDVVVQLQPNGERDYNIANTV*
>ENSG00000254647|ENST00000381330
MALWMRLLPLLALLALWGPDPAAAFVNQHLCGSHLVEALYLVCGERGFFYTPKTRREAED
LQVGQVELGGGPGAGSLQPLALEGSLQKRGIVEQCCTSICSLYQLENYCN*
>ENSG00000197461|ENST00000354513
MRTLACLLLLGCGYLAHVLAEEAEIPREVIERLARSQIHSIRDLQRLLEIDSVGSEDSLD
TSLRAHGVHATKHVPEKRPLPIRRKRSIEEAVPAVCKTRTVIYEIPRSQVDPTSANFLIW
PPCVEVKRCTGCCNTSSVKCQPSRVHHRSVKVAKVEYVRKKPKLKEVQVRLEEHLECACA
TTSLNPDYREEDTGRPRESGKKRKRKRLKPT*
>ENSG00000169241|ENST00000368404
MEAGGFLDSLIYGACVVFTLGMFSAGLSDLRHMRMTRSVDNVQFLPFLTTEVNNLGWLSY
GALKGDGILIVVNTVGAALQTLYILAYLHYCPRKRVVLLQTATLLGVLLLGYGYFWLLVP
NPEARLQQLGLFCSVFTISMYLSPLADLAKVIQTKSTQCLSYPLTIATLLTSASWCLYGF
RLRDPYIMVSNFPGIVTSFIRFWLFWKYPQEQDRNYWLLQT*
>ENSG00000174775|ENST00000397594
MTEYKLVVVGAGGVGKSALTIQLIQNHFVDEYDPTIEDSYRKQVVIDGETCLLDILDTAG
QEEYSAMRDQYMRTGEGFLCVFAINNTKSFEDIHQYREQIKRVKDSDDVPMVLVGNKCDL
AARTVESRQAQDLARSYGIPYIETSAKTRQGSRSGSSSSSGTLWDPPGPM*
>ENSG00000143545|ENST00000368575
MAKAYDHLFKLLLIGDSGVGKTCLIIRFAEDNFNNTYISTIGIDFKIRTVDIEGKKIKLQ
VWDTAGQERFKTITTAYYRGAMGIILVYDITDEKSFENIQNWMKSIKENASAGVERLLLG
NKCDMEAKRKVQKEQADKLAREHGIRFFETSAKSSMNVDEAFSSLARDILLKSGGRRSGN
GNKPPSTDLKTCDKKNTNKCSLG*
>ENSG00000136935|ENST00000373555
MFAKLKKKIAEETAVAQRPGGATRIPRSVSKESVASMGADSGDDFASDGSSSREDLSSQL
LRRNEQIRKLEARLSDYAEQVRNLQKIKEKLEIALEKHQDSSMRKFQEQNETFQANRAKM
AEGLALALARKDQEWSEKMDQLEKEKNILTAQLQEMKNQSMNLFQRRDEMDELEGFQQQE
LSKIKHMLLKKEESLGKMEQELEARTRELSRTQEELMNSNQMSSDLSQKLEELQRHYSTL
EEQRDHVIASKTGAESKITALEQKEQELQALIQQLSIDLQKVTAETQEKEDVITHLQEKV
ASLEKRLEQNLSGEEHLQELLKEKTLAEQNLEDTRQQLLAARSSQAKAINTLETRVRELE
QTLQASEEQLQQSKGIVAAQETQIQELAAANQESSHVQQQALALEQQFLERTQALEAQIV
ALERTRAADQTTAEQGMRQLEQENAALKECRNEYERSLQNHQFELKKLKEEWSQREIVSV
AMAQALEEVRKQREEFQQQAANLTAIIDEKEQNLREKTEVLLQKEQEILQLERGHNSALL
QIHQLQAELEALRTLKAEEAAVVAEQEDLLRLRGPLQAEALSVNESHVTSRAMQDPVFQL
PTAGRTPNGEVGAMDLTQLQKEKQDLEQQLLEKNKTIKQMQQRMLELRKTLQKELKIRPD
NELFEVREKPGPEMANMAPSVTNNTDLTDAREINFEYLKHVVLKFMSCRESEAFHLIKAV
SVLLNFSQEEENMLKETLEYKMSWFGSKPAPKGSIRPSISNPRIPWS*
>ENSG00000071073|ENST00000393487

MRLRNGTVATALAFITSFLTLSWYTTWQNGKEKLIAYQREFLALKERLRIAEHRISQRSS
ELNTIVQQFKRVGAETNGSKDALNKFSDNTLKLLKELTSKKSLQVPSIYYHLPHLLKNEG
SLQPAVQIGNGRTGVSIVMGIPTVKREVKSYLIETLHSLIDNLYPEEKLDCVIVVFIGET
DIDYVHGVVANLEKEFSKEISSGLVEVISPPESYYPDLTNLKETFGDSKERVRWRTKQNL
DYCFLMMYAQEKGIYYIQLEDDIIVKQNYFNTIKNFALQLSSEEWMILEFSQLGFIGKMF
QAPDLTLIVEFIFMFYKEKPIDWLLDHILWVKVCNPEKDAKHCDRQKANLRIRFRPSLFQ
HVGLHSSLSGKIQKLTDKDYMKPLLLKIHVNPPAEVSTSLKVYQGHTLEKTYMGEDFFWA
ITPIAGDYILFKFDKPVNVESYLFHSGNQEHPGDILLNTTVEVLPFKSEGLEISKETKDK
RLEDGYFRIGKFENGVAEGMVDPSLNPISAFRLSVIQNSAVWAILNEIHIKKATN*
>ENSG00000154451|ENST00000370459
MALEIHMSDPMCLIENFNEQLKVNQEALEILSAITQPVVVVAIVGLYRTGKSYLMNKLAG
KNKGFSVASTVQSHTKGIWIWCVPHPNWPNHTLVLLDTEGLGDVEKADNKNDIQIFALAL
LLSSTFVYNTVNKIDQGAIDLLHNVTELTDLLKARNSPDLDRVEDPADSASFFPDLVWTL
RDFCLGLEIDGQLVTPDEYLENSLRPKQGSDQRVQNFNLPRLCIQKFFPKKKCFIFDLPA
HQKKLAQLETLPDDELEPEFVQQVTEFCSYIFSHSMTKTLPGGIMVNGSRLKNLVLTYVN
AISSGDLPCIENAVLALAQRENSAAVQKAIAHYDQQMGQKVQLPMETLQELLDLHRTSER
EAIEVFMKNSFKDVDQSFQKELETLLDAKQNDICKRNLEASSDYCSALLKDIFGPLEEAV
KQGIYSKPGGHNLFIQKTEELKAKYYREPRKGIQAEEVLQKYLKSKESVSHAILQTDQAL
TETEKKKKEAQVKAEAEKAEAQRLAAIQRQNEQMMQERERLHQEQVRQMEIAKQNWLAEQ
QKMQEQQMQEQAAQLSTTFQAQNRSLLSELQHAQRTVNNDDPCVLL*
>ENSG00000164125|ENST00000393807
MTCPDKPGQLINWFICSLCVPRVRKLWSSRRPRTRRNLLLGTACAIYLGFLVSQVGRASL
QHGQAAEKGPHRSRDTAEPSFPEIPLDGTLAPPESQGNGSTLQPNVVYITLRSKRSKPAN
IRGTVKPKRRKKHAVASAAPGQEALVGPSLQPQEAAREADAVAPGYAQGANLVKIGERPW
RLVRGPGVRAGGPDFLQPSSRESNIRIYSESAPSWLSKDDIRRMRLLADSAVAGLRPVSS
RSGARLLVLEGGAPGAVLRCGPSPCGLLKQPLDMSEVFAFHLDRILGLNRTLPSVSRKAE
FIQAAAAACLSMRLAFFIFPAWEDMRVPGKCCIGHARLTWGTYQQLLKQKCWQNGRVPKP
ESGCTEIHHHEWSKMALFDFLLQIYNRLDTNCCGFRPRKEDACVQNGLRPKCDDQGSAAL
AHIIQRKHDPRHLVFIDNKGFFDRSEDNLNFKLLEGIKEFPASAVSVLKSQHLRQKLLQS
LFLDKVYWESQGGRQGIEKLIDVIEHRAKILITYINAHGVKVLPMNE*
>ENSG00000204301|ENST00000375023
MQPPSLLLLLLLLLLLCVSVVRPRGLLCGSFPEPCANGGTCLSLSLGQGTCQCAPGFLGE
TCQFPDPCQNAQLCQNGGSCQALLPAPLGLPSSPSPLTPSFLCTCLPGFTGERCQAKLED
PCPPSFCSKRGRCHIQASGRPQCSCMPGWTGEQCQLRDFCSANPCVNGGVCLATYPQIQC
HCPPGFEGHACERDVNECFQDPGPCPKGTSCHNTLGSFQCLCPVGQEGPRCELRAGPCPP
RGCSNGGTCQLMPEKDSTFHLCLCPPGFIGPDCEVNPDNCVSHQCQNGGTCQDGLDTYTC
LCPETWTGWDCSEDVDECETQGPPHCRNGGTCQNSAGSFHCVCVSGWGGTSCEENLDDCI
AATCAPGSTCIDRVGSFSCLCPPGRTGLLCHLEDMCLSQPCHGDAQCSTNPLTGSTLCLC
QPGYSGPTCHQDLDECLMAQQGPSPCEHGGSCLNTPGSFNCLCPPGYTGSRCEADHNECL
SQPCHPGSTCLDLLATFHCLCPPGLEGQLCEVETNECASAPCLNHADCHDLLNGFQCICL
PGFSGTRCEEDIDECRSSPCANGGQCQDQPGAFHCKCLPGFEGPRCQTEVDECLSDPCPV
GASCLDLPGAFFCLCPSGFTGQLCEVPLCAPNLCQPKQICKDQKDKANCLCPDGSPGCAP
PEDNCTCHHGHCQRSSCVCDVGWTGPECEAELGGCISAPCAHGGTCYPQPSGYNCTCPTG
YTGPTCSEEMTACHSGPCLNGGSCNPSPGGYYCTCPPSHTGPQCQTSTDYCVSAPCFNGG

TCVNRPGTFSCLCAMGFQGPRCEGKLRPSCADSPCRNRATCQDSPQGPRCLCPTGYTGGS
CQTLMDLCAQKPCPRNSHCLQTGPSFHCLCLQGWTGPLCNLPLSSCQKAALSQGIDVSSL
CHNGGLCVDSGPSYFCHCPPGFQGSLCQDHVNPCESRPCQNGATCMAQPSGYLCQCAPGY
DGQNCSKELDACQSQPCHNHGTCTPKPGGFHCACPPGFVGLRCEGDVDECLDQPCHPTGT
AACHSLANAFYCQCLPGHTGQWCEVEIDPCHSQPCFHGGTCEATAGSPLGFICHCPKGFE
GPTCSHRAPSCGFHHCHHGGLCLPSPKPGFPPRCACLSGYGGPDCLTPPAPKGCGPPSPC
LYNGSCSETTGLGGPGFRCSCPHSSPGPRCQKPGAKGCEGRSGDGACDAGCSGPGGNWDG
GDCSLGVPDPWKGCPSHSRCWLLFRDGQCHPQCDSEECLFDGYDCETPPACTPAYDQYCH
DHFHNGHCEKGCNTAECGWDGGDCRPEDGDPEWGPSLALLVVLSPPALDQQLFALARVLS
LTLRVGLWVRKDRDGRDMVYPYPGARAEEKLGGTRDPTYQERAAPQTQPLGKETDSLSAG
FVVVMGVDLSRCGPDHPASRCPWDPGLLLRFLAAMAAVGALEPLLPGPLLAVHPHAGTAP
PANQLPWPVLCSPVAGVILLALGALLVLQLIRRRRREHGALWLPPGFTRRPRTQSAPHRR
RPPLGEDSIGLKALKPKAEVDEDGVVMCSGPEEGEEVGQAEETGPPSTCQLWSLSGGCGA
LPQAAMLTPPQESEMEAPDLDTRGPDGVTPLMSAVCCGEVQSGTFQGAWLGCPEPWEPLL
DGGACPQAHTVGTGETPLHLAARFSRPTAARRLLEAGANPNQPDRAGRTPLHAAVAADAR
EVCQLLLRSRQTAVDARTEDGTTPLMLAARLAVEDLVEELIAAQADVGARDKWGKTALHW
AAAVNNARAARSLLQAGADKDAQDNREQTPLFLAAREGAVEVAQLLLGLGAARELRDQAG
LAPADVAHQRNHWDLLTLLEGAGPPEARHKATPGREAGPFPRARTVSVSVPPHGGGALPR
CRTLSAGAGPRGGGACLQARTWSVDLAARGGGAYSHCRSLSGVGAGGGPTPRGRRFSAGM
RGPRPNPAIMRGRYGVAAGRGGRVSTDDWPCDWVALGACGSASNIPIPPPCLTPSPERGS
PQLDCGPPALQEMPINQGGEGKK*
>ENSG00000198794|ENST00000361900
MAEKVNNFPPLPKFIPLKPCFYQDFEADIPPQHVSMTKRLYYLWMLNSVTLAVNLVGCLA
WLIGGGGATNFGLAFLWLILFTPCSYVCWFRPIYKAFKTDSSFSFMAFFFTFMAQLVISI
IQAVGIPGWGVCGWIATISFFGTNIGSAVVMLIPTVMFTVMAVFSFIALSMVHKFYRGSG
GSFSKAQEEWTTGAWKNPHVQQAAQNAAMGAAQGAMNQPQTQYSATPNYTYSNEM*
>ENSG00000126231|ENST00000375547
MAGCVPLLQGLVLVLALHRVEPSVFLPASKANDVLVRWKRAGSYLLEELFEGNLEKECYE
EICVYEEAREVFENEVVTDEFWRRYKGGSPCISQPCLHNGSCQDSIWGYTCTCSPGYEGS
NCELAKNECHPERTDGCQHFCLPGQESYTCSCAQGYRLGEDHKQCVPHDQCACGVLTSEK
RAPDLQDLPWQVKLTNSEGKDFCGGVIIRENFVLTTAKCSLLHRNITVKTYFNRTSQDPL
MIKITHVHVHMRYDADAGENDLSLLELEWPIQCPGAGLPVCTPEKDFAEHLLIPRTRGLL
SGWARNGTDLGNSLTTRPVTLVEGEECGQVLNVTVTTRTYCERSSVAAMHWMDGSVVTRE
HRGSWFLTGVLGSQPVGGQAHMVLVTKVSRYSLWFKQIMN*
>ENSG00000138069|ENST00000398529
MSSMNPEYDYLFKLLLIGDSGVGKSCLLLRFADDTYTESYISTIGVDFKIRTIELDGKTI
KLQIEFADSLGIPFLETSAKNATNVEQSFMTMAAEIKKRMGPGATAGGAEKSNVKIQSTP
VKQSGGGCC*
>ENSG00000143578|ENST00000368603
MDLGIPDLLDAWLEPPEDIFSTGSVLELGLHCPPPEVPVTRLQEQGLQGWKSGGDRGCGL
QESEPEDFLKLFIDPNEVYCSEASPGSDSGISEDPCHPDSPPAPRATSSPMLYEVVYEAG
ALERMQGETGPNVGLISIQLDQWSPAFMVPDSCMVSELPFDAHAHILPRAGTVAPVPCTT
LLPCQTLFLTDEEKRLLGQEGVSLPSHLPLTKAEERVLKKVRRKIRNKQSAQDSRRRKKE
YIDGLESRVAACSAQNQELQKKVQELERHNISLVAQLRQLQTLIAQTSNKAAQTSTCVLI

LLFSLALIILPSFSPFQSRPEAGSEDYQPHGVTSRNILTHKDVTENLETQVVESRLREPP
GAKDANGSTRTLLEKMGGKPRPSGRIRSVLHADEM*
>ENSG00000118217|ENST00000367942
MGEPAGVAGTMESPFSPGLFHRLDEDWDSALFAELGYFTDTDELQLEAANETYENNFDNL
DFDLDLMPWESDIWDINNQICTVKDIKAEPQPLSPASSSYSVSSPRSVDSYSSTQHVPEE
LDLSSSSQMSPLSLYGENSNSLSSAEPLKEDKPVTGPRNKTENGLTPKKKIQVNSKPSIQ
PKPLLLPAAPKTQTNSSVPAKTIIIQTVPTLMPLAKQQPIISLQPAPTKGQTVLLSQPTV
VQLQAPGVLPSAQPVLAVAGGVTQLPNHVVNVVPAPSANSPVNGKLSVTKPVLQSTMRNV
GSDIAVLRRQQRMIKNRESACQSRKKKKEYMLGLEARLKAALSENEQLKKENGTLKRQLD
EVVSENQRLKVPSPKRRVVCVMIVLAFIILNYGPMSMLEQDSRRMNPSVSPANQRRHLLG
FSAKEAQDTSDGIIQKNSYRYDHSVSNDKALMVLTEEPLLYIPPPPCQPLINTTESLRLN
HELRGWVHRHEVERTKSRRMTNNQQKTRILQGALEQGSNSQLMAVQYTETTSSISRNSGS
ELQVYYASPRSYQDFFEAIRRRGDTFYVVSFRRDHLLLPATTHNKTTRPKMSIVLPAINI
NENVINGQDYEVMMQIDCQVMDTRILHIKSSSVPPYLRDQQRNQTNTFFGSPPAATEATH
VVSTIPESLQ*
>ENSG00000163539|ENST00000359576
MEPRSMEYFCAQVQQKDVGGRLQVGQELLLYLGAPGAISDLEEDLGRLGKTVDALTGWVG
SSNYRVSLMGLEILSAFVDRLSTRFKSYVAMVIVALIDRMGDAKDKVRDEAQTLILKLMD
QVAPPMYIWEQLASGFKHKNFRSREGVCLCLIETLNIFGAQPLVISKLIPHLCILFGDSN
SQVRDAAILAIVEIYRHVGEKVRMDLYKRGIPPARLEMIFAKFDEVQSSGGMILSVCKDK
SFDDEESVDGNRPSSAASAFKVPAPKTSGNPANSARKPGSAGGPKVGAGASKEGGAGAVD
EDDFIKAFTDVPSIQIYSSRELEETLNKIREILSDDKHDWDQRANALKKIRSLLVAGAAQ
YDCFFQHLRLLDGALKLSAKDLRSQVVREACITVAHLSTVLGNKFDHGAEAIVPTLFNLV
PNSAKVMATSGCAAIRFIIRHTHVPRLIPLITSNCTSKSVPVRRRSFEFLDLLLQEWQTH
SLERHAAVLVETIKKGIHDADAEARVEARKTYMGLRNHFPGEAETLYNSLEPSYQKSLQT
YLKSSGSVASLPQSDRSSSSSQESLNRPFSSKWSTANPSTVAGRVSAGSSKASSLPGSLQ
RSRSDIDVNAAAGAKAHHAAGQSVRSGRLGAGALNAGSYASLEDTSDKLDGTASEDGRVR
AKLSAPLAGMGNAKADSRGRSRTKMVSQSQPGSRSGSPGRVLTTTALSTVSSGVQRVLVN
SASAQKRSKIPRSQGCSREASPSRLSVARSSRIPRPSVSQGCSREASRESSRDTSPVRSF
QPLGPGYGISQSSRLSSSVSAMRVLNTGSDVEEAVADALLLGDIRTKKKPARRRYESYGM
HSDDDANSDASSACSERSYSSRNGSIPTYMRQTEDVAEVLNRCASSNWSERKEGLLGLQN
LLKNQRTLSRVELKRLCEIFTRMFADPHGKRVFSMFLETLVDFIQVHKDDLQDWLFVLLT
QLLKKMGADLLGSVQAKVQKALDVTRESFPNDLQFNILMRFTVDQTQTPSLKVKVAILKY
IETLAKQMDPGDFINSSETRLAVSRVITWTTEPKSSDVRKAAQSVLISLFELNTPEFTML
LGALPKTFQDGATKLLHNHLRNTGNGTQSSMGSPLTRPTPRSPANWSSPLTSPTNTSQNT
LSPSAFDYDTENMNSEDIYSSLRGVTEAIQNFSFRSQEDMNEPLKRDSKKDDGDSMCGGP
GMSDPRAGGDATDSSQTALDNKASLLHSMPTHSSPRSRDYNPYNYSDSISPFNKSALKEA
MFDDDADQFPDDLSLDHSDLVAELLKELSNHNERVEERKIALYELMKLTQEESFSVWDEH
FKTILLLLLETLGDKEPTIRALALKVLREILRHQPARFKNYAELTVMKTLEAHKDPHKEV
VRSAEEAASVLATSISPEQCIKVLCPIIQTADYPINLAAIKMQTKVIERVSKETLNLLLP
EIMPGLIQGYDNSESSVRKACVFCLVAVHAVIGDELKPHLSQLTGSKMKLLNLYIKRAQT
GSGGADPTTDVSGQS*
>ENSG00000158488|ENST00000368164
MLLLFLLFEGLCCPGENTAVKPEAWLSCGPSPGPGRLQLVCHVSGFYPKPVWVMWMRGEQ

EQRGTQRGDVLPNADETWWIFHLSHPDLFDCDSYPGHIGCS*
>ENSG00000106086|ENST00000396257
MEGVLYKWTNYLSGWQPRWFLLCGGILSYYDSPEDAWKGCKGSIQMAVCEIQVHSVDNTR
MDLIIPGEQYFYLKARSVAERQRWLVALGSAKACLTDSRTQKEKEFAENTENLKTKMSEL
RLYCDLLVQQVDKTKEVTTTGVSNSEEGIDVGTLLKSTCNTFLKTLEECMQIANAAFTSE
LLYRTPPGSPQLAMLKSSKMKHPIIPIHNSLERQMELSTCENGSLNMEINGEEEILMKNK
NSLYLKSAEIDCSISSEENTDDNITVQGEIRKEDGMENLKNHDNNLTQSGSDSSCSPECL
WEEGKEVIPTFFSTMNTSFSDIELLEDSGIPTEAFLASCYAVVPVLDKLGPTVFAPVKMD
LVGNIKKVNQKYITNKEEFTTLQKIVLHEVEADVAQVRNSATEALLWLKRGLKFLKGFLT
EVKNGEKDIQTALNNAYGKTLRQHHGWVVRGVFAV*
>ENSG00000011009|ENST00000374514
MCGNTMSVPLLTDAATVSGAERETAAVIFLHGLGDTGHSWADALSTIRLPHVKYICPHAP
RIPVTLNMKMVMPSWFDLMGLSPDAPEDEAGIKKAAENIKALIEHEMKNGIPANRIVLGG
FSQGGALSLYTALTCPHPLAGIVALSCWLPLHRAFPQAANGSAKDLAILQCHGELDPMVP
VRFGALTAEKLRSVVTPARVQFKTYPGVMHSSCPQEMAAVKEFLEKLLPPV*
>ENSG00000116729|ENST00000370976
MAGAIIENMSTKKLCIVGGILLVFQIIAFLVGGLIGENAEVSMDVSLAYRDDAFAEWTEM
AHERVPRKLKCTFTSPKTPEHEGRYYECDVLPFMEIGSVAHKFYLLNIRLPVNEKKKINV
GIGEIKDIRLVGIHQNGGFTKVWFAMKTFLTPSIFIIMVWYWRRITMMSRPPVLLEKVIF
ALGISMTFINIPVEWFSIGFDWTWMLLFGDIRQGIFYAMLLSFWIIFCGEHMMDQHERNH
IAGYWKQVGPIAVGSFCLFIFDMCERGVQLTNPFYSIWTTDIGTELAMAFIIVAGICLCL
YFLFLCFMVFQVFRNISGKQSSLPAMSKVRRLHYEGLIFRFKFLMLITLACAAMTVIFFI
VSQVTEGHWKWGGVTVQVNSAFFTGIYGMWNLYVFALMFLYAPSHKNYGEDQSNGDLGVH
SGEELQLTTTITHVDGPTEIYKLTRKEAQE*
>ENSG00000249967|ENST00000370649
MLGPQVWSSVRQGLSRSLSRNVGVWASGEGKKVDIAGIYPPVTTPFTATAEVDYGKLEEN
LHKLGTFPFRGAVGGVCALANVLGAQVCQLERLCCTGQWEDAQKLQHRLIEPNAARIIAV
FKPKNEEPYGHLNPKWTKWLQKLCCPCCFGRDCLVLNQGYLSEAGASLVDQKLELNIVPR
TKVVYLASETFNYSAIDRVKSRGKRLALEKVPKVGQRFNRIGLPPKVGSFQLFVEGYKDA
DYWLRRFEAEPLPENTNRQLLLQFERLVVLDYIIRNTDRGNDNWLIKYDCPMDSSSSRDT
DWVVVKEPVIKVAAIDNGLAFPLKHPDSWRAYPFYWAWLPQAKVPFSQEIKDLILPKISD
PNFVKDLEEDLYELFKKDPGFDRGQFHKQIAVMRGQILNLTQALKDNKSPLHLVQMPPVI
VETARSHQRSSSESYTQSFQSRKPFFSWW*
>ENSG00000085998|ENST00000371992
MDDWKPSPLIKPFGARKKRSWYLTWKYKLTNQRALRRFCQTGAVLFLLVTVIVNIKLILD
TRRAISEANEDPEPEQDYDEALGRLEPPRRRGSGPRRVLDVEVYSSRSKVYVAVDGTTVL
EDEAREQGRGIHVIVLNQATGHVMAKRVFDTYSPHEDEAMVLFLNMVAPGRVLICTVKDE
GSFHLKDTAKALLRSLGSQAGPALGWRDTWAFVGRKGGPVFGEKHSKSPALSSWGDPVLL
KTDVPLSSAEEAECHWADTELNRRRRRFCSKVEGYGSVCSCKDPTPIEFSPDPLPDNKVL
NVPVAVIAGNRPNYLYRMLRSLLSAQGVSPQMITVFIDGYYEEPMDVVALFGLRGIQHTP
ISIKNARVSQHYKASLTATFNLFPEAKFAVVLEEDLDIAVDFFSFLSQSIHLLEEDDSLY
CISAWNDQGYEHTAEDPALLYRVETMPGLGWVLRRSLYKEELEPKWPTPEKLWDWDMWMR
MPEQRRGRECIIPDVSRSYHFGIVGLNMNGYFHEAYFKKHKFNTVPGVQLRNVDSLKKEA
YEVEVHRLLSEAEVLDHSKNPCEDSFLPDTEGHTYVAFIRMEKDDDFTTWTQLAKCLHIW

DLDVRGNHRGLWRLFRKKNHFLMSEEATLSHPNFPGATPKGGGSPRSPRTDMRPPPGPCG
AGPGSESNLFIDCPEGLENRPNLEGLDFFLGWNAALRVGLALTQETAVPNPWTGPAGAHM
LTQTHSETLRHWTRPPLSLLFVQISKAG*
>ENSG00000206047|ENST00000382679
MRTLAILAAILLVALQAQAEPLQARADEVAAAPEQIAADIPEVVVSLAWDESLAPKHPGS
RKNMACYCRIPACIAGERRYGTCIYQGRLWAFCC*
>ENSG00000112493|ENST00000374572
MKSLSLLLAVALGLATAVSAGPAVIECWFVEDASGKGLAKRPGALLLRQGPGEPPPRPDL
DPELYLSVHDPAGALQAAFRRYPRGAPAPHCEMSRFVPLPASAKWASGLTPAQNCPRALD
GAWLMVSISSPVLSLSSLLRPQPEPQQEPVLITMATVVLTVLTHTPAPRVRLGQDALLDL
SFAYMPPTSEAASSLAPGPPPFGLEWRRQHLGKGHLLLAATPGLNGQMPAAQEGAVAFAA
WDDDEPWGPWTGNGTFWLPRVQPFQEGTYLATIHLPYLQGQVTLELAVYKPPKVSLMPAT
LARAAPGEAPPELLCLVSHFYPSGGLEVEWELRGGPGGRSQKAEGQRWLSALRHHSDGSV
SLSGHLQPPPVTTEQHGARYACRIHHPSLPASGRSAEVTLEVAGLSGPSLEDSVGLFLSA
FLLLGLFKALGWAAVYLSTCKDSKKKAE*
>ENSG00000177692|ENST00000381947
MNTMYVMMAQILRSHLIKATVIPNRVKMLPYFGIIRNRMMSTHKSKKKIREYYRLLNVEE
GCSADEVRESFHKLAKQYHPDSGSNTADSATFIRIEKAYRKVLSHVIEQTNASQSKGEEE
EDVEKFKYKTPQHRHYLSFEGIGFGTPTQREKHYRQFRADRAAEQVMEYQKQKLQSQYFP
DSVIVKNIRQSKQQKITQAIERLVEDLIQESMAKGDFDNLSGKGKPLKKFSDCSYIDPMT
HNLNRILIDNGYQPEWILKQKEISDTIEQLREAILVSRKKLGNPMTPTEKKQWNHVCEQF
QENIRKLNKRINDFNLIVPILTRQKVHFDAQKEIVRAQKIYETLIKTKEVTDRNPNNLDQ
GEGEKTPEIKKGFLNWMNLWKFIKIRSF*
>ENSG00000206452|ENST00000400394
MRVMAPRTLILLLSGALALTETWACSHSMRYFYTAVSRPGRGEPRFIAVGYVDDTQFVQF
DSDAASPRGEPRAPWVEQEGPEYWDRETQKYKRQAQTDRVNLRKLRGYYNQSEAGSHTLQ
RMYGCDLGPDGRLLRGYNQFAYDGKDYIALNEDLRSWTAADKAAQITQRKWEAAREAEQR
RAYLEGTCVEWLRRYLENGKKTLQRAEHPKTHVTHHPVSDHEATLRCWALGFYPAEITLT
WQRDGEDQTQDTELVETRPAGDGTFQKWAAVVVPSGEEQRYTCHVQHEGLPEPLTLRWGP
SSQPTIPIVGIVAGLAVLAVLAVLGAVMAVVMCRRKSSGGKGGSCSQAASSNSAQGSDES
LIACKA*
>ENSG00000110195|ENST00000393676
MAQRMTTQLLLLLVWVAVVGEAQTRIAWARTELLNVCMNAKHHKEKPGPEDKLHEQCRPW
RKNACCSTNTSQEAHKDVSYLYRFNWNHCGEMAPACKRHFIQDTCLYECSPNLGPWIQQV
DQSWRKERVLNVPLCKEDCEQWWEDCRTSYTCKSNWHKGWNWTSGFNKCAVGAACQPFHF
YFPTPTVLCNEIWTHSYKVSNYSRGSGRCIQMWFDPAQGNPNEEVARFYAAAMSGAGPWA
AWPFLLSLALMLLWLLS*
>ENSG00000158470|ENST00000371711
MRARRGLLRLPRRSLLAALFFFSLSSSLLYFVYVAPGIVNTYLFMMQAQGILIRDNVRTI
GAQVYEQVLRSAYAKRNSSVNDSDYPLDLNHSETFLQTTTFLPEDFTYFANHTCPERLPS
MKGPIDINMSEIGMDYIHELFSKDPTIKLGGHWKPSDCMPRWKVAILIPFRNRHEHLPVL
FRHLLPMLQRQRLQFAFYVVEQVGTQPFNRAMLFNVGFQEAMKDLDWDCLIFHDVDHIPE
SDRNYYGCGQMPRHFATKLDKYMYLLPYTEFFGGVSGLTVEQFRKINGFPNAFWGWGGED
DDLWNRVQNAGYSVSRPEGDTGKYKSIPHHHRGEVQFLGRYALLRKSKERQGLDGLNNLN

YFANITYDALYKNITVNLTPELAQVNEY*
>ENSG00000182533|ENST00000397368
MMAEEHTDLEAQIVKDIHCKEIDLVNRDPKNINEDIVKVDFEDVIAEPVGTYSFDGVWKV
SYTTFTVSKYWCYRLLSTLLGVPLALLWGFLFACISFCHIWAVVPCIKSYLIEIQCISHI
YSLCIRTFCNPLFAALGQVCSSIKVVLRKEV*
>ENSG00000124406|ENST00000381668
MPTMRRTVSEIRSRAEGYEKTDDVSEKTSLADQEEVRTIFINQPQLTKFCNNHVSTAKYN
IITFLPRFLYSQFRRAANSFFLFIALLQQIPDVSPTGRYTTLVPLLFILAVAAIKEIIED
IKRHKADNAVNKKQTQVLRNGAWEIVHWEKVAVGEIVKVTNGEHLPADLISLSSSEPQAM
CYIETSNLDGETNLKIRQGLPATSDIKDVDSLMRISGRIECESPNRHLYDFVGNIRLDGH
GTVPLGADQILLRGAQLRNTQWVHGIVVYTGHDTKLMQNSTSPPLKLSNVERITNVQILI
LFCILIAMSLVCSVGSAIWNRRHSGKDWYLNLNYGGASNFGLNFLTFIILFNNLIPISLL
VTLEVVKFTQAYFINWDLDMHYEPTDTAAMARTSNLNEELGQVKYIFSDKTGTLTCNVMQ
FKKCTIAGVAYGHVPEPEDYGCSPDEWQNSQFGDEKTFSDSSLLENLQNNHPTAPIICEF
LTMMAVCHTAVPEREGDKIIYQAASPDEGALVRAAKQLNFVFTGRTPDSVIIDSLGQEER
YELLNVLEFTSARKRMSVIVRTPSGKLRLYCKGADTVIYDRLAETSKYKEITLKHLEQFA
TEGLRTLCFAVAEISESDFQEWRAVYQRASTSVQNRLLKLEESYELIEKNLQLLGATAIE
DKLQDQVPETIETLMKADIKIWILTGDKQETAINIGHSCKLLKKNMGMIVINEGSLDGTR
ETLSRHCTTLGDALRKENDFALIIDGKTLKYALTFGVRQYFLDLALSCKAVICCRVSPLQ
KSEVVEMVKKQVKVVTLAIGDGANDVSMIQTAHVGVGISGNEGLQAANSSDYSIAQFKYL
KNLLMIHGAWNYNRVSKCILYCFYKNIVLYIIEIWFAFVNGFSGQILFERWCIGLYNVMF
TAMPPLTLGIFERSCRKENMLKYPELYKTSQNALDFNTKVFWVHCLNGLFHSVILFWFPL
KALQYGTAFGNGKTSDYLLLGNFVYTFVVITVCLKAGLETSYWTWFSHIAIWGSIALWVV
FFGIYSSLWPAIPMAPDMSGEAAMLFSSGVFWMGLLFIPVASLLLDVVYKVIKRTAFKTL
VDEVQELEAKSQDPGAVVLGKSLTERAQLLKNVFKKNHVNLYRSESLQQNLLHGYAFSQD
ENGIVSQSEVIRAYDTTKQRPDEW*
>ENSG00000085063|ENST00000395850
MGIQGGSVLFGLLLVLAVFCHSGHSLQCYNCPNPTADCKTAVNCSSDFDACLITKAGLQV
YNKCWKFEHCNFNDVTTRLRENELTYYCCKKDLCNFNEQLENGGTSLSEKTVLLLVTPFL
AAAWSLHP*
>ENSG00000133424|ENST00000402320
MLGICRGRRKFLAASLSLLCIPAITWIYLFSGSFEDGKPVSLSPLESQAHSPRYTASSQR
ERESLEVRMREVEEENRALRRQLSLAQGRAPSHRRGNHSKTYSMEEGTGDSENLRAGIVA
GNSSECGQQPVVEKCETIHVAIVCAGYNASRDVVTLVKSVLFHRRNPLHFHLIADSIAEQ
ILATLFQTWMVPAVRVDFYNADELKSEVSWIPNKHYSGIYGLMKLVLTKTLPANLERVIV
LDTDITFATDIAELWAVFHKFKGQQVLGLVENQSDWYLGNLWKNHRPWPALGRGYNTGVI
LLLLDKLRKMKWEQMWRLTAERELMGMLSTSLADQDIFNAVIKQNPFLVYQLPCFWNVQL
SDHTRSEQCYRDVSDLKLQKQLSELDEDDLCYEFRRERFTVHRTHLYFLHYEYEPAADST
DVTLVAQLSMDRLQMLEAICKHWEGPISLALYLSDAEAQQFLRYAQGSEVLMSRHNVGYH
IVYKEGQFYPVNLLRNVAMKHISTPYMFLSDIDFLPMYGLYEYLRKSVIQLDLANTKKAM
IVPAFETLRYRLSFPKSKAELLSMLDMGTLFTFRYHVWTKGHAPTNFAKWRTATTPYRVE
WEADFEPYVVVRRDCPEYDRRFVGFGWNKVAHIMELDVQEYEFIVLPNAYMIHMPHAPSF
DITKFRSNKQYRICLKTLKEEFQQDMSRRYGFAALKYLTAENNS*
>ENSG00000126091|ENST00000353126

MGLLVFVRNLLLALCLFLVLGFLYYSAWKLHLLQWEEDSNSVVLSFDSAGQTLGSEYDRL
GFLLNLDSKLPAELATKYANFSEGACKPGYASALMTAIFPRFSKPAPMFLDDSFRKWARI
REFVPPFGIKGQDNLIKAILSVTKEYRLTPALDSLRCRRCIIVGNGGVLANKSLGSRIDD
YDIVVRLNSAPVKGFEKDVGSKTTLRITYPEGAMQRPEQYERDSLFVLAGFKWQDFKWLK
YIVYKERVSWTHNIQREKEFLRKLVKARVITDLSSGI*
>ENSG00000143801|ENST00000366783
MLTFMASDSEEEVCDERTSLMSAESPTPRSCQEGRQGPEDGENTAQWRSQENEEDGEEDP
DRYVCSGVPGRPPGLEEELTLKYGAKHVIMLFVPVTLCMIVVVATIKSVRFYTEKNGQLI
YTPFTEDTPSVGQRLLNSVLNTLIMISVIVVMTIFLVVLYKYRCYKFIHGWLIMSSLMLL
FLFTYIYLGEVLKTYNVAMDYPTLLLTVWNFGAVGMVCIHWKGPLVLQQAYLIMISALMA
LVFIKYLPEWSAWVILGAISVYDLVAVLCPKGPLRMLVETAQERNEPIFPALIYSSAMVW
TVGMAKLDPSSQGALQLPYDPEMEEDSYDSFGEPSYPEVFEPPLTGYPGEELEEEEERGV
KLGLGDFIFYSVLVGKAAATGSGDWNTTLACFVAILIGLCLTLLLLAVFKKALPALPISI
TFGLIFYFSTDNLVRPFMDTLASHQLYI*
>ENSG00000147642|ENST00000399066
MSNFEKEHRVQHHDKEISRSRIPRLILRPHMPQQQHKVSPASESPFSEEESREFNPSSSG
RSARTVSSNSFCSDDTGCPSSQSVSPVKTPSDAGNSPIGFCPGSDEGFTRKKCTIGMVGE
GSIQSSRYKKESKSGLVKPGSEADFSSSSSTGSISAPEVHMSTAGSKRSSSSRNRGPHGR
SNGASSHKPGSSPSSPREKDLLSMLCRNQLSPVNIHPSYAPSSPSSSNSGSYKGSDCSPI
MRRSGRYMSCGENHGVRPPNPEQYLTPLQQKEVTVRHLKTKLKESERRLHERESEIVELK
SQLARMREDWIEEECHRVEAQLALKEARKEIKQLKQVIETMRSSLADKDKGIQKYFVDIN
IQNKKLESLLQSMEMAHSGSLRDELCLDFPCDSPEKSLTLNPPLDTMADGLSLEEQVTGE
GADRELLVGDSIANSTDLFDEIVTATTTESGDLELVHSTPGANVLELLPIVMGQEEGSVV
VERAVQTDVVPYSPAISELIQSVLQKLQDPCPSSLASPDESEPDSMESFPESLSALVVDL
TPRNPNSAILLSPVETPYANVDAEVHANRLMRELDFAACVEERLDGVIPLARGGVVRQYW
SSSFLVDLLAVAAPVVPTVLWAFSTQRGGTDPVYNIGALLRGCCVVALHSLRRTAFRIKT
*
>ENSG00000152683|ENST00000379343
MGTIHLFRKPQRSFFGKLLREFRLVAADRRSWKILLFGVINLICTGFLLMWCSSTNSIAL
TAYTYLTIFDLFRDGVSPFWLGWSQTPDLKWSTHLGLPKCWDNRRELPCLSNSLMTCLIS
YWVTLRKPSPVYSFGFERLEVLAVFASTVLAQLGALFILKESAERFLEQPEIHTGRLLVG
TFVALCFNLFTMLSIRNKPFAYVSEAASTSWLQEHVADLSRSLCGIIPGLSSIFLPRMNP
FVLIDLAGAFALCITYMLIEINNYFAVDTASAIAIALMTFGTMYPMSVYSGKVLLQTTPP
HVIGQLDKLIREVSTLDGVLEVRNEHFWTLGFGSLAGSVHVRIRRDANEQMVLAHVTNRL
YTLVSTLTVQIFKDDWIRPALLSGPVAANVLNFSDHHVIPMPLLKGTDDLNPVTSTPAKP
SSPPPEFSFNTPGKNVNPVILLNTQTRPYGFGLNHGHTPYSSMLNQGLGVPGIGATQGLR
TGFTNIPSRYGTNNRIGQPRP*
>ENSG00000117620|ENST00000370155
MFANLKYVSLGILVFQTTSLVLTMRYSRTLKEEGPRYLSSTAVVVAELLKIMACILLVYK
DSSRM*
>ENSG00000155957|ENST00000358230
MADPDPRYPRSSIEDDFNYGSSVASATVHIRMAFLRKVYSILSLQVLLTTVTSTVFLYFE
SVRTFVHESPALILLFALGSLGLIFALILNRHKYPLNLYLLFGFTLLEALTVAVVVTFYD
VYIILQAFILTTTVFFGLTVYTLQSKKDFSKFGAGLFALLWILCLSGFLKFFFYSEIMEL

VLAAAGALLFCGFIIYDTHSLMHKLSPEEYVLAAISLYLDIINLFLHLLRFLEAVNKK*
>ENSG00000109956|ENST00000392580
MPKRRDILAIVLIVLPWTLLITVWHQSTLAPLLAVHKDEGSDPRRETPPGADPREYCTSD
RDIVEVVRTEYVYTRPPPWSDTLPTIHVVTPTYSRPVQKAELTRMANTLLHVPNLHWLVV
EDAPRRTPLTARLLRDTGLNYTHLHVETPRNYKLRGDARDPRIPRGTMQRNLALRWLRET
FPRNSSQPGVVYFADDDNTYSLELFEEMRSTRRVSVWPVAFVGGLRYEAPRVNGAGKVVG
WKTVFDPHRPFAIDMAGFAVNLRLILQRSQAYFKLRGVKGGYQESSLLRELVTLNDLEPK
AANCTKILVWHTRTEKPVLVNEGKKGFTDPSVEI*
>ENSG00000070610|ENST00000378094
MGTQDPGNMGTGVPASEQISCAKEDPQVYCPEETGGTKDVQVTDCKSPEDSRPPKETDCC
NPEDSGQLMVSYEGKAMGYQVPPFGWRICLAHEFTEKRKPFQANNVSLSNMIKHIGMGLR
YLQWWYRKTHVEKKTPFIDMINSVPLRQIYGCPLGGIGGGTITRGWRGQFCRWQLNPGMY
QHRTVIADQFTVCLRREGQTVYQQVLSLERPSVLRSWNWGLCGYFAFYHALYPRAWTVYQ
LPGQNVTLTCRQITPILPHDYQDSSLPVGVFVWDVENEGDEALDVSIMFSMRNGLGGGDD
APGGLWNEPFCLERSGETVRGLLLHHPTLPNPYTMAVAARVTAATTVTHITAFDPDSTGQ
QVWQDLLQDGQLDSPTGQSTPTQKGVGIAGAVCVSSKLRPRGQCRLEFSLAWDMPRIMFG
AKGQVHYRRYTRFFGQDGDAAPALSHYALCRYAEWEERISAWQSPVLDDRSLPAWYKSAL
FNELYFLADGGTVWLEVLEDSLPEELGRNMCHLRPTLRDYGRFGYLEGQEYRMYNTYDVH
FYASFALIMLWPKLELSLQYDMALATLREDLTRRRYLMSGVMAPVKRRNVIPHDIGDPDD
EPWLRVNAYLIHDTADWKDLNLKFVLQVYRDYYLTGDQNFLKDMWPVCLAVMESEMKFDK
DHDGLIENGGYADQTYDGWVTTGPSAYCGGLWLAAVAVMVQMAALCGAQDIQDKFSSILS
RGQEAYERLLWNGRYYNYDSSSRPQSRSVMSDQCAGQWFLKACGLGEGDTEVFPTQHVVR
ALQTIFELNVQAFAGGAMGAVNGMQPHGVPDKSSVQSDEVWVGVVYGLAATMIQELLPSG
FCLWVIVISSTCWELLEGKDSTASIYPVEVALQRVPS*
>ENSG00000137486|ENST00000360025
MGDKGTRVFKKASPNGKLTVYLGKRDFVDHIDLVDPVDGVVLVDPEYLKERRVYVTLTCA
FRYGREDLDVLGLTFRKDLFVANVQSFPPAPEDKKPLTRLQERLIKKLGEHAYPFTFEIP
PNLPCSVTLQPGPEDTGKACGVDYEVKAFCAENLEEKIHKRNSVRLVIRKVQYAPERPGP
QPTAETTRQFLMSDKPLHLEASLDKEIYYHGEPISVNVHVTNNTNKTVKKIKISVRQYAD
ICLFNTAQYKCPVAMEEADDTVAPSSTFCKVYTLTPFLANNREKRGLALDGKLKHEDTNL
ASSTLLREGANREILGIIVSYKVKVKLVVSRGGDVAVELPFTLMHPKPKEEPPHREVPEN
ETPVDTNLIELDTNDDDIVFEDFARQRLKGMKDDKEEEEDGTGSPQLNNR*
>ENSG00000169255|ENST00000392781
MASALWTVLPSRMSLRSLKWSLLLLSLLSFFVMWYLSLPHYNVIERVNWMYFYEYEPIYR
QDFHFTLREHSNCSHQNPFLVILVTSHPSDVKARQAIRVTWGEKKSWWGYEVLTFFLLGQ
EAEKEDKMLALSLEDEHLLYGDIIRQDFLDTYNNLTLKTIMAFRWVTEFCPNAKYVMKTD
TDVFINTGNLVKYLLNLNHSEKFFTGYPLIDNYSYRGFYQKTHISYQEYPFKVFPPYCSG
LGYIMSRDLVPRIYEMMGHVKPIKFEDVYVGICLNLLKVNIHIPEDTNLFFLYRIHLDVC
QLRRVIAAHGFSSKEIITFWQVMLRNTTCHY*
>ENSG00000118508|ENST00000367495
MAGGGAGDPGLGAAAAPAPETREHLFKVLVIGELGVGKTSIIKRYVHQLFSQHYRATIGV
DFALKVLNWDSRTLVRLQLWDIAGQERFGNMTRVYYKEAVGAFVVFDISRSSTFEAVLKW
KSDLDSKVHLPNGSPIPAVLLANKCDQNKDSSQSPSQVDQFCKEHGFAGWFETSAKDNIN
IEEAARFLVEKILVNHQSFPNEENDVDKIKLDQETLRAENKSQCC*

>ENSG00000169826|ENST00000374466
MPRRGLILHTRTHWLLLGLALLCSLVLFMYLLECAPQTDGNASLPGVVGENYGKEYYQAL
LQEQEEHYQTRATSLKRQIAQLKQELQEMSEKMRSLQERRNVGANGIGYQSNKEQAPSDL
LEFLHSQIDKAEVSIGAKLPSEYGVIPFESFTLMKVFQLEMGLTRHPEEKPVRKDKRDEL
VEVIEAGLEVINNPDEDDEQEDEEGPLGEKLIFNENDFVEGYYRTERDKGTQYELFFKKA
DLTEYRHVTLFRPFGPLMKVKSEMIDITRSIINIIVPLAERTEAFVQFMQNFRDVCIHQD
KKIHLTVVYFGKEGLSKVKSILESVTSESNFHNYTLVSLNEEFNRGRGLNVGARAWDKGE
VLMFFCDVDIYFSAEFLNSCRLNAEPGKKVFYPVVFSLYNPAIVYANQEVPPPVEQQLVH
KKDSGFWRDFGFGMTCQYRSDFLTIGGFDMEVKGWGGEDVHLYRKYLHGDLIVIRTPVPG
LFHLWHEKRCADELTPEQYRMCIQSKAMNEASHSHLGMLVFREEIETHLHKQAYRTNSEA
VG*
>ENSG00000161395|ENST00000378011
MAGLAARLVLLAGAAALASGSQGDREPVYRDCVLQCEEQNCSGGALNHFRSRQPIYMSLA
GWTCRDDCKYECMWVTVGLYLQEGHKVPQFHGKVSLNAWFWSTVFHTRDTDLTEKMDYFC
ASTVILHSIYLCCVRTVGLQHPAVVSAFRALLLLMLTVHVSYLSLIRFDYGYNLVANVAI
GLVNVVWWLAWCLWNQRRLPHVRKCVVVVLLLQGLSLLELLDFPPLFWVLDAHAIWHIST
IPVHVLFFSFLEDDSLYLLKESEDKFKLD*
>ENSG00000165905|ENST00000401752
MLPRGRPRALGAAALLLLLLLLGFLLFGGDLGCERREPGGRAGAPGCFPGPLMPRVPPDG
RLRRAAALDGDPGAGPGDHNRSDCGPQPPPPPKCELLHVAIVCAGHNSSRDVITLVKSML
FYRKNPLHLHLVTDAVARNILETLFHTWMVPAVRVSFYHADQLKPQVSWIPNKHYSGLYG
LMKLVLPSALPAELARVIVLDTDVTFASDISELWALFAHFSDTQAIGLVENQSDWYLGNL
WKNHRPWPALGRGFNTGVILLRLDRLRQAGWEQMWRLTARRELLSLPATSLADQDIFNAV
IKEHPGLVQRLPCVWNVQLSDHTLAERCYSEASDLKVIHWNSPKKLRVKNKHVEFFRNFY
LTFLEYDGNLLRRELFVCPSQPPPGAEQLQQALAQLDEEDPCFEFRQQQLTVHRVHVTFL
PHEPPPPRPHDVTLVAQLSMDRLQMLEALCRHWPGPMSLALYLTDAEAQQFLHFVEASPV
LAARQDVAYHVVYREGPLYPVNQLRNVALAQALTPYVFLSDIDFLPAYSLYDYLRASIEQ
LGLGSRRKAALVVPAFETLRYRFSFPHSKVELLALLDAGTLYTFRYHEWPRGHAPTDYAR
WREAQAPYRVQWAANYEPYVVVPRDCPRYDPRFVGFGWNKVAHIVELDAQEYELLVLPEA
FTIHLPHAPSLDISRFRSSPTYRDCLQALKDEFHQDLSRHHGAAALKYLPALQQPQSPAR
G*
>ENSG00000128294|ENST00000398110
MRLSVRRVLLAAGCALVLVLAVQLGQQVLECRAVLAGLRSPRGAMRPEQEELVMVGTNHV
EYRYGKAMPLIFVGGVPRSGTTLMRAMLDAHPEVRCGEETRIIPRVLAMRQAWSKSGREK
LRLDEAGVTDEVLDAAMQAFILEVIAKHGEPARVLCNKDPFTLKSSVYLSRLFPNSKFLL
MVRDGRASVHSMITRKVTIAGFDLSSYRDCLTKWNKAIEVMYAQCMEVGKEKCLPVYYEQ
LVLHPRRSLKLILDFLGIAWSDAVLHHEDLIGKPGGVSLSKIERSTDQVIKPVNLEALSK
WTGHIPGDVVRDMAQIAPMLAQLGYDPYANPPNYGNPDPFVINNTQRVLKGDYKTPANLK
GYFQVNQNSTSSHLGSS*
>ENSG00000183778|ENST00000380620
MAFPKMRLMYICLLVLGALCLYFSMYSLNPFKEQSFVYKKDGNFLKLPDTDCRQTPPFLV
LLVTSSHKQLAERMAIRQTWGKERMVKGKQLKTFFLLGTTSSAAETKEVDQESQRHGDII
QKDFLDVYYNLTLKTMMGIEWVHRFCPQAAFVMKTDSDMFINVDYLTELLLKKNRTTRFF
TGFLKLNEFPIRQPFSKWFVSKSEYPWDRYPPFCSGTGYVFSGDVASQVYNVSKSVPYIK

LEDVFVGLCLERLNIRLEELHSQPTFFPGGLRFSVCLFRRIVACHFIKPRTLLDYWQALE
NSRGEDCPPV*
>ENSG00000144278|ENST00000392825
MRRFVYCKVVLATSLMWVLVDVFLLLYFSECNKCDDKKERSLLPALRAVISRNQEGPGEM
GKAVLIPKDDQEKMKELFKINQFNLMASDLIALNRSLPDVRLEGCKTKVYPDELPNTSVV
IVFHNEAWSTLLRTVYSVINRSPHYLLSEVILVDDASERDFLKLTLENYVKNLEVPVKII
RMEERSGLIRARLRGAAASKGQVITFLDAHCECTLGWLEPLLARIKEDRKTVVCPIIDVI
SDDTFEYMAGSDMTYGGFNWKLNFRWYPVPQREMDRRKGDRTLPVRTPTMAGGLFSIDRN
YFEEIGTYDAGMDIWGGENLEMSFRIWQCGGSLEIVTCSHVGHVFRKATPYTFPGGTGHV
INKNNRRLAEVWMDEFKDFFYIISPGVVKVDYGDVSVRKTLRENLKCKPFSWYLENIYPD
SQIPRRYYSLGEIRNVETNQCLDNMGRKENEKVGIFNCHGMGGNQVFSYTADKEIRTDDL
CLDVSRLNGPVIMLKCHHMRGNQLWEYDAERLTLRHVNSNQCLDEPSEEDKMVPTMQDCS
GSRSQQWLLRNMTLGT*
>ENSG00000164597|ENST00000393603
MGWVGGRRRDSASPPGRSRSAADDINPAPANMEGGGGSVAVAGLGARGSGAAAATVRELL
QDGCYSDFLNEDFDVKTYTSQSIHQAVIAEQLAKLAQGISQLDRELHLQVVARHEDLLAQ
ATGIESLEGVLQMMQTRIGALQGAVDRIKAKIVEPYNKIVARTAQLARLQVACDLLRRII
RILNLSKRLQGQLQGGSREITKAAQSLNELDYLSQGIDLSGIEVIENDLLFIARARLEVE
NQAKRLLEQGLETQNPTQVGTALQVFYNLGTLKDTITSVVDGYCATLEENINSALDIKVL
TQPSQSAVRGGPGRSTMPTPGNTAALRASFWTNMEKLMDHIYAVCGQVQHLQKVLAKKRD
PVSHICFIEEIVKDGQPEIFYTFWNSVTQALSSQFHMATNSSMFLKQAFEGEYPKLLRLY
NDLWKRLQQYSQHIQGNFNASGTTDLYVDLQHMEDDAQDIFIPKKPDYDPEKALKDSLQP
YEAAYLSKSLSRLFDPINLVFPPGGRNPPSSDELDGIIKTIASELNVAAVDTNLTLAVSK
NVAKTIQLYSVKSEQLLSTQGDASQVIGPLTEGQRRNVAVVNSLYKLHQSVTKVVSSQSS
FPLAAEQTIISALKAIHALMENAVQPLLTSVGDAIEAIIITMHQEDFSGSLSSSGKPDVP
CSLYMKELQGFIARVMSDYFKHFECLDFVFDNTEAIAQRAVELFIRHASLIRPLGEGGKM
RLAADFAQMELAVGPFCRRVSDLGKSYRMLRSFRPLLFQASEHVASSPALGDVIPFSIII
QFLFTRAPAELKSPFQRAEWSHTRFSQWLDDHPSEKDRLLLIR*
>ENSG00000206281|ENST00000383197
MKSLSLLLAVALGLATAVSAGPAVIECWFVEDASGKGLAKRPGALLLRQGPGEPPPRPDL
DPELYLSVHDPAGALQAAFRRYPRGAPAPHCEMSRFVPLPASAKWASGLTPAQNCPRALD
GAWLMVSISSPVLSLSSLLRPQPEPQQEPVLITMATVVLTVLTHTPAPRVRLGQDALLDL
SFAYMPPTSEAASSLAPGPPPFGLEWRRQHLGKGHLLLAATPGLNGQMPAAQEGAVAFAA
WDDDEPWGPWTGNGTFWLPRVQPFQEGTYLATIHLPYLQGQVTLELAVYKPPKVSLMPAT
LARAAPGEAPPELLCLVSHFYPSGGLEVEWELRGGPGGRSQKAEGQRWLSALRHHSDGSV
SLSGHLQPPPVTTEQHGARYACRIHHPSLPASGRSAEVTLEVAGLSGPSLEDSVGLFLSA
FLLLGLFKALGWAAVYLSTCKDSKKVQCSTSLYLSLVTLSPHPISKPMEGGCWCGRQNLG
LEFTLIWVKTWHYILTVGLFEHAT*
>ENSG00000158488|ENST00000368154
MLLLFLLFEGLCCPGENTAVKPEAWLSCGPSPGPGRLQLVCHVSGFYPKPVWVMWMRGGY
SIFLILICLTVIVTLVILVVVDSRLKKQSSNKNILSPHTPSPVFLMGANTQDTKNSRHQF
CLAQVSWIKNRVLKKWKTRLNQLW*
>ENSG00000110080|ENST00000392669
MVSKSRWKLLAMLALVLVVMVWYSISREDRYIELFYFPIPEKKEPCLQGEAESKASKLFG

NYSRDQPIFLRLEDYFWVKTPSAYELPYGTKGSEDLLLRVLAITSSSIPKNIQSLRCRRC
VVVGNGHRLRNSSLGDAINKYDVVIRLNNAPVAGYEGDVGSKTTMRLFYPESAHFDPKVE
NNPDTLLVLVAFKAMDFHWIETILSDKKRVRKGFWKQPPLIWDVNPKQIRILNPFFMEIA
ADKLLSLPMQQPRKIKQKPTTGLLAITLALHLCDLVHIAGFGYPDAYNKKQTIHYYEQIT
LKSMAGSGHNVSQEALAIKRMLEMGAIKNLTSF*
>ENSG00000129472|ENST00000397762
MTYAYLFKYIIIGDTGVGKSCLLLQFTDKRFQPVHDLTIGVEFGARMVNIDGKQIKLQIW
DTAGQESFRSITRSYYRGAAGALLVYDITRRETFNHLTSWLEDARQHSSSNMVIMLIGNK
SDLESRRDVKREEGEAFAREHGLIFMETSAKTACNVEEAFINTAKEIYRKIQQGLFDVHN
EANGIKIGPQQSISTSVGPSASQRNSRDIGSNSGCC*
>ENSG00000136631|ENST00000369128
MVYTQSEILQKEVYLFERIDSQNREIMKHLKAICFLRPTKENVDYIIQELRRPKYTIYFI
YFSNVISKSDVKSLAEADEQEVVAEVQQVITKEYELFEFRRTEVPPLLLILDRCDDAITP
LLNQWTYQAMVHELLGINNNRIDLSRVPGISKDLREVVLSAENDEFYANNMYLNFAEIGS
NIKNLMEDFQKKKPKEQQKLESIADMKAFVENYPQFKKMSGTVSKHVTVVGELSRLVSER
NLLEVSEVEQELACQNDHSSALQNIKRLLQNPKVTEFDAARLVMLYALHYERHSSNSLPG
LMMDLRNKGVSEKYRKLVSAVVEYGGKRVRGSDLFSPKDAVAITKQFLKGLKGVENVYTQ
HQPFLHETLDHLIKGRLKENLYPYLGPSTLRDRPQDIIVFVIGGATYEEALTVYNLNRTT
PGVRIVLGGTTVHNTKRDGVSLCSPAWFRTPGLKRSTRLSLPKCWDYSFPRGSSGFWTAQ
PKQGELSSHIKVSEQKMKRWLGEGHSFLSCPHYRFSLLNKGVGEQLWVLCWLLELISR*
>ENSG00000088833|ENST00000353088
MAAERQEALREFVAVTGAEEDRARFFLESAGWDLQIALASFYEDGGDEDIVTISQATPSS
VSRGTAPSDNRVTSFRDLIHDQDEDEEEEEGQRFYAGGSERSGQQIVGPPRKKSPNELVD
DLFKGAKEHGAVAVERVTKSPGETSKPRVHVVLKLWKSGFSLDNGELRSYQDPSNAQFLE
SIRRGEVPAELRRLAHGGQVNLDMEDHRDEDFVKPKGAFKAFTGEGQKLGSTAPQVLSTS
SPAQQAENEAKASSSILIDESEPTTNIQIRLADGGRLVQKFNHSHRISDIRLFIVDARPA
MAATSFILMTTFPNKELADESQTLKEANLLNAVIVQRLT*
>ENSG00000036054|ENST00000394144
MAEGEDVPPLPTSSGDGWEKDLEEALEAGGCDLETLRNIIQGRPLPADLRAKVWKIALNV
AGKGDSLASWDGILDLPEQNTIHKDCLQFIDQLSVPEEKAAELLLDIESVITFYCKSRNI
KYSTSLSWIHLLKPLVHLQLPRSDLYNCFYAIMNKYIPRDCSQKGRPFHLFRLLIQYHEP
ELCSYLDTKKITPDSYALNWLGSLFACYCSTEVTQAIWDGYLQQADPFFIYFLMLIILVN
AKEVILTQESDSKEEVIKFLENTPSSLNIEDIEDLFSLAQYYCSKTPASFRKDNHHLFGS
TLLGIKDDDADLSQALCLAISVSEILQANQLQGEGVRFFVVDCRPAEQYNAGHLSTAFHL
DSDLMLQNPSEFAQSVKSLLEAQKQSIESGSIAGGEHLCFMGSGREEEDMYMNMVLAHFL
QKNKEYVSIASGGFMALQQHLADINVDGPENGYGHWIASTSGSRSSINSVDGESPNGSSD
RGMKSLVNKMTVALKTKSVNVREKVISFIENTSTPVDRMSFNLPWPDRSCTERHVSSSDR
VGKPYRGVKPVFSIGDEEEYDTDEIDSSSMSDDDRKEVVNIQTWINKPDVKHHFPCKEVK
ESGHMFPSHLLVTATHMYCLREIVSRKGLAYIQSRQALNSVVKITSKKKHPELITFKYGN
SSASGIEILAIERYLIPNAGDATKAIKQQIMKVLDALES*
>ENSG00000109083|ENST00000395418
MAKDILGEAGLHFDELNKLRVLDPEVTQQTIELKEECKDFVDKIGQFQKIVGGLIELVDQ
LAKEAENEKMKAIGARNLLKSIAKQREAQQQQLQALIAEKKMQLERYRVEYEALCKVEAE
QNEFIDQFIFQK*

>ENSG00000215048|ENST00000399500
MMVLQVSAAPRTVALTALLMVLLTSVVQGRATPENYLFQGRQECYAFNGTQRFLERYIYN
REELVRFDSDVGEFRAVTELGRPEAEYWNSQKDILEEERAVPDRMCRHNYELGGPMTLQR
RVQPRVNVSPSKKGPLQHHNLLVCHVTDFYPGSIQVRWFLNGQEETAGVVSTNLIRNGDW
TFQILVMLEMTPQQGDVYTCQVEHTSLDSPVTVEWKAQSDSARSKTLTGAGGFVLGLIIC
GVGIFMHRRSKKVQRGSA*
>ENSG00000142192|ENST00000354192
MLPGLALLLLAAWTARALEVYPELQITNVVEANQPVTIQNWCKRGRKQCKTHPHFVIPYR
CLVGEFVSDALLVPDKCKFLHQERMDVCETHLHWHTVAKETCSEKSTNLHDYGMLLPCGI
DKFRGVEFVCCPLAEESDNVDSADAEEDDSDVWWGGADTDYADGSEDKVVEVAEEEEVAE
VEEEEADDDEDDEDGDEVEEEAEEPYEEATERTTSIATTTTTTTESVEEVVRVPTTAAST
PDAVDKYLETPGDENEHAHFQKAKERLEAKHRERMSQVMREWEEAERQAKNLPKADKKAV
IQHFQEKVESLEQEAANERQQLVETHMARVEAMLNDRRRLALENYITALQAVPPRPRHVF
NMLKKYVRAEQKDRQHTLKHFEHVRMVDPKKAAQIRSQVMTHLRVIYERMNQSLSLLYNV
PAVAEEIQDEVDELLQKEQNYSDDVLANMISEPRISYGNDALMPSLTETKTTVELLPVNG
EFSLDDLQPWHSFGADSVPANTENEVEPVDARPAADRGLTTRPGSGLTNIKTEEISEVKM
DAEFRHDSGYEVHHQKLVFFAEDVGSNKGAIIGLMVGGVVIATVIVITLVMLKKKQYTSI
HHGVVEVDAAVTPEERHLSKMQQNGYENPTYKFFEQMQN*
>ENSG00000188706|ENST00000357166
MSVMVVRKKVTRKWEKLPGRNTFCCDGRVMMARQKGIFYLTLFLILGTCTLFFAFECRYL
AVQLSPAIPVFAAMLFLFSMATLLRTSFSDPGVIPRALPDEAAFIEMEIEATNGAVPQGQ
RPPPRIKNFQINNQIVKLKYCYTCKIFRPPRASHCSICDNCVERFDHHCPWVGNCVGKRN
YRYFYLFILSLSLLTIYVFAFNIVYVALKSLKIGFLETLKETPGTVLEVLICFFTLWSVV
GLTGFHTFLVALNQTTNEDIKGSWTGKNRVQNPYSHGNIVKNCCEVLCGPLPPSVLDRRG
ILPLEESGSRPPSTQETSSSLLPQSPAPTEHLNSNEMPEDSSTPEEMPPPEPPEPPQEAA
EAEK*
>ENSG00000206506|ENST00000383621
MVVMAPRTLFLLLSGALTLTETWAGSHSMRYFSAAVSRPGRGEPRFIAMGYVDDTQFVRF
DSDSACPRMEPRAPWVEQEGPEYWEEETRNTKAHAQTDRMNLQTLRGYYNQSEASSHTLQ
WMIGCDLGSDGRLLRGYEQYAYDGKDYLALNEDLRSWTAADTAAQISKRKCEAANVAEQR
RAYLEGTCVEWLHRYLENGKEMLQRADPPKTHVTHHPVFDYEATLRCWALGFYPAEIILT
WQRDGEDQTQDVELVETRPAGDGTFQKWAAVVVPSGEEQRYTCHVQHEGLPEPLMLRWKQ
SSLPTIPIMGIVAGLVVLAAVVTGAAVAAVLWRKKSSD*
>ENSG00000089063|ENST00000379279
MMPSRTNLATGIPSSKVKYSRLSSTDDGYIDLQFKKTPPKIPYKAIALATVLFLIGAFLI
IIGSLLLSGYISKGGADRAVPVLIIGILVFLPGFYHLRIAYYASKGYRGYSYDDIPDFDD
*
>ENSG00000172780|ENST00000393304
MAGPGPGPGDPDEQYDFLFKLVLVGDASVGKTCVVQRFKTGAFSERQGSTIGVDFTMKTL
EIQGKRVKLQIWDTAGQERFRTITQSYYRSANGAILAYDITKRSSFLSVPHWIEDVRKYA
GSNIVQLLIGNKSDLSELREVSLAEAQSLAEHYDILCAIETSAKDSSNVEEAFLRVATEL
IMRHGGPLFSEKSPDHIQLNSKDIGEGWGCGC*
>ENSG00000165861|ENST00000394207
MAHSSFFPDEYFTCSSLCLSCGVGCKKSMNHGKEGVPHEAKSRCRYSHQYDNRVYTCKAC

YERGEEVSVVPKTSASTDSPWMGLAKYAWSGYVIECPNCGVVYRSRQYWFGNQDPVDTVV
RTEIVHVWPGTDGFLKDNNNAAQRLLDGMNFMAQSVSELSLGPTKAVTSWLTDQIAPAYW
RPNSQILSCNKCATSFKDNDTKHHCRACGEGFCDSCSSKTRPVPERGWGPAPVRVCDNCY
EARNVQLAVTEAQVDDEGGTLIARKVGEAVQNTLGAVVTAIDIPLGLVKDAARPAYWVPD
HEILHCHNCRKEFSIKLSKHHCRACGQGFCDECSHDRRAVPSRGWDHPVRVCFNCNKKPG
DL*
>ENSG00000126091|ENST00000372374
MGLLVFVRNLLLALCLFLVLGFLYYSAWKLHLLQWEEDSNSVVLSFDSAGQTLGSEYDRL
GFLLNLDSKLFSKPAPMFLDDSFRKWARIREFVPPFGIKGQDNLIKAILSVTKEYRLTPA
LDSLRCRRCIIVGNGGVLANKSLGSRIDDYDIVVRLNSAPVKGFEKDVGSKTTLRITYPE
GAMQRPEQYERDSLFVLAGFKWQDFKWLKYIVYKERVSASDGFWKSVATRVPKEPPEIRI
LNPYFIQEAAFTLIGLPFNNGLMGRGNIPTLGSVAVTMALHGCDEVAVAGFGYDMSTPNA
PLHYYETVRMAAIKESWTHNIQREKEFLRKLVKARVITDLSSGI*
>ENSG00000142920|ENST00000373441
MAGYLSESDFVMVEEGFSTRDLLKELTLGASQATTDEVAAFFVADLGAIVRKHFCFLKCL
PRVRPFYAVKCNSSPGVLKVLAQLGLGFSCANKAEMELVQHIGIPASKIICANPCKQIAQ
IKYAAKHGIQLLSFDNEMELAKVVKSHPSAKMVLCIATDDSHSLSCLSLKFGVSLKSCRH
LLENAKKHHVEVVGVSFHIGSGCPDPQAYAQSIADARLVFEMGTELGHKMHVLDLGGGFP
GTEGAKVRFEEIASVINSALDLYFPEGCGVDIFAELGRYYVTSAFTVAVSIIAKKEVLLD
QPGREAPLPPPHIATCAASEPSPPAEENGSTSKTIVYHLDEGVYGIFNSVLFDNICPTPI
LQKKPSTEQPLYSSSLWGPAVDGCDCVAEGLWLPQLHVGDWLVFDNMGAYTVGMGSPFWG
TQACHITYAMSRVAWEALRRQLMAAEQEDDVEGVCKPLSCGWEITDTLCVGPVFTPASIM
*
>ENSG00000157978|ENST00000374338
MDALKSAGRALIRSPSLAKQSWGGGGRHRKLPENWTDTRETLLEGMLFSLKYLGMTLVEQ
PKGEELSAAAIKRIVATAKASGKKLQKVTLKVSPRGIILTDNLTNQLIENVSIYRISYCT
ADKMHDKVFAYIAQSQHNQSLECHAFLCTKRKMAQAVTLTVAQAFKVAFEFWQVSKEEKE
KRDKASQEGGDVLGARQDCTPSLKSLVATGNLLDLEETAKAPLSTVSANTTNMDEVPRPQ
ALSGSSVVWELDDGLDEAFSRLAQSRTNPQVLDTGLTAQDMHYAQCLSPVDWDKPDSSGT
EQDDLFSF*
>ENSG00000134851|ENST00000381334
MAAAAPGNGRASAPRLLLLFLVPLLWAPAAVRAGPDEDLSHRNKEPPAPAQQLQPQPVAV
QGPEPARVEKIFTPAAPVHTNKEDPATQTNLGFIHAFVAAISVIIVSELGDKTFFIAAIM
AMRYNRLTVLAGAMLALGLMTCLSVLFGYATTVIPRVYTYYVSTVLFAIFGIRMLREGLK
MSPDEGQEELEEVQAELKKKDEEFQRTKLLNGPGDVETGTSITVPQKKWLHFISPIFVQA
LTLTFLAEWGDRSQLTTIVLAAREDPYGVAVGGTVGHCLCTGLAVIGGRMIAQKISVRTV
TIIGGIVFLAFAFSALFISPDSGF*
>ENSG00000132958|ENST00000390680
MNESPQTNEFKGTTEEAPAKESPHTSEFKGAALVSPISKRIFGVFLVLLDVTLLLADLIF
TDSKLYIPLEYRSISLAIGLFFLMDVLLRVFVEGWTHLVRLLRLIILIRIFHLLHQKRQL
EKLMRRLVSENKRRYTRDGFDLDLTYVTERIIAMSFPSSGRQSFYRNPIEEVVRFLDKKH
RNHYRVYNLCSERAYDPKHFHNRVSRIMIDDHNVPTLHEMVVFTKEVNEWMAQDLENIVA
IHCKGGKGRTGTMVCALLIASEIFLTAEESLYYFGERRTNKTHSNKFQGVETPSQNRYVG
YFAQVKHLYNWNLPPRRILFIKRFIIYSIRGDVCDLKVQVVMEKKVVFSSTSLGNCSILH

DIETDKILINVYDGPPLYDDVKVQFFSSNLPKYYDNCPFFFWFNTSFIQNNRLCLPRNEL
DNPHKQKAWKIYPPEFAVEILFGEK*
>ENSG00000123989|ENST00000373891
MRASLLLSVLRPAGPVAVGISLGFTLSLLSVTWVEEPCGPGPPQPGDSELPPRGNTNAAR
RPNSVQPGAEREKPGAGEGAGENWEPRVLPYHPAQPGQAAKKAVRTRYISTELGIRQRLL
VAVLTSQTTLPTLGVAVNRTLGHRLERVVFLTGARGRRAPPGMAVVTLGEERPIGHLHLA
LRHLLEQHGDDFDWFFLVPDTTYTEAHGLARLTGHLSLASAAHLYLGRPQDFIGGEPTPG
RYCHGGFGVLLSRMLLQQLRPHLEGCRNDIVSARPDEWLGRCILDATGVGCTGDHEVG*
>ENSG00000171365|ENST00000376088
MAMWQGAMDNRGFQQGSFSSFQNSSSDEDLMDIPATAMDFSMRDDVPPLDREVGEDKSYN
GGGIGSSNRIMDFLEEPIPGVGTYDDFNTIDWVREKSRDRDRHREITNKSKESTWALIHS
VSDAFSGWLLMLLIGLLSGSLAGLIDISAHWMTDLKEGICTGGFWFNHEHCCWNSEHVTF
EERDKCPEWNSWSQLIISTDEGAFAYIVNYFMYVLWALLFAFLAVSLVKVFAPYACGSGI
PEIKTILSGFIIRGYLGKWTLVIKTITLVLAVSSGLSLGKEGPLVHVACCCGNILCHCFN
KYRKNEAKRREVLSAAAAAGVSVAFGAPIGGVLFSLEEVSYYFPLKTLWRSFFAALVAAF
TLRSINPFGNSRLVLFYVEFHTPWHLFELVPFILLGIFGGLWGALFIRTNIAWCRKRKTT
QLGKYPVIEVLVVTAITAILAFPNEYTRMSTSELISELFNDCGLLDSSKLCDYENRFNTS
KGGELPDRPAGVGVYSAMWQLALTLILKIVITIFTFGMKIPSGLFIPSMAVGAIAGRLLG
VGMEQLAYYHQEWTVFNSWCSQGADCITPGLYAMVGAAACLGGVTRMTVSLVVIMFELTG
GLEYIVPLMAAAMTSKWVADALGREGIYDAHIRLNGYPFLEAKEEFAHKTLAMDVMKPRR
NDPLLTVLTQDSMTVEDVETIISETTYSGFPVVVSRESQRLVGFVLRRDLIISIENARKK
QDGVVSTSIIYFTEHSPPLPPYTPPTLKLRNILDLSPFTVTDLTPMEIVVDIFRKLGLRQ
CLVTHNGRLLGIITKKDVLKHIAQMANQDPDSILFN*
>ENSG00000196459|ENST00000359680
MSGSFYFVIVGHHDNPVFEMEFLPAGKAESKDDHRHLNQFIAHAALDLVDENMWLSNNMY
LKTVDKFNEWFVSAFVTAGHMRFIMLHDIRQEDGIKNFFTDVYDLYIKFSMNPFYEPNSP
IRSSAFDRKVQFLGKKHLLS*
>ENSG00000103528|ENST00000355377
MAYIQLEPLNEGFLSRISGLLLCRWTCRHCCQKCYESSCCQSSEDEVEILGPFPAQTPPW
LMASRSSDKDGDSVHTASEVPLTPRTNSPDGRRSSSDTSKSTYSLTRRISSLESRRPSSP
LIDIKPIEFGVLSAKKEPIQPSVLRRTYNPDDYFRKFEPHLYSLDSNSDDVDSLTDEEIL
SKYQLGMLHFSTQYDLLHNHLTVRVIEARDLPPPISHDGSRQDMAHSNPYVKICLLPDQK
NSKQTGVKRKTQKPVFEERYTFEIPFLEAQRRTLLLTVVDFDKFSRHCVIGKVSVPLCEV
DLVKGGHWWKALIPSSQNEVELGELLLSLNYLPSAGRLNVDVIRAKQLLQTDVSQGSDPF
VKIQLVHGLKLVKTKKTSFLRGTIDPFYNESFSFKVPQEELENASLVFTVFGHNMKSSND
FIGRIVIGQYSSGPSETNHWRRMLNTHRTAVEQWHSLRSRAECDRVSPASLEVT*
>ENSG00000147654|ENST00000395785
MAITQFRLFKFCTCLATVFSFLKRLICRSGRGRKLSGDQITLPTTVDYSSVPKQTDVEEW
TSWDEDAPTSVKIEGGNGNVATQQNSLEQLEPDYFKDMTPTIRKTQKIVIKKREPLNFGI
PDGSTGFSSRLAATQDLPFIHQSSELGDLDTWQENTNAWEEEEDAAWQAEEVLRQQKLAD
REKRAAEQQRKKMEKEAQRLMKKEQNKIGVKLS*
>ENSG00000079332|ENST00000373238
MSFIFEWIYNGFSSVLQFLGLYKKSGKLVFLGLDNAGKTTLLHMLKDDRLGQHVPTLHPT
SEELTIAGMTFTTFDLGGHEQARRVWKNYLPAINGIVFLVDCADHSRLVESKVELNALMT

DETISNVPILILGNKIDRTDAISEEKLREIFGLYGQTTGKGNVTLKELNARPMEVFMCSV
LKRQGYGEGFRWLSQYID*
>ENSG00000125875|ENST00000354200
MALRSAQGDGPTSGHWDGGAEKADFNAKRKKKVAEIHQALNSDPTDVAALRRMAISEGGL
LTDEIRRKVWPKLLNVNANDPPPISGKNLRQMSKDYQQVLLDVRRSLRRFPPGMPEEQRE
GLQEELIDIILLILERNPQLHYYQGYHDIVVTFLLVVGERLATSLVEKLSTHHLRDFMDP
TMDNTKHILNYLMPIIDQVNPELHDFMQSAEVGTIFALSWLITWFGHVLSDFRHVVRLYD
FFLACHPLMPIYFAAVIVLYREQEVLDCDCDMASVHHLLSQIPQDLPYETLISRAGDLFV
QFPPSELAREAAAQQQAERTAASTFKDFELASAQQRPDMVLRQRFRGLLRPEDRTKDVLT
KPRTNRFVKLAVMGLTVALGAAALAVVKSALEWAPKFQLQLFP*
>ENSG00000122218|ENST00000368069
MLTKFETKSARVKGLSFHPKRPWILTSLHNGVIQLWDYRMCTLIDKFDEHDGPVRGIDFH
KQQPLFVSGGDDYKIKVWNYKLRRCLFTLLGHLDYIRTTFFHHEYPWILSASDDQTIRVW
NWQSRTCVCVLTGHNHYVMCAQFHPTEDLVVSASLDQTVRVWDISGLRKKNLSPGAVESD
VRGITGVDLFGTTDAVVKHVLEGHDRGVNWAAFHPTMPLIVSGADDRQVKIWRMNESKAW
EVDTCRGHYNNVSCAVFHPRQELILSNSEDKSIRVWDMSKRTGVQTFRRDHDRFWVLAAH
PNLNLFAAGHDGGMIVFKLERERPAYAVHGNMLHYVKDRFLRQLDFNSSKDVAVMQLRSG
SKFPVFNMSYNPAENAVLLCTRASNLENSTYDLYTIPKDADSQNPDAPEGKRSSGLTAVW
VARNRFAVLDRMHSLLIKNLKNEITKKVQVPNCDEIFYAGTGNLLLRDADSITLFDVQQK
RTLASVKISKVKYVIWSADMSHVALLAKHEHSCPLPLTAIVICNRKLDALCNIHENIRVK
SGAWDESGVFIYTTSNHIKYAVTTGDHGIIRTLDLPIYVTRVKGNNVYCLDRECRPRVLT
IDPTEFKFKLALINRKYDEVLHMVRNAKLVGQSIIAYLQKKGYPEVALHFVKDEKTRFSL
ALECGNIEIALEAAKALDDKNCWEKLGEVALLQGNHQIVEMCYQRTKNFDKLSFLYLITG
NLEKLRKMMKIAEIRKDMSGHYQNALYLGDVSERVRILKNCGQKSLAYLTAATHGLDEEA
ESLKETFDPEKETIPDIDPNAKLLQPPAPIMPLDTNWPLLTVSKGFFEGTIASKGKGGAL
AADIDIDTVGTEGWGEDAELQLDEDGFVEATEGLGDDALGKGQEEGGGWDVEEDLELPPE
LDISPGAAGGAEDGFFVPPTKGTSPTQIWCNNSQLPVDHILAGSFETAMRLLHDQVGVIQ
FGPYKQLFLQTYARGRTTYQALPCLPSMYGYPNRNWKDAGLKNGVPAVGLKLNDLIQRLQ
LCYQLTTVGKFEEAVEKFRSILLSVPLLVVDNKQEIAEAQQLITICREYIVGLSVETERK
KLPKETLEQQKRICEMAAYFTHSNLQPVHMILVLRTALNLFFKLKNFKTAATFARRLLEL
GPKPEVAQQTRKILSACEKNPTDAYQLNYDMHNPFDICAASYRPIYRGKPVEKCPLSGAC
YSPEFKGQICRVTTVTEIGKDVIGLRISPLQFR*
>ENSG00000135775|ENST00000366669
MEKSRMNLPKGPDTLCFDKDEFMKEDFDVDHFVSDCRKRVQLEELRDDLELYYKLLKTAM
VELINKDYADFVNLSTNLVGMDKALNQLSVPLGQLREEVLSLRSSVSEGIRAVDERMSKQ
EDIRKKKMCVLRLIQVIRSVEKIEKILNSQSSKETSALEASSPLLTGQILERIATEFNQL
QFHAVQSKGMPLLDKVRPRIAGITAMLQQSLEGLLLEGLQTSDVDIIRHCLRTYATIDKT
RDAEALVGQVLVKPYIDEVIIEQFVESHPNGLQVMYNKLLEFVPHHCRLLREVTGGAISS
EKGNTVPGYDFLVNSVWPQIVQGLEEKLPSLFNPGNPDAFHEKYTISMDFVRRLERQCGS
QASVKRLRAHPAYHSFNKKWNLPVYFQIRFREIAGSLEAALTDVLEDAPAESPYCLLASH
RTWSSLRRCWSDEMFLPLLVHRLWRLTLQILARYSVFVNELSLRPISNESPKEIKKPLVT
GSKEPSITQGNTEDQGSGPSETKPVVSISRTQLVYVVADLDKLQEQLPELLEIIKPKLEM
IGFKNFSSISAALEDSQSSFSACVPSLSSKIIQDLSDSCFGFLKSALEVPRLYRRTNKEV
PTTASSYVDSALKPLFQLQSGHKDKLKQAIIQQWLEGTLSESTHKYYETVSDVLNSVKKM

EESLKRLKQARKTTPANPVGPSGGMSDDDKIRLQLALDVEYLGEQIQKLGLQASDIKSFS
ALAELVAAAKDQATAEQP*
>ENSG00000115963|ENST00000375734
MKERRASQKLSSKSIMDPNQNVKCKIVVVGDSQCGKTALLHVFAKDCFPENYVPTVFENY
TASFEIDTQRIELSLWDTSGSPYYDNVRPLSYPDSDAVLICFDISRPETLDSVLKKWKGE
IQEFCPNTKMLLVGCKSDLRTDVSTLVELSNHRQTPVSYDQGANMAKQIGAATYIECSAL
QSENSVRDIFHVATLACVNKTNKNVKRNKSQRATKRISHMPSRPELSAVATDLRKDKAKS
CTVM*
>ENSG00000092108|ENST00000396629
MPTEENIDRMCQDLRNQLYESYYLNFISAISRSKLEDIANAALAASAVTQVAKVFDQYLN
FITLEDDMFVLCNQNKELVSYRAINRPDITDTEMETVMDTIVDSLFCFFVTLGAVPIIRC
SRGTAAEMVAVKLDKKLRENLRDARNSLFTGDTLGAGQFSFQRPLLVLVDRNIDLATPLH
HTWTYQALVHDVLDFHLNRVNLEESSGVENSPAGARPKRKNKKSYDLTPVDKFWQKHKGS
PFPEVAESVQQELESYRAQEDEVKRLKSIMGLEGEDEGAISMLSDNTAKLTSAVSSLPEL
LEKKRLIDLHTNVATAVLEHIKARKLDVYFEYEEKIMSKTTLDKSLLDIISDPDAGTPED
KMRLFLIYYISTQQAPSEADLEQYKKALTDAGCNLNPLQYIKQWKAFTKMASAPASYGST
TTKPMGLLSRVMNTGSQFVMEGVKNLVLKQQNLPVTRILDNLMEMKSNPETDDYRYFDPK
MLRGNDSSVPRNKNPFQEAIVFVVGGGNYIEYQNLVDYIKGKQGKHILYGCSELFNATQF
IKQLSQLGQK*
>ENSG00000128274|ENST00000401850
MSKPPDLLLRLLRGAPRQRVCTLFIIGFKFTFFVSIMIYWHVVGEPKEKGQLYNLPAEIP
CPTLTPPTPPSHGPTPGNIFFLETSDRTNPNFLFMCSVESAARTHPESHVLVLMKGLPGG
NASLPRHLGISLLSCFPNVQMLPLDLRELFRDTPLADWYAAVQGRWEPYLLPVLSDASRI
ALMWKFGGIYLDTDFIVLKNLRNLTNVLGTQSRYVLNGAFLAFERRHEFMALCMRDFVDH
YNGWIWGHQGPQLLTRVFKKWCSIRSLAESRACRGVTTLPPEAFYPIPWQDWKKYFEDIN
PEELPRLLSATYAVHVWNKKSQGTRFEATSRALLAQLHARYCPTTHEAMKMYL*
>ENSG00000106392|ENST00000402468
MASKSWLNFLTFLCGSAIGFLLCSQLFSILLGEKVDTQPNVLHNDPHARHSDDNGQNHLE
GQMNFNADSSQHKDENTDIAENLYQKVRILCWVMTGPQNLEKKAKHVKATWAQRCNKVLF
MSSEENKDFPAVGLKTKEGRDQLYWKTIKAFQYVHEHYLEDADWFLKADDDTYVILDNLR
WLLSKYDPEEPIYFGRRFKPYVKQGYMSGGAGYVLSKEALKRFVDAFKTDKCTHSSSIED
LALGRCMEIMNVEAGDSRDTIGKETFHPFVPEHHLIKGYLPRTFWYWNYNYYPPVEVSLE
ILLLCQYLD*
>ENSG00000126091|ENST00000372366
MGLLVFVRNLLLALCLFLVLGFLYYSAWKLHLLQWEEDSSKYSHSSSPQEKPVAEYDRLG
FLLNLDSKLPAELATKYANFSEGACKPGYASALMTAIFPRFSKPAPMFLDDSFRKWARIR
EFVPPFGIKGQDNLIKAILSVTKEYRLTPALDSLRCRRCIIVGNGGVLANKSLGSRIDDY
DIVVR*
>ENSG00000105669|ENST00000349893
MAPPAPGPASGGSGEVDELFDVKNAFYIGSYQQCINEAQRVKLSSPERDVERDVFLYRAY
LAQRKFGVVLDEIKPSSAPELQAVRMFADYLAHESRRDSIVAELDREMSRSVDVTNTTFL
LMAASIYLHDQNPDAALRALHQGDSLECTAMTVQILLKLDRLDLARKELKRMQDLDEDAT
LTQLATAWVSLATDSGYPETLVNLIVLSQHLGKPPEVTNRYLSQLKDAHRSHPFIKEYQA
KENDFDRLVLQYAPSA*

>ENSG00000204842|ENST00000377617
MRSAAAAPRSPAVATESRRFAAARWPGWRSLQRPARRSGRGGGGAAPGPYPSAAPPPPGP
GPPPSRQSSPPSASDCFGSNGNGGGAFRPGSRRLLGLGGPPRPFVVLLLPLASPGAPPAA
PTRASPLGARASPPRSGVSLARPAPGCPRPACEPVYGPLTMSLKPQQQQQQQQQQQQQQQ
QQQQQQQQPPPAAANVRKPGGSGLLASPAAAPSPSSSSVSSSSATAPSSVVAATSGGGRP
GLGRGRNSNKGLPQSTISFDGIYANMRMVHILTSVVGSKCEVQVKNGGIYEGVFKTYSPK
CDLVLDAAHEKSTESSSGPKREEIMESILFKCSDFVVVQFKDMDSSYAKRDAFTDSAISA
KVNGEHKEKDLEPWDAGELTANEELEALENDVSNGWDPNDMFRYNEENYGVVSTYDSSLS
SYTVPLERDNSEEFLKREARANQLAEEIESSAQYKARVALENDDRSEEEKYTAVQRNSSE
REGHSINTRENKYIPPGQRNREVISWGSGRQNSPRMGQPGSGSMPSRSTSHTSDFNPNSG
SDQRVVNGGVPWPSPCPSPSSRPPSRYQSGPNSLPPRAATPTRPPSRPPSRPSRPPSHPS
AHGSPAPVSTMPKRMSSEGPPRMSPKAQRHPRNHRVSAGRGSISSGLEFVSHNPPSEAAT
PPVARTSPSGGTWSSVVSGVPRLSPKTHRPRSPRQNSIGNTPSGPVLASPQAGIIPTEAV
AMPIPAASPTPASPASNRAVTPSSEAKDSRLQDQRQNSPAGNKENIKPNETSPSFSKAEN
KGISPVVSEHRKQIDDLKKFKNDFRLQPSSTSESMDQLLNKNREGEKSRDLIKDKIEPSA
KDSFIENSSSNCTSGSSKPNSPSISPSILSNTEHKRGPEVTSQGVQTSSPACKQEKDDKE
EKKDAAEQVRKSTLNPNAKEFNPRSFSQPKPSTTPTSPRPQAQPSPSMVGHQQPTPVYTQ
PVCFAPNMMYPVPVSPGVQPLYPIPMTPMPVNQAKTYRAVPNMPQQRQDQHHQSAMMHPA
SAAGPPIAATPPAYSTQYVAYSPQQFPNQPLVQHVPHYQSQHPHVYSPVIQGNARMMAPP
THAQPGLVSSSATQYGAHEQTHAMYACPKLPYNKETSPSFYFAISTGSLAQQYAHPNATL
HPHTPHPQPSATPTGQQQSQHGGSHPAPSPVQHHQHQAAQALHLASPQQQSAIYHAGLAP
TPPSMTPASNTQSPQNSFPAAQQTVFTIHPSHVQPAYTNPPHMAHVPQAHVQSGMVPSHP
TAHAPMMLMTTQPPGGPQAALAQSALQPIPVSTTAHFPYMTHPSVQAHHQQQL*
>ENSG00000206305|ENST00000399675
MILNKALMLGALALTTVMSPCGGEDIVADHVASYGVNLYQSYGPSGQYTHEFDGDEQFYV
DLGRKETVWCLPVLRQFRFDPQFALTNIAVLKHNLNSLIKRSNSTAATNEVPEVTVFSKS
PVTLGQPNILICLVDNIFPPVVNITWLSNGHSVTEGVSETSFLSKSDHSFFKISYLTLLP
SAEESYDCKVEHWGLDKPLLKHWEPEIPAPMSELTETVVCALGLSVGLVGIVVGTVFIIR
GLRSVGASRHQGPL*
>ENSG00000126091|ENST00000372367
MGLLVFVRNLLLALCLFLVLGFLYYSAWKLHLLQWEEDSSKYSHSSSPQEKPVAEYDRLG
FLLNLDSKLPAELATKYANFSEGACKPGYASALMTAIFPRFSKPAPMFLDDSFRKWARIR
EFVPPFGIKGQDNLIKAILSVTKEYRLTPALDSLRCRRCIIVGNGGVLANKSLGSRIDDY
DIVVSPGRTISSERKSFCGSW*
>ENSG00000157350|ENST00000393640
MKCSLRVWFLSVAFLLVFIMSLLFTYSHHSMATLPYLDSGALDGTHRVKLVPGYAGLQRL
SKERLSGKSCACRRCMGDAGASDWFDSHFDGNISPVWTRENMDLPPDVQRWWMMLQPQFK
SHNTNEVLEKLFQIVPGENPYRFRDPHQCRRCAVVGNSGNLRGSGYGQDVDGHNFIMRMN
QAPTVGFEQDVGSRTTHHFMYPESAKNLPANVSFVLVPFKVLDLLWIASALSTGQIRFTY
APVKSFLRVDKEKVQIYNPAFFKYIHDRWTEHHGRYPSTGMLVLFFALHVCDEVNVYGFG
ADSRGNWHHYWENNRYAGEFRKTGVHDADFEAHIIDMLAKASKIEVYRGN*
>ENSG00000188603|ENST00000355477
MGGCAGSRRRFSDSEGEETVPEPRLPLLDHQGAHWKNAVGFWLLGLCNNFSYVVMLSAAH
DILSHKRTSGNQSHVDPGPTPIPHNSSSRFDCNSVSTAAVLLADILPTLVIKLLAPLGLH

LLPYSPRVLVSGICAAGSFVLVAFSHSVGTSLCGVVFASISSGLGEVTFLSLTAFYPSYF
LLLTSPEAQDPGGEEEAESAARQPLIRTEAPESKPGSSSSLSLRERWTVFKGLLWYIVPL
VVVYFAEYFINQGLFELLFFWNTSLSHAQQYRWYQMLYQAGVFASRSSLRCCRIRFTWAL
ALLQCLNLVFLLADVWFGFLPSIYLVFLIILYEGLLGGAAYVNTFHNIALETSDEHREFA
MAATCISDTLGISLSGLLALPLHDFLCQLS*
>ENSG00000197586|ENST00000376652
MKKGIRYETSRKTSYIFQQPQHGPWQTRMRKISNHGSLRVAKVAYPLGLCVGVFIYVAYI
KWHRATATQAFFSITRAAPGARWGQQAHSPLGTAADGHEVFYGIMFDAGSTGTRVHVFQF
TRPPRETPTLTHETFKALKPGLSAYADDVEKSAQGIRELLDVAKQDIPFDFWKATPLVLK
ATAGLRLLPGEKAQKLLQKVKKVFKASPFLVGDDCVSIMNGTDEGVSAWITINFLTGSLK
TPGGSSVGMLDLGGGSTQIAFLPRVEGTLQASPPGYLTALRMFNRTYKLYSYSYLGLGLM
SARLAILGGVEGQPAKDGKELVSPCLSPSFKGEWEHAEVTYRVSGQKAAASLHELCAARV
SEVLQNRVHRTEEVKHVDFYAFSYYYDLAAGVGLIDAEKGGSLVVGDFEIAAKYVCRTLE
TQPQSSPFSCMDLTYVSLLLQEFGFPRSKVLKLTRKIDNVETSWALGAIFHYIDSLNRQK
SPAS*
>ENSG00000142192|ENST00000357903
MLPGLALLLLAAWTARALEVPTDGNAGLLAEPQIAMFCGRLNMHMNVQNGKWDSDPSGTK
TCIDTKEGILQYCQEVYPELQITNVVEANQPVTIQNWCKRGRKQCKTHPHFVIPYRCLVG
EFVSDALLVPDKCKFLHQERMDVCETHLHWHTVAKETCSEKSTNLHDYGMLLPCGIDKFR
GVEFVCCPLAEESDNVDSADAEEDDSDVWWGGADTDYADGSEDKVVEVAEEEEVAEVEEE
EADDDEDDEDGDEVEEEAEEPYEEATERTTSIATTTTTTTESVEEVVREVCSEQAETGPC
RAMISRWYFDVTEGKCAPFFYGGCGGNRNNFDTEEYCMAVCGSAIPTTAASTPDAVDKYL
ETPGDENEHAHFQKAKERLEAKHRERMSQVMREWEEAERQAKNLPKADKKAVIQHFQEKV
ESLEQEAANERQQLVETHMARVEAMLNDRRRLALENYITALQAVPPRPRHVFNMLKKYVR
AEQKDRQHTLKHFEHVRMVDPKKAAQIRSQVMTHLRVIYERMNQSLSLLYNVPAVAEEIQ
DEVDELLQKEQNYSDDVLANMISEPRISYGNDALMPSLTETKTTVELLPVNGEFSLDDLQ
PWHSFGADSVPANTENEVEPVDARPAADRGLTTRPGSGLTNIKTEEISEVKMDAEFRHDS
GYEVHHQKLVFFAEDVGSNKGAIIGLMVGGVVIATVIVITLVMLKKKQYTSIHHGVVEVD
AAVTPEERHLSKMQQNGYENPTYKFFEQMQN*
>ENSG00000101928|ENST00000370777
MHQQKRQPELVEGNLPVFVFPTELIFYADDQSTHKQVLTLYNPYEFALKFKVLCTTPNKY
VVVDAAGAVKPQCCVDIVIRHRDVRSCHYGVIDKFRLQVSEQSQRKALGRKEVVATLLPS
AKEQQKEEEEKRLKEHLTESLFFEQSFQPGKNRAVSSGPSLLTVFLGVVCIAALMLPTLG
DVESLVPLYLHLSVNQKLVAAYILGLITMAILRT*
>ENSG00000141551|ENST00000392334
MELRVGNRYRLGRKIGSGSFGDIYLGTDIAAGEEVAIKLECVKTKHPQLHIESKIYKMMQ
GGVGIPTIRWCGAEGDYNVMVMELLGPSLEDLFNFCSRKFSLKTVLLLADQMISRIEYIH
SKNFIHRDVKPDNFLMGLGKKGNLVYIIDFGLAKKYRDARTHQHIPYRENKNLTGTARYA
SINTHLGIEQSRRDDLESLGYVLMYFNLGSLPWQGLKAATKRQKYERISEKKMSTPIEVL
CKGYPSEFATYLNFCRSLRFDDKPDYSYLRQLFRNLFHRQGFSYDYVFDWNMLKFGASRA
ADDAERERRDREERLRHSRNPATRGLPSTASGRLRGTQEVAPPTPLTPTSHTANTSPRPV
SGMERERKVSMRLHRGAPVNISSSDLTGRQDTSRMSTSQNSIPFEHHGK*
>ENSG00000204482|ENST00000376096
MLSRNDEAASAQQ*

>ENSG00000144747|ENST00000398559
MSWFNASQLSSFAKQALSQAQKSIDRVLDIQEEEPSIWAETIPYGEPGISSPVSGGWDTS
TWGLKSNTEPQSPPIASPKAITKPVRRTVVDESENFFSAFLSPTDVQTIQKSPVVSKPPA
KSQRPEEEVKSSLHESLHIGQSRTPETTESQVKDSSLCVSGETLAAGTSSPKTEGKHEET
VNKESDMKVPTVSLKVSESVIDVKTTMESISNTSTQSLTAETKDIALEPKEQKHEDRQSN
TPSPPVSTFSSGTSTTSDIEVLDHESVISESSASSRQETTDSKSSLHLMQTSFQLLSASA
CPEYNRLDDFQKLTESCCSSDAFERIDSFSVQSLDSRSVSEINSDDELSGKGYALVPIIV
NSSTPKSKTVESAEGKSEEVNETLVIPTEEAEMEESGRSATPVNCEQPDILVSSTPINEG
QTVLDKVAEQCEPAESQPEALSEKEDVCKTVEFLNEKLEKREAQLLSLSKEKALLEEAFD
NLKDEMFRVKEESSSISSLKDEFTQRIAEAEKKVQLACKERDAAKKEIKNIKEELATRLN
SSETADLLKEKDEQIRGLMEEGEKLSKQQLHNSNIIKKLRAKDKENENMVAKLNKKVKEL
EEELQHLKQVLDGKEEVEKQHRENIKKLNSMVERQEKDLGRLQVDMDELEEKNRSIQAAL
DSAYKELTDLHKANAAKDSEAQEAALSREMKAKEELSAALEKAQEEARQQQETLAIQVGD
LRLALQRTEQAAARKEDYLRHEIGELQQRLQEAENRNQELSQSVSSTTRPLLRQIENLQA
TLGSQTSSWEKLEKNLSDRLGESQTLLAAAVERERAATEELLANKIQMSSMESQNSLLRQ
ENSRFQAQLESEKNRLCKLEDENNRYQVELENLKDEYVRTLEETRKEKTLLNSQLEMERM
KVEQERKKAIFTQETIKEKERKPFSVSSTPTMSRSSSISGVDMAGLQTSFLSQDESHDHS
FGPMPISANGSNLYDAVRMGAGSSIIENLQSQLKLREGEITHLQLEIGNLEKTRSIMAEE
LVKLTNQNDELEEKVKEIPKLRTQLRDLDQRYNTILQMYGEKAEEAEELRLDLEDVKNMY
KTQIDELLRQSLS*
>ENSG00000206281|ENST00000383198
MKSLSLLLAVALGLATAVSAGPAVIECWFVEDASGKGLAKRPGALLLRQGPGEPPPRPDL
DPELYLSVHDPAGALQAAFRRYPRGAPAPHCEMSRFVPLPASAKWASGLTPAQNCPRALD
GAWLMVSISSPVLSLSSLLRPQPEPQQEPVLITMATVVLTVLTHTPAPRVRLGQDALLDL
SFAYMPPTSEAASSLAPGPPPFGLEWRRQHLGKGHLLLAATPGLNGQMPAAQEGAVAFAA
WDDDEPWGPWTGNGTFWLPRVQPFQEGTYLATIHLPYLQGQVTLELAVYKPPKVSLMPAT
LARAAPGEAPPELLCLVSHFYPSGGLEVEWELRGGPGGRSQKAEGQRWLSALRHHSDGSV
SLSGHLQPPPVTTEQHGARYACRIHHPSLPASGRSAEVTLEVAGLSGPSLEDSVGLFLSA
FLLLGLFKALGWAAVYLSTCKDSKKKAE*
>ENSG00000019582|ENST00000353334
MHRRRSRSCREDQKPVMDDQRDLISNNEQLPMLGRRPGAPESKCSRGALYTGFSILVTLL
LAGQATTAYFLYQQQGRLDKLTVTSQNLQLENLRMKLPKPPKPVSKMRMATPLLMQALPM
GALPQGPMQNATKYGNMTEDHVMHLLQNADPLKVYPPLKGSFPENLRHLKNTMETIDWKV
FESWMHHWLLFEMSRHSLEQKPTDAPPKESLELEDPSSGLGVTKQDLGPVPM*
>ENSG00000229698|ENST00000376848
MAPRSLLLLLSGALALTDTWAGSHSLRYFSTAVSRPGRGEPRYIAVEYVDDTQFLRFDSD
AAIPRMEPREPWVEQEGPQYWEWTTGYAKANAQTDRVALRNLLRRYNQSEAGSHTLQGMN
GCDMGPDGRLLRGYHQHAYDGKDYISLNEDLRSWTAADTVAQITQRFYEAEEYAEEFRTY
LEGECLELLRRYLENGKETLQRAEQSPQPTIPIVGIVAGLVVLGAVVTGAVVAAVMWRKK
SSDRNRGSYSQAAV*
>ENSG00000079332|ENST00000373241
MSFIFEWIYNGFSSVLQFLGLYKKSGKLVFLGLDNAGKTTLLHMLKDDRLGQHVPTLHPT
SEELTIAGMTFTTFDLGGHEQARRVWKNYLPAINGIVFLVDCADHSRLVESKVELNALMT
DETISNVPILILGNKIDRTDAISEEKLREIFGLYGQTTGKGNVTLKELNARPMEVFMCSV

LKRQGYGEGFRWLSQYID*
>ENSG00000204642|ENST00000376861
MAPRSLLLLLSGALALTDTWAGSHSLRYFSTAVSRPGRGEPRYIAVEYVDDTQFLRFDSD
AAIPRMEPREPWVEQEGPQYWEWTTGYAKANAQTDRVALRNLLRRYNQSEAGSHTLQGMN
GCDMGPDGRLLRGYHQHAYDGKDYISLNEDLRSWTAADTVAQITQRFYEAEEYAEEFRTY
LEGECLELLRRYLENGKETLQRADPPKAHVAHHPISDHEATLRCWALGFYPAEITLTWQR
DGEEQTQDTELVETRPAGDGTFQKWAAVVVPPGEEQRYTCHVQHEGLPQPLILRWEQSPQ
PTIPIVGIVAGLVVLGAVVTGAVVAAVMWRKKSSDRNRGSYSQAAV*
>ENSG00000182870|ENST00000397325
MEVLPCSRVAHIERTRKPYNNDIDYYAKRNALRAAEVWMDDFKSHVYMAWNIPMSNPGVD
FGDVSERLALRQRLKCRSFKWYLENVYPEMRVYNNTLTYGEVRNSKASAYCLDQGAEDGD
RAILYPCHGMSSQLVRYSADGLLQLGPLGSTAFLPDSKCLVDDGTGRMPTLKKCEDVARP
TQRLWDFTQSGPIVSRATGRCLEVEMSKDANFGLRLVVQRCSGQKWMIRNWIKHARH*
>ENSG00000115525|ENST00000393808
MASVPMPSEYTYVKLRSDCSRPSLQWYTRAQSKMRRPSLLLKDILKCTLLVFGVWILYIL
KLNYTTEECDMKKMHYVDPDHVKRAQKYAQQVLQKECRPKFAKTSMALLFEHRYSVDLLP
FVQKAPKDSEAESKYDPPFGFRKFSSKVQTLLELLPEHDLPEHLKAKTCRRCVVIGSGGI
LHGLELGHTLNQFDVVIRLNSAPVEGYSEHVGNKTTIRMTYPEGAPLSDLEYYSNDLFVA
VLFKSVDFNWLQAMVKKETLPFWVRLFFWKQVAEKIPLQPKHFRILNPVIIKETAFDILQ
YSEPQSRFWGRDKNVPTIGVIAVVLATHLCDEVSLAGFGYDLNQPRTPLHYFDSQCMAAM
NFQTMHNVTTETKFLLKLVKEGVVKDLSGGIDREF*
>ENSG00000107854|ENST00000371627
MSGRRCAGGGAACASAAAEAVEPAARELFEACRNGDVERVKRLVTPEKVNSRDTAGRKST
PLHFAAGFGRKDVVEYLLQNGANVQARDDGGLIPLHNACSFGHAEVVNLLLRHGADPNAR
DNWNYTPLHEAAIKGKIDVCIVLLQHGAEPTIRNTDGRTALDLADPSAKAVLTGEYKKDE
LLESARSGNEEKMMALLTPLNVNCHASDGRKSTPLHLAAGYNRVKIVQLLLQHGADVHAK
DKGDLVPLHNACSYGHYEVTELLVKHGACVNAMDLWQFTPLHEAASKNRVEVCSLLLSYG
ADPTLLNCHNKSAIDLAPTPQLKERLAYEFKGHSLLQAAREADVTRIKKHLSLEMVNFKH
PQTHETALHCAAASPYPKRKQICELLLRKGANINEKTKEFLTPLHVASEKAHNDVVEVVV
KHEAKVNALDNLGQTSLHRAAYCGHLQTCRLLLSYGCDPNIISLQGFTALQMGNENVQQL
LQEGISLGNSEADRQLLEAAKAGDVETVKKLCTVQSVNCRDIEGRQSTPLHFAAGYNRVS
VVEYLLQHGADVHAKDKGGLVPLHNACSYGHYEVAELLVKHGAVVNVADLWKFTPLHEAA
AKGKYEICKLLLQHGADPTKKNRDGNTPLDLVKDGDTDIQDLLRGDAALLDAAKKGCLAR
VKKLSSPDNVNCRDTQGRHSTPLHLAAGYNNLEVAEYLLQHGADVNAQDKGGLIPLHNAA
SYGHVDVAALLIKYNACVNATDKWAFTPLHEAAQKGRTQLCALLLAHGADPTLKNQEGQT
PLDLVSADDVSALLTAAMPPSALPSCYKPQVLNGVRSPGATADALSSGPSSPSSLSAASS
LDNLSGSFSELSSVVSSSGTEGASSLEKKEVPGVDFSITQFVRNLGLEHLMDIFEREQIT
LDVLVEMGHKELKEIGINAYGHRHKLIKGVERLISGQQGLNPYLTLNTSGSGTILIDLSP
DDKEFQSVEEEMQSTVREHRDGGHAGGIFNRYNILKIQKVCNKKLWERYTHRRKEVSEEN
HNHANERMLFHGSPFVNAIIHKGFDERHAYIGGMFGAGIYFAENSSKSNQYVYGIGGGTG
CPVHKDRSCYICHRQLLFCRVTLGKSFLQFSAMKMAHSPPGHHSVTGRPSVNGLALAEYV
IYRGEQAYPEYLITYQIMRPEGMVDG*
>ENSG00000179399|ENST00000377067
MDAQTWPVGFRCLLLLALVGSARSEGVQTCEEVRKLFQWRLLGAVRGLPDSPRAGPDLQV

CISKKPTCCTRKMEERYQIAARQDMQQFLQTSSSTLKFLISRNAAAFQETLETLIKQAEN
YTSILFCSTYRNMALEAAASVQEFFTDVGLYLFGADVNPEEFVNRFFDSLFPLVYNHLIN
PGVTDSSLEYSECIRMARRDVSPFGNIPQRVMGQMGRSLLPSRTFLQALNLGIEVINTTD
YLHFSKECSRALLKMQYCPHCQGLALTKPCMGYCLNVMRGCLAHMAELNPHWHAYIRSLE
ELSDAMHGTYDIGHVLLNFHLLVNDAVLQAHLNGQKLLEQVNRICGRPVRTPTQSPRCSF
DQSKEKHGMKTTTRNSEETLANRRKEFINSLRLYRSFYGGLADQLCANELAAADGLPCWN
GEDIVKSYTQRVVGNGIKAQSGNPEVKVKGIDPVINQIIDKLKHVVQLLQGRSPKPDKWE
LLQLGSGGGMVEQVSGDCDDEDGCGGSGSGEVKRTLKITDWMPDDMNFSDVKQIHQTDTG
STLDTTGAGCAVATESMTFTLISVVMLLPGIW*
>ENSG00000152700|ENST00000402673
MSFIFDWIYSGFSSVLQFLGLYKKTGKLVFLGLDNAGKTTLLHMLKDDRLGQHVPTLHPT
SEELTIAGMTFTTFDLGGHVQARRVWKNYLPAINGIVFLVDCADHERLLESKEELDSLMT
DETIANVPILILGNKIDRPEAISEERLREMFGLYGQTTGKGSISLKELNARPLEVFMCSV
LKRQGYGEGFRWMAQYID*
>ENSG00000137497|ENST00000366394
MTLHATRGAALLSWVNSLHVADPVEAVLQLQDCSIFIKIIDRIHGTEEGQQILKQPVSER
LDFVCSFLQKNRKHP
>ENSG00000198925|ENST00000396761
MAQFDTEYQRLEASYSDSPPGEEDLLVHVAEGSKSPWHHIENLDLFFSRVYNLHQKNGFT
CMLIGEIFELMQFLFVVAFTTFLVSCVDYDILFANKMVNHSLHPTEPVKVTLPDAFLPAQ
VCSARIQENGSLITILVIAGVFWIHRLIKFIYNICCYWEIHSFYLHALRIPMSALPYCTW
QEVQARIVQTQKEHQICIHKRELTELDIYHRILRFQNYMVALVNKSLLPLRFRLPGLGEA
VFFTRGLKYNFELILFWGPGSLFLNEWSLKAEYKRGGQRLELAQRLSNRILWIGIANFLL
CPLILIWQILYAFFSYAEVLKREPGALGARCWSLYGRCYLRHFNELEHELQSRLNRGYKP
ASKYMNCFLSPLLTLLAKNGAFFAGSILAVLIALTIYDEDVLAVEHVLTTVTLLGVTVTV
CRSFIPDQHMVFCPEQLLRVILAHIHYMPDHWQGNAHRSQTRDEFAQLFQYKAVFILEEL
LSPIVTPLILIFCLRPRALEIIDFFRNFTVEVVGVGDTCSFAQMDVRQHGHPQWLSAGQT
EASVYQQAEDGKTELSLMHFAITNPGWQPPRESTAFLGFLKEQVQRDGAAASLAQGGLLP
ENALFTSIQSLQSESEPLSLIANVVAGSSCRGPPLPRDLQGSRHRAEVASALRSFSPLQP
GQAPTGRAHSTMTGSGVDARTASSGSSVWEGQLQSLVLSEYASTEMSLHALYMHQLHKQQ
AQAEPERHVWHRRESDESGESAPDEGGEGARAPQSIPRSASYPCAAPRPGAPETTALHGG
FQRRYGGITDPGTVPRVPSHFSRLPLGGWAEDGQSASRHPEPVPEEGSEDELPPQVHKV*
>ENSG00000164023|ENST00000359079
MDIIETAKLEEHLENQPSDPTNTYARPAEPVEEENKNGNGKPKSLSSGLRKGTKKYPDYI
QIAMPTESRNKFPLEWWKTGIAFIYAVFNLVLTTVMITVVHERVPPKELSPPLPDKFFDY
IDRVKWAFSVSEINGIILVGLWITQWLFLRYKSIVGRRFCFIIGTLYLYRCITMYVTTLP
VPGMHFQCAPKLNGDSQAKVQRILRLISGGGLSITGSHILCGDFLFSGHTVTLTLTYLFI
KEYSPRHFWWYHLICWLLSAAGIICILVAHEHYTIDVIIAYYITTRLFWWYHSMANEKNL
KVSSQTNFLSRAWWFPIFYFFEKNVQGSIPCCFSWPLSWPPGCFKSSCKKYSRVQKIGED
NEKST*
>ENSG00000158488|ENST00000368165
MLLLFLLFEGLCCPGENTAAPQALQSYHLAAEEQLSFRMLQTSSFANHSWAHSEGSGWLG
DLQTHGWDTVLGTIRFLKPWSHGNFSKQELKNLQSLFQLYFHSFIQIVQASAGQFQLELK
PEAWLSCGPSPGPGRLQLVCHVSGFYPKPVWVMWMRGEQEQRGTQRGDVLPNADETWYLR

ATLDVAAGEAAGLSCRVKHSSLGGHDLIIHWGGYSIFLILICLTVIVTLVILVVVDSRLK
KQSSNKNILSPHTPSPVFLMGANTQDTKNSRHQFCLAQVSWIKNRVLKKWKTRLNQLW*
>ENSG00000097033|ENST00000370558
MNIMDFNVKKLAADAGTFLSRAVQFTEEKLGQAEKTELDAHLENLLSKAECTKIWTEKIM
KQTEVLLQPNPNARIEEFVYEKLDRKAPSRINNPELLGQYMIDAGTEFGPGTAYGNALIK
CGETQKRIGTADRELIQTSALNFLTPLRNFIEGDYKTIAKERKLLQNKRLDLDAAKTRLK
KAKAAETRNSSEQELRITQSEFDRQAEITRLLLEGISSTHAHHLRCLNDFVEAQMTYYAQ
CYQYMLDLQKQLGSFPSNYLSNNNQTSVTPVPSVLPNAIGSSAMASTSGLVITSPSNLSD
LKECSGSRKARVLYDYDAANSTELSLLADEVITVFSVVGMDSDWLMGERGNQKGKVPITY
LELLN*
>ENSG00000198925|ENST00000361242
MAQFDTEYQRLEASYSDSPPGEEDLLVHVAEGSKSPWHHIENLDLFFSRVYNLHQKNGFT
CMLIGEIFELMQFLFVVAFTTFLVSCVDYDILFANKMVNHSLHPTEPVKVTLPDAFLPAQ
VCSARIQENGSLITILVIAGVFWIHRLIKFIYNICCYWEIHSFYLHALRIPMSALPYCTW
QEVQARIVQTQKEHQICIHKRELTELDIYHRILRFQNYMVALVNKSLLPLRFRLPGLGEA
VFFTRGLKYNFELILFWGPGSLFLNEWSLKAEYKRGGQRLELAQRLSNRILWIGIANFLL
CPLILIWQILYAFFSYAEVLKREPGALGARCWSLYGRCYLRHFNELEHELQSRLNRGYKP
ASKYMNCFLSPLLTLLAKNGAFFAGSILAVLIALTIYDEDVLAVEHVLTTVTLLGVTVTV
CRSFIPDQHMVFCPEQLLRVILAHIHYMPDHWQGNAHRSQTRDEFAQLFQYKAVFILEEL
LSPIVTPLILIFCLRPRALEIIDFFRNFTVEVVGVGDTCSFAQMDVRQHGHPQWLSAGQT
EASVYQQAEDGKTELSLMHFAITNPGWQPPRESTAFLGFLKEQVQRDGAAASLAQGGLLP
ENALFTSIQSLQSESEPLSLIANVVAGSSCRGPPLPRDLQGSRHRAEVASALRSFSPLQP
GQAPTGRAHSTMTGSGVDARTASSGSSVWEGQLQSLVLSEYASTEMSLHALYMHQLHKQQ
AQAEPERHVWHRRESDESGESAPDEGGEGARAPQSIPRSASYPCAAPRPGAPETTALHGG
FQRRYGGITDPGTVPRVPSHFSRLPLGGWAEDGQSASRHPEPVPEEGSEDELPPQVHKV*
>ENSG00000126091|ENST00000361746
MGLLVFVRNLLLALCLFLVLGFLYYSAWKLHLLQWEEDSNSVVLSFDSAGQTLGSEYDRL
GFLLNLDSKLPAELATKYANFSEGACKPGYASALMTAIFPRFSKPAPMFLDDSFRKWARI
REFVPPFGIKGQETGFNHVAQAGLKLLSSSNPPSLASQSAGITDNLIKAILSVTKEYRLT
PALDSLRCRRCIIVGNGGVLANKSLGSRIDDYDIVVRLNSAPVKGFEKDVGSKTTLRITY
PEGAMQRPEQYERDSLFVLAGFKWQDFKWLKYIVYKERVSASDGFWKSVATRVPKEPPEI
RILNPYFIQEAAFTLIGLPFNNGLMGRGNIPTLGSVAVTMALHGCDEVAVAGFGYDMSTP
NAPLHYYETVRMAAIKESWTHNIQREKEFLRKLVKARVITDLSSGI*
>ENSG00000204482|ENST00000376110
MLSRNDVKRLERSWRLPVPSSEGPDLRGRDKRGTKEDPRADYACIAENKPT*
>ENSG00000188603|ENST00000357806
MGGCAGSRRRFSDSEGEETVPEPRLPLLDHQGAHWKNAVGFWLLGLCNNFSYVVMLSAAH
DILSHKRTSGNQSHVDPGPTPIPHNSSSRFDCNSVSTAPPGSRQWDLCCWKLRPGCLFSF
CGDQPVCYFLLLTSPEAQDPGGEEEAESAARQPLIRTEAPESKPGSSSSLSLRERWTVFK
GLLWYIVPLVVVYFAEYFINQGLFELLFFWNTSLSHAQQYRWYQMLYQAGVFASRSSLRC
CRIRFTWALALLQCLNLVFLLADVWFGFLPSIYLVFLIILYEGLLGGAAYVNTFHNIALE
TSDEHREFAMAATCISDTLGISLSGLLALPLHDFLCQLS*
>ENSG00000108669|ENST00000361101
MEEDDSYVPSDLTAEERQELENIRRRKQELLADIQRLKDEIAEVANEIENLGSTEERKNM

QRNKQVAMGRKKFNMDPKKGIQFLIENDLLKNTCEDIAQFLYKGEGLNKTAIGDYLGERD
EFNIQVLHAFVELHEFTDLNLVQALRQFLWSFRLPGEAQKIDRMMEAFAQRYCQCNNGVF
QSTDTCYVLSFAIIMLNTSLHNPNVKDKPTVERFIAMNRGINDGGDLPEELLRNLYESIK
NEPFKIPEDDGNDLTHTFFNPDREGWLLKLGGGRVKTWKRRWFILTDNCLYYFEYTTDKE
PRGIIPLENLSIREVEDSKKPNCFELYIPDNKDQVIKACKTEADGRVVEGNHTVYRISAP
TPEEKEEWIKCIKAAISRDPFYEMLAARKKKVSSTKRH*
>ENSG00000206240|ENST00000399450
MVCLRLPGGSCMAVLTVTLMVLSSPLALAGDTRPRFLEYSTSECHFFNGTERVRYLDRYF
HNQEENVRFDSDVGEFRAVTELGRPDAEYWNSQKDLLEQKRGRVDNYCRHNYGVVESFTV
QRRVHPKVTVYPSKTQPLQHHNLLVCSVSGFYPGSIEVRWFRNGQEEKTGVVSTGLIHNG
DWTFQTLVMLETVPRSGEVYTCQVEHPSVTSPLTVEWRARSESAQSKMLSGVGGFVLGLL
FLGAGLFIYFRNQKGHSGLQPRGFLS*
>ENSG00000087460|ENST00000371095
MGCLGNSKTEDQRNEEKAQREANKKIEKQLQKDKQVYRATHRLLLLGAGESGKSTIVKQM
RILHVNGFNGDSEKATKVQDIKNNLKEAIETIVAAMSNLVPPVELANPENQFRVDYILSV
MNVPDFDFPPEFYEHAKALWEDEGVRACYERSNEYQLIDCAQYFLDKIDVIKQADYVPSD
QDLLRCRVLTSGIFETKFQVDKVNFHMFDVGGQRDERRKWIQCFNDVTAIIFVVASSSYN
MVIREDNQTNRLQEALNLFKSIWNNRWLRTISVILFLNKQDLLAEKVLAGKSKIEDYFPE
FARYTTPEDATPEPGEDPRVTRAKYFIRDEFLRISTASGDGRHYCYPHFTCAVDTENIRR
VFNDCRDIIQRMHLRQYELL*
>ENSG00000068001|ENST00000357750
MRAGPGPTVTLALVLAVSWAMELKPTAPPIFTGRPFVVAWDVPTQDCGPRLKVPLDLNAF
DVQASPNEGFVNQNITIFYRDRLGLYPRFDSAGRSVHGGVPQNVSLWAHRKMLQKRVEHY
IRTQESAGLAVIDWEDWRPVWVRNWQDKDVYRRLSRQLVASRHPDWPPDRIVKQAQYEFE
FAAQQFMLETLRYVKAVRPRHLWGFYLFPDCYNHDYVQNWESYTGRCPDVEVARNDQLAW
LWAESTALFPSVYLDETLASSRHGRNFVSFRVQEALRVARTHHANHALPVYVFTRPTYSR
RLTGLSEMDLISTIGESAALGAAGVILWGDAGYTTSTETCQYLKDYLTRLLVPYVVNVSW
ATQYCSRAQCHGHGRCVRRNPSASTFLHLSTNSFRLVPGHAPGEPQLRPVGELSWADIDH
LQTHFRCQCYLGWSGEQCQWDHRQAAGGASEAWAGSHLTSLLALAALAFTWTL*
>ENSG00000160282|ENST00000397746
MSQLVECVPNFSEGKNQEVIDAISGAITQTPGCVLLDVDAGPSTNRTVYTFVGPPECVVE
GALNAARVASRLIDMSRHQGEHPRMGALDVCPFIPVRGVSVDECVLCAQAFGQRLAEELD
VPVYLYGEAARMDSRRTLPAIRAGEYEALPKKLQQADWAPDFGPSSFVPSWGATATGARK
FLIAFNINLLGTKEQAHRIALNLREQGRGKDQPGRLKKVQGIGWYLDEKNLAQVSTNLLD
FEVTALHTVYEETCREAQELSLPVVGSQLVGLVPLKALLDAAAFYCEKENLFILEEEQRI
RLVVSRLGLDSLCPFSPKERIIEYLVPERGPERGLGSKSLRAFVGEVGARSAAPGGGSVA
AAAAAMGAALGSMVGLMTYGRRQFQSLDTTMRRLIPPFREASAKLTTLVDADAEAFTAYL
EAMRLPKNTPEEKDRRTAALQEGLRRAVSVPLTLAETVASLWPALQELARCGNLACRSDL
QVAAKALEMGVFGAYFNVLINLRDITDEAFKDQIHHRVSSLLQEAKTQAALVLDCLETRQ
E*
>ENSG00000017260|ENST00000359644
MKVARFQKIPNGENETMIPVLTSKKASELPVSEVASILQADLQNGLNKCEVSHRRAFHGW
NEFDISEDEPLWKKYISQFKNPLIMLLLASAVISVLMHQFDDAVSITVAILIVVTVAFVQ
EYRSEKSLEELSKLVPPECHCVREGKLEHTLARDLVPGDTVCLSVGDRVPADLRLFEAVD

LSIDESSLTGETTPCSKVTAPQPAATNGDLASRSNIAFMGTLVRCGKAKGVVIGTGENSE
FGEVFKMMQAEEAPKTPLQKSMDLLGKQLSFYSFGIIGIIMLVGWLLGKDILEMFTISVS
LAVAAIPEGLPIVVTVTLALGVMRMVKKRAIVKKLPIVETLGCCNVICSDKTGTLTKNEM
TVTHIFTSDGLHAEVTGVGYNQFGEVIVDGDVVHGFYNPAVSRIVEAGCVCNDAVIRNNT
LMGKPTEGALIALAMKMGLDGLQQDYIRKAEYPFSSEQKWMAVKCVHRTQQDRPEICFMK
GAYEQVIKYCTTYQSKGQTLTLTQQQRDVYQQEKARMGSAGLRVLALASGPELGQLTFLG
LVGIIDPPRTGVKEAVTTLIASGVSIKMITGDSQETAVAIASRLGLYSKTSQSVSGEEID
AMDVQQLSQIVPKVAVFYRASPRHKMKIIKSLQKNGSVVAMTGDGVNDAVALKAADIGVA
MGQTGTDVCKEAADMILVDDDFQTIMSAIEEGKGIYNNIKNFVRFQLSTSIAALTLISLA
TLMNFPNPLNAMQILWINIIMDGPPAQSLGVEPVDKDVIRKPPRNWKDSILTKNLILKIL
VSSIIIVCGTLFVFWRELRDNVITPRDTTMTFTCFVFFDMFNALSSRSQTKSVFEIGLCS
NRMFCYAVLGSIMGQLLVIYFPPLQKVFQTESLSILDLLFLLGLTSSVCIVAEIIKKVER
SREKIQKHVSSTSSSFLEVWLWERSGQQLVEIHPHLETGLPLTEDVSCV*
>ENSG00000183166|ENST00000395275
MRLPEQPGEGKPENEKKGDGGALGGGEEPPRSQAPDFPTWEKMPFHHVTAGLLYKGNYLN
RSLSAGSDSEQLANISVEELDEIREAFRVLDRDGNGFISKQELGMAMRSLGYMPSEVELA
IIMQRLDMDGDGQVDFDEFMTILGPKLVSSEGRDGFLGNTIDSIFWQFDMQRITLEELKH
ILYHAFRDHLTMKDIENIIINEEESLNETSGNCQTEFEGVHSQKQNRQTCVRKSLICAFA
MAFIISVMLIAANQILRSGME*
>ENSG00000143641|ENST00000366672
MRRRSRMLLCFAFLWVLGIAYYMYSGGGSALAGGAGGGAGRKEDWNEIDPIKKKDLHHSN
GEEKAQSMETLPPGKVRWPDFNQEAYVGGTMVRSGQDPYARNKFNQVESDKLRMDRAIPD
TRHDQCQRKQWRVDLPATSVVITFHNEARSALLRTVVSVLKKSPPHLIKEIILVDDYSND
PEDGALLGKIEKVRVLRNDRREGLMRSRVRGADAAQAKVLTFLDSHCECNEHWLEPLLER
VAEDRTRVVSPIIDVINMDNFQYVGASADLKGGFDWNLVFKWDYMTPEQRRSRQGNPVAP
IKTPMIAGGLFVMDKFYFEELGKYDMMMDVWGGENLEISFRVWQCGGSLEIIPCSRVGHV
FRKQHPYTFPGGSGTVFARNTRRAAEVWMDEYKNFYYAAVPSARNVPYGNIQSRLELRKK
LSCKPFKWYLENVYPELRVPDHQDIAFGALQQGTNCLDTLGHFADGVVGVYECHNAGGNQ
EWALTKEKSVKHMDLCLTVVDRAPGSLIKLQGCRENDSRQKWEQIEGNSKLRHVGSNLCL
DSRTAKSGGLSVEVCGPALSQQWKFTLNLQQ*
>ENSG00000157214|ENST00000394622
MESISMMGSPKSLSETFLPNGINGIKDARKVTVGVIGSGDFAKSLTIRLIRCGYHVVIGS
RNPKFASEFFPHVVDVTHHEDALTKTNIIFVAIHREHYTSLWDLRHLLVGKILIDVSNNM
RINQYPESNAEYLASLFPDSLIVKGFNVVSAWALQLGPKDASRQVYICSNNIQARQQVIE
LARQLNFIPIDLGSLSSAREIENLPLRLFTLWRGPVVVAISLATFFFLYSFVRDVIHPYA
RNQQSDFYKIPIEIVNKTLPIVAITLLSLVYLAGLLAAAYQLYYGTKYRRFPPWLETWLQ
CRKQLGLLSFFFAMVHVAYSLCLPMRRSERYLFLNMAYQQVHANIENSWNEEEVWRIEMY
ISFGIMSLGLLSLLAVTSIPSVSNALNWREFSFIQSTLGYVALLISTFHVLIYGWKRAFE
EEYYRFYTPPNFVLALVLPSIVILGKIILFLPCISRKLKRIKKGWEKSQFLEEGMGGTIP
HVSPERVTVM*
>ENSG00000227993|ENST00000383127
MAISGVPVLGFFIIAVLMSAQESWAIKEEHVIIQAEFYLNPDQSGEFMFDFDGDEIFHVD
MAKKETVWRLEEFGRFASFEAQGALANIAVDKANLEIMTKRSNYTPITNVPPEVTVLTNS
PVELREPNVLICFIDKFTPPVVNVTWLRNGKPVTTGVSETVFLPREDHLFRKFHYLPFLP

STEDVYDCRVEHWGLDEPLLKHWEFDAPSPLPETTENVVCALGLTVGLVGIIIGTIFIIK
GLRKSNAAERRGPL*
>ENSG00000117758|ENST00000373943
MSYGPLDMYRNPGPSGPQLRDFSSIIQTCSGNIQRISQATAQIKNLMSQLGTKQDSSKLQ
ENLQQLQHSTNQLAKETNELLKELGSLPLPLSTSEQRQQRLQKERLMNDFSAALNNFQAV
QRRVSEKEKESIARARAGSRLSAEERQREEQLVSFDSHEEWNQMQSQEDEVAITEQDLEL
IKERETAIRQLEADILDVNQIFKDLAMMIHDQGDLIDSIEANVESSEVHVERATEQLQRA
AYYQKKSRKKMCILVLVLSVIILILGLIIWLVYKTK*
>ENSG00000008513|ENST00000399640
MVTLRKRTLKVLTFLVLFIFLTSFFLNYSHTMVATTWFPKQMVLELSENLKRLIKHRPCT
CTHCIGQRKLSAWFDERFNQTMQPLLTAQNALLEDDTYRWWLRLQREKKPNNLNDTIKEL
FRVVPGNVDPMLEKRSVGCRRCAVVGNSGNLRESSYGPEIDSHDFVLRMNKAPTAGFEAD
VGTKTTHHLVYPESFRELGDNVSMILVPFKTIDLEWVVSAITTGTISHTYIPVPAKIRVK
QDKILIYHPAFIKYVFDNWLQGHGRYPSTGILSVIFSMHVCDEVDLYGFGADSKGNWHHY
WENNPSAGAFRKTGVHDADFESNVTATLASINKIRIFKGR*
>ENSG00000206433|ENST00000400265
MLSRNDVKRLERSWRLPVPSSEGPDLRGRDKRGTKEDPRADYACIAENKPT*
>ENSG00000173230|ENST00000393667
MLSRLSGLANVVLHELSGDDDTDQNMRAPLDPELHQESDMEFNNTTQEDVQERLAYAEQL
VVELKDIIRQKDVQLQQKDEALQEERKAADNKIKKLKLHAKAKLTSLNKYIEEMKAQGGT
VLPTEPQSEEQLSKHDKSSTEEEMEIEKIKHKLQEKEELISTLQAQLTQAQAEQPAQSST
EMEEFVMMKQQLQEKEEFISTLQAQLSQTQAEQAAQLSSMQQVVREKDARFETQVRLHED
ELLQLVTQADVETEMQQKLRVLQRKLEEHEESLVGRAQVVDLLQQELTAAEQRNQILSQQ
LQQMEAEHNTLRNTVETEREESKILLEKMELEVAERKLSFHNLQEEMHHLLEQFEQAGQA
QAELESRYSALEQKHKAEMEEKTSHILSLQKTGQELQSACDALKDQNSKLLQDKNEQAVQ
SAQTIQQLEDQLQQKSKEISQFLNRLPLQQHETASQTSFPDVYNEGTQAVTEENIASLQK
RVVELENEKGALLLSSIELEELKAENEKLSSQITLLEAQNRTGEADREVSEISIVDIANK
RSSSAEESGQDVLENTFSQKHKELSVLLLEMKEAQEEIAFLKLQLQGKRAEEADHEVLDQ
KEMKQMEGEGIAPIKMKVFLEDTGQDFPLMPNEESSLPAVEKEQASTEHQSRTSEEISLN
DAGVELKSTKQDGDKSLSAVPDIGQCHQDELERLKSQILELELNFHKAQEIYEKNLDEKA
KEISNLNQLIEEFKKNADNNSSAFTALSEERDQLLSQVKELSMVTELRAQVKQLEMNLAE
AERQRRLDYESQTAHDNLLTEQIHSLSIEAKSKDVKIEVLQNELDDVQLQFSEQSTLIRS
LQSQLQNKESEVLEGAERVRHISSKVEELSQALSQKELEITKMDQLLLEKKRDVETLQQT
IEEKDQQVTEISFSMTEKMVQLNEEKFSLGVEIKTLKEQLNLLSRAEEAKKEQVEEDNEV
SSGLKQNYDEMSPAGQISKEELQHEFDLLKKENEQRKRKLQAALINRKELLQRVSRLEEE
LANLKDESKKEIPLSETERGEVEEDKENKEYSEKCVTSKCQEIEIYLKQTISEKEVELQH
IRKDLEEKLAAEEQFQALVKQMNQTLQDKTNQIDLLQAEISENQAIIQKLITSNTDASDG
DSVALVKETVVISPPCTGSSEHWKPELEEKILALEKEKEQLQKKLQEALTSRKAILKKAQ
EKERHLREELKQQKDDYNRLQEQFDEQSKENENIGDQLRQLQIQVRESIDGKLPSTDQQE
SCSSTPGLEEPLFKATEQHHTQPVLESNLCPDWPSHSEDASALQGGTSVAQIKAQLKEIE
AEKVELELKVSSTTSELTKKSEEVFQLQEQINKQGLEIESLKTVSHEAEVHAESLQQKLE
SSQLQIAGLEHLRELQPKLDELQKLISKKEEDVSYLSGQLSEKEAALTKIQTEIIEQEDL
IKALHTQLEMQAKEHDERIKQLQVELCEMKQKPEEIGEESRAKQQIQRKLQAALISRKEA
LKENKSLQEELSLARGTIERLTKSLADVESQVSAQNKEKDTVLGRLALLQEERDKLITEM

DRSLLENQSLSSSCESLKLALEGLTEDKEKLVKEIESLKSSKIAESTEWQEKHKELQKEY
EILLQSYENVSNEAERIQHVVEAVRQEKQELYGKLRSTEANKKETEKQLQEAEQEMEEMK
EKMRKFAKSKQQKILELEEENDRLRAEVHPAGDTAKECMETLLSSNASMKEELERVKMEY
ETLSKKFQSLMSEKDSLSEEVQDLKHQIEGNVSKQANLEATEKHDNQTNVTEEGTQSIPG
ETEEQDSLSMSTRPTCSESVPSAKSANPAVSKDFSSHDEINNYLQQIDQLKERIAGLEEE
KQKNKEFSQTLENEKNTLLSQISTKDGELKMLQEEVTKMNLLNQQIQEELSRVTKLKETA
EEEKDDLEERLMNQLAELNGSIGNYCQDVTDAQIKNELLESEMKNLKKCVSELEEEKQQL
VKEKTKVESEIRKEYLEKIQGAQKEPGNKSHAKELQELLKEKQQEVKQLQKDCIRYQEKI
SALERTVKALEFVQTESQKDLEITKENLAQAVEHRKKAQAELASFKVLLDDTQSEAARVL
ADNLKLKKELQSNKESVKSQMKQKDEDLERRLEQAEEKHLKEKKNMQEKLDALRREKVHL
EETIGEIQVTLNKKDKEVQQLQENLDSTVTQLAAFTKSMSSLQDDRDRVIDEAKKWERKF
SDAIQSKEEEIRLKEDNCSVLKDQLRQMSIHMEELKINISRLEHDKQIWESKAQTEVQLQ
QKVCDTLQGENKELLSQLEETRHLYHSSQNELAKLESELKSLKDQLTDLSNSLEKCKEQK
GNLEGIIRQQEADIQNSKFSYEQLETDLQASRELTSRLHEEINMKEQKIISLLSGKEEAI
QVAIAELRQQHDKEIKELENLLSQEEEENIVLEEENKKAVDKTNQLMETLKTIKKENIQQ
KAQLDSFVKSMSSLQNDRDRIVGDYQQLEERHLSIILEKDQLIQEAAAENNKLKEEIRGL
RSHMDDLNSENAKLDAELIQYREDLNQVITIKDSQQKQLLEVQLQQNKELENKYAKLEEK
LKESEEANEDLRRSFNALQEEKQDLSKEIESLKVSISQLTRQVTALQEEGTLGLYHAQLK
VKEEEVHRLSALFSSSQKRIAELEEELVCVQKEAAKKVGEIEDKLKKELKHLHHDAGIMR
NETETAEERVAELARDLVEMEQKLLMVTKENKGLTAQIQSFGRSMSSLQNSRDHANEELD
ELKRKYDASLKELAQLKEQGLLNRERDALLSETAFSMNSTEENSLSHLEKLNQQLLSKDE
QLLHLSSQLEDSYNQVQSFSKAMASLQNERDHLWNELEKFRKSEEGKQRSAAQPSTSPAE
VQSLKKAMSSLQNDRDRLLKELKNLQQQYLQINQEITELHPLKAQLQEYQDKTKAFQIMQ
EELRQENLSWQHELHQLRMEKSSWEIHERRMKEQYLMAISDKDQQLSHLQNLIRELRSSS
SQTQPLKVQYQRQASPETSASPDGSQNLVYETELLRTQLNDSLKEIHQKELRIQQLNSNF
SQLLEEKNTLSIQLCDTSQSLRENQQHYGDLLNHCAVLEKQVQELQAVSKEKGPLNIDVA
PGAPQEKNGVHRKSDPEELREPQQSFSEAQQQLCNTRQEVNELRKLLEEERDQRVAAENA
LSVAEEQIRRLEHSEWDSSRTPIIGSCGTQEQALLIDLTSNSCRRTRSGVGWKRVLRSLC
HSRTRVPLLAAIYFLMIHVLLILCFTGHL*
>ENSG00000070831|ENST00000400259
MQTIKCVVVGDGAVGKTCLLISYTTNKFPSEYVPTVFDNYAVTVMIGGEPYTLGLFDTAG
QEDYDRLRPLSYPQTDVFLVCFSVVSPSSFENVKEKWVPEITHHCPKTPFLLVGTQIDLR
DDPSTIEKLAKNKQKPITPETAEKLARDLKAVKYVECSALTQKGLKNVFDEAILAALEPP
EPKKSRRCVLL*
>ENSG00000167632|ENST00000389328
MVPAGDQDRAPHRGKPAQAGARTSRASRALRSWRRSQAARATVTHPRGGHDRGSHGGYRE
GHRGCRRDPQWASAGPPPLSFTEEVKFELRALKDWDFKMSVPDYMQCAEDHQTLLVVVQP
VGIVSEENFFRIYKRICSVSQISVRDSQRVLYIRYRHHYPPENNEWGDFQTHRKVVGLIT
ITDCFSAKDWPQTFEKFHVQKEIYGSTLYDSRLFVFGLQGEIVEQPRTDVAFYPNYEDCQ
TVEKRIEDFIESLFIVLESKRLDRATDKSGDKIPLLCVPFEKKDFVGLDTDSRHYKKRCQ
GRMRKHVGDLCLQAGMLQDSLVHYHMSVELLRSVNDFLWLGAALEGLCSASVIYHYPGGT
GGKSGARRFQGSTLPAEAANRHRPGAQEVLIDPGALTTNGINPDTSTEIGRAKNCLSPED
IIDKYKEAISYYSKYKNAGVIELEACIKAVRVLAIQKRSMEASEFLQNAVYINLRQLSEE
EKIQRYSILSELYELIGFHRKSAFFKRVAAMQCVAPSIAEPGWRACYKLLLETLPGYSLS

LDPKDFSRGTHRGWAAVQMRLLHELVYASRRMGNPALSVRHLSFLLQTMLDFLSDQEKKD
VAQSLENYTSKCPGTMEPIALPGGLTLPPVPFTKLPIVRHVKLLNLPASLRPHKMKSLLG
QNVSTKSPFIYSPIIAHNRGEERNKKIDFQWVQGDVCEVQLMVYNPMPFELRVENMGLLT
SGVEFESLPAALSLPAESGLYPVTLVGVPQTTGTITVNGYHTTVFGVFSDCLLDNLPGIK
TSGSTVEVIPALPRLQISTSLPRSAHSLQPSSGDEISTNVSVQLYNGESQQLIIKLENIG
MEPLEKLEVTSKVLTTKEKLYGDFLSWKLEETLAQFPLQPGKVATFTINIKVKLDFSCQE
NLLQDLSDDGISVSGFPLSSPFRQVVRPRVEGKPVNPPESNKAGDYSHVKTLEAVLNFKY
SGGPGHTEGYYRNLSLGLHVEVEPSVFFTRVSTLPATSTRQCHLLLDVFNSTEHELTVST
RSSEALILHAGECQRMAIQVDKFNFESFPESPGEKGQFANPKQLEEERREARGLEIHSKL
GICWRIPSLKRSGEASVEGLLNQLVLEHLQLAPLQWDVLVDGQPCDREAVAACQVGDPVR
LEVRLTNRSPRSVGPFALTVVPFQDHQNGVHNYDLHDTVSFVGSSTFYLDAVQPSGQSAC
LGALLFLYTGDFFLHIRFHEDSTSKELPPSWFCLPSVHVCALEAQA*
>ENSG00000169894|ENST00000379458
MQLLGLLGLLWMLKASPWATGTLSTATSISQVPFPRAEAASAVLSNSPHSRDLAGWPLGV
PQLASPAPGHRENAPMTLTTSPHDTLISETLLNSPVSSNTSTTPTSKFAFKVETTPPTVL
VYSATTECVYPTSFIITISHPTSICVTTTQVAFTSSYTSTPVTQKPVTTVTSTYSMTTTE
KGTSAMTSSPSTTTARETPIVTVTPSSVSATDTTFHTTISSTTRTTERTPLPTGSIHTTT
SPTPVFTTLKTAVTSTSPITSSITSTNTVTSMTTTASQPTATNTLSSPTRTILSSTPVLS
TETITSGITNTTPLSTLVTTLPTTISRSTPTSETTYTTSPTSTVTDSTTKIAYSTSMTGT
LSTETSLPPTSSSLPTTETATTPMTNLVTTTTEISSHSTPSFSSSTIYSTVSTSTTAISS
LPPTSGTMVTSTTMTPSSLSTDIPFTTPTTITHHSVGSTGFLTTATDLTSTFTVSSSSAM
STSVIPSSPSIQNTETSSLVSMTSATTPNVRPTFVSTLSTPTSSLLTTFPATYSFSSSMS
ASSAGTTHTESISSPPASTSTLHTTAESTLAPTTTTSFTTSTTMEPPSTTAATTGTGQTT
FTSSTATFPETTTPTPTTDMSTESLTTAMTSPPITSSVTSTNTVTSMTTTTSPPTTTNSF
TSLTSMPLSSTPVPSTEVVTSGTINTIPPSILVTTLPTPNASSMTTSETTYPNSPTGPGT
NSTTEITYPTTMTETSSTATSLPPTSPLVSTAKTAKTPTTNLVTTTTKTTSHSTTSFTSS
TVYSTASTYTTAITSVPTTLGTMVTSTSMISSTVSTGIPTSQPTTITPSSVGISGSLPMM
TDLTSVYTVSNMSARPTTVIPSSPTVQNTEISISVSMTSATTPSGGPTFTSTENTPTRSL
LTSFPMTHSFSSSMSESSAGTTHTESISSPRGTTSTLHTTVESTPSPTTTTSFTTSTMME
PPSSTVSTTGRGQTTFPSSTATFPETTTLTPTTDISTVSLTTAMTSPPPVSSSITPTNTM
TSMRTTTYWPTATNTLSPLTSSILSSTPVPSTEMITSHTTNTTPLSTLVTTLLTTITRST
PTSETTYPTSPTSIVSDSTTEITYSTSITGTLSTATTLPPTSSSLPTTETATMTPTTTLI
TTTPNTTSLSTPSFTSSTIYSTVSTSTTAISSASPTSGTMVTSTTMTPSSLSTDTPSTTP
TTITYPSVGSTGFLTTATDLTSTFTVSSSSAMSTSVIPSSPSIQNTETSSLVSMTSATTP
SLRPTITSTDSTLTSSLLTTFPSTYSFSSSMSASSAGTTHTETISSLPASTNTIHTTAES
ALAPTTTTSFTTSPTMEPPSTTVATTGTGQTTFPSSTATFLETTTLTPTTDFSTESLTTA
MTSTPPITSSITPTDTMTSMRTTTSWPTATNTLSPLTSSILSSTPVPSTEVTTSHTTNTN
PVSTLVTTLPITITRSTLTSETAYPSSPTSTVTESTTEITYPTTMTETSSTATSLPPTSS
LVSTAETAKTPTTNLVTTTTKTTSHSTTSFTSSTIYSTASTPTTAITSVPTTLGTMVTST
SMIPSTVSTGIPTSQPTTITPSSVGISGSLPMMTDLTSVYTVSSMSARPTSVIPSSPTVQ
NTETSIFVSMMSATTPSGGPTFTSTENTPTRSLLTSFPVTHSFSSSMSASSVGTTHTQSI
SSPPAITSTLHTTAESTPSPTTTMSFTTFTKMETPSSTVATTGTGQTTFTSSTATSPKTT
TLTPTSDISTGSFKTAVSSTPPITSSITSTYTVTSMTTTTPLGPTATNTLPSFTSSVSSS
TPVPSTEAITSGTTNTTPLSTLVTTFSNSDTSSTPTSETTYPTSLTSALTDSTTRTTYST

NMTGTLSTVTSLRPTSSSLLTTVTATVPTTNLVTTTTKITSHSTPSFTSSIATTETPSHS
TPRFTSSITTTETPSHSTPRFTSSITNTKTTSHSSPSFTSSITTTETTSHNTPSLTSSIT
TTKTTSHSTPSYTSLITTTTTTSHSTPSFTSSITTTETTSHNTPSLTSSITTTETTSHST
PSFTSSITTETTSHSTPSFTSLITITEITSHSTLSYTTSITTTETPSHSTLSFTSSITTT
ETTSHSTPSFTSSITTSEMPSHSTPSFTSSITTTENATHSTPNFTSSITTTETTSHSTPS
FTSLITTTETTSHRWGTTETTSYSTPSFTSSNTITETTSHSTPSYITSITTTETPSSSTP
SFSSSITTTETTSHSTPGFTSSITTTETTSHSTPSFTSSITTTETTSHDTPSFTSSITTS
ETPSHSTPSSTSLITTTKTTSHSTPSFTSSITTTETTSHSAHSFTSSITTTETTSHNTRS
FTSSITTTETNSHSTTSFTSSITTTETTSHSTPSFSSSITTTETPLHSTPGLTSWVTTTK
TTSHITPGLTSSITTTETTSHSTPGFTSSITTTETTSESTPSLSSSTIYSTVSTSTTAIT
SHFTTSETAVTPTPVTPSSLSTDIPTTSLRTLTPSSVGTSTSLTTTTDFPSIPTDISTLP
TRTHIISSSPSIQSTETSSLVGTTSPTMSTVRMTLRITENTPISSFSTSIVVIPETPTQT
PPVLTSATGTQTSPAPTTVTFGSTDSSTSTLHTLTPSTALSTIVSTSQVPIPSTHSSTLQ
TTPSTPSLQTSLTSTSEFTTESFTRGSTSTNAILTSFSTIIWSSTPTIIMSSSPSSASIT
PVFSTTIHSVPSSPYIFSTENVGSASITGFPSLSSSATTSTSSTSSSLTTALTEITPFSY
ISLPSTTPCPGTITITIVPASPTDPCVEMDPSTEATSPPTTPLTVFPFTTEMVTCPTSIS
IQTTLTTYMDTSSMMPESESSISPNASSSTGTGTVPTNTVFTSTRLPTSETWLSNSSVIP
LPLPGVSTIPLTMKPSSSLPTILRTSSKSTHPSPPTTRTSETPVATTQTPTTLTSRRTTR
ITSQMTTQSTLTTTAGTCDNGGTWEQGQCACLPGFSGDRCQLQTRCQNGGQWDGLKCQCP
STFYGSSCEFAVEQVDLDVVETEVGMEVSVDQQFSPDLNDNTSQAYRDFNKTFWNQMQKI
FADMQGFTFKGVEILSLRNGSIVVDYLVLLEMPFSPQLESEYEQVKTTLKEGLQNASQDV
NSCQDSQTLCFKPDSIKVNNNSKTELTPAAICRRAAPTGYEEFYFPLVEATRLRCVTKCT
SGVDNAIDCHQGQCVLETSGPTCRCYSTDTHWFSGPRCEVAVHWRALVGGLTAGAALLVL
LLLALGVRAVRSGWWGGQRRGRSWDQDRKWFETWDEEVVGTFSNWGFEDDGTDKDTNFYV
ALENVDTTMKVHIKRPEMTSSSV*
>ENSG00000164023|ENST00000394686
MDIIETAKLEEHLENQPSDPTNTYARPAEPVEEENKNGNGKPKSLSSGLRKGTKKYPDYI
QIAMPTESRNKFPLEWWKTGIAFIYAVFNLVLTTVMITVVHERVPPKELSPPLPDKFFDY
IDRVKWAFSVSEINGIILVGLWITQWLFLRYKSIVGRRFCFIIGTLYLYRCITMYVTTLP
VPGMHFQCAPKLNGDSQAKVQRILRLISGGGLSITGSHILCGDFLFSGHTVTLTLTYLFI
KEYSPRHFWWYHLICWLLSAAGIICILVAHEHYTIDVIIAYYITTRLFWWYHSMANEKNL
KVSSQTNFLSRAWWFPIFYFFEKNVQGSIPCCFSWPLSWPPGCFKSSCKKYSRVQKIGED
NEKST*
>ENSG00000057593|ENST00000375581
MVSQALRLLCLLLGLQGCLAAGGVAKASGGETRDMPWKPGPHRVFVTQEEAHGVLHRRRR
ANAFLEELRPGSLERECKEEQCSFEEAREIFKDAERTKLFWISYSDGDQCASSPCQNGGS
CKDQLQSYICFCLPAFEGRNCETHKDDQLICVNENGGCEQYCSDHTGTKRSCRCHEGYSL
LADGVSCTPTVEYPCGKIPILEKRNASKPQGRIVGGKVCPKGECPWQVLLLVNGAQLCGG
TLINTIWVVSAAHCFDKIKNWRNLIAVLGEHDLSEHDGDEQSRRVAQVIIPSTYVPGTTN
HDIALLRLHQPVVLTDHVVPLCLPERTFSERTLAFVRFSLVSGWGQLLDRGATALELMVL
NVPRLMTQDCLQQSRKVGDSPNITEYMFCAGYSDGSKDSCKGDSGGPHATHYRGTWYLTG
IVSWGQGCATVGHFGVYTRVSQYIEWLQKLMRSEPRPGVLLRAPFP*
>ENSG00000117533|ENST00000367740
MPPKFKRHLNDDDVTGSVKSERRNLLEDDSDEEEDFFLGPSGPRFGPRNDKIKHVQNQVD

EVIDVMQENITKVIERGERLDELQDKSESLSDNATAFSNRSKQLRRQMWWRGCKIKAIMA
LVAAILLLVIIILIVMKYRT*
>ENSG00000181143|ENST00000397910
MLKPSGLPGSSSPTRSLMTGSRSTKATPEMDSGLTGATLSPKTSTGAIVVTEHTLPFTSP
DKTLASPTSSVVGRTTQSLGVMSSALPESTSRGMTHSEQRTSPSLSPQVNGTPSRNYPAT
SMVSGLSSPRTRTSSTEGNFTKEASTYTLTVETTSGPVTEKYTVPTETSTTEGDSTETPW
DTRYIPVKITSPMKTFADSTASKENAPVSMTPAETTVTDSHTPGRTNPSFGTLYSSFLDL
SPKGTPNSRGETSLELILSTTGYPFSSPEPGSAGHSRISTSAPLSSSASVLDNKISETSI
FSGQSLTSPLSPGVPEARASTMPNSAIPFSMTLSNAETSAERVRSTISSLGTPSISTKQT
AETILTFHAFAETMDIPSTHIAKTLASEWLGSPGTLGGTSTSALTTTSPSTTLVSEETNT
HHSTSGKETEGTLNTSMTPLETSAPGEESEMTATLVPTLGFTTLDSKIRSPSQVSSSHPT
RELRTTGSTSGRQSSSTAAHGSSDILRATTSSTSKASSWTSESTAQQFSEPQHTQWVETS
PSMKTERPPASTSVAAPITTSVPSVVSGFTTLKTSSTKGIWLEETSADTLIGESTAGPTT
HQFAVPTGISMTGGSSTRGSQGTTHLLTRATASSETSADLTLATNGVPVSVSPAVSKTAA
GSSPPGGTKPSYTMVSSVIPETSSLQSSAFREGTSLGLTPLNTRHPFSSPEPDSAGHTKI
STSIPLLSSASVLEDKVSATSTFSHHKATSSITTGTPEISTKTKPSSAVLSSMTLSNAAT
SPERVRNATSPLTHPSPSGEETAGSVLTLSTSAETTDSPNIHPTGTLTSESSESPSTLSL
PSVSGVKTTFSSSTPSTHLFTSGEETEETSNPSVSQPETSVSRVRTTLASTSVPTPVFPT
MDTWPTRSAQFSSSHLVSELRATSSTSVTNSTGSALPKISHLTGTATMSQTNRDTFNDSA
APQSTTWPETSPRFKTGLPSATTTVSTSATSLSATVMVSKFTSPATSSMEATSIREPSTT
ILTTETTNGPGSMAVASTNIPIGKGYITEGRLDTSHLPIGTTASSETSMDFTMAKESVSM
SVSPSQSMDAAGSSTPGRTSQFVDTFSDDVYHLTSREITIPRDGTSSALTPQMTATHPPS
PDPGSARSTWLGILSSSPSSPTPKVTMSSTFSTQRVTTSMIMDTVETSRWNMPNLPSTTS
LTPSNIPTSGAIGKSTLVPLDTPSPATSLEASEGGLPTLSTYPESTNTPSIHLGAHASSE
SPSTIKLTMASVVKPGSYTPLTFPSIETHIHVSTARMAYSSGSSPEMTAPGETNTGSTWD
PTTYITTTDPKDTSSAQVSTPHSVRTLRTTENHPKTESATPAAYSGSPKISSSPNLTSPA
TKAWTITDTTEHSTQLHYTKLAEKSSGFETQSAPGPVSVVIPTSPTIGSSTLELTSDVPG
EPLVLAPSEQTTITLPMATWLSTSLTEEMASTDLDISSPSSPMSTFAIFPPMSTPSHELS
KSEADTSAIRNTDSTTLDQHLGIRSLGRTGDLTTVPITPLTTTWTSVIEHSTQAQDTLSA
TMSPTHVTQSLKDQTSIPASASPSHLTEVYPELGTQGRSSSEATTFWKPSTDTLSREIET
GPTNIQSTPPMDNTTTGSSSSGVTLGIAHLPIGTSSPAETSTNMALERRSSTATVSMAGT
MGLLVTSAPGRSISQSLGRVSSVLSESTTEGVTDSSKGSSPRLNTQGNTALSSSLEPSYA
EGSQMSTSIPLTSSPTTPDVEFIGGSTFWTKEVTTVMTSDISKSSARTESSSATLMSTAL
GSTENTGKEKLRTASMDLPSPTPSMEVTPWISLTLSNAPNTTDSLDLSHGVHTSSAGTLA
TDRSLNTGVTRASRLENGSDTSSKSLSMGNSTHTSMTYTEKSEVSSSIHPRPETSAPGAE
TTLTSTPGNRAISLTLPFSSIPVEEVISTGITSGPDINSAPMTHSPITPPTIVWTSTGTI
EQSTQPLHAVSSEKVSVQTQSTPYVNSVAVSASPTHENSVSSGSSTSSPYSSASLESLDS
TISRRNAITSWLWDLTTSLPTTTWPSTSLSEALSSGHSGVSNPSSTTTEFPLFSAASTSA
AKQRNPETETHGPQNTAASTLNTDASSVTGLSETPVGASISSEVPLPMAITSRSDVSGLT
SESTANPSLGTASSAGTKLTRTISLPTSESLVSFRMNKDPWTVSIPLGSHPTTNTETSIP
VNSAGPPGLSTVASDVIDTPSDGAESIPTVSFSPSPDTEVTTISHFPEKTTHSFRTISSL
THELTSRVTPIPGDWMSSAMSTKPTGASPSITLGERRTITSAAPTTSPIVLTASFTETST
VSLDNETTVKTSDILDARKTNELPSDSSSSSDLINTSIASSTMDVTKTASISPTSISGMT
ASSSPSLFSSDRPQVPTSTTETNTATSPSVSSNTYSLDGGSNVGGTPSTLPPFTITHPVE

TSSALLAWSRPVRTFSTMVSTDTASGENPTSSNSVVTSVPAPGTWTSVGSTTDLPAMGFL
KTSPAGEAHSLLASTIEPATAFTPHLSAAVVTGSSATSEASLLTTSESKAIHSSPQTPTT
PTSGANWETSATPESLLVVTETSDTTLTSKILVTDTILFSTVSTPPSKFPSTGTLSGASF
PTLLPDTPAIPLTATEPTSSLATSFDSTPLVTIASDSLGTVPETTLTMSETSNGDALVLK
TVSNPDRSIPGITIQGVTESPLHPSSTSPSKIVAPRNTTYEGSITVALSTLPAGTTGSLV
FSQSSENSETTALVDSSAGLERASVMPLTTGSQGMASSGGIRSGSTHSTGTKTFSSLPLT
MNPGEVTAMSEITTNRLTATQSTAPKGIPVKPTSAESGLLTPVSASSSPSKAFASLTTAP
PTWGIPQSTLTFEFSEVPSLDTKSASLPTPGQSLNTIPDSDASTASSSLSKSPEKNPRAR
MMTSTKAISASSFQSTGFTETPEGSASPSMAGHEPRVPTSGTGDPRYASESMSYPDPSKA
SSAMTSTSLASKLTTLFSTGQAARSGSSSSPISLSTEKETSFLSPTASTSRKTSLFLGPS
MARQPNILVHLQTSALTLSPTSTLNMSQEEPPELTSSQTIAEEEGTTAETQTLTFTPSET
PTSLLPVSSPTEPTARRKSSPETWASSISVPAKTSLVETTDGTLVTTIKMSSQAAQGNST
WPAPAEETGSSPAGTSPGSPEMSTTLKIMSSKEPSISPEIRSTVRNSPWKTPETTVPMET
TVEPVTLQSTALGSGSTSISHLPTGTTSPTKSPTENMLATERVSLSPSPPEAWTNLYSGT
PGGTRQSLATMSSVSLESPTARSITGTGQQSSPELVSKTTGMEFSMWHGSTGGTTGDTHV
SLSTSSNILEDPVTSPNSVSSLTDKSKHKTETWVSTTAIPSTVLNNKIMAAEQQTSRSVD
EAYSSTSSWSDQTSGSDITLGASPDVTNTLYITSTAQTTSLVSLPSGDQGITSLTNPSGG
KTSSASSVTSPSIGLETLRANVSAVKSDIAPTAGHLSQTSSPAEVSILDVTTAPTPGIST
TITTMGTNSISTTTPNPEVGMSTMDSTPATERRTTSTEHPSTWSSTAASDSWTVTDMTSN
LKVARSPGTISTMHTTSFLASSTELDSMSTPHGRITVIGTSLVTPSSDASAVKTETSTSE
RTLSPSDTTASTPISTFSRVQRMSISVPDILSTSWTPSSTEAEDVPVSMVSTDHASTKTD
PNTPLSTFLFDSLSTLDWDTGRSLSSATATTSAPQGATTPQELTLETMISPATSQLPFSI
GHITSAVTPAAMARSSGVTFSRPDPTSKKAEQTSTQLPTTTSAHPGQVPRSAATTLDVIP
HTAKTPDATFQRQGQTALTTEARATSDSWNEKEKSTPSAPWITEMMNSVSEDTIKEVTSS
SSVLRTLNTLDINLESGTTSSPSWKSSPYERIAPSESTTDKEAIHPSTNTVETTGWVTSS
EHASHSTIPAHSASSKLTSPVVTTSTREQAIVSMSTTTWPESTRARTEPNSFLTIELRDV
SPYMDTSSTTQTSIISSPGSTAITKGPRTEITSSKRISSSFLAQSMRSSDSPSEAITRLS
NFPAMTESGGMILAMQTSPPGATSLSAPTLDTSATASWTGTPLATTQRFTYSEKTTLFSK
GPEDTSQPSPPSVEETSSSSSLVPIHATTSPSNILLTSQGHSPSSTPPVTSVFLSETSGL
GKTTDMSRISLEPGTSLPPNLSSTAGEALSTYEASRDTKAIHHSADTAVTNMEATSSEYS
PIPGHTKPSKATSPLVTSHIMGDITSSTSVFGSSETTEIETVSSVNQGLQERSTSQVASS
ATETSTVITHVSSGDATTHVTKTQATFSSGTSISSPHQFITSTNTFTDVSTNPSTSLIMT
ESSGVTITTQTGPTGAATQGPYLLDTSTMPYLTETPLAVTPDFMQSEKTTLISKGPKDVS
WTSPPSVAETSYPSSLTPFLVTTIPPATSTLQGQHTSSPVSATSVLTSGLVKTTDMLNTS
MEPVTNSPQNLNNPSNEILATLAATTDIETIHPSINKAVTNMGTASSAHVLHSTLPVSSE
PSTATSPMVPASSMGDALASISIPGSETTDIEGEPTSSLTAGRKENSTLQEMNSTTESNI
ILSNVSVGAITEATKMEVPSFDATFIPTPAQSTKFPDIFSVASSRLSNSPPMTISTHMTT
TQTGSSGATSKIPLALDTSTLETSAGTPSVVTEGFAHSKITTAMNNDVKDVSQTNPPFQD
EASSPSSQAPVLVTTLPSSVAFTPQWHSTSSPVSMSSVLTSSLVKTAGKVDTSLETVTSS
PQSMSNTLDDISVTSAATTDIETTHPSINTVVTNVGTTGSAFESHSTVSAYPEPSKVTSP
NVTTSTMEDTTISRSIPKSSKTTRTETETTSSLTPKLRETSISQEITSSTETSTVPYKEL
TGATTEVSRTDVTSSSSTSFPGPDQSTVSLDISTETNTRLSTSPIMTESAEITITTQTGP
HGATSQDTFTMDPSNTTPQAGIHSAMTHGFSQLDVTTLMSRIPQDVSWTSPPSVDKTSSP
SSFLSSPAMTTPSLISSTLPEDKLSSPMTSLLTSGLVKITDILRTRLEPVTSSLPNFSST

SDKILATSKDSKDTKEIFPSINTEETNVKANNSGHESHSPALADSETPKATTQMVITTTV
GDPAPSTSMPVHGSSETTNIKREPTYFLTPRLRETSTSQESSFPTDTSFLLSKVPTGTIT
EVSSTGVNSSSKISTPDHDKSTVPPDTFTGEIPRVFTSSIKTKSAEMTITTQASPPESAS
HSTLPLDTSTTLSQGGTHSTVTQGFPYSEVTTLMGMGPGNVSWMTTPPVEETSSVSSLMS
SPAMTSPSPVSSTSPQSIPSSPLPVTALPTSVLVTTTDVLGTTSPESVTSSPPNLSSITH
ERPATYKDTAHTEAAMHHSTNTAVTNVGTSGSGHKSQSSVLADSETSKATPLMSTTSTLG
DTSVSTSTPNISQTNQIQTEPTASLSPRLRESSTSEKTSSTTETNTAFSYVPTGAITQAS
RTEISSSRTSISDLDRPTIAPDISTGMITRLFTSPIMTKSAEMTVTTQTTTPGATSQGIL
PWDTSTTLFQGGTHSTVSQGFPHSEITTLRSRTPGDVSWMTTPPVEETSSGFSLMSPSMT
SPSPVSSTSPESIPSSPLPVTALLTSVLVTTTNVLGTTSPEPVTSSPPNLSSPTQERLTT
YKDTAHTEAMHASMHTNTAVANVGTSISGHESQSSVPADSHTSKATSPMGITFAMGDTSV
STSTPAFFETRIQTESTSSLIPGLRDTRTSEEINTVTETSTVLSEVPTTTTTEVSRTEVI
TSSRTTISGPDHSKMSPYISTETITRLSTFPFVTGSTEMAITNQTGPIGTISQATLTLDT
SSTASWEGTHSPVTQRFPHSEETTTMSRSTKGVSWQSPPSVEETSSPSSPVPLPAITSHS
SLYSAVSGSSPTSALPVTSLLTSGRRKTIDMLDTHSELVTSSLPSASSFSGEILTSEAST
NTETIHFSENTAETNMGTTNSMHKLHSSVSIHSQPSGHTPPKVTGSMMEDAIVSTSTPGS
PETKNVDRDSTSPLTPELKEDSTALVMNSTTESNTVFSSVSLDAATEVSRAEVTYYDPTF
MPASAQSTKSPDISPEASSSHSNSPPLTISTHKTIATQTGPSGVTSLGQLTLDTSTIATS
AGTPSARTQDFVDSETTSVMNNDLNDVLKTSPFSAEEANSLSSQAPLLVTTSPSPVTSTL
QEHSTSSLVSVTSVPTPTLAKITDMDTNLEPVTRSPQNLRNTLATSEATTDTHTMHPSIN
TAVANVGTTSSPNEFYFTVSPDSDPYKATSAVVITSTSGDSIVSTSMPRSSAMKKIESET
TFSLIFRLRETSTSQKIGSSSDTSTVFDKAFTAATTEVSRTELTSSSRTSIQGTEKPTMS
PDTSTRSVTMLSTFAGLTKSEERTIATQTGPHRATSQGTLTWDTSITTSQAGTHSAMTHG
FSQLDLSTLTSRVPEYISGTSPPSVEKTSSSSSLLSLPAITSPSPVPTTLPESRPSSPVH
LTSLPTSGLVKTTDMLASVASLPPNLGSTSHKIPTTSEDIKDTEKMYPSTNIAVTNVGTT
TSEKESYSSVPAYSEPPKVTSPMVTSFNIRDTIVSTSMPGSSEITRIEMESTFSLAHGLK
GTSTSQDPIVSTEKSAVLHKLTTGATETSRTEVASSRRTSIPGPDHSTESPDISTEVIPS
LPISLGITESSNMTIITRTGPPLGSTSQGTFTLDTPTTSSRAGTHSMATQEFPHSEMTTV
MNKDPEILSWTIPPSIEKTSFSSSLMPSPAMTSPPVSSTLPKTIHTTPSPMTSLLTPSLV
MTTDTLGTSPEPTTSSPPNLSSTSHEILTTDEDTTAIEAMHPSTSTAATNVETTSSGHGS
QSSVLADSEKTKATAPMDTTSTMGHTTVSTSMSVSSETTKIKRESTYSLTPGLRETSISQ
NASFSTDTSIVLSEVPTGTTAEVSRTEVTSSGRTSIPGPSQSTVLPEISTRTMTRLFASP
TMTESAEMTIPTQTGPSGSTSQDTLTLDTSTTKSQAKTHSTLTQRFPHSEMTTLMSRGPG
DMSWQSSPSLENPSSLPSLLSLPATTSPPPISSTLPVTISSSPLPVTSLLTSSPVTTTDM
LHTSPELVTSSPPKLSHTSDERLTTGKDTTNTEAVHPSTNTAASNVEIPSSGHESPSSAL
ADSETSKATSPMFITSTQEDTTVAISTPHFLETSRIQKESISSLSPKLRETGSSVETSSA
IETSAVLSEVSIGATTEISRTEVTSSSRTSISGSAESTMLPEISTTRKIIKFPTSPILAE
SSEMTIKTQTSPPGSTSESTFTLDTSTTPSLVITHSTMTQRLPHSEITTLVSRGAGDVPR
PSSLPVEETSPPSSQLSLSAMISPSPVSSTLPASSHSSSASVTSLLTPGQVKTTEVLDAS
AEPETSSPPSLSSTSVEILATSEVTTDTEKIHPFSNTAVTKVGTSSSGHESPSSVLPDSE
TTKATSAMGTISIMGDTSVSTLTPALSNTRKIQSEPASSLTTRLRETSTSEETSLATEAN
TVLSKVSTGATTEVSRTEAISFSRTSMSGPEQSTMSQDISIGTIPRISASSVLTESAKMT
ITTQTGPSESTLESTLNLNTATTPSWVETHSIVIQGFPHPEMTTSMGRGPGGVSWPSPPF
VKETSPPSSPLSLPAVTSPHPVSTTFLAHIPPSPLPVTSLLTSGPATTTDILGTSTEPGT

SSSSSLSTTSHERLTTYKDTAHTEAVHPSTNTGGTNVATTSSGYKSQSSVLADSSPMCTT
STMGDTSVLTSTPAFLETRRIQTELASSLTPGLRESSGSEGTSSGTKMSTVLSKVPTGAT
TEISKEDVTSIPGPAQSTISPDISTRTVSWFSTSPVMTESAEITMNTHTSPLGATTQGTS
TLDTSSTTSLTMTHSTISQGFSHSQMSTLMRRGPEDVSWMSPPLLEKTRPSFSLMSSPAT
TSPSPVSSTLPESISSSPLPVTSLLTSGLAKTTDMLHKSSEPVTNSPANLSSTSVEILAT
SEVTTDTEKTHPSSNRTVTDVGTSSSGHESTSFVLADSQTSKVTSPMVITSTMEDTSVST
STPGFFETSRIQTEPTSSLTLGLRKTSSSEGTSLATEMSTVLSGVPTGATAEVSRTEVTS
SSRTSISGFAQLTVSPETSTETITRLPTSSIMTESAEMMIKTQTDPPGSTPESTHTVDIS
TTPNWVETHSTVTQRFSHSEMTTLVSRSPGDMLWPSQSSVEETSSASSLLSLPATTSPSP
VSSTLVEDFPSASLPVTSLLNPGLVITTDRMGISREPGTSSTSNLSSTSHERLTTLEDTV
DTEDMQPSTHTAVTNVRTSISGHESQSSVLSDSETPKATSPMGTTYTMGETSVSISTSDF
FETSRIQIEPTSSLTSGLRETSSSERISSATEGSTVLSEVPSGATTEVSRTEVISSRGTS
MSGPDQFTISPDISTEAITRLSTSPIMTESAESAITIETGSPGATSEGTLTLDTSTTTFW
SGTHSTASPGFSHSEMTTLMSRTPGDVPWPSLPSVEEASSVSSSLSSPAMTSTSFFSTLP
ESISSSPHPVTALLTLGPVKTTDMLRTSSEPETSSPPNLSSTSAEILATSEVTKDREKIH
PSSNTPVVNVGTVIYKHLSPSSVLADLVTTKPTSPMATTSTLGNTSVSTSTPAFPETMMT
QPTSSLTSGLREISTSQETSSATERSASLSGMPTGATTKVSRTEALSLGRTSTPGPAQST
ISPEISTETITRISTPLTTTGSAEMTITPKTGHSGASSQGTFTLDTSSRASWPGTHSAAT
HRSPHSGMTTPMSRGPEDVSWPSRPSVEKTSPPSSLVSLSAVTSPSPLYSTPSESSHSSP
LRVTSLFTPVMMKTTDMLDTSLEPVTTSPPSMNITSDESLATSKATMETEAIQLSENTAV
TQMGTISARQEFYSSYPGLPEPSKVTSPVVTSSTIKDIVSTTIPASSEITRIEMESTSTL
TPTPRETSTSQEIHSATKPSTVPYKALTSATIEDSMTQVMSSSRGPSPDQSTMSQDISTE
VITRLSTSPIKTESTEMTITTQTGSPGATSRGTLTLDTSTTFMSGTHSTASQGFSHSQMT
ALMSRTPGDVPWLSHPSVEEASSASFSLSSPVMTSSSPVSSTLPDSIHSSSLPVTSLLTS
GLVKTTELLGTSSEPETSSPPNLSSTSAEILAITEVTTDTEKLEMTNVVTSGYTHESPSS
VLADSVTTKATSSMGITYPTGDTNVLTSTPAFSDTSRIQTKSKLSLTPGLMETSISEETS
SATEKSTVLSSVPTGATTEVSRTEAISSSRTSIPGPAQSTMSSDTSMETITRISTPLTRK
ESTDMAITPKTGPSGATSQGTFTLDSSSTASWPGTHSATTQRFPQSVVTTPMSRGPEDVS
WPSPLSVEKNSPPSSLVSSSSVTSPSPLYSTPSGSSHSSPVPVTSLFTSIMMKATDMLDA
SLEPETTSAPNMNITSDESLAASKATTETEAIHVFENTAASHVETTSATEELYSSSPGFS
EPTKVISPVVTSSSIRDNMVSTTMPGSSGITRIEIESMSSLTPGLRETRTSQDITSSTET
STVLYKMPSGATPEVSRTEVMPSSRTSIPGPAQSTMSLDISDEVVTRLSTSPIMTESAEI
TITTQTGYSLATSQVTLPLGTSMTFLSGTHSTMSQGLSHSEMTNLMSRGPESLSWTSPRF
VETTRSSSSLTSLPLTTSLSPVSSTLLDSSPSSPLPVTSLILPGLVKTTEVLDTSSEPKT
SSSPNLSSTSVEIPATSEIMTDTEKIHPSSNTAVAKVRTSSSVHESHSSVLADSETTITI
PSMGITSAVDDTTVFTSNPAFSETRRIPTEPTFSLTPGFRETSTSEETTSITETSAVLYG
VPTSATTEVSMTEIMSSNRIHIPDSDQSTMSPDIITEVITRLSSSSMMSESTQMTITTQK
SSPGATAQSTLTLATTTAPLARTHSTVPPRFLHSEMTTLMSRSPENPSWKSSLFVEKTSS
SSSLLSLPVTTSPSVSSTLPQSIPSSSFSVTSLLTPGMVKTTDTSTEPGTSLSPNLSGTS
VEILAASEVTTDTEKIHPSSSMAVTNVGTTSSGHELYSSVSIHSEPSKATYPVGTPSSMA
ETSISTSMPANFETTGFEAEPFSHLTSGFRKTNMSLDTSSVTPTNTPSSPGSTHLLQSSK
TDFTSSAKTSSPDWPPASQYTEIPVDIITPFNASPSITESTGITSFPESRFTMSVTESTH
HLSTDLLPSAETISTGTVMPSLSEAMTSFATTGVPRAISGSGSPFSRTESGPGDATLSTI
AESLPSSTPVPFSSSTFTTTDSSTIPALHEITSSSATPYRVDTSLGTESSTTEGRLVMVS

TLDTSSQPGRTSSSPILDTRMTESVELGTVTSAYQVPSLSTRLTRTDGIMEHITKIPNEA
AHRGTIRPVKGPQTSTSPASPKGLHTGGTKRMETTTTALKTTTTALKTTSRATLTTSVYT
PTLGTLTPLNASMQMASTIPTEMMITTPYVFPDVPETTSSLATSLGAETSTALPRTTPSV
FNRESETTASLVSRSGAERSPVIQTLDVSSSEPDTTASWVIHPAETIPTVSKTTPNFFHS
ELDTVSSTATSHGADVSSAIPTNISPSELDALTPLVTISGTDTSTTFPTLTKSPHETETR
TTWLTHPAETSSTIPRTIPNFSHHESDATPSIATSPGAETSSAIPIMTVSPGAEDLVTSQ
VTSSGTDRNMTIPTLTLSPGEPKTIASLVTHPEAQTSSAIPTSTISPAVSRLVTSMVTSL
AAKTSTTNRALTNSPGEPATTVSLVTHPAQTSPTVPWTTSIFFHSKSDTTPSMTTSHGAE
SSSAVPTPTVSTEVPGVVTPLVTSSRAVISTTIPILTLSPGEPETTPSMATSHGEEASSA
IPTPTVSPGVPGVVTSLVTSSRAVTSTTIPILTFSLGEPETTPSMATSHGTEAGSAVPTV
LPEVPGMVTSLVASSRAVTSTTLPTLTLSPGEPETTPSMATSHGAEASSTVPTVSPEVPG
VVTSLVTSSSGVNSTSIPTLILSPGELETTPSMATSHGAEASSAVPTPTVSPGVSGVVTP
LVTSSRAVTSTTIPILTLSSSEPETTPSMATSHGVEASSAVLTVSPEVPGMVTSLVTSSR
AVTSTTIPTLTISSDEPETTTSLVTHSEAKMISAIPTLAVSPTVQGLVTSLVTSSGSETS
AFSNLTVASSQPETIDSWVAHPGTEASSVVPTLTVSTGEPFTNISLVTHPAESSSTLPRT
TSRFSHSELDTMPSTVTSPEAESSSAISTTISPGIPGVLTSLVTSSGRDISATFPTVPES
PHESEATASWVTHPAVTSTTVPRTTPNYSHSEPDTTPSIATSPGAEATSDFPTITVSPDV
PDMVTSQVTSSGTDTSITIPTLTLSSGEPETTTSFITYSETHTSSAIPTLPVSPGASKML
TSLVISSGTDSTTTFPTLTETPYEPETTAIQLIHPAETNTMVPRTTPKFSHSKSDTTLPV
AITSPGPEASSAVSTTTISPDMSDLVTSLVPSSGTDTSTTFPTLSETPYEPETTATWLTH
PAETSTTVSGTIPNFSHRGSDTAPSMVTSPGVDTRSGVPTTTIPPSIPGVVTSQVTSSAT
DTSTAIPTLTPSPGEPETTASSATHPGTQTGFTVPIRTVPSSEPDTMASWVTHPPQTSTP
VSRTTSSFSHSSPDATPVMATSPRTEASSAVLTTISPGAPEMVTSQITSSGAATSTTVPT
LTHSPGMPETTALLSTHPRTETSKTFPASTVFPQVSETTASLTIRPGAETSTALPTQTTS
SLFTLLVTGTSRVDLSPTASPGVSAKTAPLSTHPGTETSTMIPTSTLSLGLLETTGLLAT
SSSAETSTSTLTLTVSPAVSGLSSASITTDKPQTVTSWNTETSPSVTSVGPPEFSRTVTG
TTMTLIPSEMPTPPKTSHGEGVSPTTILRTTMVEATNLATTGSSPTVAKTTTTFNTLAGS
LFTPLTTPGMSTLASESVTSRTSYNHRSWISTTSSYNRRYWTPATSTPVTSTFSPGISTS
SIPSSTAATVPFMVPFTLNFTITNLQYEEDMRHPGSRKFNATERELQGLLKPLFRNSSLE
YLYSGCRLASLRPEKDSSATAVDAICTHRPDPEDLGLDRERLYWELSNLTNGIQELGPYT
LDRNSLYVNGFTHRSSMPTTSTPGTSTVDVGTSGTPSSSPSPTTAGPLLMPFTLNFTITN
LQYEEDMRRTGSRKFNTMESVLQGLLKPLFKNTSVGPLYSGCRLTLLRPEKDGAATGVDA
ICTHRLDPKSPGLNREQLYWELSKLTNDIEELGPYTLDRNSLYVNGFTHQSSVSTTSTPG
TSTVDLRTSGTPSSLSSPTIMAAGPLLVPFTLNFTITNLQYGEDMGHPGSRKFNTTERVL
QGLLGPIFKNTSVGPLYSGCRLTSLRSEKDGAATGVDAICIHHLDPKSPGLNRERLYWEL
SQLTNGIKELGPYTLDRNSLYVNGFTHRTSVPTSSTPGTSTVDLGTSGTPFSLPSPATAG
PLLVLFTLNFTITNLKYEEDMHRPGSRKFNTTERVLQTLLGPMFKNTSVGLLYSGCRLTL
LRSEKDGAATGVDAICTHRLDPKSPGVDREQLYWELSQLTNGIKELGPYTLDRNSLYVNG
FTHWIPVPTSSTPGTSTVDLGSGTPSSLPSPTTAGPLLVPFTLNFTITNLKYEEDMHCPG
SRKFNTTERVLQSLLGPMFKNTSVGPLYSGCRLTLLRSEKDGAATGVDAICTHRLDPKSP
GVDREQLYWELSQLTNGIKELGPYTLDRNSLYVNGFTHQTSAPNTSTPGTSTVDLGTSGT
PSSLPSPTSAGPLLVPFTLNFTITNLQYEEDMHHPGSRKFNTTERVLQGLLGPMFKNTSV
GLLYSGCRLTLLRPEKNGAATGMDAICSHRLDPKSPGLNREQLYWELSQLTHGIKELGPY
TLDRNSLYVNGFTHRSSVAPTSTPGTSTVDLGTSGTPSSLPSPTTAVPLLVPFTLNFTIT

NLQYGEDMRHPGSRKFNTTERVLQGLLGPLFKNSSVGPLYSGCRLISLRSEKDGAATGVD
AICTHHLNPQSPGLDREQLYWQLSQMTNGIKELGPYTLDRNSLYVNGFTHRSSGLTTSTP
WTSTVDLGTSGTPSPVPSPTTTGPLLVPFTLNFTITNLQYEENMGHPGSRKFNITESVLQ
GLLKPLFKSTSVGPLYSGCRLTLLRPEKDGVATRVDAICTHRPDPKIPGLDRQQLYWELS
QLTHSITELGPYTLDRDSLYVNGFTQRSSVPTTSTPGTFTVQPETSETPSSLPGPTATGP
VLLPFTLNFTITNLQYEEDMRRPGSRKFNTTERVLQGLLMPLFKNTSVSSLYSGCRLTLL
RPEKDGAATRVDAVCTHRPDPKSPGLDRERLYWKLSQLTHGITELGPYTLDRHSLYVNGF
THQSSMTTTRTPDTSTMHLATSRTPASLSGPMTASPLLVLFTINFTITNLRYEENMHHPG
SRKFNTTERVLQGLLRPVFKNTSVGPLYSGCRLTLLRPKKDGAATKVDAICTYRPDPKSP
GLDREQLYWELSQLTHSITELGPYTLDRDSLYVNGFTQRSSVPTTSIPGTPTVDLGTSGT
PVSKPGPSAASPLLVLFTLNFTITNLRYEENMQHPGSRKFNTTERVLQGLLRSLFKSTSV
GPLYSGCRLTLLRPEKDGTATGVDAICTHHPDPKSPRLDREQLYWELSQLTHNITELGPY
ALDNDSLFVNGFTHRSSVSTTSTPGTPTVYLGASKTPASIFGPSAASHLLILFTLNFTIT
NLRYEENMWPGSRKFNTTERVLQGLLRPLFKNTSVGPLYSGCRLTLLRPEKDGEATGVDA
ICTHRPDPTGPGLDREQLYLELSQLTHSITELGPYTLDRDSLYVNGFTHRSSVPTTSTGV
VSEEPFTLNFTINNLRYMADMGQPGSLKFNITDNVMQHLLSPLFQRSSLGARYTGCRVIA
LRSVKNGAETRVDLLCTYLQPLSGPGLPIKQVFHELSQQTHGITRLGPYSLDKDSLYLNG
YNEPGPDEPPTTPKPATTFLPPLSEATTAMGYHLKTLTLNFTISNLQYSPDMGKGSATFN
STEGVLQHLLRPLFQKSSMGPFYLGCQLISLRPEKDGAATGVDTTCTYHPDPVGPGLDIQ
QLYWELSQLTHGVTQLGFYVLDRDSLFINGYAPQNLSIRGEYQINFHIVNWNLSNPDPTS
SEYITLLRDIQDKVTTLYKGSQLHDTFRFCLVTNLTMDSVLVTVKALFSSNLDPSLVEQV
FLDKTLNASFHWLGSTYQLVDIHVTEMESSVYQPTSSSSTQHFYLNFTITNLPYSQDKAQ
PGTTNYQRNKRNIEDALNQLFRNSSIKSYFSDCQVSTFRSVPNRHHTGVDSLCNFSPLAR
RVDRVAIYEEFLRMTRNGTQLQNFTLDRSSVLVDGYSPNRNEPLTGNSDLPFWAVILIGL
AGLLGVITCLICGVLVTTRRRKKEGEYNVQQQCPGYYQSHLDLEDLQ*
>ENSG00000054116|ENST00000373166
MSRQANRGTESKKMSSELFTLTYGALVTQLCKDYENDEDVNKQLDKMGFNIGVRLIEDFL
ARSNVGRCHDFRETADVIAKVAFKMYLGITPSITNWSPAGDEFSLILENNPLVDFVELPD
NHSSLIYSNLLCGVLRGALEMVQMAVEAKFVQDTLKGDGVTEIRMRFIRRIEDNLPAGEE
*
>ENSG00000033170|ENST00000394586
MRPWTGSWRWIMLILFAWGTLLFYIGGHLVRDNDHPDHSSRELSKILAKLERLKQQNEDL
RRMAESLRIPEGPIDQGPAIGRVRVLEEQLVKAKEQIENYKKQTRNGLGKDHEILRRRIE
NGAKELWFFLQSELKKLKNLEGNELQRHADEFLLDLGHHERSIMTDLYYLSQTDGAGDWR
EKEAKDLTELVQRRITYLQNPKDCSKAKKLVCNINKGCGYGCQLHHVVYCFMIAYGTQRT
LILESQNWRYATGGWETVFRPVSETCTDRSGISTGHWSGEVKDKNVQVVELPIVDSLHPR
PPYLPLAVPEDLADRLVRVHGDPAVWWVSQFVKYLIRPQPWLEKEIEEATKKLGFKHPVI
GVHVRRTDKVGTEAAFHPIEEYMVHVEEHFQLLARRMQVDKKRVYLATDDPSLLKEAKTK
YPNYEFISDNSISWSAGLHNRYTENSLRGVILDIHFLSQADFLVCTFSSQVCRVAYEIMQ
TLHPDASANFHSLDDIYYFGGQNAHNQIAIYAHQPRTADEIPMEPGDIIGVAGNHWDGYS
KGVNRKLGRTGLYPSYKVREKIETVKYPTYPEAEK*
>ENSG00000185090|ENST00000397631
MARRRRRACIALFLVLLFAFGTLMGLRTLKAPDGLPALGPGLELAPFERRPEGAPAPAAR
APAAPAAPPPPPPPPRTADPGGSPGPAPAEAEPAPVQSLRVYSDLHAFYYSWYGSPRREG

HYIHWDHVMVPHWDPKISASYPRGRHSPPDDLGSSFYPELGPYSSRDPEVLREHMTQLKE
AAIGVLVLSWYPPGMADDNGEPSDDLVPAILDTAHQYSIQVAFHIQPYKGRDDITVHDNI
KYIIDTTGKL*
>ENSG00000171603|ENST00000361311
MLRRPAPALAPAARLLLAGLLCGGGVWAARVNKHKPWLEPTYHGIVTENDNTVLLDPPLI
ALDKDAPLRFAGEICGFKIHGQNVPFDAVVVDKSTGEGVIRSKEKLDCELQKDYSFTIQA
YDCGKGPDGTNVKKSHKATVHIQVNDVNEYAPVFKEKSYKATVIEGKQYDSILRVEAVDA
DCSPQFSQICSYEIITPDVPFTVDKDGYIKNTEKLNYGKEHQYKLTVTAYDCGKKRATED
VLVKISIKPTCTPGWQGWNNRIEYEPGTGALAVFPNIHLETCDEPVASVQATVELETSHI
GKGCDRDTYSEKSLHRLCGAAAGTAELLPSPSGSLNWTMGLPTDNGHDSDQVFEFNGTQA
VRIPDGVVSVSPKEPFTISVWMRHGPFGRKKETILCSSDKTDMNRHHYSLYVHGCRLIFL
FRQDPSEEKKYRPAEFHWKLNQVCDEEWHHYVLNVEFPSVTLYVDGTSHEPFSVTEDYPL
HPSKIETQLVVGACWQEFSGVENDNETEPVTVASAGGDLHMTQFFRGNLAGLTLRSGKLA
DKKVIDCLYTCKEGLDLQVLEDSGRGVQIQAHPSQLVLTLEGEDLGELDKAMQHISYLNS
RQFPTPGIRRLKITSTIKCFNEATCISVPPVDGYVMVLQPEEPKISLSGVHHFARAASEF
ESSEGVFLFPELRIISTITREVEPEGDGAEDPTVQESLVSEEIVHDLDTCEVTVEGEELN
HEQESLEVDMARLQQKGIEVSSSELGMTFTGVDTMASYEEVLHLLRYRNWHARSLLDRKF
KLICSELNGRYISNEFKVEVNVIHTANPMEHANHMAAQPQFVHPEHRSFVDLSGHNLANP
HPFAVVPSTATVVIVVCVSFLVFMIILGVFRIRAAHRRTMRDQDTGKENEMDWDDSALTI
TVNPMETYEDQHSSEEEEEEEEEEEESEDGEEEDDITSAESESSEEEEGEQGDPQNATRQQ
QLEWDDSTLSY*
>ENSG00000101558|ENST00000400000
MASASGAMAKHEQILVLDPPTDLKFKGPFTDVVTTNLKLRNPSDRKVCFKVKTTAPRRYC
VRPNSGIIDPGSTVTVSVMLQPFDYDPNEKSKHKFMVQTIFAPPNTSDMEAVWKEAKPDE
LMDSKLRCVFEMPNENDKLNDMEPSKAVPLNASKQDGPMPKPHSVSLNDTETRKLMEECK
RLQGEMMKLSEENRHLRDEGLRLRKVAHSDKPGSTSTASFRDNVTSPLPSLLVVIAAIFI
GFFLGKFIL*
>ENSG00000137497|ENST00000368959
MTLHATRGAALLSWVNSLHVADPVEAVLQLQDCSIFIKIIDRIHGTEEGQQILKQPVSER
LDFVCSFLQKNRKHPSSPECLVSAQKVLEGS
>ENSG00000107651|ENST00000369075
MAERKPNGGSGGASTSSSGTNLLFSSSATEFSFNVPFIPVTQASASPASLLLPGEDSTDV
GEEDSFLGQTSIHTSAPQTFSYFSQVSSSSDPFGNIGQSPLTTAATSVGQSGFPKPLTAL
PFTTGSQDVSNAFSPSISKAQPGAPPSSLMGINSYLPSQPSSLPPSYFGNQPQGIPQPGY
NPYRHTPGSSRANPYIAPPQLQQCQTPGPPAHPPPSGPPVQMYQMPPGSLPPVPSSVQSP
AQQQVPARPGAPSVQVPSPFLLQNQYEPVQPHWFYCKEVEYKQLWMPFSVFDSLNLEEIY
NSVQPDPESVVLGTDGGRYDVYLYDRIRKAAYWEEEPAEVRRCTWFYKGDTDSRFIPYTE
EFSEKLEAEYKKAVTTNQWHRRLEFPSGETIVMHNPKVIVQFQPSSVPDEWGTTQDGQTR
PRVVKRGIDDNLDEIPDGEMPQVDHLVFVVHGIGPVCDLRFRSIIECVDDFRVVSLKLLR
THFKKSLDDGKVSRVEFLPVHWHSSLGGDATGVDRNIKKITLPSIGRFRHFTNETLLDIL
FYNSPTYCQTIVEKVGMEINHLHALFMSRNPDFKGGVSVAGHSLGSLILFDILSNQKDLN
LSKCPGPLAVANGVVKQLHFQEKQMPEEPKLTLDESYDLVVENKEVLTLQETLEALSLSE
YFSTFEKEKIDMESLLMCTVDDLKEMGIPLGPRKKIANFVEHKAAKLKKAASEKKAVAAT
STKGQEQSAQKTKDMASLPSESNEPKRKLPVGACVSSVCVNYESFEVGAGQVSVAYNSLD

FEPEIFFALGSPIAMFLTIRGVDRIDENYSLPTCKGFFNIYHPLDPVAYRLEPMIVPDLD
LKAVLIPHHKGRKRLHLELKESLSRMGSDLKQGFISSLKSAWQTLNEFARAHTSSTQLQE
ELEKVANQIKEEEEKQVVEAEKVVESPDFSKDEDYLGKVGMLNGGRRIDYVLQEKPIESF
NEYLFALQSHLCYWESEDTALLLLKEIYRTMNISPEQPQH*
>ENSG00000132958|ENST00000400230
MNESPQTNEFKGTTEEAPAKESPHTSEFKGAALVSPISKSMLERLSKFEVEDAENVASYD
SKIKKIVHSIVSSFAFGIFGVFLVLLDVTLLLADLIFTDSKLYIPLEYRSISLAIGLFFL
MDVLLRVFVEGRQQYFSDLFNILDTAIIVIPLLVDVIYIFFDIKLLRNIPRWTHLVRLLR
LIILIRIFHLLHQKRQLEKLMRRLVSENKRRYTRDGFDLDLTYVTERIIAMSFPSSGRQS
FYRNPIEEVVRFLDKKHRNHYRVYNLCSERAYDPKHFHNRVSRIMIDDHNVPTLHEMVVF
TKEVNEWMAQDLENIVAIHCKGGKGRTGTMVCALLIASEIFLTAEESLYYFGERRTNKTH
SNKFQGVETPSQNRYVGYFAQVKHLYNWNLPPRRILFIKRFIIYSIRGDVCDLKVQVVME
KKVVFSSTSLGNCSILHDIETDKILINVYDGPPLYDDVKVQFFSSNLPKYYDNCPFFFWF
NTSFIQNNRLCLPRNELDNPHKQKAWKIYPPEFAVEILFGEK*
>ENSG00000142798|ENST00000374695
MGWRAAGALLLALLLHGRLLAVTHGLRAYDGLSLPEDIETVTASQMRWTHSYLSDDEDML
ADSISGDDLGSGDLGSGDFQMVYFRALVNFTRSIEYSPQLEDAGSREFREVSEAVVDTLE
SEYLKIPGDQVVSVVFIKELDGWVFVELDVGSEGNADGAQIQEMLLRVISSGSVASYVTS
PQGFQFRRLGTVPQFPRACTEAEFACHSYNECVALEYRCDRRPDCRDMSDELNCEEPVLG
ISPTFSLLVETTSLPPRPETTIMRQPPVTHAPQPLLPGSVRPLPCGPQEAACRNGHCIPR
DYLCDGQEDCEDGSDELDCGPPPPCEPNEFPCGNGHCALKLWRCDGDFDCEDRTDEANCP
TKRPEEVCGPTQFRCVSTNMCIPASFHCDEESDCPDRSDEFGCMPPQVVTPPRESIQASR
GQTVTFTCVAIGVPTPIINWRLNWGHIPSHPRVTVTSEGGRGTLIIRDVKESDQGAYTCE
AMNARGMVFGIPDGVLELVPQRGPCPDGHFYLEHSAACLPCFCFGITSVCQSTRRFRDQI
RLRFDQPDDFKGVNVTMPAQPGTPPLSSTQLQIDPSLHEFQLVDLSRRFLVHDSFWALPE
QFLGNKVDSYGGSLRYNVRYELARGMLEPVQRPDVVLMGAGYRLLSRGHTPTQPGALNQR
QVQFSEEHWVHESGRPVQRAELLQVLQSLEAVLIQTVYNTKMASVGLSDIAMDTTVTHAT
SHGRAHSVEECRCPIGYSGLSCESCDAHFTRVPGGPYLGTCSGCNCNGHASSCDPVYGHC
LNCQHNTEGPQCNKCKAGFFGDAMKATATSCRPCPCPYIDASRRFSDTCFLDTDGQATCD
ACAPGYTGRRCESCAPGYEGNPIQPGGKCRPVNQEIVRCDERGSMGTSGEACRCKNNVVG
RLCNECADGSFHLSTRNPDGCLKCFCMGVSRHCTSSSWSRAQLHGASEEPGHFSLTNAAS
THTTNEGIFSPTPGELGFSSFHRLLSGPYFWSLPSRFLGDKVTSYGGELRFTVTQRSQPG
STPLHGQPLVVLQGNNIILEHHVAQEPSPGQPSTFIVPFREQAWQRPDGQPATREHLLMA
LAGIDTLLIRASYAQQPAESRVSGISMDVAVPEETGQDPALEVEQCSCPPGYRGPSCQDC
DTGYTRTPSGLYLGTCERCSCHGHSEACEPETGACQGCQHHTEGPRCEQCQPGYYGDAQR
GTPQDCQLCPCYGDPAAGQAAHTCFLDTDGHPTCDACSPGHSGRHCERCAPGYYGNPSQG
QPCQRDSQVPGPIGCNCDPQGSVSSQCDAAGGQCQCKAQVEGLTCSHCRPHHFHLSASNPD
GCLPCFCMGITQQCASSAYTRHLISTHFAPGDFQGFALVNPQRNSRLTGEFTVEPVPEGA
QLSFGNFAQLGHESFYWQLPETYQGDKVAAYGGKLRYTLSYTAGPQGSPLSDPDVQITGN
NIMLVASQPALQGPERRSYEIMFREEFWRRPDGQPATREHLLMALADLDELLIRATFSSV
PLAASISAVSLEVAQPGPSNRPRALEVEECRCPPGYIGLSCQDCAPGYTRTGSGLYLGHC
ELCECNGHSDLCHPETGACSQCQHNAAGEFCELCAPGYYGDATAGTPEDCQPCACPLTNP
ENMFSRTCESLGAGGYRCTACEPGYTGQYCEQCGPGYVGNPSVQGGQCLPETNQAPLVVE
VHPARSIVPQGGSHSLRCQVSGSPPHYFYWSREDGRPVPSGTQQRHQGSELHFPSVQPSD

AGVYICTCRNLHQSNTSRAELLVTEAPSKPITVTVEEQRSQSVRPGADVTFICTAKSKSP
AYTLVWTRLHNGKLPTRAMDFNGILTIRNVQLSDAGTYVCTGSNMFAMDQGTATLHVQAS
GTLSAPVVSIHPPQLTVQPGQLAEFRCSATGSPTPTLEWTGGPGGQLPAKAQIHGGILRL
PAVEPTDQAQYLCRAHSSAGQQVARAVLHVHGGGGPRVQVSPERTQVHAGRTVRLYCRAA
GVPSATITWRKEGGSLPPQARSERTDIATLLIPAITTADAGFYLCVATSPAGTAQARIQV
VVLSASDASPPPVKIESSSPSVTEGQTLDLNCVVAGSAHAQVTWYRRGGSLPPHTQVHGS
RLRLPQVSPADSGEYVCRVENGSGPKEASITVSVLHGTHSGPSYTPVPGSTRPIRIEPSS
SHVAEGQTLDLNCVVPGQAHAQVTWHKRGGSLPARHQTHGSLLRLHQVTPADSGEYVCHV
VGTSGPLEASVLVTIEASVIPGPIPPVRIESSSSTVAEGQTLDLSCVVAGQAHAQVTWYK
RGGSLPARHQVRGSRLYIFQASPADAGQYVCRASNGMEASITVTVTGTQGANLAYPAGST
QPIRIEPSSSQVAEGQTLDLNCVVPGQSHAQVTWHKRGGSLPVRHQTHGSLLRLYQASPA
DSGEYVCRVLGSSVPLEASVLVTIEPAGSVPALGVTPTVRIESSSSQVAEGQTLDLNCLV
AGQAHAQVTWHKRGGSLPARHQVHGSRLRLLQVTPADSGEYVCRVVGSSGTQEASVLVTI
QQRLSGSHSQGVAYPVRIESSSASLANGHTLDLNCLVASQAPHTITWYKRGGSLPSRHQI
VGSRLRIPQVTPADSGEYVCHVSNGAGSRETSLIVTIQGSGSSHVPSVSPPIRIESSSPT
VVEGQTLDLNCVVARQPQAIITWYKRGGSLPSRHQTHGSHLRLHQMSVADSGEYVCRANN
NIDALEASIVISVSPSAGSPSAPGSSMPIRIESSSSHVAEGETLDLNCVVPGQAHAQVTW
HKRGGSLPSHHQTRGSRLRLHHVSPADSGEYVCRVMGSSGPLEASVLVTIEASGSSAVHV
PAPGGAPPIRIEPSSSRVAEGQTLDLKCVVPGQAHAQVTWHKRGGNLPARHQVHGPLLRL
NQVSPADSGEYSCQVTGSSGTLEASVLVTIEPSSPGPIPAPGLAQPIYIEASSSHVTEGQ
TLDLNCVVPGQAHAQVTWYKRGGSLPARHQTHGSQLRLHLVSPADSGEYVCRAASGPGPE
QEASFTVTVPPSEGSSYRLRSPVISIDPPSSTVQQGQDASFKCLIHDGAAPISLEWKTRN
QELEDNVHISPNGSIITIVGTRPSNHGTYRCVASNAYGVAQSVVNLSVHGPPTVSVLPEG
PVWVKVGKAVTLECVSAGEPRSSARWTRISSTPAKLEQRTYGLMDSHAVLQISSAKPSDA
GTYVCLAQNALGTAQKQVEVIVDTGAMAPGAPQVQAEEAELTVEAGHTATLRCSATGSPA
PTIHWSKLRSPLPWQHRLEGDTLIIPRVAQQDSGQYICNATSPAGHAEATIILHVESPPY
ATTVPEHASVQAGETVQLQCLAHGTPPLTFQWSRVGSSLPGRATARNELLHFERAAPEDS
GRYRCRVTNKVGSAEAFAQLLVQGPPGSLPATSIPAGSTPTVQVTPQLETKSIGASVEFH
CAVPSDRGTQLRWFKEGGQLPPGHSVQDGVLRIQNLDQSCQGTYICQAHGPWGKAQASAQ
LVIQALPSVLINIRTSVQTVVVGHAVEFECLALGDPKPQVTWSKVGGHLRPGIVQSGGVV
RIAHVELADAGQYRCTATNAAGTTQSHVLLLVQALPQISMPQEVRVPAGSAAVFPCIASG
YPTPDISWSKLDGSLPPDSRLENNMLMLPSVRPQDAGTYVCTATNRQGKVKAFAHLQVPE
RVVPYFTQTPYSFLPLPTIKDAYRKFEIKITFRPDSADGMLLYNGQKRVPGSPTNLANRQ
PDFISFGLVGGRPEFRFDAGSGMATIRHPTPLALGHFHTVTLLRSLTQGSLIVGDLAPVN
GTSQGKFQGLDLNEELYLGGYPDYGAIPKAGLSSGFIGCVRELRIQGEEIVFHDLNLTAH
GISHCPTCRDRPCQNGGQCHDSESSSYVCVCPAGFTGSRCEHSQALHCHPEACGPDATCV
NRPDGRGYTCRCHLGRSGLRCEEGVTVTTPSLSGAGSYLALPALTNTHHELRLDVEFKPL
APDGVLLFSGGKSGPVEDFVSLAMVGGHLEFRYELGSGLAVLRSAEPLALGRWHRVSAER
LNKDGSLRVNGGRPVLRSSPGKSQGLNLHTLLYLGGVEPSVPLSPATNMSAHFRGCVGEV
SVNGKRLDLTYSFLGSQGIGQCYDSSPCERQPCQHGATCMPAGEYEFQCLCRDGFKGDLC
EHEENPCQLREPCLHGGTCQGTRCLCLPGFSGPRCQQGSGHGIAESDWHLEGSGGNDAPG
QYGAYFHDDGFLAFPGHVFSRSLPEVPETIELEVRTSTASGLLLWQGVEVGEAGQGKDFI
SLGLQDGHLVFRYQLGSGEARLVSEDPINDGEWHRVTALREGRRGSIQVDGEELVSGRSP
GPNVAVNAKGSVYIGGAPDVATLTGGRFSSGITGCVKNLVLHSARPGAPPPQPLDLQHRA

QAGANTRPCPS*
>ENSG00000152683|ENST00000357055
MSWKILLFGVINLICTGFLLMWCSSTNSIALTAYTYLTIFDLFSLMTCLISYWVTLRKPS
PVYSFGFERLEVLAVFASTVLAQLGALFILKESAERFLEQPEIHTGRLLVGTFVALCFNL
FTMLSIRNKPFAYVSEAASTSWLQEHVADLSRSLCGIIPGLSSIFLPRMNPFVLIDLAGA
FALCITYMLIEINNYFAVDTASAIAIALMTFGTMYPMSVYSGKVLLQTTPPHVIGQLDKL
IREVSTLDGVLEVRNEHFWTLGFGSLAGSVHVRIRRDANEQMVLAHVTNRLYTLVSTLTV
QIFKDDWIRPALLSGPVAANVLNFSDHHVIPMPLLKGTDDLNPVTSTPAKPSSPPPEFSF
NTPGKNVNPVILLNTQTRPYGFGLNHGHTPYSSMLNQGLGVPGIGATQGLRTGFTNIPSR
YGTNNRIGQPRP*
>ENSG00000138193|ENST00000371385
MVSEGSAAGRDFAGMEEVRQLHVRFCKGIKIWHQAWFLCSLLGREPQEREAGCQLWLCTL
SAVLKVGWLFPLSEVPNFTLLKDGCGCWRLKEDQIYNAVRREETENTVGSLLHFLTKLPA
SETAHGRISVGPCLKQCVRDTVCEYRATLQRTSISQYITGSLLEATTSLGARSGLLSTFG
GSTGRMMLKERQPGPSVANSNALPSSSAGISKELIDLQPLIQFPEEVASILMEQEQTIYR
RVLPVDYLCFLTRDLGTPECQSSLPCLKASISASILTTQNGEHNALEDLVMRFNEVSSWV
TWLILTAGSMEEKREVFSYLVHVAKCCWNMGNYNAVMEFLAGLRSRKVLKMWQFMDQSDI
ETMRSLKDAMAQHESSCEYRKVVTRALHIPGCKVVPFCGVFLKELCEVLDGASGLMKLCP
RYNSQEETLEFVADYSGQDNFLQRVGQNGLKNSEKESTVNSIFQVIRSCNRSLETDEEDS
PSEGNSSRKSSLKDKSRWQFIIGDLLDSDNDIFEQSKEYDSHGSEDSQKAFDHGTELIPW
YVLSIQADVHQFLLQGATVIHYDQDTHLSARCFLQLQPDNSTLTWVKPTTASPASSKAKL
GVLNNTAEPGKFPLLGNAGLSSLTEGVLDLFAVKAVYMGHPGIDIHTVCVQNKLGSMFLS
ETGVTLLYGLQTTDNRLLHFVAPKHTAKMLFSGLLELTRAVRKMRKFPDQRQQWLRKQYV
SLYQEDGRYEGPTLAHAVELFGGRRWSARNPSPGTSAKNAEKPNMQRNNTLGISTTKKKK
KILMRGESGEVTDDEMATRKAKMHKECRSRSGSDPQDINEQEESEVNAIANPPNPLPSRR
AHSLTTAGSPNLAAGTSSPIRPVSSPVLSSSNKSPSSAWSSSSWHGRIKGGMKGFQSFMV
SDSNMSFVEFVELFKSFSVRSRKDLKDLFDVYAVPCNRSGSESAPLYTNLTIDENTSDLQ
PDLDLLTRNVSDLGLFIKSKQQLSDNQRQISDAIAAASIVTNGTGIESTSLGIFGVGILQ
LNDFLVNCQGEHCTYDEILSIIQKFEPSISMCHQGLMSFEGFARFLMDKENFASKNDESQ
ENIKELQLPLSYYYIESSHNTYLTGHQLKGESSVELYSQVLLQGCRSVELDCWDGDDGMP
IIYHGHTLTTKIPFKEVVEAIDRSAFINSDLPIIISIENHCSLPQQRKMAEIFKTVFGEK
LVTKFLFETDFSDDPMLPSPDQLRKKVLLKNKKLKAHQTPVDILKQKAHQLASMQVQAYN
GGNANPRPANNEEEEDEEDEYDYDYESLSDDNILEDRPENKSCNDKLQFEYNEEIPKRIK
KADNSACNKGKVYDMELGEEFYLDQNKKESRQIAPELSDLVIYCQAVKFPGLSTLNASGS
SRGKERKSRKSIFGNNPGRMSPGETASFNKTSGKSSCEGIRQTWEESSSPLNPTTSLSAI
IRTPKCYHISSLNENAAKRLCRRYSQKLTQHTACQLLRTYPAATRIDSSNPNPLMFWLHG
IQLVALNYQTDDLPLHLNAAMFEANGGCGYVLKPPVLWDKNCPMYQKFSPLERDLDSMDP
AVYSLTIVSGQNVCPSNSMGSPCIEVDVLGMPLDSCHFRTKPIHRNTLNPMWNEQFLFHV
HFEDLVFLRFAVVENNSSAVTAQRIIPLKALKRGYRHLQLRNLHNEVLEISSLFINSRRM
EENSSGNTMSASSMFNTEERKCLQTHRVTVHGVPGPEPFTVFTINGGTKAKQLLQQILTN
EQDIKPVTTDYFLMEEKYFISKEKNECRKQPFQRAIGPEEEIMQILSSWFPEEGYMGRIV
LKTQQENLEEKNIVQDDKEVILSSEEESFFVQVHDVSPEQPRTVIKAPRVSTAQDVIQQT
LCKAKYSYSILSNPNPSDYVLLEEVVKDTTNKKTTTPKSSQRVLLDQECVFQAQSKWKGA
GKFILKLKEQVQASREDKKKGISFASELKKLTKSTKQPRGLTSPSQLLTSESIQTKEEKP

VGGLSSSDTMDYRQ*
>ENSG00000196735|ENST00000395363
MILNKALLLGALALTTVMSPCGGEDIVADHVASCGVNLYQFYGPSGQYTHEFDGDEQFYV
DLERKETAWRWPEFSKFGGFDPQGALRNMAVAKHNLNIMIKRYNSTAATNEVPEVTVFSK
SPVTLGQPNTLICLVDNIFPPVVNITWLSNGQSVTEGVSETSFLSKSDHSFFKISYLTFL
PSADEIYDCKVEHWGLDQPLLKHWEPEIPAPMSELTETVVCALGLSVGLMGIVVGTVFII
QGLRSVGASRHQGPL*
>ENSG00000123240|ENST00000378757
MSHQPLSCLTEKEDSPSESTGNGPPHLAHPNLDTFTPEELLQQMKELLTENHQLKEAMKL
NNQAMKGRFEELSAWTEKQKEERQFFEIQSKEAKERLMALSHENEKLKEELGKLKGKSER
SSEDPTDDSRLPRAEAEQEKDQLRTQVVRLQAEKADLLGIVSELQLKLNSSGSSEDSFVE
IRMAEGEAEGSVKEIKHSPGPTRTVSTGTALSKYRSRSADGAKNYFEHEELTVSQLLLCL
REGNQKVERLEVALKEAKERVSDFEKKTSNRSEIETQTEGSTEKENDEEKGPETVGSEVE
ALNLQVTSLFKELQEAHTKLSEAELMKKRLQEKCQALERKNSAIPSELNEKQELVYTNKK
LELQVESMLSEIKMEQAKTEDEKSKLTVLQMTHNKLLQEHNNALKTIEELTRKESEKVDR
AVLKELSEKLELAEKALASKQLQMDEMKQTIAKQEEDLETMTILRAQMEVYCSDFHAERA
AREKIHEEKEQLALQLAVLLKENDAFEDGGRQSLMEMQSRHGARTSDSDQQAYLVQRGAE
DRDWRQQRNIPIHSCPKCGEVLPDIDTLQIHVMDCII*
>ENSG00000118276|ENST00000383131
MSVLRRMMRVSNRSLLAFIFFFSLSSSCLYFIYVAPGIANTYLFMVQARGIMLRENVKTI
GHMIRLYTNKNSTLNGTDYPEGNNSSDYLVQTTTYLPENFTYSPYLPCPEKLPYMRGFLN
VNVSEVSFDEIHQLFSKDLDIEPGGHWRPKDCKPRWKTGTQPFNRAMLFNVGFKEAMKDS
VWDCVIFHDVDHLPENDRNYYGCGEMPRHFAAKLDKYMYILPYKEFFGGVSGLTVEQFRK
INGFPNAFWGWGGEDDDLWNRVHYAGYNVTRPEGDLGKYKSIPHHHRGEVQFLGRYKLLR
YSKERQYIDGLNNLIYRPKILVDRLYTNISVNLMPELAPIEDY*
>ENSG00000003147|ENST00000396675
MSGHKCSYPWDLQDRYAQDKSVVNKMQQKYWETKQAFIKATGKKEDEHVVASDADLDAKL
ELFHSIQRTCLDLSKAIVLYQKRICFLSQEENELGKFLRSQGFQDKTRAGKMMQATGKAL
CFSSQQRLALRNPLCRFHQEVETFRHRAISDTWLTVNRMEQCRTEYRGALLWMKDVSQEL
DPDLYKQMEKFRKVQTQVRLAKKNFDKLKMDVCQKVDLLGASRCNLLSHMLATYQTTLLH
FWEKTSHTMAAIHESFKGYQPYEFTTLKSLQDPMKKLVEKEEKKKINQQESTDAAVQEPS
QLISLEEENQRKESSSFKTEDGKSILSALDKGSTHTACSGPIDELLDMKSEEGACLGPVA
GTPEPEGADKDDLLLLSEIFNASSLEEGEFSKEWAAVFGDGQVKEPVPTMALGEPDPKAQ
TGSGFLPSQLLDQNMKDLQASLQEPAKAASDLTAWFSLFADLDPLSNPDAVGKTDKEHEL
LNA*
>ENSG00000157593|ENST00000393812
MDARWWAVVVLAAFPSLGAGGETPEAPPESWTQLWFFRFVVNAAGYASFMVPGYLLVQYF
RRKNYLETGRGLCFPLVKACVFGNEPKASDEVPLAPRTEAAETTPMWQALKLLFCATGLQ
VSYLTWGVLQERVMTRSYGATATSPGERFTDSQFLVLMNRVLALIVAGLSCVLCKQPRHG
APMYRYSFASLSNVLSSWCQYEALKFVSFPTQVLAKASKVIPVMLMGKLVSRRSYEHWEY
LTATLISIGVSMFLLSSGPEPRSSPATTLSGLILLAGYIAFDSFTSNWQDALFAYKMSSV
QMMFGVNFFSCLFTVGSLLEQGALLEGTRFMGRHSEFAAHALLLSICSACGQLFIFYTIG
QFGAAVFTIIMTLRQAFAILLSCLLYGHTVTVVGGLGVAVVFAALLLRVYARGRLKQRGK
KAVPVESPVQKV*

>ENSG00000206435|ENST00000400341
MRVMAPRTLILLLSGALALTETWACSHSMRYFYTAVSRPGRGEPRFIAVGYVDDTQFVQF
DSDAASPRGEPRAPWVEQEGPEYWDRETQKYKRQAQTDRVNLRKLRGYYNQSEAGSHTLQ
RMYGCDLGPDGRLLRGYNQFAYDGKDYIALNEDLRSWTAADKAAQITQRKWEAAREAEQR
RAYLEGTCVEWLRRYLENGKKTLQRAEHPKTHVTHHPVSDHEATLRCWALGFYPAEITLT
WQRDGEDQTQDTELVETRPAGDGTFQKWAAVVVPSGEEQRYTCHVQHEGLPEPLTLRWGP
SSQPTIPIVGIVAGLAVLAVLAVLGAVMAVVMCRRKSSGGKGGSCSQAASSNSAQGSDES
LIACKA*
>ENSG00000122694|ENST00000377960
MGKSASKQFHNEVLKAHNEYRQKHGVPPLKLCKNLNREAQQYSEALASTRILKHSPESSR
GQCGENLAWASYDQTGKEVADRWYSEIKNYNFQQPGFTSGTGHFTAMVWKNTKKMGVGKA
SASDGSSFVVARYFPAGNVVNEGFFEENVLPPKK*
>ENSG00000182197|ENST00000378204
MQAKKRYFILLSAGSCLALLFYFGGLQFRASRSHSRREEHSGRNGLHHPSPDHFWPRFPD
ALRPFVPWDQLENEDSSVHISPRQKRDANSSIYKGKKCRMESCFDFTLCKKNGFKVYVYP
QQKGEKIAESYQNILAAIEGSRFYTSDPSQACLFVLSLDTLDRDQLSPQYVHNLRSKVQS
LHLWNNGRNHLIFNLYSGTWPDYTEDVGFDIGQAMLAKASISTENFRPNFDVSIPLFSKD
HPRTGGERGFLKFNTIPPLRKYMLVFKGKRYLTGIGSDTRNALYHVHNGEDVVLLTTCKH
GKDWQKHKDSRCDRDNTEYEKYDYREMLHNATFCLVPRGRRLGSFRFLEALQAACVPVML
SNGWELPFSEVINWNQAAVIGDERLLLQIPSTIRSIHQDKILALRQQTQFLWEAYFSSVE
KIVLTTLEIIQDRIFKHISRNSLIWNKHPGGLFVLPQYSSYLGDFPYYYANLGLKPPSKF
TAVIHAVTPLVSQSQPVLKLLVAAAKSQYCAQIIVLWNCDKPLPAKHRWPATAVPVVVIE
GESKVMSSRFLPYDNIITDAVLSLDEDTVLSTTEVDFAFTVWQSFPERIVGYPARSHFWD
NSKERWGYTSKWTNDYSMVLTGAAIYHKYYHYLYSHYLPASLKNMVDQLANCEDILMNFL
VSAVTKLPPIKVTQKKQYKETMMGQTSRASRWADPDHFAQRQSCMNTFASWFGYMPLIHS
QMRLDPVLFKDQVSILRKKYRDIERL*
>ENSG00000160216|ENST00000398061
MGLLAFLKTQFVLHLLVGFVFVVSGLVINFVQLCTLALWPVSKQLYRRLNCRLAYSLWSQ
LVMLLEWWSCTECTLFTDQATVERFGKEHAVIILNHNFEIDFLCGWTMCERFGVLGSSKV
LAKKELLYVPLIGWTWYFLEIVFCKRKWEEDRDTVVEGLRRLSDYPEYMWFLLYCEGTRF
TETKHRVSMEVAAAKGLPVLKYHLLPRTKGFTTAVKCLRGTVAAVYDVTLNFRGNKNPSL
LGILYGKKYEADMCVRRFPLEDIPLDEKEAAQWLHKLYQEKDALQEIYNQKGMFPGEQFK
PARRPWTLLNFLSWATILLSPLFSFVLGVFASGSPLLILTFLGFVGAASFGVRRLIGVTE
IEKGSSYGNQEFKKKE*
>ENSG00000171302|ENST00000392446
MPVQLSEHPEWNESMHSLRISVGGLPVLASMTKAADPRFRPRWKVILTFFVGAAILWLLC
SHRPAPGRPPTHNAHNWRLGQAPANWYNDTYPLSPPQRTPAGIRYRIAVIADLDTESRAQ
EENTWFSYLKKGYLTLSDSGDKVAVEWDKDHGVLESHLAEKGRGMELSDLIVFNGKLYSV
DDRTGVVYQIEGSKAVPWVILSDGDGTVEKGFKAEWLAVKDERLYVGGLGKEWTTTTGDV
VNENPEWVKVVGYKGSVDHENWVSNYNALRAAAGIQPPGYLIHESACWSDTLQRWFFLPR
RASQERYSEKDDERKGANLLLSASPDFGDIAVSHVGAVVPTHGFSSFKFIPNTDDQIIVA
LKSEEDSGRVASYIMAFTLDGRFLLPETKIGSVKYEGIEFI*
>ENSG00000058799|ENST00000371399
MWRNSKVMNIVSYSFLEIVCVYGYSLFIYIPTAILWIIPQKAVRWILVMIALGISGSLLA

MTFWPAVREDNRRVALATIVTIVLLHMLLSVGCLAYFFDAPEMDHLPTTTATPNQTVAAA
KSS*
>ENSG00000110075|ENST00000393801
MFWKFDLHSSSHIDTLLEREDVTLKELMDEEDVLQECKAQNRKLIEFLLKAECLEDLVSF
IIEEPPQDMDEKIRYKYPNISCELLTSDVSQMNDRLGEDESLLMKLYSFLLNDSPLNPLL
ASFFSKVLSILISRKPEQIVDFLKKKHDFVDLIIKHIGTSAIMDLLLRLLTCIEPPQPRQ
DVLNWLNEEKIIQRLVEIVHPSQEEDRHSNASQSLCEIVRLSRDQMLQIQNSTEPDPLLA
TLEKQEIIEQLLSNIFHKEKNESAIVSAIQILLTLLETRRPTFEGHIEICPPGMSHSACS
VNKSVLEAIRGRLGSFHELLLEPPKKSVMKTTWGVLDPPVGNTRLNVIRLISSLLQTNTS
SINGDLMELNSIGVILNMFFKYTWNNFLHTQVEICIALILASPFENTENATITDQDSTGD
NLLLKHLFQKCQLIERILEAWEMNEKKQAEGGRRHGYMGHLTRIANCIVHSTDKGPNSAL
VQQLIKDLPDEVRERWETFCTSSLGETNKRNTVDLVTTCHIHSSSDDEIDFKETGFSQDS
SLQQAFSDYQMQQMTSNFIDQFGFNDEKFADQDDIGNVSFDRVSDINFTLNTNESGNIAL
FEACCKERIQQFDDGGSDEEDIWEEKHIAFTPESQRRSSSGSTDSEESTDSEEEDGAKQD
LFEPSSANTEDKMEVDLSEPPNWSANFDVPMETTHGAPLDSVGSDVWSTEEPMPTKETGW
ASFSEFTSSLSTKDSLRSNSPVEMETSTEPMDPLTPSAAALAVQPEAAGSVAMEASSDGE
EDAESTDKVTETVMNGGMKETLSLTVDAKTETAVFKRVLKSYREEGKLSTSQDAACKDAE
ECPETAEAKCAAPRPPSSSPEQRTGQPSAPGDTSVNGPV*
>ENSG00000089063|ENST00000379286
MMPSRTNLATGIPSSKVKYSRLSSTDDGYIDLQFKKTPPKIPYKAIALATVLFLIGAFLI
IIGSLLLSGYISKGGADRAVPVLIIGILVFLPGFYHLRIAYYASKGYRGYSYDDIPDFDD
*
>ENSG00000003147|ENST00000401396
MDRYAQDKSVVNKMQQKYWETKQAFIKATGKKEDEHVVASDADLDAKLELFHSIQRTCLD
LSKAIVLYQKRICFLSQEENELGKFLRSQGFQDKTRAGKMMQATGKALCFSSQQRLALRN
PLCRFHQEVETFRHRAISDTWLTVNRMEQCRTEYRGALLWMKDVSQELDPDLYKQMEKFR
KVQTQVRLAKKNFDKLKMDVCQKVDLLGASRCNLLSHMLATYQTTLLHFWEKTSHTMAAI
HESFKGYQPYEFTTLKSLQDPMKKLVEKEEKKKINQQESTDAAVQEPSQLISLEEENQRK
ESSSFKTEDGKSILSALDKGSTHTACSGPIDELLDMKSEEGACLGPVAGTPEPEGADKDD
LLLLSEIFNASSLEEGEFSKEWAAVFGDGQVKEPVPTMALGEPDPKAQTGSGFLPSQLLD
QNMKDLQASLQEPAKAASDLTAWFSLFADLDPLSNPDAVGKTDKEHELLNA*
>ENSG00000087460|ENST00000371085
MGCLGNSKTEDQRNEEKAQREANKKIEKQLQKDKQVYRATHRLLLLGAGESGKSTIVKQM
RILHVNGFNGEGGEEDPQAARSNSDGEKATKVQDIKNNLKEAIETIVAAMSNLVPPVELA
NPENQFRVDYILSVMNVPDFDFPPEFYEHAKALWEDEGVRACYERSNEYQLIDCAQYFLD
KIDVIKQADYVPSDQDLLRCRVLTSGIFETKFQVDKVNFHMFDVGGQRDERRKWIQCFND
VTAIIFVVASSSYNMVIREDNQTNRLQEALNLFKSIWNNRWLRTISVILFLNKQDLLAEK
VLAGKSKIEDYFPEFARYTTPEDATPEPGEDPRVTRAKYFIRDEFLRISTASGDGRHYCY
PHFTCAVDTENIRRVFNDCRDIIQRMHLRQYELL*
>ENSG00000164292|ENST00000379982
MSIHIVALGNEGDTFHQDNRPSGLIRTYLGRSPLVSGDESSLLLNAASTVARPVFTEYQA
SAFGNVKLVVHDCPVWDIFDSDWYTSRNLIGGADIIVIKYNVNDKFSFHEVKDNYIPVIK
RALNSVPVIIAAVGTRQNEELPCTCPLCTSDRGSCVSTTEGIQLAKELGATYLELHSLDD
FYIGKYFGGVLEYFMIQALNQKTSEKMKKRKMSNSFHGIRPPQLEQPEKMPVLKAEASHY

NSDLNNLLFCCQCVDVVFYNPNLKKVVEAHKIVLCAVSHVFMLLFNVKSPTDIQDSSIIR
TTQDLFAINRDTAFPGASHESSGNPPLRVIVKDALFCSCLSDILRFIYSGAFQWEELEED
IRKKLKDSGDVSNVIEKVKCILKTPGKINCLRNCKTYQARKPLWFYNTSLKFFLNKPMLA
DVVFEIQGTTVPAHRAILVARCEVMAAMFNGNYMEAKSVLIPVYGVSKETFLSFLEYLYT
DSCCPAGIFQAMCLLICAEMYQVSRLQHICELFIITQLQSMPSRELASMNLDIVDLLKKA
KFHHSDCLSTWLLHFIATNYLIFSQKPEFQDLSVEERSFVEKHRWPSNMYLKQLAEYRKY
IHSRKCRCLVM*
>ENSG00000116299|ENST00000369939
MAEPGHSHHLSARVRGRTERRIPRLWRLLLWAGTAFQVTQGTGPELHACKESEYHYEYTA
CDSTGSRWRVAVPHTPGLCTSLPDPIKGTECSFSCNAGEFLDMKDQSCKPCAEGRYSLGT
GIRFDEWDELPHGFASLSANMELDDSAAESTGNCTSSKWVPRGDYIASNTDECTATLMYA
VNLKQSGTVNFEYYYPDSSIIFEFFVQNDQCQPNADDSRWMKTTEKGWEFHSVELNRGNN
VLYWRTTAFSVWTKVPKPVLVRNIAITGVAYTSECFPCKPGTYADKQGSSFCKLCPANSY
SNKGETSCHQCDPDKYSEKGSSSCNVRPACTDKDYFYTHTACDANGETQLMYKWAKPKIC
SEDLEGAVKLPASGVKTHCPPCNPGFFKTNNSTCQPCPYGSYSNGSDCTRCPAGTEPAVG
FEYKWWNTLPTNMETTVLSGINFEYKGMTGWEVAGDHIYTAAGASDNDFMILTLVVPGFR
PPQSVMADTENKEVARITFVFETLCSVNCELYFMVGVNSRTNTPVETWKGSKGKQSYTYI
IEENTTTSFTWAFQRTTFHEASRKYTNDVAKIYSINVTNVMNGVASYCRPCALEASDVGS
SCTSCPAGYYIDRDSGTCHSCPTNTILKAHQPYGVQACVPCGPGTKNNKIHSLCYNDCTF
SRNTPTRTFNYNFSALANTVTLAGGPSFTSKGLKYFHHFTLSLCGNQGRKMSVCTDNVTD
LRIPEGESGFSKSITAYVCQAVIIPPEVTGYKAGVSSQPVSLADRLIGVTTDMTLDGITS
PAELFHLESLGIPDVIFFYRSNDVTQSCSSGRSTTIRVRCSPQKTVPGSLLLPGTCSDGT
CDGCNFHFLWESAAACPLCSVADYHAIVSSCVAGIQKTTYVWREPKLCSGGISLPEQRVT
ICKTIDFWLKVGISAGTCTAILLTVLTCYFWKKNQKLEYKYSKLVMNATLKDCDLPAADS
CAIMEGEDVEDDLIFTSKKSLFGKIKSFTSKRTPDGFDSVPLKTSSGGLDMDL*
>ENSG00000080815|ENST00000357710
MTELPAPLSYFQNAQMSEDNHLSNTNDNRERQEHNDRRSLGHPEPLSNGRPQGNSRQVVE
QDEEEDEELTLKYGAKHVIMLFVPVTLCMVVVVATIKSVSFYTRKDGQLIYTPFTEDTET
VGQRALHSILNAAIMISVIVVMTILLVVLYKYRCYKVIHAWLIISSLLLLFFFSFIYLGE
VFKTYNVAVDYITVALLIWNFGVVGMISIHWKGPLRLQQAYLIMISALMALVFIKYLPEW
TAWLILAVISVYDLVAVLCPKGPLRMLVETAQERNETLFPALIYSSTMVWLVNMAEGDPE
AQRRVSKNSKYNAESTERESQDTVAENDDGGFSEEWEAQRDSHLGPHRSTPESRAAVQEL
SSSILAGEDPEERGVKLGLGDFIFYSVLVGKASATASGDWNTTIACFVAILIGLCLTLLL
LAIFKKALPALPISITFGLVFYFATDYLVQPFMDQLAFHQFYI*
>ENSG00000136297|ENST00000401401
MFAPRLLDFQKTKYARFMNHRVPAHKRYQPTEYEHAANCATHAFWIIPSILGSSNLYFLS
DDDWETISAWIYGLGLCGLFVVSTVFHTISWKKSHLRMVEHCLHMFDRMVIYFFIAASYA
PWLNLRELGPWASHMRWLVWIMASVGTIYVFFFHERYKLVELLCYVVMGFFPALVILSMP
NTEGIWELVTGGVFYCLGMVFFKSDGRIPFAHAIWHLFVAFGAGTHYYAIWRYLYLPSTL
QTKVSK*
>ENSG00000164144|ENST00000353617
MAQESPKNSAAEIPVTSNGEVDDSREHSFNRDLKHSLPSGLGLSETQITSHGFDNTKEGV
IEAGAFQGSPAPPLPSVMSPSRVAASRLAQQGSDLIVPAGGQRTQTKSGPVILADEIKNP
AMEKLELVRKWSLNTYKCTRQIISEKLGRGSRTVDLELEAQIDILRDNKKKYENILKLAQ

TLSTQLFQMVHTQRQLGDAFADLSLKSLELHEEFGYNADTQKLLAKNGETLLGAINFFIA
SVNTLVNKTIEDTLMTVKQYESARIEYDAYRTDLEELNLGPRDANTLPKIEQSQHLFQAH
KEKYDKMRNDVSVKLKFLEENKVKVLHNQLVLFHNAIAAYFAGNQKQLEQTLKQFHIKLK
TPGVDAPSWLEEQ*
>ENSG00000127954|ENST00000380079
MEKTCIDALPLTMNSSEKQETVCIFGTGDFGRSLGLKMLQCGYSVVFGSRNPQKTTLLPS
GAEVLSYSEAAKKSGIIIIAIHREHYDFLTELTEVLNGKILVDISNNLKINQYPESNAEY
LAHLVPGAHVVKAFNTISAWALQSGALDASRQVFVCGNDSKAKQRVMDIVRNLGLTPMDQ
GSLMAAKEIEKYPLQLFPMWRFPFYLSAVLCVFLFFYCVIRDVIYPYVYEKKDNTFRMAI
SIPNRIFPITALTLLALVYLPGVIAAILQLYRGTKYRRFPDWLDHWMLCRKQLGLVALGF
AFLHVLYTLVIPIRYYVRWRLGNLTVTQAILKKENPFSTSSAWLSDSYVALGILGFFLFV
LLGITSLPSVSNAVNWREFRFVQSKLGYLTLILCTAHTLVYGGKRFLSPSNLRWYLPAAY
VLGLIIPCTVLVIKFVLIMPCVDNTLTRIRQGWERNSKH*
>ENSG00000206433|ENST00000400262
MLSRNDVKRLERSWAQGSSEQELHYASLQRLPVPSSEGPDLRGRDKRGTKEDPRADYACI
AENKPT*
>ENSG00000183098|ENST00000377047
MPSWIGAVILPLLGLLLSLPAGADVKARSCGEVRQAYGAKGFSLADIPYQEIAGEHLRIC
PQEYTCCTTEMEDKLSQQSKLEFENLVEETSHFVRTTFVSRHKKFDEFFRELLENAEKSL
NDMFVRTYGMLYMQNSEVFQDLFTELKRYYTGGNVNLEEMLNDFWARLLERMFQLINPQY
HFSEDYLECVSKYTDQLKPFGDVPRKLKIQVTRAFIAARTFVQGLTVGREVANRVSKVSP
TPGCIRALMKMLYCPYCRGLPTVRPCNNYCLNVMKGCLANQADLDTEWNLFIDAMLLVAE
RLEGPFNIESVMDPIDVKISEAIMNMQENSMQVSAKVFQGCGQPKPAPALRSARSAPENF
NTRFRPYNPEERPTTAAGTSLDRLVTDIKEKLKLSKKVWSALPYTICKDESVTAGTSNEE
ECWNGHSKARYLPEIMNDGLTNQINNPEVDVDITRPDTFIRQQIMALRVMTNKLKNAYNG
NDVNFQDTSDESSGSGSGSGCMDDVCPTEFEFVTTEAPAVDPDRREVDSSAAQRGHSLLS
WSLTCIVLALQRLCR*
>ENSG00000133318|ENST00000354497
MAEPSAATQSHSISSSSFGAEPSAPGGGGSPGACPALGTKSCSSSCAVHDLIFWRDVKKT
GFVFGTTLIMLLSLAAFSVISVVSYLILALLSVTISFRIYKSVIQAVQKSEEGHPFKPRL
ITMLASPEIRPSQLLKRSKQNSLESPKKRQNKYMETRNATVTKTPFNSYNVVTCTMKENT
QCQLEPAFQAFFLIWCFLPSFPFNPQYQAQKLMD*
>ENSG00000168384|ENST00000374808
MRPEDRMFHIRAVILRALSLAFLLSLRGAGAIKADHVSTYAAFVQTHRPTGEFMFEFDED
EMFYVDLDKKETVWHLEEFGQAFSFEAQGGLANIAILNNNLNTLIQRSNHTQATNDPPEV
TVFPKEPVELGQPNTLICHIDKFFPPVLNVTWLCNGELVTEGVAESLFLPRTDYSFHKFH
YLTFVPSAEDFYDCRVEHWGLDQPLLKHWEAQEPIQMPETTETVLCALGLVLGLVGIIVG
TVLIIKSLRSGHDPRAQGTL*
>ENSG00000206450|ENST00000359635
MRVTAPRTLLLLLWGAVALTETWAGSHSMRYFHTSVSRPGRGEPRFISVGYVDGTQFVRF
DSDAASPRTEPRAPWIEQEGPEYWDRNTQISKTNTQTYRESLRNLRGYYNQSEAGSHTLQ
RMYGCDVGPDGRLLRGHDQSAYDGKDYIALNEDLSSWTAADTAAQITQRKWEAARVAEQL
RAYLEGTCVEWLRRHLENGKETLQRADPPKTHVTHHPISDHEATLRCWALGFYPAEITLT
WQRDGEDQTQDTELVETRPAGDRTFQKWAAVVVPSGEEQRYTCHVQHEGLPKPLTLRWEP

SSQSTIPIVGIVAGLAVLAVVVIGAVVATVMCRRKSSGGKGGSYSQAASSDSAQGSDVSL
TA*
>ENSG00000079805|ENST00000355667
MGNRGMEELIPLVNKLQDAFSSIGQSCHLDLPQIAVVGGQSAGKSSVLENFVGRDFLPRG
SGIVTRRPLILQLIFSKTEHAEFLHCKSKKFTDFDEVRQEIEAETDRVTGTNKGISPVPI
NLRVYSPHVLNLTLIDLPGITKVPVGDQPPDIEYQIKDMILQFISRESSLILAVTPANMD
LANSDALKLAKEVDPQGLRTIGVITKLDLMDEGTDARDVLENKLLPLRRGYIGVVNRSQK
DIEGKKDIRAALAAERKFFLSHPAYRHMADRMGTPHLQKTLNQQLTNHIRESLPALRSKL
QSQLLSLEKEVEEYKNFRPDDPTRKTKALLQMVQQFGVDFEKRIEGSGDQVDTLELSGGA
RINRIFHERFPFELVKMEFDEKDLRREISYAIKNIHGVRTGLFTPDLAFEAIVKKQVVKL
KEPCLKCVDLVIQELINTVRQCTSKLSSYPRLREETERIVTTYIREREGRTKDQILLLID
IEQSYINTNHEDFIGFANAQQRSTQLNKKRAIPNQGEILVIRRGWLTINNISLMKGGSKE
YWFVLTAESLSWYKDEEEKEKKYMLPLDNLKIRDVEKGFMSNKHVFAIFNTEQRNVYKDL
RQIELACDSQEDVDSWKASFLRAGVYPEKDQAENEDGAQENTFSMDPQLERQVETIRNLV
DSYVAIINKSIRDLMPKTIMHLMINNTKAFIHHELLAYLYSSADQSSLMEESADQAQRRD
DMLRMYHALKEALNIIGDISTSTVSTPVPPPVDDTWLQSASSHSPTPQRRPVSSIHPPGR
PPAVRGPTPGPPLIPVPVGAAASFSAPPIPSRPGPQSVFANSDLFPAPPQIPSRPVRIPP
GIPPGVPSRRPPAAPSRPTIIRPAEPSLLD*
>ENSG00000133424|ENST00000397394
MLGICRGRRKFLAASLSLLCIPAITWIYLFSGSFEDGKPVSLSPLESQAHSPRYTASSQR
ERESLEVRMREVEEENRALRRQLSLAQGRAPSHRRGNHSKTYSMEEGTGDSENLRAGIVA
GNSSECGQQPVVEKCETIHVAIVCAGYNASRDVVTLVKSVLFHRRNPLHFHLIADSIAEQ
ILATLFQTWMVPAVRVDFYNADELKSEVSWIPNKHYSGIYGLMKLVLTKTLPANLERVIV
LDTDITFATDIAELWAVFHKFKGQQVLGLVENQSDWYLGNLWKNHRPWPALGRGYNTGVI
LLLLDKLRKMKWEQMWRLTAERELMGMLSTSLADQDIFNAVIKQNPFLVYQLPCFWNVQL
SDHTRSEQCYRDVSDLKVIHWNSPKKLRVKNKHVEFFRNLYLTFLEYDGNLLRRELFGCP
SEADVNSENLQKQLSELDEDDLCYEFRRERFTVHRTHLYFLHYEYEPAADSTDVTLVAQL
SMDRLQMLEAICKHWEGPISLALYLSDAEAQQFLRYAQGSEVLMSRHNVGYHIVYKEGQF
YPVNLLRNVAMKHISTPYMFLSDIDFLPMYGLYEYLRKSVIQLDLANTKKAMIVPAFETL
RYRLSFPKSKAELLSMLDMGTLFTFRYHVWTKGHAPTNFAKWRTATTPYRVEWEADFEPY
VVVRRDCPEYDRRFVGFGWNKVAHIMELDVQEYEFIVLPNAYMIHMPHAPSFDITKFRSN
KQYRICLKTLKEEFQQDMSRRYGFAALKYLTAENNS*
>ENSG00000164850|ENST00000401670
MDVTSQARGVGLEMYPGTAQPAAPNTTSPELNLSHPLLGTALANGTGELSEHQQYVIGLF
LSCLYTIFLFPIGFVGNILILVVNISFREKMTIPDLYFINLAVADLILVADSLIEVFNLH
ERYYDIAVLCTFMSLFLQVNMYSSVFFLTWMSFDRYIALARAMRCSLFRTKHHARLSCGL
IWMASVSATLVPFTAVHLQHTDEACFCFADVREVQWLEVTLGFIVPFAIIGLCYSLIVRV
LVRAHRHRGLRPRRQKALRMILAVVLVFFVCWLPENVFISVHLLQRTQPGAAPCKQSFRH
AHPLTGHIVNLAAFSNSCLNPLIYSFLGETFRDKLRLYIEQKTNLPALNRFCHAALKAVI
PDSTEQSDVRFSSAV*
>ENSG00000083097|ENST00000369739
MNTEELELLSDSKYRNYVAAIDKALKNFEYSSEWADLISALGKLNKVLQNNAKYQVVPKK
LTIGKRLAQCLHPALPGGVHRKALETYEIIFKIIGPKRLAKDLFLYSSGLFPLLANAAMS
VKPTLLSLYEIYYLPLGKTLKPGLQGLLTGILPGLEEGSEYYERTNMLLEKVAAAVDQSA

FYSALWGSLLTSPAVRLPGITYVLAHLNRKLSMEDQLYIIGSDIELMVEAVSTSVQDSSV
LVQRSTLDLILFCFPFHMSQATRPDMIRILSAALHVVLRRDMSLNRRLYAWLLGPRSTRH
SNPEEHATYYFTTFSKELLVQAMVGILQVNGFGEENTLMQDLKPFRILISLLDKPELGPV
ILEDVLIEVFRTLYSQCKAELDLQTEPPFSKDHAQLSSKLRENKKTAELIKTANLLFNSF
EPYYMWDYVARWFEECCRRTLHVRLQIGPGDSNDSSELQLTNFCLLVDFLLDIVSLPTRS
MRVLCQETYIEIQTEHLPQLLLRMISALTSHLQTLHLSELTDSLRLCSKILSKVQPPLLS
ASTGGVLQFPSGQNNSVKEWEDKKVSSVSHENPTEVFEDGENPPSSRSSESGFTEFIQYQ
ADRTDDIDRELSEGQGAAAIPIGSTSSETETASTVGSEETIIQTPSVVTQGTATRSRKTA
QKTAMQCCLEYVQQFLTRLINLYIIQNNSFSQSLATEHQGDLGREQGETSKWDRNSQGDV
KEKNISKQKTSKEYLSAFLAACQLFLECSSFPVYIAEGNHTSELRSEKLETDCEHVQPPQ
WLQTLMNACSQASDFSVQSVAISLVMDLVGLTQSVAMVTGENINSVEPAQPLSPNQGRVA
VVIRPPLTQGNLRYIAEKTEFFKHVALTLWDQLGDGTPQHHQKSVELFYQLHNLVPSSSI
CEDVISQQLTHKDKKIRMEAHAKFAVLWHLTRDLHINKSSSFVRSFDRSLFIMLDSLNSL
DGSTSSVGQAWLNQVLQRHDIARVLEPLLLLLLHPKTQRVSVQRVQAERYWNKSPCYPGE
ESDKHFMQNFACSNVSQVQLITSKGNGEKPLTMDEIENFSLTVNPLSDRLSLLSTSSETI
PMVVSDFDLPDQQIEILQSSDSGCSQSSAGDNLSYEVDPETVNAQEDSQMPKESSPDDDV
QQVVFDLICKVVSGLEVESASVTSQLEIEAMPPKCSDIDPDEETIKIEDDSIQQSQNALL
SNESSQFLSVSAEGGHECVANGISRNSSSPCISGTTHTLHDSSVASIETKSRQRSHSSIQ
FSFKEKLSEKVSEKETIVKESGKQPGAKPKVKLARKKDDDKKKSSNEKLKQTSVFFSDGL
DLENWYSCGEGDISEIESDMGSPGSRKSPNFNIHPLYQHVLLYLQLYDSSRTLYAFSAIK
AILKTNPIAFVNAISTTSVNNAYTPQLSLLQNLLARHRISVMGKDFYSHIPVDSNHNFRS
SMYIEILISLCLYYMRSHYPTHVKVTAQDLIGNRNMQMMSIEILTLLFTELAKVIESSAK
GFPSFISDMLSKCKVQKVILHCLLSSIFSAQKWHSEKMAGKNLVAVEEGFSEDSLINFSE
DEFDNGSTLQSQLLKVLQRLIVLEHRVMTIPEENETGFDFVVSDLEHISPHQPMTSLQYL
HAQPITCQGMFLCAVIRALHQHCACKMHPQWIGLITSTLPYMGKVLQRVVVSVTLQLCRN
LDNLIQQYKYETGLSDSRPLWMASIIPPDMILTLLEGITAIIHYCLLDPTTQYHQLLVSV
DQKHLFEARSGILSILHMIMSSVTLLWSILHQADSSEKMTIAASASLTTINLGATKNLRQ
QILELLGPISMNHGVHFMAAIAFVWNERRQNKTTTRTKVIPAASEEQLLLVELVRSISVM
RAETVIQTVKEVLKQPPAIAKDKKHLSLEVCMLQFFYAYIQRIPVPNLVDSWASLLILLK
DSIQLSLPAPGQFLILGVLNEFIMKNPSLENKKDQRDLQDVTHKIVDAIGAIAGSSLEQT
TWLRRNLEVKPSPKIMVDGTNLESDVEDMLSPAMETANITPSVYSVHALTLLSEVLAHLL
DMVFYSDEKERVIPLLVNIMHYVVPYLRNHSAHNAPSYRACVQLLSSLSGYQYTRRAWKK
EAFDLFMDPSFFQMDASCVNHWRAIMDNLMTHDKTTFRDLMTRVAVAQSSSLNLFANRDV
ELEQRAMLLKRLAFAIFSSEIDQYQKYLPDIQERLVESLRLPQVPTLHSQVFLFFRVLLL
RMSPQHLTSLWPTMITELVQVFLLMEQELTADEDISRTSGPSVAGLETTYTGGNGFSTSY
NSQRWLNLYLSACKFLDLALALPSENLPQFQMYRWAFIPEASDDSGLEVRRQGIHQREFK
PYVVRLAKLLRKRAKDKEEDFKTVILEGLEMAKHQKNPEEDNSGRTLGWEPGHLLLTICT
VRSMEQLLPFFNVLSQVFNSKVTSRCGGHSGSPILYSNAFPNKDMKLENHKPCSSKARQK
IEEMVEKDFLEGMIKT*
>ENSG00000198663|ENST00000355190
MDFILEDMDLAANEISIYDKLSETVDLVRQTGHQCGMSEKAIEKFIRQLLEKNEPQRPPP
QYPLLIVVYKVLATLGLILLTAYFVIQPFSPLAPEPVLSGAHTWRSLIHHIRLMSLPIAK
KYMSENKGVPLHGGDEDRPFPDFDPWWTNDCEQNESEPIPANCTGCAQKHLKVMLLEDAP
RKFERLHPLVIKTGKPLLEEEIQHFLCQYPEATEGFSEGFFAKWWRCFPERWFPFPYPWR

RPLNRSQMLRELFPVFTHLPFPKDASLNKCSFLHPEPVVGSKMHKMPDLFIIGSGEAMLQ
LIPPFQCRRHCQSVAMPIEPGDIGYVDTTHWKVYVIARGVQPLVICDGTAFSEL*
>ENSG00000157782|ENST00000351200
MGNCVKYPLRNLSRKDRSLRPEEIEELREAFREFDKDKDGYINCRDLGNCMRTMGYMPTE
MELIELSQQINMNLGGHVDFDDFVELMGPKLLAETADMIGVKELRDAFREFDTNGDGEIS
TSELREAMRKLLGHQVGHRDIEEIIRDVDLNGDGRVDFEEFVRMMSR*
>ENSG00000188706|ENST00000371064
MSVMVVRKKVTRKWEKLPGRNTFCCDGRVMMARQKGIFYLTLFLILGTCTLFFAFECRYL
AVQLSPAIPVFAAMLFLFSMATLLRTSFSDPGVIPRALPDEAAFIEMEIEATNGAVPQGQ
RPPPRIKNFQINNQIVKLKYCYTCKIFRPPRASHCSICDNCVERFDHHCPWVGNCVGKRN
YRYFYLFILSLSLLTIYVFAFNIVYVALKSLKIGFLETLKETPGTVLEVLICFFTLWSVV
GLTGFHTFLVALNQTTNEDIKGSWTGKNRVQNPYSHGNIVKNCCEVLCGPLPPSVLDRRG
ILPLEESGSRPPSTQETSSSLLPQSPAPTEHLNSNEMPEDSSTPEEMPPPEPPEPPQEAA
EAEK*
>ENSG00000132259|ENST00000379936
MSQDTKVKTTESSPPAPSKARKLLPVLDPSGDYYYWWLNTMVFPVMYNLIILVCRACFPD
LQHGYLVAWLVLDYTSDLLYLLDMVVRFHTGFLEQGILVVDKGRISSRYVRTWSFFLDLA
SLMPTDVVYVRLGPHTPTLRLNRFLRAPRLFEAFDRTETRTAYPNAFRIAKLMLYIFVVI
HWNSCLYFALSRYLGFGRDAWVYPDPAQPGFERLRRQYLYSFYFSTLILTTVGDTPPPAR
EEEYLFMVGDFLLAVMGFATIMGSMSSVIYNMNTADAAFYPDHALVKKYMKLQHVNRKLE
RRVIDWYQHLQINKKMTNEVAILQHLPERLRAEVAVSVHLSTLSRVQIFQNCEASLLEEL
VLKLQPQTYSPGEYVCRKGDIGQEMYIIREGQLAVVADDGITQYAVLGAGLYFGEISIIN
IKGNMSGNRRTANIKSLGYSDLFCLSKEDLREVLSEYPQAQTIMEEKGREILLKMNKLDV
NAEAAEIALQEATESRLRGLDQQLDDLQTKFARLLAELESSALKIAYRIERLEWQTREWP
MPEDLAEADDEGEPEEGTSKDEEGRASQEGPPGPE*
>ENSG00000198663|ENST00000359359
MSLPIAKKYMSENKGVPLHGGDEDRPFPDFDPWWTNDCEQNESEPIPANCTGCAQKHLKV
MLLEDAPRKFERLHPLVIKTGKPLLEEEIQHFLCQYPEATEGFSEGFFAKWWRCFPERWF
PFPYPWRRPLNRSQMLRELFPVFTHLPFPKDASLNKCSFLHPEPVVGSKMHKMPDLFIIG
SGEAMLQLIPPFQCRRHCQSVAMPIEPGDIGYVDTTHWKVYVIARGVQPLVICDGTAFSE
L*
>ENSG00000142920|ENST00000373443
MAGYLSESDFVMVEEGFSTRDLLKELTLGASQATTDEVAAFFVADLGAIVRKHFCFLKCL
PRVRPFYAVKCNSSPGVLKVLAQLGLGFSCANKAEMELVQHIGIPASKIICANPCKQIAQ
IKYAAKHGIQLLSFDNEMELAKVVKSHPSAKMVLCIATDDSHSLSCLSLKFGVSLKSCRH
LLENAKKHHVEVVGVSFHIGSGCPDPQAYAQSIADARLVFEMGTELGHKMHVLDLGGGFP
GTEGAKVRFEEIASVINSALDLYFPEGCGVDIFAELGRYYVTSAFTVAVSIIAKKEVLLD
QPGREEENGSTSKTIVYHLDEGVYGIFNSVLFDNICPTPILQKKPSTEQPLYSSSLWGPA
VDGCDCVAEGLWLPQLHVGDWLVFDNMGAYTVGMGSPFWGTQACHITYAMSRVAWEALRR
QLMAAEQEDDVEGVCKPLSCGWEITDTLCVGPVFTPASIM*
>ENSG00000008256|ENST00000350796
MDEDGGGEGGGVPEDLSLEEREELLDIRRRKKELIDDIERLKYEIAEVMTEIDNLTSVEE
SKTTQRNKQIAMGRKKFNMDPKKGIQFLIENDLLQSSPEDVAQFLYKGEGLNKTVIGDYL
GERDEFNIKVLQAFVELHEFADLNLVQALRQFLWSFRLPGEAQKIDRMMEAFASRYCLCN

PGVFQSTDTCYVLSFAIIMLNTSLHNHNVRDKPTAERFIAMNRGINEGGDLPEELLRNLY
ESIKNEPFKIPEDDGNDLTHTFFNPDREGWLLKLGGRVKTWKRRWFILTDNCLYYFEYTT
DKEPRGIIPLENLSIREVEDPRKPNCFELYNPSHKGQVIKACKTEADGRVVEGNHVVYRI
SAPSPEEKEEWMKSIKASISRDPFYDMLATRKRRIANKK*
>ENSG00000114988|ENST00000377079
MAATLGPLGSWQQWRRCLSARDGSRMLLLLLLLGSGQGPQQVGAGQTFEYLKREHSLSKP
YQGVGTGSSSLWNLMGNAMVMTQYIRLTPDMQSKQGALWNRVPCFLRDWELQVHFKIHGQ
GKKNLHGDGLAIWYTKDRMQPGPVFGNMDKFVGLGVFVDTYPNEEKQQEAQKRRYSPGVQ
RVFPYISAMVNNGSLSYDHERDGRPTELGGCTAIVRNLHYDTFLVIRYVKRHLTIMMDID
GKHEWRDCIEVPGVRLPRGYYFGTSSITGDLSDNHDVISLKLFELTVERTPEEEKLHRDV
FLPSVDNMKLPEMTAPLPPLSGLALFLIVFFSLVFSVFAIVIGIILYNKWQEQSRKRFY*
>ENSG00000116574|ENST00000366691
MPPQQGDPAFPDRCEAPPVPPRRERGGRGGRGPGEPGGRGRAGGAEGRGVKCVLVGDGAV
GKTSLVVSYTTNGYPTEYIPTAFDNFSAVVSVDGRPVRLQLCDTAGQDEFDKLRPLCYTN
TDIFLLCFSVVSPSSFQNVSEKWVPEIRCHCPKAPIILVGTQSDLREDVKVLIELDKCKE
KPVPEEAAKLCAEEIKAASYIECSALTQKNLKEVFDAAIVAGIQYSDTQQQPKKSKSRTP
DKMKNLSKSWWKKYCCFV*
>ENSG00000188175|ENST00000394468
MGQDAFMEPFGDTLGVFQCKIYLLLFGACSGLKVTVPSHTVHGVRGQALYLPVHYGFHTP
ASDIQIIWLFERPHTMPKYLLGSVNKSVVPDLEYQHKFTMMPPNASLLINPLQFPDEGNY
IVKVNIQGNGTLSASQKIQVTVDDPVTKPVVQIHPPSGAVEYVGNMTLTCHVEGGTRLAY
QWLKNGRPVHTSSTYSFSPQNNTLHIAPVTKEDIGNYSCLVRNPVSEMESDIIMPIIYYG
PYGLQVNSDKGLKVGEVFTVDLGEAILFDCSADSHPPNTYSWIRRTDNTTYIIKHGPRLE
VASEKVAQKTMDYVCCAYNNITGRQDETHFTVIITSVGLEKLAQKGKSLSPLASITGISL
FLIISMCLLFLWKKYQPYKVIKQKLEGRPETEYRKAQTFSGHEDALDDFGIYEFVAFPDV
SGVSRIPSRSVPASDCVSGQDLHSTVYEVIQHIPAQQQDHPE*
>ENSG00000124786|ENST00000379660
MDLTQQAKDIQNITVQETNKNNSESIECSKITMDLKFNNSRKYISITVPSKTQTMSPHIK
SVDDVVVLGMNLSKFNKLTQFFICVAGVFVFYLIYGYLQELIFSVEGFKSCGWYLTLVQF
AFYSIFGLIELQLIQDKRRRIPGKTYMIIAFLTVGTMGLSNTSLGYLNYPTQVIFKCCKL
IPVMLGGVFIQGKRYNVADVSAAICMSLGLIWFTLADSTTAPNFNLTGVVLISLALCADA
VIGNVQEKAMKLHNASNSEMVLYSYSIGFVYILLGLTCTSGLGPAVTFCAKNPVRTYGYA
FLFSLTGYFGISFVLALIKIFGALIAVTVTTGRKAMTIVLSFIFFAKPFTFQYVWSGLLV
VLGIFLNVYSKNMDKIRLPSLYDLINKSVEARKSRTLAQTV*
>ENSG00000206286|ENST00000383210
MAAAATMAAAARELVLRAGTSDMEEEEGPLAGGPGLQEPLQLGELDITSDEFILDEVDVH
IQANLEDELVKEALKTGVDLRHYSKQVELELQQIEQKSIRDYIQESENIASLHNQITACD
AVLERMEQMLGAFQSDLSSISSEIRTLQEQSGAMNIRLRNRQAVRGKLGELVDGLVVPSA
LVTAILEAPVTEPRFLEQLQELDAKAAAVREQEARGTAACADVRGVLDRLRVKAVTKIRE
FILQKIYSFRKPMTNYQIPQTALLKYRFFYQFLLGNERATAKEIRDEYVETLSKIYLSYY
RSYLGRLMKVQYEEVAEKDDLMGVEDTAKKGFFSKPSLRSRNTIFTLGTRGSVISPTELE
APILVPHTAQRGEQRYPFEALFRSQHYALLDNSCREYLFICEFFVVSGPAAHDLFHAVMG
RTLSMTLKHLDSYLADCYDAIAVFLCIHIVLRFRNIAAKRDVPALDRYWEQVLALLWPRF
ELILEMNVQSVRSTDPQRLGGLDTRPHYITRRYAEFSSALVSINQTIPNERTMQLLGQLQ

VEVENFVLRVAAEFSSRKEQLVFLINNYDMMLGVLMERAADDSKEVESFQQLLNARTQEF
IEELLSPPFGGLVAFVKEAEALIERGQAERLRGEEARVTQLIRGFGSSWKSSVESLSQDV
MRSFTNFRNGTSIIQGALTQLIQLYHRFHRVLSQPQLRALPARAELINIHHLMVELKKHK
PNF*
>ENSG00000107175|ENST00000353704
MELELDAGDQDLLAFLLEESGDLGTAPDEAVRAPLDWALPLSEVPSDWEVDDLLCSLLSP
PASLNILSSSNPCLVHHDHTYSLPRETVSMDLESESCRKEGTQMTPQHMEELAEQEIARL
VLTDEEKSLLEKEGLILPETLPLTKTEEQILKRVRRKIRNKRSAQESRRKKKVYVGGLES
RVLKYTAQNMELQNKVQLLEEQNLSLLDQLRKLQAMVIEISNKTSSSSTCILVLLVSFCL
LLVPAMYSSDTRGSLPAEHGVLSRQLRALPSEDPYQLELPALQSEVPKDSTHQWLDGSDC
VLQAPGNTSCLLHYMPQAPSAEPPLEWPFPDLFSEPLCRGPILPLQANLTRKGGWLPTGS
PSVILQDRYSG*
>ENSG00000102100|ENST00000376529
MAAVGAGGSTAAPGPGAVSAGALEPGTASAAHRRLKYISLAVLVVQNASLILSIRYARTL
PGDRFFATTAVVMAEVLKGLTCLLLLFAQKRGNVKHLVLFLHEAVLVQYVDTLKLAVPSL
IYTLQNNLQYVAISNLPAATFQPSPRCSQSHSLCLCLRLRALRSPAASRAATTTAAVFPP
WRPHHGALSAKVAHQGEGFLAAGIEDIGLASFSLLALGPAGTKL*
>ENSG00000133424|ENST00000354992
MLGICRGRRKFLAASLSLLCIPAITWIYLFSGSFEDGKPVSLSPLESQAHSPRYTASSQR
ERESLEVRMREVEEENRALRRQLSLAQGRAPSHRRGNHSKTYSMEEGTGDSENLRAGIVA
GNSSECGQQPVVEKCETIHVAIVCAGYNASRDVVTLVKSVLFHRRNPLHFHLIADSIAEQ
ILATLFQTWMVPAVRVDFYNADELKSEVSWIPNKHYSGIYGLMKLVLTKTLPANLERVIV
LDTDITFATDIAELWAVFHKFKGQQVLGLVENQSDWYLGNLWKNHRPWPALGRGYNTGVI
LLLLDKLRKMKWEQMWRLTAERELMGMLSTSLADQDIFNAVIKQNPFLVYQLPCFWNVQL
SDHTRSEQCYRDVSDLKVIHWNSPKKLRVKNKHVEFFRNLYLTFLEYDGNLLRRELFGCP
SEADVNSENLQKQLSELDEDDLCYEFRRERFTVHRTHLYFLHYEYEPAADSTDVTLVAQL
SMDRLQMLEAICKHWEGPISLALYLSDAEAQQFLRYAQGSEVLMSRHNVGYHIVYKEGQF
YPVNLLRNVAMKHISTPYMFLSDIDFLPMYGLYEYLRKSVIQLDLANTKKAMIVPAFETL
RYRLSFPKSKAELLSMLDMGTLFTFRYHVWTKGHAPTNFAKWRTATTPYRVEWEADFEPY
VVVRRDCPEYDRRFVGFGWNKVAHIMELDVQEYEFIVLPNAYMIHMPHAPSFDITKFRSN
KQYRICLKTLKEEFQQDMSRRYGFAALKYLTAENNS*
>ENSG00000167645|ENST00000392124
MADPHQLFDDTSSAQSRGYGAQRAPGGLSYPAASPTPHAAFLADPVSNMAMAYGSSLAAQ
GKELVDKNIDRFIPITKLKYYFAVDTMYVGRKLGLLFFPYLHQDWEVQYQQDTPVAPRFD
VNAPDLYIPAMAFITYVLVAGLALGTQDRFSPDLLGLQASSALAWLTLEVLAILLSLYLV
TVNTDLTTIDLVAFLGYKYVGMIGGVLMGLLFGKIGYYLVLGWCCVAIFVFMIRTLRLKI
LADAAAEGVPVRGARNQLRMYLTMAVAAAQPMLMYWLTFHLVR*
>ENSG00000112493|ENST00000395114
MKSLSLLLAVALGLATAVSAGPAVIECWFVEDASGKGLAKRPGALLLRQGPGEPPPRPDL
DPELYLSVHDPAGALQAAFRRYPRGAPAPHCEMSRFVPLPASAKWASGLTPAQNCPRALD
GAWLMVSISSPVLSLSSLLRPQPEPQQEPVLITMATVVLTVLTHTPAPRVRLGQDALLDL
SFAYMPPTSEAASSLAPGPPPFGLEWRRQHLGKGHLLLAATPGLNGQMPAAQEGAVAFAA
WDDDEPWGPWTGNGTFWLPRVQPFQEGTYLATIHLPYLQGQVTLELAVYKPPKVSLMPAT
LARAAPGEAPPELLCLVSHFYPSGGLEVEWELRGGPGGRSQKAEGQRWLSALRHHSDGSV

SLSGHLQPPPVTTEQHGARYACRIHHPSLPASGRSAEVTLEVAGLSGPSLEDSVGLFLSA
FLLLGLFKALGWAAVYLSTCKDSKKVQCSTSLYLSLVTLSPHPISKPMEGGCWCGRQNLG
LEFTLIWVKTWHYILTVGLFEHAT*
>ENSG00000196961|ENST00000359032
MPAVSKGDGMRGLAVFISDIRNCKSKEAEIKRINKELANIRSKFKGDKALDGYSKKKYVC
KLLFIFLLGHDIDFGHMEAVNLLSSNKYTEKQIGYLFISVLVNSNSELIRLINNAIKNDL
ASRNPTFMCLALHCIANVGSREMGEAFAADIPRILVAGDSMDSVKQSAALCLLRLYKASP
DLVPMGEWTARVVHLLNDQHMGVVTAAVSLITCLCKKNPDDFKTCVSLAVSRLSRIVSSA
STDLQDYTYYFVPAPWLSVKLLRLLQCYPPPEDAAVKGRLVECLETVLNKAQEPPKSKKV
QHSNAKNAILFETISLIIHYDSEPNLLVRACNQLGQFLQHRETNLRYLALESMCTLASSE
FSHEAVKTHIDTVINALKTERDVSVRQRAADLLYAMCDRSNAKQIVSEMLRYLETADYAI
REEIVLKVAILAEKYAVDYSWYVDTILNLIRIAGDYVSEEVWYRVLQIVTNRDDVQGYAA
KTVFEALQAPACHENMVKVGGYILGEFGNLIAGDPRSSPPVQFSLLHSKFHLCSVATRAL
LLSTYIKFINLFPETKATIQGVLRAGSQLRNADVELQQRAVEYLTLSSVASTDVLATVLE
EMPPFPERESSILAKLKRKKGPGAGSALDDGRRDPSSNDINGGMEPTPSTVSTPSPSADL
LGLRAAPPPAAPPASAGAGNLLVDVFDGPAAQPSLGPTPEEAFLSELEPPAPESPMALLA
DPAPAADPGPEDIGPPIPEADELLNKFVCKNNGVLFENQLLQIGVKSEFRQNLGRMYLFY
GNKTSVQFQNFSPTVVHPGDLQTQLAVQTKRVAAQVDGGAQVQQVLNIECLRDFLTPPLL
SVRFRYGGAPQALTLKLPVTINKFFQPTEMAAQDFFQRWKQLSLPQQEAQKIFKANHPMD
AEVTKAKLLGFGSALLDNVDPNPENFVGAGIIQTKALQVGCLLRLEPNAQAQMYRLTLRT
SKEPVSRHLCELLAQQF*
>ENSG00000136933|ENST00000373538
MKQLPVLEPGDKPRKATWYTLTVPGDSPCARVGHSCSYLPPVGNAKRGKVFIVGGANPNR
SFSDVHTMDLGKHQWDLDTCKGLLPRYEHASFIPSCTPDRIWVFGGANQSGNRNCLQVLN
PETRTWTTPEVTSPPPSPRTFHTSSAAIGNQLYVFGGGERGAQPVQDTKLHVFDANTLTW
SQPETLGNPPSPRHGHVMVAAGTKLFIHGGLAGDRFYDDLHCIDISDMKWQKLNPTGAAP
AGCAAHSAVAMGKHVYIFGGMTPAGALDTMYQYHTEEQHWTLLKFDTLLPPGRLDHSMCI
IPWPVTCASEKEDSNSLTLNHEAEKEDSADKVMSHSGDSHEESQTATLLCLVFGGMNTEG
EIYDDCIVTVVD*
>ENSG00000144674|ENST00000361924
MFKKLKQKISEEQQQLQQALAPAQASSNSSTPTRMRSRTSSFTEQLDEGTPNRESGDTQS
FAQKLQLRVPSVESLFRSPIKESLFRSSSKESLVRTSSRESLNRLDLDSSTASFDPPSDM
DSEAEDLVGNSDSLNKEQLIQRLRRMERSLSSYRGKYSELVTAYQMLQREKKKLQGILSQ
SQDKSLRRIAELREELQMDQQAKKHLQEEFDASLEEKDQYISVLQTQVSLLKQRLRNGPM
NVDVLKPLPQLEPQAEVFTKEENPESDGEPVVEDGTSVKTLETLQQRVKRQENLLKRCKE
TIQSHKEQCTLLTSEKEALQEQLDERLQELEKIKDLHMAEKTKLITQLRDAKNLIEQLEQ
DKGMVIAETKRQMHETLEMKEEEIAQLRSRIKQMTTQGEELREQKEKSERAAFEELEKAL
STAQKTEEARRKLKAEMDEQIKTIEKTSEEERISLQQELSRVKQEVVDVMKKSSEEQIAK
LQKLHEKELARKEQELTKKLQTREREFQEQMKVALEKSQSEYLKISQEKEQQESLALEEL
ELQKKAILTESENKLRDLQQEAETYRTRILELESSLEKSLQENKNQSKDLAVHLEAEKNK
HNKEITVMVEKHKTELESLKHQQDALWTEKLQVLKQQYQTEMEKLREKCEQEKETLLKDK
EIIFQAHIEEMNEKTLEKLDVKQTELESLSSELSEVLKARHKLEEELSVLKDQTDKMKQE
LEAKMDEQKNHHQQQVDSIIKEHEVSIQRTEKALKDQINQLELLLKERDKHLKEHQAHVE
NLEADIKRSEGELQQASAKLDVFQSYQSATHEQTKAYEEQLAQLQQKLLDLETERILLTK

QVAEVEAQKKDVCTELDAHKIQVQDLMQQLEKQNSEMEQKVKSLTQVYESKLEDGNKEQE
QTKQILVEKENMILQMREGQKKEIEILTQKLSAKEDSIHILNEEYETKFKNQEKKMEKVK
QKAKEMQETLKKKLLDQEAKLKKELENTALELSQKEKQFNAKMLEMAQANSAGISDAVSR
LETNQKEQIESLTEVHRRELNDVISIWEKKLNQQAEELQEIHEIQLQEKEQEVAELKQKI
LLFGCEKEEMNKEITWLKEEGVKQDTTLNELQEQLKQKSAHVNSLAQDETKLKAHLEKLE
VDLNKSLKENTFLQEQLVELKMLAEEDKRKVSELTSKLKTTDEEFQSLKSSHEKSNKSLE
DKSLEFKKLSEELAIQLDICCKKTEALLEAKTNELINISSSKTNAILSRISHCQHRTTKV
KEALLIKTCTVSELEAQLRQLTEEQNTLNISFQQATHQLEEKENQIKSMKADIESLVTEK
EALQKEGGNQQQAASEKESCITQLKKELSENINAVTLMKEELKEKKVEISSLSKQLTDLN
VQLQNSISLSEKEAAISSLRKQYDEEKCELLDQVQDLSFKVDTLSKEKISALEQVDDWSN
KFSEWKKKAQSRFTQHQNTVKELQIQLELKSKEAYEKDEQINLLKEELDQQNKRFDCLKG
EMEDDKSKMEKKESNLETELKSQTARIMELEDHITQKTIEIESLNEVLKNYNQQKDIEHK
ELVQKLQHFQELGEEKDNRVKEAEEKILTLENQVYSMKAELETKKKELEHVNLSVKSKEE
ELKALEDRLESESAAKLAELKRKAEQKIAAIKKQLLSQMEEKEEQYKKGTESHLSELNTK
LQEREREVHILEEKLKSVESSQSETLIVPRSAKNVAAYTEQEEADSQGCVQKTYEEKISV
LQRNLTEKEKLLQRVGQEKEETVSSHFEMRCQYQERLIKLEHAEAKQHEDQSMIGHLQEE
LEEKNKKYSLIVAQHVEKEGGKNNIQAKQNLENVFDDVQKTLQEKELTCQILEQKIKELD
SCLVRQKEVHRVEMEELTSKYEKLQALQQMDGRNKPTELLEENTEEKSKSHLVQPKLLSN
MEAQHNDLEFKLAGAEREKQKLGKEIVRLQKDLRMLRKEHQQELEILKKEYDQEREEKIK
QEQEDLELKHNSTLKQLMREFNTQLAQKEQELEMTIKETINKAQEVEAELLESHQEETNQ
LLKKIAEKDDDLKRTAKRYEEILDAREEEMTAKVRDLQTQLEELQKKYQQKLEQEENPGN
DNVTIMELQTQLAQKTTLISDSKLKEQEFREQIHNLEDRLKKYEKNVYATTVGTPYKGGN
LYHTDVSLFGEPTEFEYLRKVLFEYMMGRETKTMAKVITTVLKFPDDQTQKILEREDARL
MFTSPRSGIF*
>ENSG00000089063|ENST00000379277
MMPSRTNLATGIPSSKVKYSRLSSTDDGYIDLQFKKTPPKIPYKAIALATVLFLIGAFLI
IIGSLLLSGYISKGGADRAVPVLIIGILVFLPGFYHLRIAYYASKGYRGYSYDDIPDFDD
*
>ENSG00000082805|ENST00000360905
MYGSARSVGKVEPSSQSPGRSPRLPRSPRLGHRRTNSTGGSSGSSVGGGSGKTLSMENIQ
SLNAAYATSGPMYLSDHENVGSETPKSTMTLGRSGGRLPYGVRMTAMGSSPNIASSGVAS
DTIAFGEHHLPPVSMASTVPHSLRQARDNTIMDLQTQLKEVLRENDLLRKDVEVKESKLS
SSMNSIKTFWSPELKKERALRKDEASKITIWKEQYRVVQEENQHMQMTIQALQDELRIQR
DLNQLFQQDSSSRTGEPCVAELTEENFQRLHAEHERQAKELFLLRKTLEEMELRIETQKQ
TLNARDESIKKLLEMLQSKGLSAKATEEDHERTRRLAEAEMHVHHLESLLEQKEKENSML
REEMHRRFENAPDSAKTKALQTVIEMKDSKISSMERGLRDLEEEIQMLKSNGALSTEERE
EEMKQMEVYRSHSKFMKNKVEQLKEELSSKEAQWEELKKKAAGLQAEIGQVKQELSRKDT
ELLALQTKLETLTNQFSDSKQHIEVLKESLTAKEQRAAILQTEVDALRLRLEEKETMLNK
KTKQIQDMAEEKGTQAGEIHDLKDMLDVKERKVNVLQKKIENLQEQLRDKEKQMSSLKER
VKSLQADTTNTDTALTTLEEALAEKERTIERLKEQRDRDEREKQEEIDNYKKDLKDLKEK
VSLLQGDLSEKEASLLDLKEHASSLASSGLKKDSRLKTLEIALEQKKEECLKMESQLKKA
HEAALEARASPEMSDRIQHLEREITRYKDESSKAQAEVDRLLEILKEVENEKNDKDKKIA
ELERQVKDQNKKVANLKHKEQVEKKKSAQMLEEARRREDNLNDSSQQLQDSLRKKDDRIE
ELEEALRESVQITAEREMVLAQEESARTNAEKQVEELLMAMEKVKQELESMKAKLSSTQQ

SLAEKETHLTNLRAERRKHLEEVLEMKQEALLAAISEKDANIALLELSSSKKKTQEEVAA
LKREKDRLVQQLKQQTQNRMKLMADNYEDDHFKSSHSNQTNHKPSPDQIIQPLLELDQNR
SKLKLYIGHLTTLCHDRDPLILRGLTPPASYNLDDDQAAWENELQKMTRGQLQDELEKGE
RDNAELQEFANAILQQIADHCPDILEQVVNALEESS*
>ENSG00000197217|ENST00000358689
MGRIGISCLFPASWHFSISPVGCPRILNTNLRQIMVISVLAAAVSLLYFSVVIIRNKYGR
LTRDKKFQRYLARVTDIEATDTNNPNVNYGIVVDCGSSGSRVFVYCWPRHNGNPHDLLDI
RQMRDKNRKPVVMKIKPGISEFATSPEKVSDYISPLLNFAAEHVPRAKHKETPLYILCTA
GMRILPESQQKAILEDLLTDIPVHFDFLFSDSHAEVISGKQEGVYAWIGINFVLGRFEHI
EDDDEAVVEVNIPGSESSEAIVRKRTAGILDMGGVSTQIAYEVPKTVSFASSQQEEVAKN
LLAEFNLGCDVHQTEHVYRVYVATFLGFGGNAARQRYEDRIFANTIQKNRLLGKQTGLTP
DMPYLDPCLPLDIKDEIQQNGQTIYLRGTGDFDLCRETIQPFMNKTNETQTSLNGVYQPP
IHFQNSEFYGFSEFYYCTEDVLRMGGDYNAAKFTKAAKDYCATKWSILRERFDRGLYASH
ADLHRLKYQCFKSAWMFEVFHRGFSFPVNYKSLKTALQVYDKEVQWTLGAILYRTRFLPL
RDIQQEAFRASHTHWRGVSFVYNHYLFSGCFLVVLLAILLYLLRLRRIHRRTPRSSSAAA
LWMEEGLPAQNAPGTL*
>ENSG00000198911|ENST00000361204
MDDSGELGGLETMETLTELGDELTLGDIDEMLQFVSNQVGEFPDLFSEQLCSSFPGSGGS
GSSSGSSGSSSSSSNGRGSSSGAVDPSVQRSFTQVTLPSFSPSAASPQAPTLQVKVSPTS
VPTTPRATPILQPRPQPQPQPQTQLQQQTVMITPTFSTTPQTRIIQQPLIYQNAATSFQV
LQPQVQSLVTSSQVQPVTIQQQVQTVQAQRVLTQTANGTLQTLAPATVQTVAAPQVQQVP
VLVQPQIIKTDSLVLTTLKTDGSPVMAAVQNPALTALTTPIQTAALQVPTLVGSSGTILT
TMPVMMGQEKVPIKQVPGGVKQLEPPKEGERRTTHNIIEKRYRSSINDKIIELKDLVMGT
DAKMHKSGVLRKAIDYIKYLQQVNHKLRQENMVLKLANQKNKLLKGIDLGSLVDNEVDLK
IEDFNQNVLLMSPPASDSGSQAGFSPYSIDSEPGSPLLDDAKVKDEPDSPPVALGMVDRS
RILLCVLTFLCLSFNPLTSLLQWGGAHDSDQHPHSGSGRSVLSFESGSGGWFDWMMPTLL
LWLVNGVIVLSVFVKLLVHGEPVIRPHSRSSVTFWRHRKQADLDLARGDFAAAAGNLQTC
LAVLGRALPTSRLDLACSLSWNVIRYSLQKLRLVRWLLKKVFQCRRATPATEAGFEDEAK
TSARDAALAYHRLHQLHITGKLPAGSACSDVHMALCAVNLAECAEEKIPPSTLVEIHLTA
AMGLKTRCGGKLGFLASYFLSRAQSLCGPEHSAVPDSLRWLCHPLGQKFFMERSWSVKSA
AKESLYCAQRNPADPIAQVHQAFCKNLLERAIESLVKPQAKKKAGDQEEESCEFSSALEY
LKLLHSFVDSVGVMSPPLSRSSVLKSALGPDIICRWWTSAITVAISWLQGDDAAVRSHFT
KVERIPKALEVTESPLVKAIFHACRAMHASLPGKADGQQSSFCHCERASGHLWSSLNVSG
ATSDPALNHVVQLLTCDLLLSLRTALWQKQASASQAVGETYHASGAELAGFQRDLGSLRR
LAHSFRPAYRKVFLHEATVRLMAGASPTRTHQLLEHSLRRRTTQSTKHGEVDAWPGQRER
ATAILLACRHLPLSFLSSPGGQRAVLLAEAARTLEKVGDRRSCNDCQQMIVKLGGGTAIAA
S*
>ENSG00000182827|ENST00000366812
MAAVLNAERLEVSVDGLTLSPDPEERPGAEGAPLLPPPLPPPSPPGSGRGPGASGEQPEP
GEAAAGGAAEEARRLEQRWGFGLEELYGLALRFFKEKDGKAFHPTYEEKLKLVALHKQVL
MGPYNPDTCPEVGFFDVLGNDRRREWAALGNMSKEDAMVEFVKLLNRCCHLFSTYVASHK
IEKEEQEKKRKEEEERRRREEEERERLQKEEEKRRREEEERLRREEEERRRIEEERLRLE
QQKQQIMAALNSQTAVQFQQYAAQQYPGNYEQQQILIRQLQEQHYQQYMQQLYQVQLAQQ
QAALQKQQEVVVAGSSLPTSSKVNATVPSNMMSVNGQAKTHTDSSEKELEPEAAEEALEN

GPKESLPVIAAPSMWTRPQIKDFKEKIQQDADSVITVGRGEVVTVRVPTHEEGSYLFWEF
ATDNYDIGFGVYFEWTDSPNTAVSVHVSESSDDDEEEEENIGCEEKAKKNANKPLLDEIV
PVYRRDCHEEVYAGSHQYPGRGVYLLKFDNSYSLWRSKSVYYRVYYTR*
>ENSG00000132958|ENST00000382978
MNESPQTNEFKGTTEEAPAKESPHTSEFKGAALVSPISKSMLERLSKFEVEDAENVASYD
SKIKKIVHSIVSSFAFGIFGVFLVLLDVTLLLADLIFTDSKLYIPLEYRSISLAIGLFFL
MDVLLRVFVEGWTHLVRLLRLIILIRIFHLLHQKRQLEKLMRRLVSENKRRYTRDGFDLD
LTYVTERIIAMSFPSSGRQSFYRNPIEEVVRFLDKKHRNHYRVYNLCSERAYDPKHFHNR
VSRIMIDDHNVPTLHEMVVFTKEVNEWMAQDLENIVAIHCKGGKGRTGTMVCALLIASEI
FLTAEESLYYFGERRTNKTHSNKFQGVETPSQNRYVGYFAQVKHLYNWNLPPRRILFIKR
FIIYSIRGDVCDLKVQVVMEKKVVFSSTSLGNCSILHDIETDKILINVYDGPPLYDDVKV
QFFSSNLPKYYDNCPFFFWFNTSFIQNNRLCLPRNELDNPHKQKAWKIYPPEFAVEILFG
EK*
>ENSG00000206285|ENST00000383209
MQLRLFRRLLLAALLLVIVWTLFGPSGLGEELLSLSLASLLPAPASPGPPLALPRLLIPN
QEACSGPGAPPFLLILVCTAPENLNQRNAIRASWGGLREARGLRVQTLFLLGEPNAQHPV
WGSQGSDLASESAAQGDILQAAFQDSYRNLTLKTLSGLNWAEKHCPMARYVLKTDDDVYV
NVPELVSELVLRGGRWGQWERSTEPQREAEQEGGQVLHSEEVPLLYLGRVHWRVNPSRTP
GGRHRVSEEQWPHTWGPFPPYASGTGYVLSASAVQLILKVASRAPLLPLEDVFVGVSARR
GGLAPTQCVKLAGATHYPLDRCCYGKFLLTSHRLDPWKMQEAWKLVGGSDGERTAPFCSW
FQGVLGILRCRAIAWLQS*
>ENSG00000204525|ENST00000376228
MRVMAPRALLLLLSGGLALTETWACSHSMRYFDTAVSRPGRGEPRFISVGYVDDTQFVRF
DSDAASPRGEPRAPWVEQEGPEYWDRETQKYKRQAQADRVSLRNLRGYYNQSEDGSHTLQ
RMSGCDLGPDGRLLRGYDQSAYDGKDYIALNEDLRSWTAADTAAQITQRKLEAARAAEQL
RAYLEGTCVEWLRRYLENGKETLQRAEPPKTHVTHHPLSDHEATLRCWALGFYPAEITLT
WQRDGEDQTQDTELVETRPAGDGTFQKWAAVVVPSGQEQRYTCHMQHEGLQEPLTLSWEP
SSQPTIPIMGIVAGLAVLVVLAVLGAVVTAMMCRRKSSGGKGGSCSQAACSNSAQGSDES
LITCKA*
>ENSG00000204482|ENST00000376086
MLSRNDVKRLERSWHLLSWSQAQGSSEQELHYASLQRLPVPSSEGPDLRGRDKRGTKEDP
RADYACIAENKPT*
>ENSG00000160408|ENST00000373144
MSSNKEQRSAVFVILFALITILILYSSNSANEVFHYGSLRGRSRRPVNLKKWSITDGYVP
ILGNKTLPSRCHQCVIVSSSSHLLGTKLGPEIERAECTIRMNDAPTTGYSADVGNKTTYR
VVAHSSVFRVLRRPQEFVNRTPETVFIFWGPPSKMQKPQGSLVRVIQRAGLVFPNMEAYA
VSPGRMRQFDDLFRGETGKDREKSHSWLSTGWFTMVIAVELCDHVHVYGMVPPNYCSQRP
RLQRMPYHYYEPKGPDECVTYIQNEHSRKGNHHRFITEKRVFSSWAQLYGITFSHPSWT*
>ENSG00000160360|ENST00000392944
MDDQRCPLDDGQAGAAEATAAPTLEDRIAQPSMTASPQTEEFFDLIASSQSRRLDDQRAS
VGSLPGLRITHSNAGHLRGHGEPQEPGDDFFNMLIKYQSSRIDDQRCPPPDVLPRGPTMP
DEDFFSLIQRVQAKRMDEQRVDLAGGPEQGAGGPPEPQQQCQPGAS*
>ENSG00000105974|ENST00000393467
MADELSEKQVYDAHTKEIDLVNRDPKHLNDDVVKIDFEDVIAEPEGTHSFDGIWKASFTT

FTVTKYWFYRLLSALFGIPMALIWGIYFAILSFLHIWAVVPCIKSFLIEIQCISRVYSIY
VHTVCDPLFEAVGKIFSNVRINLQKEI*
>ENSG00000162517|ENST00000373703
MASYPYRQGCPGAAGQAPGAPPGSYYPGPPNSGGQYGSGLPPGGGYGGPAPGGPYGPPAG
GGPYGHPNPGMFPSGTPGGPYGGAAPGGPYGQPPPSSYGAQQPGLYGQGGAPPNVDPEAY
SWFQSVDSDHSGYISMKELKQALVNCNWSSFNDETCLMMINMFDKTKSGRIDVYGFSALW
KFIQQWKNLFQQYDRDRSGSISYTELQQALSQMGYNLSPQFTQLLVSRYCPRSANPAMQL
DRFIQVCTQLQVLTEAFREKDTAVQGNIRLSFEDFVTMTASRML*
>ENSG00000136933|ENST00000394125
MKQLPVLEPGDKPRKATWYTLTVPGDSPCARVGHSCSYLPPVGNAKRGKVFIVGGANPNR
SFSDVHTMDLGKHQWDLDTCKGLLPRYEHASFIPSCTPDRIWVFGGANQSGNRNCLQVLN
PETRTWTTPEVTSPPPSPRTFHTSSAAIGNQLYVFGGGERGAQPVQDTKLHVFDANTLTW
SQPETLGNPPSPRHGHVMVAAGTKLFIHGGLAGDRFYDDLHCIDISDMKWQKLNPTGAAP
AGCAAHSAVAMGKHVYIFGGMTPAGALDTMYQYHTEEQHWTLLKFDTLLPPGRLDHSMCI
IPWPVTCASEKEDSNSLTLNHEAEKEDSADKVMSHSGDSHEESQTATLLCLVFGGMNTEG
EIYDDCIVTVVD*
>ENSG00000106003|ENST00000402506
MDEQTGRLRLDTYCMSAKQIWAWSKCSGRLWDEHMKWMEGWTDRWTDGWMDGWMDEWSPT
PALRSYGGGLSQQTFIFTDGEDEALARHTGNVVITNCSAAHSRQALSCKMAVEYDRFIES
GRKWFCHVDDDNYVNLRALLRLLASYPHTRDVYVGKPSLDRPIQAMERVSENKVRPVHFW
FATGGAGFCISRGLALKMSPWASGGHFMNTAERIRLPDDCTIGYIVEALLGVPLIRSGLF
HSHLENLQQVPTSELHEQVTLSYGMFENKRNAVHVKGPFSVEADPSRFRSIHCHLYPDTP
WCPRTAIF*
>ENSG00000206506|ENST00000383622
MVVMAPRTLFLLLSGALTLTETWAGSHSMRYFSAAVSRPGRGEPRFIAMGYVDDTQFVRF
DSDSACPRMEPRAPWVEQEGPEYWEEETRNTKAHAQTDRMNLQTLRGYYNQSEAKQSSLP
TIPIMGIVAGLVVLAAVVTGAAVAAVLWRKKSSD*
>ENSG00000137497|ENST00000351960
MTLHATRGAALLSWVNSLHVADPVEAVLQLQDCSIFIKIIDRIHGTEEGQQILKQPVSER
LDFVCSFLQKNRKHPSSPECLVSAQKVLEGSELELAKMTMLLLYHSTMSSKSPRDWEQFE
YKIQAELAVILKFVLDHEDGLNLNEDLENFLQKAPVPSTCSSTFPEELSPPSHQAKREIR
FLELQKVASSSSGNNFLSGSPASPMGDILQTPQFQMRRLKKQLADERSNRDELELELAEN
RKLLTEKDAQIAMMQQRIDRLALLNEKQAASPLEPKELEELRDKNESLTMRLHETLKQCQ
DLKTEKSQMDRKINQLSEENGDLSFKLREFASHLQQLQDALNELTEEHSKATQEWLEKQA
QLEKELSAALQDKKCLEEKNEILQGKLSQLEEHLSQLQDNPPQEKGEVLGDVLQVEELSK
KLADSDQASKVQQQKLKAVQAQGGESQQEAQRLQAQLNELQAQLSQKEQAAEHYKLQMEK
AKTHYDAKKQQNQELQEQLRSLEQLQKENKELRAEAERLGHELQQAGLKTKEAEQTCRHL
TAQVRSLEAQVAHADQQLRDLGKFQVATDALKSREPQAKPQLDLSIDSLDLSCEEGTPLS
ITSKLPRTQPDGTSVPGEPASPISQRLPPKVESLESLYFTPIPARSQAPLESSLDSLGDV
FLDSGRKTRSARRRTTQIINITMTKKLDVEEPDSANSSFYSTRSAPASQASLRATSSTQS
LARLGSPDYGNSALLSLPGYRPTTRSSARRSQAGVSSGAPPGRNSFYMGTCQDEPEQLDD
WNRIAELQQRNRVCPPHLKTCYPLESRPSLSLGTITDEEMKTGDPQETLRRASMQPIQIA
EGTGITTRQQRKRVSLEPHQGPGTPESKKATSCFPRPMTPRDRHEGRKQSTTEAQKKAAP
ASTKQADRRQSMAFSILNTPKKLGNSLLRRGASKKALSKASPNTRSGTRRSPRIATTTAS

AATAAAIGATPRAKGKAKH*
>ENSG00000019582|ENST00000377795
MHRRRSRSCREDQKPVMDDQRDLISNNEQLPMLGRRPGAPESKCSRGALYTGFSILVTLL
LAGQATTAYFLYQQQGRLDKLTVTSQNLQLENLRMKLPKPPKPVSKMRMATPLLMQALPM
GALPQGPMQNATKYGNMTEDHVMHLLQSHWNWRTRLLGWV*
>ENSG00000170962|ENST00000393158
MHRLIFVYTLICANFCSCRDTSATPQSASIKALRNANLRRDESNHLTDLYRRDETIQVKG
NGYVQSPRFPNSYPRNLLLTWRLHSQENTRIQLVFDNQFGLEEAENDICRYDFVEVEDIS
ETSTIIRGRWCGHKEVPPRIKSRTNQIKITFKSDDYFVAKPGFKIYYSLLEDFQPAAASE
TNWESVTSSISGVSYNSPSVTDPTLIADALDKKIAEFDTVEDLLKYFNPESWQEDLENMY
LDTPRYRGRSYHDRKSKVDLDRLNDDAKRYSCTPRNYSVNIREELKLANVVFFPRCLLVQ
RCGGNCGCGTVNWRSCTCNSGKTVKKYHEVLQFEPGHIKRRGRAKTMALVDIQLDHHERC
DCICSSRPPR*
>ENSG00000171365|ENST00000376091
MAMWQGAMDNRGFQQGSFSSFQNSSSDEDLMDIPATAMDFSMRDDVPPLDREVGEDKSYN
GGGIGSSNRIMDFLEEPIPGVGTYDDFNTIDWVREKSRDRDRHREITNKSKESTWALIHS
VSDAFSGWLLMLLIGLLSGSLAGLIDISAHWMTDLKEGICTGGFWFNHEHCCWNSEHVTF
EERDKCPEWNSWSQLIISTDEGAFAYIVNYFMYVLWALLFAFLAVSLVKVFAPYACGSGI
PEIKTILSGFIIRGYLGKWTLVIKTITLVLAVSSGLSLGKEGPLVHVACCCGNILCHCFN
KYRKNEAKRREVLSAAAAAGVSVAFGAPIGGVLFSLEEVSYYFPLKTLWRSFFAALVAAF
TLRSINPFGNSRLVLFYVEFHTPWHLFELVPFILLGIFGGLWGALFIRTNIAWCRKRKTT
QLGKYPVIEVLVVTAITAILAFPNEYTRMSTSELISELFNDCGLLDSSKLCDYENRFNTS
KGGELPDRPAGVGVYSAMWQLALTLILKIVITIFTFGMKIPSGLFIPSMAVGAIAGRLLG
VGMEQLAYYHQEWTVFNSWCSQGADCITPGLYAMVGAAACLGGVTRMTVSLVVIMFELTG
GLEYIVPLMAAAMTSKWVADALGREGIYDAHIRLNGYPFLEAKEEFAHKTLAMDVMKPRR
NDPLLTVLTQDSMTVEDVETIISETTYSGFPVVVSRESQRLVGFVLRRDLIISIENARKK
QDGVVSTSIIYFTEHSPPLPPYTPPTLKLRNILDLSPFTVTDLTPMEIVVDIFRKLGLRQ
CLVTHNGRLLGIITKKDVLKHIAQMANQDPDSILFN*
>ENSG00000198168|ENST00000354193
MGLCFPCPGESAPPTPDLEEKRAKLAEAAERRQKEAASRGILDVQSVQEKRKKKEKIEKQ
IATSGPPPEGGLRWTVS*
>ENSG00000125967|ENST00000375238
MACAGLLTVCLLRPPAPQPQPQTPRHPQLAPDPGPAGHTLFQDVFRRADKNDDGKLSFEE
FQNYFADGVLSLGELQELFSGIDGHLTDNLETEKLCDYFSEHLGVYRPVLAALESLNRAV
LAAMDATKLEYERASKVDQFVTRFLLRETVSQLQALQSSLEGASDTLEAQAHGWRSDAES
VEAQSRLCGSRRAGRRALRSVSRSSTWSPGSSDTGRSSEAEMQWRLQVNRLQELIDQLEC
KAPRLEPLREEDLAKGPDLHILMAQRQVQVAEEGLQDFHRALRCYVDFTGAQSHCLHVSA
QKMLDGASFTLYEFWQDEASWRRHQQSPGSKAFQRILIDHLRAPDTLTTVFFPASWWIMN
NN*
>ENSG00000003147|ENST00000402384
MSGHKCSYPWDLQDRYAQDKSVVNKMQQKYWETKQAFIKATGKKEDEHVVASDADLDAKL
ELFHSIQRTCLDLSKAIVLYQKRICFLSQEENELGKFLRSQGFQDKTRAGKMMQATGKAL
CFSSQQRLALRNPLCRFHQEVETFRHRAISDTWLTVNRMEQCRTEYRGALLWMKDVSQEL
DPDLYKQMEKFRKVQTQVRLAKKNFDKLKMDVCQKVDLLGASRCNLLSHMLATYQTTLLH

FWEKTSHTMAAIHESFKGYQPYEFTTLKSLQDPMKKLVEKEEKKKINQQESTDAAVQEPS
QLISLEEENQRKESSSFKTEDGKSILSALDKGSTHTACSGPIDELLDMKSEEGACLGPVA
GTPEPEGADKDDLLLLSEIFNASSLEEGEFSKEWAAVFGDGQVKEPVPTMALGEPDPKAQ
TGSGFLPSQLLDQNMKDLQASLQEPAKAASDLTAWFSLFADLDPLSNPDAVGKTDKEHEL
LNA*
>ENSG00000110080|ENST00000356132
MVSKSRWKLLAMLALVLVVMVWYSISREDRYIELFYFPIPEKKEPCLQGEAESKASKLFG
KLSPLCSYSRDQPIFLRLEDYFWVKTPSAYELPYGTKGSEDLLLRVLAITSSSIPKNIQS
LRCRRCVVVGNGHRLRNSSLGDAINKYDVVIRLNNAPVAGYEGDVGSKTTMRLFYPESAH
FDPKVENNPDTLLVLVAFKAMDFHWIETILSDKKRVRKGFWKQPPLIWDVNPKQIRILNP
FFMEIAADKLLSLPMQQPRKIKQKPTTGLLAITLALHLCDLVHIAGFGYPDAYNKKQTIH
YYEQITLKSMAGSGHNVSQEALAIKRMLEMGAIKNLTSF*
>ENSG00000114209|ENST00000392750
MRMTMEEMKNEAETTSMVSMPLYAVMYPVFNELERVNLSAAQTLRAAFIKAEKENPGLTQ
DIIMKILEKKSVEVNFTESLLRMAADDVEEYMIERPEPEFQDLNEKARALKQILSKIPDE
INDRVRFLQTIKDIASAIKELLDTVNNVFKKYQYQNRRALEHQKKEFVKYSKSFSDTLKT
YFKDGKAINVFVSANRLIHQTNLILQTFKTVA*
>ENSG00000104490|ENST00000395923
MGKQNSKLRPEVMQDLLESTDFTEHEIQEWYKGFLRDCPSGHLSMEEFKKIYGNFFPYGD
ASKFAEHVFRTFDANGDGTIDFREFIIALSVTSRGKLEQKLKWAFSMYDLDGNGYISKAE
MLEIVQAIYKMVSSVMKMPEDESTPEKRTEKIFRQMDTNRDGKLSLEEFIRGAKSDPSIV
RLLQCDPSSAGQF*
>ENSG00000143183|ENST00000392129
MSTMFADTLLIVFISVCTALLAEGITWVLVYRTDKYKRLKAEVEKQSKKLEKKKETITES
AGRQQKKKIERQEEKLKNNNRDLSMVRMKSMFAIGFCFTALMGMFNSIFDGRVVAKLPFT
PLSYIQGLSHRNLLGDDTTDCSFIFLYILCTMSIRQNIQKILGLAPSRAATKQAGGFLGP
PPPSGKFS*
>ENSG00000106809|ENST00000375561
MKTLQSTLLLLLLVPLIKPAPPTQQDSRIIYDYGTDNFEESIFSQDYEDKYLDGKNIKEK
ETVIIPNEKSLQLQKDEAITPLPPKKENDEMPTCLLCVCLSGSVYCEEVDIDAVPPLPKE
SAYLYARFNKIKKLTAKDFADIPNLRRLDFTGNLIEDIEDGTFSKLSLLEELSLAENQLL
KLPVLPPKLTLFNAKYNKIKSRGIKANAFKKLNNLTFLYLDHNALESVPLNLPESLRVIH
LQFNNIASITDDTFCKANDTSYIRDRIEEIRLEGNPIVLGKHPNSFICLKRLPIGSYF*
>ENSG00000116133|ENST00000371269
MEPAVSLAVCALLFLLWVRLKGLEFVLIHQRWVFVCLFLLPLSLIFDIYYYVRAWVVFKL
SSAPRLHEQRVRDIQKQVREWKEQGSKTFMCTGRPGWLTVSLRVGKYKKTHKNIMINLMD
ILEVDTKKQIVRVEPLVTMGQVTALLTSIGWTLPVLPELDDLTVGGLIMGTGIESSSHKY
GLFQHICTAYELVLADGSFVRCTPSENSDLFYAVPWSCGTLGFLVAAEIRIIPAKKYVKL
RFEPVRGLEAICAKFTHESQRQENHFVEGLLYSLDEAVIMTGVMTDEAEPSKLNSIGNYY
KPWFFKHVENYLKTNREGLEYIPLRHYYHRHTRSIFWELQDIIPFGNNPIFRYLFGWMVP
PKISLLKLTQGETLRKLYEQHHVVQDMLVPMKCLQQALHTFQNDIHVYPIWLCPFILPSQ
PGLVHPKGNEAELYIDIGAYGEPRVKHFEARSCMRQLEKFVRSVHGFQMLYADCYMNREE
FWEMFDGSLYHKLREKLGCQDAFPEVYDKICKAARH*
>ENSG00000076864|ENST00000374765

MIEKMQGSRMDEQRCSFPPPLKTEEDYIPYPSVHEVLGREGPFPLILLPQFGGYWIEGTN
HEITSIPETEPLQSPTTKVKLECNPTARIYRKHFLGKEHFNYYSLDAALGHLVFSLKYDV
IGDQEHLRLLLRTKCRTYHDVIPISCLTEFPNVVQMAKLVCEDVNVDRFYPVLYPKASRL
IVTFDEHVISNNFKFGVIYQKLGQTSEEELFSTNEESPAFVEFLEFLGQKVKLQDFKGFR
GGLDVTHGQTGTESVYCNFRNKEIMFHVSTKLPYTEGDAQQLQRKRHIGNDIVAVVFQDE
NTPFVPDMIASNFLHAYVVVQAEGGGPDGPLYKVSVTARDDVPFFGPPLPDPAVFRKGPE
FQEFLLTKLINAEYACYKAEKFAKLEERTRAALLETLYEELHIHSQSMMGLGGDEDKMEN
GSGGGGFFESFKRVIRSRSQSMDAMGLSNKKPNTVSTSHSGSFAPNNPDLAKAAGISLIV
PGKSPTRKKSGPFGSRRSSAIGIENIQEVQEKRESPPAGQKTPDSGHVSQEPKSENSSTQ
SSPEMPTTKNRAETAAQRAEALKDFSRSSSSASSFASVVEETEGVDGEDTGLESVSSSGT
PHKRDSFIYSTWLEDSVSTTSGGSSPGPSRSPHPDAGKLGDPACPEIKIQLEASEQHMPQ
LGC*
>ENSG00000070610|ENST00000378103
MGTQDPGNMGTGVPASEQISCAKEDPQVYCPEETGGTKDVQVTDCKSPEDSRPPKETDCC
NPEDSGQLMVSYEGKAMGYQVPPFGWRICLAHEFTEKRKPFQANNVSLSNMIKHIGMGLR
YLQWWYRKTHVEKKTPFIDMINSVPLRQIYGCPLGGIGGGTITRGWRGQFCRWQLNPGMY
QHRTVIADQFTVCLRREGQTVYQQVLSLERPSVLRSWNWGLCGYFAFYHALYPRAWTVYQ
LPGQNVTLTCRQITPILPHDYQDSSLPVGVFVWDVENEGDEALDVSIMFSMRNGLGGGDD
APGGLWNEPFCLERSGETVRGLLLHHPTLPNPYTMAVAARVTAATTVTHITAFDPDSTGQ
QVWQDLLQDGQLDSPTGQSTPTQKGVGIAGAVCVSSKLRPRGQCRLEFSLAWDMPRIMFG
AKGQVHYRRYTRFFGQDGDAAPALSHYALCRYAEWEERISAWQSPVLDDRSLPAWYKSAL
FNELYFLADGGTVWLEVLEDSLPEELGRNMCHLRPTLRDYGRFGYLEGQEYRMYNTYDVH
FYASFALIMLWPKLELSLQYDMALATLREDLTRRRYLMSGVMAPVKRRNVIPHDIGDPDD
EPWLRVNAYLIHDTADWKDLNLKFVLQVYRDYYLTGDQNFLKDMWPVCLAVMESEMKFDK
DHDGLIENGGYADQTYDGWVTTGPSAYCGGLWLAAVAVMVQMAALCGAQDIQDKFSSILS
RGQEAYERLLWNGRYYNYDSSSRPQSRSVMSDQCAGQWFLKACGLGEGDTEVFPTQHVVR
ALQTIFELNVQAFAGGAMGAVNGMQPHGVPDKSSVQSDEVWVGVVYGLAATMIQEGLTWE
GFQTAEGCYRTVWERLGLAFQTPEAYCQQRVFRSLAYMRPLSIWAMQLALQQQQHKKASW
PKVKQGTGLRTGPMFGPKEAMANLSPE*
>ENSG00000089063|ENST00000379299
MMPSRTNLATGIPSSKVKYSRLSSTDDGYIDLQFKKTPPKIPYKAIALATVLFLIGAFLI
IIGSLLLSGYISKGGADRAVPVLIIGILVFLPGFYHLRIAYYASKGYRGYSYDDIPDFDD
*
>ENSG00000126091|ENST00000361812
MGLLVFVRNLLLALCLFLVLGFLYYSAWKLHLLQWEEDSSKYSHSSSPQEKPVADSVVLS
FDSAGQTLGSEYDRLGFLLNLDSKLPAELATKYANFSEGACKPGYASALMTAIFPRFSKP
APMFLDDSFRKWARIREFVPPFGIKGQVLDAQYPARERVSAEAGESSRHH*
>ENSG00000186318|ENST00000392937
MVPFIYLQAHFTLCSGWSSTYRDLRKGVYVPYTQGKWEGELGTDLVSIPHGPNVTVRANI
AAITESDKFFINGSNWEGILGLAYAEIARPDDSLEPFFDSLVKQTHVPNLFSLQLCGAGF
PLNQSEVLASVGGSMIIGGIDHSLYTGSLWYTPIRREWYYEVIIVRVEINGQDLKMDCKE
YNYDKSIVDSGTTNLRLPKKVFEAAVKSIKAASSTEKFPDGFWLGEQLVCWQAGTTPWNI
FPVISLYLMGEVTNQSFRITILPQQYLRPVEDVATSQDDCYKFAISQSSTGTVMGAVIME
GFYVVFDRARKRIGFAVSACHVHDEFRTAAVEGPFVTLDMEDCGYNIPQTDESTLMTIAY

VMAAICALFMLPLCLMVCQWRCLRCLRQQHDDFADDISLLK*
>ENSG00000143207|ENST00000367669
MSGSRQAGSGSAGTSPGSSAASSVTSASSSLSSSPSPPSVAVSAAALVSGGVAQAAGSGG
LGGPVRPVLVAPAVSGSGGGAVSTGLSRHSCAARPSAGVGGSSSSLGSGSRKRPLLAPLC
NGLINSYEDKSNDFVCPICFDMIEEAYMTKCGHSFCYKCIHQSLEDNNRCPKCNYVVDNI
DHLYPNFLVNELILKQKQRFEEKRFKLDHSVSSTNGHRWQIFQDWLGTDQDNLDLANVNL
MLELLVQKKKQLEAESHAAQLQILMEFLKVARRNKREQLEQIQKELSVLEEDIKRVEEMS
GLYSPVSEDSTVPQFEAPSPSHSSIIDSTEYSQPPGFSGSSQTKKQPWYNSTLASRRKRL
TAHFEDLEQCYFSTRMSRISDDSRTASQLDEFQECLSKFTRYNSVRPLATLSYASDLYNG
SSIVSSIEFDRDCDYFAIAGVTKKIKVYEYDTVIQDAVDIHYPENEMTCNSKISCISWSS
YHKNLLASSDYEGTVILWDGFTGQRSKVYQEHEKRCWSVDFNLMDPKLLASGSDDAKVKL
WSTNLDNSVASIEAKANVCCVKFSPSSRYHLAFGCADHCVHYYDLRNTKQPIMVFKGHRK
AVSYAKFVSGEEIVSASTDSQLKLWNVGKPYCLRSFKGHINEKNFVGLASNGDYIACGSE
NNSLYLYYKGLSKTLLTFKFDTVKSVLDKDRKEDDTNEFVSAVCWRALPDGESNVLIAAN
SQGTIKVLELV*
>ENSG00000078808|ENST00000360001
MVWPWVAMASRWGPLIGLAPCCLWLLGAVLLMDASARPANHSSTRERVANREENEILPPD
HLNGVKLEMDGHLNRGFHQEVFLGKDLGGFDEDAEPRRSRRKLMVIFSKVDVNTDRKISA
KEMQRWIMEKTAEHFQEAMEESKTHFRAVDPDGDGHVSWDEYKVKFLASKGHSEKEVADA
IRLNEELKVDEETQEVLENLKDRWYQADSPPADLLLTEEEFLSFLHPEHSRGMLRFMVKE
IVRDLDQDGDKQLSVPEFISLPVGTVENQQGQDIDDNWVKDRKKEFEELIDSNHDGIVTA
EELESYMDPMNEYNALNEAKQMIAVADENQNHHLEPEEVLKYSEFFTGSKLVDYARSVHE
EF*
>ENSG00000196610|ENST00000399661
MSWKMALQIPGGFWAAAVTVMLVMLSTPVAEARDFPKDFLVQFKGMCYFTNGTERVRGVA
RYIYNREEYGRFDSDVGEFQAVTELGRSIEDWNNYKDFLEQERAAVDKVCRHNYEAELRT
TLQRQVEPTVTISPSRTEALNHHNLLVCSVTDFYPAQIKVQWFRNDQEETAGVVSTSLIR
NGDWTFQILVMLEITPQRGDIYTCQVEHPSLQSPITVEWRAQSESAQSKMLGGVGGFVLG
LIFLGLGLIIRHRGQKGPRGPPPAGLLH*
>ENSG00000106692|ENST00000357998
MSRINKNVVLALLTLTSSAFLLFQLYYYKHYLSTKNGAGLSKSKGSRIGFDSTQWRAVKK
FIMLTSNQNVPVFLIDPLILELINKNFEQVKNTSHGSTSQCKFFCVPRDFTAFALQYHLW
KNEEGWFRIAENMGFQCLKIESKDPRLDGIDSLSGTEIPLHYICKLATHAIHLVVFHERS
GNYLWHGHLRLKEHIDRKFVPFRKLQFGRYPGAFDRPELQQVTVDGLEVLIPKDPMHFVE
EVPHSRFIECRYKEARAFFQQYLDDNTVEAVAFRKSAKELLQLAAKTLNKLGVPFWLSSG
TCLGWYRQCNIIPYSKDVDLGIFIQDYKSDIILAFQDAGLPLKHKFGKVEDSLELSFQGK
DDVKLDVFFFYEETDHMWNGGTQAKTGKKFKYLFPKFTLCWTEFVDMKVHVPCETLEYIE
ANYGKNQQGA*
>ENSG00000174775|ENST00000397596
MTEYKLVVVGAGGVGKSALTIQLIQNHFVDEYDPTIEDSYRKQVVIDGETCLLDILDTAG
QEEYSAMRDQYMRTGEGFLCVFAINNTKSFEDIHQYREQIKRVKDSDDVPMVLVGNKCDL
AARTVESRQAQDLARSYGIPYIETSAKTRQGVEDAFYTLVREIRQHKLRKLNPPDESGPG
CMSCKCVLS*
>ENSG00000110697|ENST00000356404

MLIKEYHILLPMSLDEYQVAQLYMIQKKSREESSGEGSGVEILANRPYTDGPGGSGQYTH
KVYHVGSHIPGWFRALLPKAALQVEEESWNAYPYTRTRYTCPFVEKFSIEIETYYLPDGG
QQPNVFNLSGAERRQRILDTIDIVRDAVAPGEYKAEEDPRLYHSVKTGRGPLSDDWARTA
AQTGPLMCAYKLCKVEFRYWGMQAKIEQFIHDVGLRRVMLRAHRQAWCWQDEWTELSMAD
IRALEEETARMLAQRMAKCNTGSEGSEAQPPGKPSTEARSAASNTGTPDGPEAPPGPDAS
PDASFGKQWSSSSRSSYSSQHGGAVSPQSLSEWRMQNIARDSENSSEEEFFDAHEGFSDS
EEVFPKEMTKWNSNDFIDAFASPVEAEGTPEPGAEAAKGIEDGAQAPRDSEGLDGAGELG
AEACAVHALFLILHSGNILDSGPGDANSKQADVQTLSSAFEAVTRIHFPEALGHVALRLV
PCPPICAAAYALVSNLSPYSHDGDSLSRSQDHIPLAALPLLATSSSRYQGAVATVIARTN
QAYSAFLRSPEGAGFCGQVALIGDGVGGILGFDALCHSANAGTGSRGSSRRGSMNNELLS
PEFGPVRDPLADGVEGLGRGSPEPSALPPQRIPSDMASPEPEGSQNSLQAAPATTSSWEP
RRASTAFCPPAASSEAPDGPSSTARLDFKVSGFFLFGSPLGLVLALRKTVMPALEAAQMR
PACEQIYNLFHAADPCASRLEPLLAPKFQAIAPLTVPRYQKFPLGDGSSLLLADTLQTHS
SLFLEELEMLVPSTPTSTSGAFWKGSELATDPPAQPAAPSTTSEVVKILERWWGTKRIDY
SLYCPEALTAFPTVTLPHLFHASYWESADVVAFILRQVIEKERPQLAECEEPSIYSPAFP
REKWQRKRTQVKIRNVTSNHRASDTVVCEGRPQVLSGRFMYGPLDVVTLTGEKVDVYIMT
QPLSGKWIHFGTEVTNSSGRLTFPVPPERALGIGVYPVRMVVRGDHTYAECCLTVVARGT
EAVVFSIDGSFTASVSIMGSDPKVRAGAVDVVRHWQDSGYLIVYVTGRPDMQKHRVVAWL
SQHNFPHGVVSFCDGLTHDPLRQKAMFLQSLVQEVELNIVAGYGSPKDVAVYAALGLSPS
QTYIVGRAVRKLQAQCQFLSDGYVAHLGQLEAGSHSHASSGPPRAALGKSSYGVAAPVDF
LRKQSQLLRSRGPSQAEREGPGTPPTTLARGKARSISLKLDSEE*
>ENSG00000171004|ENST00000370833
MALPACAVREFEPPRQPERGAPVRTTCPRRHSRVEAELAASRPGSVAASVRAGPPRGVSH
GFHTRPLLDKPRKASSSLAGAACAPLFALLSRGRRRRMHVLRRRWDLGSLCRALLTRGLA
ALGHSLKHVLGAIFSKIFGPMASVGNMDEKSNKLLLALVMLFLFAVIVLQYVCPGTECQL
LRLQAFSSPVPDPYRSEDESSARFVPRYNFTRGDLLRKVDFDIKGDDLIVFLHIQKTGGT
TFGRHLVRNIQLEQPCECRVGQKKCTCHRPGKRETWLFSRFSTGWSCGLHADWTELTSCV
PSVVDGKRDARLRPSRWRIFQILDAASKDKRGSPNTNAGANSPSSTKTRNTSKSGKNFHY
ITILRDPVSRYLSEWRHVQRGATWKASLHVCDGRPPTSEELPSCYTGDDWSGCPLKEFMD
CPYNLANNRQVRMLSDLTLVGCYNLSVMPEKQRNKVLLESAKSNLKHMAFFGLTEFQRKT
QYLFEKTFNMNFISPFTQYNTTRASSVEINEEIQKRIEGLNFLDMELYSYAKDLFLQRYQ
FMRQKEHQEARRKRQEQRKFLKGRLLQTHFQSQGQGQSQNPNQNQSQNPNPNANQNLTQN
LMQNLTQSLSQKENRESPKQNSGKEQNDNTSNGTNDYIGSVEKWR*
>ENSG00000008256|ENST00000396741
MDEDGGGEGGGVPEDLSLEEREELLDIRRRKKELIDDIERLKYEIAEVMTEIDNLTSVEE
SKTTQRNKQIAMGRKKFNMDPKKGIQFLIENDLLQSSPEDVAQFLYKGEGLNKTVIGDYL
GERDEFNIKVLQAFVELHEFADLNLVQALRQFLWSFRLPGEAQKIDRMMEAFASRYCLCN
PGVFQSTDTCYVLSFAIIMLNTSLHNHNVRDKPTAERFIAMNRGINEGGDLPEELLRNLY
ESIKNEPFKIPEDDGNDLTHTFFNPDREGWLLKLGGGRVKTWKRRWFILTDNCLYYFEYT
TDKEPRGIIPLENLSIREVEDPRKPNCFELYNPSHKGQVIKACKTEADGRVVEGNHVVYR
ISAPSPEEKEEWMKSIKASISRDPFYDMLATRKRRIANKK*
>ENSG00000204632|ENST00000376815
MVVMAPRTLFLLLSGALTLTETWAGSHSMRYFSAAVSRPGRGEPRFIAMGYVDDTQFVRF
DSDSACPRMEPRAPWVEQEGPEYWEEETRNTKAHAQTDRMNLQTLRGYYNQSEAKQSSLP

TIPIMGIVAGLVVLAAVVTGAAVAAVLWRKKSSD*
>ENSG00000122705|ENST00000396603
MAELDPFGAPAGAPGGPALGNGVAGAGEEDPAAAFLAQQESEIAGIENDEAFAILDGGAP
GPQPHGEPPGGPDAVDGVMNGEYYQESNGPTDSYAAISQVDRLQSEPESIRKWREEQMER
LEALDANSRKQEAEWKEKAIKELEEWYARQDEQLQKTKANNRVADEAFYKQPFADVIGYV
AAEEAFVNDIDESSPGTEWERVARLCDFNPKSSKQAKDVSRMRSVLISLKQAPLVH*
>ENSG00000136238|ENST00000356142
MQAIKCVVVGDGAVGKTCLLISYTTNAFPGEYIPTVFDNYSANVMVDGKPVNLGLWDTAG
QEDYDRLRPLSYPQTVGETYGKDITSRGKDKPIADVFLICFSLVSPASFENVRAKWYPEV
RHHCPNTPIILVGTKLDLRDDKDTIEKLKEKKLTPITYPQGLAMAKEIGAVKYLECSALT
QRGLKTVFDEAIRAVLCPPPVKKRKRKCLLL*
>ENSG00000197249|ENST00000393087
MPSSVSWGILLLAGLCCLVPVSLAEDPQGDAAQKTDTSHHDQDHPTFNKITPNLAEFAFS
LYRQLAHQSNSTNIFFSPVSIATAFAMLSLGTKADTHDEILEGLNFNLTEIPEAQIHEGF
QELLRTLNQPDSQLQLTTGNGLFLSEGLKLVDKFLEDVKKLYHSEAFTVNFGDTEEAKKQ
INDYVEKGTQGKIVDLVKELDRDTVFALVNYIFFKGKWERPFEVKDTEEEDFHVDQVTTV
KVPMMKRLGMFNIQHCKKLSSWVLLMKYLGNATAIFFLPDEGKLQHLENELTHDIITKFL
ENEDRRSASLHLPKLSITGTYDLKSVLGQLGITKVFSNGADLSGVTEEAPLKLSKAVHKA
VLTIDEKGTEAAGAMFLEAIPMSIPPEVKFNKPFVFLMIEQNTKSPLFMGKVVNPTQK*
>ENSG00000143578|ENST00000368607
MDLGIPDLLDAWLEPPEDIFSTGSVLELGLHCPPPEVPVTRLQEQGLQGWKSGGDRGCGL
QESEPEDFLKLFIDPNEVYCSEASPGSDSGISEDPCHPDSPPAPRATSSPMLYEVVYEAG
ALERMQGETGPNVGLISIQLDQWSPAFMVPDSCMVSELPFDAHAHILPRAGTVAPVPCTT
LLPCQTLFLTDEEKRLLGQEGVSLPSHLPLTKAEERVLKKVRRKIRNKQSAQDSRRRKKE
YIDGLESRVAACSAQNQELQKKVQELERHNISLVAQLRQLQTLIAQTSNKAAQTSTCVLI
LLFSLALIILPSFSPFQSRPEAGSEDYQPHGVTSRNILTHKDVTENLETQVVESRLREPP
GAKDANGSTRTLLEKMGGKPRPSGRIRSVLHADEM*
>ENSG00000257923|ENST00000393824
MAANVGSMFQYWKRFDLQQLQDLRKQVAPLLKSFQGEIDALSKRSKEAEAAFLNVYKRLI
DVPDPVPALDLGQQLQLKVQRLHDIETENQKLRETLEEYNKEFAEVKNQEVTIKALKEKI
REYEQTLKNQAETIALEKEQKLQNDFAEKERKLQETQMSTTSKLEEAEHKVQSLQTALEK
TRTELFDLKTKYDEETTAKADEIEMIMTDLERANQRAEVAQREAETLREQLSSANHSLQL
ASQIQKAPDVAIEVLTRSSLEVELAAKEREIAQLVEDVQRLQASLTKLRENSASQISQLE
QQLSAKNSTLKQLEEKLKGQADYEEVKKELNILKSMEFAPSEGAGTQDAAKPLEVLLLEK
NRSLQSENAALRISNSDLSGRCAELQVRITEAVATATEQRELIARLEQDLSIIQSIQRPD
AEGAAEHRLEKIPEPIKEATALFYGPAAPASGALPEGQVDSLLSIISSQRERFRARNQEL
EAENRLAQHTLQALQSELDSLRADNIKLFEKIKFLQSYPGRGSGSDDTELRYSSQYEERL
DPFSSFSKRERQRKYLSLSPWDKATLSMGRLVLSNKMARTIGFFYTLFLHCLVFLVLYKL
AWSESMERDCATFCAKKFADHLHKFHENDNGAAAGDLWQ*
>ENSG00000109572|ENST00000360642
MESEQLFHRGYYRNSYNSITSASSDEELLDGAGVIMDFQTSEDDNLLDGDTAVGTHYTMT
NGGSINSSTHLLDLLDEPIPGVGTYDDFHTIDWVREKCKDRERHRRINSKKKESAWEMTK
SLYDAWSGWLVVTLTGLASGALAGLIDIAADWMTDLKEGICLSALWYNHEQCCWGSNETT
FEERDKCPQWKTWAELIIGQAEGPGSYIMNYIMYIFWALSFAFLAVSLVKVFAPYACGSG

IPEIKTILSGFIIRGYLGKWTLMIKTITLVLAVASGLSLGKEGPLVHVACCCGNIFSYLF
PKYSTNEAKKREVSYYFPLKTLWRSFFAALVAAFVLRSINPFGNSRLVLFYVEYHTPWYL
FELFPFILLGVFGGLWGAFFIRANIAWCRRRKSTKFGKYPVLEVIIVAAITAVIAFPNPY
TRLNTSELIKELFTDCGPLESSSLCDYRNDMNASKIVDDIPDRPAGIGVYSAIWQLCLAL
IFKIIMTVFTFGIKVPSGLFIPSMAIGAIAGRIVGIAVEQLAYYHHDWFIFKEWCEVGAD
CITPGLYAMVGAAACLGGVTRMTVSLVVIVFELTGGLEYIVPLMAAVMTSKWVGDAFGRE
GIYEAHIRLNGYPFLDAKEEFTHTTLAADVMRPRRNDPPLAVLTQDNMTVDDIENMINET
SYNGFPVIMSKESQRLVGFALRRDLTIAIESARKKQEGIVGSSRVCFAQHTPSLPAESPR
PLKLRSILDMSPFTVTDHTPMEIVVDIFRKLGLRQCLVTHNGRLLGIITKKDILRHMAQT
ANQDPASIMFN*
>ENSG00000142920|ENST00000373440
MAGYLSESDFVMVEEGFSTRDLLKELTLGASQATTDEVAAFFVADLGAIVRKHFCFLKCL
PRVRPFYAVKCNSSPGVLKVLAQLGLGFSCANKIASVINSALDLYFPEGCGVDIFAELGR
YYVTSAFTVAVSIIAKKEVLLDQPGREEENGSTSKTIVYHLDEGVYGIFNSVLFDNICPT
PILQKSKNHSPCYMSLESIHFIAV*
>ENSG00000110075|ENST00000393800
MFWKFDLHSSSHIDTLLEREDVTLKELMDEEDVLQECKAQNRKLIEFLLKAECLEDLVSF
IIEEPPQDMDEKIRYKYPNISCELLTSDVSQMNDRLGEDESLLMKLYSFLLNDSPLNPLL
ASFFSKVLSILISRKPEQIVDFLKKKHDFVDLIIKHIGTSAIMDLLLRLLTCIEPPQPRQ
DVLNWLNEEKIIQRLVEIVHPSQEEDRHSNASQSLCEIVRLSRDQMLQIQNSTEPDPLLA
TLEKQEIIEQLLSNIFHKEKNESAIVSAIQILLTLLETRRPTFEGHIEICPPGMSHSACS
VNKSVLEAIRGRLGSFHELLLEPPKKSVMKTTWGVLDPPVGNTRLNVIRLISSLLQTNTS
SINGDLMELNSIGVILNMFFKYTWNNFLHTQVEICIALILASPFENTENATITDQDSTGD
NLLLKHLFQKCQLIERILEAWEMNEKKQAEGGRRHGYMGHLTRIANCIVHSTDKGPNSAL
VQQLIKDLPDEVRERWETFCTSSLGETNKRNTVDLVTTCHIHSSSDDEIDFKETGFSQDS
SLQQAFSDYQMQQMTSNFIDQFGFNDEKFADQDDIGNVSFDRVSDINFTLNTNESGNIAL
FEACCKERIQQFDDGGSDEEDIWEEKHIAFTPESQRRSSSGSTDSEESTDSEEEDGAKQD
LFEPSSANTEDKMEVDLSEPPNWSANFDVPMETTHGAPLDSVGSDVWSTEEPMPTKETGW
ASFSEFTSSLSTKDSLRSNSPVEMETSTEPMDPLTPSAAALAVQPEAAGSVAMEASSDGE
EDAESTDKVTETVMNGGMKETLSLTVDAKTETAVFKSEEGKLSTSQDAACKDAEECPETA
EAKCAAPRPPSSSPEQRTGQPSAPGDTSVNGPV*
>ENSG00000122390|ENST00000360862
MIVAEIKNRTKIHKEDGDILASNFSVDTQVAYILSLGVVKEFRKHGIGSLLLESLKDHIS
TTAQDHCKAIYLHVLTTNNTAINFYENRDFKQHHYLPYYYSIRGVLKDGFTYVLYINGGH
PPWTILDYIQHLGSALASLSPCSIPHRVYRQAHSLLCSFLPWSGISSKSGIEYSRTM*
>ENSG00000134470|ENST00000379971
MAPRRARGCRTLGLPALLLLLLLRPPATREPAASSPSSNNTAATTAAIVPGSQLMPSKSP
STGTTEISSHESSHGTPSQTTAKNWELTASASHQPPGVYPQGHSDTTVAISTSTVLLCGL
SAVSLLACYLKSRASVCSCHPRSAGHTCSVGSVC*
>ENSG00000206509|ENST00000359076
MAPRSLLLLLSGALALTDTWAGSHSLRYFSTAVSRPGRGEPRYIAVEYVDDTQFLRFDSD
AAIPRMEPREPWVEQEGPQYWEWTTGYAKANAQTDRVALRNLLRRYNQSEAGSHTLQGMN
GCDMGPDGRLLRGYHQHAYDGKDYISLNEDLRSWTAADTVAQITQRFYEAEEYAEEFRTY
LEGECLELLRRYLENGKETLQRAEQSPQPTIPIVGIVAGLVVLGAVVTGAVVAAVMWRKK

SSDRNRGSYSQAAV*
>ENSG00000124222|ENST00000355957
MATRRLTDAFLLLRNNSIQNRQLLAEQELDELADDRMALVSGISLDPEAAIGVTKRPPPK
WVDGVDEIQYDVGRIKQKMKELASLHDKHLNRPTLDDSSEEEHAIEITTQEITQLFHRCQ
RAVQALPSRARACSEQEGRLLGNVVASLAQALQELSTSFRHAQSGYLKRMKNREERSQHF
FDTSVPLMDDGDDNTLYHRGFTEDQLVLVEQNTLMVEEREREIRQIVQSISDLNEIFRDL
GAMIVEQGTVLDRIDYNVEQSCIKTEDGLKQLHKAEQYQKKNRKMLVILILFVIIIVLIV
VLVGVKSR*
>ENSG00000188157|ENST00000379370
MAGRSHPGPLRPLLPLLVVAACVLPGAGGTCPERALERREEEANVVLTGTVEEILNVDPV
QHTYSCKVRVWRYLKGKDLVARESLLDGGNKVVISGFGDPLICDNQVSTGDTRIFFVNPA
PPYLWPAHKNELMLNSSLMRITLRNLEEVEFCVEDKPGTHFTPVPPTPPDACRGMLCGFG
AVCEPNAEGPGRASCVCKKSPCPSVVAPVCGSDASTYSNECELQRAQCSQQRRIRLLSRG
PCGSRDPCSNVTCSFGSTCARSADGLTASCLCPATCRGAPEGTVCGSDGADYPGECQLLR
RACARQENVFKKFDGPCDPCQGALPDPSRSCRVNPRTRRPEMLLRPESCPARQAPVCGDD
GVTYENDCVMGRSGAARGLLLQKVRSGQCQGRDQCPEPCRFNAVCLSRRGRPRCSCDRVT
CDGAYRPVCAQDGRTYDSDCWRQQAECRQQRAIPSKHQGPCDQAPSPCLGVQCAFGATCA
VKNGQAACECLQACSSLYDPVCGSDGVTYGSACELEATACTLGREIQVARKGPCDRCGQC
RFGALCEAETGRCVCPSECVALAQPVCGSDGHTYPSECMLHVHACTHQISLHVASAGPCE
TCGDAVCAFGAVCSAGQCVCPRCEHPPPGPVCGSDGVTYGSACELREAACLQQTQIEEAR
AGPCEQAECGSGGSGSGEDGDCEQELCRQRGGIWDEDSEDGPCVCDFSCQSVPGSPVCGS
DGVTYSTECELKKARCESQRGLYVAAQGACRGPTFAPLPPVAPLHCAQTPYGCCQDNITA
ARGVGLAGCPSACQCNPHGSYGGTCDPATGQCSCRPGVGGLRCDRCEPGFWNFRGIVTDG
RSGCTPCSCDPQGAVRDDCEQMTGLCSCKPGVAGPKCGQCPDGRALGPAGCEADASAPAT
CAEMRCEFGARCVEESGSAHCVCPMLTCPEANATKVCGSDGVTYGNECQLKTIACRQGLQ
ISIQSLGPCQEAVAPSTHPTSASVTVTTPGLLLSQALPAPPGALPLAPSSTAHSQTTPPP
SSRPRTTASVPRTTVWPVLTVPPTAPSPAPSLVASAFGESGSTDGSSDEELSGDQEASGG
GSGGLEPLEGSSVATPGPPVERASCYNSALGCCSDGKTPSLDAEGSNCPATKVFQGVLEL
EGVEGQELFYTPEMADPKSELFGETARSIESTLDDLFRNSDVKKDFRSVRLRDLGPGKSV
RAIVDVHFDPTTAFRAPDVARALLRQIQVSRRRSLGVRRPLQEHVRFMDFDWFPAFITGA
TSGAIAAGATARATTASRLPSSAVTPRAPHPSHTSQPVAKTTAAPTTRRPPTTAPSRVPG
RRPPAPQQPPKPCDSQPCFHGGTCQDWALGGGFTCSCPAGRGGAVCEKVLGAPVPAFEGR
SFLAFPTLRAYHTLRLALEFRALEPQGLLLYNGNARGKDFLALALLDGRVQLRFDTGSGP
AVLTSAVPVEPGQWHRLELSRHWRRGTLSVDGETPVLGESPSGTDGLNLDTDLFVGGVPE
DQAAVALERTFVGAGLRGCIRLLDVNNQRLELGIGPGAATRGSGVGECGDHPCLPNPCHG
GAPCQNLEAGRFHCQCPPGRVGPTCADEKSPCQPNPCHGAAPCRVLPEGGAQCECPLGRE
GTFCQTASGQDGSGPFLADFNGFSHLELRGLHTFARDLGEKMALEVVFLARGPSGLLLYN
GQKTDGKGDFVSLALRDRRLEFRYDLGKGAAVIRSREPVTLGAWTRVSLERNGRKGALRV
GDGPRVLGESPVPHTVLNLKEPLYVGGAPDFSKLARAAAVSSGFDGAIQLVSLGGRQLLT
PEHVLRQVDVTSFAGHPCTRASGHPCLNGASCVPREAAYVCLCPGGFSGPHCEKGLVEKS
AGDVDTLAFDGRTFVEYLNAVTESEKALQSNHFELSLRTEATQGLVLWSGKATERADYVA
LAIVDGHLQLSYNLGSQPVVLRSTVPVNTNRWLRVVAHREQREGSLQVGNEAPVTGSSPL
GATQLDTDGALWLGGLPELPVGPALPKAYGTGFVGCLRDVVVGRHPLHLLEDAVTKPELR
PCPTP*

>ENSG00000120942|ENST00000376804
MAASQVLGEKINILSGETVKAGDRDPLGNDCPEQDRLPQRSWRQKCASYVLALRPWSFSA
SLTPVALGSALAYRSHGVLDPRLLVGCAVAVLAVHGAGNLVNTYYDFSKGIDHKKSDDRT
LVDRILEPQDVVRFGVFLYTLGCVCAACLYYLSPLKLEHLALIYFGGLSGSFLYTGVLI*
>ENSG00000174851|ENST00000376901
MAYHSGYGAHGSKHRARAAPDPPPLFDDTSGGYSSQPGGYPATGADVAFSVNHLLGDPMA
NVAMAYGSSIASHGKDMVHKELHRFVSVSKLKYFFAVDTAYVAKKLGLLVFPYTHQNWEV
QYSRDAPLPPRQDLNAPDLYIPTMAFITYVLLAGMALGIQKRFSPEVLGLCASTALVWVV
MEVLALLLGLYLATVRSDLSTFHLLAYSGYKYVGMILSVLTGLLFGSDGYYVALAWTSSA
LMYFIVRSLRTAALGPDSMGGPVPRQRLQLYLTLGAAAFQPLIIYWLTFHLVR*
>ENSG00000204070|ENST00000372727
MAGQFRSYVWDPLLILSQIVLMQTVYYGSLGLWLALVDGLVRSSPSLDQMFDAEILGFST
PPGRLSMMSFILNALTCALGLLYFIRRGKQCLDFTVTVHFFHLLGCWFYSSRFPSALTWW
LVQAVCIALMAVIGEYLCMRTELKEIPLNSAPKSNV*
>ENSG00000206433|ENST00000383482
MLSRNDAPSVLVPGPGLLRAGTPLCISAEAASAQQ*
>ENSG00000122966|ENST00000392521
MLKFKYGARNPLDAGAAEPIASRASRLNLFFQGKPPFMTQQQMSPLSREGILDALFVLFE
ECSQPALMKIKHVSNFVRKYSDTIAELQELQPSAKDFEVRSLVGCGHFAEVQVVREKATG
DIYAMKVMKKKALLAQEQVSFFEEERNILSRSTSPWIPQLQYAFQDKNHLYLVMEYQPGG
DLLSLLNRYEDQLDENLIQFYLAELILAVHSVHLMGYVHRDIKPENILVDRTGHIKLVDF
GSAAKMNSNKMVNAKLPIGTPDYMAPEVLTVMNGDGKGTYGLDCDWWSVGVIAYEMIYGR
SPFAEGTSARTFNNIMNFQRFLKFPDDPKVSSDFLDLIQSLLCGQKERLKFEGLCCHPFF
SKIDWNNIRNSPPPFVPTLKSDDDTSNFDEPEKNSWVSSSPCQLSPSGFSGEELPFVGFS
YSKALGILGRSESVVSGLDSPAKTSSMEKKLLIKSKELQDSQDKCHKMEQEMTRLHRRVS
EVEAVLSQKEVELKASETQRSLLEQDLATYITECSSLKRSLEQARMEVSQEDDKALQLLH
DIREQSRKLQEIKEQEYQAQVEEMRLMMNQLEEDLVSARRRSDLYESELRESRLAAEEFK
RKATECQHKLLKAKDQGKPEVGEYAKLEKINAEQQLKIQELQEKLEKAVKASTEATELLQ
NIRQAKERAERELEKLQNREDSSEGIRKKLVEAEERRHSLENKVKRLETMERRENRLKDD
IQTKSQQIQQMADKILELEEKHREAQVSAQHLEVHLKQKEQHYEEKIKVLDNQIKKDLAD
KETLENMMQRHEEEAHEKGKILSEQKAMINAMDSKIRSLEQRIVELSEANKLAANSSLFT
QRNMKAQEEMISELRQQKFYLETQAGKLEAQNRKLEEQLEKISHQDHSDKNRLLELETRL
REVSLEHEEQKLELKRQLTELQLSLQERESQLTALQAARAALESQLRQAKTELEETTAEA
EEEIQALTAHRDEIQRKFDALRNSCTVITDLEEQLNQLTEDNAELNNQNFYLSKQLDEAS
GANDEIVQLRSEVDHLRREITEREMQLTSQKQTMEALKTTCTMLEEQVMDLEALNDELLE
KERQWEAWRSVLGDEKSQFECRVRELQRMLDTEKQSRARADQRITESRQVVELAVKEHKA
EILALQQALKEQKLKAESLSDKLNDLEKKHAMLEMNARSLQQKLETERELKQRLLEEQAK
LQQQMDLQKNHIFRLTQGLQEALDRADLLKTERSDLEYQLENIQVLYSHEKVKMEGTISQ
QTKLIDFLQAKMDQPAKKKKGLFSRRKEDPALPTQVPLQYNELKLALEKEKARCAELEEA
LQKTRIELRSAREEAAHRKATDHPHPSTPATARQQIAMSAIVRSPEHQPSAMSLLAPPSS
RRKESSTPEEFSRRLKERMHHNIPHRFNVGLNMRATKCAVCLDTVHFGRQASKCLECQVM
CHPKCSTCLPATCGLPAEYATHFTEAFCRDKMNSPGLQTKEPSSSLHLEGWMKVPRNNKR
GQQGWDRKYIVLEGSKVLIYDNEAREAGQRPVEEFELCLPDGDVSIHGAVGASELANTAK
ADVPYILKMESHPHTTCWPGRTLYLLAPSFPDKQRWVTALESVVAGGRVSREKAEADAKL

LGNSLLKLEGDDRLDMNCTLPFSDQVVLVGTEEGLYALNVLKNSLTHVPGIGAVFQIYII
KDLEKLLMIAGEERALCLVDVKKVKQSLAQSHLPAQPDISPNIFEAVKGCHLFGAGKIEN
GLCICAAMPSKVVILRYNENLSKYCIRKEIETSEPCSCIHFTNYSILIGTNKFYEIDMKQ
YTLEEFLDKNDHSLAPAVFAASSNSFPVSIVQVNSAGQREEYLLCFHEFGVFVDSYGRRS
RTDDLKWSRLPLAFAYREPYLFVTHFNSLEVIEIQARSSAGTPARAYLDIPNPRYLGPAI
SSGAIYLASSYQDKLRVICCKGNLVKESGTEHHRGPSTSRSSPNKRGPPTYNEHITKRVA
SSPAPPEGPSHPREPSTPHRYREGRTELRRDKSPGRPLEREKSPGRMLSTRRERSPGRLF
EDSSRGRLPAGAVRTPLSQVNKVWDQSSV*
>ENSG00000151348|ENST00000358681
MCASVKYNIRGPALIPRMKTKHRIYYITLFSIVLLGLIATGMFQFWPHSIESSNDWNVEK
RSIRDVPVVRLPADSPIPERGDLSCRMHTCFDVYRCGFNPKNKIKVYIYALKKYVDDFGV
SVSNTISREYNELLMAISDSDYYTDDINRACLFVPSIDVLNQNTLRIKETAQAMAQLSRW
DRGTNHLLFNMLPGGPPDYNTALDVPRDRALLAGGGFSTWTYRQGYDVSIPVYSPLSAEV
DLPEKGPGPRQYFLLSSQVGLHPEYREDLEALQVKHGESVLVLDKCTNLSEGVLSVRKRC
HKHQVFDYPQVLQEATFCVVLRGARLGQAVLSDVLQAGCVPVVIADSYILPFSEVLDWKR
ASVVVPEEKMSDVYSILQSIPQRQIEEMQRQLFMEPARRENWSAANHQMNSLIWPREQWD
SQIINDRIYPYAAISYEEWNDPPAVKWGSVSNPLFLPLIPPQSQGFTAIVLTYDRVESLF
RVITEVSKVPSLSKLLVVWNNQNKNPPEDSLWPKIRVPLKVVRTAENKLSNRFFPYDEIE
TEAVLAIDDDIIMLTSDELQFGYEVWREFPDRLVGYPGRLHLWDHEMNKWKYESEWTNEV
SMVLTGAAFYHKYFNYLYTYKMPGDIKNWVDAHMNCEDIAMNFLVANVTGKAVIKVTPRK
KFKCPECTAIDGLSLDQTHMVERSECINKFASVFGTMPLKVVEHRADPVLYKDDFPEKLK
SFPNIGSL*
>ENSG00000115446|ENST00000357765
MLPSTSVNSLVQGNGVLNSRDAARHTAGAKRYKYLRRLFRFRQMDFEFAAWQMLYLFTSP
QRVYRNFHYRKQTKDQWARDDPAFLVLLSIWLCVSTIGFGFVLDMGFFETIKLLLWVVLI
DCVGVGLLIATLMWFISNKYLVKRQSRDYDVEWGYAFDVHLNAFYPLLVILHFIQLFFIN
HVILTDTFIGYLVGNTLWLVAVGYYIYVTFLGYSALPFLKNTVILLYPFAPLILLYGLSL
ALGWNFTHTLCSFYKYRVK*
>ENSG00000206433|ENST00000400263
MLSRNDVKRLERSWAQGSSEQELHYASLQRLPVPSSEGPDLRGRDKRGTKEDPRADYACI
AENKPT*
>ENSG00000164023|ENST00000394684
MDIIETAKLEEHLENQPSDPTNTYARPAEPVEEENKNGNGKPKSLSSGLRKGTKKYPDYI
QIAMPTESRNKFPLEWWKTGIAFIYAVFNLVLTTVMITVVHERVPPKELSPPLPDKFFDY
IDRVKWAFSVSEINGIILVGLWITQWLFLRYKSIVGRRFCFIIGTLYLYRCITMYVTTLP
VPGMHFQCAPKLNGDSQAKVQRILRLISGGGLSITGSHILCGDFLFSGHTVTLTLTYLFI
KEYSPRHFWWYHLICWLLSAAGIICILVAHEHYTIDVIIAYYITTRLFWWYHSMANEKNL
KVSSQTNFLSRAWWFPIFYFFEKNVQGSIPCCFSWPLSWPPGCFKSSCKKYSRVQKIGED
NEKST*
>ENSG00000146352|ENST00000368438
MIEDPELQVNGFVLIIDWSNFTFKQASKLTPSMLRLAIEGLQDSFPARFGGIHFVNQPWY
IHALYTVIRPFLKEKTRKRIFLHGNNLNSLHQLIHPEILPSEFGGMLPPYDMGTWARTLL
DHEYDDDSEYNVDSYSMPVKEVEKELSPKSMKRSQSVVDPTVLKRMDKNEEENMQPLLSL
D*

>ENSG00000165915|ENST00000354884
MPGCPCPGCGMAGPRLLFLTALALELLERAGGSQPALRSRGTATACRLDNKESESWGALL
SGERLDTWICSLLGSLMVGLSGVFPLLVIPLEMGTMLRSEAGAWRLKQLLSFALGGLLGN
VFLHLLPEAWAYTCSASPGGEGQSLQQQQQLGLWVIAGILTFLALEKMFLDSKEEGTSQA
PNKDPTAAAAALNGGHCLAQPAAEPGLGAVVRSIKVSGYLNLLANTIDNFTHGLAVAASF
LVSKKIGLLTTMAILLHEIPHEVGDFAILLRAGFDRWSAAKLQLSTALGGLLGAGFAICT
QSPKGVEETAAWVLPFTSGGFLYIALVNVLPDLLEEEDPWRSLQQLLLLCAGIVVMVLFS
LFVD*
>ENSG00000168824|ENST00000397958
MVKLGNNFAEKGTKQPLLEDGFDTIPLMTPLDVNQLQFPPPDKVVVKTKTEYEPDRKKGK
ARPPQIAEFTVSITEGVTERFKVSVLVLFALAFLTCVVFLVVYKVYKYDRACPDGFVLKN
TQCIPEGLESYYAEQDSSAREKFYTVINHYNLAKQSITRSVSPWMSVLSEEKLSEQETEA
AEKSA*
>ENSG00000164144|ENST00000356064
MAQESPKNSAAEIPVTSNGEVDDSREHSFNRDLKHSLPSGLGLSETQITSHGFDNTKEGV
IEAGAFQGGQRTQTKSGPVILADEIKNPAMEKLELVRKWSLNTYKCTRQIISEKLGRGSR
TVDLELEAQIDILRDNKKKYENILKLAQTLSTQLFQMVHTQRQLGDAFADLSLKSLELHE
EFGYNADTQKLLAKNGETLLGAINFFIASVNTLVNKTIEDTLMTVKQYESARIEYDAYRT
DLEELNLGPRDANTLPKIEQSQHLFQAHKEKYDKMRNDVSVKLKFLEENKVKVLHNQLVL
FHNAIAAYFAGNQKQLEQTLKQFHIKLKTPGVDAPSWLEEQ*
>ENSG00000160179|ENST00000398457
MLGTQGWTKQRKPCPQNASSYSAEMTEPKSVCVSVDEVVSSNMEATETDLLNGHLKKVDN
NLTEAQRFSSLPRRAAVNIEFRDLSYSVPEGPWWRKKGYKTLLKGISGKFNSGELVAIMG
PSGAGKSTLMNILAGYRETGMKGAVLINGLPRDLRCFRKVSCYIMQDDMLLPHLTVQEAM
MVSAHLKLQEKDEGRREMVKEILTALGLLSCANTRTGSLSGGQRKRLAIALELVNNPPVM
FFDEPTSGLDSASCFQVVSLMKGLAQGGRSIICTIHQPSAKLFELFDQLYVLSQGQCVYR
GKVCNLVPYLRDLGLNCPTYHNPADFVMEVASGEYGDQNSRLVRAVREGMCDSDHKRDLG
GDAEVNPFLWHRPSEEDSSSMEGCHSFSASCLTQFCILFKRTFLSIMRDSVLTHLRITSH
IGIGLLIGLLYLGIGNEAKKVLSNSGFLFFSMLFLMFAALMPTVLTFPLEMGVFLREHLN
YWYSLKAYYLAKTMADVPFQIMFPVAYCSIVYWMTSQPSDAVRFVLFAALGTMTSLVAQS
LGLLIGAASTSLQVATFVGPVTAIPVLLFSGFFVSFDTIPTYLQWMSYISYVRYGFEGVI
LSIYGLDREDLHCDIDETCHFQKSEAILRELDVENAKLYLDFIVLGIFFISLRLIAYFVL
RYKIRAER*
>ENSG00000119514|ENST00000375011
MWGRTARRRCPRELRRGREALLVLLALLALAGLGSVLRAQRGAGAGAAEPGPPRTPRPGR
REPVMPRPPVPANALGARGEAVRLQLQGEELRLQEESVRLHQINIYLSDRISLHRRLPER
WNPLCKEKKYDYDNLPRTSVIIAFYNEAWSTLLRTVYSVLETSPDILLEEVILVDDYSDR
EHLKERLANELSGLPKVRLIRANKREGLVRARLLGASAARGDVLTFLDCHCECHEGWLEP
LLQRIHEEESAVVCPVIDVIDWNTFEYLGNSGEPQIGGFDWRLVFTWHTVPERERIRMQS
PVDVIRSPTMAGGLFAVSKKYFEYLGSYDTGMEVWGGENLEFSFRIWQCGGVLETHPCSH
VGHVFPKQAPYSRNKALANSVRAAEVWMDEFKELYYHRNPRARLEPFGDVTERKQLRDKL
QCKDFKWFLETVYPELHVPEDRPGFFGMLQNKGLTDYCFDYNPPDENQIVGHQVILYLCH
GMGQNQFFEYTSQKEIRYNTHQPEGCIAVEAGMDTLIMHLCEETAPENQKFILQEDGSLF
HEQSKKCVQAARKESSDSFVPLLRDCTNSDHQKWFFKERML*

>ENSG00000231286|ENST00000399088
MSWKKALRIPGGLRAATVTLMLAMLSTPVAEGRDSPEDFVYQFKAMCYFTNGTERVRYVT
RYIYNREEYARFDSDVEVYRAVTPLGPPDAEYWNSQKEVLERTRAELDTVCRHNYQLELR
TTLQRRVEPTVTISPSRTEALNHHNLLVCSVTDFYPAQIKVRWFRNDQEETTGVVSTPLI
RNGDWTFQILVMLEMTPQHGDVYTCHVEHPSLQNPITVEWRAQSESAQSKMLSGIGGFVL
GLIFLGLGLIIHHRSQKGLLH*
>ENSG00000116260|ENST00000367602
MRRCNSGSGPPPSLLLLLLWLLAVPGANAAPRSALYSPSDPLTLLQADTVRGAVLGSRSA
WAVEFFASWCGHCIAFAPTWKALAEDVKAWRPALYLAALDCAEETNSAVCRDFNIPGFPT
VRFFKAFTKNGSGAVFPVAGADVQTLRERLIDALESHHDTWPPACPPLEPAKLEEIDGFF
ARNNEEYLALIFEKGGSYLGREVALDLSQHKGVAVRRVLNTEANVVRKFGVTDFPSCYLL
FRNGSVSRVPVLMESRSFYTAYLQRLSGLTREAAQTTVAPTTANKIAPTVWKLADRSKIY
MADLESALHYILRIEVGRFPVLEGQRLVALKKFVAVLAKYFPGRPLVQNFLHSVNEWLKR
QKRNKIPYSFFKTALDDRKEGAVLAKKVNWIGCQGSEPHFRGFPCSLWVLFHFLTVQAAR
QNVDHSQEAAKAKEVLPAIRGYVHYFFGCRDCASHFEQMAAASMHRVGSPNAAVLWLWSS
HNRVNARLAGAPSEDPQFPKVQWPPRELCSACHNERLDVPVWDVEATLNFLKAHFSPSNI
ILDFPAAGSAARRDVQNVAAAPELAMGALELESRNSTLDPGKPEMMKSPTNTTPHVPAEG
PEASRPPKLHPGLRAAPGQEPPEHMAELQRNEQEQPLGQWHLSKRDTGAALLAESRAEKN
RLWGPLEVRRVGRSSKQLVDIPEGQLEARAGRGRGQWLQVLGGGFSYLDISLCVGLYSLS
FMGLLAMYTYFQAKIRALKGHAGHPAA*
>ENSG00000152291|ENST00000377386
MRFVVALVLLNVAAAGAVPLLATESVKQEEAGVRPSAGNVSTHPSLSQRPGGSTKSHPEP
QTPKDSPSKSSAEAQTPEDTPNKSGAEAKTQKDSSNKSGAEAKTQKGSTSKSGSEAQTTK
DSTSKSHPELQTPKDSTGKSGAEAQTPEDSPNRSGAEAKTQKDSPSKSGSEAQTTKDVPN
KSGADGQTPKDGSSKSGAEDQTPKDVPNKSGAEKQTPKDGSNKSGAEEQGPIDGPSKSGA
EEQTSKDSPNKVVPEQPSRKDHSKPISNPSDNKELPKADTNQLADKGKLSPHAFKTESGE
ETDLISPPQEEVKSSEPTEDVEPKEAEDDDTGPEEGSPPKEEKEKMSGSASSENREGTLS
DSTGSEKDDLYPNGSGNGSAESSHFFAYLVTAAILVAVLYIAHHNKRKIIAFVLEGKRSK
VTRRPKASDYQRLDQKS*
>ENSG00000079332|ENST00000373242
MSFIFEWIYNGFSSVLQFLGLYKKSGKLVFLGLDNAGKTTLLHMLKDDRLGQHVPTLHPT
SEELTIAGMTFTTFDLGGHEQARRVWKNYLPAINGIVFLVDCADHSRLVESKVELNALMT
DETISNVPILILGNKIDRTDAISEEKLREIFGLYGQTTGKGNVTLKELNARPMEVFMCSV
LKRQGYGEGFRWLSQYID*
>ENSG00000134259|ENST00000369512
MSMLFYTLITAFLIGIQAEPHSESNVPAGHTIPQAHWTKLQHSLDTALRRARSAPAAAIA
ARVAGQTRNITVDPRLFKKRRLRSPRVLFSTQPPREAADTQDLDFEVGGAAPFNRTHRSK
RSSSHPIFHRGEFSVCDSVSVWVGDKTTATDIKGKEVMVLGEVNINNSVFKQYFFETKCR
DPNPVDSGCRGIDSKHWNSYCTTTHTFVKALTMDGKQAAWRFIRIDTACVCVLSRKAVRR
A*
>ENSG00000107862|ENST00000369983
MVDKNIYIIQGEINIVVGAIKRNARWSTHTPLDEERDPLLHSFGHLKEVLNSITELSEIE
PNVFLRPFLEVIRSEDTTGPITGLALTSVNKFLSYALIDPTHEGTAEGMENMADAVTHAR
FVGTDPASDEVVLMKILQVLRTLLLTPVGAHLTNESVCEIMQSCFRICFEMRLSELLRKS

AEHTLVDMVQLLFTRLPQFKEEPKNYVGTNMKKLKMRAGGMSDSSKWKKQKRSPRPPRHM
TKVTPGSELPTPNGTTLSSNLTGGMPFIDVPTPISSASSEAASAVVSPSTDSGLEFSSQT
TSKEDLTDLEQPGSPGYSTATEPGSSELGVPEQPDLQEGTHVEKSQSASVESIPEVLEEC
TSPADHSDSASVHDMDYVNPRGVRFTQSSQKEGTALVPYGLPCIRELFRFLISLTNPHDR
HNSEVMIHMGLHLLTVALESAPVAQCQTLLGLIKDEMCRHLFQLLSIERLNLYAASLRVC
FLLFESMREHLKFQMEMYIKKLMEIITVENPKMPYEMKEMALEAIVQLWRIPSFVTELYI
NYDCDYYCSNLFEELTKLLSKNAFPVSGQLYTTHLLSLDALLTVIDSTEAHCQAKVLNSL
TQQEKKETARPSCEIVDGTREASNTERTASDGKAVGMASDIPGLHLPGGGRLPPEHGKSG
CSDLEEAVDSGADKKFARKPPRFSCLLPDPRELIEIKNKKKLLITGTEQFNQKPKKGIQF
LQEKGLLTIPMDNTEVAQWLRENPRLDKKMIGEFVSDRKNIDLLESFVSTFSFQGLRLDE
ALRLYLEAFRLPGEAPVIQRLLEAFTERWMNCNGSPFANSDACFSLAYAVIMLNTDQHNH
NVRKQNAPMTLEEFRKNLKGVNGGKDFEQDILEDMYHAIKNEEIVMPEEQTGLVRENYVW
NVLLHRGATPEGIFLRVPTASYDLDLFTMTWGPTIAALSYVFDKSLEETIIQKAISGFRK
CAMISAHYGLSDVFDNLIISLCKFTALSSESIENLPSVFGSNPKAHIAAKTVFHLAHRHG
DILREGWKNIMEAMLQLFRAQLLPKAMIEVEDFVDPNGKISLQREETPSNRGESTVLSFV
SWLTLSGPEQSSVRGPSTENQEAKRVALECIKQCDPEKMITESKFLQLESLQELMKALVS
VTPDEETYDEEDAAFCLEMLLRIVLENRDRVGCVWQTVRDHLYHLCVQAQDFCFLVERAV
VGLLRLAIRLLRREEISAQVLLSLRILLLMKPSVLSRVSHQVAYGLHELLKTNAANIHSG
DDWATLFTLLECIGSGVKPPAALQATARADAPDAGAQSDSELPSYHQNDVSLDRGYTSDS
EVYTDHGRPGKIHRSATDADVVNSGWLVVGKDDVDNSKPGPSRPGPSPLINQYSLTVGLD
LGPHDTKSLLKCVESLSFIVRDAAHITPDNFELCVKTLRIFVEASLNGGCKSQEKRGKSH
KYDSKGNRFKKKSKEGSMLRRPRTSSQHASRGGQSDDDEDEGVPASYHTVSLQVSQDLLD
LMHTLHTRAASIYSSWAEEQRHLETGGQKIEADSRTLWAHCWCPLLQGIACLCCDARRQV
RMQALTYLQRALLVHDLQKLDALEWESCFNKVLFPLLTKLLENISPADVGGMEETRMRAS
TLLSKVFLQHLSPLLSLSTFAALWLTILDFMDKYMHAGSSDLLSEAIPESLKNMLLVMDT
AEIFHSADARGGGPSALWEITWERIDCFLPHLRDELFKQTVIQDPMPMEPQGQKPLASAH
LTSAAGDTRTPGHPPPPEIPSELGACDFEKPESPRAASSSSPGSPVASSPSRLSPTPDGP
PPLAQPPLILQPLASPLQVGVPPMTLPIILNPALIEATSPVPLLATPRPTDPIPTSEVN*
>ENSG00000154265|ENST00000392676
MSTAIREVGVWRQTRTLLLKNYLIKCRTKKSSVQEILFPLFFLFWLILISMMHPNKKYEE
VPNIELNPMDKFTLSNLILGYTPVTNITSSIMQKVSTDHLPDVIITEEYTNEKEMLTSSL
SKPSNFVGVVFKDSMSYELRFFPDMIPVSSIYMDSRAGCSKSCEAAQYWSSGFTVLQASI
DAAIIQLKTNVSLWKELESTKAVIMGETAVVEIDTFPRGVILIYLVIAFSPFGYFLAIHI
VAEKEKKIKEFLKIMGLHDTAFWLSWVLLYTSLIFLMSLLMAVIATASLLFPQSSSIVIF
LLFFLYGLSSVFFALMLTPLFKKSKHVGIVEFFVTVAFGFIGLMIILIESFPKSLVWLFS
PFCHCTFVIGIAQVMHLEDFNEGASFSNLTAGPYPLIITIIMLTLNSIFYVLLAVYLDQV
IPGEFGLRRSSLYFLKPSYWSKSKRNYEELSEGNVNGNISFSEIIEPVSSEFVGKEAIRI
SGIQKTYRKKGENVEALRNLSFDIYEGQITALLGHSGTGKSTLMNILCGLCPPSDGFASI
YGHRVSEIDEMFEARKMIGICPQLDIHFDVLTVEENLSILASIKGIPANNIIQEVQKVLL
DLDMQTIKDNQAKKLSGGQKRKLSLGIAVLGNPKILLLDEPTAGMDPCSRHIVWNLLKYR
KANRVTVFSTHFMDEADILADRKAVISQGMLKCVGSSMFLKSKWGIGYRLSMYIDKYCAT
ESLSSLVKQHIPGATLLQQNDQQLVYSLPFKDMDKFSGLFSALDSHSNLGVISYGVSMTT
LEDVFLKLEVEAEIDQADYSVFTQQPLEEEMDSKSFDEMEQSLLILSETKAALVSTMSLW
KQQMYTIAKFHFFTLKRESKSVRSVLLLLLIFFTVQIFMFLVHHSFKNAVVPIKLVPDLY

FLKPGDKPHKYKTSLLLQNSADSDISDLISFFTSQNIMVTMINDSDYVSVAPHSAALNVM
HSEKDYVFAAVFNSTMVYSLPILVNIISNYYLYHLNVTETIQIWSTPFFQEITDIVFKIE
LYFQAALLGIIVTAMPPYFAMENAENHKIKAYTQLKLSGLLPSAYWIGQAVVDIPLFFII
LILMLGSLLAFHYGLYFYTVKFLAVVFCLIGYVPSVILFTYIASFTFKKILNTKEFWSFI
YSVAALACIAITEITFFMGYTIATILHYAFCIIIPIYPLLGCLISFIKISWKNVRKNVDT
YNPWDRLSVAVISPYLQCVLWIFLLQYYEKKYGGRSIRKDPFFRNLSTKSKNRKLPEPPD
NEDEDEDVKAERLKVKELMGCQCCEEKPSIMVSNLHKEYDDKKDFLLSRKVKKVATKYIS
FCVKKGEILGLLGPNGAGKSTIINILVGDIEPTSGQVFLGDYSSETSEDDDSLKCMGYCP
QINPLWPDTTLQEHFEIYGAVKGMSASDMKEVISRITHALDLKEHLQKTVKKLPAGIKRK
LCFALSMLGNPQITLLDEPSTGMDPKAKQHMWRAIRTAFKNRKRAAILTTHYMEEAEAVC
DRVAIMVSGQLRCIGTVQHLKSKFGKGYFLEIKLKDWIENLEVDRLQREIQYIFPNASRQ
ESFSSILAYKIPKEDVQSLSQSFFKLEEAKHAFAIEEYSFSQATLEQVFVELTKEQEEED
NSCGTLNSTLWWERTQEDRVVF*
>ENSG00000137403|ENST00000383515
MAPRSLLLLLSGALALTDTWAGSHSLRYFSTAVSRPGRGEPRYIAVEYVDDTQFLRFDSD
AAIPRMEPREPWVEQEGPQYWEWTTGYAKANAQTDRVALRNLLRRYNQSEAGSHTLQGMN
GCDMGPDGRLLRGYHQHAYDGKDYISLNEDLRSWTAADTVAQITQRFYEAEEYAEEFRTY
LEGECLELLRRYLENGKETLQRAEQSPQPTIPIVGIVAGLVVLGAVVTGAVVAAVMWRKK
SSDRNRGSYSQAAV*
>ENSG00000185090|ENST00000373045
MARRRRRACIALFLVLLFAFGTLMGLRTLKAPDGLPALGPGLELAPFERRPEGAPAPAAR
APAAPAAPPPPPPPPRTADPGGSPGPAPAEAEPAPVQSLRVYSDLHAFYYSWYGSPRREG
HYIHWDHVMVPHWDPKISASYPRGRHSPPDDLGSSFYPELGPYSSRDPEVLREHMTQLKE
AAIGVLVLSWYPPGMADDNGEPSDDLVPAILDTAHQYSIQVAFHIQPYKGRDDITVHDNI
KYIIDTYGSHGAFYRYKNSMGKSLPLFYIYDSYLTSPEAWAHLLTPNGPHSIRNTPYDGV
FIALLVEEGHTHDILAAGFDGMYTYFASNGFSFGSSHQNWKAVKNFCDANNLMFIPSVGP
GYIDTSIRPWNNHNTRNRVNGKYYETALQAALTVRPEIVSITSFNEWHEGTQIEKAIPKK
TPTRLYLDYLPHQPSLYLELTRRWAEHFIKEKEQWLM*
>ENSG00000157020|ENST00000397109
MIHDAQMDYYGTRLATCSSDRSVKIFDVRNGGQILIADLRGHEGPVWQVAWAHPMYGNIL
ASCSYDRKVIIWREENGTWEKSHEHAGHDSSVNSVCWAPHDYGLILACGSSDGAISLLTY
TGEGQWEVKKINNAHTIGCNAVSWAPAVVPGSLIDHPSGQKPNYIKRFASGGCDNLIKLW
KEEEDGQWKEEQKLEAHSDWVRDVAWAPSIGLPTSTIASCSQDGRVFIWTCDDASSNTWS
PKLLHKFNDVVWHVSWSITANILAVSGGDNKVTLWKESVDGQWVCISDVNKGQGSVSASV
TEGQQNEQ*
>ENSG00000133110|ENST00000379742
MIPFLPMFSLLLLLIVNPINANNHYDKILAHSRIRGRDQGPNVCALQQILGTKKKYFSTC
KNWYKKSICGQKTTVLYECCPGYMRMEGMKGCPAVLPIDHVYGTLGIVGATTTQRYSDAS
KLREEIEGKGSFTYFAPSNEAWDNLDSDIRRGLESNVNVELLNALHSHMINKRMLTKDLK
NGMIIPSMYNNLGLFINHYPNGVVTVNCARIIHGNQIATNGVVHVIDRVLTQIGTSIQDF
IEAEDDLSSFRAAAITSDILEALGRDGHFTLFAPTNEAFEKLPRGVLERIMGDKVASEAL
MKYHILNTLQCSESIMGGAVFETLEGNTIEIGCDGDSITVNGIKMVNKKDIVTNNGVIHL
IDQVLIPDSAKQVIELAGKQQTTFTDLVAQLGLASALRPDGEYTLLAPVNNAFSDDTLSM
DQRLLKLILQNHILKVKVGLNELYNGQILETIGGKQLRVFVYRTAVCIENSCMEKGSKQG

RNGAIHIFREIIKPAEKSLHEKLKQDKRFSTFLSLLEAADLKELLTQPGDWTLFVPTNDA
FKGMTSEEKEILIRDKNALQNIILYHLTPGVFIGKGFEPGVTNILKTTQGSKIFLKEVND
TLLVNELKSKESDIMTTNGVIHVVDKLLYPADTPVGNDQLLEILNKLIKYIQIKFVRGST
FKEIPVTVYKPIIKKYTKIIDGVPVEITEKETREERIITGPEIKYTRISTGGGETEETLK
KLLQEEVTKVTKFIEGGDGHLFEDEEIKRLLQGDTPVRKLQANKKVQGSRRRLREGRSQ*
>ENSG00000124222|ENST00000361830
MALVSGISLDPEAAIGVTKRPPPKWVDGVDEIQYDVGRIKQKMKELASLHDKHLNRPTLD
DSSEEEHAIEITTQEITQLFHRCQRAVQALPSRARACSEQEGRLLGNVVASLAQALQELS
TSFRHAQSGYLKRMKNREERSQHFFDTSVPLMDDGDDNTLYHRGFTEDQLVLVEQNTLMV
EEREREIRQIVQSISDLNEIFRDLGAMIVEQGTVLDRIDYNVEQSCIKTEDGLKQLHKAE
QYQKKNRKMLVILILFVIIIVLIVVLVGVKSR*
>ENSG00000140297|ENST00000396065
MVQWKRLCQLHYLWALGCYMLLATVALKLSFRLKCDSDHLGLESRESQSQYCRNILYNFL
KLPAKRSINCSGVTRGDQEAVLQAILNNLEVKKKREPFTDTHYLSLTRDCEHFKAERKFI
QFPLSKEEVEFPIAYSMVIHEKIENFERLLRAVYAPQNIYCVHVDEKSPETFKEAVKAII
SCFPNVFIASKLVRVVYASWSRVQADLNCMEDLLQSSVPWKYFLNTCGTDFPIKSNAEMV
QALKMLNGRNSMESEVPPKHKETRWKYHFEVVRDTLHLTNKKKDPPPYNLTMFTGNAYIV
ASRDFVQHVLKNPKSQQLIEWVKDTYSPDEHLWATLQRARWMPGSVPNHPKYDISDMTSI
ARLVKWQGHEGDIDKGAPYAPCSGIHQRAICVYGAGDLNWMLQNHHLLANKFDPKVDDNA
LQCLEEYLRYKAIYGTEL*
>ENSG00000123240|ENST00000378747
MSHQPLSCLTEKEDSPSESTGNGPPHLAHPNLDTFTPEELLQQMKELLTENHQLKEAMKL
NNQAMKGRFEELSAWTEKQKEERQFFEIQSKEAKERLMALSHENEKLKEELGKLKGKSER
SSEDPTDDSRLPRAEAEQEKDQLRTQVVRLQAEKADLLGIVSELQLKLNSSGSSEDSFVE
IRMAEGEAEGSVKEIKHSPGPTRTVSTGTALSKYRSRSADGAKNYFEHEELTVSQLLLCL
REGNQKVERLEVALKEAKERVSDFEKKTSNRSEIETQTEGSTEKENDEEKGPETVGSEVE
ALNLQVTSLFKELQEAHTKLSEAELMKKRLQEKCQALERKNSAIPSELNEKQELVYTNKK
LELQVESMLSEIKMEQAKTEDEKSKLTVLQMTHNKLLQEHNNALKTIEELTRKESEKVDR
AVLKELSEKLELAEKALASKQLQMDEMKQTIAKQEEDLETMTILRAQMEVYCSDFHAERA
AREKIHEEKEQLALQLAVLLKENDAFEDGGRQSLMEMQSRHGARTSDSDQQAYLVQRGAE
DRDWRQQRNIPIHSCPKCGEVLPDIDTLQIHVMDCII*
>ENSG00000128242|ENST00000402321
MLPPQKKPWESMAKGLVLGALFTSFLLLVYSYAVPPLHAGLASTTPEAAASCSPPALEPE
AVIRANGSAGECQPRRNIVFLKTHKTASSTLLNILFRFGQKHRLKFAFPNGRNDFDYPTF
FARSLVQDYRPGACFNIICNHMRFHYDEVRGLVPTNAIFITVLRDPARLFESSFHYFGPV
VPLTWKLSAGDKLTEFLQDPDRYYDPNGFNAHYLRNLLFFDLGYDNSLDPSSPQVQEHIL
EVERRFHLVLLQEYFDESLVLLKDLLCWELEDVLYFKLNARRDSPVPRLSGELYGRATAW
NMLDSHLYRHFNASFWRKVEAFGRERMAREVAALRHANERMRTICIDGGHAVDAAAIQDE
AMQPWQPLGTKSILGYNLKKSIGQRHAQLCRRMLTPEIQYLMDLGANLWVTKLWKFIRDF
LRW*
>ENSG00000213625|ENST00000371065
MAGVKALVALSFSGAIGLTFLMLGCALEDYGVYWPLFVLIFHAISPIPHFIAKRVTYDSD
ATSSACRELAYFFTTGIVVSAFGFPVILARVAVIKWGACGLVLAGNAVIFLTIQGFFLIF
GRGDDFSWEQW*

>ENSG00000198774|ENST00000361228
MAPFGRNLLKTRHKNRSPTKDMDSEEKEIVVWVCQEEKLVCGLTKRTTSADVIQALLEEH
EATFGEKRFLLGKPSDYCIIEKWRGSERVLPPLTRILKLWKAWGDEQPNMQFVLVKADAF
LPVPLWRTAEAKLVQNTEKLWELSPANYMKTLPPDKQKRIVRKTFRKLAKIKQDTVSHDR
DNMETLVHLIISQDHTIHQQVKRMKELDLEIEKCEAKFHLDRVENDGENYVQDAYLMPSF
SEVEQNLDLQYEENQTLEDLSESDGIEQLEERLKYYRILIDKLSAEIEKEVKSVCIDINE
DAEGEAASELESSNLESVKCDLEKSMKAGLKIHSHLSGIQKEIKYSDSLLQMKAKEYELL
AKEFNSLHISNKDGCQLKENRAKESEVPSSNGEIPPFTQRVFSNYTNDTDSDTGISSNHS
QDSETTVGDVVLLST*
>ENSG00000111885|ENST00000368468
MPVGGLLPLFSSPAGGVLGGGLGGGGGRKGSGPAALRLTEKFVLLLVFSAFITLCFGAIF
FLPDSSKLLSGVLFHSSPALQPAADHKPGPGARAEDAAEGRARRREEGAPGDPEAALEDN
LARIRENHERALREAKETLQKLPEEIQRDILLEKKKVAQDQLRDKAPFRGLPPVDFVPPI
GVESREPADAAIREKRAKIKEMMKHAWNNYKGYAWGLNELKPISKGGHSSSLFGNIKGAT
IVDALDTLFIMEMKHEFEEAKSWVEENLDFNVNAEISVFEVNIRFVGGLLSAYYLSGEEI
FRKKAVELGVKLLPAFHTPSGIPWALLNMKSGIGRNWPWASGGSSILAEFGTLHLEFMHL
SHLSGNPIFAEKVMNIRTVLNKLEKPQGLYPNYLNPSSGQWGQHHVSVGGLGDSFYEYLL
KAWLMSDKTDLEAKKMYFDAVQAIETHLIRKSSSGLTYIAEWKGGLLEHKMGHLTCFAGG
MFALGADAAPEGMAQHYLELGAEIARTCHESYNRTFMKLGPEAFRFDGGVEAIATRQNEK
YYILRPEVMETYMYMWRLTHDPKYRKWAWEAVEALENHCRVNGGYSGLRDVYLLHESYDD
VQQSFFLAETLKYLYLIFSDDDLLPLEHWIFNSEAHLLPILPKDKKEVEIREE*
>ENSG00000130958|ENST00000375259
MTAGGQAEAEGAGGEPGAARLPSRVARLLSALFYGTCSFLIVLVNKALLTTYGFPSPIFL
GIGQMAATIMILYVSKLNKIIHFPDFDKKIPVKLFPLPLLYVGNHISGLSSTSKLSLPMF
TVLRKFTIPLTLLLETIILGKQYSLNIILSVFAIILGAFIAAGFLLMYSTVLCSYYNSAL
TTAVVGAIKNVSVAYIGILIGGDYIFSLLNFVGLNICMAGGLRYSFLTLSSQLKPKPVGE
ENICLDLKS*
>ENSG00000196352|ENST00000367064
MTVARPSVPAALPLLGELPRLLLLVLLCLPAVWGDCGLPPDVPNAQPALEGRTSFPEDTV
ITYKCEESFVKIPGEKDSVICLKGSQWSDIEEFCNRSCEVPTRLNSASLKQPYITQNYFP
VGTVVEYECRPGYRREPSLSPKLTCLQNLKWSTAVEFCKKKSCPNPGEIRNGQIDVPGGI
LFGATISFSCNTGYKLFGSTSSFCLISGSSVQWSDPLPECREIYCPAPPQIDNGIIQGER
DHYGYRQSVTYACNKGFTMIGEHSIYCTVNNDEGEWSGPPPECRGKSLTSKVPPTVQKPT
TVNVPTTEVSPTSQKTTTKTTTPNAQATRSTPVSRTTKHFHETTPNKGSGTTSGTTRLLS
GHTCFTLTGLLGTLVTMGLLT*
>ENSG00000124222|ENST00000371132
MATRRLTDAFLLLRNNSIQNRQLLAEQLADDRMALVSGISLDPEAAIGVTKRPPPKWVDG
VDEIQYDVGRIKQKMKELASLHDKHLNRPTLDDSSEEEHAIEITTQEITQLFHRCQRAVQ
ALPSRARACSEQEGRLLGNVVASLAQALQELSTSFRHAQSGYLKRMKNREERSQHFFDTS
VPLMDDGDDNTLYHRGFTEDQLVLVEQNTLMVEEREREIRQIVQSISDLNEIFRDLGAMI
VEQGTVLDRIDYNVEQSCIKTEDGLKQLHKAEQYQKKNRKMLVILILFVIIIVLIVVLVG
VKSR*
>ENSG00000110195|ENST00000393681
MAQRMTTQLLLLLVWVAVVGEAQTRIAWARTELLNVCMNAKHHKEKPGPEDKLHEQCRPW

RKNACCSTNTSQEAHKDVSYLYRFNWNHCGEMAPACKRHFIQDTCLYECSPNLGPWIQQV
DQSWRKERVLNVPLCKEDCEQWWEDCRTSYTCKSNWHKGWNWTSGFNKCAVGAACQPFHF
YFPTPTVLCNEIWTHSYKVSNYSRGSGRCIQMWFDPAQGNPNEEVARFYAAAMSGAGPWA
AWPFLLSLALMLLWLLS*
>ENSG00000079805|ENST00000389253
MGNRGMEELIPLVNKLQDAFSSIGQSCHLDLPQIAVVGGQSAGKSSVLENFVGRDFLPRG
SGIVTRRPLILQLIFSKTEHAEFLHCKSKKFTDFDEVRQEIEAETDRVTGTNKGISPVPI
NLRVYSPHVLNLTLIDLPGITKVPVGDQPPDIEYQIKDMILQFISRESSLILAVTPANMD
LANSDALKLAKEVDPQGLRTIGVITKLDLMDEGTDARDVLENKLLPLRRGYIGVVNRSQK
DIEGKKDIRAALAAERKFFLSHPAYRHMADRMGTPHLQKTLNQQLTNHIRESLPALRSKL
QSQLLSLEKEVEEYKNFRPDDPTRKTKALLQMVQQFGVDFEKRIEGSGDQVDTLELSGGA
RINRIFHERFPFELVKMEFDEKDLRREISYAIKNIHGVRTGLFTPDMAFEAIVKKQIVKL
KEPSLKCVDLVVSELATVIKKCAEKLSSYPRLREETERIVTTYIREREGRTKDQILLLID
IEQSYINTNHEDFIGFANAQQRSTQLNKKRAIPNQGEILVIRRGWLTINNISLMKGGSKE
YWFVLTAESLSWYKDEEEKEKKYMLPLDNLKIRDVEKGFMSNKHVFAIFNTEQRNVYKDL
RQIELACDSQEDVDSWKASFLRAGVYPEKDQAENEDGAQENTFSMDPQLERQVETIRNLV
DSYVAIINKSIRDLMPKTIMHLMINNTKAFIHHELLAYLYSSADQSSLMEESADQAQRRD
DMLRMYHALKEALNIIGDISTSTVSTPVPPPVDDTWLQSASSHSPTPQRRPVSSIHPPGR
PPAVRGPTPGPPLIPVPVGAAASFSAPPIPSRPGPQSVFANSDLFPAPPQIPSRPVRIPP
GIPPGVPSRRPPAAPSRPTIIRPAEPSLLD*
>ENSG00000134602|ENST00000394334
MAHSPVAVQVPGMQNNIADPEELFTKLERIGKGSFGEVFKGIDNRTQQVVAIKIIDLEEA
EDEIEDIQQEITVLSQCDSSYVTKYYGSYLKGSKLWIIMEYLGGGSALDLLRAGPFDEFQ
IATMLKEILKGLDYLHSEKKIHRDIKAANVLLSEQGDVKLADFGVAGQLTDTQIKRNTFV
GTPFWMAPEVIQQSAYDSKADIWSLGITAIELAKGEPPNSDMHPMRVLFLIPKNNPPTLV
GDFTKSFKEFIDACLNKDPSFRPTAKELLKHKFIVKNSKKTSYLTELIDRFKRWKAEGHS
DDESDSEGSDSESTSRENNTHPEWSFTTVRKKPDPKKVQNGAEQDLVQTLSCLSMIITPA
FAELKQQDENNASRNQAIEELEKSIAVAEAACPGITDKMVKKLIEKFQKCSADESP*
>ENSG00000138107|ENST00000369905
MESYDVIANQPVVIDNGSGVIKAGFAGDQIPKYCFPNYVGRPKHVRVMAGALEGDIFIGP
KAEEHRGLLSIRYPMEHGIVKDWNDMERIWQYVYSKDQLQTFSEEHPVLLTEAPLNPRKN
RERAAEVFFETFNVPALFISMQAVLSLYATGRTTGVVLDSGDGVTHAVPIYEGFAMPHSI
MRIDIAGRDVSRFLRLYLRKEGYDFHSSSEFEIVKAIKERACYLSINPQKDETLETEKAQ
YYLPDGSTIEIGPSRFRAPELLFRPDLIGEESEGIHEVLVFAIQKSDMDLRRTLFSNIVL
SGGSTLFKGFGDRLLSEVKKLAPKDVKIRISAPQERLYSTWIGGSILASLDTFKKMWVSK
KEYEEDGARSIHRKTF*
>ENSG00000100060|ENST00000356998
MQCRLPRGLAGALLTLLCMGLLCLRYHLNLSPQRVQGTPELSQPNPGPPKLQLHDVFIAV
KTTRAFHRLRLELLLDTWVSRTREQTFVFTDSPDKGLQERLGSHLVVTNCSAEHSHPALS
CKMAAEFDTFLASGLRWFCHVDDDNYVNPRALLQLLRAFPLARDVYVGRPSLNRPIHASE
PQPHNRTRLVQFWFATGGAGFCINRKLALKMAPWASGSRFMDTSALIRLPDDCTMGYIIE
CKLGGRLQPSPLFHSHLETLQLLRTAQLPEQVTLSYGVFEGKLNVIKLQGPFSPEEDPSR
FRSLHCLLYPDTPWCPQLGAR*
>ENSG00000124222|ENST00000371141

MATRRLTDAFLLLRNNSIQNRQLLAEQVSSHITSSPLHSRSIAAELDELADDRMALVSGI
SLDPEAAIGVTKRPPPKWVDGVDEIQYDVGRIKQKMKELASLHDKHLNRPTLDDSSEEEH
AIEITTQEITQLFHRCQRAVQALPSRARACSEQEGRLLGNVVASLAQALQELSTSFRHAQ
SGYLKRMKNREERSQHFFDTSVPLMDDGDDNTLYHRGFTEDQLVLVEQNTLMVEEREREI
RQIVQSISDLNEIFRDLGAMIVEQGTVLDRIDYNVEQSCIKTEDGLKQLHKAEQYQKKNR
KMLVILILFVIIIVLIVVLVGVKSR*
>ENSG00000153936|ENST00000370550
MGLLRIMMPPKLQLLAVVAFAVAMLFLENQIQKLEESRSKLERAIARHEVREIEQRHTMD
GPRQDATLDEEEDMVIIYNRVPKTASTSFTNIAYDLCAKNKYHVLHINTTKNNPVMSLQD
QVRFVKNITSWKEMKPGFYHGHVSYLDFAKFGVKKKPIYINVIRDPIERLVSYYYFLRFG
DDYRPGLRRRKQGDKKTFDECVAEGGSDCAPEKLWLQIPFFCGHSSECWNVGSRWAMDQA
KYNLINEYFLVGVTEELEDFIMLLEAALPRFFRGATELYRTGKKSHLRKTTEKKLPTKQT
IAKLQQSDIWKMENEFYEFALEQFQFIRAHAVREKDGDLYILAQNFFYEKIYPKSN*
>ENSG00000134470|ENST00000397255
MAPRRARGCRTLGLPALLLLLLLRPPATRGITCPPPMSVEHADIWVKSYSLYSRERYICN
SGFKRKAGTSSLTECVLNKATNVAHWTTPSLKCIRDPALVHQRPAPPSTVTTAGVTPQPE
SLSPSGKEPAASSPSSNNTAATTAAIVPGSQLMPSKSPSTGTTEISSHESSHGTPSQTTA
KNWELTASASHQPPGVYPQGHSDTTVAISTSTVLLCGLSAVSLLACYLKSRASVCSCHPR
SAGHTCSVGSVC*
>ENSG00000136750|ENST00000376261
MASPGSGFWSFGSEDGSGDSENPGTARAWCQVAQKFTGGIGNKLCALLYGDAEKPAESGG
SQPPRAAARKAACACDQKPCSCSKVDVNYAFLHATDLLPACDGERPTLAFLQDVMNILLQ
YVVKSFDRSTKVIDFHYPNELLQEYNWELADQPQNLEEILMHCQTTLKYAIKTGHPRYFN
QLSTGLDMVGLAADWLTSTANTNMFTYEIAPVFVLLEYVTLKKMREIIGWPGGSGDGIFS
PGGAISNMYAMMIARFKMFPEVKEKGMAALPRLIAFTSEHSHFSLKKGAAALGIGTDSVI
LIKCDERGKMIPSDLERRILEAKQKGFVPFLVSATAGTTVYGAFDPLLAVADICKKYKIW
MHVDAAWGGGLLMSRKHKWKLSGVERANSVTWNPHKMMGVPLQCSALLVREEGLMQNCNQ
MHASYLFQQDKHYDLSYDTGDKALQCGRHVDVFKLWLMWRAKGTTGFEAHVDKCLELAEY
LYNIIKNREGYEMVFDGKPQHTNVCFWYIPPSLRTLEDNEERMSRLSKVAPVIKARMMEY
GTTMVSYQPLGDKVNFFRMVISNPAATHQDIDFLIEEIERLGQDL*
>ENSG00000185499|ENST00000368396
MTPGTQSPFFLLLLLTVLTATTAPKPATVVTGSGHASSTPGGEKETSATQRSSVPSSTEK
NAFNSSLEDPSTDYYQELQRDISEMAVCQCRRKNYGQLDIFPARDTYHPMSEYPTYHTHG
RYVPPSSTDRSPYEKVSAGNGGSSLSYTNPAVAATSANL*
>ENSG00000116260|ENST00000367600
MRRCNSGSGPPPSLLLLLLWLLAVPGANAAPRSALYSPSDPLTLLQADTVRGAVLGSRSA
WAVEFFASWCGHCIAFAPTWKALAEDVKAWRPALYLAALDCAEETNSAVCRDFNIPGFPT
VRFFKAFTKNGSGAVFPVAGADVQTLRERLIDALESHHDTWPPACPPLEPAKLEEIDGFF
ARNNEEYLALIFEKGGSYLGREVALDLSQHKGVAVRRVLNTEANVVRKFGVTDFPSCYLL
FRNGSVSRVPVLMESRSFYTAYLQRLSGLTREAAQTTVAPTTANKIAPTVWKLADRSKIY
MADLESALHYILRIEVGRFPVLEGQRLVALKKFVAVLAKYFPGRPLVQNFLHSVNEWLKR
QKRNKIPYSFFKTALDDRKEGAVLAKKVNWIGCQGSEPHFRGFPCSLWVLFHFLTVQAAR
QNVDHSQEAAKAKEVLPAIRGYVHYFFGCRDCASHFEQMAAASMHRVGSPNAAVLWLWSS
HNRVNARLAGAPSEDPQFPKVQWPPRELCSACHNERLDVPVWDVEATLNFLKAHFSPSNI

ILDFPAAGSAARRDVQNVAAAPELAMGALELESRNSTLDPGKPEMMKSPTNTTPHVPAEG
PELI*
>ENSG00000101928|ENST00000370779
MHQQKRQPELVEGNLPVFVFPTELIFYADDQSTHKQVLTLYNPYEFALKFKVLCTTPNKY
VVVDAAGAVKPQCCVDIVIRHRDVRSCHYGVIDKFRLQVSEQSQRKALGRKEVVATLLPS
AKEQQKEEEEKRLKEHLTESLFFEQSFQPGLITMAILRT*
>ENSG00000137221|ENST00000372445
MTSAAPAKKPYRKAPPEHRELRLEIPGSRLEQEEPLTDAERMKLLQEENEELRRRLASAT
RRTEALERELEIGQDCLELELGQSREELDKFKDKFRRLQNSYTASQRTNQELEDKLHTLA
SLSHSWIFAIKKAEMDRKTLDWEIVELTNKLLDAKNTINKLEELNERYRLDCNLAVQLLK
CNKSHFRNHKFADLPCELQDMVRKHLHSGQEAASPGPAPSLAPGAVVPTSVIARVLEKPE
SLLLNSAQSGSAGRPLAEDVFVHVDMSEGVPGDPASPPAPGSPTPQPNGECHSLGTARGS
PEEELPLPAFEKLNPYPTPSPPHPLYPGRRVIEFSEDKVRIPRNSPLPNCTYATRQAISL
SLVEEGSERARPSPVPSTPASAQASPHHQPSPAPLTLSAPASSASSEEDLLVSWQRAFVD
RTPPPAAVAQRTAFGRDALPELQRHFAHSPADRDEVVQAPSARPEESELLLPTEPDSGFP
REEEELNLPISPEEERQSLLPINRGTEEGPGTSHTEGRAWPLPSSSRPQRSPKRMGVHHL
HRKDSLTQAQEQGNLLN*
>ENSG00000239732|ENST00000360658
MGFCRSALHPLSLLVQAIMLAMTLALGTLPAFLPCELQPHGLVNCNWLFLKSVPHFSMAA
PRGNVTSLSLSSNRIHHLHDSDFAHLPSLRHLNLKWNCPPVGLSPMHFPCHMTIEPSTFL
AVPTLEELNLSYNNIMTVPALPKSLISLSLSHTNILMLDSASLAGLHALRFLFMDGNCYY
KNPCRQALEVAPGALLGLGNLTHLSLKYNNLTVVPRNLPSSLEYLLLSYNRIVKLAPEDL
ANLTALRVLDVGGNCRRCDHAPNPCMECPRHFPQLHPDTFSHLSRLEGLVLKDSSLSWLN
ASWFRGLGNLRVLDLSENFLYKCITKTKAFQGLTQLRKLNLSFNYQKRVSFAHLSLAPSF
GSLVALKELDMHGIFFRSLDETTLRPLARLPMLQTLRLQMNFINQAQLGIFRAFPGLRYV
DLSDNRISGASELTATMGEADGGEKVWLQPGDLAPAPVDTPSSEDFRPNCSTLNFTLDLS
RNNLVTVQPEMFAQLSHLQCLRLSHNCISQAVNGSQFLPLTGLQVLDLSHNKLDLYHEHS
FTELPRLEALDLSYNSQPFGMQGVGHNFSFVAHLRTLRHLSLAHNNIHSQVSQQLCSTSL
RALDFSGNALGHMWAEGDLYLHFFQGLSGLIWLDLSQNRLHTLLPQTLRNLPKSLQVLRL
RDNYLAFFKWWSLHFLPKLEVLDLAGNQLKALTNGSLPAGTRLRRLDVSCNSISFVAPGF
FSKAKELRELNLSANALKTVDHSWFGPLASALQILDVSANPLHCACGAAFMDFLLEVQAA
VPGLPSRVKCGSPGQLQGLSIFAQDLRLCLDEALSWDCFALSLLAVALGLGVPMLHHLCG
WDLWYCFHLCLAWLPWRGRQSGRDEDALPYDAFVVFDKTQSAVADWVYNELRGQLEECRG
RWALRLCLEERDWLPGKTLFENLWASVYGSRKTLFVLAHTDRVSGLLRASFLLAQQRLLE
DRKDVVVLVILSPDGRRSRYVRLRQRLCRQSVLLWPHQPSGQRSFWAQLGMALTRDNHHF
YNRNFCQGPTAE*
>ENSG00000157020|ENST00000383801
MREPVLTWCVPLELLCSHPLPLSAFLKSQVKLYTYRACAGKDEMGKMVSVINTVDTSHED
MIHDAQMDYYGTRLATCSSDRSVKIFDVRNGGQILIADLRGHEGPVWQVAWAHPMYGNIL
ASCSYDRKVIIWREENGTWEKSHEHAGHDSSVNSVCWAPHDYGLILACGSSDGAISLLTY
TGEGQWEVKKINNAHTIGCNAVSWAPAVVPGSLIDHPSGQKPNYIKRFASGGCDNLIKLW
KEEEDGQWKEEQKLEAHSDWVRDVAWAPSIGLPTSTIASCSQDGRVFIWTCDDASSNTWS
PKLLHKFNDVVWHVSWSITANILAVSGGDNKVTLWKESVDGQWVCISDVNKGQGSVSASV
TEGQQNEQ*

>ENSG00000131446|ENST00000393340
MLKKQSAGLVLWGAILFVAWNALLLLFFWTRPAPGRPPSVSALDGDPASLTREVIRLAQD
AEVELERQRGLLQQIGDALSSQRGRVPTAAPPAQPRVPVTPAPAVIPILVIACDRSTVRR
CLDKLLHYRPSAELFPIIVSQDCGHEETAQAIASYGSAVTHIRQPDLSSIAVPPDHRKFQ
GYYKIARHYRWALGQVFRQFRFPAAVVVEDDLEVAPDFFEYFRATYPLLKADPSLWCVSA
WNDNGKEQMVDASRPELLYRTDFFPGLGWLLLAELWAELEPKWPKAFWDDWMRRPEQRQG
RACIRPEISRTMTFGRKGVSHGQFFDQHLKFIKLNQQFVHFTQLDLSYLQREAYDRDFLA
RVYGAPQLQVEKVRTNDRKELGEVRVQYTGRDSFKAFAKALGVMDDLKSGVPRAGYRGIV
TFQFRGRRVHLAPPLTWEGYDPSWN*
>ENSG00000124225|ENST00000395816
MMVMVVVITCLLSHYKLSARSFISRHSQGRRREDALSSEGCLWPSESTVSGNGIPEPQVY
APPRPTDRLAVPPFAQRERFHRFQPTYPYLQHEIDLPPTISLSDGEEPPPYQGPCTLQLR
DPEQQLELNRESVRAPPNRTIFDSDLMDSARLGGPCPPSSNSGISATCYGSGGRMEGPPP
TYSEVIGHYPGSSFQHQQSSGPPSLLEGTRLHHTHIAPLESAAIWSKEKDKQKGHPL*
>ENSG00000111846|ENST00000379597
MMGSWKHCLFSASLISALIFVFVYNTELWENKRFLRAALSNASLLAEACHQIFEGKVFYP
TENALKTTLDEATCYEYMVRSHYVTETLSEEEAGFPLAYTVTIHKDFGTFERLFRAIYMP
QNVYCVHLDQKATDAFKGAVKQLLSCFPNAFLASKKESVVYGGISRLQADLNCLEDLVAS
EVPWKYVINTCGQDFPLKTNREIVQYLKGFKGKNITPGVLPPDHAVGRTKYVHQELLNHK
NSYVIKTTKLKTPPPHDMVIYFGTAYVALTRDFANFVLQDQLALDLLSWSKDTYSPDEHF
WVTLNRIPGVPGSMPNASWTGNLRAIKWSDMEDRHGGCHGHYVHGICIYGNGDLKWLVNS
PSLFANKFELNTYPLTVECLELRHRERTLNQSETAIQPSWYF*
>ENSG00000205277|ENST00000379442
MLVIWILTLALRLCASVTTVTPEGSAVHKAISQQGTLWTGEVLEKQTVEQGKSTLRRQKN
HFHRSAGELRCRNALKDEGASAGWSVMFAGESVVVLVHLWMTGARVKNLGLVEFASPGDD
GDGRAEGFSLGLPLSEQARAAGAREKERQETVINHSTFSGFSQITGSTVNTSIGGNTTSA
STPSSSDPFTTFSDYGVSVTFITGSTATKHFLDSSTNSGHSEESTVSHSGPGATGTTLFP
SHSATSVFVGEPKTSPITSASMETTALPGSTTTAGLSEKSTTFYSSPRSPDRTLSPARTT
SSGVSEKSTTSHSRPGPTHTIAFPDSTTMPGVSQESTASHSIPGSTDTTLSPGTTTPSSL
GPESTTFHSSPGYTKTTRLPDNTTTSGLLEASTPVHSSTGSPHTTLSPSSSTTHEGEPTT
FQSWPSSKDTSPAPSGTTSAFVKLSTTYHSSPSSTPTTHFSASSTTLGHSEESTPVHSSP
VATATTPPPARSATSGHVEESTAYHRSPGSTQTMHFPESSTTSGHSEESATFHGSTTHTK
SSTPSTTAALAHTSYHSSLGSTETTHFRDSSTISGRSEESKASHSSPDAMATTVLPAGST
PSVLVGDSTPSPISSGSMETTALPGSTTKPGLSEKSTTFYSSPRSPDTTHLPASMTSSGV
SEESTTSHSRPGSTHTTAFPGSTTMPGLSQESTASHSSPGPTDTTLSPGSTTASSLGPEY
TTFHSRPGSTETTLLPDNTTASGLLEASMPVHSSTRSPHTTLSPAGSTTRQGESTTFHSW
PSSKDTRPAPPTTTSAFVEPSTTSHGSPSSIPTTHISARSTTSGLVEESTTYHSSPGSTQ
TMHFPESDTTSGRGEESTTSHSSTTHTISSAPSTTSALVEEPTSYHSSPGSTATTHFPDS
STTSGRSEESTASHSSQDATGTIVLPARSTTSVLLGESTTSPISSGSMETTALPGSTTTP
GLSERSTTFHSSPRSPATTLSPASTTSSGVSEESTTSRSRPGSTHTTAFPDSTTTPGLSR
HSTTSHSSPGSTDTTLLPASTTTSGPSQESTTSHSSSGSTDTALSPGSTTALSFGQESTT
FHSNPGSTHTTLFPDSTTSSGIVEASTRVHSSTGSPRTTLSPASSTSPGLQGESTAFQTH
PASTHTTPSPPSTATAPVEESTTYHRSPGSTPTTHFPASSTTSGHSEKSTIFHSSPDASG
TTPSSAHSTTSGRGESTTSRISPGSTEITTLPGSTTTPGLSEASTTFYSSPRSPTTTLSP

ASMTSLGVGEESTTSRSQPGSTHSTVSPASTTTPGLSEESTTVYSSSRGSTETTVFPHST
TTSVHGEEPTTFHSRPASTHTTLFTEDSTTSGLTEESTAFPGSPASTQTGLPATLTTADL
GEESTTFPSSSGSTGTKLSPARSTTSGLVGESTPSRLSPSSTETTTLPGSPTTPSLSEKS
TTFYTSPRSPDATLSPATTTSSGVSEESSTSHSQPGSTHTTAFPDSTTTSDLSQEPTTSH
SSQGSTEATLSPGSTTASSLGQQSTTFHSSPGDTETTLLPDDTITSGLVEASTPTHSSTG
SLHTTLTPASSTSAGLQEESTTFQSWPSSSDTTPSPPGTTAAPVEVSTTYHSRPSSTPTT
HFSASSTTLGRSEESTTVHSSPGATGTALFPTRSATSVLVGEPTTSPISSGSTETTALPG
STTTAGLSEKSTTFYSSPRSPDTTLSPASTTSSGVSEESTTSHSRPGSTHTTAFPGSTTM
PGVSQESTASHSSPGSTDTTLSPGSTTASSLGPESTTFHSSPGSTETTLLPDNTTASGLL
EASTPVHSSTGSPHTTLSPAGSTTRQGESTTFQSWPSSKDTMPAPPTTTSAFVELSTTSH
GSPSSTPTTHFSASSTTLGRSEESTTVHSSPVATATTPSPARSTTSGLVEESTAYHSSPG
STQTMHFPESSTASGRSEESRTSHSSTTHTISSPPSTTSALVEEPTSYHSSPGSTATTHF
PDSSTTSGRSEESTASHSSQDATGTIVLPARSTTSVLLGESTTSPISSGSMETTALPGST
TTPGLSEKSTTFHSSPRSPATTLSPASTTSSGVSEESTTSHSRPGSTHTTAFPDSTTTPG
LSRHSTTSHSSPGSTDTTLLPASTTTSGPSQESTTSHSSPGSTDTALSPGSTTALSFGQE
STTFHSSPGSTHTTLFPDSTTSSGIVEASTRVHSSTGSPRTTLSPASSTSPGLQGESTAF
QTHPASTHTTPSPPSTATAPVEESTTYHRSPGSTPTTHFPASSTTSGHSEKSTIFHSSPD
ASGTTPSSAHSTTSGRGESTTSRISPGSTEITTLPGSTTTPGLSEASTTFYSSPRSPTTT
LSPASMTSLGVGEESTTSRSQPGSTHSTVSPASTTTPGLSEESTTVYSSSPGSTETTVFP
RTPTTSVRGEEPTTFHSRPASTHTTLFTEDSTTSGLTEESTAFPGSPASTQTGLPATLTT
ADLGEESTTFPSSSGSTGTTLSPARSTTSGLVGESTPSRLSPSSTETTTLPGSPTTPSLS
EKSTTFYTSPRSPDATLSPATTTSSGVSEESSTSHSQPGSTHTTAFPDSTTTPGLSRHST
TSHSSPGSTDTTLLPASTTTSGPSQESTTSHSSPGSTDTALSPGSTTALSFGQESTTFHS
SPGSTHTTLFPDSTTSSGIVEASTRVHSSTGSPRTTLSPASSTSPGLQGESTTFQTHPAS
THTTPSPPSTATAPVEESTTYHRSPGSTPTTHFPASSTTSGHSEKSTIFHSSPDASGTTP
SSAHSTTSGRGESTTSRISPGSTEITTLPGSTTTPGLSEASTTFYSSPRSPTTTLSPASM
TSLGVGEESTTSRSQPGSTHSTVSPASTTTPGLSEESTTVYSSSPGSTETTVFPRSTTTS
VRGEEPTTFHSRPASTHTTLFTEDSTTSGLTEESTAFPGSPASTQTGLPATLTTADLGEE
STTFPSSSGSTGTTLSPARSTTSGLVGESTPSRLSPSSTETTTLPGSPTTPSLSEKSTTF
YTSPRSPDATLSPATTTSSGVSEESSTSHSQPGSTHTTAFPDSTTTSGLSQEPTASHSSQ
GSTEATLSPGSTTASSLGQQSTTFHSSPGDTETTLLPDDTITSGLVEASTPTHSSTGSLH
TTLTPASSTSAGLQEESTTFQSWPSSSDTTPSPPGTTAAPVEVSTTYHSRPSSTPTTHFS
ASSTTLGRSEESTTVHSSPGATGTALFPTRSATSVLVGEPTTSPISSGSTETTALPGSTT
TAGLSEKSTTFYSSPRSPDTTLSPASTTSSGVSEESTTSHSRPGSTHTTAFPGSTTMPGV
SQESTASHSSPGSTDTTLSPGSTTASSLGPESTTFHSGPGSTETTLLPDNTTASGLLEAS
TPVHSSTGSPHTTLSPAGSTTRQGESTTFQSWPNSKDTTPAPPTTTSAFVELSTTSHGSP
SSTPTTHFSASSTTLGRSEESTTVHSSPVATATTPSPARSTTSGLVEESTTYHSSPGSTQ
TMHFPESDTTSGRGEESTTSHSSTTHTISSAPSTTSALVEEPTSYHSSPGSTATTHFPDS
STTSGRSEESTASHSSQDATGTIVLPARSTTSVLLGESTTSPISSGSMETTALPGSTTTP
GLSEKSTTFHSSPRSPATTLSPASTTSSGVSEESTTSHSRPGSTHTTAFPDSTTTPGLSR
HSTTSHSSPGSTDTTLLPASTTTSGSSQESTTSHSSSGSTDTALSPGSTTALSFGQESTT
FHSSPGSTHTTLFPDSTTSSGIVEASTRVHSSTGSPRTTLSPASSTSPGLQGESTAFQTH
PASTHTTPSPPSTATAPVEESTTYHRSPGSTPTTHFPASSTTSGHSEKSTIFHSSPDASG
TTPSSAHSTTSGRGESTTSRISPGSTEITTLPGSTTTPGLSEASTTFYSSPRSPTTTLSP

ASMTSLGVGEESTTSRSQPGSTHSTVSPASTTTPGLSEESTTVYSSSPGSTETTVFPRST
TTSVRREEPTTFHSRPASTHTTLFTEDSTTSGLTEESTAFPGSPASTQTGLPATLTTADL
GEESTTFPSSSGSTGTKLSPARSTTSGLVGESTPSRLSPSSTETTTLPGSPTTPSLSEKS
TTFYTSPRSPDATLSPATTTSSGVSEESSTSHSQPGSTHTTAFPDSTTTSGLSQEPTTSH
SSQGSTEATLSPGSTTASSLGQQSTTFHSSPGDTETTLLPDDTITSGLVEASTPTHSSTG
SLHTTLTPASSTSTGLQEESTTFQSWPSSSDTTPSPPSTTAVPVEVSTTYHSRPSSTPTT
HFSASSTTLGRSEESTTVHSSPGATGTALFPTRSATSVLVGEPTTSPISSGSTETTALPG
STTTAGLSEKSTTFYSSPRSPDTTLSPASTTSSGVSEESTTSHSRPGSMHTTAFPSSTTM
PGVSQESTASHSSPGSTDTTLSPGSTTASSLGPESTTFHSSPGSTETTLLPDNTTASGLL
EASTPVHSSTGSPHTTLSPAGSTTRQGESTTFQSWPNSKDTTPAPPTTTSAFVELSTTSH
GSPSSTPTTHFSASSTTLGRSEESTTVHSSPVATATTPSPARSTTSGLVEESTTYHSSPG
STQTMHFPESNTTSGRGEESTTSHSSTTHTISSAPSTTSALVEEPTSYHSSPGSTATTHF
PDSSTTSGRSEESTASHSSQDATGTIVLPARSTTSVLLGESTTSPISSGSMETTALPGST
TTPGLSEKSTTFHSSPSSTPTTHFSASSTTLGRSEESTTVHSSPVATATTPSPARSTTSG
LVEESTAYHSSPGSTQTMHFPESSTASGRSEESRTSHSSTTHTISSPPSTTSALVEEPTS
YHSSPGSIATTHFPESSTTSGRSEESTASHSSPDTNGITPLPAHFTTSGRIAESTTFYIS
PGSMETTLASTATTPGLSAKSTILYSSSRSPDQTLSPASMTSSSISGEPTSLYSQAESTH
TTAFPASTTTSGLSQESTTFHSKPGSTETTLSPGSITTSSFAQEFTTPHSQPGSALSTVS
PASTTVPGLSEESTTFYSSPGSTETTAFSHSNTMSIHSQQSTPFPDSPGFTHTVLPATLT
TTDIGQESTAFHSSSDATGTTPLPARSTASDLVGEPTTFYISPSPTYTTLFPASSSTSGL
TEESTTFHTSPSFTSTIVSTESLETLAPGLCQEGQIWNGKQCVCPQGYVGYQCLSPLESF
PVETPEKLNATLGMTVKVTYRNFTEKMNDASSQEYQNFSTLFKNRMDVVLKGDNLPQYRG
VNIRRLLNGSIVVKNDVILEADYTLEYEELFENLAEIVKAKIMNETRTTLLDPDSCRKAI
LCYSEEDTFVDSSVTPGFDFQEQCTQKAAEGYTQFYYVDVLDGKLACVNKCTKGTKSQMN
CNLGTCQLQRSGPRCLCPNTNTHWYWGETCEFNIAKSLVYGIVGAVMAVLLLALIILIIL
FSLSQRKRHREQYDVPQEWRKEGTPGIFQKTAIWEDQNLRESRFGLENAYNNFRPTLETV
DSGTELHIQRPEMVASTV*

>ENSG00000197081|ENST00000356956

MGAAAGRSPHLGPAPARRPQRSLLLLQLLLLVAAPGSTQAQAAPFPELCSYTWEAVDTKN
NVLYKINICGSVDIVQCGPSSAVCMHDLKTRTYHSVGDSVLRSATRSLLEFNTTVSCDQQ
GTNHRVQSSIAFLCGKTLGTPEFVTATECVHYFEWRTTAACKKDIFKANKEVPCYVFDEE
LRKHDLNPLIKLSGAYLVDDSDPDTSLFINVCRDIDTLRDPGSQLRACPPGTAACLVRGH
QAFDVGQPRDGLKLVRKDRLVLSYVREEAGKLDFCDGHSPAVTITFVCPSERREGTIPKL
TAKSNCRYEIEWITEYACHRDYLESKTCSLSGEQQDVSIDLTPLAQSGGSSYISDGKEYL
FYLNVCGETEIQFCNKKQAAVCQVKKSDTSQVKAAGRYHNQTLRYSDGDLTLIYFGGDEC
SSGFQRMSVINFECNKTAGNDGKGTPVFTGEVDCTYFFTWDTEYACVKEKEDLLCGATDG
KKRYDLSALVRHAEPEQNWEAVDGSQTETEKKHFFINICHRVLQEGKARGCPEDAAVCAV
DKNGSKNLGKFISSPMKEKGNIQLSYSDGDDCGHGKKIKTNITLVCKPGDLESAPVLRTS
GEGGCFYEFEWHTAAACVLSKTEGENCTVFDSQAGFSFDLSPLTKKNGAYKVETKKYDFY
INVCGPVSVSPCQPDSGACQVAKSDEKTWNLGLSNAKLSYYDGMIQLNYRGGTPYNNERH
TPRATLITFLCDRDAGVGFPEYQEEDNSTYNFRWYTSYACPEEPLECVVTDPSTLEQYDL
SSLAKSEGGLGGNWYAMDNSGEHVTWRKYYINVCRPLNPVPGCNRYASACQMKYEKDQGS
FTEVVSISNLGMAKTGPVVEDSGSLLLEYVNGSACTTSDGRQTTYTTRIHLVCSRGRLNS
HPIFSLNWECVVSFLWNTEAACPIQTTTDTDQACSIRDPNSGFVFNLNPLNSSQGYNVSG

IGKIFMFNVCGTMPVCGTILGKPASGCEAETQTEELKNWKPARPVGIEKSLQLSTEGFIT
LTYKGPLSAKGTADAFIVRFVCNDDVYSGPLKFLHQDIDSGQGIRNTYFEFETALACVPS
PVDCQVTDLAGNEYDLTGLSTVRKPWTAVDTSVDGRKRTFYLSVCNPLPYIPGCQGSAVG
SCLVSEGNSWNLGVVQMSPQAAANGSLSIMYVNGDKCGNQRFSTRITFECAQISGSPAFQ
LQDGCEYVFIWRTVEACPVVRVEGDNCEVKDPRHGNLYDLKPLGLNDTIVSAGEYTYYFR
VCGKLSSDVCPTSDKSKVVSSCQEKREPQGFHKVAGLLTQKLTYENGLLKMNFTGGDTCH
KVYQRSTAIFFYCDRGTQRPVFLKETSDCSYLFEWRTQYACPPFDLTECSFKDGAGNSFD
LSSLSRYSDNWEAITGTGDPEHYLINVCKSLAPQAGTEPCPPEAAACLLGGSKPVNLGRV
RDGPQWRDGIIVLKYVDGDLCPDGIRKKSTTIRFTCSESQVNSRPMFISAVEDCEYTFAW
PTATACPMKSNEHDDCQVTNPSTGHLFDLSSLSGRAGFTAAYSEKGLVYMSICGENENCP
PGVGACFGQTRISVGKANKRLRYVDQVLQLVYKDGSPCPSKSGLSYKSVISFVCRPEARP
TNRPMLISLDKQTCTLFFSWHTPLACEQATECSVRNGSSIVDLSPLIHRTGGYEAYDESE
DDASDTNPDFYINICQPLNPMHGVPCPAGAAVCKVPIDGPPIDIGRVAGPPILNPIANEI
YLNFESSTPCLADKHFNYTSLIAFHCKRGVSMGTPKLLRTSECDFVFEWETPVVCPDEVR
MDGCTLTDEQLLYSFNLSSLSTSTFKVTRDSRTYSVGVCTFAVGPEQGGCKDGGVCLLSG
TKGASFGRLQSMKLDYRHQDEAVVLSYVNGDRCPPETDDGVPCVFPFIFNGKSYEECIIE
SRAKLWCSTTADYDRDHEWGFCRHSNSYRTSSIIFKCDEDEDIGRPQVFSEVRGCDVTFE
WKTKVVCPPKKLECKFVQKHKTYDLRLLSSLTGSWSLVHNGVSYYINLCQKIYKGPLGCS
ERASICRRTTTGDVQVLGLVHTQKLGVIGDKVVVTYSKGYPCGGNKTASSVIELTCTKTV
GRPAFKRFDIDSCTYYFSWDSRAACAVKPQEVQMVNGTITNPINGKSFSLGDIYFKLFRA
SGDMRTNGDNYLYEIQLSSITSSRNPACSGANICQVKPNDQHFSRKVGTSDKTKYYLQDG
DLDVVFASSSKCGKDKTKSVSSTIFFHCDPLVEDGIPEFSHETADCQYLFSWYTSAVCPL
GVGFDSENPGDDGQMHKGLSERSQAVGAVLSLLLVALTCCLLALLLYKKERRETVISKLT
TCCRRSSNVSYKYSKVNKEEETDENETEWLMEEIQLPPPRQGKEGQENGHITTKSVKALS
SLHGDDQDSEDEVLTIPEVKVHSGRGAGAESSHPVRNAQSNALQEREDDRVGLVRGEKAR
KGKSSSAQQKTVSSTKLVSFHDDSDEDLLHI*
>ENSG00000158488|ENST00000368167
MLLLFLLFEGLCCPGENTAAPQALQSYHLAAEEQLSFRMLQTSSFANHSWAHSEGSGWLG
DLQTHGWDTVLGTIRFLKPWSHGNFSKQELKNLQSLFQLYFHSFIQIVQASAGQFQLEYP
FEIQILAGCRMNAPQIFLNMAYQGSDFLSFQGISWEPSPGAGIRAQNICKVLNRYLDIKE
ILQSLLGHTCPRFLAGLMEAGESELKRKVKPEAWLSCGPSPGPGRLQLVCHVSGFYPKPV
WVMWMRGEQEQRGTQRGDVLPNADETWYLRATLDVAAGEAAGLSCRVKHSSLGGHDLIIH
WGGYSIFLILICLTVIVTLVILVVVDSRLKKQSSNKNILSPHTPSPVFLMGANTQDTKNS
RHQFCLAQVSWIKNRVLKKWKTRLNQLW*
>ENSG00000162645|ENST00000370466
MAPEINLPGPMSLIDNTKGQLVVNPEALKILSAITQPVVVVAIVGLYRTGKSYLMNKLAG
KKNGFSLGSTVKSHTKGIWMWCVPHPKKPEHTLVLLDTEGLGDIEKGDNENDSWIFALAI
LLSSTFVYNSMGTINQQAMDQLHYVTELTDRIKANSSPGNNSVDDSADFVSFFPAFVWTL
RDFTLELEVDGEPITADDYLELSLKLRKGTDKKSKSFNDPRLCIRKFFPKRKCFVFDWPA
PKKYLAHLEQLKEEELNPDFIEQVAEFCSYILSHSNVKTLSGGIPVNGPRLESLVLTYVN
AISSGDLPCMENAVLALAQIENSAAVEKAIAHYEQQMGQKVQLPTETLQELLDLHRDSER
EAIEVFMKNSFKDVDQMFQRKLGAQLEARRDDFCKQNSKASSDCCMALLQDIFGPLEEDV
KQGTFSKPGGYRLFTQKLQELKNKYYQVPRKGIQAKEVLKKYLESKEDVADALLQTDQSL
SEKEKAIEVERIKAESAEAAKKMLEEIQKKNEEMMEQKEKSYQEHVKQLTEKMERDRAQL

MAEQEKTLALKLQEQERLLKEGFENESKRLQKDIWDIQMRSKSLEPICNIL*
>ENSG00000123240|ENST00000378764
MSHQPLSCLTEKEDSPSESTGNGPPHLAHPNLDTFTPEELLQQMKELLTENHQLKEAMKL
NNQAMKGRFEELSAWTEKQKEERQFFEIQSKEAKERLMALSHENEKLKEELGKLKGKSER
SSEDPTDDSRLPRAEAEQEKDQLRTQVVRLQAEKADLLGIVSELQLKLNSSGSSEDSFVE
IRMAEGEAEGSVKEIKHSPGPTRTVSTGTSRSADGAKNYFEHEELTVSQLLLCLREGNQK
VERLEVALKEAKERVSDFEKKTSNRSEIETQTEGSTEKENDEEKGPETVGSEVEALNLQV
TSLFKELQEAHTKLSEAELMKKRLQEKCQALERKNSAIPSELNEKQELVYTNKKLELQVE
SMLSEIKMEQAKTEDEKSKLTVLQMTHNKLLQEHNNALKTIEELTRKESEKVDRAVLKEL
SEKLELAEKALASKQLQMDEMKQTIAKQEEDLETMTILRAQMEVYCSDFHAERAAREKIH
EEKEQLALQLAVLLKENDAFEDGGRQSLMEMQSRHGARTSDSDQQAYLVQRGAEDRDWRQ
QRNIPIHSCPKCGEVLPDIDTLQIHVMDCII*
>ENSG00000124225|ENST00000395814
MMVMVVVITCLLSHYKLSARSFISRHSQGRRREDALSSEGCLWPSESTVSGNGIPEPQVY
APPRPTDRLAVPPFAQRERFHRFQPTYPYLQHEIDLPPTISLSDGEEPPPYQGPCTLQLR
DPEQQLELNRESVRAPPNRTIFDSDLMDSARLGGPCPPSSNSGISATCYGSGGRMEGPPP
TYSEVIGHYPGSSFQHQQSSGPPSLLEGTRLHHTHIAPLESAAIWSKEKDKQKGHPL*
>ENSG00000157020|ENST00000350697
MVSVINTVDTSHEDMIHDAQMDYYGTRLATCSSDRSVKIFDVRNGGQILIADLRGHEGPV
WQVAWAHPMYGNILASCSYDRKVIIWREENGTWEKSHEHAGHDSSVNSVCWAPHDYGLIL
ACGSSDGAISLLTYTGEGQWEVKKINNAHTIGCNAVSWAPAVVPGSLIDHPSGQKPNYIK
RFASGGCDNLIKLWKEEEDGQWKEEQKLEAHSDWVRDVAWAPSIGLPTSTIASCSQDGRV
FIWTCDDASSNTWSPKLLHKFNDVVWHVSWSITANILAVSGGDNKVTLWKESVDGQWVCI
SDVNKGQGSVSASVTEGQQNEQ*
>ENSG00000107185|ENST00000378078
MIEVVAELSRGPVFLAGEALECVVTVTNPLPPTATSASSEALAWASAQIHCQFHASESRV
ALPPPDSSQPDVQPDSQTVFLPHRGERGQCILSTPPKILFCDLRLDPGESKSYSYSEVLP
IEGPPSFRGQSVKYVYKLTIGCQRVNSPITLLRVPLRVLVLTGLQDVRFPQDEAVAPSSP
FLEEDEGGKKDSWLAELAGERLMAATSCRSLHLYNISDGRGKVGTFGIFKSVYRLGEDVV
GTLNLGEGTVACLQFSVSLQTEERVQPEYQRRRGAGGVPSVSHVTHARHQESCLHTTRTS
FSLPIPLSSTPGFCTAIVSLKWRLHFEFVTSREPGLVLLPPVEQPEPTTWTGPEQVPVDT
FSWDLPIKVLPTSPTLASYAAPGPSTSTITI*
>ENSG00000148288|ENST00000372043
MHRRRLALGLGFCLLAGTSLSVLWVYLENWLPVSYVPYYLPCPEIFNMKLHYKREKPLQP
VVWSQYPQPKLLEHRPTQLLTLTPWLAPIVSEGTFNPELLQHIYQPLNLTIGVTVFAVGN
PSWSQPRSSSCVGTGCTTTSSLTTLQPFPGSRWVPTGFSAPSPSRVTPTGRRHPCAGWRP
SASTLLRGLTGRWTTSSALMWTWCFGTRGALRPWETWWLPFTQATTPFPASSSPMSAGVF
PLPLWQTAKGTSIMVGQSSGGRWPGYMSLLGAATWPSWRTRPMASWLPGGRKAT*
>ENSG00000130340|ENST00000392185
MATKARVMYDFAAEPGNNELTVNEGEIITITNPDVGGGWLEGRNIKGERGLVPTDYVEIL
PSDGKDQFSCGNSVADQAFLDSLSASTAQASSSAASNNHQVGSGNDPWSAWSASKSGNWE
SSEGWGAQPEGAGAQRNTNTPNNWDTAFGHPQAYQGPATGDDDDWDEDWDGPKSSSYFKD
SESADAGGAQRGNSRASSSSMKIPLNKFPGFAKPGTEQYLLAKQLAKPKEKIPIIVGDYG
PMWVYPTSTFDCVVADPRKGSKMYGLKSYIEYQLTPTNTNRSVNHRYKHFDWLYERLLVK

FGSAIPIPSLPDKQVTGRFEEEFIKMRMERLQAWMTRMCRHPVISESEVFQQFLNFRDEK
EWKTGKRKAERDELAGVMIFSTMEPEAPDLDLVEIEQKCEAVGKFTKAMDDGVKELLTVG
QEHWKRCTGPLPKEYQKIGKALQSLATVFSSSGYQGETDLNDAITEAGKTYEEIASLVAE
QPKKDLHFLMECNHEYKGFLGCFPDIIGTHKGAIEKVKESDKLVATSKITLQDKQNMVKR
VSIMSYALQAEMNHFHSNRIYDYNSVIRLYLEQQVQFYETIAEKLRQALSRFPVM*
>ENSG00000187714|ENST00000374115
MESAEPAGQARAAATKLSEAVGAALQEPRRQRRLVLVIVCVALLLDNMLYMVIVPIVPDY
IAHMRGGGEGPTRTPEVWEPTLPLPTPANASAYTANTSASPTAAWPAGSALRPRYPTESE
DVKIGVLFASKAILQLLVNPLSGPFIDRMSYDVPLLIGLGVMFASTVLFAFAEDYATLFA
ARSLQGLGSAFADTSGIAMIADKYPEEPERSRALGVALAFISFGSLVAPPFGGILYEFAG
KRVPFLVLAAVSLFDALLLLAVAKPFSAAARARANLPVGTPIHRLMLDPYIAVVAGALTT
CNIPLAFLEPTIATWMKHTMAASEWEMGMAWLPAFVPHVLGVYLTVRLAARYPHLQWLYG
ALGLAVIGASSCIVPACRSFAPLVVSLCGLCFGIALVDTALLPTLAFLVDVRHVSVYGSV
YAIADISYSVAYALGPIVAGHIVHSLGFEQLSLGMGLANLLYAPVLLLLRNVGLLTRSRS
ERDVLLDEPPQGLYDAVRLRERPVSGQDGEPRSPPGPFDACEDDYNYYYTRS*
>ENSG00000177674|ENST00000376629
MELPAVNLKVILLGHWLLTTWGCIVFSGSYAWANFTILALGVWAVAQRDSIDAISMFLGG
LLATIFLDIVHISIFYPRVSLTDTGRFGVGMAILSLLLKPLSCCFVYHMYRERGGFLGSS
QDRSAYQTIDSAEAPADPFAVPEGRSQDARGY*
>ENSG00000122176|ENST00000354955
MQWTSLLLLAGLFSLSQAQYEDDPHWWFHYLRSQQSTYYDPYDPYPYETYEPYPYGVDEG
PAYTYGSPSPPDPRDCPQECDCPPNFPTAMYCDNRNLKYLPFVPSRMKYVYFQNNQITSI
QEGVFDNATGLLWIALHGNQITSDKVGRKVFSKLRHLERLYLDHNNLTRMPGPLPRSLRE
LHLDHNQISRVPNNALEGLENLTALYLQHNEIQEVGSSMRGLRSLILLDLSYNHLRKVPD
GLPSALEQLYMEHNNVYTVPDSYFRGAPKLLYVRLSHNSLTNNGLASNTFNSSSLLELDL
SYNQLQKIPPVNTNLENLYLQGNRINEFSISSFCTVVDVVNFSKLQVLRLDGNEIKRSAM
PADAPLCLRLASLIEI*
>ENSG00000123191|ENST00000400366
MPEQERQITAREGASRKILSKLSLPTRAWEPAMKKSFAFDNVGYEGGLDGLGPSSQVATS
TVRILGMTCQSCVKSIEDRISNLKGIISMKVSLEQGSATVKYVPSVVCLQQVCHQIGDMG
FEASIAEGKAASWPSRSLPAQEAVVKLRVEGMTCQSCVSSIEGKVRKLQGVVRVKVSLSN
QEAVITYQPYLIQPEDLRDHVNDMGFEAAIKSKVAPLSLGPIDIERLQSTNPKRPLSSAN
QNFNNSETLGHQGSHVVTLQLRIDGMHCMISQLEGVQQISVSLAEGTATVLYNPSVISPE
ELRAAIEDMGFEASVVSESCSTNPLGNHSAGNSMVQTTDGTPTSVQEVAPHTGRLPANHA
PDILAKSPQSTRAVAPQKCFLQIKGMTCASCVSNIERNLQKEAGVLSVLVALMAGKAEIK
YDPEVIQPLEIAQFIQDLGFEAAVMEDYAGSDGNIELTITGMTCASCVHNIESKLTRTNG
ITYASVALATSKALVKFDPEIIGPRDIIKIIEEIGFHASLAQRNPNAHHLDHKMEIKQWK
KSFLCSLVFGIPVMALMIYMLIPSNEPHQSMVLDHNIIPGLSILNLIFFILCTFVQLLGG
WYFYVQAYKSLRHRSANMDVLIVLATSIAYVYSLVILVVAVAEKAERSPVTFFDTPPMLF
VFIALGRWLEHLAKSKTSEALAKLMSLQATEATVVTLGEDNLIIREEQVPMELVQRGDIV
KVVPGGKFPVDGKVLEGNTMADESLITGEAMPVTKKPGSTVIAGSINAHGSVLIKATHVG
NDTTLAQIVKLVEEAQMSKAPIQQLADRFSGYFVPFIIIMSTLTLVVWIVIGFIDFGVVQ
RYFPNPNKHISQTEVIIRFAFQTSITVLCIACPCSLGLATPTAVMVGTGVAAQNGILIKG
GKPLEMAHKIKTVMFDKTGTITHGVPRVMRVLLLGDVATLPLRKVLAVVGTAEASSEHPL

GVAVTKYCKEELGTETLGYCTDFQAVPGCGIGCKVSNVEGILAHSERPLSAPASHLNEAG
SLPAEKDAVPQTFSVLIGNREWLRRNGLTISSDVSDAMTDHEMKGQTAILVAIDGVLCGM
IAIADAVKQEAALAVHTLQSMGVDVVLITGDNRKTARAIATQVGINKVFAEVLPSHKVAK
VQELQNKGKKVAMVGDGVNDSPALAQADMGVAIGTGTDVAIEAADVVLIRNDLLDVVASI
HLSKRTVRRIRINLVLALIYNLVGIPIAAGVFMPIGIVLQPWMGSAAMAASSVSVVLSSL
QLKCYKKPDLERYEAQAHGHMKPLTASQVSVHIGMDDRWRDSPRATPWDQVSYVSQVSLS
SLTSDKPSRHSAAADDDGDKWSLLLNGRDEEQYI*
>ENSG00000164850|ENST00000397092
MDVTSQARGVGLEMYPGTAQPAAPNTTSPELNLSHPLLGTALANGTGELSEHQQYVIGLF
LSCLYTIFLFPIGFVGNILILVVNISFREKMTIPDLYFINLAVADLILVADSLIEVFNLH
ERYYDIAVLCTFMSLFLQVNMYSSVFFLTWMSFDRYIALARAMRCSLFRTKHHARLSCGL
IWMASVSATLVPFTAVHLQHTDEACFCFADVREVQWLEVTLGFIVPFAIIGLCYSLIVRV
LVRAHRHRGLRPRRQKALRMILAVVLVFFVCWLPENVFISVHLLQRTQPGAAPCKQSFRH
AHPLTGHIVNLAAFSNSCLNPLIYSFLGETFRDKLRLYIEQKTNLPALNRFCHAALKAVI
PDSTEQSDVRFSSAV*
>ENSG00000117280|ENST00000367139
MGSRDHLFKVLVVGDAAVGKTSLVQRYSQDSFSKHYKSTVGVDFALKVLQWSDYEIVRLQ
LWDIAGQERFTSMTRLYYRDASACVIMFDVTNATTFSNSQRWKQDLDSKLTLPNGEPVPC
LLLANKCDLSPWAVSRDQIDRFSKENGFTGWTETSVKENKNINEAMRVLIEKMMRNSTED
IMSLSTQGDYINLQTKSSSWSCC*
>ENSG00000155893|ENST00000393010
MLFRNRFLLLLALAALLAFVSLSLQFFHLIPVSTPKNGMSSKSRKRIMPDPVTEPPVTDP
VYEALLYCNIPSVAERSMEGHAPHHFKLVSVHVFIRHGDRYPLYVIPKTKRPEIDCTLVA
NRKPYHPKLEAFISHMSKGSGASFESPLNSLPLYPNHPLCEMGELTQTGVVQHLQNGQLL
RDIYLKKHKLLPNDWSADQLYLETTGKSRTLQSGLALLYGFLPDFDWKKIYFRHQPSALF
CSGSCYCPVRNQYLEKEQRRQYLLRLKNSQLEKTYGEMAKIVDVPTKQLRAANPIDSMLC
HFCHNVSFPCTRNGCVDMEHFKVIKTHQIEDERERREKKLYFGYSLLGAHPILNQTIGRM
QRATEGRKEELFALYSAHDVTLSPVLSALGLSEARFPRFAARLIFELWQDREKPSEHSVR
ILYNGVDVTFHTSFCQDHHKRSPKPMCPLENLVRFVKRDMFVALGGSGTNYYDACHREGF
*
>ENSG00000151348|ENST00000395673
MSCASGSGGGLRHPLRCQKPWDEECEEEAVCVIMCASVKYNIRGPALIPRMKTKHRIYYI
TLFSIVLLGLIATGMFQFWPHSIESSNDWNVEKRSIRDVPVVRLPADSPIPERGDLSCRM
HTCFDVYRCGFNPKNKIKVYIYALKKYVDDFGVSVSNTISREYNELLMAISDSDYYTDDI
NRACLFVPSIDVLNQNTLRIKETAQAMAQLSRWDRGTNHLLFNMLPGGPPDYNTALDVPR
DRALLAGGGFSTWTYRQGYDVSIPVYSPLSAEVDLPEKGPGPRQYFLLSSQVGLHPEYRE
DLEALQVKHGESVLVLDKCTNLSEGVLSVRKRCHKHQVFDYPQVLQEATFCVVLRGARLG
QAVLSDVLQAGCVPVVIADSYILPFSEVLDWKRASVVVPEEKMSDVYSILQSIPQRQIEE
MQRQARWFWEAYFQSIKAIALATLQIINDRIYPYAAISYEEWNDPPAVKWGSVSNPLFLP
LIPPQSQGFTAIVLTYDRVESLFRVITEVSKVPSLSKLLVVWNNQNKNPPEDSLWPKIRV
PLKVVRTAENKLSNRFFPYDEIETEAVLAIDDDIIMLTSDELQFGYEVWREFPDRLVGYP
GRLHLWDHEMNKWKYESEWTNEVSMVLTGAAFYHKYFNYLYTYKMPGDIKNWVDAHMNCE
DIAMNFLVANVTGKAVIKVTPRKKFKCPECTAIDGLSLDQTHMVERSECINKFASVFGTM
PLKVVEHRADPVLYKDDFPEKLKSFPNIGSL*

>ENSG00000124222|ENST00000359617
MALVSGISLDPEAAIGVTKRPPPKWVDGVDEIQYDVGRIKQKMKELASLHDKHLNRPTLD
DSSEEEHAIEITTQEITQLFHRCQRAVQALPSRARACSEQEGRLLGNVVASLAQALQELS
TSFRHAQSGYLKRMKNREERSQHFFDTSVPLMDDGDDNTLYHRGFTEDQLVLVEQNTLMV
EEREREIRQIVQSISDLNEIFRDLGAMIVEQGTVLDRIDYNVEQSCIKTEDGLKQLHKAE
QYQKKNRKMLVILILFVIIIVLIVVLVGVKSR*
>ENSG00000198663|ENST00000373685
MDLAANEISIYDKLSETVDLVRQTGHQCGMSEKAIEKFIRQLLEKNEPQRPPPQYPLLIV
VYKVLATLGLILLTAYFVIQPFSPLAPEPVLSGAHTWRSLIHHIRLMSLPIAKKYMSENK
GVPLHGGDEDRPFPDFDPWWTNDCEQNESEPIPANCTGCAQKHLKVMLLEDAPRKFERLH
PLVIKTGKPLLEEEIQHFLCQYPEATEGFSEGFFAKWWRCFPERWFPFPYPWRRPLNRSQ
MLRELFPVFTHLPFPKDASLNKCSFLHPEPVVGSKMHKMPDLFIIGSGEAMLQLIPPFQC
RRHCQSVAMPIEPGDIGYVDTTHWKVYVIARGVQPLVICDGTAFSEL*
>ENSG00000089063|ENST00000379283
MMPSRTNLATGIPSSKVKYSRLSSTDDGYIDLQFKKTPPKIPYKAIALATVLFLIGAFLI
IIGSLLLSGYISKGGADRAVPVLIIGILVFLPGFYHLRIAYYASKGYRGYSYDDIPDFDD
*
>ENSG00000109113|ENST00000395245
MNILAPVRRDRVLAELPQCLRKEAALHGHKDFHPRVTCACQEHRTGTVGFKISKVIVVGD
LSVGKTCLINRFCKDTFDKNYKATIGVDFEMERFEVLGIPFSLQLWDTAGQERFKCIAST
YYRGAQAIIIVFNLNDVASLEHTKQWLADALKENDPSSVLLFLVGSKKDLSTPAQYALME
KDALQVAQEMKAEYWAVSSLTGENVREFFFRVAALTFEANVLAELEKSGARRIGDVVRIN
SDDSNLYLTASKKKPTCCP*
>ENSG00000133110|ENST00000379747
MIPFLPMFSLLLLLIVNPINANNHYDKILAHSRIRGRDQGPNVCALQQILGTKKKYFSTC
KNWYKKSICGQKTTVLYECCPGYMRMEGMKGCPAVLPIDHVYGTLGIVGATTTQRYSDAS
KLREEIEGKGSFTYFAPSNEAWDNLDSDIRRGLESNVNVELLNALHSHMINKRMLTKDLK
NGMIIPSMYNNLGLFINHYPNGVVTVNCARIIHGNQIATNGVVHVIDRVLTQIGTSIQDF
IEAEDDLSSFRAAAITSDILEALGRDGHFTLFAPTNEAFEKLPRGVLERIMGDKVASEAL
MKYHILNTLQCSESIMGGAVFETLEGNTIEIGCDGDSITVNGIKMVNKKDIVTNNGVIHL
IDQVLIPDSAKQVIELAGKQQTTFTDLVAQLGLASALRPDGEYTLLAPVNNAFSDDTLSM
DQRLLKLILQNHILKVKVGLNELYNGQILETIGGKQLRVFVYRTAVCIENSCMEKGSKQG
RNGAIHIFREIIKPAEKSLHEKLKQDKRFSTFLSLLEAADLKELLTQPGDWTLFVPTNDA
FKGMTSEEKEILIRDKNALQNIILYHLTPGVFIGKGFEPGVTNILKTTQGSKIFLKEVND
TLLVNELKSKESDIMTTNGVIHVVDKLLYPADTPVGNDQLLEILNKLIKYIQIKFVRGST
FKEIPVTVYTTKIITKVVEPKIKVIEGSLQPIIKTEGPTLTKVKIEGEPEFRLIKEGETI
TEVIHGEPIIKKYTKIIDGVPVEITEKETREERIITGPEIKYTRISTGGGETEETLKKLL
QEEVTKVTKFIEGGDGHLFEDEEIKRLLQGDTPVRKLQANKKVQGSRRRLREGRSQ*
>ENSG00000204544|ENST00000376296
MKMQKGNVLLMFGLLLHLEAATNSNETSTSANTGSSVISSGASTATNSGSSVTSSGVSTA
TISGSSVTSNGVSIVTNSEFHTTSSGISTATNSEFSTVSSGISIATNSESSTTSSGASTA
TNSESSTPSSGASTATNSDSSTTSSGASTATNSDSSTTSSEASTATNSESSTTSSGASTA
TNSESSTVSSRASTATNSESSTTSSGASTATNSESRTTSNGAGTATNSESSTTSSGASTA
TNSESSTPSSGAGTATNSESSTTSSGAGTATNSESSTVSSGISTVTNSESSTPSSGANTA

TNSESSTTSSGANTATNSDSSTTSSGASTATNSESSTTSSGASTATNSESSTTSSGASTA
TNSGSSTTSSGTSTATNSESSTVSSGASTATTSESSTTSSGASTATNSESSTVSSGASTA
TNSESSTTSSGANTATNSGSSVTSAGSGTAALTGMHTTSHSASTAVSEAKPGGSLVPWEI
FLITLVSVVAAVGLFAGLFFCVRNSLSLRNTFNTAVYHPHGLNHGLGPGPGGNHGAPHRP
RWSPNWFWRRPVSSIAMEMSGRNSGP*
>ENSG00000148396|ENST00000371706
MPGLDRPLSRQNPHDGVVTPAASPSLPQPGLQMPGQWGPVQGGPQPSGQHRSPCPEGPVP
SGVPCATSVPHFPTPSILHQGPGHEQHSPLVAPPAALPSDGRDEVSHLQSGSHLANNSDP
ESTFRQNPRIVNHWASPELRQNPGVKNEHRPASALVNPLARGDSPENRTHHPLGAGAGSG
CAPLEADSGASGALAMFFQGGETENEENLSSEKAGLSGQADFDDFCSSPGLGRPPAPTHV
GAGSLCQALLPGPSNEAAGDVWGDTASTGVPDASGSQYENVENLEFVQNQEVLPSEPLNL
DPSSPSDQFRYGPLPGPAVPRHGAVCHTGAPDATLHTVHPDSVSSSYSSRSHGRLSGSAR
PQELVGTFIQQEVGKPEDEASGSFFKQIDSSPVGGETDETTVSQNYRGSVSQPSTPSPPK
PTGIFQTSANSSFEPVKSHLVGVKPFEADRANVVGEVRETCVRQKQCRPAAALPDASPGN
LEQPPDNMETLCAPQVCPLPLNSTTEAVHMLPHAGAPPLDTVYPAPEKRPSARTQGPVKC
ESPATTLWAQSELPDFGGNVLLAPAAPALYVCAKPQPPVVQPPEEAMSGQQSRNPSSAAP
VQSRGGIGASENLENPPKMGEEEALQSQASSGYASLLSSPPTESLQNPPVLIAQPDHSYN
LAQPINFSVSLSNSHEKNQSWREALVGDRPAVSSWALGGDSGENTSLSGIPTSSVLSLSL
PSSVAQSNFPQGSGASEMVSNQPANLLVQPPSQPVPENLVPESQKDRKAGSALPGFANSP
AGSTSVVLVPPAHGTLVPDGNKANHSSHQEDTYGALDFTLSRTLENPVNVYNPSHSDSLA
SQQSVASHPRQSGPGAPNLDRFYQQVTKDAQGQPGLERAQQELVPPQQQASPPQLPKAMF
SELSNPESLPAQGQAQNSAQSPASLVLVDAGQQLPPRPPQSSSVSLVSSGSGQAAVPSEQ
PWPQPVPALAPGPPPQDLAAYYYYRPLYDAYQPQYSLPYPPEPGAASLYYQDVYSLYEPR
YRPYDGAASAYAQNYRYPEPERPSSRASHSSERPPPRQGYPEGYYSSKSGWSSQSDYYAS
YYSSQYDYGDPGHWDRYHYSARVRDPRTYDRRYWCDAEYDAYRREHSAFGDRPEKRDNNW
RYDPRFTGSFDDDPDPHRDPYGEEVDRRSVHSEHSARSLHSAHSLASRRSSLSSHSHQSQ
IYRSHNVAAGSYEAPLPPGSFHGDFAYGTYRSNFSSGPGFPEYGYPADTVWPAMEQVSSR
PTSPEKFSVPHVCARFGPGGQLIKVIPNLPSEGQPALVEVHSMEALLQHTSEQEEMRAFP
GPLAKDDTHKVDVINFAQNKAMKCLQNENLIDKESASLLWNFIVLLCRQNGTVVGTDIAE
LLLRDHRTVWLPGKSPNEANLIDFTNEAVEQVEEEESGEAQLSFLTGGPAAAASSLERET
ERFRELLLYGRKKDALESAMKNGLWGHALLLASKMDSRTHARVMTRFANSLPINDPLQTV
YQLMSGRMPAASTCCGDEKWGDWRPHLAMVLSNLNNNMDVESRTMATMGDTLASRGLLDA
AHFCYLMAQAGFGVYTKKTTKLVLIGSNHSLPFLKFATNEAIQRTEAYEYAQSLGAETCP
LPSFQVFKFIYSCRLAEMGLATQAFHYCEAIAKSILTQPHLYSPVLISQLVQMASQLRLF
DPQLKEKPEEESLAAPTWLVHLQQVERQIKEGAGVWHQDGALPQQCPGTPSSEMEQLDRP
GLSQPGALGIANPLLAVPAPSPEHSSPSVRLLPSAPQTLPDGPLASPARVPMFPVPLPPG
PLEPGPGCVTPGPALGFLEPSGPGLPPGVPPLQERRHLLQEARSPDPGIVPQEAPVGNSL
SELSEENFDGKFANLTPSRTVPDSEAPPGWDRADSGPTQPPLSLSPAPETKRPGQAAKKE
TKEPKKGESWFFRWLPGKKKTEAYLPDDKNKSIVWDEKKNQWVNLNEPEEEKKAPPPPPT
SMPKTVQAAPPALPGPPGAPVNMYSRRAAGTRARYVDVLNPSGTQRSEPALAPADFVAPL
APLPIPSNLFVPTPDAEEPQLPDGTGREGPAAARGLANPEPAPEPKAPGDLPAAGGPPSG
AMPFYNPAQLAQACATSGSSRLGRIGQRKHLVLN*
>ENSG00000124164|ENST00000395802
MAKVEQVLSLEPQHELKFRGPFTDVVTTNLKLGNPTDRNVCFKVKTTAPRRYCVRPNSGI

IDAGASINVSGRRWTADEEDSAEQQPHFSISPNWEGRRP*
>ENSG00000165915|ENST00000362021
MPGCPCPGCGMAGPRLLFLTALALELLERAGGSQPALRSRGTATACRLDNKESESWGALL
SGERLDTWICSLLGSLMVGLSGVFPLLVIPLEMGTMLRSEAGAWRLKQLLSFALGGLLGN
VFLHLLPEAWAYTCSASPGGEGQSLQQQQQLGLWVIAGILTFLALEKMFLDSKEEGTSQA
PNKDPTAAAAALNGGHCLAQPAAEPGLGAVVRSIKVSGYLNLLANTIDNFTHGLAVAASF
LVSKKIGLLTTMAILLHEIPHEVGDFAILLRAGFDRWSAAKLQLSTALGGLLGAGFAICT
QSPKGVVGCSPAAEETAAWVLPFTSGGFLYIALVNVLPDLLEEEDPWRSLQQLLLLCAGI
VVMVLFSLFVD*
>ENSG00000117228|ENST00000370473
MASEIHMTGPMCLIENTNGRLMANPEALKILSAITQPMVVVAIVGLYRTGKSYLMNKLAG
KKKGFSLGSTVQSHTKGIWMWCVPHPKKPGHILVLLDTEGLGDVEKGDNQNDSWIFALAV
LLSSTFVYNSIGTINQQAMDQLYYVTELTHRIRSKSSPDENENEVEDSADFVSFFPDFVW
TLRDFSLDLEADGQPLTPDEYLTYSLKLKKGTSQKDETFNLPRLCIRKFFPKKKCFVFDR
PVHRRKLAQLEKLQDEELDPEFVQQVADFCSYIFSNSKTKTLSGGIQVNGPRLESLVLTY
VNAISSGDLPCMENAVLALAQIENSAAVQKAIAHYEQQMGQKVQLPTETLQELLDLHRDS
EREAIEVFIRSSFKDVDHLFQKELAAQLEKKRDDFCKQNQEASSDRCSALLQVIFSPLEE
EVKAGIYSKPGGYRLFVQKLQDLKKKYYEEPRKGIQAEEILQTYLKSKESMTDAILQTDQ
TLTEKEKEIEVERVKAESAQASAKMLQEMQRKNEQMMEQKERSYQEHLKQLTEKMENDRV
QLLKEQERTLALKLQEQEQLLKEGFQKESRIMKNEIQDLQTKMRRRKACTIS*
>ENSG00000105971|ENST00000393480
MGLETEKADVQLFMDDDSYSHHSGLEYADPEKFADSDQDRDPHRLNSHLKLGFEDVIAEP
VTTHSFDKVWICSHALFEISKYVMYKFLTVFLAIPLAFIAGILFATLSCLHIW*
>ENSG00000143952|ENST00000354504
MLPTKNRIKREKIHERCKNICPPKDTFERTLLHTHDKSRTDLEQVPKIFMKPDFALDDSL
TFNSVLPWSHFNTAGGKGNRDAASSKLLQEKLSHYLDIVEVNIAHQISLRSEAFFHAMTS
QHELQDYLRKTSQAVKMLRDKIAQIDKVMCEGSLHILRLALTRNNCVKVYNKLKLMATVH
QTQPTVQVLLSTSEFVGALDLIATTQEVLQQELQGIHSFRHLGSQLCELEKLIDKMMIAE
FSTYSHSDLNRPLEDDCQVLEECVINKVSQTEEIDTDVVVKLADQMRMLNFPQWFDLLKD
IFSKFTIFLQRVKATLNIIHSVVLSVLDKNQRTRELEEISQQKNAAKDNSLDTEVAYLIH
EGMFISDAFGEGELTPIAVDTTSQRNASPNSEPCSSDSVSEPECTTDSSSSKEHTSSSAI
PGGVDIMVSEDMKLTDSELGKLANNIQELLYSASDICHDRAVKFLMSRAKDGFLEKLNSM
EFITLSRLMETFILDTEQICGRKSTSLLGALQSQAIKFVNRFHEERKTKLSLLLDNERWK
QADVPAEFQDLVDSLSDGKIALPEKKSGATEERKPAEVLIVEGQQYAVVGTVLLLIRIIL
EYCQCVDNIPSVTTDMLTRLSDLLKYFNSRSCQLVLGAGALQVVGLKTITTKNLALSSRC
LQLIVHYIPVIRAHFEARLPPKQYSMLRHFDHITKDYHDHIAEISAKLVAIMDSLFDKLL
SKYEVKAPVPSACFRNICKQMTKMHEAIFDLLPEEQTQMLFLRINASYKLHLKKQLSHLN
VINDGGPQNGLVTADVAFYTGNLQALKGLKDLDLNMAEIWEQKR*
>ENSG00000067182|ENST00000366159
MGLSTVPDLLLPLVLLELLVGIYPSGVIGLVPHLGDREKRDSVCPQGKYIHPQNNSICCT
KCHKGTYLYNDCPGPGQDTDCRECESGSFTASENHLRHCLSCSKCRKEMGQVEISSCTVD
RDTVCGCRKNQYRHYWSENLFQCFNCSLCLNGTVHLSCQEKQNTVCTCHAGFFLRENECV
SCSKVLLCRPGWNAVARSRLTATSASQIQAILLLQPPK*
>ENSG00000177674|ENST00000376637

MELPAVNLKGLHCILRLLCLGQLHHPGLGRVGCGSAGLHRRHKHVSGWLAGHHLPGHRAH
QHLLPAGQPHGHGPLWRGHGHPQLAAQAALLLLRLPHVPGARGFPWVFSGP*
>ENSG00000185499|ENST00000368390
MTPGTQSPFFLLLLLTVLTVVTGSGHASSTPGGEKETSATQRSSVPSSTEKNAFNSSLED
PSTDYYQELQRDISEMFLQIYKQGGFLGLSNIKFRPGSVVVQLTLAFREGTINVHDVETQ
FNQYKTEAASRYNLTISDVSVSDVPFPFSAQSGAGVPGWGIALLVLVCVLVALAIVYLIA
LAVCQCRRKNYGQLDIFPARDTYHPMSEYPTYHTHGRYVPPSSTDRSPYEKVSAGNGGSS
LSYTNPAVAATSANL*
>ENSG00000160218|ENST00000380221
MDASEEPLPPVIYTMENKPIVTCAGDQNLFTSVYPTLSQQLPREPMEWRRSYGRAPKMIH
LESNFVQFKEELLPKEGNKALLTFPFLHIYWTECCDTEVYKATVKDDLTKWQNVLKAHSS
VDWLIVIVENDAKKKNKTNILPRTSIVDKIRNDFCNKQSDRCVVLSDPLKDSSRTQESWN
AFLTKLRTLLLMSFTKNLGKFEDDMRTLREKRTEPGWSFCEYFMVQEELAFVFEMLQQFE
DALVQYDELDALFSQYVVNFGAGGIKCPFHNSVACW*
>ENSG00000162885|ENST00000366600
MRNWLVLLCPCVLGAALHLWLRLRSPPPACASGAGPADQLALFPQWKSTHYDVVVGVLSA
RNNHELRNVIRSTWMRHLLQHPTLSQRVLVKFIIGAHGCEVPVEDREDPYSCKLLNITNP
VLNQEIEAFSLSEDTSSGLPEDRVVSVSFRVLYPIVITSLGVFYDANDVGFQRNITVKLY
QAEQEEALFIARFSPPSCGVQVNKLWYKPVEQFILPESFEGTIVWESQDLHGLVSRNLHK
VTVNDGGGVLRVITAGEGALPHEFLEGVEGVAGGFIYTIQEGDALLHNLHSRPQRLIDHI
RNLHEEDALLKEESSIYDDIVFVDVVDTYRNVPAKLLNFYRWTVETTSFNLLLKTDDDCY
IDLEAVFNRIVQKNLDGPNFWWGNFRLNWAVDRTGKWQELEYPSPAYPAFACGSGYVISK
DIVKWLASNSGRLKTYQGEDVSMGIWMAAIGPKRYQDSLWLCEKTCETGMLSSPQYSPWE
LTELWKLKERCGDPCRCQAR*
>ENSG00000153130|ENST00000394205
MDGSRKEEEEDSTFTNISLADDIDHSSRILYPRPKSLLPKMMNADMDAVDAENQVELEEK
TRLINQVLELQHTLEDLSARVDAVKEENLKLKSENQVLGQYIENLMSASSVFQTTDTKSK
RK*
>ENSG00000107863|ENST00000396432
MMATRRTGLSEGDGDKLKACEVSKNKDGKEQSETVSLSEDETFSWPGPKTVTLKRTSQGF
GFTLRHFIVYPPESAIQFSYKDEENGNRGGKQRNRLEPMDTIFVKQVKEGGPAFEAGLCT
GDRIIKVNGESVIGKTYSQVIALIQNSDTTLELSVMPKDEDILQVLQFTKDVTALAYSQD
AYLKGNEAYSGNARNIPEPPPICYPWLPSAPSAMAQPVEISPPDSSLSKQQTSTPVLTQP
GRAYRMEIQVPPSPTDVAKSNTAVCVCNESVRTVIVPSEKVVDLLSNRNNHTGPSHRTEE
VRYGVSEQTSLKTVSRTTSPPLSIPTTHLIHQPAGSRSLEPSGILLKSGNYSGHSDGISS
SRSQAVEAPSVSVNHYSPNSHQHIDWKNYKTYKEYIDNRRLHIGCRTIQERLDSLRAASQ
STTDYNQVVPNRTTLQGRRRSTSHDRVPQSVQIRQRSVSQERLEDSVLMKYCPRSASQGA
LTSPSVSFSNHRTRSWDYIEGQDETLENVNSGTPIPDSNGEKKQTYKWSGFTEQDDRRGI
CERPRQQEIHKSFRGSNFTVAPSVVNSDNRRMSGRGVGSVSQFKKIPPDLKTLQSNRNFQ
TTCGMSLPRGISQDRSPLVKVRSNSLKAPSTHVTKPSFSQKSFVSIKDQRPVNHLHQNSL
LNQQTWVRTDSAPDQQVETGKSPSLSGASAKPAPQSSENAGTSDLELPVSQRNQDLSLQE
AETEQSDTLDNKEAVILREKPPSGRQTPQPLRHQSYILAVNDQETGSDTTCWLPNDARRE
VHIKRMEERKASSTSPPGDSLASIPFIDEPTSPSIDHDIAHIPASAVISASTSQVPSIAT
VPPCLTTSAPLIRRQLSHDHESVGPPSLDAQPNSKTERSKSYDEGLDDYREDAKLSFKHV

SSLKGIKIADSQKSSEDSGSRKDSSSEVFSDAAKEGWLHFRPLVTDKGKRVGGSIRPWKQ
MYVVLRGHSLYLYKDKREQTTPSEEEQPISVNACLIDISYSETKRKNVFRLTTSDCECLF
QAEDRDDMLAWIKTIQESSNLNEEDTGVTNRDLISRRIKEYNNLMSKAEQLPKTPRQSLS
IRQTLLGAKSEPKTQSPHSPKEESERKLLSKDDTSPPKDKGTWRKGIPSIMRKTFEKKPT
ATGTFGVRLDDCPPAHTNRYIPLIVDICCKLVEERGLEYTGIYRVPGNNAAISSMQEELN
KGMADIDIQDDKWRDLNVISSLLKSFFRKLPEPLFTNDKYADFIEANRKEDPLDRLKTLK
RLIHDLPEHHYETLKFLSAHLKTVAENSEKNKMEPRNLAIVFGPTLVRTSEDNMTHMVTH
MPDQYKIVETLIQHHDWFFTEEGAEEPLTTVQEESTVDSQPVPNIDHLLTNIGRTGVSPG
DVSDSATSDSTKSKGSWGSGKDQYSRELLVSSIFAAASRKRKKPKEKAQPSSSEDELDNV
FFKKENVEQCHNDTKEESKKESETLGRKQKIIIAKENSTRKDPSTTKDEKISLGKESTPS
EEPSPPHNSKHNKSPTLSCRFAILKESPRSLLAQKSSHLEETGSDSGTLLSTSSQASLAR
FSMKKSTSPETKHSEFLANVSTITSDYSTTSSATYLTSLDSSRLSPEVQSVAESKGDEAD
DERSELISEGRPVETDSESEFPVFPTALTSERLFRGKLQEVTKSSRRNSEGSELSCTEGS
LTSSLDSRRQLFSSHKLIECDTLSRKKSARFKSDSGSLGDAKNEKEAPSLTKVFDVMKKG
KSTGSLLTPTRGESEKQEPTWKTKIADRLKLRPRAPADDMFGVGNHKVNAETAKRKSIRR
RHTLGGHRDATEISVLNFWKVHEQSGERESELSAVNRLKPKCSAQDLSISDWLARERLRT
STSDLSRGEIGDPQTENPSTREIATTDTPLSLHCNTGSSSSTLASTNRPLLSIPPQSPDQ
INGESFQNVSKNASSAANAQPHKLSETPGSKAEFHPCL*
>ENSG00000176920|ENST00000391876
MLVVQMPFSFPMAHFILFVFTVSTIFHVQQRLAKIQAMWELPVQIPVLASTSKALGPSQL
RGMWTINAIGRLGNQMGEYATLYALAKMNGRPAFIPAQMHSTLAPIFRITLPVLHSATAS
RIPWQNYHLNDWMEEEYRHIPGEYVRFTGYPCSWTFYHHLRQEILQEFTLHDHVREEAQK
FLRGLQVNGSRPGTFVGVHVRRGDYVHVMPKVWKGVVADRRYLQQALDWFRARYSSLIFV
VTSNGMAWCRENIDTSHGDVVFAGDGIEGSPAKDFALLTQCNHTIMTIGTFGIWAAYLTG
GDTIYLANYTLPDSPFLKIFKPEAAFLPEWTGIAADLSPLLKH*
>ENSG00000167080|ENST00000393354
MTSGGSRFLWLLKILVIILVLGIVGFMFGSMFLQAVFSSPKPELPSPAPGVQKLKLLPEE
RLRNLFSYDGIWLFPKNQCKCEANKEQGGYNFQDAYGQSDLPAVKARRQAEFEHFQRREG
LPRPLPLLVQPNLPFGYPVHGVEVMPLHTVPIPGLQFEGPDAPVYEVTLTASLGTLNTLA
DVPDSVVQGRGQKQLIISTSDRKLLKFILQHVTYTSTGYQHQKVDIVSLESRSSVAKFPV
TIRHPVIPKLYDPGPERKLRNLVTIATKTFLRPHKLMIMLRSIREYYPDLTVIVADDSQK
PLEIKDNHVEYYTMPFGKGWFAGRNLAISQVTTKYVLWVDDDFLFNEETKIEVLVDVLEK
TELDVVGGSVLGNVFQFKLLLEQSENGACLHKRMGFFQPLDGFPSCVVTSGVVNFFLAHT
ERLQRVGFDPRLQRVAHSEFFIDGLGTLLVGSCPEVIIGHQSRSPVVDSELAALEKTYNT
YRSNTLTRVQFKLALHYFKNHLQCAA*
>ENSG00000105669|ENST00000351079
MAPPAPGPASGGSGEVDELFDVKNAFYIGSYQQCINEAQRVKLSSPERDVERDVFLYRAY
LAQRKFGVVLDEIKPSSAPELQAVRMFADYLAHESRSTAMTVQILLKLDRLDLARKELKR
MQDLDEDATLTQLATAWVSLATGGEKLQDAYYIFQEMADKCSPTLLLLNGQAACHMAQGR
WEAAEGLLQEALDKDSGYPETLVNLIVLSQHLGKPPEVTNRYLSQLKDAHRSHPFIKEYQ
AKENDFDRLVLQYAPSA*
>ENSG00000120215|ENST00000381477
MPREDAHFIYGYPKKGHGHSYTTAEEAAGIGILTVILGVLLLIGCWYCRRRNGYRALMDK
SLHVGTQCALTRRCPQEGFDHRDSKVSLQEKNCEPVVPNAPPAYEKLSAEQSPPPYSP*

>ENSG00000206503|ENST00000376809
MAVMAPRTLLLLLSGALALTQTWAGSHSMRYFFTSVSRPGRGEPRFIAVGYVDDTQFVRF
DSDAASQRMEPRAPWIEQEGPEYWDQETRNVKAQSQTDRVDLGTLRGYYNQSEAGSHTIQ
IMYGCDVGSDGRFLRGYRQDAYDGKDYIALNEDLRSWTAADMAAQITKRKWEAAHEAEQL
RAYLDGTCVEWLRRYLENGKETLQRTDPPKTHMTHHPISDHEATLRCWALGFYPAEITLT
WQRDGEDQTQDTELVETRPAGDGTFQKWAAVVVPSGEEQRYTCHVQHEGLPKPLTLRWEL
SSQPTIPIVGIIAGLVLLGAVITGAVVAAVMWRRKSSDRKGGSYTQAASSDSAQGSDVSL
TACKV*
>ENSG00000143801|ENST00000366782
MLAGTVRFARHCLKFFPAQKPACVDFGASRGRAMLTFMASDSEEEVCDERTSLMSAESPT
PRSCQEGRQGPEDGENTAQWRSQENEEDGEEDPDRYVCSGVPGRPPGLEEELTLKYGAKH
VIMLFVPVTLCMIVVVATIKSVRFYTEKNGQLIYTPFTEDTPSVGQRLLNSVLNTLIMIS
VIVVMTIFLVVLYKYRCYKFIHGWLIMSSLMLLFLFTYIYLGEVLKTYNVAMDYPTLLLT
VWNFGAVGMVCIHWKGPLVLQQAYLIMISALMALVFIKYLPEWSAWVILGAISVYDLVAV
LCPKGPLRMLVETAQERNEPIFPALIYSSAMVWTVGMAKLDPSSQGALQLPYDPEMEEDS
YDSFGEPSYPEVFEPPLTGYPGEELEEEEERGVKLGLGDFIFYSVLVGKAAATGSGDWNT
TLACFVAILIGLCLTLLLLAVFKKALPALPISITFGLIFYFSTDNLVRPFMDTLASHQLY
I*
>ENSG00000177239|ENST00000371589
MAACEGRRSGALGSSQSDFLTPPVGGAPWAVATTVVMYPPPPPPPHRDFISVTLSFGENY
DNSKSWRRRSCWRKWKQLSRLQRNMILFLLAFLLFCGLLFYINLADHWKALAFRLEEEQK
MRPEIAGLKPANPPVLPAPQKADTDPENLPEISSQKTQRHIQRGPPHLQIRPPSQDLKDG
TQEEATKRQEAPVDPRPEGDPQRTVISWRGAVIEPEQGTELPSRRAEVPTKPPLPPARTQ
GTPVHLNYRQKGVIDVFLHAWKGYRKFAWGHDELKPVSRSFSEWFGLGLTLIDALDTMWI
LGLRKEFEEARKWVSKKLHFEKDVDVNLFESTIRILGGLLSAYHLSGDSLFLRKAEDFGN
RLMPAFRTPSKIPYSDVNIGTGVAHPPRWTSDSTVAEVTSIQLEFRELSRLTGDKKFQEA
VEKVTQHIHGLSGKKDGLVPMFINTHSGLFTHLGVFTLGARADSYYEYLLKQWIQGGKQE
TQLLEDYVEAIEGVRTHLLRHSEPSKLTFVGELAHGRFSAKMDHLVCFLPGTLALGVYHG
LPASHMELAQELMETCYQMNRQMETGLSPEIVHFNLYPQPGRRDVEVKPADRHNLLRPET
VESLFYLYRVTGDRKYQDWGWEILQSFSRFTRVPSGGYSSINNVQDPQKPEPRDKMESFF
LGETLKYLFLLFSDDPNLLSLDAYVFNTEAHPLPIWTPA*
>ENSG00000155265|ENST00000370602
MATEVHNLQELRRSASLATKVFIQRDYSDGTICQFQTKFPPELDSRIERQLFEETVKTLN
GFYAEAEKIGGSSYLEGCLACATAYFIFLCMETHYEKVLKKISRYIQEQNEKIFAPRGLL
LTDPVERGMRVIEISIYEDRCSSGSSSSGSSSGSGSSSGGGGGAGAR*
>ENSG00000254647|ENST00000397262
MALWMRLLPLLALLALWGPDPAAAFVNQHLCGSHLVEALYLVCGERGFFYTPKTRREAED
LQVGQVELGGGPGAGSLQPLALEGSLQKRGIVEQCCTSICSLYQLENYCN*
>ENSG00000109321|ENST00000395748
MRAPLLPPAPVVLSLLILGSGHYAAGLDLNDTYSGKREPFSGDHSADGFEVTSRSEMSSG
SEISPVSEMPSSSEPSSGADYDYSEEYDNEPQIPGYIVDDSVRVEQVVKPPQNKTESENT
SDKPKRKKKGGKNGKNRRNRKKKNPCNAEFQNFCIHGECKYIEHLEAVTCKCQQEYFGER
CGEKSMKTHSMIDSSLSKIALAAIAAFMSAVILTAVAVITVQLRRQYVRKYEGEAEERKK
LRQENGNVHAIA*

>ENSG00000082805|ENST00000397203
MYGSARSVGKVEPSSQSPGRSPRLPRSPRLGHRRTNSTGGSSGSSVGGGSGKTLSMENIQ
SLNAAYATSGPMYLSDHENVGSETPKSTMTLGRSGGRLPYGVRMTAMGSSPNIASSGVAS
DTIAFGEHHLPPVSMASTVPHSLRQARDNTIMDLQTQLKEVLRENDLLRKDVEVKESKLS
SSMNSIKTFWSPELKKERALRKDEASKITIWKEQYRVVQEENQHMQMTIQALQDELRIQR
DLNQLFQQDSSSRTGEPCVAELTEENFQRLHAEHERQAKELFLLRKTLEEMELRIETQKQ
TLNARDESIKKLLEMLQSKGLSAKATEEDHERTRRLAEAEMHVHHLESLLEQKEKENSML
REEMHRRFENAPDSAKTKALQTVIEMKDSKISSMERGLRDLEEEIQMLKSNGALSTEERE
EEMKQMEVYRSHSKFMKNKIGQVKQELSRKDTELLALQTKLETLTNQFSDSKQHIEVLKE
SLTAKEQRAAILQTEVDALRLRLEEKETMLNKKTKQIQDMAEEKGTQAGEIHDLKDMLDV
KERKVNVLQKKIENLQEQLRDKEKQMSSLKERVKSLQADTTNTDTALTTLEEALAEKERT
IERLKEQRDRDEREKQEEIDNYKKDLKDLKEKVSLLQGDLSEKEASLLDLKEHASSLASS
GLKKDSRLKTLEIALEQKKEECLKMESQLKKAHEAALEARASPEMSDRIQHLEREITRYK
DESSKAQAEVDRLLEILKEVENEKNDKDKKIAELERQVKDQNKKVANLKHKEQVEKKKSA
QMLEEARRREDNLNDSSQQLQDSLRKKDDRIEELEEALRESVQITAEREMVLAQEESART
NAEKQVEELLMAMEKVKQELESMKAKLSSTQQSLAEKETHLTNLRAERRKHLEEVLEMKQ
EALLAAISEKDANIALLELSSSKKKTQEEVAALKREKDRLVQQLKQQTQNRMKLMADNYE
DDHFKSSHSNQTNHKPSPDQIIQPLLELDQNRSKLKLYIGHLTTLCHDRDPLILRGLTPP
ASYNLDDDQAAWENELQKMTRGQLQDELEKGERDNAELQEFANAILQQIADHCPDILEQV
VNALEESS*
>ENSG00000121578|ENST00000393765
MGFNLTFHLSYKFRLLLLLTLCLTVVGWATSNYFVGAIQEIPKAKEFMANFHKTLILGKG
KTLTNEASTKKVELDNCPSVSPYLRGQSKLIFKPDLTLEEVQAENPKVSRGRYRPQECKA
LQRVAILVPHRNREKHLMYLLEHLHPFLQRQQLDYGIYVIHQAEGKKFNRAKLLNVGYLE
ALKEENWDCFIFHDVDLVPENDFNLYKCEEHPKHLVVGRNSTGYRLRYSGYFGGVTALSR
EQFFKVNGFSNNYWGWGGEDDDLRLRVELQRMKISRPLPEVGKYTMVFHTRDKGNEVNAE
RMKLLHQVSRVWRTDGLSSCSYKLVSVEHNPLYINITVDFWFGA*
>ENSG00000164574|ENST00000377661
MRRKEKRLLQAVALVLAALVLLPNVGLWALYRERQPDGTPGGSGAAVAPAAGQGSHSRQK
KTFFLGDGQKLKDWHDKEAIRRDAQRVGNGEQGRPYPMTDAERVDQAYRENGFNIYVSDK
ISLNRSLPDIRHPNCNSKRYLETLPNTSIIIPFHNEGWSSLLRTVHSVLNRSPPELVAEI
VLVDDFSDRDRIARNRKTIVCPMIDVIDHDDFRYETQAGDAMRGAFDWEMYYKRIPIPPE
LQKADPSDPFESPVMAGGLFAVDRKWFWELGGYDPGLEIWGGEQYEISFKVWMCGGRMED
IPCSRVGHIYRKYVPYKVPAGVSLARNLKRVAEVWMDEYAEYIYQRRPEYRHLSAGDVAV
QKKLRSSLNCKSFKWFMTKIAWDLPKFYPPVEPPAAAWGEIRNVGTGLCADTKHGALGSP
LRLEGCVRGRGEAAWNNMQVFTFTWREDIRPGDPQHTKKFCFDAISHTSPVTLYDCHSMK
GNQLWKYRKDKTLYHPVSGSCMDCSESDHRIFMNTCNPSSLTQQWLFEHTNSTVLEKFNR
N*
>ENSG00000136156|ENST00000378549
MVKVTFNSALAQKEAKKDEPKSGEEALIIPPDAVAVDCKDPDDVVPVGQRRAWCWCMCFG
LAFMLAGVILGGAYLYKYFALQAGTYLPQSYLIHEHMVITDRIENIDHLGFFIYRLCHDK
ETYKLQRRETIKGIQKREASNCFAIRHFENKFAVETLICS*
>ENSG00000171365|ENST00000376108
MDFLEEPIPGVGTYDDFNTIDWVREKSRDRDRHREITNKSKESTWALIHSVSDAFSGWLL

MLLIGLLSGSLAGLIDISAHWMTDLKEGICTGGFWFNHEHCCWNSEHVTFEERDKCPEWN
SWSQLIISTDEGAFAYIVNYFMYVLWALLFAFLAVSLVKVFAPYACGSGIPEIKTILSGF
IIRGYLGKWTLVIKTITLVLAVSSGLSLGKEGPLVHVACCCGNILCHCFNKYRKNEAKRR
EVLSAAAAAGVSVAFGAPIGGVLFSLEEVSYYFPLKTLWRSFFAALVAAFTLRSINPFGN
SRLVLFYVEFHTPWHLFELVPFILLGIFGGLWGALFIRTNIAWCRKRKTTQLGKYPVIEV
LVVTAITAILAFPNEYTRMSTSELISELFNDCGLLDSSKLCDYENRFNTSKGGELPDRPA
GVGVYSAMWQLALTLILKIVITIFTFGMKIPSGLFIPSMAVGAIAGRLLGVGMEQLAYYH
QEWTVFNSWCSQGADCITPGLYAMVGAAACLGGVTRMTVSLVVIMFELTGGLEYIVPLMA
AAMTSKWVADALGREGIYDAHIRLNGYPFLEAKEEFAHKTLAMDVMKPRRNDPLLTVLTQ
DSMTVEDVETIISETTYSGFPVVVSRESQRLVGFVLRRDLIISIENARKKQDGVVSTSII
YFTEHSPPLPPYTPPTLKLRNILDLSPFTVTDLTPMEIVVDIFRKLGLRQCLVTHNGRLL
GIITKKDVLKHIAQMANQDPDSILFN*
>ENSG00000173947|ENST00000369738
MCFSRADAADNYPFGTCQQRKLFPHFHPPNLIGNKFVPLRGSPHRGPGCYFSDGYGLAYD
LSKIPTSIKGYTLGARTAVRFKPIQKEMTPHAGRYQKVSPQQEKHKQNFAPFNVLVPRFK
NYPKDTYYPSPGAYNPEKKPPPKIAWPMKFGSPDWAQVPCLQKRTLKAELSTDKDFRKHR
NRVAYLSLYYN*
>ENSG00000162236|ENST00000394690
MSCRDRTQEFLSACKSLQTRQNGIQTNKPALRAVRQRSEFTLMAKRIGKDLSNTFAKLEK
LTILAKRKSLFDDKAVEIEELTYIIKQDINSLNKQIAQLQDFVRAKGSQSGRHLQTHSNT
IVVSLQSKLASMSNDFKSVLEVRTENLKQQRSRREQFSRAPVSALPLAPNHLGGGAVVLG
AESHASKDVAIDMMDSRTSQQLQLIDEQDSYIQSRADTMQNIESTIVELGSIFQQLAHMV
KEQEETIQRIDENVLGAQLDVEAAHSEILKYFQSVTSNRWLMVKIFLILIVFFIIFVVFL
A*
>ENSG00000172780|ENST00000393307
MAGPGPGPGDPDEQYDFLFKLVLVGDASVGKTCVVQRFKTGAFSERQGSTIGVDFTMKTL
EIQGKRVKLQIWDTAGQERFRTITQSYYRSANGAILAYDITKRSSFLSVPHWIEDVRKYA
GSNIVQLLIGNKSDLSELREVSLAEAQSLAEHYDILCAIETSAKDSSNVEEAFLRVATEL
IMRHGGPLFSEKSPDHIQLNSKDIGEGWGCGC*
>ENSG00000128274|ENST00000381278
MSKPPDLLLRLLRGAPRQRVCTLFIIGFKFTFFVSIMIYWHVVGEPKEKGQLYNLPAEIP
CPTLTPPTPPSHGPTPGNIFFLETSDRTNPNFLFMCSVESAARTHPESHVLVLMKGLPGG
NASLPRHLGISLLSCFPNVQMLPLDLRELFRDTPLADWYAAVQGRWEPYLLPVLSDASRI
ALMWKFGGIYLDTDFIVLKNLRNLTNVLGTQSRYVLNGAFLAFERRHEFMALCMRDFVDH
YNGWIWGHQGPQLLTRVFKKWCSIRSLAESRACRGVTTLPPEAFYPIPWQDWKKYFEDIN
PEELPRLLSATYAVHVWNKKSQGTRFEATSRALLAQLHARYCPTTHEAMKMYL*
>ENSG00000122126|ENST00000357121
MEPPLPVGAQPLATVEGMEMKGPLREPCALTLAQRNGQYELIIQLHEKEQHVQDIIPINS
HFRCVQEAEETLLIDIASNSGCKIRVQGDWIRERRFEIPDEEHCLKFLSAVLAAQKAQSQ
LLVPEQKDSSSWYQKLDTKDKPSVFSGLLGFEDNFSSMNLDKKINSQNQPTGIHREPPPP
PFSVNKMLPREKEASNKEQPKVTNTMRKLFVPNTQSGQREGLIKHILAKREKEYVNIQTF
RFFVGTWNVNGQSPDSGLEPWLNCDPNPPDIYCIGFQELDLSTEAFFYFESVKEQEWSMA
VERGLHSKAKYKKVQLVRLVGMMLLIFARKDQCRYIRDIATETVGTGIMGKMGNKGGVAV
RFVFHNTTFCIVNSHLAAHVEDFERRNQDYKDICARMSFVVPNQTLPQLNIMKHEVVIWL

GDLNYRLCMPDANEVKSLINKKDLQRLLKFDQLNIQRTQKKAFVDFNEGEIKFIPTYKYD
SKTDRWDSSGKCRVPAWCDRILWRGTNVNQLNYRSHMELKTSDHKPVSALFHIGVKVVDE
RRYRKVFEDSVRIMDRMENDFLPSLELSRREFVFENVKFRQLQKEKFQISNNGQVPCHFS
FIPKLNDSQYCKPWLRAEPFEGYLEPNETVDISLDVYVSKDSVTILNSGEDKIEDILVLH
LDRGKDYFLTISGNYLPSCFGTSLEALCRMKRPIREVPVTKLIDLEKSLLQMVPLDEGAS
ERPLQVPKEIWLLVDHLFKYACHQEDLFQTPGMQEELQQIIDCLDTSIPETIPGSNHSVA
EALLIFLEALPEPVICYELYQRCLDSAYDPRICRQVISQLPRCHRNVFRYLMAFLRELLK
FSEYNSVNANMIATLFTSLLLRPPPNLMARQTPSDRQRAIQFLLGFLLGSEED*
>ENSG00000175893|ENST00000380916
MGLRIHFVVDPHGWCCMGLIVFVWLYNIVLIPKIVLFPHYEEGHIPGILIIIFYGISIFC
LVALVRASITDPGRLPENPKIPHGEREFWELCNKCNLMRPKRSHHCSRCGHCVRRMDHHC
PWINNCVGEDNHWLFLQLCFYTELLTCYALMFSFCHYYYFLPLKKRNLDLFVFRHELAIM
RLAAFMGITMLVGITGLFYTQLIGIITDTTSIEKMSNCCEDISRPRKPWQQTFSEVFGTR
WKILWFIPFRQRQPLRVPYHFANHV*
>ENSG00000237541|ENST00000374940
MILNKALLLGALALTAVMSPCGGEDIVADHVASYGVNFYQSHGPSGQYTHEFDGDEEFYV
DLETKETVWQLPMFSKFISFDPQSALRNMAVGKHTLEFMMRQSNSTAATNEVPEVTVFSK
FPVTLGQPNTLICLVDNIFPPVVNITWLSNGHSVTEGVSETSFLSKSDHSFFKISYLTFL
PSADEIYDCKVEHWGLDEPLLKHWEPEIPAPMSELTETLVCALGLSVGLMGIVVGTVFII
QGLRSVGASRHQGLL*
>ENSG00000162654|ENST00000355754
MGERTLHAAVPTPGYPESESIMMAPICLVENQEEQLTVNSKALEILDKISQPVVVVAIVG
LYRTGKSYLMNRLAGKRNGFPLGSTVQSETKGIWMWCVPHLSKPNHTLVLLDTEGLGDVE
KSNPKNDSWIFALAVLLSSSFVYNSVSTINHQALEQLHYVTELAELIRAKSCPRPDEAED
SSEFASFFPDFIWTVRDFTLELKLDGNPITEDEYLENALKLIPGKNPKIQNSNMPRECIR
HFFRKRKCFVFDRPTNDKQYLNHMDEVPEENLERHFLMQSDNFCSYIFTHAKTKTLREGI
IVTGKRLGTLVVTYVDAINSGAVPCLENAVTALAQLENPAAVQRAADHYSQQMAQQLRLP
TDTLQELLDVHAACEREAIAVFMEHSFKDENHEFQKKLVDTIEKKKGDFVLQNEEASAKY
CQAELKRLSEHLTESILRGIFSVPGGHNLYLEEKKQVEWDYKLVPRKGVKANEVLQNFLQ
SQVVVEESILQSDKALTAGEKAIAAERAMKEAAEKEQELLREKQKEQQQMMEAQERSFQE
YMAQMEKKLEEERENLLREHERLLKHKLKVQEEMLKEEFQKKSEQLNKEINQLKEKIEST
KNEQLRLLKILDMASNIMIVTLPGASKLLGVGTKYLGSRI*
>ENSG00000160282|ENST00000397748
MSQLVECVPNFSEGKNQEVIDAISGAITQTPGCVLLDVDAGPSTNRTVYTFVGPPECVVE
GALNAARVASRLIDMSRHQGEHPRMGALDVCPFIPVRGVSVDECVLCAQAFGQRLAEELD
VPVYLYGEAARMDSRRTLPAIRAGEYEALPKKLQQADWAPDFGPSSFVPSWGATATGARK
FLIAFNINLLGTKEQAHRIALNLREQGRGKDQPGRLKKVQGIGWYLDEKNLAQVSTNLLD
FEVTALHTVYEETCREAQELSLPVVGSQLVGLVPLKALLDAAAFYCEKENLFILEEEQRI
RLVVSRLGLDSLCPFSPKERIIEYLVPERGPERGLGSKSLRAFVGEVGARSAAPGGGSVA
AAAAAMGAALGSMVGLMTYGRRQFQSLDTTMRRLIPPFREASAKLTTLVDADAEAFTAYL
EAMRLPKNTPEEKDRRTAALQEGLRRAVSVPLTLAETVASLWPALQELARCGNLACRSDL
QVAAKALEMGVFGAYFNVLINLRDITDEAFKDQPPAGSQDPGCTGAGLLGDPAGVTVREA
SPGSVAPPSPIPRGQSCDLETPGTAGPSTLEG*
>ENSG00000240247|ENST00000382689

MRTLAILAAILLVALQAQAEPLQARADEVAAAPEQIAADIPEVVVSLAWDESLAPKHPGS
RKNMACYCRIPACIAGERRYGTCIYQGRLWAFCC*
>ENSG00000197586|ENST00000354989
MQPQHGPWQTRMRKISNHGSLRVAKVAYPLGLCVGVFIYVAYIKWHRATATQAFFSITRA
APGARWGQQAHSPLGTAADGHEVFYGIMFDAGSTGTRVHVFQFTRPPRETPTLTHETFKA
LKPGLSAYADDVEKSAQGIRELLDVAKQDIPFDFWKATPLVLKATAGLRLLPGEKAQKLL
QKVKKVFKASPFLVGDDCVSIMNGTDEGVSAWITINFLTGSLKTPGGSSVGMLDLGGGST
QIAFLPRVEGTLQASPPGYLTALRMFNRTYKLYSYSYLGLGLMSARLAILGGVEGQPAKD
GKELVSPCLSPSFKGEWEHAEVTYRVSGQKAAASLHELCAARVSEVLQNRVHRTEEVKHV
DFYAFSYYYDLAAGVGLIDAEKGGSLVVGDFEIAAKYVCRTLETQPQSSPFSCMDLTYVS
LLLQEFGFPRSKVLKLTRKIDNVETSWALGAIFHYIDSLNRQKSPAS*
>ENSG00000177674|ENST00000400895
MELPAVNLKVILLGHWLLTTCFWRHFSAKPRLETIELTCALCKLRSAAHRATAGLHCILR
LLCLGQLHHPGLGRVGCGSAGLHRRHKHVSGWLAGHHLPGHRAHQHLLPAGQPHGHGPLW
RGHGHPQLAAQAALLLLRLPHVPGARG*
>ENSG00000231679|ENST00000383126
MVCLKLPGGSSLAALTVTLMVLSSRLAFAGDTRPRFLELRKSECHFFNGTERVRYLDRYF
HNQEEFLRFDSDVGEYRAVTELGRPVAESWNSQKDLLEQKRGRVDNYCRHNYGVGESFTV
QRRVHPQVTVYPAKTQPLQHHNLLVCSVSGFYPGSIEVRWFRNGQEEKAGVVSTGLIQNG
DWTFQTLVMLETVPRSGEVYTCQVEHPSVTSALTVEWRARSESAQSKMLSGVGGFVLGLL
FLGAGLFIYFRNQKGHSGLQPTGFLS*
>ENSG00000123106|ENST00000381259
MDDDDFGGFEAAETFDGGSGETQTTSPAIPWAAFPAVSGVHLSPSSPEIVLDRDHSSSIG
CLSSDAIISSPENTHAANSIVSQTIPKAQIQQSTHTHLDISLFPLGLTDEKSNGTIALVD
DSEDPGANVSNIQLQQKISSLEIKLKVSEEEKQRIKQDVESLMEKHNVLEKGFLKEKEQE
AISFQDRYKELQEKHKQELEDMRKAGHEALSIIVDEYKALLQSSVKQQVEAIEKQYISAI
EKQAHKCEELLNAQHQRLLEMLDTEKELLKEKIKEALIQQSQEQKEILEKCLEEERQRNK
EALVSAAKLEKEAVKDAVLKVVEEERKNLEKAHAEERELWKTEHAKDQEKVSQEIQKAIQ
EQRKISQETVKAAIIEEQKRSEKAVEEAVKRTRDELIEYIKEQKRLDQVIRQRSLSSLEL
FLSCAQKQLSALIATEPVDIE*
>ENSG00000169359|ENST00000392845
MSPTISHKDSSRQRRPGNFSHSLDMKSGPLPPGGWDDSHLDSAGREGDREALLGDTGTGD
FLKAPQSFRAELSSILLLLFLYVLQGIPLGLAGSIPLILQSKNVSYTDQAFFSFVFWPFS
LKLLWAPLVDAVYVKNFGRRKSWLVPTQYILGLFMIYLSTQVDRLLGNTDDRTPDVIALT
VAFFLFEFLAATQDIAVDGWALTMLSRENVGYASTCNSVGQTAGYFLGNVLFLALESADF
CNKYLRFQPQPRGIVTLSDFLFFWGTVFLITTTLVALLKKENEVSVVKEETQGITDTYKL
LFAIIKMPAVLTFCLLILTAKIGFSAADAVTGLKLVEEGVPKEHLALLAVPMVPLQIILP
LIISKYTAGPQPLNTFYKAMPYRLLLGLEYALLVWWTPKVEHQGGFPIYYYIVVLLSYAL
HQVTVYSMYVSIMAFNAKVSDPLIGGTYMTLLNTVSNLGGNWPSTVALWLVDPLTVKECV
GASNQNCRTPDAVELCKKLGGSCVTALDGYYVESIICVFIGFGWWFFLGPKFKKLQDEGS
SSWKCKRNN*
>ENSG00000132692|ENST00000361588
MAQLFLPLLAALVLAQAPAALADVLEGDSSEDRAFRVRIAGDAPLQGVLGGALTIPCHVH
YLRPPPSRRAVLGSPRVKWTFLSRGREAEVLVARGVRVKVNEAYRFRVALPAYPASLTDV

SLALSELRPNDSGIYRCEVQHGIDDSSDAVEVKVKGVVFLYREGSARYAFSFSGAQEACA
RIGAHIATPEQLYAAYLGGYEQCDAGWLSDQTVRYPIQTPREACYGDMDGFPGVRNYGVV
DPDDLYDVYCYAEDLNGELFLGDPPEKLTLEEARAYCQERGAEIATTGQLYAAWDGGLDH
CSPGWLADGSVRYPIVTPSQRCGGGLPGVKTLFLFPNQTGFPNKHSRFNVYCFRDSAQPS
AIPEASNPASNPASDGLEAIVTVTETLEELQLPQEATESESRGAIYSIPIMEDGGGGSST
PEDPAEAPRTLLEFETQSMVPPTGFSEEEGKALEEEEKYEDEEEKEEEEEEEEEVEDEALW
AWPSELSSPGPEASLPTEPAAQEESLSQAPARAVLQPGASPLPDGESEASRPPRVHGPPT
ETLPTPRERNLASPSPSTLVEAREVGEATGGPELSGVPRGESEETGSSEGAPSLLPATRA
PEGTRELEAPSEDNSGRTAPAGTSVQAQPVLPTDSASRGGVAVVPASGNSAQGSTALSIL
LLFFPLQLWVT*
>ENSG00000111728|ENST00000396037
MSPCGRARRQTSRGAMAVLAWKFPRTRLPMGASALCVVVLCWLYIFPVYRLPNEKEIVQG
VLQQGTAWRRNQTAARAFRKQMEDCCDPAHLFAMTKMNSPMGKSMWYDGEFLYSFTIDNS
TYSLFPQATPFQLPLKKCAVVGNGGILKKSGCGRQIDEANFVMRCNLPPLSSEYTKDVGS
KSQLVTANPSIIRQRFQNLLWSRKTFVDNMKIYNHSYIYMPAFSMKTGTEPSLRVYYTLS
DVGANQTVLFANPNFLRSIGKFWKSRGIHAKRLSTGLFLVSAALGLCEEVAIYGFWPFSV
NMHEQPISHHYYDNVLPFSGFHAMPEEFLQLWYLHKIGALRMQLDPCEDTSLQPTS*
>ENSG00000114646|ENST00000383738
MGRAGGGGPGRGPPPLLLFLGAALVLASGAVPAREAGSAVEAEELVKGSPAWEPPANDTR
EEAGPPAAGEDEASWTAPGGELAGPEEVLQESAAVTGTAWLEADSPGLGGVTAEAGSGDA
QALPATLQAPHEVLGQSIMPPAIPEATEASGPPSPTPGDKLSPASELPKESPLEVWLNLG
GSTPDPQGPELTYPFQGTLEPQPASDIIDIDYFEGLDGEGRGADLGSFPGSPGTSENHPD
TEGETPSWSLLDLYDDFTPFDESDFYPTTSFYDDLDEEEEEEEDDKDAVGGGDLEDENEL
LVPTGKPGLGPGTGQPTSRWHAVPPQHTLGSVPGSSIALRPRPGEPGRDLASSENGTECR
SGFVRHNGSCRSVCDLFPSYCHNGGQCYLVENIGAFCRCNTQDYIWHKGMRCESIITDFQ
VMCVAVGSAALVLLLLFMMTVFFAKKLYLLKTENTKLRRTNKFRTPSELHNDNFSLSTIA
EGSHPNVRKLCNTPRTSSPHARALAHYDNVICQDDPSAPHKIQEVLKSCLKEEESFNIQN
SMSPKLEGGKGDQADLDVNCLQNNLT*
>ENSG00000126091|ENST00000372372
MGLLVFVRNLLLALCLFLVLGFLYYSAWKLHLLQWEEDSKYDRLGFLLNLDSKLSPRTLC
TVVFGLDCILESPGEPKKLLMPASHPLEILKSLSEDTAFALGFLKLPRPAELATKYANFS
EGACKPGYASALMTAIFPRFSKPAPMFLDDSFRKWARIREFVPPFGIKGQDNLIKAILSV
TKEYRLTPALDSLRCRRCIIVGNGGVLANKSLGSRIDDYDIVVRLNSAPVKGFEKDVGSK
TTLRITYPEGAMQRPEQYERDSLFVLAGFKWQDFKWLKYIVYKERVSASDGFWKSVATRV
PKEPPEIRILNPYFIQEAAFTLIGLPFNNGLMGRGNIPTLGSVAVTMALHGCDEVAVAGF
GYDMSTPNAPLHYYETVRMAAIKESWTHNIQREKEFLRKLVKARVITDLSSGI*
>ENSG00000162236|ENST00000377897
MIPRKRYGSKNTDQGVYLGLSKTQVLSPATAGSSSSDIAPLPPPVTLVPPPPDTMSCRDR
TQEFLSACKSLQTRQNGIQTNKPALRAVRQRSEFTLMAKRIGKDLSNTFAKLEKLTILAK
RKSLFDDKAVEIEELTYIIKQDINSLNKQIAQLQDFVRAKGSQSGRHLQTHSNTIVVSLQ
SKLASMSNDFKSVLEVRTENLKQQRSRREQFSRAPVSALPLAPNHLGGGAVVLGAESHAS
KDVAIDMMDSRTSQQLQLIDEQDSYIQSRADTMQNIESTIVELGSIFQQLAHMVKEQEET
IQSVLLFPLLPALSPGSTRTC*
>ENSG00000183166|ENST00000395276

MPFHHVTAGLLYKGNYLNRSLSAGSDSEQLANISVEELDEIREAFRVLDRDGNGFISKQE
LGMAMRSLGYMPSEVELAIIMQRLDMDGDGQVDFDEFMTILGPKLVSSEGRDGFLGNTID
SIFWQFDMQRITLEELKHILYHAFRDHLTMKDIENIIINEEESLNETSGNCQTEFEGVHS
QKQNRQTCVRKSLICAFAMAFIISVMLIAANQILRSGME*
>ENSG00000172915|ENST00000400445
MASEKPGPGPGLEPQPVGLIAVGAAGGGGGGSGGGGTGGSGMGELRGASGSGSVMLPAGM
INPSVPIRNIRMKFAVLIGLIQVGEVSNRDIVETVLNLLVGGEFDLEMNFIIQDAESITC
MTELLEHCDVTCQAEIWSMFTAILRKSVRNLQTSTEVGLIEQVLLKMSAVDDMIADLLVD
MLGVLASYSITVKELKLLFSMLRGESGIWPRHAVKLLSVLNQMPQRHGPDTFFNFPGCSA
AAIALPPIAKWPYQNGFTLNTWFRMDPLNNINVDKDKPYLYCFRTSKGVGYSAHFVGNCL
IVTSLKSKGKGFQHCVKYDFQPRKWYMISIVHIYNRWRNSEIRCYVNGQLVSYGDMAWHV
NTNDSYDKCFLGSSETADANRVFCGQLGAVYVFSEALNPAQIFAIHQLGPGYKSTFKFKS
ESDIHLAEHHKQVLYDGKLASSIAFTYNAKATDAQLCLESSPKENASIFVHSPHALMLQD
VKAIVTHSIHSAIHSIGGIQVLFPLFAQLDNRQLNDSQVETTVCATLLAFLVELLKSSVA
MQEQMLGGKGFLVIGYLLEKSSRVHITRAVLEQFLSFAKYLDGLSHGAPLLKQLCDHILF
NPAIWIHTPAKVQLSLYTYLSAEFIGTATIYTTIRRVGTVLQLMHTLKYYYWVINPADSS
GITPKGLDGPRPSQKEIISLRAFMLLFLKQLILKDRGVKEDELQSILNYLLTMHEDENIH
DVLQLLVALMSEHPASMIPAFDQRNGIRVIYKLLASKSESIWVQALKVLGYFLKHLGHKR
KVEIMHTHSLFTLLGERLMLHTNTVTVTTYNTLYEILTEQVCTQVVHKPHPEPDSTVKIQ
NPMILKVVATLLKNSTPSAELMEVRRLFLSDMIKLFSNSRENRRCLLQCSVWQDWMFSLG
YINPKNSEEQKITEMVYNIFRILLYHAIKYEWGGWRVWVDTLSIAHSKVTYEAHKEYLAK
MYEEYQRQEEENIKKGKKGNVSTISGLSSQTTGAKGGMEIREIEDLSQSQSPESETDYPV
STDTRDLLMSTKVSDDILGNSDRPGSGVHVEVHDLLVDIKAEKVEATEVKLDDMDLSPET
LVGGENGALVEVESLLDNVYSAAVEKLQNNVHGSVGIIKKNEEKDNGPLITLADEKEDLP
NSSTSFLFDKIPKQEEKLLPELSSNHIIPNIQDTQVHLGVSDDLGLLAHMTGSVDLTCTS
SIIEEKEFKIHTTSDGMSSISERDLASSTKGLEYAEMTATTLETESSSSKIVPNIDAGSI
ISDTERSDDGKESGKEIRKIQTTTTTQAVQGRSITQQDRDLRVDLGFRGMPMTEEQRRQF
SPGPRTTMFRIPEFKWSPMHQRLLTDLLFALETDVHVWRSHSTKSVMDFVNSNENIIFVH
NTIHLISQMVDNIIIACGGILPLLSAATSPTGSKTELENIEVTQGMSAETAVTFLSRLMA
MVDVLVFASSLNFSEIEAEKNMSSGGLMRQCLRLVCCVAVRNCLECRQRQRDRGNKSSHG
SSKPQEVPQSVTATAASKTPLENVPGNLSPIKDPDRLLQDVDINRLRAVVFRDVDDSKQA
QFLALAVVYFISVLMVSKYRDILEPQRETTRTGSQPGRNIRQEINSPTSTVVVIPSIPHP
SLNHGFLAKLIPEQSFGHSFYKETPAAFPDTIKEKETPTPGEDIQVESSIPHTDSGIGEE
QVASILNGAELETSTGPDAMSELLSTLSSEVKKSQESLTENPSETLKPATSISSISQTKG
INVKEILKSLVAAPVEIAECGPEPIPYPDPALKRETQAILPMQFHSFDRSVVVPVKKPPP
GSLAVTTVGATTAGSGLPTGSTSNIFAATGATPKSMINTTGAVDSGSSSSSSSSSFVNGA
TSKNLPAVQTVAPMPEDSAENMSITAKLERALEKVAPLLREIFVDFAPFLSRTLLGSHGQ
ELLIEGLVCMKSSTSVVELVMLLCSQEWQNSIQKNAGLAFIELINEGRLLCHAMKDHIVR
VANEAEFILNRQRAEDVHKHAEFESQCAQYAADRREEEKMCDHLISAAKHRDHVTANQLK
QKILNILTNKHGAWGAVSHSQLHDFWRLDYWEDDLRRRRRFVRNAFGSTHAEALLKAAIE
YGTEEDVVKSKKTFRSQAIVNQNAETELMLEGDDDAVSLLQEKEIDNLAGPVVLSTPAQL
IAPVVVAKGTLSITTTEIYFEVDEDDSAFKKIDTKVLAYTEGLHGKWMFSEIRAVFSRRY
LLQNTALEVFMANRTSVMFNFPDQATVKKVVYSLPRVGVGTSYGLPQARRISLATPRQLY
KSSNMTQRWQRREISNFEYLMFLNTIAGRTYNDLNQYPVFPWVLTNYESEELDLTLPGNF

RDLSKPIGALNPKRAVFYAERYETWEDDQSPPYHYNTHYSTATSTLSWLVRIEPFTTFFL
NANDGKFDHPDRTFSSVARSWRTSQRDTSDVKELIPEFYYLPEMFVNSNGYNLGVREDEV
VVNDVDLPPWAKKPEDFVRINRMALESEFVSCQLHQWIDLIFGYKQRGPEAVRALNVFHY
LTYEGSVNLDSITDPVLREAMEAQIQNFGQTPSQLLIEPHPPRSSAMHLCFLPQSPLMFK
DQMQQDVIMVLKFPSNSPVTHVAANTLPHLTIPAVVTVTCSRLFAVNRWHNTVGLRGAPG
YSLDQAHHLPIEMDPLIANNSGVNKRQITDLVDQSIQINAHCFVVTADNRYILICGFWDK
SFRVYSTETGKLTQIVFGHWDVVTCLARSESYIGGDCYIVSGSRDATLLLWYWSGRHHII
GDNPNSSDYPAPRAVLTGHDHEVVCVSVCAELGLVISGAKEGPCLVHTITGDLLRALEGP
ENCLFPRLISVSSEGHCIIYYERGRFSNFSINGKLLAQMEINDSTRAILLSSDGQNLVTG
GDNGVVEVWQACDFKQLYIYPGCDAGIRAMDLSHDQRTLITGMASGSIVAFNIDFNRWHY
EHQNRY*
>ENSG00000134262|ENST00000369569
MPYLGSEDVVKELKKALCNPHIQADRLRYRNVIQRVIRYMTQGLDMSGVFMEMVKASATV
DIVQKKLVYLYMCTYAPLKPDLALLAINTLCKDCSDPNPMVRGLALRSMCSLRMPGVQEY
IQQPILNGLRDKASYVRRVAVLGCAKMHNLHGDSEVDGALVNELYSLLRDQDPIVVVNCL
RSLEEILKQEGGVVINKPIAHHLLNRMSKLDQWGQAEVLNFLLRYQPRSEEELFDILNLL
DSFLKSSSPGVVMGATKLFLILAKMFPHVQTDVLVRVKGPLLAACSSESRELCFVALCHV
RQILHSLPGHFSSHYKKFFCSYSEPHYIKLQKVEVLCELVNDENVQQVLEELRGYCTDVS
ADFAQAAIFAIGGIARTYTDQCVQILTELLGLRQEHITTVVVQTFRDLVWLCPQCTEAVC
QALPGCEENIQDSEGKQALIWLLGVHGERIPNAPYVLEDFVENVKSETFPAVKMELLTAL
LRLFLSRPAECQDMLGRLLYYCIEEEKDMAVRDRGLFYYRLLLVGIDEVKRILCSPKSDP
TLGLLEDPAERPVNSWASDFNTLVPVYGKAHWATISKCQGAERCDPELPKTSSFAASGPL
IPEENKERVQELPDSGALMLVPNRQLTADYFEKTWLSLKVAHQQVLPWRGEFHPDTLQMA
LQVVNIQTIAMSRAGSRPWKAYLSAQDDTGCLFLTELLLEPGNSEMQISVKQNEARTETL
NSFISVLETVIGTIEEIKS*
>ENSG00000204632|ENST00000360323
MVVMAPRTLFLLLSGALTLTETWAGSHSMRYFSAAVSRPGRGEPRFIAMGYVDDTQFVRF
DSDSACPRMEPRAPWVEQEGPEYWEEETRNTKAHAQTDRMNLQTLRGYYNQSEASSHTLQ
WMIGCDLGSDGRLLRGYEQYAYDGKDYLALNEDLRSWTAADTAAQISKRKCEAANVAEQR
RAYLEGTCVEWLHRYLENGKEMLQRADPPKTHVTHHPVFDYEATLRCWALGFYPAEIILT
WQRDGEDQTQDVELVETRPAGDGTFQKWAAVVVPSGEEQRYTCHVQHEGLPEPLMLRWKQ
SSLPTIPIMGIVAGLVVLAAVVTGAAVAAVLWRKKSSD*
>ENSG00000275066|ENST00000614941
MALRPGAGSGGGGAAGAGAGSAGGGGFMFPVAGGIRPPQAGLMPMQQQGFPMVSVMQPNM
QGIMGMNYSSQMSQGPIAMQAGIPMGPMPAAGMPYLGQAPFLGMRPPGPQYTPDMQKQFA
EEQQKRFEQQQKLLEEERKRRQFEEQKQKLRLLSSVKPKTGEKSRDDALEAIKGNLDGFS
RDAKMHPTPASHPKKPGVGVFPSQDPAQPRMPPWIYNESLVPDAYKKILETTMTPTGIDT
AKLYPILMSSGLPRETLGQIWALANRTTPGKLTKEELYTVLAMIAVTQVVKPEEDDFQDF
QDASKSGSLDDSFSDFQELPASSKTSNSQHGNSAPSLLMPLPGTKALPSMDKYAVFKGIA
ADKSSENTVPPGDPGDKYSAFRELEQTAENKPLGESFAEFRSAGTDDGFTDFKTADSVSP
LEPPTKDKTFPPSFPSGTIQQKQQTQVKNPLNLADLDMFSSVNCSSEKPLSFSAVFSTSK
SVSTPQSTGSAATMTALAATKTSSLADDFGEFSLFGEYSGLAPVGEQDDFADFMAFSNSS
ISSEQKPDDKYDALKEEASPVPLTSNVGSTVKGGQNSTAASTKYDVFRQLSLEGSGLGVE
DLKDNTPSGKSDDDFADFHSSKFSSINSDKSLGEKAVAFRHTKEDSASVKSLDLPSIGGS

SVGKEDSEDALSVQFDMKLADVGGDLKHVMSDSSLDLPTVSGQHPPAAAGSGSPSATSIL
QKKETSFGSSENITMTSLSKVTTFVSEDALPETTFPALASFKDTIPQTSEQKEYENRDYK
DFTKQDLPTAERSQEATCPSPASSGASQETPNECSDDFGEFQSEKPKISKFDFLVATSQS
KMKSSEEMIKSELATFDLSVQGSHKRSLSLGDKEISRSSPSPALEQPFRDRSNTLNEKPA
LPVIRDKYKDLTGEVEENERYAYEWQRCLGSALNVIKKANDTLNGISSSSVCTEVIQSAQ
GMEYLLGVVEVYRVTKRVELGIKATAVCSEKLQQLLKDIDKVWNNLIGFMSLATLTPDEN
SLDFSSCMLRPGIKNAQELACGVCLLNVDSRSRKEEKPAEEHPKKAFNSETDSFKLAYGG
HQYHASCANFWINCVEPKPPGLVLPDLL*
>ENSG00000134909|ENST00000527272
MKSRPTKQKLKQRGILKERVFGCDLGEHLLNSGFEVPQVLQSCTAFIERYGIVDGIYRLS
GVASNIQRLRHEFDSEHVPDLTKEPYVQDIHSVGSLCKLYFRELPNPLLTYQLYEKFSDA
VSAATDEERLIKIHDVIQQLPPPHYRTLEFLMRHLSLLADYCSITNMHAKNLAIVWAPNL
LRSKQIESACFSGTAAFMEVRIQSVVVEFILNHVDVLFSGRISMAMQEGAASLSRPKSLL
VSSPSTKLLTLEEAQARTQAQVNSPIVTENKYIEVGEGPAALQGKFHTIIEFPLERKRPQ
NKMKKSPVGSWRSFFNLGKSSSVSKRKLQRNESEPSEMKAMALKGGRAEGTLRSAKSEES
LTSLHAVDGDSKLFRPRRPRSSSDALSASFNGEMLGNRCNSYDNLPHDNESEEEGGLLHI
PALMSPHSAEDVDLSPPDIGVASLDFDPMSFQCSPPKAESECLESGASFLDSPGYSKDKP
SANKKDAETGSSQCQTPGSTASSEPVSPLQEKLSPFFTLDLSPTEDKSSKPSSFTEKVVY
AFSPKIGRKLSKSPSMSISEPISVTLPPRVSEVIGTVSNTTAQNASSSTWDKCVEERDAT
NRSPTQIVKMKTNETVAQEAYESEVQPLDQVAAEEVELPGKEDQSVSSSQSKAVASGQTQ
TGAVTHDPPQDSVPVSSVSLIPPPPPPKNVARMLALALAESAQQASTQSLKRPGTSQAGY
TNYGDIAVATTEDNLSSSYSAVALDKAYFQTDRPAEQFHLQNNAPGNCDHPLPETTATGD
PTHSNTTESGEQHHQVDLTGNQPHQAYLSGDPEKARITSVPLDSEKSDDHVSFPEDQSGK
NSMPTVSFLDQDQSPPRFYSGDQPPSYLGASVDKLHHPLEFADKSPTPPNLPSDKIYPPS
GSPEENTSTATMTYMTTTPATAQMSTKEASWDVAEQPTTADFAAATLQRTHRTNRPLPPP
PSQRSAEQPPVVGQVQAATNIGLNNSHKVQGVVPVPERPPEPRAMDDPASAFISDSGAAA
AQCPMATAVQPGLPEKVRDGARVPLLHLRAESVPAHPCGFPAPLPPTRMMESKMIAAIHS
SSADATSSSNYHSFVTASSTSVDDALPLPLPVPQPKHASQKTVYSSFARPDVTTEPFGPD
NCLHFNMTPNCQYRPQSVPPHHNKLEQHQVYGARSEPPASMGLRYNTYVAPGRNASGHHS
KPCSRVEYVSSLSSSVRNTCYPEDIPPYPTIRRVQSLHAPPSSMIRSVPISRTEVPPDDE
PAYCPRPLYQYKPYQSSQARSDYHVTQLQPYFENGRVHYRYSPYSSSSSSYYSPDGALCD
VDAYGTVQLRPLHRLPNRDFAFYNPRLQGKSLYSYAGLAPRPRANVTGYFSPNDHNVVSM
PPAADVKHTYTSWDLEDMEKYRMQSIRRESRARQKVKGPVMSQYDNMTPAVQDDLGGIYV
IHLRSKSDPGKTGLLSVAEGKESRHAAKAISPEGEDRFYRRHPEAEMDRAHHHGGHGSTQ
PEKPSLPQKQSSLRSRKLPDMGCSLPEHRAHQEASHRQFCESKNGPPYPQGAGQLDYGSK
GIPDTSEPVSYHNSGVKYAASGQESLRLNHKEVRLSKEMERPWVRQPSAPEKHSRDCYKE
EEHLTQSIVPPPKPERSHSLKLHHTQNVERDPSVLYQYQPHGKRQSSVTVVSQYDNLEDY
HSLPQHQRGVFGGGGMGTYVPPGFPHPQSRTYATALGQGAFLPAELSLQHPETQIHAE*
>ENSG00000284369|ENST00000639557
MGWVGGRRRDSASPPGRSRSAADDINPAPANMEGGGGSVAVAGLGARGSGAAAATVRELL
QDGCYSDFLNEDFDVKTYTSQSIHQAVIAEQLAKLAQGISQLDRELHLQVVARHEDLLAQ
ATGIESLEGVLQMMQTRIGALQGAVDRIKAKIVEPYNKIVARTAQLARLQVACDLLRRII
RILNLSKRLQGQLQGGSREITKAAQSLNELDYLSQGIDLSGIEVIENDLLFIARARLEVE
NQAKRLLEQGLETQNPTQVGTALQVFYNLGTLKDTITSVVDGYCATLEENINSALDIKVL

TQPSQSAVRGGPGRSTMPTPGNTAALRASLWTNMEKLMDHIYAVCGQVQHLQKVLAKKRD
PVSHICFIEEIVKDGQPEIFYTFWNSVTQALSSQFHMATNSSMFLKQAFEGEYPKLLRLY
NDLWKRLQQYSQHIQGNFNASGTTDLYVDLQHMEDDAQDIFIPKKPDYDPEKALKDSLQP
YEAAYLSKSLSRLFDPINLVFPPGGRNPPSSDELDGIIKTIASELNVAAVDTNLTLAVSK
NVAKTIQLYSVKSEQLLSTQGDASQVIGPLTEGQRRNVAVVNSLYKLHQSVTKVVSSQSS
FPLAAEQTIISALKAIHALMENAVQPLLTSVGDAIEAIIITMHQEDFSGSLSSSGKPDVP
CSLYMKELQGFIARVMSDYFKHFECLDFVFDNTEAIAQRAVELFIRHASLIRPLGEGGKM
RLAADFAQMELAVGPFCRRVSDLGKSYRMLRSFRPLLFQASEHVASSPALGDVIPFSIII
QFLFTRAPAELKSPFQRAEWSHTRFSQWLDDHPSEKDRLLLIRGALEAYVQSVRSREGKE
FAPVYPIMVQLLQKAMSALQ*
>ENSG00000169255|ENST00000473285
MASALWTVLPSRMSLRSLKWSLLLLSLLSFFVMWYLSLPHYNVIERVNWMYFYEYEPIYR
QDFHFTLREHSNCSHQNPFLVILVTSHPSDVKARQAIRVTWGEKKSWWGYEVLTFFLLGQ
EAEKEDKMLALSLEDEHLLYGDIIRQDFLDTYNNLTLKTIMAFRWVTEFCPNAKYVMKTD
TDVFINTGNLVKYLLNLNHSEKFFTGYPLIDNYSYRGFYQKTHISYQEYPFKVFPPYCSG
LGYIMSRDLVPRIYEMMGHVKPIKFEDVYVGICLNLLKVNIHIPEDTNLFFLYRIHLDVC
QLRRVIAAHGFSSKEIITFWQVMLRNTTCHY*
>ENSG00000188175|ENST00000453812
MGQDAFMEPFGDTLGVFQCKIYLLLFDIVANCLLLRFKLSVSSYEIWKKGACSGLKVTVP
SHTVHGVRGQALYLPVHYGFHTPASDIQIIWLFERPHTMPKYLLGSVNKSVVPDLEYQHK
FTMMPPNASLLINPLQFPDEGNYIVKVNIQGNGTLSASQKIQVTVDDPVTKPVVQIHPPS
GAVEYVGNMTLTCHVEGGTRLAYQWLKNGRPVHTSSTYSFSPQNNTLHIAPVTKEDIGNY
SCLVRNPVSEMESDIIMPIIYYGPYGLQVNSDKGLKVGEVFTVDLGEAILFDCSADSHPP
NTYSWIRRTDNTTYIIKHGPRLEVASEKVAQKTMDYVCCAYNNITGRQDETHFTVIITSV
GLEKLAQKGKSLSPLASITGISLFLIISMCLLFLWKKYQPYKVIKQKLEGRPETEYRKAQ
TFSGHEDALDDFGIYEFVAFPDVSGVSRIPSRSVPASDCVSGQDLHSTVYEVIQHIPAQQ
QDHPE*
>ENSG00000282725|ENST00000614702
MNRFRVSKFRHTEARPPRRESWISDIRAGTAPSCRNHIKSSCSLIAFNSDRPGVLGIVPL
QGQGEDKRRVAHLGCHSDLVTDLDFSPFDDFLLATGSADRTVKLWRLPGPGQALPSAPGV
VLGPEDLPVEVLQFHPTSDGILVSAAGTTVKVWDAAKQQPLTELAAHGDLVQSAVWSRDG
ALVGTACKDKQLRIFDPRTKPRASQSTQAHENSRDSRLAWMGTWEHLVSTGFNQMREREV
KLWDTRFFSSALASLTLDTSLGCLVPLLDPDSGLLVLAGKGERQLYCYEVVPQQPALSPV
TQCVLESVLRGAALVPRQALAVMSCEVLRVLQLSDTAIVPIGYHVPRKAVEFHEDLFPDT
AGCVPATDPHSWWAGDNQQVQKVSLNPACRPHPSFTSCLVPPAEPLPDTAQPAVMETPVG
DADASEGFSSPPSSLTSPSTPSSLGPSLSSTSGIGTSPSLRSLQSLLGPSSKFRHAQGTV
LHRDSHITNLKGLNLTTPGESDGFCANKLRVAVPLLSSGGQVAVLELRKPGRLPDTALPT
LQNGAAVTDLAWDPFDPHRLAVAGEDARIRLWRVPAEGLEEVLTTPETVLTGHTEKICSL
RFHPLAANVLASSSYDLTVRIWDLQAGADRLKLQGHQDQIFSLAWSPDGQQLATVCKDGR
VRVYRPRSGPEPLQEGPGPKGGRGARIVWVCDGRCLLVSGFDSQSERQLLLYEAEALAGG
PLAVLGLDVAPSTLLPSYDPDTGLVLLTGKGDTRVFLYELLPESPFFLECNSFTSPDPHK
GLVLLPKTECDVREVELMRCLRLRQSSLEPVAFRLPRVRKEFFQDDVFPDTAVIWEPVLS
AEAWLQGANGQPWLLSLQPPDMSPVSQAPREAPARRAPSSAQYLEEKSDQQKKEELLNAM
VAKLGNREDPLPQDSFEGVDEDEWD*

>ENSG00000169550|ENST00000455601
MLALAKILLISTLFYSLLSGSHGKENQDINTTQNIAEVFKTMENKPISLESEANLNSDKE
NITTSNLKASHSPPLNLPNNSHGITDFSSNSSAEHSLGSLKPTSTISTSPPLIHSFVSKV
PWNAPIADEDLLPISAHPNATPALSSENFTWSLVNDTVKTPDNSSITVSILSSEPTSPSV
TPLIVEPSGWLTTNSDSFTGFTPYQEKTTLQPTLKFTNNSKLFPNTSDPQKENRNTGIVF
GAILGAILGVSLLTLVGYLLCGKRKTDSFSHRRLYDDRNEPVLRLDNAPEPYDVSFGNSS
YYNPTLNDSAMPESEENARDGIPMDDIPPLRTSV*
>ENSG00000149541|ENST00000531383
MKLKLKNVFLAYFLVSIAGLLYALVQLGQPCDCLPPLRAAAEQLRQKDLRISQLQAELRR
PPPAPAQPPEPEALPTIYVVTPTYARLVQKAELVRLSQTLSLVPRLHWLLVEDAEGPTPL
VSGLLAASGLLFTHLVVLTPKAQRLREGEPGWVHPRGVEQRNKALDWLRGRGGAVGGEKD
PPPPGTQGVVYFADDDNTYSRELFEEMRWTRGVSVWPVGLVGGLRFEGPQVQDGRVVGFH
TAWEPSRPFPVDMAGFAVALPLLLDKPNAQFDSTAPRGHLESSLLSHLVDPKDLEPRAAN
CTRSLAVSPRLECSSAILA*
>ENSG00000172575|ENST00000559830
MGTLGKAREAPRKPSHGCRAASKARLEAKPANSPFPSHPSLAHITQFRMMVSLGHLAKGA
SLDDLIDSCIQSFDADGNLCRSNQLLQVMLTMHRIVISSAELLQKVITLYKDALAKNSPG
LCLKICYFVRYWITEFWVMFKMDASLTDTMEEFQELVKAKGEELHCRLIDTTQINARDWS
RKLTQRIKSNTSKKRKVSLLFDHLEPEELSEHLTYLEFKSFRRISFSDYQNYLVNSCVKE
NPTMERSIALCNGISQWVQLMVLSRPTPQLRAEVFIKFIQVAQKLHQLQNFNTLMAVIGG
LCHSSISRLKETSSHVPHEINKVLGEMTELLSSSRNYDNYRRAYGECTDFKIPILGVHLK
DLISLYEAMPDYLEDGKVNVHKLLALYNHISELVQLQEVAPPLEANKDLVHLLTLSLDLY
YTEDEIYELSYAREPRNHRAPSVFKNYDHDQDGYISQEEFEKIAASFPFSFCVMDKDREG
LISRDEITAYFMRASSIYSKLGLGFPHNFQETTYLKPTFCDNCAGFLWGVIKQGYRCKDC
GMNCHKQCKDLVVFECKKRAKNPVAPTENNTSVGPVSNLCSLGAKDLLHGNKYSESR*
>ENSG00000154080|ENST00000618847
MQPSEMVMNPKQVFLSVLIFGVAGLLLFMYLQVWIEEQHTGRVEKRREQKVTSGWGPVKY
LRPVPRIMSTEKIQEHITNQNPKFHMPEDVREKKENLLLNSERSTRLLTKTSHSQGGDQA
LSKSTGSPTEKLIEKRQGAKTVFNKFSNMNWPVDIHPLNKSLVKDNKWKKTEETQEKRRS
FLQEFCKKYGGVSHHQSHLFHTVSRIYVEDKHKILYCEVPKAGCSNWKRILMVLNGLASS
AYNISHNAVHYGKHLKKLDSFDLKGIYTRLNTYTKAVFVRDPMERLVSAFRDKFEHPNSY
YHPVFGKAIIKKYRPNACEEALINGSGVKFKEFIHYLLDSHRPVGMDIHWEKVSKLCYPC
LINYDFVGKFETLEEDANYFLQMIGAPKELKFPNFKDRHSSDERTNAQVVRQYLKDLTRT
ERQLIYDFYYLDYLMFNYTTPFL*
>ENSG00000143801|ENST00000472139
MISVIVVMTIFLVVLYKYRCYKFIHGWLIMSSLMLLFLFTYIYLGEVLKTYNVAMDYPTL
LLTVWNFGAVGMVCIHWKGPLVLQQAYLIMISALMALVFIKYLPEWSAWVILGAISVYDL
VAVLCPKGPLRMLVETAQERNEPIFPALIYSSAMVWTVGMAKLDPSSQGALQLPYDPEME
EDSYDSFGEPSYPEVFEPPLTGYPGEELEEEEERGVKLGLGDFIFYSVLVGKAAATGSGD
WNTTLACFVAILIGLCLTLLLLAVFKKALPALPISITFGLIFYFSTDNLVRPFMDTLASH
QLYI*
>ENSG00000183778|ENST00000615480
MAFPKMRLMYICLLVLGALCLYFSMYSLNPFKEQSFVYKKDGNFLKLPDTDCRQTPPFLV
LLVTSSHKQLAERMAIRQTWGKERMVKGKQLKTFFLLGTTSSAAETKEVDQESQRHGDII

QKDFLDVYYNLTLKTMMGIEWVHRFCPQAAFVMKTDSDMFINVDYLTELLLKKNRTTRFF
TGFLKLNEFPIRQPFSKWFVSKSEYPWDRYPPFCSGTGYVFSGDVASQVYNVSKSVPYIK
LEDVFVGLCLERLNIRLEELHSQPTFFPGGLRFSVCLFRRIVACHFIKPRTLLDYWQALE
NSRGEDCPPV*
>ENSG00000257923|ENST00000547394
MAANVGSMFQYWKRFDLQQLQRELDATATVLANRQDESEQSRKRLIEQSREFKKNTPEID
ALSKRSKEAEAAFLNVYKRLIDVPDPVPALDLGQQLQLKVQRLHDIETENQKLRETLEEY
NKEFAEVKNQEVTIKALKEKIREYEQTLKNQAETIALEKEQKLQNDFAEKERKLQETQMS
TTSKLEEAEHKVQSLQTALEKTRTELFDLKTKYDEETTAKADEIEMIMTDLERANQRAEV
AQREAETLREQLSSANHSLQLASQIQKAPDVEQAIEVLTRSSLEVELAAKEREIAQLVED
VQRLQASLTKLRENSASQISQLEQQLSAKNSTLKQLEEKLKGQADYEEVKKELNILKSME
FAPSEGAGTQDAAKPLEVLLLEKNRSLQSENAALRISNSDLSGRCAELQVRITEAVATAT
EQRELIARLEQDLSIIQSIQRPDAEGAAEHRLEKIPEPIKEATALFYGPAAPASGALPEG
QVDSLLSIISSQRERFRARNQELEAENRLAQHTLQALQSELDSLRADNIKLFEKIKFLQS
YPGRGSGSDDTELRYSSQYEERLDPFSSFSKRERQRKYLSLSPWDKATLSMGRLVLSNKM
ARTIGFFYTLFLHCLVFLVLYKLAWSESMERDCATFCAKKFADHLHKFHENDNGAAAGDL
WQ*
>ENSG00000235657|ENST00000547271
MAVMAPRTLVLLLSGALALTQTWAGSHSMRYFFTSVSRPGRGEPRFIAVGYVDDTQFVRF
DSDAASQRMEPRAPWIEQEGPEYWDGETRKVKAHSQTHRVDLGTLRGYYNQSEAGSHTVQ
RMYGCDVGSDWRFLRGYHQYAYDGKDYIALKEDLRSWTAADMAAQTTKHKWEAAHVAEQL
RAYLEGTCVEWLRRYLENGKETLQRTDAPKTHMTHHAVSDHEATLRCWALSFYPAEITLT
WQRDGEDQTQDTELVETRPAGDGTFQKWAAVVVPSGQEQRYTCHVQHEGLPKPLTLRWEP
SSQPTIPIVGIIAGLVLFGAVITGAVVAAVMWRRKSSDRKGGSYSQAASSDSAQGSDVSL
TACKV*
>ENSG00000223465|ENST00000414266
MLSRNDVKRLERSWAQGSSEQELHYASLQRLPVPSSEGPDLRGRDKRGTKEDPRADYACI
AENKPT*
>ENSG00000117983|ENST00000529681
MGAPSACRTLVLALAAMLVVPQAETQGPVEPSWENAGHTMDGGAPTSSPTRRVSFVPPVT
VFPSLSPLNPAHNGRVCSTWGDFHYKTFDGDVFRFPGLCNYVFSEHCRAAYEDFNVQLRR
GLVGSRPVVTRVVIKAQGLVLEASNGSVLINGQREELPYSRTGLLVEQSGDYIKVSIRLV
LTFLWNGEDSALLELDPKYANQTCGLCGDFNGLPAFNEFYAHNARLTPLQFGNLQKLDGP
TEQCPDPLPLPAGNCTDEEGICHRTLLGPAFAECHALVDSTAYLAACAQDLCRCPTCPCA
TFVEYSRQCAHAGGQPRNWRCPELCPRTCPLNMQHQECGSPCTDTCSNPQRAQLCEDHCV
DGCFCPPGTVLDDITHSGCLPLGQCPCTHGGRTYSPGTSFNTTCSSCTCSGGLWQCQDLP
CPGTCSVQGGAHISTYDEKLYDLHGDCSYVLSKKCADSSFTVLAELRKCGLTDNENCLKA
VTLSLDGGDTAIRVQADGGVFLNSIYTQLPLSAANITLFTPSSFFIVVQTGLGLQLLVQL
VPLMQVFVRLDPAHQGQMCGLCGNFNQNQADDFTALSGVVEATGAAFANTWKAQAACANA
RNSFEDPCSLSVENENYARHWCSRLTDPNSAFSRCHSIINPKPFHSNCMFDTCNCERSED
CLCAALSSYVHACAAKGVQLSDWRDGVCTKYMQNCPKSQRYAYVVDACQPTCRGLSEADV
TCSVSFVPVDGCTCPAGTFLNDAGACVPAQECPCYAHGTVLAPGEVVHDEGAVCSCTGGK
LSCLGASLQKSTGCAAPMVYLDCSNSSAGTPGAECLRSCHTLDVGCFSTHCVSGCVCPPG
LVSDGSGGCIAEEDCPCVHNEATYKPGETIRVDCNTCTCRNRRWECSHRLCLGTCVAYGD

GHFITFDGDRYSFEGSCEYILAQDYCGDNTTHGTFRIVTENIPCGTTGTTCSKAIKLFVE
SYELILQEGTFKAVARGPGGDPPYKIRYMGIFLVIETHGMAVSWDRKTSVFIRLHQDYKG
RVCGLCGNFDDNAINDFATRSRSVVGDALEFGNSWKLSPSCPDALAPKDPCTANPFRKSW
AQKQCSILHGPTFAACRSQVDSTKYYEACVNDACACDSGGDCECFCTAVAAYAQACHDAG
LCVSWRTPDTCPLFCDFYNPHGGCEWHYQPCGAPCLKTCRNPSGHCLVDLPGLEGCYPKC
PPSQPFFNEDQMKCVAQCGCYDKDGNYYDVGARVPTAENCQSCNCTPSGIQCAHSLEACT
CTYEDRTYSYQDVIYNTTDGLGACLIAICGSNGTIIRKAVACPGTPATTPFTFTTAWVPH
STTSPALPVSTVCVREVCRWSSWYNGHRPEPGLGGGDFETFENLRQRGYQVCPVLADIEC
RAAQLPDMPLEELGQQVDCDRMRGLMCANSQQSPPLCHDYELRVLCCEYVPCGPSPAPGT
SPQPSLSASTEPAVPTPTQTTATEKTTLWVTPSIRSTAALTSQTGSSSGPVTVTPSAPGT
TTCQPRCQWTEWFDEDYPKSEQLGGDVESYDKIRAAGGHLCQQPKDIECQAESFPNWTLA
QVGQKVHCDVHFGLVCRNWEQEGVFKMCYNYRIRVLCCSDDHCRGRATTPPPTTELETAT
TTTTQALFSTPQPTSSPGLTRAPPASTTAVPTLSEGLTSPRYTSTLGTATTGGPTTPAGS
TEPTVPGVATSTLPTRSALPGTTGSLGTWRPSQPPTLAPTTMATSRARPTGTASTASKEP
LTTSLAPTLTSELSTSQAETSTPRTETTMSPLTNTTTSQGTTRCQPKCEWTEWFDVDFPT
SGVAGGDMETFENIRAAGGKMCWAPKSIECRAENYPEVSIDQVGQVLTCSLETGLTCKNE
DQTGRFNMCFNYNVRVLCCDDYSHCPSTPATSSTATPSSTPGTTWILTKPTTTATTTAST
GSTATPTSTLRTAPPPKVLTTTATTPTVTSSKATPSSSPGTATALPALRSTATTPTATSV
TPIPSSSLGTTWTRLSQTTTPTATMSTATPSSTPETAHTSTVLTATATTTGATGSVATPS
STPGTAHTTKVPTTTTTGFTATPSSSPGTALTPPVWISTTTTPTTRGSTVTPSSIPGTTH
TATVLTTTTTTVATGSMATPSSSTQTSGTPPSLTTTATTITATGSTTNPSSTPGTTPIPP
VLTTTATTPAATSNTVTPSSALGTTHTPPVPNTMATTHGRSLPPSSPHTVRTAWTSATSG
ILGTTHITEPSTVTSHTLAATTGTTQHSTPALSSPHPSSRTTESPPSPGTTTPGHTTATS
RTTATATPSKTRTSTLLPSSPTSAPITTVVTMGCEPQCAWSEWLDYSYPMPGPSGGDFDT
YSNIRAAGGAVCEQPLGLECRAQAQPGVPLRELGQVVECSLDFGLVCRNREQVGKFKMCF
NYEIRVFCCNYGHCPSTPATSSTAMPSSTPGTTWILTELTTTATTTESTGSTATPSSTPG
TTWILTEPSTTATVTVPTGSTATASSTQATAGTPHVSTTATTPTVTSSKATPFSSPGTAT
ALPALRSTATTPTATSFTAIPSSSLGTTWTRLSQTTTPTATMSTATPSSTPETVHTSTVL
TTTATTTGATGSVATPSSTPGTAHTTKVLTTTTTGFTATPSSSPGTARTLPVWISTTTTP
TTRGSTVTPSSIPGTTHTPTVLTTTTTTVATGSMATPSSSTQTSGTPPSLTTTATTITAT
GSTTNPSSTPGTTPIPPVLTTTATTPAATSSTVTPSSALGTTHTPPVPNTTATTHGRSLS
PSSPHTVRTAWTSATSGTLGTTHITEPSTGTSHTPAATTGTTQHSTPALSSPHPSSRTTE
SPPSPGTTTPGHTRATSRTTATATPSKTRTSTLLPSSPTSAPITTVVTMGCEPQCAWSEW
LDYSYPMPGPSGGDFDTYSNIRAAGGAVCEQPLGLECRAQAQPGVPLRELGQVVECSLDF
GLVCRNREQVGKFKMCFNYEIRVFCCNYGHCPSTPATSSTATPSSTPGTTWILTEQTTAA
TTTATTGSTAIPSSTPGTAPPPKVLTSTATTPTATSSKATSSSSPRTATTLPVLTSTATK
STATSFTPIPSFTLGTTGTLPEQTTTPMATMSTIHPSSTPETTHTSTVLTTKATTTRATS
SMSTPSSTPGTTWILTELTTAATTTAATGPTATPSSTPGTTWILTEPSTTATVTVPTGST
ATASSTRATAGTLKVLTSTATTPTVISSRATPSSSPGTATALPALRSTATTPTATSVTAI
PSSSLGTAWTRLSQTTTPTATMSTATPSSTPETVHTSTVLTTTTTTTRATGSVATPSSTP
GTAHTTKVPTTTTTGFTATPSSSPGTALTPPVWISTTTTPTTRGSTVTPSSIPGTTHTAT
VLTTTTTTVATGSMATPSSSTQTSGTPPSLTTTATTITATGSTTNPSSTPGTTPIPPVLT
TTATTPAATSSTVTPSSALGTTHTPPVPNTTATTHGRSLPPSSPHTVRTAWTSATSGILG
TTHITEPSTVTSHTPAATTSTTQHSTPALSSPHPSSRTTESPPSPGTTTPGHTRGTSRTT

ATATPSKTRTSTLLPSSPTSAPITTVVTTGCEPQCAWSEWLDYSYPMPGPSGGDFDTYSN
IRAAGGAVCEQPLGLECRAQAQPGVPLRELGQVVECSLDFGLVCRNREQVGKFKMCFNYE
IRVFCCNYGHCPSTPATSSTATPSSTPGTTWILTKLTTTATTESTGSTATPSSTPGTTW
ILTEPSTTATVTVPTGSTATASSTQATAGTPHVSTTATTPTVTSSKATPFSSPGTATALP
ALRSTATTPTATSFTAIPSSSLGTTWTRLSQTTTPTATMSTATPSSTPETAHTSTVLTTT
ATTTRATGSVATPSSTPGTAHTTKVPTTTTTGFTVTPSSSPGTARTPPVWISTTTTPTTS
GSTVTPSSVPGTTHTPTVLTTTTTTVATGSMATPSSSTQTSGTPPSLITTATTITATGST
TNPSSTPGTTPIPPVLTTTATTPAATSSTVTPSSALGTTHTPPVPNTTATTHGRSLSPSS
PHTVRTAWTSATSGTLGTTHITEPSTGTSHTPAATTGTTQHSTPALSSPHPSSRTTESPP
SPGTTTPGHTTATSRTTATATPSKTRTSTLLPSSPTSAPITTVVTTGCEPQCAWSEWLDY
SYPMPGPSGGDFDTYSNIRAAGGAVCEQPLGLECRAQAQPGVPLGELGQVVECSLDFGLV
CRNREQVGKFKMCFNYEIRVFCCNYGHCPSTPATSSTAMPSSTPGTTWILTELTTTATTT
ASTGSTATPSSTPGTAPPPKVLTSPATTPTATSSKATSSSSPRTATTLPVLTSTATKSTA
TSVTPIPSSTLGTTGTLPEQTTTPVATMSTIHPSSTPETTHTSTVLTTKATTTRATSSTS
TPSSTPGTTWILTELTTAATTTAATGPTATPSSTPGTTWILTELTTTATTASTGSTATP
SSTPGTTWILTEPSTTATVTVPTGSTATASSTQATAGTPHVSTTATTPTVTSSKATPSSS
PGTATALPALRSTATTPTATSFTAIPSSSLGTTWTRLSQTTTPTATMSTATPSSTPETVH
TSTVLTATATTTGATGSVATPSSTPGTAHTTKVPTTTTTGFTATPSSSPGTALTPPVWIS
TTTTPTTTTPTTSGSTVTPSSIPGTTHTARVLTTTTTTVATGSMATPSSSTQTSGTPPSL
TTTATTITATGSTTNPSSTPGTTPITPVLTSTATTPAATSSKATSSSSPRTATTLPVLTS
TATKSTATSFTPIPSSTLWTTWTVPAQTTTPMSTMSTIHTSSTPETTHTSTVLTTTATMT
RATNSTATPSSTLGTTRILTELTTTATTTAATGSTATLSSTPGTTWILTEPSTIATVMVP
TGSTATASSTLGTAHTPKVVTTMATMPTATASTVPSSSTVGTTRTPAVLPSSLPTFSVST
VSSSVLTTLRPTGFPSSHFSTPCFCRAFGQFFSPGEVIYNKTDRAGCHFYAVCNQHCDID
RFQGACPTSPPPVSSAPLSSPSPAPGCDNAIPLRQVNETWTLENCTVARCVGDNRVVLLD
PKPVANVTCVNKHLPIKVSDPSQPCDFHYECECICSMWGGSHYSTFDGTSYTFRGNCTYV
LMREIHARFGNLSLYLDNHYCTASATAAAARCPRALSIHYKSMDIVLTVTMVHGKEEGLI
LFDQIPVSSGFSKNGVLVSVLGTTTMRVDIPALGVSVTFNGQVFQARLPYSLFHNNTEGQ
CGTCTNNQRDDCLQRDGTTAASCKDMAKTWLVPDSRKDGCWAPTGTPPTASPAAPVSSTP
TPTPCPPQPLCDLMLSQVFAECHNLVPPGPFFNACISDHCRGRLEVPCQSLEAYAELCRA
RGVCSDWRGATGGLCDLTCPPTKVYKPCGPIQPATCNSRNQSPQLEGMAEGCFCPEDQIL
FNAHMGICVQACPCVGPDGFPKFPGERWVSNCQSCVCDEGSVSVQCKPLPCDAQGQPPPC
NRPGFVTVTRPRAENPCCPETVCVCNTTTCPQSLPVCPPGQESICTQEEGDCCPTFRCRP
QLCSYNGTFYGVGATFPGALPCHMCTCLSGDTQDPTVQCQEDACNNTTCPQGFEYKRVAG
QCCGECVQTACLTPDGQPVQLNETWVNSHVDNCTVYLCEAEGGVHLLTPQPASCPDVSSC
RGSLRKTGCCYSCEEDSCQVRINTTILWHQGCETEVNITFCEGSCPGASKYSAEAQAMQH
QCTCCQERRVHEETVPLHCPNGSAILHTYTHVDECGCTPFCVPAPMAPPHTRGFPAQEAT
AV*
>ENSG00000158104|ENST00000543163
MGFEPLAYRGLETGSREVVSHVIKQGKIVFVLSSALNPWNKEMGDHLVKHGDGVKDIAFE
VEDCDYIVQKARERGAKIMREPWVEQDKFGKVKFAVLQTYGDTTHTLVEKMNYIGQFLPG
YEAPAFMDPLLPKLPKCSLEMIDHIVGNQPDQEMVSASEWYLKNLQFHRFWSVDDTQVHT
EYSSLRSIVVANYEESIKMPINEPAPGKKKSQIQEYVDYNGGAGVQHIALKTEDIITAIR
HLRERGLEFLSVPSTYYKQLREKLKTAKIKVKENIDALEELKILVDYDEKGYLLQIFTKP

VQDRPTLFLEVIQRHNHQGFGAGNFNSLFKAFEEEQNLRGNLTNMETNGVVPGM*
>ENSG00000237693|ENST00000522154
MEAMNVEKASADGNLPEVISNIKETLKIVSRTPVNITMAGDSGNGMSTFISALRNTGHEG
KASPPTELVKATQRCASYFSSHFSNVVLWDLPGTGSATTTLENYLMEMQFNRYDFIMVAS
AQFSMNHVMLAKTAEDMGKKFYIVWTKLDMDLSTGALPEVQLLQIRENVLENLQKERVCE
Y*
>ENSG00000236177|ENST00000515317
MRPEDRMFHIRAVILRALSLAFLLSLRGAGAIKADHVSTYAAFVQTHRPTGEFMFEFDED
EMFYVDLDKKETVWHLEEFGQAFSFEAQGGLANIAILNNNLNTLIQRSNHTQATNDPPEV
TVFPKEPVELGQPNTLICHIDKFFPPVLNVTWLCNGELVTEGVAESLFLPRTDYSFHKFH
YLTFVPSAEDFYDCRVEHWGLDQPLLKHWEAQEPIQMPETTETVLCALGLVLGLVGIIVG
TVLIIKSLRSGHDPRAQGTL*
>ENSG00000054116|ENST00000616395
MSRQANRGTESKKMSSELFTLTYGALVTQLCKDYENDEDVNKQLDKILFSNFLPRGFNIG
VRLIEDFLARSNVGRCHDFRETADVIAKVAFKMYLGITPSITNWSPAGDEFSLILENNPL
VDFVELPDNHSSLIYSNLLCGVLRGALEMVQMAVEAKFVQDTLKGDGVTEIRMRFIRRIE
DNLPAGEE*
>ENSG00000123595|ENST00000618931
MAGKSSLFKVILLGDGGVGKSSLMNRYVTNKFDTQLFHTIGVEFLNKDLEVDGHFVTMQI
WDTAGQERFRSLRTPFYRGSDCCLLTFSVDDSQSFQNLSNWKKEFIYYADVKEPESFPFV
ILGNKIDISERQVSTEEAQAWCRDNGDYPYFETSAKDATNVAAAFEEAVRRVLATEDRSD
HLIQTDTVNLHRKPKPSSSCC*
>ENSG00000229493|ENST00000457432
MSWKMALQIPGGFWAAAVTVMLVMLSTPVAEARDFPKDFLVQFKGMCYFTNGTERVRGVA
RYIYNREEYGRFDSDVGEFQAVTELGRSIEDWNNYKDFLEQERAAVDKVCRHNYEAELRT
TLQRQVEPTVTISPSRTEALNHHNLLVCSVTDFYPAQIKVQWFRNDQEETAGVVSTSLIR
NGDWTFQILVMLEITPQRGDIYTCQVEHPSLQSPITVEWRAQSESAQSKMLGGVGGFVLG
LIFLGLGLIIRHRGQKGPRGPPPAGLLH*
>ENSG00000230791|ENST00000429874
MLSRNDDICIYGGLGLGGLLLLAVVLLSACLCWLHRRVKRLERSWAQGSSEQELHYASLQ
RLPVPSSEGPDLRGRDKRGTKEDPRADYACIAENKPT*
>ENSG00000171195|ENST00000456088
MKTLPLFVCICALSACFSFSEGRERDHELRHRRHHHQSPKSHFELPHYPGLLAHQKPFIR
KSYKCLHKRCRPKLPPSPNNPPKFPNPHQPPKHPDKNSSVVNPTLVATTQIPSVTFPSAS
TKITTLPNVTFLPQNATTISSRENVNTSSSVATLAPVNSPAPQDTTAAPPTPSATTPAPP
SSSAPPETTAAPPTPSATTQAPPSSSAPPETTAAPPTPPATTPAPPSSSAPPETTAAPPT
PSATTPAPLSSSAPPETTAVPPTPSATTLDPSSASAPPETTAAPPTPSATTPAPPSSPAP
QETTAAPITTPNSSPTTLAPDTSETSAAPTHQTTTSVTTQTTTTKQPTSAPGQNKISRFL
LYMKNLLNRIIDDMVEQ*
>ENSG00000176597|ENST00000464191
MRMLVSGRRVKKWQLIIQLFATCFLASLMFFWEPIDNHIVSHMKSYSYRYLINSYDFVND
TL
>ENSG00000175265|ENST00000432566
MLPVDGEERKSEGSDTEGDRTSPCAVSSATLKDLEVGGSGRRCSDPAGQPSNLLPQRGLG

APLPAETAHTQPSPNDRSLYLSPKSSSASSSLHARQSPCQEQAAVLNSRSIKISRLNDTI
KSLKQQKKQVEHQLEEEKKANNEKQKAERELEGQIQRLNTEKKKLNTDLYHMKHSLRYFE
EESKDLAGRLQRSSQRIGELEWSLCAVAATQKKKPDGFSSRSKALLKRQLEQSIREQILL
KGHVTQLKESLKEVQLERDQYAEQIKGERAQWQQRMRKMSQEVCTLKEEKKHDTHRVEEL
ERSLSRLKNQMAEPLPPDAPAVSSEVELQDLRKELERVAGELQAQVENNQCISLLNRGQK
ERLREQEERLQEQQERLREREKRLQQLAEPQSDLEELKHENKSALQLEQQVKELQEKLGQ
VMETLTSAEKEPEAAVPASGTGGESSGLMDLLEEKADLREHVEKLELGFIQYRRERCHQK
VHRLLTEPGDSAKDASPGGGHHQAGPGQGGEEGEAAGAAGDGVAACGSYSEGHGKFLAAA
RNPAAEPSPGAPAPQELGAADKHGDLCEASLTNSVEPAQGEAREGSSQDNPTAQPVVQLL
GEMQDHQEHPGLGSNCCVPCFCWAWLPRRRR*
>ENSG00000177830|ENST00000528581
MRTLFNLLWLALACSPVHTTLSKSDAKKAASKTLLEKVKFCSCCPGWSAMARSWLTATSA
TQSQFSDKPVQDRGLVVTDLKAESVVLEHRSYCSAKARDRHFAGDVLGYVTPWNSHGYDV
TKVFGSKFTQISPVWLQLKRRGREMFEVTGLHDVDQGWMRAVRKHAKGLHIVPRLLFEDW
TYDDFRNVLDSEDEIEELSKTVVQVAKNQHFDGFVVEVWNQLLSQKRVGLIHMLTHLAEA
LHQARLLALLVIPPAITPGTDQLGMFTHKEFEQLAPVLDGFSLMTYDYSTAHQPGPNAPL
SWVRACVQVLDPKSKWRSKILLGLNFYGMDYATSKDAREPVVGARYIQTLKDHRPRMVWD
SQASEHFFEYKKSRSGRHVVFYPTLKSLQVRLELARELGVGVSIWELGQGLDYFYDLL*
>ENSG00000198925|ENST00000409422
MLIGEIFELMQFLFVVAFTTFLVSCVDYDILFANKMVNHSLHPTEPVKVTLPDAFLPAQV
CSARIQENGSLITILVIAGVFWIHRLIKFIYNICCYWEIHSFYLHALRIPMSALPYCTWQ
EVQARIVQTQKEHQICIHKRELTELDIYHRILRFQNYMVALVNKSLLPLRFRLPGLGEAV
FFTRGLKYNFELILFWGPGSLFLNEWSLKAEYKRGGQRLELAQRLSNRILWIGIANFLLC
PLILIWQILYAFFSYAEVLKREPGALGARCWSLYGRCYLRHFNELEHELQSRLNRGYKPA
SKYMNCFLSPLLTLLAKNGAFFAGSILAVLIALTIYDEDVLAVEHVLTTVTLLGVTVTVC
RSFIPDQHMVFCPEQLLRVILAHIHYMPDHWQGNAHRSQTRDEFAQLFQYKAVFILEELL
SPIVTPLILIFCLRPRALEIIDFFRNFTVEVVGVGDTCSFAQMDVRQHGHPQWLSAGQTE
ASVYQQAEDGKTELSLMHFAITNPGWQPPRESTAFLGFLKEQVQRDGAAASLAQGGLLPE
NALFTSIQSLQSESEPLSLIANVVAGSSCRGPPLPRDLQGSRHRAEVASALRSFSPLQPG
QAPTGRAHSTMTGSGVDARTASSGSSVWEGQLQSLVLSEYASTEMSLHALYMHQLHKQQA
QAEPERHVWHRRESDESGESAPDEGGEGARAPQSIPRSASYPCAAPRPGAPETTALHGGF
QRRYGGITDPGTVPRVPSHFSRLPLGGWAEDGQSASRHPEPVPEEGSEDELPPQVHKV*
>ENSG00000181744|ENST00000495414
MKSENGTVVGSFPSDEGWPFAKYLGACGRMVAVNYVGEELWSYFNAPWEKRVDLAWQLME
IAEQLTNNDFEFALYLLDVSFDNFAVGPRDGKVIIVDAENVLVADKRLIRQNKPENWDVW
YESKFDDCDKEACLSFSKEILCARATVDHNYYAVCQNLLSRHATWRGTSGGLLHDPPSEI
AKDGRLEALLDECANPKKRYGRFQAAKELREYLAQLSNNVR*
>ENSG00000144730|ENST00000640796
MESQPFLNMKFETDYFVKVVPFPSIKNESNYHPFFFRTRACDLLLQPDNLACKPFWKPRN
LNISQHGSDMQVSFDHAPHNFGFRFFYLHYKLKHEGPFKRKTCKQEQTTETTSCLLQNVS
PGDYIIELVDDTNTTRKVMHYALKPVHSPWAGPIRAVAITVPLVVISAFATLFTVMCRKK
QQENIYSHLDEESSESSTYTAALPRERLRPRPKVFLCYSSKDGQNHMNVVQCFAYFLQDF
CGCEVALDLWEDFSLCREGQREWVIQKIHESQFIIVVCSKGMKYFVDKKNYKHKGGGRGS
GKGELFLVAVSAIAEKLRQAKQSSSAALSKFIAVYFDYSCEGDVPGILDLSTKYRLMDNL

PQLCSHLHSRDHGLQEPGQHTRQGSRRNYFRSKSGRSLYVAICNMHQFIDEEPDWFEKQF
VPFHPPPLRYREPVLEKFDSGLVLNDVMCKPGPESDFCLKVEAAVLGATGPADSQHESQH
GGLDQDGEARPALDGSAALQPLLHTVKAGSPSDMPRDSGIYDSSVPSSELSLPLMEGLST
DQTETSSLTESVSSSSGLGEEEPPALPSKLLSSGSCKADLGCRSYTDELHAVAPL*
>ENSG00000284369|ENST00000639445
MEGGGGSVAVAGLGARGSGAAAATVRELLQDGCYSDFLNEDFDVKTYTSQSIHQAVIAEQ
LAKLAQGISQLDRELHLQVVARHEDLLAQATGIESLEGVLQMMQTRIGALQGAVDRIKAK
IVEPYNKIVARTAQLARLQVACDLLRRIIRILNLSKRLQGQLQGGSREITKAAQSLNEL
>ENSG00000173402|ENST00000539901
MRMSVGLSLLLPLSGRTFLLLLSVVMAQSHWPSEPSEAVRDWENQLEASMHSVLSDLHEA
VPTVVGIPDGTAVVGRSFRVTIPTDLIASSGDIIKVSAAGKEALPSWLHWDSQSHTLEGL
PLDTDKGVHYISVSATRLGANGSHIPQTSSVFSIEVYPEDHSELQSVRTASPDPGEVVSS
ACAADEPVTVLTVILDADLTKMTPKQRIDLLHRMRSFSEVELHNMKLVPVVNNRLFDMSA
FMAGPGNAKKVVENGALLSWKLGCSLNQNSVPDIHGVEAPAREGAMSAQLGYPVVGWHIA
NKKPPLPKRVRRQIHATPTPVTAIGPPTTAIQEPPSRIVPTPTSPAIAPPTETMAPPVRD
PVPGKPTVTIRTRGAIIQTPTLGPIQPTRVSEAGTTVPGQIRPTMTIPGYVEPTAVATPP
TTTTKKPRVSTPKPATPSTDSTTTTTRRPTKKPRTPRPVPRVTTKVSITRLETASPPTRI
RTTTSGVPRGGEPNQRPELKNHIDRVDAWVGTYFEVKIPSDTFYDHEDTTTDKLKLTLKL
REQQLVGEKSWVQFNSNSQLMYGLPDSSHVGKHEYFMHATDKGGLSAVDAFEIHVHRRPQ
GDRAPARFKAKFVGDPALVLNDIHKKIALVKKLAFAFGDRNCSTITLQNITRGSIVVEWT
NNTLPLEPCPKEQIAGLSRRIAEDDGKPRPAFSNALEPDFKATSITVTGSGSCRHLQFIP
VVPPRRVPSEAPPTEVPDRDPEKSSEDDVYLHTVIPAVVVAAILLIAGIIAMICYRKKRK
GKLTLEDQATFIKKGVPIIFADELDDSKPPPSSSMPLILQEEKAPLPPPEYPNQSVPETT
PLNQDTMGEYTPLRDEDPNAPPYQPPPPFTAPMEGKGSRPKNMTPYRSPPPYVPP*
>ENSG00000236693|ENST00000411749
MMVLQVSAAPRTVALTALLMVLLTSVVQGRATPENYLFQGRQECYAFNGTQRFLERYIYN
REEFVRFDSDVGEFRAVTELGRPDEEYWNSQKDILEEKRAVPDRMCRHNYELGGPMTLQR
RVQPRVNVSPSKKGPLQHHNLLVCHVTDFYPGSIQVRWFLNGQEETAGVVSTNLIRNGDW
TFQILVMLEMTPQQGDVYTCQVEHTSMDSPVTVEWKAQSDSARSKTLTGAGGFVLGLIIC
GVGIFMHRRSKKVQRGSA*
>ENSG00000115446|ENST00000409975
MFLQNTCKRNVFLPSRKMLPSTSVNSLVQGNGVLNSRDAARHTAGAKRYKYLRRLFRFRQ
MDFEFAAWQMLYLFTSPQRVYRNFHYRKQTKDQWARDDPAFLVLLSIWLCVSTIGFGFVL
DMGFFETIKLLLWVVLIDCVGVGLLIATLMWFISNKYLVKRQSRDYDVEWGYAFDVHLNA
FYPLLVILHFIQLFFINHVILTDTFIGYLVGNTLWLVAVGYYIYVTFLGYSALPFLKNTV
ILLYPFAPLILLYGLSLALGWNFTHTLCSFYKYRVK*
>ENSG00000230254|ENST00000427936
MVDGTLLLLLSEALALTQTWAGSHSLKYFHTSVSRPGRGEPRFISVGYVDDTQFVRFDND
AASPRMVPRAPWMEQEGSEYWDRETRSARDTAQIFRVNLRTLRGYYNQSEAGSHTLQWMH
GCELGPDRRFLRGYEQFAYDGKDYLTLNEDLRSWTAVDTAAQISEQKSNDASEAEHQRAY
LEDTCVEWLHKYLEKGKETLLHLEPPKTHVTHHPISDHEATLRCWALGFYPAEITLTWQQ
DGEGHTQDTELVETRPAGDGTFQKWAAVVVPSGEEQRYTCHVQHEGLPEPVTLRWKPASQ
PTIPIVGIIAGLVLLGSVVSGAVVAAVIWRKKSSGGKGGSYSKAEWSDSAQGSESHSL*
>ENSG00000117620|ENST00000465289

MFANLKYVSLGILVFQTTSLVLTMRYSRTLKEEGPRYLSSTAVVVAELLKIMACILLVYK
DSKCSLRALNRVLHDEILNKPMETLKLAIPSGIYTLQNNLLYVALSNLDAATYQVTYQLK
ILTTALFSVSMLSKKLGVYQWLSLVILMTGVAFVQWPSDSQLDSKELSAGSQFVGLMAVL
TACFSSGFAGVYFEKILKETKQSVWIRNIQLGFFGSIFGLMGVYIYDGELVSKNGFFQGY
NRLTWIVVVLQALGGLVIAAVIKYADNILKGFATSLSIILSTLISYFWLQDFVPTSVFFL
GAILVITATFLYGYDPKPAGNPTKA*
>ENSG00000180398|ENST00000409105
MTMRSLLRTPFLCGLLWAFCAPGARAEEPAASFSQPGSMGLDKNTVHDQEHIMEHLEGVI
NKPEAEMSPQELQLHYFKMHDYDGNNLLDGLELSTAITHVHKEEGSEQAPLMSEDELINI
IDGVLRDDDKNNDGYIDYAEFAKSLQ*
>ENSG00000234514|ENST00000458456
MLSRNDEAASAQQ*
>ENSG00000262795|ENST00000576463
XPLSQLPAPQHPKIRLYNAEQVLSWEPVALSNSTRPVVYQVQFKYTDSKWFTADIMSIGV
NCTQITATECDFTAASPSAGFPMDFNVTLRLRAELGALHSAWVTMPWFQHYRNVTVGPPE
NIEVTPGEGSLIIRFSSPFDIADTSTAFFCYYVHYWEKGGIQQVKGPFRSNSISLDNLKP
SRVYCLQVQAQLLWNKSNIFRVGHLSNISCYETMADASTELQQVILISVGTFSLLSVLAG
ACFFLVLKYRGLIKYWFHTPPSIPLQIEEYLKDPTQPILEALDKDSSPKDDVWDSVSIIS
FPEKEQEDVLQTL*
>ENSG00000230791|ENST00000420333
MLSRNDDICIYGGLGLGGLLLLAVVLLSACLCWLHRRGPGLLRAGTPLCISAEAASAQQ*
>ENSG00000167632|ENST00000438773
MSVPDYMQCAEDHQTLLVVVQPVGIVSEENFFRIYKRICSVSQISVRDSQRVLYIRYRHH
YPPENNEWGDFQTHRKVVGLITITDCFSAKDWPQTFEKFHVQKEIYGSTLYDSRLFVFGL
QGEIVEQPRTDVAFYPNYEDCQTVEKRIEDFIESLFIVLESKRLDRATDKSGDKIPLLCV
PFEKKDFVGLDTDSRHYKKRCQGRMRKHVGDLCLQAGMLQDSLVHYHMSVELLRSVNDFL
WLGAALEGLCSASVIYHYPGGTGGKSGARRFQGSTLPAEAANRHRPGAQEVLIDPGALTT
NGINPDTSTEIGRAKNCLSPEDIIDKYKEAISYYSKYKNAGVIELEACIKAVRVLAIQKR
SMEASEFLQNAVYINLRQLSEEEKIQRYSILSELYELIGFHRKSAFFKRVAAMQCVAPSI
AEPGWRACYKLLLETLPGYSLSLDPKDFSRGTHRGWAAVQMRLLHELVYASRRMGNPALS
VRHLSFLLQTMLDFLSDQEKKDVAQSLENYTSKCPGTMEPIALPGGLTLPPVPFTKLPIV
RHVKLLNLPASLRPHKMKSLLGQNVSTKSPFIYSPIIAHNRGEERNKKIDFQWVQGDVCE
VQLMVYNPMPFELRVENMGLLTSGVEFESLPAALSLPAESGLYPVTLVGVPQTTGTITVN
GYHTTVFGVFSDCLLDNLPGIKTSGSTVEVIPALPRLQISTSLPRSAHSLQPSSGDEIST
NVSVQLYNGESQQLIIKLENIGMEPLEKLEVTSKVLTTKEKLYGDFLSWKLEETLAQFPL
QPGKVATFTINIKVKLDFSCQENLLQDLSDDGISVSGFPLSSPFRQVVRPRVEGKPVNPP
ESNKAGDYSHVKTLEAVLNFKYSGGPGHTEGYYRNLSLGLHVEVEPSVFFTRVSTLPATS
TRQCHLLLDVFNSTEHELTVSTRSSEALILHAGECQRMAIQVDKFNFESFPESPGEKGQF
ANPKQLEEERREARGLEIHSKLGICWRIPSLKRSGEASVEGLLNQLVLEHLQLAPLQWDV
LVDGQPCDREAVAACQVGDPVRLEVRLTNRSPRSVGPFALTVVPFQDHQNGVHNYDLHDT
VSFVGSSTFYLDAVQPSGQSACLGALLFLYTGDFFLHIRFHEDSTSKELPPSWFCLPSVH
VCALEAQA*
>ENSG00000232629|ENST00000411527
MALQIPGGFWAAAVTVMLVMLSTPVAEARDFPKDFLVQFKGMCYFTNGTERVRGVARYIY

NREEYGRFDSDVGEFQAVTELGRSIEDWNNYKDFLEQERAAVDKVCRHNYEAELRTTLQR
QVEPTVTISPSRTEALNHHNLLVCSVTDFYPAQIKVRWFRNDQEETAGVVSTSLIRNGDW
TFQILVMLEITPQRGDIYTCQVEHPSLQSPITVEWRPRGPPPAGLLH*
>ENSG00000141367|ENST00000621829
MAQILPIRFQEHLQLQNLGINPANIGFSTLTMESDKFICIREKVGEQAQVVIIDMNDPSN
PIRRPISADSAIMNPASKVIALKGIKESGKTLQIFNIEMKSKMKAHTMTDDVTFWKWISL
NTVALVTDNAVYHWSMEGESQPVKMFDRHSSLAGCQIINYRTDAKQKWLLLTGISAQQNR
VVGAMQLYSVDRKVSQPIEGHAASFAQFKMEGNAEESTLFCFAVRGQAGGKLHIIEVGTP
PTGNQPFPKKAVDVFFPPEAQNDFPVAMQISEKHDVVFLITKYGYIHLYDLETGTCIYMN
RISGETIFVTAPHEATAGIIGVNRKGQVLSVCVEEENIIPYITNVLQNPDLALRMAVRNN
LAGAEELFARKFNALFAQGNYSEAAKVAANAPKGILRTPDTIRRFQSVPAQPGQTSPLLQ
YFGILLDQGQLNKYESLELCRPVLQQGRKQLLEKWLKEDKLECSEELGDLVKSVDPTLAL
SVYLRANVPNKVIQCFAETGQVQKIVLYAKKVGYTPDWIFLLRNVMRISPDQGQQFAQML
VQDEEPLADITQIVDVFMEYNLIQQCTAFLLDALKNNRPSEGPLQTRLLEMNLMHAPQVA
DAILGNQMFTHYDRAHIAQLCEKAGLLQRALEHFTDLYDIKRAVVHTHLLNPEWLVNYFG
SLSVEDSLECLRAMLSANIRQNLQICVQVASKYHEQLSTQSLIELFESFKSFEGLFYFLG
SIVNFSQDPDVHFKYIQAACKTGQIKEVERICRESNCYDPERVKNFLKEAKLTDQLPLII
VCDRFDFVHDLVLYLYRNNLQKYIEIYVQKVNPSRLPVVIGGLLDVDCSEDVIKNLILVV
RGQFSTDELVAEVEKRNRLKLLLPWLEARIHEGCEEPATHNALAKIYIDSNNNPERFLRE
NPYYDSRVVGKYCEKRDPHLACVAYERGQCDLELINVCNENSLFKSLSRYLVRRKDPELW
GSVLLESNPYRRPLIDQVVQTALSETQDPEEVSVTVKAFMTADLPNELIELLEKIVLDNS
VFSEHRNLQNLLILTAIKADRTRVMEYINRLDNYDAPDIANIAISNELFEEAFAIFRKFD
VNTSAVQVLIEHIGNLDRAYEFAERCNEPAVWSQLAKAQLQKGMVKEAIDSYIKADDPSS
YMEVVQAANTSGNWEELVKYLQMARKKARESYVETELIFALAKTNRLAELEEFINGPNNA
HIQQVGDRCYDEKMYDAAKLLYNNVSNFGRLASTLVHLGEYQAAVDGARKANSTRTWKEV
CFACVDGKEFRLAQMCGLHIVVHADELEELINYYQDRGYFEELITMLEAALGLERAHMGM
FTELAILYSKFKPQKMREHLELFWSRVNIPKVLRAAEQAHLWAELVFLYDKYEEYDNAII
TMMNHPTDAWKEGQFKDIITKVANVELYYRAIQFYLEFKPLLLNDLLMVLSPRLDHTRAV
NYFSKVKQLPLVKPYLRSVQNHNNKSVNESLNNLFITEEDYQALRTSIDAYDNFDNISLA
QRLEKHELIEFRRIAAYLFKGNNRWKQSVELCKKDSLYKDAMQYASESKDTELAEELLQW
FLQEEKRECFGACLFTCYDLLRPDVVLETAWRHNIMDFAMPYFIQVMKEYLTKVDKLDAS
ESLRKEEEQATETQPIVYGQPQLMLTAGPSVAVPPQAPFGYGYTAPPYGQPQPGFGYSM*
>ENSG00000156413|ENST00000524754
MDPLGPAKPQWSWRCCLTTLLFQLLMAVCFFSYLRVSQDDPTVYPNGSRFPDSTGTPAHS
IPLILLWTWPFNKPIALPRCSEMVPGTADCNITADRKVYPQADAVIVHHREVMYNPSAQL
PRSPRRQGQRWIWFSMESPSHCWQLKAMDGYFNLTMSYRSDSDIFTPYGWLEPWSGQPAH
PPLNLSAKTELVAWAVSNWGPNSARVRYYQSLQAHLKVDVYGRSHKPLPQGTMMETLSRY
KFYLAFENSLHPDYITEKLWRNALEAWAVPVVLGPSRSNYERFLPPDAFIHVDDFQSPKD
LARYLQELDKDHARYLSYFRWRETLRPRSFSWALAFCKACWKLQEESRYQTRGIAAWFT*
>ENSG00000136152|ENST00000617493
MAEAALLLLPEAAAERDAREKLALWDRRPDTTAPLTDRQTDSVLELKAAAENLPVPAELP
IEDLCSLTSQSLPIELTSVVPESTEDILLKGFTSLGMEEERIETAQQFFSWFAKLQTQMD
QDEGTKYRQMRDYLSGFQEQCDAILNDVNSALQHLESLQKQYLFVSNKTGTLHEACEQLL
KEQSELVDLAENIQQKLSYFNELETINTKLNSPTLSVNSDGFIPMLAKLDDCITYISSHP

NFKDYPIYLLKFKQCLSKALHLMKTYTVNTLQTLTSQLLKRDPSSVPNADNAFTLFYVKF
RAAAPKVRTLIEQIELRSEKIPEYQQLLNDIHQCYLDQRELLLGPSIACTVAELTSQNNR
DHCALVRSGCAFMVHVCQDEHQLYNEFFTKPTSKLDELLEKLCVSLYDVFRPLIIHVIHL
ETLSELCGILKNEVLEDHVQNNGK*
>ENSG00000149541|ENST00000534026
MKLKLKNVFLAYFLVSIAGLLYALVQLGQPCDCLPPLRAAAEQLRQKDLRISQLQAELRR
PPPAPAQPPEPEALPTIYVVTPTYARLVQKAELVRLSQTLSLVPRLHWLLVEDAEGPTPL
VSGLLAASGLLFTHLVVLTPKAQRLREGEPGWVHPRGVEQRNKALDWLRGRGGAVGGEKD
PPPPGTQGVVYFADDDNTYSRELFEEMRWTRGVSVWPVGLVGGLRFEGPQVQDGRVVGFH
TAWEPSRPFPVDMAGFAVALPLLLDKPNAQFDSTAPRGHLESSLLSHLVDPKDLEPRAAN
CTRTESRCVTQAGVQ*
>ENSG00000147642|ENST00000528647
MGPLRESKEHRVQHHDKEISRSRIPRLILRPHMPQQQHKVSPASESPFSEEESREFNPSS
SGRSARTVSSNSFCSDDTGCPSSQSVSPVKTPSDAGNSPIGFCPGSDEGFTRKKCTIGMV
GEGSIQSSRYKKESKSGLVKPGSEADFSSSSSTGSISAPEVHMSTAGSKRSSSSRNRGPH
GRSNGASSHKPGSSPSSPREKDLLSMLCRNQLSPVNIHPSYAPSSPSSSNSGSYKGSDCS
PIMRRSGRYMSCGENHGVRPPNPEQYLTPLQQKEVTVRHLKTKLKESERRLHERESEIVE
LKSQLARMREDWIEEECHRVEAQLALKEARKEIKQLKQVIETMRSSLADKDKGIQKYFVD
INIQNKKLESLLQSMEMAHSGSLRDELCLDFPCDSPEKSLTLNPPLDTMADGLSLEEQVT
GEGADRELLVGDSIANSTDLFDEIVTATTTESGDLELVHSTPGANVLELLPIVMGQEEGS
VVVERAVQTDVVPYSPAISELIQSVLQKLQDPCPSSLASPDESEPDSMESFPESLSALVV
DLTPRNPNSAILLSPVETPYANVDAEVHANRLMRELDFAACVEERLDGVIPLARGGVVRQ
YWSSSFLVDLLAVAAPVVPTVLWAFSTQRGGTDPVYNIGALLRGCCVVALHSLRRTAFRI
KT*
>ENSG00000147642|ENST00000440310
MGPLRESKKEHRVQHHDKEISRSRIPRLILRPHMPQQQHKVSPASESPFSEEESREFNPS
SSGRSARTVSSNSFCSDDTGCPSSQSVSPVKTPSDAGNSPIGFCPGSDEGFTRKKCTIGM
VGEGSIQSSRYKKESKSGLVKPGSEADFSSSSSTGSISAPEVHMSTAGSKRSSSSRNRGP
HGRSNGASSHKPGSSPSSPREKDLLSMLCRNQLSPVNIHPSYAPSSPSSSNSGSYKGSDC
SPIMRRSGRYMSCGENHGVRPPNPEQYLTPLQQKEVTVRHLKTKLKESERRLHERESEIV
ELKSQLARMREDWIEEECHRVEAQLALKEARKEIKQLKQVIETMRSSLADKDKGIQKYFV
DINIQNKKLESLLQSMEMAHSGSLRDELCLDFPCDSPEKSLTLNPPLDTMADGLSLEEQV
TGEGADRELLVGDSIANSTDLFDEIVTATTTESGDLELVHSTPGANVLELLPIVMGQEEG
SVVVERAVQTDVVPYSPAISELIQSVLQKLQDPCPSSLASPDESEPDSMESFPESLSALV
VDLTPRNPNSAILLSPVETPYANVDAEVHANRLMRELDFAACVEERLDGVIPLARGGVVR
QYWSSSFLVDLLAVAAPVVPTVLWAFSTQRGGTDPVYNIGALLRGCCVVALHSLRRTAFR
IKT*
>ENSG00000230791|ENST00000448236
MLSRNDDICIYGGLGLGGLLLLAVVLLSACLCWLHRRVKRLERSWHLLSWSQAQGSSEQE
LHYASLQRLPVPSSEGPDLRGRDKRGTKEDPRADYACIAENKPT*
>ENSG00000231526|ENST00000415898
MILNKALLLGALALTAVMSPCGGEDIVADHVASYGVNFYQSHGPSGQYTHEFDGDEEFYV
DLETKETVWQLPMFSKFISFDPQSALRNMAVGKHTLEFMMGQSNSTAATNEVPEVTVFSK
FPVTLGQPNTLICLVDNIFPPVVNITWLSNGHSVTEGVSETSFLSKSDHSFFKISYLTFL

PSADEIYDCKVEHWGLDEPLLKHWEPEIPAPMSELTETLVCALGLSVGLMGIVVGTVFII
QGLRSVGASRHQGLL*
>ENSG00000111481|ENST00000549043
MGGFRTEGMFVSLQEPSLYTVKAILILDNDGDRLFAKYYDDTYPSVKEQKAFEKNIFNKT
HRTDSEIALLEGLTVVYKSSIDLYFYVIGSSYENELMLMAVLNCLFDSLSQMLRKNVEKR
ALLENMEGLFLAVDEIVDGGVILESDPQQVVHRVALRGEDVPLTEQTVSQVLQSAKEQIK
WSLLR*
>ENSG00000011465|ENST00000456569
MKATIILLLLAQVSWAGPFQQRGLFDFMLEDEASGIGPEVPDDRDFEPSLGPVCPFRCQC
HLRVVQCSDLGCLPS*
>ENSG00000231834|ENST00000456012
MAVMAPRTLLLLLLGALALTQTWAGSHSMRYFTTSVSRPGRGEPRFIAVGYVDDTQFVRF
DSDAASQRMEPRAPWIEQEGPEYWDLQTRNVKAQSQTDRANLGTLRGYYNQSEAGSHTIQ
MMYGCDVGSDGRFLRGYRQDAYDGKDYIALNEDLRSWTAADMAAQITQRKWEAARVAEQL
RAYLEGTCVEWLRRYLENGKETLQRTDAPKTHMTHHAVSDHEATLRCWALSFYPAEITLT
WQRDGEDQTQDTELVETRPAGDGTFQKWASVVVPSGQEQRYTCHVQHEGLPKPLTLRWEP
SSQPTIPIVGIIAGLVLFGAVFAGAVVAAVRWRRKSSDRKGGSYSQAASSDSAQGSDMSL
TACKV*
>ENSG00000173402|ENST00000541308
MRMSVGLSLLLPLSGRTFLLLLSVVMAQSHWPSEPSEAVRDWENQLEASMHSVLSDLHEA
VPTVVGIPDGTAVVGRSFRVTIPTDLIASSGDIIKVSAAGKEALPSWLHWDSQSHTLEGL
PLDTDKGVHYISVSATRLGANGSHIPQTSSVFSIEVYPEDHSELQSVRTASPDPGEVVSS
ACAADEPVTVLTVILDADLTKMTPKQRIDLLHRMRSFSEVELHNMKLVPVVNNRLFDMSA
FMAGPGNAKKVVENGALLSWKLGCSLNQNSVPDIHGVEAPAREGAMSAQLGYPVVGWHIA
NKKPPLPKRVRRQIHATPTPVTAIGPPTTAIQEPPSRIVPTPTSPAIAPPTETMAPPVRD
PVPGKPTVTIRTRGAIIQTPTLGPIQPTRVSEAGTTVPGQIRPTMTIPGYVEPTAVATPP
TTTTKKPRVSTPKPATPSTDSTTTTTRRPTKKPRTPRPVPRVTTKVSITRLETASPPTRI
RTTTSGVPRGGEPNQRPELKNHIDRVDAWVGTYFEVKIPSDTFYDHEDTTTDKLKLTLKL
REQQLVGEKSWVQFNSNSQLMYGLPDSSHVGKHEYFMHATDKGGLSAVDAFEIHVHRRPQ
GDRAPARFKAKFVGDPALVLNDIHKKIALVKKLAFAFGDRNCSTITLQNITRGSIVVEWT
NNTLPLEPCPKEQIAGLSRRIAEDDGKPRPAFSNALEPDFKATSITVTGSGSCRHLQFIP
VVPPRRVPSEAPPTEVPDRDPEKSSEDDVYLHTVIPAVVVAAILLIAGIIAMICYRKKRK
GKLTLEDQATFIKKGVPIIFADELDDSKPPPSSSMPLILQEEKAPLPPPEYPNQSVPETT
PLNQDTMGEYTPLRDEDPNAPPYQPPPPFTAPMEGKGSRPKNMTPYRSPPPYVPP*
>ENSG00000137497|ENST00000535947
MTLHATRGAALLSWVNSLHVADPVEAVLQLQDCSIFIKIIDRIHGTEEGQQILKQPVSER
LDFVCSFLQKNRKHPSSPECLVSAQKVLEGSELELAKMTMLLLYHSTMSSKSPRDWEQFE
YKIQAELAVILKFVLDHEDGLNLNED
>ENSG00000100626|ENST00000553471
MRKIRANAIAILTVAWILGTFYYLWQDNRAHAASSGGRGAQRAGRRSEQLREDRTIPLIV
TGTPSKGFDEKAYLSAKQLKAGEDPYRQHAFNQLESDKLSPDRPIRDTRHYSCPSVSYSS
DLPATSVIITFHNEARSTLLRTVKSVLNRTPANLIQEIILVDDFSSDPEDCLLLTRIPKV
KCLRNDRREGLIRSRVRGADVAAATVLTFLDSHCEVNTEWLPPMLQRVKEDHTRVVSPII
DVISLDNFAYLAASADLRGGFDWSLHFKWEQIPLEQKMTRTDPTRPIRTPVIAGGIFVID

KSWFNHLGKYDAQMDIWGGENFELSFRVWMCGGSLEIVPCSRVGHVFRKRHPYNFPEGNA
LTYIRNTKRTAEVWMDEYKQYYYEARPSAIGKAFGSVATRIEQRKKMNCKSFRWYLENVY
PELTVPVKEALPGIIKQGVNCLESQGQNTAGDFLLGMGICRGSAKNPQPAQAWLFSDHLI
QQQGKCLAATSTLMSSPGSPVILQMCNPREGKQVSLLASGPEAQQPEGPCLRVADLGRRA
PD*
>ENSG00000280951|ENST00000629578
MGQNDLMGTAEDFADQFLRVTKQYLPHVARLCLISTFLEDGIRMWFQWSEQRDYIDTTWN
CGYLLASSFVFLNLLGQLTGCVLVLSRNFVQYACFGLFGIIALQTIAYSILWDLKFLMRN
LALGGGLLLLLAESRSEGKSMFAGVPTMRESSPKQYMQLGGRVLLVLMFMTLLHFDASFF
SIVQNIVGTALMILVAIGFKTKLAALTLVVWLFAINVYFNAFWTIPVYKPMHDFLKYDFF
QTMSVIGGLLLVVALGPGGVSMDEKKKEW*
>ENSG00000224305|ENST00000438757
MALQIPGGFWAAAVTVMLVMLSTPVAEARDFPKDFLVQFKGMCYFTNGTERVRGVARYIY
NREEYGRFDSDVGEFQAVTELGRSIEDWNNYKDFLEQERAAVDKVCRHNYEAELRTTLQR
QVEPTVTISPSRTEALNHHNLLVCSVTDFYPAQIKVRWFRNDQEETAGVVSTSLIRNGDW
TFQILVMLEITPQRGDIYTCQVEHPSLQSPITVEWRPRGPPPAGLLH*
>ENSG00000158488|ENST00000444681
MLLLFLLFEGLCCPGENTADPFEIQILAGCRMNAPQIFLNMAYQGSDFLSFQGISWEPSP
GAGIRAQNICKVLNRYLDIKEILQSLLGHTCPRFLAGLMEAGESELKRKVKPEAWLSCGP
SPGPGRLQLVCHVSGFYPKPVWVMWMRGEQEQRGTQRGDVLPNADETWYLRATLDVAAGE
AAGLSCRVKHSSLGGHDLIIHWGGYSIFLILICLTVIVTLVILVVVDSRLKKQSSNKNIL
SPHTPSPVFLMGANTQDTKNSRHQFCLAQVSWIKNRVLKKWKTRLNQLW*
>ENSG00000007255|ENST00000585934
MADTVLFEFLHTEMVAELWAHDPDPGPGGQKMSLSVLEGMGFRVGQALGERLPRETLAFR
EELDVLKFLCKDLWVAVFQKQMDSLRTNHQGTYVLQDNSFPLLLPMASGLQYLEEAPKFL
AFTCGLLRGALYTLGIESVVTASVAALPVCKFQVVIPKS*
>ENSG00000164144|ENST00000429148
MAQESPKNSAAEIPVTSNGEVDDSREHSFNREEFGYNADTQKLLAKNGETLLGAINFFIA
SVNTLVNKTIEDTLMTVKQYESARIEYDAYRTDLEELNLGPRDANTLPKIEQSQHLFQAH
KEKYDKMRNDVSVKLKFLEENKVKVLHNQLVLFHNAIAAYFAGNQKQLEQTLKQFHIKLK
TPGVDAPSWLEEQ*
>ENSG00000213380|ENST00000562595
XVEDEGLLASLFRDRFPEAQWRERPDVGRYLRELSGSGLERLRREPERLAEERAQLLQQT
RDLAFANYKTFIRGAECTERIHRLFGDVEASLGRLLDRLPSFQQSCRNFVKEAEEISSNR
RMNSLTLNRHTEILEILEIPQLMDTCVRNSYYEEALELAAYVRRLERKYSSIPVIQGIVN
EVRHAGITGGSHGARPGAFLAAHPQHQPQGQTLARMARARLCR*
>ENSG00000173402|ENST00000545947
MRMSVGLSLLLPLSGRTFLLLLSVVMAQSHWPSEPSEAVRDWENQLEASMHSVLSDLHEA
VPTVVGIPDGTAVVGRSFRVTIPTDLIASSGDIIKVSAAGKEALPSWLHWDSQSHTLEGL
PLDTDKGVHYISVSATRLGANGSHIPQTSSVFSIEVYPEDHSELQSVRTASPDPGEVVSS
ACAADEPVTVLTVILDADLTKMTPKQRIDLLHRMRSFSEVELHNMKLVPVVNNRLFDMSA
FMAGPGNAKKVVENGALLSWKLGCSLNQNSVPDIHGVEAPAREGAMSAQLGYPVVGWHIA
NKKPPLPKRVRRQIHATPTPVTAIGPPTTAIQEPPSRIVPTPTSPAIAPPTETMAPPVRD
PVPGKPTVTIRTRGAIIQTPTLGPIQPTRVSEAGTTVPGQIRPTMTIPGYVEPTAVATPP

TTTTKKPRVSTPKPATPSTDSTTTTTRRPTKKPRTPRPVPRVTTKVSITRLETASPPTRI
RTTTSGVPRGGEPNQRPELKNHIDRVDAWVGTYFEVKIPSDTFYDHEDTTTDKLKLTLKL
REQQLVGEKSWVQFNSNSQLMYGLPDSSHVGKHEYFMHATDKGGLSAVDAFEIHVHRRPQ
GDRAPARFKAKFVGDPALVLNDIHKKIALVKKLAFAFGDRNCSTITLQNITRGSIVVEWT
NNTLPLEPCPKEQIAGLSRRIAEDDGKPRPAFSNALEPDFKATSITVTGSGSCRHLQFIP
VVPPRRVPSEAPPTEVPDRDPEKSSEDDVYLHTVIPAVVVAAILLIAGIIAMICYRKKRK
GKLTLEDQATFIKKGVPIIFADELDDSKPPPSSSMPLILQEEKAPLPPPEYPNQSVPETT
PLNQDTMGEYTPLRDEDPNAPPYQPPPPFTAPMEGKGSRPKNMTPYRSPPPYVPP*
>ENSG00000231048|ENST00000449236
MLSRNDVKRLERSWAQGSSEQELHYASLQRLPVPSSEGPDLRGRDKRGTKEDPRADYACI
AENKPT*
>ENSG00000125447|ENST00000582376
MAEAEGESLESWLNKATNPSNRQEDWEYIIGFCDQINKELEGPQIAVRLLAHKIQSPQEW
EALQALTVLEACMKNCGRRFHNEVGKFRFLNELIKVVSPKRARPWR*
>ENSG00000154080|ENST00000581714
MQPSEMVMNPKQVFLSVLIFGVAGLLLFMYLQVWIEEQHTGRVEKRREQKVTSGWGPVKY
LRPVPRIMSTEKIQEHITNQNPKFHMPEDVREKKENLLLNSERSTRLLTKTSHSQGGDQA
LSKSTGSPTEKLIEKRQGAKTVFNKFSNMNWPVDIHPLNKSLVKDNKWKKTEETQEKRRS
FLQEFCKKYGGVSHHQSHLFHTVSRIYVEDKHKILYCEVPKAGCSNWKRILMVLNGLASS
AYNISHNAVHYGKHLKKLDSFDLKGIYTRLNTYTKAVFVRDPMERLVSAFRDKFEHPNSY
YHPVFGKAIIKKYRPNACEEALINGSGVKFKEFIHYLLDSHRPVGMDIHWEKVSKLCYPC
LINYDFVGKFETLEEDANYFLQMIGAPKELKFPNFKDRHSSDERTNAQVVRQYLKDLTRT
ERQLIYDFYYLDYLMFNYTTPFL*
>ENSG00000235915|ENST00000430997
MLSRNDDICIYGGLGLGGLLLLAVVLLSACLCWLHRRGPGLLRAGTPLCISAEAASAQQ*
>ENSG00000177830|ENST00000454838
MRTLFNLLWLALACSPVHTTLSKSDAKKAASKTLLEKVKFCSCCPGWSAMARSWLTATSA
TQSQFSDKPVQDRGLVVTDLKAESVVLEHRSYCSAKARDRHFAGDVLGYVTPWNSHGYDV
TKVFGSKFTQISPVWLQLKRRGREMFEVTGLHDVDQGWMRAVRKHAKGLHIVPRLLFEDW
TYDDFRNVLDSEDEIEELSKTVVQVAKNQHFDGFVVEVWNQLLSQKRVGLIHMLTHLAEA
LHQARLLALLVIPPAITPGTDQLGMFTHKEFEQLAPVLDGFSLMTYDYSTAHQPGPNAPL
SWVRACVQVLDPKSKWRSKILLGLNFYGMDYATSKDAREPVVGARYIQTLKDHRPRMVWD
SQASEHFFEYKKSRSGRHVVFYPTLKSLQVRLELARELGVGVSIWELGQGLDYFYDLL*
>ENSG00000113532|ENST00000451528
MRSIRKRWTICTISLLLIFYKTKEIARTEEHQETQLIGDGELSLSRSLVNSSDKIIRKAG
SSIFQHNVEGWKINSSLVLEIRKNILRFLDAERDVSVVKSSFKPGDVIHYVLDRRRTLNI
SHDLHSLLPEVSPMKNRRFKTCAVVGNSGILLDSECGKEIDSHNFVIR*
>ENSG00000152700|ENST00000439578
MSFIFDWIYSGFSSVLQFLGLYKKTGKLVFLGLDNAGKTTLLHMLKDDRLGQHVPTLHPT
SEELTIAGMTFTTFDLGGHVQARRVWKNYLPAINGIVFLVDCADHERLLESKEELDSLMT
DETIANVPILILGNKIDRPEAISEERLREMFGLYGQTTGKGSISLKELNARPLEVFMCSV
LKRQGYGEGFRWMAQYID*
>ENSG00000185825|ENST00000458587
MGAEASSSWCPGTALPEERLSVKRASEISGFLGQGSSGEAALDVLTHVLEGAGNKLTSSC

GKPSSNRMSLQWTAVATFLYAEVFVVLLLCIPFISPKRWQKIFKSRLVELLVSYGNTFFV
VLIVILVLLVIDAVREIRKYDDVTEKVNLQNNPGAMEHFHMKLFRAQRNLYIAGFSLLLS
FLLRRLVTLISQQATLLASNEAFKKQAESASEAAKKYMEENDQLKKGAAVDGGKLDVGNA
EVKLEEENRSLKADLQKLKDELASTKQKLEKAENQVLAMRKQSEGLTKEYDRLLEEHAKL
QAAVDGPMDKKEE*
>ENSG00000176597|ENST00000464923
MRMLVSGRRVKKWQLIIQLFATCFLASLMFFWEPIDNHIVSHMKSYSYRYLINSYDFVND
TLSLKHTSAGPRYQYLINHKE
>ENSG00000237710|ENST00000454006
MMVLQVSAAPRTVALTALLMVLLTSVVQGRATPENYLFQGRQECYAFNGTQRFLERYIYN
REEFARFDSDVGEFRAVTELGRPAAEYWNSQKDILEEKRAVPDRMCRHNYELGGPMTLQR
RVQPRVNVSPSKKGPLQHHNLLVCHVTDFYPGSIQVRWFLNGQEETAGVVSTNLIRNGDW
TFQILVMLEMTPQQGDVYTCQVEHTSLDSPVTVEWKAQSDSARSKTLTGAGGFVLGLIIC
GVGIFMHRRSKKVQRGSA*
>ENSG00000140297|ENST00000560585
MVQWKRLCQLHYLWALGCYMLLATVALKLSFRLKCDSDHLGLESRESQSQYCRNILYNFL
KLPAKRSINCSGVTRGDQEAVLQAILNNLEVKKKREPFTDTHYLSLTRDCEHFKAERKFI
QFPLSKEEVEFPIAYSMVIHEKIENFERLLRAVYAPQNIYCVHVDEKSPETFKEAVKAII
SCFPNVFIASKLVRVVYASWSRVQADLNCMEDLLQSSVPWKYFLNTCGTDFPIKSNAEMV
QALKMLNGRNSMESEVPPKHKETRWKYHFEVVRDTLHLTNKKKDPPPYNLTMFTGNAYIV
ASRDFVQHVLKNPKSQQLIEWVKDTYSPDEHLWATLQRARWMPGSVPNHPKYDISDMTSI
ARLVKWQGHEGDIDKGAPYAPCSGIHQRAICVYGAGDLNWMLQNHHLLANKFDPKVDDNA
LQCLEEYLRYKAIYGTEL*
>ENSG00000149823|ENST00000530773
MAAAAAAGPSPGSGPGDSPEGPEGEAPERRRKAHGMLKLYYGLSEGEAAGRPAGPDPLDP
TDLNGAHFDPEVYLDKLRRECPLAQLMDSETDMVRQIRALDSDMQTLVYENYNKFISATG
VHALLRKLQFLFELPSRLTKCVELGAYGQAVRYQGRAQAVLQQYQHLPSFRAIQDDCQVI
TARLAQQLRQRFREGGSGAPEQAECVELLLALGEPAEELCEEFLAHARGRLEK
>ENSG00000153395|ENST00000475622
MRLRGCGPRAAPASSAGASDARLLAPPGRNPFVHELRLSALQKAQVALMTLTLFPVRLLV
AAAMMLLAWPLALVASLGSAEKEPEQPPALWRKVVDFLLKAIMRTMWFAGGFHRVAVKGR
QALPTEAAILTLAPHSSYFDAIPVTMTMSSIVMKAESRDIPIWGTLIQYIRPVFVSRSDQ
DSRRKTVEEIKRRAQSNGKWPQIMIFPEGTCTNRTCLITFKPGAFIPGAPVQPVVLRYPN
KLDTITWTWQGPGALEILWLTLCQFHNQVEIEFLPVYSPSEEEKRNPALYASNVRRVMAE
ALGVSVTDYTFEDCQLALAEGQLRLPADTCLLEFARLVRGLGLKPEKLEKDLDRYSERAR
MKGGEKIGIAEFAASLEVPVSDLLEDMFSLFDESGSGEVDLRECVVALSVVCRPARTLDT
IQLAFKMYGAQEDGSVGEGDLSCILKTALGVAELTVTDLFRAIDQEEKGKITFADFHRFA
EMYPAFAEEYLYPDQTHFESCAETSPAPIPNGFCADFSPENSDAGRKPVRKKLD*
>ENSG00000102100|ENST00000446885
MAEVLKGLTCLLLLFAQKRGNVKHLVLFLHEAVLVQYVDTLKLAVPSLIYTLQNNLQYVA
ISNLPAATFQVTYQLKILTTALFSVLMLNRSLSRLQWASLLLLFTGVAIVQAQQAGGGGP
RPLDQNPGAGLAAVVASCLSSGFAGVYFEKILKGSSGSVWLRNLQLGLFGTALGLV
>ENSG00000284369|ENST00000639330
MGWVGGRRRDSASPPGRSRSAADDINPAPANMEGGGGSVAVAGLGARGSGAAAATVRELL

QDGCYSDFLNEDFDVKTYTSQSIHQAVIAEQLAKLAQGISQLDRELHLQVVARHEDLLAQ
ATGIESLEGVLQMMQTRIGALQGAVDRIKAKIVEPYNKIVARTAQLARLQVACDLLRRII
RILNLSKRLQGQLQGGSREITKAAQSLNELDYLSQGIDLSGIEVIENDLLFIARARLEVE
NQAKRLLEQGLETQNPTQVGTALQVFYNLGTLKDTITSVVDGYCATLEENINSALDIKVL
TQPSQSAVRGGPGRSTMPTPGNTAALRASLWTNMEKLMDHIYAVCGQVQHLQKVLAKKRD
PVSHICFIEEIVKDGQPEIFYTFWNSVTQALSSQFHMATNSSMFLKQAFEGEYPKLLRLY
NDLWKRLQQYSQHIQGNFNASGTTDLYVDLQHMEDDAQDIFIPKKPDYDPEKALKDSLQP
YEAAYLSKSLSRLFDPINLVFPPGGRNPPSSDELDGIIKTIASELNVAAVDTNLTLAVSK
NVAKTIQLYSVKSEQLLSTQGDASQVIGPLTEGQRRNVAVVNSLYKLHQSVTKVVSSQSS
FPLAAEQTIISALKAIHALMENAVQPLLTSVGDAIEAIIITMHQEDFSGSLSSSGKPDVP
CSLYMKELQGFIARVMSDYFKHFECLDFVFDNTEAIAQRAVELFIRHASLIRPLGEGGKM
RLAADFAQMELAVGPFCRRVSDLGKSYRMLRSFRPLLFQASEHVASSPALGDVIPFSIII
QFLFTRAPAELKSPFQRAEWSHTRFSQWLDDHPSEKDRLLLIR*
>ENSG00000033100|ENST00000495645
MLSLARPPLPPTGLRTSLSLLRVSWIQGEGEDPCVEAVGERGGPQNPDSRARLDQSDEDF
KPRIVPYYRDPNKPYKKVLRTRYIQTELGSRERLLVAVLTSRATLSTLAVAVNRTVAHHF
PRLLYFTGQRGARAPAGMQVVSHGDERPAWLMSETLRHLHTHFGADYDWFFIMQDDTYVQ
APRLAALAGHLSINQDLYLGRAEEFIGAGEQARYCHGGFGYLLSRSLLLRLRPHLDGCRG
DILSARPDEWLGRCLIDSLGVGCVSQHQGQQYRSFELAKNRDPEKEGSSAFLSAFAVHPV
SEGTLMYRLHKRFSALELERAYSEIEQLQAQIRNLTVLTPEGEAGLSWPVGLPAPFTPHS
RFEVLGWDYFTEQHTFSCADGAPKCPLQGASRADVGDALETALEQLNRRYQPRLRFQKQR
LLNGYRRFDPARGMEYTLDLLLECVTQRGHRRALARRVSLLRPLSRVEILPMPYVTEATR
VQLVLPLLVAEAAAAPAFLEAFAANVLEPREHALLTLLLVYGPREGGRGAPDPFLGVKAA
AAELERRYPGTRLAWLAVRAEAPSQVRLMDVVSKKHPVDTLFFLTTVWTRPGPEVLNRCR
MNAISGWQAFFPVHFQEFNPALSPQRSPPGPPGAGPDPPSPPGADPSRGAPIGGRFDRQA
SAEGCFYNADYLAARARLAGELAGQEEEEALEGLEVMDVFLRFSGLHLFRAVEPGLVQKF
SLRDCSPRLSEELYHRCRLSNLEGLGGRAQLAMALFEQEQANST*
>ENSG00000224103|ENST00000415247
MRPEDRMFHIRAVILRALSLAFLLSLRGAGAIKADHVSTYAAFVQTHRPTGEFMFEFDED
EMFYVDLDKKETVWHLEEFGQAFSFEAQGGLANIAILNNNLNTLIQRSNHTQATNDPPEV
TVFPKEPVELGQPNTLICHIDKFFPPVLNVTWLCNGELVTEGVAESLFLPRTDYSFHKFH
YLTFVPSAEDFYDCRVEHWGLDQPLLKHWEAQEPIQMPETTETVLCALGLVLGLVGIIVG
TVLIIKSLRSGHDPRAQGTL*
>ENSG00000282880|ENST00000635195
MRNWLVLLCPCVLGAALHLWLRLRSPPPACASGAGPADQLALFPQWKSTHYDVVVGVLSA
RNNHELRNVIRSTWMRHLLQHPTLSQRFESGN*
>ENSG00000226182|ENST00000432009
MLSRNDVKRLERSWAQGSSEQELHYASLQRLPVPSSEGPDLRGRDKRGTKEDPRADYACI
AENKPT*
>ENSG00000132872|ENST00000590752
MAPITTSREEFVSLFAWICCQRKSSKSNKTPPYKFVHVLKGVDIYPENLNSKKKFGADDK
NEVKNKPAVPKNSLHLDLEKRDLNGNFPKTNLKPGSPSDLENATPKLFLEGEKESVSPES
LKSSTSLTSEEKQEKLGTLFFSLEYNFERKAFVVNIKEARGLPAMDEQSMTSDPYIKMTI
LPEKKHKVKTRVLRKTLDPAFDETFTFYGIPYTQIQELALHFTILSFDRFSRDDIIGEVL

IPLSGIELSEGKMLMNREIIKRNVRKSSGRGELLISLCYQSTTNTLTVVVLKARHLPKSD
VSGLSDPYVKVNLYHAKKRISKKKTHVKKCTPNAVFNELFVFDIPCEGLEDISVEFLVLD
SERGSRNEVIGQLVLGAAAEGTGGEHWKEICDYPRRQIAKWHVLCDG*
>ENSG00000225824|ENST00000424806
MSWKKALRIPGGLRAATVTLMLSMLSTPVAEGRDSPEDFVYQFKGMCYFTNGTERVRLVS
RSIYNREEIVRFDSDVGEFRAVTLLGLPAAEYWNSQKDILERKRAAVDRVCRHNYQLELR
TTLQRRVEPTVTISPSRTEALNHHNLLVCSVTDFYPAQIKVRWFRNGQEETAGVVSTPLI
RNGDWTFQILVMLEMTPQRGDVYTCHVEHPSLQSPITVEWRAQSESAQSKMLSGIGGFVL
GLIFLGLGLIIHHRSQKGLLH*
>ENSG00000085063|ENST00000415002
MGIQGGSVLFGLLLVLAVFCHSGHSLQCYNCPNPTADCKTAVNCSSDFDACLITKAGLQV
YNKCWKFEHCNFNDVTTRLRENELTYYCCKKDLCNFNEQLENGGTSLSEKTVLLLVTPFL
AAAWSLHP*
>ENSG00000082805|ENST00000543086
MYGSARSVGKVEPSSQSPGRSPRLPRSPRLGHRRTNSTGGSSGSSVGGGSGKTLSMENIQ
SLNAAYATSGPMYLSDHENVGSETPKSTMTLGRSGGRLPYGVRMTAMGSSPNIASSGVAS
DTIAFGEHHLPPVSMASTVPHSLRQARDNTIMDLQTQLKEVLRENDLLRKDVEVKESKLS
SSMNSIKTFWSPELKKERALRKDEASKITIWKEQYRVVQEENQHMQMTIQALQDELRIQR
DLNQLFQQDSSSRTGEPCVAELTEENFQRLHAEHERQAKELFLLRKTLEEMELRIETQKQ
TLNARDESIKKLLEMLQSKGLSAKATEEDHERTRRLAEAEMHVHHLESLLEQKEKENSML
REEMHRRFENAPDSAKTKALQTVIEMKDSKISSMERGLRDLEEEIQMLKSNGALSTEERE
EEMKQMEVYRSHSKFMKNKIGQVKQELSRKDTELLALQTKLETLTNQFSDSKQHIEVLKE
SLTAKEQRAAILQTEVDALRLRLEEKETMLNKKTKQIQDMAEEKGTQAGEIHDLKDMLDV
KERKVNVLQKKIENLQEQLRDKEKQMSSLKERVKSLQADTTNTDTALTTLEEALAEKERT
IERLKEQRDRDEREKQEEIDNYKKDLKDLKEKVSLLQGDLSEKEASLLDLKEHASSLASS
GLKKDSRLKTLEIALEQKKEECLKMESQLKKAHEAALEARASPEMSDRIQHLEREITRYK
DESSKAQAEVDRLLEILKEVENEKNDKDKKIAELERQVKDQNKKVANLKHKEQVEKKKSA
QMLEEARRREDNLNDSSQQLQDSLRKKDDRIEELEEALRESVQITAEREMVLAQEESART
NAEKQVEELLMAMEKVKQELESMKAKLSSTQQSLAEKETHLTNLRAERRKHLEEVLEMKQ
EALLAAISEKDANIALLELSSSKKKTQEEVAALKREKDRLVQQLKQQTQNRMKLMADNYE
DDHFKSSHSNQTNHKPSPDQIIQPLLELDQNRSKLKLYIGHLTTLCHDRDPLILRGLTPP
ASYNLDDDQAAWENELQKMTRGQLQDELEKGERDNAELQEFANAILQQIADHCPDILEQV
VNALEESS*
>ENSG00000175229|ENST00000527878
MPPILQRLQQATKMMSRRKILLLVLGCSTVSLLIHQGAQLSWYPKLFPLSCPPLRNSPPR
PKHMTVAFLKTHKTAGTTVQNILFRFAERHNLTVALPHPSCEHQFCYPRNFSAHFVHPAT
RPPHVLASHLRFDRAELERLMPPSTVYVTILREPAAMFESLFSYYNQYCPAFRRVPNASL
EAFLRAPEAYYRAGEHFAMFAHNTLAYDLGGDNERSPRDDAAYLAGLIRQVEEVFSLVMI
AEYFDESLVLLRRLLAWDLDDVLYAKLNARAASSRLAAIPAALARAARTWNALDAGLYDH
FNATFWRHVARAGRACVEREARELREARQRLLRRCFGDEPLLRPAAQIRTKQLQPWQPSR
KVDIMGYDLPGGGAGPATEACLKLAMPEVQYSNYLLRKQKRRGGARARPEPVLDNPPPRP
IRVLPRGPQGP*
>ENSG00000257923|ENST00000550008
MLCVAGARLKRELDATATVLANRQDESEQSRKRLIEQSREFKKNTPEDLRKQVAPLLKSF

QGEIDALSKRSKEAEAAFLNVYKRLIDVPDPVPALDLGQQLQLKVQRLHDIETENQKLRE
TLEEYNKEFAEVKNQEVTIKALKEKIREYEQTLKNQAETIALEKEQKLQNDFAEKERKLQ
ETQMSTTSKLEEAEHKVQSLQTALEKTRTELFDLKTKYDEETTAKADEIEMIMTDLERAN
QRAEVAQREAETLREQLSSANHSLQLASQIQKAPDVEQAIEVLTRSSLEVELAAKEREIA
QLVEDVQRLQASLTKLRENSASQISQLEQQLSAKNSTLKQLEEKLKGQADYEEVKKELNI
LKSMEFAPSEGAGTQDAAKPLEVLLLEKNRSLQSENAALRISNSDLSGSARRKGKDQPES
RRPGSLPAPPPSQLPRNPGEQASNTNGTHQFSPAGLSQDFFSSSLASPSLPLASTGKFAL
NSLLQRQLMQSFYSKAMQEAGSTSMIFSTGPYSTNSISSQSPLQQSPDVNGMAPSPSQSE
SAGSVSEGEEMDTAEIARQVKEQLIKHNIGQRIFGHYVLGLSQGSVSEILARPKPWNKLT
VRGKEPFHKMKQFLSDEQNILALRSIQGRQRAEPAQPSSASGSGNSDDAIRSILQQARRE
MEAQQAALDPALKQAPLSQSDITILTPKLLSTSPMPTVSSYPPLAISLKKPSAAPEAGAS
ALPNPPALKKEAQDAPGLDPQGAADCAQGVLRQVKNEVGRSGAWKDHWWSAVQPERRNAA
SSEEAKAEETGGGKEKGSGGSGGGSQPRAERSQLQGPSSSEYWKEWPSAESPYSQSSELS
LTGASRSETPQNSPLPSSPIVPMSKPTKPSVPPLTPEQYEVYMYQEVDTIELTRQVKEKL
AKNGICQRIFGEKVLGLSQGSVSDMLSRPKPWSKLTQKGREPFIRMQLWLNGELGQGVLP
VQGQQQGPVLHSVTSLQDPLQQGCVSSESTPKTSASCSPAPESPMSSSESVKSLTELVQQ
PCPPIEASKDSKPPEPSDPPASDSQPTTPLPLSGHSALSIQELVAMSPELDTYGITKRVK
EVLTDNNLGQRLFGETILGLTQGSVSDLLARPKPWHKLSLKGREPFVRMQLWLNDPNNVE
KLMDMKRMEKKAYMKRRHSSVSDSQPCEPPSVGTEYSQGASPQPQHQLKKPRVVLAPEEK
EALKRAYQQKPYPSPKTIEDLATQLNLKTSTVINWFHNYRSRIRRELFIEEIQAGSQGQA
GASDSPSARSGRAAPSSEGDSCDGVEATEGPGSADTEEPKSQGEAEREEVPRPAEQTEPP
PSGTPGPDDARDDDHEGGPVEGPGPLPSPASATATAAPAAPEDAATSAAAAPGEGPAAPS
SAPPPSNSSSSSAPRRPSSLQSLFGLPEAAGARDSRDNPLRKKKAANLNSIIHRLEKAAS
REEPIEWEF*
>ENSG00000182050|ENST00000611864
MFKFHQMKHIFEILDKMRCLRKRSTVSFLGVLVIFLLFMNLYIEDSYVLEGDKQLIRETS
THQLNSERYVHTFKDLSNFSGAINVTYRYLAATPLQRKRYLTIGLSSVKRKKGNYLLETI
KSIFEQSSYEELKEISVVVHLADFNSSWRDAMVQDITQKFAHHIIAGRLMVIHAPEEYYP
ILDGLKRNYNDPEDRVKFRSKQNVDYAFLLNFCANTSDYYVMLEDDVRCSKNFLTAIKKV
IASLEGTYWVTLEFSKLGYIGKLYHSHDLPRLAHFLLMFYQEMPCDWLLTHFRGLLAQKN
VIRFKPSLFQHMGYYSSYKGTENKLKDDDFEEESFDIPDNPPASLYTNMNVFENYEASKA
YSSVDEYFWGKPPSTGDVFVIVFENPIIIKKIKVNTGTEDRQNDILHHGALDVGENVMPS
KQRRQCSTYLRLGEFKNGNFEMSGVNQKIPFDIHCMRIYVTKTQKEWLIIRSISIWTS*
>ENSG00000257923|ENST00000546411
MLCVAGARLKRELDATATVLANRQDESEQSRKRLIEQSREFKKNTPEDLRKQVAPLLKSF
QGEIDALSKRSKEAEAAFLNVYKRLIDVPDPVPALDLGQQLQLKVQRLHDIETENQKLRE
TLEEYNKEFAEVKNQEVTIKALKEKIREYEQTLKNQAETIALEKEQKLQNDFAEKERKLQ
ETQMSTTSKLEEAEHKVQSLQTALEKTRTELFDLKTKYDEETTAKADEIEMIMTDLERAN
QRAEVAQREAETLREQLSSANHSLQLASQIQKAPDVEQAIEVLTRSSLEVELAAKEREIA
QLVEDVQRLQASLTKLRENSASQISQLEQQLSAKNSTLKQLEEKLKGQADYEEVKKELNI
LKSMEFAPSEGAGTQDAAKPLEVLLLEKNRSLQSENAALRISNSDLSGSARRKGKDQPES
RRPGSLPAPPPSQLPRNPGEQASNTNGTHQFSPAGLSQDFFSSSLASPSLPLASTGKFAL
NSLLQRQLMQSFYSKAMQEAGSTSMIFSTGPYSTNSISSQSPLQQSPDVNGMAPSPSQSE
SAGSVSEGEEMDTAEIARQVKEQLIKHNIGQRIFGHYVLGLSQGSVSEILARPKPWNKLT

VRGKEPFHKMKQFLSDEQNILALRSIQGRQRENPGQSLNRLFQEVPKRRNGSEGNITTRI
RASETGSDEAIKSILEQAKRELQVQKTAEPAQPSSASGSGNSDDAIRSILQQARREMEAQ
QAALDPALKQAPLSQSDITILTPKLLSTSPMPTVSSYPPLAISLKKPSAAPEAGASALPN
PPALKKEAQDAPGLDPQGAADCAQGVLRQVKNEVGRSGAWKDHWWSAVQPERRNAASSEE
AKAEETGGGKEKGSGGSGGGSQPRAERSQLQGPSSSEYWKEWPSAESPYSQSSELSLTGA
SRSETPQNSPLPSSPIVPMSKPTKPSVPPLTPEQYEVYMYQEVDTIELTRQVKEKLAKNG
ICQRIFGEKVLGLSQGSVSDMLSRPKPWSKLTQKGREPFIRMQLWLNGELGQGVLPVQGQ
QQGPVLHSVTSLQDPLQQGCVSSESTPKTSASCSPAPESPMSSSESVKSLTELVQQPCPP
IEASKDSKPPEPSDPPASDSQPTTPLPLSGHSALSIQELVAMSPELDTYGITKRVKEVLT
DNNLGQRLFGETILGLTQGSVSDLLARPKPWHKLSLKGREPFVRMQLWLNDPNNVEKLMD
MKRMEKKAYMKRRHSSVSDSQPCEPPSVGTEYSQGASPQPQHQLKKPRVVLAPEEKEALK
RAYQQKPYPSPKTIEDLATQLNLKTSTVINWFHNYRSRIRRELFIEEIQAGSQGQAGASD
SPSARSGRAAPSSEGDSCDGVEATEGPGSADTEEPKSQGEAEREEVPRPAEQTEPPPSGT
PGPDDARDDDHEGGPVEGPGPLPSPASATATAAPAAPEDAATSAAAAPGEGPAAPSSAPP
PSNSSSSSAPRRPSSLQSLFGLPEAAGARDSRDNPLRKKKAANLNSIIHRLEKAASREEP
IEWEF*
>ENSG00000185274|ENST00000618959
GLSKSLGLIEGYGGRGKGGLPATLSPAEEEKAKGPHEKYGYNSYLSEKISLDRSIPDYRP
TKCKELKYSKDLPQISIIFIFVNEALSVILRSVHSAVNHTPTHLLKEIILVDDNSDEEEL
KVPLEEYVHKRYPGLVKVVRNQKREGLIRARIEGWKVATGQVTGFFDAHVEFTAGWAEPV
LSRIQENRKRVILPSIDNIKQDNFEVQRYENSAHGYSWELWCMYISPPKDWWDAGDPSLP
IRTPAMIGCSFVVNRKFFGEIGLLDPGMDVYGGENIELGIKVWLCGGSMEVLPCSRVAHI
ERKKKPYNSNIGFYTKRNALRVAEVWMDDYKSHVYIAWNLPLENPGIDIGDVSERRALRK
SLKCKNFQWYLDHVYPEMRRYNNTVAYGELRNNKAKDVCLDQGPLENHTAILYPCHGWGP
QLARYTKEGFLHLGALGTTTLLPDTRCLVDNSKSRLPQLLDCDKVKSSLYKRWNFIQNGA
IMNKGTGRCLEVENRGLAGIDLILRSCTGQRWTIKNSIK*
>ENSG00000174775|ENST00000417302
MTEYKLVVVGAGGVGKSALTIQLIQNHFVDEYDPTIEDSYRKQVVIDGETCLLDILDTAG
QEEYSAMRDQYMRTGEGFLCVFAINNTKSFEDIHQYREQIKRVKDSDDVPMVLVGNKCDL
AARTVESRQAQDLARSYGIPYIETSAKTRQGSRSGSSSSSGTLWDPPGPM*
>ENSG00000108587|ENST00000427274
MAAGTSSYWEDLRKQARQLENELDLKLVSFSKLCTSYSHSSTRDGRRDSSDTTPLLNGSS
QDRMFETMAIEIEQLLARLTGVNDKMAEYTNSAGVPSLNAALMHTLQRHRDILQDYTHEF
HKTKANFMAIRERENLMGSVRKDIESYKSGSGVNNRRTELFLKEHDHLRKGFQW*
>ENSG00000110075|ENST00000529710
MFWKFDLHSSSHIDTLLEREDVTLKELMDEEDVLQECKAQNRKLIEFLLKAECLEDLVSF
IIEEPPQDMDEKIRYKYPNISCELLTSDVSQMNDRLGEDESLLMKLYSFLLNDSPLNPLL
ASFFSKVLSILISRKPEQIVDFLKKKHDFVDLIIKHIGTSAIMDLLLRLLTCIEPPQPRQ
DVLNWLNEEKIIQRLVEIVHPSQEEDRHSNASQSLCEIVRLSRDQMLQIQNSTEPDPLLA
TLEKQEIIEQLLSNIFHKEKNESAIVSAIQILLTLLETRRPTFEGHIEICPPGMSHSACS
VNKSVLEAIRGRLGSFHELLLEPPKNMFFKYTWNNFLHTQVEICIALILASPFENTENAT
ITDQDSTGDNLLLKHLFQKCQLIERILEAWEMNEKKQAEGGRRHGYMGHLTRIANCIVHS
TDKGPNSALVQQLIKDLPDEVRERWETFCTSSLGETNKRNTVDLAFSDYQMQQMTSNFID
QFGFNDEKFADQDDIGNVSFDRVSDINFTLNTNESGNIALFEACCKERIQQFDDGGSDEE

DIWEEKHIAFTPESQRRSSSGSTDSEESTDSEEEDGAKQDLFEPSSANTEDKMEVDLSEP
PNWSANFDVPMETTHGAPLDSVGSDVWSTEEPMPTKETGWASFSEFTSSLSTKDSLRSNS
PVEMETSTEPMDPLTPSAAALAVQPEAAGSVAMEASSDGEEDAESTDKVTETVMNGGMKE
TLSLTVDAKTETAVFKSEEGKLSTSQDAACKDAEECPETAEAKCAAPRPPSSSPEQSGVE
IPALPGQWSQQ*
>ENSG00000102100|ENST00000635460
XAAVGAGGSTAAPGPGAVSAGALEPGTASAAHRRLKYISLAVLVVQNASLILSIRYARTL
PGDRFFATTAVVMAEVLKGLTCLLLLFAQKRGNVKHLVLFLHEAVLVQYVDTLKLAVPSL
IYTLQNNLQYVAISNLPAATFQVAHQGEGFLAAGIEDIGLASFSLLALGPAGTKL*
>ENSG00000284501|ENST00000639538
MSRVAGTRTDVNELLQRWTPRCVRWHTGGARRVALDRPLVTACLPPAGDVVDVYQREFLA
LRDRLHAAEQESLKRSKELNLVLDEIKRAVSERQALRDGDGNRTWGRLTEDPRLKPWNGS
HRHVLHLPTVFHHLPHLLAKESSLQPAVRVGQGRTGVSVVMGIPSVRREVHSYLTDTLHS
LISELSPQEKEDSVIVVLIAETDSQYTSAVTENIKALFPTEIHSGLLEVISPSPHFYPDF
SRLRESFGDPKERVRWRTKQNLDYCFLMMYAQSKGIYYVQLEDDIVAKPNYLSTMKNFAL
QQPSEDWMILEFSQLGFIGKMFKSLDLSLIVEFILMFYRDKPIDWLLDHILWVKVCNPEK
DAKHCDRQKANLRIRFKPSLFQHVGTHSSLAGKIQKLKDKDFGKQALRKEHVNPPAEVST
SLKTYQHFTLEKAYLREDFFWAFTPAAGDFIRFRFFQPLRLERFFFRSGNIEHPEDKLFN
TSVEVLPFDNPQSDKEALQEGRTATLRYPRSPDGYLQIGSFYKGVAEGEVDPAFGPLEAL
RLSIQTDSPVWVILSEIFLKKAD*
>ENSG00000249915|ENST00000505221
MAAYSYRPGPGAGPGPAAGAALPDQSFLWNVFQRVDKDRSGVISDTELQQALSNGTWTPF
NPVTVRSII*
>ENSG00000182050|ENST00000552808
MFKFHQMKHIFEILDKMRCLRKRSTVSFLGVLVIFLLFMNLYIEDSYVLEGDKQLIRETS
THQLNSERYVHTFKDLSNFSGAINVTYRYLAATPLQRKRYLTIGLSSVKRKKGNYLLETI
KSIFEQSSYEELKEISVVVHLADFNSSWRDAMVQDITQKFAHHIIAGRLMVIHAPEEYYP
ILDGLKRNYNDPEDRVKFRSKQNVDYAFLLNFCANTSDYYVMLEDDVRCSKNFLTAIKKV
IASLEGTYWVTLEFSKLGYIGKLYHSHDLPRLAHFLLMFYQEMPCDWLLTHFRGLLAQKN
VIRFKPSLFQHMGYYSSYKGTENKLKDDDFEEESFDIPDNPPASLYTNMNVFENYEASKA
YSSVDEYFWGKPPSTGDVFVIVFENPIIIKKIKVNTGTEDRQNDILHHGALDVGENVMPS
KQRRQCSTYLRLGEFKNGNFEMSGVNQKIPFDIHCMRIYVTKTQKEWLIIRSISIWTS*
>ENSG00000138069|ENST00000409892
MSSMNPEYDYLFKLLLIGDSGVGKSCLLLRFAESFNNVKQWLQEIDRYASENVNKLLVGN
KCDLTTKKVVDYTTAKEFADSLGIPFLETSAKNATNVEQSFMTMAAEIKKRMGPGATAGG
AEKSNVKIQSTPVKQSGGGCC*
>ENSG00000078328|ENST00000547338
MNCEREQLRGNQEAAAAPDTMAQPYASAQFAPPQNGIPAEYTAPHPHPAPEYTGQTTVPE
HTLNLYPPAQTHSEQSPADTSAQTVSGTATQTDDAAPTDGQPQTQPSENTENKSQPKRLH
VSNIPFRFRDPDLRQMFGQFGKILDVEIIFNERGSKGFGFVTFENSADADRAREKLHGTV
VEGRKIEVNNATARVMTNKKTVNPYTNGWKLNPVVGAVYSPEFYAGTVLLCQANQEGSSM
YSAPSSLVYTSAMPGFPYPAATAAAAYRGAHLRGRGRTVYNTFRAAAPPPPIPAYGGVVY
QDGFYGADIYGGYAAYRYAQPTPATAAAYSDSYGRVYAADPYHHALAPAPTYGVGAMNAF
APLTDAKTRSHADDVGLVLSSLQASIYRGGYNRFAPY*

>ENSG00000226182|ENST00000434314
MLSRNDDICIYGGLGLGGLLLLAVVLLSACLCWLHRRVKRLERSWAQGSSEQELHYASLQ
RLPVPSSEGPDLRGRDKRGTKEDPRADYACIAENKPT*
>ENSG00000172780|ENST00000476465
MAGPGPGPGDPDEQYDFLFKLVLVGDASVGKTCVVQRFKTGAFSERQGSTIGVDFTMKTL
EIQGKRVKLQIWDTAGQERFRTITQSYYRSANGAILAYDITKRSSFLSVPHWIEDVRKYA
GSNIVQLLIEMQSCYVAQADLELLASSNPPASTSK*
>ENSG00000197093|ENST00000413800
MGPLSPARTLRLWGPRSLGVALGVFMTIGFALQLLGGPFQRRLPGLQLRQPSAPSLRPAL
PSCPPRQRLVFLKTHKSGSSSVLSLLHRYGDQHGLRFALPARYQFGYPKLFQASRVKGYR
PQGGGTQLPFHILCHHMRFNLKEVLQVMPSDSFFFSIVRDPAALARSAFSYYKSTSSAFR
KSPSLAAFLANPRGFYRPGARGDHYARNLLWFDFGLPFPPEKRAKRGNIHPPRDPNPPQL
QVLPSGAGPRAQTLNPNALIHPVSTVTDHRSQISSPASFDLGSSSFIQWGLAWLDSVFDL
VMVAEYFDESLVLLADALCWGLDDVVGFMHNAQAGHKQGLSTVSNSGLTAEDRQLTARAR
AWNNLDWALYVHFNRSLWARIEKYGQGRLQTAVAELRARREALAKHCLVGGEASDPKYIT
DRRFRPFQFGSAKVLGYILRSGLSPQDQEECERLATPELQYKDKLDAKQFPPTVSLPLKT
SRPLSP*
>ENSG00000151746|ENST00000551848
MAAEEVLQTVDHYKTEIERLTKELTETTHEKIQAAEYGLVVLEEKLTLKQQYDELEAEYD
SLKQELEQLKEVSCLSPLPFLALTPPTRKAHSS*
>ENSG00000101246|ENST00000612157
MYTLLSGLYKYMFQKDEYCILILGLDNAGKTTFLEQSKTRFNKNYKGMSLSKITTTVGLN
IGTVDVGKARLMFWDLGGQEELQSLWDKYYAECHGVIYVIDSTDEERLAESKQAFEKVVT
SEALCGVPVLVLANKQDVETCLSIPDIKTAFSDCTSKIGRRDCLTQACSALTG*
>ENSG00000090615|ENST00000541013
XDLTEQQGRKELEGLQQLLQNVKSELEMAQEDLSMTQKDKFMLQAKVSELKNNMKTLLQQ
NQQLKLDLRRGAAKTVLRPASLPG*
>ENSG00000273686|ENST00000617605
MSRSVALAVLALLSLSGLEAIQRTPKIQVYSRHPAENGKSNFLNCYVSGFHPSDIEVDLL
KNGERIEKVEHSDLSFSKDWSFYLLYYTEFTPTEKDEYACRVNHVTLSQPKIVKWDRDM*
>ENSG00000154917|ENST00000486858
MWHMTRNWPLVGKTSLITRFMYDSFDNTYQATIGIDFLSKTMYLEDRTVRLQLWDTAGQE
RFRSLIPSYIRDSTVAVVVYDITNLNSFQQTSKWIDDVRTERGSDVIIMLVGNKTDLADK
RQITIEEGEQRAKELSVMFIETSAKTGYNVKQLFRRVASALPGMENVQEKSKEGMIDIKL
DKPQEPPASEGGCSC*
>ENSG00000137573|ENST00000419716
MKYSCCALVLAVLGTELLGSLCSTVRSPRFRGRIQQERKNIRPNIILVLTDDQDVELGSL
QVMNKTRKIMEHGGATFINAFVTTPMCCPSRSSMLTGKYVHNHNVYTNNENCSSPSWQAM
HEPRTFAVYLNNTGYRTAFFGKYLNEYNGSYIPPGWREWLGLIKNSRFYNYTVCRNGIKE
KHGFDYAKDYFTDLITNESINYFKMSKRMYPHRPVMMVISHAAPHGPEDSAPQFSKLYPN
ASQHITPSYNYAPNMDKHWIMQYTGPMLPIHMEFTNILQRKRLQTLMSVDDSVERLYNML
VETGELENTYIIYTADHGYHIGQFGLVKGKSMPYDFDIRVPFFIRGPSVEPGSIVPQIVL
NIDLAPTILDIAGLDTPPDVDGKSVLKLLDPEKPGNRFRTNKKAKIWRDTFLVERGKFLR
KKEESSKNIQQSNHLPKYERVKELCQQARYQTACEQPGQKWQCIEDTSGKLRIHKCKGPS

DLLTVRQSTRNLYARGFHDKDKECSCRESGYRASRSQRKSQRQFLRNQGTPKYKPRFVHT
RQTRSLSVEFEGEIYDINLEEEEELQVLQPRNIAKRHDEGHKGPRDLQASSGGNRGRMLA
DSSNAVGPPTTVRVTHKCFILPNDSIHCERELYQSARAWKDHKAYIDKEIEALQDKIKNL
REVRGHLKRRKPEECSCSKQSYYNKEKGVKKQEKLKSHLHPFKEAAQEVDSKLQLFKENN
RRRKKERKEKRRQRKGEECSLPGLTCFTHDNNHWQTAPFWNLGSFCACTSSNNNTYWCLR
TVNETHNFLFCEFATGFLEYFDMNTDPYQLTNTVHTVERGILNQLHVQLMELRSCQGYKQ
CNPRPKNLDVGNKDGGSYDLHRGQLWDGWEG*
>ENSG00000276536|ENST00000631404
MTEYKLVVVGAGGVGKSALTIQLIQNHFVDEYDPTIEDSYRKQVVIDGETCLLDILDTAG
QEEYSAMRDQYMRTGEGFLCVFAINNTKSFEDIHQYREQIKRVKDSDDVPMVLVGNKCDL
AARTVESRQAQDLARSYGIPYIETSAKTRQGVEDAFYTLVREIRQHKLRKLNPPDESGPG
CMSCKCVLS*
>ENSG00000138459|ENST00000468642
MEKQCCSHPVICSLSTMYTFLLGAIFIALSSSRILLVKYSANEENKYDYLPTTVNVCSEL
VKLVFCVLVSFCVIKKDHQSRNLKYASW
>ENSG00000070371|ENST00000621271
MAQILPVRFQEHFQLQNLGINPANIGFSTLTMESDKFICIREKVGEQAQVTIIDMSDPMA
PIRRPISAESAIMNPASKVIALKAGKTLQIFNIEMKSKMKAHTMAEEVIFWKWVSVNTVA
LVTETAVYHWSMEGDSQPMKMFDRHTSLVGCQVIHYRTDEYQKWLLLVGISAQQNRVVGA
MQLYSVDRKVSQPIEGHAAAFAEFKMEGNAKPATLFCFAVRNPTGGKLHIIEVGQPAAGN
QPFVKKAVDVFFPPEAQNDFPVAMQIGAKHGVIYLITKYGYLHLYDLESGVCICMNRISA
DTIFVTAPHKPTSGIIGVNKKGQVLSVCVEEDNIVNYATNVLQNPDLGLRLAVRSNLAGA
EKLFVRKFNTLFAQGSYAEAAKVAASAPKGILRTRETVQKFQSIPAQSGQASPLLQYFGI
LLDQGQLNKLESLELCHLVLQQGRKQLLEKWLKEDKLECSEELGDLVKTTDPMLALSVYL
RANVPSKVIQCFAETGQFQKIVLYAKKVGYTPDWIFLLRGVMKISPEQGLQFSRMLVQDE
EPLANISQIVDIFMENSLIQQCTSFLLDALKNNRPAEGLLQTWLLEMNLVHAPQVADAIL
GNKMFTHYDRAHIAQLCEKAGLLQQALEHYTDLYDIKRAVVHTHLLNPEWLVNFFGSLSV
EDSVECLHAMLSANIRQNLQLCVQVASKYHEQLGTQALVELFESFKSYKGLFYFLGSIVN
FSQDPDVHLKYIQAACKTGQIKEVERICRESSCYNPERVKNFLKEAKLTDQLPLIIVCDR
FGFVHDLVLYLYRNNLQRYIEIYVQKVNPSRTPAVIGGLLDVDCSEEVIKHLIMAVRGQF
STDELVAEVEKRNRLKLLLPWLESQIQEGCEEPATHNALAKIYIDSNNSPECFLRENAYY
DSSVVGRYCEKRDPHLACVAYERGQCDLELIKVCNENSLFKSEARYLVCRKDPELWAHVL
EETNPSRRQLIDQVVQTALSETRDPEEISVTVKAFMTADLPNELIELLEKIVLDNSVFSE
HRNLQNLLILTAIKADRTRVMEYISRLDNYDALDIASIAVSSALYEEAFTVFHKFDMNAS
AIQVLIEHIGNLDRAYEFAERCNEPAVWSQLAQAQLQKDLVKEAINSYIRGDDPSSYLEV
VQSASRSNNWEDLVKFLQMARKKGRESYIETELIFALAKTSRVSELEDFINGPNNAHIQQ
VGDRCYEEGMYEAAKLLYSNVSNFARLASTLVHLGEYQAAVDNSRKASSTRTWKEVCFAC
MDGQEFRFAQLCGLHIVIHADELEELMCYYQDRGYFEELILLLEAALGLERAHMGMFTEL
AILYSKFKPQKMLEHLELFWSRVNIPKVLRAAEQAHLWAELVFLYDKYEEYDNAVLTMMS
HPTEAWKEGQFKDIITKVANVELCYRALQFYLDYKPLLINDLLLVLSPRLDHTWTVSFFS
KAGQLPLVKPYLRSVQSHNNKSVNEALNHLLTEEEDYQDAMQHAAESRDAELAQKLLQWF
LEEGKRECFAACLFTCYDLLRPDMVLELAWRHNLVDLAMPYFIQVMREYLSKVDKLDALE
SLRKQEEHVTEPAPLVFDFDGHE*
>ENSG00000114209|ENST00000470131

MRMTMEEMKNEAETTSMVSMPLYAVMYPVFNELERVNLSAAQTLRAAFIKAEKENPGLTQ
DIIMKILEKKSVEVNFTESLLRMAADDVEEYMIERPEPEFQDLNEKARALKQILSKIPDE
INDRVRFLQTIKDIASAIKELLDTVNNVFKKYQYQNRRALEHQKKEFVKYSKSFSDTLKT
YFKDGKAINVFVSANRLIHQTNLILQTFKTVA*
>ENSG00000156413|ENST00000592563
MDPLGPAKPQWSWRCCLTTLLFQLLMAVCFFSYLRVSQDDPTVYPNGSRFPDSTGTPAHS
IPLILLWTWPFNKPIALPRCSEMVPGTADCNITADRKVYPQADAVIVHHREVMYNPSAQL
PRSPRRQGQRWIWFSMESPSHCWQLKAMDGYFNLTMSYRSDSDIFTPYGWLEPWSGQPAH
PPLNLSAKTELVAWAVSNWGPNSARVRYYQSLQAHLKVDVYGRSHKPLPQGTMMETLSRY
KFYLAFENSLHPDYITEKLWRNALEAWAVPVVLGPSRSNYERFLPPDAFIHVDDFQSPKD
LARYLQELDKDHARYLSYFRWRETLRPRSFSWALAFCKACWKLQEESSGGLIYLRTRLPE
ASPA*
>ENSG00000275501|ENST00000620530
MGCLWGLALPLFFFCWEVGVSGSSAGPSTRRADTAMTTDDTEVPAMTLAPGHAALETQTL
SAETSSRASTPAGPIPEAETRGAKRISPARETRSFTKTSPNFMVLIATSVETSAASGSPE
GAGMTTVQTITGSDPREAIFDTLCTDDSSEEAKTLTMDILTLAHTSTEAKGLSSESSASS
DSPHPVITPSRASESSASSDGPHPVITPSRASESSASSDGPHPVITPSRASESSASSDGP
HPVITPSRASESSASSDGPHPVITPSRASESSASSDGPHPVITPSRASESSASSDGPHPV
ITPSRASESSASSDGPHPVITPSRASESSASSDGPHPVITPSRASESSASSDGLHPVITP
SRASESSASSDGPHPVITPSRASESSASSDGPHPVITPSWSPGSDVTLLAEALVTVTNIE
VINCSITEIETTTSSIPGASDTDLIPTEGVKASSTSDPPALPDSTEAKPHITEVTASAET
LSTAGTTESAAPDATVGTPLPTNSATEREVTAPGATTLSGALVTVSRNPLEETSALSVET
PSYVKVSGAAPVSIEAGSAVGKTTSFAGSSASSYSPSEAALKNFTPSETPTMDIATKGPF
PTSRDPLPSVPPTTTNSSRGTNSTLAKITTSAKTTMKPPTATPTTARTRPTTDVSAGENG
GFLLLRLSVASPEDLTDPRVAERLMQQLHRELHAHAPHFQVSLLRVRRG*
>ENSG00000169255|ENST00000498216
MASALWTVLPSRMSLRSLKWSLLLLSLLSFFVMWYLSLPHYNVIERVNWMYFYE
>ENSG00000141198|ENST00000575333
MAFGKSHRDPYATSVGHLIEKATFAGVQTEDWGQFMHICDIINTTQDGPKDAVKALKKRI
SKNYNHKEIQLTLSLIDMCVQNCGPSFQSLIVKKEFVKENLVKLLNPRYNLPLDIQNRIL
NFIKTWSQGFPGGVDVSEVKEVYLDLVKKGVQFPPSEAEAETARQETAQISSNPPTSVPT
APALSSVIAPKNSTVTLVPEQIGKLHSELDMVKMNVRVMSAILMENTPGSENHEDIELLQ
KLYKTGREMQERIMDLLVVVENEDVTVELIQVNEDLNNAILGYERFTRNQQRILEQNKNQ
KEATNTTSEPSAPSQDLLDLSPSPRMPRATLGELNTMNNQLSGLSK*
>ENSG00000109586|ENST00000505308
XNLLTSSVVIVFHNEGWSTLMRTVHSVIKRTPRKYLAEIVLIDDFSNKEHLKEKLDEYIK
LWNGLVKVFRNERREGLIQARSIGAQKAKLGQVLIYLDAHCEVAVNWYAPLVAPISKDRT
TCTVPLIDYIDGNDYSIEPQQGGDEDGFARGAWDWSLLWKRIPLSHKEKAKRKHKTEPYR
SPAMAGGLFAIEREFFFELGLYDPGLQIWGGENFEISYKIWQCGGKLLFVPCSRVGHIYR
LEGWQGNPPPIYVGSSPTLKNYVRVVEVWWDEYKDYFYASRPESQALPYGDISELKKFRE
DHNCKSFKWFMEEIAYDITSHYPLPPKNVDWGEIRGFETAYCIDSMGKTNGGFVELGPCH
RMGGNQLFRINEANQLMQYDQCLTKGADGSKVMITHCNLNEFKEWQYFKNLHRFTHIPSG
KCLDRSEVLHQVFISNCDSSKTTQKWEMNNIHSV*
>ENSG00000168824|ENST00000621129

MVKLGNNFAEKGTKQPLLEDGFDTIPLMTPLDVNQLQFPPPDKVVVKTKTEYEPDRKKGK
ARPPQIAEFTVSITEGVTERFKVSVLVLFALAFLTCVVFLVVYKVYKYDRACPDGFVLKN
TQCIPEGLESYYAEQDSSAREKFYTVINHYNLAKQSITRSVSPWMSVLSEEKLSEQETEA
AEKSA*
>ENSG00000283883|ENST00000639801
MMEEEELEFVEELEAVLQLTPEVQLAIEQVFPSQDPLDRADFNAVEYINTLFPTEQSLAN
IDEVVNKIRLKIRRLDDNIRTVVRGQTNVGQDGRQALEEAQKAIQQLFGKIKDIKDKAEK
SEQMVKEITRDIKQLDHAKRHLTTSITTLNHLHMLAGGVDSLEAMTRRRQYGEVANLLQG
VMNVLEHFHKYMGIPQIRQLSERVKAAQTELGQQILADFEEAFPSQGTKRPGGPSNVLRD
ACLVANILDPRIKQEIIKKFIKQHLSEYLVLFQENQDVAWLDKIDRRYAWIKRQLVDYEE
KYGRMFPREWCMAERIAVEFCHVTRAELAKIMRTRAKEIEVKLLLFAIQRTTNFEGFLAK
RFSGCTLTDGTLKKLESPPPSTNPFLEDEPTPEMEELATEKGDLDQPKKPKAPDNPFHGI
VSKCFEPHLYVYIESQDKNLGELIDRFVADFKAQGPPKPNTDEGGAVLPSCADLFVYYKK
CMVQCSQLSTGEPMIALTTIFQKYLREYAWKILSGNLPKTTTSSGGLTISSLLKEKEGSE
VAKFTLEELCLICNILSTAEYCLATTQQLEEKLKEKVDVSLIERINLTGEMDTFSTVISS
SIQLLVQDLDAACDPALTAMSKMQWQNVEHVGDQSPYVTSVILHIKQNVPIIRDNLASTR
KYFTQFCVKFANSFIPKFITHLFKCKPISMVGAEQLLLDTHSLKMVLLDLPSISSQVVRK
APASYTKIVVKGMTRAEMILKVVMAPHEPLVVFVDNYIKLLTDCNTETFQKILDMKGLKR
SEQSSMLELLRQRLPAPPSGAESSGSLSLTAPTPEQESSRIRKLEKLIKKRL*
>ENSG00000138069|ENST00000409784
MSSMNPEYDYLFKLLLIGDSGVGKSCLLLRFADDTYTESYISTIGVDFKIRTIELDGKTI
KLQIWDTAGQERFRTITSSYYRGAHGIIVVYDVTDQESFNNVKQWLQEIDRYASENVNKL
LVGNKCDLTTKKVVDYTTAKEFADSLGIPFLETSAKNATNVEQSFMTMAAEIKKRMGPGA
TAGGAEKSNVKIQSTPVKQSGGGCC*
>ENSG00000176087|ENST00000514199
MSVEDGGMPGLGRPRQARWTLMLLLSTAMYGAHAPLLALCHVDGRVPFRPSSAVLLTELT
KLLLCAFSLLVGWQAWPQGPPPWRQAAPFALSALLYGANNNLVIYLQRYMDPSTYQVLSN
LKIGSTAVLYCLCLRHRLSVRQGLALLLLMAAGACYAAGGLQVPGNTLPSPPPAAAASPM
PLHITPLGLLLLILYCLISGLSSVYTELLMKRQRLPLALQNLFLYTFGVLLNLGLHAGGG
SGPGLLEGFSGWAALVVLSQALNGLLMSAVMKHGSSITRLFVVSCSLVVNAVLSAVLLRL
QLTAAFFLATLLIGLAMRLYYGSR*
>ENSG00000070371|ENST00000617103
MAQILPVRFQEHFQLQNLGINPANIGFSTLTMESDKFICIREKVGEQAQVTIIDMSDPMA
PIRRPISAESAIMNPASKVIALKAGKTLQIFNIEMKSKMKAHTMAEEVIFWKWVSVNTVA
LVTETAVYHWSMEGDSQPMKMFDRHTSLVGCQVIHYRTDEYQKWLLLVGISAQQNRVVGA
MQLYSVDRKVSQPIEGHAAAFAEFKMEGNAKPATLFCFAVRNPTGGKLHIIEVGQPAAGN
QPFVKKAVDVFFPPEAQNDFPVAMQIGAKHGVIYLITKYGYLHLYDLESGVCICMNRISA
DTIFVTAPHKPTSGIIGVNKKGQVLSVCVEEDNIVNYATNVLQNPDLGLRLAVRSNLAGA
EKLFVRKFNTLFAQGSYAEAAKVAASAPKGILRTRETVQKFQSIPAQSGQASPLLQYFGI
LLDQGQLNKLESLELCHLVLQQGRKQLLEKWLKEDKLECSEELGDLVKTTDPMLALSVYL
RANVPSKVIQCFAETGQFQKIVLYAKKVGYTPDWIFLLRGVMKISPEQGLQFSRMLVQDE
EPLANISQIVDIFMENSLIQQCTSFLLDALKNNRPAEGLLQTWLLEMNLVHAPQVADAIL
GNKMFTHYDRAHIAQLCEKAGLLQQALEHYTDLYDIKRAVVHTHLLNPEWLVNFFGSLSV
EDSVECLHAMLSANIRQNLQLCVQVASKYHEQLGTQALVELFESFKSYKGLFYFLGSIVN

FSQDPDVHLKYIQAACKTGQIKEVERICRESSCYNPERVKNFLKEAKLTDQLPLIIVCDR
FGFVHDLVLYLYRNNLQRYIEIYVQKVNPSRTPAVIGGLLDVDCSEEVIKHLIMAVRGQF
STDELVAEVEKRNRLKLLLPWLESQIQEGCEEPATHNALAKIYIDSNNSPECFLRENAYY
DSSVVGRYCEKRDPHLACVAYERGQCDLELIKVCNENSLFKSEARYLVCRKDPELWAHVL
EETNPSRRQLIDQVVQTALSETRDPEEISVTVKAFMTADLPNELIELLEKIVLDNSVFSE
HRNLQNLLILTAIKADRTRVMEYISRLDNYDALDIASIAVSSALYEEAFTVFHKFDMNAS
AIQVLIEHIGNLDRAYEFAERCNEPAVWSQLAQAQLQKDLVKEAINSYIRGDDPSSYLEV
VQSASRSNNWEDLVKFLQMARKKGRESYIETELIFALAKTSRVSELEDFINGPNNAHIQQ
VGDRCYEEGMYEAAKLLYSNVSNFARLASTLVHLGVLCLHGWTRVPLRTAVWSSHRHSCR
*
>ENSG00000229215|ENST00000431930
MAVMAPRTLLLLLSGALALTQTWAGSHSMRYFFTSVSRPGRGEPRFIAVGYVDDTQFVRF
DSDAASQKMEPRAPWIEQEGPEYWDQETRNMKAHSQTDRANLGTLRGYYNQSEDGSHTIQ
IMYGCDVGPDGRFLRGYRQDAYDGKDYIALNEDLRSWTAADMAAQITKRKWEAVHAAEQR
RVYLEGRCVDGLRRYLENGKETLQRTDPPKTHMTHHPISDHEATLRCWALGFYPAEITLT
WQRDGEDQTQDTELVETRPAGDGTFQKWAAVVVPSGEEQRYTCHVQHEGLPKPLTLRWEL
SSQPTIPIVGIIAGLVLLGAVITGAVVAAVMWRRKSSDRKGGSYTQAASSDSAQGSDVSL
TACKV*
>ENSG00000188603|ENST00000569430
MGGCAGSRRRFSDSEGEETVPEPRLPLLDHQGAHWKNAVGFWLLGLCNNFSYVVMLSAAH
DILSHKRTSGNQSHVDPGPTPIPHNSSSRFDCNSVSTAAVLLADILPTLVIKLLAPLGLH
LLPYSPRVLVSGICAAGSFVLVAFSHSVGTSLCGVVFASISSGLGEVTFLSLTAFYPRAV
ISWWSSGTGGAGLLGALSYLGLTQAGLSPQQTLLSMLGIPALLLASYFLLLTSPEAQDPG
GEEEAESAARQPLIRTEAPESKPGSSSSLSLRERWTVFKGLLWYIVPLVVVYFAEYFINQ
GLFELLFFWNTSLSHAQQYRWYQMLYQAGVFASRSSLRCCRIRFTWALALLQCLNLVFLL
ADVWFGFLPSIYLVFLIILYEGLLGGAAYVNTFHNIALETSDEHREFAMAATCISDTLGI
SLSGLLALPLHDFLCQLS*
>ENSG00000033170|ENST00000557164
MTDLYYLSQTDGAGDWREKEAKDLTELVQRRITYLQNPKDCSKAKKLVCNINKGCGYGCQ
LHHVVYCFMIAYGTQRTLILESQNWRYATGGWETVFRPVSETCTDRSGISTGHWSGEVKD
KNVQVVELPIVDSLHPRPPYLPLAVPEDLADRLVRVHGDPAVWWVSQFVKYLIRPQPWLE
KEIEEATKKLGFKHPVIGVHVRRTDKVGTEAAFHPIEEYMVHVEEHFQLLARRMQVDKKR
VYLATDDPSLLKEAKTKYPNYEFISDNSISWSAGLHNRYTENSLRGVILDIHFLSQADFL
VCTFSSQVCRVAYEIMQTLHPDASANFHSLDDIYYFGGQNAHNQIAIYAHQPRTADEIPM
EPGDIIGVAGNHWDGYSKGVNRKLGRTGLYPSYKVREKIETVKYPTYPEAEK*
>ENSG00000126091|ENST00000533212
MGLLVFVRNLLLALCLFLVLGFLYYSAWKLHLLQWEEDSNSVVLSFDSAGQTLGSEYDRL
GFLLNLDSKLPAELATKYANFSEGACKPGYASALMTAIFPRFSKPAPMFLDDSFRKWARI
REFVPPFGIKGQECIGWLLEICGHSSAQGAP*
>ENSG00000005206|ENST00000613503
MAAAVAAALARLLAAFLLLAAQVACEYGMVHVVSQAGGPEGKDYCILYNPQWAHLPHDLS
KASFLQLRNWTASLLCSAADLPARGFSNQIPLVARGNCTFYEKVRLAQGSGARGLLIVSR
ERLVPPGGNKTQYDEIGIPVALLSYKDMLDIFTRFGRTVRAALYAPKEPVLDYNMVIIFI
MAVGTVAIGGYWAGSRDVKKRYMKHKRDDGPEKQEDEAVDVTPVMTCVFVVMCCSMLVLL

YYFYDLLVYVVIGIFCLASATGLYSCLAPCVRRLPFGKCRIPNNSLPYFHKRPQARMLLL
ALFCVAVSVVWGVFRNEDQWAWVLQDALGIAFCLYMLKTIRLPTFKACTLLLLVLFLYDI
FFVFITPFLTKSGSSIMVEVATGPSDSATREKLPMVLKVPRLNSSPLALCDRPFSLLGFG
DILVPGLLVAYCHRFDIQVQSSRVYFVACTIAYGVGLLVTFVALALMQRGQPALLYLVPC
TLVTSCAVALWRRELGVFWTGSGFAKVLPPSPWAPAPADGPQPPKDSATPLSPQPPSEEP
ATSPWPAEQSPKSRTSEEMGAGAPMREPGSPAESEGRDQAQPSPVTQPGASA*
>ENSG00000283017|ENST00000634945
MYGSARSVGKVEPSSQSPGRSPRLPRSPRLGHRRTNSTGGSSGSSVGGGSGKTLSMENIQ
SLNAAYATSGPMYLSDHENVGSETPKSTMTLGRSGGRLPYGVRMTAMGSSPNIASSGVAS
DTIAFGEHHLPPVSMASTVPHSLRQARDNTIMDLQTQLKEVLRENDLLRKDVEVKESKLS
SSMNSIKTFWSPELKKERALRKDEASKITIWKEQYRVVQEENQHMQMTIQALQDELRIQR
DLNQLFQQDSSSRTGEPCVAELTEENFQRLHAEHERQAKELFLLRKTLEEMELRIETQKQ
TLNARDESIKKLLEMLQSKGLSAKATEEDHERTRRLAEAEMHVHHLESLLEQKEKENSML
REEMHRRFENAPDSAKTKALQTVIEMKDSKISSMERGLRDLEEEIQMLKSNGALSTEERE
EEMKQMEVYRSHSKFMKNKIGQVKQELSRKDTELLALQTKLETLTNQFSDSKQHIEVLKE
SLTAKEQRAAILQTEVDALRLRLEEKETMLNKKTKQIQDMAEEKGTQAGEIHDLKDMLDV
KERKVNVLQKKIENLQEQLRDKEKQMSSLKERVKSLQADTTNTDTALTTLEEALAEKERT
IERLKEQRDRDEREKQEEIDNYKKDLKDLKEKVSLLQGDLSEKEASLLDLKEHASSLASS
GLKKDSRLKTLEIALEQKKEECLKMESQLKKAHEAALEARASPEMSDRIQHLEREITRYK
DESSKAQAEVDRLLEILKEVENEKNDKDKKIAELERQVKDQNKKVANLKHKEQVEKKKSA
QMLEEARRREDNLNDSSQQLQDSLRKKDDRIEELEEALRESVQITAEREMVLAQEESART
NAEKQVEELLMAMEKVKQELESMKAKLSSTQQSLAEKETHLTNLRAERRKHLEEVLEMKQ
EALLAAISEKDANIALLELSSSKKKTQEEVAALKREKDRLVQQLKQQTQNRMKLMADNYE
DDHFKSSHSNQTNHKPSPDQIIQPLLELDQNRSKLKLYIGHLTTLCHDRDPLILRGLTPP
ASYNLDDDQAAWENELQKMTRGQLQDELEKGERDNAELQEFANAILQQIADHCPDILEQV
VNALEESS*
>ENSG00000198488|ENST00000528622
MAFPCRRSLTAKTLACLLVGVSFLALQQWFLQAPRSPREERSPQEETPEGPTDAPAADEP
PSELVPGPPCVANASANATADFEQLPARIQDFLRYRHCRHFPLLWDAPAKCAGGRGVFLL
L
>ENSG00000206306|ENST00000415796
XLLKLTRDFPQRHEKYQSWAGTFHLCLKKNKYPDSRSTFIKHAGSYLKRGEFLAIHTAIT
CFFRQISARFLEYSTSECHFFNGTERVRYLDRYFHNQEENVRFDSDVGEFRAVTELGRPD
AEYWNSQKDLLEQKRGRVDNYCRHNYGVVESFTVQRRVHPKVTVYPSKTQPLQHHNLLVC
SVSGFYPGSIEVRWFRNGQEEKTGVVSTGLIHNGDWTFQTLVMLETVPRSGEVYTCQVEH
PSVTSPLTVEW
>ENSG00000282725|ENST00000631534
MNRFRVSKFRHTEARPPRRESWISDIRAGTAPSCRNHIKSSCSLIAFNSDRPGVLGIVPL
QGQGEDKRRVAHLGCHSELAAHGDLVQSAVWSRDGALVGTACKDKQLRIFDPRTKPRASQ
STQAHENSRDSRLAWMGTWEHLVSTGFNQMREREVKLWDTRFFSSALASLTLDTSLGCLV
PLLDPDSGLLVLAGKGERQLYCYEVVPQQPALSPVTQCVLESVLRGAALVPRQALAVMSC
EVLRVLQLSDTAIVPIGYHVPRKAVEFHEDLFPDTAGCVPATDPHSWWAGDNQQVQKVSL
NPACRPHPSFTSCLVPPAEPLPDTAQPAVMETPVGDADASEGFSSPPSSLTSPSTPSSLG
PSLSSTSGIGTSPSLRSLQSLLGPSSKFRHAQGTVLHRDSHITNLKGLNLTTPGESDGFC

ANKLRVAVPLLSSGGQVAVLELRKPGRLPDTALPTLQNGAAVTDLAWDPFDPHRLAVAGE
DARIRLWRVPAEGLEEVLTTPETVLTGHTEKICSLRFHPLAANVLASSSYDLTVRIWDLQ
AGADRLKLQGHQDQIFSLAWSPDGQQLATVCKDGRVRVYRPRSGPEPLQEGPGPKGGRGA
RIVWVCDGRCLLVSGFDSQSERQLLLYEAEALAGGPLAVLGLDVAPSTLLPSYDPDTGLV
LLTGKGDTRVFLYELLPESPFFLECNSFTSPDPHKGLVLLPKTECDVREVELMRCLRLRQ
SSLEPVAFRLPRVRKEFFQDDVFPDTAVIWEPVLSAEAWLQGANGQPWLLSLQPPDMSPV
SQAPREAPARRAPSSAQYLEEKSDQQKKEELLNAMVAKLGNREDPLPQDSFEGVDEDEWD
*
>ENSG00000163291|ENST00000512760
MHQKLLKSAHYIELGSYQYWPVLVPRGIRLYTYEQIPGSLKDNPYITDGYRAYLPSRLCI
KRSVCFALWAIIFFPAIGQKKHVEDGWH*
>ENSG00000275066|ENST00000621136
MALRPGAGSGGGGAAGAGAGSAGGGGFMFPVAGGIRPPQAGLMPMQQQGFPMVSVMQPNM
QGIMGMNYSSQMSQGPIAMQAGIPMGPMPAAGMPYLGQAPFLGMRPPGPQYTPDMQKQFA
EEQQKRFEQQQKLLEEERKRRQFEEQKQKLRLLSSVKPKTGEKSRDDALEAIKGNLDGFS
RDAKMHPTPASHPKKPGVGVFPSQDPAQPRMPPWIYNESLVPDAYKKILETTMTPTGIDT
AKLYPILMSSGLPRETLGQIWALANRTTPGKLTKEELYTVLAMIAVTQRGVPAMSPDALN
QFPAAPIPTLSGFSMTLPTPVSQPTVIPSGPAGSMPLSLGQPVMGINLVGPVGGAAAQAS
SGFIPTYPANQVVKPEEDDFQDFQDASKSGSLDDSFSDFQELPASSKTSNSQHGNSAPSL
LMPLPGTKALPSMDKYAVFKGIAADKSSENTVPPGDPGDKYSAFRELEQTAENKPLGESF
AEFRSAGTDDGFTDFKTADSVSPLEPPTKDKTFPPSFPSGTIQQKQQTQVKNPLNLADLD
MFSSVNCSSEKPLSFSAVFSTSKSVSTPQSTGSAATMTALAATKTSSLADDFGEFSLFGE
YSGLAPVGEQDDFADFMAFSNSSISSEQKPDDKYDALKEEASPVPLTSNVGSTVKGGQNS
TAASTKYDVFRQLSLEGSGLGVEDLKDNTPSGKSDDDFADFHSSKFSSINSDKSLGEKAV
AFRHTKEDSASVKSLDLPSIGGSSVGKEDSEDALSVQFDMKLADVGGDLKHVMSDSSLDL
PTVSGQHPPAADIEDLKYAAFGSYSSNFAVSTLTSYDWSDRDDATQGRKLSPFVLSAGSG
SPSATSILQKKETSFGSSENITMTSLSKVTTFVSEDALPETTFPALASFKDTIPQTSEQK
EYENRDYKDFTKQDLPTAERSQEATCPSPASSGASQETPNECSDDFGEFQSEKPKISKFD
FLVATSQSKMKSSEEMIKSELATFDLSVQGSHKRSLSLGDKEISRSSPSPALEQPFRDRS
NTLNEKPALPVIRDKYKDLTGEVEENERYAYEWQRCLGSALNVIKKANDTLNGISSSSVC
TEVIQSAQGMEYLLGVVEVYRVTKRVELGIKATAVCSEKLQQLLKDIDKVWNNLIGFMSL
ATLTPDENSLDFSSCMLRPGIKNAQELACGVCLLNVDSRSRAFNSETDSFKLAYGGHQYH
ASCANFWINCVEPKPPGLVLPDLL*
>ENSG00000108587|ENST00000467337
MFETMAIEIEQLLARLTGVNDKMAEYTNSAGVPSLNAALMHTLQRHRDILQDYTHEFHKT
KANFMAIRERENLMGSVRKDIESYKSGSGVNNRRTELFLKEHDHLRNSDRLIEETISIAM
ATKENMTSQRGMLKSIHSKMNTLANRFPAVNSLIQRINLRKRRDSLILGGVIGICTILLL
LYAFH*
>ENSG00000101246|ENST00000607873
MSLSKITTTVGLNIGTVDVGKARLMFWDLGGQEELQSLWDKYYAECHGVIYVIDSTDEER
LAESKQAFEKVVTSEALCGVPVLVLANKQDVETCLSIPDIKTAFSDCTSKIGRRDCLTQA
CSALTGKGVREGIEWMVKCVVRNVHRPPRQRDIT*
>ENSG00000147408|ENST00000519222
MMMVRRGLLAWISRVVVLLVLLCCAISVLYMLACTPKGDEEQLALPRANSPTGKEGYQAV

LQEWEEQHRNYVSSLKRQIAQLKEELQERSEQLRNGQYQASDAAGLGLDRSPPEKTQADL
LAFLHSQVDKAEVNAGVKLATEYAAVPFDSFTLQKVYQLETGLTRHPEEKPVRKDKRDEL
VEAIESALETLNSPAENSPNHRPYTASDFIEGIYRTERDKGTLYELTFKGDHKHEFKRLI
LFRPFGPIMKVKNEKLNMANTLINVIVPLAKRVDKFRQFMQNFRPADEVFRCVPLSP*
>ENSG00000007255|ENST00000592647
MADTVLFEFLHTEMVAELWAHDPDPGPGVSAGLRGEEAGATKAAPGDAGLQGGAGCPQVL
VQRPVGGGVPEADGQPAHQSPGDLRPARQQLPPPPPDGLWPAVSGGSTQVPGLHLRPPAR
RPLYPGH*
>ENSG00000106086|ENST00000449726
MEGVLYKWTNYLSGWQPRWFLLCGGILSYYDSPEDAWKGCKGSIQMAVCEIQVHSVDNTR
MDLIIPGEQYFYLKARSVAERQRWLVALGSAKACLTDSRTQKEKEFAENTENLKTKMSEL
RLYCDLLVQQVDKTKEVTTTGVSNSEEGIDVGTLLKSTCNTFLKTLEECMQIANAAFTSE
LLYRTPPGSPQLAMLKSSKMKHPIIPIHNSLERQMELSTCENGSLNMEINGEEEILMKNK
NSLYLKSAEIDCSISSEENTDDNITVQGEIRKEDGMENLKNHDNNLTQSGSDSSCSPECL
WEEGKEVIPTFFSTMNTSFSDIELLEDSGIPTEAFLASCYAVVPVLDKLGPTVFAPVKMD
LVGNIKKVNQKYITNKEEFTTLQKIVLHEVEADVAQVRNSATEALLWLKRGLKFLKGFLT
EVKNGEKDIQTALNNAYGKTLRQHHGWVVRGVFALALRAAPSYEDFVAALTVKEGDHQKE
AFSIGMQRDLSLYLPAMEKQLAILDTLYEVHGLESDEVV*
>ENSG00000147408|ENST00000454498
MMMVRRGLLAWISRVVVLLVLLCCAISVLYMLACTPKGDEEQLALPRANSPTGKEGYQAV
LQEWEEQHRNYVSSLKRQIAQLKEELQERSEQLRNGQYQASDAAGLGLDRSPPEKTQADL
LAFLHSQVDKAEVNAGVKLATEYAAVPFDSFTLQKVYQLETGLTRHPEEKPVRKDKRDEL
VEAIESALETLNSPAENSPNHRPYTASDFIEGIYRTERDKGTLYELTFKGDHKHEFKRLI
LFRPFGPIMKVKNEKLNMANTLINVIVPLAKRVDKFRQFMQNFREMCIEQDGRVHLTVVY
FGKEEINEVKGILENTSKAANFRNFTFIQLNGEFSRGKGLDVGARFWKGSNVLLFFCDVD
IYFTSEFLNTCRLNTQPGKKVFYPVLFSQYNPGIIYGHHDAVPPLEQQLVIKKETGFWRD
FGFGMTCQYRSDFINIGGFDLDIKGWGGEDVHLYRKYLHSNLIVVRTPVRGLFHLWHEKR
CMDELTPEQYKMCMQSKAMNEASHGQLGMLVFRHEIEAHLRKQKQKTSSKKT*
>ENSG00000235346|ENST00000457132
MVVMAPRTLFLLLSGALTLTETWAGSHSMRYFSAAVSRPGRGEPRFIAMGYVDDTQFVRF
DSDSACPRMEPRAPWVEQEGPEYWEEETRNTKAHAQTDRMNLQTLRGYYNQSEAKQSSLP
TIPIMGIVAGLVVLAAVVTGAAVAAVLWRKKSSD*
>ENSG00000100478|ENST00000622409
MIKFFLMVNKQGQTRLSKYYEHVDINKRTLLETEVIKSCLSRSNEQCSFIEYKDFKLIYR
QYAALFIVVGVNDTENEMAIYEFIHNFVEVLDEYFSRVSELDIMFNLDKVHIILDEMVLN
GCIVETNRARILAPLLILDKMSES*
>ENSG00000137502|ENST00000612684
MSMEDYDFLFKIVLIGNAGVGKTCLVRRFTQGLFPPGQGATIGVDFMIKTVEINGEKVKL
QIWDTAGQERFRSITQSYYRSANALILTYDITCEESFRCLPEWLREIEQYASNKVITVLV
GNKIDLAERREVSQQRAEEFSEAQDMYYLETSAKESDNVEKLFLDLACRLISEARQNTLV
NNVSSPLPGEGKSISYLTCCNFN*
>ENSG00000110080|ENST00000444328
MVSKSRWKLLAMLALVLVVMVWYSISREDRYIELFYFPIPEKKEPCLQGEAESKASKLFG
NYSRDQPIFLRLEDYFWVKTPSAYELPYGTKGSEDLLLRVLAITSSSIPKNIQSLRCRRC

VVVGNGHRLRNSSLGDAINKYDVVIRLNNAPVAGYEGDVGSKTTMRLFYPESAHFDPKVE
NNPDTLLVLVAFKAMDFHWIETILSDKKRVRKGFWKQPPLIWDVNPKQIRILNPFFMEIA
ADKLLSLPMQQPRKIKQKPTTGLLAITLALHLCDLVHIAGFGYPDAYNKKQTIHYYEQIT
LKSMAGSGHNVSQEALAIKRMLEMGAIKNLTSF*
>ENSG00000204642|ENST00000434407
MAPRSLLLLLSGALALTDTWAGSHSLRYFSTAVSRPGRGEPRYIAVEYVDDTQFLRFDSD
AAIPRMEPREPWVEQEGPQYWEWTTGYAKANAQTDRVALRNLLRRYNQSEAGSHTLQGMN
GCDMGPDGRLLRGYHQHAYDGKDYISLNEDLRSWTAADTVAQITQRFYEAEEYAEEFRTY
LEGECLELLRRYLENGKETLQRAEQSPQPTIPIVGIVAGLVVLGAVVTGAVVAAVMWRKK
SSDRNRGSYSQAAV*
>ENSG00000206433|ENST00000443340
MLSRNDDICIYGGLGLGGLLLLAVVLLSACLCWLHRRVKRLERSWAQGSSEQELHYASLQ
RLPVPSSEGPDLRGRDKRGTKEDPRADYACIAENKPT*
>ENSG00000204482|ENST00000438075
MLSRNDDICIYGGLGLGGLLLLAVVLLSACLCWLHRRVKRLERSWAQGSSEQELHYASLQ
RLPVPSSEGPDLRGRDKRGTKEDPRADYACIAENKPT*
>ENSG00000197217|ENST00000417069
MGRIGISCLFPASWHFSISPVGCPRILNTNLRQIMVISVLAAAVSLLYFSVVIIRNKYGR
LTRDKKFQRYLARVTDIEATDTNNPNVNYGIVVDCGSSGSRVFVYCWPRHNGNPHDLLDI
RQMRDKNRKPVVMKIKPGISEFATSPEKVSDYISPLLNFAAEHVPRAKHKETPLYILCTA
GMRILPESQQKAILEDLLTDIPVHFDFLFSDSHAEVISGKQEGVYAWIGINFVLGRFEHI
EDDDEAVVEVNIPGSESSEAIVRKRTAGILDMGGVSTQIAYEVPKTEEVAKNLLAEFNLG
CDVHQTEHVYRVYVATFLGFGGNAARQRYEDRIFANTIQKNRLLGKQTGLTPDMPYLDPC
LPLDIKDEIQQNGQTIYLRGTGDFDLCRETIQPFMNKTNETQTSLNGVYQPPIHFQNSEF
YGFSEFYYCTEDVLRMGGDYNAAKFTKAAKDYCATKWSILRERFDRGLYASHADLHRLKY
QCFKSAWMFEVFHRGFSFPVNYKSLKTALQVYDKEVQWTLGAILYRTRFLPLRDIQQEAF
RASHTHWRGVSFVYNHYLFSGCFLVVLLAILLYLLRLRRIHRRTPRSSSAAALWMEEGLP
AQNAPGTL*
>ENSG00000125447|ENST00000537686
MAEAEGESLESWLNKATNPSNRQEDWEYIIGFCDQINKELEGPQIAVRLLAHKIQSPQEW
EALQALTVLEACMKNCGRRFHNEVGKFRFLNELIKVVSPKYLGDRVSEKVKTKVIELLYS
WTMALPEEAKIKDAYHMLKRQGIVQSDPPIPVDRTLIPSPPPRPKNPVFDDEEKSKLLAK
LLKSKNPDDLQEANKLIKSMVKEDEARIQKVTKRLHTLEEVNNNVRLLSEMLLHYSQEDS
SDGDRELMKELFDQCENKRRTLFKLASETEDNDNSLGDILQASDNLSRVINSYKTIIEGQ
VINGEVATLTLPDSEGNSQCSNQGTLIDLAELDTTNSLSSVLAPAPTPPSSGIPILPPPP
QASGPPRSRSSSQAEATLGPSSTSNALSWLDEELLCLGLADPAPNVPPKESAGNSQWHLL
QREQSDLDFFSPRPGTAACGASDAPLLQPSAPSSSSSQAPLPPPFPAPVVPASVPAPSAG
SSLFSTGVAPALAPKVEPAVPGHHGLALGNSALHHLDALDQLLEEAKVTSGLVKPTTSPL
IPTTTPARPLLPFSTGPGSPLFQPLSFQSQGSPPKGPELSLASIHVPLESIKPSSALPVT
AYDKNGFRILFHFAKECPPGRPDVLVVVVSMLNTAPLPVKSIVLQAAVPKSMKVKLQPPS
GTELSPFSPIQPPAAITQVMLLANPLKEKVRLRYKLTFALGEQLSTEVGEVDQFPPVEQW
GNL*
>ENSG00000108669|ENST00000585509
MQRNKQVAMGRKKFNMDPKKGIQFLIENDLLKNTCEDIAQFLYKGEGLNKTAIGDYLGER

DEFNIQVLHAFVELHEFTDLNLVQALRQFLWSFRLPGEAQKIDRMMEAFAQRYCQCNNGV
FQSTDTCYVLSFAIIMLNTSLHNPNVKDKPTVERFIAMNRGINDGGDLPEELLRNLYESI
KNEPFKIPEDDGNDLTHTFFNPDREGWLLKLGGGRVKTWKRRWFILTDNCLYYFEYTTDK
EPRGIIPLENLSIREVEDSKKPNCFELYIPDNKDQVIKACKTEADGRVVEGNHTVYRISA
PTPEEKEEWIKCIKAAISRDPFYEMLAARKKKVSSTKRH*
>ENSG00000101246|ENST00000618568
MYTLLSGLYKYMFQKDEYCILILGLDNAGKTTFLEQSKTRFNKNYKGMSLSKITTTVGLN
IGTVDVGKARLMFWDLGGQEELQSLWDKYYAECHGVIYVIDSTDEERLAESKQAFDVPLN
P*
>ENSG00000154265|ENST00000588877
MSTAIREVGVWRQTRTLLLKNYLIKCRTKKSSVQEILFPLFFLFWLILISMMHPNKKYEE
VPNIELNPMDKFTLSNLILGYTPVTNITSSIMQKVSTDHLPDVIITEEYTNEKEMLTSSL
SKPSNFVGVVFKDSMSYELRFFPDMIPVSSIYMDSRAGCSKSCEAAQYWSSGFTVLQASI
DAAIIQLKTNVSLWKELESTKAVIMGETAVVEIDTFPRGVILIYLVIAFSPFGYFLAIHI
VAEKEKKIKEFLKIMGLHDTAFWLSWVLLYTSLIFLMSLLMAVIATASLLFPQSSSIVIF
LLFFLYGLSSVFFALMLTPLFKKSKHVGIVEFFVTVAFGFIGLMIILIESFPKSLVWLFS
PFCHCTFVIGIAQVMHLEDFNEGASFSNLTAGPYPLIITIIMLTLNSIFYVLLAVYLDQV
IPGEFGLRRSSLYFLKPSYWSKSKRNYEELSEGNVNGNISFSEIIEPVSSEFVGKEAIRI
SGIQKTYRKKGENVEALRNLSFDIYEGQITALLGHSGTGKSTLMNILCGLCPPSDGFASI
YGHRVSEIDEMFEARKMIGICPQLDIHFDVLTVEENLSILASIKGIPANNIIQEVQKVLL
DLDMQTIKDNQAKKLSGGQKRKLSLGIAVLGNPKILLLDEPTAGMDPCSRHIVWNLLKYR
KANRVTVFSTHFMDEADILADRKAVISQGMLKCVGSSMFLKSKWGIGYRLSMYIDKYCAT
ESLSSLVKQHIPGATLLQQNDQQLVYSLPFKDMDKFSGLFSALDSHSNLGVISYGVSMTT
LEDVFLKLEVEAEIDQADYSVFTQQPLEEEMDSKSFDEMEQSLLILSETKAALVSTMSLW
KQQMYTIAKFHFFTLKRESKSVRSVLLLLLIFFTVQIFMFLVHHSFKNAVVPIKLVPDLY
FLKPGDKPHKYKTSLLLQNSADSDISDLISFFTSQNIMVTMINDSDYVSVAPHSAALNVM
HSEKDYVFAAVFNSTMVYSLPILVNIISNYYLYHLNVTETIQIWSTPFFQEITDIVFKIE
LYFQAALLGIIVTAMPPYFAMENAENHKIKAYTQLKLSGLLPSAYWIGQAVVDIPLFFII
LILMLGSLLAFHYGLYFYTVKFLAVVFCLIGYVPSVILFTYIASFTFKKILNTKEFWSFI
YSVAALACIAITEITFFMGYTIATILHYAFCIIIPIYPLLGCLISFIKISWKNVRKNVDT
YNPWDRLSVAVISPYLQCVLWIFLLQYYEKKYGGRSIRKDPFFRNLSTKSKNRKLPEPPD
NEDEDEDVKAERLKVKELMGCQCCEEKPSIMVSNLHKEYDDKKDFLLSRKVKKVATKYIS
FCVKKGEILGLLGPNGAGKSTIINILVGDIEPTSGQVFLGDYSSETSEDDDSLKCMGYCP
QINPLWPDTTLQEHFEIYGAVKGMSASDMKEVISRITHALDLKEHLQKTVKKLPAGIKRK
LCFALSMLGNPQITLLDEPSTGMDPKAKQHMWRAIRTAFKNRKRAAILTTHYMEEAEAVC
DRVAIMVSGQLRCIGTVQHLKSKFGKGYFLEIKLKDWIENLEVDRLQREIQYIFPNASRQ
ESFSSILAYKIPKEDVQSLSQSFFKLEEAKHAFAIEEYSFSQATLEQVFVELTKEQEEED
NSCGTLNSTLWWERTQEDRVVF*
>ENSG00000068001|ENST00000447092
MRAGPGPTVTLALVLAVSWAMELKPTAPPIFTGRPFVVAWDVPTQDCGPRLKVPLDLNAF
DVQASPNEGFVNQNITIFYRDRLGLYPRFDSAGRSVHGGVPQNVSLWAHRKMLQKRVEHY
IRTQESAGLAVIDWEDWRPVWVRNWQDKDVYRRLSRQLVASRHPDWPPDRIVKQAQYEFE
FAAQQFMLETLRYVKAVRPRHLWGFYLFPDCYNHDYVQNWESYTGRCPDVEVARNDQLAW
LWAESTALFPSVYLDETLASSRHGRNFVSFRVQEALRVARTHHANHALPVYVFTRPTYSR

RLTGLSEMDLISTIGESAALGAAGVILWGDAGYTTSTETCQYLKDYLTRLLVPYVVNVSW
ATQYCSRAQCHGHGRCVRRNPSASTFLHLSTNSFRLVPGHAPGEPQLRPVGELSWADIDH
LQTHFRCQCYLGWSGEQCQWDHRQAAGGASEAWAGSHLTSLLALAALAFTWTL*
>ENSG00000136699|ENST00000431183
MTTFGAVAEWRLPSLRRATLWIPQWFAKKAIFNSPLEAAMAFPHLQQPSFLLASLKADSI
NKPFAQQCQDLVKVIEDFPAKGPVKASIQECILPDSPLYHNKVQFTPTGGLGLNLALNPF
EYYIFFFALSLITQKPLPVSLHVRTSDCAYFILVDRYLSWFLPTEGSVPPPLSSSPGGTS
PSPPPRTPAIPFASYGLHHTSLLKRHISHQTSVNADPASHEIWRSETLLQVFVEMWLHHY
SLEMYQKMQSPHAKESFTPTEEHVLVVRLLLKHLHAFANSLKPEQASPSAHSHATSPLEE
FKRAAVPRFVQQKLYLFLQHCFGHWPLDASFRAVLEMWLSYLQPWRYAPDKQAPGSDSQP
RCVSEKWAPFVQENLLMYTKLFVGFLNRALRTDLVSPKHALMVFRVAKVFAQPNLAEMIQ
KGEQLFLEPELVIPHRQHRLFTAPTFTGSFLSPWPPAVTDASFKVKSHVYSLEGQDCKYT
PMFGPEARTLVLRLAQLITQAKHTAKSISDQCAESPAGHSFLSWLGFSSMDTNGSYTAND
LDEMGQDSVRKTDEYLEKALEYLRQIFRLSEAQLRQFTLALGTTQDENGKKQLPDCIVGE
DGLILTPLGRYQIINGLRRFEIEYQGDPELQPIRSYEIASLVRTLFRLSSAINHRFAGQM
AALCSRDDFLGSFCRYHLTEPGLASRHLLSPVGRRQVAGHTRGPRLSLRFLGSYRTLVSL
LLAFFVASLFCVGPLPCTLLLTLGYVLYASAMTLLTERGKLHQP*
>ENSG00000163291|ENST00000515853
MHQKLLKSAHYIELGSYQYWPVLVPRGIRLYTYEQIPGSLKDNPYITDGYRAYLPSRLCI
KRSVCFALWAIIFFPAIGQKKHVEDGWH*
>ENSG00000226182|ENST00000422035
MLSRNDVKRLERSWRLPVPSSEGPDLRGRDKRGTKEDPRADYACIAENKPT*
>ENSG00000232126|ENST00000450871
MRVTAPRTVLLLLSGALALTETWAGSHSMRYFYTAMSRPGRGEPRFIAVGYVDDTQFVRF
DSDAASPRMAPRAPWIEQEGPEYWDRETQISKTNTQTYRESLRNLRGYYNQSEAGSHTLQ
RMYGCDVGPDGRLLRGHDQSAYDGKDYIALNEDLSSWTAADTAAQITQRKWEAAREAEQW
RAYLEGLCVEWLRRYLENGKETLQRADPPKTHVTHHPISDHEATLRCWALGFYPAEITLT
WQRDGEDQTQDTELVETRPAGDRTFQKWAAVVVPSGEEQRYTCHVQHEGLPKPLTLRWEP
SSQSTIPIVGIVAGLAVLAVVVIGAVVATVMCRRKSSGGKGGSYSQAASSDSAQGSDVSL
TA*
>ENSG00000008513|ENST00000522652
MVTLRKRTLKVLTFLVLFIFLTSFFLNYSHTMVATTWFPKQMVLELSENLKRLIKHRPCT
CTHCIGQRKLSAWFDERFNQTMQPLLTAQNALLEDDTYRWWLRLQREKKPNNLNDTIKEL
FRVVPGNVDPMLEKRSVGCRRCAVVGNSGNLRESSYGPEIDSHDFVLRMNKAPTAGFEAD
VGTKTTHHLVYPESFRELGDNVSMILVPFKTIDLEWVVSAITTGTISHTYIPVPAKIRVK
QDKILIYHPAFIKYVFDNWLQGHGRYPSTGILSVIFSMHVCDEVDLYGFGADSKGNWHHY
WENNPSAGAFRKTGVHDADFESNVTATLASINKIRIFKGR*
>ENSG00000139182|ENST00000537408
MVLGCELSGSTRVVVGVEALLTGASSPLPGVGPANKHKPWIEAEYQGIVMENDNTVLLNP
PLFALDKDAPLRYAGEICGFRLHGSGVPFEAVILDKATGEGLIRAKEPVDCEAQKEHTFT
IQAYDCGEGPDGANTKKSHKATVHVRVNDVNEFAPVFVERLYRAAVTEGKLYDRILRVEA
IDGDCSPQYSQICYYEILTPNTPFLIDNDGNIENTEKLQYSGERLYKFTVTAYDCGKKRA
ADDAEVEIQVKPTCKPSWQGWNKRIEYAPGAGSLALFPGIRLETCDEPLWNIQATIELQT
SHVAKGCDRDNYSERALRKLCGAATGEVDLLPMPGPNANWTAGLSVHYSQDSSLIYWFNG

TQAVQVPLGGPSGLGSGPQDSLSDHFTLSFWMKHGVTPNKGKKEEETIVCNTVQNEDGFS
HYSLTVHGCRIAFLYWPLLESARPVKFLWKLEQVCDDEWHHYALNLEFPTVTLYTDGISF
DPALIHDNGLIHPPRREPALMIGACWTEEKNKEKEKGDNSTDTTQGDPLSIHHYFHGYLA
GFSVRSGRLESREVIECLYACREGLDYRDFESLGKGMKVHVNPSQSLLTLEGDDVETFNH
ALQHVAYMNTLRFATPGVRPLRLTTAVKCFSEESCVSIPEVEGYVVVLQPDAPQILLSGT
AHFARPAVDFEGTNGVPLFPDLQITCSISHQVEAKKDESWQGTVTDTRMSDEIVHNLDGC
EISLVGDDLDPERESLLLDTTSLQQRGLELTNTSAYLTIAGVESITVYEEILRQARYRLR
HGAALYTRKFRLSCSEMNGRYSSNEFIVEVNVLHSMNRVAHPSHVLSSQQFLHRGHQPPP
EMAGHSLASSHRNSMIPSAATLIIVVCVGFLVLMVVLGLVRIHSLHRRVSGAGGPPGASS
DPKDPDLFWDDSALTIIVNPMESYQNRQSCVTGAVGGQQEDEDSSDSEVADSPSSDERRI
IETPPHRY*
>ENSG00000175416|ENST00000510734
MADDFGFFSSSESGAPEAAEEDPAAAFLAQQESEIAGIENDEGFGAPAGSHAAPAQPGPT
SGAGSEDMGTTVNGDVFQPVHHQRVQLQTQLPIQPKDEPAHNYSSSIWHSQGQGQKGPAG
LRLPTSAQILSPFP*
>ENSG00000117620|ENST00000639994
MFANLKYVSLGILVFQTTSLVLTMRYSRTLKEEGPRYLSSTAVVVAELLKIMACILLVYK
DSKCSLRALNRVLHDEILNKPMETLKLAIPSGIYTLQNNLLYVALSNLDAATYQVTYQLK
ILTTALFSVSMLSKKLGVYQWLSLVILMTGVAFVQWPSDSQLDSKELSAGSQFVGLMAVL
TACFSSGFAGVYFEKILKETKQSVWIRNIQLGFFGSIFGLMGVYIYDGELVSKNGFFQGY
NRLTWIVVVLQALGGLVIAAVIKYADNILKGFATSLSIILSTLISYFWLQDFVPTR*
>ENSG00000182050|ENST00000621808
MFKFHQMKHIFEILDKMRCLRKRSTVSFLGVLVIFLLFMNLYIEDSYVLEGDKQLIRETS
THQLNSERYVHTFKDLSNFSGAINVTYRYLAATPLQRKRYLTIGLSSVKRKKGNYLLETI
KSIFEQSSYEELKEISVVVHLADFNSSWRDAMVQDITQKFAHHIIAGRLMVIHAPEEYYP
ILDGLKRNYNDPEDRVKFRSKQNVDYAFLLNFCANTSDYYVMLEDDVRCSKNFLTAIKKV
IASLEGTYWVTLEFSKLGYIGKLYHSHDLPRLAHFLLMFYQEMPCDWLLTHFRGLLAQKN
VIRFKPSLFQHMGYYSSYKGTENKLKDDDFEEESFDIPDNPPASLYTNMNVFENYEASKA
YSSVDEYFWGKPPSTGDVFVIVFENPIIIKKIKVNTGTEDRQNDILHHGALDVGENVMPS
KQRRQCSTYLRLGEFKNGNFEMSGVNQKIPFDIHCMRIYVTKTQKEWLIIRSISIWTS*
>ENSG00000114650|ENST00000495603
MTLTERLREKISRAFYNHGLLCASYPIPIILFTGFCILACCYPLLKLPLPGTGPVEFTTP
VKDYSPPPVDSDRKQGEPTEQPEWYVGAPVAYVQQIFVKSSVFPWHKNLLAVDVFRSPLS
RAFQLVEEIRNH
>ENSG00000111846|ENST00000495262
MMGSWKHCLFSASLISALIFVFVYNTELWENKRFLRAALSNASLLAEACHQIFEGKVFYP
TENALKTTLDEATCYEYMVRSHYVTETLSEEEAGFPLAYTVTIHKDFGTFERLFRAIYMP
QNVYCVHLDQKATDAFKGAVKQLLSCFPNAFLASKKESVVYGGISRLQADLNCLEDLVAS
EVPWKYVINTCGQDFPLKTNREIVQYLKGFKGKNITPGVLPPDHAVGRTKYVHQELLNHK
NSYVIKTTKLKTPPPHDMVIYFGTAYVALTRDFANFVLQDQLALDLLSWSKDTYSPDEHF
WVTLNRIPGVPGSMPNASWTGNLRAIKWSDMEDRHGGCHGHYVHGICIYGNGDLKWLVNS
PSLFANKFELNTYPLTVECLELRHRERTLNQSETAIQPSWYF*
>ENSG00000237022|ENST00000438171
MRVMAPRTLILLLSGALALTETWACSHSMRYFYTAVSRPGRGEPRFIAVGYVDDTQFVRF

DSDAASPRGEPRAPWVEQEGPEYWDRETQKYKRQAQTDRVSLRNLRGYYNQSEAGSHTLQ
WMYGCDLGPDGRLLRGYDQSAYDGKDYIALNEDLRSWTAADTAAQITQRKWEAARAAEQQ
RAYLEGTCVEWLRRYLENGKETLQRAEHPKTHVTHHLVSDHEATLRCWALGFYPAEITLT
WQRDGEDQTQDTELVETRPAGDGTFQKWAAVVVPSGEEQRYTCHVQHEGLPEPLTLRWEP
SSQPTIPIVGIVAGLAVLAVLAVLGAVVAVVMCRRKSSGGKGGSCSQAASSNSAQGSDES
LIACKA*
>ENSG00000223865|ENST00000418931
MMVLQVSAAPRTVALTALLMVLLTSVVQGRATPENYLFQGRQECYAFNGTQRFLERYIYN
REEFARFDSDVGEFRAVTELGRPAAEYWNSQKDILEEKRAVPDRMCRHNYELGGPMTLQR
RVQPRVNVSPSKKGPLQHHNLLVCHVTDFYPGSIQVRWFLNGQEETAGVVSTNLIRNGDW
TFQILVMLEMTPQQGDVYTCQVEHTSLDSPVTVEWKAQSDSARSKTLTGAGGFVLGLIIC
GVGIFMHRRSKKVQRGSA*
>ENSG00000283017|ENST00000635703
MYGSARSVGKVEPSSQSPGRSPRLPRSPRLGHRRTNSTGGSSGSSVGGGSGKTLSMENIQ
SLNAAYATSGPMYLSDHENVGSETPKSTMTLGRSGGRLPYGVRMTAMGSSPNIASSGVAS
DTIAFGEHHLPPVSMASTVPHSLRQARDNTIMDLQTQLKEVLRENDLLRKDVEVKESKLS
SSMNSIKTFWSPELKKERALRKDEASKITIWKEQYRVVQEENQHMQMTIQALQDELRIQR
DLNQLFQQDSSSRTGEPCVAELTEENFQRLHAEHERQAKELFLLRKTLEEMELRIETQKQ
TLNARDESIKKLLEMLQSKGLSAKATEEDHERTRRLAEAEMHVHHLESLLEQKEKENSML
REEMHRRFENAPDSAKTKALQTVIEMKDSKISSMERGLRDLEEEIQMLKSNGALSTEERE
EEMKQMEVYRSHSKFMKNKIGQVKQELSRKDTELLALQTKLETLTNQFSDSKQHIEVLKE
SLTAKEQRAAILQTEVDALRLRLEEKETMLNKKTKQIQDMAEEKGTQAGEIHDLKDMLDV
KERKVNVLQKKIENLQEQLRDKEKQMSSLKERVKSLQADTTNTDTALTTLEEALAEKERT
IERLKEQRDRDEREKQEEIDNYKKDLKDLKEKVSLLQGDLSEKEASLLDLKEHASSLASS
GLKKDSRLKTLEIALEQKKEECLKMESQLKKAHEAALEARASPEMSDRIQHLEREITRYK
DESSKAQAEVDRLLEILKEVENEKNDKDKKIAELERQVKDQNKKVANLKHKEQVEKKKSA
QMLEEARRREDNLNDSSQQLQDSLRKKDDRIEELEEALRESVQITAEREMVLAQEESART
NAEKQVEELLMAMEKVKQELESMKAKLSSTQQSLAEKETHLTNLRAERRKHLEEVLEMKQ
EALLAAISEKDANIALLELSSSKKKTQEEVAALKREKDRLVQQLKQQTQNRMKLMADNYE
DDHFKSSHSNQTNHKPSPDQIIQPLLELDQNRSKLKLYIGHLTTLCHDRDPLILRGLTPP
ASYNLDDDQAAWENELQKMTRGQLQDELEKGERDNAELQEFANAILQQIADHCPDILEQV
VNALEESS*
>ENSG00000254806|ENST00000458187
MAGQFRSYVWDPLLILSQIVLMQTVYYGSLGLWLALVDGLVRSSPSLDQMFDAEILGFST
PPGRLSMMSFILNALTWHFSLLLPHEEGRVCFPFHHD*
>ENSG00000166448|ENST00000416379
MAQAVWSRLGRILWLACLLPWAPAGVAAGLYELNLTTDSPATTGAVVTISASLVAKDNGS
LALPADAHLYRFHWIHTPLVLTGKMEKGLSSTIRVVGHVPGEFPVSVWVTAADCWMCQPV
ARGFVVLPITEFLVGDLVVTQNTSLPWPSSYLTKTVLKVSFLLHDPSNFLKTALFLYSWD
FGDGTQMVTEDSVVYYNYSIIGTFTVKLKVVAEWEEVEPDATRAVKQKTGDFSASLKLQE
TLRGIQVLGPTLIQTFQKMTVTLNFLGSPPLTVCWRLKPECLPLEEGECHPVSVASTAYN
LTHTFRDPGDYCFSIRAENIISKTHQYHKIQVWPSRIQPAVFAFPCATLITVMLAFIMYM
TLRNATQQKDMVEVADFDFSPMSDKNPEPPSGVRCCCQMCCGPFLLETPSEYLEIVRENH
GLLPPLYKSVKTYTV*

>ENSG00000064687|ENST00000433129
MAFWTQLMLLLWKNFMYRRRQPVQLLVELLWPLFLFFILVAVRHSHPPLEHHECHFPNKP
LPSAGTVPWLQGLICNVNNTCFPQLTPGEEPGRLSNFNDSLVSRLLADARTVLGGASAHR
TLAGLGKLIATLRAARSTAQPQPTKQSPLEPPMLDVAELLTSLLRTESLGLALGQAQEPL
HSLLEAAEDLAQELLALRSLVELRALLQRPRGTSGPLELLSEALCSVRGPSSTVGPSLNW
YEASDLMELVGQEPESALPDSSLSPACSELIGALDSHPLSRLLWRRLKPLILGKLLFAPD
TPFTRKLMAQVNRTFEELTLLRDVREVWEMLGPRIFTFMNDSSNVAMLQRLLQMQDEGRR
QPRPGGRDHMEALRSFLDPGSGGYSWQDAHADVGHLVGTLGRVTECLSLDKLEAAPSEAA
LVSRALQLLAEHRFWAGVVFLGPEDSSDPTEHPTPDLGPGHVRIKIRMDIDVVTRTNKIR
DRFWDPGPAADPLTDLRYVWGGFVYLQDLVERAAVRVLSGANPRAGLYLQQMPYPCYVDD
VFLRVLSRSLPLFLTLAWIYSVTLTVKAVVREKETRLRDTMRAMGLSRAVLWLGWFLSCL
GPFLLSAALLVLVLKLGDILPYSHPGVVFLFLAAFAVATVTQSFLLSAFFSRANLAAACG
GLAYFSLYLPYVLCVAWRDRLPAGGRVAASLLSPVAFGFGCESLALLEEQGEGAQWHNVG
TRPTADVFSLAQVSGLLLLDAALYGLATWYLEAVCPGQYGIPEPWNFPFRRSYWCGPRPP
KSPAPCPTPLDPKVLVEEAPPGLSPGVSVRSLEKRFPGSPQPALRGLSLDFYQGHITAFL
GHNGAGKTTTLSILSGLFPPSGGSAFILGHDVRSSMAAIRPHLGVCPQYNVLFDMLTVDE
HVWFYGRLKGLSAAVVGPEQDRLLQDVGLVSKQSVQTRHLSGGMQRKLSVAIAFVGGSQV
VILDEPTAGVDPASRRGIWELLLKYREGRTLILSTHHLDEAELLGDRVAVVAGGRLCCCG
SPLFLRRHLGSGYYLTLVKARLPLTTNEKADTDMEGSVDTRQEKKNGSQGSRVGTPQLLA
LVQHWVPGARLVEELPHELVLVLPYTGAHDGSFATLFRELDTRLAELRLTGYGISDTSLE
EIFLKVVEECAADTDMEDGSCGQHLCTGIAGLDVTLRLKMPPQETALENGEPAGSAPETD
QGSGPDAVGRVQGWALTRQQLQALLLKRFLLARRSRRGLFAQIVLPALFVGLALVFSLIV
PPFGHYPALRLSPTMYGAQVSFFSEDAPGDPGRARLLEALLQEAGLEEPPVQHSSHRFSA
PEVPAEVAKVLASGNWTPESPSPACQCSRPGARRLLPDCPAAAGGPPPPQAVTGSGEVVQ
NLTGRNLSDFLVKTYPRLVRQGLKTKKWVNEVRYGGFSLGGRDPGLPSGQELGRSVEELW
ALLSPLPGGALDRVLKNLTAWAHSLDAQDSLKIWFNNKGWHSMVAFVNRASNAILRAHLP
PGPARHAHSITTLNHPLNLTKEQLSEGALMASSVDVLVSICVVFAMSFVPASFTLVLIEE
RVTRAKHLQLMGGLSPTLYWLGNFLWDMCNYLVPACIVVLIFLAFQQRAYVAPANLPALL
LLLLLYGWSITPLMYPASFFFSVPSTAYVVLTCINLFIGINGSMATFVLELFSDQKLQEV
SRILKQVFLIFPHFCLGRGLIDMVRNQAMADAFERLGDRQFQSPLRWEVVGKNLLAMVIQ
GPLFLLFTLLLQHRSQLLPQPRVRSLPLLGEEDEDVARERERVVQGATQGDVLVLRNLTK
VYRGQRMPAVDRLCLGIPPGECFGLLGVNGAGKTSTFRMVTGDTLASRGEAVLAGHSVAR
EPSAAHLSMGYCPQSDAIFELLTGREHLELLARLRGVPEAQVAQTAGSGLARLGLSWYAD
RPAGTYSGGNKRKLATALALVGDPAVVFLDEPTTGMDPSARRFLWNSLLAVVREGRSVML
TSHSMEECEALCSRLAIMVNGRFRCLGSPQHLKGRFAAGHTLTLRVPAARSQPAAAFVAA
EFPGAELREAHGGRLRFQLPPGGRCALARVFGELAVHGAEHGVEDFSVSQTMLEEVFLYF
SKDQGKDEDTEEQKEAGVGVDPAPGLQHPKRVSQFLDDPSTAETVL*
>ENSG00000115540|ENST00000448447
MVMAEGTAVLRRNRPGTKAQYIQQNIRADCSNIDKILEPPEGQDEGVWKYEHLRQFCLEL
NGLAVKLQSECHPDTCTQMTATEQWIFLCAAHKTPKECPAIDYTRHTLDGAACLLNSNKY
FPSRVSIKESSVAKLGSVCRRIYRIFSHAYFHHRQIFDEYENETFLCHRFTKFVMKYNLM
SKDNLIVPILEEEVQNSVSGESEA*
>ENSG00000005243|ENST00000621465
MQRPEAWPRPHPGEGAAAAQAGGPAPPARAGEPSGLRLQEPSLYTIKAVFILDNDGRRLL

AKYYDDTFPSMKEQMVFEKNVFNKTSRTESEIAFFGGMTIVYKNSIDLFLYVVGSSYENE
LMLMSVLTCLFESLNHMLRKNVEKRWLLENMDGAFLVLDEIVDGGVILESDPQQVIQKVN
FRADDGGLTEQSVAQVLQSAKEQIKWSLLK*
>ENSG00000117069|ENST00000477717
MKTLMRHGLAVCLALTTMCTSLLLVYSSLGGQKERPPQQQQQQQQQQQQASATGSSQPAA
ESSTQQRPGVPAGPRPLDGYLGVADHKPLKMHCRDCALVTSSGHLLHSRQGSQIDQTECV
IRMNDAPTRGYGRDVGNRTSLRVIAHSSIQRILRNRHDLLNVSQGTVFIFWGPSSYMRRD
GKGQVYNNLHLLSQVLPRLKAFMITRHKMLQFDELFKQETGKDRKISNTWLSTGWFTMTI
ALELCDRINVYGMVPPDFCRDPNHPSVPYHYYEPFGPDECTMYLSHERGRKGSHHRFITE
KRVFKNWARTFNIHFFQPDWKPESLAINHPENKPVF*
>ENSG00000226182|ENST00000412623
MLSRNDDICIYGGLGLGGLLLLAVVLLSACLCWLHRRVKRLERSWHLLSWSQAQGSSEQE
LHYASLQRLPVPSSEGPDLRGRDKRGTKEDPRADYACIAENKPT*
>ENSG00000164597|ENST00000605888
MEGGGGSVAVAGLGARGSGAAAATVRELLQDGCYSDFLNEDFDVKTYTSQSIHQAVIAEQ
LAKLAQGISQLDRELHLQVVARHEDLLAQATGIESLEGVLQMMQTRIGALQGAVDRIKAK
IVEPYNKIVARTAQLARLQVACDLLRRIIRILNLSKRLQGQLQGGSREITKAAQSLNEL
>ENSG00000137497|ENST00000535087
MTLHATRGAALLSWVNSLHVADPVEAVLQLQDCSIFIKIIDRIHGTEEGQQILKQPVSER
LDFVCSFLQKNRKHPSSPECLVSAQKVLEGSELELAKMTMLLLYHSTMS
>ENSG00000137497|ENST00000543009
MTLHATRGAALLSWVNSLHVADPVEAVLQLQDCSIFIKIIDRIHGTEEGQQILKQPVSER
LDFVCSFLQKNRKHPSSPECLVSAQKVLEGSELELAKMTMLLLYHSTMSSKSPRDWEQFE
YKIQAELAVILKFVLDHEDGLNLNEDLENFLQKAPVPSTCSSTFPEELSPPSHQ
>ENSG00000145431|ENST00000502773
MSLFGLLLLTSALAGQRQGTQAESNLSSKFQFSSNKEQNGVQDPQHERIITVSTNGSIHS
PRFPHTYPRNTVLVWRLVAVEENVWIQLTFDERFGLEDPEDDICKYDFVEVEEPSDGTIL
GRWCGSGTVPGKQISKGNQIRIRFVSDEYFPSEPGFCIHYNIVMPQFTEAVSPSVLPPSA
LPLDLLNNAITAFSTLEDLIRYLEPERWQLDLEDLYRPTWQLLGKAFVFGRKSRVVDLNL
LTEEVRLYSCTPRNFSVSIREELKRTDTIFWPGCLLVKRCGGNCACCLHNCNECQCVPSK
VTKKYHEVLQLRPKTGVRGLHKSLTDVALEHHEECDCVCRGSTGG*
>ENSG00000228080|ENST00000412634
LLKLTRDFPQRHEKYQSWAGTFHLCLKKNKYPDSRSTFIKHAGSYLKRGEFLSIHTAITC
FFLQISARFLEQVKHECHFFNGTERVRFLDRYFYHQEEYVRFDSDVGEYRAVTELGRPDA
EYWNSQKDLLEQRRAEVDTYCRHNYGVVESFTVQRRVYPEVTVYPAKTQPLQHHNLLVCS
VNGFYPGSIEVRWFRNGQEEKTGVVSTGLIQNGDWTFQTLVMLETVPRSGEVYTCQVEHP
SLTSPLTVEW
>ENSG00000122705|ENST00000470744
MAELDPFGAPAGAPGGPALGNGVAGAGEEDPAAAFLAQQESEIAGIENDEAFAILDGGAP
GPQPHGEPPGGPDAVDGVMNGEYYQESNGPTDSYAAISQVDRLQSEPESIRKWREEQMER
LEALDANSRKQEAEWKEKAIKELEEWYARQDEQLQKTKANNSTNINHPCYSLEQAAEEAF
VNDIDESSPGTEWERVARLCDFNPKSSKQAKDVSRMRSVLISLKQAPLVH*
>ENSG00000277626|ENST00000611547
MEPHLLGLLLGLLLGGTRVLAGYPIWWSLALGQQYTSLGSQPLLCGSIPGLVPKQLRFCR

NYIEIMPSVAEGVKLGIQECQHQFRGRRWNCTTIDDSLAIFGPVLDKATRESAFVHAIAS
AGVAFAVTRSCAEGTSTICGCDSHHKGPPGEGWKWGGCSEDADFGVLVSREFADARENRP
DARSAMNKHNNEAGRTTILDHMHLKCKCHGLSGSCEVKTCWWAQPDFRAIGDFLKDKYDS
ASEMVVEKHRESRGWVETLRAKYSLFKPPTERDLVYYENSPNFCEPNPETGSFGTRDRTC
NVTSHGIDGCDLLCCGRGHNTRTEKRKEKCHCIFHWCCYVSCQECIRIYDVHTCK*
>ENSG00000234745|ENST00000412585
MLVMAPRTVLLLLSAALALTETWAGSHSMRYFYTSVSRPGRGEPRFISVGYVDDTQFVRF
DSDAASPREEPRAPWIEQEGPEYWDRNTQIYKAQAQTDRESLRNLRGYYNQSEAGSHTLQ
SMYGCDVGPDGRLLRGHDQYAYDGKDYIALNEDLRSWTAADTAAQITQRKWEAAREAEQR
RAYLEGECVEWLRRYLENGKDKLERADPPKTHVTHHPISDHEATLRCWALGFYPAEITLT
WQRDGEDQTQDTELVETRPAGDRTFQKWAAVVVPSGEEQRYTCHVQHEGLPKPLTLRWEP
SSQSTVPIVGIVAGLAVLAVVVIGAVVAAVMCRRKSSGGKGGSYSQAACSDSAQGSDVSL
TA*
>ENSG00000166377|ENST00000586366
MADQIPLYPVRSAAAAAANRKRAAYYSAAGPRPGADRHSRYQLEDESAHLDEMPLMMSEE
GFENEESDYHTLPRARIMQRKRGLEWFVCDGWKFLCTSCCGWLINICRRKKELKARTVWL
GCPEKCEEKHPRNSIKNQKYNVFTFIPGVLYEQFKFFLNLYFLVISCSQFVPALKIGYLY
TYWAPLGFVLAVTMTREAIDEFRRFQRDKEVNSQLYSKLTVRGKVQVKSSDIQVGDLIIV
EKNQRIPSDMVFLRTSEKAGPFFYQCLCLCSETTNGHSQFRRHIYQGRQ*
>ENSG00000196562|ENST00000484875
MGPPSLVLCLLSATVFSLLGGSSAFLSHHRLKGRFQRDRRNIRPNIILVLTDDQDVELGS
MQVMNKTRRIMEQGGAHFINAFVTTPMCCPSRSSILTGKYVHNHNTYTNNENCSSPSWQA
QHESRTFAVYLNSTGYRTAFFGKYLNEYNGSYVPPGWKEWVGLLKNSRFYNYTLCRNGVK
EKHGSDYSKDYLTDLITNDSVSFFRTSKKMYPHRPVLMVISHAAPHGPEDSAPQYSRLFP
NASQHITPSYNYAPNPDKHWIMRYTGPMKPIHMEFTNMLQRKRLQTLMSVDDSMETIYNM
LVETGELDNTYIVYTADHGYHIGQFGLVKGKSMPYEFDIRVPFYVRGPNVEAGCLNPHIV
LNIDLAPTILDIAGLDIPADMDGKSILKLLDTERPVNRFHLKKKMRVWRDSFLVERGKLL
HKRDNDKVDAQEENFLPKYQRVKDLCQRAEYQTACEQLGQKWQCVEDATGKLKLHKCKGP
MRLGGSRALSNLVPKYYGQGSEACTCDSGDYKLSLAGRRKKLFKKKYKASYVRSRSIRSV
AIEVDGRVYHVGLGDAAQPRNLTKRHWPGAPEDQDDKDGGDFSGTGGLPDYSAANPIKVT
HRCYILENDTVQCDLDLYKSLQAWKDHKLHIDHEIETLQNKIKNLREVRGHLKKKRPEEC
DCHKISYHTQHKGRLKHRGSSLHPFRKGLQEKDKVWLLREQKRKKKLRKLLKRLQNNDTC
SMPGLTCFTHDNQHWQTAPFWTLGPFCACTSANNNTYWCMRTINETHNFLFCEFATGFLE
YFDLNTDPYQLMNAVNTLDRDVLNQLHVQLMELRSCKGYKQCNPRTRNMDLGLKDGGSYE
QYRQFQRRKWPEMKRPSSKSLGQLWEGWEG*
>ENSG00000137177|ENST00000502704
MSDTKVKVAVRVRPMNRRELELNTKCVVEMEGNQTVLHPPPSNTKQGERLVTVAHISNSS
TLGGQGKRIT*
>ENSG00000163235|ENST00000445399
MVPSAGQLALFALGIVLAACQALENSTSPLSDPPVAAAVVSHFNDCPDSHTQFCFHGTCR
FLVQEDKPACVCHSGYVGARCEHADLLAVVAASQKKQAITALVVVSIVALAVLIITCVLI
HCCQVRKHCEWCRALICRHEKPSALLKGRTACCHSETATLG*
>ENSG00000228813|ENST00000430849
MSWKMALQIPGGFWAAAVTVMLVMLSTPVAEARDFPKDFLVQFKGMCYFTNGTERVRGVA

RYIYNREEYGRFDSDVGEFQAVTELGRSIEDWNNYKDFLEQERAAVDKVCRHNYEAELRT
TLQRQVEPTVTISPSRTEALNHHNLLVCSVTDFYPAQIKVQWFRNDQEETAGVVSTSLIR
NGDWTFQILVMLEITPQRGDIYTCQVEHPSLQSPITVEWRAQSESAQSKMLGGVGGFVLG
LIFLGLGLIIRHRGQKGPRGPPPAGLLH*
>ENSG00000120805|ENST00000551828
MRILILGLDGAGKTTILYRLQVGEVVTTIPTIGFNVETVTYKNLKFQVWDLGGQTSIRPY
WRCYYSNTDAVIYVVDSCDRDRIGISKSELVAMLEEEELRKAILVVFANKQDMEQAMTSS
EMANSLGLPALKDRKWQIFKTSATKGTGLDEAMEWLVETLKSRQ*
>ENSG00000138802|ENST00000504968
MSAPAGSSHPAASARIPPKFGGAAVSGAAAPAGPGAGPAPHQQNETGFHHVAQASLELLD
PSNLPASASQIAGSTGPAQNQMQVPSGYGLHHQNYIAPSGHYSQGPGKMTSLPLDTQCGD
YYSALYTVPTQNVTPNTVNQQPGAQQLYSRGPPAPHIVGSTLGSFQGAASSASHLHTSAS
QPYSSFVNHYNSPAMYSASSSVASQGFPSTCGHYAMSTVSNAAYPSVSYPSLPAGDTYGQ
MFTSQNAPTVRPVKDNSFSGQNTAISHPSPLPPLPSQQHHQQQSLSGYSTLTWSSPGLPS
TQDNLIRNHTGSLAVANNNPTITVADSLSCPVMQNVQPPKSSPVVSTVLSGSSGSSSTRT
PPTANHPVEPVTSVTQPSELLQQKGVQYGEYVNNQASSAPTPLSSTSDDEEEEEEDEEAG
VDSSSSTTSSASPMPNSYDALEGGSYPDMLSSSASSPAPDPAPEPDPASAPAPASAPAPVV
PQPSKMAKPFGYGYPTLQPGYQNATAPLISGVQPSNPVYSGFQQYPQYPGVNQLSSSIGG
LSLQSSPQPESLRPVNLTQERNILPMTPVWAPVPNLNADLKKLNCSPDSFRCTLTNIPQT
QALLNKAKLPLGLLLHPFRDLTQLPVITSNTIVRCRSCRTYINPFVSFIDQRRWKCNLCY
RVNDVPEEFMYNPLTRSYGEPHKRPEVQNSTVEFIASSDYMLRPPQPAVYLFVLDVSHNA
VEAGYLTILCQSLLENLDKLPGDSRTRIGFMTFDSTIHFYNLQEGLSQPQMLIVSDIDDV
FLPTPDSLLVNLYESKELIKDLLNALPNMFTNTRETHSALGPALQAAFKLMSPTGGRVSV
FQTQLPSLGAGLLQSREDPNQRSSTKVVQHLGPATDFYKKLALDCSGQQTAVDLFLLSSQ
YSDLASLACMSKYSAGCIYYYPSFHYTHNPSQAEKLQKDLKRYLTRKIGFEAVMRIRCTK
GLSMHTFHGNFFVRSTDLLSLANINPDAGFAVQLSIEESLTDTSLVCFQTALLYTSSKGE
RRIRVHTLCLPVVSSLADVYAGVDVQAAICLLANMAVDRSVSSSLSDARDALVNAVVDSL
SAYGSTVSNLQHSALMAPSSLKLFPLYVLALLKQKAFRTGTSTRLDDRVYAMCQIKSQPL
VHLMKMIHPNLYRIDRLTDEGAVHVNDRIVPQPPLQKLSAEKLTREGAFLMDCGSVFYIW
VGKGCDNNFIEDVLGYTNFASIPQKMTHLPELDTLSSERARSFITWLRDSRPLSPILHIV
KDESPAKAEFFQHLIEDRTEAAFSYYEFLLHVQQQICK*
>ENSG00000176383|ENST00000546192
MLCRLCWLVSYSLAVLLLGCLLFLRKAAKPAGDPTAHQPFWAPPTPRHSRCPPNHTVSSA
SLSLPSRHRLFLTYRHCRNFSILLEPSGCSKDTFLLLAIKSQPGHVERRAAIRSTWGRVG
GWARGRQLKLVFLLGVAGSAPPAQLLAYESREFDDILQWDFTEDFFNLTLKELHLQRWVV
AACPQAHFMLKGDDDVFVHVPNVLEFLDGWDPAQDLLVGDVIRQALPNRNTKVKYFIPPS
MYRATHYPPYAGGGGYVMSRATVRRLQAIMEDAELFPIDDVFVGMCLRRLGLSPMHHAGF
KTFGIRRPLDPLDPCLYRGLLLVHRLSPLEMWTMWALVTDEGLKCAAGPIPQR*
>ENSG00000114650|ENST00000545718
MTLTERLREKISRAFYNHGLLCASYPIPIILFGYFTLVPAIQEFCLFAVVGLVSDFFLQM
LFFTTVLSIDIRRMELADLNKRLPPEACLPSAKPVGQPTRYERQLAVRPSTPHTITLQPS
SFRNLRLPKRLRVVYFLARTRLAQRLIMAGTVVWIGILVYTDPAGLRNYLAAQVTEQSPL
GEGALAPMPVPSGMLPPSHPDPAFSIFPPDAPKLPENQTSPGESPERGGPAEVVHDSPVP
EVTWGPEDEELWRKLSFRHWPTLFSYYNITLAKRYISLLPVIPVTLRLNPREALEGRHPQ

DGRSAWPPPGPIPAGHWEAGPKGPGGVQAHGDVTLYKVAALGLATGIVLVLLLLCLYRVL
CPRNYGQLGGGPGRRRRGELPCDDYGYAPPETEIVPLVLRGHLMDIECLASDGMLLVSCC
LAGHVCVWDAQTGDCLTRIPRPGQRRDSGVGSGLEAQESWERLSDGGKAGPEEPGDSPPL
RHRPRGPPPPSLFGDQPDLTCLIDTNFSAQPRSSQPTQPEPRHRAVCGRSRDSPGYDFSC
LVQRVYQEEGLAAVCTPALRPPSPGPVLSQAPEDEGGSPEKGSPSLAWAPSAEGSIWSLE
LQGNLIVVGRSSGRLEVWDAIEGVLCCSSEEVSSGITALVFLDKRIVAARLNGSLDFFSL
ETHTALSPLQFRGTPGRGSSPASPVYSSSDTVACHLTHTVPCAHQKPITALKAAAGRLVT
GSQDHTLRVFRLEDSCCLFTLQGHSGAITTVYIDQTMVLASGGQDGAICLWDVLTGSRVS
HVFAHRGDVTSLTCTTSCVISSGLDDLISIWDRSTGIKFYSIQQDLGCGASLGVISDNLL
VTGGQGCVSFWDLNYGDLLQTVYLGKNSEAQPARQILVLDNAAIVCNFGSELSLVYVPSV
LEKLD*
>ENSG00000282725|ENST00000617235
MNRFRVSKFRHTEARPPRRESWISDIRAGTAPSCRNHIKSSCSLIAFNSDRPGVLGIVPL
QGQGEDKRRVAHLGCHSGSADRTVKLWRLPGPGQALPSAPGVVLGPEDLPVEVLQFHPTS
DGILVSAAGTTVKVWDAAKQQPLTELAAHGDLVQSAVWSRDGALVGTACKDKQLRIFDPR
TKPRASQSTQAHENSRDSRLAWMGTWEHLVSTGFNQMREREVKLWDTRFFSSALASLTLD
TSLGCLVPLLDPDSGLLVLAGKGERQLYCYEVVPQQPALSPVTQCVLESVLRGAALVPRQ
ALAVMSCEVLRVLQLSDTAIVPIGYHVPRKAVEFHEDLFPDTAGCVPATDPHSWWAGDNQ
QVQKVSLNPACRPHPSFTSCLVPPAEPLPDTAQPAVMETPVGDADASEGFSSPPSSLTSP
STPSSLGPSLSSTSGIGTSPSLRSLQSLLGPSSKFRHAQGTVLHRDSHITNLKGLNLTTP
GESDGFCANKLRVAVPLLSSGGQVAVLELRKPGRLPDTALPTLQNGAAVTDLAWDPFDPH
RLAVAGEDARIRLWRVPAEGLEEVLTTPETVLTGHTEKICSLRFHPLAANVLASSSYDLT
VRIWDLQAGADRLKLQGHQDQIFSLAWSPDGQQLATVCKDGRVRVYRPRSGPEPLQEGPG
PKGGRGARIVWVCDGRCLLVSGFDSQSERQLLLYEAEALAGGPLAVLGLDVAPSTLLPSY
DPDTGLVLLTGKGDTRVFLYELLPESPFFLECNSFTSPDPHKGLVLLPKTECDVREVELM
RCLRLRQSSLEPVAFRLPRVRKEFFQDDVFPDTAVIWEPVLSAEAWLQGANGQPWLLSLQ
PPDMSPVSQAPREAPARRAPSSAQYLEEKSDQQKKEELLNAMVAKLGNREDPLPQDSFEG
VDEDEWD*
>ENSG00000138459|ENST00000492406
MEKQCCSHPVICSLSTMYTFLLGAIFIALSSSRILLVKYSANEENKYDYLPTTVNVCSEL
VKLVFCVLVSFCVIKKDHQSRNLKYASWKEFSDFMKWSIPAFLYFLDNLIVFYVLSYLQP
AMAVIFSNFSIITTALLFRIVLKRRLNWIQWASLLTLFLSIVALTAGTKTLQHNLAGRGF
HHDAFFSPSNSCLLFRSECPRKDNCTAKEWTFPEAKWNTTARVFSHIRLGMGHVLIIVQC
FISSMANIYNEKILKEGNQLTESIFIQNSKLYFFGILFNGLTLGLQRSNRDQIKNCGFFY
GHSAFSVALIFVTAFQGLSVAFILKFLDNMFHVLMAQVTTVIITTVSVLVFDFRPSLEFF
LEAPSVLLSIFIYNASKPQVPEYAPRQERIRDLSGNLWERSSGDGEELERLTKPKSDESD
EDTF*
>ENSG00000147654|ENST00000531677
MAITQFRLFKFCTCLATVFSFLKRLICRSGRGRKLSGDQITLPTTVDYSSVPKQTDVEEW
TSWDEDAPTSVKIEGGNGNVATQQNSLEQLEPDYFKDMTPTIRKTQKIVIKKREPLNFGI
PDGSTGFSSRLAATQDLPFIHQSSELGDLDTWQENTNAWEEEEDAAWQAEEVLRSRTNVC
LLCSLLFHHPTPTSTPYINQSVKIERVSLGQWSYGKSKEQQKLADREKRAAEQQRKKMEK
EAQRLMKKEQNKIGVKLS*
>ENSG00000100023|ENST00000626352

MGKRQHQKDKMYITCAEYTHFYGGKKPDLPQTNFRRLPFDHCSLSLQPFVYPVCTPDGIV
FDLLNIVPWLKKYGTNPSNGEKLDGRSLIKLNFSKNSEGKYHCPVLFTVFTNNTHIVAVR
TTGNVYAYEAVEQLNIKAKNFRDLLTDEPFSRQDIITLQDPTNLDKFNVSNFYHVKNNMK
IIDPDEEKAKQDPSYYLKNTNAETRETLQELYKEFKGDEILAATMKAPEKKKVDKLNAAH
YSTGKVSASFTSTAMVPETTHEAAAIDEDVLRYQFVKKKGYVRLHTNKGDLNLELHCDLT
PKTCENFIRLCKKHYYDGTIFHRSIRNFVIQGGDPTGTGTGGESYWGKPFKDEFRPNLSH
TGRGILSMANSGPNSNRSQFFITFRSCAYLDKKHTIFGRVVGGFDVLTAMENVESDPKTD
RPKEEIRIDATTVFVDPYEEADAQIAQERKTQLKVAPETKVKSSQPQAGSQGPQTFRQGV
GKYINPAATEQQRKSPQPVPLSPCPRRSPVGVLGTSAPGSSRLPDDH*
>ENSG00000111186|ENST00000537031
MPSLLLLFTAALLSSWAQLLTDANSWWSLALNPVQRPEMFIIGAQPVCSQLPGLSPGQRK
LCQLYQEHMAYIGEGAKTGIKECQHQFRQRRWNCSTADNASVFGRVMQIGSRETAFTHAV
SAAGVVNAISRACREGELSTCGCSRTARPKDLPRDWLWGGCGDNVEYGYRFAKEFVDARE
REKNFAKGSEEQGRVLMNLQNNEAGRRAVYKMADVACKCHGVSGSCSLKTCWLQLAEFRK
VGDRLKEKYDSAAAMRVTRKGRLELVNSRFTQPTPEDLVYVDPSPDYCLRNESTGSLGTQ
GRLCNKTSEGMDGCELMCCGRGYNQFKSVQVERCHCKFHWCCFVRCKKCTEIVDQYICK*
>ENSG00000168824|ENST00000513555
MVKLGNNFAEKGTKQPLLEDGFDTIPLMTPLDVNQLQFPPPDKVVVKTKTEYEPDRKKGK
ARPPQIAEFTVSITEGVTERFKVSVLVLFALAFLTCVVFLVVYKVYKYDRACPDGFVLKN
TQCIPEGLESYYAEQDSSAREKFYTVINHYNLAKQSITRSVSPWMSVLSEEKLSEQETEA
AEKSA*
>ENSG00000274047|ENST00000610513
MALRPGAGSGGGGAAGAGAGSAGGGGFMFPVAGGIRPPQAGLMPMQQQGFPMVSVMQPNM
QGIMGMNYSSQMSQGPIAMQAGIPMGPMPAAGMPYLGQAPFLGMRPPGPQYTPDMQKQFA
EEQQKRFEQQQKLLEEERKRRQFEEQKQKLRLLSSVKPKTGEKSRDDALEAIKGNLDGFS
RDAKMHPTPASHPKKPGVGVFPSQDPAQPRMPPWIYNESLVPDAYKKILETTMTPTGIDT
AKLYPILMSSGLPRETLGQIWALANRTTPGKLTKEELYTVLAMIAVTQVVKPEEDDFQDF
QDASKSGSLDDSFSDFQELPASSKTSNSQHGNSAPSLLMPLPGTKALPSMDKYAVFKGIA
ADKSSENTVPPGDPGDKYSAFRELEQTAENKPLGESFAEFRSAGTDDGFTDFKTADSVSP
LEPPTKDKTFPPSFPSGTIQQKQQTQVKNPLNLADLDMFSSVNCSSEKPLSFSAVFSTSK
SVSTPQSTGSAATMTALAATKTSSLADDFGEFSLFGEYSGLAPVGEQDDFADFMAFSNSS
ISSEQKPDDKYDALKEEASPVPLTSNVGSTVKGGQNSTAASTKYDVFRQLSLEGSGLGVE
DLKDNTPSGKSDDDFADFHSSKFSSINSDKSLGEKAVAFRHTKEDSASVKSLDLPSIGGS
SVGKEDSEDALSVQFDMKLADVGGDLKHVMSDSSLDLPTVSGQHPPAAAGSGSPSATSIL
QKKETSFGSSENITMTSLSKVTTFVSEDALPETTFPALASFKDTIPQTSEQKEYENRDYK
DFTKQDLPTAERSQEATCPSPASSGASQETPNECSDDFGEFQSEKPKISKFDFLVATSQS
KMKSSEEMIKSELATFDLSVQGSHKRSLSLGDKEISRSSPSPALEQPFRDRSNTLNEKPA
LPVIRDKYKDLTGEVEENERYAYEWQRCLGSALNVIKKANDTLNGISSSSVCTEVIQSAQ
GMEYLLGVVEVYRVTKRVELGIKATAVCSEKLQQLLKDIDKVWNNLIGFMSLATLTPDEN
SLDFSSCMLRPGIKNAQELACGVCLLNVDSRSRKEEKPAEEHPKKAFNSETDSFKLAYGG
HQYHASCANFWINCVEPKPPGLVLPDLL*
>ENSG00000147533|ENST00000520817
MRPQQAPVSGKVFIQRDYSSGTRCQFQTKFPAELENRIDRQQFEETVRTLNNLYAEAEKL
GGQSYLEGCLACLTAYTIFLCMETHYEKVLKKVSKYIQEQNEKIYAPQGLLLTDPIERGL

RVIEITIYEDRGMSSGR*
>ENSG00000142192|ENST00000440126
MDQLEDLLVLFINYVPTDGNAGLLAEPQIAMFCGRLNMHMNVQNGKWDSDPSGTKTCIDT
KEGILQYCQEVYPELQITNVVEANQPVTIQNWCKRGRKQCKTHPHFVIPYRCLVGEFVSD
ALLVPDKCKFLHQERMDVCETHLHWHTVAKETCSEKSTNLHDYGMLLPCGIDKFRGVEFV
CCPLAEESDNVDSADAEEDDSDVWWGGADTDYADGSEDKVVEVAEEEEVAEVEEEEADDD
EDDEDGDEVEEEAEEPYEEATERTTSIATTTTTTTESVEEVVREVCSEQAETGPCRAMIS
RWYFDVTEGKCAPFFYGGCGGNRNNFDTEEYCMAVCGSAIPTTAASTPDAVDKYLETPGD
ENEHAHFQKAKERLEAKHRERMSQVMREWEEAERQAKNLPKADKKAVIQHFQEKVESLEQ
EAANERQQLVETHMARVEAMLNDRRRLALENYITALQAVPPRPRHVFNMLKKYVRAEQKD
RQHTLKHFEHVRMVDPKKAAQIRSQVMTHLRVIYERMNQSLSLLYNVPAVAEEIQDEVDE
LLQKEQNYSDDVLANMISEPRISYGNDALMPSLTETKTTVELLPVNGEFSLDDLQPWHSF
GADSVPANTENEVEPVDARPAADRGLTTRPGSGLTNIKTEEISEVKMDAEFRHDSGYEVH
HQKLVFFAEDVGSNKGAIIGLMVGGVVIATVIVITLVMLKKKQYTSIHHGVVEVDAAVTP
EERHLSKMQQNGYENPTYKFFEQMQN*
>ENSG00000136279|ENST00000448521
MAANLSRNGPALQEAYVRVVTEKSPTDWALFTYEGNSNDIRVAGTGEGGLEEMVEELNSG
KVMYAFCRVKDPNSGLPKFVLINWTGEGVNDVRKGACASHVSTMASFLKGAHVTINARAE
EDVEPECIMEKVAKASGANYSFHKESGRFQDVGPQAPVGSVYQKTNAVSEIKRVGKDSFW
AKAEKEEENRRLEEKRRAEEAQRQLEQERRERELREAARREQRYQEQGGEASPQRTWEQQ
QEVVSRNRNEQESAVHPREIFKQKERAMSTTSISSPQPGKLRSPFLQKQLTQPETHFGRE
PAAAISRPRADLPAEEPAPSTPPCLVQAEEEAVYEEPPEQETFYEQPPLVQQQGAGSEHI
DHHIQGQGLSGQGLCARALYDYQAADDTEISFDPENLITGIEVIDEGWWRGYGPDGHFGM
FPANYVELIE*
>ENSG00000226826|ENST00000433800
MMVLQVSAAPRTVALTALLMVLLTSVVQGRATPENYLFQGRQECYAFNGTQRFLERYIYN
REEFARFDSDVGEFRAVTELGRPAAEYWNSQKDILEEKRAVPDRMCRHNYELGGPMTLQR
RVQPRVNVSPSKKGPLQHHNLLVCHVTDFYPGSIQVRWFLNGQEETAGVVSTNLIRNGDW
TFQILVMLEMTPQQGDVYTCQVEHTSLDSPVTVEWKAQSDSARSKTLTGAGGFVLGLIIC
GVGIFMHRRSKKVQRGSA*
>ENSG00000103502|ENST00000570016
MPDENIFLFVPNLIGYARIVFAIISFYFMPCCPLTASSFYLLSGLLDAFDGHAARALNQG
TRFGAMLDMLTDRCSTMCLLVNLALLYPGATLFFQISMSLDVASHWLHLHSSVVRGSESH
KMIDLSGNPVLRIYYTSRPALFTLCAGNELFYCLLYLFHFSEGPLVGSVGLFRMGLWVTA
PIALLKSLISVIHLITAARNMAALDAADRAKKK*
>ENSG00000168824|ENST00000506380
MVKLGNNFAEKGTKQPLLEDGFDTIPLMTPLDVNQLQFPPPDKVVVKTKTEYEPDRKKGK
ARPPQIAEFTVSITEGVTERFKVSVLVLFALAFLTCVVFLVVYKVYKYDRACPDGFVLKN
TQCIPEGLESYYAEQDSSAREKFYTVINHYNLAKQSITRSVSPWMSVLSEEKLSEQETEA
AEKSA*
>ENSG00000137497|ENST00000541584
XRAERDSALETLQGQLEEKAQELGHSQSALASAQRELAAFRTKVQDHSKAEDEWKAQVAR
GRQEAERKNSLISSLEEEVSILNRQVLEKEGESKELKRLVMAESEKSQKLEERLRLLQAE
TASNSARAAERSSALREEVQSLREEAEKQRVASENLRQELTSQAERAEELGQELKAWQEK

FFQKEQALSTLQLEHTSTQALVSELLPAKHLCQQLQAEQAAAEKRHREELEQSKQAAGGL
RAELLRAQRELGELIPLRQKVAEQERTAQQLRAEKASYAEQLSMLKKAHGLLAEENRGLG
ERANLGRQFLEVELDQAREKYVQELAAVRADAETRLAEVQREAQSTARELEVMTAKYEGA
KVKVLEERQRFQEERQKLTAQVEELSKKLADSDQASKVQQQKLKAVQAQGGESQQEAQRL
QAQLNELQAQLSQKEQAAEHYKLQMEKAKTHYDAKKQQNQELQEQLRSLEQLQKENKELR
AEAERLGHELQQAGLKTKEAEQTCRHLTAQVRSLEAQVAHADQQLRDLGKFQVATDALKS
REPQAKPQLDLSIDSLDLSCEEGTPLSITRHKALMTIIPDLSPNNPLSKLPRTQPDGTSV
PGEPASPISQRLPPKVESLESLYFTPIPARSQAPLESSLDSLGDVFLDSGRKTRSARRRT
TQIINITMTKKLDVEEPDSANSSFYSTRSAPASQASLRATSSTQSLARLGSPDYGNSALL
SLPGYRPTTRSSARRSQAGVSSGAPPGRNSFYMGTCQDEPEQLDDWNRIAELQQRNRVCP
PHLKTCYPLESRPSLSLGTITDEEMKTGDPQETLRRASMQPIQIAEGTGITTRQQRKRVS
LEPHQGPGTPESKKATSCFPRPMTPRDRHEGRKQSTTEAQKKAAPASTKQADRRQSMAFS
ILNTPKKLGNSLLRRGASKKALSKASPNTRSGTRRSPRIATTTASAATAAAIGATPRAKG
KAKH*
>ENSG00000172575|ENST00000414708
MGTLGKAREAPRKPSHGCRAASKARLEAKPANSPFPSHPSLAHITQFRMMVSLGHLAKGA
SLDDLIDSCIQSFDADGNLCRSNQLLQVMLTMHRIVISSAELLQKVITLYKDALAKNSPG
LCLKICYFVRYWITEFWVMFKMDASLTDTMEEFQELVKAKGEELHCRLIDTTQINARDWS
RKLTQRIKSNTSKKRKVSLLFDHLEPEELSEHLTYLEFKSFRRISFSDYQNYLVNSCVKE
NPTMERSIALCNGISQWVQLMVLSRPTPQLRAEVFIKFIQVAQKLHQLQNFNTLMAVIGG
LCHSSISRLKETSSHVPHEINKVLGEMTELLSSSRNYDNYRRAYGECTDFKIPILGVHLK
DLISLYEAMPDYLEDGKVNVHKLLALYNHISELVQLQEVAPPLEANKDLVHLLTLSLDLY
YTEDEIYELSYAREPRNHRAPSVFKNYDHDQDGYISQEEFEKIAASFPFSFCVMDKDSSG
E*
>ENSG00000108587|ENST00000414833
XGPAVTGKVRARRPPDLRKQARQLENELDLKLVSFSKLCTSYSHSSTRDGRRDRYSSDTT
PLLNGSSQDRMFETMAIEIEQLLARLTGVNDKMAEYTNSAGVPSLNAALMHTLQRHRDIL
QDYTHEFHKTKANFMAIRERENLMGSVRKDIESYKSGSGVNNRRTELFLKEHDHLRNSDR
LIEETISIAMATKENMTSQRGMLKSIHSKMNTLANRFPAVNSLIQRINLRKRRDSLILGG
VIGICTILLLLYAFH*
>ENSG00000152056|ENST00000415298
MIHFILLFSRQGKLRLQKWYITLPDKERKKITREIVQIILSRGHRTSSFVDWKELKLVYK
RYASLYFCCAIENQDNELLTLEIVHRYVELLDKYFGNTWPFARA*
>ENSG00000157593|ENST00000537814
MTRSYGATATSPGERFTDSQFLVLMNRVLALIVAGLSCVLCKQPRHGAPMYRYSFASLSN
VLSSWCQYEALKFVSFPTQVLAKASKVIPVMLMGKLVSRRSYEHWEYLTATLISIGVSMF
LLSSGPEPRSSPATTLSGLILLAGYIAFDSFTSNWQDALFAYKMSSVQMMFGVNFFSCLF
TVGSLLEQGALLEGTRFMGRHSEFAAHALLLSICSACGQLFIFYTIGQFGAAVFTIIMTL
RQAFAILLSCLLYGHTVTVVGGLGVAVVFAALLLRVYARGRLKQRGKKAVPVESPVQKV*
>ENSG00000011465|ENST00000441303
MKATIILLLLAQVSWAGPFQQRGLFDFMLEDEASGIGPEVPDDRDFEPSLGPVCPFRCQC
HLRVVQCSDLGLDKVPKDLPPDTTLLDLQNNKITEIKDGDFKNLKNLHVVYLHNNNISVV
GSSDFCPPGHNTKKASYSGVSLFSNPVQYWEIQPSTFRCVYYVRSAIQLGNYK*
>ENSG00000178234|ENST00000434507

MGSVTVRYFCYGCLFTSATWTVLLFVYFNFSEVTQPLKNVPVKGSGPHGPSPKKFYPRFT
RGPSRVLEPQFKANKIDDVIDSRVEDPEEGHLKFSSELGMIFNERDQELRDLGYQKHAFN
MLISDRLGYHRDVPDTRNAACKEKFYPPDLPAASVVICFYNEAFSALLRTVHSVIDRTPA
HLLHEIILVDDDSDFDDLKGELDEYVQKYLPGKIKVIRNTKREGLIRGRMIGAAHATGEV
LVFLDSHCEVNVMWLQPLLAAIREDRHTVVCPVIDIISADTLAYSSSPVVRGGFNWGLHF
KWDLVPLSELGRAEGATAPIKSPTMAGGLFAMNRQYFHELGQYDSGMDIWGGENLEISFR
IWMCGGKLFIIPCSRVGHIFRKRRPYGSPEGQDTMTHNSLRLAHVWLDEYKEQYFSLRPD
LKTKSYGNISERVELRKKLGCKSFKWYLDNVYPEMQISGSHAKPQQPIFVNRGPKRPKVL
QRGRLYHLQTNKCLVAQGRPSQKGGLVVLKACDYSDPNQIWIYNEEHELVLNSLLCLDMS
ETRSSDPPRLMKCHGSGGSQQWTFGKNNRLYQVSVGQCLRAVDPLGQKGSVAMAICDGSS
SQQWHLEG*
>ENSG00000060566|ENST00000602257
MNTDLAAGKMASAACSMDPIDSFELLDLLFDRQDGILRHVELGEGWGHVKDQQVLPNPDS
DDFLSSILGSGDSLPSSPLWSPEGSDSGISEDLPSDPQDTPPRSGPATSPAGCHPAQPGK
GPCLSYHPGNSCSTTTPGPVIQVPEASVTIDLEMWSPGGRICAEKPADPVDLSPRCNLTV
KDLLLSGSSGDLHHLGASYLLRPGAGHCQELVLTEDEKKLLAKEGITLPTQLPLTKYEER
VLKKIRRKIRNKQSAQESRKKKKEYIDGLETRMSACTAQNQELQRKVLHLEKQNLSLLEQ
LKKLQAIVVQSTSKSAQTGTCVAVLLLSFALIILPSISPFGPNKTESPGDFAPVRVFSRT
LHNDAASRVAADAVPGSEAPGPRPEADTTREESPGSPGADWGFQDTANLTNSTEELDNAT
LVLRNATEGLGQVALLDWVAPGPSTGSGRAGLEAAGDEL*
>ENSG00000134109|ENST00000445686
MGNSSEFQKAVKLVINTVSFDKDSTVQVFEATIRVLGSLLSAHRIITDSKQPFGDMTIKD
YDNELLYMAHDLAVRLLPAFENTKTGIPYPRVNLKTGVPPDTNNETCTAGAGSLLVEFGI
LSRLLGDSTFEWVARRAVKALWNLRSNDTGLLGNVVNIQTGHWVGKQSGLGAGLDSFYEY
LLKSYILFGEKEDLEMFNAAYQSIQNYLRRGREACNEGEGDPPLYVNVNMFSGQLMNTWI
DSLQAFFPGLQVLIGDVEDAICLHAFYYAIWKRYGALPERYNWQLQAPDVLFYPLRPELV
ESTYLLYQATKNPFYLHVGMDILQSLEKYTKVKCGYATLHHVIDKSTEDRMESFFLSETC
KYLYLVCVLQDEPRNI*
>ENSG00000109956|ENST00000524765
MPKRRDILAIVLIVLPWTLLITVWHQSTLAPLLAVHKDEGSDPRRETPPGADPREYCTSD
RDIVEVVRTEYVYTRPPPWSDTLPTIHVVTPTYSRPVQKAELTRMANTLLHVPNLHWLVV
EDAPRRTPLTARLLRDTGLNYTHLHVETPRNYKLRGDARDPRIPRGTMQRNLALRWLRET
FPRNSSQPGVVYFADDDNTYSLELFEEMRSTRRVSVWPVAFVGGLRYEAPRVNGAGKVVG
WKTVFDPHRPFAIDMAGFAVNLRLILQRSQAYFKLRGVKGGYQESSLLRELVTLNDLEPK
AANCTKILVWHTRTEKPVLVNEGKKGFTDPSVEI*
>ENSG00000080815|ENST00000557511
MTELPAPLSYFQNAQMSEDNHLSNTVRSQNDNRERQEHNDRRSLGHPEPLSNGRPQGNSR
QVVEQDEEEDEELTLKYGAKHVIMLFVPVTLCMVVVVATIKSVSFYTRKDGQLIYTPFTE
DTETVGQRALHSILNAAIMISVIVVMTILLVVLYKYRCYKVIHAWLIISSLLLLFFFSFI
YLGEVFKTYNVAVDYITVALLIWNFGVVGMISIHWKGPLRLQQAYLIMISALMALVFIKY
LPEWTAWLILAVISVYDLVAVLCPKGPLRMLVETAQERNETLFPALIYSSTMVWLVNMAE
GDPEAQRRVSKNSKYNAERGVKLGLGDFIFYSVLVGKASATASGDWNTTIACFVAILIGL
CLTLLLLAIFKKALPALPISITFGLVFYFATDYLVQPFMDQLAFHQFYI*
>ENSG00000102100|ENST00000635015

MAAVGAGGSTAAPGPGAVSAGALEPGTASAGETVCPSSRMGGGAHRRLKYISLAVLVVQN
ASLILSIRYARTLPGDRFFATTAVVMAEVLKGLTCLLLLFAQKRGNVKHLVLFLHEAVLV
QYVDTLKLAVPSLIYTLQNNLQYVAISNLPAATFQVSPKPSMAPEELTGLGTGVWE*
>ENSG00000147654|ENST00000614147
MAITQFRLFKFCTCLATVFSFLKRLICRSGRGRKLSGDQITLPTTVDYSSVPKQTDVEEW
TSWDEDAPTSVKIEGGNGNVATQQNSLEQLEPDYFKDMTPTIRKTQKIVIKKREPLNFGI
PDGSTGFSSRLAATQDLPFIHQSSELGDLDTWQENTNAWEEEEDAAWQAEEVLRSRTNVC
LLCSLLFHHPTPTSTPYINQSVKIERVSLGQWSYGKSKEQQKLADREKRAAEQQRKKMEK
EAQRLMKKEQNKIGVKLS*
>ENSG00000161395|ENST00000429199
MAGLAARLVLLAGAAALASGSQGDREPVYRDCVLQCEEQNCSGGALNHFRSRQPIYMSLA
GWTCRDDCKYECMWVTVGLYLQEGHKVPQFHGKWPFSRFLFFQEPASAVASFLNGLASLV
MLCRYRTFVPASSPMYHTCVAFAWKMDYFCASTVILHSIYLCCVRTVGLQHPAVVSAFRA
LLLLMLTVHVSYLSLIRFDYGYNLVANVAIGLVNVVWWLAWCLWNQRRLPHVRKCVVVVL
LLQGLSLLELLDFPPLFWVLDAHAIWHISTIPVHVLFFSFLEDDSLYLLKESEDKFKLD*
>ENSG00000181789|ENST00000514478
XMEPTEAYEVLCYVPARSLPYNQPGTCYTLVALPKEDPTAVACTFSCMMKFTVKDCDPTT
GETDDEGYEDEYVSHS*
>ENSG00000117620|ENST00000533028
MFANLKYVSLGILVFQTTSLVLTMRYSRTLKEEGPRYLSSTAVVVAELLKIMACILLVYK
DSKCSLRALNRVLHDEILNKPMETLKLAIPSGIYTLQNNLLYVALSNLDAATYQVTYQLK
ILTTALFSVSMLSKKLGVYQWLSLVILMTGVAFVQWPSDSQLDSKELSAGSQFVGLMAVL
TACFSSGFAGVYFEKILKETKQSVWIRNIQLGFFGSIFGLMGVYIYDGELVSKNGFFQGY
NRLTWIVVVLQALGGLVIAAVIKYADNILKGFATSLSIILSTLISYFWLQDFVPTSVFFL
GAILVITATFLYGYDPKPAGNPTKA*
>ENSG00000283017|ENST00000635430
MYGSARSVGKVEPSSQSPGRSPRLPRSPRLGHRRTNSTGGSSGSSVGGGSGKTLSMENIQ
SLNAAYATSGPMYLSDHENVGSETPKSTMTLGRSGGRLPYGVRMTAMGSSPNIASSGVAS
DTIAFGEHHLPPVSMASTVPHSLRQARDNTIMDLQTQLKEVLRENDLLRKDVEVKESKLS
SSMNSIKTFWSPELKKERALRKDEASKITIWKEQYRVVQEENQHMQMTIQALQDELRIQR
DLNQLFQQDSSSRTGEPCVAELTEENFQRLHAEHERQAKELFLLRKTLEEMELRIETQKQ
TLNARDESIKKLLEMLQSKGLSAKATEEDHERTRRLAEAEMHVHHLESLLEQKEKENSML
REEMHRRFENAPDSAKTKALQTVIEMKDSKISSMERGLRDLEEEIQMLKSNGALSTEERE
EEMKQMEVYRSHSKFMKNKVEQLKEELSSKEAQWEELKKKAAGLQAEIGQVKQELSRKDT
ELLALQTKLETLTNQFSDSKQHIEVLKESLTAKEQRAAILQTEVDALRLRLEEKETMLNK
KTKQIQDMAEEKGTQAGEIHDLKDMLDVKERKVNVLQKKIENLQEQLRDKEKQMSSLKER
VKSLQADTTNTDTALTTLEEALAEKERTIERLKEQRDRDEREKQEEIDNYKKDLKDLKEK
VSLLQGDLSEKEASLLDLKEHASSLASSGLKKDSRLKTLEIALEQKKEECLKMESQLKKA
HEAALEARASPEMSDRIQHLEREITRYKDESSKAQAEVDRLLEILKEVENEKNDKDKKIA
ELERQVKDQNKKVANLKHKEQVEKKKSAQMLEEARRREDNLNDSSQQLQDSLRKKDDRIE
ELEEALRESVQITAEREMVLAQEESARTNAEKQVEELLMAMEKVKQELESMKAKLSSTQQ
SLAEKETHLTNLRAERRKHLEEVLEMKQEALLAAISEKDANIALLELSSSKKKTQEEVAA
LKREKDRLVQQLKQQTQNRMKLMADNYEDDHFKSSHSNQTNHKPSPDQIIQPLLELDQNR
SKLKLYIGHLTTLCHDRDPLILRGLTPPASYNLDDDQAAWENELQKMTRGQLQDELEKGE

RDNAELQEFANAILQQIADHCPDILEQVVNALEESS*
>ENSG00000152291|ENST00000409232
MRFVVALVLLNVAAAGAVPLLATESVKQEEAGVRPSAGNVSTHPSLSQRPGGSTKSHPEP
QTPKDSPSKSSAEAQTPEDTPNKSGAEAKTQKDSSNKSGAEAKTQKGSTSKSGSEAQTTK
DSTSKSHPELQTPKDSTGKSGAEAQTPEDSPNRSGAEAKTQKDSPSKSGSEAQTTKDVPN
KSGADGQTPKDGSSKSGAEDQTPKDVPNKSGAEKQTPKDGSNKSGAEEQGPIDGPSKSGA
EEQTSKDSPNKVVPEQPSRKDHSKPISNPSDNKELPKADTNQLADKGKLSPHAFKTESGE
ETDLISPPQEEVKSSEPTEDVEPKEAEDDDTGPEEGSPPKEEKEKMSGSASSENREGTLS
DSTGSEKDDLYPNGSGNGSAESSHFFAYLVTAAILVAVLYIAHHNKRKIIAFVLEGKRSK
VTRRPKASDYQRLDQKIFFPLSPNRMVYSSGKR*
>ENSG00000164574|ENST00000425427
MRRKEKRLLQAVALVLAALVLLPNVGLWALYRERQPDGTPGGSGAAVAPAAGQGSHSRQK
KTFFLGDGQKLKDWHDKEAIRRDAQRVGNGEQGRPYPMTDAERVDQAYRENGFNIYVSDK
ISLNRSLPDIRHPNCNSKRYLETLPNTSIIIPFHNEGWSSLLRTVHSVLNRSPPELVAEI
VLVDDFSDREHLKKPLEDYMALFPSVRILRTKKREGLIRTRMLGASVATGDVITFLDSHC
EANVNWLPPLLDRIARNRKTIVCPMIDVIDHDDFRYETQAGDAMRGAFDWEMYYKRIPIP
PELQKADPSDPFESPVMAGGLFAVDRKWFWELGGYDPGLEIWGGEQYEISFKVSQLSRRP
VLGTAS*
>ENSG00000078328|ENST00000550418
MNCEREQLRGNQEAAAAPDTMAQPYASAQFAPPQNGIPAEYTAPHPHPAPEYTGQTTVPE
HTLNLYPPAQTHSEQSPADTSAQTVSGTATQTDDAAPTDGQPQTQPSENTENKSQPKRLH
VSNIPFRFRDPDLRQMFGQFGKILDVEIIFNERGSKGFGFVTFENSADADRAREKLHGTV
VEGRKIEVNNATARVMTNKKTVNPYTNGWKLNPVVGAVYSPEFYAGTVLLCQANQEGSSM
YSAPSSLVYTSAMPGFPYPAATAAAAYRGAHLRGRGRTVYNTFRAAAPPPPIPAYGGVVY
QDGFYGADIYGGYAAYRYAQPTPATAAAYSDSYGRVYAADPYHHALAPAPTYGVGAMNAF
APLTDAKTRSHADDVGLVLSSLQASIYRGGYNRFAPY*
>ENSG00000226182|ENST00000455003
MLSRNDDICIYGGLGLGGLLLLAVVLLSACLCWLHRRVKRLERSWAQGSSEQELHYASLQ
RLPVPSSEGPDLRGRDKRGTKEDPRADYACIAENKPT*
>ENSG00000114354|ENST00000490574
MNGQLDLSGKLIIKAQLGEDIRRIPIHNEDITYDELVLMMQRVFRGKLLSNDEVTIKYKD
EDGDLITIFDSSDLSFAIQCSRILKLTLFVNGQPRPLESSQVKYLRRELIELRNKVNRLL
DSLEPPGEPGPSTNIPENDTVDGREEKSASDSSGKQSTQVMAASMSAFDPLKNQDEINKN
VMSAFGLTDDQVSGPPSAPAEDRSGTPDSIASSSSAAHPPGVQPQQPPYTGAQTQAGQIE
GQMYQQYQQQAGYGAQQPQAPPQQPQQYGIQYSASYSQQTGPQQPQQFQGYGQQPTSQAP
APAFSGQPQQLPAQPPQQYQASNYPAQTYTAQTSQPTNYTVAPASQPGMAPSQPGAYQPR
PGFTSLPGSTMTPPPSGPNPYARNRPPFGQGYTQPGPGYR*
>ENSG00000097033|ENST00000535010
MIDAGTEFGPGTAYGNALIKCGETQKRIGTADRELIQTSALNFLTPLRNFIEGDYKTIAK
ERKLLQNKRLDLDAAKTRLKKAKAAETRNSSEQELRITQSEFDRQAEITRLLLEGISSTH
AHHLRCLNDFVEAQMTYYAQCYQYMLDLQKQLGSFPSNYLSNNNQTSVTPVPSVLPNAIG
SSAMASTSGLVITSPSNLSDLKECSGSRKARVLYDYDAANSTELSLLADEVITVFSVVGM
DSDWLMGERGNQKGKVPITYLELLN*
>ENSG00000100934|ENST00000545328

MTTYLEFIQQNEERDGVRFSWNVWPSSRLEATRMVVPVAALFTPLKERPDLPPIQYEPVL
CSRTTCRAVLNPLCQVDYRAKLWACNFCYQRNQRGPQMPLIFLYVVDTCMEDEDLQALKE
SMQMSLSLLPPTALVGLITFGRMVQVHELGCEGISKSYVFRGTKDLSAKQLQEMLGLSKV
PLTQATRGPQVQQPPPSNRFLQPVQKIDMNLTDLLGELQRDPWPVPQGKRPLRSSGVALS
IAVGLLECTFPNTGARIMMFIGGPATQGPGMVVGDELKTPIRSWHDIDKDNAKYVKKGTK
HFEALANRAATTGHVIDIYACALDQTGLLEMKCCPNLTGGYMVMGDSFNTSLFKQTFQRV
FTKDMHGQFKMGFGGTLEIKTSREIKISGAIGPCVSLNSKGPCVSENEIGTGGTCQWKIC
GLSPTTTLAIYFEVVNQHNAPIPQGGRGAIQFVTQYQHSSGQRRIRVTTIARNWADAQTQ
IQNIAASFDQEAAAILMARLAIYRAETEEGPDVLRWLDRQLIRLCQKFGEYHKDDPSSFR
FSETFSLYPQFMFHLRRSSFLQVFNNSPDESSYYRHHFMRQDLTQSLIMIQPILYAYSFS
GPPEPVLLDSSSILADRILLMDTFFQILIYHGETIAQWRKSGYQDMPEYENFRHLLQAPV
DDAQEILHSRFPMPRYIDTEHGGSQARFLLSKVNPSQTHNNMYAWGQESGAPILTDDVSL
QVFMDHLKKLAVSSAA*
>ENSG00000231925|ENST00000434618
MKSLSLLLAVALGLATAVSAGPAVIECWFVEDASGKGLAKRPGALLLRQGPGEPPPRPDL
DPELYLSVHDPAGALQAAFRRYPRGAPAPHCEMSRFVPLPASAKWASGLTPAQNCPRALD
GAWLMVSISSPVLSLSSLLRPQPEPQQEPVLITMATVVLTVLTHTPAPRVRLGQDALLDL
SFAYMPPTSEAASSLAPGPPPFGLEWRRQHLGKGHLLLAATPGLNGQMPAAQEGAVAFAA
WDDDEPWGPWTGNGTFWLPTVQPFQEGTYLATIHLPYLQGQVTLELAVYKPPKVSLMPAT
LARAAPGEAPPELLCLVSHFYPSGGLEVEWELRGGPGGRSQKAEGQRWLSALRHHSDGSV
SLSGHLQPPPVTTEQHGARYACRIHHPSLPASGRSAEVTLEVAGLSGPSLEDSVGLFLSA
FLLLGLFKALGWAAVYLSTCKDSKKKAE*
>ENSG00000276536|ENST00000634098
MTEYKLVVVGAGGVGKSALTIQLIQNHFVDEYDPTIEDSYRKQVVIDGETCLLDILDTAG
QEEYSAMRDQYMRTGEGFLCVFAINNTKSFEDIHQYREQIKRVKDSDDVPMVLVGNKCDL
AARTVESRQAQDLARSYGIPYIETSAKTRQGSRSGSSSSSGTLWDPPGPM*
>ENSG00000232062|ENST00000444296
MILNKALMLGALALTTVMSPCGGEDIVADHVASYGVNLYQSYGPSGQYSHEFDGDEEFYV
DLERKETVWQLPLFRRFRRFDPQFALTNIAVLKHNLNIVIKRSNSTAATNEVPEVTVFSK
SPVTLGQPNTLICLVDNIFPPVVNITWLSNGHSVTEGVSETSFLSKSDHSFFKISYLTFL
PSADEIYDCKVEHWGLDEPLLKHWEPEIPTPMSELTETVVCALGLSVGLVGIVVGTVLII
RGLRSVGASRHQGPL*
>ENSG00000142546|ENST00000596358
MTRHGKNCTAGAVYTYHEKKKDTAASGYGTQNIRLSRDAVKDFDCCCLSLQPCHDPVVTP
DGYLYEREAILEYILHQKKEIARQMKAYEKQRGTRREEQKELQRAASQDHVRGFLEKESA
IVSRPLNPFTAKALSGTSPDDVQPGPSVGPPSKDKDKVLPSFWIPSLTPEAKATKLEKPS
RTVTCPMSGKPLRMSDLTPVHFTPLDSSVDRVGLITRSERYVCAVTRDSLSNATPCAVLR
PSGAVVTLECVEKLIRKDMVDPVTGDKLTDRDIIVLQRGGTGFAGSGVKLQAEKSRPVMQ
A*
>ENSG00000221838|ENST00000421755
MISQFFILSSKGDPLIYKDFRGDSGGRDVAELFYRKLTGLPGDESPVVMHHHGRHFIHIR
HSGLYLVVTTSENVSPFSLLELLSRLATLLGDYCGSLGEGTISRNVALVYELLDEVLDYG
YVQTTSTEMLRNFIQTEAVVSKPFSLFDLSSVGLFGAETQQSKVAPSSAASRPVLSSRSD
QSQKNEVFLDVVERLSVLIASNGSLLKVDVQGEIRLKSFLPSGSEMRIGLTEEFCVGKSE

LRGYGPGIRVDEVSFHSSVNLDEFESHRILRLQPPQGELTVMRYQLSDDLPSPLPFRLFP
SVQWDRGSGRLQVYLKLRCDLLSKSQALNVRLHLPLPRGVVSLSQELSSPEQKAELAEGA
LRWDLPRVQGGSQLSGLFQMDVPGPPGPPSHGLSTSASPLGLGPASLSFELPRHTCSGLQ
VRFLRLAFRPCGNANPHKWVRHLSHSDAYVIRI*
>ENSG00000223355|ENST00000439349
MQPPSLLLLLLLLLLLCVSVVRPRGLLCGSFPEPCANGGTCLSLSLGQGTCQCAPGFLGE
TCQFPDPCQNAQLCQNGGSCQALLPAPLGLPSSPSPLTPSFLCTCLPGFTGERCQAQLED
PCPPSFCSKRGRCHIQASGRPQCSCMPGWTGEQCQLRDFCSANPCVNGGVCLATYPQIQC
HCPPGFEGHACERDVNECFQDPGPCPKGTSCHNTLGSFQCLCPVGQEGPRCELRAGPCPP
RGCSNGGTCQLMPEKDSTFHLCLCPPGFIGPDCEVNPDNCVSHQCQNGGTCQDGLDTYTC
LCPETWTGWDCSEDVDECETQGPPHCRNGGTCQNSAGSFHCVCVSGWGGTSCEENLDDCI
AATCAPGSTCIDRVGSFSCLCPPGRTGLLCHLEDMCLSQPCHGDAQCSTNPLTGSTLCLC
QPGYSGPTCHQDLDECLMAQQGPSPCEHGGSCLNTPGSFNCLCPPGYTGSRCEADHNECL
SQPCHPGSTCLDLLATFHCLCPPGLEGQLCEVETNECASAPCLNHADCHDLLNGFQCICL
PGFSGTRCEEDIDECRSSPCANGGQCQDQPGAFHCKCLPGFEGPRCQTEVDECLSDPCPV
GASCLDLPGAFFCLCPSGFTGQLCEVPLCAPNLCQPKQICKDQKDKANCLCPDGSPGCAP
PEDNCTCHHGHCQRSSCVCDVGWTGPECEAELGGCISAPCAHGGTCYPQPSGYNCTCPTG
YTGPTCSEEMTACHSGPCLNGGSCNPSPGGYYCTCPPSHTGPQCQTSTDYCVSAPCFNGG
TCVNRPGTFSCLCAMGFQGPRCEGKLRPSCADSPCRNRATCQDSPQGPRCLCPTGYTGGS
CQTLMDLCAQKPCPRNSHCLQTGPSFHCLCLQGWTGPLCNLPLSSCQKAALSQGIDVSSL
CHNGGLCVDSGPSYFCHCPPGFQGSLCQDHVNPCESRPCQNGATCMAQPSGYLCQCAPGY
DGQNCSKELDACQSQPCHNHGTCTPKPGGFHCACPPGFVGLRCEGDVDECLDQPCHPTGT
AACHSLANAFYCQCLPGHTGQWCEVEIDPCHSQPCFHGGTCEATAGSPLGFICHCPKGFE
GPTCSHRAPSCGFHHCHHGGLCLPSPKPGFPPRCACLSGYGGPDCLTPPAPKGCGPPSPC
LYNGSCSETTGLGGPGFRCSCPHSSPGPRCQKPGAKGCEGRSGDGACDAGCSGPGGNWDG
GDCSLGVPDPWKGCPSHSRCWLLFRDGQCHPQCDSEECLFDGYDCETPPACTPAYDQYCH
DHFHNGHCEKGCNTAECGWDGGDCRPEDGDPEWGPSLALLVVLSPPALDQQLFALARVLS
LTLRVGLWVRKDRDGRDMVYPYPGARAEEKLGGTRDPTYQERAAPQTQPLGKETDSLSAG
FVVVMGVDLSRCGPDHPASRCPWDPGLLLRFLAAMAAVGALEPLLPGPLLAVHPHAGTAP
PANQLPWPVLCSPVAGVILLALGALLVLQLIRRRRREHGALWLPPGFTRRPRTQSAPHRR
RPPLGEDSIGLKALKPKAEVDEDGVVMCSGPEEGEEVGQAEETGPPSTCQLWSLSGGCGA
LPQAAMLTPPQESEMEAPDLDTRGPDGVTPLMSAVCCGEVQSGTFQGAWLGCPEPWEPLL
DGGACPQAHTVGTGETPLHLAARFSRPTAARRLLEAGANPNQPDRAGRTPLHAAVAADAR
EVCQLLLRSRQTAVDARTEDGTTPLMLAARLAVEDLVEELIAAQADVGARDKWGKTALHW
AAAVNNARAARSLLQAGADKDAQDNREQTPLFLAAREGAVEVAQLLLGLGAARELRDQAG
LAPADVAHQRNHWDLLTLLEGAGPPEARHKATPGREAGPFPRARTVSVSVPPHGGGALPR
CRTLSAGAGPRGGGACLQARTWSVDLAARGGGAYSHCRSLSGVGAGGGPTPRGRRFSAGM
RGPRPNPAIMRGRYGVAAGRGGRVSTDDWPCDWVALGACGSASNIPIPPPCLTPSPERGS
PQLDCGPPALQEMPINQGGEGKK*
>ENSG00000101246|ENST00000612256
MSLSKITTTVGLNIGTVDVGKARLMFWDLGGQEELQSLWDKYYAECHGVIYVIDSTDEER
LAESKQAFEKVVTSEALCGVPVLVLANKQDVETCLSIPDIKTAFSDCTSKIGRRDCLTQA
CSALTGKGVREGIEWMVKCVVRNVHRPPRQRDIT*
>ENSG00000177830|ENST00000436108

MRTLFNLLWLALACSPVHTTLSKSDAKKAASKTLLEKSQFSDKPVQDRGLVVTDLKAESV
VLEHRSYCSAKARDRHFAGDVLGYVTPWNSHGYDVTKVFGSKFTQISPVWLQLKRRGREM
FEVTGLHDVDQGWMRAVRKHAKGLHIVPRLLFEDWTYDDFRNVLDSEDEIEELSKTVVQV
AKNQHFDGFVVEVWNQLLSQKRVGLIHMLTHLAEALHQARLLALLVIPPAITPGTDQLGM
FTHKEFEQLAPVLDGFSLMTYDYSTAHQPGPNAPLSWVRACVQVLDPKSKWRSKILLGLN
FYGMDYATSKDAREPVVGARYIQTLKDHRPRMVWDSQASEHFFEYKKSRSGRHVVFYPTL
KSLQVRLELARELGVGVSIWELGQGLDYFYDLL*
>ENSG00000109083|ENST00000585313
MAKDILGEAGLHFDELNKLRVLDPEVTQQTIELKEECKDFVDKIGQFQKIVGGLIELVDQ
LAKEAENEKMKAIGARNLLKSIAKQREAQQQQLQALIAEKKMQLERYRVEYEALCKVEAE
QNEFIDQFIFQK*
>ENSG00000147642|ENST00000433638
MGPLRESKKEHRVQHHDKEISRSRIPRLILRPHMPQQQHKVSPASESPFSEEESREFNPS
SSGRSARTVSSNSFCSDDTGCPSSQSVSPVKTPSDAGNSPIGFCPGSDEGFTRKKCTIGM
VGEGSIQSSRYKKESKSGLVKPGSEADFSSSSSTGSISAPEVHMSTAGSKRSSSSRNRGP
HGRSNGASSHKPGSSPSSPREKDLLSMLCRNQLSPVNIHPSYAPSSPSSSNSGSYKGSDC
SPIMRRSGRYMSCGENHGVRPPNPEQYLTPLQQKEVTVRHLKTKLKESERRLHERESEIV
ELKSQLARMREDWIEEECHRVEAQLALKEARKEIKQLKQVIETMRSSLADKDKGIQKYFV
DINIQNKKLESLLQSMEMAHSGSLRDELCLDFPCDSPEKSLTLNPPLDTMADGLSLEEQV
TGEGADRELLVGDSIANSTDLFDEIVTATTTESGDLELVHSTPGANVLELLPIVMGQEEG
SVVVERAVQTDVVPYSPAISELIQSVLQKLQDPCPSSLASPDESEPDSMESFPESLSALV
VDLTPRNPNSAILLSPVETPYANVDAEVHANRLMRELDFAACVEERLDGVIPLARGGVVR
QYWSSSFLVDLLAVAAPVVPTVLWAFSTQRGGTDPVYNIGALLRGCCVVALHSLRRTAFR
IKT*
>ENSG00000173702|ENST00000616727
MKAIIHLTLLALLSVNTATNQGNSADAVTTTETATSGPTVAAADTTETNFPETASTTANT
PSFPTATSPAPPIISTHSSSTIPTPAPPIISTHSSSTIPIPTAADSESTTNVNSLATSDI
ITASSPNDGLITMVPSETQSNNEMSPTTEDNQSSGPPTGTALLETSTLNSTGPSNPCQDD
PCADNSLCVKLHNTSFCLCLEGYYYNSSTCKKGKVFPGKISVTVSETFDPEEKHSMAYQD
LHSEITSLFKDVFGTSVYGQTVILTVSTSLSPRSEMRADDKFVNVTIVTILAETTSDNEK
TVTEKINKAIRSSSSNFLNYDLTLRCDYYGCNQTADDCLNGLACDCKSDLQRPNPQSPFC
VASSLKCPDACNAQHKQCLIKKSGGAPECACVPGYQEDANGNCQKCAFGYSGLDCKDKFQ
LILTIVGTIAGIVILSMIIALIVTARSNNKTKHIEEENLIDEDFQNLKLRSTGFTNLGAE
GSVFPKVRITASRDSQMQNPYSRHSSMPRPDY*
>ENSG00000137502|ENST00000527633
MSMEDYDFLFKIVLIGNAGVGKTCLVRRFTQGLFPPGQGATIGVDFMIKTVEINGEKVKL
QIWDTAGQERFRSITQSYYRSANALILTYDITCEESFRCLPEWLREIEQYASNKVITVLV
GNKIDLAERREVSQQRAEEFSEAQDMYYLETSAKESDNVEKLFLDLACRLISEARQNTLV
NNVSSPLPGEGKSISYLTCCNFN*
>ENSG00000090487|ENST00000416889
MGEIKVSPDYNWFRGTVPLKKIIVDDDDSKIWSLYDAGPRSIRCPLIFLPPVSGTADVFF
RQILALTGWGYRVIAVHLFGASLGGFLAQKFAEYTHKSPRVHSLILCNSFSDTSIFNQTW
TANSFWLMPAFMLKKIVLGNFSSGPVDPMMADAIDFMVDRLESLGQSELASRLTLNCQNS
YVEPHKIRDIPVTIMDVFDQSALSTEAKEEMYKLYPNARRAHLKTGGNFPYLCRSAEVNL

YVQIHLLQFHGTKYAAIDPSMVSAEELEVQKGSLGISQEEQ*
>ENSG00000119844|ENST00000409933
MEPDIIRMYSSSPPPLDNGAEDDDDDEFGEFGGFSEVSPSGVGFVDFDTPDYTRPKEEFV
PSNHFMPIHEFSENVDSLTSFKSIKNGNDKDITAELSAPVKGQSDVLLSTTSKEIISSEM
LATSIDGMERPGNLNKVVEQRQNVGTLESFSPGDFRTNMNVVHQNKQLESCNGEKPPCLE
ILTNGFAVLETVNPQGTDDLDNVADSKGRKPLSTHSTEYNLDSVPSPAEEFADFATFSKK
ERIQLEEIECAVLNDREALTIRENNKINRVNELNSVKEVALGRSLDNKGDTDGEDQVCVS
EISIVTNRGFSVEKQGLPTLQQDEFLQSGVQSKAWSLVDSADNSEAIRREQCKTEEKLDL
LTSKCAHLCMDSVKTSDDEVGSPKEESRKFTNFQSPNIDPTEENDLDDSLSVKNGDSSND
FVTCNDINEDDFGDFGDFGSASGSTPPFVTGTQDSMSDATFEESSEHFPHFSEPGDDFGE
FGDINAVSCQEETILTKSDLKQTSDNLSEECQLARKSSGTGTEPVAKLKNGQEGEIGHFD
SVPNIQDDCNGFQDSDDFADFSSAGPSQVVDWNAFEDEQKDSCSWAAFGDQQATESHHRK
EAWQSHRTDENIDTPGTPKTHSVPSATSKGAVASGHLQESATSVQTALLNRLERIFEACF
PSILVPDAEEEVTSLKHLLETSTLPIKTREALPESGELLDVWTELQDIHDAHGLRYQWGG
SHSNKKLLSSLGIDTRNILFTGNKKQPVIVPMYAAGLGMLEPTKEPLKPLSAAEKIASIG
QTATMSPDMNTCTSDQFQESLPPVQFDWSSSGLTNPLDASGGSTLLNLDFFGPVDDSSSS
SSTTIPGVDPELYELTTSKLEISTSSLKVTDAFARLMSTVEKTSTSTRKPKREEHLSEEA
IKVIAGLPDLTFMHAKVLMFPATLTPSTSSQEKADG*
>ENSG00000280951|ENST00000626303
MGQNDLMGTAEDFADQFLRVTKQYLPHVARLCLISTFLEDGIRMWFQWSEQRDYIDTTWN
CGYLLASSFVFLNLLGQLTGCVLVLSRNFVQYACFGLFGIIALQTIAYSILWDLKFLMRN
LALGGGLLLLLAESRSEGKSMFAGVPTMRESSPKQYLPG*
>ENSG00000090615|ENST00000450791
MDGASAEQDGLQEDRSHSGPSSLPEAPLKPPGPLVPPDQQDKVQCAEVNRASTEGESPDG
PGQGGLCQNGPTPPFPDPPSSLDPTTSPVGPDASPGVAGFHDNLRKSQGTSAEGSVRKEA
LQSLRLSLPMQETQLCSTDSPLPLEKEEQVRLQARKWLEEQLKQYRVKRQQERSSQPATK
TRLFSTLDPELMLNPENLPRASTLAMTKEYSFLRTSVPRGPKVGSLGLPAHPREKKTSKS
SKIRSLADYRTEDSNAGNSGGNVPAPDSTKGSLKQNRSSAASVVSEISLSPDTDDRLENT
SLAGDSVSEVDGNDSDSSSYSSASTRGTYGILSKTVGTQDTPYMVNGQEIPADTLGQFPS
IKDVLQAAAAEHQDQGQEVNGEVRSRRDSICSSVSLESSAAETQEEMLQVLKEKMRLEGQ
LEALSLEASQALKEKAELQAQLAALSTKLQAQVECSHSSQQRQDSLSSEVDTLKQSCWDL
ERAMTDLQNMLEAKNASLASSNNDLQVAEEQYQRLMAKVEDMQRSMLSKDNTVHDLRQQM
TALQSQLQQVQLERTTLTSKLKASQAEISSLQSVRQWYQQQLALAQEARVRLQGEMAHIQ
VGQMTQAGLLEHLKLENVSLSQQLTETQHRSMKEKGRIAAQLQGIEADMLDQEAAFMQIQ
EAKTMVEEDLQRRLEEFEGERERLQRMADSAASLEQQLEQVKLTLLQRDQQLEALQQEHL
DLMKQLTLTQEALQSREQSLDALQTHYDELQARLGELQGEAASREDTICLLQNEKIILEA
ALQAAKSGKEELDRGARRLEEGTEETSETLEKLREELAIKSGQVEHLQQETAALKKQMQK
IKEQFLQQKVMVEAYRRDATSKDQLISELKATRKRLDSELKELRQELMQVHGEKRTAEAE
LSRLHREVAQVRQHMADLEGHLQSAQKERDEMETHLQSLQFDKEQMVAVTEANEALKKQI
EELQQEARKAITEQKQKMRRLGSDLTSAQKEMKTKHKAYENAVGILSRRLQEALAAKEAA
DAELGQLRAQGGSSDSSLALHERIQALEAELQAVSHSKTLLEKELQEVIALTSQELEESR
EKVLELEDELQESRGFRKKIKRLEESNKKLALELEHEKGKLTGLGQSNAALREHNSILET
ALAKREADLVQLNLQVQAVLQRKEEEDRQMKHLVQALQASLEKEKEKVNSLKEQVAAAKV
EAGHNRRHFKAASLELSEVKKELQAKEHLVQKLQAEADDLQIREGKHSQEIAQFQAELAE

ARAQLQLLQKQLDEQLSKQPVGNQEMENLKWEVDQKEREIQSLKQQLDLTEQQGRKELEG
LQQLLQNVKSELEMAQEDLSMTQKDKFMLQAKVSELKNNMKTLLQQNQQLKLDLRRGAAK
TRKEPKGEASSSNPATPIKIPDCPVPASLLEELLRPPPAVSKEPLKNLNSCLQQLKQEMD
SLQRQMEEHALTVHESLSSWTPLEPATASPVPPGGHAGPRGDPQRHSQSRASKEGPGE*
>ENSG00000166377|ENST00000588895
XCGNMAREGLRTLVVAKKALTEEQYQDFEVPQGGRGSREPGEGDGTAVPHRRGGPAAGRR
AAHAGDAAQRRDQDMDANRR*
>ENSG00000283017|ENST00000635345
MYGSARSVGKVEPSSQSPGRSPRLPRSPRLGHRRTNSTGGSSGSSVGGGSGKTLSMENIQ
SLNAAYATSGPMYLSDHENVGSETPKSTMTLGRSGGRLPYGVRMTAMGSSPNIASSGVAS
DTIAFGEHHLPPVSMASTVPHSLRQARDNTIMDLQTQLKEVLRENDLLRKDVEVKESKLS
SSMNSIKTFWSPELKKERALRKDEASKITIWKEQYRVVQEENQHMQMTIQALQDELRIQR
DLNQLFQQDSSSRTGEPCVAELTEENFQRLHAEHERQAKELFLLRKTLEEMELRIETQKQ
TLNARDESIKKLLEMLQSKGLSAKATEEDHERTRRLAEAEMHVHHLESLLEQKEKENSML
REEMHRRFENAPDSAKTKALQTVIEMKDSKISSMERGLRDLEEEIQMLKSNGALSTEERE
EEMKQMEVYRSHSKFMKNKIGQVKQELSRKDTELLALQTKLETLTNQFSDSKQHIEVLKE
SLTAKEQRAAILQTEVDALRLRLEEKETMLNKKTKQIQDMAEEKGTQAGEIHDLKDMLDV
KERKVNVLQKKIENLQEQLRDKEKQMSSLKERVKSLQADTTNTDTALTTLEEALAEKERT
IERLKEQRDRDEREKQEEIDNYKKDLKDLKEKVSLLQGDLSEKEASLLDLKEHASSLASS
GLKKDSRLKTLEIALEQKKEECLKMESQLKKAHEAALEARASPEMSDRIQHLEREITRYK
DESSKAQAEVDRLLEILKEVENEKNDKDKKIAELESLTSRQVKDQNKKVANLKHKEQVEK
KKSAQMLEEARRREDNLNDSSQQLQDSLRKKDDRIEELEEALRESVQITAEREMVLAQEE
SARTNAEKQVEELLMAMEKVKQELESMKAKLSSTQQSLAEKETHLTNLRAERRKHLEEVL
EMKQEALLAAISEKDANIALLELSSSKKKTQEEVAALKREKDRLVQQLKQQTQNRMKLMA
DNYEDDHFKSSHSNQTNHKPSPDQDEEEGIWA*
>ENSG00000188603|ENST00000569030
MGGCAGSRRRFSDSEGEETVPEPRLPLLDHQGAHWKNAVGFWLLGLCNNFSYVVMLSAAH
DILSHKRTSGNQSHVDPGPTPIPHNSSSRFDCNSVSTAAVLLADILPTLVIKLLAPLGLH
LLPYSPRVLVSGICAAGSFVLVAFSHSVGTSLCGSSSSLSLRERWTVFKGLLWYIVPLVV
VYFAEYFINQGLFELLFFWNTSLSHAQQYRWYQMLYQAGVFASRSSLRCCRIRFTWALAL
LQMESRSVAQAGM*
>ENSG00000117620|ENST00000640732
MFANLKYVSLGILVFQTTSLVLTMRYSRTLKEEGPRYLSSTAVVVAELLKIMACILLVYK
DSKCSLRALNRVLHDEILNKPMETLKLAIPSGIYTLQNNLLYVALSNLDAATYQWPSDSQ
LDSKELSAGSQFVGLMAVLTACFSSGFAGVYFEKILKETKQSVWIRNIQLGFFGSIFGLM
GVYIYDGELVSKNGFFQGYNRLTWIVVVLQALGGLVIAAVIKYADNILKGFATSLSIILS
TLISYFWLQDFVPTSVFFLGAILVITATFLYGYDPKPAGNPTKA*
>ENSG00000180767|ENST00000615522
MGRRCCRRRVLAAACLGAALLLLCAAPRSLRPAFGNRALGSSWLGGEKRSPLQKLYDLDQ
DPRSTLAKVHRQRRDLLNSACSRHSRRQRLLQPEDLRHVLVDDAHGLLYCYVPKVACTNW
KRVLLALSGQARGDPRAISAQEAHAPGRLPSLADFSPAEINRRLRAYLAFLFVREPFERL
ASAYRNKLARPRSATRVASATTSWASSRRWRRTRPSCWAWRAHPT*
>ENSG00000273686|ENST00000631728
MSRSVALAVLALLSLSGLEAIQRTPKIQVYSRHPAENGKSNFLNCYVSGFHPSDIEVDLL

KNGERIEKVEHSDLSFSKDWSFYLLYYTEFTPTEKDEYACRVNHVTLSQPKIVKWDRDM*
>ENSG00000275501|ENST00000630334
MTTDDTEVPAMTLAPGHAALETQTLSAETSSRASTPAGPIPEAETRGAKRISPARETRSF
TKTSPNFMVLIATSVETSAASGSPEGAGMTTVQTITGSDPREAIFDTLCTDDSSEEAKTL
TMDILTLAHTSTEAKGLSSESSASSDSPHPVITPSRASESSASSDGPHPVITPSRASESS
ASSDGPHPVITPSRASESSASSDGPHPVITPSRASESSASSDGPHPVITPSRASESSASS
DGPHPVITPSRASESSASSDGPHPVITPSRASESSASSDGPHPVITPSRASESSASSDGP
HPVITPSRASESSASSDGLHPVITPSRASESSASSDGPHPVITPSRASESSASSDGPHPV
ITPSWSPGSDVTLLAEALVTVTNIEVINCSITEIETTTSSIPGASDTDLIPTEGVKASST
SDPPALPDSTEAKPHITEVTASAETLSTAGTTESAAPDATVGTPLPTNSATEREVTAPGA
TTLSGALVTVSRNPLEETSALSVETPSYVKVSGAAPVSIEAGSAVGKTTSFAGSSASSYS
PSEAALKNFTPSETPTMDIATKGPFPTSRDPLPSVPPTTTNSSRGTNSTLAKITTSAKTT
MKPPTATPTTARTRPTTDVSAGENGGFLLLRLSVASPEDLTDPRVAERLMQQLHRELHAH
APHFQVSLLRVRRG*
>ENSG00000176087|ENST00000612662
MSVEDGGMPGLGRPRQARWTLMLLLSTAMYGAHAPLLALCHVDGRVPFRPSSAVLLTELT
KLLLCAFSLLVGWQAWPQGPPPWRQAAPFALSALLYGANNNLVIYLQRYMDPSTYQVLSN
LKIGSTAVLYCLCLRHRLSVRQGLALLLLMAAGACYAAGGLQVPGNTLPSPPPAAAASPM
PLHITPLGLLLLILYCLISGLSSVYTELLMKRQRLPLALQNLFLYTFGVLLNLGLHAGGG
SGPGLLEGFSGWAALVVLSQALNGLLMSAVMKHGSSITRLFVVSCSLVVNAVLSAVLLRL
QLTAAFFLATLLIGLAMRLYYGSR*
>ENSG00000148248|ENST00000485435
MGQNDLMGTAEDFADQFLRVTKQYLPHVARLCLISTFLEDGIRMWFQWSEQRDYIDTTWN
CGYLLASSFVFLNLLGQLTGCVLVLSRNFVQYACFGLFGIIALQTIAYSILWDLKFLMRN
LALGGGLLLLLAESRSEGKSMFAGVPTMRESSPKQYLPG*
>ENSG00000174473|ENST00000506823
MKRKQKRFLQMTLLFTVALIFLPNVGLWSLYKDKHLVKSAEPGEQQTFPLGLGDGQFYSW
TDGLRRKDWHDYESIQKEAMRSGKGEHGKPYPLTEEDHDDSAYRENGFNIFVSNNIALER
SLPDIRHANCKHKMYLERLPNTSIIIPFHNEGWTSLLRTIHSIINRTPGSLIAEIILVDD
FSEREHLKDKLEEYMARFSKVRIVRTKKREGLIRTRLLGASMARGEVLTFLDSHCEVNVN
WLPPLLNQIALNHKTIVCPMIDVIDHNHFGYEAQAGDAMRGAFDWEMYYKRIPIPPELQR
ADPSDPFESPVMAGGLFAVDRKWFWELGGYDPGLEIWGGEQYEISFKVWMCGGEMFDVPC
SRVGHIYRKYVPYKVPSGTSLARNLKRVAETWMDEFAEYIYQRRPEYRHLSTGDISAQKE
LRKQLKCKDFKWFMAAVAWDVPKYYPPVEPPPAAWGEIRNVAANLCVDSKHGATGTELRL
DICVKDGSERTWSHEQLFTFGWREDIRPGEPLHTRKFCFDAISHNSPVTLYDCHGMKGNQ
LWGYRKDRTLFHPVSNSCMDCNPAEKKIFMARCDPLSETQQWIFEHINMTVLEKFNHHAN
S*
>ENSG00000011465|ENST00000425043
MKATIILLLLAQVSWAGPFQQRGLFDFMLEDEASGIGPEVPDDRDFEPSLGPVCPFRCQC
HLRVVQCSDLGLPPSLTELHLDGNKISRVDAASLKGLNNLAKLGLSFNSISAVDNGSLAN
TPHLRELHLDNNKLTRVPGGLAEHKYIQVVYLHNNNISVVGSSDFCPPGHNTKKASYSGV
SLFSNPVQYWEIQPSTFRCVYVRSAIQLGNYK*
>ENSG00000166377|ENST00000458297
MPLMMSEEGFENEESDYHTLPRARIMQRKRGLEWFVCDGWKFLCTSCCGWLINICRRKKE

LKARTVWLGCPEKCEEKHPRNSIKNQKYNVFTFIPGVLYEQFKFFLNLYFLVISCSQFVP
ALKIGYLYTYWAPLGFVLAVTMTREAIDEFRRFQRDKEVNSQLYSKLTVRGKVQVKSSDI
QVGDLIIVEKNQRIPSDMVFLRTSEKAGSCFIRTDQLDGETDWKLKVAVSCTQQLPALGD
LFSISAYVYAQKPQMDIHSFEGTFTREDSDPPIHESLSIENTLWASTIVASGTVIGVVIY
TGKETRSVMNTSNPKNKVGLLDLELNRLTKALFLALVALSIVMVTLQGFVGPWYRNLFRF
LLLFSYIIPISLRVNLDMGKAVYGWMMMKDENIPGTVVRTSTIPEELGRLVYLLTDKTGA
SPLLFFLFLYCQNCISILKQLEFLLKMSPNYFTITVKGSSYSGILRIRLKV*
>ENSG00000103723|ENST00000620652
MSAAPAYSEDKGGSAGPGEPEYGHDPASGGIFSSDYKRHDDLKEMLDTNKDSLKLEAMKR
IVAMIARGKNASDLFPAVVKNVACKNIEVKKLVYVYLVRYAEEQQDLALLSISTFQRGLK
DPNQLIRASALRVLSSIRVPIIVPIMMLAIKEAASDMSPYVRKTAAHAIPKLYSLDSDQK
DQLIEVIEKLLADKTTLVAGSVVMAFEEVCPERIDLIHKNYRKLCNLLIDVEEWGQVVII
SMLTRYARTQFLSPTQNESLLEENAEKAFYGSEEDEAKGAGSEETAAAAAPSRKPYVMDP
DHRLLLRNTKPLLQSRSAAVVMAVAQLYFHLAPKAEVGVIAKALVRLLRSHSEVQYVVLQ
NVATMSIKRRGMFEPYLKSFYIRSTDPTQIKILKLEVLTNLANETNIPTVLREFQTYIRS
MDKDFVAATIQAIGRCATNIGRVRDTCLNGLVQLLSNRDELVVAESVVVIKKLLQMQPAQ
HGEIIKHLAKLTDNIQVPMARASILWLIGEYCEHVPRIAPDVLRKMAKSFTAEEDIVKLQ
VINLAAKLYLTNSKQTKLLTQYVLSLAKYDQNYDIRDRARFTRQLIVPSEQGGALSRHAK
KLFLAPKPAPVLESSFKDRDHFQLGSLSHLLNAKATGYQELPDWPEEAPDPSVRNVEVPE
WTKCSNREKRKEKEKPFYSDSEGESGPTESADSDPESESESDSKSSSESGSGESSSESDN
EDQDEDEEKGRGSESEQSEEDGKRKTKKKVPERKGEASSSDEGSDSSSSSSSESEMTSESE
EEQLEPASWSRKTPPSSKSAPATKEISLLDLEDFTPPSVQPVSPPAIVSTSLAADLEGLT
LTDSTLVPSLLSPVSGVGRQELLHRVAGEGLAVDYTFSRQPFSGDPHMVSVHIHFSNSSD
TPIKGLHVGTPKLPAGISIQEFPEIESLAPGESATAVMGINFCDSTQAANFQLCTQTRQF
YVSIQPPVGELMAPVFMSENEFKKEQGKLMGMNEITEKLMLPDTCRSDHIVVQKVTATAN
LGRVPCGTSDEYRFAGRTLTGGSLVLLTLDARPAGAAQLTVNSEKMVIGTMLVKDVIQAL
TQ*
>ENSG00000109113|ENST00000436730
MNILAPVRRDRVLAELPQCLRKEAALHGHKDFHPRVTCACQEHRTGTVGFKISKVIVVGD
LSVGKTCLINRFCKDTFDKNYKATIGVDFEMERFEVLGIPFSLQLWDTAGQERFKCIAST
YYRGAQAIIIVFNLNDVASLEHTKQWLADALKENDPSSVLLFLVGSKKDLSTPAQYALME
KDALQVAQEMKAEYWAVSSLTGENVREFFFRVAALTFEANVLAELEKSGARRIGDVVRIN
SDDSNLYLTASKKKPTCCP*
>ENSG00000231389|ENST00000419277
MRPEDRMFHIRAVILRALSLAFLLSLRGAGAIKADHVSTYAAFVQTHRPTGEFMFEFDED
EMFYVDLDKKETVWHLEEFGQAFSFEAQGGLANIAILNNNLNTLIQRSNHTQATNDPPEV
TVFPKEPVELGQPNTLICHIDKFFPPVLNVTWLCNGELVTEGVAESLFLPRTDYSFHKFH
YLTFVPSAEDFYDCRVEHWGLDQPLLKHWEAQEPIQMPETTETVLCALGLVLGLVGIIVG
TVLIIKSLRSGHDPRAQGTL*
>ENSG00000141198|ENST00000575882
MAFGKSHRDPYATSVGHLIEKATFAGVQTEDWGQFMHICDIINTTQDGPKDAVKALKKRI
SKNYNHKEIQLTLSLIDMCVQNCGPSFQSLIVKKEFVKENLVKLLNPRYNLPLDIQNRIL
NFIKTWSQGFPGGVDVSEVKEVYLDLVKKGVQFPPSEAEAETARQETAQISSNPPTSVPT
APALSSVIAPKNSTVTLVPEQIGKLHSELDMVKMNVRVMSAILMENTPGSENHEDIELLQ

KLYKTGREMQERIMDLLVVVENEDVTVELIQVNEDLNNAILGYERFTRNQQRILEQNKNQ
KEATNTTSEPSAPSQDLLDLSPSPRMPRATLGELNTMNNQLSGLNFSLPSSDVTNNLKPS
LHPQMNLLALENTEIPPFAQRTSQNLTSSHAYDNFLEHSNSVFLQPVSLQTIAAAPSNQS
LPPLPSNHPAMTKSDLQPPNYYEVMEFDPLAPAVTTEAIYEEIDAHQHKGAQNDGD*
>ENSG00000138674|ENST00000513858
MKLKEVDRTAMQAWSPAQNHPIYLATGTSAQQLDATFSTNASLEIFELDLSDPSLDMKSC
ATFSSSHRYHKLIWGPYKMDSKGDVSGVLIAGGENGNIILYDPSKIIAGDKEVVIAQNDK
HTGPVRALDVNIFQTNLVASGANESEIYIWDLNNFATPMTPGAKTQPPEDISCIAWNRQV
QHILASASPSGRATVWDLRKNEPIIKVSDHSNRMHCSGLAWHPDVATQMVLASEDDRLPV
IQMWDLRFASSPLRVLENHARGILAIAWSMADPELLLSCGKDAKILCSNPNTGEVLYELP
TNTQWCFDIQWCPRNPAVLSAASFDGRISVYSIMGGSTDGLRQKQVDKLSSSFGNLDPFG
TGQPLPPLQIPQQTAQHSIVLPLKKPPKWIRRPVGASFSFGGKLVTFENVRMPSHQGAEQ
QQQQHHVFISQVVTEKEFLSRSDQLQQAVQSQGFINYCQKKIDASQTEFEKNVWSFLKVN
FEDDSRGKYLELLGYRKEDLGKKHIKEEKEESEFLPSSGGTFNISVSGDIDGLITQALLT
GNFESAVDLCLHDNRMADAIILAIAGGQELLARTQKKYFAKSQSKITRLITAVVMKNWKE
IVESCDLKNWREALAAVLTYAKPDEFSALCDLLGTRLENEGDSLLQTQACLCYICAGNVE
KLVACWTKAQDGSHPLSLQDLIEKVVILRKAVQLTQAMDTSTVGVLLAAKMSQYANLLAA
QGSIAAALAFLPDNTNQPNIMQLRDRLCRAQGEPVAGHESPKIPYEKQQLPKGRPGPVAG
HHQMPRVQTQQYYPHGENPPPPGFIMHGNVNPNAAGQLPTSPGHMHTQVPPYPQPQRPQN
GWNDPPALNRVPKKKKMPENFMPPVPITSPIMNPLGDPQSQMLQQQPSAPVPLSSQSSFP
QPHLPGGQPFHGVQQPLGQTGMPPSFSKPNIEGAPGAPIGNTFQHVQSLPTKKITKKPIP
DEHLILKTTFEDLIQRCLSSATDPQTKRKLDDASKRLEFLYDKLREQTLSPTITSGLHNI
ARSIETRNYSEGLTMHTHIVSTSNFSETSAFMPVLKVVLTQANKLGV*
>ENSG00000104886|ENST00000589097
MRYNEKELQALSRQPAEMAAELGMRGPKKGSVLKRRLVKLVVNFLFYFRTDEAEPVGALL
LERCRVVREEPGTFSISFIEDPERKYHFECSSEEQCQEWMEALRRASYEFMRRSLIFYRN
EIRKVTGKDPLEQFGISEEARFQLSGLQA*
>ENSG00000151532|ENST00000432306
MSSDFEGYEQDFAVLTAEITSKIARVPRLPPDEKKQMVANVEKQLEEAKELLEQMDLEVR
EIPPQSRGMYSNRMRSYKQEMGKLETDFKRSRIAYSDEVRNELLGDDGNSSENQRAHLLD
NTERLERSSRRLEAGYQIAVETEQIGQEMLENLSHDREKIQRARERLRETDANLGKSSRI
LTGMLRRGCSVKKQCNLSLAPKA*
>ENSG00000129354|ENST00000590923
MSASAVFILDVKGKPLISRNYKGDVAMSKIEHFMPLLVQREEEGALAPLLSHGQVHFLWI
KHSNLYLVATTSKNANASLVYSFLYKTIEVFCEYFKELEEESIRDNFVIVYELLDELMDF
GFPQTTDSKILQEYITQQSNKLETGKSRVPPTVTNAVSWRSEGIKYKKNEVFIDVIESVN
LLVNANGSVLLSEIVGTIKLKVFLSGMPELRLGLNDRVLFELTGLSGSKNKSVELEDVKF
HQCVRLSRFDNDRTISFIPPDGDFELMSYRLSTQVKPLIWIESVIEKFSHSRVEIMVKAK
GQFKKQSVANGVEISVPVPSDADSPRFKTSVGSAKYVPERNVVIWSIKSFPGGKEYLMRA
HFGLPSVEKEEVEGRPPIGVKFEIPYFTVSGIQVRYMKIIEKSGYQALPWVRYITQSGDY
QLRTS*
>ENSG00000003147|ENST00000407906
MSGHKCSYPWDLQDRYAQDKSVVNKMQQKYWETKQAFIKATGKKEDEHVVASDADLDAKL
ELFHSIQRTCLDLSKAIVLYQKRICFLSQEENELGKFLRSQGFQDKTRAGKMMQATGKAL

CFSSQQRLALRNPLCRFHQEVETFRHRAISDTWLTVNRMEQCRTEYRGALLWMKDVSQEL
DPDLYKQMEKFRKVQTQVRLAKKNFDKLKMDVCQKVDLLGASRCNLLSHMLATYQTTLLH
FWEKTSHTMAAIHESFKGYQPYEFTTLKVWKLEGPLENLEKSKVLFISQAKSIAESIIIP
GLRMLKRELCPQQSLFLSFFLSFFFFFFFFTASGFNTMRIYCNPKARIEP*
>ENSG00000206433|ENST00000448441
MLSRNDVKRLERSWAQGSSEQELHYASLQRLPVPSSEGPDLRGRDKRGTKEDPRADYACI
AENKPT*
>ENSG00000166710|ENST00000559916
MSRSVALAVLALLSLSGLEAIQRTPKIQVYSRHPAENGKSNFLNCYVSGFHPSDIEVDLL
KNGERIEKVEHSDLSFSKDWSFYLLYYTEFTPTEKDEYACRVNHVTLSQPKIVKWDRDM*
>ENSG00000188467|ENST00000449382
MQTKGGQTWARRALLLGILWATAHLPLSGTSLPQRLPRATALGLSQDVAGTTFMAAGSSA
PELVTAFLGVFITKGDIGISTILGSAIYNLLGICAACGLLSNTVSTLSCWPLFRDCAAYT
ISAAAVLGIIYDNQVYWYEGALLLLIYGLYVLVLCFDIKINQYIIKKCSPCCACLAKAME
RSEQQPLMGWEDEGQPFIRRQSRTDSGIFYEDSGYSQLSISLHGLSQVSEDPPSVFNMPE
ADLKRIFWVLSLPIITLLFLTTPDCRKKFWKNYFVITFFMSAIWISAFTYILVWMVTITG
ETLEIPDTVMGLTLLAAGTSIPDTIASVLVARKGKGDMAMSNIVGSNVFDMLCLGIPWFI
KTAFINGSAPAEVNSRGLTYITISLNISIIFLFLAVHFNGWKLDRKLGIVCLLSYLGLAT
LSVLYELGIIGNNKIRGCGG*
>ENSG00000138674|ENST00000448323
MKLKEVDRTAMQAWSPAQNHPIYLATGTSAQQLDATFSTNASLEIFELDLSDPSLDMKSC
ATFSSSHRYHKLIWGPYKMDSKGDVSGVLIAGGENGNIILYDPSKIIAGDKEVVIAQNDK
HTGPVRALDVNIFQTNLVASGANESEIYIWDLNNFATPMTPGAKTQPPEDISCIAWNRQV
QHILASASPSGRATVWDLRKNEPIIKVSDHSNRMHCSGLAWHPDVATQMVLASEDDRLPV
IQMWDLRFASSPLRVLENHARGILAIAWSMADPELLLSCGKDAKILCSNPNTGEVLYELP
TNTQWCFDIQWCPRNPAVLSAASFDGRISVYSIMGGSTDGLRQKQVDKLSSSFGNLDPFG
TGQPLPPLQIPQQTAQHSIVLPLKKPPKWIRRPVGASFSFGGKLVTFENVRMPSHQGAEQ
QQQQHHVFISQVVTEKEFLSRSDQLQQAVQSQGFINYCQKKIDASQTEFEKNVWSFLKVN
FEDDSRGKYLELLGYRKEDLGKKIALALNKVDGANVALKDSDQVAQSDGEESPAAEEQLL
GEHIKEEKEESEFLPSSGGTFNISVSGDIDGLITQALLTGNFESAVDLCLHDNRMADAII
LAIAGGQELLARTQKKYFAKSQSKITRLITAVVMKNWKEIVESCDLKNWREALAAVLTYA
KPDEFSALCDLLGTRLENEGDSLLQTQACLCYICAGNVEKLVACWTKAQDGSHPLSLQDL
IEKVVILRKAVQLTQAMDTSTVGVLLAAKMSQYANLLAAQGSIAAALAFLPDNTNQPNIM
QLRDRLCRAQGEPVAGHESPKIPYEKQQLPKGRPGPVAGHHQMPRVQTQQYYPHGENPPP
PGFIMHGNVNPNAAGQLPTSPGHMHTQVPPYPQPQPYQPAQPYPFGTGGSAMYRPQQPVA
PPTSNAYPNTPYISSASSYTGQSQLYAAQHQASSPTSSPATSFPPPPSSGASFQHGGPGA
PPSSSAYALPPGTTGTLPAASELPASQRTGPQNGWNDPPALNRVPKKKKMPENFMPPVPI
TSPIMNPLGDPQSQMLQQQPSAPVPLSSQSSFPQPHLPGGQPFHGVQQPLGQTGMPPSFS
KPNIEGAPGAPIGNTFQHVQSLPTKKITKKPIPDEHLILKTTFEDLIQRCLSSATDPQTK
RKLDDASKRLEFLYDKLREQTLSPTITSGLHNIARSIETRNYSEGLTMHTHIVSTSNFSE
TSAFMPVLKVVLTQANKLGV*
>ENSG00000158850|ENST00000622395
MLRRLLERPCTLALLVGSQLAVMMYLSLGGFRSLSALFGRDQGPTFDYSHPRDVYSNLSH
LPGAPGGPPAPQGLPYCPERSPLLVGPVSVSFSPVPSLAEIVERNPRVEPGGRYRPAGCE

PRSRTAIIVPHRAREHHLRLLLYHLHPFLQRQQLAYGIYVIHQAGNGTFNRAKLLNVGVR
EALRDEEWDCLFLHDVDLLPENDHNLYVCDPRGPRHVAVAMNKFGYSLPYPQYFGGVSAL
TPDQYLKMNGFPNEYWGWGGEDDDIATRVRLAGMKISRPPTSVGHYKMVKHRGDKGNEEN
PHRFDLLVRTQNSWTQDGMNSLTYQLLARELGPLYTNITADIGTDPRGPRAPSGPRYPPG
SSQAFRQEMLQRRPPARPGPLSTANHTALRGSH*
>ENSG00000226260|ENST00000442960
MAISGVPVLGFFIIAVLMSAQESWAIKEEHVIIQAEFYLNPDQSGEFMFDFDGDEIFHVD
MAKKETVWRLEEFGRFASFEAQGALANIAVDKANLEIMTKRSNYTPITNVPPEVTVLTNS
PVELREPNVLICFIDKFTPPVVNVTWLRNGKPVTTGVSETVFLPREDHLFRKFHYLPFLP
STEDVYDCRVEHWGLDEPLLKHWEFDAPSPLPETTENVVCALGLTVGLVGIIIGTIFIIK
GVRKSNAAERRGPL*
>ENSG00000164125|ENST00000592057
MTCPDKPGQLINWFICSLCVPRVRKLWSSRRPRTRRNLLLGTACAIYLGFLVSQVGRASL
QHGQAAEKGPHRSRDTAEPSFPEIPLDGTLAPPESQGNGSTLQPNVVYITLRSKRSKPAN
IRGTVKPKRRKKHAVASAAPGQEALVGPSLQPQEAAREADAVAPGYAQGANLVKIGERPW
RLVRGPGVRAGGPDFLQPSSRESNIRIYSESAPSWLSKDDIRRMRLLADSAVAGLRPVSS
RSGARLLVLEGGAPGAVLRCGPSPCGLLKQPLDMSEVFAFHLDRILGLNRTLPSVSRKAE
FIQGNRFTCFVICSCWGFWFIFFFPSLSFSK*
>ENSG00000158488|ENST00000452291
MLLLFLLFEGLCCPGENTAVKPEAWLSCGPSPGPGRLQLVCHVSGFYPKPVWVMWMRGEQ
EQRGTQRGDVLPNADETWYLRATLDVAAGEAAGLSCRVKHSSLGGHDLIIHWGGYSIFLI
LICLTVIVTLVILVVVDSRLKKQSSNKNILSPHTPSPVFLMGANTQDTKNSRHQFCLAQV
SWIKNRVLKKWKTRLNQLW*
>ENSG00000076864|ENST00000495204
MSGRKRSFTFGAYGGVDKSFTSRRSVWRSDGQNQHFPQALDLSRVNLVPSYTPSLYPKNT
DLFEMIEKMQGSRMDEQRCSFPPPLKTEEDYIPYPSVHEVLGREGPFPLILLPQFGGYWI
EGTNHEITSIPETEPLQSPTTKVKLECNPTARIYRKHFLGKEHFNYYSLDAALGHLVFSL
KYDVIGDQEHLRLLLRTKCRTYHDVIPISCLTEFPNVVQMAKLVCEDVNVDRFYPVLYPK
ASRLIVTFDEHVISNNFKFGVIYQKLGQTSEEELFSTNEESPAFVEFLEFLGQKVKLQDF
KGFRGGLDVTHGQTGTESVYCNFRNKEIMFHVSTKLPYTEGDAQQLQRKRHIGNDIVAVV
FQDENTPFVPDMIASNFLHAYVVVQAEGGGPDGPLYKVSVTARDDVPFFGPPLPDPAVFR
KGPEFQEFLLTKLINAEYACYKAEKFAKLEERTRAALLETLYEELHIHSQSMMGLGGDED
KMENGSGGGGFFESFKRVIRSRSQSMDAMGLSNKKPNTVSTSHSGSFAPNNPDLAKAAGI
SLIVPGKSPTRKKSGPFGSRRSSAIGIENIQEVQEKRESPPAGQKTPDSGHVSQEPKSEN
SSTQSSPEMPTTKNRAETAAQRAEALKDFSRSSSSASSFASVVEETEGVDGEDTGLESVS
SSGTPHKRDSFIYSTWLEDSVSTTSGGSSPGPSRSPHPDAGKLGDPACPEIKIQLEASEQ
HMPQLGC*
>ENSG00000198488|ENST00000622824
MAFPCRRSLTAKTLACLLVGVSFLALQQWFLQAPRSPREERSPQEETPEGPTDAPAADEP
PSELVPGPPCVANASANATADFEQLPARIQDFLRYRHCRHFPLLWDAPAKCAGGRGVFLL
LAVKSAPEHYERRELIRRTWGQERSYGGRPVRRLFLLGTPGPEDEARAERLAELVALEAR
EHGDVLQWAFADTFLNLTLKHLHLLDWLAARCPHARFLLSGDDDVFVHTANVVRFLQAQP
PGRHLFSGQLMEGSVPIRDSWSKYFVPPQLFPGSAYPVYCSGGGFLLSGPTARALRAAAR
HTPLFPIDDAYMGMCLERAGLAPSGHEGIRPFGVQLPGAQQSSFDPCMYRELLLVHRFAP

YEMLLMWKALHSPALSCDRGHRVS*
>ENSG00000169255|ENST00000484127
MASALWTVLPSRMSLRSLKWSLLLLSLLSFFVMWYLSLPHY
>ENSG00000122390|ENST00000424546
MFPRRRTERRLGDTGTRKKIAYRKAVPGGRKCGASLSWEKSSREYPDSWYRDITSNKKFF
SLAATYRGAIVGMIVAEIKNRTKIHKEDGDILASNFSVDTQVAYILSLGVVKEFRKHGIG
SLLLESLKDHISTTAQDHCKAIYLHVLTTNNTAINFYENRDFKQHHYLPYYYSIRGVLKD
GFTYVLYINGGHPPWTILDYIQHLGSALASLSPCSIPHRVYRQAHSLLCSFLPWSGISSK
SGIEYSRTM*
>ENSG00000137497|ENST00000544238
MTLHATRGAALLSWVNSLHVADPVEAVLQLQDCSIFIKIIDRIHGTEEGQQILKQPVSER
LDFVCSFLQKNRKHPSSPECLVSAQKVLEGSELELAKMTMLLLYHSTMSSKSPRDWEQFE
YKIQAELAVILKFVLDHEDGLNLNEDLENFLQKAPVPSTCSSTFPEELSPPSHQAKREIR
FLELQKVASSSSGNNFLSGSPASPMGDILQTPQFQMRRLK
>ENSG00000263160|ENST00000571778
MAGFLDNFRWPECECIDWSERRNAVASVVAGILFFTGWWIMIDAAVVYPKPEQLNHAFHT
CGVFSTLAFFMINAVSNAQVRGDSYESGCLGRTGARVWLFIGFMLMFGSLIASMWILFGA
YVTQNTDVYPGLAVFFQNALIFFSTLIYKFGRTEELWT*
>ENSG00000127564|ENST00000440027
MLERPPALAMPMPTEGTPPPLSGTPIPVPAYFRHAEPGFSLKRPRGLSRSLPPPPPAKGS
IPISRLFPPRTPGWHQLQPRRVSFRGEASETLQSPGYDPSRPESFFQQSFQRLSRLGHGS
YGEVFKVRSKEDGRLYAVKRSMSPFRGPKDRARKLAEVGSHEKVGQHPCCVRLEQAWEEG
GILYLQTELCGPSLQQHCEAWGASLPEAQVWGYLRDTLLALAHLHSQGLVHLDVKPANIF
LGPRGRCKLGDFGLLVELGTAGAGEVQEGDPRYMAPELLQGSYGTAADVFSLGLTILEVA
CNMELPHGGEGWQQLRQGYLPPEFTAGLSSELRSVLVMMLEPDPKLRATAEALLALPVLR
QPRAWGVLWCMAAEALSRGWALWQALLALLCWLWHGLAHPASWLQPLGPPATPPGSPPCS
LLLDSSLSSNWDDDSLGPGHPPCLACPPAGLHSPLRLSWPGLWGAPPPPGAGAHPGMPWT
*
>ENSG00000114354|ENST00000418917
MNGQLDLSGKLIIKAQLGEDIRRIPIHNEDITYDELVLMMQRVFRGKLLSNDEVTIKYKD
EDGDLITIFDSSDLSFAIQCSRILKLTLFVNGQPRPLESSQVKYLRRELIELRNKVNRLL
DSLEPPGEPGPSTNIPENDTVDGREEKSASDSSGKQSTQVMAASMSAFDPLKNQDEINKN
VMSAFGLTDDQVSGPPSAPAEDRSGTPDSIASSSSAAHPPGVQPQQPPYTGAQTQAGQMY
QQYQQQAGYGAQQPQAPPQQPQQYGIQYSASYSQQTGPQQPQQFQGYGQQPTSQAPAPAF
SGQPQQLPAQPPQQYQASNYPAQTYTAQTSQPTNYTVAPASQPGMAPSQPGAYQPRPGFT
SLPGSTMTPPPSGPNPYARNRPPFGQGYTQPGPGYR*
>ENSG00000284501|ENST00000638266
MRLRNGTFLTLLLFCLCAFLSLSWYAALSGGQKGDVVDVYQREFLALRDRLHAAEQESLKR
SKELNLVLDEIKRAVSERQALRDGDGNRTWGRLTEDPRLKPWNGSHRHVLHLPTVFHHLP
HLLAKESSLQPAVRVGQGRTGVSVVMGIPSVRREVHSYLTDTLHSLISELSPQEKEDSVI
VVLIAETDSQYTSAVTENIKALFPTEIHSGLLEVISPSPHFYPDFSRLRESFGDPKERVR
WRTKQNLDYCFLMMYAQSKGIYYVQLEDDIVAKPNYLSTMKNFALQQPSEDWMILEFSQL
GFIGKMFKSLDLSLIVEFILMFYRDKPIDWLLDHILWVKVCNPEKDAKHCDRQKANLRIR
FKPSLFQHVGTHSSLAGKIQKLKDKDFGKQALRKEHVNPPAEVSTSLKTYQHFTLEKAYL

REDFFWAFTPAAGDFIRFRFFQPLRLERFFFRSGNIEHPEDKLFNTSVEVLPFDNPQSDK
EALQEGRTATLRYPRSPDGYLQIGSFYKGVAEGEVDPAFGPLEALRLSIQTDSPVWVILS
EIFLKKAD*
>ENSG00000134470|ENST00000525219
MSVEHADIWVKSYSLYSRERYICNSGFKRKAGTSSLTECVLNKATNVAHWTTPSLKCIRD
PALVHQRPAPPSTVTTAGVTPQPESLSPSGKEPAASSPSSNNTAATTAAIVPGSQLMPSK
SPSTGTTEISSHESSHGTPSQTTAKNWELTASASHQPPGVYPQGHSDTTVAISTSTVLLC
GLSAVSLLACYLKSRQTPPLASVEMEAMEALPVTWGTSSRDEDLENCSHHL*
>ENSG00000135241|ENST00000422087
MSINLTVDIYIYLLSNARSVCGKQRSKQLYFLFSPKHYWRISHISLQRGFHTNIIRCKWT
KSEAHSCSKHCYSPSNHGLHIGILKLSTSAPKGLTKVNICMSRIKSTLNSVSKAVFGNQN
EMISRLAQFKPSSQILRKVSDSGWLKQKNIKQAIKSLKKYSDKSAEKSPFPEEKSHIIDK
EEDIGKRSLFHYTSSITTKFGDSFYFLSNHINSYFKRKEKMSQQKENEHFRDKSELEDKK
VEEGKLRSPDPGILAYKPGSESVHTVDKPTSPSAIPDVLQVSTKQSIANFLSRPTEGVQA
LVGGYIGGLVPKLKYDSKSQSEEQEEPAKTDQAVSKDRNAEEKKRLSLQREKIIARVSID
NRTRALVQALRRTTDPKLCITRVEELTFHLLEFPEGKGVAVKERIIPYLLRLRQIKDETL
QAAVREILALIGYVDPVKGRGIRILSIDGGGTRGVVALQTLRKLVELTQKPVHQLFDYIC
GVSTGAILAFMLGLFHMPLDECEELYRKLGSDVFSQNVIVGTVKMSWSHAFYDSQTWENI
LKDRMGSALMIETARNPTCPKVAAVSTIVNRGITPKAFVFRNYGHFPGINSHYLGGCQYK
MWQAIRASSAAPGYFAEYALGNDLHQDGGLLLNNPSALAMHECKCLWPDVPLECIVSLGT
GRYESDVRNTVTYTSLKTKLSNVINSATDTEEVHIMLDGLLPPDTYFRFNPVMCENIPLD
ESRNEKLDQLQLEGLKYIERNEQKMKKVAKILSQEKTTLQKINDWIKLKTDMYEGLPFFS
KL*
>ENSG00000164125|ENST00000585682
MTCPDKPGQLINWFICSLCVPRVRKLWSSRRPRTRRNLLLGTACAIYLGFLVSQVGRASL
QHGQAAEKGPHRSRDTAEPSFPEIPLDGTLAPPESQGNGSTLQPNVVYITLRSKRSKPAN
IRGTVKPKRRKKHAVASAAPGQEALVGPSLQPQEAAREADAVAPGYAQGANLVKIGERPW
RLVRGPGVRAGGPDFLQPSSRESNIRIYSESAPSWLSKDDIRRMRLLADSAVAGLRPVSS
RSGARLLVLEGGAPGAVLRCGPSPCGLLKQPLDMSEVFAFHLDRILGLNRTLPSVSRKAE
FIQDGRPCPIILWDASLSSASNDTHSSVKLTWGTYQQLLKQKCWQNGRVPKPESGCTEIH
HHEWSKMALFDFLLQIYNRLDTNCCGFRPRKEDACVQNGLRPKCDDQGSAALAHIIQRKH
DPRHLVFIDNKGFFDRSEDNLNFKLLEGIKEFPASAVSVLKSQHLRQKLLQSLFLDKVYW
ESQGGRQGIEKLIDVIEHRAKILITYINAHGVKVLPMNE*
>ENSG00000036257|ENST00000409096
MDEKYVNSIWDLLKNAIQEIQRKNNSGLSFEELYRNAYTMVLHKHGEKLYTGLREVVTEH
LINKVREDVLNSLNNNFLQTLNQAWNDHQTAMVMIRDILMYMDRVYVQQNNVENVYNLGL
IIFRDQVVRYGCIRDHLRQTLLDMIARERKGEVVDRGAIRNACQMLMILGLEGRSVYEED
FEAPFLEMSAEFFQMESQKFLAENSASVYIKKVEARINEEIERVMHCLDKSTEEPIVKVV
ERELISKHMKTIVEMENSGLVHMLKNGKTEDLGCMYKLFSRVPNGLKTMCECMSSYLREQ
GKALVSEEGEGKNPVDYIQGLLDLKSRFDRFLLESFNNDRLFKQTIAGDFEYFLNLNSRS
PEYLSLFIDDKLKKGVKGLTEQEVETILDKAMVLFRFMQEKDVFERYYKQHLARRLLTNK
SVSDDSEKNMISKLKTECGCQFTSKLEGMFRDMSISNTTMDEFRQHLQATGVSLGGVDLT
VRVLTTGYWPTQSATPKCNIPPAPRHAFEIFRRFYLAKHSGRQLTLQHHMGSADLNATFY
GPVKKEDGSEVGVGGAQVTGSNTRKHILQVSTFQMTILMLFNNREKYTFEEIQQETDIPE

RELVRALQSLACGKPTQRVLTKEPKSKEIENGHIFTVNDQFTSKLHRVKIQTVAAKQGES
DPERKETRQKVDDDRKHEIEAAIVRIMKSRKKMQHNVLVAEVTQQLKARFLPSPVVIKKR
IEGLIEREYLARTPEDRKVYTYVA*
>ENSG00000117620|ENST00000638876
MFANLKYVSLGILVFQTTSLVLTMRYSRTLKEEGPRYLSSTAVVVAELLKIMACILLVYK
DSSHVSVKNSYNSIIFCVYA*
>ENSG00000232062|ENST00000418023
MILNKALMLGALALTTVMSPCGGEDIVADHVASYGVNLYQSYGPSGQYSHEFDGDEEFYV
DLERKETVWQLPLFRRFRRFDPQFALTNIAVLKHNLNIVIKRSNSTAATNEVPEVTVFSK
SPVTLGQPNTLICLVDNIFPPVVNITWLSNGHSVTEGVSETSFLSKSDHSFFKISYLTFL
PSADEIYDCKVEHWGLDEPLLKHWEPEIPTPMSELTETVVCALGLSVGLVGIVVGTVLII
RGLRSVGASRHQGPL*
>ENSG00000168434|ENST00000563164
XDSALELALHAGKLPFPPEQGDELPELDNMADNWLGSIARATMQTYCDAILQIPELSPHS
AKQLATDIGGSPQDRRWEGQLLPAASVSFSFALQESHPKSSLSFSCGFGSEAG*
>ENSG00000142235|ENST00000600059
MPAPGALILLAAVSASGCLASPAHPDGFALGRAPLAPPYAVVLISCSGLLAFIFLLLTCL
CCKRGDVGFKEFENPEGEDCSGEYTPPAEETSSSQSLPDVYILPLAEVSLPMPAPQPSHS
DMTTPLGLSRQHLSYLQEIGSGWFGKVILGEIFSDYTPAQVVVKELRASAGPLEQRKFIS
EAQPYRSLQHPNVLQCLGLCVETLPFLLIMEFCQLGDLKRYLRAQRPPEGLSPELPPRDL
RTLQRMGLEIARGLAHLHSHNYVHSDLALRNCLLTSDLTVRIGDYGLAHSNYKEDYYLTP
ERLWIPLRWAAPELLGELHGTFMVVDQSRESNIWSLGVTLWELFEFGAQPYRHLSDEEVL
AFVVRQQHVKLARPRLKLPYADYWYDILQSCWRPPAQRPSASDLQLQLTYLLSERPPRPP
PPPPPPRDGPFPWPWPPAHSAPRPGTLSSPFPLLDGFPGADPDDVLTVTESSRGLNLECL
WEKARRGAGRGGGAPAWQPASAPPAPHANPSNPFYEALSTPSVLPVISARSPSVSSEYYI
RLEEHGSPPEPLFPNDWDPLDPGVPAPQAPQAPSEVPQLVSETWASPLFPAPRPFPAQSS
ASGSFLLSGWDPEGRGAGETLAGDPAEVLGERGTAPWVEEEEEEEEGSSPGEDSSSLGGG
PSRRGPLPCPLCSREGACSCLPLERGDAVAGWGGHPALGCPHPPEDDSSLRAERGSLADL
PMAPPASAPPEFLDPLMGAAAPQYPGRGPPPAPPPPPPPPRAPADPAASPDPPSAVASPG
SGLSSPGPKPGDSGYETETPFSPEGAFPGGGAAEEEGVPRPRAPPEPPDPGAPRPPPDPG
PLPLPGPREKPTFVVQVSTEQLLMSLREDVTRNLLGEKGATARETGPRKAGRGPGNREKV
PGLNRDPTVLGNGKQAPSLSLPVNGVTVLENGDQRAPGIEEKAAENGALGSPEREEKVLE
NGELTPPRREEKALENGELRSPEAGEKVLVNGGLTPPKSEDKVSENGGLRFPRNTERPPE
TGPWRAPGPWEKTPESWGPAPTIGEPAPETSLERAPAPSAVVSSRNGGETAPGPLGPAPK
NGTLEPGTERRAPETGGAPRAPGAGRLDLGSGGRAPVGTGTAPGGGPGSGVDAKAGWVDN
TRPQPPPPPLPPPPEAQPRRLEPAPPRARPEVAPEGEPGAPDSRAGGDTALSGDGDPPKP
ERKGPEMPRLFLDLGPPQGNSEQIKARLSRLSLALPPLTLTPFPGPGPRRPPWEGADAGA
AGGEAGGAGAPGPAEEDGEDEDEDEEEDEEAAAPGAAAGPRGPGRARAAPVPVVVSSADA
DAARPLRGLLKSPRGADEPEDSELERKRKMVSFHGDVTVYLFDQETPTNELSVQAPPEGD
TDPSTPPAPPTPPHPATPGDGFPSNDSGFGGSFEWAEDFPLLPPPGPPLCFSRFSVSPAL
ETPGPPARAPDARPAGPVEN*
>ENSG00000125447|ENST00000584243
XAEAEGESLESWLNKATNPSNRQEDWEYIIGFCDQINKELEGPQIAVRLLAHKIQSPQEW
EALQALTVLEACMKNCGRRFHNEVGKFRFLNELIKVVSPKYLGDRVSEKVKTKVIELLYS

WTMALPEEAKIKDAYHMLKRQGLDHRDVALGV*
>ENSG00000223465|ENST00000451641
MLSRNDDICIYGGLGLGGLLLLAVVLLSACLCWLHRRAPSVLVPGPGLLRAGTPLCISAE
AASAQQ*
>ENSG00000224608|ENST00000435618
MRVTAPRTLLLLLWGAVALTETWAGSHSMRYFYTAMSRPGRGEPRFITVGYVDDTLFVRF
DSDATSPRKEPRAPWIEQEGPEYWDRETQISKTNTQTYRENLRTALRYYNQSEAGSHIIQ
RMYGCDVGPDGRLLRGYDQDAYDGKDYIALNEDLSSWTAADTAAQITQRKWEAARVAEQL
RAYLEGLCVESLRRYLENGKETLQRADPPKTHVTHHPISDHEVTLRCWALGFYPAEITLT
WQRDGEDQTQDTELVETRPAGDRTFQKWAAVVVPSGEEQRYTCHVQHEGLPKPLTLRWEP
SSQSTVPIVGIVAGLAVLAVVVIGAVVAAVMCRRKSSGGKGGSYSQAACSDSAQGSDVSL
TA*
>ENSG00000206505|ENST00000549224
MAVMAPRTLVLLLSGALALTQTWAGSHSMRYFYTSVSRPGRGEPRFIAVGYVDDTQFVRF
DSDAASQRMEPRAPWIEQEGPEYWDRNTRNVKAHSQTDRANLGTLRGYYNQSEDGSHTIQ
RMYGCDVGPDGRFLRGYQQDAYDGKDYIALNEDLRSWTAADMAAQITQRKWETAHEAEQW
RAYLEGRCVEWLRRYLENGKETLQRTDAPKTHMTHHAVSDHEATLRCWALSFYPAEITLT
WQRDGEDQTQDTELVETRPAGDGTFQKWASVVVPSGQEQRYTCHVQHEGLPKPLTLRWEP
SSQPTIPIVGIIAGLVLFGAVIAGAVVAAVMWRRKSSDRKGGSYSQAASSDSAQGSDMSL
TACKV*
>ENSG00000124302|ENST00000438847
MTLRPGTMRLACMFSSILLFGAAGLLLFISLQDPTELAPQQVPGIKFNIRPRQPHHDLPP
GGSQDGDLKEPTERVTRDLSSGAPRGRNLPAPDQPQPPLQRGTRLRLRQRRRRLLIKKMP
AAATIPANSSDAPFIRPGPGTLDGRWVSLHRSQQERKRVMQEACAKYRASSSRRAVTPRH
VSRIFVEDRHRVLYCEVPKAGCSNWKRVLMVLAGLASSTADIQHNTVHYGSALKRLDTFD
RQGILHRLSTYTKMLFVREPFERLVSAFRDKFEHPNSYYHPVFGKAILARYRANASREAL
RTGSGVRFPEFVQYLLDVHRPVGMDIHWDHVSRLCSPCLIDYDFVGKFESMEDDANFFLS
LIRAPRNLTFPRFKDRHSQEARTTARIAHQYFAQLSALQRQRTYDFYYMDYLMFNYSKPF
ADLY*
>ENSG00000149294|ENST00000621850
MLQTKDLIWTLFFLGTAVSLQVDIVPSQGEISVGESKFFLCQVAGDAKDKDISWFSPNGE
KLTPNQQRISVVWNDDSSSTLTIYNANIDDAGIYKCVVTGEDGSESEATVNVKIFQKLMF
KNAPTPQEFREGEDAVIVCDVVSSLPPTIIWKHKGRDVILKKDVRFIVLSNNYLQIRGIK
KTDEGTYRCEGRILARGEINFKDIQVIVNVPPTIQARQNIVNATANLGQSVTLVCDAEGF
PEPTMSWTKDGEQIEQEEDDEKYIFSDDSSQLTIKKVDKNDEAEYICIAENKAGEQDATI
HLKVFAKPKITYVENQTAMELEEQVTLTCEASGDPIPSITWRTSTRNISSEEKTLDGHMV
VRSHARVSSLTLKSIQYTDAGEYICTASNTIGQDSQSMYLEVQYAPKLQGPVAVYTWEGN
QVNITCEVFAYPSATISWFRDGQLLPSSNYSNIKIYNTPSASYLEVTPDSENDFGNYNCT
AVNRIGQESLEFILVQADTPSSPSIDQVEPYSSTAQVQFDEPEATGGVPILKYKAEWRAV
GEEVWHSKWYDAKEASMEGIVTIVGLKPETTYAVRLAALNGKGLGEISAASEFKTQPVHS
PPPPASASSSTPVPLSPPDTTWPLPALATTEPAKGEPSAPKLEGQMGEDGNSIKVNLIKQ
DDGGSPIRHYLVRYRALSSEWKPEIRLPSGSDHVMLKSLDWNAEYEVYVVAENQQGKSKA
AHFVFRTSAQPTAIPATLGGNSASYTFVSLLFSAVTLLLLC*
>ENSG00000132376|ENST00000421807

MSSRKLSGPKGRRLSIHVVTWNVASAAPPLDLSDLLQLNNRNLNLDIYVIGLQELNSGII
SLLSDAAFNDSWSSFLMDVLSPLSFIKVSHVRMQGILLLVFAKYQHLPYIQILSTKSTPT
GLFGYWGNKGGVNICLKLYGYYVSIINCHLPPHISNNYQRLEHFDRILEMQNCEGRDIPN
ILDHDLIIWFGDMNFRIEDFGLHFVRESIKNRCYGGLWEKDQLSIAKKHDPLLREFQEGR
LLFPPTYKFDRNSNDYDTSEKKRKPAWTDRILWRLKRQPCAGPDTPIPPASHFSLSLRGY
SSHMTYGISDHKPVSGTFDLELKPLVSAPLIVLMPEDLWTVENDMMVSYSSTSDFPSSPW
DWIGLYKVGLRDVNDYVSYAWVGDSKVSCSDNLNQVYIDISNIPTTEDEFLLCYYSNSLR
SVVGISRPFQIPPGSLREDPLGEAQPQI*
>ENSG00000231048|ENST00000437137
MLSRNDDICIYGGLGLGGLLLLAVVLLSACLCWLHRRVKRLERSWAQGSSEQELHYASLQ
RLPVPSSEGPDLRGRDKRGTKEDPRADYACIAENKPT*
>ENSG00000135241|ENST00000426128
MSINLTVDIYIYLLSNARSVCGKQRSKQLYFLFSPKHYWRISHISLQRGFHTNIIRCKWT
KSEAHSCSKHCYSPSNHGLHIGILKLSTSAPKGLTKVNICMSRIKSTLNSVSKAVFGNQN
EMISRLAQFKPSSQILRKVSDSGWLKQKNIKQAIKSLKKYSDKSAEKSPFPEEKSHIIDK
EEDIGKRSLFHYTSSITTKFGDSFYFLSNHINSYFKRKEKMSQQKENEHFRDKSELEDKK
VEEGKLRSPDPGILAYKPGSESVHTVDKPTSPSAIPDVLQVSTKQSIANFLSRPTEGVQA
LVGGYIGGLVPKLKYDSKSQSEEQEEPAKTDQAVSKDRNAEEKKRLSLQREKIIARVSID
NRTRALVQALRRTTDPKLCITRVEELTFHLLEFPEGKGVAVKERIIPYLLRLRQIKDETL
QAAVREILALIGYVDPVKGRGIRILSIDGGGTRGVVALQTLRKLVELTQKPVHQLFDYIC
GVSTGAILAFMLGLFHMPLDECEELYRKLGSDVFSQNVIVGTVKMSWSHAFYDSQTWENI
LKDRMGSALMIETARNPTCPKVAADGGLLLNNPSALAMHECKCLWPDVPLECIVSLGTGR
YESDVRNTVTYTSLKTKLSNVINSATDTEEVHIMLDGLLPPDTYFRFNPVMCENIPLDES
RNEKLDQLQLEGLKYIERNEQKMKKVAKILSQEKTTLQKINDWIKLKTDMYEGLPFFSKL
*
>ENSG00000120341|ENST00000464631
MELWAPQRLPQTRGKATAPSKDPDRGFRRDGHHRPVPHSWHNGERFHQWQDNRGSPQPQQ
EPRADHQQQPHYASRPGDWHQPVSGVDYYEGGYRNQLYSRPGYENSYQSYQSPTMREEYA
YGSYYYHGHPQWLQEERVPRQRSPYIWHEDYREQKYLDEHHYENQHSPFGTNSETHFQSN
SRNPCKDSPASNSGQEWPGELFPGSLLAEAQKNKPSLASESNLLQQRESGLSSSSYELSQ
YIRDAPERDDPPASAAWSPVQAEDVSSAGPKAPMKFYIPHVPVSFGPGGQLVHVGPSSPT
DGQAALVELHSMEVILNDSEEQEEMRSFSGPLIREDVHKVDIMTFCQQKAAQSCKSETLG
SRDSALLWQLLVLLCRQNGSMVGSDIAELLMQDCKKLEKYKRQPPVANLINLTDEDWPVL
SSGTPNLLTGEIPPSVETPAQIVEKFTRLLYYGRKKEALEWAMKNHLWGHALFLSSKMDP
QTYSWVMSGFTSTLALNDPLQTLFQLMSGRIPQAATCCGEKQWGDWRPHLAVILSNQAGD
PELYQRAIVAIGDTLAGKGLVEAAHFCYLMAHVPFGHYTVKTDHLVLLGSSHRYATWEKG
NSKDIFQGTVLALVGFYGSIFHFLM*
>ENSG00000102100|ENST00000634461
MAAVGAGGSTAAPGPGAVSAGALEPGTASAGNVKHLVLFLHEAVLVQYVDTLKLAVPSLI
YTLQNNLQYVAISNLPAATFQVSPKPSMAPEELTGLGTGVWE*
>ENSG00000173230|ENST00000614479
MEFNNTTQEDVQERLAYAEQLVVELKDIIRQKDVQLQQKDEALQEERKAADNKIKKLKLH
AKAKLTSLNKYIEEMKAQGGTVLPTEPQSEEQLSKHDKSSTEEEMEIEKIKHKLQEKEEL
ISTLQAQLTQAQAEQPAQSSTEMEEFVMMKQQLQEKEEFISTLQAQLSQTQAEQAAQKLR

VLQRKLEEHEESLVGRAQVVDLLQQELTAAEQRNQILSQQLQQMEAEHNTLRNTVETERE
ESKILLEKMELEVAERKLSFHNLQEEMHHLLEQFEQAGQAQAELESRYSALEQKHKAEME
EKTSHILSLQKTGQELQSACDALKDQNSKLLQDKNEQAVQSAQTIQQLEDQLQQKSKEIS
QFLNRLPLQQHETASQTSFPDVYNEGTQAVTEENIASLQKRVVELENEKGALLLSSIELE
ELKAENEKLSSQITLLEAQNRTGEADREVSEISIVDIANKRSSSAEESGQDVLENTFSQK
HKELSVLLLEMKEAQEEIAFLKLQLQGKRAEEADHEVLDQKEMKQMEGEGIAPIKMKVFL
EDTGQDFPLMPNEESSLPAVEKEQASTEHQSRTSEEISLNDAGVELKSTKQDGDKSLSAV
PDIGQCHQDELERLKSQILELELNFHKAQEIYEKNLDEKAKEISNLNQLIEEFKKNADNN
SSAFTALSEERDQLLSQVKELSMVTELRAQVKQLEMNLAEAERQRRLDYESQTAHDNLLT
EQIHSLSIEAKSKDVKIEVLQNELDDVQLQFSEQSTLIRSLQSQLQNKESEVLEGAERVR
HISSKVEELSQALSQKELEITKMDQLLLEKKRDVETLQQTIEEKDQQVTEISFSMTEKMV
QLNEEKFSLGVEIKTLKEQLNLLSRAEEAKKEQVEEDNEVSSGLKQNYDEMSPAGQISKE
ELQHEFDLLKKENEQRKRKLQAALINRKELLQRVSRLEEELANLKDESKKEIPLSETERG
EVEEDKENKEYSEKCVTSKCQEIEIYLKQTISEKEVELQHIRKDLEEKLAAEEQFQALVK
QMNQTLQDKTNQIDLLQAEISENQAIIQKLITSNTDASDGDSVALVKETVVISPPCTGSS
EHWKPELEEKILALEKEKEQLQKKLQEALTSRKAILKKAQEKERHLREELKQQKDDYNRL
QEQFDEQSKENENIGDQLRQLQIQVRESIDGKLPSTDQQESCSSTPGLEEPLFKATEQHH
TQPVLESNLCPDWPSHSEDASALQGGTSVAQIKAQLKEIEAEKVELELKVSSTTSELTKK
SEEVFQLQEQINKQGLEIESLKTVSHEAEVHAESLQQKLESSQLQIAGLEHLRELQPKLD
ELQKLISKKEEDVSYLSGQLSEKEAALTKIQTEIIEQEDLIKALHTQLEMQAKEHDERIK
QLQVELCEMKQKPEEIGEESRAKQQIQRKLQAALISRKEALKENKSLQEELSLARGTIER
LTKSLADVESQVSAQNKEKDTVLGRLALLQEERDKLITEMDRSLLENQSLSSSCESLKLA
LEGLTEDKEKLVKEIESLKSSKIAESTEWQEKHKELQKEYEILLQSYENVSNEAERIQHV
VEAVRQEKQELYGKLRSTEANKKETEKQLQEAEQEMEEMKEKMRKFAKSKQQKILELEEE
NDRLRAEVHPAGDTAKECMETLLSSNASMKEELERVKMEYETLSKKFQSLMSEKDSLSEE
VQDLKHQIEGNVSKQANLEATEKHDNQTNVTEEGTQSIPGETEEQDSLSMSTRPTCSESV
PSAKSANPAVSKDFSSHDEINNYLQQIDQLKERIAGLEEEKQKNKEFSQTLENEKNTLLS
QISTKDGELKMLQEEVTKMNLLNQQIQEELSRVTKLKETAEEEKDDLEERLMNQLAELNG
SIGNYCQDVTDAQIKNELLESEMKNLKKCVSELEEEKQQLVKEKTKVESEIRKEYLEKIQ
GAQKEPGNKSHAKELQELLKEKQQEVKQLQKDCIRYQEKISALERTVKALEFVQTESQKD
LEITKENLAQAVEHRKKAQAELASFKVLLDDTQSEAARVLADNLKLKKELQSNKESVKSQ
MKQKDEDLERRLEQAEEKHLKEKKNMQEKLDALRREKVHLEETIGEIQVTLNKKDKEVQQ
LQENLDSTVTQLAAFTKSMSSLQDDRDRVIDEAKKWERKFSDAIQSKEEEIRLKEDNCSV
LKDQLRQMSIHMEELKINISRLEHDKQIWESKAQTEVQLQQKVCDTLQGENKELLSQLEE
TRHLYHSSQNELAKLESELKSLKDQLTDLSNSLEKCKEQKGNLEGIIRQQEADIQNSKFS
YEQLETDLQASRELTSRLHEEINMKEQKIISLLSGKEEAIQVAIAELRQQHDKEIKELEN
LLSQEEEENIVLEEENKKAVDKTNQLMETLKTIKKENIQQKAQLDSFVKSMSSLQNDRDR
IVGDYQQLEERHLSIILEKDQLIQEAAAENNKLKEEIRGLRSHMDDLNSENAKLDAELIQ
YREDLNQVITIKDSQQKQLLEVQLQQNKELENKYAKLEEKLKESEEANEDLRRSFNALQE
EKQDLSKEIESLKVSISQLTRQVTALQEEGTLGLYHAQLKVKEEEVHRLSALFSSSQKRI
AELEEELVCVQKEAAKKVGEIEDKLKKELKHLHHDAGIMRNETETAEERVAELARDLVEM
EQKLLMVTKENKGLTAQIQSFGRSMSSLQNSRDHANEELDELKRKYDASLKELAQLKEQG
LLNRERDALLSETAFSMNSTEENSLSHLEKLNQQLLSKDEQLLHLSSQLEDSYNQVQSFS
KAMASLQNERDHLWNELEKFRKSEEGKQRSAAQPSTSPAEVQSLKKAMSSLQNDRDRLLK

ELKNLQQQYLQINQEITELHPLKAQLQEYQDKTKAFQIMQEELRQENLSWQHELHQLRME
KSSWEIHERRMKEQYLMAISDKDQQLSHLQNLIRELRSSSSQTQPLKVQYQRQASPETSA
SPDGSQNLVYETELLRTQLNDSLKEIHQKELRIQQLNSNFSQLLEEKNTLSIQLCDTSQS
LRENQQHYGDLLNHCAVLEKQVQELQAGPLNIDVAPGAPQEKNGVHRKSDPEELREPQQS
FSEAQQQLCNTRQEVNELRKLLEEERDQRVAAENALSVAEEQIRRLEHSEWDSSRTPIIG
SCGTQEQALLIDLTSNSCRRTRSGVGWKRVLRSLCHSRTRVPLLAAIYFLMIHVLLILCF
TGHL*
>ENSG00000157593|ENST00000619636
MVPGYLLVQYFRRKNYLETGRGLCFPLVKACVFGNEPKASDEVPLAPRTEAAETTPMWQA
LKLLFCATGLQVSYLTWGVLQERVMTRSYGATATSPGERFTDSQFLVLMNRVLALIVAGL
SCVLCKQPRHGAPMYRYSFASLSNVLSSWCQYEALKFVSFPTQVLAKASKVIPVMLMGKL
VSRRSYEHWEYLTATLISIGVSMFLLSSGPEPRSSPATTLSGLILLAGYIAFDSFTSNWQ
DALFAYKMSSVQMMFGVNFFSCLFTVGSLLEQGALLEGTRFMGRHSEFAAHALLLSICSA
CGQLFIFYTIGQFGAAVFTIIMTLRQAFAILLSCLLYGHTVTVVGGLGVAVVFAALLLRV
YARGRLKQRGKKAVPVESPVQKV*
>ENSG00000171124|ENST00000458379
MDPLGAAKPQWPWRRCLAALLFQLLVAVCFFSYLRVSRDDATGSPRAPSGSSRQDTTPTR
PTLLILLRTWPFHIPVALSRCSEMVPGTADCHITADRKVYPQADMVIVHHWDIMSNPKSR
LPPSPRPQGQRWIWFNLEPPPNCQHLEALDRYFNLTMSYRSDSDIFTPYGWLEPWSGQPA
HPPLNLSAKTELVAWAVSNWKPDSARVRYYQSLQAHLKVDVYGRSHKPLPKGTMMETLSR
YKFYLAFENSLHPDYITEKLWRNALEAWAVPVVLGPSRSNYERFLPPDAFIHVDDFQSPK
DLARYLQELDKDHARYLSYFRWRETLRPRSFSWALDFCKACWKLQQESRYQTVRSIAAWF
T*
>ENSG00000173402|ENST00000538711
MRMSVGLSLLLPLSGRTFLLLLSVVMAQSHWPSEPSEAVRDWENQLEASMHSVLSDLHEA
VPTVVGIPDGTAVVGRSFRVTIPTDLIASSGDIIKVSAAGKEALPSWLHWDSQSHTLEGL
PLDTDKGVHYISVSATRLGANGSHIPQTSSVFSIEVYPEDHSELQSVRTASPDPGEVVSS
ACAADEPVTVLTVILDADLTKMTPKQRIDLLHRMRSFSEVELHNMKLVPVVNNRLFDMSA
FMAGPGNAKKVVENGALLSWKLGCSLNQNSVPDIHGVEAPAREGAMSAQLGYPVVGWHIA
NKKPPLPKRVRRQIHATPTPVTAIGPPTTAIQEPPSRIVPTPTSPAIAPPTETMAPPVRD
PVPGKPTVTIRTRGAIIQTPTLGPIQPTRVSEAGTTVPGQIRPTMTIPGYVEPTAVATPP
TTTTKKPRVSTPKPATPSTDSTTTTTRRPTKKPRTPRPVPRVTTKVSITRLETASPPTRI
RTTTSGVPRGGEPNQRPELKNHIDRVDAWVGTYFEVKIPSDTFYDHEDTTTDKLKLTLKL
REQQLVGEKSWVQFNSNSQLMYGLPDSSHVGKHEYFMHATDKGGLSAVDAFEIHVHRRPQ
GDRAPARFKAKFVGDPALVLNDIHKKIALVKKLAFAFGDRNCSTITLQNITRGSIVVEWT
NNTLPLEPCPKEQIAGLSRRIAEDDGKPRPAFSNALEPDFKATSITVTGSGSCRHLQFIP
VVPPRRVPSEAPPTEVPDRDPEKSSEDDVYLHTVIPAVVVAAILLIAGIIAMICYRKKRK
GKLTLEDQATFIKKGVPIIFADELDDSKPPPSSSMPLILQEEKAPLPPPEYPNQSVPETT
PLNQDTMGEYTPLRDEDPNAPPYQPPPPFTAPMEGKGSRPKNMTPYRSPPPYVPP*
>ENSG00000164144|ENST00000451320
MAQESPKNSAAEIPVTSNGEVDDSREHSFNRDLKHSLPSGLGLSETQITSHGFDNTKEGV
IEAGAFQGSPAPPLPSVMSPSRVAASRLAQQGSDLIVPAGGQRTQTKSGPVILADEIKNP
AMEKLELVRKWSLNTYKCTRQIISEKLGRGSRTVDLELEAQIDILRDNKKKYENILKLAQ
TLSTQLFQMVHTQRQLGDAFADLSLKSLELHEEFGYNADTQKLLAKNGETLLGAINFFIA

SVNTLVNKTIEDTLMTVKQYESARIEYDAYRTDLEELNLGPRDANTLPKIEQSQHLFQAH
KEKYDKMRNDVSVKLKFLEENKVKVLHNQLVLFHNAIAAYFAGNQKQLEQTLKQFHIKLK
TPGVDAPSWLEEQ*
>ENSG00000108669|ENST00000589297
MQRNKQVAMGRKKFNMDPKKGIQFLIENDLLKNTCEDIAQFLYKGEGLNKTAIGDYLGER
DEFNIQVLHAFVELHEFTDLNLVQALRQFLWSFRLPGEAQKIDRMMEAFAQRYCQCNNGV
FQSTDTCYVLSFAIIMLNTSLHNPNVKDKPTVERFIAMNRGINDGGDLPEELLRNLYESI
KNEPFKIPEDDGNDLTHTFFNPDREGWLLKLGGGRVKTWKRRWFILTDNCLYYFEYTTDK
EPRGIIPLENLSIREVEDSKKPNCFELYIPDNKDQVIKACKTEADGRVVEGNHTVYRISA
PTPEEKEEWIKCIKAAISRDPFYEMLAARKKKVSSTKRH*
>ENSG00000206506|ENST00000553052
MVVMAPRTLFLLLSGALTLTETWAGSHSMRYFSAAVSRPGRGEPRFIAMGYVDDTQFVRF
DSDSACPRMEPRAPWVEQEGPEYWEEETRNTKAHAQTDRMNLQTLRGYYNQSEASSHTLQ
WMIGCDLGSDGRLLRGYEQYAYDGKDYLALNEDLRSWTAADTAAQISKRKCEAANVAEQR
RAYLEGTCVEWLHRYLENGKEMLQRADPPKTHVTHHPVFDYEATLRCWALGFYPAEIILT
WQRDGEDQTQDVELVETRPAGDGTFQKWAAVVVPSGEEQRYTCHVQHEGLPEPLMLRWKQ
SSLPTIPIMGIVAGLVVLAAVVTGAAVAAVLWRKKSSD*
>ENSG00000154917|ENST00000543906
MSAGGDFGNPLRKFKLVFLGEQSVGKTSLITRFMYDSFDNTYQATIGIDFLSKTMYLEDR
TVRLQLWDTAGQERFRSLIPSYIRDSTVAVVVYDITNLNSFQQTSKWIDDVRTERGSDVI
IMLVGNKTDLADKRQITIEEGEQRAKELSVMFIETSAKTGYNVKQLFRRVASALPGMENV
QEKSKEGMIDIKLDKPQEPPASEGGCSC*
>ENSG00000135823|ENST00000542060
MKDQMSTSSVQALAERKNRQALLGDSGSQNWSTGTTDKYGRLDRELQRANSHFIEEQQAQ
QQLIVEQQDEQLELVSGSIGVLKNMSQRIGGELEEQAVMLEDFSHELESTQSRLDNVMKK
LAKVSHMTSDRRQWCAIAILFAVLLVVLILFLVL*
>ENSG00000169105|ENST00000559991
MFPRPLTPLAAPNGAEPLGRALRRAPLGRARAGLGGPPLLLPSMLMFAVIVASSGLLLMI
ERGILAEMKPLPLHPPGREGTAWRGKAPKPGGLSLRAGDADLQVRQDVRNRTLRAVCGQP
GMPRDPWDLPVGQRRTLLRHILVLAGVLDSVDVRLKMDHRSDLVFLADLRPEEIRYRLQH
YFKFLFVREPLERLLSAYRNKFGEIREYQQRYGAEIVRRYRAGAGPSPAGDDVTFPEFLR
YLVDEDPERMNEHWMPVYHLCQPCAVHYDFVGSYERLEADANQVLEWVRAPPHVRFPARQ
AWYRPASPESLHYHLCSAPRALLQDVLPKYILDFSLFAYPLPNVTKEACQQ*
>ENSG00000229685|ENST00000454805
MRPEDRMFHIRAVILRALSLAFLLSLRGAGAIKADHVSTYAAFVQTHRPTGEFMFEFDED
EMFYVDLDKKETVWHLEEFGQAFSFEAQGGLANIAILNNNLNTLIQRSNHTQATNDPPEV
TVFPKEPVELGQPNTLICHIDKFFPPVLNVTWLCNGELVTEGVAESLFLPRTDYSFHKFH
YLTFVPSAEDFYDCRVEHWGLDQPLLKHWEAQEPIQMPETTETVLCALGLVLGLVGIIVG
TVLIIKSLRSGHDPRAQGTL*
>ENSG00000115760|ENST00000421745
MVTGGGAAPPGTVTEPLPSVIVLSAGRKMAAAAAAASGPGCSSAAGAGAAGVSEWLVLRD
GCMHCDADGLHSLSYHPALNAILAVTSRGTIKVIDGTSGATLQASALSAKPGGQVKCQYI
SAVDKVIFVDDYAVGCRKDLNGILLLDTALQTPVSKQDDVVQLELPVTEAQQLLSACLEK
VDISSTEGYDLFITQLKDGLKNTSHETAANHKVAKWATVTFHLPHHVLKSIASAIVNELK

KINQNVAALPVASSVMDRLSYLLPSARPELGVGPGRSVDRSLMYSEANRRETFTSWPHVG
YRWAQPDPMAQAGFYHQPASSGDDRAMCFTCSVCLVCWEPTDEPWSEHERHSPNCPFVKG
EHTQNVPLSVTLATSPAQFPCTDGTDRISCFGSGSCPHFLAAATKRGKICIWDVSKLMKV
HLKFEINAYDPAIVQQLILSGDPSSGVDSRRPTLAWLEDSSSCSDIPKLEGDSDDLLEDS
DSEEHSRSDSVTGHTSQKEAMEVSLDITALSILQQPEKLQWEIVANVLEDTVKDLEELGA
NPCLTNSKSEKTKEKHQEQHNIPFPCLLAGGLLTYKSPATSPISSNSHRSLDGLSRTQGE
SISEQGSTDNESCTNSELNSPLVRRTLPVLLLYSIKESDEKAGKIFSQMNNIMSKSLHDD
GFTVPQIIEMELDSQEQLLLQDPPVTYIQQFADAAANLTSPDSEKWNSVFPKPGTLVQCL
RLPKFAEEENLCIDSITPCADGIHLLVGLRTCPVESLSAINQVEALNNLNKLNSALCNRR
KGELESNLAVVNGANISVIQHESPADVQTPLIIQPEQRNVSGGYLVLYKMNYATRIVTLE
EEPIKIQHIKDPQDTITSLILLPPDILDNREDDCEEPIEDMQLTSKNGFEREKTSDISTL
GHLVITTQGGYVKILDLSNFEILAKVEPPKKEGTEEQDTFVSVIYCSGTDRLCACTKGGE
LHFLQIGGTCDDIDEADILVDGSLSKGIEPSSEGSKPLSNPSSPGISGVDLLVDQPFTLE
ILTSLVELTRFETLTPRFSATVPPCWVEVQQEQQQRRHPQHLHQQHHGDAAQHTRTWKLQ
TDSNSWDEHVFELVLPKACMVGHVDFKFVLNSNITNIPQIQVTLLKNKAPGLGKVNALNI
EVEQNGKPSLVDLNEEMQHMDVEESQCLRLCPFLEDHKEDILCGPVWLASGLDLSGHAGM
LTLTSPKLVKGMAGGKYRSFLIHVKAVNERGTEEICNGGMRPVVRLPSLKHQSNKGYSLA
SLLAKVAAGKEKSSNVKNENTSGTRKSENLRGCDLLQEVSVTIRRFKKTSISKERVQRCA
MLQFSEFHEKLLNTLCRKTDDGQITEHAQSLVLDTLCWLAGVHSNGPGSSKEGNENLLSK
TRKFLSDIVRVCFFEAGRSIAHKCARFLALCISNGKCDPCQPAFGPVLLKALLDNMSFLP
AATTGGSVYWYFVLLNYVKDEDLAGCSTACASLLTAVSRQLQDRLTPMEALLQTRYGLYS
SPFDPVLFDLEMSGSSCKNVYNSSIGVQSDEIDLSDVLSGNGKVSSCTAAEGSFTSLTGL
LEVEPLHFTCVSTSDGTRIERDDAMSSFGVTPAVGGLSSGTVGEASTALSSAAQVALQSL
SHAMASAEQQLQVLQEKQQQLLKLQQQKAKLEAKLHQTTAAAAAAASAVGPVHNSVPSNP
VAAPGFFIHPSDVIPPTPKTTPLFMTPPLTPPNEAVSVVINAELAQLFPGSVIDPPAVNL
AAHNKNSNKSRMNPLGSGLALAISHASHFLQPPPHQSIIIERMHSGARRFVTLDFGRPIL
LTDVLIPTCGDLASLSIDIWTLGEEVDGRRLVVATDISTHSLILHDLIPPPVCRFMKITV
IGRYGSTNARAKIPLGFYYGHTYILPWESELKLMHDPLKGEGESANQPEIDQHLAMMVAL
QEDIQCRYNLACHRLETLLQSIDLPPLNSANNAQYFLRKPDKAVEEDSRVFSAYQDCIQL
QLQLNLAHNAVQRLKVALGASRKMLSETSNPEDLIQTSSTEQLRTIIRYLLDTLLSLLHA
SNGHSVPAVLQSTFHAQACEELFKHLCISGTPKIRLHTGLLLVQLCGGERWWGQFLSNVL
QELYNSEQLLIFPQDRVFMLLSCIGQRSLSNSGVLESLLNLLDNLLSPLQPQLPMHRRTE
GVLDIPMISWVVMLVSRLLDYVATVEDEAAAAKKPLNGNQWSFINNNLHTQSLNRSSKGS
SSLDRLYSRKIRKQLVHHKQQLNLLKAKQKALVEQMEKEKIQSNKGSSYKLLVEQAKLKQ
ATSKHFKDLIRLRRTAEWSRSNLDTEVTTAKESPEIEPLPFTLAHERCISVVQKLVLFLL
SMDFTCHADLLLFVCKVLARIANATRPTIHLCEIVNEPQLERLLLLLVGTDFNRGDISWG
GAWAQYSLTCMLQDILAGELLAPVAAEAMEEGTVGDDVGATAGDSDDSLQQSSVQLLETI
DEPLTHDITGAPPLSSLEKDKEIDLELLQDLMEVDIDPLDIDLEKDPLAAKVFKPISSTW
YDYWGADYGTYNYNPYIGGLGIPVAKPPANTEKNGSQTVSVSVSQALDARLEVGLEQQAE
LMLKMMSTLEADSILQALTNTSPTLSQSPTGTDDSLLGGLQAANQTSQLIIQLSSVPMLN
VCFNKLFSMLQVHHVQLESLLQLWLTLSLNSSSTGNKENGADIFLYNANRIPVISLNQAS
ITSFLTVLAWYPNTLLRTWCLVLHSLTLMTNMQLNSGSSSAIGTQESTAHLLVSDPNLIH
VLVKFLSGTSPHGTNQHSPQVGPTATQAMQEFLTRLQVHLSSTCPQIFSEFLLKLIHILS
TERGAFQTGQGPLDAQVKLLEFTLEQNFEVVSVSTISAVIESVTFLVHHYITCSDKVMSR

SGSDSSVGARACFGGLFANLIRPGDAKAVCGEMTRDQLMFDLLKLVNILVQLPLSGNREY
SARVSVTTNTTDSVSDEEKVSGGKDGNGSSTSVQGSPAYVADLVLANQQIMSQILSALGL
CNSSAMAMIIGASGLHLTKHENFHGGLDAISVGDGLFTILTTLSKKASTVHMMLQPILTY
MACGYMGRQGSLATCQLSEPLLWFILRVLDTSDALKAFHDMGGVQLICNNMVTSTRAIVN
TARSMVSTIMKFLDSGPNKAVDSTLKTRILASEPDNAEGIHNFAPLGTITSSSPTAQPAE
VLLQATPPHRRARSAAWSYIFLPEEAWCDLTIHLPAAVLLKEIHIQPHLASLATCPSSVS
VEVSADGVNMLPLSTPVVTSGLTYIKIQLVKAEVASAVCLRLHRPRDASTLGLSQIKLLG
LTAFGTTSSATVNNPFLPSEDQVSKTSIGWLRLLHHCLTHISDLEGMMASAAAPTANLLQ
TCAALLMSPYCGMHSPNIEVVLVKIGLQSTRIGLKLIDILLRNCAASGSDPTDLNSPLLF
GRLNGLSSDSTIDILYQLGTTQDPGTKDRIQALLKWVSDSARVAAMKRSGRMNYMCPNSS
TVEYGLLMPSPSHLHCVAAILWHSYELLVEYDLPALLDQELFELLFNWSMSLPCNMVLKK
AVDSLLCSMCHVHPNYFSLLMGWMGITPPPVQCHHRLSMTDDSKKQDLSSSLTDDSKNAQ
APLALTESHLATLASSSQSPEAIKQLLDSGLPSLLVRSLASFCFSHISSSESIAQSIDIS
QDKLRRHHVPQQCNKMPITADLVAPILRFLTEVGNSHIMKDWLGGSEVNPLWTALLFLLC
HSGSTSGSHNLGAQQTSARSASLSSAATTGLTTQQRTAIENATVAFFLQCISCHPNNQKL
MAQVLCELFQTSPQRGNLPTSGNISGFIRRLFLQLMLEDEKVTMFLQSPCPLYKGRINAT
SHVIQHPMYGAGHKFRTLHLPVSTTLSDVLDRVSDTPSITAKLISEQKDDKEKKNHEEKE
KVKAENGFQDNYSVVVASGLKSQSKRAVSATPPRPPSRRGRTIPDKIGSTSGAEAANKII
TVPVFHLFHKLLAGQPLPAEMTLAQLLTLLYDRKLPQGYRSIDLTVKLGSRVITDPSLSK
TDSYKRLHPEKDHGDLLASCPEDEALTPGDECMDGILDESLLETCPIQSPLQVFAGMGGL
ALIAERLPMLYPEVIQQVSAPVVTSTTQEKPKDSDQFEWVTIEQSGELVYEAPETVAAEP
PPIKSAVQTMSPIPAHSLAAFGLFLRLPGYAEVLLKERKHAQCLLRLVLGVTDDGEGSHI
LQSPSANVLPTLPFHVLRSLFSTTPLTTDDGVLLRRMALEIGALHLILVCLSALSHHSPR
VPNSSVNQTEPQVSSSHNPTSTEEQQLYWAKGTGFGTGSTASGWDVEQALTKQRLEEEHV
TCLLQVLASYINPVSSAVNGEAQSSHETRGQNSNALPSVLLELLSQSCLIPAMSSYLRND
SVLDMARHVPLYRALLELLRAIASCAAMVPLLLPLSTENGEEEEEQSECQTSVGTLLAKM
KTCVDTYTNRLRSKRENVKTGVKPDASDQEPEGLTLLVPDIQKTAEIVYAATTSLRQANQ
EKKLGEYSKKAAMKPKPLSVLKSLEEKYVAVMKKLQFDTFEMVSEDEDGKLGFKVNYHYM
SQVKNANDANSAARARRLAQEAVTLSTSLPLSSSSSVFVRCDEERLDIMKVLITGPADTP
YANGCFEFDVYFPQDYPSSPPLVNLETTGGHSVRFNPNLYNDGKVCLSILNTWHGRPEEK
WNPQTSSFLQVLVSVQSLILVAEPYFNEPGYERSRGTPSGTQSSREYDGNIRQATVKWAM
LEQIRNPSPCFKEVIHKHFYLKRVEIMAQCEEWIADIQQYSSDKRVGRTMSHHAAALKRH
TAQLREELLKLPCPEGLDPDTDDAPEVCRATTGAEETLMHDQVKPSSSKELPSDFQL*
>ENSG00000206240|ENST00000428566
XLLKLTRDFPQRHEKYQSWAGTFHLCLKKNKYPDSRSTFIKHAGSYLKRGEFLAIHTAIT
CFFRQISARFLEYSTSECHFFNGTERVRYLDRYFHNQEENVRFDSDVGEFRAVTELGRPD
AEYWNSQKDLLEQKRGRVDNYCRHNYGVVESFTVQRRVHPKVTVYPSKTQPLQHHNLLVC
SVSGFYPGSIEVRWFRNGQEEKTGVVSTGLIHNGDWTFQTLVMLETVPRSGEVYTCQVEH
PSVTSPLTVEW
>ENSG00000127564|ENST00000574385
MPMPTEGTPPPLSGTPIPVPAYFRHAEPGFSLKRPRGLSRSLPPPPPAKGSIPISRLFPP
RTPGWHQLQPRRVSFRGEASETLQSPGYDPSRPESFFQQSFQRLSRLGHGSYGEVFKVRS
KEDGRLYAVKRSMSPFRGPKDRARKLAEVGSHEKVGQHPCCVRLEQAWEEGGILYLQTEL
CGPSLQQHCEAWGASLPEAQVWGYLRDTLLALAHLHSQGLVHLDVKPANIFLGPRGRCKL

GDFGLLVELGTAGAGEVQEGDPRYMAPELLQGSYGTAADVFSLGLTILEVACNMELPHGG
EGWQQLRQGYLPPEFTAGLSSELRSVLVMMLEPDPKLRATAEALLALPVLRQPRAWGVLW
CMAAEALSRGWALWQALLALLCWLWHGLAHPASWLQPLGPPATPPGSPPCSLLLDSSLSS
NWDDDSLGPSLSPEAVLARTVGSTSTPRSRCTPRDALDLSDINSEPPRGSFPSFEPRNLL
SLFEDTLDPT*
>ENSG00000257923|ENST00000556210
MLCVAGARLKRELDATATVLANRQDESEQSRKRLIEQSREFKKNTPEDLRKQVAPLLKSF
QGEIDALSKRSKEAEAAFLNVYKRLIDVPDPVPALDLGQQLQLKVQRLHDIETENQKLRE
TLEEYNKEFAEVKNQEVTIKALKEKIREYEQTLKNQAETIALEKEQKLQNDFAEKERKLQ
ETQMSTTSKLEEAEHKVQSLQTALEKTRTELFDLKTKYDEETTAKADEIEMIMTDLERAN
QRAEVAQREAETLREQLSSANHSLQLASQIQKAPDVEQAIEVLTRSSLEVELAAKEREIA
QLVEDVQRLQASLTKLRENSASQISQLEQQLSAKNSTLKQLEEKLKGQADYEEVKKELNI
LKSMEFAPSEGAGTQDAAKPLEVLLLEKNRSLQSENAALRISNSDLSGPYSTNSISSQSP
LQQSPDVNGMAPSPSQSESAGSVSEGEEMDTAEIARQVKEQLIKHNIGQRIFGHYVLGLS
QGSVSEILARPKPWNKLTVRGKEPFHKMKQFLSDEQNILALRSIQGRQRAEPAQPSSASG
SGNSDDAIRSILQQARREMEAQQAALDPALKQAPLSQSDITILTPKLLSTSPMPTVSSYP
PLAISLKKPSAAPEAGASALPNPPALKKEAQDAPGLDPQGAADCAQGVLRQVKNEVGRSG
AWKDHWWSAVQPERRNAASSEEAKAEETGGGKEKGSGGSGGGSQPRAERSQLQGPSSSEY
WKEWPSAESPYSQSSELSLTGASRSETPQNSPLPSSPIVPMSKPTKPSVPPLTPEQYEVY
MYQEVDTIELTRQVKEKLAKNGICQRIFGEKVLGLSQGSVSDMLSRPKPWSKLTQKGREP
FIRMQLWLNGELGQGVLPVQGQQQGPVLHSVTSLQDPLQQGCVSSESTPKTSASCSPAPE
SPMSSSESVKSLTELVQQPCPPIEASKDSKPPEPSDPPASDSQPTTPLPLSGHSALSIQE
LVAMSPELDTYGITKRVKEVLTDNNLGQRLFGETILGLTQGSVSDLLARPKPWHKLSLKG
REPFVRMQLWLNDPNNVEKLMDMKRMEKKAYMKRRHSSVSDSQPCEPPSVGTEYSQGASP
QPQHQLKKPRVVLAPEEKEALKRAYQQKPYPSPKTIEDLATQLNLKTSTVINWFHNYRSR
IRRELFIEEIQAGSQGQAGASDSPSARSGRAAPSSEGDSCDGVEATEGPGSADTEEPKSQ
GEAEREEVPRPAEQTEPPPSGTPGPDDARDDDHEGGPVEGPGPLPSPASATATAAPAAPE
DAATSAAAAPGEGPAAPSSAPPPSNSSSSSAPRRPSSLQSLFGLPEAAGARDSRDNPLRK
KKAANLNSIIHRLEKAASREEPIEWEF*
>ENSG00000231048|ENST00000455426
MLSRNDVKRLERSWAQGSSEQELHYASLQRLPVPSSEGPDLRGRDKRGTKEDPRADYACI
AENKPT*
>ENSG00000237216|ENST00000547931
MVVMAPRTLFLLLSGALTLTETWAGSHSMRYFSAAVSRPGRGEPRFIAMGYVDDTQFVRF
DSDSACPRMEPRAPWVEQEGPEYWEEETRNTKAHAQTDRMNLQTLRGYYNQSEASSHTLQ
WMIGCDLGSDGRLLRGYEQYAYDGKDYLALNEDLRSWTAADTAAQISKRKCEAANVAEQR
RAYLEGTCVEWLHRYLENGKEMLQRADPPKTHVTHHPVFDYEATLRCWALGFYPAEIILT
WQRDGEDQTQDVELVETRPAGDGTFQKWAAVVVPSGEEQRYTCHVQHEGLPEPLMLRWKQ
SSLPTIPIMGIVAGLVVLAAVVTGAAVAAVLWRKKSSD*
>ENSG00000196547|ENST00000559717
MKLKKQVTVCGAAIFCVAVFSLYLMLDRVQHDPTRHQNGGNFPRSQISVLQNRIEQLEQL
LEENHEIISHIKDSVLELTANAEGPPAMLPYYTVNGSWVVPPEPRPSFFSISPQDCQFAL
GGRGQKPELQMLTVSEELPFDNVDGGVWRQGFDISYDPHDWDAEDLQVFVVPHSHNDPGW
IKTFDKYYTEQTQHILNSMVSKLQEDPRRRFLWAEVSFFAKWWDNINVQKRAAVRRLVGN

GQLEIATGGWVMPDEANSHYFALIDQLIEGHQWLERNLGATPRSGWAVDPFGYSSTMPYL
LRRANLTSMLIQRVHYAIKKHFAATHSLEFMWRQTWDSDSSTDIFCHMMPFYSYDVPHTC
GPDPKICCQFDFKRLPGGRINCPWKVPPRAITEANVAERAALLLDQYRKKSQLFRSNVLL
VPLGDDFRYDKPQEWDAQFFNYQRLFDFFNSRPNLHVQAQFGTLSDYFDALYKRTGVEPG
ARPPGFPVLSGDFFSYADREDHYWTGYYTSRPFYKSLDRVLEAHLRGAEVLYSLAAAHAR
RSGLAGRYPLSDFTLLTEARRTLGLFQHHDAITGTAKEAVVVDYGVRLLRSLVNLKQVII
HAAHYLVLGDKETYHFDPEAPFLQVDDTRLSHDALPERTVIQLDSSPRFVVLFNPLEQER
FSMVSLLVNSPRVRVLSEEGQPLAVQISAHWSSATEAVPDVYQVSVPVRLPALGLGVLQL
QLGLDGHRTLPSSVRIYLHGRQLSVSRHEAFPLRVIDSGTSDFALSNRYMQVWFSGLTGL
LKSIRRVDEEHEQQVDMQVLVYGTRTSKDKSGAYLFLPDGEAKPYVPKEPPVLRVTEGPF
FSEVVAYYEHIHQAVRLYNLPGVEGLSLDISSLVDIRDYVNKELALHIHTDIDSQGIFFT
DLNGFQVQPRRYLKKLPLQANFYPMPVMAYIQDAQKRLTLHTAQALGVSSLKDGQLEVIL
DRRLMQDDNRGLGQGLKDNKRTCNRFRLLLERRTVGSEVQDSHSTSYPSLLSHLTSMYLN
APALALPVARMQLPGPGLRSFHPLASSLPCDFHLLNLRTLQAEEDTLPSAETALILHRKG
FDCGLEAKNLGFNCTTSQGKVALGSLFHGLDVVFLQPTSLTLLYPLASPSNSTDVYLEPM
EIATFRLRLG*
>ENSG00000223465|ENST00000417428
MLSRNDDICIYGGLGLGGLLLLAVVLLSACLCWLHRRVKRLERSWAQGSSEQELHYASLQ
RLPVPSSEGPDLRGRDKRGTKEDPRADYACIAENKPT*
>ENSG00000106692|ENST00000602661
MSRINKNVVLALLTLTSSAFLLFQLYYYKHYLSTKNGAGLSKSKGSRIGFDSTQWRAVKK
FIMLTSNQNVPVFLIDPLILELINKNFEQVKNTSHGSTSQCKFFCVPRDFTAFALQYHLW
KNEEGWFRIAENMGFQCLKIESKDPRLDGIDSLSGTEIPLHYICKLATHAIHLVVFHERS
GNYLWHGHLRLKEHIDRKFVPFRKLQFGRYPGAFDRPELQQVTVDGLEVLIPKDPMHFVE
EVPHSRFIECRYKEARAFFQQYLDDNTVEAVAFRKSAKELLQLAAKTLNKLGVPFWLSSG
TCLGWYRQCNIIPYSKDVDLGIFIQDYKSDIILAFQDAGLPLKHKFGKVEDSLELSFQGK
DDVKLDVFFFYEETDHMWNGGTQAKTGKKFKYLFPKFTLCWTEFVDMKVHVPCETLEYIE
ANYGKTWKIPVKTWDWKRSPPNVQPNGIWPISEWDEVIQLY*
>ENSG00000122390|ENST00000414063
MTEVVPSSALSEVSLRLLCHDDIDTVKHLCGDWFPIEYPDSWYRDITSNKKFFSLAATYR
GAIVGMIVAEIKNRTKIHKEDGDILASNFSVDTQVAYILSLGVVKEFRKHGIGSLLLESL
KDHISTTAQDHCKAIYLHVLTTNNTAINFYENRDFKQHHYLPYYYSIRGVLKDGFTYVLY
INGGHPPWTILDYIQHLGSALASLSPCSIPHRVYRQAHSLLCSFLPWSGISSKSGIEYSR
TM*
>ENSG00000280759|ENST00000627250
XKSKEAEIKRINKELANIRSKFKGDKALDGYSKKKYVCKLLFIFLLGHDIDFGHMEAVNL
LSSNRYTEKQIGYLFISVLVNSNSELIRLINNAIKNDLASRNPTFMGLALHCIASVGSRE
MAEAFAGEIPKVLVAGDTMDSVKQSAALCLLRLYRTSPDLVPMGDWTSRVVHLLNDQHLG
VVTAATSLITTLAQKNPEEFKTSVSLAVSRLSRIVTSASTDLQDYTYYFVPAPWLSVKLL
RLLQCYPPPEDPAVRGRLTECLETILNKAQEPPKSKKVQHSNAKNAVLFEAISLIIHHDS
EPNLLVRACNQLGQFLQHRETNLRYLALESMCTLASSEFSHEAVKTHIETVINALKTERD
VSVRQRAVDLLYAMCDRSNAPQIVAEMLSYLETADYSIREEIVLKVAILAEKYAVDYTWY
VDTILNLIRIAGDYVSEEVWYRVIQIVINRDDVQGYAAKTVFEALQAPACHENLVKVGGY
ILGEFGNLIAGDPRSSPLIQFHLLHSKFHLCSVPTRALLLSTYIKFVNLFPEVKPTIQDV

LRSDSQLRNADVELQQRAVEYLRLSTVASTDILATVLEEMPPFPERESSILAKLKKKKGP
STVTDLEDTKRDRSVDVNGGPEPAPASTSAVVCL*
>ENSG00000164144|ENST00000405727
MAQESPKNSAAEIPVTSNGEVDDSREHSFNRDLKHSLPSGLGLSETQITSHGFDNTKEGV
IEAGAFQGGQRTQTKSGPVILADEIKNPAMEKLELVRKWSLNTYKCTRQIISEKLGRGSR
TVDLELEAQIDILRDNKKKYENILKLAQTLSTQLFQMVHTQRQLGDAFADLSLKSLELHE
EFGYNADTQKLLAKNGETLLGAINFFIASVNTLVNKTIEDTLMTVKQYESARIEYDAYRT
DLEELNLGPRDANTLPKIEQSQHLFQAHKEKYDKMRNDVSVKLKFLEENKVKVLHNQLVL
FHNAIAAYFAGNQKQLEQTLKQFHIKLKTPGVDAPSWLEEQ*
>ENSG00000080815|ENST00000406768
MVVVVATIKSVSFYTRKDGQLIYTPFTEDTETVGQRALHSILNAAIMISVIVVMTILLVV
LYKYRCYKVIHAWLIISSLLLLFFFSFIYLGEVFKTYNVAVDYITVALLIWNFGVVGMIS
IHWKGPLRLQQAYLIMISALMALVFIKYLPEWTAWLILAVISVYDLVAVLCPKGPLRMLV
ETAQERNETLFPALIYSSTMVWLVNMAEGDPEAQRRVSKNSKYNAESTERESQDTVAEND
DGGFSEEWEAQRDSHLGPHRSTPESRAAVQELSSSILAGEDPEERGVKLGLGDFIFYSVL
VGKASATASGDWNTTIACFVAILIGLCLTLLLLAIFKKALPALPISITFGLVFYFATDYL
VQPFMDQLAFHQFYI*
>ENSG00000128242|ENST00000406361
MLPPQKKPWESMAKGLVLGALFTSFLLLVYSYAVPPLHAGLASTTPEAAASCSPPALEPE
AVIRANGSAGECQPRRNIVFLKTHKTASSTLLNILFRFGQKHRLKFAFPNGRNDFDYPTF
FARSLVQDYRPGACFNIICNHMRFHYDEVRGLVPTNAIFITVLRDPARLFESSFHYFGPV
VPLTWKLSAGDKLTEFLQDPDRYYDPNGFNAHYLRNLLFFDLGYDNSLDPSSPQVQEHIL
EVERRFHLVLLQEYFDESLVLLKDLLCWELEDVLYFKLNARRDSPVPRLSGELYGRATAW
NMLDSHLYRHFNASFWRKVEAFGRERMAREVAALRHANERMRTICIDGGHAVDAAAIQDE
AMQPWQPLGTKSILGYNLKKSIGQRHAQLCRRMLTPEIQYLMDLGANLWVTKLWKFIRDF
LRW*
>ENSG00000215182|ENST00000621226
MSVGRRKLALLWALALALACTRHTGHAQDGSSESSYKHHPALSPIARGPSGVPLRGATVF
PSLRTIPVVRASNPAHNGRVCSTWGSFHYKTFDGDVFRFPGLCNYVFSEHCGAAYEDFNI
QLRRSQESAAPTLSRVLMKVDGVVIQLTKGSVLVNGHPVLLPFSQSGVLIQQSSSYTKVE
ARLGLVLMWNHDDSLLLELDTKYANKTCGLCGDFNGMPVVSELLSHNTKLTPMEFGNLQK
MDDPTDQCQDPVPEPPRNCSTGFGICEELLHGQLFSGCVALVDVGSYLEACRQDLCFCED
TDLLSCVCHTLAEYSRQCTHAGGLPQDWRGPDFCPQKCPNNMQYHECRSPCADTCSNQEH
SRACEDHCVAGCFCPEGTVLDDIGQTGCVPVSKCACVYNGAAYAPGATYSTDCTNCTCSG
GRWSCQEVPCPGTCSVLGGAHFSTFDGKQYTVHGDCSYVLTKPCDSSAFTVLAELRRCGL
TDSETCLKSVTLSLDGAQTVVVIKASGEVFLNQIYTQLPISAANVTIFRPSTFFIIAQTS
LGLQLNLQLVPTMQLFMQLAPKLRGQTCGLCGNFNSIQADDFRTLSGVVEATAAAFFNTF
KTQAACPNIRNSFEDPCSLSVENEKYAQHWCSQLTDADGPFGRCHAAVKPGTYYSNCMFD
TCNCERSEDCLCAALSSYVHACAAKGVQLGGWRDGVCTKPMTTCPKSMTYHYHVSTCQPT
CRSLSEGDITCSVGFIPVDGCICPKGTFLDDTGKCVQASNCPCYHRGSMIPNGESVHDSG
AICTCTHGKLSCIGGQAPAPVCAAPMVFFDCRNATPGDTGAGCQKSCHTLDMTCYSPQCV
PGCVCPDGLVADGEGGCITAEDCPCVHNEASYRAGQTIRVGCNTCTCDSRMWRCTDDPCL
ATCAVYGDGHYLTFDGQSYSFNGDCEYTLVQNHCGGKDSTQDSFRVVTENVPCGTTGTTC
SKAIKIFLGGFELKLSHGKVEVIGTDESQEVPYTIRQMGIYLVVDTDIGLVLLWDKKTSI

FINLSPEFKGRVCGLCGNFDDIAVNDFATRSRSVVGDVLEFGNSWKLSPSCPDALAPKDP
CTANPFRKSWAQKQCSILHGPTFAACHAHVEPARYYEACVNDACACDSGGDCECFCTAVA
AYAQACHEVGLCVSWRTPSICPLFCDYYNPEGQCEWHYQPCGVPCLRTCRNPRGDCLRDV
RGLEGCYPKCPPEAPIFDEDKMQCVATCPTPPLPPRCHVHGKSYRPGAVVPSDKNCQSCL
CTERGVECTYKAEACVCTYNGQRFHPGDVIYHTTDGTGGCISARCGANGTIERRVYPCSP
TTPVPPTTFSFSTPPLVVSSTHTPSNGPSSAHTGPPSSAWPTTAGTSPRTRLPTASASLP
PVCGEKCLWSPWMDVSRPGRGTDSGDFDTLENLRAHGYRVCESPRSVECRAEDAPGVPLR
ALGQRVQCSPDVGLTCRNREQASGLCYNYQIRVQCCTPLPCSTSSSPAQTTPPTTSKTTE
TRASGSSAPSSTPGTVSLSTARTTPAPGTATSVKKTFSTPSPPPVPATSTSSMSTTAPGT
SVVSSKPTPTEPSTSSCLQELCTWTEWIDGSYPAPGINGGDFDTFQNLRDEGYTFCESPR
SVQCRAESFPNTPLADLGQDVICSHTEGLICLNKNQLPPICYNYEIRIQCCETVNVCRDI
TRLPKTVATTRPTPHPTGAQTQTTFTTHMPSASTEQPTATSRGGPTATSVTQGTHTTLVT
RNCHPRCTWTKWFDVDFPSPGPHGGDKETYNNIIRSGEKICRRPEEITRLQCRAKSHPEV
SIEHLGQVVQCSREEGLVCRNQDQQGPFKMCLNYEVRVLCCETPRGCHMTSTPGSTSSSP
AQTTPSTTSKTTETQASGSSAPSSTPGTVSLSTARTTPAPGTATSVKKTFSTPSPPPVPA
TSTSSMSTTAPGTSVVSSKPTPTEPSTSSCLQELCTWTEWIDGSYPAPGINGGDFDTFQN
LRDEGYTFCESPRSVQCRAESFPNTPLADLGQDVICSHTEGLICLNKNQLPPICYNYEIR
IQCCETVNVCRDITRPPKTVATTRPTPHPTGAQTQTTFTTHMPSASTEQPTATSRGGPTA
TSVTQGTHTTPVTRNCHPRCTWTTWFDVDFPSPGPHGGDKETYNNIIRSGEKICRRPEEI
TRLQCRAKSHPEVSIEHLGQVVQCSREEGLVCRNQDQQGPFKMCLNYEVRVLCCETPKGC
PVTSTPVTAPSTPSGRATSPTQSTSSWQKSRTTTLVTTSTTSTPQTSTTYAHTTSTTSAP
TARTTSAPTTRTTSASPASTTSGPGNTPSPVPTTSTISAPTTSITSAPTTSTTSAPTSST
TSGPGTTPSPVPTTSITSAPTTSTTSAPTTSTTSARTSSTTSATTTSRISGPETTPSPVP
TTSTTSATTTSTTSAPTTSTTSAPTSSTTSSPQTSTTSAPTTSTTSGPGTTPSPVPTTST
TSAPTTRTTSAPKSSTTSAATTSTTSGPETTPRPVPTTSTTSSPTTSTTSAPTTSTTSAS
TTSTTSGAGTTPSPVPTTSTTSAPTTSTTSAPISSTTSATTTSTTSGPGTTPSPVPTTST
TSAPTTSTTSGPGTTPSAVPTTSITSAPTTSTNSAPISSTTSATTTSRISGPETTPSPVP
TASTTSASTTSTTSGPGTTPSPVPTTSTISVPTTSTTSASTTSTTSASTTSTTSGPGTTP
SPVPTTSTTSAPTTSTTSAPTTSTISAPTTSTTSATTTSTTSAPTPRRTSAPTTSTISAS
TTSTTSATTTSTTSATTTSTISAPTTSTTLSPTTSTTSTTITSTTSAPISSTTSTPQTST
TSAPTTSTTSGPGTTSSPVPTTSTTSAPTTSTTSAPTTRTTSVPTSSTTSTATTSTTSGP
GTTPSPVPTTSTTSAPTTRTTSAPTTSTTSAPTTSTTSAPTSSTTSATTTSTISVPTTST
TSVPGTTPSPVPTTSTISVPTTSTTSASTTSTTSGPGTTPSPVPTTSTTSAPTTSTTSAP
TTSTISAPTTSTPSAPTTSTTLAPTTSTTSAPTTSTTSTPTSSTTSSPQTSTTSASTTSI
TSGPGTTPSPVPTTSTTSAPTTSTTSAATTSTISAPTTSTTSAPTTSTTSASTASKTSGL
GTTPSPIPTTSTTSPPTTSTTSASTASKTSGPGTTPSPVPTTSTIFAPRTSTTSASTTST
TPGPGTTPSPVPTTSTASVSKTSTSHVSISKTTHSQPVTRDCHLRCTWTKWFDIDFPSPG
PHGGDKETYNNIIRSGEKICRRPEEITRLQCRAESHPEVSIEHLGQVVQCSREEGLVCRN
QDQQGPFKMCLNYEVRVLCCETPKGCPVTSTPVTAPSTPSGRATSPTQSTSSWQKSRTTT
LVTTSTTSTPQTSTTSAPTTSTTSAPTTSTTSAPTTSTTSTPQTSISSAPTSSTTSAPTS
STISARTTSIISAPTTSTTSSPTTSTTSATTTSTTSAPTSSTTSTPQTSKTSAATSSTTS
GSGTTPSPVTTTSTASVSKTSTSHVSVSKTTHSQPVTRDCHPRCTWTKWFDVDFPSPGPH
GGDKETYNNIIRSGEKICRRPEEITRLQCRAKSHPEVSIEHLGQVVQCSREEGLVCRNQD
QQGPFKMCLNYEVRVLCCETPKGCPVTSTSVTAPSTPSGRATSPTQSTSSWQKSRTTTLV

TSSITSTTQTSTTSAPTTSTTPASIPSTTSAPTTSTTSAPTTSTTSAPTTSTTSTPQTTT
SSAPTSSTTSAPTTSTISAPTTSTISAPTTSTTSAPTASTTSAPTSTSSAPTTNTTSAPT
TSTTSAPITSTISAPTTSTTSTPQTSTISSPTTSTTSTPQTSTTSSPTTSTTSAPTTSTT
SAPTTSTTSTPQTSISSAPTSSTTSAPTASTISAPTTSTTSFHTTSTTSPPTSSTSSTPQ
TSKTSAATSSTTSGSGTTPSPVPTTSTASVSKTSTSHVSVSKTTHSQPVTRDCHPRCTWT
KWFDVDFPSPGPHGGDKETYNNIIRSGEKICRRPEEITRLQCRAESHPEVSIEHLGQVVQ
CSREEGLVCRNQDQQGPFKMCLNYEVRVLCCETPKGCPVTSTPVTAPSTPSGRATSPTQS
TSSWQKSRTTTLVTTSTTSTPQTSTTSAPTTSTIPASTPSTTSAPTTSTTSAPTTSTTSA
PTHRTTSGPTTSTTLAPTTSTTSAPTTSTNSAPTTSTISASTTSTISAPTTSTISSPTSS
TTSTPQTSKTSAATSSTTSGSGTTPSPVPTTSTTSASTTSTTSAPTTSTTSGPGTTPSPV
PSTSTTSAATTSTTSAPTTRTTSAPTSSMTSGPGTTPSPVPTTSTTSAPTTSTTSGPGTT
PSPVPTTSTTSAPITSTTSGPGSTPSPVPTTSTTSAPTTSTTSASTASTTSGPGTTPSPV
PTTSTTSAPTTRTTSASTASTTSGPGSTPSPVPTTSTTSAPTTRTTPASTASTTSGPGTT
PSPVPTTSTTSASTTSTISLPTTSTTSAPITSMTSGPGTTPSPVPTTSTTSAPTTSTTSA
STASTTSGPGTTPSPVPTTSTTSAPTTSTTSASTASTTSGPGTSLSPVPTTSTTSAPTTS
TTSGPGTTPSPVPTTSTTSAPTTSTTSGPGTTPSPVPTTSTTPVSKTSTSHLSVSKTTHS
QPVTSDCHPLCAWTKWFDVDFPSPGPHGGDKETYNNIIRSGEKICRRPEEITRLQCRAES
HPEVNIEHLGQVVQCSREEGLVCRNQDQQGPFKMCLNYEVRVLCCETPRGCPVTSVTPYG
TSPTNALYPSLSTSMVSASVASTSVASSSVASSSVAYSTQTCFCNVADRLYPAGSTIYRH
RDLAGHCYYALCSQDCQVVRGVDSDCPSTTLPPAPATSPSISTSEPVTELGCPNAVPPRK
KGETWATPNCSEATCEGNNVISLRPRTCPRVEKPTCANGYPAVKVADQDGCCHHYQCQCV
CSGWGDPHYITFDGTYYTFLDNCTYVLVQQIVPVYGHFRVLVDNYFCGAEDGLSCPRSII
LEYHQDRVVLTRKPVHGVMTNEIIFNNKVVSPGFRKNGIVVSRIGVKMYATIPELGVQVM
FSGLIFSVEVPFSKFANNTEGQCGTCTNDRKDECRTPRGTVVASCSEMSGLWNVSIPDQP
ACHRPHPTPTTVGPTTVGSTTVGPTTVGSTTVGPTTPPAPCLPSPICQLILSKVFEPCHT
VIPPLLFYEGCVFDRCHMTDLDVVCSSLELYAALCASHDICIDWRGRTGHMCPFTCPADK
VYQPCGPSNPSYCYGNDSASLGALPEAGPITEGCFCPEGMTLFSTSAQVCVPTGCPRCLG
PHGEPVKVGHTVGMDCQECTCEAATWTLTCRPKLCPLPPACPLPGFVPVPAAPQAGQCCP
QYSCACNTSRCPAPVGCPEGARAIPTYQEGACCPVQNCSWTVCSINGTLYQPGAVVSSSL
CETCRCELPGGPPSDAFVVSCETQICNTHCPVGFEYQEQSGQCCGTCVQVACVTNTSKSP
AHLFYPGETWSDAGNHCVTHQCEKHQDGLVVVTTKKACPPLSCSLDEARMSKDGCCRFCP
PPPPPYQNQSTCAVYHRSLIIQQQGCSSSEPVRLAYCRGNCGDSSSMYSLEGNTVEHRCQ
CCQELRTSLRNVTLHCTDGSSRAFSYTEVEECGCMGRRCPAPGDTQHSEEAEPEPSQEAE
SGSWERGVPVSPMH*
>ENSG00000157593|ENST00000538577
MLLAMPALWYLATSWCSTSGGRTTWRPVSYLTWGVLQERVMTRSYGATATSPGERFTDSQ
FLVLMNRVLALIVAGLSCVLCKQPRHGAPMYRYSFASLSNVLSSWCQYEALKFVSFPTQV
LAKASKVIPVMLMGKLVSRRSYEHWEYLTATLISIGVSMFLLSSGPEPRSSPATTLSGLI
LLAGYIAFDSFTSNWQDALFAYKMSSVQMMFGVNFFSCLFTVGSLLEQGALLEGTRFMGR
HSEFAAHALLLSICSACGQLFIFYTIGQFGAAVFTIIMTLRQAFAILLSCLLYGHTVTVV
GGLGVAVVFAALLLRVYARGRLKQRGKKAVPVESPVQKV*
>ENSG00000087470|ENST00000553257
MEALIPVINKLQDVFNTVGADIIQLPQIVVVGTQSSGKSSVLESLVGRDLLPRGTGIVTR
RPLILQLVHVSQEDKRKTTGEENDPATWKNSRHLSKGVEAEEWGKFLHTKNKLYTDFDEI

RQEIENETERISGNNKGVSPEPIHLKIFSPNVVNLTLVDLPGMTKVPVGDQPKDIELQIR
ELILRFISNPNSIILAVTAANTDMATSEALKISREVDPDGRRTLAVITKLDLMDAGTDAM
DVLMGRVIPVKLGIIGVVNRSQLDINNKKSVTDSIRDEYAFLQKKYPSLANRNGTKYLAR
TLNRLLMHHIRDCLPELKTRINVLAAQYQSLLNSYGEPVDDKSATLLQLITKFATEYCNT
IEGTAKYIETSELCGGARICYIFHETFGRTLESVDPLGGLNTIDILTAIRNATGPRPALF
VPEVSFELLVKRQIKRLEEPSLRCVELVHEEMQRIIQHCSNYSTQELLRFPKLHDAIVEV
VTCLLRKRLPVTNEMVHNLVAIELAYINTKHPDFADACGLMNNNIEEQRRNRLARELPSA
VSRDKSSKVPSALAPASQEPSPAASAEADGKLIQDSRRETKNVASGGGGVGDGVQEPTTG
NWRGMLKTSKAEELLAEEKSKPIPIMPASPQKGHAVNLLDVPVPVARKLSAREQRDCEVI
ERLIKSYFLIVRKNIQDSVPKAVMHFLVNHVKDTLQSELVGQLYKSSLLDDLLTESEDMA
QRRKEAADMLKALQGASQIIAEIRETHLW*
>ENSG00000172728|ENST00000524021
MVELGKFERKEFKSSSLQDGHTKMEEAPTHLNSFLKKEGLTFNRKRKWELDSYPIMLWWS
PLTGETGRLGQCGADACFFTINRTYLHHHMTKAFLFYGTDFNIDSLPLPRKAHHDWAVFH
EESPKNNYKLFHKPVITLFNYTATFSRHSHLPLTTQYLESIEVLKSLRYLVPLQSKNKLR
KRLAPLVYVQSDCDPPSDRDSYVRELMTYIEVDSYGECLRNKDLPQQLKNPASMDADGFY
RIIAQYKFILAFENAVCDDYITEKFWRPLKLGVVPVYYGSPSITDWLPSNKSAILVSEFS
HPRELASYIRRLDSDDRLYEAYVEWKLKGEISNQRLLTALRERKWGVQDVNQDNYIDAFE
CMVCTKVWANIRLQEKGLPPKRWEAEDTHLSCPEPTVFAFSPLRTPPLSSLREMWISSFE
QSKKEAQALRWLVDRNQNFSSQEFWGLVFKD*
>ENSG00000233192|ENST00000449560
MILNKALLLGALALTAVMSPCGGEDIVADHVASYGVNFYQSHGPSGQYTHEFDGDEEFYV
DLETKETVWQLPMFSKFISFDPQSALRNMAVGKHTLEFMMRQSNSTAATNEVPEVTVFSK
FPVTLGQPNTLICLVDNIFPPVVNITWLSNGHSVTEGVSETSFLSKSDHSFFKISYLTFL
PSADEIYDCKVEHWGLDEPLLKHWEPEIPAPMSELTETLVCALGLSVGLMGIVVGTVFII
QGLRSVGASRHQGLL*
>ENSG00000231048|ENST00000412041
MLSRNDDICIYGGLGLGGLLLLAVVLLSACLCWLHRRVKRLERSWHLLSWSQAQGSSEQE
LHYASLQRLPVPSSEGPDLRGRDKRGTKEDPRADYACIAENKPT*
>ENSG00000152291|ENST00000409015
MRFVVALVLLNVAAAGAVPLLATESVKQEEAGVRPSAGNVSTHPSLSQRPGGSTKSHPEP
QTPKDSPSKSSAEAQTPEDTPNKSGAEAKTQKDSSNKSGAEAKTQKGSTSKSGSEAQTTK
DSTSKSHPELQTPKDSTGKSGAEAQTPEDSPNRSGAEAKTQKDSPSKSGSEAQTTKDVPN
KSGADGQTPKDGSSKSGAEDQTPKDVPNKSGAEKQTPKDGSNKSGAEEQGPIDGPSKSGA
EEQTSKDSPNKVVPEQPSRKDHSKPISNPSDNKELPKADTNQLADKGKLSPHAFKTESGE
ETDLISPPQEEVKSSEPTEDVEPKEAEDDDTGPEEGSPPKEEKEKMSGSASSENREGTLS
DSTGSEKDDLYPNGSGNGSAESSHFFAYLVTAAILVAVLYIAHHNKRKIIAFVLEGKRSK
VTRRPKASDYQRLDQKYVLILNVFPAPPKRSFFP*
>ENSG00000176542|ENST00000478658
MPEMTENETPTKKQHRKKNRETHNAVERHRKKKINAGINRIGELIPCSPALKQSKNMILD
QAFKYITELKRQNDELLLNGGNNEQAEEIKKLRKQLEEIQKENGRYIELLKANDICLYDD
PTIHWKGNLKNSKVSVVIPSDQVQKKIIVYSNGNQPGGNSQGTAVQGITFNVSHNLQKQT
ANVVPVQRTCNLVTPVSISGVYPSENKPWHQTTVPALATNQPVPLCLPAAISAQSILELP
TSESESNVLGATSGSLIAVSIESEPHQHHSLHTCLNDQNSSENKNGQENPKVLKKMTPCV

TNIPHSSSATATKVHHGNKSCLSIQDFRGDFQNTFVVSVTTTVCSQPPRTAGDSSPMSIS
KSADLTSTATVVASSAPGVGKATIPISTLSGNPLDNGWTLSCSLPSSSVSTSDLKNINSL
TRISSAGNTQTTWTTLQLAGNTIQPLSQTPSSAVTPVLNESGTSPTTSNHSRYVATDINL
NNSFPADGQPVEQVVVTLPSCPSLPMQPLIAQPQVKSQPPKNILPLNSAMQVIQMAQPVG
SAVNSAPTNQNVIILQPPSTTPCPTVMRAEVSNQTVGQQIVIIQAANQNPLPLLPAPPPG
SVRLPINGANTVIGSNNSVQNVPTPQTFGGKHLVHILPRPSSLSASNSTQTFSVTMSNQQ
PQTISLNGQLFALQPVMSSSGTTNQTPMQIIQPTTSEDPNTNVALNTFGALASLNQSISQ
MAGQSCVQLSISQPANSQTAANSQTTTANCVSLTTTAAPPVTTDSSATLASTYNLVSTSS
MNTVACLPNMKSKRLNKKPGGRKHLAANKSACPLNSVRDVSKLDCPNTEGSAEPPCNDGL
LESFPAVLPSVSVSQANSVSVSASHSLGVLSSESLIPESVSKSKSAEKSSPPSQESVTSE
HFAMAAAKSKDSTPNLQQETSQDKPPSSLALSDAAKPCASANVLIPSPSDPHILVSQVPG
LSSTTSTTSTDCVSEVEIIAEPCRVEQDSSDTMQTTGLLKGQGLTTLLSDLAKKKNPQKS
SLSDQMDHPDFSSENPKIVDSSVNLHPKQELLLMNNDDRDPPQHHSCLPDQEVINGSLIN
GRQADSPMSTSSGSSRSFSVASMLPETTREDVTSNATTNTCDSCTFVEQTDIVALAARAI
FDQENLEKGRVGLQADIREVASKPSEASLLEGDPPFKSQIPKESGTGQAEATPNEFNSQG
SIEATMERPLEKPSCSLGIKTSNASLQDSTSQPPSITSLSVNNLIHQSSISHPLASCAGL
SPTSEQTTVPATVNLTVSSSSYGSQPPGPSLMTEYSQEQLNTMTSTIPNSQIQEPLLKPS
HESRKDSAKRAVQDDLLLSSAKRQKHCQPAPLRLESMSLMSRTPDTISDQTQMMVSQIPP
NSSNSVVPVSNPAHGDGLTRLFPPSNNFVTPALRQTEVQCGSQPSVAEQQQTQASQHLQA
LQQHVPAQGVSHLHSNHLYIKQQQQQQQQQQQQQQQQQQAGQLRERHHLYQMQHHVPHAES
SVHSQPHNVHQQRTLQQEVQMQKKRNLVQGTQTSQLSLQPKHHGTDQSRSKTGQPHPHHQ
QMQQQMQQHFGSSQTEKSCENPSTSRNHHNHPQNHLNQDIMHQQQDVGSRQQGSGVSSEH
VSGHNPMQRLLTSRGLEQQMVSQPSIVTRSSDMTCTPHRPERNRVSSYSAEALIGKTSSN
SEQRMGISIQGSRVSDQLEMRSYLDVPRNKSLAIHNMQGRVDHTVASDIRLSDCQTFKPS
GASQQPQSNFEVQSSRNNEIGNPVSSLRSMQSQAFRISQNTGPPPIDRQKRLSYPPVQSI
PTGNGIPSRDSENTCHQSFMQSLLAPHLSDQVIGSQRSLSEHQRNTQCGPSSAIEYNCPP
THENVHIRRESESQNRESCDMSLGAINTRNSTLNIPFSSSSSSGDIQGRNTSPNVSVQKS
NPMRITESHATKGHMNPPVTTNMHGVARPALPHPSVSHGNGDQGPAVRQANSSVPQRSRH
PLQDSSGSKIRQPERNRSGNQRQSTVFDPSLPHLPLSTGGSMILGRQQPATEKRGSIVRF
MPDSPQVPNDNSGPDQHTLSQNFGFSFIPEGGMNPPINANASFIPQVTQPSATRTPALIP
VDPQNTLPSFYPPYSPAHPTLSNDISIPYFPNQMFSNPSTEKVNSGSLNNRFGSILSPPR
PVGFAQPSFPLLPDMPPMHMTNSHLSNFNMTSLFPEIATALPDGSAMSPLLTIANSSASD
SSKQSSNRPAHNISHILGHDCSSAV*
>ENSG00000283883|ENST00000639896
MMEEEELEFVEELEAVLQLTPEVQLAIEQVFPSQDPLDRADFNAVEYINTLFPTEQSLAN
IDEVVNKIRLKIRRLDDNIRTVVRGQTNVGQDGRQALEEAQKAIQQLFGKIKDIKDKAEK
SEQMVKEITRDIKQLDHAKRHLTTSITTLNHLHMLAGGVDSLEAMTRRRQYGEVANLLQG
VMNVLEHFHKYMGIPQIRQLSERVKAAQTELGQQILADFEEAFPSQGTKRPGGPSNVLRD
ACLVANILDPRIKQEIIKKFIKQHLSEYLVLFQENQDVAWLDKIDRRYAWIKRQLVDYEE
KYGRMFPREWCMAERIAVEFCHVTRAELAKIMRTRAKEIEVKLLLFAIQRTTNFEGFLAK
RFSGCTLTDGTLKKLESPPPSTNPFLEDEPTPEMEELATEKGDLDQPKKPKAPDNPFHGI
VSKCFEPHLYVYIESQDKNLGELIDRFVADFKAQGPPKPNTDEGGAVLPSCADLFVYYKK
CMVQCSQLSTGEPMIALTTIFQKYLREYAWKILSGNLPKTTTSSGGLTISSLLKEKEGSE
VAKFTLEELCLICNILSTAEYCLATTQQLEEKLKEKVDVSLIERINLTGEMDTFSTVISS

SIQLLVQDLDAACDPALTAMSKMQWQNVEHVGDQSPYVTSVILHIKQNVPIIRDNLASTR
KYFTQFCVKFANSFIPKFITHLFKCKPISMVGAEQVRWT*
>ENSG00000105974|ENST00000456473
MADELSEKQVYDAHTKEIDLVNRDPKHLNDDVVKIDFEDVIAEPEGTHSFDGIWKASFTT
FTVTKYWFYRLLSALFGIPMALIWGIYFAILSFLHIWAVVPCIKSFLIEIQCISRVYSIY
VHTVCDPLFEAVGKIFSN
>ENSG00000110080|ENST00000534457
MCPAGWKLLAMLALVLVVMVWYSISREDSFYFPIPEKKEPCLQGEAESKASKLFGNYSRD
QPIFLRLEDYFWVKTPSAYELPYGTKGSEDLLLRVLAITSSSIPKNIQSLRCRRCVVVGN
GHRLRNSSLGDAINKYDVVIRLNNAPVAGYEGDVGSKTTMRLFYPESAHFDPKVENNPDT
LLVLVAFKAMDFHWIETILSDKKRVRKGFWKQPPLIWDVNPKQIRILNPFFMEIAADKLL
SLPMQQPRKIKQKPTTGLLAITLALHLCDLVHIAGFGYPDAYNKKQTIHYYEQITLKSMA
GSGHNVSQEALAIKRMLEMGAIKNLTSF*
>ENSG00000152127|ENST00000409645
MALFTPWKLSSQKLGFFLVTFGFIWGMMLLHFTIQQRTQPESSSMLREQILDLSKRYIKA
LAEENRNVVDGPYAGVMTAYDLKKTLAVLLDNILQRIGKLESKVDNLVVNGTGTNSTNST
TAVPSLVALEKINVADIINGAQEKCVLPPMDGYPHCEGKIKWMKDMWRSDPCYADYGVDG
STCSFFIYLSEVENWCPHLPWRAKNPYEEADHNSLAEIRTDFNILYSMMKKHEEFRWMRL
RIRRMADAWIQAIKSLAEKQNLEKRKRKKVLVHLGLLTKESGFKIAETAFSGGPLGELVQ
WSDLITSLYLLGHDIRISASLAELKEIMKKVVGNRSGCPTVGDRIVELIYIDIVGLAQFK
KTLGPSWVHYQCMLRVLDSFGTEPEFNHANYAQSKGHKTPWGKWNLNPQQFYTMFPHTPD
NSFLGFVVEQHLNSSDIHHINEIKRQNQSLVYGKVDSFWKNKKIYLDIIHTYMEVHATVY
GSSTKNIPSYVKNHGILSGRDLQFLLRETKLFVGLGFPYEGPAPLEAIANGCAFLNPKFN
PPKSSKNTDFFIGKPTLRELTSQHPYAEVFIGRPHVWTVDLNNQEEVEDAVKAILNQKIE
PYMPYEFTCEGMLQRINAFIEKQDFCHGQVMWPPLSALQVKLAEPGQSCKQVCQESQLIC
EPSFFQHLNKDKDMLKYKVTCQSSELAKDILVPSFDPKNKHCVFQGDLLLFSCAGAHPRH
QRVCPCRDFIKGQVALCKDCL*
>ENSG00000283761|ENST00000638792
MFANLKYVSLGILVFQTTSLVLTMRYSRTLKEEGPRYLSSTAVVVAELLKIMACILLVYK
DSKCSLRALNRVLHDEILNKPMETLKLAIPSGIYTLQNNLLYVALSNLDAATYQVTYQLK
ILTTALFSVSMLSKKLGVYQWLSLVILMTGVAFVQWPSDSQLDSKELSAGSQFVGLMAVL
TACFSSGFAGVYFEKILKETKQSVWIRNIQLGFFGSIFGLMGVYIYDGELVSKNGFFQGY
NRLTWIVVVLQPQGIGSPSVYHAVIVIFLEFFAWGLLTAPTLVVLHETFPKHTFLMNGLI
QGVKGLLSFLSAPLIGALSDVWGRKSFLLLTVFFTCAPIPLMKISPWWYFAVISVSGVFA
VTFSVVFAYVADITQEHERSMAYGLVSATFAASLVTSPAIGAYLGRVYGDSLVVVLATAI
ALLDICFILVAVPESLPEKMRPASWGAPISWEQADPFASLKKVGQDSIVLLICITVFLSY
LPEAGQYSSFFLYLRQTIVLSLLMRSIGNKNTILLGLGFQILQLAWYGFGSEPWMMWAAG
AVAAMSSITFPAVSALVSRTADADQQGVVQGMITGIRGLCNGLGPALYGFIFYIFHVELK
ELPITGTDLGTNTSPQHHFEQNSIIPGPPFLFGACSVLLALLVALFIPEHTNLSLRSSSW
RKHCGSHSHPHNTQAPGEAKEPLLQDTNV*
>ENSG00000125447|ENST00000583282
SALPVTAYDKNGFRILFHFAKECPPGRPDVLVVVVSMLNTAPLPVKSIVLQAAVPKSMKV
KLQPPSGTELSPFSPIQPPAAITQVMLLANPLKLLLPSPGRWPRKVPGPE*
>ENSG00000111670|ENST00000549940

MLFKLLQRQTYTCLSHRYGLYVCFLGVVVTIVSAFQFGEVVLEWSRDQYHVLFDSYRDNI
AGKSFQNRLCLPMPIDVVYTWVNGTDLELLKELQQVREQMEEEQKAMREILGKNTTEPTK
KSEKQLECLLTHCIKVPMLVLDPALPANITLKDLPSLYPSFHSASDIFNVAKPKNPSTNV
SVVVFDSTKDVEDAHSGLLKGNSRQTVWRGYLTTDKEVPGLVLMQDLAFLSGFPPTFKET
NQLKTKLPENLSSKVKLLQLYSEASVALLKLNNPKDFQELNKQTKKNMTIDGKELTISPA
YLLWDLSAISQSKQDEDISASRFEDNEELRYSLRSIERHAPWVRNIFIVTNGQIPSWLNL
DNPRVTIVTHQDVFRNLSHLPTFSSPAIESHIHRIEGLSQKFIYLNDDVMFGKDVWPDDF
YSHSKGQKVYLTWPVPNCAEGCPGSWIKDGYCDKACNNSACDWDGGDCSGKDVLNCNSFI
FMEYFLLNHY*
>ENSG00000283530|ENST00000637362
MFKFHQMKHIFEILDKMRCLRKRSTVSFLGVLVIFLLFMNLYIEDSYVLEGDKQLIRETS
THQLNSERYVHTFKDLSNFSGAINVTYRYLAATPLQRKRYLTIGLSSVKRKKGNYLLETI
KSIFEQSSYEELKEISVVVHLADFNSSWRDAMVQDITQKFAHHIIAGRLMVIHAPEEYYP
ILDGLKRNYNDPEDRVKFRSKQNVDYAFLLNFCANTSDYYVMLEDDVRCSKNFLTAIKKV
IASLEGTYWVTLEFSKLGYIGKLYHSHDLPRLAHFLLMFYQEMPCDWLLTHFRGLLAQKN
VIRFKPSLFQHMGYYSSYKGTENKLKDDDFEEESFDIPDNPPASLYTNMNVFENYEASKA
YSSVDEYFWGKPPSTGDVFVIVFENPIIIKKIKVNTGTEDRQNDILHHGALDVGENVMPS
KQRRQCSTYLRLGEFKNGNFEMSGVNQKIPFDIHCMRIYVTKTQKEWLIIRSISIWTS*
>ENSG00000283350|ENST00000636545
MVQRWLLLSCCGALLSAGLANTSYTSPGLQRLKDSPQTAPDKGQCSTWGAGHFSTFDHHV
YDFSGTCNYIFAATCKDAFPTFSVQLRRGPDGSISRIIVELGASVVTVSEAIISVKDIGV
ISLPYTSNGLQITPFGQSVRLVAKQLELELEVVWGPDSHLMVLVERKYMGQMCGLCGNFD
GKVTNEFVSEEGKFLEPHKFAALQKLDDPGEICTFQDIPSTHVRQAQHARICTQLLTLVA
PECSVSKEPFVLSCQADVAAAPQPGPQNSSCATLSEYSRQCSMVGQPVRRWRSPGLCSVG
QCPANQVYQECGSACVKTCSNPQHSCSSSCTFGCFCPEGTVLNDLSNNHTCVPVTQCPCV
LHGAMYAPGEVTIAACQTCRCTLGRWVCTERPCPGHCSLEGGSFVTTFDARPYRFHGTCT
YILLQSPQLPEDGALMAVYDKSGVSHSETSLVAVVYLSRQDKIVISQDEVVTNNGEAKWL
PYKTRNITVFRQTSTHLQMATSFGLELVVQLRPIFQAYVTVGPQFRGQTRGLCGNFNGDT
TDDFTTSMGIAEGTASLFVDSWRAGNCPAALERETDPCSMSQLNKVCAETHCSMLLRTGT
VFERCHATVNPAPFYKRCVYQACNYEETFPHICAALGDYVHACSLRGVLLWGWRSSVDNC
TIPCTGNTTFSYNSQACERTCLSLSDRATECHHSAVPVDGCNCPDGTYLNQKGECVRKAQ
CPCILEGYKFILAEQSTVINGITCHCINGRLSCPQRPQMFLASCQAPKTFKSCSQSSENK
FGAACAPTCQMLATGVACVPTKCEPGCVCAEGLYENADGQCVPPEECPCEFSGVSYPGGA
ELHTDCRTCSCSRGRWACQQGTHCPSTCTLYGEGHVITFDGQRFVFDGNCEYILATDVCG
VNDSQPTFKILTENVICGNSGVTCSRAIKIFLGGLSVVLADRNYTVTGEEPHVQLGVTPG
ALSLVVDISIPGRYNLTLIWNRHMTILIRIARASQDPLCGLCGNFNGNMKDDFETRSRYV
ASSELELVNSWKESPLCGDVSFVTDPCSLNAFRRSWAERKCSVINSQTFATCHSKVYHLP
YYEACVRDACGCDSGGDCECLCDAVAAYAQACLDKGVCVDWRTPAFCPIYCGFYNTHTQD
GHGEYQYTQEANCTWHYQPCLCPSQPQSVPGSNIEGCYNCSQDEYFDHEEGVCVPCMPPT
TPQPPTTPQLPTTGSRPTQVWPMTGTSTTIGLLSSTGPSPSSNHTPASPTQTPLLPATLT
SSKPTASSGEPPRPTTAVTPQATSGLPPTATLRSTATKPTVTQATTRATASTASPATTST
AQSTTRTTMTLPTPATSGTSPTLPKSTNQELPGTTATQTTGPRPTPASTTGPTTPQPGQP
TRPTATETTQTRTTTEYTTPQTPHTTHSPPTAGSPVPSTGPVTATSFHATTTYPTPSHPE
TTLPTHVPPFSTSLVTPSTHTVITPTHAQMATSASNHSAPTGTIPPPTTLKATGSTHTAP

PITPTTSGTSQAHSSFSTNKTPTSLHSHTSSTHHPEVTPTSTTTITPNPTSTRTRTPVAH
TNSATSSRPPPPFTTHSPPTGSSPFSSTGPMTATSFKTTTTYPTPSHPQTTLPTHVPPFS
TSLVTPSTHTVITPTHAQMATSASIHSMPTGTIPPPTTLKATGSTHTAPTMTLTTSGTSQ
ALSSLNTAKTSTSLHSHTSSTHHAEATSTSTTNITPNPTSTGTPPMTVTTSGTSQSRSSF
STAKTSTSLHSHTSSTHHPEVTSTSTTSITPNHTSTGTRTPVAHTTSATSSRLPTPFTTH
SPPTGTTPISSTGPVTATSFQTTTTYPTPSHPHTTLPTHVPSFSTSLVTPSTHTVIIPTH
TQMATSASIHSMPTGTIPPPTTIKATGSTHTAPPMTPTTSGTSQSPSSFSTAKTSTSLPY
HTSSTHHPEVTPTSTTNITPKHTSTGTRTPVAHTTSASSSRLPTPFTTHSPPTGSSPFSS
TGPMTATSFQTTTTYPTPSHPQTTLPTHVPPFSTSLVTPSTHTVIITTHTQMATSASIHS
TPTGTVPPPTTLKATGSTHTAPPMTVTTSGTSQTHSSFSTATASSSFISSSSWLPQNSSS
RPPSSPITTQLPHLSSATTPVSTTNQLSSSFSPSPSAPSTVSSYVPSSHSSPQTSSPSVG
TSSSFVSAPVHSTTLSSGSHSSLSTHPTTASVSASPLFPSSPAASTTIRATLPHTISSPF
TLSALLPISTVTVSPTPSSHLASSTIAFPSTPRTTASTHTAPAFSSQSTTSRSTSLTTRV
PTSGFVSLTSGVTGIPTSPVTNLTTRHPGPTLSPTTRFLTSSLTAHGSTPASAPVSSLGT
PTPTSPGVCSVREQQEEITFKGCMANVTVTRCEGACISAASFNIITQQVDARCSCCRPLH
SYEQQLELPCPDPSTPGRRLVLTLQVFSHCVCSSVACGD*
>ENSG00000182050|ENST00000548651
MFKFHQMKHIFEILDKMRCLRKRSTVSFLGVLVIFLLFMNLYIEDSYVLEGDKQLIRETS
THQLNSERYVHTFKDLSNFSGAINVTYRYLAATPLQRKRYLTIGLSSVKRKKGNYLLETI
KSIFEQSSYEELKEISVVVHLADFNSSWRDAMVQDITQKFAHHIIAGRLMVIHAPEEYYP
ILDGLKRNYNDPEDRVKFRSKQNVDYAFLLNFCANTSDYYVMLEDDVRCSKNFLTAIKKV
IASLEGTYWVTLEFSKLGYIGKLYHSHDLPRLAHFLLMFYQEMPCDWLLTHFRGLLAQKN
VIRFKPSLFQHMGYYSSYKGTENKLKDDDFEEESFDIPDNPPASLYTNMNVFENYEASKA
YSSVDEYFWGKPPSTGDVFVIVFENPIIIKKIKVNTGTEDRQNDILHHGALDVGENVMPS
KQRRQCSTYLRLGEFKNGNFEMSGVNQKIPFDIHCMRIYVTKTQKEWLIIRSISIWTS*
>ENSG00000122390|ENST00000575076
MTEVVPSSALSEVSLRLLCHDDIDTVKHLCGDWFPIEYPDSWYRDITSNKKFFSLAATYR
GAIVGMIVAEIKNRTKIHKEDGDILASNFSVDTQVAYILSLGVVKEFRKHGIGSLLLESL
KDHISTTAQDHCKAIYLHVLTTNNTAINFYENRDFKQHHYLPYYYSIRGVLKDGFTYVLY
INGGHPPWTILDYIQHLGSALASLSPCSIPHRVYRQAHSLLCSFLPWSGISSKSGIEYSR
TM*
>ENSG00000092108|ENST00000458591
MAAAAAATAAAAASIRERQTVALKRMLNFNVPHIKNSTGEPVWKVLIYDRFGQDIISPLL
SVKELRDMGITLHLLLHSDRDPIPDVPAVYFVMPTEENIDRMCQDLRNQLYESYYLNFIS
AISRSKLEDIANAALAASAVTQVAKVFDQYLNFITLEDDMFVLCNQNKELVSYRAINRPD
ITDTEMETVMDTIVDSLFCFFVTLGAVPIIRCSRGTAAEMVAVKLDKKLRENLRDARNSL
FTGDTLGAGQFSFQRPLLVLVDRNIDLATPLHHTWTYQALVHDVLDFHLNRVNLEESSGV
ENSPAGARPKRKNKKSYDLTPVDKFWQKHKGSPFPEVAESVQQELESYRAQEDEVKRLKS
IMGLEGEDEGAISMLSDNTAKLTSAVSSLPELLEKKRLIDLHTNVATAVLEHIKARKLDV
YFEYEEKIMSKTTLDKSLLDIISDPDAGTPEDKMRLFLIYYISTQQAPSEADLEQYKKAL
TDAGCNLNPLQYIKQWKAFTKMASAPASYGSTTTKPMGLLSRVMNTGSQFVMEGVKNLVL
KQQNLPVTRILDNLMEMKSNPETDDYRYFDPKMLRGNDSSVPRNKNPFQEAIVFVVGGGN
YIEYQNLVDYIKGKQGKHILYGCSELFNATQFIKQLSQLGQK*
>ENSG00000137502|ENST00000534141

MSMEDYDFLFKIVLIGNAGVGKTCLVRRFTQGLFPPGQGATIGVDFMIKTVEINGEKVKL
QIWDTAGQERFRSITQSYYRSANALILTYDITCEESFRCLPEWLREIEQYASNKVITVLA
TRLTWLKGERFPSSELKNSQKLRTCIIWRPQPRNLIMWRNSSLT*
>ENSG00000166676|ENST00000575135
MKQALVDDTEDVSLDFGNEEELAFRKAKIRHPLATFFHLFFRVSAIVTYVSCDWFSKSFV
GCFVMVLLLLSLDFWSVKNVTGRLLVGLRWWNQIDEDGKSHWIFEARKVSPNSIAATEAE
ARIFWLGLIICPMIWIVFFFSTLFSLKLKWLALVVAGISLQAANLYGYILCKMGGNSDIG
KVTASFLSQTVFQTACPGDFQKPGLEGLEIHQH*
>ENSG00000223465|ENST00000432164
MLSRNDAPSVLVPGPGLLRAGTPLCISAEAASAQQ*
>ENSG00000079805|ENST00000585892
MGNRGMEELIPLVNKLQDAFSSIGQSCHLDLPQIAVVGGQSAGKSSVLENFVGRDFLPRG
SGIVTRRPLILQLIFSKTEHAEFLHCKSKKFTDFDEVRQEIEAETDRVTGTNKGISPVPI
NLRVYSPHVLNLTLIDLPGITKVPVGDQPPDIEYQIKDMILQFISRESSLILAVTPANMD
LANSDALKLAKEVDPQGLRTIGVITKLDLMDEGTDARDVLENKLLPLRRGYIGVVNRSQK
DIEGKKDIRAALAAERKFFLSHPAYRHMADRMGTPHLQKTLNQQLTNHIRESLPALRSKL
QSQLLSLEKEVEEYKNFRPDDPTRKTKALLQMVQQFGVDFEKRIEGSGDQVDTLELSGGA
RINRIFHERFPFELVKMEFDEKDLRREISYAIKNIHGVRTGLFTPDLAFEAIVKKQVVKL
KEPCLKCVDLVIQELINTVRQCTSKLSSYPRLREETERIVTTYIREREGRTKDQILLLID
IEQSYINTNHEDFIGFANAQQRSTQLNKKRAIPNQGEILVIRRGWLTINNISLMKGGSKE
YWFVLTAESLSWYKDEEEKEKKYMLPLDNLKIRDVEKGFMSNKHVFAIFNTEQRNVYKDL
RQIELACDSQEDVDSWKASFLRAGVYPEKDQAENEDGAQENTFSMDPQLERQVETIRNLV
DSYVAIINKSIRDLMPKTIMHLMINNTKAFIHHELLAYLYSSADQSSLMEESADQAQRRD
DMLRMYHALKEALNIIGDISTSTVSTPVPPPVDDTWLQSASSHSPTPQRRPVSSIHPPGR
PPAVRGPTPGPPLIPVPVGAAASFSAPPIPSRPGPQSVFANSDLFPAPPQIPSRPVRIPP
GIPPGVPRRPPAAPSRPTIIRPAEPSLLD*
>ENSG00000278114|ENST00000625274
MTTDDTEVPAMTLAPGHAALETQTLSAETSSRASTPAGPIPEAETRGAKRISPARETRSF
TKTSPNFMVLIATSVETSAASGSPEGAGMTTVQTITGSDPREAIFDTLCTDDSSEEAKTL
TMDILTLAHTSTEAKGLSSESSASSDSPHPVITPSRASESSASSDGPHPVITPSRASESS
ASSDGPHPVITPSRASESSASSDGPHPVITPSRASESSASSDGPHPVITPSRASESSASS
DGPHPVITPSRASESSASSDGPHPVITPSRASESSASSDGPHPVITPSRASESSASSDGP
HPVITPSRASESSASSDGLHPVITPSRASESSASSDGPHPVITPSRASESSASSDGPHPV
ITPSWSPGSDVTLLAEALVTVTNIEVINCSITEIETTTSSIPGASDTDLIPTEGVKASST
SDPPALPDSTEAKPHITEVTASAETLSTAGTTESAAPDATVGTPLPTNSATEREVTAPGA
TTLSGALVTVSRNPLEETSALSVETPSYVKVSGAAPVSIEAGSAVGKTTSFAGSSASSYS
PSEAALKNFTPSETPTMDIATKGPFPTSRDPLPSVPPTTTNSSRGTNSTLAKITTSAKTT
MKPPTATPTTARTRPTTDVSAGENGGFLLLRLSVASPEDLTDPRVAERLMQQLHRELHAH
APHFQVSLLRVRRG*
>ENSG00000230413|ENST00000444098
MVVMAPRTLFLLLSGALTLTETWAGSHSMRYFSAAVSRPGRGEPRFIAMGYVDDTQFVRF
DSDSACPRMEPRAPWVEQEGPEYWEEETRNTKAHAQTDRMNLQTLRGYYNQSEASSHTLQ
WMIGCDLGSDGRLLRGYEQYAYDGKDYLALNEDLRSWTAADTAAQISKRKCEAANVAEQR
RAYLEGTCVEWLHRYLENGKEMLQRADPPKTHVTHHPVFDYEATLRCWALGFYPAEIILT

WQRDGEDQTQDVELVETRPAGDGTFQKWAAVVVPSGEEQRYTCHVQHEGLPEPLMLRWKQ
SSLPTIPIMGIVAGLVVLAAVVTGAAVAAVLWRKKSSD*
>ENSG00000117262|ENST00000534502
MRQLFKDYEIRQYVVQVIFSVTFAFSCTMFELIIFEILGVLNSSSRYFHWKMNLCVILLI
LVFMVPFYIGYFIVSNIRLLHKQRLLFSCLLWLTFMYFFWKLGDPFPILSPKHGILSIEQ
LISRVGVIGVTLMALLSGFGAVNCPYTYMSYFLRNVTDTDILALERRLLQTMDMIISKKK
RMAMARRTMFQKGEVHNKPSGFWGMIKSVTTSASGSENLTLIQQEVDALEELSRQLFLET
ADLYATKERIEYSKTFKGKYFNFLGYFFSIYCVWKIFMATINIVFDRVGKTDPVTRGIEI
TVNYLGIQFDVKFWSQHISFILVGIIIVTSIRGLLITLTKFFYAISSSKSSNVIVLLLAQ
IMGMYFVSSVLLIRMSMPLEYRTIITEVLGELQFNFYHRWFDVIFLVSALSSILFLYLAH
KQAPEKQMAP*
>ENSG00000234514|ENST00000455052
MLSRNDVKRLERSWAQGSSEQELHYASLQRLPVPSSEGPDLRGRDKRGTKEDPRADYACI
AENKPT*
>ENSG00000167645|ENST00000591784
MADPHQLFDDTSSAQSRGYGAQRAPGGLSYPAASPTPHAAFLADPVSNMAMAYGSSLAAQ
GKELVDKNIDRFIPITKLKYYFAVDTMYVGRKLGLLFFPYLHQDWEVQYQQDTPVAPRFD
VNAPDLYIPAMAFITYVLVAGLALGTQDRFSPDLLGLQASSALAWLTLEVLAILLSLYLV
TVNTDLTTIDLVAFLGYKYVGMIGGVLMGLLFGKIGYYLVLGWCCVAIFVFMIRTLRLKI
LADAAAEGVPVRGARNQLRMYLTMAVAAAQPMLMYWLTFHLVR*
>ENSG00000274047|ENST00000618402
MALRPGAGSGGGGAAGAGAGSAGGGGFMFPVAGGIRPPQAGLMPMQQQGFPMVSVMQPNM
QGIMGMNYSSQMSQGPIAMQAGIPMGPMPAAGMPYLGQAPFLGMRPPGPQYTPDMQKQFA
EEQQKRFEQQQKLLEEERKRRQFEEQKQKLRLLSSVKPKTGEKSRDDALEAIKGNLDGFS
RDAKMHPTPASHPKKPGPSLEEKFLVSCDISTSGQEQIKLNTSEVGHKALGPGSSKKYPS
LMASNGVAVDGCVSGTTTAEAENTSDQNLSIEESGVGVFPSQDPAQPRMPPWIYNESLVP
DAYKKILETTMTPTGIDTAKLYPILMSSGLPRETLGQIWALANRTTPGKLTKEELYTVLA
MIAVTQRGVPAMSPDALNQFPAAPIPTLSGFSMTLPTPVSQPTVIPSGPAGSMPLSLGQP
VMGINLVGPVGGAAAQASSGFIPTYPANQVVKPEEDDFQDFQDASKSGSLDDSFSDFQEL
PASSKTSNSQHGNSAPSLLMPLPGTKALPSMDKYAVFKGIAADKSSENTVPPGDPGDKYS
AFRELEQTAENKPLGESFAEFRSAGTDDGFTDFKTADSVSPLEPPTKDKTFPPSFPSGTI
QQKQQTQVKNPLNLADLDMFSSVNCSSEKPLSFSAVFSTSKSVSTPQSTGSAATMTALAA
TKTSSLADDFGEFSLFGEYSGLAPVGEQDDFADFMAFSNSSISSEQKPDDKYDALKEEAS
PVPLTSNVGSTVKGGQNSTAASTKYDVFRQLSLEGSGLGVEDLKDNTPSGKSDDDFADFH
SSKFSSINSDKSLGEKAVAFRHTKEDSASVKSLDLPSIGGSSVGKEDSEDALSVQFDMKL
ADVGGDLKHVMSDSSLDLPTVSGQHPPAADIEDLKYAAFGSYSSNFAVSTLTSYDWSDRD
DATQGRKLSPFVLSAGSGSPSATSILQKKETSFGSSENITMTSLSKVTTFVSEDALPETT
FPALASFKDTIPQTSEQKEYENRDYKDFTKQDLPTAERSQEATCPSPASSGASQETPNEC
SDDFGEFQSEKPKISKFDFLVATSQSKMKSSEEMIKSELATFDLSVQGSHKRSLSLGDKE
ISRSSPSPALEQPFRDRSNTLNEKPALPVIRDKYKDLTGEVEENERYAYEWQRCLGSALN
VIKKANDTLNGISSSSVCTEVIQSAQGMEYLLGVVEVYRVTKRVELGIKATAVCSEKLQQ
LLKDIDKVWNNLIGFMSLATLTPDENSLDFSSCMLRPGIKNAQELACGVCLLNVDSRSRK
EEKPAEEHPKKAFNSETDSFKLAYGGHQYHASCANFWINCVEPKPPGLVLPDLL*
>ENSG00000177182|ENST00000518592

MGTWARTLLGPDYSDENDYTHTSYNAMHVKHTSSNLERECSPKLMKRSQSVVEAGTLKHE
EKGENENTQPLLALD*
>ENSG00000230413|ENST00000445373
MVVMAPRTLFLLLSGALTLTETWAGSHSMRYFSAAVSRPGRGEPRFIAMGYVDDTQFVRF
DSDSACPRMEPRAPWVEQEGPEYWEEETRNTKAHAQTDRMNLQTLRGYYNQSEAKQSSLP
TIPIMGIVAGLVVLAAVVTGAAVAAVLWRKKSSD*
>ENSG00000259075|ENST00000548729
MASATEDPVLERYFKGHKAAITSLDLSPNGKQLGAGRARELGSRRLSDLQKNTEDLSRPL
YKKPPADSRALGEWGKASKLQLNEDELKQQEELIERYAINIYLSDRISLHRHIEDKRMYE
CKSQKFNYRTLPTTSVIIAFYNEAWSTLLRTIHSVLETSPAVLLKEIILVDDLSDRVYLK
TQLETYISNLDRVRLIRTNKREGLVRARLIGATFATGDVLTFLDCHCECNSGWLEPLLER
IGRDETAVVCPVIDTIDWNTFEFYMQIGEPMIGGFDWRLTFQWHSVPKQERDRRISRIDP
IRSPTMAGGLFAVSKKYFQYLGTYDTGMEVWGGENLELSFRVWQCGGKLEIHPCSHVGHV
FPKRAPYARPNFLQNTARAAEVWMDEYKEHFYNRNPPARKEAYGDISERKLLRERLRCKS
FDWYLKNVFPNLHVPEDRPGWHGAIRSRGISSECLDYNSPDNNPTGANLSLFGCHGQGGN
QFFEYTSNKEIRFNSVTELCAEVPEQKNYVGMQNCPKDGFPVPANIIWHFKEDGTIFHPH
SGLCLSAYRTPEGRPDVQMRTCDALDKNQIWSFEK*
>ENSG00000147642|ENST00000446070
MGPLRESKEHRVQHHDKEISRSRIPRLILRPHMPQQQHKVSPASESPFSEEESREFNPSS
SGRSARTVSSNSFCSDDTGCPSSQSVSPVKTPSDAGNSPIGFCPGSDEGFTRKKCTIGMV
GEGSIQSSRYKKESKSGLVKPGSEADFSSSSSTGSISAPEVHMSTAGSKRSSSSRNRGPH
GRSNGASSHKPGSSPSSPREKDLLSMLCRNQLSPVNIHPSYAPSSPSSSNSGSYKGSDCS
PIMRRSGRYMSCGENHGVRPPNPEQYLTPLQQKEVTVRHLKTKLKESERRLHERESEIVE
LKSQLARMREDWIEEECHRVEAQLALKEARKEIKQLKQVIETMRSSLADKDKGIQKYFVD
INIQNKKLESLLQSMEMAHSGSLRDELCLDFPCDSPEKSLTLNPPLDTMADGLSLEEQVT
GEGADRELLVGDSIANSTDLFDEIVTATTTESGDLELVHSTPGANVLELLPIVMGQEEGS
VVVERAVQTDVVPYSPAISELIQSVLQKLQDPCPSSLASPDESEPDSMESFPESLSALVV
DLTPRNPNSAILLSPVETPYANVDAEVHANRLMRELDFAACVEERLDGVIPLARGGVVRQ
YWSSSFLVDLLAVAAPVVPTVLWAFSTQRGGTDPVYNIGALLRGCCVVALHSLRRTAFRI
KT*
>ENSG00000105669|ENST00000600932
MAPPAPGPASGGSGEVDELFDVKNAFYIGSYQQCINEAQRVKLSSPERDVERDVFLYRAY
LAQRKFGVVLDEIKPSSAPELQAVRMFADYLAHESRRDSIVAELDREMSRSVDVTNTTFL
LMAASIYLHDQNPDAALRALHQGDSLEWVSSPGRALHLEAQPMEAASPDGRTAMTVQILL
KLDRLDLARKELKRMQDLDEDATLTQLATAWVSLATGGEKLQDAYYIFQEMADKCSPTLL
LLNGQAACHMAQGRWEAAEGLLQEALDKDSGYPETLVNLIVLSQHLGKPPEVTNRYLSQL
KDAHRSHPFIKEYQAKENDFDRLVLQYAPSA*
>ENSG00000148429|ENST00000609104
MNSDQDVALKLAQERAEIVAKYDRGREGAEIEPWEDADYLVYKVTDRFGFLHEEELPDHN
VAVERQKHLEIERTTKWLKMLKGWEKYKNTEKFHRRIYKGIPLQLRGEVWALLLEIPKMK
EETRDLYSKLKHRARGCSPDIRQIDLDVNRTFRDHIMFRDRYGVKQQSLFHVLAAYSIYN
TEVGYCQGMSQITALLLMYMNEEDAFWALVKLFSGPKHAMHGFFVQGFPKLLRFQEHHEK
ILNKFLSKLKQHLDSQEIYTSFYTMKWFFQCFLDRTPFTLNLRIWDIYIFEGERVLTAMS
YTILKLHKKHLMKLSMEELVEFFQETLAKDFFFEDDFVIEQLQISMTELKRAKLDLPEPG

KEDEYPKKPLGQLPPELQSWGVHHLSNGGQRSVGRPSPLASGRRESGAPHRRHEHSPHPQS
RTGTPERAQPPRRKSVEEESKKLKDEADFQRKLPSGPQDSSRQYNHAAANQNSNATSNIR
KEFVPKWNKPSDVSATERTAKYTMEGKGRAAHPALAVTVPGPAEVRVSNVRPKMKALDAE
DGKRGSTASQYDNVPGPELDSGASVEEALERAYSQSPRHALYPPSPRKHAEPSSSPSKVS
NKFTFKVQPPSHARYPSQLDGEARGLAHPPSYSNPPVYHGNSPKHFPTANSSFASPQFSP
GTQLNPSRRPHGSTLSVSASPEKSYSRPSPLVLPSSRIEVLPVDTGAGGYSGNSGSPKNG
KLIIPPVDYLPDNRTWSEVSYTYRPETQGQSWTRDASRGNLPKYSAFQLAPFQDHGLPAV
SVDSPVRYKASPAAEDASPSGYPYSGPPPPAYHYRNRDGLSIQESVLL*
>ENSG00000231048|ENST00000413426
MLSRNDEAASAQQ*
>ENSG00000017260|ENST00000533801
MKVARFQKIPNGENETMIPVLTSKKASELPVSEVASILQADLQNGLNKCEVSHRRAFHGW
NEFDISEDEPLWKKYISQFKNPLIMLLLASAVISVLMHQFDDAVSITVAILIVVTVAFVQ
EYRSEKSLEELSKLVPPECHCVREGKLEHTLARDLVPGDTVCLSVGDRVPADLRLFEAVD
LSIDESSLTGETTPCSKVTAPQPAATNGDLASRSNIAFMGTLVRCGKAKGVVIGTGENSE
FGEVFKMMQAEEAPKTPLQKSMDLLGKQLSFYSFGIIGIIMLVGWLLGKDILEMFTISVS
LAVAAIPEGLPIVVTVTLALGVMRMVKKRAIVKKLPIVETLGCCNVICSDKTGTLTKNEM
TVTHIFTSDGLHAEVTGVGYNQFGEVIVDGDVVHGFYNPAVSRIVEAGCVCNDAVIRNNT
LMGKPTEGALIALAMKMGLDGLQQDYIRKAEYPFSSEQKWMAVKCVHRTQQDRPEICFMK
GAYEQVIKYCTTYQSKGQTLTLTQQQRDVYQQEKARMGSAGLRVLALASGPELGQLTFLG
LVGIIDPPRTGVKEAVTTLIASGVSIKMITGDSQETAVAIASRLGLYSKTSQSVSGEEID
AMDVQQLSQIVPKVAVFYRASPRHKMKIIKSLQKNGSVVAMTGDGVNDAVALKAADIGVA
MGQTGTDVCKEAADMILVDDDFQTIMSAIEEGKGIYNNIKNFVRFQLSTSIAALTLISLA
TLMNFPNPLNAMQILWINIIMDGPPAQSLGVEPVDKDVIRKPPRNWKDSILTKNLILKIL
VSSIIIVCGTLFVFWRELRDNVITPRDTTMTFTCFVFFDMFNALSSRSQTKSVFEIGLCS
NRMFCYAVLGSIMGQLLVIYFPPLQKVFQTESLSILGLALGEEWTAAG*
>ENSG00000011465|ENST00000552962
MKATIILLLLAQVSWAGPFQQRGLFDFMLEDEASGIGPEVPDDRDFEPSLGPVCPFRCQC
HLRVVQCSDLGLDKVPKDLPPDTTLLDLQNNKITEIKDGDFKNLKNLHALILVNNKISKV
SPGAFTPLVKLERLYLSKNQLKELPEKMPKTLQELRAHENEITKVRKVTFNGLNQMIVIE
LGTNPLKSSGIENGAFQGMKKLSYIRIADTNITSIPQGLPPSLTELHLDGNKISRVDAAS
LKGLNNLAKLGLSFNSISAVDNGSLANTPHLRELHLDNNKLTRVPGGLAEHKYIQVVYLH
NNNISVVGSSDFCPPGHNTKKASYSGVSLFSNPVQYWEIQPSTFRCVYVRSAIQLGNYK*
>ENSG00000138674|ENST00000509142
MKLKEVDRTAMQAWSPAQNHPIYLATGTSAQQLDATFSTNASLEIFELDLSDPSLDMKSC
ATFSSSHRYHKLIWGPYKMDSKGDVSGVLIAGGENGNIILYDPSKIIAGDKEVVIAQNDK
HTGPVRALDVNIFQTNLVASGANESEIYIWDLNNFATPMTPGAKTQPPEDISCIAWNRQV
QHILASASPSGRATVWDLRKNEPIIKVSDHSNRMHCSGLAWHPDVATQMVLASEDDRLPV
IQMWDLRFASSPLRVLENHARGILAIAWSMADPELLLSCGKDAKILCSNPNTGEVLYELP
TNTQWCFDIQWCPRNPAVLSAASFDGRISVYSIMGGSTDGLRQKQVDKLSSSFGNLDPFG
TGQPLPPLQIPQQTAQHSIVLPLKKPPKWIRRPVGASFSFGGKLVTFENVRMPSHQGAEQ
QQQQHHVFISQVVTEKEFLSRSDQLQQAVQSQGFINYCQKKIDASQTEFEKNVWSFLKVN
FEDDSRGKYLELLGYRKEDLGKKIALALNKVDGANVALKDSDQVAQSDGEESPAAEEQLL
GEHIKEEKEESEFLPSSGGTFNISVSGDIDGLITQALLTGNFESAVDLCLHDNRMADAII

LAIAGGQELLARTQKKYFAKSQSKITRLITAVVMKNWKEIVESCDLKNWREALAAVLTYA
KPDEFSALCDLLGTRLENEGDSLLQTQACLCYICAGNVEKLVACWTKAQDGSHPLSLQDL
IEKVVILRKAVQLTQAMDTSTVGVLLAAKMSQYANLLAAQGSIAAALAFLPDNTNQPNIM
QLRDRLCRAQGEPVAGHESPKIPYEKQQLPKGRPGPVAGHHQMPRVQTQQYYPHGENPPP
PGFIMHGNVNPNAAGQLPTSPGHMHTQVPPYPQPQRPQNGWNDPPALNRVPKKKKMPENF
MPPVPITSPIMNPLGDPQSQMLQQQPSAPVPLSSQSSFPQPHLPGGQPFHGVQQPLGQTG
MPPSFSKPNIEGAPGAPIGNTFQHVQSLPTKKITKKPIPDEHLILKTTFEDLIQRCLSSA
TDPQTKRKLDDASKRLEFLYDKLREQTLSPTITSGLHNIARSIETRNYSEGLTMHTHIVS
TSNFSETSAFMPVLKVVLTQANKLGV*
>ENSG00000166747|ENST00000569748
MPAPIRLRELIRTIRTARTQAEEREMIQKECAAIRSSFREEDNTYRCRNVAKLLYMHMLG
YPAHFGQLECLKLIASQKFTDKRIGYLGAMLLLDERQDVHLLMTNCIKNDLNHSTQFVQG
LALCTLGCMGSSEMCRDLAGEVEKLLKTSNSYLRKKAALCAVHVIRKVPELMEMFLPATK
NLLNEKNHGVLHTSVVLLTEMCERSPDMLAHFRKLVPQLVRILKNLIMSGYSPEHDVSGI
SDPFLQVRILRLLRILGRNDDDSSEAMNDILAQVATNTETSKNVGNAILYETVLTIMDIK
SESGLRVLAINILGRFLLNNDKNIRYVALTSLLKTVQTDHNAVQRHRSTIVDCLKDLDVS
IKRRAMELSFALVNGNNIRGMMKELLYFLDSCEPEFKADCASGIFLAAEKYAPSKRWHID
TIMRVLTTAGSYVRDDAVPNLIQLITNSVEMHAYTVQRLYKAILGDYSQQPLVQVAAWCI
GEYGDLLVSGQCEEEEPIQVTEDEVLDILESVLISNMSTSVTRGYALTAIMKLSTRFTCT
VNRIKKVVSIYGSSIDVELQQRAVEYNALFKKYDHMRSALLERMPVMEKVTTNGPTEIVQ
TNGETEPAPLETKPPPSGPQPTSQANDLLDLLGGNDITPVIPTAPTSKPSSAGGELLDLL
GDINLTGAPAAAPAPASVPQISQPPFLLDGLSSQPLFNDIAAGIPSITAYSKNGLKIEFT
FERSNTNPSVTVITIQASNSTELDMTDFVFQAAVPKTFQLQLLSPSSSIVPAFNTGTITQ
VIKVLNPQKQQLRMRIKLTYNHKGSAMQDLAEVNNFPPQSWQ*
>ENSG00000147408|ENST00000522854
MMMVRRGLLAWISRVVVLLVLLCCAISVLYMLACTPKGDEEQLALPRANSPTGKEGYQAV
LQEWEEQHRNYVSSLKRQIAQLKEELQERSEQLRNGQYQASDAAGLGLDRSPPEKTQADL
LAFLHSQVDKAEVNAGVKLATEYAAVPFDSFTLQKVYQLETGLTRHPEEKPVRKDKRDEL
VEAIESALETLNSPAENSPNHRPYTASDFIEGIYRTERDKGTLYELTFKGDHKHEFKRLI
LFRPFGPIMKVKNEKLNMANTLINVIVPLAKRVDKFRQFMQNFREMCIEQDGRVHLTVVY
FGKEEINEVKGILENTSKAANFRNFTFIQLNGEFSRGKGLDVGARFWKGSNVLLFFCDVD
IYFTSEFLNTCRLNTQPGKKVFYPVLFSQYNPGIIYGHHDAVPPLEQQLVIKKETGFWRD
FGFGMTCQYRSDFINIGGFDLDIKGWGGEDVHLYRKYLHSNLIVVRTPVRGLFHLWHEKR
CMDELTPEQYKMCMQSKAMNEASHGQLGMLVFRHEIEAHLRKQKQKTSSKKT*
>ENSG00000114745|ENST00000452389
MGLGVSAEQPAGGAEGFHLHGVQENSPAQQAGLEPYFDFIITIGHSRLNKENDTLKALLK
ANVEKPVKLEVFNMKTMRVREVEVVPSNMWGGQGLLGASVRFCSFRRASEQVWHVLDVEP
SSPAALAGLRPYTDYVVGSDQILQESEDFFTLIESHEGKPLKLMVYNSKSDSCRESGMWH
WLWVSTPDPNSAPQLPQEATWHPTTFCSTTWCPTT*
>ENSG00000180398|ENST00000409973
MTMRSLLRTPFLCGLLWAFCAPGARAEEPAASFSQPGSMGLDKNTVHDQEHIMEHLEGVI
NKPEAEMSPQELQLHYFKMHDYDGNNLLDGLELSTAITHVHKEEGSEQAPLMSEDELINI
IDGVLRDDDKNNDGYIDYAEFAKSLQ*
>ENSG00000082805|ENST00000611180

MYGSARSVGKVEPSSQSPGRSPRLPRSPRLGHRRTNSTGGSSGSSVGGGSGKTLSMENIQ
SLNAAYATSGPMYLSDHENVGSETPKSTMTLGRSGGRLPYGVRMTAMGSSPNIASSGVAS
DTIAFGEHHLPPVSMASTVPHSLRQARDNTIMDLQTQLKEVLRENDLLRKDVEVKESKLS
SSMNSIKTFWSPELKKERALRKDEASKITIWKEQYRVVQEENQVDALRLRLEEKETMLNK
KTKQIQDMAEEKGTQAGEIHDLKDMLDVKERKVNVLQKKIENLQEQLRDKEKQMSSLKER
VKSLQADTTNTDTALTTLEEALAEKERTIERLKEQRDRDEREKQEEIDNYKKDLKDLKEK
VSLLQGDLSEKEASLLDLKEHASSLASSGLKKDSRLKTLEIALEQKKEECLKMESQLKKA
HEAALEARASPEMSDRIQHLEREITRYKDESSKAQAEVDRLLEILKEVENEKNDKDKKIA
ELESLTSRQVKDQNKKVANLKHKEQVEKKKSAQMLEEARRREDNLNDSSQQLQDSLRKKD
DRIEELEEALRESVQITAEREMVLAQEESARTNAEKQVEELLMAMEKVKQELESMKAKLS
STQQSLAEKETHLTNLRAERRKHLEEVLEMKQEALLAAISEKDANIALLELSSSKKKTQE
EVAALKREKDRLVQQLKQQTQNRMKLMADNYEDDHFKSSHSNQTNHKPSPDQDEEEGIWA
*
>ENSG00000153130|ENST00000614192
MRRRVFSSQDWRASGWDGMGFFSRRTFCGRSGRSCRGQLVQVSRPEVSAGSLLLPAPQAE
DHSSRILYPRPKSLLPKMMNADMDAVDAENQVELEEKTRLINQVLELQHTLEDLSARVDA
VKEENLKLKSENQVLGQYIENLMSASSVFQTTDTKSKRK*
>ENSG00000125875|ENST00000461304
MALRSAQGDGPTSGHWDGGAEKADFNAKRKKKVAEIHQALNSDPTDVAALRRMAISEGGL
LTDEIRRKVWPKLLNVNANDPPPISGKNLRQMSKDYQQVLLDVRRSLRRFPPGMPEEQRE
GLQEELIDIILLILERNPQLHYYQGYHDIVVTFLLVVGERLATSLVEKLSTHHLRDFMDP
TMDNTKHILNYLMPIIDQVNPELHDFMQSAEVGTIFALSWLITWFGHVLSDFRHVVRLYD
FFLACHPLMPIYFAAVIVLYREQEVLDCDCDMASVHHLLSQIPQDLPYETLISRAGDLFV
QFPPSELAREAAAQQQAERTAASTFKDFELASAQQRPDMVLRQRFRGLLRPEDRTKDVLT
KPRTNRFVKLAVMGLTVALGAAALAVVKSALEWAPKFQLQLFP*
>ENSG00000172780|ENST00000615093
MAGPGPGPGDPDEQYDFLFKLVLVGDASVGKTCVVQRFKTGAFSERQGSTIGVDFTMKTL
EIQGKRVKLQIWDTAGQERFRTITQSYYRSANGAILAYDITKRSSFLSVPHWIEDVRKYA
GSNIVQLLIEMQSCYVAQADLELLASSNPPASTSK*
>ENSG00000176920|ENST00000522966
MLVVQMPFSFPMAHFILFVFTVSTIFHVQQRLAKIQAMWELPVQIPVLASTSKALGPSQL
RGMWTINAIGRLGNQMGEYATLYALAKMNGRPAFIPAQMHSTLAPIFRITLPVLHSATAS
RIPWQNYHLNDWMEEEYRHIPGEYVRFTGYPCSWTFYHHLRQEILQEFTLHDHVREEAQK
FLRGLQVNGSRPGTFVGVHVRRGDYVHVMPKVWKGVVADR
>ENSG00000262911|ENST00000572540
MNTMYVMMAQILRSHLIKATVIPNRVKMLPYFGIIRNRMMSTHKSKKKIREYYRLLNVEE
GCSADEVRESFHKLAKQYHPDSGSNTADSATFIRIEKAYRKVLSHVIEQTNASQSKGEEE
EDVEKFKYKTPQHRHYLSFEGIGFGTPTQREKHYRQFRADRAAEQVMEYQKQKLQSQYFP
DSVIVKNIRQSKQQKITQAIERLVEDLIQESMAKGDFDNLSGKGKPLKKFSDCSYIDPMT
HNLNRILIDNGYQPEWILKQKEISDTIEQLREAILVSRKKLGNPMTPTEKKQWNHVCEQF
QENIRKLNKRINDFNLIVPILTRQKVHFDAQKEIVRAQKIYETLIKTKEVTDRNPNNLDQ
GEGEKTPEIKKGFLNWMNLWKFIKIRSF*
>ENSG00000225590|ENST00000448042
MAAAATMAAAARELVLRAGTSDMEEEEGPLAGGPGLQEPLQLGELDITSDEFILDEVDVH

IQANLEDELVKEALKTGVDLRHYSKQVELELQQIEQKSIRDYIQESENIASLHNQITACD
AVLERMEQMLGAFQSDLSSISSEIRTLQEQSGAMNIRLRNRQAVRGKLGELVDGLVVPSA
LVTAILEAPVTEPRFLEQLQELDAKAAAVREQEARGTAACADVRGVLDRLRVKAVTKIRE
FILQKIYSFRKPMTNYQIPQTALLKYRFFYQFLLGNERATAKEIRDEYVETLSKIYLSYY
RSYLGRLMKVQYEEVAEKDDLMGVEDTAKKGFFSKPSLRSRNTIFTLGTRGSVISPTELE
APILVPHTAQRGEQRYPFEALFRSQHYALLDNSCREYLFICEFFVVSGPAAHDLFHAVMG
RTLSMTLKHLDSYLADCYDAIAVFLCIHIVLRFRNIAAKRDVPALDRYWEQVLALLWPRF
ELILEMNVQSVRSTDPQRLGGLDTRPHYITRRYAEFSSALVSINQTIPNERTMQLLGQLQ
VEVENFVLRVAAEFSSRKEQLVFLINNYDMMLGVLMERAADDSKEVESFQQLLNARTQEF
IEELLSPPFGGLVAFVKEAEALIERGQAERLRGEEARVTQLIRGFGSSWKSSVESLSQDV
MRSFTNFRNGTSIIQGALTQLIQLYHRFHRVLSQPQLRALPARAELINIHHLMVELKKHK
PNF*
>ENSG00000123106|ENST00000539107
MDDDDFGGFEAAETFDGGSGETQTTSPAIPWAAFPAVSGVHLSPSSPEIVLDRDHSSSIG
CLSSDAIISSPENTHAANSIVSQTIPKAQIQQSTHTHLDISLFPLGLTDEKSNGTIALVD
DSEDPGANVSNIQLQQKISSLEIKLKVSEEEKQRIKQDVESLMEKHNVLEKGFLKEKEQE
AISFQDRYKELQEKHKQELEDMRKAGHEALSIIVDEYKHQRLLEMLDTEKELLKEKIKEA
LIQQSQEQKEILEKCLEEERQRNKEALVSAAKLEKEAVKDAVLKVVEEERKNLEKAHAEE
RELWKTEHAKDQEKVSQEIQKAIQEQRKISQETVKAAIIEEQKRSEKAVEEAVKRTRDEL
IEYIKEQKRLDQVIRQRSLSSLELFLSCAQKQLSALIATEPVDIE*
>ENSG00000238196|ENST00000425600
CAPGYDGQNCSKELDACQSQPCHNHGTCTPKPGGFHCACPPGFVGLRCEGDVDECLDQPC
HPTGTAACHSLANAFYCQCLPGHTGQWCEVEIDPCHSQPCFHGGTCEATAGSPLGFICHC
PKGFEGPTCSHRAPSCGFHHCHHGGLCLPSPKPGFPPRCACLSGYGGPDCLTPPAPKGCG
PPSPCLYNGSCSETTGLGGPGFRCSCPHSSPGPRCQKPGAKGCEGRSGDGACDAGCSGPG
GNWDGGDCSLGVPDPWKGCPSHSRCWLLFRDGQCHPQCDSEECLFDGYDCETPPACTPAY
DQYCHDHFHNGHCEKGCNTAECGWDGGDCRPEDGDPEWGPSLALLVVLSPPALDQQLFAL
ARVLSLTLRVGLWVRKDRDGRDMVYPYPGARAEEKLGGTRDPTYQERAAPQTQPLGKETD
SLSAGFVVVMGVDLSRCGPDHPASRCPWDPGLLLRFLAAMAAVGALEPLLPGPLLAVHPH
AGTAPPANQLPWPVLCSPVAGVILLALGALLVLQLIRRRRREHGALWLPPGFTRRPRTQS
APHRRRPPLGEDSIGLKALKPKAEVDEDGVVMCSGPEEGEEVGQAEETGPPSTCQLWSLS
GGCGALPQAAMLTPPQESEMEAPDLDTRGPDGVTPLMSAVCCGEVQSGTFQGAWLGCPEP
WEPLLDGGACPQAHTVGTGETPLHLAARFSRPTAARRLLEAGANPNQPDRAGRTPLHAAV
AADAREVCQLLLRSRQTAVDARTEDGTTPLMLAARLAVEDLVEELIAAQADVGARDKWGK
TALHWAAAVNNARAARSLLQAGADKDAQDNREQTPLFLAAREGAVEVAQLLLGLGAAREL
RDQAGLAPADVAHQRNHWDLLTLLEGAGPPEARHKATPGREAGPFPRARTVSVSVPPHGG
GALPRCRTLSAGAGPRGGGACLQARTWSVDLAARGGGAYSHCRSLSGVGAGGGPTPRGRR
FSAGMRGPRPNPAIMRGRYGVAAGRGGRVSTDDWPCDWVALGACGSASNIPIPPPCLTPS
PERGSPQLDCGPPALQEMPINQGGEGKK*
>ENSG00000123595|ENST00000464506
MAGKSSLFKVILLGDGGVGKSSLMNRYVTNKFDTQLFHTIGVEFLNKDLEVDGHFVTMQI
WDTAGQERFRSLRTPFYRGSDCCLLTFSVDDSQSFQNLSNWKKEFIYYADVKEPESFPFV
ILGNKIDISERQVSTEEAQAWCRDNGDYPYFETSAKDATNVAAAFEEAVRRVLATEDRSD
HLIQTDTVNLHRKPKPSSSCC*

>ENSG00000180398|ENST00000409147
MEHLEGVINKPEAEMSPQELQLHYFKMHDYDGNNLLDGLELSTAITHVHKEEGSEQAPLM
SEDELINIIDGVLRDDDKNNDGYIDYAEFAKSLQ*
>ENSG00000168824|ENST00000505246
MVKLGNNFAEKGTKQPLLEDGFDTIPLMTPLDVNQLQFPPPDKVVVKTKTEYEPDRKKGK
ARPPQIAEFTVSITEGVTERFKVSVLVLFALAFLTCVVFLVVYKVYKYDRACPDGFVLKN
TQCIPEGLESYYAEQDSSAREKFYTVINHYNLAKQSITRSVSPWMSVLSEEKLSEQETEA
AEKSA*
>ENSG00000100478|ENST00000554345
MIKFFLMVNKQGQTRLSKYYEHVDINKRTLLETEVIKSCLSRSNEQCSFIEYKDFKLIYR
QYAALFIVVGVNDTENEMAIYEFIHNFVEVLDEYFSRVEPIDELPKICSALEPQQTCFSP
DSSSFKGAASTTPIY*
>ENSG00000128294|ENST00000403880
MRLSVRRVLLAAGCALVLVLAVQLGQQVLECRAVLAGLRSPRGAMRPEQEELVMVGTNHV
EYRYGKAMPLIFVGGVPRSGTTLMRAMLDAHPEVRCGEETRIIPRVLAMRQAWSKSGREK
LRLDEAGVTDEVLDAAMQAFILEVIAKHGEPARVLCNKDPFTLKSSVYLSRLFPNSKFLL
MVRDGRASVHSMITRKVTIAGFDLSSYRDCLTKWNKAIEVMYAQCMEVGKEKCLPVYYEQ
LVLHPRRSLKLILDFLGIAWSDAVLHHEDLIGKPGGVSLSKIERSTDQVIKPVNLEALSK
WTGHIPGDVVRDMAQIAPMLAQLGYDPYANPPNYGNPDPFVINNTQRVLKGDYKTPANLK
GYFQVNQNSTSSHLGSS*
>ENSG00000235680|ENST00000429890
MVVMAPRTLFLLLSGALTLTETWAGSHSMRYFSAAVSRPGRGEPRFIAMGYVDDTQFVRF
DSDSACPRMEPRAPWVEQEGPEYWEEETRNTKAHAQTDRMNLQTLRGYYNQSEAKQSSLP
TIPIMGIVAGLVVLAAVVTGAAVAAVLWRKKSSD*
>ENSG00000184304|ENST00000415220
MSAPPVLRPPSPLLPVAAAAAAAAAALVPGSGPGPAPFLAPVAAPVGGISFHLQIGLSRE
PVLLLQDSSGDYSLAHVREMACSIVDQKFPECGFYGMYDKILLFRHDPTSENILQLVKAA
SDIQEGDLIEVVLSASATFEDFQIRPHALFVHSYRAPAFCDHCGEMLWGLVRQGLKCEGC
GLNYHKRCAFKIPNNCSGVRRRRLSNVSLTGVSTIRTSSAELSTSAPDEPLLSPVSPGFE
QKSPSESFIGREKRSNSQSYIGRPIHLDKILMSKVKVPHTFVIHSYTRPTVCQYCKKLLK
GLFRQGLQCKDCRFNCHKRCAPKVPNNCLGEVTINGDLLSPGAESDVVMEEGSDDNDSER
NSGLMDDMEEAMVQDAEMAMAECQNDSGEMQDPDPDHEDANRTISPSTSNNIPLMRVVQS
VKHTKRKSSTVMKEGWMVHYTSKDTLRKRHYWRLDSKCITLFQNDTGSRYYKEIPLSEIL
SLEPVKTSALIPNGANPHCFEITTANVVYYVGENVVNPSSPSPNNSVLTSGVGADVARMW
EIAIQHALMPVIPKGSSVGTGTNLHRDISVSISVSNCQIQENVDISTVYQIFPDEVLGSG
QFGIVYGGKHRKTGRDVAIKIIDKLRFPTKQESQLRNEVAILQNLHHPGVVNLECMFETP
ERVFVVMEKLHGDMLEMILSSEKGRLPEHITKFLITQILVALRHLHFKNIVHCDLKPENV
LLASADPFPQVKLCDFGFARIIGEKSFRRSVVGTPAYLAPEVLRNKGYNRSLDMWSVGVI
IYVSLSGTFPFNEDEDIHDQIQNAAFMYPPNPWKEISHEAIDLINNLLQVKMRKRYSVDK
TLSHPWLQDYQTWLDLRELECKIGERYITHESDDLRWEKYAGEQGLQYPTHLINPSASHS
DTPETEETEMKALGERVSIL*
>ENSG00000233095|ENST00000448306
MVVMAPRTLFLLLSGALTLTETWAGSHSMRYFSAAVSRPGRGEPRFIAMGYVDDTQFVRF
DSDSACPRMEPRAPWVEQEGPEYWEEETRNTKAHAQTDRMNLQTLRGYYNQSEAKQSSLP

TIPIMGIVAGLVVLAAVVTGAAVAAVLWRKKSSD*
>ENSG00000169255|ENST00000468268
MASALWTVLPSRMSLRSLKWSLLLLSLLSFFVMWYLSLPHYNVIERVNWMYFYEYEPIYR
QDFHFTLREHSNCSHQNPFLVILVTSHPSDVKARQAIRVTWGEKKSWWGYE
>ENSG00000085365|ENST00000621999
MSDFDSNPFADPDLNNPFKDPSVTQVTRNVPPGLDEYNPFSDSRTPPPGGVKMPNVPNTQ
PAIMKPTEEHPAYTQIAKEHALAQAELLKRQEELERKAAELDRREREMQNLSQHGRKNNW
PPLPSNFPVGPCFYQDFSVDIPVEFQKTVKLMYYLWMFHAVTLFLNIFGCLAWFCVDSAR
AVDFGLSILWFLLFTPCSFVCWYRPLYGAFRSDSSFRFFVFFFVYICQFAVHVLQAAGFH
NWGNCGWISSLTGLNQNIPVGIMMIIIAALFTASAVISLVMFKKVHGLYRTTGASFEKAQ
QEFATGVMSNKTVQTAAANAASTAASSAAQNAFKGNQI*
>ENSG00000115526|ENST00000435960
MHHQWLLLAACFWVIFMFMVASKFITLTFKDPD
>ENSG00000139266|ENST00000548358
MVSDKCHILDLIKAQRRQMLILPEGFREGSWSTLLFRATHHLALHPFLGLFGALSPTQWQ
AISLTVIEKVQIAAIVLGSLFLVASISWLIWSSLSPSAKWQRQDLLFQICYGMYGFMDVV
CIGLIIHEGSSVYRIFKRWQAVNQQWKVLNYDKTKDIGGDAGGGTAGKSGPRNSRTGPTS
GATSRPPAAQRMRTLLPQRCGYTILHLLGQLRPPDARSSSHSGREVVMRVTTV*
>ENSG00000117533|ENST00000474047
MPPKFKRHLNDDDVTGSVKSERRNLLEDDSDEEEDFFLRGPSGPRFGPRNDKIKHVQNQV
DEVIDVMQENITKVIERGERLDELQDKSESLSDNATAFSNRSKQLRRQMWWRGCKIKAIM
ALVAAILLLVIIILIVMKYRT*
>ENSG00000177830|ENST00000429789
MRTLFNLLWLALACSPVHTTLSKSDAKKAASKTLLEKSQFSDKPVQDRGLVVTDLKAESV
VLEHRSYCSAKARDRHFAGDVLGYVTPWNSHGYDVTKVFGSKFTQISPVWLQLKRRGREM
FEVTGLHDVDQGWMRAVRKHAKGLHIVPRLLFEDWTYDDFRNVLDSEDEIEELSKTVVQV
AKNQHFDGFVVEVWNQLLSQKRVTDQLGMFTHKEFEQLAPVLDGFSLMTYDYSTAHQPGP
NAPLSWVRACVQVLDPKSKWRSKILLGLNFYGMDYATSKDAREPVVGARYIQTLKDHRPR
MVWDSQASEHFFEYKKSRSGRHVVFYPTLKSLQVRLELARELGVGVSIWELGQGLDYFYD
LL*
>ENSG00000148985|ENST00000493547
MYQVPLPLDRDGTLVRLRFTMVALVTVCCPLVAFLFCILWSLLFHFKETTATHCGVPNYL
PSVSSAIGGEVPQRYVWRFCIGLHSAPRFLVAFAYWNHYLSCTSPCSCYRPLCRLNFGLN
VVENLALLVLTYVSSSEDFTIHENAFIVFIASSLGHMLLTCILWRLTKKHTVSQEVRSIP
SGGSKAAQKKIKDICPQDSGSQVLQLETAALHHQLHLLLLGAGCLLSAQHVL*
>ENSG00000114209|ENST00000473645
MRMTMEEMKNEAETTSMVSMPLYAVMYPVFNELERVNLSAAQTLRAAFIKAEKENPGLTQ
DIIMKILEKKSVEVNFTESLLRMAADDVEEYMIERPEPEFQDLNEKARALKQILSKIPDE
INDRVRFLQTIKDIASAIKELLDTVNNVFKKYQYQNRRALEHQKKEFVKYSKSFSDTLKT
YFKDGKAINVFVSANRLIHQTNLILQTFKTVA*
>ENSG00000235680|ENST00000550897
MVVMAPRTLFLLLSGALTLTETWAGSHSMRYFSAAVSRPGRGEPRFIAMGYVDDTQFVRF
DSDSACPRMEPRAPWVEQEGPEYWEEETRNTKAHAQTDRMNLQTLRGYYNQSEASSHTLQ
WMIGCDLGSDGRLLRGYEQYAYDGKDYLALNEDLRSWTAADTAAQISKRKCEAANVAEQR

RAYLEGTCVEWLHRYLENGKEMLQRADPPKTHVTHHPVFDYEATLRCWALGFYPAEIILT
WQRDGEDQTQDVELVETRPAGDGTFQKWAAVVVPSGEEQRYTCHVQHEGLPEPLMLRWKQ
SSLPTIPIMGIVAGLVVLAAVVTGAAVAAVLWRKKSSD*
>ENSG00000204842|ENST00000535949
MRMVHILTSVVCDLVLDAAHEKSTESSSGPKREEIMESILFKCSDFVVVQFKDMDSSYAK
RDAFTDSAISAKVNGEHKEKDLEPWDAGELTANEELEALENDVSNGWDPNDMFRYNEENY
GVVSTYDSSLSSYTVPLERDNSEEFLKREARANQLAEEIESSAQYKARVALENDDRSEEE
KYTAVQRNSSEREGHSINTRENKYIPPGQRNREVISWGSGRQNSPRMGQPGSGSMPSRST
SHTSDFNPNSGSDQRVVNGGVPWPSPCPSPSSRPPSRYQSGPNSLPPRAATPTRPPSRPP
SRPSRPPSHPSAHGSPAPVSTMPKRMSSEGPPRMSPKAQRHPRNHRVSAGRGSISSGLEF
VSHNPPSEAATPPVARTSPSGGTWSSVVSGVPRLSPKTHRPRSPRQNSIGNTPSGPVLAS
PQAGIIPTEAVAMPIPAASPTPASPASNRAVTPSSEAKDSRLQDQRQNSPAGNKENIKPN
ETSPSFSKAENKGISPVVSEHRKQIDDLKKFKNDFRLQPSSTSESMDQLLNKNREGEKSR
DLIKDKIEPSAKDSFIENSSSNCTSGSSKPNSPSISPSILSNTEHKRGPEVTSQGVQTSS
PACKQEKDDKEEKKDAAEQVRKSTLNPNAKEFNPRSFSQPKPSTTPTSPRPQAQPSPSMV
GHQQPTPVYTQPVCFAPNMMYPVPVSPGVQPLYPIPMTPMPVNQAKTYRAVPNMPQQRQD
QHHQSAMMHPASAAGPPIAATPPAYSTQYVAYSPQQFPNQPLVQHVPHYQSQHPHVYSPV
IQGNARMMAPPTHAQPGLVSSSATQYGAHEQTHAMYVSTGSLAQQYAHPNATLHPHTPHP
QPSATPTGQQQSQHGGSHPAPSPVQHHQHQAAQALHLASPQQQSAIYHAGLAPTPPSMTP
ASNTQSPQNSFPAAQQTVFTIHPSHVQPAYTNPPHMAHVPQAHVQSGMVPSHPTAHAPMM
LMTTQPPGGPQAALAQSALQPIPVSTTAHFPYMTHPSVQAHHQQQL*
>ENSG00000102100|ENST00000616181
MAAVGAGGSTAAPGPGAVSAGALEPGTASAGETVCPSSRMGGGAHRRLKYISLAVLVVQN
ASLILSIRYARTLPGDRFFATTAVVMAEVLKGLTCLLLLFAQKRGNVKHLVLFLHEAVLV
QYVDTLKLAVPSLIYTLQNNLQYVAISNLPAATFQVTYQLKILTTALFSVLMLNRSLSRL
QWASLLLLFTGVAIVQAQQAGGGGPRPLDQNPGAGLAAVVASCLSSGFAGVYFEKILKGS
SGSVWLRNLQLGLFGTALGLVGLWWAEGTAVATRGFFFGYTPAVWGVVLNQAFGGLLVAV
VVKYADNILKGFATSLSIVLSTVASIRLFGFHVDPLFALGAGLVIGAVYLYSLPRGAAKA
IASASASASGPCVHQQPPGQPPPPQLSSHRGDLITEPFLPKSVLVK*
>ENSG00000147642|ENST00000533171
MGPLRESKKEHRVQHHDKEISRSRIPRLILRPHMPQQQHKVSPASESPFSEEESREFNPS
SSGRSARTVSSNSFCSDDTGCPSSQSVSPVKTPSDAGNSPIGFCPGSDEGFTRKKCTIGM
VGEGSIQSSRYKKESKSGLVKPGSEADFSSSSSTGSISAPEVHMSTAGSKRSSSSRNRGP
HGRSNGASSHKPGSSPSSPREKDLLSMLCRNQLSPVNIHPSYAPSSPSSSNSGSYKGSDC
SPIMRRSGRYMSCGENHGVRPPNPEQYLTPLQQKEVTVRHLKTKLKESERRLHERESEIV
ELKSQLARMREDWIEEECHRVEAQLALKEARKEIKQLKQVIETMRSSLADKDKGIQKYFV
DINIQNKKLESLLQSMEMAHSGSLRDELCLDFPCDSPEKSLTLNPPLDTMADGLSLEEQV
TGEGADRELLVGDSIANSTDLFDEIVTATTTESGDLELVHSTPGANVLELLPIVMGQEEG
SVVVERAVQTDVVPYSPAISELIQSVLQKLQDPCPSSLASPDESEPDSMESFPESLSALV
VDLTPRNPNSAILLSPVETPYANVDAEVHANRLMRELDFAACVEERLDGVIPLARGGVVR
QYWSSSFLVDLLAVAAPVVPTVLWAFSTQRGGTDPVYNIGALLRGCCVVALHSLRRTAFR
IKT*
>ENSG00000122705|ENST00000493185
AQQESEIAGIENDEAFAILDGGAPGPQPHGEPPGGPDAVDGVMNGEYYQMPILGSKKQSG

KKRQ*
>ENSG00000123892|ENST00000526372
XQAALPWSGTARLAGRQDAGPAQGAPVQVAGDWRPGRGEDQYHQALRAPELLFALPGHNR
RGLRAQGQERFGNMTRVYYREAMGAFIVFDVTRPATFEAVAKWKNDLDSKLSLPNGKPVS
VVLLANKCDQGKDVLMNNGLKMDQFCKEHGFVGWFETSAKENINIDEASRCLVKHILANE
CDLMESIEPDVVKPHLTSTKVASCSGCAKS*
>ENSG00000141252|ENST00000571805
MMEEEELEFVEELEAVLQLTPEVQLAIEQVFPSQDPLDRADFNAVEYINTLFPTEQSLAN
IDEVVNKIRLKIRRLDDNIRTVVRGQTNVGQDGRQALEEAQKAIQQLFGKIKDIKDKAEK
SEQMVKEITRDIKQLDHAKRHLTTSITTLNHLHMLAGGVDSLEAMTRRRQYGEVANLLQG
VMNVLEHFHKYMGIPQIRQLSERVKAAQTELGQQILADFEEAFPSQGTKRPGGPSNVLRD
ACLVANILDPRIKQEIIKKFIKQHLSEYLVLFQENQDVAWLDKIDRRYAWIKRQLVDYEE
KYGRMFPREWCMAERIAVEFCHVTRAELAKIMRTRAKEIEVKLLLFAIQRTTNFEGFLAK
RFSGCTLTDGTLKKLESPPPSTNPFLEDEPTPEMEELATEKGDLDQPKKPKAPDNPFHGI
VSKCFEPHLYVYIESQDKNLGELIDRFVADFKAQGPPKPNTDEGGAVLPSCADLFVYYKK
CMVQCSQLSTGEPMIALTTIFQKYLREYAWKILSGNLPKTTTSSGGLTISSLLKEKEGSE
VAKFTLEELCLICNILSTAEYCLATTQQLEEKLKEKVDVSLIERINLTGEMDTFSTVISS
SIQLLVQDLDAACDPALTAMSKMQWQNVEHVGDQSPYVTSVILHIKQNVPIIRDNLASTR
KYFTQFCVKFANSFIPKFITHLFKCKPISMVGAEQVRWT*
>ENSG00000102100|ENST00000413561
XMAEVLKGLTCLLLLFAQKRGNVKHLVLFLHEAVLVQYVDTLKLAVPSLIYTLQNNLQYV
AISNLPAATFQVSTLRRSSKAAQAPCGCATCNWASSAQHWAWWGSGGLRVPPWPPVVSFL
GTHLLSGAWCSTRPSAGYWWLWLSSTLTISSRALPPPCPLCCPLLPPFASLASTWTHYLP
LALDSSLVLSTSTAFPEVQPKP*
>ENSG00000173402|ENST00000515359
MRMSVGLSLLLPLSGRTFLLLLSVVMAQSHWPSEPSEAVRDWENQLEASMHSVLSDLHEA
VPTVVGIPDGTAVVGRSFRVTIPTDLIASSGDIIKVSAAGKEALPSWLHWDSQSHTLEGL
PLDTDKGVHYISVSATRLGANGSHIPQTSSVFSIEVYPEDHSELQSVRTASPDPGEVVSS
ACAADEPVTVLTVILDADLTKMTPKQRIDLLHRMRSFSEVELHNMKLVPVVNNRLFDMSA
FMAGPGNAKKVVENGALLSWKLGCSLNQNSVPDIHGVEAPAREGAMSAQLGYPVVGWHIA
NKKPPLPKRVRRQIHATPTPVTAIGPPTTAIQEPPSRIVPTPTSPAIAPPTETMAPPVRD
PVPGKPTVTIRTRGAIIQTPTLGPIQPTRVSEAGTTVPGQIRPTMTIPGYVEPTAVATPP
TTTTKKPRVSTPKPATPSTDSTTTTTRRPTKKPRTPRPVPRVTTKVSITRLETASPPTRI
RTTTSGVPRGGEPNQRPELKNHIDRVDAWVGTYFEVKIPSDTFYDHEDTTTDKLKLTLKL
REQQLVGEKSWVQFNSNSQLMYGLPDSSHVGKHEYFMHATDKGGLSAVDAFEIHVHRRPQ
GDRAPARFKAKFVGDPALVLNDIHKKIALVKKLAFAFGDRNCSTITLQNITRGSIVVEWT
NNTLPLEPCPKEQIAGLSRRIAEDDGKPRPAFSNALEPDFKATSITVTGSGSCRHLQFIP
VVPPRRVPSEAPPTEVPDRDPEKSSEDDVYLHTVIPAVVVAAILLIAGIIAMICYRKKRK
GKLTLEDQATFIKKGVPIIFADELDDSKPPPSSSMPLILQEEKAPLPPPEYPNQSVPETT
PLNQDTMGEYTPLRDEDPNAPPYQPPPPFTAPMEGKGSRPKNMTPYRSPPPYVPP*
>ENSG00000163472|ENST00000405535
MTEQETLALLEVKRSDSPEKSSPQALVPNGRQPEGEGGAESPGAESLRVGSSAGSPTAIE
GAEDGLDSTVSEAATLPWGTGPQPSAPFPDPPGWRDIEPEPPESEPLTKLEELPEDDANL
LPEKAARAFVPIDLQCIERQPQEDLIVRCEAGEGECRTFMPPRVTHPDPTERKWAEAVVR

PPGCSCGGCGSCGDREWLRAVASVGAALILFPCLLYGAYAFLPFDVPRLPTMSSRLIYTL
RCGVFATFPIVLGILVYGLSLLCFSALRPFGEPRREVEIHRRYVAQSVQLFILYFFNLAV
LSTYLPQDTLKLLPLLTGLFAVSRLIYWLTFAVGRSFRGFGYGLTFLPLLSMLMWNLYYM
FVVEPERMLTATESRLDYPDHARSASDYRPRPWG*
>ENSG00000148985|ENST00000463452
MYQVPLPLDRDGTLVRLRFTMVALVTVCCPLVAFLFCILWSLLFHFKETTATHCGVPNYL
PSVSSAIGGEVPQRYVWRFCIGLHSAPRFLVAFAYWNHYLSCTSPCSCYRPLCRLNFGLN
VVENLALLVLTYVSSSEDFTIHENAFIVFIASSLGHMLLTCILWRLTKKHTVSQEDRKSY
SWKQRLFIINFISFFSALAVYFRHNMYCEAGVYTIFAILEYTVVLTNMAFHMTAWWDFGN
KELLITSQPEEKRF*
>ENSG00000237216|ENST00000415687
MVVMAPRTLFLLLSGALTLTETWAGSHSMRYFSAAVSRPGRGEPRFIAMGYVDDTQFVRF
DSDSACPRMEPRAPWVEQEGPEYWEEETRNTKAHAQTDRMNLQTLRGYYNQSEAKQSSLP
TIPIMGIVAGLVVLAAVVTGAAVAAVLWRKKSSD*
>ENSG00000231048|ENST00000452169
MLSRNDDICIYGGLGLGGLLLLAVVLLSACLCWLHRRAPSVLVPGPGLLRAGTPLCISAE
AASAQQ*
>ENSG00000103769|ENST00000569896
MGTRDDEYDYLFKVVLIGDSGVGKSNLLSRFTRNEFNLESKSTIGVEFATRSIQVDGKTI
KAQIWDTAGQERYRAITSAYYRGAVGALLVYDIAKHLTYENVERWLKELRDHADSNIVIM
LVGNKSDLRHLRAVPTDEARAFAEKNEANVRQTRK*
>ENSG00000170340|ENST00000405767
MSVGRRRIKLLGILMMANVFIYFIMEVSKSSSQEKNGKGEVIIPKEKFWKISTPPEAYWN
REQEKLNRQYNPILSMLTNQTGEAGRLSNISHLNYCEPDLRVTSVVTGFNNLPDRFKDFL
LYLRCRNYSLLIDQPDKCAKKPFLLLAIKSLTPHFARRQAIRESWGQESNAGNQTVVRVF
LLGQTPPEDNHPDLSDMLKFESEKHQDILMWNYRDTFFNLSLKEVLFLRWVSTSCPDTEF
VFKGDDDVFVNTHHILNYLNSLSKTKAKDLFIGDVIHNAGPHRDKKLKYYIPEVVYSGLY
PPYAGGGGFLYSGHLALRLYHITDQVHLYPIDDVYTGMCLQKLGLVPEKHKGFRTFDIEE
KNKNNICSYVDLMLVHSRKPQEMIDIWSQLQSAHLKC*
>ENSG00000115652|ENST00000409032
MYNPIKTLKTNTIGTLNMLGLAKRVGARLLLASTSEVYGDPEVHPQSEDYWGHVNPIGPR
ACYDEGKRVAETMCYAYMKQEGVEVRVARIFNTFGPRMHMNDGRVVSNFILQALQGEPLT
VYGSGSQTRAFQYVSDLVNGLVALMNSNVSSPVNLGNPEEHTILEFAQLIKNLVGSGSEI
QFLSEAQDDPQKRKPDIKKAKLMLGWEPVVPLEEGLNKAIHYFRKELEYQANNQYIPKPK
PARIKKGRTRHS*
>ENSG00000095139|ENST00000614498
MVLLAAAVCTKAGKAIVSRQFVEMTRTRIEGLLAAFPKLMNTGKQHTFVETESVRYVYQP
MEKLYMVLITTKNSNILEDLETLRLFSRVIPEYCRALEENEISEHCFDLIFAFDEIVALG
YRENVNLAQIRTFTEMDSHEEKVFRAVRETQEREAKAEMRRKAKELQQAQSVGITGMSHH
AQLLLYFNP*
>ENSG00000144057|ENST00000409382
MKPHLKQWRQRMLFGIFAWGLLFLLIFIYFTDSNPAEPVPSSLSFLETRRLLPVQGKQRA
IMGAAHEPSPPGGLDARQALPRAHPAGSFHAGPGDLQKWAQSQDGFEHKEFFSSQVGRKS
QSAFYPEDDDYFFAAGQPGWHSHTQGTLGFPSPGEPGPREGAFPAAQVQRRRVKKRHRRQ

RRSHVLEEGDDGDRLYSSMSRAFLYRLWKGNVSSKMLNPRLQKAMKDYLTANKHGVRFRG
KREAGLSRAQLLCQLRSRARVRTLDGTEAPFSALGWRRLVPAVPLSQLHPRGLRSCAVVM
SAGAILNSSLGEEIDSHDAVLRFNSAPTRGYEKDVGNKTTIRIINSQILTNPSHHFIDSS
LYKDVILVAWDPAPYSANLNLWYKKPDYNLFTPYIQHRQRNPNQPFYILHPKFIWQLWDI
IQENTKEKIQPNPPSSGFIGILIMMSMCREVHVYEYIPSVRQTELCHYHELYYDAACTLG
AYHPLLYEKLLVQRLNMGTQGDLHRKGKVVLPGFQAVHCPAPSPVIPHS*
>ENSG00000223465|ENST00000437812
MLSRNDDICIYGGLGLGGLLLLAVVLLSACLCWLHRRVKRLERSWHLLSWSQAQGSSEQE
LHYASLQRLPVPSSEGPDLRGRDKRGTKEDPRADYACIAENKPT*
>ENSG00000117262|ENST00000460277
MSFLIDSSIMITSQILFFGFGWLFFMRQLFKDYEIRQYVVQVIFSVTFAFSCTMFELIIF
EILGVLNSSSRYFHWKMNLCVILLILVFMVPFYIGYFIVSNIRLLLSPLSQCINNDCFFP
VSYG*
>ENSG00000170091|ENST00000519717
MVKLNSNPSEKGTKPPSVEDGFQTVPLITPLEVNHLQLPAPEKVIVKTRTEYQPEQKNKG
KFRVPKIAEFTVTILVSLALAFLACIVFLVVYKAFTYDHSCPEGFVYKHKRCIPASLDAY
YSSQDPNSRSRFYTVISHYSVAKQSTARAIGPWLSAAAVIHEPKPPKTQGH*
>ENSG00000128965|ENST00000446533
MGGAQLELPSGARPGVCVRRSFRAHAGDQPRRPPGPIPVPGTMKQESAAPNTPPTSQSPT
PSAQFPRNDGDPQALWIFGYGSLVWRPDFAYSDSRVGFVRGYSRRFWQGDTFHRGSDKMP
GRVVTLLEDHEGCTWGVAYQVQGEQVSKALKYLNVREAVLGGYDTKEVTFYPQDAPDQPL
KALAYVATPQNPGYLGPAPEEAIATQILACRGFSGHNLEYLLRLADFMQLCGPQAQDEHL
AAIVDAVGTMLPCFCPTEQALALV*
>ENSG00000068001|ENST00000442581
MRAGPGPTVTLALVLAVSWAMELKPTAPPIFTGRPFVVAWDVPTQDCGPRLKVPLDLNAF
DVQASPNEGFVNQNITIFYRDRLGLYPRFDSAGRSVHGGVPQNVSLWAHRKMLQKRVEHY
IRTQESAGLAVIDWEDWRPVWVRNWQDKDVYRRLSRQLVASRHPDWPPDRIVKQAQYEFE
FAAQQFMLETLRYVKAVRPRHLWGFYLFPDCYNHDYVQNWESYTGRCPDVEVARNDQLAW
LWAESTALFPSVYLDETLASSRHGRNFVSFRVQEALRVARTHHANHALPVYVFTRPTYSR
RLTGLSEMDLISTIGESAALGAAGVILWGDAGYTTSTETCQYLKDYLTRLLVPYVVNVSW
ATQYCSRAQCHGHGRCVRRNPSASTFLHLSTNSFRLVPGHAPGEPQLRPVGELSWADIDH
LQTHFRCQCYLGWSGEQCQWDHRQAAGGASEAWAGSHLTSLLALAALAFTWTL*
>ENSG00000163291|ENST00000511594
MHQKLLKSAHYIELGSYQYWPVLVPRGIRLYTYEQIPGSLKDNPYITDGYRAYLPSRLCI
KSLFILSNETVNIWSHLLGFFLFFTLGIYDMTSVLPSASASREDFVICSICLFCFQVCML
CSVGYHLFSCHRSEKTCRRWMALDYAGISIGILGCYVSGVFYAFYCNNYWRQVYLITVLA
MILAVFFAQIHPNYLTQQWQRLRSIIFCSVSGYGVIPTLHWVWLNGGIGAPIVQDFAPRV
IVMYMIALLAFLFYISKVPERYFPGQLNYLGSSHQIWHILAVVMLYWWHQSTVYVMQYRH
SKPCPDYVSHL*
>ENSG00000136213|ENST00000618655
MTKARLFRLWLVLGSVFMILLIIVYWDSAGAAHFYLHTSFSRPHTGPPLPTPGPDRDREL
TADSDVDEFLDKFLSAGVKQSDLPRKETEQPPAPGSMEESVRGYDWSPRDARRSPDQGRQ
QAERRSVLRGFCANSSLAFPTKERAFDDIPNSELSHLIVDDRHGAIYCYVPKVACTNWKR
VMIVLSGSLLHRGAPYRDPLRIPREHVHNASAHLTFNKFWRRYGKLSRHLMKVKLKKYTK

FLFVRDPFVRLISAFRSKFELENEEFYRKFAVPMLRLYANHTSLPASAREAFRAGLKVSF
ANFIQYLLDPHTEKLAPFNEHWRQVYRLCHPCQIDYDFVGKLETLDEDAAQLLQLLQVDR
QLRFPPSYRNRTASSWEEDWFAKIPLAWRQQLYKLYEADFVLFGYPKPENLLRD*
>ENSG00000105270|ENST00000593074
MTKTDPAPMAPPPRGEEEEEEEEEDEPVPEAPSPTQERRQKPVVHPSAPAPLPKDYAFTFF
DPNDPACQEILFDPQTTIPELFAIVRQWVPQVQHKIDVIGNEILRRGCHVNDRDGLTDMT
LLHYACKAGAHGVGDPAAAVRLSQQLLALGADVTLRSRWTNMNALHYAAYFDVPDLVRVL
LKGARPRVVNSTCSDFNHGSALHIAASSLCLGAAKCLLEHGANPALRNRKGQVPAEVVPD
PMDMSLDKAEAALVAKELRTLLEEAVPLSCALPKVTLPNYDNVPGNLMLSALGLRLGDRV
LLDGQKTGTLRFCGTTEFASGQWVGVELDEPEGKNDGSVGGVRYFICPPKQGLFASVSKI
SKAVDAPPSSVTSTPRTPRMDFSRVTGKGRREHKGKKKTPSSPSLGSLQQRDGAKAEVGD
QVLVAGQKQGIVRFYGKTDFAPGYWYGIELDQPTGKHDGSVFGVRYFTCPPRHGVFAPAS
RIQRIGGSTDSPGDSVGAKKVHQVTMTQPKRTFTTVRTPKDIASENSISRLLFCCWFPWM
LRAEMQS*
>ENSG00000274047|ENST00000633429
MALRPGAGSGGGGAAGAGAGSAGGGGFMFPVAGGIRPPQGLMPMQQQGFPMVSVMQPNMQ
GIMGMNYSSQMSQGPIAMQAGIPMGPMPAAGMPYLGQAPFLGMRPPGPQYTPDMQKQFAE
EQQKRFEQQQKLLEEERKRRQFEEQKQKLRLLSSVKPKTGEKSRDDALEAIKGNLDGFSR
DAKMHPTPASHPKKPGVGVFPSQDPAQPRMPPWIYNESLVPDAYKKILETTMTPTGIDTA
KLYPILMSSGLPRETLGQIWALANRTTPGKLTKEELYTVLAMIAVTQRGVPAMSPDALNQ
FPAAPIPTLSGFSMTLPTPVSQPTVIPSGPAGSMPLSLGQPVMGINLVGPVGGAAAQASS
GFIPTYPANQVVKPEEDDFQDFQDASKSGSLDDSFSDFQELPASSKTSNSQHGNSAPSLL
MPLPGTKALPSMDKYAVFKGIAADKSSENTVPPGDPGDKYSAFRELEQTAENKPLGESFA
EFRSAGTDDGFTDFKTADSVSPLEPPTKDKTFPPSFPSGTIQQKQQTQVKNPLNLADLDM
FSSVNCSSEKPLSFSAVFSTSKSVSTPQSTGSAATMTALAATKTSSLADDFGEFSLFGEY
SGLAPVGEQDDFADFMAFSNSSISSEQKPDDKYDALKEEASPVPLTSNVGSTVKGGQNST
AASTKYDVFRQLSLEGSGLGVEDLKDNTPSGKSDDDFADFHSSKFSSINSDKSLGEKAVA
FRHTKEDSASVKSLDLPSIGGSSVGKEDSEDALSVQFDMKLADVGGDLKHVMSDSSLDLP
TVSGQHPPAADIEDLKYAAFGSYSSNFAVSTLTSYDWSDRDDATQGRKLSPFVLSAGSGS
PSATSILQKKETSFGSSENITMTSLSKVTTFVSEDALPETTFPALASFKDTIPQTSEQKE
YENRDYKDFTKQDLPTAERSQEATCPSPASSGASQETPNECSDDFGEFQSEKPKISKFDF
LVATSQSKMKSSEEMIKSELATFDLSVQGSHKRSLSLGDKEISRSSPSPALEQPFRDRSN
TLNEKPALPVIRDKYKDLTGEVEENERYAYEWQRCLGSALNVIKKANDTLNGISSSSVCT
EVIQSAQGMEYLLGVVEVYRVTKRVELGIKATAVCSEKLQQLLKDIDKVWNNLIGFMSLA
TLTPDENSLDFSSCMLRPGIKNAQELACGVCLLNVDSRSRKEEKPAEEHPKKAFNSETDS
FKLAYGGHQYHASCANFWINCVEPKPPGLVLPDLL*
>ENSG00000184389|ENST00000442999
MALKEGLRAWKRIFWRQILLTLGLLGLFLYGLPKFRHLEALIPMGVCPSATMSQLRDNFT
GALRPWARPEVLTCTPWGAPIIWDGSFDPDVAKQEARQQNLTIGLTIFAVGRYLEKYLER
FLETAEQHFMAGQSVMYYVFTELPGAVPRVALGPGRRLPVERVARERRWQDVSMARMRTL
HAALGGLPGREAHFMFCMDVDQHFSGTFGPEALAESVAQLHSWHYHWPSWLLPFERDAHS
AAAMAWGQGDFYNHAAVFGGSVAALRGLTAHCAGGLDWDRARGLEARWHDESHLNKFFWL
HKPAKVLSPEFCWSPDIGPRAEIRRPRLLWAPKGYRLLRN*
>ENSG00000117620|ENST00000638371

MFANLKYVSLGILVFQTTSLVLTMRYSRTLKEEGPRYLSSTAVVVAELLKIMACILLVYK
DSKCSLRALNRVLHDEILNKPMETLKLAIPSGIYTLQNNLLYVALSNLDAATYQVTYQLK
ILTTALFSVSMLSKKLGVYQWLSLVILMTGVAFVQWPSDSQLDSKELSAGSQFVGLMAVL
TACFSSGFAGVYFEKILKETKQSVWIRNIQLGFFGSIFGLMGVYIYDGELVSKNGFFQGY
NRLTWIVVVLQALGGLVIAAVIKYADNILKGFATSLSIILSTLISYFWLQDFVPTSPYSS
MKYWVGQVQWLIPVIPTL*
>ENSG00000167110|ENST00000611957
MWPQPRLPPRPAMSEETRQSKLAAAKKKLREYQQRNSPGVPTGAKKKKKIKNGSNPETTT
SGGCHSPEDTPKDNAATLQPSDDTVLPGGVPSPGASLTSMAASQNHDADNVPNLMDETKT
FSSTESLRQLSQQLNGLVCESATCVNGEGPASSANLKDLESRYQQLAVALDSSYVTNKQL
NITIEKLKQQNQEITDQLEEEKKECHQKQGALREQLQVHIQTIGILVSEKAELQTALAHT
QHAARQKEGESEDLASRLQYSRRRVGELERALSAVSTQQKKADRYNKELTKERDALRLEL
YKNTQSNEDLKQEKSELEEKLRVLVTEKAGMQLNLEELQKKLEMTELLLQQFSSRCEAPD
ANQQLQQAMEERAQLEAHLGQVMESVRQLQMERDKYAENLKGESAMWRQRMQQMSEQVHT
LREEKECSMSRVQELETSLAELRNQMAEPPPPEPPAGPSEVEQQLQAEAEHLRKELEGLA
GQLQAQVQDNEGLSRLNREQEERLLELERAAELWGEQAEARRQILETMQNDRTTISRALS
QNRELKEQLAELQSGFVKLTNENMEITSALQSEQHVKRELGKKLGELQEKLSELKETVEL
KSQEAQSLQQQRDQYLGHLQQYVAAYQQLTSEKEVLHNQLLLQTQLVDQLQQQEAQGKAV
AEMARQELQETQERLEAATQQNQQLRAQLSLMAHPGEGDGLDREEEEDEEEEEEEAVAVP
QPMPSIPEDLESREAMVAFFNSAVASAEEEQARLRGQLKEQRVRCRRLAHLLASAQKEPE
AAAPAPGTGGDSVCGETHRALQGAMEKLQSRFMELMQEKADLKERVEELEHRCIQLSGET
DTIGEYIALYQSQRAVLKERHREKEEYISRLAQDKEEMKVKLLELQELVLRLVGDRNEWH
GRFLAAAQNPADEPTSGAPAPQELGAANQQGDLCEVSLAGSVEPAQGEAREGSPRDNPTA
QQIMQLLREMQNPRERPGLGSNPCIPFFYRADENDEVKITVI*
>ENSG00000166676|ENST00000456096
MKQALVDDTEDVSLDFGNEEELAFRKAKIRDRVSKLLASVILLPWPPKVLGLKNVTGRLL
VGLRWWNQIDEDGKSHWIFEARKVSPNSIAATEAEARIFWLGLIICPMIWIVFFFSTLFS
LKLKWLALVVAGISLQAANLYGYILCKMGGNSDIGKVTASFLSQTVFQTACPGDFQKPGL
EGLEIHQH*
>ENSG00000166377|ENST00000589732
FSDENRTYQASSPDEVALVQWTESVGLTLVSRDLTSMQLKTPSGQVLSFCILQLFPFTSE
SKRMGVIVRDESTAEITFYMKGADVAMSPIVQYNDWLEEEETGRDTAVGCIKKGARPTPL
LQPFLPSLQPPVQRQIQSTG*
>ENSG00000085063|ENST00000445143
MGIQGGSVLFGLLLVLAVFCHSGHSLQCYNCPNPTADCKTAVNCSSDFDACLITKAGLQV
YNKCWKFEHCNFNDVTTRLRENELTYYCCKKDLCNFNEQLENGGTSLSEKTVLLLVTPFL
AAAWSLHP*
>ENSG00000090863|ENST00000567951
MAACGRVRRMFRLSAALHLLLLFAAGAEKLPGQGVHSQGQGPGANFVSFVGQAGGGGPAG
QQLPQLPQSSQLQQQQQQQQQQQPQPPQPPFPAGGPPARRGGAGAGGGWKLAEEESCRE
DVTRVCPKHTWSNNLAVLECLQDVREPENEISSDCNHLLWNYKLNLTTDPKFESVAREVC
KSTITEIKECADEPVGKGYMVSCLVDHRGNITEYQCHQYITKMTAIIFSDYRLICGFMDD
CKNDINILKCGSIRLGEKDAHSQDTPTALRNREGGSHGSAELKSKGSYTSVPWMSSWILP
SRISA*

>ENSG00000071073|ENST00000409391
MRLRNGTVATALAFITSFLTLSWYTTWQNGKEKLIAYQREFLALKERLRIAEHRISQRSS
ELNTIVQQFKRVGAETNGSKDALNKFSDNTLKLLKELTSKKSLQVPSIYYHLPHLLKNEG
SLQPAVQIGNGRTGVSIVMGIPTVKREVKSYLIETLHSLIDNLYPEEKLDCVIVVFIGET
DIDYVHGVVANLEKEFSKEISSGLVEVISPPESYYPDLTNLKETFGDSKERVRWRTKQNL
DYCFLMMYAQEKGIYYIQLEDDIIVKQNYFNTIKNFALQLSSEEWMILEFSQLGFIGKMF
QAPDLTLIVEFIFMFYKEKPIDWLLDHILWVKVCNPEKDAKHCDRQKANLRIRFRPSLFQ
HVGLHSSLSGKIQKLTDKDYMKPLLLKIHVNPPAEVSTSLKVYQGHTLEKTYMGEDFFWA
ITPIAGDYILFKFDKPVNVESYLFHSGNQEHPGDILLNTTVEVLPFKSEGLEISKETKDK
RLEDGYFRIGKFENGVAEGMVDPSLNPISAFRLSVIQNSAVWAILNEIHIKKATN*
>ENSG00000085063|ENST00000426650
MGIQGGSVLFGLLLVLAVFCHSGHSLQCYNCPNPTADCKTAVNCSSDFDACLITKAGLQV
YNKCWKFEHCNFNDVTTRLRENELTYYCCKKDLCNFNEQLENGGTSLSEKTVLLLVTPFL
AAAWSLHP*
>ENSG00000140835|ENST00000539698
MLLPKKMKLLLFLVSQMAILALFFHMYSHNISSLSMKAQPERMHVLVLSSWRSGSSFVGQ
LFGQHPDVFYLMEPAWHVWMTFKQSTAWMLHMAVRDLIRAVFLCDMSVFDAYMEPGPRRQ
SSLFQWENSRALCSAPACDIIPQDEIIPRAHCRLLCSQQPFEVVEKACRSYSHVVLKEVR
FFNLQSLYPLLKDPSLNLHIVHLVRDPRAVFRSRERTKGDLMIDSRIVMGQHEQKLKKED
QPYYVMQVICQSQLEIYKTIQSLPKALQERYLLVRYEDLARAPVAQTSRMYEFVGLEFLP
HLQTWVHNITRGKGMGDHAFHTNARDALNVSQAWRWSLPYEKVSRLQKACGDAMNLLGYR
HVRSEQEQRNLLLDLLSTWTVPEQIH*
>ENSG00000135241|ENST00000436062
MSINLTVDIYIYLLSNARSVCGKQRSKQLYFLFSPKHYWRISHISLQRGFHTNIIRCKWT
KSEAHSCSKHCYSPSNHGLHIGILKLSTSAPKGLTKVNICMSRIKSTLNSVSKAVFGNQN
EMISRLAQFKPSSQILRKVSDSGWLKQKNIKQAIKSLKKYSDKSAEKSPFPEEKSHIIDK
EEDIGKRSLFHYTSSITTKFGDSFYFLSNHINSYFKRKEKMSQQKENEHFRDKSELEDKK
VEEGKLRSPDPGILAYKPGSESVHTVDKPTSPSAIPDVLQVSTKQSIANFLSRPTEGVQA
LVGGYIGGLVPKLKYDSKSQSEEQEEPAKTDQAVSKDRNAEEKKRLSLQREKIIARVSID
NRTRALVQALRRTTDPKLCITRVEELTFHLLEFPEGKGVAVKERIIPYLLRLRQIKDETL
QAAVREILALIGYVDPVKGRGIRILSIDGGGTRGVVALQTLRKLVELTQKPVHQLFDYIC
GVSTGAILAFMLGLFHMPLDECEELYRKLGSDVFSQNVIVGTVKMSWSHAFYDSQTWENI
LKDRMGSALMIETARNPTCPKVAAVSTIVNRGITPKAFVFRNYGHFPGINSHYLGGCQYK
MWQAIRASSAAPGYFAEYALGNDLHQDGGLLLNNPSALAMHECKCLWPDVPLECIVSLGT
GRYESDVRNTVTYTSLKTKLSNVINSATDTEEVHIMLDGLLPPDTYFRFNPVMCENIPLD
ESRNEKLDQLQLEGLKYIERNEQKMKKVAKILSQEKTTLQKINDWIKLKTDMYEGLPFFS
KL*
>ENSG00000226936|ENST00000415322
MQLRLFRRLLLAALLLVIVWTLFGPSGLGEELLSLSLASLLPAPASPGPPLALPRLLIPN
QEACSGPGAPPFLLILVCTAPENLNQRNAIRASWGGLREARGLRVQTLFLLGEPNAQHPV
WGSQGSDLASESAAQGDILQAAFQDSYRNLTLKTLSGLNWAEKHCPMARYVLKTDDDVYV
NVPELVSELVLRGGRWGQWERSTEPQREAEQEGGQVLHSEEVPLLYLGRVHWRVNPSRTP
GGRHRVSEEQWPHTWGPFPPYASGTGYVLSASAVQLILKVASRAPLLPLEDVFVGVSARR
GGLAPTQCVKLAGATHYPLDRCCYGKFLLTSHRLDPWKMQEAWKLVGGSDGERTAPFCSW

FQGVLGILRCRAIAWLQS*
>ENSG00000104805|ENST00000405315
MPPSGPRGTLLLLPLLLLLLLRAVLAVPLERGAPNKEETPATESPDTGLYYHRYLQEVID
VLETDGHFREKLQAANAEDIKSGKLSRELDFVSHHVRTKLDELKRQEVSRLRMLLKAKMD
AEQDPNVQVDHLNLLKQFEHLDPQNQHTFEARDLELLIQTATRDLAQYDAAHHEEFKRYE
MLKEHERRRYLESLGEEQRKEAERKLEEQQRRHREHPKVNVPGSQAQLKEVWEELDGLDP
NRFNPKTFFILHDINSDGVLDEQELEALFTKELEKVYDPKNEEDDMREMEEERLRMREHV
MKNVDTNQDRLVTLEEFLASTQRKEFGDTGEGWETVEMHPAYTEEELRRFEEELAAREAE
LNAKAQRLSQETEALGRSQGRLEAQKRELQQAVLHMEQRKQQQQQQQGHKAPAAHPEGQL
KFHPDTDDVPVPAPAGDQKEVDTSEKKLLERLPEVEVPQHL*
>ENSG00000143952|ENST00000409558
MASSHSSSPVPQGSSSDVFFKIEVDPSKHIRPVPSLPDVCPKEPTVTDQHRWTVYHSKVN
LPAALNDPRLAKRESDFFTKTWGLDFVDTEVIPSFYLPQISKEHFTVYQQEISQREKIHE
RCKNICPPKDTFERTLLHTHDKSRTDLEQVPKIFMKPDFALDDSLTFNSVLPWSHFNTAG
GKGNRDAASSKLLQEKLSHYLDIVEVNIAHQISLRSEAFFHAMTSQHELQDYLRKTSQAV
KMLRDKIAQIDKVMCEGSLHILRLALTRNNCVKVYNKLKLMATVHQTQPTVQVLLSTSEF
VGALDLIATTQEVLQQELQGIHSFRHLGSQLCELEKLIDKMMIAEFSTYSHSDLNRPLED
DCQVLEEERLISLVFGLLKQRKLNFLEIYGEKMVITAKNIIKQCVINKVSQTEEIDTDVV
VKLADQMRMLNFPQWFDLLKDIFSKFTIFLQRVKATLNIIHSVVLSVLDKNQRTRELEEI
SQQKNAAKDNSLDTEVAYLIHEGMFISDAFGEGELTPIAVDTTSQRNASPNSEPCSSDSV
SEPECTTDSSSSKEHTSSSAIPGGVDIMVSEDMKLTDSELGKLANNIQELLYSASDICHD
RAVKFLMSRAKDGFLEKLNSMEFITLSRLMETFILDTEQICGRKSTSLLGALQSQAIKFV
NRFHEERKTKLSLLLDNERWKQADVPAEFQDLVDSLSDGKIALPEKKSGATEERKPAEVL
IVEGQQYAVVGTVLLLIRIILEYCQCVDNIPSVTTDMLTRLSDLLKYFNSRSCQLVLGAG
ALQVVGLKTITTKNLALSSRCLQLIVHYIPVIRAHFEARLPPKQYSMLRHFDHITKDYHD
HIAEISAKLVAIMDSLFDKLLSKYEVKAPVPSACFRNICKQMTKMHEAIFDLLPEEQTQM
LFLRINASYKLHLKKQLSHLNVINDGGPQNGLVTADVAFYTGNLQALKGLKDLDLNMAEI
WEQKR*
>ENSG00000228987|ENST00000416883
MAISGVPVLGFFIIAVLMSAQESWAIKEEHVIIQAEFYLNPDQSGEFMFDFDGDEIFHVD
MAKKETVWRLEEFGRFASFEAQGALANIAVDKANLEIMTKRSNYTPITNVPPEVTVLTNS
PVELREPNVLICFIDKFTPPVVNVTWLRNGKPVTTGVSETVFLPREDHLFRKFHYLPFLP
STEDVYDCRVEHWGLDEPLLKHWEFDAPSPLPETTENVVCALGLTVGLVGIIIGTIFIIK
GVRKSNAAERRGPL*
>ENSG00000167080|ENST00000504681
MFGSMFLQAVFSSPKPELPSPAPGVQKLKLLPEERLRNLFSYDGIWLFPKNQCKCEANKE
QGGYNFQDAYGQSDLPAVKARRQAEFEHFQRREGLPRPLPLLVQPNLPFGYPVHGVEVMP
LHTVPIPGLQFEGPDAPVYEVTLTASLGTLNTLADVPDSVVQGRGQKQLIISTSDRKLLK
FILQHVTYTSTGYQHQKVDIVSLESRSSVAKFPVTIRHPVIPKLYDPGPERKLRNLVTIA
TKTFLRPHKLMIMLRSIREYYPDLTVIVADDSQKPLEIKDNHVEYYTMPFGKGWFAGRNL
AISQVTTKYVLWVDDDFLFNEETKIEVLVDVLEKTELDVVGGSVLGNVFQFKLLLEQSEN
GACLHKRMGFFQPLDGFPSCVVTSGVVNFFLAHTERLQRVGFDPRLQRVAHSEFFIDGLG
TLLVGSCPEVIIGHQSRSPVVDSELAALEKTYNTYRSNTLTRVQFKLALHYFKNHLQCAA
*

>ENSG00000283017|ENST00000635106
MYGSARSVGKVEPSSQSPGRSPRLPRSPRLGHRRTNSTGGSSGSSVGGGSGKTLSMENIQ
SLNAAYATSGPMYLSDHENVGSETPKSTMTLGRSGGRLPYGVRMTAMGSSPNIASSGVAS
DTIAFGEHHLPPVSMASTVPHSLRQARDNTIMDLQTQLKEVLRENDLLRKDVEVKESKLS
SSMNSIKTFWSPELKKERALRKDEASKITIWKEQYRVVQEENQVDALRLRLEEKETMLNK
KTKQIQDMAEEKGTQAGEIHDLKDMLDVKERKVNVLQKKIENLQEQLRDKEKQMSSLKER
VKSLQADTTNTDTALTTLEEALAEKERTIERLKEQRDRDEREKQEEIDNYKKDLKDLKEK
VSLLQGDLSEKEASLLDLKEHASSLASSGLKKDSRLKTLEIALEQKKEECLKMESQLKKA
HEAALEARASPEMSDRIQHLEREITRYKDESSKAQAEVDRLLEILKEVENEKNDKDKKIA
ELESLTSRQVKDQNKKVANLKHKEQVEKKKSAQMLEEARRREDNLNDSSQQLQDSLRKKD
DRIEELEEALRESVQITAEREMVLAQEESARTNAEKQVEELLMAMEKVKQELESMKAKLS
STQQSLAEKETHLTNLRAERRKHLEEVLEMKQEALLAAISEKDANIALLELSSSKKKTQE
EVAALKREKDRLVQQLKQQTQNRMKLMADNYEDDHFKSSHSNQTNHKPSPDQDEEEGIWA
*
>ENSG00000165905|ENST00000531526
MLPRGRPRALGAAALLLLLLLLLGFLLFGGDLGCERREPGGRAGAPGCFPGPLMPRVPPDG
RLRRAAALDGDPGAGPGDHNRSDCGPQPPPPPKCELLHVAIVCAGHNSSRDVITLVKSML
FYRKNPLHLHLVTDAVARNILETLFHTWMVPAVRVSFYHADQLKPQVSWIPNKHYSGLYG
LMKLVLPSALPAELARVIVLDTDVTFASDISELWALFAHFSDTQAIGLVENQSDWYLGNL
WKNHRPWPALGRGFNTGVILLRLDRLRQAGWEQMWRLTARRELLSLPATSLADQDIFNAV
IKEHPGLVQRLPCVWNVQLSDHTLAERCYSEASDLKVIHWNSPKKLRVKNKHVEFFRNFY
LTFLEYDGNLLRRELFVCPSQPPPGAEQLQQALAQLDEEDPCFEFRQQQLTVHRVHVTFL
PHEPPPPRPHDVTLVAQLSMDRLQMLEALCRHWPGPMSLALYLTDAEAQQFLHFVEASPV
LAARQDVAYHVVYREGPLYPVNQLRNVALAQALTPYVFLSDIDFLPAYSLYDYLRASIEQ
LGLGSRRKAALVVPAFETLRYRFSFPHSKVELLALLDAGTLYTFRYHEWPRGHAPTDYAR
WREAQAPYRVQWAANYEPYVVVPRDCPRYDPRFVGFGWNKVAHIVELDAQEYELLVLPEA
FTIHLPHAPSLDISRFRSSPTYRDCLQALKDEFHQDLSRHHGAAALKYLPALQQPQSPAR
G*
>ENSG00000138653|ENST00000613194
MWSHMQPHLFHNESSLVEQMILNKEFALEHGIPINMGYAVAPHHSGVYPVHIQLYAAWKK
VWGIQVTSTEEYPHLKPARYRKGFIHNSIMVLPRQTCGLFTHTIFYKEYPGGPQELDKSI
RGGELFLTILLNPISIFMTHLSNYGNDRLGLYTFVNLVNFVQSWTNLKLQTLPPVQLAHQ
YFELFPEQKDPLWQNPCDDKRHKDIWSREKTCDHLPKFLVIGPQKTGTTALYLFLLMHPS
IISNLPSPKTFEEVQFFNGNNYHKGIDWYMDFFPTPSNTTSDFLFEKSANYFHSEEAPRR
AASLVPKAKIITILIDPSDRAYSWYQVGVQWRDLGSLQPSPPRFMPFSCLSLLSSWDYST
NDHMKIQLL*
>ENSG00000152056|ENST00000443700
MIHFILLFSRQGKLRLQKWYITLPDKERKKITREIVQIILSRGHRTSSFVDWKELKLVYK
RYASLYFCCAIENQDNELLTLEIVHRYVELLDKYFGNVCELDIIFNFEKAYFILDEFIIG
GEIQETSKKIAVKAIEDSDMLQENRLSPRGRDCSEPRSCHCTLA*
>ENSG00000186635|ENST00000429686
MTKKEEPPPSRVPRAVRVASLLSEGEELSGDDQGDEEEDDHAYEGVPNGGWHTSSLSLSL
PSTIAAPHPMDGPPGGSTPVTPVIKAGWLDKNPPQGSYIYQKRWVRLDTDHLRYFDSNKD
AYSKRFISVACISHVAAIGDQKFEVITNNRTFAFRAESDVERKEWMQALQQAMAEQRARA

RLSSAYLLGVPGSEQPDRAGSLELRGFKNKLYVAVVGDKVQLYKNLEEYHLGIGITFIDM
SVGNVKEVDRRSFDLTTPYRIFSFSADSELEKEQWLEAMQGAIAEALSTSEVAERIWAAA
PNRFCADCGAPQPDWASINLCVVICKRCAGEHRGLGAGVSKVRSLKMDRKVWTETLIEAL
CAAVTTTDLAETQALLGCGAGINCFSGDPEAPTPLALAEQAGQTLQMEFLRNNRTTEVPR
LDSMKPLEKHYSVVLPTVSHSGFLYKTASAGKLLQDRRAREEFSRRWCVLGDGVLSYFEN
ERAVTPNGEIRASEIVCLAVPPPDTHGFEHTFEVYTEGERLYLFGLESAEQAHEWVKCIA
KAFVPPLAEDLLARDFERLGRLPYKAGLSLQRAQEGWFSLSGSELRAVFPEGPCEEPLQL
RKLQELSIQGDSENQVLVLVERRRTLYIQGERRLDFMGWLGAIQKAAASMGDTLSEQQLG
DSDIPVIVYRCVDYITQCGLTSEGIYRKCGQTSKTQRLLESLRQDARSVHLKEGEQHVDD
VSSALKRFLRDLPDGLFTRAQRLTWLEASEIEDEEEKVSRYRELLVRLPPVNRATVKALI
SHLYCVQCFSDTNQMNVHNLAIVFGPTLFQTDGQDYKAGRVVEDLINHYVVVFSVDEEEL
RKQREEITAIVKMRVAGTASGTQHAGDFICTVYLEEKKAETEQHIKVPASMTAEELTLEI
LDRRNVGIREKDYWTCFEVNEREEAERPLHFAEKVLPILHGLGTDSHLVVKKHQAMEAML
LYLASRVGDTKHGMMKFREDRSLLGLGLPSGGFHDRYFILNSSCLRLYKEVRSHRPEKEW
PIKSLKVYLGVKKKLRPPTCWGFTVVHETEKHEKQQWYLCCDTQMELREWFATFLFVQHD
GLVWPSEPSRVSRAVPEVRLGSVSLIPLRGSENEMRRSVAAFTADPLSLLRNV*
>ENSG00000149294|ENST00000621128
MLQTKDLIWTLFFLGTAVSLQVDIVPSQGEISVGESKFFLCQVAGDAKDKDISWFSPNGE
KLTPNQQRISVVWNDDSSSTLTIYNANIDDAGIYKCVVTGEDGSESEATVNVKIFQKLMF
KNAPTPQEFREGEDAVIVCDVVSSLPPTIIWKHKGRDVILKKDVRFIVLSNNYLQIRGIK
KTDEGTYRCEGRILARGEINFKDIQVIVNVPPTIQARQNIVNATANLGQSVTLVCDAEGF
PEPTMSWTKDGEQIEQEEDDEKYIFSDDSSQLTIKKVDKNDEAEYICIAENKAGEQDATI
HLKVFAKPKITYVENQTAMELEEQVTLTCEASGDPIPSITWRTSTRNISSEEKTLDGHMV
VRSHARVSSLTLKSIQYTDAGEYICTASNTIGQDSQSMYLEVQYAPKLQGPVAVYTWEGN
QVNITCEVFAYPSATISWFRDGQLLPSSNYSNIKIYNTPSASYLEVTPDSENDFGNYNCT
AVNRIGQESLEFILVQADTPSSPSIDQVEPYSSTAQVQFDEPEATGGVPILKYKAEWRAV
GEEVWHSKWYDAKEASMEGIVTIVGLKPETTYAVRLAALNGKGLGEISAASEFKTQPVQG
EPSAPKLEGQMGEDGNSIKVNLIKQDDGGSPIRHYLVRYRALSSEWKPEIRLPSGSDHVM
LKSLDWNAEYEVYVVAENQQGKSKAAHFVFRTSAQPTAIPATLGGNSASYTFVSLLFSAV
TLLLLC*
>ENSG00000135454|ENST00000550764
MWLGRRALCALVLLLACASLGLLYASTRDAPGLRLPLAPWAPPQSPRRPELPDLAPEPRY
AHIPVRIKEQVVGLLAWNNCSCESSGGGLPLPFQKQVRAIDLTKAFDPAELRAASATREQ
EFQAFLSRSQSPADQLLIAPANSPLQYPLQGVEVQPLRSILVPGLSLQAASGQEVYQVNL
TASLGTWDVAGEVTGVTLTGEGQADLTLVSPGLDQLNRQLQLVTYSSRSYQTNTADTGAR
PGWRDGQAGQTEKNQKGWSGQMAEGMGGIWAMARAVQPHNGCFNWTSRARGRKGAFVHLG
LEQARGKPEPWVCLPFRPTVGGPRKRLV*
>ENSG00000147642|ENST00000408908
MGPLRESKKEHRVQHHDKEISRSRIPRLILRPHMPQQQHKVSPASESPFSEEESREFNPS
SSGRSARTVSSNSFCSDDTGCPSSQSVSPVKTPSDAGNSPIGFCPGSDEGFTRKKCTIGM
VGEGSIQSSRYKKESKSGLVKPGSEADFSSSSSTGSISAPEVHMSTAGSKRSSSSRNRGP
HGRSNGASSHKPGSSPSSPREKDLLSMLCRNQLSPVNIHPSYAPSSPSSSNSGSYKGSDC
SPIMRRSGRYMSCGENHGVRPPNPEQYLTPLQQKEVTVRHLKTKLKESERRLHERESEIV
ELKSQLARMREDWIEEECHRVEAQLALKEARKEIKQLKQVIETMRSSLADKDKGIQKYFV

DINIQNKKLESLLQSMEMAHSGSLRDELCLDFPCDSPEKSLTLNPPLDTMADGLSLEEQV
TGEGADRELLVGDSIANSTDLFDEIVTATTTESGDLELVHSTPGANVLELLPIVMGQEEG
SVVVERAVQTDVVPYSPAISELIQSVLQKLQDPCPSSLASPDESEPDSMESFPESLSALV
VDLTPRNPNSAILLSPVETPYANVDAEVHANRLMRELDFAACVEERLDGVIPLARGGVVR
QYWSSSFLVDLLAVAAPVVPTVLWAFSTQRGGTDPVYNIGALLRGCCVVALHSLRRTAFR
IKT*
>ENSG00000174775|ENST00000493230
MTEYKLVVVGAGGVGKSALTIQLIQNHFVDEYDPTIEDSYRKQVVIDGETCLLDILDTAG
QEEYSAMRDQYMRTGEGFLCVFAINNTKSFEDIHQYREQIKRVKDSDDVPMVLVGNKCDL
AARTVESRQAQDLARSYGIPYIETSAKTRQGSRSGSSSSSGTLWDPPGPM*
>ENSG00000276536|ENST00000615062
MTEYKLVVVGAGGVGKSALTIQLIQNHFVDEYDPTIEDSYRKQVVIDGETCLLDILDTAG
QEEYSAMRDQYMRTGEGFLCVFAINNTKSFEDIHQYREQIKRVKDSDDVPMVLVGNKCDL
AARTVESRQAQDLARSYGIPYIETSAKTRQGVEDAFYTLVREIRQHKLRKLNPPDESGPG
CMSCKCVLS*
>ENSG00000114209|ENST00000461494
MRMTMEEMKNEAETTSMVSMPLYAVMYPVFNELERVNLSAAQTLRAAFIKAEKENPGLTQ
DIIMKILEKKSVEVNFTESLLRMAADDVEEYMIERPEPEFQDLNEKARALKQILSKIPDE
INDRVRFLQTIKDIASAIKELLDTVNNVFKKYQYQNRRALEHQKKEFVKYSKSFSDTLKT
YFKDGKAINVFVSANRLIHQTNLILQTFKTVA*
>ENSG00000104490|ENST00000519508
MGKQNSKLRPEVMQDLLESTDFTEHEIQEWYKGFLRDCPSGHLSMEEFKKIYGNFFPYGD
ASKFAEHVFRTFDANGDGTIDFREFIIALSVTSRGKLEQKLKWAFSMYDLDGNGYISKAE
MLEIVQAIYKMVSSVMKMPEDESTPEKRTEKIFRQMDTNRDGKLSLEEFIRGAKSDPSIV
RLLQCDPSSAGQF*
>ENSG00000137497|ENST00000541719
MTLHATRGAALLSWVNSLHVADPVEAVLQLQDCSIFIKIIDRIHGTEEGQQILKQPVSER
LDFVCSFLQKNRKHPSSPECLVS
>ENSG00000276051|ENST00000622601
MVVMAPRTLFLLLSGALTLTETWAGSHSMRYFSAAVSRPGRGEPRFIAMGYVDDTQFVRF
DSDSACPRMEPRAPWVEQEGPEYWEEETRNTKAHAQTDRMNLQTLRGYYNQSEAKQSSLP
TIPIMGIVAGLVVLAAVVTGAAVAAVLWRKKSSD*
>ENSG00000011465|ENST00000420120
MKATIILLLLAQVSWAGPFQQRGLFDFMLEDEASGIGPEVPDDRDFEPSLGPVCPFRCQC
HLRVVQCSDLELGTNPLKSSGIENGAFQGMKKLSYIRIADTNITSIPQGLPPSLTELHLD
GNKISRVDAASLKGLNNLAKLGLSFNSISAVDNGSLANTPHLRELHLDNNKLTRVPGGLA
EHKYIQVVYLHNNNISVVGSSDFCPPGHNTKKASYSGVSLFSNPVQYWEIQPSTFRCVYV
RSAIQLGNYK*
>ENSG00000223802|ENST00000429504
MAAAGPAAGPTGPEPMPSYAQLVQRGWGSALAAARGCTDCGWGLARRGLAEHAHLAPPEL
LLLALGALGWTALRSAATARLFRPLAKRCCLQPRDAAKMPESAWKFLFYLGSWSYSAYLL
FGTDYPFFHDPPSVFYDWTPGMAVPRDIAAAYLLQGSFYGHSIYATLYMDTWRKDSVVML
LHHVVTLILIVSSYAFRYHNVGILVLFLHDISDVQLEFTKLNIYFKSRGGSYHRLHALAA
DLGCLSFGFSWFWFRLYWFPLKVLYATSHCSLRTVPDIPFYFFFNALLLLLTLMNLYWFL

YIVAFAAKVLTGQVHELKDLREYDTAEAQSLKPSKAE*
>ENSG00000153786|ENST00000564466
MQPSGHRLRDVEHHPLLAENDNYDSSSSSSSEADVADRVWFIRDGCGMICAVMTWLLVAY
ADFVVTFVMLLPSKDFWYSVVNGVIFNCLAVLALSSHLRTMLTDPEKSSDCRPSACTVKT
GLDPTLVGICGEGTESVQSLLLGAVPKGNATKEYMESLQLKPGEVIYKCPKCCCIKPERA
HHCSICKRCIRKMDHHCPWVNNCVGEKNQRFFVLFTMYIALSSVHALILCGFQFISCVRG
QWTECSDFSPPITVILLIFLCLEGLLFFTFTAVMFGTQIHSICNDETEIERLKSEKPTWE
RRLRWEGMKSVFGGPPSLLWMNPFVGFRFRRLPTRPRKGGPEFSV*
>ENSG00000180398|ENST00000444761
MLSVCSCRTSSGMRSQWPSARQRSSSLSTFRHIMEHLEGVINKPEAEMSPQELQLHYFKM
HDYDGNNLLDGLELSTAITHVHKEEGSEQAPLMSEDELINIIDGVLRDDDKNNDGYIDYA
EFAKSLQ*
>ENSG00000276536|ENST00000631967
MTEYKLVVVGAGGVGKSALTIQLIQNHFVDEYDPTIEDSYRKQVVIDGETCLLDILDTAG
QEEYSAMRDQYMRTGEGFLCVFAINNTKSFEDIHQYREQIKRVKDSDDVPMVLVGNKCDL
AARTVESRQAQDLARSYGIPYIETSAKTRQGSRSGSSSSSGTLWDPPGPM*
>ENSG00000177182|ENST00000519846
MGPVSLLPKYQKLNTWNGDLAKMTHLQAGLSPETIEKARLELNENPDVLHQDIQQVRDMI
ITRPDIGFLRTDDAFILRFLRARKFHQADAFRLLAQYFQYRQLNLDMFKNFKADDPGIKR
ALIDGFPGVLENRDHYGRKILLLFAANWDQSRNSFTDILRAILLSLEVLIEDPELQINGF
ILIIDWSNFSFKQASKLTPSILKLAIEGLQDSFPARFGGVHFVNQPWYIHALYTLIKPFL
KDKTRKRIFLHGNNLNSLHQLIHPEFLPSEFGGTLPPYDMGTWARTLLGPDYSDENDYTH
TSYNAMHVKHTSSNLERECSPKLMKRSQSVVEAGTLKHEEKGENENTQPLLALD*
>ENSG00000080815|ENST00000555386
MTELPAPLSYFQNAQMSEDNHLSNTNDNRERQEHNDRRSLGHPEPLSNGRPQGNSRQVVE
QDEEEDEELTLKYGAKHVIMLFVPVTLCMVVVVATIKSVSFYTRKDGQLIYTPFTEDTET
VGQRALHSILNAAIMISVIVVMTILLVVLYKYRCYKVIHAWLIISSLLLLFFFSFIYLGE
VFKTYNVAVDYITVALLIWNFGVVGMISIHWKGPLRLQQAYLIMISALMALVFIKYLPEW
TAWLILAVISVYDLVAVLCPKGPLRMLVETAQERNETLFPALIYSSTMVWLVNMAEGDPE
AQRRVSKNSKYNAERACLPPAAINLLSIAPMAPRLFMPKGACRPTAQKGSHKTLLQRMMM
AGSVRNGKPRGTVI*
>ENSG00000163235|ENST00000418333
MVPSAGQLALFALGIVLAACQALENSTSPLSDPPVAAAVVSHFNDCPDSHTQFCFHGTCR
FLVQEDKPACVCHSGYVGARCEHADLLAVVAASQKKQAITALVVVSIVALAVLIITCVLI
HCCQVRKHCEWCRALICRHEKPSALLKGRTACCHSETVV*
>ENSG00000147642|ENST00000422135
MGPLRESKKEHRVQHHDKEISRSRIPRLILRPHMPQQQHKVSPASESPFSEEESREFNPS
SSGRSARTVSSNSFCSDDTGCPSSQSVSPVKTPSDAGNSPIGFCPGSDEGFTRKKCTIGM
VGEGSIQSSRYKKESKSGLVKPGSEADFSSSSSTGSISAPEVHMSTAGSKRSSSSRNRGP
HGRSNGASSHKPGSSPSSPREKDLLSMLCRNQLSPVNIHPSYAPSSPSSSNSGSYKGSDC
SPIMRRSGRYMSCGENHGVRPPNPEQYLTPLQQKEVTVRHLKTKLKESERRLHERESEIV
ELKSQLARMREDWIEEECHRVEAQLALKEARKEIKQLKQVIETMRSSLADKDKGIQKYFV
DINIQNKKLESLLQSMEMAHSGSLRDELCLDFPCDSPEKSLTLNPPLDTMADGLSLEEQV
TGEGADRELLVGDSIANSTDLFDEIVTATTTESGDLELVHSTPGANVLELLPIVMGQEEG

SVVVERAVQTDVVPYSPAISELIQSVLQKLQDPCPSSLASPDESEPDSMESFPESLSALV
VDLTPRNPNSAILLSPVETPYANVDAEVHANRLMRELDFAACVEERLDGVIPLARGGVVR
QYWSSSFLVDLLAVAAPVVPTVLWAFSTQRGGTDPVYNIGALLRGCCVVALHSLRRTAFR
IKT*
>ENSG00000136279|ENST00000468694
MAANLSRNGPALQEAYVRVVTEKSPTDWALFTYEGNSNDIRVAGTGEGGLEEMVEELNSG
KVMYAFCRVKDPNSGLPKFVLINWTGEGVNDVRKGACASHVSTMASFLKGAHVTINARAE
EDVEPECIMEKVAKASGANYSFHKESGRFQDVGPQAPVGSVYQKTNAVSEIKRVGKDSFW
AKAEKEEENRRLEEKRRAEEAQRQLEQERRERELREAARREQRYQEQGGEASPQSRTWEQ
QQEVVSRNRNEQGSTCASLQESAVHPREIFKQKERAMSTTSISSPQPGKLRSPFLQKQLT
QPETHFGREPAAAISRPRADLPAEEPAPSTPPCLVQAEEEAVYEEPPEQETFYEQPPLVQ
QQGAGSEHIDHHIQGQGLSGQGLCARALYDYQAADDTEISFDPENLITGIEVIDEGWWRG
YGPDGHFGMFPANYVELIE*
>ENSG00000136279|ENST00000440166
MASFLKGAHVTINARAEEDVEPECIMEKVAKASGANYSFHKESGRFQDVGPQAPVGSVYQ
KTNAVSEIKRVGKDSFWAKAEKEEENRRLEEKRRAEEAQRQLEQERRERELREAARREQR
YQEQGGEASPQRTWEQQQEVVSRNRNEQESAVHPREIFKQKERAMSTTSISSPQPGKLRS
PFLQKQLTQPETHFGREPAAAISRPRADLPAEEPAPSTPPCLVQAEEEAVYEEPPEQETF
YEQPPLVQQQGAGSEHIDHHIQGQGLSGQGLCARALYDYQAADDTEISFDPENLITGIEV
IDEGWWRGYGPDGHFGMFPANYVELIE*
>ENSG00000103978|ENST00000448392
MAHSDTVVDGEPCDLSLNITWYLKSADCYNEIYNFKAEEVELYLEKLKEKRGLSGKYQTS
SKLFQNCSELFKTQTFSGDFMHRLPLLGEKQEAKENGTNLTFIGDKTAMHEPLQTWQDAP
YIFIVHIGISSSKESSKENSLSNLFTMTVEVKGPYEYLTLEDYPLMIFFMVMCIVYVLFG
VLWLAWSACYWRDLLRIQFWIGAVIFLGMLEKAVFYAEFQNIRYKGESVQGALILAELLS
AVKRSLARTLVIIVSLGYGIVKPRLGVTLHKVVVAGALYLLFSGMEGVLRVTGAQTDLAS
LAFIPLAFLDTALCWWIFISLTQTMKLLKLRRNIVKLSLYRHFTNTLILAVAASIVFIIW
TTMKFRIVTCQSDWRELWVDDAIWRLLFSMILFVIMVLWRPSANNQRFAFSPLSEEEEED
EQKEPMLKESFEGMKMRSTKQEPNGNSKVNKAQEDDLKWVEENVPSSVTDVALPALLDSD
EERMITHFERSKME*
>ENSG00000234514|ENST00000415490
MLSRNDAPSVLVPGPGLLRAGTPLCISAEAASAQQ*
>ENSG00000122705|ENST00000466396
MAELDPFGAPAGAPGGPALGNGVAGAGEEDPAAAFLAQQESEIAGIENDEAFAILDGGAP
GPQPHGEPPGGPDANSRKQEAEWKEKAIKELEEWYARQDEQLQKTKANNRVADEAFYKQP
FADVIGYVTNINHPCYSLEQAAEEAFVNDIDESSPGTEWERVARLCDFNPKSSKQAKDVS
RMRSVLISLKQAPLVH*
>ENSG00000189067|ENST00000576036
MSVPGPYQAATGPSSAPSAPPSYEETVAVNSYYPTPPAPMPGPTTGLVTGPDGKGMNPPS
YYTQPAPIPNNNPITVQTVYVQHPITFLDRPIQMCCPSCNKMIVSQLSYNAGALTWLSCG
SLCLLGCIAGCCFIPFCVDALQDVDHYCPNCRALLGTYKRL*
>ENSG00000090863|ENST00000447066
MAACGRVRRMFRLSAALHLLLLFAAGAEKLPGQGVHSQGQGPGANFVSFVGQAGGGGPAG
QQLPQLPQSSQLQQQQQQQQQQQPQPPQPPFPAGGPPARRGGAGAGGGWKLAEEESCRE

DVTRVCPKHTWSNNLAVLECLQDVRELLWNYKLNLTTDPKFESVAREVCKSTITEIKECA
DEPVGKGYMVSCLVDHRGNITEYQCHQYITKMTAIIFSDYRLICGFMDDCKNDINILKCG
SIRLGEKDAHSQGEVVSCLEKGLVKEAEEREPKIQVSELCKKAILRVAELSSDDFHLDRH
LYFACRDDRERFCENTQAGEGRVYKCLFNHKFEESMSEKCREALTTRQKLIAQDYKVSYS
LAKSCKSDLKKYRCNVENLPRSREARLSYLLMCLESAVHRGRQVSSECQGEMLDYRRMLM
EDFSLSPEIILSCRGEIEHHCSGLHRKGRTLHCLMKVVRGEKGNLGMNCQQALQTLIQET
DPGADYRIDRALNEACESVIQTACKHIRSGDPMILSCLMEHLYTEKMVEDCEHRLLELQY
FISRDWKLDPVLYRKCQGDASRLCHTHGWNETSEFMPQGAVFSCLYRHAYRTEEQGRRLS
RECRAEVQRILHQRAMDVKLDPALQDKCLIDLGKWCSEKTETGQELECLQDHLDDLVVEC
RDIVGNLTELESEDIQIEALLMRACEPIIQNFCHDVADNQIDSGDLMECLIQNKHQKDMN
EKCAIGVTHFQLVQMKDFRFSYKFKMACKEDVLKLCPNIKKKVDVVICLSTTVRNDTLQE
AKEHRVSLKCRRQLRVEELEMTEDIRLEPDLYEACKSDIKNFCSAVQYGNAQIIECLKEN
KKQLSTRCHQKVFKLQETEMMDPELDYTLMRVCKQMIKRFCPEADSKTMLQCLKQNKNSE
LMDPKCKQMITKRQITQNTDYRLNPMLRKACKADIPKFCHGILTKAKDDSELEGQVISCL
KLRYADQRLSSDCEDQIRIIIQESALDYRLDPQLQLHCSDEISSLCAEEAAAQEQTGQVE
ECLKVNLLKIKTELCKKEVLNMLKESKADIFVDPVLHTACALDIKHHCAAITPGRGRQMS
CLMEALEDKRVRLQPECKKRLNDRIEMWSYAAKVAPADGFSDLAMQVMTSPSKNYILSVI
SGSICILFLIGLMCGRITKRVTRELKDRLQYRSETMAYKGLVWSQDVTGSPA*
>ENSG00000145431|ENST00000422544
MSLFGLLLLTSALAGQRQGTQAESNLSSKFQFSSNKEQNGVQDPQHERIITVSTNGSIHS
PRFPHTYPRNTVLVWRLVAVEENVWIQLTFDERFGLEDPEDDICKYDFVEVEEPSDGTIL
GRWCGSGTVPGKQISKGNQIRIRFVSDEYFPSEPGFCIHYNIVMPQFTEAVSPSVLPPSA
LPLDLLNNAITAFSTLEDLIRYLEPERWQLDLEDLYRPTWQLLGKAFVFGRKSRVVDLNL
LTEEVLQLRPKTGVRGLHKSLTDVALEHHEECDCVCRGSTGG*
>ENSG00000233095|ENST00000547241
MVVMAPRTLFLLLSGALTLTETWAGSHSMRYFSAAVSRPGRGEPRFIAMGYVDDTQFVRF
DSDSACPRMEPRAPWVEQEGPEYWEEETRNTKAHAQTDRMNLQTLRGYYNQSEASSHTLQ
WMIGCDLGSDGRLLRGYEQYAYDGKDYLALNEDLRSWTAADTAAQISKRKCEAANVAEQR
RAYLEGTCVEWLHRYLENGKEMLQRADPPKTHVTHHPVFDYEATLRCWALGFYPAEIILT
WQRDGEDQTQDVELVETRPAGDGTFQKWAAVVVPSGEEQRYTCHVQHEGLPEPLMLRWKQ
SSLPTIPIMGIVAGLVVLAAVVTGAAVAAVLWRKKSSD*
>ENSG00000166685|ENST00000618996
MATAATSPALKRLDLRDPAALFETHGAEEIRGLERQVRAEIEHKKEELRQMVGERYRDLI
EAADTIGQMRRCAVGLVDAVKATDQYCARLRQAGSAAPRPPRAQQPQQPSQEKFYSMAAQ
IKLLLEIPEKIWSSMEASQCLHATQLYLLCCHLHSLLQLDSSSSRYSPVLSRFPILIRQV
AAASHFRSTILHESKMLLKCQGVSDQAVAEALCSIMLLEESSPRQALTDFLLARKATIQK
LLNQPHHGAGIKAQICSLVELLATTLKQAHALFYTLPEGLLPDPALPCGLLFSTLETITG
QHPAGKGTGVLQEEMKLCSWFKHLPASIVEFQPTLRTLAHPISQEYLKDTLQKWIHMCNE
DIKNGITNLLMYVKSMKGLAGIRDAMWELLTNESTNHSWDVLCRRLLEKPLLFWEDMMQQ
LFLDRLQTLTKEGFDSISSSSKELLVSALQELESSTSNSPSNKHIHFEYNMSLFLWSESP
NDLPSDAAWVSVANRGQFASSGLSMKAQAISPCVQNFCSALDSKLKVKLDDLLAYLPSDD
SSLPKDVSPTQAKSSAFDRYADAGTVQEMLRTQSVACIKHIVDCIRAELQSIEEGVQGQQ
DALNSAKLHSVLFMARLCQSLGELCPHLKQCILGKSESSEKPAREFRALRKQGKVKTQEI
IPTQAKWQEVKEVLLQQSVMGYQVWSSAVVKVLIHGFTQSLLLDDAGSVLATATSWDELE

IQEEAESGSSVTSKIRLPAQPSWYVQSFLFSLCQEINRVGGHALPKVTLQEMLKSCMVQV
VAAYEKLSEEKQIKKEGAFPVTQNRALQLLYDLRYLNIVLTAKGDEVKSGRSKPDSRIEK
VTDHLEALIDPFDLDVFTPHLNSNLHRLVQRTSVLFGLVTGTENQLAPRSSTFNSQEPHN
ILPLASSQIRFGLLPLSMTSTRKAKSTRNIETKAQYDANC*
>ENSG00000133103|ENST00000416691
MAEGSGEVVAVSATGAANGLNNGAGGTSATTCNPLSRKLHKILETRLDNDKEMLEALKAL
STFFVENSLRTRRNLRGDIERKSLAINEEFVSIFKEVKEELESISEDVQAMSNCCQDMTS
RLQAAKEQTQDLIVKTTKLQSESQKLEIRAQVADAFLSKFQLTSDEMSLLRGTREGPITE
DFFKALGRVKQIHNDVKVLLRTNQQTAGLEIMEQMALLQETAYERLYRWAQSECRTLTQE
SCDVSPVLTQAMEALQDRPVLYKYTLDEFGTARRSTVVRGFIDALTRGGPGGTPRPIEMH
SHDPLRYVGDMLAWLHQATASEKEHLEALLKHVTTQGVEENIQEVVGHITEGVCRPLKVR
IEQVIVAEPGAVLLYKISNLLKFYHHTISGIVGNSATALLTTIEEMHLLSKKIFFNSLSL
HASKLMDKVELPPPDLGPSSALNQTLMLLREVLASHDSSVVPLDARQADFVQVLSCVLDP
LLQMCTVSASNLGTADMATFMVNSLYMMKTTLALFEFTDRRLEMLQFQIEAHLDTLINEQ
ASYVLTRVGLSYIYNTVQQHKPEQGSLANMPNLDSVTLKAAMVQFDRYLSAPDNLLIPQL
NFLLSATVKRPPNGP*
>ENSG00000262102|ENST00000572659
MPKGGAPPWIMALMFTGHLLFLALLMFAFSTFEESVSNYSEWAVFTDDIDQFKTQKVQDF
RPNQKLKKSMLHPSLYFDAGEIQAMRQKSRASHLHLFRAIRSAVTVMLSNPTYYLPPPKH
ADFAAKWNEIYGNNLPPLALYCLLCPEDKVAFEFVLEYMDRMVGYKDWLVENAPGDEVPI
GHSLTGFATAFDFLYNLLDNHRRQKYLEKIWVITEEMYEYSKVRSWGKQLLHNHQATNMI
ALLTGALVTGVDKGSKANIWKQAVVDVMEKTMFLLNHIVDGSLDEGVAYGSYTAKSVTQY
VFLAQRHFNINNLDNNWLKMHFWFYYATLLPGFQRTVGIADSNYNWFYGPESQLVFLDKF
ILKNGAGNWLAQQIRKHRPKDGPMVPSTAQRWSTLHTEYIWYDPQLTPQPPADYGTAKIH
TFPNWGVVTYGAGLPNTQTNTFVSFKSGKLGGRAVYDIVHFQPYSWIDGWRSFNPGHEHP
DQNSFTFAPNGQVFVSEALYGPKLSHLNNVLVFAPSPSSQCNKPWEGQLGECAQWLKWTG
EEVGDAAGEIITASQHGEMVFVSGEAVSAYSSAMRLKSVYRALLLLNSQTLLVVDHIERQ
EDSPINSVSAFFHNLDIDFKYIPYKFMNRYNGAMMDVWDAHYKMFWFDHHGNSPMASIQE
AEQAAEFKKRWTQFVNVTFQMEPTITRIAYVFYGPYINVSSCRFIDSSNPGLQISLNVNN
TEHVVSIVTDYHNLKTRFNYLGFGGFASVADQGQITRFGLGTQAIVKPVRHDRIIFPFGF
KFNIAVGLILCISLVILTFQWRFYLSFRKLMRWILILVIALWFIELLDVWSTCSQPICAK
WTRTEAEGSKKSLSSEGHHMDLPDVVITSLPGSGAEILKQLFFNSSDFLYIRVPTAYIDI
PETELEIDSFVDACEWKVSDIRSGHFRLLRGWLQSLVQDTKLHLQNIHLHEPNRGKLAQY
FAMNKDKKRKFKRRESLPEQRSQMKGAFDRDAEYIRALRRHLVYYPSARPVLSLSSGSWT
LKLHFFQEVLGASMRALYIVRDPRAWIYSMLYNSKPSLYSLKNVPEHLAKLFKIEGGKGK
CNLNSGYAFEYEPLRKELSKSKSNAVSLLSHLWLANTAAALRINTDLLPTSYQLVKFEDI
VHFPQKTTERIFAFLGIPLSPASLNQILFATSTNLFYLPYEGEISPTNTNVWKQNLPRDE
IKLIENICWTLMDRLGYPKFMD*
>ENSG00000100196|ENST00000409006
MNVFRILGDLSHLLAMILLLGKIWRSKCCKGISGKSQILFALVFTTRYLDLFTNFISIYN
TVMKVVFLLCAYVTVYMIYGKFRKTFDSENDTFRLEFLLVPVIGLSFLENYSFTLLEILW
TFSIYLESVAILPQLFMISKTGEAETITTHYLFFLGLYRALYLANWIRRYQTENFYDQIA
VVSGVVQTIFYCDFFYLYVTKGRSWDDSNADTGLRSYSSI*
>ENSG00000137573|ENST00000458141

MKYSCCALVLAVLGTELLGSLCSTVRSPRFRGRIQQERKNIRPNIILVLTDDQDVELGSL
QVMNKTRKIMEHGGATFINAFVTTPMCCPSRSSMLTGKYVHNHNVYTNNENCSSPSWQAM
HEPRTFAVYLNNTGYRTAFFGKYLNEYNGSYIPPGWREWLGLIKNSRFYNYTVCRNGIKE
KHGFDYAKDYFTDLITNESINYFKMSKRMYPHRPVMMVISHAAPHGPEDSAPQFSKLYPN
ASQHITPSYNYAPNMDKHWIMQYTGPMLPIHMEFTNILQRKRLQTLMSVDDSVERLYNML
VETGELENTYIIYTADHGYHIGQFGLVKGKSMPYDFDIRVPFFIRGPSVEPGSIVPQIVL
NIDLAPTILDIAGLDTPPDVDGKSVLKLLDPEKPGNRFRTNKKAKIWRDTFLVERGKFLR
KKEESSKNIQQSNHLPKYERVKELCQQARYQTACEQPGQKWQCIEDTSGKLRIHKCKGPS
DLLTVRQSTRNLYARGFHDKDKECSCRESGYRASRSQRKSQRQFLRNQGTPKYKPRFVHT
RQTRSLSVEFEGEIYDINLEEEEELQVLQPRNIAKRHDEGHKGPRDLQASSGGNRGRMLA
DSSNAVGPPTTVRVTHKCFILPNDSIHCERELYQSARAWKDHKAYIDKEIEALQDKIKNL
REVRGHLKRRKPEECSCSKQSYYNKEKGVKKQEKLKSHLHPFKEAAQEVDSKLQLFKENN
RRRKKERKEKRRQRKGEECSLPGLTCFTHDNNHWQTAPFWNLGSFCACTSSNNNTYWCLR
TVNETHNFLFCEFATGFLEYFDMNTDPYQLTNTVHTVERGILNQLHVQLMELRSCQGYKQ
CNPRPKNLDVGNKDGGSYDLHRGQLWDGWEG*
>ENSG00000230413|ENST00000546545
MVVMAPRTLFLLLSGALTLTETWAGSHSMRYFSAAVSRPGRGEPRFIAMGYVDDTQFVRF
DSDSACPRMEPRAPWVEQEGPEYWEEETRNTKAHAQTDRMNLQTLRGYYNQSEASSHTLQ
WMIGCDLGSDGRLLRGYEQYAYDGKDYLALNEDLRSWTAADTAAQISKRKCEAANVAEQR
RAYLEGTCVEWLHRYLENGKEMLQRADPPKTHVTHHPVFDYEATLRCWALGFYPAEIILT
WQRDGEDQTQDVELVETRPAGDGTFQKWAAVVVPSGEEQRYTCHVQHEGLPEPLMLRWKQ
SSLPTIPIMGIVAGLVVLAAVVTGAAVAAVLWRKKSSD*
>ENSG00000126091|ENST00000545417
MGLLVFVRNLLLALCLFLVLGFLYYSAWKLHLLQWEEDSSKYSHSSSPQEKPVADSVVLS
FDSAGQTLGSEYDRLGFLLNLDSKLPAELATKYANFSEGACKPGYASALMTAIFPRFSKP
APMFLDDSFRKWARIREFVPPFGIKGQVLDAQYPARERVSAEAGESSRHH*
>ENSG00000141252|ENST00000437048
MMEEEELEFVEELEAVLQLTPEVQLAIEQVFPSQDPLDRADFNAVEYINTLFPTEQSLAN
IDEVVNKIRLKIRRLDDNIRTVVRGQTNVGQDGRQALEEAQKAIQQLFGKIKDIKDKAEK
SEQMVKEITRDIKQLDHAKRHLTTSITTLNHLHMLAGGVDSLEAMTRRRQYGEVANLLQG
VMNVLEHFHKYMGIPQIRQLSERVKAAQTELGQQILADFEEAFPSQGTKRPGGPSNVLRD
ACLVANILDPRIKQEIIKKFIKQHLSEYLVLFQENQDVAWLDKIDRRYAWIKRQLVDYEE
KYGRMFPREWCMAERIAVEFCHVTRAELAKIMRTRAKEIEVKLLLFAIQRTTNFEGFLAK
RFSGCTLTDGTLKKLESPPPSTNPFLEDEPTPEMEELATEKGDLDQPKKPKAPDNPFHGI
VSKCFEPHLYVYIESQDKNLGELIDRFVADFKAQGPPKPNTDEGGAVLPSCADLFVYYKK
CMVQCSQLSTGEPMIALTTIFQKYLREYAWKILSGNLPKTTTSSGGLTISSLLKEKEGSE
VAKFTLEELCLICNILSTAEYCLATTQQLEEKLKEKVDVSLIERINLTGEMDTFSTVISS
SIQLLVQDLDAACDPALTAMSKMQWQNVEHVGDQSPYVTSVILHIKQNVPIIRDNLASTR
KYFTQFCVKFANSFIPKFITHLFKCKPISMVGAEQLLLDTHSLKMVLLDLPSISSQVVRK
APASYTKIVVKGMTRAEMILKVVMAPHEPLVVFVDNYIKLLTDCNTETFQKILDMKGLKR
SEQSSMLELLRQRLPAPPSGAESSGSLSLTAPTPEQESSRIRKLEKLIKKRL*
>ENSG00000127564|ENST00000574730
MLERWHQLQPRRVSFRGEASETLQSPGYDPSRPESFFQQSFQRLSRLGHGSYGEVFKVRS
KEDGRLYAVKRSMSPFRGPKDRARKLAEVGSHEKVGQHPCCVRLEQAWEEGGILYLQTEL

CGPSLQQHCEAWGASLPEAQVWGYLRDTLLALAHLHSQGLVHLDVKPANIFLGPRGRCKL
GDFGLLVELGTAGAGEVQEGDPRYMAPELLQGSYGTAADVFSLGLTILEVACNMELPHGG
EGWQQLRQGYLPPEFTAGLSSELRSVLVMMLEPDPKLRATAEALLALPVLRQPRAWGVLW
CMAAEALSRGWALWQALLALLCWLWHGLAHPASWLQPLGPPATPPGSPPCSLLLDSSLSS
NWDDDSLGPSLSPEAVLARTVGSTSTPRSRCTPRDALDLSDINSEPPRGSFPSFEPRNLL
SLFEDTLDPT*
>ENSG00000134287|ENST00000541959
MGNIFGNLLKSLIGKKEMRILMVGLDAAGKTTILYKLKLGEIVTTIPTIGFNVETVEYKN
ISFTVWDVGGQDKIRPLWRHYFQNTQGLIFVVDSNDRERVNEAREELMRMLAEDELRDAV
LLVFANKQDLPNAMNAAEITDKLGLHSLRHRNWYIQATCATSGDGLYEGLDWLANQLKNK
K*
>ENSG00000078124|ENST00000532485
MAPAADREGYWGPTTSTLDWCEENYSVTWYIAEFWNTVSNLIMIIPPMFGAVQSVRDGLE
KRYIASYLALTVVGMGSWCFHMTLKYEMQLLDELPMIYSCCIFVYCMFECFKIKNSVNYH
LLFTLVLFSLIVTTVYLKVKEPIFHQVMYGMLVFTLVLRSIYIVTWVYPWLRGLGYTSLG
IFLLGFLFWNIDNIFCESLRNFRKKVPPIIGITTQFHAWWHILTGLGSYLHILFSLYTRT
LYLRYRPKVKFLFGIWPVILFEPLRKH*
>ENSG00000283761|ENST00000638968
MFANLKYVSLGILVFQTTSLVLTMRYSRTLKEEGPRYLSSTAVVVAELLKIMACILLVYK
DSKCSLRALNRVLHDEILNKPMETLKLAIPSGIYTLQNNLLYVALSNLDAATYQVTYQLK
ILTTALFSVSMLSKKLGVYQWLSLVILMTGVAFVQWPSDSQLDSKELSAGSQFVGLMAVL
TACFSSGFAGVYFEKILKETKQSVWIRNIQLGFFGSIFGLMGVYIYDGELVSKNGFFQGY
NRLTWIVVVLQALGGLVIAAVIKYADNILKGFATSLSIILSTLISYFWLQDFVPTRWLYA
SSSSSCS*
>ENSG00000187210|ENST00000444201
MLRTLLRRRLFSYPTKYYFMVLVLSLITFSVLRIHQKPEFVSVRHLELAGENPSSDINCT
KVLQGDVNEIQKVKLEILTVKFKKRPRWTPDDYINMTSDCSSFIKRRKYIVEPLSKEEAE
FPIAYSIVVHHKIEMLDRLLRAIYMPQNFYCIHVDTKSEDSYLAAVMGIASCFSNVFVAS
RLESVVYASWSRVQADLNCMKDLYAMSANWKYLINLCGMDFPIKTNLEIVRKLKLLMGEN
NLETERMPSHKEERWKKRYEVVNGKLTNTGTVKMLPPLETPLFSGSAYFVVSREYVGYVL
QNEKIQKLMEWAQDTYSPDEYLWATIQRIPEVPGSLPASHKYDLSDMQAVARFVKWQYFE
GDVSKGAPYPPCDGVHVRSVCIFGAGDLNWMLRKHHLFANKFDVDVDLFAIQCLDEHLRH
KALETLKH*
>ENSG00000275066|ENST00000612223
MALRPGAGSGGGGAAGAGAGSAGGGGFMFPVAGGIRPPQAGLMPMQQQGFPMVSVMQPNM
QGIMGMNYSSQMSQGPIAMQAGIPMGPMPAAGMPYLGQAPFLGMRPPGPQYTPDMQKQFA
EEQQKRFEQQQKLLEEERKRRQFEEQKQKLRLLSSVKPKTGEKSRDDALEAIKGNLDGFS
RDAKMHPTPASHPKKPGPSLEEKFLVSCDISTSGQEQIKLNTSEVGHKALGPGSSKKYPS
LMASNGVAVDGCVSGTTTAEAENTSDQNLSIEESGVGVFPSQDPAQPRMPPWIYNESLVP
DAYKKILETTMTPTGIDTAKLYPILMSSGLPRETLGQIWALANRTTPGKLTKEELYTVLA
MIAVTQRGVPAMSPDALNQFPAAPIPTLSGFSMTLPTPVSQPTVIPSGPAGSMPLSLGQP
VMGINLVGPVGGAAAQASSGFIPTYPANQVVKPEEDDFQDFQDASKSGSLDDSFSDFQEL
PASSKTSNSQHGNSAPSLLMPLPGTKALPSMDKYAVFKGIAADKSSENTVPPGDPGDKYS
AFRELEQTAENKPLGESFAEFRSAGTDDGFTDFKTADSVSPLEPPTKDKTFPPSFPSGTI

QQKQQTQVKNPLNLADLDMFSSVNCSSEKPLSFSAVFSTSKSVSTPQSTGSAATMTALAA
TKTSSLADDFGEFSLFGEYSGLAPVGEQDDFADFMAFSNSSISSEQKPDDKYDALKEEAS
PVPLTSNVGSTVKGGQNSTAASTKYDVFRQLSLEGSGLGVEDLKDNTPSGKSDDDFADFH
SSKFSSINSDKSLGEKAVAFRHTKEDSASVKSLDLPSIGGSSVGKEDSEDALSVQFDMKL
ADVGGDLKHVMSDSSLDLPTVSGQHPPAADIEDLKYAAFGSYSSNFAVSTLTSYDWSDRD
DATQGRKLSPFVLSAGSGSPSATSILQKKETSFGSSENITMTSLSKVTTFVSEDALPETT
FPALASFKDTIPQTSEQKEYENRDYKDFTKQDLPTAERSQEATCPSPASSGASQETPNEC
SDDFGEFQSEKPKISKFDFLVATSQSKMKSSEEMIKSELATFDLSVQGSHKRSLSLGDKE
ISRSSPSPALEQPFRDRSNTLNEKPALPVIRDKYKDLTGEVEENERYAYEWQRCLGSALN
VIKKANDTLNGISSSSVCTEVIQSAQGMEYLLGVVEVYRVTKRVELGIKATAVCSEKLQQ
LLKDIDKVWNNLIGFMSLATLTPDENSLDFSSCMLRPGIKNAQELACGVCLLNVDSRSRK
EEKPAEEHPKKAFNSETDSFKLAYGGHQYHASCANFWINCVEPKPPGLVLPDLL*
>ENSG00000160216|ENST00000546158
MGLLAFLKTQFVLHLLVGFVFVVSGLVINFVQLCTLALWPVSKQLYRRLNCRLAYSLWSQ
LVMLLEWWSCTECTLFTDQATVERFGKEHAVIILNHNFEIDFLCGWTMCERFGVLGSSKV
LAKKELLYVPLIGWTWYFLEIVFCKRKWEEDRDTVVEGLRRLSDYPEYMWFLLYCEGTRF
TETKHRVSMEVAAAKGLPVLKYHLLPRTKGFTTAVKCLRGTVAAVYDVTLNFRGNKNPSL
LGILYGKKYEADMCVRRFPLEDIPLDEKEAAQWLHKLYQEKDALQEIYNQKGMFPGEQFK
PARRPWTLLNFLSWATILLSPLFSFVLGVFASGSPLLILTFLGFVGAASFGVRRLIGVTE
IEKGSSYGNQEFKKKE*
>ENSG00000105974|ENST00000405348
MADELSEKQVYDAHTKEIDLVNRDPKHLNDDVVKIDFEDVIAEPEGTHSFDGIWKASFTT
FTVTKYWFYRLLSALFGIPMALIWGIYFAILSFLHIWAVVPCIKSFLIEIQCISRVYSIY
VHTVCDPLFEAVGKIFSNVRINLQKEI*
>ENSG00000163291|ENST00000512733
MHQKLLKSAHYIELGSYQYWPVLVPRGIRLYTYEQIPGSLKDNPYITDGYRAYLPSRLCI
KSLFILSNETVNIWSHLLGFFLFFTLGIYDMTSVLPSASASREDFVICSICLFCFQVCML
CSVGYHLFSCHRSEKTCRRWMALDYAGISIGILGCYVSGVFYAFYCNNYWRQVYLITVLA
MILAVFFAQIHPNYLTQQWQRLRSIIFCSVSGYGVIPTLHWVWLNGGIGAPIVQDFAPRV
IVMYMIALLAFLFYISKVPERYFPGQLNYLGSSHQIWHILAVVMLYWWHQSTVYVMQYRH
SKPCPDYVSHL*
>ENSG00000257923|ENST00000549414
MLCVAGARLKRELDATATVLANRQDESEQSRKRLIEQSREFKKNTPEDLRKQVAPLLKSF
QGEIDALSKRSKEAEAAFLNVYKRLIDVPDPVPALDLGQQLQLKVQRLHDIETENQKLRE
TLEEYNKEFAEVKNQEVTIKALKEKIREYEQTLKNQAETIALEKEQKLQNDFAEKERKLQ
ETQMSTTSKLEEAEHKVQSLQTALEKTRTELFDLKTKYDEETTAKADEIEMIMTDLERAN
QRAEVAQREAETLREQLSSANHSLQLASQIQKAPDVEQAIEVLTRSSLEVELAAKEREIA
QLVEDVQRLQASLTKLRENSASQISQLEQQLSAKNSTLKQLEEKLKGQADYEEVKKELNI
LKSMEFAPSEGAGTQDAAKPLEVLLLEKNRSLQSENAALRISNSDLSGSARRKGKDQPES
RRPGSLPAPPPSQLPRNPGEQASNTNGTHQFSPAGLSQDFFSSSLASPSLPLASTGKFAL
NSLLQRQLMQSFYSKAMQEAGSTSMIFSTGPYSTNSISSQSPLQQSPDVNGMAPSPSQSE
SAGSVSEGEEMDTAEIARQVKEQLIKHNIGQRIFGHYVLGLSQGSVSEILARPKPWNKLT
VRGKEPFHKMKQFLSDEQNILALRSIQGRQRGNITTRIRASETGSDEAIKSILEQAKREL
QVQKTAEPAQPSSASGSGNSDDAIRSILQQARREMEAQQAALDPALKQAPLSQSDITILT

PKLLSTSPMPTVSSYPPLAISLKKPSAAPEAGASALPNPPALKKEAQDAPGLDPQGAADC
AQGVLRQVKNEVGRSGAWKDHWWSAVQPERRNAASSEEAKAEETGGGKEKGSGGSGGGSQ
PRAERSQLQGPSSSEYWKEWPSAESPYSQSSELSLTGASRSETPQNSPLPSSPIVPMSKP
TKPSVPPLTPEQYEVYMYQEVDTIELTRQVKEKLAKNGICQRIFGEKVLGLSQGSVSDML
SRPKPWSKLTQKGREPFIRMQLWLNGELGQGVLPVQGQQQGPVLHSVTSLQDPLQQGCVS
SESTPKTSASCSPAPESPMSSSESVKSLTELVQQPCPPIEASKDSKPPEPSDPPASDSQP
TTPLPLSGHSALSIQELVAMSPELDTYGITKRVKEVLTDNNLGQRLFGETILGLTQGSVS
DLLARPKPWHKLSLKGREPFVRMQLWLNDPNNVEKLMDMKRMEKKAYMKRRHSSVSDSQP
CEPPSVGTEYSQGASPQPQHQLKKPRVVLAPEEKEALKRAYQQKPYPSPKTIEDLATQLN
LKTSTVINWFHNYRSRIRRELFIEEIQAGSQGQAGASDSPSARSGRAAPSSEGDSCDGVE
ATEGPGSADTEEPKSQGEAEREEVPRPAEQTEPPPSGTPGPDDARDDDHEGGPVEGPGPL
PSPASATATAAPAAPEDAATSAAAAPGEGPAAPSSAPPPSNSSSSSAPRRPSSLQSLFGL
PEAAGARDSRDNPLRKKKAANLNSIIHRLEKAASREEPIEWEF*
>ENSG00000204842|ENST00000616825
MRMVHILTSVVCDLVLDAAHEKSTESSSGPKREEIMESILFKCSDFVVVQFKDMDSSYAK
RDAFTDSAISAKVNGEHKEKDLEPWDAGELTANEELEALENDVSNGWDPNDMFRYNEENY
GVVSTYDSSLSSYTVPLERDNSEEFLKREARANQLAEEIESSAQYKARVALENDDRSEEE
KYTAVQRNSSEREGHSINTRENKYIPPGQRNREVISWGSGRQNSPRMGQPGSGSMPSRST
SHTSDFNPNSGSDQRVVNGGVPWPSPCPSPSSRPPSRYQSGPNSLPPRAATPTRPPSRPP
SRPSRPPSHPSAHGSPAPVSTMPKRMSSEGPPRMSPKAQRHPRNHRVSAGRGSISSGLEF
VSHNPPSEAATPPVARTSPSGGTWSSVVSGVPRLSPKTHRPRSPRQNSIGNTPSGPVLAS
PQAGIIPTEAVAMPIPAASPTPASPASNRAVTPSSEAKDSRLQDQRQNSPAGNKENIKPN
ETSPSFSKAENKGISPVVSEHRKQIDDLKKFKNDFRLQPSSTSESMDQLLNKNREGEKSR
DLIKDKIEPSAKDSFIENSSSNCTSGSSKPNSPSISPSILSNTEHKRGPEVTSQGVQTSS
PACKQEKDDKEEKKDAAEQVRKSTLNPNAKEFNPRSFSQPKPSTTPTSPRPQAQPSPSMV
GHQQPTPVYTQPVCFAPNMMYPVPVSPGVQPLYPIPMTPMPVNQAKTYRAVPNMPQQRQD
QHHQSAMMHPASAAGPPIAATPPAYSTQYVAYSPQQFPNQPLVQHVPHYQSQHPHVYSPV
IQGNARMMAPPTHAQPGLVSSSATQYGAHEQTHAMYVSTGSLAQQYAHPNATLHPHTPHP
QPSATPTGQQQSQHGGSHPAPSPVQHHQHQAAQALHLASPQQQSAIYHAGLAPTPPSMTP
ASNTQSPQNSFPAAQQTVFTIHPSHVQPAYTNPPHMAHVPQAHVQSGMVPSHPTAHAPMM
LMTTQPPGGPQAALAQSALQPIPVSTTAHFPYMTHPSVQAHHQQQL*
>ENSG00000172915|ENST00000537702
MFSEIRAVFSRRYLLQNTALEVFMANRTSVMFNFPDQATVKKVVYSLPRVGVGTSYGLPQ
ARRISLATPRQLYKSSNMTQRWQRREISNFEYLMFLNTIAGRTYNDLNQYPVFPWVLTNY
ESEELDLTLPGNFRDLSKPIGALNPKRAVFYAERYETWEDDQSPPYHYNTHYSTATSTLS
WLVRIEPFTTFFLNANDGKFDHPDRTFSSVARSWRTSQRDTSDVKELIPEFYYLPEMFVN
SNGYNLGVREDEVVVNDVDLPPWAKKPEDFVRINRMALESEFVSCQLHQWIDLIFGYKQR
GPEAVRALNVFHYLTYEGSVNLDSITDPVLREAMEAQIQNFGQTPSQLLIEPHPPRSSAM
HLCFLPQSPLMFKDQMQQDVIMVLKFPSNSPVTHVAANTLPHLTIPAVVTVTCSRLFAVN
RWHNTVGLRGAPGYSLDQAHHLPIEMDPLIANNSGVNKRQITDLVDQSIQINAHCFVVTA
DNRYILICGFWDKSFRVYSTETGKLTQIVFGHWDVVTCLARSESYIGGDCYIVSGSRDAT
LLLWYWSGRHHIIGDNPNSSDYPAPRAVLTGHDHEVVCVSVCAELGLVISGAKEGPCLVH
TITGDLLRALEGPENCLFPRLISVSSEGHCIIYYERGRFSNFSINGKLLAQMEINDSTRA
ILLSSDGQNLVTGGDNGVVEVWQACDFKQLYIYPGCDAGIRAMDLSHDQRTLITGMASGS

IVAFNIDFNRWHYEHQNRY*
>ENSG00000128965|ENST00000617768
MGGAQLELPSGARPGVCVRRSFRAHAGDQPRRPPGPIPVPGTMKQESAAPNTPPTSQSPT
PSAQFPRNDGDPQALWIFGYGSLVWRPDFAYSDSRVGFVRGYSRRFWQGDTFHRGSDKMP
GRVVTLLEDHEGCTWGVAYQVQGEQVSKALKYLNVREAVLGGYDTKEVTFYPQDAPDQPL
KALAYVATPQNPGYLGPAPEEAIATQILACRGFSGHNLEYLLRLADFMQLCGPQAQDEHL
AAIVDAVGTMLPCFCPTEQALALV*
>ENSG00000106692|ENST00000448551
MSRINKNVVLALLTLTSSAFLLFQLYYYKHYLSTKNGAGLSKSKGSRIGFDSTQWRAVKK
FIMLTSNQNVPVFLIDPLILELINKNFEQVKNTSHGSTSQCKFFCVPRDFTAFALQYHLW
KNEEGWFRIAENMGFQCLKIESKDPRLDGIDSLSGTEIPLHYICKLATHAIHLVVFHERS
GNYLWHGHLRLKEHIDRKFVPFRKLQFGRYPGAFDRPELQQVTVDGLEVLIPKDPMHFVE
EVPHSRFIECRYKEARAFFQQYLDDNTVEAVAFRKSAKELLQLAAKTLNKLGVPFWLSSG
TCLGWYRQCNIIPYSKDVDLGIFIQDYKSDIILAFQDAGLPLKHKFGKVEDSLELSFQGK
DDVKLDVFFFYEETDHMWNGGTQAKTGKKFKYLFPKFTLCWTEFVDMKVHVPCETLEYIE
ANYGKNQQGA*
>ENSG00000143801|ENST00000460775
MSSLMLLFLFTYIYLGEVLKTYNVAMDYPTLLLTVWNFGAVGMVCIHWKGPLVLQQAYLI
MISALMALVFIKYLPEWSAWVILGAISVYDLVAVLCPKGPLRMLVETAQERNEPIFPALI
YSSAMVWTVGMAKLDPSSQGALQLP
>ENSG00000148396|ENST00000431893
MPGLDRPLSRQNPHDGVVTPAASPSLPQPGLQMPGQWGPVQGGPQPSGQHRSPCPEGPVP
SGVPCATSVPHFPTPSILHQGPGHEQHSPLVAPPAALPSDGRDEVSHLQSGSHLANNSDP
ESTFRQNPRIVNHWASPELRQNPGVKNEHRPASALVNPLARGDSPENRTHHPLGAGAGSG
CAPLEADSGASGALAMFFQGGETENEENLSSEKAGLSGQADFDDFCSSPGLGRPPAPTHV
GAGSLCQALLPGPSNEAAGDVWGDTASTGVPDASGSQYENVENLEFVQNQEVLPSEPLNL
DPSSPSDQFRYGPLPGPAVPRHGAVCHTGAPDATLHTVHPDSVSSSYSSRSHGRLSGSAR
PQELVGTFIQQEVGKPEDEASGSFFKQIDSSPVGGETDETTVSQNYRGSVSQPSTPSPPK
PTGIFQTSANSSFEPVKSHLVGVKPFEADRANVVGEVRETCVRQKQCRPAAALPDASPGN
LEQPPDNMETLCAPQVCPLPLNSTTEAVHMLPHAGAPPLDTVYPAPEKRPSARTQGPVKC
ESPATTLWAQSELPDFGGNVLLAPAAPALYVCAKPQPPVVQPPEEAMSGQQSRNPSSAAP
VQSRGGIGASENLENPPKMGEEEALQSQASSGYASLLSSPPTESLQNPPVLIAQPDHSYN
LAQPINFSVSLSNSHEKNQSWREALVGDRPAVSSWALGGDSGENTSLSGIPTSSVLSLSL
PSSVAQSNFPQGSGASEMVSNQPANLLVQPPSQPVPENLVPESQKDRKAGSALPGFANSP
AGSTSVVLVPPAHGTLVPDGNKANHSSHQEDTYGALDFTLSRTLENPVNVYNPSHSDSLA
SQQSVASHPRQSGPGAPNLDRFYQQVTKDAQGQPGLERAQQELVPPQQQASPPQLPKAMF
SELSNPESLPAQGQAQNSAQSPASLVLVDAGQQLPPRPPQSSSVSLVSSGSGQAAVPSEQ
PWPQPVPALAPGPPPQDLAAYYYYRPLYDAYQPQYSLPYPPEPGAASLYYQDVYSLYEPR
YRPYDGAASAYAQNYRYPEPERPSSRASHSSERPPPRQGYPEGYYSSKSGWSSQSDYYAS
YYSSQYDYGDPGHWDRYHYSARVRDPRTYDRRYWCDAEYDAYRREHSAFGDRPEKRDNNW
RYDPRFTGSFDDDPDPHRDPYGEEVDRRSVHSEHSARSLHSAHSLASRRSSLSSHSHQSQ
IYRSHNVAAGSYEAPLPPGSFHGDFAYGTYRSNFSSGPGFPEYGYPADTVWPAMEQVSSR
PTSPEKFSVPHVCARFGPGGQLIKVIPNLPSEGQPALVEVHSMEALLQHTSEQEEMRAFP
GPLAKDDTHKVDVINFAQNKAMKCLQNENLIDKESASLLWNFIVLLCRQNGTVVGTDIAE

LLLRDHRTVWLPGKSPNEANLIDFTNEAVEQVEEEESGEAQLSFLTGGPAAAASSLERET
ERFRELLLYGRKKDALESAMKNGLWGHALLLASKMDSRTHARVMTRFANSLPINDPLQTV
YQLMSGRMPAASTCCGDEKWGDWRPHLAMVLSNLNNNMDVESRTMATMGDTLASRGLLDA
AHFCYLMAQAGFGVYTKKTTKLVLIGSNHSLPFLKFATNEAIQRTEAYEYAQSLGAETCP
LPSFQVFKFIYSCRLAEMGLATQAFHYCEAIAKSILTQPHLYSPVLISQLVQMASQLRLF
DPQLKEKPEEESLAAPTWLVHLQQVERQIKEGAGVWHQDGALPQQCPGTPSSEMEQLDRP
GLSQPGALGIANPLLAVPAPSPEHSSPSVRLLPSAPQTLPDGPLASPARVPMFPVPLPPG
PLEPGPGCVTPGPALGFLEPSGPGLPPGVPPLQERRHLLQEARSPDPGIVPQEAPVGNSL
SELSEENFDGKFANLTPSRTVPDSEAPPGWDRADSGPTQPPLSLSPAPETKRPGQAAKKE
TKEPKKGESWFFRWLPGKKKTEAYLPDDKNKSIVWDEKKNQWVNLNEPEEEKKAPPPPPT
SMPKTVQAAPPALPGPPGAPVNMYSRRAAGTRARYVDVLNPSGTQRSEPALAPADFVAPL
APLPIPSNLFVPTPDAEEPQLPDGTGREGPAAARGLANPEPAPEPKLSRCSSMSSLSREV
SQHFNQAPGDLPAAGGPPSGAMPFYNPAQLAQACATSGSSRLGRIGQRKHLVLN*
>ENSG00000181029|ENST00000426877
MEARFTRGKSALLERALARPRTEVSLSAFALLFSELVQHCQSRVFSVAELQSRLAALGRQ
VGARVLDALVAREKGARRETKVLGALLFVKGAVWKALFGKEADKLEQANDDARTFYIIER
EPLINTYISVPKENSTLNCASFTAGIVEAVLTHSGFPAKVTAHWHKGTTLMIKFEEAVIA
RDRALEGR*
>ENSG00000115540|ENST00000409360
MDSTLAVQQYIQQNIRADCSNIDKILEPPEGQDEGVWKYEHLRQFCLELNGLAVKLQSEC
HPDTCTQMTATEQWIFLCAAHKTPKECPAIDYTRHTLDGAACLLNSNKYFPSRVSIKESS
VAKLGSVCRRIYRIFSHAYFHHRQIFDEYENETFLCHRFTKFVMKYNLMSKDNLIVPILE
EEVQNSVSGESEA*
>ENSG00000178234|ENST00000422997
MGSVTVRYFCYGCLFTSATWTVLLFVYFNFSEVTQPLKNVPVKGSGPHGPSPKKFYPRFT
RGPSRVLEPQFKANKIDDVIDSRVEDPEEGHLKFSSELGMIFNERDQELRDLGYQKHAFN
MLISDRLGYHRDVPDTRNAACKEKFYPPDLPAASVVICFYNEAFSALLRTVHSVIDRTPA
HLLHEIILVDDDSDFGKECCTW*
>ENSG00000230675|ENST00000456529
MALQIPGGFWAAAVTVMLVMLSTPVAEARDFPKDFLVQFKGMCYFTNGTERVRGVARYIY
NREEYGRFDSDVGEFQAVTELGRSIEDWNNYKDFLEQERAAVDKVCRHNYEAELRTTLQR
QVEPTVTISPSRTEALNHHNLLVCSVTDFYPAQIKVRWFRNDQEETAGVVSTSLIRNGDW
TFQILVMLEITPQRGDIYTCQVEHPSLQSPITVEWRPRGPPPAGLLH*
>ENSG00000113262|ENST00000517717
MARPRRAREPLLVALLPLAWLAQAGLARAAGSVRLAGGLTLGGLFPVHARGAAGRACGQL
KKEQGVHRLEAMLYALDRVNADPELLPGVRLGARLLDTCSRDTYALEQALSFVQALIRGR
GDGDEVGVRCPGGVPPLRPAPPERVVAVVGASASSVSIMVANVLRLFAIPQISYASTAPE
LSDSTRYDFFSRVVPPDSYQAQAMVDIVRALGWNYVSTLASEGNYGESGVEAFVQISREA
GGVCIAQSIKIPREPKPGEFSKVIRRLMETPNARGIIIFANEDDIRRVLEAARQANLTGH
FLWVGSDSWGAKTSPILSLEDVAVGAITILPKRASIDGFDQYFMTRSLENNRRNIWFAEF
WEENFNCKLTSSGTQSDDSTRKCTGEERIGRDSTYEQEGKVQFVIDAVYAIAHALHSMHQ
ALCPGHTGLCPAMEPTDGRMLLQYIRAVRFNGSAGTPVMFNENGDAPGRYDIFQYQATNG
SASSGGYQAVGQWAETLRLDVEALQWSGDPHEVPSSLCSLPCGPGERKKMVKGVPCCWHC
EACDGYRFQVDEFTCEACPGDMRPTPNHTGCRPTPVVRLSWSSPWAAPPLLLAVLGIVAT

TTVVATFVRYNNTPIVRASGRELSYVLLTGIFLIYAITFLMVAEPGAAVCAARRLFLGLG
TTLSYSALLTKTNRIYRIFEQGKRSVTPPPFISPTSQLVITFSLTSLQVVGMIAWLGARP
PHSVIDYEEQRTVDPEQARGVLKCDMSDLSLIGCLGYSLLLMVTCTVYAIKARGVPETFN
EAKPIGFTMYTTCIIWLAFVPIFFGTAQSAEKIYIQTTTLTVSLSLSASVSLGMLYVPKT
YVILFHPEQNVQKRKRSLKATSTVAAPPKGEDAEAHK*
>ENSG00000100626|ENST00000448469
MRKIRANAIAILTVAWILGTFYYLWQDNRAHAASSGGRGAQRAGRRSEQLREDRTIPLIV
TGTPSKGFDEKAYLSAKQLKAGEDPYRQHAFNQLESDKLSPDRPIRDTRHYSCPSVSYSS
DLPATSVIITFHNEARSTLLRTVKSVLNRTPANLIQEIILVDDFSSDPEDCLLLTRIPKV
KCLRNDRREGLIRSRVRGADVAAATVLTFLDSHCEVNTEWLPPMLQRVKEDHTRVVSPII
DVISLDNFAYLAASADLRGGFDWSLHFKWEQIPLEQKMTRTDPTRPIRTPVIAGGIFVID
KSWFNHLGKYDAQMDIWGGENFELSFRVWMCGGSLEIVPCSRVGHVFRKRHPYNFPEGNA
LTYIRNTKRTAEVWMDEYKQYYYEARPSAIGKAFGSVATRIEQRKKMNCKSFRWYLENVY
PELTVPVKEALPGIIKQGVNCLESQGQNTAGDFLLGMGICRGSAKNPQPAQAWLFSDHLI
QQQGKCLAATSTLMSSPGSPVILQMCNPREGKQKWRRKGSFIQHSVSGLCLETKPAQLVT
SKCQADAQAQQWQLLPHT*
>ENSG00000115525|ENST00000638986
MPSEYTYVKLRSDCSRPSLQWYTRAQSKMRRPSLLLKDILKCTLLVFGVWILYILKLNYT
TEECDMKKMHYVDPDHVKRAQKYAQQVLQKECRPKFAKTSMALLFEHRYSVDLLPFVQKA
PKDSEAESKYDPPFGFRKFSSKVQTLLELLPEHDLPEHLKAKTCRRCVVIGSGGILHGLE
LGHTLNQFDVVIRLNSAPVEGYSEHVGNKTTIRMTYPEGAPLSDLEYYSNDLFVAVLFKS
VDFNWLQAMVKKETLPFWVRLFFWKQVAEKIPLQPKHFRILNPVIIKETAFDILQYSEPQ
SRFWGRDKNVPTIGVIAVVLATHLCDEVSLAGFGYDLNQPRTPLHYFDSQCMAAMNFQTM
HNVTTETKFLLKLVKEGVVKDLSGGIDREF*
>ENSG00000116299|ENST00000529753
MAEPGHSHHLSARVRGRTERRIPRLWRLLLWAGTAFQVTQGTGPELHACKESEYHYEYTA
CDSTGSRWRVAVPHTPGLCTSLPDPIKGTECSFSCNAGEFLDMKDQSCKPCAEGRYSLGT
GIRFDEWDELPHGFASLSANMELDDSAAESTGNCTSSKWVPRGDYIASNTDECTATLMYA
VNLKQSGTVNFEYYYPDSSIIFEFFVQNDQCQPNADDSRWMKTTEKGWEFHSVELNRGNN
VLYWRTTAFSVWTKVPKPVLTQLMYKWAKPKICSEDLEGAVKLPASGVKTHCPPCNPGFF
KTNNSTCQPCPYGSYSNGSDCTRCPAGTEPAVGFEYKWWNTLPTNMETTVLSGINFEYKG
MTGWEVAGDHIYTAAGASDNDFMILTLVVPGFRPPQSVMADTENKEVARITFVFETLCSV
NCELYFMVGVNSRTNTPVETWKGSKGKQSYTYIIEENTTTSFTWAFQRTTFHEASRKYTN
DVAKIYSINVTNVMNGVASYCRPCALEASDVGSSCTSCPAGYYIDRDSGTCHSCPTNTIL
KAHQPYGVQACVPCGPGTKNNKIHSLCYNDCTFSRNTPTRTFNYNFSALANTVTLAGGPS
FTSKGLKYFHHFTLSLCGNQGRKMSVCTDNVTDLRIPEGESGFSKSITAYVCQAVIIPPE
VTGYKAGVSSQPVSLADRLIGVTTDMTLDGITSPAELFHLESLGIPDVIFFYRSNDVTQS
CSSGRSTTIRVRCSPQKTVPGSLLLPGTCSDGTCDGCNFHFLWESAAACPLCSVADYHAI
VSSCVAGIQKTTYVWREPKLCSGGISLPEQRVTICKTIDFWLKVGISAGTCTAILLTVLT
CYFWKKNQKLEYKYSKLVMNATLKDCDLPAADSCAIMEGEDVEDDLIFTSKKSLFGKIKS
FTSKRTPDGFDSVPLKTSSGGLDMDL*
>ENSG00000144730|ENST00000463523
MESQPFLNMKFETDYFVKVVPFPSIKNESNYHPFFFRTRACDLLLQPDNLACKPFWKPRN
LNISQHGSDMQVSFDHAPHNFGFRFFYLHYKLKHEGPFKRKTCKQEQTTETTSCLLQNVS

PGDYIIELVDDTNTTRKVMHYALKPVHSPWAGPIRAVAITVPLVVISAFATLFTVMCRKK
QQENIYSHLDEESSESSTYTAALPRERLRPRPKVFLCYSSKDGQNHMNVVQCFAYFLQDF
CGCEVALDLWEDFSLCREGQREWVIQKIHESQFIIVVCSKGMKYFVDKKNYKHKGGGRGS
GKGELFLVAVSAIAEKLRQAKQSSSAALSKFIAVYFDYSCEGDVPGILDLSTKYRLMDNL
PQLCSHLHSRDHGLQEPGQHTRQGSRRNYFRSKSGRSLYVAICNMHQFIDEEPDWFEKQF
VPFHPPPLRYREPVLEKFDSGLVLNDVMCKPGPESDFCLKVEAAVLGATGPADSQHESQH
GGLDQDGEARPALDGSAALQPLLHTVKAGSPSDMPRDSGIYDSSVPSSELSLPLMEGLST
DQTETSSLTESVSSSSGLGEEEPPALPSKLLSSGSCKADLGCRSYTDELHAVAPL*
>ENSG00000184014|ENST00000530044
MSGGGGGGGSAPSRFADYFVICGLDTETGLEPDELSALCQYIQASKARDGASPFISSTTE
GENFEQTPLRRTFKSKVLARYPENVEWNPFDQDAVGMLCMPKGLAFKTQADPREPQFHAF
IITREDGSRTFGFALTFYEEVTSKQICSAMQTLYHMHNAEYDVLHAPPADDRDQSSMEDG
EDTPVTKLQRFNSYDISRDTLYVSKCICLITPMSFMKACRSVLEQLHQAVTSPQPPPLPL
ESYIYNVLYEVPLPPPGRSLKFSGVYGPIICQRPSTNELPLFDFPVKEVFELLGVENVFQ
LFTCALLEFQILLYSQHYQRLMTVAETITALMFPFQWQHVYVPILPASLLHFLDAPVPYL
MGLHSNGLDDRSKLELPQEANLCFVDIDNHFIELPEDLPQFPNKLEFVQEVSEILMAFGI
PPEGNLHCSESASKLKRLRASELVSDKRNGNIAGSPLHSYELLKENETIARLQALVKRTG
VSLEKLEVREDPSSNKDLKVQCDEEELRIYQLNIQIREVFANRFTQMFADYEVFVIQPSQ
DKESWFTNREQMQNFDKASFLSDQPEPYLPFLSRFLETQMFASFIDNKIMCHDDDDKDPV
LRVFDSRVDKIRLLNVRTPTLRTSMYQKCTTVDEAEKAIELRLAKIDHTAIHPHLLDMKI
GQGKYEPGFFPKLQSDVLSTGPASNKWTKRNAPAQWRRKDRQKQHTEHLRLDNDQREKYI
QEARTMGSTIRQPKLSNLSPSVIAQTNWKFVEGLLKECRNKTKRMLVEKMGREAVELGHG
EVNITGVEENTLIASLCDLLERIWSHGLQVKQGKSALWSHLLHYQDNRQRKLTSGSLSTS
GILLDSERRKSDASSLMPPLRISLIQDMRHIQNIGEIKTDVGKARAWVRLSMEKKLLSRH
LKQLLSDHELTKKLYKRYAFLRCDDEKEQFLYHLLSFNAVDYFCFTNVFTTILIPYHILI
VPSKKLGGSMFTANPWICISGELGETQIMQIPRNVLEMTFECQNLGKLTTVQIGHDNSGL
YAKWLVEYVMVRNEITGHTYKFPCGRWLGKGMDDGSLERILVGELLTSQPEVDERPCRTP
PLQQSPSVIRRLVTISPNNKPKLNTGQIQESIGEAVNGIVKHFHKPEKERGSLTLLLCGE
CGLVSALEQAFQHGFKSPRLFKNVFIWDFLEKAQTYYETLEKNEVVPEENWHTRARNFCR
FVTAINNTPRNIGKDEITSYTTGLPCWLTAPSLHTCMRMWH*
>ENSG00000101638|ENST00000538168
MRYADPSANRDLLGSRTLLFIFICAFALVTLLQQILYGRNYIKRGLQFGWQSGDQLTNWT
GLFNVTDDPAVQSGSDSSSRYFEFYEGPFEYNSTRCLELRHEILEVKVLSMVKQSELFDR
WKSLQMCKWAMNISEANQFKSTLSRCCNAPAFLFTTQKNTPLGTKLKYEVDTSGIYHINQ
EIFRMFPKDMPYYRSQFKKCAVVGNGGILKNSRCGREINSADFVFRCNLPPISEKYTMDV
GVKTDVVTVNPSIITERFHKLEKWRRPFYRVLQVYENASVLLPAFYNTRNTDVSIRVKYV
LDDFESPQAVYYFHPQYLVNVSRYWLSLGVRAKRISTGLILVTAALELCEEVHLFGFWAF
PMNPSGLYITHHYYDNVKPRPGFHAMPSEIFNFLHLHSRGILRVHTGTCSCC*
>ENSG00000228299|ENST00000420206
MRVMAPRTLILLLSGALALTETWACSHSMRYFDTAVSRPGRGEPRFISVGYVDDTQFVRF
DSDAASPRGEPRAPWVEQEGPEYWDRETQKYKRQAQADRVNLRKLRGYYNQSEDGSHTLQ
WMYGCDLGPDGRLLRGYDQSAYDGKDYIALNEDLRSWTAADTAAQITQRKWEAAREAEQW
RAYLEGTCVEWLRRYLENGKETLQRAEHPKTHVTHHPVSDHEATLRCWALGFYPAEITLT
WQRDGEDQTQDTELVETRPAGDGTFQKWAAVVVPSGEEQRYTCHVQHEGLPEPLTLRWEP

SSQPTIPIVGIVAGLAVLAVLAVLGAVMAVVMCRRKSSGGKGGSCSQAASSNSAQGSDES
LIACKA*
>ENSG00000265808|ENST00000578049
MVLLTMIARVADGLPLAASMQEDEQSGRDLQQYQSQAKQLFRKLNEQSPTRCTLEAGAMT
FHYIIEQGVCYLVLCEAAFPKKLAFAYLEDLHSEFDEQHGKKVPTVSRPYSFIEFDTFIQ
KTKKLYIDSRARRNLGSINTELQDVQRIMVANIEEVLQRGEALSALDSKANNLSSLSKKY
RQDAKYLNMRSTYAKLAAVAVFFIMLIVYVRFWWL*
>ENSG00000115526|ENST00000448989
MQRKAGKKEHVQLRDKRNQEPQFRGNTARKCEPQAAEGRVSEWLRCDNMHHQWLLLAACF
WVIFMFMVASKFITLTFKDPDVYSAKQEFLFLTTMPEVRKLPEEKHIPEELKPTGKELPD
SQLVQPLVYMERLELIRNVCRD
>ENSG00000006125|ENST00000618940
MTDSKYFTTNKKGEIFELKAELNNEKKEKRKEAVKKVIAAMTVGKDVSSLFPDVVNCMQT
DNLELKKLVYLYLMNYAKSQPDMAIMAVNSFVKDCEDPNPLIRALAVRTMGCIRVDKITE
YLCEPLRKCLKDEDPYVRKTAAVCVAKLHDINAQMVEDQGFLDSLRDLIADSNPMVVANA
VAALSEISESHPNSNLLDLNPQNINKLLTALNECTEWGQIFILDCLSNYNPKDDREAQSI
CERVTPRLSHANSAVVLSAVKVLMKFLELLPKDSDYYNMLLKKLAPPLVTLLSGEPEVQY
VALRNINLIVQKRPEILKQEIKVFFVKYNDPIYVKLEKLDIMIRLASQANIAQVLAELKE
YATEVDVDFVRKAVRAIGRCAIKVEQSAERCVSTLLDLIQTKVNYVVQEAIVVIRDIFRK
YPNKYESIIATLCENLDSLDEPDARAAMIWIVGEYAERIDNADELLESFLEGFHDESTQV
QLTLLTAIVKLFLKKPSETQELVQQVLSLATQDSDNPDLRDRGYIYWRLLSTDPVTAKEV
VLSEKPLISEETDLIEPTLLDELICHIGSLASVYHKPPNAFVEGSHGIHRKHLPIHHGST
DAGDSPVGTTTATNLEQPQVIPSQGDLLGDLLNLDLGPPVNVPQVSSMQMGAVDLLGGGL
DSLLGSDLGGGIGGSPAVGQSFIPSSVPATFAPSPTPAVVSSGLNDLFELSTGIGMAPGG
YVAPKAVWLPAVKAKGLEISGTFTHRQGHIYMEMNFTNKALQHMTDFAIQFNKNSFGVIP
STPLAIHTPLMPNQSIDVSLPLNTLGPVMKMEPLNNLQVAVKNNIDVFYFSCLIPLNVLF
VEDGKMERQVFLATWKDIPNENELQFQIKECHLNADTVSSKLQNNNVYTIAKRNVEGQDM
LYQSLKLTNGIWILAELRIQPGNPNYTLSLKCRAPEVSQYIYQVYDSILKN*
>ENSG00000166377|ENST00000589326
CHNVTPVYESRAGVTEETEFAEADQDFSDENRTYQASSPDEVALVQWTESVGLTLVSRDL
TSMQLKTPSGQVLSFCILQLFPFTSESKRMGVIVRDESTAEITFYMKGADVAMSPIVQYN
DWLEEERSLFQFLNRRREETQLLDA*
>ENSG00000189067|ENST00000413364
MSVPGPYQAATGPSSAPSAPPSYEETVAVNSYYPTPPAPMPGPTTGLVTGPDGKGMNPPS
YYTQPAPIPNNNPITVQTVYVQHPITFLDRPIQMCCPSCNKMIVSQLSYNAGALTWLSCG
SLCLLGQECSGTIVALRSFDLLGSCNPPSSAS*
>ENSG00000147642|ENST00000532779
MGPFWARKDDTGCPSSQSVSPVKTPSDAGNSPIGFCPGSDEGFTRKKCTIGMVGEGSIQS
SRYKKESKSGLVKPGSEADFSSSSSTGSISAPEVHMSTAGSKRSSSSRNRGPHGRSNGAS
SHKPGSSPSSPREKDLLSMLCRNQLSPVNIHPSYAPSSPSSSNSGSYKGSDCSPIMRRSG
RYMSCGENHGVRPPNPEQYLTPLQQKEVTVRHLKTKLKESERRLHERESEIVELKSQLAR
MREDWIEEECHRVEAQLALKEARKEIKQLKQVIETMRSSLADKDKGIQKYFVDINIQNKK
LESLLQSMEMAHSGSLRDELCLDFPCDSPEKSLTLNPPLDTMADGLSLEEQVTGEGADRE
LLVGDSIANSTDLFDEIVTATTTESGDLELVHSTPGANVLELLPIVMGQEEGSVVVERAV

QTDVVPYSPAISELIQSVLQKLQDPCPSSLASPDESEPDSMESFPESLSALVVDLTPRNP
NSAILLSPVETPYANVDAEVHANRLMRELDFAACVEERLDGVIPLARGGVVRQYWSSSFL
VDLLAVAAPVVPTVLWAFSTQRGGTDPVYNIGALLRGCCVVALHSLRRTAFRIKT*
>ENSG00000100280|ENST00000432560
MTDSKYFTTTKKGEIFELKAELNSDKKEKKKEAVKKVIASMTVGKDVSALFPDVVNCMQT
DNLELKKLVYLYLMNYAKSQPDMAIMAVNTFVKDCEDPNPLIRALAVRTMGCIRVDKITE
YLCEPLRKCLKDEDPYVRKTAAVCVAKLHDINAQLVEDQGFLDTLKDLISDSNPMVVANA
VAALSEIAESHPSSNLLDLNPQSINKLLTALNECTEWGQIFILDCLANYMPKDDREAQSI
CERVTPRLSHANSAVVLSAVKVLMKFMEMLSKDLDYYGTLLKKLAPPLVTLLSAEPELQY
VALRNINLIVQKRPEILKHEMKVFFVKYNDPIYVKLEKLDIMIRLASQANIAQVLAELKE
YATEVDVDFVRKAVRAIGRCAIKVEQSAERCVSTLLDLIQTKVNYVVQEAIVVIKDIFRK
YPNKYESVIATLCENLDSLDEPEARAAMIWIVGEYAERIDNADELLESFLEGFHDESTQV
QLQLLTAIVKLFLKKPTETQELVQQVLSLATQDSDNPDLRDRGYIYWRLLSTDPVAAKEV
VLAEKPLISEETDLIEPTLLDELICYIGTLASVYHKPPSAFVEGGRGVVHKSLPPRTASS
ESAESPETAPTGAPPGEQPDVIPAQGDLLGDLLNLDLGPPVSGPPLATSSVQMGAVDLLG
GGLDSLIGGTNFVAPPTAAVPANLGAPIGSGLSDLFDLTSGVGTLSGSYVAPKAVWLPAM
KAKGLEISGTFTRQVGSISMDLQLTNKALQVMTDFAIQFNRNSFGLAPAAPLQVHAPLSP
NQTVEISLPLSTVGSVMKMEPLNNLQVAVKNNIDVFYFSTLYPLHILFVEDGKMDRQMFL
ATWKDIPNENEAQFQIRDCPLNAEAASSKLQSSNIFTVAKRNVEGQDMLYQSLKLTNGIW
VLAELRIQPGNPSCTLSLKCRAPEVSQHVYQAYETILKN*
>ENSG00000198794|ENST00000562212
MAEKVNNFPPLPKFIPLKPCFYQDFEADIPPQHVSMTKRLYYLWMLNSVTLAVNLVGCLA
WLIGGGGATNFGLAFLWLILFTPCSYVCWFRPIYKAFKTDSSFSFMAFFFTFMAQLVISI
IQAVGIPGWGVCPTLASSCSGWIATISFFGTNIGSAVVMLIPTVMFTVMAVFSFIALSMV
HKFYRGSGGSFSKAQEEWTTGAWKNPHVQQAAQNAAMGAAQGAMNQPQTQYSATPNYTYS
NEM*
>ENSG00000262179|ENST00000573382
MPTPLLPLLLRLLLSCLLLPAARLARQYLLPLLRRLARRLGSQDMREALLGCLLFILSQR
HSPDAGEASRVDRLERRERLGPQK*
>ENSG00000117620|ENST00000638336
MFANLKYVSLGILVFQTTSLVLTMRYSRTLKEEGPRYLSSTAVVVAELLKIMACILLVYK
DSKCSLRALNRVLHDEILNKPMETLKLAIPSGIYTLQNNLLYVALSNLDAATYQVTYQLK
ILTTALFSVSMLSKKLGVYQWLSLVILMTGVAFVQWPSDSQLDSKELSAGSQFVGLMAVL
TACFSSGFAGVYFEKILKETKQSVWIRNIQLVSFSLEPSL*
>ENSG00000085365|ENST00000614488
MSDFDSNPFADPDLNNPFKDPSVTQVTRNVPPGLDEYNPFSDSRTPPPGGVKMPNVPNTQ
PAIMKPTEEHPAYTQIAKEHALAQAELLKRQEELERKAAELDRREREMQNLSQHGRKNNW
PPLPSNFPVGPCFYQDFSVDIPVEFQKTVKLMYYLWM*
>ENSG00000196459|ENST00000458511
MSSWKQDRSGLRSTELNVLEYQPLCAVRSHILKTMSGSFYFVIVGHHDNPVFEMEFLPAG
KAESKDDHRHLNQFIAHAALDLVDENMWLSNNMYLKTVDKFNEWFVSAFVTAGHMRFIML
HDIRQEDGIKNFFTDVYDLYIKFSMNPFYEPNSPIRSSAFDRKVQFLGKKHLLS*
>ENSG00000175582|ENST00000541588
MSTGGDFGNPLRKFKLVFLGEQSVGKTSLITRFMYDSFDNTYQATIGIDFLSKTMYLEDR

TLFRRVAAALPGMESTQDRSREDMIDIKLEKPQEQPVSEGGCSC*
>ENSG00000138653|ENST00000504854
MWSHMQPHLFHNESSLVEQMILNKEFALEHGIPINMGYAVAPHHSGVYPVHIQLYAAWKK
VWGIQVTSTEEYPHLKPARYRKGFIHNSIMVLPRQTCGLFTHTIFYKEYPGGPQELDKSI
RGGELFLTILLNPISIFMTHLSNYGNDRLGLYTFVNLVNFVQSWTNLKLQTLPPVQLAHQ
YFELFPEQKDPLWQNPCDDKRHKDIWSREKTCDHLPKFLVIGPQKTGTTALYLFLLMHPS
IISNLPSPKTFEEVQFFNGNNYHKGIDWYMDFFPTPSNTTSDFLFEKSANYFHSEEAPRR
AASLVPKAKIITILIDPSDRAYSWYQVGVQWRDLGSLQPSPPRFMPFSCLSLLSSWDYST
NDHMKIQLL*
>ENSG00000226182|ENST00000420157
MLSRNDVKRLERSWAQGSSEQELHYASLQRLPVPSSEGPDLRGRDKRGTKEDPRADYACI
AENKPT*
>ENSG00000110075|ENST00000524845
MFWKFDLHSSSHIDTLLEREDVTLKELMDEEDVLQECKAQNRKLIEFLLKAECLEDLVSF
IIEEPPQDMDEKIRYKYPNISCELLTSDVSQMNDRLGEDESLLMKLYSFLLNDSPLNPLL
ASFFSKVLSILISRKPEQIVDFLKKKHDFVDLIIKHIGTSAIMDLLLRLLTCIEPPQPRQ
DVLNWLNEEKIIQRLVEIVHPSQEEDRHSNASQSLCEIVRLSRDQMLQIQNSTEPDPLLA
TLEKQEIIEQLLSNIFHKEKNESAIVSAIQILLTLLETRRPTFEGHIEICPPGMSHSACS
VNKSVLEAIRGRLGSFHELLLEPPKKSVMKTTWGVLDPPVGNTRLNVIRLISSLLQTNTS
SINGDLMELNSIGVILNMFFKYTWNNFLHTQVEICIALILASPFENTENATITDQDSTGD
NLLLKHLFQKCQLIERILEAWEMNEKKQAEGGRRHGYMGHLTRIANCIVHSTDKGPNSAL
VQQLIKDLPDEVRERWETFCTSSLGETNKRNTVDLAFSDYQMQQMTSNFIDQFGFNDEKF
ADQDDIGNVSFDRVSDINFTLNTNESGNIALFEACCKERIQQFDDGGSDEEDIWEEKHIA
FTPESQRRSSSGSTDSEESTDSEEEDGAKQDLFEPSSANTEDKMEVDLSEPPNWSANFDV
PMETTHGAPLDSVGSDVWSTEEPMPTKETGWASFSEFTSSLSTKDSLRSNSPVEMETSTE
PMDPLTPSAAALAVQPEAAGSVAMEASSDGEEDAESTDKVTETVMNGGMKETLSLTVDAK
TETAVFKSEEGKLSTSQDAACKDAEECPETAEAKCAAPRPPSSSPEQRTGQPSAPGDTSV
NGPV*
>ENSG00000102471|ENST00000612570
MARRRSQRVCASGPSMLNSARGAPELLRGTATNAEVSAAAAGATGSEELPPGDRGCRNGG
GRGPAATTSSTGVAVGAEHGEDSLSRKPDPEPGRMDHHQPGTGRYQVLLNEEDNSESSAI
EQPPTSNPAPQIVQAASSAPALETDSSPPPYSSITVEVPTTSDTEVYGEFYPVPPPYSVA
TSLPTYDEAEKAKAAAMAAAAAETSQRIQEEECPPRDDFSDADQLRVGNDGIFMLAFFMA
FIFNWLGFCLSFCITNTIAGRYGAICGFGLSLIKWILIVRFSDYFTGYFNGQYWLWWIFL
VLGLLLFFRGFVNYLKVRNMSESMAAAHRTRYFFLL*
>ENSG00000105443|ENST00000452733
MEDGVYEPPDLTPEERMELENIRRRKQELLVEIQRLREELSEAMSEVEGLEANEGSKTLQ
RNRKMAMGRKKFNMDPKKGIQFLVENELLQNTPEEIARFLYKGEGLNKTAIGDYLGEREE
LNLAVLHAFVDLHEFTDLNLVQALRQFLWSFRLPGEAQKIDRMMEAFAQRYCLCNPGVFQ
STDTCYVLSFAVIMLNTSLHNPNVRDKPGLERFVAMNRGINEGGDLPEELLRNLYDSIRN
EPFKIPEDDGNDLTHTFFNPDREGWLLKLGGRVKTWKRRWFILTDNCLYYFEYTTDKEPR
GIIPLENLSIREVDDPRKPNCFELYIPNNKGQLIKACKTEADGRVVEGNHMVYRISAPTQ
EEKDEWIKSIQAAVSVDPFYEMLAARKKRISVKKKQEQP*
>ENSG00000109861|ENST00000529974

MGAGPSLLLAALLLLLSGDGAVRCDTPANCTYLDLLGTWVFQVGSSGSQRDVNCSVMGPQ
EKKVVVYLQKLDTAYDDLGNSGHFTIIYNQGFEIVLNDYKWFAFFKDVTDFISHLFMQLG
TVGIYDLPHLRNKLAMNRRWG*
>ENSG00000168434|ENST00000561854
XNLEPFVTQEDSALELALHAGKLPFPPEQGDELPELDNMADNWLGSIARATMQTYCDAIL
QIPELSPHSAKQLATDIGSPGPAPEILT*
>ENSG00000141429|ENST00000591924
MRKFAYCKVVLATSLIWVLLDMFLLLYFSECNKCDEKKERGLPAGDVLEPVQKPHEGPGE
MGKPVVIPKEDQEKMKEMFKINQFNLMASEMIALNRSLPDVRLEG
>ENSG00000137497|ENST00000620566
MTLHATRGAALLSWVNSLHVADPVEAVLQLQDCSIFIKIIDRIHGTEEGQQILKQPVSER
LDFVCSFLQKNRKHPSSPECLVSAQKVLEGSELELAKMTMLLLYHSTMSSKSPRDWEQFE
YKIQAELAVILKFVLDHEDGLNLNEDLENFLQKAPVPSTCSSTFPEELSPPSHQAKREIR
FLELQKVASSSSGNNFLSGSPASPMGDILQTPQFQMRRLKKQLADERSNRDELELELAEN
RKLLTEKDAQIAMMQQRIDRLALLNEKQAASPLEPKELEELRDKNESLTMRLHETLKQCQ
DLKTEKSQMDRKINQLSEENGDLSFKLREFASHLQQLQDALNELTEEHSKATQEWLEKQA
QLEKELSAALQDKKCLEEKNEILQGKLSQLEEHLSQLQDNPPQEKGEVLGDVLQLETLKQ
EAATLAANNTQLQARVEMLETERGQQEAKLLAERGHFEEEKQQLSSLITDLQSSISNLSQ
AKEELEQASQAHGARLTAQVASLTSELTTLNATIQQQDQELAGLKQQAKEKQAQLAQTLQ
QQEQASQGLRHQVEQLSSSLKQKEQQLKEVAEKQEATRQDHAQQLATAAEEREASLRERD
AALKQLEALEKEKAAKLEILQQQLQVANEARDSAQTSVTQAQREKAELSRKVEELQACVE
TARQEQHEAQAQVAELELQLRSEQQKATEKERVAQEKDQLQEQLQALKESLKVTKGSLEE
EKRRAADALEEQQRCISELKAETRSLVEQHKRERKELEEERAGRKGLEARLQQLGEAHQA
ETEVLRRELAEAMAAQHTAESECEQLVKEVAAWRERYEDSQQEEAQYGAMFQEQLMTLKE
ECEKARQELQEAKEKVAGIESHSELQISRQQNELAELHANLARALQQVQEKEVRAQKLAD
DLSTLQEKMAATSKEVARLETLVRKAGEQQETASRELVKEPARAGDRQPEWLEEQQGRQF
CSTQAALQAMEREAEQMGNELERLRAALMESQGQQQEERGQQEREVARLTQERGRAQADL
ALEKAARAELEMRLQNALNEQRVEFATLQEALAHALTEKEGKDQELAKLRGLEAAQIKEL
EELRQTVKQLKEQLAKKEKEHASGSGAQSEAAGRTEPTGPKLEALRAEVSKLEQQCQKQQ
EQADSLERSLEAERASRAERDSALETLQGQLEEKAQELGHSQSALASAQRELAAFRTKVQ
DHSKAEDEWKAQVARGRQEAERKNSLISSLEEEVSILNRQVLEKEGESKELKRLVMAESE
KSQKLEERLRLLQAETASNSARAAERSSALREEVQSLREEAEKQRVASENLRQELTSQAE
RAEELGQELKAWQEKFFQKEQALSTLQLEHTSTQALVSELLPAKHLCQQLQAEQAAAEKR
HREELEQSKQAAGGLRAELLRAQRELGELIPLRQKVAEQERTAQQLRAEKASYAEQLSML
KKAHGLLAEENRGLGERANLGRQFLEVELDQAREKYVQELAAVRADAETRLAEVQREAQS
TARELEVMTAKYEGAKVKVLEERQRFQEERQKLTAQVEELSKKLADSDQASKVQQQKLKA
VQAQGGESQQEAQRLQAQLNELQAQLSQKEQAAEHYKLQMEKAKTHYDAKKQQNQELQEQ
LRSLEQLQKENKELRAEAERLGHELQQAGLKTKEAEQTCRHLTAQVRSLEAQVAHADQQL
RDLGKFQVATDALKSREPQAKPQLDLSIDSLDLSCEEGTPLSITSKLPRTQPDGTSVPGE
PASPISQRLPPKVESLESLYFTPIPARSQAPLESSLDSLGDVFLDSGRKTRSARRRTTQI
INITMTKKLDVEEPDSANSSFYSTRSAPASQASLRATSSTQSLARLGSPDYGNSALLSLP
GYRPTTRSSARRSQAGVSSGAPPGRNSFYMGTCQDEPEQLDDWNRIAELQQRNRVCPPHL
KTCYPLESRPSLSLGTITDEEMKTGDPQETLRRASMQPIQIAEGTGITTRQQRKRVSLEP
HQGPGTPESKKATSCFPRPMTPRDRHEGRKQSTTEAQKKAAPASTKQADRRQSMAFSILN

TPKKLGNSLLRRGASKKALSKASPNTRSGTRRSPRIATTTASAATAAAIGATPRAKGKAK
H*
>ENSG00000111481|ENST00000552362
MEALILEPSLYTVKAILILDNDGDRLFAKYYDDTYPSVKEQKAFEKNIFNKTHRTDSEIA
LLEGLTVVYKSSIDLYFYVIGSSYENELMLMAVLNCLFDSLSQMLRKNVEKRALLENMEG
LFLAVDEIVDGGVILESDPQQVVHRVLQSAKEQIKWSLLR*
>ENSG00000260371|ENST00000563634
MNFVKEAEEISSNRRMNSLTLNRHTEILEILEIPQLMDTCVRNSYYEEALELAAYVRRLE
RKYSSIPVIQGIVNEVRQSMQLMLSQLIQQLRTNIQLPACLRVIGYLRRMDVFTEAELRV
KFLQARDAWLRSILTAIPNDDPYFHITKTIEASRVH
>ENSG00000115526|ENST00000420858
MHHQWLLLAACFWVIFMFMVASKFITLTFKDPDVYSAKQEFLFLTTMPEVRKLPEEKHIP
EELKPTGKELPDSQLVQPLVYMERLELIRNVCRDDALKNLSHTPVSKFVLDRIFVCDKHK
ILFCQTPKVGNTQ
>ENSG00000104388|ENST00000531289
MAYAYLFKYIIIGDTGVEFGARMITIDGKQIKLQIWDTAGQESFRSITRSYYRGAAGALL
VYDITRRDTFNHLTTWLEDARQHSNSNMVIMLIGNKSDLESRREVKKEEGEAFAREHGLI
FMETSAKTASNVEEAFINTAKEIYEKIQEGVFDINNEANGIKIGPQHAATNATHAGNQGG
QQAGGGCC*
>ENSG00000197249|ENST00000449399
MPSSVSWGILLLAGLCCLVPVSLAEDPQGDAAQKTDTSHHDQDHPTFNKITPNLAEFAFS
LYRQLAHQSNSTNIFFSPVSIATAFAMLSLGTKADTHDEILEGLNFNLTEIPEAQIHEGF
QELLRTLNQPDSQLQLTTGNGLFLSEGLKLVDKFLEDVKKLYHSEAFTVNFGDTEEAKKQ
INDYVEKGTQGKIVDLVKELDRDTVFALVNYIFFKGKWERPFEVKDTEEEDFHVDQVTTV
KVPMMKRLGMFNIQHCKKLSSWVLLMKYLGNATAIFFLPDEGKLQHLENELTHDIITKFL
ENEDRRSASLHLPKLSITGTYDLKSVLGQLGITKVFSNGADLSGVTEEAPLKLSKAVHKA
VLTIDEKGTEAAGAMFLEAIPMSIPPEVKFNKPFVFLMIEQNTKSPLFMGKVVNPTQK*
>ENSG00000262911|ENST00000615701
MNTMYVMMAQILRSHLIKATVIPNRVKMLPYFGIIRNRMMSTHKSKKKIREYYRLLNVEE
GCSADEVRESFHKLAKQYHPDSGSNTADSATFIRIEKAYRKVLSHVIEQTNASQSKGEEE
EDVEKFKYKTPQHRHYLSFEGIGFGTPTQREKHYRQFRADRAAEQVMEYQKQKLQSQYFP
DSVIVKNIRQSKQQKITQAIERLVEDLIQESMAKGDFDNLSGKGKPLKKFSDCSYIDPMT
HNLNRILIDNGYQPEWILKQKEISDTIEQLREAILVSRKKLGNPMTPTEKKQWNHVCEQF
QENIRKLNKRINDFNLIVPILTRQKVHFDAQKEIVRAQKIYETLIKTKEVTDRNPNNLDQ
GEGEKTPEIKKGFLNWMNLWKFIKIRSF*
>ENSG00000003147|ENST00000447326
MSGHKCSYPWDLQDRYAQDKSVVNKMQQKYWETKQAFIKATGKKEDEHVVASDADLDAKL
ELFHSIQRTCLDLSKAIVLYQKRICFLSQEENELGKFLRSQGFQDKTRAGKMMQATGKAL
CFSSQQRLALRNPLCRFHQEVETFRHRAISDTWLTVNRMEQC
>ENSG00000101246|ENST00000614942
MYTLLSGLYKYMFQKDEYCILILGLDNAGKTTFLEQSKTRFNKNYKGMSLSKITTTVGLN
IGTVDVGKARLMFWDLGGQEELQSLWDKYYAECHGVIYVIDSTDEERLAESKQAFDVPLN
P*
>ENSG00000115526|ENST00000409701

MHHQWLLLAACFWVIFMFMVASKFITLTFKDPDVYSAKQEFLFLTTMPEVRKLPEEKHIP
EELKPTGKELPDSQLVQPLVYMERLELIRNVCRDDALKNLSHTPVSKFVLDRIFVCDKHK
ILFCQTPKVGNTQWKKVLIVLNGAFSSIEEIPENVVHDHEKNGLPRLSSFSDAEIQKRLK
TYFKFFIVRDPFERLISAFKDKFVHNPRFEPWYRHEIAPGIIRKYRRNRTETRGIQFEDF
VRYLGDPNHRWLDLQFGDHIIHWVTYVELCAPCEIMYSVIGHHETLEDDAPYILKEAGID
HLVSYPTIPPGITVYNRTKVEHYFLGISKRDIRRLYARFEGDFKLFGYQKPDFLLN*
>ENSG00000235915|ENST00000415419
MLSRNDVKRLERSWRLPVPSSEGPDLRGRDKRGTKEDPRADYACIAENKPT*
>ENSG00000182022|ENST00000628426
MRHCINCCIQLLPDGAHKQQVNCQGGPHHGHQACPTCKGENKILFRVDSKQMNLLAVLEV
RTEGNENWGGFLRFKKGKRCSLVFGLIIMTLVMASYILSGAHQELLISSPFHYGGFPSNP
SLMDSENPSDTKEHHHQSSVNNISYMKDYPSIKLIINSITTRIEFTTRQLPDLEDLKKQE
LHMFSVIPNKFLPNSKSPCWYEEFSGQNTTDPYLTNSYVLYSKRFRSTFDALRKAFWGHL
AHAHGKHFRLRCLPHFYIIGQPKCGTTDLYDRLRLHPEVKFSAIKEPHWWTRKRFGIVRL
RDGLRDRYPVEDYLDLFDLAAHQIHQGLQASSAKEQSKMNTIIIGEASASTMWDNNAWTF
FYDNSTDGEPPFLTQDFIHAFQPNARLIVMLRDPVERLYSDYLYFASSNKSADDFHEKVT
EALQLFENCMLDYSLRACVYNNTLNNAMPVCTPPPRTPRAGPWQKELVCCYYASGIVGLR
FSIGTERSVLMCKCCSPLFMDVKAEN*
>ENSG00000138674|ENST00000508502
MKLKEVDRTAMQAWSPAQNHPIYLATGTSAQQLDATFSTNASLEIFELDLSDPSLDMKSC
ATFSSSHRYHKLIWGPYKMDSKGDVSGVLIAGGENGNIILYDPSKIIAGDKEVVIAQNDK
HTGPVRALDVNIFQTNLVASGANESEIYIWDLNNFATPMTPGAKTQPPEDISCIAWNRQV
QHILASASPSGRATVWDLRKNEPIIKVSDHSNRMHCSGLAWHPDVATQMVLASEDDRLPV
IQMWDLRFASSPLRVLENHARGILAIAWSMADPELLLSCGKDAKILCSNPNTGEVLYELP
TNTQWCFDIQWCPRNPAVLSAASFDGRISVYSIMGGSTDGLRQKQVDKLSSSFGNLDPFG
TGQPLPPLQIPQQTAQHSIVLPLKKPPKWIRRPVGASFSFGGKLVTFENVRMPSHQGAEQ
QQQQHHVFISQVVTEKEFLSRSDQLQQAVQSQGFINYCQKKIDASQTEFEKNVWSFLKVN
FEDDSRGKYLELLGYRKEDLGKKIALALNKVDGANVALKDSDQVAQSDGEESPAAEEQLL
GEHIKEEKEESEFLPSSGGTFNISVSGDIDGLITQALLTGNFESAVDLCLHDNRMADAII
LAIAGGQELLARTQKKYFAKSQSKITRLITAVVMKNWKEIVESCDLKNWREALAAVLTYA
KPDEFSALCDLLGTRLENEGDSLLQTQACLCYICAGNVEKLVACWTKAQDGSHPLSLQDL
IEKVVILRKAVQLTQAMDTSTVGVLLAAKMSQYANLLAAQGSIAAALAFLPDNTNQPNIM
QLRDRLCRAQGEPVAGHESPKIPYEKQQLPKGRPGPVAGHHQMPRVQTQQYYPHGENPPP
PGFIMHGNVNPNAAGQLPTSPGHMHTQVPPYPQPQPYQPAQPYPFGTGGSAMYRPQQPVA
PPTSNAYPNTPYISSASSYTGQSQLYAAQHQASSPTSSPATSFPPPPSSGASFQHGGPGA
PPSSSAYALPPGTTGPQNGWNDPPALNRVPKKKKMPENFMPPVPITSPIMNPLGDPQSQM
LQQQPSAPVPLSSQSSFPQPHLPGGQPFHGVQQPLGQTGMPPSFSKPNIEGAPGAPIGNT
FQHVQSLPTKKITKKPIPDEHLILKTTFEDLIQRCLSSATDPQTKRKLDDASKRLEFLYD
KLREQTLSPTITSGLHNIARSIETRNYSEGLTMHTHIVSTSNFSETSAFMPVLKVVLTQA
NKLGV*
>ENSG00000109861|ENST00000524463
MGAGPSLLLAALLLLLSGDGAVRCDTPANCTYLDLLGTWVFQVGSSGSQRDVNCSVMGPQ
EKKVVVYLQKLDTAYDDLGNSGHFTIIYNQGFEIVLNDYKWFAFFKDVTDFISHLFMQLG
TVGIYDLPHLRNKLVIK*

>ENSG00000198925|ENST00000409618
MAQFDTEYQRLEASYSDSPPGEEDLLVHVAEGSKSPWHHIENLDLFFSRVYNLHQKNGFT
CMLIGEIFELMQFLFVVAFTTFLVSCVDYDILFANKMVNHSLHPTEPVKVTLPDAFLPAQ
VCSARIQENGSLITILVIAGVFWIHRLIKFIYNICCYWEIHSFYLHALRIPMSALPYCTW
QEVQARIVQTQKEHQICIHKRELTELDIYHRILRFQNYMVALVNKSLLPLRFRLPGLGEA
VFFTRGLKYNFELILFWGPGSLFLNEWSLKAEYKRGGQRLELAQRLSNRILWIGIANFLL
CPLILIWQILYAFFSYAEVLKREPGALGARCWSLYGRCYLRHFNELEHELQSRLNRGYKP
ASKYMNCFLSPLLTLLAKNGAFFAGSILAVLIALTIYDEDVLAVEHVLTTVTLLGVTVTV
CRSFIPDQHMVFCPEQLLRVILAHIHYMPDHWQGNAHRSQTRDEFAQLFQYKAVFILEEL
LSPIVTPLILIFCLRPRALEIIDFFRNFTVEVVGVGDTCSFAQMDVRQHGHPQWLSAGQT
EASVYQQAEDGKTELSLMHFAITNPGWQPPRESTAFLGFLKEQVQRDGAAASLAQGGLLP
ENALFTSIQSLQSESEPLSLIANVVAGSSCRGPPLPRDLQGSRHRAEVASALRSFSPLQP
GQAPTGRAHSTMTGSGVDARTASSGSSVWEGQLQSLVLSEYASTEMSLHALYMHQLHKQQ
AQAEPERHVWHRRESDESGESAPDEGGEGARAPQSIPRSASYPCAAPRPGAPETTALHGG
FQRRYGGITDPGTVPRVPSHFSRLPLGGWAEDGQSASRHPEPVPEEGSEDELPPQVHKV*
>ENSG00000147642|ENST00000533895
MGPLRESKEHRVQHHDKEISRSRIPRLILRPHMPQQQHKVSPASESPFSEEESREFNPSS
SGRSARTVSSNSFCSDDTGCPSSQSVSPVKTPSDAGNSPIGFCPGSDEGFTRKKCTIGMV
GEGSIQSSRYKKESKSGLVKPGSEADFSSSSSTGSISAPEVHMSTAGSKRSSSSRNRGPH
GRSNGASSHKPGSSPSSPREKDLLSMLCRNQLSPVNIHPSYAPSSPSSSNSGSYKGSDCS
PIMRRSGRYMSCGENHGVRPPNPEQYLTPLQQKEVTVRHLKTKLKESERRLHERESEIVE
LKSQLARMREDWIEEECHRVEAQLALKEARKEIKQLKQVIETMRSSLADKDKGIQKYFVD
INIQNKKLESLLQSMEMAHSGSLRDELCLDFPCDSPEKSLTLNPPLDTMADGLSLEEQVT
GEGADRELLVGDSIANSTDLFDEIVTATTTESGDLELVHSTPGANVLELLPIVMGQEEGS
VVVERAVQTDVVPYSPAISELIQSVLQKLQDPCPSSLASPDESEPDSMESFPESLSALVV
DLTPRNPNSAILLSPVETPYANVDAEVHANRLMRELDFAACVEERLDGVIPLARGGVVRQ
YWSSSFLVDLLAVAAPVVPTVLWAFSTQRGGTDPVYNIGALLRGCCVVALHSLRRTAFRI
KT*
>ENSG00000117411|ENST00000434555
MPSTQLLAAAAAAATAPGPTPPPLAPGSLRSPVPCPVPRLPRCHPVLTRHLVLRVQRENP
GVLMGGRYTPPDCTPAQTVAVIIPFRHREHHLRYWLHYLHPILRRQRLRYGVYVINQHGE
DTFNRAKLLNVGFLEALKEDAAYDCFIFSDVDLVPMDDRNLYRCGDQPRHFAIAMDKFGF
RLPYAGYFGGVSGLSKAQFLRINGFPNEYWGWGGEDDDIFNRISLTGMKISRPDIRIGRY
RMIKHDRDKHNEPNPQRFTKIQNTKLTMKRDGIGSVRYQVLEVSRQPLFTNITVDIGRPP
SWPPRG*
>ENSG00000167889|ENST00000428789
MHSFVKHLCSRYVVERQGTMALPALLTRLLPLRRLFVLGIGFFTLCFLMTSLGGQFSARR
LGDSPFTIRTEVMGGPESRGVLRKMSDLLELMVKRMDALARLENSSELHRAGGDLHFPAD
RMPPGAGLMERIQAIAQNVSDIAVKVDQILRHSLLLHSKVSEGRRDQCEAPSDPKFPDCS
GKVEWMRARWTSDPCYAFFGVDGTECSFLIYLSEVEWFCPPLPWRNQTAAQRAPKPLPKV
QAVFRSNLSHLLDLMGSGKESLIFMKKRTKRLTAQWALAAQRLAQKLGATQRDQKQILVH
IGFLTEESGDVFSPRVLKGGPLGEMVQWADILTALYVLGHGLRVTVSLKELQSNLGVPPG
RGSCPLTMPLPFDLIYTDYHGLQQMKRHMGLSFKKYRCRIRVIDTFGTEPAYNHEEYATL
HGYRTNWGYWNLNPKQFMTMFPHTPDNSFMGFVSEELNETEKRLIKGGKASNMAVVYGKE

ASIWKGKEKFLGILNKYMEIHGTVYYESQRPPEVPAFVKNHGLLPQPEFQQLLRKAKLFI
GFGFPYEGPAPLEAIANGCIFLQSRFSPPHSSLNHEFFRGKPTSREVFSQHPYAENFIGK
PHVWTVDYNNSEEFEAAIKAIMRTQVDPYLPYEYTCEGMLERIHAYIQHQDFCRAPDPAL
PEAHAPQSPFVLAPNATHLEWARNTSLAPGAWPPAHALRAWLAVPGRACTDTCLDHGLIC
EPSFFPFLNSQDAFLKLQVPCDSTESEMNHLYPAFAQPGQECYLQKEPLLFSCAGSNTKY
RRLCPCRDFRKGQVALCQGCL*
>ENSG00000135454|ENST00000552350
MWLGRRALCALVLLLACASLGLLYASTRDAPGLRLPLAPWAPPQSPRRPELPDLAPEPRY
AHIPVRIKEQVVGLLAWNNCSCESSGGGLPLPFQKQVRAIDLTKAFDPAELRAASATREQ
EFQAFLSRSQSPADQLLIAPANSPLQYPLQGVEVQPLRSILVPGLSLQAASGQEVYQVNL
TASLGTWDVAGEVTGVTLTGEGQADLTLVSPGLDQLNRQLQLVTYSSRSYQTNTADTGAR
PGWRDGQAGQTEKNQKGWSGQMAEGMGGIWAMARAVQPHNGCFNWTSRARGRKGAFVHLG
LEQARGKPEPWVCLPFRPTVGGPRKRLV*
>ENSG00000206560|ENST00000412318
MAFLKLRDQPSLVQAIFNGDPDEVRALIFKKEDVNFQDNEKRTPLHAAAYLGDAEIIELL
ILSGARVNAKDSKWLTPLHRAVASCSEEAVQVLLKHSADVNARDKNWQTPLHIAAANKAV
KCAEALVPLLSNVNVSDRAGRTALHHAAFSGHGEMVKLLLSRGANINAFDKKDRRAIHWA
AYMGHIEVVKLLVSHGAEVTCKDKKSYTPLHAAASSGMISVVKYLLDLGVDMNEPNAYGN
TPLHVACYNGQDVVVNELIDCGAIVNQKNEKGFTPLHFAAASTHGALCLELLVGNGADVN
MKSKDGKTPLHMTALHGRFSRSQTIIQSGAVIDCEDKNGNTPLHIAARYGHELLINTLIT
SGADTAKRGIHGMFPLHLAALSGFSDCCRKLLSSGFDIDTPDDFGRTCLHAAAAGGNLEC
LNLLLNTGADFNKKDKFGRSPLHYAAANCNYQCLFALVGSGASVNDLDERGCTPLHYAAT
SDTDGKCLEYLLRNDANPGIRDKQGYNAVHYSAAYGHRLCLQLIASETPLDVLMETSGTD
MLSDSDNRATISPLHLAAYHGHHQALEVLVQSLLDLDVRNSSGRTPLDLAAFKGHVECVD
VLINQGASILVKDYILKRTPIHAAATNGHSECLRLLIGNAEPQNAVDIQDGNGQTPLMLS
VLNGHTDCVYSLLNKGANVDAKDKWGRTALHRGAVTGHEECVDALLQHGAKCLLRDSRGR
TPIHLSAACGHIGVLGALLQSAASMDANPATADNHGYTALHWACYNGHETCVELLLEQEV
FQKTEGNAFSPLHCAVINDNEGAAEMLIDTLGASIVNATDSKGRTPLHAAAFTDHVECLQ
LLLSHNAQVNSVDSTGKTPLMMAAENGQTNTVEMLVSSASAELTLQDNSKNTALHLACSK
GHETSALLILEKITDRNLINATNAALQTPLHVAARNGLTMVVQELLGKGASVLAVDENGY
TPALACAPNKDVADCLALILATMMPVSSSSPLSSLTFNAINRYTNTSKTVSFEALPIMRN
EPSSYCSFNNIGGEQEYLYTDVDELNDSDSETY*
>ENSG00000138459|ENST00000494706
MKWSIPAFLYFLDNLIVFYVLSYLQPAMAVIFSNFSIITTALLFRIVLKRRLNWIQWASL
LTLFLSIVALTAGTKTLQHNLAGRGFHHDAFFSPSNSCLLFRTSLKFRNTHLGKKGSEI*
>ENSG00000125975|ENST00000444723
MKRWQIFVLWVFWVLILWLMTPYLDLTPESAPQEKRMYLVPQHCDCPWFSSGKCGCPSET
LNCSSCHHTADEWNWLDACSRKTMGYLMRTRESMTSDTVLWWLGMNSGSELGKLWRKLFK
GIPRLSVSHFDFYCGTCVLLGRPQIPQGSSLGNDIDQYPVVFRNASDQGSWMQLEMLLRK
LSDLVWTSDALSDKILEDGLVP*
>ENSG00000262911|ENST00000573306
MNTMYVMMAQILRSHLIKATVIPNRVKMLPYFGIIRNRMMSTHKSKKKIREYYRLLNVEE
GCSADEVRESFHKLAKQYHPDSGSNTADSATFIRIEKAYRKVLSHVIEQTNASQSKGEEE
EDVEKFKYKTPQHRHYLSFEGIGFGTPTQREKHYRQFRADRAAEQVMEYQKQKLQSQYFP

DSVIVKNIRQSKQQKITQAIERLVEDLIQESMAKGDFDNLSGKGKPLKKFSDCSYIDPMT
HNLNRILIDNGYQPEWILKQKEISDTIEQLREAILVSRKKLGNPMTPTEKKQWNHVCEQF
QENIRKLNKRINDFNLIVPILTRQKVHFDAQKEIVRAQKIYETLIKTKEVTDRNPNNLDQ
GEGEKTPEIKKGFLNWMNLWKFIKIRSF*
>ENSG00000226182|ENST00000422533
MLSRNDDICIYGGLGLGGLLLLAVVLLSACLCWLHRRGPGLLRAGTPLCISAEAASAQQ*
>ENSG00000143801|ENST00000626989
MLAGTVRFARHCLKFFPAQKPACVDFGASRGRAMLTFMASDSEEEVCDERTSLMSAESPT
PRSCQEGRQGPEDGENTAQWRSQENEEDGEEDPDRYVCSGVPGRPPGLEEELTLKYGAKH
VIMLFVPVTLCMIVVVATIKSVRFYTEKNGQLIYTPFTEDTPSVGQRLLNSVLNTLIMIS
VIVVMTIFLVVLYKYRCYKFIHGWLIMSSLMLLFLFTYIYLGEVLKTYNVAMDYPTLLLT
VWNFGAVGMVCIHWKGPLVLQQAYLIMISALMALVFIKYLPEWSAWVILGAISVYDLVAV
LCPKGPLRMLVETAQERNEPIFPALIYSSAMVWTVGMAKLDPSSQGALQLPYDPEMEEDS
YDSFGEPSYPEVFEPPLTGYPGEELEEEEERGVKLGLGDFIFYSVLVGKAAATGSGDWNT
TLACFVAILIGLCLTLLLLAVFKKALPALPISITFGLIFYFSTDNLVRPFMDTLASHQLY
I*
>ENSG00000128965|ENST00000444189
MGGAQLELPSGARPGVCVRRSFRAHAGDQPRRPPGPIPVPGTMKQESAAPNTPPTSQSPT
PSAQFPRNDGDPQALWIFGYGSLVWRPDFAYSDSRVGFVRGYSRRFWQGDTFHRGSDKMP
GRVVTLLEDHEGCTWGVAYQVQGEQNPGYLGPAPEEAIATQILACRGFSGHNLEYLLRLA
DFMQLCGPQAQDEHLAAIVDAVGTMLPCFCPTEQALALV*
>ENSG00000162885|ENST00000612859
MRNWLVLLCPCVLGAALHLWLRLRSPPPACASGAGPADQLALFPQWKSTHYDVVVGVLSA
RNNHELRNVIRSTWMRHLLQHPTLSQRFESGN*
>ENSG00000261949|ENST00000576655
MKSFSRILFLVFLLAGLRSKAAPSAPLPLGCGFPDMAHPSETSPLKGASENSKRDRLNPE
FPGTPYPEPSKLPHTVSLETFPLDFTEPLNPDLRETPHPESPETPKADSLTTSISESLDM
PKTNLSKMAHPESSETPTPGPTEMPHPGSPETPKPNFSKTSRPEFPETPNTDLMQTTPQE
SPEILQLNATEVSQAELPETSNTNPTKTPDPKSPEKHDLNSTETPNSEFLQALHPDPSKT
PHPESHVTHNPSPTEISQTEFPTTYYQNATDVPRTSDPQISTSLYPETPVPFKDDATALN
ELSLNPKPGTPAAIQPDSPKLPTSDSPGMVELKAPQNSGPKESNVPPPSARIAGPPALPG
RPSQLAPATLRAPQRHSRGEGVNTIIVVERVKETGVTLVGRPRGAAGGALCLFFAGTALL
IGIFVLLWCLYRRAARQRPFAHHRLPDDGDEPVLHLDAPKDPYDLYFYAPDTWVPSHIAT
KQPPPTPPLPPKLPPPPRGGRPQRLEALSPATLPNNFV*
>ENSG00000003147|ENST00000446305
MSGHKCYPWDLQDRYAQDKSVVNKMQQKYWETKQAFIKATGKKEDEHVVASDADLDAKLE
LFHSIQRTCL
>ENSG00000282880|ENST00000635453
MRNWLVLLCPCVLGAALHLWLRLRSPPPACASGAGPADQLALFPQWKSTHYDVVVGVLSA
RNNHELRNVIRSTWMRHLLQHPTLSQRVLVKFIIGAHGCEVPVEDREDPYSCKLLNITNP
VLNQEIEAFSLSEDTSSGLPEDRVVSVSFRVLYPIVITSLGVFYDANDVGFQRNITVKLY
QAEQEEALFIARFSPPSCGVQVNKLWYKPVEQFILPESFEGTIVWESQDLHGLVSRNLHK
VTVNDGGGVLRVITAGEGALPHEFLEGVEGVAGGFIYTIQEGDALLHNLHSRPQRLIDHI
RNLHEEDALLKEESSIYDDIVFVDVVDTYRNVPAKLLNFYRWTVETTSFNLLLKTDDDCY

IDLEAVFNRIVQKNLDGPNFWWGNFRLNWAVDRTGKWQELEYPSPAYPAFACGSGYVISK
DIVKWLASNSGRLKTYQGEDVSMGIWMAAIGPKRYQDSLWLCEKTCETGMLSSPQYSPWE
LTELWKLKERCGDPCRCQAR*
>ENSG00000115526|ENST00000418201
MHHQWLLLAACFWVIFMFMVASKFITLTFKDPDVYSAKQEFLFLTTMPEVRKLPEEKHIP
EELK
>ENSG00000003147|ENST00000406470
MSGHKCSYPWDLQDRYAQDKSVVNKMQQKYWETKQAFIKATGKKEDEHVVASDADLDAKL
ELFHSIQRTCLDLSKAIVLYQKRICFLSQEENELGKFLRSQGFQDKTRAGKMMQATGKAL
CFSSQQRLALRNPLCRFHQEVETFRHRAISDTWLTVNRMEQCRTEYRGALLWMKDVSQEL
DPDLYKQMEKFRKVQTQVRLAKKNFDKLKMDVCQKVDLLGASRCNLLSHMLATYQTTLLH
FWEKTSHTMAAIHESFKGYQPYEFTTLKSLQDPMKKLVEKEEKKKINQQESTDAAVQEPS
QLISLEEENQRKESSSFKTEDGKSILSALDKGSTHTACSGPIDELLDMKSEEGACLGPVA
GTPEPEGADKDDLLLLSEIFNASSLEEGEFSKEWAAVFGDGQVKEPVPTMALGEPDPKAQ
TGSGFLPSQLLDQNMKDLQASLQEPAKAASDLTAWFSLFADLDPLSNPDAVGKTDKEHEL
LNA*
>ENSG00000114646|ENST00000456150
MPPAIPEATEASGPPSPTPGDKLSPASELPKESPLEVWLNLGGSTPDPQGPELTYPFQGT
LEPQPASDIIDIDYFEGLDGEGRGADLGSFPGSPGTSENHPDTEGETPSWSLLDLYDDFT
PFDESDFYPTTSFYDDLDEEEEEEEDDKDAVGGGDLEDENELLVPTGKPGLGPGTGQPTS
RWHAVPPQHTLGSVPGSSIALRPRPGEPGRDLASSENGTECRSGFVRHNGSCRSVCDLFP
SYCHNGGQCYLVENIGAFCRCNTQDYIWHKGMRCESIITDFQVMCVAVGSAALVLLLLFM
MTVFFAKKLYLLKTENTKLRRTNKFRTPSELHNDNFSLSTIAEGSHPNDDPSAPHKIQEV
LKSCLKEEESFNIQNSMSPKLEGGKGDQADLDVNCLQNNLT*
>ENSG00000227506|ENST00000549059
MKMQKGNVLLMFGLLLHLEAATNSNETSTSANTGSSVISSGASTATNSGSSVTSSGVSTA
TISGSSVTSNGVSIVTNSEFHTTSSGISTATNSEFSTVSSGISIATNSESSTTSSGASTA
TNSESSTPSSGASTATNSDSSTTSSGASTATNSDSSTTSSEASTATNSESSTTSSGASTA
TNSESSTTSSGASTATNSESSTTSSGASTATNSGSSTTSSGTSTATNSESSTVSSGASTA
TTSESSTTSSGASTATNSESSTVSSGASTATNSESSTTSSGANTATNSGSSVTSAGSGTA
ALTGMHTTSHSASTAVSEAKPGGSLVPWEIFLITLVSVVAAVGLFAGLFFCVRNSLSLRN
TFNTAVYHPHGLNHGLGPGPGGNHGAPHRPRWSPNWFWRRPVSSIAMEMSGRNSGP*
>ENSG00000136213|ENST00000432336
MTKARLFRLWLVLGSVFMILLIIVYWDSAGAAHFYLHTSFSRPHTGPPLPTPGPDRDREL
TADSDVDEFLDKFLSAGVKQSDLPRKETEQPPAPGSMEESVRGYDWSPRDARRSPDQGRQ
QAERRSVLRGFCANSSLAFPTKERAFDDIPNSELSHLIVDDRHGAIYCYVPKVACTNWKR
VMIVLSGSLLHRGAPYRDPLRIPREHVHNASAHLTFNKFWRRYGKLSRHLMKVKLKKYTK
FLFVR
>ENSG00000166405|ENST00000419822
MAYSTVQRVALASGLVLALSLLLPKAFLSRGKRQEPPPTPEGKLGRFPPMMHHHQAPSDG
QTPGARFQRSHLAEAFAKAKGSGGGAGGGGSGRGLMGQIIPIYGFGIFLYILYILFKVSR
IILIILHQ*
>ENSG00000109113|ENST00000415040
MNILAPVRRDRVLAELPQCLRKEAALHGHKDFHPRVTCACQEHRTGTVGFCKDTFDKNYK

ATIGVDFEMERFEVLGIPFSLQLWDTAGQERFKCIASTYYRGAQAIIIVFNLNDVASLEH
TKQWLADALKENDPSSVLLFLVGSKKDLSTPAQYALMEKDALQVAQEMKAEYWAVSSLTG
ENVREFFFRVAALTFEANVLAELEKSGARRIGDVVRINSDDSNLYLTASKKKPTCCP*
>ENSG00000106392|ENST00000436587
MASKSWLNFLTFLCGSAIGFLLCSQLFSILLGEKVDTQPNVLHNDPHARHSDDNGQNHLE
GQMNFNADSSQHKDENTDIAENLYQKVRILCWVMTGPQNLEKKAKHVKATWAQRCNKVLF
MSSEENKDFPAVGLKTKEGRDQLYWKTIKAFQYVHEHYLEDADWFLKADDDTYVILDNLR
WLLSKYDPEEPIYFGRRFKPYVKQGYMSGGAGYVLSKEALKRFVDAFKTDKCTHSSSIED
LALGRCMEIMNVEAGDSRDTIGKETFHPFVPEHHLIKGYLPRTFWYWNYNYYPPVEGPGC
CSDLAVSFHYVDSTTMYELEYLVYHLRPYGYLYRYQPTLPERILKEISQANKNEDTKVKL
GNP*
>ENSG00000100023|ENST00000406385
MGKRQHQKDKMYITCAEYTHFYGGKKPDLPQTNFRRLPFDHCSLSLQPFVYPVCTPDGIV
FDLLNIVPWLKKYGTNPSNGEKLDGRSLIKLNFSKNSEGKYHCPVLFTVFTNNTHIVAVR
TTGNVYAYEAVEQLNIKAKNFRDLLTDEPFSRQDIITLQDPTNLDKFNVSNFYHVKNNMK
IIDPDEEKAKQDPSYYLKNTNAETRETLQELYKEFKGDEILAATMKAPEKKKVDKLNAAH
YSTGKVSASFTSTAMVPETTHEAAAIDEDVLRYQFVKKKGYVRLHTNKGDLNLELHCDLT
PKTCENFIRLCKKHYYDGTIFHRSIRNFVIQGGDPTGTGTGGESYWGKPFKDEFRPNLSH
TGRGILSMANSGPNSNRSQFFITFRSCAYLDKKHTIFGRVVGGFDVLTAMENVESDPKTD
RPKEEIRIDATTVFVDPYEEADAQIAQERKTQLKVAPETKVKSSQPQAGSQGPQTFRQGV
GKYINPAATKRAAEEEPSTSATVPMSKKKPSRGFGDFSSW*
>ENSG00000017260|ENST00000513801
MIPVLTSKKASELPVSEVASILQADLQNGLNKCEVSHRRAFHGWNEFDISEDEPLWKKYI
SQFKNPLIMLLLASAVISVLMHQFDDAVSITVAILIVVTVAFVQEYRSEKSLEELSKLVP
PECHCVREGKLEHTLARDLVPGDTVCLSVGDRVPADLRLFEAVDLSIDESSLTGETTPCS
KVTAPQPAATNGDLASRSNIAFMGTLVRCGKAKGVVIGTGENSEFGEVFKMMQAEEAPKT
PLQKSMDLLGKQLSFYSFGIIGIIMLVGWLLGKDILEMFTISVSLAVAAIPEGLPIVVTV
TLALGVMRMVKKRAIVKKLPIVETLGCCNVICSDKTGTLTKNEMTVTHIFTSDGLHAEVT
GVGYNQFGEVIVDGDVVHGFYNPAVSRIVEAGCVCNDAVIRNNTLMGKPTEGALIALAMK
MGLDGLQQDYIRKAEYPFSSEQKWMAVKCVHRTQQDRPEICFMKGAYEQVIKYCTTYQSK
GQTLTLTQQQRDVYQQEKARMGSAGLRVLALASGPELGQLTFLGLVGIIDPPRTGVKEAV
TTLIASGVSIKMITGDSQETAVAIASRLGLYSKTSQSVSGEEIDAMDVQQLSQIVPKVAV
FYRASPRHKMKIIKSLQKNGSVVAMTGDGVNDAVALKAADIGVAMGQTGTDVCKEAADMI
LVDDDFQTIMSAIEEGKGIYNNIKNFVRFQLSTSIAALTLISLATLMNFPNPLNAMQILW
INIIMDGPPAQSLGVEPVDKDVIRKPPRNWKDSILTKNLILKILVSSIIIVCGTLFVFWR
ELRDNVITPRDTTMTFTCFVFFDMFNALSSRSQTKSVFEIGLCSNRMFCYAVLGSIMGQL
LVIYFPPLQKVFQTESLSILDLLFLLGLTSSVCIVAEIIKKVERSREKIQKHVWLWERSG
QQLVEIHPHLETGLPLTEDVSCV*
>ENSG00000136297|ENST00000404774
MFAPRLLDFQKTKYARFMNHRVPAHKRYQPTEYEHAANCATHAFWIIPSILGSSNLYFLS
DDDWETISAWIYGLGLCGLFVVSTVFHTISWKKSHLRMVEHCLHMFDRMVIYFFIAASYA
PWLNLRELGPWASHMRWLVWIMASVGTIYVFFFHERTGSCVQFLRGEACPKAGTACLPAR
YKLVELLCYVVMGFFPALVILSMPNTEGIWELVTGGVFYCLGMVFFKSDGRIPFAHAIWH
LFVAFGAGTHYYAIWRYLYLPSTLQTKVSK*

>ENSG00000172270|ENST00000545507
MGTANIQLHGPPRVKAVKSSEHINEGETAMLVCKSESVPPVTDWAWYKITDSEDKALMNG
SESRFFVSSSQGRSELHIENLNMEADPGQYRCNGTSSKGSDQAIITLRVRSHLAALWPFL
GIVAEVLVLVTIIFIYEKRRKPEDVLDDDDAGSAPLKSSGQHQNDKGKNVRQRNSS*
>ENSG00000233095|ENST00000423011
MVVMAPRTLFLLLSGALTLTETWAGSHSMRYFSAAVSRPGRGEPRFIAMGYVDDTQFVRF
DSDSACPRMEPRAPWVEQEGPEYWEEETRNTKAHAQTDRMNLQTLRGYYNQSEASSHTLQ
WMIGCDLGSDGRLLRGYEQYAYDGKDYLALNEDLRSWTAADTAAQISKRKCEAANVAEQR
RAYLEGTCVEWLHRYLENGKEMLQRADPPKTHVTHHPVFDYEATLRCWALGFYPAEIILT
WQRDGEDQTQDVELVETRPAGDGTFQKWAAVVVPSGEEQRYTCHVQHEGLPEPLMLRWKQ
SSLPTIPIMGIVAGLVVLAAVVTGAAVAAVLWRKKSSD*
>ENSG00000137497|ENST00000537930
MTLHATRGAALLSWVNSLHVADPVEAVLQLQDCSIFIKIIDRIHGTEEGQQILKQPVSER
LDFVCSFLQKNRKHPSSPECLVSAQKVLEGSELELAKMTMLLLYHSTMSSKSPRDWEQFE
YKIQAELAVILKFVLDHEDGLNLNEDLENFLQKAPVPST
>ENSG00000170043|ENST00000540486
MTVHNLYLFDRNGVCLHYSEWHRKKQAGIPKEEEYKLMYGMLFSIRSFVSKMSPLDMKDG
FLAFQTSRYKLHYYETPTGIKVVMNTDLGVGPIRDVLHHIYSALYVELVVKNPLCPLGQT
VQSELFRSRLDSYVRSLPFFSARAG*
>ENSG00000100280|ENST00000415447
MTDSKYFTTTKKGEIFELKAELNSDKKEKKKEAVKKVIASMTVGKDVSALFPDVVNCMQT
DNLELKKLVYLYLMNYAKSQPDMAIMAVNTFVKDCEDPNPLIRALAVRTMGCIRVDKITE
YLCEPLRKCLKDEDPYVRKTAAVCVAKLHDINAQLVEDQGFLDTLKDLISDSNPMVVANA
VAALSEIAESHPSSNLLDLNPQSINKLLTALNECTEWGQIFILDCLANYMPKDDREAQSI
CERVTPRLSHANSAVVLSAVKVLMKFMEMLSKDLDYYGTLLKKLAPPLVTLLSAEPELQY
VALRNINLIVQKRPEILKHEMKVFFVKYNDPIYVKLEKLDIMIRLASQANIAQVLAELKE
YATEVDVDFVRKAVRAIGRCAIKVEQSAERCVSTLLDLIQTKVNYVVQEAIVVIKDIFRK
YPNKYESVIATLCENLDSLDEPEARAAMIWIVGEYAERIDNADELLESFLEGFHDESTQV
QLQLLTAIVKLFLKKPTETQELVQQVLSLATQDSDNPDLRDRGYIYWRLLSTDPVAAKEV
VLAEKPLISEETDLIEPTLLDELICYIGTLASVYHKPPSAFVEGGRGVVHKSLPPRTASS
ESAESPETAPTGAPPGEQPDVIPAQGDLLGDLLNLDLGPPVSGPPLATSSVQMGAVDLLG
GGLDSLIGGTNFVAPPTAAVPANLGAPIGSGLSDLFDLTSGVGTLSGSYVAPKAVWLPAM
KAKGLEISGTFTRQVGSISMDLQLTNKALQVMTDFAIQFNRNSFGLAPAAPLQVHAPLSP
NQTVEISLPLSTVGSVMKMEPLNNLQVAVKNNIDVFYFSTLYPLHILFVEDGKMDRQMFL
ATWKDIPNENEAQFQIRDCPLNAEAASSKLQSSNIFTVAKRNVEGQDMLYQSLKLTNGIW
VLAELRIQPGNPSCTLSLKCRAPEVSQHVYQAYETILKN*
>ENSG00000166428|ENST00000540372
MPGCPVQMLKPLWKAAVAPTWPCSMPPRRPWDREAGTLQVLGALAVLWLGSVALICLLWQ
VPRPPTWGQVQPKDVPRSWEHGSSPAWEPLEAEARQQRDSCQLVLVESIPQDLPSAAGSP
SAQPLGQAWLQLLDTAQESVHVASYYWSLTGPDIGVNDSSSQLGEALLQKLQQLLGRNIS
LAVATSSPTLARTSTDLQVLAARGAHVRQVPMGRLTRGVLHSKFWVVDGRHIYMGSANMD
WRSLTQVKELGAVIYNCSHLAQDLEKTFQTYWVLGVPKAVLPKTWPQNFSSHFNRFQPFH
GLFDGVPTTAYFSASPPALCPQGRTRDLEALLAVMGSAQEFIYASVMEYFPTTRFSHPPR
YWPVLDNALRAAAFGKGVRVRLLVGCGLNTDPTMFPYLRSLQALSNPAANVSVDVKVFIV

PVGNHSNIPFSRVNHSKFMVTEKAAYIGTSNWSEDYFSSTAGVGLVVTQSPGAQPAGATV
QEQLRQLFERDWSSRYAVGLDGQAPGQDCVWQG*
>ENSG00000276262|ENST00000620290
MAGRSMQAARCPTDELSLTNCAVVNEKDFQSGQHVIVRTSPNHRYTFTLKTHPSVVPGSI
AFSLPQRKWAGLSIGQEIEVSLYTFDKAKQCIGTMTIEIDFLQKKSIDSNPYDTDKMAAE
FIQQFNNQAFSVGQQLVFSFNEKLFGLLVKDIEAMDPSILKGEPATGKRQKIEVGLVVGN
SQVAFEKAENSSLNLIGKAKTKENRQSIINPDWNFEKMGIGGLDKEFSDIFRRAFASRVF
PPEIVEQMGCKHVKGILLYGPPGCGKTLLARQIGKMLNAREPKVVNGPEILNKYVGESEA
NIRKLFADAEEEQRRLGANSGLHIIIFDEIDAICKQRGSMAGSTGVHDTVVNQLLSKIDG
VEQLNNILVIGMTNRPDLIDEALLRPGRLEVKMEIGLPDEKGRLQILHIHTARMRGHQLL
SADVDIKELAVETKNFSGAELEGLVRAAQSTAMNRHIKASTKVEVDMEKAESLQVTRGDF
LASLENDIKPAFGTNQEDYASYIMNGIIKWGDPVTRVLDDGELLVQQTKNSDRTPLVSVL
LEGPPHSGKTALAAKIAEESNFPFIKICSPDKMIGFSETAKCQAMKKIFDDAYKSQLSCV
VVDDIERLLDYVPIGPRFSNLVLQALLVLLKKAPPQGRKLLIIGTTSRKDVLQEMEMLNA
FSTTIHVPNIATGEQLLEALELLGNFKDKERTTIAQQVKGKKVWIGIKKLLMLIEMSLQM
DPEYRVRKFLALLREEGASPLDFD*
>ENSG00000196562|ENST00000467815
MGPPSLVLCLLSATVFSLLGGSSAFLSHHRLKGRFQRDRRNIRPNIILVLTDDQDVELGS
MQVMNKTRRIMEQGGAHFINAFVTTPMCCPSRSSILTGKYVHNHNTYTNNENCSSPSWQA
QHESRTFAVYLNSTGYRTAFFGKYLNEYNGSYVPPGWKEWVGLLKNSRFYNYTLCRNGVK
EKHGSDYSKDYLTDLITNDSVSFFRTSKKMYPHRPVLMVISHAAPHGPEDSAPQYSRLFP
NASQHITPSYNYAPNPDKHWIMRYTGPMKPIHMEFTNMLQRKRLQTLMSVDDSMETIYNM
LVETGELDNTYIVYTADHGYHIGQFGLVKGKSMPYEFDIRVPFYVRGPNVEAGCLNPHIV
LNIDLAPTILDIAGLDIPADMDGKSILKLLDTERPVNRFHLKKKMRVWRDSFLVERGKLL
HKRDNDKVDAQEENFLPKYQRVKDLCQRAEYQTACEQLGQKWQCVEDATGKLKLHKCKGP
MRLGGSRALSNLVPKYYGQGSEACTCDSGDYKLSLAGRRKKLFKKKYKASYVRSRSIRSV
AIEVDGRVYHVGLGDAAQPRNLTKRHWPGAPEDQDDKDGGDFSGTGGLPDYSAANPIKVT
HRCYILENDTVQCDLDLYKSLQAWKDHKLHIDHEIETLQNKIKNLREVRGHLKKKRPEEC
DCHKISYHTQHKGRLKHRGSSLHPFRKGLQEKDKVWLLREQKRKKKLRKLLKRLQNNDTC
SMPGLTCFTHDNQHWQTAPFWTLGPFCACTSANNNTYWCMRTINETHNFLFCEFATGFLE
YFDLNTDPYQLMNAVNTLDRDVLNQLHVQLMELRSCKGYKQCNPRTRNMDLDGGSYEQYR
QFQRRKWPEMKRPSSKSLGQLWEGWEG*
>ENSG00000114354|ENST00000615993
MNGQLDLSGKLIIKAQLGEDIRRIPIHNEDITYDELVLMMQRVFRGKLLSNDEVTIKYKD
EDGDLITIFDSSDLSFAIQCSRILKLTLFVNGQPRPLESSQVKYLRRELIELRNKVNRLL
DSLEPPGEPGPSTNIPENDTVDGREEKSASDSSGKQSTQVMAASMSAFDPLKNQDEINKN
VMSAFGLTDDQVSGPPSAPAEDRSGTPDSIASSSSAAHPPGVQPQQPPYTGAQTQAGQIE
GQMYQQYQQQAGYGAQQPQAPPQQPQQYGIQYSGFQSMERFHCK*
>ENSG00000283017|ENST00000634346
MYGSARSVGKVEPSSQSPGRSPRLPRSPRLGHRRTNSTGGSSGSSVGGGSGKTLSMENIQ
SLNAAYATSGPMYLSDHENVGSETPKSTMTLGRSGGRLPYGVRMTAMGSSPNIASSGVAS
DTIAFGEHHLPPVSMASTVPHSLRQARDNTIMDLQTQLKEVLRENDLLRKDVEVKESKLS
SSMNSIKTFWSPELKKERALRKDEASKITIWKEQYRVVQEENQHMQMTIQALQDELRIQR
DLNQLFQQDSSSRTGEPCVAELTEENFQRLHAEHERQAKELFLLRKTLEEMELRIETQKQ

TLNARDESIKKLLEMLQSKGLSAKATEEDHERTRRLAEAEMHVHHLESLLEQKEKENSML
REEMHRRFENAPDSAKTKALQTVIEMKDSKISSMERGLRDLEEEIQMLKSNGALSTEERE
EEMKQMEVYRSHSKFMKNKIGQVKQELSRKDTELLALQTKLETLTNQFSDSKQHIEVLKE
SLTAKEQRAAILQTEVDALRLRLEEKETMLNKKTKQIQDMAEEKGTQAGEIHDLKDMLDV
KERKVNVLQKKIENLQEQLRDKEKQMSSLKERVKSLQADTTNTDTALTTLEEALAEKERT
IERLKEQRDRDEREKQEEIDNYKKDLKDLKEKVSLLQGDLSEKEASLLDLKEHASSLASS
GLKKDSRLKTLEIALEQKKEECLKMESQLKKAHEAALEARASPEMSDRIQHLEREITRYK
DESSKAQAEVDRLLEILKEVENEKNDKDKKIAELESLTSRQVKDQNKKVANLKHKEQVEK
KKSAQMLEEARRREDNLNDSSQQLQVEELLMAMEKVKQELESMKAKLSSTQQSLAEKETH
LTNLRAERRKHLEEVLEMKQEALLAAISEKDANIALLELSSSKKKTQEEVAALKREKDRL
VQQLKQQTQNRMKLMADNYEDDHFKSSHSNQTNHKPSPDQDEEEGIWA*
>ENSG00000198513|ENST00000441560
MAKNRRDRNSWGGFSEKTYEWSSEEEEPVKKAGPVQVLIVKDDHSFELDETALNRILLSE
AVRDKEVVAVSVAGAFRKGKSFLMDFMLRYMYNQESVDWVGDYNEPLTGFSWRGGSERET
TGIQIWSEIFLINKPDGKKVAVLLMDTQGTFDSQSTLRDSATVFALSTMISSIQVYNLSQ
NVQEDDLQHLQLFTEYGRLAMEETFLKPFQSLIFLVRDWSFPYEFSYGADGGAKFLEKRL
KVSGNQHEELQNVRKHIHSCFTNISCFLLPHPGLKVATNPNFDGKLKEIDDEFIKNLKIL
IPWLLSPESLDIKEINGNKITCRGLVEYFKAYIKIYQGEELPHPKSMLQATAEANNLAAV
ATAKDTYNKKMEEICGGDKPFLAPNDLQTKHLQLKEESVKLFRGVKKMGGEEFSRRYLQQ
LESEIDELYIQYIKHNDSKNIFHAARTPATLFVVIFITYVIAGVTGFIGLDIIASLCNMI
MGLTLITLCTWAYIRYSGEYRELGAVIDQVAAALWDQALYKLYSAAATHRHLYHQAFPTP
KSESTEQSEKKKM*
>ENSG00000169255|ENST00000492353
MASALWTVLPSRMSLRSLKWSLLLLSLLSFFVMWYLSLPHYNVIERVNWMYFYEYEPIYR
QDFHF
>ENSG00000122390|ENST00000572942
MTEVVPSSALSEVSLRLLCHDDIDTVKHLCGDWFPIEYPDSWYRDITSNKKFFSLAATYR
GAIVGMIVAEIKNRTKIHKEDGDILASNFSVDTQVAYILSLGVVKEFRKHGIESTARPTA
CSAASCHGRASLPRVASSTAGPCDVGWAAATRPHPSAARRARLPVHLTPSVFCKELPAI*
>ENSG00000225103|ENST00000446482
MILNKALLLGALALTAVMSPCGGEDIVADHVASYGVNFYQSHGPSGQYTHEFDGDEEFYV
DLETKETVWQLPMFSKFISFDPQSALRNMAVGKHTLEFMMRQSNSTAATNEVPEVTVFSK
FPVTLGQPNTLICLVDNIFPPVVNITWLSNGHSVTEGVSETSFLSKSDHSFFKISYLTFL
PSADEIYDCKVEHWGLDEPLLKHWEPEIPAPMSELTETLVCALGLSAGLMGIVVGTVFII
QGLRSVGASRHQGLL*
>ENSG00000102100|ENST00000634665
MAAVGAGGSTAAPGPGAVSAGALEPGTASAAHRRLKYISLAVLVVQNASLILSIRYARTL
PGDRFFATTAVVMAEVLKGLTCLLLLFAQKRGCPGAVCGHAQARSALSHLHLAE*
>ENSG00000114650|ENST00000428413
MVKSKWGLALAAVVTVLSSLLMSVGLCTLFGLTPTLNGGEIFPYLVVVIGLENVLVLTKS
VVSTPVDLEVKLRIAQGLSSESWSIMKNMATELGIILIGYFTLVPAIQEFCLFAVVGLVS
DFFLQMLFFTTVLSIDIRRMELADLNKRLPPEACLPSAKPVGQPTRYERQLAVRPSTPHT
ITLQPSSFRNLRLPKRLRVVYFLAPTGAHTCWALGSRTQGPRWGAGPWRRHAVQGGGAGP
GHRHRLGAAAALPLPRAMPAQLRAAGWWARAAEARGAALRRLRLCATRDGDRAACAARPP

HGHRVPGQRRHAAGELLPGRPRLRVGRADRGLPNAHSAPRQAAPGQWRGQRA*
>ENSG00000223465|ENST00000440242
MLSRNDVKRLERSWHLLSWSQAQGSSEQELHYASLQRLPVPSSEGPDLRGRDKRGTKEDP
RADYACIAENKPT*
>ENSG00000141198|ENST00000536554
MCVQNCGPSFQSLIVKKEFVKENLVKLLNPRYNLPLDIQNRILNFIKTWSQGFPGGVDVS
EVKEVYLDLVKKGVQFPPSEAEAETARQETAQISSNPPTSVPTAPALSSVIAPKNSTVTL
VPEQIGKLHSELDMVKMNVRVMSAILMENTPGSENHEDIELLQKLYKTGREMQERIMDLL
VVVENEDVTVELIQVNEDLNNAILGYERFTRNQQRILEQNKNQKEATNTTSEPSAPSQDL
LDLSPSPRMPRATLGELNTMNNQLSGLNFSLPSSDVTNNLKPSLHPQMNLLALENTEIPP
FAQRTSQNLTSSHAYDNFLEHSNSVFLQPVSLQTIAAAPSNQSLPPLPSNHPAMTKSDLQ
PPNYYEVMEFDPLAPAVTTEAIYEEIDAHQHKGAQNDGD*
>ENSG00000126091|ENST00000528371
MGLLVFVRNLLLALCLFLVLGFLYYSAWKLHLLQWEEDSKYDRLGFLLNLDSKLPAELAT
KYANFSEGACKPGYASALMTAIFPRFSKPAPMFLDDSFRKWARIREFVPPFGIKGQDNLI
KAILSVTKEYRLTPALDSLRCRRCIIVGNGGVLANKSLGSRIDDYDIVVSPGRTISSERK
SFCGSW*
>ENSG00000080815|ENST00000553855
MTELPAPLSYFQNAQMSEDNHLSNTVRSQNDNRERQEHNDRRSLGHPEPLSNGRPQGNSR
QVVEQDEEEDEELTLKYGAKHVIMLFVPVTLCMVVVVATIKSVSFYTRKDGQLIYTPFTE
DTETVGQRALHSILNAAIMISVIVVMTILLVVLYKYRCYKVIHAWLIISSLLLLFFFSFI
YLGEVFKTYNVAVDYITVALLIWNFGVVGMISIHWKGPLRLQQAYLIMISALMALVFIKY
LPEWTAWLILAVISVYDLVAVLCPKGPLRMLVETAQERNETLFPALIYSSTMVWLVNMAE
GDPEAQRRVSKNSKYNAERACLPPAAINLLSIAPMAPRLFMPKGACRPTAQKGSHKTLLQ
RMMMAGSVRNGKPRGTVI*
>ENSG00000166557|ENST00000424155
MGSTVPRSASVLLLLLLLLRRAEQPCGAELTFELPDNAKQCFHEEVEQGVKFSLDYQVITG
GHYDVDCYVEDPQGNTIYRETKKQYDSFTYRAEVKGVYQFCFSNEFSTFSHKTVYFDFQV
GDEPPILPDMGNRVTALTQRFRGAYWKEVDKMVDYMQPGGTPATEGLGRLAPS*
>ENSG00000174775|ENST00000451590
MTEYKLVVVGAGGVGKSALTIQLIQNHFVDEYDPTIEDSYRKQVVIDGETCLLDILDTAG
QEEYSAMRDQYMRTGEGFLCVFAINNTKSFEDIHQYREQIKRVKDSDDVPMVLVGNKCDL
AARTVESRQAQDLARSYGIPYIETSAKTRQGVEDAFYTLVREIRQHKLRKLNPPDESGPG
CMSCKCVLS*
>ENSG00000196535|ENST00000531253
MFNLMKKDKDKDGGRKEKKEKKEKKERMSAAELRSLEEMSLRRGFFNLNRSSKRESKTRL
EISNPIPIKVASGSDLHLTDIDSDSNRGSVILDSGHLSTASSSDDLKGEEGSFRGSVLQR
AAKFGSLAKQNSQMIVKRFSFSQRSRDESASETSTPSEHSAAPSPQVEVRTLEGQLVQHP
GPGIPRPGHRSRAPELVTKKFPVDLRLPPVVPLPPPTLRELELQRRPTGDFGFSLRRTTM
LDRGPEGQACRRVVHFAEPGAGTKDLALGLVPGDRLVEINGHNVESKSRDEIVEMIRQSG
DSVRLKVQPIPELSELSRSWLRSGEGPRREPSDAKTEEQIAAEEAWNETEKVWLVHRDGF
SLASQLKSEELNLPEGKVRVKLDHDGAILDVDEDDVEKANAPSCDRLEDLASLVYLNESS
VLHTLRQRYGASLLHTYAGPSLLVLGPRGAPAVYSEKVMHMFKGCRREDMAPHIYAVAQT
AYRAMLMSRQDQSIILLGSSGSGKTTSCQHLVQYLATIAGISGNKVFSVEKWQALYTLLE

AFGNSPTIINGNATRFSQILSLDFDQAGQVASASIQTMLLEKLRVARRPASEATFNVFYY
LLACGDGTLRTELHLNHLAENNVFGIVPLAKPEEKQKAAQQFSKLQAAMKVLGISPDEQK
ACWFILAAIYHLGAAGATKEAAEAGRKQFARHEWAQKAAYLLGCSLEELSSAIFKHQHKG
GTLQRSTSFRQGPEESGLGDGTGPKLSALECLEGMAAGLYSELFTLLVSLVNRALKSSQH
SLCSMMIVDTPGFQNPEQGGSARGASFEELCHNYTQDRLQRLFHERTFVQELERYKEENI
ELAFDDLEPPTDDSVAAVDQASHQSLVRSLARTDEARGLLWLLEEEALVPGASEDTLLER
LFSYYGPQEGDKKGQSPLLHSSKPHHFLLGHSHGTNWVEYNVTGWLNYTKQNPATQNAPR
LLQDSQKKIISNLFLGRAGSATVLSGSIAGLEGGSQLALRRATSMRKTFTTGMAAVKKKS
LCIQMKLQVDALIDTIKKSKLHFVHCFLPVAEGWAGEPRSASSRRVSSSSELDLPSGDHC
EAGLLQLDVPLLRTQLRGSRLLDAMRMYRQGYPDHMVFSEFRRRFDVLAPHLTKKHGRNY
IVVDERRAVEELLECLDLEKSSCCMGLSRVFFRAGTLARLEEQRDEQTSRNLTLFQAACR
GYLARQHFKKRKIQDLAIRCVQKNIKKNKGVKDWPWWKLFTTVRPLIEVQLSEEQIRNKD
EEIQQLRSKLEKAEKERNELRLNSDRLESRISELTSELTDERNTGESASQLLDAETAERL
RAEKEMKELQTQYDALKKQMEVMEMEVMEARLIRAAEINGEVDDDDAGGEWRLKYERAVR
EVDFTKKRLQQEFEDKLEVEQQNKRQLERRLGDLQADSEESQRALQQLKKKCQRLTAELQ
DTKLHLEGQQVRNHELEKKQRRFDSELSQAHEEAQREKLQREKLQREKDMLLAEAFSLKQ
QLEEKDMDIAGFTQKVVSLEAELQDISSQESKDEASLAKVKKQLRDLEAKVKDQEEELDE
QAGTIQMLEQAKLRLEMEMERMRQTHSKEMESRDEEVEEARQSCQKKLKQMEVQLEEEYE
DKQKVLREKRELEGKLATLSDQVNRRDFESEKRLRKDLKRTKALLADAQLMLDHLKNSAP
SKREIAQLKNQLEESEFTCAAAVKARKAMEVEIEDLHLQIDDIAKAKTALEEQLSRLQRE
KNEIQNRLEEDQEDMNELMKKHKAAVAQASRDLAQINDLQAQLEEANKEKQELQEKLQAL
QSQVEFLEQSMVDKSLVSRQEAKIRELETRLEFERTQVKRLESLASRLKENMEKLTEERD
QRIAAENREKEQNKRLQRQLRDTKEEMGELARKEAEASRKKHELEMDLESLEAANQSLQA
DLKLAFKRIGDLQAAIEDEMESDENEDLINSEGDSDVDSELEDRVDGVKSWLSKNKGPSK
AASDDGSLKSSSPTSYWKSLAPDRSDDEHDPLDNTSRPRYSHSYLSDSDTEAKLTETNA*
>ENSG00000134470|ENST00000528354
MAPRRARGCRTLGLPALLLLLLLRPPATRGITCPPPMSVEHADIWVKSYSLYSRERYICN
SGFKRKAGTSSLTECVLNKATNVAHWTTPSLKCIKPAASSPSSNNTAATTAAIVPGSQLM
PSKSPSTGTTEISSHESSHGTPSQTTAKNWELTASASHQPPGVYPQGHSDTTVAISTSTV
LLCGLSAVSLLACYLKSRQTPPLASVEMEAMEALPVTWGTSSRDEDLENCSHHL*
>ENSG00000109572|ENST00000513761
MESEQLFHRGYYRNSYNSITSASSDEELLDGAGVIMDFQTSEDDNLLDGDTAVGTHYTMT
NGGSINSSTHLLDLLDEPIPGVGTYDDFHTIDWVREKCKDRERHRRINSKKKESAWEMTK
SLYDAWSGWLVVTLTGLASGALAGLIDIAADWMTDLKEGICLSALWYNHEQCCWGSNETT
FEERDKCPQWKTWAELIIGQAEGPGSYIMNYIMYIFWALSFAFLAVSLVKVFAPYACGSG
IPEIKTILSGFIIRGYLGKWTLMIKTITLVLAVASGLSLGKEGPLVHVACCCGNIFSYLF
PKYSTNEAKKREVLSAASAAGVSVAFGAPIGGVLFSLEEVSYYFPLKTLWRSFFAALVAA
FVLRSINPFGNSRLVLFYVEYHTPWYLFELFPFILLGVFGGLWGAFFIRANIAWCRRRKS
TKFGKYPVLEVIIVAAITAVIAFPNPYTRLNTSELIKELFTDCGPLESSSLCDYRNDMNA
SKIVDDIPDRPAGIGVYSAIWQLCLALIFKIIMTVFTFGIKVPSGLFIPSMAIGAIAGRI
VGIAVEQLAYYHHDWFIFKEWCEVGADCITPGLYAMVGAAACLGGVTRMTVSLVVIVFEL
TGGLEYIVPLMAAVMTSKWVGDAFGREGIYEAHIRLNGYPFLDAKEEFTHTTLAADVMRP
RRNDPPLAVLTQDNMTVDDIENMINETSYNGFPVIMSKESQRLVGFALRRDLTIAIESAR
KKQEGIVGSSRVCFAQHTPSLPAESPRPLKLRSILDMSPFTVTDHTPMEIVVDIFRKLGL

RQCLVTHNGRLLGIITKKDILRHMAQTANQDPASIMFN*
>ENSG00000282880|ENST00000635244
MRNWLVLLCPCVLGAALHLWLRLRSPPPACASGAGPAGGVSLLLPRLECNGAVSAHPNLH
LPGSRDSPASASQVAGITDQLALFPQWKSTHYDVVVGVLSARNNHELRNVIRSTWMRHLL
QHPTLSQRVLVKFIIGAHGCEVPVEDREDPYSCKLLNITNPVLNQEIEAFSLSEDTSSGL
PEDRVVSVSFRVLYPIVITSLGVFYDANDVGFQRNITVKLYQAEQEEALFIARFSPPSCG
VQVNKLWYKPVEQFILPESFEGTIVWESQDLHGLVSRNLHKVTVNDGGGVLRVITAGEGA
LPHEFLEGVEGVAGGFIYTIQGKFAS*
>ENSG00000103723|ENST00000535359
MSAAPAYSEDKGGSAGPGEPEYGHDPASGGIFSSDYKRHDDLKEMLDTNKDSLKLEAMKR
IVAMIARGKNASDLFPAVVKNVACKNIEVKKLVYVYLVRYAEEQQDLALLSISTFQRGLK
DPNQLIRASALRVLSSIRVPIIVPIMMLAIKEAASDMSPYVRKTAAHAIPKLYSLDSDQK
DQLIEVIEKLLADKTTLVAGSVVMAFEEVCPERIDLIHKNYRKLCNLLIDVEEWGQVVII
SMLTRYARTQFLSPTQNESLLEENAEKAFYGSEEDEAKGAGSEETAAAAAPSRKPYVMDP
DHRLLLRNTKPLLQSRSAAVVMAVAQLYFHLAPKAEVGVIAKALVRLLRSHSEVQYVVLQ
NVATMSIKRRGMFEPYLKSFYIRSTDPTQIKILKLEVLTNLANETNIPTVLREFQTYIRS
MDKDFVAATIQAIGRCATNIGRVRDTCLNGLVQLLSNRDELVVAESVVVIKKLLQMQPAQ
HGEIIKHLAKLTDNIQVPMARASILWLIGEYCEHVPRIAPDVLRKMAKSFTAEEDIVKLQ
VINLAAKLYLTNSKQTKLLTQYVLSLAKYDQNYDIRDRARFTRQLIVPSEQGGALSRHAK
KLFLAPKPAPVLESSFKDRDHFQLGSLSHLLNAKATGYQELPDWPEEAPDPSVRNVEEED
LSLIETHVGLLGEYTEVPEWTKCSNREKRKEKEKPFYSDSEGESGPTESADSDPESESES
DSKSSSESGSGESSSESDNEDQDEDEEKGRGSESEQSEEDGKRKTKKKVPERKGEASSSD
EGSDSSSSSSESEMTSESEEEQLEPASWSRKTPPSSKSAPATKEISLLDLEDFTPPSVQP
VSPPAIVSTSLAADLEGLTLTDSTLVPSLLSPVSGVGRQELLHRVAGEGLAVDYTFSRQP
FSGDPHMVSVHIHFSNSSDTPIKGLHVGTPKLPAGISIQEFPEIESLAPGESATAVMGIN
FCDSTQAANFQLCTQTRQFYVSIQPPVGELMAPVFMSENEFKKEQGKLMGMNEITEKLML
PDTCRSDHIVVQKVTATANLGRVPCGTSDEYRFAGRTLTGGSLVLLTLDARPAGAAQLTV
NSEKMVIGTMLVKDVIQALTQ*
>ENSG00000126091|ENST00000489897
MGLLVFVRNLLLALCLFLVLGFLYYSAWKLHLLQWEEDSNSVVLSFDSAGQTLGSEYDRL
GFLLNLDSKLPAELATKYANFSEGACKPGYASALMTAIFPRAKRNGAWRQKHIQAYVLRQ
R*
>ENSG00000120708|ENST00000442011
MALFVRLLALALALALGPAATLAGPAKSPYQLVLQHSRLRGRQHGPNVCAVQKVIGTNRK
YFTNCKQWYQRKICGKSTVISYECCPGYEKVPGEKGCPAALPLSNLYETLGVVGSTTTQL
YTDRTEKLRPEMEGPGSFTIFAPSNEAWASLPAEVLDSLVSNVNIELLNALRYHMVGRRV
LTDELKHGMTLTSMYQNSNIQIHHYPNGIVTVNCARLLKADHHATNGVVHLIDKVISTIT
NNIQQIIEIEDTFETLRAAVAASGLNTMLEGNGQYTLLAPTNEAFEKIPSETLNRILGDP
EALRDLLNNHILKSAMCAEAIVAGLSVETLEGTTLEVGCSGDMLTINGKAIISNKDILAT
NGVIHYIDELLIPDSAKTLFELAAESDVSTAIDLFRQAGLGNHLSGSERLTLLAPLNSVF
KDGTPPIDAHTRNLLRNHIIKDQLASKYLYHGQTLETLGGKKLRVFVYRNSLCIENSCIA
AHDKRGRYGTLFTMDRVLTPPMGTVMDVLKGDNRFSMLVAAIQSAGLTETLNREGVYTVF
APTNEAFRALPPRERSRLLGDAKELANILKYHIGDEILVSGGIGALVRLKSLQGDKLEVS
LKNNVVSVNKEPVAEPDIMATNGVVHVITNVLQPPANRPQERGDELADSALEIFKQASAF

SRASQRSVRLAPVYQKLLERMKH*
>ENSG00000122705|ENST00000433436
MAELDPFGAPAGAPGGPALGNGVAGAGEEDPAAAFLAQQESEIAGIENDEAFAILDGGAP
GPQPHGEPPGGPDAVDGVMNGEYYQESNGPTDSYAAISQVDRLQSEPESIRKWREEQMER
LEALDANSRKQEAEWKEKAIKELEEWYARQDEQLQKTKANNRVADEAFYKQPFADVIGYV
TNINHPCYSLEQAAEEAFVNDIDESSPGTEWERVARLCDFNPKSSKQAKDVSRMRSVLIS
LKQAPLVH*
>ENSG00000213853|ENST00000536829
MLVLLAFIIAFHITSAALLFIATVDNAWWVGDEFFADVWRICTNNTNCTVINDSFQEYST
LQAVQATMILSTILCCIAFFIFVLQLFRLKQGERFVLTSIIQLMSCLCVMIAASIYTDRR
EDIHDKNAKFYPVTREGSYGYSYILAWVAFACTFISGMMYLILRKRK*
>ENSG00000226165|ENST00000426733
MALQIPGGFWAAAVTVMLVMLSTPVAEARDFPKDFLVQFKGMCYFTNGTERVRGVARYIY
NREEYGRFDSDVGEFQAVTELGRSIEDWNNYKDFLEQERAAVDKVCRHNYEAELRTTLQR
QVEPTVTISPSRTEALNHHNLLVCSVTDFYPAQIKVRWFRNDQEETAGVVSTSLIRNGDW
TFQILVMLEITPQRGDIYTCQVEHPSLQSPITVEWRPRGPPPAGLLH*
>ENSG00000051825|ENST00000606320
MEEFDLVKTLHKTSSSVGSDENSLHSLGLNLNTDRSSPHLSTNGVSSFSGKTRPSVIQGT
VEVLTSLMQELQNSGKTDSELWKNCETRWLQLFNLVEKQCQEQIVAQQEQFHNQIQHIQE
EIKNLVKLQTSSASLASCEGNSSNKQVSSESQMGFFSLSSERNESVIHYPESTEPEIQQE
MSTSQPDCNVDSCSVSSGYGTFCISELNLYKSKDPKEFMEHIDVPKGQYVAPAVPAESLV
DGVKNENFYIQTPEECHVSLKEDVSISPGEFEHNFLGENKVSEVYSGKTNSNAITSWAQK
LKQNQPKRAHVEDGGSRSKQGNEQSKKTPIEKSDFAAATHPRAFYLSKPDETPNAWMSDS
GTGLTYWKLEEKDMHHSLPETLEKTFISLSSTDVSPNQSNTSNEMKLPSLKDIYYKKQRE
NKQLPERNLTSASNPNHPPEVLTLDPTLHMKPKQQISGIQPHGLPNALDDRISFSPDSVL
EPSMSSPSDIDSFSQASNVTSQLPGFPKYPSHTKASPVDSWKNQTFQNESRTSSTFPSVY
TITSNDISVNTVDEENTVMVASASVSQSQLPGTANSVPECISLTSLEDPVILSKIRQNLK
EKHARHIADLRAYYESEINSLKQKLEAKEISGVEDWKITNQILVDRCGQLDSALHEATSR
VRTLENKNNLLEIEVNDLRERFSAASSASKILQERIEEMRTSSKEKDNTIIRLKSRLQDL
EEAFENAYKLSDDKEAQLKQENKMFQDLLGEYESLGKEHRRVKDALNTTENKLLDAYTQI
SDLKRMISKLEAQVKQVEHENMLSLRHNSRIHVRPSRANTLATSDVSRRKWLIPGAEYSI
FTGQPLDTQDSNVDNQLEETCSLGHRSPLEKDSSPGSSSTSLLIKKQRETSDTPIMRALK
ELDEGKIFKNWGTQTEKEDTSNINPRQTETSVNASRSPEKCAQQRQKRLNSASQRSSSLP
PSNRKSSTPTKREIMLTPVTVAYSPKRSPKENLSPGFSHLLSKNESSPIRFDILLDDLDT
VPVSTLQRTNPRKQLQFLPLDDSEEKTYSEKATDNHVNHSSCPEPVPNGVKKVSVRTAWE
KNKSVSYEQCKPVSVTPQGNDFEYTAKIRTLAETERFFDELTKEKDQIEAALSRMPSPGG
RITLQTRLNQEALEDRLERINRELGSVRMTLKKFHVLRTSANL*
>ENSG00000280495|ENST00000629243
MAIFSVYVVNKAGGLIYQLDSYAPRAEAEKTFSYPLDLLLKLHDERVLVAFGQRDGIRVG
HAVLAINGMDVNGRYTADGKEVLEYLGNPANYPVSIRFGRPRLTSNEKLMLASMFHSLFA
IGSQLSPEQGSSGIEMLETDTFKLHCYQTLTGIKFVVLADPRQAGIDSLLRKIYEIYSDF
ALKNPFYSLEMPIRCELFDQNLKLALEVAEKAGTFGPGS*
>ENSG00000117262|ENST00000528944
MSFLIDSSIMITSQILFFGFGWLFFMRQLFKDYEIRQYVVQVIFSVTFAFSCTMFELIIF

EILGVLNSSSRYFHWKMNLCVILLILVFMVPFYIGYFIVSNIRLLLSPLSQCINNDCFFP
VSYG*
>ENSG00000189067|ENST00000571688
MSVPGPYQAATGPSSAPSAPPSYEETVAVNSYYPTPPAPMPGPTTGLVTGPDGKGMNPPS
YYTQPAPIPNNNPITVQTVYVQHPITFLDRPIQMCCPSCNKMIVSQLSYNAGALTWLSCG
SLCLLGCIAGCCFIPFCVDALQDVDHYCPNCRALLGTYKRL*
>ENSG00000142186|ENST00000420247
MWFFARDPVRDFPFELIPEPPEGGLPGPWALHRGRKKATGSPVSIFVYDVKPGAEEQTQV
AKAAFKRFKTLRHPNILAYIDGLETEKCLHVVTEAVTPLGIYLKARVEAGGLKELEISWG
LHQIVKALSFLVNDCSLIHNNVCMAAVFVDRAGEWKLGGLDYMYSAQGNGGGPPRKGIPE
LEQYDPPELADSSGRVVREKWSADMWRLGCLIWEVFNGPLPRAAALRNPGKIPKTLVPHY
CELVGANPKVRPNPARFLQNCRAPGGFMSNRFVETNLFLEEIQIKEPAEKQKFFQELSKS
LDAFPEDFCRHKVLPQLLTAFEFGNAGAVVLTPLFKVGKFLSAEEYQQKIIPVVVKMFSS
TDRAMRIRLLQQMEQFIQYLDEPTVNTQIFPHVVHGFLDTNPAIREQTVKSMLLLAPKLN
EANLNVELMKHFARLQAKDEQGPIRCNTTVCLGKIGSYLSASTRHRVLTSAFSRATRDPF
APSRVAGVLGFAATHNLYSMNDCAQKILPVLCGLTVDPEKSVRDQAFKAIRSFLSKLESV
SEDPTQLEEVEKDVHAASSPGMGGAAASWAGWAVTGVSSLTSKLIRSHPTTAPTETNIPQ
RPTPEGHWETQEEDKDTAEDSSTADRWDDEDWGSLEQEAESVLAQQDDWSTGGQVSRASQ
VSNSDHKSSKSPESDWSSWEAEGSWEQGWQEPSSQEPPPDGTRLASEYNWGGPESSDKGD
PFATLSARPSTQPRPDSWGEDNWEGLETDSRQVKAELARKKREERRREMEAKRAERKVAK
GPMKLGARKLD*
>ENSG00000148985|ENST00000464906
MYQVPLPLDRDGTLVRLRFTMVALVTVCCPLVAFLFCILWSLLFHFKETTATHCGATPCR
MFSAASQPLDPDGTLFRLRFTAMVWWAITFPVFGFFFCIIWSLVFHFEYTVATDCGVPNY
LPSVSSAIGGEVPQRYVWRFCIGLHSAPRFLVAFAYWNHYLSCTSPCSCYRPLCRLNFGL
NVVENLALLVLTYVSSSEDFTIHENAFIVFIASSLGHMLLTCILWRLTKKHTVSQEDRKS
YSWKQRLFIINFISFFSALAVYFRHNMYCEAGVYTIFAILEYTVVLTNMAFHMTAWWDFG
NKELLITSQPEEKRF*
>ENSG00000175106|ENST00000518321
MLQQDSNDDTEDVSLFDAEEETTNRPRKAKIRHPVASFFHLFFRVSAIIVCLLCELLSSS
FITCMVTIILLLSCDFWAVKNVTGRLMVGLRWWNHIDEDGKSHWVFESRKESSQENKTVS
EAESRIFWLGLIACSVLWVIFAFSALFSFTVKWLAVVIMGVVLQGANLYGYIRCKVRSRK
HLTSMATSYFGKQFLRQVSVFWM*
>ENSG00000137497|ENST00000535111
MTLHATRGAALLSWVNSLHVADPVEAVLQLQDCSIFIKIIDRIHGTEEGQQILKQPVSER
L
>ENSG00000125447|ENST00000621217
MAEAEGESLESWLNKATNPSNRQEDWEYIIGFCDQINKELEGPQIAVRLLAHKIQSPQEW
EALQALTVLEACMKNCGRRFHNEVGKFRFLNELIKVVSPKYLGDRVSEKVKTKVIELLYS
WTMALPEEAKIKDAYHMLKRQAFSQAAEKQKPR*
>ENSG00000171195|ENST00000413702
MKTLPLFVCICALSACFSFSEGRERDHELRHRRHHHQSPKSHFELPHYPGLLAHQKPFIR
KSYKCLHKRCRPKLPPSPNNPPKFPNPHQPPKHPDKNSSVVNPTLVATTQIPSVTFPSAS
TKITTLPNVTFLPQNATTISSRENVNTSSSVATLAPVNSPAPQDTTAAPPTPSATTPAPP

SSSAPPETTAAPPTPSATTQAPPSSSAPPETTAAPPTPPATTPAPPSSSAPPETTAAPPT
PSATTPAPLSSSAPPETTAVPPTPSATTLDPSSASAPPETTAAPPTPSATTPAPPSSPAP
QETTAAPITTPNSSPTTLAPDTSETSAAPTHQTTTSVTTQTTTTKQPTSAPGQNKISRFL
LYMKNLLNRIIDDMVEQ*
>ENSG00000180398|ENST00000409800
MEHLEGVINKPEAEMSPQELQLHYFKMHDYDGNNLLDGLELSTAITHVHKEEGSEQAPLM
SEDELINIIDGVLRDDDKNNDGYIDYAEFAKSLQ*
>ENSG00000175582|ENST00000541973
MYDSFDNTYQATIGIDFLSKTMYLEDRTIRLQLWDTAGQERFRSLIPSYIRDSAAAVVVY
DITNVNSFQQTTKWIDDVRTERGSDVIIMLVGNKTDLADKRQVSIEEGERKAKELNVMFI
ETSAKAGYNVKQLFRRVAAALPGMESTQDRSREDMIDIKLEKPQEQPVSEGGCSC*
>ENSG00000231048|ENST00000451922
MLSRNDVKRLERSWHLLSWSQAQGSSEQELHYASLQRLPVPSSEGPDLRGRDKRGTKEDP
RADYACIAENKPT*
>ENSG00000169255|ENST00000488170
MASALWTVLPSRMSLRSLKWSLLLLSLLSFFVMWYLSLPHYNVIERVNWMYFYEYEPIYR
QDFHFTLREHSNCSHQNPFLVILVTSHPSDVKARQAIRVTWGEKKSWWGYEVLTFFLLGQ
EAEKEDKMLALSLEDEHLLYGDIIRQDFLDTYNNLTLKTIMAFRWVTEFCPNAKYVMKTD
TDVFINTGNLVKYLLNLNHSEKFFTGYPLIDNYSYRGFYQKTHISYQEYPFKVFPPYCSG
LGYIMSRDLVPRIYEMMGHVKPIKFEDVYVGICLNLLKVNIHIPEDTNLFFLYRIHLDVC
QLRRVIAAHGFSSKEIITFWQVMLRNTTCHY*
>ENSG00000102531|ENST00000492622
MAEHPPLLDTTQILSSDISLLSAPIVSADGTQQVILVQVNPGEAFTIRREDGQFQCITGP
AQVPMMSPNGSVPPIYVPPGYAPQVIEDNGVRRVVVVPQAPEFHPGSHTVLHRSPHPPLP
GFIPVPTMMPPPPRHMYSPVTGAGDMTTQYMPQYQSSQVYGDVDAHSTHGRSNFRDERSS
KTYERLQKKLKDRQGTQKDKMSSPPSSPQKCPSPINEHNGLIKGQIAGGINTGSAKIKSG
KGKGGTQVDTEIEEKDEETKAFEALLSNIVKPVASDIQARTVVLTWSPPSSLINGETDES
SVPELYGYEVLISSTGKDGKYKSVYVGEETNITLNDLKPAMDYHAKVQAEYNSIKGTPSE
AEIFTTLSCEPDIPNPPRIANRTKNSLTLQWKAPSDNGSKIQNFVLEWDEGKGNGEFCQC
YMGSQKQFKITKLSPAMGCKFRLSARNDYGTSGFSEEVLYYTSGCAPSMPASPVLTKAGI
TWLSLQWSKPSGTPSDEGISYILEMEEETSGYGFKPKYDGEDLAYTVKNLRRSTKYKFKV
IAYNSEGKSNPSEVVEFTTCPDKPGIPVKPSVKGKIHSHSFKITWDPPKDNGGATINKYV
VEMAEGSNGNKWEMIYSGATREHLCDRLNPGCFYRLRVYCISDGGQSAVSESLLVQTPAV
PPGPCLPPRLQGRPKAKEIQLRWGPPLVDGGSPISCYSVEMSPIEKDEPREVYQGSEVEC
TVSSLLPGKTYSFRLRAANKMGFGPFSEKCDITTAPGPPDQCKPPQVTCRSATCAQVNWE
VPLSNGTDVTEYRLEWGGVEGSMQICYCGPGLSYEIKGLSPATTYYCRVQALSVVGAGPF
SEVVACVTPPSVPGIVTCLQEISDDEIENPHYSPSTCLAISWEKPCDHGSEILAYSIDFG
DKQSLTVGKVTSYIINNLQPDTTYRIRIQALNSLGAGPFSHMIKLKTKPLPPDPPRLECV
AFSHQNLKLKWGEGTPKTLSTDSIQYHLQMEDKNGRFVSLYRGPCHTYKVQRLNESTSYK
FCIQACNEAGEGPLSQEYIFTTPKSVPAALKAPKIEKVNDHICEITWECLQPMKGDPVIY
SLQVMLGKDSEFKQIYKGPDSSFRYSSLQLNCEYRFRVCAIRQCQDSLGHQDLVGPYSTT
VLFISQRTEPPASTNRDTVESTRTRRALSDEQCAAVILVLFAFFSILIAFIIQYFVIK*
>ENSG00000124164|ENST00000475243
MAKVEQVLSLEPQHELKFRGPFTDVVTTNLKLGNPTDRNVCFKVKTTAPRRYCVRPNSGI

IDAGASINVSVMLQPFDYDPNEKSKHKFMVQSMFAPTDTSDMEAVWKEAKPEDLMDSKLR
CVFELPAENDKPHDVEINKIISTTASKTETPIVSKSLSSSLDDTEVKKVMEECKRLQGEV
QRLREENKQFKEEDGLRMRKTVQSNSPISALAPTGKEEGLSTRLLALVVLFFIVGVIIGK
IAL*
>ENSG00000283530|ENST00000637661
MFKFHQMKHIFEILDKMRCLRKRSTVSFLGVLVIFLLFMNLYIEDSYVLEGDKQLIRETS
THQLNSERYVHTFKDLSNFSGAINVTYRYLAATPLQRKRYLTIGLSSVKRKKGNYLLETI
KSIFEQSSYEELKEISVVVHLADFNSSWRDAMVQDITQKFAHHIIAGRLMVIHAPEEYYP
ILDGLKRNYNDPEDRVKFRSKQNVDYAFLLNFCANTSDYYVMLEDDVRCSKNFLTAIKKV
IASLEGTYWVTLEFSKLGYIGKLYHSHDLPRLAHFLLMFYQEMPCDWLLTHFRGLLAQKN
VIRFKPSLFQHMGYYSSYKGTENKLKDDDFEEESFDIPDNPPASLYTNMNVFENYEASKA
YSSVDEYFWGKPPSTGDVFVIVFENPIIIKKIKVNTGTEDRQNDILHHGALDVGENVMPS
KQRRQCSTYLRLGEFKNGNFEMSGVNQKIPFDIHCMRIYVTKTQKEWLIIRSISIWTS*
>ENSG00000276583|ENST00000627739
MTTDDTEVPAMTLAPGHAALETQTLSAETSSRASTPAGPIPEAETRGAKRISPARETRSF
TKTSPNFMVLIATSVETSAASGSPEGAGMTTVQTITGSDPREAIFDTLCTDDSSEEAKTL
TMDILTLAHTSTEAKGLSSESSASSDSPHPVITPSRASESSASSDGPHPVITPSRASESS
ASSDGPHPVITPSRASESSASSDGPHPVITPSRASESSASSDGPHPVITPSRASESSASS
DGPHPVITPSRASESSASSDGPHPVITPSRASESSASSDGPHPVITPSRASESSASSDGP
HPVITPSRASESSASSDGLHPVITPSRASESSASSDGPHPVITPSRASESSASSDGPHPV
ITPSWSPGSDVTLLAEALVTVTNIEVINCSITEIETTTSSIPGASDTDLIPTEGVKASST
SDPPALPDSTEAKPHITEVTASAETLSTAGTTESAAPDATVGTPLPTNSATEREVTAPGA
TTLSGALVTVSRNPLEETSALSVETPSYVKVSGAAPVSIEAGSAVGKTTSFAGSSASSYS
PSEAALKNFTPSETPTMDIATKGPFPTSRDPLPSVPPTTTNSSRGTNSTLAKITTSAKTT
MKPPTATPTTARTRPTTDVSAGENGGFLLLRLSVASPEDLTDPRVAERLMQQLHRELHAH
APHFQVSLLRVRRG*
>ENSG00000131446|ENST00000427865
MLKKQSAGLVLWGAILFVAWNALLLLFFWTRPAPGRPPSVSALDGDPASLTREVIRLAQD
AEVELERQRGLLQQIGDALSSQRGRVPTAAPPAQPRVPVTPAPAVIPILVIACDRSTVRR
CLDKLLHYRPSAELFPIIVSQDCGHEETAQAIASYGSAVTHIRQPDLSSIAVPPDHRKFQ
GYYKIARHYRWALGQVFRQFRFPAAVVVEDDLEVAPDFFEYFRATYPLLKADPSLWCVSA
WNDNGKEQMVDASRPELLYRTDFFPGLGWLLLAELWAELEPKWPKAFWDDWMRRPEQRQG
RACIRPEISRTMTFGRKGVSHGQFFDQHLKFIKLNQQFVHFTQLDLSYLQREAYDRDFLA
RVYGAPQLQVEKVRTNDRKELGEVRVQYTGRDSFKAFAKALGVMDDLKSGVPRAGYRGIV
TFQFRGRRVHLAPPLTWEGYDPSWN*
>ENSG00000122390|ENST00000573580
MIVAEIKNRTKIHKEDGDILASNFSVDTQVAYILSLGVVKEFRKHGIGSLLLESLKDHIS
TTAQDHCKAIYLHVLTTNNTAINFYENRDFKQHHYLPYYYSIRGVLKDGFTYVLYINGGH
PPWTILDYIQHLGSALASLSPCSIPHRVYRQAHSLLCSFLPWSGISSKSGIEYSRTM*
>ENSG00000171310|ENST00000549016
MRRNPFGVDICCRKGSRSPLQELYNPIQLELSNTAVLHQMRRDQVTDTCRANSATSRKRR
VLTPNDLKHLVVDEDHELIYCYVPKVACTNWKRLMMVLTGRGKYSDPMEIPANEAHVSAN
LKTLNQYSIPEINHRLKSYMKFLFVREPFERLVSAYRN
>ENSG00000198663|ENST00000480824

MDLAANEISIYDKLSETVDLVRQTGHQCGMSEKAIEKFIRQLLEKNEPQRPPPQYPLLIV
VYKVLATLGLILLTAYFVIQPFSPLAPEPVLSGAHTWRSLIHHIRLMSLPIAKKYMSENK
GVPLHGGDEDRPFPDFDPWWTNDCEQNESEPIPANCTGCAQKHLKVMLLEDAPRKFERLH
PLVIKTGKPLLEEEIQHFLCQYPEATEGFSEGFFAKWWRCFPERWFPFPYPWRRPLNRSQ
MLRELFPVFTHLPFPKDASLNKCSFLHPEPVVGSKMHKMPDLFIIGSGEAMLQLIPPFQC
RRHCQSVAMPIEPGDIGYVDTTHWKVYVIARGVQPLVICDGTAFSEL*
>ENSG00000186318|ENST00000445823
MAQALPWLLLWMGAGVLPAHGTQHGIRLPLRSGLGGAPLGLRLPRETDEEPEEPGRRGSF
VEMVDNLRGKSGQGYYVEMTVGSPPQTLNILVDTGSSNFAVGAAPHPFLHRYYQRQLSST
YRDLRKGVYVPYTQGKWEGELGTDLPDDSLEPFFDSLVKQTHVPNLFSLQLCGAGFPLNQ
SEVLASVGGSMIIGGIDHSLYTGSLWYTPIRREWYYEVIIVRVEINGQDLKMDCKEYNYD
KSIVDSGTTNLRLPKKVFEAAVKSIKAASSTEKFPDGFWLGEQLVCWQAGTTPWNIFPVI
SLYLMGEVTNQSFRITILPQQYLRPVEDVATSQDDCYKFAISQSSTGTVMGAVIMEGFYV
VFDRARKRIGFAVSACHVHDEFRTAAVEGPFVTLDMEDCGYNIPQTDESTLMTIAYVMAA
ICALFMLPLCLMVCQWRCLRCLRQQHDDFADDISLLK*
>ENSG00000106636|ENST00000496112
MKLYSLSVLYKGEAKVVLLKAAYDVSSFSFFQRSSVQEFMTFTSQLIVERSSKGTRASVK
EQDYLCHVYVRNDSLAGVVIADNEYPSRVAFTLLEKVLDEFSKQVDRIDWPVGSPATIHY
PALDGHLSRYQNPREADPMTKVQAELDETKIILARKQNSCCAIM*
>ENSG00000171310|ENST00000546689
MKPALLEVMRMNRICRMVLATCLGSFILVIFYFQIMRRNPFGVDICCRKGSRSPLQELYN
PIQVSFKHSVSM*
>ENSG00000064225|ENST00000483910
MRGYLVAIFLSAVFLYYVLHCILWGTNVYWVAPVEMKRRNKIQPCLSKPAFASLLRFHQF
HPFLCAADFRKIASLYGSDKFDLPYGMRTSAEYFRLALSKLQSCDLFDEFDNIPCKKCVV
VGNGGVLKNKTLGEKIDSYDVIIRMNNGPVLGHEEEVGRRTTFRLFYPESVFSDPIHNDP
NTTVILTAFKPHDLRWLLELLMGDKINTNGFWKKPALNLIYKPYQIRILDPFIIRTAAYE
LLHFPKVFPKNQKPKHPTTGIIAITLAFYICHEVHLAGFKYNFSDLKSPLHYYGNATMSL
MNKNAYHNVTAEQLFLKDIIEKNLVINLTQD*
>ENSG00000054116|ENST00000617904
MGFNIGVRLIEDFLARSNVGRCHDFRETADVIAKVAFKMYLGITPSITNWSPAGDEFSLI
LENNPLVDFVELPDNHSSLIYSNLLCGVLRGALEMVQMAVEAKFVQDTLKGDGVTEIRMR
FIRRIEDNLPAGEE*
>ENSG00000114650|ENST00000448217
MTLTERLREKISRAFYNHGLLCASYPIPIILFTGFCILACCYPLLKLPLPGTGPVEFTTP
VKDYSPPPVDSDRKQGEPTEQPEWVGTR
>ENSG00000003147|ENST00000430867
MSGHKCYPWDLQDRYAQDKSVVNKMQQKYWETKQAFIKATGKKEDEHVVASDADLDAKLE
LFHSIQRTCLDLSKAIVLYQKRICFLSQEENELGKFLRSQGFQDKTRAGKMMQATGKALC
FSSQQRLALRNPLC
>ENSG00000257923|ENST00000437600
MAANVGSMFQYWKRFDLQQLQRELDATATVLANRQDESEQSRKRLIEQSREFKKNTPEDL
RKQVAPLLKSFQGEIDALSKRSKEAEAAFLNVYKRLIDVPDPVPALDLGQQLQLKVQRLH
DIETENQKLRETLEEYNKEFAEVKNQEVTIKALKEKIREYEQTLKNQAETIALEKEQKLQ

NDFAEKERKLQETQMSTTSKLEEAEHKVQSLQTALEKTRTELFDLKTKYDEETTAKADEI
EMIMTDLERANQRAEVAQREAETLREQLSSANHSLQLASQIQKAPDVAIEVLTRSSLEVE
LAAKEREIAQLVEDVQRLQASLTKLRENSASQISQLEQQLSAKNSTLKQLEEKLKGQADY
EEVKKELNILKSMEFAPSEGAGTQDAAKPLEVLLLEKNRSLQSENAALRISNSDLSGRCA
ELQVRITEAVATATEQRELIARLEQDLSIIQSIQRPDAEGAAEHRLEKIPEPIKEATALF
YGPAAPASGALPEGQVDSLLSIISSQRERFRARNQELEAENRLAQHTLQALQSELDSLRA
DNIKLFEKIKFLQSYPGRGSGSDDTELRYSSQYEERLDPFSSFSKRERQRKYLSLSPWDK
ATLSMGRLVLSNKMARTIGFFYTLFLHCLVFLVLYKLAWSESMERDCATFCAKKFADHLH
KFHENDNGAAAGDLWQ*
>ENSG00000114209|ENST00000497056
MRMTMEEMKNEAETTSMVSMPLYAVMYPVFNELERVNLSAAQTLRAAFIKAEKENPGLTQ
DIIMKILEKKSVEVNFTESLLRMAADDVEEYMIERPEPEFQDLNEKARALKQILSKIPDE
INDRVRFLQTIKDIASAIKELLDTVNNVFKKYQYQNRRALEHQKKEFVKYSKSFSDTLKT
YFKDGKAINVFVSANRLIHQTNLILQTFKTVA*
>ENSG00000174473|ENST00000616459
EHLKDKLEEYMARFSKVRIVRTKKREGLIRTRLLGASMARGEVLTFLDSHCEVNVNWLPP
LLNQIALNHKTIVCPMIDVIDHNHFGYEAQAGDAMRGAFDWEMYYKRIPIPPELQRADPS
DPFESPVMAGGLFAVDRKWFWELGGYDPGLEIWGGEQYEISFKVWMCGGEMFDVPCSRVG
HIYRKYVPYKVPSGTSLARNLKRVAETWMDEFAEYIYQRRPEYRHLSTGDISAQKELRKQ
LKCKDFKWFMAAVAWDVPKYYPPVEPPPAAWGEIRNVAANLCVDSKHGATGTELRLDICV
KDGSERTWSHEQLFTFGWREDIRPGEPLHTRKFCFDAISHNSPVTLYDCHGMKGNQLWGY
RKDRTLFHPVSNSCMDCNPAEKKIFMARCDPLSETQQWIFEHINMTVLEKFNHHANS*
>ENSG00000105974|ENST00000614113
MADELSEKQVYDAHTKEIDLVNRDPKHLNDDVVKIDFEDVIAEPEGTHSFDGIWKASFTT
FTVTKYWFYRLLSALFGIPMALIWGIYFAILSFLHIWAVVPCIKSFLIEIQCISRVYSIY
VHTVCDPLFEAVGKIFSNVRINLQKEI*
>ENSG00000204632|ENST00000428701
MVVMAPRTLFLLLSGALTLTETWAGSHSMRYFSAAVSRPGRGEPRFIAMGYVDDTQFVRF
DSDSACPRMEPRAPWVEQEGPEYWEEETRNTKAHAQTDRMNLQTLRGYYNQSEASSHTLQ
WMIGCDLGSDGRLLRGYEQYAYDGKDYLALNEDLRSWTAADTAAQISKRKCEAANVAEQR
RAYLEGTCVEWLHRYLENGKEMLQRADPPKTHVTHHPVFDYEATLRCWALGFYPAEIILT
WQRDGEDQTQDVELVETRPAGDGTFQKWAAVVVPSGEEQRYTCHVQHEGLPEPLMLRWKQ
SSLPTIPIMGIVAGLVVLAAVVTGAAVAAVLWRKKSSD*
>ENSG00000017260|ENST00000428331
MKVARFQKIPNGENETMIPVLTSKKASELPVSEVASILQADLQNGLNKCEVSHRRAFHGW
NEFDISEDEPLWKKYISQFKNPLIMLLLASAVISVLMHQFDDAVSITVAILIVVTVAFVQ
EYRSEKSLEELSKLVPPECHCVREGKLEHTLARDLVPGDTVCLSVGDRVPADLRLFEAVD
LSIDESSLTGETTPCSKVTAPQPAATNGDLASRSNIAFMGTLVRCGKAKGVVIGTGENSE
FGEVFKMMQAEEAPKTPLQKSMDLLGKQLSFYSFGIIGIIMLVGWLLGKDILEMFTISVS
LAVAAIPEGLPIVVTVTLALGVMRMVKKRAIVKKLPIVETLGCCNVICSDKTGTLTKNEM
TVTHIFTSDGLHAEVTGVGYNQFGEVIVDGDVVHGFYNPAVSRIVEAGCVCNDAVIRNNT
LMGKPTEGALIALAMKMGLDGLQQDYIRKAEYPFSSEQKWMAVKCVHRTQQDRPEICFMK
GAYEQVIKYCTTYQSKGQTLTLTQQQRDVYQQEKARMGSAGLRVLALASGPELGQLTFLG
LVGIIDPPRTGVKEAVTTLIASGVSIKMITGDSQETAVAIASRLGLYSKTSQSVSGEEID

AMDVQQLSQIVPKVAVFYRASPRHKMKIIKSLQKNGSVVAMTGDGVNDAVALKAADIGVA
MGQTGTDVCKEAADMILVDDDFQTIMSAIEEGKGIYNNIKNFVRFQLSTSIAALTLISLA
TLMNFPNPLNAMQILWINIIMDGPPAQSLGVEPVDKDVIRKPPRNWKDSILTKNLILKIL
VSSIIIVCGTLFVFWRELRDNVITPRDTTMTFTCFVFFDMFNALSSRSQTKSVFEIGLCS
NRMFCYAVLGSIMGQLLVIYFPPLQKVFQTESLSILDLLFLLGLTSSVCIVAEIIKKVER
SREKIQKHVSSTSSSFLEV*
>ENSG00000142188|ENST00000420455
MAGFLDNFRWPECECIDWSERRNAVASVVAGILFFTGWWIMIDAAVVYPKPEQLNHAFHT
CGVFSTLAFFMINAVSNAQVRGDSYESGCLGRTGARVWLFIGFMLMFGSLIASMWILFGA
YVTQNTDVYPGLAVFFQNALIFFSTLIYKFGRTEELWT*
>ENSG00000017260|ENST00000422190
MKVARFQKIPNGENETMIPVLTSKKASELPVSEVASILQADLQNGLNKCEVSHRRAFHGW
NEFDISEDEPLWKKYISQFKNPLIMLLLASAVISVLMHQFDDAVSITVAILIVVTVAFVQ
EYRSEKSLEELSKLVPPECHCVREGKLEHTLARDLVPGDTVCLSVGDRVPADLRLFEAVD
LSIDESSLTGETTPCSKVTAPQPAATNGDLASRSNIAFMGTLVRCGKAKGVVIGTGENSE
FGEVFKMMQAEEAPKTPLQKSMDLLGKQLSFYSFGIIGIIMLVGWLLGKDILEMFTISVS
LAVAAIPEGLPIVVTVTLALGVMRMVKKRAIVKKLPIVETLGCCNVICSDKTGTLTKNEM
TVTHIFTSDGLHAEVTGVGYNQFGEVIVDGDVVHGFYNPAVSRIVEAGCVCNDAVIRNNT
LMGKPTEGALIALAMKMGLDGLQQDYIRKAEYPFSSEQKWMAVKCVHRTQQDRPEICFMK
GAYEQVIKYCTTYQSKGQTLTLTQQQRDVYQQEKARMGSAGLRVLALASGPELGQLTFLG
LVGIIDPPRTGVKEAVTTLIASGVSIKMITGDSQETAVAIASRLGLYSKTSQSVSGEEID
AMDVQQLSQIVPKVAVFYRASPRHKMKIIKSLQKNGSVVAMTGDGVNDAVALKAADIGVA
MGQTGTDVCKEAADMILVDDDFQTIMSAIEEGKGIYNNIKNFVRFQLSTSIAALTLISLA
TLMNFPNPLNAMQILWINIIMDGPPAQSLGVEPVDKDVIRKPPRNWKDSILTKNLILKIL
VSSIIIVCGTLFVFWRELRDNVITPRDTTMTFTCFVFFDMFNALSSRSQTKSVFEIGLCS
NRMFCYAVLGSIMGQLLVIYFPPLQKVFQTESLSILDLLFLLGLTSSVCIVAEIIKKVER
SREKIQKHVWLWERSGQQLVEIHPHLETGLPLTEDVSCV*
>ENSG00000276600|ENST00000617991
MNPRKKVDLKLIIVGAIGVGKTSLLHQYVHKTFYEEYQTTLGASILSKIIILGDTTLKLQ
IWDTGGQERFRSMVSTFYKGSDGCILAFDVTDLESFEALDIWRGDVLAKIVPMEQSYPMV
LLGNKIDLADRKVPQEVAQGWCREKDIPYFEVSAKNDINVVQAFEMLASRALSRYQSILE
NHLTESIKLSPDQSRSRCC*
>ENSG00000262246|ENST00000574025
MNRFRVSKFRHTEARPPRRESWISDIRAGTAPSCRNHIKSSCSLIAFNSDRPGVLGIVPL
QGQGEDKRRVAHLGCHSELAAHGDLVQSAVWSRDGALVGTACKDKQLRIFDPRTKPRASQ
STQAHENSRDSRLAWMGTWEHLVSTGFNQMREREVKLWDTRFFSSALASLTLDTSLGCLV
PLLDPDSGLLVLAGKGERQLYCYEVVPQQPALSPVTQCVLESVLRGAALVPRQALAVMSC
EVLRVLQLSDTAIVPIGYHVPRKAVEFHEDLFPDTAGCVPATDPHSWWAGDNQQVQKVSL
NPACRPHPSFTSCLVPPAEPLPDTAQPAVMETPVGDADASEGFSSPPSSLTSPSTPSSLG
PSLSSTSGIGTSPSLRSLQSLLGPSSKFRHAQGTVLHRDSHITNLKGLNLTTPGESDGFC
ANKLRVAVPLLSSGGQVAVLELRKPGRLPDTALPTLQNGAAVTDLAWDPFDPHRLAVAGE
DARIRLWRVPAEGLEEVLTTPETVLTGHTEKICSLRFHPLAANVLASSSYDLTVRIWDLQ
AGADRLKLQGHQDQIFSLAWSPDGQQLATVCKDGRVRVYRPRSGPEPLQEGPGPKGGRGA
RIVWVCDGRCLLVSGFDSQSERQLLLYEAEALAGGPLAVLGLDVAPSTLLPSYDPDTGLV

LLTGKGDTRVFLYELLPESPFFLECNSFTSPDPHKGLVLLPKTECDVREVELMRCLRLRQ
SSLEPVAFRLPRVRKEFFQDDVFPDTAVIWEPVLSAEAWLQGANGQPWLLSLQPPDMSPV
SQAPREAPARRAPSSAQYLEEKSDQQKKEELLNAMVAKLGNREDPLPQDSFEGVDEDEWD
*
>ENSG00000164342|ENST00000504367
MLDLSYNNLNVVGNDSFAWLPQLEYFFLEYNNIQHLFSHSLHGLFNVRYLNLKRSFTKQS
ISLASLPKIDDFSFQWLKCLEHLNMEDNDIPGIKSNMFTGLINLKYLSLSNSFTSLRTLT
NETFVSLAHSPLHILNLTKNKISKIESDAFSWLGHLEVLDLGLNEIGQELTGQEWRGLEN
IFEIYLSYNKYLQLTRNSFALVPSLQRLMLRRVALKNVDSSPSPFQPLRNLTILDLSNNN
IANINDDMLEGLEKLEILDLQHNNLARLWKHANPGGPIYFLKGLSHLHILNLESNGFDEI
PVEVFKDLFELKIIDLGLNNLNTLPASVFNNQVSLKSLNLQKNLITSVEKKVFGPAFRNL
TELDMRFNPFDCTCESIAWFVNWINETHTNIPELSSHYLCNTPPHYHGFPVRLFDTSSCK
DSAPFELFFMINTSILLIFIFIVLLIHFEGWRISFYWNVSVHRVLGFKEIDRQTEQFEYA
AYIIHAYKDKDWVWEHFSSMEKEDQSLKFCLEERDFEAGVFELEAIVNSIKRSRKIIFVI
THHLLKDPLCKRFKVHHAVQQAIEQNLDSIILVFLEEIPDYKLNHALCLRRGMFKSHCIL
NWPVQKERIGAFRHKLQVALGSKNSVH*
>ENSG00000140479|ENST00000618548
MPPRAPPAPGPRPPPRAAAATDTAAGAGGAGGAGGAGGPGFRPLAPRPWRWLLLLALPAA
CSAPPPRPVYTNHWAVQVLGGPAEADRVAAAHGYLNLGQIGNLEDYYHFYHSKTFKRSTL
SSRGPHTFLRMDPQVKWLQQQEVKRRVKRQVRSDPQALYFNDPIWSNMWYLHCGDKNSRC
RSEMNVQAAWKRGYTGKNVVVTILDDGIERNHPDLAPNYDSYASYDVNGNDYDPSPRYDA
SNENKHGTRCAGEVAASANNSYCIVGIAYNAKIGGIRMLDGDVTDVVEAKSLGIRPNYID
IYSASWGPDDDGKTVDGPGRLAKQAFEYGIKKGRQGLGSIFVWASGNGGREGDYCSCDGY
TNSIYTISVSSATENGYKPWYLEECASTLATTYSSGAFYERKIVTTDLRQRCTDGHTGTS
VSAPMVAGIIALALEANSQLTWRDVQHLLVKTSRPAHLKASDWKVNGAGHKVSHFYGFGL
VDAEALVVEAKKWTAVPSQHMCVAASDKRPRSIPLVQVLRTTALTSACAEHSDQRVVYLE
HVVVRTSISHPRRGDLQIYLVSPSGTKSQLLAKRLLDLSNEGFTNWEFMTVHCWGEKAEG
QWTLEIQDLPSQVRNPEKQGKLKEWSLILYGTAEHPYHTFSAHQSRSRMLELSAPELEPP
KAALSPSQVEVPEDEEDYTGVCHPECGDKGCDGPNADQCLNCVHFSLGSVKTSRKCVSVC
PLGYFGDTAARRCRRCHKGCETCSSRAATQCLSCRRGFYHHQEMNTCVTLCPAGFYADES
QKNCLKCHPSCKKCVDEPEKCTVCKEGFSLARGSCIPDCEPGTYFDSELIRCGECHHTCG
TCVGPGREECIHCAKNFHFHDWKCVPACGEGFYPEEMPGLPHKVCRRCDENCLSCAGSSR
NCSRCKTGFTQLGTSCITNHTCSNADETFCEMVKSNRLCERKLFIQFCCRTCLLAG*
>ENSG00000138768|ENST00000514213
MNFLRGVMGGQSAGPQHTEAETIQKLCDRVASSTLLDDRRNAVRALKSLSKKYRLEVGIQ
AMEHLIHVLQTDRSDSEIIGYALDTLYNIISNEEEEEVEENSTRQSEDLGSQFTEIFIKQ
QENVTLLLSLLEEFDFHVRWPGVKLLTSLLKQLGPQVQQIILVSPMGVSRLMDLLADSRE
VIRNDGVLLLQALTRSNGAIQKIVAFENAFERLLDIISEEGNSDGGIVVEDCLILLQNLL
KNNNSNQNFFKEGSYIQRMKPWFEVGDENSGWSAQKVTNLHLMLQLVRVLVSPTNPPGAT
SSCQKAMFQCGLLQQLCTILMATGVPADILTETINTVSEVIRGCQVNQDYFASVNAPSNP
PRPAIVVLLMSMVNERQPFVLRCAVLYCFQCFLYKNQKGQGEIVSTLLPSTIDATGNSVS
AGQLLCGGLFSTDSLSNWCAAVALAHALQENATQKEQLLRVQLATSIGNPPVSLLQQCTN
ILSQGSKIQTRVGLLMLLCTWLSNCPIAVTHFLHNSANVPFLTGQIAENLGEEEQLVQGL
CALLLGISIYFNDNSLESYMKEKLKQLIEKRIGKENFIEKLGFISKHELYSRASQKPQPN

FPSPEYMIFDHEFTKLVKELEGVITKAIYKSSEEDKKEEEVKKTLEQHDNIVTHYKNMIR
EQDLQLEELRQQVSTLKCQNEQLQTAVTQQVSQIQQHKDQYNLLKIQLGKDNQHQGSYSE
GAQMNGIQPEEIGRLREEIEELKRNQELLQSQLTEKDSMIENMKSSQTSGTNEQSSAIVS
ARDSEQVAELKQELATLKSQLNSQSVEITKLQTEKQELLQKTEAFAKSVEVQGETETIIA
TKTTDVEGRLSALLQETKELKNEIKALSEERTAIKEQLDSSNSTIAILQTEKDKLELEIT
DSKKEQDDLLVLLADQDQKILSLKNKLKDLGHPVEEEDELESGDQEDEDDESEDPGKDLD
HI*
>ENSG00000249853|ENST00000411826
MLFKQQAWLRQKLLVLGSLAVGSLLYLVARVGSLDRLQPICPIEGRLGGARTQAEFPLRA
LQFKRGLLHEFRKGNASKEQVRLHDLVQQLPKAIIIGVRKGGTRALLEMLNLHPAVVKAS
QEIHFFDNDENYGKGIEWYRKKMPFSYPQQITIEKSPAYFITEEVPERIYKMNSSIKLLI
IVREPTTRAISDYTQVLEGKERKNKTYYKFEKLAIDPNTCEVNTKYKAVRTSIYTKHLER
WLKYFPIEQFHVVDGDRLITEPLPELQLVEKFLNLPPRISQYNLYFNATRGFYCLRFNII
FNKCLAGSKGRIHPEVDPSVITKLRKFFHPFNQKFYQITGRTLNWP*
>ENSG00000224320|ENST00000443552
MAVMAPRTLLLLLSGALALTQTWAGSHSMRYFFTSVSRPGRGEPRFIAVGYVDDTQFVRF
DSDAASQKMEPRAPWIEQEGPEYWDQETRNMKAHSQTDRANLGTLRGYYNQSEDGSHTIQ
IMYGCDVGPDGRFLRGYRQDAYDGKDYIALNEDLRSWTAADMAAQITKRKWEAVHAAEQR
RVYLEGRCVDGLRRYLENGKETLQRTDPPKTHMTHHPISDHEATLRCWALGFYPAEITLT
WQRDGEDQTQDTELVETRPAGDGTFQKWAAVVVPSGEEQRYTCHVQHEGLPKPLTLRWEL
SSQPTIPIVGIIAGLVLLGAVITGAVVAAVMWRRKSSDRKGGSYTQAASSDSAQGSDVSL
TACKV*
>ENSG00000235915|ENST00000420797
MLSRNDVKRLERSWAQGSSEQELHYASLQRLPVPSSEGPDLRGRDKRGTKEDPRADYACI
AENKPT*
>ENSG00000234514|ENST00000455901
MLSRNDDICIYGGLGLGGLLLLAVVLLSACLCWLHRRVKRLERSWAQGSSEQELHYASLQ
RLPVPSSEGPDLRGRDKRGTKEDPRADYACIAENKPT*
>ENSG00000196535|ENST00000527372
MFNLMKKDKDKDGGRKEKKEKKEKKERMSAAELRSLEEMSLRRGFFNLNRSSKRESKTRL
EISNPIPIKVASGSDLHLTDIDSDSNRGSVILDSGHLSTASSSDDLKGEEGSFRGSVLQR
AAKFGSLAKQNSQMIVKRFSFSQRSRDESASETSTPSEHSAAPSPQVEVRTLEGQLVQHP
GPGIPRPGHRSRAPELVTKKFPVDLRLPPVVPLPPPTLRELELQRRPTGDFGFSLRRTTM
LDRGPEGQACRRVVHFAEPGAGTKDLALGLVPGDRLVEINGHNVESKSRDEIVEMIRQSG
DSVRLKVQPIPELSELSRSWLRSGEGPRREPSDAKTEEQIAAEEAWNETEKVWLVHRDGF
SLASQLKSEELNLPEGKVRVKLDHDGAILDVDEDDVEKANAPSCDRLEDLASLVYLNESS
VLHTLRQRYGASLLHTYAGPSLLVLGPRGAPAVYSEKVMHMFKGCRREDMAPHIYAVAQT
AYRAMLMSRQDQSIILLGSSGSGKTTSCQHLVQYLATIAGISGNKVFSVEKWQALYTLLE
AFGNSPTIINGNATRFSQILSLDFDQAGQVASASIQTMLLEKLRVARRPASEATFNVFYY
LLACGDGTLRTELHLNHLAENNVFGIVPLAKPEEKQKAAQQFSKLQAAMKVLGISPDEQK
ACWFILAAIYHLGAAGATKEAAEAGRKQFARHEWAQKAAYLLGCSLEELSSAIFKHQHKG
GTLQRSTSFRQGPEESGLGDGTGPKLSALECLEGMAAGLYSELFTLLVSLVNRALKSSQH
SLCSMMIVDTPGFQNPEQGGSARGASFEELCHNYTQDRLQRLFHERTFVQELERYKEENI
ELAFDDLEPPTDDSVAAVDQASHQSLVRSLARTDEARGLLWLLEEEALVPGASEDTLLER

LFSYYGPQEGDKKGQSPLLHSSKPHHFLLGHSHGTNWVEYNVTGWLNYTKQNPATQNAPR
LLQDSQKKIISNLFLGRAGSATVLSGSIAGLEGGSQLALRRATSMRKTFTTGMAAVKKKS
LCIQMKLQVDALIDTIKKSKLHFVHCFLPVAEGWAGEPRSASSRRVSSSSELDLPSGDHC
EAGLLQLDVPLLRTQLRGSRLLDAMRMYRQGYPDHMVFSEFRRRFDVLAPHLTKKHGRNY
IVVDERRAVEELLECLDLEKSSCCMGLSRVFFRAGTLARLEEQRDEQTSRNLTLFQAACR
GYLARQHFKKRKIQDLAIRCVQKNIKKNKGVKDWPWWKLFTTVRPLIEVQLSEEQIRNKD
EEIQQLRSKLEKAEKERNELRLNSDRLESRISELTSELTDERNTGESASQLLDAETAERL
RAEKEMKELQTQYDALKKQMEVMEMEVMEARLIRAAEINGEVDDDDAGGEWRLKYERAVR
EVDFTKKRLQQEFEDKLEVEQQNKRQLERRLGDLQADSEESQRALQQLKKKCQRLTAELQ
DTKLHLEGQQVRNHELEKKQRRFDSELSQAHEEAQREKLQREKLQREKDMLLAEAFSLKQ
QLEEKDMDIAGFTQKVVSLEAELQDISSQESKDEASLAKVKKQLRDLEAKVKDQEEELDE
QAGTIQMLEQAKLRLEMEMERMRQTHSKEMESRDEEVEEARQSCQKKLKQMEVQLEEEYE
DKQKVLREKRELEGKLATLSDQVNRRDFESEKRLRKDLKRTKALLADAQLMLDHLKNSAP
SKREIAQLKNQLEESEFTCAAAVKARKAMEVEIEDLHLQIDDIAKAKTALEEQLSRLQRE
KNEIQNRLEEDQEDMNELMKKHKAAVAQASRDLAQINDLQAQLEEANKEKQELQEKLQAL
QSQVEFLEQSMVDKSLVSRQEAKIRELETRLEFERTQVKRLESLASRLKENMEKLTEERD
QRIAAENREKEQNKRLQRQLRDTKEEMGELARKEAEASRKKHELEMDLESLEAANQSLQA
DLKLAFKRIGDLQAAIEDEMESDENEDLINSLQDMVTKYQKRKNKLEGDSDVDSELEDRV
DGVKSWLSKNKGPSKAASDDGSLKSSSPTSYWKSLAPDRSDDEHDPLDNTSRPRYSHSYL
SDSDTEAKLTETNA*
>ENSG00000223465|ENST00000450425
MLSRNDVKRLERSWAQGSSEQELHYASLQRLPVPSSEGPDLRGRDKRGTKEDPRADYACI
AENKPT*
>ENSG00000110080|ENST00000526727
MVSKSRWKLLAMLALVLVVMVWYSISREDRYIELFYFPIPEKKEPCLQGEAESKASKLFG
NYSRDQPIFLRLEDYFWVKTPSAYELPYGTKGSEDLLLRVLAITSSSIPKNIQSLRCRRC
VVVGNGHRLRNSSLGDAINKYDVVIRLNNAPVAGYEGDVGSKTTMRLFYPESAHFDPKVE
NNPDTLLVLVAFKAMDFHWIETILSDKKRVRKGFWKQPPLIWDVNPKQIRILNPFFMEIA
ADKLLSLPMQQPRKIKQKPTTGLLAITLALHLCDLVHIAGFGYPDAYNKKQTIHYYEQIT
LKSMAGSGHNVSQEALAIKRMLEMGAIKNLTSF*
>ENSG00000090615|ENST00000545875
MDGASAEQDGLQEDRSHSGPSSLPEAPLKPPGPLVPPDQQDKVQCAEVNRASTEGESPDG
PGQGGLCQNGPTPPFPDPPSSLDPTTSPVGPDASPGVAGFHDNLRKSQGTSAEGSVRKEA
LQSLRLSLPMQETQLCSTDSPLPLEKEEQVRLQARKWLEEQLKQYRVKRQQERSSQPATK
TRLFSTLDPELMLNPENLPRASTLAMTKEYSFLRTSVPRGPKVGSLGLPAHPREKKTSKS
SKIRSLADYRTEDSNAGNSGGNVPAPDSTKGSLKQNRSSAASVVSEISLSPDTDDRLENT
SLAGDSVSEVDGNDSDSSSYSSASTRGTYGILSKTVGTQDTPYMVNGQEIPADTLGQFPS
IKDVLQAAAAEHQDQGQEVNGEVRSRRDSICSSVSLESSAAETQEEMLQVLKEKMRLEGQ
LEALSLEASQALKEKAELQAQLAALSTKLQAQVECSHSSQQRQDSLSSEVDTLKQSCWDL
ERAMTDLQNMLEAKNASLASSNNDLQVAEEQYQRLMAKVEDMQRSMLSKDNTVHDLRQQM
TALQSQLQQVQLERTTLTSKLKASQAEISSLQSVRQWYQQQLALAQEARVRLQGEMAHIQ
VGQMTQAGLLEHLKLENVSLSQQLTETQHRSMKEKGRIAAQLQGIEADMLDQEAAFMQIQ
EAKTMVEEDLQRRLEEFEGERERLQRMADSAASLEQQLEQVKLTLLQRDQQLEALQQEHL
DLMKQLTLTQEALQSREQSLDALQTHYDELQARLGELQGEAASREDTICLLQNEKIILEA

ALQAAKSGKEELDRGARRLEEGTEETSETLEKLREELAIKSGQVEHLQQETAALKKQMQK
IKEQFLQQKVMVEAYRRDATSKDQLISELKATRKRLDSELKELRQELMQVHGEKRTAEAE
LSRLHREVAQVRQHMADLEGHLQSAQKERDEMETHLQSLQFDKEQMVAVTEANEALKKQI
EELQQEARKAITEQKQKMRRLGSDLTSAQKEMKTKHKAYENAVGILSRRLQEALAAKEAA
DAELGQLRAQGGSSDSSLALHERIQALEAELQAVSHSKTLLEKELQEVIALTSQELEESR
EKVLELEDEVRPGHLLWRQRGAGHVSPGHAATRETRRTKLHRVPSVATFGVATF*
>ENSG00000152056|ENST00000446015
MIHFILLFSRQGKLRLQKWYITLPDKERKKITREIVQIILSRGHRTSSFVDWKELKLVYK
RYASLYFCCAIENQDNELLTLEIVHRYVELLDKYFGNVCELDIIFNFEKAYFILDEFIIG
GEIQETSKKIAVKAIEDSDMLQEVSTVSQTMGER*
>ENSG00000228163|ENST00000422504
MRPEDRMFHIRAVILRALSLAFLLSLRGAGAIKADHVSTYAAFVQTHRPTGEFMFEFDED
EMFYVDLDKKETVWHLEEFGQAFSFEAQGGLANIAILNNNLNTLIQRSNHTQATNDPPEV
TVFPKEPVELGQPNTLICHIDKFFPPVLNVTWLCNGELVTEGVAESLFLPRTDYSFHKFH
YLTFVPSAEDFYDCRVEHWGLDQPLLKHWEAQEPIQMPETTETVLCALGLVLGLVGIIVG
TVLIIKSLRSGHDPRAQGTL*
>ENSG00000133318|ENST00000537981
MAEPSAATQSHSISSSSFGAEPSAPGGGGSPGACPALGTKSCSSSCAVHDLIFWRDVKKT
GFVFGTTLIMLLSLAAFSVISVVSYLILALLSVTISFRIYKSVIQAVQKSEEGHPFKAYL
DVDITLSSEAFHNYMNAAMVHINRALKLIIRLFLVEDLVDSLKLAVFMWLMTYVGAVFNG
ITLLILAELLIFSVPIVYEKYKTQIDHYVGIARDQTKSIVEKIQAKLPGIAKKKAE*
>ENSG00000166685|ENST00000582512
XLNIVLTAKGDEVKSGRSKPDSRIEKVTDHLEALIDPFDLDVFTPHLNSNLHRLVQRTSV
LFGLVTGTENQLAPRSSTFNSQEPHNILPLASSQIRFGLLPLSMTSTRKAKSTRNIETKA
QYDANC*
>ENSG00000117620|ENST00000639221
MFANLKYVSLGILVFQTTSLVLTMRYSRTLKEEGPRYLSSTAVVVAELLKIMACILLVYK
DSKCSLRALNRVLHDEILNKPMETLKLAIPSGIYTLQNNLLYVALSNLDAATYQWPSDSQ
LDSKELSAGSQFVGLMAVLTACFSSGFAGVYFEKILKETKQSVWIRNIQLGFFGSIFGLM
GVYIYDGELVSKNGFFQGYNRLTWIVVVLQCLFPWSHPCNNSYFFVWL*
>ENSG00000158258|ENST00000458420
MLPGRLCWVPLLLALGVGSGSGGGGDSRQRRLLAAKVNKHKPWIETSYHGVITENNDTVI
LDPPLVALDKDAPVPFAGEICAFKIHGQELPFEAVVLNKTSGEGRLRAKSPIDCELQKEY
TFIIQAYDCGAGPHETAWKKSHKAVVHIQVKDVNEFAPTFKEPAYKAVVTEGKIYDSILQ
VEAIDEDCSPQYSQICNYEIVTTDVPFAIDRNGNIRNTEKLSYDKQHQYEILVTAYDCGQ
KPAAQDTLVQVDVKPVCKPGWQDWTKRIEYQPGSGSMPLFPSIHLETCDGAVSSLQIVTE
LQTNYIGKGCDRETYSEKSLQKLCGASSGIIDLLPSPSAATNWTAGLLVDSSEMIFKFDG
RQGAKVPDGIVPKNLTDQFTITMWMKHGPSPGVRAEKETILCNSDKTEMNRHHYALYVHN
CRLVFLLRKDFDQADTFRPAEFHWKLDQICDKEWHYYVINVEFPVVTLYMDGATYEPYLV
TNDWPIHPSHIAMQLTVGACWQGGEVTKPQFAQFFHGSLASLTIRPGKMESQKVISCLQA
CKEGLDINSLESLGQGIKYHFNPSQSILVMEGDDIGNINRALQKVSYINSRQFPTAGVRR
LKVSSKVQCFGEDVCISIPEVDAYVMVLQAIEPRITLRGTDHFWRPAAQFESARGVTLFP
DIKIVSTFAKTEAPGDVKTTDPKSEVLEEMLHNLDFCDILVIGGDLDPRQECLELNHSEL
HQRHLDATNSTAGYSIYGVGSMSRYEQVLHHIRYRNWRPASLEARRFRIKCSELNGRYTS

NEFNLEVSILHEDQVSDKEHVNHLIVQPPFLQSVHHPESRSSIQHSSVVPSIATVVIIIS
VCMLVFVVAMGVYRVRIAHQHFIQETEAAKESEMDWDDSALTITVNPMEKHEGPGHGEDE
TEGEEEEEAEEEMSSSSGSDDSEEEEEEEGMGRGRHGQNGARQAQLEWDDSTLPY*
>ENSG00000115525|ENST00000640982
MPSEYTYVKLRSDCSRPSLQWYTRAQSKMRRPSLLLKDILKCTLLVFGVWILYILKLNYT
TEECDMKKMHYVDPDHVKRAQKYAQQVLQKECRPKFAKTSMALLFEHRYSVDLLPFVQKA
PKDSEAESKYDPPFGFRKFSSKVQTLLELLPEHDLPEHLKAKTCRRCVVIGSGGILHGLE
LGHTLNQFDVVIRLNSAPVEGYSEHVGNKTTIRMTYPEGAPLSDLEYYSNDLFVAVLFKS
VDFNWLQAMVKKETLPFWVRLFFWKQVAEKIPLQPKHFRILNPVIIKETAFDILQYSEPQ
SRFWGRDKNVPTIGVIAVVLATHLCDEVSLAGFGYDLNQPRTPLHYFDSQCMAAMNFQTM
HNVTTETKFLLKLVKEGVVKDLSGGIDREF*
>ENSG00000105438|ENST00000597017
MKVVYIACSFTTVWLIYSKFKATYDGNHDTFRVEFLVVPTAILAFLVNHDFTPLEILWTF
SIYLESVAILPQLFMVSKTGEAETITSHYLFALGVYRTLYLFNWIWRYHFEGFFDLIAIV
AGLVQTVLYCDFFYLYITKVLKGKKLSLPA*
>ENSG00000163956|ENST00000500728
MAPRRVRSFLRGLPALLLLLLFLGPWPAASHGGKYSREKNQPKPSPKRESGEEFRMEKLN
QLWEKAQRLHLPPVRLAELHADLKIQERDELAWKKLKLDGLDEDGEKEARLIRNLNVILA
KYGLDGKKDARQVTSNSLSGTQEDGLDDPRLEKLWHKAKTSGKFSGEELDKLWREFLHHK
EKVHEYNVLLETLSRTEEIHENVISPSDLSDIKGSVLHSRHTELKEKLRSINQGLDRLRR
VSHQGYSTEAEFEEPRVIDLWDLAQSANLTDKELEAFREELKHFEAKIEKHNHYQKQLEI
AHEKLRHAESVGDGERVSRSREKHALLEGRTKELGYTVKKHLQDLSGRISRARHNEL*
>ENSG00000187210|ENST00000442371
MLRTLLRRRLFSYPTKYYFMVLVLSLITFSVLRIHQKPEFVSVRHLELAGENPSSDINCT
KVLQGDVNEIQKVKLEILTVKFKKRPRWTPDDYINMTSDCSSFIKRRKYIVEPLSKEEAE
FPIAYSIVVHHKIEMLDRLLRAIYMPQNFYCIHVDTKSEDSYLAAVMGIASCFSNVFVAS
RLESVVYASWSRVQADLNCMKDLYAMSANWKYLINLCGMDFPIKTNLEIVRKLKLLMGEN
NLETERMPSHKEERWKKRYEVVNGKLTNTGTVKMLPPLETPLFSGSAYFVVSREYVGYVL
QNEKIQKLMEWAQDTYSPDEYLWATIQRIPEVPGSLPASHKYDLSDMQAVARFVKWQYFE
GDVSKGAPYPPCDGVHVRSVCIFGAGDLNWMLRKHHLFANKFDVDVDLFAIQCLDEHLRH
KALETLKH*
>ENSG00000144057|ENST00000409087
MKPHLKQWRQRMLFGIFAWGLLFLLIFIYFTDSNPAEPVPSSLSFLETRRLLPVQGKQRA
IMGAAHEPSPPGGLDARQALPRAHPAGSFHAGPGDLQKWAQSQDGFEHKEFFSSQVGRKS
QSAFYPEDDDYFFAAGQPGWHSHTQGTLGFPSPGEPGPREGAFPAAQVQRRRVKKRHRRQ
RRSHVLEEGDDGDRLYSSMSRAFLYRLWKGNVSSKMLNPRLQKAMKDYLTANKHGVRFRG
KREAGLSRAQLLCQLRSRARVRTLDGTEAPFSALGWRRLVPAVPLSQLHPRGLRSCAVVM
SAGAILNSSLGEEIDSHDAVLRFNSAPTRGYEKDVGNKTTIRIINSQILTNPSHHFIDSS
LYKDVILVAWDPAPYSANLNLWYKKPDYNLFTPYIQHRQRNPNQPFYILHPKFIWQLWDI
IQENTKEKIQPNPPSSGFIGSFVKIGHIRACSEPRSRDCTPAWTTE*
>ENSG00000223465|ENST00000423871
MLSRNDVKRLERSWRLPVPSSEGPDLRGRDKRGTKEDPRADYACIAENKPT*
>ENSG00000167964|ENST00000541451
MLVGDSGVGKTCLLVRFKDGAFLAGTFISTVGIDFRNKVLDVDGVKVKLQMWDTAGQERF

RSVTHAYYRDAHALLLLYDVTNKASFDNIQAWLTEIHEYAQHDVALMLLGNKVDSAHERV
VKREDGEKLAKEYGLPFMETSAKTGLNVDLAFTAIAKELKQRSMKAPSEPRFRLHDYVKR
EGRGASCCRP*
>ENSG00000165861|ENST00000555072
MAHSSFFPDEYFTCSSLCLSCGVGCKKSMNHGKEGVPHEAKSRCRYSHQYDNRVYTCKAC
YERGEEVSVVPKTSASTDSPWMGLAKYAWSGYVIECPNCGVVYRSRQYWFGNQDPVDTVV
RTEIVHVWPGTDGFLKDNNNAAQRLLDGMNFMAQSVSELSLGPTKAVTSWLTDQIAPAYW
RPNSQILSCNKCATSFKDNDTKHHCRACGEGFCDSCSSKTRPVPERGWGPAPVRVCDNCY
EARNVQLAVTEAQVDDEGGTLIARKVGEAVQNTLGAVVTAIDIPLGLVKDAARPAYWVPD
HEILHCHNCRKEFSIKLSKHHCRACGQGFCDECSHDRRAVPSRGWDHPVRVCFNCNKKPG
DL*
>ENSG00000175416|ENST00000502877
MADDFGFFSSSESGAPEAAEEDPAAAFLAQQESEIAGIENDEGFGAPAGSHAAPAQPGPT
SGAGSEDMGTTVNGDVFQEAFVKESKEETPGTEWEKVAQLCDFNPKSSKQCKDVSRLRSV
LMSLK
>ENSG00000071073|ENST00000414521
MNYSITVSIVMGIPTVKREVKSYLIETLHSLIDNLYPEEKLDCVIVVFIGETDIDYVHGV
VANLEKEFSKEISSGLVEVISPPESYYPDLTNLKETFGDSKERVRWRTKQNLDYCFLMMY
AQEKGIYYIQLEDDIIVKQNYFNTIKNFALQLSSEEWMILEFSQLGFIGKMFQAPDLTLI
VEFIFMFYKEKPIDWLLDHILWVKVCNPEKDAKHCDRQKANLRIRFRPSLFQHVGLHSSL
SGKIQKLTDKDYMKPLLLKIHVNPPAEVSTSLKVYQGHTLEKTYMGEDFFWAITPIAGDY
ILFKFDKPVNVESYLFHSGNQEHPGDILLNTTVEVLPFKSEGLEISKETKDKRLEDGYFR
IGKFENGVAEGMVDPSLNPISAFRLSVIQNSAVWAILNEQRVFSGWWQKSRDKAEGPQAP
LLF*
>ENSG00000196655|ENST00000533632
MAIFSVYVVNKAGGLIYQLDSYAPRAEAEKTFSYPLDLLLKLHDERVLVAFGQRDGIRVG
HAVLAINGMDVNGRYTADGKEVLEYLGNPANYPVSIRFGRPRLTSNEKLMLASMFHSLFA
IGSQLSPEQGSSGIEMLETDTFKLHCYQTLTGIKFVVLADPRQAGIDSLLRKIYEIYSDF
ALKNPFYSLEMPIRCELFDQNLKLALEVAEKAGTFGPGS*
>ENSG00000235346|ENST00000546634
MVVMAPRTLFLLLSGALTLTETWAGSHSMRYFSAAVSRPGRGEPRFIAMGYVDDTQFVRF
DSDSACPRMEPRAPWVEQEGPEYWEEETRNTKAHAQTDRMNLQTLRGYYNQSEASSHTLQ
WMIGCDLGSDGRLLRGYEQYAYDGKDYLALNEDLRSWTAADTAAQISKRKCEAANVAEQR
RAYLEGTCVEWLHRYLENGKEMLQRADPPKTHVTHHPVFDYEATLRCWALGFYPAEIILT
WQRDGEDQTQDVELVETRPAGDGTFQKWAAVVVPSGEEQRYTCHVQHEGLPEPLMLRWKQ
SSLPTIPIMGIVAGLVVLAAVVTGAAVAAVLWRKKSSD*
>ENSG00000133110|ENST00000541481
MIPFLPMFSLLLLLIVNPINANNHYDKILAHSRIRGRDQGPNVCALQQILGTKKKYFSTC
KNWYKKSICGQKTTVLYECCPGYMRMEGMKGCPAVLPIDHVYGTLGIVGATTTQRYSDAS
KLREEIEGKGSFTYFAPSNEAWDNLDSDIRRGLESNVNVELLNALHSHMINKRMLTKDLK
NGMIIPSMYNNLGLFINHYPNGVVTVNCARIIHGNQIATNGVVHVIDRVLTQIGTSIQDF
IEAEDDLSSFRAAAITSDILEALGRDGHFTLFAPTNEAFEKLPRGVLERIMGDKVASEAL
MKYHILNTLQCSESIMGGAVFETLEGNTIEIGCDGDSITVNGIKMVNKKDIVTNNGVIHL
IDQVLIPDSAKQVIELAGKQQTTFTDLVAQLGLASALRPDGEYTLLAPVNNAFSDDTLSM

DQRLLKLILQNHILKVKVGLNELYNGQILETIGGKQLRVFVYRTAVCIENSCMEKGSKQG
RNGAIHIFREIIKPAEKSLHEKLKQDKRFSTFLSLLEAADLKELLTQPGDWTLFVPTNDA
FKGMTSEEKEILIRDKNALQNIILYHLTPGVFIGKGFEPGVTNILKTTQGSKIFLKEVND
TLLVNELKSKESDIMTTNGVIHVVDKLLYPADTPVGNDQLLEILNKLIKYIQIKFVRGST
FKEIPVTVYSPEIKYTRISTGGGETEETLKKLLQEEVTKVTKFIEGGDGHLFEDEEIKRL
LQGDTPVRKLQANKKVQGSRRRLREGRSQ*
>ENSG00000225691|ENST00000457903
MRVMAPRTLILLLSGALALTETWAGSHSMRYFYTAVSRPGRGEPHFIAVGYVDDTQFVRF
DSDAASPRGEPRAPWVEQEGPEYWDRETQKYKRQAQTDRVSLRNLRGYYNQSEAGSHIIQ
RMYGCDVGPDGRLLRGYDQYAYDGKDYIALNEDLRSWTAADTAAQITQRKWEAAREAEQL
RAYLEGLCVEWLRRYLKNGKETLQRAEHPKTHVTHHPVSDHEATLRCWALGFYPAEITLT
WQWDGEDQTQDTELVETRPAGDGTFQKWAAVVVPSGEEQRYTCHVQHEGLPEPLTLRWEP
SSQPTIPIVGIVAGLAVLAVLAVLGAVVAVVMCRRKSSGGKGGSCSQAASSNSAQGSDES
LIACKA*
>ENSG00000136297|ENST00000406755
MFAPRLLDFQKTKYARFMNHRVPAHKRYQPTEYEHAANCATHAFWIIPSILGSSNLYFLS
DDDWETISAWIYGLGLCGLFVVSTVFHTISWKKSHLRMVEHCLHMFDRMVIYFFIAASYA
PWLNLRELGPWASHMRWLVWIMASVGTIYVFFFHERYKLVELLCYVVMGFFPALVILSMA
GKLRPGMTCLKRQ*
>ENSG00000117620|ENST00000639088
MFANLKYVSLGILVFQTTSLVLTMRYSRTLKEEGPRYLSSTAVVVAELLKIMACILLVYK
DSSHVSVKNSYNSIIFCVYA*
>ENSG00000236802|ENST00000430971
MQLRLFRRLLLAALLLVIVWTLFGPSGLGEELLSLSLASLLPAPASPGPPLALPRLLIPN
QEACSGPGAPPFLLILVCTAPENLNQRNAIRASWGGLREARGLRVQTLFLLGEPNAQHPV
WGSQGSDLASESAAQGDILQAAFQDSYRNLTLKTLSGLNWAEKHCPMARYVLKTDDDVYV
NVPELVSELVLRGGRWGQWERSTEPQREAEQEGGQVLHSEEVPLLYLGRVHWRVNPSRTP
GGRHRVSEEQWPHTWGPFPPYASGTGYVLSASAVQLILKVASRAPLLPLEDVFVGVSARR
GGLAPTQCVKLAGATHYPLDRCCYGKFLLTSHRLDPWKMQEAWKLVGGSDGERTAPFCSW
FQGVLGILRCRAIAWLQS*
>ENSG00000115525|ENST00000640322
MPSEYTYVKLRSDCSRPSLQWYTRAQSKMRRPSLLLKDILKCTLLVFGVWILYILKLNYT
TEECDMKKMHYVDPDHVKRAQKYAQQVLQKECRPKFAKTSMALLFEHRYSVDLLPFVQKA
PKDSEAESKYDPPFGFRKFSSKVQTLLELLPEHDLPEHLKAKTCRRCVVIGSGGILHGLE
LGHTLNQFDVVIRLNSAPVEGYSEHVGNKTTIRMTYPEGAPLSDLEYYSNDLFVAVLFKS
VDFNWLQAMVKKETLPFWVRLFFWKQVAEKIPLQPKHFRILNPVIIKETAFDILQYSEPQ
SRFWGRDKNVPTIGVIAVVLATHLCDEVSLAGFGYDLNQPRTPLHYFDSQCMAAMNFQTM
HNVTTETKFLLKLVKEGVVKDLSGGIDREF*
>ENSG00000280674|ENST00000630389
MMLIPTHHFRNIERKPEYLQPEKCVPPPYPGPVGTMWFIRDGCGIACAIVTWFLVLYAEF
VVLFVMLIPSRDYVYSIINGIVFNLLAFLALASHCRAMLTDPGAVPKGNATKEFIESLQL
KPGQVVYKCPKCCSIKPDRAHHCSVCKRCIRKMDHHCPWVNNCVGENNQKYFVLFTMYIA
LISLHALIMVGFHFLHCFEEDWTTYGLNREEMAETGISLHEKMQPLNFSSTECSSFSPPT
TVILLILLCFEGLLFLIFTSVMFGTQVHSICTDETGIEQLKKEERRWAKKTKWMNMKAVF

GHPFSLGWASPFATPDQGKADPYQYVV*
>ENSG00000278114|ENST00000610415
MGCLWGLALPLFFFCWEVGVSGSSAGPSTRRADTAMTTDDTEVPAMTLAPGHAALETQTL
SAETSSRASTPAGPIPEAETRGAKRISPARETRSFTKTSPNFMVLIATSVETSAASGSPE
GAGMTTVQTITGSDPREAIFDTLCTDDSSEEAKTLTMDILTLAHTSTEAKGLSSESSASS
DSPHPVITPSRASESSASSDGPHPVITPSRASESSASSDGPHPVITPSRASESSASSDGP
HPVITPSRASESSASSDGPHPVITPSRASESSASSDGPHPVITPSRASESSASSDGPHPV
ITPSRASESSASSDGPHPVITPSRASESSASSDGPHPVITPSRASESSASSDGLHPVITP
SRASESSASSDGPHPVITPSRASESSASSDGPHPVITPSWSPGSDVTLLAEALVTVTNIE
VINCSITEIETTTSSIPGASDTDLIPTEGVKASSTSDPPALPDSTEAKPHITEVTASAET
LSTAGTTESAAPDATVGTPLPTNSATEREVTAPGATTLSGALVTVSRNPLEETSALSVET
PSYVKVSGAAPVSIEAGSAVGKTTSFAGSSASSYSPSEAALKNFTPSETPTMDIATKGPF
PTSRDPLPSVPPTTTNSSRGTNSTLAKITTSAKTTMKPPTATPTTARTRPTTDVSAGENG
GFLLLRLSVASPEDLTDPRVAERLMQQLHRELHAHAPHFQVSLLRVRRG*
>ENSG00000197249|ENST00000636712
MPSSVSWGILLLAGLCCLVPVSLAEDPQGDAAQKTDTSHHDQDHPTFNKITPNLAEFAFS
LYRQLAHQSNSTNIFFSPVSIATAFAMLSLGTKADTHDEILEGLNFNLTEIPEAQIHEGF
QELLRTLNQPDSQLQLTTGNGLFLSEGLKLVDKFLEDVKKLYHSEAFTVNFGDTEEAKKQ
INDYVEKGTQGKIVDLVKELDRDTVFALVNYIFFKGKWERPFEVKDTEEEDFHVDQVTTV
KVPMMKRLGMFNIQHCKKLSSWVLLMKYLGNATAIFFLPDEGKLQHLENELTHDIITKFL
ENEDRRSASLHLPKLSITGTYDLKSVLGQLGITKVFSNGADLSGVTEEAPLKLSKAVHKA
VLTIDEKGTEAAGAMFLEAIPMSIPPEVKFNKPFVFLMIEQNTKSPLFMGKVVNPTQK*
>ENSG00000273641|ENST00000613448
MRIHYLLFALLFLFLVPVPGHGGIINTLQKYYCRVRGGRCAVLSCLPKEEQIGKCSTRGR
KCCRRKK*
>ENSG00000236490|ENST00000456807
MKSLSLLLAVALGLATAVSAGPAVIECWFVEDASGKGLAKRPGALLLRQGPGEPPPRPDL
DPELYLSVHDPAGALQAAFRRYPRGAPAPHCEMSRFVPLPASAKWASGLTPAQNCPRALD
GAWLMVSISSPVLSLSSLLRPQPEPQQEPVLITMATVVLTVLTHTPAPRVRLGQDALLDL
SFAYMPPTSEAASSLAPGPPPFGLEWRRQHLGKGHLLLAATPGLNGQMPAAQEGAVAFAA
WDDDEPWGPWTGNGTFWLPRVQPFQEGTYLATIHLPYLQGQVTLELAVYKPPKVSLMPAT
LARAAPGEAPPELLCLVSHFYPSGGLEVEWELRGGPGGRSQKAEGQRWLSALRHHSDGSV
SLSGHLQPPPVTTEQHGARYACRIHHPSLPASGRSAEVTLEVAGLSGPSLEDSVGLFLSA
FLLLGLFKALGWAAVYLSTCKDSKKVQCSTSLYLSLVTLSPHPISKPMEGGCWCGRQNLG
LEFTLIWVKTWHYILTVGLFEHAT*
>ENSG00000234514|ENST00000426938
MLSRNDDICIYGGLGLGGLLLLAVVLLSACLCWLHRRVKRLERSWAQGSSEQELHYASLQ
RLPVPSSEGPDLRGRDKRGTKEDPRADYACIAENKPT*
>ENSG00000205060|ENST00000470969
MRPALAVGLVFAGCCSNVIFLELLARKHPGCGNIVTFAQFLFIAVEGFLFEADLGRKPPA
IPIRYYAIMVTMFFTVSVVNNYALNLNIAMPLHMIFRSGSLIANMILGIIILKKRYSIFK
YTSIALVSVGIFICTFMSAKQVTSQSSLSENDGFQAFVWWLLGIGALTFALLMSARMGIF
QETLYKRFGKHSKEALFYNHALPLPGFVFLASDIYDHAVLFNKSGEFGVRQ*
>ENSG00000197249|ENST00000489769

MPSSVSWGILLLAGLCCLVPVSLAEDPQGDAAQKTDTSHHDQDHPTFNKITPNLAEFAFS
LYRQLAHQSNSTNIFFSPVSIATAFAMLSLGTKADTHDEILEGLNFNLTEIPEAQIHEGF
QELLRTLNQPDSQLQLTTGNGLFLSEGLKLVDKFLEDVKKLYHSEAFTVNFGDTEEAKKQ
INDYVEKGTQGKIVDLVKELDRDTVFALVNYIFFKGKWERPFEVKDTEEEDFHVDQVTTV
KVPMMKRLGMFNIQHCKKLSSWVLLMKYLGNATAIFFLPDEGKLQHLENELTHDIITKFL
ENEDRR*
>ENSG00000235346|ENST00000423373
MVVMAPRTLFLLLSGALTLTETWAGSHSMRYFSAAVSRPGRGEPRFIAMGYVDDTQFVRF
DSDSACPRMEPRAPWVEQEGPEYWEEETRNTKAHAQTDRMNLQTLRGYYNQSEASSHTLQ
WMIGCDLGSDGRLLRGYEQYAYDGKDYLALNEDLRSWTAADTAAQISKRKCEAANVAEQR
RAYLEGTCVEWLHRYLENGKEMLQRADPPKTHVTHHPVFDYEATLRCWALGFYPAEIILT
WQRDGEDQTQDVELVETRPAGDGTFQKWAAVVVPSGEEQRYTCHVQHEGLPEPLMLRWKQ
SSLPTIPIMGIVAGLVVLAAVVTGAAVAAVLWRKKSSD*
>ENSG00000231286|ENST00000413089
MSWKKALRIPGGLRAATVTLMLAMLSTPVAEGRDSPEDFVYQFKAMCYFTNGTERVRYVT
RYIYNREEYARFDSDVEVYRAVTPLGPPDAEYWNSQKEVLERTRAELDTVCRHNYQLELR
TTLQRRVEPTVTISPSRTEALNHHNLLVCSVTDFYPAQIKVRWFRNDQEETTGVVSTPLI
RNGDWTFQILVMLEMTPQHGDVYTCHVEHPSLQNPITVEWRAQSESAQSKMLSGIGGFVL
GLIFLGLGLIIHHRSQKGLLH*
>ENSG00000177054|ENST00000446113
MEGPGLGSQCRNHSHGPHPPGFGRYGICAHENKELANAREALPLIEDSSNCDIVKATQYG
IFERCKELVEAGYDVRQPDKENVSLLHWAAINNRLDLVKFYISKGAVVDQLGGDLNSTPL
HWAIRQGHLPMVILLLQHGADPTLIDGEGFSSIHLAVLFQHMPIIAYLISKGQSVNMTDV
NGQTPLMLSAHKVIGPEPTGFLLKFNPSLNVVDKIHQNTPLHWAVAAGNVNAVDKLLEAG
SSLDIQNVKGETPLDMALQNKNQLIIHMLKTEAKMRANQKFRLWRWLQKCELFLLLMLSV
ITMWAIGYILDFNSDSWLLKGCLLVTLFFLTSLFPRFLVGYKNLVYLPTAFLLSSVFWIF
MTWFILFFPDLAGAPFYFSFIFSIVAFLYFFYKTWATDPGFTKASEEEKKVNIITLAETG
SLDFRTFCTSCLIRKPLRSLHCHVCNCCVARYDQHCLWTGRCIGFGNHHYYIFFLFFLSM
VCGWIIYGSFIYLSSHCATTFKEDGLWTYLNQIVACSPWVLYILMLATFHFSWSTFLLLN
QLFQIAFLGLTSHERISLQKQSKHMKQTLSLRKTPYNLGFMQNLADFFQCGCFGLVKPCV
VDWTSQYTMVFHPAREKVLRSV*
>ENSG00000114251|ENST00000474267
MKKSIGILSPGVALGMAGSAMSSKFFLVALAIFFSFAQVVIEANSWWSLGMNNPVQMSEV
YIIGAQPLCSQLAGLSQGQKKLCHLYQDHMQYIGEGAKTGIKECQYQFRHRRWNCSTVDN
TSVFGRVMQIGSRETAFTYAVSAAGVVNAMSRACREGELSTCGCSRAARPKDLPRDWLWG
GCGDNIDYGYRFAKEFVDARERERIHAKGSYESARILMNLHNNEAGRRTVYNLADVACKC
HGVSGSCSLKTCWLQLADFRKVGDALKEKYDSAAAMRLNSRGKLVQVNSRFNSPTTQDLV
YIDPSPDYCVRNESTGSLGTQGRLCNKTSEGMDGCELMCCGRGYDQFKTVQTERCHCKFH
WCCYVKCKKCTEIVDQFVCK*
>ENSG00000197818|ENST00000417961
MGEKMAEEERFPNTTHEGFNVTLHTTLVVTTKLVLPTPGKPILPVQTGEQAQQEEQSSGM
TIFFSLLVLAICIILVHLLIRYRLHFLPESVAVVSLGILMGAVIKIIEFKKLANWKEEEM
FRPNMFFLLLLPPIIFESGYSLHKGNFFQNIGSITLFAVFGTAISAFVVGGGIYFLGQGF
FFVCVCVFVCFILQADVISKLNMTDSFAFGSLISAVDPVATIAIFNALHVDPVLNMLVFG

ESILNDAVSIVLTNTAEGLTRKNMSDVSGWQTFLQALDYFLKMFFGSAALGTLTGLISAL
VLKHIDLRKTPSLEFGMMIIFAYLPYGLAEGISLSGIMAILFSGIVMSHYTHHNLSPVTQ
ILMQQTLRTVAFLCETCVFAFLGLSIFSFPHKFEISFVIWCIVLVLFGRAVNIFPLSYLL
NFFRDHKITPKMMFIMWFSGLRGAIPYALSLHLDLEPMEKRQLIGTTTIVIVLFTILLLG
GSTMPLIRLMDIEDAKAHRRNKKDVNLSKTEKMGNTVESEHLSELTEEEYEAHYIRRQDL
KGFVWLDAKYLNPFFTRRLTQEDLHHGRIQMKTLTNKWYEEVRQGPSGSEDDEQELL*
>ENSG00000283883|ENST00000638350
MMEEEELEFVEELEAVLQLTPEVQLAIEQVFPSQDPLDRADFNAVEYINTLFPTEQSLAN
IDEVVNKIRLKIRRLDDNIRTVVRGQTNVGQDGRQVKEITRDIKQLDHAKRHLTTSITTL
NHLHMLAGGVDSLEAMTRRRQYGEVANLLQGVMNVLEHFHKYMGIPQIRQLSERVKAAQT
ELGQQILADFEEAFPSQGTKRPGGPSNVLRDACLVANILDPRIKQEIIKKFIKQHLSEYL
VLFQENQDVAWLDKIDRRYAWIKRQLVDYEEKYGRMFPREWCMAERIAVEFCHVTRAELA
KIMRTRAKEIEVKLLLFAIQRTTNFEGFLAKRFSGCTLTDGTLKKLESPPPSTNPFLEDE
PTPEMEELATEKGDLDQPKKPKAPDNPFHGIVSKCFEPHLYVYIESQDKNLGELIDRFVA
DFKAQGPPKPNTDEGGAVLPSCADLFVYYKKCMVQCSQLSTGEPMIALTTIFQKYLREYA
WKILSGNLPKTTTSSGGLTISSLLKEKEGSEVAKFTLEELCLICNILSTAEYCLATTQQL
EEKLKEKVDVSLIERINLTGEMDTFSTVISSSIQLLVQDLDAACDPALTAMSKMQWQNVE
HVGDQSPYVTSVILHIKQNVPIIRDNLASTRKYFTQFCVKFANSFIPKFITHLFKCKPIS
MVGAEQVRWT*
>ENSG00000138674|ENST00000500777
MKLKEVDRTAMQAWSPAQNHPIYLATGTSAQQLDATFSTNASLEIFELDLSDPSLDMKSC
ATFSSSHRYHKLIWGPYKMDSKGDVSGVLIAGGENGNIILYDPSKIIAGDKEVVIAQNDK
HTGPVRALDVNIFQTNLVASGANESEIYIWDLNNFATPMTPGAKTQPPEDISCIAWNRQV
QHILASASPSGRATVWDLRKNEPIIKVSDHSNRMHCSGLAWHPDVATQMVLASEDDRLPV
IQMWDLRFASSPLRVLENHARGILAIAWSMADPELLLSCGKDAKILCSNPNTGEVLYELP
TNTQWCFDIQWCPRNPAVLSAASFDGRISVYSIMGGSTDGLRQKQVDKLSSSFGNLDPFG
TGQPLPPLQIPQQTAQHSIVLPLKKPPKWIRRPVGASFSFGGKLVTFENVRMPSHQGAEQ
QQQQHHVFISQVVTEKEFLSRSDQLQQAVQSQGFINYCQKKIDASQTEFEKNVWSFLKVN
FEDDSRGKYLELLGYRKEDLGKKHIKEEKEESEFLPSSGGTFNISVSGDIDGLITQALLT
GNFESAVDLCLHDNRMADAIILAIAGGQELLARTQKKYFAKSQSKITRLITAVVMKNWKE
IVESCDLKNWREALAAVLTYAKPDEFSALCDLLGTRLENEGDSLLQTQACLCYICAGNVE
KLVACWTKAQDGSHPLSLQDLIEKVVILRKAVQLTQAMDTSTVGVLLAAKMSQYANLLAA
QGSIAAALAFLPDNTNQPNIMQLRDRLCRAQGEPVAGHESPKIPYEKQQLPKGRPGPVAG
HHQMPRVQTQQYYPHGENPPPPGFIMHGNVNPNAAGQLPTSPGHMHTQVPPYPQPQRPQN
GWNDPPALNRVPKKKKMPENFMPPVPITSPIMNPLGDPQSQMLQQQPSAPVPLSSQSSFP
QPHLPGGQPFHGVQQPLGQTGMPPSFSKPNIEGAPGAPIGNTFQHVQSLPTKKITKKPIP
DEHLILKTTFEDLIQRCLSSATDPQTKRKLDDASKRLEFLYDKLREQTLSPTITSGLHNI
ARSIETRNYSEGLTMHTHIVSTSNFSETSAFMPVLKVVLTQANKLGV*
>ENSG00000007255|ENST00000588062
MADTVLFEFLHTEMVAELWAHDPDPGPGAAPGDAGLQGGAGCPQVLVQRPVGGGVPEADG
QPAHQSPGDLRPARQQLPPPPPDGLWPAVSGGSTQVPGLHLRPPARRPLYPGH*
>ENSG00000172575|ENST00000558164
MGTLGKAREAPRKPSHGCRAASKARLEAKPANSPFPSHPSLAHITQFRMMVSLGHLAKGA
SLDDLIDSCIQSFDADGNLCRSNQLLQVMLTMHRIVISSAELLQKVITLYKDALAKNSPG

LCLKICYFVRYWITEFWVMFKMDASLTDTMEEFQELVKAKGEELHCRLIDTTQINARDWS
RKLTQRIKSNTSKKRKVSLLFDHLEPEELSEHLTYLEFKSFRRISFSDYQNYLVNSCVKE
NPTMERSIALCNGISQWVQLMVLSRPTPQLRAEVFIKFIQVAQKLHQLQNFNTLMAVIGG
LCHSSISRLKETSSHVPHEINKVLGEMTELLSSSRNYDNYRRAYGECTDFKIPILGVHLK
DLISLYEAMPDYLEDGKVNVHKLLALYNHISELVQLQEVAPPLEANKDLVHLLTLSLDLY
YTEDEIYELSYAREPRNHRAPSVFKNYDHDQDGYISQEEFEKIAASFPFSFCVMDKDREG
LISRDEITAYFMRASSIYSKLGLGFPHNFQETTYLKPTFCDNCAGFLWGVIKQGYRCKGN
KYSESR*
>ENSG00000005206|ENST00000610743
MAAAVAAALARLLAAFLLLAAQVACEYGMVHVVSQAGGPEGKDYCILYNPQWAHLPHDLS
KASFLQLRNWTASLLCSAADLPARGFSNQIPLVARGNCTFYEKVRLAQGSGARGLLIVSR
ERLVPPGGNKTQYDEIGIPVALLSYKDMLDIFTRFGRTVRAALYAPKEPVLDYNMVIIFI
MAVGTVAIGGYWAGSRDVKKRYMKHKRDDGPEKQEDEAVDVTPVMTCVFVVMCCSMLVLL
YYFYDLLVYVVIGIFCLASATGLYSCLAPCVRRLPFGKCRIPNNSLPYFHKRPQARMLLL
ALFCVAVSVVWGVFRNEDQWAWVLQDALGIAFCLYMLKTIRLPTFKACTLLLLVLFLYDI
FFVFITPFLTKSGSSIMVEVATGPSDSATREKLPMVLKVPRLNSSPLALCDRPFSLLGFG
DILVPGLLVAYCHRFDIQVQSSRVYFVACTIAYGVGLLVTFVALALMQRGQPALLYLVPC
TLVTSCAVALWRRELGVFWTGSGFAVNTSLL*
>ENSG00000136279|ENST00000490734
MKATAMTSAWLAQGGAHVTINARAEEDVEPECIMEKVAKASGANYSFHKESGRFQDVGPQ
APVGSVYQKTNAVSEIKRVGKDSFWAKAEKEEENRRLEEKRRAEEAQRQLEQERRERELR
EAARREQRYQEQGGEASPQSRTWEQQQEVVSRNRNEQESAVHPREIFKQKERAMSTTSIS
SPQPGKLRSPFLQKQLTQPETHFGREPAAAISRPRADLPAEEPAPSTPPCLVQAEEEAVY
EEPPEQETFYEQPPLVQQQGAGSEHIDHHIQGQGLSGQGLCARALYDYQAADDTEISFDP
ENLITGIEVIDEGWWRGYGPDGHFGMFPANYVELIE*
>ENSG00000167645|ENST00000591755
MHPAGLAAAAAGTPRLPSKRRIPVSQPGMADPHQLFDDTSSAQSRGYGAQRAPGGLSYPA
ASPTPHAAFLADPVSNMAMAYGSSLAAQGKELVDKNIDRFIPITKLKYYFAVDTMYVGRK
LGLLFFPYLHQDWEVQYQQDTPVAPRFDVNAPDLYIPAMAFITYVLVAGLALGTQDRFSP
DLLGLQASSALAWLTLEVLAILLSLYLVTVNTDLTTIDLVAFLGYKYVGMIGGVLMGLLF
GKIGYYLVLGWCCVAIFVFMFPLLPGAVAHACNPSTLGGRGGRITRSGDQDHPG*
>ENSG00000234514|ENST00000428227
MLSRNDVKRLERSWAQGSSEQELHYASLQRLPVPSSEGPDLRGRDKRGTKEDPRADYACI
AENKPT*
>ENSG00000134470|ENST00000530685
MAPRRARGCRTLGLPALLLLLLLRPPATRGITCPPPMSVEHADIWVKSYSLYSRERYICN
SGFKRKAGTSSLTECVLNKATNVAHWTTPSLKCIKPAASSPSSNNTAATTAAIVPGSQLM
PSKSPSTGTTEISSHESSHGTPSQTTAKNWELTASASHQPPGVYPQGHSDTTVAISTSTV
LLCGLSAVSLLACYLKSRASVCSCHPRSAGHTCSVGSVC*
>ENSG00000142188|ENST00000542230
MAGFLDNFRWPECECIDWSERRNAVASVVAGILFFTGWWIMIDAAVVYPKPEQLNHAFHT
CGVFSTLAFFMINAVSNAQVRGDSYESGCLGRTGARVWLFIGFMLMFGSLIASMWILFGA
YVTQNTDVYPGLAVFFQNALIFFSTLIYKFGRTEELWT*
>ENSG00000171004|ENST00000406696

MALPACAVREFEPPRQPERGAPVRTTCPRRHSRVEAELAASRPGSVAASVRAGPPRGVSH
GFHTRPLLDKPRKASSSLAGAACAPLFALLSRGRRRRMHVLRRRWDLGSLCRALLTRGLA
ALGHSLKHVLGAIFSKIFGPMASVGNMDEKSNKLLLALVMLFLFAVIVLQYVCPGTECQL
LRLQAFSSPVPDPYRSEDESSARFVPRYNFTRGDLLRKVDFDIKGDDLIVFLHIQKTGGT
TFGRHLVRNIQLEQPCECRVGQKKCTCHRPGKRETWLFSRFSTGWSCGLHADWTELTSCV
PSVVDGKRDARLRPSRNFHYITILRDPVSRYLSEWRHVQRGATWKASLHVCDGRPPTSEE
LPSCYTGDDWSGCPLKEFMDCPYNLANNRQVRMLSDLTLVGCYNLSVMPEKQRNKVLLES
AKSNLKHMAFFGLTEFQRKTQYLFEKTFNMNFISPFTQYNTTRASSVEINEEIQKRIEGL
NFLDMELYSYAKDLFLQRYQFMRQKEHQEARRKRQEQRKFLKGRLLQTHFQSQGQGQSQN
PNQNQSQNPNPNANQNLTQNLMQNLTQSLSQKENRESPKQNSGKEQNDNTSNGTNDYIGS
VEKWR*
>ENSG00000003147|ENST00000455539
MSGHKCSYPWDLQDRYAQDKSVVNKMQQKYWETKQAFIKATGKKEDEHVVASDADLDAKL
ELFHSIQRTCLDLSKAIVLYQKRICSF*
>ENSG00000130383|ENST00000588525
MDPLGPAKPQWLWRRCLAGLLFQLLVAVCFFSYLRVSRDDATGSPRPGLMAVEPVTGAPN
GSRCQDSMATPAHPTLLILLWTWPFNTPVALPRCSEMVPGAADCNITADSSVYPQADAVI
VHHWDIMYNPSANLPPPTRPQGQRWIWFSMESPSNCRHLEALDGYFNLTMSYRSDSDIFT
PYGWLEPWSGQPAHPPLNLSAKTELVAWAVSNWKPDSARVRYYQSLQAHLKVDVYGRSHK
PLPKGTMMETLSRYKFYLAFENSLHPDYITEKLWRNALEAWAVPVVLGPSRSNYERFLPP
DAFIHVDDFQSPKDLARYLQELDKDHARYLSYFHWRETLRPRSFSWALAFCKACWKLQQE
SRYQTVRSIAAWFT*
>ENSG00000273686|ENST00000631573
MSRSVALAVLALLSLSGLEAIQRTPKIQVYSRHPAENGKSNFLNCYVSGFHPSDIEVDLL
KNGERIEKVEHSDLSFSKDWSFYLLYYTEFTPTEKDEYACRVNHVTLSQPKIVKWDRDM*
>ENSG00000147642|ENST00000408889
MVGEGSIQSSRYKKESKSGLVKPGSEADFSSSSSTGSISAPEVHMSTAGSKRSSSSRNRG
PHGRSNGASSHKPGSSPSSPREKDLLSMLCRNQLSPVNIHPSYAPSSPSSSNSGSYKGSD
CSPIMRRSGRYMSCGENHGVRPPNPEQYLTPLQQKEVTVRHLKTKLKESERRLHERESEI
VELKSQLARMREDWIEEECHRVEAQLALKEARKEIKQLKQVIETMRSSLADKDKGIQKYF
VDINIQNKKLESLLQSMEMAHSGSLRDELCLDFPCDSPEKSLTLNPPLDTMADGLSLEEQ
VTGEGADRELLVGDSIANSTDLFDEIVTATTTESGDLELVHSTPGANVLELLPIVMGQEE
GSVVVERAVQTDVVPYSPAISELIQSVLQKLQDPCPSSLASPDESEPDSMESFPESLSAL
VVDLTPRNPNSAILLSPVETPYANVDAEVHANRLMRELDFAACVEERLDGVIPLARGGVV
RQYWSSSFLVDLLAVAAPVVPTVLWAFSTQRGGTDPVYNIGALLRGCCVVALHSLRRTAF
RIKT*
>ENSG00000132254|ENST00000423813
MVSGPNLNETSIVSGGYGGSGDGLIPTGSGRHPSHSTTPSGPGDEVARGIAGEKFDIVKK
WGINTYKCTKQLLSERFGRGSRTVDLELELQIELLRETKRKYESVLQLGRALTAHLYSLL
QTQHALGDAFADLSQKSPELQEEFGYNAETQKLLCKNGETLLGAVNFFVSSINTLVTKTM
EDTLMTVKQYEAARLEYDAYRTDLEELSLGPRDAGTRGRLESAQATFQAHRDKYEKLRGD
VAIKLKFLEENKIKVMHKQLLLFHNAVSAYFAGNQKQLEQTLQQFNIKLRPPGAEKPSWL
EEQ*
>ENSG00000234514|ENST00000449273

MLSRNDDICIYGGLGLGGLLLLAVVLLSACLCWLHRRGPGLLRAGTPLCISAEAASAQQ*
>ENSG00000126091|ENST00000531451
MGLLVFVRNLLLALCLFLVLGFLYYSAWKLHLLQWEEDSKYDRLGFLLNLDSKLPAELAT
KYANFSEGACKPGYASALMTAIFPRFSKPAPMFLDDSFRKWARIREFVPPFGIKGQVLDA
QYPARERVSAEAGESSRHH*
>ENSG00000188603|ENST00000565316
MGGCAGSRRRFSDSEGEETVPEPRLPLLDHQGAHWKNAVGFWLLGLCNNFSYVVMLSAAH
DILSHKRTSGNQSHVDPGPTPIPHNSSSRFDCNSVSTAAVLLADILPTLVIKLLAPLGLH
LLPYSPRVLVSGICAAGSFVLVAFSHSVGTSLCGVVFASISSGLGEVTFLSLTAFYPRAV
ISWWSSGTGGAGLLGALSYLGLTQAGLSPQQTLLSMLGIPALLLASYFLLLTSPEAQDPG
GEEEAESAARQPLIRTEAPESKPGSSSSLSLRERWTVFKVRMMAGFELLFFWNTSLSHAQ
QYRWYQMLYQAGVFASRSSLRCCRIRFTWALALLQCLNLVFLLADVWFGFLPSIYLVFLI
ILYEGLLGGAAYVNTFHNIALETSDEHREFAMAATCISDTLGISLSGLLALPLHDFLCQL
S*
>ENSG00000137497|ENST00000546131
MTLHATRGAALLSWVNSLHVADPVEAVLQLQDCSIFIKIIDRIHGTEEGQQILKQP
>ENSG00000102100|ENST00000452555
MAAVGAGGSTAAPGPGAVSAGALEPGTASAELLLTWEEAEARGQGLPQPLPDTSVRIPAH
RRLKYISLAVLVVQNASLILSIRYARTLPGDRFFATTAVVMAEVLKGLTCLLLLFAQKRG
NVKHLVLFLHEAVLVQYVDTLKLAVPSLIYTLQNNLQYVAISNLPAATFQVTYQLKILTT
ALFSVLMLNRSLSRLQWASLLLLFTGVAIVQAQQAGGGGPRPLDQNPGAGLAAVVASCLS
SGFAGVYFEKILKGSSGSVWLRNLQLGLFGTALGLVGLWWAEGTAVATRGFFFGYTPAVW
GVVLNQAFGGLLVAVVVKYADNILKGFATSLSIVLSTVASIRLFGFHVDPLFALGAGLVI
GAVYLYSLPRGAAKAIASASASASGPCVHQQPPGQPPPPQLSSHRGDLITEPFLPKSVLV
K*
>ENSG00000134602|ENST00000496850
MAHSPVAVQVPGMQNNIADPEELFTKLERIGKGSFGEVFKGIDNRTQQVVAIKIIDLEEA
EDEIEDIQQEITVLSQCDSSYVTKYYGSYLKGSKLWIIMEYLGGGSALDLLRAGPFDEFQ
IATMLKEILKGLDYLHSEKKIHRDIKAANVLLSEQGDVKLADFGVAGQLTDTQIKRNTFV
GTPFWMAPEVIQQSAYDSKRPTAKELLKHKFIVKNSKKTSYLTELIDRFKRWKAEGHSDD
ESDSEGSDSESTSRENNTHPEWSFTTVRKKPDPKKVQNGAEQDLVQTLSCLSMIITPAFA
ELKQQDENNASRNQAIEELEKSIAVAEAACPGITDKMVKKLIEKFQKCSADESP*
>ENSG00000115525|ENST00000638572
MRTKAAGCAERRPLQPRTEAAAAPAGRAMPSEYTYVKLRSDCSRPSLQWYTRAQSKMRRP
SLLLKDILKCTLLVFGVWILYILKLNYTTEECDMKKMHYVDPDHVKRAQKYAQQVLQKEC
RPKFAKTSMALLFEHRYSVDLLPFVQKAPKDSEAESKYDPPFGFRKFSSKVQTLLELLPE
HDLPEHLKAKTCRRCVVIGSGGILHGLELGHTLNQFDVVIRLNSAPVEGYSEHVGNKTTI
RMTYPEGAPLSDLEYYSNDLFVAVLFKSVDFNWLQAMVKKETLPFWVRLFFWKQVAEKIP
LQPKHFRILNPVIIKETAFDILQYSEPQSRFWGRDKNVPTIGVIAVVLATHLCDEVSLAG
FGYDLNQPRTPLHYFDSQCMAAMNFQTMHNVTTETKFLLKLVKEGVVKDLSGGIDREF*
>ENSG00000223465|ENST00000429910
MLSRNDDICIYGGLGLGGLLLLAVVLLSACLCWLHRRGPGLLRAGTPLCISAEAASAQQ*
>ENSG00000235915|ENST00000443741
MLSRNDDICIYGGLGLGGLLLLAVVLLSACLCWLHRRVKRLERSWAQGSSEQELHYASLQ

RLPVPSSEGPDLRGRDKRGTKEDPRADYACIAENKPT*
>ENSG00000140479|ENST00000615296
MPPRAPPAPGPRPPPRAAAATDTAAGAGGAGGAGGAGGPGFRPLAPRPWRWLLLLALPAA
CSAPPPRPVYTNHWAVQVLGGPAEADRVAAAHGYLNLGQIGNLEDYYHFYHSKTFKRSTL
SSRGPHTFLRMDPQVKWLQQQEVKRRVKRQVRSDPQALYFNDPIWSNMWYLHCGDKNSRC
RSEMNVQAAWKRGYTGKNVVVTILDDGIERNHPDLAPNYDSYASYDVNGNDYDPSPRYDA
SNENKHGTRCAGEVAASANNSYCIVGIAYNAKIGGIRMLDGDVTDVVEAKSLGIRPNYID
IYSASWGPDDDGKTVDGPGRLAKQAFEYGIKKGRQGLGSIFVWASGNGGREGDYCSCDGY
TNSIYTISVSSATENGYKPWYLEECASTLATTYSSGAFYERKIVTTDLRQRCTDGHTGTS
VSAPMVAGIIALALEANSQLTWRDVQHLLVKTSRPAHLKASDWKVNGAGHKVSHFYGFGL
VDAEALVVEAKKWTAVPSQHMCVAASDKRPRSIPLVQVLRTTALTSACAEHSDQRVVYLE
HVVVRTSISHPRRGDLQIYLVSPSGTKSQLLAKRLLDLSNEGFTNWEFMTVHCWGEKAEG
QWTLEIQDLPSQVRNPEKQGNLD*
>ENSG00000227357|ENST00000411565
MVCLKLPGGSCMAALTVTLTVLSSPLALAGDTQPRFLEQAKCECHFLNGTERVWNLIRYI
YNQEEYARYNSDLGEYQAVTELGRPDAEYWNSQKDLLERRRAEVDTYCRYNYGVVESFTV
QRRVQPKVTVYPSKTQPLQHHNLLVCSVNGFYPGSIEVRWFRNGQEEKAGVVSTGLIQNG
DWTFQTLVMLETVPRSGEVYTCQVEHPSMMSPLTVQWSARSESAQSKMLSGVGGFVLGLL
FLGTGLFIYFRNQKGHSGLQPTGLLS*
>ENSG00000181029|ENST00000596148
MEARFTRGKSALLERALARPRTEVSLSAFALLFSELVQHCQSRVFSVAELQSRLAALGRQ
VGARVLDALVAREKGARRETKVLGALLFVKGAVWKALFGKEADKLEQANDDARTFYIIER
EPLINTYISVPKENSTLNCASFTAGIVEAVLTHSGFPAKVTAHWHKGTTLMIKFEEAVIA
RDRALEGR*
>ENSG00000229074|ENST00000437784
MVCLKLPGGSCMAALTVTLMVLSSPLALAGDTQPRFLWQGKYKCHFFNGTERVQFLERLF
YNQEEFVRFDSDVGEYRAVTELGRPVAESWNSQKDILEDRRGQVDTVCRHNYGVGESFTV
QRRVHPEVTVYPAKTQPLQHHNLLVCSVSGFYPGSIEVRWFRNGQEEKAGVVSTGLIQNG
DWTFQTLVMLETVPRSGEVYTCQVEHPSVMSPLTVEWRARSESAQSKMLSGVGGFVLGLL
FLGAGLFIYFRNQKGHSGLQPTGFLS*
>ENSG00000230791|ENST00000450291
MLSRNDVKRLERSWHLLSWSQAQGSSEQELHYASLQRLPVPSSEGPDLRGRDKRGTKEDP
RADYACIAENKPT*
>ENSG00000167889|ENST00000569840
MITVNPDGKIMVRRCLVTLRPFRLFVLGIGFFTLCFLMTSLGGQFSARRLGDSPFTIRTE
VMGGPESRGVLRKMSDLLELMVKRMDALARLENSSELHRAGGDLHFPADRMPPGAGLMER
IQAIAQNVSDIAVKVDQILRHSLLLHSKVSEGRRDQCEAPSDPKFPDCSGKVEWMRARWT
SDPCYAFFGVDGTECSFLIYLSEVEWFCPPLPWRNQTAAQRAPKPLPKVQAVFRSNLSHL
LDLMGSGKESLIFMKKRTKRLTAQWALAAQRLAQKLGATQRDQKQILVHIGFLTEESGDV
FSPRVLKGGPLGEMVQWADILTALYVLGHGLRVTVSLKELQSNLGVPPGRGSCPLTMPLP
FDLIYTDYHGLQQMKRHMGLSFKKYRCRIRVIDTFGTEPAYNHEEYATLHGYRTNWGYWN
LNPKQFMTMFPHTPDNSFMGFVSEELNETEKRLIKGGKASNMAVVYGKEASIWKLQGKEK
FLGILNKYMEIHGTVYYESQRPPEVPAFVKNHGLLPQPEFQQLLRKAKLFIGFGFPYEGP
APLEAIANGCIFLQSRFSPPHSSLNHEFFRGKPTSREVFSQHPYAENFIGKPHVWTVDYN

NSEEFEAAIKAIMRTQVDPYLPYEYTCEGMLERIHAYIQHQDFCRAPDPALPEAHAPQSP
FVLAPNATHLEWARNTSLAPGAWPPAHALRAWLAVPGRACTDTCLDHGLICEPSFFPFLN
SQDAFLKLQVPCDSTESEMNHLYPAFAQPGQECYLQKEPLLFSCAGSNTKYRRLCPCRDF
RKGQVALCQGCL*
>ENSG00000126091|ENST00000531993
MGLLVFVRNLLLALCLFLVLGFLYYSAWKLHLLQWEEDSKYDRLGFLLNLDSKLFSKPAP
MFLDDSFRKWARIREFVPPFGIKGQDNLIKAILSVTKEYRLTPALDSLRCRRCIIVGNGG
VLANKSLGSRIDDYDIVVRLNSAPVKGFEKDVGSKTTLRITYPEGAMQRPEQYERDSLFV
LAGFKWQDFKWLKYIVYKERVSWTHNIQREKEFLRKLVKARVITDLSSGI*
>ENSG00000237508|ENST00000440590
MAPRSLLLLLSGALALTDTWAGSHSLRYFSTAVSRPGRGEPRYIAVEYVDDTQFLRFDSD
AAIPRMEPREPWVEQEGPQYWEWTTGYAKANAQTDRVALRNLLRRYNQSEAGSHTLQGMN
GCDMGPDGRLLRGYHQHAYDGKDYISLNEDLRSWTAADTVAQITQRFYEAEEYAEEFRTY
LEGECLELLRRYLENGKETLQRAEQSPQPTIPIVGIVAGLVVLGAVVTGAVVAAVMWRKK
SSDRNRGSYSQAAV*
>ENSG00000169255|ENST00000417187
MASALWTVLPSRMSLRSLKWSLLLLSLLSFFVMWYLSLPHYNVIERVNWMYFYEIHLDVC
QLRRVIAAHGFSSKEIITFWQVMLRNTTCHY*
>ENSG00000114650|ENST00000416847
MTLTERLREKISRAFYNHGLLCASYPIPIILFTGFCILACCYPLLKLPLPGTGPVEFTTP
VKDYSPPPVDSDRKQGEPTEQPEWYVGAPVAYVQQIFVKSSVFPWHKNLLAVDVFRSPLS
RAFQLVEEIRNHVLRD
>ENSG00000229252|ENST00000415289
MVDGTLLLLLSEALALTQTWAGSHSLKYFHTSVSRPGRGEPRFISVGYVDDTQFVRFDND
AASPRMVPRAPWMEQEGSEYWDRETRSARDTAQIFRVNLRTLRGYYNQSEAGSHTLQWMH
GCELGPDRRFLRGYEQFAYDGKDYLTLNEDLRSWTAVDTAAQISEQKSNDASEAEHQRAY
LEDTCVEWLHKYLEKGKETLLHLEPPKTHVTHHPISDHEATLRCWALGFYPAEITLTWQQ
DGEGHTQDTELVETRPAGDGTFQKWAAVVVPSGEEQRYTCHVQHEGLPEPVTLRWKPASQ
PTIPIVGIIAGLVLLGSVVSGAVVAAVIWRKKSSGGKGGSYSKAEWSDSAQGSESHSL*
>ENSG00000110697|ENST00000436757
MLIKEYHILLPMSLDEYQVAQLYMIQKKSREESSGEGSGVEILANRPYTDGPGGSGQYTH
KVYHVGSHIPGWFRALLPKAALQVEEESWNAYPYTRTRYTCPFVEKFSIEIETYYLPDGG
QQPNVFNLSGAERRQRILDTIDIVRDAVAPGEYKAEEDPRLYHSVKTGRGPLSDDWARTA
AQTGPLMCAYKLCKVEFRYWGMQAKIEQFIHDVGLRRVMLRAHRQAWCWQDEWTELSMAD
IRALEEETARMLAQRMAKCNTGSEGSEAQPPGKPSTEARSAASNTGTPDGPEAPPGPDAS
PDASFGKQWSSSSRSSYSSQHGGAVSPQSLSEWRMQNIARDSENSSEEEFFDAHEGFSDS
EEVFPKEMTKWNSNDFIDAFASPVEAEGTPEPGAEAAKGIEDGAQAPRDSEGLDGAGELG
AEACAVHALFLILHSGNILDSGPGDANSKQADVQTLSSAFEAVTRIHFPEALGHVALRLV
PCPPICAAAYALVSNLSPYSHDGDSLSRSQDHIPLAALPLLATSSSRYQGAVATVIARTN
QAYSAFLRSPEGAGFCGQVALIGDGVGGILGFDALCHSANAGTGSRGSSRRGSMNNELLS
PEFGPVRDPLADGVEGLGRGSPEPSALPPQRIPSDMASPEPEGSQNSLQAAPATTSSWEP
RRASTAFCPPAASSEAPDGPSSTARLDFKVSGFFLFGSPLGLVLALRKTVMPALEAQMRP
ACEQIYNLFHAADPCASRLEPLLAPKFQAIAPLTVPRYQKFPLGDGSSLLLADTLQTHSS
LFLEELEMLVPSTPTSTSGAFWKGSELATDPPAQPAAPSTTSEVVKILERWWGTKRIDYS

LYCPEALTAFPTVTLPHLFHASYWESADVVAFILRQVIEKERPQLAECEEPSIYSPAFPR
EKWQRKRTQVKIRNVTSNHRASDTVVCEGRPQVLSGRFMYGPLDVVTLTGEKVDVYIMTQ
PLSGKWIHFGTEVTNSSGRLTFPVPPERALGIGVYPVRMVVRGDHTYAECCLTVVARGTE
AVVFSIDGSFTASVSIMGSDPKVRAGAVDVVRHWQDSGYLIVYVTGRPDMQKHRVVAWLS
QHNFPHGVVSFCDGLTHDPLRQKAMFLQSLVQEVELNIVAGYGSPKDVAVYAALGLSPSQ
TYIVGRAVRKLQAQCQFLSDGYVAHLGQLEAGSHSHASSGPPRAALGKSSYGVAAPVDFL
RKQSQLLRSRGPSQAEREGPGTPPTTLARGKARSISLKLDSEE*
>ENSG00000180398|ENST00000409218
MTMRSLLRTPFLCGLLWAFCAPGARAEEPAASFSQPGSMGLDKNTVHDQEHIMEHLEGVI
NKPEAEMSPQELQLHYFKMHDYDGNNLLDGLELSTAITHVHKEEGSEQAPLMSEDELINI
IDGVLRDDDKNNDGYIDYAEFAKSLQ*
>ENSG00000103502|ENST00000569956
MPDENIFLFVPNLIGYARIVFAIISFYFMPCCPLTASSFYLLSGLLDAFDGHAARALNQG
TRFGAMLDMLTDRCSTMCLLVNLALLYPGATLFFQISMSLDVASHWLHLHSSVVRGSESH
KMIDLSGNPVLRIYYTSRPALFTLCAGNELFYCLLYLFHFSEGPLVGSVGLFRMGLWVTA
PIALLKSLISVIHLITAARNMAALDAADRAKKK*
>ENSG00000182050|ENST00000620241
MFKFHQMKHIFEILDKMRCLRKRSTVSFLGVLVIFLLFMNLYIEDSYVLEGDKQLIRETS
THQLNSERYVHTFKDLSNFSGAINVTYRYLAATPLQRKRYLTIGLSSVKRKKGNYLLETI
KSIFEQSSYEELKEISVVVHLADFNSSWRDAMVQDITQKFAHHIIAGRLMVIHAPEEYYP
ILDGLKRNYNDPEDRVKFRSKQNVDYAFLLNFCANTSDYYVMLEDDVRCSKNFLTAIKKV
IASLEGTYWVTLEFSKLGYIGKLYHSHDLPRLAHFLLMFYQEMPCDWLLTHFRGLLAQKN
VIRFKPSLFQHMGYYSSYKGTENKLKDDDFEEESFDIPDNPPASLYTNMNVFENYEASKA
YSSVDEYFWGKPPSTGDVFVIVFENPIIIKKIKVNTGTEDRQNDILHHGALDVGENVMPS
KQRRQCSTYLRLGEFKNGNFEMSGVNQKIPFDIHCMRIYVTKTQKEWLIIRSISIWTS*
>ENSG00000134287|ENST00000447318
MGNIFGNLLKSLIGKKEMRILMVGLDAAGKTTILYKLKLGEIVTTIPTIGLIFVVDSNDR
ERVNEAREELMRMLAEDELRDAVLLVFANKQDLPNAMNAAEITDKLGLHSLRHRNWYIQA
TCATSGDGLYEGLDWLANQLKNKK*
>ENSG00000136240|ENST00000490996
MNIFRLTGDLSHLAAIVILLLKIWKTRSCAGISGKSQLLFALVFTTRYLDLFTSFISLYN
TSMKVIYLACSYATVYLIYLKFKATYDGNHDTFRVEFLVVPVGGLSFLVNHDFSPLEYSR
ERSSVCQHKCQRPSPASVLQGARTEFLPQQRHKMLDTENQKLNSFVADSHQWLCKNAEEK
SQKVSV*
>ENSG00000233209|ENST00000422950
MSWKKALRIPGGLRVATVTLMLAMLSTPVAEGRDSPEDFVYQFKGMCYFTNGTERVRLVT
RYIYNREEYARFDSDVGVYRAVTPLGPPDAEYWNSQKEVLERTRAELDTVCRHNYQLELR
TTLQRRVEPTVTISPSRTEALNHHNLLVCSVTDFYPAQIKVRWFRNDQEETTGVVSTPLI
RNGDWTFQILVMLEMTPQRGDVYTCHVEHPSLQNPIIVEWRAQSESAQSKMLSGIGGFVL
GLIFLGLGLIIHHRSQKGLLH*
>ENSG00000136279|ENST00000494774
MAANLSRNGPALQEAYVRVVTEKSPTDWALFTYEGNSNDIRVAGTGEGGLEEMVEELNSG
KVMYAFCRVKDPNSGLPKFVLINWTGEGVNDVRKGACASHVSTMASFLKGAHVTINARAE
EDVEPECIMEKVAKASGANYSFHKESGRFQDVGPQAPVGSVYQKTNAVSEIKRVGKDSFW

AKAEKEEENRRLEEKRRAEEAQRQLEQERRERELREAARREQRYQEQGGEASPQSRTWEQ
QQEVVSRNRNEQESAVHPREIFKQKERAMSTTSISSPQPGKLRSPFLQKQLTQPETHFGR
EPAAAISRPRADLPAEEPAPSTPPCLVQAEEEAVYEEPPEQETFYEQPPLVQQQGAGSEH
IDHHIQGQGLSGQGLCARALYDYQAADDTEISFDPENLITGIEVIDEGWWRGYGPDGHFG
MFPANYVELIE*
>ENSG00000017260|ENST00000510168
MKVARFQKIPNGENETMIPVLTSKKASELPVSEVASILQADLQNGLNKCEVSHRRAFHGW
NEFDISEDEPLWKKYISQFKNPLIMLLLASAVISVLMHQFDDAVSITVAILIVVTVAFVQ
EYRSEKSLEELSKLVPPECHCVREGKLEHTLARDLVPGDTVCLSVGDRVPADLRLFEAVD
LSIDESSLTGETTPCSKVTAPQPAATNGDLASRSNIAFMGTLVRCGKAKGVVIGTGENSE
FGEVFKMMQAEEAPKTPLQKSMDLLGKQLSFYSFGIIGIIMLVGWLLGKDILEMFTISVS
LAVAAIPEGLPIVVTVTLALGVMRMVKKRAIVKKLPIVETLGCCNVICSDKTGTLTKNEM
TVTHIFTSDGLHAEVTGVGYNQFGEVIVDGDVVHGFYNPAVSRIVEAGCVCNDAVIRNNT
LMGKPTEGALIALAMKMGLDGLQQDYIRKAEYPFSSEQKWMAVKCVHRTQQDRPEICFMK
GAYEQVIKYCTTYQSKGQTLTLTQQQRDVYQQEKARMGSAGLRVLALASGPELGQLTFLG
LVGIIDPPRTGVKEAVTTLIASGVSIKMITGDSQETAVAIASRLGLYSKTSQSVSGEEID
AMDVQQLSQIVPKVAVFYRASPRHKMKIIKSLQKNGSVVAMTGDGVNDAVALKAADIGVA
MGQTGTDVCKEAADMILVDDDFQTIMSAIEEGKGIYNNIKNFVRFQLSTSIAALTLISLA
TLMNFPNPLNAMQILWINIIMDGPPAQSLGVEPVDKDVIRKPPRNWKDSILTKNLILKIL
VSSIIIVCGTLFVFWRELRDNVITPRDTTMTFTCFVFFDMFNALSSRSQTKSVFEIGLCS
NRMFCYAVLGSIMGQLLVIYFPPLQKVFQTESLSILDLLFLLGLTSSVCIVAEIIKKVER
SREKIQKHVSSTSSSFLEV*
>ENSG00000283761|ENST00000640238
MFANLKYVSLGILVFQTTSLVLTMRYSRTLKEEGPRYLSSTAVVVAELLKIMACILLVYK
DSSRM*
>ENSG00000196655|ENST00000434101
MAIFSVYVVNKAGGLIYQLDSYAPRAEAEKTFSYPLDLLLKLHDERVLVAFGQRDGIRVG
HAVLAINGMDVNGRYTADGKEMLETDTFKLHCYQTLTGIKFVVLADPRQAGIDSLLRKIY
EIYSDFALKNPFYSLEMPIRCELFDQNLKLALEVAEKAGTFGPGS*
>ENSG00000276583|ENST00000615394
MGCLWGLALPLFFFCWEVGVSGSSAGPSTRRADTAMTTDDTEVPAMTLAPGHAALETQTL
SAETSSRASTPAGPIPEAETRGAKRISPARETRSFTKTSPNFMVLIATSVETSAASGSPE
GAGMTTVQTITGSDPREAIFDTLCTDDSSEEAKTLTMDILTLAHTSTEAKGLSSESSASS
DSPHPVITPSRASESSASSDGPHPVITPSRASESSASSDGPHPVITPSRASESSASSDGP
HPVITPSRASESSASSDGPHPVITPSRASESSASSDGPHPVITPSRASESSASSDGPHPV
ITPSRASESSASSDGPHPVITPSRASESSASSDGPHPVITPSRASESSASSDGLHPVITP
SRASESSASSDGPHPVITPSRASESSASSDGPHPVITPSWSPGSDVTLLAEALVTVTNIE
VINCSITEIETTTSSIPGASDTDLIPTEGVKASSTSDPPALPDSTEAKPHITEVTASAET
LSTAGTTESAAPDATVGTPLPTNSATEREVTAPGATTLSGALVTVSRNPLEETSALSVET
PSYVKVSGAAPVSIEAGSAVGKTTSFAGSSASSYSPSEAALKNFTPSETPTMDIATKGPF
PTSRDPLPSVPPTTTNSSRGTNSTLAKITTSAKTTMKPPTATPTTARTRPTTDVSAGENG
GFLLLRLSVASPEDLTDPRVAERLMQQLHRELHAHAPHFQVSLLRVRRG*
>ENSG00000258881|ENST00000606025
MWFMYLLSWLSLFIQVAFITLAVAAGLYYLAELIEEYTVATSRIIKYMIWFSTAVLIGLY

VFERFPTSMIGVGLFTNLVYFGLLQTFPFIMLTSPNFILSCGLVVVNHYLAFQFFAEEYY
PFSEVLAYFTFCLWIIPFAFFVSLSAGENVLPSTMQPGGLGLGRSTGHGDSCTDFYKDPP
SVTTIWKTPGLEDSQRR*
>ENSG00000275066|ENST00000616179
MALRPGAGSGGGGAAGAGAGSAGGGGFMFPVAGGIRPPQAGLMPMQQQGFPMVSVMQPNM
QGIMGMNYSSQMSQGPIAMQAGIPMGPMPAAGMPYLGQAPFLGMRPPGPQYTPDMQKQFA
EEQQKRFEQQQKLLEEERKRRQFEEQKQKLRLLSSVKPKTGEKSRDDALEAIKGNLDGFS
RDAKMHPTPASHPKKPGVGVFPSQDPAQPRMPPWIYNESLVPDAYKKILETTMTPTGIDT
AKLYPILMSSGLPRETLGQIWALANRTTPGKLTKEELYTVLAMIAVTQRGVPAMSPDALN
QFPAAPIPTLSGFSMTLPTPVSQPTVIPSGPAGSMPLSLGQPVMGINLVGPVGGAAAQAS
SGFIPTYPANQVVKPEEDDFQDFQDASKSGSLDDSFSDFQELPASSKTSNSQHGNSAPSL
LMPLPGTKALPSMDKYAVFKGIAADKSSENTVPPGDPGDKYSAFRELEQTAENKPLGESF
AEFRSAGTDDGFTDFKTADSVSPLEPPTKDKTFPPSFPSGTIQQKQQTQVKNPLNLADLD
MFSSVNCSSEKPLSFSAVFSTSKSVSTPQSTGSAATMTALAATKTSSLADDFGEFSLFGE
YSGLAPVGEQDDFADFMAFSNSSISSEQKPDDKYDALKEEASPVPLTSNVGSTVKGGQNS
TAASTKYDVFRQLSLEGSGLGVEDLKDNTPSGKSDDDFADFHSSKFSSINSDKSLGEKAV
AFRHTKEDSASVKSLDLPSIGGSSVGKEDSEDALSVQFDMKLADVGGDLKHVMSDSSLDL
PTVSGQHPPAAAGSGSPSATSILQKKETSFGSSENITMTSLSKVTTFVSEDALPETTFPA
LASFKDTIPQTSEQKEYENRDYKDFTKQDLPTAERSQEATCPSPASSGASQETPNECSDD
FGEFQSEKPKISKFDFLVATSQSKMKSSEEMIKSELATFDLSVQGSHKRSLSLGDKEISR
SSPSPALEQPFRDRSNTLNEKPALPVIRDKYKDLTGEVEENERYAYEWQRCLGSALNVIK
KANDTLNGISSSSVCTEVIQSAQGMEYLLGVVEVYRVTKRVELGIKATAVCSEKLQQLLK
DIDKVWNNLIGFMSLATLTPDENSLDFSSCMLRPGIKNAQELACGVCLLNVDSRSRAFNS
ETDSFKLAYGGHQYHASCANFWINCVEPKPPGLVLPDLL*
>ENSG00000283017|ENST00000635459
MYGSARSVGKVEPSSQSPGRSPRLPRSPRLGHRRTNSTGGSSGSSVGGGSGKTLSMENIQ
SLNAAYATSGPMYLSDHENVGSETPKSTMTLGRSGGRLPYGVRMTAMGSSPNIASSGVAS
DTIAFGEHHLPPVSMASTVPHSLRQARDNTIMDLQTQLKEVLRENDLLRKDVEVKESKLS
SSMNSIKTFWSPELKKERALRKDEASKITIWKEQYRVVQEENQVDALRLRLEEKETMLNK
KTKQIQDMAEEKGTQAGEIHDLKDMLDVKERKVNVLQKKIENLQEQLRDKEKQMSSLKER
VKSLQADTTNTDTALTTLEEALAEKERTIERLKEQRDRDEREKQEEIDNYKKDLKDLKEK
VSLLQGDLSEKEASLLDLKEHASSLASSGLKKDSRLKTLEIALEQKKEECLKMESQLKKA
HEAALEARASPEMSDRIQHLEREITRYKDESSKAQAEVDRLLEILKEVENEKNDKDKKIA
ELESLTSRQVKDQNKKVANLKHKEQVEKKKSAQMLEEARRREDNLNDSSQQLQDSLRKKD
DRIEELEEALRESVQITAEREMVLAQEESARTNAEKQVEELLMAMEKVKQELESMKAKLS
STQQSLAEKETHLTNLRAERRKHLEEVLEMKQEALLAAISEKDANIALLELSSSKKKTQE
EVAALKREKDRLVQQLKQQTQNRMKLMADNYEDDHFKSSHSNQTNHKPSPDQDEEEGIWA
*
>ENSG00000115661|ENST00000409638
MGHALCVCSRGTVIIDNKRYLFIQKLGEGGFSYVDLVEGLHDGHFYALKRILCHEQQDRE
EAQREADMHRLFNHPNILRLVAYCLRERGAKHEAWLLLPFFKRGTLWNEIERLKDKGNFL
TEDQILWLLLGICRGLEAIHAKGYAHRDLKPTNILLGDEGQPVLMDLGSMNQACIHVEGS
RQALTLQDWAAQRCTISYRAPELFSVQSHCVIDERTDVWSLGCVLYAMMFGEGPYDMVFQ
KGDSVALAVQNQLSIPQSPRHSSALRQLLNSMMTVDPHQRPHIPLLLSQLEALQPPAPGQ

HTTQI*
>ENSG00000011465|ENST00000547568
MKATIILLLLAQVSWAGPFQQRGLFDFMLEDEASGIGPEVPDDRDFEPSLGPVCPFRCQC
HLRVVQCSDLGLPPSLTELHLDGNKISRVDAASLKGLNNLAKLGLSFNSISAVDNGSLAN
TPHLRELHLDNNKLTRVPGGLAEHKYIQVVYLHNNNISVVGSSDFCPPGHNTKKASYSGV
SLFSNPVQYWEIQPSTFRCVYVRSAIQLGNYK*
>ENSG00000171124|ENST00000589620
MDPLGAAKPQWPWRRCLAALLFQLLVAVCFFSYLRVSRDDATGSPRAPSGSSRQDTTPTR
PTLLILLRTWPFHIPVALSRCSEMVPGTADCHITADRKVYPQADMVIVHHWDIMSNPKSR
LPPSPRPQGQRWIWFNLEPPPNCQHLEALDRYFNLTMSYRSDSDIFTPYGWLEPWSGQPA
HPPLNLSAKTELVAWAVSNWKPDSARVRYYQSLQAHLKVDVYGRSHKPLPKGTMMETLSR
YKFYLAFENSLHPDYITEKLWRNALEAWAVPVVLGPSRSNYERFLPPDAFIHVDDFQSPK
DLARYLQELDKDHARYLSYFRWRETLRPRSFSWALDFCKACWKLQQESRYQTVRSIAAWF
T*
>ENSG00000106367|ENST00000443943
MMRFMLLFSRQGKLRLQKWYLATSDKERKKMVRELMQVVLARKPKMCSFLEWRDLKVVYK
RYASLYFCCAIEGQDNELITLELIHRYVELLDKYFGSVCELDIIFNFEKAYFILDEFLMG
GDVQDTSKKSVLKAIEQADLLQEEDESPRSVLEEMGLA*
>ENSG00000262911|ENST00000571432
MNTMYVMMAQILRSHLIKATVIPNRVKMLPYFGIIRNRMMSTHKSKKKIREYYRLLNVEE
GCSADEVRESFHKLAKQYHPDSGSNTADSATFIRIEKAYRKVLSHVIEQTNASQSKGEEE
EDVEKFKYKTPQHRHYLSFEGIGFGTPTQREKHYRQFRADRAAEQVMEYQKQKLQSQYFP
DSVIVKNIRQSKQQKITQAIERLVEDLIQESMAKGDFDNLSGKGKPLKKFSDCSYIDPMT
HNLNRILIDNGYQPEWILKQKEISDTIEQLREAILVSRKKLGNPMTPTEKKQWNHVCEQF
QENIRKLNKRINDFNLIVPILTRQKVHFDAQKEIVRAQKIYETLIKTKEVTDRNPNNLDQ
GEGEKTPEIKKGFLNWMNLWKFIKIRSF*
>ENSG00000283761|ENST00000639037
MFANLKYVSLGILVFQTTSLVLTMRYSRTLKEEGPRYLSSTAVVVAELLKIMACILLVYK
DSKCSLRALNRVLHDEILNKPMETLKLAIPSGIYTLQNNLLYVALSNLDAATYQVTYQLK
ILTTALFSVSMLSKKLGVYQWLSLVILMTGVAFVQWPSDSQLDSKELSAGSQFVGLMAVL
TACFSSGFAGVYFEKILKETKQSVWIRNIQLGFFGSIFGLMGVYIYDGELVSKNGFFQGY
NRLTWIVVVLQPQGIGSPSVYHAVIVIFLEFFAWGLLTAPTLVVLHETFPKHTFLMNGLI
QGVKGLLSFLSAPLIGALSDVWGRKSFLLLTVFFTCAPIPLMKISPWWYFAVISVSGVFA
VTFSVVFAYVADITQEHERSMAYGLVSATFAASLVTSPAIGAYLGRVYGDSLVVVLATAI
ALLDICFILVAVPESLPEKMRPASWGAPISWEQADPFASLKKVGQDSIVLLICITVFLSY
LPEAGQYSSFFLYLRQIMKFSPESVAAFIAVLGILSIIAQTIVLSLLMRSIGNKNTILLG
LGFQILQLAWYGFGSEPWMMWAAGAVAAMSSITFPAVSALVSRTADADQQGVVQGMITGI
RGLCNGLGPALYGFIFYIFHVELKELPITGTDLGTNTSPQHHFEQNSIIPGPPFLFGACS
VLLALLVALFIPEHTNLSLRSSSWRKHCGSHSHPHNTQAPGEAKEPLLQDTNV*
>ENSG00000283761|ENST00000639040
MFANLKYVSLGILVFQTTSLVLTMRYSRTLKEEGPRYLSSTAVVVAELLKIMACILLVYK
DSKCSLRALNRVLHDEILNKPMETLKLAIPSGIYTLQNNLLYVALSNLDAATYQVTYQLK
ILTTALFSVSMLSKKLGVYQWLSLVILMTGVAFVQWPSDSQLDSKELSAGSQFVGLMAVL
TACFSSGFAGVYFEKILKETKQSVWIRNIQLASRNRFS*

>ENSG00000140479|ENST00000611967
MPPRAPPAPGPRPPPRAAAATDTAAGAGGAGGAGGAGGPGFRPLAPRPWRWLLLLALPAA
CSAPPPRPVYTNHWAVQVLGGPAEADRVAAAHGYLNLGQIGNLEDYYHFYHSKTFKRSTL
SSRGPHTFLRMDPQVKWLQQQEVKRRVKRQVRSDPQALYFNDPIWSNMWYLHCGDKNSRC
RSEMNVQAAWKRGYTGKNVVVTILDDGIERNHPDLAPNYDSYASYDVNGNDYDPSPRYDA
SNENKHGTRCAGEVAASANNSYCIVGIAYNAKIGGIRMLDGDVTDVVEAKSLGIRPNYID
IYSASWGPDDDGKTVDGPGRLAKQAFEYGIKKGRQGLGSIFVWASGNGGREGDYCSCDGY
TNSIYTISVSSATENGYKPWYLEECASTLATTYSSGAFYERKIVTTDLRQRCTDGHTGTS
VSAPMVAGIIALALEANSQLTWRDVQHLLVKTSRPAHLKASDWKVNGAGHKVSHFYGFGL
VDAEALVVEAKKWTAVPSQHMCVAASDKRPRSIPLVQVLRTTALTSACAEHSDQRVVYLE
HVVVRTSISHPRRGDLQIYLVSPSGTKSQLLAKRLLDLSNEGFTNWEFMTVHCWGEKAEG
QWTLEIQDLPSQVRNPEKQGDLETPVANQLTTEEREPGLKHVFRWQIEQELW*
>ENSG00000274047|ENST00000613407
MALRPGAGSGGGGAAGAGAGSAGGGGFMFPVAGGIRPPQAGLMPMQQQGFPMVSVMQPNM
QGIMGMNYSSQMSQGPIAMQAGIPMGPMPAAGMPYLGQAPFLGMRPPGPQYTPDMQKQFA
EEQQKRFEQQQKLLEEERKRRQFEEQKQKLRLLSSVKPKTGEKSRDDALEAIKGNLDGFS
RDAKMHPTPASHPKKPGVGVFPSQDPAQPRMPPWIYNESLVPDAYKKILETTMTPTGIDT
AKLYPILMSSGLPRETLGQIWALANRTTPGKLTKEELYTVLAMIAVTQRGVPAMSPDALN
QFPAAPIPTLSGFSMTLPTPVSQPTVIPSGPAGSMPLSLGQPVMGINLVGPVGGAAAQAS
SGFIPTYPANQVVKPEEDDFQDFQDASKSGSLDDSFSDFQELPASSKTSNSQHGNSAPSL
LMPLPGTKALPSMDKYAVFKGIAADKSSENTVPPGDPGDKYSAFRELEQTAENKPLGESF
AEFRSAGTDDGFTDFKTADSVSPLEPPTKDKTFPPSFPSGTIQQKQQTQVKNPLNLADLD
MFSSVNCSSEKPLSFSAVFSTSKSVSTPQSTGSAATMTALAATKTSSLADDFGEFSLFGE
YSGLAPVGEQDDFADFMAFSNSSISSEQKPDDKYDALKEEASPVPLTSNVGSTVKGGQNS
TAASTKYDVFRQLSLEGSGLGVEDLKDNTPSGKSDDDFADFHSSKFSSINSDKSLGEKAV
AFRHTKEDSASVKSLDLPSIGGSSVGKEDSEDALSVQFDMKLADVGGDLKHVMSDSSLDL
PTVSGQHPPAADIEDLKYAAFGSYSSNFAVSTLTSYDWSDRDDATQGRKLSPFVLSAGSG
SPSATSILQKKETSFGSSENITMTSLSKVTTFVSEDALPETTFPALASFKDTIPQTSEQK
EYENRDYKDFTKQDLPTAERSQEATCPSPASSGASQETPNECSDDFGEFQSEKPKISKFD
FLVATSQSKMKSSEEMIKSELATFDLSVQGSHKRSLSLGDKEISRSSPSPALEQPFRDRS
NTLNEKPALPVIRDKYKDLTGEVEENERYAYEWQRCLGSALNVIKKANDTLNGISSSSVC
TEVIQSAQGMEYLLGVVEVYRVTKRVELGIKATAVCSEKLQQLLKDIDKVWNNLIGFMSL
ATLTCCWEKMTVITKHLSPYHELLEEKPDENSLDFSSCMLRPGIKNAQELACGVCLLNVD
SRSRKEEKPAEEHPKKAFNSETDSFKLAYGGHQYHASCANFWINCVEPKPPGLVLPDLL*
>ENSG00000143801|ENST00000422240
MLTFMASDSEEEVCDERTSLMSAESPTPRSCQEGRQGPEDGENTAQWRSQENEEDGEEDP
DRYVCSGVPGRPPGLEEELTLKYGAKHVIMLFVPVTLCMIVVVATIKSVRFYTEKNGQLI
YTPFTEDTPSVGQRLLNSVLNTLIMISVIVVMTIFLVVLYKYRCYKFIHGWLIMSSLMLL
FLFTYIYLGEVLKTYNVAMDYPTLLLTVWNFGAVGMVCIHWKGPLVLQQAYLIMISALMA
LVFIKYLPEWSAWVILGAISVYDLVAVLCPKGPLRMLVETAQERNEPIFPALIYSSAMVW
TVGMAKLDPSSQGALQLPYDPEMEDSYDSFGEPSYPEVFEPPLTGYPGEELEEEEERGVK
LGLGDFIFYSVLVGKAAATGSGDWNTTLACFVAILIGLCLTLLLLAVFKKALPALPISIT
FGLIFYFSTDNLVRPFMDTLASHQLYI*
>ENSG00000230791|ENST00000428405

MLSRNDVKRLERSWAQGSSEQELHYASLQRLPVPSSEGPDLRGRDKRGTKEDPRADYACI
AENKPT*
>ENSG00000078328|ENST00000436368
MLASQGVLLHPYGVPMIVPAAPYLPGLIQGNQEAAAAPDTMAQPYASAQFAPPQNGIPAE
YTAPHPHPAPEYTGQTTVPEHTLNLYPPAQTHSEQSPADTSAQTVSGTATQTDDAAPTDG
QPQTQPSENTENKSQPKRLHVSNIPFRFRDPDLRQMFGQFGKILDVEIIFNERGSKGFGF
VTFENSADADRAREKLHGTVVEGRKIEVNNATARVMTNKKTVNPYTNGWKLNPVVGAVYS
PEFYAGTVLLCQANQEGSSMYSAPSSLVYTSAMPGFPYPAATAAAAYRGAHLRGRGRTVY
NTFRAAAPPPPIPAYGGVVYQEPVYGNKLLQGGYAAYRYAQPTPATAAAYSDSYGRVYAA
DPYHHALAPAPTYGVGAMASIYRGGYNRFAPY*
>ENSG00000108433|ENST00000640621
MDPLFQQTHKQVHEIQSCMGRLETADKQSVHIVENEIQASIDQIFSRLERLEILSSKEPP
NKRQNARLRVDQLKYDVQHLQTALRNFQHRRHAREQQERQREELLSRTFTTNDSDTTIPM
DESLQFNSSLQKVHNGMDDLILDGHNILDGLRTQRLTLKVGSLLGDREKASCFSLIQQFS
NCVYILITCPQIVIF*
>ENSG00000230763|ENST00000425130
MMVLQVSAAPRTVALTALLMVLLTSVVQGRATPENYLFQGRQECYAFNGTQRFLERYIYN
REEFVRFDSDVGEFRAVTELGRPDEEYWNSQKDILEEKRAVPDRMCRHNYELGGPMTLQR
RVQPRVNVSPSKKGPLQHHNLLVCHVTDFYPGSIQVRWFLNGQEETAGVVSTNLIRNGDW
TFQILVMLEMTPQQGDVYTCQVEHTSMDSPVTVEWKAQSDSARSKTLTGAGGFVLGLIIC
GVGIFMHRRSKKVQRGSA*
>ENSG00000151348|ENST00000533608
MCASVKYNIRGPALIPRMKTKHRIYYITLFSIVLLGLIATGMFQFWPHSIESSNDWNVEK
RSIRDVPVVRLPADSPIPERGDLSCRMHTCFDVYRCGFNPKNKIKVYIYALKKYVDDFGV
SVSNTISREYNELLMAISDSDYYTDDINRACLFVPSIDVLNQNTLRIKETAQAMAQLSRW
DRGTNHLLFNMLPGGPPDYNTALDVPRDRALLAGGGFSTWTYRQGYDVSIPVYSPLSAEV
DLPEKGPGPRQYFLLSSQVGLHPEYREDLEALQVKHGESVLVLDKCTNLSEGVLSVRKRC
HKHQVFDYPQVLQEATFCVVLRGARLGQAVLSDVLQAGCVPVVIADSYILPFSEVLDWKR
ASVVVPEEKMSDVYSILQSIPQRQIEEMQRQARWFWEAYFQSIKAIALATLQIINDRIYP
YAAISYEEWNDPPAVKWGSVSNPLFLPLIPPQSQGFTAIVLTYDRVESLFRVITEVSKVP
SLSKLLVVWNNQNKNPPEDSLWPKIRVPLKVVRTAENKLSNRFFPYDEIETEAVLAIDDD
IIMLTSDELQFGYEVWREFPDRLVGYPGRLHLWDHEMNKWKYESEWTNEVSMVLTGAAFY
HKYFNYLYTYKMPGDIKNWVDAHMNCEDIAMNFLVANVTGKAVIKVTPRKKFKCPECTAI
DGLSLDQTHMVERSECINKFASVFGTMPLKVVEHRADPVLYKDDFPEKLKSFPNIGSL*
>ENSG00000111817|ENST00000452085
MRTHTRGAPSVFFIYLLCFVSAYITDENPEVMIPFTNANYDSHPMLYFSRAEVAELQLRA
ASSHEHIAARLTEAVHTMLSSPLEYLPPWDPKDYSARWNEIFGNNLGALAMFCVLYPENI
EARDMAKDYMERMAAQPSWLVKDAPWDEVPLAHSLVGFATAYDFLYNYLSKTQQEKFLEV
IANASGYMYETSYRRGWGFQYLHNHQPTNCMALLTGSLVLMNQGYLQEAYLWTKQVLTIM
EKSLVLLREVTDGSLYEGVAYGSYTTRSLFQYMFLVQRHFNINHFGHPWLKQHFAFMYRT
ILPGFQRTVAIADSNYNWFYGPESQLVFLDKFVMRNGSGNWLADQIRRNRVVEGPGTPSK
GQRWCTLHTEFLWYDGSLKSVPPPDFGTPTLHYFEDWGVVTYGSALPAEINRSFLSFKSG
KLGGRAIYDIVHRNKYKDWIKGWRNFNAGHEHPDQNSFTFAPNGVPFITEALYGPKYTFF
NNVLMFSPAVSKSCFSPWVGQVTEDCSSKWSKYKHDLAASCQGRVVAAEEKNGVVFIRGE

GVGAYNPQLNLKNVQRNLILLHPQLLLLVDQIHLGEESPLETAASFFHNVDVPFEETVVD
GVHGAFIRQRDGLYKMYWMDDTGYSEKATFASVTYPRGYPYNGTNYVNVTMHLRSPITRA
AYLFIGPSIDVQSFTVHGDSQQLDVFIATSKHAYATYLWTGEATGQSAFAQVIADRHKIL
FDRNSAIKSSIVPEVKDYAAIVEQNLQHFKPVFQLLEKQILSRVRNTASFRKTAERLLRF
SDKRQTEEAIDRIFAISQQQQQQSKSKKNRRAGKRYKFVDAVPDIFAQIEVNEKKIRQKA
QILAQKELPIDEDEEMKDLLDFADVTYEKHKNGGLIKGRFGQARMVTTTHSRAPSLSASY
TRLFLILNIAIFFVMLAMQLTYFQRAQSLHGQRCLYAVLLIDSCILLWLYSSCSQSQC*
>ENSG00000152683|ENST00000435660
MGTIHLFRKPQRSFFGKLLREFRLVAADRRSWKILLFGVINLICTGFLLMWCSSTNSIAL
TAYTYLTIFDLFSLMTCLISYWVTLRKPSPVYSFGFERLEVLAVFASTVLAQLGALFILK
ESAERFLEQPEIHTGRLLVGTFVALCFNLFTMLSIRNKPFAYVSEAASTSWLQEHVADLS
RSLCGIIPGLSSIFLPRMNPFVLIDLAGAFALCITYMLIEINNYFAVDTASAIAIALMTF
GTMYPMSVYSGKVLLQTTPPHVIGQLDKLIREAGSVHVRIRRDANEQMVLAHVTNRLYTL
VSTLTVQIFKDDWIRPALLSGPVAANVLNFSDHHVIPMPLLKGTDDLNPVTSTPAKPSSP
PPEFSFNTPGKNVNPVILLNTQTRPYGFGLNHGHTPYSSMLNQGLGVPGIGATQGLRTGF
TNIPSRYGTNNRIGQPRP*
>ENSG00000164414|ENST00000622775
MTLMAAVYTIALRYTRTSDKELYFSTTAVCITEVIKLLLSVGILAKETGSLGRFKASLRE
NVLGSPKELLKLSVPSLVYAVQNNMAFLALSNLDAAVYQVTYQLKIPCTALCTVLMLNRT
LSKLQWVSVFMLCAGVTLVQWKPAQATKVVEYILKKFKEFRYFSLGEKHSNVSIRDYCDI
SWRLLVRWS*
>ENSG00000226182|ENST00000420676
MLSRNDVKRLERSWHLLSWSQAQGSSEQELHYASLQRLPVPSSEGPDLRGRDKRGTKEDP
RADYACIAENKPT*
>ENSG00000137497|ENST00000543937
MTLHATRGAALLSWVNSLHVADPVEAVLQLQDCSIFIKIIDRIHGTEEGQQILKQPVSER
LDFVCSFLQKNRKHPSSPECLVSAQKVLEGSELELAKMTMLLLYHSTMSSKSPRDWEQFE
YKIQAELAVILKFVLDHEDGLNLNEDLENFLQKAPVPSTCSSTFPEELSPPSHQAKREIR
FLELQKVASSSSGNNFLSGSP
>ENSG00000143761|ENST00000541182
MGNIFANLFKGLFGKKEMRILMVGLDAAGKTTILYKLKLGEIVTTIPTIGFNVETVEYKN
ISFTVWDVGGQDKIRPLWRHYFQNTQGLIFVVDSNDRERVNEAREELMRMLAEDELRDAV
LLVFANKQDLPNAMNAAEITDKLGLHSLRHRNWYIQATCATSGDGLYEGLDWLSNQLRNQ
K*
>ENSG00000213380|ENST00000567493
MNSLTLNRHTEILEILEIPQLMDTCVRNSYYEEALELAAYVRRLERKYSSIPVIQGIVNE
VRQSMQLMLSQLIQQLRTNIQLPACLRVIGYLRRMDVFT
>ENSG00000230791|ENST00000446239
MLSRNDVKRLERSWRLPVPSSEGPDLRGRDKRGTKEDPRADYACIAENKPT*
>ENSG00000235915|ENST00000433676
MLSRNDVKRLERSWAQGSSEQELHYASLQRLPVPSSEGPDLRGRDKRGTKEDPRADYACI
AENKPT*
>ENSG00000280951|ENST00000629119
MGQNDLMGTAEDFADQFLRVTKQYLPHVARLCLISTFLEDGIRMWFQWSEQRDYIDTTWN

CGYLLASSFVFLNLLGQLTGCVLVLSRNFVQYACFGLFGIIALQTIAYSILWDLKFLMRL
SRTSWAQL*
>ENSG00000133110|ENST00000541179
MIPFLPMFSLLLLLIVNPINANNHYDKILAHSRIRGRDQGPNVCALQQILGTKKKYFSTC
KNWYKKSICGQKTTVLYECCPGYMRMEGMKGCPAVLPIDHVYGTLGIVGATTTQRYSDAS
KLREEIEGKGSFTYFAPSNEAWDNLDSDIRRGLESNVNVELLNALHSHMINKRMLTKDLK
NGMIIPSMYNNLGLFINHYPNGVVTVNCARIIHGNQIATNGVVHVIDRVLTQIGTSIQDF
IEAEDDLSSFRAAAITSDILEALGRDGHFTLFAPTNEAFEKLPRGVLERIMGDKVASEAL
MKYHILNTLQCSESIMGGAVFETLEGNTIEIGCDGDSITVNGIKMVNKKDIVTNNGVIHL
IDQVLIPDSAKQVIELAGKQQTTFTDLVAQLGLASALRPDGEYTLLAPVNNAFSDDTLSM
DQRLLKLILQNHILKVKVGLNELYNGQILETIGGKQLRVFVYRTAVCIENSCMEKGSKQG
RNGAIHIFREIIKPAEKSLHEKLKQDKRFSTFLSLLEAADLKELLTQPGDWTLFVPTNDA
FKGMTSEEKEILIRDKNALQNIILYHLTPGVFIGKGFEPGVTNILKTTQGSKIFLKEVND
TLLVNELKSKESDIMTTNGVIHVVDKLLYPADTPVGNDQLLEILNKLIKYIQIKFVRGST
FKEIPVTVYRPTLTKVKIEGEPEFRLIKEGETITEVIHGEPIIKKYTKIIDGVPVEITEK
ETREERIITGPEIKYTRISTGGGETEETLKKLLQEDTPVRKLQANKKVQGSRRRLREGRS
Q*
>ENSG00000168461|ENST00000578921
MMAIRELKVCLLGDTGVGKSSIVCRFVQDHFDHNISPTIGASFMTKTVPCGNELHKFLIW
DTAGQERFHSLAPMYYRGSAAAVIVYDITKQDSFYTLKKWVKELKEHGPENIVMAIAGNK
CDLSDIREVPLKDAKEYAESIGAIVVETSAKNAINIEELFQGISRQIPPLDPHENGNNGT
IKVEKPTMQASRRCC*
>ENSG00000140564|ENST00000618099
MELRPWLLWVVAATGTLVLLAADAQGQKVFTNTWAVRIPGGPAVANSVARKHGFLNLGQI
FGDYYHFWHRGVTKRSLSPHRPRHSRLQREPQVQWLEQQVAKRRTKRDVYQEPTDPKFPQ
QWYLSGVTQRDLNVKAAWAQGYTGHGIVVSILDDGIEKNHPDLAGNYDPGASFDVNDQDP
DPQPRYTQMNDNRHGTRCAGEVAAVANNGVCGVGVAYNARIGGVRMLDGEVTDAVEARSL
GLNPNHIHIYSASWGPEDDGKTVDGPARLAEEAFFRGVSQGRGGLGSIFVWASGNGGREH
DSCNCDGYTNSIYTLSISSATQFGNVPWYSEACSSTLATTYSSGNQNEKQIVTTDLRQKC
TESHTGTSASAPLAAGIIALTLEANKNLTWRDMQHLVVQTSKPAHLNANDWATNGVGRKV
SHSYGYGLLDAGAMVALAQNWTTVAPQRKCIIDILTEPKDIGKRLEVRKTVTACLGEPNH
ITRLEHAQARLTLSYNRRGDLAIHLVSPMGTRSTLLAARPHDYSADGFNDWAFMTTHSWD
EDPSGEWVLEIENTSEANNYGTLTKFTLVLYGTAPEGLPVPPESSGCKTLTSSQACVVCE
EGFSLHQKSCVQHCPPGFAPQVLDTHYSTENDVETIRASVCAPCHASCATCQGPALTDCL
SCPSHASLDPVEQTCSRQSQSSRESPPQQQPPRLPPEVEAGQRLRAGLLPSHLPEVVAGL
SCAFIVLVFVTVFLVLQLRSGFSFRGVKVYTMDRGLISYKGLPPEAWQEECPSDSEEDEG
RGERTAFIKDQSAL*
>ENSG00000144278|ENST00000409237
MRRFVYCKVVLATSLMWVLVDVFLLLYFSECNKCDDKKERSLLPALRAVISRNQEGPGEM
GKAVLIPKDDQEKMKELFKINQFNLMASDLIALNRSLPDVRLEGCKTKVYPDELPNTSVV
IVFHNEAWSTLLRTVYSVINRSPHYLLSEVILVDDASERDFLKLTLENYVKNLEVPVKII
RMEERSGLIRARLRGAAASKGQVITFLDAHCECTLGWLEPLLARIKEDRKTVVCPIIDVI
SDDTFEYMAGSDMTYGGFNWKLNFRWYPVPQREMDRRKGDRTLPVRTPTMAGGLFSIDRN
YFEEIGTYDAGMDIWGGENLEMSFRIWQCGGSLEIVTCSHVGHVFRKATPYTFPGGTGHV

INKNNRRLAEVWMDEFKDFFYIISPGVVKVDYGDVSVRKTLRENLKCKPFSWYLENIYPD
SQIPRRYYSLGEIRNVETNQCLDNMGRKENEKVGIFNCHGMGGNQVFSYTADKEIRTDDL
CLDVSRLNGPVIMLKCHHMRGNQLWEYDAESCLSVNKVADGSQHPTVETCNDSTLQKWLL
RNYTRMEIFRNIFGNSTDYIL*
>ENSG00000249915|ENST00000507528
MAAYSYRPGPGAGPGPAAGAALPDQSFLWNVFQRVDKDRSGVISDTELQQALSNGTWTPF
NPVTVRSIISMFDRENKAGVNFSEFTGVWKYITDWQNVFRTYDRDNSGMIDKNELKQALS
GYRLSDQFHDILIRKFDRQGRGQIAFDDFIQGCIVLQRLTDIFRRYDTDQDGWIQVSYEQ
YLSMVFSIV*
>ENSG00000175416|ENST00000508425
IAGIENDEGFGAPAGSHAAPAQPGPTSGAGSEDMGTTVNGDVFQEANGPADGYAAIAQAD
RLTQEPESIRKWREEQRKRLS*
>ENSG00000099139|ENST00000545128
MGWGSRCCCPGRLDLLCVLALLGGCLLPVCRTRVYTNHWAVKIAGGFPEANRIASKYGFI
NIGQIGALKDYYHFYHSRTIKRSVISSRGTHSFISMEPKVEWIQQQVVKKRTKRDYDFSR
AQSTYFNDPKWPSMWYMHCSDNTHPCQSDMNIEGAWKRGYTGKNIVVTILDDGIERTHPD
LMQNYDALASCDVNGNDLDPMPRYDASNENKHGTRCAGEVAAAANNSHCTVGIAFNAKIG
GVRMLDGDVTDMVEAKSVSFNPQHVHIYSASWGPDDDGKTVDGPAPLTRQAFENGVRMGR
RGLGSVFVWASGNGGRSKDHCSCDGYTNSIYTISISSTAESGKKPWYLEECSSTLATTYS
SGESYDKKIITTDLRQRCTDNHTGTSASAPMAAGIIALALEANPFLTWRDVQHVIVRTSR
AGHLNANDWKTNAAGFKVSHLYGFGLMDAEAMVMEAEKWTTVPRQHVCVESTDRQIKTIR
PNSAVRSIYKASGCSDNPNRHVNYLEHVVVRITITHPRRGDLAIYLTSPSGTRSQLLANR
LFDHSMEGFKNWEFMTIHCWGERAAGDWVLEVYDTPSQLRNFKTPGKLKEWSLVLYGTSV
QPYSPTNEFPKVERFRYSRVEDPTDDYGTEDYAGPCDPECSEVGCDGPGPDHCNDCLHYY
YKLKNNTRICVSSCPPGHYHADKKRCRKCAPNCESCFGSHGDQCMSCKYGYFLNEETNSC
VTHCPDGSYQDTKKNLCRKCSENCKTCTEFHNCTECRDGLSLQGSRCSVSCEDGRYFNGQ
DCQPCHRFCATCAGAGADGCINCTEGYFMEDGRCVQSCSISYYFDHSSENGYKSCKKCDI
SCLTCNGPGFKNCTSCPSGYLLDLGMCQMGAICKDGEYVDEHGHCQTCEASCAKCQGPTQ
EDCTTCPMTRIFDDGRCVSNCPSWKFEFENQCHPCHHTCQRCQGSGPTHCTSCGADNYGR
EHFLYQGECGDSCPEGHYATEGNTCLPCPDNCELCHSVHVCTRCMKGYFIAPTNHTCQKL
ECGQGEVQDPDYEECVPCEEGCLGCSLDDPGTCTSCAMGYYRFDHHCYKTCPEKTYSEEV
ECKACDSNCGSCDQNGCYWCEEGFFLLGGSCVRKCGPGFYGDQEMGECESCHRACETCTG
PGHDECSSCQEGLQLLRGMCVHATKTQEEGKFWNDILRKLQPCHSSCKTCNGSATLCTSC
PKGAYLLAQACVSSCPQGTWPSVRSGSCENCTEACAICSGADLCKKCQMQPGHPLFLHEG
RCYSKCPEGSYAEDGICERCSSPCRTCEGNATNCHSCEGGHVLHHGVCQENCPERHVAVK
GVCKHCPEMCQDCIHEKTCKECTPEFFLHDDMCHQSCPRGFYADSRHCVPCHKDCLECSG
PKADDCELCLESSWVLYDGLCLEECPAGTYYEKETKECRDCHKSCLTCSSSGTCTTCQKG
LIMNPRGSCMANEKCSPSEYWDEDAPGCKPCHVKCFHCMGPAEDQCQTCPMNSLLLNTTC
VKDCPEGYYADEDSNRCAHCHSSCRTCEGRHSRQCHSCRPGWFQLGKECLLQCREGYYAD
NSTGRCERCNRSCKGCQGPRPTDCLSCDRFFFLLRSKGECHRSCPDHYYVEQSTQTCERC
HPTCDQCKGKGALNCLSCVWSYHLMGGICTSDCLVGEYRVGEGEKFNCEKCHESCMECKG
PGAKNCTLCPANLVLHMDDSHCLHCCNTSDPPSAQECCDCQDTTDECILRTSKVRPATEH
FKTALFITSSMMLVLLLGAAVVVWKKSRGRVQPAAKAGYEKLADPNKSYSSYKSSYREST
SFEEDQVIEYRDRDYDEDDDDDIVYMGQDGTVYRKFKYGLLDDDDIDELEYDDESYSYYQ

*
>ENSG00000137573|ENST00000402687
MKYSCCALVLAVLGTELLGSLCSTVRSPRFRGRIQQERKNIRPNIILVLTDDQDVELGSL
QVMNKTRKIMEHGGATFINAFVTTPMCCPSRSSMLTGKYVHNHNVYTNNENCSSPSWQAM
HEPRTFAVYLNNTGYRTAFFGKYLNEYNGSYIPPGWREWLGLIKNSRFYNYTVCRNGIKE
KHGFDYAKDYFTDLITNESINYFKMSKRMYPHRPVMMVISHAAPHGPEDSAPQFSKLYPN
ASQHITPSYNYAPNMDKHWIMQYTGPMLPIHMEFTNILQRKRLQTLMSVDDSVERLYNML
VETGELENTYIIYTADHGYHIGQFGLVKGKSMPYDFDIRVPFFIRGPSVEPGSIVPQIVL
NIDLAPTILDIAGLDTPPDVDGKSVLKLLDPEKPGNRFRTNKKAKIWRDTFLVERGKFLR
KKEESSKNIQQSNHLPKYERVKELCQQARYQTACEQPGQKWQCIEDTSGKLRIHKCKGPS
DLLTVRQSTRNLYARGFHDKDKECSCRESGYRASRSQRKSQRQFLRNQGTPKYKPRFVHT
RQTRSLSVEFEGEIYDINLEEEEELQVLQPRNIAKRHDEGHKGPRDLQASSGGNRGRMLA
DSSNAVGPPTTVRVTHKCFILPNDSIHCERELYQSARAWKDHKAYIDKEIEALQDKIKNL
REVRGHLKRRKPEECSCSKQSYYNKEKGVKKQEKLKSHLHPFKEAAQEVDSKLQLFKENN
RRRKKERKEKRRQRKGEECSLPGLTCFTHDNNHWQTAPFWNLGSFCACTSSNNNTYWCLR
TVNETHNFLFCEFATGFLEYFDMNTDPYQLTNTVHTVERGILNQLHVQLMELRSCQGYKQ
CNPRPKNLDVGNKDGGSYDLHRGQLWDGWEG*
>ENSG00000197249|ENST00000437397
MPSSVSWGILLLAGLCCLVPVSLAEDPQGDAAQKTDTSHHDQDHPTFNKITPNLAEFAFS
LYRQLAHQSNSTNIFFSPVSIATAFAMLSLGTKADTHDEILEGLNFNLTEIPEAQIHEGF
QELLRTLNQPDSQLQLTTGNGLFLSEGLKLVDKFLEDVKKLYHSEAFTVNFGDTEEAKKQ
INDYVEKGTQGKIVDLVKELDRDTVFALVNYIFFKGKWERPFEVKDTEEEDFHVDQVTTV
KVPMMKRLGMFNIQHCKKLSSWVLLMKYLGNATAIFFLPDEGKLQHLENELTHDIITKFL
ENEDRRSASLHLPKLSITGTYDLKSVLGQLGITKVFSNGADLSGVTEEAPLKLSKAVHKA
VLTIDEKGTEAAGAMFLEAIPMSIPPEVKFNKPFVFLMIEQNTKSPLFMGKVVNPTQK*
>ENSG00000137497|ENST00000544129
MTLHATRGAALLSWVNSLHVADPVEAVLQLQDCSIFIKIIDRIHGTEEGQQILKQPVSER
LDFVCSFLQKNRKHPSSPECLVSAQKVLEGSELELAKMTMLLLYHSTMSSKSPRDWEQFE
YKIQAELAVILKFVLDHEDGLNLNEDLENFLQKAPVPST
>ENSG00000226182|ENST00000442052
MLSRNDDICIYGGLGLGGLLLLAVVLLSACLCWLHRRAPSVLVPGPGLLRAGTPLCISAE
AASAQQ*
>ENSG00000090615|ENST00000456883
MDGASAEQDGLQEDRSHSGPSSLPEAPLKPPGPLVPPDQQDKVQCAEVNRASTEGESPDG
PGQGGLCQNGPTPPFPDPPSSLDPTTSPVGPDASPGVAGFHDNLRKSQGTSAEGSVRKEA
LQSLRLSLPMQETQLCSTDSPLPLEKEEQVRLQARKWLEEQLKQYRVKRQQERSSQPATK
TRLFSTLDPELMLNPENLPRASTLAMTKEYSFLRTSVPRGPKVGSLGLPAHPREKKTSKS
SKIRSLADYRTEDSNAGNSGGNVPAPDSTKGSLKQNRSSAASVVSEISLSPDTDDRLENT
SLAGDSVSEVDGNDSDSSSYSSASTRGTYGILSKTVGTQDTPYMVNGQEIPADTLGQFPS
IKDVLQAAAAEHQDQGQEVNGEVRSRRDSICSSVSLESSAAETQEEMLQVLKEKMRLEGQ
LEALSLEASQALKEKAELQAQLAALSTKLQAQVECSHSSQQRQDSLSSEVDTLKQSCWDL
ERAMTDLQNMLEAKNASLASSNNDLQVAEEQYQRLMAKVEDMQRSMLSKDNTVHDLRQQM
TALQSQLQQVQLERTTLTSKLKASQAEISSLQSVRQWYQQQLALAQEARVRLQGEMAHIQ
VGQMTQAGLLEHLKLENVSLSQQLTETQHRSMKEKGRIAAQLQGIEADMLDQEAAFMQIQ

EAKTMVEEDLQRRLEEFEGERERLQRMADSAASLEQQLEQVKLTLLQRDQQLEALQQEHL
DLMKQLTLTQEALQSREQSLDALQTHYDELQARLGELQGEAASREDTICLLQNEKIILEA
ALQAAKSGKEELDRGARRLEEGTEETSETLEKLREELAIKSGQVEHLQQETAALKKQMQK
IKEQFLQQKVMVEAYRRDATSKDQLISELKATRKRLDSELKELRQELMQVHGEKRTAEAE
LSRLHREVAQVRQHMADLEGHLQSAQKERDEMETHLQSLQFDKEQMVAVTEANEALKKQI
EELQQEARKAITEQKQKMRRLGSDLTSAQKEMKTKHKAYENAVGILSRRLQEALAAKEAA
DAELGQLRAQGGSSDSSLALHERIQALEAELQAVSHSKTLLEKELQEVIALTSQELEESR
EKVLELEDELQESRGFRKKIKRLEESNKKLALELEHEKGKLTGLGQSNAALREHNSILET
ALAKREADLVQLNLQVQAVLQRKEEEDRQMKHLVQALQASLEKEKEKVNSLKEQVAAAKV
EAGHNRRHFKAASLELSEVKKELQAKEHLVQKLQAEADDLQIREGKHSQEIAQFQAELAE
ARAQLQLLQKQLDEQLSKQPVGNQEMENLKWEVDQKEREIQSLKQQLDLTEQQGRKELEG
LQQLLQNVKSELEMAQEDLSMTQKDKFMLQAKVSELKNNMKTLLQQNQQLKLDLRRGAAK
TVLRPASLPG*
>ENSG00000181704|ENST00000462683
MAEAEESPGDPGTASPRPLFAGLSDISISQDIPVEGEITIPMRSRIREFDSSTLNESVRN
TIMRDLKAVGKKFMHVLYPRKSNTLLRDWDLWGPLILCVTLALMLQRDSADSEKDGGPQF
AEVFVIVWFGAVTITLNSKLLGGNISFFQSLCVLGYCILPLTVAMLICRLVLLADPGPVN
FMVRLFVVIVMFAWSIVASTAFLADSQPPNRRALAVYPVFLFYFVISWMILTFTPQ*
>ENSG00000171310|ENST00000547956
MKPALLEVMRMNRICRMVLATCLGSFILVIFYFQSMLHPVMRRNPFGVDICCRKGSRSPL
QELYNPIQVSFKHSVSM*
>ENSG00000162645|ENST00000464839
MAPEINLPGPMSLIDNTKGQLVVNPEALKILSAITQPVVVVAIVGLYRTGKSYLMNKLAG
KKNGFSLGSTVKSHTKGIWMWCVPHPKKPEHTLVLLDTEGLGDIEKGDNENDSWIFALAI
LLSSTFVYNSMGTINQQAMDQLHYVTELTDRIKANSSPGNNSVDDSADFVSFFPAFVWTL
RDFTLELEVDGEPITADDYLELSLKLRKGTDKKSKSFNDPRLCIRKFFPKRKCFVFDWPA
PKKYLAHLEQLKEEELNPDFIEQVAEFCSYILSHSNVKTLSGGIPVNGPRLESLVLTYVN
AISSGDLPCMENAVLALAQIENSAAVEKAIAHYEQQMGQKVQLPTETLQELLDLHRDSER
EAIEVFMKNSFKDVDQMFQRKLGAQLEARRDDFCKQNSKASSDCCMALLQDIFGPLEEDV
KQGTFSKPGGYRLFTQKLQELKNKYYQVPRKGIQAKEVLKKYLESKEDVADALLQTDQSL
SEKEKAIEVERIKAESAEAAKKMLEEIQKKNEEMMEQKEKSYQEHVKQLTEKMERDRAQL
MAEQEKTLALKLQEQERLLKEGFENESKRLQKDIWDIQMRSKSLEPICNIL*
>ENSG00000151746|ENST00000548411
MAAEEVLQTVDHYKTEIERLTKELTETTHEKIQAAEYGLVVLEEKLTLKQQYDELEAEYD
SLKQELEQLKEAFGQSFSIHRKVAEDGETREETLLQESASKEAYYLGKILEMQNELKQSR
AVVTNVQAENERLTAVVQDLKENNEMVELQRIRMKDEIREYKFREARLLQDYTELEEENI
TLQKLVSTLKQNQVEYEGLKHEIKRFEEETVLLNSQLEDAIRLKEIAEHQLEEALETLKN
EREQKNNLRKELSQYISLNDNHISISVDGLKFAEDGSEPNNDDKMNGHIHGPLVKLNGDY
RTPTLRKGESLNPVSDLFSELNISEIQKLKQQLMQVEREKAILLANLQESQTQLEHTKGA
LTEQHERVHRLTEHVNAMRGLQSSKELKAELDGEKGRDSGEEAHDYEVDINGLEILECKY
RVAVTEVIDLKAEIKALKEKYNKSVENYTDEKAKYESKIQMYDEQVTSLEKTTKESGEKM
AHMEKELQKMTSIANENHSTLNTAQDELVTFSEELAQLYHHVCLCNNETPNRVMLDYYRQ
SRVTRSGSLKGPDDPRGLLSPRLARRGVSSPVETRTSSEPVAKESTEASKEPSPTKTPTI
SPVITAPPSSPVLDTSDIRKEPMNIYNLNAIIRDQIKHLQKAVDRSLQLSRQRAAARELA

PMIDKDKEALMEEILKLKSLLSTKREQIATLRAVLKANKQTAEVALANLKNKYENEKAMV
TETMTKLRNELKALKEDAATFSSLRAMFATRCDEYVTQLDEMQRQLAAAEDEKKTLNTLL
RMAIQQKLALTQRLEDLEFDHEQSRRSKGKLGKSKIGSPKIVSSLLPPYRHSAHN*
>ENSG00000230791|ENST00000453995
MLSRNDDICIYGGLGLGGLLLLAVVLLSACLCWLHRRVKRLERSWAQGSSEQELHYASLQ
RLPVPSSEGPDLRGRDKRGTKEDPRADYACIAENKPT*
>ENSG00000090863|ENST00000562090
MAACGRVRRMFRLSAALHLLLLFAAGAEKLPGQGVHSQGQGPGANFVSFVGQAGGGGPAG
QQLPQLPQSSQLQQQQQQQQQQQQPQPPQPPFPAGGPPARRGGAGAGGGWKLAEEESCRE
DVTRVCPKHTWSNNLAVLECLQDVREPENEISSDCNHLLWNYKLNLTTDPKFESVAREVC
KSTITEIKECADEPVGKGYMVSCLVDHRGNITEYQCHQYITKMTAIIFSDYRLICGFMDD
CKNDINILKCGSIRLGEKDAHSQGEVVSCLEKGLVKEAEEREPKIQVSELCKKAILRVAE
LSSDDFHLDRHLYFACRDDRERFCENTQAGEGRVYKCLFNHKFEESMSEKCREALTTRQK
LIAQDYKVSYSLAKSCKSDLKKYRCNVENLPRSREARLSYLLMCLESAVHRGRQVSSECQ
GEMLDYRRMLMEDFSLSPEIILSCRGEIEHHCSGLHRKGRTLHCLMKVVRGEKGNLGMNC
QQALQTLIQETDPGADYRIDRALNEACESVIQTACKHIRSGDPMILSCLMEHLYTEKMVE
DCEHRLLELQYFISRDWKLDPVLYRKCQGDASRLCHTHGWNETSEFMPQGAVFSCLYRHA
YRTEEQGRRLSRECRAEVQRILHQRAMDVKLDPALQDKCLIDLGKWCSEKTETGQELECL
QDHLDDLVVECRDIVGNLTELESEAEREYVFKNLPFKV*
>ENSG00000284137|ENST00000640494
MMKFRFRRQGADPQREKLKQELFAFNKTVEHGFPNQPSALAFDPELRIMAIGTRSGAVKI
YGAPGVEFTGLHRDAATVTQMHFLTGQGRLLSLLDDSSLHLWEIVHHNGCAHLEEALSFQ
LPSRPGFDGASAPLSLTRVTVVLLVAASDIAALGTEGSSVFFLDVTTLTLLEGQTLAPGE
VLRSVPDDYRCGKALGPVESLQGHLRDPTKILIGYSRGLLVIWNQASQCVDHIFLGNQQL
ESLCWGRDSSTVVSSHSDGSYAVWSVDAGSFPTLQPTVATTPYGPFPCKAINKILWRNCE
SGGHFIIFSGGMPRASYGDRHCVSVLRAETLVTLDFTSRIIDFFTVHSTRPEDEFDDPQA
LAVLLEEELVVLDLQTPGWPAVPAPYLAPLHSSAITCSAHVASVPAKLWARIVSAGEQQS
PQPVSSALSWPITGGRNLAQEPSQRGLLLTGHEDGTVRFWDASGVALRPLYKLSTAGLFQ
TDCEHADSLAQAAEDDWPPFRKVGCFDPYSDDPRLGVQKVALCKYTAQMVVAGTAGQVLV
LELSDVPVEQAVSVAIIDLLQDREGFTWKGHERLSPRTGPLPWPAGFQPRVLVQCLPPAA
VTAVTLHTEWSLVAFGTSHGFGLFDYQRKSPVLARCTLHPNDSLAMEGPLSRVKSLKKSL
RQSFRRIRKSRVSGKKRAANASSKLQEANAQLAEQACPHDVEMTPVQRRIEPRSADDSLS
GVVRCLYFADTFLRDGAHHGPTMWAGTNSGSVFAYALEVPAAAVGGEKRPEQAVEAVLGK
EVQLMHRAPVVAIAVLDGRGRPLPEPYEASRDLAQAPDMQGGHAVLIASEEQFKVFTLPK
VSAKTKFKLTAHEGCRVRKVALATFASVACEDYAETCLACLTNLGDVHVFSVPGLRPQVH
YSCIRKEDISGIASCVFTRHGQGFYLISPSEFERFSLSARNITEPLCSLDINWPRDATQA
SYRIRESPKLSQANGTPSILLAPQSLDGSPDPAHSMGPDTPEPPEAALSPMSIDSATSAD
TTLDTTGDVTVEDVKDFLGSSEESEKNLRNLAEDEAHACAILIK*
>ENSG00000228425|ENST00000441058
MAAAATMAAAARELVLRAGTSDMEEEEGPLAGGPGLQEPLQLGELDITSDEFILDEVDVH
IQANLEDELVKEALKTGVDLRHYSKQVELELQQIEQKSIRDYIQESENIASLHNQITACD
AVLERMEQMLGAFQSDLSSISSEIRTLQEQSGAMNIRLRNRQAVRGKLGELVDGLVVPSA
LVTAILEAPVTEPRFLEQLQELDAKAAAVREQEARGTAACADVRGVLDRLRVKAVTKIRE
FILQKIYSFRKPMTNYQIPQTALLKYRFFYQFLLGNERATAKEIRDEYVETLSKIYLSYY

RSYLGRLMKVQYEEVAEKDDLMGVEDTAKKGFFSKPSLRSRNTIFTLGTRGSVISPTELE
APILVPHTAQRGEQRYPFEALFRSQHYALLDNSCREYLFICEFFVVSGPAAHDLFHAVMG
RTLSMTLKHLDSYLADCYDAIAVFLCIHIVLRFRNIAAKRDVPALDRYWEQVLALLWPRF
ELILEMNVQSVRSTDPQRLGGLDTRPHYITRRYAEFSSALVSINQTIPNERTMQLLGQLQ
VEVENFVLRVAAEFSSRKEQLVFLINNYDMMLGVLMERAADDSKEVESFQQLLNARTQEF
IEELLSPPFGGLVAFVKEAEALIERGQAERLRGEEARVTQLIRGFGSSWKSSVESLSQDV
MRSFTNFRNGTSIIQGALTQLIQLYHRFHRVLSQPQLRALPARAELINIHHLMVELKKHK
PNF*
>ENSG00000073849|ENST00000448044
MIHTNLKKKFSCCVLVFLLFAVICVWKEKKKGSYYDSFKLQTKEFQVLKSLGKLAMGSDS
QSVSSSSTQDPHRGRQTLGSLRGLAKAKPEASFQVWNKDSSSKNLIPRLQKIWKNYLSMN
KYKVSYKGPGPGIKFSAEALRCHLRDHVNVSMVEVTDFPFNTSEWEGYLPKESIRTKAGP
WGRCAVVSSAGSLKSSQLGREIDDHDAVLRFNGAPTANFQQDVGTKTTIRLMNSQLVTTE
KRFLKDSLYNEGILIVWDPSVYHSDIPKWYQNPDYNFFNNYKTYRKLHPNQPFYILKPQM
PWELWDILQEISPEEIQPNPPSSGMLGIIIMMTLCDQVDIYEFLPSKRKTDVCYYYQKFF
DSACTMGAYHPLLYEKNLVKHLNQGTDEDIYLLGKATLPGFRTIHC*
>ENSG00000132254|ENST00000445086
MKPALCLVAMGALVMDSSPQCTKQLLSERFGRGSRTVDLELELQIELLRETKRKYESVLQ
LGRALTAHLYSLLQTQHALGDAFADLSQKSPELQEEFGYNAETQKLLCKNGETLLGAVNF
FVSSINTLVTKTMEDTLMTVKQYEAARLEYDAYRTDLEELSLGPRDAGTRGRLESAQATF
QAHRDKYEKLRGDVAIKLKFLEENKIKVMHKQLLLFHNAVSAYFAGNQKQLEQTLQQFNI
KLRPPGAEKPSWLEEQ*
>ENSG00000223802|ENST00000623882
MAAAGPAAGPTGPEPMPSYAQLVQRGWGSALAAARGCTDCGWGLARRGLAEHAHLAPPEL
LLLALGALGWTALRSAATARLFRPLAKRCCLQPRDAAKMPESAWKFLFYLGSWSYSAYLL
FGTDYPFFHDPPSVFYDWTPGMAVPRDIAAAYLLQGSFYGHSIYATLYMDTWRKDSVVML
LHHVVTLILIVSSYAFRYHNVGILVLFLHDISDVQLEFTKLNIYFKSRGGSYHRLHALAA
DLGCLSFGFSWFWFRLYWFPLKVLYATSHCSLRTVPDIPFYFFFNALLLLLTLMNLYWFL
YIVAFAAKVLTGQVHELKDLREYDTAEAQSLKPSKAEKPLRNGLVKDKRF*
>ENSG00000070371|ENST00000427926
MAQILPVRFQEHFQLQNLGINPANIGFSTLTMESDKFICIREKVGEQAQVTIIDMSDPMA
PIRRPISAESAIMNPASKVIALKAGKTLQIFNIEMKSKMKAHTMAEEVIFWKWVSVNTVA
LVTETAVYHWSMEGDSQPMKMFDRHTSLVGCQVIHYRTDEYQKWLLLVGISAQQNRVVGA
MQLYSVDRKVSQPIEGHAAAFAEFKMEGNAKPATLFCFAVRNPTGGKLHIIEVGQPAAGN
QPFVKKAVDVFFPPEAQNDFPVAMQIGAKHGVIYLITKYGYLHLYDLESGVCICMNRISA
DTIFVTAPHKPTSGIIGVNKKGQVLSVCVEEDNIVNYATNVLQNPDLGLRLAVRSNLAGA
EKLFVRKFNTLFAQGSYAEAAKVAASAPKGILRTRETVQKFQSIPAQSGQASPLLQYFGI
LLDQGQLNKLESLELCHLVLQQGRKQLLEKWLKEDKLECSEELGDLVKTTDPMLALSVYL
RANVPSKVIQCFAETGQFQKIVLYAKKVGYTPDWIFLLRGVMKISPEQGLQFSRMLVQDE
EPLANISQIVDIFMENSLIQQCTSFLLDALKNNRPAEGLLQTWLLEMNLVHAPQVADAIL
GNKMFTHYDRAHIAQLCEKAGLLQQALEHYTDLYDIKRAVVHTHLLNPEWLVNFFGSLSV
EDSVECLHAMLSANIRQNLQLCVQVASKYHEQLGTQALVELFESFKSYKGLFYFLGSIVN
FSQDPDVHLKYIQAACKTGQIKEVERICRESSCYNPERVKNFLKEAKLTDQLPLIIVCDR
FGFVHDLVLYLYRNNLQRYIEIYVQKVNPSRTPAVIGGLLDVDCSEEVIKHLIMAVRGQF

STDELVAEVEKRNRLKLLLPWLESQIQEGCEEPATHNALAKIYIDSNNSPECFLRENAYY
DSSVVGRYCEKRDPHLACVAYERGQCDLELIKVCNENSLFKSEARYLVCRKDPELWAHVL
EETNPSRRQLIDQVVQTALSETRDPEEISVTVKAFMTADLPNELIELLEKIVLDNSVFSE
HRNLQNLLILTAIKADRTRVMEYISRLDNYDALDIASIAVSSALYEEAFTVFHKFDMNAS
AIQVLIEHIGNLDRAYEFAERCNEPAVWSQLAQAQLQKDLVKEAINSYIRGDDPSSYLEV
VQSASRSNNWEDLVKFLQMARKKGRESYIETELIFALAKTSRVSELEDFINGPNNAHIQQ
VGDRCYEEGMYEAAKLLYSNVSNFARLASTLVHLGEYQAAVDNSRKASSTRTWKEVCFAC
MDGQEFRFAQLCGLHIVIHADELEELMCYYQDRGYFEELILLLEAALGLERAHMGMFTEL
AILYSKFKPQKMLEHLELFWSRVNIPKVLRAAEQAHLWAELVFLYDKYEEYDNAVLTMMS
HPTEAWKEGQFKDIITKVANVELCYRALQFYLDYKPLLINDLLLVLSPRLDHTWTVSFFS
KAGQLPLVKPYLRSVQSHNNKSVNEALNHLLTEEEDYQGLRASIDAYDNFDNISLAQQLE
KHQLMEFRCIAAYLYKGNNWWAQSVELCKKDHLYKDAMQHAAESRDAELAQKLLQWFLEE
GKRECFAACLFTCYDLLRPDMVLELAWRHNLVDLAMPYFIQVMREYLSKVDKLDALESLR
KQEEHVTEPAPLVFDFDGHE*
>ENSG00000149182|ENST00000524782
MAAEPNKTEIQTLFKRLRAVPTNKACFDCGAKNPSWASITYGVFLCIDCSGVHRSLGVHL
SFIRSTELDSNWNWFQLRCMQVGGNANATAFFRQHGCTANDANTKYNSRAAQMYREKIRQ
LGSAALARHGTDLWIDNMSSAVPNHSPEKKDSDFFTEHTQPPAWDAPATEPSGTQQPAPS
TESSGLAQPEHGPNTDLLGTSPKASLELKSSIIGKKKPAAAKKGLGAKKGLGAQKVSSQS
FSEIERQAQVAEKLREQQAADAKKQAEESMVASMRLAYQELQIDRKKEEKKLQNLEGKKR
EQAERLGMGLVSRSSVSHSVLSEMQVIEQETPVSAKSSRSQLDLFDDVGTFASGPPKYKD
NPFSLGESFGSRWDTDAAWGMDRVEEKEPEVTISSIRPISERATNRREVESRSSGLESSE
ARQKFAGAKAISSDMFFGREVDAEYEARSRLQQLSGSSAISSSDLFGDMDGAHGAGSVSL
GNVLPTADIAQFKQGVKSVAGKMAVLANGVMNSLQDRYGSY*
>ENSG00000135968|ENST00000482325
MEDLVQDGVASPATPGTGKSKNWRKKLKNSDQNLLLKELVILLRH*
>ENSG00000180398|ENST00000412438
MTMRSLLRTPFLCGLLWAFCAPGARAEEPAASFSQPGSMGLDKNTVHDQEHIMEHLEGVI
NKPEAEMSPQELQLHYFKMHDYDGNNLLDGLELSTAITHVHKEEGSEQAPLMSEDELINI
IDGVLRDDDKNNDGYIDYAEFAKSLQ
>ENSG00000234514|ENST00000423107
MLSRNDVKRLERSWAQGSSEQELHYASLQRLPVPSSEGPDLRGRDKRGTKEDPRADYACI
AENKPT*
>ENSG00000073849|ENST00000457772
MNSQLVTTEKRFLKDSLYNEGILIVWDPSVYHSDIPKWYQNPDYNFFNNYKTYRKLHPNQ
PFYILKPQMPWELWDILQEISPEEIQPNPPSSGMLGIIIMMTLCDQVDIYEFLPSKRKTD
VCYYYQKFFDSACTMGAYHPLLYEKNLVKHLNQGTDEDIYLLGKATLPGFRTIHC*
>ENSG00000162040|ENST00000454677
MAGSGGLGGGAGGGQGAGAGQGAALRASRAPMLLVALVLGAYCLCALPGRCPPAARAPAP
APAPSEPSSSVHRPGAPGLPLASGPGRRRFPQALIVGVKKGGTRALLEFLRLHPDVRALG
SEPHFFDRCYERGLAWYRSLMPRTLDGQITMEKTPSYFVTREAPRRIHAMSPDTKLIVVV
RNPVTRAISDYAQTLSKTPGLPSFRALAFRHGLGPVDTAWSAVRIGLYAQHLDHWLRYFP
LSHFLFVSGERLVSDPAGEVGRVQDFLGLKRVVTDKHFYFNATKGFPCLKKAQGGSRPRC
LGKSKGRPHPRVPQALVRRLQEFYRPFNRRFYQMTGQDFGWG*

>ENSG00000177692|ENST00000617313
MNTMYVMMAQILRSHLIKATVIPNRVKMLPYFGIIRNRMMSTHKSKKKIREYYRLLNVEE
GCSADEVRESFHKLAKQYHPDSGSNTADSATFIRIEKAYRKVLSHVIEQTNASQSKGEEE
EDVEKFKYKTPQHRHYLSFEGIGFGTPTQREKHYRQFRADRAAEQVMEYQKQKLQSQYFP
DSVIVKNIRQSKQQKITQAIERLVEDLIQESMAKGDFDNLSGKGKPLKKFSDCSYIDPMT
HNLNRILIDNGYQPEWILKQKEISDTIEQLREAILVSRKKLGNPMTPTEKKQWNHVCEQF
QENIRKLNKRINDFNLIVPILTRQKVHFDAQKEIVRAQKIYETLIKTKEVTDRNPNNLDQ
GEGEKTPEIKKGFLNWMNLWKFIKIRSF*
>ENSG00000136279|ENST00000456905
MAANLSRNGPALQEAYVRVVTEKSPTDWALFTYEGNSNDIRVAGTGEGGLEEMVEELNSG
KVMYAFCRVKDPNSGLPKFVLINWTGEGVNDVRKGACASHVSTMASFLKGSVYQKTNAVS
EIKRVGKDSFWAKAEKEEENRRLEEKRRAEEAQRQLEQERRERELREAARREQRYQEQGG
EASPQSRTWEQQQEVVSRNRNEQESAVHPREIFKQKERAMSTTSISSPQPGKLRSPFLQK
QLTQPETHFGREPAAAISRPRADLPAEEPAPSTPPCLVQAEEEAVYEEPPEQETFYEQPP
LVQQQGAGSEHIDHHIQGQGLSGQGLCARALYDYQAADDTEISFDPENLITGIEVIDEGW
WRGYGPDGHFGMFPANYVELIE*
>ENSG00000223980|ENST00000454091
MAVMAPRTLLLLLLGALALTQTWAGSHSMRYFFTSVSRPGRGEPRFIAVGYVDDTQFVRF
DSDAASQRMEPRAPWIEQEGPEYWDQETRNVKAHSQTDRESLRIALRYYNQSEAGSHTIQ
MMYGCDVGPDGRLLRGYQQDAYDGKDYIALNEDLRSWTAADMAAQITQRKWEAARVAEQL
RAYLEGTCVEWLRRYLENGKETLQRTDAPKTHMTHHAVSDHEATLRCWALSFYPAEITLT
WQRDGEDQTQDTELVETRPAGDGTFQKWASVVVPSGQEQRYTCHVQHEGLPKPLTLRWEP
SSQPTIPIVGIIAGLVLFGAMFAGAVVAAVRWRRKSSDRKGGSYSQAASSDSAQGSDMSL
TACKV*
>ENSG00000205277|ENST00000536621
MLVIWILTLALRLCASVTTVTPGSTVNTSIGGNTTSASTPSSSDPFTTFSDYGVSVTFIT
GSTATKHFLDSSTNSGHSEESTVSHSGPGATGTTLFPSHSATSVFVGEPKTSPITSASME
TTALPGSTTTAGLSEKSTTFYSSPRSPDRTLSPARTTSSGVSEKSTTSHSRPGPTHTIAF
PDSTTMPGVSQESTASHSIPGSTDTTLSPGTTTPSSLGPESTTFHSSPGYTKTTRLPDNT
TTSGLLEASTPVHSSTGSPHTTLSPSSSTTHEGEPTTFQSWPSSKDTSPAPSGTTSAFVK
LSTTYHSSPSSTPTTHFSASSTTLGHSEESTPVHSSPVATATTPPPARSATSGHVEESTA
YHRSPGSTQTMHFPESSTTSGHSEESATFHGSTTHTKSSTPSTTAALAHTSYHSSLGSTE
TTHFRDSSTISGRSEESKASHSSPDAMATTVLPAGSTPSVLVGDSTPSPISSGSMETTAL
PGSTTKPGLSEKSTTFYSSPRSPDTTHLPASMTSSGVSEESTTSHSRPGSTHTTAFPGST
TMPGLSQESTASHSSPGPTDTTLSPGSTTASSLGPEYTTFHSRPGSTETTLLPDNTTASG
LLEASMPVHSSTRSPHTTLSPAGSTTRQGESTTFHSWPSSKDTRPAPPTTTSAFVEPSTT
SHGSPSSIPTTHISARSTTSGLVEESTTYHSSPGSTQTMHFPESDTTSGRGEESTTSHSS
TTHTISSAPSTTSALVEEPTSYHSSPGSTATTHFPDSSTTSGRSEESTASHSSQDATGTI
VLPARSTTSVLLGESTTSPISSGSMETTALPGSTTTPGLSERSTTFHSSPRSPATTLSPA
STTSSGVSEESTTSRSRPGSTHTTAFPDSTTTPGLSRHSTTSHSSPGSTDTTLLPASTTT
SGPSQESTTSHSSSGSTDTALSPGSTTALSFGQESTTFHSNPGSTHTTLFPDSTTSSGIV
EASTRVHSSTGSPRTTLSPASSTSPGLQGESTAFQTHPASTHTTPSPPSTATAPVEESTT
YHRSPGSTPTTHFPASSTTSGHSEKSTIFHSSPDASGTTPSSAHSTTSGRGESTTSRISP
GSTEITTLPGSTTTPGLSEASTTFYSSPRSPTTTLSPASMTSLGVGEESTTSRSQPGSTH

STVSPASTTTPGLSEESTTVYSSSRGSTETTVFPHSTTTSVHGEEPTTFHSRPASTHTTL
FTEDSTTSGLTEESTAFPGSPASTQTGLPATLTTADLGEESTTFPSSSGSTGTKLSPARS
TTSGLVGESTPSRLSPSSTETTTLPGSPTTPSLSEKSTTFYTSPRSPDATLSPATTTSSG
VSEESSTSHSQPGSTHTTAFPDSTTTSDLSQEPTTSHSSQGSTEATLSPGSTTASSLGQQ
STTFHSSPGDTETTLLPDDTITSGLVEASTPTHSSTGSLHTTLTPASSTSAGLQEESTTF
QSWPSSSDTTPSPPGTTAAPVEVSTTYHSRPSSTPTTHFSASSTTLGRSEESTTVHSSPG
ATGTALFPTRSATSVLVGEPTTSPISSGSTETTALPGSTTTAGLSEKSTTFYSSPRSPDT
TLSPASTTSSGVSEESTTSHSRPGSTHTTAFPGSTTMPGVSQESTASHSSPGSTDTTLSP
GSTTASSLGPESTTFHSSPGSTETTLLPDNTTASGLLEASTPVHSSTGSPHTTLSPAGST
TRQGESTTFQSWPSSKDTMPAPPTTTSAFVELSTTSHGSPSSTPTTHFSASSTTLGRSEE
STTVHSSPVATATTPSPARSTTSGLVEESTAYHSSPGSTQTMHFPESSTASGRSEESRTS
HSSTTHTISSPPSTTSALVEEPTSYHSSPGSTATTHFPDSSTTSGRSEESTASHSSQDAT
GTIVLPARSTTSVLLGESTTSPISSGSMETTALPGSTTTPGLSEKSTTFHSSPRSPATTL
SPASTTSSGVSEESTTSHSRPGSTHTTAFPDSTTTPGLSRHSTTSHSSPGSTDTTLLPAS
TTTSGPSQESTTSHSSPGSTDTALSPGSTTALSFGQESTTFHSSPGSTHTTLFPDSTTSS
GIVEASTRVHSSTGSPRTTLSPASSTSPGLQGESTAFQTHPASTHTTPSPPSTATAPVEE
STTYHRSPGSTPTTHFPASSTTSGHSEKSTIFHSSPDASGTTPSSAHSTTSGRGESTTSR
ISPGSTEITTLPGSTTTPGLSEASTTFYSSPRSPTTTLSPASMTSLGVGEESTTSRSQPG
STHSTVSPASTTTPGLSEESTTVYSSSPGSTETTVFPRTPTTSVRGEEPTTFHSRPASTH
TTLFTEDSTTSGLTEESTAFPGSPASTQTGLPATLTTADLGEESTTFPSSSGSTGTTLSP
ARSTTSGLVGESTPSRLSPSSTETTTLPGSPTTPSLSEKSTTFYTSPRSPDATLSPATTT
SSGVSEESSTSHSQPGSTHTTAFPDSTTTPGLSRHSTTSHSSPGSTDTTLLPASTTTSGP
SQESTTSHSSPGSTDTALSPGSTTALSFGQESTTFHSSPGSTHTTLFPDSTTSSGIVEAS
TRVHSSTGSPRTTLSPASSTSPGLQGESTTFQTHPASTHTTPSPPSTATAPVEESTTYHR
SPGSTPTTHFPASSTTSGHSEKSTIFHSSPDASGTTPSSAHSTTSGRGESTTSRISPGST
EITTLPGSTTTPGLSEASTTFYSSPRSPTTTLSPASMTSLGVGEESTTSRSQPGSTHSTV
SPASTTTPGLSEESTTVYSSSPGSTETTVFPRSTTTSVRGEEPTTFHSRPASTHTTLFTE
DSTTSGLTEESTAFPGSPASTQTGLPATLTTADLGEESTTFPSSSGSTGTTLSPARSTTS
GLVGESTPSRLSPSSTETTTLPGSPTTPSLSEKSTTFYTSPRSPDATLSPATTTSSGVSE
ESSTSHSQPGSTHTTAFPDSTTTSGLSQEPTASHSSQGSTEATLSPGSTTASSLGQQSTT
FHSSPGDTETTLLPDDTITSGLVEASTPTHSSTGSLHTTLTPASSTSAGLQEESTTFQSW
PSSSDTTPSPPGTTAAPVEVSTTYHSRPSSTPTTHFSASSTTLGRSEESTTVHSSPGATG
TALFPTRSATSVLVGEPTTSPISSGSTETTALPGSTTTAGLSEKSTTFYSSPRSPDTTLS
PASTTSSGVSEESTTSHSRPGSTHTTAFPGSTTMPGVSQESTASHSSPGSTDTTLSPGST
TASSLGPESTTFHSGPGSTETTLLPDNTTASGLLEASTPVHSSTGSPHTTLSPAGSTTRQ
GESTTFQSWPNSKDTTPAPPTTTSAFVELSTTSHGSPSSTPTTHFSASSTTLGRSEESTT
VHSSPVATATTPSPARSTTSGLVEESTTYHSSPGSTQTMHFPESDTTSGRGEESTTSHSS
TTHTISSAPSTTSALVEEPTSYHSSPGSTATTHFPDSSTTSGRSEESTASHSSQDATGTI
VLPARSTTSVLLGESTTSPISSGSMETTALPGSTTTPGLSEKSTTFHSSPRSPATTLSPA
STTSSGVSEESTTSHSRPGSTHTTAFPDSTTTPGLSRHSTTSHSSPGSTDTTLLPASTTT
SGSSQESTTSHSSSGSTDTALSPGSTTALSFGQESTTFHSSPGSTHTTLFPDSTTSSGIV
EASTRVHSSTGSPRTTLSPASSTSPGLQGESTAFQTHPASTHTTPSPPSTATAPVEESTT
YHRSPGSTPTTHFPASSTTSGHSEKSTIFHSSPDASGTTPSSAHSTTSGRGESTTSRISP
GSTEITTLPGSTTTPGLSEASTTFYSSPRSPTTTLSPASMTSLGVGEESTTSRSQPGSTH

STVSPASTTTPGLSEESTTVYSSSPGSTETTVFPRSTTTSVRREEPTTFHSRPASTHTTL
FTEDSTTSGLTEESTAFPGSPASTQTGLPATLTTADLGEESTTFPSSSGSTGTKLSPARS
TTSGLVGESTPSRLSPSSTETTTLPGSPTTPSLSEKSTTFYTSPRSPDATLSPATTTSSG
VSEESSTSHSQPGSTHTTAFPDSTTTSGLSQEPTTSHSSQGSTEATLSPGSTTASSLGQQ
STTFHSSPGDTETTLLPDDTITSGLVEASTPTHSSTGSLHTTLTPASSTSTGLQEESTTF
QSWPSSSDTTPSPPSTTAVPVEVSTTYHSRPSSTPTTHFSASSTTLGRSEESTTVHSSPG
ATGTALFPTRSATSVLVGEPTTSPISSGSTETTALPGSTTTAGLSEKSTTFYSSPRSPDT
TLSPASTTSSGVSEESTTSHSRPGSMHTTAFPSSTTMPGVSQESTASHSSPGSTDTTLSP
GSTTASSLGPESTTFHSSPGSTETTLLPDNTTASGLLEASTPVHSSTGSPHTTLSPAGST
TRQGESTTFQSWPNSKDTTPAPPTTTSAFVELSTTSHGSPSSTPTTHFSASSTTLGRSEE
STTVHSSPVATATTPSPARSTTSGLVEESTTYHSSPGSTQTMHFPESNTTSGRGEESTTS
HSSTTHTISSAPSTTSALVEEPTSYHSSPGSTATTHFPDSSTTSGRSEESTASHSSQDAT
GTIVLPARSTTSVLLGESTTSPISSGSMETTALPGSTTTPGLSEKSTTFHSSPSSTPTTH
FSASSTTLGRSEESTTVHSSPVATATTPSPARSTTSGLVEESTAYHSSPGSTQTMHFPES
STASGRSEESRTSHSSTTHTISSPPSTTSALVEEPTSYHSSPGSIATTHFPESSTTSGRS
EESTASHSSPDTNGITPLPAHFTTSGRIAESTTFYISPGSMETTLASTATTPGLSAKSTI
LYSSSRSPDQTLSPASMTSSSISGEPTSLYSQAESTHTTAFPASTTTSGLSQESTTFHSK
PGSTETTLSPGSITTSSFAQEFTTPHSQPGSALSTVSPASTTVPGLSEESTTFYSSPGST
ETTAFSHSNTMSIHSQQSTPFPDSPGFTHTVLPATLTTTDIGQESTAFHSSSDATGTTPL
PARSTASDLVGEPTTFYISPSPTYTTLFPASSSTSGLTEESTTFHTSPSFTSTIVSTESL
ETLAPGLCQEGQIWNGKQCVCPQGYVGYQCLSPLESFPVETPEKLNATLGMTVKVTYRNF
TEKMNDASSQEYQNFSTLFKNRMDVVLKGDNLPQYRGVNIRRLLNGSIVVKNDVILEADY
TLEYEELFENLAEIVKAKIMNETRTTLLDPDSCRKAILCYSEEDTFVDSSVTPGFDFQEQ
CTQKAAEGYTQFYYVDVLDGKLACVNKCTKGTKSQMNCNLGTCQLQRSGPRCLCPNTNTH
WYWGETCEFNIAKSLVYGIVGAVMAVLLLALIILIILFSLSQRKRHREQYDVPQEWRKEG
TPGIFQKTAIWEDQNLRESRFGLENAYNNFRPTLETVDSGTELHIQRPEMVASTV*
>ENSG00000124507|ENST00000620693
MSSSYDEASLAPEETTDSFWEVGNYKRTVKRIDDGHRLCNDLMNCVQERAKIEKAYGQQL
TDWAKRWRQLIEKGPQYGSLERAWGAIMTEADKVSELHQEVKNNLLNEDLEKVKNWQKDA
YHKQIMGGFKETKEAEDGFRKAQKPWAKKMKELEAAKKAYHLACKEEKLAMTREMNSKTE
QSVTPEQQKKLQDKVDKCKQDVQKTQEKYEKVLEDVGKTTPQYMENMEQVFEQCQQFEEK
RLVFLKEVLLDIKRHLNLAENSSYIHVYRELEQAIRGADAQEDLRWFRSTSGPGMPMNWP
QFEEWNPDLPHTTTKKEKQPKKAEGVALTNATGAVESTSQAGDRGSVSSYDRGQPYATEW
SDDESGNPFGGSETNGGANPFEDDSKGVRVRALYDYDGQEQDELSFKAGDELTKLGEEDE
QGWCRGRLDSGQLGLYPANYVEAI*
>ENSG00000117262|ENST00000462900
MRQLFKDYEIRQYVVQVIFSVTFAFSCTMFELIIFEILGVLNSSSRYFHWKMNLCVILLI
LVFMVPFYIGYFIVSNIRLLHKQRLLFSCLLWLTFMYFFWKLGDPFPILSPKHGILSIEQ
LISRVGVIGVTLMALLSGFGAVNCPYTYMSYFLRNVTDTDILALERRLLQTMDMIISKKK
RMAMARRTMFQKGEVHNKPSGFWGMIKSVTTSASGSENLTLIQQEVDALEELSRQLFLET
ADLYATKERIEYSKTFKGKYFNFLGYFFSIYCVWKIFMATINIVFDRVGKTDPVTRGIEI
TVNYLGIQFDVKFWSQHISFILVGIIIVTSIRGLLITLTKFFYAISSSKSSNVIVLLLAQ
IMGMYFVSSVLLIRMSMPLEYRTIITEVLGELQFNFYHRWFDVIFLVSALSSILFLYLAH
KQAPEKQMAP*

>ENSG00000078328|ENST00000553186
MNCEREQLRGNQEAAAAPDTMAQPYASAQFAPPQNGIPAEYTAPHPHPAPEYTGQTTVPE
HTLNLYPPAQTHSEQSPADTSAQTVSGTATQTDDAAPTDGQPQTQPSENTENKSQPKRLH
VSNIPFRFRDPDLRQMFGQFGKILDVEIIFNERGSKGFGFVTFENSADADRAREKLHGTV
VEGRKIEVNNATARVMTNKKTVNPYTNGWKLNPVVGAVYSPEFYAVPGFPYPAATAAAAY
RGAHLRGRGRTVYNTFRAAAPPPPIPAYGGVVYQDGFYGADIYGGYAAYRYAQPTPATAA
AYSDSYGRVYAADPYHHALAPAPTYGVGAMNAFAPLTDAKTRSHADDVGLVLSSLQASIY
RGGYNRFAPY*
>ENSG00000223465|ENST00000455493
MLSRNDEAASAQQ*
>ENSG00000188467|ENST00000482911
MQTKGGQTWARRALLLGILWATAHLPLSGTSLPQRLPRATGNSTQCVISPSSEFPEGFFT
RQERRDGGIIIYFLIIVYMFMAISIVCDEYFLPSLEIISECKWLESCPVTFWESVPHRYD
CL*
>ENSG00000257473|ENST00000443184
MILNKALLLGALALTAVMSPCGGEDIVADHVASYGVNFYQSHGPSGQYTHEFDGDEEFYV
DLETKETVWQLPMFSKFISFDPQSALRNMAVGKHTLEFMMRQSNSTAATNEVPEVTVFSK
FPVTLGQPNTLICLVDNIFPPVVNITWLSNGHSVTEGVSETSFLSKSDHSFFKISYLTFL
PSADEIYDCKVEHWGLDEPLLKHWEPEIPAPMSELTETLVCALGLSVGLMGIVVGTVFII
QGLRSVGASRHQGLL*
>ENSG00000157916|ENST00000488353
MSEGDSVGESVHGKPSVVYRFFTRLGQIYQSWLDKSTPYTAVRWVVTLGLSFVYMIRVYL
LQGWYIVTYALGIYHLNLFIAFLSPKVDPSLMEDSDDGPSLPTKQNEEFRPFIRRLPEFK
FWHAATKGILVAMVCTFFDAFNVPVFWPILVMYFIMLFCITMKRQIKHMIKYRYIPFTHG
KRRYRGKEDAGKAFAS*
>ENSG00000104490|ENST00000521599
MGKQNSKLRPEVMQDLLESTDFTEHEIQEWYKGFLRDCPSGHLSMEEFKKIYGNFFPYGD
ASKFAEHVFRTFDANGDGTIDFREFIIALSVTSRGKLEQKLKWAFSMYDLDGNGYISKAE
MLEIVQAIYKMVSSVMKMPEDESTPEKRTEKIFRQMDTNRDGKLSLEEFIRGAKSDPSIV
RLLQCDPSSAGQF*
>ENSG00000198794|ENST00000425597
MAEKVNNFPPLPKFIPLKPCFYQDFEADIPPQHVSMTKRLYYLWMLNSVTLAVNLVGCLA
WLIGGGGATNFGLAFLWLILFTPCSYVCWFRPIYKAFKTDSSFSFMAFFFTFMAQLVISI
IQAVGIPGWGVCGWIATISFFGTNIGSAVVMLIPTVMFTVMAVFSFIALSMVHKFYRGSG
GSFSKAQEEWTTGAWKNPHVQQAAQNAAMGAAQGAMNQPQTQYSATPNYTYSNEM*
>ENSG00000167110|ENST00000421699
MWPQPRLPPRPAMSEETRQSKLAAAKKKLREYQQRNSPGVPTGAKKKKKIKNGSNPETTT
SGGCHSPEDTPKDNAATLQPSDDTVLPGGVPSPGASLTSMAASQNHDADNVPNLMDETKT
FSSTESLRQLSQQLNGLVCESATCVNGEGPASSANLKDLESRYQQLAVALDSSYVTNKQL
NITIEKLKQQNQEITDQLEEEKKECHQKQGALREQLQVHIQTIGILVSEKAELQTALAHT
QHAARQKEGESEDLASRLQYSRRRVGELERALSAVSTQQKKADRYNKELTKERDALRLEL
YKNTQSNEDLKQEKSELEEKLRVLVTEKAGMQLNLEELQKKLEMTELLLQQFSSRCEAPD
ANQQLQQAMEERAQLEAHLGQVMESVRQLQMERDKYAENLKGESAMWRQRMQQMSEQVHT
LREEKECSMSRVQELETSLAELRNQMAEPPPPEPPAGPSEVEQQLQAEAEHLRKELEGLA

GQLQAQVQDNEGLSRLNREQEERLLELERAAELWGEQAEARRQILETMQNDRTTISRALS
QNRELKEQLAELQSGFVKLTNENMEITSALQSEQHVKRELGKKLGELQEKLSELKETVEL
KSQEAQSLQQQRDQYLGHLQQYVAAYQQLTSEKEVLHNQLLLQTQLVDQLQQQEAQGKAV
AEMARQELQETQERLEAATQQNQQLRAQLSLMAHPGEGDGLDREEEEDEEEEEEEAVAVP
QPMPSIPEDLESREAMVAFFNSAVASAEEEQARLRGQLKEQRVRCRRLAHLLASAQKEPE
AAAPAPGTGGDSVCGETHRALQGAMEKLQSRFMELMQEKADLKERVEELEHRCIQLSGET
DTIGEYIALYQSQRAVLKERHREKEEYISRLAQDKEEMKVKLLELQELVLRLVGDRNEWH
GRFLAAAQNPADEPTSGAPAPQELGAANQQGDLCEVSLAGSVEPAQGEAREGSPRDNPTA
QQIMQLLREMQNPRERPGLGSNPCIPFFYRADENDEVKITVI*
>ENSG00000153130|ENST00000506597
MRRRVFSSQDWRASGWDGMGFFSRRTFCGRSGRSCRGQLVQVSRPEVSAGSLLLPAPQAE
DHSSRILYPRPKSLLPKMMNADMDDLSARVDAVKEENLKLKSENQVLGQYIENLMSASSV
FQTTDTKSKRK*
>ENSG00000126091|ENST00000531816
MGLLVFVRNLLLALCLFLVLGFLYYSAWKLHLLQWEEDSKYDRLGFLLNLDSKLPAELAT
KYANFSEGACKPGYASALMTAIFPRPGRTISSERKSFCGSW*
>ENSG00000183166|ENST00000431984
MPFHHVTAGLLYKGNYLNRSLSAGSDSEQLANISVEELDEIREAFRVLDRDGNGFISKQE
LGMAMRSLGYMPSEVELAIIMQRLDMDGDGQVDFDEFMTILGPKLVSSEGRDGFLGNTID
SIFWQFDMQRITLEELKHILYHAFRDHLTMKDIENIIINEEESLNETSGNCQTEFEGVHS
QKQNRQTCVRKSLICAFAMAFIISVMLIAANQILRSGME*
>ENSG00000134970|ENST00000456936
MPRPGSAQRWAAVAGRWGCRLLALLLLVPGPGGASEITFELPDNAKQCFYEDIAQGTKCT
LEFQVITGGHYDVDCRLEDPDGKVLYKEMKKQYDSFTFTASKNGTYKFCFSNEFSTFTHK
TVYFDFQVGEDPPLFPSENRVSALTQMESACVSIHEALKSVIDYQTHFRLREAQGRSRAE
DLNTRVAYWSVGEALILLVVSIGQVFLLKSFFSDKRTTTTRVGS*
>ENSG00000280674|ENST00000628351
MMLIPTHHFRNIERKPEYLQPEKCVPPPYPGPVGTMWFIRDGCGIACAIVTWFLVLYAEF
VVLFVMLIPSRDYVYSIINGIVFNLLAFLALASHCRAMLTDPGAVPKGNATKEFIESLQL
KPGQVVYKCPKCCSIKPDRAHHCSVCKRCIRKMDHHCPWVNNCVGENNQKYFVLFTMYIA
LISLHALIMVGFHFLHCFEEDWTKCSSFSPPTTVILLILLCFEGLLFLIFTSVMFGTQVH
SICTDETGIEQLKKEERRWAKKTKWMNMKAVFGHPFSLGWASPFATPDQGKADPYQYVV*
>ENSG00000272617|ENST00000562949
SFSRVGADFRGQLAPVFQRVAISTFQKAIQETVEKFQEEMNSYMLISAPAILGTSNMPAA
VPATQPGTLQPPMVLLDFPPLACFLNNILVAFNDLRLCCPVALAQDVTGALEDALAKGWT
PMENRWCGRRAGGQPASSSTRWTTCRAACLLTKWTAGRSQTSIG*
>ENSG00000230791|ENST00000455655
MLSRNDVKRLERSWAQGSSEQELHYASLQRLPVPSSEGPDLRGRDKRGTKEDPRADYACI
AENKPT*
>ENSG00000119396|ENST00000451303
MATAPYNYSYIFKYIIIGDMGVGKSCLLHQFTEKKFMADCPHTIGVEFGTRIIEVSGQKI
KLQIWDTAGQERFRAVTRSYYRGAAGALMVYDITRRSTYNHLSSWLTDARNLTNPNTVII
LIGNKADLEAQRDVTYEEAKQFAEENGLLFLEASAKTGENVEDAFLEAAKKIYQNIQDGS
L

>ENSG00000147654|ENST00000527709
MAITQFRLFKFCTCLATVFSFLKRLICRSGRGRKLSGDQITLPTTVDYSSVPKQTDVEEW
TSWDEDAPTSVKIEGGNGNVATQQNSLEQLEPDYFKDMTPTIRKTQKIVIKKREPLNFGI
PDGSTGFSSRLAATQDLPFIHQSTSHWVGATII*
>ENSG00000157593|ENST00000615337
MVPGYLLVQYFRRKNYLETGRGLCFPLVKACVFGNEPKASDEVPLAPRTEAAETTPMWQA
LKLLFCATGLQVSYLTWGVLQERVMTRSYGATATSPGERFTDSQFLVLMNRVLALIVAGL
SCVLCKQPRHGAPMYRYSFASLSNVLSSWCQYEALKFVSFPTQVLAKASKVIPVMLMGKL
VSRRSYEHWEYLTATLISIGVSMFLLSSGPEPRSSPATTLSGLILLAGYIAFDSFTSNWQ
DALFAYKMSSVQMMFGVNFFSCLFTVGSLLEQGALLEGTRFMGRHSEFAAHALLLSICSA
CGQLFIFYTIGQFGAAVFTIIMTLRQAFAILLSCLLYGHTVTVVGGLGVAVVFAALLLRV
YARGRLKQRGKKAVPVESPVQKV*
>ENSG00000156671|ENST00000542569
MAGPNQLCIRRWTTKHVAVWLKDEGFFEYVDILCNKHRLDGITLLTLTEYDLRSPPLEIK
VLGDIKRLMLSVRKLQKIHIDVLEEMGYNSDSPMGSMTPFISALQSTDWLCNGELSHDCD
GPITDLNSDQYQYMNGKNKHSVRRLDPEYWKTILSCIYVFIVFGFTSFIMVIVHERVPDM
QTYPPLPDIFLDSVPRIPWAFAMTEVCGMILCYIWLLVLLLHKHRSILLRRLCSLMGTVF
LLRCFTMFVTSLSVPGQHLQCTGKIYGSVWEKLHRAFAIWSGFGMTLTGVHTCGDYMFSG
HTVVLTMLNFFVTEYTPRSWNFLHTLSWVLNLFGIFFILAAHEHYSIDVFIAFYITTRLF
LYYHTLANTRAYQQSRRARIWFPMFSFFECNVNGTVPNEYCWPFSKPAIMKRLIG*
>ENSG00000131446|ENST00000446023
MLKKQSAGLVLWGAILFVAWNALLLLFFWTRPAPGRPPSVSALDGDPASLTREVIRLAQD
AEVELERQRGLLQQIGDALSSQRGRVPTAAPPAQPRVPVTPAPAVIPILVIACDRSTVRR
CLDKLLHYRPSAELFPIIVSQDCGHEETAQAIASYGSAVTHIRQPDLSSIAVPPDHRKFQ
GYYKIARHYRWALGQVFRQFRFPAAVVVEDDLEVAPDFFEYFRATYPLLKADPSLWCVSA
WNDNGKEQMVDASRPELLYRTDFFPGLGWLLLAELWAELEPKWPKAFWDDWMRRPEQRQG
RACIRPEISRTMTFGRKGVSHGQFFDQHLKFIKLNQQFVHFTQLDLSYLQREAYDRDFLA
RVYGAPQLQVEKVRTNDRKELGEVRVQYTGRDSFKAFAKALGVMDDLKSGVPRAGYRGIV
TFQFRGRRVHLAPPLTWEGYDPSWN*
>ENSG00000064687|ENST00000435683
MVCLGTGQSAGPLVSVQNHCPPCGLSPQESLGLALGQAQEPLHSLLEAAEDLAQELLALR
SLVELRALLQRPRGTSGPLELLSEALCSVRGPSSTVGPSLNWYEASDLMELVGQEPESAL
PDSSLSPACSELIGALDSHPLSRLLWRRLKPLILGKLLFAPDTPFTRKLMAQVNRTFEEL
TLLRDVREVWEMLGPRIFTFMNDSSNVAMLQRLLQMQDEGRRQPRPGGRDHMEALRSFLD
PGSGGYSWQDAHADVGHLVGTLGRVTECLSLDKLEAAPSEAALVSRALQLLAEHRFWAGV
VFLGPEDSSDPTEHPTPDLGPGHVRIKIRMDIDVVTRTNKIRDRFWDPGPAADPLTDLRY
VWGGFVYLQDLVERAAVRVLSGANPRAGLYLQQMPYPCYVDDVFLRVLSRSLPLFLTLAW
IYSVTLTVKAVVREKETRLRDTMRAMGLSRAVLWLGWFLSCLGPFLLSAALLVLVLKLGD
ILPYSHPGVVFLFLAAFAVATVTQSFLLSAFFSRANLAAACGGLAYFSLYLPYVLCVAWR
DRLPAGGRVAASLLSPVAFGFGCESLALLEEQGEGAQWHNVGTRPTADVFSLAQVSGLLL
LDAALYGLATWYLEAVCPGQYGIPEPWNFPFRRSYWCGPRPPKSPAPCPTPLDPKVLVEE
APPGLSPGVSVRSLEKRFPGSPQPALRGLSLDFYQGHITAFLGHNGAGKTTTLSILSGLF
PPSGGSAFILGHDVRSSMAAIRPHLGVCPQYNVLFDMLTVDEHVWFYGRLKGLSAAVVGP
EQDRLLQDVGLVSKQSVQTRHLSGGMQRKLSVAIAFVGGSQVVILDEPTAGVDPASRRGI

WELLLKYREGRTLILSTHHLDEAELLGDRVAVVAGGRLCCCGSPLFLRRHLGSGYYLTLV
KARLPLTTNEKADTDMEGSVDTRQEKKNGSQGSRVGTPQLLALVQHWVPGARLVEELPHE
LVLVLPYTGAHDGSFATLFRELDTRLAELRLTGYGISDTSLEEIFLKVVEECAADTDMED
GSCGQHLCTGIAGLDVTLRLKMPPQETALENGEPAGSAPETDQGSGPDAVGRVQGWALTR
QQLQALLLKRFLLARRSRRGLFAQIVLPALFVGLALVFSLIVPPFGHYPALRLSPTMYGA
QVSFFSEDAPGDPGRARLLEALLQEAGLEEPPVQHSSHRFSAPEVPAEVAKVLASGNWTP
ESPSPACQCSRPGARRLLPDCPAAAGGPPPPQAVTGSGEVVQNLTGRNLSDFLVKTYPRL
VRQGLKTKKWVNEVRYGGFSLGGRDPGLPSGQELGRSVEELWALLSPLPGGALDRVLKNL
TAWAHSLDAQDSLKIWFNNKGWHSMVAFVNRASNAILRAHLPPGPARHAHSITTLNHPLN
LTKEQLSEGALMASSVDVLVSICVVFAMSFVPASFTLVLIEERVTRAKHLQLMGGLSPTL
YWLGNFLWDMCNYLVPACIVVLIFLAFQQRAYVAPANLPALLLLLLLYGWSITPLMYPAS
FFFSVPSTAYVVLTCINLFIGINGSMATFVLELFSDQKLQEVSRILKQVFLIFPHFCLGR
GLIDMVRNQAMADAFERLGDRQFQSPLRWEVVGKNLLAMVIQGPLFLLFTLLLQHRSQLL
PQPRVRSLPLLGEEDEDVARERERVVQGATQGDVLVLRNLTKVYRGQRMPAVDRLCLGIP
PGECFGLLGVNGAGKTSTFRMVTGDTLASRGEAVLAGHSVAREPSAAHLSMGYCPQSDAI
FELLTGREHLELLARLRGVPEAQVAQTAGSGLARLGLSWYADRPAGTYSGGNKRKLATAL
ALVGDPAVVFLDEPTTGMDPSARRFLWNSLLAVVREGRSVMLTSHSMEECEALCSRLAIM
VNGRFRCLGSPQHLKGRFAAGHTLTLRVPAARSQPAAAFVAAEFPGAELREAHGGRLRFQ
LPPGGRCALARVFGELAVHGAEHGVEDFSVSQTMLEEVFLYFSKDQGKDEDTEEQKEAGV
GVDPAPGLQHPKRVSQFLDDPSTAETVL*
>ENSG00000109083|ENST00000585089
MTHLLLTATVTPSEQNSSREPGWETAMAKDILGEAGLHFDELNKLRVLDPEVTQQTIELK
EECKDFVDKIGQFQKIVGGLIELVDQLAKEAENEKMKAIGARNLLKSIAKQREAQQQQLQ
ALIAEKKMQLERYRVEYEALCKVEAEQNEFIDQFIFQK*
>ENSG00000225824|ENST00000419914
MSWKKALRIPGGLRAATVTLMLSMLSTPVAEGRDSPEDFVYQFKGMCYFTNGTERVRLVS
RSIYNREEIVRFDSDVGEFRAVTLLGLPAAEYWNSQKDILERKRAAVDRVCRHNYQLELR
TTLQRRVEPTVTISPSRTEALNHHNLLVCSVTDFYPAQIKVRWFRNGQEETAGVVSTPLI
RNGDWTFQILVMLEMTPQRGDVYTCHVEHPSLQSPITVEWRAQSESAQSKMLSGIGGFVL
GLIFLGLGLIIHHRSQKGLLH*
>ENSG00000079332|ENST00000431664
MSFIFEWIYNGFSSVLQFLGLYKKSGKLVFLGLDNAGKTTLLHMLKDDRLGQHVPTLHPT
SEELTIAGMTFTTFDLGGHEQARRVWKNYLPAINGIVFLVDCADHSRLVESKVELNALMT
DETISNVPILILGNKIDRTDAISEEKLREIFGLYGQTTGKGNVTLKELNARPMEVFMCSV
LKRQGYGEGFRWLSQYID*
>ENSG00000108669|ENST00000591455
MEEDDSYVPSDLTAEERQELENIRRRKQELLADIQRLKDEIAEVANEIENLGSTEERKNM
QRNKQVAMGRKKFNMDPKKGIQFLIENDLLKNTCEDIAQFLYKGEGLNKTAIGDYLGERD
EFNIQVLHAFVELHEFTDLNLVQALRQFLWSFRLPGEAQKIDRMMEAFAQRYCQCNNGVF
QSTDTCYVLSFAIIMLNTSLHNPNVKDKPTVERFIAMNRGINDGGDLPEELLRNLYESIK
NEPFKIPEDDGNDLTHTFFNPDREGWLLKLGGRVKTWKRRWFILTDNCLYYFEYTTDKEP
RGIIPLENLSIREVEDSKKPNCFELYIPDNKDQVIKACKTEADGRVVEGNHTVYRISAPT
PEEKEEWIKCIKAAISRDPFYEMLAARKKKVSSTKRH*
>ENSG00000167461|ENST00000586682

MAKTYDYLFKLLLIGDSGVGKTCVLFRFSEDAFNSTFISTIGIDFKIRTIELDGKRIKLQ
IWDTAGQERFRTITTAYYRGAMGIMLVYDITNEKSFDNIRNWIRNIEEHASADVEKMILG
NKCDVNDKRQVSKERGEKLALDYGIKFMETSAKANINVENRYQSKNGQKIGRQQPPGEQP
GSQNHTGPAEEEQLFPMCSSVRNTALL*
>ENSG00000198324|ENST00000547838
MKLNERSLAFYATCDAPVDNAGFLYKKGGRHAAYHRRWFVLRGNMLFYFEDAASREPVGV
IILEGCTVELVEAAEEFAFAVRFAGTRARTYVLAAESQDAMEGWVKALSRASFDYLRLVV
RELEQQLAAVRGGGGMALPQPQPQSLPLPPSLPSALAPVPSLPSAPAPVPALPLPRRPSA
LPPKENGCAVWSTEATFRPGPEPPPPPPRRRASAPHGPLDMAPFARLHECYGQEIRALRG
QWLSSRVQP*
>ENSG00000176597|ENST00000460419
MRMLVSGRRVKKWQLIIQLFATCFLASLMFFWEPIDNHIVSHMKSYSYRYLINSYDFVND
TLSLKHTSAGPRYQYLINHKEKCQAQDVLLLLFVKTAPENYDRRSGIRRTWGNENYVRSQ
LNANIKTLFALGTPNPLEGEELQRKLAWEDQRYNDIIQQDFVDSFYNLTLKLLMQFSWAN
TYCPHAKFLMTADDDIFIHMPNLIEYLQSLEQIGVQDFWIGRVHRGAPPIRDKSSKYYVS
YEMYQWPAYPDYTAGAAYVISGDVAAKVYEASQTLNSSLYIDDVFMGLCANKIGIVPQDH
VFFSGEGKTPYHPCIYEKMMTSHGHLEDLQDLWKNATDPKVKTISKGFFGQIYCRLMKII
LLCKISYVDTYPCRAAFI*
>ENSG00000136297|ENST00000612910
MFAPRLLDFQKTKYARFMNHRVPAHKRYQPTEYEHAANCATHAFWIIPSILGSSNLYFLS
DDDWETISAWIYGLGLCGLFVVSTVFHTISWKKSHLRMVEHCLHMFDRMVIYFFIAASYA
PWLNLRELGPWASHMRWLVWIMASVGTIYVFFFHERYKLVELLCYVVMGFFPALVILSMA
GKLRPGMTCLKRQ*
>ENSG00000235155|ENST00000419471
MQLRLFRRLLLAALLLVIVWTLFGPSGLGEELLSLSLASLLPAPASPGPPLALPRLLIPN
QEACSGPGAPPFLLILVCTAPENLNQRNAIRASWGGLREARGLRVQTLFLLGEPNAQHPV
WGSQGSDLASESAAQGDILQAAFQDSYRNLTLKTLSGLNWAEKHCPMARYVLKTDDDVYV
NVPELVSELVLRGGRWGQWERSTEPQREAEQEGGQVLHSEEVPLLYLGRVHWRVNPSRTP
GGRHRVSEEQWPHTWGPFPPYASGTGYVLSASAVQLILKVASRAPLLPLEDVFVGVSARR
GGLAPTQCVKLAGATHYPLDRCCYGKFLLTSHRLDPWKMQEAWKLVGGSDGERTAPFCSW
FQGVLGILRCRAIAWLQS*
>ENSG00000235844|ENST00000443117
MRPEDRMFHIRAVILRALSLAFLLSLRGAGAIKADHVSTYAAFVQTHRPTGEFMFEFDED
EMFYVDLDKKETVWHLEEFGQAFSFEAQGGLANIAILNNNLNTLIQRSNHTQATNDPPEV
TVFPKEPVELGQPNTLICHIDKFFPPVLNVTWLCNGELVTEGVAESLFLPRTDYSFHKFH
YLTFVPSAEDFYDCRVEHWGLDQPLLKHWEAQEPIQMPETTETVLCALGLVLGLVGIIVG
TVLIIKSLRSGHDPRAQGTL*
>ENSG00000137497|ENST00000537217
MTLHATRGAALLSWVNSLHVADPVEAVLQLQDCSIFIKIIDRIHGTEEGQQILKQPVSER
LDFVCSFLQKNRKHPSSPECLVSAQKVLEGSELELAKMTMLLLYHSTMSSKSPRDWEQFE
YKIQAELAVILKFVLDHEDGLNLNEDLENFLQKAPVPSTCSSTFPEELSPPSHQAKREIR
FLELQKVASSSSGNNFLSGSPASPMGDILQTPQFQMRRLKKQLADERSNRDELELELAEN
RKLLTEKDAQIAMMQQRIDRLALLNEKQAASPLEPKELEELRDKNESLTMRLHETLKQCQ
DLKTEKSQMDRKINQLSEENGDLSFKLREFASHLQQLQDALNELTEEHSKATQEWLEKQA

QLEKELSAALQDKKCLEEKNEILQGKLSQLEEHLSQLQDNPPQEKGEVLGDVLQLETLKQ
EAATLAANNTQLQARVEMLETERGQQEAKLLAERGHFEEEKQQLSSLITDLQSSISNLSQ
AKEELEQASQAHGARLTAQVASLTSELTTLNATIQQQDQELAGLKQQAKEKQAQLAQTLQ
QQEQASQGLRHQVEQLSSSLKQKEQQLKEVAEKQEATRQDHAQQLATAAEEREASLRERD
AALKQLEALEKEKAAKLEILQQQLQVANEARDSAQTSVTQAQREKAELSRKVEELQACVE
TARQEQHEAQAQVAELELQLRSEQQKATEKE
>ENSG00000226182|ENST00000440328
MLSRNDAPSVLVPGPGLLRAGTPLCISAEAASAQQ*
>ENSG00000133103|ENST00000455146
MAEGSGEVVAVSATGAANGLNNGAGGTSATTCNPLSRKLHKILETRLDNDKEMLEALKAL
STFFVENSLRTRRNLRGDIERKSLAINEEFVSIFKEVKEELESISEDVQAMSNCCQDMTS
RLQAAKEQTQDLIVKTTKLQSESQKLEIRAQVADAFLSKFQLTSDEMSLLRGTREGPITE
DFFKALGRVKQIHNDVKVLLRTNQQTAGLEIMEQMALLQETAYERLYRWAQSECRTLTQE
SCDVSPVLTQAMEALQDRPVLYKYTLDEFGTARRSTVVRGFIDALTRGGPGGTPRPIEMH
SHDPLRYVGDMLAWLHQATASEKEHLEALLKHVTTQGVEENIQEVVGHITEGVCRPLKVR
IEQVIVAEPGAVLLYKISNLLKFYHHTISGIVGNSATALLTTIEEMHLLSKKIFFNSLSL
HASKLMDKVELPPPDLGPSSALNQTLMLLREVLASHDSSVVPLDARQADFVQVLSCVLDP
LLQMCTVSASNLGTADMATFMVNSLYMMKTTLALFEFTDRRLEMLQFQIEAHLDTLINEQ
ASYVLTRVGLSYIYNTVQQHKPEQGSLANMPNLDSVTLKAAMVQFDRYLSAPDNLLIPQL
NFLLSATVKEQIVKQSTELVCRAYGEVYAAVMNPINEYKDPENILHRSPQQVQTLLS*
>ENSG00000230791|ENST00000438381
MLSRNDDICIYGGLGLGGLLLLAVVLLSACLCWLHRRAPSVLVPGPGLLRAGTPLCISAE
AASAQQ*
>ENSG00000123989|ENST00000535926
MAVVTLGEERPIGHLHLALRHLLEQHGDDFDWFFLVPDTTYTEAHGLARLTGHLSLASAA
HLYLGRPQDFIGGEPTPGRYCHGGFGVLLSRMLLQQLRPHLEGCRNDIVSARPDEWLGRC
ILDATGVGCTGDHEGVHYSHLELSPGEPVQEGDPHFRSALTAHPVRDPVHMYQLHKAFAR
AELERTYQEIQELQWEIQNTSHLAVDGDQAAAWPVGIPAPSRPASRFEVLRWDYFTEQHA
FSCADGSPRCPLRGADRADVADVLGTALEELNRRYHPALRLQKQQLVNGYRRFDPARGME
YTLDLQLEALTPQGGRRPLTRRVQLLRPLSRVEILPVPYVTEASRLTVLLPLAAAERDLA
PGFLEAFATAALEPGDAAAALTLLLLYEPRQAQRVAHADVFAPVKAHVAELERRFPGARV
PWLSVQTAAPSPLRLMDLLSKKHPLDTLFLLAGPDTVLTPDFLNRCRMHAISGWQAFFPM
HFQAFHPAVAPPQGPGPPELGRDTGRFDRQAASEACFYNSDYVAARGRLAAASEQEEELL
ESLDVYELFLHFSSLHVLRAVEPALLQRYRAQTCSARLSEDLYHRCLQSVLEGLGSRTQL
AMLLFEQEQGNST*
>ENSG00000114354|ENST00000620299
MNGQLDLSGKLIIKAQLGEDIRRIPIHNEDITYDELVLMMQRVFRGKLLSNDEVTIKYKD
EDGDLITIFDSSDLSFAIQCSRILKLTLFVNGQPRPLESSQVKYLRRELIELRNKVNRLL
DSLEPPGEPGPSTNIPENDTVDGREEKSASDSSGKQSTQVMAASMSAFDPLKNQDEINKN
VMSAFGLTDDQVSGPPSAPAEDRSGTPDSIASSSSAAHPPGVQPQQPPYTGAQTQAGQIE
GQMYQQYQQQAGYGAQQPQAPPQQPQQYGIQYSGFQSMERFHCK*
>ENSG00000168824|ENST00000513829
MVKLGNNFAEKGTKQPLLEDGFDTIPLMTPLDVNQLQFPPPDKVVVKTKTEYEPDRKKGK
ARPPQIAEFTVSITEGVTERFKVSVLVLFALAFLTCVVFLVVYKVYKYDRACPDGFVLKN

TQCIPEGLESYYAEQDSSAREKFYTVINHYNLAKQSITRSVSPWMSVLSEEKLSEQETEA
AEKSA*
>ENSG00000103043|ENST00000536184
MADILLREEDLKFASTMVHALNTILLTSTELFQLRNQLKDLKTLESQNLFCCLYRSWCHN
PVTTVSLCFLTQNYRHAYDLIQKFGDLEVTVDFLAEVDKLVQLIECPIFTYLRLQLLDVK
NNPYLIKALYGLLMLLPQSSAFQLLSHRLQCVPNPELLQTEDSLKAAPKSQKADSPSIDY
AELLQHFEKVQNKHLEVRHQRSGRGDHLDRRVVL*
>ENSG00000234514|ENST00000454168
MLSRNDVKRLERSWHLLSWSQAQGSSEQELHYASLQRLPVPSSEGPDLRGRDKRGTKEDP
RADYACIAENKPT*
>ENSG00000176945|ENST00000447234
MGCLWGLALPLFFFCWEVGVSGSSAGPSTRRADTAMTTDDTEVPAMTLAPGHAALETQTL
SAETSSRASTPAGPIPEAETRGAKRISPARETRSFTKTSPNFMVLIATSVETSAASGSPE
GAGMTTVQTITGSDPREAIFDTLCTDDSSEEAKTLTMDILTLAHTSTEAKGLSSESSASS
DSPHPVITPSRASESSASSDGPHPVITPSRASESSASSDGPHPVITPSRASESSASSDGP
HPVITPSRASESSASSDGPHPVITPSRASESSASSDGPHPVITPSRASESSASSDGPHPV
ITPSRASESSASSDGPHPVITPSRASESSASSDGPHPVITPSRASESSASSDGLHPVITP
SRASESSASSDGPHPVITPSRASESSASSDGPHPVITPSWSPGSDVTLLAEALVTVTNIE
VINCSITEIETTTSSIPGASDTDLIPTEGVKASSTSDPPALPDSTEAKPHITEVTASAET
LSTAGTTESAAPDATVGTPLPTNSATEREVTAPGATTLSGALVTVSRNPLEETSALSVET
PSYVKVSGAAPVSIEAGSAVGKTTSFAGSSASSYSPSEAALKNFTPSETPTMDIATKGPF
PTSRDPLPSVPPTTTNSSRGTNSTLAKITTSAKTTMKPPTATPTTARTRPTTDVSAGENG
GFLLLRLSVASPEDLTDPRVAERLMQQLHRELHAHAPHFQVSLLRVRRG*
>ENSG00000275066|ENST00000619541
MALRPGAGSGGGGAAGAGAGSAGGGGFMFPVAGGIRPPQGLMPMQQQGFPMVSVMQPNMQ
GIMGMNYSSQMSQGPIAMQAGIPMGPMPAAGMPYLGQAPFLGMRPPGPQYTPDMQKQFAE
EQQKRFEQQQKLLEEERKRRQFEEQKQKLRLLSSVKPKTGEKSRDDALEAIKGNLDGFSR
DAKMHPTPASHPKKPGVGVFPSQDPAQPRMPPWIYNESLVPDAYKKILETTMTPTGIDTA
KLYPILMSSGLPRETLGQIWALANRTTPGKLTKEELYTVLAMIAVTQRGVPAMSPDALNQ
FPAAPIPTLSGFSMTLPTPVSQPTVIPSGPAGSMPLSLGQPVMGINLVGPVGGAAAQASS
GFIPTYPANQVVKPEEDDFQDFQDASKSGSLDDSFSDFQELPASSKTSNSQHGNSAPSLL
MPLPGTKALPSMDKYAVFKGIAADKSSENTVPPGDPGDKYSAFRELEQTAENKPLGESFA
EFRSAGTDDGFTDFKTADSVSPLEPPTKDKTFPPSFPSGTIQQKQQTQVKNPLNLADLDM
FSSVNCSSEKPLSFSAVFSTSKSVSTPQSTGSAATMTALAATKTSSLADDFGEFSLFGEY
SGLAPVGEQDDFADFMAFSNSSISSEQKPDDKYDALKEEASPVPLTSNVGSTVKGGQNST
AASTKYDVFRQLSLEGSGLGVEDLKDNTPSGKSDDDFADFHSSKFSSINSDKSLGEKAVA
FRHTKEDSASVKSLDLPSIGGSSVGKEDSEDALSVQFDMKLADVGGDLKHVMSDSSLDLP
TVSGQHPPAADIEDLKYAAFGSYSSNFAVSTLTSYDWSDRDDATQGRKLSPFVLSAGSGS
PSATSILQKKETSFGSSENITMTSLSKVTTFVSEDALPETTFPALASFKDTIPQTSEQKE
YENRDYKDFTKQDLPTAERSQEATCPSPASSGASQETPNECSDDFGEFQSEKPKISKFDF
LVATSQSKMKSSEEMIKSELATFDLSVQGSHKRSLSLGDKEISRSSPSPALEQPFRDRSN
TLNEKPALPVIRDKYKDLTGEVEENERYAYEWQRCLGSALNVIKKANDTLNGISSSSVCT
EVIQSAQGMEYLLGVVEVYRVTKRVELGIKATAVCSEKLQQLLKDIDKVWNNLIGFMSLA
TLTPDENSLDFSSCMLRPGIKNAQELACGVCLLNVDSRSRKEEKPAEEHPKKAFNSETDS

FKLAYGGHQYHASCANFWINCVEPKPPGLVLPDLL*
>ENSG00000177182|ENST00000520712
MGTWARTLLGPDYSDENDYTHTSYNAMHVKHTSSNLERECSPKLMKRSQSVVEAGTLKH
>ENSG00000142186|ENST00000533862
MWFFARDPVRDFPFELIPEPPEGGLPGPWALHRGRKKATGSPVSIFVYDVKPGAEEQTQV
AKAAFKRFKTLRHPNILAYIDGLETEKCLHVVTEAVTPLGIYLKARVEAGGLKELEISWG
LHQIVKALSFLVNDCSLIHNNVCMAAVFVDRAGEWKLGGLDYMYSAQGNGGGPPRKGIPE
LEQYDPPELADSSGRVVREKWSADMWRLGCLIWEVFNGPLPRAAALRNPGKIPKTLVPHY
CELVGANPKVRPNPARFLQNCRAPGGFMSNRFVETNLFLEEIQIKEPAEKQKFFQELSKS
LDAFPEDFCRHKVLPQLLTAFEFGNAGAVVLTPLFKVGKFLSAEEYQQKIIPVVVKMFSS
TDRAMRIRLLQQMEQFIQYLDEPTVNTQIFPHVVHGFLDTNPAIREQTVKSMLLLAPKLN
EANLNVELMKHFARLQAKDEQGPIRCNTTVCLGKIGSYLSASTRHRVLTSAFSRATRDPF
APSRVAGVLGFAATHNLYSMNDCAQKILPVLCGLTVDPEKSVRDQAFKAIRSFLSKLESV
SEDPTQLEEVEKDVHAASSPGMGGAAASWAGWAVTGVSSLTSKLIRSHPTTAPTETNIPQ
RPTPEGVPAPAPTPVPATPTTSGHWETQEEDKDTAEDSSTADRWDDEDWGSLEQEAESVL
AQQDDWSTGGQVSRASQSPTGAAGKLRAPGNRAGRSQAPRSHLLTVHGWPASITGVAQSP
ATRATPSLPCLHVPAPSRGQTLGVRTTGRASRLTVDRSRLSWPGRSARSGGGRWRPNAPR
GRWPRAP*
>ENSG00000085063|ENST00000527577
MGIQGGSVLFGLLLVLAVFCHSGHSLQCYNCPNPTADCKTAVNCSSDFDACLITKAGLQV
YNKCWKFEHCNFNDVTTRLRENELTYYCCKKDLCNFNEQLENGGTSLSEKTVLLLVTPFL
AAAWSLHP*
>ENSG00000060566|ENST00000595923
MNTDLAAGKMASAACSMDPIDSFELLDLLFDRQDGILRHVELGEGWGHVKDQVLPNPDSD
DFLSSILGSGDSLPSSPLWSPEGSDSGISEDLPSDPQDTPPRSGPATSPAGCHPAQPGKG
PCLSYHPGNSCSTTTPGPVIQVPEASVTIDLEMWSPGGRICAEKPADPVDLSPRCNLTVK
DLLLSGSSGDLQQHHLGASYLLRPGAGHCQELVLTEDEKKLLAKEGITLPTQLPLTKYEE
RVLKKIRRKIRNKQSAQESRKKKKEYIDGLETRMSACTAQNQELQRKVLHLEKQNLSLLE
QLKKLQAIVVQSTSKSAQTGTCVAVLLLSFALIILPSISPFGPNKTESPGDFAPVRVFSR
TLHNDAASRVAADAVPGSEAPGPRPEADTTREESPGSPGADWGFQDTANLTNSTEELDNA
TLVLRNATEGLGQVALLDWVAPGPSTGSGRAGLEAAGDEL*
>ENSG00000233904|ENST00000415649
MVDGTLLLLLSEALALTQTWAGSHSLKYFHTSVSRPGRGEPRFISVGYVDDTQFVRFDND
AASPRMVPRAPWMEQEGSEYWDRETRSARDTAQIFRVNLRTLRGYYNQSEAGSHTLQWMH
GCELGPDRRFLRGYEQFAYDGKDYLTLNEDLRSWTAVDTAAQISEQKSNDASEAEHQRAY
LEDTCVEWLHKYLEKGKETLLHLEPPKTHVTHHPISDHEATLRCWALGFYPAEITLTWQQ
DGEGHTQDTELVETRPAGDGTFQKWAAVVVPSGEEQRYTCHVQHEGLPEPVTLRWKPASQ
PTIPIVGIIAGLVLLGSVVSGAVVAAVIWRKKSSGGKGGSYSKAEWSDSAQGSESHSL*
>ENSG00000143761|ENST00000540651
MGNIFANLFKGLFGKKEMRILMVGLDAAGKTTILYKLKLGEIVTTIPTIGFNVETVEYKN
ISFTVWDVGGQDKIRPLWRHYFQNTQGLIFVVDSNDRERVNEAREELMRMLAEDELRDAV
LLVFANKQDLPNAMNAAEITDKLGLHSLRHRNWYIQATCATSGDGLYEGLDWLSNQLRNQ
K*
>ENSG00000235915|ENST00000438335

MLSRNDDICIYGGLGLGGLLLLAVVLLSACLCWLHRRVKRLERSWHLLSWSQAQGSSEQE
LHYASLQRLPVPSSEGPDLRGRDKRGTKEDPRADYACIAENKPT*
>ENSG00000189067|ENST00000570904
MSVPGPYQAATGPSSAPSAPPSYEETVAVNSYYPTPPAPMPGPTTGLVTGPDGKGMNPPS
YYTQPAPIPNNNPITVQTVYVQHPITFLDRPIQMCCPSCNKMIVSQLSYNAGALTWLSCG
SLCLLGCIAGCCFIPFCVDALQDVDHYCPNCRALLGTYKRL*
>ENSG00000278174|ENST00000611398
MAGRSMQAARCPTDELSLTNCAVVNEKDFQSGQHVIVRTSPNHRYTFTLKTHPSVVPGSI
AFSLPQRKWAGLSIGQEIEVSLYTFDKAKQCIGTMTIEIDFLQKKSIDSNPYDTDKMAAE
FIQQFNNQAFSVGQQLVFSFNEKLFGLLVKDIEAMDPSILKGEPATGKRQKIEVGLVVGN
SQVAFEKAENSSLNLIGKAKTKENRQSIINPDWNFEKMGIGGLDKEFSDIFRRAFASRVF
PPEIVEQMGCKHVKGILLYGPPGCGKTLLARQIGKMLNAREPKVVNGPEILNKYVGESEA
NIRKLFADAEEEQRRLGANSGLHIIIFDEIDAICKQRGSMAGSTGVHDTVVNQLLSKIDG
VEQLNNILVIGMTNRPDLIDEALLRPGRLEVKMEIGLPDEKGRLQILHIHTARMRGHQLL
SADVDIKELAVETKNFSGAELEGLVRAAQSTAMNRHIKASTKVEVDMEKAESLQVTRGDF
LASLENDIKPAFGTNQEDYASYIMNGIIKWGDPVTRVLDDGELLVQQTKNSDRTPLVSVL
LEGPPHSGKTALAAKIAEESNFPFIKICSPDKMIGFSETAKCQAMKKIFDDAYKSQLSCV
VVDDIERLLDYVPIGPRFSNLVLQALLVLLKKAPPQGRKLLIIGTTSRKDVLQEMEMLNA
FSTTIHVPNIATGEQLLEALELLGNFKDKERTTIAQQVKGKKVWIGIKKLLMLIEMSLQM
DPEYRVRKFLALLREEGASPLDFD*
>ENSG00000283017|ENST00000635644
MYGSARSVGKVEPSSQSPGRSPRLPRSPRLGHRRTNSTGGSSGSSVGGGSGKTLSMENIQ
SLNAAYATSGPMYLSDHENVGSETPKSTMTLGRSGGRLPYGVRMTAMGSSPNIASSGVAS
DTIAFGEHHLPPVSMASTVPHSLRQARDNTIMDLQTQLKEVLRENDLLRKDVEVKESKLS
SSMNSIKTFWSPELKKERALRKDEASKITIWKEQYRVVQEENQHMQMTIQALQDELRIQR
DLNQLFQQDSSSRTGEPCVAELTEENFQRLHAEHERQAKELFLLRKTLEEMELRIETQKQ
TLNARDESIKKLLEMLQSKGLSAKATEEDHERTRRLAEAEMHVHHLESLLEQKEKENSML
REEMHRRFENAPDSAKTKALQTVIEMKDSKISSMERGLRDLEEEIQMLKSNGALSTEERE
EEMKQMEVYRSHSKFMKNKVEQLKEELSSKEAQWEELKKKAAGLQAEIGQVKQELSRKDT
ELLALQTKLETLTNQFSDSKQHIEVLKESLTAKEQRAAILQTEVDALRLRLEEKETMLNK
KTKQIQDMAEEKGTQAGEIHDLKDMLDVKERKVNVLQKKIENLQEQLRDKEKQMSSLKER
VKSLQADTTNTDTALTTLEEALAEKERTIERLKEQRDRDEREKQEEIDNYKKDLKDLKEK
VSLLQGDLSEKEASLLDLKEHASSLASSGLKKDSRLKTLEIALEQKKEECLKMESQLKKA
HEAALEARASPEMSDRIQHLEREITRYKDESSKAQAEVDRLLEILKEVENEKNDKDKKIA
ELERQVKDQNKKVANLKHKEQVEKKKSAQMLEEARRREDNLNDSSQQLQDSLRKKDDRIE
ELEEALRESVQITAEREMVLAQEESARTNAEKQVEELLMAMEKVKQELESMKAKLSSTQQ
SLAEKETHLTNLRAERRKHLEEVLEMKQEALLAAISEKDANIALLELSSSKKKTQEEVAA
LKREKDRLVQQLKQQTQNRMKLMADNYEDDHFKSSHSNQTNHKPSPDQIIQPLLELDQNR
SKLKLYIGHLTTLCHDRDPLILRGLTPPASYNLDDDQAAWENELQKMTRGQLQDELEKGE
RDNAELQEFANAILQQIADHCPDILEQVVNALEESS*
>ENSG00000155511|ENST00000518142
MQHIFAFFCTGFLGAVVGANFPNNIQIGGLFPNQQSQEHAAFRFALSQLTEPPKLLPQID
IVNISDSFEMTYRCLSVLQKVLDTAAEKNWQVTAVNILTTTEEGYRMLFQDLEKKKERLV
VVDCESERLNAILGQIIKLEKNGIGYHYILANLGFMDIDLNKFKESGANVTGFQLVNYTD

TIPAKIMQQWKNSDARDHTRVDWKRPKYTSALTYDGVKVMAEAFQSLRRQRIDISRRGNA
GDCLANPAVPWGQGIDIQRALQQVRFEGLTGNVQFNEKGRRTNYTLHVIEMKHDGIRKIG
YWNEDDKFVPAATDAQAGGDNSSVQNRTYIVTTILEDPYVMLKKNANQFEGNDRYEGYCV
ELAAEIAKHVGYSYRLEIVSDGKYGARDPDTKAWNGMVGELVYGRADVAVAPLTITLVRE
EVIDFSKPFMSLGISIMIKKPQKSKPGVFSFLDPLAYEIWMCIVFAYIGVSVVLFLVSRF
SPYEWHSEEFEEGRDQTTSDQSNEFGIFNSLWFSLGAFMQQGCDISPRSLSGRIVGGVWW
FFTLIIISSYTANLAAFLTVERMVSPIESAEDLAKQTEIAYGTLEAGSTKEFFRRSKIAV
FEKMWTYMKSAEPSVFVRTTEEGMIRVRKSKGKYAYLLESTMNEYIEQRKPCDTMKVGGN
LDSKGYGIATPKGSALRNPVNLAVLKLNEQGLLDKLKNKWWYDKGECGSGGGDSKDKTSA
LSLSNVAGVFYILIGGLGLAMLVALIEFCYKSRSESKRMKGFCLIPQQSINEAIRTSTLP
RNSGAGASSGGSGENGRVVSHDFPKSMQSIPCMSHSSGMPLGATGL*
>ENSG00000101246|ENST00000622789
MYTLLSGLYKYMFQKDEYCILILGLDNAGKTTFLEQSKTRFNKNYKGMSLSKITTTVGLN
IGTVDVGKARLMFWDLGGQEELQSLWDKYYAECHGVIYVIDSTDEERLAESKQAFEKVVT
SEALCGVPVLVLANKQDVETCLSIPDIKTAFSDCTSKIGRRDCLTQACSALTGKGVREGI
EWMVKCVVRNVHRPPRQRDIT*
>ENSG00000114251|ENST00000497027
MAGSAMSSKFFLVALAIFFSFAQVVIEANSWWSLGMNNPVQMSEVYIIGAQPLCSQLAGL
SQGQKKLCHLYQDHMQYIGEGAKTGIKECQYQFRHRRWNCSTVDNTSVFGRVMQIGSRET
AFTYAVSAAGVVNAMSRACREGELSTCGCSRAARPKDLPRDWLWGGCGDNIDYGYRFAKE
FVDARERERIHAKGSYESARILMNLHNNEAGRRTVYNLADVACKCHGVSGSCSLKTCWLQ
LADFRKVGDALKEKYDSAAAMRLNSRGKLVQVNSRFNSPTTQDLVYIDPSPDYCVRNEST
GSLGTQGRLCNKTSEGMDGCELMCCGRGYDQFKTVQTERCHCKFHWCCYVKCKKCTEIVD
QFVCK*
>ENSG00000115526|ENST00000421474
MHHQWLLLAACFWVIFMFMVASKFITLTFKDPDVYSAKQEFLFLTTMPEVRKLPEEKHIP
EELKPTGKELPDSQLVQPLVYMERL
>ENSG00000058799|ENST00000464950
MAAVDDLQFEEFGNAATSLTANPDATTVNIEDPGETPKHQPGSPRGSGREEDDELLGNDD
SDKTELLAGQKKSSPFWTFEYYQTFFDVDTYQVFDRIKGSLLPIPGKNFVRLYIRSNPDL
YGPFWICATLVFAIAISGNLSNFLIHLGEKTYHYVPEFRKVSIAATIIYAYAWLVPLALW
GFLMWRNSKVMNIVSYSFLEIVCVYGYSLFIYIPTAILWIIPQKAVRWILVMIALGISGS
LLAMTFWPAVREDNRRVALATIVTIVLLHMLLSVGCLAYFFDAPEMDHLPTTTATPNQTV
AAAKSS*
>ENSG00000169894|ENST00000483366
MQLLGLLGLLWMLKASPWATGTLSTATSISQVPFPRAEAASAVLSNSPHSRDLAGWPLGV
PQLASPAPGHRENAPMTLTTSPHDTLISETLLNSPVSSNTSTTPTSKFAFKVETTPPTVL
VYSATTECVYPTSFIITISHPTSICVTTTQVAFTSSYTSTPVTQKPVTTVTSTYSMTTTE
KGTSAMTSSPSTTTARETPIVTVTPSSVSATDTTFHTTISSTTRTTERTPLPTGSIHTTT
SPTPVFTTLKTAVTSTSPITSSITSTNTVTSMTTTASQPTATNTLSSPTRTILSSTPVLS
TETITSGITNTTPLSTLVTTLPTTISRSTPTSETTYTTSPTSTVTDSTTKIAYSTSMTGT
LSTETSLPPTSSSLPTTETATTPMTNLVTTTTEISSHSTPSFSSSTIYSTVSTSTTAISS
LPPTSGTMVTSTTMTPSSLSTDIPFTTPTTITHHSVGSTGFLTTATDLTSTFTVSSSSAM
STSVIPSSPSIQNTETSSLVSMTSATTPNVRPTFVSTLSTPTSSLLTTFPATYSFSSSMS

ASSAGTTHTESISSPPASTSTLHTTAESTLAPTTTTSFTTSTTMEPPSTTAATTGTGQTT
FTSSTATFPETTTPTPTTDMSTESLTTAMTSPPITSSVTSTNTVTSMTTTTSPPTTTNSF
TSLTSMPLSSTPVPSTEVVTSGTINTIPPSILVTTLPTPNASSMTTSETTYPNSPTGPGT
NSTTEITYPTTMTETSSTATSLPPTSPLVSTAKTAKTPTTNLVTTTTKTTSHSTTSFTSS
TVYSTASTYTTAITSVPTTLGTMVTSTSMISSTVSTGIPTSQPTTITPSSVGISGSLPMM
TDLTSVYTVSNMSARPTTVIPSSPTVQNTEISISVSMTSATTPSGGPTFTSTENTPTRSL
LTSFPMTHSFSSSMSESSAGTTHTESISSPRGTTSTLHTTVESTPSPTTTTSFTTSTMME
PPSSTVSTTGRGQTTFPSSTATFPETTTLTPTTDISTVSLTTAMTSPPPVSSSITPTNTM
TSMRTTTYWPTATNTLSPLTSSILSSTPVPSTEMITSHTTNTTPLSTLVTTLLTTITRST
PTSETTYPTSPTSIVSDSTTEITYSTSITGTLSTATTLPPTSSSLPTTETATMTPTTTLI
TTTPNTTSLSTPSFTSSTIYSTVSTSTTAISSASPTSGTMVTSTTMTPSSLSTDTPSTTP
TTITYPSVGSTGFLTTATDLTSTFTVSSSSAMSTSVIPSSPSIQNTETSSLVSMTSATTP
SLRPTITSTDSTLTSSLLTTFPSTYSFSSSMSASSAGTTHTETISSLPASTNTIHTTAES
ALAPTTTTSFTTSPTMEPPSTTVATTGTGQTTFPSSTATFLETTTLTPTTDFSTESLTTA
MTSTPPITSSITPTDTMTSMRTTTSWPTATNTLSPLTSSILSSTPVPSTEVTTSHTTNTN
PVSTLVTTLPITITRSTLTSETAYPSSPTSTVTESTTEITYPTTMTETSSTATSLPPTSS
LVSTAETAKTPTTNLVTTTTKTTSHSTTSFTSSTIYSTASTPTTAITSVPTTLGTMVTST
SMIPSTVSTGIPTSQPTTITPSSVGISGSLPMMTDLTSVYTVSSMSARPTSVIPSSPTVQ
NTETSIFVSMMSATTPSGGPTFTSTENTPTRSLLTSFPVTHSFSSSMSASSVGTTHTQSI
SSPPAITSTLHTTAESTPSPTTTMSFTTFTKMETPSSTVATTGTGQTTFTSSTATSPKTT
TLTPTSDISTGSFKTAVSSTPPITSSITSTYTVTSMTTTTPLGPTATNTLPSFTSSVSSS
TPVPSTEAITSGTTNTTPLSTLVTTFSNSDTSSTPTSETTYPTSLTSALTDSTTRTTYST
NMTGTLSTVTSLRPTSSSLLTTVTATVPTTNLVTTTTKITSHSTPSFTSSIATTETPSHS
TPRFTSSITTTETPSHSTPRFTSSITNTKTTSHSSPSFTSSITTTETTSHNTPSLTSSIT
TTKTTSHSTPSYTSLITTTTTTSHSTPSFTSSITTTETTSHNTPSLTSSITTTETTSHST
PSFTSSITTETTSHSTPSFTSLITITEITSHSTLSYTTSITTTETPSHSTLSFTSSITTT
ETTSHSTPSFTSSITTSEMPSHSTPSFTSSITTTENATHSTPNFTSSITTTETTSHSTPS
FTSLITTTETTSHRWGTTETTSYSTPSFTSSNTITETTSHSTPSYITSITTTETPSSSTP
SFSSSITTTETTSHSTPGFTSSITTTETTSHSTPSFTSSITTTETTSHDTPSFTSSITTS
ETPSHSTPSSTSLITTTKTTSHSTPSFTSSITTTETTSHSAHSFTSSITTTETTSHNTRS
FTSSITTTETNSHSTTSFTSSITTTETTSHSTPSFSSSITTTETPLHSTPGLTSWVTTTK
TTSHITPGLTSSITTTETTSHSTPGFTSSITTTETTSESTPSLSSSTIYSTVSTSTTAIT
SHFTTSETAVTPTPVTPSSLSTDIPTTSLRTLTPSSVGTSTSLTTTTDFPSIPTDISTLP
TRTHIISSSPSIQSTETSSLVGTTSPTMSTVRMTLRITENTPISSFSTSIVVIPETPTQT
PPVLTSATGTQTSPAPTTVTFGSTDSSTSTLHTLTPSTALSTIVSTSQVPIPSTHSSTLQ
TTPSTPSLQTSLTSTSEFTTESFTRGSTSTNAILTSFSTIIWSSTPTIIMSSSPSSASIT
PVFSTTIHSVPSSPYIFSTENVGSASITGFPSLSSSATTSTSSTSSSLTTALTEITPFSY
ISLPSTTPCPGTITITIVPASPTDPCVEMDPSTEATSPPTTPLTVFPFTTEMVTCPTSIS
IQTTLTTYMDTSSMMPESESSISPNASSSTGTGTVPTNTVFTSTRLPTSETWLSNSSVIP
LPLPGVSTIPLTMKPSSSLPTILRTSSKSTHPSPPTTRTSETPVATTQTPTTLTSRRTTR
ITSQMTTQSTLTTTAGTCDNGGTWEQGQCACLPGFSGDRCQLQTRCQNGGQWDGLKCQCP
STFYGSSCEFAVEQVDLDVVETEVGMEVSVDQQFSPDLNDNTSQAYRDFNKTFWNQMQKI
FADMQGFTFKGVEILSLRNGSIVVDYLVLLEMPFSPQLESEYEQVKTTLKEGLQNASQDV
NSCQDSQTLCFKPDSIKVNNNSKTELTPAAICRRAAPTGYEEFYFPLVEATRLRCVTKCT

SGVDNAIDCHQGQCVLETSGPTCRSWDQDRKWFETWDEEVVGTFSNWGFEDDGTDKDTNF
YVALENVDTTMKVHIKRPEMTSSSV*
>ENSG00000188603|ENST00000636147
MGGCAGSRRRFSDSEGEETVPEPRLPLLDHQGAHWKNAVGFWLLGLCNNFSYVVMLSAAH
DILSHKRTSGNQSHVDPGPTPIPHNSSSRFDCNSVSTAAVLLADILPTLVIKLLAPLGLH
LLPYSPRVLVSGICAAGSFVLVAFSHSVGTSLCGVVFASISSGLGEVTFLSLTAFYPRAV
ISWWSSGTGGAGLLGALSYLGLTQAGLSPQQTLLSMLGIPALLLASYFLLLTSPEAQDPG
GEEEAESAARQPLIRTEAPESKPGSSSSLSLRERWTVFKGLLWYIVPLVVVYFAEYFINQ
GLFELLFFWNTSLSHAQQYRWYQMLYQAGVFASRSSLRCCRIRFTWALALLQCLNLVFLL
ADVWFGFLPSIYLVFLIILYEGLLGGAAYVNTFHNIALETSDEHREFAMAATCISDTLGI
SLSGLLALPLHDFLCQLS*
>ENSG00000283530|ENST00000636626
MFKFHQMKHIFEILDKMRCLRKRSTVSFLGVLVIFLLFMNLYIEDSYVLEGDKQLIRETS
THQLNSERYVHTFKDLSNFSGAINVTYRYLAATPLQRKRYLTIGLSSVKRKKGNYLLETI
KSIFEQSSYEELKEISVVVHLADFNSSWRDAMVQDITQKFAHHIIAGRLMVIHAPEEYYP
ILDGLKRNYNDPEDRVKFRSKQNVDYAFLLNFCANTSDYYVMLEDDVRCSKNFLTAIKKV
IASLEGTYWVTLEFSKLGYIGKLYHSHDLPRLAHFLLMFYQEMPCDWLLTHFRGLLAQKN
VIRFKPSLFQHMGYYSSYKGTENKLKDDDFEEESFDIPDNPPASLYTNMNVFENYEASKA
YSSVDEYFWGKPPSTGDVFVIVFENPIIIKKIKVNTGTEDRQNDILHHGALDVGENVMPS
KQRRQCSTYLRLGEFKNGNFEMSGVNQKIPFDIHCMRIYVTKTQKEWLIIRSISIWTS*
>ENSG00000137497|ENST00000542977
MTLHATRGAALLSWVNSLHVADPVEAVLQLQDCSIFIKIIDRIHGTEEGQQILKQPVSER
LDFVCSFLQKNRKHPSSPECLVSAQKVLEGSELELAKMTMLLLYHSTMSSKSPRDWEQFE
YKIQAELAVILKFVLDHEDGLNLNEDLENFLQKAPVPSTCSSTFPEELSPPSHQAKREIR
FLELQKVASSSSGNNFLSGSPASPMGDILQTPQFQMRRLKKQLADERSNRDELELELAEN
RKLLTEKDAQIAMMQQRIDRLALLNEKQAASPLEPKELEELRDKNESLTMRLHETLKQCQ
DLKTEKSQMDRKINQLSEENGDLSFKLREFASHLQQLQDALNELTEEHSKATQEWLEKQA
QLEKELSAALQDKKCLEEKNEILQGKLSQLEEHLSQLQDNPPQEKGEVLGDVLQLETLKQ
EAATLAANNTQLQARVEMLETERGQQEAKLLAERGHFEEEKQQLSSLITDLQSSISNLSQ
AKEELEQASQAHGARLTAQVASLTSELTTLNATIQQQDQELAGLKQQAKEKQAQLAQTLQ
QQEQASQGLRHQVEQLSSSLKQKEQQLKEVAEKQEATRQDHAQQLATAAEEREASLRERD
AALKQLEALEKEKAAKLEILQQQLQVANEARDSAQTSVTQAQREKAELSRKVEELQACVE
TARQEQHEAQAQVAELELQLRSEQQKATEKERVAQEKDQLQEQLQALKESLKVTKGSLEE
EKRRAADALEEQQRCISELKAETRSLVEQHKRERKELEEERAGRKGLEARLQQLGEAHQA
ETEVLRRELAEAMAAQHTAESECEQLVKEVAAWRERYEDSQQEEAQYGAMFQEQLMTLKE
ECEKARQELQEA
>ENSG00000008513|ENST00000521180
MVTLRKRTLKVLTFLVLFIFLTSFFLNYSHTMVATTWFPKQMVLELSENLKRLIKHRPCT
CTHCIGQRKLSAWFDERFNQTMQPLLTAQNALLEDDTYRWWLRLQREKKPNNLNDTIKEL
FRVVPGNVDPMLEKRSVGCRRCAVVGNSGNLRESSYGPEIDSHDFVLRMNKAPTAGFEAD
VGTKTTHHLVYPESFRELGDNVSMILVPFKTIDLEWVVSAITTGTISHTYIPVPAKIRVK
QDKILIYHPAFIKYVFDNWLQGHGRYPSTGILSVIFSMHVCDEVDLYGFGADSKGNWHHY
WENNPSAGAFRKTGVHDADFESNVTATLASINKIRIFKGR*
>ENSG00000179913|ENST00000600777

MKYLRHRRPNATLILAIGAFTLLLFSLLVSPPTCKVQEQPPAIPEALAWPTPPTRPAPAP
CHANTSMVTHPDFATQPQHVQNFLLYRHCRHFPLLQDVPPSKCAQPVFLLLVIKSSPSNY
VRRELLR
>ENSG00000017260|ENST00000507488
MDSLLPPSRFSYFKKYPLHAIRRYLSTLRNQRAEEQVARFQKIPNGENETMIPVLTSKKA
SELPVSEVASILQADLQNGLNKCEVSHRRAFHGWNEFDISEDEPLWKKYISQFKNPLIML
LLASAVISVLMHQFDDAVSITVAILIVVTVAFVQEYRSEKSLEELSKLVPPECHCVREGK
LEHTLARDLVPGDTVCLSVGDRVPADLRLFEAVDLSIDESSLTGETTPCSKVTAPQPAAT
NGDLASRSNIAFMGTLVRCGKAKGVVIGTGENSEFGEVFKMMQAEEAPKTPLQKSMDLLG
KQLSFYSFGIIGIIMLVGWLLGKDILEMFTISVSLAVAAIPEGLPIVVTVTLALGVMRMV
KKRAIVKKLPIVETLGCCNVICSDKTGTLTKNEMTVTHIFTSDGLHAEVTGVGYNQFGEV
IVDGDVVHGFYNPAVSRIVEAGCVCNDAVIRNNTLMGKPTEGALIALAMKMGLDGLQQDY
IRKAEYPFSSEQKWMAVKCVHRTQQDRPEICFMKGAYEQVIKYCTTYQSKGQTLTLTQQQ
RDVYQQEKARMGSAGLRVLALASGPELGQLTFLGLVGIIDPPRTGVKEAVTTLIASGVSI
KMITGDSQETAVAIASRLGLYSKTSQSVSGEEIDAMDVQQLSQIVPKVAVFYRASPRHKM
KIIKSLQKNGSVVAMTGDGVNDAVALKAADIGVAMGQTGTDVCKEAADMILVDDDFQTIM
SAIEEGKGIYNNIKNFVRFQLSTSIAALTLISLATLMNFPNPLNAMQILWINIIMDGPPA
QSLGVEPVDKDVIRKPPRNWKDSILTKNLILKILVSSIIIVCGTLFVFWRELRDNVITPR
DTTMTFTCFVFFDMFNALSSRSQTKSVFEIGLCSNRMFCYAVLGSIMGQLLVIYFPPLQK
VFQTESLSILDLLFLLGLTSSVCIVAEIIKKVERSREKIQKHVWLWERSGQQLVEIHPHL
ETGLPLTEDVSCV*
>ENSG00000117620|ENST00000640715
MFANLKYVSLGILVFQTTSLVLTMRYSRTLKEEGPRYLSSTAVVVAELLKIMACILLVYK
DSKCSLRALNRVLHDEILNKPMETLKLAIPSGIYTLQNNLLYVALSNLDAATYQVTYQLK
ILTTALFSVSMLSKKLGVYQWLSLVILMTGVAFVQWPSDSQLDSKELSAGSQFVGLMAVL
TACFSSGFAGVYFEKILKETKQSVWIRNIQLGFFGSIFGLMGVYIYDGELVSKNGFFQGY
NRLTWIVVVLQCLFPWSHPCNNSYFFVWL*
>ENSG00000283158|ENST00000636567
MSVGRRKLALLWALALALACTRHTGHAQDGSSESSYKHHPALSPIARGPSGVPLRGATVF
PSLRTIPVVRASNPAHNGRVCSTWGSFHYKTFDGDVFRFPGLCNYVFSEHCGAAYEDFNI
QLRRSQESAAPTLSRVLMKVDGVVIQLTKGSVLVNGHPVLLPFSQSGVLIQQSSSYTKVE
ARLGLVLMWNHDDSLLLELDTKYANKTCGLCGDFNGMPVVSELLSHNTKLTPMEFGNLQK
MDDPTDQCQDPVPEPPRNCSTGFGICEELLHGQLFSGCVALVDVGSYLEACRQDLCFCED
TDLLSCVCHTLAEYSRQCTHAGGLPQDWRGPDFCPQKCPNNMQYHECRSPCADTCSNQEH
SRACEDHCVAGCFCPEGTVLDDIGQTGCVPVSKCACVYNGAAYAPGATYSTDCTNCTCSG
GRWSCQEVPCPGTCSVLGGAHFSTFDGKQYTVHGDCSYVLTKPCDSSAFTVLAELRRCGL
TDSETCLKSVTLSLDGAQTVVVIKASGEVFLNQIYTQLPISAANVTIFRPSTFFIIAQTS
LGLQLNLQLVPTMQLFMQLAPKLRGQTCGLCGNFNSIQADDFRTLSGVVEATAAAFFNTF
KTQAACPNIRNSFEDPCSLSVENEKYAQHWCSQLTDADGPFGRCHAAVKPGTYYSNCMFD
TCNCERSEDCLCAALSSYVHACAAKGVQLGGWRDGVCTKPMTTCPKSMTYHYHVSTCQPT
CRSLSEGDITCSVGFIPVDGCICPKGTFLDDTGKCVQASNCPCYHRGSMIPNGESVHDSG
AICTCTHGKLSCIGGQAPAPVCAAPMVFFDCRNATPGDTGAGCQKSCHTLDMTCYSPQCV
PGCVCPDGLVADGEGGCITAEDCPCVHNEASYRAGQTIRVGCNTCTCDSRMWRCTDDPCL
ATCAVYGDGHYLTFDGQSYSFNGDCEYTLVQNHCGGKDSTQDSFRVVTENVPCGTTGTTC

SKAIKIFLGGFELKLSHGKVEVIGTDESQEVPYTIRQMGIYLVVDTDIGLVLLWDKKTSI
FINLSPEFKGRVCGLCGNFDDIAVNDFATRSRSVVGDVLEFGNSWKLSPSCPDALAPKDP
CTANPFRKSWAQKQCSILHGPTFAACHAHVEPARYYEACVNDACACDSGGDCECFCTAVA
AYAQACHEVGLCVSWRTPSICPLFCDYYNPEGQCEWHYQPCGVPCLRTCRNPRGDCLRDV
RGLEGCYPKCPPEAPIFDEDKMQCVATCPTPPLPPRCHVHGKSYRPGAVVPSDKNCQSCL
CTERGVECTYKAEACVCTYNGQRFHPGDVIYHTTDGTGGCISARCGANGTIERRVYPCSP
TTPVPPTTFSFSTPPLVVSSTHTPSNGPSSAHTGPPSSAWPTTAGTSPRTRLPTASASLP
PVCGEKCLWSPWMDVSRPGRGTDSGDFDTLENLRAHGYRVCESPRSVECRAEDAPGVPLR
ALGQRVQCSPDVGLTCRNREQASGLCYNYQIRVQCCTPLPCSTSSSPAQTTPPTTSKTTE
TRASGSSAPSSTPGTVSLSTARTTPAPGTATSVKKTFSTPSPPPVPATSTSSMSTTAPGT
SVVSSKPTPTEPSTSSCLQELCTWTEWIDGSYPAPGINGGDFDTFQNLRDEGYTFCESPR
SVQCRAESFPNTPLADLGQDVICSHTEGLICLNKNQLPPICYNYEIRIQCCETVNVCRDI
TRLPKTVATTRPTPHPTGAQTQTTFTTHMPSASTEQPTATSRGGPTATSVTQGTHTTLVT
RNCHPRCTWTKWFDVDFPSPGPHGGDKETYNNIIRSGEKICRRPEEITRLQCRAKSHPEV
SIEHLGQVVQCSREEGLVCRNQDQQGPFKMCLNYEVRVLCCETPRGCHMTSTPGSTSSSP
AQTTPSTTSKTTETQASGSSAPSSTPGTVSLSTARTTPAPGTATSVKKTFSTPSPPPVPA
TSTSSMSTTAPGTSVVSSKPTPTEPSTSSCLQELCTWTEWIDGSYPAPGINGGDFDTFQN
LRDEGYTFCESPRSVQCRAESFPNTPLADLGQDVICSHTEGLICLNKNQLPPICYNYEIR
IQCCETVNVCRDITRPPKTVATTRPTPHPTGAQTQTTFTTHMPSASTEQPTATSRGGPTA
TSVTQGTHTTPVTRNCHPRCTWTTWFDVDFPSPGPHGGDKETYNNIIRSGEKICRRPEEI
TRLQCRAKSHPEVSIEHLGQVVQCSREEGLVCRNQDQQGPFKMCLNYEVRVLCCETPKGC
PVTSTPVTAPSTPSGRATSPTQSTSSWQKSRTTTLVTTSTTSTPQTSTTYAHTTSTTSAP
TARTTSAPTTRTTSASPASTTSGPGNTPSPVPTTSTISAPTTSITSAPTTSTTSAPTSST
TSGPGTTPSPVPTTSITSAPTTSTTSAPTTSTTSARTSSTTSATTTSRISGPETTPSPVP
TTSTTSATTTSTTSAPTTSTTSAPTSSTTSSPQTSTTSAPTTSTTSGPGTTPSPVPTTST
TSAPTTRTTSAPKSSTTSAATTSTTSGPETTPRPVPTTSTTSSPTTSTTSAPTTSTTSAS
TTSTTSGAGTTPSPVPTTSTTSAPTTSTTSAPISSTTSATTTSTTSGPGTTPSPVPTTST
TSAPTTSTTSGPGTTPSAVPTTSITSAPTTSTNSAPISSTTSATTTSRISGPETTPSPVP
TASTTSASTTSTTSGPGTTPSPVPTTSTISVPTTSTTSASTTSTTSASTTSTTSGPGTTP
SPVPTTSTTSAPTTSTTSAPTTSTISAPTTSTTSATTTSTTSAPTPRRTSAPTTSTISAS
TTSTTSATTTSTTSATTTSTISAPTTSTTLSPTTSTTSTTITSTTSAPISSTTSTPQTST
TSAPTTSTTSGPGTTSSPVPTTSTTSAPTTSTTSAPTTRTTSVPTSSTTSTATTSTTSGP
GTTPSPVPTTSTTSAPTTRTTSAPTTSTTSAPTTSTTSAPTSSTTSATTTSTISVPTTST
TSVPGTTPSPVPTTSTISVPTTSTTSASTTSTTSGPGTTPSPVPTTSTTSAPTTSTTSAP
TTSTISAPTTSTPSAPTTSTTLAPTTSTTSAPTTSTTSTPTSSTTSSPQTSTTSASTTSI
TSGPGTTPSPVPTTSTTSAPTTSTTSAATTSTISAPTTSTTSAPTTSTTSASTASKTSGL
GTTPSPIPTTSTTSPPTTSTTSASTASKTSGPGTTPSPVPTTSTIFAPRTSTTSASTTST
TPGPGTTPSPVPTTSTASVSKTSTSHVSISKTTHSQPVTRDCHLRCTWTKWFDIDFPSPG
PHGGDKETYNNIIRSGEKICRRPEEITRLQCRAESHPEVSIEHLGQVVQCSREEGLVCRN
QDQQGPFKMCLNYEVRVLCCETPKGCPVTSTPVTAPSTPSGRATSPTQSTSSWQKSRTTT
LVTTSTTSTPQTSTTSAPTTSTTSAPTTSTTSAPTTSTTSTPQTSISSAPTSSTTSAPTS
STISARTTSIISAPTTSTTSSPTTSTTSATTTSTTSAPTSSTTSTPQTSKTSAATSSTTS
GSGTTPSPVTTTSTASVSKTSTSHVSVSKTTHSQPVTRDCHPRCTWTKWFDVDFPSPGPH
GGDKETYNNIIRSGEKICRRPEEITRLQCRAKSHPEVSIEHLGQVVQCSREEGLVCRNQD

QQGPFKMCLNYEVRVLCCETPKGCPVTSTSVTAPSTPSGRATSPTQSTSSWQKSRTTTLV
TSSITSTTQTSTTSAPTTSTTPASIPSTTSAPTTSTTSAPTTSTTSAPTTSTTSTPQTTT
SSAPTSSTTSAPTTSTISAPTTSTISAPTTSTTSAPTASTTSAPTSTSSAPTTNTTSAPT
TSTTSAPITSTISAPTTSTTSTPQTSTISSPTTSTTSTPQTSTTSSPTTSTTSAPTTSTT
SAPTTSTTSTPQTSISSAPTSSTTSAPTASTISAPTTSTTSFHTTSTTSPPTSSTSSTPQ
TSKTSAATSSTTSGSGTTPSPVPTTSTASVSKTSTSHVSVSKTTHSQPVTRDCHPRCTWT
KWFDVDFPSPGPHGGDKETYNNIIRSGEKICRRPEEITRLQCRAESHPEVSIEHLGQVVQ
CSREEGLVCRNQDQQGPFKMCLNYEVRVLCCETPKGCPVTSTPVTAPSTPSGRATSPTQS
TSSWQKSRTTTLVTTSTTSTPQTSTTSAPTTSTIPASTPSTTSAPTTSTTSAPTTSTTSA
PTHRTTSGPTTSTTLAPTTSTTSAPTTSTNSAPTTSTISASTTSTISAPTTSTISSPTSS
TTSTPQTSKTSAATSSTTSGSGTTPSPVPTTSTTSASTTSTTSAPTTSTTSGPGTTPSPV
PSTSTTSAATTSTTSAPTTRTTSAPTSSMTSGPGTTPSPVPTTSTTSAPTTSTTSGPGTT
PSPVPTTSTTSAPITSTTSGPGSTPSPVPTTSTTSAPTTSTTSASTASTTSGPGTTPSPV
PTTSTTSAPTTRTTSASTASTTSGPGSTPSPVPTTSTTSAPTTRTTPASTASTTSGPGTT
PSPVPTTSTTSASTTSTISLPTTSTTSAPITSMTSGPGTTPSPVPTTSTTSAPTTSTTSA
STASTTSGPGTTPSPVPTTSTTSAPTTSTTSASTASTTSGPGTSLSPVPTTSTTSAPTTS
TTSGPGTTPSPVPTTSTTSAPTTSTTSGPGTTPSPVPTTSTTPVSKTSTSHLSVSKTTHS
QPVTSDCHPLCAWTKWFDVDFPSPGPHGGDKETYNNIIRSGEKICRRPEEITRLQCRAES
HPEVNIEHLGQVVQCSREEGLVCRNQDQQGPFKMCLNYEVRVLCCETPRGCPVTSVTPYG
TSPTNALYPSLSTSMVSASVASTSVASSSVASSSVAYSTQTCFCNVADRLYPAGSTIYRH
RDLAGHCYYALCSQDCQVVRGVDSDCPSTTLPPAPATSPSISTSEPVTELGCPNAVPPRK
KGETWATPNCSEATCEGNNVISLRPRTCPRVEKPTCANGYPAVKVADQDGCCHHYQCQCV
CSGWGDPHYITFDGTYYTFLDNCTYVLVQQIVPVYGHFRVLVDNYFCGAEDGLSCPRSII
LEYHQDRVVLTRKPVHGVMTNEIIFNNKVVSPGFRKNGIVVSRIGVKMYATIPELGVQVM
FSGLIFSVEVPFSKFANNTEGQCGTCTNDRKDECRTPRGTVVASCSEMSGLWNVSIPDQP
ACHRPHPTPTTVGPTTVGSTTVGPTTVGSTTVGPTTPPAPCLPSPICQLILSKVFEPCHT
VIPPLLFYEGCVFDRCHMTDLDVVCSSLELYAALCASHDICIDWRGRTGHMCPFTCPADK
VYQPCGPSNPSYCYGNDSASLGALPEAGPITEGCFCPEGMTLFSTSAQVCVPTGCPRCLG
PHGEPVKVGHTVGMDCQECTCEAATWTLTCRPKLCPLPPACPLPGFVPVPAAPQAGQCCP
QYSCACNTSRCPAPVGCPEGARAIPTYQEGACCPVQNCSWTVCSINGTLYQPGAVVSSSL
CETCRCELPGGPPSDAFVVSCETQICNTHCPVGFEYQEQSGQCCGTCVQVACVTNTSKSP
AHLFYPGETWSDAGNHCVTHQCEKHQDGLVVVTTKKACPPLSCSLDEARMSKDGCCRFCP
PPPPPYQNQSTCAVYHRSLIIQQQGCSSSEPVRLAYCRGNCGDSSSMYSLEGNTVEHRCQ
CCQELRTSLRNVTLHCTDGSSRAFSYTEVEECGCMGRRCPAPGDTQHSEEAEPEPSQEAE
SGSWERGVPVSPMH*
>ENSG00000148288|ENST00000540636
MHRRRLALGLGFCLLAGTSLSVLWVYLENWLPVSYVPYYLPCPEILSQYPQPKLLEHRPT
QLLTLTPWLAPIVSEGTFNPELLQHIYQPLNLTIGVTVFAVGKYTHFIQSFLESAEEFFM
RGYRVHYYIFTDNPAAVPGVPLGPHRLLSSIPIQGHSHWEETSMRRMETISQHIAKRAHR
EVDYLFCLDVDMVFRNPWGPETLGDLVAAIHPSYYAVPRQQFPYERRRVSTAFVADSEGD
FYYGGAVFGGQVARVYEFTRGCHMAILADKANGIMAAWREESHLNRHFISNKPSKVLSPE
YLWDDRKPQPPSLKLIRFSTLDKDISCLRS*
>ENSG00000082805|ENST00000545948
MYGSARSVGKVEPSSQSPGRSPRLPRSPRLGHRRTNSTGGSSGSSVGGGSGKTLSMENIQ

SLNAAYATSGPMYLSDHENVGSETPKSTMTLGRSGGRLPYGVRMTAMGSSPNIASSGVAS
DTIAFGEHHLPPVSMASTVPHSLRQARDNTIMDLQTQLKEVLRENDLLRKDVEVKESKLS
SSMNSIKTFWSPELKKERALRKDEASKITIWKEQYRVVQEENQVDALRLRLEEKETMLNK
KTKQIQDMAEEKGTQAGEIHDLKDMLDVKERKVNVLQKKIENLQEQLRDKEKQMSSLKER
VKSLQADTTNTDTALTTLEEALAEKERTIERLKEQRDRDEREKQEEIDNYKKDLKDLKEK
VSLLQGDLSEKEASLLDLKEHASSLASSGLKKDSRLKTLEIALEQKKEECLKMESQLKKA
HEAALEARASPEMSDRIQHLEREITRYKDESSKAQAEVDRLLEILKEVENEKNDKDKKIA
ELESLTSRQVKDQNKKVANLKHKEQVEKKKSAQMLEEARRREDNLNDSSQQLQDSLRKKD
DRIEELEEALRESVQITAEREMVLAQEESARTNAEKQVEELLMAMEKVKQELESMKAKLS
STQQSLAEKETHLTNLRAERRKHLEEVLEMKQEALLAAISEKDANIALLELSSSKKKTQE
EVAALKREKDRLVQQLKQQTQNRMKLMADNYEDDHFKSSHSNQTNHKPSPDQDEEEGIWA
*
>ENSG00000097033|ENST00000482504
MNIMDFNVKKLAADAGTFLSRAVQFTEEKLGQAEKTELDAHLENLLSKAECTKIWTEKIM
KQTEVLLQPNPNARIEEFVYEKLDRKAPSRINNPELLGQYMIDAGTEFGPGTAYGNALIK
CGETQKRIGTADRELIQTSALNFLTPLRNFIEGDYKTIAKERKLLQNKRLDLDAAKTRLK
KAKAAETRNSQLNSARLEGDNIMIWAEEVTKSEQELRITQSEFDRQAEITRLLLEGISST
HAHHLRCLNDFVEAQMTYYAQCYQYMLDLQKQLGSFPSNYLSNNNQTSVTPVPSVLPNAI
GSSAMASTSGLVITSPSNLSDLKECSGSRKARVLYDYDAANSTELSLLADEVITVFSVVG
MDSDWLMGERGNQKGKVPITYLELLN*
>ENSG00000186908|ENST00000426126
MQREEGFNTKMADGPDEYDTEAGCVPLLHPEEIKPQSHYNHGYGEPLGRKTHIDDYSTWD
IVKATQYGIYERCRELVEAGYDVRQPDKENVTLLHWAAINNRIDLVKYYISKGAIVDQLG
GDLNSTPLHWATRQGHLSMVVQLMKYGADPSLIDGEGCSCIHLAAQFGHTSIVAYLIAKG
QDVDMMDQNGMTPLMWAAYRTHSVDPTRLLLTFNVSVNLGDKYHKNTALHWAVLAGNTTV
ISLLLEAGANVDAQNIKGESALDLAKQRKNVWMINHLQEARQAKGYDNPSFLRKLKADKE
FRQKVMLGTPFLVIWLVGFIADLNIDSWLIKGLMYGGVWATVQFLSKSFFDHSMHSALPL
GIYLATKFWMYVTWFFWFWNDLNFLFIHLPFLANSVALFYNFGKSWKSDPGIIKATEEQK
KKTIVELAETGSLDLSIFCSTCLIRKPVRSKHCGVCNRCIAKFDHHCPWVGNCVGAGNHR
YFMGYLFFLLFMICWMIYGCISYWGLHCETTYTKDGFWTYITQIATCSPWMFWMFLNSVF
HFMWVAVLLMCQMYQISCLGITTNERMNARRYKHFKVTTTSIESPFNHGCVRNIIDFFEF
RCCGLFRPVIVDWTRQYTIEYDQISGSGYQLV*
>ENSG00000171302|ENST00000620915
MPVQLSEHPEWNESMHSLRISVGGLPVLASMTKAADPRFRPRWKVILTFFVGAAILWLLC
SHRPAPGRPPTHNAHNWRLGQAPANWYNDTYPLSPPQRTPAGIRYRIAVIADLDTESRAQ
EENTWFSYLKKGYLTLSDSGDKVAVEWDKDHGVLESHLAEKGRGMELSDLIVFNGKLYSV
DDRTGVVYQIEGSKAVPWVILSDGDGTVEKGFKAEWLAVKDERLYVGGLGKEWTTTTGDV
VNENPEWVKVVGYKGSVDHENWVSNYNALRAAAGIQPPGYLIHESACWSDTLQRWFFLPR
RASQERYSEKDDERKGANLLLSASPDFGDIAVSHVGAVVPTHGFSSFKFIPNTDDQIIVA
LKSEEDSGRVASYIMAFTLDGRFLLPETKIGSVKYEGIEFI*
>ENSG00000005206|ENST00000618220
MAAAVAAALARLLAAFLLLAAQVACEYGMVHVVSQAGGPEGKDYCILYNPQWAHLPHDLS
KASFLQLRNWTASLLCSAADLPARGFSNQIPLVARGNCTFYEKVRLAQGSGARGLLIVSR
ERLVPPGGNKTQYDEIGIPVALLSYKDMLDIFTRFGRTVRAALYAPKEPVLDYNMVIIFI

MAVGTVAIGGYWAGSRDVKKRYMKHKRDDGPEKQEDEAVDVTPVMTCVFVVMCCSMLVLL
YYFYDLLVYVVIGIFCLASATGLYSCLAPCVRRLPFGKCRIPNNSLPYFHKRPQARMLLL
ALFCVAVSVVWGVFRNEDQ*
>ENSG00000189067|ENST00000622633
MSVPGPYQAATGPSSAPSAPPSYEETVAVNSYYPTPPAPMPGPTTGLVTGPDGKGMNPPS
YYTQPAPIPNNNPITVQTVYVQHPITFLDRPIQMCCPSCNKMIVSQLSYNAGALTWLSCG
SLCLLGCIAGCCFIPFCVDALQDVDHYCPNCRALLGTYKRL*
>ENSG00000224320|ENST00000549869
MAVMAPRTLLLLLSGALALTQTWAGSHSMRYFFTSVSRPGRGEPRFIAVGYVDDTQFVRF
DSDAASQKMEPRAPWIEQEGPEYWDQETRNMKAHSQTDRANLGTLRGYYNQSEDGSHTIQ
IMYGCDVGPDGRFLRGYRQDAYDGKDYIALNEDLRSWTAADMAAQITKRKWEAVHAAEQR
RVYLEGRCVDGLRRYLENGKETLQRTDPPKTHMTHHPISDHEATLRCWALGFYPAEITLT
WQRDGEDQTQDTELVETRPAGDGTFQKWAAVVVPSGEEQRYTCHVQHEGLPKPLTLRWEL
SSQPTIPIVGIIAGLVLLGAVITGAVVAAVMWRRKSSDRKGGSYTQAASSDSAQGSDVSL
TACKV*
>ENSG00000003147|ENST00000422063
MSGHKCSYPWDLQDRYAQDKSVVNKMQQKYWETKQAFIKATGKKEDEHVVASDADLDAKL
ELFHSIQRTCLDLSKAIVLYQKRICFLSQEENELGKFLRSQGFQDKTRAGKMMQATGKAL
CFSSQQRLALRNPLCRFHQEVETFRHRAISDTWLTVNRMEQCRTEYRGALLWMKDVSQEL
DPDLYKQMEKFRKVQTQVRLAKKNFDKLKMDVCQKVDLLGASRCNLLSHMLATYQTTLLH
FWEKTSHTMAAIHESFKGYQPYEFTTLKSLQDPMKKLVEKEEKKKINQQESTDAAVQEPS
QYLQNICLEMKANSDIKMLQKGKEGQQIGILISLEEENQRKESSSFKTEDGKSILSALDK
GSTHTACSGPIDELLDMKSEEGACLGPVAGTPEPEGADKDDLLLLSEIFNASSLEEGEFS
KEWAAVFGDGQVKEPVPTMALGEPDPKAQTGSGFLPSQLLDQNMKDLQASLQEPAKAASD
LTAWFSLFADLDPLSNPDAVGKTDKEHELLNA*
>ENSG00000136631|ENST00000535106
MVYTQSEILQKEVYLFERIDSQNREIMKHLKAICFLRPTKENVDYIIQELRRPKYTIYFI
YFSNVISKSDVKSLAEADEQEVVAEVQQVITKEYELFEFRRTEVPPLLLILDRCDDAITP
LLNQWTYQAMVHELLGINNNRIDLSRVPGISKDLREVVLSAENDEFYANNMYLNFAEIGS
NIKNLMEDFQKKKPKEQQKLESIADMKAFVENYPQFKKMSGTVSKHVTVVGELSRLVSER
NLLEVSEVEQELACQNDHSSALQNIKRLLQNPKVTEFDAARLVMLYALHYERHSSNSLPG
LMMDLRNKGVSEKYRKLVSAVVEYGGKRVRGSDLFSPKDAVAITKQFLKGLKGVENVYTQ
HQPFLHETLDHLIKGRLKENLYPYLGPSTLRDRPQDIIVFVIGGATYEEALTVYNLNRTT
PGVRIVLGGTTVHNTKRDGVSLCSPAWFRTPGLKRSTRLSLPKCWDYSFPRGSSGFWTAQ
PKQGELSSHIKVSEQKMKRWLGEGHSFLSCPHYRFSLLNKGVGEQLWVLCWLLELISR*
>ENSG00000131386|ENST00000437509
MLLRKRYRHRPCRLQFLLLLLMLGCVLMMVAMLHPPHHTLHQTVTAQASKHSPEARYRLD
FGESQDWVLEAEDEGEEYSPLEGLPPFISLREDQLLVAVALPQARRNQSQGRRGGSYRLI
KQPRRQDKEAPKRDWGADEDGEVSEEEELTPFSLDPRGLQEALSARIPLQRALPEVRHPL
CLQQHPQDSLPTASVILCFHDEAWSTLLRTVHSILDTVPRAFLKEIILVDDLSQQGQLKS
ALSEYVARLEGVKLLRSNKRLGAIRARMLGATRATGDVLVFMDAHCECHPGWLEPLLSRI
AGDRSRVVSPVIDVIDWKTFQYYPSKDLQRGVLDWKLDFHWEPLPEHVRKALQSPISPIR
SPVVPGEVVAMDRHYFQNTGAYDSLMSLRGGENLELSFKAWLCGGSVEILPCSRVGHIYQ
NQDSHSPLDQEATLRNRVRIAETWLGSFKETFYKHSPEAFSLSKAEKPDCMERLQLQRRL

GCRTFHWFLANVYPELYPSEPRPSFSGKLHNTGLGLCADCQAEGDILGCPMVLAPCSDSR
QQQYLQHTSRKEIHFGSPQHLCFAVRQEQVILQNCTEEGLAIHQQHWDFQEDENRANSAA
AEAYQRPLTFQQIVNNG*
>ENSG00000166377|ENST00000586672
MADQIPLYPVRSAAAAAANRKRAAYYSAAGPRPGADRHSSCCGWLINICRRKKELKARTV
WLGCPEKCEEKHPRNSIKNQKYNVFTFIPGVLYEQFKFFLNLYFLVISCSQFVPALKIGY
LYTYWAPLGFVLAVTMTREAIDEFRRFQRDKEVNSQLYSKLTVRGKVQVKSSDIQVGDLI
IVEKNQRIPSDMV
>ENSG00000160360|ENST00000429455
MDDQRCPLDDGQAGAAEATAAPTLEDRIAQPSMTASPQTEEFFDLIASSQSRRLDDQRAS
VGSLPGLRITHSNAGHLRGHGEPQEPGDDFFNMLIKYQSSRIDDQRCPPPDVLPRGPTMP
DEDFFSLIQRVQAKRMDEQRVDLAGGPEQGAGGPPEPQQQCQPGAS*
>ENSG00000283761|ENST00000639171
MFANLKYVSLGILVFQTTSLVLTMRYSRTLKEEGPRYLSSTAVVVAELLKIMACILLVYK
DSKCSLRALNRVLHDEILNKPMETLKLAIPSGIYTLQNNLLYVALSNLDAATYQVTYQLK
ILTTALFSVSMLSKKLGVYQWLSLVILMTGVAFVQWPSDSQLDSKELSAGSQFVGLMAVL
TACFSSGFAGVYFEKILKETKQSVWIRNIQLDGYMHPVHPHVVRRLAYQHNQTVCRNSPT
VEM*
>ENSG00000158623|ENST00000425248
MIKKFDKKDEESGSGSNPFQHLEKSAVLQEARIFNETPINPRRCLHILTKILYLLNQGEH
FGTTEATEAFFAMTRLFQSNDQTLRRMCYLTIKEMATISEDVIIVTSSLTKDMTGKEDVY
RGPAIRALCRITDGTMLQAIERYMKQAIVDKVSSVSSSALVSSLHMMKISYDVVKRWINE
AQEAASSDNIMVQYHALGVLYHLRKNDRLAVSKMLNKFTKSGLKSQFAYCMLIRIASRLL
KETEDGHESPLFDFIESCLRNKHEMVIYEAASAIIHLPNCTARELAPAVSVLQLFCSSPK
PALRYAAVRTLNKVAMKHPSAVTACNLDLENLITDSNRSIATLAITTLLKTGSESSVDRL
MKQISSFVSEISDEFKVVVVQAISALCQKYPRKHSVMMTFLSNMLRDDGGFEYKRAIVDC
IISIVEENPESKEAGLAHLCEFIEDCEHTVLATKILHLLGKEGPRTPVPSKYIRFIFNRV
VLENEAVRAAAVSALAKFGAQNESLLPSILVLLQRCMMDTDDEVRDRATFYLNVLQQRQM
ALNATYIFNGLTVSVPGMEKALHQYTLEPSEKPFDMKSIPLAMAPVFEQKAEITLVATKP
EKLAPSRQDIFQEQLAAIPEFLNIGPLFKSSEPVQLTEAETEYFVRCIKHMFTNHIVFQF
DCTNTLNDQLLEKVTVQMEPSDSYEVLSCIPAPSLPYNQPGICYTLVRLPDDDPTAVAGS
FSCTMKFTVRDCDPNTGVPDEDGYDDEYVLEDLEVTVSDHIQKVLKPNFAAAWEEVGDTF
EKEETFALSSTKTLEEAVNNIITFLGMQPCERSDKVPENKNSHSLYLAGIFRGGYDLLVR
SRLALADGVTMQVTVRSKERTPVDVILASVG*
>ENSG00000280433|ENST00000623998
TLYNVKAEIFPPSGMEYCRTGSLCSLEVLITRLSDLLEVDKDEALTESDEHFSTKLMYEV
VDNSSNWAVCGKSCGVISMPVAARATHRVHMEVMPLFAGYLPLPDVRLFKYLPHHSAHSS
QLDADSWIENDSLSVDKHGDDQPDSSSLKSRGSVHSACSSEHKGLPMPRLQALPAGQVFN
SSSGTQVLVIPSQDDHVLEVSVT*
>ENSG00000100478|ENST00000542754
MIKFFLMVNKQGQTRLSKYYEHVDINKRTLLETEVIKSCLSRSNEQCSFIEYKDFKLIYR
QYAALFIVVGVNDTENEMAIYEFIHNFVEVLDEYFSRVSELDIMFNLDKVHIILDEMVLN
GCIVETNRARILAPLLILDKMSES*
>ENSG00000284969|ENST00000525763

MGIQGGSVLFGLLLVLAVFCHSGHSLQCYNCPNPTADCKTAVNCSSDFDACLITKAGLQV
YNKCWKFEHCNFNDVTTRLRENELTYYCCKKDLCNFNEQLENGGTSLSEKTVLLLVTPFL
AAAWSLHP*
>ENSG00000235915|ENST00000447141
MLSRNDEAASAQQ*
>ENSG00000110075|ENST00000524904
MFWKFDLHSSSHIDTLLEREDVTLKELMDEEDVLQECKAQNRKLIEFLLKAECLEDLVSF
IIEEPPQDMDEKIRYKYPNISCELLTSDVSQMNDRLGEDESLLMKLYSFLLNDSPLNPLL
ASFFSKVLSILISRKPEQIVDFLKKKHDFVDLIIKHIGTSAIMDLLLRLLTCIEPPQPRQ
DVLNWLNEEKIIQRLVEIVHPSQEEDRHSNASQSLCEIVRLSRDQMLQIQNSTEPDPLLA
TLEKQEIIEQLLSNIFHKEKNESAIVSAIQILLTLLETRRPTFEGHIEICPPGMSHSACS
VNKSVLEAIRGRLGSFHELLLEPPKKSVMKTTWGVLDPPVGNTRLNVIRLISSLLQTNTS
SINGDLMELNSIGVILNMFFKYTWNNFLHTQVEICIALILASPFENTENATITDQDSTGD
NLLLKHLFQKCQLIERILEAWEMNEKKQAEGGRRHGYMGHLTRIANCIVHSTDKGPNSAL
VQQLIKDLPDEVRERWETFCTSSLGETNKRNTVDLVTTCHIHSSSDDEIDFKETGFSQDS
SLQQMQQMTSNFIDQFGFNDEKFADQDDIGNVSFDRVSDINFTLNTNESGNIALFEACCK
ERIQQFDDGGSDEEDIWEEKHIAFTPESQRRSSSGSTDSEESTDSEEEDGAKQDLFEPSS
ANTEDKMEVDLSEPPNWSANFDVPMETTHGAPLDSVGSDVWSTEEPMPTKETGWASFSEF
TSSLSTKDSLRSNSPVEMETSTEPMDPLTPSAAALAVQPEAAGSVAMEASSDGEEDAEST
DKVTETVMNGGMKETLSLTVDAKTETAVFKSEEGKLSTSQDAACKDAEECPETAEAKCAA
PRPPSSSPEQRTGQPSAPGDTSVNGPV*
>ENSG00000003147|ENST00000457755
MSGHKCSYPWDLQDRYAQDKSVVNKMQQKYWETKQAFIKATGKKEDEHVVASDADLDAKL
ELFHSIQRTCLDLSKAIVLYQKRIC*
>ENSG00000117280|ENST00000446390
MGSRDHLFKVLVVGDAAVGKTSLVQRYSQDSFSKHYKSTVGGQERFTSMTRLYYRDASAC
VIMFDVTNATTFSNSQRWKQDLDSKLTLPNGEPVPCLLLANKCDLSPWAVSRDQIDRFSK
ENGFTGWTETSVKENKNINEAMRVLIEKMMRNSTEDIMSLSTQGDYINLQTKSSSWSCC*
>ENSG00000132376|ENST00000406424
MDVLSPLSFIKVSHVRMQGILLLVFAKYQHLPYIQILSTKSTPTGLFGYWGNKGGVNICL
KLYGYYVSIINCHLPPHISNNYQRLEHFDRILEMQNCEGRDIPNILDHDLIIWFGDMNFR
IEDFGLHFVRESIKNRCYGGLWEKDQLSIAKKHDPLLREFQEGRLLFPPTYKFDRNSNDY
DTSEKKRKPAWTDRILWRLKRQPCAGPDTPIPPASHFSLSLRGYSSHMTYGISDHKPVSG
TFDLELKPLVSAPLIVLMPEDLWTVENDMMVSYSSTSDFPSSPWDWIGLYKVGLRDVNDY
VSYAWVGDSKVSCSDNLNQVYIDISNIPTTEDEFLLCYYSNSLRSVVGISRPFQIPPGSL
REDPLGEAQPQI*
>ENSG00000234794|ENST00000411524
MAISGVPVLGFFIIAVLMSAQESWAIKEEHVIIQAEFYLNPDQSGEFMFDFDGDEIFHVD
MAKKETVWRLEEFGRFASFEAQGALANIAVDKANLEIMTKRSNYTPITNVPPEVTVLTNS
PVELREPNVLICFIDKFTPPVVNVTWLRNGKPVTTGVSETVFLPREDHLFRKFHYLPFLP
STEDVYDCRVEHWGLDEPLLKHWEFDAPSPLPETTENVVCALGLTVGLVGIIIGTIFIIK
GVRKSNAAERRGPL*
>ENSG00000230791|ENST00000433305
MLSRNDAPSVLVPGPGLLRAGTPLCISAEAASAQQ*

>ENSG00000180398|ENST00000409207
MTMRSLLRTPFLCGLLWAFCAPGARAEEPAASFSQPGSMGLDKNTVHDQEHIMEHLEGVI
NKPEAEMSPQELQLHYFKMHDYDGNNLLDGLELSTAITHVHKEEGSEQAPLMSEDELINI
IDGVLRDDDKNNDGYIDYAEFAKSLQ*
>ENSG00000108433|ENST00000640051
MDPLFQQTHKQVHEIQSCMGRLETADKQSVHIVENEIQASIDQIFSRLERLEILSSKEPP
NKRQNARLRVDQLKYDVQHLQTALRNFQHRRHAREQQERQREELLSRTFTTNDSDTTIPM
DESLQFNSSLQKVHNGMDDLILDGHNILDGLRTQRLTLKGTQKKILDIANMLGLSNTVMR
LIEKRAFQDKYFMIGGMLLTCVVMFLVVQYLT*
>ENSG00000125447|ENST00000582486
MKNCGRRFHNEVGKFRFLNELIKVVSPKYLGDRVSEKVKTKVIELLYSWTMALPEEAKIK
DAYHMLKRQGIVQSDPPIPVDRTLIPSPPPRPKNPVFDDEEKSKLLAKLLKSKNPDDLQE
ANKLIKSMVKEDEARIQKVTKRLHTLEEVNNNVRLLSEMLLHYSQEDSSDGDRELMKELF
DQCENKRRTLFKLASETEDNDNSLGDILQASDNLSRVINSYKTIIEGQVINGEVATLTLP
DSEGNSQCSNQGTLIDLAELDTTNSLSSVLAPAPTPPSSGIPILPPPPQASGPPRSRSSS
QAEATLGPSSTSNALSWLDEELLCLGLADPAPNVPPKESAGNSQWHLLQREQSDLDFFSP
RPGTAACGASDAPLLQPSAPSSSSSQAPLPPPFPAPVVPASVPAPSAGSSLFSTGVAPAL
APKVEPAVPGHHGLALGNSALHHLDALDQLLEEAKVTSGLVKPTTSPLIPTTTPARPLLP
FSTGPGSPLFQPLSFQSQGSPPKGPELSLASIHVPLESIKPSSALPVTAYDKNGFRILFH
FAKECPPGRPDVLVVVVSMLNTAPLPVKSIVLQAAVPKSMKVKLQPPSGTELSPFSPIQP
PAAITQVMLLANPLKWQAVSYGHLGRTWNLAS*
>ENSG00000176945|ENST00000445522
MTTDDTEVPAMTLAPGHAALETQTLSAETSSRASTPAGPIPEAETRGAKRISPARETRSF
TKTSPNFMVLIATSVETSAASGSPEGAGMTTVQTITGSDPREAIFDTLCTDDSSEEAKTL
TMDILTLAHTSTEAKGLSSESSASSDSPHPVITPSRASESSASSDGPHPVITPSRASESS
ASSDGPHPVITPSRASESSASSDGPHPVITPSRASESSASSDGPHPVITPSRASESSASS
DGPHPVITPSRASESSASSDGPHPVITPSRASESSASSDGPHPVITPSRASESSASSDGP
HPVITPSRASESSASSDGLHPVITPSRASESSASSDGPHPVITPSRASESSASSDGPHPV
ITPSWSPGSDVTLLAEALVTVTNIEVINCSITEIETTTSSIPGASDTDLIPTEGVKASST
SDPPALPDSTEAKPHITEVTASAETLSTAGTTESAAPDATVGTPLPTNSATEREVTAPGA
TTLSGALVTVSRNPLEETSALSVETPSYVKVSGAAPVSIEAGSAVGKTTSFAGSSASSYS
PSEAALKNFTPSETPTMDIATKGPFPTSRDPLPSVPPTTTNSSRGTNSTLAKITTSAKTT
MKPPTATPTTARTRPTTDVSAGENGGFLLLRLSVASPEDLTDPRVAERLMQQLHRELHAH
APHFQVSLLRVRRG*
>ENSG00000017260|ENST00000504381
MDSLLPPSRFSYFKKYPLHAIRRYLSTLRNQRAEEQVARFQKIPNGENETMIPVLTSKKA
SELPVSEVASILQFKNPLIMLLLASAVISVLMHQFDDAVSITVAILIVVTVAFVQEYRSE
KSLEELSKLVPPECHCVREGKLEHTLARDLVPGDTVCLSVGDRVPADLRLFEAVDLSIDE
SSLTGETTPCSKVTAPQPAATNGDLASRSNIAFMGTLVRCGKAKGVVIGTGENSEFGEVF
KMMQAEEAPKTPLQKSMDLLGKQLSFYSFGIIGIIMLVGWLLGKDILEMFTISVSLAVAA
IPEGLPIVVTVTLALGVMRMVKKRAIVKKLPIVETLGCCNVICSDKTGTLTKNEMTVTHI
FTSDGLHAEVTGVGYNQFGEVIVDGDVVHGFYNPAVSRIVEAGCVCNDAVIRNNTLMGKP
TEGALIALAMKMGLDGLQQDYIRKAEYPFSSEQKWMAVKCVHRTQQDRPEICFMKGAYEQ
VIKYCTTYQSKGQTLTLTQQQRDVYQQEKARMGSAGLRVLALASGPELGQLTFLGLVGII

DPPRTGVKEAVTTLIASGVSIKMITGDSQETAVAIASRLGLYSKTSQSVSGEEIDAMDVQ
QLSQIVPKVAVFYRASPRHKMKIIKSLQKNGSVVAMTGDGVNDAVALKAADIGVAMGQTG
TDVCKEAADMILVDDDFQTIMSAIEEGKGIYNNIKNFVRFQLSTSIAALTLISLATLMNF
PNPLNAMQILWINIIMDGPPAQSLGVEPVDKDVIRKPPRNWKDSILTKNLILKILVSSII
IVCGTLFVFWRELRDNVITPRDTTMTFTCFVFFDMFNALSSRSQTKSVFEIGLCSNRMFC
YAVLGSIMGQLLVIYFPPLQKVFQTESLSILDLLFLLGLTSSVCIVAEIIKKVERSREKI
QKHVSSTSSSFLEVWLWERSGQQLVEIHPHLETGLPLTEDVSCV*
>ENSG00000105696|ENST00000600490
MAAVALMPPPLLLLLLLASPPAASAPSARDPFAPQLGDTQNCQLRCRDRDLGPQPSQAGL
EGASESPYDRAVLISACERGCRLFSICRFVARSSKPNATQTECEAACVEAYVKEAEQQAC
SHGCWSQPAEPEPEQKRKVLEAPSGALSLLDLFSTLCNDLVNSAQGFVSSTWTYYLQTDN
GKVVVFQTQPIVESLGFQGGRLQRVEVTWRGSHPEALEVHVDPVGPLDKVRKAKIRVKTS
SKAKVESEEPQDNDFLSCMSRRSGLPRWILACCLFLSVLVMLWLSCSTLVTAPGQHLKFQ
PLTLEQHKGFMMEPDWPLYPPPSHACEDSLPPYKLKLDLTKL*
>ENSG00000102100|ENST00000635238
MAAVGAGGSTAAPGPGAVSAGALEPGTASAAHRRLKYISLAVLVVQNASLILSIRYARTL
PGDRFFATTAVVMAEVLKGNVKHLVLFLHEAVLVQYVDTLKLAVPSLIYTLQNNLQYVAI
SNLPAATFQVTYQLKILTTALFSVLMLNRSLSRLQWASLLLLFTGVAIVQAQQAGGGGPR
PLDQNPGAGLA
>ENSG00000284369|ENST00000640820
MGWVGGRRRDSASPPGRSRSAADDINPAPANMEGGGGSVAVAGLGARGSGAAAATVRELL
QDGCYSDFLNEDFDVKTYTSQSIHQAVIAEQLAKLAQGISQLDRELHLQVVARHEDLLAQ
ATGIESLEGVLQMMQTRIGALQGAVDRIKAKIVEPYNKIVARTAQLARLQVACDLLRRII
RILNLSKRLQGQLQGGSREITKAAQSLNELDYLSQGIDLSGIEVIENDLLFIARARLEVE
NQAKRLLEQGLETQNPTQVGTALQVFYNLGTLKDTITSVVDGYCATLEENINSALDIKVL
TQPSQSAVRGGPGRSTMPTPGNTAALRASLWTNMEKLMDHIYAVCGQVQHLQKVLAKKRD
PVSHICFIEEIVKDGQPEIFYTFWNSVTQALSSQFHMATNSSMFLKQAFEGEYPKLLRLY
NDLWKRLQQYSQHIQGNFNASGTTDLYVDLQHMEDDAQDIFIPKKPDYDPEKALKDSLQP
YEAAYLSKSLSRLFDPINLVFPPGGRNPPSSDELDGIIKTIASELNVAAVDTNLTLAVSK
NVAKTIQLYSVKSEQLLSTQGDASQVIGPLTEGQRRNVAVVNSLYKLHQSVTKAIHALME
NAVQPLLTSVGDAIEAIIITMHQEDFSGSLSSSGKPDVPCSLYMKELQGFIARVMSDYFK
HFECLDFVFDNTEAIAQRAVELFIRHASLIRPLGEGGKMRLAADFAQMELAVGPFCRRVS
DLGKSYRMLRSFRPLLFQASEHVASSPALGDVIPFSIIIQFLFTRAPAELKSPFQRAEWS
HTRFSQWLDDHPSEKDRLLLIRGALEAYVQSVRSREGKEFAPVYPIMVQLLQKAMSALQ*
>ENSG00000137486|ENST00000420843
MGDKGTRVFKKASPNGKLTVYLGKRDFVDHIDLVDPVDGVVLVDPEYLKERRVYVTLTCA
FRYGREDLDVLGLTFRKDLFVANVQSFPPAPEDKKPLTRLQERLIKKLGEHAYPFTFEIP
PNLPCSVTLQPGPEDTGKACGVDYEVKAFCAENLEEKIHKRNSVRLVIRKVQYAPERPGP
QPTAETTRQFLMSDKPLHLEASLDKEIYYHGEPISVNVHVTNNTNKTVKKIKISVRQYAD
ICLFNTAQYKCPVAMEEADDTVAPSSTFCKVYTLTPFLANNREKRGLALDGKLKHEDTNL
ASSTLLREGANREILGIIVSYKVKVKLVVSRGGLLGDLASSDVAVELPFTLMHPKPKEEP
PHREVPENETPVDTNLIELDTNDDDIVFEDFARQRLKGMKDDKEEEEDGTGSPQLNNR*
>ENSG00000236490|ENST00000417059
MKSLSLLLAVALGLATAVSAGPAVIECWFVEDASGKGLAKRPGALLLRQGPGEPPPRPDL

DPELYLSVHDPAGALQAAFRRYPRGAPAPHCEMSRFVPLPASAKWASGLTPAQNCPRALD
GAWLMVSISSPVLSLSSLLRPQPEPQQEPVLITMATVVLTVLTHTPAPRVRLGQDALLDL
SFAYMPPTSEAASSLAPGPPPFGLEWRRQHLGKGHLLLAATPGLNGQMPAAQEGAVAFAA
WDDDEPWGPWTGNGTFWLPRVQPFQEGTYLATIHLPYLQGQVTLELAVYKPPKVSLMPAT
LARAAPGEAPPELLCLVSHFYPSGGLEVEWELRGGPGGRSQKAEGQRWLSALRHHSDGSV
SLSGHLQPPPVTTEQHGARYACRIHHPSLPASGRSAEVTLEVAGLSGPSLEDSVGLFLSA
FLLLGLFKALGWAAVYLSTCKDSKKKAE*
>ENSG00000204070|ENST00000414310
MQTVYYGSLGLWLALVDGLVRSSPSLDQMFDAEILGFSTPPGRLSMMSFILNALTCALGL
LYFIRRGKQCLDFTVTVHFFHLLGCWFYSSRFPSALTWWLVQAVCIALMAVIGEYLCMRT
ELKEIPLNSAPKSNV*
>ENSG00000117280|ENST00000414729
MGSRDHLFKVLVVGDAAVGKTSLVQRYSQDSFSKHYKSTVGVDFALKVLQWSDYEIVRLQ
LWDIAGQERFTSMTRLYYRDASACVIMFDVTNATTFSNSQRWKQDLDSKLTLPNGEPVPC
LLLANKCDLSPWAVSRDQIDRFSKENGFTGWTETSVKENKNINEAMRVLIEKMMRNSTED
IMSLSTQGDYINLQTKSSSWSCC*
>ENSG00000124333|ENST00000460621
MAILFAVVARGTTILAKHAWCGGNFLEDFERSRAFNFLNEIKKRFQTTYGSRAQTALPYA
MNSEFSSVLAAQLKHHSENKGLDKVMETQAQVDELKGIMVRNIDLVAQRGERLELLIDKT
ENLVDSSVTFKTTSRNLARAMCMKNLKLTIIIIIVSIVFIYIIVSPLCGGFTWPSCVKK*
>ENSG00000101236|ENST00000432261
MLNKSGESRYPALFPVLGGSSMSSDFPHYNFRMPNIGFQNLPLNIYIVVFGTAIFVFILS
LLFCCYLIRLRHQAHKEFYAYKQVILKEKVKELNLHELCAVCLEDFKPRDELGICPCKHA
FHRKCLIKWLEVRKVCPLCNMPVLQLAQLHSKQDRGPPQGPLPGAENIV*
>ENSG00000153130|ENST00000608372
MRRRVFSSQDWRASGWDGMGFFSRRTFCGRSGRSCRGQLVQVSRPEVSAGSLLLPAPQAE
DHSSRILYPRPKSLLPKMMNADMDAVDAENQVELEEKTRLINQVLELQHTLEDLSARVDA
VKEENLKLKSENQVLGQYIENLMSASSVFQTTDTKSKRK*
>ENSG00000100280|ENST00000405198
MTDSKYFTTTKKGEIFELKAELNSDKKEKKKEAVKKVIASMTVGKDVSALFPDVVNCMQT
DNLELKKLVYLYLMNYAKSQPDMAIMAVNTFVKDCEDPNPLIRALAVRTMGCIRVDKITE
YLCEPLRKCLKDEDPYVRKTAAVCVAKLHDINAQLVEDQGFLDTLKDLISDSNPMVVANA
VAALSEIAESHPSSNLLDLNPQSINKLLTALNECTEWGQIFILDCLANYMPKDDREAQSI
CERVTPRLSHANSAVVLSAVKVLMKFMEMLSKDLDYYGTLLKKLAPPLVTLLSAEPELQY
VALRNINLIVQKRPEILKHEMKVFFVKYNDPIYVKLEKLDIMIRLASQANIAQVLAELKE
YATEVDVDFVRKAVRAIGRCAIKVEQSAERCVSTLLDLIQTKVNYVVQEAIVVIKDIFRK
YPNKYESVIATLCENLDSLDEPEARAAMIWIVGEYAERIDNADELLESFLEGFHDESTQV
QLQLLTAIVKLFLKKPTETQELVQQVLSLATQDSDNPDLRDRGYIYWRLLSTDPVAAKEV
VLAEKPLISEETDLIEPTLLDELICYIGTLASVYHKPPSAFVEGGRGVVHKSLPPRTASS
ESAESPETAPTGAPPGEQPDVIPAQGDLLGDLLNLDLGPPVSGPPLATSSVQMGAVDLLG
GGLDSLMGDEPEGIGGTNFVAPPTAAVPANLGAPIGSGLSDLFDLTSGVGTLSGSYVAPK
AVWLPAMKAKGLEISGTFTRQVGSISMDLQLTNKALQVMTDFAIQFNRNSFGLAPAAPLQ
VHAPLSPNQTVEISLPLSTVGSVMKMEPLNNLQVAVKNNIDVFYFSTLYPLHILFVEDGK
MDRQMFLATWKDIPNENEAQFQIRDCPLNAEAASSKLQSSNIFTVAKRNVEGQDMLYQSL

KLTNGIWVLAELRIQPGNPSCTDLELSLKCRAPEVSQHVYQAYETILKN*
>ENSG00000229215|ENST00000552193
MAVMAPRTLLLLLSGALALTQTWAGSHSMRYFFTSVSRPGRGEPRFIAVGYVDDTQFVRF
DSDAASQKMEPRAPWIEQEGPEYWDQETRNMKAHSQTDRANLGTLRGYYNQSEDGSHTIQ
IMYGCDVGPDGRFLRGYRQDAYDGKDYIALNEDLRSWTAADMAAQITKRKWEAVHAAEQR
RVYLEGRCVDGLRRYLENGKETLQRTDPPKTHMTHHPISDHEATLRCWALGFYPAEITLT
WQRDGEDQTQDTELVETRPAGDGTFQKWAAVVVPSGEEQRYTCHVQHEGLPKPLTLRWEL
SSQPTIPIVGIIAGLVLLGAVITGAVVAAVMWRRKSSDRKGGSYTQAASSDSAQGSDVSL
TACKV*
>ENSG00000237216|ENST00000449127
MVVMAPRTLFLLLSGALTLTETWAGSHSMRYFSAAVSRPGRGEPRFIAMGYVDDTQFVRF
DSDSACPRMEPRAPWVEQEGPEYWEEETRNTKAHAQTDRMNLQTLRGYYNQSEASSHTLQ
WMIGCDLGSDGRLLRGYEQYAYDGKDYLALNEDLRSWTAADTAAQISKRKCEAANVAEQR
RAYLEGTCVEWLHRYLENGKEMLQRADPPKTHVTHHPVFDYEATLRCWALGFYPAEIILT
WQRDGEDQTQDVELVETRPAGDGTFQKWAAVVVPSGEEQRYTCHVQHEGLPEPLMLRWKQ
SSLPTIPIMGIVAGLVVLAAVVTGAAVAAVLWRKKSSD*
>ENSG00000070729|ENST00000564448
MLGWVQRVLPQPPGTPRKTKMQEEEEVEPEPEMEAEVEPEPNPEEAETESESMPPEESFK
EEEVAVADPSPQETKEAALTSTISLRAQGAEISEMNSPSRRVLTWLMKGVEKVIPQPVHS
ITEDPAQILGHGSTGDTGCTDEPNEALEAQDTRPGLRLLLWLEQNLERVLPQPPKSSEVW
RDEPAVATAPPGRPQEMGPKLQARETPSLPTPIPLQPKEEPKEAPAPEPQPGSQAQTSSL
PPTRDPARLVAWVLHRLEMALPQPVLHGKIGEQEPDSPGICDVQTISILPGGQVEPDLVL
EEVEPPWEDAHQDVSTSPQGTEVVPAYEEENKAVEKMPRELSRIEEEKEDEEEEEEEEEE
EEEEEVTEVLLDSCVVSQVGVGQSEEDGTRPQSTSDQKLWEEVGEEAKKEAEEKAKEEAE
EVAEEEAEKEPQDWAETKEEPEAEAEAASSGVPATKQHPEVQVEDTDADSCPLMAEENPP
STVLPPPSPAKSDTLIVPSSASGTHRKKLPSEDDEAEELKALSPAESPVVAWSDPTTPKD
TDGQDRAASTASTNSAIINDRLQELVKLFKERTEKVKEKLIDPDVTSDEESPKPSPAKKA
PEPAPDTKPAEAEPVEEEHYCDMLCCKFKHRPWKKYQFPQSIDPLTNLMYVLWLFFVVMA
WNWNCWLIPVRWAFPYQTPDNIHHWLLMDYLCDLIYFLDITVFQTRLQFVRGGDIITDKK
DMRNNYLKSRRFKMDLLSLLPLDFLYLKVGVNPLLRLPRCLKYMAFFEFNSRLESILSKA
YVYRVIRTTAYLLYSLHLNSCLYYWASAYQGLGSTHWVYDGVGNSYIRCYYFAVKTLITI
GGLPDPKTLFEIVFQLLNYFTGVFAFSVMIGQMRDVVGAATAGQTYYRSCMDSTVKYMNF
YKIPKSVQNRVKTWYEYTWHSQGMLDESELMVQLPDKMRLDLAIDVNYNIVSKVALFQGC
DRQMIFDMLKRLRSVVYLPNDYVCKKGEIGREMYIIQAGQVQVLGGPDGKSVLVTLKAGS
VFGEISLLAVGGGNRRTANVVAHGFTNLFILDKKDLNEILVHYPESQKLLRKKARRMLRS
NNKPKEEKSVLILPPRAGTPKLFNAALAMTGKMGGKGAKGGKLAHLRARLKELAALEAAA
KQQELVEQAKSSQDVKGEEGSAAPDQHTHPKEAATDPPAPRTPPEPPGSPPSSPPPASLG
RPEGEEEGPAEPEEHSVRICMSPGPEPGEQILSVKMPEEREEKAE*
>ENSG00000038427|ENST00000502527
MFINIKSILWMCSTLIVTHALHKVKVGKSPPVRGSLSGKVSLPCHFSTMPTLPPSYNTSE
FLRIKWSKIEVDKNGKDLKETTVLVAQNGNIKIGQDYKGRVSVPTHPEAVGDASLTVVKL
LASDAGLYRCDVMYGIEDTQDTVSLTVDGVVFHYRAATSRYTLNFEAAQKACLDVGAVIA
TPEQLFAAYEDGFEQCDAGWLADQTVRYPIRAPRVGCYGDKMGKAGVRTYGFRSPQETYD
VYCYVDHLDGDVFHLTVPSKFTFEEAAKECENQDARLATVGELQAAWRNGFDQCDYGWLS

DASVRHPVTVARAQCGGGLLGVRTLYRFENQTGFPPPDSRFDAYCFKRPDRCKMNPCLNG
GTCYPTETSYVCTCVPGYSGDQCELDFDECHSNPCRNGATCVDGFNTFRCLCLPSYVGAL
CEQDTETCDYGWHKFQGQCYKYFAHRRTWDAAERECRLQGAHLTSILSHEEQMFVNRVGH
DYQWIGLNDKMFEHDFRWTDGSTLQYENWRPNQPDSFFSAGEDCVVIIWHENGQWNDVPC
NYHLTYTCKKGTVACGQPPVVENAKTFGKMKPRYEINSLIRYHCKDGFIQRHLPTIRCLG
NGRWAIPKITCMNPSAYQRTYSMKYFKNSSSAKDNSINTSKHDHRWSRRWQESRR*
>ENSG00000198925|ENST00000409033
MAQFDTEYQRLEASYSDSPPGEEDLLVHVAEGSKSPWHHIENLDLFFSRVYNLHQKNGFT
CMLIGEIFELMQFLFVVAFTTFLVSCVDYDILFANKMVNHSLHPTEPVKVTLPDAFLPAQ
VCSARIQENGSLITILVIAGVFWIHRLIKFIYNICCYWEIHSFYLHALRIPMSALPYCTW
QEVQARIVQTQKEHQICIHKRELTELDIYHRILRFQNYMVALVNKSLLPLRFRLPGLGEA
VFFTRGLKYNFELILFWGPGSLFLNEWSLKAEYKRGGQRLELAQRLSNRILWIGIANFLL
CPLILIWQILYAFFSYAEVLKREPGALGARCWSLYGRCYLRHFNELEHELQSRLNRGYKP
ASKYMNCFLSPLLTLLAKNGAFFAGSILAVLIALTIYDEDVLAVEHVLTTVTLLGVTVTV
CRSFIPDQHMVFCPEQLLRVILAHIHYMPDHWQGVHFGRVAEPHCHTPHPHLLPAPTGPG
DYRLLPKLHRGGRWCGRYLLLCSDGCSPAWSSPVAICWADRGLSVPAS*
>ENSG00000111666|ENST00000552351
MAAGAGAGSAPRWLRALSEPLSAAQLRRLEEHRYSAAGVSLLEPPLQLYWTWLLQWIPLW
MAPNSITLLGLAVNVVTTLVLISYCPTATEEAPYWTYLLCALGLFIYQSLDAIDGKQARR
TNSCSPLGELFDHGCDSLSTVFMAVGASIAARLGTYPDWFFFCSFIGMFVFYCAHWQTYV
SGMLRFGKVDVTEIQIALVIVFVLSAFGGATMWDYTFS*
>ENSG00000204070|ENST00000426004
MAGQFRSYVWDPLLILSQIVLMQTVYYGSLGLWLALVDGLVRSSPSLDQMFDAEILGFST
PPGRLSMMSFILNALT*
>ENSG00000036257|ENST00000409777
MDEKYVNSIWDLLKNAIQEIQRKNNSGLSFEELYRNAYTMVLHKHGEKLYTGLREVVTEH
LINKVREDVLNSLNNNFLQTLNQAWNDHQTAMVMIRDILMYMDRVYVQQNNVENVYNLGL
IIFRDQVVRYGCIRDHLRQTLLDMIARERKGEVVDRGAIRNACQMLMILGLEGRSVYEED
FEAPFLEMSAEFFQMESQKFLAENSASVYIKKVEARINEEIERVMHCLDKSTEEPIVKVV
ERELISKHMKTIVEMENSGLVHMLKNGKTEDLGCMYKLFSRVPNGLKTMCECMSSYLREQ
GKALVSEEGEGKNPVDYIQGLLDLKSRFDRFLLESFNNDRLFKQTIAGDFEYFLNLNSRS
PEYLSLFIDDKLKKGVKGLTEQEVETILDKAMVLFRFMQEKDVFERYYKQHLARRLLTNK
SVSDDSEKNMISKLKTECGCQFTSKLEGMFRDMSISNTTMDEFRQHLQATGVSLGGVDLT
VRVLTTGYWPTQSATPKCNIPPAPRHAFEIFRRFYLAKHSGRQLTLQHHMGSADLNATFY
GPVKKEDGSEVGVGGAQVTGSNTRKHILQVSTFQMTILMLFNNREKYTFEEIQQETDIPE
RELVRALQSLACGKPTQRVLTKEPKSKEIENGHIFTVNDQFTSKLHRVKIQTVAAKQGES
DPERKETRQKVDDDRKHEIEAAIVRIMKSRKKMQHNVLVAEVTQQLKARFLPSPVVIKKR
IEGLIEREYLARTPEDRKVYTYVA*
>ENSG00000283761|ENST00000638779
MFANLKYVSLGILVFQTTSLVLTMRYSRTLKEEGPRYLSSTAVVVAELLKIMACILLVYK
DSKCSLRALNRVLHDEILNKPMETLKLAIPSGIYTLQNNLLYVALSNLDAATYQWPSDSQ
LDSKELSAGSQFVGLMAVLTACFSSGFAGVYFEKILKETKQSVWIRNIQLGFFGSIFGLM
GVYIYDGELVSKNGFFQGYNRLTWIVVVLQALGGLVIAAVIKYADNILKGFATSLSIILS
TLISYFWLQDFVPTRWLYASSSSSCS*

>ENSG00000108587|ENST00000451249
MAAGTSSYWEDLRKQARQLENELDLKLVSFSKLCTSYSHSSTRDGRRDSSDTTPLLNGSS
QDRMFETMAIEIEQLLARLTGVNDKMAEYTNSAGVPSLNAALMHTLQRHRDILQDYTHEF
HKTKANFMAIRERENLMGSVRKDIESYKSGSGVNNRRTELFLKEHDHLRNSDRLIEETIS
IAMATKENMTSQRGMLKSIHSKMNTLANRFPAVNSLIQRINLRKRRDSLILGGVIGICTI
LLLLYAFH*
>ENSG00000127564|ENST00000573944
MPMPTEGTPPPLSGTPIPVPAYFRHAEPGFSLKRPRGLSRSLPPPPAKGSIPISRLFPP
RTPGWHQLQPRRVSFRGEASETLQSPGYDPSRPESFFQQSFQRLSRLGHGSYGEVFKVRS
KEDGRLYAVKRSMSPFRGPKDRARKLAEVGSHEKVGQHPCCVRLEQAWEEGGILYLQTEL
CGPSLQQHCEAWGASLPEAQVWGYLRDTLLALAHLHSQGLVHLDVKPANIFLGPRGRCKL
GDFGLLVELGTAGAGEVQEGDPRYMAPELLQGSYGTAADVFSLGLTILEVACNMELPHGG
EGWQQLRQGYLPPEFTAGLSSELRSVLVMMLEPDPKLRATAEALLALPVLRQPRAWGVLW
CMAAEALSRGWALWQALLALLCWLWHGLAHPASWLQPLGPPATPPGSPPCSLLLDSSLSS
NWDDDSLGPSLSPEAVLARTVGSTSTPRSRCTPRDALDLSDINSEPPRGSFPSFEPRNLL
SLFEDTLDPT*
>ENSG00000237172|ENST00000449549
MRRRLRLRRDALLTLLLGASLGLLLYAQRDGAAPTASAPRGRGRAAPRPTPGPRAFQLPD
AGAAPPAYEGDTPAPPTPTGPFDFARYLRAKDQRRFPLLINQPHKCRGDGAPGGRPDLLI
AVKSVAEDFERRQAVRQTWGAEGRVQGALVRRVFLLGVPRGAGSGGADEVGEGARTHWRA
LLRAESLAYADILLWAFDDTFFNLTLKEIHFLAWASAFCPDVRFVFKGDADVFVNVGNLL
EFLAPRDPAQDLLAGDVIVHARPIRTRASKYYIPEAVYGLPAYPAYAGGGGFVLSGATLH
RLAGACAQVELFPIDDVFLGMCLQRLRLTPEPHPAFRTFGIPQPSAAPHLSTFDPCFYRE
LVVVHGLSAADIWLMWRLLHGPHGPACAHPQPVAAGPFQWDS*
>ENSG00000257594|ENST00000529983
MAVRWTWAGKSCLLLAFLTVAYIFVELLVSTFHASAGAGRARELGSRRLSDLQKNTEDLS
RPLYKKPPADSRALGEWGKASKLQLNEDELKQQEELIERYAINIYLSDRISLHRHIEDKR
MYECKSQKFNYRTLPTTSVIIAFYNEAWSTLLRTIHSVLETSPAVLLKEIILVDDLSDRV
YLKTQLETYISNLDRVRLIRTNKREGLVRARLIGATFATGDVLTFLDCHCECNSGWLEPL
LERIGRDETAVVCPVIDTIDWNTFEFYMQIGEPMIGGFDWRLTFQWHSVPKQERDRRISR
IDPIRSPTMAGGLFAVSKKYFQYLGTYDTGMEVWGGENLELSFRVWQCGGKLEIHPCSHV
GHVFPKRAPYARPNFLQNTARAAEVWMDEYKEHFYNRNPPARKEAYGDISERKLLRERLR
CKSFDWYLKNVFPNLHVPEDRPGWHGAIRSRGISSECLDYNSPDNNPTGANLSLFGCHGQ
GGNQFFEYTSNKEIRFNSVTELCAEVPEQKNYVGMQNCPKDGFPVPANIIWHFKEDGTIF
HPHSGLCLSAYRTPEGRPDVQMRTCDALDKNQIWSFEK*
>ENSG00000115526|ENST00000409046
MHHQWLLLAACFWVIFMFMVASKFITLTFKDPDVYSAKQEFLFLTTMPEVRKLPEEKHIP
EELKPTGKELPDSQLVQPLVYMERLELIRNVCRDDALKNLSHTPVSKFVLDRIFVCDKHK
ILFCQTPKVGNTQWKKVLIVL
>ENSG00000117620|ENST00000639807
MFANLKYVSLGILVFQTTSLVLTMRYSRTLKEEGPRYLSSTAVVVAELLKIMACILLVYK
DSKCSLRALNRVLHDEILNKPMETLKLAIPSGIYTLQNNLLYVALSNLDAATYQWPSDSQ
LDSKELSAGSQFVGLMAVLTACFSSGFAGVYFEKILKETKQSVWIRNIQLGFFGSIFGLM
GVYIYDGELVSKNGFFQGYNRLTWIVVVLQALGGLVIAAVIKYADNILKGFATSLSIILS

TLISYFWLQDFVPTRVSLCRQAGVLWRDLGLLQPPTPWFKGFSCLSL*
>ENSG00000184956|ENST00000421673
MVQRWLLLSCCGALLSAGLANTSYTSPGLQRLKDSPQTAPDKGQCSTWGAGHFSTFDHHV
YDFSGTCNYIFAATCKDAFPTFSVQLRRGPDGSISRIIVELGASVVTVSEAIISVKDIGV
ISLPYTSNGLQITPFGQSVRLVAKQLELELEVVWGPDSHLMVLVERKYMGQMCGLCGNFD
GKVTNEFVSEEGKFLEPHKFAALQKLDDPGEICTFQDIPSTHVRQAQHARICTQLLTLVA
PECSVSKEPFVLSCQADVAAAPQPGPQNSSCATLSEYSRQCSMVGQPVRRWRSPGLCSVG
QCPANQVYQECGSACVKTCSNPQHSCSSSCTFGCFCPEGTVLNDLSNNHTCVPVTQCPCV
LHGAMYAPGEVTIAACQTCRCTLGRWVCTERPCPGHCSLEGGSFVTTFDARPYRFHGTCT
YILLQSPQLPEDGALMAVYDKSGVSHSETSLVAVVYLSRQDKIVISQDEVVTNNGEAKWL
PYKTRNITVFRQTSTHLQMATSFGLELVVQLRPIFQAYVTVGPQFRGQTRGLCGNFNGDT
TDDFTTSMGIAEGTASLFVDSWRAGNCPAALERETDPCSMSQLNKVCAETHCSMLLRTGT
VFERCHATVNPAPFYKRCVYQACNYEETFPHICAALGDYVHACSLRGVLLWGWRSSVDNC
TIPCTGNTTFSYNSQACERTCLSLSDRATECHHSAVPVDGCNCPDGTYLNQKGECVRKAQ
CPCILEGYKFILAEQSTVINGITCHCINGRLSCPQRPQMFLASCQAPKTFKSCSQSSENK
FGAACAPTCQMLATGVACVPTKCEPGCVCAEGLYENADGQCVPPEECPCEFSGVSYPGGA
ELHTDCRTCSCSRGRWACQQGTHCPSTCTLYGEGHVITFDGQRFVFDGNCEYILATDVCG
VNDSQPTFKILTENVICGNSGVTCSRAIKIFLGGLSVVLADRNYTVTGEEPHVQLGVTPG
ALSLVVDISIPGRYNLTLIWNRHMTILIRIARASQDPLCGLCGNFNGNMKDDFETRSRYV
ASSELELVNSWKESPLCGDVSFVTDPCSLNAFRRSWAERKCSVINSQTFATCHSKVYHLP
YYEACVRDACGCDSGGDCECLCDAVAAYAQACLDKGVCVDWRTPAFCPIYCGFYNTHTQD
GHGEYQYTQEANCTWHYQPCLCPSQPQSVPGSNIEGCYNCSQDEYFDHEEGVCVPCMPPT
TPQPPTTPQLPTTGSRPTQVWPMTGTSTTIGLLSSTGPSPSSNHTPASPTQTPLLPATLT
SSKPTASSGEPPRPTTAVTPQATSGLPPTATLRSTATKPTVTQATTRATASTASPATTST
AQSTTRTTMTLPTPATSGTSPTLPKSTNQELPGTTATQTTGPRPTPASTTGPTTPQPGQP
TRPTATETTQTRTTTEYTTPQTPHTTHSPPTAGSPVPSTGPVTATSFHATTTYPTPSHPE
TTLPTHVPPFSTSLVTPSTHTVITPTHAQMATSASNHSAPTGTIPPPTTLKATGSTHTAP
PITPTTSGTSQAHSSFSTNKTPTSLHSHTSSTHHPEVTPTSTTTITPNPTSTRTRTPVAH
TNSATSSRPPPPFTTHSPPTGSSPFSSTGPMTATSFKTTTTYPTPSHPQTTLPTHVPPFS
TSLVTPSTHTVITPTHAQMATSASIHSMPTGTIPPPTTLKATGSTHTAPTMTLTTSGTSQ
ALSSLNTAKTSTSLHSHTSSTHHAEATSTSTTNITPNPTSTGTPPMTVTTSGTSQSRSSF
STAKTSTSLHSHTSSTHHPEVTSTSTTSITPNHTSTGTRTPVAHTTSATSSRLPTPFTTH
SPPTGTTPISSTGPVTATSFQTTTTYPTPSHPHTTLPTHVPSFSTSLVTPSTHTVIIPTH
TQMATSASIHSMPTGTIPPPTTIKATGSTHTAPPMTPTTSGTSQSPSSFSTAKTSTSLPY
HTSSTHHPEVTPTSTTNITPKHTSTGTRTPVAHTTSASSSRLPTPFTTHSPPTGSSPFSS
TGPMTATSFQTTTTYPTPSHPQTTLPTHVPPFSTSLVTPSTHTVIITTHTQMATSASIHS
TPTGTVPPPTTLKATGSTHTAPPMTVTTSGTSQTHSSFSTATASSSFISSSSWLPQNSSS
RPPSSPITTQLPHLSSATTPVSTTNQLSSSFSPSPSAPSTVSSYVPSSHSSPQTSSPSVG
TSSSFVSAPVHSTTLSSGSHSSLSTHPTTASVSASPLFPSSPAASTTIRATLPHTISSPF
TLSALLPISTVTVSPTPSSHLASSTIAFPSTPRTTASTHTAPAFSSQSTTSRSTSLTTRV
PTSGFVSLTSGVTGIPTSPVTNLTTRHPGPTLSPTTRFLTSSLTAHGSTPASAPVSSLGT
PTPTSPGVCSVREQQEEITFKGCMANVTVTRCEGACISAASFNIITQQVDARCSCCRPLH
SYEQQLELPCPDPSTPGRRLVLTLQVFSHCVCSSVACGD*
>ENSG00000177830|ENST00000449825

MRTLFNLLWLALACSPVHTTLSKSDAKKAASKTLLEKSQFSDKPVQDRGLVVTDLKAESV
VLEHRSYCSAKARDRHFAGDVLGYVTPWNSHGYDVTKVFGSKFTQISPVWLQLKRRGREM
FEVTGLHDVDQGWMRAVRKHAKGLHIVPRLLFEDWTYDDFRNVLDSEDEIEELSKTVVQV
AKNQHFDGFVVEVWNQLLSQKRVGLIHMLTHLAEALHQARLLALLVIPPAITPGTDQLGM
FTHKEFEQLAPVLDGFSLMTYDYSTAHQPGPNAPLSWVRACVQVLDPKSKWRSKILLGLN
FYGMDYATSKDAREPVVGARYIQTLKDHRPRMVWDSQASEHFFEYKKSRSGRHVVFYPTL
KSLQVRLELARELGVGVSIWELGQGLDYFYDLL*
>ENSG00000105063|ENST00000412770
MFWKFDLHTSSHLDTLLEREDLSLPELLDEEDVLQECKVVNRKLLDFLLQPPHLQAMVAW
VTQEPPDSGEERLRYKYPSVACEILTSDVPQINDALGADESLLNRLYGFLQSTGSLNPLL
ASFFSKVMGILINRKTDQLVSFLRKKDDFVDLLLQHIGTSAIMDLLLRLLTCVERPQLRQ
DVVNWLNEEKIVQRLIEQIHPSKDENQHSNASQSLCDIIRLSREQMIQVQDSPEPDQLLA
TLEKQETIEQLLSNMFEGEQSQSVIVSGIQVLLTLLEPRRPRSESVTVNSFFSSVDGQLE
LLAQGALESTVSSVGALHALRPRLSCFHQLLLEPPKLEPLQMTWGMLAPPLGNTRLHVVK
LLASALSANDAALTHELLALDVPNTMLDLFFHYVFNNFLHAQVEGCVSTMLSLGPPPDSS
PETPIQNPVVKHLLQQCRLVERILTSWEENDRVQCAGGPRKGYMGHLTRVAGALVQNTEK
GPNAEQLRQLLKELPSEQQEQWEAFVSGPLAETNKKNMVDLVNTHHLHSSSDDEDDRLKE
FNFPEEAVLQQAFMDFQMQRMTSAFIDHFGFNDEEFGEQEESVNAPFDKTANITFSLNAD
DENPNANLLEICYKDRIQQFDDDEEEEDEEEAQGSGESDGEDGAWQGSQLARGARLGQPP
GVRSGGSTDSEDEEEEDEEEEEDEEGIGCAARGGATPLSYPSPGPQPPGPSWTATFDPVP
TDAPTSPRVSGEEELHTGPPAPQGPLSVPQGLPTQSLASPPARDALQLRSQDPTPPSAPQ
EATEGSKVTEPSAPCQALVSIGDLQATFHGIRSAPSSSDSATRDPSTSVPASGAHQPPQT
TEGEKSPEPLGLPQSQSAQALTPPPIPNGSAPEGPASPGSQ*
>ENSG00000117620|ENST00000640600
MFANLKYVSLGILVFQTTSLVLTMRYSRTLKEEGPRYLSSTAVVVAELLKIMACILLVYK
DSKCSLRALNRVLHDEILNKPMETLKLAIPSGIYTLQNNLLYVALSNLDAATYQVTYQLK
ILTTALFSVSMLSKKLGVYQWLSLVILMTGVAFVQWPSDSQLDSKELSAGSQFVGLMAVL
TACFSSGFAGVYFEKILKETKQSVWIRNIQLGFFGSIFGLMGVYIYDGELVSKNGFFQGY
NRLTWIVVVLQALGGLVIAAVIKYADNILKGFATSLSIILSTLISYFWLQDFVPTRVSLC
RQAGVLWRDLGLLQPPTPWFKGFSCLSL*
>ENSG00000117620|ENST00000638338
MFANLKYVSLGILVFQTTSLVLTMRYSRTLKEEGPRYLSSTAVVVAELLKIMACILLVYK
DSKCSLRALNRVLHDEILNKPMETLKLAIPSGIYTLQNNLLYVALSNLDAATYQVTYQLK
ILTTALFSVSMLSKKLGVYQWLSLVILMTGVAFVQWPSDSQLDSKELSAGSQFVGLMAVL
TACFSSGFAGVYFEKILKETKQSVWIRNIQLGFFGSIFGLMGVYIYDGELVSKNGFFQGY
NRLTWIVVVLQSLTLSPGWSAVARSRLTATSHSLVQGILLPQPLSSWDCRREPLRPAYTV
YIFKDCSNRYKNCKK*
>ENSG00000126091|ENST00000530581
MGLLVFVRNLLLALCLFLVLGFLYYSAWKLHLLQWEEDSKYDRLGFLLNLDSKLPAELAT
KYANFSEGACKPGYASALMTAIFPRFSKPAPMFLDDSFRKWARIREFVPPFGIKGQDNLI
KAILSVTKEYRLTPALDSLRCRRCIIVGNGGVLANKSLGSRIDDYDIVVRVHRMASGNLW
PLECPRSPLRFESSTHISSRRPPSPSLACPSTMASWAGGTSLPLAVWQ*
>ENSG00000092108|ENST00000544052
MGITLHLLLHSDRDPIPDVPAVYFVMPTEENIDRMCQDLRNQLYESYYLNFISAISRSKL

EDIANAALAASAVTQVAKVFDQYLNFITLEDDMFVLCNQNKELVSYRAINRPDITDTEME
TVMDTIVDSLFCFFVTLGAVPIIRCSRGTAAEMVAVKLDKKLRENLRDARNSLFTGDTLG
AGQFSFQRPLLVLVDRNIDLATPLHHTWTYQALVHDVLDFHLNRVNLEESSGVENSPAGA
RPKRKNKKSYDLTPVDKFWQKHKGSPFPEVAESVQQELESYRAQEDEVKRLKSIMGLEGE
DEGAISMLSDNTAKLTSAVSSLPELLEKKRLIDLHTNVATAVLEHIKARKLDVYFEYEEK
IMSKTTLDKSLLDIISDPDAGTPEDKMRLFLIYYISTQQAPSEADLEQYKKALTDAGCNL
NPLQYIKQWKAFTKMASAPASYGSTTTKPMGLLSRVMNTGSQFVMEGVKNLVLKQQNLPV
TRILDNLMEMKSNPETDDYRYFDPKMLRGNDSSVPRNKNPFQEAIVFVVGGGNYIEYQNL
VDYIKGKQGKHILYGCSELFNATQFIKQLSQLGQK*
>ENSG00000235680|ENST00000428952
MVVMAPRTLFLLLSGALTLTETWAGSHSMRYFSAAVSRPGRGEPRFIAMGYVDDTQFVRF
DSDSACPRMEPRAPWVEQEGPEYWEEETRNTKAHAQTDRMNLQTLRGYYNQSEASSHTLQ
WMIGCDLGSDGRLLRGYEQYAYDGKDYLALNEDLRSWTAADTAAQISKRKCEAANVAEQR
RAYLEGTCVEWLHRYLENGKEMLQRADPPKTHVTHHPVFDYEATLRCWALGFYPAEIILT
WQRDGEDQTQDVELVETRPAGDGTFQKWAAVVVPSGEEQRYTCHVQHEGLPEPLMLRWKQ
SSLPTIPIMGIVAGLVVLAAVVTGAAVAAVLWRKKSSD*
>ENSG00000102100|ENST00000635285
MAAVGAGGSTAAPGPGAVSAGALEPGTASAAHRRLKYISLAVLVVQNASLILSIRYARTL
PGDRFFATTAVVMAEVLKGLTCLLLLFAQKRGNVKHLVLFLHEAVLVQYVDTLKLAVPSL
IYTLQNNLQYVAISNLPAATFQVTYQLKILTTALFSVLMLNRSLSRLQWASLLLLFTGVA
IVQAQQAGGGGPRPLDQNPGAGLAAVVASCLSSGFAGVYFEKILKGSSGSVWLRNLQLGL
FGTALGLVGLWWAEGTAVATRGFFFGYTPAVWGVVLNQAFGGLLVAVVVKYADNILKGFA
TSLSIVLSTVASIRLFGFHVDPLFALGAGLVIGAVYLYSLPRGAAKAIASASASASGPCV
HQQPPGQPPPPQLSSHRGDLITEPFLPKSVLVK*
>ENSG00000110697|ENST00000534749
MLIKEYHILLPMSLDEYQVAQLYMIQKKSREESSGEGSGVEILANRPYTDGPGGSGQYTH
KVYHVGSHIPGWFRALLPKAALQVEEESWNAYPYTRTRYTCPFVEKFSIEIETYYLPDGG
QQPNVFNLSGAERRQRILDTIDIVRDAVAPGEYKAEEDPRLYHSVKTGRGPLSDDWARTA
AQTGPLMCAYKLCKVEFRYWGMQAKIEQFIHDVGLRRVMLRAHRQAWCWQDEWTELSMAD
IRALEEETARMLAQRMAKCNTGSEGSEAQPPGKPSTEARSAASNTGTPDGPEAPPGPDAS
PDASFGKQWSSSSRSSYSSQHGGAVSPQSLSEWRMQNIARDSENSSEEEFFDAHEGFSDS
EEVFPKEMTKWNSNDFIDAFASPVEAEGTPEPGAEAAKGIEDGAQAPRDSEGLDGAGELG
AEACAVHALFLILHSGNILDSGPGDANSKQADVQTLSSAFEAVTRIHFPEALGHVALRLV
PCPPICAAAYALVSNLSPYSHDGDSLSRSQDHIPLAALPLLATSSSRYQGAVATVIARTN
QAYSAFLRSPEGAGFCGQVALIGDGVGGILGFDALCHSANAGTGSRGSSRRGSMNNELLS
PEFGPVRDPLADGVEGLGRGSPEPSALPPQRIPSDMASPEPEGSQNSLQAAPATTSSWEP
RRASTAFCPPAASSEAPDGPSSTARLDFKVSGFFLFGSPLGLVLALRKTVMPALEAAQMR
PACEQIYNLFHAADPCASRLEPLLAPKFQAIAPLTVPRYQKFPLGDGSSLLLADTLQTHS
SLFLEELEMLVPSTPTSTSGAFWKGSELATDPPAQPAAPSTTSEVVKILERWWGTKRIDY
SLYCPEALTAFPTVTLPHLFHASYWESADVVAFILRQVIEKERPQLAECEEPSIYSPAFP
REKWQRKRTQVKIRNVTSNHRASDTVVCEGRPQVLSGRFMYGPLDVVTLTGEKVDVYIMT
QPLSGKWIHFGTEVTNSSGRLTFPVPPERALGIGVYPVRMVVRGDHTYAECCLTVVARGT
EAVVFSIDGSFTASVSIMGSDPKVRAGAVDVVRHWQDSGYLIVYVTGRPDMQKHRVVAWL
SQHNFPHGVVSFCDGLTHDPLRQKAMFLQSLVQEVELNIVAGYGSPKDVAVYAALGLSPS

QTYIVGRAVRKLQAQCQFLSDGYVAHLGQLEAGSHSHASSGPPRAALGKSSYGVAAPVDF
LRKQSQLLRSRGPSQAEREGPGTPPTTLARGKARSISLKLDSEE*
>ENSG00000171302|ENST00000591773
MPVQLSEHPEWNESMHSLRISVGGLPVLASMTKAADPRFRPRWKVILTFFVGAAILWLLC
SHRPAPGRPPTHNAHNWRLGQAPANWYNDTYPLSPPQRTPAGIRYRIAVIADLDTESRAQ
EENTWFSYLKKGYLTLSDSGDKVAVEWDKDHGVLESHLAEKGRGMELSDLIVFNGKLYSV
DDRTGVVYQIEGSKAVPWVILSDGDGTVEKGFKAEWLAVKDERLYVGGLGKEWTTTTGDV
VNENPEWVKVVGYKGSVDHENWVSNYNALRAAAGIQPPGYLIHESACWSDTLQRWFFLPR
RASQERYSEKDDERKGANLLLSASPDFGDIAVSHVGAVVPTHGFSSFKFIPNTDDQIIVA
LKSEEDSGRVASYIMAFTLDGRFLLPETKIGSVKYEGIEFI*
>ENSG00000100060|ENST00000416983
MQCRLPRGLAGALLTLLCMGLLCLRYHLNLSPQRVQGTPELSQPNPGPPKLQLHDVFIAV
KTTRAFHRLRLELLLDTWVSRTREQVTRSHLVVTNCSAEHSHPALSCKMAAEFDTFLASG
LRWFCHVDDDNYVNPRALLQLLRAFPLARDVYVGRPSLNRPIHASEPQPHNRTRLVQFWF
ATGGAGFCINRKLALKMAPWASGSRFMDTSALIRLPDDCTMGYIIECKLGGRLQPSPLFH
SHLETLQLLRTAQLPEQVTLSYGVFEGKLNVIKLQGPFSPEEDPSRFRSLHCLLYPDTPW
CPQLGAR*
>ENSG00000166377|ENST00000586722
MADQIPLYPVRSAAAAAANRKRAAYYSAAGPRPGADRHSRYQLEDESAHLDEMPLMMSEE
GFENEESDYHTLPRARIMQRKRGLEWFVCDGWKFLCTSCCGWLINICRRKKELKARTVWL
GCPEKCEEKHPRNSIKNQKYNVFTFIPGVLYEQFKFFLNLYFLVISCSQFVPALKIGYLY
TYWAPLMT*
>ENSG00000235915|ENST00000411490
MLSRNDDICIYGGLGLGGLLLLAVVLLSACLCWLHRRVKRLERSWAQGSSEQELHYASLQ
RLPVPSSEGPDLRGRDKRGTKEDPRADYACIAENKPT*
>ENSG00000105443|ENST00000641098
MEDGVYEPPDLTPEERMELENIRRRKQELLVEIQRLREELSEAMSEVEGLEANEGSKTLQ
RNRKMAMGRKKFNMDPKKGIQFLVENELLQNTPEEIARFLYKGEGLNKTAIGDYLGEREE
LNLAVLHAFVDLHEFTDLNLVQALRQFLWSFRLPGEAQKIDRMMEAFAQRYCLCNPGVFQ
STDTCYVLSFAVIMLNTSLHNPNVRDKPGLERFVAMNRGINEGGDLPEELLRNLYDSIRN
EPFKIPEDDGNDLTHTFFNPDREGWLLKLGGGRVKTWKRRWFILTDNCLYYFEYTTDKEP
RGIIPLENLSIREVDDPRKPNCFELYIPNNKGQLIKACKTEADGRVVEGNHMVYRISAPT
QEEKDEWIKSIQAAVSVDPFYEMLAARKKRISVKKKQEQP*
>ENSG00000148396|ENST00000453963
ARVRDPRTYDRRYWCDAEYDAYRREHSAFGDRPEKRDNNWRYDPRFTGSFDDDPDPHRDP
YGEEVDRRSVHSEHSARSLHSAHSLASRRSSLSSHSHQSQIYRSHNVAAGSYEAPLPPGS
FHGDFAYGTYRSNFSSGPGFPEYGYPADTVWPAMEQVSSRPTSPEKFSVPHVCARFGPGG
QLIKVIPNLPSEGQPALVEVHSMEALLQHTSEQEEMRAFPGPLAKDDTHKVDVINFAQNK
AMKCLQNENLIDKESASLLWNFIVLLCRQNGTVVGTDIAELLLRDHRTVWLPGKSPNEAN
LIDFTNEAVEQVEEEESGEAQLSFLTGGPAAAASSLERETERFRELLLYGRKKDALESAM
KNGLWGHALLLASKMDSRTHARVMTRFANSLPINDPLQTVYQLMSGRMPAASTCCGDEKW
GDWRPHLAMVLSNLNNNMDVESRTMATMGDTLASRGLLDAAHFCYLMAQAGFGVYTKKTT
KLVLIGSNHSLPFLKFATNEAIQRTEAYEYAQSLGAETCPLPSFQVFKFIYSCRLAEMGL
ATQAFHYCEAIAKSILTQPHLYSPVLISQLVQMASQLRLFDPQLKEKPEEESLAAPTWLV

HLQQVERQIKEGAGVWHQDGALPQQCPGTPSSEMEQLDRPGLSQPGALGIANPLLAVPAP
SPEHSSPSVRLLPSAPQTLPDGPLASPARVPMFPVPLPPGPLEPGPGCVTPGPALGFLEP
SGPGLPPGVPPLQERRHLLQEARSPDPGIVPQEAPVGNSLSELSEENFDGKFANLTPSRT
VPDSEAPPGWDRADSGPTQPPLSLSPAPETKRPGQAAKKETKEPKKGESWFFRWLPGKKK
TEAYLPDDKNKSIVWDEKKNQWVNLNEPEEEKKAPPPPPTSMPKTVQAAPPALPGPPGAP
VNMYSRRAAGTRARYVDVLNPSGTQRSEPALAPADFVAPLAPLPIPSNLFVPTPDAEEPQ
LPDGTGREGPAAARGLANPEPAPEPKVLSSAASLPGSELPSSRPEGSQGGEAPGDLPAAG
GPPSGAMPFYNPAQLAQACATSGSSRLGRIGQRKHLVLN*
>ENSG00000109113|ENST00000450529
MNILAPVRRDRVLAELPQCLRKEAALHGHKDFHPRVTCACQEHRTGTVGFKISKVIVVGD
LSVGKTCLINRFCKDTFDKNYKATIGVDFEMERFEVLGIPFSLQLWDTAGQERFKCIAST
YYRGAQAIIIVFNLNDVASLEHTKQWLADALKENDPSSVLLFLTPAQYALMEKDALQVAQ
EMKAEYWAVSSLTGENVREFFFRVAALTFEANVLAELEKSGARRIGDVVRINSDDSNLYL
TASKKKPTCCP*
>ENSG00000128242|ENST00000406955
MLPPQKKPWESMAKGLVLGALFTSFLLLVYSYAVPPLHAGLASTTPEAAASCSPPALEPE
AVIRANGSAGECQPRRNIVFLKTHKTASSTLLNILFRFGQKHRLKFAFPNGRNDFDYPTF
FARSLVQDYRPGACFNIICNHMRFHYDEVRGLVPTNAIFITVLRDPARLFESSFHYFGPV
VPLTWKLSAGDKLTEFLQDPDRYYDPNGFNAHYLRNLLFFDLGYDNSLDPSSPQVQEHIL
EVERRFHLVLLQEYFDESLVLLKDLLCWELEDVLYFKLNARRDSPVPRLSGELYGRATAW
NMLDSHLYRHFNASFWRKVEAFGRERMAREVAALRHANERMRTICIDGGHAVDAAAIQDE
AMQPWQPLGTKSILGYNLKKSIGQRHAQLCRRMLTPEIQYLMDLGANLWVTKLWKFIRDF
LRW*
>ENSG00000114650|ENST00000416208
MTLTERLREKISRAFYNHGLLCASYPIPIILFTGFCILACCYPLLKLPLPGTGPVEFTTP
VKDYSPPPVDSDRKQGEPTEQPEWA*
>ENSG00000104915|ENST00000587230
MSLEDPFFVVRGEVQKAVNTARGLYQRWCELLQESAAVGREELDWTTNELRNGLRSIEWD
LEDLEETIGIVEANPGKFKLPAGDLQERKVFVERMREAVQEMKDHMVSPTAVAFLERNNR
EILAGKPAAQKSPSDLLDASAVSATSRYIEEQQATQQLIMDEQDQQLEMVSGSIQVLKHM
SGRVGEELDEQGIMLDAFAQEMDHTQSRMDGVLRKLAKVSHMTSDRRQWCAIAVLVGVLL
LVLILLFSL*
>ENSG00000140479|ENST00000611716
MPPRAPPAPGPRPPPRAAAATDTAAGAGGAGGAGGAGGPGFRPLAPRPWRWLLLLALPAA
CSAPPPRPVYTNHWAVQVLGGPAEADRVAAAHGYLNLGQIGNLEDYYHFYHSKTFKRSTL
SSRGPHTFLRMDPQVKWLQQQEVKRRVKRQVRSDPQALYFNDPIWSNMWYLHCGDKNSRC
RSEMNVQAAWKRGYTGKNVVVTILDDGIERNHPDLAPNYDSYASYDVNGNDYDPSPRYDA
SNENKHGTRCAGEVAASANNSYCIVGIAYNAKIGGIRMLDGDVTDVVEAKSLGIRPNYID
IYSASWGPDDDGKTVDGPGRLAKQAFEYGIKKGRQGLGSIFVWASGNGGREGDYCSCDGY
TNSIYTISVSSATENGYKPWYLEECASTLATTYSSGAFYERKIVTTDLRQRCTDGHTGTS
VSAPMVAGIIALALEANSQLTWRDVQHLLVKTSRPAHLKASDWKVNGAGHKVSHFYGFGL
VDAEALVVEAKKWTAVPSQHMCVAASDKRPRSIPLVQVLRTTALTSACAEHSDQRVVYLE
HVVVRTSISHPRRGDLQIYLVSPSGTKSQLLAKRLLDLSNEGFTNWEFMTVHCWGEKAEG
QWTLEIQDLPSQVRNPEKQGKLKEWSLILYGTAEHPYHTFSAHQSRSRMLELSAPELEPP

KAALSPSQVEVPEDEEDYTAQSTPGSANILQTSVCHPECGDKGCDGPNADQCLNCVHFSL
GSVKTSRKCVSVCPLGYFGDTAARRCRRCHKGCETCSSRAATQCLSCRRGFYHHQEMNTC
VTLCPAGFYADESQKNCLKCHPSCKKCVDEPEKCTVCKEGFSLARGSCIPDCEPGTYFDS
ELIRCGECHHTCGTCVGPGREECIHCAKNFHFHDWKCVPACGEGFYPEEMPGLPHKVCRR
CDENCLSCAGSSRNCSRCKTGFTQLGTSCITNHTCSNADETFCEMVKSNRLCERKLFIQF
CCRTCLLAG*
>ENSG00000280495|ENST00000629713
MAIFSVYVVNKAGGLIYQLDSYAPRAEAEKTFSYPLDLLLKLHDERVLVAFGQRDGIRVG
HAVLAINGMDVNGRYTADGKEMLETDTFKLHCYQTLTGIKFVVLADPRQAGIDSLLRKIY
EIYSDFALKNPFYSLEMPIRCELFDQNLKLALEVAEKAGTFGPGS*
>ENSG00000110080|ENST00000534083
MVSKSRWKLLAMLALVLVVMVWYSISREDRYIELFYFPIPEKKEPCLQGEAESKASKLFG
NYSRDQPIFLRLEDYFWVKTPSAYELPYGTKGSEDLLLRVLAITSSSIPKNIQSLRCRRC
VVVGNGHRLRNSSLGDAINKYDVVIRLNNAPVAGYEGDVGSKTTMRLFYPESAHFDPKVE
NNPDTLLVLVAFKAMDFHWIETILSDKKRVRKGFWKQPPLIWDVNPKQIRILNPFFMEIA
ADKLLSLPMQQPRKIKQKPTTGLLAITLALHLCDLVHIAGFGYPDAYNKKQTIHYYEQIT
LKSMAGSGHNVSQEALAIKRMLEMGAIKNLTSF*
>ENSG00000085063|ENST00000437761
MGIQGGSVLFGLLLVLAVFCHSGHSLQCYNCPNPTADCKTAVNCSSDFDACLITKAGLQV
YNKCWKFEHCNFNDVTTRLRENELTYYCCKKDLCNFNEQLENGGTSLSEKTVLLLVTPFL
AAAWSLHP*
>ENSG00000156413|ENST00000527106
MDPLGPAKPQWSWRCCLTTLLFQLLMAVCFFSYLRVSQDDPTVYPNGSRFPDSTGTPAHS
IPLILLWTWPFNKPIALPRCSEMVPGTADCNITADRKVYPQADAVIVHHREVMYNPSAQL
PRSPRRQGQRWIWFSMESPSHCWQLKAMDGYFNLTMSYRSDSDIFTPYGWLEPWSGQPAH
PPLNLSAKTELVAWAVSNWGPNSARVRYYQSLQAHLKVDVYGRSHKPLPQGTMMETLSRY
KFYLAFENSLHPDYITEKLWRNALEAWAVPVVLGPSRSNYERFLPPDAFIHVDDFQSPKD
LARYLQELDKDHARYLSYFRWRETLRPRSFSWALAFCKACWKLQEESRYQTRGIAAWFT*
>ENSG00000095139|ENST00000534182
MVLLAAAVCTKAGKAIVSRQFVEMTRTRIEGLLAAFPKLMNTGKQHTFVETESIRMSSVL
FPYQFIPSSLCQVVFLSGPVLPV*
>ENSG00000174695|ENST00000502346
MSAIFNFQSLLTVILLLICTCAYIRSLAPSLLDRNKTGLLGIFWKCARIGERKSPYVAVC
CIVMAFSILFIQ*
>ENSG00000107036|ENST00000418622
MYFLSGWPKRLLCPLGSPAEAPFHVQSDPQRAFFAVLAAARLSIWYSRPSVLIVTYKEPA
KSSTQFGSYKQAEWRPDSTMIAVSTANGYILFFHITSTRGDKYLYEPVYPKGSPQMKGTP
HFKEEQCAPALNLEMRKILDLQAPIMSLQSVLEDLLVATSDGLLHLIHWEGMTNGRKAIN
LCTVPFSVDLQSSRVGSFLGFTDVHIRDMEYCATLDGFAVVFNDGKVGFITPVSSRFTAE
QLHGVWPQDVVDGTCVAVNNKYRLMAFGCVSGSVQVYTIDNSTGAMLLSHKLELTAKQYP
DIWNKTGAVKLMRWSPDNSVVIVTWEYGGLSLWSVFGAQLICTLGGDFAYRSDGTKKDPL
KINSMSWGAEGYHLWVISGFGSQNTEIESDLRSVVKQPSILLFQFIKSVLTVNPCMSNQE
QVLLQGEDRLYLNCGEASQTQNPRSSSTHSEHKPSREKSPFADGGLESQGLSTLLGHRHW
HVVQISSTYLESNWPIREQNMIVTGGLAWWNDFMVLACYNINDRQEELRVYLRTSNLDNA

FAHVTKAQAETLLLSVFQDMVIVFRADCSICLYSIERKSDGPNTTAGIQVLQEVSMSRYI
PHPFLVVSVTLTSVSTENGITLKMPQQARGAESIMLNLAGQLIMMQRDRSGPQIREKDSN
PNNQRKLLPFCPPVVLAQSVENVWTTCRANKQKRHLLEALWLSCGGAGMKVWLPLFPRDH
RKPHSFLSQRIMLPFHINIYPLAVLFEDALVLGAVNDTLLYDSLYTRNNAREQLEVLFPF
CVVERTSQIYLHHILRQLLVRNLGEQALLLAQSCATLPYFPHVLELMLHEVLEEEATSRE
PIPDPLLPTVAKFITEFPLFLQTVVHCARKTEYALWNYLFAAVGNPKDLFEECLMAQDLD
TAASYLIILQNMEVPAVSRQHATLLFNTALEQGKWDLCRHMIRFLKAIGSGESETPPSTP
TAQEPSSSGGFEFFRNRSISLSQSAENVPASKFSLQKTLSMPSGPSGKRWSKDSDCAENM
YIDMMLWRHARRLLEDVRLKDLGCFAAQLGFELISWLCKERTRAARVDNFVIALKRLHKD
FLWPLPIIPASSISSPFKNGKYRTVGEQLLKSQSADPFLNLEMDAGISNIQRSQSWLSNI
GPTHHEIDTASSHGPQMQDAFLSPLSNKGDECSIGSATDLTESSSMVDGDWTMVDENFST
LSLTQSELEHISMELASKGPHKSQVQLRYLLHIFMEAGCLDWCIVIGLILRESSIINQIL
VITQSSEVDGEMLQNIKTGLHAVDRWASTDCPGYKPFLNIIKPQLQKLSEITEEQVQPDA
FQPITMGKTPEQTSPRAEESRGSSSHGSIPQGEVGSSNMVSRKEEDTAQAEEEEPFQDGT
YDCSVS*
>ENSG00000137497|ENST00000545721
XELLRAQRELGELIPLRQKVAEQERTAQQLRAEKASYAEQLSMLKKAHGLLAEENRGLGE
RANLGRQFLEVELDQAREKYVQELAAVRADAETRLAEVQREAQSTARELEVMTAKYEGAK
VKVLEERQRFQEERQKLTAQVEELSKKLADSDQASKVQQQKLKAVQAQGGESQQEAQRLQ
AQLNELQAQLSQKEQAAEHYKLQMEKAKTHYDAKKQQNQELQEQLRSLEQLQKENKELRA
EAERLGHELQQAGLKTKEAEQTCRHLTAQVRSLEAQVAHADQQLRDLGKFQVATDALKSR
EPQAKPQLDLSIDSLDLSCEEGTPLSITRSGGSLPPYVCLWSACCLSGCILVR*
>ENSG00000119414|ENST00000451402
MAPLDLDKYVEIARLCKYLPENDLKVSPICGLAPSGCGAPAGRPFLSPGPPPVFHFLRFL
KERLCDYVCDLLLEESNVQPVSTPVTVCGDIHGQFYDLCELFRTGGQVPDTNYIFMGDFV
DRGYYSLETFTYLLALKAKWPDRITLLRGNHESRQITQVYGFYDECQTKYGNANAWRYCT
KVFDMLTVAALIDEQILCVHGGLSPDIKTLDQIRTIERNQEIPHKGAFCDLVWSDPEDVD
TWAISPRGAGWLFGAKVTNEFVHINNLKLICRAHQLVHEGYKFMFDEKLVTVWSAPNYCY
RCGNIASIMVFKDVNTREPKLFRAVPDSERVIPPRTTTPYFL*
>ENSG00000275444|ENST00000617136
MRVLYLLFSFLFIFLMPLPGVFGGIGDPVTCLKSGAICHPVFCPRRYKQIGTCGLPGTKC
CKKP*
>ENSG00000230791|ENST00000447391
MLSRNDVKRLERSWAQGSSEQELHYASLQRLPVPSSEGPDLRGRDKRGTKEDPRADYACI
AENKPT*
>ENSG00000234514|ENST00000420785
MLSRNDDICIYGGLGLGGLLLLAVVLLSACLCWLHRRAPSVLVPGPGLLRAGTPLCISAE
AASAQQ*
>ENSG00000158089|ENST00000406653
MSFPMSLARSSVPFADSGLSSSQPSDADWDDLWDQFDERRYLNAKKWRVGDDPYKLYAFN
QRESERISSNRAIPDTRHLRCTLLVYCTDLPPTSIIITFHNEARSTLLRTIRSVLNRTPT
HLIREIILVDDFSNDPDDCKQLIKLPKVKCLRNNERQGLVRSRIRGADIAQGTTLTFLDS
HCEVNRDWLQPLLHRVKEDYTRVVCPVIDIINLDTFTYIESASELRGGFDWSLHFQWEQL
SPEQKARRLDPTEPIRTPIIAGGLFVIDKAWFDYLGKYDMDMDIWGGENFEISFRVWMCG

GSLEIVPCSRVGHVFRKKHPYVFPDGNANTYIKNTKRTAEVWMDEYKQYYYAARPFALER
PFGNVESRLDLRKNLRCQSFKWYLENIYPELSIPKESSIQKGNIRQRQKCLESQRQNNQE
TPNLKLSPCAKVKGEDAKSQVWAFTYTQQILQEELCLSVITLFPGAPVVLVLCKNGDDRQ
QWTKTGSHIEHIASHLCLDTDMFGDGTENGKEIVVNPCESSLMSQHWDMVSS*
>ENSG00000228964|ENST00000421349
MRVTAPRTLLLLLWGAVALTETWAGSHSMRYFYTAMSRPGRGEPRFITVGYVDDTLFVRF
DSDATSPRKEPRAPWIEQEGPEYWDRETQISKTNTQTYRENLRTALRYYNQSEAGSHIIQ
RMYGCDVGPDGRLLRGYDQDAYDGKDYIALNEDLSSWTAADTAAQITQRKWEAARVAEQD
RAYLEGLCVESLRRYLENGKETLQRADPPKTHVTHHPISDHEVTLRCWALGFYPAEITLT
WQRDGEDQTQDTELVETRPAGDRTFQKWAAVVVPSGEEQRYTCHVQHEGLPKPLTLRWEP
SSQSTVPIVGIVAGLAVLAVVVIGAVVAAVMCRRKSSGGKGGSYSQAACSDSAQGSDVSL
TA*
>ENSG00000276600|ENST00000617070
MNPRKKVDLKLIIVGAIGVGKTSLLHQYVHKTFYEEYQTTLGASILSKIIILGDTTLKLQ
IWDTGGQERFRSMVSTFYKGSDGCILAFDVTDLESFEALDIWRGDVLAKIVPMEQSYPMV
LLGNKIDLADRKVPQEVAQGWCREKDIPYFEVSAKNDINVVQAFEMLASRALSRYQSILE
NHLTESIKLSPDQSRSRCC*
>ENSG00000206560|ENST00000624145
MVKLLLSRGANINAFDKKDRRAIHWAAYMGHIEVVKLLVSHGAEVTCKDKKSYTPLHAAA
SSGMISVVKYLLDLGVDMNEPNAYGNTPLHVACYNGQDVVVNELIDCGAIVNQKNEKGFT
PLHFAAASTHGALCLELLVGNGADVNMKSKDGKTPLHMTALHGRFSRSQTIIQSGAVIDC
EDKNGNTPLHIAARYGHELLINTLITSGADTAKRGIHGMFPLHLAALSGFSDCCRKLLSS
GFDIDTPDDFGRTCLHAAAAGGNLECLNLLLNTGADFNKKDKFGRSPLHYAAANCNYQCL
FALVGSGASVNDLDERGCTPLHYAATSDTDGKCLEYLLRNDANPGIRDKQGYNAVHYSAA
YGHRLCLQLIASETPLDVLMETSGTDMLSDSDNRATISPLHLAAYHGHHQALEVLVQSLL
DLDVRNSSGRTPLDLAAFKGHVECVDVLINQGASILVKDYILKRTPIHAAATNGHSECLR
LLIGNAEPQNAVDIQDGNGQTPLMLSVLNGHTDCVYSLLNKGANVDAKDKWGRTALHRGA
VTGHEECVDALLQHGAKCLLRDSRGRTPIHLSAACGHIGVLGALLQSAASMDANPATADN
HGYTALHWACYNGHETCVELLLEQEVFQKTEGNAFSPLHCAVINDNEGAAEMLIDTLGAS
IVNATDSKGRTPLHAAAFTDHVECLQLLLSHNAQVNSVDSTGKTPLMMAAENGQTNTVEM
LVSSASAELTLQDNSKNTALHLACSKGHETSALLILEKITDRNLINATNAALQTPLHVAA
RNGLTMVVQELLGKGASVLAVDENGYTPALACAPNKDVADCLALILATMMPVSSSSPLSS
LTFNAINRYTNTSKTVSFEALPIMRNEPSSYCSFNNIGGEQEYLYTDVDELNDSDSETY*
>ENSG00000103502|ENST00000566113
MPDENIFLFVPNLIGTRFGAMLDMLTDRCSTMCLLVNLALLYPGATLFFQISMSLDVASH
WLHLHSSVVRGSESHKMIDLSGNPVLRIYYTSRPALFTLCAGNELFYCLLYLFHFSEGPL
VGSVGLFRMGLWVTAPIALLKSLISVIHLITAARNMAALDAADRAKKK*
>ENSG00000112309|ENST00000615536
MKSALFTRFFILLPWILIVIIMLDVDTRRPVPPLTPRPYFSPYAVGRGGARLPLRRGGPA
HGTQKRNQSRPQPQPEPQLPTIYAITPTYSRPVQKAELTRLANTFRQVAQLHWILVEDAA
ARSELMRTTRKVSVWPVGLVGGRRYERPLVENGKVVGWYTGWRADRPFAIDMAGFAVSLQ
VILSNPKAVFKRRGSQPGMQESDFLKQITTVEELEPKANNCTKVLVWHTRTEKVNLANEP
KYHLDTVKIEV*
>ENSG00000186318|ENST00000510630

MVPFIYLQAHFTLCSGWSSTYRDLRKGVYVPYTQGKWEGELGTDLVSIPHGPNVTVRANI
AAITESDKFFINGSNWEGILGLAYAEIARLCGAGFPLNQSEVLASVGGSMIIGGIDHSLY
TGSLWYTPIRREWYYEVIIVRVEINGQDLKMDCKEYNYDKSIVDSGTTNLRLPKKVFEAA
VKSIKAASSTEKFPDGFWLGEQLVCWQAGTTPWNIFPVISLYLMGEVTNQSFRITILPQQ
YLRPVEDVATSQDDCYKFAISQSSTGTVMGAVIMEGFYVVFDRARKRIGFAVSACHVHDE
FRTAAVEGPFVTLDMEDCGYNIPQTDESTLMTIAYVMAAICALFMLPLCLMVCQWRCLRC
LRQQHDDFADDISLLK*
>ENSG00000109083|ENST00000579419
MAKDILGEAGLHFDELNKLRVLDPEVTQQTIELKEECKDFVDKIGQFQKIVGGLIELVDQ
LAKEAENEKMKAIGARNLLKSIAKQREAQQQQLQALIAEKKMQLERNELKISLL*
>ENSG00000115652|ENST00000409501
MVSKALLRLVSAVNRRRMKLLLGIALLAYVASVWGNFVNMRSIQENGELKIESKIEEMVE
PLREKIRDLEKSFTQKYPPVKFLSEKDRKRILITGGAGFVGSHLTDKLMMDGHEVTVVDN
FFTGRKRNVEHWIGHENFELINHDVVEPLYIEVDQIYHLASPASPPNYMYNPIKTLKTNT
IGTLNMLGLAKRVGARLLLASTSEVYGDPEVHPQSEDYWGHVNPIGPRACYDEGKRVAET
MCYAYMKQEGVEVRVARIFNTFGPRMHMNDGRVVSNFILQALQGEPLTVYGSGSQTRAFQ
YVSDLVNGLVALMNSNVSSPVNLGNPEEHTILEFAQLIKNLVGSGSEIQFLSEAQDDPQK
RKPDIKKAKLMLGWEPVVPLEEGLNKAIHYFRKELEYQANNQYIPKPKPARIKKGRTRHS
*
>ENSG00000174473|ENST00000508122
MRAKFRAGAGHQRNPSISADHGVHELVYQTFPLGLGDGQFYSWTDGLRRKDWHDYESIQK
EAMRSGKGEHGKPYPLTEEDHDDSAYRENGFNIFVSNNIALERSLPDIRHANCKHKMYLE
RLPNTSIIIPFHNEGWTSLLRTIHSIINRTPGSLIAEIILVDDFSEREHLKDKLEEYMAR
FSKVRIVRTKKREGLIRTRLLGASMARGEVLTFLDSHCEVNVNWLPPLLNQIALNHKTIV
CPMIDVIDHNHFGYEAQAGDAMRGAFDWEMYYKRIPIPPELQRADPSDPFESPVMAGGLF
AVDRKWFWELGGYDPGLEIWGGEQYEISFKVWMCGGEMFDVPCSRVGHIYRKYVPYKVPS
GTSLARNLKRVAETWMDEFAEYIYQRRPEYRHLSTGDISAQKELRKQLKCKDFKWFMAAV
AWDVPKYYPPVEPPPAAWGEIRNVAANLCVDSKHGATGTELRLDICVKDGSERTWSHEQL
FTFGWREDIRPGEPLHTRKFCFDAISHNSPVTLYDCHGMKGNQLWGYRKDRTLFHPVSNS
CMDCNPAEKKIFMARCDPLSETQQWIFEHINMTVLEKFNHHANS*
>ENSG00000060566|ENST00000602147
MNTDLAAGKMASAACSMDPIDSFELLDLLFDRQDGILRHVELGEGWGHVKDQQVLPNPDS
DDFLSSILGSGDSLPSSPLWSPEGSDSGISEDLPSDPQDTPPRSGPATSPAGCHPAQPGK
GPCLSYHPGNSCSTTTPGPVIQVPEASVTIDLEMWSPGGRICAEKPADPVDLSPRCNLTV
KDLLLLSGSSGDLQQHHLGASYLLRPGAGHCQELVLTEDEKKLLAKEGITLPTQLPLTKDV
SLHCSESGVTEESLASREAKPVPLGATEETPGHCGAVHQQVSPDRHLCRSPVAVLCPHHP
PLHQPFWPQQNREPWGLCACTSVLQNFAQRCCLPRGC*
>ENSG00000242247|ENST00000437119
MGDPSKQDILTIFKRLRSVPTNKVCFDCGAKNPSWASITYGVFLCIDCSGSHRSLGVHLS
FIRSTELDSNWSWFQLRCMQVGGNASALWLDSCVVPPLSPPPKEEDFFASHVSPEVSDTA
WASAIAEPSSLTSRPVETTLENNEGGQEQGPSVEGLNVPTKATLEVSSIIKKKPNQAKKG
LGAKKGSLGAQKLANTCFNEIEKQAQAADKMKEQEDLAKVVSKEESIVSSLRLAYKDLEI
QMKKDEKMNISGKKNVDSDRLGMGFGNCRSVISHSVTSDMQTIEQESPIMAKPRKKYNDD
SDDSYFTSSSSYFDEPVELRSSSFSSWDDSSDSYWKKETSKDTETVLKTTGYSDRPTARR

KPDYEPVENTDEAQKKFGNVKAISSDMYFGRQSQADYETRARLERLSASSSISSADLFEE
PRKQPAGNYSLSSVLPNAPDMAQFKQGVRSVAGKLSVFANGVVTSIQDRYGS*
>ENSG00000143393|ENST00000529142
MAIGKRLATLPTKEQKTQRLISELSLLNHKLPARVWLPTAGFDHHVVRVPHTQAVVLNSK
DKAPYLIYVEVLECENFDTTSVPARIPENRIRSTRSVENLPECGITHEQRAGSFSTVPNY
DNDDEAWSVDDIGELQVELPEVHTNSCDNISQFSVDSITSQESKEPVFIAAGDIRRRLSE
QLAHTPTAFKRDPEDPSAVALKEPWQEKVRRIREGSPYGHLPNWRLLSVIVKCGDDLRQE
LLAFQVLKQLQSIWEQERVPLWIKPYKILVISADSGMIEPVVNAVSIHQVKKQSQLSLLD
YFLQEHGSYTTEAFLSAQRNFVQSCAGYCLVCYLLQVKDRHNGNILLDAEGHIIHIDFGF
ILSSSPRNLGFETSAFKLTTEFVDVMGGLDGDMFNYYKMLMLQGLIAARKHMDKVVQIVE
IMQQGSQLPCFHGSSTIRNLKERFHMSMTEEQLQLLVEQMVDGSMRSITTKLYDGFQYLT
NGIM*
>ENSG00000235915|ENST00000430680
MLSRNDDICIYGGLGLGGLLLLAVVLLSACLCWLHRRAPSVLVPGPGLLRAGTPLCISAE
AASAQQ*
>ENSG00000124302|ENST00000434302
MTLRPGTMRLACMFSSILLFGAAGLLLFISLQDPTELAPQQVPGIKFNIRPRQPHHDLPP
GGSQDGDLKEPTERVTRDLSSGAPRGRNLPAPDQPQPPLQRGTRLRLRQRRRRLLIKKMP
AAATIPANSSDAPFIRPGPGTLDGRWVSLHRSQQERKRVMQEACAKYRASSSRRAVTPRH
VSRIFVEDRHRVLYCEVPKAGCSNWKRVLMVLAGLASSTADIQHNTVHYGSALKRLDTFD
RQGILHRLSTYTKMLFVREPFERLVSAFRDKFEHPNSYYHPVFGKAILARYRANASREAL
RTGSGVRFPEFVQYLLDVHRPVGMDIHWDHVSRLCSPCLIDYDFVGKFESMEDDANFFLS
LIRAPRNLTFPRFKDRHSQEARTTARIAHQYFAQLSALQRQRTYDFYYMDYLMFNYSKPF
ADLY*
>ENSG00000197249|ENST00000404814
MPSSVSWGILLLAGLCCLVPVSLAEDPQGDAAQKTDTSHHDQDHPTFNKITPNLAEFAFS
LYRQLAHQSNSTNIFFSPVSIATAFAMLSLGTKADTHDEILEGLNFNLTEIPEAQIHEGF
QELLRTLNQPDSQLQLTTGNGLFLSEGLKLVDKFLEDVKKLYHSEAFTVNFGDTEEAKKQ
INDYVEKGTQGKIVDLVKELDRDTVFALVNYIFFKGKWERPFEVKDTEEEDFHVDQVTTV
KVPMMKRLGMFNIQHCKKLSSWVLLMKYLGNATAIFFLPDEGKLQHLENELTHDIITKFL
ENEDRRSASLHLPKLSITGTYDLKSVLGQLGITKVFSNGADLSGVTEEAPLKLSKAVHKA
VLTIDEKGTEAAGAMFLEAIPMSIPPEVKFNKPFVFLMIEQNTKSPLFMGKVVNPTQK*
>ENSG00000204482|ENST00000418507
MLSRNDVKRLERSWAQGSSEQELHYASLQRLPVPSSEGPDLRGRDKRGTKEDPRADYACI
AENKPT*
>ENSG00000122705|ENST00000540080
MAELDPFGAPAGAPGGPALGNGVAGAGEEDPAAAFLAQQESEIAGIENDEAFAILDGGAP
GPQPHGEPPGGPDANSRKQEAEWKEKAIKELEEWYARQDEQLQKTKANNRAAEEAFVNDI
DESSPGTEWERVARLCDFNPKSSKQAKDVSRMRSVLISLKQAPLVH*
>ENSG00000231048|ENST00000440323
MLSRNDVKRLERSWAQGSSEQELHYASLQRLPVPSSEGPDLRGRDKRGTKEDPRADYACI
AENKPT*
>ENSG00000154080|ENST00000580774
MQPSEMVMNPKQVFLSVLIFGVAGLLLFMYLQVWIEEQHTGRVEKRREQKVTSGWGPVKY

LRPVPRIKPQVSHA*
>ENSG00000117620|ENST00000640178
MFANLKYVSLGILVFQTTSLVLTMRYSRTLKEEGPRYLSSTAVVVAELLKIMACILLVYK
DSSHVSVKNSYNSIIFCVYA*
>ENSG00000231048|ENST00000443655
MLSRNDDICIYGGLGLGGLLLLAVVLLSACLCWLHRRGPGLLRAGTPLCISAEAASAQQ*
>ENSG00000134243|ENST00000538502
MTFGQSKLYRSEDYGKNFKDITDLINNTFIRTEFGMAIGPENSGKVVLTAEVSGGSRGGR
IFRSSDFAKNFVQTDLPFHPLTQMMYSPQNSDYLLALSTENGLWVSKNFGGKWEEIHKAV
CLAKWGSDNTIFFTTYANGSCTDLGALELWRTSDLGKSFKTIGVKIYSFGLGGRFLFASV
MADKDTTRRIHVSTDQGDTWSMAQLPSVGQEQFYSILAANDDMVFMHVDEPGDTGFGTIF
TSDDRGIVYSKSLDRHLYTTTGGETDFTNVTSLRGVYITSVLSEDNSIQTMITFDQGGRW
THLRKPENSECDATAKNKNECSLHIHASYSISQKLNVPMAPLSEPNAVGIVIAHGSVGDA
ISVMVPDVYISDDGGYSWTKMLEGPHYYTILDSGGIIVAIEHSSRPINVIKFSTDEGQCW
QTYTFTRDPIYFTGLASEPGARSMNISIWGFTESFLTSQWVSYTIDFKDILERNCEEKDY
TIWLAHSTDPEDYEDGCILGYKEQFLRLRKSSVCQNGRDYVVTKQPSICLCSLEDFLCDF
GYYRPENDSKCVEQPELKGHDLEFCLYGREEHLTTNGYRKIPGDKCQGGVNPVREVKDLK
KKCTSNFLSPEKQNSKSNSVPIILAIVGLMLVTVVAGVLIVKKYVCGGRFLVHRYSVLQQ
HAEANGVDGVDALDTASHTNKSGYHDDSDEDLLE*
>ENSG00000135346|ENST00000627148
MDYYRKYAAIFLVTLSVFLHVLHSAPDVQDCPECTLQENPFFSQPGAPILQCMGCCFSRA
YPTPLRSKKTMLVQKNVTSESTCCVAKSYNRVTVMGGFKVENHTACHCSTCYYHKS*
>ENSG00000110080|ENST00000449406
MLALVLVVMVWYSISREDRYIELFYFPIPEKKEPCLQGEAESKASKLFGNYSRDQPIFLR
LEDYFWVKTPSAYELPYGTKGSEDLLLRVLAITSSSIPKNIQSLRCRRCVVVGNGHRLRN
SSLGDAINKYDVVIRLNNAPVAGYEGDVGSKTTMRLFYPESAHFDPKVENNPDTLLVLVA
FKAMDFHWIETILSDKKRVRKGFWKQPPLIWDVNPKQIRILNPFFMEIAADKLLSLPMQQ
PRKIKQKPTTGLLAITLALHLCDLVHIAGFGYPDAYNKKQTIHYYEQITLKSMAGSGHNV
SQEALAIKRMLEMGAIKNLTSF*
>ENSG00000176597|ENST00000481531
MRMLVSGRRVKKWQLIIQLFATCFLASLMFFWEPIDNHIVSHMKSYSYRY
>ENSG00000223532|ENST00000425848
MLVMAPRTVLLLLSAALALTETWAGSHSMRYFDTAMSRPGRGEPRFISVGYVDDTQFVRF
DSDAASPREEPRAPWIEQEGPEYWDRNTQIFKTNTQTDRESLRNLRGYYNQSEAGSHTLQ
SMYGCDVGPDGRLLRGHNQYAYDGKDYIALNEDLRSWTAADTAAQITQRKWEAARVAEQD
RAYLEGTCVEWLRRYLENGKDTLERADPPKTHVTHHPISDHEATLRCWALGFYPAEITLT
WQRDGEDQTQDTELVETRPAGDRTFQKWAAVVVPSGEEQRYTCHVQHEGLPKPLTLRWEP
SSQSTVPIVGIVAGLAVLAVVVIGAVVAAVMCRRKSSGGKGGSYSQAACSDSAQGSDVSL
TA*
>ENSG00000114354|ENST00000479672
MNGQLDLSGKLIIKAQLGEDIRRIPIHNEDITYDELVLMMQRVFRGKLLSNDEVTIKYKD
EDGDLITIFDSSDLSFAIQCSRILKLTLFVNGQPRPLESSQVKYLRRELIELRNKVNRLL
DSLEPPGE
>ENSG00000155511|ENST00000521843

MTYRFCSQFSKGVYAIFGFYERRTVNMLTSFCGALHVCFITPSFPVDTSNQFVLQLRPEL
QDALISIIDHYKWQKFVYIYDADRGLSVLQKVLDTAAEKNWQVTAVNILTTTEEGYRMLF
QDLEKKKERLVVVDCESERLNAILGQIIKLEKNGIGYHYILANLGFMDIDLNKFKESGAN
VTGFQLVNYTDTIPAKIMQQWKNSDARDHTRVDWKRPKYTSALTYDGVKVMAEAFQSLRR
QRIDISRRGNAGDCLANPAVPWGQGIDIQRALQQVRFEGLTGNVQFNEKGRRTNYTLHVI
EMKHDGIRKIGYWNEDDKFVPAATDAQAGGDNSSVQNRTYIVTTILEDPYVMLKKNANQF
EGNDRYEGYCVELAAEIAKHVGYSYRLEIVSDGKYGARDPDTKAWNGMVGELVYGRADVA
VAPLTITLVREEVIDFSKPFMSLGISIMIKKPQKSKPGVFSFLDPLAYEIWMCIVFAYIG
VSVVLFLVSRFSPYEWHSEEFEEGRDQTTSDQSNEFGIFNSLWFSLGAFMQQGCDISPRS
LSGRIVGGVWWFFTLIIISSYTANLAAFLTVERMVSPIESAEDLAKQTEIAYGTLEAGST
KEFFRRSKIAVFEKMWTYMKSAEPSVFVRTTEEGMIRVRKSKGKYAYLLESTMNEYIEQR
KPCDTMKVGGNLDSKGYGIATPKGSALRNPVNLAVLKLNEQGLLDKLKNKWWYDKGECGS
GGGDSKDKTSALSLSNVAGVFYILIGGLGLAMLVALIEFCYKSRSESKRMKGFCLIPQQS
INEAIRTSTLPRNSGAGASSGGSGENGRVVSHDFPKSMQSIPCMSHSSGMPLGATGL*
>ENSG00000122390|ENST00000570819
MTEVVPSSALSEVSLRLLCHDDIDTVKHLCGDWFPIEYPDSWYRDITSNKKFFSLAATYR
GAIVGMIVAEIKNRTKIHKEDGDILASNFSVDTQVAYILSLGVVKEFRKHGIESTARPTA
CSAASCHGRASLPRVASSTAGPCDVGWAAATRPHPSAARRARLPVHLTPSVFCKELPAI*
>ENSG00000138674|ENST00000443462
MLGESDERCTNAGSGCRRSSPGTSAQQLDATFSTNASLEIFELDLSDPSLDMKSCATFSS
SHRYHKLIWGPYKMDSKGDVSGVLIAGGENGNIILYDPSKIIAGDKEVVIAQNDKHTGPV
RALDVNIFQTNLVASGANESEIYIWDLNNFATPMTPGAKTQPPEDISCIAWNRQVQHILA
SASPSGRATVWDLRKNEPIIKVSDHSNRMHCSGLAWHPDVATQMVLASEDDRLPVIQMWD
LRFASSPLRVLENHARGILAIAWSMADPELLLSCGKDAKILCSNPNTGEVLYELPTNTQW
CFDIQWCPRNPAVLSAASFDGRISVYSIMGGSTDGLRQKQVDKLSSSFGNLDPFGTGQPL
PPLQIPQQTAQHSIVLPLKKPPKWIRRPVGASFSFGGKLVTFENVRMPSHQGAEQQQQQH
HVFISQVVTEKEFLSRSDQLQQAVQSQGFINYCQKKIDASQTEFEKNVWSFLKVNFEDDS
RGKYLELLGYRKEDLGKKIALALNKVDGANVALKDSDQVAQSDGEESPAAEEQLLGEHIK
EEKEESEFLPSSGGTFNISVSGDIDGLITQALLTGNFESAVDLCLHDNRMADAIILAIAG
GQELLARTQKKYFAKSQSKITRLITAVVMKNWKEIVESCDLKNWREALAAVLTYAKPDEF
SALCDLLGTRLENEGDSLLQTQACLCYICAGNVEKLVACWTKAQDGSHPLSLQDLIEKVV
ILRKAVQLTQAMDTSTVGVLLAAKMSQYANLLAAQGSIAAALAFLPDNTNQPNIMQLRDR
LCRAQGEPVAGHESPKIPYEKQQLPKGRPGPVAGHHQMPRVQTQQYYPHGENPPPPGFIM
HGNVNPNAAGQLPTSPGHMHTQVPPYPQPQPYQPAQPYPFGTGGSAMYRPQQPVAPPTSN
AYPNTPYISSASSYTGQSQLYAAQHQASSPTSSPATSFPPPPSSGASFQHGGPGAPPSSS
AYALPPGTTGPQNGWNDPPALNRVPKKKKMPENFMPPVPITSPIMNPLGDPQSQMLQQQP
SAPVPLSSQSSFPQPHLPGGQPFHGVQQPLGQTGMPPSFSKPNIEGAPGAPIGNTFQHVQ
SLPTKKITKKPIPDEHLILKTTFEDLIQRCLSSATDPQTKRKLDDASKRLEFLYDKLREQ
TLSPTITSGLHNIARSIETRNYSEGLTMHTHIVSTSNFSETSAFMPVLKVVLTQANKLGV
*
>ENSG00000130733|ENST00000586748
MASADELTFHEFEEATNLLADTPDAATTSRSDQLTPQGHVAVAVGSGGSYGAEDEVEEES
DKAALLQEQQQQQQPGFWTFSYYQSFFDVDTSQVLDRIKGSLLPRPGHNFVRHHLRNRPD
LYGPFWICATLAFVLAVTGNLTLVLAQRRDPSIHYSPQFHKVTVAGISIYCYAWLVPLAL

WGFLRWRKGVQERMGPYTFLETVCIYGYSLFVFIPMVVLWLIPVPWLQWLFGALALGLSA
AGLVFTLWPVVREDTRLVATVLLSVVVLLHALLAMGCKLYFFQSLPPENVAPPPQITSLP
SNIALSPTLPQSLAPS*
>ENSG00000283530|ENST00000638103
MFKFHQMKHIFEILDKMRCLRKRSTVSFLGVLVIFLLFMNLYIEDSYVLEGDKQLIRETS
THQLNSERYVHTFKDLSNFSGAINVTYRYLAATPLQRKRYLTIGLSSVKRKKGNYLLETI
KSIFEQSSYEELKEISVVVHLADFNSSWRDAMVQDITQKFAHHIIAGRLMVIHAPEEYYP
ILDGLKRNYNDPEDRVKFRSKQNVDYAFLLNFCANTSDYYVMLEDDVRCSKNFLTAIKKV
IASLEGTYWVTLEFSKLGYIGKLYHSHDLPRLAHFLLMFYQEMPCDWLLTHFRGLLAQKN
VIRFKPSLFQHMGYYSSYKGTENKLKDDDFEEESFDIPDNPPASLYTNMNVFENYEASKA
YSSVDEYFWGKPPSTGDVFVIVFENPIIIKKIKVNTGTEDRQNDILHHGALDVGENVMPS
KQRRQCSTYLRLGEFKNGNFEMSGVNQKIPFDIHCMRIYVTKTQKEWLIIRSISIWTS*
>ENSG00000231048|ENST00000425760
MLSRNDAPSVLVPGPGLLRAGTPLCISAEAASAQQ*
>ENSG00000114354|ENST00000476228
MNGQLDLSGKLIIKAQLGEDIRRIPIHNEDITYDELVLMMQRVFRGKLLSNDEVTIKYKD
EDGDLITIFDSSDLSFAIQCSRILKLTLFVNGQPRPLESSQVKYLRRELIELRNKVNRLL
DSLEPPGEPGPSTNIPENDTVDGREEKSASDSSGKQSTQVMAASMSAFDPLKNQDEINKN
VMSAFGLTDDQVSGPPSAPAEDRSGTPDSIASSSSAAHPPGVQPQQPPYTGAQTQAGQMY
QQYQQQAGYGAQQPQAPPQQPQQYGIQYSASYSQQTGPQQPQQFQGYGQQPTSQAPAPAF
SGQPQQLPAQPPQQYQASNYPAQTYTAQTSQPTNYTVAPASQPGMAPSQPGAYQPRPGFT
SLPGSTMTPPPSGPNPYARNRPPFGQGYTQPGPGYR*
>ENSG00000214654|ENST00000464488
MQVTFCRLRTHQWCFILFNVILFHALLFGTDFVEEYFLHSLPYIDVKVLEIKNKARKLNI
EPLRSNLSKYYVLSQSEICKGKNIFLLSLIFSSPGNGTRRDLIRKTWGNVTSVQGHPILT
LFALGMPVSVTTQKEINKESCKNNDIIEGIFLDSSENQTLKIIAMIQWAVAFCPNALFIL
KVDEETFVNLPSLVDYLLNLKEHLEDIYVGRVLHQVTPNRDPQNRDFVPLSEYPEKYYPD
YCSGEAFIMSQDVARMMYVVFKEVPMMVPADVFVGICAKFIGLIPIHSSRFSGKRHIRYN
RCCYKFIFTSSEIADPEMPLAWKEINDGKECTLFETSYELISCKLLTYLDSFKRFHMGTI
KNNLMYFAD*
>ENSG00000173905|ENST00000470487
MGNGMCSRKQKRIFQTLLLLTVVFGFLYGAMLYYELQTQLRKAEAVALKYQQHQESLSAQ
LQVVYEHRSRLEKSLQKERLEHKKAKEDFLVYKLEAQETLNKGRQDSNSRYSALNVQHQM
LKSQHEELKKQHSDLEEEHRKQGEDFSRTFNDHKQKYLQLQQEKEQELSKLKETVYNLRE
ENRQLRKAHQDIHTQLQDVKQQHKNLLSEHEQLVVTLEDHKSALAAAQTQVAEYKQLKDT
LNRIPSLRKPDPAEQQNVTQVAHSPQGYNTAREKPTREVQEVSRNNDVWQNHEAVPGRAE
DTKLYAPTHKEAEFQAPPEPIQQEVERREPEEHQVEEEHRKALEEEEMEQVGQAEHLEEE
HDPSPEEQDREWKEQHEQREAANLLEGHARAEVYPSAKPMIKFQSPYEEQLEQQRLAVQQ
VEEAQQLREHQEALHQQRLQGHLLRQQEQQQQQVAREMALQRQAELEEGRPQHQEQLRQQ
AHYDAMDNDIVQGAEDQGIQGEEGAYERDNQHQDEAEGDPGNRHEPREQGPREADPESEA
DRAAVEDINPADDPNNQGEDEFEEAEQVREENLPDENEEQKQSNQKQENTEVEEHLVMAG
NPDQQEDNVDEQYQEEAEEEVQEDLTEEKKRELEHNAEETYGENDENTDDKNNDGEEQEV
RDDNRPKGREEHYEEEEEEEEDGAAVAEKSHRRAEM*
>ENSG00000090487|ENST00000433215

MGEIKVSPDYNWFRGTVPLKKIIVDDDDSKIWSLYDAGPRSIRCPLIFLPPVSGTADVFF
RQILALTGWGYRVIALQYPVYWDHLEFCDGFRKLLDHLQLDKVHLFGASLGGFLAQKFAE
YTHKSPRVHSLILCNSFSDTSIFNQTWTANSFWLMPAFMLKKIVLGNFSSGPVDPMMADA
IDFMVDRLESLGQSELASRLTLNCQNSYVEPHKIRDIPVTIMDVFDQSALSTEAKEEMYK
LYPNARRAHLKTGGNFPYLCRSAEVNLYVQIHLLQFHGTKYAAIDPSMVSAEELEVQKGS
LGISQEEQ*
>ENSG00000135454|ENST00000418555
MWLGRRALCALVLLLACASLGLLYASTRDAPGLRLPLAPWAPPQSPRRPELPDLAPEPRY
AHIPVRIKEQVVGSQSPADQLLIAPANSPLQYPLQGVEVQPLRSILVPGLSLQAASGQEV
YQVNLTASLGTWDVAGEVTGVTLTGEGQADLTLVSPGLDQLNRQLQLVTYSSRSYQTNTA
DTVRFSTEGHEAAFTIRIRHPPNPRLYPPGSLPQGAQYNISALVTIATKTFLRYDRLRAL
ITSIRRFYPTVTVVIADDSDKPERVSGPYVEHYLMPFGKGWFAGRNLAVSQVTTKYVLWV
DDDFVFTARTRLERLVDVLERTPLDLVGGAVREISGFATTYRQLLSVEPGAPGLGNCLRQ
RRGFHHELVGFPGCVVTDGVVNFFLARTDKVREVGFDPRLSRVAHLEFFLDGLGSLRVGS
CSDVVVDHASKLKLPWTSRDAGAETYARYRYPGSLDESQMAKHRLLFFKHRLQCMTSQ*
>ENSG00000166377|ENST00000590477
LVISGDSLEVCLKYYEHEFVELACQCPAVVCCRCSPTQKARIVTLLQQHTGRRTCAIGDG
GNDVSMIQAADCGIGIEGKEGKQASLAADFSITQFRHIGRLLMVHGRNSYKRSAALGQFV
MHRGLIISTMQVCHHIHHVPSVLLSAGPGREARDGDALPGAVQGPHQGKILVLQNLPHLG
FNKYLPRRHPHVWGPGALRV*
>ENSG00000117620|ENST00000640726
MFANLKYVSLGILVFQTTSLVLTMRYSRTLKEEGPRYLSSTAVVVAELLKIMACILLVYK
DSKCSLRALNRVLHDEILNKPMETLKLAIPSGIYTLQNNLLYVALSNLDAATYQVTYQLK
ILTTALFSVSMLSKKLGVYQWLSLVILMTGVAFVQ*
>ENSG00000088833|ENST00000476071
MAAERQEALREFVAVTGAEEDRARFFLESAGWDLQIALASFYEDGGDEDIVTISQATPSS
VSRGTAPSDNRVTSFRDLIHDQDEDEEEEEGQRSRFYAGGSERSGQQIVGPPRKKSPNEL
VDDLFKGAKEHGAVAVERVTKSPGETSKPRPFAGGGYRLGAAPEEESAYVAGEKRQHSSQ
DVHVVLKLWKSGFSLDNGELRSYQDPSNAQFLESIRRGEVPAELRRLAHGGQVNLDMEDH
RDEDFVKPKGAFKAFTGEGQKLGSTAPQVLSTSSPAQQAENEAKASSSILIDESEPTTNI
QIRLADGGRLVQKFNHSHRISDIRLFIVDARPAMAATSFILMTTFPNKELADESQTLKEA
NLLNAVIVQRLT*
>ENSG00000039068|ENST00000422392
MGPWSRSLSALLLLLQVSSWLCQEPEPCHPGFDAESYTFTVPRRHLERGRVLGRVNFEDC
TGRQRTAYFSLDTRFKVGTDGVITVKRPLRFHNPQIHFLVYAWDSTYRKFSTKVTLNTVG
HHHRPPPHQASVSGIQAELLTFPNSSPGLRRQKRDWVIPPISCPENEKGPFPKNLVQIKS
NKDKEGKVFYSITGQGADTPPVGVFIIERETGWLKVTEPLDRERIATYTLFSHAVSSNGN
AVEDPMEILITVTDQNDNKPEFTQEVFKGSVMEGALPGTSVMEVTATDADDDVNTYNAAI
AYTILSQDPELPDKNMFTINRNTGVISVVTTGLDRESFPTYTLVVQAADLQGEGLSTTAT
AVITVTDTNDNPPIFNPTTGLDFEAKQQYILHVAVTNVVPFEVSLTTSTATVTVDVLDVN
EAPIFVPPEKRVEVSEDFGVGQEITSYTAQEPDTFMEQKITYRIWRDTANWLEINPDTGA
ISTRAELDREDFEHVKNSTYTALIIATDNGSPVATGTGTLLLILSDVNDNAPIPEPRTIF
FCERNPKPQVINIIDADLPPNTSPFTAELTHGASANWTIQYNDPTQESIILKPKMALEVG
DYKINLKLMDNQNKDQVTTLEVSVCDCEGAAGVCRKAQPVEAGLQIPAILGILGGILALL

ILILLLLLFLRRRAVVKEPLLPPEDDTRDNVYYYDEEGGGEEDQDFDLSQLHRGLDARPE
VTRNDVAPTLMSVPRYLPRPANPDEIGNFIDENLKAADTDPTAPPYDSLLVFDYEGSGSE
AASLSSLNSSESDKDQDYDYLNEWGNRFKKLADMYGGGEDD*
>ENSG00000101246|ENST00000619493
MYTLLSGLYKYMFQKDEYCILILGLDNAGKTTFLEQSKTRFNKNYKGMSLSKITTTVGLN
IGTVDVGKARLMFWDLGGQEELQSLWDKYYAECHGVIYVIDSTDEERLAESKQAFEKVVT
SEALCGVPVLVLANKQDVETCLSIPDIKTAFSDCTSKIGRRDCLTQACSALTGKGVREGI
EWMVKCVVRNVHRPPRQRDIT*
>ENSG00000166377|ENST00000588921
XIISTMQAVFSSVFYFASVPLYQGFLMVGYATIYTMFPVFSLVLDQDVKPEMAMLYPELY
KDLTKSQQDGRSNRRQHERLTSGTSGHVWDPGSFFRIRVSGKGARNAGKILVLQNLPHLG
FNKYLPRRHPHVWGPGALRV*
>ENSG00000226182|ENST00000433004
MLSRNDVKRLERSWAQGSSEQELHYASLQRLPVPSSEGPDLRGRDKRGTKEDPRADYACI
AENKPT*
>ENSG00000082805|ENST00000440394
MYGSARSVGKVEPSSQSPGRSPRLPRSPRLGHRRTNSTGGSSGSSVGGGSGKTLSMENIQ
SLNAAYATSGPMYLSDHENVGSETPKSTMTLGRSGGRLPYGVRMTAMGSSPNIASSGVAS
DTIAFGEHHLPPVSMASTVPHSLRQARDNTIMDLQTQLKEVLRENDLLRKDVEVKESKLS
SSMNSIKTFWSPELKKERALRKDEASKITIWKEQYRVVQEENQHMQMTIQALQDELRIQR
DLNQLFQQDSSSRTGEPCVAELTEENFQRLHAEHERQAKELFLLRKTLEEMELRIETQKQ
TLNARDESIKKLLEMLQSKGLSAKATEEDHERTRRLAEAEMHVHHLESLLEQKEKENSML
REEMHRRFENAPDSAKTKALQTVIEMKDSKISSMERGLRDLEEEIQMLKSNGALSTEERE
EEMKQMEVYRSHSKFMKNKIGQVKQELSRKDTELLALQTKLETLTNQFSDSKQHIEVLKE
SLTAKEQRAAILQTEVDALRLRLEEKETMLNKKTKQIQDMAEEKGTQAGEIHDLKDMLDV
KERKVNVLQKKIENLQEQLRDKEKQMSSLKERVKSLQADTTNTDTALTTLEEALAEKERT
IERLKEQRDRDEREKQEEIDNYKKDLKDLKEKVSLLQGDLSEKEASLLDLKEHASSLASS
GLKKDSRLKTLEIALEQKKEECLKMESQLKKAHEAALEARASPEMSDRIQHLEREITRYK
DESSKAQAEVDRLLEILKEVENEKNDKDKKIAELESLTSRQVKDQNKKVANLKHKEQVEK
KKSAQMLEEARRREDNLNDSSQQLQDSLRKKDDRIEELEEALRESVQITAEREMVLAQEE
SARTNAEKQVEELLMAMEKVKQELESMKAKLSSTQQSLAEKETHLTNLRAERRKHLEEVL
EMKQEALLAAISEKDANIALLELSSSKKKTQEEVAALKREKDRLVQQLKQQTQNRMKLMA
DNYEDDHFKSSHSNQTNHKPSPDQDEEEGIWA*
>ENSG00000079805|ENST00000408974
MGNRGMEELIPLVNKLQDAFSSIGQSCHLDLPQIAVVGGQSAGKSSVLENFVGRDFLPRG
SGIVTRRPLILQLIFSKTEHAEFLHCKSKKFTDFDEVRQEIEAETDRVTGTNKGISPVPI
NLRVYSPHVLNLTLIDLPGITKVPVGDQPPDIEYQIKDMILQFISRESSLILAVTPANMD
LANSDALKLAKEVDPQGLRTIGVITKLDLMDEGTDARDVLENKLLPLRRGYIGVVNRSQK
DIEGKKDIRAALAAERKFFLSHPAYRHMADRMGTPHLQKTLNQQLTNHIRESLPALRSKL
QSQLLSLEKEVEEYKNFRPDDPTRKTKALLQMVQQFGVDFEKRIEGSGDQVDTLELSGGA
RINRIFHERFPFELVKMEFDEKDLRREISYAIKNIHGVRTGLFTPDMAFEAIVKKQIVKL
KEPSLKCVDLVVSELATVIKKCAEKLSSYPRLREETERIVTTYIREREGRTKDQILLLID
IEQSYINTNHEDFIGFANAQQRSTQLNKKRAIPNQVIRRGWLTINNISLMKGGSKEYWFV
LTAESLSWYKDEEEKEKKYMLPLDNLKIRDVEKGFMSNKHVFAIFNTEQRNVYKDLRQIE

LACDSQEDVDSWKASFLRAGVYPEKDQAENEDGAQENTFSMDPQLERQVETIRNLVDSYV
AIINKSIRDLMPKTIMHLMINNTKAFIHHELLAYLYSSADQSSLMEESADQAQRRDDMLR
MYHALKEALNIIGDISTSTVSTPVPPPVDDTWLQSASSHSPTPQRRPVSSIHPPGRPPAV
RGPTPGPPLIPVPVGAAASFSAPPIPSRPGPQSVFANSDLFPAPPQIPSRPVRIPPGIPP
GVPSRRPPAAPSRPTIIRPAEPSLLD*
>ENSG00000186318|ENST00000428381
MAQALPWLLLWMGAGVLPAHGTQHGIRLPLRSGLGGAPLGLRLPRETDEEPEEPGRRGSF
VEMVDNLRGKSGQGYYVEMTVGSPPQTLNILVDTGSSNFAVGAAPHPFLHRYYQRQLSST
YRDLRKGVYVPYTQGKWEGELGTDLLCGAGFPLNQSEVLASVGGSMIIGGIDHSLYTGSL
WYTPIRREWYYEVIIVRVEINGQDLKMDCKEYNYDKSIVDSGTTNLRLPKKVFEAAVKSI
KAASSTEKFPDGFWLGEQLVCWQAGTTPWNIFPVISLYLMGEVTNQSFRITILPQQYLRP
VEDVATSQDDCYKFAISQSSTGTVMGAVIMEGFYVVFDRARKRIGFAVSACHVHDEFRTA
AVEGPFVTLDMEDCGYNIPQTDESTLMTIAYVMAAICALFMLPLCLMVCQWRCLRCLRQQ
HDDFADDISLLK*
>ENSG00000125447|ENST00000580799
MALPEEAKIKDAYHMLKRQGIVQSDPPIPVDRTLIPSPPPRPKNPVFDDEEKSKLLAKLL
KSKNPDDLQEANKLIKSMVKEDEARIQKVTKRLHTLEEVNNNVRLLSEMLLHYSQEDSSD
GDRELMKELFDQCENK
>ENSG00000231048|ENST00000430632
MLSRNDDICIYGGLGLGGLLLLAVVLLSACLCWLHRRVKRLERSWAQGSSEQELHYASLQ
RLPVPSSEGPDLRGRDKRGTKEDPRADYACIAENKPT*
>ENSG00000147533|ENST00000405786
MRPQQAPVSGKVFIQRDYSSGTRCQFQTKFPAELENRIDRQQFEETVRTLNNLYAEAEKL
GGQSYLEGCLACLTAYTIFLCMETHYEKVLKKVSKYIQEQNEKIYAPQGLLLTDPIERGL
RVFRLKLPFMKTEA*
>ENSG00000223980|ENST00000552493
MAVMAPRTLLLLLLGALALTQTWAGSHSMRYFFTSVSRPGRGEPRFIAVGYVDDTQFVRF
DSDAASQRMEPRAPWIEQEGPEYWDQETRNVKAHSQTDRESLRIALRYYNQSEAGSHTIQ
MMYGCDVGPDGRLLRGYQQDAYDGKDYIALNEDLRSWTAADMAAQITQRKWEAARVAEQL
RAYLEGTCVEWLRRYLENGKETLQRTDAPKTHMTHHAVSDHEATLRCWALSFYPAEITLT
WQRDGEDQTQDTELVETRPAGDGTFQKWASVVVPSGQEQRYTCHVQHEGLPKPLTLRWEP
SSQPTIPIVGIIAGLVLFGAMFAGAVVAAVRWRRKSSDRKGGSYSQAASSDSAQGSDMSL
TACKV*
>ENSG00000276536|ENST00000616241
MTEYKLVVVGAGGVGKSALTIQLIQNHFVDEYDPTIEDSYRKQVVIDGETCLLDILDTAG
QEEYSAMRDQYMRTGEGFLCVFAINNTKSFEDIHQYREQIKRVKDSDDVPMVLVGNKCDL
AARTVESRQAQDLARSYGIPYIETSAKTRQGSRSGSSSSSGTLWDPPGPM*
>ENSG00000125447|ENST00000580646
XLIPTTTPARPLLPFSTGPGSPLFQPLSFQSQGSPPKGPELSLASIHVPLESIKPSSALP
VTAYDKNGFRILFHFAKECPPGRPDVLVVVVSMLNTAPLPVKSIVLQAAVPKSMKVKLQP
PSGTELSPFSPIQPPAAITQVMLLANPLKDLFLSTCVT*
>ENSG00000117620|ENST00000640360
MFANLKYVSLGILVFQTTSLVLTMRYSRTLKEEGPRYLSSTAVVVAELLKIMACILLVYK
DSSHVSVKNSYNSIIFCVYA*

>ENSG00000123106|ENST00000545336
MDDDDFGGFEAAETFDGGSGETQTTSPAIPWAAFPAVSGVHLSPSSPEIVLDRDHSSSIG
CLSSDAIISSPENTHAANSIVSQTIPKAQIQQSTHTHLDISLFPLGLTDEKSNGTIALVD
DSEDPGANVSNIQLQQKISSLEIKLKVSEEEKQRIKQDVESLMEKHNVLEKGFLKEKEQE
AISFQDRYKELQEKHKQELEDMRKAGHEALSIIVDEYKALLQSSVKQQVEAIEKQYISAI
EKQAHKCEELLNAQHQRLLEMLDTEKELLKEKIKEALIQQSQEQKEILEKCLEEERQRNK
EALVSAAKLEKEAVKDAVLKVVEEERKNLEKAHAEERELWKTEHAKDQEKVSQEIQKAIQ
EQRKISQETVKAAIIEEQKRSEKAVEEAVKRTRDELIEYIKEQKRLDQVIRQRSLSSLEL
FLSCAQKQLSALIATEPVDIE*
>ENSG00000179913|ENST00000595387
MKYLRHRRPNATLILAIGAFTLLLFSLLVSPPTCKVQEQPPAIPEALAWPTPPTRPAPAP
CHANTSMVTHPDFATQPQHVQNFLLYRHCRHFPLLQDVPPSKCAQPVFLLLVIKSSPSNY
VRRELLRRTWGRERKVRGLQLRLLFLVGTASNPHEARKVNRLLELEAQTHGDILQWDFHD
SFFNLTLKQVLFLQWQETRCANASFVLNGDDDVFAHTDNMVFYLQDHDPGRHLFVGQLIQ
NVGPIRAFWSKYYVPEVVTQNERYPPYCGGGGFLLSRFTAAALRRAAHVLDIFPIDDVFL
GMCLELEGLKPASHSGIRTSGVRAPSQRLSSFDPCFYRDLLLVHRFLPYEMLLMWDALNQ
PNLTCGNQTQIY*
>ENSG00000277641|ENST00000616347
MEPHLLGLLLGLLLGGTRVLAGYPIWWSLALGQQYTSLGSQPLLCGSIPGLVPKQLRFCR
NYIEIMPSVAEGVKLGIQECQHQFRGRRWNCTTIDDSLAIFGPVLDKATRESAFVHAIAS
AGVAFAVTRSCAEGTSTICGCDSHHKGPPGEGWKWGGCSEDADFGVLVSREFADARENRP
DARSAMNKHNNEAGRTTILDHMHLKCKCHGLSGSCEVKTCWWAQPDFRAIGDFLKDKYDS
ASEMVVEKHRESRGWVETLRAKYSLFKPPTERDLVYYENSPNFCEPNPETGSFGTRDRTC
NVTSHGIDGCDLLCCGRGHNTRTEKRKEKCHCIFHWCCYVSCQECIRIYDVHTCK*
>ENSG00000117280|ENST00000437324
MTRLYYRDASACVIMFDVTNATTFSNSQRWKQDLDSKLTLPNGEPVPCLLLANKCDLSPW
AVSRDQIDRFSKENGFTGWTETSVKENKNINEAMRVLIEKMMRNSTEDIMSLSTQGDYIN
LQTKSSSWSCC*
>ENSG00000184432|ENST00000507777
MLASLYNGSVCVWNHETQTLVKTFEVCDLPVRAAKFVARKNWVVTGADDMQIRVFNYNTL
ERVHMFEAHSDYIRCIAVHPTQPFILTSSDDMLIKLWDWDKKWSCSQVFEGHTHYVMQIV
INPKDNNQFASASLDRTIKVWQLGSSSPNFTLEGHEKGVNCIDYYSGGDKPYLISGADDR
LVKIWDYQNKTCVQTLEGHAQNVSCASFHPELPIIITGSEDGTVRIWHSSTYRLESTLNY
GMERVWCVASLRGSNNVALGYDEGSIIVKLGREEPAMSMDANGKIIWAKHSEVQQANLKA
MGDAEIKDGERLPLAVKDMGSCEIYPQTIQHNPNGRFVVVCGDGEYIIYTAMALRNKSFG
SAQEFAWAHDSSEYAIRESNSIVKIFKNFKEKKSFKPDFGAESIYGGFLLGVRSVNGLAF
YDWDNTELIRRIEIQPKHIFWSDSGELVCIATEESFFILKYLSEKVLAAQETHEGVTEDG
IEDAFEVLGEIQEIVKTGLWVGDCFIYTSSVNRLNYYVGGEIVTIAHLDRTMYLLGYIPK
DNRLYLGDKELNIISYSLLVSVLEYQTAVMRRDFSMADKVLPTIPKEQRTRVAHFLEKQG
FKQQALTVSTDPEHRFELALQLGELKIAYQLAVEAESEQKWKQLAELAISKCQFGLAQEC
LHHAQDYGGLLLLATASGNANMVNKLAEGAERDGKNNVAFMSYFLQGKVDACLELLIRTG
RLPEAAFLARTYLPSQVSRVVKLWRENLSKVNQKAAESLADPTEYENLFPGLKEAFVVEE
WVKETHADLWPAKQYPLVTPNEERNVMEEGKDFQPSRSTAQQELDGKPASPTPVIVASHT
ANKEEKSLLELEVDLDNLELEDIDTTDINLDEDILDD*

>ENSG00000081014|ENST00000560508
MMKECMVRLIYCEMLGYDASFGYIHAIKLAQQGNLLEKRVGYLAVSLFLHESHELLLLLV
NTVVKDLQSTNLVEVCMALTVVSQIFPCEMIPAVLPLIEDKLQHSKEIVRRKAVLALYKF
HLIAPNQVQHIHIKFRKALCDRDVGVMAASLHIYLRMIKENSSGYKDLTGSFVTILKQVV
GGKLPVEFNYHSVPAPWLQIQLLRILGLLGKDDQRTSELMYDVLDESLRRAELNHNVTYA
ILFECVHTVYSIYPKSELLEKAAKCIGKFVLSPKINLKYLGLKALTYVIQQDPTLALQHQ
MTIIECLDHPDPIIKRETLELLYRITNAQNITVIVQKMLEYLHQSKEEYVIVNLVGKIAE
LAEKYAPDNAWFIQTMNAVFSVGGDVMHPDIPNNFLRLLAEGFDDETEDQQLRLYAVQSY
LTLLDMENVFYPQRFLQVMSWVLGEYSYLLDKETPEEVIAKLYKLLMNDSVSSETKAWLI
AAVTKLTSQAHSSNTVERLIHEFTISLDTCMRQHAFELKHLHENVELMKSLLPVDRSCED
LVVDASLSFLDGFVAEGLSQGAAPYKPPHQRQEEKLSQEKVLNFEPYGLSFSSSGFTGRQ
SPAGISLGSDVSGNSAETGLKETNSLKLEGIKKLWGKEGYLPKKESKTGDESGALPVPQE
SIMENVDQAITKKDQSQVLTQSKEEKEKQLLASSLFVGLGSESTINLLGKADTVSHKFRR
KSKVKEAKSGETTSTHNMTCSSFSSLSNVAYEDDYYSNTLHDTGDKELKKFSLTSELLDS
ESLTELPLVEKFSYCSLSTPSLFANNNMEIFHPPQSTAASVAKESSLASSFLEETTEYIH
SNAMEVCNNETISVSSYKIWKDDCLLMVWSVTNKSGLELKSADLEIFPAENFKVTEQPGC
CLPVMEAESTKSFQYSVQIEKPFTEGNLTGFISYHMMDTHSAQLEFSVNLSLLDFIRPLK
ISSDDFGKLWLSFANDVKQNVKMSESQAALPSALKTLQQKLRLHIIEIIGNEGLLACQLL
PSIPCLLHCRVHADVLALWFRSSCSTLPDYLLYQCQKVMEGS*
>ENSG00000149294|ENST00000531044
MLQTKDLIWTLFFLGTAVSLQVDIVPSQGEISVGESKFFLCQVAGDAKDKDISWFSPNGE
KLTPNQQRISVVWNDDSSSTLTIYNANIDDAGIYKCVVTGEDGSESEATVNVKIFQKLMF
KNAPTPQEFREGEDAVIVCDVVSSLPPTIIWKHKGRDVILKKDVRFIVLSNNYLQIRGIK
KTDEGTYRCEGRILARGEINFKDIQVIVNVPPTIQARQNIVNATANLGQSVTLVCDAEGF
PEPTMSWTKDGEQIEQEEDDEKYIFSDDSSQLTIKKVDKNDEAEYICIAENKAGEQDATI
HLKVFAKPKITYVENQTAMELEEQVTLTCEASGDPIPSITWRTSTRNISSEEKTLDGHMV
VRSHARVSSLTLKSIQYTDAGEYICTASNTIGQDSQSMYLEVQYAPKLQGPVAVYTWEGN
QVNITCEVFAYPSATISWFRDGQLLPSSNYSNIKIYNTPSASYLEVTPDSENDFGNYNCT
AVNRIGQESLEFILVQADTPSSPSIDQVEPYSSTAQVQFDEPEATGGVPILKYKAEWRAV
GEEVWHSKWYDAKEASMEGIVTIVGLKPETTYAVRLAALNGKGLGEISAASEFKTQPVQG
EPSAPKLEGQMGEDGNSIKVNLIKQDDGGSPIRHYLVRYRALSSEWKPEIRLPSGSDHVM
LKSLDWNAEYEVYVVAENQQGKSKAAHFVFRTSAQPTAIPANGSPTSGLSTGAIVGILIV
IFVLLLVVVDITCYFLNKCGLFMCIAVNLCGKAGPGAKGKDMEEGKAAFSKDESKEPIVE
VRTEEERTPNHDGGKHTEPNETTPLTEPEKGPVEAKPECQETETKPAPAEVKTVPNDATQ
TKENESKA*
>ENSG00000235915|ENST00000436185
MLSRNDVKRLERSWAQGSSEQELHYASLQRLPVPSSEGPDLRGRDKRGTKEDPRADYACI
AENKPT*
>ENSG00000108587|ENST00000581721
MAAGTSSYWEDLRKQARQLENELDLKLVSFSKLCTSYSHSSTRDGRRDRYSSDTTPLLNG
SSQDRMFETMAIEIEQLLARLTGVNDKMAEYTNSAGVPSLNAALMHTLQRHRDILQDYTH
EFHKTKANFMAIRERENLMGSVRKDIDGSGVNNRRTELFLKEHDHLRNIAMATKENMTSQ
RGMLKSIHSKMNTLANRFPAVNSLIQRINLRKRRDSLILGGVIGICTILLLLLYAFH*
>ENSG00000147533|ENST00000518270

MRPQQAPVSGKVFIQRDYSSGTRCQFQTKFPAELENRIDRQQFEETVRTLNNLYAEAEKL
GGQSYLEGCLACLTAYTIFLCMETHYEKVLKKVSKYIQEQNEKIYAPQGLLLTDPIERGL
RVIEITIYEDRGMSSGR*
>ENSG00000090863|ENST00000422840
MAACGRVRRMFRLSAALHLLLLFAAGAEKLPGQGVHSQGQGPGANFVSFVGQAGGGGPAG
QQLPQLPQSSQLQQQQQQQQQQQPQPPQPPFPAGGPPARRGGAGAGGGWKLAEEESCRE
DVTRVCPKHTWSNNLAVLECLQDVREPENEISSDCNHLLWNYKLNLTTDPKFESVAREVC
KSTITEIKECADEPVGKGYMVSCLVDHRGNITEYQCHQYITKMTAIIFSDYRLICGFMDD
CKNDINILKCGSIRLGEKDAHSQGEVVSCLEKGLVKEAEEREPKIQVSELCKKAILRVAE
LSSDDFHLDRHLYFACRDDRERFCENTQAGEGRVYKCLFNHKFEESMSEKCREALTTRQK
LIAQDYKVSYSLAKSCKSDLKKYRCNVENLPRSREARLSYLLMCLESAVHRGRQVSSECQ
GEMLDYRRMLMEDFSLSPEIILSCRGEIEHHCSGLHRKGRTLHCLMKVVRGEKGNLGMNC
QQALQTLIQETDPGADYRIDRALNEACESVIQTACKHIRSGDPMILSCLMEHLYTEKMVE
DCEHRLLELQYFISRDWKLDPVLYRKCQGDASRLCHTHGWNETSEFMPQGAVFSCLYRHA
YRTEEQGRRLSRECRAEVQRILHQRAMDVKLDPALQDKCLIDLGKWCSEKTETGQELECL
QDHLDDLVVECRDIVGNLTELESEDIQIEALLMRACEPIIQNFCHDVADNQIDSGDLMEC
LIQNKHQKDMNEKCAIGVTHFQLVQMKDFRFSYKFKMACKEDVLKLCPNIKKKVDVVICL
STTVRNDTLQEAKEHRVSLKCRRQLRVEELEMTEDIRLEPDLYEACKSDIKNFCSAVQYG
NAQIIECLKENKKQLSTRCHQKVFKLQETEMMDPELDYTLMRVCKQMIKRFCPEADSKTM
LQCLKQNKNSELMDPKCKQMITKRQITQNTDYRLNPMLRKACKADIPKFCHGILTKAKDD
SELEGQVISCLKLRYADQRLSSDCEDQIRIIIQESALDYRLDPQLQLHCSDEISSLCAEE
AAAQEQTGQVEECLKVNLLKIKTELCKKEVLNMLKESKADIFVDPVLHTACALDIKHHCA
AITPGRGRQMSCLMEALEDKRVRLQPECKKRLNDRIEMWSYAAKVAPADGFSDLAMQVMT
SPSKNYILSVISGSICILFLIGLMCGRITKRVTRELKDR*
>ENSG00000125447|ENST00000578348
MALPEEAKIKDAYHMLKRQGIVQSDPPIPVDRTLIPSPPPRPKNPVFDDEEKSKLLAKLL
KSKNPDDLQEANKLIKSMVKEDEARIQKVTKRLHTLEEVNNNVRLLSEMLLHYSQEDSSD
GDRELMKELFDQCENKRRTLFKLASETEDNDNSLGDILQASDNLSRVINSYKTIIEGQVI
NGEVATLTLPDSEGNSQCSNQGTLIDLAELDTTNSLSSVLAPAPTPPSSGIPILPPPPQA
SGPPRSRSSSQAEATLGPSSTSNALSWLDEELLCLGLADPAPNVPPKESAGNSQWHLLQR
EQSDLDFFSPRPGTAACGASDAPLLQPSAPSSSSSQAPLPPPFPAPVVPASVPAPSAGSS
LFSTGVAPALAPKVEPAVPGHHGLALGNSALHHLDALDQLLEEAKVTSGLVKPTTSPLIP
TTTPARPLLPFSTGPGSPLFQPLSFQSQGSPPKGPELSLASIHVPLESIKPSSALPVTAY
DKNGFRILFHFAKECPPGRPDVLVVVVSMLNTAPLPVKSIVLQAAVPKSMKVKLQPPSGT
ELSPFSPIQPPAAITQVMLLANPLKKQVLSFLGKACLQPWGQAILLTTSCLA*
>ENSG00000177674|ENST00000452018
MELPAVNLKVILLGHWLLTTCFWRHFSAKPRLETIELTCALCKLRSAAHRATAGLHCILR
LLCLGQLHHPGLGRVGCGSAGLHRRHKHVSGWLAGHHLPGHRAHQHLLPAGQPHGHGPLW
RGHGHPQLAAQAALLLLRLPHVPGARGFPWVFSGP*
>ENSG00000117394|ENST00000426263
MEPSSKKLTGRLMLAVGGAVLGSLQFGYNTGVINAPQKVIEEFYNQTWVHRYGESILPTT
LTTLWSLSVAIFSVGGMIGSFSVGLFVNRFGRRNSMLMMNLLAFVSAVLMGFSKLGKSFE
MLILGRFIIGVYCGLTTGFVPMYVGEVSPTALRGALGTLHQLGIVVGILIAQVFGLDSIM
GNKDLWPLLLSIIFIPALLQCIVLPFCPESPRFLLINRNEENRAKSVLKKLRGTADVTHD

LQEMKEESRQMMREKKVTILELFRSPAYRQPILIAVVLQLSQQLSGINAVFYYSTSIFEK
AGVQQPVYATIGSGIVNTAFTVVSLFVVERAGRRTLHLIGLAGMAGCAILMTIALALLEQ
LPWMSYLSIVAIFGFVAFFEVGPGPIPWFIVAELFSQGPRPAAIAVAGFSNWTSNFIVGM
CFQYVEQLCGPYVFIIFTVLLVLFFIFTYFKVPETKGRTFDEIASGFRQGGASQSDKTPE
ELFHPLGADSQV*
>ENSG00000147257|ENST00000631057
MAGTVRTACLVVAMLLSLDFPGQAQPPPPPPDATCHQVRSFFQRLQPGLKWVPETPVPEA
FEIVVRHAKNYTNAMFKNNYPSLTPQAFEFVGEFFTDVSLYILGSDINVDDMVNELFDSL
FPVIYTQLMNPGLPDSALDINECLRGARRDLKVFGNFPKLIMTQVSKSLQVTRIFLQALN
LGIEVINTTDHLKFSKDCGRMLTRMWYCSYCQGLMMVKPCGGYCNVVMQGCMAGVVEIDK
YWREYILSLEELVNGMYRIYDMENVLLGLFSTIHDSIQYVQKNAGKLTTTIGKLCAHSQQ
RQYRSAYYPEDLFIDKKVLKVAHVEHEETLSSRRRELIQKLKSFISFYSALPGYICSHSP
VAENDTLCWNGQELVERYSQKAARNGMKNQFNLHELKMKGPEPVVSQIIDKLKHINQLLR
TMSMPKGRVLDKNLDEEGFESGDCGDDEDECIGGSGDGMIKVKNQLRFLAELAYDLDVDD
APGNSQQATPKDNEISTFHNLGNVHSPLKLLTSMAISVVCFFFLVH*
>ENSG00000141198|ENST00000570371
MAFGKSHRDPYATSVGHLIEKATFAGVQTEDWGQFMHICDIINTTQDGPKDAVKALKKRI
SKNYNHKEIQLTLSLIDMCVQNCGPSFQSLIVKKEFVKENLVKLLNPRYNLPLDIQNRIL
NFIKTWSQGFPGGVDVSEVKEVYLDLVKKGVQFPPSEAEAETARQETAQISSNPPTSVPT
APALSSVIAPKNSTVTLVPEQIGKLHSELDMVKMNVRVMSAILMENTPGSENHEDIELLQ
KLYKTGREMQERIMDLLVVVENEDVTVELIQVNEDLNNAILGYERFTRNQQRILEQNKNQ
KEATNTTSEPSAPSQDLLDLSPSPRMPRATLGELNTMNNQLSGLSK*
>ENSG00000169344|ENST00000570689
MGQPSLTWMLMVVVASWFITTAATDTSEARWCSECHSNATCTEDEAVTTCTCQEGFTGDG
LTCVDLDECAIPGAHNCSANSSCVNTPGSFSCVCPEGFRLSPGLGCTDVDECAEPGLSHC
HALATCVNVVGSYLCVCPAGYRGDGWHCECSPGSCGPGLDCVPEGDALVCADPCQAHRTL
DEYWRSTEYGEGYACDTDLRGWYRFVGQGGARMAETCVPVLRCNTAAPMWLNGTHPSSDE
GIVSRKACAHWSGHCCLWDASVQVKACAGGYYVYNLTAPPECHLAYCTDPSSVEGTCEEC
SIDEDCKSNNGRWHCQCKQDFNITDISLLEHRLECGANDMKVSLGKCQLKSLGFDKVFMY
LSDSRCSGFNDRDNRDWVSVVTPARDGPCGTVLTRNETHATYSNTLYLADEIIIRDLNIK
INFACSYPLDMKVSLKTALQPMVSALNIRVGGTGMFTVRMALFQTPSYTQPYQGSSVTLS
TEAFLYVGTMLDGGDLSRFALLMTNCYATPSSNATDPLKYFIIQDRCPHTRDSTIQVVEN
GESSQGRFSVQMFRFAGNYDLVYLHCEVYLCDTMNEKCKPTCSGTRFRSGSVIDQSRVLN
LGPITRKGVQATVSRAFSSLGLLKVWLPLLLSATLTLTFQ*
>ENSG00000236884|ENST00000444645
MVCLKLPGGSCMAALTVTLMVLSSPLALAGDTQPRFLWQGKYKCHFFNGTERVQFLERLF
YNQEEFVRFDSDVGEYRAVTELGRPVAESWNSQKDILEDRRGQVDTVCRHNYGVGESFTV
QRRVHPEVTVYPAKTQPLQHHNLLVCSVSGFYPGSIEVRWFRNGQEEKAGVVSTGLIQNG
DWTFQTLVMLETVPRSGEVYTCQVEHPSVMSPLTVEWRARSESAQSKMLSGVGGFVLGLL
FLGAGLFIYFRNQKGHSGLQPTGFLS*
>ENSG00000228080|ENST00000419393
MVCLKFPGGSCMAALTVTLMVLSSPLALAGDTRPRFLEQVKHECHFFNGTERVRFLDRYF
YHQEEYVRFDSDVGEYRAVTELGRPDAEYWNSQKDLLEQRRAEVDTYCRHNYGVVESFTV
QRRVYPEVTVYPAKTQPLQHHNLLVCSVNGFYPGSIEVRWFRNGQEEKTGVVSTGLIQNG

DWTFQTLVMLETVPRSGEVYTCQVEHPSLTSPLTVEWRARSESAQSKMLSGVGGFVLGLL
FLGAGLFIYFRNQKGHSGLQPTGFLS*
>ENSG00000235915|ENST00000427186
MLSRNDAPSVLVPGPGLLRAGTPLCISAEAASAQQ*
>ENSG00000169255|ENST00000473142
MASALWTVLPSRMSLRSLKWSLLLLSLLSFFVMWYLSLPHYNVIERVNWMYFYEYEPI
>ENSG00000230726|ENST00000414698
MAISGVPVLGFFIIAVLMSAQESWAIKEEHVIIQAEFYLNPDQSGEFMFDFDGDEIFHVD
MAKKETVWRLEEFGRFASFEAQGALANIAVDKANLEIMTKRSNYTPITNVPPEVTVLTNS
PVELREPNVLICFIDKFTPPVVNVTWLRNGKPVTTGVSETVFLPREDHLFRKFHYLPFLP
STEDVYDCRVEHWGLDEPLLKHWEFDAPSPLPETTENVVCALGLTVGLVGIIIGTIFIIK
GVRKSNAAERRGPL*
>ENSG00000117620|ENST00000639148
MFANLKYVSLGILVFQTTSLVLTMRYSRTLKEEGPRYLSSTAVVVAELLKIMACILLVYK
DSMALRFSA*
>ENSG00000103056|ENST00000563226
MVLYTTPFPNSCLSALHCVSWALIFPCYWLVDRLAASFIPTTYEKRQRADDPCCLQLLCT
ALFTPIYLALLVASLPFAFLGFLFWSPLQSARRPYIYSRLEDKGLAGGAALLSEWKGTGP
GKSFCFATANVCLLPDSLARVNNLFNTQARAKEIGQRIRNGAARPQIKIYIDSPTNTSIS
AASFSSLVSPQGGDGVARAVPGSIKRTASVEYKGDGGRHPGDEAANGPASGDPVDSSSPE
DACIVRIGGEEGGRPPEADDPVPGGQARNGAGGGPRGQTPNHNQQDGDSGSLGSPSASRE
SLVKGRAGPDTSASGEPGANSKLLYKASVVKKAAARRRRHPDEAFDHEVSAFFPANLDFL
CLQEVFDKRAATKLKEQLHGYFEYILYDVGVYGCQGCCSFKCLNSGLLFASRYPIMDVAY
HCYPNKCNDDALASKGALFLKVQVGSTPQDQRIVGYIACTHLHAPQEDSAIRCGQLDLLQ
DWLADFRKSTSSSSAANPEELVAFDVVCGDFNFDNCSSDDKLEQQHSLFTHYRDPCRLGP
GTLLDTNGLYDEDVCTPDNLQKVLESEEGRREYLAFPTSKSSGQKGRKELLKGNGRRIDY
MLHAEEGLCPDWKAEVEEFSFITQLSGLTDHLPVAMRLMVSSGEEEA*
>ENSG00000166685|ENST00000582587
XALKRLDLRDPAALFETHGAEEIRGLERQVRAEIEHKKEELRQMVGERYRDLIEAADTIG
QMRRCAVGLVDAVKATDQYCARLRQAGSAAPRPPRAQQFCAFPCSHSSHPRRSSTAWLPR
SSYS*
>ENSG00000108669|ENST00000446868
MEEDDSYVPSDLTAEERQELENIRRRKQELLADIQRLKDEIAEVANEIENLGSTEERKNM
QRNKQVAMGRKKFNMDPKKGIQFLIENDLLKNTCEDIAQFLYKGEGLNKTAIGDYLGERD
EFNIQVLHAFVELHEFTDLNLVQALRQFLWSFRLPGEAQKIDRMMEAFAQRYCQCNNGVF
QSTDTCYVLSFAIIMLNTSLHNPNVKDKPTVERFIAMNRGINDGGDLPEELLRNLYESIK
NEPFKIPEDDGNDLTHTFFNPDREGWLLKLGGGRVKTWKRRWFILTDNCLYYFEYTTDKE
PRGIIPLENLSIREVEDSKKPNCFELYIPDNKDQVIKACKTEADGRVVEGNHTVYRISAP
TPEEKEEWIKCIKAAISRDPFYEMLAARKKKVSSTKRH*
>ENSG00000257923|ENST00000425244
MAANVGSMFQYWKRFDLQQLQRELDATATVLANRQDESEQSRKRLIEQSREFKKNTPEDL
RKQVAPLLKSFQGEIDALSKRSKEAEAAFLNVYKRLIDVPEVTIKALKEKIREYEQTLKN
QAETIALEKEQKLQNDFAEKERKLQETQMSTTSKLEEAEHKVQSLQTALEKTRTELFDLK
TKYDEETTAKADEIEMIMTDLERANQRAEVAQREAETLREQLSSANHSLQLASQIQKAPD

VEQAIEVLTRSSLEVELAAKEREIAQLVEDVQRLQASLTKLRENSASQISQLEQQLSAKN
STLKQLEEKLKGQADYEEVKKELNILKSMEFAPSEGAGTQDAAKPLEVLLLEKNRSLQSE
NAALRISNSDLSGRCAELQVRITEAVATATEQRELIARLEQDLSIIQSIQRPDAEGAAEH
RLEKIPEPIKEATALFYGPAAPASGALPEGQVDSLLSIISSQRERFRARNQELEAENRLA
QHTLQALQSELDSLRADNIKLFEKIKFLQSYPGRGSGSDDTELRYSSQYEERLDPFSSFS
KRERQRKYLSLSPWDKATLSMGRLVLSNKMARTIGFFYTLFLHCLVFLVLYKLAWSESME
RDCATFCAKKFADHLHKFHENDNGAAAGDLWQ*
>ENSG00000283761|ENST00000640357
MFANLKYVSLGILVFQTTSLVLTMRYSRTLKEEGPRYLSSTAVVVAELLKIMACILLVYK
DSKCSLRALNRVLHDEILNKPMETLKLAIPSGIYTLQNNLLYVALSNLDAATYQVTYQLK
ILTTALFSVSMLSKKLGVYQWLSLVILMTGVAFVQWPSDSQLDSKELSAGSQFVGLMAVL
TACFSSGFAGVYFEKILKETKQSVWIRNIQLGFFGSIFGLMGVYIYDGELVSKNGFFQGY
NRLTWIVVVLQALGGLVIAAVIKYADNILKGFATSLSIILSTLISYFWLQDFVPTSLKE*
>ENSG00000006118|ENST00000453848
MCARMAGRTTAAPRGPYGPWLCLLVALALDVVRVDCGQAPLDPVYLPAALELLDAPEHFR
VQQVGHYPPANSSLSSRSETFLLLQPWPRAQPLLRASYPPFATQQVVPPRVTEPHQRPVP
WDVRAVSVEAAVTPAEPYARVLFHLKGQDWPPGSGSLPCARLHATHPAGTAHQACRFQPS
LGACVVELELPSHWFSQASTTRAELAYTLEPAAEGPGGCGSGEENDPGEQALPVGGVELR
PADPPQYQEVPLDEAVTLRVPDMPVRPGQLFSATLLLRHNFTASLLTLRIKVKKGLHVTA
ARPAQPTLWTAKLDRFKGSRHHTTLITCHRAGLTEPDSSPLELSEFLWVDFVVENSTGGG
VAVTRPVTWQLEYPGQAPEAEKDKMVWEILVSERDIRALIPLAKAEELVNTAPLTGVPQH
VPVRLVTVDGGGALVEVTEHVGCESANTQVLQVSEACDAVFVAGKESRGARGVRVDFWWR
RLRASLRLTVWAPLLPLRIELTDTTLEQVRGWRVPGPAEGPAEPAAEASDEAERRARGCH
LQYQRAGVRFLAPFAAHPLDGGRRLTHLLGPDWLLDVSHLVAPHARVLDSRVASLEGGRV
VVGREPGVTSIEVRSPLSDSILGEQALAVTDDKVSVLELRVQPVMGISLTLSRGTAHPGE
VTATCWAQSALPAPKQEVALSLWLSFSDHTVAPAELYDRRDLGLSVSAEEPGAILPAEEQ
GAQLGVVVSGAGAEGLPLHVALHPPEPCRRGRHRVPLASGTAWLGLPPASTPAPALPSSP
AWSPPATEATMGGKRQVAGSVGGNTGVRGKFERAEEEARKEETEAREEEEEEEEEMVPAP
QHVTELELGMYALLGVFCVAIFIFLVNGVVFVLRYQRKEPPDSATDPTSPQPHNWVWLGT
DQEELSRQLDRQSPGPPKGEGSCPCESGGGGEAPTLAPGPPGGTTSSSSTLARKEAGGRR
KRVEFVTFAPAPPAQSPEEPVGAPAVQSILVAGEEDIRWVCEDMGLKDPEELRNYMERIR
GSS*
>ENSG00000235863|ENST00000451237
MQLRLFRRLLLAALLLVIVWTLFGPSGLGEELLSLSLASLLPAPASPGPPLALPRLLIPN
QEACSGPGAPPFLLILVCTAPENLNQRNAIRASWGGLREARGLRVQTLFLLGEPNAQHPV
WGSQGSDLASESAAQGDILQAAFQDSYRNLTLKTLSGLNWAEKHCPMARYVLKTDDDVYV
NVPELVSELVLRGGRWGQWERSTEPQREAEQEGGQVLHSEEVPLLYLGRVHWRVNPSRTP
GGRHRVSEEQWPHTWGPFPPYASGTGYVLSASAVQLILKVASRAPLLPLEDVFVGVSARR
GGLAPTQCVKLAGATHYPLDRCCYGKFLLTSHRLDPWKMQEAWKLVGGSDGERTAPFCSW
FQGVLGILRCRAIAWLQS*
>ENSG00000223501|ENST00000445902
MAAAATMAAAARELVLRAGTSDMEEEEGPLAGGPGLQEPLQLGELDITSDEFILDEVDVH
IQANLEDELVKEALKTGVDLRHYSKQVELELQQIEQKSIRDYIQESENIASLHNQITACD
AVLERMEQMLGAFQSDLSSISSEIRTLQEQSGAMNIRLRNRQAVRGKLGELVDGLVVPSA

LVTAILEAPVTEPRFLEQLQELDAKAAAVREQEARGTAACADVRGVLDRLRVKAVTKIRE
FILQKIYSFRKPMTNYQIPQTALLKYRFFYQFLLGNERATAKEIRDEYVETLSKIYLSYY
RSYLGRLMKVQYEEVAEKDDLMGVEDTAKKGFFSKPSLRSRNTIFTLGTRGSVISPTELE
APILVPHTAQRGEQRYPFEALFRSQHYALLDNSCREYLFICEFFVVSGPAAHDLFHAVMG
RTLSMTLKHLDSYLADCYDAIAVFLCIHIVLRFRNIAAKRDVPALDRYWEQVLALLWPRF
ELILEMNVQSVRSTDPQRLGGLDTRPHYITRRYAEFSSALVSINQTIPNERTMQLLGQLQ
VEVENFVLRVAAEFSSRKEQLVFLINNYDMMLGVLMERAADDSKEVESFQQLLNARTQEF
IEELLSPPFGGLVAFVKEAEALIERGQAERLRGEEARVTQLIRGFGSSWKSSVESLSQDV
MRSFTNFRNGTSIIQGALTQLIQLYHRFHRVLSQPQLRALPARAELINIHHLMVELKKHK
PNF*
>ENSG00000115020|ENST00000407449
MATDDKTSPTLDSANDLPRSPTSPSHLTHFKPLTPDQDEPPFKSAYSSFVNLFRFNKERA
EGGQGEQQPLSGSWTSPQLPSRTQSVRSPTPYKKQLNEELQRRSSALDTRRKAEPTFGGH
DPRTAVQLRSLSTVLKRLKEIMEGKSQDSDLKQYWMPDSQCKECYDCSEKFTTFRRRHHC
RLCGQIFCSRCCNQEIPGKFMGYTGDLRACTYCRKIALSYAHSTDSNSIGEDLNALSDSA
CSVSVLDPSEPRTPVGSRKASRNIFLEDDLAWQSLIHPDSSNTPLSTRLVSVQEDAGKSP
ARNRSASITNLSLDRSGSPMVPSYETSVSPQANRTYVRTETTEDERKILLDSVQLKDLWK
KICHHSSGMEFQDHRYWLRTHPNCIVGKELVNWLIRNGHIATRAQAIAIGQAMVDGRWLD
CVSHHDQLFRDEYALYRPLQSTEFSETPSPDSDSVNSVEGHSEPSWFKDIKFDDSDTEQI
AEEGDDNLANSASPSKRTSVSSFQSTVDSDSAASISLNVELDNVNFHIKKPSKYPHVPPH
PADQKGRR*
>ENSG00000231048|ENST00000437436
MLSRNDVKRLERSWRLPVPSSEGPDLRGRDKRGTKEDPRADYACIAENKPT*
>ENSG00000100626|ENST00000553669
MRKIRANAIAILTVAWILGTFYYLWQDNRAHAASSGGRGAQRAGRRSEQLREDRTIPLIV
TGTPSKGFDEKAYLSAKQLKAGEDPYRQHAFNQLESDKLSPDRPIRDTRHYSCPSVSYSS
DLPATSVIITFHNEARSTLLRTVKSVLNRTPANLIQEIILVDDFSSDPEDCLLLTRIPKV
KCLRNDRREGLIRSRVRGADVAAATVLTFLDSHCEVNTEWLPPMLQRVKEDHTRVVSPII
DVISLDNFAYLAASADLRGGFDWSLHFKWEQIPLEQKMTRTDPTRPIRTPVIAGGIFVID
KSWFNHLGKYDAQMDIWGGENFELSFRVWMCGGSLEIVPCSRVGHVFRKRHPYNFPEGNA
LTYIRNTKRTAEVWMDEYKQYYYEARPSAIGKAFGSVATRIEQRKKMNCKSFRWYLENVY
PELTVPVKEALPGIIKQGVNCLESQGGQNTAGDFLLGMGICRGSAKNPQPAQAWLFSDHLI
QQQGKCLAATSTLMSSPGSPVILQMCNPREGKQVSLLASGPEAQQPEGPCLRVADLGRRA
PD*
>ENSG00000114745|ENST00000431601
MKTMRVREVEVVPSNMWGGQGLLGASVRFCSFRRASEQVWHVLDVEPSSPAALAGLRPYT
DYVVGSDQILQESEDFFTLIESHEGKPLKLMVYNSKSDSCREVTVTPNAAWGGEGSLGCG
IGYGYLHRIPTQPPSYHKKPPGTPPPSALPLGAPPPDALPPGPTPEDSPSLETGSRQSDY
MEALLQAPGSSMEDPLPGPGSPSHSAPDPDGLPHFMETPLQPPPPVQRVMDPAMPVCGPA
CPLPQN*
>ENSG00000076864|ENST00000542643
MIEKMQGSRMDEQRCSFPPPLKTEEDYIPYPSVHEVLGREGPFPLILLPQFGGYWIEGTN
HEITSIPETEPLQSPTTKVKLECNPTARIYRKHFLGKEHFNYYSLDAALGHLVFSLKYDV
IGDQEHLRLLLRTKCRTYHDVIPISCLTEFPNVVQMAKLVCEDVNVDRFYPVLYPKASRL

IVTFDEHVISNNFKFGVIYQKLGQTSEEELFSTNEESPAFVEFLEFLGQKVKLQDFKGFR
GGLDVTHGQTGTESVYCNFRNKEIMFHVSTKLPYTEGDAQQLQRKRHIGNDIVAVVFQDE
NTPFVPDMIASNFLHAYVVVQAEGGGPDGPLYKVSVTARDDVPFFGPPLPDPAVFRKGPE
FQEFLLTKLINAEYACYKAEKFAKLEERTRAALLETLYEELHIHSQSMMGLGGDEDKMEN
GSGGGGFFESFKRVIRSRSQSMDAMGLSNKKPNTVSTSHSGSFAPNNPDLAKAAGISLLI
PGKSASRFGRRGSAIGIGTVEESLIVPGKSPTRKKSGPFGSRRSSAIGIENIQEVQEKRE
SPPAGQKTPDSGHVSQEPKSENSSTQSSPEMPTTKNRAETAAQRAEALKDFSRSSSSASS
FASVVEETEGVDGEDTGLESVSSSGTPHKRDSFIYSTWLEDSVSTTSGGSSPDAGKLGDP
ACPEIKIQLEASEQHMPQLGC*
>ENSG00000125430|ENST00000466596
MGQRLSGGRSCLDVPGRLLPQPPPPPPPVRRKLALLFAMLCVWLYMFLYSCAGSCAAAPG
LLLLGSGSRAAHDPPALATAPDGTPPRLPFRAPPATPLASGKEMAEGAASPEEQSPEVPD
SPSPISSFFSGSGSKQLPQAIIIGVKKGGTRALLEFLRVHPDVRAVGAEPHFFDRSYDKG
LAWYRDLMPRTLDGQITMEKTPSYFVTREAPARISAMSKDTKLIVVVRDPVTRAISDYTQ
TLSKRPDIPTFESLTFKNRTAGLIDTSWSAIQIGIYAKHLEHWLRHFPIRQMLFVSGERL
ISDPAGELGRVQDFLGLKRIITDKHFYFNKTKGFPCLKKAEGSSRPHCLGKTKGRTHPEI
DREVVRRLREFYRPFNLKFYQMTGHDFGWD*
>ENSG00000124302|ENST00000591231
MTLRPGTMRLACMFSSILLFGAAGLLLFISLQDPTELAPQQVPGIKFNIRPRQPHHDLPP
GGSQDGDLKEPTERVTRDLSSGAPRGRNLPAPDQPQPPLQRGTRLRLRQRRRRLLIKKMP
AAATIPANSSDAPFIRPGPGTLDGRWVSLHRSQQERKRVMQEACAKYRASSSRRAVTP
>ENSG00000166710|ENST00000561424
MSRSVALAVLALLSLSGLEAIQRTPKIQVYSRHPAENGKSNFLNCYVSGFHPSDIEVDLL
KNGERIEKVEHSDLSFSKDWSFYLLYYTEFTPTEKDEYACRVNHVTLSQPKIVKWDRDM*
>ENSG00000138760|ENST00000452464
MGRCCFYTAGTLSLLLLVTSVTLLVARVFQKAVDQSIEKKIVLRNGTEAFDSWEKPPLPV
YTQFYFFNVTNPEEILRGETPRVEEVGPYTYRSLDWWITDKCNMINGTDGDSFHPLITKD
EVLYVFPSDFCRSVYITFSDYESVQGLPAFRYKVPAEILANTSDNAGFCIPEGNCLGSGV
LNVSICKNGAPIIMSFPHFYQADERFVSAIEGMHPNQEDHETFVDINPLTGIILKAAKRF
QINIYVKKLDDFVETGDIRTMVFPVMYLNESVHIDKETASRLKSMINTTLIITNIPYIIM
ALGVFFGLVFTWLACKGQGSMDEGTADERAPLIRT*
>ENSG00000234514|ENST00000436037
MLSRNDDICIYGGLGLGGLLLLAVVLLSACLCWLHRRVKRLERSWHLLSWSQAQGSSEQE
LHYASLQRLPVPSSEGPDLRGRDKRGTKEDPRADYACIAENKPT*
>ENSG00000263160|ENST00000573374
MAGFLDNFRWPECECIDWSERRNAVASVVAGILFFTGWWIMIDAAVVYPKPEQLNHAFHT
CGVFSTLAFFMINAVSNAQVRGDSYESGCLGRTGARVWLFIGFMLMFGSLIASMWILFGA
YVTQNTDVYPGLAVFFQNALIFFSTLIYKFGRTEELWT*
>ENSG00000121578|ENST00000483209
MGFNLTFHLSYKFRLLLLLTLCLTVVGWATSNYFVGAIQEIPKAKEFMANFHKTLILGKG
KTLTNEASTKKVELDNCPSVSPYLRGQSKLIFKPDLTLEEVQAENPKVSRGRYRPQECKA
LQRVAILVPHRNREKHLMYLLEHLHPFLQRQQLDYGIYVIHQAEGKKFNRAKLLNVGYLE
ALKEENWDCFIFHDVDLVPENDFNLYKCEEHPKHLVVGRNSTGYRLRYSGYFGGVTALSR
EQFFKVNGFSNNYWGWGGEDDDLRLRVELQRMKISRPLPEVGKYTMVFHTRDKGNEVNAE

RMKLLHQVSRVWRTDGLSSCSYKLVSVEHNPLYINITVDFWFGA*
>ENSG00000129083|ENST00000439561
MTAAENVCYTLINVPMDSEPPSEISLKNDLEKGDVKSKTEALKKVIIMILNGEKLPGLLM
TIIRFVLPLQDHTIKKLLLVFWEIVPKTTPDGRLLHEMILVCDAYRKDLQHPNEFIRGST
LRFLCKLKEAELLEPLMPAIRACLEHRHSYVRRNAVLAIYTIYRNFEHLIPDAPELIHDF
LVNEKDASCKRNAFMMLIHADQDRALDYLSTCIDQVQTFGDILQLVIVELIYKVCHANPS
ERARFIRCIYNLLQSSSPAVKYEAAGTLVTLSSAPTAIKAAAQCYIDLIIKESDNNVKLI
VLDRLIELKEHPAHERVLQDLVMDILRVLSTPDLEVRKKTLQLALDLVSSRNVEELVIVL
KKEVIKTNNVSEHEDTDKYRQLLVRTLHSCSVRFPDMAANVIPVLMEFLSDNNEAAAADV
LEFVREAIQRFDNLRMLIVEKMLEVFHAIKSVKIYRGALWILGEYCSTKEDIQSVMTEIR
RSLGEIPIVESEIKKEAGELKPEEEITVGPVQKLVTEMGTYATQSALSSSRPTKKEEDRP
PLRGFLLDGDFFVAASLATTLTKIALRYVALVQEKKKQNSFVAEAMLLMATILHLGKSSL
PKKPITDDDVDRISLCLKVLSECSPLMNDIFNKECRQSLSHMLSAKLEEEKLSQKKESEK
RNVTVQPDDPISFMQLTAKNEMNCKEDQFQLSLLAAMGNTQRKEAADPLASKLNKVTQLT
GFSDPVYAEAYVHVNQYDIVLDVLVVNQTSDTLQNCTLELATLGDLKLVEKPSPLTLAPH
DFANIKANVKVASTENGIIFGNIVYDVSGAASDRNCVVLSDIHIDIMDYIQPATCTDAEF
RQMWAEFEWENKVTVNTNMVDLNDYLQHILKSTNMKCLTPEKALSGYCGFMAANLYARSI
FGEDALANVSIEKPIHQGPDAAVTGHIRIRAKSQGMALSLGDKINLSQKKTSI*
>ENSG00000197249|ENST00000440909
MPSSVSWGILLLAGLCCLVPVSLAEDPQGDAAQKTDTSHHDQDHPTFNKITPNLAEFAFS
LYRQLAHQSNSTNIFFSPVSIATAFAMLSLGTKADTHDEILEGLNFNLTEIPEAQIHEGF
QELLRTLNQPDSQLQLTTGNGLFLSEGLKLVDKFLEDVKKLYHSEAFTVNFGDTEEAKKQ
INDYVEKGTQGKIVDLVKELDRDTVFALVNYIFFKGKWERPFEVKDTEEEDFHVDQVTTV
KVPMMKRLGMFNIQHCKKLSSWVLLMKYLGNATAIFFLPDEGKLQHLENELTHDIITKFL
ENEDRRSASLHLPKLSITGTYDLKSVLGQLGITKVFSNGADLSGVTEEAPLKLSKAVHKA
VLTIDEKGTEAAGAMFLEAIPMSIPPEVKFNKPFVFLMIEQNTKSPLFMGKVVNPTQK*
>ENSG00000223465|ENST00000440253
MLSRNDVKRLERSWAQGSSEQELHYASLQRLPVPSSEGPDLRGRDKRGTKEDPRADYACI
AENKPT*
>ENSG00000126091|ENST00000469715
MGLLVFVRNLLLALCLFLVLGFLYYSAWKLHLLQWEEDSSSPSQHPCSWMTPFASGLESG
SSCRLLGSKVKTI*
>ENSG00000226182|ENST00000415781
MLSRNDEAASAQQ*
>ENSG00000172728|ENST00000518672
MVELGKFERKEFKSSSLQDGHTKMEEAPTHLNSFLKKEGLTFNRKRKWELDSYPIMLWWS
PLTGETGRLGQCGADACFFTINRTYLHHHMTKAFLFYGTDFNIDSLPLPRKAHHDWAVFH
EESPKNNYKLFHKPVITLFNYTATFSRHSHLPLTTQYLESIEVLKSLRYLVPLQSKNKLR
KRLAPLVYVQSDCDPPSDRDSYVRELMTYIEVDSYGECLRNKDLPQQLKNPASMDADGFY
RIIAQYKFILAFENAVCDDYITEKFWRPLKLGVVPVYYGSPSITDWLPSNKSAILVSEFS
HPRELASYIRRLDSDDRLYEAYVEWKLKGEISNQRLLTALRERKWGVQDVNQDNYIDAFE
CMVCTKVWANIRLQEKGLPPKRWEAEDTHLSCPEPTVFAFSPLRTPPLSSLREMWISSFE
QSKKEAQALRWLVDRNQNFSSQEFWGLVFKD*
>ENSG00000114354|ENST00000487505

MNGQLDLSGKLIIKAQLGEDIRRIPIHNEDITYDELVLMMQRVFRGKLLSNDEVTIKYKD
EDGDLITIFDSSDLSFAIQCSRILKLTLFVNGQPRPLESSQVKYLRRELIELRNKVNRLL
DSLEPPGEPGPSTNIPENDTVDGREEKSASDSSGKQSTQVMAASMSAFDPLKNQDEINKN
V
>ENSG00000139292|ENST00000540815
MDTSRLGVLLSLPVLLQLATGGSSPRSGVLLRGCPTHCHCEPDGRMLLRVDCSDLGLSEL
PSNLSVFTSYLDLSMNNISQLLPNPLPSLRFLEELRLAGNALTYIPKGAFTGLYSLKVLM
LQNNQLRHVPTEALQNLRSLQSLRLDANHISYVPPSCFSGLHSLRHLWLDDNALTEIPVQ
AFRSLSALQAMTLALNKIHHIPDYAFGNLSSLVVLHLHNNRIHSLGKKCFDGLHSLETLD
LNYNNLDEFPTAIRTLSNLKELHFYDNPIQFVGRSAFQHLPELRTLTLNGASQITEFPDL
TGTANLESLTLTGAQISSLPQTVCNQLPNLQVLDLSYNLLEDLPSFSVCQKLQKIDLRHN
EIYEIKVDTFQQLLSLRSLNLAWNKIAIIHPNAFSTLPSLIKLDLSSNLLSSFPITGLHG
LTHLKLTGNHALQSLISSENFPELKVIEMPYAYQCCAFGVCENAYKISNQWNKGDNSSMD
DLHKKDAGMFQAQDERDLEDFLLDFEEDLKALHSVQCSPSPGPFKPCEHLLDGWLIRIGV
WTIAVLALTCNALVTSTVFRSPLYISPIKLLIGVIAAVNMLTGVSSAVLAGVDAFTFGSF
ARHGAWWENGVGCHVIGFLSIFASESSVFLLTLAALERGFSVKYSAKFETKAPFSSLKVI
ILLCALLALTMAAVPLLGGSKYGASPLCLPLPFGEPSTMGYMVALILLNSLCFLMMTIAY
TKLYCNLDKGDLENIWDCSMVKHIALLLFTNCILNCPVAFLSFSSLINLTFISPEVIKFI
LLVVVPLPACLNPLLYILFNPHFKEDLVSLRKQTYVWTRSKHPSLMSINSDDVEKQSCDS
TQALVTFTSSSITYDLPPSSVPSPAYPVTESCHLSSVAFVPCL*
>ENSG00000166377|ENST00000426216
MADQIPLYPVRSAAAAAANRKRAAYYSAAGPRPGADRHSRYQLEDESAHLDEMPLMMSEE
GFENEESDYHTLPRARIMQRKRGLEWFVCDGWKFLCTSCCGWLINICRRKKELKARTVWL
GCPEKCEEKHPRNSIKNQKYNVFTFIPGVLYEQFKFFLNLYFLVISCSQFVPALKIGYLY
TYWAPLGFVLAVTMTREAIDEFRRFQRDKEVNSQLYSKLTVRGKVQVKSSDIQVGDLIIV
EKNQRIPSDMVFLRTSEKAGSCFIRTDQLDGETDWKLKVAVSCTQQLPALGDLFSISAYV
YAQKPQMDIHSFEGTFTREDSDPPIHESLSIENTLWASTIVASGTVIGVVIYTGKETRSV
MNTSNPKNKVGLLDLELNRLTKALFLALVALSIVMVTLQGFVGPWYRNLFRFLLLFSYII
PISLRVNLDMGKAVYGWMMMKDENIPGTVVRTSTIPEELGRLVYLLTDKTGTLTQNEMIF
KRLHLGTVSYGADTMDEIQSHVRDSYSQMQSQAGGNNTGSTPLRKAQSSAPKVRKSVSSR
IHEAVKAIVLCHNVTPVYESRAGVTEETEFAEADQDFSDENRTYQASSPDEVALVQWTES
VGLTLVSRDLTSMQLKTPSGQVLSFCILQLFPFTSESKRMGVIVRDESTAEITFYMKGAD
VAMSPIVQYNDWLEEECGNMAREGLRTLVVAKKALTEEQYQDFESRYTQAKLSMHDRSLK
VAAVVESLEREMELLCLTGVEDQLQADVRPTLEMLRNAGIKIWMLTGDKLETATCIAKSS
HLVSRTQDIHIFRQVTSRGEAHLELNAFRRKHDCALVISGDSLEVCLKYYEHEFVELACQ
CPAVVCCRCSPTQKARIVTLLQQHTGRRTCAIGDGGNDVSMIQAADCGIGIEGKEGKQAS
LAADFSITQFRHIGRLLMVHGRNSYKRSAALGQFVMHRGLIISTMQAVFSSVFYFASVPL
YQGFLMVGYATIYTMFPVFSLVLDQDVKPEMAMLYPELYKDLTKGRSLSFKTFLIWVLIS
IYQGGILMYGALVLFESEFVHVVAISFTALILTELLMVALTVRTWHWLMVVAEFLSLGCY
VSSLAFLNEYFGIGRVSFGAFLDVAFITTVTFLWKVSAITVVSCLPLYVLKYLRRKLSPP
SYCKLAS*
>ENSG00000184304|ENST00000616995
MSAPPVLRPPSPLLPVAAAAAAAAAALVPGSGPGPAPFLAPVAAPVGGISFHLQIGLSRE
PVLLLQDSSGDYSLAHVREMACSIVDQKFPECGFYGMYDKILLFRHDPTSENILQLVKAA

SDIQEGDLIEVVLSASATFEDFQIRPHALFVHSYRAPAFCDHCGEMLWGLVRQGLKCEGC
GLNYHKRCAFKIPNNCSGVRRRRLSNVSLTGVSTIRTSSAELSTSAPDEPLLQKSPSESF
IGREKRSNSQSYIGRPIHLDKILMSKVKVPHTFVIHSYTRPTVCQYCKKLLKGLFRQGLQ
CKDCRFNCHKRCAPKVPNNCLGEVTINGDLLSPGAESDVVMEEGSDDNDSERNSGLMDDM
EEAMVQDAEMAMAECQNDSGEMQDPDPDHEDANRTISPSTSNNIPLMRVVQSVKHTKRKS
STVMKEGWMVHYTSKDTLRKRHYWRLDSKCITLFQNDTGSRYYKEIPLSEILSLEPVKTS
ALIPNGANPHCFEITTANVVYYVGENVVNPSSPSPNNSVLTSGVGADVARMWEIAIQHAL
MPVIPKGSSVGTGTNLHRDISVSISVSNCQIQENVDISTVYQIFPDEVLGSGQFGIVYGG
KHRKTGRDVAIKIIDKLRFPTKQESQLRNEVAILQNLHHPGVVNLECMFETPERVFVVME
KLHGDMLEMILSSEKGRLPEHITKFLITQILVALRHLHFKNIVHCDLKPENVLLASADPF
PQVKLCDFGFARIIGEKSFRRSVVGTPAYLAPEVLRNKGYNRSLDMWSVGVIIYVSLSGT
FPFNEDEDIHDQIQNAAFMYPPNPWKEISHEAIDLINNLLQVKMRKRYSVDKTLSHPWLQ
DYQTWLDLRELECKIGERYITHESDDLRWEKYAGEQGLQYPTHLINPSASHSDTPETEET
EMKALGERVSIL*
>ENSG00000105063|ENST00000587283
MFWKFDLHTSSHLDTLLEREDLSLPELLDEEDVLQECKVVNRKLLDFLLQPPHLQAMVAW
VTQEPPDSGEERLRYKYPSVACEILTSDVPQINDALGADESLLNRLYGFLQSTGSLNPLL
ASFFSKVMGILINRKTDQLVSFLRKKDDFVDLLLQHIGTSAIMDLLLRLLTCVERPQLRQ
DVVNWLNEEKIVQRLIEQIHPSKDENQHSNASQSLCDIIRLSREQMIQVQDSPEPDQLLA
TLEKQETIEQLLSNMFEGEQSQSVIVSGIQVLLTLLEPRRPRSESVTVNSFFSSVDGQLE
LLAQGALESTVSSVGALHALRPRLSCFHQLLLEPPKLEPLQMTWGMLAPPLGNTRLHVVK
LLASALSANDAALTHELLALDVPNTMLDLFFHYVFNNFLHAQVEGCVSTMLSLGPPPDSS
PETPIQNPVVKHLLQQCRLVERILTSWEENDRVQCAGGPRKGYMGHLTRVAGALVQNTEK
GPNAEQLRQLLKELPSEQQEQWEAFVSGPLAETNKKNMVDLVNTHHLHSSSDDEDDRLKE
FNFPEEAVLQQAFMDFQMQRMTSAFIDHFGFNDEEFGEQEESVNAPFDKTANITFSLNAD
DENPNANLLEICYKDRIQQFDDDEEEEDEEEAQGSGESDGEDGAWQGSQLARGARLGQPP
GVRSGGSTDSEDEEEEDEEEEEDEEGIGCAARGGATPLSYPSPGPQPPGPSWTATFDPVP
TDAPTSPRVSGEEELHTGPPAPQGPLSVPQGLPTQSLASPPARDALQLRSQDPTPPSAPQ
EATEGSKVTEPSAPCQALVSIGDLQATFHGIRSAPSSSDSATRDPSTSVPASGAHQPPQT
TEGEKSPEPLGLPQSQSAQALTPPPIPNGSAPEGPASPGSQ*
>ENSG00000103174|ENST00000562746
MATSTGRWLLLRLALFGFLWEASGGLDSGASRDDDLLLPYPRARARLPRDCTRVRAGNRE
HESWPPPPATPGAGGLAVRTFVSHFRDRAVAGHLTRAVEPLRTFSVLEPGGPGGCAARRR
ATVEETARAADCRVAQNGGFFRMNSGECLGNVVSDERRVSSSGGLQNAQFGIRRDGTLVT
GYLSEEEVLDTENPFVQLLSGVVWLIRNGSIYINESQATECDETQETGSFSKFVNVISAR
TAIGHDRKGQLVLFHADGQTEQRGINLWEMAEFLLKQDVVNAINLDGGGSATFVLNGTLA
SYPSDHWQA*
>ENSG00000171310|ENST00000549260
MKPALLEVMRMNRICRMVLATCLGSFILVIFYFQIMRRNPFGVDICCRKGSRSPLQELYN
PIQLELSNTAVLHQMRRDQVTDTCRANSATSRKRRVLTPNDLKHLVVDEDHELIYCYVPK
VACTNWKRLMMVLTGRGKYSDPMEIPANEAHVSANLKTLNQYSIPEINHRLKSYMKFLFV
REPFERLVSAYRNKFTQKYNISFHKRYGTKIIKRQRKNATQEALRKGDDVKFEEFVAYLI
DPHTQREEPFNEHWQTVYSLCHPCHIHYDLVGKYETLEEDSNYVLQLAGVGSYLKFPTYA
KSTRTTDEMTTEFFQNISSEHQTQLYEVYKLDFLMFNYSVPSYLKLE*

>ENSG00000276600|ENST00000623597
MNPRKKVDLKLIIVGAIGVGKTSLLHQYVHKTFYEEYQTTLGASILSKIIILGDTTLKLQ
IWDTGGQERFRSMVSTFYKGSDGCILAFDVTDLESFEALDIWRGDVLAKIVPMEQSYPMV
LLGNKIDLADRKVPQEVAQGWCREKDIPYFEVSAKNDINVVQAFEMLASRALSRYQSILE
NHLTESIKLSPDQSRSRCC*
>ENSG00000122705|ENST00000464497
MAELDPFGAPAGAPGGPALGNGVAGAGEEDPAAAFLAQQESEIAGIENDEAFAILDGGAP
GPQPHGEPPGGPDAVDGVMNGEYYQESNGPTDSYAAISQVDRLQSEPESIRKWREEQMER
LEALDANSRKQEAEWKEKAIKELEEWYARQDEQLQKTKANNRVSLLPRLECNGVISAHCN
LCLPGSSNSPASASRGAGITDMCHRARLIFVFLVEMGFHHVGQAGLKILTSNDLPASARA
RITGRWKGVKDVAMYVPSLP*
>ENSG00000157916|ENST00000605895
MSEGDSVGESVHGKPSVVYRFFTRLGQIYQSWLDKSTPYTAVRWVVTLGLSFVYMIRVYL
LQGWYIVTYALGIYHLNLFIAFLSPKVDPSLMEDSDDGPSLPTKQNEEFRPFIRRLPEFK
FWHAATKGILVAMVCTFFDAFNVPVFWPILVMYFIMLFCITMKRQIKHMIKYRYIPFTHG
KRRYRGKEDAGKAFAS*
>ENSG00000223793|ENST00000551533
MILNKALLLGALALTAVMSPCGGEDIVADHVASYGVNFYQSHGPSGQYTHEFDGDEEFYV
DLETKETVWQLPMFSKFISFDPQSALRNMAVGKHTLEFMMRQSNSTAATNEVPEVTVFSK
FPVTLGQPNTLICLVDNIFPPVVNITWLSNGHSVTEGVSETSFLSKSDHSFFKISYLTFL
PSADEIYDCKVEHWGLDEPLLKHWEPEIPAPMSELTETLVCALGLSVGLMGIVVGTVFII
QGLRSVGASRHQGLL*
>ENSG00000110080|ENST00000530591
MVSKSRWKLLAMLALVLVVMVWYSISREDSFYFPIPEKKEPCLQGEAESKASKLFGNYSR
DQPIFLRLEDYFWVKTPSAYELPYGTKGSEDLLLRVLAITSSSIPKNIQSLRCRRCVVVG
NGHRLRNSSLGDAINKYDVVIRLNNAPVAGYEGDVGSKTTMRLFYPESAHFDPKVENNPD
TLLVLVAFKAMDFHWIETILSDKKRVRKGFWKQPPLIWDVNPKQIRILNPFFMEIAADKL
LSLPMQQPRKIKQKPTTGLLAITLALHLCDLVHIAGFGYPDAYNKKQTIHYYEQITLKSM
AGSGHNVSQEALAIKRMLEMGAIKNLTSF*
>ENSG00000283530|ENST00000636211
MFKFHQMKHIFEILDKMRCLRKRSTVSFLGVLVIFLLFMNLYIEDSYVLEGDKQLIRETS
THQLNSERYVHTFKDLSNFSGAINVTYRYLAATPLQRKRYLTIGLSSVKRKKGNYLLETI
KSIFEQSSYEELKEISVVVHLADFNSSWRDAMVQDITQKFAHHIIAGRLMVIHAPEEYYP
ILDGLKRNYNDPEDRVKFRSKQNVDYAFLLNFCANTSDYYVMLEDDVRCSKNFLTAIKKV
IASLEGTYWVTLEFSKLGYIGKLYHSHDLPRLAHFLLMFYQEMPCDWLLTHFRGLLAQKN
VIRFKPSLFQHMGYYSSYKGTENKLKDDDFEEESFDIPDNPPASLYTNMNVFENYEASKA
YSSVDEYFWGKPPSTGDVFVIVFENPIIIKKIKVNTGTEDRQNDILHHGALDVGENVMPS
KQRRQCSTYLRLGEFKNGNFEMSGVNQKIPFDIHCMRIYVTKTQKEWLIIRSISIWTS*
>ENSG00000146648|ENST00000420316
MRPSGTAGAALLALLAALCPASRALEEKKVCQGTSNKLTQLGTFEDHFLSLQRMFNNCEV
VLGNLEITYVQRNYDLSFLKTIQEVAGYVLIALNTVERIPLENLQIIRGNMYYENSYALA
VLSNYDANKTGLKELPMRNLQEILHGAVRFSNNPALCNVESIQWRDIVSSDFLSNMSMDF
QNHLGSCQKCDPSCPNGSCWGAGEENCQKLTKIICAQQCSGRCRGKSPSDCCHNQCAAGC
TGPRESDCLVCRKFRDEATCKDTCPPLMLYNPTTYQMDVNPEGKYSFGATCVKKCPRNYV

VTDHGSCVRACGADSYEMEEDGVRKCKKCEGPCRKVCNGIGIGEFKDSLSINATNIKHFK
NCTSISGDLHILPVAFRGDSFTHTPPLDPQELDILKTVKEITGLS*
>ENSG00000138834|ENST00000610761
MMEIQMDEGGGVVVYQDDYCSGSVMSERVSGLAGSIYREFERLIHCYDEEVVKELMPLVV
NVLENLDSVLSENQEHEVELELLREDNEQLLTQYEREKALRRQAEEKFIEFEDALEQEKK
ELQIQVEHYEFQTRQLELKAKNYADQISRLEERESEMKKEYNALHQRHTEMIQTYVEHIE
RSKMQQVGGNSQTESSLPGRSRKERPTSLNVFPLADGTVRAQIGGKLVPAGDHWHLSDLG
QLQSSSSYQCPQDEMSESGQSSAAATPSTTGTKSNTPTSSVPSAAVTPLNESLQPLGDYG
VGSKNSKRAREKRDSRNMEVQVTQEMRNVSIGMGSSDEWSDVQDIIDSTPELDMCPETRL
DRTGSSPTQGIVNKAFGINTDSLYHELSTAGSEVIGDVDEGADLLGEFSVRDDFFGMGKE
VGNLLLENSQLLETKNALNVVKNDLIAKVDQLSGEQEVLRGELEAAKQAKVKLENRIKEL
EEELKRVKSEAIIARREPKEEAEDVSSYLCTESDKIPMAQRRRFTRVEMARVLMERNQYK
ERLMELQEAVRWTEMIRASREHPSVQEKKKSTIWQFFSRLFSSSSSPPPAKRPYPSVNIH
YKSPTTAGFSQRRNHAMCPISAGSRPLEFFPDDDCTSSARREQKREQYRQVREHVRNDDG
RLQACGWSLPAKYKQLSPNGGQEDTRMKNVPVPVYCRPLVEKDPTMKLWCAAGVNLSGWR
PNEDDAGNGVKPAPGRDPLTCDREGDGEPKSAHTSPEKKKAKELPEMDATSSRVWILTST
LTTSKVVIIDANQPGTVVDQFTVCNAHVLCISSIPAASDSDYPPGEMFLDSDVNPEDPGA
DGVLAGITLVGCATRCNVPRSNCSSRGDTPVLDKGQGEVATIANGKVNPSQSTEEATEAT
EVPDPGPSEPETATLRPGPLTEHVFTDPAPTPSSGPQPGSENGPEPDSSSTRPEPEPSGD
PTGAGSSAAPTMWLGAQNGWLYVHSAVANWKKCLHSIKLKDSVLSLVHVKGRVLVALADG
TLAIFHRGEDGQWDLSNYHLMDLGHPHHSIRCMAVVYDRVWCGYKNKVHVIQPKTMQIEK
SFDAHPRRESQVRQLAWIGDGVWVSIRLDSTLRLYHAHTHQHLQDVDIEPYVSKMLGTGK
LGFSFVRITALLVAGSRLWVGTGNGVVISIPLTETVVLHRGQLLGLRANKTSPTSGEGAR
PGGIIHVYGDDSSDRAASSFIPYCSMAQAQLCFHGHRDAVKFFVSVPGNVLATLNGSVLD
SPAEGPGPAAPASEVEGQKLRNVLVLSGGEGYIDFRIGDGEDDETEEGAGDMSQVKPVLS
KAERSHIIVWQVSYTPE*
>ENSG00000114354|ENST00000463568
MNGQLDLSGKLIIKAQLGEDIRRIPIHNEDITYDELVLMMQRVFRGKLLSNDEVTIKYKD
EDGDLITIFDSSDLSFAIQCSRILKLTLFVNGQPRPLESSQVKYLRRELIELRNKVNRLL
DSLEPPGEPGPSTNIPENDTVDGREEKSASDSSGKQS
>ENSG00000126091|ENST00000533933
LGFLYYSAWKLHLLQWEEDSNSVVLSFDSAGQTLGSEYDRLGFLLNLDSKLPAELATKYA
NFSEGACKPGYASALMTAIFPRFSKPAPMFLDDSFRKWARIREFVPPFGIKGQDNLIKAI
LSVTKEYRLTPALDSLRCRRCIIVGNGGVLANKSLGSRIDDYDIVVRLNSAPVKGFEKDV
GSKTTLRITYPEGAMQRPEQYERDSLFVLAGFKWQDFKWLKYIVYKERVSASDGFWKSVA
TRVPKEPPEIRILNPYFIQEAAFTLIGLPFNNGLMGRGSWTHNIQREKEFLRKLVKARVI
TDLSSGI*
>ENSG00000137497|ENST00000613205
MTLHATRGAALLSWVNSLHVADPVEAVLQLQDCSIFIKIIDRIHGTEEGQQILKQPVSER
LDFVCSFLQKNRKHPSSPECLVSAQKVLEGSELELAKMTMLLLYHSTMSSKSPRDWEQFE
YKIQAELAVILKFVLDHEDGLNLNEDLENFLQKAPVPSTCSSTFPEELSPPSHQAKREIR
FLELQKVASSSSGNNFLSGSPASPMGDILQTPQFQMRRLKKQLADERSNRDELELELAEN
RKLLTEKDAQIAMMQQRIDRLALLNEKQAASPLEPKELEELRDKNESLTMRLHETLKQCQ
DLKTEKSQMDRKINQLSEENGDLSFKLREFASHLQQLQDALNELTEEHSKATQEWLEKQA

QLEKELSAALQDKKCLEEKNEILQGKLSQLEEHLSQLQDNPPQEKGEVLGDVLQVEELSK
KLADSDQASKVQQQKLKAVQAQGGESQQEAQRLQAQLNELQAQLSQKEQAAEHYKLQMEK
AKTHYDAKKQQNQELQEQLRSLEQLQKENKELRAEAERLGHELQQAGLKTKEAEQTCRHL
TAQVRSLEAQVAHADQQLRDLGKFQVATDALKSREPQAKPQLDLSIDSLDLSCEEGTPLS
ITSKLPRTQPDGTSVPGEPASPISQRLPPKVESLESLYFTPIPARSQAPLESSLDSLGDV
FLDSGRKTRSARRRTTQIINITMTKKLDVEEPDSANSSFYSTRSAPASQASLRATSSTQS
LARLGSPDYGNSALLSLPGYRPTTRSSARRSQAGVSSGAPPGRNSFYMGTCQDEPEQLDD
WNRIAELQQRNRVCPPHLKTCYPLESRPSLSLGTITDEEMKTGDPQETLRRASMQPIQIA
EGTGITTRQQRKRVSLEPHQGPGTPESKKATSCFPRPMTPRDRHEGRKQSTTEAQKKAAP
ASTKQADRRQSMAFSILNTPKKLGNSLLRRGASKKALSKASPNTRSGTRRSPRIATTTAS
AATAAAIGATPRAKGKAKH*
>ENSG00000101236|ENST00000545616
MLNKSGESRYPALFPVLGGSSMSSDFPHYNFRMPNIGFQNLPLNIYIVVFGTAIFVFILS
LLFCCYLIRLRHQAHKEFYAYKQVILKEKVKELNLHELCAVCLEDFKPRDELGICPCKHA
FHRKCLIKWLEVRKVCPLCNMPVLQLAQLHSKQDRGPPQGPLPGAENIV*
>ENSG00000275365|ENST00000612388
MAPAAWLRSAAARALLPPMLLLLLQPPPLLARALPPDAHHLHAERRGPQPWHAALPSSPA
PAPATQEAPRPASSLRPPRCGVPDPSDGLSARNRQKRFVLSGGRWEKTDLTYRILRFPWQ
LVQEQVRQTMAEALKVWSDVTPLTFTEVHEGRADIMIDFARYWHGDDLPFDGPGGILAHA
FFPKTHREGDVHFDYDETWTIGDDQGTDLLQVAAHEFGHVLGLQHTTAAKALMSAFYTFR
YPLSLSPDDCRGVQHLYGQPWPTVTSRTPALGPQAGIDTNEIAPLEPDAPPDACEASFDA
VSTIRGELFFFKAGFVWRLRGGQLQPGYPALASRHWQGLPSPVDAAFEDAQGHIWFFQGA
QYWVYDGEKPVLGPAPLTELGLVRFPVHAALVWGPEKNKIYFFRGRDYWRFHPSTRRVDS
PVPRRATDWRGVPSEIDAAFQDADGYAYFLRGRLYWKFDPVKVKALEGFPRLVGPDFFGC
AEPANTFL*
>ENSG00000102531|ENST00000541916
MAEHPPLLDTTQILSSDISLLSAPIVSADGTQQVILVQVNPGEAFTIRREDGQFQCITGP
AQVPMMSPNGSVPPIYVPPGYAPQVIEDNGVRRVVVVPQAPEFHPGSHTVLHRSPHPPLP
GFIPVPTMMPPPPRHMYSPVTGAGDMTTQYMPQYQSSQVYGDVDAHSTHGRSNFRDERSS
KTYERLQKKLKDRQGTQKDKMSSPPSSPQKCPSPINEHNGLIKGQIAGGINTGSAKIKSG
KGKGGTQVDTEIEEKDEETKAFEALLSNIVKPVASDIQARTVVLTWSPPSSLINGETDES
SVPELYGYEVLISSTGKDGKYKSVYVGEETNITLNDLKPAMDYHAKVQAEYNSIKGTPSE
AEIFTTLSCEPDIPNPPRIANRTKNSLTLQWKAPSDNGSKIQNFVLEWDEGKGNGEFCQC
YMGSQKQFKITKLSPAMGCKFRLSARNDYGTSGFSEEVLYYTSGCAPSMPASPVLTKAGI
TWLSLQWSKPSGTPSDEGISYILEMEEETSGYGFKPKYDGEDLAYTVKNLRRSTKYKFKV
IAYNSEGKSNPSEVVEFTTCPDKPGIPVKPSVKGKIHSHSFKITWDPPKDNGGATINKYV
VEMAEGSNGNKWEMIYSGATREHLCDRLNPGCFYRLRVYCISDGGQSAVSESLLVQTPAV
PPGPCLPPRLQGRPKAKEIQLRWGPPLVDGGSPISCYSVEMSPIEKDEPREVYQGSEVEC
TVSSLLPGKTYSFRLRAANKMGFGPFSEKCDITTAPGPPDQCKPPQVTCRSATCAQVNWE
VPLSNGTDVTEYRLEWGGVEGSMQICYCGPGLSYEIKGLSPATTYYCRVQALSVVGAGPF
SEVVACVTPPSVPGIVTCLQEISDDEIENPHYSPSTCLAISWEKPCDHGSEILAYSIDFG
DKQSLTVGKVTSYIINNLQPDTTYRIRIQALNSLGAGPFSHMIKLKTKPLPPDPPRLECV
AFSHQNLKLKWGEGTPKTLSTDSIQYHLQMEDKNGRFVSLYRGPCHTYKVQRLNESTSYK
FCIQACNEAGEGPLSQEYIFTTPKSVPAALKAPKIEKVNDHICEITWECLQPMKGDPVIY

SLQVMLGKDSEFKQIYKGPDSSFRYSSLQLNCEYRFRVCAIRQCQDSLGHQDLVGPYSTT
VLFISQRTEPPASTNRDTVESTRTRRALSDEQCAAVILVLFAFFSILIAFIIQYFVIK*
>ENSG00000181789|ENST00000509889
XITEWSWSMRSLPYNQPGTCYTLVALPKEDPTAVACTFSCMMKFTVKDCDPTTGETDDEG
YEDEYVLEDLEVTVADHIQKVMKLNFEAAWDEVGDEFEKEETFTLSTIKTLEEAVGNIVK
FLGMHPCERSDKVPDNKNTHTLLLAGVFRGGHDILVRSRLLLLDTVTMQVTARSLEELPV
DIILASVG*
>ENSG00000167645|ENST00000592694
MADPHQLFDDTSSAQSRGYGAQRAPGGLSYPAASPTPHAAFLADPVSNMAMAYGSSLAAQ
GKELVDKNIDRFIPITKLKYYFAVDTMYVGRKLGLLFFPYLHQDWEVQYQQDTPVAPRFD
VNAPDLYIPAMAFITYVLVAGLALGTQDRFSPDLLGLQASSALAWLTLEVLAILLSLYLV
TVNTDLTTIDLVAFLGYKYVGMIGGVLMGLLFGKIGYYLVLGWCCVAIFVFMIRTLRLKI
LADAAAEGVPVRGARNQLRMYLTMAVAAAQPMLMYWLTFHLVR*
>ENSG00000180957|ENST00000634017
MGDLLMEKCRVVLPCSVQEYQVGQLYSVAEASKNETGGGEGIEVLKNEPYEKDGEKGQYT
HKIYHLKSKVPAFVRMIAPEGSLVFHEKAWNAYPYCRTIVTNEYMKDDFFIKIETWHKPD
LGTLENVHGLDPNTWKTVEIVHIDIADRSQVEPADYKADEDPALFQSVKTKRGPLGPNWK
KELANSPDCPQMCAYKLVTIKFKWWGLQSKVENFIQKQEKRIFTNFHRQLFCWIDKWIDL
TMEDIRRMEDETQKELETMRKRGSVRGTSAADV*
>ENSG00000126091|ENST00000490541
MGLLVFVRNLLLALCLFLVLGFLYYSAWKLHLLQWEEDSSLLN*
>ENSG00000133103|ENST00000536488
XFFVENSLRTRRNLRGDIERKSLAINEEFVSIFKEVKEELESISEDVQAMSNCCQDMTSR
LQAAKEQTQDLIVKTTKLQSESQKLEIRAQVADAFLSKFQLTSDEMSLLRGTREGPITEF
RNYGTDGLTSRNGL*
>ENSG00000231823|ENST00000453672
MILNKALLLGALALTAVMSPCGGEDIVADHVASYGVNFYQSHGPSGQYTHEFDGDEEFYV
DLETKETVWQLPMFSKFISFDPQSALRNMAVGKHTLEFMMRQSNSTAATNEVPEVTVFSK
FPVTLGQPNTLICLVDNIFPPVVNITWLSNGHSVTEGVSETSFLSKSDHSFFKISYLTFL
PSADEIYDCKVEHWGLDEPLLKHWEPEIPAPMSELTETLVCALGLSAGLMGIVVGTVFII
QGLRSVGASRHQGLL*
>ENSG00000224455|ENST00000443860
MAAAATMAAAARELVLRAGTSDMEEEEGPLAGGPGLQEPLQLGELDITSDEFILDEVDVH
IQANLEDELVKEALKTGVDLRHYSKQVELELQQIEQKSIRDYIQESENIASLHNQITACD
AVLERMEQMLGAFQSDLSSISSEIRTLQEQSGAMNIRLRNRQAVRGKLGELVDGLVVPSA
LVTAILEAPVTEPRFLEQLQELDAKAAAVREQEARGTAACADVRGVLDRLRVKAVTKIRE
FILQKIYSFRKPMTNYQIPQTALLKYRFFYQFLLGNERATAKEIRDEYVETLSKIYLSYY
RSYLGRLMKVQYEEVAEKDDLMGVEDTAKKGFFSKPSLRSRNTIFTLGTRGSVISPTELE
APILVPHTAQRGEQRYPFEALFRSQHYALLDNSCREYLFICEFFVVSGPAAHDLFHAVMG
RTLSMTLKHLDSYLADCYDAIAVFLCIHIVLRFRNIAAKRDVPALDRYWEQVLALLWPRF
ELILEMNVQSVRSTDPQRLGGLDTRPHYITRRYAEFSSALVSINQTIPNERTMQLLGQLQ
VEVENFVLRVAAEFSSRKEQLVFLINNYDMMLGVLMERAADDSKEVESFQQLLNARTQEF
IEELLSPPFGGLVAFVKEAEALIERGQAERLRGEEARVTQLIRGFGSSWKSSVESLSQDV
MRSFTNFRNGTSIIQGALTQLIQLYHRFHRVLSQPQLRALPARAELINIHHLMVELKKHK

PNF*
>ENSG00000257923|ENST00000622516
MAANVGSMFQYWKRFDLQQLQRELDATATVLANRQDESEQSRKRLIEQSREFKKNTPEDL
RKQVAPLLKSFQGEIDALSKRSKEAEAAFLNVYKRLIDVPDPVPALDLGQQLQLKVQRLH
DIETENQKLRETLEEYNKEFAEVKNQEVTIKALKEKIREYEQTLKNQAETIALEKEQKLQ
NDFAEKERKLQETQMSTTSKLEEAEHKVQSLQTALEKTRTELFDLKTKYDEETTAKADEI
EMIMTDLERANQRAEVAQREAETLREQLSSANHSLQLASQIQKAPDVEQAIEVLTRSSLE
VELAAKEREIAQLVEDVQRLQASLTKLRENSASQISQLEQQLSAKNSTLKQLEEKLKGQA
DYEEVKKELNILKSMEFAPSEGAGTQDAAKPLEVLLLEKNRSLQSENAALRISNSDLSGR
CAELQVRITEAVATATEQRELIARLEQDLSIIQSIQRPDAEGAAEHRLEKIPEPIKEATA
LFYGPAAPASGALPEGQVDSLLSIISSQRERFRARNQELEAENRLAQHTLQALQSELDSL
RADNIKLFEKIKFLQSYPGRGSGSDDTELRYSSQYEERLDPFSSFSKRERQRKYLSLSPW
DKATLSMGRLVLSNKMARTIGFFYTLFLHCLVFLVLYKLAWSESMERDCATFCAKKFADH
LHKFHENDNGAAAGDLWQ*
>ENSG00000133067|ENST00000439764
MRLEGEGRSARAGQNLSRAGSARRGAPRDLSMNNLTELQPGLFHHLRFLEELRLSGNHLS
HIPGQAFSGLYSLKILMLQNNQLGGIPAEALWELPSLQSLDLNYNKLQEFPVAIRTLGRL
QELGFHNNNIKAIPEKAFMGNPLLQTIHFYDNPIQFVGRSAFQYLPKLHTLSLNGAMDIQ
EFPDLKGTTSLEILTLTRAGIRLLPSGMCQQLPRLRVLELSHNQIEELPSLHRCQKLEEI
GLQHNRIWEIGADTFSQLSSLQALDLSWNAIRSIHPEAFSTLHSLVKLDLTDNQLTTLPL
AGLGGLMHLKLKGNLALSQAFSKDSFPKLRILEVPYAYQCCPYGMCASFFKASGQWEAED
LHLDDEESSKRPLGLLARQAENHYDQDLDELQLEMEDSKPHPSVQCSPTPGPFKPCEYLF
ESWGIRLAVWAIVLLSVLCNGLVLLTVFAGGPVPLPPVKFVVGAIAGANTLTGISCGLLA
SVDALTFGQFSEYGARWETGLGCRATGFLAVLGSEASVLLLTLAAVQCSVSVSCVRAYGK
SPSLGSVRAGVLGCLALAGLAAALPLASVGEYGASPLCLPYAPPEGQPAALGFTVALVMM
NSFCFLVVAGAYIKLYCDLPRGDFEAVWDCAMVRHVAWLIFADGLLYCPVAFLSFASMLG
LFPVTPEAVKSVLLVVLPLPACLNPLLYLLFNPHFRDDLRRLRPRAGDSGPLAYAAAGEL
EKSSCDSTQALVAFSDVDLILEASEAGRPPGLETYGFPSVTLISCQQPGAPRLEGSHCVE
PEGNHFGNPQPSMDGELLLRAEGSTPAGGGLSGGGGFQPSGLAFASHV*
>ENSG00000147654|ENST00000620557
MAITQFRLFKFCTCLATVFSFLKRLICRSGRGRKLSGDQITLPTTVDYSSVPKQTDVEEW
TSWDEDAPTSVKIEGGNGNVATQQNSLEQLEPDYFKDMTPTIRKTQKIVIKKREPLNFGI
PDGSTGFSSRLAATQDLPFIHQSSELGDLDTWQENTNAWEEEEDAAWQAEEVLRQQKLAD
REKRAAEQQRKKMEKEAQRLMKKEQNKIGVKLS*
>ENSG00000275066|ENST00000622045
MALRPGAGSGGGGAAGAGAGSAGGGGFMFPVAGGIRPPQAGLMPMQQQGFPMVSVMQPNM
QGIMGMNYSSQMSQGPIAMQAGIPMGPMPAAGMPYLGQAPFLGMRPPGPQYTPDMQKQFA
EEQQKRFEQQQKLLEEERKRRQFEEQKQKLRLLSSVKPKTGEKSRDDALEAIKGNLDGFS
RDAKMHPTPASHPKKPGVGVFPSQDPAQPRMPPWIYNESLVPDAYKKILETTMTPTGIDT
AKLYPILMSSGLPRETLGQIWALANRTTPGKLTKEELYTVLAMIAVTQRGVPAMSPDALN
QFPAAPIPTLSGFSMTLPTPVSQPTVIPSGPAGSMPLSLGQPVMGINLVGPVGGAAAQAS
SGFIPTYPANQVVKPEEDDFQDFQDASKSGSLDDSFSDFQELPASSKTSNSQHGNSAPSL
LMPLPGTKALPSMDKYAVFKGIAADKSSENTVPPGDPGDKYSAFRELEQTAENKPLGESF
AEFRSAGTDDGFTDFKTADSVSPLEPPTKDKTFPPSFPSGTIQQKQQTQVKNPLNLADLD

MFSSVNCSSEKPLSFSAVFSTSKSVSTPQSTGSAATMTALAATKTSSLADDFGEFSLFGE
YSGLAPVGEQDDFADFMAFSNSSISSEQKPDDKYDALKEEASPVPLTSNVGSTVKGGQNS
TAASTKYDVFRQLSLEGSGLGVEDLKDNTPSGKSDDDFADFHSSKFSSINSDKSLGEKAV
AFRHTKEDSASVKSLDLPSIGGSSVGKEDSEDALSVQFDMKLADVGGDLKHVMSDSSLDL
PTVSGQHPPAADIEDLKYAAFGSYSSNFAVSTLTSYDWSDRDDATQGRKLSPFVLSAGSG
SPSATSILQKKETSFGSSENITMTSLSKVTTFVSEDALPETTFPALASFKDTIPQTSEQK
EYENRDYKDFTKQDLPTAERSQEATCPSPASSGASQETPNECSDDFGEFQSEKPKISKFD
FLVATSQSKMKSSEEMIKSELATFDLSVQGSHKRSLSLGDKEISRSSPSPALEQPFRDRS
NTLNEKPALPVIRDKYKDLTGEVEENERYAYEWQRCLGSALNVIKKANDTLNGISSSSVC
TEVIQSAQGMEYLLGVVEVYRVTKRVELGIKATAVCSEKLQQLLKDIDKVWNNLIGFMSL
ATLTCCWEKMTVITKHLSPYHELLEEKPDENSLDFSSCMLRPGIKNAQELACGVCLLNVD
SRSRKEEKPAEEHPKKAFNSETDSFKLAYGGHQYHASCANFWINCVEPKPPGLVLPDLL*
>ENSG00000135241|ENST00000453144
MSRIKSTLNSVSKAVFGNQNEMISRLAQFKPSSQILRKVSDSGWLKQKNIKQAIKSLKKY
SDKSAEKSPFPEEKSHIIDKEEDIGKRSLFHYTSSITTKFGDSFYFLSNHINSYFKRKEK
MSQQKENEHFRDKSELEDKKVEEGKLRSPDPGILAYKPGSESVHTVDKPTSPSAIPDVLQ
VSTKQSIANFLSRPTEGVQALVGGYIGGLVPKLKYDSKSQSEEQEEPAKTDQAVSKDRNA
EEKKRLSLQREKIIARVSIDNRTRALVQALRRTTDPKLCITRVEELTFHLLEFPEGKGVA
VKERIIPYLLRLRQIKDETLQAAVREILALIGYVDPVKGRGIRILSIDGGGTRGVVALQT
LRKLVELTQKPVHQLFDYICGVSTGAILAFMLGLFHMPLDECEELYRKLGSDVFSQNVIV
GTVKMSWSHAFYDSQTWENILKDRMGSALMIETARNPTCPKVAAVSTIVNRGITPKAFVF
RNYGHFPGINSHYLGGCQYKMWQAIRASSAAPGYFAEYALGNDLHQDGGLLLNNPSALAM
HECKCLWPDVPLECIVSLGTGRYESDVRNTVTYTSLKTKLSNVINSATDTEEVHIMLDGL
LPPDTYFRFNPVMCENIPLDESRNEKLDQLQLEGLKYIERNEQKMKKVAKILSQEKTTLQ
KINDWIKLKTDMYEGLPFFSKL*
>ENSG00000235220|ENST00000420067
MAPRSLLLLLSGALALTDTWAGSHSLRYFSTAVSRPGRGEPRYIAVEYVDDTQFLRFDSD
AAIPRMEPREPWVEQEGPQYWEWTTGYAKANAQTDRVALRNLLRRYNQSEAGSHTLQGMN
GCDMGPDGRLLRGYHQHAYDGKDYISLNEDLRSWTAADTVAQITQRFYEAEEYAEEFRTY
LEGECLELLRRYLENGKETLQRAEQSPQPTIPIVGIVAGLVVLGAVVTGAVVAAVMWRKK
SSDRNRGSYSQAAV*
>ENSG00000125447|ENST00000579743
MKNCGRRFHNEVGKFRFLNELIKVVSPKYLGDRVSEKVKTKVIELL
>ENSG00000235915|ENST00000457549
MLSRNDVKRLERSWHLLSWSQAQGSSEQELHYASLQRLPVPSSEGPDLRGRDKRGTKEDP
RADYACIAENKPT*
>ENSG00000213380|ENST00000562081
MATAATIPSVATATAAALGEVEDEGLLASLFRDRFPEAQWRERPDVGRYLRELSGSGLER
LRREPERLAEERAQLLQQTRDLAFANYKTFIRGAECTERIHRLFGDVEASLGRLLDRLPS
FQQSCRNFVKEAEEISSNRRMNSLTLNRHTEILEILEIPQLMDTCVRNSYYEEALELAAY
VRRLERKYSSIPVIQGIVNEVRQSMQLMLSQLIQQLRTNIQLPACLRVIGYLRRMDVFTE
AELRVKFLQARDAWLRSILTAIPNDDPYFHITKTIEASRVHLFDIITQYRAIFSDEDPLL
PPAMGEHTVNESAIFHGWVLQKVSQFLQVLETDLYRGIGGHLDSLLGQCMYFGLSFSRVG
ADFRGQLAPVFQRVAISTFQKAIQETVEKFQEEMNSYMLISAPAILGTSNMPAAVPATQP

GTLQPPMVLLDFPPLACFLNNILVAFNDLRLCCPVALAQDVTGALEDALAKVTKIILAFH
RAEEAAFSSGEQELFVQFCTVFLEDLVPYLNRCLQVLFPPAQIAQTLGKRMKIL*
>ENSG00000223465|ENST00000427507
MLSRNDDICIYGGLGLGGLLLLAVVLLSACLCWLHRRVKRLERSWAQGSSEQELHYASLQ
RLPVPSSEGPDLRGRDKRGTKEDPRADYACIAENKPT*
>ENSG00000117362|ENST00000414276
MGAAVFFGCTFVAFGPAFALFLITVAGDPLRVIILVAGRCSALPTTSCLISGLSFGIISG
VFSVINILADALGPGVVGIHGDSPYYFLTSAFLTAAIILLHTFWGVVFFDACERRRYWAL
GLVVGSHLLTSGLTFLNPWYEASLLPIYAVTVSMGLWAFITAGGSLRSIQRSLLCRRQED
SRVMVYSALRIPPED*
>ENSG00000090863|ENST00000627032
MAACGRVRRMFRLSAALHLLLLFAAGAEKLPGQGVHSQGQGPGANFVSFVGQAGGGGPAG
QQLPQLPQSSQLQQQQQQQQQQQPQPPQPPFPAGGPPARRGGAGAGGGWKLAEEESCRE
DVTRVCPKHTWSNNLAVLECLQDVREPENEISSDCNHLLWNYKLNLTTDPKFESVAREVC
KSTITEIKECADEPVGKGYMVSCLVDHRGNITEYQCHQYITKMTAIIFSDYRLICGFMDD
CKNDINILKCGSIRLGEKDAHSQGEVVSCLEKGLVKEAEEREPKIQVSELCKKAILRVAE
LSSDDFHLDRHLYFACRDDRERFCENTQAGEGRVYKCLFNHKFEESMSEKCREALTTRQK
LIAQDYKVSYSLAKSCKSDLKKYRCNVENLPRSREARLSYLLMCLESAVHRGRQVSSECQ
GEMLDYRRMLMEDFSLSPEIILSCRGEIEHHCSGLHRKGRTLHCLMKVVRGEKGNLGMNC
QQALQTLIQETDPGADYRIDRALNEACESVIQTACKHIRSGDPMILSCLMEHLYTEKMVE
DCEHRLLELQYFISRDWKLDPVLYRKCQGDASRLCHTHGWNETSEFMPQGAVFSCLYRHA
YRTEEQGRRLSRECRAEVQRILHQRAMDVKLDPALQDKCLIDLGKWCSEKTETGQELECL
QDHLDDLVVECRDIVGNLTELESEAEREYVFKNLPFKV*
>ENSG00000234876|ENST00000457094
MQPPSLLLLLLLLLLLCVSVVRPRGLLCGSFPEPCANGGTCLSLSLGQGTCQCAPGFLGE
TCQFPDPCQNAQLCQNGGSCQALLPAPLGLPSSPSPLTPSFLCTCLPGFTGERCQAKLED
PCPPSFCSKRGRCHIQASGRPQCSCMPGWTGEQCQLRDFCSANPCVNGGVCLATYPQIQC
HCPPGFEGHACERDVNECFQDPGPCPKGTSCHNTLGSFQCLCPVGQEGPRCELRAGPCPP
RGCSNGGTCQLMPEKDSTFHLCLCPPGFIGPDCEVNPDNCVSHQCQNGGTCQDGLDTYTC
LCPETWTGWDCSEDVDECETQGPPHCRNGGTCQNSAGSFHCVCVSGWGGTSCEENLDDCI
AATCAPGSTCIDRVGSFSCLCPPGRTGLLCHLEDMCLSQPCHGDAQCSTNPLTGSTLCLC
QPGYSGPTCHQDLDECLMAQQGPSPCEHGGSCLNTPGSFNCLCPPGYTGSRCEADHNECL
SQPCHPGSTCLDLLATFHCLCPPGLEGQLCEVETNECASAPCLNHADCHDLLNGFQCICL
PGFSGTRCEEDIDECRSSPCANGGQCQDQPGAFHCKCLPGFEGPRCQTEVDECLSDPCPV
GASCLDLPGAFFCLCPSGFTGQLCEVPLCAPNLCQPKQICKDQKDKANCLCPDGSPGCAP
PEDNCTCHHGHCQRSSCVCDVGWTGPECEAELGGCISAPCAHGGTCYPQPSGYNCTCPTG
YTGPTCSEEMTACHSGPCLNGGSCNPSPGGYYCTCPPSHTGPQCQTSTDYCVSAPCFNGG
TCVNRPGTFSCLCAMGFQGPRCEGKLRPSCADSPCRNRATCQDSPQGPRCLCPTGYTGGS
CQTLMDLCAQKPCPRNSHCLQTGPSFHCLCLQGWTGPLCNLPLSSCQKAALSQGIDVSSL
CHNGGLCVDSGPSYFCHCPPGFQGSLCQDHVNPCESRPCQNGATCMAQPSGYLCQCAPGY
DGQNCSKELDACQSQPCHNHGTCTPKPGGFHCACPPGFVGLRCEGDVDECLDQPCHPTGT
AACHSLANAFYCQCLPGHTGQWCEVEIDPCHSQPCFHGGTCEATAGSPLGFICHCPKGFE
GPTCSHRAPSCGFHHCHHGGLCLPSPKPGFPPRCACLSGYGGPDCLTPPAPKGCGPPSPC
LYNGSCSETTGLGGPGFRCSCPHSSPGPRCQKPGAKGCEGRSGDGACDAGCSGPGGNWDG

GDCSLGVPDPWKGCPSHSRCWLLFRDGQCHPQCDSEECLFDGYDCETPPACTPAYDQYCH
DHFHNGHCEKGCNTAECGWDGGDCRPEDGDPEWGPSLALLVVLSPPALDQQLFALARVLS
LTLRVGLWVRKDRDGRDMVYPYPGARAEEKLGGTRDPTYQERAAPQTQPLGKETDSLSAG
FVVVMGVDLSRCGPDHPASRCPWDPGLLLRFLAAMAAVGALEPLLPGPLLAVHPHAGTAP
PANQLPWPVLCSPVAGVILLALGALLVLQLIRRRRREHGALWLPPGFTRRPRTQSAPHRR
RPPLGEDSIGLKALKPKAEVDEDGVVMCSGPEEGEEVGQAEETGPPSTCQLWSLSGGCGA
LPQAAMLTPPQESEMEAPDLDTRGPDGVTPLMSAVCCGEVQSGTFQGAWLGCPEPWEPLL
DGGACPQAHTVGTGETPLHLAARFSRPTAARRLLEAGANPNQPDRAGRTPLHAAVAADAR
EVCQLLLRSRQTAVDARTEDGTTPLMLAARLAVEDLVEELIAAQADVGARDKWGKTALHW
AAAVNNARAARSLLQAGADKDAQDNREQTPLFLAAREGAVEVAQLLLGLGAARELRDQAG
LAPADVAHQRNHWDLLTLLEGAGPPEARHKATPGREAGPFPRARTVSVSVPPHGGGALPR
CRTLSAGAGPRGGGACLQARTWSVDLAARGGGAYSHCRSLSGVGAGGGPTPRGRRFSAGM
RGPRPNPAIMRGRYGVAAGRGGRVSTDDWPCDWVALGACGSASNIPIPPPCLTPSPERGS
PQLDCGPPALQEMPINQGGEGKK*
>ENSG00000171124|ENST00000589918
MDPLGAAKPQWPWRRCLAALLFQLLVAVCFFSYLRVSRDDATGSPRAPSGSSRQDTTPTR
PTLLILLRTWPFHIPVALSRCSEMVPGTADCHITADRKVYPQADMVIVHHWDIMSNPKSR
LPPSPRPQGQRWIWFNLEPPPNCQHLEALDRYFNLTMSYRSDSDIFTPYGWLEPWSGQPA
HPPLNLSAKTELVAWAVSNWKPDSARVRYYQSLQAHLKVDVYGRSHKPLPKGTMMETLSR
YKFYLAFENSLHPDYITEKLWRNALEAWAVPVVLGPSRSNYERFLPPDAFIHVDDFQSPK
DLARYLQELDKDHARYLSYFRWRETLRPRSFSWALDFCKACWKLQQESRYQTVRSIAAWF
T*
>ENSG00000122390|ENST00000572584
MTEVVPSSALSEVSLRLLCHDDIDTVKHLCGDWFPIEYPDSWYRDITSNKKFFSLAATYR
GAIVGMIVAEIKNRTKIHKEDGDILASNFSVDTQVAYILSLGVVKEFRKHGIGSLLLESL
KDHISTTAQDHCKAIYLHVLTTNNTAINFYENRDFKQHHYLPYYYSIRGVLKDGFTYVLY
INGGHPPWTILDYIQHLGSALASLSPCSIPHRVYRQAHSLLCSFLPWSGISSKSGIEYSR
TM*
>ENSG00000122390|ENST00000421765
MTEVVPSSALSEVSLRLLCHDDIDTVKHLCGDWFPIEYPDSWYRDITSNKKFFSLAATYR
GAIVGMIVAEIKNRTKIHKEDGDILASNFSVDTQVAYILSLGVVKEFRKHGIEPHGLHPA
PGLCTSQPEPLLHSAQSLPPGPQPALQLPAMVGHLFQEWHRVQPDHVMSAGQPPPGPTLR
PPAEPAFLSI*
>ENSG00000122390|ENST00000407558
MTEVVPSSALSEVSLRLLCHDDIDTVKHLCGDWFPIEYPDSWYRDITSNKKFFSLAATYR
GAIVGMIVAEIKNRTKIHKEDGDILASNFSVDTQVAYILSLGVVKEFRKHGIGSLLLESL
KDHISTTAQDHCKAIYLHVLTTNNTAINFYENRDFKQHHYLPYYYSIRGVLKDGFTYVLY
INGGHPPWTILDYIQHLGSALASLSPCSIPHRVYRQAHSLLCSFLPWSGISSKSGIEYSR
TM*
>ENSG00000166405|ENST00000425599
MAYSTVQRVALASGLVLALSLLLPKAFLSRGKRQEPPPTPEGKLGRFPPMMHHHQAPSDG
QTPGARFQRSHLAEAFAKAKGSGGGAGGGGSGRGLMGQIIPIYGFGIFLYILYILFKLSK
GKTTAEDGKCYTAMPGNTHRKISYPEETYPIYDLSDCIKRRQETILVDYPDPKELSAEEI
AERMGMIEEEEESDHLGWESLPTDPRAQEDNSVTSCDPKPETCSCCFHEDEDPAVLAENAG

FSADSYPEQEETTKEEWSQDFKDEGLGISTDKAYTGSMLRKRNPQGLE*
>ENSG00000102100|ENST00000445167
MAAVGAGGSTAAPGPGAVSAGALEPGTASAAHRRLKYISLAVLVVQNASLILSIRYARTL
PGDRFFATTAVVMAEVLKGLTCLLLLFAQKRGNVKHLVLFLHEAVLVQYVDTLKLAVPSL
IYTLQNNLQYVAISNLPAATFQPSPRCSQSHSLCLCLRLRALRSPAASRAATTTAAVFPP
WRPHHGALSAKVSAGEVRAGSNGGTQGRGTGVEGVGHLQDPSRHPPGPGSSGFGRWSFLP
GH*
>ENSG00000155511|ENST00000518783
MCCSTHLFQPLQLAGGLEWPWSNLLCFLTPVKLHPEVWGLFPNQQSQEHAAFRFALSQLT
EPPKLLPQIDIVNISDSFEMTYRFCSQFSKGVYAIFGFYERRTVNMLTSFCGALHVCFIT
PSFPVDTSNQFVLQLRPELQDALISIIDHYKWQKFVYIYDADRGLSVLQKVLDTAAEKNW
QVTAVNILTTTEEGYRMLFQDLEKKKERLVVVDCESERLNAILGQIIKLEKNGIGYHYIL
ANLGFMDIDLNKFKESGANVTGFQLVNYTDTIPAKIMQQWKNSDARDHTRVDWKRPKYTS
ALTYDGVKVMAEAFQSLRRQRIDISRRGNAGDCLANPAVPWGQGIDIQRALQQVRFEGLT
GNVQFNEKGRRTNYTLHVIEMKHDGIRKIGYWNEDDKFVPAATDAQAGGDNSSVQNRTYI
VTTILEDPYVMLKKNANQFEGNDRYEGYCVELAAEIAKHVGYSYRLEIVSDGKYGARDPD
TKAWNGMVGELVYGRADVAVAPLTITLVREEVIDFSKPFMSLGISIMIKKPQKSKPGVFS
FLDPLAYEIWMCIVFAYIGVSVVLFLVSRFSPYEWHSEEFEEGRDQTTSDQSNEFGIFNS
LWFSLGAFMQQGCDISPRSLSGRIVGGVWWFFTLIIISSYTANLAAFLTVERMVSPIESA
EDLAKQTEIAYGTLEAGSTKEFFRRSKIAVFEKMWTYMKSAEPSVFVRTTEEGMIRVRKS
KGKYAYLLESTMNEYIEQRKPCDTMKVGGNLDSKGYGIATPKGSALRNPVNLAVLKLNEQ
GLLDKLKNKWWYDKGECGSGGGDSKDKTSALSLSNVAGVFYILIGGLGLAMLVALIEFCY
KSRSESKRMKGFCLIPQQSINEAIRTSTLPRNSGAGASSGGSGENGRVVSHDFPKSMQSI
PCMSHSSGMPLGATGL*
>ENSG00000108587|ENST00000537788
MAAGTSSYWEDLRKQARQLENELDLKLVSFSKLCTSYSHSSTRDGRRDRYRKNYLENISQ
EILPQTETGFVELLQPNSPSK*
>ENSG00000235657|ENST00000457879
MAVMAPRTLVLLLSGALALTQTWAGSHSMRYFFTSVSRPGRGEPRFIAVGYVDDTQFVRF
DSDAASQRMEPRAPWIEQEGPEYWDGETRKVKAHSQTHRVDLGTLRGYYNQSEAGSHTVQ
RMYGCDVGSDWRFLRGYHQYAYDGKDYIALKEDLRSWTAADMAAQTTKHKWEAAHVAEQL
RAYLEGTCVEWLRRYLENGKETLQRTDAPKTHMTHHAVSDHEATLRCWALSFYPAEITLT
WQRDGEDQTQDTELVETRPAGDGTFQKWAAVVVPSGQEQRYTCHVQHEGLPKPLTLRWEP
SSQPTIPIVGIIAGLVLFGAVITGAVVAAVMWRRKSSDRKGGSYSQAASSDSAQGSDVSL
TACKV*
>ENSG00000103723|ENST00000535348
MSAAPAYSEDKGGSAGPGEPEYGHDPASGGIFSSDYKRHDDLKEMLDTNKDSLKLEAMKR
IVAMIARGKNASDLFPAVVKNVACKNIEDPNQLIRASALRVLSSIRVPIIVPIMMLAIKE
AASDMSPYVRKTAAHAIPKLYSLDSDQKDQLIEVIEKLLADKTTLVAGSVVMAFEEVCPE
RIDLIHKNYRKLCNLLIDVEEWGQVVIISMLTRYARTQFLSPTQNESLLEENAEKAFYGS
EEDEAKGAGSEETAAAAAPSRKPYVMDPDHRLLLRNTKPLLQSRSAAVVMAVAQLYFHLA
PKAEVGVIAKALVRLLRSHSEVQYVVLQNVATMSIKRRGMFEPYLKSFYIRSTDPTQIKI
LKLEVLTNLANETNIPTVLREFQTYIRSMDKDFVAATIQAIGRCATNIGRVRDTCLNGLV
QLLSNRDELVVAESVVVIKKLLQMQPAQHGEIIKHLAKLTDNIQVPMARASILWLIGEYC

EHVPRIAPDVLRKMAKSFTAEEDIVKLQVINLAAKLYLTNSKQTKLLTQYVLSLAKYDQN
YDIRDRARFTRQLIVPSEQGGALSRHAKKLFLAPKPAPVLESSFKDRDHFQLGSLSHLLN
AKATGYQELPDWPEEAPDPSVRNVEVPEWTKCSNREKRKEKEKPFYSDSEGESGPTESAD
SDPESESESDSKSSSESGSGESSSESDNEDQDEDEEKGRGSESEQSEEDGKRKTKKKVPE
RKGEASSSDEGSDSSSSSSESEMTSESEEEQLEPASWSRKTPPSSKSAPATKEISLLDLE
DFTPPSVQPVSPPAIVSTSLAADLEGLTLTDSTLVPSLLSPVSGVGRQELLHRVAGEGLA
VDYTFSRQPFSGDPHMVSVHIHFSNSSDTPIKGLHVGTPKLPAGISIQEFPEIESLAPGE
SATAVMGINFCDSTQAANFQLCTQTRQFYVSIQPPVGELMAPVFMSENEFKKEQGKLMGM
NEITEKLMLPDTCRSDHIVVQKVTATANLGRVPCGTSDEYRFAGRTLTGGSLVLLTLDAR
PAGAAQLTVNSEKMVIGTMLVKDVIQALTQ*
>ENSG00000274047|ENST00000632841
MALRPGAGSGGGGAAGAGAGSAGGGGFMFPVAGGIRPPQAGLMPMQQQGFPMVSVMQPNM
QGIMGMNYSSQMSQGPIAMQAGIPMGPMPAAGMPYLGQAPFLGMRPPGPQYTPDMQKQFA
EEQQKRFEQQQKLLEEERKRRQFEEQKQKLRLLSSVKPKTGEKSRDDALEAIKGNLDGFS
RDAKMHPTPASHPKKPGVGVFPSQDPAQPRMPPWIYNESLVPDAYKKILETTMTPTGIDT
AKLYPILMSSGLPRETLGQIWALANRTTPGKLTKEELYTVLAMIAVTQRGVPAMSPDALN
QFPAAPIPTLSGFSMTLPTPVSQPTVIPSGPAGSMPLSLGQPVMGINLVGPVGGAAAQAS
SGFIPTYPANQVVKPEEDDFQDFQDASKSGSLDDSFSDFQELPASSKTSNSQHGNSAPSL
LMPLPGTKALPSMDKYAVFKGIAADKSSENTVPPGDPGDKYSAFRELEQTAENKPLGESF
AEFRSAGTDDGFTDFKTADSVSPLEPPTKDKTFPPSFPSGTIQQKQQTQVKNPLNLADLD
MFSSVNCSSEKPLSFSAVFSTSKSVSTPQSTGSAATMTALAATKTSSLADDFGEFSLFGE
YSGLAPVGEQDDFADFMAFSNSSISSEQKPDDKYDALKEEASPVPLTSNVGSTVKGGQNS
TAASTKYDVFRQLSLEGSGLGVEDLKDNTPSGKSDDDFADFHSSKFSSINSDKSLGEKAV
AFRHTKEDSASVKSLDLPSIGGSSVGKEDSEDALSVQFDMKLADVGGDLKHVMSDSSLDL
PTVSGQHPPAAAGSGSPSATSILQKKETSFGSSENITMTSLSKVTTFVSEDALPETTFPA
LASFKDTIPQTSEQKEYENRDYKDFTKQDLPTAERSQEATCPSPASSGASQETPNECSDD
FGEFQSEKPKISKFDFLVATSQSKMKSSEEMIKSELATFDLSVQGSHKRSLSLGDKEISR
SSPSPALEQPFRDRSNTLNEKPALPVIRDKYKDLTGEVEENERYAYEWQRCLGSALNVIK
KANDTLNGISSSSVCTEVIQSAQGMEYLLGVVEVYRVTKRVELGIKATAVCSEKLQQLLK
DIDKVWNNLIGFMSLATLTPDENSLDFSSCMLRPGIKNAQELACGVCLLNVDSRSRAFNS
ETDSFKLAYGGHQYHASCANFWINCVEPKPPGLVLPDLL*
>ENSG00000105829|ENST00000433727
MRRAGLGEGVPPGNYGNYGYANSGYSACEEENERLTESLRSKVTAIKSLSIEIGHEVKTQ
NKLLAEMDSQFDSTTGFLGLCPM*
>ENSG00000166676|ENST00000612195
MKQALVDDTEDVSLDFGNEEELAFRKAKIRHPLATFFHLFFRVSAIVTYVSCDWFSKSFV
GCFVMVLLLLSLDFWSVKNVTGRLLVGLRWWNQIDEDGKSHWIFEARKVSPNSIAATEAE
ARIFWLGLIICPMIWIVFFFSTLFSLKLKWLALVVAGISLQAANLYGYILCKMGGNSDIG
KVTASFLSQTVFQTACPGDFQKPGLEGLEIHQH*
>ENSG00000274047|ENST00000618176
MALRPGAGSGGGGAAGAGAGSAGGGGFMFPVAGGIRPPQAGLMPMQQQGFPMVSVMQPNM
QGIMGMNYSSQMSQGPIAMQAGIPMGPMPAAGMPYLGQAPFLGMRPPGPQYTPDMQKQFA
EEQQKRFEQQQKLLEEERKRRQFEEQKQKLRLLSSVKPKTGEKSRDDALEAIKGNLDGFS
RDAKMHPTPASHPKKPGVGVFPSQDPAQPRMPPWIYNESLVPDAYKKILETTMTPTGIDT

AKLYPILMSSGLPRETLGQIWALANRTTPGKLTKEELYTVLAMIAVTQRGVPAMSPDALN
QFPAAPIPTLSGFSMTLPTPVSQPTVIPSGPAGSMPLSLGQPVMGINLVGPVGGAAAQAS
SGFIPTYPANQVVKPEEDDFQDFQDASKSGSLDDSFSDFQELPASSKTSNSQHGNSAPSL
LMPLPGTKALPSMDKYAVFKGIAADKSSENTVPPGDPGDKYSAFRELEQTAENKPLGESF
AEFRSAGTDDGFTDFKTADSVSPLEPPTKDKTFPPSFPSGTIQQKQQTQVKNPLNLADLD
MFSSVNCSSEKPLSFSAVFSTSKSVSTPQSTGSAATMTALAATKTSSLADDFGEFSLFGE
YSGLAPVGEQDDFADFMAFSNSSISSEQKPDDKYDALKEEASPVPLTSNVGSTVKGGQNS
TAASTKYDVFRQLSLEGSGLGVEDLKDNTPSGKSDDDFADFHSSKFSSINSDKSLGEKAV
AFRHTKEDSASVKSLDLPSIGGSSVGKEDSEDALSVQFDMKLADVGGDLKHVMSDSSLDL
PTVSGQHPPAADIEDLKYAAFGSYSSNFAVSTLTSYDWSDRDDATQGRKLSPFVLSAGSG
SPSATSILQKKETSFGSSENITMTSLSKVTTFVSEDALPETTFPALASFKDTIPQTSEQK
EYENRDYKDFTKQDLPTAERSQEATCPSPASSGASQETPNECSDDFGEFQSEKPKISKFD
FLVATSQSKMKSSEEMIKSELATFDLSVQGSHKRSLSLGDKEISRSSPSPALEQPFRDRS
NTLNEKPALPVIRDKYKDLTGEVEENERYAYEWQRCLGSALNVIKKANDTLNGISSSSVC
TEVIQSAQGMEYLLGVVEVYRVTKRVELGIKATAVCSEKLQQLLKDIDKVWNNLIGFMSL
ATLTPDENSLDFSSCMLRPGIKNAQELACGVCLLNVDSRSRAFNSETDSFKLAYGGHQYH
ASCANFWINCVEPKPPGLVLPDLL*
>ENSG00000111481|ENST00000455864
MEALILYYDDTYPSVKEQKAFEKNIFNKTHRTDSEIALLEGLTVVYKSSIDLYFYVIGSS
YENELMLMAVLNCLFDSLSQMLRKNVEKRALLENMEGLFLAVDEIVDGGVILESDPQQVV
HRVALRGEDVPLTEQTVSQVLQSAKEQIKWSLLR*
>ENSG00000179913|ENST00000599265
MKYLRHRRPNATLILAIGAFTLLLFSLLVSPPTCKVQEQPPAIPEALAWPTPPTRPAPAP
CHANTSMVTHPDFATQPQHVQNFLLYRHCRHFPLLQDVPPSKCAQPVFLLLVIKSSPSNY
VRRELLRRTWGRERKVRGLQLRLLFLVGTASNPHEARKVNRLLELEAQTHGDILQWDFHD
SFFNLTLKQVLFLQWQETRCANASFVLNGDDDVFAHTDNMVFYLQDHDPGRHLFVGQLIQ
NVGPIRAFWS
>ENSG00000213380|ENST00000615447
XALGEVEDEGLLASLFRDRFPEAQWRERPDVGRYLRELSGSGLERLRREPERLAEERAQL
LQQTRDLAFANYKTFIRGAECTERIHRLFGDVEASLGRLLDRLPSFQQSCRCDAPGAKGV
L*
>ENSG00000145416|ENST00000503008
MLGWCEAIARNPHRIPNNTRTPEISGDLADASQTSTLNEKSPGRSASRSSNISKASSPTT
GTAPRSQSRLSVCPSTQDICRICHCEGDEESPLITPCRCTGTLRFVHQSCLHQWIKSSDT
RCCELCKYDFIMETKLKPLRKWEKLQMTTSERRKIFCSVTFHVIAITCVVWSLYVLIDRT
AEEIKQGNDNGVLEWPFWTKLVVVAIGFTGGLVFMYVQCKVYVQLWRRLKAYNRVIFVQN
CPDTAKKLEKNFSCNVNTDIKDAVVVPVPQTGANSLPSAEGGPPEVVSV*
>ENSG00000072958|ENST00000444449
MSASAVYVLDLKGKVLICRNYRGDVDMSEVEHFMPILMEKEEEGMLSPILAHGGVRFMWI
KHNNLYLVATSKKNACVSLVFSFLYKVVQVFSEYFKELEEESIRDNFVIIYELLDELMDF
GYPQTTDSKILQEYITQEGHKLETGAPRPPATVTNAVSWRSEGIKYRKNEVFLDVIESVN
LLGKYPGVGWLGHTVSANGNVLRSEIVGSIKMRVFLSGMPELRLGLNDKVLFDNTGRGKS
KSVELEDVKFHQCVRLSRFENDRTISFIPPDGEFELMSYRLNTHVKPLIWIESVIEKHSH
SRIEYMIKAKSQFKRRSTANNVEIHIPVPNDADSPKFKTTVGSVKWVPENSEIVWSIKSF

PGGKEYLMRAHFGLPSVEAEDKEGKPPISVKFEIPYFTTSGIQVRYLKIIEKSGYQALPW
VRYITQNGDYQLRTQ*
>ENSG00000102100|ENST00000635589
MAAVGAGGSTAAPGPGAVSAGALEPGTASAGNVKHLVLFLHEAVLVQYVDTLKLAVPSLI
YTLQNNLQYVAISNLPAATFQVTYQLKILTTALFSVLMLNRSLSRLQWASLLLLFTGVAI
VQAQQAGGGGPRPLDQNPGAGLAAVVASCLSSGFAGVYFEKILKGSSGSVWLRNLQLGLF
GTALGLVGLWWAEGTAVATRGFFFGYTPAVWGVVLNQAFGGLLVAVVVKYADNILKGFAT
SLSIVLSTVASIRLFGFHVDPLFALGAGLVIGAVYLYSLPRGAAKAIASASASASGPCVH
QQPPGQPPPPQLSSHRGDLITEPFLPKSVLVK*
>ENSG00000115525|ENST00000639432
MPSEYTYVKLRSDCSRPSLQWYTRAQSKMRRPSLLLKDILKCTLLVFGVWILYILKLNYT
TEECDMKKMHYVDPDHVKRAQKYAQQVLQKECRPKFAKTSMALLFEHRYSVDLLPFVQKA
PKDSEAESKYDPPFGFRKFSSKVQTLLELLPEHDLPEHLKAKTCRRCVVIGSGGILHGLE
LGHTLNQFDVVIRLNSAPVEGYSEHVGNKTTIRMTYPEGAPLSDLEYYSNDLFVAVLFKS
VDFNWLQAMVKKETLPFWVRLFFWKQVAEKIPLQPKHFRILNPVIIKETAFDILQYSEPQ
SRFWGRDKNVPTIGVIAVVLATHLCDEVSLAGFGYDLNQPRTPLHYFDSQCMAAMNFQTM
HNVTTETKFLLKLVKEGVVKDLSGGIDREF*
>ENSG00000085063|ENST00000528700
MGIQGGSVLFGLLLVLAVFCHSGHSLQCYNCPNPTADCKTAVNCSSDFDACLITKAGLQV
YNKCWKFEHCNFNDVTTRLRENELTYYCCKKDLCNFNEQLENGGTSLSEKTVLLLVTPFL

>ENSG00000017260|ENST00000504948
MIPVLTSKKASELPVSEVASILQADLQNGLNKCEVSHRRAFHGWNEFDISEDEPLWKKYI
SQFKNPLIMLLLASAVISVLMHQFDDAVSITVAILIVVTVAFVQEYRSEKSLEELSKLVP
PECHCVREGKLEHTLARDLVPGDTVCLSVGDRVPADLRLFEAVDLSIDESSLTGETTPCS
KVTAPQPAATNGDLASRSNIAFMGTLVRCGKAKGVVIGTGENSEFGEVFKMMQAEEAPKT
PLQKSMDLLGKQLSFYSFGIIGIIMLVGWLLGKDILEMFTISVSLAVAAIPEGLPIVVTV
TLALGVMRMVKKRAIVKKLPIVETLGCCNVICSDKTGTLTKNEMTVTHIFTSDGLHAEVT
GVGYNQFGEVIVDGDVVHGFYNPAVSRIVEAGCVCNDAVIRNNTLMGKPTEGALIALAMK
MGLDGLQQDYIRKAEYPFSSEQKWMAVKCVHRTQQDRPEICFMKGAYEQVIKYCTTYQSK
GQTLTLTQQQRDVYQQEKARMGSAGLRVLALASGPELGQLTFLGLVGIIDPPRTGVKEAV
TTLIASGVSIKMITGDSQETAVAIASRLGLYSKTSQSVSGEEIDAMDVQQLSQIVPKVAV
FYRASPRHKMKIIKSLQKNGSVVAMTGDGVNDAVALKAADIGVAMGQTGTDVCKEAADMI
LVDDDFQTIMSAIEEGKGIYNNIKNFVRFQLSTSIAALTLISLATLMNFPNPLNAMQILW
INIIMDGPPAQSLGVEPVDKDVIRKPPRNWKDSILTKNLILKILVSSIIIVCGTLFVFWR
ELRDNVITPRDTTMTFTCFVFFDMFNALSSRSQTKSVFEIGLCSNRMFCYAVLGSIMGQL
LVIYFPPLQKVFQTESLSILDLLFLLGLTSSVCIVAEIIKKVERSREKIQKHVSSTSSSF
LEV*
>ENSG00000133318|ENST00000540798
MAEPSAATQSHSISSSSFGAEPSAPGGGGSPGACPALGTKSCSSSCAASFPEHPAFLSKK
IGQVEEQIDKETKNPNGVSSREAKTALDADDRFTLLTAQKPPTEYSKVEGIYTYSLSPSK
VSGDDVIEKDSPESPFFEVIIDKAAFDKEFKDSYKESTDDFGSWSVHTDKESSEDISETND
KLFPLRNKEAGRYPMSALLSRQFSHTNAALEEVSRCVNDMHNFTNEILTWDLVPQVKQQT
DKSSDCITKTTGLDMSEYNSEIPVVNLKTSTHQKTPVCSIDGSTPITKSTGDWAEASLQQ

ENAITGKPVPDSLNSTKEFSIKGVQGNMQKQDDTLAELPGSPPEKCDSLGSGVATVKVVL
PDDHLKDEMDWQSSALGEITEADSSGESDDTVIEDITADTSFENNKIQAEKPVSIPSAVV
KTGEREIKEIPSCEREEKTSKNFEELVSDSELHQDQPDILGRSPASEAACSKVPDTNVSL
EDVSEVAPEKPITTENPKLPSTVSPNVFNETEFSLNVTTSAYLESLHGKNVKHIDDSSPE
DLIAAFTETRDKGIVDSERNAFKAISEKMTDFKTTPPVEVLHENESGGSEIKDIGSKYSE
QSKETNGSEPLGVFPTQGTPVASLDLEQEQLTIKALKELGERQVEKSTSAQRDAELPSEE
VLKQTFTFAPESWPQRSYDILERNVKNGSDLGISQKPITIRETTRVDAVSSLSKTELVKK
HVLARLLTDFSVHDLIFWRDVKKTGFVFGTTLIMLLSLAAFSVISVVSYLILALLSVTIS
FRIYKSVIQAVQKSEEGHPFKAYLDVDITLSSEAFHNYMNAAMVHINRALKLIIRLFLVE
DLVDSLKLAVFMWLMTYVGAVFNGITLLILAELLIFSVPIVYEKYKTQIDHYVGIARDQT
KSIVEKIQAKLPGIAKKKAE*
>ENSG00000223793|ENST00000447735
MILNKALLLGALALTAVMSPCGGEDIVADHVASYGVNFYQSHGPSGQYTHEFDGDEEFYV
DLETKETVWQLPMFSKFISFDPQSALRNMAVGKHTLEFMMRQSNSTAATNEVPEVTVFSK
FPVTLGQPNTLICLVDNIFPPVVNITWLSNGHSVTEGVSETSFLSKSDHSFFKISYLTFL
PSADEIYDCKVEHWGLDEPLLKHWEPEIPAPMSELTETLVCALGLSVGLMGIVVGTVFII
QGLRSVGASRHQGLL*
>ENSG00000176920|ENST00000425340
MLVVQMPFSFPMAHFILFVFTVSTIFHVQQRLAKIQAMWELPVQIPVLASTSKALGPSQL
RGMWTINAIGRLGNQMGEYATLYALAKMNGRPAFIPAQMHSTLAPIFRITLPVLHSATAS
RIPWQNYHLNDWMEEEYRHIPGEYVRFTGYPCSWTFYHHLRQEILQEFTLHDHVREEAQK
FLRGLQVNGSRPGTFVGVHVRRGDYVHVMPKVWKGVVADRRYLQQALDWFRARYSSLIFV
VTSNGMAWCRENIDTSHGDVVFAGDGIEGSPAKDFALLTQCNHTIMTIGTFGIWAAYLTG
GDTIYLANYTLPDSPFLKIFKPEAAFLPEWTGIAADLSPLLKH*
>ENSG00000137502|ENST00000533486
MSMEDYDFLFKIVLIGNAGVGKTCLVRRFTQGLFPPGQGATIGVDFMIKTVEINGEKVKL
QIWDTAGQERFRSITQSYYRSANALILTYDITCEESFRCLPEWLREIEQYASNKVITVLV
GNKIDLAERREVSQQRAEEFSEAQDMYYLETSAKESDNVEKLFLDLACRLISEARQNTLV
NNVSSPLPGEGKSISYLTCCNFN*
>ENSG00000120805|ENST00000536227
MRILILGLDGAGKTTILYRLQVGEVVTTIPTIGFNVETVTYKNLKFQVWDLGGQTSIRPY
WRCYYSNTDAVIYVVDSCDRDRIGISKSELVAMLEEEELRKAILVVFANKQDMEQAMTSS
EMANSLGLPALKDRKWQIFKTSATKGTGLDEAMEWLVETLKSRQ*
>ENSG00000165861|ENST00000556143
MSAQTSPAEKGLNPGLMCQESYACSGTDEAIFECDECCSLQCLRCEEELHRQERLRNHER
IRLKPGHVPYCDLCKGLSGHLPGVRQRAIVRCQTCKINLCLECQKRTHSGGNKRRHPVTV
YNVSNLQESLEAEEMDEETKRKKMTEKVVSFLLVDENEEIQVTNEEDFIRKLDCKPDQHL
KVVSIFGNTGDGKSHTLNHTFFYGREVFKTSPTQESCTVGVWAAYDPVHKVAVIDTEGLL
GATVNLSQRTRLLLKVLAISDLVIYRTHADRLHNDLFKFLGDASEAYLKHFTKELKATTA
RCGLDVPLSTLGPAVIIFHETVHTQLLGSDHPSEVPEKLIQDRFRKLGRFPEAFSSIHYK
GTRTYNPPTDFSGLRRALEQLLENNTTRSPRHPGVIFKALKALSDRFSGEIPDDQMAHSS
FFPDEYFTCSSLCLSCGVGCKKSMNHGKEGVPHEAKSRCRYSHQYDNRVYTCKACYERGE
EVSVVPKTSASTDSPWMGLAKYAWSGYVIECPNCGVVYRSRQYWFGNQDPVDTVVRTEIV
HVWPGTDGFLKDNNNAAQRLLDGMNFMAQSVSELSLGPTKAVTSWLTDQIAPAYWRPNSQ

ILSCNKCATSFKDNDTKHHCRACGEGFCDSCSSKTRPVPERGWGPAPVRVCDNCYEARNV
QLAVTEAQVDDEGGTLIARKVGEAVQNTLGAVVTAIDIPLGLVKDAARPAYWVPDHEILH
CHNCRKEFSIKLSKHHCRACGQGFCDECSHDRRAVPSRGWDHPVRVCFNCNKKPGDL*
>ENSG00000137497|ENST00000541641
MTLHATRGAALLSWVNSLHVADPVEAVLQLQDCSIFIKIIDRIHGTEEGQQILKQPVSER
LDFVCSFLQKNR
>ENSG00000172575|ENST00000450598
MGTLGKAREAPRKPSHGCRAASKARLEAKPANSPFPSHPSLAHITQFRMMVSLGHLAKGA
SLDDLIDSCIQSFDADGNLCRSNQLLQVMLTMHRIVISSAELLQKVITLYKDALAKNSPG
LCLKICYFVRYWITEFWVMFKMDASLTDTMEEFQELVKAKGEELHCRLIDTTQINARDWS
RKLTQRIKSNTSKKRKVSLLFDHLEPEELSEHLTYLEFKSFRRISFSDYQNYLVNSCVKE
NPTMERSIALCNGISQWVQLMVLSRPTPQLRAEVFIKFIQVAQKLHQLQNFNTLMAVIGG
LCHSSISRLKETSSHVPHEINKVLGEMTELLSSSRNYDNYRRAYGECTDFKIPILGVHLK
DLISLYEAMPDYLEDGKVNVHKLLALYNHISELVQLQEVAPPLEANKDLVHLLTLSLDLY
YTEDEIYELSYAREPRNHRAPSVFKNYDHDQDGYISQEEFEKIAASFPFSFCVMDKDREG
LISRDEITAYFMRASSIYSKLGLGFPHNFQETTYLKPTFCDNCAGFLWGVIKQGYRCKDC
GMNCHKQCKDLVVFECKKRAKNPVAPTENNTSVGPVSNLCSLGAKDLLHAPEEGPFTFPN
GEAVEHGEESKDRTIMLMGVSSQKISLRLKRAVAHKATQTESQPWIGSEGPSGPFVLSSP
RKTAQDTLYVLPSPTSPCPSPVLVRKRAFVKWENKDSLIKSKEELRHLRLPTYQELEQEI
NTLKADNDALKIQLKYAQKKIESLQLEKSNHVLAQMEQGDCS*
>ENSG00000137497|ENST00000616538
MTLHATRGAALLSWVNSLHVADPVEAVLQLQDCSIFIKIIDRIHGTEEGQQILKQPVSER
LDFVCSFLQKNRKHPSSPECLVSAQKVLEGSELELAKMTMLLLYHSTMSSKSPRDWEQFE
YKIQAELAVILKFVLDHEDGLNLNEDLENFLQKAPVPSTCSSTFPEELSPPSHQAKREIR
FLELQKVASSSSGNNFLSGSPASPMGDILQTPQFQMRRLKKQLADERSNRDELELELAEN
RKLLTEKDAQIAMMQQRIDRLALLNEKQAASPLEPKELEELRDKNESLTMRLHETLKQCQ
DLKTEKSQMDRKINQLSEENGDLSFKLREFASHLQQLQDALNELTEEHSKATQEWLEKQA
QLEKELSAALQDKKCLEEKNEILQGKLSQLEEHLSQLQDNPPQEKGEVLGDVLQLETLKQ
EAATLAANNTQLQARVEMLETERGQQEAKLLAERGHFEEEKQQLSSLITDLQSSISNLSQ
AKEELEQASQAHGARLTAQVASLTSELTTLNATIQQQDQELAGLKQQAKEKQAQLAQTLQ
QQEQASQGLRHQVEQLSSSLKQKEQQLKEVAEKQEATRQDHAQQLATAAEEREASLRERD
AALKQLEALEKEKAAKLEILQQQLQVANEARDSAQTSVTQAQREKAELSRKVEELQACVE
TARQEQHEAQAQVAELELQLRSEQQKATEKERVAQEKDQLQEQLQALKESLKVTKGSLEE
EKRRAADALEEQQRCISELKAETRSLVEQHKRERKELEEERAGRKGLEARLQQLGEAHQA
ETEVLRRELAEAMAAQHTAESECEQLVKEVAAWRERYEDSQQEEAQYGAMFQEQLMTLKE
ECEKARQELQEAKEKVAGIESHSELQISRQQNELAELHANLARALQQVQEKEVRAQKLAD
DLSTLQEKMAATSKEVARLETLVRKAGEQQETASRELVKEPARAGDRQPEWLEEQQGRQF
CSTQAALQAMEREAEQMGNELERLRAALMESQGQQQEERGQQEREVARLTQERGRAQADL
ALEKAARAELEMRLQNALNEQRVEFATLQEALAHALTEKEGKDQELAKLRGLEAAQIKEL
EELRQTVKQLKEQLAKKEKEHASGSGAQSEAAGRTEPTGPKLEALRAEVSKLEQQCQKQQ
EQADSLERSLEAERASRAERDSALETLQGQLEEKAQELGHSQSALASAQRELAAFRTKVQ
DHSKAEDEWKAQVARGRQEAERKNSLISSLEEEVSILNRQVLEKEGESKELKRLVMAESE
KSQKLEEGCACCRQRQPATVPELQNAALLCGRRCRASGREKQRVASENLRQELTSQAERA
EELGQELKAWQEKFFQKEQALSTLQLEHTSTQALVSELLPAKHLCQQLQAEQAAAEKRHR

EELEQSKQAAGGLRAELLRAQRELGELIPLRQKVAEQERTAQQLRAEKASYAEQLSMLKK
AHGLLAEENRGLGERANLGRQFLEVELDQAREKYVQELAAVRADAETRLAEVQREAQSTA
RELEVMTAKYEGAKVKVLEERQRFQEERQKLTAQVEELSKKLADSDQASKVQQQKLKAVQ
AQGGESQQEAQRLQAQLNELQAQLSQKEQAAEHYKLQMEKAKTHYDAKKQQNQELQEQLR
SLEQLQKENKELRAEAERLGHELQQAGLKTKEAEQTCRHLTAQVRSLEAQVAHADQQLRD
LGKFQVATDALKSREPQAKPQLDLSIDSLDLSCEEGTPLSITSKLPRTQPDGTSVPGEPA
SPISQRLPPKVESLESLYFTPIPARSQAPLESSLDSLGDVFLDSGRKTRSARRRTTQIIN
ITMTKKLDVEEPDSANSSFYSTRSAPASQASLRATSSTQSLARLGSPDYGNSALLSLPGY
RPTTRSSARRSQAGVSSGAPPGRNSFYMGTCQDEPEQLDDWNRIAELQQRNRVCPPHLKT
CYPLESRPSLSLGTITDEEMKTGDPQETLRRASMQPIQIAEGTGITTRQQRKRVSLEPHQ
GPGTPESKKATSCFPRPMTPRDRHEGRKQSTTEAQKKAAPASTKQADRRQSMAFSILNTP
KKLGNSLLRRGASKKALSKASPNTRSGTRRSPRIATTTASAATAAAIGATPRAKGKAKH*
>ENSG00000152291|ENST00000444342
MRFVVALVLLNVAAAGAVPLLATESVKQEEAGVRPSAGNVSTHPSLSQRPGGSTKSHPEP
QTPKDSPSKSSAEAQTPEDTPNKSGAEAKTQKDSSNKSGAEAKTQKGSTSKSGSEAQTTK
DSTSKSHPELQTPKDSTGKSGAEAQTPEDSPNRSGAEAKTQKDSPSKSGSEAQTTKDVPN
KSGADGQTPKDGSSKSGAEDQTPKDVPNKSGAEKQTPKDGSNKSGAEEQGPIDGPSKSGA
EEQTSKDSPNKVVPEQPSRKDHSKPISNPSDNKELPKADTNQLADKGKLSPHAFKTESGE
ETDLISPPQEEVKSSEPTEDVEPKEAEDDDTGPEEGSPPKEEKEKMSGSASSENREGTLS
DSTGSEKDDLYPNGSGNGSAESSHFFAYLVTAAILVAVLYIAHHNKRKIIAFVLEGKRSK
VTRRPKASDYQRLDQKVRKEEPGPWEG*
>ENSG00000166685|ENST00000438720
XATAATSPALKRLDLRDPAALFETHGAEEIRGLERQVRAEIEHKKEELRQMVGERYRDLI
EAADTIGQMRRCAVGLVDAVKATDQYCARLRQAGSAAPRPPRAQQPQQPSQEKFYSMAAQ
IKLLLEIPEKIWSSMEASQCLHATQLYLLCCHLHSLLQLDSSSSRYSPVLSRFPILIRQV
AAASHFRSTILHESKMLLKCQGVSDQAVAEALCSIMLLEESSPRQALTDFLLARKATIQK
LLNQPHHGAGIKAQICSLVELLATTLKQAHALFYTLPEGLLPDPALPCGLLFSTLETITG
QHPAGKGTGVLQEEMKLCSWFKHLPASIVEFQPTLRTLAHPISQEYLKDTLQKWIHMCNE
DIKNGITNLLMYVKSMKGLAGIRDAMWELLTNESTNHSWDVLCRRLLEKPLLFWEDMMQQ
LFLDRLQTLTKEGFDSISSSSKELLVSALQELESSTSNSPSNKHIHFEYNMSLFLWSESP
NDLPSDAAWVSVANRGQFASSGLSMKAQAISPCVQNFCSALDSKLKVKLDDLLAYLPSDD
SSLPKDVSPTQAKSSAFDRYADAGTVQEMLRTQSVACIKHIVDCIRAELQSIEEGVQGQQ
DALNSAKLHSVLFMARLCQSLGELCPHLKQCILGKSESSEKPAREFRALRKQGKVKTQEI
IPTQAKWQEVKEVLLQQSVMGYQVWSSAVVKVLIHGFTQSLLLDDAGSVLATATSWDELE
IQEEAESGSSVTSKIRLPAQPSWYVQSFLFSLCQEINRVGGHALPKVTLQEMLKSCMVQV
VAAYEKLSEEKQIKKEGAFPVTQNRALQLLYDLRYLNIVLTAKGDEVKSGRSKPDSRIEK
VTDHLEALIDPFDLDVFTPHLNSNLHRLVQRTSVLFGLVTGTENQLAPRSSTFNSQEPHN
ILPLASSQIRFGLLPLSMTSTRKAKSTRNIETKAQVGAKSKRLIRGWVPTSHRATHDQLP
FK*
>ENSG00000189067|ENST00000620789
MSVPGPYQAATGPSSAPSAPPSYEETVAVNSYYPTPPAPMPGPTTGLVTGPDGKGMNPPS
YYTQPAPIPNNNPITVQTVYVQHPITFLDRPIQMCCPSCNKMIVSQLSYNAGALTWLSCG
SLCLLGQECSGTIVALRSFDLLGSCNPPSSAS*
>ENSG00000281385|ENST00000625871

XKSKEAEIKRINKELANIRSKFKGDKALDGYSKKKYVCKLLFIFLLGHDIDFGHMEAVNL
LSSNRYTEKQIGYLFISVLVNSNSELIRLINNAIKNDLASRNPTFMGLALHCIASVGSRE
MAEAFAGEIPKVLVAGDTMDSVKQSAALCLLRLYRTSPDLVPMGDWTSRVVHLLNDQHLG
VVTAATSLITTLAQKNPEEFKTSVSLAVSRLSRIVTSASTDLQDYTYYFVPAPWLSVKLL
RLLQCYPPPEDPAVRGRLTECLETILNKAQEPPKSKKVQHSNAKNAVLFEAISLIIHHDS
EPNLLVRACNQLGQFLQHRETNLRYLALESMCTLASSEFSHEAVKTHIETVINALKTERD
VSVRQRAVDLLYAMCDRSNAPQIVAEMLSYLETADYSIREEIVLKVAILAEKYAVDYTWY
VDTILNLIRIAGDYVSEEVWYRVIQIVINRDDVQGYAAKTVFEALQAPACHENLVKVGGY
ILGEFGNLIAGDPRSSPLIQFHLLHSKFHLCSVPTRALLLSTYIKFVNLFPEVKPTIQDV
LRSDSQLRNADVELQQRAVEYLRLSTVASTDILATVLEEMPPFPERESSILAKLKKKKGP
STVTDLEDTKRDRSVDVNGGPEPAPASTSAVVCL*
>ENSG00000234514|ENST00000438349
MLSRNDVKRLERSWRLPVPSSEGPDLRGRDKRGTKEDPRADYACIAENKPT*
>ENSG00000017260|ENST00000508532
MKVARFQKIPNGENETMIPVLTSKKASELPVSEVASILQADLQNGLNKCEVSHRRAFHGW
NEFDISEDEPLWKKYISQFKNPLIMLLLASAVISVLMHQFDDAVSITVAILIVVTVAFVQ
EYRSEKSLEELSKLVPPECHCVREGKLEHTLARDLVPGDTVCLSVGDRVPADLRLFEAVD
LSIDESSLTGETTPCSKVTAPQPAATNGDLASRSNIAFMGTLVRCGKAKGVVIGTGENSE
FGEVFKMMQAEEAPKTPLQKSMDLLGKQLSFYSFGIIGIIMLVGWLLGKDILEMFTISVS
LAVAAIPEGLPIVVTVTLALGVMRMVKKRAIVKKLPIVETLGCCNVICSDKTGTLTKNEM
TVTHIFTSDGLHAEVTGVGYNQFGEVIVDGDVVHGFYNPAVSRIVEAGCVCNDAVIRNNT
LMGKPTEGALIALAMKMGLDGLQQDYIRKAEYPFSSEQKWMAVKCVHRTQQDRPEICFMK
GAYEQVIKYCTTYQSKGQTLTLTQQQRDVYQQEKARMGSAGLRVLALASGPELGQLTFLG
LVGIIDPPRTGVKEAVTTLIASGVSIKMITGDSQETAVAIASRLGLYSKTSQSVSGEEID
AMDVQQLSQIVPKVAVFYRASPRHKMKIIKSLQKNGSVVAMTGDGVNDAVALKAADIGVA
MGQTGTDVCKEAADMILVDDDFQTIMSAIEEGKGIYNNIKNFVRFQLSTSIAALTLISLA
TLMNFPNPLNAMQILWINIIMDGPPAQSLGVEPVDKDVIRKPPRNWKDSILTKNLILKIL
VSSIIIVCGTLFVFWRELRDNVITPRDTTMTFTCFVFFDMFNALSSRSQTKSVFEIGLCS
NRMFCYAVLGSIMGQLLVIYFPPLQKVFQTESLSILDLLFLLGLTSSVCIVAEIIKKVER
SREKIQKHVSSTSSSFLEV*
>ENSG00000082805|ENST00000589028
MYGSARSVGKVEPSSQSPGRSPRLPRSPRLGHRRTNSTGGSSGSSVGGGSGKTLSMENIQ
SLNAAYATSGPMYLSDHENVGSETPKSTMTLGRSGGRLPYGVRMTAMGSSPNIASSGVAS
DTIAFGEHHLPPVSMASTVPHSLRQARDNTIMDLQTQLKEVLRENDLLRKDVEVKESKLS
SSMNSIKTFWSPELKKERALRKDEASKITIWKEQYRVVQEENQHMQMTIQALQDELRIQR
DLNQLFQQDSSSRTGEPCVAELTEENFQRLHAEHERQAKELFLLRKTLEEMELRIETQKQ
TLNARDESIKKLLEMLQSKGLSAKATEEDHERTRRLAEAEMHVHHLESLLEQKEKENSML
REEMHRRFENAPDSAKTKALQTVIEMKDSKISSMERGLRDLEEEIQMLKSNGALSTEERE
EEMKQMEVYRSHSKFMKNKVEQLKEELSSKEAQWEELKKKAAGLQAEIGQVKQELSRKDT
ELLALQTKLETLTNQFSDSKQHIEVLKESLTAKEQRAAILQTEVDALRLRLEEKETMLNK
KTKQIQDMAEEKGTQAGEIHDLKDMLDVKERKVNVLQKKIENLQEQLRDKEKQMSSLKER
VKSLQADTTNTDTALTTLEEALAEKERTIERLKEQRDRDEREKQEEIDNYKKDLKDLKEK
VSLLQGDLSEKEASLLDLKEHASSLASSGLKKDSRLKTLEIALEQKKEECLKMESQLKKA
HEAALEARASPEMSDRIQHLEREITRYKDESSKAQAEVDRLLEILKEVENEKNDKDKKIA

ELERQVKDQNKKVANLKHKEQVEKKKSAQMLEEARRREDNLNDSSQQLQDSLRKKDDRIE
ELEEALRESVQITAEREMVLAQEESARTNAEKQVEELLMAMEKVKQELESMKAKLSSTQQ
SLAEKETHLTNLRAERRKHLEEVLEMKQEALLAAISEKDANIALLELSSSKKKTQEEVAA
LKREKDRLVQQLKQQTQNRMKLMADNYEDDHFKSSHSNQTNHKPSPDQIIQPLLELDQNR
SKLKLYIGHLTTLCHDRDPLILRGLTPPASYNLDDDQAAWENELQKMTRGQLQDELEKGE
RDNAELQEFANAILQQIADHCPDILEQVVNALEESS*
>ENSG00000233192|ENST00000546801
MILNKALLLGALALTAVMSPCGGEDIVADHVASYGVNFYQSHGPSGQYTHEFDGDEEFYV
DLETKETVWQLPMFSKFISFDPQSALRNMAVGKHTLEFMMRQSNSTAATNEVPEVTVFSK
FPVTLGQPNTLICLVDNIFPPVVNITWLSNGHSVTEGVSETSFLSKSDHSFFKISYLTFL
PSADEIYDCKVEHWGLDEPLLKHWEPEIPAPMSELTETLVCALGLSVGLMGIVVGTVFII
QGLRSVGASRHQGLL*
>ENSG00000114650|ENST00000441517
MTLTERLREKISRAFYNHGLLCASYPIPIILFTGFCILACCYPLLKLPLPGTGPVEFTTP
VKDYSPPPVDSDRKQGEPTEQPEWVPGQPACPPDASAPQPQLQPSGGEPGPRALQGGDWC
R*
>ENSG00000149294|ENST00000529356
MLQTKDLIWTLFFLGTAVSLQVDIVPSQGEISVGESKFFLCQVAGDAKDKDISWFSPNGE
KLTPNQQRISVVWNDDSSSTLTIYNANIDDAGIYKCVVTGEDGSESEATVNVKIFQKLMF
KNAPTPQEFREGEDAVIVCDVVSSLPPTIIWKHKGRDVILKKDVRFIVLSNNYLQIRGIK
KTDEGTYRCEGRILARGEINFKDIQVIVNVPPTIQARQNIVNATANLGQSVTLVCDAEGF
PEPTMSWTKDGEQIEQEEDDEKYIFSDDSSQLTIKKVDKNDEAEYICIAENKAGEQDATI
HLKVFAKPKITYVENQTAMELEEQVTLTCEASGDPIPSITWRTSTRNISSEEKASWTRPE
KQEV*
>ENSG00000228254|ENST00000432486
MSWKMALQIPGGFWAAAVTVMLVMLSTPVAEARDFPKDFLVQFKGMCYFTNGTERVRGVA
RYIYNREEYGRFDSDVGEFQAVTELGRSIEDWNNYKDFLEQERAAVDKVCRHNYEAELRT
TLQRQVEPTVTISPSRTEALNHHNLLVCSVTDFYPAQIKVQWFRNDQEETAGVVSTSLIR
NGDWTFQILVMLEITPQRGDIYTCQVEHPSLQSPITVEWRAQSESAQSKMLGGVGGFVLG
LIFLGLGLIIRHRGQKGPRGPPPAGLLH*
>ENSG00000236014|ENST00000428608
MAAAATMAAAARELVLRAGTSDMEEEEGPLAGGPGLQEPLQLGELDITSDEFILDEVDVH
IQANLEDELVKEALKTGVDLRHYSKQVELELQQIEQKSIRDYIQESENIASLHNQITACD
AVLERMEQMLGAFQSDLSSISSEIRTLQEQSGAMNIRLRNRQAVRGKLGELVDGLVVPSA
LVTAILEAPVTEPRFLEQLQELDAKAAAVREQEARGTAACADVRGVLDRLRVKAVTKIRE
FILQKIYSFRKPMTNYQIPQTALLKYRFFYQFLLGNERATAKEIRDEYVETLSKIYLSYY
RSYLGRLMKVQYEEVAEKDDLMGVEDTAKKGFFSKPSLRSRNTIFTLGTRGSVISPTELE
APILVPHTAQRGEQRYPFEALFRSQHYALLDNSCREYLFICEFFVVSGPAAHDLFHAVMG
RTLSMTLKHLDSYLADCYDAIAVFLCIHIVLRFRNIAAKRDVPALDRYWEQVLALLWPRF
ELILEMNVQSVRSTDPQRLGGLDTRPHYITRRYAEFSSALVSINQTIPNERTMQLLGQLQ
VEVENFVLRVAAEFSSRKEQLVFLINNYDMMLGVLMERAADDSKEVESFQQLLNARTQEF
IEELLSPPFGGLVAFVKEAEALIERGQAERLRGEEARVTQLIRGFGSSWKSSVESLSQDV
MRSFTNFRNGTSIIQGALTQLIQLYHRFHRVLSQPQLRALPARAELINIHHLMVELKKHK
PNF*

>ENSG00000124222|ENST00000467096
MATRRLTDAFLLLRNNSIQNRQLLAEQELDELADDRMALVSGISLDPEAAIGVTKRPPPK
WVDGVDEIQYDVGRIKQKMKELASLHDKHLNRPTLDDSSEEEHAIEITTQEITQA*
>ENSG00000149541|ENST00000534715
MKLKLKNVFLAYFLVSIAGLLYALVQLAKGMDRTLPLMQRFRDSATALSSGQPCDCLPPL
RAAAEQLRQKDLRISQLQAELRRPPPAPAQPPEPEALPTIYVVTPTYARLVQKAELVRLS
QTLSLVPRLHWLLVEDAEGPTPLVSGLLAASGLLFTHLVVLTPKAQRLREGEPGWVHPRG
VEQRNKALDWLRGRGGAVGGEKDPPPPGTQGVVYFADDDNTYS
>ENSG00000138459|ENST00000484995
MEKQCCSHPVICSLSTMYTFLLGAIFIALSSSRILLVKYSANEENKYDYLPTTVNVCSEL
VKLVFCVLVSFCVIKKDHQSRNLKYASWKEFSDFMKWSIPAFLYFLDNLIVFYVLSYLQP
AMAVIFSNFSIITTALLFRIVLKRRLN
>ENSG00000122705|ENST00000538225
MAELDPFGAPAGAPGGPALGNGVAGAGEEDPAAAFLAQQESEIAGIENDEAFAILDGGAP
GPQPHGEPPGGPDAVDGVMNGEYYQESNGPTDSYAAISQVDRLQSEPESIRKWREEQMER
LEALDANSRKQEAEWKEKAIKELEEWYARQDEQLQKTKANNSTNINHPCYSLEQAAEEAF
VNDIDESSPGTEWERVARLCDFNPKSSKQAKDVSRMRSVLISLKQAPLVH*
>ENSG00000008710|ENST00000423118
MPPAAPARLALALGLGLWLGALAGGPGRGCGPCEPPCLCGPAPGAACRVNCSGRGLRTLG
PALRIPADATALDVSHNLLRALDVGLLANLSALAELDISNNKISTLEEGIFANLFNLSEI
NLSGNPFECDCGLAWLPRWAEEQQVRVVQPEAATCAGPGSLAGQPLLGIPLLDSGCGEEY
VACLPDNSSGTVAAVSFSAAHEGLLQPEACSAFCFSTGQGLAALSEQGWCLCGAAQPSSA
SFACLSLCSGPPPPPAPTCRGPTLLQHVFPASPGATLVGPHGPLASGQLAAFHIAAPLPV
TATRWDFGDGSAEVDAAGPAASHRYVLPGRYHVTAVLALGAGSALLGTDVQVEAAPAALE
LVCPSSVQSDESLDLSIQNRGGSGLEAAYSIVALGEEPARAVHPLCPSDTEIFPGNGHCY
RLVVEKAAWLQAQEQCQAWAGAALAMVDSPAVQRFLVSRVTRSLDVWIGFSTVQGVEVGP
APQGEAFSLESCQNWLPGEPHPATAEHCVRLGPTGWCNTDLCSAPHSYVCELQPGGPVQD
AENLLVGAPSGDLQGPLTPLAQQDGLSAPHEPVEVMVFPGLRLSREAFLTTAEFGTQELR
RPAQLRLQVYRLLSTAGTPENGSEPESRSPDNRTQLAPACMPGGRWCPGANICLPLDASC
HPQACANGCTSGPGLPGAPYALWREFLFSVPAGPPAQYSVTLHGQDVLMLPGDLVGLQHD
AGPGALLHCSPAPGHPGPRAPYLSANASSWLPHLPAQLEGTWACPACALRLLAATEQLTV
LLGLRPNPGLRLPGRYEVRAEVGNGVSRHNLSCSFDVVSPVAGLRVIYPAPRDGRLYVPT
NGSALVLQVDSGANATATARWPGGSVSARFENVCPALVATFVPGCPWETNDTLFSVVALP
WLSEGEHVVDVVVENSASRANLSLRVTAEEPICGLRATPSPEARVLQGVLVRYSPVVEAG
SDMVFRWTINDKQSLTFQNVVFNVIYQSAAVFKLSLTASNHVSNVTVNYNVTVERMNRMQ
GLQVSTVPAVLSPNATLALTAGVLVDSAVEVAFLWTFGDGEQALHQFQPPYNESFPVPDP
SVAQVLVEHNVMHTYAAPGEYLLTVLASNAFENLTQQVPVSVRASLPSVAVGVSDGVLVA
GRPVTFYPHPLPSPGGVLYTWDFGDGSPVLTQSQPAANHTYASRGTYHVRLEVNNTVSGA
AAQADVRVFEELRGLSVDMSLAVEQGAPVVVSAAVQTGDNITWTFDMGDGTVLSGPEATV
EHVYLRAQNCTVTVGAASPAGHLARSLHVLVFVLEVLRVEPAACIPTQPDARLTAYVTGN
PAHYLFDWTFGDGSSNTTVRGCPTVTHNFTRSGTFPLALVLSSRVNRAHYFTSICVEPEV
GNVTLQPERQFVQLGDEAWLVACAWPPFPYRYTWDFGTEEAAPTRARGPEVTFIYRDPGS
YLVTVTASNNISAANDSALVEVQEPVLVTSIKVNGSLGLELQQPYLFSAVGRGRPASYLW
DLGDGGWLEGPEVTHAYNSTGDFTVRVAGWNEVSRSEAWLNVTVKRRVRGLVVNASRTVV

PLNGSVSFSTSLEAGSDVRYSWVLCDRCTPIPGGPTISYTFRSVGTFNIIVTAENEVGSA

QDSIFVYVLQLIEGLQVVGGGRYFPTNHTVQLQAVVRDGTNVSYSWTAWRDRGPALAGSG

KGFSLTVLEAGTYHVQLRATNMLGSAWADCTMDFVEPVGWLMVAASPNPAAVNTSVTLSA

ELAGGSGVVYTWSLEEGLSWETSEPFTTHSFPTPGLHLVTMTAGNPLGSANATVEVDVQV

PVSGLSIRASEPGGSFVAAGSSVPFWGQLATGTNVSWCWAVPGGSSKRGPHVTMVFPDAG

TFSIRLNASNAVSWVSATYNLTAEEPIVGLVLWASSKVVAPGQLVHFQILLAAGSAVTFR

LQVGGANPEVLPGPRFSHSFPRVGDHVVSVRGKNHVSWAQAQVRIVVLEAVSGLQVPNCC

EPGIATGTERNFTARVQRGSRVAYAWYFSLQKVQGDSLVILSGRDVTYTPVAAGLLEIQV

RAFNALGSENRTLVLEVQDAVQYVALQSGPCFTNRSAQFEAATSPSPRRVAYHWDFGDGS

PGQDTDEPRAEHSYLRPGDYRVQVNASNLVSFFVAQATVTVQVLACREPEVDVVLPLQVL

MRRSQRNYLEAHVDLRDCVTYQTEYRWEVYRTASCQRPGRPARVALPGVDVSRPRLVLPR

LALPVGHYCFVFVVSFGDTPLTQSIQANVTVAPERLVPIIEGGSYRVWSDTRDLVLDGSE

SYDPNLEDGDQTPLSFHWACVASTQREAGGCALNFGPRGSSTVTIPRERLAAGVEYTFSL

TVWKAGRKEEATNQTVLIRSGRVPIVSLECVSCKAQAVYEVSRSSYVYLEGRCLNCSSGS

KRGRWAARTFSNKTLVLDETTTSTGSAGMRLVLRRGVLRDGEGYTFTLTVLGRSGEEEGC

ASIRLSPNRPPLGGSCRLFPLGAVHALTTKVHFECTGWHDAEDAGAPLVYALLLRRCRQG

HCEEFCVYKGSLSSYGAVLPPGFRPHFEVGLAVVVQDQLGAAVVALNRSLAITLPEPNGS

ATGLTVWLHGLTASVLPGLLRQADPQHVIEYSLALVTVLNEYERALDVAAEPKHERQHRA

QIRKNITETLVSLRVHTVDDIQQIAAALAQCMGPSRELVCRSCLKQTLHKLEAMMLILQA

ETTAGTVTPTAIGDSILNITGDLIHLASSDVRAPQPSELGAESPSRMVASQAYNLTSALM

RILMRSRVLNEEPLTLAGEEIVAQGKRSDPRSLLCYGGAPGPGCHFSIPEAFSGALANLS

DVVQLIFLVDSNPFPFGYISNYTVSTKVASMAFQTQAGAQIPIERLASERAITVKVPNNS

DWAARGHRSSANSANSVVVQPQASVGAVVTLDSSNPAAGLHLQLNYTLLDGHYLSEEPEP

YLAVYLHSEPRPNEHNCSASRRIRPESLQGADHRPYTFFISPGSRDPAGSYHLNLSSHFR

WSALQVSVGLYTSLCQYFSEEDMVWRTEGLLPLEETSPRQAVCLTRHLTAFGASLFVPPS

HVRFVFPEPTADVNYIVMLTCAVCLVTYMVMAAILHKLDQLDASRGRAIPFCGQRGRFKY

EILVKTGWGRGSGTTAHVGIMLYGVDSRSGHRHLDGDRAFHRNSLDIFRIATPHSLGSVW

KIRVWHDNKGLSPAWFLQHVIVRDLQTARSAFFLVNDWLSVETEANGGLVEKEVLAASDA

ALLRFRRLLVAELQRGFFDKHIWLSIWDRPPRSRFTRIQRATCCVLLICLFLGANAVWYG

AVGDSAYSTGHVSRLSPLSVDTVAVGLVSSVVVYPVYLAILFLFRMSRSKVAGSPSPTPA

GQQVLDIDSCLDSSVLDSSFLTFSGLHAEAFVGQMKSDLFLDDSKSLVCWPSGEGTLSWP

DLLSDPSIVGSNLRQLARGQAGHGLGPEEDGFSLASPYSPAKSFSASDEDLIQQVLAEGV

SSPAPTQDTHMETDLLSSLSSTPGEKTETLALQRLGELGPPSPGLNWEQPQAARLSRTGL

VEGLRKRLLPAWCASLAHGLSLLLVAVAVAVSGWVGASFPPGVSVAWLLSSSASFLASFL

GWEPLKVLLEALYFSLVAKRLHPDEDDTLVESPAVTPVSARVPRVRPPHGFALFLAKEEA

RKVKRLHGMLRSLLVYMLFLLVTLLASYGDASCHGHAYRLQSAIKQELHSRAFLAITRSE

ELWPWMAHVLLPYVHGNQSSPELGPPRLRQVRLQEALYPDPPGPRVHTCSAAGGFSTSDY

DVGWESPHNGSGTWAYSAPDLLGAWSWGSCAVYDSGGYVQELGLSLEESRDRLRFLQLHN

WLDNRSRAVFLELTRYSPAVGLHAAVTLRLEFPAAGRALAALSVRPFALRRLSAGLSLPL

LTSVCLLLFAVHFAVAEARTWHREGRWRVLRLGAWARWLLVALTAATALVRLAQLGAADR

QWTRFVRGRPRRFTSFDQVAQLSSAARGLAASLLFLLLVKAAQQLRFVRQWSVFGKTLCR

ALPELLGVTLGLVVLGVAYAQLAILLVSSCVDSLWSVAQALLVLCPGTGLSTLCPAESWH

LSPLLCVGLWALRLWGALRLGAVILRWRYHALRGELYRPAWEPQDYEMVELFLRRLRLWM

GLSKVKEFRHKVRFEGMEPLPSRSSRGSKVSPDVPPPSAGSDASHPSTSSSQLDGLSVSL

GRLGTRCEPEPSRLQAVFEALLTQFDRLNQATEDVYQLEQQLHSLQGRRSSRAPAGSSRG
PSPGLRPALPSRLARASRGVDLATGPSRTPLRAKNKVHPSST*
>ENSG00000117620|ENST00000638988
MFANLKYVSLGILVFQTTSLVLTMRYSRTLKEEGPRYLSSTAVVVAELLKIMACILLVYK
DSKCSLRALNRVLHDEILNKPMETLKLAIPSGIYTLQNNLLYVALSNLDAATYQVTYQLK
ILTTALFSVSMLSKKLGVYQWLSLVILMTGVAFVQWPSDSQLDSKELSAGSQFVGLMAVL
TACFSSGFAGVYFEKILKETKQSVWIRNIQLGFFGSIFGLMGVYIYDGELVSKNGFFQGY
NRLTWIVVVLQVKHLKS*
>ENSG00000100478|ENST00000616371
MIKFFLMVNKQGQTRLSKYYEHVDINKRTLLETEVIKSCLSRSNEQCSFIEYKDFKLIYR
QYAALFIVVGVNDTENEMAIYEFIHNFVEVLDEYFSRVSELDIMFNLDKVHIILDEMVLN
GCIVETNRARILAPLLILDKMSES*
>ENSG00000160408|ENST00000622357
MSSNKEQRSAVFVILFALITILILYSSNSANEVFHYGSLRGRSRRPVNLKKWSITDGYVP
ILGNKTLPSRCHQCVIVSSSSHLLGTKLGPEIERAECTIRMNDAPTTGYSADVGNKTTYR
VVAHSSVFRVLRRPQEFVNRTPETVFIFWGPPSKMQKPQGSLVRVIQRAGLVFPNMEAYA
VSPGRMRQFDDLFRGETGKDREKSHSWLSTGWFTMVIAVELCDHVHVYGMVPPNYCSQRP
RLQRMPYHYYEPKGPDECVTYIQNEHSRKGNHHRFITEKRVFSSWAQLYGITFSHPSWT*
>ENSG00000139629|ENST00000543196
MRLLRRRHMPLRLAMVGCAFVLFLFLLHRDVSSREEATEKPWLKSLVSRKDHVLDLMLEA
MNNLRDSMPKLQIRAPEAQQTLFSINQSCLPGFYTPAELKPFWERPPQDPNAPGADGKAF
QKSKWTPLETQEKEEGYKKHCFNAFASDRISLQRSLGPDTRPPECVDQKFRRCPPLATTS
VIIVFHNEAWSTLLRTVYSVLHTTPAILLKEIILVDDASTEEHLKEKLEQYVKQLQVVRV
VRQEERKGLITARLLGASVAQAEVLTFLDAHCECFHGWLEPLLARIAEDKTVVVSPDIVT
IDLNTFEFAKPVQRGRVHSRGNFDWSLTFGWETLPPHEKQRRKDETYPIKSPTFAGGLFS
ISKSYFEHIGTYDNQMEIWGGENVEMSFRVWQCGGQLEIIPCSVVGHVFRTKSPHTFPKG
TSVIARNQVRLAEVWMDSYKKIFYRRNLQAAKMAQEKSFGDISERLQLREQLHCHNFSWY
LHNVYPEMFVPDLTPTFYGAIKNLGTNQCLDVGENNRGGKPLIMYSCHGLGGNQYFEYTT
QRDLRHNIAKQLCLHVSKGALGLGSCHFTGKNSQVPKDEEWELAQDQLIRNSGSGTCLTS
QDKKPAMAPCNPSDPHQLWLFV*
>ENSG00000178234|ENST00000415421
MGSVTVRYFCYGCLFTSATWTVLLFVVYFNFSEVTQPLKNVPVKGSGPHGPSPKKFYPRFT
RGPSRVLEPQFKANKIDDVIDSRVEDPEEGHLKFSSELGMIFNERDQELRDLGYQKHAFN
MLISDRLGYHRDVPDTRNAACKEKFYPPDLPAASVVICFYNEAFSALLRTVHSVIDRTPA
HLLHEIILVDDDSDFGKECCTW*
>ENSG00000141429|ENST00000591081
MRKFAYCKVVLATSLIWVLLDMFLLLYFSECNKCDEKKERGLPAGDVLEPVQKPHEGPGE
MGKPVVIPKEDQEKMKEMFKINQFNLMASEMIALNRSLPDVRLEG*
>ENSG00000033100|ENST00000465601
CHGGFGYLLSRSLLLRLRPHLDGCRGDILSARPDEWLGRCLIDSLGVGCVSQHQGQQYRS
FELAKNRDPEKEGSSAFLSAFAVHPVSEGTLMYRLHKRFSALELERAYSEIEQLQNCPKK
AQG*
>ENSG00000182870|ENST00000541995
MEVLPCSRVAHIERTRKPYNNDIDYYAKRNALRAAEVWMDDFKSHVYMAWNIPMSNPGVD

FGDVSERLALRQRLKCRSFKWYLENVYPEMRVYNNTLTYGEVRNSKASAYCLDQGAEDGD
RAILYPCHGMSSQLVRYSADGLLQLGPLGSTAFLPDSKCLVDDGTGRMPTLKKCEDVARP
TQRLWDFTQSGPIVSRATGRCLEVEMSKDANFGLRLVVQRCSGQKWMIRNWIKHARH*
>ENSG00000125447|ENST00000538886
MAEAEGESLESWLNKATNPSNRQEDWEYIIGFCDQINKELEGPQIAVRLLAHKIQSPQEW
EALQALTYLGDRVSEKVKTKVIELLYSWTMALPEEAKIKDAYHMLKRQGIVQSDPPIPVD
RTLIPSPPPRPKNPVFDDEEKSKLLAKLLKSKNPDDLQEANKLIKSMVKEDEARIQKVTK
RLHTLEEVNNNVRLLSEMLLHYSQEDSSDGDRELMKELFDQCENKRRTLFKLASETEDND
NSLGDILQASDNLSRVINSYKTIIEGQVINGEVATLTLPDSEGNSQCSNQGTLIDLAELD
TTNSLSSVLAPAPTPPSSGIPILPPPPQASGPPRSRSSSQAEATLGPSSTSNALSWLDEE
LLCLGLADPAPNVPPKESAGNSQWHLLQREQSDLDFFSPRPGTAACGASDAPLLQPSAPS
SSSSQAPLPPPFPAPVVPASVPAPSAGSSLFSTGVAPALAPKVEPAVPGHHGLALGNSAL
HHLDALDQLLEEAKVTSGLVKPTTSPLIPTTTPARPLLPFSTGPGSPLFQPLSFQSQGSP
PKGPELSLASIHVPLESIKPSSALPVTAYDKNGFRILFHFAKECPPGRPDVLVVVVSMLN
TAPLPVKSIVLQAAVPKSMKVKLQPPSGTELSPFSPIQPPAAITQVMLLANPLKEKVRLR
YKLTFALGEQLSTEVGEVDQFPPVEQWGNL*
>ENSG00000176383|ENST00000535274
MLCRLCWLVSYSLAVLLLGCLLFLRKAAKPAGDPTAHQPFWAPPTPRHSRCPPNHTVSSA
SLSLPSRHRLFLTYRHCRNFSILLEPSGCSKDTFLLLAIKSQPGHVERRAAIRSTWGRVG
GWARGRQLKLVFLLGVAGSAPPAQLLAYESREFDDILQWDFTEDFFNLTLKELHLQRWVV
AACPQAHFMLKGDDDVFVHVPNVLEFLDGWDPAQDLLVGDVIRQALPNRNTKVKYFIPPS
MYRATHYPPYAGGGGYVMSRATVRRLQAIMEDAELFPIDDVFVGMCLRRLGLSPMHHAGF
KTFGIRRPLDPLDPCLYRGLLLVHRLSPLEMWTMWALVTDEGLKCAAGPIPQR*
>ENSG00000103051|ENST00000482252
MADLDSPPKLSGVQQPSEGVGGGRCSEISAELIRSLTELQELEAVYERLCGEEKVVEREL
DALLEQQNTIESKMVTLHRMGPNLQLIEGDAKQLAGMITFTCNLAENVSSKVRQLDLAKN
RLYQAIQRADDILDLKFCMDGVQTALRSEDYEQAAAHTHRYLCLDKSVIELSRQGKEGSM
IDANLKLLQEAEQRLKAIVAEKFAIATKEGDLPQVERFFKIFPLLGLHEEGLRKFSEYLC
KQVASKAEENLLMVLGTDMSDRRAAVIFADTLTLLFEGIARIVETHQPIVETYYGPGRLY
TLIKYLQVECDRQVEKVVDKFIKQRDYHQQNFVFSFF*
>ENSG00000181830|ENST00000442528
MALTGASDPSAEAEANGEKPFLLRALQIALVVSLYWVTSISMVFLNKYLLDSPSLRLDTP
IFVTFYQCLVTTLLCKGLSALAACCPGAVDFPSLRLDLRVARSVLPLSVVFIGMITFNNL
CLKYVGVAFYNVGRSLTTVFNVLLSYLLLKQTTSFYALLTCGIIIGGFWLGVDQEGAEGT
LSWLGTVFGVLASLCVSLNAIYTTKVLPAVDGSIWRLTFYNNVNACILFLPLLLLLGELQ
ALRDFAQLGSAHFWGMMTLGGLFGFAIGYVTGLQIKFTSPLTHNVSGTAKACAQTVLAVL
YYEETKSFLWWTSNMMVLGGSSAYTWVRGWEMKKTPEEPSPKDSEKSAMGV*
>ENSG00000276536|ENST00000610977
MTEYKLVVVGAGGVGKSALTIQLIQNHFVDEYDPTIEDSYRKQVVIDGETCLLDILDTAG
QEEYSAMRDQYMRTGEGFLCVFAINNTKSFEDIHQYREQIKRVKDSDDVPMVLVGNKCDL
AARTVESRQAQDLARSYGIPYIETSAKTRQGVEDAFYTLVREIRQHKLRKLNPPDESGPG
CMSCKCVLS*
>ENSG00000164867|ENST00000484524
MGNLKSVAQEPGPPCGLGLGLGLGLCGKQGPATPAPEPSRAPASLLPPAPEHSPPSSPLT

QPPEGPKFPRVKNWEVGSITYDTLSAQAQQDGPCTPRRCLGSLVFPRKLQGRPSPGPPAP
EQLLSQARDFINQYYSSIKRSGSQAHEQRLQEVEAEVAATGTYQLRESELVFGAKQAWRN
APRCVGRIQWGKLQVFDARDCRSAQEMFTYICNHIKYATNRGNLRSAITVFPQRCPGRGD
FRIWNSQLVRYAGYRQQDGSVRGDPANVEITELCIQHGWTPGNGRFDVLPLLLQAPDDPP
ELFLLPPELVLEVPLEHPTLEWFAALGLRWYALPAVSNMLLEIGGLEFPAAPFSGWYMST
EIGTRNLCDPHRYNILEDVAVCMDLDTRTTSSLWKDKAAVEINVAVLHSYQLAKVTIVDH
HAATASFMKHLENEQKARGGCPADWAWIVPPISGSLTPVFHQEMVNYFLSPAFRYQPDPW
KGSAAKGTGITRKKTFKEVANAVKISASLMGTVMAKRVKATILYGSETGRAQSYAQQLGR
LFRKAFDPRVLCMDEYDVVSLEHETLVLVVTSTFGNGDPPENGEGLTLWPRLECSSTITA
HCSLNLLDSSNPPTSTSQVVGTTGACHDA*
>ENSG00000148248|ENST00000545297
MGQNDLMGTAEDFADQFLRVTKQYLPHVARLCLISTFLEDGIRMWFQWSEQRDYIDTTWN
CGYLLASSFVFLNLLGQLTGCVLVLSRNFVQYACFGLFGIIALQTIAYSILWDLKFLMRL
SRTSWAQL*
>ENSG00000138674|ENST00000505984
MKLKEVDRTAMQAWSPAQNHPIYLATGTSAQQLDATFSTNASLEIFELDLSDPSLDMKSC
ATFSSSHRYHKLIWGPYKMDSKGDVSGVLIAGGENGNIILYDPSKIIAGDKEVVIAQNDK
HTGPVRALDVNIFQTNLVASGANESEIYIWDLNNFATPMTPGAKTQPPEDISCIAWNRQV
QHILASASPSGRATVWDLRKNEPIIKVSDHSNRMHCSGLAWHPDVATQMVLASEDDRLPV
IQMWDLRFASSPLRVLENHARGILAIAWSMADPELLLSCGKDAKILCSNPNTGEVLYELP
TNTQWCFDIQWCPRNPAVLSAASFDGRISVYSIMGGSTDGLRQKQVDKLSSSFGNLDPFG
TGQPLPPLQIPQQTAQHSIVLPLKKPPKWIRRPVGASFSFGGKLVTFENVRMPSHQGAEQ
QQQQHHVFISQVVTEKEFLSRSDQLQQAVQSQGFINYCQKKIDASQTEFEKNVWSFLKVN
FEDDSRGKYLELLGYRKEDLGKKHIKEEKEESEFLPSSGGTFNISVSGDIDGLITQALLT
GNFESAVDLCLHDNRMADAIILAIAGGQELLARTQKKYFAKSQSKITRLITAVVMKNWKE
IVESCDLKNWREALAAVLTYAKPDEFSALCDLLGTRLENEGDSLLQTQACLCYICAGNVE
KLVACWTKAQDGSHPLSLQDLIEKVVILRKAVQLTQAMDTSTVGVLLAAKMSQYANLLAA
QGSIAAALAFLPDNTNQPNIMQLRDRLCRAQGEPVAGHESPKIPYEKQQLPKGRPGPVAG
HHQMPRVQTQQYYPHGENPPPPGFIMHGNVNPNAAGQLPTSPGHMHTQVPPYPQPQPYQP
AQPYPFGTGGSAMYRPQQPVAPPTSNAYPNTPYISSASSYTGQSQLYAAQHQASSPTSSP
ATSFPPPPSSGASFQHGGPGAPPSSSAYALPPGTTGPQNGWNDPPALNRVPKKKKMPENF
MPPVPITSPIMNPLGDPQSQMLQQQPSAPVPLSSQSSFPQPHLPGGQPFHGVQQPLGQTG
MPPSFSKPNIEGAPGAPIGNTFQHVQSLPTKKITKKPIPDEHLILKTTFEDLIQRCLSSA
TDPQTKRKLDDASKRLEFLYDKLREQTLSPTITSGLHNIARSIETRNYSEGLTMHTHIVS
TSNFSETSAFMPVLKVVLTQANKLGV*
>ENSG00000167861|ENST00000425042
MGSTDSKLNFRKAVIQLTTKTQPVEATDDAFWDQFWADTATSVQDVFALVPAAEIRAVRE
ESPSNLATLCYKAVEKLVQGAESGCHSEKEKQIVLNCSRLLTRVLPYIFEDPDWRGFFWS
TVPGAGRGGQGEEDDEHARPLAESLLLAIADLLFCPDFTVQSHRRSTVDSAEDVHSLDSC
EYIWEAGVGFAHSPQPNYIHDMNRMELLKLLLTCFSEAMYLPPAPESGSTNPWVQFFCST
ENRHALPLFTSLLNTVCAYDPVGYGIPYNHLLFSDYREPLVEEAAQVLIVTLDHDSASSA
SPTVDGTTTGTAMDDADPPGPENLFVNYLSRIHREEDFQFILKGIARLLSNPLLQTYLPN
STKKIQFHQELLVLFWKLCDFNKKFLFFVLKSSDVLDILVPILFFLNDARADQSRVGLMH
IGVFILLLLSGERNFGVRLNKPYSIRVPMDIPVFTGTHADLLIVVFHKIITSGHQRLQPL

FDCLLTIVVNVSPYLKSLSMVTANKLLHLLEAFSTTWFLFSAAQNHHLVFFLLEVFNNII
QYQFDGNSNLVYAIIRKRSIFHQLANLPTDPPTIHKALQRRRRTPEPLSRTGSQEGTSME
GSRPAAPAEPGTLKTSLVATPGIDKLTEKSQVSEDGTLRSLEPEPQQSLEDGSPAKGEPS
QAWREQRRPSTSSASGQWSPTPEWVLSWKSKLPLQTIMRLLQVLVPQVEKICIDKGLTDE
SEILRFLQHGTLVGLLPVPHPILIRKYQANSGTAMWFRTYMWGVIYLRNVDPPVWYDTDV
KLFEIQRV*
>ENSG00000117620|ENST00000427993
MFANLKYVSLGILVFQTTSLVLTMRYSRTLKEEGPRYLSSTAVVVAELLKIMACILLVYK
DSKCSLRALNRVLHDEILNKPMETLKLAIPSGIYTLQNNLLYVALSNLDAATYQVTYQLK
ILTTALFSVSMLSKKLGVYQWLSLVILMTGVAFVQWPSDSQLDSKELSAGSQFVGLMAVL
TACFSSGFAGVYFEKILKETKQSVWIRNIQLGFFGSIFGLMGVYIYDGELVSKNGFFQGY
NRLTWIVVVLQALGGLVIAAVIKYADNILKGFATSLSIILSTLISYFWLQDFVPTSVFFL
GAILVITATFLYGYDPKPAGNPTKA*
>ENSG00000261949|ENST00000610896
MKSFSRILFLVFLLAGLRSKAAPSAPLPLGCGFPDMAHPSETSPLKGASENSKRDRLNPE
FPGTPYPEPSKLPHTVSLETFPLDFTEPLNPDLRETPHPESPETPKADSLTTSISESLDM
PKTNLSKMAHPESSETPTPGPTEMPHPGSPETPKPNFSKTSRPEFPETPNTDLMQTTPQE
SPEILQLNATEVSQAELPETSNTNPTKTPDPKSPEKHDLNSTETPNSEFLQALHPDPSKT
PHPESHVTHNPSPTEISQTEFPTTYYQNATDVPRTSDPQISTSLYPETPVPFKDDATALN
ELSLNPKPGTPAAIQPDSPKLPTSDSPGMVELKAPQNSGPKESNVPPPSARIAGPPALPG
RPSQLAPATLRAPQRHSRGEGVNTIIVVERVKETGVTLVGRPRGAAGGALCLFFAGTALL
IGIFVLLWCLYRRAARQRPFAHHRLPDDGDEPVLHLDAPKDPYDLYFYAPDTWVPSHIAT
KQPPPTPPLPPKLPPPPRGGRPQRLEALSPATLPNNFV*
>ENSG00000230791|ENST00000428898
MLSRNDEAASAQQ*
>ENSG00000166710|ENST00000558401
MSRSVALAVLALLSLSGLEAIQRTPKIQVYSRHPAENGKSNFLNCYVSGFHPSDIEVDLL
KNGERIEKVEHSDLSFSKDWSFYLLYYTEFTPTEKDEYACRVNHVTLSQPKIVKWDRDM*
>ENSG00000168824|ENST00000421177
MVKLGNNFAEKGTKQPLLEDGFDTIPLMTPLDVNQLQFPPPDKVVVKTKTEYEPDRKKGK
ARPPQIAEFTVSITEGVTERFKVSVLVLFALAFLTCVVFLVVYKVYKYDRACPDGFVLKN
TQCIPEGLESYYAEQDSSAREKFYTVINHYNLAKQSITRSVSPWMSVLSEEKLSEQETEA
AEKSA*
>ENSG00000197249|ENST00000448921
MPSSVSWGILLLAGLCCLVPVSLAEDPQGDAAQKTDTSHHDQDHPTFNKITPNLAEFAFS
LYRQLAHQSNSTNIFFSPVSIATAFAMLSLGTKADTHDEILEGLNFNLTEIPEAQIHEGF
QELLRTLNQPDSQLQLTTGNGLFLSEGLKLVDKFLEDVKKLYHSEAFTVNFGDTEEAKKQ
INDYVEKGTQGKIVDLVKELDRDTVFALVNYIFFKGKWERPFEVKDTEEEDFHVDQVTTV
KVPMMKRLGMFNIQHCKKLSSWVLLMKYLGNATAIFFLPDEGKLQHLENELTHDIITKFL
ENEDRRSASLHLPKLSITGTYDLKSVLGQLGITKVFSNGADLSGVTEEAPLKLSKAVHKA
VLTIDEKGTEAAGAMFLEAIPMSIPPEVKFNKPFVFLMIEQNTKSPLFMGKVVNPTQK*
>ENSG00000233209|ENST00000416192
MSWKKALRIPGGLRVATVTLMLAMLSTPVAEGRDSPEDFVYQFKGMCYFTNGTERVRLVT
RYIYNREEYARFDSDVGVYRAVTPLGPPDAEYWNSQKEVLERTRAELDTVCRHNYQLELR

TTLQRRVEPTVTISPSRTEALNHHNLLVCSVTDFYPAQIKVRWFRNDQEETTGVVSTPLI
RNGDWTFQILVMLEMTPQRGDVYTCHVEHPSLQNPIIVEWRAQSESAQSKMLSGIGGFVL
GLIFLGLGLIIHHRSQKGLLH*
>ENSG00000147642|ENST00000528331
MVGEGSIQSSRYKKESKSGLVKPGSEADFSSSSSTGSISAPEVHMSTAGSKRSSSSRNRG
PHGRSNGASSHKPGSSPSSPREKDLLSMLCRNQLSPVNIHPSYAPSSPSSSNSGSYKGSD
CSPIMRRSGRYMSCGENHGVRPPNPEQYLTPLQQKEVTVRHLKTKLKESERRLHERESEI
VELKSQLARMREDWIEEECHRVEAQLALKEARKEIKQLKQVIETMRSSLADKDKGIQKYF
VDINIQNKKLESLLQSMEMAHSGSLRDELCLDFPCDSPEKSLTLNPPLDTMADGLSLEEQ
VTGEGADRELLVGDSIANSTDLFDEIVTATTTESGDLELVHSTPGANVLELLPIVMGQEE
GSVVVERAVQTDVVPYSPAISELIQSVLQKLQDPCPSSLASPDESEPDSMESFPESLSAL
VVDLTPRNPNSAILLSPVETPYANVDAEVHANRLMRELDFAACVEERLDGVIPLARGGVV
RQYWSSSFLVDLLAVAAPVVPTVLWAFSTQRGGTDPVYNIGALLRGCCVVALHSLRRTAF
RIKT*
>ENSG00000180398|ENST00000409913
MEHLEGVINKPEAEMSPQELQLHYFKMHDYDGNNLLDGLELSTAITHVHKEEGSEQAPLM
SEDELINIIDGVLRDDDKNNDGYIDYAEFAKSLQ*
>ENSG00000101246|ENST00000618838
MYTLLSGLYKYMFQKDEYCILILGLDNAGKTTFLEQSKTRFNKNYKGMSLSKITTTVGLN
IGTVDVGKARLMFWDLGGQEELQSLWDKYYAECHGVIYVIDSTDEERLAESKQAFEKVVT
SEALCGVPVLVLANKQDVETCLSIPDIKTAFSDCTSKIGRRDCLTQACSALTGKGVREGI
EWMVKCVVRNVHRPPRQRDIT*
>ENSG00000166685|ENST00000582672
XKVTDHLEALIDPFDLDVFTPHLNSNLHRLVQRTSVLFGLVTGTENQLAPRSSTFNSQEP
HNILPLASSQIRFGLLPLSMTSTRKAKSTRNIETKAQYDANC*
>ENSG00000119414|ENST00000415905
MAPLDLDKYVEIARLCKYLPENDLKRLCDYVCDLLLEESNVQPVSTPVTVCGDIHGQGDF
VDRGYYSLETFTYLLALKAKWPDRITLLRGNHESRQITQVYGFYDECQTKYGNANAWRYC
TKVFDMLTVAALIDEQILCVHGGLSPDIKTLDQIRTIERNQEIPHKGAFCDLVWSDPEDV
DTWAISPRGAGWLFGAKVTNEFVHINNLKLICRAHQLVHEGYKFMFDEKLVTVWSAPNYC
YRCGNIASIMVFKDVNTREPKLFRAVPDSERVIPPRTTTPYFL*
>ENSG00000178234|ENST00000430044
MGSVTVRYFCYGCLFTSATWTVLLFVYFNFSEVTQPLKNVPVKGSGPHGPSPKKFYPRFT
RGPSRVLEPQFKANKIDDVIDSRVEDPEEGHLKFSSELGMIFNERDQELRDLGYQKHAFN
MLISDRLGYHRDVPDTRNAACKEKFYPPDLPAASVVICFYNEAFSALLRTVHSVIDRTPA
HLLHEIILVDDDSDFDDLKGELDEYVQKYLPGKIKVIRNTKREGLIRGRMIGAAHATGEV
LVFLDSHCEVNVMWLQPLLAAIREDRHTVVCPVIDIISADTLAYSSSPVVRGGFNWGLHF
KWDLVPLSELGRAEGATAPIKSPTMAGGLFAMNRQYFHELGQYDSGMDIWGGENLEISFR
IWMCGGKLFIIPCSRVGHIFRKRRPYGSPEGQDTMTHNSLRLAHVWLDEYKEQYFSLRPD
LKTKSYGNISERVELRKKLGCKSFKWYLDNVYPEMQISGSHAKPQQPIFVNRGPKRPKVL
QRGRLYHLQTNKCLVAQGRPSQKGGLVVLKACDYSDPNQIWIYNEEHELVLNSLLCLDMS
ETRSSDPPRLMKCHGSGGSQQWTFGKNNRLYQVSVGQCLRAVDPLGQKGSVAMAICDGSS
SQQWHLEG*
>ENSG00000171004|ENST00000521489

MALPACAVREFEPPRQPERGAPVRTTCPRRHSRVEAELAASRPGSVAASVRAGPPRGVSH
GFHTRPLLDKPRKASSSLAGAACAPLFALLSRGRRRRMHVLRRRWDLGSLCRALLTRGLA
ALGHSLKHVLGAIFSKIFGPMASVGNMDEKSNKLLLALVMLFLFAVIVLQYVCPGTECQL
LRLQAFSSPVPDPYRSEDESSARFVPRYNFTRGDLLRKVDFDIKGDDLIVFLHIQKTGGT
TFGRHLVRNIQLEQPCECRVGQKKCTCHRPGKRETWLFSRFSTGWSCGLHADWTELTSCV
PSVVDGKRDARLRPSRWRIFQILDAASKDKRGSPNTNAGANSPSSTKTRNTSKSGKNFHY
ITILRDPVSRYLSEWRHVQRGATWKASLHVCDGRPPTSEELPSCYTGDDWSGCPLKEFMD
CPYNLANNRQVRMLSDLTLVGCYNLSVMPEKQRNKVLLESAKSNLKHMAFFGLTEFQRKT
QYLFEKTFNMNFISPFTQYNTTRASSVEINEEIQKRIEGLNFLDMELYSYAKDLFLQRYQ
FMRQKEHQEARRKRQEQRKFLKGRLLQTHFQSQGQGQSQNPNQNQSQNPNPNANQNLTQN
LMQNLTQSLSQKENRESPKQNSGKEQNDNTSNGTNDYIGSVEKWR*
>ENSG00000189067|ENST00000574763
MSVPGPYQAATGPSSAPSAPPSYEETVAVNSYYPTPPAPMPGPTTGLVTGPDGKGMNPPS
YYTQPAPIPNNNPITVQTVYVQHPITFLDRPIQMCCPSCNKMIVSQLSYNAGALTWLSCG
SLCLLGCIAGCCFIPFCVDALQDVDHYCPNCRALLGTYKRL*
>ENSG00000115525|ENST00000640992
MPSEYTYVKLRSDCSRPSLQWYTRAQSKMRRPSLLLKDILKCTLLVFGVWILYILKLNYT
TEECDMKKMHYVDPDHVKRAQKYAQQVLQKECRPKFAKTSMALLFEHRYSVDLLPFVQKA
PKDSEAESKYDPPFGFRKFSSKVQTLLELLPEHDLPEHLKAKTCRRCVVIGSGGILHGLE
LGHTLNQFDVVIRLNSAPVEGYSEHVGNKTTIRMTYPEGAPLSDLEYYSNDLFVAVLFKS
VDFNWLQAMVKKETLPFWVRLFFWKQVAEKIPLQPKHFRILNPVIIKETAFDILQYSEPQ
SRFWGRDKNVPTIGVIAVVLATHLCDEVSLAGFGYDLNQPRTPLHYFDSQCMAAMNFQTM
HNVTTETKFLLKLVKEGVVKDLSGGIDREF*
>ENSG00000196535|ENST00000533112
MFNLMKKDKDKDGGRKEKKEKKEKKERMSAAELRSLEEMSLRRGFFNLNRSSKRESKTRL
EISNPIPIKVASGSDLHLTDIDSDSNRGSVILDSGHLSTASSSDDLKGEEGSFRGSVLQR
AAKFGSLAKQNSQMIVKRFSFSQRSRDESASETSTPSEHSAAPSPQVEVRTLEGQLVQHP
GPGIPRPGHRSRAPELVTKKFPVDLRLPPVVPLPPPTLRELELQRRPTGDFGFSLRRTTM
LDRGPEGQACRRVVHFAEPGAGTKDLALGLVPGDRLVEINGHNVESKSRDEIVEMIRQSG
DSVRLKVQPIPELSELSRSWLRSGEGPRREPSDAKTEEQIAAEEAWNETEKVWLVHRDGF
SLASQLKSEELNLPEGKVRVKLDHDGAILDVDEDDVEKANAPSCDRLEDLASLVYLNESS
VLHTLRQRYGASLLHTYAGPSLLVLGPRGAPAVYSEKVMHMFKGCRREDMAPHIYAVAQT
AYRAMLMSRQDQSIILLGSSGSGKTTSCQHLVQYLATIAGISGNKVFSVEKWQALYTLLE
AFGNSPTIINGNATRFSQILSLDFDQAGQVASASIQTMLLEKLRVARRPASEATFNVFYY
LLACGDGTLRTELHLNHLAENNVFGIVPLAKPEEKQKAAQQFSKLQAAMKVLGISPDEQK
ACWFILAAIYHLGAAGATKEAAEAGRKQFARHEWAQKAAYLLGCSLEELSSAIFKHQHKG
GTLQRSTSFRQGPEESGLGDGTGPKLSALECLEGMAAGLYSELFTLLVSLVNRALKSSQH
SLCSMMIVDTPGFQNPEQGGSARGASFEELCHNYTQDRLQRLFHERTFVQELERYKEENI
ELAFDDLEPPTDDSVAAVDQASHQSLVRSLARTDEARGLLWLLEEEALVPGASEDTLLER
LFSYYGPQEGDKKGQSPLLHSSKPHHFLLGHSHGTNWVEYNVTGWLNYTKQNPATQNAPR
LLQDSQKKIISNLFLGRAGSATVLSGSIAGLEGGSQLALRRATSMRKTFTTGMAAVKKKS
LCIQMKLQVDALIDTIKKSKLHFVHCFLPVAEGWAGEPRSASSRRVSSSSELDLPSGDHC
EAGLLQLDVPLLRTQLRGSRLLDAMRMYRQGYPDHMVFSEFRRRFDVLAPHLTKKHGRNY
IVVDERRAVEELLECLDLEKSSCCMGLSRVFFRAGTLARLEEQRDEQTSRNLTLFQAACR

GYLARQHFKKRKIQDLAIRCVQKNIKKNKGVKDWPWWKLFTTVRPLIEVQLSEEQIRNKD
EEIQQLRSKLEKAEKERNELRLNSDRLESRISELTSELTDERNTGESASQLLDAETAERL
RAEKEMKELQTQYDALKKQMEVMEMEVMEARLIRAAEINGEVDDDDAGGEWRLKYERAVR
EVDFTKKRLQQEFEDKLEVEQQNKRQLERRLGDLQADSEESQRALQQLKKKCQRLTAELQ
DTKLHLEGQQVRNHELEKKQRRFDSELSQAHEEAQREKLQREKLQREKDMLLAEAFSLKQ
QLEEKDMDIAGFTQKVVSLEAELQDISSQESKDEASLAKVKKQLRDLEAKVKDQEEELDE
QAGTIQMLEQLKQMEVQLEEEYEDKQKVLREKRELEGKLATLSDQVNRRDFESEKRLRKD
LKRTKALLADAQLMLDHLKNSAPSKREIAQLKNQLEESEFTCAAAVKARKAMEVEIEDLH
LQIDDIAKAKTALEEQLSRLQREKNEIQNRLEEDQEDMNELMKKHKAAVAQASRDLAQIN
DLQAQLEEANKEKQELQEKLQALQSQVEFLEQSMVDKSLVSRQEAKIRELETRLEFERTQ
VKRLESLASRLKENMEKLTEERDQRIAAENREKEQNKRLQRQLRDTKEEMGELARKEAEA
SRKKHELEMDLESLEAANQSLQADLKLAFKRIGDLQAAIEDEMESDENEDLINSEGDSDV
DSELEDRVDGVKSWLSKNKGPSKAASDDGSLKSSSPTSYWKSLAPDRSDDEHDPLDNTSR
PRYSHSYLSDSDTEAKLTETNA*
>ENSG00000186318|ENST00000513780
MAQALPWLLLWMGAGVLPAHGTQHGIRLPLRSGLGGAPLGLRLPRETDEEPEEPGRRGSF
VEMVDNLRGKSGQGYYVEMTVGSPPQTLNILVDTGSSNFAVGAAPHPFLHRYYQRQLSST
YRDLRKGVYVPYTQGKWEGELGTDLVSIPHGPNVTVRANIAAITESDKFFINGSNWEGIL
GLAYAEIARLCGAGFPLNQSEVLASVGGSMIIGGIDHSLYTGSLWYTPIRREWYYEVIIV
RVEINGQDLKMDCKEYNYDKSIVDSGTTNLRLPKKVFEAAVKSIKAASSTEKFPDGFWLG
EQLVCWQAGTTPWNIFPVISLYLMGEVTNQSFRITILPQQYLRPVEDVATSQDDCYKFAI
SQSSTGTVMGAVIMEGFYVVFDRARKRIGFAVSACHVHDEFRTAAVEGPFVTLDMEDCGY
NIPQTDESTLMTIAYVMAAICALFMLPLCLMVCQWRCLRCLRQQHDDFADDISLLK*
>ENSG00000198324|ENST00000548163
MKLNERSLAFYATCDAPVDNAGFLYKKGGRHAAYHRRWFVLRGNMLFYFEDAASREPVGV
IILEGCTVELVEAAEEFAFAVRFAGTRARTYVLAAESQDAMEGWVKALSRASFDYLRLVV
RELEQQLAAVRGGGGMALPQPQPQSLPLPPSLPSALAPVPSLPSAPAPVPALPLPRRPSA
LPPKENGCAVWSTEATFRPGPEPPPPPPRRRASAPHGPLDMAPFARLHECYGQEIRALRG
QWLSSRVQP*
>ENSG00000262246|ENST00000537233
MNRFRVSKFRHTEARPPRRESWISDIRAGTAPSCRNHIKSSCSLIAFNSDRPGVLGIVPL
QGQGEDKRRVAHLGCHSGSADRTVKLWRLPGPGQALPSAPGVVLGPEDLPVEVLQFHPTS
DGILVSAAGTTVKVWDAAKQQPLTELAAHGDLVQSAVWSRDGALVGTACKDKQLRIFDPR
TKPRASQSTQAHENSRDSRLAWMGTWEHLVSTGFNQMREREVKLWDTRFFSSALASLTLD
TSLGCLVPLLDPDSGLLVLAGKGERQLYCYEVVPQQPALSPVTQCVLESVLRGAALVPRQ
ALAVMSCEVLRVLQLSDTAIVPIGYHVPRKAVEFHEDLFPDTAGCVPATDPHSWWAGDNQ
QVQKVSLNPACRPHPSFTSCLVPPAEPLPDTAQPAVMETPVGDADASEGFSSPPSSLTSP
STPSSLGPSLSSTSGIGTSPSLRSLQSLLGPSSKFRHAQGTVLHRDSHITNLKGLNLTTP
GESDGFCANKLRVAVPLLSSGGQVAVLELRKPGRLPDTALPTLQNGAAVTDLAWDPFDPH
RLAVAGEDARIRLWRVPAEGLEEVLTTPETVLTGHTEKICSLRFHPLAANVLASSSYDLT
VRIWDLQAGADRLKLQGHQDQIFSLAWSPDGQQLATVCKDGRVRVYRPRSGPEPLQEGPG
PKGGRGARIVWVCDGRCLLVSGFDSQSERQLLLYEAEALAGGPLAVLGLDVAPSTLLPSY
DPDTGLVLLTGKGDTRVFLYELLPESPFFLECNSFTSPDPHKGLVLLPKTECDVREVELM
RCLRLRQSSLEPVAFRLPRVRKEFFQDDVFPDTAVIWEPVLSAEAWLQGANGQPWLLSLQ

PPDMSPVSQAPREAPARRAPSSAQYLEEKSDQQKKEELLNAMVAKLGNREDPLPQDSFEG
VDEDEWD*
>ENSG00000137497|ENST00000535838
MTLHATRGAALLSWVNSLHVADPVEAVLQLQDCSIFIKIIDRIHGTEEGQQILKQPVSE
>ENSG00000163812|ENST00000424952
MMLIPTHHFRNIERKPEYLQPEKCVPPPYPGPVGTMWFIRDGCGIACAIVTWFLVLYAEF
VVLFVMLIPSRDYVYSIINGIVFNLLAFLALASHCRAMLTDPGAVPKGNATKEFIESLQL
KPGQVVYKCPKCCSIKPDRAHHCSVCKRCIRKMDHHCPWVNNCVGENNQKYFVLFTMYIA
LISLHALIMVGFHFLHCFEEDWTKCSSFSPPTTVILLILLCFEGLLFLIFTSVMFGTQVH
SICTDETGIEQLKKEERRWAKKTKWMNMKAVFGHPFSLGWASPFATPDQGKADPYQYVV*
>ENSG00000125447|ENST00000582717
MKNCGRRFHNEVGKFRFLNELIKVVSPKYLGDRVSEKVKTKVIELLYSWTMALPEEAKIK
DAYHMLKRQGIVQSDPPIPVDRTLIPSPPPRPKNPVFDDEEKSKLLAKLLKSKNPDDLQE
ANKLIKSMVKEDEARIQKVTKRLHTLEEVNNNVRLLSEMLLHYSQEDSSDGDRELMKELF
DQCENKRRTLFKLASETEDNDNSLGDILQASDNLSRVINSYKTIIEGQVINGEVATLTLP
DSEGNSQCSNQGTLIDLAELDTTNSLSSVLAPAPTPPSSGIPILPPPPQASGPPRSRSSS
QAEATLGPSSTSNALSWLDEELLCLGLADPAPNVPPKESAGNSQWHLLQREQSDLDFFSP
RPGTAACGASDAPLLQPSAPSSSSSQAPLPPPFPAPVVPASVPAPSAGSSLFSTGVAPAL
APKVEPAVPGHHGLALGNSALHHLDALDQLLEEAKVTSGLVKPTTSPLIPTTTPARPLLP
FSTGPGSPLFQPLSFQSQGSPPKGPELSLASIHVPLESIKPSSALPVTAYDKNGFRILFH
FAKECPPGRPDVLVVVVSMLNTAPLPVKSIVLQAAVPKSMKVKLQPPSGTELSPFSPIQP
PAAITQVMLLANPLKEKVRLRYKLTFALGEQLSTEVGEVDQFPPVEQWGNL*
>ENSG00000167434|ENST00000586876
MRMLLALLALSAARPSASAESHWCYEVQAESSNYPCLVPVKWGGNCQKDRQSPINIVTTK
AKVDKKLGRFFFSGYDKKQTWTVQNNGHSGWNPGERGLPATGGGTV*
>ENSG00000130649|ENST00000463117
MSALGVTVALLVWAAFLLLVSMWRQVHSSWNLPPGPFPLPIIGNLFQLELKNIPKSFTRL
AQRFGPVFTLYVGSQRMVVMHGYKAVKEALLDYKDEFSGRGDLPAFHAHRDRGIIFNNGP
TWKDIRRFSLTTLRNYGMGKQGNESRIQREAHFLLEALRKTQGQPFDPTFLIGCAPCNVI
ADILFRKHFDYNDEKFLRLMYLFNENFHLLSTPWLQLYNNFPSFLHYLPGSHRKVIKNVA
EVKEYVSERVKEHHQSLDPNCPRDLTDCLLVEMEKEKHSAERLYTMDGITVTVADLFFAG
TETTSTTLRYGLLILMKYPEIEEKLHEEIDRVIGPSRIPAIKDRQEMPYMDAVVHEIQRF
ITLVPSNLPHEATRDTIFRGYLIPKGTVVVPTLDSVLYDNQEFPDPEKFKPEHFLNENGK
FKYSDYFKPFSTGKRVCAGEGLARMELFLLLCAILQHFNLKPLVDPKDIDLSPIHIGFGC
IPPRYKLCVIPRS*
>ENSG00000205301|ENST00000511113
MRTKQVNLLITLVAVALFSFSCFSIYRITQTNNQLINCRNHILEFKENMLHLRNKTEKNT
QEMMKVLNRMKYEITKREILSGNLVAQKADILNKNETVSNTFEDLKFFFPHLRKEGRIYP
DVIIGKGKTGVSFALGISTVNRGNYSYLKQTLTSVVSRMTLSQEKDSVVIVLVADSNEDY
LHSVVKMITKKFKRQVRSGSLEVISIPAFLYSSMLNAKHLAEASQKLASWRIKQVLDFCI
LLLYAQPKAKYYLQLEDDIIAKEMYFTKITDFVGNISSNNWFFIEFSMLGFIGKLFRSED
LTHFVRFFLMFYKEKPIDWLLNDIFQVKVCDAGEDLRNCMKRKKQIRIQYKPSLFQHVGI
HSSFPRKEQYEKKI*
>ENSG00000176597|ENST00000465010

MRMLVSGRRVKKWQLIIQLFATCFLASLMFFWEPIDNHIVSHMKSYSYRYLINSYDFVND
TLSLKHTSAGPRYQYLINHKEKCQAQDVLLLLFVKTAPENYDRRSGIRRTWGNENYVRSQ
LNANIKTLFALGTPNPLEGEELQRKLAWEDQRYNDIIQQDFVDSFYNLTLKLLMQFSWAN
TYCPHAKFLMTADDDIFIHMPNLIEYLQSLEQIGVQDFWIGRVHRGAPPIRDKSSKYYVS
YEMYQWPAYPDYTAGAAYVISGDVAAKVYEASQTLNSSLYIDDVFMGLCANKIGIVPQDH
VFFSGEGKTPYHPCIYEKMMTSHGHLEDLQDLWKNATDPKVKTISKGFFGQIYCRLMKII
LLCKISYVDTYPCRAAFI*
>ENSG00000147642|ENST00000533065
MVGEGSIQSSRYKKESKSGLVKPGSEADFSSSSSTGSISAPEVHMSTAGSKRSSSSRNRG
PHGRSNGASSHKPGSSPSSPREKDLLSMLCRNQLSPVNIHPSYAPSSPSSSNSGSYKGSD
CSPIMRRSGRYMSCGENHGVRPPNPEQYLTPLQQKEVTVRHLKTKLKESERRLHERESEI
VELKSQLARMREDWIEEECHRVEAQLALKEARKEIKQLKQVIETMRSSLADKDKGIQKYF
VDINIQNKKLESLLQSMEMAHSGSLRDELCLDFPCDSPEKSLTLNPPLDTMADGLSLEEQ
VTGEGADRELLVGDSIANSTDLFDEIVTATTTESGDLELVHSTPGANVLELLPIVMGQEE
GSVVVERAVQTDVVPYSPAISELIQSVLQKLQDPCPSSLASPDESEPDSMESFPESLSAL
VVDLTPRNPNSAILLSPVETPYANVDAEVHANRLMRELDFAACVEERLDGVIPLARGGVV
RQYWSSSFLVDLLAVAAPVVPTVLWAFSTQRGGTDPVYNIGALLRGCCVVALHSLRRTAF
RIKT*
>ENSG00000177182|ENST00000522621
MGPVSLLPKYQKLNTWNGDLAKMTHLQAGLSPETIEKARLELNENPDVLHQDIQQVRDMI
ITRPDIGFLRTDDAFILRF
>ENSG00000197461|ENST00000402802
MRTLACLLLLGCGYLAHVLAEEAEIPREVIERLARSQIHSIRDLQRLLEIDSVGSEDSLD
TSLRAHGVHATKHVPEKRPLPIRRKRSIEEAVPAVCKTRTVIYEIPRSQVDPTSANFLIW
PPCVEVKRCTGCCNTSSVKCQPSRVHHRSVKVAKVEYVRKKPKLKEVQVRLEEHLECACA
TTSLNPDYREEDTDVR*
>ENSG00000139292|ENST00000536515
MDTSRLGVLLSLPVLLQLATGGSSPRSGVLLRGCPTHCHCEPDGRMLLRVDCSDLGLSEL
PSNLSVFTSYLDLSMNNISQLLPNPLPSLRFLEELRLAGNALTYIPKGAFTGLYSLKVLM
LQNNQLRHVPTEALQNLRSLQSLHLHNNRIHSLGKKCFDGLHSLETLDLNYNNLDEFPTA
IRTLSNLKELGFHSNNIRSIPEKAFVGNPSLITIHFYDNPIQFVGRSAFQHLPELRTLTL
NGASQITEFPDLTGTANLESLTLTGAQISSLPQTVCNQLPNLQVLDLSYNLLEDLPSFSV
CQKLQKIDLRHNEIYEIKVDTFQQLLSLRSLNLAWNKIAIIHPNAFSTLPSLIKLDLSSN
LLSSFPITGLHGLTHLKLTGNHALQSLISSENFPELKVIEMPYAYQCCAFGVCENAYKIS
NQWNKGDNSSMDDLHKKDAGMFQAQDERDLEDFLLDFEEDLKALHSVQCSPSPGPFKPCE
HLLDGWLIRIGVWTIAVLALTCNALVTSTVFRSPLYISPIKLLIGVIAAVNMLTGVSSAV
LAGVDAFTFGSFARHGAWWENGVGCHVIGFLSIFASESSVFLLTLAALERGFSVKYSAKF
ETKAPFSSLKVIILLCALLALTMAAVPLLGGSKYGASPLCLPLPFGEPSTMGYMVALILL
NSLCFLMMTIAYTKLYCNLDKGDLENIWDCSMVKHIALLLFTNCILNCPVAFLSFSSLIN
LTFISPEVIKFILLVVVPLPACLNPLLYILFNPHFKEDLVSLRKQTYVWTRSKHPSLMSI
NSDDVEKQSCDSTQALVTFTSSSITYDLPPSSVPSPAYPVTESCHLSSVAFVPCL*
>ENSG00000182022|ENST00000435907
MRHCINCCIQLLPDGAHKQQVNCQGGPHHGHQACPTCKGENKILFRVDSKQMNLLAVLEV
RTEGNENWGGFLRFKKGKRCSLVFGLIIMTLVMASYILSGAHQELLISSPFHYGGFPSNP

SLMDSENPSDTKEHHHQSSVNNISYMKDYPSIKLIINSITTRIEFTTRQLPDLEDLKKQE
LHMFSVIPNKFLPNSKSPCWYEEFSGQNTTDPYLTNSYVLYSKRFRSTFDALRKAFWGHL
AHAHGKHFRLRCLPHFYIIGQPKCGTTDLYDRLRLHPEVKFSAIKEPHWWTRKRFGIVRL
RDGLRDRYPVEDYLDLFDLAAHQIHQGLQASSAKEQSKMNTIIIGEASASTMWDNNAWTF
FYDNSTDGEPPFLTQDFIHAFQPNARLIVMLRDPVERLYSDYLYFASSNKSADDFHEKVT
EALQLFENCMLDYSLRACVYNNTLNNAMPVRLQVGLYAVYLLDWLSVFDKQQFLILRLED
HASNVKYTMHKVFQFLNLGPLSEKQEALMTKSPASNARRPEDRNLGPMWPITQKILRDFY
RPFNARLAQVLADEAFAWKTT*
>ENSG00000110080|ENST00000532243
MCPAGWKLLAMLALVLVVMVWYSISREDRYIELFYFPIPEKKEPCLQGEAESKASKLFGN
YSRDQPIFLRLEDYFWVKTPSAYELPYGTKGSEDLLLRVLAITSSSIPKNIQSLRCRRCV
VVGNGHRLRNSSLGDAINKYDVVIRLNNAPVAGYEGDVGSKTTMRLFYPESAHFDPKVEN
NPDTLLVLVAFKAMDFHWIETILSDKKRVRKGFWKQPPLIWDVNPKQIRILNPFFMEIAA
DKLLSLPMQQPRKIKQKPTTGLLAITLALHLCDLVHIAGFGYPDAYNKKQTIHYYEQITL
KSMAGSGHNVSQEALAIKRMLEMGAIKNLTSF*
>ENSG00000205155|ENST00000587708
MNLERVSNEEKLNLCRKYYLGGFAFLPFLWLVNIFWFFREAFLVPAYTEQSQIKGYVWRS
AVGFLFWVIVLTSWITIFQIYRPRWGALGDYLSFTIPLGTP*
>ENSG00000168824|ENST00000433139
MVKLGNNFAEKGTKQPLLEDGFDTIPLMTPLDVNQLQFPPPDKVVVKTKTEYEPDRKKGK
ARPPQIAEFTVSITEGVTERFKVSVLVLFALAFLTCVVFLVVYKVYKYDRACPDGFVLKN
TQCIPEGLESYYAEQDSSAREKFYTVINHYNLAKQSITRSVSPWMSVLSEEKLSEQETEA
AEKSA*
>ENSG00000140564|ENST00000610579
MELRPWLLWVVAATGTLVLLAADAQGQKVFTNTWAVRIPGGPAVANSVARKHGFLNLGQI
FGDYYHFWHRGVTKRSLSPHRPRHSRLQREPQVQWLEQQVAKRRTKRDVYQEPTDPKFPQ
QWYLSGVTQRDLNVKAAWAQGYTGHGIVVSILDDGIEKNHPDLAGNYDPGASFDVNDQDP
DPQPRYTQMNDNRHGTRCAGEVAAVANNGVCGVGVAYNARIGGVRMLDGEVTDAVEARSL
GLNPNHIHIYSASWGPEDDGKTVDGPARLAEEAFFRGVSQGRGGLGSIFVWASGNGGREH
DSCNCDGYTNSIYTLSISSATQFGNVPWYSEACSSTLATTYSSGNQNEKQIVTTDLRQKC
TESHTGTSASAPLAAGIIALTLEANKNLTWRDMQHLVVQTSKPAHLNANDWATNGVGRKV
SHSYGYGLLDAGAMVALAQNWTTVAPQRKCIIDILTEPKDIGKRLEVRKTVTACLGEPNH
ITRLEHAQARLTLSYNRRGDLAIHLVSPMGTRSTLLAARPHDYSADGFNDWAFMTTHSWD
EDPSGEWVLEIENTSEANNYGTLTKFTLVLYGTAPEGLPVPPESSGCKTLTSSQACVVCE
EGFSLHQKSCVQHCPPGFAPQVLDTHYSTENDVETIRASVCAPCHASCATCQGPALTDCL
SCPSHASLDPVEQTCSRQSQSSRESPPQQQPPRLPPEVEAGQRLRAGLLPSHLPEVVAGL
SCAFIVLVFVTVFLVLQLRSGFSFRGVKVYTMDRGLISYKGLPPEAWQEECPSDSEEDEG
RGERTAFIKDQSAL*
>ENSG00000107036|ENST00000414202
MYFLSGWPKRLLCPLGSPAEAPFHVQSDPQRAFFAVLAAARLSIWYSRPSVLIVTYKEPA
KSSTQFGSYKQAEWRPDSTMIAVSTANGYILFFHITSTRGDKYLYEPVYPKGSPQMKGTP
HFKEEQCAPALNLEMRKILDLQAPIMSLQSVLEDLLVATSDGLLHLIHWEGMTNGRKAIN
LCTVPFSVDLQSSRVGSFLGFTDVHIRDMEYCATLDGFAVVFNDGKVGFITPVSSRFTAE
QLHGVWPQDVVDGTCVAVNNKYRLMAFGCVSGSVQVYTIDNSTGAMLLSHKLELTAKQYP

DIWNKTGAVKLMRWSPDNSVVIVTWEYGGLSLWSVFGAQLICTLGGDFAYRSDGTKKDPL
KINSMSWGAEGYHLWVISGFGSQNTEIESDLRSVVKQPSILLFQFIKSVLTVNPCMSNQE
QVLLQGEDRLYLNCGEASQTQNPRSSSTHSEHKPSREKSPFADGGLESQGLSTLLGHRHW
HVVQISSTYLESNWPIRFSAIDKLGQNIAVVGKFGFAHYSLLTKKWKLFGNITQEQNMIV
TGGLAWWNDFMVLACYNINDRQEELRVYLRTSNLDNAFAHVTKAQAETLLLSVFQDMVIV
FRADCSICLYSIERKSDGPNTTAGIQVLQEVSMSRYIPHPFLVVSVTLTSVSTENGITLK
MPQQARGAESIMLNLAGQLIMMQRDRSGPQIREKDSNPNNQRKLLPFCPPVVLAQSVENV
WTTCRANKQKRHLLEALWLSCGGAGMKVWLPLFPRDHRKPHSFLSQRIMLPFHINIYPLA
VLFEDALVLGAVNDTLLYDSLYTRNNAREQLEVLFPFCVVERTSQIYLHHILRQLLVRNL
GEQALLLAQSCATLPYFPHVLELMLHEVLEEEATSREPIPDPLLPTVAKFITEFPLFLQT
VVHCARKTEYALWNYLFAAVGNPKDLFEECLMAQDLDTAASYLIILQNMEVPAVSRQHAT
LLFNTALEQGKWDLCRHMIRFLKAIGSGESETPPSTPTAQEPSSSGGFEFFRNRSISLSQ
SAENVPASKFSLQKTLSMPSGPSGKRWSKDSDCAENMYIDMMLWRHARRLLEDVRLKDLG
CFAAQLGFELISWLCKERTRAARVDNFVIALKRLHKDFLWPLPIIPASSISSPFKNGKYR
TVGEQLLKSQSADPFLNLEMDAGISNIQRSQSWLSNIGPTHHEIDTASSHGPQMQDAFLS
PLSNKGDECSIGSATDLTESSSMVDGDWTMVDENFSTLSLTQSELEHISMELASKGPHKS
QVQLRYLLHIFMEAGCLDWCIVIGLILRESSIINQILVITQSSEVDGEMLQNIKTGLHAV
DRWASTDCPGYKPFLNIIKPQLQKLSEITEEQVQPDAFQPITMGKTPEQTSPRAEESRGS
SSHGSIPQGEVGSSNMVSRKEEDTAQAEEEEPFQDGTYDCSVS*
>ENSG00000155511|ENST00000448073
MCCSTHLFQPLQLAGGLEWPWSNLLCFLTPVKLHPEVWGLFPNQQSQEHAAFRFALSQLT
EPPKLLPQIDIVNISDSFEMTYRFCSQFSKGVYAIFGFYERRTVNMLTSFCGALHVCFIT
PSFPVDTSNQFVLQLRPELQDALISIIDHYKWQKFVYIYDADRGLSVLQKVLDTAAEKNW
QVTAVNILTTTEEGYRMLFQDLEKKKERLVVVDCESERLNAILGQIIKLEKNGIGYHYIL
ANLGFMDIDLNKFKESGANVTGFQLVNYTDTIPAKIMQQWKNSDARDHTRVDWKRPKYTS
ALTYDGVKVMAEAFQSLRRQRIDISRRGNAGDCLANPAVPWGQGIDIQRALQQVRFEGLT
GNVQFNEKGRRTNYTLHVIEMKHDGIRKIGYWNEDDKFVPAATDAQAGGDNSSVQNRTYI
VTTILEDPYVMLKKNANQFEGNDRYEGYCVELAAEIAKHVGYSYRLEIVSDGKYGARDPD
TKAWNGMVGELVYGRADVAVAPLTITLVREEVIDFSKPFMSLGISIMIKKPQKSKPGVFS
FLDPLAYEIWMCIVFAYIGVSVVLFLVSRFSPYEWHSEEFEEGRDQTTSDQSNEFGIFNS
LWFSLGAFMQQGCDISPRSLSGRIVGGVWWFFTLIIISSYTANLAAFLTVERMVSPIESA
EDLAKQTEIAYGTLEAGSTKEFFRRSKIAVFEKMWTYMKSAEPSVFVRTTEEGMIRVRKS
KGKYAYLLESTMNEYIEQRKPCDTMKVGGNLDSKGYGIATPKGSALRGPVNLAVLKLSEQ
GVLDKLKSKWWYDKGECGSKDSGSKDKTSALSLSNVAGVFYILIGGLGLAMLVALIEFCY
KSRSESKRMKGFCLIPQQSINEAIRTSTLPRNSGAGASSGGSGENGRVVSHDFPKSMQSI
PCMSHSSGMPLGATGL*
>ENSG00000204842|ENST00000550104
MRSAAAAPRSPAVATESRRFAAARWPGWRSLQRPARRSGRGGGAAPGPYPSAAPPPPGP
GPPPSRQSSPPSASDCFGSNGNGGGAFRPGSRRLLGLGGPPRPFVVLLLPLASPGAPPAA
PTRASPLGARASPPRSGVSLARPAPGCPRPACEPVYGPLTMSLKPQQQQQQQQQQQQQQQ
QQQQQQQQPPPAAANVRKPGGSGLLASPAAAPSPSSSSVSSSSATAPSSVVAATSGGGRP
GLGRGRNSNKGLPQSTISFDGIYANMRMVHILTSVVGSKCEVQVKNGGIYEGVFKTYSPK
CDLVLDAAHEKSTESSSGPKREEIMESILFKCSDFVVVQFKDMDSSYAKRDAFTDSAISA
KVNGEHKEKDLEPWDAGELTANEELEALENDVSNGWDPNDMFRYNEENYGVVSTYDSSLS

SYTVPLERDNSEEFLKREARANQLAEEIESSAQYKARVALENDDRSEEEKYTAVQRNSSE
REGHSINTRENKYIPPGQRNREVISWGSGRQNSPRMGQPGSGSMPSRSTSHTSDFNPNSG
SDQRVVNGGVPWPSPCPSPSSRPPSRYQSGPNSLPPRAATPTRPPSRPPSRPSRPPSHPS
AHGSPAPVSTMPKRMSSEGPPRMSPKAQRHPRNHRVSAGRGSISSGLEFVSHNPPSEAAT
PPVARTSPSGGTWSSVVSGVPRLSPKTHRPRSPRQNSIGNTPSGPVLASPQAGIIPTEAV
AMPIPAASPTPASPASNRAVTPSSEAKDSRLQDQRQNSPAGNKENIKPNETSPSFSKAEN
KGISPVVSEHRKQIDDLKKFKNDFRLQPSSTSESMDQLLNKNREGEKSRDLIKDKIEPSA
KDSFIENSSSNCTSGSSKPNSPSISPSILSNTEHKRGPEVTSQGVQTSSPACKQEKDDKE
EKKDAAEQVRKSTLNPNAKEFNPRSFSQPKPSTTPTSPRPQAQPSPSMVGHQQPTPVYTQ
PVCFAPNMMYPVPVSPGVQPLYPIPMTPMPVNQAKTYRAVPNMPQQRQDQHHQSAMMHPA
SAAGPPIAATPPAYSTQYVAYSPQQFPNQPLVQHVPHYQSQHPHVYSPVIQGNARMMAPP
THAQPGLVSSSATQYGAHEQTHAMYACPKLPYNKETSPSFYFAISTGSLAQQYAHPNATL
HPHTPHPQPSATPTGQQQSQHGGSHPAPSPVQHHQHQAAQALHLASPQQQSAIYHAGLAP
TPPSMTPASNTQSPQNSFPAAQQTVFTIHPSHVQPAYTNPPHMAHVPQAHVQSGMVPSHP
TAHAPMMLMTTQPPGGPQAALAQSALQPIPVSTTAHFPYMTHPSVQAHHQQQL*
>ENSG00000126091|ENST00000533997
MGLLVFVRNLLLALCLFLVLGFLYYSAWKLHLLQWEEDSNSVVLSFDSAGQTLGSEYDRL
GFLLNLDSKLPAELATKYANFSEGACKPGYASALMTAIFPREKVRTFMAWLAWYG*
>ENSG00000174951|ENST00000645652
MWLRSHRQLCLAFLLVCVLSVIFFLHIHQDSFPHGLGLSILCPDRRLVTPPVAIFCLPGT
AMGPNASSSCPQHPASLSGTWTVYPNGRFGNQMGQYATLLALAQLNGRRAFILPAMHAAL
APVFRITLPVLAPEVDSRTPWRELQLHDWMSEEYADLRDPFLKLSGFPCSWTFFHHLREQ
IRREFTLHDHLREEAQSVLGQLRLGRTGDRPRTFVGVHVRRGDYLQVMPQRWKGVVGDSA
YLRQAMDWFRARHEAPVFVVTSNGMEWCKENIDTSQGDVTFAGDGQEATPWKDFALLTQC
NHTIMTIGTFGFWAAYLAGGDTVYLANFTLPDSEFLKIFKPEAAFLPEWVGINADLSPLW
TLAKP*
