# Supplementary material for: The Amino Acid Composition of Quadruplex Binding Proteins Reveals a Shared Motif and Predicts New Potential Quadruplex Interactors
Source: Molecules. 2018 Sep 13;23(9):2341. doi: 10.3390/molecules23092341 (PMC6225207; doi:10.3390/molecules23092341)
Supplement: Supplementary file 1 [file molecules-23-02341-s001.zip › molecules-355581-supplementary/Supplementary material 5.pdf]

Supplementary material 5. Short patterns and homo repeats of amino acid residues in analysed QBPs (according to HRaP database)

QBP	Patterns	Homo repeats (3 AA and more)
CNBP	-	E3
IGF-2	-	-
MAZ	PPPPP, GGGGG, PPAPP, PAPPP, AAPPA	A3, A5, A13, A14, G3, G5, P3, P7
HNRNPA1	GGGGSGG, GGGGG	G3, G4, G5, S3, S6
NUCL	EDEREE, EEEEEEE, DDDDEDD, RGEGGFG, EDDEDED, EEDDD, DSDEE	V3, A3, A5, G3, G4, E3, E10, D3, D4, D5, P3
NPM	EDEREE, DDDDEDD, EEEED, EEDDD	G3, E4, D3, D6
TLS/FUS	GSHM, GGGGSGG, GGGGG, GGSGGGGSGGG, RGGGGSG	G3, G4, G7, G10, S3, Q3,
POT1	-	L3, D3
BLM	KKTSS	G3, T3, S3, S5, Q3, D4, D5, K3, P3
FANCJ	-	C3, L3, N3
DHX36	GGGGSGG, GGGGG	G3, G10, E3, R3, K3
VEGF	-	L3, G3
SP1	QQQQQQQ	I3, A3, G3, G4, T5, S3, S4, Q4, K3
BRCA1	RRGKKK	L3, V3, A3, S3, E3, K4
TOPO	PSPPP, EEEED, KKPKNK, KKEKK	I3, Q3, E3, E4, K3, K4, P3
PARP1	KKSKK	K3, P3
p53	APAPA	V3, S3, E3, K3, P3
IFI16	PPAPAG	F3, I4, V3, S3, K3
RECQ4	EEEEG	I3, V3, A3, G3, G4, S3, E4, R3
DPOE1	EEEEEEE	L3, A3, S3, E3, E4, E5, R3, K3, P3
POLH	-	L3, G3, S3
DSRAD	RGRPRG, PPAPAG, GVPRG	L3, V3
NF2L2	-	S3, R3, P3
ERCC3	-	Q3, E3, E6, K3
ERCC2	-	I3, L3, V3
TERF1	EEEEEEE, EEEED	A3, Q3, E8, K4
TINF2	DSDEE	V3, A4, Q3, E3, R3
ACD	-	L3, L4, S3, P3
SPAST	APAPA, PAPPP	L3, L4, K3, P3, P4
CAMP	-	L4
ROA2	-	G3, G4
RFA2	-	S3, D3
RFA1	-	S3, E3
NDKB	-	-
MYOD1	-	L3, A3, A4, R3
MYF5	-	T3, S3, R3
MYF6	-	M3, S3, R3
MYOG	-	G4, R3
TERT	PPAAP	L3, A3, S3, E3, R3, P3
TNR4	-	L4, G3

EGR1	GGGGSGG, GGGGG, NSSSS, GGGGSGGGGS, GGHNSS	A3, G9, S4, S6, Q3, P3
YBOX1	PPAAP, AAPPA	R3, R4
TE2IP	EEEEEEE	V3, G3, E3, E8, D3
NEIL1	-	K3
NEIL2	-	G4, S3
NEIL3	-	V3, A3, T3
TOPRS	KKKKK	Y4, T3, S3, R3, K3, K6, P3
ATRX	KKKKK, KKTSS, EEEEEEE, EEEED, KKGKS, KKSKK	C3, L3, G3, S3, S4, S5, Q3, Q6, E3, E4, E6, E15, D3, D6, R3, K3, K4, K5, P3
DNM3A	RGRPRG	A3, S3, N3, E3, D3, R3, P4
DNM3B	-	L3, S3, N3, D3, R3
DNMT1	EDEREE	V3, A3, T3, S3, E3, D3, R3, K4, P3
FMR1	MGRGS	G3, G4, E3, R3, P3
ADA10	CGYSD, SDGKDD	L3, N3, R3, K3, P3
EFHD2	-	A3, A6, G3, E3
SRSF1	GGGGSGG, GGGGG	V3, G3, G9
U2AF2	-	V3, R4, K4, P3
NOA1	Not found in database	
HNRPF	-	-
EBNA1BP2	Not found in database	
ROA3	GGGGSGG, GGGGG, RPQLDS	G3, G4, G5, G6, Q3, R6, P3
SFPQ	GGGGSGG, PPPPP, GGGGG, QQQQQP, PPPPQ	A3, G3, G4, G5, G6, T3, S3, Q4, Q5, E3, R4, P3, P5
SF3B3	-	F3, I3, A3, D3, R3, K3
ELAV1	-	-
ILF3	GGGGSGG, GGGGG, KKKKK, SKKKK, RGRPRG, GGGSSSG, GGGGSGGGGS	L3, G3, G5, G9, Q3, K3, K5, P4
NONO	PPPPP, QQQQQP	Q3, Q6, E3, H3, R3, R4, P5
HNRPR	RGRPRG	Y3, G3, Q3, E4, R3, K3, P3, P4
TERF2	-	G4
HNRPL	GGGGSGG, PPPPP, GGGGG	V3, A3, G3, G6, G7, G9, S3, N3, R3, R4, P4, P8
SAFB1	-	A3, S3, E3, D3, R3, P4
IF2B1	-	V3, S3, Q3, P4
SAFB2	GHMA	G3, E3, R3, P4
DHX15	LDNGED, EEEEG	L3, V3, Q4, E4, P4
ILF2	RGRPRG	G3, S3, E4, E5
DHX30	EDEREE, EEEEEEE, EEEED, REEEE	L3, V3, A3, A5, S3, E4, D3, R3, K4, P3
HNRPK	PPPPP	E3, P6
DDX21	-	A3, G3, E3, E4, K3
TADBP	-	A3, G3

Most frequent patterns:

GGGGG … 10 (MAZ, HNRNPA1, TLS/FUS, DHX36, EGR1, SRSF1, ROA3, SFPQ, ILF3, HNRPL)

GGGGSGG … 9 (HNRNPA1, TLS/FUS, DHX36, EGR1, SRSF1, ROA3, SFPQ, ILF3, HNRPL)

EEEEEEE … 6 (NUCL, DPOE1, TERF1, TE2IP, ATRX, DHX30)

RGRPRG … 5 (DSRAD, DNM3A, ILF3, HNRPR, ILF2)
