# Supplementary figures and images for: The Amino Acid Composition of Quadruplex Binding Proteins Reveals a Shared Motif and Predicts New Potential Quadruplex Interactors
Source: Molecules. 2018 Sep 13;23(9):2341. doi: 10.3390/molecules23092341 (PMC6225207; doi:10.3390/molecules23092341)

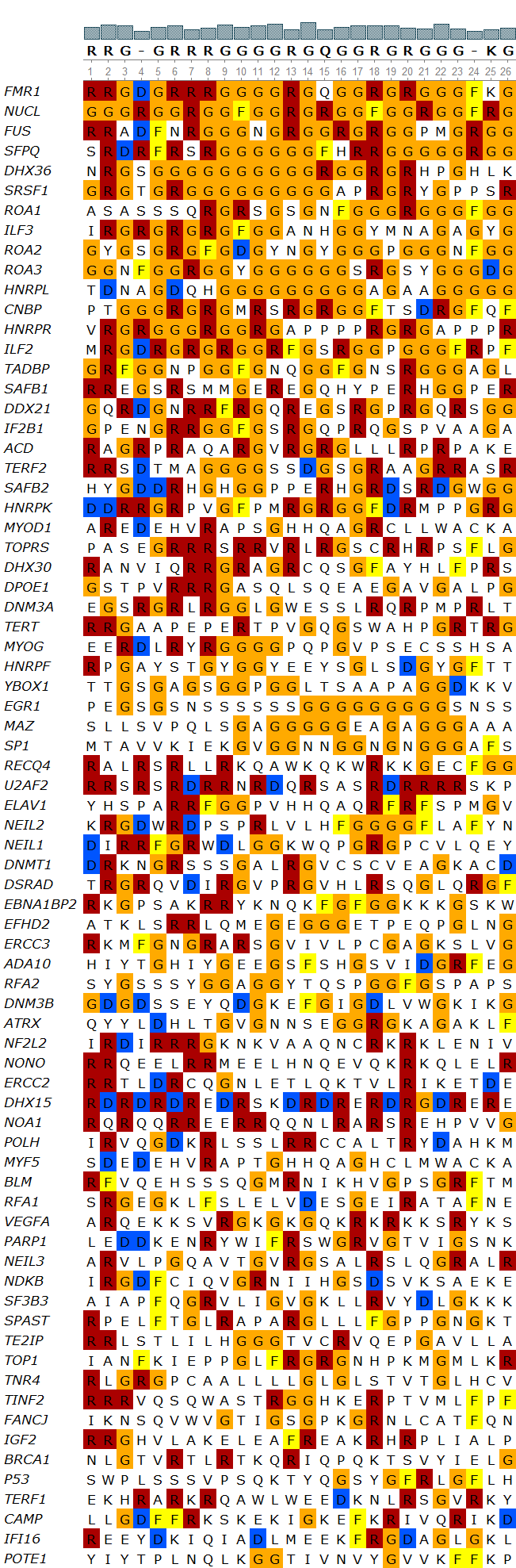

Supplement: Supplementary file 1 [file molecules-23-02341-s001.zip › molecules-355581-supplementary/Supplementary material 14.tiff]

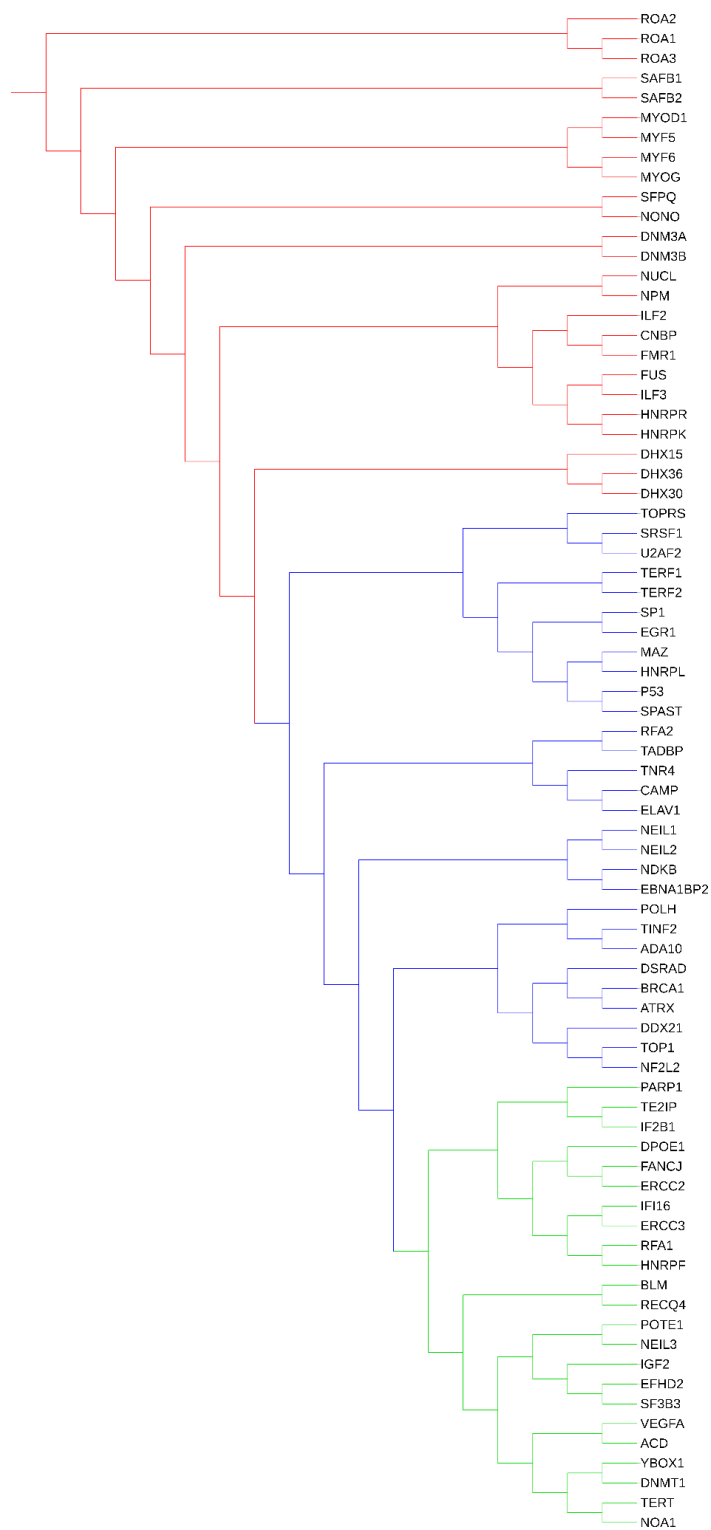

**Supplementary material 8: CLAP tree of 77 quadruplex binding proteins**

Supplement: Supplementary file 1 [file molecules-23-02341-s001.zip › molecules-355581-supplementary/Supplementary material 8.pdf]
