# Supplementary material for: The Amino Acid Composition of Quadruplex Binding Proteins Reveals a Shared Motif and Predicts New Potential Quadruplex Interactors
Source: Molecules. 2018 Sep 13;23(9):2341. doi: 10.3390/molecules23092341 (PMC6225207; doi:10.3390/molecules23092341)
Supplement: Supplementary file 1 [file Supplementary material 3A - all human protein sequences.pdf › molecules-355581-supplementary/Supplementary material 3A - all human protein sequences.pdf]

>sp|P31947|1433S_HUMAN 14-3-3 protein sigma OS=Homo sapiens GN=SFN PE=1 SV=1
MERASLIQKAKLAEQAERYEDMAAFMKGAVEKGEELSCEERNLLSVAYKNVVGGQRAAWR
VLSSIEQKSNEEGSEEKGPEVREYREKVETELQGVCDTVLGLLDSHLIKEAGDAESRVFY
LKMKGDYYRYLAEVATGDDKKRIIDSARSAYQEAMDISKKEMPPTNPIRLGLALNFSVFH
YEIANSPEEAISLAKTTFDEAMADLHTLSEDSYKDSTLIMQLLRDNLTLWTADNAGEEGG
EAPQEPQS
>sp|P63104|1433Z_HUMAN 14-3-3 protein zeta/delta OS=Homo sapiens GN=YWHAZ PE=1 SV=1
MDKNELVQKAKLAEQAERYDDMAACMKSVTEQGAELSNEERNLLSVAYKNVVGARRSSWR
VVSSIEQKTEGAEKKQQMAREYREKIETELRDICNDVLSLLEKFLIPNASQAESKVFYLK
MKGDYYRYLAEVAAGDDKKGIVDQSQQAYQEAFEISKKEMQPTHPIRLGLALNFSVFYYE
ILNSPEKACSLAKTAFDEAIAELDTLSEESYKDSTLIMQLLRDNLTLWTSDTQGDEAEAG
EGGEN
>sp|P18462|1A25_HUMAN HLA class I histocompatibility antigen, A-25 alpha chain OS=Homo sapiens GN=HLA-A PE=1 SV=1
MAVMAPRTLVLLLSGALALTQTWAGSHSMRYFYTSVSRPGRGEPRFIAVGYVDDTQFVRF
DSDAASQRMEPRAPWIEQEGPEYWDRNTRNVKAHSQTDRESLRIALRYYNQSEDGSHTIQ
RMYGCDVGPDGRFLRGYQQDAYDGKDYIALNEDLRSWTAADMAAQITQRKWETAHEAEQW
RAYLEGRCVEWLRRYLENGKETLQRTDAPKTHMTHHAVSDHEATLRCWALSFYPAEITLT
WQRDGEDQTQDTELVETRPAGDGTFQKWASVVVPSGQEQRYTCHVQHEGLPKPLTLRWEP
SSQPTIPIVGIIAGLVLFGAVIAGAVVAAVMWRRKSSDRKGGSYSQAASSDSAQGSDMSL
TACKV
>sp|P30512|1A29_HUMAN HLA class I histocompatibility antigen, A-29 alpha chain OS=Homo sapiens GN=HLA-A PE=1 SV=2
MAVMAPRTLLLLLLGALALTQTWAGSHSMRYFTTSVSRPGRGEPRFIAVGYVDDTQFVRF
DSDAASQRMEPRAPWIEQEGPEYWDLQTRNVKAQSQTDRANLGTLRGYYNQSEAGSHTIQ
MMYGCHVGSDGRFLRGYRQDAYDGKDYIALNEDLRSWTAADMAAQITQRKWEAARVAEQL
RAYLEGTCVEWLRRYLENGKETLQRTDAPKTHMTHHAVSDHEATLRCWALSFYPAEITLT
WQRDGEDQTQDTELVETRPAGDGTFQKWASVVVPSGQEQRYTCHVQHEGLPKPLTLRWEP
SSQPTIPIVGIIAGLVLFGAVFAGAVVAAVRWRRKSSDRKGGSYSQAASSDSAQGSDMSL
TACKV
>sp|P16189|1A31_HUMAN HLA class I histocompatibility antigen, A-31 alpha chain OS=Homo sapiens GN=HLA-A PE=1 SV=2
MAVMAPRTLLLLLLGALALTQTWAGSHSMRYFTTSVSRPGRGEPRFIAVGYVDDTQFVRF
DSDAASQRMEPRAPWIEQERPEYWDQETRNVKAHSQIDRVDLGTLRGYYNQSEAGSHTIQ
MMYGCDVGSDGRFLRGYQQDAYDGKDYIALNEDLRSWTAADMAAQITQRKWEAARVAEQL
RAYLEGTCVEWLRRYLENGKETLQRTDPPKTHMTHHAVSDHEATLRCWALSFYPAEITLT
WQRDGEDQTQDTELVETRPAGDGTFQKWASVVVPSGQEQRYTCHVQHEGLPKPLTLRWEP
SSQPTIPIVGIIAGLVLFGAVFAGAVVAAVRWRRKSSDRKGGSYSQAASSDSAQGSDMSL
TACKV
>sp|P16190|1A33_HUMAN HLA class I histocompatibility antigen, A-33 alpha chain OS=Homo sapiens GN=HLA-A PE=1 SV=3
MAVMAPRTLLLLLLGALALTQTWAGSHSMRYFTTSVSRPGRGEPRFIAVGYVDDTQFVRF
DSDAASQRMEPRAPWIEQEGPEYWDRNTRNVKAHSQIDRVDLGTLRGYYNQSEAGSHTIQ
MMYGCDVGSDGRFLRGYQQDAYDGKDYIALNEDLRSWTAADMAAQITQRKWEAARVAEQL

RAYLEGTCVEWLRRHLENGKETLQRTDPPKTHMTHHAVSDHEATLRCWALSFYPAEITLT
WQRDGEDQTQDTELVETRPAGDGTFQKWASVVVPSGQEQRYTCHVQHEGLPKPLTLRWEP
SSQPTIPIVGIIAGLVLFGAVFAGAVVAAVRWRRKSSDRKGGSYSQAASSDSAQGSDMSL
TACKV
>sp|P30453|1A34_HUMAN HLA class I histocompatibility antigen, A-34 alpha chain OS=Homo sapiens GN=HLA-A PE=1 SV=1
MAIMAPRTLVLLLSGALALTQTWAGSHSMRYFYTSVSRPGRGEPRFIAVGYVDDTQFVRF
DSDAASQRMEPRAPWIEQEGPEYWDRNTRKVKAQSQTDRVDLGTLRGYYNQSEDGSHTIQ
RMYGCDVGPDGRFLRGYQQDAYDGKDYIALNEDLRSWTAADMAAQITQRKWETAHEAEQW
RAYLEGTCVEWLRRYLENGKETLQRTDAPKTHMTHHAVSDHEATLRCWALSFYPAEITLT
WQRDGEDQTQDTELVETRPAGDGTFQKWASVVVPSGQEQRYTCHVQHEGLPKPLTLRWEP
SSQPTIPIVGILAGLVLFGAVIAGAVVAAVMWRRKSSDRKGGSYSQAASSDSAQGSDMSL
TACKV
>sp|P01891|1A68_HUMAN HLA class I histocompatibility antigen, A-68 alpha chain OS=Homo sapiens GN=HLA-A PE=1 SV=4
MAVMAPRTLVLLLSGALALTQTWAGSHSMRYFYTSVSRPGRGEPRFIAVGYVDDTQFVRF
DSDAASQRMEPRAPWIEQEGPEYWDRNTRNVKAQSQTDRVDLGTLRGYYNQSEAGSHTIQ
MMYGCDVGSDGRFLRGYRQDAYDGKDYIALKEDLRSWTAADMAAQTTKHKWEAAHVAEQW
RAYLEGTCVEWLRRYLENGKETLQRTDAPKTHMTHHAVSDHEATLRCWALSFYPAEITLT
WQRDGEDQTQDTELVETRPAGDGTFQKWVAVVVPSGQEQRYTCHVQHEGLPKPLTLRWEP
SSQPTIPIVGIIAGLVLFGAVITGAVVAAVMWRRKSSDRKGGSYSQAASSDSAQGSDVSL
TACKV
>sp|Q09160|1A80_HUMAN HLA class I histocompatibility antigen, A-80 alpha chain OS=Homo sapiens GN=HLA-A PE=1 SV=1
MAVMPPRTLLLLLSGALALTQTWAGSHSMRYFFTSVSRPGRGEPRFIAVGYVDDSQFVQF
DSDAASQRMEPRAPWIEQEEPEYWDEETRNVKAHSQTNRANLGTLRGYYNQSEDGSHTIQ
IMYGCDVGSDGRFLRGYRQDAYDGKDYIALNEDLRSWTAADMAAQITKRKWEAARRAEQL
RAYLEGECVDGLRRYLENGKETLQRTDPPKTHMTHHPISDHEATLRCWALSFYPAEITLT
WQRDGEDQTQDTELVETRPAGDGTFQKWAAVVVPSGKEKRYTCHVQHEGLPEPLTLRWEP
SSQPTIPIVGIIAGLVLLGAVIAGAVVAAVMWRKKSSVRKGGSYSQAASSDSAQGSDVSL
TACKV
>sp|P30461|1B13_HUMAN HLA class I histocompatibility antigen, B-13 alpha chain OS=Homo sapiens GN=HLA-B PE=1 SV=1
MRVTAPRTLLLLLWGAVALTETWAGSHSMRYFYTAMSRPGRGEPRFITVGYVDDTQFVRF
DSDATSPRMAPRAPWIEQEGPEYWDRETQISKTNTQTYRENLRTALRYYNQSEAGSHTWQ
TMYGCDLGPDGRLLRGHNQLAYDGKDYIALNEDLSSWTAADTAAQITQLKWEAARVAEQL
RAYLEGECVEWLRRYLENGKETLQRADPPKTHVTHHPISDHEATLRCWALGFYPAEITLT
WQRDGEDQTQDTELVETRPAGDRTFQKWAAVVVPSGEEQRYTCHVQHEGLPKPLTLRWEP
SSQSTVPIVGIVAGLAVLAVVVIGAVVAAVMCRRKSSGGKGGSYSQAACSDSAQGSDVSL
TA
>sp|P03989|1B27_HUMAN HLA class I histocompatibility antigen, B-27 alpha chain OS=Homo sapiens GN=HLA-B PE=1 SV=2
MRVTAPRTLLLLLWGAVALTETWAGSHSMRYFHTSVSRPGRGEPRFITVGYVDDTLFVRF
DSDAASPREEPRAPWIEQEGPEYWDRETQICKAKAQTDREDLRTLLRYYNQSEAGSHTLQ

NMYGCDVGPDGRLLRGYHQDAYDGKDYIALNEDLSSWTAADTAAQITQRKWEAARVAEQL
RAYLEGECVEWLRRYLENGKETLQRADPPKTHVTHHPISDHEATLRCWALGFYPAEITLT
WQRDGEDQTQDTELVETRPAGDRTFQKWAAVVVPSGEEQRYTCHVQHEGLPKPLTLRWEP
SSQSTVPIVGIVAGLAVLAVVVIGAVVAAVMCRRKSSGGKGGSYSQAACSDSAQGSDVSL
TA
>sp|P30479|1B41_HUMAN HLA class I histocompatibility antigen, B-41 alpha chain OS=Homo sapiens GN=HLA-B PE=1 SV=1
MRVTAPRTVLLLLSAALALTETWAGSHSMRYFHTAMSRPGRGEPRFITVGYVDDTLFVRF
DSDATSPRKEPRAPWIEQEGPEYWDRETQISKTNTQTYRESLRNLRGYYNQSEAGSHTWQ
RMYGCDVGPDGRLLRGHNQYAYDGKDYIALNEDLRSWTAADTAAQITQRKWEAARVAEQD
RAYLEGTCVEWLRRYLENGKDTLERADPPKTHVTHHPISDHEATLRCWALGFYPAEITLT
WQRDGEDQTQDTELVETRPAGDRTFQKWAAVVVPSGEEQRYTCHVQHEGLPKPLTLRWEP
SSQSTVPIVGIVAGLAVLAVVVIGAVVAAVMCRRKSSGGKGGSYSQAACSDSAQGSDVSL
TA
>sp|P30481|1B44_HUMAN HLA class I histocompatibility antigen, B-44 alpha chain OS=Homo sapiens GN=HLA-B PE=1 SV=1
MRVTAPRTLLLLLWGAVALTETWAGSHSMRYFYTAMSRPGRGEPRFITVGYVDDTLFVRF
DSDATSPRKEPRAPWIEQEGPEYWDRETQISKTNTQTYRENLRTALRYYNQSEAGSHIIQ
RMYGCDVGPDGRLLRGYDQDAYDGKDYIALNEDLSSWTAADTAAQITQRKWEAARVAEQD
RAYLEGLCVESLRRYLENGKETLQRADPPKTHVTHHPISDHEVTLRCWALGFYPAEITLT
WQRDGEDQTQDTELVETRPAGDRTFQKWAAVVVPSGEEQRYTCHVQHEGLPKPLTLRWEP
SSQSTVPIVGIVAGLAVLAVVVIGAVVAAVMCRRKSSGGKGGSYSQAACSDSAQGSDVSL
TA
>sp|P78314|3BP2_HUMAN SH3 domain-binding protein 2 OS=Homo sapiens GN=SH3BP2 PE=1 SV=2
MAAEEMHWPVPMKAIGAQNLLTMPGGVAKAGYLHKKGGTQLQLLKWPLRFVIIHKRCVYY
FKSSTSASPQGAFSLSGYNRVMRAAEETTSNNVFPFKIIHISKKHRTWFFSASSEEERKS
WMALLRREIGHFHEKKDLPLDTSDSSSDTDSFYGAVERPVDISLSPYPTDNEDYEHDDED
DSYLEPDSPEPGRLEDALMHPPAYPPPPVPTPRKPAFSDMPRAHSFTSKGPGPLLPPPPP
KHGLPDVGLAAEDSKRDPLCPRRAEPCPRVPATPRRMSDPPLSTMPTAPGLRKPPCFRES
ASPSPEPWTPGHGACSTSSAAIMATATSRNCDKLKSFHLSPRGPPTSEPPPVPANKPKFL
KIAEEDPPREAAMPGLFVPPVAPRPPALKLPVPEAMARPAVLPRPEKPQLPHLQRSPPDG
QSFRSFSFEKPRQPSQADTGGDDSDEDYEKVPLPNSVFVNTTESCEVERLFKATSPRGEP
QDGLYCIRNSSTKSGKVLVVWDETSNKVRNYRIFEKDSKFYLEGEVLFVSVGSMVEHYHT
HVLPSHQSLLLRHPYGYTGPR
>sp|P11171|41_HUMAN Protein 4.1 OS=Homo sapiens GN=EPB41 PE=1 SV=4
MTTEKSLVTEAENSQHQQKEEGEEAINSGQQEPQQEESCQTAAEGDNWCEQKLKASNGDT
PTHEDLTKNKERTSESRGLSRLFSSFLKRPKSQVSEEEGKEVESDKEKGEGGQKEIEFGT
SLDEEIILKAPIAAPEPELKTDPSLDLHSLSSAETQPAQEELREDPDFEIKEGEGLEECS
KIEVKEESPQSKAETELKASQKPIRKHRNMHCKVSLLDDTVYECVVEKHAKGQDLLKRVC
EHLNLLEEDYFGLAIWDNATSKTWLDSAKEIKKQVRGVPWNFTFNVKFYPPDPAQLTEDI
TRYYLCLQLRQDIVAGRLPCSFATLALLGSYTIQSELGDYDPELHGVDYVSDFKLAPNQT
KELEEKVMELHKSYRSMTPAQADLEFLENAKKLSMYGVDLHKAKDLEGVDIILGVCSSGL
LVYKDKLRINRFPWPKVLKISYKRSSFFIKIRPGEQEQYESTIGFKLPSYRAAKKLWKVC
VEHHTFFRLTSTDTIPKSKFLALGSKFRYSGRTQAQTRQASALIDRPAPHFERTASKRAS

RSLDGAAAVDSADRSPRPTSAPAITQGQVAEGGVLDASAKKTVVPKAQKETVKAEVKKED
EPPEQAEPEPTEAWKVEKTHIEVTVPTSNGDQTQKLAEKTEDLIRMRKKKRERLDGENIY
IRHSNLMLEDLDKSQEEIKKHHASISELKKNFMESVPEPRPSEWDKRLSTHSPFRTLNIN
GQIPTGEGPPLVKTQTVTISDNANAVKSEIPTKDVPIVHTETKTITYEAAQTDDNSGDLD
PGVLLTAQTITSETPSSTTTTQITKTVKGGISETRIEKRIVITGDADIDHDQVLVQAIKE
AKEQHPDMSVTKVVVHQETEIADE
>sp|P41595|5HT2B_HUMAN 5-hydroxytryptamine receptor 2B OS=Homo sapiens GN=HTR2B PE=1 SV=1
MALSYRVSELQSTIPEHILQSTFVHVISSNWSGLQTESIPEEMKQIVEEQGNKLHWAALL
ILMVIIPTIGGNTLVILAVSLEKKLQYATNYFLMSLAVADLLVGLFVMPIALLTIMFEAM
WPLPLVLCPAWLFLDVLFSTASIMHLCAISVDRYIAIKKPIQANQYNSRATAFIKITVVW
LISIGIAIPVPIKGIETDVDNPNNITCVLTKERFGDFMLFGSLAAFFTPLAIMIVTYFLT
IHALQKKAYLVKNKPPQRLTWLTVSTVFQRDETPCSSPEKVAMLDGSRKDKALPNSGDET
LMRRTSTIGKKSVQTISNEQRASKVLGIVFFLFLLMWCPFFITNITLVLCDSCNQTTLQM
LLEIFVWIGYVSSGVNPLVYTLFNKTFRDAFGRYITCNYRATKSVKTLRKRSSKIYFRNP
MAENSKFFKKHGIRNGINPAMYQSPMRLRSSTIQSSSIILLDTLLLTENEGDKTEEQVSY
V
>sp|P28335|5HT2C_HUMAN 5-hydroxytryptamine receptor 2C OS=Homo sapiens GN=HTR2C PE=1 SV=1
MVNLRNAVHSFLVHLIGLLVWQCDISVSPVAAIVTDIFNTSDGGRFKFPDGVQNWPALSI
VIIIIMTIGGNILVIMAVSMEKKLHNATNYFLMSLAIADMLVGLLVMPLSLLAILYDYVW
PLPRYLCPVWISLDVLFSTASIMHLCAISLDRYVAIRNPIEHSRFNSRTKAIMKIAIVWA
ISIGVSVPIPVIGLRDEEKVFVNNTTCVLNDPNFVLIGSFVAFFIPLTIMVITYCLTIYV
LRRQALMLLHGHTEEPPGLSLDFLKCCKRNTAEEENSANPNQDQNARRRKKKERRPRGTM
QAINNERKASKVLGIVFFVFLIMWCPFFITNILSVLCEKSCNQKLMEKLLNVFVWIGYVC
SGINPLVYTLFNKIYRRAFSNYLRCNYKVEKKPPVRQIPRVAATALSGRELNVNIYRHTN
EPVIEKASDNEPGIEMQVENLELPVNPSSVVSERISSV
>sp|P46098|5HT3A_HUMAN 5-hydroxytryptamine receptor 3A OS=Homo sapiens GN=HTR3A PE=1 SV=1
MLLWVQQALLALLLPTLLAQGEARRSRNTTRPALLRLSDYLLTNYRKGVRPVRDWRKPTT
VSIDVIVYAILNVDEKNQVLTTYIWYRQYWTDEFLQWNPEDFDNITKLSIPTDSIWVPDI
LINEFVDVGKSPNIPYVYIRHQGEVQNYKPLQVVTACSLDIYNFPFDVQNCSLTFTSWLH
TIQDINISLWRLPEKVKSDRSVFMNQGEWELLGVLPYFREFSMESSNYYAEMKFYVVIRR
RPLFYVVSLLLPSIFLMVMDIVGFYLPPNSGERVSFKITLLLGYSVFLIIVSDTLPATAI
GTPLIGVYFVVCMALLVISLAETIFIVRLVHKQDLQQPVPAWLRHLVLERIAWLLCLREQ
STSQRPPATSQATKTDDCSAMGNHCSHMGGPQDFEKSPRDRCSPPPPPREASLAVCGLLQ
ELSSIRQFLEKRDEIREVARDWLRVGSVLDKLLFHIYLLAVLAYSITLVMLWSIWQYA
>sp|Q70Z44|5HT3D_HUMAN 5-hydroxytryptamine receptor 3D OS=Homo sapiens GN=HTR3D PE=1 SV=3
MQKHSPGPPALALLSQSLLTTGNGDTLIINCPGFGQHRVDPAAFQAVFDRKAIGPVTNYS
VATHVNISFTLSAIWNCYSRIHTFNCHHARPWHNQFVQWNPDECGGIKKSGMATENLWLS
DVFIEESVDQTPAGLMASMSIVKATSNTISQCGWSASANWTPSISPSMDRARAWRRMSRS
FQIHHRTSFRTRREWVLLGIQKRTIKVTVATNQYEQAIFHVAIRRRCRPSPYVVNFLVPS
GILIAIDALSFYLPLESGNCAPFKMTVLLGYSVFLLMMNDLLPATSTSSHASLVAPLALM
QTPLPAGVYFALCLSLMVGSLLETIFITHLLHVATTQPLPLPRWLHSLLLHCTGQGRCCP
TAPQKGNKGPGLTPTHLPGVKEPEVSAGQMPGPGEAELTGGSEWTRAQREHEAQKQHSVE
LWVQFSHAMDALLFRLYLLFMASSIITVICLWNT

>sp|Q969T7|5NT3B_HUMAN 7-methylguanosine phosphate-specific 5'-nucleotidase OS=Homo sapiens GN=NT5C3B PE=1 SV=4
MAEEVSTLMKATVLMRQPGRVQEIVGALRKGGGDRLQVISDFDMTLSRFAYNGKRCPSSY
NILDNSKIISEECRKELTALLHHYYPIEIDPHRTVKEKLPHMVEWWTKAHNLLCQQKIQK
FQIAQVVRESNAMLREGYKTFFNTLYHNNIPLFIFSAGIGDILEEIIRQMKVFHPNIHIV
SNYMDFNEDGFLQGFKGQLIHTYNKNSSACENSGYFQQLEGKTNVILLGDSIGDLTMADG
VPGVQNILKIGFLNDKVEERRERYMDSYDIVLEKDETLDVVNGLLQHILCQGVQLEMQGP
>sp|P49902|5NTC_HUMAN Cytosolic purine 5'-nucleotidase OS=Homo sapiens GN=NT5C2 PE=1 SV=1
MSTSWSDRLQNAADMPANMDKHALKKYRREAYHRVFVNRSLAMEKIKCFGFDMDYTLAVY
KSPEYESLGFELTVERLVSIGYPQELLSFAYDSTFPTRGLVFDTLYGNLLKVDAYGNLLV
CAHGFNFIRGPETREQYPNKFIQRDDTERFYILNTLFNLPETYLLACLVDFFTNCPRYTS
CETGFKDGDLFMSYRSMFQDVRDAVDWVHYKGSLKEKTVENLEKYVVKDGKLPLLLSRMK
EVGKVFLATNSDYKYTDKIMTYLFDFPHGPKPGSSHRPWQSYFDLILVDARKPLFFGEGT
VLRQVDTKTGKLKIGTYTGPLQHGIVYSGGSSDTICDLLGAKGKDILYIGDHIFGDILKS
KKRQGWRTFLVIPELAQELHVWTDKSSLFEELQSLDIFLAELYKHLDSSSNERPDISSIQ
RRIKKVTHDMDMCYGMMGSLFRSGSRQTLFASQVMRYADLYAASFINLLYYPFSYLFRAA
HVLMPHESTVEHTHVDINEMESPLATRNRTSVDFKDTDYKRHQLTRSISEIKPPNLFPLA
PQEITHCHDEDDDEEEEEEEE
>sp|P21589|5NTD_HUMAN 5'-nucleotidase OS=Homo sapiens GN=NT5E PE=1 SV=1
MCPRAARAPATLLLALGAVLWPAAGAWELTILHTNDVHSRLEQTSEDSSKCVNASRCMGG
VARLFTKVQQIRRAEPNVLLLDAGDQYQGTIWFTVYKGAEVAHFMNALRYDAMALGNHEF
DNGVEGLIEPLLKEAKFPILSANIKAKGPLASQISGLYLPYKVLPVGDEVVGIVGYTSKE
TPFLSNPGTNLVFEDEITALQPEVDKLKTLNVNKIIALGHSGFEMDKLIAQKVRGVDVVV
GGHSNTFLYTGNPPSKEVPAGKYPFIVTSDDGRKVPVVQAYAFGKYLGYLKIEFDERGNV
ISSHGNPILLNSSIPEDPSIKADINKWRIKLDNYSTQELGKTIVYLDGSSQSCRFRECNM
GNLICDAMINNNLRHTDEMFWNHVSMCILNGGGIRSPIDERNNGTITWENLAAVLPFGGT
FDLVQLKGSTLKKAFEHSVHRYGQSTGEFLQVGGIHVVYDLSRKPGDRVVKLDVLCTKCR
VPSYDPLKMDEVYKVILPNFLANGGDGFQMIKDELLRHDSGDQDINVVSTYISKMKVIYP
AVEGRIKFSTGSHCHGSFSLIFLSLWAVIFVLYQ
>sp|Q9NQ94|A1CF_HUMAN APOBEC1 complementation factor OS=Homo sapiens GN=A1CF PE=1 SV=1
MESNHKSGDGLSGTQKEAALRALVQRTGYSLVQENGQRKYGGPPPGWDAAPPERGCEIFI
GKLPRDLFEDELIPLCEKIGKIYEMRMMMDFNGNNRGYAFVTFSNKVEAKNAIKQLNNYE
IRNGRLLGVCASVDNCRLFVGGIPKTKKREEILSEMKKVTEGVVDVIVYPSAADKTKNRG
FAFVEYESHRAAAMARRKLLPGRIQLWGHGIAVDWAEPEVEVDEDTMSSVKILYVRNLML
STSEEMIEKEFNNIKPGAVERVKKIRDYAFVHFSNREDAVEAMKALNGKVLDGSPIEVTL
AKPVDKDSYVRYTRGTGGRGTMLQGEYTYSLGQVYDPTTTYLGAPVFYAPQTYAAIPSLH
FPATKGHLSNRAIIRAPSVREIYMNVPVGAAGVRGLGGRGYLAYTGLGRGYQVKGDKRED
KLYDILPGMELTPMNPVTLKPQGIKLAPQILEEICQKNNWGQPVYQLHSAIGQDQRQLFL
YKITIPALASQNPAIHPFTPPKLSAFVDEAKTYAAEYTLQTLGIPTDGGDGTMATAAAAA
TAFPGYAVPNATAPVSAAQLKQAVTLGQDLAAYTTYEVYPTFAVTARGDGYGTF
>sp|Q8WTS1|ABHD5_HUMAN 1-acylglycerol-3-phosphate O-acyltransferase ABHD5 OS=Homo sapiens GN=ABHD5 PE=1 SV=1
MAAEEEEVDSADTGERSGWLTGWLPTWCPTSISHLKEAEEKMLKCVPCTYKKEPVRISNG
NKIWTLKFSHNISNKTPLVLLHGFGGGLGLWALNFGDLCTNRPVYAFDLLGFGRSSRPRF

DSDAEEVENQFVESIEEWRCALGLDKMILLGHNLGGFLAAAYSLKYPSRVNHLILVEPWG
FPERPDLADQDRPIPVWIRALGAALTPFNPLAGLRIAGPFGLSLVQRLRPDFKRKYSSMF
EDDTVTEYIYHCNVQTPSGETAFKNMTIPYGWAKRPMLQRIGKMHPDIPVSVIFGARSCI
DGNSGTSIQSLRPHSYVKTIAILGAGHYVYADQPEEFNQKVKEICDTVD
>sp|Q9UKU7|ACAD8_HUMAN Isobutyryl-CoA dehydrogenase, mitochondrial OS=Homo sapiens GN=ACAD8 PE=1 SV=1
MLWSGCRRFGARLGCLPGGLRVLVQTGHRSLTSCIDPSMGLNEEQKEFQKVAFDFAAREM
APNMAEWDQKELFPVDVMRKAAQLGFGGVYIQTDVGGSGLSRLDTSVIFEALATGCTSTT
AYISIHNMCAWMIDSFGNEEQRHKFCPPLCTMEKFASYCLTEPGSGSDAASLLTSAKKQG
DHYILNGSKAFISGAGESDIYVVMCRTGGPGPKGISCIVVEKGTPGLSFGKKEKKVGWNS
QPTRAVIFEDCAVPVANRIGSEGQGFLIAVRGLNGGRINIASCSLGAAHASVILTRDHLN
VRKQFGEPLASNQYLQFTLADMATRLVAARLMVRNAAVALQEERKDAVALCSMAKLFATD
ECFAICNQALQMHGGYGYLKDYAVQQYVRDSRVHQILEGSNEVMRILISRSLLQE
>sp|Q9H845|ACAD9_HUMAN Acyl-CoA dehydrogenase family member 9, mitochondrial OS=Homo sapiens GN=ACAD9 PE=1 SV=1
MSGCGLFLRTTAAARACRGLVVSTANRRLLRTSPPVRAFAKELFLGKIKKKEVFPFPEVS
QDELNEINQFLGPVEKFFTEEVDSRKIDQEGKIPDETLEKLKSLGLFGLQVPEEYGGLGF
SNTMYSRLGEIISMDGSITVTLAAHQAIGLKGIILAGTEEQKAKYLPKLASGEHIAAFCL
TEPASGSDAASIRSRATLSEDKKHYILNGSKVWITNGGLANIFTVFAKTEVVDSDGSVKD
KITAFIVERDFGGVTNGKPEDKLGIRGSNTCEVHFENTKIPVENILGEVGDGFKVAMNIL
NSGRFSMGSVVAGLLKRLIEMTAEYACTRKQFNKRLSEFGLIQEKFALMAQKAYVMESMT
YLTAGMLDQPGFPDCSIEAAMVKVFSSEAAWQCVSEALQILGGLGYTRDYPYERILRDTR
ILLIFEGTNEILRMYIALTGLQHAGRILTTRIHELKQAKVSTVMDTVGRRLRDSLGRTVD
LGLTGNHGVVHPSLADSANKFEENTYCFGRTVETLLLRFGKTIMEEQLVLKRVANILINL
YGMTAVLSRASRSIRIGLRNHDHEVLLANTFCVEAYLQNLFSLSQLDKYAPENLDEQIKK
VSQQILEKRAYICAHPLDRTC
>sp|Q8NC06|ACBD4_HUMAN Acyl-CoA-binding domain-containing protein 4 OS=Homo sapiens GN=ACBD4 PE=1 SV=2
MGTEKESPEPDCQKQFQAAVSVIQNLPKNGSYRPSYEEMLRFYSYYKQATMGPCLVPRPG
FWDPIGRYKWDAWNSLGKMSREEAMSAYITEMKLVAQKVIDTVPLGEVAEDMFGYFEPLY
QVIPDMPRPPETFLRRVTGWKEQVVNGDVGAVSEPPCLPKEPAPPSPESHSPRDLDSEVF
CDSLEQLEPELSSGQHLEESVIPGTAPCPPQRKRGCGAARRGPRSWTCGCWGQFEHYRRA
CRRCRRGCRAWRACPGPLSSLTLSVRLE
>sp|Q5T8D3|ACBD5_HUMAN Acyl-CoA-binding domain-containing protein 5 OS=Homo sapiens GN=ACBD5 PE=1 SV=1
MFQFHAGSWESWCCCCLIPADRPWDRGQHWQLEMADTRSVHETRFEAAVKVIQSLPKNGS
FQPTNEMMLKFYSFYKQATEGPCKLSRPGFWDPIGRYKWDAWSSLGDMTKEEAMIAYVEE
MKKIIETMPMTEKVEELLRVIGPFYEIVEDKKSGRSSDITSVRLEKISKCLEDLGNVLTS
TPNAKTVNGKAESSDSGAESEEEEAQEEVKGAEQSDNDKKMMKKSADHKNLEVIVTNGYD
KDGFVQDIQNDIHASSSLNGRSTEEVKPIDENLGQTGKSAVCIHQDINDDHVEDVTGIQH
LTSDSDSEVYCDSMEQFGQEESLDSFTSNNGPFQYYLGGHSSQPMENSGFREDIQVPPGN
GNIGNMQVVAVEGKGEVKHGGEDGRNNSGAPHREKRGGETDEFSNVRRGRGHRMQHLSEG
TKGRQVGSGGDGERWGSDRGSRGSLNEQIALVLMRLQEDMQNVLQRLQKLETLTALQAKS
STSTLQTAPQPTSQRPSWWPFEMSPGVLTFAIIWPFIAQWLVYLYYQRRRRKLN

>sp|Q9BR61|ACBD6_HUMAN Acyl-CoA-binding domain-containing protein 6 OS=Homo sapiens GN=ACBD6 PE=1 SV=1
MASSFLPAGAITGDSGGELSSGDDSGEVEFPHSPEIEETSCLAELFEKAAAHLQGLIQVA
SREQLLYLYARYKQVKVGNCNTPKPSFFDFEGKQKWEAWKALGDSSPSQAMQEYIAVVKK
LDPGWNPQIPEKKGKEANTGFGGPVISSLYHEETIREEDKNIFDYCRENNIDHITKAIKS
KNVDVNVKDEEGRALLHWACDRGHKELVTVLLQHRADINCQDNEGQTALHYASACEFLDI
VELLLQSGADPTLRDQDGCLPEEVTGCKTVSLVLQRHTTGKA
>sp|Q5FVE4|ACBG2_HUMAN Long-chain-fatty-acid--CoA ligase ACSBG2 OS=Homo sapiens GN=ACSBG2 PE=1 SV=2
MTGTPKTQEGAKDLEVDMNKTEVTPRLWTTCRDGEVLLRLSKHGPGHETPMTIPEFFRES
VNRFGTYPALASKNGKKWEILNFNQYYEACRKAAKSLIKLGLERFHGVGILGFNSAEWFI
TAVGAILAGGLCVGIYATNSAEVCQYVITHAKVNILLVENDQQLQKILSIPQSSLEPLKA
IIQYRLPMKKNNNLYSWDDFMELGRSIPDTQLEQVIESQKANQCAVLIYTSGTTGIPKGV
MLSHDNITWIAGAVTKDFKLTDKHETVVSYLPLSHIAAQMMDIWVPIKIGALTYFAQADA
LKGTLVSTLKEVKPTVFIGVPQIWEKIHEMVKKNSAKSMGLKKKAFVWARNIGFKVNSKK
MLGKYNTPVSYRMAKTLVFSKVKTSLGLDHCHSFISGTAPLNQETAEFFLSLDIPIGELY
GLSESSGPHTISNQNNYRLLSCGKILTGCKNMLFQQNKDGIGEICLWGRHIFMGYLESET
ETTEAIDDEGWLHSGDLGQLDGLGFLYVTGHIKEILITAGGENVPPIPVETLVKKKIPII
SNAMLVGDKLKFLSMLLTLKCEMNQMSGEPLDKLNFEAINFCRGLGSQASTVTEIVKQQD
PLVYKAIQQGINAVNQEAMNNAQRIEKWVILEKDFSIYGGELGPMMKLKRHFVAQKYKKQ
IDHMYH
>sp|P07108|ACBP_HUMAN Acyl-CoA-binding protein OS=Homo sapiens GN=DBI PE=1 SV=2
MSQAEFEKAAEEVRHLKTKPSDEEMLFIYGHYKQATVGDINTERPGMLDFTGKAKWDAWN
ELKGTSKEDAMKAYINKVEELKKKYGI
>sp|P02708|ACHA_HUMAN Acetylcholine receptor subunit alpha OS=Homo sapiens GN=CHRNA1 PE=1 SV=2
MEPWPLLLLFSLCSAGLVLGSEHETRLVAKLFKDYSSVVRPVEDHRQVVEVTVGLQLIQL
INVDEVNQIVTTNVRLKQGDMVDLPRPSCVTLGVPLFSHLQNEQWVDYNLKWNPDDYGGV
KKIHIPSEKIWRPDLVLYNNADGDFAIVKFTKVLLQYTGHITWTPPAIFKSYCEIIVTHF
PFDEQNCSMKLGTWTYDGSVVAINPESDQPDLSNFMESGEWVIKESRGWKHSVTYSCCPD
TPYLDITYHFVMQRLPLYFIVNVIIPCLLFSFLTGLVFYLPTDSGEKMTLSISVLLSLTV
FLLVIVELIPSTSSAVPLIGKYMLFTMVFVIASIIITVIVINTHHRSPSTHVMPNWVRKV
FIDTIPNIMFFSTMKRPSREKQDKKIFTEDIDISDISGKPGPPPMGFHSPLIKHPEVKSA
IEGIKYIAETMKSDQESNNAAAEWKYVAMVMDHILLGVFMLVCIIGTLAVFAGRLIELNQ
QG
>sp|Q9UKV3|ACINU_HUMAN Apoptotic chromatin condensation inducer in the nucleus OS=Homo sapiens GN=ACIN1 PE=1 SV=2
MWRRKHPRTSGGTRGVLSGNRGVEYGSGRGHLGTFEGRWRKLPKMPEAVGTDPSTSRKMA
ELEEVTLDGKPLQALRVTDLKAALEQRGLAKSGQKSALVKRLKGALMLENLQKHSTPHAA
FQPNSQIGEEMSQNSFIKQYLEKQQELLRQRLEREAREAAELEEASAESEDEMIHPEGVA
SLLPPDFQSSLERPELELSRHSPRKSSSISEEKGDSDDEKPRKGERRSSRVRQARAAKLS
EGSQPAEEEEDQETPSRNLRVRADRNLKTEEEEEEEEEEEEDDEEEEGDDEGQKSREAPI
LKEFKEEGEEIPRVKPEEMMDERPKTRSQEQEVLERGGRFTRSQEEARKSHLARQQQEKE
MKTTSPLEEEEREIKSSQGLKEKSKSPSPPRLTEDRKKASLVALPEQTASEEETPPPLLT

KEASSPPPHPQLHSEEEIEPMEGPAPAVLIQLSPPNTDADTRELLVSQHTVQLVGGLSPL
SSPSDTKAESPAEKVPEESVLPLVQKSTLADYSAQKDLEPESDRSAQPLPLKIEELALAK
GITEECLKQPSLEQKEGRRASHTLLPSHRLKQSADSSSSRSSSSSSSSSRSRSRSPDSSG
SRSHSPLRSKQRDVAQARTHANPRGRPKMGSRSTSESRSRSRSRSRSASSNSRKSLSPGV
SRDSSTSYTETKDPSSGQEVATPPVPQLQVCEPKERTSTSSSSVQARRLSQPESAEKHVT
QRLQPERGSPKKCEAEEAEPPAATQPQTSETQTSHLPESERIHHTVEEKEEVTMDTSENR
PENDVPEPPMPIADQVSNDDRPEGSVEDEEKKESSLPKSFKRKISVVSATKGVPAGNSDT
EGGQPGRKRRWGASTATTQKKPSISITTESLKSLIPDIKPLAGQEAVVDLHADDSRISED
ETERNGDDGTHDKGLKICRTVTQVVPAEGQENGQREEEEEEKEPEAEPPVPPQVSVEVAL
PPPAEHEVKKVTLGDTLTRRSISQQKSGVSITIDDPVRTAQVPSPPRGKISNIVHISNLV
RPFTLGQLKELLGRTGTLVEEAFWIDKIKSHCFVTYSTVEEAVATRTALHGVKWPQSNPK
FLCADYAEQDELDYHRGLLVDRPSETKTEEQGIPRPLHPPPPPPVQPPQHPRAEQREQER
AVREQWAEREREMERRERTRSEREWDRDKVREGPRSRSRSRDRRRKERAKSKEKKSEKKE
KAQEEPPAKLLDDLFRKTKAAPCIYWLPLTDSQIVQKEAERAERAKEREKRRKEQEEEEQ
KEREKEAERERNRQLEREKRREHSRERDREREREREREDRGDRDRDRERDRERGRERDRRD
TKRHSRSRSRSTPVRDRGGRR
>sp|O96019|ACL6A_HUMAN Actin-like protein 6A OS=Homo sapiens GN=ACTL6A PE=1 SV=1
MSGGVYGGDEVGALVFDIGSYTVRAGYAGEDCPKVDFPTAIGMVVERDDGSTLMEIDGDK
GKQGGPTYYIDTNALRVPRENMEAISPLKNGMVEDWDSFQAILDHTYKMHVKSEASLHPV
LMSEAPWNTRAKREKLTELMFEHYNIPAFFLCKTAVLTAFANGRSTGLILDSGATHTTAI
PVHDGYVLQQGIVKSPLAGDFITMQCRELFQEMNIELVPPYMIASKEAVREGSPANWKRK
EKLPQVTRSWHNYMCNCVIQDFQASVLQVSDSTYDEQVAAQMPTVHYEFPNGYNCDFGAE
RLKIPEGLFDPSNVKGLSGNTMLGVSHVVTTSVGMCDIDIRPGLYGSVIVAGGNTLIQSF
TDRLNRELSQKTPPSMRLKLIANNTTVERRFSSWIGGSILASLGTFQQMWISKQEYEEGG
KQCVERKCP
>sp|Q8WYK0|ACO12_HUMAN Acyl-coenzyme A thioesterase 12 OS=Homo sapiens GN=ACOT12 PE=1 SV=1
MERPAPGEVVMSQAIQPAHATARGELSAGQLLKWIDTTACLAAEKHAGVSCVTASVDDIQ
FEETARVGQVITIKAKVTRAFSTSMEISIKVMVQDMLTGIEKLVSVAFSTFVAKPVGKEK
IHLKPVTLLTEQDHVEHNLAAERRKVRLQHEDTFNNLMKESSKFDDLIFDEEEGAVSTRG
TSVQSIELVLPPHANHHGNTFGGQIMAWMETVATISASRLCWAHPFLKSVDMFKFRGPST
VGDRLVFTAIVNNTFQTCVEVGVRVEAFDCQEWAEGRGRHINSAFLIYNAADDKENLITF
PRIQPISKDDFRRYRGAIARKRIRLGRKYVISHKEEVPLCIHWDISKQASLSDSNVEALK
KLAAKRGWEVTSTVEKIKIYTLEEHDVLSVWVEKHVGSPAHLAYRLLSDFTKRPLWDPHF
VSCEVIDWVSEDDQLYHITCPILNDDKPKDLVVLVSRRKPLKDGNTYTVAVKSVILPSVP
PSPQYIRSEIICAGFLIHAIDSNSCIVSYFNHMSASILPYFAGNLGGWSKSIEETAASCI
QFLENPPDDGFVSTF
>sp|Q99424|ACOX2_HUMAN Peroxisomal acyl-coenzyme A oxidase 2 OS=Homo sapiens GN=ACOX2 PE=1 SV=1
MGSPVHRVSLGDTWSRQMHPDIESERYMQSFDVERLTNILDGGAQNTALRRKVESIIHSY
PEFSCKDNYFMTQNERYKAAMRRAFHIRLIARRLGWLEDGRELGYAYRALSGDVALNIHR
VFVRALRSLGSEEQIAKWDPLCKNIQIIATYAQTELGHGTYLQGLETEATYDAATQEFVI
HSPTLTATKWWPGDLGRSATHALVQAQLICSGARRGMHAFIVPIRSLQDHTPLPGIIIGD
IGPKMDFDQTDNGFLQLNHVRVPRENMLSRFAQVLPDGTYVKLGTAQSNYLPMVVVRVEL

LSGEILPILQKACVIAMRYSVIRRQSRLRPSDPEAKVLDYQTQQQKLFPQLAISYAFHFL
AVSLLEFFQHSYTAILNQDFSFLPELHALSTGMKAMMSEFCTQGAEMCRRACGGHGYSKL
SGLPSLVTKLSASCTYEGENTVLYLQVARFLVKSYLQTQMSPGSTPQRSLSPSVAYLTAP
DLARCPAQRAADFLCPELYTTAWAHVAVRLIKDSVQHLQTLTQSGADQHEAWNQTTVIHL
QAAKVHCYYVTVKGFTEALEKLENEPAIQQVLKRLCDLHAIHGILTNSGDFLHDAFLSGA
QVDMARTAYLDLLRLIRKDAILLTDAFDFTDQCLNSALGCYDGNVYERLFQWAQKSPTNT
QENPAYEEYIRPLLQSWRSKL
>sp|O15254|ACOX3_HUMAN Peroxisomal acyl-coenzyme A oxidase 3 OS=Homo sapiens GN=ACOX3 PE=1 SV=2
MASTVEGGDTALLPEFPRGPLDAYRARASFSWKELALFTEGEGMLRFKKTIFSALENDPL
FARSPGADLSLEKYRELNFLRCKRIFEYDFLSVEDMFKSPLKVPALIQCLGMYDSSLAAK
YLLHSLVFGSAVYSSGSERHLTYIQKIFRMEIFGCFALTELSHGSNTKAIRTTAHYDPAT
EEFIIHSPDFEAAKFWVGNMGKTATHAVVFAKLCVPGDQCHGLHPFIVQIRDPKTLLPMP
GVMVGDIGKKLGQNGLDNGFAMFHKVRVPRQSLLNRMGDVTPEGTYVSPFKDVRQRFGAS
LGSLSSGRVSIVSLAILNLKLAVAIALRFSATRRQFGPTEEEEIPVLEYPMQQWRLLPYL
AAVYALDHFSKSLFLDLVELQRGLASGDRSARQAELGREIHALASASKPLASWTTQQGIQ
ECREACGGHGYLAMNRLGVLRDDNDPNCTYEGDNNILLQQTSNYLLGLLAHQVHDGACFR
SPLKSVDFLDAYPGILDQKFEVSSVADCLDSAVALAAYKWLVCYLLRETYQKLNQEKRSG
SSDFEARNKCQVSHGRPLALAFVELTVVQRFHEHVHQPSVPPSLRAVLGRLSALYALWSL
SRHAALLYRGGYFSGEQAGEVLESAVLALCSQLKDDAVALVDVIAPPDFVLDSPIGRADG
ELYKNLWGAVLQESKVLERASWWPEFSVNKPVIGSLKSKL
>sp|P45381|ACY2_HUMAN Aspartoacylase OS=Homo sapiens GN=ASPA PE=1 SV=1
MTSCHIAEEHIQKVAIFGGTHGNELTGVFLVKHWLENGAEIQRTGLEVKPFITNPRAVKK
CTRYIDCDLNRIFDLENLGKKMSEDLPYEVRRAQEINHLFGPKDSEDSYDIIFDLHNTTS
NMGCTLILEDSRNNFLIQMFHYIKTSLAPLPCYVYLIEHPSLKYATTRSIAKYPVGIEVG
PQPQGVLRADILDQMRKMIKHALDFIHHFNEGKEFPPCAIEVYKIIEKVDYPRDENGEIA
AIIHPNLQDQDWKPLHPGDPMFLTLDGKTIPLGGDCTVYPVFVNEAAYYEKKEAFAKTTK
LTLNAKSIRCCLH
>sp|Q96HD9|ACY3_HUMAN N-acyl-aromatic-L-amino acid amidohydrolase (carboxylate-forming) OS=Homo sapiens GN=ACY3 PE=1 SV=1
MCSLPVPREPLRRVAVTGGTHGNEMSGVYLARHWLHAPAELQRASFSAVPVLANPAATSG
CRRYVDHDLNRTFTSSFLNSRPTPDDPYEVTRARELNQLLGPKASGQAFDFVLDLHNTTA
NMGTCLIAKSSHEVFAMHLCRHLQLQYPELSCQVFLYQRSGEESYNLDSVAKNGLGLELG
PQPQGVLRADIFSRMRTLVATVLDFIELFNQGTAFPAFEMEAYRPVGVVDFPRTEAGHLA
GTVHPQLQDRDFQPLQPGAPIFQMFSGEDLLYEGESTVYPVFINEAAYYEKGVAFVQTEK
FTFTVPAMPALTPAPSPAS
>sp|O14672|ADA10_HUMAN Disintegrin and metalloproteinase domain-containing protein 10 OS=Homo sapiens GN=ADAM10 PE=1 SV=1
MVLLRVLILLLSWAAGMGGQYGNPLNKYIRHYEGLSYNVDSLHQKHQRAKRAVSHEDQFL
RLDFHAHGRHFNLRMKRDTSLFSDEFKVETSNKVLDYDTSHIYTGHIYGEEGSFSHGSVI
DGRFEGFIQTRGGTFYVEPAERYIKDRTLPFHSVIYHEDDINYPHKYGPQGGCADHSVFE
RMRKYQMTGVEEVTQIPQEEHAANGPELLRKKRTTSAEKNTCQLYIQTDHLFFKYYGTRE
AVIAQISSHVKAIDTIYQTTDFSGIRNISFMVKRIRINTTADEKDPTNPFRFPNIGVEKF
LELNSEQNHDDYCLAYVFTDRDFDDGVLGLAWVGAPSGSSGGICEKSKLYSDGKKKSLNT

GIITVQNYGSHVPPKVSHITFAHEVGHNFGSPHDSGTECTPGESKNLGQKENGNYIMYAR
ATSGDKLNNNKFSLCSIRNISQVLEKKRNNCFVESGQPICGNGMVEQGEECDCGYSDQCK
DECCFDANQPEGRKCKLKPGKQCSPSQGPCCTAQCAFKSKSEKCRDDSDCAREGICNGFT
ALCPASDPKPNFTDCNRHTQVCINGQCAGSICEKYGLEECTCASSDGKDDKELCHVCCMK
KMDPSTCASTGSVQWSRHFSGRTITLQPGSPCNDFRGYCDVFMRCRLVDADGPLARLKKA
IFSPELYENIAEWIVAHWWAVLLMGIALIMLMAGFIKICSVHTPSSNPKLPPPKPLPGTL
KRRRPPQPIQQPQRQRPRESYQMGHMRR
>sp|O43506|ADA20_HUMAN Disintegrin and metalloproteinase domain-containing protein 20 OS=Homo sapiens GN=ADAM20 PE=2 SV=2
MAVGEPLVHIRVTLLLLWFGMFLSISGHSQARPSQYFTSPEVVIPLKVISRGRGAKAPGW
LSYSLRFGGQRYIVHMRVNKLLFAAHLPVFTYTEQHALLQDQPFIQDDCYYHGYVEGVPE
SLVALSTCSGGFLGMLQINDLVYEIKPISVSATFEHLVYKIDSDDTQFPPMRCGLTEEKI
AHQMELQLSYNFTLKQSSFVGWWTHQRFVELVVVVDNIRYLFSQSNATTVQHEVFNVVNI
VDSFYHPLEVDVILTGIDIWTASNPLPTSGDLDNVLEDFSIWKNYNLNNRLQHDVAHLFI
KDTQGMKLGVAYVKGICQNPFNTGVDVFEDNRLVVFAITLGHELGHNLGMQHDTQWCVCE
LQWCIMHAYRKVTTKFSNCSYAQYWDSTISSGLCIQPPPYPGNIFRLKYCGNLVVEEGEE
CDCGTIRQCAKDPCCLLNCTLHPGAACAFGICCKDCKFLPSGTLCRQQVGECDLPEWCNG
TSHQCPDDVYVQDGISCNVNAFCYEKTCNNHDIQCKEIFGQDARSASQSCYQEINTQGNR
FGHCGIVGTTYVKCWTPDIMCGRVQCENVGVIPNLIEHSTVQQFHLNDTTCWGTDYHLGM
AIPDIGEVKDGTVCGPEKICIRKKCASMVHLSQACQPKTCNMRGICNNKQHCHCNHEWAP
PYCKDKGYGGSADSGPPPKNNMEGLNVMGKLRYLSLLCLLPLVAFLLFCLHVLFKKRTKS
KEDEEG
>sp|O75077|ADA23_HUMAN Disintegrin and metalloproteinase domain-containing protein 23 OS=Homo sapiens GN=ADAM23 PE=1 SV=1
MKPPGSSSRQPPLAGCSLAGASCGPQRGPAGSVPASAPARTPPCRLLLVLLLLPPLAASS
RPRAWGAAAPSAPHWNETAEKNLGVLADEDNTLQQNSSSNISYSNAMQKEITLPSRLIYY
INQDSESPYHVLDTKARHQQKHNKAVHLAQASFQIEAFGSKFILDLILNNGLLSSDYVEI
HYENGKPQYSKGGEHCYYHGSIRGVKDSKVALSTCNGLHGMFEDDTFVYMIEPLELVHDE
KSTGRPHIIQKTLAGQYSKQMKNLTMERGDQWPFLSELQWLKRRKRAVNPSRGIFEEMKY
LELMIVNDHKTYKKHRSSHAHTNNFAKSVVNLVDSIYKEQLNTRVVLVAVETWTEKDQID
ITTNPVQMLHEFSKYRQRIKQHADAVHLISRVTFHYKRSSLSYFGGVCSRTRGVGVNEYG
LPMAVAQVLSQSLAQNLGIQWEPSSRKPKCDCTESWGGCIMEETGVSHSRKFSKCSILEY
RDFLQRGGGACLFNRPTKLFEPTECGNGYVEAGEECDCGFHVECYGLCCKKCSLSNGAHC
SDGPCCNNTSCLFQPRGYECRDAVNECDITEYCTGDSGQCPPNLHKQDGYACNQNQGRCY
NGECKTRDNQCQYIWGTKAAGSDKFCYEKLNTEGTEKGNCGKDGDRWIQCSKHDVFCGFL
LCTNLTRAPRIGQLQGEIIPTSFYHQGRVIDCSGAHVVLDDDTDVGYVEDGTPCGPSMMC
LDRKCLQIQALNMSSCPLDSKGKVCSGHGVCSNEATCICDFTWAGTDCSIRDPVRNLHPP
KDEGPKGPSATNLIIGSIAGAILVAAIVLGGTGWGFKNVKKRRFDPTQQGPI
>sp|Q9UKF5|ADA29_HUMAN Disintegrin and metalloproteinase domain-containing protein 29 OS=Homo sapiens GN=ADAM29 PE=1 SV=3
MKMLLLLHCLGVFLSCSGHIQDEHPQYHSPPDVVIPVRITGTTRGMTPPGWLSYILPFGG
QKHIIHIKVKKLLFSKHLPVFTYTDQGAILEDQPFVQNNCYYHGYVEGDPESLVSLSTCF
GGFQGILQINDFAYEIKPLAFSTTFEHLVYKMDSEEKQFSTMRSGFMQNEITCRMEFEEI
DNSTQKQSSYVGWWIHFRIVEIVVVIDNYLYIRYERNDSKLLEDLYVIVNIVDSILDVIG

VKVLLFGLEIWTNKNLIVVDDVRKSVHLYCKWKSENITPRMQHDTSHLFTTLGLRGLSGI
GAFRGMCTPHRSCAIVTFMNKTLGTFSIAVAHHLGHNLGMNHDEDTCRCSQPRCIMHEGN
PPITKFSNCSYGDFWEYTVERTKCLLETVHTKDIFNVKRCGNGVVEEGEECDCGPLKHCA
KDPCCLSNCTLTDGSTCAFGLCCKDCKFLPSGKVCRKEVNECDLPEWCNGTSHKCPDDFY
VEDGIPCKERGYCYEKSCHDRNEQCRRIFGAGANTASETCYKELNTLGDRVGHCGIKNAT
YIKCNISDVQCGRIQCENVTEIPNMSDHTTVHWARFNDIMCWSTDYHLGMKGPDIGEVKD
GTECGIDHICIHRHCVHITILNSNCSPAFCNKRGICNNKHHCHCNYLWDPPNCLIKGYGG
SVDSGPPPKRKKKKKFCYLCILLLIVLFILLCCLYRLCKKSKPIKKQQDVQTPSAKEEEK
IQRRPHELPPQSQPWVMPSQSQPPVTPSQSHPQVMPSQSQPPVTPSQSQPRVMPSQSQPP
VMPSQSHPQLTPSQSQPPVTPSQRQPQLMPSQSQPPVTPS
>sp|P08913|ADA2A_HUMAN Alpha-2A adrenergic receptor OS=Homo sapiens GN=ADRA2A PE=1 SV=3
MGSLQPDAGNASWNGTEAPGGGARATPYSLQVTLTLVCLAGLLMLLTVFGNVLVIIAVFT
SRALKAPQNLFLVSLASADILVATLVIPFSLANEVMGYWYFGKAWCEIYLALDVLFCTSS
IVHLCAISLDRYWSITQAIEYNLKRTPRRIKAIIITVWVISAVISFPPLISIEKKGGGGG
PQPAEPRCEINDQKWYVISSCIGSFFAPCLIMILVYVRIYQIAKRRTRVPPSRRGPDAVA
APPGGTERRPNGLGPERSAGPGGAEAEPLPTQLNGAPGEPAPAGPRDTDALDLEESSSSD
HAERPPGPRRPERGPRGKGKARASQVKPGDSLPRRGPGATGIGTPAAGPGEERVGAAKAS
RWRGRQNREKRFTFVLAVVIGVFVVCWFPFFFTYTLTAVGCSVPRTLFKFFFWFGYCNSS
LNPVIYTIFNHDFRRAFKKILCRGDRKRIV
>sp|P18089|ADA2B_HUMAN Alpha-2B adrenergic receptor OS=Homo sapiens GN=ADRA2B PE=1 SV=3
MDHQDPYSVQATAAIAAAITFLILFTIFGNALVILAVLTSRSLRAPQNLFLVSLAAADIL
VATLIIPFSLANELLGYWYFRRTWCEVYLALDVLFCTSSIVHLCAISLDRYWAVSRALEY
NSKRTPRRIKCIILTVWLIAAVISLPPLIYKGDQGPQPRGRPQCKLNQEAWYILASSIGS
FFAPCLIMILVYLRIYLIAKRSNRRGPRAKGGPGQGESKQPRPDHGGALASAKLPALASV
ASAREVNGHSKSTGEKEEGETPEDTGTRALPPSWAALPNSGQGQKEGVCGASPEDEAEEE
EEEEEECEPQAVPVSPASACSPPLQQPQGSRVLATLRGQVLLGRGVGAIGGQWWRRRAQL
TREKRFTFVLAVVIGVFVLCWFPFFFSYSLGAICPKHCKVPHGLFQFFFWIGYCNSSLNP
VIYTIFNQDFRRAFRRILCRPWTQTAW
>sp|Q9UKF2|ADA30_HUMAN Disintegrin and metalloproteinase domain-containing protein 30 OS=Homo sapiens GN=ADAM30 PE=1 SV=2
MRSVQIFLSQCRLLLLLVPTMLLKSLGEDVIFHPEGEFDSYEVTIPEKLSFRGEVQGVVS
PVSYLLQLKGKKHVLHLWPKRLLLPRHLRVFSFTEHGELLEDHPYIPKDCNYMGSVKESL
DSKATISTCMGGLRGVFNIDAKHYQIEPLKASPSFEHVVYLLKKEQFGNQVCGLSDDEIE
WQMAPYENKARLRDFPGSYKHPKYLELILLFDQSRYRFVNNNLSQVIHDAILLTGIMDTY
FQDVRMRIHLKALEVWTDFNKIRVGYPELAEVLGRFVIYKKSVLNARLSSDWAHLYLQRK
YNDALAWSFGKVCSLEYAGSVSTLLDTNILAPATWSAHELGHAVGMSHDEQYCQCRGRLN
CIMGSGRTGFSNCSYISFFKHISSGATCLNNIPGLGYVLKRCGNKIVEDNEECDCGSTEE
CQKDRCCQSNCKLQPGANCSIGLCCHDCRFRPSGYVCRQEGNECDLAEYCDGNSSSCPND
VYKQDGTPCKYEGRCFRKGCRSRYMQCQSIFGPDAMEAPSECYDAVNLIGDQFGNCEITG
IRNFKKCESANSICGRLQCINVETIPDLPEHTTIISTHLQAENLMCWGTGYHLSMKPMGI
PDLGMINDGTSCGEGRVCFKKNCVNSSVLQFDCLPEKCNTRGVCNNRKNCHCMYGWAPPF
CEEVGYGGSIDSGPPGLLRGAIPSSIWVVSIIMFRLILLILSVVFVFFRQVIGNHLKPKQ
EKMPLSKAKTEQEESKTKTVQEESKTKTGQEESEAKTGQEESKAKTGQEESKANIESKRP
KAKSVKKQKK

>sp|Q9BZ11|ADA33_HUMAN Disintegrin and metalloproteinase domain-containing protein 33 OS=Homo sapiens GN=ADAM33 PE=1 SV=2
MGWRPRRARGTPLLLLLLLLLLWPVPGAGVLQGHIPGQPVTPHWVLDGQPWRTVSLEEPV
SKPDMGLVALEAEGQELLLELEKNHRLLAPGYIETHYGPDGQPVVLAPNHTDHCHYQGRV
RGFPDSWVVLCTCSGMSGLITLSRNASYYLRPWPPRGSKDFSTHEIFRMEQLLTWKGTCG
HRDPGNKAGMTSLPGGPQSRGRREARRTRKYLELYIVADHTLFLTRHRNLNHTKQRLLEV
ANYVDQLLRTLDIQVALTGLEVWTERDRSRVTQDANATLWAFLQWRRGLWAQRPHDSAQL
LTGRAFQGATVGLAPVEGMCRAESSGGVSTDHSELPIGAAATMAHEIGHSLGLSHDPDGC
CVEAAAESGGCVMAAATGHPFPRVFSACSRRQLRAFFRKGGGACLSNAPDPGLPVPPALC
GNGFVEAGEECDCGPGQECRDLCCFAHNCSLRPGAQCAHGDCCVRCLLKPAGALCRQAMG
DCDLPEFCTGTSSHCPPDVYLLDGSPCARGSGYCWDGACPTLEQQCQQLWGPGSHPAPEA
CFQVVNSAGDAHGNCGQDSEGHFLPCAGRDALCGKLQCQGGKPSLLAPHMVPVDSTVHLD
GQEVTCRGALALPSAQLDLLGLGLVEPGTQCGPRMVCQSRRCRKNAFQELQRCLTACHSH
GVCNSNHNCHCAPGWAPPFCDKPGFGGSMDSGPVQAENHDTFLLAMLLSVLLPLLPGAGL
AWCCYRLPGAHLQRCSWGCRRDPACSGPKDGPHRDHPLGGVHPMELGPTATGQPWPLDPE
NSHEPSSHPEKPLPAVSPDPQADQVQMPRSCLW
>sp|Q6DHV7|ADAL_HUMAN Adenosine deaminase-like protein OS=Homo sapiens GN=ADAL PE=2 SV=2
MIEAEEQQPCKTDFYSELPKVELHAHLNGSISSHTMKKLIAQKPDLKIHDQMTVIDKGKK
RTLEECFQMFQTIHQLTSSPEDILMVTKDVIKEFADDGVKYLELRSTPRRENATGMTKKT
YVESILEGIKQSKQENLDIDVRYLIAVDRRGGPLVAKETVKLAEEFFLSTEGTVLGLDLS
GDPTVGQAKDFLEPLLEAKKAGLKLALHLSEIPNQKKETQILLDLLPDRIGHGTFLNSGE
GGSLDLVDFVRQHRIPLELCLTSNVKSQTVPSYDQHHFGFWYSIAHPSVICTDDKGVFAT
HLSQEYQLAAETFNLTQSQVWDLSYESINYIFASDSTRSELRKKWNHLKPRVLHI
>sp|Q13443|ADAM9_HUMAN Disintegrin and metalloproteinase domain-containing protein 9 OS=Homo sapiens GN=ADAM9 PE=1 SV=1
MGSGARFPSGTLRVRWLLLLGLVGPVLGAARPGFQQTSHLSSYEIITPWRLTRERREAPR
PYSKQVSYVIQAEGKEHIIHLERNKDLLPEDFVVYTYNKEGTLITDHPNIQNHCHYRGYV
EGVHNSSIALSDCFGLRGLLHLENASYGIEPLQNSSHFEHIIYRMDDVYKEPLKCGVSNK
DIEKETAKDEEEEPPSMTQLLRRRRAVLPQTRYVELFIVVDKERYDMMGRNQTAVREEMI
LLANYLDSMYIMLNIRIVLVGLEIWTNGNLINIVGGAGDVLGNFVQWREKFLITRRRHDS
AQLVLKKGFGGTAGMAFVGTVCSRSHAGGINVFGQITVETFASIVAHELGHNLGMNHDDG
RDCSCGAKSCIMNSGASGSRNFSSCSAEDFEKLTLNKGGNCLLNIPKPDEAYSAPSCGNK
LVDAGEECDCGTPKECELDPCCEGSTCKLKSFAECAYGDCCKDCRFLPGGTLCRGKTSEC
DVPEYCNGSSQFCQPDVFIQNGYPCQNNKAYCYNGMCQYYDAQCQVIFGSKAKAAPKDCF
IEVNSKGDRFGNCGFSGNEYKKCATGNALCGKLQCENVQEIPVFGIVPAIIQTPSRGTKC
WGVDFQLGSDVPDPGMVNEGTKCGAGKICRNFQCVDASVLNYDCDVQKKCHGHGVCNSNK
NCHCENGWAPPNCETKGYGGSVDSGPTYNEMNTALRDGLLVFFFLIVPLIVCAIFIFIKR
DQLWRSYFRKKRSQTYESDGKNQANPSRQPGSVPRHVSPVTPPREVPIYANRFAVPTYAA
KQPQQFPSRPPPPQPKVSSQGNLIPARPAPAPPLYSSLT
>sp|O75689|ADAP1_HUMAN Arf-GAP with dual PH domain-containing protein 1 OS=Homo sapiens GN=ADAP1 PE=1 SV=2
MAKERRRAVLELLQRPGNARCADCGAPDPDWASYTLGVFICLSCSGIHRNIPQVSKVKSV
RLDAWEEAQVEFMASHGNDAARARFESKVPSFYYRPTPSDCQLLREQWIRAKYERQEFIY
PEKQEPYSAGYREGFLWKRGRDNGQFLSRKFVLTEREGALKYFNRNDAKEPKAVMKIEHL

NATFQPAKIGHPHGLQVTYLKDNSTRNIFIYHEDGKEIVDWFNALRAARFHYLQVAFPGA
GDADLVPKLSRNYLKEGYMEKTGPKQTEGFRKRWFTMDDRRLMYFKDPLDAFARGEVFIG
SKESGYTVLHGFPPSTQGHHWPHGITIVTPDRKFLFACETESDQREWVAAFQKAVDRPML
PQEYAVEAHFKHKP
>sp|Q96EY9|ADAT3_HUMAN Probable inactive tRNA-specific adenosine deaminase-like protein 3 OS=Homo sapiens GN=ADAT3 PE=1 SV=1
MEPAPGLVEQPKCLEAGSPEPEPAPWQALPVLSEKQSGDVELVLAYAAPVLDKRQTSRLL
KEVSALHPLPAQPHLKRVRPSRDAGSPHALEMLLCLAGPASGPRSLAELLPRPAVDPRGL
GQPFLVPVPARPPLTRGQFEEARAHWPTSFHEDKQVTSALAGRLFSTQERAAMQSHMERA
VWAARRAAARGLRAVGAVVVDPASDRVLATGHDCSCADNPLLHAVMVCVDLVARGQGRGT
YDFRPFPACSFAPAAAPQAVRAGAVRKLDADEDGLPYLCTGYDLYVTREPCAMCAMALVH
ARILRVFYGAPSPDGALGTRFRIHARPDLNHRFQVFRGVLEEQCRWLDPDT
>sp|P00813|ADA_HUMAN Adenosine deaminase OS=Homo sapiens GN=ADA PE=1 SV=3
MAQTPAFDKPKVELHVHLDGSIKPETILYYGRRRGIALPANTAEGLLNVIGMDKPLTLPD
FLAKFDYYMPAIAGCREAIKRIAYEFVEMKAKEGVVYVEVRYSPHLLANSKVEPIPWNQA
EGDLTPDEVVALVGQGLQEGERDFGVKARSILCCMRHQPNWSPKVVELCKKYQQQTVVAI
DLAGDETIPGSSLLPGHVQAYQEAVKSGIHRTVHAGEVGSAEVVKEAVDILKTERLGHGY
HTLEDQALYNRLRQENMHFEICPWSSYLTGAWKPDTEHAVIRLKNDQANYSLNTDDPLIF
KSTLDTDYQMTKRDMGFTEEEFKRLNINAAKSSFLPEDEKRELLDLLYKAYGMPPSASAG
QNL
>sp|Q13542|4EBP2_HUMAN Eukaryotic translation initiation factor 4E-binding protein 2 OS=Homo sapiens GN=EIF4EBP2 PE=1 SV=1
MSSSAGSGHQPSQSRAIPTRTVAISDAAQLPHDYCTTPGGTLFSTTPGGTRIIYDRKFLL
DRRNSPMAQTPPCHLPNIPGVTSPGTLIEDSKVEVNNLNNLNNHDRKHAVGDDAQFEMDI
>sp|P04217|A1BG_HUMAN Alpha-1B-glycoprotein OS=Homo sapiens GN=A1BG PE=1 SV=4
MSMLVVFLLLWGVTWGPVTEAAIFYETQPSLWAESESLLKPLANVTLTCQAHLETPDFQL
FKNGVAQEPVHLDSPAIKHQFLLTGDTQGRYRCRSGLSTGWTQLSKLLELTGPKSLPAPW
LSMAPVSWITPGLKTTAVCRGVLRGVTFLLRREGDHEFLEVPEAQEDVEATFPVHQPGNY
SCSYRTDGEGALSEPSATVTIEELAAPPPPVLMHHGESSQVLHPGNKVTLTCVAPLSGVD
FQLRRGEKELLVPRSSTSPDRIFFHLNAVALGDGGHYTCRYRLHDNQNGWSGDSAPVELI
LSDETLPAPEFSPEPESGRALRLRCLAPLEGARFALVREDRGGRRVHRFQSPAGTEALFE
LHNISVADSANYSCVYVDLKPPFGGSAPSERLELHVDGPPPRPQLRATWSGAVLAGRDAV
LRCEGPIPDVTFELLREGETKAVKTVRTPGAAANLELIFVGPQHAGNYRCRYRSWVPHTF
ESELSDPVELLVAES
>sp|A7E2S9|A30BL_HUMAN Putative ankyrin repeat domain-containing protein 30B-like OS=Homo sapiens GN=ANKRD30BL PE=2 SV=3
MERLSAAPVKGQTGPERPSPFSQLVYTNNDSYVIHHGDLRKIHKAASRGQAWKLERMMKK
TTMDLNIRDAKKRTALYWACANGHAEVVTLLVDRKCQLDVLDGENRTILMKALQCQREAC
ANILIDSGADPNIVDVYGNTAVHYAVNSENLSVVAKLLSCGADIEVKNKAGHTPLLLAIR
KRSEEIVEFLLTKNANANAVDKFKCVHQQLLEYKQKISKNSQNSNPEGTSEGTPDEAAPL
AERTPDTAESLVERTPDE
>sp|U3KPV4|A3LT2_HUMAN Alpha-1,3-galactosyltransferase 2 OS=Homo sapiens GN=A3GALT2 PE=2 SV=1
MALKEGLRAWKRIFWRQILLTLGLLGLFLYGLPKFRHLEALIPMGVCPSATMSQLRDNFT

GALRPWARPEVLTCTPWGAPIIWDGSFDPDVAKQEARQQNLTIGLTIFAVGRYLEKYLER
FLETAEQHFMAGQSVMYYVFTELPGAVPRVALGPGRRLPVERVARERRWQDVSMARMRTL
HAALGGLPGREAHFMFCMDVDQHFSGTFGPEALAESVAQLHSWHYHWPSWLLPFERDAHS
AAAMAWGQGDFYNHAAVFGGSVAALRGLTAHCAGGLDWDRARGLEARWHDESHLNKFFWL
HKPAKVLSPEFCWSPDIGPRAEIRRPRLLWAPKGYRLLRN
>sp|P0DMS8|AA3R_HUMAN Adenosine receptor A3 OS=Homo sapiens GN=ADORA3 PE=2 SV=1
MPNNSTALSLANVTYITMEIFIGLCAIVGNVLVICVVKLNPSLQTTTFYFIVSLALADIA
VGVLVMPLAIVVSLGITIHFYSCLFMTCLLLIFTHASIMSLLAIAVDRYLRVKLTVRYKR
VTTHRRIWLALGLCWLVSFLVGLTPMFGWNMKLTSEYHRNVTFLSCQFVSVMRMDYMVYF
SFLTWIFIPLVVMCAIYLDIFYIIRNKLSLNLSNSKETGAFYGREFKTAKSLFLVLFLFA
LSWLPLSIINCIIYFNGEVPQLVLYMGILLSHANSMMNPIVYAYKIKKFKETYLLILKAC
VVCHPSDSLDTSIEKNSE
>sp|Q9NS82|AAA1_HUMAN Asc-type amino acid transporter 1 OS=Homo sapiens GN=SLC7A10 PE=2 SV=1
MAGHTQQPSGRGNPRPAPSPSPVPGTVPGASERVALKKEIGLLSACTIIIGNIIGSGIFI
SPKGVLEHSGSVGLALFVWVLGGGVTALGSLCYAELGVAIPKSGGDYAYVTEIFGGLAGF
LLLWSAVLIMYPTSLAVISMTFSNYVLQPVFPNCIPPTTASRVLSMACLMLLTWVNSSSV
RWATRIQDMFTGGKLLALSLIIGVGLLQIFQGHFEELRPSNAFAFWMTPSVGHLALAFLQ
GSFAFSGWNFLNYVTEEMVDARKNLPRAIFISIPLVTFVYTFTNIAYFTAMSPQELLSSN
AVAVTFGEKLLGYFSWVMPVSVALSTFGGINGYLFTYSRLCFSGAREGHLPSLLAMIHVR
HCTPIPALLVCCGATAVIMLVGDTYTLINYVSFINYLCYGVTILGLLLLRWRRPALHRPI
KVNLLIPVAYLVFWAFLLVFSFISEPMVCGVGVIIILTGVPIFFLGVFWRSKPKCVHRLT
ESMTHWGQELCFVVYPQDAPEEEENGPCPPSLLPATDKPSKPQ
>sp|Q7RTV5|AAED1_HUMAN Thioredoxin-like protein AAED1 OS=Homo sapiens GN=AAED1 PE=2 SV=1
MAAPAPVTRQVSGAAALVPAPSGPDSGQPLAAAVAELPVLDARGQRVPFGALFRERRAVV
VFVRHFLCYICKEYVEDLAKIPRSFLQEANVTLIVIGQSSYHHIEPFCKLTGYSHEIYVD
PEREIYKRLGMKRGEEIASSGQSPHIKSNLLSGSLQSLWRAVTGPLFDFQGDPAQQGGTL
ILGPGNNIHFIHRDRNRLDHKPINSVLQLVGVQHVNFTNRPSVIHV
>sp|Q9UDR5|AASS_HUMAN Alpha-aminoadipic semialdehyde synthase, mitochondrial OS=Homo sapiens GN=AASS PE=1 SV=1
MLQVHRTGLGRLGVSLSKGLHHKAVLAVRREDVNAWERRAPLAPKHIKGITNLGYKVLIQ
PSNRRAIHDKDYVKAGGILQEDISEACLILGVKRPPEEKLMSRKTYAFFSHTIKAQEANM
GLLDEILKQEIRLIDYEKMVDHRGVRVVAFGQWAGVAGMINILHGMGLRLLALGHHTPFM
HIGMAHNYRNSSQAVQAVRDAGYEISLGLMPKSIGPLTFVFTGTGNVSKGAQAIFNELPC
EYVEPHELKEVSQTGDLRKVYGTVLSRHHHLVRKTDAVYDPAEYDKHPERYISRFNTDIA
PYTTCLINGIYWEQNTPRLLTRQDAQSLLAPGKFSPAGVEGCPALPHKLVAICDISADTG
GSIEFMTECTTIEHPFCMYDADQHIIHDSVEGSGILMCSIDNLPAQLPIEATECFGDMLY
PYVEEMILSDATQPLESQNFSPVVRDAVITSNGTLPDKYKYIQTLRESRERAQSLSMGTR
RKVLVLGSGYISEPVLEYLSRDGNIEITVGSDMKNQIEQLGKKYNINPVSMDICKQEEKL
GFLVAKQDLVISLLPYVLHPLVAKACITNKVNMVTASYITPALKELEKSVEDAGITIIGE
LGLDPGLDHMLAMETIDKAKEVGATIESYISYCGGLPAPEHSNNPLRYKFSWSPVGVLMN
VMQSATYLLDGKVVNVAGGISFLDAVTSMDFFPGLNLEGYPNRDSTKYAEIYGISSAHTL
LRGTLRYKGYMKALNGFVKLGLINREALPAFRPEANPLTWKQLLCDLVGISPSSEHDVLK
EAVLKKLGGDNTQLEAAEWLGLLGDEQVPQAESILDALSKHLVMKLSYGPEEKDMIVMRD

SFGIRHPSGHLEHKTIDLVAYGDINGFSAMAKTVGLPTAMAAKMLLDGEIGAKGLMGPFS
KEIYGPILERIKAEGIIYTTQSTIKP
>sp|Q8NHS2|AATC2_HUMAN Putative aspartate aminotransferase, cytoplasmic 2 OS=Homo sapiens GN=GOT1L1 PE=2 SV=2
MPTLSVFMDVPLAHKLEGSLLKTYKQDDYPNKIFLAYRVCMTNEGHPWVSLVVQKTRLQI
SQDPSLNYEYLPTMGLKSFIQASLALLFGKHSQAIVENRVGGVHTVGDSGAFQLGVQFLR
AWHKDARIVYIISSQKELHGLVFQDMGFTVYEYSVWDPKKLCMDPDILLNVVEQIPHGCV
LVMGNIIDCKLTPSGWAKLMSMIKSKQIFPFFDIPCQGLYTSDLEEDTRILQYFVSQGFE
FFCSQSLSKNFGIYDEGVGMLVVVAVNNQQLLCVLSQLEGLAQALWLNPPNTGARVITSI
LCNPALLGEWKQSLKEVVENIMLTKEKVKEKLQLLGTPGSWGHITEQSGTHGYLGLNSQQ
VEYLVRKKHIYIPKNGQINFSCINANNINYITEGINEAVLLTESSEMCLPKEKKTLIGIK
L
>sp|Q7Z5R6|AB1IP_HUMAN Amyloid beta A4 precursor protein-binding family B member 1-interacting protein OS=Homo sapiens GN=APBB1IP PE=1 SV=1
MGESSEDIDQMFSTLLGEMDLLTQSLGVDTLPPPDPNPPRAEFNYSVGFKDLNESLNALE
DQDLDALMADLVADISEAEQRTIQAQKESLQNQHHSASLQASIFSGAASLGYGTNVAATG
ISQYEDDLPPPPADPVLDLPLPPPPPEPLSQEEEEAQAKADKIKLALEKLKEAKVKKLVV
KVHMNDNSTKSLMVDERQLARDVLDNLFEKTHCDCNVDWCLYEIYPELQIERFFEDHENV
VEVLSDWTRDTENKILFLEKEEKYAVFKNPQNFYLDNRGKKESKETNEKMNAKNKESLLE
ESFCGTSIIVPELEGALYLKEDGKKSWKRRYFLLRASGIYYVPKGKTKTSRDLACFIQFE
NVNIYYGTQHKMKYKAPTDYCFVLKHPQIQKESQYIKYLCCDDTRTLNQWVMGIRIAKYG
KTLYDNYQRAVAKAGLASRWTNLGTVNAAAPAQPSTGPKTGTTQPNGQIPQATHSVSAVL
QEAQRHAETSKDKKPALGNHHDPAVPRAPHAPKSSLPPPPPVRRSSDTSGSPATPLKAKG
TGGGGLPAPPDDFLPPPPPPPPLDDPELPPPPPDFMEPPPDFVPPPPPSYAGIAGSELPP
PPPPPPAPAPAPVPDSARPPPAVAKRPPVPPKRQENPGHPGGAGGGEQDFMSDLMKALQK
KRGNVS
>sp|Q9NP78|ABCB9_HUMAN ATP-binding cassette sub-family B member 9 OS=Homo sapiens GN=ABCB9 PE=1 SV=1
MRLWKAVVVTLAFMSVDICVTTAIYVFSHLDRSLLEDIRHFNIFDSVLDLWAACLYRSCL
LLGATIGVAKNSALGPRRLRASWLVITLVCLFVGIYAMVKLLLFSEVRRPIRDPWFWALF
VWTYISLGASFLLWWLLSTVRPGTQALEPGAATEAEGFPGSGRPPPEQASGATLQKLLSY
TKPDVAFLVAASFFLIVAALGETFLPYYTGRAIDGIVIQKSMDQFSTAVVIVCLLAIGSS
FAAGIRGGIFTLIFARLNIRLRNCLFRSLVSQETSFFDENRTGDLISRLTSDTTMVSDLV
SQNINVFLRNTVKVTGVVVFMFSLSWQLSLVTFMGFPIIMMVSNIYGKYYKRLSKEVQNA
LARASNTAEETISAMKTVRSFANEEEEAEVYLRKLQQVYKLNRKEAAAYMYYVWGSGLTL
LVVQVSILYYGGHLVISGQMTSGNLIAFIIYEFVLGDCMESVGSVYSGLMQGVGAAEKVF
EFIDRQPTMVHDGSLAPDHLEGRVDFENVTFTYRTRPHTQVLQNVSFSLSPGKVTALVGP
SGSGKSSCVNILENFYPLEGGRVLLDGKPISAYDHKYLHRVISLVSQEPVLFARSITDNI
SYGLPTVPFEMVVEAAQKANAHGFIMELQDGYSTETGEKGAQLSGGQKQRVAMARALVRN
PPVLILDEATSALDAESEYLIQQAIHGNLQKHTVLIIAHRLSTVEHAHLIVVLDKGRVVQ
QGTHQQLLAQGGLYAKLVQRQMLGLQPAADFTAGHNEPVANGSHKA
>sp|Q9NRK6|ABCBA_HUMAN ATP-binding cassette sub-family B member 10, mitochondrial OS=Homo sapiens GN=ABCB10 PE=1 SV=2
MRGPPAWPLRLLEPPSPAEPGRLLPVACVWAAASRVPGSLSPFTGLRPARLWGAGPALLW

GVGAARRWRSGCRGGGPGASRGVLGLARLLGLWARGPGSCRCGAFAGPGAPRLPRARFPG
GPAAAAWAGDEAWRRGPAAPPGDKGRLRPAAAGLPEARKLLGLAYPERRRLAAAVGFLTM
SSVISMSAPFFLGKIIDVIYTNPTVDYSDNLTRLCLGLSAVFLCGAAANAIRVYLMQTSG
QRIVNRLRTSLFSSILRQEVAFFDKTRTGELINRLSSDTALLGRSVTENLSDGLRAGAQA
SVGISMMFFVSPNLATFVLSVVPPVSIIAVIYGRYLRKLTKVTQDSLAQATQLAEERIGN
VRTVRAFGKEMTEIEKYASKVDHVMQLARKEAFARAGFFGATGLSGNLIVLSVLYKGGLL
MGSAHMTVGELSSFLMYAFWVGISIGGLSSFYSELMKGLGAGGRLWELLEREPKLPFNEG
VILNEKSFQGALEFKNVHFAYPARPEVPIFQDFSLSIPSGSVTALVGPSGSGKSTVLSLL
LRLYDPASGTISLDGHDIRQLNPVWLRSKIGTVSQEPILFSCSIAENIAYGADDPSSVTA
EEIQRVAEVANAVAFIRNFPQGFNTVVGEKGVLLSGGQKQRIAIARALLKNPKILLLDEA
TSALDAENEYLVQEALDRLMDGRTVLVIAHRLSTIKNANMVAVLDQGKITEYGKHEELLS
KPNGIYRKLMNKQSFISA
>sp|O60706|ABCC9_HUMAN ATP-binding cassette sub-family C member 9 OS=Homo sapiens GN=ABCC9 PE=1 SV=2
MSLSFCGNNISSYNINDGVLQNSCFVDALNLVPHVFLLFITFPILFIGWGSQSSKVQIHH
NTWLHFPGHNLRWILTFALLFVHVCEIAEGIVSDSRRESRHLHLFMPAVMGFVATTTSIV
YYHNIETSNFPKLLLALFLYWVMAFITKTIKLVKYCQSGLDISNLRFCITGMMVILNGLL
MAVEINVIRVRRYVFFMNPQKVKPPEDLQDLGVRFLQPFVNLLSKATYWWMNTLIISAHK
KPIDLKAIGKLPIAMRAVTNYVCLKDAYEEQKKKVADHPNRTPSIWLAMYRAFGRPILLS
STFRYLADLLGFAGPLCISGIVQRVNETQNGTNNTTGISETLSSKEFLENAYVLAVLLFL
ALILQRTFLQASYYVTIETGINLRGALLAMIYNKILRLSTSNLSMGEMTLGQINNLVAIE
TNQLMWFLFLCPNLWAMPVQIIMGVILLYNLLGSSALVGAAVIVLLAPIQYFIATKLAEA
QKSTLDYSTERLKKTNEILKGIKLLKLYAWEHIFCKSVEETRMKELSSLKTFALYTSLSI
FMNAAIPIAAVLATFVTHAYASGNNLKPAEAFASLSLFHILVTPLFLLSTVVRFAVKAII
SVQKLNEFLLSDEIGDDSWRTGESSLPFESCKKHTGVQPKTINRKQPGRYHLDSYEQSTR
RLRPAETEDIAIKVTNGYFSWGSGLATLSNIDIRIPTGQLTMIVGQVGCGKSSLLLAILG
EMQTLEGKVHWSNVNESEPSFEATRSRNRYSVAYAAQKPWLLNATVEENITFGSPFNKQR
YKAVTDACSLQPDIDLLPFGDQTEIGERGINLSGGQRQRICVARALYQNTNIVFLDDPFS
ALDIHLSDHLMQEGILKFLQDDKRTLVLVTHKLQYLTHADWIIAMKDGSVLREGTLKDIQ
TKDVELYEHWKTLMNRQDQELEKDMEADQTTLERKTLRRAMYSREAKAQMEDEDEEEEEE
EDEDDNMSTVMRLRTKMPWKTCWRYLTSGGFFLLILMIFSKLLKHSVIVAIDYWLATWTS
EYSINNTGKADQTYYVAGFSILCGAGIFLCLVTSLTVEWMGLTAAKNLHHNLLNKIILGP
IRFFDTTPLGLILNRFSADTNIIDQHIPPTLESLTRSTLLCLSAIGMISYATPVFLVALL
PLGVAFYFIQKYFRVASKDLQELDDSTQLPLLCHFSETAEGLTTIRAFRHETRFKQRMLE
LTDTNNIAYLFLSAANRWLEVRTDYLGACIVLTASIASISGSSNSGLVGLGLLYALTITN
YLNWVVRNLADLEVQMGAVKKVNSFLTMESENYEGTMDPSQVPEHWPQEGEIKIHDLCVR
YENNLKPVLKHVKAYIKPGQKVGICGRTGSGKSSLSLAFFRMVDIFDGKIVIDGIDISKL
PLHTLRSRLSIILQDPILFSGSIRFNLDPECKCTDDRLWEALEIAQLKNMVKSLPGGLDA
VVTEGGENFSVGQRQLFCLARAFVRKSSILIMDEATASIDMATENILQKVVMTAFADRTV
VTIAHRVSSIMDAGLVLVFSEGILVECDTVPNLLAHKNGLFSTLVMTNK
>sp|Q96J66|ABCCB_HUMAN ATP-binding cassette sub-family C member 11 OS=Homo sapiens GN=ABCC11 PE=1 SV=1
MTRKRTYWVPNSSGGLVNRGIDIGDDMVSGLIYKTYTLQDGPWSQQERNPEAPGRAAVPP
WGKYDAALRTMIPFRPKPRFPAPQPLDNAGLFSYLTVSWLTPLMIQSLRSRLDENTIPPL

SVHDASDKNVQRLHRLWEEEVSRRGIEKASVLLVMLRFQRTRLIFDALLGICFCIASVLG
PILIIPKILEYSEEQLGNVVHGVGLCFALFLSECVKSLSFSSSWIINQRTAIRFRAAVSS
FAFEKLIQFKSVIHITSGEAISFFTGDVNYLFEGVCYGPLVLITCASLVICSISSYFIIG
YTAFIAILCYLLVFPLAVFMTRMAVKAQHHTSEVSDQRIRVTSEVLTCIKLIKMYTWEKP
FAKIIEDLRRKERKLLEKCGLVQSLTSITLFIIPTVATAVWVLIHTSLKLKLTASMAFSM
LASLNLLRLSVFFVPIAVKGLTNSKSAVMRFKKFFLQESPVFYVQTLQDPSKALVFEEAT
LSWQQTCPGIVNGALELERNGHASEGMTRPRDALGPEEEGNSLGPELHKINLVVSKGMML
GVCGNTGSGKSSLLSAILEEMHLLEGSVGVQGSLAYVPQQAWIVSGNIRENILMGGAYDK
ARYLQVLHCCSLNRDLELLPFGDMTEIGERGLNLSGGQKQRISLARAVYSDRQIYLLDDP
LSAVDAHVGKHIFEECIKKTLRGKTVVLVTHQLQYLEFCGQIILLENGKICENGTHSELM
QKKGKYAQLIQKMHKEATSDMLQDTAKIAEKPKVESQALATSLEESLNGNAVPEHQLTQE
EEMEEGSLSWRVYHHYIQAAGGYMVSCIIFFFVVLIVFLTIFSFWWLSYWLEQGSGTNSS
RESNGTMADLGNIADNPQLSFYQLVYGLNALLLICVGVCSSGIFTKVTRKASTALHNKLF
NKVFRCPMSFFDTIPIGRLLNCFAGDLEQLDQLLPIFSEQFLVLSLMVIAVLLIVSVLSP
YILLMGAIIMVICFIYYMMFKKAIGVFKRLENYSRSPLFSHILNSLQGLSSIHVYGKTED
FISQFKRLTDAQNNYLLLFLSSTRWMALRLEIMTNLVTLAVALFVAFGISSTPYSFKVMA
VNIVLQLASSFQATARIGLETEAQFTAVERILQYMKMCVSEAPLHMEGTSCPQGWPQHGE
IIFQDYHMKYRDNTPTVLHGINLTIRGHEVVGIVGRTGSGKSSLGMALFRLVEPMAGRIL
IDGVDICSIGLEDLRSKLSVIPQDPVLLSGTIRFNLDPFDRHTDQQIWDALERTFLTKAI
SKFPKKLHTDVVENGGNFSVGERQLLCIARAVLRNSKIILIDEATASIDMETDTLIQRTI
REAFQGCTVLVIAHRVTTVLNCDHILVMGNGKVVEFDRPEVLRKKPGSLFAALMATATSS
LR
>sp|Q8NE71|ABCF1_HUMAN ATP-binding cassette sub-family F member 1 OS=Homo sapiens GN=ABCF1 PE=1 SV=2
MPKAPKQQPPEPEWIGDGESTSPSDKVVKKGKKDKKIKKTFFEELAVEDKQAGEEEKVLK
EKEQQQQQQQQQKKKRDTRKGRRKKDVDDDGEEKELMERLKKLSVPTSDEEDEVPAPKP
RGGKKTKGGNVFAALIQDQSEEEEEEEKHPPKPAKPEKNRINKAVSEEQQPALKGKKGKE
EKSKGKAKPQNKFAALDNEEEDKEEEIIKEKEPPKQGKEKAKKAEQGSEEEGEGEEEEEE
GGESKADDPYAHLSKKEKKKLKKQMEYERQVASLKAANAAENDFSVSQAEMSSRQAMLEN
ASDIKLEKFSISAHGKELFVNADLYIVAGRRYGLVGPNGKGKTTLLKHIANRALSIPPNI
DVLLCEQEVVADETPAVQAVLRADTKRLKLLEEERRLQGQLEQGDDTAAERLEKVYEELR
ATGAAAAEAKARRILAGLGFDPEMQNRPTQKFSGGWRMRVSLARALFMEPTLLMLDEPTN
HLDLNAVIWLNNYLQGWRKTLLIVSHDQGFLDDVCTDIIHLDAQRLHYYRGNYMTFKKMY
QQKQKELLKQYEKQEKKLKELKAGGKSTKQAEKQTKEALTRKQQKCRRKNQDEESQEAPE
LLKRPKEYTVRFTFPDPPPLSPPVLGLHGVTFGYQGQKPLFKNLDFGIDMDSRICIVGPN
GVGKSTLLLLLTGKLTPTHGEMRKNHRLKIGFFNQQYAEQLRMEETPTEYLQRGFNLPYQ
DARKCLGRFGLESHAHTIQICKLSGGQKARVVFAELACREPDVLILDEPTNNLDIESIDA
LGEAINEYKGAVIVVSHDARLITETNCQLWVVEEQSVSQIDGDFEDYKREVLEALGEVMV
SRPRE
>sp|Q9UNQ0|ABCG2_HUMAN ATP-binding cassette sub-family G member 2 OS=Homo sapiens GN=ABCG2 PE=1 SV=3
MSSSNVEVFIPVSQGNTNGFPATASNDLKAFTEGAVLSFHNICYRVKLKSGFLPCRKPVE
KEILSNINGIMKPGLNAILGPTGGGKSSLLDVLAARKDPSGLSGDVLINGAPRPANFKCN
SGYVVQDDVVMGTLTVRENLQFSAALRLATTMTNHEKNERINRVIQELGLDKVADSKVGT

QFIRGVSGGERKRTSIGMELITDPSILFLDEPTTGLDSSTANAVLLLLKRMSKQGRTIIF
SIHQPRYSIFKLFDSLTLLASGRLMFHGPAQEALGYFESAGYHCEAYNNPADFFLDIING
DSTAVALNREEDFKATEIIEPSKQDKPLIEKLAEIYVNSSFYKETKAELHQLSGGEKKKK
ITVFKEISYTTSFCHQLRWVSKRSFKNLLGNPQASIAQIIVTVVLGLVIGAIYFGLKNDS
TGIQNRAGVLFFLTTNQCFSSVSAVELFVVEKKLFIHEYISGYYRVSSYFLGKLLSDLLP
MRMLPSIIFTCIVYFMLGLKPKADAFFVMMFTLMMVAYSASSMALAIAAGQSVVSVATLL
MTICFVFMMIFSGLLVNLTTIASWLSWLQYFSIPRYGFTALQHNEFLGQNFCPGLNATGN
NPCNYATCTGEEYLVKQGIDLSPWGLWKNHVALACMIVIFLTIAYLKLLFLKKYS
>sp|Q9H221|ABCG8_HUMAN ATP-binding cassette sub-family G member 8 OS=Homo sapiens GN=ABCG8 PE=1 SV=1
MAGKAAEERGLPKGATPQDTSGLQDRLFSSESDNSLYFTYSGQPNTLEVRDLNYQVDLAS
QVPWFEQLAQFKMPWTSPSCQNSCELGIQNLSFKVRSGQMLAIIGSSGCGRASLLDVITG
RGHGGKIKSGQIWINGQPSSPQLVRKCVAHVRQHNQLLPNLTVRETLAFIAQMRLPRTFS
QAQRDKRVEDVIAELRLRQCADTRVGNMYVRGLSGGERRRVSIGVQLLWNPGILILDEPT
SGLDSFTAHNLVKTLSRLAKGNRLVLISLHQPRSDIFRLFDLVLLMTSGTPIYLGAAQHM
VQYFTAIGYPCPRYSNPADFYVDLTSIDRRSREQELATREKAQSLAALFLEKVRDLDDFL
WKAETKDLDEDTCVESSVTPLDTNCLPSPTKMPGAVQQFTTLIRRQISNDFRDLPTLLIH
GAEACLMSMTIGFLYFGHGSIQLSFMDTAALLFMIGALIPFNVILDVISKCYSERAMLYY
ELEDGLYTTGPYFFAKILGELPEHCAYIIIYGMPTYWLANLRPGLQPFLLHFLLVWLVVF
CCRIMALAAAALLPTFHMASFFSNALYNSFYLAGGFMINLSSLWTVPAWISKVSFLRWCF
EGLMKIQFSRRTYKMPLGNLTIAVSGDKILSVMELDSYPLYAIYLIVIGLSGGFMVLYYV
SLRFIKQKPSQDW
>sp|Q8TB40|ABHD4_HUMAN Protein ABHD4 OS=Homo sapiens GN=ABHD4 PE=2 SV=1
MADDLEQQSQGWLSSWLPTWRPTSMSQLKNVEARILQCLQNKFLARYVSLPNQNKIWTVT
VSPEQNDRTPLVMVHGFGGGVGLWILNMDSLSARRTLHTFDLLGFGRSSRPAFPRDPEGA
EDEFVTSIETWRETMGIPSMILLGHSLGGFLATSYSIKYPDRVKHLILVDPWGFPLRPTN
PSEIRAPPAWVKAVASVLGRSNPLAVLRVAGPWGPGLVQRFRPDFKRKFADFFEDDTISE
YIYHCNAQNPSGETAFKAMMESFGWARRPMLERIHLIRKDVPITMIYGSDTWIDTSTGKK
VKMQRPDSYVRDMEIKGASHHVYADQPHIFNAVVEEICDSVD
>sp|Q9NUJ1|ABHDA_HUMAN Mycophenolic acid acyl-glucuronide esterase, mitochondrial OS=Homo sapiens GN=ABHD10 PE=1 SV=1
MAVARLAAVAAWVPCRSWGWAAVPFGPHRGLSVLLARIPQRAPRWLPACRQKTSLSFLNR
PDLPNLAYKKLKGKSPGIIFIPGYLSYMNGTKALAIEEFCKSLGHACIRFDYSGVGSSDG
NSEESTLGKWRKDVLSIIDDLADGPQILVGSSLGGWLMLHAAIARPEKVVALIGVATAAD
TLVTKFNQLPVELKKEVEMKGVWSMPSKYSEEGVYNVQYSFIKEAEHHCLLHSPIPVNCP
IRLLHGMKDDIVPWHTSMQVADRVLSTDVDVILRKHSDHRMREKADIQLLVYTIDDLIDK
LSTIVN
>sp|Q9H3Z7|ABHGB_HUMAN Protein ABHD16B OS=Homo sapiens GN=ABHD16B PE=3 SV=1
MCVICFVKALVRVFKIYLTASYTYPFRGWPVAFRWDDVRAVGRSSSHRALTCAAAAAGVW
LLRDETLGGDALGRPPRGARSQAQCLLQQLRELPGQLASYALAHSLGRWLVYPGSVSLMT
RALLPLLQQGQERLVERYHGRRAKLVACDGNEIDTMFMDRRQHPGSHVHGPRLVICCEGN
AGFYEMGCLSAPLEAGYSVLGWNHPGFGSSTGVPFPQHDANAMDVVVEYALHRLHFPPAH
LVVYGWSVGGFTATWATMTYPELGALVLDATFDDLVPLALKVMPHSWKGLVVRTVREHFN
LNVAEQLCCYPGPVLLLRRTQDDVVSTSGRLRPLSPGDVEGNRGNELLLRLLEHRYPVVM

AREGRAVVTRWLRAGSLAQEAAFYARYRVDEDWCLALLRSYRARCEEELEGEEALGPHGP
AFPWLVGQGLSSRRRRRLALFLARKHLKNVEATHFSPLEPEEFQLPWRL
>sp|Q68CK6|ACS2B_HUMAN Acyl-coenzyme A synthetase ACSM2B, mitochondrial OS=Homo sapiens GN=ACSM2B PE=1 SV=2
MHWLRKVQGLCTLWGTQMSSRTLYINSRQLVSLQWGHQEVPAKFNFASDVLDHWADMEKA
GKRLPSPALWWVNGKGKELMWNFRELSENSQQAANILSGACGLQRGDRVAVMLPRVPEWW
LVILGCIRAGLIFMPGTIQMKSTDILYRLQMSKAKAIVAGDEVIQEVDTVASECPSLRIK
LLVSEKSCDGWLNFKKLLNEASTTHHCVETGSQEASAIYFTSGTSGLPKMAEHSYSSLGL
KAKMDAGWTGLQASDIMWTISDTGWILNILGSLLESWTLGACTFVHLLPKFDPLVILKTL
SSYPIKSMMGAPIVYRMLLQQDLSSYKFPHLQNCLAGGESLLPETLENWRAQTGLDIREF
YGQTETGLTCMVSKTMKIKPGYMGTAASCYDVQVIDDKGNVLPPGTEGDIGIRVKPIRPI
GIFSGYVENPDKTAANIRGDFWLLGDRGIKDEDGYFQFMGRADDIINSSGYRIGPSEVEN
ALMKHPAVVETAVISSPDPVRGEVVKAFVILASQFLSHDPEQLTKELQQHVKSVTAPYKY
PRKIEFVLNLPKTVTGKIQRTKLRDKEWKMSGKARAQ
>sp|P33121|ACSL1_HUMAN Long-chain-fatty-acid--CoA ligase 1 OS=Homo sapiens GN=ACSL1 PE=1 SV=1
MQAHELFRYFRMPELVDFRQYVRTLPTNTLMGFGAFAALTTFWYATRPKPLKPPCDLSMQ
SVEVAGSGGARRSALLDSDEPLVYFYDDVTTLYEGFQRGIQVSNNGPCLGSRKPDQPYEW
LSYKQVAELSECIGSALIQKGFKTAPDQFIGIFAQNRPEWVIIEQGCFAYSMVIVPLYDT
LGNEAITYIVNKAELSLVFVDKPEKAKLLLEGVENKLIPGLKIIVVMDAYGSELVERGQR
CGVEVTSMKAMEDLGRANRRKPKPPAPEDLAVICFTSGTTGNPKGAMVTHRNIVSDCSAF
VKATENTVNPCPDDTLISFLPLAHMFERVVECVMLCHGAKIGFFQGDIRLLMDDLKVLQP
TVFPVVPRLLNRMFDRIFGQANTTLKRWLLDFASKRKEAELRSGIIRNNSLWDRLIFHKV
QSSLGGRVRLMVTGAAPVSATVLTFLRAALGCQFYEGYGQTECTAGCCLTMPGDWTAGHV
GAPMPCNLIKLVDVEEMNYMAAEGEGEVCVKGPNVFQGYLKDPAKTAEALDKDGWLHTGD
IGKWLPNGTLKIIDRKKHIFKLAQGEYIAPEKIENIYMRSEPVAQVFVHGESLQAFLIAI
VVPDVETLCSWAQKRGFEGSFEELCRNKDVKKAILEDMVRLGKDSGLKPFEQVKGITLHP
ELFSIDNGLLTPTMKAKRPELRNYFRSQIDDLYSTIKV
>sp|O60488|ACSL4_HUMAN Long-chain-fatty-acid--CoA ligase 4 OS=Homo sapiens GN=ACSL4 PE=1 SV=2
MKLKLNVLTIILLPVHLLITIYSALIFIPWYFLTNAKKKNAMAKRIKAKPTSDKPGSPYR
SVTHFDSLAVIDIPGADTLDKLFDHAVSKFGKKDSLGTREILSEENEMQPNGKVFKKLIL
GNYKWMNYLEVNRRVNNFGSGLTALGLKPKNTIAIFCETRAEWMIAAQTCFKYNFPLVTL
YATLGKEAVVHGLNESEASYLITSVELLESKLKTALLDISCVKHIIYVDNKAINKAEYPE
GFEIHSMQSVEELGSNPENLGIPPSRPTPSDMAIVMYTSGSTGRPKGVMMHHSNLIAGMT
GQCERIPGLGPKDTYIGYLPLAHVLELTAEISCFTYGCRIGYSSPLTLSDQSSKIKKGSK
GDCTVLKPTLMAAVPEIMDRIYKNVMSKVQEMNYIQKTLFKIGYDYKLEQIKKGYDAPLC
NLLLFKKVKALLGGNVRMMLSGGAPLSPQTHRFMNVCFCCPIGQGYGLTESCGAGTVTEV
TDYTTGRVGAPLICCEIKLKDWQEGGYTINDKPNPRGEIVIGGQNISMGYFKNEEKTAED
YSVDENGQRWFCTGDIGEFHPDGCLQIIDRKKDLVKLQAGEYVSLGKVEAALKNCPLIDN
ICAFAKSDQSYVISFVVPNQKRLTLLAQQKGVEGTWVDICNNPAMEAEILKEIREAANAM
KLERFEIPIKVRLSPEPWTPETGLVTDAFKLKRKELRNHYLKDIERMYGGK
>sp|Q9H6R3|ACSS3_HUMAN Acyl-CoA synthetase short-chain family member 3, mitochondrial OS=Homo sapiens GN=ACSS3 PE=1 SV=1

MKPSWLQCRKVTSAGGLGGPLPGSSPARGAGAALRALVVPGPRGGLGGRGCRALSSGSGS
EYKTHFAASVTDPERFWGKAAEQISWYKPWTKTLENKHSPSTRWFVEGMLNICYNAVDRH
IENGKGDKIAIIYDSPVTNTKATFTYKEVLEQVSKLAGVLVKHGIKKGDTVVIYMPMIPQ
AMYTMLACARIGAIHSLIFGGFASKELSSRIDHVKPKVVVTASFGIEPGRRVEYVPLVEE
ALKIGQHKPDKILIYNRPNMEAVPLAPGRDLDWDEEMAKAQSHDCVPVLSEHPLYILYTS
GTTGLPKGVIRPTGGYAVMLHWSMSSIYGLQPGEVWWAASDLGWVVGHSYICYGPLLHGN
TTVLYEGKPVGTPDAGAYFRVLAEHGVAALFTAPTAIRAIRQQDPGAALGKQYSLTRFKT
LFVAGERCDVETLEWSKNVFRVPVLDHWWQTETGSPITASCVGLGNSKTPPPGQAGKSVP
GYNVMILDDNMQKLKARCLGNIVVKLPLPPGAFSGLWKNQEAFKHLYFEKFPGYYDTMDA
GYMDEEGYLYVMSRVDDVINVAGHRISAGAIEESILSHGTVADCAVVGKEDPLKGHVPLA
LCVLRKDINATEEQVLEEIVKHVRQNIGPVAAFRNAVFVKQLPKTRSGKIPRSALSAIVN
GKPYKITSTIEDPSIFGHVEEMLKQA
>sp|Q562R1|ACTBL_HUMAN Beta-actin-like protein 2 OS=Homo sapiens GN=ACTBL2 PE=1 SV=2
MTDNELSALVVDNGSGMCKAGFGGDDAPRAVFPSMIGRPRHQGVMVGMGQKDCYVGDEAQ
SKRGVLTLKYPIEHGVVTNWDDMEKIWYHTFYNELRVAPDEHPILLTEAPLNPKINREKM
TQIMFEAFNTPAMYVAIQAVLSLYASGRTTGIVMDSGDGVTHIVPIYEGYALPHAILRLD
LAGRDLTDYLMKILTERGYNFTTTAEREIVRDVKEKLCYVALDFEQEMVRAAASSSPERS
YELPDGQVITIGNERFRCPEAIFQPSFLGIESSGIHETTFNSIMKCDVDIRKDLYANTVL
SGGSTMYPGIADRMQKEIITLAPSTMKIKIIAPPERKYSVWIGGSILASLSTFQQMWISK
QEYDEAGPPIVHRKCF
>sp|P68032|ACTC_HUMAN Actin, alpha cardiac muscle 1 OS=Homo sapiens GN=ACTC1 PE=1 SV=1
MCDDEETTALVCDNGSGLVKAGFAGDDAPRAVFPSIVGRPRHQGVMVGMGQKDSYVGDEA
QSKRGILTLKYPIEHGIITNWDDMEKIWHHTFYNELRVAPEEHPTLLTEAPLNPKANREK
MTQIMFETFNVPAMYVAIQAVLSLYASGRTTGIVLDSGDGVTHNVPIYEGYALPHAIMRL
DLAGRDLTDYLMKILTERGYSFVTTAEREIVRDIKEKLCYVALDFENEMATAASSSSLEK
SYELPDGQVITIGNERFRCPETLFQPSFIGMESAGIHETTYNSIMKCDIDIRKDLYANNV
LSGGTTMYPGIADRMQKEITALAPSTMKIKIIAPPERKYSVWIGGSILASLSTFQQMWIS
KQEYDEAGPSIVHRKCF
>sp|P12814|ACTN1_HUMAN Alpha-actinin-1 OS=Homo sapiens GN=ACTN1 PE=1 SV=2
MDHYDSQQTNDYMQPEEDWDRDLLLDPAWEKQQRKTFTAWCNSHLRKAGTQIENIEEDFR
DGLKLMLLLEVISGERLAKPERGKMRVHKISNVNKALDFIASKGVKLVSIGAEEIVDGNV
KMTLGMIWTIILRFAIQDISVEETSAKEGLLLWCQRKTAPYKNVNIQNFHISWKDGLGFC
ALIHRHRPELIDYGKLRKDDPLTNLNTAFDVAEKYLDIPKMLDAEDIVGTARPDEKAIMT
YVSSFYHAFSGAQKAETAANRICKVLAVNQENEQLMEDYEKLASDLLEWIRRTIPWLENR
VPENTMHAMQQKLEDFRDYRRLHKPPKVQEKCQLEINFNTLQTKLRLSNRPAFMPSEGRM
VSDINNAWGCLEQVEKGYEEWLLNEIRRLERLDHLAEKFRQKASIHEAWTDGKEAMLRQK
DYETATLSEIKALLKKHEAFESDLAAHQDRVEQIAAIAQELNELDYYDSPSVNARCQKIC
DQWDNLGALTQKRREALERTEKLLETIDQLYLEYAKRAAPFNNWMEGAMEDLQDTFIVHT
IEEIQGLTTAHEQFKATLPDADKERLAILGIHNEVSKIVQTYHVNMAGTNPYTTITPQEI
NGKWDHVRQLVPRRDQALTEEHARQQHNERLRKQFGAQANVIGPWIQTKMEEIGRISIEM
HGTLEDQLSHLRQYEKSIVNYKPKIDQLEGDHQLIQEALIFDNKHTNYTMEHIRVGWEQL
LTTIARTINEVENQILTRDAKGISQEQMNEFRASFNHFDRDHSGTLGPEEFKACLISLGY
DIGNDPQGEAEFARIMSIVDPNRLGVVTFQAFIDFMSRETADTDTADQVMASFKILAGDK
NYITMDELRRELPPDQAEYCIARMAPYTGPDSVPGALDYMSFSTALYGESDL

>sp|P35609|ACTN2_HUMAN Alpha-actinin-2 OS=Homo sapiens GN=ACTN2 PE=1 SV=1
MNQIEPGVQYNYVYDEDEYMIQEEEWDRDLLLDPAWEKQQRKTFTAWCNSHLRKAGTQIE
NIEEDFRNGLKLMLLLEVISGERLPKPDRGKMRFHKIANVNKALDYIASKGVKLVSIGAE
EIVDGNVKMTLGMIWTIILRFAIQDISVEETSAKEGLLLWCQRKTAPYRNVNIQNFHTSW
KDGLGLCALIHRHRPDLIDYSKLNKDDPIGNINLAMEIAEKHLDIPKMLDAEDIVNTPKP
DERAIMTYVSCFYHAFAGAEQAETAANRICKVLAVNQENERLMEEYERLASELLEWIRRT
IPWLENRTPEKTMQAMQKKLEDFRDYRRKHKPPKVQEKCQLEINFNTLQTKLRISNRPAF
MPSEGKMVSDIAGAWQRLEQAEKGYEEWLLNEIRRLERLEHLAEKFRQKASTHETWAYGK
EQILLQKDYESASLTEVRALLRKHEAFESDLAAHQDRVEQIAAIAQELNELDYHDAVNVN
DRCQKICDQWDRLGTLTQKRREALERMEKLLETIDQLHLEFAKRAAPFNNWMEGAMEDLQ
DMFIVHSIEEIQSLITAHEQFKATLPEADGERQSIMAIQNEVEKVIQSYNIRISSSNPYS
TVTMDELRTKWDKVKQLVPIRDQSLQEELARQHANERLRRQFAAQANAIGPWIQNKMEEI
ARSSIQITGALEDQMNQLKQYEHNIINYKNNIDKLEGDHQLIQEALVFDNKHTNYTMEHI
RVGWELLLTTIARTINEVETQILTRDAKGITQEQMNEFRASFNHFDRRKNGLMDHEDFRA
CLISMGYDLGEAEFARIMTLVDPNGQGTVTFQSFIDFMTRETADTDTAEQVIASFRILAS
DKPYILAEELRRELPPDQAQYCIKRMPAYSGPGSVPGALDYAAFSSALYGESDL
>sp|O43707|ACTN4_HUMAN Alpha-actinin-4 OS=Homo sapiens GN=ACTN4 PE=1 SV=2
MVDYHAANQSYQYGPSSAGNGAGGGGSMGDYMAQEDDWDRDLLLDPAWEKQQRKTFTAWC
NSHLRKAGTQIENIDEDFRDGLKLMLLLEVISGERLPKPERGKMRVHKINNVNKALDFIA
SKGVKLVSIGAEEIVDGNAKMTLGMIWTIILRFAIQDISVEETSAKEGLLLWCQRKTAPY
KNVNVQNFHISWKDGLAFNALIHRHRPELIEYDKLRKDDPVTNLNNAFEVAEKYLDIPKM
LDAEDIVNTARPDEKAIMTYVSSFYHAFSGAQKAETAANRICKVLAVNQENEHLMEDYEK
LASDLLEWIRRTIPWLEDRVPQKTIQEMQQKLEDFRDYRRVHKPPKVQEKCQLEINFNTL
QTKLRLSNRPAFMPSEGKMVSDINNGWQHLEQAEKGYEEWLLNEIRRLERLDHLAEKFRQ
KASIHEAWTDGKEAMLKHRDYETATLSDIKALIRKHEAFESDLAAHQDRVEQIAAIAQEL
NELDYYDSHNVNTRCQKICDQWDALGSLTHSRREALEKTEKQLEAIDQLHLEYAKRAAPF
NNWMESAMEDLQDMFIVHTIEEIEGLISAHDQFKSTLPDADREREAILAIHKEAQRIAES
NHIKLSGSNPYTTVTPQIINSKWEKVQQLVPKRDHALLEEQSKQQSNEHLRRQFASQANV
VGPWIQTKMEEIGRISIEMNGTLEDQLSHLKQYERSIVDYKPNLDLLEQQHQLIQEALIF
DNKHTNYTMEHIRVGWEQLLTTIARTINEVENQILTRDAKGISQEQMQEFRASFNHFDKD
HGGALGPEEFKACLISLGYDVENDRQGEAEFNRIMSLVDPNHSGLVTFQAFIDFMSRETT
DTDTADQVIASFKVLAGDKNFITAEELRRELPPDQAEYCIARMAPYQGPDAVPGALDYKS
FSTALYGESDL
>sp|P37023|ACVL1_HUMAN Serine/threonine-protein kinase receptor R3 OS=Homo sapiens GN=ACVRL1 PE=1 SV=2
MTLGSPRKGLLMLLMALVTQGDPVKPSRGPLVTCTCESPHCKGPTCRGAWCTVVLVREEG
RHPQEHRGCGNLHRELCRGRPTEFVNHYCCDSHLCNHNVSLVLEATQPPSEQPGTDGQLA
LILGPVLALLALVALGVLGLWHVRRRQEKQRGLHSELGESSLILKASEQGDSMLGDLLDS
DCTTGSGSGLPFLVQRTVARQVALVECVGKGRYGEVWRGLWHGESVAVKIFSSRDEQSWF
RETEIYNTVLLRHDNILGFIASDMTSRNSSTQLWLITHYHEHGSLYDFLQRQTLEPHLAL
RLAVSAACGLAHLHVEIFGTQGKPAIAHRDFKSRNVLVKSNLQCCIADLGLAVMHSQGSD
YLDIGNNPRVGTKRYMAPEVLDEQIRTDCFESYKWTDIWAFGLVLWEIARRTIVNGIVED
YRPPFYDVVPNDPSFEDMKKVVCVDQQTPTIPNRLAADPVLSGLAQMMRECWYPNPSARL
TALRIKKTLQKISNSPEKPKVIQ

>sp|Q9H013|ADA19_HUMAN Disintegrin and metalloproteinase domain-containing protein 19 OS=Homo sapiens GN=ADAM19 PE=1 SV=3
MPGGAGAARLCLLAFALQPLRPRAAREPGWTRGSEEGSPKLQHELIIPQWKTSESPVREK
HPLKAELRVMAEGRELILDLEKNEQLFAPSYTETHYTSSGNPQTTTRKLEDHCFYHGTVR
ETELSSVTLSTCRGIRGLITVSSNLSYVIEPLPDSKGQHLIYRSEHLKPPPGNCGFEHSK
PTTRDWALQFTQQTKKRPRRMKREDLNSMKYVELYLVADYLEFQKNRRDQDATKHKLIEI
ANYVDKFYRSLNIRIALVGLEVWTHGNMCEVSENPYSTLWSFLSWRRKLLAQKYHDNAQL
ITGMSFHGTTIGLAPLMAMCSVYQSGGVNMDHSENAIGVAATMAHEMGHNFGMTHDSADC
CSASAADGGCIMAAATGHPFPKVFNGCNRRELDRYLQSGGGMCLSNMPDTRMLYGGRRCG
NGYLEDGEECDCGEEEECNNPCCNASNCTLRPGAECAHGSCCHQCKLLAPGTLCREQARQ
CDLPEFCTGKSPHCPTNFYQMDGTPCEGGQAYCYNGMCLTYQEQCQQLWGPGARPAPDLC
FEKVNVAGDTFGNCGKDMNGEHRKCNMRDAKCGKIQCQSSEARPLESNAVPIDTTIIMNG
RQIQCRGTHVYRGPEEEGDMLDPGLVMTGTKCGYNHICFEGQCRNTSFFETEGCGKKCNG
HGVCNNNQNCHCLPGWAPPFCNTPGHGGSIDSGPMPPESVGPVVAGVLVAILVLAVLMLM
YYCCRQNNKLGQLKPSALPSKLRQQFSCPFRVSQNSGTGHANPTFKLQTPQGKRKVINTP
EILRKPSQPPPRPPPDYLRGGSPPAPLPAHLSRAARNSPGPGSQIERTESSRRPPPSRPI
PPAPNCIVSQDFSRPRPPQKALPANPVPGRRSLPRPGGASPLRPPGAGPQQSRPLAALAP
KVSPREALKVKAGTRGLQGGRCRVEKTKQFMLLVVWTELPEQKPRAKHSCFLVPA
>sp|Q13541|4EBP1_HUMAN Eukaryotic translation initiation factor 4E-binding protein 1 OS=Homo sapiens GN=EIF4EBP1 PE=1 SV=3
MSGGSSCSQTPSRAIPATRRVVLGDGVQLPPGDYSTTPGGTLFSTTPGGTRIIYDRKFLM
ECRNSPVTKTPPRDLPTIPGVTSPSSDEPPMEASQSHLRNSPEDKRAGGEESQFEMDI
>sp|P28566|5HT1E_HUMAN 5-hydroxytryptamine receptor 1E OS=Homo sapiens GN=HTR1E PE=1 SV=1
MNITNCTTEASMAIRPKTITEKMLICMTLVVITTLTTLLNLAVIMAIGTTKKLHQPANYL
ICSLAVTDLLVAVLVMPLSIIYIVMDRWKLGYFLCEVWLSVDMTCCTCSILHLCVIALDR
YWAITNAIEYARKRTAKRAALMILTVWTISIFISMPPLFWRSHRRLSPPPSQCTIQHDHV
IYTIYSTLGAFYIPLTLILILYYRIYHAAKSLYQKRGSSRHLSNRSTDSQNSFASCKLTQ
TFCVSDFSTSDPTTEFEKFHASIRIPPFDNDLDHPGERQQISSTRERKAARILGLILGAF
ILSWLPFFIKELIVGLSIYTVSSEVADFLTWLGYVNSLINPLLYTSFNEDFKLAFKKLIR
CREHT
>sp|Q8WXA8|5HT3C_HUMAN 5-hydroxytryptamine receptor 3C OS=Homo sapiens GN=HTR3C PE=1 SV=2
MEGGWPARQSALLCLTVSLLLQGRGDAFTINCSGFDQHGVDPAVFQAVFDRKAFRPFTNY
SIPTRVNISFTLSAILGVDAQLQLLTSFLWMDLVWDNPFINWNPKECVGINKLTVLAENL
WLPDIFIVESMDVDQTPSGLTAYISSEGRIKYDKPMRVTSICNLDIFYFPFDQQNCTFTF
SSFLYTVDSMLLGMDKEVWEITDTSRKVIQTQGEWELLGINKATPKMSMGNNLYDQIMFY
VAIRRRPSLYIINLLVPSSFLVAIDALSFYLPAESENRAPFKITLLLGYNVFLLMMNDLL
PASGTPLISVYFALCLSLMVVSLLETVFITYLLHVATTQPPPMPRWLHSLLLHCTSPGRC
CPTAPQKGNKGLGLTLTHLPGPKEPGELAGKKLGPRETEPDGGSGWTKTQLMELWVQFSH
AMDTLLFRLYLLFMASSILTVIVLWNT
>sp|A5X5Y0|5HT3E_HUMAN 5-hydroxytryptamine receptor 3E OS=Homo sapiens GN=HTR3E PE=1 SV=1
MEGSWFHRKRFSFYLLLGFLLQGRGVTFTINCSGFGQHGADPTALNSVFNRKPFRPVTNI
SVPTQVNISFAMSAILDVNEQLHLLSSFLWLEMVWDNPFISWNPEECEGITKMSMAAKNL
WLPDIFIIELMDVDKTPKGLTAYVSNEGRIRYKKPMKVDSICNLDIFYFPFDQQNCTLTF
SSFLYTVDSMLLDMEKEVWEITDASRNILQTHGEWELLGLSKATAKLSRGGNLYDQIVFY

VAIRRRPSLYVINLLVPSGFLVAIDALSFYLPVKSGNRVPFKITLLLGYNVFLLMMSDLL
PTSGTPLIGVYFALCLSLMVGSLLETIFITHLLHVATTQPPPLPRWLHSLLLHCNSPGRC
CPTAPQKENKGPGLTPTHLPGVKEPEVSAGQMPGPAEAELTGGSEWTRAQREHEAQKQHS
VELWLQFSHAMDAMLFRLYLLFMASSIITVICLWNT
>sp|P0DKL9|A14EL_HUMAN ARL14 effector protein-like OS=Homo sapiens GN=ARL14EPL PE=4 SV=1
MNEQSEKNNSIQERHTDHSFPEKNCQIGQKQLQQIERQLKCLAFRNPGPQVADFNPETRQ
QKKKARMSKMNEYFSTKYKIMRKYDKSGRLICNDADLCDCLEKNCLGCFYPCPKCNSNKC
GPECRCNRRWVYDAIVTESGEVISTLPFNVPD
>sp|P01009|A1AT_HUMAN Alpha-1-antitrypsin OS=Homo sapiens GN=SERPINA1 PE=1 SV=3
MPSSVSWGILLLAGLCCLVPVSLAEDPQGDAAQKTDTSHHDQDHPTFNKITPNLAEFAFS
LYRQLAHQSNSTNIFFSPVSIATAFAMLSLGTKADTHDEILEGLNFNLTEIPEAQIHEGF
QELLRTLNQPDSQLQLTTGNGLFLSEGLKLVDKFLEDVKKLYHSEAFTVNFGDTEEAKKQ
INDYVEKGTQGKIVDLVKELDRDTVFALVNYIFFKGKWERPFEVKDTEEEDFHVDQVTTV
KVPMMKRLGMFNIQHCKKLSSWVLLMKYLGNATAIFFLPDEGKLQHLENELTHDIITKFL
ENEDRRSASLHLPKLSITGTYDLKSVLGQLGITKVFSNGADLSGVTEEAPLKLSKAVHKA
VLTIDEKGTEAAGAMFLEAIPMSIPPEVKFNKPFVFLMIEQNTKSPLFMGKVVNPTQK
>sp|Q8NF67|A2012_HUMAN Putative ankyrin repeat domain-containing protein 20A12 pseudogene OS=Homo sapiens GN=ANKRD20A12P PE=5 SV=3
MKEMYENAEDKVNNSTGKWSCVEERICHLQHENPCIEQQLDDVHQKEDHKEIVTNIQRGF
IESGKKDLMLEEKNKKLMNECDHLKESLFQYEREKAERVVVVRQLQQEAADSLKKLTMLE
SPLEGISHYHINLDETQVPKKKLFQVESQFDDLMVEKEAVSSKCVNLAKENQVFQQKLLS
MKKVQQECEKLEEDKKMLEEEILNLKTHMENSMVELSKLQEYKSELDERAMQAVEKLEEI
HLQEQAQYKKQLEQLNKDIIQLH
>sp|Q9NPC4|A4GAT_HUMAN Lactosylceramide 4-alpha-galactosyltransferase OS=Homo sapiens GN=A4GALT PE=2 SV=1
MSKPPDLLLRLLRGAPRQRVCTLFIIGFKFTFFVSIMIYWHVVGEPKEKGQLYNLPAEIP
CPTLTPPTPPSHGPTPGNIFFLETSDRTNPNFLFMCSVESAARTHPESHVLVLMKGLPGG
NASLPRHLGISLLSCFPNVQMLPLDLRELFRDTPLADWYAAVQGRWEPYLLPVLSDASRI
ALMWKFGGIYLDTDFIVLKNLRNLTNVLGTQSRYVLNGAFLAFERRHEFMALCMRDFVDH
YNGWIWGHQGPQLLTRVFKKWCSIRSLAESRACRGVTTLPPEAFYPIPWQDWKKYFEDIN
PEELPRLLSATYAVHVWNKKSQGTRFEATSRALLAQLHARYCPTTHEAMKMYL
>sp|Q96AP0|ACD_HUMAN Adrenocortical dysplasia protein homolog OS=Homo sapiens GN=ACD PE=1 SV=3
MPGRCQSDAAMRVNGPASRAPAGWTSGSLHTGPRAGRPRAQARGVRGRGLLLRPRPAKEL
PLPRKGGAWAPAGNPGPLHPLGVAVGMAGSGRLVLRPWIRELILGSETPSSPRAGQLLEV
LQDAEAAVAGPSHAPDTSDVGATLLVSDGTHSVRCLVTREALDTSDWEEKEFGFRGTEGR
LLLLQDCGVHVQVAEGGAPAEFYLQVDRFSLLPTEQPRLRVPGCNQDLDVQKKLYDCLEE
HLSESTSSNAGLSLSQLLDEMREDQEHQGALVCLAESCLTLEGPCTAPPVTHWAASRCKA
TGEAVYTVPSSMLCISENDQLILSSLGPCQRTQGPELPPPDPALQDLSLTLIASPPSSPS
SSGTPALPGHMSSEESGTSISLLPALSLAAPDPGQRSSSQPSPAICSAPATLTPRSPHAS
RTPSSPLQSCTPSLSPRSHVPSPHQALVTRPQKPSLEFKEFVGLPCKNRPPFPRTGATRG
AQEPCSVWEPPKRHRDGSAFQYEYEPPCTSLCARVQAVRLPPQLMAWALHFLMDAQPGSE
PTPM
>sp|Q9BYF1|ACE2_HUMAN Angiotensin-converting enzyme 2 OS=Homo sapiens GN=ACE2 PE=1 SV=2

MSSSSWLLLSLVAVTAAQSTIEEQAKTFLDKFNHEAEDLFYQSSLASWNYNTNITEENVQ
NMNNAGDKWSAFLKEQSTLAQMYPLQEIQNLTVKLQLQALQQNGSSVLSEDKSKRLNTIL
NTMSTIYSTGKVCNPDNPQECLLLEPGLNEIMANSLDYNERLWAWESWRSEVGKQLRPLY
EEYVVLKNEMARANHYEDYGDYWRGDYEVNGVDGYDYSRGQLIEDVEHTFEEIKPLYEHL
HAYVRAKLMNAYPSYISPIGCLPAHLLGDMWGRFWTNLYSLTVPFGQKPNIDVTDAMVDQ
AWDAQRIFKEAEKFFVSVGLPNMTQGFWENSMLTDPGNVQKAVCHPTAWDLGKGDFRILM
CTKVTMDDFLTAHHEMGHIQYDMAYAAQPFLLRNGANEGFHEAVGEIMSLSAATPKHLKS
IGLLSPDFQEDNETEINFLLKQALTIVGTLPFTYMLEKWRWMVFKGEIPKDQWMKKWWEM
KREIVGVVEPVPHDETYCDPASLFHVSNDYSFIRYYTRTLYQFQFQEALCQAAKHEGPLH
KCDISNSTEAGQKLFNMLRLGKSEPWTLALENVVGAKNMNVRPLLNYFEPLFTWLKDQNK
NSFVGWSTDWSPYADQSIKVRISLKSALGDKAYEWNDNEMYLFRSSVAYAMRQYFLKVKN
QMILFGEEDVRVANLKPRISFNFFVTAPKNVSDIIPRTEVEKAIRMSRSRINDAFRLNDN
SLEFLGIQPTLGPPNQPPVSIWLIVFGVVMGVIVVGIVILIFTGIRDRKKKNKARSGENP
YASIDISKGENNPGFQNTDDVQTSF
>sp|Q9NUN7|ACER3_HUMAN Alkaline ceramidase 3 OS=Homo sapiens GN=ACER3 PE=1 SV=3
MAPAADREGYWGPTTSTLDWCEENYSVTWYIAEFWNTVSNLIMIIPPMFGAVQSVRDGLE
KRYIASYLALTVVGMGSWCFHMTLKYEMQLLDELPMIYSCCIFVYCMFECFKIKNSVNYH
LLFTLVLFSLIVTTVYLKVKEPIFHQVMYGMLVFTLVLRSIYIVTWVYPWLRGLGYTSLG
IFLLGFLFWNIDNIFCESLRNFRKKVPPIIGITTQFHAWWHILTGLGSYLHILFSLYTRT
LYLRYRPKVKFLFGIWPVILFEPLRKH
>sp|Q05901|ACHB3_HUMAN Neuronal acetylcholine receptor subunit beta-3 OS=Homo sapiens GN=CHRNB3 PE=2 SV=2
MLPDFMLVLIVLGIPSSATTGFNSIAENEDALLRHLFQGYQKWVRPVLHSNDTIKVYFGL
KISQLVDVDEKNQLMTTNVWLKQEWTDHKLRWNPDDYGGIHSIKVPSESLWLPDIVLFEN
ADGRFEGSLMTKVIVKSNGTVVWTPPASYKSSCTMDVTFFPFDRQNCSMKFGSWTYDGTM
VDLILINENVDRKDFFDNGEWEILNAKGMKGNRRDGVYSYPFITYSFVLRRLPLFYTLFL
IIPCLGLSFLTVLVFYLPSDEGEKLSLSTSVLVSLTVFLLVIEEIIPSSSKVIPLIGEYL
LFIMIFVTLSIIVTVFVINVHHRSSSTYHPMAPWVKRLFLQKLPKLLCMKDHVDRYSSPE
KEESQPVVKGKVLEKKKQKQLSDGEKVLVAFLEKAADSIRYISRHVKKEHFISQVVQDWK
FVAQVLDRIFLWLFLIVSVTGSVLIFTPALKMWLHSYH
>sp|Q07001|ACHD_HUMAN Acetylcholine receptor subunit delta OS=Homo sapiens GN=CHRND PE=1 SV=1
MEGPVLTLGLLAALAVCGSWGLNEEERLIRHLFQEKGYNKELRPVAHKEESVDVALALTL
SNLISLKEVEETLTTNVWIEHGWTDNRLKWNAEEFGNISVLRLPPDMVWLPEIVLENNND
GSFQISYSCNVLVYHYGFVYWLPPAIFRSSCPISVTYFPFDWQNCSLKFSSLKYTAKEIT
LSLKQDAKENRTYPVEWIIIDPEGFTENGEWEIVHRPARVNVDPRAPLDSPSRQDITFYL
IIRRKPLFYIINILVPCVLISFMVNLVFYLPADSGEKTSVAISVLLAQSVFLLLISKRLP
ATSMAIPLIGKFLLFGMVLVTMVVVICVIVLNIHFRTPSTHVLSEGVKKLFLETLPELLH
MSRPAEDGPSPGALVRRSSSLGYISKAEEYFLLKSRSDLMFEKQSERHGLARRLTTARRP
PASSEQAQQELFNELKPAVDGANFIVNHMRDQNNYNEEKDSWNRVARTVDRLCLFVVTPV
MVVGTAWIFLQGVYNQPPPQPFPGDPYSYNVQDKRFI
>sp|P07510|ACHG_HUMAN Acetylcholine receptor subunit gamma OS=Homo sapiens GN=CHRNG PE=1 SV=2
MHGGQGPLLLLLLLAVCLGAQGRNQEERLLADLMQNYDPNLRPAERDSDVVNVSLKLTLT

NLISLNEREEALTTNVWIEMQWCDYRLRWDPRDYEGLWVLRVPSTMVWRPDIVLENNVDG
VFEVALYCNVLVSPDGCIYWLPPAIFRSACSISVTYFPFDWQNCSLIFQSQTYSTNEIDL
QLSQEDGQTIEWIFIDPEAFTENGEWAIQHRPAKMLLDPAAPAQEAGHQKVVFYLLIQRK
PLFYVINIIAPCVLISSVAILIHFLPAKAGGQKCTVAINVLLAQTVFLFLVAKKVPETSQ
AVPLISKYLTFLLVVTILIVVNAVVVLNVSLRSPHTHSMARGVRKVFLRLLPQLLRMHVR
PLAPAAVQDTQSRLQNGSSGWSITTGEEVALCLPRSELLFQQWQRQGLVAAALEKLEKGP
ELGLSQFCGSLKQAAPAIQACVEACNLIACARHQQSHFDNGNEEWFLVGRVLDRVCFLAM
LSLFICGTAGIFLMAHYNRVPALPFPGDPRPYLPSPD
>sp|Q07912|ACK1_HUMAN Activated CDC42 kinase 1 OS=Homo sapiens GN=TNK2 PE=1 SV=3
MQPEEGTGWLLELLSEVQLQQYFLRLRDDLNVTRLSHFEYVKNEDLEKIGMGRPGQRRLW
EAVKRRKALCKRKSWMSKVFSGKRLEAEFPPHHSQSTFRKTSPAPGGPAGEGPLQSLTCL
IGEKDLRLLEKLGDGSFGVVRRGEWDAPSGKTVSVAVKCLKPDVLSQPEAMDDFIREVNA
MHSLDHRNLIRLYGVVLTPPMKMVTELAPLGSLLDRLRKHQGHFLLGTLSRYAVQVAEGM
GYLESKRFIHRDLAARNLLLATRDLVKIGDFGLMRALPQNDDHYVMQEHRKVPFAWCAPE
SLKTRTFSHASDTWMFGVTLWEMFTYGQEPWIGLNGSQILHKIDKEGERLPRPEDCPQDI
YNVMVQCWAHKPEDRPTFVALRDFLLEAQPTDMRALQDFEEPDKLHIQMNDVITVIEGRA
ENYWWRGQNTRTLCVGPFPRNVVTSVAGLSAQDISQPLQNSFIHTGHGDSDPRHCWGFPD
RIDELYLGNPMDPPDLLSVELSTSRPPQHLGGVKKPTYDPVSEDQDPLSSDFKRLGLRKP
GLPRGLWLAKPSARVPGTKASRGSGAEVTLIDFGEEPVVPALRPCAPSLAQLAMDACSLL
DETPPQSPTRALPRPLHPTPVVDWDARPLPPPPAYDDVAQDEDDFEICSINSTLVGAGVP
AGPSQGQTNYAFVPEQARPPPPLEDNLFLPPQGGGKPPSSAQTAEIFQALQQECMRQLQA
PAGSPAPSPSPGGDDKPQVPPRVPIPPRPTRPHVQLSPAPPGEEETSQWPGPASPPRVPP
REPLSPQGSRTPSPLVPPGSSPLPPRLSSSPGKTMPTTQSFASDPKYATPQVIQAPGPRA
GPCILPIVRDGKKVSSTHYYLLPERPSYLERYQRFLREAQSPEEPTPLPVPLLLPPPSTP
APAAPTATVRPMPQAALDPKANFSTNNSNPGARPPPPRATARLPQRGCPGDGPEAGRPAD
KIQMAMVHGVTTEECQAALQCHGWSVQRAAQYLKVEQLFGLGLRPRGECHKVLEMFDWNL
EQAGCHLLGSWGPAHHKR
>sp|Q16570|ACKR1_HUMAN Atypical chemokine receptor 1 OS=Homo sapiens GN=ACKR1 PE=1 SV=3
MGNCLHRAELSPSTENSSQLDFEDVWNSSYGVNDSFPDGDYGANLEAAAPCHSCNLLDDS
ALPFFILTSVLGILASSTVLFMLFRPLFRWQLCPGWPVLAQLAVGSALFSIVVPVLAPGL
GSTRSSALCSLGYCVWYGSAFAQALLLGCHASLGHRLGAGQVPGLTLGLTVGIWGVAALL
TLPVTLASGASGGLCTLIYSTELKALQATHTVACLAIFVLLPLGLFGAKGLKKALGMGPG
PWMNILWAWFIFWWPHGVVLGLDFLVRSKLLLLSTCLAQQALDLLLNLAEALAILHCVAT
PLLLALFCHQATRTLLPSLPLPEGWSSHLDTLGSKS
>sp|P25106|ACKR3_HUMAN Atypical chemokine receptor 3 OS=Homo sapiens GN=ACKR3 PE=1 SV=3
MDLHLFDYSEPGNFSDISWPCNSSDCIVVDTVMCPNMPNKSVLLYTLSFIYIFIFVIGMI
ANSVVVWVNIQAKTTGYDTHCYILNLAIADLWVVLTIPVWVVSLVQHNQWPMGELTCKVT
HLIFSINLFGSIFFLTCMSVDRYLSITYFTNTPSSRRKKMVRRVVCILVWLLAFCVSLPDT
YYLKTVTSASNNETYCRSFYPEHSIKEWLIGMELVSVVLGFAVPFSIIAVFYFLLARAIS
ASSDQEKHSSRKIIFSYVVVFLVCWLPYHVAVLLDIFSILHYIPFTCRLEHALFTALHVT
QCLSLVHCCVNPVLYSFINRNYRYELMKAFIFKYSAKTGLTKLIDASRVSETEYSALEQS
TK
>sp|Q3I5F7|ACOT6_HUMAN Putative acyl-coenzyme A thioesterase 6 OS=Homo sapiens GN=ACOT6
PE=1 SV=1

MLQHPKVKGPSIALLGFSKGGDLCLSMASFLKGITATVLINACVANTVAPLHYKDMIIPK
LVDDLGKVKITKSGFLTFMDTWSNPLEEHNHQSLVPLEKAQVPFLFIVGMDDQSWKSEFY
AQIASERLQAHGKERPQIICYPETGHCIDPPYFPPSRASVHAVLGEAIFYGGEPKAHSKA
QVDAWQQIQTFFHKHLNGKKSVKHSKI
>sp|Q15067|ACOX1_HUMAN Peroxisomal acyl-coenzyme A oxidase 1 OS=Homo sapiens GN=ACOX1 PE=1 SV=3
MNPDLRRERDSASFNPELLTHILDGSPEKTRRRREIENMILNDPDFQHEDLNFLTRSQRY
EVAVRKSAIMVKKMREFGIADPDEIMWFKKLHLVNFVEPVGLNYSMFIPTLLNQGTTAQK
EKWLLSSKGLQIIGTYAQTEMGHGTHLRGLETTATYDPETQEFILNSPTVTSIKWWPGGL
GKTSNHAIVLAQLITKGKCYGLHAFIVPIREIGTHKPLPGITVGDIGPKFGYDEIDNGYL
KMDNHRIPRENMLMKYAQVKPDGTYVKPLSNKLTYGTMVFVRSFLVGEAARALSKACTIA
IRYSAVRHQSEIKPGEPEPQILDFQTQQYKLFPLLATAYAFQFVGAYMKETYHRINEGIG
QGDLSELPELHALTAGLKAFTSWTANTGIEACRMACGGHGYSHCSGLPNIYVNFTPSCTF
EGENTVMMLQTARFLMKSYDQVHSGKLVCGMVSYLNDLPSQRIQPQQVAVWPTMVDINSP
ESLTEAYKLRAARLVEIAAKNLQKEVIHRKSKEVAWNLTSVDLVRASEAHCHYVVVKLFS
EKLLKIQDKAIQAVLRSLCLLYSLYGISQNAGDFLQGSIMTEPQITQVNQRVKELLTLIR
SDAVALVDAFDFQDVTLGSVLGRYDGNVYENLFEWAKNSPLNKAEVHESYKHLKSLQSKL
>sp|Q9NUZ1|ACOXL_HUMAN Acyl-coenzyme A oxidase-like protein OS=Homo sapiens GN=ACOXL PE=2 SV=3
MRALTVQRVKFAMDLPLLKRAGQDLAEKTKNFVSRSLVIGEVLSMADMATGVKCGIIYWL
FGGAIRNLGSPEHVTKWFQPLQEQKYTGMFAMTERGHGSNARGIQTEATFDLSAQEFVID
TPCENAEKMYIGNAMYGNYAAVFAQLIIDGRSQGPHCFIVPVRDENGSLYPGVTAIDMMY
KEGLHGVDNGILIFDKVRIPRENLLDKFGSVAPDGQYHSPIRNKSARFNAMLAALTPSRL
AVAFQAMGAMKLGLTIAIRYSHSRRQFGPKTKEEVKIIEHQTQTLRLMPHLATALALTFV
SRYAGALLDEDVFQGKELVNSRSLQALVAGLKAYSTWENIRCLQDCRECTGGMGYMMENR
ISGLKCDTDVFATFEGDDVVMLQVVGRELLAQYTKQYEEKPLFGLLQNWAESVGDKLRTS
FLAFNMDTVDDLAFLLKAVKFRERVLQRGLVARIYYKVKTKKEDFFHAWNSCLHHVASLS
LAHTHRVTLEQFSLAVKSCPDQEDQTLLMKFCLLYGTKLVFQERAWYLEHKYLTPMASTR
IRNQERC
>sp|Q08AH3|ACS2A_HUMAN Acyl-coenzyme A synthetase ACSM2A, mitochondrial OS=Homo sapiens GN=ACSM2A PE=1 SV=2
MHWLRKVQGLCTLWGTQMSSRTLYINSRQLVSLQWGHQEVPAKFNFASDVLDHWADMEKA
GKRLPSPALWWVNGKGKELMWNFRELSENSQQAANVLSGACGLQRGDRVAVVLPRVPEWW
LVILGCIRAGLIFMPGTIQMKSTDILYRLQMSKAKAIVAGDEVIQEVDTVASECPSLRIK
LLVSEKSCDGWLNFKKLLNEASTTHHCVETGSQEASAIYFTSGTSGLPKMAEHSYSSLGL
KAKMDAGWTGLQASDIMWTISDTGWILNILCSLMEPWALGACTFVHLLPKFDPLVILKTL
SSYPIKSMMGAPIVYRMLLQQDLSSYKFPHLQNCVTVGESLLPETLENWRAQTGLDIRES
YGQTETGLTCMVSKTMKIKPGYMGTAASCYDVQIIDDKGNVLPPGTEGDIGIRVKPIRPI
GIFSGYVDNPDKTAANIRGDFWLLGDRGIKDEDGYFQFMGRANDIINSSGYRIGPSEVEN
ALMEHPAVVETAVISSPDPVRGEVVKAFVVLASQFLSHDPEQLTKELQQHVKSVTAPYKY
PRKIEFVLNLPKTVTGKIQRAKLRDKEWKMSGKARAQ
>sp|Q4G176|ACSF3_HUMAN Acyl-CoA synthetase family member 3, mitochondrial OS=Homo sapiens GN=ACSF3 PE=1 SV=3
MLPHVVLTFRRLGCALASCRLAPARHRGSGLLHTAPVARSDRSAPVFTRALAFGDRIALV

DQHGRHTYRELYSRSLRLSQEICRLCGCVGGDLREERVSFLCANDASYVVAQWASWMSGG
VAVPLYRKHPAAQLEYVICDSQSSVVLASQEYLELLSPVVRKLGVPLLPLTPAIYTGAVE
EPAEVPVPEQGWRNKGAMIIYTSGTTGRPKGVLSTHQNIRAVVTGLVHKWAWTKDDVILH
VLPLHHVHGVVNALLCPLWVGATCVMMPEFSPQQVWEKFLSSETPRINVFMAVPTIYTKL
MEYYDRHFTQPHAQDFLRAVCEEKIRLMVSGSAALPLPVLEKWKNITGHTLLERYGMTEI
GMALSGPLTTAVRLPGSVGTPLPGVQVRIVSENPQREACSYTIHAEGDERGTKVTPGFEE
KEGELLVRGPSVFREYWNKPEETKSAFTLDGWFKTGDTVVFKDGQYWIRGRTSVDIIKTG
GYKVSALEVEWHLLAHPSITDVAVIGVPDMTWGQRVTAVVTLREGHSLSHRELKEWARNV
LAPYAVPSELVLVEEIPRNQMGKIDKKALIRHFHPS
>sp|O95573|ACSL3_HUMAN Long-chain-fatty-acid--CoA ligase 3 OS=Homo sapiens GN=ACSL3 PE=1 SV=3
MNNHVSSKPSTMKLKHTINPILLYFIHFLISLYTILTYIPFYFFSESRQEKSNRIKAKPV
NSKPDSAYRSVNSLDGLASVLYPGCDTLDKVFTYAKNKFKNKRLLGTREVLNEEDEVQPN
GKIFKKVILGQYNWLSYEDVFVRAFNFGNGLQMLGQKPKTNIAIFCETRAEWMIAAQACF
MYNFQLVTLYATLGGPAIVHALNETEVTNIITSKELLQTKLKDIVSLVPRLRHIITVDGK
PPTWSEFPKGIIVHTMAAVEALGAKASMENQPHSKPLPSDIAVIMYTSGSTGLPKGVMIS
HSNIIAGITGMAERIPELGEEDVYIGYLPLAHVLELSAELVCLSHGCRIGYSSPQTLADQ
SSKIKKGSKGDTSMLKPTLMAAVPEIMDRIYKNVMNKVSEMSSFQRNLFILAYNYKMEQI
SKGRNTPLCDSFVFRKVRSLLGGNIRLLLCGGAPLSATTQRFMNICFCCPVGQGYGLTES
AGAGTISEVWDYNTGRVGAPLVCCEIKLKNWEEGGYFNTDKPHPRGEILIGGQSVTMGYY
KNEAKTKADFFEDENGQRWLCTGDIGEFEPDGCLKIIDRKKDLVKLQAGEYVSLGKVEAA
LKNLPLVDNICAYANSYHSYVIGFVVPNQKELTELARKKGLKGTWEELCNSCEMENEVLK
VLSEAAISASLEKFEIPVKIRLSPEPWTPETGLVTDAFKLKRKELKTHYQADIERMYGRK
>sp|Q6P461|ACSM6_HUMAN Acyl-coenzyme A synthetase ACSM6, mitochondrial OS=Homo sapiens GN=ACSM6 PE=2 SV=3
MLGRFQPFSLVRSFRLGFEACCYPNQKCATQTIRPPDSRCLVQAVSQNFNFAKDVLDQWS
QLEKDGLRGPYPALWKVSAKGEEDKWSFERMTQLSKKAASILSDTCALSHGDRLMIILPP
TPEAYWICLACVRLGITFVPGSPQLTAKKIRYQLRMSKAQCIVANEAMAPVVNSAVSDCP
TLKTKLLVSDKSYDGWLDFKKLIQVAPPKQTYMRTKSQDPMAIFFTKGTTGAPKMVEYSQ
YGLGMGFSQASRRWMDLQPTDVLWSLGDAFGGSLSLSAVLGTWFQGACVFLCHMPTFCPE
TVLNVLSRFPITTLSANPEMYQELLQHKCFTSYRFKSLKQCVAAGGPISPGVIEDWKRIT
KLDIYEGYGQTETGLLCATSKTIKLKPSSLGKPLPPYIVQIVDENSNLLPPGEEGNIAIR
IKLNQPASLYCPHMVSWEEYASARGHMLYLTGDRGIMDEDGYFWWSGRVDDVANALGQRL
>sp|P04439|1A03_HUMAN HLA class I histocompatibility antigen, A-3 alpha chain OS=Homo sapiens GN=HLA-A PE=1 SV=2
MAVMAPRTLLLLLSGALALTQTWAGSHSMRYFFTSVSRPGRGEPRFIAVGYVDDTQFVRF
DSDAASQRMEPRAPWIEQEGPEYWDQETRNVKAQSQTDRVDLGTLRGYYNQSEAGSHTIQ
IMYGCDVGSDGRFLRGYRQDAYDGKDYIALNEDLRSWTAADMAAQITKRKWEAAHEAEQL
RAYLDGTCVEWLRRYLENGKETLQRTDPPKTHMTHHPISDHEATLRCWALGFYPAEITLT
WQRDGEDQTQDTELVETRPAGDGTFQKWAAVVVPSGEEQRYTCHVQHEGLPKPLTLRWEL
SSQPTIPIVGIIAGLVLLGAVITGAVVAAVMWRRKSSDRKGGSYTQAASSDSAQGSDVSL
TACKV
>sp|Q96QU6|1A1L1_HUMAN 1-aminocyclopropane-1-carboxylate synthase-like protein 1 OS=Homo sapiens GN=ACCS PE=1 SV=1

MFTLPQKDFRAPTTCLGPTCMQDLGSSHGEDLEGECSRKLDQKLPELRGVGDPAMISSDT
SYLSSRGRMIKWFWDSAEEGYRTYHMDEYDEDKNPSGIINLGTSENKLCFDLLSWRLSQR
DMQRVEPSLLQYADWRGHLFLREEVAKFLSFYCKSPVPLRPENVVVLNGGASLFSALATV
LCEAGEAFLIPTPYYGAITQHVCLYGNIRLAYVYLDSEVTGLDTRPFQLTVEKLEMALRE
AHSEGVKVKGLILISPQNPLGDVYSPEELQEYLVFAKRHRLHVIVDEVYMLSVFEKSVGY
RSVLSLERLPDPQRTHVMWATSKDFGMSGLRFGTLYTENQDVATAVASLCRYHGLSGLVQ
YQMAQLLRDRDWINQVYLPENHARLKAAHTYVSEELRALGIPFLSRGAGFFIWVDLRKYL
PKGTFEEEMLLWRRFLDNKVLLSFGKAFECKEPGWFRFVFSDQVHRLCLGMQRVQQVLAG
KSQVAEDPRPSQSQEPSDQRR
>sp|P05534|1A24_HUMAN HLA class I histocompatibility antigen, A-24 alpha chain OS=Homo sapiens GN=HLA-A PE=1 SV=2
MAVMAPRTLVLLLSGALALTQTWAGSHSMRYFSTSVSRPGRGEPRFIAVGYVDDTQFVRF
DSDAASQRMEPRAPWIEQEGPEYWDEETGKVKAHSQTDRENLRIALRYYNQSEAGSHTLQ
MMFGCDVGSDGRFLRGYHQYAYDGKDYIALKEDLRSWTAADMAAQITKRKWEAAHVAEQQ
RAYLEGTCVDGLRRYLENGKETLQRTDPPKTHMTHHPISDHEATLRCWALGFYPAEITLT
WQRDGEDQTQDTELVETRPAGDGTFQKWAAVVVPSGEEQRYTCHVQHEGLPKPLTLRWEP
SSQPTVPIVGIIAGLVLLGAVITGAVVAAVMWRRNSSDRKGGSYSQAASSDSAQGSDVSL
TACKV
>sp|P30450|1A26_HUMAN HLA class I histocompatibility antigen, A-26 alpha chain OS=Homo sapiens GN=HLA-A PE=1 SV=2
MAVMAPRTLVLLLSGALALTQTWAGSHSMRYFYTSVSRPGRGEPRFIAVGYVDDTQFVRF
DSDAASQRMEPRAPWIEQEGPEYWDRNTRNVKAHSQTDRANLGTLRGYYNQSEDGSHTIQ
RMYGCDVGPDGRFLRGYQQDAYDGKDYIALNEDLRSWTAADMAAQITQRKWETAHEAEQW
RAYLEGRCVEWLRRYLENGKETLQRTDAPKTHMTHHAVSDHEATLRCWALSFYPAEITLT
WQRDGEDQTQDTELVETRPAGDGTFQKWASVVVPSGQEQRYTCHVQHEGLPKPLTLRWEP
SSQPTIPIVGIIAGLVLFGAVIAGAVVAAVMWRRKSSDRKGGSYSQAASSDSAQGSDMSL
TACKV
>sp|P30457|1A66_HUMAN HLA class I histocompatibility antigen, A-66 alpha chain OS=Homo sapiens GN=HLA-A PE=1 SV=1
MAVMAPRTLVLLLSGALALTQTWAGSHSMRYFYTSVSRPGRGEPRFIAVGYVDDTQFVRF
DSDAASQRMEPRAPWIEQEGPEYWDRNTRNVKAQSQTDRVDLGTLRGYYNQSEDGSHTIQ
RMYGCDVGPDGRFLRGYQQDAYDGKDYIALNEDLRSWTAADMAAQITQRKWETAHEAEQW
RAYLEGRCVEWLRRYLENGKETLQRTDAPKTHMTHHAVSDHEATLRCWALSFYPAEITLT
WQRDGEDQTQDTELVETRPAGDGTFQKWASVVVPSGQEQRYTCHVQHEGLPKPLTLRWEP
SSQPTIPIVGIIAGLVLFGAVIAGAVVAAVMWRRKSSDRKGGSYSQAASSDSAQGSDMSL
TACKV
>sp|P10316|1A69_HUMAN HLA class I histocompatibility antigen, A-69 alpha chain OS=Homo sapiens GN=HLA-A PE=1 SV=2
MAVMAPRTLVLLLSGALALTQTWAGSHSMRYFYTSVSRPGRGEPRFIAVGYVDDTQFVRF
DSDAASQRMEPRAPWIEQEGPEYWDRNTRNVKAQSQTDRVDLGTLRGYYNQSEAGSHTVQ
RMYGCDVGSDWRFLRGYHQYAYDGKDYIALKEDLRSWTAADMAAQTTKHKWEAAHVAEQL
RAYLEGTCVEWLRRYLENGKETLQRTDAPKTHMTHHAVSDHEATLRCWALSFYPAEITLT
WQRDGEDQTQDTELVETRPAGDGTFQKWAAVVVPSGQEQRYTCHVQHEGLPKPLTLRWEP
SSQPTIPIVGIIAGLVLFGAVITGAVVAAVMWRRKSSDRKGGSYSQAASSDSAQGSDVSL

TACKV
>sp|Q95365|1B38_HUMAN HLA class I histocompatibility antigen, B-38 alpha chain OS=Homo sapiens GN=HLA-B PE=1 SV=1
MLVMAPRTVLLLLSAALALTETWAGSHSMRYFYTSVSRPGRGEPRFISVGYVDDTQFVRF
DSDAASPREEPRAPWIEQEGPEYWDRNTQICKTNTQTYRENLRIALRYYNQSEAGSHTLQ
RMYGCDVGPDGRLLRGHNQFAYDGKDYIALNEDLSSWTAADTAAQITQRKWEAARVAEQL
RTYLEGTCVEWLRRYLENGKETLQRADPPKTHVTHHPISDHEATLRCWALGFYPAEITLT
WQRDGEDQTQDTELVETRPAGDRTFQKWAAVVVPSGEEQRYTCHVQHEGLPKPLTLRWEP
SSQSTVPIVGIVAGLAVLAVVVIGAVVAAVMCRRKSSGGKGGSYSQAASSDSAQGSDVSL
TA
>sp|P30480|1B42_HUMAN HLA class I histocompatibility antigen, B-42 alpha chain OS=Homo sapiens GN=HLA-B PE=1 SV=1
MLVMAPRTVLLLLSAALALTETWAGSHSMRYFYTSVSRPGRGEPRFISVGYVDDTQFVRF
DSDAASPREEPRAPWIEQEGPEYWDRNTQIYKAQAQTDRESLRNLRGYYNQSEAGSHTLQ
SMYGCDVGPDGRLLRGHNQYAYDGKDYIALNEDLRSWTAADTAAQITQRKWEAARVAEQD
RAYLEGTCVEWLRRYLENGKDTLERADPPKTHVTHHPISDHEATLRCWALGFYPAEITLT
WQRDGEDQTQDTELVETRPAGDRTFQKWAAVVVPSGEEQRYTCHVQHEGLPKPLTLRWEP
SSQSTVPIVGIVAGLAVLAVVVIGAVVAAVMCRRKSSGGKGGSYSQAACSDSAQGSDVSL
TA
>sp|P30484|1B46_HUMAN HLA class I histocompatibility antigen, B-46 alpha chain OS=Homo sapiens GN=HLA-B PE=1 SV=1
MRVTAPRTVLLLLSGALALTETWAGSHSMRYFYTAMSRPGRGEPRFIAVGYVDDTQFVRF
DSDAASPRMAPRAPWIEQEGPEYWDRETQKYKRQAQTDRVSLRNLRGYYNQSEAGSHTLQ
RMYGCDVGPDGRLLRGHDQSAYDGKDYIALNEDLSSWTAADTAAQITQRKWEAAREAEQW
RAYLEGLCVEWLRRYLENGKETLQRADPPKTHVTHHPISDHEATLRCWALGFYPAEITLT
WQRDGEDQTQDTELVETRPAGDRTFQKWAAVVVPSGEEQRYTCHVQHEGLPKPLTLRWEP
SSQSTIPIVGIVAGLAVLAVVVIGAVVATVMCRRKSSGGKGGSYSQAASSDSAQGSDVSL
TA
>sp|Q04826|1B40_HUMAN HLA class I histocompatibility antigen, B-40 alpha chain OS=Homo sapiens GN=HLA-B PE=1 SV=1
MRVTAPRTLLLLLWGAVALTETWAGSHSMRYFHTSVSRPGRGEPRFITVGYVDDTLFVRF
DSDATSPRKEPRAPWIEQEGPEYWDRETQISKTNTQTYRESLRNLRGYYNQSEAGSHTLQ
SMYGCDVGPDGRLLRGHNQYAYDGKDYIALNEDLRSWTAADTAAQITQRKWEAARVAEQL
RAYLEGECVEWLRRYLENGKETLQRADPPKTHVTHHPISDHEATLRCWALGFYPAEITLT
WQRDGEDQTQDTELVETRPAGDRTFQKWAAVVVPSGEEQRYTCHVQHEGLPKPLTLRWEP
SSQSTVPIVGIVAGLAVLAVVVIGAVVAAVMCRRKSSGGKGGSYSQAACSDSAQGSDVSL
TA
>sp|P30492|1B54_HUMAN HLA class I histocompatibility antigen, B-54 alpha chain OS=Homo sapiens GN=HLA-B PE=1 SV=1
MRVTAPRTLLLLLWGALALTETWAGSHSMRYFYTAMSRPGRGEPRFIAVGYVDDTQFVRF
DSDAASPRGEPRAPWVEQEGPEYWDRNTQIYKAQAQTDRESLRNLRGYYNQSEAGSHTWQ
TMYGCDLGPDGRLLRGHNQLAYDGKDYIALNEDLSSWTAADTAAQITQRKWEAARVAEQL
RAYLEGTCVEWLRRYLENGKETLQRADPPKTHVTHHPISDHEATLRCWALGFYPAEITLT
WQRDGEDQTQDTELVETRPAGDRTFQKWAAVVVPSGEEQRYTCHVQHEGLPKPLTLRWEP

SSQSTIPIVGIVAGLAVLAVVVIGAVVATVMCRRKSSGGKGGSYSQAASSDSAQGSDVSL
TA
>sp|P30495|1B56_HUMAN HLA class I histocompatibility antigen, B-56 alpha chain OS=Homo sapiens GN=HLA-B PE=1 SV=1
MRVTAPRTLLLLLWGALALTETWAGSHSMRYFYTAMSRPGRGEPRFIAVGYVDDTQFVRF
DSDAASPREEPRAPWIEQEGPEYWDRNTQIYKAQAQTDRESLRNLRGYYNQSEAGSHTWQ
TMYGCDLGPDGRLLRGHNQLAYDGKDYIALNEDLSSWTAADTAAQITQRKWEAARVAEQL
RAYLEGLCVEWLRRYLENGKETLQRADPPKTHVTHHPISDHEATLRCWALGFYPAEITLT
WQRDGEDQTQDTELVETRPAGDRTFQKWAAVVVPSGEEQRYTCHVQHEGLPKPLTLRWEP
SSQSTIPIVGIVAGLAVLAVVVIGAVVATVMCRRKSSGGKGGSYSQAASSDSAQGSDVSL
TA
>sp|Q29718|1B82_HUMAN HLA class I histocompatibility antigen, B-82 alpha chain OS=Homo sapiens GN=HLA-B PE=1 SV=1
MRVTAPRTLLLLLWGALALTETWAGSHSMRYFYTAMSRPGRGEPRFISVGYVDDTQFVRF
DSDAASPREEPRAPWIEQEGPEYWDRNTQIYKAQAQTDRESLRNLRGYYNQSEAGSHTLQ
RMFGCDLGPDGRLLRGHNQLAYDGKDYIALNEDLSSWTAADTAAQITQRKWEAARVAEQD
RAYLEDLCVESLRRYLENGKETLQRADPPKTHVTHHPISDHEATLRCWALGFYPAEITLT
WQRDGEDQTQDTELVETRPAGDRTFQKWAAVVVPSGEEQRYTCHVQHEGLPKPLTLRWEP
SSQSTIPIVGIVAGLAVLAVVVIGAVVATVMCRRKSSGGKGGSYSQAASSDSAQGSDVSL
TA
>sp|Q29865|1C18_HUMAN HLA class I histocompatibility antigen, Cw-18 alpha chain OS=Homo sapiens GN=HLA-C PE=1 SV=1
MRVMAPRALLLLLSGGLALTETWACSHSMRYFDTAVSRPGRGEPRFISVGYVDDTQFVRF
DSDAASPRGEPRAPWVEQEGPEYWDRETQKYKRQAQADRVNLRKLRGYYNQSEDGSHTLQ
RMFGCDLGPDGRLLRGYNQFAYDGKDYIALNEDLRSWTAADTAAQITQRKWEAAREAEQR
RAYLEGTCVEWLRRYLENGKETLQRAEHPKTHVTHHPVSDHEATLRCWALGFYPAEITLT
WQWDGEDQTQDTELVETRPAGDGTFQKWAAVVVPSGEEQRYTCHVQHEGLPEPLTLRWKP
SSQPTIPIVGIVAGLAVLVVLAVLGAVVAVVMCRRKSSGGKGGSCSQAASSNSAQGSDES
LIACKA
>sp|Q15172|2A5A_HUMAN Serine/threonine-protein phosphatase 2A 56 kDa regulatory subunit alpha isoform OS=Homo sapiens GN=PPP2R5A PE=1 SV=1
MSSSSPPAGAASAAISASEKVDGFTRKSVRKAQRQKRSQGSSQFRSQGSQAELHPLPQLK
DATSNEQQELFCQKLQQCCILFDFMDSVSDLKSKEIKRATLNELVEYVSTNRGVIVESAY
SDIVKMISANIFRTLPPSDNPDFDPEEDEPTLEASWPHIQLVYEFFLRFLESPDFQPSIA
KRYIDQKFVQQLLELFDSEDPRERDFLKTVLHRIYGKFLGLRAFIRKQINNIFLRFIYET
EHFNGVAELLEILGSIINGFALPLKAEHKQFLMKVLIPMHTAKGLALFHAQLAYCVVQFL
EKDTTLTEPVIRGLLKFWPKTCSQKEVMFLGEIEEILDVIEPTQFKKIEEPLFKQISKCV
SSSHFQVAERALYFWNNEYILSLIEENIDKILPIMFASLYKISKEHWNPTIVALVYNVLK
TLMEMNGKLFDDLTSSYKAERQREKKKELEREELWKKLEELKLKKALEKQNSAYNMHSIL
SNTSAE
>sp|P30153|2AAA_HUMAN Serine/threonine-protein phosphatase 2A 65 kDa regulatory subunit A alpha isoform OS=Homo sapiens GN=PPP2R1A PE=1 SV=4
MAAADGDDSLYPIAVLIDELRNEDVQLRLNSIKKLSTIALALGVERTRSELLPFLTDTIY
DEDEVLLALAEQLGTFTTLVGGPEYVHCLLPPLESLATVEETVVRDKAVESLRAISHEHS

PSDLEAHFVPLVKRLAGGDWFTSRTSACGLFSVCYPRVSSAVKAELRQYFRNLCSDDTPM
VRRAAASKLGEFAKVLELDNVKSEIIPMFSNLASDEQDSVRLLAVEACVNIAQLLPQEDL
EALVMPTLRQAAEDKSWRVRYMVADKFTELQKAVGPEITKTDLVPAFQNLMKDCEAEVRA
AASHKVKEFCENLSADCRENVIMSQILPCIKELVSDANQHVKSALASVIMGLSPILGKDN
TIEHLLPLFLAQLKDECPEVRLNIISNLDCVNEVIGIRQLSQSLLPAIVELAEDAKWRVR
LAIIEYMPLLAGQLGVEFFDEKLNSLCMAWLVDHVYAIREAATSNLKKLVEKFGKEWAHA
TIIPKVLAMSGDPNYLHRMTTLFCINVLSEVCGQDITTKHMLPTVLRMAGDPVANVRFNV
AKSLQKIGPILDNSTLQSEVKPILEKLTQDQDVDVKYFAQEALTVLSLA
>sp|P63151|2ABA_HUMAN Serine/threonine-protein phosphatase 2A 55 kDa regulatory subunit B alpha isoform OS=Homo sapiens GN=PPP2R2A PE=1 SV=1
MAGAGGGNDIQWCFSQVKGAVDDDVAEADIISTVEFNHSGELLATGDKGGRVVIFQQEQE
NKIQSHSRGEYNVYSTFQSHEPEFDYLKSLEIEEKINKIRWLPQKNAAQFLLSTNDKTIK
LWKISERDKRPEGYNLKEEDGRYRDPTTVTTLRVPVFRPMDLMVEASPRRIFANAHTYHI
NSISINSDYETYLSADDLRINLWHLEITDRSFNIVDIKPANMEELTEVITAAEFHPNSCN
TFVYSSSKGTIRLCDMRASALCDRHSKLFEEPEDPSNRSFFSEIISSISDVKFSHSGRYM
MTRDYLSVKIWDLNMENRPVETYQVHEYLRSKLCSLYENDCIFDKFECCWNGSDSVVMTG
SYNNFFRMFDRNTKRDITLEASRENNKPRTVLKPRKVCASGKRKKDEISVDSLDFNKKIL
HTAWHPKENIIAVATTNNLYIFQDKVN
>sp|Q00005|2ABB_HUMAN Serine/threonine-protein phosphatase 2A 55 kDa regulatory subunit B beta isoform OS=Homo sapiens GN=PPP2R2B PE=1 SV=1
MEEDIDTRKINNSFLRDHSYATEADIISTVEFNHTGELLATGDKGGRVVIFQREQESKNQ
VHRRGEYNVYSTFQSHEPEFDYLKSLEIEEKINKIRWLPQQNAAYFLLSTNDKTVKLWKV
SERDKRPEGYNLKDEEGRLRDPATITTLRVPVLRPMDLMVEATPRRVFANAHTYHINSIS
VNSDYETYMSADDLRINLWNFEITNQSFNIVDIKPANMEELTEVITAAEFHPHHCNTFVY
SSSKGTIRLCDMRASALCDRHTKFFEEPEDPSNRSFFSEIISSISDVKFSHSGRYIMTRD
YLTVKVWDLNMENRPIETYQVHDYLRSKLCSLYENDCIFDKFECVWNGSDSVIMTGSYNN
FFRMFDRNTKRDVTLEASRENSKPRAILKPRKVCVGGKRRKDEISVDSLDFSKKILHTAW
HPSENIIAVAATNNLYIFQDKVN
>sp|P04229|2B11_HUMAN HLA class II histocompatibility antigen, DRB1-1 beta chain OS=Homo sapiens GN=HLA-DRB1 PE=1 SV=2
MVCLKLPGGSCMTALTVTLMVLSSPLALAGDTRPRFLWQLKFECHFFNGTERVRLLERCI
YNQEESVRFDSDVGEYRAVTELGRPDAEYWNSQKDLLEQRRAAVDTYCRHNYGVGESFTV
QRRVEPKVTVYPSKTQPLQHHNLLVCSVSGFYPGSIEVRWFRNGQEEKAGVVSTGLIQNG
DWTFQTLVMLETVPRSGEVYTCQVEHPSVTSPLTVEWRARSESAQSKMLSGVGGFVLGLL
FLGAGLFIYFRNQKGHSGLQPTGFLS
>sp|P20039|2B1B_HUMAN HLA class II histocompatibility antigen, DRB1-11 beta chain OS=Homo sapiens GN=HLA-DRB1 PE=1 SV=1
MVCLRLPGGSCMAVLTVTLMVLSSPLALAGDTRPRFLEYSTSECHFFNGTERVRFLDRYF
YNQEEYVRFDSDVGEFRAVTELGRPDEEYWNSQKDFLEDRRAAVDTYCRHNYGVGESFTV
QRRVHPKVTVYPSKTQPLQHHNLLVCSVSGFYPGSIEVRWFRNGQEEKTGVVSTGLIHNG
DWTFQTLVMLETVPRSGEVYTCQVEHPSVTSPLTVEWRARSESAQSKMLSGVGGFVLGLL
FLGAGLFIYFRNQKGHSGLQPRGFLS
>sp|Q9GIY3|2B1E_HUMAN HLA class II histocompatibility antigen, DRB1-14 beta chain OS=Homo sapiens GN=HLA-DRB1 PE=1 SV=1

MVCLRLPGGSCMAVLTVTLMVLSSPLALAGDTRPRFLEYSTSECHFFNGTERVRFLDRYF
HNQEEFVRFDSDVGEYRAVTELGRPAAEHWNSQKDLLERRRAEVDTYCRHNYGVVESFTV
QRRVHPKVTVYPSKTQPLQHYNLLVCSVSGFYPGSIEVRWFRNGQEEKTGVVSTGLIHNG
DWTFQTLVMLETVPRSGEVYTCQVEHPSVTSPLTVEWRARSESAQSKMLSGVGGFVLGLL
FLGAGLFIYFRNQKGHSGLQPRGFLS
>sp|Q5TYW2|A20A1_HUMAN Ankyrin repeat domain-containing protein 20A1 OS=Homo sapiens GN=ANKRD20A1 PE=2 SV=1
MKLFGFGSRRGQTAQGSIDHVYTGSGYRIRDSELQKIHRAAVKGDAAEVERCLARRSGDL
DALDKQHRTALHLACTSGHVQVVTLLVNRKCQIDVCDKENRTPLIQAVHCQEEACAVILL
EHGANPNLKDIYGNTALHYAVYSESTSLAEKLLSHGAHIEALDKDNNTPLLFAIICKKEK
MVEFLLKKKASSHAVDRLRRSALMLAVYYDSPGIVNILLKQNIDVFAQDMCGRDAEDYAI
SHHLTKIQQQILEHKKKILKKEKSDVGSSDESAVSIFHELRVDSLPASDDKDLNVATKQC
VPEKVSEPLPGSSHEKGNRIVNGQGEGPPAKHPSLKPSTEVEDPAVKGAVQRKNVQTLRA
EQALPVASEEEQERHERSEKKQPQVKEGNNTNKSEKIQLSENICDSTSSAAAGRLTQQRK
IGKTYPQQFPKKLKEEHDRCTLKQENEEKTNVNMLYKKNREELERKEKQYKKEVEAKQLE
PTVQSLEMKSKTARNTPNWDFHNHEEMKGLMDENCILKADIAILRQEICTMKNDNLEKEN
KYLKDIKIVKETNAALEKYIKLNEEMITETAFRYQQELNDLKAENTRLNAELLKEKESKK
RLEADIESYQSRLAAAISKHSESVKTERNLKLALERTRDVSVQVEMSSAISKVKAENEFL
TEQLSETQIKFNALKDKFRKTRDSLRKKSLALETVQNDLSQTQQQTQEMKEMYQNAEAKV
NNSTGKWNCVEERICHLQRENAWLVQQLDDVHQKEDHKEIVTNIQRGFIESGKKDLVLEE
KSKKLMNECDHLKESLFQYEREKTEGVVSIKEDKYFQTSRKTI
>sp|A0PJZ0|A20A5_HUMAN Putative ankyrin repeat domain-containing protein 20A5 OS=Homo sapiens GN=ANKRD20A5P PE=5 SV=1
MKLFGFRSRRGQTVLGSIDHLYTGSGYRIRYSELQKIHKAAVKGDAAEMERCLARRSGDL
DALDKQHRTALHLACASGHVKVVTLLVNRKCQIDIYDKENRTPLIQAVHCQEEACAVILL
EHGANPNLKDIYGNTALHYAVYSESTSLAEKLLFHGENIEALDKV
>sp|Q6PD74|AAGAB_HUMAN Alpha- and gamma-adaptin-binding protein p34 OS=Homo sapiens GN=AAGAB PE=1 SV=1
MAAGVPCALVTSCSSVFSGDQLVQHILGTEDLIVEVTSNDAVRFYPWTIDNKYYSADINL
CVVPNKFLVTAEIAESVQAFVVYFDSTQKSGLDSVSSWLPLAKAWLPEVMILVCDRVSED
GINRQKAQEWCIKHGFELVELSPEELPEEDDDFPESTGVKRIVQALNANVWSNVVMKNDR
NQGFSLLNSLTGTNHSIGSADPCHPEQPHLPAADSTESLSDHRGGASNTTDAQVDSIVDP
MLDLDIQELASLTTGGGDVENFERLFSKLKEMKDKAATLPHEQRKVHAEKVAKAFWMAIG
GDRDEIEGLSSDEEH
>sp|Q9Y312|AAR2_HUMAN Protein AAR2 homolog OS=Homo sapiens GN=AAR2 PE=1 SV=2
MAAVQMDPELAKRLFFEGATVVILNMPKGTEFGIDYNSWEVGPKFRGVKMIPPGIHFLHY
SSVDKANPKEVGPRMGFFLSLHQRGLTVLRWSTLREEVDLSPAPESEVEAMRANLQELDQ
FLGPYPYATLKKWISLTNFISEATVEKLQPENRQICAFSDVLPVLSMKHTKDRVGQNLPR
CGIECKSYQEGLARLPEMKPRAGTEIRFSELPTQMFPEGATPAEITKHSMDLSYALETVL
NKQFPSSPQDVLGELQFAFVCFLLGNVYEAFEHWKRLLNLLCRSEAAMMKHHTLYINLIS
ILYHQLGEIPADFFVDIVSQDNFLTSTLQVFFSSACSIAVDATLRKKAEKFQAHLTKKFR
WDFAAEPEDCAPVVVELPEGIEMG
>sp|P31941|ABC3A_HUMAN DNA dC->dU-editing enzyme APOBEC-3A OS=Homo sapiens GN=APOBEC3A PE=1 SV=3

MEASPASGPRHLMDPHIFTSNFNNGIGRHKTYLCYEVERLDNGTSVKMDQHRGFLHNQAK
NLLCGFYGRHAELRFLDLVPSLQLDPAQIYRVTWFISWSPCFSWGCAGEVRAFLQENTHV
RLRIFAARIYDYDPLYKEALQMLRDAGAQVSIMTYDEFKHCWDTFVDHQGCPFQPWDGLD
EHSQALSGRLRAILQNQGN
>sp|Q9HC16|ABC3G_HUMAN DNA dC->dU-editing enzyme APOBEC-3G OS=Homo sapiens GN=APOBEC3G PE=1 SV=1
MKPHFRNTVERMYRDTFSYNFYNRPILSRRNTVWLCYEVKTKGPSRPPLDAKIFRGQVYS
ELKYHPEMRFFHWFSKWRKLHRDQEYEVTWYISWSPCTKCTRDMATFLAEDPKVTLTIFV
ARLYYFWDPDYQEALRSLCQKRDGPRATMKIMNYDEFQHCWSKFVYSQRELFEPWNNLPK
YYILLHIMLGEILRHSMDPPTFTFNFNNEPWVRGRHETYLCYEVERMHNDTWVLLNQRRG
FLCNQAPHKHGFLEGRHAELCFLDVIPFWKLDLDQDYRVTCFTSWSPCFSCAQEMAKFIS
KNKHVSLCIFTARIYDDQGRCQEGLRTLAEAGAKISIMTYSEFKHCWDTFVDHQGCPFQP
WDGLDEHSQDLSGRLRAILQNQEN
>sp|Q6NTF7|ABC3H_HUMAN DNA dC->dU-editing enzyme APOBEC-3H OS=Homo sapiens GN=APOBEC3H PE=1 SV=3
MALLTAETFRLQFNNKRRLRRPYYPRKALLCYQLTPQNGSTPTRGYFENKKKCHAEICFI
NEIKSMGLDETQCYQVTCYLTWSPCSSCAWELVDFIKAHDHLNLGIFASRLYYHWCKPQQ
KGLRLLCGSQVPVEVMGFPKFADCWENFVDHEKPLSFNPYKMLEELDKNSRAIKRRLERI
KIPGVRAQGRYMDILCDAEV
>sp|O75027|ABCB7_HUMAN ATP-binding cassette sub-family B member 7, mitochondrial OS=Homo sapiens GN=ABCB7 PE=1 SV=2
MALLAMHSWRWAAAAAAFEKRRHSAILIRPLVSVSGSGPQWRPHQLGALGTARAYQIPES
LKSITWQRLGKGNSGQFLDAAKALQVWPLIEKRTCWHGHAGGGLHTDPKEGLKDVDTRKI
IKAMLSYVWPKDRPDLRARVAISLGFLGGAKAMNIVVPFMFKYAVDSLNQMSGNMLNLSD
APNTVATMATAVLIGYGVSRAGAAFFNEVRNAVFGKVAQNSIRRIAKNVFLHLHNLDLGF
HLSRQTGALSKAIDRGTRGISFVLSALVFNLLPIMFEVMLVSGVLYYKCGAQFALVTLGT
LGTYTAFTVAVTRWRTRFRIEMNKADNDAGNAAIDSLLNYETVKYFNNERYEAQRYDGFL
KTYETASLKSTSTLAMLNFGQSAIFSVGLTAIMVLASQGIVAGTLTVGDLVMVNGLLFQL
SLPLNFLGTVYRETRQALIDMNTLFTLLKVDTQIKDKVMASPLQITPQTATVAFDNVHFE
YIEGQKVLSGISFEVPAGKKVAIVGGSGSGKSTIVRLLFRFYEPQKGSIYLAGQNIQDVS
LESLRRAVGVVPQDAVLFHNTIYYNLLYGNISASPEEVYAVAKLAGLHDAILRMPHGYDT
QVGERGLKLSGGEKQRVAIARAILKDPPVILYDEATSSLDSITEETILGAMKDVVKHRTS
IFIAHRLSTVVDADEIIVLDQGKVAERGTHHGLLANPHSIYSEMWHTQSSRVQNHDNPKW
EAKKENISKEEERKKLQEEIVNSVKGCGNCSC
>sp|P33897|ABCD1_HUMAN ATP-binding cassette sub-family D member 1 OS=Homo sapiens GN=ABCD1 PE=1 SV=2
MPVLSRPRPWRGNTLKRTAVLLALAAYGAHKVYPLVRQCLAPARGLQAPAGEPTQEASGV
AAAKAGMNRVFLQRLLWLLRLLFPRVLCRETGLLALHSAALVSRTFLSVYVARLDGRLAR
CIVRKDPRAFGWQLLQWLLIALPATFVNSAIRYLEGQLALSFRSRLVAHAYRLYFSQQTY
YRVSNMDGRLRNPDQSLTEDVVAFAASVAHLYSNLTKPLLDVAVTSYTLLRAARSRGAGT
AWPSAIAGLVVFLTANVLRAFSPKFGELVAEEARRKGELRYMHSRVVANSEEIAFYGGHE
VELALLQRSYQDLASQINLILLERLWYVMLEQFLMKYVWSASGLLMVAVPIITATGYSES
DAEAVKKAALEKKEEELVSERTEAFTIARNLLTAAADAIERIMSSYKEVTELAGYTARVH
EMFQVFEDVQRCHFKRPRELEDAQAGSGTIGRSGVRVEGPLKIRGQVVDVEQGIICENIP

IVTPSGEVVVASLNIRVEEGMHLLITGPNGCGKSSLFRILGGLWPTYGGVLYKPPPQRMF
YIPQRPYMSVGSLRDQVIYPDSVEDMQRKGYSEQDLEAILDVVHLHHILQREGGWEAMCD
WKDVLSGGEKQRIGMARMFYHRPKYALLDECTSAVSIDVEGKIFQAAKDAGIALLSITHR
PSLWKYHTHLLQFDGEGGWKFEKLDSAARLSLTEEKQRLEQQLAGIPKMQRRLQELCQIL
GEAVAPAHVPAPSPQGPGGLQGAST
>sp|Q9UBJ2|ABCD2_HUMAN ATP-binding cassette sub-family D member 2 OS=Homo sapiens GN=ABCD2 PE=1 SV=1
MTHMLNAAADRVKWTRSSAAKRAACLVAAAYALKTLYPIIGKRLKQSGHGKKKAAAYPAA
ENTEILHCTETICEKPSPGVNADFFKQLLELRKILFPKLVTTETGWLCLHSVALISRTFL
SIYVAGLDGKIVKSIVEKKPRTFIIKLIKWLMIAIPATFVNSAIRYLECKLALAFRTRLV
DHAYETYFTNQTYYKVINMDGRLANPDQSLTEDIMMFSQSVAHLYSNLTKPILDVMLTSY
TLIQTATSRGASPIGPTLLAGLVVYATAKVLKACSPKFGKLVAEEAHRKGYLRYVHSRII
ANVEEIAFYRGHKVEMKQLQKSYKALADQMNLILSKRLWYIMIEQFLMKYVWSSSGLIMV
AIPIITATGFADGEDGQKQVMVSERTEAFTTARNLLASGADAIERIMSSYKEVTELAGYT
ARVYNMFWVFDEVKRGIYKRTAVIQESESHSKNGAKVELPLSDTLAIKGKVIDVDHGIIC
ENVPIITPAGEVVASRLNFKVEEGMHLLITGPNGCGKSSLFRILSGLWPVYEGVLYKPPP
QHMFYIPQRPYMSLGSLRDQVIYPDSVDDMHDKGYTDQDLERILHNVHLYHIVQREGGWD
AVMDWKDVLSGGEKQRMGMARMFYHKPKYALLDECTSAVSIDVEGKIFQAAKGAGISLLS
ITHRPSLWKYHTHLLQFDGEGGWRFEQLDTAIRLTLSEEKQKLESQLAGIPKMQQRLNEL
CKILGEDSVLKTIKNEDETS
>sp|Q6UXT9|ABH15_HUMAN Protein ABHD15 OS=Homo sapiens GN=ABHD15 PE=1 SV=2
MPPWGAALALILAVLALLGLLGPRLRGPWGRAVGERTLPGAQDRDDGEEADGGGPADQFS
DGREPLPGGCSLVCKPSALAQCLLRALRRSEALEAGPRSWFSGPHLQTLCHFVLPVAPGP
ELAREYLQLADDGLVALDWVVGPCVRGRRITSAGGLPAVLLVIPNAWGRLTRNVLGLCLL
ALERGYYPVIFHRRGHHGCPLVSPRLQPFGDPSDLKEAVTYIRFRHPAAPLFAVSEGSGS
ALLLSYLGECGSSSYVTGAACISPVLRCREWFEAGLPWPYERGFLLHQKIALSRYATALE
DTVDTSRLFRSRSLREFEEALFCHTKSFPISWDTYWDRNDPLRDVDEAAVPVLCICSADD
PVCGPPDHTLTTELFHSNPYFFLLLSRHGGHCGFLRQEPLPAWSHEVILESFRALTEFFR
TEERIKGLSRHRASFLGGRRRGGALQRREVSSSSNLEEIFNWKRSYTR
>sp|Q96SE0|ABHD1_HUMAN Protein ABHD1 OS=Homo sapiens GN=ABHD1 PE=1 SV=2
MLSSFLSPQNGTWADTFSLLLALAVALYLGYYWACVLQRPRLVAGPQFLAFLEPHCSITT
ETFYPTLWCFEGRLQSIFQVLLQSQPLVLYQSDILQTPDGGQLLLDWAKQPDSSQDPDPT
TQPIVLLLPGITGSSQDTYVLHLVNQALRDGYQAVVFNNRGCRGEELRTHRAFCASNTED
LETVVNHIKHRYPQAPLLAVGISFGGILVLNHLAQARQAAGLVAALTLSACWDSFETTRS
LETPLNSLLFNQPLTAGLCQLVERNRKVIEKVVDIDFVLQARTIRQFDERYTSVAFGYQD
CVTYYKAASPRTKIDAIRIPVLYLSAADDPFSPVCALPIQAAQHSPYVALLITARGGHIG
FLEGLLPWQHWYMSRLLHQYAKAIFQDPEGLPDLRALLPSEDRNS
>sp|P08910|ABHD2_HUMAN Monoacylglycerol lipase ABHD2 OS=Homo sapiens GN=ABHD2 PE=1 SV=1
MNAMLETPELPAVFDGVKLAAVAAVLYVIVRCLNLKSPTAPPDLYFQDSGLSRFLLKSCP
LLTKEYIPPLIWGKSGHIQTALYGKMGRVRSPHPYGHRKFITMSDGATSTFDLFEPLAEH
CVGDDITMVICPGIANHSEKQYIRTFVDYAQKNGYRCAVLNHLGALPNIELTSPRMFTYG
CTWEFGAMVNYIKKTYPLTQLVVVGFSLGGNIVCKYLGETQANQEKVLCCVSVCQGYSAL
RAQETFMQWDQCRRFYNFLMADNMKKIILSHRQALFGDHVKKPQSLEDTDLSRLYTATSL
MQIDDNVMRKFHGYNSLKEYYEEESCMRYLHRIYVPLMLVNAADDPLVHESLLTIPKSLS

EKRENVMFVLPLHGGHLGFFEGSVLFPEPLTWMDKLVVEYANAICQWERNKLQCSDTEQV
EADLE
>sp|P00519|ABL1_HUMAN Tyrosine-protein kinase ABL1 OS=Homo sapiens GN=ABL1 PE=1 SV=4
MLEICLKLVGCKSKKGLSSSSSCYLEEALQRPVASDFEPQGLSEAARWNSKENLLAGPSE
NDPNLFVALYDFVASGDNTLSITKGEKLRVLGYNHNGEWCEAQTKNGQGWVPSNYITPVN
SLEKHSWYHGPVSRNAAEYLLSSGINGSFLVRESESSPGQRSISLRYEGRVYHYRINTAS
DGKLYVSSESRFNTLAELVHHHSTVADGLITTLHYPAPKRNKPTVYGVSPNYDKWEMERT
DITMKHKLGGGQYGEVYEGVWKKYSLTVAVKTLKEDTMEVEEFLKEAAVMKEIKHPNLVQ
LLGVCTREPPFYIITEFMTYGNLLDYLRECNRQEVNAVVLLYMATQISSAMEYLEKKNFI
HRDLAARNCLVGENHLVKVADFGLSRLMTGDTYTAHAGAKFPIKWTAPESLAYNKFSIKS
DVWAFGVLLWEIATYGMSPYPGIDLSQVYELLEKDYRMERPEGCPEKVYELMRACWQWNP
SDRPSFAEIHQAFETMFQESSISDEVEKELGKQGVRGAVSTLLQAPELPTKTRTSRRAAE
HRDTTDVPEMPHSKGQGESDPLDHEPAVSPLLPRKERGPPEGGLNEDERLLPKDKKTNLF
SALIKKKKKTAPTPPKRSSSFREMDGQPERRGAGEEEGRDISNGALAFTPLDTADPAKSP
KPSNGAGVPNGALRESGGSGFRSPHLWKKSSTLTSSRLATGEEEGGGSSSKRFLRSCSAS
CVPHGAKDTEWRSVTLPRDLQSTGRQFDSSTFGGHKSEKPALPRKRAGENRSDQVTRGTV
TPPPRLVKKNEEAADEVFKDIMESSPGSSPPNLTPKPLRRQVTVAPASGLPHKEEAGKGS
ALGTPAAAEPVTPTSKAGSGAPGGTSKGPAEESRVRRHKHSSESPGRDKGKLSRLKPAPP
PPPAASAGKAGGKPSQSPSQEAAGEAVLGAKTKATSLVDAVNSDAAKPSQPGEGLKKPVL
PATPKPQSAKPSGTPISPAPVPSTLPSASSALAGDQPSSTAFIPLISTRVSLRKTRQPPE
RIASGAITKGVVLDSTEALCLAISRNSEQMASHSAVLEAGKNLYTFCVSYVDSIQQMRNK
FAFREAINKLENNLRELQICPATAGSGPAATQDFSKLLSSVKEISDIVQR
>sp|Q9P1F3|ABRAL_HUMAN Costars family protein ABRACL OS=Homo sapiens GN=ABRACL PE=1 SV=1
MNVDHEVNLLVEEIHRLGSKNADGKLSVKFGVLFRDDKCANLFEALVGTLKAAKRRKIVT
YPGELLLQGVHDDVDIILLQD
>sp|Q12979|ABR_HUMAN Active breakpoint cluster region-related protein OS=Homo sapiens GN=ABR PE=2 SV=2
MEPLSHRGLPRLSWIDTLYSNFSYGTDEYDGEGNEEQKGPPEGSETMPYIDESPTMSPQL
SARSQGGGDGVSPTPPEGLAPGVEAGKGLEMRKLVLSGFLASEEIYINQLEALLLPMKPL
KATATTSQPVLTIQQIETIFYKIQDIYEIHKEFYDNLCPKVQQWDSQVTMGHLFQKLASQ
LGVYKAFVDNYKVALETAEKCSQSNNQFQKISEELKVKGPKDSKDSHTSVTMEALLYKPI
DRVTRSTLVLHDLLKHTPVDHPDYPLLQDALRISQNFLSSINEDIDPRRTAVTTPKGETR
QLVKDGFLVEVSESSRKLRHVFLFTDVLLCAKLKKTSAGKHQQYDCKWYIPLADLVFPSP
EESEASPQVHPFPDHELEDMKMKISALKSEIQKEKANKGQSRAIERLKKKMFENEFLLLL
NSPTIPFRIHNRNGKSYLFLLSSDYERSEWREAIQKLQKKDLQAFVLSSVELQVLTGSCF
KLRTVHNIPVTSNKDDDESPGLYGFLHVIVHSAKGFKQSANLYCTLEVDSFGYFVSKAKT
RVFRDTAEPKWDEEFEIELEGSQSLRILCYEKCYDKTKVNKDNNEIVDKIMGKGQIQLDP
QTVETKNWHTDVIEMNGIKVEFSMKFTSRDMSLKRTPSKKQTGVFGVKISVVTKRERSKV
PYIVRQCVEEVEKRGIEEVGIYRISGVATDIQALKAVFDANNKDILLMLSDMDINAIAGT
LKLYFRELPEPLLTDRLYPAFMEGIALSDPAAKENCMMHLLRSLPDPNLITFLFLLEHLK
RVAEKEPINKMSLHNLATVFGPTLLRPSEVESKAHLTSAADIWSHDVMAQVQVLLYYLQH
PPISFAELKRNTLYFSTDV
>sp|Q96P50|ACAP3_HUMAN Arf-GAP with coiled-coil, ANK repeat and PH domain-containing protein 3 OS=Homo sapiens GN=ACAP3 PE=1 SV=2

MTVEFEECVKDSPRFRATIDEVETDVVEIEAKLDKLVKLCSGMVEAGKAYVSTSRLFVSG
VRDLSQQCQGDTVISECLQRFADSLQEVVNYHMILFDQAQRSVRQQLQSFVKEDVRKFKE
TKKQFDKVREDLELSLVRNAQAPRHRPHEVEEATGALTLTRKCFRHLALDYVLQINVLQA
KKKFEILDSMLSFMHAQSSFFQQGYSLLHQLDPYMKKLAAELDQLVIDSAVEKREMERKH
AAIQQRTLLQDFSYDESKVEFDVDAPSGVVMEGYLFKRASNAFKTWNRRWFSIQNSQLVY
QKKLKDALTVVVDDLRLCSVKPCEDIERRFCFEVLSPTKSCMLQADSEKLRQAWVQAVQA
SIASAYRESPDSCYSERLDRTASPSTSSIDSATDTRERGVKGESVLQRVQSVAGNSQCGD
CGQPDPRWASINLGVLLCIECSGIHRSLGVHCSKVRSLTLDSWEPELLKLMCELGNSAVN
QIYEAQCEGAGSRKPTASSSRQDKEAWIKDKYVEKKFLRKAPMAPALEAPRRWRVQKCLR
PHSSPRAPTARRKVRLEPVLPCVAALSSVGTLDRKFRRDSLFCPDELDSLFSYFDAGAAG
AGPRSLSSDSGLGGSSDGSSDVLAFGSGSVVDSVTEEEGAESEESSGEADGDTEAEAWGL
ADVRELHPGLLAHRAARARDLPALAAALAHGAEVNWADAEDEGKTPLVQAVLGGSLIVCE
FLLQNGADVNQRDSRGRAPLHHATLLGRTGQVCLFLKRGADQHALDQEQRDPLAIAVQAA
NADIVTLLRLARMAEEMREAEAAPGPPGALAGSPTELQFRRCIQEFISLHLEES
>sp|Q8TDN7|ACER1_HUMAN Alkaline ceramidase 1 OS=Homo sapiens GN=ACER1 PE=2 SV=1
MPSIFAYQSSEVDWCESNFQYSELVAEFYNTFSNIPFFIFGPLMMLLMHPYAQKRSRYIY
VVWVLFMIIGLFSMYFHMTLSFLGQLLDEIAILWLLGSGYSIWMPRCYFPSFLGGNRSQF
IRLVFITTVVSTLLSFLRPTVNAYALNSIALHILYIVCQEYRKTSNKELRHLIEVSVVLW
AVALTSWISDRLLCSFWQRIHFFYLHSIWHVLISITFPYGMVTMALVDANYEMPGETLKV
RYWPRDSWPVGLPYVEIRGDDKDC
>sp|Q5QJU3|ACER2_HUMAN Alkaline ceramidase 2 OS=Homo sapiens GN=ACER2 PE=1 SV=2
MGAPHWWDQLQAGSSEVDWCEDNYTIVPAIAEFYNTISNVLFFILPPICMCLFRQYATCF
NSGIYLIWTLLVVVGIGSVYFHATLSFLGQMLDELAVLWVLMCALAMWFPRRYLPKIFRN
DRGRFKVVVSVLSAVTTCLAFVKPAINNISLMTLGVPCTALLIAELKRCDNMRVFKLGLF
SGLWWTLALFCWISDRAFCELLSSFNFPYLHCMWHILICLAAYLGCVCFAYFDAASEIPE
QGPVIKFWPNEKWAFIGVPYVSLLCANKKSSVKIT
>sp|P22303|ACES_HUMAN Acetylcholinesterase OS=Homo sapiens GN=ACHE PE=1 SV=1
MRPPQCLLHTPSLASPLLLLLLWLLGGGVGAEGREDAELLVTVRGGRLRGIRLKTPGGPV
SAFLGIPFAEPPMGPRRFLPPEPKQPWSGVVDATTFQSVCYQYVDTLYPGFEGTEMWNPN
RELSEDCLYLNVWTPYPRPTSPTPVLVWIYGGGFYSGASSLDVYDGRFLVQAERTVLVSM
NYRVGAFGFLALPGSREAPGNVGLLDQRLALQWVQENVAAFGGDPTSVTLFGESAGAASV
GMHLLSPPSRGLFHRAVLQSGAPNGPWATVGMGEARRRATQLAHLVGCPPGGTGGNDTEL
VACLRTRPAQVLVNHEWHVLPQESVFRFSFVPVVDGDFLSDTPEALINAGDFHGLQVLVG
VVKDEGSYFLVYGAPGFSKDNESLISRAEFLAGVRVGVPQVSDLAAEAVVLHYTDWLHPE
DPARLREALSDVVGDHNVVCPVAQLAGRLAAQGARVYAYVFEHRASTLSWPLWMGVPHGY
EIEFIFGIPLDPSRNYTAEEKIFAQRLMRYWANFARTGDPNEPRDPKAPQWPPYTAGAQQ
YVSLDLRPLEVRRGLRAQACAFWNRFLPKLLSATDTLDEAERQWKAEFHRWSSYMVHWKN
QFDHYSKQDRCSDL
>sp|P12821|ACE_HUMAN Angiotensin-converting enzyme OS=Homo sapiens GN=ACE PE=1 SV=1
MGAASGRRGPGLLLPLPLLLLLPPQPALALDPGLQPGNFSADEAGAQLFAQSYNSSAEQV
LFQSVAASWAHDTNITAENARRQEEAALLSQEFAEAWGQKAKELYEPIWQNFTDPQLRRI
IGAVRTLGSANLPLAKRQQYNALLSNMSRIYSTAKVCLPNKTATCWSLDPDLTNILASSR
SYAMLLFAWEGWHNAAGIPLKPLYEDFTALSNEAYKQDGFTDTGAYWRSWYNSPTFEDDL
EHLYQQLEPLYLNLHAFVRRALHRRYGDRYINLRGPIPAHLLGDMWAQSWENIYDMVVPF

PDKPNLDVTSTMLQQGWNATHMFRVAEEFFTSLELSPMPPEFWEGSMLEKPADGREVVCH
ASAWDFYNRKDFRIKQCTRVTMDQLSTVHHEMGHIQYYLQYKDLPVSLRRGANPGFHEAI
GDVLALSVSTPEHLHKIGLLDRVTNDTESDINYLLKMALEKIAFLPFGYLVDQWRWGVFS
GRTPPSRYNFDWWYLRTKYQGICPPVTRNETHFDAGAKFHVPNVTPYIRYFVSFVLQFQF
HEALCKEAGYEGPLHQCDIYRSTKAGAKLRKVLQAGSSRPWQEVLKDMVGLDALDAQPLL
KYFQPVTQWLQEQNQQNGEVLGWPEYQWHPPLPDNYPEGIDLVTDEAEASKFVEEYDRTS
QVVWNEYAEANWNYNTNITTETSKILLQKNMQIANHTLKYGTQARKFDVNQLQNTTIKRI
IKKVQDLERAALPAQELEEYNKILLDMETTYSVATVCHPNGSCLQLEPDLTNVMATSRKY
EDLLWAWEGWRDKAGRAILQFYPKYVELINQAARLNGYVDAGDSWRSMYETPSLEQDLER
LFQELQPLYLNLHAYVRRALHRHYGAQHINLEGPIPAHLLGNMWAQTWSNIYDLVVPFPS
APSMDTTEAMLKQGWTPRRMFKEADDFFTSLGLLPVPPEFWNKSMLEKPTDGREVVCHAS
AWDFYNGKDFRIKQCTTVNLEDLVVAHHEMGHIQYFMQYKDLPVALREGANPGFHEAIGD
VLALSVSTPKHLHSLNLLSSEGGSDEHDINFLMKMALDKIAFIPFSYLVDQWRWRVFDGS
ITKENYNQEWWSLRLKYQGLCPPVPRTQGDFDPGAKFHIPSSVPYIRYFVSFIIQFQFHE
ALCQAAGHTGPLHKCDIYQSKEAGQRLATAMKLGFSRPWPEAMQLITGQPNMSASAMLSY
FKPLLDWLRTENELHGEKLGWPQYNWTPNSARSEGPLPDSGRVSFLGLDLDAQQARVGQW
LLLFLGIALLVATLGLSQRLFSIRHRSLHRHSHGPQFGSEVELRHS

>sp|P43681|ACHA4_HUMAN Neuronal acetylcholine receptor subunit alpha-4 OS=Homo sapiens GN=CHRNA4 PE=1 SV=2
MELGGPGAPRLLPPLLLLLGTGLLRASSHVETRAHAEERLLKKLFSGYNKWSRPVANISD
VVLVRFGLSIAQLIDVDEKNQMMTTNVWVKQEWHDYKLRWDPADYENVTSIRIPSELIWR
PDIVLYNNADGDFAVTHLTKAHLFHDGRVQWTPPAIYKSSCSIDVTFFPFDQQNCTMKFG
SWTYDKAKIDLVNMHSRVDQLDFWESGEWVIVDAVGTYNTRKYECCAEIYPDITYAFVIR
RLPLFYTINLIIPCLLISCLTVLVFYLPSECGEKITLCISVLLSLTVFLLLITEIIPSTS
LVIPLIGEYLLFTMIFVTLSIVITVFVLNVHHRSPRTHTMPTWVRRVFLDIVPRLLLMKR
PSVVKDNCRRLIESMHKMASAPRFWPEPEGEPPATSGTQSLHPPSPSFCVPLDVPAEPGP
SCKSPSDQLPPQQPLEAEKASPHPSPGPCRPPHGTQAPGLAKARSLSVQHMSSPGEAVEG
GVRCRSRSIQYCVPRDDAAPEADGQAAGALASRNTHSAELPPPDQPSPCKCTCKKEPSSV
SPSATVKTRSTKAPPPHLPLSPALTRAVEGVQYIADHLKAEDTDFSVKEDWKYVAMVIDR
IFLWMFIIVCLLGTVGLFLPPWLAGMI

>sp|P17787|ACHB2_HUMAN Neuronal acetylcholine receptor subunit beta-2 OS=Homo sapiens GN=CHRNB2 PE=1 SV=1
MARRCGPVALLLGFGLLRLCSGVWGTDTEERLVEHLLDPSRYNKLIRPATNGSELVTVQL
MVSLAQLISVHEREQIMTTNVWLTQEWEDYRLTWKPEEFDNMKKVRLPSKHIWLPDVVLY
NNADGMYEVSFYSNAVVSYDGSIFWLPPAIYKSACKIEVKHFPFDQQNCTMKFRSWTYDR
TEIDLVLKSEVASLDDFTPSGEWDIVALPGRRNENPDDSTYVDITYDFIIRRKPLFYTIN
LIIPCVLITSLAILVFYLPSDCGEKMTLCISVLLALTVFLLLISKIVPPTSLDVPLVGKY
LMFTMVLVTFSIVTSVCVLNVHHRSPTTHTMAPWVKVVFLEKLPALLFMQQPRHHCARQR
LRLRRRQREREGAGALFFREAPGADSCTCFVNRASVQGLAGAFGAEPAPVAGPGRSGEPC
GCGLREAVDGVRFIADHMRSEDDDQSVSEDWKYVAMVIDRLFLWIFVFVCVFGTIGMFLQ
PLFQNYTTTTFLHSDHSAPSSK

>sp|P11230|ACHB_HUMAN Acetylcholine receptor subunit beta OS=Homo sapiens GN=CHRNB1 PE=1 SV=3
MTPGALLMLLGALGAPLAPGVRGSEAEGRLREKLFSGYDSSVRPAREVGDRVRVSVGLIL

AQLISLNEKDEEMSTKVYLDLEWTDYRLSWDPAEHDGIDSLRITAESVWLPDVVLLNNND
GNFDVALDISVVVSSDGSVRWQPPGIYRSSCSIQVTYFPFDWQNCTMVFSSYSYDSSEVS
LQTGLGPDGQGHQEIHIHEGTFIENGQWEIIHKPSRLIQPPGDPRGGREGQRQEVIFYLI
IRRKPLFYLVNVIAPCILITLLAIFVFYLPPDAGEKMGLSIFALLTLTVFLLLLADKVPE
TSLSVPIIIKYLMFTMVLVTFSVILSVVVLNLHHRSPHTHQMPLWVRQIFIHKLPLYLRL
KRPKPERDLMPEPPHCSSPGSGWGRGTDEYFIRKPPSDFLFPKPNRFQPELSAPDLRRFI
DGPNRAVALLPELREVVSSISYIARQLQEQEDHDALKEDWQFVAMVVDRLFLWTFIIFTS
VGTLVIFLDATYHLPPPDPFP
>sp|P08172|ACM2_HUMAN Muscarinic acetylcholine receptor M2 OS=Homo sapiens GN=CHRM2 PE=1 SV=1
MNNSTNSSNNSLALTSPYKTFEVVFIVLVAGSLSLVTIIGNILVMVSIKVNRHLQTVNNY
FLFSLACADLIIGVFSMNLYTLYTVIGYWPLGPVVCDLWLALDYVVSNASVMNLLIISFD
RYFCVTKPLTYPVKRTTKMAGMMIAAAWVLSFILWAPAILFWQFIVGVRTVEDGECYIQF
FSNAAVTFGTAIAAFYLPVIIMTVLYWHISRASKSRIKKDKKEPVANQDPVSPSLVQGRI
VKPNNNNMPSSDDGLEHNKIQNGKAPRDPVTENCVQGEEKESSNDSTSVSAVASNMRDDE
ITQDENTVSTSLGHSKDENSKQTCIRIGTKTPKSDSCTPTNTTVEVVGSSGQNGDEKQNI
VARKIVKMTKQPAKKKPPPSREKKVTRTILAILLAFIITWAPYNVMVLINTFCAPCIPNT
VWTIGYWLCYINSTINPACYALCNATFKKTFKHLLMCHYKNIGATR
>sp|Q8TDX5|ACMSD_HUMAN 2-amino-3-carboxymuconate-6-semialdehyde decarboxylase OS=Homo sapiens GN=ACMSD PE=1 SV=1
MKIDIHSHILPKEWPDLKKRFGYGGWVQLQHHSKGEAKLLKDGKVFRVVRENCWDPEVRI
REMDQKGVTVQALSTVPVMFSYWAKPEDTLNLCQLLNNDLASTVVSYPRRFVGLGTLPMQ
APELAVKEMERCVKELGFPGVQIGTHVNEWDLNAQELFPVYAAAERLKCSLFVHPWDMQM
DGRMAKYWLPWLVGMPAETTIAICSMIMGGVFEKFPKLKVCFAHGGGAFPFTVGRISHGF
SMRPDLCAQDNPMNPKKYLGSFYTDALVHDPLSLKLLTDVIGKDKVILGTDYPFPLGELE
PGKLIESMEEFDEETKNKLKAGNALAFLGLERKQFE
>sp|Q9Y305|ACOT9_HUMAN Acyl-coenzyme A thioesterase 9, mitochondrial OS=Homo sapiens GN=ACOT9 PE=1 SV=2
MRRAALRLCALGKGQLTPGRGLTQGPQNPKKQGIFHIHEVRDKLREIVGASTNWRDHVKA
MEERKLLHSFLAKSQDGLPPRRMKDSYIEVLLPLGSEPELREKYLTVQNTVRFGRILEDL
DSLGVLICYMHNKIHSAKMSPLSIVTALVDKIDMCKKSLSPEQDIKFSGHVSWVGKTSME
VKMQMFQLHGDEFCPVLDATFVMVARDSENKGPAFVNPLIPESPEEEELFRQGELNKGRR
IAFSSTSLLKMAPSAEERTTIHEMFLSTLDPKTISFRSRVLPSNAVWMENSKLKSLEICH
PQERNIFNRIFGGFLMRKAYELAWATACSFGGSRPFVVAVDDIMFQKPVEVGSLLFLSSQ
VCFTQNNYIQVRVHSEVASLQEKQHTTTNVFHFTFMSEKEVPLVFPKTYGESMLYLDGQR
HFNSMSGPATLRKDYLVEP
>sp|P62258|1433E_HUMAN 14-3-3 protein epsilon OS=Homo sapiens GN=YWHAE PE=1 SV=1
MDDREDLVYQAKLAEQAERYDEMVESMKKVAGMDVELTVEERNLLSVAYKNVIGARRASW
RIISSIEQKEENKGGEDKLKMIREYRQMVETELKLICCDILDVLDKHLIPAANTGESKVF
YYKMKGDYHRYLAEFATGNDRKEAAENSLVAYKAASDIAMTELPPTHPIRLGLALNFSVF
YYEILNSPDRACRLAKAAFDDAIAELDTLSEESYKDSTLIMQLLRDNLTLWTSDMQGDGE
EQNKEALQDVEDENQ
>sp|Q9BTE6|AASD1_HUMAN Alanyl-tRNA editing protein Aarsd1 OS=Homo sapiens GN=AARSD1 PE=1 SV=2

MAFWCQRDSYAREFTTTVVSCCPAELQTEGSNGKKEVLSGFQVVLEDTVLFPEGGGQPDD
RGTINDISVLRVTRRGEQADHFTQTPLDPGSQVLVRVDWERRFDHMQQHSGQHLITAVAD
HLFKLKTTSWELGRFRSAIELDTPSMTAEQVAAIEQSVNEKIRDRLPVNVRELSLDDPEV
EQVSGRGLPDDHAGPIRVVNIEGVDSNMCCGTHVSNLSDLQVIKILGTEKGKKNRTNLIF
LSGNRVLKWMERSHGTEKALTALLKCGAEDHVEAVKKLQNSTKILQKNNLNLLRDLAVHI
AHSLRNSPDWGGVVILHRKEGDSEFMNIIANEIGSEETLLFLTVGDEKGGGLFLLAGPPA
SVETLGPRVAEVLEGKGAGKKGRFQGKATKMSRRMEAQALLQDYISTQSAKE
>sp|P00505|AATM_HUMAN Aspartate aminotransferase, mitochondrial OS=Homo sapiens GN=GOT2 PE=1 SV=3
MALLHSGRVLPGIAAAFHPGLAAAASARASSWWTHVEMGPPDPILGVTEAFKRDTNSKKM
NLGVGAYRDDNGKPYVLPSVRKAEAQIAAKNLDKEYLPIGGLAEFCKASAELALGENSEV
LKSGRFVTVQTISGTGALRIGASFLQRFFKFSRDVFLPKPTWGNHTPIFRDAGMQLQGYR
YYDPKTCGFDFTGAVEDISKIPEQSVLLLHACAHNPTGVDPRPEQWKEIATVVKKRNLFA
FFDMAYQGFASGDGDKDAWAVRHFIEQGINVCLCQSYAKNMGLYGERVGAFTMVCKDADE
AKRVESQLKILIRPMYSNPPLNGARIAAAILNTPDLRKQWLQEVKVMADRIIGMRTQLVS
NLKKEGSTHNWQHITDQIGMFCFTGLKPEQVERLIKEFSIYMTKDGRISVAGVTSSNVGY
LAHAIHQVTK
>sp|Q9NRW3|ABC3C_HUMAN DNA dC->dU-editing enzyme APOBEC-3C OS=Homo sapiens GN=APOBEC3C PE=1 SV=2
MNPQIRNPMKAMYPGTFYFQFKNLWEANDRNETWLCFTVEGIKRRSVVSWKTGVFRNQVD
SETHCHAERCFLSWFCDDILSPNTKYQVTWYTSWSPCPDCAGEVAEFLARHSNVNLTIFT
ARLYYFQYPCYQEGLRSLSQEGVAVEIMDYEDFKYCWENFVYNDNEPFKPWKGLKTNFRL
LKRRLRESLQ
>sp|O95477|ABCA1_HUMAN ATP-binding cassette sub-family A member 1 OS=Homo sapiens GN=ABCA1 PE=1 SV=3
MACWPQLRLLLWKNLTFRRRQTCQLLLEVAWPLFIFLILISVRLSYPPYEQHECHFPNKA
MPSAGTLPWVQGIICNANNPCFRYPTPGEAPGVVGNFNKSIVARLFSDARRLLLYSQKDT
SMKDMRKVLRTLQQIKKSSSNLKLQDFLVDNETFSGFLYHNLSLPKSTVDKMLRADVILH
KVFLQGYQLHLTSLCNGSKSEEMIQLGDQEVSELCGLPREKLAAAERVLRSNMDILKPIL
RTLNSTSPFPSKELAEATKTLLHSLGTLAQELFSMRSWSDMRQEVMFLTNVNSSSSSTQI
YQAVSRIVCGHPEGGGLKIKSLNWYEDNNYKALFGGNGTEEDAETFYDNSTTPYCNDLMK
NLESSPLSRIIWKALKPLLVGKILYTPDTPATRQVMAEVNKTFQELAVFHDLEGMWEELS
PKIWTFMENSQEMDLVRMLLDSRDNDHFWEQQLDGLDWTAQDIVAFLAKHPEDVQSSNGS
VYTWREAFNETNQAIRTISRFMECVNLNKLEPIATEVWLINKSMELLDERKFWAGIVFTG
ITPGSIELPHHVKYKIRMDIDNVERTNKIKDGYWDPGPRADPFEDMRYVWGGFAYLQDVV
EQAIIRVLTGTEKKTGVYMQQMPYPCYVDDIFLRVMSRSMPLFMTLAWIYSVAVIIKGIV
YEKEARLKETMRIMGLDNSILWFSWFISSLIPLLVSAGLLVVILKLGNLLPYSDPSVVFV
FLSVFAVVTILQCFLISTLFSRANLAAACGGIIYFTLYLPYVLCVAWQDYVGFTLKIFAS
LLSPVAFGFGCEYFALFEEQGIGVQWDNLFESPVEEDGFNLTTSVSMMLFDTFLYGVMTW
YIEAVFPGQYGIPRPWYFPCTKSYWFGEESDEKSHPGSNQKRISEICMEEEPTHLKLGVS
IQNLVKVYRDGMKVAVDGLALNFYEGQITSFLGHNGAGKTTTMSILTGLFPPTSGTAYIL
GKDIRSEMSTIRQNLGVCPQHNVLFDMLTVEEHIWFYARLKGLSEKHVKAEMEQMALDVG
LPSSKLKSKTSQLSGGMQRKLSVALAFVGGSKVVILDEPTAGVDPYSRRGIWELLLKYRQ
GRTIILSTHHMDEADVLGDRIAIISHGKLCCVGSSLFLKNQLGTGYYLTLVKKDVESSLS

SCRNSSSTVSYLKKEDSVSQSSSDAGLGSDHESDTLTIDVSAISNLIRKHVSEARLVEDI
GHELTYVLPYEAAKEGAFVELFHEIDDRLSDLGISSYGISETTLEEIFLKVAEESGVDAE
TSDGTLPARRNRRAFGDKQSCLRPFTEDDAADPNDSDIDPESRETDLLSGMDGKGSYQVK
GWKLTQQQFVALLWKRLLIARRSRKGFFAQIVLPAVFVCIALVFSLIVPPFGKYPSLELQ
PWMYNEQYTFVSNDAPEDTGTLELLNALTKDPGFGTRCMEGNPIPDTPCQAGEEEWTTAP
VPQTIMDLFQNGNWTMQNPSPACQCSSDKIKKMLPVCPPGAGGLPPPQRKQNTADILQDL
TGRNISDYLVKTYVQIIAKSLKNKIWVNEFRYGGFSLGVSNTQALPPSQEVNDAIKQMKK
HLKLAKDSSADRFLNSLGRFMTGLDTKNNVKVWFNNKGWHAISSFLNVINNAILRANLQK
GENPSHYGITAFNHPLNLTKQQLSEVALMTTSVDVLVSICVIFAMSFVPASFVVFLIQER
VSKAKHLQFISGVKPVIYWLSNFVWDMCNYVVPATLVIIIFICFQQKSYVSSTNLPVLAL
LLLLYGWSITPLMYPASFVFKIPSTAYVVLTSVNLFIGINGSVATFVLELFTDNKLNNIN
DILKSVFLIFPHFCLGRGLIDMVKNQAMADALERFGENRFVSPLSWDLVGRNLFAMAVEG
VVFFLITVLIQYRFFIRPRPVNAKLSPLNDEDEDVRRERQRILDGGGQNDILEIKELTKI
YRRKRKPAVDRICVGIPPGECFGLLGVNGAGKSSTFKMLTGDTTVTRGDAFLNKNSILSN
IHEVHQNMGYCPQFDAITELLTGREHVEFFALLRGVPEKEVGKVGEWAIRKLGLVKYGEK
YAGNYSGGNKRKLSTAMALIGGPPVVFLDEPTTGMDPKARRFLWNCALSVVKEGRSVVLT
SHSMEECEALCTRMAIMVNGRFRCLGSVQHLKNRFGDGYTIVVRIAGSNPDLKPVQDFFG
LAFPGSVLKEKHRNMLQYQLPSSLSSLARIFSILSQSKKRLHIEDYSVSQTTLDQVFVNF
AKDQSDDDHLKDLSLHKNQTVVDVAVLTSFLQDEKVKESYV
>sp|Q8WWZ7|ABCA5_HUMAN ATP-binding cassette sub-family A member 5 OS=Homo sapiens GN=ABCA5 PE=2 SV=2
MSTAIREVGVWRQTRTLLLKNYLIKCRTKKSSVQEILFPLFFLFWLILISMMHPNKKYEE
VPNIELNPMDKFTLSNLILGYTPVTNITSSIMQKVSTDHLPDVIITEEYTNEKEMLTSSL
SKPSNFVGVVFKDSMSYELRFFPDMIPVSSIYMDSRAGCSKSCEAAQYWSSGFTVLQASI
DAAIIQLKTNVSLWKELESTKAVIMGETAVVEIDTFPRGVILIYLVIAFSPFGYFLAIHI
VAEKEKKIKEFLKIMGLHDTAFWLSWVLLYTSLIFLMSLLMAVIATASLLFPQSSSIVIF
LLFFLYGLSSVFFALMLTPLFKKSKHVGIVEFFVTVAFGFIGLMIILIESFPKSLVWLFS
PFCHCTFVIGIAQVMHLEDFNEGASFSNLTAGPYPLIITIIMLTLNSIFYVLLAVYLDQV
IPGEFGLRRSSLYFLKPSYWSKSKRNYEELSEGNVNGNISFSEIIEPVSSEFVGKEAIRI
SGIQKTYRKKGENVEALRNLSFDIYEGQITALLGHSGTGKSTLMNILCGLCPPSDGFASI
YGHRVSEIDEMFEARKMIGICPQLDIHFDVLTVEENLSILASIKGIPANNIIQEVQKVLL
DLDMQTIKDNQAKKLSGGQKRKLSLGIAVLGNPKILLLDEPTAGMDPCSRHIVWNLLKYR
KANRVTVFSTHFMDEADILADRKAVISQGMLKCVGSSMFLKSKWGIGYRLSMYIDKYCAT
ESLSSLVKQHIPGATLLQQNDQQLVYSLPFKDMDKFSGLFSALDSHSNLGVISYGVSMTT
LEDVFLKLEVEAEIDQADYSVFTQQPLEEEMDSKSFDEMEQSLLILSETKAALVSTMSLW
KQQMYTIAKFHFFTLKRESKSVRSVLLLLLIFFTVQIFMFLVHHSFKNAVVPIKLVPDLY
FLKPGDKPHKYKTSLLLQNSADSDISDLISFFTSQNIMVTMINDSDYVSVAPHSAALNVM
HSEKDYVFAAVFNSTMVYSLPILVNIISNYYLYHLNVTETIQIWSTPFFQEITDIVFKIE
LYFQAALLGIIVTAMPPYFAMENAENHKIKAYTQLKLSGLLPSAYWIGQAVVDIPLFFII
LILMLGSLLAFHYGLYFYTVKFLAVVFCLIGYVPSVILFTYIASFTFKKILNTKEFWSFI
YSVAALACIAITEITFFMGYTIATILHYAFCIIIPIYPLLGCLISFIKISWKNVRKNVDT
YNPWDRLSVAVISPYLQCVLWIFLLQYYEKKYGGRSIRKDPFFRNLSTKSKNRKLPEPPD
NEDEDEDVKAERLKVKELMGCQCCEEKPSIMVSNLHKEYDDKKDFLLSRKVKKVATKYIS
FCVKKGEILGLLGPNGAGKSTIINILVGDIEPTSGQVFLGDYSSETSEDDDSLKCMGYCP

QINPLWPDTTLQEHFEIYGAVKGMSASDMKEVISRITHALDLKEHLQKTVKKLPAGIKRK
LCFALSMLGNPQITLLDEPSTGMDPKAKQHMWRAIRTAFKNRKRAAILTTHYMEEAEAVC
DRVAIMVSGQLRCIGTVQHLKSKFGKGYFLEIKLKDWIENLEVDRLQREIQYIFPNASRQ
ESFSSILAYKIPKEDVQSLSQSFFKLEEAKHAFAIEEYSFSQATLEQVFVELTKEQEEED
NSCGTLNSTLWWERTQEDRVVF
>sp|Q8N139|ABCA6_HUMAN ATP-binding cassette sub-family A member 6 OS=Homo sapiens GN=ABCA6 PE=1 SV=2
MNMKQKSVYQQTKALLCKNFLKKWRMKRESLLEWGLSILLGLCIALFSSSMRNVQFPGMA
PQNLGRVDKFNSSSLMVVYTPISNLTQQIMNKTALAPLLKGTSVIGAPNKTHMDEILLEN
LPYAMGIIFNETFSYKLIFFQGYNSPLWKEDFSAHCWDGYGEFSCTLTKYWNRGFVALQT
AINTAIIEITTNHPVMEELMSVTAITMKTLPFITKNLLHNEMFILFFLLHFSPLVYFISL
NVTKERKKSKNLMKMMGLQDSAFWLSWGLIYAGFIFIISIFVTIIITFTQIIVMTGFMVI
FILFFLYGLSLVALVFLMSVLLKKAVLTNLVVFLLTLFWGCLGFTVFYEQLPSSLEWILN
ICSPFAFTTGMIQIIKLDYNLNGVIFPDPSGDSYTMIATFSMLLLDGLIYLLLALYFDKI
LPYGDERHYSPLFFLNSSSCFQHQRTNAKVIEKEIDAEHPSDDYFEPVAPEFQGKEAIRI
RNVKKEYKGKSGKVEALKGLLFDIYEGQITAILGHSGAGKSSLLNILNGLSVPTEGSVTI
YNKNLSEMQDLEEIRKITGVCPQFNVQFDILTVKENLSLFAKIKGIHLKEVEQEVQRILL
ELDMQNIQDNLAKHLSEGQKRKLTFGITILGDPQILLLDEPTTGLDPFSRDQVWSLLRER
RADHVILFSTQSMDEADILADRKVIMSNGRLKCAGSSMFLKRRWGLGYHLSLHRNEICNP
EQITSFITHHIPDAKLKTENKEKLVYTLPLERTNTFPDLFSDLDKCSDQGVTGYDISMST
LNEVFMKLEGQSTIEQDFEQVEMIRDSESLNEMELAHSSFSEMQTAVSDMGLWRMQVFAM
ARLRFLKLKRQTKVLLTLLLVFGIAIFPLIVENIMYAMLNEKIDWEFKNELYFLSPGQLP
QEPRTSLLIINNTESNIEDFIKSLKHQNILLEVDDFENRNGTDGLSYNGAIIVSGKQKDY
RFSVVCNTKRLHCFPILMNIISNGLLQMFNHTQHIRIESSPFPLSHIGLWTGLPDGSFFL
FLVLCSISPYITMGSISDYKKNAKSQLWISGLYTSAYWCGQALVDVSFFILILLLMYLIF
YIENMQYLLITSQIVFALVIVTPGYAASLVFFIYMISFIFRKRRKNSGLWSFYFFFASTI
MFSITLINHFDLSILITTMVLVPSYTLLGFKTFLEVRDQEHYREFPEANFELSATDFLVC
FIPYFQTLLFVFVLRCMELKCGKKRMRKDPVFRISPQSRDAKPNPEEPIDEDEDIQTERI
RTATALTTSILDEKPVIIASCLHKEYAGQKKSCFSKRKKKIAARNISFCVQEGEILGLLG
PNGAGKSSSIRMISGITKPTAGEVELKGCSSVLGHLGYCPQENVLWPMLTLREHLEVYAA
VKGLRKADARLAIARLVSAFKLHEQLNVPVQKLTAGITRKLCFVLSLLGNSPVLLLDEPS
TGIDPTGQQQMWQAIQAVVKNTERGVLLTTHNLAEAEALCDRVAIMVSGRLRCIGSIQHL
KNKLGKDYILELKVKETSQVTLVHTEILKLFPQAAGQERYSSLLTYKLPVADVYPLSQTF
HKLEAVKHNFNLEEYSLSQCTLEKVFLELSKEQEVGNFDEEIDTTMRWKLLPHSDEP
>sp|Q8IZY2|ABCA7_HUMAN ATP-binding cassette sub-family A member 7 OS=Homo sapiens GN=ABCA7 PE=1 SV=3
MAFWTQLMLLLWKNFMYRRRQPVQLLVELLWPLFLFFILVAVRHSHPPLEHHECHFPNKP
LPSAGTVPWLQGLICNVNNTCFPQLTPGEEPGRLSNFNDSLVSRLLADARTVLGGASAHR
TLAGLGKLIATLRAARSTAQPQPTKQSPLEPPMLDVAELLTSLLRTESLGLALGQAQEPL
HSLLEAAEDLAQELLALRSLVELRALLQRPRGTSGPLELLSEALCSVRGPSSTVGPSLNW
YEASDLMELVGQEPESALPDSSLSPACSELIGALDSHPLSRLLWRRLKPLILGKLLFAPD
TPFTRKLMAQVNRTFEELTLLRDVREVWEMLGPRIFTFMNDSSNVAMLQRLLQMQDEGRR
QPRPGGRDHMEALRSFLDPGSGGYSWQDAHADVGHLVGTLGRVTECLSLDKLEAAPSEAA
LVSRALQLLAEHRFWAGVVFLGPEDSSDPTEHPTPDLGPGHVRIKIRMDIDVVTRTNKIR

DRFWDPGPAADPLTDLRYVWGGFVYLQDLVERAAVRVLSGANPRAGLYLQQMPYPCYVDD
VFLRVLSRSLPLFLTLAWIYSVTLTVKAVVREKETRLRDTMRAMGLSRAVLWLGWFLSCL
GPFLLSAALLVLVLKLGDILPYSHPGVVFLFLAAFAVATVTQSFLLSAFFSRANLAAACG
GLAYFSLYLPYVLCVAWRDRLPAGGRVAASLLSPVAFGFGCESLALLEEQGEGAQWHNVG
TRPTADVFSLAQVSGLLLLDAALYGLATWYLEAVCPGQYGIPEPWNFPFRRSYWCGPRPP
KSPAPCPTPLDPKVLVEEAPPGLSPGVSVRSLEKRFPGSPQPALRGLSLDFYQGHITAFL
GHNGAGKTTTLSILSGLFPPSGGSAFILGHDVRSSMAAIRPHLGVCPQYNVLFDMLTVDE
HVWFYGRLKGLSAAVVGPEQDRLLQDVGLVSKQSVQTRHLSGGMQRKLSVAIAFVGGSQV
VILDEPTAGVDPASRRGIWELLLKYREGRTLILSTHHLDEAELLGDRVAVVAGGRLCCCG
SPLFLRRHLGSGYYLTLVKARLPLTTNEKADTDMEGSVDTRQEKKNGSQGSRVGTPQLLA
LVQHWVPGARLVEELPHELVLVLPYTGAHDGSFATLFRELDTRLAELRLTGYGISDTSLE
EIFLKVVEECAADTDMEDGSCGQHLCTGIAGLDVTLRLKMPPQETALENGEPAGSAPETD
QGSGPDAVGRVQGWALTRQQLQALLLKRFLLARRSRRGLFAQIVLPALFVGLALVFSLIV
PPFGHYPALRLSPTMYGAQVSFFSEDAPGDPGRARLLEALLQEAGLEEPPVQHSSHRFSA
PEVPAEVAKVLASGNWTPESPSPACQCSRPGARRLLPDCPAAAGGPPPPQAVTGSGEVVQ
NLTGRNLSDFLVKTYPRLVRQGLKTKKWVNEVRYGGFSLGGRDPGLPSGQELGRSVEELW
ALLSPLPGGALDRVLKNLTAWAHSLDAQDSLKIWFNNKGWHSMVAFVNRASNAILRAHLP
PGPARHAHSITTLNHPLNLTKEQLSEGALMASSVDVLVSICVVFAMSFVPASFTLVLIEE
RVTRAKHLQLMGGLSPTLYWLGNFLWDMCNYLVPACIVVLIFLAFQQRAYVAPANLPALL
LLLLLYGWSITPLMYPASFFFSVPSTAYVVLTCINLFIGINGSMATFVLELFSDQKLQEV
SRILKQVFLIFPHFCLGRGLIDMVRNQAMADAFERLGDRQFQSPLRWEVVGKNLLAMVIQ
GPLFLLFTLLLQHRSQLLPQPRVRSLPLLGEEDEDVARERERVVQGATQGDVLVLRNLTK
VYRGQRMPAVDRLCLGIPPGECFGLLGVNGAGKTSTFRMVTGDTLASRGEAVLAGHSVAR
EPSAAHLSMGYCPQSDAIFELLTGREHLELLARLRGVPEAQVAQTAGSGLARLGLSWYAD
RPAGTYSGGNKRKLATALALVGDPAVVFLDEPTTGMDPSARRFLWNSLLAVVREGRSVML
TSHSMEECEALCSRLAIMVNGRFRCLGSPQHLKGRFAAGHTLTLRVPAARSQPAAAFVAA
EFPGAELREAHGGRLRFQLPPGGRCALARVFGELAVHGAEHGVEDFSVSQTMLEEVFLYF
SKDQGKDEDTEEQKEAGVGVDPAPGLQHPKRVSQFLDDPSTAETVL
>sp|O94911|ABCA8_HUMAN ATP-binding cassette sub-family A member 8 OS=Homo sapiens GN=ABCA8 PE=1 SV=3
MRKRKISVCQQTWALLCKNFLKKWRMKRESLMEWLNSLLLLLCLYIYPHSHQVNDFSSLL
TMDLGRVDTFNESRFSVVYTPVTNTTQQIMNKVASTPFLAGKEVLGLPDEESIKEFTANY
PEEIVRVTFTNTYSYHLKFLLGHGMPAKKEHKDHTAHCYETNEDVYCEVSVFWKEGFVAL
QAAINAAIIEITTNHSVMEELMSVTGKNMKMHSFIGQSGVITDLYLFSCIISFSSFIYYA
SVNVTRERKRMKALMTMMGLRDSAFWLSWGLLYAGFIFIMALFLALVIRSTQFIILSGFM
VVFSLFLLYGLSLVALAFLMSILVKKSFLTGLVVFLLTVFWGCLGFTSLYRHLPASLEWI
LSLLSPFAFMLGMAQLLHLDYDLNSNAFPHPSDGSNLIVATNFMLAFDTCLYLALAIYFE
KILPNEYGHRRPPLFFLKSSFWSQTQKTDHVALEDEMDADPSFHDSFEQAPPEFQGKEAI
RIRNVTKEYKGKPDKIEALKDLVFDIYEGQITAILGHSGAGKSTLLNILSGLSVPTKGSV
TIYNNKLSEMADLENLSKLTGVCPQSNVQFDFLTVRENLRLFAKIKGILPQEVDKEIFLL
DEPTAGLDPFSRHQVWNLLKERKTDRVILFSTQFMDEADILADRKVFLSQGKLKCAGSSL
FLKKKWGIGYHLSLQLNEICVEENITSLVKQHIPDAKLSAKSEGKLIYTLPLERTNKFPE
LYKDLDSYPDLGIENYGVSMTTLNEVFLKLEGKSTINESDIAILGEVQAEKADDTERLVE
MEQVLSSLNKMRKTIGGVALWRQQICAIARVRLLKLKHERKALLALLLILMAGFCPLLVE

YTMVKIYQNSYTWELSPHLYFLAPGQQPHDPLTQLLIINKTGASIDDFIQSVEHQNIALE
VDAFGTRNGTDDPSYNGAITVCCNEKNYSFSLACNAKRLNCFPVLMDIVSNGLLGMVKPS
VHIRTERSTFLENGQDNPIGFLAYIMFWLVLTSSCPPYIAMSSIDDYKNRARSQLRISGL
SPSAYWFGQALVDVSLYFLVFVFIYLMSYISNFEDMLLTIIHIIQIPCAVGYSFSLIFMT
YVISFIFRKGRKNSGIWSFCFYVVTVFSVAGFAFSIFESDIPFIFTFLIPPATMIGCLFL
SSHLLFSSLFSEERMDVQPFLVFLIPFLHFIIFLFTLRCLEWKFGKKSMRKDPFFRISPR
SSDVCQNPEEPEGEDEDVQMERVRTANALNSTNFDEKPVIIASCLRKEYAGKRKGCFSKR
KNKIATRNVSFCVRKGEVLGLLGHNGAGKSTSIKVITGDTKPTAGQVLLKGSGGGDALEF
LGYCPQENALWPNLTVRQHLEVYAAVKGLRKGDAEVAITRLVDALKLQDQLKSPVKTLSE
GIKRKLCFVLSILGNPSVVLLDEPSTGMDPEGQQQMWQAIRATFRNTERGALLTTHYMAE
AEAVCDRVAIMVSGRLRCIGSIQHLKSKFGKDYLLEMKVKNLAQVEPLHAEILRLFPQAA
RQERYSSLMVYKLPVEDVQPLAQAFFKLEKVKQSFDLEEYSLSQSTLEQVFLELSKEQEL
GDFEEDFDPSVKWKLLPQEEP
>sp|Q4W5N1|ABCAB_HUMAN Putative ATP-binding cassette sub-family A member 11 OS=Homo sapiens GN=ABCA11P PE=2 SV=1
MKYGNEIMNKDPVFRISPRSRGTHTNPEEPEEDVQAERVQAANALTTPNLEEEPVITASC
LHKEYYETKKVAFQQQRRKQPSEMFRFVLKSEVLGLLGHNGAGKSTSIKMITGCTVPTAG
VVVLQGNRASVRQQRDNSLKFLGTALRRTHCVPNLQ
>sp|Q86UK0|ABCAC_HUMAN ATP-binding cassette sub-family A member 12 OS=Homo sapiens GN=ABCA12 PE=1 SV=3
MASLFHQLQILVWKNWLGVKRQPLWTLVLILWPVIIFIILAITRTKFPPTAKPTCYLAPR
NLPSTGFFPFLQTLLCDTDSKCKDTPYGPQDLLRRKGIDDALFKDSEILRKSSNLDKDSS
LSFQSTQVPERRHASLATVFPSPSSDLEIPGTYTFNGSQVLARILGLEKLLKQNSTSEDI
RRELCDSYSGYIVDDAFSWTFLGRNVFNKFCLSNMTLLESSLQELNKQFSQLSSDPNNQK
IVFQEIVRMLSFFSQVQEQKAVWQLLSSFPNVFQNDTSLSNLFDVLRKANSVLLVVQKVY
PRFATNEGFRTLQKSVKHLLYTLDSPAQGDSDNITHVWNEDDGQTLSPSSLAAQLLILEN
FEDALLNISANSPYIPYLACVRNVTDSLARGSPENLRLLQSTIRFKKSFLRNGSYEDYFP
PVPEVLKSKLSQLRNLTELLCESETFSLIEKSCQLSDMSFGSLCEESEFDLQLLEAAELG
TEIAASLLYHDNVISKKVRDLLTGDPSKINLNMDQFLEQALQMNYLENITQLIPIIEAML
HVNNSADASEKPGQLLEMFKNVEELKEDLRRTTGMSNRTIDKLLAIPIPDNRAEIISQVF
WLHSCDTNITTPKLEDAMKEFCNLSLSERSRQSYLIGLTLLHYLNIYNFTYKVFFPRKDQ
KPVEKMMELFIRLKEILNQMASGTHPLLDKMRSLKQMHLPRSVPLTQAMYRSNRMNTPQG
SFSTISQALCSQGITTEYLTAMLPSSQRPKGNHTKDFLTYKLTKEQIASKYGIPINSTPF
CFSLYKDIINMPAGPVIWAFLKPMLLGRILYAPYNPVTKAIMEKSNVTLRQLAELREKSQ
EWMDKSPLFMNSFHLLNQAIPMLQNTLRNPFVQVFVKFSVGLDAVELLKQIDELDILRLK
LENNIDIIDQLNTLSSLTVNISSCVLYDRIQAAKTIDEMEREAKRLYKSNELFGSVIFKL
PSNRSWHRGYDSGNVFLPPVIKYTIRMSLKTAQTTRSLRTKIWAPGPHNSPSHNQIYGRA
FIYLQDSIERAIIELQTGRNSQEIAVQVQAIPYPCFMKDNFLTSVSYSLPIVLMVAWVVF
IAAFVKKLVYEKDLRLHEYMKMMGVNSCSHFFAWLIESVGFLLVTIVILIIILKFGNILP
KTNGFILFLYFSDYSFSVIAMSYLISVFFNNTNIAALIGSLIYIIAFFPFIVLVTVENEL
SYVLKVFMSLLSPTAFSYASQYIARYEEQGIGLQWENMYTSPVQDDTTSFGWLCCLILAD
SFIYFLIAWYVRNVFPGTYGMAAPWYFPILPSYWKERFGCAEVKPEKSNGLMFTNIMMQN
TNPSASPEYMFSSNIEPEPKDLTVGVALHGVTKIYGSKVAVDNLNLNFYEGHITSLLGPN
GAGKTTTISMLTGLFGASAGTIFVYGKDIKTDLHTVRKNMGVCMQHDVLFSYLTTKEHLL

LYGSIKVPHWTKKQLHEEVKRTLKDTGLYSHRHKRVGTLSGGMKRKLSISIALIGGSRVV
ILDEPSTGVDPCSRRSIWDVISKNKTARTIILSTHHLDEAEVLSDRIAFLEQGGLRCCGS
PFYLKEAFGDGYHLTLTKKKSPNLNANAVCDTMAVTAMIQSHLPEAYLKEDIGGELVYVL
PPFSTKVSGAYLSLLRALDNGMGDLNIGCYGISDTTVEEVFLNLTKESQKNSAMSLEHLT
QKKIGNSNANGISTPDDLSVSSSNFTDRDDKILTRGERLDGFGLLLKKIMAILIKRFHHT
RRNWKGLIAQVILPIVFVTTAMGLGTLRNSSNSYPEIQISPSLYGTSEQTAFYANYHPST
EALVSAMWDFPGIDNMCLNTSDLQCLNKDSLEKWNTSGEPITNFGVCSCSENVQECPKFN
YSPPHRRTYSSQVIYNLTGQRVENYLISTANEFVQKRYGGWSFGLPLTKDLRFDITGVPA
NRTLAKVWYDPEGYHSLPAYLNSLNNFLLRVNMSKYDAARHGIIMYSHPYPGVQDQEQAT
ISSLIDILVALSILMGYSVTTASFVTYVVREHQTKAKQLQHISGIGVTCYWVTNFIYDMV
FYLVPVAFSIGIIAIFKLPAFYSENNLGAVSLLLLLFGYATFSWMYLLAGLFHETGMAFI
TYVCVNLFFGINSIVSLSVVYFLSKEKPNDPTLELISETLKRIFLIFPQFCFGYGLIELS
QQQSVLDFLKAYGVEYPNETFEMNKLGAMFVALVSQGTMFFSLRLLINESLIKKLRLFFR
KFNSSHVRETIDEDEDVRAERLRVESGAAEFDLVQLYCLTKTYQLIHKKIIAVNNISIGI
PAGECFGLLGVNGAGKTTIFKMLTGDIIPSSGNILIRNKTGSLGHVDSHSSLVGYCPQED
ALDDLVTVEEHLYFYARVHGIPEKDIKETVHKLLRRLHLMPFKDRATSMCSYGTKRKLST
ALALIGKPSILLLDEPSSGMDPKSKRHLWKIISEEVQNKCSVILTSHSMEECEALCTRLA
IMVNGKFQCIGSLQHIKSRFGRGFTVKVHLKNNKVTMETLTKFMQLHFPKTYLKDQHLSM
LEYHVPVTAGGVANIFDLLETNKTALNITNFLVSQTTLEEVFINFAKDQKSYETADTSSQ
GSTISVDSQDDQMES
>sp|Q9BV23|ABHD6_HUMAN Monoacylglycerol lipase ABHD6 OS=Homo sapiens GN=ABHD6 PE=1 SV=1
MDLDVVNMFVIAGGTLAIPILAFVASFLLWPSALIRIYYWYWRRTLGMQVRYVHHEDYQF
CYSFRGRPGHKPSILMLHGFSAHKDMWLSVVKFLPKNLHLVCVDMPGHEGTTRSSLDDLS
IDGQVKRIHQFVECLKLNKKPFHLVGTSMGGQVAGVYAAYYPSDVSSLCLVCPAGLQYST
DNQFVQRLKELQGSAAVEKIPLIPSTPEEMSEMLQLCSYVRFKVPQQILQGLVDVRIPHN
NFYRKLFLEIVSEKSRYSLHQNMDKIKVPTQIIWGKQDQVLDVSGADMLAKSIANCQVEL
LENCGHSVVMERPRKTAKLIIDFLASVHNTDNNKKLD
>sp|Q9BUJ0|ABHEA_HUMAN Protein ABHD14A OS=Homo sapiens GN=ABHD14A PE=2 SV=2
MVGALCGCWFRLGGARPLIPLGPTVVQTSMSRSQVALLGLSLLLMLLLYVGLPGPPEQTS
CLWGDPNVTVLAGLTPGNSPIFYREVLPLNQAHRVEVVLLHGKAFNSHTWEQLGTLQLLS
QRGYRAVALDLPGFGNSAPSKEASTEAGRAALLERALRDLEVQNAVLVSPSLSGHYALPF
LMRGHHQLHGFVPIAPTSTQNYTQEQFWAVKTPTLILYGELDHILARESLRQLRHLPNHS
VVKLRNAGHACYLHKPQDFHLVLLAFLDHLP
>sp|Q9P2A4|ABI3_HUMAN ABI gene family member 3 OS=Homo sapiens GN=ABI3 PE=1 SV=2
MAELQQLQEFEIPTGREALRGNHSALLRVADYCEDNYVQATDKRKALEETMAFTTQALAS
VAYQVGNLAGHTLRMLDLQGAALRQVEARVSTLGQMVNMHMEKVARREIGTLATVQRLPP
GQKVIAPENLPPLTPYCRRPLNFGCLDDIGHGIKDLSTQLSRTGTLSRKSIKAPATPASA
TLGRPPRIPEPVHLPVVPDGRLSAASSAFSLASAGSAEGVGGAPTPKGQAAPPAPPLPSS
LDPPPPPAAVEVFQRPPTLEELSPPPPDEELPLPLDLPPPPPLDGDELGLPPPPPGFGPD
EPSWVPASYLEKVVTLYPYTSQKDNELSFSEGTVICVTRRYSDGWCEGVSSEGTGFFPGN
YVEPSC
>sp|O94929|ABLM3_HUMAN Actin-binding LIM protein 3 OS=Homo sapiens GN=ABLIM3 PE=1 SV=3
MNTSIPYQQNPYNPRGSSNVIQCYRCGDTCKGEVVRVHNNHFHIRCFTCQVCGCGLAQSG
FFFKNQEYICTQDYQQLYGTRCDSCRDFITGEVISALGRTYHPKCFVCSLCRKPFPIGDK

VTFSGKECVCQTCSQSMASSKPIKIRGPSHCAGCKEEIKHGQSLLALDKQWHVSCFKCQT
CSVILTGEYISKDGVPYCESDYHAQFGIKCETCDRYISGRVLEAGGKHYHPTCARCVRCH
QMFTEGEEMYLTGSEVWHPICKQAARAEKKLKHRRTSETSISPPGSSIGSPNRVICAKVD
NEILNYKDLAALPKVKSIYEVQRPDLISYEPHSRYMSDEMLERCGYGESLGTLSPYSQDI
YENLDLRQRRASSPGYIDSPTYSRQGMSPTFSRSPHHYYRSGPESGRSSPYHSQLDVRSS
TPTSYQAPKHFHIPAGDSNIYRKPPIYKRHGDLSTATKSKTSEDISQTSKYSPIYSPDPY
YASESEYWTYHGSPKVPRARRFSSGGEEDDFDRSMHKLQSGIGRLILKEEMKARSSSYAD
PWTPPRSSTSSREALHTAGYEMSLNGSPRSHYLADSDPLISKSASLPAYRRNGLHRTPSA
DLFHYDSMNAVNWGMREYKIYPYELLLVTTRGRNRLPKDVDRTRLERHLSQEEFYQVFGM
TISEFDRLALWKRNELKKQARLF
>sp|Q9NPB9|ACKR4_HUMAN Atypical chemokine receptor 4 OS=Homo sapiens GN=ACKR4 PE=1 SV=1
MALEQNQSTDYYYEENEMNGTYDYSQYELICIKEDVREFAKVFLPVFLTIVFVIGLAGNS
MVVAIYAYYKKQRTKTDVYILNLAVADLLLLFTLPFWAVNAVHGWVLGKIMCKITSALYT
LNFVSGMQFLACISIDRYVAVTKVPSQSGVGKPCWIICFCVWMAAILLSIPQLVFYTVND
NARCIPIFPRYLGTSMKALIQMLEICIGFVVPFLIMGVCYFITARTLMKMPNIKISRPLK
VLLTVVIVFIVTQLPYNIVKFCRAIDIIYSLITSCNMSKRMDIAIQVTESIALFHSCLNP
ILYVFMGASFKNYVMKVAKKYGSWRRQRQSVEEFPFDSEGPTEPTSTFSI
>sp|Q9Y615|ACL7A_HUMAN Actin-like protein 7A OS=Homo sapiens GN=ACTL7A PE=1 SV=1
MWAPPAAIMGDGPTKKVGNQAPLQTQALQTASLRDGPAKRAVWVRHTSSEPQEPTESKAA
KERPKQEVTKAVVVDLGTGYCKCGFAGLPRPTHKISTTVGKPYMETAKTGDNRKETFVGQ
ELNNTNVHLKLVNPLRHGIIVDWDTVQDIWEYLFRQEMKIAPEEHAVLVSDPPLSPHTNR
EKYAEMLFEAFNTPAMHIAYQSRLSMYSYGRTSGLVVEVGHGVSYVVPIYEGYPLPSITG
RLDYAGSDLTAYLLGLLNSAGNEFTQDQMGIVEDIKKKCCFVALDPIEEKKVPLSEHTIR
YVLPDGKEIQLCQERFLCSEMFFKPSLIKSMQLGLHTQTVSCLNKCDIALKRDLMGNILL
CGGSTMLSGFPNRLQKELSSMCPNDTPQVNVLPERDSAVWTGGSILASLQGFQPLWVHRF
EYEEHGPFFLYRRCF
>sp|P08173|ACM4_HUMAN Muscarinic acetylcholine receptor M4 OS=Homo sapiens GN=CHRM4 PE=1 SV=2
MANFTPVNGSSGNQSVRLVTSSSHNRYETVEMVFIATVTGSLSLVTVVGNILVMLSIKVN
RQLQTVNNYFLFSLACADLIIGAFSMNLYTVYIIKGYWPLGAVVCDLWLALDYVVSNASV
MNLLIISFDRYFCVTKPLTYPARRTTKMAGLMIAAAWVLSFVLWAPAILFWQFVVGKRTV
PDNQCFIQFLSNPAVTFGTAIAAFYLPVVIMTVLYIHISLASRSRVHKHRPEGPKEKKAK
TLAFLKSPLMKQSVKKPPPGEAAREELRNGKLEEAPPPALPPPPRPVADKDTSNESSSGS
ATQNTKERPATELSTTEATTPAMPAPPLQPRALNPASRWSKIQIVTKQTGNECVTAIEIV
PATPAGMRPAANVARKFASIARNQVRKKRQMAARERKVTRTIFAILLAFILTWTPYNVMV
LVNTFCQSCIPDTVWSIGYWLCYVNSTINPACYALCNATFKKTFRHLLLCQYRNIGTAR
>sp|Q96M93|ADAD1_HUMAN Adenosine deaminase domain-containing protein 1 OS=Homo sapiens GN=ADAD1 PE=2 SV=1
MASNNHWFQSSQVPSFAQMLKKNLPVQPATKTITTPTGWSSESYGLSKMASKVTQVTGNF
PEPLLSKNLSSISNPVLPPKKIPKEFIMKYKRGEINPVSALHQFAQMQRVQLDLKETVTT
GNVMGPYFAFCAVVDGIQYKTGLGQNKKESRSNAAKLALDELLQLDEPEPRILETSGPPP
FPAEPVVLSELAYVSKVHYEGRHIQYAKISQIVKERFNQLISNRSEYLKYSSSLAAFIIE
RAGQHEVVAIGTGEYNYSQDIKPDGRVLHDTHAVVTARRSLLRYFYRQLLLFYSKNPAMM
EKSIFCTEPTSNLLTLKQNINICLYMNQLPKGSAQIKSQLRLNPHSISAFEANEELCLHV

AVEGKIYLTVYCPKDGVNRISSMSSSDKLTRWEVLGVQGALLSHFIQPVYISSILIGDGN
CSDTRGLEIAIKQRVDDALTSKLPMFYLVNRPHISLVPSAYPLQMNLEYKFLSLNWAQGD
VSLEIVDGLSGKITESSPFKSGMSMASRLCKAAMLSRFNLLAKEAKKELLEAGTYHAAKC
MSASYQEAKCKLKSYLQQHGYGSWIVKSPCIEQFNM
>sp|Q6NVV9|ADAM5_HUMAN Putative disintegrin and metalloproteinase domain-containing protein 5 OS=Homo sapiens GN=ADAM5 PE=5 SV=2
MQTSILIKSSCRPQFQRRFHHRMQKQIQNIISILSSASVINSYDENDIRHSKPLLVQMDC
NYNGYVAGIPNSLVTLSVCSGLRGTMQLKNISYGIEPMEAVSGFIHKIYEEKYADTNILL
EENDTYTWFNSEYQVRKSSEKTDFIKLFPRYIEMHIVVDKNLFKPANMICRKSVGKECDF
TEYCNGDLPYCLPDTYVRDGEYCDSGGAFCFQGKCRTFDKQCDDLIGRGSRGAPVFCYDE
INTRGDNFGNCGTAHCLFQHILCGKLVCTWEHRDLISRPNLSVIYAHVRDQTCVSTYLPR
RTPPPVNSPISITSYYSAEDRDETFVQDGSMCGPDMYCFEMHCKHVRFLMNLKLCDASNH
CDRHGVCNNFNHCHCEKGYNPPYCQPKQGAFGSIDDGHLVPPTERSYMEEGR
>sp|Q7Z6V5|ADAT2_HUMAN tRNA-specific adenosine deaminase 2 OS=Homo sapiens GN=ADAT2 PE=1 SV=1
MEAKAAPKPAASGACSVSAEETEKWMEEAMHMAKEALENTEVPVGCLMVYNNEVVGKGRN
EVNQTKNATRHAEMVAIDQVLDWCRQSGKSPSEVFEHTVLYVTVEPCIMCAAALRLMKIP
LVVYGCQNERFGGCGSVLNIASADLPNTGRPFQCIPGYRAEEAVEMLKTFYKQENPNAPK
SKVRKKECQKS
>sp|P31946|1433B_HUMAN 14-3-3 protein beta/alpha OS=Homo sapiens GN=YWHAB PE=1 SV=3
MTMDKSELVQKAKLAEQAERYDDMAAAMKAVTEQGHELSNEERNLLSVAYKNVVGARRSS
WRVISSIEQKTERNEKKQQMGKEYREKIEAELQDICNDVLELLDKYLIPNATQPESKVFY
LKMKGDYFRYLSEVASGDNKQTTVSNSQQAYQEAFEISKKEMQPTHPIRLGLALNFSVFY
YEILNSPEKACSLAKTAFDEAIAELDTLNEESYKDSTLIMQLLRDNLTLWTSENQGDEGD
AGEGEN
>sp|P18463|1B37_HUMAN HLA class I histocompatibility antigen, B-37 alpha chain OS=Homo sapiens GN=HLA-B PE=1 SV=1
MRVTAPRTLLLLLWGAVALTETWAGSHSMRYFHTSVSRPGRGEPRFISVGYVDDTQFVRF
DSDAASPRTEPRAPWIEQEGPEYWDRETQISKTNTQTYREDLRTLLRYYNQSEAGSHTIQ
RMSGCDVGPDGRLLRGYNQFAYDGKDYIALNEDLSSWTAADTAAQITQRKWEAARVAEQD
RAYLEGTCVEWLRRYLENGKETLQRADPPKTHVTHHPISDHEATLRCWALGFYPAEITLT
WQRDGEDQTQDTELVETRPAGDRTFQKWAAVVVPSGEEQRYTCHVQHEGLPKPLTLRWEP
SSQSTIPIVGIVAGLAVLAVVVIGAVVATVMCRRKSSGGKGGSYSQAASSDSAQGSDVSL
TA
>sp|P10319|1B58_HUMAN HLA class I histocompatibility antigen, B-58 alpha chain OS=Homo sapiens GN=HLA-B PE=1 SV=1
MRVTAPRTVLLLLWGAVALTETWAGSHSMRYFYTAMSRPGRGEPRFIAVGYVDDTQFVRF
DSDAASPRTEPRAPWIEQEGPEYWDGETRNMKASAQTYRENLRIALRYYNQSEAGSHIIQ
RMYGCDLGPDGRLLRGHDQSAYDGKDYIALNEDLSSWTAADTAAQITQRKWEAARVAEQL
RAYLEGLCVEWLRRYLENGKETLQRADPPKTHVTHHPVSDHEATLRCWALGFYPAEITLT
WQRDGEDQTQDTELVETRPAGDRTFQKWAAVVVPSGEEQRYTCHVQHEGLPKPLTLRWEP
SSQSTIPIVGIVAGLAVLAVVVIGAVVATVMCRRKSSGGKGGSYSQAASSDSAQGSDVSL
TA

>sp|P30498|1B78_HUMAN HLA class I histocompatibility antigen, B-78 alpha chain OS=Homo sapiens GN=HLA-B PE=1 SV=1
MRVTAPRTVLLLLWGAVALTETWAGSHSMRYFYTAMSRPGRGEPRFIAVGYVDDTQFVRF
DSDAASPRTEPRAPWIEQEGPEYWDRNTQIFKTNTQTDRESLRNLRGYYNQSEAGSHTWQ
TMYGCDVGPDGRLLRGHNQYAYDGKDYIALNEDLSSWTAADTAAQITQRKWEAAREAEQL
RAYLEGLCVEWLRRHLENGKETLQRADPPKTHVTHHPVSDHEATLRCWALGFYPAEITLT
WQRDGEDQTQDTELVETRPAGDRTFQKWAAVVVPSGEEQRYTCHVQHEGLPKPLTLRWEP
SSQSTIPIVGIVAGLAVLAVVVIGAVVATVMCRRKSSGGKGGSYSQAASSDSAQGSDVSL
TA
>sp|Q9TNN7|1C05_HUMAN HLA class I histocompatibility antigen, Cw-5 alpha chain OS=Homo sapiens GN=HLA-C PE=1 SV=1
MRVMAPRTLILLLSGALALTETWACSHSMRYFYTAVSRPGRGEPRFIAVGYVDDTQFVQF
DSDAASPRGEPRAPWVEQEGPEYWDRETQKYKRQAQTDRVNLRKLRGYYNQSEAGSHTLQ
RMYGCDLGPDGRLLRGYNQFAYDGKDYIALNEDLRSWTAADKAAQITQRKWEAAREAEQR
RAYLEGTCVEWLRRYLENGKKTLQRAEHPKTHVTHHPVSDHEATLRCWALGFYPAEITLT
WQRDGEDQTQDTELVETRPAGDGTFQKWAAVVVPSGEEQRYTCHVQHEGLPEPLTLRWGP
SSQPTIPIVGIVAGLAVLAVLAVLGAVMAVVMCRRKSSGGKGGSCSQAASSNSAQGSDES
LIACKA
>sp|Q07000|1C15_HUMAN HLA class I histocompatibility antigen, Cw-15 alpha chain OS=Homo sapiens GN=HLA-C PE=1 SV=1
MRVMAPRTLLLLLSGALALTETWACSHSMRYFYTAVSRPGRGEPHFIAVGYVDDTQFVRF
DSDAASPRGEPRAPWVEQEGPEYWDRETQNYKRQAQTDRVNLRKLRGYYNQSEAGSHIIQ
RMYGCDLGPDGRLLRGHDQLAYDGKDYIALNEDLRSWTAADTAAQITQRKWEAAREAEQL
RAYLEGTCVEWLRRYLENGKETLQRAEHPKTHVTHHPVSDHEATLRCWALGFYPAEITLT
WQRDGEDQTQDTELVETRPAGDGTFQKWAAVVVPSGEEQRYTCHVQHEGLPEPLTLRWEP
SSQPTIPIVGIVAGLAVLAVLAVLGAVMAVVMCRRKSSGGKGGSCSQAASSNSAQGSDES
LIACKA
>sp|Q14738|2A5D_HUMAN Serine/threonine-protein phosphatase 2A 56 kDa regulatory subunit delta isoform OS=Homo sapiens GN=PPP2R5D PE=1 SV=1
MPYKLKKEKEPPKVAKCTAKPSSSGKDGGGENTEEAQPQPQPQPQPQAQSQPPSSNKRPS
NSTPPPTQLSKIKYSGGPQIVKKERRQSSSRFNLSKNRELQKLPALKDSPTQEREELFIQ
KLRQCCVLFDFVSDPLSDLKFKEVKRAGLNEMVEYITHSRDVVTEAIYPEAVTMFSVNLF
RTLPPSSNPTGAEFDPEEDEPTLEAAWPHLQLVYEFFLRFLESPDFQPNIAKKYIDQKFV
LALLDLFDSEDPRERDFLKTILHRIYGKFLGLRAYIRRQINHIFYRFIYETEHHNGIAEL
LEILGSIINGFALPLKEEHKMFLIRVLLPLHKVKSLSVYHPQLAYCVVQFLEKESSLTEP
VIVGLLKFWPKTHSPKEVMFLNELEEILDVIEPSEFSKVMEPLFRQLAKCVSSPHFQVAE
RALYYWNNEYIMSLISDNAARVLPIMFPALYRNSKSHWNKTIHGLIYNALKLFMEMNQKL
FDDCTQQYKAEKQKGRFRMKEREEMWQKIEELARLNPQYPMFRAPPPLPPVYSMETETPT
AEDIQLLKRTVETEAVQMLKDIKKEKVLLRRKSELPQDVYTIKALEAHKRAEEFLTASQE
AL
>sp|Q66LE6|2ABD_HUMAN Serine/threonine-protein phosphatase 2A 55 kDa regulatory subunit B delta isoform OS=Homo sapiens GN=PPP2R2D PE=2 SV=1
MAGAGGGGCPAGGNDFQWCFSQVKGAIDEDVAEADIISTVEFNYSGDLLATGDKGGRVVI
FQREQENKSRPHSRGEYNVYSTFQSHEPEFDYLKSLEIEEKINKIRWLPQQNAAHFLLST

NDKTIKLWKISERDKRAEGYNLKDEDGRLRDPFRITALRVPILKPMDLMVEASPRRIFAN
AHTYHINSISVNSDHETYLSADDLRINLWHLEITDRSFNIVDIKPANMEELTEVITAAEF
HPHQCNVFVYSSSKGTIRLCDMRSSALCDRHSKFFEEPEDPSSRSFFSEIISSISDVKFS
HSGRYMMTRDYLSVKVWDLNMESRPVETHQVHEYLRSKLCSLYENDCIFDKFECCWNGSD
SAIMTGSYNNFFRMFDRDTRRDVTLEASRESSKPRASLKPRKVCTGGKRRKDEISVDSLD
FNKKILHTAWHPVDNVIAVAATNNLYIFQDKIN
>sp|Q95IE3|2B1C_HUMAN HLA class II histocompatibility antigen, DRB1-12 beta chain OS=Homo sapiens GN=HLA-DRB1 PE=1 SV=1
MVCLRLPGGSCMAVLTVTLMVLSSPLALAGDTRPRFLEYSTGECYFFNGTERVRLLERHF
HNQEELLRFDSDVGEFRAVTELGRPVAESWNSQKDILEDRRAAVDTYCRHNYGAVESFTV
QRRVHPKVTVYPSKTQPLQHHNLLVCSVSGFYPGSIEVRWFRNGQEEKTGVVSTGLIHNG
DWTFQTLVMLETVPRSGEVYTCQVEHPSVTSPLTVEWRARSESAQSKMLSGVGGFVLGLL
FLGAGLFIYFRNQKGHSGLQPRGFLS
>sp|P01911|2B1F_HUMAN HLA class II histocompatibility antigen, DRB1-15 beta chain OS=Homo sapiens GN=HLA-DRB1 PE=1 SV=2
MVCLKLPGGSCMTALTVTLMVLSSPLALSGDTRPRFLWQPKRECHFFNGTERVRFLDRYF
YNQEESVRFDSDVGEFRAVTELGRPDAEYWNSQKDILEQARAAVDTYCRHNYGVVESFTV
QRRVQPKVTVYPSKTQPLQHHNLLVCSVSGFYPGSIEVRWFLNGQEEKAGMVSTGLIQNG
DWTFQTLVMLETVPRSGEVYTCQVEHPSVTSPLTVEWRARSESAQSKMLSGVGGFVLGLL
FLGAGLFIYFRNQKGHSGLQPTGFLS
>sp|Q29974|2B1G_HUMAN HLA class II histocompatibility antigen, DRB1-16 beta chain OS=Homo sapiens GN=HLA-DRB1 PE=1 SV=1
MVCLKLPGGSCMTALTVTLMVLSSPLALAGDTRPRFLWQPKRECHFFNGTERVRFLDRYF
YNQEESVRFDSDVGEYRAVTELGRPDAEYWNSQKDFLEDRRAAVDTYCRHNYGVGESFTV
QRRVQPKVTVYPSKTQPLQHHNLLVCSVSGFYPGSIEVRWFLNGQEEKAGMVSTGLIQNG
DWTFQTLVMLETVPRSGEVYTCQVEHPSVTSPLTVEWRARSESAQSKMLSGVGGFVLGLL
FLGAGLFIYFRNQKGHSGLQPTGFLS
>sp|P29372|3MG_HUMAN DNA-3-methyladenine glycosylase OS=Homo sapiens GN=MPG PE=1 SV=3
MVTPALQMKKPKQFCRRMGQKKQRPARAGQPHSSSDAAQAPAEQPHSSSDAAQAPCPRER
CLGPPTTPGPYRSIYFSSPKGHLTRLGLEFFDQPAVPLARAFLGQVLVRRLPNGTELRGR
IVETEAYLGPEDEAAHSRGGRQTPRNRGMFMKPGTLYVYIIYGMYFCMNISSQGDGACVL
LRALEPLEGLETMRQLRSTLRKGTASRVLKDRELCSGPSKLCQALAINKSFDQRDLAQDE
AVWLERGPLEPSEPAVVAAARVGVGHAGEWARKPLRFYVRGSPWVSVVDRVAEQDTQA
>sp|Q5VUR7|A20A3_HUMAN Ankyrin repeat domain-containing protein 20A3 OS=Homo sapiens GN=ANKRD20A3 PE=3 SV=1
MKLFGFGSRRGQTAQGSIDHVYTGSGYRIRDSELQKIHRAAVKGDAAEVERCLARRSGDL
DALDKQHRTALHLACASGHVQVVTLLVNRKCQIDVCDKENRTPLIQAVHCQEEACAVILL
EHGANPNLKDIYGNTALHYAVYSESTSLAEKLLSHGAHIEALDKDNNTPLLFAIICKKEK
MVEFLLKRKASSHAVDRLRRSALMLAVYYDSPGIVNILLKQNIDVFAQDMCGRDAEDYAI
SHHLTKIQQQILEHKKKILKKEKSDVGSSDESAVSIFHELRVDSLPASDDKDLNVATKQC
VPEKVSEPLPGSSHEKGNRIVNGQGEGPPAKHPSLKPSTEVEDPAVKGAVQRKNVQTLRA
EQALPVASEEEQERHERSEKKQPQVKEGNNTNKSEKIQLSENICDSTSSAAAGRLTQQRK
IGKTYPQQFPKKLKEEHDRCTLKQENEEKTNVNMLYKKNREELERKEKQYKKEVEAKQLE
PTVQSLEMKSKTARNTPNRDFHNHEEMKGLMDENCILKADIAILRQEICTMKNDNLEKEN

KYLKDIKIVKETNAALEKYIKLNEEMITETAFRYQQELNYLKAENTRLNAELLKEKESKK
RLEADIESYQSRLAAAISKHSESVKTERNLKLALERTRDVSVQVEMSSAISKVKDENEFL
TEQLSETQIKFNALKDKFRKTRDSLRKKSLALETVQNDLSQTQQQTQEMKEMYQNAEAKV
NNSTGKWNCVEERICHLQRENAWLVQQLDDVHQKEDHKEIVTNIQRGFIESGKKDLVLEE
KSKKLMNECDHLKESLFQYEREKTEGVVSIKEDKYFQTSRKTI
>sp|Q9UNA3|A4GCT_HUMAN Alpha-1,4-N-acetylglucosaminyltransferase OS=Homo sapiens GN=A4GNT PE=2 SV=1
MRKELQLSLSVTLLLVCGFLYQFTLKSSCLFCLPSFKSHQGLEALLSHRRGIVFLETSER
MEPPHLVSCSVESAAKIYPEWPVVFFMKGLTDSTPMPSNSTYPAFSFLSAIDNVFLFPLD
MKRLLEDTPLFSWYNQINASAERNWLHISSDASRLAIIWKYGGIYMDTDVISIRPIPEEN
FLAAQASRYSSNGIFGFLPHHPFLWECMENFVEHYNSAIWGNQGPELMTRMLRVWCKLED
FQEVSDLRCLNISFLHPQRFYPISYREWRRYYEVWDTEPSFNVSYALHLWNHMNQEGRAV
IRGSNTLVENLYRKHCPRTYRDLIKGPEGSVTGELGPGNK
>sp|P30542|AA1R_HUMAN Adenosine receptor A1 OS=Homo sapiens GN=ADORA1 PE=1 SV=1
MPPSISAFQAAYIGIEVLIALVSVPGNVLVIWAVKVNQALRDATFCFIVSLAVADVAVGA
LVIPLAILINIGPQTYFHTCLMVACPVLILTQSSILALLAIAVDRYLRVKIPLRYKMVVT
PRRAAVAIAGCWILSFVVGLTPMFGWNNLSAVERAWAANGSMGEPVIKCEFEKVISMEYM
VYFNFFVWVLPPLLLMVLIYLEVFYLIRKQLNKKVSASSGDPQKYYGKELKIAKSLALIL
FLFALSWLPLHILNCITLFCPSCHKPSILTYIAIFLTHGNSAMNPIVYAFRIQKFRVTFL
KIWNDHFRCQPAPPIDEDLPEERPDD
>sp|P29274|AA2AR_HUMAN Adenosine receptor A2a OS=Homo sapiens GN=ADORA2A PE=1 SV=2
MPIMGSSVYITVELAIAVLAILGNVLVCWAVWLNSNLQNVTNYFVVSLAAADIAVGVLAI
PFAITISTGFCAACHGCLFIACFVLVLTQSSIFSLLAIAIDRYIAIRIPLRYNGLVTGTR
AKGIIAICWVLSFAIGLTPMLGWNNCGQPKEGKNHSQGCGEGQVACLFEDVVPMNYMVYF
NFFACVLVPLLLMLGVYLRIFLAARRQLKQMESQPLPGERARSTLQKEVHAAKSLAIIVG
LFALCWLPLHIINCFTFFCPDCSHAPLWLMYLAIVLSHTNSVVNPFIYAYRIREFRQTFR
KIIRSHVLRQQEPFKAAGTSARVLAAHGSDGEQVSLRLNGHPPGVWANGSAPHPERRPNG
YALGLVSGGSAQESQGNTGLPDVELLSHELKGVCPEPPGLDDPLAQDGAGVS
>sp|P22760|AAAD_HUMAN Arylacetamide deacetylase OS=Homo sapiens GN=AADAC PE=1 SV=5
MGRKSLYLLIVGILIAYYIYTPLPDNVEEPWRMMWINAHLKTIQNLATFVELLGLHHFMD
SFKVVGSFDEVPPTSDENVTVTETKFNNILVRVYVPKRKSEALRRGLFYIHGGGWCVGSA
ALSGYDLLSRWTADRLDAVVVSTNYRLAPKYHFPIQFEDVYNALRWFLRKKVLAKYGVNP
ERIGISGDSAGGNLAAAVTQQLLDDPDVKIKLKIQSLIYPALQPLDVDLPSYQENSNFLF
LSKSLMVRFWSEYFTTDRSLEKAMLSRQHVPVESSHLFKFVNWSSLLPERFIKGHVYNNP
NYGSSELAKKYPGFLDVRAAPLLADDNKLRGLPLTYVITCQYDLLRDDGLMYVTRLRNTG
VQVTHNHVEDGFHGAFSFLGLKISHRLINQYIEWLKENL
>sp|Q15758|AAAT_HUMAN Neutral amino acid transporter B(0) OS=Homo sapiens GN=SLC1A5 PE=1 SV=2
MVADPPRDSKGLAAAEPTANGGLALASIEDQGAAAGGYCGSRDQVRRCLRANLLVLLTVV
AVVAGVALGLGVSGAGGALALGPERLSAFVFPGELLLRLLRMIILPLVVCSLIGGAASLD
PGALGRLGAWALLFFLVTTLLASALGVGLALALQPGAASAAINASVGAAGSAENAPSKEV
LDSFLDLARNIFPSNLVSAAFRSYSTTYEERNITGTRVKVPVGQEVEGMNILGLVVFAIV
FGVALRKLGPEGELLIRFFNSFNEATMVLVSWIMWYAPVGIMFLVAGKIVEMEDVGLLFA
RLGKYILCCLLGHAIHGLLVLPLIYFLFTRKNPYRFLWGIVTPLATAFGTSSSSATLPLM

MKCVEENNGVAKHISRFILPIGATVNMDGAALFQCVAAVFIAQLSQQSLDFVKIITILVT
ATASSVGAAGIPAGGVLTLAIILEAVNLPVDHISLILAVDWLVDRSCTVLNVEGDALGAG
LLQNYVDRTESRSTEPELIQVKSELPLDPLPVPTEEGNPLLKHYRGPAGDATVASEKESV
M
>sp|Q4LEZ3|AARD_HUMAN Alanine and arginine-rich domain-containing protein OS=Homo sapiens GN=AARD PE=2 SV=1
MGPGDFRRCRERISQGLQGLPGRAELWFPPRPACDFFGDGRSTDIQEEALAASPLLEDLR
RRLTRAFQWAVQRAISRRVQEAAAAAAAREEQSWTGVEATLARLRAELVEMHFQNHQLAR
TLLDLNMKVQQLKKEYELEITSDSQSPKDDAANPE
>sp|P86434|AAS1_HUMAN Putative uncharacterized protein ADORA2A-AS1 OS=Homo sapiens GN=ADORA2A-AS1 PE=5 SV=1
MEQDWQPGEEVTPGPEPCSKGQAPLYPIVHVTELKHTDPNFPSNSNAVGTSSGWNRIGTG
CSHTWDWRFSCTQQALLPLLGAWEWSIDTEAGGGRREQSQKPCSNGGPAAAGEGRVLPSP
CFPWSTCQAAIHKVCRWQGCTRPALLAPSLATLKEHSYP
>sp|Q9BZC7|ABCA2_HUMAN ATP-binding cassette sub-family A member 2 OS=Homo sapiens GN=ABCA2 PE=1 SV=3
MGFLHQLQLLLWKNVTLKRRSPWVLAFEIFIPLVLFFILLGLRQKKPTISVKEAFYTAAP
LTSAGILPVMQSLCPDGQRDEFGFLQYANSTVTQLLERLDRVVEEGNLFDPARPSLGSEL
EALRQHLEALSAGPGTSGSHLDRSTVSSFSLDSVARNPQELWRFLTQNLSLPNSTAQALL
AARVDPPEVYHLLFGPSSALDSQSGLHKGQEPWSRLGGNPLFRMEELLLAPALLEQLTCT
PGSGELGRILTVPESQKGALQGYRDAVCSGQAAARARRFSGLSAELRNQLDVAKVSQQLG
LDAPNGSDSSPQAPPPRRLQALLGDLLDAQKVLQDVDVLSALALLLPQGACTGRTPGPPA
SGAGGAANGTGAGAVMGPNATAEEGAPSAAALATPDTLQGQCSAFVQLWAGLQPILCGNN
RTIEPEALRRGNMSSLGFTSKEQRNLGLLVHLMTSNPKILYAPAGSEVDRVILKANETFA
FVGNVTHYAQVWLNISAEIRSFLEQGRLQQHLRWLQQYVAELRLHPEALNLSLDELPPAL
RQDNFSLPSGMALLQQLDTIDNAACGWIQFMSKVSVDIFKGFHDEESIVNYTLNQAYQDN
VTVFASVIFQTRKDGSLPPHVHYKIRQNSSFTEKTNEIRRAYWRPGPNTGGRFYFLYGFV
WIQDMMERAIIDTFVGHDVVEPGSYVQMFPYPCYTRDDFLFVIEHMMPLCMVISWVYSVA
MTIQHIVAEKEHRLKEVMKTMGLNNAVHWVAWFITGFVQLSISVTALTAILKYGQVLMHS
HVVIIWLFLAVYAVATIMFCFLVSVLYSKAKLASACGGIIYFLSYVPYMYVAIREEVAHD
KITAFEKCIASLMSTTAFGLGSKYFALYEVAGVGIQWHTFSQSPVEGDDFNLLLAVTMLM
VDAVVYGILTWYIEAVHPGMYGLPRPWYFPLQKSYWLGSGRTEAWEWSWPWARTPRLSVM
EEDQACAMESRRFEETRGMEEEPTHLPLVVCVDKLTKVYKDDKKLALNKLSLNLYENQVV
SFLGHNGAGKTTTMSILTGLFPPTSGSATIYGHDIRTEMDEIRKNLGMCPQHNVLFDRLT
VEEHLWFYSRLKSMAQEEIRREMDKMIEDLELSNKRHSLVQTLSGGMKRKLSVAIAFVGG
SRAIILDEPTAGVDPYARRAIWDLILKYKPGRTILLSTHHMDEADLLGDRIAIISHGKLK
CCGSPLFLKGTYGDGYRLTLVKRPAEPGGPQEPGLASSPPGRAPLSSCSELQVSQFIRKH
VASCLLVSDTSTELSYILPSEAAKKGAFERLFQHLERSLDALHLSSFGLMDTTLEEVFLK
VSEEDQSLENSEADVKESRKDVLPGAEGPASGEGHAGNLARCSELTQSQASLQSASSVGS
ARGDEGAGYTDVYGDYRPLFDNPQDPDNVSLQEVEAEALSRVGQGSRKLDGGWLKVRQFH
GLLVKRFHCARRNSKALFSQILLPAFFVCVAMTVALSVPEIGDLPPLVLSPSQYHNYTQP
RGNFIPYANEERREYRLRLSPDASPQQLVSTFRLPSGVGATCVLKSPANGSLGPTLNLSS
GESRLLAARFFDSMCLESFTQGLPLSNFVPPPPSPAPSDSPASPDEDLQAWNVSLPPTAG
PEMWTSAPSLPRLVREPVRCTCSAQGTGFSCPSSVGGHPPQMRVVTGDILTDITGHNVSE

YLLFTSDRFRLHRYGAITFGNVLKSIPASFGTRAPPMVRKIAVRRAAQVFYNNKGYHSMP
TYLNSLNNAILRANLPKSKGNPAAYGITVTNHPMNKTSASLSLDYLLQGTDVVIAIFIIV
AMSFVPASFVVFLVAEKSTKAKHLQFVSGCNPIIYWLANYVWDMLNYLVPATCCVIILFV
FDLPAYTSPTNFPAVLSLFLLYGWSITPIMYPASFWFEVPSSAYVFLIVINLFIGITATV
ATFLLQLFEHDKDLKVVNSYLKSCFLIFPNYNLGHGLMEMAYNEYINEYYAKIGQFDKMK
SPFEWDIVTRGLVAMAVEGVVGFLLTIMCQYNFLRRPQRMPVSTKPVEDDVDVASERQRV
LRGDADNDMVKIENLTKVYKSRKIGRILAVDRLCLGVRPGECFGLLGVNGAGKTSTFKML
TGDESTTGGEAFVNGHSVLKELLQVQQSLGYCPQCDALFDELTAREHLQLYTRLRGISWK
DEARVVKWALEKLELTKYADKPAGTYSGGNKRKLSTAIALIGYPAFIFLDEPTTGMDPKA
RRFLWNLILDLIKTGRSVVLTSHSMEECEALCTRLAIMVNGRLRCLGSIQHLKNRFGDGY
MITVRTKSSQSVKDVVRFFNRNFPEAMLKERHHTKVQYQLKSEHISLAQVFSKMEQVSGV
LGIEDYSVSQTTLDNVFVNFAKKQSDNLEQQETEPPSALQSPLGCLLSLLRPRSAPTELR
ALVADEPEDLDTEDEGLISFEEERAQLSFNTDTLC
>sp|Q99758|ABCA3_HUMAN ATP-binding cassette sub-family A member 3 OS=Homo sapiens GN=ABCA3 PE=1 SV=2
MAVLRQLALLLWKNYTLQKRKVLVTVLELFLPLLFSGILIWLRLKIQSENVPNATIYPGQ
SIQELPLFFTFPPPGDTWELAYIPSHSDAAKTVTETVRRALVINMRVRGFPSEKDFEDYI
RYDNCSSSVLAAVVFEHPFNHSKEPLPLAVKYHLRFSYTRRNYMWTQTGSFFLKETEGWH
TTSLFPLFPNPGPREPTSPDGGEPGYIREGFLAVQHAVDRAIMEYHADAATRQLFQRLTV
TIKRFPYPPFIADPFLVAIQYQLPLLLLLSFTYTALTIARAVVQEKERRLKEYMRMMGLS
SWLHWSAWFLLFFLFLLIAASFMTLLFCVKVKPNVAVLSRSDPSLVLAFLLCFAISTISF
SFMVSTFFSKANMAAAFGGFLYFFTYIPYFFVAPRYNWMTLSQKLCSCLLSNVAMAMGAQ
LIGKFEAKGMGIQWRDLLSPVNVDDDFCFGQVLGMLLLDSVLYGLVTWYMEAVFPGQFGV
PQPWYFFIMPSYWCGKPRAVAGKEEEDSDPEKALRNEYFEAEPEDLVAGIKIKHLSKVFR
VGNKDRAAVRDLNLNLYEGQITVLLGHNGAGKTTTLSMLTGLFPPTSGRAYISGYEISQD
MVQIRKSLGLCPQHDILFDNLTVAEHLYFYAQLKGLSRQKCPEEVKQMLHIIGLEDKWNS
RSRFLSGGMRRKLSIGIALIAGSKVLILDEPTSGMDAISRRAIWDLLQRQKSDRTIVLTT
HFMDEADLLGDRIAIMAKGELQCCGSSLFLKQKYGAGYHMTLVKEPHCNPEDISQLVHHH
VPNATLESSAGAELSFILPRESTHRFEGLFAKLEKKQKELGIASFGASITTMEEVFLRVG
KLVDSSMDIQAIQLPALQYQHERRASDWAVDSNLCGAMDPSDGIGALIEEERTAVKLNTG
LALHCQQFWAMFLKKAAYSWREWKMVAAQVLVPLTCVTLALLAINYSSELFDDPMLRLTL
GEYGRTVVPFSVPGTSQLGQQLSEHLKDALQAEGQEPREVLGDLEEFLIFRASVEGGGFN
ERCLVAASFRDVGERTVVNALFNNQAYHSPATALAVVDNLLFKLLCGPHASIVVSNFPQP
RSALQAAKDQFNEGRKGFDIALNLLFAMAFLASTFSILAVSERAVQAKHVQFVSGVHVAS
FWLSALLWDLISFLIPSLLLLVVFKAFDVRAFTRDGHMADTLLLLLLYGWAIIPLMYLMN
FFFLGAATAYTRLTIFNILSGIATFLMVTIMRIPAVKLEELSKTLDHVFLVLPNHCLGMA
VSSFYENYETRRYCTSSEVAAHYCKKYNIQYQENFYAWSAPGVGRFVASMAASGCAYLIL
LFLIETNLLQRLRGILCALRRRRTLTELYTRMPVLPEDQDVADERTRILAPSPDSLLHTP
LIIKELSKVYEQRVPLLAVDRLSLAVQKGECFGLLGFNGAGKTTTFKMLTGEESLTSGDA
FVGGHRISSDVGKVRQRIGYCPQFDALLDHMTGREMLVMYARLRGIPERHIGACVENTLR
GLLLEPHANKLVRTYSGGNKRKLSTGIALIGEPAVIFLDEPSTGMDPVARRLLWDTVARA
RESGKAIIITSHSMEECEALCTRLAIMVQGQFKCLGSPQHLKSKFGSGYSLRAKVQSEGQ
QEALEEFKAFVDLTFPGSVLEDEHQGMVHYHLPGRDLSWAKVFGILEKAKEKYGVDDYSV
SQISLEQVFLSFAHLQPPTAEEGR

>sp|P61221|ABCE1_HUMAN ATP-binding cassette sub-family E member 1 OS=Homo sapiens GN=ABCE1 PE=1 SV=1
MADKLTRIAIVNHDKCKPKKCRQECKKSCPVVRMGKLCIEVTPQSKIAWISETLCIGCGI
CIKKCPFGALSIVNLPSNLEKETTHRYCANAFKLHRLPIPRPGEVLGLVGTNGIGKSTAL
KILAGKQKPNLGKYDDPPDWQEILTYFRGSELQNYFTKILEDDLKAIIKPQYVDQIPKAA
KGTVGSILDRKDETKTQAIVCQQLDLTHLKERNVEDLSGGELQRFACAVVCIQKADIFMF
DEPSSYLDVKQRLKAAITIRSLINPDRYIIVVEHDLSVLDYLSDFICCLYGVPSAYGVVT
MPFSVREGINIFLDGYVPTENLRFRDASLVFKVAETANEEEVKKMCMYKYPGMKKKMGEF
ELAIVAGEFTDSEIMVMLGENGTGKTTFIRMLAGRLKPDEGGEVPVLNVSYKPQKISPKS
TGSVRQLLHEKIRDAYTHPQFVTDVMKPLQIENIIDQEVQTLSGGELQRVALALCLGKPA
DVYLIDEPSAYLDSEQRLMAARVVKRFILHAKKTAFVVEHDFIMATYLADRVIVFDGVPS
KNTVANSPQTLLAGMNKFLSQLEITFRRDPNNYRPRINKLNSIKDVEQKKSGNYFFLDD
>sp|Q9H172|ABCG4_HUMAN ATP-binding cassette sub-family G member 4 OS=Homo sapiens GN=ABCG4 PE=1 SV=2
MAEKALEAVGCGLGPGAVAMAVTLEDGAEPPVLTTHLKKVENHITEAQRFSHLPKRSAVD
IEFVELSYSVREGPCWRKRGYKTLLKCLSGKFCRRELIGIMGPSGAGKSTFMNILAGYRE
SGMKGQILVNGRPRELRTFRKMSCYIMQDDMLLPHLTVLEAMMVSANLKLSEKQEVKKEL
VTEILTALGLMSCSHTRTALLSGGQRKRLAIALELVNNPPVMFFDEPTSGLDSASCFQVV
SLMKSLAQGGRTIICTIHQPSAKLFEMFDKLYILSQGQCIFKGVVTNLIPYLKGLGLHCP
TYHNPADFIIEVASGEYGDLNPMLFRAVQNGLCAMAEKKSSPEKNEVPAPCPPCPPEVDP
IESHTFATSTLTQFCILFKRTFLSILRDTVLTHLRFMSHVVIGVLIGLLYLHIGDDASKV
FNNTGCLFFSMLFLMFAALMPTVLTFPLEMAVFMREHLNYWYSLKAYYLAKTMADVPFQV
VCPVVYCSIVYWMTGQPAETSRFLLFSALATATALVAQSLGLLIGAASNSLQVATFVGPV
TAIPVLLFSGFFVSFKTIPTYLQWSSYLSYVRYGFEGVILTIYGMERGDLTCLEERCPFR
EPQSILRALDVEDAKLYMDFLVLGIFFLALRLLAYLVLRYRVKSER
>sp|Q0P651|ABD18_HUMAN Protein ABHD18 OS=Homo sapiens GN=ABHD18 PE=2 SV=1
MGVSKLDILYRRLLLTKLFIRGWGRPEDLKRLFEFRKMIGNRERCQNLVSSDYPVHIDKI
EEQSDCKILDGHFVSPMAHYVPDIMPIESVIARFQFIVPKEWNSKYRPVCIHLAGTGDHH
YWRRRTLMARPMIKEARMASLLLENPYYGCRKPKDQVRSSLKNVSDLFVMGGALVLESAA
LLHWLEREGYGPLGMTGISMGGHMASLAVSNWPKPMPLIPCLSWSTASGVFTTTDSFKMG
QEFVKHFTSSADKLTNLNLVSRTLNLDISNQVVSQKPADCHNSSKTSVSATSEGLLLQDT
SKMKRFNQTLSTNKSGYTSRNPQSYHLLSKEQSRNSLRKESLIFMKGVMDECTHVANFSV
PVDPSLIIVVQAKEDAYIPRTGVRSLQEIWPGCEIRYLEGGHISAYLFKQGLFR
>sp|Q96I13|ABHD8_HUMAN Protein ABHD8 OS=Homo sapiens GN=ABHD8 PE=2 SV=1
MLTGVTDGIFCCLLGTPPNAVGPLESVESSDGYTFVEVKPGRVLRVKHAGPAPAAAPPPP
SSASSDAAQGDLSGLVRCQRRITVYRNGRLLVENLGRAPRADLLHGQNGSGEPPAALEVE
LADPAGSDGRLAPGSAGSGSGSGSGGRRRRARRPKRTIHIDCEKRITSCKGAQADVVLFF
IHGVGGSLAIWKEQLDFFVRLGYEVVAPDLAGHGASSAPQVAAAYTFYALAEDMRAIFKR
YAKKRNVLIGHSYGVSFCTFLAHEYPDLVHKVIMINGGGPTALEPSFCSIFNMPTCVLHC
LSPCLAWSFLKAGFARQGAKEKQLLKEGNAFNVSSFVLRAMMSGQYWPEGDEVYHAELTV
PVLLVHGMHDKFVPVEEDQRMAEILLLAFLKLIDEGSHMVMLECPETVNTLLHEFLLWEP
EPSPKALPEPLPAPPEDKK
>sp|Q7L211|ABHDD_HUMAN Protein ABHD13 OS=Homo sapiens GN=ABHD13 PE=2 SV=1
MEKSWMLWNFVERWLIALASWSWALCRISLLPLIVTFHLYGGIILLLLIFISIAGILYKF

QDVLLYFPEQPSSSRLYVPMPTGIPHENIFIRTKDGIRLNLILIRYTGDNSPYSPTIIYF
HGNAGNIGHRLPNALLMLVNLKVNLLLVDYRGYGKSEGEASEEGLYLDSEAVLDYVMTRP
DLDKTKIFLFGRSLGGAVAIHLASENSHRISAIMVENTFLSIPHMASTLFSFFPMRYLPL
WCYKNKFLSYRKISQCRMPSLFISGLSDQLIPPVMMKQLYELSPSRTKRLAIFPDGTHND
TWQCQGYFTALEQFIKEVVKSHSPEEMAKTSSNVTII
>sp|Q8IZP0|ABI1_HUMAN Abl interactor 1 OS=Homo sapiens GN=ABI1 PE=1 SV=4
MAELQMLLEEEIPSGKRALIESYQNLTRVADYCENNYIQATDKRKALEETKAYTTQSLAS
VAYQINALANNVLQLLDIQASQLRRMESSINHISQTVDIHKEKVARREIGILTTNKNTSR
THKIIAPANMERPVRYIRKPIDYTVLDDVGHGVKWLKAKHGNNQPARTGTLSRTNPPTQK
PPSPPMSGRGTLGRNTPYKTLEPVKPPTVPNDYMTSPARLGSQHSPGRTASLNQRPRTHS
GSSGGSGSRENSGSSSIGIPIAVPTPSPPTIGPENISVPPPSGAPPAPPLAPLLPVSTVI
AAPGSAPGSQYGTMTRQISRHNSTTSSTSSGGYRRTPSVTAQFSAQPHVNGGPLYSQNSI
SIAPPPPPMPQLTPQIPLTGFVARVQENIADSPTPPPPPPPDDIPMFDDSPPPPPPPPVD
YEDEEAAVVQYNDPYADGDPAWAPKNYIEKVVAIYDYTKDKDDELSFMEGAIIYVIKKND
DGWYEGVCNRVTGLFPGNYVESIMHYTD
>sp|Q9GZZ6|ACH10_HUMAN Neuronal acetylcholine receptor subunit alpha-10 OS=Homo sapiens GN=CHRNA10 PE=1 SV=1
MGLRSHHLSLGLLLLFLLPAECLGAEGRLALKLFRDLFANYTSALRPVADTDQTLNVTLE
VTLSQIIDMDERNQVLTLYLWIRQEWTDAYLRWDPNAYGGLDAIRIPSSLVWRPDIVLYN
KADAQPPGSASTNVVLRHDGAVRWDAPAITRSSCRVDVAAFPFDAQHCGLTFGSWTHGGH
QLDVRPRGAAASLADFVENVEWRVLGMPARRRVLTYGCCSEPYPDVTFTLLLRRRAAAYV
CNLLLPCVLISLLAPLAFHLPADSGEKVSLGVTVLLALTVFQLLLAESMPPAESVPLIGK
YYMATMTMVTFSTALTILIMNLHYCGPSVRPVPAWARALLLGHLARGLCVRERGEPCGQS
RPPELSPSPQSPEGGAGPPAGPCHEPRCLCRQEALLHHVATIANTFRSHRAAQRCHEDWK
RLARVMDRFFLAIFFSMALVMSLLVLVQAL
>sp|P30532|ACHA5_HUMAN Neuronal acetylcholine receptor subunit alpha-5 OS=Homo sapiens GN=CHRNA5 PE=1 SV=2
MAARGSGPRALRLLLLVQLVAGRCGLAGAAGGAQRGLSEPSSIAKHEDSLLKDLFQDYER
WVRPVEHLNDKIKIKFGLAISQLVDVDEKNQLMTTNVWLKQEWIDVKLRWNPDDYGGIKV
IRVPSDSVWTPDIVLFDNADGRFEGTSTKTVIRYNGTVTWTPPANYKSSCTIDVTFFPFD
LQNCSMKFGSWTYDGSQVDIILEDQDVDKRDFFDNGEWEIVSATGSKGNRTDSCCWYPYV
TYSFVIKRLPLFYTLFLIIPCIGLSFLTVLVFYLPSNEGEKICLCTSVLVSLTVFLLVIE
EIIPSSSKVIPLIGEYLVFTMIFVTLSIMVTVFAININIHHRSSSTHNAMAPLVRKIFLHTL
PKLLCMRSHVDRYFTQKEETESGSGPKSSRNTLEAALDSIRYITRHIMKENDVREVVEDW
KFIAQVLDRMFLWTFLFVSIVGSLGLFVPVIYKWANILIPVHIGNANK
>sp|Q9UGM1|ACHA9_HUMAN Neuronal acetylcholine receptor subunit alpha-9 OS=Homo sapiens GN=CHRNA9 PE=1 SV=2
MNWSHSCISFCWIYFAASRLRAAETADGKYAQKLFNDLFEDYSNALRPVEDTDKVLNVTL
QITLSQIKDMDERNQILTAYLWIRQIWHDAYLTWDRDQYDGLDSIRIPSDLVWRPDIVLY
NKADDESSEPVNTNVVLRYDGLITWDAPAITKSSCVVDVTYFPFDNQQCNLTFGSWTYNG
NQVDIFNALDSGDLSDFIEDVEWEVHGMPAVKNVISYGCCSEPYPDVTFTLLLKRRSSFY
IVNLLIPCVLISFLAPLSFYLPAASGEKVSLGVTILLAMTVFQLMVAEIMPASENVPLIG
KYYIATMALITASTALTIMVMNIHFCGAEARPVPHWARVVILKYMSRVLFVYDVGESCLS
PHHSRERDHLTKVYSKLPESNLKAARNKDLSRKKDMNKRLKNDLGCQGKNPQEAESYCAQ

YKVLTRNIEYIAKCLKDHKATNSKGSEWKKVAKVIDRFFMWIFFIMVFVMTILIIARAD
>sp|P30926|ACHB4_HUMAN Neuronal acetylcholine receptor subunit beta-4 OS=Homo sapiens GN=CHRNB4 PE=1 SV=2
MRRAPSLVLFFLVALCGRGNCRVANAEEKLMDDLLNKTRYNNLIRPATSSSQLISIKLQL
SLAQLISVNEREQIMTTNVWLKQEWTDYRLTWNSSRYEGVNILRIPAKRIWLPDIVLYNN
ADGTYEVSVYTNLIVRSNGSVLWLPPAIYKSACKIEVKYFPFDQQNCTLKFRSWTYDHTE
IDMVLMTPTASMDDFTPSGEWDIVALPGRRTVNPQDPSYVDVTYDFIIKRKPLFYTINLI
IPCVLTTLLAILVFYLPSDCGEKMTLCISVLLALTFFLLLISKIVPPTSLDVPLIGKYLM
FTMVLVTFSIVTSVCVLNVHHRSPSTHTMAPWVKRCFLHKLPTFLFMKRPGPDSSPARAF
PPSKSCVTKPEATATSTSPSNFYGNSMYFVNPASAASKSPAGSTPVAIPRDFWLRSSGRF
RQDVQEALEGVSFIAQHMKNDDEDQSVVEDWKYVAMVVDRLFLWVFMFVCVLGTVGLFLP
PLFQTHAASEGPYAAQRD
>sp|P30443|1A01_HUMAN HLA class I histocompatibility antigen, A-1 alpha chain OS=Homo sapiens GN=HLA-A PE=1 SV=1
MAVMAPRTLLLLLSGALALTQTWAGSHSMRYFFTSVSRPGRGEPRFIAVGYVDDTQFVRF
DSDAASQKMEPRAPWIEQEGPEYWDQETRNMKAHSQTDRANLGTLRGYYNQSEDGSHTIQ
IMYGCDVGPDGRFLRGYRQDAYDGKDYIALNEDLRSWTAADMAAQITKRKWEAVHAAEQR
RVYLEGRCVDGLRRYLENGKETLQRTDPPKTHMTHHPISDHEATLRCWALGFYPAEITLT
WQRDGEDQTQDTELVETRPAGDGTFQKWAAVVVPSGEEQRYTCHVQHEGLPKPLTLRWEL
SSQPTIPIVGIIAGLVLLGAVITGAVVAAVMWRRKSSDRKGGSYTQAASSDSAQGSDVSL
TACKV
>sp|P01892|1A02_HUMAN HLA class I histocompatibility antigen, A-2 alpha chain OS=Homo sapiens GN=HLA-A PE=1 SV=1
MAVMAPRTLVLLLSGALALTQTWAGSHSMRYFFTSVSRPGRGEPRFIAVGYVDDTQFVRF
DSDAASQRMEPRAPWIEQEGPEYWDGETRKVKAHSQTHRVDLGTLRGYYNQSEAGSHTVQ
RMYGCDVGSDWRFLRGYHQYAYDGKDYIALKEDLRSWTAADMAAQTTKHKWEAAHVAEQL
RAYLEGTCVEWLRRYLENGKETLQRTDAPKTHMTHHAVSDHEATLRCWALSFYPAEITLT
WQRDGEDQTQDTELVETRPAGDGTFQKWAAVVVPSGQEQRYTCHVQHEGLPKPLTLRWEP
SSQPTIPIVGIIAGLVLFGAVITGAVVAAVMWRRKSSDRKGGSYSQAASSDSAQGSDVSL
TACKV
>sp|P30483|1B45_HUMAN HLA class I histocompatibility antigen, B-45 alpha chain OS=Homo sapiens GN=HLA-B PE=1 SV=1
MRVTAPRTVLLLLSAALALTETWAGSHSMRYFHTAMSRPGRGEPRFITVGYVDDTLFVRF
DSDATSPRKEPRAPWIEQEGPEYWDRETQISKTNTQTYRESLRNLRGYYNQSEAGSHTWQ
RMYGCDLGPDGRLLRGYNQLAYDGKDYIALNEDLSSWTAADTAAQITQRKWEAARVAEQD
RAYLEGLCVESLRRYLENGKETLQRADPPKTHVTHHPISDHEATLRCWALGFYPAEITLT
WQRDGEDQTQDTELVETRPAGDRTFQKWAAVVVPSGEEQRYTCHVQHEGLPKPLTLRWEP
SSQSTIPIVGIVAGLAVLAVVVIGAVVATVMCRRKSSGGKGGSYSQAASSDSAQGSDVSL
TA
>sp|P30487|1B49_HUMAN HLA class I histocompatibility antigen, B-49 alpha chain OS=Homo sapiens GN=HLA-B PE=1 SV=2
MRVTAPRTVLLLLSAALALTETWAGSHSMRYFHTAMSRPGRGEPRFITVGYVDDTLFVRF
DSDATSPRKEPRAPWIEQEGPEYWDRETQISKTNTQTYRENLRIALRYYNQSEAGSHTWQ
RMYGCDLGPDGRLLRGYNQLAYDGKDYIALNEDLSSWTAADTAAQITQRKWEAAREAEQL

RAYLEGLCVEWLRRYLENGKETLQRADPPKTHVTHHPISDHEATLRCWALGFYPAEITLT
WQRDGEDQTQDTELVETRPAGDRTFQKWAAVVVPSGEEQRYTCHVQHEGLPKPLTLRWEP
SSQSTIPIVGIVAGLAVLAVVVIGAVVATVMCRRKSSGGKGGSYSQAASSDSAQGSDVSL
TA
>sp|P18464|1B51_HUMAN HLA class I histocompatibility antigen, B-51 alpha chain OS=Homo sapiens GN=HLA-B PE=1 SV=1
MRVTAPRTVLLLLWGAVALTETWAGSHSMRYFYTAMSRPGRGEPRFIAVGYVDDTQFVRF
DSDAASPRTEPRAPWIEQEGPEYWDRNTQIFKTNTQTYRENLRIALRYYNQSEAGSHTWQ
TMYGCDVGPDGRLLRGHNQYAYDGKDYIALNEDLSSWTAADTAAQITQRKWEAAREAEQL
RAYLEGLCVEWLRRHLENGKETLQRADPPKTHVTHHPVSDHEATLRCWALGFYPAEITLT
WQRDGEDQTQDTELVETRPAGDRTFQKWAAVVVPSGEEQRYTCHVQHEGLPKPLTLRWEP
SSQSTIPIVGIVAGLAVLAVVVIGAVVATVMCRRKSSGGKGGSYSQAASSDSAQGSDVSL
TA
>sp|P18465|1B57_HUMAN HLA class I histocompatibility antigen, B-57 alpha chain OS=Homo sapiens GN=HLA-B PE=1 SV=1
MRVTAPRTVLLLLWGAVALTETWAGSHSMRYFYTAMSRPGRGEPRFIAVGYVDDTQFVRF
DSDAASPRMAPRAPWIEQEGPEYWDGETRNMKASAQTYRENLRIALRYYNQSEAGSHIIQ
VMYGCDVGPDGRLLRGHDQSAYDGKDYIALNEDLSSWTAADTAAQITQRKWEAARVAEQL
RAYLEGLCVEWLRRYLENGKETLQRADPPKTHVTHHPISDHEATLRCWALGFYPAEITLT
WQRDGEDQTQDTELVETRPAGDRTFQKWAAVVVPSGEEQRYTCHVQHEGLPKPLTLRWEP
SSQSTVPIVGIVAGLAVLAVVVIGAVVAAVMCRRKSSGGKGGSYSQAACSDSAQGSDVSL
TA
>sp|Q29836|1B67_HUMAN HLA class I histocompatibility antigen, B-67 alpha chain OS=Homo sapiens GN=HLA-B PE=1 SV=1
MLVMAPRTVLLLLSAALALTETWAGSHSMRYFYTSVSRPGRGEPRFISVGYVDDTQFVRF
DSDAASPREEPRAPWIEQEGPEYWDRNTQIYKAQAQTDRESLRNLRGYYNQSEAGSHTLQ
RMYGCDVGPDGRLLRGHNQFAYDGKDYIALNEDLSSWTAADTAAQITQRKWEAARVAEQL
RTYLEGTCVEWLRRYLENGKETLQRADPPKTHVTHHPISDHEATLRCWALGFYPAEITLT
WQRDGEDQTQDTELVETRPAGDRTFQKWAAVVVPSGEEQRYTCHVQHEGLPKPLTLRWEP
SSQSTVPIVGIVAGLAVLAVVVIGAVVAAVMCRRKSSGGKGGSYSQAASSDSAQGSDVSL
TA
>sp|Q29940|1B59_HUMAN HLA class I histocompatibility antigen, B-59 alpha chain OS=Homo sapiens GN=HLA-B PE=1 SV=1
MRVTAPRTLLLLLWGALALTETWAGSHSMRYFYTAMSRPGRGEPRFIAVGYVDDTQFVRF
DSDAASPREEPRAPWIEQEGPEYWDRNTQIFKTNTQTYRENLRIALRYYNQSEAGSHTWQ
TMYGCDLGPDGRLLRGHNQLAYDGKDYIALNEDLSSWTAADTAAQITQRKWEAARVAEQL
RAYLEGTCVEWLRRYLENGKETLQRADPPKTHVTHHPISDHEATLRCWALGFYPAEITLT
WQRDGEDQTQDTELVETRPAGDRTFQKWAAVVVPSGEEQRYTCHVQHEGLPKPLTLRWEP
SSQSTIPIVGIVAGLAVLAVVVIGAVVATVMCRRKSSGGKGGSYSQAASSDSAQGSDVSL
TA
>sp|P04222|1C03_HUMAN HLA class I histocompatibility antigen, Cw-3 alpha chain OS=Homo sapiens GN=HLA-C PE=1 SV=2
MRVMAPRTLILLLSGALALTETWAGSHSMRYFYTAVSRPGRGEPHFIAVGYVDDTQFVRF
DSDAASPRGEPRAPWVEQEGPEYWDRETQKYKRQAQTDRVSLRNLRGYYNQSEAGSHIIQ

RMYGCDVGPDGRLLRGYDQYAYDGKDYIALNEDLRSWTAADTAAQITQRKWEAAREAEQL
RAYLEGLCVEWLRRYLKNGKETLQRAEHPKTHVTHHPVSDHEATLRCWALGFYPAEITLT
WQWDGEDQTQDTELVETRPAGDGTFQKWAAVVVPSGEEQRYTCHVQHEGLPEPLTLRWEP
SSQPTIPIVGIVAGLAVLAVLAVLGAVVAVVMCRRKSSGGKGGSCSQAASSNSAQGSDES
LIACKA
>sp|P10321|1C07_HUMAN HLA class I histocompatibility antigen, Cw-7 alpha chain OS=Homo sapiens GN=HLA-C PE=1 SV=3
MRVMAPRALLLLLSGGLALTETWACSHSMRYFDTAVSRPGRGEPRFISVGYVDDTQFVRF
DSDAASPRGEPRAPWVEQEGPEYWDRETQKYKRQAQADRVSLRNLRGYYNQSEDGSHTLQ
RMSGCDLGPDGRLLRGYDQSAYDGKDYIALNEDLRSWTAADTAAQITQRKLEAARAAEQL
RAYLEGTCVEWLRRYLENGKETLQRAEPPKTHVTHHPLSDHEATLRCWALGFYPAEITLT
WQRDGEDQTQDTELVETRPAGDGTFQKWAAVVVPSGQEQRYTCHMQHEGLQEPLTLSWEP
SSQPTIPIMGIVAGLAVLVVLAVLGAVVTAMMCRRKSSGGKGGSCSQAACSNSAQGSDES
LITCKA
>sp|P30508|1C12_HUMAN HLA class I histocompatibility antigen, Cw-12 alpha chain OS=Homo sapiens GN=HLA-C PE=1 SV=2
MRVMAPRTLILLLSGALALTETWACSHSMRYFYTAVSRPGRGEPRFIAVGYVDDTQFVRF
DSDAASPRGEPRAPWVEQEGPEYWDRETQKYKRQAQADRVSLRNLRGYYNQSEAGSHTLQ
RMYGCDLGPDGRLLRGYDQSAYDGKDYIALNEDLRSWTAADTAAQITQRKWEAAREAEQW
RAYLEGTCVEWLRRYLENGKETLQRAEHPKTHVTHHPVSDHEATLRCWALGFYPAEITLT
WQRDGEDQTQDTELVETRPAGDGTFQKWAAVVVPSGEEQRYTCHVQHEGLPEPLTLRWEP
SSQPTIPIVGIVAGLAVLAVLAVLGAVMAVVMCRRKSSGGKGGSCSQAASSNSAQGSDES
LIACKA
>sp|Q29960|1C16_HUMAN HLA class I histocompatibility antigen, Cw-16 alpha chain OS=Homo sapiens GN=HLA-C PE=1 SV=1
MRVMAPRTLILLLSGALALTETWACSHSMRYFYTAVSRPGRGEPRFIAVGYVDDTQFVRF
DSDAASPRGEPRAPWVEQEGPEYWDRETQKYKRQAQTDRVSLRNLRGYYNQSEAGSHTLQ
WMYGCDLGPDGRLLRGYDQSAYDGKDYIALNEHLRSCTAADTAAQITQRKWEAARAAEQQ
RAYLEGTCVEWLRRYLENGKETLQRAEHPKTHVTHHLVSDHEATLRCWALGFYPAEITLT
WQRDGEDQTQDTELVETRPAGDGTFQKWAAVVVPSGEEQRYTCHVQHEGLPEPLTLRWEP
SSQPTIPIVGIVAGLAVLAVLAVLGAVVAVVMCRRKSSGGKGGSCSQAASSNSAQGSDES
LIACKA
>sp|O60516|4EBP3_HUMAN Eukaryotic translation initiation factor 4E-binding protein 3 OS=Homo sapiens GN=EIF4EBP3 PE=1 SV=1
MSTSTSCPIPGGRDQLPDCYSTTPGGTLYATTPGGTRIIYDRKFLLECKNSPIARTPPCC
LPQIPGVTTPPTAPLSKLEELKEQETEEEIPDDAQFEMDI
>sp|P28221|5HT1D_HUMAN 5-hydroxytryptamine receptor 1D OS=Homo sapiens GN=HTR1D PE=1 SV=1
MSPLNQSAEGLPQEASNRSLNATETSEAWDPRTLQALKISLAVVLSVITLATVLSNAFVL
TTILLTRKLHTPANYLIGSLATTDLLVSILVMPISIAYTITHTWNFGQILCDIWLSSDIT
CCTASILHLCVIALDRYWAITDALEYSKRRTAGHAATMIAIVWAISICISIPPLFWRQAK
AQEEMSDCLVNTSQISYTIYSTCGAFYIPSVLLIILYGRIYRAARNRILNPPSLYGKRFT
TAHLITGSAGSSLCSLNSSLHEGHSHSAGSPLFFNHVKIKLADSALERKRISAARERKAT
KILGIILGAFIICWLPFFVVSLVLPICRDSCWIHPALFDFFTWLGYLNSLINPIIYTVFN
EEFRQAFQKIVPFRKAS

>sp|O95264|5HT3B_HUMAN 5-hydroxytryptamine receptor 3B OS=Homo sapiens GN=HTR3B PE=1 SV=1
MLSSVMAPLWACILVAAGILATDTHHPQDSALYHLSKQLLQKYHKEVRPVYNWTKATTVY
LDLFVHAILDVDAENQILKTSVWYQEVWNDEFLSWNSSMFDEIREISLPLSAIWAPDIII
NEFVDIERYPDLPYVYVNSSGTIENYKPIQVVSACSLETYAFPFDVQNCSLTFKSILHTV
EDVDLAFLRSPEDIQHDKKAFLNDSEWELLSVSSTYSILQSSAGGFAQIQFNVVMRRHPL
VYVVSLLIPSIFLMLVDLGSFYLPPNCRARIVFKTSVLVGYTVFRVNMSNQVPRSVGSTP
LIGHFFTICMAFLVLSLAKSIVLVKFLHDEQRGGQEQPFLCLRGDTDADRPRVEPRAQRA
VVTESSLYGEHLAQPGTLKEVWSQLQSISNYLQTQDQTDQQEAEWLVLLSRFDRLLFQSY
LFMLGIYTITLCSLWALWGGV
>sp|P47898|5HT5A_HUMAN 5-hydroxytryptamine receptor 5A OS=Homo sapiens GN=HTR5A PE=2 SV=1
MDLPVNLTSFSLSTPSPLETNHSLGKDDLRPSSPLLSVFGVLILTLLGFLVAATFAWNLL
VLATILRVRTFHRVPHNLVASMAVSDVLVAALVMPLSLVHELSGRRWQLGRRLCQLWIAC
DVLCCTASIWNVTAIALDRYWSITRHMEYTLRTRKCVSNVMIALTWALSAVISLAPLLFG
WGETYSEGSEECQVSREPSYAVFSTVGAFYLPLCVVLFVYWKIYKAAKFRVGSRKTNSVS
PISEAVEVKDSAKQPQMVFTVRHATVTFQPEGDTWREQKEQRAALMVGILIGVFVLCWIP
FFLTELISPLCSCDIPAIWKSIFLWLGYSNSFFNPLIYTAFNKNYNSAFKNFFSRQH
>sp|P50406|5HT6R_HUMAN 5-hydroxytryptamine receptor 6 OS=Homo sapiens GN=HTR6 PE=1 SV=1
MVPEPGPTANSTPAWGAGPPSAPGGSGWVAAALCVVIALTAAANSLLIALICTQPALRNT
SNFFLVSLFTSDLMVGLVVMPPAMLNALYGRWVLARGLCLLWTAFDVMCCSASILNLCLI
SLDRYLLILSPLRYKLRMTPLRALALVLGAWSLAALASFLPLLLGWHELGHARPPVPGQC
RLLASLPFVLVASGLTFFLPSGAICFTYCRILLAARKQAVQVASLTTGMASQASETLQVP
RTPRPGVESADSRRLATKHSRKALKASLTLGILLGMFFVTWLPFFVANIVQAVCDCISPG
LFDVLTWLGYCNSTMNPIIYPLFMRDFKRALGRFLPCPRCPRERQASLASPSLRTSHSGP
RPGLSLQQVLPLPLPPDSDSDSDAGSGGSSGLRLTAQLLLPGEATQDPPLPTRAAAAVNF
FNIDPAEPELRPHPLGIPTN
>sp|P52209|6PGD_HUMAN 6-phosphogluconate dehydrogenase, decarboxylating OS=Homo sapiens GN=PGD PE=1 SV=3
MAQADIALIGLAVMGQNLILNMNDHGFVVCAFNRTVSKVDDFLANEAKGTKVVGAQSLKE
MVSKLKKPRRIILLVKAGQAVDDFIEKLVPLLDTGDIIIDGGNSEYRDTTRRCRDLKAKG
ILFVGSGVSGGEEGARYGPSLMPGGNKEAWPHIKTIFQGIAAKVGTGEPCCDWVGDEGAG
HFVKMVHNGIEYGDMQLICEAYHLMKDVLGMAQDEMAQAFEDWNKTELDSFLIEITANIL
KFQDTDGKHLLPKIRDSAGQKGTGKWTAISALEYGVPVTLIGEAVFARCLSSLKDERIQA
SKKLKGPQKFQFDGDKKSFLEDIRKALYASKIISYAQGFMLLRQAATEFGWTLNYGGIAL
MWRGGCIIRSVFLGKIKDAFDRNPELQNLLLDDFFKSAVENCQDSWRRAVSTGVQAGIPM
PCFTTALSFYDGYRHEMLPASLIQAQRDYFGAHTYELLAKPGGQFIHTNWTGHGGTVSSS
YNA
>sp|Q5SQ80|A20A2_HUMAN Ankyrin repeat domain-containing protein 20A2 OS=Homo sapiens GN=ANKRD20A2 PE=3 SV=1
MKLFGFGSRRGQTAQGSIDHVYTGSGYRIRDSELQKIHRAAVKGDAAEVERCLARRSGDL
DALDKQHRTALHLACASGHVQVVTLLVNRKCQIDVCDKENRTPLIQAVHCQEEACAVILL
EHGANPNLKDIYGNTALHYAVYSESTSLAEKLLSHGAHIEALDKDNNTPLLFAIICKKEK
MVEFLLKRKASSHAVDRLRRSALMLAVYYDSPGIVNILLKQNIDVFAQDMCGRDAEDYAI
SHHLTKIQQQILEHKKKILKKEKSDVGSSDESAVSIFHELRVDSLPASDDKDLNVATKQC
VPEKVSEPLPGSSHEKGNRIVNGQGEGPPAKHPSLKPSTEVEDPAVKGAVQRKNVQTLRA

EQALPVASEEEQERHERSEKKQPQVKEGNNTNKSEKIQLSENICDSTSSAAAGRLTQQRK
IGKTYPQQFPKKLKEEHDRCTLKQENEEKTNVNMLYKKNREELERKEKQYKKEVEAKQLE
PTVQSLEMKSKTARNTPNRDFHNHEEMKGLMDENCILKADIAILRQEICTMKNDNLEKEN
KYLKDIKIVKETNAALEKYIKLNEEMITETAFRYQQELNDLKAENTRLNAELLKEKESKK
RLEADIESYQSRLAAAISKHSESVKTERNLKLALERTRDVSVQVEMSSAISKVKDENEFL
TEQLSETQIKFNALKDKFRKTRDSLRKKSLALETVQNDLSQTQQQTQEMKEMYQNAEAKV
NNSTGKWNCVEERICHLQRENAWLVQQLDDVHQKEDHKEIVTNIQRGFIESGKKDLVLEE
KSKKLMNECDHLKESLFQYEREKTEGVVSIKEDKYFQTSRKKI
>sp|P02750|A2GL_HUMAN Leucine-rich alpha-2-glycoprotein OS=Homo sapiens GN=LRG1 PE=1 SV=2
MSSWSRQRPKSPGGIQPHVSRTLFLLLLLAASAWGVTLSPKDCQVFRSDHGSSISCQPPA
EIPGYLPADTVHLAVEFFNLTHLPANLLQGASKLQELHLSSNGLESLSPEFLRPVPQLRV
LDLTRNALTGLPPGLFQASATLDTLVLKENQLEVLEVSWLHGLKALGHLDLSGNRLRKLP
PGLLANFTLLRTLDLGENQLETLPPDLLRGPLQLERLHLEGNKLQVLGKDLLLPQPDLRY
LFLNGNKLARVAAGAFQGLRQLDMLDLSNNSLASVPEGLWASLGQPNWDMRDGFDISGNP
WICDQNLSDLYRWLQAQKDKMFSQNDTRCAGPEAVKGQTLLAVAKSQ
>sp|Q5T5F5|A4AS1_HUMAN Uncharacterized protein ADAMTSL4-AS1 OS=Homo sapiens GN=ADAMTSL4-AS1 PE=2 SV=1
MWLWQDIQCCPAPPSAPPRALEPGRAPPPPGEGLGAGIPSLSPPQKKPQSVGICVRQKGR
QKAGLEKGNRKKELRQANCPSLRPQRKGADTRRLPRETRPTKKRTAAAQPFLQLWNPAPH
TSNGRTGDL
>sp|P05067|A4_HUMAN Amyloid beta A4 protein OS=Homo sapiens GN=APP PE=1 SV=3
MLPGLALLLLAAWTARALEVPTDGNAGLLAEPQIAMFCGRLNMHMNVQNGKWDSDPSGTK
TCIDTKEGILQYCQEVYPELQITNVVEANQPVTIQNWCKRGRKQCKTHPHFVIPYRCLVG
EFVSDALLVPDKCKFLHQERMDVCETHLHWHTVAKETCSEKSTNLHDYGMLLPCGIDKFR
GVEFVCCPLAEESDNVDSADAEEDDSDVWWGGADTDYADGSEDKVVEVAEEEEVAEVEEE
EADDDEDDEDGDEVEEEAEEPYEEATERTTSIATTTTTTTESVEEVVREVCSEQAETGPC
RAMISRWYFDVTEGKCAPFFYGGCGGNRNNFDTEEYCMAVCGSAMSQSLLKTTQEPLARD
PVKLPTTAASTPDAVDKYLETPGDENEHAHFQKAKERLEAKHRERMSQVMREWEEAERQA
KNLPKADKKAVIQHFQEKVESLEQEAANERQQLVETHMARVEAMLNDRRRLALENYITAL
QAVPPRPRHVFNMLKKYVRAEQKDRQHTLKHFEHVRMVDPKKAAQIRSQVMTHLRVIYER
MNQSLSLLYNVPAVAEEIQDEVDELLQKEQNYSDDVLANMISEPRISYGNDALMPSLTET
KTTVELLPVNGEFSLDDLQPWHSFGADSVPANTENEVEPVDARPAADRGLTTRPGSGLTN
IKTEEISEVKMDAEFRHDSGYEVHHQKLVFFAEDVGSNKGAIIGLMVGGVVIATVIVITL
VMLKKKQYTSIHHGVVEVDAAVTPEERHLSKMQQNGYENPTYKFFEQMQN
>sp|Q9UGJ0|AAKG2_HUMAN 5'-AMP-activated protein kinase subunit gamma-2 OS=Homo sapiens GN=PRKAG2 PE=1 SV=1
MGSAVMDTKKKKDVSSPGGSGGKKNASQKRRSLRVHIPDLSSFAMPLLDGDLEGSGKHSS
RKVDSPFGPGSPSKGFFSRGPQPRPSSPMSAPVRPKTSPGSPKTVFPFSYQESPPRSPRR
MSFSGIFRSSSKESSPNSNPATSPGGIRFFSRSRKTSGLSSSPSTPTQVTKQHTFPLESY
KHEPERLENRIYASSSPPDTGQRFCPSSFQSPTRPPLASPTHYAPSKAAALAAALGPAEA
GMLEKLEFEDEAVEDSESGVYMRFMRSHKCYDIVPTSSKLVVFDTTLQVKKAFFALVANG
VRAAPLWESKKQSFVGMLTITDFINILHRYYKSPMVQIYELEEHKIETWRELYLQETFKP
LVNISPDASLFDAVYSLIKNKIHRLPVIDPISGNALYILTHKRILKFLQLFMSDMPKPAF
MKQNLDELGIGTYHNIAFIHPDTPIIKALNIFVERRISALPVVDESGKVVDIYSKFDVIN

LAAEKTYNNLDITVTQALQHRSQYFEGVVKCNKLEILETIVDRIVRAEVHRLVVVNEADS
IVGIISLSDILQALILTPAGAKQKETETE
>sp|Q9NY61|AATF_HUMAN Protein AATF OS=Homo sapiens GN=AATF PE=1 SV=1
MAGPQPLALQLEQLLNPRPSEADPEADPEEATAARVIDRFDEGEDGEGDFLVVGSIRKLA
SASLLDTDKRYCGKTTSRKAWNEDHWEQTLPGSSDEEISDEEGSGDEDSEGLGLEEYDED
DLGAAEEQECGDHRESKKSRSHSAKTPGFSVQSISDFEKFTKGMDDLGSSEEEEDEESGM
EEGDDAEDSQGESEEDRAGDRNSEDDGVVMTFSSVKVSEEVEKGRAVKNQIALWDQLLEG
RIKLQKALLTTNQLPQPDVFPLFKDKGGPEFSSALKNSHKALKALLRSLVGLQEELLFQY
PDTRYLVDGTKPNAGSEEISSEDDELVEEKKQQRRRVPAKRKLEMEDYPSFMAKRFADFT
VYRNRTLQKWHDKTKLASGKLGKGFGAFERSILTQIDHILMDKERLLRRTQTKRSVYRVL
GKPEPAAQPVPESLPGEPEILPQAPANAHLKDLDEEIFDDDDFYHQLLRELIERKTSSLD
PNDQVAMGRQWLAIQKLRSKIHKKVDRKASKGRKLRFHVLSKLLSFMAPIDHTTMNDDAR
TELYRSLFGQLHPPDEGHGD
>sp|Q96AK3|ABC3D_HUMAN DNA dC->dU-editing enzyme APOBEC-3D OS=Homo sapiens GN=APOBEC3D PE=1 SV=1
MNPQIRNPMERMYRDTFYDNFENEPILYGRSYTWLCYEVKIKRGRSNLLWDTGVFRGPVL
PKRQSNHRQEVYFRFENHAEMCFLSWFCGNRLPANRRFQITWFVSWNPCLPCVVKVTKFL
AEHPNVTLTISAARLYYYRDRDWRWVLLRLHKAGARVKIMDYEDFAYCWENFVCNEGQPF
MPWYKFDDNYASLHRTLKEILRNPMEAMYPHIFYFHFKNLLKACGRNESWLCFTMEVTKH
HSAVFRKRGVFRNQVDPETHCHAERCFLSWFCDDILSPNTNYEVTWYTSWSPCPECAGEV
AEFLARHSNVNLTIFTARLCYFWDTDYQEGLCSLSQEGASVKIMGYKDFVSCWKNFVYSD
DEPFKPWKGLQTNFRLLKRRLREILQ
>sp|Q8IUX4|ABC3F_HUMAN DNA dC->dU-editing enzyme APOBEC-3F OS=Homo sapiens GN=APOBEC3F PE=1 SV=3
MKPHFRNTVERMYRDTFSYNFYNRPILSRRNTVWLCYEVKTKGPSRPRLDAKIFRGQVYS
QPEHHAEMCFLSWFCGNQLPAYKCFQITWFVSWTPCPDCVAKLAEFLAEHPNVTLTISAA
RLYYYWERDYRRALCRLSQAGARVKIMDDEEFAYCWENFVYSEGQPFMPWYKFDDNYAFL
HRTLKEILRNPMEAMYPHIFYFHFKNLRKAYGRNESWLCFTMEVVKHHSPVSWKRGVFRN
QVDPETHCHAERCFLSWFCDDILSPNTNYEVTWYTSWSPCPECAGEVAEFLARHSNVNLT
IFTARLYYFWDTDYQEGLRSLSQEGASVEIMGYKDFKYCWENFVYNDDEPFKPWKGLKYN
FLFLDSKLQEILE
>sp|Q9NUT2|ABCB8_HUMAN ATP-binding cassette sub-family B member 8, mitochondrial OS=Homo sapiens GN=ABCB8 PE=1 SV=3
MLVHLFRVGIRGGPFPGRLLPPLRFQTFSAVRNTWRNGKTGQLHKAEGEYSDGYRSSSLL
RAVAHLRSQLWAHLPRAPLAPRWSPSAWCWVGGALLGPMVLSKHPHLCLVALCEAEEAPP
ASSTPHVVGSRFNWKLFWQFLHPHLLVLGVAVVLALGAALVNVQIPLLLGQLVEVVAKYT
RDHVGSFMTESQNLSTHLLILYGVQGLLTFGYLVLLSHVGERMAVDMRRALFSSLLRQDI
TFFDANKTGQLVSRLTTDVQEFKSSFKLVISQGLRSCTQVAGCLVSLSMLSTRLTLLLMV
ATPALMGVGTLMGSGLRKLSRQCQEQIARAMGVADEALGNVRTVRAFAMEQREERYGAE
LEACRCRAEELGRGIALFQGLSNIAFNCMVLGTLFIGGSLVAGQQLTGGDLMSFLVASQT
VQRSMANLSVLFGQVVRGLSAGARVFEYMALNPCIPLSGGCCVPKEQLRGSVTFQNVCFS
YPCRPGFEVLKDFTLTLPPGKIVALVGQSGGGKTTVASLLERFYDPTAGVVMLDGRDLRT
LDPSWLRGQVVGFISQEPVLFGTTIMENIRFGKLEASDEEVYTAAREANAHEFITSFPEG
YNTVVGERGTTLSGGQKQRLAIARALIKQPTVLILDEATSALDAESERVVQEALDRASAG

RTVLVIAHRLSTVRGAHCIVVMADGRVWEAGTHEELLKKGGLYAELIRRQALDAPRTAAP
PPKKPEGPRSHQHKS
>sp|Q9NUQ8|ABCF3_HUMAN ATP-binding cassette sub-family F member 3 OS=Homo sapiens GN=ABCF3 PE=1 SV=2
MATCAEILRSEFPEIDGQVFDYVTGVLHSGSADFESVDDLVEAVGELLQEVSGDSKDDAG
IRAVCQRMYNTLRLAEPQSQGNSQVLLDAPIQLSKITENYDCGTKLPGLLKREQSSTVNA
KKLEKAEARLKAKQEKRSEKDTLKTSNPLVLEEASASQAGSRKESRLESSGKNKSYDVRI
ENFDVSFGDRVLLAGADVNLAWGRRYGLVGRNGLGKTTLLKMLATRSLRVPAHISLLHVE
QEVAGDDTPALQSVLESDSVREDLLRRERELTAQIAAGRAEGSEAAELAEIYAKLEEIEA
DKAPARASVILAGLGFTPKMQQQPTREFSGGWRMRLALARALFARPDLLLLDEPTNMLDV
RAILWLENYLQTWPSTILVVSHDRNFLNAIATDIIHLHSQRLDGYRGDFETFIKSKQERL
LNQQREYEAQQQYRQHIQVFIDRFRYNANRASQVQSKLKMLEKLPELKPVDKESEVVMKF
PDGFEKFSPPILQLDEVDFYYDPKHVIFSRLSVSADLESRICVVGENGAGKSTMLKLLLG
DLAPVRGIRHAHRNLKIGYFSQHHVEQLDLNVSAVELLARKFPGRPEEEYRHQLGRYGIS
GELAMRPLASLSGGQKSRVAFAQMTMPCPNFYILDEPTNHLDMETIEALGRALNNFRGGV
ILVSHDERFIRLVCRELWVCEGGGVTRVEGGFDQYRALLQEQFRREGFL
>sp|P45844|ABCG1_HUMAN ATP-binding cassette sub-family G member 1 OS=Homo sapiens GN=ABCG1 PE=1 SV=3
MACLMAAFSVGTAMNASSYSAEMTEPKSVCVSVDEVVSSNMEATETDLLNGHLKKVDNNL
TEAQRFSSLPRRAAVNIEFRDLSYSVPEGPWWRKKGYKTLLKGISGKFNSGELVAIMGPS
GAGKSTLMNILAGYRETGMKGAVLINGLPRDLRCFRKVSCYIMQDDMLLPHLTVQEAMMV
SAHLKLQEKDEGRREMVKEILTALGLLSCANTRTGSLSGGQRKRLAIALELVNNPPVMFF
DEPTSGLDSASCFQVVSLMKGLAQGGRSIICTIHQPSAKLFELFDQLYVLSQGQCVYRGK
VCNLVPYLRDLGLNCPTYHNPADFVMEVASGEYGDQNSRLVRAVREGMCDSDHKRDLGGD
AEVNPFLWHRPSEEVKQTKRLKGLRKDSSSMEGCHSFSASCLTQFCILFKRTFLSIMRDS
VLTHLRITSHIGIGLLIGLLYLGIGNEAKKVLSNSGFLFFSMLFLMFAALMPTVLTFPLE
MGVFLREHLNYWYSLKAYYLAKTMADVPFQIMFPVAYCSIVYWMTSQPSDAVRFVLFAAL
GTMTSLVAQSLGLLIGAASTSLQVATFVGPVTAIPVLLFSGFFVSFDTIPTYLQWMSYIS
YVRYGFEGVILSIYGLDREDLHCDIDETCHFQKSEAILRELDVENAKLYLDFIVLGIFFI
SLRLIAYFVLRYKIRAER
>sp|Q9H222|ABCG5_HUMAN ATP-binding cassette sub-family G member 5 OS=Homo sapiens GN=ABCG5 PE=1 SV=1
MGDLSSLTPGGSMGLQVNRGSQSSLEGAPATAPEPHSLGILHASYSVSHRVRPWWDITSC
RQQWTRQILKDVSLYVESGQIMCILGSSGSGKTTLLDAMSGRLGRAGTFLGEVYVNGRAL
RREQFQDCFSYVLQSDTLLSSLTVRETLHYTALLAIRRGNPGSFQKKVEAVMAELSLSHV
ADRLIGNYSLGGISTGERRRVSIAAQLLQDPKVMLFDEPTTGLDCMTANQIVVLLVELAR
RNRIVVLTIHQPRSELFQLFDKIAILSFGELIFCGTPAEMLDFFNDCGYPCPEHSNPFDF
YMDLTSVDTQSKEREIETSKRVQMIESAYKKSAICHKTLKNIERMKHLKTLPMVPFKTKD
SPGVFSKLGVLLRRVTRNLVRNKLAVITRLLQNLIMGLFLLFFVLRVRSNVLKGAIQDRV
GLLYQFVGATPYTGMLNAVNLFPVLRAVSDQESQDGLYQKWQMMLAYALHVLPFSVVATM
IFSSVCYWTLGLHPEVARFGYFSAALLAPHLIGEFLTLVLLGIVQNPNIVNSVVALLSIA
GVLVGSGFLRNIQEMPIPFKIISYFTFQKYCSEILVVNEFYGLNFTCGSSNVSVTTNPMC
AFTQGIQFIEKTCPGATSRFTMNFLILYSFIPALVILGIVVFKIRDHLISR
>sp|Q8N2K0|ABD12_HUMAN Monoacylglycerol lipase ABHD12 OS=Homo sapiens GN=ABHD12 PE=1 SV=2

MRKRTEPVALEHERCAAAGSSSSGSAAAALDADCRLKQNLRLTGPAAAEPRCAADAGMKR
ALGRRKGVWLRLRKILFCVLGLYIAIPFLIKLCPGIQAKLIFLNFVRVPYFIDLKKPQDQ
GLNHTCNYYLQPEEDVTIGVWHTVPAVWWKNAQGKDQMWYEDALASSHPIILYLHGNAGT
RGGDHRVELYKVLSSLGYHVVTFDYRGWGDSVGTPSERGMTYDALHVFDWIKARSGDNPV
YIWGHSLGTGVATNLVRRLCERETPPDALILESPFTNIREEAKSHPFSVIYRYFPGFDWF
FLDPITSSGIKFANDENVKHISCPLLILHAEDDPVVPFQLGRKLYSIAAPARSFRDFKVQ
FVPFHSDLGYRHKYIYKSPELPRILREFLGKSEPEHQH
>sp|Q969K4|ABTB1_HUMAN Ankyrin repeat and BTB/POZ domain-containing protein 1 OS=Homo sapiens GN=ABTB1 PE=1 SV=1
MDTSDLFASCRKGDVGRVRYLLEQRDVEVNVRDKWDSTPLYYACLCGHEELVLYLLANGA
RCEANTFDGERCLYGALSDPIRRALRDYKQVTASCRRRDYYDDFLQRLLEQGIHSDVVFV
VHGKPFRVHRCVLGARSAYFANMLDTKWKGKSVVVLRHPLINPVAFGALLQYLYTGRLDI
GVEHVSDCERLAKQCQLWDLLSDLEAKCEKVSEFVASKPGTCVKVLTIEPPPADPRLRED
MALLADCALPPELRGDLWELPFPCPDGFNSCPDICFRVAGCSFLCHKAFFCGRSDYFRAL
LDDHFRESEEPATSGGPPAVTLHGISPDVFTHVLYYMYSDHTELSPEAAYDVLSVADMYL
LPGLKRLCGRSLAQMLDEDTVVGVWRVAKLFRLARLEDQCTEYMAKVIEKLVEREDFVEA
VKEEAAAVAARQETDSIPLVDDIRFHVASTVQTYSAIEEAQQRLRALEDLLVSIGLDC
>sp|Q15027|ACAP1_HUMAN Arf-GAP with coiled-coil, ANK repeat and PH domain-containing protein 1 OS=Homo sapiens GN=ACAP1 PE=1 SV=1
MTVKLDFEECLKDSPRFRASIELVEAEVSELETRLEKLLKLGTGLLESGRHYLAASRAFV
VGICDLARLGPPEPMMAECLEKFTVSLNHKLDSHAELLDATQHTLQQQIQTLVKEGLRGF
REARRDFWRGAESLEAALTHNAEVPRRRAQEAEEAGAALRTARAGYRGRALDYALQINVI
EDKRKFDIMEFVLRLVEAQATHFQQGHEELSRLSQYRKELGAQLHQLVLNSAREKRDMEQ
RHVLLKQKELGGEEPEPSLREGPGGLVMEGHLFKRASNAFKTWSRRWFTIQSNQLVYQKK
YKDPVTVVVDDLRLCTVKLCPDSERRFCFEVVSTSKSCLLQADSERLLQLWVSAVQSSIA
SAFSQARLDDSPRGPGQGSGHLAIGSAATLGSGGMARGREPGGVGHVVAQVQSVDGNAQC
CDCREPAPEWASINLGVTLCIQCSGIHRSLGVHFSKVRSLTLDSWEPELVKLMCELGNVI
INQIYEARVEAMAVKKPGPSCSRQEKEAWIHAKYVEKKFLTKLPEIRGRRGGRGRPRGQP
PVPPKPSIRPRPGSLRSKPEPPSEDLGSLHPGALLFRASGHPPSLPTMADALAHGADVNW
VNGGQDNATPLIQATAANSLLACEFLLQNGANVNQADSAGRGPLHHATILGHTGLACLFL
KRGADLGARDSEGRDPLTIAMETANADIVTLLRLAKMREAEAAQGQAGDETYLDIFRDFS
LMASDDPEKLSRRSHDLHTL
>sp|O00400|ACATN_HUMAN Acetyl-coenzyme A transporter 1 OS=Homo sapiens GN=SLC33A1 PE=1 SV=1
MSPTISHKDSSRQRRPGNFSHSLDMKSGPLPPGGWDDSHLDSAGREGDREALLGDTGTGD
FLKAPQSFRAELSSILLLLFLYVLQGIPLGLAGSIPLILQSKNVSYTDQAFFSFVFWPFS
LKLLWAPLVDAVYVKNFGRRKSWLVPTQYILGLFMIYLSTQVDRLLGNTDDRTPDVIALT
VAFFLFEFLAATQDIAVDGWALTMLSRENVGYASTCNSVGQTAGYFLGNVLFLALESADF
CNKYLRFQPQPRGIVTLSDFLFFWGTVFLITTTLVALLKKENEVSVVKEETQGITDTYKL
LFAIIKMPAVLTFCLLILTAKIGFSAADAVTGLKLVEEGVPKEHLALLAVPMVPLQIILP
LIISKYTAGPQPLNTFYKAMPYRLLLGLEYALLVWWTPKVEHQGGFPIYYYIVVLLSYAL
HQVTVYSMYVSIMAFNAKVSDPLIGGTYMTLLNTVSNLGGNWPSTVALWLVDPLTVKECV
GASNQNCRTPDAVELCKKLGGSCVTALDGYYVESIICVFIGFGWWFFLGPKFKKLQDEGS
SSWKCKRNN

>sp|P30486|1B48_HUMAN HLA class I histocompatibility antigen, B-48 alpha chain OS=Homo sapiens GN=HLA-B PE=1 SV=1
MLVMAPRTVLLLLSAALALTETWAGSHSMRYFYTSVSRPGRGEPRFISVGYVDDTQFVRF
DSDAASPREEPRAPWIEQEGPEYWDRETQISKTNTQTYRESLRNLRGYYNQSEAGSHTLQ
SMYGCDVGPDGRLLRGHNQYAYDGKDYIALNEDLRSWTAADTAAQISQRKLEAARVAEQL
RAYLEGECVEWLRRYLENGKDKLERADPPKTHVTHHPISDHEATLRCWALGFYPAEITLT
WQRDGEDQTQDTELVETRPAGDRTFQKWTAVVVPSGEEQRYTCHVQHEGLPKPLTLRWEP
SSQSTVPIVGIVAGLAVLAVVVIGAVVAAVMCRRKSSGGKGGSYSQAACSDSAQGSDVSL
TA
>sp|P30488|1B50_HUMAN HLA class I histocompatibility antigen, B-50 alpha chain OS=Homo sapiens GN=HLA-B PE=1 SV=1
MRVTAPRTVLLLLSAALALTETWAGSHSMRYFHTAMSRPGRGEPRFITVGYVDDTLFVRF
DSDATSPRKEPRAPWIEQEGPEYWDRETQISKTNTQTYRESLRNLRGYYNQSEAGSHTWQ
RMYGCDLGPDGRLLRGYNQLAYDGKDYIALNEDLSSWTAADTAAQITQRKWEAAREAEQL
RAYLEGLCVEWLRRYLENGKETLQRADPPKTHVTHHPISDHEATLRCWALGFYPAEITLT
WQRDGEDQTQDTELVETRPAGDRTFQKWAAVVVPSGEEQRYTCHVQHEGLPKPLTLRWEP
SSQSTIPIVGIVAGLAVLAVVVIGAVVATVMCRRKSSGGKGGSYSQAASSDSAQGSDVSL
TA
>sp|P30490|1B52_HUMAN HLA class I histocompatibility antigen, B-52 alpha chain OS=Homo sapiens GN=HLA-B PE=1 SV=1
MRVTAPRTVLLLLWGAVALTETWAGSHSMRYFYTAMSRPGRGEPRFIAVGYVDDTQFVRF
DSDAASPRTEPRAPWIEQEGPEYWDRETQISKTNTQTYRENLRIALRYYNQSEAGSHTWQ
TMYGCDVGPDGRLLRGHNQYAYDGKDYIALNEDLSSWTAADTAAQITQRKWEAAREAEQL
RAYLEGLCVEWLRRHLENGKETLQRADPPKTHVTHHPVSDHEATLRCWALGFYPAEITLT
WQRDGEDQTQDTELVETRPAGDRTFQKWAAVVVPSGEEQRYTCHVQHEGLPKPLTLRWEP
SSQSTIPIVGIVAGLAVLAVVVIGAVVATVMCRRKSSGGKGGSYSQAASSDSAQGSDVSL
TA
>sp|Q13362|2A5G_HUMAN Serine/threonine-protein phosphatase 2A 56 kDa regulatory subunit gamma isoform OS=Homo sapiens GN=PPP2R5C PE=1 SV=3
MLTCNKAGSRMVVDAANSNGPFQPVVLLHIRDVPPADQEKLFIQKLRQCCVLFDFVSDPL
SDLKWKEVKRAALSEMVEYITHNRNVITEPIYPEVVHMFAVNMFRTLPPSSNPTGAEFDP
EEDEPTLEAAWPHLQLVYEFFLRFLESPDFQPNIAKKYIDQKFVLQLLELFDSEDPRERD
FLKTTLHRIYGKFLGLRAYIRKQINNIFYRFIYETEHHNGIAELLEILGSIINGFALPLK
EEHKIFLLKVLLPLHKVKSLSVYHPQLAYCVVQFLEKDSTLTEPVVMALLKYWPKTHSPK
EVMFLNELEEILDVIEPSEFVKIMEPLFRQLAKCVSSPHFQVAERALYYWNNEYIMSLIS
DNAAKILPIMFPSLYRNSKTHWNKTIHGLIYNALKLFMEMNQKLFDDCTQQFKAEKLKEK
LKMKEREEAWVKIENLAKANPQYTVYSQASTMSIPVAMETDGPLFEDVQMLRKTVKDEAH
QAQKDPKKDRPLARRKSELPQDPHTKKALEAHCRADELASQDGR
>sp|P30154|2AAB_HUMAN Serine/threonine-protein phosphatase 2A 65 kDa regulatory subunit A beta isoform OS=Homo sapiens GN=PPP2R1B PE=1 SV=3
MAGASELGTGPGAAGGDGDDSLYPIAVLIDELRNEDVQLRLNSIKKLSTIALALGVERTR
SELLPFLTDTIYDEDEVLLALAEQLGNFTGLVGGPDFAHCLLPPLENLATVEETVVRDKA
VESLRQISQEHTPVALEAYFVPLVKRLASGDWFTSRTSACGLFSVCYPRASNAVKAEIRQ
QFRSLCSDDTPMVRRAAASKLGEFAKVLELDSVKSEIVPLFTSLASDEQDSVRLLAVEAC

VSIAQLLSQDDLETLVMPTLRQAAEDKSWRVRYMVADRFSELQKAMGPKITLNDLIPAFQ
NLLKDCEAEVRAAAAHKVKELGENLPIEDRETIIMNQILPYIKELVSDTNQHVKSALASV
IMGLSTILGKENTIEHLLPLFLAQLKDECPDVRLNIISNLDCVNEVIGIRQLSQSLLPAI
VELAEDAKWRVRLAIIEYMPLLAGQLGVEFFDEKLNSLCMAWLVDHVYAIREAATNNLMK
LVQKFGTEWAQNTIVPKVLVMANDPNYLHRMTTLFCINALSEACGQEITTKQMLPIVLKM
AGDQVANVRFNVAKSLQKIGPILDTNALQGEVKPVLQKLGQDEDMDVKYFAQEAISVLAL
A
>sp|Q9Y2T4|2ABG_HUMAN Serine/threonine-protein phosphatase 2A 55 kDa regulatory subunit B gamma isoform OS=Homo sapiens GN=PPP2R2C PE=1 SV=4
MGEDTDTRKINHSFLRDHSYVTEADIISTVEFNHTGELLATGDKGGRVVIFQREPESKNA
PHSQGEYDVYSTFQSHEPEFDYLKSLEIEEKINKIKWLPQQNAAHSLLSTNDKTIKLWKI
TERDKRPEGYNLKDEEGKLKDLSTVTSLQVPVLKPMDLMVEVSPRRIFANGHTYHINSIS
VNSDCETYMSADDLRINLWHLAITDRSFNIVDIKPANMEDLTEVITASEFHPHHCNLFVY
SSSKGSLRLCDMRAAALCDKHSKLFEEPEDPSNRSFFSEIISSVSDVKFSHSGRYMLTRD
YLTVKVWDLNMEARPIETYQVHDYLRSKLCSLYENDCIFDKFECAWNGSDSVIMTGAYNN
FFRMFDRNTKRDVTLEASRESSKPRAVLKPRRVCVGGKRRRDDISVDSLDFTKKILHTAW
HPAENIIAIAATNNLYIFQDKVNSDMH
>sp|P13760|2B14_HUMAN HLA class II histocompatibility antigen, DRB1-4 beta chain OS=Homo sapiens GN=HLA-DRB1 PE=1 SV=1
MVCLKFPGGSCMAALTVTLMVLSSPLALAGDTRPRFLEQVKHECHFFNGTERVRFLDRYF
YHQEEYVRFDSDVGEYRAVTELGRPDAEYWNSQKDLLEQKRAAVDTYCRHNYGVGESFTV
QRRVYPEVTVYPAKTQPLQHHNLLVCSVNGFYPGSIEVRWFRNGQEEKTGVVSTGLIQNG
DWTFQTLVMLETVPRSGEVYTCQVEHPSLTSPLTVEWRARSESAQSKMLSGVGGFVLGLL
FLGAGLFIYFRNQKGHSGLQPTGFLS
>sp|Q30134|2B18_HUMAN HLA class II histocompatibility antigen, DRB1-8 beta chain OS=Homo sapiens GN=HLA-DRB1 PE=1 SV=2
MVCLRLPGGSCMAVLTVTLMVLSSPLALAGDTRPRFLEYSTGECYFFNGTERVRFLDRYF
YNQEEYVRFDSDVGEYRAVTELGRPSAEYWNSQKDFLEDRRALVDTYCRHNYGVGESFTV
QRRVHPKVTVYPSKTQPLQHHNLLVCSVSGFYPGSIEVRWFRNGQEEKTGVVSTGLIHNG
DWTFQTLVMLETVPRSGEVYTCQVEHPSVTSPLTVEWSARSESAQSKMLSGVGGFVLGLL
FLGAGLFIYFRNQKGHSGLQPTGFLS
>sp|Q5Y7A7|2B1D_HUMAN HLA class II histocompatibility antigen, DRB1-13 beta chain OS=Homo sapiens GN=HLA-DRB1 PE=1 SV=1
MVCLRLPGGSCMAVLTVTLMVLSSPLALAGDTRPRFLEYSTSECHFFNGTERVRFLDRYF
HNQEENVRFDSDVGEFRAVTELGRPDAEYWNSQKDILEDERAAVDTYCRHNYGVVESFTV
QRRVHPKVTVYPSKTQPLQHHNLLVCSVSGFYPGSIEVRWFRNGQEEKTGVVSTGLIHNG
DWTFQTLVMLETVPRSGEVYTCQVEHPSVTSPLTVEWRARSESAQSKMLSGVGGFVLGLL
FLGAGLFIYFRNQKGHSGLQPRGFLS
>sp|Q8IZ83|A16A1_HUMAN Aldehyde dehydrogenase family 16 member A1 OS=Homo sapiens GN=ALDH16A1 PE=1 SV=2
MAATRAGPRAREIFTSLEYGPVPESHACALAWLDTQDRCLGHYVNGKWLKPEHRNSVPCQ
DPITGENLASCLQAQAEDVAAAVEAARMAFKGWSAHPGVVRAQHLTRLAEVIQKHQRLLW
TLESLVTGRAVREVRDGDVQLAQQLLHYHAIQASTQEEALAGWEPMGVIGLILPPTFSFL
EMMWRICPALAVGCTVVALVPPASPAPLLLAQLAGELGPFPGILNVLSGPASLVPILASQ

PGIRKVAFCGAPEEGRALRRSLAGECAELGLALGTESLLLLTDTADVDSAVEGVVDAAWS
DRGPGGLRLLIQESVWDEAMRRLQERMGRLRSGRGLDGAVDMGARGAAACDLVQRFVREA
QSQGAQVFQAGDVPSERPFYPPTLVSNLPPASPCAQVEVPWPVVVASPFRTAKEALLVAN
GTPRGGSASVWSERLGQALELGYGLQVGTVWINAHGLRDPSVPTGGCKESGCSWHGGPDG
LYEYLRPSGTPARLSCLSKNLNYDTFGLAVPSTLPAGPEIGPSPAPPYGLFVGGRFQAPG
ARSSRPIRDSSGNLHGYVAEGGAKDIRGAVEAAHQAFPGWAGQSPGARAALLWALAAALE
RRKSTLASRLERQGAELKAAEAEVELSARRLRAWGARVQAQGHTLQVAGLRGPVLRLREP
LGVLAVVCPDEWPLLAFVSLLAPALAYGNTVVMVPSAACPLLALEVCQDMATVFPAGLAN
VVTGDRDHLTRCLALHQDVQAMWYFGSAQGSQFVEWASAGNLKPVWASRGCPRAWDQEAE
GAGPELGLRVARTKALWLPMGD
>sp|Q8NAA4|A16L2_HUMAN Autophagy-related protein 16-2 OS=Homo sapiens GN=ATG16L2 PE=1 SV=2
MAGPGVPGAPAARWKRHIVRQLRLRDRTQKALFLELVPAYNHLLEKAELLDKFSKKLQPE
PNSVTPTTHQGPWEESELDSDQVPSLVALRVKWQEEEEGLRLVCGEMAYQVVEKGAALGT
LESELQQRQSRLAALEARVAQLREARAQQAQQVEEWRAQNAVQRAAYEALRAHVGLREAA
LRRLQEEARDLLERLVQRKARAAAERNLRNERRERAKQARVSQELKKAAKRTVSISEGPD
TLGDGMRERRETLALAPEPEPLEKEACEKWKRPFRSASATSLTLSHCVDVVKGLLDFKKR
RGHSIGGAPEQRYQIIPVCVAARLPTRAQDVLDAHLSEVNAVRFGPNSSLLATGGADRLI
HLWNVVGSRLEANQTLEGAGGSITSVDFDPSGYQVLAATYNQAAQLWKVGEAQSKETLSG
HKDKVTAAKFKLTRHQAVTGSRDRTVKEWDLGRAYCSRTINVLSYCNDVVCGDHIIISGH
NDQKIRFWDSRGPHCTQVIPVQGRVTSLSLSHDQLHLLSCSRDNTLKVIDLRVSNIRQVF
RADGFKCGSDWTKAVFSPDRSYALAGSCDGALYIWDVDTGKLESRLQGPHCAAVNAVAWC
YSGSHMVSVDQGRKVVLWQ
>sp|Q96IX9|A26L1_HUMAN Putative ankyrin repeat domain-containing protein 26-like 1 OS=Homo sapiens GN=ANKRD36BP1 PE=5 SV=1
MGTRTLQFEISDSHEKEEDLLHKNHLMQDEIARLRLEIHTIKNQILEKKYLKDIEIIKRK
HEDLQKALKQNGEKSTKTIAHYSGQLTALTDENTMLRSKLEKEKQSRQRLTKWNHTIVD
>sp|P08697|A2AP_HUMAN Alpha-2-antiplasmin OS=Homo sapiens GN=SERPINF2 PE=1 SV=3
MALLWGLLVLSWSCLQGPCSVFSPVSAMEPLGRQLTSGPNQEQVSPLTLLKLGNQEPGGQ
TALKSPPGVCSRDPTPEQTHRLARAMMAFTADLFSLVAQTSTCPNLILSPLSVALALSHL
ALGAQNHTLQRLQQVLHAGSGPCLPHLLSRLCQDLGPGAFRLAARMYLQKGFPIKEDFLE
QSEQLFGAKPVSLTGKQEDDLANINQWVKEATEGKIQEFLSGLPEDTVLLLLNAIHFQGF
WRNKFDPSLTQRDSFHLDEQFTVPVEMMQARTYPLRWFLLEQPEIQVAHFPFKNNMSFVV
LVPTHFEWNVSQVLANLSWDTLHPPLVWERPTKVRLPKLYLKHQMDLVATLSQLGLQELF
QAPDLRGISEQSLVVSGVQHQSTLELSEVGVEAAAATSIAMSRMSLSSFSVNRPFLFFIF
EDTTGLPLFVGSVRNPNPSAPRELKEQQDSPGNKDFLQSLKGFPRGDKLFGPDLKLVPPM
EEDYPQFGSPK
>sp|P01023|A2MG_HUMAN Alpha-2-macroglobulin OS=Homo sapiens GN=A2M PE=1 SV=3
MGKNKLLHPSLVLLLLVLLPTDASVSGKPQYMVLVPSLLHTETTEKGCVLLSYLNETVTV
SASLESVRGNRSLFTDLEAENDVLHCVAFAVPKSSSNEEVMFLTVQVKGPTQEFKKRTTV
MVKNEDSLVFVQTDKSIYKPGQTVKFRVVSMDENFHPLNELIPLVYIQDPKGNRIAQWQS
FQLEGGLKQFSFPLSSEPFQGSYKVVVQKKSGGRTEHPFTVEEFVLPKFEVQVTVPKIIT
ILEEEMNVSVCGLYTYGKPVPGHVTVSICRKYSDASDCHGEDSQAFCEKFSGQLNSHGCF
YQQVKTKVFQLKRKEYEMKLHTEAQIQEEGTVVELTGRQSSEITRTITKLSFVKVDSHFR

QGIPFFGQVRLVDGKGVPIPNKVIFIRGNEANYYSNATTDEHGLVQFSINTTNVMGTSLT
VRVNYKDRSPCYGYQWVSEEHEEAHHTAYLVFSPSKSFVHLEPMSHELPCGHTQTVQAHY
ILNGGTLLGLKKLSFYYLIMAKGGIVRTGTHGLLVKQEDMKGHFSISIPVKSDIAPVARL
LIYAVLPTGDVIGDSAKYDVENCLANKVDLSFSPSQSLPASHAHLRVTAAPQSVCALRAV
DQSVLLMKPDAELSASSVYNLLPEKDLTGFPGPLNDQDNEDCINRHNVYINGITYTPVSS
TNEKDMYSFLEDMGLKAFTNSKIRKPKMCPQLQQYEMHGPEGLRVGFYESDVMGRGHARL
VHVEEPHTETVRKYFPETWIWDLVVVNSAGVAEVGVTVPDTITEWKAGAFCLSEDAGLGI
SSTASLRAFQPFFVELTMPYSVIRGEAFTLKATVLNYLPKCIRVSVQLEASPAFLAVPVE
KEQAPHCICANGRQTVSWAVTPKSLGNVNFTVSAEALESQELCGTEVPSVPEHGRKDTVI
KPLLVEPEGLEKETTFNSLLCPSGGEVSEELSLKLPPNVVEESARASVSVLGDILGSAMQ
NTQNLLQMPYGCGEQNMVLFAPNIYVLDYLNETQQLTPEIKSKAIGYLNTGYQRQLNYKH
YDGSYSTFGERYGRNQGNTWLTAFVLKTFAQARAYIFIDEAHITQALIWLSQRQKDNGCF
RSSGSLLNNAIKGGVEDEVTLSAYITIALLEIPLTVTHPVVRNALFCLESAWKTAQEGDH
GSHVYTKALLAYAFALAGNQDKRKEVLKSLNEEAVKKDNSVHWERPQKPKAPVGHFYEPQ
APSAEVEMTSYVLLAYLTAQPAPTSEDLTSATNIVKWITKQQNAQGGFSSTQDTVVALHA
LSKYGAATFTRTGKAAQVTIQSSGTFSSKFQVDNNNRLLLQQVSLPELPGEYSMKVTGEG
CVYLQTSLKYNILPEKEEFPFALGVQTLPQTCDEPKAHTSFQISLSVSYTGSRSASNMAI
VDVKMVSGFIPLKPTVKMLERSNHVSRTEVSSNHVLIYLDKVSNQTLSLFFTVLQDVPVR
DLKPAIVKVYDYYETDEFAIAEYNAPCSKDLGNA
>sp|A8K2U0|A2ML1_HUMAN Alpha-2-macroglobulin-like protein 1 OS=Homo sapiens GN=A2ML1 PE=1 SV=3
MWAQLLLGMLALSPAIAEELPNYLVTLPARLNFPSVQKVCLDLSPGYSDVKFTVTLETKD
KTQKLLEYSGLKKRHLHCISFLVPPPAGGTEEVATIRVSGVGNNISFEEKKKVLIQRQGN
GTFVQTDKPLYTPGQQVYFRIVTMDSNFVPVNDKYSMVELQDPNSNRIAQWLEVVPEQGI
VDLSFQLAPEAMLGTYTVAVAEGKTFGTFSVEEYVLPKFKVEVVEPKELSTVQESFLVKI
CCRYTYGKPMLGAVQVSVCQKANTYWYREVEREQLPDKCRNLSGQTDKTGCFSAPVDMAT
FDLIGYAYSHQINIVATVVEEGTGVEANATQNIYISPQMGSMTFEDTSNFYHPNFPFSGK
IRVRGHDDSFLKNHLVFLVIYGTNGTFNQTLVTDNNGLAPFTLETSGWNGTDVSLEGKFQ
MEDLVYNPEQVPRYYQNAYLHLRPFYSTTRSFLGIHRLNGPLKCGQPQEVLVDYYIDPAD
ASPDQEISFSYYLIGKGSLVMEGQKHLNSKKKGLKASFSLSLTFTSRLAPDPSLVIYAIF
PSGGVVADKIQFSVEMCFDNQVSLGFSPSQQLPGAEVELQLQAAPGSLCALRAVDESVLL
LRPDRELSNRSVYGMFPFWYGHYPYQVAEYDQCPVSGPWDFPQPLIDPMPQGHSSQRSII
WRPSFSEGTDLFSFFRDVGLKILSNAKIKKPVDCSHRSPEYSTAMGAGGGHPEAFESSTP
LHQAEDSQVRQYFPETWLWDLFPIGNSGKEAVHVTVPDAITEWKAMSFCTSQSRGFGLSP
TVGLTAFKPFFVDLTLPYSVVRGESFRLTATIFNYLKDCIRVQTDLAKSHEYQLESWADS
QTSSCLCADDAKTHHWNITAVKLGHINFTISTKILDSNEPCGGQKGFVPQKGRSDTLIKP
VLVKPEGVLVEKTHSSLLCPKGKVASESVSLELPVDIVPDSTKAYVTVLGDIMGTALQNL
DGLVQMPSGCGEQNMVLFAPIIYVLQYLEKAGLLTEEIRSRAVGFLEIGYQKELMYKHSN
GSYSAFGERDGNGNTWLTAFVTKCFGQAQKFIFIDPKNIQDALKWMAGNQLPSGCYANVG
NLLHTAMKGGVDDEVSLTAYVTAALLEMGKDVDDPMVSQGLRCLKNSATSTTNLYTQALL
AYIFSLAGEMDIRNILLKQLDQQAIISGESIYWSQKPTPSSNASPWSEPAAVDVELTAYA
LLAQLTKPSLTQKEIAKATSIVAWLAKQHNAYGGFSSTQDTVVALQALAKYATTAYMPSE
EINLVVKSTENFQRTFNIQSVNRLVFQQDTLPNVPGMYTLEASGQGCVYVQTVLRYNILP
PTNMKTFSLSVEIGKARCEQPTSPRSLTLTIHTSYVGSRSSSNMAIVEVKMLSGFSPMEG

TNQLLLQQPLVKKVEFGTDTLNIYLDELIKNTQTYTFTISQSVLVTNLKPATIKVYDYYL
PDEQATIQYSDPCE
>sp|Q2M2I8|AAK1_HUMAN AP2-associated protein kinase 1 OS=Homo sapiens GN=AAK1 PE=1 SV=3
MKKFFDSRREQGGSGLGSGSSGGGGSTSGLGSGYIGRVFGIGRQQVTVDEVLAEGGFAIV
FLVRTSNGMKCALKRMFVNNEHDLQVCKREIQIMRDLSGHKNIVGYIDSSINNVSSGDVW
EVLILMDFCRGGQVVNLMNQRLQTGFTENEVLQIFCDTCEAVARLHQCKTPIIHRDLKVE
NILLHDRGHYVLCDFGSATNKFQNPQTEGVNAVEDEIKKYTTLSYRAPEMVNLYSGKIIT
TKADIWALGCLLYKLCYFTLPFGESQVAICDGNFTIPDNSRYSQDMHCLIRYMLEPDPDK
RPDIYQVSYFSFKLLKKECPIPNVQNSPIPAKLPEPVKASEAAAKKTQPKARLTDPIPTT
ETSIAPRQRPKAGQTQPNPGILPIQPALTPRKRATVQPPPQAAGSSNQPGLLASVPQPKP
QAPPSQPLPQTQAKQPQAPPTPQQTPSTQAQGLPAQAQATPQHQQQLFLKQQQQQQQPPP
AQQQPAGTFYQQQQAQTQQFQAVHPATQKPAIAQFPVVSQGGSQQQLMQNFYQQQQQQQQ
QQQQQQLATALHQQQLMTQQAALQQKPTMAAGQQPQPQPAAAPQPAPAQEPAIQAPVRQQ
PKVQTTPPPAVQGQKVGSLTPPSSPKTQRAGHRRILSDVTHSAVFGVPASKSTQLLQAAA
AEASLNKSKSATTTPSGSPRTSQQNVYNPSEGSTWNPFDDDNFSKLTAEELLNKDFAKLG
EGKHPEKLGGSAESLIPGFQSTQGDAFATTSFSAGTAEKRKGGQTVDSGLPLLSVSDPFI
PLQVPDAPEKLIEGLKSPDTSLLLPDLLPMTDPFGSTSDAVIEKADVAVESLIPGLEPPV
PQRLPSQTESVTSNRTDSLTGEDSLLDCSLLSNPTTDLLEEFAPTAISAPVHKAAEDSNL
ISGFDVPEGSDKVAEDEFDPIPVLITKNPQGGHSRNSSGSSESSLPNLARSLLLVDQLID
L
>sp|O43741|AAKB2_HUMAN 5’-AMP-activated protein kinase subunit beta-2 OS=Homo sapiens GN=PRKAB2 PE=1 SV=1
MGNTTSDRVSGERHGAKAARSEGAGGHAPGKEHKIMVGSTDDPSVFSLPDSKLPGDKEFV
SWQQDLEDSVKPTQQARPTVIRWSEGGKEVFISGSFNNWSTKIPLIKSHNDFVAILDLPE
GEHQYKFFVDGQWVHDPSEPVVTSQLGTINNLIHVKKSDFEVFDALKLDSMESSETSCRD
LSSSPPGPYGQEMYAFRSEERFKSPPILPPHLLQVILNKDTNISCDPALLPEPNHVMLNH
LYALSIKDSVMVLSATHRYKKKYVTTLLYKPI
>sp|P54619|AAKG1_HUMAN 5’-AMP-activated protein kinase subunit gamma-1 OS=Homo sapiens GN=PRKAG1 PE=1 SV=1
METVISSDSSPAVENEHPQETPESNNSVYTSFMKSHRCYDLIPTSSKLVVFDTSLQVKKA
FFALVTNGVRAAPLWDSKKQSFVGMLTITDFINILHRYYKSALVQIYELEEHKIETWREV
YLQDSFKPLVCISPNASLFDAVSSLIRNKIHRLPVIDPESGNTLYILTHKRILKFLKLFI
TEFPKPEFMSKSLEELQIGTYANIAMVRTTTPVYVALGIFVQHRVSALPVVDEKGRVVDI
YSKFDVINLAAEKTYNNLDVSVTKALQHRSHYFEGVLKCYLHETLETIINRLVEAEVHRL
VVVDENDVVKGIVSLSDILQALVLTGGEKKP
>sp|Q96GS6|AB17A_HUMAN Protein ABHD17A OS=Homo sapiens GN=ABHD17A PE=1 SV=1
MNGLSLSELCCLFCCPPCPGRIAAKLAFLPPEATYSLVPEPEPGPGGAGAAPLGTLRASS
GAPGRWKLHLTERADFQYSQRELDTIEVFPTKSARGNRVSCMYVRCVPGARYTVLFSHGN
AVDLGQMSSFYIGLGSRLHCNIFSYDYSGYGASSGRPSERNLYADIDAAWQALRTRYGIS
PDSIILYGQSIGTVPTVDLASRYECAAVVLHSPLTSGMRVAFPDTKKTYCFDAFPNIEKV
SKITSPVLIIHGTEDEVIDFSHGLALYERCPKAVEPLWVEGAGHNDIELYSQYLERLRRF
ISQELPSQRA
>sp|Q5VST6|AB17B_HUMAN Protein ABHD17B OS=Homo sapiens GN=ABHD17B PE=1 SV=1
MNNLSFSELCCLFCCPPCPGKIASKLAFLPPDPTYTLMCDESGSRWTLHLSERADWQYSS

REKDAIECFMTRTSKGNRIACMFVRCSPNAKYTLLFSHGNAVDLGQMSSFYIGLGSRINC
NIFSYDYSGYGASSGKPTEKNLYADIEAAWLALRTRYGIRPENVIIYGQSIGTVPSVDLA
ARYESAAVILHSPLTSGMRVAFPDTKKTYCFDAFPNIDKISKITSPVLIIHGTEDEVIDF
SHGLALFERCQRPVEPLWVEGAGHNDVELYGQYLERLKQFVSQELVNL
>sp|Q9NSE7|ABCCD_HUMAN Putative ATP-binding cassette sub-family C member 13 OS=Homo sapiens GN=ABCC13 PE=2 SV=2
MLSSTQNAGGSYQRVRGALDTQKCSPEKSASFFSKVTYSWFSRVITLGYKRPLEREDLFE
LKESDSFCTACPIFEKQWRKEVLRNQERQKVKVSCYKEAHIKKPSLLYALWNTFKSILIQ
VALFKVFADILSFTSPLIMKQIIIFCEHSSDFGWNGYGYAVALLVVVFLQTLILQQYQRF
NMLTSAKVKTAVNGLIYKKALLLSNVSRQKFSTGEIINLMSATHGLDSKPQSPLVCPFSN
PNGRISPLARAGSSSVSRGGSPCVCYTNKCFSCN
>sp|Q8NFV4|ABHDB_HUMAN Protein ABHD11 OS=Homo sapiens GN=ABHD11 PE=1 SV=1
MRAGQQLASMLRWTRAWRLPREGLGPHGPSFARVPVAPSSSSGGRGGAEPRPLPLSYRLL
DGEAALPAVVFLHGLFGSKTNFNSIAKILAQQTGRRVLTVDARNHGDSPHSPDMSYEIMS
QDLQDLLPQLGLVPCVVVGHSMGGKTAMLLALQRPELVERLIAVDISPVESTGVSHFATY
VAAMRAINIADELPRSRARKLADEQLSSVIQDMAVRQHLLTNLVEVDGRFVWRVNLDALT
QHLDKILAFPQRQESYLGPTLFLLGGNSQFVHPSHHPEIMRLFPRAQMQTVPNAGHWIHA
DRPQDFIAAIRGFLV
>sp|Q96IU4|ABHEB_HUMAN Protein ABHD14B OS=Homo sapiens GN=ABHD14B PE=1 SV=1
MAASVEQREGTIQVQGQALFFREALPGSGQARFSVLLLHGIRFSSETWQNLGTLHRLAQA
GYRAVAIDLPGLGHSKEAAAPAPIGELAPGSFLAAVVDALELGPPVVISPSLSGMYSLPF
LTAPGSQLPGFVPVAPICTDKINAANYASVKTPALIVYGDQDPMGQTSFEHLKQLPNHRV
LIMKGAGHPCYLDKPEEWHTGLLDFLQGLQ
>sp|O95870|ABHGA_HUMAN Protein ABHD16A OS=Homo sapiens GN=ABHD16A PE=1 SV=3
MAKLLSCVLGPRLYKIYRERDSERAPASVPETPTAVTAPHSSSWDTYYQPRALEKHADSI
LALASVFWSISYYSSPFAFFYLYRKGYLSLSKVVPFSHYAGTLLLLLAGVACLRGIGRWT
NPQYRQFITILEATHRNQSSENKRQLANYNFDFRSWPVDFHWEEPSSRKESRGGPSRRGV
ALLRPEPLHRGTADTLLNRVKKLPCQITSYLVAHTLGRRMLYPGSVYLLQKALMPVLLQG
QARLVEECNGRRAKLLACDGNEIDTMFVDRRGTAEPQGQKLVICCEGNAGFYEVGCVSTP
LEAGYSVLGWNHPGFAGSTGVPFPQNEANAMDVVVQFAIHRLGFQPQDIIIYAWSIGGFT
ATWAAMSYPDVSAMILDASFDDLVPLALKVMPDSWRGLVTRTVRQHLNLNNAEQLCRYQG
PVLLIRRTKDEIITTTVPEDIMSNRGNDLLLKLLQHRYPRVMAEEGLRVVRQWLEASSQL
EEASIYSRWEVEEDWCLSVLRSYQAEHGPDFPWSVGEDMSADGRRQLALFLARKHLHNFE
ATHCTPLPAQNFQMPWHL
>sp|Q9NYB9|ABI2_HUMAN Abl interactor 2 OS=Homo sapiens GN=ABI2 PE=1 SV=1
MAELQMLLEEEIPGGRRALFDSYTNLERVADYCENNYIQSADKQRALEETKAYTTQSLAS
VAYLINTLANNVLQMLDIQASQLRRMESSINHISQTVDIHKEKVARREIGILTTNKNTSR
THKIIAPANLERPVRYIRKPIDYTILDDIGHGVKWLLRFKVSTQNMKMGGLPRTTPPTQK
PPSPPMSGKGTLGRHSPYRTLEPVRPPVVPNDYVPSPTRNMAPSQQSPVRTASVNQRNRT
YSSSGSSGGSHPSSRSSSRENSGSGSVGVPIAVPTPSPPSVFPAPAGSAGTPPLPATSAS
APAPLVPATVPSSTAPNAAAGGAPNLADGFTSPTPPVVSSTPPTGHPVQFYSMNRPASRH
TPPTIGGSLPYRRPPSITSQTSLQNQMNGGPFYSQNPVSDTPPPPPPVEEPVFDESPPPP
PPPEDYEEEEAAVVEYSDPYAEEDPPWAPRSYLEKVVAIYDYTKDKEDELSFQEGAIIYV
IKKNDDGWYEGVMNGVTGLFPGNYVESIMHYSE

>sp|Q6H8Q1|ABLM2_HUMAN Actin-binding LIM protein 2 OS=Homo sapiens GN=ABLIM2 PE=1 SV=2
MSAVSQPQAAPSPLEKSPSTAILCNTCGNVCKGEVLRVQDKYFHIKCFVCKACGCDLAEG
GFFVRQGEYICTLDYQRLYGTRCFSCDQFIEGEVVSALGKTYHPDCFVCAVCRLPFPPGD
RVTFNGKECMCQKCSLPVSVGSSAHLSQGLRSCGGCGTEIKNGQALVALDKHWHLGCFKC
KSCGKLLNAEYISKDGLPYCEADYHAKFGIRCDSCEKYITGRVLEAGEKHYHPSCALCVR
CGQMFAEGEEMYLQGSSIWHPACRQAARTEDRNKETRTSSESIISVPASSTSGSPSRVIY
AKLGGEILDYRDLAALPKSKAIYDIDRPDMISYSPYISHSAGDRQSYGEGDQDDRSYKQC
RTSSPSSTGSVSLGRYTPTSRSPQHYSRPGSESGRSTPSLSVLSDSKPPPSTYQQAPRHF
HVPDTGVKDNIYRKPPIYRQHAARRSDGEDGSLDQDNRKKSSWLMLKGDADTRTNSPDLD
TQSLSHSSGTDRDPLQRMAGDSFHSRFPYSKSDPLPGHGKNGLDQRNANLAPCGADPDAS
WGMREYKIYPYDSLIVTNRIRVKLPKDVDRTRLERHLSPEEFQEVFGMSIEEFDRLALWK
RNDLKKKALLF
>sp|Q8N0Z2|ABRA_HUMAN Actin-binding Rho-activating protein OS=Homo sapiens GN=ABRA PE=2 SV=1
MAPGEKESGEGPAKSALRKIRTATLVISLARGWQQWANENSIRQAQEPTGWLPGGTQDSP
QAPKPITPPTSHQKAQSAPKSPPRLPEGHGDGQSSEKAPEVSHIKKKEVSKTVVSKTYER
GGDVSHLSHRYERDAGVLEPGQPENDIDRILHSHGSPTRRRKCANLVSELTKGWRVMEQE
EPTWRSDSVDTEDSGYGGEAEERPEQDGVQVAVVRIKRPLPSQVNRFTEKLNCKAQQKYS
PVGNLKGRWQQWADEHIQSQKLNPFSEEFDYELAMSTRLHKGDEGYGRPKEGTKTAERAK
RAEEHIYREMMDMCFIICTMARHRRDGKIQVTFGDLFDRYVRISDKVVGILMRARKHGLV
DFEGEMLWQGRDDHVVITLLK
>sp|P28330|ACADL_HUMAN Long-chain specific acyl-CoA dehydrogenase, mitochondrial OS=Homo sapiens GN=ACADL PE=1 SV=2
MAARLLRGSLRVLGGHRAPRQLPAARCSHSGGEERLETPSAKKLTDIGIRRIFSPEHDIF
RKSVRKFFQEEVIPHHSEWEKAGEVSREVWEKAGKQGLLGVNIAEHLGGIGGDLYSAAIV
WEEQAYSNCSGPGFSIHSGIVMSYITNHGSEEQIKHFIPQMTAGKCIGAIAMTEPGAGSD
LQGIKTNAKKDGSDWILNGSKVFISNGSLSDVVIVVAVTNHEAPSPAHGISLFLVENGMK
GFIKGRKLHKMGLKAQDTAELFFEDIRLPASALLGEENKGFYYIMKELPQERLLIADVAI
SASEFMFEETRNYVKQRKAFGKTVAHLQTVQHKLAELKTHICVTRAFVDNCLQLHEAKRL
DSATACMAKYWASELQNSVAYDCVQLHGGWGYMWEYPIAKAYVDARVQPIYGGTNEIMKE
LIAREIVFDK
>sp|P11310|ACADM_HUMAN Medium-chain specific acyl-CoA dehydrogenase, mitochondrial OS=Homo sapiens GN=ACADM PE=1 SV=1
MAAGFGRCCRVLRSISRFHWRSQHTKANRQREPGLGFSFEFTEQQKEFQATARKFAREEI
IPVAAEYDKTGEYPVPLIRRAWELGLMNTHIPENCGGLGLGTFDACLISEELAYGCTGVQ
TAIEGNSLGQMPIIIAGNDQQKKKYLGRMTEEPLMCAYCVTEPGAGSDVAGIKTKAEKKG
DEYIINGQKMWITNGGKANWYFLLARSDPDPKAPANKAFTGFIVEADTPGIQIGRKELNM
GQRCSDTRGIVFEDVKVPKENVLIGDGAGFKVAMGAFDKTRPVVAAGAVGLAQRALDEAT
KYALERKTFGKLLVEHQAISFMLAEMAMKVELARMSYQRAAWEVDSGRRNTYYASIAKAF
AGDIANQLATDAVQILGGNGFNTEYPVEKLMRDAKIYQIYEGTSQIQRLIVAREHIDKYK
N
>sp|P16219|ACADS_HUMAN Short-chain specific acyl-CoA dehydrogenase, mitochondrial OS=Homo sapiens GN=ACADS PE=1 SV=1
MAAALLARASGPARRALCPRAWRQLHTIYQSVELPETHQMLLQTCRDFAEKELFPIAAQV

DKEHLFPAAQVKKMGGLGLLAMDVPEELGGAGLDYLAYAIAMEEISRGCASTGVIMSVNN
SLYLGPILKFGSKEQKQAWVTPFTSGDKIGCFALSEPGNGSDAGAASTTARAEGDSWVLN
GTKAWITNAWEASAAVVFASTDRALQNKGISAFLVPMPTPGLTLGKKEDKLGIRGSSTAN
LIFEDCRIPKDSILGEPGMGFKIAMQTLDMGRIGIASQALGIAQTALDCAVNYAENRMAF
GAPLTKLQVIQFKLADMALALESARLLTWRAAMLKDNKKPFIKEAAMAKLAASEAATAIS
HQAIQILGGMGYVTEMPAERHYRDARITEIYEGTSEIQRLVIAGHLLRSYRS
>sp|P49748|ACADV_HUMAN Very long-chain specific acyl-CoA dehydrogenase, mitochondrial OS=Homo sapiens GN=ACADVL PE=1 SV=1
MQAARMAASLGRQLLRLGGGSSRLTALLGQPRPGPARRPYAGGAAQLALDKSDSHPSDAL
TRKKPAKAESKSFAVGMFKGQLTTDQVFPYPSVLNEEQTQFLKELVEPVSRFFEEVNDPA
KNDALEMVEETTWQGLKELGAFGLQVPSELGGVGLCNTQYARLVEIVGMHDLGVGITLGA
HQSIGFKGILLFGTKAQKEKYLPKLASGETVAAFCLTEPSSGSDAASIRTSAVPSPCGKY
YTLNGSKLWISNGGLADIFTVFAKTPVTDPATGAVKEKITAFVVERGFGGITHGPPEKKM
GIKASNTAEVFFDGVRVPSENVLGEVGSGFKVAMHILNNGRFGMAAALAGTMRGIIAKAV
DHATNRTQFGEKIHNFGLIQEKLARMVMLQYVTESMAYMVSANMDQGATDFQIEAAISKI
FGSEAAWKVTDECIQIMGGMGFMKEPGVERVLRDLRIFRIFEGTNDILRLFVALQGCMDK
GKELSGLGSALKNPFGNAGLLLGEAGKQLRRRAGLGSGLSLSGLVHPELSRSGELAVRAL
EQFATVVEAKLIKHKKGIVNEQFLLQRLADGAIDLYAMVVVLSRASRSLSEGHPTAQHEK
MLCDTWCIEAAARIREGMAALQSDPWQQELYRNFKSISKALVERGGVVTSNPLGF
>sp|Q96GR2|ACBG1_HUMAN Long-chain-fatty-acid--CoA ligase ACSBG1 OS=Homo sapiens GN=ACSBG1 PE=2 SV=2
MPRNSGAGYGCPHGDPSMLDSRETPQESRQDMIVRTTQEKLKTSSLTDRQPLSKESLNHA
LELSVPEKVNNAQWDAPEEALWTTRADGRVRLRIDPSCPQLPYTVHRMFYEALDKYGDLI
ALGFKRQDKWEHISYSQYYLLARRAAKGFLKLGLKQAHSVAILGFNSPEWFFSAVGTVFA
GGIVTGIYTTSSPEACQYIAYDCCANVIMVDTQKQLEKILKIWKQLPHLKAVVIYKEPPP
NKMANVYTMEEFMELGNEVPEEALDAIIDTQQPNQCCVLVYTSGTTGNPKGVMLSQDNIT
WTARYGSQAGDIRPAEVQQEVVVSYLPLSHIAAQIYDLWTGIQWGAQVCFAEPDALKGSL
VNTLREVEPTSHMGVPRVWEKIMERIQEVAAQSGFIRRKMLLWAMSVTLEQNLTCPGSDL
KPFTTRLADYLVLAKVRQALGFAKCQKNFYGAAPMMAETQHFFLGLNIRLYAGYGLSETS
GPHFMSSPYNYRLYSSGKLVPGCRVKLVNQDAEGIGEICLWGRTIFMGYLNMEDKTCEAI
DEEGWLHTGDAGRLDADGFLYITGRLKELIITAGGENVPPVPIEEAVKMELPIISNAMLI
GDQRKFLSMLLTLKCTLDPDTSDQTDNLTEQAMEFCQRVGSRATTVSEIIEKKDEAVYQA
IEEGIRRVNMNAAARPYHIQKWAILERDFSISGGELGPTMKLKRLTVLEKYKGIIDSFYQ
EQKM
>sp|P13746|1A11_HUMAN HLA class I histocompatibility antigen, A-11 alpha chain OS=Homo sapiens GN=HLA-A PE=1 SV=1
MAVMAPRTLLLLLSGALALTQTWAGSHSMRYFYTSVSRPGRGEPRFIAVGYVDDTQFVRF
DSDAASQRMEPRAPWIEQEGPEYWDQETRNVKAQSQTDRVDLGTLRGYYNQSEDGSHTIQ
IMYGCDVGPDGRFLRGYRQDAYDGKDYIALNEDLRSWTAADMAAQITKRKWEAAHAAEQQ
RAYLEGRCVEWLRRYLENGKETLQRTDPPKTHMTHHPISDHEATLRCWALGFYPAEITLT
WQRDGEDQTQDTELVETRPAGDGTFQKWAAVVVPSGEEQRYTCHVQHEGLPKPLTLRWEL
SSQPTIPIVGIIAGLVLLGAVITGAVVAAVMWRRKSSDRKGGSYTQAASSDSAQGSDVSL
TACKV

>sp|P30460|1B08_HUMAN HLA class I histocompatibility antigen, B-8 alpha chain OS=Homo sapiens GN=HLA-B PE=1 SV=1
MLVMAPRTVLLLLSAALALTETWAGSHSMRYFDTAMSRPGRGEPRFISVGYVDDTQFVRF
DSDAASPREEPRAPWIEQEGPEYWDRNTQIFKTNTQTDRESLRNLRGYYNQSEAGSHTLQ
SMYGCDVGPDGRLLRGHNQYAYDGKDYIALNEDLRSWTAADTAAQITQRKWEAARVAEQD
RAYLEGTCVEWLRRYLENGKDTLERADPPKTHVTHHPISDHEATLRCWALGFYPAEITLT
WQRDGEDQTQDTELVETRPAGDRTFQKWAAVVVPSGEEQRYTCHVQHEGLPKPLTLRWEP
SSQSTVPIVGIVAGLAVLAVVVIGAVVAAVMCRRKSSGGKGGSYSQAACSDSAQGSDVSL
TA
>sp|P30464|1B15_HUMAN HLA class I histocompatibility antigen, B-15 alpha chain OS=Homo sapiens GN=HLA-B PE=1 SV=2
MRVTAPRTVLLLLSGALALTETWAGSHSMRYFYTAMSRPGRGEPRFIAVGYVDDTQFVRF
DSDAASPRMAPRAPWIEQEGPEYWDRETQISKTNTQTYRESLRNLRGYYNQSEAGSHTLQ
RMYGCDVGPDGRLLRGHDQSAYDGKDYIALNEDLSSWTAADTAAQITQRKWEAAREAEQW
RAYLEGLCVEWLRRYLENGKETLQRADPPKTHVTHHPISDHEATLRCWALGFYPAEITLT
WQRDGEDQTQDTELVETRPAGDRTFQKWAAVVVPSGEEQRYTCHVQHEGLPKPLTLRWEP
SSQSTIPIVGIVAGLAVLAVVVIGAVVATVMCRRKSSGGKGGSYSQAASSDSAQGSDVSL
TA
>sp|Q31612|1B73_HUMAN HLA class I histocompatibility antigen, B-73 alpha chain OS=Homo sapiens GN=HLA-B PE=1 SV=1
MLVMAPRTVLLLLSAALALTETWAGSHSMRYFHTSVSRPGRGEPRFITVGYVDDTQFVRF
DSDAASPREEPRAPWIEQEGPEYWDRNTQICKAKAQTDRVGLRNLRGYYNQSEDGSHTWQ
TMYGCDMGPDGRLLRGYNQFAYDGKDYIALNEDLRSWTAADTAAQITQRKWEAARVAEQL
RAYLEGECVEWLRRHLENGKETLQRADPPKTHVTHHPISDHEATLRCWALGFYPAEITLT
WQRDGEDQTQDTELVETRPAGDGTFQKWAAVVVPSGQEQRYTCHVQHEGLQEPCTLRWKP
SSQSTIPIVGIVAGLAVLVVTVAVVAVVAAVMCRRKSSGGKGGSYSQAASSDSAQGSDVS
LTA
>sp|Q31610|1B81_HUMAN HLA class I histocompatibility antigen, B-81 alpha chain OS=Homo sapiens GN=HLA-B PE=1 SV=1
MLVMAPRTVLLLLWGAVALTETWAGSHSMRYFYTSVSRPGRGEPRFISVGYVDDTQFVRF
DSDAASPREEPRAPWIEQEGPEYWDRNTQIYKAQAQTDRESLRNLRGYYNQSEAGSHTLQ
SMYGCDVGPDGRLLRGHNQYAYDGKDYIALNEDLRSWTAADTAAQISQRKLEAARVAEQL
RAYLEGECVEWLRRYLENGKDKLERADPPKTHVTHHPISDHEATLRCWALGFYPAEITLT
WQRDGEDQTQDTELVETRPAGDRTFQKWTAVVVPSGEEQRYTCHVQHEGLPKPLTLRWEP
SSQSTVPIVGIVAGLAVLAVVVIGAVVAAVMCRRKSSGGKGGSYSQAACSDSAQGSDVSL
TA
>sp|P30499|1C01_HUMAN HLA class I histocompatibility antigen, Cw-1 alpha chain OS=Homo sapiens GN=HLA-C PE=1 SV=1
MRVMEPRTLILLLSGALALTETWACSHSMKYFFTSVSRPGRGEPRFISVGYVDDTQFVRF
DSDAASPRGEPRAPWVEQEGPEYWDRETQKYNRQAQTDRVSLRNLRGYYNQSEAGSHTLQ
WMCGCDLGPDGRLLRGYDQYAYDGKDYIALNEDLRSWTAADTAAQITQRKWEAAREAEER
RAYLEGTCVEWLRRYLENGKESLQRAEHPKTHVTHHPVSDHEATLRCWALGFYPAEITLT
WQWDGEDQTQDTELVETRPAGDGTFQKWAAVMVPSGEEQRYTCHVQHEGLPEPLTLRWEP
SSQPTIPIVGIVAGLAVLAVLAVLGAVVAVVMCRRKSSGGKGGSCSQAASSNSAQGSDES

LIASKA
>sp|P30501|1C02_HUMAN HLA class I histocompatibility antigen, Cw-2 alpha chain OS=Homo sapiens GN=HLA-C PE=1 SV=1
MRVMEPRTLILLLSGALALTETWACSHSMRYFYTAVSRPSRGEPHFIAVGYVDDTQFVRF
DSDAASPRGEPRGRWVEQEGPEYWDRETQKYNRQAQTDRVNLRKLRGYYNQSEAGSHTLQ
RMYGCDLGPDGRLLRGYDQSAYDGKDYIALNEDLRSWTAADTAAQITQRKWEAAREAEEW
RAYLEGECVEWLRRYLENGKEKLQRAEHPKTHVTHHPVSDHEATLRCWALGFYPTEITLT
WQRDGEDQTQDTELVETRPAGDGTFQKWAAVVVPSGEEQRYTCHVQHEGLPEPLTLRWEP
SSQPTIPIVGIVAGLAVLAVLAVLGAVVAVVMCRRKSSGGKGGSCSQAASSNSAQGSDES
LIASKA
>sp|P30504|1C04_HUMAN HLA class I histocompatibility antigen, Cw-4 alpha chain OS=Homo sapiens GN=HLA-C PE=1 SV=1
MRVMAPRTLILLLSGALALTETWAGSHSMRYFSTSVSWPGRGEPRFIAVGYVDDTQFVRF
DSDAASPRGEPREPWVEQEGPEYWDRETQKYKRQAQADRVNLRKLRGYYNQSEDGSHTLQ
RMFGCDLGPDGRLLRGYNQFAYDGKDYIALNEDLRSWTAADTAAQITQRKWEAAREAEQR
RAYLEGTCVEWLRRYLENGKETLQRAEHPKTHVTHHPVSDHEATLRCWALGFYPAEITLT
WQWDGEDQTQDTELVETRPAGDGTFQKWAAVVVPSGEEQRYTCHVQHEGLPEPLTLRWKP
SSQPTIPIVGIVAGLAVLAVLAVLGAMVAVVMCRRKSSGGKGGSCSQAASSNSAQGSDES
LIACKA
>sp|Q29963|1C06_HUMAN HLA class I histocompatibility antigen, Cw-6 alpha chain OS=Homo sapiens GN=HLA-C PE=1 SV=2
MRVMAPRTLILLLSGALALTETWACSHSMRYFDTAVSRPGRGEPRFISVGYVDDTQFVRF
DSDAASPRGEPRAPWVEQEGPEYWDRETQKYKRQAQADRVNLRKLRGYYNQSEDGSHTLQ
WMYGCDLGPDGRLLRGYDQSAYDGKDYIALNEDLRSWTAADTAAQITQRKWEAAREAEQW
RAYLEGTCVEWLRRYLENGKETLQRAEHPKTHVTHHPVSDHEATLRCWALGFYPAEITLT
WQRDGEDQTQDTELVETRPAGDGTFQKWAAVVVPSGEEQRYTCHVQHEGLPEPLTLRWEP
SSQPTIPIVGIVAGLAVLAVLAVLGAVMAVVMCRRKSSGGKGGSCSQAASSNSAQGSDES
LIACKA
>sp|P30505|1C08_HUMAN HLA class I histocompatibility antigen, Cw-8 alpha chain OS=Homo sapiens GN=HLA-C PE=1 SV=1
MRVMAPRTLILLLSGALALTETWACSHSMRYFYTAVSRPGRGEPRFIAVGYVDDTQFVQF
DSDAASPRGEPRAPWVEQEGPEYWDRETQKYKRQAQTDRVSLRNLRGYYNQSEAGSHTLQ
RMYGCDLGPDGRLLRGYNQFAYDGKDYIALNEDLRSWTAADTAAQITQRKWEAARTAEQL
RAYLEGTCVEWLRRYLENGKKTLQRAEHPKTHVTHHPVSDHEATLRCWALGFYPAEITLT
WQRDGEDQTQDTELVETRPAGDGTFQKWAAVVVPSGEEQRYTCHVQHEGLPEPLTLRWGP
SSQPTIPIVGIVAGLAVLAVLAVLGAVMAVVMCRRKSSGGKGGSCSQAASSNSAQGSDES
LIACKA
>sp|P30510|1C14_HUMAN HLA class I histocompatibility antigen, Cw-14 alpha chain OS=Homo sapiens GN=HLA-C PE=1 SV=2
MRVMAPRTLILLLSGALALTETWACSHSMRYFSTSVSRPGRGEPRFIAVGYVDDTQFVRF
DSDAASPRGEPRAPWVEQEGPEYWDRETQKYKRQAQTDRVSLRNLRGYYNQSEAGSHTLQ
WMFGCDLGPDGRLLRGYDQSAYDGKDYIALNEDLRSWTAADTAAQITQRKWEAAREAEQR
RAYLEGTCVEWLRRYLENGKETLQRAEHPKTHVTHHPVSDHEATLRCWALGFYPAEITLT
WQWDGEDQTQDTELVETRPAGDGTFQKWAAVVVPSGEEQRYTCHVQHEGLPEPLTLRWEP

SSQPTIPIVGIVAGLAVLAVLAVLGAVVAVVMCRRKSSGGKGGSCSQAASSNSAQGSDES
LIACKA
>sp|Q95604|1C17_HUMAN HLA class I histocompatibility antigen, Cw-17 alpha chain OS=Homo sapiens GN=HLA-C PE=1 SV=1
MRVMAPQALLLLLSGALALIETWAGSHSMRYFYTAVSRPGRGEPRFIAVGYVDDTQFVRF
DSDAASPRGEPRAPWVEQEGPEYWDRETQKYKRQAQADRVNLRKLRGYYNQSEAGSHTIQ
RMYGCDLGPDGRLLRGYNQFAYDGKDYIALNEDLRSWTAADTAAQISQRKLEAAREAEQL
RAYLEGECVEWLRGYLENGKETLQRAERPKTHVTHHPVSDHEATLRCWALGFYPAEITLT
WQRDGEDQTQDTELVETRPAGDGTFQKWAAVVVPSGQEQRYTCHVQHEGLQEPCTLRWKP
SSQPTIPNLGIVSGPAVLAVLAVLAVLAVLGAVVAAVIHRRKSSGGKGGSCSQAASSNSA
QGSDESLIACKA
>sp|Q15173|2A5B_HUMAN Serine/threonine-protein phosphatase 2A 56 kDa regulatory subunit beta isoform OS=Homo sapiens GN=PPP2R5B PE=1 SV=1
METKLPPASTPTSPSSPGLSPVPPPDKVDGFSRRSLRRARPRRSHSSSQFRYQSNQQELT
PLPLLKDVPASELHELLSRKLAQCGVMFDFLDCVADLKGKEVKRAALNELVECVGSTRGV
LIEPVYPDIIRMISVNIFRTLPPSENPEFDPEEDEPNLEPSWPHLQLVYEFFLRFLESPD
FQPSVAKRYVDQKFVLMLLELFDSEDPREREYLKTILHRVYGKFLGLRAYIRKQCNHIFL
RFIYEFEHFNGVAELLEILGSIINGFALPLKTEHKQFLVRVLIPLHSVKSLSVFHAQLAY
CVVQFLEKDATLTEHVIRGLLKYWPKTCTQKEVMFLGEMEEILDVIEPSQFVKIQEPLFK
QVARCVSSPHFQVAERALYFWNNEYILSLIEDNCHTVLPAVFGTLYQVSKEHWNQTIVSL
IYNVLKTFMEMNGKLFDELTASYKLEKQQEQQKAQERQELWQGLEELRLRRLQGTQGAKE
APLQRLTPQVAASGGQS
>sp|Q16537|2A5E_HUMAN Serine/threonine-protein phosphatase 2A 56 kDa regulatory subunit epsilon isoform OS=Homo sapiens GN=PPP2R5E PE=1 SV=1
MSSAPTTPPSVDKVDGFSRKSVRKARQKRSQSSSQFRSQGKPIELTPLPLLKDVPSSEQP
ELFLKKLQQCCVIFDFMDTLSDLKMKEYKRSTLNELVDYITISRGCLTEQTYPEVVRMVS
CNIFRTLPPSDSNEFDPEEDEPTLEASWPHLQLVYEFFIRFLESQEFQPSIAKKYIDQKF
VLQLLELFDSEDPRERDYLKTVLHRIYGKFLGLRAFIRKQINNIFLRFVYETEHFNGVAE
LLEILGSIINGFALPLKAEHKQFLVKVLIPLHTVRSLSLFHAQLAYCIVQFLEKDPSLTE
PVIRGLMKFWPKTCSQKEVMFLGELEEILDVIEPSQFVKIQEPLFKQIAKCVSSPHFQVA
ERALYYWNNEYIMSLIEENSNVILPIMFSSLYRISKEHWNPAIVALVYNVLKAFMEMNST
MFDELTATYKSDRQREKKKEKEREELWKKLEDLELKRGLRRDGIIPT
>sp|Q9TQE0|2B19_HUMAN HLA class II histocompatibility antigen, DRB1-9 beta chain OS=Homo sapiens GN=HLA-DRB1 PE=1 SV=1
MVCLKLPGGSCMAALTVTLMVLSSPLALAGDTQPRFLKQDKFECHFFNGTERVRYLHRGI
YNQEENVRFDSDVGEYRAVTELGRPVAESWNSQKDFLERRRAEVDTVCRHNYGVGESFTV
QRRVHPEVTVYPAKTQPLQHHNLLVCSVSGFYPGSIEVRWFRNGQEEKAGVVSTGLIQNG
DWTFQTLVMLETVPRSGEVYTCQVEHPSVMSPLTVEWRARSESAQSKMLSGVGGFVLGLL
FLGAGLFIYFRNQKGHSGLQPTGFLS
>sp|Q9Y3L3|3BP1_HUMAN SH3 domain-binding protein 1 OS=Homo sapiens GN=SH3BP1 PE=1 SV=3
MMKRQLHRMRQLAQTGSLGRTPETAEFLGEDLLQVEQRLEPAKRAAHNIHKRLQACLQGQ
SGADMDKRVKKLPLMALSTTMAESFKELDPDSSMGKALEMSCAIQNQLARILAEFEMTLE
RDVLQPLSRLSEEELPAILKHKKSLQKLVSDWNTLKSRLSQATKNSGSSQGLGGSPGSHS
HTTMANKVETLKEEEEELKRKVEQCRDEYLADLYHFVTKEDSYANYFIRLLEIQADYHRR

SLSSLDTALAELRENHGQADHSPSMTATHFPRVYGVSLATHLQELGREIALPIEACVMML
LSEGMKEEGLFRLAAGASVLKRLKQTMASDPHSLEEFCSDPHAVAGALKSYLRELPEPLM
TFDLYDDWMRAASLKEPGARLQALQEVCSRLPPENLSNLRYLMKFLARLAEEQEVNKMTP
SNIAIVLGPNLLWPPEKEGDQAQLDAASVSSIQVVGVVEALIQSADTLFPGDINFNVSGL
FSAVTLQDTVSDRLASEELPSTAVPTPATTPAPAPAPAPAPAPALASAATKERTESEVPP
RPASPKVTRSPPETAAPVEDMARRTKRPAPARPTMPPPQVSGSRSSPPAPPLPPGSGSPG
TPQALPRRLVGSSLRAPTVPPPLPPTPPQPARRQSRRSPASPSPASPGPASPSPVSLSNP
AQVDLGAATAEGGAPEAISGVPTPPAIPPQPRPRSLASETN
>sp|O60239|3BP5_HUMAN SH3 domain-binding protein 5 OS=Homo sapiens GN=SH3BP5 PE=1 SV=2
MDAALKRSRSEEPAEILPPARDEEEEEEEGMEQGLEEEEEVDPRIQGELEKLNQSTDDIN
RRETELEDARQKFRSVLVEATVKLDELVKKIGKAVEDSKPYWEARRVARQAQLEAQKATQ
DFQRATEVLRAAKETISLAEQRLLEDDKRQFDSAWQEMLNHATQRVMEAEQTKTRSELVH
KETAARYNAAMGRMRQLEKKLKRAINKSKPYFELKAKYYVQLEQLKKTVDDLQAKLTLAK
GEYKMALKNLEMISDEIHERRRSSAMGPRGCGVGAEGSSTSVEDLPGSKPEPDAISVASE
AFEDDSCSNFVSEDDSETQSVSSFSSGPTSPSEMPDQFPAVVRPGSLDLPSPVSLSEFGM
MFPVLGPRSECSGASSPECEVERGDRAEGAENKTSDKANNNRGLSSSSGSGGSSKSQSST
SPEGQALENRMKQLSLQCSKGRDGIIADIKMVQIG
>sp|Q9NRA8|4ET_HUMAN Eukaryotic translation initiation factor 4E transporter OS=Homo sapiens GN=EIF4ENIF1 PE=1 SV=2
MDRRSMGETESGDAFLDLKKPPASKCPHRYTKEELLDIKELPHSKQRPSCLSEKYDSDGV
WDPEKWHASLYPASGRSSPVESLKKELDTDRPSLVRRIVDPRERVKEDDLDVVLSPQRRS
FGGGCHVTAAVSSRRSGSPLEKDSDGLRLLGGRRIGSGRIISARTFEKDHRLSDKDLRDL
RDRDRERDFKDKRFRREFGDSKRVFGERRRNDSYTEEEPEWFSAGPTSQSETIELTGFDD
KILEEDHKGRKRTRRRTASVKEGIVECNGGVAEEDEVEVILAQEPAADQEVPRDAVLPEQ
SPGDFDFNEFFNLDKVPCLASMIEDVLGEGSVSASRFSRWFSNPSRSGSRSSSLGSTPHE
ELERLAGLEQAILSPGQNSGNYFAPIPLEDHAENKVDILEMLQKAKVDLKPLLSSLSANK
EKLKESSHSGVVLSVEEVEAGLKGLKVDQQVKNSTPFMAEHLEETLSAVTNNRQLKKDGD
MTAFNKLVSTMKASGTLPSQPKVSRNLESHLMSPAEIPGQPVPKNILQELLGQPVQRPAS
SNLLSGLMGSLEPTTSLLGQRAPSPPLSQVFQTRAASADYLRPRIPSPIGFTPGPQQLLG
DPFQGMRKPMSPITAQMSQLELQQAALEGLALPHDLAVQAANFYQPGFGKPQVDRTRDGF
RNRQQRVTKSPAPVHRGNSSSPAPAASITSMLSPSFTPTSVIRKMYESKEKSKEEPASGK
AALGDSKEDTQKASEENLLSSSSVPSADRDSSPTTNSKLSALQRSSCSTPLSQANRYTKE
QDYRPKATGRKTPTLASPVPTTPFLRPVHQVPLVPHVPMVRPAHQLHPGLVQRMLAQGVH
PQHLPSLLQTGVLPPGMDLSHLQGISGPILGQPFYPLPAASHPLLNPRPGTPLHLAMVQQ
QLQRSVLHPPGSGSHAAAVSVQTTPQNVPSRSGLPHMHSQLEHRPSQRSSSPVGLAKWFG
SDVLQQPLPSMPAKVISVDELEYRQ
>sp|P08195|4F2_HUMAN 4F2 cell-surface antigen heavy chain OS=Homo sapiens GN=SLC3A2 PE=1 SV=3
MELQPPEASIAVVSIPRQLPGSHSEAGVQGLSAGDDSELGSHCVAQTGLELLASGDPLPS
ASQNAEMIETGSDCVTQAGLQLLASSDPPALASKNAEVTGTMSQDTEVDMKEVELNELEP
EKQPMNAASGAAMSLAGAEKNGLVKIKVAEDEAEAAAAAKFTGLSKEELLKVAGSPGWVR
TRWALLLLFWLGWLGMLAGAVVIIVRAPRCRELPAQKWWHTGALYRIGDLQAFQGHGAGN
LAGLKGRLDYLSSLKVKGLVLGPIHKNQKDDVAQTDLLQIDPNFGSKEDFDSLLQSAKKK
SIRVILDLTPNYRGENSWFSTQVDTVATKVKDALEFWLQAGVDGFQVRDIENLKDASSFL

AEWQNITKGFSEDRLLIAGTNSSDLQQILSLLESNKDLLLTSSYLSDSGSTGEHTKSLVT
QYLNATGNRWCSWSLSQARLLTSFLPAQLLRLYQLMLFTLPGTPVFSYGDEIGLDAAALP
GQPMEAPVMLWDESSFPDIPGAVSANMTVKGQSEDPGSLLSLFRRLSDQRSKERSLLHGD
FHAFSAGPGLFSYIRHWDQNERFLVVLNFGDVGLSAGLQASDLPASASLPAKADLLLSTQ
PGREEGSPLELERLKLEPHEGLLLRFPYAA
>sp|P08908|5HT1A_HUMAN 5-hydroxytryptamine receptor 1A OS=Homo sapiens GN=HTR1A PE=1 SV=3
MDVLSPGQGNNTTSPPAPFETGGNTTGISDVTVSYQVITSLLLGTLIFCAVLGNACVVAA
IALERSLQNVANYLIGSLAVTDLMVSVLVLPMAALYQVLNKWTLGQVTCDLFIALDVLCC
TSSILHLCAIALDRYWAITDPIDYVNKRTPRRAAALISLTWLIGFLISIPPMLGWRTPED
RSDPDACTISKDHGYTIYSTFGAFYIPLLLMLVLYGRIFRAARFRIRKTVKKVEKTGADT
RHGASPAPQPKKSVNGESGSRNWRLGVESKAGGALCANGAVRQGDDGAALEVIEVHRVGN
SKEHLPLPSEAGPTPCAPASFERKNERNAEAKRKMALARERKTVKTLGIIMGTFILCWLP
FFIVALVLPFCESSCHMPTLLGAIINWLGYSNSLLNPVIYAYFNKDFQNAFKKIIKCKFC
RQ
>sp|Q13639|5HT4R_HUMAN 5-hydroxytryptamine receptor 4 OS=Homo sapiens GN=HTR4 PE=1 SV=2
MDKLDANVSSEEGFGSVEKVVLLTFLSTVILMAILGNLLVMVAVCWDRQLRKIKTNYFIV
SLAFADLLVSVLVMPFGAIELVQDIWIYGEVFCLVRTSLDVLLTTASIFHLCCISLDRYY
AICCQPLVYRNKMTPLRIALMLGGCWVIPTFISFLPIMQGWNNIGIIDLIEKRKFNQNSN
STYCVFMVNKPYAITCSVVAFYIPFLLMVLAYYRIYVTAKEHAHQIQMLQRAGASSESRP
QSADQHSTHRMRTETKAAKTLCIIMGCFCLCWAPFFVTNIVDPFIDYTVPGQVWTAFLWL
GYINSGLNPFLYAFLNKSFRRAFLIILCCDDERYRRPSILGQTVPCSTTTINGSTHVLRD
AVECGGQWESQCHPPATSPLVAAQPSDT
>sp|P34969|5HT7R_HUMAN 5-hydroxytryptamine receptor 7 OS=Homo sapiens GN=HTR7 PE=1 SV=2
MMDVNSSGRPDLYGHLRSFLLPEVGRGLPDLSPDGGADPVAGSWAPHLLSEVTASPAPTW
DAPPDNASGCGEQINYGRVEKVVIGSILTLITLLTIAGNCLVVISVCFVKKLRQPSNYLI
VSLALADLSVAVAVMPFVSVTDLIGGKWIFGHFFCNVFIAMDVMCCTASIMTLCVISIDR
YLGITRPLTYPVRQNGKCMAKMILSVWLLSASITLPPLFGWAQNVNDDKVCLISQDFGYT
IYSTAVAFYIPMSVMLFMYYQIYKAARKSAAKHKFPGFPRVEPDSVIALNGIVKLQKEVE
ECANLSRLLKHERKNISIFKREQKAATTLGIIVGAFTVCWLPFFLLSTARPFICGTSCSC
IPLWVERTFLWLGYANSLINPFIYAFFNRDLRTTYRSLLQCQYRNINRKLSAAGMHEALK
LAERPERPEFVLRACTRRVLLRPEKRPPVSVWVLQSPDHHNWLADKMLTTVEKKVMIHD
>sp|Q96P26|5NT1B_HUMAN Cytosolic 5'-nucleotidase 1B OS=Homo sapiens GN=NT5C1B PE=2 SV=2
MSQTSLKQKKNEPGMRSSKESLEAEKRKESDKTGVRLSNQMRRAVNPNHSLRCCPFQGHS
SCRRCLCAAEGTALGPCHTIRIYIHMCLLWEQGQQITMMRGSQESSLRKTDSRGYLVRSQ
WSRISRSPSTKAPSIDEPRSRNTSAKLPSSSTSSRTPSTSPSLHDSSPPPLSGQPSLQPP
ASPQLPRSLDSRPPTPPEPDPGSRRSTKMQENPEAWAQGIVREIRQTRDSQPLEYSRTSP
TEWKSSSQRRGIYPASTQLDRNSLSEQQQQQREDEDDYEAAYWASMRSFYEKNPSCSRPW
PPKPKNAITIALSSCALFNMVDGRKIYEQEGLEKYMEYQLTNENVILTPGPAFRFVKALQ
YVNARLRDLYPDEQDLFDIVLMTNNHAQVGVRLINSVNHYGLLIDRFCLTGGKDPIGYLK
AYLTNLYIAADSEKVQEAIQEGIASATMFDGAKDMAYCDTQLRVAFDGDAVLFSDESEHF
TKEHGLDKFFQYDTLCESKPLAQGPLKGFLEDLGRLQKKFYAKNERLLCPIRTYLVTARS
AASSGARVLKTLRRWGLEIDEALFLAGAPKSPILVKIRPHIFFDDHMFHIEGAQRLGSIA
AYGFNKKFSS
>sp|Q9H0P0|5NT3A_HUMAN Cytosolic 5'-nucleotidase 3A OS=Homo sapiens GN=NT5C3A PE=1 SV=3

MRAPSMDRAAVARVGAVASASVCALVAGVVLAQYIFTLKRKTGRKTKIIEMMPEFQKSSV
RIKNPTRVEEIICGLIKGGAAKLQIITDFDMTLSRFSYKGKRCPTCHNIIDNCKLVTDEC
RKKLLQLKEKYYAIEVDPVLTVEEKYPYMVEWYTKSHGLLVQQALPKAKLKEIVAESDVM
LKEGYENFFDKLQQHSIPVFIFSAGIGDVLEEVIRQAGVYHPNVKVVSNFMDFDETGVLK
GFKGELIHVFNKHDGALRNTEYFNQLKDNSNIILLGDSQGDLRMADGVANVEHILKIGYL
NDRVDELLEKYMDSYDIVLVQDESLEVANSILQKIL
>sp|P05408|7B2_HUMAN Neuroendocrine protein 7B2 OS=Homo sapiens GN=SCG5 PE=1 SV=2
MVSRMVSTMLSGLLFWLASGWTPAFAYSPRTPDRVSEADIQRLLHGVMEQLGIARPRVEY
PAHQAMNLVGPQSIEGGAHEGLQHLGPFGNIPNIVAELTGDNIPKDFSEDQGYPDPPNPC
PVGKTADDGCLENTPDTAEFSREFQLHQHLFDPEHDYPGLGKWNKKLLYEKMKGGERRKR
RSVNPYLQGQRLDNVVAKKSVPHFSDEDKDPE
>sp|P29275|AA2BR_HUMAN Adenosine receptor A2b OS=Homo sapiens GN=ADORA2B PE=2 SV=1
MLLETQDALYVALELVIAALSVAGNVLVCAAVGTANTLQTPTNYFLVSLAAADVAVGLFA
IPFAITISLGFCTDFYGCLFLACFVLVLTQSSIFSLLAVAVDRYLAICVPLRYKSLVTGT
RARGVIAVLWVLAFGIGLTPFLGWNSKDSATNNCTEPWDGTTNESCCLVKCLFENVVPMS
YMVYFNFFGCVLPPLLIMLVIYIKIFLVACRQLQRTELMDHSRTTLQREIHAAKSLAMIV
GIFALCWLPVHAVNCVTLFQPAQGKNKPKWAMNMAILLSHANSVVNPIVYAYRNRDFRYT
FHKIISRYLLCQADVKSGNGQAGVQPALGVGL
>sp|Q9Y478|AAKB1_HUMAN 5'-AMP-activated protein kinase subunit beta-1 OS=Homo sapiens GN=PRKAB1 PE=1 SV=4
MGNTSSERAALERHGGHKTPRRDSSGGTKDGDRPKILMDSPEDADLFHSEEIKAPEKEEF
LAWQHDLEVNDKAPAQARPTVFRWTGGGKEVYLSGSFNNWSKLPLTRSHNNFVAILDLPE
GEHQYKFFVDGQWTHDPSEPIVTSQLGTVNNIIQVKKTDFEVFDALMVDSQKCSDVSELS
SSPPGPYHQEPYVCKPEERFRAPPILPPHLLQVILNKDTGISCDPALLPEPNHVMLNHLY
ALSIKDGVMVLSATHRYKKKYVTTLLYKPI
>sp|Q9UGI9|AAKG3_HUMAN 5'-AMP-activated protein kinase subunit gamma-3 OS=Homo sapiens GN=PRKAG3 PE=1 SV=3
MEPGLEHALRRTPSWSSLGGSEHQEMSFLEQENSSSWPSPAVTSSSERIRGKRRAKALRW
TRQKSVEEGEPPGQGEGPRSRPAAESTGLEATFPKTTPLAQADPAGVGTPPTGWDCLPSD
CTASAAGSSTDDVELATEFPATEAWECELEGLLEERPALCLSPQAPFPKLGWDDELRKPG
AQIYMRFMQEHTCYDAMATSSKLVIFDTMLEIKKAFFALVANGVRAAPLWDSKKQSFVGM
LTITDFILVLHRYYRSPLVQIYEIEQHKIETWREIYLQGCFKPLVSISPNDSLFEAVYTL
IKNRIHRLPVLDPVSGNVLHILTHKRLLKFLHIFGSLLPRPSFLYRTIQDLGIGTFRDLA
VVLETAPILTALDIFVDRRVSALPVVNECGQVVGLYSRFDVIHLAAQQTYNHLDMSVGEA
LRQRTLCLEGVLSCQPHESLGEVIDRIAREQVHRLVLVDETQHLLGVVSLSDILQALVLS
PAGIDALGA
>sp|Q9H7C9|AAMDC_HUMAN Mth938 domain-containing protein OS=Homo sapiens GN=AAMDC PE=1 SV=1
MTSPEIASLSWGQMKVKGSNTTYKDCKVWPGGSRTWDWRETGTEHSPGVQPADVKEVVEK
GVQTLVIGRGMSEALKVPSSTVEYLKKHGIDVRVLQTEQAVKEYNALVAQGVRVGGVFHS
TC
>sp|Q9Y235|ABEC2_HUMAN C->U-editing enzyme APOBEC-2 OS=Homo sapiens GN=APOBEC2 PE=1 SV=1
MAQKEEAAVATEAASQNGEDLENLDDPEKLKELIELPPFEIVTGERLPANFFKFQFRNVE
YSSGRNKTFLCYVVEAQGKGGQVQASRGYLEDEHAAAHAEEAFFNTILPAFDPALRYNVT

WYVSSSPCAACADRIIKTLSKTKNLRLLILVGRLFMWEEPEIQAALKKLKEAGCKLRIMK
PQDFEYVWQNFVEQEEGESKAFQPWEDIQENFLYYEEKLADILK
>sp|P42684|ABL2_HUMAN Abelson tyrosine-protein kinase 2 OS=Homo sapiens GN=ABL2 PE=1 SV=1
MGQQVGRVGEAPGLQQPQPRGIRGSSAARPSGRRRDPAGRTTETGFNIFTQHDHFASCVE
DGFEGDKTGGSSPEALHRPYGCDVEPQALNEAIRWSSKENLLGATESDPNLFVALYDFVA
SGDNTLSITKGEKLRVLGYNQNGEWSEVRSKNGQGWVPSNYITPVNSLEKHSWYHGPVSR
SAAEYLLSSLINGSFLVRESESSPGQLSISLRYEGRVYHYRINTTADGKVYVTAESRFST
LAELVHHHSTVADGLVTTLHYPAPKCNKPTVYGVSPIHDKWEMERTDITMKHKLGGGQYG
EVYVGVWKKYSLTVAVKTLKEDTMEVEEFLKEAAVMKEIKHPNLVQLLGVCTLEPPFYIV
TEYMPYGNLLDYLRECNREEVTAVVLLYMATQISSAMEYLEKKNFIHRDLAARNCLVGEN
HVVKVADFGLSRLMTGDTYTAHAGAKFPIKWTAPESLAYNTFSIKSDVWAFGVLLWEIAT
YGMSPYPGIDLSQVYDLLEKGYRMEQPEGCPPKVYELMRACWKWSPADRPSFAETHQAFE
TMFHDSSISEEVAEELGRAASSSSVVPYLPRLPILPSKTRTLKKQVENKENIEGAQDATE
NSASSLAPGFIRGAQASSGSPALPRKQRDKSPSSLLEDAKETCFTRDRKGGFFSSFMKKR
NAPTPPKRSSSFREMENQPHKKYELTGNFSSVASLQHADGFSFTPAQQEANLVPPKCYGG
SFAQRNLCNDDGGGGGGSGTAGGGWSGITGFFTPRLIKKTLGLRAGKPTASDDTSKPFPR
SNSTSSMSSGLPEQDRMAMTLPRNCQRSKLQLERTVSTSSQPEENVDRANDMLPKKSEES
AAPSRERPKAKLLPRGATALPLRTPSGDLAITEKDPPGVGVAGVAAAPKGKEKNGGARLG
MAGVPEDGEQPGWPSPAKAAPVLPTTHNHKVPVLISPTLKHTPADVQLIGTDSQGNKFKL
LSEHQVTSSGDKDRPRRVKPKCAPPPPPVMRLLQHPSICSDPTEEPTALTAGQSTSETQE
GGKKAALGAVPISGKAGRPVMPPPQVPLPTSSISPAKMANGTAGTKVALRKTKQAAEKIS
ADKISKEALLECADLLSSALTEPVPNSQLVDTGHQLLDYCSGYVDCIPQTRNKFAFREAV
SKLELSLQELQVSSAAAGVPGTNPVLNNLLSCVQEISDVVQR
>sp|O14639|ABLM1_HUMAN Actin-binding LIM protein 1 OS=Homo sapiens GN=ABLIM1 PE=1 SV=3
MPAFLGLKCLGKLCSSEKSKVTSSERTSARGSNRKRLIVEDRRVSGTSFTAHRRATITHL
LYLCPKDYCPRGRVCNSVDPFVAHPQDPHHPSEKPVIHCHKCGEPCKGEVLRVQTKHFHI
KCFTCKVCGCDLAQGGFFIKNGEYLCTLDYQRMYGTRCHGCGEFVEGEVVTALGKTYHPN
CFACTICKRPFPPGDRVTFNGRDCLCQLCAQPMSSSPKETTFSSNCAGCGRDIKNGQALL
ALDKQWHLGCFKCKSCGKVLTGEYISKDGAPYCEKDYQGLFGVKCEACHQFITGKVLEAG
DKHYHPSCARCSRCNQMFTEGEEMYLQGSTVWHPDCKQSTKTEEKLRPTRTSSESIYSRP
GSSIPGSPGHTIYAKVDNEILDYKDLAAIPKVKAIYDIERPDLITYEPFYTSGYDDKQER
QSLGESPRTLSPTPSAEGYQDVRDRMIHRSTSQGSINSPVYSRHSYTPTTSRSPQHFHRP
GNEPSSGRNSPLPYRPDSRPLTPTYAQAPKHFHVPDQGINIYRKPPIYKQHAALAAQSKS
SEDIIKFSKFPAAQAPDPSETPKIETDHWPGPPSFAVVGPDMKRRSSGREEDDEELLRRR
QLQEEQLMKLNSGLGQLILKEEMEKESRERSSLLASRYDSPINSASHIPSSKTASLPGYG
RNGLHRPVSTDFAQYNSYGDVSGGVRDYQTLPDGHMPAMRMDRGVSMPNMLEPKIFPYEM
LMVTNRGRNKILREVDRTRLERHLAPEVFREIFGMSIQEFDRLPLWRRNDMKKKAKLF
>sp|Q9ULW3|ABT1_HUMAN Activator of basal transcription 1 OS=Homo sapiens GN=ABT1 PE=1 SV=1
MEAEESEKAATEQEPLEGTEQTLDAEEEQEESEEAACGSKKRVVPGIVYLGHIPPRFRPL
HVRNLLSAYGEVGRVFFQAEDRFVRRKKKAAAAAGGKKRSYTKDYTEGWVEFRDKRIAKR
VAASLHNTPMGARRRSPFRYDLWNLKYLHRFTWSHLSEHLAFERQVRRQRLRAEVAQAKR
ETDFYLQSVERGQRFLAADGDPARPDGSWTFAQRPTEQELRARKAARPGGRERARLATAQ
DKARSNKGLLARIFGAPPPSESMEGPSLVRDS

>sp|Q15057|ACAP2_HUMAN Arf-GAP with coiled-coil, ANK repeat and PH domain-containing protein 2 OS=Homo sapiens GN=ACAP2 PE=1 SV=3
MKMTVDFEECLKDSPRFRAALEEVEGDVAELELKLDKLVKLCIAMIDTGKAFCVANKQFM
NGIRDLAQYSSNDAVVETSLTKFSDSLQEMINFHTILFDQTQRSIKAQLQNFVKEDLRKF
KDAKKQFEKVSEEKENALVKNAQVQRNKQHEVEEATNILTATRKCFRHIALDYVLQINVL
QSKRRSEILKSMLSFMYAHLAFFHQGYDLFSELGPYMKDLGAQLDRLVVDAAKEKREMEQ
KHSTIQQKDFSSDDSKLEYNVDAANGIVMEGYLFKRASNAFKTWNRRWFSIQNNQLVYQK
KFKDNPTVVVEDLRLCTVKHCEDIERRFCFEVVSPTKSCMLQADSEKLRQAWIKAVQTSI
ATAYREKGDESEKLDKKSSPSTGSLDSGNESKEKLLKGESALQRVQCIPGNASCCDCGLA
DPRWASINLGITLCIECSGIHRSLGVHFSKVRSLTLDTWEPELLKLMCELGNDVINRVYE
ANVEKMGIKKPQPGQRQEKEAYIRAKYVERKFVDKYSISLSPPEQQKKFVSKSSEEKRLS
ISKFGPGDQVRASAQSSVRSNDSGIQQSSDDGRESLPSTVSANSLYEPEGERQDSSMFLD
SKHLNPGLQLYRASYEKNLPKMAEALAHGADVNWANSEENKATPLIQAVLGGSLVTCEFL
LQNGANVNQRDVQGRGPLHHATVLGHTGQVCLFLKRGANQHATDEEGKDPLSIAVEAANA
DIVTLLRLARMNEEMRESEGLYGQPGDETYQDIFRDFSQMASNNPEKLNRFQQDSQKF
>sp|Q8N6N7|ACBD7_HUMAN Acyl-CoA-binding domain-containing protein 7 OS=Homo sapiens GN=ACBD7 PE=1 SV=1
MALQADFDRAAEDVRKLKARPDDGELKELYGLYKQAIVGDINIACPGMLDLKGKAKWEAW
NLKKGLSTEDATSAYISKAKELIEKYGI
>sp|Q04917|1433F_HUMAN 14-3-3 protein eta OS=Homo sapiens GN=YWHAH PE=1 SV=4
MGDREQLLQRARLAEQAERYDDMASAMKAVTELNEPLSNEDRNLLSVAYKNVVGARRSSW
RVISSIEQKTMADGNEKKLEKVKAYREKIEKELETVCNDVLSLLDKFLIKNCNDFQYESK
VFYLKMKGDYYRYLAEVASGEKKNSVVEASEAAYKEAFEISKEQMQPTHPIRLGLALNFS
VFYYEIQNAPEQACLLAKQAFDDAIAELDTLNEDSYKDSTLIMQLLRDNLTLWTSDQQDE
EAGEGN
>sp|P61981|1433G_HUMAN 14-3-3 protein gamma OS=Homo sapiens GN=YWHAG PE=1 SV=2
MVDREQLVQKARLAEQAERYDDMAAAMKNVTELNEPLSNEERNLLSVAYKNVVGARRSSW
RVISSIEQKTSADGNEKKIEMVRAYREKIEKELEAVCQDVLSLLDNYLIKNCSETQYESK
VFYLKMKGDYYRYLAEVATGEKRATVVESSEKAYSEAHEISKEHMQPTHPIRLGLALNYS
VFYYEIQNAPEQACHLAKTAFDDAIAELDTLNEDSYKDSTLIMQLLRDNLTLWTSDQQDD
DGGEGNN
>sp|P27348|1433T_HUMAN 14-3-3 protein theta OS=Homo sapiens GN=YWHAQ PE=1 SV=1
MEKTELIQKAKLAEQAERYDDMATCMKAVTEQGAELSNEERNLLSVAYKNVVGGRRSAWR
VISSIEQKTDTSDKKLQLIKDYREKVESELRSICTTVLELLDKYLIANATNPESKVFYLK
MKGDYFRYLAEVACGDDRKQTIDNSQGAYQEAFDISKKEMQPTHPIRLGLALNFSVFYYE
ILNNPELACTLAKTAFDEAIAELDTLNEDSYKDSTLIMQLLRDNLTLWTSDSAGEECDAA
EGAEN
>sp|Q4AC99|1A1L2_HUMAN Probable inactive 1-aminocyclopropane-1-carboxylate synthase-like protein 2 OS=Homo sapiens GN=ACCSL PE=2 SV=3
MSHRSDTLPVPSGQRRGRVPRDHSIYTQLLEITLHLQQAMTEHFVQLTSRQGLSLEERRH
TEAICEHEALLSRLICRMINLLQSGAASGLELQVPLPSEDSRGDVRYGQRAQLSGQPDPV
PQLSDCEAAFVNRDLSIRGIDISVFYQSSFQDYNAYQKDKYHKDKNTLGFINLGTSENKL
CMDLMTERLQESDMNCIEDTLLQYPDWRGQPFLREEVARFLTYYCRAPTRLDPENVVVLN
GCCSVFCALAMVLCDPGEAFLVPAPFYGGFAFSSRLYAKVELIPVHLESEVTVTNTHPFQ

LTVDKLEEALLEARLEGKKVRGLVLINPQNPLGDIYSPDSLMKYLEFAKRYNLHVIIDEI
YMLSVFDESITFHSILSMKSLPDSNRTHVIWGTSKDFGISGFRFGALYTHNKEVASAVSA
FGYLHSISGITQHKLCQLLQNTEWIDKVYLPTNCYRLREAHKYITAELKALEIPFHNRSS
GLYVWINLKKYLDPCTFEEERLLYCRFLDNKLLLSRGKTYMCKEPGWFCLIFADELPRLK
LAMRRFCDVLQEQKEALIVKQLEDAMRE
>sp|P30447|1A23_HUMAN HLA class I histocompatibility antigen, A-23 alpha chain OS=Homo sapiens GN=HLA-A PE=1 SV=1
MAVMAPRTLVLLLSGALALTQTWAGSHSMRYFSTSVSRPGRGEPRFIAVGYVDDTQFVRF
DSDAASQRMEPRAPWIEQEGPEYWDEETGKVKAHSQTDRENLRIALRYYNQSEAGSHTLQ
MMFGCDVGSDGRFLRGYHQYAYDGKDYIALKEDLRSWTAADMAAQITQRKWEAARVAEQL
RAYLEGTCVDGLRRYLENGKETLQRTDPPKTHMTHHPISDHEATLRCWALGFYPAEITLT
WQRDGEDQTQDTELVETRPAGDGTFQKWAAVVVPSGEEQRYTCHVQHEGLPKPLTLRWEP
SSQPTVHIVGIIAGLVLLGAVITGAVVAAVMWRRNSSDRKGGSYSQAASSDSAQGSDVSL
TACKV
>sp|P16188|1A30_HUMAN HLA class I histocompatibility antigen, A-30 alpha chain OS=Homo sapiens GN=HLA-A PE=1 SV=2
MAVMAPRTLLLLLSGALALTHTWAGSHSMRYFSTSVSRPGSGEPRFIAVGYVDDTQFVRF
DSDAASQRMEPRAPWIEQERPEYWDQETRNVKAQSQTDRVDLGTLRGYYNQSEAGSHTIQ
IMYGCDVGSDGRFLRGYEQHAYDGKDYIALNEDLRSWTAADMAAQITQRKWEAARWAEQL
RAYLEGTCVEWLRRYLENGKETLQRTDPPKTHMTHHPISDHEATLRCWALGFYPAEITLT
WQRDGEDQTQDTELVETRPAGDGTFQKWAAVVVPSGEEQRYTCHVQHEGLPKPLTLRWEL
SSQPTIPIVGIIAGLVLLGAVITGAVVAAVMWRRKSSDRKGGSYTQAASSDSAQGSDVSL
TACKV
>sp|P10314|1A32_HUMAN HLA class I histocompatibility antigen, A-32 alpha chain OS=Homo sapiens GN=HLA-A PE=1 SV=2
MAVMAPRTLLLLLLGALALTQTWAGSHSMRYFFTSVSRPGRGEPRFIAVGYVDDTQFVRF
DSDAASQRMEPRAPWIEQEGPEYWDQETRNVKAHSQTDRESLRIALRYYNQSEAGSHTIQ
MMYGCDVGPDGRLLRGYQQDAYDGKDYIALNEDLRSWTAADMAAQITQRKWEAARVAEQL
RAYLEGTCVEWLRRYLENGKETLQRTDAPKTHMTHHAVSDHEATLRCWALSFYPAEITLT
WQRDGEDQTQDTELVETRPAGDGTFQKWASVVVPSGQEQRYTCHVQHEGLPKPLTLRWEP
SSQPTIPIVGIIAGLVLFGAMFAGAVVAAVRWRRKSSDRKGGSYSQAASSDSAQGSDMSL
TACKV
>sp|P30455|1A36_HUMAN HLA class I histocompatibility antigen, A-36 alpha chain OS=Homo sapiens GN=HLA-A PE=1 SV=1
MAVMAPRTLLLLLSGALALTQTWAGSHSMRYFFTSVSRPGRGEPRFIAVGYVDDTQFVRF
DSDAASQKMEPRAPWIEQEGPEYWDQETRNMKAHSQTDRANLGTLRGYYNQSEDGSHTIQ
IMYGCDVGPDGRFLRGYRQDAYDGKDYIALNEDLRSWTAADMAAQITKRKWEAVHAAEQR
RVYLEGTCVEWLRRYLENGKETLQRTDPPKTHMTHHPISDHEATLRCWALGFYPAEITLT
WQRDGEDQTQDTELVETRPAGDGTFQKWAAVVVPSGEEQRYTCHVQHEGLPKPLTLRWEL
SSQPTIPIVGIIAGLVLLGAVITGAVVAAVMWRRKSSDRKGGSYTQAASSDSAQGSDVSL
TACKV
>sp|P30456|1A43_HUMAN HLA class I histocompatibility antigen, A-43 alpha chain OS=Homo sapiens GN=HLA-A PE=1 SV=1
MAVMAPRTLVLLLSGALALTQTWAGSHSMRYFYTSVSRPGRGEPRFIAVGYVDDTQFVRF

DSDAASQRMEPRAPWIEQEGPEYWDLQTRNVKAHSQTDRANLGTLRGYYNQSEDGSHTIQ
RMYGCDVGPDGRFLRGYQQDAYDGKDYIALNEDLRSWTAADMAAQITQRKWETAHEAEQW
RAYLEGRCVEWLRRYLENGKETLQRTDAPKTHMTHHAVSDHEATLRCWALSFYPAEITLT
WQRDGEDQTQDTELVETRPAGDGTFQKWASVVVPSGQEQRYTCHVQHEGLPKPLTLRWEP
SSQPTIPIVGIIAGLVLFGAVIAGAVVAAVMWRRKSSDRKGGSYSQAASSDSAQGSDMSL
TACKV
>sp|P30459|1A74_HUMAN HLA class I histocompatibility antigen, A-74 alpha chain OS=Homo sapiens GN=HLA-A PE=1 SV=1
MAVMAPRTLLLLLLGALALTQTRAGSHSMRYFFTSVSRPGRGEPRFIAVGYVDDTQFVRF
DSDAASQRMEPRAPWIEQEGPEYWDQETRNVKAHSQTDRVDLGTLRGYYNQSEAGSHTIQ
MMYGCDVGPDGRLLRGYQQDAYDGKDYIALNEDLRSWTAADMAAQITQRKWEAARVAEQL
RAYLEGTCVEWLRRYLENGKETLQRTDAPKTHMTHHAVSDHEATLRCWALSFYPAEITLT
WQRDGEDQTQDTELVETRPAGDGTFQKWASVVVPSGQEQRYTCHVQHEGLPKPLTLRWEP
SSQPTIPIVGIIAGLVLFGAMFAGAVVAAVRWRRKSSDRKGGSYSQAASSDSAQGSDMSL
TACKV
>sp|P01889|1B07_HUMAN HLA class I histocompatibility antigen, B-7 alpha chain OS=Homo sapiens GN=HLA-B PE=1 SV=3
MLVMAPRTVLLLLSAALALTETWAGSHSMRYFYTSVSRPGRGEPRFISVGYVDDTQFVRF
DSDAASPREEPRAPWIEQEGPEYWDRNTQIYKAQAQTDRESLRNLRGYYNQSEAGSHTLQ
SMYGCDVGPDGRLLRGHDQYAYDGKDYIALNEDLRSWTAADTAAQITQRKWEAAREAEQR
RAYLEGECVEWLRRYLENGKDKLERADPPKTHVTHHPISDHEATLRCWALGFYPAEITLT
WQRDGEDQTQDTELVETRPAGDRTFQKWAAVVVPSGEEQRYTCHVQHEGLPKPLTLRWEP
SSQSTVPIVGIVAGLAVLAVVVIGAVVAAVMCRRKSSGGKGGSYSQAACSDSAQGSDVSL
TA
>sp|P30462|1B14_HUMAN HLA class I histocompatibility antigen, B-14 alpha chain OS=Homo sapiens GN=HLA-B PE=1 SV=1
MLVMAPRTVLLLLSAALALTETWAGSHSMRYFYTSVSRPGRGEPRFISVGYVDDTQFVRF
DSDAASPREEPRAPWIEQEGPEYWDRNTQICKTNTQTDRESLRNLRGYYNQSEAGSHTLQ
WMYGCDVGPDGRLLRGYNQFAYDGKDYIALNEDLSSWTAADTAAQITQRKWEAAREAEQL
RAYLEGTCVEWLRRHLENGKETLQRADPPKTHVTHHPISDHEATLRCWALGFYPAEITLT
WQRDGEDQTQDTELVETRPAGDRTFQKWAAVVVPSGEEQRYTCHVQHEGLPKPLTLRWEP
SSQSTVPIVGIVAGLAVLAVVVIGAVVAAVMCRRKSSGGKGGSYSQAASSDSAQGSDVSL
TA
>sp|P30466|1B18_HUMAN HLA class I histocompatibility antigen, B-18 alpha chain OS=Homo sapiens GN=HLA-B PE=1 SV=1
MRVTAPRTLLLLLWGAVALTETWAGSHSMRYFHTSVSRPGRGEPRFISVGYVDGTQFVRF
DSDAASPRTEPRAPWIEQEGPEYWDRNTQISKTNTQTYRESLRNLRGYYNQSEAGSHTLQ
RMYGCDVGPDGRLLRGHDQSAYDGKDYIALNEDLSSWTAADTAAQITQRKWEAARVAEQL
RAYLEGTCVEWLRRHLENGKETLQRADPPKTHVTHHPISDHEATLRCWALGFYPAEITLT
WQRDGEDQTQDTELVETRPAGDRTFQKWAAVVVPSGEEQRYTCHVQHEGLPKPLTLRWEP
SSQSTIPIVGIVAGLAVLAVVVIGAVVATVMCRRKSSGGKGGSYSQAASSDSAQGSDVSL
TA
>sp|P30685|1B35_HUMAN HLA class I histocompatibility antigen, B-35 alpha chain OS=Homo sapiens GN=HLA-B PE=1 SV=1

MRVTAPRTVLLLLWGAVALTETWAGSHSMRYFYTAMSRPGRGEPRFIAVGYVDDTQFVRF
DSDAASPRTEPRAPWIEQEGPEYWDRNTQIFKTNTQTYRESLRNLRGYYNQSEAGSHIIQ
RMYGCDLGPDGRLLRGHDQSAYDGKDYIALNEDLSSWTAADTAAQITQRKWEAARVAEQL
RAYLEGLCVEWLRRYLENGKETLQRADPPKTHVTHHPVSDHEATLRCWALGFYPAEITLT
WQRDGEDQTQDTELVETRPAGDRTFQKWAAVVVPSGEEQRYTCHVQHEGLPKPLTLRWEP
SSQSTIPIVGIVAGLAVLAVVVIGAVVATVMCRRKSSGGKGGSYSQAASSDSAQGSDVSL
TA
>sp|P30475|1B39_HUMAN HLA class I histocompatibility antigen, B-39 alpha chain OS=Homo sapiens GN=HLA-B PE=1 SV=1
MLVMAPRTVLLLLSAALALTETWAGSHSMRYFYTSVSRPGRGEPRFISVGYVDDTQFVRF
DSDAASPREEPRAPWIEQEGPEYWDRNTQICKTNTQTDRESLRNLRGYYNQSEAGSHTLQ
RMYGCDVGPDGRLLRGHNQFAYDGKDYIALNEDLSSWTAADTAAQITQRKWEAARVAEQL
RTYLEGTCVEWLRRYLENGKETLQRADPPKTHVTHHPISDHEATLRCWALGFYPAEITLT
WQRDGEDQTQDTELVETRPAGDRTFQKWAAVVVPSGEEQRYTCHVQHEGLPKPLTLRWEP
SSQSTVPIVGIVAGLAVLAVVVIGAVVAAVMCRRKSSGGKGGSYSQAASSDSAQGSDVSL
TA
>sp|P30485|1B47_HUMAN HLA class I histocompatibility antigen, B-47 alpha chain OS=Homo sapiens GN=HLA-B PE=1 SV=1
MRVTAPRTLLLLLWGAVALTETWAGSHSMRYFYTAMSRPGRGEPRFITVGYVDDTLFVRF
DSDATSPRKEPRAPWIEQEGPEYWDRETQISKTNTQTYREDLRTLLRYYNQSEAGSHTLQ
RMFGCDVGPDGRLLRGYHQDAYDGKDYIALNEDLSSWTAADTAAQITQRKWEAARVAEQL
RAYLEGECVEWLRRYLENGKETLQRADPPKTHVTHHPISDHEATLRCWALGFYPAEITLT
WQRDGEDQTQDTELVETRPAGDRTFQKWAAVVVPSGEEQRYTCHVQHEGLPKPLTLRWEP
SSQSTVPIVGIVAGLAVLAVVVIGAVVAAVVCRRKSSGGKGGSYSQAACSDSAQGSDVSL
TA
>sp|P30491|1B53_HUMAN HLA class I histocompatibility antigen, B-53 alpha chain OS=Homo sapiens GN=HLA-B PE=1 SV=1
MRVTAPRTVLLLLWGAVALTETWAGSHSMRYFYTAMSRPGRGEPRFIAVGYVDDTQFVRF
DSDAASPRTEPRAPWIEQEGPEYWDRNTQIFKTNTQTYRENLRIALRYYNQSEAGSHIIQ
RMYGCDLGPDGRLLRGHDQSAYDGKDYIALNEDLSSWTAADTAAQITQRKWEAARVAEQL
RAYLEGLCVEWLRRYLENGKETLQRADPPKTHVTHHPVSDHEATLRCWALGFYPAEITLT
WQRDGEDQTQDTELVETRPAGDRTFQKWAAVVVPSGEEQRYTCHVQHEGLPKPLTLRWEP
SSQSTIPIVGIVAGLAVLAVVVIGAVVATVMCRRKSSGGKGGSYSQAASSDSAQGSDVSL
TA
>sp|P30493|1B55_HUMAN HLA class I histocompatibility antigen, B-55 alpha chain OS=Homo sapiens GN=HLA-B PE=1 SV=1
MRVTAPRTLLLLLWGALALTETWAGSHSMRYFYTAMSRPGRGEPRFIAVGYVDDTQFVRF
DSDAASPREEPRAPWIEQEGPEYWDRNTQIYKAQAQTDRESLRNLRGYYNQSEAGSHTWQ
TMYGCDLGPDGRLLRGHNQLAYDGKDYIALNEDLSSWTAADTAAQITQRKWEAAREAEQL
RAYLEGTCVEWLRRYLENGKETLQRADPPKTHVTHHPISDHEATLRCWALGFYPAEITLT
WQRDGEDQTQDTELVETRPAGDRTFQKWAAVVVPSGEEQRYTCHVQHEGLPKPLTLRWEP
SSQSTIPIVGIVAGLAVLAVVVIGAVVATVMCRRKSSGGKGGSYSQAASSDSAQGSDVSL
TA

>sp|P01912|2B13_HUMAN HLA class II histocompatibility antigen, DRB1-3 chain OS=Homo sapiens GN=HLA-DRB1 PE=1 SV=2
MVCLRLPGGSCMAVLTVTLMVLSSPLALAGDTRPRFLEYSTSECHFFNGTERVRYLDRYF
HNQEENVRFDSDVGEFRAVTELGRPDAEYWNSQKDLLEQKRGRVDNYCRHNYGVVESFTV
QRRVHPKVTVYPSKTQPLQHHNLLVCSVSGFYPGSIEVRWFRNGQEEKTGVVSTGLIHNG
DWTFQTLVMLETVPRSGEVYTCQVEHPSVTSPLTVEWRARSESAQSKMLSGVGGFVLGLL
FLGAGLFIYFRNQKGHSGLQPRGFLS
>sp|P13761|2B17_HUMAN HLA class II histocompatibility antigen, DRB1-7 beta chain OS=Homo sapiens GN=HLA-DRB1 PE=1 SV=1
MVCLKLPGGSCMAALTVTLMVLSSPLALAGDTQPRFLWQGKYKCHFFNGTERVQFLERLF
YNQEEFVRFDSDVGEYRAVTELGRPVAESWNSQKDILEDRRGQVDTVCRHNYGVGESFTV
QRRVHPEVTVYPAKTQPLQHHNLLVCSVSGFYPGSIEVRWFRNGQEEKAGVVSTGLIQNG
DWTFQTLVMLETVPRSGEVYTCQVEHPSVMSPLTVEWRARSESAQSKMLSGVGGFVLGLL
FLGAGLFIYFRNQKGHSGLQPTGFLS
>sp|Q30167|2B1A_HUMAN HLA class II histocompatibility antigen, DRB1-10 beta chain OS=Homo sapiens GN=HLA-DRB1 PE=1 SV=2
MVCLRLPGGSCMAVLTVTLMVLSSPLALAGDTRPRFLEEVKFECHFFNGTERVRLLERRV
HNQEEYARYDSDVGEYRAVTELGRPDAEYWNSQKDLLERRRAAVDTYCRHNYGVGESFTV
QRRVQPKVTVYPSKTQPLQHHNLLVCSVNGFYPGSIEVRWFRNGQEEKTGVVSTGLIQNG
DWTFQTLVMLETVPQSGEVYTCQVEHPSVMSPLTVEWRARSESAQSKMLSGVGGFVLGLL
FLGAGLFIYFRNQKGHSGLPPTGFLS
>sp|Q7L8J4|3BP5L_HUMAN SH3 domain-binding protein 5-like OS=Homo sapiens GN=SH3BP5L PE=1 SV=1
MAELRQVPGGRETPQGELRPEVVEDEVPRSPVAEEPGGGGSSSSEAKLSPREEEELDPRI
QEELEHLNQASEEINQVELQLDEARTTYRRILQESARKLNTQGSHLGSCIEKARPYYEAR
RLAKEAQQETQKAALRYERAVSMHNAAREMVFVAEQGVMADKNRLDPTWQEMLNHATCKV
NEAEEERLRGEREHQRVTRLCQQAEARVQALQKTLRRAIGKSRPYFELKAQFSQILEEHK
AKVTELEQQVAQAKTRYSVALRNLEQISEQIHARRRGGLPPHPLGPRRSSPVGAEAGPED
MEDGDSGIEGAEGAGLEEGSSLGPGPAPDTDTLSLLSLRTVASDLQKCDSVEHLRGLSDH
VSLDGQELGTRSGGRRGSDGGARGGRHQRSVSL
>sp|P46952|3HAO_HUMAN 3-hydroxyanthranilate 3,4-dioxygenase OS=Homo sapiens GN=HAAO PE=1 SV=2
MERRLGVRAWVKENRGSFQPPVCNKLMHQEQLKVMFIGGPNTRKDYHIEEGEEVFYQLEG
DMVLRVLEQGKHRDVVIRQGEIFLLPARVPHSPQRFANTVGLVVERRRLETELDGLRYYV
GDTMDVLFEKWFYCKDLGTQLAPIIQEFFSSEQYRTGKPIPDQLLKEPPFPLSTRSIMEP
MSLDAWLDSHHRELQAGTPLSLFGDTYETQVIAYGQGSSEGLRQNVDVWLWQLEGSSVVT
MGGRRLSLAPDDSLLVLAGTSYAWERTQGSVALSVTQDPACKKPLG
>sp|O95336|6PGL_HUMAN 6-phosphogluconolactonase OS=Homo sapiens GN=PGLS PE=1 SV=2
MAAPAPGLISVFSSSQELGAALAQLVAQRAACCLAGARARFALGLSGGSLVSMLARELPA
AVAPAGPASLARWTLGFCDERLVPFDHAESTYGLYRTHLLSRLPIPESQVITINPELPVE
EAAEDYAKKLRQAFQGDSIPVFDLLILGVGPDGHTCSLFPDHPLLQEREKIVAPISDSPK
PPPQRVTLTLPVLNAARTVIFVATGEGKAAVLKRILEDQEENPLPAALVQPHTGKLCWFL
DEAAARLLTVPFEKHSTL

>sp|Q676U5|A16L1_HUMAN Autophagy-related protein 16-1 OS=Homo sapiens GN=ATG16L1 PE=1 SV=2
MSSGLRAADFPRWKRHISEQLRRRDRLQRQAFEEIILQYNKLLEKSDLHSVLAQKLQAEK
HDVPNRHEISPGHDGTWNDNQLQEMAQLRIKHQEELTELHKKRGELAQLVIDLNNQMQRK
DREMQMNEAKIAECLQTISDLETECLDLRTKLCDLERANQTLKDEYDALQITFTALEGKL
RKTTEENQELVTRWMAEKAQEANRLNAENEKDSRRRQARLQKELAEAAKEPLPVEQDDDI
EVIVDETSDHTEETSPVRAISRAATKRLSQPAGGLLDSITNIFGRRSVSSFPVPQDNVDT
HPGSGKEVRVPATALCVFDAHDGEVNAVQFSPGSRLLATGGMDRRVKLWEVFGEKCEFKG
SLSGSNAGITSIEFDSAGSYLLAASNDFASRIWTVDDYRLRHTLTGHSGKVLSAKFLLDN
ARIVSGSHDRTLKLWDLRSKVCIKTVFAGSSCNDIVCTEQCVMSGHFDKKIRFWDIRSES
IVREMELLGKITALDLNPERTELLSCSRDDLLKVIDLRTNAIKQTFSAPGFKCGSDWTRV
VFSPDGSYVAAGSAEGSLYIWSVLTGKVEKVLSKQHSSSINAVAWSPSGSHVVSVDKGCK
AVLWAQY
>sp|P20848|A1ATR_HUMAN Putative alpha-1-antitrypsin-related protein OS=Homo sapiens GN=SERPINA2 PE=1 SV=1
MPFSVSWGVLLLAGLCCLVPSSLVEDPQGDAAQKTDTSHHDQGDWEDLACQKISYNVTDL
AFDLYKSWLIYHNQHVLVTPTSVAMAFRMLSLGTKADTRTEILEGLNVNLTETPEAKIHE
CFQQVLQALSRPDTRLQLTTGSSLFVNKSMKLVDTFLEDTKKLYHSEASSINFRDTEEAK
EQINNYVEKRTGRKVVDLVKHLKKDTSLALVDYISFHGKWKDKFKAERIMVEGFHVDDKT
IIRVPMINHLGRFDIHRDRELSSWVLAQHYVGNATAFFILPDPKKMWQLEEKLTYSHLEN
IQRAFDIRSINLHFPKLSISGTYKLKRVPRNLGITKIFSNEADLSGVSQEAPLKLSKAVH
VAVLTIDEKGTEATGAPHLEEKAWSKYQTVMFNRPFLVIIKEYITNFPLFIGKVVNPTQK
>sp|Q4UJ75|A20A4_HUMAN Ankyrin repeat domain-containing protein 20A4 OS=Homo sapiens GN=ANKRD20A4 PE=3 SV=1
MKLFGFGSRRGQTAQGSIDHVYTGSGYRIRDSELQKIHRAAVKGDAAEVERCLARRSGEL
DALDKQHRTALHLACASGHVQVVTLLVNRKCQIDVCDKENRTPLIQAVHCQEEACAVILL
EHGANPNLKDIYGNTALHYAVYSESTSLAEKLLSHGAHIEALDKDNNTPLLFAIICKKEK
MVEFLLKKKASSHAVDRLRRSALMLAVYYDSPGIVNILLKQNIDVFAQDMCGRDAEDYAI
SHHLTKIQQQILEHKKKILKKEKSDVGSSDESAVSIFHELRVDSLPASDDKDLNVATKQC
VPEKVSEPLPGSSHEKGNRIVNGQGEGPPAKHPSLKPSTEVEDPAVKGAVQRKNVQTLRA
EQALPVASEEEQQRHERSEKKQPQVKEGNNTNKSEKIQLSENICDSTSSAAAGRLTQQRK
IGKTYPQQFPKKLKEEHDRCTLKQENEEKTNVNMLYKKNREELERKEKQYKKEVEAKQLE
PTVQSLEMKSKTARNTPNWDFHNHEEMKGLMDENCILKADIAILRQEICTMKNDNLEKEN
KYLKDIKIVKETNAALEKYIKLNEEMITETAFRYQQELNDLKAENTRLNAELLKEKESKK
RLEADIESYQSRLAAAISKHSESVKTERNLKLALERTQDVSVQVEMSSAISKVKDENEFL
TEQLSETQIKFNALKDKFRKTRDSLRKKSLALETVQNNLSQTQQQTQEMKEMYQNAEAKV
NNSTGKWNCVEERICHLQRENAWLVQQLDDVHQKEDHKEIVTNIQRGFIESGKKDFVLEE
KSKKLMNECDHLKESLFQYEREKTEVVVSIKEDKYFQTSRKKI
>sp|Q9NRG9|AAAS_HUMAN Aladin OS=Homo sapiens GN=AAAS PE=1 SV=1
MCSLGLFPPPPPRGQVTLYEHNNELVTGSSYESPPPDFRGQWINLPVLQLTKDPLKTPGR
LDHGTRTAFIHHREQVWKRCINIWRDVGLFGVLNEIANSEEEVFEWVKTASGWALALCRW
ASSLHGSLFPHLSLRSEDLIAEFAQVTNWSSCCLRVFAWHPHTNKFAVALLDDSVRVYNA
SSTIVPSLKHRLQRNVASLAWKPLSASVLAVACQSCILIWTLDPTSLSTRPSSGCAQVLS
HPGHTPVTSLAWAPSGGRLLSASPVDAAIRVWDVSTETCVPLPWFRGGGVTNLLWSPDGS

KILATTPSAVFRVWEAQMWTCERWPTLSGRCQTGCWSPDGSRLLFTVLGEPLIYSLSFPE
RCGEGKGCVGGAKSATIVADLSETTIQTPDGEERLGGEAHSMVWDPSGERLAVLMKGKPR
VQDGKPVILLFRTRNSPVFELLPCGIIQGEPGAQPQLITFHPSFNKGALLSVGWSTGRIA
HIPLYFVNAQFPRFSPVLGRAQEPPAGGGGSIHDLPLFTETSPTSAPWDPLPGPPPVLPH
SPHSHL
>sp|Q86V21|AACS_HUMAN Acetoacetyl-CoA synthetase OS=Homo sapiens GN=AACS PE=1 SV=1
MSKEERPGREEILECQVMWEPDSKKNTQMDRFRAAVGAACGLALESYDDLYHWSVESYSD
FWAEFWKFSGIVFSRVYDEVVDTSKGIADVPEWFKGSRLNYAENLLRHKENDRVALYIAR
EGKEEIVKVTFEELRQEVALFAAAMRKMGVKKGDRVVGYLPNSEHAVEAMLAAASIGAIW
SSTSPDFGVNGVLDRFSQIQPKLIFSVEAVVYNGKEHNHMEKLQQVVKGLPDLKKVVVIP
YVSSRENIDLSKIPNSVFLDDFLATGTSEQAPQLEFEQLPFSHPLFIMFSSGTTGAPKCM
VHSAGGTLIQHLKEHLLHGNMTSSDILLCYTTVGWMMWNWMVSLLATGAAMVLYDGSPLV
PTPNVLWDLVDRIGITVLVTGAKWLSVLEEKAMKPVETHSLQMLHTILSTGSPLKAQSYE
YVYRCIKSSILLGSISGGTDIISCFMGHNFSLPVYKGEIQARNLGMAVEAWNEEGKAVWG
ESGELVCTKPIPCQPTHFWNDENGNKYRKAYFSKFPGIWAHGDYCRINPKTGGIVMLGRS
DGTLNPNGVRFGSSEIYNIVESFEEVEDSLCVPQYNKYREERVILFLKMASGHAFQPDLV
KRIRDAIRMGLSARHVPSLILETKGIPYTLNGKKVEVAVKQIIAGKAVEQGGAFSNPETL
DLYRDIPELQGF
>sp|Q13685|AAMP_HUMAN Angio-associated migratory cell protein OS=Homo sapiens GN=AAMP PE=1 SV=2
MESESESGAAADTPPLETLSFHGDEEIIEVVELDPGPPDPDDLAQEMEDVDFEEEEEEEG
NEEGWVLEPQEGVVGSMEGPDDSEVTFALHSASVFCVSLDPKTNTLAVTGGEDDKAFVWR
LSDGELLFECAGHKDSVTCAGFSHDSTLVATGDMSGLLKVWQVDTKEEVWSFEAGDLEWM
EWHPRAPVLLAGTADGNTWMWKVPNGDCKTFQGPNCPATCGRVLPDGKRAVVGYEDGTIR
IWDLKQGSPIHVLKGTEGHQGPLTCVAANQDGSLILTGSVDCQAKLVSATTGKVVGVFRP
ETVASQPSLGEGEESESNSVESLGFCSVMPLAAVGYLDGTLAIYDLATQTLRHQCQHQSG
IVQLLWEAGTAVVYTCSLDGIVRLWDARTGRLLTDYRGHTAEILDFALSKDASLVVTTSG
DHKAKVFCVQRPDR
>sp|Q13131|AAPK1_HUMAN 5’-AMP-activated protein kinase catalytic subunit alpha-1 OS=Homo sapiens GN=PRKAA1 PE=1 SV=4
MRRLSSWRKMATAEKQKHDGRVKIGHYILGDTLGVGTFGKVKVGKHELTGHKVAVKILNR
QKIRSLDVVGKIRREIQNLKLFRHPHIIKLYQVISTPSDIFMVMEYVSGGELFDYICKNG
RLDEKESRRLFQQILSGVDYCHRHMVVHRDLKPENVLLDAHMNAKIADFGLSNMMSDGEF
LRTSCGSPNYAAPEVISGRLYAGPEVDIWSSGVILYALLCGTLPFDDDHVPTLFKKICDG
IFYTPQYLNPSVISLLKHMLQVDPMKRATIKDIREHEWFKQDLPKYLFPEDPSYSSTMID
DEALKEVCEKFECSEEEVLSCLYNRNHQDPLAVAYHLIIDNRRIMNEAKDFYLATSPPDS
FLDDHHLTRPHPERVPFLVAETPRARHTLDELNPQKSKHQGVRKAKWHLGIRSQSRPNDI
MAEVCRAIKQLDYEWKVVNPYYLRVRRKNPVTSTYSKMSLQLYQVDSRTYLLDFRSIDDE
ITEAKSGTATPQRSGSVSNYRSCQRSDSDAEAQGKSSEVSLTSSVTSLDSSPVDLTPRPG
SHTIEFFEMCANLIKILAQ
>sp|P54646|AAPK2_HUMAN 5’-AMP-activated protein kinase catalytic subunit alpha-2 OS=Homo sapiens GN=PRKAA2 PE=1 SV=2
MAEKQKHDGRVKIGHYVLGDTLGVGTFGKVKIGEHQLTGHKVAVKILNRQKIRSLDVVGK
IKREIQNLKLFRHPHIIKLYQVISTPTDFFMVMEYVSGGELFDYICKHGRVEEMEARRLF

QQILSAVDYCHRHMVVHRDLKPENVLLDAHMNAKIADFGLSNMMSDGEFLRTSCGSPNYA
APEVISGRLYAGPEVDIWSCGVILYALLCGTLPFDDEHVPTLFKKIRGGVFYIPEYLNRS
VATLLMHMLQVDPLKRATIKDIREHEWFKQDLPSYLFPEDPSYDANVIDDEAVKEVCEKF
ECTESEVMNSLYSGDPQDQLAVAYHLIIDNRRIMNQASEFYLASSPPSGSFMDDSAMHIP
PGLKPHPERMPPLIADSPKARCPLDALNTTKPKSLAVKKAKWHLGIRSQSKPYDIMAEVY
RAMKQLDFEWKVVNAYHLRVRRKNPVTGNYVKMSLQLYLVDNRSYLLDFKSIDDEVVEQR
SGSSTPQRSCSAAGLHRPRSSFDSTTAESHSLSGSLTGSLTGSTLSSVSPRLGSHTMDFF
EMCASLITTLAR
>sp|P17174|AATC_HUMAN Aspartate aminotransferase, cytoplasmic OS=Homo sapiens GN=GOT1 PE=1 SV=3
MAPPSVFAEVPQAQPVLVFKLTADFREDPDPRKVNLGVGAYRTDDCHPWVLPVVKKVEQK
IANDNSLNHEYLPILGLAEFRSCASRLALGDDSPALKEKRVGGVQSLGGTGALRIGADFL
ARWYNGTNNKNTPVYVSSPTWENHNAVFSAAGFKDIRSYRYWDAEKRGLDLQGFLNDLEN
APEFSIVVLHACAHNPTGIDPTPEQWKQIASVMKHRFLFPFFDSAYQGFASGNLERDAWA
IRYFVSEGFEFFCAQSFSKNFGLYNERVGNLTVVGKEPESILQVLSQMEKIVRITWSNPP
AQGARIVASTLSNPELFEEWTGNVKTMADRILTMRSELRARLEALKTPGTWNHITDQIGM
FSFTGLNPKQVEYLVNEKHIYLLPSGRINVSGLTTKNLDYVATSIHEAVTKIQ
>sp|Q7Z5M8|AB12B_HUMAN Protein ABHD12B OS=Homo sapiens GN=ABHD12B PE=2 SV=1
MDAQDCQAAASPEPPGPPARSCVAAWWDMVDRNLRYFPHSCSMLGRKIAALYDSFTSKSL
KEHVFLPLIDMLIYFNFFKAPFLVDLKKPELKIPHTVNFYLRVEPGVMLGIWHTVPSCRG
EDAKGKDCCWYEAALRDGNPIIVYLHGSAEHRAASHRLKLVKVLSDGGFHVLSVDYRGFG
DSTGKPTEEGLTTDAICVYEWTKARSGITPVCLWGHSLGTGVATNAAKVLEEKGCPVDAI
VLEAPFTNMWVASINYPLLKIYRNIPGFLRTLMDALRKDKIIFPNDENVKFLSSPLLILH
GEDDRTVPLEYGKKLYEIARNAYRNKERVKMVIFPPGFQHNLLCKSPTLLITVRDFLSKQ
WS
>sp|Q6PCB6|AB17C_HUMAN Protein ABHD17C OS=Homo sapiens GN=ABHD17C PE=2 SV=2
MPEPGPRMNGFSLGELCWLFCCPPCPSRIAAKLAFLPPEPTYTVLAPEQRGAGASAPAPA
QATAAAAAAQPAPQQPEEGAGAGPGACSLHLSERADWQYSQRELDAVEVFFSRTARDNRL
GCMFVRCAPSSRYTLLFSHGNAVDLGQMCSFYIGLGSRINCNIFSYDYSGYGVSSGKPSE
KNLYADIDAAWQALRTRYGVSPENIILYGQSIGTVPTVDLASRYECAAVILHSPLMSGLR
VAFPDTRKTYCFDAFPSIDKISKVTSPVLVIHGTEDEVIDFSHGLAMYERCPRAVEPLWV
EGAGHNDIELYAQYLERLKQFISHELPNS
>sp|Q09428|ABCC8_HUMAN ATP-binding cassette sub-family C member 8 OS=Homo sapiens GN=ABCC8 PE=1 SV=6
MPLAFCGSENHSAAYRVDQGVLNNGCFVDALNVVPHVFLLFITFPILFIGWGSQSSKVHI
HHSTWLHFPGHNLRWILTFMLLFVLVCEIAEGILSDGVTESHHLHLYMPAGMAFMAAVTS
VVYYHNIETSNFPKLLIALLVYWTLAFITKTIKFVKFLDHAIGFSQLRFCLTGLLVILYG
MLLLVEVNVIRVRRYIFFKTPREVKPPEDLQDLGVRFLQPFVNLLSKGTYWWMNAFIKTA
HKKPIDLRAIGKLPIAMRALTNYQRLCEAFDAQVRKDIQGTQGARAIWQALSHAFGRRLV
LSSTFRILADLLGFAGPLCIFGIVDHLGKENDVFQPKTQFLGVYFVSSQEFLANAYVLAV
LLFLALLLQRTFLQASYYVAIETGINLRGAIQTKIYNKIMHLSTSNLSMGEMTAGQICNL
VAIDTNQLMWFFFLCPNLWAMPVQIIVGVILLYYILGVSALIGAAVIILLAPVQYFVATK
LSQAQRSTLEYSNERLKQTNEMLRGIKLLKLYAWENIFRTRVETTRRKEMTSLRAFAIYT
SISIFMNTAIPIAAVLITFVGHVSFFKEADFSPSVAFASLSLFHILVTPLFLLSSVVRST

VKALVSVQKLSEFLSSAEIREEQCAPHEPTPQGPASKYQAVPLRVVNRKRPAREDCRGLT
GPLQSLVPSADGDADNCCVQIMGGYFTWTPDGIPTLSNITIRIPRGQLTMIVGQVGCGKS
SLLLAALGEMQKVSGAVFWSSLPDSEIGEDPSPERETATDLDIRKRGPVAYASQKPWLLN
ATVEENIIFESPFNKQRYKMVIEACSLQPDIDILPHGDQTQIGERGINLSGGQRQRISVA
RALYQHANVVFLDDPFSALDIHLSDHLMQAGILELLRDDKRTVVLVTHKLQYLPHADWII
AMKDGTIQREGTLKDFQRSECQLFEHWKTLMNRQDQELEKETVTERKATEPPQGLSRAMS
SRDGLLQDEEEEEEEAAESEEDDNLSSMLHQRAEIPWRACAKYLSSAGILLLSLLVFSQL
LKHMVLVAIDYWLAKWTDSALTLTPAARNCSLSQECTLDQTVYAMVFTVLCSLGIVLCLV
TSVTVEWTGLKVAKRLHRSLLNRIILAPMRFFETTPLGSILNRFSSDCNTIDQHIPSTLE
CLSRSTLLCVSALAVISYVTPVFLVALLPLAIVCYFIQKYFRVASRDLQQLDDTTQLPLL
SHFAETVEGLTTIRAFRYEARFQQKLLEYTDSNNIASLFLTAANRWLEVRMEYIGACVVL
IAAVTSISNSLHRELSAGLVGLGLTYALMVSNYLNWMVRNLADMELQLGAVKRIHGLLKT
EAESYEGLLAPSLIPKNWPDQGKIQIQNLSVRYDSSLKPVLKHVNALIAPGQKIGICGRT
GSGKSSFSLAFFRMVDTFEGHIIIDGIDIAKLPLHTLRSRLSIILQDPVLFSGTIRFNLD
PERKCSDSTLWEALEIAQLKLVVKALPGGLDAIITEGGENFSQGQRQLFCLARAFVRKTS
IFIMDEATASIDMATENILQKVVMTAFADRTVVTIAHRVHTILSADLVIVLKRGAILEFD
KPEKLLSRKDSVFASFVRADK
>sp|P28288|ABCD3_HUMAN ATP-binding cassette sub-family D member 3 OS=Homo sapiens GN=ABCD3 PE=1 SV=1
MAAFSKYLTARNSSLAGAAFLLLCLLHKRRRALGLHGKKSGKPPLQNNEKEGKKERAVVD
KVFFSRLIQILKIMVPRTFCKETGYLVLIAVMLVSRTYCDVWMIQNGTLIESGIIGRSRK
DFKRYLLNFIAAMPLISLVNNFLKYGLNELKLCFRVRLTKYLYEEYLQAFTYYKMGNLDN
RIANPDQLLTQDVEKFCNSVVDLYSNLSKPFLDIVLYIFKLTSAIGAQGPASMMAYLVVS
GLFLTRLRRPIGKMTITEQKYEGEYRYVNSRLITNSEEIAFYNGNKREKQTVHSVFRKLV
EHLHNFILFRFSMGFIDSIIAKYLATVVGYLVVSRPFLDLSHPRHLKSTHSELLEDYYQS
GRMLLRMSQALGRIVLAGREMTRLAGFTARITELMQVLKDLNHGKYERTMVSQQEKGIEG
VQVIPLIPGAGEIIIADNIIKFDHVPLATPNGDVLIRDLNFEVRSGANVLICGPNGCGKS
SLFRVLGELWPLFGGRLTKPERGKLFYVPQRPYMTLGTLRDQVIYPDGREDQKRKGISDL
VLKEYLDNVQLGHILEREGGWDSVQDWMDVLSGGEKQRMAMARLFYHKPQFAILDECTSA
VSVDVEGYIYSHCRKVGITLFTVSHRKSLWKHHEYYLHMDGRGNYEFKQITEDTVEFGS
>sp|P41238|ABEC1_HUMAN C->U-editing enzyme APOBEC-1 OS=Homo sapiens GN=APOBEC1 PE=1 SV=3
MTSEKGPSTGDPTLRRRIEPWEFDVFYDPRELRKEACLLYEIKWGMSRKIWRSSGKNTTN
HVEVNFIKKFTSERDFHPSMSCSITWFLSWSPCWECSQAIREFLSRHPGVTLVIYVARLF
WHMDQQNRQGLRDLVNSGVTIQIMRASEYYHCWRNFVNYPPGDEAHWPQYPPLWMMLYAL
ELHCIILSLPPCLKISRRWQNHLTFFRLHLQNCHYQTIPPHILLATGLIHPSVAWR
>sp|Q8WW27|ABEC4_HUMAN Putative C->U-editing enzyme APOBEC-4 OS=Homo sapiens GN=APOBEC4 PE=2 SV=1
MEPIYEEYLANHGTIVKPYYWLSFSLDCSNCPYHIRTGEEARVSLTEFCQIFGFPYGTTF
PQTKHLTFYELKTSSGSLVQKGHASSCTGNYIHPESMLFEMNGYLDSAIYNNDSIRHIIL
YSNNSPCNEANHCCISKMYNFLITYPGITLSIYFSQLYHTEMDFPASAWNREALRSLASL
WPRVVLSPISGGIWHSVLHSFISGVSGSHVFQPILTGRALADRHNAYEINAITGVKPYFT
DVLLQTKRNPNTKAQEALESYPLNNAFPGQFFQMPSGQLQPNLPPDLRAPVVFVLVPLRD
LPPMHMGQNPNKPRNIVRHLNMPQMSFQETKDLGRLPTGRSVEIVEITEQFASSKEADEK
KKKKGKK

>sp|Q8WU67|ABHD3_HUMAN Phospholipase ABHD3 OS=Homo sapiens GN=ABHD3 PE=1 SV=2
MQRLAMDLRMLSRELSLYLEHQVRVGFFGSGVGLSLILGFSVAYAFYYLSSIAKKPQLVT
GGESFSRFLQDHCPVVTETYYPTVWCWEGRGQTLLRPFITSKPPVQYRNELIKTADGGQI
SLDWFDNDNSTCYMDASTRPTILLLPGLTGTSKESYILHMIHLSEELGYRCVVFNNRGVA
GENLLTPRTYCCANTEDLETVIHHVHSLYPSAPFLAAGVSMGGMLLLNYLGKIGSKTPLM
AAATFSVGWNTFACSESLEKPLNWLLFNYYLTTCLQSSVNKHRHMFVKQVDMDHVMKAKS
IREFDKRFTSVMFGYQTIDDYYTDASPSPRLKSVGIPVLCLNSVDDVFSPSHAIPIETAK
QNPNVALVLTSYGGHIGFLEGIWPRQSTYMDRVFKQFVQAMVEHGHELS
>sp|Q6JQN1|ACD10_HUMAN Acyl-CoA dehydrogenase family member 10 OS=Homo sapiens GN=ACAD10 PE=1 SV=1
MCVRSCFQSPRLQWVWRTAFLKHTQRRHQGSHRWTHLGGSTYRAVIFDMGGVLIPSPGRV
AAEWEVQNRIPSGTILKALMEGGENGPWMRFMRAEITAEGFLREFGRLCSEMLKTSVPVD
SFFSLLTSERVAKQFPVMTEAITQIRAKGLQTAVLSNNFYLPNQKSFLPLDRKQFDVIVE
SCMEGICKPDPRIYKLCLEQLGLQPSESIFLDDLGTNLKEAARLGIHTIKVNDPETAVKE
LEALLGFTLRVGVPNTRPVKKTMEIPKDSLQKYLKDLLGIQTTGPLELLQFDHGQSNPTY
YIRLANRDLVLRKKPPGTLLPSAHAIEREFRIMKALANAGVPVPNVLDLCEDSSVIGTPF
YVMEYCPGLIYKDPSLPGLEPSHRRAIYTAMNTVLCKIHSVDLQAVGLEDYGKQGDYIPR
QVRTWVKQYRASETSTIPAMERLIEWLPLHLPRQQRTTVVHGDFRLDNLVFHPEEPEVLA
VLDWELSTLGDPLADVAYSCLAHYLPSSFPVLRGINDCDLTQLGIPAAEEYFRMYCLQMG
LPPTENWNFYMAFSFFRVAAILQGVYKRSLTGQASSTYAEQTGKLTEFVSNLAWDFAVKE
GFRVFKEMPFTNPLTRSYHTWARPQSQWCPTGSRSYSSVPEASPAHTSRGGLVISPESLS
PPVRELYHRLKHFMEQRVYPAEPELQSHQASAARWSPSPLIEDLKEKAKAEGLWNLFLPL
EADPEKKYGAGLTNVEYAHLCELMGTSLYAPEVCNCSAPDTGNMELLVRYGTEAQKARWL
IPLLEGKARSCFAMTEPQVASSDATNIEASIREEDSFYVINGHKWWITGILDPRCQLCVF
MGKTDPHAPRHRQQSVLLVPMDTPGIKIIRPLTVYGLEDAPGGHGEVRFEHVRVPKENMV
LGPGRGFEIAQGRLGPGRIHHCMRLIGFSERALALMKARVKSRLAFGKPLVEQGTVLADI
AQSRVEIEQARLLVLRAAHLMDLAGNKAAALDIAMIKMVAPSMASRVIDRAIQAFGAAGL
SSDYPLAQFFTWARALRFADGPDEVHRATVAKLELKHRI
>sp|Q709F0|ACD11_HUMAN Acyl-CoA dehydrogenase family member 11 OS=Homo sapiens GN=ACAD11 PE=1 SV=2
MKPGATGESDLAEVLPQHKFDSKSLEAYLNQHLSGFGAEREATLTIAQYRAGKSNPTFYL
QKGFQTYVLRKKPPGSLLPKAHQIDREFKVQKALFSIGFPVPKPILYCSDTSVIGTEFYV
MEHVQGRIFRDLTIPGLSPAERSAIYVATVETLAQLRSLNIQSLQLEGYGIGAGYCKRQV
STWTKQYQAAAHQDIPAMQQLSEWLMKNLPDNDNEENLIHGDFRLDNIVFHPKECRVIAV
LDWELSTIGHPLSDLAHFSLFYFWPRTVPMINQGSYSENSGIPSMEELISIYCRCRGINS
ILPNWNFFLALSYFKMAGIAQGVYSRYLLGNNSSEDSFLFANIVQPLAETGLQLSKRTFS
TVLPQIDTTGQLFVQTRKGQEVLIKVKHFMKQHILPAEKEVTEFYVQNENSVDKWGKPLV
IDKLKEMAKVEGLWNLFLPAVSGLSHVDYALIAEETGKCFFAPDVFNCQAPDTGNMEVLH
LYGSEEQKKQWLEPLLQGNITSCFCMTEPDVASSDATNIECSIQRDEDSYVINGKKWWSS
GAGNPKCKIAIVLGRTQNTSLSRHKQHSMILVPMNTPGVKIIRPLSVFGYTDNFHGGHFE
IHFNQVRVPATNLILGEGRGFEISQGRLGPGRIHHCMRTVGLAERALQIMCERATQRIAF
KKKLYAHEVVAHWIAESRIAIEKIRLLTLKAAHSMDTLGSAGAKKEIAMIKVAAPRAVSK
IVDWAIQVCGGAGVSQDYPLANMYAITRVLRLADGPDEVHLSAIATMELRDQAKRLTAKI

>sp|P45954|ACDSB_HUMAN Short/branched chain specific acyl-CoA dehydrogenase, mitochondrial OS=Homo sapiens GN=ACADSB PE=1 SV=1
MEGLAVRLLRGSRLLRRNFLTCLSSWKIPPHVSKSSQSEALLNITNNGIHFAPLQTFTDE
EMMIKSSVKKFAQEQIAPLVSTMDENSKMEKSVIQGLFQQGLMGIEVDPEYGGTGASFLS
TVLVIEELAKVDASVAVFCEIQNTLINTLIRKHGTEEQKATYLPQLTTEKVGSFCLSEAG
AGSDSFALKTRADKEGDYYVLNGSKMWISSAEHAGLFLVMANVDPTIGYKGITSFLVDRD
TPGLHIGKPENKLGLRASSTCPLTFENVKVPEANILGQIGHGYKYAIGSLNEGRIGIAAQ
MLGLAQGCFDYTIPYIKERIQFGKRLFDFQGLQHQVAHVATQLEAARLLTYNAARLLEAG
KPFIKEASMAKYYASEIAGQTTSKCIEWMGGVGYTKDYPVEKYFRDAKIGTIYEGASNIQ
LNTIAKHIDAEY
>sp|Q04844|ACHE_HUMAN Acetylcholine receptor subunit epsilon OS=Homo sapiens GN=CHRNE PE=1 SV=2
MARAPLGVLLLLGLLGRGVGKNEELRLYHHLFNNYDPGSRPVREPEDTVTISLKVTLTNL
ISLNEKEETLTTSVWIGIDWQDYRLNYSKDDFGGIETLRVPSELVWLPEIVLENNIDGQF
GVAYDANVLVYEGGSVTWLPPAIYRSVCAVEVTYFPFDWQNCSLIFRSQTYNAEEVEFTF
AVDNDGKTINKIDIDTEAYTENGEWAIDFCPGVIRRHHGGATDGPGETDVIYSLIIRRKP
LFYVINIIVPCVLISGLVLLAYFLPAQAGGQKCTVSINVLLAQTVFLFLIAQKIPETSLS
VPLLGRFLIFVMVVATLIVMNCVIVLNVSQRTPTTHAMSPRLRHVLLELLPRLLGSPPPP
EAPRAASPPRRASSVGLLLRAEELILKKPRSELVFEGQRHRQGTWTAAFCQSLGAAAPEV
RCCVDAVNFVAESTRDQEATGEEVSDWVRMGNALDNICFWAALVLFSVGSSLIFLGAYFN
RVPDLPYAPCIQP
>sp|Q5JWF8|ACL10_HUMAN Actin-like protein 10 OS=Homo sapiens GN=ACTL10 PE=2 SV=1
MASTALLALCSTGAFSGLAVEAGAGVCHATPIYAGHSWHQATFRLNVAGSTLSRYLRDLL
VAANPDLLQQALPRKAITHLKKRSCYVSLDFEGDLRDPARHHPASFSVGNGCCVCLSSER
FRCPEPIFQPGLLGQAEQGLPALAFRALQKMPKTLRTRLADTVVLAGGSTLFPGFAERLD
KELEAQCRRHGYAALRPHLVAKHGRGMAVWTGGSMVASLHSFQRRWITRAMYQECGSRLL
YDVFN
>sp|O94805|ACL6B_HUMAN Actin-like protein 6B OS=Homo sapiens GN=ACTL6B PE=1 SV=1
MSGGVYGGDEVGALVFDIGSFSVRAGYAGEDCPKADFPTTVGLLAAEEGGGLELEGDKEK
KGKIFHIDTNALHVPRDGAEVMSPLKNGMIEDWECFRAILDHTYSKHVKSEPNLHPVLMS
EAPWNTRAKREKLTELMFEQYNIPAFFLCKTAVLTAFANGRSTGLVLDSGATHTTAIPVH
DGYVLQQGIVKSPLAGDFISMQCRELFQEMAIDIIPPYMIAAKEPVREGAPPNWKKKEKL
PQVSKSWHNYMCNEVIQDFQASVLQVSDSPYDEQVAAQMPTVHYEMPNGYNTDYGAERLR
IPEGLFDPSNVKGLSGNTMLGVGHVVTTSIGMCDIDIRPGLYGSVIVTGGNTLLQGFTDR
LNRELSQKTPPSMRLKLIASNSTMERKFSPWIGGSILASLGTFQQMWISKQEYEEGGKQC
VERKCP
>sp|P08912|ACM5_HUMAN Muscarinic acetylcholine receptor M5 OS=Homo sapiens GN=CHRM5 PE=2 SV=2
MEGDSYHNATTVNGTPVNHQPLERHRLWEVITIAAVTAVVSLITIVGNVLVMISFKVNSQ
LKTVNNYYLLSLACADLIIGIFSMNLYTTYILMGRWALGSLACDLWLALDYVASNASVMN
LLVISFDRYFSITRPLTYRAKRTPKRAGIMIGLAWLISFILWAPAILCWQYLVGKRTVPL
DECQIQFLSEPTITFGTAIAAFYIPVSVMTILYCRIYRETEKRTKDLADLQGSDSVTKAE
KRKPAHRALFRSCLRCPRPTLAQRERNQASWSSSRRSTSTTGKPSQATGPSANWAKAEQL
TTCSSYPSSEDEDKPATDPVLQVVYKSQGKESPGEEFSAEETEETFVKAETEKSDYDTPN

YLLSPAAAHRPKSQKCVAYKFRLVVKADGNQETNNGCHKVKIMPCPFPVAKEPSTKGLNP
NPSHQMTKRKRVVLVKERKAAQTLSAILLAFIITWTPYNIMVLVSTFCDKCVPVTLWHLG
YWLCYVNSTVNPICYALCNRTFRKTFKMLLLCRWKKKKVEEKLYWQGNSKLP
>sp|Q8WXI4|ACO11_HUMAN Acyl-coenzyme A thioesterase 11 OS=Homo sapiens GN=ACOT11 PE=1 SV=1
MIQNVGNHLRRGLASVFSNRTSRKSALRAGNDSAMADGEGYRNPTEVQMSQLVLPCHTNQ
RGELSVGQLLKWIDTTACLSAERHAGCPCVTASMDDIYFEHTISVGQVVNIKAKVNRAFN
SSMEVGIQVASEDLCSEKQWNVCKALATFVARREITKVKLKQITPRTEEEKMEHSVAAER
RRMRLVYADTIKDLLANCAIQGDLESRDCSRMVPAEKTRVESVELVLPPHANHQGNTFGG
QIMAWMENVATIAASRLCRAHPTLKAIEMFHFRGPSQVGDRLVLKAIVNNAFKHSMEVGV
CVEAYRQEAETHRRHINSAFMTFVVLDADDQPQLLPWIRPQPGDGERRYREASARKKIRL
DRKYIVSCKQTEVPLSVPWDPSNQVYLSYNNVSSLKMLVAKDNWVLSSEISQVRLYTLED
DKFLSFHMEMVVHVDAAQAFLLLSDLRQRPEWDKHYRSVELVQQVDEDDAIYHVTSPALG
GHTKPQDFVILASRRKPCDNGDPYVIALRSVTLPTHRETPEYRRGETLCSGFCLWREGDQ
LTKCCWVRVSLTELVSASGFYSWGLESRSKGRRSDGWNGKLAGGHLSTLKAIPVAKINSR
FGYLQDT
>sp|Q8N1Q8|ACO15_HUMAN Acyl-coenzyme A thioesterase THEM5 OS=Homo sapiens GN=THEM5 PE=1 SV=2
MIRRCFQVAARLGHHRGLLEAPRILPRLNPASAFGSSTDSMFSRFLPEKTDLKDYALPNA
SWCSDMLSLYQEFLEKTKSSGWIKLPSFKSNRDHIRGLKLPSGLAVSSDKGDCRIFTRCI
QVEGQGFEYVIFFQPTQKKSVCLFQPGSYLEGPPGFAHGGSLAAMMDETFSKTAFLAGEG
LFTLSLNIRFKNLIPVDSLVVMDVELDKIEDQKLYMSCIAHSRDQQTVYAKSSGVFLQLQ
LEEESPQ
>sp|Q99798|ACON_HUMAN Aconitate hydratase, mitochondrial OS=Homo sapiens GN=ACO2 PE=1 SV=2
MAPYSLLVTRLQKALGVRQYHVASVLCQRAKVAMSHFEPNEYIHYDLLEKNINIVRKRLN
RPLTLSEKIVYGHLDDPASQEIERGKSYLRLRPDRVAMQDATAQMAMLQFISSGLSKVAV
PSTIHCDHLIEAQVGGEKDLRRAKDINQEVYNFLATAGAKYGVGFWKPGSGIIHQIILEN
YAYPGVLLIGTDSHTPNGGGLGGICIGVGGADAVDVMAGIPWELKCPKVIGVKLTGSLSG
WSSPKDVILKVAGILTVKGGTGAIVEYHGPGVDSISCTGMATICNMGAEIGATTSVFPYN
HRMKKYLSKTGREDIANLADEFKDHLVPDPGCHYDQLIEINLSELKPHINGPFTPDLAHP
VAEVGKVAEKEGWPLDIRVGLIGSCTNSSYEDMGRSAAVAKQALAHGLKCKSQFTITPGS
EQIRATIERDGYAQILRDLGGIVLANACGPCIGQWDRKDIKKGEKNTIVTSYNRNFTGRN
DANPETHAFVTSPEIVTALAIAGTLKFNPETDYLTGTDGKKFRLEAPDADELPKGEFDPG
QDTYQHPPKDSSGQHVDVSPTSQRLQLLEPFDKWDGKDLEDLQILIKVKGKCTTDHISAA
GPWLKFRGHLDNISNNLLIGAINIENGKANSVRNAVTQEFGPVPDTARYYKKHGIRWVVI
GDENYGEGSSREHAALEPRHLGGRAIITKSFARIHETNLKKQGLLPLTFADPADYNKIHP
VDKLTIQGLKDFTPGKPLKCIIKHPNGTQETILLNHTFNETQIEWFRAGSALNRMKELQQ
>sp|P49753|ACOT2_HUMAN Acyl-coenzyme A thioesterase 2, mitochondrial OS=Homo sapiens GN=ACOT2 PE=1 SV=6
MSNKLLSPHPHSVVLRSEFKMASSPAVLRASRLYQWSLKSSAQFLGSPQLRQVGQIIRVP
ARMAATLILEPAGRCCWDEPVRIAVRGLAPEQPVTLRASLRDEKGALFQAHARYRADTLG
ELDLERAPALGGSFAGLEPMGLLWALEPEKPLVRLVKRDVRTPLAVELEVLDGHDPDPGR
LLCQTRHERYFLPPGVRREPVRVGRVRGTLFLPPEPGPFPGIVDMFGTGGGLLEYRASLL

AGKGFAVMALAYYNYEDLPKTMETLHLEYFEEAMNYLLSHPEVKGPGVGLLGISKGGELC
LSMASFLKGITAAVVINGSVANVGGTLHYKGETLPPVGVNRNRIKVTKDGYADIVDVLNS
PLEGPDQKSFIPVERAESTFLFLVGQDDHNWKSEFYANEACKRLQAHGRRKPQIICYPET
GHYIEPPYFPLCRASLHALVGSPIIWGGEPRAHAMAQVDAWKQLQTFFHKHLGGHEGTIP
SKV
>sp|Q96QF7|ACRC_HUMAN Acidic repeat-containing protein OS=Homo sapiens GN=ACRC PE=2 SV=1
MDGCKKELPRLQEPEEDEDCYILNVQSSSDDTSGSSVARRAPKRQASCILNVQSRSGDTS
GSSVARRAPKRQASSVVVIDSDSDEECHTHEEKKAKLLEINSDDESPECCHVKPAIQEPP
IVISDDDNDDDNGNDLEVPDDNSDDSEAPDDNSDDSEAPDDNSDDSEAPDDNSDDSEAPD
DNSDDSDVPDDNSDDSSDDNSDDSSDDNSDDSDVPDDKSDDSDVPDDSSDDSDVPDDSSD
DSEAPDDSSDDSEAPDDSSDDSEAPDDSSDDSEAPDDSSDDSEASDDSSDDSEASDDSSD
DSEAPDDKSDDSDVPEDKSDDSDVPDDNSDDLEVPVPAEDLCNEGQIASDEEELVEAAAA
VSQHDSSDDAGEQDLGENLSKPPSDPEANPEVSERKLPTEEEPAPVVEQSGKRKSKTKTI
VEPPRKRQTKTKNIVEPPRKRQTKTKNIVEPLRKRKAKTKNVSVTPGHKKRGPSKKKPGA
AKVEKRKTRTPKCKVPGCFLQDLEKSKKYSGKNLKRNKDELVQRIYDLFNRSVCDKKLPE
KLRIGWNNKMVKTAGLCSTGEMWYPKWRRFAKIQIGLKVCDSADRIRDTLIHEMCHAASW
LIDGIHDSHGDAWKYYARKSNRIHPELPRVTRCHNYKINYKVHYECTGCKTRIGCYTKSL
DTSRFICAKCKGSLVMVPLTQKDGTRIVPHV
>sp|P10323|ACRO_HUMAN Acrosin OS=Homo sapiens GN=ACR PE=2 SV=4
MVEMLPTAILLVLAVSVVAKDNATCDGPCGLRFRQNPQGGVRIVGGKAAQHGAWPWMVSL
QIFTYNSHRYHTCGGSLLNSRWVLTAAHCFVGKNNVHDWRLVFGAKEITYGNNKPVKAPL
QERYVEKIIIHEKYNSATEGNDIALVEITPPISCGRFIGPGCLPHFKAGLPRGSQSCWVA
GWGYIEEKAPRPSSILMEARVDLIDLDLCNSTQWYNGRVQPTNVCAGYPVGKIDTCQGDS
GGPLMCKDSKESAYVVVGITSWGVGCARAKRPGIYTATWPYLNWIASKIGSNALRMIQSA
TPPPPTTRPPPIRPPFSHPISAHLPWYFQPPPRPLPPRPPAAQPRPPPSPPPPPPPPASP
LPPPPPPPPPTPSSTTKLPQGLSFAKRLQQLIEVLKGKTYSDGKNHYDMETTELPELTST
S
>sp|Q9NR19|ACSA_HUMAN Acetyl-coenzyme A synthetase, cytoplasmic OS=Homo sapiens GN=ACSS2 PE=1 SV=1
MGLPEERVRSGSGSRGQEEAGAGGRARSWSPPPEVSRSAHVPSLQRYRELHRRSVEEPRE
FWGDIAKEFYWKTPCPGPFLRYNFDVTKGKIFIEWMKGATTNICYNVLDRNVHEKKLGDK
VAFYWEGNEPGETTQITYHQLLVQVCQFSNVLRKQGIQKGDRVAIYMPMIPELVVAMLAC
ARIGALHSIVFAGFSSESLCERILDSSCSLLITTDAFYRGEKLVNLKELADEALQKCQEK
GFPVRCCIVVKHLGRAELGMGDSTSQSPPIKRSCPDVQISWNQGIDLWWHELMQEAGDEC
EPEWCDAEDPLFILYTSGSTGKPKGVVHTVGGYMLYVATTFKYVFDFHAEDVFWCTADIG
WITGHSYVTYGPLANGATSVLFEGIPTYPDVNRLWSIVDKYKVTKFYTAPTAIRLLMKFG
DEPVTKHSRASLQVLGTVGEPINPEAWLWYHRVVGAQRCPIVDTFWQTETGGHMLTPLPG
ATPMKPGSATFPFFGVAPAILNESGEELEGEAEGYLVFKQPWPGIMRTVYGNHERFETTY
FKKFPGYYVTGDGCQRDQDGYYWITGRIDDMLNVSGHLLSTAEVESALVEHEAVAEAAVV
GHPHPVKGECLYCFVTLCDGHTFSPKLTEELKKQIREKIGPIATPDYIQNAPGLPKTRSG
KIMRRVLRKIAQNDHDLGDMSTVADPSVISHLFSHRCLTIQ
>sp|Q53FZ2|ACSM3_HUMAN Acyl-coenzyme A synthetase ACSM3, mitochondrial OS=Homo sapiens GN=ACSM3 PE=1 SV=2
MLARVTRKMLRHAKCFQRLAIFGSVRALHKDNRTATPQNFSNYESMKQDFKLGIPEYFNF

AKDVLDQWTDKEKAGKKPSNPAFWWINRNGEEMRWSFEELGSLSRKFANILSEACSLQRG
DRVILILPRVPEWWLANVACLRTGTVLIPGTTQLTQKDILYRLQSSKANCIITNDVLAPA
VDAVASKCENLHSKLIVSENSREGWGNLKELMKHASDSHTCVKTKHNEIMAIFFTSGTSG
YPKMTAHTHSSFGLGLSVNGRFWLDLTPSDVMWNTSDTGWAKSAWSSVFSPWIQGACVFT
HHLPRFEPTSILQTLSKYPITVFCSAPTVYRMLVQNDITSYKFKSLKHCVSAGEPITPDV
TEKWRNKTGLDIYEGYGQTETVLICGNFKGMKIKPGSMGKPSPAFDVKIVDVNGNVLPPG
QEGDIGIQVLPNRPFGLFTHYVDNPSKTASTLRGNFYITGDRGYMDKDGYFWFVARADDV
ILSSGYRIGPFEVENALNEHPSVAESAVVSSPDPIRGEVVKAFVVLNPDYKSHDQEQLIK
EIQEHVKKTTAPYKYPRKVEFIQELPKTISGKTKRNELRKKEWKTI
>sp|Q6NUN0|ACSM5_HUMAN Acyl-coenzyme A synthetase ACSM5, mitochondrial OS=Homo sapiens GN=ACSM5 PE=1 SV=2
MRPWLRHLVLQALRNSRAFCGSHGKPAPLPVPQKIVATWEAISLGRQLVPEYFNFAHDVL
DVWSRLEEAGHRPPNPAFWWVNGTGAEIKWSFEELGKQSRKAANVLGGACGLQPGDRMML
VLPRLPEWWLVSVACMRTGTVMIPGVTQLTEKDLKYRLQASRAKSIITSDSLAPRVDAIS
AECPSLQTKLLVSDSSRPGWLNFRELLREASTEHNCMRTKSRDPLAIYFTSGTTGAPKMV
EHSQSSYGLGFVASGRRWVALTESDIFWNTTDTGWVKAAWTLFSAWPNGSCIFVHELPRV
DAKVILNTLSKFPITTLCCVPTIFRLLVQEDLTRYQFQSLRHCLTGGEALNPDVREKWKH
QTGVELYEGYGQSETVVICANPKGMKIKSGSMGKASPPYDVQIVDDEGNVLPPGEEGNVA
VRIRPTRPFCFFNCYLDNPEKTAASEQGDFYITGDRARMDKDGYFWFMGRNDDVINSSSY
RIGPVEVESALAEHPAVLESAVVSSPDPIRGEVVKAFIVLTPAYSSHDPEALTRELQEHV
KRVTAPYKYPRKVAFVSELPKTVSGKIQRSKLRSQEWGK
>sp|P62736|ACTA_HUMAN Actin, aortic smooth muscle OS=Homo sapiens GN=ACTA2 PE=1 SV=1
MCEEEDSTALVCDNGSGLCKAGFAGDDAPRAVFPSIVGRPRHQGVMVGMGQKDSYVGDEA
QSKRGILTLKYPIEHGIITNWDDMEKIWHHSFYNELRVAPEEHPTLLTEAPLNPKANREK
MTQIMFETFNVPAMYVAIQAVLSLYASGRTTGIVLDSGDGVTHNVPIYEGYALPHAIMRL
DLAGRDLTDYLMKILTERGYSFVTTAEREIVRDIKEKLCYVALDFENEMATAASSSSLEK
SYELPDGQVITIGNERFRCPETLFQPSFIGMESAGIHETTYNSIMKCDIDIRKDLYANNV
LSGGTTMYPGIADRMQKEITALAPSTMKIKIIAPPERKYSVWIGGSILASLSTFQQMWIS
KQEYDEAGPSIVHRKCF
>sp|Q9BYX7|ACTBM_HUMAN Putative beta-actin-like protein 3 OS=Homo sapiens GN=POTEKP PE=5 SV=1
MDDDTAVLVIDNGSGMCKAGFAGDDAPQAVFPSIVGRPRHQGMMEGMHQKESYVGKEAQS
KRGMLTLKYPMEHGIITNWDDMEKIWHHTFYNELRVAPEEHPILLTEAPLNPKANREKMT
QIMFETFNTPAMYVAIQAVLSLYTSGRTTGIVMDSGDGFTHTVPIYEGNALPHATLRLDL
AGRELTDYLMKILTERGYRFTTTAEQEIVRDIKEKLCYVALDSEQEMAMAASSSSVEKSY
ELPDGQVITIGNERFRCPEALFQPCFLGMESCGIHKTTFNSIVKSDVDIRKDLYTNTVLS
GGTTMYPGIAHRMQKEITALAPSIMKIKIIAPPKRKYSVWVGGSILASLSTFQQMWISKQ
EYDESGPSIVHRKCF
>sp|Q08043|ACTN3_HUMAN Alpha-actinin-3 OS=Homo sapiens GN=ACTN3 PE=1 SV=2
MMMVMQPEGLGAGEGRFAGGGGGGEYMEQEEDWDRDLLLDPAWEKQQRKTFTAWCNSHLR
KAGTQIENIEEDFRNGLKLMLLLEVISGERLPRPDKGKMRFHKIANVNKALDFIASKGVK
LVSIGAEEIVDGNLKMTLGMIWTIILRFAIQDISVEETSAKEGLLLWCQRKTAPYRNVNV
QNFHTSWKDGLALCALIHRHRPDLIDYAKLRKDDPIGNLNTAFEVAEKYLDIPKMLDAED
IVNTPKPDEKAIMTYVSCFYHAFAGAEQAETAANRICKVLAVNQENEKLMEEYEKLASEL

LEWIRRTVPWLENRVGEPSMSAMQRKLEDFRDYRRLHKPPRIQEKCQLEINFNTLQTKLR
LSHRPAFMPSEGKLVSDIANAWRGLEQVEKGYEDWLLSEIRRLQRLQHLAEKFRQKASLH
EAWTRGKEEMLSQRDYDSALLQEVRALLRRHEAFESDLAAHQDRVEHIAALAQELNELDY
HEAASVNSRCQAICDQWDNLGTLTQKRRDALERMEKLLETIDRLQLEFARRAAPFNNWLD
GAVEDLQDVWLVHSVEETQSLLTAHDQFKATLPEADRERGAIMGIQGEIQKICQTYGLRP
CSTNPYITLSPQDINTKWDMVRKLVPSCDQTLQEELARQQVNERLRRQFAAQANAIGPWI
QAKVEEVGRLAAGLAGSLEEQMAGLRQQEQNIINYKTNIDRLEGDHQLLQESLVFDNKHT
VYSMEHIRVGWEQLLTSIARTINEVENQVLTRDAKGLSQEQLNEFRASFNHFDRKQNGMM
EPDDFRACLISMGYDLGEVEFARIMTMVDPNAAGVVTFQAFIDFMTRETAETDTTEQVVA
SFKILAGDKNYITPEELRRELPAKQAEYCIRRMVPYKGSGAPAGALDYVAFSSALYGESD
L
>sp|Q8TDG2|ACTT1_HUMAN Actin-related protein T1 OS=Homo sapiens GN=ACTRT1 PE=2 SV=2
MFNPHALDVPAVIFDNGSGLCKAGLSGEIGPRHVISSVLGHCKFNVPLARLNQKYFVGQE
ALYKYEALHLHYPIERGLVTGWDDMEKLWKHLFERELGVKPSQQPVLMTEPSLNPREIRE
KLAEMMFETFSVPGFYLSNHAVAALYASACVTGLVVDSGDGVTCTVPIFEGYSLPHAVTK
LCMAGRDITEHLTRLLFASGFNFPCILNKAVVNNIKEKLCYIALEPEKELRKSRGEVLGA
YRLPDGHVIHFGDELYQVPEVLFAPDQLGIHSPGLSKMVSSSIMKCDTDIQNKLYADIVL
SGGTTLLPGLEERLMKEVEQLASKGTPIKITASPDRCFSAWIGASIMTSMSSFKQMWVTS
ADFKEYGTSVVQRRCF
>sp|P61163|ACTZ_HUMAN Alpha-centractin OS=Homo sapiens GN=ACTR1A PE=1 SV=1
MESYDVIANQPVVIDNGSGVIKAGFAGDQIPKYCFPNYVGRPKHVRVMAGALEGDIFIGP
KAEEHRGLLSIRYPMEHGIVKDWNDMERIWQYVYSKDQLQTFSEEHPVLLTEAPLNPRKN
RERAAEVFFETFNVPALFISMQAVLSLYATGRTTGVVLDSGDGVTHAVPIYEGFAMPHSI
MRIDIAGRDVSRFLRLYLRKEGYDFHSSSEFEIVKAIKERACYLSINPQKDETLETEKAQ
YYLPDGSTIEIGPSRFRAPELLFRPDLIGEESEGIHEVLVFAIQKSDMDLRRTLFSNIVL
SGGSTLFKGFGDRLLSEVKKLAPKDVKIRISAPQERLYSTWIGGSILASLDTFKKMWVSK
KEYEEDGARSIHRKTF
>sp|P07311|ACYP1_HUMAN Acylphosphatase-1 OS=Homo sapiens GN=ACYP1 PE=1 SV=2
MAEGNTLISVDYEIFGKVQGVFFRKHTQAEGKKLGLVGWVQNTDRGTVQGQLQGPISKVR
HMQEWLETRGSPKSHIDKANFNNEKVILKLDYSDFQIVK
>sp|P14621|ACYP2_HUMAN Acylphosphatase-2 OS=Homo sapiens GN=ACYP2 PE=1 SV=2
MSTAQSLKSVDYEVFGRVQGVCFRMYTEDEARKIGVVGWVKNTSKGTVTGQVQGPEDKVN
SMKSWLSKVGSPSSRIDRTNFSNEKTISKLEYSNFSIRY
>sp|Q7Z695|ADCK2_HUMAN Uncharacterized aarF domain-containing protein kinase 2 OS=Homo sapiens GN=ADCK2 PE=2 SV=1
MVAPWRVSVRVCLSHLRCFELRQGLSLLRPSECPRDARLCWLLLGTLPKVVSLCGDVGEG
APDVLSRRRVRCSGAAGAGPAESLPRAGPLGGVFLHLRLWLRAGALLVKFFPLLLLYPLT
YLAPSVSTLWLHLLLKATETSGPTYIKLGQWASTRRDLFSEAFCAQFSKLHVRVTPHPWT
HTERFLRQAFGDDWGSILSFENREPVGSGCVAQVYKAYANTAFLETDSVQRLGRASCLPP
FSHTGAVGGLRELFGYLGNGRKPPENLADQSFLERLLLPKADLVGSNAGVSRAQVPGHQP
EATNLISVAVKVLHPGLLAQVHMDLLLMKIGSRVLGVLPGIKWLSLPEIVEEFEKLMVQQ
IDLRYEAQNLEHFQVNFRNVKAVKFPTPLRPFVTREVLVETYEESVPVSSYQQAGIPVDL
KRKIARLGINMLLKMIFVDNFVHADLHPGNILVQGANGLSSSQEAQLQQADICDTLVVAV
PSSLCPLRLVLLDAGIVAELQAPDLRNFRAVFMAVVMGQGQRVAELILHHARASECRDVE

GFKTEMAMLVTQARKNTITLEKLHVSSLLSSVFKLLMTHKVKLESNFASIVFAIMVLEGL
GRSLDPKLDILEAARPFLLTGPVCPP
>sp|P35612|ADDB_HUMAN Beta-adducin OS=Homo sapiens GN=ADD2 PE=1 SV=3
MSEETVPEAASPPPPQGQPYFDRFSEDDPEYMRLRNRAADLRQDFNLMEQKKRVTMILQS
PSFREELEGLIQEQMKKGNNSSNIWALRQIADFMASTSHAVFPTSSMNVSMMTPINDLHT
ADSLNLAKGERLMRCKISSVYRLLDLYGWAQLSDTYVTLRVSKEQDHFLISPKGVSCSEV
TASSLIKVNILGEVVEKGSSCFPVDTTGFCLHSAIYAARPDVRCIIHLHTPATAAVSAMK
WGLLPVSHNALLVGDMAYYDFNGEMEQEADRINLQKCLGPTCKILVLRNHGVVALGDTVE
EAFYKIFHLQAACEIQVSALSSAGGVENLILLEQEKHRPHEVGSVQWAGSTFGPMQKSRL
GEHEFEALMRMLDNLGYRTGYTYRHPFVQEKTKHKSEVEIPATVTAFVFEEDGAPVPALR
QHAQKQQKEKTRWLNTPNTYLRVNVADEVQRSMGSPRPKTTWMKADEVEKSSSGMPIRIE
NPNQFVPLYTDPQEVLEMRNKIREQNRQDVKSAGPQSQLLASVIAEKSRSPSTESQLMSK
GDEDTKDDSEETVPNPFSQLTDQELEEYKKEVERKKLELDGEKETAPEEPGSPAKSAPAS
PVQSPAKEAETKSPLVSPSKSLEEGTKKTETSKAATTEPETTQPEGVVVNGREEEQTAEE
ILSKGLSQMTTSADTDVDTSKDKTESVTSGPMSPEGSPSKSPSKKKKKFRTPSFLKKSKK
KEKVES
>sp|Q9H0C2|ADT4_HUMAN ADP/ATP translocase 4 OS=Homo sapiens GN=SLC25A31 PE=2 SV=1
MHREPAKKKAEKRLFDASSFGKDLLAGGVAAAVSKTAVAPIERVKLLLQVQASSKQISPE
ARYKGMVDCLVRIPREQGFFSFWRGNLANVIRYFPTQALNFAFKDKYKQLFMSGVNKEKQ
FWRWFLANLASGGAAGATSLCVVYPLDFARTRLGVDIGKGPEERQFKGLGDCIMKIAKSD
GIAGLYQGFGVSVQGIIVYRASYFGAYDTVKGLLPKPKKTPFLVSFFIAQVVTTCSGILS
YPFDTVRRRMMMQSGEAKRQYKGTLDCFVKIYQHEGISSFFRGAFSNVLRGTGGALVLVL
YDKIKEFFHIDIGGR
>sp|Q8IUX7|AEBP1_HUMAN Adipocyte enhancer-binding protein 1 OS=Homo sapiens GN=AEBP1 PE=1 SV=1
MAAVRGAPLLSCLLALLALCPGGRPQTVLTDDEIEEFLEGFLSELEPEPREDDVEAPPPP
EPTPRVRKAQAGGKPGKRPGTAAEVPPEKTKDKGKKGKKDKGPKVPKESLEGSPRPPKKG
KEKPPKATKKPKEKPPKATKKPKEKPPKATKKPKEKPPKATKKPPSGKRPPILAPSETLE
WPLPPPPSPGPEELPQEGGAPLSNNWQNPGEETHVEAREHQPEPEEETEQPTLDYNDQIE
REDYEDFEYIRRQKQPRPPPSRRRRPERVWPEPPEEKAPAPAPEERIEPPVKPLLPPLPP
DYGDGYVIPNYDDMDYYFGPPPPQKPDAERQTDEEKEELKKPKKEDSSPKEETDKWAVEK
GKDHKEPRKGEELEEEWTPTEKVKCPPIGMESHRIEDNQIRASSMLRHGLGAQRGRLNMQ
TGATEDDYYDGAWCAEDDARTQWIEVDTRRTTRFTGVITQGRDSSIHDDFVTTFFVGFSN
DSQTWVMYTNGYEEMTFHGNVDKDTPVLSELPEPVVARFIRIYPLTWNGSLCMRLEVLGC
SVAPVYSYYAQNEVVATDDLDFRHHSYKDMRQLMKVVNEECPTITRTYSLGKSSRGLKIY
AMEISDNPGEHELGEPEFRYTAGIHGNEVLGRELLLLLMQYLCREYRDGNPRVRSLVQDT
RIHLVPSLNPDGYEVAAQMGSEFGNWALGLWTEEGFDIFEDFPDLNSVLWGAEERKWVPY
RVPNNNLPIPERYLSPDATVSTEVRAIIAWMEKNPFVLGANLNGGERLVSYPYDMARTPT
QEQLLAAAMAAARGEDEDEVSEAQETPDHAIFRWLAISFASAHLTLTEPYRGGCQAQDYT
GGMGIVNGAKWNPRTGTINDFSYLHTNCLELSFYLGCDKFPHESELPREWENNKEALLTF
MEQVHRGIKGVVTDEQGIPIANATISVSGINHGVKTASGGDYWRILNPGEYRVTAHAEGY
TPSAKTCNVDYDIGATQCNFILARSNWKRIREIMAMNGNRPIPHIDPSRPMTPQQRRLQQ
RRLQHRLRLRAQMRLRRLNATTTLGPHTVPPTLPPAPATTLSTTIEPWGLIPPTTAGWEE
SETETYTEVVTEFGTEVEPEFGTKVEPEFETQLEPEFETQLEPEFEEEEEEEKEEEIATG

QAFPFTTVETYTVNFGDF
>sp|Q8TED9|AF1L1_HUMAN Actin filament-associated protein 1-like 1 OS=Homo sapiens GN=AFAP1L1 PE=1 SV=2
MDRGQVLEQLLPELTGLLSLLDHEYLSDTTLEKKMAVASILQSLQPLPAKEVSYLYVNTA
DLHSGPSFVESLFEEFDCDLSDLRDMPEDDGEPSKGASPELAKSPRLRNAADLPPPLPNK
PPPEDYYEEALPLGPGKSPEYISSHNGCSPSHSIVDGYYEDADSSYPATRVNGELKSSYN
DSDAMSSSYESYDEEEEEGKSPQPRHQWPSEEASMHLVRECRICAFLLRKKRFGQWAKQL
TVIREDQLLCYKSSKDRQPHLRLALDTCSIIYVPKDSRHKRHELRFTQGATEVLVLALQS
REQAEEWLKVIREVSKPVGGAEGVEVPRSPVLLCKLDLDKRLSQEKQTSDSDSVGVGDNC
STLGRRETCDHGKGKKSSLAELKGSMSRAAGRKITRIIGFSKKKTLADDLQTSSTEEEVP
CCGYLNVLVNQGWKERWCRLKCNTLYFHKDHMDLRTHVNAIALQGCEVAPGFGPRHPFAF
RILRNRQEVAILEASCSEDMGRWLGLLLVEMGSRVTPEALHYDYVDVETLTSIVSAGRNS
FLYARSCQNQWPEPRVYDDVPYEKMQDEEPERPTGAQVKRHASSCSEKSHRVDPQVKVKR
HASSANQYKYGKNRAEEDARRYLVEKEKLEKEKETIRTELIALRQEKRELKEAIRSSPGA
KLKALEEAVATLEAQCRAKEERRIDLELKLVAVKERLQQSLAGGPALGLSVSSKPKSGET
ANKPQNSVPEQPLPVNCVSELRKRSPSIVASNQGRVLQKAKEWEMKKT
>sp|P42568|AF9_HUMAN Protein AF-9 OS=Homo sapiens GN=MLLT3 PE=1 SV=2
MASSCAVQVKLELGHRAQVRKKPTVEGFTHDWMVFVRGPEHSNIQHFVEKVVFHLHESFP
RPKRVCKDPPYKVEESGYAGFILPIEVYFKNKEEPRKVRFDYDLFLHLEGHPPVNHLRCE
KLTFNNPTEDFRRKLLKAGGDPNRSIHTSSSSSSSSSSSSSSSSSSSSSSSSSSSSSSSS
SSSSSSSSSSTSFSKPHKLMKEHKEKPSKDSREHKSAFKEPSRDHNKSSKESSKKPKENK
PLKEEKIVPKMAFKEPKPMSKEPKPDSNLLTITSGQDKKAPSKRPPISDSEELSAKKRKK
SSSEALFKSFSSAPPLILTCSADKKQIKDKSHVKMGKVKIESETSEKKKSTLPPFDDIVD
PNDSDVEENISSKSDSEQPSPASSSSSSSSSFTPSQTRQQGPLRSIMKDLHSDDNEEESD
EVEDNDNDSEMERPVNRGGSRSRRVSLSDGSDSESSSASSPLHHEPPPPLLKTNNNQILE
VKSPIKQSKSDKQIKNGECDKAYLDELVELHRRLMTLRERHILQQIVNLIEETGHFHITN
TTFDFDLCSLDKTTVRKLQSYLETSGTS
>sp|P51816|AFF2_HUMAN AF4/FMR2 family member 2 OS=Homo sapiens GN=AFF2 PE=1 SV=4
MDLFDFFRDWDLEQQCHYEQDRSALKKREWERRNQEVQQEDDLFSSGFDLFGEPYKVAEY
TNKGDALANRVQNTLGNYDEMKNLLTNHSNQNHLVGIPKNSVPQNPNNKNEPSFFPEQKN
RIIPPHQDNTHPSAPMPPPSVVILNSTLIHSNRKSKPEWSRDSHNPSTVLASQASGQPNK
MQTLTQDQSQAKLEDFFVYPAEQPQIGEVEESNPSAKEDSNPNSSGEDAFKEIFQSNSPE
ESEFAVQAPGSPLVASSLLAPSSGLSVQNFPPGLYCKTSMGQQKPTAYVRPMDGQDQAPD
ISPTLKPSIEFENSFGNLSFGTLLDGKPSAASSKTKLPKFTILQTSEVSLPSDPSCVEEI
LREMTHSWPTPLTSMHTAGHSEQSTFSIPGQESQHLTPGFTLQKWNDPTTRASTKSVSFK
SMLEDDLKLSSDEDDLEPVKTLTTQCTATELYQAVEKAKPRNNPVNPPLATPQPPPAVQA
SGGSGSSSESESSSESDSDTESSTTDSESNEAPRVATPEPEPPSTNKWQLDKWLNKVTSQ
NKSFICGQNETPMETISLPPPIIQPMEVQMKVKTNASQVPAEPKERPLLSLIREKARPRP
TQKIPETKALKHKLSTTSETVSQRTIGKKQPKKVEKNTSTDEFTWPKPNITSSTPKEKES
VELHDPPRGRNKATAHKPAPRKEPRPNIPLAPEKKKYRGPGKIVPKSREFIETDSSTSDS
NTDQEETLQIKVLPPCIISGGNTAKSKEICGASLTLSTLMSSSGSNNNLSISNEEPTFSP
IPVMQTEILSPLRDHENLKNLWVKIDLDLLSRVPGHSSLHAAPAKPDHKETATKPKRQTA
VTAVEKPAPKGKRKHKPIEVAEKIPEKKQRLEEATTICLLPPCISPAPPHKPPNTRENNS
SRRANRRKEEKLFPPPLSPLPEDPPRRRNVSGNNGPFGQDKNIAMTGQITSTKPKRTEGK

FCATFKGISVNEGDTPKKASSATITVTNTAIATATVTATAIVTTTVTATATATATTTTTT
TTISTITSTITTGLMDSSHLEMTSWAALPLLSSSSTNVRRPKLTFDDSVHNADYYMQEAK
KLKHKADALFEKFGKAVNYADAALSFTECGNAMERDPLEAKSPYTMYSETVELLRYAMRL
KNFASPLASDGDKKLAVLCYRCLSLLYLRMFKLKKDHAMKYSRSLMEYFKQNASKVAQIP
SPWVSNGKNTPSPVSLNNVSPINAMGNCNNGPVTIPQRIHHMAASHVNITSNVLRGYEHW
DMADKLTRENKEFFGDLDTLMGPLTQHSSMTNLVRYVRQGLCWLRIDAHLL
>sp|Q9Y4W6|AFG32_HUMAN AFG3-like protein 2 OS=Homo sapiens GN=AFG3L2 PE=1 SV=2
MAHRCLRLWGRGGCWPRGLQQLLVPGGVGPGEQPCLRTLYRFVTTQARASRNSLLTDIIA
AYQRFCSRPPKGFEKYFPNGKNGKKASEPKEVMGEKKESKPAATTRSSGGGGGGGGKRGG
KKDDSHWWSRFQKGDIPWDDKDFRMFFLWTALFWGGVMFYLLLKRSGREITWKDFVNNYL
SKGVVDRLEVVNKRFVRVTFTPGKTPVDGQYVWFNIGSVDTFERNLETLQQELGIEGENR
VPVVYIAESDGSFLLSMLPTVLIIAFLLYTIRRGPAGIGRTGRGMGGLFSVGETTAKVLK
DEIDVKFKDVAGCEEAKLEIMEFVNFLKNPKQYQDLGAKIPKGAILTGPPGTGKTLLAKA
TAGEANVPFITVSGSEFLEMFVGVGPARVRDLFALARKNAPCILFIDEIDAVGRKRGRGN
FGGQSEQENTLNQLLVEMDGFNTTTNVVILAGTNRPDILDPALLRPGRFDRQIFIGPPDI
KGRASIFKVHLRPLKLDSTLEKDKLARKLASLTPGFSGADVANVCNEAALIAARHLSDSI
NQKHFEQAIERVIGGLEKKTQVLQPEEKKTVAYHEAGHAVAGWYLEHADPLLKVSIIPRG
KGLGYAQYLPKEQYLYTKEQLLDRMCMTLGGRVSEEIFFGRITTGAQDDLRKVTQSAYAQ
IVQFGMNEKVGQISFDLPRQGDMVLEKPYSEATARLIDDEVRILINDAYKRTVALLTEKK
ADVEKVALLLLEKEVLDKNDMVELLGPRPFAEKSTYEEFVEGTGSLDEDTSLPEGLKDWN
KEREKEKEEPPGEKVAN
>sp|Q53H12|AGK_HUMAN Acylglycerol kinase, mitochondrial OS=Homo sapiens GN=AGK PE=1 SV=2
MTVFFKTLRNHWKKTTAGLCLLTWGGHWLYGKHCDNLLRRAACQEAQVFGNQLIPPNAQV
KKATVFLNPAACKGKARTLFEKNAAPILHLSGMDVTIVKTDYEGQAKKLLELMENTDVII
VAGGDGTLQEVVTGVLRRTDEATFSKIPIGFIPLGETSSLSHTLFAESGNKVQHITDATL
AIVKGETVPLDVLQIKGEKEQPVFAMTGLRWGSFRDAGVKVSKYWYLGPLKIKAAHFFST
LKEWPQTHQASISYTGPTERPPNEPEETPVQRPSLYRRILRRLASYWAQPQDALSQEVSP
EVWKDVQLSTIELSITTRNNQLDPTSKEDFLNICIEPDTISKGDFITIGSRKVRNPKLHV
EGTECLQASQCTLLIPEGAGGSFSIDSEEYEAMPVEVKLLPRKLQFFCDPRKREQMLTSP
TQ
>sp|Q9H9G7|AGO3_HUMAN Protein argonaute-3 OS=Homo sapiens GN=AGO3 PE=1 SV=2
MEIGSAGPAGAQPLLMVPRRPGYGTMGKPIKLLANCFQVEIPKIDVYLYEVDIKPDKCPR
RVNREVVDSMVQHFKVTIFGDRRPVYDGKRSLYTANPLPVATTGVDLDVTLPGEGGKDRP
FKVSIKFVSRVSWHLLHEVLTGRTLPEPLELDKPISTNPVHAVDVVLRHLPSMKYTPVGR
SFFSAPEGYDHPLGGGREVWFGFHQSVRPAMWKMMLNIDVSATAFYKAQPVIQFMCEVLD
IHNIDEQPRPLTDSHRVKFTKEIKGLKVEVTHCGTMRRKYRVCNVTRRPASHQTFPLQLE
NGQTVERTVAQYFREKYTLQLKYPHLPCLQVGQEQKHTYLPLEVCNIVAGQRCIKKLTDN
QTSTMIKATARSAPDRQEEISRLVRSANYETDPFVQEFQFKVRDEMAHVTGRVLPAPMLQ
YGGRNRTVATPSHGVWDMRGKQFHTGVEIKMWAIACFATQRQCREEILKGFTDQLRKISK
DAGMPIQGQPCFCKYAQGADSVEPMFRHLKNTYSGLQLIIVILPGKTPVYAEVKRVGDTL
LGMATQCVQVKNVIKTSPQTLSNLCLKINVKLGGINNILVPHQRPSVFQQPVIFLGADVT
HPPAGDGKKPSIAAVVGSMDAHPSRYCATVRVQRPRQEIIQDLASMVRELLIQFYKSTRF
KPTRIIFYRDGVSEGQFRQVLYYELLAIREACISLEKDYQPGITYIVVQKRHHTRLFCAD
RTERVGRSGNIPAGTTVDTDITHPYEFDFYLCSHAGIQGTSRPSHYHVLWDDNCFTADEL

QLLTYQLCHTYVRCTRSVSIPAPAYYAHLVAFRARYHLVDKEHDSAEGSHVSGQSNGRDP
QALAKAVQIHQDTLRTMYFA
>sp|O60241|AGRB2_HUMAN Adhesion G protein-coupled receptor B2 OS=Homo sapiens GN=ADGRB2 PE=1 SV=2
MENTGWMGKGHRMTPACPLLLSVILSLRLATAFDPAPSACSALASGVLYGAFSLQDLFPT
IASGCSWTLENPDPTKYSLYLRFNRQEQVCAHFAPRLLPLDHYLVNFTCLRPSPEEAVAQ
AESEVGRPEEEEAEAAAGLELCSGSGPFTFLHFDKNFVQLCLSAEPSEAPRLLAPAALAF
RFVEVLLINNNNSSQFTCGVLCRWSEECGRAAGRACGFAQPGCSCPGEAGAGSTTTTSPG
PPAAHTLSNALVPGGPAPPAEADLHSGSSNDLFTTEMRYGEEPEEEPKVKTQWPRSADEP
GLYMAQTGDPAAEEWSPWSVCSLTCGQGLQVRTRSCVSSPYGTLCSGPLRETRPCNNSAT
CPVHGVWEEWGSWSLCSRSCGRGSRSRMRTCVPPQHGGKACEGPELQTKLCSMAACPVEG
QWLEWGPWGPCSTSCANGTQQRSRKCSVAGPAWATCTGALTDTRECSNLECPATDSKWGP
WNAWSLCSKTCDTGWQRRFRMCQATGTQGYPCEGTGEEVKPCSEKRCPAFHEMCRDEYVM
LMTWKKAAAGEIIYNKCPPNASGSASRRCLLSAQGVAYWGLPSFARCISHEYRYLYLSLR
EHLAKGQRMLAGEGMSQVVRSLQELLARRTYYSGDLLFSVDILRNVTDTFKRATYVPSAD
DVQRFFQVVSFMVDAENKEKWDDAQQVSPGSVHLLRVVEDFIHLVGDALKAFQSSLIVTD
NLVISIQREPVSAVSSDITFPMRGRRGMKDWVRHSEDRLFLPKEVLSLSSPGKPATSGAA
GSPGRGRGPGTVPPGPGHSHQRLLPADPDESSYFVIGAVLYRTLGLILPPPRPPLAVTSR
VMTVTVRPPTQPPAEPLITVELSYIINGTTDPHCASWDYSRADASSGDWDTENCQTLETQ
AAHTRCQCQHLSTFAVLAQPPKDLTLELAGSPSVPLVIGCAVSCMALLTLLAIYAAFWRF
IKSERSIILLNFCLSILASNILILVGQSRVLSKGVCTMTAAFLHFFFLSSFCWVLTEAWQ
SYLAVIGRMRTRLVRKRFLCLGWGLPALVVAVSVGFTRTKGYGTSSYCWLSLEGGLLYAF
VGPAAVIVLVNMLIGIIVFNKLMARDGISDKSKKQRAGSERCPWASLLLPCSACGAVPSP
LLSSASARNAMASLWSSCVVLPLLALTWMSAVLAMTDRRSVLFQALFAVFNSAQGFVITA
VHCFLRREVQDVVKCQMGVCRADESEDSPDSCKNGQLQILSDFEKDVDLACQTVLFKEVN
TCNPSTITGTLSRLSLDEDEEPKSCLVGPEGSLSFSPLPGNILVPMAASPGLGEPPPPQE
ANPVYMCGEGGLRQLDLTWLRPTEPGSEGDYMVLPRRTLSLQPGGGGGGGEDAPRARPEG
TPRRAAKTVAHTEGYPSFLSVDHSGLGLGPAYGSLQNPYGMTFQPPPPTPSARQVPEPGE
RSRTMPRTVPGSTMKMGSLERKKLRYSDLDFEKVMHTRKRHSELYHELNQKFHTFDRYRS
QSTAKREKRWSVSSGGAAERSVCTDKPSPGERPSLSQHRRHQSWSTFKSMTLGSLPPKPR
ERLTLHRAAAWEPTEPPDGDFQTEV
>sp|O60242|AGRB3_HUMAN Adhesion G protein-coupled receptor B3 OS=Homo sapiens GN=ADGRB3 PE=1 SV=2
MKAVRNLLIYIFSTYLLVMFGFNAAQDFWCSTLVKGVIYGSYSVSEMFPKNFTNCTWTLE
NPDPTKYSIYLKFSKKDLSCSNFSLLAYQFDHFSHEKIKDLLRKNHSIMQLCNSKNAFVF
LQYDKNFIQIRRVFPTNFPGLQKKGEEDQKSFFEFLVLNKVSPSQFGCHVLCTWLESCLK
SENGRTESCGIMYTKCTCPQHLGEWGIDDQSLILLNNVVLPLNEQTEGCLTQELQTTQVC
NLTREAKRPPKEEFGMMGDHTIKSQRPRSVHEKRVPQEQADAAKFMAQTGESGVEEWSQW
STCSVTCGQGSQVRTRTCVSPYGTHCSGPLRESRVCNNTALCPVHGVWEEWSPWSLCSFT
CGRGQRTRTRSCTPPQYGGRPCEGPETHHKPCNIALCPVDGQWQEWSSWSQCSVTCSNGT
QQRSRQCTAAAHGGSECRGPWAESRECYNPECTANGQWNQWGHWSGCSKSCDGGWERRIR
TCQGAVITGQQCEGTGEEVRRCNEQRCPAPYEICPEDYLMSMVWKRTPAGDLAFNQCPLN
ATGTTSRRCSLSLHGVAFWEQPSFARCISNEYRHLQHSIKEHLAKGQRMLAGDGMSQVTK
TLLDLTQRKNFYAGDLLMSVEILRNVTDTFKRASYIPASDGVQNFFQIVSNLLDEENKEK

WEDAQQIYPGSIELMQVIEDFIHIVGMGMMDFQNSYLMTGNVVASIQKLPAASVLTDINF
PMKGRKGMVDWARNSEDRVVIPKSIFTPVSSKELDESSVFVLGAVLYKNLDLILPTLRNY
TVINSKIIVVTIRPEPKTTDSFLEIELAHLANGTLNPYCVLWDDSKTNESLGTWSTQGCK
TVLTDASHTKCLCDRLSTFAILAQQPREIIMESSGTPSVTLIVGSGLSCLALITLAVVYA
ALWRYIRSERSIILINFCLSIISSNILILVGQTQTHNKSICTTTTAFLHFFFLASFCWVL
TEAWQSYMAVTGKIRTRLIRKRFLCLGWGLPALVVATSVGFTRTKGYGTDHYCWLSLEGG
LLYAFVGPAAAVVLVNMVIGILVFNKLVSRDGILDKKLKHRAGQMSEPHSGLTLKCAKCG
VVSTTALSATTASNAMASLWSSCVVLPLLALTWMSAVLAMTDKRSILFQILFAVFDSLQG
FVIVMVHCILRREVQDAFRCRLRNCQDPINADSSSSFPNGHAQIMTDFEKDVDIACRSVL
HKDIGPCRAATITGTLSRISLNDDEEEKGTNPEGLSYSTLPGNVISKVIIQQPTGLHMPM
SMNELSNPCLKKENSELRRTVYLCTDDNLRGADMDIVHPQERMMESDYIVMPRSSVNNQP
SMKEESKMNIGMETLPHERLLHYKVNPEFNMNPPVMDQFNMNLEQHLAPQEHMQNLPFEP
RTAVKNFMASELDDNAGLSRSETGSTISMSSLERRKSRYSDLDFEKVMHTRKRHMELFQE
LNQKFQTLDRFRDIPNTSSMENPAPNKNPWDTFKNPSEYPHYTTINVLDTEAKDALELRP
AEWEKCLNLPLDVQEGDFQTEV
>sp|Q6QNK2|AGRD1_HUMAN Adhesion G-protein coupled receptor D1 OS=Homo sapiens GN=ADGRD1 PE=1 SV=1
MEKLLRLCCWYSWLLLFYYNFQVRGVYSRSQDHPGFQVLASASHYWPLENVDGIHELQDT
TGDIVEGKVNKGIYLKEEKGVTLLYYGRYNSSCISKPEQCGPEGVTFSFFWKTQGEQSRP
IPSAYGGQVISNGFKVCSSGGRGSVELYTRDNSMTWEASFSPPGPYWTHVLFTWKSKEGL
KVYVNGTLSTSDPSGKVSRDYGESNVNLVIGSEQDQAKCYENGAFDEFIIWERALTPDEI
AMYFTAAIGKHALLSSTLPSLFMTSTASPVMPTDAYHPIITNLTEERKTFQSPGVILSYL
QNVSLSLPSKSLSEQTALNLTKTFLKAVGEILLLPGWIALSEDSAVVLSLIDTIDTVMGH
VSSNLHGSTPQVTVEGSSAMAEFSVAKILPKTVNSSHYRFPAHGQSFIQIPHEAFHRHAW
STVVGLLYHSMHYYLNNIWPAHTKIAEAMHHQDCLLFATSHLISLEVSPPPTLSQNLSGS
PLITVHLKHRLTRKQHSEATNSSNRVFVYCAFLDFSSGEGVWSNHGCALTRGNLTYSVCR
CTHLTNFAILMQVVPLELARGHQVALSSISYVGCSLSVLCLVATLVTFAVLSSVSTIRNQ
RYHIHANLSFAVLVAQVLLLISFRLEPGTTPCQVMAVLLHYFFLSAFAWMLVEGLHLYSM
VIKVFGSEDSKHRYYYGMGWGFPLLICIISLSFAMDSYGTSNNCWLSLASGAIWAFVAPA
LFVIVVNIGILIAVTRVISQISADNYKIHGDPSAFKLTAKAVAVLLPILGTSWVFGVLAV
NGCAVVFQYMFATLNSLQGLFIFLFHCLLNSEVRAAFKHKTKVWSLTSSSARTSNAKPFH
SDLMNGTRPGMASTKLSPWDKSSHSAHRVDLSAV
>sp|Q14246|AGRE1_HUMAN Adhesion G protein-coupled receptor E1 OS=Homo sapiens GN=ADGRE1 PE=2 SV=3
MRGFNLLLFWGCCVMHSWEGHIRPTRKPNTKGNNCRDSTLCPAYATCTNTVDSYYCACKQ
GFLSSNGQNHFKDPGVRCKDIDECSQSPQPCGPNSSCKNLSGRYKCSCLDGFSSPTGNDW
VPGKPGNFSCTDINECLTSSVCPEHSDCVNSMGSYSCSCQVGFISRNSTCEDVDECADPR
ACPEHATCNNTVGNYSCFCNPGFESSSGHLSFQGLKASCEDIDECTEMCPINSTCTNTPG
SYFCTCHPGFAPSNGQLNFTDQGVECRDIDECRQDPSTCGPNSICTNALGSYSCGCIAGF
HPNPEGSQKDGNFSCQRVLFKCKEDVIPDNKQIQQCQEGTAVKPAYVSFCAQINNIFSVL
DKVCENKTTVVSLKNTTESFVPVLKQISTWTKFTKEETSSLATVFLESVESMTLASFWKP
SANITPAVRTEYLDIESKVINKECSEENVTLDLVAKGDKMKIGCSTIEESESTETTGVAF
VSFVGMESVLNERFFKDHQAPLTTSEIKLKMNSRVVGGIMTGEKKDGFSDPIIYTLENIQ
PKQKFERPICVSWSTDVKGGRWTSFGCVILEASETYTICSCNQMANLAVIMASGELTMDF

SLYIISHVGIIISLVCLVLAIATFLLCRSIRNHNTYLHLHLCVCLLLAKTLFLAGIHKTD
NKMGCAIIAGFLHYLFLACFFWMLVEAVILFLMVRNLKVVNYFSSRNIKMLHICAFGYGL
PMLVVVISASVQPQGYGMHNRCWLNTETGFIWSFLGPVCTVIVINSLLLTWTLWILRQRL
SSVNAEVSTLKDTRLLTFKAFAQLFILGCSWVLGIFQIGPVAGVMAYLFTIINSLQGAFI
FLIHCLLNGQVREEYKRWITGKTKPSSQSQTSRILLSSMPSASKTG
>sp|Q9UHX3|AGRE2_HUMAN Adhesion G protein-coupled receptor E2 OS=Homo sapiens GN=ADGRE2 PE=1 SV=2
MGGRVFLVFLAFCVWLTLPGAETQDSRGCARWCPQDSSCVNATACRCNPGFSSFSEIITT
PMETCDDINECATLSKVSCGKFSDCWNTEGSYDCVCSPGYEPVSGAKTFKNESENTCQDV
DECQQNPRLCKSYGTCVNTLGSYTCQCLPGFKLKPEDPKLCTDVNECTSGQNPCHSSTHC
LNNVGSYQCRCRPGWQPIPGSPNGPNNTVCEDVDECSSGQHQCDSSTVCFNTVGSYSCRC
RPGWKPRHGIPNNQKDTVCEDMTFSTWTPPPGVHSQTLSRFFDKVQDLGRDYKPGLANNT
IQSILQALDELLEAPGDLETLPRLQQHCVASHLLDGLEDVLRGLSKNLSNGLLNFSYPAG
TELSLEVQKQVDRSVTLRQNQAVMQLDWNQAQKSGDPGPSVVGLVSIPGMGKLLAEAPLV
LEPEKQMLLHETHQGLLQDGSPILLSDVISAFLSNNDTQNLSSPVTFTFSHRSVIPRQKV
LCVFWEHGQNGCGHWATTGCSTIGTRDTSTICRCTHLSSFAVLMAHYDVQEEDPVLTVIT
YMGLSVSLLCLLLAALTFLLCKAIQNTSTSLHLQLSLCLFLAHLLFLVAIDQTGHKVLCS
IIAGTLHYLYLATLTWMLLEALYLFLTARNLTVVNYSSINRFMKKLMFPVGYGVPAVTVA
ISAASRPHLYGTPSRCWLQPEKGFIWGFLGPVCAIFSVNLVLFLVTLWILKNRLSSLNSE
VSTLRNTRMLAFKATAQLFILGCTWCLGILQVGPAARVMAYLFTIINSLQGVFIFLVYCL
LSQQVREQYGKWSKGIRKLKTESEMHTLSSSAKADTSKPSTVN
>sp|Q86SQ3|AGRE4_HUMAN Putative adhesion G protein-coupled receptor E4P OS=Homo sapiens GN=ADGRE4P PE=5 SV=1
MGSRFLLVLLSGASCPPCPKYASCHNSTHCTCEDGFRARSGRTYFHDSSEKCEDINECET
GLAKCKYKAYCRNKVGGYICSCLVKYTLFNFLAGIIDYDHPDCYENNSQGTTQSNVDIWV
SGVKPGFGKQLPGDKRTKHICVYWEGSEGGWSTEGCSHVHSNGSYTKCKCFHLSSFAVLV
ALAPKEDPVLTVITQVGLTISLLCLFLAILTFLLCRPIQNTSTSLHLELSLCLFLAHLLF
LTGINRTEPEVLCSIIAGLLHFLYLACFTWMLLEGLHLFLTVRNLKVANYTSTGRFKKRF
MYPVGYGIPAVIIAVSAIVGPQNYGTFTCWLKLDKGFIWSFMGPVAVIILINLVFYFQVL
WILRSKLSSLNKEVSTIQDTRVMTFKAISQLFILGCSWGLGFFMVEEVGKTIGSIIAYSF
TIINTLQGVLLFVVHCLLNRQVRLIILSVISLVPKSN
>sp|Q5T601|AGRF1_HUMAN Adhesion G-protein coupled receptor F1 OS=Homo sapiens GN=ADGRF1 PE=1 SV=2
MKVGVLWLISFFTFTDGHGGFLGKNDGIKTKKELIVNKKKHLGPVEEYQLLLQVTYRDSK
EKRDLRNFLKLLKPPLLWSHGLIRIIRAKATTDCNSLNGVLQCTCEDSYTWFPPSCLDPQ
NCYLHTAGALPSCECHLNNLSQSVNFCERTKIWGTFKINERFTNDLLNSSSAIYSKYANG
IEIQLKKAYERIQGFESVQVTQFRNGSIVAGYEVVGSSSASELLSAIEHVAEKAKTALHK
LFPLEDGSFRVFGKAQCNDIVFGFGSKDDEYTLPCSSGYRGNITAKCESSGWQVIRETCV
LSLLEELNKNFSMIVGNATEAAVSSFVQNLSVIIRQNPSTTVGNLASVVSILSNISSLSL
ASHFRVSNSTMEDVISIADNILNSASVTNWTVLLREEKYASSRLLETLENISTLVPPTAL
PLNFSRKFIDWKGIPVNKSQLKRGYSYQIKMCPQNTSIPIRGRVLIGSDQFQRSLPETII
SMASLTLGNILPVSKNGNAQVNGPVISTVIQNYSINEVFLFFSKIESNLSQPHCVFWDFS
HLQWNDAGCHLVNETQDIVTCQCTHLTSFSILMSPFVPSTIFPVVKWITYVGLGISIGSL
ILCLIIEALFWKQIKKSQTSHTRRICMVNIALSLLIADVWFIVGATVDTTVNPSGVCTAA

VFFTHFFYLSLFFWMLMLGILLAYRIILVFHHMAQHLMMAVGFCLGYGCPLIISVITIAV
TQPSNTYKRKDVCWLNWSNGSKPLLAFVVPALAIVAVNFVVVLLVLTKLWRPTVGERLSR
DDKATIIRVGKSLLILTPLLGLTWGFGIGTIVDSQNLAWHVIFALLNAFQGFFILCFGIL
LDSKLRQLLFNKLSALSSWKQTEKQNSSDLSAKPKFSKPFNPLQNKGHYAFSHTGDSSDN
IMLTQFVSNE
>sp|Q8IZF5|AGRF3_HUMAN Adhesion G-protein coupled receptor F3 OS=Homo sapiens GN=ADGRF3 PE=2 SV=1
MVCSAAPLLLLATTLPLLGSPVAQASQPVSETGVRPREGLQRRQWGPLIGRDKAWNERID
RPFPACPIPLSSSFGRWPKGGQTMWAQTSTLTLTEEELGQSQAGGESGSGQLLDQENGAGE
SALVSVYVHLDFPDKTWPPELSRTLTLPAASASSSPRPLLTGLRLTTECNVNHKGNFYCA
CLSGYQWNTSICLHYPPCQSLHNHQPCGCLVFSHPEPGYCQLLPPGSPVTCLPAVPGILN
LNSQLQMPGDTLSLTLHLSQEATNLSWFLRHPGSPSPILLQPGTQVSVTSSHGQAALSVS
NMSHHWAGEYMSCFEAQGFKWNLYEVVRVPLKATDVARLPYQLSISCATSPGFQLSCCIP
STNLAYTAAWSPGEGSKASSFNESGSQCFVLAVQRCPMADTTYACDLQSLGLAPLRVPIS
ITIIQDGDITCPEDASVLTWNVTKAGHVAQAPCPESKRGIVRRLCGADGVWGPVHSSCTD
ARLLALFTRTKLLQAGQGSPAEEVPQILAQLPGQAAEASSPSDLLTLLSTMKYVAKVVAE
ARIQLDRRALKNLLIATDKVLDMDTRSLWTLAQARKPWAGSTLLLAVETLACSLCPQDHP
FAFSLPNVLLQSQLFGPTFPADYSISFPTRPPLQAQIPRHSLAPLVRNGTEISITSLVLR
KLDHLLPSNYGQGLGDSLYATPGLVLVISIMAGDRAFSQGEVIMDFGNTDGSPHCVFWDH
SLFQGRGGWSKEGCQAQVASASPTAQCLCQHLTAFSVLMSPHTVPEEPALALLTQVGLGA
SILALLVCLGVYWLVWRVVVRNKISYFRHAALLNMVFCLLAADTCFLGAPFLSPGPRSPL
CLAAAFLCHFLYLATFFWMLAQALVLAHQLLFVFHQLAKHRVLPLMVLLGYLCPLGLAGV
TLGLYLPQGQYLREGECWLDGKGGALYTFVGPVLAIIGVNGLVLAMAMLKLLRPSLSEGP
PAEKRQALLGVIKALLILTPIFGLTWGLGLATLLEEVSTVPHYIFTILNTLQGVFILLFG
CLMDRKIQEALRKRFCRAQAPSSTISLVSCCLQILSCASKSMSEGIPWPSSEDMGTARS
>sp|Q86Y34|AGRG3_HUMAN Adhesion G protein-coupled receptor G3 OS=Homo sapiens GN=ADGRG3 PE=1 SV=1
MATPRGLGALLLLLLLPTSGQEKPTEGPRNTCLGSNNMYDIFNLNDKALCFTKCRQSGSD
SCNVENLQRYWLNYEAHLMKEGLTQKVNTPFLKALVQNLSTNTAEDFYFSLEPSQVPRQV
MKDEDKPPDRVRLPKSLFRSLPGNRSVVRLAVTILDIGPGTLFKGPRLGLGDGSGVLNNR
LVGLSVGQMHVTKLAEPLEIVFSHQRPPPNMTLTCVFWDVTKGTTGDWSSEGCSTEVRPE
GTVCCCDHLTFFALLLRPTLDQSTVHILTRISQAGCGVSMIFLAFTIILYAFLRLSRERF
KSEDAPKIHVALGGSLFLLNLAFLVNVGSGSKGSDAACWARGAVFHYFLLCAFTWMGLEA
FHLYLLAVRVFNTYFGHYFLKLSLVGWGLPALMVIGTGSANSYGLYTIRDRENRTSLELC
WFREGTTMYALYITVHGYFLITFLFGMVVLALVVWKIFTLSRATAVKERGKNRKKVLTLL
GLSSLVGVTWGLAIFTPLGLSTVYIFALFNSLQGVFICCWFTILYLPSQSTTVSSSTARL
DQAHSASQE
>sp|Q8IZF6|AGRG4_HUMAN Adhesion G-protein coupled receptor G4 OS=Homo sapiens GN=ADGRG4 PE=2 SV=2
MKEHIIYQKLYGLILMSSFIFLSDTLSLKGKKLDFFGRGDTYVSLIDTIPELSRFTACID
LVFMDDNSRYWMAFSYITNNALLGREDIDLGLAGDHQQLILYRLGKTFSIRHHLASFQWH
TICLIWDGVKGKLELFLNKERILEVTDQPHNLTPHGTLFLGHFLKNESSEVKSMMRSFPG
SLYYFQLWDHILENEEFMKCLDGNIVSWEEDVWLVNKIIPTVDRTLRCFVPENMTIQEKS
TTVSQQIDMTTPSQITGVKPQNTAHSSTLLSQSIPIFATDYTTISYSNTTSPPLETMTAQ

KILKTLVDETATFAVDVLSTSSAISLPTQSISIDNTTNSMKKTKSPSSESTKTTKMVEAM
ATEIFQPPTPSNFLSTSRFTKNSVVSTTSAIKSQSAVTKTTSLFSTIESTSMSTTPCLKQ
KSTNTGALPISTAGQEFIESTAAGTVPWFTVEKTSPASTHVGTASSFPPEPVLISTAAPV
DSVFPRNQTAFPLATTDMKIAFTVHSLTLPTRLIETTPAPRTAETELTSTNFQDVSLPRV
EDAMSTSMSKETSSKTFSFLTSFSFTGTESVQTVIDAEATRTALTPEITLASTVAETMLS
STITGRVYTQNTPTADGHLLTLMSTRSASTSKAPESGPTSTTDEAAHLFSSNETIWTSRP
DQALLASMNTTTILTFVPNENFTSAFHENTTYTEYLSATTNITPLKASPEGKGTTANDAT
TARYTTAVSKLTSPWFANFSIVSGTTSITNMPEFKLTTLLLKTIPMSTKPANELPLTPRE
TVVPSVDIISTLACIQPNFSTEESASETTQTEINGAIVFGGTTTPVPKSATTQRLNATVT
RKEATSHYLMRKSTIAAVAEVSPFSTMLEVTDESAQRVTASVTVSSFPDIEKLSTPLDNK
TATTEVRESWLLTKLVKTTPRSSYNEMTEMFNFNHTYVAHWTSETSEGISAGSPTSGSTH
IFGEPLGASTTRISETSFSTTPTDRTATSLSDGILPPQPTAAHSSATPVPVTHMFSLPVN
GSSVVAEETEVTMSEPSTLARAFSTSVLSDVSNLSSTTMTTALVPPLDQTASTTIVIVPT
HGDLIRTTSEATVISVRKTSMAVPSLTETPFHSLRLSTPVTAKAETTLFSTSVDTVTPST
HTLVCSKPPPDNIPPASSTHVISTTSTPEATQPISQVEETSTYALSFPYTFSGGGVVASL
ATGTTETSVVDETTPSHISANKLTTSVNSHISSSATYRVHTPVSIQLVTSTSVLSSDKDQ
MTISLGKTPRTMEVTEMSPSKNSFISYSRGTPSLEMTDTGFPETTKISSHQTHSPSEIPL
GTPSDGNLASSPTSGSTQITPTLTSSNTVGVHIPEMSTSLGKTALPSQALTITTFLCPEK
ESTSALPAYTPRTVEMIVNSTYVTHSVSYGQDTSFVDTTTSSSTRISNPMDINTTFSHLH
SLRTQPEVTSVASFISESTQTFPESLSLSTAGLYNDGFTVLSDRITTAFSVPNVPTMLPR
ESSMATSTPIYQMSSLPVNVTAFTSKKVSDTPPIVITKSSKTMHPGCLKSPCTATSGPMS
EMSSIPVNNSAFTPATVSSDTSTRVGLFSTLLSSVTPRTTMTMQTSTLDVTPVIYAGATS
KNKMVSSAFTTEMIEAPSRITPTTFLSPTEPTLPFVKTVPTTIMAGIVTPFVGTTAFSPL
SSKSTGAISSIPKTTFSPFLSATQQSSQADEATTLGILSGITNRSLSTVNSGTGVALTDT
YSRITVPENMLSPTHADSLHTSFNIQVSPSLTSFKSASGPTKNVKTTTNCFSSNTRKMTS
LLEKTSLTNYATSLNTPVSYPPWTPSSATLPSLTSFVYSPHSTEAEISTPKTSPPPTSQM
VEFPVLGTRMTSSNTQPLLMTSWNIPTAEGSQFPISTTINVPTSNEMETETLHLVPGPLS
TFTASQTGLVSKDVMAMSSIPMSGILPNHGLSENPSLSTSLRAITSTLADVKHTFEKMTT
SVTPGTTLPSILSGATSGSVISKSPILTWLLSSLPSGSPPATVSNAPHVMTSSTVEVSKS
TFLTSDMISAHPFTNLTTLPSATMSTILTRTIPTPTLGGITTGFPTSLPMSINVTDDIVY
ISTHPEASSRTTITANPRTVSHPSSFSRKTMSPSTTDHTLSVGAMPLPSSTITSSWNRIP
TASSPSTLIIPKPTLDSLLNIMTTTSTVPGASFPLISTGVTYPFTATVSSPISSFFETTW
LDSTPSFLSTEASTSPTATKSTVSFYNVEMSFSVFVEEPRIPITSVINEFTENSLNSIFQ
NSEFSLATLETQIKSRDISEEEMVMDRAILEQREGQEMATISYVPYSCVCQVIIKASSSL
ASSELMRKIKSKIHGNFTHGNFTQDQLTLLVNCEHVAVKKLEPGNCKADETASKYKGTYK
WLLTNPTETAQTRCIKNEDGNATRFCSISINTGKSQWEKPKFKQCKLLQELPDKIVDLAN
ITISDENAEDVAEHILNLINESPALGKEETKIIVSKISDISQCDEISMNLTHVMLQIINV
VLEKQNNSASDLHEISNEILRIIERTGHKMEFSGQIANLTVAGLALAVLRGDHTFDGMAF
SIHSYEEGTDPEIFLGNVPVGGILASIYLPKSLTERIPLSNLQTILFNFFGQTSLFKTKN
VTKALTTYVVSASISDDMFIQNLADPVVITLQHIGGNQNYGQVHCAFWDFENNNGLGGWN
SSGCKVKETNVNYTICQCDHLTHFGVLMDLSRSTVDSVNEQILALITYTGCGISSIFLGV
AVVTYIAFHKLRKDYPAKILINLCTALLMLNLVFLINSWLSSFQKVGVCITAAVALHYFL
LVSFTWMGLEAVHMYLALVKVFNIYIPNYILKFCLVGWGIPAIMVAITVSVKKDLYGTLS
PTTPFCWIKDDSIFYISVVAYFCLIFLMNLSMFCTVLVQLNSVKSQIQKTRRKMILHDLK

GTMSLTFLLGLTWGFAFFAWGPMRNFFLYLFAIFNTLQGFFIFVFHCVMKESVREQWQIH
LCCGWLRLDNSSDGSSRCQIKVGYKQEGLKKIFEHKLLTPSLKSTATSSTFKSLGSAQGT
PSEISFPNDDFDKDPYCSSP
>sp|Q86SQ4|AGRG6_HUMAN Adhesion G-protein coupled receptor G6 OS=Homo sapiens GN=ADGRG6 PE=1 SV=3
MMFRSDRMWSCHWKWKPSPLLFLFALYIMCVPHSVWGCANCRVVLSNPSGTFTSPCYPND
YPNSQACMWTLRAPTGYIIQITFNDFDIEEAPNCIYDSLSLDNGESQTKFCGATAKGLSF
NSSANEMHVSFSSDFSIQKKGFNASYIRVAVSLRNQKVILPQTSDAYQVSVAKSISIPEL
SAFTLCFEATKVGHEDSDWTAFSYSNASFTQLLSFGKAKSGYFLSISDSKCLLNNALPVK
EKEDIFAESFEQLCLVWNNSLGSIGVNFKRNYETVPCDSTISKVIPGNGKLLLGSNQNEI
VSLKGDIYNFRLWNFTMNAKILSNLSCNVKGNVVDWQNDFWNIPNLALKAESNLSCGSYL
IPLPAAELASCADLGTLCQATVNSPSTTPPTVTTNMPVTNRIDKQRNDGIIYRISVVIQN
ILRHPEVKVQSKVAEWLNSTFQNWNYTVYVVNISFHLSAGEDKIKVKRSLEDEPRLVLWA
LLVYNATNNTNLEGKIIQQKLLKNNESLDEGLRLHTVNVRQLGHCLAMEEPKGYYWPSIQ
PSEYVLPCPDKPGFSASRICFYNATNPLVTYWGPVDISNCLKEANEVANQILNLTADGQN
LTSANITNIVEQVKRIVNKEENIDITLGSTLMNIFSNILSSSDSDLLESSSEALKTIDEL
AFKIDLNSTSHVNITTRNLALSVSSLLPGTNAISNFSIGLPSNNESYFQMDFESGQVDPL
ASVILPPNLLENLSPEDSVLVRRAQFTFFNKTGLFQDVGPQRKTLVSYVMACSIGNITIQ
NLKDPVQIKIKHTRTQEVHHPICAFWDLNKNKSFGGWNTSGCVAHRDSDASETVCLCNHF
THFGVLMDLPRSASQLDARNTKVLTFISYIGCGISAIFSAATLLTYVAFEKLRRDYPSKI
LMNLSTALLFLNLLFLLDGWITSFNVDGLCIAVAVLLHFFLLATFTWMGLEAIHMYIALV
KVFNTYIRRYILKFCIIGWGLPALVVSVVLASRNNNEVYGKESYGKEKGDEFCWIQDPVI
FYVTCAGYFGVMFFLNIAMFIVVMVQICGRNGKRSNRTLREEVLRNLRSVVSLTFLLGMT
WGFAFFAWGPLNIPFMYLFSIFNSLQGLFIFIFHCAMKENVQKQWRQHLCCGRFRLADNS
DWSKTATNIIKKSSDNLGKSLSSSSIGSNSTYLTSKSKSSSTTYFKRNSHTDNVSYEHSF
NKSGSLRQCFHGQVLVKTGPC
>sp|O00468|AGRIN_HUMAN Agrin OS=Homo sapiens GN=AGRN PE=1 SV=5
MAGRSHPGPLRPLLPLLVVAACVLPGAGGTCPERALERREEEANVVLTGTVEEILNVDPV
QHTYSCKVRVWRYLKGKDLVARESLLDGGNKVVISGFGDPLICDNQVSTGDTRIFFVNPA
PPYLWPAHKNELMLNSSLMRITLRNLEEVEFCVEDKPGTHFTPVPPTPPDACRGMLCGFG
AVCEPNAEGPGRASCVCKKSPCPSVVAPVCGSDASTYSNECELQRAQCSQQRRIRLLSRG
PCGSRDPCSNVTCSFGSTCARSADGLTASCLCPATCRGAPEGTVCGSDGADYPGECQLLR
RACARQENVFKKFDGPCDPCQGALPDPSRSCRVNPRTRRPEMLLRPESCPARQAPVCGDD
GVTYENDCVMGRSGAARGLLLQKVRSGQCQGRDQCPEPCRFNAVCLSRRGRPRCSCDRVT
CDGAYRPVCAQDGRTYDSDCWRQQAECRQQRAIPSKHQGPCDQAPSPCLGVQCAFGATCA
VKNGQAACECLQACSSLYDPVCGSDGVTYGSACELEATACTLGREIQVARKGPCDRCGQC
RFGALCEAETGRCVCPSECVALAQPVCGSDGHTYPSECMLHVHACTHQISLHVASAGPCE
TCGDAVCAFGAVCSAGQCVCPRCEHPPPGPVCGSDGVTYGSACELREAACLQQTQIEEAR
AGPCEQAECGSGGSGSGEDGDCEQELCRQRGGIWDEDSEDGPCVCDFSCQSVPGSPVCGS
DGVTYSTECELKKARCESQRGLYVAAQGACRGPTFAPLPPVAPLHCAQTPYGCCQDNITA
ARGVGLAGCPSACQCNPHGSYGGTCDPATGQCSCRPGVGGLRCDRCEPGFWNFRGIVTDG
RSGCTPCSCDPQGAVRDDCEQMTGLCSCKPGVAGPKCGQCPDGRALGPAGCEADASAPAT
CAEMRCEFGARCVEESGSAHCVCPMLTCPEANATKVCGSDGVTYGNECQLKTIACRQGLQ
ISIQSLGPCQEAVAPSTHPTSASVTVTTPGLLLSQALPAPPGALPLAPSSTAHSQTTPPP

SSRPRTTASVPRTTVWPVLTVPPTAPSPAPSLVASAFGESGSTDGSSDEELSGDQEASGG
GSGGLEPLEGSSVATPGPPVERASCYNSALGCCSDGKTPSLDAEGSNCPATKVFQGVLEL
EGVEGQELFYTPEMADPKSELFGETARSIESTLDDLFRNSDVKKDFRSVRLRDLGPGKSV
RAIVDVHFDPTTAFRAPDVARALLRQIQVSRRRSLGVRRPLQEHVRFMDFDWFPAFITGA
TSGAIAAGATARATTASRLPSSAVTPRAPHPSHTSQPVAKTTAAPTTRRPPTTAPSRVPG
RRPPAPQQPPKPCDSQPCFHGGTCQDWALGGGFTCSCPAGRGGAVCEKVLGAPVPAFEGR
SFLAFPTLRAYHTLRLALEFRALEPQGLLLYNGNARGKDFLALALLDGRVQLRFDTGSGP
AVLTSAVPVEPGQWHRLELSRHWRRGTLSVDGETPVLGESPSGTDGLNLDTDLFVGGVPE
DQAAVALERTFVGAGLRGCIRLLDVNNQRLELGIGPGAATRGSGVGECGDHPCLPNPCHG
GAPCQNLEAGRFHCQCPPGRVGPTCADEKSPCQPNPCHGAAPCRVLPEGGAQCECPLGRE
GTFCQTASGQDGSGPFLADFNGFSHLELRGLHTFARDLGEKMALEVVFLARGPSGLLLYN
GQKTDGKGDFVSLALRDRRLEFRYDLGKGAAVIRSREPVTLGAWTRVSLERNGRKGALRV
GDGPRVLGESPKSRKVPHTVLNLKEPLYVGGAPDFSKLARAAAVSSGFDGAIQLVSLGGR
QLLTPEHVLRQVDVTSFAGHPCTRASGHPCLNGASCVPREAAYVCLCPGGFSGPHCEKGL
VEKSAGDVDTLAFDGRTFVEYLNAVTESELANEIPVPETLDSGALHEKALQSNHFELSLR
TEATQGLVLWSGKATERADYVALAIVDGHLQLSYNLGSQPVVLRSTVPVNTNRWLRVVAH
REQREGSLQVGNEAPVTGSSPLGATQLDTDGALWLGGLPELPVGPALPKAYGTGFVGCLR
DVVVGRHPLHLLEDAVTKPELRPCPTP
>sp|P14060|3BHS1_HUMAN 3 beta-hydroxysteroid dehydrogenase/Delta 5-->4-isomerase type 1 OS=Homo sapiens GN=HSD3B1 PE=1 SV=2
MTGWSCLVTGAGGFLGQRIIRLLVKEKELKEIRVLDKAFGPELREEFSKLQNKTKLTVLE
GDILDEPFLKRACQDVSVIIHTACIIDVFGVTHRESIMNVNVKGTQLLLEACVQASVPVF
IYTSSIEVAGPNSYKEIIQNGHEEEPLENTWPAPYPHSKKLAEKAVLAANGWNLKNGGTL
YTCALRPMYIYGEGSRFLSASINEALNNNGILSSVGKFSTVNPVYVGNVAWAHILALRAL
QDPKKAPSIRGQFYYISDDTPHQSYDNLNYTLSKEFGLRLDSRWSFPLSLMYWIGFLLEI
VSFLLRPIYTYRPPFNRHIVTLSNSVFTFSYKKAQRDLAYKPLYSWEEAKQKTVEWVGSL
VDRHKETLKSKTQ
>sp|P26439|3BHS2_HUMAN 3 beta-hydroxysteroid dehydrogenase/Delta 5-->4-isomerase type 2 OS=Homo sapiens GN=HSD3B2 PE=1 SV=2
MGWSCLVTGAGGLLGQRIVRLLVEEKELKEIRALDKAFRPELREEFSKLQNRTKLTVLEG
DILDEPFLKRACQDVSVVIHTACIIDVFGVTHRESIMNVNVKGTQLLLEACVQASVPVFI
YTSSIEVAGPNSYKEIIQNGHEEEPLENTWPTPYPYSKKLAEKAVLAANGWNLKNGDTLY
TCALRPTYIYGEGGPFLSASINEALNNNGILSSVGKFSTVNPVYVGNVAWAHILALRALR
DPKKAPSVRGQFYYISDDTPHQSYDNLNYILSKEFGLRLDSRWSLPLTLMYWIGFLLEVV
SFLLSPIYSYQPPFNRHTVTLSNSVFTFSYKKAQRDLAYKPLYSWEEAKQKTVEWVGSLV
DRHKETLKSKTQ
>sp|Q9H2F3|3BHS7_HUMAN 3 beta-hydroxysteroid dehydrogenase type 7 OS=Homo sapiens GN=HSD3B7 PE=1 SV=2
MADSAQAQKLVYLVTGGCGFLGEHVVRMLLQREPRLGELRVFDQHLGPWLEELKTGPVRV
TAIQGDVTQAHEVAAAVAGAHVVIHTAGLVDVFGRASPKTIHEVNVQGTRNVIEACVQTG
TRFLVYTSSMEVVGPNTKGHPFYRGNEDTPYEAVHRHPYPCSKALAEWLVLEANGRKVRG
GLPLVTCALRPTGIYGEGHQIMRDFYRQGLRLGGWLFRAIPASVEHGRVYVGNVAWMHVL
AARELEQRATLMGGQVYFCYDGSPYRSYEDFNMEFLGPCGLRLVGARPLLPYWLLVFLAA
LNALLQWLLRPLVLYAPLLNPYTLAVANTTFTVSTDKAQRHFGYEPLFSWEDSRTRTILW

VQAATGSAQ
>sp|P31937|3HIDH_HUMAN 3-hydroxyisobutyrate dehydrogenase, mitochondrial OS=Homo sapiens GN=HIBADH PE=1 SV=2
MAASLRLLGAASGLRYWSRRLRPAAGSFAAVCSRSVASKTPVGFIGLGNMGNPMAKNLMK
HGYPLIIYDVFPDACKEFQDAGEQVVSSPADVAEKADRIITMLPTSINAIEAYSGANGIL
KKVKKGSLLIDSSTIDPAVSKELAKEVEKMGAVFMDAPVSGGVGAARSGNLTFMVGGVED
EFAAAQELLGCMGSNVVYCGAVGTGQAAKICNNMLLAISMIGTAEAMNLGIRLGLDPKLL
AKILNMSSGRCWSSDTYNPVPGVMDGVPSANNYQGGFGTTLMAKDLGLAQDSATSTKSPI
LLGSLAHQIYRMMCAKGYSKKDFSSVFQFLREEETF
>sp|P28222|5HT1B_HUMAN 5-hydroxytryptamine receptor 1B OS=Homo sapiens GN=HTR1B PE=1 SV=1
MEEPGAQCAPPPPAGSETWVPQANLSSAPSQNCSAKDYIYQDSISLPWKVLLVMLLALIT
LATTLSNAFVIATVYRTRKLHTPANYLIASLAVTDLLVSILVMPISTMYTVTGRWTLGQV
VCDFWLSSDITCCTASILHLCVIALDRYWAITDAVEYSAKRTPKRAAVMIALVWVFSISI
SLPPFFWRQAKAEEEVSECVVNTDHILYTVYSTVGAFYFPTLLLIALYGRIYVEARSRIL
KQTPNRTGKRLTRAQLITDSPGSTSSVTSINSRVPDVPSESGSPVYVNQVKVRVSDALLE
KKKLMAARERKATKTLGIILGAFIVCWLPFFIISLVMPICKDACWFHLAIFDFFTWLGYL
NSLINPIIYTMSNEDFKQAFHKLIRFKCTS
>sp|P30939|5HT1F_HUMAN 5-hydroxytryptamine receptor 1F OS=Homo sapiens GN=HTR1F PE=2 SV=1
MDFLNSSDQNLTSEELLNRMPSKILVSLTLSGLALMTTTINSLVIAAIIVTRKLHHPANY
LICSLAVTDFLVAVLVMPFSIVYIVRESWIMGQVVCDIWLSVDITCCTCSILHLSAIALD
RYRAITDAVEYARKRTPKHAGIMITIVWIISVFISMPPLFWRHQGTSRDDECIIKHDHIV
STIYSTFGAFYIPLALILILYYKIYRAAKTLYHKRQASRIAKEEVNGQVLLESGEKSTKS
VSTSYVLEKSLSDPSTDFDKIHSTVRSLRSEFKHEKSWRRQKISGTRERKAATTLGLILG
AFVICWLPFFVKELVVNVCDKCKISEEMSNFLAWLGYLNSLINPLIYTIFNEDFKKAFQK
LVRCRC
>sp|P28223|5HT2A_HUMAN 5-hydroxytryptamine receptor 2A OS=Homo sapiens GN=HTR2A PE=1 SV=2
MDILCEENTSLSSTTNSLMQLNDDTRLYSNDFNSGEANTSDAFNWTVDSENRTNLSCEGC
LSPSCLSLLHLQEKNWSALLTAVVIILTIAGNILVIMAVSLEKKLQNATNYFLMSLAIAD
MLLGFLVMPVSMLTILYGYRWPLPSKLCAVWIYLDVLFSTASIMHLCAISLDRYVAIQNP
IHHSRFNSRTKAFLKIIAVWTISVGISMPIPVFGLQDDSKVFKEGSCLLADDNFVLIGSF
VSFFIPLTIMVITYFLTIKSLQKEATLCVSDLGTRAKLASFSFLPQSSLSSEKLFQRSIH
REPGSYTGRRTMQSISNEQKACKVLGIVFFLFVVMWCPFFITNIMAVICKESCNEDVIGA
LLNVFVWIGYLSSAVNPLVYTLFNKTYRSAFSRYIQCQYKENKKPLQLILVNTIPALAYK
SSQLQMGQKKNSKQDAKTTDNDCSMVALGKQHSEEASKDNSDGVNEKVSCV
>sp|Q9BXI3|5NT1A_HUMAN Cytosolic 5'-nucleotidase 1A OS=Homo sapiens GN=NT5C1A PE=1 SV=1
MEPGQPREPQEPREPGPGAETAAAPVWEEAKIFYDNLAPKKKPKSPKPQNAVTIAVSSRA
LFRMDEEQQIYTEQGVEEYVRYQLEHENEPFSPGPAFPFVKALEAVNRRLRELYPDSEDV
FDIVLMTNNHAQVGVRLINSINHYDLFIERFCMTGGNSPICYLKAYHTNLYLSADAEKVR
EAIDEGIAAATIFSPSRDVVVSQSQLRVAFDGDAVLFSDESERIVKAHGLDRFFEHEKAH
ENKPLAQGPLKGFLEALGRLQKKFYSKGLRLECPIRTYLVTARSAASSGARALKTLRSWG
LETDEALFLAGAPKGPLLEKIRPHIFFDDQMFHVAGAQEMGTVAAHVPYGVAQTPRRTAP
AKQAPSAQ
>sp|P56378|68MP_HUMAN 6.8 kDa mitochondrial proteolipid OS=Homo sapiens GN=MP68 PE=1 SV=1
MLQSIIKNIWIPMKPYYTKVYQEIWIGMGLMGFIVYKIRAADKRSKALKASAPAPGHH

>sp|P36639|8ODP_HUMAN 7,8-dihydro-8-oxoguanine triphosphatase OS=Homo sapiens GN=NUDT1 PE=1 SV=3
MYWSNQITRRLGERVQGFMSGISPQQMGEPEGSWSGKNPGTMGASRLYTLVLVLQPQRVL
LGMKKRGFGAGRWNGFGGKVQEGETIEDGARRELQEESGLTVDALHKVGQIVFEFVGEPE
LMDVHVFCTDSIQGTPVESDEMRPCWFQLDQIPFKDMWPDDSYWFPLLLQKKKFHGYFKF
QGQDTILDYTLREVDTV
>sp|P02763|A1AG1_HUMAN Alpha-1-acid glycoprotein 1 OS=Homo sapiens GN=ORM1 PE=1 SV=1
MALSWVLTVLSLLPLLEAQIPLCANLVPVPITNATLDQITGKWFYIASAFRNEEYNKSVQ
EIQATFFYFTPNKTEDTIFLREYQTRQDQCIYNTTYLNVQRENGTISRYVGGQEHFAHLL
ILRDTKTYMLAFDVNDEKNWGLSVYADKPETTKEQLGEFYEALDCLRIPKSDVVYTDWKK
DKCEPLEKQHEKERKQEEGES
>sp|P19652|A1AG2_HUMAN Alpha-1-acid glycoprotein 2 OS=Homo sapiens GN=ORM2 PE=1 SV=2
MALSWVLTVLSLLPLLEAQIPLCANLVPVPITNATLDRITGKWFYIASAFRNEEYNKSVQ
EIQATFFYFTPNKTEDTIFLREYQTRQNQCFYNSSYLNVQRENGTVSRYEGGREHVAHLL
FLRDTKTLMFGSYLDDEKNWGLSFYADKPETTKEQLGEFYEALDCLCIPRSDVMYTDWKK
DKCEPLEKQHEKERKQEEGES
>sp|Q96GX2|A7L3B_HUMAN Putative ataxin-7-like protein 3B OS=Homo sapiens GN=ATXN7L3B PE=3 SV=2
MEEISLANLDTNKLEAIAQEIYVDLIEDSCLGFCFEVHRAVKCGYFYLEFAETGSVKDFG
IQPVEDKGACRLPLCSLPGEPGNGPDQQLQRSPPEFQ
>sp|P01011|AACT_HUMAN Alpha-1-antichymotrypsin OS=Homo sapiens GN=SERPINA3 PE=1 SV=2
MERMLPLLALGLLAAGFCPAVLCHPNSPLDEENLTQENQDRGTHVDLGLASANVDFAFSL
YKQLVLKAPDKNVIFSPLSISTALAFLSLGAHNTTLTEILKGLKFNLTETSEAEIHQSFQ
HLLRTLNQSSDELQLSMGNAMFVKEQLSLLDRFTEDAKRLYGSEAFATDFQDSAAAKKLI
NDYVKNGTRGKITDLIKDLDSQTMMVLVNYIFFKAKWEMPFDPQDTHQSRFYLSKKKWVM
VPMMSLHHLTIPYFRDEELSCTVVELKYTGNASALFILPDQDKMEEVEAMLLPETLKRWR
DSLEFREIGELYLPKFSISRDYNLNDILLQLGIEEAFTSKADLSGITGARNLAVSQVVHK
AVLDVFEEGTEASAATAVKITLLSALVETRTIVRFNRPFLMIIVPTDTQNIFFMSKVTNP
KQA
>sp|Q8N5Z0|AADAT_HUMAN Kynurenine/alpha-aminoadipate aminotransferase, mitochondrial OS=Homo sapiens GN=AADAT PE=1 SV=2
MNYARFITAASAARNPSPIRTMTDILSRGPKSMISLAGGLPNPNMFPFKTAVITVENGKT
IQFGEEMMKRALQYSPSAGIPELLSWLKQLQIKLHNPPTIHYPPSQGQMDLCVTSGSQQG
LCKVFEMIINPGDNVLLDEPAYSGTLQSLHPLGCNIINVASDESGIVPDSLRDILSRWKP
EDAKNPQKNTPKFLYTVPNGNNPTGNSLTSERKKEIYELARKYDFLIIEDDPYYFLQFNK
FRVPTFLSMDVDGRVIRADSFSKIISSGLRIGFLTGPKPLIERVILHIQVSTLHPSTFNQ
LMISQLLHEWGEEGFMAHVDRVIDFYSNQKDAILAAADKWLTGLAEWHVPAAGMFLWIKV
KGINDVKELIEEKAVKMGVLMLPGNAFYVDSSAPSPYLRASFSSASPEQMDVAFQVLAQL
IKESL
>sp|Q9UH17|ABC3B_HUMAN DNA dC->dU-editing enzyme APOBEC-3B OS=Homo sapiens GN=APOBEC3B PE=1 SV=1
MNPQIRNPMERMYRDTFYDNFENEPILYGRSYTWLCYEVKIKRGRSNLLWDTGVFRGQVY
FKPQYHAEMCFLSWFCGNQLPAYKCFQITWFVSWTPCPDCVAKLAEFLSEHPNVTLTISA
ARLYYYWERDYRRALCRLSQAGARVTIMDYEEFAYCWENFVYNEGQQFMPWYKFDENYAF

LHRTLKEILRYLMDPDTFTFNFNNDPLVLRRRQTYLCYEVERLDNGTWVLMDQHMGFLCN
EAKNLLCGFYGRHAELRFLDLVPSLQLDPAQIYRVTWFISWSPCFSWGCAGEVRAFLQEN
THVRLRIFAARIYDYDPLYKEALQMLRDAGAQVSIMTYDEFEYCWDTFVYRQGCPFQPWD
GLEEHSQALSGRLRAILQNQGN
>sp|P78363|ABCA4_HUMAN Retinal-specific ATP-binding cassette transporter OS=Homo sapiens GN=ABCA4 PE=1 SV=3
MGFVRQIQLLLWKNWTLRKRQKIRFVVELVWPLSLFLVLIWLRNANPLYSHHECHFPNKA
MPSAGMLPWLQGIFCNVNNPCFQSPTPGESPGIVSNYNNSILARVYRDFQELLMNAPESQ
HLGRIWTELHILSQFMDTLRTHPERIAGRGIRIRDILKDEETLTLFLIKNIGLSDSVVYL
LINSQVRPEQFAHGVPDLALKDIACSEALLERFIIFSQRRGAKTVRYALCSLSQGTLQWI
EDTLYANVDFFKLFRVLPTLLDSRSQGINLRSWGGILSDMSPRIQEFIHRPSMQDLLWVT
RPLMQNGGPETFTKLMGILSDLLCGYPEGGGSRVLSFNWYEDNNYKAFLGIDSTRKDPIY
SYDRRTTSFCNALIQSLESNPLTKIAWRAAKPLLMGKILYTPDSPAARRILKNANSTFEE
LEHVRKLVKAWEEVGPQIWYFFDNSTQMNMIRDTLGNPTVKDFLNRQLGEEGITAEAILN
FLYKGPRESQADDMANFDWRDIFNITDRTLRLVNQYLECLVLDKFESYNDETQLTQRALS
LLEENMFWAGVVFPDMYPWTSSLPPHVKYKIRMDIDVVEKTNKIKDRYWDSGPRADPVED
FRYIWGGFAYLQDMVEQGITRSQVQAEAPVGIYLQQMPYPCFVDDSFMIILNRCFPIFMV
LAWIYSVSMTVKSIVLEKELRLKETLKNQGVSNAVIWCTWFLDSFSIMSMSIFLLTIFIM
HGRILHYSDPFILFLFLLAFSTATIMLCFLLSTFFSKASLAAACSGVIYFTLYLPHILCF
AWQDRMTAELKKAVSLLSPVAFGFGTEYLVRFEEQGLGLQWSNIGNSPTEGDEFSFLLSM
QMMLLDAAVYGLLAWYLDQVFPGDYGTPLPWYFLLQESYWLGGEGCSTREERALEKTEPL
TEETEDPEHPEGIHDSFFEREHPGWVPGVCVKNLVKIFEPCGRPAVDRLNITFYENQITA
FLGHNGAGKTTTLSILTGLLPPTSGTVLVGGRDIETSLDAVRQSLGMCPQHNILFHHLTV
AEHMLFYAQLKGKSQEEAQLEMEAMLEDTGLHHKRNEEAQDLSGGMQRKLSVAIAFVGDA
KVVILDEPTSGVDPYSRRSIWDLLLKYRSGRTIIMSTHHMDEADLLGDRIAIIAQGRLYC
SGTPLFLKNCFGTGLYLTLVRKMKNIQSQRKGSEGTCSCSSKGFSTTCPAHVDDLTPEQV
LDGDVNELMDVVLHHVPEAKLVECIGQELIFLLPNKNFKHRAYASLFRELEETLADLGLS
SFGISDTPLEEIFLKVTEDSDSGPLFAGGAQQKRENVNPRHPCLGPREKAGQTPQDSNVC
SPGAPAAHPEGQPPPEPECPGPQLNTGTQLVLQHVQALLVKRFQHTIRSHKDFLAQIVLP
ATFVFLALMLSIVIPPFGEYPALTLHPWIYGQQYTFFSMDEPGSEQFTVLADVLLNKPGF
GNRCLKEGWLPEYPCGNSTPWKTPSVSPNITQLFQKQKWTQVNPSPSCRCSTREKLTMLP
ECPEGAGGLPPPQRTQRSTEILQDLTDRNISDFLVKTYPALIRSSLKSKFWVNEQRYGGI
SIGGKLPVVPITGEALVGFLSDLGRIMNVSGGPITREASKEIPDFLKHLETEDNIKVWFN
NKGWHALVSFLNVAHNAILRASLPKDRSPEEYGITVISQPLNLTKEQLSEITVLTTSVDA
VVAICVIFSMSFVPASFVLYLIQERVNKSKHLQFISGVSPTTYWVTNFLWDIMNYSVSAG
LVVGIFIGFQKKAYTSPENLPALVALLLLYGWAVIPMMYPASFLFDVPSTAYVALSCANL
FIGINSSAITFILELFENNRTLLRFNAVLRKLLIVFPHFCLGRGLIDLALSQAVTDVYAR
FGEEHSANPFHWDLIGKNLFAMVVEGVVYFLLTLLVQRHFFLSQWIAEPTKEPIVDEDDD
VAEERQRIITGGNKTDILRLHELTKIYPGTSSPAVDRLCVGVRPGECFGLLGVNGAGKTT
TFKMLTGDTTVTSGDATVAGKSILTNISEVHQNMGYCPQFDAIDELLTGREHLYLYARLR
GVPAEEIEKVANWSIKSLGLTVYADCLAGTYSGGNKRKLSTAIALIGCPPLVLLDEPTTG
MDPQARRMLWNVIVSIIREGRAVVLTSHSMEECEALCTRLAIMVKGAFRCMGTIQHLKSK
FGDGYIVTMKIKSPKDDLLPDLNPVEQFFQGNFPGSVQRERHYNMLQFQVSSSSLARIFQ
LLLSHKDSLLIEEYSVTQTTLDQVFVNFAKQQTESHDLPLHPRAAGASRQAQD

>sp|Q8IUA7|ABCA9_HUMAN ATP-binding cassette sub-family A member 9 OS=Homo sapiens GN=ABCA9 PE=1 SV=1
MSKRRMSVGQQTWALLCKNCLKKWRMKRQTLLEWLFSFLLVLFLYLFFSNLHQVHDTPQM
SSMDLGRVDSFNDTNYVIAFAPESKTTQEIMNKVASAPFLKGRTIMGWPDEKSMDELDLN
YSIDAVRVIFTDTFSYHLKFSWGHRIPMMKEHRDHSAHCQAVNEKMKCEGSEFWEKGFVA
FQAAINAAIIEIATNHSVMEQLMSVTGVHMKILPFVAQGGVATDFFIFFCIISFSTFIYY
VSVNVTQERQYITSLMTMMGLRESAFWLSWGLMYAGFILIMATLMALIVKSAQIVVLTGF
VMVFTLFLLYGLSLITLAFLMSVLIKKPFLTGLVVFLLIVFWGILGFPALYTRLPAFLEW
TLCLLSPFAFTVGMAQLIHLDYDVNSNAHLDSSQNPYLIIATLFMLVFDTLLYLVLTLYF
DKILPAEYGHRCSPLFFLKSCFWFQHGRANHVVLENETDSDPTPNDCFEPVSPEFCGKEA
IRIKNLKKEYAGKCERVEALKGVVFDIYEGQITALLGHSGAGKTTLLNILSGLSVPTSGS
VTVYNHTLSRMADIENISKFTGFCPQSNVQFGFLTVKENLRLFAKIKGILPHEVEKEVQR
VVQELEMENIQDILAQNLSGGQNRKLTFGIAILGDPQVLLLDEPTAGLDPLSRHRIWNLL
KEGKSDRVILFSTQFIDEADILADRKVFISNGKLKCAGSSLFLKKKWGIGYHLSLHLNER
CDPESITSLVKQHISDAKLTAQSEEKLVYILPLERTNKFPELYRDLDRCSNQGIEDYGVS
ITTLNEVFLKLEGKSTIDESDIGIWGQLQTDGAKDIGSLVELEQVLSSFHETRKTISGVA
LWRQQVCAIAKVRFLKLKKERKSLWTILLLFGISFIPQLLEHLFYESYQKSYPWELSPNT
YFLSPGQQPQDPLTHLLVINKTGSTIDNFLHSLRRQNIAIEVDAFGTRNGTDDPSYNGAI
IVSGDEKDHRFSIACNTKRLNCFPVLLDVISNGLLGIFNSSEHIQTDRSTFFEEHMDYEY
GYRSNTFFWIPMAASFTPYIAMSSIGDYKKKAHSQLRISGLYPSAYWFGQALVDVSLYFL
ILLLMQIMDYIFSPEEIIFIIQNLLIQILCSIGYVSSLVFLTYVISFIFRNGRKNSGIWS
FFFLIVVIFSIVATDLNEYGFLGLFFGTMLIPPFTLIGSLFIFSEISPDSMDYLGASESE
IVYLALLIPYLHFLIFLFILRCLEMNCRKKLMRKDPVFRISPRSNAIFPNPEEPEGEEED
IQMERMRTVNAMAVRDFDETPVIIASCLRKEYAGKKKNCFSKRKKKIATRNVSFCVKKGE
VIGLLGHNGAGKSTTIKMITGDTKPTAGQVILKGSGGGEPLGFLGYCPQENALWPNLTVR
QHLEVYAAVKGLRKGDAMIAITRLVDALKLQDQLKAPVKTLSEGIKRKLCFVLSILGNPS
VVLLDEPSTGMDPEGQQQMWQVIRATFRNTERGALLTTHYMAEAEAVCDRVAIMVSGRLR
CIGSIQHLKSKFGKDYLLEMKLKNLAQMEPLHAEILRLFPQAAQQERFSSLMVYKLPVED
VRPLSQAFFKLEIVKQSFDLEEYSLSQSTLEQVFLELSKEQELGDLEEDFDPSVKWKLLL
QEEP
>sp|Q8WWZ4|ABCAA_HUMAN ATP-binding cassette sub-family A member 10 OS=Homo sapiens GN=ABCA10 PE=2 SV=3
MNKMALASFMKGRTVIGTPDEETMDIELPKKYHEMVGVIFSDTFSYRLKFNWGYRIPVIK
EHSEYTEHCWAMHGEIFCYLAKYWLKGFVAFQAAINAAIIEVTTNHSVMEELTSVIGINM
KIPPFISKGEIMNEWFHFTCLVSFSSFIYFASLNVARERGKFKKLMTVMGLRESAFWLSW
GLTYICFIFIMSIFMALVITSIPIVFHTGFMVIFTLYSLYGLSLIALAFLMSVLIRKPML
AGLAGFLFTVFWGCLGFTVLYRQLPLSLGWVLSLLSPFAFTAGMAQITHLDNYLSGVIFP
DPSGDSYKMIATFFILAFDTLFYLIFTLYFERVLPDKDGHGDSPLFFLKSSFWSKHQNTH
HEIFENEINPEHSSDDSFEPVSPEFHGKEAIRIRNVIKEYNGKTGKVEALQGIFFDIYEG
QITAILGHNGAGKSTLLNILSGLSVSTEGSATIYNTQLSEITDMEEIRKNIGFCPQFNFQ
FDFLTVRENLRVFAKIKGIQPKEVEQEVKRIIMELDMQSIQDIIAKKLSGGQKRKLTLGI
AILGDPQVLLLDEPTAGLDPFSRHRVWSLLKEHKVDRLILFSTQFMDEADILADRKVFLS
NGKLKCAGSSLFLKRKWGIGYHLSLHRNEMCDTEKITSLIKQHIPDAKLTTESEEKLVYS
LPLEKTNKFPDLYSDLDKCSDQGIRNYAVSVTSLNEVFLNLEGKSAIDEPDFDIGKQEKI

HVTRNTGDESEMEQVLCSLPETRKAVSSAALWRRQIYAVATLRFLKLRRERRALLCLLLV
LGIAFIPIILEKIMYKVTRETHCWEFSPSMYFLSLEQIPKTPLTSLLIVNNTGSNIEDLV
HSLKCQDIVLEIDDFRNRNGSDDPSYNGAIIVSGDQKDYRFSVACNTKKLNCFPVLMGIV
SNALMGIFNFTELIQMESTSFSRDDIVLDLGFIDGSIFLLLITNCVSPFIGMSSISDYKK
NVQSQLWISGLWPSAYWCGQALVDIPLYFLILFSIHLIYYFIFLGFQLSWELMFVLVVCI
IGCAVSLIFLTYVLSFIFRKWRKNNGFWSFGFFIILICVSTIMVSTQYEKLNLILCMIFI
PSFTLLGYVMLLIQLDFMRNLDSLDNRINEVNKTILLTTLIPYLQSVIFLFVIRCLEMKY
GNEIMNKDPVFRISPRSRETHPNPEEPEEEDEDVQAERVQAANALTAPNLEEEPVITASC
LHKEYYETKKSCFSTRKKKIAIRNVSFCVKKGEVLGLLGHNGAGKSTSIKMITGCTKPTA
GVVVLQGSRASVRQQHDNSLKFLGYCPQENSLWPKLTMKEHLELYAAVKGLGKEDAALSI
SRLVEALKLQEQLKAPVKTLSEGIKRKLCFVLSILGNPSVVLLDEPFTGMDPEGQQQMWQ
ILQATVKNKERGTLLTTHYMSEAEAVCDRMAMMVSGTLRCIGSIQHLKNKFGRDYLLEIK
MKEPTQVEALHTEILKLFPQAAWQERYSSLMAYKLPVEDVHPLSRAFFKLEAMKQTFNLE
EYSLSQATLEQVFLELCKEQELGNVDDKIDTTVEWKLLPQEDP
>sp|Q86UQ4|ABCAD_HUMAN ATP-binding cassette sub-family A member 13 OS=Homo sapiens GN=ABCA13 PE=2 SV=3
MGHAGCQFKALLWKNWLCRLRNPVLFLAEFFWPCILFVILTVLRFQEPPRYRDICYLQPR
DLPSCGVIPFVQSLLCNTGSRCRNFSYEGSMEHHFRLSRFQTAADPKKVNNLAFLKEIQD
LAEEIHGMMDKAKNLKRLWVERSNTPDSSYGSSFFTMDLNKTEEVILKLESLHQQPHIWD
FLLLLPRLHTSHDHVEDGMDVAVNLLQTILNSLISLEDLDWLPLNQTFSQVSELVLNVTI
STLTFLQQHGVAVTEPVYHLSMQNIVWDPQKVQYDLKSQFGFDDLHTEQILNSSAELKEI
PTDTSLEKMVCSVLSSTSEDEAEKWGHVGGCHPKWSEAKNYLVHAVSWLRVYQQVFVQWQ
QGSLLQKTLTGMGHSLEALRNQFEEESKPWKVVEALHTALLLLNDSLSADGPKDNHTFPK
ILQHLWKLQSLLQNLPQWPALKRFLQLDGALRNAIAQNLHFVQEVLICLETSANDFKWFE
LNQLKLEKDVFFWELKQMLAKNAVCPNGRFSEKEVFLPPGNSSIWGGLQGLLCYCNSSET
SVLNKLLGSVEDADRILQEVITWHKNMSVLIPEEYLDWQELEMQLSEASLSCTRLFLLLG
ADPSPENDVFSSDCKHQLVSTVIFHTLEKTQFFLEQAYYWKAFKKFIRKTCEVAQYVNMQ
ESFQNRLLAFPEESPCFEENMDWKMISDNYFQFLNNLLKSPTASISRALNFTKHLLMMEK
KLHTLEDEQMNFLLSFVEFFEKLLLPNLFDSSIVPSFHSLPSLTEDILNISSLWTNHLKS
LKRDPSATDAQKLLEFGNEVIWKMQTLGSHWIRKEPKNLLRFIELILFEINPKLLELWAY
GISKGKRAKLENFFTLLNFSVPENEILSTSFNFSQLFHSDWPKSPAMNIDFVRLSEAIIT
SLHEFGFLEQEQISEALNTVYAIRNASDLFSALSEPQKQEVDKILTHIHLNVFQDKDSAL
LLQIYSSFYRYIYELLNIQSRGSSLTFLTQISKHILDIIKQFNFQNISKAFAFLFKTAEV
LGGISNVSYCQQLLSIFNFLELQAQSFMSTEGQELEVIHTTLTGLKQLLIIDEDFRISLF
QYMSQFFNSSVEDLLDNKCLISDNKHISSVNYSTSEESSFVFPLAQIFSNLSANVSVFNK
FMSIHCTVSWLQMWTEIWETISQLFKFDMNVFTSLHHGFTQLLDELEDDVKVSKSCQGIL
PTHNVARLILNLFKNVTQANDFHNWEDFLDLRDFLVALGNALVSVKKLNLEQVEKSLFTM
EAALHQLKTFPFNESTSREFLNSLLEVFIEFSSTSEYIVRNLDSINDFLSNNLTNYGEKF
ENIITELREAIVFLRNVSHDRDLFSCADIFQNVTECILEDGFLYVNTSQRMLRILDTLNS
TFSSENTISSLKGCIVWLDVINHLYLLSNSSFSQGHLQNILGNFRDIENKMNSILKIVTW
VLNIKKPLCSSNGSHINCVNIYLKDVTDFLNIVLTTVFEKEKKPKFEILLALLNDSTKQV
RMSINNLTTDFDFASQSNWRYFTELILRPIEMSDEIPNQFQNIWLHLITLGKEFQKLVKG
IYFNILENNSSSKTENLLNIFATSPKEKDVNSVGNSIYHLASYLAFSLSHDLQNSPKIII
SPEIMKATGLGIQLIRDVFNSLMPVVHHTSPQNAGYMQALKKVTSVMRTLKKADIDLLVD

QLEQVSVNLMDFFKNISSVGTGNLVVNLLVGLMEKFADSSHSWNVNHLLQLSRLFPKDVV
DAVIDVYYVLPHAVRLLQGVPGKNITEGLKDVYSFTLLHGITISNITKEDFAIVIKILLD
TIELVSDKPDIISEALACFPVVWCWNHTNSGFRQNSKIDPCNVHGLMSSSFYGKVASILD
HFHLSPQGEDSPCSNESSRMEITRKVVCIIHELVDWNSILLELSEVFHVNISLVKTVQKF
WHKILPFVPPSINQTRDSISELCPSGSIKQVALQIIEKLKNVNFTKVTSGENILDKLSSL
NKILNINEDTETSVQNIISSNLERTVQLISEDWSLEKSTHNLLSLFMMLQNANVTGSSLE
ALSSFIEKSETPYNFEELWPKFQQIMKDLTQDFRIRHLLSEMNKGIKSINSMALQKITLQ
FAHFLEILDSPSLKTLEIIEDFLLVTKNWLQEYANEDYSRMIETLFIPVTNESSTEDIAL
LAKAIATFWGSLKNISRAGNFDVAFLTHLLNQEQLTNFSVVQLLFENILINLINNLAGNS
QEAAWNLNDTDLQIMNFINLILNHMQSETSRKTVLSLRSIVDFTEQFLKTFFSLFLKEDS
ENKISLLLKYFHKDVIAEMSFVPKDKILEILKLDQFLTLMIQDRLMNIFSSLKETIYHLM
KSSFILDNGEFYFDTHQGLKFMQDLFNALLRETSMKNKTENNIDFFTVVSQLFFHVNKSE
DLFKLNQDLGSALHLVRECSTEMARLLDTILHSPNKDFYALYPTLQEVILANLTDLLFFI
NNSFPLRNRATLEITKRLVGAISRASEESHVLKPLLEMSGTLVMLLNDSADLRDLATSMD
SIVKLLKLVKKVSGKMSTVFKTHFISNTKDSVKFFDTLYSIMQQSVQNLVKEIATLKKID
HFTFEKINDLLVPFLDLAFEMIGVEPYISSNSDIFSMSPSILSYMNQSKDFSDILEEIAE
FLTSVKMNLEDMRSLAVAFNNETQTFSMDSVNLREEILGCLVPINNITNQMDFLYPNPIS
THSGPQDIKWEIIHEVIPFLDKILSQNSTEIGSFLKMVICLTLEALWKNLKKDNWNVSNV
LMTFTQHPNNLLKTIETVLEASSGIKSDYEGDLNKSLYFDTPLSQNITHHQLEKAIHNVL
SRIALWRKGLLFNNSEWITSTRTLFQPLFEIFIKATTGKNVTSEKEERTKKEMIDFPYSF
KPFFCLEKYLGGLFVLTKYWQQIPLTDQSVVEICEVFQQTVKPSEAMEMLQKVKMMVVRV
LTIVAENPSWTKDILCATLSCKQNGIRHLILSAIQGVTLAQDHFQEIEKIWSSPNQLNCE
SLSKNLSSTLESFKSSLENATGQDCTSQPRLETVQQHLYMLAKSLEETWSSGNPIMTFLS
NFTVTEDVKIKDLMKNITKLTEELRSSIQISNETIHSILEANISHSKVLFSALTVALSGK
CDQEILHLLLTFPKGEKSWIAAEELCSLPGSKVYSLIVLLSRNLDVRAFIYKTLMPSEAN
GLLNSLLDIVSSLSALLAKAQHVFEYLPEFLHTFKITALLETLDFQQVSQNVQARSSAFG
SFQFVMKMVCKDQASFLSDSNMFINLPRVKELLEDDKEKFNIPEDSTPFCLKLYQEILQL
PNGALVWTFLKPILHGKILYTPNTPEINKVIQKANYTFYIVDKLKTLSETLLEMSSLFQR
SGSGQMFNQLQEALRNKFVRNFVENQLHIDVDKLTEKLQTYGGLLDEMFNHAGAGRFRFL
GSILVNLSSCVALNRFQALQSVDILETKAHELLQQNSFLASIIFSNSLFDKNFRSESVKL
PPHVSYTIRTNVLYSVRTDVVKNPSWKFHPQNLPADGFKYNYVFAPLQDMIERAIILVQT
GQEALEPAAQTQAAPYPCHTSDLFLNNVGFFFPLIMMLTWMVSVASMVRKLVYEQEIQIE
EYMRMMGVHPVIHFLAWFLENMAVLTISSATLAIVLKTSGIFAHSNTFIVFLFLLDFGMS
VVMLSYLLSAFFSQANTAALCTSLVYMISFLPYIVLLVLHNQLSFVNQTFLCLLSTTAFG
QGVFFITFLEGQETGIQWNNMYQALEQGGMTFGWVCWMILFDSSLYFLCGWYLSNLIPGT
FGLRKPWYFPFTASYWKSVGFLVEKRQYFLSSSLFFFNENFDNKGSSLQNREGELEGSAP
GVTLVSVTKEYEGHKAVVQDLSLTFYRDQITALLGTNGAGKTTIISMLTGLHPPTSGTII
INGKNLQTDLSRVRMELGVCPQQDILLDNLTVREHLLLFASIKAPQWTKKELHQQVNQTL
QDVDLTQHQHKQTRALSGGLKRKLSLGIAFMGMSRTVVLDEPTSGVDPCSRHSLWDILLK
YREGRTIIFTTHHLDEAEALSDRVAVLQHGRLRCCGPPFCLKEAYGQGLRLTLTRQPSVL
EAHDLKDMACVTSLIKIYIPQAFLKDSSGSELTYTIPKDTDKACLKGLFQALDENLHQLH
LTGYGISDTTLEEVFLMLLQDSNKKSHIALGTESELQNHRPTGHLSGYCGSLARPATVQG
VQLLRAQVAAILARRLRRTLRAGKSTLADLLLPVLFVALAMGLFMVRPLATEYPPLRLTP
GHYQRAETYFFSSGGDNLDLTRVLLRKFRDQDLPCADLNPRQKNSSCWRTDPFSHPEFQD

SCGCLKCPNRSASAPYLTNHLGHTLLNLSGFNMEEYLLAPSEKPRLGGWSFGLKIPSEAG
GANGNISKPPTLAKVWYNQKGFHSLPSYLNHLNNLILWQHLPPTVDWRQYGITLYSHPYG
GALLNKDKILESIRQCGVALCIVLGFSILSASIGSSVVRDRVIGAKRLQHISGLGYRMYW
FTNFLYDMLFYLVSVCLCVAVIVAFQLTAFTFRKNLAATALLLSLFGYATLPWMYLMSRI
FSSSDVAFISYVSLNFIFGLCTMLITIMPRLLAIISKAKNLQNIYDVLKWVFTIFPQFCL
GQGLVELCYNQIKYDLTHNFGIDSYVSPFEMNFLGWIFVQLASQGTVLLLLRVLLHWDLL
RWPRGHSTLQGTVKSSKDTDVEKEEKRVFEGRTNGDILVLYNLSKHYRRFFQNIIAVQDI
SLGIPKGECFGLLGVNGAGKSTTFKMLNGEVSLTSGHAIIRTPMGDAVDLSSAGTAGVLI
GYCPQQDALDELLTGWEHLYYYCSLRGIPRQCIPEVAGDLIRRLHLEAHADKPVATYSGG
TKRKLSTALALVGKPDILLLDEPSSGMDPCSKRYLWQTIMKEVREGCAAVLTSHSMEECE
ALCTRLAIMVNGSFKCLGSPQHIKNRFGDGYTVKVWLCKEANQHCTVSDHLKLYFPGIQF
KGQHLNLLEYHVPKRWGCLADLFKVIENNKTFLNIKHYSINQTTLEQVFINFASEQQQTL
QSTLDPSTDSHHTHHLPI
>sp|Q2M3G0|ABCB5_HUMAN ATP-binding cassette sub-family B member 5 OS=Homo sapiens GN=ABCB5 PE=1 SV=4
MENSERAEEMQENYQRNGTAEEQPKLRKEAVGSIEIFRFADGLDITLMILGILASLVNGA
CLPLMPLVLGEMSDNLISGCLVQTNTTNYQNCTQSQEKLNEDMTLLTLYYVGIGVAALIF
GYIQISLWIITAARQTKRIRKQFFHSVLAQDIGWFDSCDIGELNTRMTDDIDKISDGIGD
KIALLFQNMSTFSIGLAVGLVKGWKLTLVTLSTSPLIMASAAACSRMVISLTSKELSAYS
KAGAVAEEVLSSIRTVIAFRAQEKELQRYTQNLKDAKDFGIKRTIASKVSLGAVYFFMNG
TYGLAFWYGTSLILNGEPGYTIGTVLAVFFSVIHSSYCIGAAVPHFETFAIARGAAFHIF
QVIDKKPSIDNFSTAGYKPESIEGTVEFKNVSFNYPSRPSIKILKGLNLRIKSGETVALV
GLNGSGKSTVVQLLQRLYDPDDGFIMVDENDIRALNVRHYRDHIGVVSQEPVLFGTTISN
NIKYGRDDVTDEEMERAAREANAYDFIMEFPNKFNTLVGEKGAQMSGGQKQRIAIARALV
RNPKILILDEATSALDSESKSAVQAALEKASKGRTTIVVAHRLSTIRSADLIVTLKDGML
AEKGAHAELMAKRGLYYSLVMSQDIKKADEQMESMTYSTERKTNSLPLHSVKSIKSDFID
KAEESTQSKEISLPEVSLLKILKLNKPEWPFVVLGTLASVLNGTVHPVFSIIFAKIITMF
GNNDKTTLKHDAEIYSMIFVILGVICFVSYFMQGLFYGRAGEILTMRLRHLAFKAMLYQD
IAWFDEKENSTGGLTTILAIDIAQIQGATGSRIGVLTQNATNMGLSVIISFIYGWEMTFL
ILSIAPVLAVTGMIETAAMTGFANKDKQELKHAGKIATEALENIRTIVSLTREKAFEQMY
EEMLQTQHRNTSKKAQIIGSCYAFSHAFIYFAYAAGFRFGAYLIQAGRMTPEGMFIVFTA
IAYGAMAIGETLVLAPEYSKAKSGAAHLFALLEKKPNIDSRSQEGKKPDTCEGNLEFREV
SFFYPCRPDVFILRGLSLSIERGKTVAFVGSSGCGKSTSVQLLQRLYDPVQGQVLFDGVD
AKELNVQWLRSQIAIVPQEPVLFNCSIAENIAYGDNSRVVPLDEIKEAANAANIHSFIEG
LPEKYNTQVGLKGAQLSGGQKQRLAIARALLQKPKILLLDEATSALDNDSEKVVQHALDK
ARTGRTCLVVTHRLSAIQNADLIVVLHNGKIKEQGTHQELLRNRDIYFKLVNAQSVQ
>sp|Q9NP58|ABCB6_HUMAN ATP-binding cassette sub-family B member 6, mitochondrial OS=Homo sapiens GN=ABCB6 PE=1 SV=1
MVTVGNYCEAEGPVGPAWMQDGLSPCFFFTLVPSTRMALGTLALVLALPCRRRERPAGAD
SLSWGAGPRISPYVLQLLLATLQAALPLAGLAGRVGTARGAPLPSYLLLASVLESLAGAC
GLWLLVVERSQARQRLAMGIWIKFRHSPGLLLLWTVAFAAENLALVSWNSPQWWWARADL
GQQVQFSLWVLRYVVSGGLFVLGLWAPGLRPQSYTLQVHEEDQDVERSQVRSAAQQSTWR
DFGRKLRLLSGYLWPRGSPALQLVVLICLGLMGLERALNVLVPIFYRNIVNLLTEKAPWN
SLAWTVTSYVFLKFLQGGGTGSTGFVSNLRTFLWIRVQQFTSRRVELLIFSHLHELSLRW

HLGRRTGEVLRIADRGTSSVTGLLSYLVFNVIPTLADIIIGIIYFSMFFNAWFGLIVFLC
MSLYLTLTIVVTEWRTKFRRAMNTQENATRARAVDSLLNFETVKYYNAESYEVERYREAI
IKYQGLEWKSSASLVLLNQTQNLVIGLGLLAGSLLCAYFVTEQKLQVGDYVLFGTYIIQL
YMPLNWFGTYYRMIQTNFIDMENMFDLLKEETEVKDLPGAGPLRFQKGRIEFENVHFSYA
DGRETLQDVSFTVMPGQTLALVGPSGAGKSTILRLLFRFYDISSGCIRIDGQDISQVTQA
SLRSHIGVVPQDTVLFNDTIADNIRYGRVTAGNDEVEAAAQAAGIHDAIMAFPEGYRTQV
GERGLKLSGGEKQRVAIARTILKAPGIILLDEATSALDTSNERAIQASLAKVCANRTTIV
VAHRLSTVVNADQILVIKDGCIVERGRHEALLSRGGVYADMWQLQQGQEETSEDTKPQTM
ER
>sp|O95342|ABCBB_HUMAN Bile salt export pump OS=Homo sapiens GN=ABCB11 PE=1 SV=2
MSDSVILRSIKKFGEENDGFESDKSYNNDKKSRLQDEKKGDGVRVGFFQLFRFSSSTDIW
LMFVGSLCAFLHGIAQPGVLLIFGTMTDVFIDYDVELQELQIPGKACVNNTIVWTNSSLN
QNMTNGTRCGLLNIESEMIKFASYYAGIAVAVLITGYIQICFWVIAAARQIQKMRKFYFR
RIMRMEIGWFDCNSVGELNTRFSDDINKINDAIADQMALFIQRMTSTICGFLLGFFRGWK
LTLVIISVSPLIGIGAATIGLSVSKFTDYELKAYAKAGVVADEVISSMRTVAAFGGEKRE
VERYEKNLVFAQRWGIRKGIVMGFFTGFVWCLIFLCYALAFWYGSTLVLDEGEYTPGTLV
QIFLSVIVGALNLGNASPCLEAFATGRAAATSIFETIDRKPIIDCMSEDGYKLDRIKGEI
EFHNVTFHYPSRPEVKILNDLNMVIKPGEMTALVGPSGAGKSTALQLIQRFYDPCEGMVT
VDGHDIRSLNIQWLRDQIGIVEQEPVLFSTTIAENIRYGREDATMEDIVQAAKEANAYNF
IMDLPQQFDTLVGEGGGQMSGGQKQRVAIARALIRNPKILLLDMATSALDNESEAMVQEV
LSKIQHGHTIISVAHRLSTVRAADTIIGFEHGTAVERGTHEELLERKGVYFTLVTLQSQG
NQALNEEDIKDATEDDMLARTFSRGSYQDSLRASIRQRSKSQLSYLVHEPPLAVVDHKST
YEEDRKDKDIPVQEEVEPAPVRRILKFSAPEWPYMLVGSVGAAVNGTVTPLYAFLFSQIL
GTFSIPDKEEQRSQINGVCLLFVAMGCVSLFTQFLQGYAFAKSGELLTKRLRKFGFRAML
GQDIAWFDDLRNSPGALTTRLATDASQVQGAAGSQIGMIVNSFTNVTVAMIIAFSFSWKL
SLVILCFFPFLALSGATQTRMLTGFASRDKQALEMVGQITNEALSNIRTVAGIGKERRFI
EALETELEKPFKTAIQKANIYGFCFAFAQCIMFIANSASYRYGGYLISNEGLHFSYVFRV
ISAVVLSATALGRAFSYTPSYAKAKISAARFFQLLDRQPPISVYNTAGEKWDNFQGKIDF
VDCKFTYPSRPDSQVLNGLSVSISPGQTLAFVGSSGCGKSTSIQLLERFYDPDQGKVMID
GHDSKKVNVQFLRSNIGIVSQEPVLFACSIMDNIKYGDNTKEIPMERVIAAAKQAQLHDF
VMSLPEKYETNVGSQGSQLSRGEKQRIAIARAIVRDPKILLLDEATSALDTESEKTVQVA
LDKAREGRTCIVIAHRLSTIQNADIIAVMAQGVVIEKGTHEELMAQKGAYYKLVTTGSPI
S
>sp|O14678|ABCD4_HUMAN ATP-binding cassette sub-family D member 4 OS=Homo sapiens GN=ABCD4 PE=1 SV=1
MAVAGPAPGAGARPRLDLQFLQRFLQILKVLFPSWSSQNALMFLTLLCLTLLEQFVIYQV
GLIPSQYYGVLGNKDLEGFKTLTFLAVMLIVLNSTLKSFDQFTCNLLYVSWRKDLTEHLH
RLYFRGRAYYTLNVLRDDIDNPDQRISQDVERFCRQLSSMASKLIISPFTLVYYTYQCFQ
STGWLGPVSIFGYFILGTVVNKTLMGPIVMKLVHQEKLEGDFRFKHMQIRVNAEPAAFYR
AGHVEHMRTDRRLQRLLQTQRELMSKELWLYIGINTFDYLGSILSYVVIAIPIFSGVYGD
LSPAELSTLVSKNAFVCIYLISCFTQLIDLSTTLSDVAGYTHRIGQLRETLLDMSLKSQD
CEILGESEWGLDTPPGWPAAEPADTAFLLERVSISAPSSDKPLIKDLSLKISEGQSLLIT
GNTGTGKTSLLRVLGGLWTSTRGSVQMLTDFGPHGVLFLPQKPFFTDGTLREQVIYPLKE
VYPDSGSADDERILRFLELAGLSNLVARTEGLDQQVDWNWYDVLSPGEMQRLSFARLFYL

QPKYAVLDEATSALTEEVESELYRIGQQLGMTFISVGHRQSLEKFHSLVLKLCGGGRWEL
MRIKVE
>sp|Q9UG63|ABCF2_HUMAN ATP-binding cassette sub-family F member 2 OS=Homo sapiens GN=ABCF2 PE=1 SV=2
MPSDLAKKKAAKKKEAAKARQRPRKGHEENGDVVTEPQVAEKNEANGRETTEVDLLTKEL
EDFEMKKAAARAVTGVLASHPNSTDVHIINLSLTFHGQELLSDTKLELNSGRRYGLIGLN
GIGKSMLLSAIGKREVPIPEHIDIYHLTREMPPSDKTPLHCVMEVDTERAMLEKEAERLA
HEDAECEKLMELYERLEELDADKAEMRASRILHGLGFTPAMQRKKLKDFSGGWRMRVALA
RALFIRPFMLLLDEPTNHLDLDACVWLEEELKTFKRILVLVSHSQDFLNGVCTNIIHMHN
KKLKYYTGNYDQYVKTRLELEENQMKRFHWEQDQIAHMKNYIARFGHGSAKLARQAQSKE
KTLQKMMASGLTERVVSDKTLSFYFPPCGKIPPPVIMVQNVSFKYTKDGPCIYNNLEFGI
DLDTRVALVGPNGAGKSTLLKLLTGELLPTDGMIRKHSHVKIGRYHQHLQEQLDLDLSPL
EYMMKCYPEIKEKEEMRKIIGRYGLTGKQQVSPIRNLSDGQKCRVCLAWLAWQNPHMLFL
DEPTNHLDIETIDALADAINEFEGGMMLVSHDFRLIQQVAQEIWVCEKQTITKWPGDILA
YKEHLKSKLVDEEPQLTKRTHNV
>sp|Q8N961|ABTB2_HUMAN Ankyrin repeat and BTB/POZ domain-containing protein 2 OS=Homo sapiens GN=ABTB2 PE=2 SV=2
MAGTYSSTLKTLEDLTLDSGYGAGDSCRSLSLSSSKSNSQALNSSAQQHRGAAWWCYSGS
MNSRHNSWDTVNTVLPEDPEVADLFSRCPRLPELEEFPWTEGDVARVLRKGAGGRRLPQF
SAEAVRRLAGLLRRALIRVAREAQRLSVLHAKCTRFEVQSAVRLVHSWALAESCALAAVK
ALSLYSMSAGDGLRRGKSARCGLTFSVGRFFRWMVDTRISVRIHEYAAISLTACMENLVE
EIRARVMASHSPDGGGAGGGEVSAEALEMVINNDAELWGVLQPYEHLICGKNANGVLSLP
AYFSPYNGGSLGHDERADAYAQLELRTLEQSLLATCVGSISELSDLVSRAMHHMQGRHPL
CPGASPARQARQPPQPITWSPDALHTLYYFLRCPQMESMENPNLDPPRMTLNNERPFMLL
PPLMEWMRVAITYAEHRRSLTVDSGDIRQAARLLLPGLDCEPRQLKPEHCFSSFRRLDAR
AATEKFNQDLGFRMLNCGRTDLINQAIEALGPDGVNTMDDQGMTPLMYACAAGDEAMVQM
LIDAGANLDIQVPSNSPRHPSIHPDSRHWTSLTFAVLHGHISVVQLLLDAGAHVEGSAVN
GGEDSYAETPLQLASAAGNYELVSLLLSRGADPLLSMLEAHGMGSSLHEDMNCFSHSAAH
GHRNVLRKLLTQPQQAKADVLSLEEILAEGVEESDASSQGSGSEGPVRLSRTRTKALQEA
MYYSAEHGYVDITMELRALGVPWKLHIWIESLRTSFSQSRYSVVQSLLRDFSSIREEEYN
EELVTEGLQLMFDILKTSKNDSVIQQLATIFTHCYGSSPIPSIPEIRKTLPARLDPHFLN
NKEMSDVTFLVEGKLFYAHKVLLVTASNRFKTLMTNKSEQDGDSSKTIEISDMKYHIFQM
MMQYLYYGGTESMEIPTTDILELLSAASLFQLDALQRHCEILCSQTLSMESAVNTYKYAK
IHNAPELALFCEGFFLKHMKALLEQDAFRQLIYGRSSKVQGLDPLQDLQNTLAERVHSVY
ITSRV
>sp|Q13085|ACACA_HUMAN Acetyl-CoA carboxylase 1 OS=Homo sapiens GN=ACACA PE=1 SV=2
MDEPSPLAQPLELNQHSRFIIGSVSEDNSEDEISNLVKLDLLEEKEGSLSPASVGSDTLS
DLGISSLQDGLALHIRSSMSGLHLVKQGRDRKKIDSQRDFTVASPAEFVTRFGGNKVIEK
VLIANNGIAAVKCMRSIRRWSYEMFRNERAIRFVVMVTPEDLKANAEYIKMADHYVPVPG
GPNNNNYANVELILDIAKRIPVQAVWAGWGHASENPKLPELLLKNGIAFMGPPSQAMWAL
GDKIASSIVAQTAGIPTLPWSGSGLRVDWQENDFSKRILNVPQELYEKGYVKDVDDGLQA
AEEVGYPVMIKASEGGGGKGIRKVNNADDFPNLFRQVQAEVPGSPIFVMRLAKQSRHLEV
QILADQYGNAISLFGRDCSVQRRHQKIIEEAPATIATPAVFEHMEQCAVKLAKMVGYVSA
GTVEYLYSQDGSFYFLELNPRLQVEHPCTEMVADVNLPAAQLQIAMGIPLYRIKDIRMMY

GVSPWGDSPIDFEDSAHVPCPRGHVIAARITSENPDEGFKPSSGTVQELNFRSNKNVWGY
FSVAAAGGLHEFADSQFGHCFSWGENREEAISNMVVALKELSIRGDFRTTVEYLIKLLET
ESFQMNRIDTGWLDRLIAEKVQAERPDTMLGVVCGALHVADVSLRNSVSNFLHSLERGQV
LPAHTLLNTVDVELIYEGVKYVLKVTRQSPNSYVVIMNGSCVEVDVHRLSDGGLLLSYDG
SSYTTYMKEEVDRYRITIGNKTCVFEKENDPSVMRSPSAGKLIQYIVEDGGHVFAGQCYA
EIEVMKMVMTLTAVESGCIHYVKRPGAALDPGCVLAKMQLDNPSKVQQAELHTGSLPRIQ
STALRGEKLHRVFHYVLDNLVNVMNGYCLPDPFFSSKVKDWVERLMKTLRDPSLPLLELQ
DIMTSVSGRIPPNVEKSIKKEMAQYASNITSVLCQFPSQQIANILDSHAATLNRKSEREV
FFMNTQSIVQLVQRYRSGIRGHMKAVVMDLLRQYLRVETQFQNGHYDKCVFALREENKSD
MNTVLNYIFSHAQVTKKNLLVTMLIDQLCGRDPTLTDELLNILTELTQLSKTTNAKVALR
ARQVLIASHLPSYELRHNQVESIFLSAIDMYGHQFCIENLQKLILSETSIFDVLPNFFYH
SNQVVRMAALEVYVRRAYIAYELNSVQHRQLKDNTCVVEFQFMLPTSHPNRGNIPTLNRM
SFSSNLNHYGMTHVASVSDVLLDNSFTPPCQRMGGMVSFRTFEDFVRIFDEVMGCFSDSP
PQSPTFPEAGHTSLYDEDKVPRDEPIHILNVAIKTDCDIEDDRLAAMFREFTQQNKATLV
DHGIRRLTFLVAQKDFRKQVNYEVDRRFHREFPKFFTFRARDKFEEDRIYRHLEPALAFQ
LELNRMRNFDLTAIPCANHKMHLYLGAAKVEVGTEVTDYRFFVRAIIRHSDLVTKEASFE
YLQNEGERLLLEAMDELEVAFNNTNVRTDCNHIFLNFVPTVIMDPSKIEESVRSMVMRYG
SRLWKLRVLQAELKINIRLTPTGKAIPIRLFLTNESGYYLDISLYKEVTDSRTAQIMFQA
YGDKQGPLHGMLINTPYVTKDLLQSKRFQAQSLGTTYIYDIPEMFRQSLIKLWESMSTQA
FLPSPPLPSDMLTYTELVLDDQGQLVHMNRLPGGNEIGMVAWKMTFKSPEYPEGRDIIVI
GNDITYRIGSFGPQEDLLFLRASELARAEGIPRIYVSANSGARIGLAEEIRHMFHVAWVD
PEDPYKGYRYLYLTPQDYKRVSALNSVHCEHVEDEGESRYKITDIIGKEEGIGPENLRGS
GMIAGESSLAYNEIITISLVTCRAIGIGAYLVRLGQRTIQVENSHLILTGAGALNKVLGR
EVYTSNNQLGGIQIMHNNGVTHCTVCDDFEGVFTVLHWLSYMPKSVHSSVPLLNSKDPID
RIIEFVPTKTPYDPRWMLAGRPHPTQKGQWLSGFFDYGSFSEIMQPWAQTVVVGRARLGG
IPVGVVAVETRTVELSIPADPANLDSEAKIIQQAGQVWFPDSAFKTYQAIKDFNREGLPL
MVFANWRGFSGGMKDMYDQVLKFGAYIVDGLRECCQPVLVYIPPQAELRGGSWVVIDSSI
NPRHMEMYADRESRGSVLEPEGTVEIKFRRKDLVKTMRRVDPVYIHLAERLGTPELSTAE
RKELENKLKEREEFLIPIYHQVAVQFADLHDTPGRMQEKGVISDILDWKTSRTFFYWRLR
RLLLEDLVKKKIHNANPELTDGQIQAMLRRWFVEVEGTVKAYVWDNNKDLAEWLEKQLTE
EDGVHSVIEENIKCISRDYVLKQIRSLVQANPEVAMDSIIHMTQHISPTQRAEVIRILST
MDSPST
>sp|O00763|ACACB_HUMAN Acetyl-CoA carboxylase 2 OS=Homo sapiens GN=ACACB PE=1 SV=3
MVLLLCLSCLIFSCLTFSWLKIWGKMTDSKPITKSKSEANLIPSQEPFPASDNSGETPQR
NGEGHTLPKTPSQAEPASHKGPKDAGRRRNSLPPSHQKPPRNPLSSSDAAPSPELQANGT
GTQGLEATDTNGLSSSARPQGQQAGSPSKEDKKQANIKRQLMTNFILGSFDDYSSDEDSV
AGSSRESTRKGSRASLGALSLEAYLTTGEAETRVPTMRPSMSGLHLVKRGREHKKLDLHR
DFTVASPAEFVTRFGGDRVIEKVLIANNGIAAVKCMRSIRRWAYEMFRNERAIRFVVMVT
PEDLKANAEYIKMADHYVPVPGGPNNNNYANVELIVDIAKRIPVQAVWAGWGHASENPKL
PELLCKNGVAFLGPPSEAMWALGDKIASTVVAQTLQVPTLPWSGSGLTVEWTEDDLQQGK
RISVPEDVYDKGCVKDVDEGLEAAERIGFPLMIKASEGGGGKGIRKAESAEDFPILFRQV
QSEIPGSPIFLMKLAQHARHLEVQILADQYGNAVSLFGRDCSIQRRHQKIVEEAPATIAP
LAIFEFMEQCAIRLAKTVGYVSAGTVEYLYSQDGSFHFLELNPRLQVEHPCTEMIADVNL
PAAQLQIAMGVPLHRLKDIRLLYGESPWGVTPISFETPSNPPLARGHVIAARITSENPDE

GFKPSSGTVQELNFRSSKNVWGYFSVAATGGLHEFADSQFGHCFSWGENREEAISNMVVA
LKELSIRGDFRTTVEYLINLLETESFQNNDIDTGWLDYLIAEKVQAEKPDIMLGVVCGAL
NVADAMFRTCMTDFLHSLERGQVLPADSLLNLVDVELIYGGVKYILKVARQSLTMFVLIM
NGCHIEIDAHRLNDGGLLLSYNGNSYTTYMKEEVDSYRITIGNKTCVFEKENDPTVLRSP
SAGKLTQYTVEDGGHVEAGSSYAEMEVMKMIMTLNVQERGRVKYIKRPGAVLEAGCVVAR
LELDDPSKVHPAEPFTGELPAQQTLPILGEKLHQVFHSVLENLTNVMSGFCLPEPVFSIK
LKEWVQKLMMTLRHPSLPLLELQEIMTSVAGRIPAPVEKSVRRVMAQYASNITSVLCQFP
SQQIATILDCHAATLQRKADREVFFINTQSIVQLVQRYRSGIRGYMKTVVLDLLRRYLRV
EHHFQQAHYDKCVINLREQFKPDMSQVLDCIFSHAQVAKKNQLVIMLIDELCGPDPSLSD
ELISILNELTQLSKSEHCKVALRARQILIASHLPSYELRHNQVESIFLSAIDMYGHQFCP
ENLKKLILSETTIFDVLPTFFYHANKVVCMASLEVYVRRGYIAYELNSLQHRQLPDGTCV
VEFQFMLPSSHPNRMTVPISITNPDLLRHSTELFMDSGFSPLCQRMGAMVAFRRFEDFTR
NFDEVISCFANVPKDTPLFSEARTSLYSEDDCKSLREEPIHILNVSIQCADHLEDEALVP
ILRTFVQSKKNILVDYGLRRITFLIAQEKEFPKFFTFRARDEFAEDRIYRHLEPALAFQL
ELNRMRNFDLTAVPCANHKMHLYLGAAKVKEGVEVTDHRFFIRAIIRHSDLITKEASFEY
LQNEGERLLLEAMDELEVAFNNTSVRTDCNHIFLNFVPTVIMDPFKIEESVRYMVMRYGS
RLWKLRVLQAEVKINIRQTTTGSAVPIRLFITNESGYYLDISLYKEVTDSRSGNIMFHSF
GNKQGPQHGMLINTPYVTKDLLQAKRFQAQTLGTTYIYDFPEMFRQALFKLWGSPDKYPK
DILTYTELVLDSQGQLVEMNRLPGGNEVGMVAFKMRFKTQEYPEGRDVIVIGNDITFRIG
SFGPGEDLLYLRASEMARAEGIPKIYVAANSGARIGMAEEIKHMFHVAWVDPEDPHKGFK
YLYLTPQDYTRISSLNSVHCKHIEEGGESRYMITDIIGKDDGLGVENLRGSGMIAGESSL
AYEEIVTISLVTCRAIGIGAYLVRLGQRVIQVENSHIILTGASALNKVLGREVYTSNNQL
GGVQIMHYNGVSHITVPDDFEGVYTILEWLSYMPKDNHSPVPIITPTDPIDREIEFLPSR
APYDPRWMLAGRPHPTLKGTWQSGFFDHGSFKEIMAPWAQTVVTGRARLGGIPVGVIAVE
TRTVEVAVPADPANLDSEAKIIQQAGQVWFPDSAYKTAQAVKDFNREKLPLMIFANWRGF
SGGMKDMYDQVLKFGAYIVDGLRQYKQPILIYIPPYAELRGGSWVVIDATINPLCIEMYA
DKESRGGVLEPEGTVEIKFRKKDLIKSMRRIDPAYKKLMEQLGEPDLSDKDRKDLEGRLK
AREDLLLPIYHQVAVQFADFHDTPGRMLEKGVISDILEWKTARTFLYWRLRRLLLEDQVK
QEILQASGELSHVHIQSMLRRWFVETEGAVKAYLWDNNQVVVQWLEQHWQAGDGPRSTIR
ENITYLKHDSVLKTIRGLVEENPEVAVDCVIYLSQHISPAERAQVVHLLSTMDSPAST
>sp|Q8NCV1|ADAD2_HUMAN Adenosine deaminase domain-containing protein 2 OS=Homo sapiens GN=ADAD2 PE=2 SV=1
MASASQGADDDGSRRKPRLAASLQISPQPRPWRPLPAQAQSAWGPAPAPATYRAEGGWPQ
VSVLRDSGPGAGAGVGELGAARAWENLGEQMGKAPRVPVPPAGLSLPLKDPPASQAVSLL
TEYAASLGIFLLFREDQPPGPCFPFSVSAELDGVVCPAGTANSKTEAKQQAALSALCYIR
SQLENPESPQTSSRPPLAPLSVENILTHEQRCAALVSAGFDLLLDERSPYWACKGTVAGV
ILEREIPRARGHVKEIYKLVALGTGSSCCAGWLEFSGQQLHDCHGLVIARRALLRFLFRQ
LLLATQGGPKGKEQSVLAPQPGPGPPFTLKPRVFLHLYISNTPKGAARDIYLPPTSEGGL
PHSPPMRLQAHVLGQLKPVCYVAPSLCDTHVGCLSASDKLARWAVLGLGGALLAHLVSPL
YSTSLILADSCHDPPTLSRAIHTRPCLDSVLGPCLPPPYVRTALHLFAGPPVAPSEPTPD
TCRGLSLNWSLGDPGIEVVDVATGRVKANAALGPPSRLCKASFLRAFHQAARAVGKPYLL
ALKTYEAAKAGPYQEARRQLSLLLDQQGLGAWPSKPLVGKFRN
>sp|Q9H2U9|ADAM7_HUMAN Disintegrin and metalloproteinase domain-containing protein 7 OS=Homo sapiens GN=ADAM7 PE=1 SV=3

MLPGCIFLMILLIPQVKEKFILGVEGQQLVRPKKLPLIQKRDTGHTHDDDILKTYEEELL
YEIKLNRKTLVLHLLRSREFLGSNYSETFYSMKGEAFTRHPQIMDHCFYQGSIVHEYDSA
ASISTCNGLRGFFRINDQRYLIEPVKYSDEGEHLVFKYNLRVPYGANYSCTELNFTRKTV
PGDNESEEDSKIKGIHDEKYVELFIVADDTVYRRNGHPHNKLRNRIWGMVNFVNMIYKTL
NIHVTLVGIEIWTHEDKIELYSNIETTLLRFSFWQEKILKTRKDFDHVVLLSGKWLYSHV
QGISYPGGMCLPYYSTSIIKDLLPDTNIIANRMAHQLGHNLGMQHDEFPCTCPSGKCVMD
SDGSIPALKFSKCSQNQYHQYLKDYKPTCMLNIPFPYNFHDFQFCGNKKLDEGEECDCGP
AQECTNPCCDAHTCVLKPGFTCAEGECCESCQIKKAGSICRPAKDECDFPEMCTGHSPAC
PKDQFRVNGFPCKNSEGYCFMGKCPTREDQCSELFDDEAIESHDICYKMNTKGNKFGYCK
NKENRFLPCEEKDVRCGKIYCTGGELSSLLGEDKTYHLKDPQKNATVKCKTIFLYHDSTD
IGLVASGTKCGEGMVCNNGECLNMEKVYISTNCPSQCNENPVDGHGLQCHCEEGQAPVAC
EETLHVTNITILVVVLVLVIVGIGVLILLVRYRKCIKLKQVQSPPTETLGVENKGYFGDE
QQIRTEPILPEIHFLNKPASKDSRGIADPNQSAK
>sp|P78325|ADAM8_HUMAN Disintegrin and metalloproteinase domain-containing protein 8 OS=Homo sapiens GN=ADAM8 PE=1 SV=2
MRGLGLWLLGAMMLPAIAPSRPWALMEQYEVVLPWRLPGPRVRRALPSHLGLHPERVSYV
LGATGHNFTLHLRKNRDLLGSGYTETYTAANGSEVTEQPRGQDHCFYQGHVEGYPDSAAS
LSTCAGLRGFFQVGSDLHLIEPLDEGGEGGRHAVYQAEHLLQTAGTCGVSDDSLGSLLGP
RTAAVFRPRPGDSLPSRETRYVELYVVVDNAEFQMLGSEAAVRHRVLEVVNHVDKLYQKL
NFRVVLVGLEIWNSQDRFHVSPDPSVTLENLLTWQARQRTRRHLHDNVQLITGVDFTGTT
VGFARVSAMCSHSSGAVNQDHSKNPVGVACTMAHEMGHNLGMDHDENVQGCRCQERFEAG
RCIMAGSIGSSFPRMFSDCSQAYLESFLERPQSVCLANAPDLSHLVGGPVCGNLFVERGE
QCDCGPPEDCRNRCCNSTTCQLAEGAQCAHGTCCQECKVKPAGELCRPKKDMCDLEEFCD
GRHPECPEDAFQENGTPCSGGYCYNGACPTLAQQCQAFWGPGGQAAEESCFSYDILPGCK
ASRYRADMCGVLQCKGGQQPLGRAICIVDVCHALTTEDGTAYEPVPEGTRCGPEKVCWKG
RCQDLHVYRSSNCSAQCHNHGVCNHKQECHCHAGWAPPHCAKLLTEVHAASGSLPVFVVV
VLVLLAVVLVTLAGIIVYRKARSRILSRNVAPKTTMGRSNPLFHQAASRVPAKGGAPAPS
RGPQELVPTTHPGQPARHPASSVALKRPPPAPPVTVSSPPFPVPVYTRQAPKQVIKPTFA
PPVPPVKPGAGAANPGPAEGAVGPKVALKPPIQRKQGAGAPTAP
>sp|Q9NPF8|ADAP2_HUMAN Arf-GAP with dual PH domain-containing protein 2 OS=Homo sapiens GN=ADAP2 PE=1 SV=1
MGDRERNKKRLLELLRAPDTGNAHCADCGAADPDWASYKLGIFICLNCCGVHRNFPDISR
VKSVRLDFWDDSIVEFMIHNGNLRVKAKFEARVPAFYYIPQANDCLVLKEQWIRAKYERR
EFMADGETISLPGNREGFLWKRGRDNSQFLRRKFVLLAREGLLKYFTKEQGKSPKAVISI
KDLNATFQTEKIGHPHGLQITYRRDGHTRNLFVYHESGKEIVDWFNALRAARLQYLKMAF
PELPESELVPFLTRNYLKQGFMEKTGPKQKEPFKKRWFALDCHERRLLYYKNPLDAFEQG
QVFLGNKEQGYEAYEDLPKGIRGNRWKAGLTIVTPERRFVLTCPSEKEQQEWLESLRGVL
SSPLTPLNRLTASTESGRSSR
>sp|A8MUL3|ADAS1_HUMAN Putative uncharacterized protein ADARB2-AS1 OS=Homo sapiens GN=ADARB2-AS1 PE=5 SV=2
MKQLFPPPPGTSLTHALGAWRGRERAQAATSLLASSASQFPTAVEDALMSVLTSHCAPST
PAATRAQQTGTRGHIHPACPCQQSCVGASRPPGRPQIFLPLTTALSLEAYAADTCSAADF
LHNPSSWGKVWYLNEASFDLYSYHYFW

>sp|Q5VUY0|ADCL3_HUMAN Arylacetamide deacetylase-like 3 OS=Homo sapiens GN=AADACL3 PE=2 SV=4
MIFEKLRICSMPQFFCFMQDLPPLKYDPDVVVTDFRFGTIPVKLYQSKASTCTLKPGIVY
YHGGGGVMGSLKTHHGICSRLCKESDSVVLAVGYRKLPKHKFPVPVRDCLVATIHFLKSL
DAYGVDPARVVVCGDSFGGAIAAVVCQQLVDRPDLPRIRAQILIYAILQALDLQTPSFQQ
RKNIPLLTWSFICYCFFQNLDFSSSWQEVIMKGAHLPAEVWEKYRKWLGPENIPERFKER
GYQLKPHEPMNEAAYLEVSVVLDVMCSPLIAEDDIVSQLPETCIVSCEYDALRDNSLLYK
KRLEDLGVPVTWHHMEDGFHGVLRTIDMSFLHFPCSMRILSALVQFVKGL
>sp|P40145|ADCY8_HUMAN Adenylate cyclase type 8 OS=Homo sapiens GN=ADCY8 PE=1 SV=1
MELSDVRCLTGSEELYTIHPTPPAGDGRSASRPQRLLWQTAVRHITEQRFIHGHRGGSGS
GSGGSGKASDPAGGGPNHHAPQLSGDSALPLYSLGPGERAHSTCGTKVFPERSGSGSASG
SGGGGDLGFLHLDCAPSNSDFFLNGGYSYRGVIFPTLRNSFKSRDLERLYQRYFLGQRRK
SEVVMNVLDVLTKLTLLVLHLSLASAPMDPLKGILLGFFTGIEVVICALVVVRKDTTSHT
YLQYSGVVTWVAMTTQILAAGLGYGLLGDGIGYVLFTLFATYSMLPLPLTWAILAGLGTS
LLQVILQVVIPRLAVISINQVVAQAVLFMCMNTAGIFISYLSDRAQRQAFLETRRCVEAR
LRLETENQRQERLVLSVLPRFVVLEMINDMTNVEDEHLQHQFHRIYIHRYENVSILFADV
KGFTNLSTTLSAQELVRMLNELFARFDRLAHEHHCLRIKILGDCYYCVSGLPEPRQDHAH
CCVEMGLSMIKTIRYVRSRTKHDVDMRIGIHSGSVLCGVLGLRKWQFDVWSWDVDIANKL
ESGGIPGRIHISKATLDCLNGDYNVEEGHGKERNEFLRKHNIETYLIKQPEDSLLSLPED
IVKESVSSSDRRNSGATFTEGSWSPELPFDNIVGKQNTLAALTRNSINLLPNHLAQALHV
QSGPEEINKRIEHTIDLRSGDKLRREHIKPFSLMFKDSSLEHKYSQMRDEVFKSNLVCAF
IVLLFITAIQSLLPSSRVMPMTIQFSILIMLHSALVLITTAEDYKCLPLILRKTCCWINE
TYLARNVIIFASILINFLGAILNILWCDFDKSIPLKNLTFNSSAVFTDICSYPEYFVFTG
VLAMVTCAVFLRLNSVLKLAVLLIMIAIYALLTETVYAGLFLRYDNLNHSGEDFLGTKEV
SLLLMAMFLLAVFYHGQQLEYTARLDFLWRVQAKEEINEMKELREHNENMLRNILPSHVA
RHFLEKDRDNEELYSQSYDAVGVMFASIPGFADFYSQTEMNNQGVECLRLLNEIIADFDE
LLGEDRFQDIEKIKTIGSTYMAVSGLSPEKQQCEDKWGHLCALADFSLALTESIQEINKH
SFNNFELRIGISHGSVVAGVIGAKKPQYDIWGKTVNLASRMDSTGVSGRIQVPEETYLIL
KDQGFAFDYRGEIYVKGISEQEGKIKTYFLLGRVQPNPFILPPRRLPGQYSLAAVVLGLV
QSLNRQRQKQLLNENNNTGIIKGHYNRRTLLSPSGTEPGAQAEGTDKSDLP
>sp|P35611|ADDA_HUMAN Alpha-adducin OS=Homo sapiens GN=ADD1 PE=1 SV=2
MNGDSRAAVVTSPPPTTAPHKERYFDRVDENNPEYLRERNMAPDLRQDFNMMEQKKRVSM
ILQSPAFCEELESMIQEQFKKGKNPTGLLALQQIADFMTTNVPNVYPAAPQGGMAALNMS
LGMVTPVNDLRGSDSIAYDKGEKLLRCKLAAFYRLADLFGWSQLIYNHITTRVNSEQEHF
LIVPFGLLYSEVTASSLVKINLQGDIVDRGSTNLGVNQAGFTLHSAIYAARPDVKCVVHI
HTPAGAAVSAMKCGLLPISPEALSLGEVAYHDYHGILVDEEEKVLIQKNLGPKSKVLILR
NHGLVSVGESVEEAFYYIHNLVVACEIQVRTLASAGGPDNLVLLNPEKYKAKSRSPGSPV
GEGTGSPPKWQIGEQEFEALMRMLDNLGYRTGYPYRYPALREKSKKYSDVEVPASVTGYS
FASDGDSGTCSPLRHSFQKQQREKTRWLNSGRGDEASEEGQNGSSPKSKTKWTKEDGHRT
STSAVPNLFVPLNTNPKEVQEMRNKIREQNLQDIKTAGPQSQVLCGVVMDRSLVQGELVT
ASKAIIEKEYQPHVIVSTTGPNPFTTLTDRELEEYRREVERKQKGSEENLDEAREQKEKS
PPDQPAVPHPPPSTPIKLEEDLVPEPTTGDDSDAATFKPTLPDLSPDEPSEALGFPMLEK
EEEAHRPPSPTEAPTEASPEPAPDPAPVAEEAAPSAVEEGAAADPGSDGSPGKSPSKKKK
KFRTPSFLKKSKKKSDS

>sp|Q9BQI0|AIF1L_HUMAN Allograft inflammatory factor 1-like OS=Homo sapiens GN=AIF1L PE=1 SV=1
MSGELSNRFQGGKAFGLLKARQERRLAEINREFLCDQKYSDEENLPEKLTAFKEKYMEFD
LNNEGEIDLMSLKRMMEKLGVPKTHLEMKKMISEVTGGVSDTISYRDFVNMMLGKRSAVL
KLVMMFEGKANESSPKPVGPPPERDIASLP
>sp|P55008|AIF1_HUMAN Allograft inflammatory factor 1 OS=Homo sapiens GN=AIF1 PE=1 SV=1
MSQTRDLQGGKAFGLLKAQQEERLDEINKQFLDDPKYSSDEDLPSKLEGFKEKYMEFDLN
GNGDIDIMSLKRMLEKLGVPKTHLELKKLIGEVSSGSGETFSYPDFLRMMLGKRSAILKM
ILMYEEKAREKEKPTGPPAKKAISELP
>sp|O95831|AIFM1_HUMAN Apoptosis-inducing factor 1, mitochondrial OS=Homo sapiens GN=AIFM1 PE=1 SV=1
MFRCGGLAAGALKQKLVPLVRTVCVRSPRQRNRLPGNLFQRWHVPLELQMTRQMASSGAS
GGKIDNSVLVLIVGLSTVGAGAYAYKTMKEDEKRYNERISGLGLTPEQKQKKAALSASEG
EEVPQDKAPSHVPFLLIGGGTAAFAAARSIRARDPGARVLIVSEDPELPYMRPPLSKELW
FSDDPNVTKTLRFKQWNGKERSIYFQPPSFYVSAQDLPHIENGGVAVLTGKKVVQLDVRD
NMVKLNDGSQITYEKCLIATGGTPRSLSAIDRAGAEVKSRTTLFRKIGDFRSLEKISREV
KSITIIGGGFLGSELACALGRKARALGTEVIQLFPEKGNMGKILPEYLSNWTMEKVRREG
VKVMPNAIVQSVGVSSGKLLIKLKDGRKVETDHIVAAVGLEPNVELAKTGGLEIDSDFGG
FRVNAELQARSNIWVAGDAACFYDIKLGRRRVEHHDHAVVSGRLAGENMTGAAKPYWHQS
MFWSDLGPDVGYEAIGLVDSSLPTVGVFAKATAQDNPKSATEQSGTGIRSESETESEASE
ITIPPSTPAVPQAPVQGEDYGKGVIFYLRDKVVVGIVLWNIFNRMPIARKIIKDGEQHED
LNEVAKLFNIHED
>sp|C9JRZ8|AK1BF_HUMAN Aldo-keto reductase family 1 member B15 OS=Homo sapiens GN=AKR1B15 PE=1 SV=2
MATFVELSTKAKMPIVGLGTWRSLLGKVKEAVKVAIDAEYRHIDCAYFYENQHEVGEAIQ
EKIQEKAVMREDLFIVSKVWPTFFERPLVRKAFEKTLKDLKLSYLDVYLIHWPQGFKTGD
DFFPKDDKGNMISGKGTFLDAWEAMEELVDEGLVKALGVSNFNHFQIERLLNKPGLKYKP
VTNQVECHPYLTQEKLIQYCHSKGITVTAYSPLGSPDRPWAKPEDPSLLEDPKIKEIAAK
HKKTTAQVLIRFHIQRNVTVIPKSMTPAHIVENIQVFDFKLSDEEMATILSFNRNWRAFD
FKEFSHLEDFPFDAEY
>sp|P42330|AK1C3_HUMAN Aldo-keto reductase family 1 member C3 OS=Homo sapiens GN=AKR1C3 PE=1 SV=4
MDSKHQCVKLNDGHFMPVLGFGTYAPPEVPRSKALEVTKLAIEAGFRHIDSAHLYNNEEQ
VGLAIRSKIADGSVKREDIFYTSKLWSTFHRPELVRPALENSLKKAQLDYVDLYLIHSPM
SLKPGEELSPTDENGKVIFDIVDLCTTWEAMEKCKDAGLAKSIGVSNFNRRQLEMILNKP
GLKYKPVCNQVECHPYFNRSKLLDFCKSKDIVLVAYSALGSQRDKRWVDPNSPVLLEDPV
LCALAKKHKRTPALIALRYQLQRGVVVLAKSYNEQRIRQNVQVFEFQLTAEDMKAIDGLD
RNLHYFNSDSFASHPNYPYSDEY
>sp|Q9UKA4|AKA11_HUMAN A-kinase anchor protein 11 OS=Homo sapiens GN=AKAP11 PE=1 SV=1
MATFRNNHMKTKASVRKSFSEDVFQSVKSLLQSQKELCSVTAEDCLQQDEHANLTEVTFL
GFNEETDAAHIQDLAAVSLELPDILNSLHFCSLNENEIICMKNINKPLDISSDPLNQSHP
SGMLCVMRVSPTSPRLRIDFIFSLLSKYATGIRYTLDTFLHQKHQLETTDEDDDDTNQSV
SSIEDDFVTAFEHLEEEETSKPYNDGMNITVLRSQCDAASQTVTGHHLETHDLKILISSG
QQKSLAKPSTSSVNVLGHKELPSVKTSVTTSISEPWTQRSFYRSSNASDKDSDLQKTFFS

SSPAYSSESECSSPSPVIFLDEEGYQKSLKAKLELPKIPVMKDDIEDSDSEVSEFFDSFD
QFDELEQTLETCLFNKDPVIGKSSQRKGHKHGKSCMNPQKFKFDRPALPANVRKPTPRKP
ESPYGNLCDAPDSPRPVKASREDSGLFSPIRSSAFSPLGGCTPAECFCQTDIGGDRIHEN
HDSVYYTYEDYAKSISCEVLGSVLRTHHTNTLSNINSIKHGENKTVTFKHGNLDQKNKSK
NKSLMIKDSIQKFAADLVEKSFGSAFKDLQKGVSSCTNALYHLAIKLTSSVLQMAFDELR
RQRAFSLKERAISGLANFLVSEALSNALKDLQYVKKQIFTNTVARFAADLAEELVFEGIM
EVCQFSYPQTPASPQCGSFDFEDKVVKLYAKDLSESVIQEAFIELSQVDVTFTTKAAVSV
STDNIKYVSAESVVPSTQAVTFSPSFHNQAIMVTKPVQEYKKEYTVQQALFCTSGIVTSI
PVPLAGSALLPYHISSTACQAKAHLSSDDSNSNGDSAQVHIATKNREEKAACLRNICLPS
EHNPGNQNDFKPTNDDIEMQSSSKLPNDPAIISNFSAAVVHTIVNETLESMTSLEVTKMV
DERTDYLTKSLKEKTPPFSHCDQAVLQCSEASSNKDMFADRLSKSIIKHSIDKSKSVIPN
IDKNAVYKESLPVSGEESQLTPEKSPKFPDSQNQLTHCSLSAAKDCVPECKVSMVHGSSL
ETLPSCPAVTGQKSDLKESAKDQPLKKHNLNSTSLEALSFGQENPFPHSHTFSSTALTCV
DGLHVEDKQKVRDRNVIPDTPPSTPLVPSRASSEWDIKKLTKKLKGELAKEFAPATPPST
PHNSSVGSLSENEQNTIEKEEFMLKLMRSLSEEVESSESGELPEVDVKSEHSGKKVQFAE
ALATHILSLATEMAASHLDNKIIQEPKVKNPCLNVQSQRSVSPTFLNPSDENLKTLCNFA
GDLAAEVITEAEKIAKVRNCMLFKQKKNSCYADGDEDYKVEEKLDIEAVVHPREVDPFIL
SLPPSSCMSGLMYKYPSCESVTDEYAGHLIQILKQEGGNSELIMDQYANRLAYRSVKSGL
QEAAKTTKVQCNSRMFPVPSSQVKTNKELLMFSNKEHHQEADKKRQSKRNEGYFCKNQTC
ERTLDPYRNEVSQLYSFSTSLVHSITKDAKEELTASLVGLPKSLTDSCLFEKSGYEEDNE
CHVTPELPKSLQPSSQNHRFYHSTGSLNGYGCGDNVVQAVEQYAKKVVDDTLELTLGSTV
FRVSETTKSADRVTYAEKLSPLTGQACRYCDLKELHNCTGNSSQHFFRQGSLASSKPASN
PKFSSRYQKSRIFHLSVPQIHVNLDKKAVLAEKIVAEAIEKAERELSSTSLAADSGIGQE
GASFAESLATETMTAAVTNVGHAVSSSKEIEDFQSTESVSSQQMNLSIGDDSTGSWSNLS
FEDEHQDESSSFHHLSESNGNSSSWSSLGLEGDLYEDNLSFPTSDSDGPDDKDEEHEDEV
EGLGQDGKTLLITNIDMEPCTVDPQLRIILQWLIASEAEVAELYFHDSANKEFMLLSKQL
QEKGWKVGDLLQAVLQYYEVMEKASSEERCKSLFDWLLENA
>sp|Q02952|AKA12_HUMAN A-kinase anchor protein 12 OS=Homo sapiens GN=AKAP12 PE=1 SV=4
MGAGSSTEQRSPEQPPEGSSTPAEPEPSGGGPSAEAAPDTTADPAIAASDPATKLLQKNG
QLSTINGVAEQDELSLQEGDLNGQKGALNGQGALNSQEEEEVIVTEVGQRDSEDVSKRDS
DKEMATKSAVVHDITDDGQEETPEIIEQIPSSESNLEELTQPTESQANDIGFKKVFKFVG
FKFTVKKDKTEKPDTVQLLTVKKDEGEGAAGAGDHKDPSLGAGEAASKESEPKQSTEKPE
ETLKREQSHAEISPPAESGQAVEECKEEGEEKQEKEPSKSAESPTSPVTSETGSTFKKFF
TQGWAGWRKKTSFRKPKEDEVEASEKKKEQEPEKVDTEEDGKAEVASEKLTASEQAHPQE
PAESAHEPRLSAEYEKVELPSEEQVSGSQGPSEEKPAPLATEVFDEKIEVHQEEVVAEVH
VSTVEERTEEQKTEVEETAGSVPAEELVEMDAEPQEAEPAKELVKLKETCVSGEDPTQGA
DLSPDEKVLSKPPEGVVSEVEMLSSQERMKVQGSPLKKLFTSTGLKKLSGKKQKGKRGGG
DEESGEHTQVPADSPDSQEEQKGESSASSPEEPEEITCLEKGLAEVQQDGEAEEGATSDG
EKKREGVTPWASFKKMVTPKKRVRRPSESDKEDELDKVKSATLSSTESTASEMQEEMKGS
VEEPKPEEPKRKVDTSVSWEALICVGSSKKRARRGSSSDEEGGPKAMGGDHQKADEAGKD
KETGTDGILAGSQEHDPGQGSSSPEQAGSPTEGEGVSTWESFKRLVTPRKKSKSKLEEKS
EDSIAGSGVEHSTPDTEPGKEESWVSIKKFIPGRRKKRPDGKQEQAPVEDAGPTGANEDD
SDVPAVVPLSEYDAVEREKMEAQQAQKSAEQPEQKAATEVSKELSESQVHMMAAAVADGT
RAATIIEERSPSWISASVTEPLEQVEAEAALLTEEVLEREVIAEEEPPTVTEPLPENREA

RGDTVVSEAELTPEAVTAAETAGPLGAEEGTEASAAEETTEMVSAVSQLTDSPDTTEEAT
PVQEVEGGVPDIEEQERRTQEVLQAVAEKVKEESQLPGTGGPEDVLQPVQRAEAERPEEQ
AEASGLKKETDVVLKVDAQEAKTEPFTQGKVVGQTTPESFEKAPQVTESIESSELVTTCQ
AETLAGVKSQEMVMEQAIPPDSVETPTDSETDGSTPVADFDAPGTTQKDEIVEIHEENEV
ASGTQSGGTEAEAVPAQKERPPAPSSFVFQEETKEQSKMEDTLEHTDKEVSVETVSILSK
TEGTQEADQYADEKTKDVPFFEGLEGSIDTGITVSREKVTEVALKGEGTEEAECKKDDAL
ELQSHAKSPPSPVEREMVVQVEREKTEAEPTHVNEEKLEHETAVTVSEEVSKQLLQTVNV
PIIDGAKEVSSLEGSPPPCLGQEEAVCTKIQVQSSEASFTLTAAAEEEKVLGETANILET
GETLEPAGAHLVLEEKSSEKNEDFAAHPGEDAVPTGPDCQAKSTPVIVSATTKKGLSSDL
EGEKTTSLKWKSDEVDEQVACQEVKVSVAIEDLEPENGILELETKSSKLVQNIIQTAVDQ
FVRTEETATEMLTSELQTQAHVIKADSQDAGQETEKEGEEPQASAQDETPITSAKEESES
TAVGQAHSDISKDMSEASEKTMTVEVEGSTVNDQQLEEVVLPSEEEGGGAGTKSVPEDDG
HALLAERIEKSLVEPKEDEKGDDVDDPENQNSALADTDASGGLTKESPDTNGPKQKEKED
AQEVELQEGKVHSESDKAITPQAQEELQKQERESAKSELTES
>sp|O43687|AKA7A_HUMAN A-kinase anchor protein 7 isoforms alpha and beta OS=Homo sapiens GN=AKAP7 PE=1 SV=4
MGQLCCFPFSRDEGKISELESSSSAVLQRYSKDIPSWSSGEKNGGEPDDAELVRLSKRLV
ENAVLKAVQQYLEETQNKNKPGEGSSVKTEAADQNGNDNENNRK
>sp|Q9Y2D5|AKAP2_HUMAN A-kinase anchor protein 2 OS=Homo sapiens GN=AKAP2 PE=1 SV=3
MEIEVSVAECKSVPGITSTPHPMDHPSAFYSPPHNGLLTDHHESLDNDVAREIRYLDEVL
EANCCDSAVDGTYNGTSSPEPGAVVLVGGLSPPVHEATQPEPTERTASRQAPPHIELSNS
SPDPMAEAERTNGHSPSQPRDALGDSLQVPVSPSSTTSSRCSSRDGEFTLTTLKKEAKFE
LRAFHEDKKPSKLFEDDEHEKEQYCIRKVRPSEEMLELEKERRELIRSQAVKKNPGIAAK
WWNPPQEKTIEEQLDEEHLESHKKYKERKERRAQQEQLLLQKQLQQQQQPPSQLCTAPA
SSHERASMIDKAKEDIVTEQIDFSAARKQFQLMENSRQAVAKGQSTPRLFSIKPFYRPLG
SVNSDKPLTNPRPPSVGGPPEDSGASAAKGQKSPGALETPSAAGSQGNTASQGKEGPYSE
PSKRGPLSKLWAEDGEFTSARAVLTVVKDDDHGILDQFSRSVNVSLTQEELDSGLDELSV
RSQDTTVLETLSNDFSMDNISDSGASNETTNALQENSLADFSLPQTPQTDNPSEGRGEGV
SKSFSDHGFYSPSSTLGDSPLVDDPLEYQAGLLVQNAIQQAIAEQVDKAVSKTSRDGAEQ
QGPEATVEEAEAAAFGSEKPQSMFEPPQVSSPVQEKRDVLPKILPAEDRALRERGPPQPL
PAVQPSGPINMEETRPEGSYFSKYSEAAELRSTASLLATQESDVMVGPFKLRSRKQRTLS
MIEEEIRAAQEREEELKRQRQVLQSTQSPRTKNAPSLPSRTCYKTAPGKIEKVKPPPSPT
TEGPSLQPDLAPEEAAGTQRPKNLMQTLMEDYETHKSKRRERMDDSSVLEATRVNRRKSA
LALRWEAGIYANQEEEDNE
>sp|Q495B1|AKD1A_HUMAN Ankyrin repeat and death domain-containing protein 1A OS=Homo sapiens GN=ANKDD1A PE=2 SV=2
MQEELAWETDGLLPLERQLHEAARQNNVGRMQELIGRRVNTRARNHVGRVALHWAAGAGH
EQAVRLLLEHEAAVDEEDAVGALTEARLCFGMNALLLSAWFGHLRILQILVNSGAKIHCE
SKDGLTLLHCAAQKGHVPVLAFIMEDLEDVALDHVDKLGRTAFHRAAEHGQLDALDFLVG
SGCDHNVKDKEGNTALHLAAGRGHMAVLQRLVDIGLDLEEQNAEGLTALHSAAGGSHPDC
VQLLLRAGSTVNALTQKNLSCLHYAALSGSEDVSRVLIHAGGCANVVDHQGASPLHLAVR
HNFPALVRLLINSDSDVNAVDNRQQTPLHLAAEHAWQDIADMLLIAGVDLNLRDKQGKTA
LAVAVRSNHVSLVDMIIKADRFYRWEKDHPSDPSGKSLSFKQDHRQETQQLRSVLWRLAS
RYLQPREWKKLAYSWEFTEAHVDAIEQQWTGTRSYQEHGHRMLLIWLHGVATAGENPSKA

LFEGLVAIGRRDLAGWSTMARSQLTATSASRVQMILVPQPPE
>sp|Q9NWT8|AKIP_HUMAN Aurora kinase A-interacting protein OS=Homo sapiens GN=AURKAIP1 PE=1 SV=1
MLLGRLTSQLLRAVPWAGGRPPWPVSGVLGSRVCGPLYSTSPAGPGRAASLPRKGAQLEL
EEMLVPRKMSVSPLESWLTARCFLPRLDTGTAGTVAPPQSYQCPPSQIGEGAEQGDEGVA
DAPQIQCKNVLKIRRRKMNHHKYRKLVKKTRFLRRKVQEGRLRRKQIKFEKDLRRIWLKA
GLKEAPEGWQTPKIYLRGK
>sp|P31751|AKT2_HUMAN RAC-beta serine/threonine-protein kinase OS=Homo sapiens GN=AKT2 PE=1 SV=2
MNEVSVIKEGWLHKRGEYIKTWRPRYFLLKSDGSFIGYKERPEAPDQTLPPLNNFSVAEC
QLMKTERPRPNTFVIRCLQWTTVIERTFHVDSPDEREEWMRAIQMVANSLKQRAPGEDPM
DYKCGSPSDSSTTEEMEVAVSKARAKVTMNDFDYLKLLGKGTFGKVILVREKATGRYYAM
KILRKEVIIAKDEVAHTVTESRVLQNTRHPFLTALKYAFQTHDRLCFVMEYANGGELFFH
LSRERVFTEERARFYGAEIVSALEYLHSRDVVYRDIKLENLMLDKDGHIKITDFGLCKEG
ISDGATMKTFCGTPEYLAPEVLEDNDYGRAVDWWGLGVVMYEMMCGRLPFYNQDHERLFE
LILMEEIRFPRTLSPEAKSLLAGLLKKDPKQRLGGGPSDAKEVMEHRFFLSINWQDVVQK
KLLPPFKPQVTSEVDTRYFDDEFTAQSITITPPDRYDSLGLLELDQRTHFPQFSYSASIR
E
>sp|Q9Y614|ACL7B_HUMAN Actin-like protein 7B OS=Homo sapiens GN=ACTL7B PE=2 SV=1
MATRNSPMPLGTAQGDPGEAGTRPGPDASLRDTGAATQLKMKPRKVHKIKAVIIDLGSQY
CKCGYAGEPRPTYFISSTVGKRCPEAADAGDTRKWTLVGHELLNTEAPLKLVNPLKHGIV
VDWDCVQDIWEYIFRTAMKILPEEHAVLVSDPPLSPSSNREKYAELMFETFGIPAMHVTS
QSLLSIYSYGKTSGLVVESGHGVSHVVPISEGDVLPGLTSRADYAGGDLTNYLMQLLNEA
GHAFTDDHLHIIEHIKKKCCYAAFLPEEELGLVPEELRVDYELPDGKLITIGQERFRCSE
MLFQPSLAGSTQPGLPELTAACLGRCQDTGFKEEMAANVLLCGGCTMLDGFPERFQRELS
LLCPGDSPAVAAAPERKTSVWTGGSILASLQAFQQLWVSKEEFEERGSVAIYSKC
>sp|Q9NPJ3|ACO13_HUMAN Acyl-coenzyme A thioesterase 13 OS=Homo sapiens GN=ACOT13 PE=1 SV=1
MTSMTQSLREVIKAMTKARNFERVLGKITLVSAAPGKVICEMKVEEEHTNAIGTLHGGLT
ATLVDNISTMALLCTERGAPGVSVDMNITYMSPAKLGEDIVITAHVLKQGKTLAFTSVDL
TNKATGKLIAQGRHTKHLGN
>sp|P21399|ACOC_HUMAN Cytoplasmic aconitate hydratase OS=Homo sapiens GN=ACO1 PE=1 SV=3
MSNPFAHLAEPLDPVQPGKKFFNLNKLEDSRYGRLPFSIRVLLEAAIRNCDEFLVKKQDI
ENILHWNVTQHKNIEVPFKPARVILQDFTGVPAVVDFAAMRDAVKKLGGDPEKINPVCPA
DLVIDHSIQVDFNRRADSLQKNQDLEFERNRERFEFLKWGSQAFHNMRIIPPGSGIIHQV
NLEYLARVVFDQDGYYYPDSLVGTDSHTTMIDGLGILGWGVGGIEAEAVMLGQPISMVLP
QVIGYRLMGKPHPLVTSTDIVLTITKHLRQVGVVGKFVEFFGPGVAQLSIADRATIANMC
PEYGATAAFFPVDEVSITYLVQTGRDEEKLKYIKKYLQAVGMFRDFNDPSQDPDFTQVVE
LDLKTVVPCCSGPKRPQDKVAVSDMKKDFESCLGAKQGFKGFQVAPEHHNDHKTFIYDNT
EFTLAHGSVVIAAITSCTNTSNPSVMLGAGLLAKKAVDAGLNVMPYIKTSLSPGSGVVTY
YLQESGVMPYLSQLGFDVVGYGCMTCIGNSGPLPEPVVEAITQGDLVAVGVLSGNRNFEG
RVHPNTRANYLASPPLVIAYAIAGTIRIDFEKEPLGVNAKGQQVFLKDIWPTRDEIQAVE
RQYVIPGMFKEVYQKIETVNESWNALATPSDKLFFWNSKSTYIKSPPFFENLTLDLQPPK
SIVDAYVLLNLGDSVTTDHISPAGNIARNSPAARYLTNRGLTPREFNSYGSRRGNDAVMA

RGTFANIRLLNRFLNKQAPQTIHLPSGEILDVFDAAERYQQAGLPLIVLAGKEYGAGSSR
DWAAKGPFLLGIKAVLAESYERIHRSNLVGMGVIPLEYLPGENADALGLTGQERYTIIIP
ENLKPQMKVQVKLDTGKTFQAVMRFDTDVELTYFLNGGILNYMIRKMAK
>sp|O00767|ACOD_HUMAN Acyl-CoA desaturase OS=Homo sapiens GN=SCD PE=1 SV=2
MPAHLLQDDISSSYTTTTTITAPPSRVLQNGGDKLETMPLYLEDDIRPDIKDDIYDPTYK
DKEGPSPKVEYVWRNIILMSLLHLGALYGITLIPTCKFYTWLWGVFYYFVSALGITAGAH
RLWSHRSYKARLPLRLFLIIANTMAFQNDVYEWARDHRAHHKFSETHADPHNSRRGFFFS
HVGWLLVRKHPAVKEKGSTLDLSDLEAEKLVMFQRRYYKPGLLMMCFILPTLVPWYFWGE
TFQNSVFVATFLRYAVVLNATWLVNSAAHLFGYRPYDKNISPRENILVSLGAVGEGFHNY
HHSFPYDYSASEYRWHINFTTFFIDCMAALGLAYDRKKVSKAAILARIKRTGDGNYKSG
>sp|Q86TX2|ACOT1_HUMAN Acyl-coenzyme A thioesterase 1 OS=Homo sapiens GN=ACOT1 PE=1 SV=1
MAATLILEPAGRCCWDEPVRIAVRGLAPEQPVTLRASLRDEKGALFQAHARYRADTLGEL
DLERAPALGGSFAGLEPMGLLWALEPEKPLVRLVKRDVRTPLAVELEVLDGHDPDPGRLL
CRVRHERYFLPPGVRREPVRAGRVRGTLFLPPEPGPFPGIVDMFGTGGGLLEYRASLLAG
KGFAVMALAYYNYEDLPKTMETLHLEYFEEAVNYLLSHPEVKGPGVGLLGISKGGELCLS
MASFLKGITAAVVINGSVANVGGTLRYKGETLPPVGVNRNRIKVTKDGYADIVDVLNSPL
EGPDQKSFIPVERAESTFLFLVGQDDHNWKSEFYANEACKRLQAHGRRKPQIICYPETGH
YIEPPYFPLCRASLHALVGSPIIWGGEPRAHAMAQVDAWKQLQTFFHKHLGGHEGTIPSK
V
>sp|O14561|ACPM_HUMAN Acyl carrier protein, mitochondrial OS=Homo sapiens GN=NDUFAB1 PE=1 SV=3
MASRVLSAYVSRLPAAFAPLPRVRMLAVARPLSTALCSAGTQTRLGTLQPALVLAQVPGR
VTQLCRQYSDMPPLTLEGIQDRVLYVLKLYDKIDPEKLSVNSHFMKDLGLDSLDQVEIIM
AMEDEFGFEIPDIDAEKLMCPQEIVDYIADKKDVYE
>sp|Q9NUB1|ACS2L_HUMAN Acetyl-coenzyme A synthetase 2-like, mitochondrial OS=Homo sapiens GN=ACSS1 PE=1 SV=2
MAARTLGRGVGRLLGSLRGLSGQPARPPCGVSAPRRAASGPSGSAPAVAAAAAQPGSYPA
LSAQAAREPAAFWGPLARDTLVWDTPYHTVWDCDFSTGKIGWFLGGQLNVSVNCLDQHVR
KSPESVALIWERDEPGTEVRITYRELLETTCRLANTLKRHGVHRGDRVAIYMPVSPLAVA
AMLACARIGAVHTVIFAGFSAESLAGRINDAKCKVVITFNQGLRGGRVVELKKIVDEAVK
HCPTVQHVLVAHRTDNKVHMGDLDVPLEQEMAKEDPVCAPESMGSEDMLFMLYTSGSTGM
PKGIVHTQAGYLLYAALTHKLVFDHQPGDIFGCVADIGWITGHSYVVYGPLCNGATSVLF
ESTPVYPNAGRYWETVERLKINQFYGAPTAVRLLLKYGDAWVKKYDRSSLRTLGSVGEPI
NCEAWEWLHRVVGDSRCTLVDTWWQTETGGICIAPRPSEEGAEILPAMAMRPFFGIVPVL
MDEKGSVVEGSNVSGALCISQAWPGMARTIYGDHQRFVDAYFKAYPGYYFTGDGAYRTEG
GYYQITGRMDDVINISGHRLGTAEIEDAIADHPAVPESAVIGYPHDIKGEAAFAFIVVKD
SAGDSDVVVQELKSMVATKIAKYAVPDEILVVKRLPKTRSGKVMRRLLRKIITSEAQELG
DTTTLEDPSIIAEILSVYQKCKDKQAAAK
>sp|P68133|ACTS_HUMAN Actin, alpha skeletal muscle OS=Homo sapiens GN=ACTA1 PE=1 SV=1
MCDEDETTALVCDNGSGLVKAGFAGDDAPRAVFPSIVGRPRHQGVMVGMGQKDSYVGDEA
QSKRGILTLKYPIEHGIITNWDDMEKIWHHTFYNELRVAPEEHPTLLTEAPLNPKANREK
MTQIMFETFNVPAMYVAIQAVLSLYASGRTTGIVLDSGDGVTHNVPIYEGYALPHAIMRL
DLAGRDLTDYLMKILTERGYSFVTTAEREIVRDIKEKLCYVALDFENEMATAASSSSLEK
SYELPDGQVITIGNERFRCPETLFQPSFIGMESAGIHETTYNSIMKCDIDIRKDLYANNV

MSGGTTMYPGIADRMQKEITALAPSTMKIKIIAPPERKYSVWIGGSILASLSTFQQMWIT
KQEYDEAGPSIVHRKCF
>sp|Q9BYD9|ACTT3_HUMAN Actin-related protein T3 OS=Homo sapiens GN=ACTRT3 PE=2 SV=1
MNHCQLPVVIDNGSGMIKAGVAGCREPQFIYPNIIGRAKGQSRAAQGGLELCVGDQAQDW
RSSLFISYPVERGLITSWEDMEIMWKHIYDYNLKLKPCDGPVLITEPALNPLANRQQITE
MFFEHLGVPAFYMSIQAVLALFAAGFTTGLVLNSGAGVTQSVPIFEGYCLPHGVQQLDLA
GLDLTNYLMVLMKNHGIMLLSASDRKIVEDIKESFCYVAMNYEEEMAKKPDCLEKVYQLP
DGKVIQLHDQLFSCPEALFSPCHMNLEAPGIDKICFSSIMKCDTGLRNSFFSNIILAGGS
TSFPGLDKRLVKDIAKVAPANTAVQVIAPPERKISVWMGGSILASLSAFQDMWITAAEFK
EVGPNIVHQRCF
>sp|P42025|ACTY_HUMAN Beta-centractin OS=Homo sapiens GN=ACTR1B PE=1 SV=1
MESYDIIANQPVVIDNGSGVIKAGFAGDQIPKYCFPNYVGRPKHMRVMAGALEGDLFIGP
KAEEHRGLLTIRYPMEHGVVRDWNDMERIWQYVYSKDQLQTFSEEHPVLLTEAPLNPSKN
REKAAEVFFETFNVPALFISMQAVLSLYATGRTTGVVLDSGDGVTHAVPIYEGFAMPHSI
MRVDIAGRDVSRYLRLLLRKEGVDFHTSAEFEVVRTIKERACYLSINPQKDEALETEKVQ
YTLPDGSTLDVGPARFRAPELLFQPDLVGDESEGLHEVVAFAIHKSDMDLRRTLFANIVL
SGGSTLFKGFGDRLLSEVKKLAPKDIKIKISAPQERLYSTWIGGSILASLDTFKKMWVSK
KEYEEDGSRAIHRKTF
>sp|O75078|ADA11_HUMAN Disintegrin and metalloproteinase domain-containing protein 11 OS=Homo sapiens GN=ADAM11 PE=2 SV=3
MRLLRRWAFAALLLSLLPTPGLGTQGPAGALRWGGLPQLGGPGAPEVTEPSRLVRESSGG
EVRKQQLDTRVRQEPPGGPPVHLAQVSFVIPAFNSNFTLDLELNHHLLSSQYVERHFSRE
GTTQHSTGAGDHCYYQGKLRGNPHSFAALSTCQGLHGVFSDGNLTYIVEPQEVAGPWGAP
QGPLPHLIYRTPLLPDPLGCREPGCLFAVPAQSAPPNRPRLRRKRQVRRGHPTVHSETKY
VELIVINDHQLFEQMRQSVVLTSNFAKSVVNLADVIYKEQLNTRIVLVAMETWADGDKIQ
VQDDLLETLARLMVYRREGLPEPSDATHLFSGRTFQSTSSGAAYVGGICSLSHGGGVNEY
GNMGAMAVTLAQTLGQNLGMMWNKHRSSAGDCKCPDIWLGCIMEDTGFYLPRKFSRCSID
EYNQFLQEGGGSCLFNKPLKLLDPPECGNGFVEAGEECDCGSVQECSRAGGNCCKKCTLT
HDAMCSDGLCCRRCKYEPRGVSCREAVNECDIAETCTGDSSQCPPNLHKLDGYYCDHEQG
RCYGGRCKTRDRQCQVLWGHAAADRFCYEKLNVEGTERGSCGRKGSGWVQCSKQDVLCGF
LLCVNISGAPRLGDLVGDISSVTFYHQGKELDCRGGHVQLADGSDLSYVEDGTACGPNML
CLDHRCLPASAFNFSTCPGSGERRICSHHGVCSNEGKCICQPDWTGKDCSIHNPLPTSPP
TGETERYKGPSGTNIIIGSIAGAVLVAAIVLGGTGWGFKNIRRGRSGGA
>sp|Q13444|ADA15_HUMAN Disintegrin and metalloproteinase domain-containing protein 15 OS=Homo sapiens GN=ADAM15 PE=1 SV=4
MRLALLWALGLLGAGSPLPSWPLPNIGGTEEQQAESEKAPREPLEPQVLQDDLPISLKKV
LQTSLPEPLRIKLELDGDSHILELLQNRELVPGRPTLVWYQPDGTRVVSEGHTLENCCYQ
GRVRGYAGSWVSICTCSGLRGLVVLTPERSYTLEQGPGDLQGPPIISRIQDLHLPGHTCA
LSWRESVHTQKPPEHPLGQRHIRRRRDVVTETKTVELVIVADHSEAQKYRDFQHLLNRTL
EVALLLDTFFRPLNVRVALVGLEAWTQRDLVEISPNPAVTLENFLHWRRAHLLPRLPHDS
AQLVTGTSFSGPTVGMAIQNSICSPDFSGGVNMDHSTSILGVASSIAHELGHSLGLDHDL
PGNSCPCPGPAPAKTCIMEASTDFLPGLNFSNCSRRALEKALLDGMGSCLFERLPSLPPM
AAFCGNMFVEPGEQCDCGFLDDCVDPCCDSLTCQLRPGAQCASDGPCCQNCQLRPSGWQC
RPTRGDCDLPEFCPGDSSQCPPDVSLGDGEPCAGGQAVCMHGRCASYAQQCQSLWGPGAQ

PAAPLCLQTANTRGNAFGSCGRNPSGSYVSCTPRDAICGQLQCQTGRTQPLLGSIRDLLW
ETIDVNGTELNCSWVHLDLGSDVAQPLLTLPGTACGPGLVCIDHRCQRVDLLGAQECRSK
CHGHGVCDSNRHCYCEEGWAPPDCTTQLKATSSLTTGLLLSLLVLLVLVMLGASYWYRAR
LHQRLCQLKGPTCQYRAAQSGPSERPGPPQRALLARGTKQASALSFPAPPSRPLPPDPVS
KRLQAELADRPNPPTRPLPADPVVRSPKSQGPAKPPPPRKPLPADPQGRCPSGDLPGPGA
GIPPLVVPSRPAPPPPTVSSLYL
>sp|Q5VUY2|ADCL4_HUMAN Arylacetamide deacetylase-like 4 OS=Homo sapiens GN=AADACL4 PE=3 SV=1
MAVPWLVLLLALPIFFLGVFVWAVFEHFLTTDIPATLQHPAKLRFLHCIFLYLVTLGNIF
EKLGICSMPKFIRFLHDSVRIKKDPELVVTDLRFGTIPVRLFQPKAASSRPRRGIIFYHG
GATVFGSLDCYHGLCNYLARETESVLLMIGYRKLPDHHSPALFQDCMNASIHFLKALETY
GVDPSRVVVCGESVGGAAVAAITQALVGRSDLPRIRAQVLIYPVVQAFCLQLPSFQQNQN
VPLLSRKFMVTSLCNYLAIDLSWRDAILNGTCVPPDVWRKYEKWLSPDNIPKKFKNRGYQ
PWSPGPFNEAAYLEAKHMLDVENSPLIADDEVIAQLPEAFLVSCENDILRDDSLLYKKRL
EDQGVRVTWYHLYDGFHGSIIFFDKKALSFPCSLKIVNAVVSYIKGI
>sp|Q15847|ADIRF_HUMAN Adipogenesis regulatory factor OS=Homo sapiens GN=ADIRF PE=1 SV=1
MASKGLQDLKQQVEGTAQEAVSAAGAAAQQVVDQATEAGQKAMDQLAKTTQETIDKTANQ
ASDTFSGIGKKFGLLK
>sp|P35318|ADML_HUMAN ADM OS=Homo sapiens GN=ADM PE=1 SV=1
MKLVSVALMYLGSLAFLGADTARLDVASEFRKKWNKWALSRGKRELRMSSSYPTGLADVK
AGPAQTLIRPQDMKGASRSPEDSSPDAARIRVKRYRQSMNNFQGLRSFGCRFGTCTVQKL
AHQIYQFTDKDKDNVAPRSKISPQGYGRRRRRSLPEAGPGRTLVSSKPQAHGAPAPPSGS
APHFL
>sp|Q6IQ32|ADNP2_HUMAN ADNP homeobox protein 2 OS=Homo sapiens GN=ADNP2 PE=1 SV=1
MFQIPVENLDNIRKVRKKVKGILVDIGLDSCKELLKDLKGFDPGEKYFHNTSWGDVSLWE
PSGKKVRYRTKPYCCGLCKYSTKVLTSFKNHLHRYHEDEIDQELVIPCPNCVFASQPKVV
GRHFRMFHAPVRKVQNYTVNILGETKSSRSDVISFTCLKCNFSNTLYYSMKKHVLVAHFH
YLINSYFGLRTEEMGEQPKTNDTVSIEKIPPPDKYYCKKCNANASSQDALMYHILTSDIH
RDLENKLRSVISEHIKRTGLLKQTHIAPKPAAHLAAPANGSAPSAPAQPPCFHLALPQNS
PSPAAGQPVTVAQGAPGSLTHSPPAAGQSHMTLVSSPLPVGQNSLTLQPPAPQPVFLSHG
VPLHQSVNPPVLPLSQPVGPVNKSVGTSVLPINQTVRPGVLPLTQPVGPINRPVGPGVLP
VSPSVTPGVLQAVSPGVLSVSRAVPSGVLPAGQMTPAGQMTPAGVIPGQTATSGVLPTGQ
MVQSGVLPVGQTAPSRVLPPGQTAPLRVISAGQVVPSGLLSPNQTVSSSAVVPVNQGVNS
GVLQLSQPVVSGVLPVGQPVRPGVLQLNQTVGTNILPVNQPVRPGASQNTTFLTSGSILR
QLIPTGKQVNGIPTYTLAPVSVTLPVPPGGLATVAPPQMPIQLLPSGAAAPMAGSMPGMP
SPPVLVNAAQSVFVQASSSAADTNQVLKQAKQWKTCPVCNELFPSNVYQVHMEVAHKHSE
SKSGEKLEPEKLAACAPFLKWMREKTVRCLSCKCLVSEEELIHHLLMHGLGCLFCPCTFH
DIKGLSEHSRNRHLGKKKLPMDYSNRGFQLDVDANGNLLFPHLDFITILPKEKLGEREVY
LAILAGIHSKSLVPVYVKVRPQAEGTPGSTGKRVSTCPFCFGPFVTTEAYELHLKERHHI
MPTVHTVLKSPAFKCIHCCGVYTGNMTLAAIAVHLVRCRSAPKDSSSDLQAQPGFIHNSE
LLLVSGEVMHDSSFSVKRKLPDGHLGAEDQRHGEEQPPILNADAAPGPEKVTSVVPFKRQ
RNESRTEGPIVKDEALQILALDPKKYEGRSYEEKKQFLKDYFHKKPYPSKKEIELLSSLF
WVWKIDVASFFGKRRYICMKAIKNHKPSVLLGFDMSELKNVKHRLNFEYEP
>sp|Q9Y6U3|ADSV_HUMAN Adseverin OS=Homo sapiens GN=SCIN PE=1 SV=4

MARELYHEEFARAGKQAGLQVWRIEKLELVPVPQSAHGDFYVGDAYLVLHTAKTSRGFTY
HLHFWLGKECSQDESTAAAIFTVQMDDYLGGKPVQNRELQGYESNDFVSYFKGGLKYKAG
GVASGLNHVLTNDLTAKRLLHVKGRRVVRATEVPLSWDSFNKGDCFIIDLGTEIYQWCGS
SCNKYERLKANQVATGIRYNERKGRSELIVVEEGSEPSELIKVLGEKPELPDGGDDDDII
ADISNRKMAKLYMVSDASGSMRVTVVAEENPFSMAMLLSEECFILDHGAAKQIFVWKGKD
ANPQERKAAMKTAEEFLQQMNYSKNTQIQVLPEGGETPIFKQFFKDWRDKDQSDGFGKVY
VTEKVAQIKQIPFDASKLHSSPQMAAQHNMVDDGSGKVEIWRVENNGRIQVDQNSYGEFY
GGDCYIILYTYPRGQIIYTWQGANATRDELTTSAFLTVQLDRSLGGQAVQIRVSQGKEPV
HLLSLFKDKPLIIYKNGTSKKGGQAPAPPTRLFQVRRNLASITRIVEVDVDANSLNSNDV
FVLKLPQNSGYIWVGKGASQEEEKGAEYVASVLKCKTLRIQEGEEPEEFWNSLGGKKDYQ
TSPLLETQAEDHPPRLYGCSNKTGRFVIEEIPGEFTQDDLAEDDVMLLDAWEQIFIWIGK
DANEVEKKESLKSAKMYLETDPSGRDKRTPIVIIKQGHEPPTFTGWFLGWDSSKW
>sp|P12235|ADT1_HUMAN ADP/ATP translocase 1 OS=Homo sapiens GN=SLC25A4 PE=1 SV=4
MGDHAWSFLKDFLAGGVAAAVSKTAVAPIERVKLLLQVQHASKQISAEKQYKGIIDCVVR
IPKEQGFLSFWRGNLANVIRYFPTQALNFAFKDKYKQLFLGGVDRHKQFWRYFAGNLASG
GAAGATSLCFVYPLDFARTRLAADVGKGAAQREFHGLGDCIIKIFKSDGLRGLYQGFNVS
VQGIIIYRAAYFGVYDTAKGMLPDPKNVHIFVSWMIAQSVTAVAGLVSYPFDTVRRRMMM
QSGRKGADIMYTGTVDCWRKIAKDEGAKAFFKGAWSNVLRGMGGAFVLVLYDEIKKYV
>sp|Q8TF27|AGA11_HUMAN Arf-GAP with GTPase, ANK repeat and PH domain-containing protein 11 OS=Homo sapiens GN=AGAP11 PE=2 SV=2
MTIISVTLEIHHHITERDADRSLTILDEQLYSFAFSTVHITKKRNGGGSLNNYSSSIPLT
PSTSQEDLYFSVPPTANTPTPICKQSMGWSNLFTSEKGSDPDKGRKALESHADTIGSGRA
IPIKQGMLLKRSGKWLKTWKKKYVTLCSNGVLTYYSSLGDYMKNIHKKEIDLRTSTIKVP
GKWPSLATSACAPISSSKSNGLSKDMEALHMSANSDIGLGDSICFSPSISSTTSPKLNLP
PSPHANKKKHLKKKSTNNLKDDGLSSTAEEEEEKFMIVSVTGQTCHFKATTYEERDAWVQ
AIQSQILASLQSCESSKSKSQLTSQSEAMALQSIQNMRGNSHCVDCETQNPKWASLNLGV
LMCIECSGIHRSLGTRLSRVRSLELDDWPVELRKVMSSIGNDLANSIWEGSSQGQTKPSI
ESTREEKERWIRSKYEHKLFLAPLPCTELSLGQHLLRATADEDLRTAILLLAHGSREEVN
ETCGEGDGCTALHLACRKGNVVLAQLLIWYGVDVMARDAHGNTALTYARQASSQECINVL
LQYGCPDECV
>sp|Q96P47|AGAP3_HUMAN Arf-GAP with GTPase, ANK repeat and PH domain-containing protein 3 OS=Homo sapiens GN=AGAP3 PE=1 SV=2
MNFQAGGGQSPQQQQQLAGGPPQQFALSNSAAIRAEIQRFESVHPNIYAIYDLIERIEDL
ALQNQIREHVISIEDSFVNSQEWTLSRSVPELKVGIVGNLSSGKSALVHRYLTGTYVQEE
SPEGGRFKKEIVVDGQSYLLLIRDEGGPPELQFAAWVDAVVFVFSLEDEISFQTVYNYFL
RLCSFRNASEVPMVLVGTQDAISAANPRVIDDSRARKLSTDLKRCTYYETCATYGLNVER
VFQDVAQKVVALRKKQQLAIGPCKSLPNSPSHSAVSAASIPAVHINQATNGGGSAFSDYS
SSVPSTPSISQRELRIETIAASSTPTPIRKQSKRRSNIFTSRKGADLDREKKAAECKVDS
IGSGRAIPIKQGILLKRSGKSLNKEWKKKYVTLCDNGLLTYHPSLHDYMQNIHGKEIDLL
RTTVKVPGKRLPRATPATAPGTSPRANGLSVERSNTQLGGGTGAPHSASSASLHSERPLS
SSAWAGPRPEGLHQRSCSVSSADQWSEATTSLPPGMQHPASGPAEVLSSSPKLDPPPSPH
SNRKKHRRKKSTGTPRPDGPSSATEEAEESFEFVVVSLTGQTWHFEASTAEERELWVQSV
QAQILASLQGCRSAKDKTRLGNQNAALAVQAVRTVRGNSFCIDCDAPNPDWASLNLGALM
CIECSGIHRHLGAHLSRVRSLDLDDWPPELLAVMTAMGNALANSVWEGALGGYSKPGPDA

CREEKERWIRAKYEQKLFLAPLPSSDVPLGQQLLRAVVEDDLRLLVMLLAHGSKEEVNET
YGDGDGRTALHLSSAMANVVFTQLLIWYGVDVRSRDARGLTPLAYARRAGSQECADILIQ
HGCPGEGCGLAPTPNREPANGTNPSAELHRSPSLL
>sp|Q9UKV8|AGO2_HUMAN Protein argonaute-2 OS=Homo sapiens GN=AGO2 PE=1 SV=3
MYSGAGPALAPPAPPPPIQGYAFKPPPRPDFGTSGRTIKLQANFFEMDIPKIDIYHYELD
IKPEKCPRRVNREIVEHMVQHFKTQIFGDRKPVFDGRKNLYTAMPLPIGRDKVELEVTLP
GEGKDRIFKVSIKWVSCVSLQALHDALSGRLPSVPFETIQALDVVMRHLPSMRYTPVGRS
FFTASEGCSNPLGGGREVWFGFHQSVRPSLWKMMLNIDVSATAFYKAQPVIEFVCEVLDF
KSIEEQQKPLTDSQRVKFTKEIKGLKVEITHCGQMKRKYRVCNVTRRPASHQTFPLQQES
GQTVECTVAQYFKDRHKLVLRYPHLPCLQVGQEQKHTYLPLEVCNIVAGQRCIKKLTDNQ
TSTMIRATARSAPDRQEEISKLMRSASFNTDPYVREFGIMVKDEMTDVTGRVLQPPSILY
GGRNKAIATPVQGVWDMRNKQFHTGIEIKVWAIACFAPQRQCTEVHLKSFTEQLRKISRD
AGMPIQGQPCFCKYAQGADSVEPMFRHLKNTYAGLQLVVVILPGKTPVYAEVKRVGDTVL
GMATQCVQMKNVQRTTPQTLSNLCLKINVKLGGVNNILLPQGRPPVFQQPVIFLGADVTH
PPAGDGKKPSIAAVVGSMDAHPNRYCATVRVQQHRQEIIQDLAAMVRELLIQFYKSTRFK
PTRIIFYRDGVSEGQFQQVLHHELLAIREACIKLEKDYQPGITFIVVQKRHHTRLFCTDK
NERVGKSGNIPAGTTVDTKITHPTEFDFYLCSHAGIQGTSRPSHYHVLWDDNRFSSDELQ
ILTYQLCHTYVRCTRSVSIPAPAYYAHLVAFRARYHLVDKEHDSAEGSHTSGQSNGRDHQ
ALAKAVQVHQDTLRTMYFA
>sp|O95994|AGR2_HUMAN Anterior gradient protein 2 homolog OS=Homo sapiens GN=AGR2 PE=1 SV=1
MEKIPVSAFLLLVALSYTLARDTTVKPGAKKDTKDSRPKLPQTLSRGWGDQLIWTQTYEE
ALYKSKTSNKPLMIIHHLDECPHSQALKKVFAENKEIQKLAEQFVLLNLVYETTDKHLSP
DGQYVPRIMFVDPSLTVRADITGRYSNRLYAYEPADTALLLDNMKKALKLLKTEL
>sp|Q7Z7M1|AGRD2_HUMAN Adhesion G-protein coupled receptor D2 OS=Homo sapiens GN=ADGRD2 PE=2 SV=1
MDAPWGAGERWLHGAAVDRSGVSLGPPPTPQVNQGTLGPQVAPVAAGEVVKTAGGVCKFS
GQRLSWWQAQESCEQQFGHLALQPPDGVLASRLRDPVWVGQREAPLRRPPQRRARTTAVL
VFDERTADRAARLRSPLPELAALTACTHVQWDCASPDPAALFSVAAPALPNALQLRAFAE
PGGVVRAALVVRGQHAPFLAAFRADGRWHHVCATWEQRGGRWALFSDGRRRAGARGLGAG
HPVPSGGILVLGQDQDSLGGGFSVRHALSGNLTDFHLWARALSPAQLHRARACAPPSEGL
LFRWDPGALDVTPSLLPTVWVRLLCPVPSEECPTWNPGPRSEGSELCLEPQPFLCCYRTE
PYRRLQDAQSWPGQDVISRVNALANDIVLLPDPLSEVHGALSPAEASSFLGLLEHVLAME
MAPLGPAALLAVVRFLKRVVALGAGDPELLLTGPWEQLSQGVVSVASLVLEEQVADTWLS
LREVIGGPMALVASVQRLAPLLSTSMTSERPRMRIQHRHAGLSGVTVIHSWFTSRVFQHT
LEGPDLEPQAPASSEEANRVQRFLSTQVGSAIISSEVWDVTGEVNVAMTFHLQHRAQSPL
FPPHPPSPYTGGAWATTGCSVAALYLDSTACFCNHSTSFAILLQIYEVQRGPEEESLLRT
LSFVGCGVSFCALTTTFLLFLVAGVPKSERTTVHKNLTFSLASAEGFLMTSEWAKANEVA
CVAVTVAMHFLFLVAFSWMLVEGLLLWRKVVAVSMHPGPGMRLYHATGWGVPVGIVAVTL
AMLPHDYVAPGHCWLNVHTNAIWAFVGPVLFVLTANTCILARVVMITVSSARRRARMLSP
QPCLQQQIWTQIWATVKPVLVLLPVLGLTWLAGILVHLSPAWAYAAVGLNSIQGLYIFLV
YAACNEEVRSALQRMAEKKVAEVLRALGVWGGAAKEHSLPFSVLPLFLPPKPSTPRHPLK
APA

>sp|Q9BY15|AGRE3_HUMAN Adhesion G protein-coupled receptor E3 OS=Homo sapiens GN=ADGRE3 PE=2 SV=2
MQGPLLLPGLCFLLSLFGAVTQKTKTSCAKCPPNASCVNNTHCTCNHGYTSGSGQKLFTF
PLETCNDINECTPPYSVYCGFNAVCYNVEGSFYCQCVPGYRLHSGNEQFSNSNENTCQDT
TSSKTTEGRKELQKIVDKFESLLTNQTLWRTEGRQEISSTATTILRDVESKVLETALKDP
EQKVLKIQNDSVAIETQAITDNCSEERKTFNLNVQMNSMDIRCSDIIQGDTQGPSAIAFI
SYSSLGNIINATFFEEMDKKDQVYLNSQVVSAAIGPKRNVSLSKSVTLTFQHVKMTPSTK
KVFCVYWKSTGQGSQWSRDGCFLIHVNKSHTMCNCSHLSSFAVLMALTSQEEDPVLTVIT
YVGLSVSLLCLLLAALTFLLCKAIRNTSTSLHLQLSLCLFLAHLLFLVGIDRTEPKVLCS
IIAGALHYLYLAAFTWMLLEGVHLFLTARNLTVVNYSSINRLMKWIMFPVGYGVPAVTVA
ISAASWPHLYGTADRCWLHLDQGFMWSFLGPVCAIFSANLVLFILVFWILKRKLSSLNSE
VSTIQNTRMLAFKATAQLFILGCTWCLGLLQVGPAAQVMAYLFTIINSLQGFFIFLVYCL
LSQQVQKQYQKWFREIVKSKSESETYTLSSKMGPDSKPSEGDVFPGQVKRKY
>sp|Q8IZP9|AGRG2_HUMAN Adhesion G-protein coupled receptor G2 OS=Homo sapiens GN=ADGRG2 PE=1 SV=2
MVFSVRQCGHVGRTEEVLLTFKIFLVIICLHVVLVTSLEEDTDNSSLSPPPAKLSVVSFA
PSSNGTPEVETTSLNDVTLSLLPSNETEKTKITIVKTFNASGVKPQRNICNLSSICNDSA
FFRGEIMFQYDKESTVPQNQHITNGTLTGVLSLSELKRSELNKTLQTLSETYFIMCATAE
AQSTLNCTFTIKLNNTMNACAVIAALERVKIRPMEHCCCSVRIPCPSSPEELEKLQCDLQ
DPIVCLADHPRGPPFSSSQSIPVVPRATVLSQVPKATSFAEPPDYSPVTHNVPSPIGEIQ
PLSPQPSAPIASSPAIDMPPQSETISSPMPQTHVSGTPPPVKASFSSPTVSAPANVNTTS
APPVQTDIVNTSSISDLENQVLQMEKALSLGSLEPNLAGEMINQVSRLLHSPPDMLAPLA
QRLLKVVDDIGLQLNFSNTTISLTSPSLALAVIRVNASSFNTTTFVAQDPANLQVSLETQ
APENSIGTITLPSSLMNNLPAHDMELASRVQFNFFETPALFQDPSLENLSLISYVISSSV
ANLTVRNLTRNVTVTLKHINPSQDELTVRCVFWDLGRNGGRGGWSDNGCSVKDRRLNETI
CTCSHLTSFGVLLDLSRTSVLPAQMMALTFITYIGCGLSSIFLSVTLVTYIAFEKIRRDY
PSKILIQLCAALLLLNLVFLLDSWIALYKMQGLCISVAVFLHYFLLVSFTWMGLEAFHMY
LALVKVFNTYIRKYILKFCIVGWGVPAVVVTIILTISPDNYGLGSYGKFPNGSPDDFCWI
NNNAVFYITVVGYFCVIFLLNVSMFIVVLVQLCRIKKKKQLGAQRKTSIQDLRSIAGLTF
LLGITWGFAFFAWGPVNVTFMYLFAIFNTLQGFFIFIFYCVAKENVRKQWRRYLCCGKLR
LAENSDWSKTATNGLKKQTVNQGVSSSSNSLQSSSNSTNSTTLLVNNDCSVHASGNGNAS
TERNGVSFSVQNGDVCLHDFTGKQHMFNEKEDSCNGKGRMALRRTSKRGSLHFIEQM
>sp|Q9HAR2|AGRL3_HUMAN Adhesion G protein-coupled receptor L3 OS=Homo sapiens GN=ADGRL3 PE=1 SV=2
MWPSQLLIFMMLLAPIIHAFSRAPIPMAVVRRELSCESYPIELRCPGTDVIMIESANYGR
TDDKICDSDPAQMENIRCYLPDAYKIMSQRCNNRTQCAVVAGPDVFPDPCPGTYKYLEVQ
YECVPYKVEQKVFLCPGLLKGVYQSEHLFESDHQSGAWCKDPLQASDKIYYMPWTPYRTD
TLTEYSSKDDFIAGRPTTTYKLPHRVDGTGFVVYDGALFFNKERTRNIVKFDLRTRIKSG
EAIIANANYHDTSPYRWGGKSDIDLAVDENGLWVIYATEQNNGKIVISQLNPYTLRIEGT
WDTAYDKRSASNAFMICGILYVVKSVYEDDDNEATGNKIDYIYNTDQSKDSLVDVPFPNS
YQYIAAVDYNPRDNLLYVWNNYHVVKYSLDFGPLDSRSGQAHHGQVSYISPPIHLDSELE
RPSVKDISTTGPLGMGSTTTSTTLRTTTLSPGRSTTPSVSGRRNRSTSTPSPAVEVLDDM
TTHLPSASSQIPALEESCEAVEAREIMWFKTRQGQIAKQPCPAGTIGVSTYLCLAPDGIW
DPQGPDLSNCSSPWVNHITQKLKSGETAANIARELAEQTRNHLNAGDITYSVRAMDQLVG

LLDVQLRNLTPGGKDSAARSLNKAMVETVNNLLQPQALNAWRDLTTSDQLRAATMLLHTV
EESAFVLADNLLKTDIVRENTDNIKLEVARLSTEGNLEDLKFPENMGHGSTIQLSANTLK
QNGRNGEIRVAFVLYNNLGPYLSTENASMKLGTEALSTNHSVIVNSPVITAAINKEFSNK
VYLADPVVFTVKHIKQSEENFNPNCSFWSYSKRTMTGYWSTQGCRLLTTNKTHTTCSCNH
LTNFAVLMAHVEVKHSDAVHDLLLDVITWVGILLSLVCLLICIFTFCFFRGLQSDRNTIH
KNLCISLFVAELLFLIGINRTDQPIACAVFAALLHFFFLAAFTWMFLEGVQLYIMLVEVF
ESEHSRRKYFYLVGYGMPALIVAVSAAVDYRSYGTDKVCWLRLDTYFIWSFIGPATLIIM
LNVIFLGIALYKMFHHTAILKPESGCLDNIKSWVIGAIALLCLLGLTWAFGLMYINESTV
IMAYLFTIFNSLQGMFIFIFHCVLQKKVRKEYGKCLRTHCCSGKSTESSIGSGKTSGSRT
PGRYSTGSQSRIRRMWNDTVRKQSESSFITGDINSSASLNREGLLNNARDTSVMDTLPLN
GNHGNSYSIASGEYLSNCVQIIDRGYNHNETALEKKILKELTSNYIPSYLNNHERSSEQN
RNLMNKLVNNLGSGREDDAIVLDDATSFNHEESLGLELIHEESDAPLLPPRVYSTENHQP
HHYTRRRIPQDHSESFFPLLTNEHTEDLQSPHRDSLYTSMPTLAGVAATESVTTSTQTEP
PPAKCGDAEDVYYKSMPNLGSRNHVHQLHTYYQLGRGSSDGFIVPPNKDGTPPEGSSKGP
AHLVTSL
>sp|O00253|AGRP_HUMAN Agouti-related protein OS=Homo sapiens GN=AGRP PE=1 SV=1
MLTAAVLSCALLLALPATRGAQMGLAPMEGIRRPDQALLPELPGLGLRAPLKKTTAEQAE
EDLLQEAQALAEVLDLQDREPRSSRRCVRLHESCLGQQVPCCDPCATCYCRFFNAFCYCR
KLGTAMNPCSRT
>sp|P35869|AHR_HUMAN Aryl hydrocarbon receptor OS=Homo sapiens GN=AHR PE=1 SV=2
MNSSSANITYASRKRRKPVQKTVKPIPAEGIKSNPSKRHRDRLNTELDRLASLLPFPQDV
INKLDKLSVLRLSVSYLRAKSFFDVALKSSPTERNGGQDNCRAANFREGLNLQEGEFLLQ
ALNGFVLVVTTDALVFYASSTIQDYLGFQQSDVIHQSVYELIHTEDRAEFQRQLHWALNP
SQCTESGQGIEEATGLPQTVVCYNPDQIPPENSPLMERCFICRLRCLLDNSSGFLAMNFQ
GKLKYLHGQKKKGKDGSILPPQLALFAIATPLQPPSILEIRTKNFIFRTKHKLDFTPIGC
DAKGRIVLGYTEAELCTRGSGYQFIHAADMLYCAESHIRMIKTGESGMIVFRLLTKNNRW
TWVQSNARLLYKNGRPDYIIVTQRPLTDEEGTEHLRKRNTKLPFMFTTGEAVLYEATNPF
PAIMDPLPLRTKNGTSGKDSATTSTLSKDSLNPSSLLAAMMQQDESIYLYPASSTSSTAP
FENNFFNESMNECRNWQDNTAPMGNDTILKHEQIDQPQDVNSFAGGHPGLFQDSKNSDLY
SIMKNLGIDFEDIRHMQNEKFFRNDFSGEVDFRDIDLTDEILTYVQDSLSKSPFIPSDYQ
QQQSLALNSSCMVQEHLHLEQQQQHHQKQVVVEPQQQLCQKMKHMQVNGMFENWNSNQFV
PFNCPQQDPQQYNVFTDLHGISQEFPYKSEMDSMPYTQNFISCNQPVLPQHSKCTELDYP
MGSFEPSPYPTTSSLEDFVTCLQLPENQKHGLNPQSAIITPQTCYAGAVSMYQCQPEPQH
THVGQMQYNPVLPGQQAFLNKFQNGVLNETYPAELNNINNTQTTTHLQPLHHPSEARPFP
DLTSSGFL
>sp|Q9GZX7|AICDA_HUMAN Single-stranded DNA cytosine deaminase OS=Homo sapiens GN=AICDA PE=1 SV=1
MDSLLMNRRKFLYQFKNVRWAKGRRETYLCYVVKRRDSATSFSLDFGYLRNKNGCHVELL
FLRYISDWDLDPGRCYRVTWFTSWSPCYDCARHVADFLRGNPNLSLRIFTARLYFCEDRK
AEPEGLRRLHRAGVQIAIMTFKDYFYCWNTFVENHERTFKAWEGLHENSVRLSRQLRRIL
LPLYEVDDLRDAFRTLGL
>sp|O14862|AIM2_HUMAN Interferon-inducible protein AIM2 OS=Homo sapiens GN=AIM2 PE=1 SV=1
MESKYKEILLLTGLDNITDEELDRFKFFLSDEFNIATGKLHTANRIQVATLMIQNAGAVS
AVMKTIRIFQKLNYMLLAKRLQEEKEKVDKQYKSVTKPKPLSQAEMSPAASAAIRNDVAK

QRAAPKVSPHVKPEQKQMVAQQESIREGFQKRCLPVMVLKAKKPFTFETQEGKQEMFHAT
VATEKEFFFVKVFNTLLKDKFIPKRIIIIARYYRHSGFLEVNSASRVLDAESDQKVNVPL
NIIRKAGETPKINTLQTQPLGTIVNGLFVVQKVTEKKKNILFDLSDNTGKMEVLGVRNED
TMKCKEGDKVRLTFFTLSKNGEKLQLTSGVHSTIKVIKAKKKT
>sp|Q16352|AINX_HUMAN Alpha-internexin OS=Homo sapiens GN=INA PE=1 SV=2
MSFGSEHYLCSSSSYRKVFGDGSRLSARLSGAGGAGGFRSQSLSRSNVASSAACSSASSL
GLGLAYRRPPASDGLDLSQAAARTNEYKIIRTNEKEQLQGLNDRFAVFIEKVHQLETQNR
ALEAELAALRQRHAEPSRVGELFQRELRDLRAQLEEASSARSQALLERDGLAEEVQRLRA
RCEEESRGREGAERALKAQQRDVDGATLARLDLEKKVESLLDELAFVRQVHDEEVAELLA
TLQASSQAAAEVDVTVAKPDLTSALREIRAQYESLAAKNLQSAEEWYKSKFANLNEQAAR
STEAIRASREEIHEYRRQLQARTIEIEGLRGANESLERQILELEERHSAEVAGYQDSIGQ
LENDLRNTKSEMARHLREYQDLLNVKMALDIEIAAYRKLLEGEETRFSTSGLSISGLNPL
PNPSYLLPPRILSATTSKVSSTGLSLKKEEEEEEASKVASKKTSQIGESFEEILEETVIS
TKKTEKSNIEETTISSQKI
>sp|Q9NZN9|AIPL1_HUMAN Aryl-hydrocarbon-interacting protein-like 1 OS=Homo sapiens GN=AIPL1 PE=1 SV=2
MDAALLLNVEGVKKTILHGGTGELPNFITGSRVIFHFRTMKCDEERTVIDDSRQVGQPMH
IIIGNMFKLEVWEILLTSMRVHEVAEFWCDTIHTGVYPILSRSLRQMAQGKDPTEWHVHT
CGLANMFAYHTLGYEDLDELQKEPQPLVFVIELLQVDAPSDYQRETWNLSNHEKMKAVPV
LHGEGNRLFKLGRYEEASSKYQEAIICLRNLQTKEKPWEVQWLKLEKMINTLILNYCQCL
LKKEEYYEVLEHTSDILRHHPGIVKAYYVRARAHAEVWNEAEAKADLQKVLELEPSMQKA
VRRELRLLENRMAEKQEEERLRCRNMLSQGATQPPAEPPTEPPAQSSTEPPAEPPTAPSA
ELSAGPPAEPATEPPPSPGHSLQH
>sp|Q9UKB5|AJAP1_HUMAN Adherens junction-associated protein 1 OS=Homo sapiens GN=AJAP1 PE=1 SV=1
MWIQQLLGLSSMSIRWPGRPLGSHAWILIAMFQLAVDLPACEALGPGPEFWLLPRSPPRP
PRLWSFRSGQPARVPAPVWSPRPPRVERIHGQMQMPRARRAHRPRDQAAALVPKAGLAKP
PAAAKSSPSLASSSSSSSSAVAGGAPEQQALLRRGKRHLQGDGLSSFDSRGSRPTTETEF
IAWGPTGDEEALESNTFPGVYGPTTVSILQTRKTTVAATTTTTTTATPMTLQTKGFTESL
DPRRRIPGGVSTTEPSTSPSNNGEVTQPPRILGEASGLAVHQIITITVSLIMVIAALITT
LVLKNCCAQSGNTRRNSHQRKTNQQEESCQNLTDFPSARVPSSLDIFTAYNETLQCSHEC
VRASVPVYTDETLHSTTGEYKSTFNGNRPSSSDRHLIPVAFVSEKWFEISC
>sp|Q15822|ACHA2_HUMAN Neuronal acetylcholine receptor subunit alpha-2 OS=Homo sapiens GN=CHRNA2 PE=1 SV=2
MGPSCPVFLSFTKLSLWWLLLTPAGGEEAKRPPPRAPGDPLSSPSPTALPQGGSHTETED
RLFKHLFRGYNRWARPVPNTSDVVIVRFGLSIAQLIDVDEKNQMMTTNVWLKQEWSDYKL
RWNPTDFGNITSLRVPSEMIWIPDIVLYNNADGEFAVTHMTKAHLFSTGTVHWVPPAIYK
SSCSIDVTFFPFDQQNCKMKFGSWTYDKAKIDLEQMEQTVDLKDYWESGEWAIVNATGTY
NSKKYDCCAEIYPDVTYAFVIRRLPLFYTINLIIPCLLISCLTVLVFYLPSDCGEKITLC
ISVLLSLTVFLLLITEIIPSTSLVIPLIGEYLLFTMIFVTLSIVITVFVLNVHHRSPSTH
TMPHWVRGALLGCVPRWLLMNRPPPPVELCHPLRLKLSPSYHWLESNVDAEEREVVVEEE
DRWACAGHVAPSVGTLCSHGHLHSGASGPKAEALLQEGELLLSPHMQKALEGVHYIADHL
RSEDADSSVKEDWKYVAMVIDRIFLWLFIIVCFLGTIGLFLPPFLAGMI

>sp|P32297|ACHA3_HUMAN Neuronal acetylcholine receptor subunit alpha-3 OS=Homo sapiens GN=CHRNA3 PE=1 SV=4
MGSGPLSLPLALSPPRLLLLLLLSLLPVARASEAEHRLFERLFEDYNEIIRPVANVSDPV
IIHFEVSMSQLVKVDEVNQIMETNLWLKQIWNDYKLKWNPSDYGGAEFMRVPAQKIWKPD
IVLYNNAVGDFQVDDKTKALLKYTGEVTWIPPAIFKSSCKIDVTYFPFDYQNCTMKFGSW
SYDKAKIDLVLIGSSMNLKDYWESGEWAIIKAPGYKHDIKYNCCEEIYPDITYSLYIRRL
PLFYTINLIIPCLLISFLTVLVFYLPSDCGEKVTLCISVLLSLTVFLLVITETIPSTSLV
IPLIGEYLLFTMIFVTLSIVITVFVLNVHYRTPTTHTMPSWVKTVFLNLLPRVMFMTRPT
SNEGNAQKPRPLYGAELSNLNCFSRAESKGCKEGYPCQDGMCGYCHHRRIKISNFSANLT
RSSSSESVDAVLSLSALSPEIKEAIQSVKYIAENMKAQNEAKEIQDDWKYVAMVIDRIFL
WVFTLVCILGTAGLFLQPLMAREDA
>sp|Q15825|ACHA6_HUMAN Neuronal acetylcholine receptor subunit alpha-6 OS=Homo sapiens GN=CHRNA6 PE=2 SV=1
MLTSKGQGFLHGGLCLWLCVFTPFFKGCVGCATEERLFHKLFSHYNQFIRPVENVSDPVT
VHFEVAITQLANVDEVNQIMETNLWLRHIWNDYKLRWDPMEYDGIETLRVPADKIWKPDI
VLYNNAVGDFQVEGKTKALLKYNGMITWTPPAIFKSSCPMDITFFPFDHQNCSLKFGSWT
YDKAEIDLLIIGSKVDMNDFWENSEWEIIDASGYKHDIKYNCCEEIYTDITYSFYIRRLP
MFYTINLIIPCLFISFLTVLVFYLPSDCGEKVTLCISVLLSLTVFLLVITETIPSTSLVV
PLVGEYLLFTMIFVTLSIVVTVFVLNIHYRTPTTHTMPRWVKTVFLKLLPQVLLMRWPLD
KTRGTGSDAVPRGLARRPAKGKLASHGEPRHLKECFHCHKSNELATSKRRLSHQPLQWVV
ENSEHSPEVEDVINSVQFIAENMKSHNETKEVEDDWKYVAMVVDRVFLWVFIIVCVFGTA
GLFLQPLLGNTGKS
>sp|P36544|ACHA7_HUMAN Neuronal acetylcholine receptor subunit alpha-7 OS=Homo sapiens GN=CHRNA7 PE=1 SV=5
MRCSPGGVWLALAASLLHVSLQGEFQRKLYKELVKNYNPLERPVANDSQPLTVYFSLSLL
QIMDVDEKNQVLTTNIWLQMSWTDHYLQWNVSEYPGVKTVRFPDGQIWKPDILLYNSADE
RFDATFHTNVLVNSSGHCQYLPPGIFKSSCYIDVRWFPFDVQHCKLKFGSWSYGGWSLDL
QMQEADISGYIPNGEWDLVGIPGKRSERFYECCKEPYPDVTFTVTMRRRTLYYGLNLLIP
CVLISALALLVFLLPADSGEKISLGITVLLSLTVFMLLVAEIMPATSDSVPLIAQYFAST
MIIVGLSVVVTVIVLQYHHHDPDGGKMPKWTRVILLNWCAWFLRMKRPGEDKVRPACQHK
QRRCSLASVEMSAVAPPPASNGNLLYIGFRGLDGVHCVPTPDSGVVCGRMACSPTHDEHL
LHGGQPPEGDPDLAKILEEVRYIANRFRCQDESEAVCSEWKFAACVVDRLCLMAFSVFTI
ICTIGILMSAPNFVEAVSKDFA
>sp|O00590|ACKR2_HUMAN Atypical chemokine receptor 2 OS=Homo sapiens GN=ACKR2 PE=1 SV=2
MAATASPQPLATEDADSENSSFYYYDYLDEVAFMLCRKDAVVSFGKVFLPVFYSLIFVLG
LSGNLLLLMVLLRYVPRRRMVEIYLLNLAISNLLFLVTLPFWGISVAWHWVFGSFLCKMV
STLYTINFYSGIFFISCMSLDKYLEIVHAQPYHRLRTRAKSLLLATIVWAVSLAVSIPDM
VFVQTHENPKGVWNCHADFGGHGTIWKLFLRFQQNLLGFLLPLLAMIFFYSRIGCVLVRL
RPAGQGRALKIAAALVVAFFVLWFPYNLTLFLHTLLDLQVFGNCEVSQHLDYALQVTESI
AFLHCCFSPILYAFSSHRFRQYLKAFLAAVLGWHLAPGTAQASLSSCSESSILTAQEEMT
GMNDLGERQSENYPNKEDVGNKSA
>sp|P53396|ACLY_HUMAN ATP-citrate synthase OS=Homo sapiens GN=ACLY PE=1 SV=3
MSAKAISEQTGKELLYKFICTTSAIQNRFKYARVTPDTDWARLLQDHPWLLSQNLVVKPD
QLIKRRGKLGLVGVNLTLDGVKSWLKPRLGQEATVGKATGFLKNFLIEPFVPHSQAEEFY

VCIYATREGDYVLFHHEGGVDVGDVDAKAQKLLVGVDEKLNPEDIKKHLLVHAPEDKKEI
LASFISGLFNFYEDLYFTYLEINPLVVTKDGVYVLDLAAKVDATADYICKVKWGDIEFPP
PFGREAYPEEAYIADLDAKSGASLKLTLLNPKGRIWTMVAGGGASVVYSDTICDLGGVNE
LANYGEYSGAPSEQQTYDYAKTILSLMTREKHPDGKILIIGGSIANFTNVAATFKGIVRA
IRDYQGPLKEHEVTIFVRRGGPNYQEGLRVMGEVGKTTGIPIHVFGTETHMTAIVGMALG
HRPIPNQPPTAAHTANFLLNASGSTSTPAPSRTASFSESRADEVAPAKKAKPAMPQDSVP
SPRSLQGKSTTLFSRHTKAIVWGMQTRAVQGMLDFDYVCSRDEPSVAAMVYPFTGDHKQK
FYWGHKEILIPVFKNMADAMRKHPEVDVLINFASLRSAYDSTMETMNYAQIRTIAIIAEG
IPEALTRKLIKKADQKGVTIIGPATVGGIKPGCFKIGNTGGMLDNILASKLYRPGSVAYV
SRSGGMSNELNNIISRTTDGVYEGVAIGGDRYPGSTFMDHVLRYQDTPGVKMIVVLGEIG
GTEEYKICRGIKEGRLTKPIVCWCIGTCATMFSSEVQFGHAGACANQASETAVAKNQALK
EAGVFVPRSFDELGEIIQSVYEDLVANGVIVPAQEVPPPTVPMDYSWARELGLIRKPASF
MTSICDERGQELIYAGMPITEVFKEEMGIGGVLGLLWFQKRLPKYSCQFIEMCLMVTADH
GPAVSGAHNTIICARAGKDLVSSLTSGLLTIGDRFGGALDAAAKMFSKAFDSGIIPMEFV
NKMKKEGKLIMGIGHRVKSINNPDMRVQILKDYVRQHFPATPLLDYALEVEKITTSKKPN
LILNVDGLIGVAFVDMLRNCGSFTREEADEYIDIGALNGIFVLGRSMGFIGHYLDQKRLK
QGLYRHPWDDISYVLPEHMSM
>sp|P11229|ACM1_HUMAN Muscarinic acetylcholine receptor M1 OS=Homo sapiens GN=CHRM1 PE=1 SV=2
MNTSAPPAVSPNITVLAPGKGPWQVAFIGITTGLLSLATVTGNLLVLISFKVNTELKTVN
NYFLLSLACADLIIGTFSMNLYTTYLLMGHWALGTLACDLWLALDYVASNASVMNLLLIS
FDRYFSVTRPLSYRAKRTPRRAALMIGLAWLVSFVLWAPAILFWQYLVGERTVLAGQCYI
QFLSQPIITFGTAMAAFYLPVTVMCTLYWRIYRETENRARELAALQGSETPGKGGGSSSS
SERSQPGAEGSPETPPGRCCRCCRAPRLLQAYSWKEEEEEDEGSMESLTSSEGEEPGSEV
VIKMPMVDPEAQAPTKQPPRSSPNTVKRPTKKGRDRAGKGQKPRGKEQLAKRKTFSLVKE
KKAARTLSAILLAFILTWTPYNIMVLVSTFCKDCVPETLWELGYWLCYVNSTINPMCYAL
CNKAFRDTFRLLLLCRWDKRRWRKIPKRPGSVHRTPSRQC
>sp|P20309|ACM3_HUMAN Muscarinic acetylcholine receptor M3 OS=Homo sapiens GN=CHRM3 PE=1 SV=1
MTLHNNSTTSPLFPNISSSWIHSPSDAGLPPGTVTHFGSYNVSRAAGNFSSPDGTTDDPL
GGHTVWQVVFIAFLTGILALVTIIGNILVIVSFKVNKQLKTVNNYFLLSLACADLIIGVI
SMNLFTTYIIMNRWALGNLACDLWLAIDYVASNASVMNLLVISFDRYFSITRPLTYRAKR
TTKRAGVMIGLAWVISFVLWAPAILFWQYFVGKRTVPPGECFIQFLSEPTITFGTAIAAF
YMPVTIMTILYWRIYKETEKRTKELAGLQASGTEAETENFVHPTGSSRSCSSYELQQQSM
KRSNRRKYGRCHFWFTTKSWKPSSEQMDQDHSSSDSWNNNDAAASLENSASSDEEDIGSE
TRAIYSIVLKLPGHSTILNSTKLPSSDNLQVPEEELGMVDLERKADKLQAQKSVDDGGSF
PKSFSKLPIQLESAVDTAKTSDVNSSVGKSTATLPLSFKEATLAKRFALKTRSQITKRKR
MSLVKEKKAAQTLSAILLAFIITWTPYNIMVLVNTFCDSCIPKTFWNLGYWLCYINSTVN
PVCYALCNKTFRTTFKMLLLCQCDKKKRRKQQYQQRQSVIFHKRAPEQAL
>sp|Q8N9L9|ACOT4_HUMAN Acyl-coenzyme A thioesterase 4 OS=Homo sapiens GN=ACOT4 PE=1 SV=2
MSATLILEPPGRCCWNEPVRIAVRGLAPEQRVTLRASLRDEKGALFRAHARYCADARGEL
DLERAPALGGSFAGLEPMGLLWALEPEKPFWRFLKRDVQIPFVVELEVLDGHDPEPGRLL
CQAQHERHFLPPGVRRQSVRAGRVRATLFLPPGPGPFPGIIDIFGIGGGLLEYRASLLAG
HGFATLALAYYNFEDLPNNMDNISLEYFEEAVCYMLQHPQVKGPGIGLLGISLGADICLS

MASFLKNVSATVSINGSGISGNTAINYKHSSIPPLGYDLRRIKVAFSGLVDIVDIRNALV
GGYKNPSMIPIEKAQGPILLIVGQDDHNWRSELYAQTVSERLQAHGKEKPQIICYPGTGH
YIEPPYFPLCPASLHRLLNKHVIWGGEPRAHSKAQEDAWKQILAFFCKHLGGTQKTAVPK
L
>sp|O14734|ACOT8_HUMAN Acyl-coenzyme A thioesterase 8 OS=Homo sapiens GN=ACOT8 PE=1 SV=1
MSSPQAPEDGQGCGDRGDPPGDLRSVLVTTVLNLEPLDEDLFRGRHYWVPAKRLFGGQIV
GQALVAAAKSVSEDVHVHSLHCYFVRAGDPKLPVLYQVERTRTGSSFSVRSVKAVQHGKP
IFICQASFQQAQPSPMQHQFSMPTVPPPEELLDCETLIDQYLRDPNLQKRYPLALNRIAA
QEVPIEIKPVNPSPLSQLQRMEPKQMFWVRARGYIGEGDMKMHCCVAAYISDYAFLGTAL
LPHQWQHKVHFMVSLDHSMWFHAPFRADHWMLYECESPWAGGSRGLVHGRLWRQDGVLAV
TCAQEGVIRVKPQVSESKL
>sp|Q6ZNF0|ACP7_HUMAN Acid phosphatase type 7 OS=Homo sapiens GN=ACP7 PE=2 SV=2
MHPLPGYWSCYCLLLLFSLGVQGSLGAPSAAPEQVHLSYPGEPGSMTVTWTTWVPTRSEV
QFGLQPSGPLPLRAQGTFVPFVDGGILRRKLYIHRVTLRKLLPGVQYVYRCGSAQGWSRR
FRFRALKNGAHWSPRLAVFGDLGADNPKAVPRLRRDTQQGMYDAVLHVGDFAYNLDQDNA
RVGDRFMRLIEPVAASLPYMTCPGNHEERYNFSNYKARFSMPGDNEGLWYSWDLGPAHII
SFSTEVYFFLHYGRHLVQRQFRWLESDLQKANKNRAARPWIITMGHRPMYCSNADLDDCT
RHESKVRKGLQGKLYGLEDLFYKYGVDLQLWAHEHSYERLWPIYNYQVFNGSREMPYTNP
RGPVHIITGSAGCEERLTPFAVFPRPWSAVRVKEYGYTRLHILNGTHIHIQQVSDDQDGK
IVDDVWVVRPLFGRRMYL
>sp|P13798|ACPH_HUMAN Acylamino-acid-releasing enzyme OS=Homo sapiens GN=APEH PE=1 SV=4
MERQVLLSEPEEAAALYRGLSRQPALSAACLGPEVTTQYGGQYRTVHTEWTQRDLERMEN
IRFCRQYLVFHDGDSVVFAGPAGNSVETRGELLSRESPSGTMKAVLRKAGGTGPGEEKQF
LEVWEKNRKLKSFNLSALEKHGPVYEDDCFGCLSWSHSETHLLYVAEKKRPKAESFFQTK
ALDVSASDDEIARLKKPDQAIKGDQFVFYEDWGENMVSKSIPVLCVLDVESGNISVLEGV
PENVSPGQAFWAPGDAGVVFVGWWHEPFRLGIRFCTNRRSALYYVDLIGGKCELLSDDSL
AVSSPRLSPDQCRIVYLQYPSLIPHHQCSQLCLYDWYTKVTSVVVDVVPRQLGENFSGIY
CSLLPLGCWSADSQRVVFDSAQRSRQDLFAVDTQVGTVTSLTAGGSGGSWKLLTIDQDLM
VAQFSTPSLPPTLKVGFLPSAGKEQSVLWVSLEEAEPIPDIHWGIRVLQPPPEQENVQYA
GLDFEAILLQPGSPPDKTQVPMVVMPHGGPHSSFVTAWMLFPAMLCKMGFAVLLVNYRGS
TGFGQDSILSLPGNVGHQDVKDVQFAVEQVLQEEHFDASHVALMGGSHGGFISCHLIGQY
PETYRACVARNPVINIASMLGSTDIPDWCVVEAGFPFSSDCLPDLSVWAEMLDKSPIRYI
PQVKTPLLLMLGQEDRRVPFKQGMEYYRALKTRNVPVRLLLYPKSTHALSEVEVESDSFM
NAVLWLRTHLGS
>sp|Q9ULC5|ACSL5_HUMAN Long-chain-fatty-acid--CoA ligase 5 OS=Homo sapiens GN=ACSL5 PE=1 SV=1
MLFIFNFLFSPLPTPALICILTFGAAIFLWLITRPQPVLPLLDLNNQSVGIEGGARKGVS
QKNNDLTSCCFSDAKTMYEVFQRGLAVSDNGPCLGYRKPNQPYRWLSYKQVSDRAEYLGS
CLLHKGYKSSPDQFVGIFAQNRPEWIISELACYTYSMVAVPLYDTLGPEAIVHIVNKADI
AMVICDTPQKALVLIGNVEKGFTPSLKVIILMDPFDDDLKQRGEKSGIEILSLYDAENLG
KEHFRKPVPPSPEDLSVICFTSGTTGDPKGAMITHQNIVSNAAAFLKCVEHAYEPTPDDV
AISYLPLAHMFERIVQAVVYSCGARVGFFQGDIRLLADDMKTLKPTLFPAVPRLLNRIYD
KVQNEAKTPLKKFLLKLAVSSKFKELQKGIIRHDSFWDKLIFAKIQDSLGGRVRVIVTGA
APMSTSVMTFFRAAMGCQVYEAYGQTECTGGCTFTLPGDWTSGHVGVPLACNYVKLEDVA

DMNYFTVNNEGEVCIKGTNVFKGYLKDPEKTQEALDSDGWLHTGDIGRWLPNGTLKIIDR
KKNIFKLAQGEYIAPEKIENIYNRSQPVLQIFVHGESLRSSLVGVVVPDTDVLPSFAAKL
GVKGSFEELCQNQVVREAILEDLQKIGKESGLKTFEQVKAIFLHPEPFSIENGLLTPTLK
AKRGELSKYFRTQIDSLYEHIQD
>sp|P0C7M7|ACSM4_HUMAN Acyl-coenzyme A synthetase ACSM4, mitochondrial OS=Homo sapiens GN=ACSM4 PE=2 SV=1
MKIFFRYQTFRFIWLTKPPGRRLHKDHQLWTPLTLADFEAINRCNRPLPKNFNFAADVLD
QWSQKEKTGERPANPALWWVNGKGDEVKWSFRELGSLSRKAANVLTKPCGLQRGDRLAVI
LPRIPEWWLVNVACIRTGIIFMPGTIQLTAKDILYRLRASKAKCIVASEEVAPAVESIVL
ECPDLKTKLLVSPQSWNGWLSFQELFQFASEEHSCVETGSQEPMTIYFTSGTTGFPKMAQ
HSQSSLGIGFTLCGRYWLDLKSSDIIWNMSDTGWVKAAIGSVFSSWLCGACVFVHRMAQF
DTDTFLDTLTTYPITTLCSPPTVYRMLVQKDLKRYKFKSLRHCLTGGEPLNPEVLEQWRV
QTGLELYEGYGQTEVGMICANQKGQEIKPGSMGKGMLPYDVQIIDENGNVLPPGKEGEIA
LRLKPTRPFCFFSKYVDNPQKTAATIRGDFYVTGDRGVMDSDGYFWFVGRADDVIISSGY
RIGPFEVESALIEHPAVVESAVVSSPDQIRGEVVKAFVVLAAPFKSYNPEKLTLELQDHV
KKSTAPYKYPRKVEFVQELPKTITGKIKRNVLRDQEWRGR
>sp|P60709|ACTB_HUMAN Actin, cytoplasmic 1 OS=Homo sapiens GN=ACTB PE=1 SV=1
MDDDIAALVVDNGSGMCKAGFAGDDAPRAVFPSIVGRPRHQGVMVGMGQKDSYVGDEAQS
KRGILTLKYPIEHGIVTNWDDMEKIWHHTFYNELRVAPEEHPVLLTEAPLNPKANREKMT
QIMFETFNTPAMYVAIQAVLSLYASGRTTGIVMDSGDGVTHTVPIYEGYALPHAILRLDL
AGRDLTDYLMKILTERGYSFTTTAEREIVRDIKEKLCYVALDFEQEMATAASSSSLEKSY
ELPDGQVITIGNERFRCPEALFQPSFLGMESCGIHETTFNSIMKCDVDIRKDLYANTVLS
GGTTMYPGIADRMQKEITALAPSTMKIKIIAPPERKYSVWIGGSILASLSTFQQMWISKQ
EYDESGPSIVHRKCF
>sp|P36896|ACV1B_HUMAN Activin receptor type-1B OS=Homo sapiens GN=ACVR1B PE=1 SV=1
MAESAGASSFFPLVVLLLAGSGGSGPRGVQALLCACTSCLQANYTCETDGACMVSIFNLD
GMEHHVRTCIPKVELVPAGKPFYCLSSEDLRNTHCCYTDYCNRIDLRVPSGHLKEPEHPS
MWGPVELVGIIAGPVFLLFLIIIIVFLVINYHQRVYHNRQRLDMEDPSCEMCLSKDKTLQ
DLVYDLSTSGSGSGLPLFVQRTVARTIVLQEIIGKGRFGEVWRGRWRGGDVAVKIFSSRE
ERSWFREAEIYQTVMLRHENILGFIAADNKDNGTWTQLWLVSDYHEHGSLFDYLNRYTVT
IEGMIKLALSAASGLAHLHMEIVGTQGKPGIAHRDLKSKNILVKKNGMCAIADLGLAVRH
DAVTDTIDIAPNQRVGTKRYMAPEVLDETINMKHFDSFKCADIYALGLVYWEIARRCNSG
GVHEEYQLPYYDLVPSDPSIEEMRKVVCDQKLRPNIPNWWQSYEALRVMGKMMRECWYAN
GAARLTALRIKKTLSQLSVQEDVKI
>sp|O43184|ADA12_HUMAN Disintegrin and metalloproteinase domain-containing protein 12 OS=Homo sapiens GN=ADAM12 PE=1 SV=3
MAARPLPVSPARALLLALAGALLAPCEARGVSLWNQGRADEVVSASVGSGDLWIPVKSFD
SKNHPEVLNIRLQRESKELIINLERNEGLIASSFTETHYLQDGTDVSLARNYTVILGHCY
YHGHVRGYSDSAVSLSTCSGLRGLIVFENESYVLEPMKSATNRYKLFPAKKLKSVRGSCG
SHHNTPNLAAKNVFPPPSQTWARRHKRETLKATKYVELVIVADNREFQRQGKDLEKVKQR
LIEIANHVDKFYRPLNIRIVLVGVEVWNDMDKCSVSQDPFTSLHEFLDWRKMKLLPRKSH
DNAQLVSGVYFQGTTIGMAPIMSMCTADQSGGIVMDHSDNPLGAAVTLAHELGHNFGMNH
DTLDRGCSCQMAVEKGGCIMNASTGYPFPMVFSSCSRKDLETSLEKGMGVCLFNLPEVRE
SFGGQKCGNRFVEEGEECDCGEPEECMNRCCNATTCTLKPDAVCAHGLCCEDCQLKPAGT

ACRDSSNSCDLPEFCTGASPHCPANVYLHDGHSCQDVDGYCYNGICQTHEQQCVTLWGPG
AKPAPGICFERVNSAGDPYGNCGKVSKSSFAKCEMRDAKCGKIQCQGGASRPVIGTNAVS
IETNIPLQQGGRILCRGTHVYLGDDMPDPGLVLAGTKCADGKICLNRQCQNISVFGVHEC
AMQCHGRGVCNNRKNCHCEAHWAPPFCDKFGFGGSTDSGPIRQADNQGLTIGILVTILCL
LAAGFVVYLKRKTLIRLLFTNKKTTIEKLRCVRPSRPPRGFQPCQAHLGHLGKGLMRKPP
DSYPPKDNPRRLLQCQNVDISRPLNGLNVPQPQSTQRVLPPLHRAPRAPSVPARPLPAKP
ALRQAQGTCKPNPPQKPLPADPLARTTRLTHALARTPGQWETGLRLAPLRPAPQYPHQVP
RSTHTAYIK
>sp|Q9Y3Q7|ADA18_HUMAN Disintegrin and metalloproteinase domain-containing protein 18 OS=Homo sapiens GN=ADAM18 PE=2 SV=1
MFLLLALLTELGRLQAHEGSEGIFLHVTVPRKIKSNDSEVSERKMIYIITIDGQPYTLHL
GKQSFLPQNFLVYTYNETGSLHSVSPYFMMHCHYQGYAAEFPNSFVTLSICSGLRGFLQF
ENISYGIEPVESSARFEHIIYQMKNNDPNVSILAVNYSHIWQKDQPYKVPLNSQIKNLSK
LLPQYLEIYIIVEKALYDYMGSEMMAVTQKIVQVIGLVNTMFTQFKLTVILSSLELWSNE
NQISTSGDADDILQRFLAWKRDYLILRPHDIAYLLVYRKHPKYVGATFPGTVCNKSYDAG
IAMYPDAIGLEGFSVIIAQLLGLNVGLTYDDITQCFCLRATCIMNHEAVSASGRKIFSNC
SMHDYRYFVSKFETKCLQKLSNLQPLHQNQPVCGNGILESNEECDCGNKNECQFKKCCDY
NTCKLKGSVKCGSGPCCTSKCELSIAGTPCRKSIDPECDFTEYCNGTSSNCVPDTYALNG
RLCKLGTAYCYNGQCQTTDNQCAKIFGKGAQGAPFACFKEVNSLHERSENCGFKNSQPLP
CERKDVLCGKLACVQPHKNANKSDAQSTVYSYIQDHVCVSIATGSSMRSDGTDNAYVADG
TMCGPEMYCVNKTCRKVHLMGYNCNATTKCKGKGICNNFGNCQCFPGHRPPDCKFQFGSP
GGSIDDGNFQKSGDFYTEKGYNTHWNNWFILSFCIFLPFFIVFTTVIFKRNEISKSCNRE
NAEYNRNSSVVSESDDVGH
>sp|P25100|ADA1D_HUMAN Alpha-1D adrenergic receptor OS=Homo sapiens GN=ADRA1D PE=1 SV=2
MTFRDLLSVSFEGPRPDSSAGGSSAGGGGGSAGGAAPSEGPAVGGVPGGAGGGGGVVGAG
SGEDNRSSAGEPGSAGAGGDVNGTAAVGGLVVSAQGVGVGVFLAAFILMAVAGNLLVILS
VACNRHLQTVTNYFIVNLAVADLLLSATVLPFSATMEVLGFWAFGRAFCDVWAAVDVLCC
TASILSLCTISVDRYVGVRHSLKYPAIMTERKAAAILALLWVVALVVSVGPLLGWKEPVP
PDERFCGITEEAGYAVFSSVCSFYLPMAVIVVMYCRVYVVARSTTRSLEAGVKRERGKAS
EVVLRIHCRGAATGADGAHGMRSAKGHTFRSSLSVRLLKFSREKKAAKTLAIVVGVFVLC
WFPFFFVLPLGSLFPQLKPSEGVFKVIFWLGYFNSCVNPLIYPCSSREFKRAFLRLLRCQ
CRRRRRRRPLWRVYGHHWRASTSGLRQDCAPSSGDAPPGAPLALTALPDPDPEPPGTPEM
QAPVASRRKPPSAFREWRLLGPFRRPTTQLRAKVSSLSHKIRAGGAQRAEAACAQRSEVE
AVSLGVPHEVAEGATCQAYELADYSNLRETDI
>sp|Q9UKJ8|ADA21_HUMAN Disintegrin and metalloproteinase domain-containing protein 21 OS=Homo sapiens GN=ADAM21 PE=2 SV=2
MAVDGTLVYIRVTLLLLWLGVFLSISGYCQAGPSQHFTSPEVVIPLKVISRGRSAKAPGW
LSYSLRFGGQKHVVHMRVKKLLVSRHLPVFTYTDDRALLEDQLFIPDDCYYHGYVEAAPE
SLVVFSACFGGFRGVLKISGLTYEIEPIRHSATFEHLVYKINSNETQFPAMRCGLTEKEV
ARQQLEFEEAENSALEPKSAGDWWTHAWFLELVVVVNHDFFIYSQSNISKVQEDVFLVVN
IVDSMYKQLGTYIILIGIEIWNQGNVFPMTSIEQVLNDFSQWKQISLSQLQHDAAHMFIK
NSLISILGLAYVAGICRPPIDCGVDNFQGDTWSLFANTVAHELGHTLGMQHDEEFCFCGE
RGCIMNTFRVPAEKFTNCSYADFMKTTLNQGSCLHNPPRLGEIFMLKRCGNGVVEREEQC
DCGSVQQCEQDACCLLNCTLRPGAACAFGLCCKDCKFMPSGELCRQEVNECDLPEWCNGT

SHQCPEDRYVQDGIPCSDSAYCYQKRCNNHDQHCREIFGKDAKSASQNCYKEINSQGNRF
GHCGINGTTYLKCHISDVFCGRVQCENVRDIPLLQDHFTLQHTHINGVTCWGIDYHLRMN
ISDIGEVKDGTVCGPGKICIHKKCVSLSVLSHVCLPETCNMKGICNNKHHCHCGYGWSPP
YCQHRGYGGSIDSGPASAKRGVFLPLIVIPSLSVLTFLFTVGLLMYLRQCSGPKETKAHS
SG
>sp|O43306|ADCY6_HUMAN Adenylate cyclase type 6 OS=Homo sapiens GN=ADCY6 PE=1 SV=2
MSWFSGLLVPKVDERKTAWGERNGQKRSRRRGTRAGGFCTPRYMSCLRDAEPPSPTPAGP
PRCPWQDDAFIRRGGPGKGKELGLRAVALGFEDTEVTTTAGGTAEVAPDAVPRSGRSCWR
RLVQVFQSKQFRSAKLERLYQRYFFQMNQSSLTLLMAVLVLLTAVLLAFHAAPARPQPAY
VALLACAAALFVGLMVVCNRHSFRQDSMWVVSYVVLGILAAVQVGGALAADPRSPSAGLW
CPVFFVYIAYTLLPIRMRAAVLSGLGLSTLHLILAWQLNRGDAFLWKQLGANVLLFLCTN
VIGICTHYPAEVSQRQAFQETRGYIQARLHLQHENRQQERLLLSVLPQHVAMEMKEDINT
KKEDMMFHKIYIQKHDNVSILFADIEGFTSLASQCTAQELVMTLNELFARFDKLAAENHC
LRIKILGDCYYCVSGLPEARADHAHCCVEMGVDMIEAISLVREVTGVNVNMRVGIHSGRV
HCGVLGLRKWQFDVWSNDVTLANHMEAGGRAGRIHITRATLQYLNGDYEVEPGRGGERNA
YLKEQHIETFLILGASQKRKEEKAMLAKLQRTRANSMEGLMPRWVPDRAFSRTKDSKAFR
QMGIDDSSKDNRGTQDALNPEDEVDEFLSRAIDARSIDQLRKDHVRRFLLTFQREDLEKK
YSRKVDPRFGAYVACALLVFCFICFIQLLIFPHSTLMLGIYASIFLLLLITVLICAVYSC
GSLFPKALQRLSRSIVRSRAHSTAVGIFSVLLVFTSAIANMFTCNHTPIRSCAARMLNLT
PADITACHLQQLNYSLGLDAPLCEGTMPTCSFPEYFIGNMLLSLLASSVFLHISSIGKLA
MIFVLGLIYLVLLLLGPPATIFDNYDLLLGVHGLASSNETFDGLDCPAAGRVALKYMTPV
ILLVFALALYLHAQQVESTARLDFLWKLQATGEKEEMEELQAYNRRLLHNILPKDVAAHF
LARERRNDELYYQSCECVAVMFASIANFSEFYVELEANNEGVECLRLLNEIIADFDEIIS
EERFRQLEKIKTIGSTYMAASGLNASTYDQVGRSHITALADYAMRLMEQMKHINEHSFNN
FQMKIGLNMGPVVAGVIGARKPQYDIWGNTVNVSSRMDSTGVPDRIQVTTDLYQVLAAKG
YQLECRGVVKVKGKGEMTTYFLNGGPSS
>sp|O15204|ADEC1_HUMAN ADAM DEC1 OS=Homo sapiens GN=ADAMDEC1 PE=1 SV=2
MLRGISQLPAVATMSWVLLPVLWLIVQTQAIAIKQTPELTLHEIVCPKKLHILHKREIKN
NQTEKHGKEERYEPEVQYQMILNGEEIILSLQKTKHLLGPDYTETLYSPRGEEITTKPEN
MEHCYYKGNILNEKNSVASISTCDGLRGYFTHHHQRYQIKPLKSTDEKEHAVFTSNQEEQ
DPANHTCGVKSTDGKQGPIRISRSLKSPEKEDFLRAQKYIDLYLVLDNAFYKNYNENLTL
IRSFVFDVMNLLNVIYNTIDVQVALVGMEIWSDGDKIKVVPSASTTFDNFLRWHSSNLGK
KIHDHAQLLSGISFNNRRVGLAASNSLCSPSSVAVIEAKKKNNVALVGVMSHELGHVLGM
PDVPFNTKCPSGSCVMNQYLSSKFPKDFSTSCRAHFERYLLSQKPKCLLQAPIPTNIMTT
PVCGNHLLEVGEDCDCGSPKECTNLCCEALTCKLKPGTDCGGDAPNHTTE
>sp|P00325|ADH1B_HUMAN Alcohol dehydrogenase 1B OS=Homo sapiens GN=ADH1B PE=1 SV=2
MSTAGKVIKCKAAVLWEVKKPFSIEDVEVAPPKAYEVRIKMVAVGICRTDDHVVSGNLVT
PLPVILGHEAAGIVESVGEGVTTVKPGDKVIPLFTPQCGKCRVCKNPESNYCLKNDLGNP
RGTLQDGTRRFTCRGKPIHHFLGTSTFSQYTVVDENAVAKIDAASPLEKVCLIGCGFSTG
YGSAVNVAKVTPGSTCAVFGLGGVGLSAVMGCKAAGAARIIAVDINKDKFAKAKELGATE
CINPQDYKKPIQEVLKEMTDGGVDFSFEVIGRLDTMMASLLCCHEACGTSVIVGVPPASQ
NLSINPMLLLTGRTWKGAVYGGFKSKEGIPKLVADFMAKKFSLDALITHVLPFEKINEGF
DLLHSGKSIRTVLTF
>sp|P11766|ADHX_HUMAN Alcohol dehydrogenase class-3 OS=Homo sapiens GN=ADH5 PE=1 SV=4

MANEVIKCKAAVAWEAGKPLSIEEIEVAPPKAHEVRIKIIATAVCHTDAYTLSGADPEGC
FPVILGHEGAGIVESVGEGVTKLKAGDTVIPLYIPQCGECKFCLNPKTNLCQKIRVTQGK
GLMPDGTSRFTCKGKTILHYMGTSTFSEYTVVADISVAKIDPLAPLDKVCLLGCGISTGY
GAAVNTAKLEPGSVCAVFGLGGVGLAVIMGCKVAGASRIIGVDINKDKFARAKEFGATEC
INPQDFSKPIQEVLIEMTDGGVDYSFECIGNVKVMRAALEACHKGWGVSVVVGVAASGEE
IATRPFQLVTGRTWKGTAFGGWKSVESVPKLVSEYMSKKIKVDEFVTHNLSFDEINKAFE
LMHSGKSIRTVVKI
>sp|Q0VDE8|ADIG_HUMAN Adipogenin OS=Homo sapiens GN=ADIG PE=3 SV=1
MKYPLMPLVNDLTFSFLVFWFCLPVGLLLLLIIWLRFLLSQDSEENDSSVCLDWEPWSKG
PAEFCWKGTLHGQEKERPCW
>sp|Q9H2P0|ADNP_HUMAN Activity-dependent neuroprotector homeobox protein OS=Homo sapiens GN=ADNP PE=1 SV=1
MFQLPVNNLGSLRKARKTVKKILSDIGLEYCKEHIEDFKQFEPNDFYLKNTTWEDVGLWD
PSLTKNQDYRTKPFCCSACPFSSKFFSAYKSHFRNVHSEDFENRILLNCPYCTFNADKKT
LETHIKIFHAPNASAPSSSLSTFKDKNKNDGLKPKQADSVEQAVYYCKKCTYRDPLYEIV
RKHIYREHFQHVAAPYIAKAGEKSLNGAVPLGSNAREESSIHCKRCLFMPKSYEALVQHV
IEDHERIGYQVTAMIGHTNVVVPRSKPLMLIAPKPQDKKSMGLPPRIGSLASGNVRSLPS
QQMVNRLSIPKPNLNSTGVNMMSSVHLQQNNYGVKSVGQGYSVGQSMRLGLGGNAPVSIP
QQSQSVKQLLPSGNGRSYGLGSEQRSQAPARYSLQSANASSLSSGQLKSPSLSQSQASRV
LGQSSSKPAAAATGPPPGNTSSTQKWKICTICNELFPENVYSVHFEKEHKAEKVPAVANY
IMKIHNFTSKCLYCNRYLPTDTLLNHMLIHGLSCPYCRSTFNDVEKMAAHMRMVHIDEEM
GPKTDSTLSFDLTLQQGSHTNIHLLVTTYNLRDAPAESVAYHAQNNPPVPPKPQPKVQEK
ADIPVKSSPQAAVPYKKDVGKTLCPLCFSILKGPISDALAHHLRERHQVIQTVHPVEKKL
TYKCIHCLGVYTSNMTASTITLHLVHCRGVGKTQNGQDKTNAPSRLNQSPSLAPVKRTYE
QMEFPLLKKRKLDDDSDSPSFFEEKPEEPVVLALDPKGHEDDSYEARKSFLTKYFNKQPY
PTRREIEKLAASLWLWKSDIASHFSNKRKKCVRDCEKYKPGVLLGFNMKELNKVKHEMDF
DAEWLFENHDEKDSRVNASKTADKKLNLGKEDDSSSDSFENLEEESNESGSPFDPVFEVE
PKISNDNPEEHVLKVIPEDASESEEKLDQKEDGSKYETIHLTEEPTKLMHNASDSEVDQD
DVVEWKDGASPSESGPGSQQVSDFEDNTCEMKPGTWSDESSQSEDARSSKPAAKKKATMQ
GDREQLKWKNSSYGKVEGFWSKDQSQWKNASENDERLSNPQIEWQNSTIDSEDGEQFDNM
TDGVAEPMHGSLAGVKLSSQQA
>sp|Q86V24|ADR2_HUMAN Adiponectin receptor protein 2 OS=Homo sapiens GN=ADIPOR2 PE=1 SV=1
MNEPTENRLGCSRTPEPDIRLRKGHQLDGTRRGDNDSHQGDLEPILEASVLSSHHKKSSE
EHEYSDEAPQEDEGFMGMSPLLQAHHAMEKMEEFVCKVWEGRWRVIPHDVLPDWLKDNDF
LLHGHRPPMPSFRACFKSIFRIHTETGNIWTHLLGCVFFLCLGIFYMFRPNISFVAPLQE
KVVFGLFFLGAILCLSFSWLFHTVYCHSEGVSRLFSKLDYSGIALLIMGSFVPWLYYSFY
CNPQPCFIYLIVICVLGIAAIIVSQWDMFATPQYRGVRAGVFLGLGLSGIIPTLHYVISE
GFLKAATIGQIGWLMLMASLYITGAALYAARIPERFFPGKCDIWFHSHQLFHIFVVAGAF
VHFHGVSNLQEFRFMIGGGCSEEDAL
>sp|P07550|ADRB2_HUMAN Beta-2 adrenergic receptor OS=Homo sapiens GN=ADRB2 PE=1 SV=3
MGQPGNGSAFLLAPNGSHAPDHDVTQERDEVWVVGMGIVMSLIVLAIVFGNVLVITAIAK
FERLQTVTNYFITSLACADLVMGLAVVPFGAAHILMKMWTFGNFWCEFWTSIDVLCVTAS
IETLCVIAVDRYFAITSPFKYQSLLTKNKARVIILMVWIVSGLTSFLPIQMHWYRATHQE
AINCYANETCCDFFTNQAYAIASSIVSFYVPLVIMVFVYSRVFQEAKRQLQKIDKSEGRF

HVQNLSQVEQDGRTGHGLRRSSKFCLKEHKALKTLGIIMGTFTLCWLPFFIVNIVHVIQD
NLIRKEVYILLNWIGYVNSGFNPLIYCRSPDFRIAFQELLCLRRSSLKAYGNGYSSNGNT
GEQSGYHVEQEKENKLLCEDLPGTEDFVGHQGTVPSDNIDSQGRNCSTNDSLL
>sp|Q96IZ2|ADTRP_HUMAN Androgen-dependent TFPI-regulating protein OS=Homo sapiens GN=ADTRP PE=2 SV=1
MTKTSTCIYHFLVLSWYTFLNYYISQEGKDEVKPKILANGARWKYMTLLNLLLQTIFYGV
TCLDDVLKRTKGGKDIKFLTAFRDLLFTTLAFPVSTFVFLAFWILFLYNRDLIYPKVLDT
VIPVWLNHAMHTFIFPITLAEVVLRPHSYPSKKTGLTLLAAASIAYISRILWLYFETGTW
VYPVFAKLSLLGLAAFFSLSYVFIASIYLLGEKLNHWKWGDMRQPRKKRK
>sp|C9J202|AG1L2_HUMAN Putative glycosyltransferase ALG1L2 OS=Homo sapiens GN=ALG1L2 PE=3 SV=1
MGATAGWAVTVVYDKPASFFKEAPLDLQHRLFMKLGSTHSPFRARSEPEDPDTERSAFTER
DSGSGLVTRLHERPALLVSSTSWTEFEQLTLDGQNLPSLVCVITGKGPLREYYSRLIHQK
HFQHIQVCIPWLEGRGLPPLLGSVDLDVCLDTSSSGLDLPMKVVDMFRCCLPACAVNFKC
LHELVKHEENRLVFEDSEELAAQLQYFADAFLKLS
>sp|P06280|AGAL_HUMAN Alpha-galactosidase A OS=Homo sapiens GN=GLA PE=1 SV=1
MQLRNPELHLGCALALRFLALVSWDIPGARALDNGLARTPTMGWLHWERFMCNLDCQEEP
DSCISEKLFMEMAELMVSEGWKDAGYEYLCIDDCWMAPQRDSEGRLQADPQRFPHGIRQL
ANYVHSKGLKLGIYADVGNKTCAGFPGSFGYYDIDAQTFADWGVDLLKFDGCYCDSLENL
ADGYKHMSLALNRTGRSIVYSCEWPLYMWPFQKPNYTEIRQYCNHWRNFADIDDSWKSIK
SILDWTSFNQERIVDVAGPGGWNDPDMLVIGNFGLSWNQQVTQMALWAIMAAPLFMSNDL
RHISPQAKALLQDKDVIAINQDPLGKQGYQLRQGDNFEVWERPLSGLAWAVAMINRQEIG
GPRSYTIAVASLGKGVACNPACFITQLLPVKRKLGFYEWTSRLRSHINPTGTVLLQLENT
MQMSLKDLL
>sp|Q9UPQ3|AGAP1_HUMAN Arf-GAP with GTPase, ANK repeat and PH domain-containing protein 1 OS=Homo sapiens GN=AGAP1 PE=1 SV=4
MNYQQQLANSAAIRAEIQRFESVHPNIYSIYELLERVEEPVLQNQIREHVIAIEDAFVNS
QEWTLSRSVPELKVGIVGNLASGKSALVHRYLTGTYVQEESPEGGRFKKEIVVDGQSYLL
LIRDEGGPPEAQFAMWVDAVIFVFSLEDEISFQTVYHYYSRMANYRNTSEIPLVLVGTQD
AISSANPRVIDDARARKLSNDLKRCTYYETCATYGLNVERVFQDVAQKIVATRKKQQLSI
GPCKSLPNSPSHSSVCSAQVSAVHISQTSNGGGSLSDYSSSVPSTPSTSQKELRIDVPPT
ANTPTPVRKQSKRRSNLFTSRKGSDPDKEKKGLESRADSIGSGRAIPIKQGMLLKRSGKS
LNKEWKKKYVTLCDNGVLTYHPSLHDYMQNVHGKEIDLLRTTVKVPGKRPPRATSACAPI
SSPKTNGLSKDMSSLHISPNSGNVTSASGSQMASGISLVSFNSRPDGMHQRSYSVSSADQ
WSEATVIANSAISSDTGLGDSVCSSPSISSTTSPKLDPPPSPHANRKKHRRKKSTSNFKA
DGLSGTAEEQEENFEFIIVSLTGQTWHFEATTYEERDAWVQAIESQILASLQSCESSKNK
SRLTSQSEAMALQSIRNMRGNSHCVDCETQNPNWASLNLGALMCIECSGIHRNLGTHLSR
VRSLDLDDWPVELIKVMSSIGNELANSVWEESSQGRTKPSVDSTREEKERWIRAKYEQKL
FLAPLPCTELSLGQHLLRATADEDLRTAILLLAHGSRDEVNETCGEGDGRTALHLACRKG
NVVLAQLLIWYGVDVTARDAHGNTALAYARQASSQECIDVLLQYGCPDERFVLMATPNLS
RRNNNRNNSSGRVPTII
>sp|Q5VW22|AGAP6_HUMAN Arf-GAP with GTPase, ANK repeat and PH domain-containing protein 6 OS=Homo sapiens GN=AGAP6 PE=2 SV=1
MGNILTCRVHPSVSLEFDQQQGSVCPSESETYEAGARDRMAGAPMAAAVQPAEVTVEVGE

DLHMHHVRDREMPEALEFNPSANPEASTIFQRNSQTDVVEIRRSNCTNHVSAVRFSQQYS
LCSTIFLDDSTAIQHYLTMTIISVTLEIPHHITQRDADRTLSIPDEQLHSFAVSTVHIMK
KRNGGGSLNNYSSSIPSTPSTSQEDPQFSVPPTANTPTPVCKRSMRWSNLFTSEKGSDPD
KERKAPENHADTIGSGRAIPIKQGMLLKRSGKWLKTWKKKYVTLCSNGMLTYYSSLGDYM
KNIHKKEIDLQTSTIKVPGKWPSLATSACTPISSSKSNGLSKDMDTGLGDSICFSPSISS
TTSPKLNPPPSPHANKKKHLKKKSTNNFMIVSATGQTWHFEATTYEERDAWVQAIQSQIL
ASLQSCESSKSKSQLTSQSEAMALQSIQNMRGNAHCVDCETQNPKWASLNLGVLMCIECS
GIHRSLGPHLSRVRSLELDDWPVELRKVMSSIVNDLANSIWEGSSQGQTKPSEKSTREEK
ERWIRSKYEEKLFLAPLPCTELSLGQQLLRATADEDLQTAILLLAHGSCEEVNETCGEGD
GCTALHLACRKGNVVLAQLLIWYGVDVMARDAHGNTALTYARQASSQECINVLLQYGCPD
ECV
>sp|Q8N302|AGGF1_HUMAN Angiogenic factor with G patch and FHA domains 1 OS=Homo sapiens GN=AGGF1 PE=1 SV=2
MASEAPSPPRSPPPPTSPEPELAQLRRKVEKLERELRSCKRQVREIEKLLHHTERLYQNA
ESNNQELRTQVEELSKILQRGRNEDNKKSDVEVQTENHAPWSISDYFYQTYYNDVSLPNK
VTELSDQQDQAIETSILNSKDHLQVENDAYPGTDRTENVKYRQVDHFASNSQEPASALAT
EDTSLEGSSLAESLRAAAEAAVSQTGFSYDENTGLYFDHSTGFYYDSENQLYYDPSTGIY
YYCDVESGRYQFHSRVDLQPYPTSSTKQSKDKKLKKKRKDPDSSATNEEKDLNSEDQKAF
SVEHTSCNEEENFANMKKKAKIGIHHKNSPPKVTVPTSGNTIESPLHENISNSTSFKDEK
IMETDSEPEEGEITDSQTEDSYDEAITSEGNVTAEDSEDEDEDKIWPPCIRVIVIRSPVL
QIGSLFIITAVNPATIGREKDMEHTLRIPEVGVSKFHAEIYFDHDLQSYVLVDQGSQNGT
IVNGKQILQPKTKCDPYVLEHGDEVKIGETVLSFHIHPGSDTCDGCEPGQVRAHLRLDKK
DESFVGPTLSKEEKELERRKELKKIRVKYGLQNTEYEDEKTLKNPKYKDRAGKRREQVGS
EGTFQRDDAPASVHSEITDSNKGRKMLEKMGWKKGEGLGKDGGGMKTPIQLQLRRTHAGL
GTGKPSSFEDVHLLQNKNKKNWDKARERFTENFPETKPQKDDPGTMPWVKGTLE
>sp|Q9H0P7|AGIT1_HUMAN Putative uncharacterized protein encoded by AGPAT4-IT1 OS=Homo sapiens GN=AGPAT4-IT1 PE=5 SV=1
MADTQCCPPPCEFISSAGTDLALGMGWDATLCLLPFTGFGKCAGIWNHMDEEPDNGDDRG
SRRTTGQGRKWAAHGTMAAPRVHTDYHPGGGSACSSVKVRSHVGHTGVFFFVDQDPLAVS
LTSQSLIPPLIKPGLLKAWGFLLLCAQPSANGHSLCCLLYTDLVSSHELSPFRALCLGPS
DAPSACASCNCLASTYYL
>sp|Q8NEB7|ACRBP_HUMAN Acrosin-binding protein OS=Homo sapiens GN=ACRBP PE=2 SV=1
MRKPAAGFLPSLLKVLLLPLAPAAAQDSTQASTPGSPLSPTEYERFFALLTPTWKAETTC
RLRATHGCRNPTLVQLDQYENHGLVPDGAVCSNLPYASWFESFCQFTHYRCSNHVYYAKR
VLCSQPVSILSPNTLKEIEASAEVSPTTMTSPISPHFTVTERQTFQPWPERLSNNVEELL
QSSLSLGGQEQAPEHKQEQGVEHRQEPTQEHKQEEGQKQEEQEEEQEEEGKQEEGQGTKE
GREAVSQLQTDSEPKFHSESLSSNPSSFAPRVREVESTPMIMENIQELIRSAQEIDEMNE
IYDENSYWRNQNPGSLLQLPHTEALLVLCYSIVENTCIITPTAKAWKYMEEEILGFGKSV
CDSLGRRHMSTCALCDFCSLKLEQCHSEASLQRQQCDTSHKTPFVSPLLASQSLSIGNQV
GSPESGRFYGLDLYGGLHMDFWCARLATKGCEDVRVSGWLQTEFLSFQDGDFPTKICDTD
YIQYPNYCSFKSQQCLMRNRNRKVSRMRCLQNETYSALSPGKSEDVVLRWSQEFSTLTLG
QFG
>sp|P63261|ACTG_HUMAN Actin, cytoplasmic 2 OS=Homo sapiens GN=ACTG1 PE=1 SV=1
MEEEIAALVIDNGSGMCKAGFAGDDAPRAVFPSIVGRPRHQGVMVGMGQKDSYVGDEAQS

KRGILTLKYPIEHGIVTNWDDMEKIWHHTFYNELRVAPEEHPVLLTEAPLNPKANREKMT
QIMFETFNTPAMYVAIQAVLSLYASGRTTGIVMDSGDGVTHTVPIYEGYALPHAILRLDL
AGRDLTDYLMKILTERGYSFTTTAEREIVRDIKEKLCYVALDFEQEMATAASSSSLEKSY
ELPDGQVITIGNERFRCPEALFQPSFLGMESCGIHETTFNSIMKCDVDIRKDLYANTVLS
GGTTMYPGIADRMQKEITALAPSTMKIKIIAPPERKYSVWIGGSILASLSTFQQMWISKQ
EYDESGPSIVHRKCF
>sp|P63267|ACTH_HUMAN Actin, gamma-enteric smooth muscle OS=Homo sapiens GN=ACTG2 PE=1 SV=1
MCEEETTALVCDNGSGLCKAGFAGDDAPRAVFPSIVGRPRHQGVMVGMGQKDSYVGDEAQ
SKRGILTLKYPIEHGIITNWDDMEKIWHHSFYNELRVAPEEHPTLLTEAPLNPKANREKM
TQIMFETFNVPAMYVAIQAVLSLYASGRTTGIVLDSGDGVTHNVPIYEGYALPHAIMRLD
LAGRDLTDYLMKILTERGYSFVTTAEREIVRDIKEKLCYVALDFENEMATAASSSSLEKS
YELPDGQVITIGNERFRCPETLFQPSFIGMESAGIHETTYNSIMKCDIDIRKDLYANNVL
SGGTTMYPGIADRMQKEITALAPSTMKIKIIAPPERKYSVWIGGSILASLSTFQQMWISK
PEYDEAGPSIVHRKCF
>sp|Q9H568|ACTL8_HUMAN Actin-like protein 8 OS=Homo sapiens GN=ACTL8 PE=1 SV=1
MAARTVIIDHGSGFLKAGTAGWNEPQMVFPNIVNYLPCKENPGPSYARRRVSLGIDICHP
DTFSYPIERGRILNWEGVQYLWSFVLENHRREQEVPPVIITETPLREPADRKKMLEILFE
LLHVPSVLLADQLQMSLYASGLLTGVVVDSGYGLTRVQPFHQGRPLPASGKTLEFAGQDL
SAYLLKSLFKEDCDRRCLFQLETVAVTQMNKCYVPQNLGEALDFRERQQSALDESNTYQL
PDGSRVELTPMQRVAPEMFFSPQVFEQPGPSIPRAIVESVESCEISLRPLLVSHVMACGG
NTLYPGFTKRLFRELMGDHVSSTKATVWEGSNRNFSVWLGASVVAHLSTYQSEWMSREEY
GEHMRM
>sp|Q08462|ADCY2_HUMAN Adenylate cyclase type 2 OS=Homo sapiens GN=ADCY2 PE=1 SV=5
MWQEAMRRRRYLRDRSEEAAGGGDGLPRSRDWLYESYYCMSQQHPLIVFLLLIVMGSCLA
LLAVFFALGLEVEDHVAFLITVPTALAIFFAIFILVCIESVFKKLLRLFSLVIWICLVAM
GYLFMCFGGTVSPWDQVSFFLFIIFVVYTMLPFNMRDAIIASVLTSSSHTIVLSVCLSAT
PGGKEHLVWQILANVIIFICGNLAGAYHKHLMELALQQTYQDTCNCIKSRIKLEFEKRQQ
ERLLLSLLPAHIAMEMKAEIIQRLQGPKAGQMENTNNFHNLYVKRHTNVSILYADIVGFT
RLASDCSPGELVHMLNELFGKFDQIAKENECMRIKILGDCYYCVSGLPISLPNHAKNCVK
MGLDMCEAIKKVRDATGVDINMRVGVHSGNVLCGVIGLQKWQYDVWSHDVTLANHMEAGG
VPGRVHISSVTLEHLNGAYKVEEGDGDIRDPYLKQHLVKTYFVINPKGERRSPQHLFRPR
HTLDGAKMRASVRMTRYLESWGAAKPFAHLHHRDSMTTENGKISTTDVPMGQHNFQNRTL
RTKSQKKRFEEELNERMIQAIDGINAQKQWLKSEDIQRISLLFYNKVLEKEYRATALPAF
KYYVTCACLIFFCIFIVQILVLPKTSVLGISFGAAFLLLAFILFVCFAGQLLQCSKKASP
LLMWLLKSSGIIANRPWPRISLTIITTAIILMMAVFNMFFLSDSEETIPPTANTTNTSFS
ASNNQVAILRAQNLFFLPYFIYSCILGLISCSVFLRVNYELKMLIMMVALVGYNTILLHT
HAHVLGDYSQVLFERPGIWKDLKTMGSVSLSIFFITLLVLGRQNEYYCRLDFLWKNKFKK
EREEIETMENLNRVLLENVLPAHVAEHFLARSLKNEELYHQSYDCVCVMFASIPDFKEFY
TESDVNKEGLECLRLLNEIIADFDDLLSKPKFSGVEKIKTIGSTYMAATGLSAVPSQEHS
QEPERQYMHIGTMVEFAFALVGKLDAINKHSFNDFKLRVGINHGPVIAGVIGAQKPQYDI
WGNTVNVASRMDSTGVLDKIQVTEETSLVLQTLGYTCTCRGIINVKGKGDLKTYFVNTEM
SRSLSQSNVAS
>sp|Q8NFM4|ADCY4_HUMAN Adenylate cyclase type 4 OS=Homo sapiens GN=ADCY4 PE=1 SV=1

MARLFSPRPPPSEDLFYETYYSLSQQYPLLLLLLGIVLCALAALLAVAWASGRELTSDPS
FLTTVLCALGGFSLLLGLASREQRLQRWTRPLSGLVWVALLALGHAFLFTGGVVSAWDQV
SYFLFVIFTAYAMLPLGMRDAAVAGLASSLSHLLVLGLYLGPQPDSRPALLPQLAANAVL
FLCGNVAGVYHKALMERALRATFREALSSLHSRRRLDTEKKHQEHLLLSILPAYLAREMK
AEIMARLQAGQGSRPESTNNFHSLYVKRHQGVSVLYADIVGFTRLASECSPKELVLMLNE
LFGKFDQIAKEHECMRIKILGDCYYCVSGLPLSLPDHAINCVRMGLDMCRAIRKLRAATG
VDINMRVGVHSGSVLCGVIGLQKWQYDVWSHDVTLANHMEAGGVPGRVHITGATLALLAG
AYAVEDAGMEHRDPYLRELGEPTYLVIDPRAEEEDEKGTAGGLLSSLEGLKMRPSLLMTR
YLESWGAAKPFAHLSHGDSPVSTSTPLPEKTLASFSTQWSLDRSRTPRGLDDELDTGDAK
FFQVIEQLNSQKQWKQSKDFNPLTLYFREKEMEKEYRLSAIPAFKYYEACTFLVFLSNFI
IQMLVTNRPPALAITYSITFLLFLLILFVCFSEDLMRCVLKGPKMLHWLPALSGLVATRP
GLRIALGTATILLVFAMAITSLFFFPTSSDCPFQAPNVSSMISNLSWELPGSLPLISVPY
SMHCCTLGFLSCSLFLHMSFELKLLLLLLWLAASCSLFLHSHAWLSECLIVRLYLGPLDS
RPGVLKEPKLMGAISFFIFFFTLLVLARQNEYYCRLDFLWKKKLRQEREETETMENLTRL
LLENVLPAHVAPQFIGQNRRNEDLYHQSYECVCVLFASVPDFKEFYSESNINHEGLECLR
LLNEIIADFDELLSKPKFSGVEKIKTIGSTYMAATGLNATSGQDAQQDAERSCSHLGTMV
EFAVALGSKLDVINKHSFNNFRLRVGLNHGPVVAGVIGAQKPQYDIWGNTVNVASRMEST
GVLGKIQVTEETAWALQSLGYTCYSRGVIKVKGKGQLCTYFLNTDLTRTGPPSATLG
>sp|P55263|ADK_HUMAN Adenosine kinase OS=Homo sapiens GN=ADK PE=1 SV=2
MAAAEEEPKPKKLKVEAPQALRENILFGMGNPLLDISAVVDKDFLDKYSLKPNDQILAED
KHKELFDELVKKFKVEYHAGGSTQNSIKVAQWMIQQPHKAATFFGCIGIDKFGEILKRKA
AEAHVDAHYYEQNEQPTGTCAACITGDNRSLIANLAAANCYKKEKHLDLEKNWMLVEKAR
VCYIAGFFLTVSPESVLKVAHHASENNRIFTLNLSAPFISQFYKESLMKVMPYVDILFGN
ETEAATFAREQGFETKDIKEIAKKTQALPKMNSKRQRIVIFTQGRDDTIMATESEVTAFA
VLDQDQKEIIDTNGAGDAFVGGFLSQLVSDKPLTECIRAGHYAASIIIRRTGCTFPEKPD
FH
>sp|Q9BRR6|ADPGK_HUMAN ADP-dependent glucokinase OS=Homo sapiens GN=ADPGK PE=1 SV=1
MALWRGSAYAGFLALAVGCVFLLEPELPGSALRSLWSSLCLGPAPAPPGPVSPEGRLAAA
WDALIVRPVRRWRRVAVGVNACVDVVLSGVKLLQALGLSPGNGKDHSILHSRNDLEEAFI
HFMGKGAAAERFFSDKETFHDIAQVASEFPGAQHYVGGNAALIGQKFAANSDLKVLLCGP
VGPKLHELLDDNVFVPPESLQEVDEFHLILEYQAGEEWGQLKAPHANRFIFSHDLSNGAM
NMLEVFVSSLEEFQPDLVVLSGLHMMEGQSKELQRKRLLEVVTSISDIPTGIPVHLELAS
MTNRELMSSIVHQQVFPAVTSLGLNEQELLFLTQSASGPHSSLSSWNGVPDVGMVSDILF
WILKEHGRSKSRASDLTRIHFHTLVYHILATVDGHWANQLAAVAAGARVAGTQACATETI
DTSRVSLRAPQEFMTSHSEAGSRIVLNPNKPVVEWHREGISFHFTPVLVCKDPIRTVGLG
DAISAEGLFYSEVHPHY
>sp|Q9NRN7|ADPPT_HUMAN L-aminoadipate-semialdehyde dehydrogenase-phosphopantetheinyl transferase OS=Homo sapiens GN=AASDHPPT PE=1 SV=2
MVFPAKRFCLVPSMEGVRWAFSCGTWLPSRAEWLLAVRSIQPEEKERIGQFVFARDAKAA
MAGRLMIRKLVAEKLNIPWNHIRLQRTAKGKPVLAKDSSNPYPNFNFNISHQGDYAVLAA
EPELQVGIDIMKTSFPGRGSIPEFFHIMKRKFTNKEWETIRSFKDEWTQLDMFYRNWALK
ESFIKAIGVGLGFELQRLEFDLSPLNLDIGQVYKETRLFLDGEEEKEWAFEESKIDEHHF
VAVALRKPDGSRHQDVPSQDDSKPTQRQFTILNFNDLMSSAVPMTPEDPSFWDCFCFTEE
IPIRNGTKS

>sp|Q3LIE5|ADPRM_HUMAN Manganese-dependent ADP-ribose/CDP-alcohol diphosphatase OS=Homo sapiens GN=ADPRM PE=1 SV=1
MDDKPNPEALSDSSERLFSFGVIADVQFADLEDGFNFQGTRRRYYRHSLLHLQGAIEDWN
NESSMPCCVLQLGDIIDGYNAQYNASKKSLELVMDMFKRLKVPVHHTWGNHEFYNFSREY
LTHSKLNTKFLEDQIVHHPETMPSEDYYAYHFVPFPKFRFILLDAYDLSVLGVDQSSPKY
EQCMKILREHNPNTELNSPQGLSEPQFVQFNGGFSQEQLNWLNEVLTFSDTNQEKVVIVS
HLPIYPDASDNVCLAWNYRDALAVIWSHECVVCFFAGHTHDGGYSEDPFGVYHVNLEGVI
ETAPDSQAFGTVHVYPDKMMLKGRGRVPDRIMNYKKERAFHC
>sp|Q08117|AES_HUMAN Amino-terminal enhancer of split OS=Homo sapiens GN=AES PE=1 SV=4
MMFPQSRHSGSSHLPQQLKFTTSDSCDRIKDEFQLLQAQYHSLKLECDKLASEKSEMQRH
YVMYYEMSYGLNIEMHKQAEIVKRLNGICAQVLPYLSQEHQQQVLGAIERAKQVTAPELN
SIIRQQLQAHQLSQLQALALPLTPLPVGLQPPSLPAVSAGTGLLSLSALGSQAHLSKEDK
NGHDGDTHQEDDGEKSD
>sp|P55197|AF10_HUMAN Protein AF-10 OS=Homo sapiens GN=MLLT10 PE=1 SV=2
MVSSDRPVSLEDEVSHSMKEMIGGCCVCSDERGWAENPLVYCDGHGCSVAVHQACYGIVQ
VPTGPWFCRKCESQERAARVRCELCPHKDGALKRTDNGGWAHVVCALYIPEVQFANVSTM
EPIVLQSVPHDRYNKTCYICDEQGRESKAATGACMTCNKHGCRQAFHVTCAQFAGLLCEE
EGNGADNVQYCGYCKYHFSKLKKSKRGSNRSYDQSLSDSSSHSQDKHHEKEKKKYKEKDK
HKQKHKKQPEPSPALVPSLTVTTEKTYTSTSNNSISGSLKRLEDTTARFTNANFQEVSAH
TSSGKDVSETRGSEGKGKKSSAHSSGQRGRKPGGGRNPGTTVSAASPFPQGSFSGTPGSV
KSSSGSSVQSPQDFLSFTDSDLRNDSYSHSQQSSATKDVHKGESGSQEGGVNSFSTLIGL
PSTSAVTSQPKSFENSPGDLGNSSLPTAGYKRAQTSGIEEETVKEKKRKGNKQSKHGPGR
PKGNKNQENVSHLSVSSASPTSSVASAAGSITSSSLQKSPTLLRNGSLQSLSVGSSPVGS
EISMQYRHDGACPTTTFSELLNAIHNGIYNSNDVAVSFPNVVSGSGSSTPVSSSHLPQQS
SGHLQQVGALSPSAVSSAAPAVATTQANTLSGSSLSQAPSHMYGNRSNSSMAALIAQSEN
NQTDQDLGDNSRNLVGRGSSPRGSLSPRSPVSSLQIRYDQPGNSSLENLPPVAASIEQLL
ERQWSEGQQFLLEQGTPSDILGMLKSLHQLQVENRRLEEQIKNLTAKKERLQLLNAQLSV
PFPTITANPSPSHQIHTFSAQTAPTTDSLNSSKSPHIGNSFLPDNSLPVLNQDLTSSGQS
TSSSSALSTPPPAGQSPAQQGSGVSGVQQVNGVTVGALASGMQPVTSTIPAVSAVGGIIG
ALPGNQLAINGIVGALNGVMQTPVTMSQNPTPLTHTTVPPNATHPMPATLTNSASGLGLL
SDQQRQILIHQQQFQQLLNSQQLTPEQHQAFLYQLMQHHHQQHHQPELQQLQIPGPTQIP
INNLLAGTQAPPLHTATTNPFLTIHGDNASQKVARLSDKTGPVAQEKS
>sp|Q8N4X5|AF1L2_HUMAN Actin filament-associated protein 1-like 2 OS=Homo sapiens GN=AFAP1L2 PE=1 SV=1
MERYKALEQLLTELDDFLKILDQENLSSTALVKKSCLAELLRLYTKSSSSDEEYIYMNKV
TINKQQNAESQGKAPEEQGLLPNGEPSQHSSAPQKSLPDLPPPKMIPERKQLAIPKTESP
EGYYEEAEPYDTSLNEDGEAVSSSYESYDEEDGSKGKSAPYQWPSPEAGIELMRDARICA
FLWRKKWLGQWAKQLCVIKDNRLLCYKSSKDHSPQLDVNLLGSSVIHKEKQVRKKEHKLK
ITPMNADVIVLGLQSKDQAEQWLRVIQEVSGLPSEGASEGNQYTPDAQRFNCQKPDIAEK
YLSASEYGSSVDGHPEVPETKDVKKKCSAGLKLSNLMNLGRKKSTSLEPVERSLETSSYL
NVLVNSQWKSRWCSVRDNHLHFYQDRNRSKVAQQPLSLVGCEVVPDPSPDHLYSFRILHK
GEELAKLEAKSSEEMGHWLGLLLSESGSKTDPEEFTYDYVDADRVSCIVSAAKNSLLLMQ
RKFSEPNTYIDGLPSQDRQEELYDDVDLSELTAAVEPTEEATPVADDPNERESDRVYLDL
TPVKSFLHGPSSAQAQASSPTLSCLDNATEALPADSGPGPTPDEPCIKCPENLGEQQLES

LEPEDPSLRITTVKIQTEQQRISFPPSCPDAVVATPPGASPPVKDRLRVTSAEIKLGKNR
TEAEVKRYTEEKERLEKKKEEIRGHLAQLRKEKRELKETLLKCTDKEVLASLEQKLKEID
EECRGEESRRVDLELSIMEVKDNLKKAEAGPVTLGTTVDTTHLENVSPRPKAVTPASAPD
CTPVNSATTLKNRPLSVVVTGKGTVLQKAKEWEKKGAS
>sp|P55196|AFAD_HUMAN Afadin OS=Homo sapiens GN=AFDN PE=1 SV=3
MSAGGRDEERRKLADIIHHWNANRLDLFEISQPTEDLEFHGVMRFYFQDKAAGNFATKCI
RVSSTATTQDVIETLAEKFRPDMRMLSSPKYSLYEVHVSGERRLDIDEKPLVVQLNWNKD
DREGRFVLKNENDAIPPKKAQSNGPEKQEKEGVIQNFKRTLSKKEKKEKKKREKEALRQA
SDKDDRPFQGEDVENSRLAAEVYKDMPETSFTRTISNPEVVMKRRRQQKLEKRMQEFRSS
DGRPDSGGTLRIYADSLKPNIPYKTILLSTTDPADFAVAEALEKYGLEKENPKDYCIARV
MLPPGAQHSDEKGAKEIILDDDECPLQIFREWPSDKGILVFQLKRRPPDHIPKKTKKHLE
GKTPKGKERADGSGYGSTLPPEKLPYLVELSPGRRNHFAYYNYHTYEDGSDSRDKPKLYR
LQLSVTEVGTEKLDDNSIQLFGPGIQPHHCDLTNMDGVVTVTPRSMDAETYVEGQRISET
TMLQSGMKVQFGASHVFKFVDPSQDHALAKRSVDGGLMVKGPRHKPGIVQETTFDLGGDI
HSGTALPTSKSTTRLDSDRVSSASSTAERGMVKPMIRVEQQPDYRRQESRTQDASGPELI
LPASIEFRESSEDSFLSAIINYTNSSTVHFKLSPTYVLYMACRYVLSNQYRPDISPTERT
HKVIAVVNKMVSMMEGVIQKQKNIAGALAFWMANASELLNFIKQDRDLSRITLDAQDVLA
HLVQMAFKYLVHCLQSELNNYMPAFLDDPEENSLQRPKIDDVLHTLTGAMSLLRRCRVNA
ALTIQLFSQLFHFINMWLFNRLVTDPDSGLCSHYWGAIIRQQLGHIEAWAEKQGLELAAD
CHLSRIVQATTLLTMDKYAPDDIPNINSTCFKLNSLQLQALLQNYHCAPDEPFIPTDLIE
NVVTVAENTADELARSDGREVQLEEDPDLQLPFLLPEDGYSCDVVRNIPNGLQEFLDPLC
QRGFCRLIPHTRSPGTWTIYFEGADYESHLLRENTELAQPLRKEPEIITVTLKKQNGMGL
SIVAAKGAGQDKLGIYVKSVVKGGAADVDGRLAAGDQLLSVDGRSLVGLSQERAAELMTR
TSSVVTLEVAKQGAIYHGLATLLNQPSPMMQRISDRRGSGKPRPKSEGFELYNNSTQNGS
PESPQLPWAEYSEPKKLPGDDRLMKNRADHRSSPNVANQPPSPGGKSAYASGTTAKITSV
STGNLCTEEQTPPPRPEAYPIPTQTYTREYFTFPASKSQDRMAPPQNQWPNYEEKPHMHT
DSNHSSIAIQRVTRSQEELREDKAYQLERHRIEAAMDRKSDSDMWINQSSSLDSSTSSQE
HLNHSSKSVTPASTLTKSGPGRWKTPAAIPATPVAVSQPIRTDLPPPPPPPPVHYAGDFD
GMSMDLPLPPPPSANQIGLPSAQVAAAERRKREEHQRWYEKEKARLEEERERKRREQERK
LGQMRTQSLNPAPFSPLTAQQMKPEKPSTLQRPQETVIRELQPQQQPRTIERRDLQYITV
SKEELSSGDSLSPDPWKRDAKEKLEKQQQMHIVDMLSKEIQELQSKPDRSAEESDRLRKL
MLEWQFQKRLQESKQKDEDDEEEEDDDVDTMLIMQRLEAERRARLQDEERRRQQQLEEMR
KREAEDRARQEEERRRQEEERTKRDAEEKRRQEEGYYSRLEAERRRQHDEAARRLLEPEA
PGLCRPPLPRDYEPPSPSPAPGAPPPPPQRNASYLKTQVLSPDSLFTAKFVAYNEEEEEE
DCSLAGPNSYPGSTGAAVGAHDACRDAKEKRSKSQDADSPGSSGAPENLTFKERQRLFSQ
GQDVSNKVKASRKLTELENELNTK
>sp|O95081|AGFG2_HUMAN Arf-GAP domain and FG repeat-containing protein 2 OS=Homo sapiens GN=AGFG2 PE=1 SV=2
MVMAAKKGPGPGGGVSGGKAEAEAASEVWCRRVRELGGCSQAGNRHCFECAQRGVTYVDI
TVGSFVCTTCSGLLRGLNPPHRVKSISMTTFTEPEVVFLQSRGNEVCRKIWLGLFDARTS
LVPDSRDPQKVKEFLQEKYEKKRWYVPPDQVKGPTYTKGSASTPVQGSIPEGKPLRTLLG
DPAPSLSVAASTSSQPVSQSHARTSQARSTQPPPHSSVKKASTDLLADIGGDPFAAPQMA
PAFAAFPAFGGQTPSQGGFANFDAFSSGPSSSVFGSLPPAGQASFQAQPTPAGSSQGTPF
GATPLAPASQPNSLADVGSFLGPGVPAAGVPSSLFGMAGQVPPLQSVTMGGGGGSSTGLA

FGAFTNPFTAPAAQSPLPSTNPFQPNGLAPGPGFGMSSAGPGFPQAVPPTGAFASSFPAP
LFPPQTPLVQQQNGSSFGDLGSAKLGQRPLSQPAGISTNPFMTGPSSSPFASKPPTTNPF
L
>sp|Q8TD06|AGR3_HUMAN Anterior gradient protein 3 OS=Homo sapiens GN=AGR3 PE=1 SV=1
MMLHSALGLCLLLVTVSSNLAIAIKKEKRPPQTLSRGWGDDITWVQTYEEGLFYAQKSKK
PLMVIHHLEDCQYSQALKKVFAQNEEIQEMAQNKFIMLNLMHETTDKNLSPDGQYVPRIM
FVDPSLTVRADIAGRYSNRLYTYEPRDLPLLIENMKKALRLIQSEL
>sp|Q86SQ6|AGRA1_HUMAN Adhesion G protein-coupled receptor A1 OS=Homo sapiens GN=ADGRA1 PE=2 SV=3
MDLKTVLSLPRYPGEFLHPVVYACTAVMLLCLLASFVTYIVHQSAIRISRKGRHTLLNFC
FHAALTFTVFAGGINRTKYPILCQAVGIVLHYSTLSTMLWIGVTARNIYKQVTKKAPLCL
DTDQPPYPRQPLLRFYLVSGGVPFIICGVTAATNIRNYGTEDEDTAYCWMAWEPSLGAFY
GPAAIITLVTCVYFLGTYVQLRRHPGRRYELRTQPEEQRRLATPEGGRGIRPGTPPAHDA
PGASVLQNEHSFQAQLRAAAFTLFLFTATWAFGALAVSQGHFLDMVFSCLYGAFCVTLGL
FVLIHHCAKREDVWQCWWACCPPRKDAHPALDANGAALGRAACLHSPGLGQPRGFAHPPG
PCKMTNLQAAQGHASCLSPATPCCAKMHCEPLTADEAHVHLQEEGAFGHDPHLHGCLQGR
TKPPYFSRHPAEEPEYAYHIPSSLDGSPRSSRTDSPPSSLDGPAGTHTLACCTQGDPFPM
VTQPEGSDGSPALYSCPTQPGREAALGPGHLEMLRRTQSLPFGGPSQNGLPKGKLLEGLP
FGTDGTGNIRTGPWKNETTV
>sp|Q96PE1|AGRA2_HUMAN Adhesion G protein-coupled receptor A2 OS=Homo sapiens GN=ADGRA2 PE=1 SV=2
MGAGGRRMRGAPARLLLPLLPWLLLLLAPEARGAPGCPLSIRSCKCSGERPKGLSGGVPG
PARRRVVCSGGDLPEPPEPGLLPNGTVTLLLSNNKITGLRNGSFLGLSLLEKLDLRNNII
STVQPGAFLGLGELKRLDLSNNRIGCLTSETFQGLPRLLRLNISGNIFSSLQPGVFDELP
ALKVVDLGTEFLTCDCHLRWLLPWAQNRSLQLSEHTLCAYPSALHAQALGSLQEAQLCCE
GALELHTHHLIPSLRQVVFQGDRLPFQCSASYLGNDTRIRWYHNRAPVEGDEQAGILLAE
SLIHDCTFITSELTLSHIGVWASGEWECTVSMAQGNASKKVEIVVLETSASYCPAERVAN
NRGDFRWPRTLAGITAYQSCLQYPFTSVPLGGGAPGTRASRRCDRAGRWEPGDYSHCLYT
NDITRVLYTFVLMPINASNALTLAHQLRVYTAEAASFSDMMDVVYVAQMIQKFLGYVDQI
KELVEVMVDMASNLMLVDEHLLWLAQREDKACSRIVGALERIGGAALSPHAQHISVNARN
VALEAYLIKPHSYVGLTCTAFQRREGGVPGTRPGSPGQNPPPEPEPPADQQLRFRCTTGR
PNVSLSSFHIKNSVALASIQLPPSLFSSLPAALAPPVPPDCTLQLLVFRNGRLFHSHSNT
SRPGAAGPGKRRGVATPVIFAGTSGCGVGNLTEPVAVSLRHWAEGAEPVAAWWSQEGPGE
AGGWTSEGCQLRSSQPNVSALHCQHLGNVAVLMELSAFPREVGGAGAGLHPVVYPCTALL
LLCLFATIITYILNHSSIRVSRKGWHMLLNLCFHIAMTSAVFAGGITLTNYQMVCQAVGI
TLHYSSLSTLLWMGVKARVLHKELTWRAPPPQEGDPALPTPSPMLRFYLIAGGIPLIICG
ITAAVNIHNYRDHSPYCWLVWRPSLGAFYIPVALILLITWIYFLCAGLRLRGPLAQNPKA
GNSRASLEAGEELRGSTRLRGSGPLLSDSGSLLATGSARVGTPGPPEDGDSLYSPGVQLG
ALVTTHFLYLAMWACGALAVSQRWLPRVVCSCLYGVAASALGLFVFTHHCARRRDVRASW
RACCPPASPAAPHAPPRALPAAAEDGSPVFGEGPPSLKSSPSGSSGHPLALGPCKLTNLQ
LAQSQVCEAGAAAGGEGEPEPAGTRGNLAHRHPNNVHHGRRAHKSRAKGHRAGEACGKNR
LKALRGGAAGALELLSSESGSLHNSPTDSYLGSSRNSPGAGLQLEGEPMLTPSEGSDTSA
APLSEAGRAGQRRSASRDSLKGGGALEKESHRRSYPLNAASLNGAPKGGKYDDVTLMGAE
VASGGCMKTGLWKSETTV

>sp|Q96Q42|ALS2_HUMAN Alsin OS=Homo sapiens GN=ALS2 PE=1 SV=2
MDSKKRSSTEAEGSKERGLVHIWQAGSFPITPERLPGWGGKTVLQAALGVKHGVLLTEDG
EVYSFGTLPWRSGPVEICPSSPILENALVGQYVITVATGSFHSGAVTDNGVAYMWGENSA
GQCAVANQQYVPEPNPVSIADSEASPLLAVRILQLACGEEHTLALSISREIWAWGTGCQL
GLITTAFPVTKPQKVEHLAGRVVLQVACGAFHSLALVQCLPSQDLKPVPERCNQCSQLLI
TMTDKEDHVIISDSHCCPLGVTLTESQAENHASTALSPSTETLDRQEEVFENTLVANDQS
VATELNAVSAQITSSDAMSSQQNVMGTTEISSARNIPSYPDTQAVNEYLRKLSDHSVRED
SEHGEKPVPSQPLLEEAIPNLHSPPTTSTSALNSLVVSCASAVGVRVAATYEAGALSLKK
VMNFYSTTPCETGAQAGSSAIGPEGLKDSREEQVKQESMQGKKSSSLVDIREEETEGGSR
RLSLPGLLSQVSPRLLRKAARVKTRTVVLTPTYSGEADALLPSLRTEVWTWGKGKEGQLG
HGDVLPRLQPLCVKCLDGKEVIHLEAGGYHSLALTAKSQVYSWGSNTFGQLGHSDFPTTV
PRLAKISSENGVWSIAAGRDYSLFLVDTEDFQPGLYYSGRQDPTEGDNLPENHSGSKTPV
LLSCSKLGYISRVTAGKDSYLALVDKNIMGYIASLHELATTERRFYSKLSDIKSQILRPL
LSLENLGTTTTVQLLQEVASRFSKLCYLIGQHGASLSSFLHGVKEARSLVILKHSSLFLD
SYTEYCTSITNFLVMGGFQLLAKPAIDFLNKNQELLQDLSEVNDENTQLMEILNTLFFLP
IRRLHNYAKVLLKLATCFEVASPEYQKLQDSSSCYECLALHLGRKRKEAEYTLGFWKTFP
GKMTDSLRKPERRLLCESSNRALSLQHAGRFSVNWFILFNDALVHAQFSTHHVFPLATLW
AEPLSEEAGGVNGLKITTPEEQFTLISSTPQEKTKWLRAISQAVDQALRGMSDLPPYGSG
SSVQRQEPPISRSAKYTFYKDPRLKDATYDGRWLSGKPHGRGVLKWPDGKMYSGMFRNGL
EDGYGEYRIPNKAMNKEDHYVGHWKEGKMCGQGVYSYASGEVFEGCFQDNMRHGHGLLRS
GKLTSSSPSMFIGQWVMDKKAGYGVFDDITRGEKYMGMWQDDVCQGNGVVVTQFGLYYEG
NFHLNKMMGNGVLLSEDDTIYEGEFSDDWTLSGKGTLTMPNGDYIEGYFSGEWGSGIKIT
GTYFKPSLYESDKDRPKVFRKLGNLAVPADEKWKAVFDECWRQLGCEGPGQGEVWKAWDN
IAVALTTSRRQHRDSPEILSRSQTQTLESLEFIPQHVGAFSVEKYDDIRKYLIKACDTPL
HPLGRLVETLVAVYRMTYVGVGANRRLLQEAVKEIKSYLKRIFQLVRFLFPELPEEGSTI
PLSAPLPTERKSFCTGKSDSRSESPEPGYVVTSSGLLLPVLLPRLYPPLFMLYALDNDRE
EDIYWECVLRLNKQPDIALLGFLGVQRKFWPATLSILGESKKVLPTTKDACFASAVECLQ
QISTTFTPSDKLKVIQQTFEEISQSVLASLHEDFLWSMDDLFPVFLYVVLRARIRNLGSE
VHLIEDLMDPYLQHGEQGIMFTTLKACYYQIQREKLN
>sp|P35858|ALS_HUMAN Insulin-like growth factor-binding protein complex acid labile subunit OS=Homo sapiens GN=IGFALS PE=1 SV=1
MALRKGGLALALLLLSWVALGPRSLEGADPGTPGEAEGPACPAACVCSYDDDADELSVFC
SSRNLTRLPDGVPGGTQALWLDGNNLSSVPPAAFQNLSSLGFLNLQGGQLGSLEPQALLG
LENLCHLHLERNQLRSLALGTFAHTPALASLGLSNNRLSRLEDGLFEGLGSLWDLNLGWN
SLAVLPDAAFRGLGSLRELVLAGNRLAYLQPALFSGLAELRELDLSRNALRAIKANVFVQ
LPRLQKLYLDRNLIAAVAPGAFLGLKALRWLDLSHNRVAGLLEDTFPGLLGLRVLRLSHN
AIASLRPRTFKDLHFLEELQLGHNRIRQLAERSFEGLGQLEVLTLDHNQLQEVKAGAFLG
LTNVAVMNLSGNCLRNLPEQVFRGLGKLHSLHLEGSCLGRIRPHTFTGLSGLRRLFLKDN
GLVGIEEQSLWGLAELLELDLTSNQLTHLPHRLFQGLGKLEYLLLSRNRLAELPADALGP
LQRAFWLDVSHNRLEALPNSLLAPLGRLRYLSLRNNSLRTFTPQPPGLERLWLEGNPWDC
GCPLKALRDFALQNPSAVPRFVQAICEGDDCQPPAYTYNNITCASPPEVVGLDLRDLSEA
HFAPC
>sp|Q15699|ALX1_HUMAN ALX homeobox protein 1 OS=Homo sapiens GN=ALX1 PE=1 SV=2
MEFLSEKFALKSPPSKNSDFYMGAGGPLEHVMETLDNESFYSKASAGKCVQAFGPLPRAE

HHVRLERTSPCQDSSVNYGITKVEGQPLHTELNRAMDNCNSLRMSPVKGMQEKGELDELG
DKCDSNVSSSKKRRHRTTFTSLQLEELEKVFQKTHYPDVYVREQLALRTELTEARVQVWF
QNRRAKWRKRERYGQIQQAKSHFAATYDISVLPRTDSYPQIQNNLWAGNASGGSVVTSCM
LPRDTSSCMTPYSHSPRTDSSYTGFSNHQNQFSHVPLNNFFTDSLLTGATNGHAFETKPE
FERRSSSIAVLRMKAKEHTANISWAM
>sp|Q99218|AMELY_HUMAN Amelogenin, Y isoform OS=Homo sapiens GN=AMELY PE=2 SV=2
MGTWILFACLVGAAFAMPLPPHPGHPGYINFSYENSHSQAINVDRIALVLTPLKWYQSMI
RPPYSSYGYEPMGGWLHHQIIPVVSQQHPLTHTLQSHHHIPVVPAQQPRVRQQALMPVPG
QQSMTPTQHHQPNLPLPAQQPFQPQPVQPQPHQPMQPQPPVQPMQPLLPQPPLPPMFPLR
PLPPILPDLHLEAWPATDKTKQEEVD
>sp|Q5JTC6|AMER1_HUMAN APC membrane recruitment protein 1 OS=Homo sapiens GN=AMER1 PE=1 SV=2
METQKDEAAQAKGAAASGSTREQTAEKGAKNKAAEATEGPTSEPSSSGPGRLKKTAMKLF
GGKKGICTLPSFFGGGRSKGSGKGSSKKGLSKSKTHDGLSEAAHGPEDVVSEGTGFSLPL
PELPCQFPSSQSAHGALETGSRCKTSVAGATEKAVAEKFPSMPKPKKGLKGFFSSIRRHR
KSKVTGAEQSEPGAKGPERVRARPHEHVSSAPQVPCFEETFQAPRKENANPQDAPGPKVS
PTPEPSPPATEKMACKDPEKPMEACASAHVQPKPAPEASSLEEPHSPETGEKVVAGEVNP
PNGPVGDPLSLLFGDVTSLKSFDSLTGCGDIIAEQDMDSMTDSMASGGQRANRDGTKRSS
CLVTYQGGGEEMALPDDDDEEEEEEEEVELEEEEEEVKEEEEDDDLEYLWETAQMYPRPN
MNLGYHPTTSPGHHGYMLLDPVRSYPGLAPGELLTPQSDQQESAPNSDEGYYDSTTPGFE
DDSGEALGLVRRDCLPRDSYSGDALYEFYEPDDSLENSPPGDDCLYDLHGRSSEMFDPFL
NFEPFLSSRPPGAMETEEERLVTIQKQLLYWELRREQLEAQEARAREAHAREAHAREAYT
REAYGREAYAREAHTWEAHGREARTREAQAREVRCRETQVRETQARQEKPVLEYQMRPLG
PSVMGLAAGVSGTSQISHRGITSAFPTTASSEPDWRDFRPLEKRYEGTCSKKDQSTCLMQ
LFQSDAMFEPDMQEANFGGSPRRAYPTYSPPEDPEEEEVEKEGNATVSFSQALVEFTSNG
NLFSSMSCSSDSDSSFTQNLPELPPMVTFDIADVERDGEGKCEENPEFHNDEDLAASLEA
FELGYYHKHAFNNYHSRFYQGLPWGVSSLPRYLGLPGLHPRPPPAAMALNRRSRSLDTAE
TLEMELSNSHLVQGYLESDELQAQQEDSDEEDEEEEEGEWSRDSPLSLYTEPPGAYDWPA
WAPCPLPVGPGPAWISPNQLDRPSSQSPYRQATCCIPPMTMSISLSVPESRAPGESGPQL
ARPSHLHLPMGPCYNLQPQASQSMRARPRDVLLPVDEPSCSSSSGGFSPSPLPQAKPVGI
THGIPQLPRVRPEHPQPQPTHYGPSSLDLSKERAEQGASLATSYSSTAMNGNLAK
>sp|Q6DCA0|AMERL_HUMAN AMMECR1-like protein OS=Homo sapiens GN=AMMECR1L PE=1 SV=1
MGKRRCVPPLEPKLAAGCCGVKKPKLSGSGTHSHGNQSTTVPGSSSGPLQNHQHVDSSSG
RENVSDLTLGPGNSPITRMNPASGALSPLPRPNGTANTTKNLVVTAEMCCYCFDVLYCHL
YGFPQPRLPRFTNDPYPLFVTWKTGRDKRLRGCIGTFSAMNLHSGLREYTLTSALKDSRF
PPLTREELPKLFCSVSLLTNFEDASDYLDWEVGVHGIRIEFINEKGVKRTATYLPEVAKE
QDWDQIQTIDSLLRKGGFKAPITSEFRKTIKLTRYRSEKVTISYAEYIASRQHCFQNGTL
HAPPLYNHYS
>sp|Q9UKV5|AMFR_HUMAN E3 ubiquitin-protein ligase AMFR OS=Homo sapiens GN=AMFR PE=1 SV=2
MPLLFLERFPWPSLRTYTGLSGLALLGTIISAYRALSQPEAGPGEPDQLTASLQPEPPAP
ARPSAGGPRARDVAQYLLSDSLFVWVLVNTACCVLMLVAKLIQCIVFGPLRVSERQHLKD
KFWNFIFYKFIFIFGVLNVQTVEEVVMWCLWFAGLVFLHLMVQLCKDRFEYLSFSPTTPM
SSHGRVLSLLVAMLLSCCGLAAVCSITGYTHGMHTLAFMAAESLLVTVRTAHVILRYVIH
LWDLNHEGTWEGKGTYVYYTDFVMELTLLSLDLMHHIHMLLFGNIWLSMASLVIFMQLRY

LFHEVQRRIRRHKNYLRVVGNMEARFAVATPEELAVNNDDCAICWDSMQAARKLPCGHLF
HNSCLRSWLEQDTSCPTCRMSLNIADNNRVREEHQGENLDENLVPVAAAEGRPRLNQHNH
FFHFDGSRIASWLPSFSVEVMHTTNILGITQASNSQLNAMAHQIQEMFPQVPYHLVLQDL
QLTRSVEITTDNILEGRIQVPFPTQRSDSIRPALNSPVERPSSDQEEGETSAQTERVPLD
LSPRLEETLDFGEVEVEPSEVEDFEARGSRFSKSADERQRMLVQRKDELLQQARKRFLNK
SSEDDAASESFLPSEGASSDPVTLRRRMLAAAAERRLQKQQTS
>sp|Q86SJ2|AMGO2_HUMAN Amphoterin-induced protein 2 OS=Homo sapiens GN=AMIGO2 PE=1 SV=1
MSLRVHTLPTLLGAVVRPGCRELLCLLMITVTVGPGASGVCPTACICATDIVSCTNKNLS
KVPGNLFRLIKRLDLSYNRIGLLDSEWIPVSFAKLNTLILRHNNITSISTGSFSTTPNLK
CLDLSSNKLKTVKNAVFQELKVLEVLLLYNNHISYLDPSAFGGLSQLQKLYLSGNFLTQF
PMDLYVGRFKLAELMFLDVSYNRIPSMPMHHINLVPGKQLRGIYLHGNPFVCDCSLYSLL
VFWYRRHFSSVMDFKNDYTCRLWSDSRHSRQVLLLQDSFMNCSDSIINGSFRALGFIHEA
QVGERLMVHCDSKTGNANTDFIWVGPDNRLLEPDKEMENFYVFHNGSLVIESPRFEDAGV
YSCIAMNKQRLLNETVDVTINVSNFTVSRSHAHEAFNTAFTTLAACVASIVLVLLYLYLT
PCPCKCKTKRQKNMLHQSNAHSSILSPGPASDASADERKAGAGKRVVFLEPLKDTAAGQN
GKVRLFPSEAVIAEGILKSTRGKSDSDSVNSVFSDTPFVAST
>sp|Q8IY45|AMN1_HUMAN Protein AMN1 homolog OS=Homo sapiens GN=AMN1 PE=2 SV=4
MPRPRRVSQLLDLCLWCFMKNISRYLTDIKPLPPNIKDRLIKIMSMQGQITDSNISEILH
PEVQTLDLRSCDISDAALLHLSNCRKLKKLNLNASKGNRVSVTSEGIKAVASSCSYLHEA
SLKRCCNLTDEGVVALALNCQLLKIIDLGGCLSITDVSLHALGKNCPFLQCVDFSATQVS
DSGVIALVSGPCAKKLEEIHMGHCVNLTDGAVEAVLTYCPQIRILLFHGCPLITDHSREV
LEQLVGPNKLKQVTWTVY
>sp|Q9BXJ7|AMNLS_HUMAN Protein amnionless OS=Homo sapiens GN=AMN PE=1 SV=2
MGVLGRVLLWLQLCALTQAVSKLWVPNTDFDVAANWSQNRTPCAGGAVEFPADKMVSVLV
QEGHAVSDMLLPLDGELVLASGAGFGVSDVGSHLDCGAGEPAVFRDSDRFSWHDPHLWRS
GDEAPGLFFVDAERVPCRHDDVFFPPSASFRVGLGPGASPVRVRSISALGRTFTRDEDLA
VFLASRAGRLRFHGPGALSVGPEDCADPSGCVCGNAEAQPWICAALLQPLGGRCPQAACH
SALRPQGQCCDLCGAVVLLTHGPAFDLERYRARILDTFLGLPQYHGLQVAVSKVPRSSRL
READTEIQVVLVENGPETGGAGRLARALLADVAENGEALGVLEATMRESGAHVWGSSAAG
LAGGVAAAVLLALLVLLVAPPLLRRAGRLRWRRHEAAAPAGAPLGFRNPVFDVTASEELP
LPRRLSLVPKAAADSTSHSYFVNPLFAGAEAEA
>sp|P00326|ADH1G_HUMAN Alcohol dehydrogenase 1C OS=Homo sapiens GN=ADH1C PE=1 SV=2
MSTAGKVIKCKAAVLWELKKPFSIEEVEVAPPKAHEVRIKMVAAGICRSDEHVVSGNLVT
PLPVILGHEAAGIVESVGEGVTTVKPGDKVIPLFTPQCGKCRICKNPESNYCLKNDLGNP
RGTLQDGTRRFTCSGKPIHHFVGVSTFSQYTVVDENAVAKIDAASPLEKVCLIGCGFSTG
YGSAVKVAKVTPGSTCAVFGLGGVGLSVVMGCKAAGAARIIAVDINKDKFAKAKELGATE
CINPQDYKKPIQEVLKEMTDGGVDFSFEVIGRLDTMMASLLCCHEACGTSVIVGVPPDSQ
NLSINPMLLLTGRTWKGAIFGGFKSKESVPKLVADFMAKKFSLDALITNILPFEKINEGF
DLLRSGKSIRTVLTF
>sp|P54922|ADPRH_HUMAN [Protein ADP-ribosylarginine] hydrolase OS=Homo sapiens GN=ADPRH PE=1 SV=1
MEKYVAAMVLSAAGDALGYYNGKWEFLQDGEKIHRQLAQLGGLDALDVGRWRVSDDTVMH
LATAEALVEAGKAPKLTQLYYLLAKHYQDCMEDMDGRAPGGASVHNAMQLKPGKPNGWRI
PFNSHEGGCGAAMRAMCIGLRFPHHSQLDTLIQVSIESGRMTHHHPTGYLGALASALFTA

YAVNSRPPLQWGKGLMELLPEAKKYIVQSGYFVEENLQHWSYFQTKWENYLKLRGILDGE
SAPTFPESFGVKERDQFYTSLSYSGWGGSSGHDAPMIAYDAVLAAGDSWKELAHRAFFHG
GDSDSTAAIAGCWWGVMYGFKGVSPSNYEKLEYRNRLEETARALYSLGSKEDTVISL
>sp|Q9UL18|AGO1_HUMAN Protein argonaute-1 OS=Homo sapiens GN=AGO1 PE=1 SV=3
MEAGPSGAAAGAYLPPLQQVFQAPRRPGIGTVGKPIKLLANYFEVDIPKIDVYHYEVDIK
PDKCPRRVNREVVEYMVQHFKPQIFGDRKPVYDGKKNIYTVTALPIGNERVDFEVTIPGE
GKDRIFKVSIKWLAIVSWRMLHEALVSGQIPVPLESVQALDVAMRHLASMRYTPVGRSFF
SPPEGYYHPLGGGREVWFGFHQSVRPAMWKMMLNIDVSATAFYKAQPVIEFMCEVLDIRN
IDEQPKPLTDSQRVRFTKEIKGLKVEVTHCGQMKRKYRVCNVTRRPASHQTFPLQLESGQ
TVECTVAQYFKQKYNLQLKYPHLPCLQVGQEQKHTYLPLEVCNIVAGQRCIKKLTDNQTS
TMIKATARSAPDRQEEISRLMKNASYNLDPYIQEFGIKVKDDMTEVTGRVLPAPILQYGG
RNRAIATPNQGVWDMRGKQFYNGIEIKVWAIACFAPQKQCREEVLKNFTDQLRKISKDAG
MPIQGQPCFCKYAQGADSVEPMFRHLKNTYSGLQLIIVILPGKTPVYAEVKRVGDTLLGM
ATQCVQVKNVVKTSPQTLSNLCLKINVKLGGINNILVPHQRSAVFQQPVIFLGADVTHPP
AGDGKKPSITAVVGSMDAHPSRYCATVRVQRPRQEIIEDLSYMVRELLIQFYKSTRFKPT
RIIFYRDGVPEGQLPQILHYELLAIRDACIKLEKDYQPGITYIVVQKRHHTRLFCADKNE
RIGKSGNIPAGTTVDTNITHPFEFDFYLCSHAGIQGTSRPSHYYVLWDDNRFTADELQIL
TYQLCHTYVRCTRSVSIPAPAYYARLVAFRARYHLVDKEHDSGEGSHISGQSNGRDPQAL
AKAVQVHQDTLRTMYFA
>sp|Q8IWK6|AGRA3_HUMAN Adhesion G protein-coupled receptor A3 OS=Homo sapiens GN=ADGRA3 PE=1 SV=2
MEPPGRRRGRAQPPLLLPLSLLALLALLGGGGGGGAAALPAGCKHDGRPRGAGRAAGAAE
GKVVCSSLELAQVLPPDTLPNRTVTLILSNNKISELKNGSFSGLSLLERLDLRNNLISSI
DPGAFWGLSSLKRLDLTNNRIGCLNADIFRGLTNLVRLNLSGNLFSSLSQGTFDYLASLR
SLEFQTEYLLCDCNILWMHRWVKEKNITVRDTRCVYPKSLQAQPVTGVKQELLTCDPPLE
LPSFYMTPSHRQVVFEGDSLPFQCMASYIDQDMQVLWYQDGRIVETDESQGIFVEKNMIH
NCSLIASALTISNIQAGSTGNWGCHVQTKRGNNTRTVDIVVLESSAQYCPPERVVNNKGD
FRWPRTLAGITAYLQCTRNTHGSGIYPGNPQDERKAWRRCDRGGFWADDDYSRCQYANDV
TRVLYMFNQMPLNLTNAVATARQLLAYTVEAANFSDKMDVIFVAEMIEKFGRFTKEEKSK
ELGDVMVDIASNIMLADERVLWLAQREAKACSRIVQCLQRIATYRLAGGAHVYSTYSPNI
ALEAYVIKSTGFTGMTCTVFQKVAASDRTGLSDYGRRDPEGNLDKQLSFKCNVSNTFSSL
ALKNTIVEASIQLPPSLFSPKQKRELRPTDDSLYKLQLIAFRNGKLFPATGNSTNLADDG
KRRTVVTPVILTKIDGVNVDTHHIPVNVTLRRIAHGADAVAARWDFDLLNGQGGWKSDGC
HILYSDENITTIQCYSLSNYAVLMDLTGSELYTQAASLLHPVVYTTAIILLLCLLAVIVS
YIYHHSLIRISLKSWHMLVNLCFHIFLTCVVFVGGITQTRNASICQAVGIILHYSTLATV
LWVGVTARNIYKQVTKKAKRCQDPDEPPPPPRPMLRFYLIGGGIPIIVCGITAAANIKNY
GSRPNAPYCWMAWEPSLGAFYGPASFITFVNCMYFLSIFIQLKRHPERKYELKEPTEEQQ
RLAANENGEINHQDSMSLSLISTSALENEHTFHSQLLGASLTLLLYVALWMFGALAVSLY
YPLDLVFSFVFGATSLSFSAFFVVHHCVNREDVRLAWIMTCCPGRSSYSVQVNVQPPNSN
GTNGEAPKCPNSSAESSCTNKSASSFKNSSQGCKLTNLQAAAAQCHANSLPLNSTPQLDN
SLTEHSMDNDIKMHVAPLEVQFRTNVHSSRHHKNRSKGHRASRLTVLREYAYDVPTSVEG
SVQNGLPKSRLGNNEGHSRSRRAYLAYRERQYNPPQQDSSDACSTLPKSSRNFEKPVSTT
SKKDALRKPAVVELENQQKSYGLNLAIQNGPIKSNGQEGPLLGTDSTGNVRTGLWKHETT
V

>sp|Q8IZF7|AGRF2_HUMAN Adhesion G-protein coupled receptor F2 OS=Homo sapiens GN=ADGRF2 PE=2 SV=1
MGLTAYGNRRVQPGELPFGANLTLIHTRAQPVICSKLLLTKRVSPISFFLSKFQNSWGED
GWVQLDQLPSPNAVSSDQVHCSAGCTHRKCGWAASKSKEKVPARPHGVCDGVCTDYSQCT
QPCPPDTQGNMGFSCRQKTWHKITDTCQTLNALNIFEEDSRLVQPFEDNIKISVYTGKSE
TITDMLLQKCPTDLSCVIRNIQQSPWIPGNIAVIVQLLHNISTAIWTGVDEAKMQSYSTI
ANHILNSKSISNWTFIPDRNSSYILLHSVNSFARRLFIDKHPVDISDVFIHTMGTTISGD
NIGKNFTFSMRINDTSNEVTGRVLISRDELRKVPSPSQVISIAFPTIGAILEASLLENVT
VNGLVLSAILPKELKRISLIFEKISKSEERRTQCVGWHSVENRWDQQACKMIQENSQQAV
CKCRPSKLFTSFSILMSPHILESLILTYITYVGLGISICSLILCLSIEVLVWSQVTKTEI
TYLRHVCIVNIAATLLMADVWFIVASFLSGPITHHKGCVAATFFVHFFYLSVFFWMLAKA
LLILYGIMIVFHTLPKSVLVASLFSVGYGCPLAIAAITVAATEPGKGYLRPEICWLNWDM
TKALLAFVIPALAIVVVNLITVTLVIVKTQRAAIGNSMFQEVRAIVRISKNIAILTPLLG
LTWGFGVATVIDDRSLAFHIIFSLLNAFQVSPDASDQVQSERIHEDVL
>sp|Q8IZF3|AGRF4_HUMAN Adhesion G protein-coupled receptor F4 OS=Homo sapiens GN=ADGRF4 PE=2 SV=3
MKMKSQATMICCLVFFLSTECSHYRSKIHLKAGDKLQSPEGKPKTGRIQEKCEGPCISSS
NCSQPCAKDFHGEIGFTCNQKKWQKSAETCTSLSVEKLFKDSTGASRLSVAAPSIPLHIL
DFRAPETIESVAQGIRKNCPFDYACITDMVKSSETTSGNIAFIVELLKNISTDLSDNVTR
EKMKSYSEVANHILDTAAISNWAFIPNKNASSDLLQSVNLFARQLHIHNNSENIVNELFI
QTKGFHINHNTSEKSLNFSMSMNNTTEDILGMVQIPRQELRKLWPNASQAISIAFPTLGA
ILREAHLQNVSLPRQVNGLVLSVVLPERLQEIILTFEKINKTRNARAQCVGWHSKKRRWD
EKACQMMLDIRNEVKCRCNYTSVVMSFSILMSSKSMTDKVLDYITCIGLSVSILSLVLCL
IIEATVWSRVVVTEISYMRHVCIVNIAVSLLTANVWFIIGSHFNIKAQDYNMCVAVTFFS
HFFYLSLFFWMLFKALLIIYGILVIFRRMMKSRMMVIGFAIGYGCPLIIAVTTVAITEPE
KGYMRPEACWLNWDNTKALLAFAIPAFVIVAVNLIVVLVVAVNTQRPSIGSSKSQDVVII
MRISKNVAILTPLLGLTWGFGIATLIEGTSLTFHIIFALLNAFQGFFILLFGTIMDHKIR
DALRMRMSSLKGKSRAAENASLGPTNGSKLMNRQG
>sp|Q8IZF2|AGRF5_HUMAN Adhesion G protein-coupled receptor F5 OS=Homo sapiens GN=ADGRF5 PE=1 SV=3
MKSPRRTTLCLMFIVIYSSKAALNWNYESTIHPLSLHEHEPAGEEALRQKRAVATKSPTA
EEYTVNIEISFENASFLDPIKAYLNSLSFPIHGNNTDQITDILSINVTTVCRPAGNEIWC
SCETGYGWPRERCLHNLICQERDVFLPGHHCSCLKELPPNGPFCLLQEDVTLNMRVRLNV
GFQEDLMNTSSALYRSYKTDLETAFRKGYGILPGFKGVTVTGFKSGSVVVTYEVKTTPPS
LELIHKANEQVVQSLNQTYKMDYNSFQAVTINESNFFVTPEIIFEGDTVSLVCEKEVLSS
NVSWRYEEQQLEIQNSSRFSIYTALFNNMTSVSKLTIHNITPGDAGEYVCKLILDIFEYE
CKKKIDVMPIQILANEEMKVMCDNNPVSLNCCSQGNVNWSKVEWKQEGKINIPGTPETDI
DSSCSRYTLKADGTQCPSGSSGTTVIYTCEFISAYGARGSANIKVTFISVANLTITPDPI
SVSEGQNFSIKCISDVSNYDEVYWNTSAGIKIYQRFYTTRRYLDGAESVLTVKTSTREWN
GTYHCIFRYKNSYSIATKDVIVHPLPLKLNIMVDPLEATVSCSGSHHIKCCIEEDGDYKV
TFHTGSSSLPAAKEVNKKQVCYKHNFNASSVSWCSKTVDVCCHFTNAANNSVWSPSMKLN
LVPGENITCQDPVIGVGEPGKVIQKLCRFSNVPSSPESPIGGTITYKCVGSQWEEKRNDC
ISAPINSLLQMAKALIKSPSQDEMLPTYLKDLSISIDKAEHEISSSPGSLGAIINILDLL
STVPTQVNSEMMTHVLSTVNVILGKPVLNTWKVLQQQWTNQSSQLLHSVERFSQALQSGD

SPPLSFSQTNVQMSSMVIKSSHPETYQQRFVFPYFDLWGNVVIDKSYLENLQSDSSIVTM
AFPTLQAILAQDIQENNFAESLVMTTTVSHNTTMPFRISMTFKNNSPSGGETKCVFWNFR
LANNTGGWDSSGCYVEEGDGDNVTCICDHLTSFSILMSPDSPDPSSLLGILLDIISYVGV
GFSILSLAACLVVEAVVWKSVTKNRTSYMRHTCIVNIAASLLVANTWFIVVAAIQDNRYI
LCKTACVAATFFIHFFYLSVFFWMLTLGLMLFYRLVFILHETSRSTQKAIAFCLGYGCPL
AISVITLGATQPREVYTRKNVCWLNWEDTKALLAFAIPALIIVVVNITITIVVITKILRP
SIGDKPCKQEKSSLFQISKSIGVLTPLLGLTWGFGLTTVFPGTNLVFHIIFAILNVFQGL
FILLFGCLWDLKVQEALLNKFSLSRWSSQHSKSTSLGSSTPVFSMSSPISRRFNNLFGKT
GTYNVSTPEATSSSLENSSSASSLLN
>sp|Q8IZF4|AGRG5_HUMAN Adhesion G-protein coupled receptor G5 OS=Homo sapiens GN=ADGRG5 PE=2 SV=3
MDHCGALFLCLCLLTLQNATTETWEELLSYMENMQVSRGRSSVFSSRQLHQLEQMLLNTS
FPGYNLTLQTPTIQSLAFKLSCDFSGLSLTSATLKRVPQAGGQHARGQHAMQFPAELTRD
ACKTRPRELRLICIYFSNTHFFKDENNSSLLNNYVLGAQLSHGHVNNLRDPVNISFWHNQ
SLEGYTLTCVFWKEGARKQPWGGWSPEGCRTEQPSHSQVLCRCNHLTYFAVLMQLSPALV
PAELLAPLTYISLVGCSISIVASLITVLLHFHFRKQSDSLTRIHMNLHASVLLLNIAFLL
SPAFAMSPVPGSACTALAAALHYALLSCLTWMAIEGFNLYLLLGRVYNIYIRRYVFKLGV
LGWGAPALLVLLSLSVKSSVYGPCTIPVFDSWENGTGFQNMSICWVRSPVVHSVLVMGYG
GLTSLFNLVVLAWALWTLRRLRERADAPSVRACHDTVTVLGLTVLLGTTWALAFFSFGVF
LLPQLFLFTILNSLYGFFLFLWFCSQRCRSEAEAKAQIEAFSSSQTTQ
>sp|Q96K78|AGRG7_HUMAN Adhesion G-protein coupled receptor G7 OS=Homo sapiens GN=ADGRG7 PE=1 SV=2
MASCRAWNLRVLVAVVCGLLTGIILGLGIWRIVIRIQRGKSTSSSSTPTEFCRNGGTWEN
GRCICTEEWKGLRCTIANFCENSTYMGFTFARIPVGRYGPSLQTCGKDTPNAGNPMAVRL
CSLSLYGEIELQKVTIGNCNENLETLEKQVKDVTAPLNNISSEVQILTSDANKLTAENIT
SATRVVGQIFNTSRNASPEAKKVAIVTVSQLLDASEDAFQRVAATANDDALTTLIEQMET
YSLSLGNQSVVEPNIAIQSANFSSENAVGPSNVRFSVQKGASSSLVSSSTFIHTNVDGLN
PDAQTELQVLLNMTKNYTKTCGFVVYQNDKLFQSKTFTAKSDFSQKIISSKTDENEQDQS
ASVDMVFSPKYNQKEFQLYSYACVYWNLSAKDWDTYGCQKDKGTDGFLRCRCNHTTNFAV
LMTFKKDYQYPKSLDILSNVGCALSVTGLALTVIFQIVTRKVRKTSVTWVLVNLCISMLI
FNLLFVFGIENSNKNLQTSDGDINNIDFDNNDIPRTDTINIPNPMCTAIAALLHYFLLVT
FTWNALSAAQLYYLLIRTMKPLPRHFILFISLIGWGVPAIVVAITVGVIYSQNGNNPQWE
LDYRQEKICWLAIPEPNGVIKSPLLWSFIVPVTIILISNVVMFITISIKVLWKNNQNLTS
TKKVSSMKKIVSTLSVAVVFGITWILAYLMLVNDDSIRIVFSYIFCLFNTTQGLQIFILY
TVRTKVFQSEASKVLMLLSSIGRRKSLPSVTRPRLRVKMYNFLRSLPTLHERFRLLETSP
STEEITLSESDNAKESI
>sp|Q9HBW9|AGRL4_HUMAN Adhesion G protein-coupled receptor L4 OS=Homo sapiens GN=ADGRL4 PE=1 SV=3
MKRLPLLVVFSTLLNCSYTQNCTKTPCLPNAKCEIRNGIEACYCNMGFSGNGVTICEDDN
ECGNLTQSCGENANCTNTEGSYYCMCVPGFRSSSNQDRFITNDGTVCIENVNANCHLDNV
CIAANINKTLTKIRSIKEPVALLQEVYRNSVTDLSPTDIITYIEILAESSSLLGYKNNTI
SAKDTLSNSTLTEFVKTVNNFVQRDTFVVWDKLSVNHRRTHLTKLMHTVEQATLRISQSF
QKTTEFDTNSTDIALKVFFFDSYNMKHIHPHMNMDGDYINIFPKRKAAYDSNGNVAVAFV
YYKSIGPLLSSSDNFLLKPQNYDNSEEEERVISSVISVSMSSNPPTLYELEKITFTLSHR

KVTDRYRSLCAFWNYSPDTMNGSWSSEGCELTYSNETHTSCRCNHLTHFAILMSSGPSIG
IKDYNILTRITQLGIIISLICLAICIFTFWFFSEIQSTRTTIHKNLCCSLFLAELVFLVG
INTNTNKLFCSIIAGLLHYFFLAAFAWMCIEGIHLYLIVVGVIYNKGFLHKNFYIFGYLS
PAVVVGFSAALGYRYYGTTKVCWLSTENNFIWSFIGPACLIILVNLLAFGVIIYKVFRHT
AGLKPEVSCFENIRSCARGALALLFLLGTTWIFGVLHVVHASVVTAYLFTVSNAFQGMFI
FLFLCVLSRKIQEEYYRLFKNVPCCFGCLR
>sp|P30556|AGTR1_HUMAN Type-1 angiotensin II receptor OS=Homo sapiens GN=AGTR1 PE=1 SV=1
MILNSSTEDGIKRIQDDCPKAGRHNYIFVMIPTLYSIIFVVGIFGNSLVVIVIYFYMKLK
TVASVFLLNLALADLCFLLTLPLWAVYTAMEYRWPFGNYLCKIASASVSFNLYASVFLLT
CLSIDRYLAIVHPMKSRLRRTMLVAKVTCIIIWLLAGLASLPAIIHRNVFFIENTNITVC
AFHYESQNSTLPIGLGLTKNILGFLFPFLIILTSYTLIWKALKKAYEIQKNKPRNDDIFK
IIMAIVLFFFFSWIPHQIFTFLDVLIQLGIIRDCRIADIVDTAMPITICIAYFNNCLNPL
FYGFLGKKFKRYFLQLLKYIPPKAKSHSNLSTKMSTLSYRPSDNVSSSTKKPAPCFEVE
>sp|P50052|AGTR2_HUMAN Type-2 angiotensin II receptor OS=Homo sapiens GN=AGTR2 PE=1 SV=1
MKGNSTLATTSKNITSGLHFGLVNISGNNESTLNCSQKPSDKHLDAIPILYYIIFVIGFL
VNIVVVTLFCCQKGPKKVSSIYIFNLAVADLLLLATLPLWATYYSYRYDWLFGPVMCKVF
GSFLTLNMFASIFFITCMSVDRYQSVIYPFLSQRRNPWQASYIVPLVWCMACLSSLPTFY
FRDVRTIEYLGVNACIMAFPPEKYAQWSAGIALMKNILGFIIPLIFIATCYFGIRKHLLK
TNSYGKNRITRDQVLKMAAAVVLAFIICWLPFHVLTFLDALAWMGVINSCEVIAVIDLAL
PFAILLGFTNSCVNPFLYCFVGNRFQQKLRSVFRVPITWLQGKRESMSCRKSSSLREMET
FVS
>sp|O43823|AKAP8_HUMAN A-kinase anchor protein 8 OS=Homo sapiens GN=AKAP8 PE=1 SV=1
MDQGYGGYGAWSAGPANTQGAYGTGVASWQGYENYNYYGAQNTSVTTGATYSYGPASWEA
AKANDGGLAAGAPAMHMASYGPEPCTDNSDSLIAKINQRLDMMSKEGGRGGSGGGGEGIQ
DRESSFRFQPFESYDSRPCLPEHNPYRPSYSYDYEFDLGSDRNGSFGGQYSECRDPARER
GSLDGFMRGRGQGRFQDRSNPGTFMRSDPFVPPAASSEPLSTPWNELNYVGGRGLGGPSP
SRPPPSLFSQSMAPDYGVMGMQGAGGYDSTMPYGCGRSQPRMRDRDRPKRRGFDRFGPDG
TGRKRKQFQLYEEPDTKLARVDSEGDFSENDDAAGDFRSGDEEFKGEDELCDSGRQRGEK
EDEDEDVKKRREKQRRRDRTRDRAADRIQFACSVCKFRSFDDEEIQKHLQSKFHKETLRF
ISTKLPDKTVEFLQEYIVNRNKKIEKRRQELMEKETAKPKPDPFKGIGQEHFFKKIEAAH
CLACDMLIPAQPQLLQRHLHSVDHNHNRRLAAEQFKKTSLHVAKSVLNNRHIVKMLEKYL
KGEDPFTSETVDPEMEGDDNLGGEDKKETPEEVAADVLAEVITAAVRAVDGEGAPAPESS
GEPAEDEGPTDTAEAGSDPQAEQLLEEQVPCGTAHEKGVPKARSEAAEAGNGAETMAAEA
ESAQTRVAPAPAAADAEVEQTDAESKDAVPTE
>sp|A6NHY2|AKD1B_HUMAN Ankyrin repeat and death domain-containing protein 1B OS=Homo sapiens GN=ANKDD1B PE=3 SV=3
MDPAGRARGQGATAGGLLLRAAAAAKGLREDLWGAAALPWRSLSRIPKREGLGEEDTAVA
GHELLLPNERSFQNAAKSNNLDLMEKLFEKKVNINVVNNMNRTALHFAVGRNHLSAVDFL
LKHKARVDVADKHGLTVIHLAAWSGSLEVMLMLVKAGADQRAKNQDGMSALHFATQSNHV
RIVEYLIQDLHLKDLNQPDEKGRKPFLLAAERGHVEMIEKLTFLNLHTSEKDKGGNTALH
LAAKHGHSPAVQVLLAQWQDINEMNELNISSLQIATRNGHASLVNFLLSENVDLHQKVEP
KESPLHLVVINNHITVVNSLLSAQHDIDILNQKQQTPLHVAADRGNVELVETLLKAGCDL
KAVDKQGKTALAVASRSNHSLVVGMLIKAERYYAWREEHHESIRDPSTGFTLTFKQDHSL
ETRHIRTLLWDLAYHQLKANEWQRLARSWNFTDDQIRAIEEQWSGNESFREHGHRALLIW

LHGTLMTQGDPAKQLYEELVHAGFPKLAEKTRHFKSKTDSNSKKCVVS
>sp|P31749|AKT1_HUMAN RAC-alpha serine/threonine-protein kinase OS=Homo sapiens GN=AKT1 PE=1 SV=2
MSDVAIVKEGWLHKRGEYIKTWRPRYFLLKNDGTFIGYKERPQDVDQREAPLNNFSVAQC
QLMKTERPRPNTFIIRCLQWTTVIERTFHVETPEEREEWTTAIQTVADGLKKQEEEEMDF
RSGSPSDNSGAEEMEVSLAKPKHRVTMNEFEYLKLLGKGTFGKVILVKEKATGRYYAMKI
LKKEVIVAKDEVAHTLTENRVLQNSRHPFLTALKYSFQTHDRLCFVMEYANGGELFFHLS
RERVFSEDRARFYGAEIVSALDYLHSEKNVVYRDLKLENLMLDKDGHIKITDFGLCKEGI
KDGATMKTFCGTPEYLAPEVLEDNDYGRAVDWWGLGVVMYEMMCGRLPFYNQDHEKLFEL
ILMEEIRFPRTLGPEAKSLLSGLLKKDPKQRLGGGSEDAKEIMQHRFFAGIVWQHVYEKK
LSPPFKPQVTSETDTRYFDEEFTAQMITITPPDQDDSMECVDSERRPHFPQFSYSASGTA
>sp|P43353|AL3B1_HUMAN Aldehyde dehydrogenase family 3 member B1 OS=Homo sapiens GN=ALDH3B1 PE=1 SV=1
MDPLGDTLRRLREAFHAGRTRPAEFRAAQLQGLGRFLQENKQLLHDALAQDLHKSAFESE
VSEVAISQGEVTLALRNLRAWMKDERVPKNLATQLDSAFIRKEPFGLVLIIAPWNYPLNL
TLVPLVGALAAGNCVVLKPSEISKNVEKILAEVLPQYVDQSCFAVVLGGPQETGQLLEHR
FDYIFFTGSPRVGKIVMTAAAKHLTPVTLELGGKNPCYVDDNCDPQTVANRVAWFRYFNA
GQTCVAPDYVLCSPEMQERLLPALQSTITRFYGDDPQSSPNLGRIINQKQFQRLRALLGC
GRVAIGGQSDESDRYIAPTVLVDVQEMEPVMQEEIFGPILPIVNVQSLDEAIEFINRREK
PLALYAFSNSSQVVKRVLTQTSSGGFCGNDGFMHMTLASLPFGGVGASGMGRYHGKFSFD
TFSHHRACLLRSPGMEKLNALRYPPQSPRRLRMLLVAMEAQGCSCTLL
>sp|Q96QP1|ALPK1_HUMAN Alpha-protein kinase 1 OS=Homo sapiens GN=ALPK1 PE=2 SV=3
MNNQKVVAVLLQECKQVLDQLLLEAPDVSEEDKSEDQRCRALLPSELRTLIQEAKEMKWP
FVPEKWQYKQAVGPEDKTNLKDVIGAGLQQLLASLRASILARDCAAAAAIVFLVDRFLYG
LDVSGKLLQVAKGLHKLQPATPIAPQVVIRQARISVNSGKLLKAEYILSSLISNNGATGT
WLYRNESDKVLVQSVCIQIRGQILQKLGMWYEAAELIWASIVGYLALPQPDKKGLSTSLG
ILADIFVSMSKNDYEKFKNNPQINLSLLKEFDHHLLSAAEACKLAAAFSAYTPLFVLTAV
NIRGTCLLSYSSSNDCPPELKNLHLCEAKEAFEIGLLTKRDDEPVTGKQELHSFVKAAFG
LTTVHRRLHGETGTVHAASQLCKEAMGKLYNFSTSSRSQDREALSQEVMSVIAQVKEHLQ
VQSFSNVDDRSYVPESFECRLDKLILHGQGDFQKILDTYSQHHTSVCEVFESDCGNNKNE
QKDAKTGVCITALKTEIKNIDTVSTTQEKPHCQRDTGISSSLMGKNVQRELRRGGRRNWT
HSDAFRVSLDQDVETETEPSDYSNGEGAVFNKSLSGSQTSSAWSNLSGFSSSASWEEVNY
HVDDRSARKEPGKEHLVDTQCSTALSEELENDREGRAMHSLHSQLHDLSLQEPNNDNLEP
SQNQPQQQMPLTPFSPHNTPGIFLAPGAGLLEGAPEGIQEVRNMGPRNTSAHSRPSYRSA
SWSSDSGRPKNMGTHPSVQKEEAFEIIVEFPETNCDVKDRQGKEQGEEISERGAGPTFKA
SPSWVDPEGETAESTEDAPLDFHRVLHNSLGNISMLPCSSFTPNWPVQNPDSRKSGGPVA
EQGIDPDASTVDEEGQLLDSMDVPCTNGHGSHRLCILRQPPGQRAETPNSSVSGNILFPV
LSEDCTTTEEGNQPGNMLNCSQNSSSSSVWWLKSPAFSSGSSEGDSPWSYLNSSGSSWVS
LPGKMRKEILEARTLQPDDFEKLLAGVRHDWLFQRLENTGVFKPSQLHRAHSALLLKYSK
KSELWTAQETIVYLGDYLTVKKKGRQRNAFWVHHLHQEEILGRYVGKDYKEQKGLWHHFT
DVERQMTAQHYVTEFNKRLYEQNIPTQIFYIPSTILLILEDKTIKGCISVEPYILGEFVK
LSNNTKVVKTEYKATEYGLAYGHFSYEFSNHRDVVVDLQGWVTGNGKGLIYLTDPQIHSV
DQKVFTTNFGKRGIFYFFNNQHVECNEICHRLSLTRPSMEKPCT
>sp|Q96L96|ALPK3_HUMAN Alpha-protein kinase 3 OS=Homo sapiens GN=ALPK3 PE=2 SV=2

MEVAWLVYVLGQQPLARQGEGQSRLVPGRGLVLWLPGLPRSSPSWPAVDLAPLAPARPRG
PLICHTGHEQAGREPGPGSSTKGPVLHDQDTRCAFLPRPPGPLQTRRYCRHQGRQGSGLG
AGPGAGTWAPAPPGVSKPRCPGRARPGEGQQQVTTARPPAINRGARQPRAGAAAAGRGPG
AGAWRTGEAAASAGPAVGEGGAMGSRRAPSRGWGAGGRSGAGGDGEDDGPVWIPSPASRS
YLLSVRPETSLSSNRLSHPSSGRSTFCSIIAQLTEETQPLFETTLKSRSVSEDSDVRFTC
IVTGYPEPEVTWYKDDTELDRYCGLPKYEITHQGNRHTLQLYRCREEDAAIYQASAQNSK
GIVSCSGVLEVGTMTEYKIHQRWFAKLKRKAAAKLREIEQSWKHEKAVPGEVDTLRKLSP
DRFQRKRRLSGAQAPGPSVPTREPEGGTLAAWQEGETETAQHSGLGLINSFASGEVTTNG
EAAPENGEDGEHGLLTYICDAMELGPQRALKEESGAKKKKKDEESKQGLRKPELEKAAQS
RRSSENCIPSSDEPDSCGTQGPVGVEQVQTQPRGRAARGPGSSGTDSTRKPASAVGTPDK
AQKAPGPGPGQEVYFSLKDMYLENTQAVRPLGEEGPQTLSVRAPGESPKGKAPLRARSEG
VPGAPGQPTHSLTPQPTRPFNRKRFAPPKPKGEATTDSKPISSLSQAPECGAQSLGKAPP
QASVQVPTPPARRRHGTRDSTLQGQAGHRTPGEVLECQTTTAPTMSASSSSDVASIGVST
SGSQGIIEPMDMETQEDGRTSANQRTGSKKNVQADGKIQVDGRTRGDGTQTAQRTRADRK
TQVDAGTQESKRPQSDRSAQKGMMTQGRAETQLETTQAGEKIQEDRKAQADKGTQEDRRM
QGEKGMQGEKGTQSEGSAPTAMEGQSEQEVATSLGPPSRTPKLPPTAGPRAPLNIECFVQ
TPEGSCFPKKPGCLPRSEEAVVTASRNHEQTVLGPLSGNLMLPAQPPHEGSVEQVGGERC
RGPQSSGPVEAKQEDSPFQCPKEERPGGVPCMDQGGCPLAGLSQEVPTMPSLPGTGLTAS
PKAGPCSTPTSQHGSTATFLPSEDQVLMSSAPTLHLGLGTPTQSHPPETMATSSEGACAQ
VPDVEGRTPGPRSCDPGLIDSLKNYLLLLLKLSSTETSGAGGESQVGAATGGLVPSATLT
PTVEVAGLSPRTSRRILERVENNHLVQSAQTLLLSPCTSRRLTGLLDREVQAGRQALAAA
RGSWGPGPSSLTVPAIVVDEEDPGLASEGASEGEGEVSPEGPGLLGASQESSMAGRLGEA
GGQAAPGQGPSAESIAQEPSQEEKFPGEALTGLPAATPEELALGARRKRFLPKVRAAGDG
EATTPEERESPTVSPRGPRKSLVPGSPGTPGRERRSPTQGRKASMLEVPRAEEELAAGDL
GPSPKAGGLDTEVALDEGKQETLAKPRKAKDLLKAPQVIRKIRVEQFPDASGSLKLWCQF
FNILSDSVLTWAKDQRPVGEVGRSAGDEGPAALAIVQASPVDCGVYRCTIHNEHGSASTD
FCLSPEVLSGFISREEGEVGEEIEMTPMVFAKGLADSGCWGDKLFGRLVSEELRGGGYGC
GLRKASQAKVIYGLEPIFESGRTCIIKVSSLLVFGPSSETSLVGRNYDVTIQGCKIQNMS
REYCKIFAAEARAAPGFGEVPEIIPLYLIYRPANNIPYATLEEDLGKPLESYCSREWGCA
EAPTASGSSEAMQKCQTFQHWLYQWTNGSFLVTDLAGVDWKMTDVQIATKLRGYQGLKES
CFPALLDRFASSHQCNAYCELLGLTPLKGPEAAHPQAKAKGSKSPSAGRKGSQLSPQPQK
KGLPSPQGTRKSAPSSKATPQASEPVTTQLLGQPPTQEEGSKAQGMR
>sp|P55789|ALR_HUMAN FAD-linked sulfhydryl oxidase ALR OS=Homo sapiens GN=GFER PE=1 SV=2
MAAPGERGRFHGGNLFFLPGGARSEMMDDLATDARGRGAGRRDAAASASTPAQAPTSDSP
VAEDASRRRPCRACVDFKTWMRTQQKRDTKFREDCPPDREELGRHSWAVLHTLAAYYPDL
PTPEQQQDMAQFIHLFSKFYPCEECAEDLRKRLCRNHPDTRTRACFTQWLCHLHNEVNRK
LGKPDFDCSKVDERWRDGWKDGSCD
>sp|Q96PN6|ADCYA_HUMAN Adenylate cyclase type 10 OS=Homo sapiens GN=ADCY10 PE=1 SV=3
MNTPKEEFQDWPIVRIAAHLPDLIVYGHFSPERPFMDYFDGVLMFVDISGFTAMTEKFSS
AMYMDRGAEQLVEILNYHISAIVEKVLIFGGDILKFAGDALLALWRVERKQLKNIITVVI
KCSLEIHGLFETQEWEEGLDIRVKIGLAAGHISMLVFGDETHSHFLVIGQAVDDVRLAQN
MAQMNDVILSPNCWQLCDRSMIEIESVPDQRAVKVNFLKPPPNFNFDEFFTKCTTFMHYY
PSGEHKNLLRLACTLKPDPELEMSLQKYVMESILKQIDNKQLQGYLSELRPVTIVFVNLM
FEDQDKAEEIGPAIQDAYMHITSVLKIFQGQINKVFMFDKGCSFLCVFGFPGEKVPDELT

HALECAMDIFDFCSQVHKIQTVSIGVASGIVFCGIVGHTVRHEYTVIGQKVNLAARMMMY
YPGIVTCDSVTYNGSNLPAYFFKELPKKVMKGVADSGPLYQYWGRTEKVMFGMACLICNR
KEDYPLLGRNKEINYFMYTMKKFLISNSSQVLMYEGLPGYGKSQILMKIEYLAQGKNHRI
IAISLNKISFHQTFYTIQMFMANVLGLDTCKHYKERQTNLRNKVMTLLDEKFYCLLNDIF
HVQFPISREISRMSTLKKQKQLEILFMKILKLIVKEERIIFIIDEAQFVDSTSWRFMEKL
IRTLPIFIIMSLCPFVNIPCAAARAVIKNRNTTYIVIGAVQPNDISNKICLDLNVSCISK
ELDSYLGEGSCGIPFYCEELLKNLEHHEVLVFQQTESEEKTNRTWNNLFKYSIKLTEKLN
MVTLHSDKESEEVCHLTSGVRLKNLSPPTSLKEISLIQLDSMRLSHQMLVRCAAIIGLTF
TTELLFEILPCWNMKMMIKTLATLVESNIFYCFRNGKELQKALKQNDPSFEVHYRSLSLK
PSEGMDHGEEEQLRELENEVIECHRIRFCNPMMQKTAYELWLKDQRKAMHLKCARFLEED
AHRCDHCRGRDFIPYHHFTVNIRLNALDMDAIKKMAMSHGFKTEEKLILSNSEIPETSAF
FPENRSPEEIREKILNFFDHVLTKMKTSDEDIIPLESCQCEEILEIVILPLAHHFLALGE
NDKALYYFLEIASAYLIFCDNYMAYMYLNEGQKLLKTLKKDKSWSQTFESATFYSLKGEV
CFNMGQIVLAKKMLRKALKLLNRIFPYNLISLFLHIHVEKNRHFHYVNRQAQESPPPGKK
RLAQLYRQTVCLSLLWRIYSYSYLFHCKYYAHLAVMMQMNTALETQNCFQIIKAYLDYSL
YHHLAGYKGVWFKYEVMAMEHIFNLPLKGEGIEIVAYVAETLVFNKLIMGHLDLAIELGS
RALQMWALLQNPNRHYQSLCRLSRCLLLNSRYPQLIQVLGRLWELSVTQEHIFSKAFFYF
VCLDILLYSGFVYRTFEECLEFIHQYENNRILKFHSGLLLGLYSSVAIWYARLQEWDNFY
KFSNRAKNLLPRRTMTLTYYDGISRYMEGQVLHLQKQIKEQSENAQASGEELLKNLENLV
AQNTTGPVFCPRLYHLMAYVCILMGDGQKCGLFLNTALRLSETQGNILEKCWLNMNKESW
YSTSELKEDQWLQTILSLPSWEKIVAGRVNIQDLQKNKFLMRANTVDNHF
>sp|Q8N7X0|ADGB_HUMAN Androglobin OS=Homo sapiens GN=ADGB PE=2 SV=3
MASKQTKKKEVHRINSAHGSDKSKDFYPFGSNVQSGSTEQKKGKFPLWPEWSEADINSEK
WDAGKGAKEKDKTGKSPVFHFFEDPEGKIELPPSLKIYSWKRPQDILFSQTPVVVKNEIT
FDLFSANEHLLCSELMRWIISEIYAVWKIFNGGILSNYFKGTSGEPPLLPWKPWEHIYSL
CKAVKGHMPLFNSYGKYVVKLYWMGCWRKITIDDFLPFDEDNNLLLPATTYEFELWPMLL
SKAIIKLANIDIHVADRRELGEFTVIHALTGWLPEVISLHPGYMDKVWELLKEILPEFKL
SDEASSESKIAVLDSKLKEPGKEGKEGKEIKDGKEVKDVKEFKPESSLTTLKAPEKSDKV
PKEKADARDIGKKRSKDGEKEKFKFSLHGSRPSSEVQYSVQSLSDCSSAIQTSHMVVYAT
FTPLYLFENKIFSLEKMADSAEKLREYGLSHICSHPVLVTRSRSCPLVAPPKPPPLPPWK
LIRQKKETVITDEAQELIVKKPERFLEISSPFLNYRMTPFTIPTEMHFVRSLIKKGIPPG
SDLPSVSETDETATHSQTDLSQITKATSQGNTASQVILGKGTDEQTDFGLGDAHQSDGLN
LEREIVSQTTATQEKSQEELPTTNNSVSKEIWLDFEDFCVCFQNIYIFHKPSSYCLNFQK
SEFKFSEERVSYYLFVDSLKPIELLVCFSALVRWGEYGALTKDSPPIEPGLLTAETFSWK
SLKPGSLVLKIHTYATKATVVRLPVGRHMLLFNAYSPVGHSIHICSMVSFVIGDEHVVLP
NFEPESCRFTEQSLLIMKAIGNVIANFKDKGKLSAALKDLQTAHYPVPFHDKELTAQHFR
VFHLSLWRLMKKVQITKPPPNFKFAFRAMVLDLELLNSSLEEVSLVEWLDVKYCMPTSDK
EYSAEEVAAAIKIQAMWRGTYVRLLMKARIPDTKENISVADTLQKVWAVLEMNLEQYAVS
LLRLMFKSKCKSLESYPCYQDEETKIAFADYTVTYQEQPPNSWFIVFRETFLVHQDMILV
PKVYTTLPICILHIVNNDTMEQVPKVFQKVVPYLYTKNKKGYTFVAEAFTGDTYVAASRW
KLRLIGSSAPLPCLSRDSPCNSFAIKEIRDYYIPNDKKILFRYSVKVLTPQPATIQVRTS
KPDAFIKLQVLENEETMVSSTGKGQAIIPAFHFLKSEKGLSSQSSKHILSFHSASKKEQE
VYVKKKAAQGIQKSPKGRAVSAIQDIGLPLVEEETTSTPTREDSSSTPLQNYKYIIQCSV
LYNSWPLTESQLTFVQALKDLKKSNTKAYGERHEELINLGSPDSHTISEGQKSSVTSKTT

RKGKEKSSEKEKTAKEKQAPRFEPQISTVHPQQEDPNKPYWILRLVTEHNESELFEVKKD
TERADEIRAMKQAWETTEPGRAIKASQARLHYLSGFIKKTSDAESPPISESQTKPKEEVE
TAARGVKEPNSKNSAGSESKEMTQTGSGSAVWKKWQLTKGLRDVAKSTSSESGGVSSPGK
EEREQSTRKENIQTGPRTRSPTILETSPRLIRKALEFMDLSQYVRKTDTDPLLQTDELNQ
QQAMQKAEEIHQFRQHRTRVLSIRNIDQEERLKLKDEVLDMYKEMQDSLDEARQKIFDIR
EEYRNKLLEAEHLKLETLAAQEAAMKLETEKMTPAPDTQKKKKGKKK
>sp|P07327|ADH1A_HUMAN Alcohol dehydrogenase 1A OS=Homo sapiens GN=ADH1A PE=1 SV=2
MSTAGKVIKCKAAVLWELKKPFSIEEVEVAPPKAHEVRIKMVAVGICGTDDHVVSGTMVT
PLPVILGHEAAGIVESVGEGVTTVKPGDKVIPLAIPQCGKCRICKNPESNYCLKNDVSNP
QGTLQDGTSRFTCRRKPIHHFLGISTFSQYTVVDENAVAKIDAASPLEKVCLIGCGFSTG
YGSAVNVAKVTPGSTCAVFGLGGVGLSAIMGCKAAGAARIIAVDINKDKFAKAKELGATE
CINPQDYKKPIQEVLKEMTDGGVDFSFEVIGRLDTMMASLLCCHEACGTSVIVGVPPDSQ
NLSMNPMLLLTGRTWKGAILGGFKSKECVPKLVADFMAKKFSLDALITHVLPFEKINEGF
DLLHSGKSIRTILMF
>sp|P28332|ADH6_HUMAN Alcohol dehydrogenase 6 OS=Homo sapiens GN=ADH6 PE=1 SV=2
MCTTGQVIRCKAAILWKPGAPFSIEEVEVAPPKAKEVRIKVVATGLCGTEMKVLGSKHLD
LLYPTILGHEGAGIVESIGEGVSTVKPGDKVITLFLPQCGECTSCLNSEGNFCIQFKQSK
TQLMSDGTSRFTCKGKSIYHFGNTSTFCEYTVIKEISVAKIDAVAPLEKVCLISCGFSTG
FGAAINTAKVTPGSTCAVFGLGGVGLSVVMGCKAAGAARIIGVDVNKEKFKKAQELGATE
CLNPQDLKKPIQEVLFDMTDAGIDFCFEAIGNLDVLAAALASCNESYGVCVVVGVLPASV
QLKISGQLFFSGRSLKGSVFGGWKSRQHIPKLVADYMAEKLNLDPLITHTLNLDKINEAV
ELMKTGKW
>sp|P40394|ADH7_HUMAN Alcohol dehydrogenase class 4 mu/sigma chain OS=Homo sapiens GN=ADH7 PE=1 SV=2
MFAEIQIQDKDRMGTAGKVIKCKAAVLWEQKQPFSIEEIEVAPPKTKEVRIKILATGICR
TDDHVIKGTMVSKFPVIVGHEATGIVESIGEGVTTVKPGDKVIPLFLPQCRECNACRNPD
GNLCIRSDITGRGVLADGTTRFTCKGKPVHHFMNTSTFTEYTVVDESSVAKIDDAAPPEK
VCLIGCGFSTGYGAAVKTGKVKPGSTCVVFGLGGVGLSVIMGCKSAGASRIIGIDLNKDK
FEKAMAVGATECISPKDSTKPISEVLSEMTGNNVGYTFEVIGHLETMIDALASCHMNYGT
SVVVGVPPSAKMLTYDPMLLFTGRTWKGCVFGGLKSRDDVPKLVTEFLAKKFDLDQLITH
VLPFKKISEGFELLNSGQSIRTVLTF
>sp|Q5T7M4|ADIPL_HUMAN Adipolin OS=Homo sapiens GN=FAM132A PE=1 SV=2
MRRWAWAAVVVLLGPQLVLLGGVGARREAQRTQQPGQRADPPNATASASSREGLPEAPKP
SQASGPEFSDAHMTWLNFVRRPDDGALRKRCGSRDKKPRDLFGPPGPPGAEVTAETLLHE
FQELLKEATERRFSGLLDPLLPQGAGLRLVGEAFHCRLQGPRRVDKRTLVELHGFQAPAA
QGAFLRGSGLSLASGRFTAPVSGIFQFSASLHVDHSELQGKARLRARDVVCVLICIESLC
QRHTCLEAVSGLESNSRVFTLQVQGLLQLQAGQYASVFVDNGSGAVLTIQAGSSFSGLLL
GT
>sp|Q15848|ADIPO_HUMAN Adiponectin OS=Homo sapiens GN=ADIPOQ PE=1 SV=1
MLLLGAVLLLLALPGHDQETTTQGPGVLLPLPKGACTGWMAGIPGHPGHNGAPGRDGRDG
TPGEKGEKGDPGLIGPKGDIGETGVPGAEGPRGFPGIQGRKGEPGEGAYVYRSAFSVGLE
TYVTIPNMPIRFTKIFYNQQNHYDGSTGKFHCNIPGLYYFAYHITVYMKDVKVSLFKKDK
AMLFTYDQYQENNVDQASGSVLLHLEVGDQVWLQVYGEGERNGLYADNDNDSTFTGFLLY
HDTN

>sp|Q7Z4H4|ADM2_HUMAN ADM2 OS=Homo sapiens GN=ADM2 PE=2 SV=1
MARIPTAALGCISLLCLQLPGSLSRSLGGDPRPVKPREPPARSPSSSLQPRHPAPRPVVW
KLHRALQAQRGAGLAPVMGQPLRDGGRQHSGPRRHSGPRRTQAQLLRVGCVLGTCQVQNL
SHRLWQLMGPAGRQDSAPVDPSSPHSYG
>sp|Q96A54|ADR1_HUMAN Adiponectin receptor protein 1 OS=Homo sapiens GN=ADIPOR1 PE=1 SV=1
MSSHKGSVVAQGNGAPASNREADTVELAELGPLLEEKGKRVIANPPKAEEEQTCPVPQEE
EEEVRVLTLPLQAHHAMEKMEEFVYKVWEGRWRVIPYDVLPDWLKDNDYLLHGHRPPMPS
FRACFKSIFRIHTETGNIWTHLLGFVLFLFLGILTMLRPNMYFMAPLQEKVVFGMFFLGA
VLCLSFSWLFHTVYCHSEKVSRTFSKLDYSGIALLIMGSFVPWLYYSFYCSPQPRLIYLS
IVCVLGISAIIVAQWDRFATPKHRQTRAGVFLGLGLSGVVPTMHFTIAEGFVKATTVGQM
GWFFLMAVMYITGAGLYAARIPERFFPGKFDIWFQSHQIFHVLVVAAAFVHFYGVSNLQE
FRYGLEGGCTDDTLL
>sp|P22570|ADRO_HUMAN NADPH:adrenodoxin oxidoreductase, mitochondrial OS=Homo sapiens GN=FDXR PE=1 SV=3
MASRCWRWWGWSAWPRTRLPPAGSTPSFCHHFSTQEKTPQICVVGSGPAGFYTAQHLLKH
PQAHVDIYEKQPVPFGLVRFGVAPDHPEVKNVINTFTQTAHSGRCAFWGNVEVGRDVTVP
ELREAYHAVVLSYGAEDHRALEIPGEELPGVCSARAFVGWYNGLPENQELEPDLSCDTAV
ILGQGNVALDVARILLTPPEHLERTDITKAALGVLRQSRVKTVWLVGRRGPLQVAFTIKE
LREMIQLPGARPILDPVDFLGLQDKIKEVPRPRKRLTELLLRTATEKPGPAEAARQASAS
RAWGLRFFRSPQQVLPSPDGRRAAGVRLAVTRLEGVDEATRAVPTGDMEDLPCGLVLSSI
GYKSRPVDPSVPFDSKLGVIPNVEGRVMDVPGLYCSGWVKRGPTGVIATTMTDSFLTGQM
LLQDLKAGLLPSGPRPGYAAIQALLSSRGVRPVSFSDWEKLDAEEVARGQGTGKPREKLV
DPQEMLRLLGH
>sp|P10109|ADX_HUMAN Adrenodoxin, mitochondrial OS=Homo sapiens GN=FDX1 PE=1 SV=1
MAAAGGARLLRAASAVLGGPAGRWLHHAGSRAGSSGLLRNRGPGGSAEASRSLSVSARAR
SSSEDKITVHFINRDGETLTTKGKVGDSLLDVVVENNLDIDGFGACEGTLACSTCHLIFE
DHIYEKLDAITDEENDMLDLAYGLTDRSRLGCQICLTKSMDNMTVRVPETVADARQSIDV
GKTS
>sp|Q96SZ5|AEDO_HUMAN 2-aminoethanethiol dioxygenase OS=Homo sapiens GN=ADO PE=1 SV=2
MPRDNMASLIQRIARQACLTFRGSGGGRGASDRDAASGPEAPMQPGFPENLSKLKSLLTQ
LRAEDLNIAPRKATLQPLPPNLPPVTYMHIYETDGFSLGVFLLKSGTSIPLHDHPGMHGM
LKVLYGTVRISCMDKLDAGGGQRPRALPPEQQFEPPLQPREREAVRPGVLRSRAEYTEAS
GPCILTPHRDNLHQIDAVEGPAAFLDILAPPYDPDDGRDCHYYRVLEPVRPKEASSSACD
LPREVWLLETPQADDFWCEGEPYPGPKVFP
>sp|Q6UXC1|AEGP_HUMAN Apical endosomal glycoprotein OS=Homo sapiens GN=MAMDC4 PE=2 SV=2
MPLSSHLLPALVLFLAGSSGWAWVPNHCRSPGQAVCNFVCDCRDCSDEAQCGYHGASPTL
GAPFACDFEQDPCGWRDISTSGYSWLRDRAGAALEGPGPHSDHTLGTDLGWYMAVGTHRG
KEASTAALRSPTLREAASSCKLRLWYHAASGDVAELRVELTHGAETLTLWQSTGPWGPGW
QELAVTTGRIRGDFRVTFSATRNATHRGAVALDDLEFWDCGLPTPQANCPPGHHHCQNKV
CVEPQQLCDGEDNCGDLSDENPLTCGRHIATDFETGLGPWNRSEGWSRNHRAGGPERPSW
PRRDHSRNSAQGSFLVSVAEPGTPAILSSPEFQASGTSNCSLVFYQYLSGSEAGCLQLFL
QTLGPGAPRAPVLLRRRRGELGTAWVRDRVDIQSAYPFQILLAGQTGPGGVVGLDDLILS
DHCRPVSEVSTLQPLPPGPRAPAPQPLPPSSRLQDSCKQGHLACGDLCVPPEQLCDFEEQ
CAGGEDEQACGTTDFESPEAGGWEDASVGRLQWRRVSAQESQGSSAAAAGHFLSLQRAWG

QLGAEARVLTPLLGPSGPSCELHLAYYLQSQPRGFLALVVVDNGSRELAWQALSSSAGIW
KVDKVLLGARRRPFRLEFVGLVDLDGPDQQGAGVDNVTLRDCSPTVTTERDREVSCNFER
DTCSWYPGHLSDTHWRWVESRGPDHDHTTGQGHFVLLDPTDPLAWGHSAHLLSRPQVPAA
PTECLSFWYHLHGPQIGTLRLAMRREGEETHLWSRSGTQGNRWHEAWATLSHQPGSHAQY
QLLFEGLRDGYHGTMALDDVAVRPGPCWAPNYCSFEDSDCGFSPGGQGLWRRQANASGHA
AWGPPTDHTTETAQGHYMVVDTSPDALPRGQTASLTSKEHRPLAQPACLTFWYHGSLRSP
GTLRVYLEERGRHQVLSLSAHGGLAWRLGSMDVQAERAWRVVFEAVAAGVAHSYVALDDL
LLQDGPCPQPGSCDFESGLCGWSHLAWPGLGGYSWDWGGGATPSRYPQPPVDHTLGTEAG
HFAFFETGVLGPGGRAAWLRSEPLPATPASCLRFWYHMGFPEHFYKGELKVLLHSAQGQL
AVWGAGGHRRHQWLEAQVEVASAKEFQIVFEATLGGQPALGPIALDDVEYLAGQHCQQPA
PSPGNTAAPGSVPAVVGSALLLLMLLVLLGLGGRRWLQKKGSCPFQSNTEATAPGFDNIL
FNADGVTLPASVTSDP
>sp|Q8WTP8|AEN_HUMAN Apoptosis-enhancing nuclease OS=Homo sapiens GN=AEN PE=1 SV=2
MVPREAPESAQCLCPSLTIPNAKDVLRKRHKRRSRQHQRFMARKALLQEQGLLSMPPEPG
SSPLPTPFGAATATEAASSGKQCLRAGSGSAPCSRRPAPGKASGPLPSKCVAIDCEMVGT
GPRGRVSELARCSIVSYHGNVLYDKYIRPEMPIADYRTRWSGITRQHMRKAVPFQVAQKE
ILKLLKGKVVVGHALHNDFQALKYVHPRSQTRDTTYVPNFLSEPGLHTRARVSLKDLALQ
LLHKKIQVGQHGHSSVEDATTAMELYRLVEVQWEQQEARSLWTCPEDREPDSSTDMEQYM
EDQYWPDDLAHGSRGGAREAQDRRN
>sp|P55198|AF17_HUMAN Protein AF-17 OS=Homo sapiens GN=MLLT6 PE=1 SV=2
MKEMVGGCCVCSDERGWAENPLVYCDGHACSVVVHQACYGIVQVPTGPWFCRKCESQERA
ARVRCELCPHKDGALKRTDNGGWAHVVCALYIPEVQFANVLTMEPIVLQYVPHDRFNKTC
YICEEQGRESKAASGACMTCNRHGCRQAFHVTCAQMAGLLCEEEVLEVDNVKYCGYCKYH
FSKMKTSRHSSGGGGGGAGGGGGSMGGGGSGFISGRRSRSASPSTQQEKHPTHHERGQKK
SRKDKERLKQKHKKRPESPPSILTPPVVPTADKVSSSASSSSHHEASTQETSESSRESKG
KKSSSHSLSHKGKKLSSGKGVSSFTSASSSSSSSSSSSGGPFQPAVSSLQSSPDFSAFPK
LEQPEEDKYSKPTAPAPSAPPSPSAPEPPKADLFEQKVVFSGFGPIMRFSTTTSSSGRAR
APSPGDYKSPHVTGSGASAGTHKRMPALSATPVPADETPETGLKEKKHKASKRSRHGPGR
PKGSRNKEGTGGPAAPSLPSAQLAGFTATAASPFSGGSLVSSGLGGLSSRTFGPSGSLPS
LSLESPLLGAGIYTSNKDPISHSGGMLRAVCSTPLSSSLLGPPGTSALPRLSRSPFTSTL
PSSSASISTTQVFSLAGSTFSLPSTHIFGTPMGAVNPLLSQAESSHTEPDLEDCSFRCRG
TSPQESLSSMSPISSLPALFDQTASAPCGGGQLDPAAPGTTNMEQLLEKQGDGEAGVNIV
EMLKALHALQKENQRLQEQILSLTAKKERLQILNVQLSVPFPALPAALPAANGPVPGPYG
LPPQAGSSDSLSTSKSPPGKSSLGLDNSLSTSSEDPHSGCPSRSSSSLSFHSTPPPLPLL
QQSPATLPLALPGAPAPLPPQPQNGLGRAPGAAGLGAMPMAEGLLGGLAGSGGLPLNGLL
GGLNGAAAPNPASLSQAGGAPTLQLPGCLNSLTEQQRHLLQQQEQQLQQLQQLLASPQLT
PEHQTVVYQMIQQIQQKRELQRLQMAGGSQLPMASLLAGSSTPLLSAGTPGLLPTASAPP
LLPAGALVAPSLGNNTSLMAAAAAAAAVAAAGGPPVLTAQTNPFLSLSGAEGSGGGPKGG
TADKGASANQEKG
>sp|Q13015|AF1Q_HUMAN Protein AF1q OS=Homo sapiens GN=MLLT11 PE=1 SV=1
MRDPVSSQYSSFLFWRMPIPELDLSELEGLGLSDTATYKVKDSSVGKMIGQATAADQEKN
PEGDGLLEYSTFNFWRAPIASIHSFELDLL
>sp|Q8N556|AFAP1_HUMAN Actin filament-associated protein 1 OS=Homo sapiens GN=AFAP1 PE=1
SV=2

MEELIVELRLFLELLDHEYLTSTVREKKAVITNILLRIQSSKGFDVKDHAQKQETANSLP
APPQMPLPEIPQPWLPPDSGPPPLPTSSLPEGYYEEAVPLSPGKAPEYITSNYDSDAMSS
SYESYDEEEEDGKGKKTRHQWPSEEASMDLVKDAKICAFLLRKKRFGQWTKLLCVIKDTK
LLCYKSSKDQQPQMELPLQGCNITYIPKDSKKKKHELKITQQGTDPLVLAVQSKEQAEQW
LKVIKEAYSGCSGPVDSECPPPPSSPVHKAELEKKLSSERPSSDGEGVVENGITTCNGKE
QVKRKKSSKSEAKGTVSKVTGKKITKIISLGKKKPSTDEQTSSAEEDVPTCGYLNVLSNS
RWRERWCRVKDNKLIFHKDRTDLKTHIVSIPLRGCEVIPGLDSKHPLTFRLLRNGQEVAV
LEASSSEDMGRWIGILLAETGSSTDPEALHYDYIDVEMSASVIQTAKQTFCFMNRRVISA
NPYLGGTSNGYAHPSGTALHYDDVPCINGSLKGKKPPVASNGVTGKGKTLSSQPKKADPA
AVVKRTGSNAAQYKYGKNRVEADAKRLQTKEEELLKRKEALRNRLAQLRKERKDLRAAIE
VNAGRKPQAILEEKLKQLEEECRQKEAERVSLELELTEVKESLKKALAGGVTLGLAIEPK
SGTSSPQSPVFRHRTLENSPISSCDTSDTEGPVPVNSAAVLKKSQAAPGSSPCRGHVLRK
AKEWELKNGT
>sp|P51826|AFF3_HUMAN AF4/FMR2 family member 3 OS=Homo sapiens GN=AFF3 PE=1 SV=2
MDSFDLALLQEWDLESLCVYEPDRNALRRKERERRNQETQQDDGTFNSSYSLFSEPYKTN
KGDELSNRIQNTLGNYDEMKDFLTDRSNQSHLVGVPKPGVPQTPVNKIDEHFVADSRAQN
QPSSICSTTTSTPAAVPVQQSKRGTMGWQKAGHPPSDGQQRATQQGSLRTLLGDGVGRQQ
PRAKQVCNVEVGLQTQERPPAMAAKHSSSGHCVQNFPPSLASKPSLVQQKPTAYVRPMDG
QDQAPDESPKLKSSSETSVHCTSYRGVPASKPEPARAKAKLSKFSIPKQGEESRSGETNS
CVEEIIREMTWLPPLSAIQAPGKVEPTKFPFPNKDSQLVSSGHNNPKKGDAEPESPDSGT
SNTSMLEDDLKLSSDEEENEQQAAQRTALRALSDSAVVQQPNCRTSVPSSKGSSSSSSSG
SSSSSSDSESSSGSDSETESSSSESEGSKPPHFSSPEAEPASSNKWQLDKWLNKVNPHKP
PILIQNESHGSESNQYYNPVKEDVQDCGKVPDVCQPSLREKEIKSTCKEEQRPRTANKAP
GSKGVKQKSPPAAVAVAVSAAAPPPAVPCAPAENAPAPARRSAGKKPTRRTERTSAGDGA
NCHRPEEPAAADALGTSVVVPPEPTKTRPCGNNRASHRKELRSSVTCEKRRTRGLSRIVP
KSKEFIETESSSSSSSSDSDLESEQEEYPLSKAQTVAASASSGNDQRLKEAAANGGSGPR
APVGSINARTTSDIAKELEEQFYTLVPFGRNELLSPLKDSDEIRSLWVKIDLTLLSRIPE
HLPQEPGVLSAPATKDSESAPPSHTSDTPAEKALPKSKRKRKCDNEDDYREIKKSQGEKD
SSSRLATSTSNTLSANHCNMNINSVAIPINKNEKMLRSPISPLSDASKHKYTSEDLTSSS
RPNGNSLFTSASSSKKPKADSQLQPHGGDLTKAAHNNSENIPLHKSRPQTKPWSPGSNGH
RDCKRQKLVFDDMPRSADYFMQEAKRMKHKADAMVEKFGKALNYAEAALSFIECGNAMEQ
GPMESKSPYTMYSETVELIRYAMRLKTHSGPNATPEDKQLAALCYRCLALLYWRMFRLKR
DHAVKYSKALIDYFKNSSKAAQAPSPWGASGKSTGTPSPMSPNPSPASSVGSQGSLSNAS
ALSPSTIVSIPQRIHQMAANHVSITNSILHSYDYWEMADNLAKENREFFNDLDLLMGPVT
LHSSMEHLVQYSQQGLHWLRNSAHLS
>sp|Q96P64|AGAP4_HUMAN Arf-GAP with GTPase, ANK repeat and PH domain-containing protein 4 OS=Homo sapiens GN=AGAP4 PE=2 SV=2
MGNILTCRVHPSVSLEFDQQQGSVCPSESEIYEAGAGDRMAGAPMAAAVQPAEVTVEVGE
DLHMHHVRDREMPEALEFNPSANPEASTIFQRNSQTDVVEIRRSNCTNHVSTVRFSQQYS
LCSTIFLDDSTAIQHYLTMTIISVTLEIPHHITQRDADRSLSIPDEQLHSFAVSTVHIMK
KRNGGGSLNNYSSSIPSTPSTSQEDPQFSVPPTANTPTPVCKRSMRWSNLFTSEKGSDPD
KERKAPENHADTIGSGRAIPIKQGMLLKRSGKWLKTWKKKYVTLCSNGVLTYYSSLGDYM
KNIHKKEIDLQTSTIKVPGKWPSLATSACTPISTSKSNGLSKDMDTGLGDSICFSPSISS
TTSPKLNPPPSPHANKKKHLKKKSTNNFMIVSATGQTWHFEATTYEERDAWVQAIQSQIL

ASLQSCESSKSKSQLTSQSKAMALQSIQNMRGNAHCVDCETQNPKWASLNLGVLMCIECS
GIHRSLGTRLSRVRSLELDDWPVELRKVMSSIGNDLANSIWEGSSQGQTKPSEKSTREEK
ERWIRSKYEEKLFLAPLPCTELSLGQQLLRATADEDLQTAILLLAHGSREEVNETCGEGD
GCTALHLACRKGNVVLAQLLIWYGVDVMARDAHGNTALTYARQASSQECINVLLQYGCPD
KCV
>sp|Q5VUJ5|AGAP7_HUMAN Putative Arf-GAP with GTPase, ANK repeat and PH domain-containing protein 7 OS=Homo sapiens GN=AGAP7P PE=5 SV=1
MGNILTCRVHPSVSLEFDQQQGSVCPSESEIYEAGAEDRMAGAPMAAAVQPAEVTVEVGE
DLHMHHVRDREMPEALEFNPSANPEASTIFQRNSQTDVVEIRRSNCTNHVSTVHFSQQYS
LCSTIFLDDSTAIQHYLTMTIISVTLEIPHHITQRDADRSLSIPDEQLHSFAVSTVHITK
NRNGGGSLNNYSSSIPSTPSTSQEDPQFSVPPTANTPTPVCKRSMRWSNLFTSEKGSDPD
KERKAPENHADTIGSGRAIPIKQGMLLKRSGKWLKTWKKKYVTLCSNGVLTYYSSLGDYM
KYIHKKEIDLQTSTIKVPGKWPSLATLACTPISSSKSDGLSKDMDTGLGDSICFSPSISS
TTIPKLNPPPSPHANKKKHLKKKSTNNFMIVSATGQTWHFEATTYEERDAWVQAIQSQIL
ASLQSCESSKSKSQLTSQSEAMALQSIQNMRGNAHCVDCETQNPKWASLNLGVLMCIECS
GIHRSLGTRLSRVRSLELDDWPVELRKVMSSIGNDLANSIWEGSSQGRTKPTEKSTREEK
ERWIRSKYEEKLFLAPLPCTELSLGQQLLRATADEDLQTAILLLAHGSREEVNETCGEGD
GCTALHLACRKGNVVLAQLLIWYGVDVMARDAHGNTALTYARQASSQECINVLLQYGCPD
ECV
>sp|Q9HCK5|AGO4_HUMAN Protein argonaute-4 OS=Homo sapiens GN=AGO4 PE=1 SV=2
MEALGPGPPASLFQPPRRPGLGTVGKPIRLLANHFQVQIPKIDVYHYDVDIKPEKRPRRV
NREVVDTMVRHFKMQIFGDRQPGYDGKRNMYTAHPLPIGRDRVDMEVTLPGEGKDQTFKV
SVQWVSVVSLQLLLEALAGHLNEVPDDSVQALDVITRHLPSMRYTPVGRSFFSPPEGYYH
PLGGGREVWFGFHQSVRPAMWNMMLNIDVSATAFYRAQPIIEFMCEVLDIQNINEQTKPL
TDSQRVKFTKEIRGLKVEVTHCGQMKRKYRVCNVTRRPASHQTFPLQLENGQAMECTVAQ
YFKQKYSLQLKYPHLPCLQVGQEQKHTYLPLEVCNIVAGQRCIKKLTDNQTSTMIKATAR
SAPDRQEEISRLVKSNSMVGGPDPYLKEFGIVVHNEMTELTGRVLPAPMLQYGGRNKTVA
TPNQGVWDMRGKQFYAGIEIKVWAVACFAPQKQCREDLLKSFTDQLRKISKDAGMPIQGQ
PCFCKYAQGADSVEPMFKHLKMTYVGLQLIVVILPGKTPVYAEVKRVGDTLLGMATQCVQ
VKNVVKTSPQTLSNLCLKINAKLGGINNVLVPHQRPSVFQQPVIFLGADVTHPPAGDGKK
PSIAAVVGSMDGHPSRYCATVRVQTSRQEISQELLYSQEVIQDLTNMVRELLIQFYKSTR
FKPTRIIYYRGGVSEGQMKQVAWPELIAIRKACISLEEDYRPGITYIVVQKRHHTRLFCA
DKTERVGKSGNVPAGTTVDSTITHPSEFDFYLCSHAGIQGTSRPSHYQVLWDDNCFTADE
LQLLTYQLCHTYVRCTRSVSIPAPAYYARLVAFRARYHLVDKDHDSAEGSHVSGQSNGRD
PQALAKAVQIHHDTQHTMYFA
>sp|Q9Y653|AGRG1_HUMAN Adhesion G-protein coupled receptor G1 OS=Homo sapiens GN=ADGRG1 PE=1 SV=2
MTPQSLLQTTLFLLSLLFLVQGAHGRGHREDFRFCSQRNQTHRSSLHYKPTPDLRISIEN
SEEALTVHAPFPAAHPASRSFPDPRGLYHFCLYWNRHAGRLHLLYGKRDFLLSDKASSLL
CFQHQEESLAQGPPLLATSVTSWWSPQNISLPSAASFTFSFHSPPHTAAHNASVDMCELK
RDLQLLSQFLKHPQKASRRPSAAPASQQLQSLESKLTSVRFMGDMVSFEEDRINATVWKL
QPTAGLQDLHIHSRQEEEQSEIMEYSVLLPRTLFQRTKGRSGEAEKRLLLVDFSSQALFQ
DKNSSQVLGEKVLGIVVQNTKVANLTEPVVLTFQHQLQPKNVTLQCVFWVEDPTLSSPGH
WSSAGCETVRRETQTSCFCNHLTYFAVLMVSSVEVDAVHKHYLSLLSYVGCVVSALACLV

TIAAYLCSRVPLPCRRKPRDYTIKVHMNLLLAVFLLDTSFLLSEPVALTGSEAGCRASAI
FLHFSLLTCLSWMGLEGYNLYRLVVEVFGTYVPGYLLKLSAMGWGFPIFLVTLVALVDVD
NYGPIILAVHRTPEGVIYPSMCWIRDSLVSYITNLGLFSLVFLFNMAMLATMVVQILRLR
PHTQKWSHVLTLLGLSLVLGLPWALIFFSFASGTFQLVVLYLFSIITSFQGFLIFIWYWS
MRLQARGGPSPLKSNSDSARLPISSGSTSSSRI
>sp|O94910|AGRL1_HUMAN Adhesion G protein-coupled receptor L1 OS=Homo sapiens GN=ADGRL1 PE=1 SV=1
MARLAAVLWNLCVTAVLVTSATQGLSRAGLPFGLMRRELACEGYPIELRCPGSDVIMVEN
ANYGRTDDKICDADPFQMENVQCYLPDAFKIMSQRCNNRTQCVVVAGSDAFPDPCPGTYK
YLEVQYDCVPYKVEQKVFVCPGTLQKVLEPTSTHESEHQSGAWCKDPLQAGDRIYVMPWI
PYRTDTLTEYASWEDYVAARHTTTYRLPNRVDGTGFVVYDGAVFYNKERTRNIVKYDLRT
RIKSGETVINTANYHDTSPYRWGGKTDIDLAVDENGLWVIYATEGNNGRLVVSQLNPYTL
RFEGTWETGYDKRSASNAFMVCGVLYVLRSVYVDDDSEAAGNRVDYAFNTNANREEPVSL
TFPNPYQFISSVDYNPRDNQLYVWNNYFVVRYSLEFGPPDPSAGPATSPPLSTTTTARPT
PLTSTASPAATTPLRRAPLTTHPVGAINQLGPDLPPATAPVPSTRRPPAPNLHVSPELFC
EPREVRRVQWPATQQGMLVERPCPKGTRGIASFQCLPALGLWNPRGPDLSNCTSPWVNQV
AQKIKSGENAANIASELARHTRGSIYAGDVSSSVKLMEQLLDILDAQLQALRPIERESAG
KNYNKMHKRERTCKDYIKAVVETVDNLLRPEALESWKDMNATEQVHTATMLLDVLEEGAF
LLADNVREPARFLAAKENVVLEVTVLNTEGQVQELVFPQEEYPRKNSIQLSAKTIKQNSR
NGVVKVVFILYNNLGLFLSTENATVKLAGEAGPGGPGGASLVVNSQVIAASINKESSRVF
LMDPVIFTVAHLEDKNHFNANCSFWNYSERSMLGYWSTQGCRLVESNKTHTTCACSHLTN
FAVLMAHREIYQGRINELLLSVITWVGIVISLVCLAICISTFCFLRGLQTDRNTIHKNLC
INLFLAELLFLVGIDKTQYEIACPIFAGLLHYFFLAAFSWLCLEGVHLYLLLVEVFESEY
SRTKYYYLGGYCFPALVVGIAAAIDYRSYGTEKACWLRVDNYFIWSFIGPVSFVIVVNLV
FLMVTLHKMIRSSSVLKPDSSRLDNIKSWALGAIALLFLLGLTWAFGLLFINKESVVMAY
LFTTFNAFQGVFIFVFHCALQKKVHKEYSKCLRHSYCCIRSPPGGTHGSLKTSAMRSNTR
YYTGTQSRIRRMWNDTVRKQTESSFMAGDINSTPTLNRGTMGNHLLTNPVLQPRGGTSPY
NTLIAESVGFNPSSPPVFNSPGSYREPKHPLGGREACGMDTLPLNGNFNNSYSLRSGDFP
PGDGGPEPPRGRNLADAAAFEKMIISELVHNNLRGSSSAAKGPPPPEPPVPPVPGGGGEE
EAGGPGGADRAEIELLYKALEEPLLLPRAQSVLYQSDLDESESCTAEDGATSRPLSSPPG
RDSLYASGANLRDSPSYPDSSPEGPSEALPPPPPAPPGPPEIYYTSRPPALVARNPLQGY
YQVRRPSHEGYLAAPGLEGPGPDGDGQMQLVTSL
>sp|O95490|AGRL2_HUMAN Adhesion G protein-coupled receptor L2 OS=Homo sapiens GN=ADGRL2 PE=1 SV=2
MVSSGCRMRSLWFIIVISFLPNTEGFSRAALPFGLVRRELSCEGYSIDLRCPGSDVIMIE
SANYGRTDDKICDADPFQMENTDCYLPDAFKIMTQRCNNRTQCIVVTGSDVFPDPCPGTY
KYLEVQYECVPYIFVCPGTLKAIVDSPCIYEAEQKAGAWCKDPLQAADKIYFMPWTPYRT
DTLIEYASLEDFQNSRQTTTYKLPNRVDGTGFVVYDGAVFFNKERTRNIVKFDLRTRIKS
GEAIINYANYHDTSPYRWGGKTDIDLAVDENGLWVIYATEQNNGMIVISQLNPYTLRFEA
TWETVYDKRAASNAFMICGVLYVVRSVYQDNESETGKNSIDYIYNTRLNRGEYVDVPFPN
QYQYIAAVDYNPRDNQLYVWNNNFILRYSLEFGPPDPAQVPTTAVTITSSAELFKTIIST
TSTTSQKGPMSTTVAGSQEGSKGTKPPPAVSTTKIPPITNIFPLPERFCEALDSKGIKWP
QTQRGMMVERPCPKGTRGTASYLCMISTGTWNPKGPDLSNCTSHWVNQLAQKIRSGENAA
SLANELAKHTKGPVFAGDVSSSVRLMEQLVDILDAQLQELKPSEKDSAGRSYNKLQKREK

TCRAYLKAIVDTVDNLLRPEALESWKHMNSSEQAHTATMLLDTLEEGAFVLADNLLEPTR
VSMPTENIVLEVAVLSTEGQIQDFKFPLGIKGAGSSIQLSANTVKQNSRNGLAKLVFIIY
RSLGQFLSTENATIKLGADFIGRNSTIAVNSHVISVSINKESSRVYLTDPVLFTLPHIDP
DNYFNANCSFWNYSERTMMGYWSTQGCKLVDTNKTRTTCACSHLTNFAILMAHREIAYKD
GVHELLLTVITWVGIVISLVCLAICIFTFCFFRGLQSDRNTIHKNLCINLFIAEFIFLIG
IDKTKYAIACPIFAGLLHFFFLAAFAWMCLEGVQLYLMLVEVFESEYSRKKYYYVAGYLF
PATVVGVSAAIDYKSYGTEKACWLHVDNYFIWSFIGPVTFIILLNIIFLVITLCKMVKHS
NTLKPDSSRLENIKSWVLGAFALLCLLGLTWSFGLLFINEETIVMAYLFTIFNAFQGVFI
FIFHCALQKKVRKEYGKCFRHSYCCGGLPTESPHSSVKASTTRTSARYSSGTQSRIRRMW
NDTVRKQSESSFISGDINSTSTLNQGMTGNYLLTNPLLRPHGTNNPYNTLLAETVVCNAP
SAPVFNSPGHSLNNARDTSAMDTLPLNGNFNNSYSLHKGDYNDSVQVVDCGLSLNDTAFE
KMIISELVHNNLRGSSKTHNLELTLPVKPVIGGSSSEDDAIVADASSLMHSDNPGLELHH
KELEAPLIPQRTHSLLYQPQKKVKSEGTDSYVSQLTAEAEDHLQSPNRDSLYTSMPNLRD
SPYPESSPDMEEDLSPSRRSENEDIYYKSMPNLGAGHQLQMCYQISRGNSDGYIIPINKE
GCIPEGDVREGQMQLVTSL
>sp|Q9BYV1|AGT2_HUMAN Alanine--glyoxylate aminotransferase 2, mitochondrial OS=Homo sapiens GN=AGXT2 PE=1 SV=1
MTLIWRHLLRPLCLVTSAPRILEMHPFLSLGTSRTSVTKLSLHTKPRMPPCDFMPERYQS
LGYNRVLEIHKEHLSPVVTAYFQKPLLLHQGHMEWLFDAEGSRYLDFFSGIVTVSVGHCH
PKVNAVAQKQLGRLWHTSTVFFHPPMHEYAEKLAALLPEPLKVIFLVNSGSEANELAMLM
ARAHSNNIDIISFRGAYHGCSPYTLGLTNVGTYKMELPGGTGCQPTMCPDVFRGPWGGSH
CRDSPVQTIRKCSCAPDCCQAKDQYIEQFKDTLSTSVAKSIAGFFAEPIQGVNGVVQYPK
GFLKEAFELVRARGGVCIADEVQTGFGRLGSHFWGFQTHDVLPDIVTMAKGIGNGFPMAA
VITTPEIAKSLAKCLQHFNTFGGNPMACAIGSAVLEVIKEENLQENSQEVGTYMLLKFAK
LRDEFEIVGDVRGKGLMIGIEMVQDKISCRPLPREEVNQIHEDCKHMGLLVGRGSIFSQT
FRIAPSMCITKPEVDFAVEVFRSALTQHMERRAK
>sp|Q9BRQ8|AIFM2_HUMAN Apoptosis-inducing factor 2 OS=Homo sapiens GN=AIFM2 PE=1 SV=1
MGSQVSVESGALHVVIVGGGFGGIAAASQLQALNVPFMLVDMKDSFHHNVAALRASVETG
FAKKTFISYSVTFKDNFRQGLVVGIDLKNQMVLLQGGEALPFSHLILATGSTGPFPGKFN
EVSSQQAAIQAYEDMVRQVQRSRFIVVVGGGSAGVEMAAEIKTEYPEKEVTLIHSQVALA
DKELLPSVRQEVKEILLRKGVQLLLSERVSNLEELPLNEYREYIKVQTDKGTEVATNLVI
LCTGIKINSSAYRKAFESRLASSGALRVNEHLQVEGHSNVYAIGDCADVRTPKMAYLAGL
HANIAVANIVNSVKQRPLQAYKPGALTFLLSMGRNDGVGQISGFYVGRLMVRLTKSRDLF
VSTSWKTMRQSPP
>sp|Q9NQ31|AKIP1_HUMAN A-kinase-interacting protein 1 OS=Homo sapiens GN=AKIP1 PE=1 SV=2
MDNCLAAAALNGVDRRSLQRSARLALEVLERAKRRAVDWHALERPKGCMGVLAREAPHLE
KQPAAGPQRVLPGEREERPPTLSASFRTMAEFMDYTSSQCGKYYSSVPEEGGATHVYRYH
RGESKLHMCLDIGNGQRKDRKKTSLGPGGSYQISEHAPEASQPAENISKDLYIEVYPGTY
SVTVGSNDLTKKTHVVAVDSGQSVDLVFPV
>sp|P30837|AL1B1_HUMAN Aldehyde dehydrogenase X, mitochondrial OS=Homo sapiens GN=ALDH1B1 PE=1 SV=3
MLRFLAPRLLSLQGRTARYSSAAALPSPILNPDIPYNQLFINNEWQDAVSKKTFPTVNPT
TGEVIGHVAEGDRADVDRAVKAAREAFRLGSPWRRMDASERGRLLNLLADLVERDRVYLA
SLETLDNGKPFQESYALDLDEVIKVYRYFAGWADKWHGKTIPMDGQHFCFTRHEPVGVCG

QIIPWNFPLVMQGWKLAPALATGNTVVMKVAEQTPLSALYLASLIKEAGFPPGVVNIITG
YGPTAGAAIAQHVDVDKVAFTGSTEVGHLIQKAAGDSNLKRVTLELGGKSPSIVLADADM
EHAVEQCHEALFFNMGQCCCAGSRTFVEESIYNEFLERTVEKAKQRKVGNPFELDTQQGP
QVDKEQFERVLGYIQLGQKEGAKLLCGGERFGERGFFIKPTVFGGVQDDMRIAKEEIFGP
VQPLFKFKKIEEVVERANNTRYGLAAAVFTRDLDKAMYFTQALQAGTVWVNTYNIVTCHT
PFGGFKESGNGRELGEDGLKAYTEVKTVTIKVPQKNS
>sp|Q60I27|AL2CL_HUMAN ALS2 C-terminal-like protein OS=Homo sapiens GN=ALS2CL PE=1 SV=1
MCNPEEAALLRLEEVFSATLAHVNSLVLQPLLPAAPDPSDPWGRECLRLLQQLHKSSQQL
WEVTEESLHSLQERLRYPDSTGLESLLLLRGADRVLQAHIEYIESYTSCMVVQAFQKAAK
RRSEYWRGQRKALRQLLSGVSSEGSVGASLGQALHQPLAHHVQQYVLLLLSLGDTIGEHH
PTRELVVNAVTLFGNLQSFMKQELDQAVATQALWHTLRGRLRDVLCTPAHRLLQDSQDVP
VTVAPLRAERVLLFDDALVLLQGHNVHTFDLKLVWVDPGQDGCTFHLLTPEEEFSFCAKD
SQGQAVWQWKVTWAVHQALHGKKDFPVLGAGLEPSQPPDCRCAEYTFQAEGRLCQATYEG
EWCRGRPHGKGTLKWPDGRNHVGNFCQGLEHGFGIRLLPQASEDKFDCYKCHWREGSMCG
YGICEYSTDEVYKGYFQEGLRHGFGVLESGPQAPQPFRYTGHWERGQRSGYGIEEDGDRG
ERYIGMWQAGQRHGPGVMVTQAGVCYQGTFQADKTVGPGILLSEDDSLYEGTFTRDLTLM
GKGKVTFPNGFTLEGSFGSGAGRGLHTQGVLDTAALPPDPSSTCKRQLGVGAFPVESRWQ
GVYSPFRDFVCAGCPRDLQEALLGFDVQSSRELRRSQDYLSCERTHPEDSVGSMEDILEE
LLQHREPKALQLYLRKALSNSLHPLGKLLRTLMLTFQATYAGVGANKHLQELAQEEVKQH
AQELWAAYRGLLRVALERKGQALEEDEDTETRDLQVHGLVLPLMLPSFYSELFTLYLLLH
EREDSFYSQGIANLSLFPDTQLLEFLDVQKHLWPLKDLTLTSNQRYSLVRDKCFLSATEC
LQKIMTTVDPREKLEVLERTYGEIEGTVSRVLGREYKLPMDDLLPLLIYVVSRARIQHLG
AEIHLIRDMMDPNHTGGLYDFLLTALESCYEHIQKEDMRLHRLPGHWHSRELW
>sp|P15121|ALDR_HUMAN Aldose reductase OS=Homo sapiens GN=AKR1B1 PE=1 SV=3
MASRLLLNNGAKMPILGLGTWKSPPGQVTEAVKVAIDVGYRHIDCAHVYQNENEVGVAIQ
EKLREQVVKREELFIVSKLWCTYHEKGLVKGACQKTLSDLKLDYLDLYLIHWPTGFKPGK
EFFPLDESGNVVPSDTNILDTWAAMEELVDEGLVKAIGISNFNHLQVEMILNKPGLKYKP
AVNQIECHPYLTQEKLIQYCQSKGIVVTAYSPLGSPDRPWAKPEDPSLLEDPRIKAIAAK
HNKTTAQVLIRFPMQRNLVVIPKSVTPERIAENFKVFDFELSSQDMTTLLSYNRNWRVCA
LLSCTSHKDYPFHEEF
>sp|Q9NP73|ALG13_HUMAN Putative bifunctional UDP-N-acetylglucosamine transferase and deubiquitinase ALG13 OS=Homo sapiens GN=ALG13 PE=1 SV=2
MKCVFVTVGTTSFDDLIACVSAPDSLQKIESLGYNRLILQIGRGTVVPEPFSTESFTLDV
YRYKDSLKEDIQKADLVISHAGAGSCLETLEKGKPLVVVINEKLMNNHQLELAKQLHKEG
HLFYCTCRVLTCPGGQAKSIASAPGKCQDSAALTSTAFSGLDFGLLSGYLHKQALVTATHP
TCTLLFPSCHAFFPLPLTPTLYKMHKGWKNYCSQKSLNEASMDEYLGSLGLFRKLTAKDA
SCLFRAISEQLFCSQVHHLEIRKACVSYMRENQQTFESYVEGSFEKYLERLGDPKESAGQ
LEIRALSLIYNRDFILYRFPGKPPTYVTDNGYEDKILLCYSSSGHYDSVYSKQFQSSAAV
CQAVLYEILYKDVFVVDEEELKTAIKLFRSGSKKNRNNAVTGSEDAHTDYKSSNQNRMEE
WGACYNAENIPEGYNKGTEETKSPENPSKMPFPYKVLKALDPEIYRNVEFDVWLDSRKEL
QKSDYMEYAGRQYYLGDKCQVCLESEGRYYNAHIQEVGNENNSVTVFIEELAEKHVVPLA
NLKPVTQVMSVPAWNAMPSRKGRGYQKMPGGYVPEIVISEMDIKQQKKMFKKIRGKEVYM
TMAYGKGDPLLPPRLQHSMHYGHDPPMHYSQTAGNVMSNEHFHPQHPSPRQGRGYGMPRN
SSRFINRHNMPGPKVDFYPGPGKRCCQSYDNFSYRSRSFRRSHRQMSCVNKESQYGFTPG

NGQMPRGLEETITFYEVEEGDETAYPTLPNHGGPSTMVPATSGYCVGRRGHSSGKQTLNL
EEGNGQSENGRYHEEYLYRAEPDYETSGVYSTTASTANLSLQDRKSCSMSPQDTVTSYNY
PQKMMGNIAAVAASCANNVPAPVLSNGAAANQAISTTSVSSQNAIQPLFVSPPTHGRPVI
ASPSYPCHSAIPHAGASLPPPPPPPPPPPPPPPPPPPPPPPPPPPPPPALDVGETSNLQPPPP
LPPPPYSCDPSGSDLPQDTKVLQYYFNLGLQCYYHSYWHSMVYVPQMQQQLHVENYPVYT
EPPLVDQTVPQCYSEVRREDGIQAEASANDTFPNADSSSVPHGAVYYPVMSDPYGQPPLP
GFDSCLPVVPDYSCVPPWHPVGTAYGGSSQIHGAINPGPIGCIAPSPPASHYVPQGM
>sp|Q9H553|ALG2_HUMAN Alpha-1,3/1,6-mannosyltransferase ALG2 OS=Homo sapiens GN=ALG2 PE=1 SV=1
MAEEQGRERDSVPKPSVLFLHPDLGVGGAERLVLDAALALQARGCSVKIWTAHYDPGHCF
AESRELPVRCAGDWLPRGLGWGGRGAAVCAYVRMVFLALYVLFLADEEFDVVVCDQVSAC
IPVFRLARRRKKILFYCHFPDLLLTKRDSFLKRLYRAPIDWIEEYTTGMADCILVNSQFT
AAVFKETFKSLSHIDPDVLYPSLNVTSFDSVVPEKLDDLVPKGKKFLLLSINRYERKKNL
TLALEALVQLRGRLTSQDWERVHLIVAGGYDERVLENVEHYQELKKMVQQSDLGQYVTFL
RSFSDKQKISLLHSCTCVLYTPSNEHFGIVPLEAMYMQCPVIAVNSGGPLESIDHSVTGF
LCEPDPVHFSEAIEKFIREPSLKATMGLAGRARVKEKFSPEAFTEQLYRYVTKLLV
>sp|Q4L235|ACSF4_HUMAN Acyl-CoA synthetase family member 4 OS=Homo sapiens GN=AASDH PE=1 SV=3
MTLQELVHKAASCYMDRVAVCFDECNNQLPVYYTYKTVVNAASELSNFLLLHCDFQGIRE
IGLYCQPGIDLPSWILGILQVPAAYVPIEPDSPPSLSTHFMKKCNLKYILVEKKQINKFK
SFHETLLNYDTFTVEHNDLVLFRLHWKNTEVNLMLNDGKEKYEKEKIKSISSEHVNEEKA
EEHMDLRLKHCLAYVLHTSGTTGIPKIVRVPHKCIVPNIQHFRVLFDITQEDVLFLASPL
TFDPSVVEIFLALSSGASLLIVPTSVKLLPSKLASVLFSHHRVTVLQATPTLLRRFGSQL
IKSTVLSATTSLRVLALGGEAFPSLTVLRSWRGEGNKTQIFNVYGITEVSSWATIYRIPE
KTLNSTLKCELPVQLGFPLLGTVVEVRDTNGFTIQEGSGQVFLGGRNRVCFLDDEVTVPL
GTMRATGDFVTVKDGEIFFLGRKDSQIKRHGKRLNIELVQQVAEELQQVESCAVTWYNQE
KLILFMVSKDASVKEYIFKELQKYLPSHAVPDELVLIDSLPFTSHGKIDVSELNKIYLNY
INLKSENKLSGKEDLWEKLQYLWKSTLNLPEDLLRVPDESLFLNSGGDSLKSIRLLSEIE
KLVGTSVPGLLEIILSSSILEIYNHILQTVVPDEDVTFRKSCATKRKLSDINQEEASGTS
LHQKAIMTFTCHNEINAFVVLSRGSQILSLNSTRFLTKLGHCSSACPSDSVSQTNIQNLK
GLNSPVLIGKSKDPSCVAKVSEEGKPAIGTQKMELHVRWRSDTGKCVDASPLVVIPTFDK
SSTTVYIGSHSHRMKAVDFYSGKVKWEQILGDRIESSACVSKCGNFIVVGCYNGLVYVLK
SNSGEKYWMFTTEDAVKSSATMDPTTGLIYIGSHDQHAYALDIYRKKCVWKSKCGGTVFS
SPCLNLIPHHLYFATLGGLLLAVNPATGNVIWKHSCGKPLFSSPQCCSQYICIGCVDGNL
LCFTHFGEQVWQFSTSGPIFSSPCTSPSEQKIFFGSHDCFIYCCNMKGHLQWKFETTSRV
YATPFAFHNYNGSNEMLLAAASTDGKVWILESQSGQLQSVYELPGEVFSSPVVLESMLII
GCRDNYVYCLDLLGGNQK
>sp|Q01718|ACTHR_HUMAN Adrenocorticotropic hormone receptor OS=Homo sapiens GN=MC2R PE=1 SV=1
MKHIINSYENINNTARNNSDCPRVVLPEEIFFTISIVGVLENLIVLLAVFKNKNLQAPMY
FFICSLAISDMLGSLYKILENILIIILRNMGYLKPRGSFETTADDIIDSLFVLSLLGSIFS
LSVIAADRYITIFHALRYHSIVTMRRTVVVLTVIWTFCTGTGITMVIFSHHVPTVITFTS
LFPLMLVFILCLYVHMFLLARSHTRKISTLPRANMKGAITLTILLGVFIFCWAPFVLHVL
LMTFCPSNPYCACYMSLFQVNGMLIMCNAVIDPFIYAFRSPELRDAFKKMIFCSRYW

>sp|Q8TC94|ACTL9_HUMAN Actin-like protein 9 OS=Homo sapiens GN=ACTL9 PE=1 SV=3
MDASRPKSSESQSSLEAPRPGPNPSPNVVNKPLQRDFPGMVADRLPPKTGVVVIDMGTGT
CKVGFAGQASPTYTVATILGCQPKKPATSGQSGLQTFIGEAARVLPELTLVQPLRSGIVV
DWDAAELIWRHLLEHDLRVATHDHPLLFSDPPFSPATNREKLVEVAFESLRSPAMYVASQ
SVLSVYAHGRVSGLVVDTGHGVTYTVPVFQGYNLLHATERLDLAGNHLTAFLAEMLLQAG
LPLGQQDLDLVENIKHHYCYVASDFQKEQARPEQEYKRTLKLPDGRTVTLGKELFQCPEL
LFNPPEVPGLSPVGLSTMAKQSLRKLSLEMRADLAQNVLLCGGSSLFTGFEGRFRAELLR
ALPAETHVVVAAQPTRNFSVWIGGSILASLRAFQSCWVLREQYEEQGPYIVYRKCY
>sp|Q8TDY3|ACTT2_HUMAN Actin-related protein T2 OS=Homo sapiens GN=ACTRT2 PE=2 SV=2
MFNPHALDSPAVIFDNGSGFCKAGLSGEFGPRHMVSSIVGHLKFQAPSAEANQKKYFVGE
EALYKQEALQLHSPFERGLITGWDDVERLWKHLFEWELGVKPSDQPLLATEPSLNPRENR
EKMAEVMFENFGVPAFYLSDQAVLALYASACVTGLVVDSGDAVTCTVPIFEGYSLPHAVT
KLHVAGRDITELLMQLLLASGHTFPCQLDKGLVDDIKKKLCYVALEPEKELSRRPEEVLR
EYKLPDGNIISLGDPLHQAPEALFVPQQLGSQSPGLSNMVSSSITKCDTDIQKILFGEIV
LSGGTTLFHGLDDRLLKELEQLASKDTPIKITAPPDRWFSTWIGASIVTSLSSFKQMWVT
AADFKEFGTSVVQRRCF
>sp|Q8NER5|ACV1C_HUMAN Activin receptor type-1C OS=Homo sapiens GN=ACVR1C PE=1 SV=1
MTRALCSALRQALLLLAAAAELSPGLKCVCLLCDSSNFTCQTEGACWASVMLTNGKEQVI
KSCVSLPELNAQVFCHSSNNVTKTECCFTDFCNNITLHLPTASPNAPKLGPMELAIIITV
PVCLLSIAAMLTVWACQGRQCSYRKKKRPNVEEPLSECNLVNAGKTLKDLIYDVTASGSG
SGLPLLVQRTIARTIVLQEIVGKGRFGEVWHGRWCGEDVAVKIFSSRDERSWFREAEIYQ
TVMLRHENILGFIAADNKDNGTWTQLWLVSEYHEQGSLYDYLNRNIVTVAGMIKLALSIA
SGLAHLHMEIVGTQGKPAIAHRDIKSKNILVKKCETCAIADLGLAVKHDSILNTIDIPQN
PKVGTKRYMAPEMLDDTMNVNIFESFKRADIYSVGLVYWEIARRCSVGGIVEEYQLPYYD
MVPSDPSIEEMRKVVCDQKFRPSIPNQWQSCEALRVMGRIMRECWYANGAARLTALRIKK
TISQLCVKEDCKA
>sp|Q04771|ACVR1_HUMAN Activin receptor type-1 OS=Homo sapiens GN=ACVR1 PE=1 SV=1
MVDGVMILPVLIMIALPSPSMEDEKPKVNPKLYMCVCEGLSCGNEDHCEGQQCFSSLSIN
DGFHVYQKGCFQVYEQGKMTCKTPPSPGQAVECCQGDWCNRNITAQLPTKGKSFPGTQNF
HLEVGLIILSVVFAVCLLACLLGVALRKFKRRNQERLNPRDVEYGTIEGLITTNVGDSTL
ADLLDHSCTSGSGSGLPFLVQRTVARQITLLECVGKGRYGEVWRGSWQGENVAVKIFSSR
DEKSWFRETELYNTVMLRHENILGFIASDMTSRHSSTQLWLITHYHEMGSLYDYLQLTTL
DTVSCLRIVLSIASGLAHLHIEIFGTQGKPAIAHRDLKSKNILVKKNGQCCIADLGLAVM
HSQSTNQLDVGNNPRVGTKRYMAPEVLDETIQVDCFDSYKRVDIWAFGLVLWEVARRMVS
NGIVEDYKPPFYDVVPNDPSFEDMRKVVCVDQQRPNIPNRWFSDPTLTSLAKLMKECWYQ
NPSARLTALRIKKTLTKIDNSLDKLKTDC
>sp|P35348|ADA1A_HUMAN Alpha-1A adrenergic receptor OS=Homo sapiens GN=ADRA1A PE=1 SV=2
MVFLSGNASDSSNCTQPPAPVNISKAILLGVILGGLILFGVLGNILVILSVACHRHLHSV
THYYIVNLAVADLLLTSTVLPFSAIFEVLGYWAFGRVFCNIWAAVDVLCCTASIMGLCII
SIDRYIGVSYPLRYPTIVTQRRGLMALLCVWALSLVISIGPLFGWRQPAPEDETICQINE
EPGYVLFSALGSFYLPLAIILVMYCRVYVVAKRESRGLKSGLKTDKSDSEQVTLRIHRKN
APAGGSGMASAKTKTHFSVRLLKFSREKKAAKTLGIVVGCFVLCWLPFFLVMPIGSFFPD
FKPSETVFKIVFWLGYLNSCINPIIYPCSSQEFKKAFQNVLRIQCLCRKQSSKHALGYTL
HPPSQAVEGQHKDMVRIPVGSRETFYRISKTDGVCEWKFFSSMPRGSARITVSKDQSSCT

TARVRSKSFLQVCCCVGPSTPSLDKNHQVPTIKVHTISLSENGEEV
>sp|P35368|ADA1B_HUMAN Alpha-1B adrenergic receptor OS=Homo sapiens GN=ADRA1B PE=1 SV=3
MNPDLDTGHNTSAPAHWGELKNANFTGPNQTSSNSTLPQLDITRAISVGLVLGAFILFAI
VGNILVILSVACNRHLRTPTNYFIVNLAMADLLLSFTVLPFSAALEVLGYWVLGRIFCDI
WAAVDVLCCTASILSLCAISIDRYIGVRYSLQYPTLVTRRKAILALLSVWVLSTVISIGP
LLGWKEPAPNDDKECGVTEEPFYALFSSLGSFYIPLAVILVMYCRVYIVAKRTTKNLEAG
VMKEMSNSKELTLRIHSKNFHEDTLSSTKAKGHNPRSSIAVKLFKFSREKKAAKTLGIVV
GMFILCWLPFFIALPLGSLFSTLKPPDAVFKVVFWLGYFNSCLNPIIYPCSSKEFKRAFV
RILGCQCRGRGRRRRRRRRRLGGCAYTYRPWTRGGSLERSQSRKDSLDDSGSCLSGSQRT
LPSASPSPGYLGRGAPPPVELCAFPEWKAPGALLSLPAPEPPGRRGRHDSGPLFTFKLLT
EPESPGTDGGASNGGCEAAADVANGQPGFKSNMPLAPGQF
>sp|Q9P0K1|ADA22_HUMAN Disintegrin and metalloproteinase domain-containing protein 22 OS=Homo sapiens GN=ADAM22 PE=1 SV=1
MQAAVAVSVPFLLLCVLGTCPPARCGQAGDASLMELEKRKENRFVERQSIVPLRLIYRSG
GEDESRHDALDTRVRGDLGGPQLTHVDQASFQVDAFGTSFILDVVLNHDLLSSEYIERHI
EHGGKTVEVKGGEHCYYQGHIRGNPDSFVALSTCHGLHGMFYDGNHTYLIEPEENDTTQE
DFHFHSVYKSRLFEFSLDDLPSEFQQVNITPSKFILKPRPKRSKRQLRRYPRNVEEETKY
IELMIVNDHLMFKKHRLSVVHTNTYAKSVVNMADLIYKDQLKTRIVLVAMETWATDNKFA
ISENPLITLREFMKYRRDFIKEKSDAVHLFSGSQFESSRSGAAYIGGICSLLKGGGVNEF
GKTDLMAVTLAQSLAHNIGIISDKRKLASGECKCEDTWSGCIMGDTGYYLPKKFTQCNIE
EYHDFLNSGGGACLFNKPSKLLDPPECGNGFIETGEECDCGTPAECVLEGAECCKKCTLT
QDSQCSDGLCCKKCKFQPMGTVCREAVNDCDIRETCSGNSSQCAPNIHKMDGYSCDGVQG
ICFGGRCKTRDRQCKYIWGQKVTASDKYCYEKLNIEGTEKGNCGKDKDTWIQCNKRDVLC
GYLLCTNIGNIPRLGELDGEITSTLVVQQGRTLNCSGGHVKLEEDVDLGYVEDGTPCGPQ
MMCLEHRCLPVASFNFSTCLSSKEGTICSGNGVCSNELKCVCNRHWIGSDCNTYFPHNDD
AKTGITLSGNGVAGTNIIIGIIAGTILVLALILGITAWGYKNYREQRQLPQGDYVKKPGD
GDSFYSDIPPGVSTNSASSSKKRSNGLSHSWSERIPDTKHISDICENGRPRSNSWQGNLG
GNKKKIRGKRFRPRSNSTETLSPAKSPSSSTGSIASSRKYPYPMPPLPDEDKKVNRQSAR
LWETSI
>sp|Q9UKQ2|ADA28_HUMAN Disintegrin and metalloproteinase domain-containing protein 28 OS=Homo sapiens GN=ADAM28 PE=2 SV=3
MLQGLLPVSLLLSVAVSAIKELPGVKKYEVVYPIRLHPLHKREAKEPEQQEQFETELKYK
MTINGKIAVLYLKKNKNLLAPGYTETYYNSTGKEITTSPQIMDDCYYQGHILNEKVSDAS
ISTCRGLRGYFSQGDQRYFIEPLSPIHRDGQEHALFKYNPDEKNYDSTCGMDGVLWAHDL
QQNIALPATKLVKLKDRKVQEHEKYIEYYLVLDNGEFKRYNENQDEIRKRVFEMANYVNM
LYKKLNTHVALVGMEIWTDKDKIKITPNASFTLENFSKWRGSVLSRRKRHDIAQLITATE
LAGTTVGLAFMSTMCSPYSVGVVQDHSDNLLRVAGTMAHEMGHNFGMFHDDYSCKCPSTI
CVMDKALSFYIPTDFSSCSRLSYDKFFEDKLSNCLFNAPLPTDIISTPICGNQLVEMGED
CDCGTSEECTNICCDAKTCKIKATFQCALGECCEKCQFKKAGMVCRPAKDECDLPEMCNG
KSGNCPDDRFQVNGFPCHHGKGHCLMGTCPTLQEQCTELWGPGTEVADKSCYNRNEGGSK
YGYCRRVDDTLIPCKANDTMCGKLFCQGGSDNLPWKGRIVTFLTCKTFDPEDTSQEIGMV
ANGTKCGDNKVCINAECVDIEKAYKSTNCSSKCKGHAVCDHELQCQCEEGWIPPDCDDSS
VVFHFSIVVGVLFPMAVIFVVVAMVIRHQSSREKQKKDQRPLSTTGTRPHKQKRKPQMVK
AVQPQEMSQMKPHVYDLPVEGNEPPASFHKDTNALPPTVFKDNPVSTPKDSNPKA

>sp|Q8TC27|ADA32_HUMAN Disintegrin and metalloproteinase domain-containing protein 32 OS=Homo sapiens GN=ADAM32 PE=1 SV=2
MFRLWLLLAGLCGLLASRPGFQNSLLQIVIPEKIQTNTNDSSEIEYEQISYIIPIDEKLY
TVHLKQRYFLADNFMIYLYNQGSMNTYSSDIQTQCYYQGNIEGYPDSMVTLSTCSGLRGI
LQFENVSYGIEPLESAVEFQHVLYKLKNEDNDIAIFIDRSLKEQPMDDNIFISEKSEPAV
PDLFPLYLEMHIVVDKTLYDYWGSDSMIVTNKVIEIVGLANSMFTQFKVTIVLSSLELWS
DENKISTVGEADELLQKFLEWKQSYLNLRPHDIAYLLIYMDYPRYLGAVFPGTMCITRYS
AGVALYPKEITLEAFAVIVTQMLALSLGISYDDPKKCQCSESTCIMNPEVVQSNGVKTFS
SCSLRSFQNFISNVGVKCLQNKPQMQKKSPKPVCGNGRLEGNEICDCGTEAQCGPASCCD
FRTCVLKDGAKCYKGLCCKDCQILQSGVECRPKAHPECDIAENCNGTSPECGPDITLING
LSCKNNKFICYDGDCHDLDARCESVFGKGSRNAPFACYEEIQSQSDRFGNCGRDRNNKYV
FCGWRNLICGRLVCTYPTRKPFHQENGDVIYAFVRDSVCITVDYKLPRTVPDPLAVKNGS
QCDIGRVCVNRECVESRIIKASAHVCSQQCSGHGVCDSRNKCHCSPGYKPPNCQIRSKGF
SIFPEEDMGSIMERASGKTENTWLLGFLIALPILIVTTAIVLARKQLKKWFAKEEEFPSS
ESKSEGSTQTYASQSSSEGSTQTYASQTRSESSSQADTSKSKSEDSAEAYTSRSKSQDST
QTQSSSN
>sp|O00116|ADAS_HUMAN Alkyldihydroxyacetonephosphate synthase, peroxisomal OS=Homo sapiens GN=AGPS PE=1 SV=1
MAEAAAAAGGTGLGAGASYGSAADRDRDPDPDRAGRRLRVLSGHLLGRPREALSTNECKA
RRAASAATAAPTATPAAQESGTIPKKRQEVMKWNGWGYNDSKFIFNKKGQIELTGKRYPL
SGMGLPTFKEWIQNTLGVNVEHKTTSKASLNPSDTPPSVVNEDFLHDLKETNISYSQEAD
DRVFRAHGHCLHEIFLLREGMFERIPDIVLWPTCHDDVVKIVNLACKYNLCIIPIGGGTS
VSYGLMCPADETRTIISLDTSQMNRILWVDENNLTAHVEAGITGQELERQLKESGYCTGH
EPDSLEFSTVGGWVSTRASGMKKNIYGNIEDLVVHIKMVTPRGIIEKSCQGPRMSTGPDI
HHFIMGSEGTLGVITEATIKIRPVPEYQKYGSVAFPNFEQGVACLREIAKQRCAPASIRL
MDNKQFQFGHALKPQVSSIFTSFLDGLKKFYITKFKGFDPNQLSVATLLFEGDREKVLQH
EKQVYDIAAKFGGLAAGEDNGQRGYLLTYVIAYIRDLALEYYVLGESFETSAPWDRVVDL
CRNVKERITRECKEKGVQFAPFSTCRVTQTYDAGACIYFYFAFNYRGISDPLTVFEQTEA
AAREEILANGGSLSHHHGVGKLRKQWLKESISDVGFGMLKSVKEYVDPNNIFGNRNLL
>sp|Q9BUB4|ADAT1_HUMAN tRNA-specific adenosine deaminase 1 OS=Homo sapiens GN=ADAT1 PE=1 SV=1
MWTADEIAQLCYEHYGIRLPKKGKPEPNHEWTLLAAVVKIQSPADKACDTPDKPVQVTKE
VVSMGTGTKCIGQSKMRKNGDILNDSHAEVIARRSFQRYLLHQLQLAATLKEDSIFVPGT
QKGVWKLRRDLIFVFFSSHTPCGDASIIPMLEFEDQPCCPVFRNWAHNSSVEASSNLEAP
GNERKCEDPDSPVTKKMRLEPGTAAREVTNGAAHHQSFGKQKSGPISPGIHSCDLTVEGL
ATVTRIAPGSAKVIDVYRTGAKCVPGEAGDSGKPGAAFHQVGLLRVKPGRGDRTRSMSCS
DKMARWNVLGCQGALLMHLLEEPIYLSAVVIGKCPYSQEAMQRALIGRCQNVSALPKGFG
VQELKILQSDLLFEQSRSAVQAKRADSPGRLVPCGAAISWSAVPEQPLDVTANGFPQGTT
KKTIGSLQARSQISKVELFRSFQKLLSRIARDKWPHSLRVQKLDTYQEYKEAASSYQEAW
STLRKQVFGSWIRNPPDYHQFK
>sp|Q3MIX3|ADCK5_HUMAN Uncharacterized aarF domain-containing protein kinase 5 OS=Homo sapiens GN=ADCK5 PE=1 SV=2
MWRPVQLCHFHSALLHRRQKPWPSPAVFFRRNVRGLPPRFSSPTPLWRKVLSTAVVGAPL
LLGARYVMAEAREKRRMRLVVDGMGRFGRSLKVGLQISLDYWWCTNVVLRGVEENSPGYL

EVMSACHQRAADALVAGAISNGGLYVKLGQGLCSFNHLLPPEYTRTLRVLEDRALKRGFQ
EVDELFLEDFQALPHELFQEFDYQPIAAASLAQVHRAKLHDGTSVAVKVQYIDLRDRFDG
DIHTLELLLRLVEVMHPSFGFSWVLQDLKGTLAQELDFENEGRNAERCARELAHFPYVVV
PRVHWDKSSKRVLTADFCAGCKVNDVEAIRSQGLAVHDIAEKLIKAFAEQIFYTGFIHSD
PHPGNVLVRKGPDGKAELVLLDHGLYQFLEEKDRAALCQLWRAIILRDDAAMRAHAAALG
VQDYLLFAEMLMQRPVRLGQLWGSHLLSREEAAYMVDMARERFEAVMAVLRELPRPMLLV
LRNINTVRAINVALGAPVDRYFLMAKRAVRGWSRLAGATYRGVYGTSLLRHAKVVWEMLK
FEVALRLETLAMRLTALLARALVHLSLVPPAEELYQYLET
>sp|Q6P093|ADCL2_HUMAN Arylacetamide deacetylase-like 2 OS=Homo sapiens GN=AADACL2 PE=2 SV=3
MGLKALCLGLLCVLFVSHFYTPMPDNIEESWKIMALDAIAKTCTFTAMCFENMRIMRYEE
FISMIFRLDYTQPLSDEYITVTDTTFVDIPVRLYLPKRKSETRRRAVIYFHGGGFCFGSS
KQRAFDFLNRWTANTLDAVVVGVDYRLAPQHHFPAQFEDGLAAVKFFLLEKILTKYGVDP
TRICIAGDSSGGNLATAVTQQVQNDAEIKHKIKMQVLLYPGLQITDSYLPSHRENEHGIV
LTRDVAIKLVSLYFTKDEALPWAMRRNQHMPLESRHLFKFVNWSILLPEKYRKDYVYTEP
ILGGLSYSLPGLTDSRALPLLANDSQLQNLPLTYILTCQHDLLRDDGLMYVTRLRNVGVQ
VVHEHIEDGIHGALSFMTSPFYLRLGLRIRDMYVSWLDKNL
>sp|Q08828|ADCY1_HUMAN Adenylate cyclase type 1 OS=Homo sapiens GN=ADCY1 PE=1 SV=2
MAGAPRGGGGGGGGAGEPGGAERAAGTSRRRGLRACDEEFACPELEALFRGYTLRLEQAA
TLKALAVLSLLAGALALAELLGAPGPAPGLAKGSHPVHCVLFLALLVVTNVRSLQVPQLQ
QVGQLALLFSLTFALLCCPFALGGPARGSAGAAGGPATAEQGVWQLLLVTFVSYALLPVR
SLLAIGFGLVVAASHLLVTATLVPAKRPRLWRTLGANALLFVGVNMYGVFVRILTERSQR
KAFLQARSCIEDRLRLEDENEKQERLLMSLLPRNVAMEMKEDFLKPPERIFHKIYIQRHD
NVSILFADIVGFTGLASQCTAQELVKLLNELFGKFDELATENHCRRIKILGDCYYCVSGL
TQPKTDHAHCCVEMGLDMIDTITSVAEATEVDLNMRVGLHTGRVLCGVLGLRKWQYDVWS
NDVTLANVMEAAGLPGKVHITKTTLACLNGDYEVEPGYGHERNSFLKTHNIETFFIVPSH
RRKIFPGLILSDIKPAKRMKFKTVCYLLVQLMHCRKMFKAEIPFSNVMTCEDDDKRRALR
TASEKLRNRSSFSTNVVYTTPGTRVNRYISRLLEARQTELEMADLNFFTLKYKHVEREQK
YHQLQDEYFTSAVVLTLILAALFGLVYLLIFPQSVVVLLLLVFCICFLVACVLYLHITRV
QCFPGCLTIQIRTVLCIFIVVLIYSVAQGCVVGCLPWAWSSKPNSSLVVLSSGGQRTALP
TLPCESTHHALLCCLVGTLPLAIFFRVSSLPKMILLSGLTTSYILVLELSGYTRTGGGAV
SGRSYEPIVAILLFSCALALHARQVDIRLRLDYLWAAQAEEEREDMEKVKLDNRRILFNL
LPAHVAQHFLMSNPRNMDLYYQSYSQVGVMFASIPNFNDFYIELDGNNMGVECLRLLNEI
IADFDELMEKDFYKDIEKIKTIGSTYMAAVGLAPTSGTKAKKSISSHLSTLADFAIEMFD
VLDEINYQSYNDFVLRVGINVGPVVAGVIGARRPQYDIWGNTVNVASRMDSTGVQGRIQV
TEEVHRLLRRCPYHFVCRGKVSVKGKGEMLTYFLEGRTDGNGSQIRSLGLDRKMCPFGRA
GLQGRRPPVCPMPGVSVRAGLPPHSPGQYLPSAAAGKEA
>sp|O60266|ADCY3_HUMAN Adenylate cyclase type 3 OS=Homo sapiens GN=ADCY3 PE=1 SV=3
MPRNQGFSEPEYSAEYSAEYSVSLPSDPDRGVGRTHEISVRNSGSCLCLPRFMRLTFVPE
SLENLYQTYFKRQRHETLLVLVVFAALFDCYVVVMCAVVFSSDKLASLAVAGIGLVLDII
LFVLCKKGLLPDRVTRRVLPYVLWLLITAQIFSYLGLNFARAHAASDTVGWQVFFVFSFF
ITLPLSLSPIVIISVVSCVVHTLVLGVTVAQQQQEELKGMQLLREILANVFLYLCAIAVG
IMSYYMADRKHRKAFLEARQSLEVKMNLEEQSQQQENLMLSILPKHVADEMLKDMKKDES
QKDQQQFNTMYMYRHENVSILFADIVGFTQLSSACSAQELVKLLNELFARFDKLAAKYHQ

LRIKILGDCYYCICGLPDYREDHAVCSILMGLAMVEAISYVREKTKTGVDMRVGVHTGTV
LGGVLGQKRWQYDVWSTDVTVANKMEAGGIPGRVHISQSTMDCLKGEFDVEPGDGGSRCD
YLEEKGIETYLIIASKPEVKKTATQNGLNGSALPNGAPASSKSSSPALIETKEPNGSAHS
SGSTSEKPEEQDAQADNPSFPNPRRRLRLQDLADRVVDASEDEHELNQLLNEALLERESA
QVVKKRNTFLLSMRFMDPEMETRYSVEKEKQSGAAFSCSCVVLLCTALVEILIDPWLMTN
YVTFMVGEILLLILTICSLAAIFPRAFPKKLVAFSTWIDRTRWARNTWAMLAIFILVMAN
VVDMLSCLQYYTGPSNATAGMETEGSCLENPKYYNYVAVLSLIATIMLVQVSHMVKLTLM
LLVAGAVATINLYAWRPVFDEYDHKRFREHDLPMVALEQMQGFNPGLNGTDRLPLVPSKY
SMTVMVFLMMLSFYYFSRHVEKLARTLFLWKIEVHDQKERVYEMRRWNEALVTNMLPEHV
ARHFLGSKKRDEELYSQTYDEIGVMFASLPNFADFYTEESINNGGIECLRFLNEIISDFD
SLLDNPKFRVITKIKTIGSTYMAASGVTPDVNTNGFASSNKEDKSERERWQHLADLADFA
LAMKDTLTNINNQSFNNFMLRIGMNKGGVLAGVIGARKPHYDIWGNTVNVASRMESTGVM
GNIQVVEETQVILREYGFRFVRRGPIFVKGKGELLTFFLKGRDKLATFPNGPSVTLPHQV
VDNS
>sp|O95622|ADCY5_HUMAN Adenylate cyclase type 5 OS=Homo sapiens GN=ADCY5 PE=1 SV=3
MSGSKSVSPPGYAAQKTAAPAPRGGPEHRSAWGEADSRANGYPHAPGGSARGSTKKPGGA
VTPQQQQRLASRWRSDDDDDPPLSGDDPLAGGFGFSFRSKSAWQERGGDDCGRGSRRQRR
GAASGGSTRAPPAGGGGGSAAAAASAGGTEVRPRSVEVGLEERRGKGRAADELEAGAVEG
GEGSGDGGSSADSGSGAGPGAVLSLGACCLALLQIFRSKKFPSDKLERLYQRYFFRLNQS
SLTMLMAVLVLVCLVMLAFHAARPPLQLPYLAVLAAAVGVILIMAVLCNRAAFHQDHMGL
ACYALIAVVLAVQVVGLLLPQPRSASEGIWWTVFFIYTIYTLLPVRMRAAVLSGVLLSAL
HLAIALRTNAQDQFLLKQLVSNVLIFSCTNIVGVCTHYPAEVSQRQAFQETRECIQARLH
SQRENQQQERLLLSVLPRHVAMEMKADINAKQEDMMFHKIYIQKHDNVSILFADIEGFTS
LASQCTAQELVMTLNELFARFDKLAAENHCLRIKILGDCYYCVSGLPEARADHAHCCVEM
GMDMIEAISLVREVTGVNVNMRVGIHSGRVHCGVLGLRKWQFDVWSNDVTLANHMEAGGK
AGRIHITKATLNYLNGDYEVEPGCGGERNAYLKEHSIETFLILRCTQKRKEEKAMIAKMN
RQRTNSIGHNPPHWGAERPFYNHLGGNQVSKEMKRMGFEDPKDKNAQESANPEDEVDEFL
GRAIDARSIDRLRSEHVRKFLLTFREPDLEKKYSKQVDDRFGAYVACASLVFLFICFVQI
TIVPHSIFMLSFYLTCSLLLTLVVFVSVIYSCVKLFPSPLQTLSRKIVRSKMNSTLVGVF
TITLVFLAAFVNMFTCNSRDLLGCLAQEHNISASQVNACHVAESAVNYSLGDEQGFCGSP
WPNCNFPEYFTYSVLLSLLACSVFLQISCIGKLVLMLAIELIYVLIVEVPGVTLFDNADL
LVTANAIDFFNNGTSQCPEHATKVALKVVTPIIISVFVLALYLHAQQVESTARLDFLWKL
QATEEKEEMEELQAYNRRLLHNILPKDVAAHFLARERRNDELYYQSCECVAVMFASIANF
SEFYVELEANNEGVECLRLLNEIIADFDEIISEDRFRQLEKIKTIGSTYMAASGLNDSTY
DKVGKTHIKALADFAMKLMDQMKYINEHSFNNFQMKIGLNIGPVVAGVIGARKPQYDIWG
NTVNVASRMDSTGVPDRIQVTTDMYQVLAANTYQLECRGVVKVKGKGEMMTYFLNGGPPL
S
>sp|C9JUS6|ADM5_HUMAN Putative adrenomedullin-5-like protein OS=Homo sapiens GN=ADM5 PE=2
SV=1
MTIHILILLLLLAFSAQGDLDTAARRGQHQVPQHRGHVCYLGVCRTHRLAEIIYWIRCLH
QGALGEGQPRAPGPLQLWAPPVARGGSPARFPGFRPAARGLAQCPARWVTSGTARPLLGF
SLPICMLELLLHISSPLTPAPETVFPSPSPGCD
>sp|P05141|ADT2_HUMAN ADP/ATP translocase 2 OS=Homo sapiens GN=SLC25A5 PE=1 SV=7
MTDAAVSFAKDFLAGGVAAAISKTAVAPIERVKLLLQVQHASKQITADKQYKGIIDCVVR

IPKEQGVLSFWRGNLANVIRYFPTQALNFAFKDKYKQIFLGGVDKRTQFWLYFAGNLASG
GAAGATSLCFVYPLDFARTRLAADVGKAGAEREFRGLGDCLVKIYKSDGIKGLYQGFNVS
VQGIIIYRAAYFGIYDTAKGMLPDPKNTHIVISWMIAQTVTAVAGLTSYPFDTVRRRMMM
QSGRKGTDIMYTGTLDCWRKIARDEGGKAFFKGAWSNVLRGMGGAFVLVLYDEIKKYT
>sp|P12236|ADT3_HUMAN ADP/ATP translocase 3 OS=Homo sapiens GN=SLC25A6 PE=1 SV=4
MTEQAISFAKDFLAGGIAAAISKTAVAPIERVKLLLQVQHASKQIAADKQYKGIVDCIVR
IPKEQGVLSFWRGNLANVIRYFPTQALNFAFKDKYKQIFLGGVDKHTQFWRYFAGNLASG
GAAGATSLCFVYPLDFARTRLAADVGKSGTEREFRGLGDCLVKITKSDGIRGLYQGFSVS
VQGIIIYRAAYFGVYDTAKGMLPDPKNTHIVVSWMIAQTVTAVAGVVSYPFDTVRRRMMM
QSGRKGADIMYTGTVDCWRKIFRDEGGKAFFKGAWSNVLRGMGGAFVLVLYDELKKVI
>sp|Q6ZN18|AEBP2_HUMAN Zinc finger protein AEBP2 OS=Homo sapiens GN=AEBP2 PE=1 SV=2
MAAAITDMADLEELSRLSPLPPGSPGSAARGRAEPPEEEEEEEEEEEEAEAEAVAALLLN
GGSGGGGGGGGGGVGGGEAETMSEPSPESASQAGEDEDEEEDDEEEEDESSSSGGGEEES
SAESLVGSSGGSSSDETRSLSPGAASSSSGDGDGKEGLEEPKGPRGSQGGGGGGSSSSSV
VSSGGDEGYGTGGGGSSATSGGRRGSLEMSSDGEPLSRMDSEDSISSTIMDVDSTISSGR
STPAMMNGQGSTTSSSKNIAYNCCWDQCQACFNSSPDLADHIRSIHVDGQRGGVFVCLWK
GCKVYNTPSTSQSWLQRHMLTHSGDKPFKCVVGGCNASFASQGGLARHVPTHFSQQNSSK
VSSQPKAKEESPSKAGMNKRRKLKNKRRRSLPRPHDFFDAQTLDAIRHRAICFNLSAHIE
SLGKGHSVVFHSTVIAKRKEDSGKIKLLLHWMPEDILPDVWVNESERHQLKTKVVHLSKL
PKDTALLLDPNIYRTMPQKRLKRTLIRKVFNLYLSKQ
>sp|P43652|AFAM_HUMAN Afamin OS=Homo sapiens GN=AFM PE=1 SV=1
MKLLKLTGFIFFLFFLTESLTLPTQPRDIENFNSTQKFIEDNIEYITIIAFAQYVQEATF
EEMEKLVKDMVEYKDRCMADKTLPECSKLPNNVLQEKICAMEGLPQKHNFSHCCSKVDAQ
RRLCFFYNKKSDVGFLPPFPTLDPEEKCQAYESNRESLLNHFLYEVARRNPFVFAPTLLT
VAVHFEEVAKSCCEEQNKVNCLQTRAIPVTQYLKAFSSYQKHVCGALLKFGTKVVHFIYI
AILSQKFPKIEFKELISLVEDVSSNYDGCCEGDVVQCIRDTSKVMNHICSKQDSISSKIK
ECCEKKIPERGQCIINSNKDDRPKDLSLREGKFTDSENVCQERDADPDTFFAKFTFEYSR
RHPDLSIPELLRIVQIYKDLLRNCCNTENPPGCYRYAEDKFNETTEKSLKMVQQECKHFQ
NLGKDGLKYHYLIRLTKIAPQLSTEELVSLGEKMVTAFTTCCTLSEEFACVDNLADLVFG
ELCGVNENRTINPAVDHCCKTNFAFRRPCFESLKADKTYVPPPFSQDLFTFHADMCQSQN
EELQRKTDRFLVNLVKLKHELTDEELQSLFTNFANVVDKCCKAESPEVCFNEESPKIGN
>sp|Q9Y6Z5|AFAS1_HUMAN Putative uncharacterized protein AFDN-AS1 OS=Homo sapiens GN=AFDN-AS1 PE=5 SV=1
MGAAGSDGRCCVRSGRAGTGGAGSKWVVMDLWTGGAGSRRAVLGPDGRWVRWAVLGSVRM
GGARSKWAAPDPMGGAGFGPDWRVRVWTGGAGSKWGCSAGSGRAVLGPNGRWVPWAVLGS
VRTGGAGSKWAALGPMGGTGSGRAVLGLDGRCSIQMGAAGSGRAVLGSVRTGGAGSKWAV
LGSAGDRWAWRHRYGAQGRWSALSEGPCAPRARCSSCPRIAPGLLRVSSRVIPFRWVTSK
PTFSSCFLPRRTRC
>sp|P51825|AFF1_HUMAN AF4/FMR2 family member 1 OS=Homo sapiens GN=AFF1 PE=1 SV=1
MAAQSSLYNDDRNLLRIREKERRNQEAHQEKEAFPEKIPLFGEPYKTAKGDELSSRIQNM
LGNYEEVKEFLSTKSHTHRLDASENRLGKPKYPLIPDKGSSIPSSSFHTSVHHQSIHTPA
SGPLSVGNISHNPKMAQPRTEPMPSLHAKSCGPPDSQHLTQDRLGQEGFGSSHHKKGDRR
ADGDHCASVTDSAPERELSPLISLPSPVPPLSPIHSNQQTLPRTQGSSKVHGSSNNSKGY
CPAKSPKDLAVKVHDKETPQDSLVAPAQPPSQTFPPPSLPSKSVAMQQKPTAYVRPMDGQ

DQAPSESPELKPLPEDYRQQTFEKTDLKVPAKAKLTKLKMPSQSVEQTYSNEVHCVEEIL
KEMTHSWPPPLTAIHTPSTAEPSKFPFPTKDSQHVSSVTQNQKQYDTSSKTHSNSQQGTS
SMLEDDLQLSDSEDSDSEQTPEKPPSSSAPPSAPQSLPEPVASAHSSSAESESTSDSDSS
SDSESESSSSDSEENEPLETPAPEPEPPTTNKWQLDNWLTKVSQPAAPPEGPRSTEPPRR
HPESKGSSDSATSQEHSESKDPPPKSSSKAPRAPPEAPHPGKRSCQKSPAQQEPPQRQTV
GTKQPKKPVKASARAGSRTSLQGEREPGLLPYGSRDQTSKDKPKVKTKGRPRAAASNEPK
PAVPPSSEKKKHKSSLPAPSKALSGPEPAKDNVEDRTPEHFALVPLTESQGPPHSGSGSR
TSGCRQAVVVQEDSRKDRLPLPLRDTKLLSPLRDTPPPQSLMVKITLDLLSRIPQPPGKG
SRQRKAEDKQPPAGKKHSSEKRSSDSSSKLAKKRKGEAERDCDNKKIRLEKEIKSQSSSS
SSSHKESSKTKPSRPSSQSSKKEMLPPPPVSSSSQKPAKPALKRSRREADTCGQDPPKSA
SSTKSNHKDSSIPKQRRVEGKGSRSSSEHKGSSGDTANPFPVPSLPNGNSKPGKPQVKFD
KQQADLHMREAKKMKQKAELMTDRVGKAFKYLEAVLSFIECGIATESESQSSKSAYSVYS
ETVDLIKFIMSLKSFSDATAPTQEKIFAVLCMRCQSILNMAMFRCKKDIAIKYSRTLNKH
FESSSKVAQAPSPCIASTGTPSPLSPMPSPASSVGSQSSAGSVGSSGVAATISTPVTIQN
MTSSYVTITSHVLTAFDLWEQAEALTRKNKEFFARLSTNVCTLALNSSLVDLVHYTRQGF
QQLQELTKTP
>sp|Q9UHB7|AFF4_HUMAN AF4/FMR2 family member 4 OS=Homo sapiens GN=AFF4 PE=1 SV=1
MNREDRNVLRMKERERRNQEIQQGEDAFPPSSPLFAEPYKVTSKEDKLSSRIQSMLGNYD
EMKDFIGDRSIPKLVAIPKPTVPPSADEKSNPNFFEQRHGGSHQSSKWTPVGPAPSTSQS
QKRSSGLQSGHSSQRTSAGSSSGTNSSGQRHDRESYNNSGSSSRKKGQHGSEHSKSRSSS
PGKPQAVSSLNSSHSRSHGNDHHSKEHQRSKSPRDPDANWDSPSRVPFSSGQHSTQSFPP
SLMSKSNSMLQKPTAYVRPMDGQESMEPKLSSEHYSSQSHGNSMTELKPSSKAHLTKLKI
PSQPLDASASGDVSCVDEILKEMTHSWPPPLTAIHTPCKTEPSKFPFPTKESQQSNFGTG
EQKRYNPSKTSNGHQSKSMLKDDLKLSSSEDSDGEQDCDKTMPRSTPGSNSEPSHHNSEG
ADNSRDDSSSHSGSESSSGSDSESESSSSDSEANEPSQSASPEPEPPPTNKWQLDNWLNK
VNPHKVSPASSVDSNIPSSQGYKKEGREQGTGNSYTDTSGPKETSSATPGRDSKTIQKGS
ESGRGRQKSPAQSDSTTQRRTVGKKQPKKAEKAAAEEPRGGLKIESETPVDLASSMPSSR
HKAATKGSRKPNIKKESKSSPRPTAEKKKYKSTSKSSQKSREIIETDTSSSDSDESESLP
PSSQTPKYPESNRTPVKPSSVEEEDSFFRQRMFSPMEEKELLSPLSEPDDRYPLIVKIDL
NLLTRIPGKPYKETEPPKGEKKNVPEKHTREAQKQASEKVSNKGKRKHKNEDDNRASESK
KPKTEDKNSAGHKPSSNRESSKQSAAKEKDLLPSPAGPVPSKDPKTEHGSRKRTISQSSS
LKSSSNSNKETSGSSKNSSSTSKQKKTEGKTSSSSKEVKEKAPSSSSNCPPSAPTLDSSK
PRRTKLVFDDRNYSADHYLQEAKKLKHNADALSDRFEKAVYYLDAVVSFIECGNALEKNA
QESKSPFPMYSETVDLIKYTMKLKNYLAPDATAADKRLTVLCLRCESLLYLRLFKLKKEN
ALKYSKTLTEHLKNSYNNSQAPSPGLGSKAVGMPSPVSPKLSPGNSGNYSSGASSASASG
SSVTIPQKIHQMAASYVQVTSNFLYATEIWDQAEQLSKEQKEFFAELDKVMGPLIFNASI
MTDLVRYTRQGLHWLRQDAKLIS
>sp|Q5BKT4|AG10A_HUMAN Dol-P-Glc:Glc(2)Man(9)GlcNAc(2)-PP-Dol alpha-1,2-glucosyltransferase OS=Homo sapiens GN=ALG10 PE=2 SV=1
MAQLEGYYFSAALSCTFLVSCLLFSAFSRALREPYMDEIFHLPQAQRYCEGHFSLSQWDP
MITTLPGLYLVSIGVIKPAIWIFGWSEHVVCSIGMLRFVNLLFSVGNFYLLYLLFCKVQP
RNKAASSIQRVLSTLTLAVFPTLYFFNFLYYTEAGSMFFTLFAYLMCLYGNHKTSAFLGF
CGFMFRQTNIIWAVFCAGNVIAQKLTEAWKTELQKKEDRLPPIKGPFAEFRKILQFLLAY
SMSFKNLSMLLLLTWPYILLGFLFCAFVVVNGGIVIGDRSSHEACLHFPQLFYFFSFTLF

FSFPHLLSPSKIKTFLSLVWKRRILFFVVTLVSVFLVWKFTYAHKYLLADNRHYTFYVWK
RVFQRYETVKYLLVPAYIFAGWSIADSLKSKSIFWNLMFFICLFTVIVPQKLLEFRYFIL
PYVIYRLNIPLPPTSRLICELSCYAVVNFITFFIFLNKTFQWPNSQDIQRFMW
>sp|O95394|AGM1_HUMAN Phosphoacetylglucosamine mutase OS=Homo sapiens GN=PGM3 PE=1 SV=1
MDLGAITKYSALHAKPNGLILQYGTAGFRTKAEHLDHVMFRMGLLAVLRSKQTKSTIGVM
VTASHNPEEDNGVKLVDPLGEMLAPSWEEHATCLANAEEQDMQRVLIDISEKEAVNLQQD
AFVVIGRDTRPSSEKLSQSVIDGVTVLGGQFHDYGLLTTPQLHYMVYCRNTGGRYGKATI
EGYYQKLSKAFVELTKQASCSGDEYRSLKVDCANGIGALKLREMEHYFSQGLSVQLFNDG
SKGKLNHLCGADFVKSHQKPPQGMEIKSNERCCSFDGDADRIVYYYHDADGHFHLIDGDK
IATLISSFLKELLVEIGESLNIGVVQTAYANGSSTRYLEEVMKVPVYCTKTGVKHLHHKA
QEFDIGVYFEANGHGTALFSTAVEMKIKQSAEQLEDKKRKAAKMLENIIDLFNQAAGDAI
SDMLVIEAILALKGLTVQQWDALYTDLPNRQLKVQVADRRVISTTDAERQAVTPPGLQEA
INDLVKKYKLSRAFVRPSGTEDVVRVYAEADSQESADHLAHEVSLAVFQLAGGIGERPQP
GF
>sp|Q5TGY3|AHDC1_HUMAN AT-hook DNA-binding motif-containing protein 1 OS=Homo sapiens GN=AHDC1 PE=1 SV=1
MRVKPQGLVVTSSAVCSSPDYLREPKYYPGGPPTPRPLLPTRPPASPPDKAFSTHAFSEN
PRPPPRRDPSTRRPPVLAKGDDPLPPRAARPVSQARCPTPVGDGSSSRRCWDNGRVNLRP
VVQLIDIMKDLTRLSQDLQHSGVHLDCGGLRLSRPPAPPPGDLQYSFFSSPSLANSIRSP
EERATPHAKSERPSHPLYEPEPEPRDSPQPGQGHSPGATAAATGLPPEPEPDSTDYSELA
DADILSELASLTCPEAQLLEAQALEPPSPEPEPQLLDPQPRFLDPQALEPLGEALELPPL
QPLADPLGLPGLALQALDTLPDSLESQLLDPQALDPLPKLLDVPGRRLEPQQPLGHCPLA
EPLRLDLCSPHGPPGPEGHPKYALRRTDRPKILCRRRKAGRGRKADAGPEGRLLPLPMPT
GLVAALAEPPPPPPPPPPALPGPGPVSVPELKPESSQTPVVSTRKGKCRGVRRMVVKMAK
IPVSLGRRNKTTYKVSSLSSSLSVEGKELGLRVSAEPTPLLKMKNNGRNVVVVFPPGEMP
IILKRKRGRPPKNLLLGPGKPKEPAVVAAEAATVAAATMAMPEVKKRRRRKQKLASPQPS
YAADANDSKAEYSDVLAKLAFLNRQSQCAGRCSPPRCWTPSEPESVHQAPDTQSISHFLH
RVQGFRRRGGKAGGFGGRGGGHAAKSARCSFSDFFEGIGKKKKVVAVAAAGVGGPGLTEL
GHPRKRGRGEVDAVTGKPKRKRRSRKNGTLFPEQVPSGPGFGEAGAEWAGDKGGGWAPHH
GHPGGQAGRNCGFQGTEARAFASTGLESGASGRGSYYSTGAPSGQTELSQERQNLFTGYF
RSLLDSDDSSDLLDFALSASRPESRKASGTYAGPPTSALPAQRGLATFPSRGAKASPVAV
GSSGAGADPSFQPVLSARQTFPPGRAASYGLTPAASDCRAAETFPKLVPPPSAMARSPTT
HPPANTYLPQYGGYGAGQSVFAPTKPFTGQDCANSKDCSFAYGSGNSLPASPSSAHSAGY
APPPTGGPCLPPSKASFFSSSEGAPFSGSAPTPLRCDSRASTVSPGGYMVPKGTTASATS
AASAASSSSSSFQPSPENCRQFAGASQWPFRQGYGGLDWASEAFSQLYNPSFDCHVSEPN
VILDISNYTPQKVKQQTAVSETFSESSSDSTQFNQPVGGGGFRRANSEASSSEGQSSLSS
LEKLMMDWNEASSAPGYNWNQSVLFQSSSKPGRGRRKKVDLFEASHLGFPTSASAAASGY
PSKRSTGPRQPRGGRGGGACSAKKERGGAAAKAKFIPKPQPVNPLFQDSPDLGLDYYSGD
SSMSPLPSQSRAFGVGERDPCDFIGPYSMNPSTPSDGTFGQGFHCDSPSLGAPELDGKHF
PPLAHPPTVFDAGLQKAYSPTCSPTLGFKEELRPPPTKLAACEPLKHGLQGASLGHAAAA
QAHLSCRDLPLGQPHYDSPSCKGTAYWYPPGSAARSPPYEGKVGTGLLADFLGRTEAACL
SAPHLASPPATPKADKEPLEMARPPGPPRGPAAAAAGYGCPLLSDLTLSPVPRDSLLPLQ
DTAYRYPGFMPQAHPGLGGGPKSGFLGPMAEPHPEDTFTVTSL
>sp|Q8N157|AHI1_HUMAN Jouberin OS=Homo sapiens GN=AHI1 PE=1 SV=1

MPTAESEAKVKTKVRFEELLKTHSDLMREKKKLKKKLVRSEENISPDTIRSNLHYMKETT
SDDPDTIRSNLPHIKETTSDDVSAANTNNLKKSTRVTKNKLRNTQLATENPNGDASVEED
KQGKPNKKVIKTVPQLTTQDLKPETPENKVDSTHQKTHTKPQPGVDHQKSEKANEGREET
DLEEDEELMQAYQCHVTEEMAKEIKRKIRKKLKEQLTYFPSDTLFHDDKLSSEKRKKKKE
VPVFSKAETSTLTISGDTVEGEQKKESSVRSVSSDSHQDDEISSMEQSTEDSMQDDTKPK
PKKTKKKTKAVADNNEDVDGDGVHEITSRDSPVYPKCLLDDDLVLGVYIHRTDRLKSDFM
ISHPMVKIHVVDEHTGQYVKKDDSGRPVSSYYEKENVDYILPIMTQPYDFKQLKSRLPEW
EEQIVFNENFPYLLRGSDESPKVILFFEILDFLSVDEIKNNSEVQNQECGFRKIAWAFLK
LLGANGNANINSKLRLQLYYPPTKPRSPLSVVEAFEWWSKCPRNHYPSTLYVTVRGLKVP
DCIKPSYRSMMALQEEKGKPVHCERHHESSSVDTEPGLEESKEVIKWKRLPGQACRIPNK
HLFSLNAGERGCFCLDFSHNGRILAAACASRDGYPIILYEIPSGRFMRELCGHLNIIYDL
SWSKDDHYILTSSSDGTARIWKNEINNTNTFRVLPHPSFVYTAKFHPAVRELVVTGCYDS
MIRIWKVEMREDSAILVRQFDVHKSFINSLCFDTEGHHMYSGDCTGVIVVWNTYVKINDL
EHSVHHWTINKEIKETEFKGIPISYLEIHPNGKRLLIHTKDSTLRIMDLRILVARKFVGA
ANYREKIHSTLTPCGTFLFAGSEDGIVYVWNPETGEQVAMYSDLPFKSPIRDISYHPFEN
MVAFCAFGQNEPILLYIYDFHVAQQEAEMFKRYNGTFPLPGIHQSQDALCTCPKLPHQGS
FQIDEFVHTESSSTKMQLVKQRLETVTEVIRSCAAKVNKNLSFTSPPAVSSQQSKLKQSN
MLTAQEILHQFGFTQTGIISIERKPCNHQVDTAPTVVALYDYTANRSDELTIHRGDIIRV
FFKDNEDWWYGSIGKGQEGYFPANHVASETLYQELPPEIKERSPPLSPEEKTKIEKSPAP
QKQSINKNKSQDFRLGSESMTHSEMRKEQSHEDQGHIMDTRMRKNKQAGRKVTLIE
>sp|Q9BV10|ALG12_HUMAN Dol-P-Man:Man(7)GlcNAc(2)-PP-Dol alpha-1,6-mannosyltransferase OS=Homo sapiens GN=ALG12 PE=1 SV=1
MAGKGSSGRRPLLLGLLVAVATVHLVICPYTKVEESFNLQATHDLLYHWQDLEQYDHLEF
PGVVPRTFLGPVVIAVFSSPAVYVLSLLEMSKFYSQLIVRGVLGLGVIFGLWTLQKEVRR
HFGAMVATMFCWVTAMQFHLMFYCTRTLPNVLALPVVLLALAAWLRHEWARFIWLSAFAI
IVFRVELCLFLGLLLLLALGNRKVSVVRALRHAVPAGILCLGLTVAVDSYFWRQLTWPEG
KVLWYNTVLNKSSNWGTSPLLWYFYSALPRGLGCSLLFIPLGLVDRRTHAPTVLALGFMA
LYSLLPHKELRFIIYAFPMLNITAARGCSYLLNNYKKSWLYKAGSLLVIGHLVVNAAYSA
TALYVSHFNYPGGVAMQRLHQLVPPQTDVLLHIDVAAAQTGVSRFLQVNSAWRYDKREDV
QPGTGMLAYTHILMEAAPGLLALYRDTHRVLASVVGTTGVSLNLTQLPPFNVHLQTKLVL
LERLPRPS
>sp|Q96F25|ALG14_HUMAN UDP-N-acetylglucosamine transferase subunit ALG14 homolog OS=Homo sapiens GN=ALG14 PE=1 SV=1
MVCVLVLAAAAGAVAVFLILRIWVVLRSMDVTPRESLSILVVAGSGGHTTEILRLLGSLS
NAYSPRHYVIADTDEMSANKINSFELDRADRDPSNMYTKYYIHRIPRSREVQQSWPSTVF
TTLHSMWLSFPLIHRVKPDLVLCNGPGTCVPICVSALLLGILGIKKVIIVYVESICRVET
LSMSGKILFHLSDYFIVQWPALKEKYPKSVYLGRIV
>sp|O95433|AHSA1_HUMAN Activator of 90 kDa heat shock protein ATPase homolog 1 OS=Homo sapiens GN=AHSA1 PE=1 SV=1
MAKWGEGDPRWIVEERADATNVNNWHWTERDASNWSTDKLKTLFLAVQVQNEEGKCEVTE
VSKLDGEASINNRKGKLIFFYEWSVKLNWTGTSKSGVQYKGHVEIPNLSDENSVDEVEIS
VSLAKDEPDTNLVALMKEEGVKLLREAMGIYISTLKTEFTQGMILPTMNGESVDPVGQPA
LKTEERKAKPAPSKTQARPVGVKIPTCKITLKETFLTSPEELYRVFTTQELVQAFTHAPA
TLEADRGGKFHMVDGNVSGEFTDLVPEKHIVMKWRFKSWPEGHFATITLTFIDKNGETEL

CMEGRGIPAPEEERTRQGWQRYYFEGIKQTFGYGARLF
>sp|Q719I0|AHSA2_HUMAN Activator of 90 kDa heat shock protein ATPase homolog 2 OS=Homo sapiens GN=AHSA2 PE=1 SV=2
MAKWGQGNPHWIVEEREDGTNVNNWRWTERDATSLSKGKFQELLVGIVVENDAGRGEINE
LKQVEGEASCSSRKGKLIFFYEWNIKLGWKGIVKESGVKHKGLIEIPNLSEENEVDDTEV
SLSKKKGDGVILKDLMKTAGTAKVREALGDYLKALKTEFTTGMILPTKAMATQELTVKRK
LSGNTLQVQASSPVALGVRIPTVALHMMELFDTTVEQLYSIFTVKELTNKKIIMKWRCGN
WPEEHYAMVALNFVPTLGQTELQLKEFLSICKEENMKFCWQKQHFEEIKGSLQLTPLNG
>sp|Q96NN9|AIFM3_HUMAN Apoptosis-inducing factor 3 OS=Homo sapiens GN=AIFM3 PE=1 SV=1
MGGCFSKPKPVELKIEVVLPEKERGKEELSASGKGSPRAYQGNGTARHFHTEERLSTPHP
YPSPQDCVEAAVCHVKDLENGQMREVELGWGKVLLVKDNGEFHALGHKCPHYGAPLVKGV
LSRGRVRCPWHGACFNISTGDLEDFPGLDSLHKFQVKIEKEKVYVRASKQALQLQRRTKV
MAKCISPSAGYSSSTNVLIVGAGAAGLVCAETLRQEGFSDRIVLCTLDRHLPYDRPKLSK
SLDTQPEQLALRPKEFFRAYGIEVLTEAQVVTVDVRTKKVVFKDGFKLEYSKLLLAPGSS
PKTLSCKGKEVENVFTIRTPEDANRVVRLARGRNVVVVGAGFLGMEVAAYLTEKAHSVSV
VELEETPFRRFLGERVGRALMKMFENNRVKFYMQTEVSELRGQEGKLKEVVLKSSKVVRA
DVCVVGIGAVPATGFLRQSGIGLDSRGFIPVNKMMQTNVPGVFAAGDAVTFPLAWRNNRK
VNIPHWQMAHAQGRVAAQNMLAQEAEMSTVPYLWTAMFGKSLRYAGYGEGFDDVIIQGDL
EELKFVAFYTKGDEVIAVASMNYDPIVSKVAEVLASGRAIRKREVELFVLHSKTGDMSWL
TGKGS
>sp|Q9NVV5|AIG1_HUMAN Androgen-induced gene 1 protein OS=Homo sapiens GN=AIG1 PE=1 SV=2
MALVPCQVLRMAILLSYCSILCNYKAIEMPSHQTYGGSWKFLTFIDLVIQAVFFGICVLT
DLSSLLTRGSGNQEQERQLKKLISLRDWMLAVLAFPVGVFVVAVFWIIYAYDREMIYPKL
LDNFIPGWLNHGMHTTVLPFILIEMRTSHHQYPSRSSGLTAICTFSVGYILWVCWVHHVT
GMWVYPFLEHIGPGARIIFFGSTTILMNFLYLLGEVLNNYIWDTQKKPPSWQDMKIKFMY
LGPSS
>sp|Q9Y4K1|AIM1_HUMAN Absent in melanoma 1 protein OS=Homo sapiens GN=AIM1 PE=1 SV=3
MEKRSSGRRSGRRRGSQKSTDSPGADAELPESAARDDAVFDDEVAPNAASDNASAEKKVK
SPRAALDGGVASAASPESKPSPGTKGQLRGESDRSKQPPPASSPTKRKGRSRALEAVPAP
PASGPRAPAKESPPKRVPDPSPVTKGTAAESGEEAARAIPRELPVKSSSLLPEIKPEHKR
GPLPNHFNGRAEGGRSRELGRAAGAPGASDADGLKPRNHFGVGRSTVTTKVTLPAKPKHV
ELNLKTPKNLDSLGNEHNPFSQPVHKGNTATKISLFENKRTNSSPRHTDIRGQRNTPASS
KTFVGRAKLNLAKKAKEMEQPEKKVMPNSPQNGVLVKETAIETKVTVSEEEILPATRGMN
GDSSENQALGPQPNQDDKADVQTDAGCLSEPVASALIPVKDHKLLEKEDSEAADSKSLVL
ENVTDTAQDIPTTVDTKDLPPTAMPKPQHTFSDSQSPAESSPGPSLSLSAPAPGDVPKDT
CVQSPISSFPCTDLKVSENHKGCVLPVSRQNNEKMPLLELGGETTPPLSTERSPEAVGSE
CPSRVLVQVRSFVLPVESTQDVSSQVIPESSEVREVQLPTCHSNEPEVVSVASCAPPQEE
VLGNEHSHCTAELAAKSGPQVIPPASEKTLPIQAQSQGSRTPLMAESSPTNSPSSGNHLA
TPQRPDQTVTNGQDSPASLLNISAGSDDSVFDSSSDMEKFTEIIKQMDSAVCMPMKRKKA
RMPNSPAPHFAMPPIHEDHLEKVFDPKVFTFGLGKKKESQPEMSPALHLMQNLDTKSKLR
PKRASAEQSVLFKSLHTNTNGNSEPLVMPEINDKENRDVTNGGIKRSRLEKSALFSSLLS
SLPQDKIFSPSVTSVNTMTTAFSTSQNGSLSQSSVSQPTTEGAPPCGLNKEQSNLLPDNS
LKVFNFNSSSTSHSSLKSPSHMEKYPQKEKTKEDLDSRSNLHLPETKFSELSKLKNDDME
KANHIESVIKSNLPNCANSDTDFMGLFKSSRYDPSISFSGMSLSDTMTLRGSVQNKLNPR

PGKVVIYSEPDVSEKCIEVFSDIQDCSSWSLSPVILIKVVRGCWILYEQPNFEGHSIPLE
EGELELSGLWGIEDILERHEEAESDKPVVIGSIRHVVQDYRVSHIDLFTEPEGLGILSSY
FDDTEEMQGFGVMQKTCSMKVHWGTWLIYEEPGFQGVPFILEPGEYPDLSFWDTEEAYIG
SMRPLKMGGRKVEFPTDPKVVVYEKPFFEGKCVELETGMCSFVMEGGETEEATGDDHLPF
TSVGSMKVLRGIWVAYEKPGFTGHQYLLEEGEYRDWKAWGGYNGELQSLRPILGDFSNAH
MIMYSEKNFGSKGSSIDVLGIVANLKETGYGVKTQSINVLSGVWVAYENPDFTGEQYILD
KGFYTSFEDWGGKNCKISSVQPICLDSFTGPRRRNQIHLFSEPQFQGHSQSFEETTSQID
DSFSTKSCRVSGGSWVVYDGENFTGNQYVLEEGHYPCLSAMGCPPGATFKSLRFIDVEFS
EPTIILFEREDFKGKKIELNAETVNLRSLGFNTQIRSVQVIGGIWVTYEYGSYRGRQFLL
SPAEVPNWYEFSGCRQIGSLRPFVQKRIYFRLRNKATGLFMSTNGNLEDLKLLRIQVMED
VGADDQIWIYQEGCIKCRIAEDCCLTIVGSLVTSGSKLGLALDQNADSQFWSLKSDGRIY
SKLKPNLVLDIKGGTQYDQNHIILNTVSKEKFTQVWEAMVLYT
>sp|Q13155|AIMP2_HUMAN Aminoacyl tRNA synthase complex-interacting multifunctional protein 2 OS=Homo sapiens GN=AIMP2 PE=1 SV=2
MPMYQVKPYHGGGAPLRVELPTCMYRLPNVHGRSYGPAPGAGHVQEESNLSLQALESRQD
DILKRLYELKAAVDGLSKMIQTPDADLDVTNIIQADEPTTLTTNALDLNSVLGKDYGALK
DIVINANPASPPLSLLVLHRLLCEHFRVLSTVHTHSSVKSVPENLLKCFGEQNKKQPRQD
YQLGFTLIWKNVPKTQMKFSIQTMCPIEGEGNIARFLFSLFGQKHNAVNATLIDSWVDIA
IFQLKEGSSKEKAAVFRSMNSALGKSPWLAGNELTVADVVLWSVLQQIGGCSVTVPANVQ
RWMRSCENLAPFNTALKLLK
>sp|O43918|AIRE_HUMAN Autoimmune regulator OS=Homo sapiens GN=AIRE PE=1 SV=1
MATDAALRRLLRLHRTEIAVAVDSAFPLLHALADHDVVPEDKFQETLHLKEKEGCPQAFH
ALLSWLLTQDSTAILDFWRVLFKDYNLERYGRLQPILDSFPKDVDLSQPRKGRKPPAVPK
ALVPPPRLPTKRKASEEARAAAPAALTPRGTASPGSQLKAKPPKKPESSAEQQRLPLGNG
IQTMSASVQRAVAMSSGDVPGARGAVEGILIQQVFESGGSKKCIQVGGEFYTPSKFEDSG
SGKNKARSSSGPKPLVRAKGAQGAAPGGGEARLGQQGSVPAPLALPSDPQLHQKNEDECA
VCRDGGELICCDGCPRAFHLACLSPPLREIPSGTWRCSSCLQATVQEVQPRAEEPRPQEP
PVETPLPPGLRSAGEEVRGPPGEPLAGMDTTLVYKHLPAPPSAAPLPGLDSSALHPLLCV
GPEGQQNLAPGARCGVCGDGTDVLRCTHCAAAFHWRCHFPAGTSRPGTGLRCRSCSGDVT
PAPVEGVLAPSPARLAPGPAKDDTASHEPALHRDDLESLLSEHTFDGILQWAIQSMARPA
APFPS
>sp|P14550|AK1A1_HUMAN Alcohol dehydrogenase [NADP(+)] OS=Homo sapiens GN=AKR1A1 PE=1 SV=3
MAASCVLLHTGQKMPLIGLGTWKSEPGQVKAAVKYALSVGYRHIDCAAIYGNEPEIGEAL
KEDVGPGKAVPREELFVTSKLWNTKHHPEDVEPALRKTLADLQLEYLDLYLMHWPYAFER
GDNPFPKNADGTICYDSTHYKETWKALEALVAKGLVQALGLSNFNSRQIDDILSVASVRP
AVLQVECHPYLAQNELIAHCQARGLEVTAYSPLGSSDRAWRDPDEPVLLEEPVVLALAEK
YGRSPAQILLRWQVQRKVICIPKSITPSRILQNIKVFDFTFSPEEMKQLNALNKNWRYIV
PMLTVDGKRVPRDAGHPLYPFNDPY
>sp|O43572|AKA10_HUMAN A-kinase anchor protein 10, mitochondrial OS=Homo sapiens GN=AKAP10 PE=1 SV=2
MRGAGPSPRQSPRTLRPDPGPAMSFFRRKVKGKEQEKTSDVKSIKASISVHSPQKSTKNH
ALLEAAGPSHVAINAISANMDSFSSSRTATLKKQPSHMEAAHFGDLGRSCLDYQTQETKS
SLSKTLEQVLHDTIVLPYFIQFMELRRMEHLVKFWLEAESFHSTTWSRIRAHSLNTVKQS

SLAEPVSPSKKHETTASFLTDSLDKRLEDSGSAQLFMTHSEGIDLNNRTNSTQNHLLLSQ
ECDSAHSLRLEMARAGTHQVSMETQESSSTLTVASRNSPASPLKELSGKLMKSIEQDAVN
TFTKYISPDAAKPIPITEAMRNDIIARICGEDGQVDPNCFVLAQSIVFSAMEQEHFSEFL
RSHHFCKYQIEVLTSGTVYLADILFCESALFYFSEYMEKEDAVNILQFWLAADNFQSQLA
AKKGQYDGQEAQNDAMILYDKYFSLQATHPLGFDDVVRLEIESNICREGGPLPNCFTTPL
RQAWTTMEKVFLPGFLSSNLYYKYLNDLIHSVRGDEFLGGNVSLTAPGSVGPPDESHPGS
SDSSASQSSVKKASIKILKNFDEAIIVDAASLDPESLYQRTYAGKMTFGRVSDLGQFIRE
SEPEPDVRKSKGSMFSQAMKKWVQGNTDEAQEELAWKIAKMIVSDIMQQAQYDQPLEKST
KL
>sp|Q9P0M2|AKA7G_HUMAN A-kinase anchor protein 7 isoform gamma OS=Homo sapiens GN=AKAP7 PE=1 SV=2
MERPEAGGINSNECENVSRKKKMSEEFEANTMDSLVDMPFATVDIQDDCGITDEPQINLK
RSQENEWVKSDQVKKRKKKRKDYQPNYFLSIPITNKEIIKGIKILQNAIIQQDERLAKAM
VSDGSFHITLLVMQLLNEDEVNIGIDALLELKPFIEELLQGKHLTLPFQGIGTFGNQVGF
VKLAEGDHVNSLLEIAETANRTFQEKGILVGESRSFKPHLTFMKLSKSPWLRKNGVKKID
PDLYEKFISHRFGEEILYRIDLCSMLKKKQSNGYYHCESSIVIGEKNGGEPDDAELVRLS
KRLVENAVLKAVQQYLEETQNKNKPGEGSSVKTEAADQNGNDNENNRK
>sp|Q9H9L7|AKIR1_HUMAN Akirin-1 OS=Homo sapiens GN=AKIRIN1 PE=1 SV=1
MACGATLKRPMEFEAALLSPGSPKRRRCAPLPGPTPGLRPPDAEPPPPFQTQTPPQSLQQ
PAPPGSERRLPTPEQIFQNIKQEYSRYQRWRHLEVVLNQSEACASESQPHSSALTAPSSP
GSSWMKKDQPTFTLRQVGIICERLLKDYEDKIREEYEQILNTKLAEQYESFVKFTHDQIM
RRYGTRPTSYVS
>sp|Q7Z591|AKNA_HUMAN AT-hook-containing transcription factor OS=Homo sapiens GN=AKNA PE=1 SV=2
MASSETEIRWAEPGLGKGPQRRRWAWAEDKRDVDRSSSQSWEEERLFPNATSPELLEDFR
LAQQHLPPLEWDPHPQPDGHQDSESGETSGEEAEAEDVDSPASSHEPLAWLPQQGRQLDM
TEEEPDGTLGSLEVEEAGESSSRLGYEAGLSLEGHGNTSPMALGHGQARGWVASGEQASG
DKLSEHSEVNPSVELSPARSWSSGTVSLDHPSDSLDSTWEGETDGPQPTALAETLPEGPS
HHLLSPDGRTGGSVARATPMEFQDSSAPPAQSPQHATDRWRRETTRFFCPQPKEHIWKQT
KTSPKPLPSRFIGSISPLNPQPRPTRQGRPLPRQGATLAGRSSSNAPKYGRGQLNYPLPD
FSKVGPRVRFPKDESYRPPKSRSHNRKPQAPARPLIFKSPAEIVQEVLLSSGEAALAKDT
PPAHPITRVPQEFQTPEQATELVHQLQEDYHRLLTKYAEAENTIDQLRLGAKVNLFSDPP
QPNHSIHTGMVPQGTKVLSFTIPQPRSAEWWPGPAEDPQASAASGWPSARGDLSPSSLTS
MPTLGWLPENRDISEDQSSAEQTQALASQASQFLAKVESFERLIQAGRLMPQDQVKGFQR
LKAAHAALEEEYLKACREQHPAQPLAGSKGTPGRFDPRRELEAEIYRLGSCLEELKEHID
QTQQEPEPPGSDSALDSTPALPCLHQPTHLPAPSGQAPMPAIKTSCPEPATTTAAASTGP
CPLHVNVEVSSGNSEVEDRPQDPLARLRHKELQMEQVYHGLMERYLSVKSLPEAMRMEEE
EEGEEEEEEEGGGDSLEVDGVAATPGKAEATRVLPRQCPVQAEKSHGAPLEEATEKMVSM
KPPGFQASLARDGHMSGLGKAEAAPPGPGVPPHPPGTKSAASHQSSMTSLEGSGISERLP
QKPLHRGGGPHLEETWMASPETDSGFVGSETSRVSPLTQTPEHRLSHISTAGTLAQPFAA
SVPRDGASYPKARGSLIPRRATEPSTPRSQAQRYLSSPSGPLRQRAPNFSLERTLAAEMA
VPGSEFEGHKRISEQPLPNKTISPPPAPAPAAAPLPCGPTETIPSFLLTRAGRDQAICEL
QEEVSRLRLRLEDSLHQPLQGSPTRPASAFDRPARTRGRPADSPATWGSHYGSKSTERLP
GEPRGEEQIVPPGRQRARSSSVPREVLRLSLSSESELPSLPLFSEKSKTTKDSPQAARDG

KRGVGSAGWPDRVTFRGQYTGHEYHVLSPKAVPKGNGTVSCPHCRPIRTQDAGGAVTGDP
LGPPPADTLQCPLCGQVGSPPEADGPGSATSGAEKATTRRKASSTPSPKQRSKQAGSSPR
PPPGLWYLATAPPAPAPPAFAYISSVPIMPYPPAAVYYAPAGPTSAQPAAKWPPTASPPP
ARRHRHSIQLDLGDLEELNKALSRAVQAAESVRSTTRQMRSSLSADLRQAHSLRGSCLF
>sp|Q12802|AKP13_HUMAN A-kinase anchor protein 13 OS=Homo sapiens GN=AKAP13 PE=1 SV=2
MKLNPQQAPLYGDCVVTVLLAEEDKAEDDVVFYLVFLGSTLRHCTSTRKVSSDTLETIAP
GHDCCETVKVQLCASKEGLPVFVVAEEDFHFVQDEAYDAAQFLATSAGNQQALNFTRFLD
QSGPPSGDVNSLDKKLVLAFRHLKLPTEWNVLGTDQSLHDAGPRETLMHFAVRLGLLRLT
WFLLQKPGGRGALSIHNQEGATPVSLALERGYHKLHQLLTEENAGEPDSWSSLSYEIPYG
DCSVRHHRELDIYTLTSESDSHHEHPFPGDGCTGPIFKLMNIQQQLMKTNLKQMDSLMPL
MMTAQDPSSAPETDGQFLPCAPEPTDPQRLSSSEETESTQCCPGSPVAQTESPCDLSSIV
EEENTDRSCRKKNKGVERKGEEVEPAPIVDSGTVSDQDSCLQSLPDCGVKGTEGLSSCGN
RNEETGTKSSGMPTDQESLSSGDAVLQRDLVMEPGTAQYSSGGELGGISTTNVSTPDTAG
EMEHGLMNPDATVWKNVLQGGESTKERFENSNIGTAGASDVHVTSKPVDKISVPNCAPAA
SSLDGNKPAESSLAFSNEETSTEKTAETETSRSREESADAPVDQNSVVIPAAAKDKISDG
LEPYTLLAAGIGEAMSPSDLALLGLEEDVMPHQNSETNSSHAQSQKGKSSPICSTTGDDK
LCADSACQQNTVTSSGDLVAKLCDNIVSESESTTARQPSSQDPPDASHCEDPQAHTVTSD
PVRDTQERADFCPFKVVDNKGQRKDVKLDKPLTNMLEVVSHPHPVVPKMEKELVPDQAVI
SDSTFSLANSPGSESVTKDDALSFVPSQKEKGTATPELHTATDYRDGPDGNSNEPDTRPL
EDRAVGLSTSSTAAELQHGMGNTSLTGLGGEHEGPAPPAIPEALNIKGNTDSSLQSVGKA
TLALDSVLTEEGKLLVVSESSAAQEQDKDKAVTCSSIKENALSSGTLQEEQRTPPPGQDT
QQFHEKSISADCAKDKALQLSNSPGASSAFLKAETEHNKEVAPQVSLLTQGGAAQSLVPP
GASLATESRQEALGAEHNSSALLPCLLPDGSDGSDALNCSQPSPLDVGVKNTQSQGKTSA
CEVSGDVTVDVTGVNALQGMAEPRRENISHNTQDILIPNVLLSQEKNAVLGLPVALQDKA
VTDPQGVGTPEMIPLDWEKGKLEGADHSCTMGDAEEAQIDDEAHPVLLQPVAKELPTDME
LSAHDDGAPAGVREVMRAPPSGRERSTPSLPCMVSAQDAPLPKGADLIEEAASRIVDAVI
EQVKAAGALLTEGEACHMSLSSPELGPLTKGLESAFTEKVSTFPPGESLPMGSTPEEATG
SLAGCFAGREEPEKIILPVQGPEPAAEMPDVKAEDEVDFRASSISEEVAVGSIAATLKMK
QGPMTQAINRENWCTIEPCPDAASLLASKQSPECENFLDVGLGRECTSKQGVLKRESGSD
SDLFHSPSDDMDSIIFPKPEEEHLACDITGSSSSTDDTASLDRHSSHGSDVSLSQILKPN
RSRDRQSLDGFYSHGMGAEGRESESEPADPGDVEEEEMDSITEVPANCSVLRSSMRSLSP
FRRHSWGPGKNAASDAEMNHRSSMRVLGDVVRRPPIHRRSFSLEGLTGGAGVGNKPSSSL
EVSSANAEELRHPFSGEERVDSLVSLSEEDLESDQREHRMFDQQICHRSKQQGFNYCTSA
ISSPLTKSISLMTISHPGLDNSRPFHSTFHNTSANLTESITEENYNFLPHSPSKKDSEWK
SGTKVSRTFSYIKNKMSSSKKSKEKEKEKDKIKEKEKDSKDKEKDKKTVNGHTFSSIPVV
GPISCSQCMKPFTNKDAYTCANCSAFVHKGCRESLASCAKVKMKQPKGSLQAHDTSSLPT
VIMRNKPSQPKERPRSAVLLVDETATTPIFANRRSQQSVSLSKSVSIQNITGVGNDENMS
NTWKFLSHSTDSLNKISKVNESTESLTDEGVGTDMNEGQLLGDFEIESKQLEAESWSRII
DSKFLKQQKKDVVKRQEVIYELMQTEFHHVRTLKIMSGVYSQGMMADLLFEQQMVEKLFP
CLDELISIHSQFFQRILERKKESLVDKSEKNFLIKRIGDVLVNQFSGENAERLKKTYGKF
CGQHNQSVNYFKDLYAKDKRFQAFVKKKMSSSVVRRLGIPECILLVTQRITKYPVLFQRI
LQCTKDNEVEQEDLAQSLSLVKDVIGAVDSKVASYEKKVRLNEIYTKTDSKSIMRMKSGQ
MFAKEDLKRKKLVRDGSVFLKNAAGRLKEVQAVLLTDILVFLQEKDQKYIFASLDQKSTV
ISLKKLIVREVAHEEKGLFLISMGMTDPEMVEVHASSKEERNSWIQIIQDTINTLNRDED

EGIPSENEEEKKMLDTRARELKEQLHQKDQKILLLLEEKEMIFRDMAECSTPLPEDCSPT
HSPRVLFRSNTEEALKGGPLMKSAINEVEILQGLVSGNLGGTLGPTVSSPIEQDVVGPVS
LPRRAETFGGFDSHQMNASKGGEKEEGDDGQDLRRTESDSGLKKGGNANLVFMLKRNSEQ
VVQSVVHLYELLSALQGVVLQQDSYIEDQKLVLSERALTRSLSRPSSLIEQEKQRSLEKQ
RQDLANLQKQQAQYLEEKRRREREWEARERELREREALLAQREEEVQQGQQDLEKEREEL
QQKKGTYQYDLERLRAAQKQLEREQEQLRREAERLSQRQTERDLCQVSHPHTKLMRIPSF
FPSPEEPPSPSAPSIAKSGSLDSELSVSPKRNSISRTHKDKGPFHILSSTSQTNKGPEGQ
SQAPASTSASTRLFGLTKPKEKKEKKKKNKTSRSQPGDGPASEVSAEGEEIFC
>sp|Q9ULX6|AKP8L_HUMAN A-kinase anchor protein 8-like OS=Homo sapiens GN=AKAP8L PE=1 SV=3
MSYTGFVQGSETTLQSTYSDTSAQPTCDYGYGTWNSGTNRGYEGYGYGYGYGQDNTTNYG
YGMATSHSWEMPSSDTNANTSASGSASADSVLSRINQRLDMVPHLETDMMQGGVYGSGGE
RYDSYESCDSRAVLSERDLYRSGYDYSELDPEMEMAYEGQYDAYRDQFRMRGNDTFGPRA
QGWARDARSGRPMASGYGRMWEDPMGARGQCMSGASRLPSLFSQNIIPEYGMFQGMRGGG
AFPGGSRFGFGFGNGMKQMRRTWKTWTTADFRTKKKKRKQGGSPDEPDSKATRTDCSDNS
DSDNDEGTEGEATEGLEGTEAVEKGSRVDGEDEEGKEDGREEGKEDPEKGALTTQDENGQ
TKRKLQAGKKSQDKQKKRQRDRMVERIQFVCSLCKYRTFYEDEMASHLDSKFHKEHFKYV
GTKLPKQTADFLQEYVTNKTKKTEELRKTVEDLDGLIHQIYRDQDLTQEIAMEHFVKKVE
AAHCAACDLFIPMQFGIIQKHLKTMDHNRNRRLMMEQSKKSSLMVARSILNNKLISKKLE
RYLKGENPFTDSPEEEKEQEEAEGGALDEGAQGEAAGISEGAEGVPAQPPVPPEPAPGAV
SPPPPPPPEEEEEGAVPLLGGALQRQIRGIPGLDVEDDEEGGGGAP
>sp|Q9H8T0|AKTIP_HUMAN AKT-interacting protein OS=Homo sapiens GN=AKTIP PE=1 SV=1
MNPFWSMSTSSVRKRSEGEEKTLTGDVKTSPPRTAPKKQLPSIPKNALPITKPTSPAPAA
QSTNGTHASYGPFYLEYSLLAEFTLVVKQKLPGVYVQPSYRSALMWFGVIFIRHGLYQDG
VFKFTVYIPDNYPDGDCPRLVFDIPVFHPLVDPTSGELDVKRAFAKWRRNHNHIWQVLMY
ARRVFYKIDTASPLNPEAAVLYEKDIQLFKSKVVDSVKVCTARLFDQPKIEDPYAISFSP
WNPSVHDEAREKMLTQKKPEEQHNKSVHVAGLSWVKPGSVQPFSKEEKTVAT
>sp|Q96B36|AKTS1_HUMAN Proline-rich AKT1 substrate 1 OS=Homo sapiens GN=AKT1S1 PE=1 SV=1
MASGRPEELWEAVVGAAERFRARTGTELVLLTAAPPPPPRPGPCAYAAHGRGALAEAARR
CLHDIALAHRAATAARPPAPPPAPQPPSPTPSPPRPTLAREDNEEDEDEPTETETSGEQL
GISDNGGLFVMDEDATLQDLPPFCESDPESTDDGSLSEETPAGPPTCSVPPASALPTQQY
AKSLPVSVPVWGFKEKRTEARSSDEENGPPSSPDLDRIAASMRALVLREAEDTQVFGDLP
RPRLNTSDFQKLKRKY
>sp|O75891|AL1L1_HUMAN Cytosolic 10-formyltetrahydrofolate dehydrogenase OS=Homo sapiens
GN=ALDH1L1 PE=1 SV=2
MKIAVIGQSLFGQEVYCHLRKEGHEVVGVFTVPDKDGKADPLGLEAEKDGVPVFKYSRWR
AKGQALPDVVAKYQALGAELNVLPFCSQFIPMEIISAPRHGSIIYHPSLLPRHRGASAIN
WTLIHGDKKGGFSIFWADDGLDTGDLLLQKECEVLPDDTVSTLYNRFLFPEGIKGMVQAV
RLIAEGKAPRLPQPEEGATYEGIQKKETAKINWDQPAEAIHNWIRGNDKVPGAWTEACEQ
KLTFFNSTLNTSGLVPEGDALPIPGAHRPGVVTKAGLILFGNDDKMLLVKNIQLEDGKMI
LASNFFKGAASSVLELTEAELVTAEAVRSVWQRILPKVLEVEDSTDFFKSGAASVDVVRL
VEEVKELCDGLELENEDVYMASTFGDFIQLLVRKLRGDDEEGECSIDYVEMAVNKRTVRM
PHQLFIGGEFVDAEGAKTSETINPTDGSVICQVSLAQVTDVDKAVAAAKDAFENGRWGKI
SARDRGRLMYRLADLMEQHQEELATIEALDAGAVYTLALKTHVGMSIQTFRYFAGWCDKI
QGSTIPINQARPNRNLTLTRKEPVGVCGIIIPWNYPLMMLSWKTAACLAAGNTVVIKPAQ

VTPLTALKFAELTLKAGIPKGVVNVLPGSGSLVGQRLSDHPDVRKIGFTGSTEVGKHIMK
SCAISNVKKVSLELGGKSPLIIFADCDLNKAVQMGMSSVFFNKGENCIAAGRLFVEDSIH
DEFVRRVVEEVRKMKVGNPLDRDTDHGPQNHHAHLVKLMEYCQHGVKEGATLVCGGNQVP
RPGFFFEPTVFTDVEDHMFIAKEESFGPVMIISRFADGDLDAVLSRANATEFGLASGVFT
RDINKALYVSDKLQAGTVFVNTYNKTDVAAPFGGFKQSGFGKDLGEAALNEYLRVKTVTF
EY
>sp|Q3SY69|AL1L2_HUMAN Mitochondrial 10-formyltetrahydrofolate dehydrogenase OS=Homo sapiens GN=ALDH1L2 PE=1 SV=2
MLRRGSQALRRFSTGRVYFKNKLKLALIGQSLFGQEVYSHLRKEGHRVVGVFTVPDKDGK
ADPLALAAEKDGTPVFKLPKWRVKGKTIKEVAEAYRSVGAELNVLPFCTQFIPMDIIDSP
KHGSIIYHPSILPRHRGASAINWTLIMGDKKAGFSVFWADDGLDTGPILLQRSCDVEPND
TVDALYNRFLFPEGIKAMVEAVQLIADGKAPRIPQPEEGATYEGIQKKENAEISWDQSAE
VLHNWIRGHDKVPGAWTEINGQMVTFYGSTLLNSSVPPGEPLEIKGAKKPGLVTKNGLVL
FGNDGKALTVRNLQFEDGKMIPASQYFSTGETSVVELTAEEVKVAETIKVIWAGILSNVP
IIEDSTDFFKSGASSMDVARLVEEIRQKCGGLQLQNEDVYMATKFEGFIQKVVRKLRGED
QEVELVVDYISKEVNEIMVKMPYQCFINGQFTDADDGKTYDTINPTDGSTICKVSYASLA
DVDKAVAAAKDAFENGEWGRMNARERGRLMYRLADLLEENQEELATIEALDSGAVYTLAL
KTHIGMSVQTFRYFAGWCDKIQGSTIPINQARPNRNLTFTKKEPLGVCAIIIPWNYPLMM
LAWKSAACLAAGNTLVLKPAQVTPLTALKFAELSVKAGFPKGVINIIPGSGGIAGQRLSE
HPDIRKLGFTGSTPIGKQIMKSCAVSNLKKVSLELGGKSPLIIFNDCELDKAVRMGMGAV
FFNKGENCIAAGRLFVEESIHDEFVTRVVEEIKKMKIGDPLDRSTDHGPQNHKAHLEKLL
QYCETGVKEGATLVYGGRQVQRPGFFMEPTVFTDVEDYMYLAKEESFGPIMVISKFQNGD
IDGVLQRANSTEYGLASGVFTRDINKAMYVSEKLEAGTVFINTYNKTDVAAPFGGVKQSG
FGKDLGEEALNEYLKTKTVTLEY
>sp|Q53TS8|AL2SA_HUMAN Amyotrophic lateral sclerosis 2 chromosomal region candidate gene 11 protein OS=Homo sapiens GN=ALS2CR11 PE=1 SV=1
MEPPQETNRPFSTLDNRSGQVQVLSATPLLQRNPYSSPDIMHIKGSEASSVPYALNQGTT
ALPKNKNQEGTGHRLLNMLRKTLKESDSEELEITQETPNLVPFGDVVGCLGIHIKNCRHF
MPKISLQHYANLFIRISINKAVKCTKMCSLLSKNDEKNTVIKFDEVKYFSVQVPRRYDDK
RNNILLELIQYDNREKRAFLLGSVQIHLYEVIQKGCFIEEVQVLHGNIFVCRLEVEFMFS
YGNFGYGFSHQLKPLQKITEPSMFMNLAPPPERTDPVTKVITPQTVEYPAFLSPDLNVTV
GTPAVQSSNQPSVVRLEKLQQQPRERLEKMKKEYRNLNTWIDKANYLESILMPKLEHKDS
EETNIDEASENTKSNHPEEELENIVGVDIPLVNEEAETTANELLDNDSEKGLTIPTLNQS
DQDNSTADASKNDESTPSPTEVHSLCTISNQETIKAGRIPPLGERQSESMPDRKMKNVFF
PLEVKLKDNYPSILKADSSLSEVAFSPKEYNSPSFRPEYIEFKPKFQDCSDKFEDLHDMT
SFTHLKKVKSRSRLLGKSSDDIHNHARHSARPYTAPEVNKQRESYSGKFTSRRMVSSGLV
HINDKTSDYEMHKMRPKKIKRGY
>sp|P30838|AL3A1_HUMAN Aldehyde dehydrogenase, dimeric NADP-preferring OS=Homo sapiens GN=ALDH3A1 PE=1 SV=3
MSKISEAVKRARAAFSSGRTRPLQFRIQQLEALQRLIQEQEQELVGALAADLHKNEWNAY
YEEVVYVLEEIEYMIQKLPEWAADEPVEKTPQTQQDELYIHSEPLGVVLVIGTWNYPFNL
TIQPMVGAIAAGNSVVLKPSELSENMASLLATIIPQYLDKDLYPVINGGVPETTELLKER
FDHILYTGSTGVGKIIMTAAAKHLTPVTLELGGKSPCYVDKNCDLDVACRRIAWGKFMNS
GQTCVAPDYILCDPSIQNQIVEKLKKSLKEFYGEDAKKSRDYGRIISARHFQRVMGLIEG

QKVAYGGTGDAATRYIAPTILTDVDPQSPVMQEEIFGPVLPIVCVRSLEEAIQFINQREK
PLALYMFSSNDKVIKKMIAETSSGGVAANDVIVHITLHSLPFGGVGNSGMGSYHGKKSFE
TFSHRRSCLVRPLMNDEGLKVRYPPSPAKMTQH
>sp|P49189|AL9A1_HUMAN 4-trimethylaminobutyraldehyde dehydrogenase OS=Homo sapiens GN=ALDH9A1 PE=1 SV=3
MSTGTFVVSQPLNYRGGARVEPADASGTEKAFEPATGRVIATFTCSGEKEVNLAVQNAKA
AFKIWSQKSGMERCRILLEAARIIREREDEIATMECINNGKSIFEARLDIDISWQCLEYY
AGLAASMAGEHIQLPGGSFGYTRREPLGVCVGIGAWNYPFQIASWKSAPALACGNAMVFK
PSPFTPVSALLLAEIYSEAGVPPGLFNVVQGGAATGQFLCQHPDVAKVSFTGSVPTGMKI
MEMSAKGIKPVTLELGGKSPLIIFSDCDMNNAVKGALMANFLTQGQVCCNGTRVFVQKEI
LDKFTEEVVKQTQRIKIGDPLLEDTRMGPLINRPHLERVLGFVKVAKEQGAKVLCGGDIY
VPEDPKLKDGYYMRPCVLTNCRDDMTCVKEEIFGPVMSILSFDTEAEVLERANDTTFGLA
AGVFTRDIQRAHRVVAELQAGTCFINNYNVSPVELPFGGYKKSGFGRENGRVTIEYYSQL
KTVCVEMGDVESAF
>sp|Q13686|ALKB1_HUMAN DNA demethylase ALKBH1 OS=Homo sapiens GN=ALKBH1 PE=1 SV=2
MGKMAAAVGSVATLATEPGEDAFRKLFRFYRQSRPGTADLEGVIDFSAAHAARGKGPGAQ
KVIKSQLNVSSVSEQNAYRAGLQPVSKWQAYGLKGYPGFIFIPNPFLPGYQWHWVKQCLK
LYSQKPNVCNLDKHMSKEETQDLWEQSKEFLRYKEATKRRPRSLLEKLRWVTVGYHYNWD
SKKYSADHYTPFPSDLGFLSEQVAAACGFEDFRAEAGILNYYRLDSTLGIHVDRSELDHS
KPLLSFSFGQSAIFLLGGLQRDEAPTAMFMHSGDIMIMSGFSRLLNHAVPRVLPNPEGEG
LPHCLEAPLPAVLPRDSMVEPCSMEDWQVCASYLKTARVNMTVRQVLATDQNFPLEPIED
EKRDISTEGFCHLDDQNSEVKRARINPDS
>sp|Q9UM73|ALK_HUMAN ALK tyrosine kinase receptor OS=Homo sapiens GN=ALK PE=1 SV=3
MGAIGLLWLLPLLLSTAAVGSGMGTGQRAGSPAAGPPLQPREPLSYSRLQRKSLAVDFVV
PSLFRVYARDLLLPPSSSELKAGRPEARGSLALDCAPLLRLLGPAPGVSWTAGSPAPAEA
RTLSRVLKGGSVRKLRRAKQLVLELGEEAILEGCVGPPGEAAVGLLQFNLSELFSWWIRQ
GEGRLRIRLMPEKKASEVGREGRLSAAIRASQPRLLFQIFGTGHSSLESPTNMPSPSPDY
FTWNLTWIMKDSFPFLSHRSRYGLECSFDFPCELEYSPPLHDLRNQSWSWRRIPSEEASQ
MDLLDGPGAERSKEMPRGSFLLLNTSADSKHTILSPWMRSSSEHCTLAVSVHRHLQPSGR
YIAQLLPHNEAAREILLMPTPGKHGWTVLQGRIGRPDNPFRVALEYISSGNRSLSAVDFF
ALKNCSEGTSPGSKMALQSSFTCWNGTVLQLGQACDFHQDCAQGEDESQMCRKLPVGFYC
NFEDGFCGWTQGTLSPHTPQWQVRTLKDARFQDHQDHALLLSTTDVPASESATVTSATFP
APIKSSPCELRMSWLIRGVLRGNVSLVLVENKTGKEQGRMVWHVAAYEGLSLWQWMVLPL
LDVSDRFWLQMVAWWGQGSRAIVAFDNISISLDCYLTISGEDKILQNTAPKSRNLFERNP
NKELKPGENSPRQTPIFDPTVHWLFTTCGASGPHGPTQAQCNNAYQNSNLSVEVGSEGPL
KGIQIWKVPATDTYSISGYGAAGGKGGKNTMMRSHGVSVLGIFNLEKDDMLYILVGQQGE
DACPSTNQLIQKVCIGENNVIEEEIRVNRSVHEWAGGGGGGGGATYVFKMKDGVPVPLII
AAGGGGRAYGAKTDTFHPERLENNSSVLGLNGNSGAAGGGGGWNDNTSLLWAGKSLQEGA
TGGHSCPQAMKKWGWETRGGFGGGGGGCSSGGGGGGYIGGNAASNNDPEMDGEDGVSFIS
PLGILYTPALKVMEGHGEVNIKHYLNCSHCEVDECHMDPESHKVICFCDHGTVLAEDGVS
CIVSPTPEPHLPLSLILSVVTSALVAALVLAFSGIMIVYRRKHQELQAMQMELQSPEYKL
SKLRTSTIMTDYNPNYCFAGKTSSISDLKEVPRKNITLIRGLGHGAFGEVYEGQVSGMPN
DPSPLQVAVKTLPEVCSEQDELDFLMEALIISKFNHQNIVRCIGVSLQSLPRFILLELMA
GGDLKSFLRETRPRPSQPSSLAMLDLLHVARDIACGCQYLEENHFIHRDIAARNCLLTCP

GPGRVAKIGDFGMARDIYRASYYRKGGCAMLPVKWMPPEAFMEGIFTSKTDTWSFGVLLW
EIFSLGYMPYPSKSNQEVLEFVTSGGRMDPPKNCPGPVYRIMTQCWQHQPEDRPNFAIIL
ERIEYCTQDPDVINTALPIEYGPLVEEEEKVPVRPKDPEGVPPLLVSQQAKREEERSPAA
PPPLPTTSSGKAAKKPTAAEISVRVPRGPAVEGGHVNMAFSQSNPPSELHKVHGSRNKPT
SLWNPTYGSWFTEKPTKKNNPIAKKEPHDRGNLGLEGSCTVPPNVATGRLPGASLLLEPS
SLTANMKEVPLFRLRHFPCGNVNYGYQQQGLPLEAATAPGAGHYEDTILKSKNSMNQPGP
>sp|Q8N6M5|ALLC_HUMAN Probable allantoicase OS=Homo sapiens GN=ALLC PE=2 SV=3
MADAPKEGRLTRFLDFTQLMDMASESVGGKILFATDDFFAPAENLIKSDSPCFKEHEYTE
FGKWMDGWETRRKRIPGHDWCVLRLGIQGVIRGFDVDVSYFTGDYAPRVSIQAANLEEDK
LPEIPERGTRTGAAATPEEFEAIAELKSDDWSYLVPMTELKPGNPASGHNYFLVNSQQRW
THIRLNIFPDGGIARLRVFGTGQKDWTATDPKEPADLVAIAFGGVCVGFSNAKFGHPNNI
IGVGGAKSMADGWETARRLDRPPILENDENGILLVPGCEWAVFRLAHPGVITRIEIDTKY
FEGNAPDSCKVDGCILTTQEEEAVIRQKWILPAHKWKPLLPVTKLSPNQSHLFDSLTLEL
QDVITHARLTIVPDGGVSRLRLRGFPSSICLLRPREKPMLKFSVSFKANP
>sp|O95076|ALX3_HUMAN Homeobox protein aristaless-like 3 OS=Homo sapiens GN=ALX3 PE=1 SV=2
MDPEHCAPFRVGPAPGPYVASGDEPPGPQGTPAAAPHLHPAPPRGPRLTRFPACGPLEPY
LPEPAKPPAKYLQDLGPGPALNGGHFYEGPAEAEEKTSKAASFPQLPLDCRGGPRDGPSN
LQGSPGPCLASLHLPLSPGLPDSMELAKNKSKKRRNRTTFSTFQLEELEKVFQKTHYPDV
YAREQLALRTDLTEARVQVWFQNRRAKWRKRERYGKIQEGRNPFTAAYDISVLPRTDSHP
QLQNSLWASPGSGSPGGPCLVSPEGIPSPCMSPYSHPHGSVAGFMGVPAPSAAHPGIYSI
HGFPPTLGGHSFEPSSDGDYKSPSLVSLRVKPKEPPGLLNWTT
>sp|Q9UHK6|AMACR_HUMAN Alpha-methylacyl-CoA racemase OS=Homo sapiens GN=AMACR PE=1 SV=2
MALQGISVVELSGLAPGPFCAMVLADFGARVVRVDRPGSRYDVSRLGRGKRSLVLDLKQP
RGAAVLRRLCKRSDVLLEPFRRGVMEKLQLGPEILQRENPRLIYARLSGFGQSGSFCRLA
GHDINYLALSGVLSKIGRSGENPYAPLNLLADFAGGGLMCALGIIMALFDRTRTGKGQVI
DANMVEGTAYLSSFLWKTQKLSLWEAPRGQNMLDGGAPFYTTYRTADGEFMAVGAIEPQF
YELLIKGLGLKSDELPNQMSMDDWPEMKKKFADVFAEKTKAEWCQIFDGTDACVTPVLTF
EEVVHHDHNKERGSFITSEEQDVSPRPAPLLLNTPAIPSFKRDPFIGEHTEEILEEFGFS
REEIYQLNSDKIIESNKVKASL
>sp|Q9Y2J4|AMOL2_HUMAN Angiomotin-like protein 2 OS=Homo sapiens GN=AMOTL2 PE=1 SV=3
MRTLEDSSGTVLHRLIQEQLRYGNLTETRTLLAIQQQALRGGAGTGGTGSPQASLEILAP
EDSQVLQQATRQEPQGQEHQGGENHLAENTLYRLCPQPSKGEELPTYEEAKAHSQYYAAQ
QAGTRPHAGDRDPRGAPGGSRRQDEALRELRHGHVRSLSERLLQLSLERNGARAPSHMSS
SHSFPQLARNQQGPPLRGPPAEGPESRGPPPQYPHVVLAHETTTAVTDPRYRARGSPHFQ
HAEVRILQAQVPPVFLQQQQQYQYLQQSQEHPPPPHPAALGHGPLSSLSPPAVEGPVSAQ
ASSATSGSAHLAQMEAVLRENARLQRDNERLQRELESSAEKAGRIEKLESEIQRLSEAHE
SLTRASSKREALEKTMRNKMDSEMRRLQDFNRDLRERLESANRRLASKTQEAQAGSQDMV
AKLLAQSYEQQQEQEKLEREMALLRGAIEDQRRRAELLEQALGNAQGRAARAEEELRKKQ
AYVEKVERLQQALGQLQAACEKREQLELRLRTRLEQELKALRAQQRQAGAPGGSSGSGGS
PELSALRLSEQLREKEEQILALEADMTKWEQKYLEERAMRQFAMDAAATAAAQRDTTLIR
HSPQPSPSSSFNEGLLTGGHRHQEMESRLKVLHAQILEKDAVIKVLQQRSRRDPGKAIQG
SLRPAKSVPSVFAAAAAGTQGWQGLSSSERQTADAPARLTTDRAPTEEPVVTAPPAAHAK
HGSRDGSTQTEGPPDSTSTCLPPEPDSLLGCSSSQRAASLDSVATSRVQDLSDMVEILI

>sp|P01160|ANF_HUMAN Natriuretic peptides A OS=Homo sapiens GN=NPPA PE=1 SV=1
MSSFSTTTVSFLLLLAFQLLGQTRANPMYNAVSNADLMDFKNLLDHLEEKMPLEDEVVPP
QVLSEPNEEAGAALSPLPEVPPWTGEVSPAQRDGGALGRGPWDSSDRSALLKSKLRALLT
APRSLRRSSCFGGRMDRIGAQSGLGCNSFRYRR
>sp|O95841|ANGL1_HUMAN Angiopoietin-related protein 1 OS=Homo sapiens GN=ANGPTL1 PE=2 SV=1
MKTFTWTLGVLFFLLVDTGHCRGGQFKIKKINQRRYPRATDGKEEAKKCAYTFLVPEQRI
TGPICVNTKGQDASTIKDMITRMDLENLKDVLSRQKREIDVLQLVVDVDGNIVNEVKLLR
KESRNMNSRVTQLYMQLLHEIIRKRDNSLELSQLENKILNVTTEMLKMATRYRELEVKYA
SLTDLVNNQSVMITLLEEQCLRIFSRQDTHVSPPLVQVVPQHIPNSQQYTPGLLGGNEIQ
RDPGYPRDLMPPPDLATSPTKSPFKIPPVTFINEGPFKDCQQAKEAGHSVSGIYMIKPEN
SNGPMQLWCENSLDPGGWTVIQKRTDGSVNFFRNWENYKKGFGNIDGEYWLGLENIYMLS
NQDNYKLLIELEDWSDKKVYAEYSSFRLEPESEFYRLRLGTYQGNAGDSMMWHNGKQFTT
LDRDKDMYAGNCAHFHKGGWWYNACAHSNLNGVWYRGGHYRSKHQDGIFWAEYRGGSYSL
RAVQMMIKPID
>sp|Q6UXH0|ANGL8_HUMAN Angiopoietin-like protein 8 OS=Homo sapiens GN=ANGPTL8 PE=1 SV=1
MPVPALCLLWALAMVTRPASAAPMGGPELAQHEELTLLFHGTLQLGQALNGVYRTTEGRL
TKARNSLGLYGRTIELLGQEVSRGRDAAQELRASLLETQMEEDILQLQAEATAEVLGEVA
QAQKVLRDSVQRLEVQLRSAWLGPAYREFEVLKAHADKQSHILWALTGHVQRQRREMVAQ
QHRLRQIQERLHTAALPA
>sp|Q15389|ANGP1_HUMAN Angiopoietin-1 OS=Homo sapiens GN=ANGPT1 PE=1 SV=2
MTVFLSFAFLAAILTHIGCSNQRRSPENSGRRYNRIQHGQCAYTFILPEHDGNCRESTTD
QYNTNALQRDAPHVEPDFSSQKLQHLEHVMENYTQWLQKLENYIVENMKSEMAQIQQNAV
QNHTATMLEIGTSLLSQTAEQTRKLTDVETQVLNQTSRLEIQLLENSLSTYKLEKQLLQQ
TNEILKIHEKNSLLEHKILEMEGKHKEELDTLKEEKENLQGLVTRQTYIIQELEKQLNRA
TTNNSVLQKQQLELMDTVHNLVNLCTKEGVLLKGGKREEEKPFRDCADVYQAGFNKSGIY
TIYINNMPEPKKVFCNMDVNGGGWTVIQHREDGSLDFQRGWKEYKMGFGNPSGEYWLGNE
FIFAITSQRQYMLRIELMDWEGNRAYSQYDRFHIGNEKQNYRLYLKGHTGTAGKQSSLIL
HGADFSTKDADNDNCMCKCALMLTGGWWFDACGPSNLNGMFYTAGQNHGKLNGIKWHYFK
GPSYSLRSTTMMIRPLDF
>sp|Q8IWZ3|ANKH1_HUMAN Ankyrin repeat and KH domain-containing protein 1 OS=Homo sapiens GN=ANKHD1 PE=1 SV=1
MLTDSGGGGTSFEEDLDSVAPRSAPAGASEPPPPGGVGLGIRTVRLFGEAGPASGVGSSG
GGGSGSGTGGGDAALDFKLAAAVLRTGGGGGASGSDEDEVSEVESFILDQEDLDNPVLKT
TSEIFLSSTAEGADLRTVDPETQARLEALLEAAGIGKLSTADGKAFADPEVLRRLTSSVS
CALDEAAAALTRMKAENSHNAGQVDTRSLAEACSDGDVNAVRKLLDEGRSVNEHTEEGES
LLCLACSAGYYELAQVLLAMHANVEDRGNKGDITPLMAASSGGYLDIVKLLLLHDADVNS
QSATGNTALTYACAGGFVDIVKVLLNEGANIEDHNENGHTPLMEAASAGHVEVARVLLDH
GAGINTHSNEFKESALTLACYKGHLDMVRFLLEAGADQEHKTDEMHTALMEACMDGHVEV
ARLLLDSGAQVNMPADSFESPLTLAACGGHVELAALLIERGANLEEVNDEGYTPLMEAAR
EGHEEMVALLLAQGANINAQTEETQETALTLACCGGFSEVADFLIKAGADIELGCSTPLM
EASQEGHLELVKYLLASGANVHATTATGDTALTYACENGHTDVADVLLQAGADLEHESEG
GRTPLMKAARAGHLCTVQFLISKGANVNRATANNDHTVVSLACAGGHLAVVELLLAHGAD
PTHRLKDGSTMLIEAAKGGHTNVVSYLLDYPNNVLSVPTTDVSQLPPPSQDQSQVPRVPT

HTLAMVVPPQEPDRTSQENSPALLGVQKGTSKQKSSSLQVADQDLLPSFHPYQPLECIVE
ETEGKLNELGQRISAIEKAQLKSLELIQGEPLNKDKIEELKKNREEQVQKKKKILKELQK
VERQLQMKTQQQFTKEYLETKGQKDTVSLHQQCSHRGVFPEGEGDGSLPEDHFSELPQVD
TILFKDNDVDDEQQSPPSAEQIDFVPVQPLSSPQCNFSSDLGSNGTNSLELQKVSGNQQI
VGQPQIAITGHDQGLLVQEPDGLMVATPAQTLTDTLDDLIAAVSTRVPTGSNSSSQTTEC
LTPESCSQTTSNVASQSMPPVYPSVDIDAHTESNHDTALTLACAGGHEELVSVLIARDAK
IEHRDKKGFTPLILAATAGHVGVVEILLDKGGDIEAQSERTKDTPLSLACSGGRQEVVDL
LLARGANKEHRNVSDYTPLSLAASGGYVNIIKILLNAGAEINSRTGSKLGISPLMLAAMN
GHVPAVKLLLDMGSDINAQIETNRNTALTLACFQGRAEVVSLLLDRKANVEHRAKTGLTP
LMEAASGGYAEVGRVLLDKGADVNAPPVPSSRDTALTIAADKGHYKFCELLIHRGAHIDV
RNKKGNTPLWLASNGGHFDVVQLLVQAGADVDAADNRKITPLMSAFRKGHVKVVQYLVKE
VNQFPSDIECMRYIATITDKELLKKCHQCVETIVKAKDQQAAEANKNASILLKELDLEKS
REESRKQALAAKREKRKEKRKKKKEEQKRKQEEDEENKPKENSELPEDEDEEENDEDVEQ
EVPIEPPSATTTTTIGISATSATFTNVFGKKRANVVTTPSTNRKNKKNKTKETPPTAHLI
LPEQHMSLAQQKADKNKINGEPRGGGAGGNSDSDNLDSTDCNSESSSGGKSQELNFVMDV
NSSKYPSLLLHSQEEKTSTATSKTQTRLEGEVTPNSLSTSYKTVSLPLSSPNIKLNLTSP
KRGQKREEGWKEVVRRSKKLSVPASVVSRIMGRGGCNITAIQDVTGAHIDVDKQKDKNGE
RMITIRGGTESTRYAVQLINALIQDPAKELEDLIPKNHIRTPASTKSIHANFSSGVGTTA
ASSKNAFPLGAPTLVTSQATTLSTFQPANKLNKNVPTNVRSSFPVSLPLAYPHPHFALLA
AQTMQQIRHPRLPMAQFGGTFSPSPNTWGPFPVRPVNPGNTNSSPKHNNTSRLPNQNGTV
LPSESAGLATASCPITVSSVVAASQQLCVTNTRTPSSVRKQLFACVPKTSPPATVISSVT
STCSSLPSVSSAPITSGQAPTTFLPASTSQAQLSSQKMESFSAVPPTKEKVSTQDQPMAN
LCTPSSTANSCSSSASNTPGAPETHPSSSPTPTSSNTQEEAQPSSVSDLSPMSMPFASNS
EPAPLTLTSPRMVAADNQDTSNLPQLAVPAPRVSHRMQPRGSFYSMVPNATIHQDPQSIF
VTNPVTLTPPQGPPAAVQLSSAVNIMNGSQMHINPANKSLPPTFGPATLFNHFSSLFDSS
QVPANQGWGDGPLSSRVATDASFTVQSAFLGNSVLGHLENMHPDNSKAPGFRPPSQRVST
SPVGLPSIDPSGSSPSSSSAPLASFSGIPGTRVFLQGPAPVGTPSFNRQHFSPHPWTSAS
NSSTSAPPTLGQPKGVSASQDRKIPPPIGTERLARIRQGGSVAQAPAGTSFVAPVGHSGI
WSFGVNAVSEGLSGWSQSVMGNHPMHQQLSDPSTFSQHQPMERDDSGMVAPSNIFHQPMA
SGFVDFSKGLPISMYGGTIIPSHPQLADVPGGPLFNGLHNPDPAWNPMIKVIQNSTECTD
AQQIWPGTWAPHIGNMHLKYVN
>sp|Q9Y2G4|ANKR6_HUMAN Ankyrin repeat domain-containing protein 6 OS=Homo sapiens GN=ANKRD6 PE=2 SV=3
MSQQDAVAALSERLLVAAYKGQTENVVQLINKGARVAVTKHGRTPLHLAANKGHLPVVQI
LLKAGCDLDVQDDGDQTALHRATVVGNTEIIAALIHEGCALDRQDKDGNTALHEASWHGF
SQSAKLLIKAGANVLAKNKAGNTALHLACQNSHSQSTRVLLLAGSRADLKNNAGDTCLHV
AARYNHLSIIRLLLTAFCSVHEKNQAGDTALHVAAALNHKKVAKILLEAGADTTIVNNAG
QTPLETARYHNNPEVALLLTKAPQVLRFSRGRSLRKKRERLKEERRAQSVPRDEVAQSKG
SVSAGDTPSSEQAVARKEEAREEFLSASPEPRAKDDRRRKSRPKVSAFSDPTPPADQQPG
HQKNLHAHNHPKKRNRHRCSSPPPPHEFRAYQLYTLYRGKDGKVMQAPINGCRCEPLINK
LENQLEATVEEIKAELGSVQDKMNTKLGQMENKTQHQMRVLDKLMVERLSAERTECLNRL
QQHSDTEKHEGEKRQISLVDELKTWCMLKIQNLEQKLSGDSRACRAKSTPSTCESSTGVD
QLVVTAGPAAASDSSPPVVRPKEKALNSTATQRLQQELSSSDCTGSRLRNVKVQTALLPM
NEAARSDQQAGPCVNRGTQTKKSGKSGPTRHRAQQPAASSTCGQPPPATGSEQTGPHIRD

TSQALELTQYFFEAVSTQMEKWYERKIEEARSQANQKAQQDKATLKEHIKSLEEELAKLR
TRVQKEN
>sp|Q92527|ANKR7_HUMAN Ankyrin repeat domain-containing protein 7 OS=Homo sapiens GN=ANKRD7 PE=2 SV=3
MNKLFSFWKRKNETRSQGYNLREKDLKKLHRAASVGDLKKLKEYLQIKKYDVNMQDKKYR
TPLHLACANGHTDVVLFLIEQQCKINVRDSENKSPLIKAVQCQNEDCATILLNFGADPDL
RDIRYNTVLHYAVCGQSLSLVEKLLEYEADLEAKNKDGYTPLLVAVINNNPKMVKFLLEK
GADVNASDNYQRTALILAVSGEPPCLVKLLLQQGVELCYEGIVDSQLRNMFISMVLLHRY
PQFTASHGKKKHAK
>sp|A9YTQ3|AHRR_HUMAN Aryl hydrocarbon receptor repressor OS=Homo sapiens GN=AHRR PE=1 SV=3
MPRTMIPPGECTYAGRKRRRPLQKQRPAVGAEKSNPSKRHRDRLNAELDHLASLLPFPPD
IISKLDKLSVLRLSVSYLRVKSFFQVVQEQSSRQPAAGAPSPGDSCPLAGSAVLEGRLLL
ESLNGFALVVSAEGTIFYASATIVDYLGFHQTDVMHQNIYDYIHVDDRQDFCRQLHWAMD
PPQVVFGQPPPLETGDDAILGRLLRAQEWGTGTPTEYSAFLTRCFICRVRCLLDSTSGFL
TMQFQGKLKFLFGQKKKAPSGAMLPPRLSLFCIAAPVLLPSAAEMKMRSALLRAKPRADT
AATADAKVKATTSLCESELHGKPNYSAGRSSRESGVLVLREQTDAGRWAQVPARAPCLCL
RGGPDLVLDPKGGSGDREEEQHRMLSRASGVTGRRETPGPTKPLPWTAGKHSEDGARPRL
QPSKNDPPSLRPMPRGSCLPCPCVQGTFRNSPISHPPSPSPSAYSSRTSRPMRDVGEDQV
HPPLCHFPQRSLQHQLPQPGAQRFATRGYPMEDMKLQGVPMPPGDLCGPTLLLDVSIKME
KDSGCEGAADGCVPSQVWLGASDRSHPATFPTRMHLKTEPDSRQQVYISHLGHGVRGAQP
HGRATAGRSRELTPFHPAHCACLEPTDGLPQSEPPHQLCARGRGEQSCTCRAAEAAPVVK
REPLDSPQWATHSQGMVPGMLPKSALATLVPPQASGCTFLP
>sp|Q9NZD4|AHSP_HUMAN Alpha-hemoglobin-stabilizing protein OS=Homo sapiens GN=AHSP PE=1 SV=1
MALLKANKDLISAGLKEFSVLLNQQVFNDPLVSEEDMVTVVEDWMNFYINYYRQQVTGEP
QERDKALQELRQELNTLANPFLAKYRDFLKSHELPSHPPPSS
>sp|Q96BJ3|AIDA_HUMAN Axin interactor, dorsalization-associated protein OS=Homo sapiens GN=AIDA PE=1 SV=1
MSEVTRSLLQRWGASFRRGADFDSWGQLVEAIDEYQILARHLQKEAQAQHNNSEFTEEQK
KTIGKIATCLELRSAALQSTQSQEEFKLEDLKKLEPILKNILTYNKEFPFDVQPVPLRRI
LAPGEEENLEFEEDEEEGGAGAGSPDSFPARVPGTLLPRLPSEPGMTLLTIRIEKIGLKD
AGQCIDPYITVSVKDLNGIDLTPVQDTPVASRKEDTYVHFNVDIELQKHVEKLTKGAAIF
FEFKHYKPKKRFTSTKCFAFMEMDEIKPGPIVIELYKKPTDFKRKKLQLLTKKPLYLHLH
QTLHKE
>sp|P51857|AK1D1_HUMAN 3-oxo-5-beta-steroid 4-dehydrogenase OS=Homo sapiens GN=AKR1D1 PE=1 SV=1
MDLSAASHRIPLSDGNSIPIIGLGTYSEPKSTPKGACATSVKVAIDTGYRHIDGAYIYQN
EHEVGEAIREKIAEGKVRREDIFYCGKLWATNHVPEMVRPTLERTLRVLQLDYVDLYIIE
VPMAFKPGDEIYPRDENGKWLYHKSNLCATWEAMEACKDAGLVKSLGVSNFNRRQLELIL
NKPGLKHKPVSNQVECHPYFTQPKLLKFCQQHDIVITAYSPLGTSRNPIWVNVSSPPLLK
DALLNSLGKRYNKTAAQIVLRFNIQRGVVVIPKSFNLERIKENFQIFDFSLTEEEMKDIE
ALNKNVRFVELLMWRDHPEYPFHDEY
>sp|Q86UN6|AKA28_HUMAN A-kinase anchor protein 14 OS=Homo sapiens GN=AKAP14 PE=1 SV=1

MSETQNSTSQKAMDEDNKAASQTMPNTQDKNYEDELTQVALALVEDVINYAVKIVEEERN
PLKNIKWMTHGEFTVEKGLKQIDEYFSKCVSKKCWAHGVEFVERKDLIHSFLYIYYVHWS
ISTADLPVARISAGTYFTMKVSKTKPPDAPIVVSYVGDHQALVHRPGMVRFRENWQKNLT
DAKYSFMESFPPFLFNRV
>sp|Q9Y243|AKT3_HUMAN RAC-gamma serine/threonine-protein kinase OS=Homo sapiens GN=AKT3 PE=1 SV=1
MSDVTIVKEGWVQKRGEYIKNWRPRYFLLKTDGSFIGYKEKPQDVDLPYPLNNFSVAKCQ
LMKTERPKPNTFIIRCLQWTTVIERTFHVDTPEEREEWTEAIQAVADRLQRQEEERMNCS
PTSQIDNIGEEEMDASTTHHKRKTMNDFDYLKLLGKGTFGKVILVREKASGKYYAMKILK
KEVIIAKDEVAHTLTESRVLKNTRHPFLTSLKYSFQTKDRLCFVMEYVNGGELFFHLSRE
RVFSEDRTRFYGAEIVSALDYLHSGKIVYRDLKLENLMLDKDGHIKITDFGLCKEGITDA
ATMKTFCGTPEYLAPEVLEDNDYGRAVDWWGLGVVMYEMMCGRLPFYNQDHEKLFELILM
EDIKFPRTLSSDAKSLLSGLLIKDPNKRLGGGPDDAKEIMRHSFFSGVNWQDVYDKKLVP
PFKPQVTSETDTRYFDEEFTAQTITITPPEKYDEDGMDCMDNERRPHFPQFSYSASGRE
>sp|Q8N8R7|AL14E_HUMAN ARL14 effector protein OS=Homo sapiens GN=ARL14EP PE=1 SV=1
MMDPCSVGVQLRTTNECHKTYYTRHTGFKTLQELSSNDMLLLQLRTGMTLSGNNTICFHH
VKIYIDRFEDLQKSCCDPFNIHKKLAKKNLHVIDLDDATFLSAKFGRQLVPGWKLCPKCT
QIINGSVDVDTEDRQKRKPESDGRTAKALRSLQFTNPGRQTEFAPETGKREKRRLTKNAT
AGSDRQVIPAKSKVYDSQGLLIFSGMDLCDCLDEDCLGCFYACPACGSTKCGAECRCDRK
WLYEQIEIEGGEIIHNKHAG
>sp|O94788|AL1A2_HUMAN Retinal dehydrogenase 2 OS=Homo sapiens GN=ALDH1A2 PE=1 SV=3
MTSSKIEMPGEVKADPAALMASLHLLPSPTPNLEIKYTKIFINNEWQNSESGRVFPVYNP
ATGEQVCEVQEADKADIDKAVQAARLAFSLGSVWRRMDASERGRLLDKLADLVERDRAVL
ATMESLNGGKPFLQAFYVDLQGVIKTFRYYAGWADKIHGMTIPVDGDYFTFTRHEPIGVC
GQIIPWNFPLLMFAWKIAPALCCGNTVVIKPAEQTPLSALYMGALIKEAGFPPGVINILP
GYGPTAGAAIASHIGIDKIAFTGSTEVGKLIQEAAGRSNLKRVTLELGGKSPNIIFADAD
LDYAVEQAHQGVFFNQGQCCTAGSRIFVEESIYEEFVRRSVERAKRRVVGSPFDPTTEQG
PQIDKKQYNKILELIQSGVAEGAKLECGGKGLGRKGFFIEPTVFSNVTDDMRIAKEEIFG
PVQEILRFKTMDEVIERANNSDFGLVAAVFTNDINKALTVSSAMQAGTVWINCYNALNAQ
SPFGGFKMSGNGREMGEFGLREYSEVKTVTVKIPQKNS
>sp|P47895|AL1A3_HUMAN Aldehyde dehydrogenase family 1 member A3 OS=Homo sapiens GN=ALDH1A3 PE=1 SV=2
MATANGAVENGQPDRKPPALPRPIRNLEVKFTKIFINNEWHESKSGKKFATCNPSTREQI
CEVEEGDKPDVDKAVEAAQVAFQRGSPWRRLDALSRGRLLHQLADLVERDRATLAALETM
DTGKPFLHAFFIDLEGCIRTLRYFAGWADKIQGKTIPTDDNVVCFTRHEPIGVCGAITPW
NFPLLMLVWKLAPALCCGNTMVLKPAEQTPLTALYLGSLIKEAGFPPGVVNIVPGFGPTV
GAAISSHPQINKIAFTGSTEVGKLVKEAASRSNLKRVTLELGGKNPCIVCADADLDLAVE
CAHQGVFFNQGQCCTAASRVFVEEQVYSEFVRRSVEYAKKRPVGDPFDVKTEQGPQIDQK
QFDKILELIESGKKEGAKLECGGSAMEDKGLFIKPTVFSEVTDNMRIAKEEIFGPVQPIL
KFKSIEEVIKRANSTDYGLTAAVFTKNLDKALKLASALESGTVWINCYNALYAQAPFGGF
KMSGNGRELGEYALAEYTEVKTVTIKLGDKNP
>sp|Q96Q35|AL2SB_HUMAN Amyotrophic lateral sclerosis 2 chromosomal region candidate gene 12 protein OS=Homo sapiens GN=ALS2CR12 PE=1 SV=2
MYPNPLIYCTCWDPWNLGPRKLIKTPQLPRKNSTGSSKLTPLVPAPKNHNYLQPTKPVVS

PKMKIHSARQEETNKSFYEVINVSPGYQLVRNREQISVTLGDEMFDRKKRWESEIPDKGR
FSRTNIISDLEEQISELTAIIEQMNRDHQSAQKLLSSEMDLRCAEMKQNFENKNRELKEA
HEAELSELENNYKAALKAEKLAAQEKLEEMGKEYKYLKNMFRTYQDSIYDEMEEKWSKQK
AKWKKDEKFERENILLQQKKKMTKKFEMESGEEDKKINESCSAVFENFIQEKEELLKQHQ
SDTLQLEELRKTKEVPWRRDQINRHWHDVLQQLLLMQVMQEELHAQALILESLNTNLYYT
QLELQKEKAIVGNLEKMLQTKFAETEEKYKHTIQILTEENIHLKQKIISKNEEICEGCSG
RLASITVSKDDSDTVQDGSKKGQES
>sp|P30038|AL4A1_HUMAN Delta-1-pyrroline-5-carboxylate dehydrogenase, mitochondrial OS=Homo sapiens GN=ALDH4A1 PE=1 SV=3
MLLPAPALRRALLSRPWTGAGLRWKHTSSLKVANEPVLAFTQGSPERDALQKALKDLKGR
MEAIPCVVGDEEVWTSDVQYQVSPFNHGHKVAKFCYADKSLLNKAIEAALAARKEWDLKP
IADRAQIFLKAADMLSGPRRAEILAKTMVGQGKTVIQAEIDAAAELIDFFRFNAKYAVEL
EGQQPISVPPSTNSTVYRGLEGFVAAISPFNFTAIGGNLAGAPALMGNVVLWKPSDTAML
ASYAVYRILREAGLPPNIIQFVPADGPLFGDTVTSSEHLCGINFTGSVPTFKHLWKQVAQ
NLDRFHTFPRLAGECGGKNFHFVHRSADVESVVSGTLRSAFEYGGQKCSACSRLYVPHSL
WPQIKGRLLEEHSRIKVGDPAEDFGTFFSAVIDAKSFARIKKWLEHARSSPSLTILAGGK
CDDSVGYFVEPCIVESKDPQEPIMKEEIFGPVLSVYVYPDDKYKETLQLVDSTTSYGLTG
AVFSQDKDVVQEATKVLRNAAGNFYINDKSTGSIVGQQPFGGARASGTNDKPGGPHYILR
WTSPQVIKETHKPLGDWSYAYMQ
>sp|Q9H2A2|AL8A1_HUMAN Aldehyde dehydrogenase family 8 member A1 OS=Homo sapiens GN=ALDH8A1 PE=1 SV=1
MAGTNALLMLENFIDGKFLPCSSYIDSYDPSTGEVYCRVPNSGKDEIEAAVKAAREAFPS
WSSRSPQERSRVLNQVADLLEQSLEEFAQAESKDQGKTLALARTMDIPRSVQNFRFFASS
SLHHTSECTQMDHLGCMHYTVRAPVGVAGLISPWNLPLYLLTWKIAPAMAAGNTVIAKPS
ELTSVTAWMLCKLLDKAGVPPGVVNIVFGTGPRVGEALVSHPEVPLISFTGSQPTAERIT
QLSAPHCKKLSLELGGKNPAIIFEDANLDECIPATVRSSFANQGEICLCTSRIFVQKSIY
SEFLKRFVEATRKWKVGIPSDPLVSIGALISKAHLEKVRSYVKRALAEGAQIWCGEGVDK
LSLPARNQAGYFMLPTVITDIKDESCCMTEEIFGPVTCVVPFDSEEEVIERANNVKYGLA
ATVWSSNVGRVHRVAKKLQSGLVWTNCWLIRELNLPFGGMKSSGIGREGAKDSYDFFTEI
KTITVKH
>sp|P04075|ALDOA_HUMAN Fructose-bisphosphate aldolase A OS=Homo sapiens GN=ALDOA PE=1 SV=2
MPYQYPALTPEQKKELSDIAHRIVAPGKGILAADESTGSIAKRLQSIGTENTEENRRFYR
QLLLTADDRVNPCIGGVILFHETLYQKADDGRPFPQVIKSKGGVVGIKVDKGVVPLAGTN
GETTTQGLDGLSERCAQYKKDGADFAKWRCVLKIGEHTPSALAIMENANVLARYASICQQ
NGIVPIVEPEILPDGDHDLKRCQYVTEKVLAAVYKALSDHHIYLEGTLLKPNMVTPGHAC
TQKFSHEEIAMATVTALRRTVPPAVTGITFLSGGQSEEEASINLNAINKCPLLKPWALTF
SYGRALQASALKAWGGKKENLKAAQEEYVKRALANSLACQGKYTPSGQAGAAASESLFVS
NHAY
>sp|P09972|ALDOC_HUMAN Fructose-bisphosphate aldolase C OS=Homo sapiens GN=ALDOC PE=1 SV=2
MPHSYPALSAEQKKELSDIALRIVAPGKGILAADESVGSMAKRLSQIGVENTEENRRLYR
QVLFSADDRVKKCIGGVIFFHETLYQKDDNGVPFVRTIQDKGIVVGIKVDKGVVPLAGTD
GETTTQGLDGLSERCAQYKKDGADFAKWRCVLKISERTPSALAILENANVLARYASICQQ

NGIVPIVEPEILPDGDHDLKRCQYVTEKVLAAVYKALSDHHVYLEGTLLKPNMVTPGHAC
PIKYTPEEIAMATVTALRRTVPPAVPGVTFLSGGQSEEEASFNLNAINRCPLPRPWALTF
SYGRALQASALNAWRGQRDNAGAATEEFIKRAEVNGLAAQGKYEGSGEDGGAAAQSLYIA
NHAY
>sp|P84996|ALEX_HUMAN Protein ALEX OS=Homo sapiens GN=GNAS PE=1 SV=1
MMARPVDPQRSPDPTFRSSTRHSGKLEPMEATAHLLRKQCPSRLNSPAWEASGLHWSSLD
SPVGSMQALRPSAQHSWSPEPSVVPDQAWEDTALHQKKLCPLSLTSLPREAAVNFSYRSQ
TLLQEAQVLQGSPELLPRSPKPSGLQRLAPEEATALPLRRLCHLSLMEKDLGTTAHPRGF
PELSHKSTAAASSRQSRPRVRSASLPPRTRLPSGSQAPSAAHPKRLSDLLLTSRAAAPGW
RSPDPRSRLAAPPLGSTTLPSTWTAPQSRLTARPSRSPEPQIRESEQRDPQLRRKQQRWK
EPLMPRREEKYPLRGTDPLPPGQPQRIPLPGQPLQPQPILTPGQPQKIPTPGQHQPILTP
GHSQPIPTPGQPLPPQPIPTPGRPLTPQPIPTPGRPLTPQPIQMPGRPLRLPPPLRLLRP
GQPMSPQLRQTQGLPLPQPLLPPGQPKSAGRPLQPLPPGPDARSISDPPAPRSRLPIRLL
RGLLARLPGGASPRAAAAAACTTMKGWPAATMTPAETSPTMGPPDASAGFSIGEIAAAES
PSATYSATFSCKPSGAASVDLRVPSPKPRALSRSRRYPWRRSADRCAKKPWRSGPRSAQR
RNAVSSSTNNSRTKRWATCVRTACCF
>sp|Q9H161|ALX4_HUMAN Homeobox protein aristaless-like 4 OS=Homo sapiens GN=ALX4 PE=1 SV=2
MNAETCVSYCESPAAAMDAYYSPVSQSREGSSPFRAFPGGDKFGTTFLSAAAKAQGFGDA
KSRARYGAGQQDLATPLESGAGARGSFNKFQPQPSTPQPQPPPQPQPQQQQPQPQPPAQP
HLYLQRGACKTPPDGSLKLQEGSSGHSAALQVPCYAKESSLGEPELPPDSDTVGMDSSYL
SVKEAGVKGPQDRASSDLPSPLEKADSESNKGKKRRNRTTFTSYQLEELEKVFQKTHYPD
VYAREQLAMRTDLTEARVQVWFQNRRAKWRKRERFGQMQQVRTHFSTAYELPLLTRAENY
AQIQNPSWLGNNGAASPVPACVVPCDPVPACMSPHAHPPGSGASSVTDFLSVSGAGSHVG
QTHMGSLFGAASLSPGLNGYELNGEPDRKTSSIAALRMKAKEHSAAISWAT
>sp|P19021|AMD_HUMAN Peptidyl-glycine alpha-amidating monooxygenase OS=Homo sapiens GN=PAM PE=1 SV=2
MAGRVPSLLVLLVFPSSCLAFRSPLSVFKRFKETTRPFSNECLGTTRPVVPIDSSDFALD
IRMPGVTPKQSDTYFCMSMRIPVDEEAFVIDFKPRASMDTVHHMLLFGCNMPSSTGSYWF
CDEGTCTDKANILYAWARNAPPTRLPKGVGFRVGGETGSKYFVLQVHYGDISAFRDNNKD
CSGVSLHLTRLPQPLIAGMYLMMSVDTVIPAGEKVVNSDISCHYKNYPMHVFAYRVHTHH
LGKVVSGYRVRNGQWTLIGRQSPQLPQAFYPVGHPVDVSFGDLLAARCVFTGEGRTEATH
IGGTSSDEMCNLYIMYYMEAKHAVSFMTCTQNVAPDMFRTIPPEANIPIPVKSDMVMMHE
HHKETEYKDKIPLLQQPKREEEEVLDQGDFYSLLSKLLGEREDVVHVHKYNPTEKAESES
DLVAEIANVVQKKDLGRSDAREGAEHERGNAILVRDRIHKFHRLVSTLRPPESRVFSLQQ
PPPGEGTWEPEHTGDFHMEEALDWPGVYLLPGQVSGVALDPKNNLVIFHRGDHVWDGNSF
DSKFVYQQIGLGPIEEDTILVIDPNNAAVLQSSGKNLFYLPHGLSIDKDGNYWVTDVALH
QVFKLDPNNKEGPVLILGRSMQPGSDQNHFCQPTDVAVDPGTGAIYVSDGYCNSRIVQFS
PSGKFITQWGEESSGSSPLPGQFTVPHSLALVPLLGQLCVADRENGRIQCFKTDTKEFVR
EIKHSSFGRNVFAISYIPGLLFAVNGKPHFGDQEPVQGFVMNFSNGEIIDIFKPVRKHFD
MPHDIVASEDGTVYIGDAHTNTVWKFTLTEKLEHRSVKKAGIEVQEIKEAEAVVETKMEN
KPTSSELQKMQEKQKLIKEPGSGVPVVLITTLLVIPVVVLLAIAIFIRWKKSRAFGDSEH
KLETSSGRVLGRFRGKGSGGLNLGNFFASRKGYSRKGFDRLSTEGSDQEKEDDGSESEEE
YSAPLPALAPSSS

>sp|P01019|ANGT_HUMAN Angiotensinogen OS=Homo sapiens GN=AGT PE=1 SV=1
MRKRAPQSEMAPAGVSLRATILCLLAWAGLAAGDRVYIHPFHLVIHNESTCEQLAKANAG
KPKDPTFIPAPIQAKTSPVDEKALQDQLVLVAAKLDTEDKLRAAMVGMLANFLGFRIYGM
HSELWGVVHGATVLSPTAVFGTLASLYLGALDHTADRLQAILGVPWKDKNCTSRLDAHKV
LSALQAVQGLLVAQGRADSQAQLLLSTVVGVFTAPGLHLKQPFVQGLALYTPVVLPRSLD
FTELDVAAEKIDRFMQAVTGWKTGCSLMGASVDSTLAFNTYVHFQGKMKGFSLLAEPQEF
WVDNSTSVSVPMLSGMGTFQHWSDIQDNFSVTQVPFTESACLLLIQPHYASDLDKVEGLT
FQQNSLNWMKKLSPRTIHLTMPQLVLQGSYDLQDLLAQAELPAILHTELNLQKLSNDRIR
VGEVLNSIFFELEADEREPTESTQQLNKPEVLEVTLNRPFLFAVYDQSATALHFLGRVAN
PLSTA
>sp|P16157|ANK1_HUMAN Ankyrin-1 OS=Homo sapiens GN=ANK1 PE=1 SV=3
MPYSVGFREADAATSFLRAARSGNLDKALDHLRNGVDINTCNQNGLNGLHLASKEGHVKM
VVELLHKEIILETTTKKGNTALHIAALAGQDEVVRELVNYGANVNAQSQKGFTPLYMAAQ
ENHLEVVKFLLENGANQNVATEDGFTPLAVALQQGHENVVAHLINYGTKGKVRLPALHIA
ARNDDTRTAAVLLQNDPNPDVLSKTGFTPLHIAAHYENLNVAQLLLNRGASVNFTPQNGI
TPLHIASRRGNVIMVRLLLDRGAQIETKTKDELTPLHCAARNGHVRISEILLDHGAPIQA
KTKNGLSPIHMAAQGDHLDCVRLLLQYDAEIDDITLDHLTPLHVAAHCGHHRVAKVLLDK
GAKPNSRALNGFTPLHIACKKNHVRVMELLLKTGASIDAVTESGLTPLHVASFMGHLPIV
KNLLQRGASPNVSNVKVETPLHMAARAGHTEVAKYLLQNKAKVNAKAKDDQTPLHCAARI
GHTNMVKLLLENNANPNLATTAGHTPLHIAAREGHVETVLALLEKEASQACMTKKGFTPL
HVAAKYGKVRVAELLLERDAHPNAAGKNGLTPLHVAVHHNNLDIVKLLLPRGGSPHSPAW
NGYTPLHIAAKQNQVEVARSLLQYGGSANAESVQGVTPLHLAAQEGHAEMVALLLSKQAN
GNLGNKSGLTPLHLVAQEGHVPVADVLIKHGVMVDATTRMGYTPLHVASHYGNIKLVKFL
LQHQADVNAKTKLGYSPLHQAAQQGHTDIVTLLLKNGASPNEVSSDGTTPLAIAKRLGYI
SVTDVLKVVTDETSFVLVSDKHRMSFPETVDEILDVSEDEGEELISFKAERRDSRDVDEE
KELLDFVPKLDQVVESPAIPRIPCAMPETVVIRSEEQEQASKEYDEDSLIPSSPATETSD
NISPVASPVHTGFLVSFMVDARGGSMRGSRHNGLRVVIPPRTCAAPTRITCRLVKPQKLS
TPPPLAEEEGLASRIIALGPTGAQFLSPVIVEIPHFASHGRGDRELVVLRSENGSVWKEH
RSRYGESYLDQILNGMDEELGSLEELEKKRVCRIITTDFPLYFVIMSRLCQDYDTIGPEG
GSLKSKLVPLVQATFPENAVTKRVKLALQAQPVPDELVTKLLGNQATFSPIVTVEPRRRK
FHRPIGLRIPLPPSWTDNPRDSGEGDTTSLRLLCSVIGGTDQAQWEDITGTTKLVYANEC
ANFTTNVSARFWLSDCPRTAEAVNFATLLYKELTAVPYMAKFVIFAKMNDPREGRLRCYC
MTDDKVDKTLEQHENFVEVARSRDIEVLEGMSLFAELSGNLVPVKKAAQQRSFHFQSFRE
NRLAMPVKVRDSSREPGGSLSFLRKAMKYEDTQHILCHLNITMPPCAKGSGAEDRRRTPT
PLALRYSILSESTPGSLSGTEQAEMKMAVISEHLGLSWAELARELQFSVEDINRIRVENP
NSLLEQSVALLNLWVIREGQNANMENLYTALQSIDRGEIVNMLEGSGRQSRNLKPDRRHT
DRDYSLSPSQMNGYSSLQDELLSPASLGCALSSPLRADQYWNEVAVLDAIPLAATEHDTM
LEMSDMQVWSAGLTPSLVTAEDSSLECSKAEDSDATGHEWKLEGALSEEPRGPELGSLEL
VEDDTVDSDATNGLIDLLEQEEGQRSEEKLPGSKRQDDATGAGQDSENEVSLVSGHQRGQ
ARITHSPTVSQVTERSQDRLQDWDADGSIVSYLQDAAQGSWQEEVTQGPHSFQGTSTMTE
GLEPGGSQEYEKVLVSVSEHTWTEQPEAESSQADRDRRQQGQEEQVQEAKNTFTQVVQGN
EFQNIPGEQVTEEQFTDEQGNIVTKKIIRKVVRQIDLSSADAAQEHEEVTVEGPLEDPSE
LEVDIDYFMKHSKDHTSTPNP
>sp|Q01484|ANK2_HUMAN Ankyrin-2 OS=Homo sapiens GN=ANK2 PE=1 SV=4

MMNEDAAQKSDSGEKFNGSSQRRKRPKKSDSNASFLRAARAGNLDKVVEYLKGGIDINTC
NQNGLNALHLAAKEGHVGLVQELLGRGSSVDSATKKGNTALHIASLAGQAEVVKVLVKEG
ANINAQSQNGFTPLYMAAQENHIDVVKYLLENGANQSTATEDGFTPLAVALQQGHNQAVA
ILLENDTKGKVRLPALHIAARKDDTKSAALLLQNDHNADVQSKMMVNRTTESGFTPLHIA
AHYGNVNVATLLLNRGAAVDFTARNGITPLHVASKRGNTNMVKLLLDRGGQIDAKTRDGL
TPLHCAARSGHDQVVELLLERGAPLLARTKNGLSPLHMAAQGDHVECVKHLLQHKAPVDD
VTLDYLTALHVAAHCGHYRVTKLLLDKRANPNARALNGFTPLHIACKKNRIKVMELLVKY
GASIQAITESGLTPIHVAAFMGHLNIVLLLLQNGASPDVTNIRGETALHMAARAGQVEVV
RCLLRNGALVDARAREEQTPLHIASRLGKTEIVQLLLQHMAHPDAATTNGYTPLHISARE
GQVDVASVLLEAGAAHSLATKKGFTPLHVAAKYGSLDVAKLLLQRRAAADSAGKNGLTPL
HVAAHYDNQKVALLLLEKGASPHATAKNGYTPLHIAAKKNQMQIASTLLNYGAETNIVTK
QGVTPLHLASQEGHTDMVTLLLDKGANIHMSTKSGLTSLHLAAQEDKVNVADILTKHGAD
QDAHTKLGYTPLIVACHYGNVKMVNFLLKQGANVNAKTKNGYTPLHQAAQQGHTHIINVL
LQHGAKPNATTANGNTALAIAKRLGYISVVDTLKVVTEEVTTTTTTITEKHKLNVPETMT
EVLDVSDEEGDDTMTGDGGEYLRPEDLKELGDDSLPSSQFLDGMNYLRYSLEGGRSDSLR
SFSSDRSHTLSHASYLRDSAVMDDSVVIPSHQVSTLAKEAERNSYRLSWGTENLDNVALS
SSPIHSGFLVSFMVDARGGAMRGCRHNGLRIIIPPRKCTAPTRVTCRLVKRHRLATMPPM
VEGEGLASRLIEVGPSGAQFLGKLHLPTAPPPLNEGESLVSRILQLGPPGTKFLGPVIVE
IPHFAALRGKERELVVLRSENGDSWKEHFCDYTEDELNEILNGMDEVLDSPEDLEKKRIC
RIITRDFPQYFAVVSRIKQDSNLIGPEGGVLSSTVVPQVQAVFPEGALTKRIRVGLQAQP
MHSELVKKILGNKATFSPIVTLEPRRRKFHKPITMTIPVPKASSDVMLNGFGGDAPTLRL
LCSITGGTTPAQWEDITGTTPLTFVNECVSFTTNVSARFWLIDCRQIQESVTFASQVYRE
IICVPYMAKFVVFAKSHDPIEARLRCFCMTDDKVDKTLEQQENFAEVARSRDVEVLEGKP
IYVDCFGNLVPLTKSGQHHIFSFFAFKENRLPLFVKVRDTTQEPCGRLSFMKEPKSTRGL
VHQAICNLNITLPIYTKESESDQEQEEEIDMTSEKNDETESTETSVLKSHLVNEVPVLAS
PDLLSEVSEMKQDLIKMTAILTTDVSDKAGSIKVKELVKAAEEEPGEPFEIVERVKEDLE
KVNEILRSGTCTRDESSVQSSRSERGLVEEEWVIVSDEEIEEARQKAPLEITEYPCVEVR
IDKEIKGKVEKDSTGLVNYLTDDLNTCVPLPKEQLQTVQDKAGKKCEALAVGRSSEKEGK
DIPPDETQSTQKQHKPSLGIKKPVRRKLKEKQKQKEEGLQASAEKAELKKGSSEESLGED
PGLAPEPLPTVKATSPLIEETPIGSIKDKVKALQKRVEDEQKGRSKLPIRVKGKEDVPKK
TTHRPHPAASPSLKSERHAPGSPSPKTERHSTLSSSAKTERHPPVSPSSKTEKHSPVSPS
AKTERHSPASSSSKTEKHSPVSPSTKTERHSPVSSTKTERHPPVSPSGKTDKRPPVSPSG
RTEKHPPVSPGRTEKRLPVSPSGRTDKHQPVSTAGKTEKHLPVSPSGKTEKQPPVSPTSK
TERIEETMSVRELMKAFQSGQDPSKHKTGLFEHKSAKQKQPQEKGKVRVEKEKGPILTQR
EAQKTENQTIKRGQRLPVTGTAESKRGVRVSSIGVKKEDAAGGKEKVLSHKIPEPVQSVP
EEESHRESEVPKEKMADEQGDMDLQISPDRKTSTDFSEVIKQELEDNDKYQQFRLSEETE
KAQLHLDQVLTSPFNTTFPLDYMKDEFLPALSLQSGALDGSSESLKNEGVAGSPCGSLME
GTPQISSEESYKHEGLAETPETSPESLSFSPKKSEEQTGETKESTKTETTTEIRSEKEHP
TTKDITGGSEERGATVTEDSETSTESFQKEATLGSPKDTSPKRQDDCTGSCSVALAKETP
TGLTEEAACDEGQRTFGSSAHKTQTDSEVQESTATSDETKALPLPEASVKTDTGTESKPQ
GVIRSPQGLELALPSRDSEVLSAVADDSLAVSHKDSLEASPVLEDNSSHKTPDSLEPSPL
KESPCRDSLESSPVEPKMKAGIFPSHFPLPAAVAKTELLTEVASVRSRLLRDPDGSAEDD
SLEQTSLMESSGKSPLSPDTPSSEEVSYEVTPKTTDVSTPKPAVIHECAEEDDSENGEKK
RFTPEEEMFKMVTKIKMFDELEQEAKQKRDYKKEPKQEESSSSSDPDADCSVDVDEPKHT

GSGEDESGVPVLVTSESRKVSSSSESEPELAQLKKGADSGLLPEPVIRVQPPSPLPSSMD
SNSSPEEVQFQPVVSKQYTFKMNEDTQEEPGKSEEEKDSESHLAEDRHAVSTEAEDRSYD
KLNRDTDQPKICDGHGCEAMSPSSSAAPVSSGLQSPTGDDVDEQPVIYKESLALQGTHEK
DTEGEELDVSRAESPQADCPSESFSSSSSLPHCLVSEGKELDEDISATSSIQKTEVTKTD
ETFENLPKDCPSQDSSITTQTDRFSMDVPVSDLAENDEIYDPQITSPYENVPSQSFFSSE
ESKTQTDANHTTSFHSSEVYSVTITSPVEDVVVASSSSGTVLSKESNFEGQDIKMESQQE
STLWEMQSDSVSSSFEPTMSATTTVVGEQISKVIITKTDVDSDSWSEIREDDEAFEARVK
EEEQKIFGLMVDRQSQGTTPDTTPARTPTEEGTPTSEQNPFLFQEGKLFEMTRSGAIDMT
KRSYADESFHFFQIGQESREETLSEDVKEGATGADPLPLETSAESLALSESKETVDDEAD
LLPDDVSEEVEEIPASDAQLNSQMGISASTETPTKEAVSVGTKDLPTVQTGDIPPLSGVK
QISCPDSSEPAVQVQLDFSTLTRSVYSDRGDDSPDSSPEEQKSVIEIPTAPMENVPFTES
KSKIPVRTMPTSTPAPPSAEYESSVSEDFLSSVDEENKADEAKPKSKLPVKVPLQRVEQQ
LSDLDTSVQKTVAPQGQDMASIAPDNRSKSESDASSLDSKTKCPVKTRSYTETETESRER
AEELELESEEGATRPKILTSRLPVKSRSTTSSCRGGTSPTKESKEHFFDLYRNSIEFFEE
ISDEASKLVDRLTQSEREQEIVSDDESSSALEVSVIENLPPVETEHSVPEDIFDTRPIWD
ESIETLIERIPDENGHDHAEDPQDEQERIEERLAYIADHLGFSWTELARELDFTEEQIHQ
IRIENPNSLQDQSHALLKYWLERDGKHATDTNLVECLTKINRMDIVHLMETNTEPLQERI
SHSYAEIEQTITLDHSEGFSVLQEELCTAQHKQKEEQAVSKESETCDHPPIVSEEDISVG
YSTFQDGVPKTEGDSSATALFPQTHKEQVQQDFSGKMQDLPEESSLEYQQEYFVTTPGTE
TSETQKAMIVPSSPSKTPEEVSTPAEEEKLYLQTPTSSERGGSPIIQEPEEPSEHREESS
PRKTSLVIVESADNQPETCERLDEDAAFEKGDDMPEIPPETVTEEEYIDEHGHTVVKKVT
RKIIRRYVSSEGTEKEEIMVQGMPQEPVNIEEGDGYSKVIKRVVLKSDTEQSEDNNE
>sp|Q96LA8|ANM6_HUMAN Protein arginine N-methyltransferase 6 OS=Homo sapiens GN=PRMT6 PE=1 SV=1
MSQPKKRKLESGGGGEGGEGTEEEDGAEREAALERPRRTKRERDQLYYECYSDVSVHEEM
IADRVRTDAYRLGILRNWAALRGKTVLDVGAGTGILSIFCAQAGARRVYAVEASAIWQQA
REVVRFNGLEDRVHVLPGPVETVELPEQVDAIVSEWMGYGLLHESMLSSVLHARTKWLKE
GGLLLPASAELFIAPISDQMLEWRLGFWSQVKQHYGVDMSCLEGFATRCLMGHSEIVVQG
LSGEDVLARPQRFAQLELSRAGLEQELEAGVGGRFRCSCYGSAPMHGFAIWFQVTFPGGE
SEKPLVLSTSPFHPATHWKQALLYLNEPVQVEQDTDVSGEITLLPSRDNPRRLRVLLRYK
VGDQEEKTKDFAMED
>sp|Q9NVM4|ANM7_HUMAN Protein arginine N-methyltransferase 7 OS=Homo sapiens GN=PRMT7 PE=1 SV=1
MKIFCSRANPTTGSVEWLEEDEHYDYHQEIARSSYADMLHDKDRNVKYYQGIRAAVSRVK
DRGQKALVLDIGTGTGLLSMMAVTAGADFCYAIEVFKPMADAAVKIVEKNGFSDKIKVIN
KHSTEVTVGPEGDMPCRANILVTELFDTELIGEGALPSYEHAHRHLVEENCEAVPHRATV
YAQLVESGRMWSWNKLFPIHVQTSLGEQVIVPPVDVESCPGAPSVCDIQLNQVSPADFTV
LSDVLPMFSIDFSKQVSSSAACHSRRFEPLTSGRAQVVLSWWDIEMDPEGKIKCTMAPFW
AHSDPEEMQWRDHWMQCVYFLPQEEPVVQGSALYLVAHHDDYCVWYSLQRTSPEKNERVR
QMRPVCDCQAHLLWNRPRFGEINDQDRTDRYVQALRTVLKPDSVCLCVSDGSLLSVLAHH
LGVEQVFTVESSAASHKLLRKIFKANHLEDKINIIEKRPELLTNEDLQGRKVSLLLGEPF
FTTSLLPWHNLYFWYVRTAVDQHLGPGAMVMPQAASLHAVVVEFRDLWRIRSPCGDCEGF
DVHIMDDMIKRALDFRESREAEPHPLWEYPCRSLSEPWQILTFDFQQPVPLQPLCAEGTV
ELRRPGQSHAAVLWMEYHLTPECTLSTGLLEPADPEGGCCWNPHCKQAVYFFSPAPDPRA

LLGGPRTVSYAVEFHPDTGDIIMEFRHADTPD
>sp|Q6VUC0|AP2E_HUMAN Transcription factor AP-2-epsilon OS=Homo sapiens GN=TFAP2E PE=2 SV=1
MLVHTYSAMERPDGLGAAAGGARLSSLPQAAYGPAPPLCHTPAATAAAEFQPPYFPPPYP
QPPLPYGQAPDAAAAFPHLAGDPYGGLAPLAQPQPPQAAWAAPRAAARAHEEPPGLLAPP
ARALGLDPRRDYATAVPRLLHGLADGAHGLADAPLGLPGLAAAPGLEDLQAMDEPGMSLL
DQSVIKKVPIPSKASSLSALSLAKDSLVGGITNPGEVFCSVPGRLSLLSSTSKYKVTVGE
VQRRLSPPECLNASLLGGVLRRAKSKNGGRCLRERLEKIGLNLPAGRRKAANVTLLTSLV
EGEAVHLARDFGYVCETEFPAKAAAEYLCRQHADPGELHSRKSMLLAAKQICKEFADLMA
QDRSPLGNSRPALILEPGVQSCLTHFSLITHGFGGPAICAALTAFQNYLLESLKGLDKMF
LSSVGSGHGETKASEKDAKHRK
>sp|O14617|AP3D1_HUMAN AP-3 complex subunit delta-1 OS=Homo sapiens GN=AP3D1 PE=1 SV=1
MALKMVKGSIDRMFDKNLQDLVRGIRNHKEDEAKYISQCIDEIKQELKQDNIAVKANAVC
KLTYLQMLGYDISWAAFNIIEVMSASKFTFKRIGYLAASQSFHEGTDVIMLTTNQIRKDL
SSPSQYDTGVALTGLSCFVTPDLARDLANDIMTLMSHTKPYIRKKAVLIMYKVFLKYPES
LRPAFPRLKEKLEDPDPGVQSAAVNVICELARRNPKNYLSLAPLFFKLMTSSTNNWVLIK
IIKLFGALTPLEPRLGKKLIEPLTNLIHSTSAMSLLYECVNTVIAVLISLSSGMPNHSAS
IQLCVQKLRILIEDSDQNLKYLGLLAMSKILKTHPKSVQSHKDLILQCLDDKDESIRLRA
LDLLYGMVSKKNLMEIVKKLMTHVDKAEGTTYRDELLTKIIDICSQSNYQYITNFEWYIS
ILVELTRLEGTRHGHLIAAQMLDVAIRVKAIRKFAVSQMSALLDSAHLLASSTQRNGICE
VLYAAAWICGEFSEHLQEPHHTLEAMLRPRVTTLPGHIQAVYVQNVVKLYASILQQKEQA
GEAEGAQAVTQLMVDRLPQFVQSADLEVQERASCILQLVKHIQKLQAKDVPVAEEVSALF
AGELNPVAPKAQKKVPVPEGLDLDAWINEPLSDSESEDERPRAVFHEEEQRRPKHRPSEA
DEEELARRREARKQEQANNPFYIKSSPSPQKRYQDTPGVEHIPVVQIDLSVPLKVPGLPM
SDQYVKLEEERRHRQKLEKDKRRKKRKEKEKKGKRRHSSLPTESDEDIAPAQQVDIVTEE
MPENALPSDEDDKDPNDPYRALDIDLDKPLADSEKLPIQKHRNTETSKSPEKDVPMVEKK
SKKPKKKEKKHKEKERDKEKKKEKEKKKSPKPKKKKHRKEKEERTKGKKKSKKQPPGSEE
AAGEPVQNGAPEEEQLPPESSYSLLAENSYVKMTCDIRGSLQEDSQVTVAIVLENRSSSI
LKGMELSVLDSLNARMARPQGSSVHDGVPVPFQLPPGVSNEAQYVFTIQSIVMAQKLKGT
LSFIAKNDEGATHEKLDFRLHFSCSSYLITTPCYSDAFAKLLESGDLSMSSIKVDGIRMS
FQNLLAKICFHHHFSVVERVDSCASMYSRSIQGHHVCLLVKKGENSVSVDGKCSDSTLLS
NLLEEMKATLAKC
>sp|Q9Y587|AP4S1_HUMAN AP-4 complex subunit sigma-1 OS=Homo sapiens GN=AP4S1 PE=2 SV=1
MIKFFLMVNKQGQTRLSKYYEHVDINKRTLLETEVIKSCLSRSNEQCSFIEYKDFKLIYR
QYAALFIVVGVNDTENEMAIYEFIHNFVEVLDEYFSRVSELDIMFNLDKVHIILDEMVLN
GCIVETNRARILAPLLILDKMSES
>sp|Q9UJX5|APC4_HUMAN Anaphase-promoting complex subunit 4 OS=Homo sapiens GN=ANAPC4 PE=1 SV=2
MLRFPTCFPSFRVVGEKQLPQEIIFLVWSPKRDLIALANTAGEVLLHRLASFHRVWSFPP
NENTGKEVTCLAWRPDGKLLAFALADTKKIVLCDVEKPESLHSFSVEAPVSCMHWMEVTV
ESSVLTSFYNAEDESNLLLPKLPTLPKNYSNTSKIFSEENSDEIIKLLGDVRLNILVLGG
SSGFIELYAYGMFKIARVTGIAGTCLALCLSSDLKSLSVVTEVSTNGASEVSYFQLETNL
LYSFLPEVTRMARKFTHISALLQYINLSLTCMCEAWEEILMQMDSRLTKFVQEKNTTTSV
QDEFMHLLLWGKASAELQTLLMNQLTVKGLKKLGQSIESSYSSIQKLVISHLQSGSESLL

YHLSELKGMASWKQKYEPLGLDAAGIEEAITAVGSFILKANELLQVIDSSMKNFKAFFRW
LYVAMLRMTEDHVLPELNKMTQKDITFVAEFLTEHFNEAPDLYNRKGKYFNVERVGQYLK
DEDDDLVSPPNTEGNQWYDFLQNSSHLKESPLLFPYYPRKSLHFVKRRMENIIDQCLQKP
ADVIGKSMNQAICIPLYRDTRSEDSTRRLFKFPFLWNNKTSNLHYLLFTILEDSLYKMCI
LRRHTDISQSVSNGLIAIKFGSFTYATTEKVRRSIYSCLDAQFYDDETVTVVLKDTVGRE
GRDRLLVQLPLSLVYNSEDSAEYQFTGTYSTRLDEQCSAIPTRTMHFEKHWRLLESMKAQ
YVAGNGFRKVSCVLSSNLRHVRVFEMDIDDEWELDESSDEEEEASNKPVKIKEEVLSESE
AENQQAGAAALAPEIVIKVEKLDPELDS
>sp|P25054|APC_HUMAN Adenomatous polyposis coli protein OS=Homo sapiens GN=APC PE=1 SV=2
MAAASYDQLLKQVEALKMENSNLRQELEDNSNHLTKLETEASNMKEVLKQLQGSIEDEAM
ASSGQIDLLERLKELNLDSSNFPGVKLRSKMSLRSYGSREGSVSSRSGECSPVPMGSFPR
RGFVNGSRESTGYLEELEKERSLLLADLDKEEKEKDWYYAQLQNLTKRIDSLPLTENFSL
QTDMTRRQLEYEARQIRVAMEEQLGTCQDMEKRAQRRIARIQQIEKDILRIRQLLQSQAT
EAERSSQNKHETGSHDAERQNEGQGVGEINMATSGNGQGSTTRMDHETASVLSSSSTHSA
PRRLTSHLGTKVEMVYSLLSMLGTHDKDDMSRTLLAMSSSQDSCISMRQSGCLPLLIQLL
HGNDKDSVLLGNSRGSKEARARASAALHNIIHSQPDDKRGRREIRVLHLLEQIRAYCETC
WEWQEAHEPGMDQDKNPMPAPVEHQICPAVCVLMKLSFDEEHRHAMNELGGLQAIAELLQ
VDCEMYGLTNDHYSITLRRYAGMALTNLTFGDVANKATLCSMKGCMRALVAQLKSESEDL
QQVIASVLRNLSWRADVNSKKTLREVGSVKALMECALEVKKESTLKSVLSALWNLSAHCT
ENKADICAVDGALAFLVGTLTYRSQTNTLAIIESGGGILRNVSSLIATNEDHRQILRENN
CLQTLLQHLKSHSLTIVSNACGTLWNLSARNPKDQEALWDMGAVSMLKNLIHSKHKMIAM
GSAAALRNLMANRPAKYKDANIMSPGSSLPSLHVRKQKALEAELDAQHLSETFDNIDNLS
PKASHRSKQRHKQSLYGDYVFDTNRHDDNRSDNFNTGNMTVLSPYLNTTVLPSSSSSRGS
LDSSRSEKDRSLERERGIGLGNYHPATENPGTSSKRGLQISTTAAQIAKVMEEVSAIHTS
QEDRSSGSTTELHCVTDERNALRRSSAAHTHSNTYNFTKSENSNRTCSMPYAKLEYKRSS
NDSLNSVSSSDGYGKRGQMKPSIESYSEDDESKFCSYGQYPADLAHKIHSANHMDDNDGE
LDTPINYSLKYSDEQLNSGRQSPSQNERWARPKHIIEDEIKQSEQRQSRNQSTTYPVYTE
STDDKHLKFQPHFGQQECVSPYRSRGANGSETNRVGSNHGINQNVSQSLCQEDDYEDDKP
TNYSERYSEEEQHEEEERPTNYSIKYNEEKRHVDQPIDYSLKYATDIPSSQKQSFSFSKS
SSGQSSKTEHMSSSSENTSTPSSNAKRQNQLHPSSAQSRSGQPQKAATCKVSSINQETIQ
TYCVEDTPICFSRCSSLSSLSSAEDEIGCNQTTQEADSANTLQIAEIKEKIGTRSAEDPV
SEVPAVSQHPRTKSSRLQGSSLSSESARHKAVEFSSGAKSPSKSGAQTPKSPPEHYVQET
PLMFSRCTSVSSLDSFESRSIASSVQSEPCSGMVSGIISPSDLPDSPGQTMPPSRSKTPP
PPPQTAQTKREVPKNKAPTAEKRESGPKQAAVNAAVQRVQVLPDADTLLHFATESTPDGF
SCSSSLSALSLDEPFIQKDVELRIMPPVQENDNGNETESEQPKESNENQEKEAEKTIDSE
KDLLDDSDDDDIEILEECIISAMPTKSSRKAKKPAQTASKLPPPVARKPSQLPVYKLLPS
QNRLQPQKHVSFTPGDDMPRVYCVEGTPINFSTATSLSDLTIESPPNELAAGEGVRGGAQ
SGEFEKRDTIPTEGRSTDEAQGGKTSSVTIPELDDNKAEEGDILAECINSAMPKGKSHKP
FRVKKIMDQVQQASASSSAPNKNQLDGKKKKPTSPVKPIPQNTEYRTRVRKNADSKNNLN
AERVFSDNKDSKKQNLKNNSKVFNDKLPNNEDRVRGSFAFDSPHHYTPIEGTPYCFSRND
SLSSLDFDDDDVDLSREKAELRKAKENKESEAKVTSHTELTSNQQSANKTQAIAKQPINR
GQPKPILQKQSTFPQSSKDIPDRGAATDEKLQNFAIENTPVCFSHNSSLSSLSDIDQENN
NKENEPIKETEPPDSQGEPSKPQASGYAPKSFHVEDTPVCFSRNSSLSSLSIDSEDDLLQ
ECISSAMPKKKKPSRLKGDNEKHSPRNMGGILGEDLTLDLKDIQRPDSEHGLSPDSENFD

WKAIQEGANSIVSSLHQAAAAACLSRQASSDSDSILSLKSGISLGSPFHLTPDQEEKPFT
SNKGPRILKPGEKSTLETKKIESESKGIKGGKKVYKSLITGKVRSNSEISGQMKQPLQAN
MPSISRGRTMIHIPGVRNSSSSTSPVSKKGPPLKTPASKSPSEGQTATTSPRGAKPSVKS
ELSPVARQTSQIGGSSKAPSRSGSRDSTPSRPAQQPLSRPIQSPGRNSISPGRNGISPPN
KLSQLPRTSSPSTASTKSSGSGKMSYTSPGRQMSQQNLTKQTGLSKNASSIPRSESASKG
LNQMNNGNGANKKVELSRMSSTKSSGSESDRSERPVLVRQSTFIKEAPSPTLRRKLEESA
SFESLSPSSRPASPTRSQAQTPVLSPSLPDMSLSTHSSVQAGGWRKLPPNLSPTIEYNDG
RPAKRHDIARSHSESPSRLPINRSGTWKREHSKHSSSLPRVSTWRRTGSSSSILSASSES
SEKAKSEDEKHVNSISGTKQSKENQVSAKGTWRKIKENEFSPTNSTSQTVSSGATNGAES
KTLIYQMAPAVSKTEDVWVRIEDCPINNPRSGRSPTGNTPPVIDSVSEKANPNIKDSKDN
QAKQNVGNGSVPMRTVGLENRLNSFIQVDAPDQKGTEIKPGQNNPVPVSETNESSIVERT
PFSSSSSSKHSSPSGTVAARVTPFNYNPSPRKSSADSTSARPSQIPTPVNNNTKKRDSKT
DSTESSGTQSPKRHSGSYLVTSV
>sp|Q9BZZ5|API5_HUMAN Apoptosis inhibitor 5 OS=Homo sapiens GN=API5 PE=1 SV=3
MPTVEELYRNYGILADATEQVGQHKDAYQVILDGVKGGTKEKRLAAQFIPKFFKHFPELA
DSAINAQLDLCEDEDVSIRRQAIKELPQFATGENLPRVADILTQLLQTDDSAEFNLVNNA
LLSIFKMDAKGTLGGLFSQILQGEDIVRERAIKFLSTKLKTLPDEVLTKEVEELILTESK
KVLEDVTGEEFVLFMKILSGLKSLQTVSGRQQLVELVAEQADLEQTFNPSDPDCVDRLLQ
CTRQAVPLFSKNVHSTRFVTYFCEQVLPNLGTLTTPVEGLDIQLEVLKLLAEMSSFCGDM
EKLETNLRKLFDKLLEYMPLPPEEAENGENAGNEEPKLQFSYVECLLYSFHQLGRKLPDF
LTAKLNAEKLKDFKIRLQYFARGLQVYIRQLRLALQGKTGEALKTEENKIKVVALKITNN
INVLIKDLFHIPPSYKSTVTLSWKPVQKVEIGQKRASEDTTSGSPPKKSSAGPKRDARQI
YNPPSGKYSSNLGNFNYEQRGAFRGSRGGRGWGTRGNRSRGRLY
>sp|P51693|APLP1_HUMAN Amyloid-like protein 1 OS=Homo sapiens GN=APLP1 PE=1 SV=3
MGPASPAARGLSRRPGQPPLPLLLPLLLLLLRAQPAIGSLAGGSPGAAEAPGSAQVAGLC
GRLTLHRDLRTGRWEPDPQRSRRCLRDPQRVLEYCRQMYPELQIARVEQATQAIPMERWC
GGSRSGSCAHPHHQVVPFRCLPGEFVSEALLVPEGCRFLHQERMDQCESSTRRHQEAQEA
CSSQGLILHGSGMLLPCGSDRFRGVEYVCCPPPGTPDPSGTAVGDPSTRSWPPGSRVEGA
EDEEEEESFPQPVDDYFVEPPQAEEEEETVPPPSSHTLAVVGKVTPTPRPTDGVDIYFGM
PGEISEHEGFLRAKMDLEERRMRQINEVMREWAMADNQSKNLPKADRQALNEHFQSILQT
LEEQVSGERQRLVETHATRVIALINDQRRAALEGFLAALQADPPQAERVLLALRRYLRAE
QKEQRHTLRHYQHVAAVDPEKAQQMRFQVHTHLQVIEERVNQSLGLLDQNPHLAQELRPQ
IQELLHSEHLGPSELEAPAPGGSSEDKGGLQPPDSKDDTPMTLPKGSTEQDAASPEKEKM
NPLEQYERKVNASVPRGFPFHSSEIQRDELAPAGTGVSREAVSGLLIMGAGGGSLIVLSM
LLLRRKKPYGAISHGVVEVDPMLTLEEQQLRELQRHGYENPTYRFLEERP
>sp|Q9HDC9|APMAP_HUMAN Adipocyte plasma membrane-associated protein OS=Homo sapiens GN=APMAP PE=1 SV=2
MSEADGLRQRRPLRPQVVTDDDGQAPEAKDGSSFSGRVFRVTFLMLAVSLTVPLLGAMML
LESPIDPQPLSFKEPPLLLGVLHPNTKLRQAERLFENQLVGPESIAHIGDVMFTGTADGR
VVKLENGEIETIARFGSGPCKTRDDEPVCGRPLGIRAGPNGTLFVADAYKGLFEVNPWKR
EVKLLLSSETPIEGKNMSFVNDLTVTQDGRKIYFTDSSSKWQRRDYLLLVMEGTDDGRLL
EYDTVTREVKVLLDQLRFPNGVQLSPAEDFVLVAETTMARIRRVYVSGLMKGGADLFVEN
MPGFPDNIRPSSSGGYWVGMSTIRPNPGFSMLDFLSERPWIKRMIFKLFSQETVMKFVPR
YSLVLELSDSGAFRRSLHDPDGLVATYISEVHEHDGHLYLGSFRSPFLCRLSLQAV

>sp|P02647|APOA1_HUMAN Apolipoprotein A-I OS=Homo sapiens GN=APOA1 PE=1 SV=1
MKAAVLTLAVLFLTGSQARHFWQQDEPPQSPWDRVKDLATVYVDVLKDSGRDYVSQFEGS
ALGKQLNLKLLDNWDSVTSTFSKLREQLGPVTQEFWDNLEKETEGLRQEMSKDLEEVKAK
VQPYLDDFQKKWQEEMELYRQKVEPLRAELQEGARQKLHELQEKLSPLGEEMRDRARAHV
DALRTHLAPYSDELRQRLAARLEALKENGGARLAEYHAKATEHLSTLSEKAKPALEDLRQ
GLLPVLESFKVSFLSALEEYTKKLNTQ
>sp|P06727|APOA4_HUMAN Apolipoprotein A-IV OS=Homo sapiens GN=APOA4 PE=1 SV=3
MFLKAVVLTLALVAVAGARAEVSADQVATVMWDYFSQLSNNAKEAVEHLQKSELTQQLNA
LFQDKLGEVNTYAGDLQKKLVPFATELHERLAKDSEKLKEEIGKELEELRARLLPHANEV
SQKIGDNLRELQQRLEPYADQLRTQVNTQAEQLRRQLTPYAQRMERVLRENADSLQASLR
PHADELKAKIDQNVEELKGRLTPYADEFKVKIDQTVEELRRSLAPYAQDTQEKLNHQLEG
LTFQMKKNAEELKARISASAEELRQRLAPLAEDVRGNLRGNTEGLQKSLAELGGHLDQQV
EEFRRRVEPYGENFNKALVQQMEQLRQKLGPHAGDVEGHLSFLEKDLRDKVNSFFSTFKE
KESQDKTLSLPELEQQQEQQQEQQQEQVQMLAPLES
>sp|P08519|APOA_HUMAN Apolipoprotein(a) OS=Homo sapiens GN=LPA PE=1 SV=1
MEHKEVVLLLLLFLKSAAPEQSHVVQDCYHGDGQSYRGTYSTTVTGRTCQAWSSMTPHQH
NRTTENYPNAGLIMNYCRNPDAVAAPYCYTRDPGVRWEYCNLTQCSDAEGTAVAPPTVTP
VPSLEAPSEQAPTEQRPGVQECYHGNGQSYRGTYSTTVTGRTCQAWSSMTPHSHSRTPEY
YPNAGLIMNYCRNPDAVAAPYCYTRDPGVRWEYCNLTQCSDAEGTAVAPPTVTPVPSLEA
PSEQAPTEQRPGVQECYHGNGQSYRGTYSTTVTGRTCQAWSSMTPHSHSRTPEYYPNAGL
IMNYCRNPDAVAAPYCYTRDPGVRWEYCNLTQCSDAEGTAVAPPTVTPVPSLEAPSEQAP
TEQRPGVQECYHGNGQSYRGTYSTTVTGRTCQAWSSMTPHSHSRTPEYYPNAGLIMNYCR
NPDAVAAPYCYTRDPGVRWEYCNLTQCSDAEGTAVAPPTVTPVPSLEAPSEQAPTEQRPG
VQECYHGNGQSYRGTYSTTVTGRTCQAWSSMTPHSHSRTPEYYPNAGLIMNYCRNPDAVA
APYCYTRDPGVRWEYCNLTQCSDAEGTAVAPPTVTPVPSLEAPSEQAPTEQRPGVQECYH
GNGQSYRGTYSTTVTGRTCQAWSSMTPHSHSRTPEYYPNAGLIMNYCRNPDAVAAPYCYT
RDPGVRWEYCNLTQCSDAEGTAVAPPTVTPVPSLEAPSEQAPTEQRPGVQECYHGNGQSY
RGTYSTTVTGRTCQAWSSMTPHSHSRTPEYYPNAGLIMNYCRNPDAVAAPYCYTRDPGVR
WEYCNLTQCSDAEGTAVAPPTVTPVPSLEAPSEQAPTEQRPGVQECYHGNGQSYRGTYST
TVTGRTCQAWSSMTPHSHSRTPEYYPNAGLIMNYCRNPDAVAAPYCYTRDPGVRWEYCNL
TQCSDAEGTAVAPPTVTPVPSLEAPSEQAPTEQRPGVQECYHGNGQSYRGTYSTTVTGRT
CQAWSSMTPHSHSRTPEYYPNAGLIMNYCRNPDAVAAPYCYTRDPGVRWEYCNLTQCSDA
EGTAVAPPTVTPVPSLEAPSEQAPTEQRPGVQECYHGNGQSYRGTYSTTVTGRTCQAWSS
MTPHSHSRTPEYYPNAGLIMNYCRNPDAVAAPYCYTRDPGVRWEYCNLTQCSDAEGTAVA
PPTVTPVPSLEAPSEQAPTEQRPGVQECYHGNGQSYRGTYSTTVTGRTCQAWSSMTPHSH
SRTPEYYPNAGLIMNYCRNPDAVAAPYCYTRDPGVRWEYCNLTQCSDAEGTAVAPPTVTP
VPSLEAPSEQAPTEQRPGVQECYHGNGQSYRGTYSTTVTGRTCQAWSSMTPHSHSRTPEY
YPNAGLIMNYCRNPDAVAAPYCYTRDPGVRWEYCNLTQCSDAEGTAVAPPTVTPVPSLEA
PSEQAPTEQRPGVQECYHGNGQSYRGTYSTTVTGRTCQAWSSMTPHSHSRTPEYYPNAGL
IMNYCRNPDAVAAPYCYTRDPGVRWEYCNLTQCSDAEGTAVAPPTVTPVPSLEAPSEQAP
TEQRPGVQECYHGNGQSYRGTYSTTVTGRTCQAWSSMTPHSHSRTPEYYPNAGLIMNYCR
NPDAVAAPYCYTRDPGVRWEYCNLTQCSDAEGTAVAPPTVTPVPSLEAPSEQAPTEQRPG
VQECYHGNGQSYRGTYSTTVTGRTCQAWSSMTPHSHSRTPEYYPNAGLIMNYCRNPDAVA
APYCYTRDPGVRWEYCNLTQCSDAEGTAVAPPTVTPVPSLEAPSEQAPTEQRPGVQECYH

GNGQSYRGTYSTTVTGRTCQAWSSMTPHSHSRTPEYYPNAGLIMNYCRNPDAVAAPYCYT
RDPGVRWEYCNLTQCSDAEGTAVAPPTVTPVPSLEAPSEQAPTEQRPGVQECYHGNGQSY
RGTYSTTVTGRTCQAWSSMTPHSHSRTPEYYPNAGLIMNYCRNPDAVAAPYCYTRDPGVR
WEYCNLTQCSDAEGTAVAPPTVTPVPSLEAPSEQAPTEQRPGVQECYHGNGQSYRGTYST
TVTGRTCQAWSSMTPHSHSRTPEYYPNAGLIMNYCRNPDAVAAPYCYTRDPGVRWEYCNL
TQCSDAEGTAVAPPTVTPVPSLEAPSEQAPTEQRPGVQECYHGNGQSYRGTYSTTVTGRT
CQAWSSMTPHSHSRTPEYYPNAGLIMNYCRNPDAVAAPYCYTRDPGVRWEYCNLTQCSDA
EGTAVAPPTVTPVPSLEAPSEQAPTEQRPGVQECYHGNGQSYRGTYSTTVTGRTCQAWSS
MTPHSHSRTPEYYPNAGLIMNYCRNPDAVAAPYCYTRDPGVRWEYCNLTQCSDAEGTAVA
PPTVTPVPSLEAPSEQAPTEQRPGVQECYHGNGQSYRGTYSTTVTGRTCQAWSSMTPHSH
SRTPEYYPNAGLIMNYCRNPDAVAAPYCYTRDPGVRWEYCNLTQCSDAEGTAVAPPTVTP
VPSLEAPSEQAPTEQRPGVQECYHGNGQSYRGTYSTTVTGRTCQAWSSMTPHSHSRTPEY
YPNAGLIMNYCRNPDAVAAPYCYTRDPGVRWEYCNLTQCSDAEGTAVAPPTVTPVPSLEA
PSEQAPTEQRPGVQECYHGNGQSYRGTYSTTVTGRTCQAWSSMTPHSHSRTPEYYPNAGL
IMNYCRNPDAVAAPYCYTRDPGVRWEYCNLTQCSDAEGTAVAPPTVTPVPSLEAPSEQAP
TEQRPGVQECYHGNGQSYRGTYSTTVTGRTCQAWSSMTPHSHSRTPEYYPNAGLIMNYCR
NPDAVAAPYCYTRDPGVRWEYCNLTQCSDAEGTAVAPPTVTPVPSLEAPSEQAPTEQRPG
VQECYHGNGQSYRGTYSTTVTGRTCQAWSSMTPHSHSRTPEYYPNAGLIMNYCRNPDAVA
APYCYTRDPGVRWEYCNLTQCSDAEGTAVAPPTVTPVPSLEAPSEQAPTEQRPGVQECYH
GNGQSYRGTYSTTVTGRTCQAWSSMTPHSHSRTPEYYPNAGLIMNYCRNPDAVAAPYCYT
RDPGVRWEYCNLTQCSDAEGTAVAPPTVTPVPSLEAPSEQAPTEQRPGVQECYHGNGQSY
RGTYSTTVTGRTCQAWSSMTPHSHSRTPEYYPNAGLIMNYCRNPDAVAAPYCYTRDPGVR
WEYCNLTQCSDAEGTAVAPPTVTPVPSLEAPSEQAPTEQRPGVQECYHGNGQSYRGTYST
TVTGRTCQAWSSMTPHSHSRTPEYYPNAGLIMNYCRNPDAVAAPYCYTRDPGVRWEYCNL
TQCSDAEGTAVAPPTVTPVPSLEAPSEQAPTEQRPGVQECYHGNGQSYRGTYSTTVTGRT
CQAWSSMTPHSHSRTPEYYPNAGLIMNYCRNPDAVAAPYCYTRDPGVRWEYCNLTQCSDA
EGTAVAPPTVTPVPSLEAPSEQAPTEQRPGVQECYHGNGQSYRGTYSTTVTGRTCQAWSS
MTPHSHSRTPEYYPNAGLIMNYCRNPDPVAAPYCYTRDPSVRWEYCNLTQCSDAEGTAVA
PPTITPIPSLEAPSEQAPTEQRPGVQECYHGNGQSYQGTYFITVTGRTCQAWSSMTPHSH
SRTPAYYPNAGLIKNYCRNPDPVAAPWCYTTDPSVRWEYCNLTRCSDAEWTAFVPPNVIL
APSLEAFFEQALTEETPGVQDCYYHYGQSYRGTYSTTVTGRTCQAWSSMTPHQHSRTPEN
YPNAGLTRNYCRNPDAEIRPWCYTMDPSVRWEYCNLTQCLVTESSVLATLTVVPDPSTEA
SSEEAPTEQSPGVQDCYHGDGQSYRGSFSTTVTGRTCQSWSSMTPHWHQRTTEYYPNGGL
TRNYCRNPDAEISPWCYTMDPNVRWEYCNLTQCPVTESSVLATSTAVSEQAPTEQSPTVQ
DCYHGDGQSYRGSFSTTVTGRTCQSWSSMTPHWHQRTTEYYPNGGLTRNYCRNPDAEIRP
WCYTMDPSVRWEYCNLTQCPVMESTLLTTPTVVPVPSTELPSEEAPTENSTGVQDCYRGD
GQSYRGTLSTTITGRTCQSWSSMTPHWHRRIPLYYPNAGLTRNYCRNPDAEIRPWCYTMD
PSVRWEYCNLTRCPVTESSVLTTPTVAPVPSTEAPSEQAPPEKSPVVQDCYHGDGRSYRG
ISSTTVTGRTCQSWSSMIPHWHQRTPENYPNAGLTENYCRNPDSGKQPWCYTTDPCVRWE
YCNLTQCSETESGVLETPTVVPVPSMEAHSEAAPTEQTPVVRQCYHGNGQSYRGTFSTTV
TGRTCQSWSSMTPHRHQRTPENYPNDGLTMNYCRNPDADTGPWCFTMDPSIRWEYCNLTR
CSDTEGTVVAPPTVIQVPSLGPPSEQDCMFGNGKGYRGKKATTVTGTPCQEWAAQEPHRH
STFIPGTNKWAGLEKNYCRNPDGDINGPWCYTMNPRKLFDYCDIPLCASSSFDCGKPQVE
PKKCPGSIVGGCVAHPHSWPWQVSLRTRFGKHFCGGTLISPEWVLTAAHCLKKSSRPSSY

KVILGAHQEVNLESHVQEIEVSRLFLEPTQADIALLKLSRPAVITDKVMPACLPSPDYMV
TARTECYITGWGETQGTFGTGLLKEAQLLVIENEVCNHYKYICAEHLARGTDSCQGDSGG
PLVCFEKDKYILQGVTSWGLGCARPNKPGVYARVSRFVTWIEGMMRNN
>sp|Q0VD83|APOBR_HUMAN Apolipoprotein B receptor OS=Homo sapiens GN=APOBR PE=1 SV=2
MDFLRLYLPGLHQALRGALDSLGTFVSYLLGDAVPTVEREAQAAEELGVVAVGKTGKIVE
EEAQEDLEGLRGSQNEGAGRLRGPGDDRRHEVGSSAVEQTWGWGDGSSHGSQAERQDSGA
GETAKAARCQEPSAHLEARKKSKAGSGACQDRSGQAQERQESHEQEVNREERLRSWEQEE
EEEEVRAREPGMARGAESEWTWHGETEGKAGAVGPKAAGDNREMEQGVREADAGETEEPG
AEGAGKGEEVVVVEKACESTRAWGTWGPGAEPEDWGILGREEARTTPGREEARAILDGEE
ARTISGGEEAETASGGEEAETASGGEEAGTASGGEEAGIASGGEAGTASGGEEAGTASGG
DEAWTTSGKEEADLLGVRQTEYGAVPGERLLEATGKVWVLEEEGDEEREAEVSPFPKQPQ
VLGTERTEEAAESQTAGREAVGGQEAGESFEGQVDLRGKEAEMRQDLGIRADRARMEELV
QAEEAQEERGSSRDPVAELPSDGEAEGTADLEATPEARPEEELTGEESEAAQTSCGLLGV
EWGGLTHSVTKGQGPELMGGAQTPTKQPEEREAGEVELMGVLALSKEEQERSLEAGPRHA
GSVKPEASEAFPGAWENRTRKDMERGNTQEDAADGEQREEEETAGGQTLAAEAEGDRESE
LSEVPEAGGEGLTTQDAGCGTEEGEASVSENQELDGSTGADAGPCPSLGEAYARETEDEE
AEADRTSRRGWRLQAVAVGLPDREDAQTGSVAAGIMGGDVVPHISAAGAGEALEGVLGQG
WDSKEKEEAAAGEHAGGQEFGLEGSAEEEVTGRGSQVEAFESREGGPWGGRVEAEESAGA
EDSCGLDPAGSQTARAEGMGAMVEAGGLLEKWTLLEEEAVGWQEREQREDSEGRCGDYHP
EGEAPRLLDAEGLMVTGGRRAEAKETEPESLEHVRGQEEQPTHQAPAEAAPESVGEAETA
EAMGSARGGAANSWSEAPLPGSLLDVSVPRSRVHLSRSSSQRRSRPSFRRTPAWEQQEEP
PAPNPPEEELSAPEQRPLQLEEPLEPSPLRHDGTPVPARRRPLGHGFGLAHPGMMQELQA
RLGRPKPQ
>sp|P04114|APOB_HUMAN Apolipoprotein B-100 OS=Homo sapiens GN=APOB PE=1 SV=2
MDPPRPALLALLALPALLLLLLAGARAEEEMLENVSLVCPKDATRFKHLRKYTYNYEAES
SSGVPGTADSRSATRINCKVELEVPQLCSFILKTSQCTLKEVYGFNPEGKALLKKTKNSE
EFAAAMSRYELKLAIPEGKQVFLYPEKDEPTYILNIKRGIISALLVPPETEEAKQVLFLD
TVYGNCSTHFTVKTRKGNVATEISTERDLGQCDRFKPIRTGISPLALIKGMTRPLSTLIS
SSQSCQYTLDAKRKHVAEAICKEQHLFLPFSYKNKYGMVAQVTQTLKLEDTPKINSRFFG
EGTKKMGLAFESTKSTSPPKQAEAVLKTLQELKKLTISEQNIQRANLFNKLVTELRGLSD
EAVTSLLPQLIEVSSPITLQALVQCGQPQCSTHILQWLKRVHANPLLIDVVTYLVALIPE
PSAQQLREIFNMARDQRSRATLYALSHAVNNYHKTNPTGTQELLDIANYLMEQIQDDCTG
DEDYTYLILRVIGNMGQTMEQLTPELKSSILKCVQSTKPSLMIQKAAIQALRKMEPKDKD
QEVLLQTFLDDASPGDKRLAAYLMLMRSPSQADINKIVQILPWEQNEQVKNFVASHIANI
LNSEELDIQDLKKLVKEALKESQLPTVMDFRKFSRNYQLYKSVSLPSLDPASAKIEGNLI
FDPNNYLPKESMLKTTLTAFGFASADLIEIGLEGKGFEPTLEALFGKQGFFPDSVNKALY
WVNGQVPDGVSKVLVDHFGYTKDDKHEQDMVNGIMLSVEKLIKDLKSKEVPEARAYLRIL
GEELGFASLHDLQLLGKLLLMGARTLQGIPQMIGEVIRKGSKNDFFLHYIFMENAFELPT
GAGLQLQISSSGVIAPGAKAGVKLEVANMQAELVAKPSVSVEFVTNMGIIIPDFARSGVQ
MNTNFFHESGLEAHVALKAGKLKFIIPSPKRPVKLLSGGNTLHLVSTTKTEVIPPLIENR
QSWSVCKQVFPGLNYCTSGAYSNASSTDSASYYPLTGDTRLELELRPTGEIEQYSVSATY
ELQREDRALVDTLKFVTQAEGAKQTEATMTFKYNRQSMTLSSEVQIPDFDVDLGTILRVN
DESTEGKTSYRLTLDIQNKKITEVALMGHLSCDTKEERKIKGVISIPRLQAEARSEILAH
WSPAKLLLQMDSSATAYGSTVSKRVAWHYDEEKIEFEWNTGTNVDTKKMTSNFPVDLSDY

PKSLHMYANRLLDHRVPQTDMTFRHVGSKLIVAMSSWLQKASGSLPYTQTLQDHLNSLKE
FNLQNMGLPDFHIPENLFLKSDGRVKYTLNKNSLKIEIPLPFGGKSSRDLKMLETVRTPA
LHFKSVGFHLPSREFQVPTFTIPKLYQLQVPLLGVLDLSTNVYSNLYNWSASYSGGNTST
DHFSLRARYHMKADSVVDLLSYNVQGSGETTYDHKNTFTLSYDGSLRHKFLDSNIKFSHV
EKLGNNPVSKGLLIFDASSSWGPQMSASVHLDSKKKQHLFVKEVKIDGQFRVSSFYAKGT
YGLSCQRDPNTGRLNGESNLRFNSSYLQGTNQITGRYEDGTLSLTSTSDLQSGIIKNTAS
LKYENYELTLKSDTNGKYKNFATSNKMDMTFSKQNALLRSEYQADYESLRFFSLLSGSLN
SHGLELNADILGTDKINSGAHKATLRIGQDGISTSATTNLKCSLLVLENELNAELGLSGA
SMKLTTNGRFREHNAKFSLDGKAALTELSLGSAYQAMILGVDSKNIFNFKVSQEGLKLSN
DMMGSYAEMKFDHTNSLNIAGLSLDFSSKLDNIYSSDKFYKQTVNLQLQPYSLVTTLNSD
LKYNALDLTNNGKLRLEPLKLHVAGNLKGAYQNNEIKHIYAISSAALSASYKADTVAKVQ
GVEFSHRLNTDIAGLASAIDMSTNYNSDSLHFSNVFRSVMAPFTMTIDAHTNGNGKLALW
GEHTGQLYSKFLLKAEPLAFTFSHDYKGSTSHHLVSRKSISAALEHKVSALLTPAEQTGT
WKLKTQFNNNEYSQDLDAYNTKDKIGVELTGRTLADLTLLDSPIKVPLLLSEPINIIDAL
EMRDAVEKPQEFTIVAFVKYDKNQDVHSINLPFFETLQEYFERNRQTIIVVLENVQRNLK
HINIDQFVRKYRAALGKLPQQANDYLNSFNWERQVSHAKEKLTALTKKYRITENDIQIAL
DDAKINFNEKLSQLQTYMIQFDQYIKDSYDLHDLKIAIANIIDEIIEKLKSLDEHYHIRV
NLVKTIHDLHLFIENIDFNKSGSSTASWIQNVDTKYQIRIQIQEKLQQLKRHIQNIDIQH
LAGKLKQHIEAIDVRVLLDQLGTTISFERINDILEHVKHFVINLIGDFEVAEKINAFRAK
VHELIERYEVDQQIQVLMDKLVELAHQYKLKETIQKLSNVLQQVKIKDYFEKLVGFIDDA
VKKLNELSFKTFIEDVNKFLDMLIKKLKSFDYHQFVDETNDKIREVTQRLNGEIQALELP
QKAEALKLFLEETKATVAVYLESLQDTKITLIINWLQEALSSASLAHMKAKFRETLEDTR
DRMYQMDIQQELQRYLSLVGQVYSTLVTYISDWWTLAAKNLTDFAEQYSIQDWAKRMKAL
VEQGFTVPEIKTILGTMPAFEVSLQALQKATFQTPDFIVPLTDLRIPSVQINFKDLKNIK
IPSRFSTPEFTILNTFHIPSFTIDFVEMKVKIIRTIDQMLNSELQWPVPDIYLRDLKVED
IPLARITLPDFRLPEIAIPEFIIPTLNLNDFQVPDLHIPEFQLPHISHTIEVPTFGKLYS
ILKIQSPLFTLDANADIGNGTTSANEAGIAASITAKGESKLEVLNFDFQANAQLSNPKIN
PLALKESVKFSSKYLRTEHGSEMLFFGNAIEGKSNTVASLHTEKNTLELSNGVIVKINNQ
LTLDSNTKYFHKLNIPKLDFSSQADLRNEIKTLLKAGHIAWTSSGKGSWKWACPRFSDEG
THESQISFTIEGPLTSFGLSNKINSKHLRVNQNLVYESGSLNFSKLEIQSQVDSQHVGHS
VLTAKGMALFGEGKAEFTGRHDAHLNGKVIGTLKNSLFFSAQPFEITASTNNEGNLKVRF
PLRLTGKIDFLNNYALFLSPSAQQASWQVSARFNQYKYNQNFSAGNNENIMEAHVGINGE
ANLDFLNIPLTIPEMRLPYTIITTPPLKDFSLWEKTGLKEFLKTTKQSFDLSVKAQYKKN
KHRHSITNPLAVLCEFISQSIKSFDRHFEKNRNNALDFVTKSYNETKIKFDKYKAEKSHD
ELPRTFQIPGYTVPVVNVEVSPFTIEMSAFGYVFPKAVSMPSFSILGSDVRVPSYTLILP
SLELPVLHVPRNLKLSLPDFKELCTISHIFIPAMGNITYDFSFKSSVITLNTNAELFNQS
DIVAHLLSSSSSVIDALQYKLEGTTRLTRKRGLKLATALSLSNKFVEGSHNSTVSLTTKN
MEVSVATTTKAQIPILRMNFKQELNGNTKSKPTVSSSMEFKYDFNSSMLYSTAKGAVDHK
LSLESLTSYFSIESSTKGDVKGSVLSREYSGTIASEANTYLNSKSTRSSVKLQGTSKIDD
IWNLEVKENFAGEATLQRIYSLWEHSTKNHLQLEGLFFTNGEHTSKATLELSPWQMSALV
QVHASQPSSFHDFPDLGQEVALNANTKNQKIRWKNEVRIHSGSFQSQVELSNDQEKAHLD
IAGSLEGHLRFLKNIILPVYDKSLWDFLKLDVTTSIGRRQHLRVSTAFVYTKNPNGYSFS
IPVKVLADKFIIPGLKLNDLNSVLVMPTFHVPFTDLQVPSCKLDFREIQIYKKLRTSSFA
LNLPTLPEVKFPEVDVLTKYSQPEDSLIPFFEITVPESQLTVSQFTLPKSVSDGIAALDL

NAVANKIADFELPTIIVPEQTIEIPSIKFSVPAGIVIPSFQALTARFEVDSPVYNATWSA
SLKNKADYVETVLDSTCSSTVQFLEYELNVLGTHKIEDGTLASKTKGTFAHRDFSAEYEE
DGKYEGLQEWEGKAHLNIKSPAFTDLHLRYQKDKKGISTSAASPAVGTVGMDMDEDDDFS
KWNFYYSPQSSPDKKLTIFKTELRVRESDEETQIKVNWEEEAASGLLTSLKDNVPKATGV
LYDYVNKYHWEHTGLTLREVSSKLRRNLQNNAEWVYQGAIRQIDDIDVRFQKAASGTTGT
YQEWKDKAQNLYQELLTQEGQASFQGLKDNVFDGLVRVTQEFHMKVKHLIDSLIDFLNFP
RFQFPGKPGIYTREELCTMFIREVGTVLSQVYSKVHNGSEILFSYFQDLVITLPFELRKH
KLIDVISMYRELLKDLSKEAQEVFKAIQSLKTTEVLRNLQDLLQFIFQLIEDNIKQLKEM
KFTYLINYIQDEINTIFSDYIPYVFKLLKENLCLNLHKFNEFIQNELQEASQELQQIHQY
IMALREEYFDPSIVGWTVKYYELEEKIVSLIKNLLVALKDFHSEYIVSASNFTSQLSSQV
EQFLHRNIQEYLSILTDPDGKGKEKIAELSATAQEIIKSQAIATKKIISDYHQQFRYKLQ
DFSDQLSDYYEKFIAESKRLIDLSIQNYHTFLIYITELLKKLQSTTVMNPYMKLAPGELT
IIL
>sp|P02654|APOC1_HUMAN Apolipoprotein C-I OS=Homo sapiens GN=APOC1 PE=1 SV=1
MRLFLSLPVLVVVLSIVLEGPAPAQGTPDVSSALDKLKEFGNTLEDKARELISRIKQSEL
SAKMREWFSETFQKVKEKLKIDS
>sp|Q9BWW9|APOL5_HUMAN Apolipoprotein L5 OS=Homo sapiens GN=APOL5 PE=2 SV=1
MPCGKQGNLQVPGSKVLPGLGEGCKEMWLRKVIYGGEVWGKSPEPEFPSLVNLCQSWKIN
NLMSTVHSDEAGMLSYFLFEELMRCDKDSMPDGNLSEEEKLFLSYFPLHKFELEQNIKEL
NTLADQVDTTHELLTKTSLVASSSGAVSGVMNILGLALAPVTAGGSLMLSATGTGLGAAA
AITNIVTNVLENRSNSAARDKASRLGPLTTSHEAFGGINWSEIEAAGFCVNKCVKAIQGI
KDLHAYQMAKSNSGFMAMVKNFVAKRHIPFWTARGVQRAFEGTTLAMTNGAWVMGAAGAG
FLLMKDMSSFLQSWKHLEDGARTETAEELRALAKKLEQELDRLTQHHRHLPQKASQTCSS
SRGRAVRGSRVVKPEGSRSPLPWPVVEHQPRLGPGVALRTPKRTVSAPRMLGHQPAPPAP
ARKGRQAPGRHRQ
>sp|Q96IL0|APOP1_HUMAN Apoptogenic protein 1, mitochondrial OS=Homo sapiens GN=APOPT1 PE=2 SV=3
MLPCAAGARGRGAMVVLRAGKKTFLPPLCRAFACRGCQLAPERGAERRDTAPSGVSRFCP
PRKSCHDWIGPPDKYSNLRPVHFYIPENESPLEQKLRKLRQETQEWNQQFWANQNLTFSK
EKEEFIHSRLKTKGLGLRTESGQKATLNAEEMADFYKEFLSKNFQKHMYYNRDWYKRNFA
ITFFMGKVALERIWNKLKQKQKKRSN
>sp|Q7Z2E3|APTX_HUMAN Aprataxin OS=Homo sapiens GN=APTX PE=1 SV=2
MSNVNLSVSDFWRVMMRVCWLVRQDSRHQRIRLPHLEAVVIGRGPETKITDKKCSRQQVQ
LKAECNKGYVKVKQVGVNPTSIDSVVIGKDQEVKLQPGQVLHMVNELYPYIVEFEEEAKN
PGLETHRKRKRSGNSDSIERDAAQEAEAGTGLEPGSNSGQCSVPLKKGKDAPIKKESLGH
WSQGLKISMQDPKMQVYKDEQVVVIKDKYPKARYHWLVLPWTSISSLKAVAREHLELLKH
MHTVGEKVIVDFAGSSKLRFRLGYHAIPSMSHVHLHVISQDFDSPCLKNKKHWNSFNTEY
FLESQAVIEMVQEAGRVTVRDGMPELLKLPLRCHECQQLLPSIPQLKEHLRKHWTQ
>sp|Q86TW2|ADCK1_HUMAN Uncharacterized aarF domain-containing protein kinase 1 OS=Homo sapiens GN=ADCK1 PE=2 SV=2
MARKALKLASWTSMALAASGIYFYSNKYLDPNDFGAVRVGRAVATTAVISYDYLTSLKSV
PYGSEEYLQLRSKSWPVFLQVHLRSARRLCELCCANRGTFIKVGQHLGALDYLLPEEYTS
TLKVLHSQAPQSSMQEIRQVIREDLGKEIHDLFQSFDDTPLGTASLAQVHKAVLHDGRTV
AVKVQHPKVRAQSSKDILLMEVLVLAVKQLFPEFEFMWLVDEAKKNLPLELDFLNEGRNA

EKVSQMLRHFDFLKVPRIHWDLSTERVLLMEFVDGGQVNDRDYMERNKIDVNEISRHLGK
MYSEMIFVNGFVHCDPHPGNVLVRKHPGTGKAEIVLLDHGLYQMLTEEFRLNYCHLWQSL
IWTDMKRVKEYSQRLGAGDLYPLFACMLTARSWDSVNRGISQAPVTATEDLEIRNNAANY
LPQISHLLNHVPRQMLLILKTNDLLRGIEAALGTRASASSFLNMSRCCIRALAEHKKKNT
CSFFRRTQISFSEAFNLWQINLHELILRVKGLKLADRVLALICWLFPAPL
>sp|P51828|ADCY7_HUMAN Adenylate cyclase type 7 OS=Homo sapiens GN=ADCY7 PE=2 SV=1
MPAKGRYFLNEGEEGPDQDALYEKYQLTSQHGPLLLTLLLVAATACVALIIIAFSQGDPS
RHQAILGMAFLVLAVFAALSVLMYVECLLRRWLRALALLTWACLVALGYVLVFDAWTKAA
CAWEQVPFFLFIVFVVYTLLPFSMRGAVAVGAVSTASHLLVLGSLMGGFTTPSVRVGLQL
LANAVIFLCGNLTGAFHKHQMQDASRDLFTYTVKCIQIRRKLRIEKRQQENLLLSVLPAH
ISMGMKLAIIERLKEHGDRRCMPDNNFHSLYVKRHQNVSILYADIVGFTQLASDCSPKEL
VVVLNELFGKFDQIAKANECMRIKILGDCYYCVSGLPVSLPTHARNCVKMGLDMCQAIKQ
VREATGVDINMRVGIHSGNVLCGVIGLRKWQYDVWSHDVSLANRMEAAGVPGRVHITEAT
LKHLDKAYEVEDGHGQQRDPYLKEMNIRTYLVIDPRSQQPPPPSQHLPRPKGDAALKMRA
SVRMTRYLESWGAARPFAHLNHRESVSSGETHVPNGRRPKSVPQRHRRTPDRSMSPKGRS
EDDSYDDEMLSAIEGLSSTRPCCSKSDDFYTFGSIFLEKGFEREYRLAPIPRARHDFACA
SLIFVCILLVHVLLMPRTAALGVSFGLVACVLGLVLGLCFATKFSRCCPARGTLCTISER
VETQPLLRLTLAVLTIGSLLTVAIINLPLMPFQVPELPVGNETGLLAASSKTRALCEPLP
YYTCSCVLGFIACSVFLRMSLEPKVVLLTVALVAYLVLFNLSPCWQWDCCGQGLGNLTKP
NGTTSGTPSCSWKDLKTMTNFYLVLFYITLLTLSRQIDYYCRLDCLWKKKFKKEHEEFET
MENVNRLLLENVLPAHVAAHFIGDKLNEDWYHQSYDCVCVMFASVPDFKVFYTECDVNKE
GLECLRLLNEIIADFDELLLKPKFSGVEKIKTIGSTYMAAAGLSVASGHENQELERQHAH
IGVMVEFSIALMSKLDGINRHSFNSFRLRVGINHGPVIAGVIGARKPQYDIWGNTVNVAS
RMESTGELGKIQVTEETCTILQGLGYSCECRGLINVKGKGELRTYFVCTDTAKFQGLGLN
>sp|O60503|ADCY9_HUMAN Adenylate cyclase type 9 OS=Homo sapiens GN=ADCY9 PE=1 SV=4
MASPPHQQLLHHHSTEVSCDSSGDSNSVRVKINPKQLSSNSHPKHCKYSISSSCSSSGDS
GGVPRRVGGGGRLRRQKKLPQLFERASSRWWDPKFDSVNLEEACLERCFPQTQRRFRYAL
FYIGFACLLWSIYFAVHMRSRLIVMVAPALCFLLVCVGFFLFTFTKLYARHYAWTSLALT
LLVFALTLAAQFQVLTPVSGRGDSSNLTATARPTDTCLSQVGSFSMCIEVLFLLYTVMHL
PLYLSLCLGVAYSVLFETFGYHFRDEACFPSPGAGALHWELLSRGLLHGCIHAIGVHLFV
MSQVRSRSTFLKVGQSIMHGKDLEVEKALKERMIHSVMPRIIADDLMKQGDEESENSVKR
HATSSPKNRKKKSSIQKAPIAFRPFKMQQIEEVSILFADIVGFTKMSANKSAHALVGLLN
DLFGRFDRLCEETKCEKISTLGDCYYCVAGCPEPRADHAYCCIEMGLGMIKAIEQFCQEK
KEMVNMRVGVHTGTVLCGILGMRRFKFDVWSNDVNLANLMEQLGVAGKVHISEATAKYLD
DRYEMEDGKVIERLGQSVVADQLKGLKTYLISGQRAKESRCSCAEALLSGFEVIDGSQVS
SGPRGQGTASSGNVSDLAQTVKTFDNLKTCPSCGITFAPKSEAGAEGGAPQNGCQDEHKN
STKASGGPNPKTQNGLLSPPQEEKLTNSQTSLCEILQEKGRWAGVSLDQSALLPLRFKNI
REKTDAHFVDVIKEDSLMKDYFFKPPINQFSLNFLDQELERSYRTSYQEEVIKNSPVKTF
ASPTFSSLLDVFLSTTVFLTLSTTCFLKYEAATVPPPPAALAVFSAALLLEVLSLAVSIR
MVFFLEDVMACTKRLLEWIAGWLPRHCIGAILVSLPALAVYSHVTSEYETNIHFPVFTGS
AALIAVVHYCNFCQLSSWMRSSLATVVGAGPLLLLYVSLCPDSSVLTSPLDAVQNFSSER
NPCNSSVPRDLRRPASLIGQEVVLVFFLLLLLVWFLNREFEVSYRLHYHGDVEADLHRTK
IQSMRDQADWLLRNIIPYHVAEQLKVSQTYSKNHDSGGVIFASIVNFSEFYEENYEGGKE
CYRVLNELIGDFDELLSKPDYSSIEKIKTIGATYMAASGLNTAQAQDGSHPQEHLQILFE

FAKEMMRVVDDFNNNMLWFNFKLRVGFNHGPLTAGVIGTTKLLYDIWGDTVNIASRMDTT
GVECRIQVSEESYRVLSKMGYDFDYRGTVNVKGKGQMKTYLYPKCTDHRVIPQHQLSISP
DIRVQVDGSIGRSPTDEIANLVPSVQYVDKTSLGSDSSTQAKDAHLSPKRPWKEPVKAEE
RGRFGKAIEKDDCDETGIEEANELTKLNVSKSV
>sp|P08319|ADH4_HUMAN Alcohol dehydrogenase 4 OS=Homo sapiens GN=ADH4 PE=1 SV=5
MGTKGKVIKCKAAIAWEAGKPLCIEEVEVAPPKAHEVRIQIIATSLCHTDATVIDSKFEG
LAFPVIVGHEAAGIVESIGPGVTNVKPGDKVIPLYAPLCRKCKFCLSPLTNLCGKISNLK
SPASDQQLMEDKTSRFTCKGKPVYHFFGTSTFSQYTVVSDINLAKIDDDANLERVCLLGC
GFSTGYGAAINNAKVTPGSTCAVFGLGGVGLSAVMGCKAAGASRIIGIDINSEKFVKAKA
LGATDCLNPRDLHKPIQEVIIELTKGGVDFALDCAGGSETMKAALDCTTAGWGSCTFIGV
AAGSKGLTIFPEELIIGRTINGTFFGGWKSVDSIPKLVTDYKNKKFNLDALVTHTLPFDK
ISEAFDLMNQGKSVRTILIF
>sp|Q9Y2D8|ADIP_HUMAN Afadin- and alpha-actinin-binding protein OS=Homo sapiens GN=SSX2IP PE=1 SV=3
MGDWMTVTDPGLSSESKTISQYTSETKMSPSSLYSQQVLCSSIPLSKNVHSFFSAFCTED
NIEQSISYLDQELTTFGFPSLYEESKGKETKRELNIVAVLNCMNELLVLQRKNLLAQENV
ETQNLKLGSDMDHLQSCYSKLKEQLETSRREMIGLQERDRQLQCKNRNLHQLLKNEKDEV
QKLQNIIASRATQYNHDMKRKEREYNKLKERLHQLVMNKKDKKIAMDILNYVGRADGKRG
SWRTGKTEARNEDEMYKILLNDYEYRQKQILMENAELKKVLQQMKKEMISLLSPQKKKPR
ERVDDSTGTVISDVEEDAGELSRESMWDLSCETVREQLTNSIRKQWRILKSHVEKLDNQV
SKVHLEGFNDEDVISRQDHEQETEKLELEIQQCKEMIKTQQQLLQQQLATAYDDDTTSLL
RDCYLLEEKERLKEEWSLFKEQKKNFERERRSFTEAAIRLGLERKAFEEERASWLKQQFL
NMTTFDHQNSENVKLFSAFSGSSDWDNLIVHSRQPQKKPHSVSNGSPVCMSKLTKSLPAS
PSTSDFCQTRSCISEHSSINVLNITAEEIKPNQVGGECTNQKWSVASRPGSQEGCYSGCS
LSYTNSHVEKDDLP
>sp|Q6ULP2|AFTIN_HUMAN Aftiphilin OS=Homo sapiens GN=AFTPH PE=1 SV=2
MEPDIIRMYSSSPPPLDNGAEDDDDDEFGEFGGFSEVSPSGVGFVDFDTPDYTRPKEEFV
PSNHFMPIHEFSENVDSLTSFKSIKNGNDKDITAELSAPVKGQSDVLLSTTSKEIISSEM
LATSIDGMERPGNLNKVVEQRQNVGTLESFSPGDFRTNMNVVHQNKQLESCNGEKPPCLE
ILTNGFAVLETVNPQGTDDLDNVADSKGRKPLSTHSTEYNLDSVPSPAEEFADFATFSKK
ERIQLEEIECAVLNDREALTIRENNKINRVNELNSVKEVALGRSLDNKGDTDGEDQVCVS
EISIVTNRGFSVEKQGLPTLQQDEFLQSGVQSKAWSLVDSADNSEAIRREQCKTEEKLDL
LTSKCAHLCMDSVKTSDDEVGSPKEESRKFTNFQSPNIDPTEENDLDDSLSVKNGDSSND
FVTCNDINEDDFGDFGDFGSASGSTPPFVTGTQDSMSDATFEESSEHFPHFSEPGDDFGE
FGDINAVSCQEETILTKSDLKQTSDNLSEECQLARKSSGTGTEPVAKLKNGQEGEIGHFD
SVPNIQDDCNGFQDSDDFADFSSAGPSQVVDWNAFEDEQKDSCSWAAFGDQQATESHHRK
EAWQSHRTDENIDTPGTPKTHSVPSATSKGAVASGHLQESATSVQTALLNRLERIFEACF
PSILVPDAEEEVTSLKHLLETSTLPIKTREALPESGELLDVWTELQDIHDAHGLRYQWGG
SHSNKKLLSSLGIDTRNILFTGNKKQPVIVPMYAAGLGMLEPTKEPLKPLSAAEKIASIG
QTATMSPDMNTCTSDQFQESLPPVQFDWSSSGLTNPLDASGGSTLLNLDFFGPVDDSSSS
SSTTIPGVDPELYELTTSKLEISTSSLKVTDAFARLMSTVEKTSTSTSRKPKREEHLSEE
AIKVIAGLPDLTFMHAKVLMFPATLTPSTSSQEKADG
>sp|A8MT82|AG14P_HUMAN Putative Arf-GAP with GTPase, ANK repeat and PH domain-containing protein 14 OS=Homo sapiens GN=AGAP14P PE=5 SV=2

MGNILTCRVHPSVSLEFDQQQGSVCPSESEIYEAGAKDRMAGAPMAAAVQPAEVTVEVGE
DLHMHHVRDREMPEALEFNPSANPEASTIFQRNSQTDVVEIRRSNCTNHVSTERFSQQYS
SCSTIFLDDSTASQHYLTMTIISVTLEIPHHITQRDADRSLSIPDEQLTHLRHSSFCSTQ
VSTVHITKNRNGGGSLNNYSSSIPSTPSTSQEDPQFSVPPTANTPTPVCKWSMCWSNLFT
SEKGSHPDKERKAPENHADTIGSSRAIPIKQGMLLKRSGKWLKTWKKKYVTLCSNGVLTY
YSSLGDYMKNIHKKEIDLRTSTIKVPGKWPSLATSACAPISSSKSNGLSKDMDTGLGDSI
CFSPSISSTTSPKLNLPPSPHANKKKHLKKKSTNNFMIVSATGQTWHFEATTYEERDAWV
QAIQSQIVASLQSCESSKSKSQLTSQSEAMALQSIQNMRGNAHCVDCETQNPKWASLNLG
VLMCIECSGIHCSLGTRLSRVRSLELDDWPVELRKVMSSIGNDLANSIWEGSSQGQTKPS
VKSTREEKERWIRSKYEKKLFLAPLPCTELSLGQHLLRATADEDLQTAILLLAHGSREEV
NETCGEGDGCTALHLACRKGNVVLAQLLIWYRVDVMARDAHGNTALTYARQASSQECINV
LLQYGCPDECV
>sp|P52594|AGFG1_HUMAN Arf-GAP domain and FG repeat-containing protein 1 OS=Homo sapiens GN=AGFG1 PE=1 SV=2
MAASAKRKQEEKHLKMLRDMTGLPHNRKCFDCDQRGPTYVNMTVGSFVCTSCSGSLRGLN
PPHRVKSISMTTFTQQEIEFLQKHGNEVCKQIWLGLFDDRSSAIPDFRDPQKVKEFLQEK
YEKKRWYVPPEQAKVVASVHASISGSSASSTSSTPEVKPLKSLLGDSAPTLHLNKGTPSQ
SPVVGRSQGQQQEKKQFDLLSDLGSDIFAAPAPQSTATANFANFAHFNSHAAQNSANADF
ANFDAFGQSSGSSNFGGFPTASHSPFQPQTTGGSAASVNANFAHFDNFPKSSSADFGTFN
TSQSHQTASAVSKVSTNKAGLQTADKYAALANLDNIFSAGQGGDQGSGFGTTGKAPVGSV
VSVPSQSSASSDKYAALAELDSVFSSAATSSNAYTSTSNASSNVFGTVPVVASAQTQPAS
SSVPAPFGATPSTNPFVAAAGPSVASSTNPFQTNARGATAATFGTASMSMPTGFGTPAPY
SLPTSFSGSFQQPAFPAQAAFPQQTAFSQQPNGAGFAAFGQTKPVVTPFGQVAAAGVSSN
PFMTGAPTGQFPTGSSSTNPFL
>sp|Q8IVF2|AHNK2_HUMAN Protein AHNAK2 OS=Homo sapiens GN=AHNAK2 PE=1 SV=2
MCDCFHMVLPTWPGTPGSVSGRQLQPGEPGAETEDDHSVTEGPADEGIRPRPQGSSPVYE
YTTEAADFGLQEDAPGRQGSAGRRRSWWKRDSGDSRTFFRMSRPEAVQEATEVTLKTEVE
AGASGYSVTGGGDQGIFVKQVLKDSSAAKLFNLREGDQLLSTTVFFENIKYEDALKILQY
SEPYKVQFKIRRQLPAPQDEEWASSDAQHGPQGKEKEDTDVADGCRETPTKTLEGDGDQE
RLISKPRVGRGRQSQRERLSWPKFQSIKSKRGPGPQRSHSSSEAYEPRDAHDVSPTSTDT
EAQLTVERQEQKAGPGSQRRRKFLNLRFRTGSGQGPSSTGQPGRGFQSGVGRAGVLEELG
PWGDSLEETGAATGSRREERAEQDREVMPAQSMPLPTELGDPRLCEGTPQEGGLRAARLH
GKTLEGQAQETAVAQRKPRAQPTPGMSREGEGEGLQSLEIGIARLSLRDTTEGGTQIGPP
EIRVRVHDLKTPKFAFSTEKEPERERRLSTPQRGKRQDASSKAGTGLKGEEVEGAGWMPG
REPTTHAEAQGDEGDGEEGLQRTRITEEQDKGREDTEGQIRMPKFKIPSLGWSPSKHTKT
GREKATEDTEQGREGEATATADRREQRRTEEGLKDKEDSDSMTNTTKIQLIHDEKRLKKE
QILTEKEVATKDSKFKMPKFKMPLFGASAPGKSMEASVDVSAPKVEADVSLLSMQGDLKT
TDLSVQTPSADLEVQDGQVDVKLPEGPLPEGASLKGHLPKVQRPSLKMPKVDLKGPKLDL
KGPKAEVTAPDVKMSLSSMEVDVQAPRAKLDGARLEGDLSLADKEVTAKDSKFKMPKFKM
PSFGVSAPGKSMEDSVDVSAPKVEADVSLSSMQGDLKATDLSIQPPSADLEVQAGQVDVK
LPEGPVPEGAGPKVHLPKVEMPSFKMPKVDLKGPQIDVKGPKLDLKGPKAEVTAPDGEVS
LPSMEVDVQAQKAKLDGAWLEGDLSLADKDVTAKDSKFKMPKFKMPSFGVSAPGKSIKAL
VDVSAPKVEADLSLPSMQGDLKTTDLSIQPASTDLKVQADQVDVKLPEGHLPEGAGLKGH
LPKVEMPSFKMPKVALKGPQVDVKGPKLDLKSPKAEVTAPDVEVSLPSVEVDVEAPGAKL

DSARLEGELSLADKDVTAKDSRFKMPKFKMPSFGASAPGKSIEASVDVSAPKVEADVSLP
SMQGDLKTTDLSIQPPSADLEVHAGQVDVKLLEGHVPEGAGFKGHLPKVQMPSLKMPKVD
LKGPQVEVRGPKLDLKGHKAEVTAHEVAVSLPSVEVDMQAPGAKLDGAQLDGDLSLADKD
VTAKDSKFKMPKFKMPSFGVSAPGKSIEASVDLSAPKVEADMSLPSMQGDLKTTDLSIQP
PSTDLELQAGQLDVKLPEGPVPEGAGLKGHLPKLQMPSFKVPKVDLKGPEIDIKGPKLDL
KDPKVEVTAPDVEVSLPSVEVDVEAPGAKLDGGRLEEDMSLADKDLTTKDSKFKMPKFKM
PSFGVSAPGKSIEASVDVSAPKVEADVSLPSMQGDLKATDLSIQPPSADLEVQAGQVDVK
LPEGPVSEGAGLKGHLPKVQMPSFKMPKVDLKGPQIDVKGPKLDLKGPKVEVTAPDVKMS
LSSMEVDVQAPRAKLDGAQLEGDLSLADKAVTAKDSKFKMPKFKMPSFGVSAPGKSIEAS
VDVSEPKVEADVSLPSMQGDLKTTDLSIQSPSADLEVQAGQVNVKLPEGPLPEGAGFKGH
LPKVQMPSLKMPKVALKGPQMDVKGPKLDLKGPKAEVMAPDVEVSLPSVEVDVEAPGAKL
DSVRLEGDLSLADKDVTAKDSKFKMPKFKMPSFGVSAPGKSIEASVDVSAPKVEAEVSLP
SMQGDLKTTDLCIPLPSADLVVQAGQVDMKLPEGQVPEGAGLKGHLPKVDMPSFKMPKVD
LKGPQTDVKGAKLDLKGPKAEVTAPDVEVSLPSMEVDVQAQKAKLDGARLEGDLSLADKD
MTAKDSKFKMPKFKMPSFGVSAPGRSIEASVDVPAPKVEADVSLPSMQGDLKTTDLSIQP
PSADLKVQTGQVDVKLPEGHVPEGAGLKGHLPKVEMPSLKMPKVDLKGPQVDIKGPKLDL
KDPKVEMRVPDVEVSLPSMEVDVQAPRAKLDSAHLQGDLTLANKDLTTKDSKFKMPKFKM
PSFGVSAPGKSIEASVDVSPPKVEADMSLPSMQGDLKTTDLSIQPLSADVKVQAGQVDVK
LLEGPVPEEVGLKGHLPKLQMPSFKVPKVDLKGPEIDIKGPKLDLKDPKVEVTAPDVEVS
LPSVEVDVKAPGAKLDGARLEGDMSLADKDVTAKDSKFKMPKFKMLSFGVSALGKSIEAS
ADVSALKVEADVSLPSMQGDLKTTDLSVQPPSADLEVQAGQVDVKLPEGPVPEGAGLKGH
LPKLQMPSFKMPKVDLKGPQIDVKGPKLDLKGPKTDVMAPDVEVSQPSVEVDVEAPGAKL
DGAWLEGDLSVADKDVTTKDSRFKIPKFKMPSFGVSAPGKSIEASVDVSAPKVEADGSLS
SMQGDLKATDLSIQPPSADLEVQAGQVDVKLPEGPVPEGAGLKGHLPKVQMPSFKMPEMD
LKGPQLDVKGPKLDLKGPKAEVTAPDVEMSLSSMEVDVQAPRAKLDGARLEGDLSLADKG
VTAKDSKFKMPKFKMPSFRVSAPGESIEALVDVSELKVEADMSLPSMQGDLKTTDISIQP
PSAQLEVQAGQVDVKLPEGHVPEGAGLKGHLPKLQMPSFKMPEVDLKGPQIDVKGPNVDL
KGPKAEVTAPDVKMSLSSMEVDVQAPRAKLDGARLEGDLSLADKGMTAKDSKFKMPKFKM
PSFGVSAPGKSIEASVDVSELKVEADGSFPSMQGDLKTTDIRIQPPSAQLEVQAGQVDVK
LPEGHVPEGAGLKGHLPKVQMPSFKMPKVDLKGPQIDVKGPKLDLKGPKAEVTAPDVEVS
LPSVEVDVEAPRAKLDGARLEGDLSLADKDVTAKDSKFKMPKFKMPSFGVSAPGKSIEVS
VDVSAPKVEAEVSLPSMQGDLKTTDISIEPPSAQLEVQAGQVDLKLPEGHVPEGAGLKGH
LPKLQMPSFKMPKVDRKGPQIDVKGPKLDLKGPKTDVTAPDVEVSQPGMEVDVEAPGAKL
DGARLEGDLSLADKDVTAKDSKFKMPKFKMPSFGVSAPGKSIEVLVDVSAPKVEADLSLP
SMQGDLKNTDISIEPPSAQLEVQAGQVDVKLPEGHVLEGAGLKGHLPKLQMPSFKMPKVD
RKGPQIDIKGPKLDLKGPKMDVTAPDVEVSQPSMEVDVEAPGAKLDGARLEGDLSLADKD
VTAKDSKFKMPKFKMPSYRASAPGKSIQASVDVSAPKAEADVSLPSMQGDLKTTDLSIQL
PSVDLEVQAGQVDVKLPEGHVPEGAGLKGHLPKVEMPSFKMPKVDLKSPQVDIKGPKLDL
KVPKAEVTVPDVEVSLPSVEVDVQAPRAKLDGARLEGDLSLAEKDVTAKDSKFKMPKFKM
PSFGVSAPGRSIEASLDVSAPKVEADVSLSSMQGDLKATDLSIQPPSADLEVQAVQVDVE
LLEGPVPEGAGLKGHLPKVEMPSLKTPKVDLKGPQIDVKGPKLDLKGPKAEVRVPDVEVS
LPSVEVDVQAPKAKLDAGRLEGDLSLADKDVTAKDSKFKMPKFKMPSFRVSAPGKSMEAS
VDVSAPKVEADVSLPSMQGDLKTTDLSIQPPSADLKVQAGQMDVKLPEGQVPEGAGLKEH
LPKVEMPSLKMPKVDLKGPQVDIKGPKLDLKVSKAEVTAPDVEVSLPSVEVDVQAPRAKL

DSAQLEGDLSLADKDVTAKDSKFKMPKFKMPSFGVSAPGKSIEASVHVSAPKVEADVSLP
SMQGDLKTTDLSIQPHSADLTVQARQVDMKLLEGHVPEEAGLKGHLPKVQMPSFKMPKVD
LKGPEIDIKGPKLDLKDPKVEVTAPDVEVSLPSVEVDVEAPGAKLDGARLEGDLSLADKD
MTAKDSKFKMPKFKMPSFGVSAPGKSMEASVDVTAPKVEADVSLPSMQGDLKATDLSVQP
PSADLEVQAGQVDVKLPEGPVPEGASLKGHLPKVQMPSFKMPKVDLKGPQIDVKGPKLDL
KGPKAEVTAPDVKMSLSSMEVDVQAPRAKLDGVQLEGDLSLADKDVTAKDSKFKMPKFKM
PSFGVSAPGKSMEASVDVSELKAKADVSLPSMQGDLKTTDLSIQSPSADLEVQAGQVDVK
LPEGPLPKGAGLKGHLPKVQMPCLKMPKVALKGPQVDVKGPKLDLKGPKADVMTPVVEVS
LPSMEVDVEAPGAKLDSVRLEGDLSLADKDMTAKDSKFKMPKFKMPSFGVSAPGKSIEAS
LDVSALKVEADVSLPSMQGDLKTTHLSIQPPSADLEVQAGQEDVKLPEGPVHEGAGLKGH
LPKLQMPSFKVPKVDLKGPQIDVNVPKLDLKGPKVEVTSPNLDVSLPSMEVDIQAPGAKL
DSTRLEGDLSLADKDVTAKDSKFKMPKFKMPSFGMLSPGKSIEVSVDVSAPKMEADMSIP
SMQGDLKTTDLRIQAPSADLEVQAGQVDLKLPEGHMPEVAGLKGHLPKVEMPSFKMPKVD
LKGPQVDVKGPKLDLKGPKAEVMAPDVEVSLPSVETDVQAPGSMLDGARLEGDLSLAHED
VAGKDSKFQGPKLSTSGFEWSSKKVSMSSSEIEGNVTFHEKTSTFPIVESVVHEGDLHDP
SRDGNLGLAVGEVGMDSKFKKLHFKVPKVSFSSTKTPKDSLVPGAKSSIGLSTIPLSSSE
CSSFELQQVSACSEPSMQMPKVGFAGFPSSRLDLTGPHFESSILSPCEDVTLTKYQVTVP
RAALAPELALEIPSGSQADIPLPKTECSTDLQPPEGVPTSQAESHSGPLNSMIPVSLGQV
SFPKFYKPKFVFSVPQMAVPEGDLHAAVGAPVMSPLSPGERVQCPLPSTQLPSPGTCVSQ
GPEELVASLQTSVVAPGEAPSEDADHEGKGSPLKMPKIKLPSFRWSPKKETGPKVDPECS
VEDSKLSLVLDKDEVAPQSAIHMDLPPERDGEKGRSTKPGFAMPKLALPKMKASKSGVSL
PQRDVDPSLSSATAGGSFQDTEKASSDGGRGGLGATASATGSEGVNLHRPQVHIPSLGFA
KPDLRSSKAKVEVSQPEADLPLPKHDLSTEGDSRGCGLGDVPVSQPCGEGIAPTPEDPLQ
PSCRKPDAEVLTVESPEEEAMTKYSQESWFKMPKFRMPSLRRSFRDRGGAGKLEVAQTQA
PAATGGEAAAKVKEFLVSGSNVEAAMSLQLPEADAEVTASESKSSTDILRCDLDSTGLKL
HLSTAGMTGDELSTSEVRIHPSKGPLPFQMPGMRLPETQVLPGEIDETPLSKPGHDLASM
EDKTEKWSSQPEGPLKLKASSTDMPSQISVVNVDQLWEDSVLTVKFPKLMVPRFSFPAPS
SEDDVFIPTVREVQCPEANIDTALCKESPGLWGASILKAGAGVPGEQPVDLNLPLEAPPI
SKVRVHIQGAQVESQEVTIHSIVTPEFVDLSVPRTFSTQIVRESEIPTSEIQTPSYGFSL
LKVKIPEPHTQARVYTTMTQHSRTQEGTEEAPIQATPGVDSISGDLQPDTGEPFEMISSS
VNVLGQQTLTFEVPSGHQLADSCSDEEPAEILEFPPDDSQEATTPLADEGRAPKDKPESK
KSGLLWFWLPNIGFSSSVDETGVDSKNDVQRSAPIQTQPEARPEAELPKKQEKAGWFRFP
KLGFSSSPTKKSKSTEDGAELEEQKLQEETITFFDARESFSPEEKEEGELIGPVGTGLDS
RVMVTSAARTELILPEQDRKADDESKGSGLGPNEG
>sp|Q09666|AHNK_HUMAN Neuroblast differentiation-associated protein AHNAK OS=Homo sapiens GN=AHNAK PE=1 SV=2
MEKEETTRELLLPNWQGSGSHGLTIAQRDDGVFVQEVTQNSPAARTGVVKEGDQIVGATI
YFDNLQSGEVTQLLNTMGHHTVGLKLHRKGDRSPEPGQTWTREVFSSCSSEVVLSGDDEE
YQRIYTTKIKPRLKSEDGVEGDLGETQSRTITVTRRVTAYTVDVTGREGAKDIDISSPEF
KIKIPRHELTEISNVDVETQSGKTVIRLPSGSGAASPTGSAVDIRAGAISASGPELQGAG
HSKLQVTMPGIKVGGSGVNVNAKGLDLGGRGGVQVPAVDISSSLGGRAVEVQGPSLESGD
HGKIKFPTMKVPKFGVSTGREGQTPKAGLRVSAPEVSVGHKGGKPGLTIQAPQLEVSVPS
ANIEGLEGKLKGPQITGPSLEGDLGLKGAKPQGHIGVDASAPQIGGSITGPSVEVQAPDI
DVQGPGSKLNVPKMKVPKFSVSGAKGEETGIDVTLPTGEVTVPGVSGDVSLPEIATGGLE

GKMKGTKVKTPEMIIQKPKISMQDVDLSLGSPKLKGDIKVSAPGVQGDVKGPQVALKGSR
VDIETPNLEGTLTGPRLGSPSGKTGTCRISMSEVDLNVAAPKVKGGVDVTLPRVEGKVKV
PEVDVRGPKVDVSAPDVEAHGPEWNLKMPKMKMPTFSTPGAKGEGPDVHMTLPKGDISIS
GPKVNVEAPDVNLEGLGGKLKGPDVKLPDMSVKTPKISMPDVDLHVKGTKVKGEYDVTVP
KLEGELKGPKVDIDAPDVDVHGPDWHLKMPKMKMPKFSVPGFKAEGPEVDVNLPKADVDI
SGPKIDVTAPDVSIEEPEGKLKGPKFKMPEMNIKVPKISMPDVDLHLKGPNVKGEYDVTM
PKVESEIKVPDVELKSAKMDIDVPDVEVQGPDWHLKMPKMKMPKFSMPGFKAEGPEVDVN
LPKADVDISGPKVGVEVPDVNIEGPEGKLKGPKFKMPEMNIKAPKISMPDVDLHMKGPKV
KGEYDMTVPKLEGDLKGPKVDVSAPDVEMQGPDWNLKMPKIKMPKFSMPSLKGEGPEFDV
NLSKANVDISAPKVDTNAPDLSLEGPEGKLKGPKFKMPEMHFRAPKMSLPDVDLDLKGPK
MKGNVDISAPKIEGEMQVPDVDIRGPKVDIKAPDVEGQGLDWSLKIPKMKMPKFSMPSLK
GEGPEVDVNLPKADVVVSGPKVDIEAPDVSLEGPEGKLKGPKFKMPEMHFKTPKISMPDV
DLHLKGPKVKGDVDVSVPKVEGEMKVPDVEIKGPKMDIDAPDVEVQGPDWHLKMPKMKMP
KFSMPGFKGEGREVDVNLPKADIDVSGPKVDVEVPDVSLEGPEGKLKGPKFKMPEMHFKA
PKISMPDVDLNLKGPKLKGDVDVSLPEVEGEMKVPDVDIKGPKVDISAPDVDVHGPDWHL
KMPKVKMPKFSMPGFKGEGPEVDVKLPKADVDVSGPKMDAEVPDVNIEGPDAKLKGPKFK
MPEMSIKPQKISIPDVGLHLKGPKMKGDYDVTVPKVEGEIKAPDVDIKGPKVDINAPDVE
VHGPDWHLKMPKVKMPKFSMPGFKGEGPEVDMNLPKADLGVSGPKVDIDVPDVNLEAPEG
KLKGPKFKMPSMNIQTHKISMPDVGLNLKAPKLKTDVDVSLPKVEGDLKGPEIDVKAPKM
DVNVGDIDIEGPEGKLKGPKFKMPEMHFKAPKISMPDVDLHLKGPKVKGDMDVSVPKVEG
EMKVPDVDIKGPKVDIDAPDVEVHDPDWHLKMPKMKMPKFSMPGFKAEGPEVDVNLPKAD
IDVSGPSVDTDAPDLDIEGPEGKLKGSKFKMPKLNIKAPKVSMPDVDLNLKGPKLKGEID
ASVPELEGDLRGPQVDVKGPFVEAEVPDVDLECPDAKLKGPKFKMPEMHFKAPKISMPDV
DLHLKGPKVKGDADVSVPKLEGDLTGPSVGVEVPDVELECPDAKLKGPKFKMPDMHFKAP
KISMPDVDLHLKGPKVKGDVDVSVPKLEGDLTGPSVGVEVPDVELECPDAKLKGPKFKMP
EMHFKTPKISMPDVDLHLKGPKVKGDMDVSVPKVEGEMKVPDVDIKGPKMDIDAPDVDVH
GPDWHLKMPKMKMPKFSMPGFKAEGPEVDVNLPKADVVVSGPKVDVEVPDVSLEGPEGKL
KGPKLKMPEMHFKAPKISMPDVDLHLKGPKVKGDVDVSLPKLEGDLTGPSVDVEVPDVEL
ECPDAKLKGPKFKMPEMHFKTPKISMPDVNLNLKGPKVKGDMDVSVPKVEGEMKVPDVDI
RGPKVDIDAPDVDVHGPDWHLKMPKMKMPKFSMPGFKGEGPEVDVNLPKADVDVSGPKVD
VEVPDVSLEGPEGKLKGPKFKMPEMHFKTPKISMPDVDFNLKGPKIKGDVDVSAPKLEGE
LKGPELDVKGPKLDADMPEVAVEGPNGKWKTPKFKMPDMHFKAPKISMPDLDLHLKSPKA
KGEVDVDVPKLEGDLKGPHVDVSGPDIDIEGPEGKLKGPKFKMPDMHFKAPNISMPDVDL
NLKGPKIKGDVDVSVPEVEGKLEVPDMNIRGPKVDVNAPDVQAPDWHLKMPKMKMPKFSM
PGFKAEGPEVDVNLPKADVDISGPKVDIEGPDVNIEGPEGKLKGPKLKMPEMNIKAPKIS
MPDFDLHLKGPKVKGDVDVSLPKVEGDLKGPEVDIKGPKVDINAPDVGVQGPDWHLKMPK
VKMPKFSMPGFKGEGPDGDVKLPKADIDVSGPKVDIEGPDVNIEGPEGKLKGPKFKMPEM
NIKAPKISMPDIDLNLKGPKVKGDVDVSLPKVEGDLKGPEVDIKGPKVDIDAPDVDVHGP
DWHLKMPKIKMPKISMPGFKGEGPDVDVNLPKADIDVSGPKVDVECPDVNIEGPEGKWKS
PKFKMPEMHFKTPKISMPDIDLNLTGPKIKGDVDVTGPKVEGDLKGPEVDLKGPKVDIDV
PDVNVQGPDWHLKMPKMKMPKFSMPGFKAEGPEVDVNLPKADVDVSGPKVDVEGPDVNIE
GPEGKLKGPKFKMPEMNIKAPKIPMPDFDLHLKGPKVKGDVDISLPKVEGDLKGPEVDIR
GPQVDIDVPDVGVQGPDWHLKMPKVKMPKFSMPGFKGEGPDVDVNLPKADLDVSGPKVDI
DVPDVNIEGPEGKLKGPKFKMPEMNIKAPKISMPDIDLNLKGPKVKGDMDVSLPKVEGDM

KVPDVDIKGPKVDINAPDVDVQGPDWHLKMPKIKMPKISMPGFKGEGPEVDVNLPKADLD
VSGPKVDVDVPDVNIEGPDAKLKGPKFKMPEMNIKAPKISMPDLDLNLKGPKMKGEVDVS
LANVEGDLKGPALDIKGPKIDVDAPDIDIHGPDAKLKGPKLKMPDMHVNMPKISMPEIDL
NLKGSKLKGDVDVSGPKLEGDIKAPSLDIKGPEVDVSGPKLNIEGKSKKSRFKLPKFNFS
GSKVQTPEVDVKGKKPDIDITGPKVDINAPDVEVQGKVKGSKFKMPFLSISSPKVSMPDV
ELNLKSPKVKGDLDIAGPNLEGDFKGPKVDIKAPEVNLNAPDVDVHGPDWNLKMPKMKMP
KFSVSGLKAEGPDVAVDLPKGDINIEGPSMNIEGPDLNVEGPEGGLKGPKFKMPDMNIKA
PKISMPDIDLNLKGPKVKGDVDISLPKLEGDLKGPEVDIKGPKVDINAPDVDVHGPDWHL
KMPKVKMPKFSMPGFKGEGPEVDVTLPKADIDISGPNVDVDVPDVNIEGPDAKLKGPKFK
MPEMNIKAPKISMPDFDLNLKGPKMKGDVVVSLPKVEGDLKGPEVDIKGPKVDIDTPDIN
IEGSEGKFKGPKFKIPEMHLKAPKISMPDIDLNLKGPKVKGDVDVSLPKMEGDLKGPEVD
IKGPKVDINAPDVDVQGPDWHLKMPKVKMPKFSMPGFKGEGPDVDVNLPKADLDVSGPKV
DIDVPDVNIEGPEGKLKGPKFKMPEMNIKAPKISMPDIDLNLKGPKVKGDMDVSLPKVEG
DMQVPDLDIKGPKVDINAPDVDVRGPDWHLKMPKIKMPKISMPGFKGEGPEVDVNLPKAD
LDVSGPKVDVDVPDVNIEGPDAKLKGPKFKMPEMNIKAPKISMPDFDLHLKGPKVKGDVD
VSLPKMEGDLKAPEVDIKGPKVDIDAPDVDVHGPDWHLKMPKVKMPKFSMPGFKGEGPEV
DVNLPKADIDVSGPKVDIDTPDIDIHGPEGKLKGPKFKMPDLHLKAPKISMPEVDLNLKG
PKMKGDVDVSLPKVEGDLKGPEVDIKGPKVDIDVPDVDVQGPDWHLKMPKVKMPKFSMPG
FKGEGPDVDVNLPKADLDVSGPKVDIDVPDVNIEGPDAKLKGPKFKMPEMNIKAPKISMP
DFDLHLKGPKVKGDVDVSLPKVEGDLKGPEVDIKGPKVDIDAPDVDVHGPDWHLKMPKVK
MPKFSMPGFKGEGPDVDVTLPKADIEISGPKVDIDAPDVSIEGPDAKLKGPKFKMPEMNI
KAPKISMPDIDFNLKGPKVKGDVDVSLPKVEGDLKGPEIDIKGPSLDIDTPDVNIEGPEG
KLKGPKFKMPEMNIKAPKISMPDFDLHLKGPKVKGDVDVSLPKVESDLKGPEVDIEGPEG
KLKGPKFKMPDVHFKSPQISMSDIDLNLKGPKIKGDMDISVPKLEGDLKGPKVDVKGPKV
GIDTPDIDIHGPEGKLKGPKFKMPDLHLKAPKISMPEVDLNLKGPKVKGDMDISLPKVEG
DLKGPEVDIRDPKVDIDVPDVDVQGPDWHLKMPKVKMPKFSMPGFKGEGPDVDVNLPKAD
IDVSGPKVDVDVPDVNIEGPDAKLKGPKFKMPEMSIKAPKISMPDIDLNLKGPKVKGDVD
VTLPKVEGDLKGPEADIKGPKVDINTPDVDVHGPDWHLKMPKVKMPKFSMPGFKGEGPDV
DVSLPKADIDVSGPKVDVDIPDVNIEGPDAKLKGPKFKMPEINIKAPKISIPDVDLDLKG
PKVKGDFDVSVPKVEGTLKGPEVDLKGPRLDFEGPDAKLSGPSLKMPSLEISAPKVTAPD
VDLHLKAPKIGFSGPKLEGGEVDLKGPKVEAPSLDVHMDSPDINIEGPDVKIPKFKKPKF
GFGAKSPKADIKSPSLDVTVPEAELNLETPEISVGGKGKKSKFKMPKIHMSGPKIKAKKQ
GFDLNVPGGEIDASLKAPDVDVNIAGPDAALKVDVKSPKTKKTMFGKMYFPDVEFDIKSP
KFKAEAPLPSPKLEGELQAPDLELSLPAIHVEGLDIKAKAPKVKMPDVDISVPKIEGDLK
GPKVQANLGAPDINIEGLDAKVKTPSFGISAPQVSIPDVNVNLKGPKIKGDVPSVGLEGP
DVDLQGPEAKIKFPKFSMPKIGIPGVKMEGGGAEVHAQLPSLEGDLRGPDVKLEGPDVSL
KGPGVDLPSVNLSMPKVSGPDLDLNLKGPSLKGDLDASVPSMKVHAPGLNLSGVGGKMQV
GGDGVKVPGIDATTKLNVGAPDVTLRGPSLQGDLAVSGDIKCPKVSVGAPDLSLEASEGS
IKLPKMKLPQFGISTPGSDLHVNAKGPQVSGELKGPGVDVNLKGPRISAPNVDFNLEGPK
VKGSLGATGEIKGPTVGGGLPGIGVQGLEGNLQMPGIKSSGCDVNLPGVNVKLPTGQISG
PEIKGGLKGSEVGFHGAAPDISVKGPAFNMASPESDFGINLKGPKIKGGADVSGGVSAPD
ISLGEGHLSVKGSGGEWKGPQVSSALNLDTSKFAGGLHFSGPKVEGGVKGGQIGLQAPGL
SVSGPQGHLESGSGKVTFPKMKIPKFTFSGRELVGREMGVDVHFPKAEASIQAGAGDGEW
EESEVKLKKSKIKMPKFNFSKPKGKGGVTGSPEASISGSKGDLKSSKASLGSLEGEAEAE

ASSPKGKFSLFKSKKPRHRSNSFSDEREFSGPSTPTGTLEFEGGEVSLEGGKVKGKHGKL
KFGTFGGLGSKSKGHYEVTGSDDETGKLQGSGVSLASKKSRLSSSSSNDSGNKVGIQLPE
VELSVSTKKE
>sp|Q96Q83|ALKB3_HUMAN Alpha-ketoglutarate-dependent dioxygenase alkB homolog 3 OS=Homo sapiens GN=ALKBH3 PE=1 SV=1
MEEKRRRARVQGAWAAPVKSQAIAQPATTAKSHLHQKPGQTWKNKEHHLSDREFVFKEPQ
QVVRRAPEPRVIDREGVYEISLSPTGVSRVCLYPGFVDVKEADWILEQLCQDVPWKQRTG
IREDITYQQPRLTAWYGELPYTYSRITMEPNPHWHPVLRTLKNRIEENTGHTFNSLLCNL
YRNEKDSVDWHSDDEPSLGRCPIIASLSFGATRTFEMRKKPPPEENGDYTYVERVKIPLD
HGTLLIMEGATQADWQHRVPKEYHSREPRVNLTFRTVYPDPRGAPW
>sp|Q6P6C2|ALKB5_HUMAN RNA demethylase ALKBH5 OS=Homo sapiens GN=ALKBH5 PE=1 SV=2
MAAASGYTDLREKLKSMTSRDNYKAGSREAAAAAAAAVAAAAAAAAAAEPYPVSGAKRKY
QEDSDPERSDYEEQQLQKEEEARKVKSGIRQMRLFSQDECAKIEARIDEVVSRAEKGLYN
EHTVDRAPLRNKYFFGEGYTYGAQLQKRGPGQERLYPPGDVDEIPEWVHQLVIQKLVEHR
VIPEGFVNSAVINDYQPGGCIVSHVDPIHIFERPIVSVSFFSDSALCFGCKFQFKPIRVS
EPVLSLPVRRGSVTVLSGYAADEITHCIRPQDIKERRAVIILRKTRLDAPRLETKSLSSS
VLPPSYASDRLSGNNRDPALKPKRSHRKADPDAAHRPRILEMDKEENRRSVLLPTHRRRG
SFSSENYWRKSYESSEDCSEAAGSPARKVKMRRH
>sp|Q3KRA9|ALKB6_HUMAN Alpha-ketoglutarate-dependent dioxygenase alkB homolog 6 OS=Homo sapiens GN=ALKBH6 PE=1 SV=2
MEEQDARVPALEPFRVEQAPPVIYYVPDFISKEEEEYLLRQVFNAPKPKWTQLSGRKLQN
WGGLPHPRGMVPERLPPWLQRYVDKVSNLSLFGGLPANHVLVNQYLPGEGIMPHEDGPLY
YPTVSTISLGSHTVLDFYEPRRPEDDDPTEQPRPPPRPTTSLLLEPRSLLVLRGPAYTRL
LHGIAAARVDALDAASSPPNAAACPSARPGACLVRGTRVSLTIRRVPRVLRAGLLLGK
>sp|Q9BT30|ALKB7_HUMAN Alpha-ketoglutarate-dependent dioxygenase alkB homolog 7, mitochondrial OS=Homo sapiens GN=ALKBH7 PE=1 SV=1
MAGTGLLALRTLPGPSWVRGSGPSVLSRLQDAAVVRPGFLSTAEEETLSRELEPELRRRR
YEYDHWDAAIHGFRETEKSRWSEASRAILQRVQAAAFGPGQTLLSSVHVLDLEARGYIKP
HVDSIKFCGATIAGLSLLSPSVMRLVHTQEPGEWLELLLEPGSLYILRGSARYDFSHEIL
RDEESFFGERRIPRGRRISVICRSLPEGMGPGESGQPPPAC
>sp|Q8TCU4|ALMS1_HUMAN Alstrom syndrome protein 1 OS=Homo sapiens GN=ALMS1 PE=1 SV=3
MEPEDLPWPGELEEEEEEEEEEEEEEEEEAAAAAAANVDDVVVVEEVEEEAGRELDSDSHY
GPQHLESIDDEEDEEAKAWLQAHPGRILPPLSPPQHRYSEGERTSLEKIVPLTCHVWQQI
VYQGNSRTQISDTNVVCLETTAQRGSGDDQKTESWHCLPQEMDSSQTLDTSQTRFNVRTE
DTEVTDFPSLEEGILTQSENQVKEPNRDLFCSPLLVIQDSFASPDLPLLTCLTQDQEFAP
DSLFHQSELSFAPLRGIPDKSEDTEWSSRPSEVSEALFQATAEVASDLASSRFSVSQHPL
IGSTAVGSQCPFLPSEQGNNEETISSVDELKIPKDCDRYDDLCSYMSWKTRKDTQWPENN
LADKDQVSVATSFDITDENIATKRSDHFDAARSYGQYWTQEDSSKQAETYLTKGLQGKVE
SDVITLDGLNENAVVCSERVAELQRKPTRESEYHSSDLRMLRMSPDTVPKAPKHLKAGDT
SKGGIAKVTQSNLKSGITTTPVDSDIGSHLSLSLEDLSQLAVSSLETTTGQHTDTLNQKT
LADTHLTEETLKVTAIPEPADQKTATPTVLSSSHSHRGKPSIFYQQGLPDSHLTEEALKV
SAAPGLADQTTGMSTLTSTSYSHREKPGTFYQQELPESNLTEEPLEVSAAPGPVEQKTGI
PTVSSTSHSHVEDLLFFYRQTLPDGHLTDQALKVSAVSGPADQKTGTATVLSTPHSHREK
PGIFYQQEFADSHQTEETLTKVSATPGPADQKTEIPAVQSSSYSQREKPSILYPQDLADS

HLPEEGLKVSAVAGPADQKTGLPTVPSSAYSHREKLLVFYQQALLDSHLPEEALKVSAVS
GPADGKTGTPAVTSTSSASSSLGEKPSAFYQQTLPNSHLTEEALKVSIVPGPGDQKTGIP
SAPSSFYSHREKPIIFSQQTLPDFLFPEEALKVSAVSVLAAQKTGTPTVSSNSHSHSEKS
SVFYQQELPDSDLPRESLKMSAIPGLTDQKTVPTPTVPSGSFSHREKPSIFYQQEWPDSY
ATEKALKVSTGPGPADQKTEIPAVQSSSYPQREKPSVLYPQVLSDSHLPEESLKVSAFPG
PADQMTDTPAVPSTFYSQREKPGIFYQQTLPESHLPKEALKISVAPGLADQKTGTPTVTS
TSYSQHREKPSIFHQQALPGTHIPEEAQKVSAVTGPGNQKTWIPRVLSTFYSQREKPGIF
YQQTLPGSHIPEEAQKVSPVLGPADQKTGTPTPTSASYSHTEKPGIFYQQVLPDNHPTEE
ALKISVASEPVDQTTGTPAVTSTSYSQYREKPSIFYQQSLPSSHLTEEAKNVSAVPGPAD
QKTVIPILPSTFYSHTEKPGVFYQQVLPHSHPTEEALKISVASEPVDQTTGTPTVTSTSY
SQHTEKPSIFYQQSLPGSHLTEEAKNVSAVPGPGDRKTGIPTLPSTFYSHTEKPGSFYQQ
VLPHSHLPEEALEVSVAPGPVDQTIGTPTVTSPSSSFGEKPIVIYKQAFPEGHLPEESLK
VSVAPGPVGQTTGAPTITSPSYSQHRAKSGSFYQLALLGSQIPEEALRVSSAPGPADQTT
GIPTITSTSYSFGEKPIVNYKQAFPDGHLPEEALKVSIVSGPTEKKTDIPAGPLGSSALG
EKPITFYRQALLDSPLNKEVVKVSAAPGPADQKTETLPVHSTSYSNRGKPVIFYQQTLSD
SHLPEEALKVPPVPGPDAQKTETPSVSSSLYSYREKPIVFYQQALPDSELTQEALKVSAV
PQPADQKTGLSTVTSSFYSHTEKPNISYQQELPDSHLTEEALKVSNVPGPADQKTGVSTV
TSTSYSHREKPIVSYQRELPHFTEAGLKILRVPGPADQKTGINILPSNSYPQREHSVISY
EQELPDLTEVTLKAIGVPGPADQKTGIQIASSSSYSNREKASIFHQQELPDVTEEALNVF
VVPGQGDRKTEIPTVPLSYYSRREKPSVISQQELPDSHLTEEALKVSPVSIPAEQKTGIP
IGLSSSYSHSHKEKLKISTVHIPDDQKTEFPAATLSSYSQIEKPKISTVIGPNDQKTPSQ
TAFHSSYSQTVKPNILFQQQLPDRDQSKGILKISAVPELTDVNTGKPVSLSSSYFHREKS
NIFSPQELPGSHVTEDVLKVSTIPGPAGQKTVLPTALPSSFSHREKPDIFYQKDLPDRHL
TEDALKISSALGQADQITGLQTVPSGTYSHGENHKLVSEHVQRLIDNLNSSDSSVSSNNV
LLNSQADDRVVINKPESAGFRDVGSEEIQDAENSAKTLKEIRTLLMEAENMALKRCNFPA
PLARFRDISDISFIQSKKVVCFKEPSSTGVSNGDLLHRQPFTEESPSSRCIQKDIGTQTN
LKCRRGIENWEFISSTTVRSPLQEAESKVSMALEETLRQYQAAKSVMRSEPEGCSGTIGN
KIIIPMMTVIKSDSSSDASDGNGSCSWDSNLPESLESVSDVLLNFFPYVSPKTSITDSRE
EEGVSESEDGGGSSVDSLAAHVKNLLQCESSLNHAKEILRNAEEEESRVRAHAWNMKFNL
AHDCGYSISELNEDDRRKVEEIKAELFGHGRTTDLSKGLQSPRGMGCKPEAVCSHIIIES
HEKGCFRTLTSEHPQLDRHPCAFRSAGPSEMTRGRQNPSSCRAKHVNLSASLDQNNSHFK
VWNSLQLKSHSPFQNFIPDEFKISKGLRMPFDEKMDPWLSELVEPAFVPPKEVDFHSSSQ
MPSPEPMKKFTTSITFSSHRHSKCISNSSVVKVGVTEGSQCTGASVGVFNSHFTEEQNPP
RDLKQKTSSPSSFKMHSNSQDKEVTILAEGRRQSQKLPVDFERSFQEEKPLERSDFTGSH
SEPSTRANCSNFKEIQISDNHTLISMGRPSSTLGVNRSSSRLGVKEKNVTITPDLPSCIF
LEQRELFEQSKAPRADDHVRKHHSPSPQHQDYVAPDLPSCIFLEQRELFEQCKAPYVDHQ
MRENHSPLPQGQDSIASDLPSPISLEQCQSKAPGVDDQMNKHHFPLPQGQDCVVEKNNQH
KPKSHISNINVEAKFNTVVSQSAPNHCTLAASASTPPSNRKALSCVHITLCPKTSSKLDS
GTLDERFHSLDAASKARMNSEFNFDLHTVSSRSLEPTSKLLTSKPVAQDQESLGFLGPKS
SLDFQVVQPSLPDSNTITQDLKTIPSQNSQIVTSRQIQVNISDFEGHSNPEGTPVFADRL
PEKMKTPLSAFSEKLSSDAVTQITTESPEKTLFSSEIFINAEDRGHEIIEPGNQKLRKAP
VKFASSSSVQQVTFSRGTDGQPLLLPYKPSGSTKMYYVPQLRQIPPSPDSKSDTTVESSH
SGSNDAIAPDFPAQVLGTRDDDLSATVNIKHKEGIYSKRVVTKASLPVGEKPLQNENADA
SVQVLITGDENLSDKKQQEIHSTRAVTEAAQAKEKESLQKDTADSSAAAAAEHSAQVGDP

EMKNLPDTKAITQKEEIHRKKTVPEEAWPNNKESLQINIEESECHSEFENTTRSVFRSAK
FYIHHPVHLPSDQDICHESLGKSVFMRHSWKDFFQHHPDKHREHMCLPLPYQNMDKTKTD
YTRIKSLSINVNLGNKEVMDTTKSQVRDYPKHNGQISDPQRDQKVTPEQTTQHTVSLNEL
WNKYRERQRQQRQPELGDRKELSLVDRLDRLAKILQNPITHSLQVSESTHDDSRGERSVK
EWSGRQQQRNKLQKKKRFKSLEKSHKNTGELKKSKVLSHHRAGRSNQIKIEQIKFDKYIL
SKQPGFNYISNTSSDCRPSEESELLTDTTTNILSGTTSTVESDILTQTDREVALHERSSS
VSTIDTARLIQAFGHERVCLSPRRIKLYSSITNQQRRYLEKRSKHSKKVLNTGHPLVTSE
HTRRRHIQVANHVISSDSISSSASSFLSSNSTFCNKQNVHMLNKGIQAGNLEIVNGAKKH
TRDVGITFPTPSSSEAKLEENSDVTSWSEEKREEKMLFTGYPEDRKLKKNKKNSHEGVSW
FVPVENVESRSKKENVPNTCGPGISWFEPITKTRPWREPLREQNCQGQHLDGRGYLAGPG
REAGRDLLRPFVRATLQESLQFHRPDFISRSGERIKRLKLIVQERKLQSMLQTERDALFN
IDRERQGHQNRMCPLPKRVFLAIQKNKPISKKEMIQRSKRIYEQLPEVQKKREEEKRKSE
YKSYRLRAQLYKKRVTNQLLGRKVPWD
>sp|Q86TB3|ALPK2_HUMAN Alpha-protein kinase 2 OS=Homo sapiens GN=ALPK2 PE=2 SV=3
MKDSEGPQRPPLCFLSTLLSQKVPEKSDAVLRCIISGQPKPEVTWYKNGQAIDGSGIISN
YEFFENQYIHVLHLSCCTKNDAAVYQISAKNSFGMICCSASVEVECSSENPQLSPNLEDD
RDRGWKHETGTHEEERANQIDEKEHPYKEEESISPGTPRSADSSPSKSNHSLSLQSLGNL
DISVSSSENPLGVKGTRHTGEAYDPSNTEEIANGLLFLNSSHIYEKQDRCCHKTVHSMAS
KFTDGDLNNDGPHDEGLRSSQQNPKVQKYISFSLPLSEATAHIYPGDSAVANKQPSPQLS
SEDSDSDYELCPEITLTYTEEFSDDDLEYLECSDVMTDYSNAVWQRNLLGTEHVFLLESD
DEEMEFGEHCLGGCEHFLSGMGCGSRVSGDAGPMVATAGFCGHHSQPQEVGVRSSRVSKH
GPSSPQTGMTLILGPHQDGTSSVTEQGRYKLPTAPEAAENDYPGIQGETRDSHQAREEFA
SDNLLNMDESVRETEMKLLSGESENSGMSQCWETAADKRVGGKDLWSKRGSRKSARVRQP
GMKGNPKKPNANLRESTTEGTLHLCSAKESAEPPLTQSDKRETSHTTAAATGRSSHADAR
ECAISTQAEQEAKTLQTSTDSVSKEGNTNCKGEGMQVNTLFETSQVPDWSDPPQVQVQET
VRETISCSQMPAFSEPAGEESPFTGTTTISFSNLGGVHKENASLAQHSEVKPCTCGPQHE
EKQDRDGNIPDNFREDLKYEQSISEANDETMSPGVFSRHLPKDARADFREPVAVSVASPE
PTDTALTLENVCDEPRDREAVCAMECFEAGDQGTCFDTIDSLVGRPVDKYSPQEICSVDT
ELAEGQNKVSDLCSSNDKTLEVFFQTQVSETSVSTCKSSKDGNSVMSPLFTSTFTLNISH
TASEGATGENLAKVENSTYPLASTVHAGGQEQPSPSNSGGLDETQLLSSENNPLVQFKEGG
DKSPSPSAADTTATPASYSSIVSFPWEKPTTLTANNECFQATRETEDTSTVTIATEVHPA
KYLAVSIPEDKHAGGTEERFPRASHEKVSQFPSQVQLDHILSGATIKSTKELLCRAPSVP
GVPHHVLQLPEGEGFCSNSPLQVDNLSGDKSQTVDRADFRSYEENFQERGSETKQGVQQQ
SLSQQGSLSAPDFQQSLPTTSAAQEERNLVPTAHSPASSREGAGQRSGWGTRVSVVAETA
GEEDSQALSNVPSLSDILLEESKEYRPGNWEAGNKLKIITLEASASEIWPPRQLTNSESK
ASDGGLIIPDKVWAVPDSLKADAVVPELAPSEIAALAHSPEDAESALADSRESHKGEEPT
ISVHWRSLSSRGFSQPRLLESSVDPVDEKELSVTDSLSAASETGGKENVNNVSQDQEEKQ
LKMDHTAFFKKFLTCPKILESSVDPIDEISVIEYTRAGKPEPSETTPQGAREGGQSNDGN
MGHEAEIQPAILQVPCLQGTILSENRISRSQEGSMKQEAEQIQPEEAKTAIWQVLQPSEG
GERIPSGCSIGQIQESSDGSLGEAEQSKKDKAELISPTSPLSSCLPIMTHASLGVDTHNS
TGQIHDVPENDIVEPRKRQYVFPVSQKRGTIENERGKPLPSSPDLTRFPCTSSPEGNVTD
FLISHKMEEPKIEVLQIGETKPPSSSSSSAKTLAFISGERELEKAPKLLQDPCQKGTLGC
AKKSREREKSLEARAGKSPGTLTAVTGSEEVKRKPEAPGSGHLAEGVKKKILSRVAALRL
KLEEKENIRKNSAFLKKMPKLETSLSHTEEKQDPKKPSCKREGRAPVLLKKIQAEMFPEH

SGNVKLSCQFAEIHEDSTICWTKDSKSIAQVQRSAGDNSTVSFAIVQASPKDQGLYYCCI
KNSYGKVTAEFNLTAEVLKQLSSRQDTKGCEEIEFSQLIFKEDFLHDSYFGGRLRGQIAT
EELHFGEGVHRKAFRSTVMHGLMPVFKPGHACVLKVHNAIAYGTRNNDELIQRNYKLAAQ
ECYVQNTARYYAKIYAAEAQPLEGFGEVPEIIPIFLIHRPENNIPYATVEEELIGEFVKY
SIRDGKEINFLRRESEAGQKCCTFQHWVYQKTSGCLLVTDMQGVGMKLTDVGIATLAKGY
KGFKGNCSMTFIDQFKALHQCNKYCKMLGLKSLQNNNQKQKQPSIGKSKVQTNSMTIKKA
GPETPGEKKT
>sp|Q99217|AMELX_HUMAN Amelogenin, X isoform OS=Homo sapiens GN=AMELX PE=1 SV=1
MGTWILFACLLGAAFAMPLPPHPGHPGYINFSYEVLTPLKWYQSIRPPYPSYGYEPMGGW
LHHQIIPVLSQQHPPTHTLQPHHHIPVVPAQQPVIPQQPMMPVPGQHSMTPIQHHQPNLP
PPAQQPYQPQPVQPQPHQPMQPQPPVHPMQPLPPQPPLPPMFPMQPLPPMLPDLTLEAWP
STDKTKREEVD
>sp|Q9H4A4|AMPB_HUMAN Aminopeptidase B OS=Homo sapiens GN=RNPEP PE=1 SV=2
MASGEHSPGSGAARRPLHSAQAVDVASASNFRAFELLHLHLDLRAEFGPPGPGAGSRGLS
GTAVLDLRCLEPEGAAELRLDSHPCLEVTAAALRRERPGSEEPPAEPVSFYTQPFSHYGQ
ALCVSFPQPCRAAERLQVLLTYRVGEGPGVCWLAPEQTAGKKKPFVYTQGQAVLNRAFFP
CFDTPAVKYKYSALIEVPDGFTAVMSASTWEKRGPNKFFFQMCQPIPSYLIALAIGDLVS
AEVGPRSRVWAEPCLIDAAKEEYNGVIEEFLATGEKLFGPYVWGRYDLLFMPPSFPFGGM
ENPCLTFVTPCLLAGDRSLADVIIHEISHSWFGNLVTNANWGEFWLNEGFTMYAQRRIST
ILFGAAYTCLEAATGRALLRQHMDITGEENPLNKLRVKIEPGVDPDDTYNETPYEKGFCF
VSYLAHLVGDQDQFDSFLKAYVHEFKFRSILADDFLDFYLEYFPELKKKRVDIIPGFEFD
RWLNTPGWPPYLPDLSPGDSLMKPAEELAQLWAAEELDMKAIEAVAISPWKTYQLVYFLD
KILQKSPLPPGNVKKLGDTYPSISNARNAELRLRWGQIVLKNDHQEDFWKVKEFLHNQGK
QKYTLPLYHAMMGGSEVAQTLAKETFASTASQLHSNVVNYVQQIVAPKGS
>sp|P28838|AMPL_HUMAN Cytosol aminopeptidase OS=Homo sapiens GN=LAP3 PE=1 SV=3
MFLLPLPAAGRVVVRRLAVRRFGSRSLSTADMTKGLVLGIYSKEKEDDVPQFTSAGENFD
KLLAGKLRETLNISGPPLKAGKTRTFYGLHQDFPSVVLVGLGKKAAGIDEQENWHEGKEN
IRAAVAAGCRQIQDLELSSVEVDPCGDAQAAAEGAVLGLYEYDDLKQKKKMAVSAKLYGS
GDQEAWQKGVLFASGQNLARQLMETPANEMTPTRFAEIIEKNLKSASSKTEVHIRPKSWI
EEQAMGSFLSVAKGSDEPPVFLEIHYKGSPNANEPPLVFVGKGITFDSGGISIKASANMD
LMRADMGGAATICSAIVSAAKLNLPINIIGLAPLCENMPSGKANKPGDVVRAKNGKTIQV
DNTDAEGRLILADALCYAHTFNPKVILNAATLTGAMDVALGSGATGVFTNSSWLWNKLFE
ASIETGDRVWRMPLFEHYTRQVVDCQLADVNNIGKYRSAGACTAAAFLKEFVTHPKWAHL
DIAGVMTNKDEVPYLRKGMTGRPTRTLIEFLLRFSQDNA
>sp|P15144|AMPN_HUMAN Aminopeptidase N OS=Homo sapiens GN=ANPEP PE=1 SV=4
MAKGFYISKSLGILGILLGVAAVCTIIALSVVYSQEKNKNANSSPVASTTPSASATTNPA
SATTLDQSKAWNRYRLPNTLKPDSYRVTLRPYLTPNDRGLYVFKGSSTVRFTCKEATDVI
IIHSKKLNYTLSQGHRVVLRGVGGSQPPDIDKTELVEPTEYLVVHLKGSLVKDSQYEMDS
EFEGELADDLAGFYRSEYMEGNVRKVVATTQMQAADARKSFPCFDEPAMKAEFNITLIHP
KDLTALSNMLPKGPSTPLPEDPNWNVTEFHTTPKMSTYLLAFIVSEFDYVEKQASNGVLI
RIWARPSAIAAGHGDYALNVTGPILNFFAGHYDTPYPLPKSDQIGLPDFNAGAMENWGLV
TYRENSLLFDPLSSSSSNKERVVTVIAHELAHQWFGNLVTIEWWNDLWLNEGFASYVEYL
GADYAEPTWNLKDLMVLNDVYRVMAVDALASSHPLSTPASEINTPAQISELFDAISYSKG
ASVLRMLSSFLSEDVFKQGLASYLHTFAYQNTIYLNLWDHLQEAVNNRSIQLPTTVRDIM

NRWTLQMGFPVITVDTSTGTLSQEHFLLDPDSNVTRPSEFNYVWIVPITSIRDGRQQQDY
WLIDVRAQNDLFSTSGNEWVLLNLNVTGYYRVNYDEENWRKIQTQLQRDHSAIPVINRAQ
IINDAFNLASAHKVPVTLALNNTLFLIEERQYMPWEAALSSLSYFKLMFDRSEVYGPMKN
YLKKQVTPLFIHFRNNTNNWREIPENLMDQYSEVNAISTACSNGVPECEEMVSGLFKQWM
ENPNNNPIHPNLRSTVYCNAIAQGGEEEWDFAWEQFRNATLVNEADKLRAALACSKELWI
LNRYLSYTLNPDLIRKQDATSTIISITNNVIGQGLVWDFVQSNWKKLFNDYGGGSFSFSN
LIQAVTRRFSTEYELQQLEQFKKDNEETGFGSGTRALEQALEKTKANIKWVKENKEVVLQ
WFTENSK
>sp|Q9UJ72|ANX10_HUMAN Annexin A10 OS=Homo sapiens GN=ANXA10 PE=1 SV=3
MFCGDYVQGTIFPAPNFNPIMDAQMLGGALQGFDCDKDMLINILTQRCNAQRMMIAEAYQ
SMYGRDLIGDMREQLSDHFKDVMAGLMYPPPLYDAHELWHAMKGVGTDENCLIEILASRT
NGEIFQMREAYCLQYSNNLQEDIYSETSGHFRDTLMNLVQGTREEGYTDPAMAAQDAMVL
WEACQQKTGEHKTMLQMILCNKSYQQLRLVFQEFQNISGQDMVDAINECYDGYFQELLVA
IVLCVRDKPAYFAYRLYSAIHDFGFHNKTVIRILIARSEIDLLTIRKRYKERYGKSLFHD
IRNFASGHYKKALLAICAGDAEDY
>sp|P50995|ANX11_HUMAN Annexin A11 OS=Homo sapiens GN=ANXA11 PE=1 SV=1
MSYPGYPPPPGGYPPAAPGGGPWGGAAYPPPPSMPPIGLDNVATYAGQFNQDYLSGMAAN
MSGTFGGANMPNLYPGAPGAGYPPVPPGGFGQPPSAQQPVPPYGMYPPPGGNPPSRMPSY
PPYPGAPVPGQPMPPPGQQPPGAYPGQPPVTYPGQPPVPLPGQQQPVPSYPGYPGSGTVT
PAVPPTQFGSRGTITDAPGFDPLRDAEVLRKAMKGFGTDEQAIIDCLGSRSNKQRQQILL
SFKTAYGKDLIKDLKSELSGNFEKTILALMKTPVLFDIYEIKEAIKGVGTDEACLIEILA
SRSNEHIRELNRAYKAEFKKTLEEAIRSDTSGHFQRLLISLSQGNRDESTNVDMSLAQRD
AQELYAAGENRLGTDESKFNAVLCSRSRAHLVAVFNEYQRMTGRDIEKSICREMSGDLEE
GMLAVVKCLKNTPAFFAERLNKAMRGAGTKDRTLIRIMVSRSETDLLDIRSEYKRMYGKS
LYHDISGDTSGDYRKILLKICGGND
>sp|Q10567|AP1B1_HUMAN AP-1 complex subunit beta-1 OS=Homo sapiens GN=AP1B1 PE=1 SV=2
MTDSKYFTTTKKGEIFELKAELNSDKKEKKKEAVKKVIASMTVGKDVSALFPDVVNCMQT
DNLELKKLVYLYLMNYAKSQPDMAIMAVNTFVKDCEDPNPLIRALAVRTMGCIRVDKITE
YLCEPLRKCLKDEDPYVRKTAAVCVAKLHDINAQLVEDQGFLDTLKDLISDSNPMVVANA
VAALSEIAESHPSSNLLDLNPQSINKLLTALNECTEWGQIFILDCLANYMPKDDREAQSI
CERVTPRLSHANSAVVLSAVKVLMKFMEMLSKDLDYYGTLLKKLAPPLVTLLSAEPELQY
VALRNINLIVQKRPEILKHEMKVFFVKYNDPIYVKLEKLDIMIRLASQANIAQVLAELKE
YATEVDVDFVRKAVRAIGRCAIKVEQSAERCVSTLLDLIQTKVNYVVQEAIVVIKDIFRK
YPNKYESVIATLCENLDSLDEPEARAAMIWIVGEYAERIDNADELLESFLEGFHDESTQV
QLQLLTAIVKLFLKKPTETQELVQQVLSLATQDSDNPDLRDRGYIYWRLLSTDPVAAKEV
VLAEKPLISEETDLIEPTLLDELICYIGTLASVYHKPPSAFVEGGRGVVHKSLPPRTASS
ESAESPETAPTGAPPGEQPDVIPAQGDLLGDLLNLDLGPPVSGPPLATSSVQMGAVDLLG
GGLDSLMGDEPEGIGGTNFVAPPTAAVPANLGAPIGSGLSDLFDLTSGVGTLSGSYVAPK
AVWLPAMKAKGLEISGTFTRQVGSISMDLQLTNKALQVMTDFAIQFNRNSFGLAPATPLQ
VHAPLSPNQTVEISLPLSTVGSVMKMEPLNNLQVAVKNNIDVFYFSTLYPLHILFVEDGK
MDRQMFLATWKDIPNENEAQFQIRDCPLNAEAASSKLQSSNIFTVAKRNVEGQDMLYQSL
KLTNGIWVLAELRIQPGNPSCTDLELSLKCRAPEVSQHVYQAYETILKN
>sp|O75843|AP1G2_HUMAN AP-1 complex subunit gamma-like 2 OS=Homo sapiens GN=AP1G2 PE=1 SV=1

MVVPSLKLQDLIEEIRGAKTQAQEREVIQKECAHIRASFRDGDPVHRHRQLAKLLYVHML
GYPAHFGQMECLKLIASSRFTDKRVGYLGAMLLLDERHDAHLLITNSIKNDLSQGIQPVQ
GLALCTLSTMGSAEMCRDLAPEVEKLLLQPSPYVRKKAILTAVHMIRKVPELSSVFLPPC
AQLLHERHHGILLGTITLITELCERSPAALRHFRKVVPQLVHILRTLVTMGYSTEHSISG
VSDPFLQVQILRLLRILGRNHEESSETMNDLLAQVATNTDTSRNAGNAVLFETVLTIMDI
RSAAGLRVLAVNILGRFLLNSDRNIRYVALTSLLRLVQSDHSAVQRHRPTVVECLRETDA
SLSRRALELSLALVNSSNVRAMMQELQAFLESCPPDLRADCASGILLAAERFAPTKRWHI
DTILHVLTTAGTHVRDDAVANLTQLIGGAQELHAYSVRRLYNALAEDISQQPLVQVAAWC
IGEYGDLLLAGNCEEIEPLQVDEEEVLALLEKVLQSHMSLPATRGYALTALMKLSTRLCG
DNNRIRQVVSIYGSCLDVELQQRAVEYDTLFRKYDHMRAAILEKMPLVERDGPQADEEAK
ESKEAAQLSEAAPVPTEPQASQLLDLLDLLDGASGDVQHPPHLDPSPGGALVHLLDLPCV
PPPPAPIPDLKVFEREGVQLNLSFIRPPENPALLLITITATNFSEGDVTHFICQAAVPKS
LQLQLQAPSGNTVPARGGLPITQLFRILNPNKAPLRLKLRLTYDHFHQSVQEIFEVNNLP
VESWQ
>sp|Q13367|AP3B2_HUMAN AP-3 complex subunit beta-2 OS=Homo sapiens GN=AP3B2 PE=1 SV=2
MSAAPAYSEDKGGSAGPGEPEYGHDPASGGIFSSDYKRHDDLKEMLDTNKDSLKLEAMKR
IVAMIARGKNASDLFPAVVKNVACKNIEVKKLVYVYLVRYAEEQQDLALLSISTFQRGLK
DPNQLIRASALRVLSSIRVPIIVPIMMLAIKEAASDMSPYVRKTAAHAIPKLYSLDSDQK
DQLIEVIEKLLADKTTLVAGSVVMAFEEVCPERIDLIHKNYRKLCNLLIDVEEWGQVVII
SMLTRYARTQFLSPTQNESLLEENAEKAFYGSEEDEAKGAGSEETAAAAAPSRKPYVMDP
DHRLLLRNTKPLLQSRSAAVVMAVAQLYFHLAPKAEVGVIAKALVRLLRSHSEVQYVVLQ
NVATMSIKRRGMFEPYLKSFYIRSTDPTQIKILKLEVLTNLANETNIPTVLREFQTYIRS
MDKDFVAATIQAIGRCATNIGRVRDTCLNGLVQLLSNRDELVVAESVVVIKKLLQMQPAQ
HGEIIKHLAKLTDNIQVPMARASILWLIGEYCEHVPRIAPDVLRKMAKSFTAEEDIVKLQ
VINLAAKLYLTNSKQTKLLTQYVLSLAKYDQNYDIRDRARFTRQLIVPSEQGGALSRHAK
KLFLAPKPAPVLESSFKDRDHFQLGSLSHLLNAKATGYQELPDWPEEAPDPSVRNVEVPE
WTKCSNREKRKEKEKPFYSDSEGESGPTESADSDPESESESDSKSSSESGSGESSSESDN
EDQDEDEEKGRGSESEQSEEDGKRKTKKKVPERKGEASSSDEGSDSSSSSSESEMTSESE
EEQLEPASWSRKTPPSSKSAPATKEISLLDLEDFTPPSVQPVSPPAIVSTSLAADLEGLT
LTDSTLVPSLLSPVSGVGRQELLHRVAGEGLAVDYTFSRQPFSGDPHMVSVHIHFSNSSD
TPIKGLHVGTPKLPAGISIQEFPEIESLAPGESATAVMGINFCDSTQAANFQLCTQTRQF
YVSIQPPVGELMAPVFMSENEFKKEQGKLMGMNEITEKLMLPDTCRSDHIVVQKVTATAN
LGRVPCGTSDEYRFAGRTLTGGSLVLLTLDARPAGAAQLTVNSEKMVIGTMLVKDVIQAL
TQ
>sp|Q96LR9|APLD1_HUMAN Apolipoprotein L domain-containing protein 1 OS=Homo sapiens GN=APOLD1 PE=2 SV=2
MFRAPCHRLRARGTRKARAGAWRGCTFPCLGKGMERPAAREPHGPDALRRFQGLLLDRRG
RLHGQVLRLREVARRLERLRRRSLVANVAGSSLSATGALAAIVGLSLSPVTLGTSLLVSA
VGLGVATAGGAVTITSDLSLIFCNSRELRRVQEIAATCQDQMREILSCLEFFCRWQGCGD
RQLLQCGRNASIALYNSVYFIVFFGSRGFLIPRRAEGDTKVSQAVLKAKIQKLAESLESC
TGALDELSEQLESRVQLCTKSSRGHDLKISADQRAGLFF
>sp|P02652|APOA2_HUMAN Apolipoprotein A-II OS=Homo sapiens GN=APOA2 PE=1 SV=1
MKLLAATVLLLTICSLEGALVRRQAKEPCVESLVSQYFQTVTDYGKDLMEKVKSPELQAE
AKSYFEKSKEQLTPLIKKAGTELVNFLSYFVELGTQPATQ

>sp|Q6Q788|APOA5_HUMAN Apolipoprotein A-V OS=Homo sapiens GN=APOA5 PE=1 SV=1
MASMAAVLTWALALLSAFSATQARKGFWDYFSQTSGDKGRVEQIHQQKMAREPATLKDSL
EQDLNNMNKFLEKLRPLSGSEAPRLPQDPVGMRRQLQEELEEVKARLQPYMAEAHELVGW
NLEGLRQQLKPYTMDLMEQVALRVQELQEQLRVVGEDTKAQLLGGVDEAWALLQGLQSRV
VHHTGRFKELFHPYAESLVSGIGRHVQELHRSVAPHAPASPARLSRCVQVLSRKLTLKAK
ALHARIQQNLDQLREELSRAFAGTGTEEGAGPDPQMLSEEVRQRLQAFRQDTYLQIAAFT
RAIDQETEEVQQQLAPPPPGHSAFAPEFQQTDSGKVLSKLQARLDDLWEDITHSLHDQGH
SHLGDP
>sp|P55056|APOC4_HUMAN Apolipoprotein C-IV OS=Homo sapiens GN=APOC4 PE=1 SV=1
MSLLRNRLQALPALCLCVLVLACIGACQPEAQEGTLSPPPKLKMSRWSLVRGRMKELLET
VVNRTRDGWQWFWSPSTFRGFMQTYYDDHLRDLGPLTKAWFLESKDSLLKKTHSLCPRLV
CGDKDQG
>sp|O00170|AIP_HUMAN AH receptor-interacting protein OS=Homo sapiens GN=AIP PE=1 SV=2
MADIIARLREDGIQKRVIQEGRGELPDFQDGTKATFHYRTLHSDDEGTVLDDSRARGKPM
ELIIGKKFKLPVWETIVCTMREGEIAQFLCDIKHVVLYPLVAKSLRNIAVGKDPLEGQRH
CCGVAQMREHSSLGHADLDALQQNPQPLIFHMEMLKVESPGTYQQDPWAMTDEEKAKAVP
LIHQEGNRLYREGHVKEAAAKYYDAIACLKNLQMKEQPGSPEWIQLDQQITPLLLNYCQC
KLVVEEYYEVLDHCSSILNKYDDNVKAYFKRGKAHAAVWNAQEAQADFAKVLELDPALAP
VVSRELQALEARIRQKDEEDKARFRGIFSH
>sp|Q96IF1|AJUBA_HUMAN LIM domain-containing protein ajuba OS=Homo sapiens GN=AJUBA PE=1 SV=1
MERLGEKASRLLEKFGRRKGESSRSGSDGTPGPGKGRLSGLGGPRKSGPRGATGGPGDEP
LEPAREQGSLDAERNQRGSFEAPRYEGSFPAGPPPTRALPLPQSLPPDFRLEPTAPALSP
RSSFASSSASDASKPSSPRGSLLLDGAGAGGAGGSRPCSNRTSGISMGYDQRHGSPLPAG
PCLFGPPLAGAPAGYSPGGVPSAYPELHAALDRLYAQRPAGFGCQESRHSYPPALGSPGA
LAGAGVGAAGPLERRGAQPGRHSVTGYGDCAVGARYQDELTALLRLTVGTGGREAGARGE
PSGIEPSGLEEPPGPFVPEAARARMREPEAREDYFGTCIKCNKGIYGQSNACQALDSLYH
TQCFVCCSCGRTLRCKAFYSVNGSVYCEEDYLFSGFQEAAEKCCVCGHLILEKILQAMGK
SYHPGCFRCIVCNKCLDGIPFTVDFSNQVYCVTDYHKNYAPKCAACGQPILPSEGCEDIV
RVISMDRDYHFECYHCEDCRMQLSDEEGCCCFPLDGHLLCHGCHMQRLNARQPPANYI
>sp|Q5JQC9|AKAP4_HUMAN A-kinase anchor protein 4 OS=Homo sapiens GN=AKAP4 PE=1 SV=1
MMAYSDTTMMSDDIDWLRSHRGVCKVDLYNPEGQQDQDRKVICFVDVSTLNVEDKDYKDA
ASSSSEGNLNLGSLEEKEIIVIKDTEKKDQSKTEGSVCLFKQAPSDPVSVLNWLLSDLQK
YALGFQHALSPSTSTCKHKVGDTEGEYHRASSENCYSVYADQVNIDYLMNRPQNLRLEMT
AAKNTNNNQSPSAPPAKPPSTQRAVISPDGECSIDDLSFYVNRLSSLVIQMAHKEIKEKL
EGKSKCLHHSICPSPGNKERISPRTPASKIASEMAYEAVELTAAEMRGTGEESREGGQKS
FLYSELSNKSKSGDKQMSQRESKEFADSISKGLMVYANQVASDMMVSLMKTLKVHSSGKP
IPASVVLKRVLLRHTKEIVSDLIDSCMKNLHNITGVLMTDSDFVSAVKRNLFNQWKQNAT
DIMEAMLKRLVSALIGEEKETKSQSLSYASLKAGSHDPKCRNQSLEFSTMKAEMKERDKG
KMKSDPCKSLTSAEKVGEHILKEGLTIWNQKQGNSCKVATKACSNKDEKGEKINASTDSL
AKDLIVSALKLIQYHLTQQTKGKDTCEEDCPGSTMGYMAQSTQYEKCGGGQSAKALSVKQ
LESHRAPGPSTCQKENQHLDSQKMDMSNIVLMLIQKLLNENPFKCEDPCEGENKCSEPRA
SKAASMSNRSDKAEEQCQEHQELDCTSGMKQANGQFIDKLVESVMKLCLIMAKYSNDGAA
LAELEEQAASANKPNFRGTRCIHSGAMPQNYQDSLGHEVIVNNQCSTNSLQKQLQAVLQW

IAASQFNVPMLYFMGDKDGQLEKLPQVSAKAAEKGYSVGGLLQEVMKFAKERQPDEAVGK
VARKQLLDWLLANL
>sp|P24588|AKAP5_HUMAN A-kinase anchor protein 5 OS=Homo sapiens GN=AKAP5 PE=1 SV=3
METTISEIHVENKDEKRSAEGSPGAERQKEKASMLCFKRRKKAAKALKPKAGSEAADVAR
KCPQEAGASDQPEPTRGAWASLKRLVTRRKRSESSKQQKPLEGEMQPAINAEDADLSKKK
AKSRLKIPCIKFPRGPKRSNHSKIIEDSDCSIKVQEEAEILDIQTQTPLNDQATKAKSTQ
DLSEGISRKDGDEVCESNVSNSTTSGEKVISVELGLDNGHSAIQTGTLILEEIETIKEKQ
DVQPQQASPLETSETDHQQPVLSDVPPLPAIPDQQIVEEASNSTLESAPNGKDYESTEIV
AEETKPKDTELSQESDFKENGITEEKSKSEESKRMEPIAIIITDTEISEFDVTKSKNVPK
QFLISAENEQVGVFANDNGFEDRTSEQYETLLIETASSLVKNAIQLSIEQLVNEMASDDN
KINNLLQ
>sp|Q13023|AKAP6_HUMAN A-kinase anchor protein 6 OS=Homo sapiens GN=AKAP6 PE=1 SV=3
MLTMSVTLSPLRSQDLDPMATDASPMAINMTPTVEQGEGEEAMKDMDSDQQYEKPPPLHT
GADWKIVLHLPEIETWLRMTSERVRDLTYSVQQDSDSKHVDVHLVQLKDICEDISDHVEQ
IHALLETEFSLKLLSYSVNVIVDIHAVQLLWHQLRVSVLVLRERILQGLQDANGNYTRQT
DILQAFSEETKEGRLDSLTEVDDSGQLTIKCSQNYLSLDCGITAFELSDYSPSEDLLSGL
GDMTSSQVKTKPFDSWSYSEMEKEFPELIRSVGLLTVAADSISTNGSEAVTEEVSQVSLS
VDDKGGCEEDNASAVEEQPGLTLGVSSSSGEALTNAAQPSSETVQQESSSSSHHDAKNQQ
PVPCENATPKRTIRDCFNYNEDSPTQPTLPKRGLFLKEETFKNDLKGNGGKRQMVDLKPE
MSRSTPSLVDPPDRSKLCLVLQSSYPNSPSAASQSYECLHKVGNGNLENTVKFHIKEISS
SLGRLNDCYKEKSRLKKPHKTSEEVPPCRTPKRGTGSGKQAKNTKSSAVPNGELSYTSKA
IEGPQTNSASTSSLEPCNQRSWNAKLQLQSETSSSPAFTQSSESSVGSDNIMSPVPLLSK
HKSKKGQASSPSHVTRNGEVVEAWYGSDEYLALPSHLKQTEVLALKLENLTKLLPQKPRG
ETIQNIDDWELSEMNSDSEIYPTYHVKKKHTRLGRVSPSSSSDIASSLGESIESGPLSDI
LSDEESSMPLAGMKKYADEKSERASSSEKNESHSATKSALIQKLMQDIQHQDNYEAIWEK
IEGFVNKLDEFIQWLNEAMETTENWTPPKAEMDDLKLYLETHLSFKLNVDSHCALKEAVE
EEGHQLLELIASHKAGLKDMLRMIASQWKELQRQIKRQHSWILRALDTIKAEILATDVSV
EDEEGTGSPKAEVQLCYLEAQRDAVEQMSLKLYSEQYTSSSKRKEEFADMSKVHSVGSNG
LLDFDSEYQELWDWLIDMESLVMDSHDLMMSEEQQQHLYKRYSVEMSIRHLKKTELLSKV
EALKKGGVLLPNDLLEKVDSINEKWELLGKTLGEKIQDTMAGHSGSSPRDLLSPESGSLV
RQLEVRIKELKGWLRDTELFIFNSCLRQEKEGTMNTEKQLQYFKSLCREIKQRRRGVASI
LRLCQHLLDDRETCNLNADHQPMQLIIVNLERRWEAIVMQAVQWQTRLQKKMGKESETLN
VIDPGLMDLNGMSEDALEWDEMDISNKLISLNEESNDLDQELQPVIPSLKLGETSNEDPG
YDEEADNHGGSQYASNITAPSSPHIYQVYSLHNVELYEDNHMPFLKNNPKVTGMTQPNVL
TKSLSKDSSFSSTKSLPDLLGGSNLVKPCACHGGDMSQNSGSESGIVSEGDTETTTNSEM
CLLNAVDGSPSNLETEHLDPQMGDAVNVLKQKFTDEGESIKLPNSSQSSISPVGCVNGKV
GDLNSITKHTPDCLGEELQGKHDVFTFYDYSYLQGSKLKLPMIMKQSQSEKAHVEDPLLR
GFYFDKKSCKSKHQTTELQPDVPPHERILASASHEMDRISYKSGNIEKTFTGMQNAKQLS
LLSHSSSIESLSPGGDLFGLGIFKNGSDSLQRSTSLESWLTSYKSNEDLFSCHSSGDISV
SSGSVGELSKRTLDLLNRLENIQSPSEQKIKRSVSDITLQSSSQKMSFTGQMSLDIASSI
NEDSAASLTELSSSDELSLCSEDIVLHKNKIPESNASFRKRLTRSVADESDVNVSMIVNV
SCTSACTDDEDDSDLLSSSTLTLTEEELCIKDEDDDSSIATDDEIYEDCTLMSGLDYIKN
ELQTWIRPKLSLTRDKKRCNVSDEMKGSKDISSSEMTNPSDTLNIETLLNGSVKRVSENN
GNGKNSSHTHELGTKRENKKTIFKVNKDPYVADMENGNIEGIPERQKGKPNVTSKVSENL

GSHGKEISESEHCKCKALMDSLDDSNTAGKEFVSQDVRHLPKKCPNHHHFENQSTASTPT
EKSFSELALETRFNNRQDSDALKSSDDAPSMAGKSAGCCLALEQNGTEENASISNISCCN
CEPDVFHQKDAEDCSVHNFVKEIIDMASTALKSKSQPENEVAAPTSLTQIKEKVLEHSHR
PIQLRKGDFYSYLSLSSHDSDCGEVTNYIEEKSSTPLPLDTTDSGLDDKEDIECFFEACV
EGDSDGEEPCFSSAPPNESAVPSEAAMPLQATACSSEFSDSSLSADDADTVALSSPSSQE
RAEVGKEVNGLPQTSSGCAENLEFTPSKLDSEKESSGKPGESGMPEEHNAASAKSKVQDL
SLKANQPTDKAALHPSPKTLTCEENLLNLHEKRHRNMHR
>sp|Q96JD6|AKCL2_HUMAN 1,5-anhydro-D-fructose reductase OS=Homo sapiens GN=AKR1E2 PE=1 SV=2
MGDIPAVGLSSWKASPGKVTEAVKEAIDAGYRHFDCAYFYHNEREVGAGIRCKIKEGAVR
REDLFIATKLWCTCHKKSLVETACRKSLKALKLNYLDLYLIHWPMGFKPPHPEWIMSCSE
LSFCLSHPRVQDLPLDESNMVIPSDTDFLDTWEAMEDLVITGLVKNIGVSNFNHEQLERL
LNKPGLRFKPLTNQIECHPYLTQKNLISFCQSRDVSVTAYRPLGGSCEGVDLIDNPVIKR
IAKEHGKSPAQILIRFQIQRNVIVIPGSITPSHIKENIQVFDFELTQHDMDNILSLNRNL
RLAMFPITKNHKDYPFHIEY
>sp|P00352|AL1A1_HUMAN Retinal dehydrogenase 1 OS=Homo sapiens GN=ALDH1A1 PE=1 SV=2
MSSSGTPDLPVLLTDLKIQYTKIFINNEWHDSVSGKKFPVFNPATEEELCQVEEGDKEDV
DKAVKAARQAFQIGSPWRTMDASERGRLLYKLADLIERDRLLLATMESMNGGKLYSNAYL
NDLAGCIKTLRYCAGWADKIQGRTIPIDGNFFTYTRHEPIGVCGQIIPWNFPLVMLIWKI
GPALSCGNTVVVKPAEQTPLTALHVASLIKEAGFPPGVVNIVPGYGPTAGAAISSHMDID
KVAFTGSTEVGKLIKEAAGKSNLKRVTLELGGKSPCIVLADADLDNAVEFAHHGVFYHQG
QCCIAASRIFVEESIYDEFVRRSVERAKKYILGNPLTPGVTQGPQIDKEQYDKILDLIES
GKKEGAKLECGGGPWGNKGYFVQPTVFSNVTDEMRIAKEEIFGPVQQIMKFKSLDDVIKR
ANNTFYGLSAGVFTKDIDKAITISSALQAGTVWVNCYGVVSAQCPFGGFKMSGNGRELGE
YGFHEYTEVKTVTVKISQKNS
>sp|P51648|AL3A2_HUMAN Fatty aldehyde dehydrogenase OS=Homo sapiens GN=ALDH3A2 PE=1 SV=1
MELEVRRVRQAFLSGRSRPLRFRLQQLEALRRMVQEREKDILTAIAADLCKSEFNVYSQE
VITVLGEIDFMLENLPEWVTAKPVKKNVLTMLDEAYIQPQPLGVVLIIGAWNYPFVLTIQ
PLIGAIAAGNAVIIKPSELSENTAKILAKLLPQYLDQDLYIVINGGVEETTELLKQRFDH
IFYTGNTAVGKIVMEAAAKHLTPVTLELGGKSPCYIDKDCDLDIVCRRITWGKYMNCGQT
CIAPDYILCEASLQNQIVWKIKETVKEFYGENIKESPDYERIINLRHFKRILSLLEGQKI
AFGGETDEATRYIAPTVLTDVDPKTKVMQEEIFGPILPIVPVKNVDEAINFINEREKPLA
LYVFSHNHKLIKRMIDETSSGGVTGNDVIMHFTLNSFPFGGVGSSGMGAYHGKHSFDTFS
HQRPCLLKSLKREGANKLRYPPNSQSKVDWGKFFLLKRFNKEKLGLLLLTFLGIVAAVLV
KAEYY
>sp|Q9BT22|ALG1_HUMAN Chitobiosyldiphosphodolichol beta-mannosyltransferase OS=Homo sapiens GN=ALG1 PE=1 SV=2
MAASCLVLLALCLLLPLLLLGGWKRWRRGRAARHVVAVVLGDVGRSPRMQYHALSLAMHG
FSVTLLGFCNSKPHDELLQNNRIQIVGLTELQSLAVGPRVFQYGVKVVLQAMYLLWKLMW
REPGAYIFLQNPPGLPSIAVCWFVGCLCGSKLVIDWHNYGYSIMGLVHGPNHPLVLLAKW
YEKFFGRLSHLNLCVTNAMREDLADNWHIRAVTVYDKPASFFKETPLDLQHRLFMKLGSM
HSPFRARSEPEDPVTERSAFTERDAGSGLVTRLRERPALLVSSTSWTEDEDFSILLAALE
KFEQLTLDGHNLPSLVCVITGKGPLREYYSRLIHQKHFQHIQVCTPWLEAEDYPLLLGSA
DLGVCLHTSSSGLDLPMKVVDMFGCCLPVCAVNFKCLHELVKHEENGLVFEDSEELAAQL

QMLFSNFPDPAGKLNQFRKNLRESQQLRWDESWVQTVLPLVMDT
>sp|Q9H6U8|ALG9_HUMAN Alpha-1,2-mannosyltransferase ALG9 OS=Homo sapiens GN=ALG9 PE=1 SV=2
MASRGARQRLKGSGASSGDTAPAADKLRELLGSREAGGAEHRTELSGNKAGQVWAPEGST
AFKCLLSARLCAALLSNISDCDETFNYWEPTHYLIYGEGFQTWEYSPAYAIRSYAYLLLH
AWPAAFHARILQTNKILVFYFLRCLLAFVSCICELYFYKAVCKKFGLHVSRMMLAFLVLS
TGMFCSSSAFLPSSFCMYTTLIAMTGWYMDKTSIAVLGVAAGAILGWPFSAALGLPIAFD
LLVMKHRWKSFFHWSLMALILFLVPVVVIDSYYYGKLVIAPLNIVLYNVFTPHGPDLYGT
EPWYFYLINGFLNFNVAFALALLVLPLTSLMEYLLQRFHVQNLGHPYWLTLAPMYIWFII
FFIQPHKEERFLFPVYPLICLCGAVALSALQKCYHFVFQRYRLEHYTVTSNWLALGTVFL
FGLLSFSRSVALFRGYHGPLDLYPEFYRIATDPTIHTVPEGRPVNVCVGKEWYRFPSSFL
LPDNWQLQFIPSEFRGQLPKPFAEGPLATRIVPTDMNDQNLEEPSRYIDISKCHYLVDLD
TMRETPREPKYSSNKEEWISLAYRPFLDASRSSKLLRAFYVPFLSDQYTVYVNYTILKPR
KAKQIRKKSGG
>sp|Q9NP70|AMBN_HUMAN Ameloblastin OS=Homo sapiens GN=AMBN PE=1 SV=1
MSASKIPLFKMKDLILILCLLEMSFAVPFFPQQSGTPGMASLSLETMRQLGSLQRLNTLS
QYSRYGFGKSFNSLWMHGLLPPHSSLPWMRPREHETQQYEYSLPVHPPPLPSQPSLKPQQ
PGLKPFLQSAAATTNQATALKEALQPPIHLGHLPLQEGELPLVQQQVAPSDKPPKPELPG
VDFADPQGPSLPGMDFPDPQGPSLPGLDFADPQGSTIFQIARLISHGPMPQNKQSPLYPG
MLYVPFGANQLNAPARLGIMSSEEVAGGREDPMAYGAMFPGFGGMRPGFEGMPHNPAMGG
DFTLEFDSPVAATKGPENEEGGAQGSPMPEANPDNLENPAFLTELEPAPHAGLLALPKDD
IPGLPRSPSGKMKGLPSVTPAAADPLMTPELADVYRTYDADMTTSVDFQEEATMDTTMAP
NSLQTSMPGNKAQEPEMMHDAWHFQEP
>sp|P02760|AMBP_HUMAN Protein AMBP OS=Homo sapiens GN=AMBP PE=1 SV=1
MRSLGALLLLLSACLAVSAGPVPTPPDNIQVQENFNISRIYGKWYNLAIGSTCPWLKKIM
DRMTVSTLVLGEGATEAEISMTSTRWRKGVCEETSGAYEKTDTDGKFLYHKSKWNITMES
YVVHTNYDEYAIFLTKKFSRHHGPTITAKLYGRAPQLRETLLQDFRVVAQGVGIPEDSIF
TMADRGECVPGEQEPEPILIPRVRRAVLPQEEEGSGGGQLVTEVTKKEDSCQLGYSAGPC
MGMTSRYFYNGTSMACETFQYGGCMGNGNNFVTEKECLQTCRTVAACNLPIVRGPCRAFI
QLWAFDAVKGKCVLFPYGGCQGNGNKFYSEKECREYCGVPGDGDEELLRFSN
>sp|Q8N7J2|AMER2_HUMAN APC membrane recruitment protein 2 OS=Homo sapiens GN=AMER2 PE=1 SV=3
METSRSRGGGGAVSERGGAGASVGVCRRKAEAGAGTGTLAADMDLHCDCAAETPAAEPPS
GKINKAAFKLFKKRKSGGTMPSIFGVKNKGDGKSSGPTGLVRSRTHDGLAEVLVLESGRK
EEPRGGGDSGGGGGGRPNPGPPRAAGPGGGSLASSSVAKSHSFFSLLKKNGRSENGKGEP
VDASKAGGKQKRGLRGLFSGMRWHRKDKRAKAEAAEGRAPGGGLILPGSLTASLECVKEE
TPRAAREPEEPSQDAPRDPAGEPAGGEEVPAPADRAPARSCREAEGLAHPGDTGARGEDA
AGHRRAEPGPGEVRTAEDASRTGAVPVKTVPLVDSEGGSGRAPAAPDPASVDPPSDPSAD
RICLMFSDVTSLKSFDSLTGCGDIIADQEEEAGPSCDKHVPGPGKPALSKKNPGVVAYQG
GGEEMASPDEVDDTYLQEFWDMLSQTEEQGPEPQEGAAKVAAALETKVVPETPKDTRCVE
AAKDASSVKRRRLNRIPIEPHPKEEPKHPEKEQQEGVPNSDEGYWDSTTPGPEEDSSSSG
KKAGIPRDSYSGDALYDLYADPDGSPATLPGGKDNEETSSLSRLKPVSPGTITCPLRTPG
SLLKDSKIPISIKHLTNLPSSHPVVHQQPSRSEMPRTKIPVSKVLVRRVSNRGLAGTTIR
ATACHDSAKKL

>sp|Q16671|AMHR2_HUMAN Anti-Muellerian hormone type-2 receptor OS=Homo sapiens GN=AMHR2 PE=1 SV=1
MLGSLGLWALLPTAVEAPPNRRTCVFFEAPGVRGSTKTLGELLDTGTELPRAIRCLYSRC
CFGIWNLTQDRAQVEMQGCRDSDEPGCESLHCDPSPRAHPSPGSTLFTCSCGTDFCNANY
SHLPPPGSPGTPGSQGPQAAPGESIWMALVLLGLFLLLLLLLGSIILALLQRKNYRVRGE
PVPEPRPDSGRDWSVELQELPELCFSQVIREGGHAVVWAGQLQGKLVAIKAFPPRSVAQF
QAERALYELPGLQHDHIVRFITASRGGPGRLLSGPLLVLELHPKGSLCHYLTQYTSDWGS
SLRMALSLAQGLAFLHEERWQNGQYKPGIAHRDLSSQNVLIREDGSCAIGDLGLALVLPG
LTQPPAWTPTQPQGPAAIMEAGTQRYMAPELLDKTLDLQDWGMALRRADIYSLALLLWEI
LSRCPDLRPDSSPPPFQLAYEAELGNTPTSDELWALAVQERRRPYIPSTWRCFATDPDGL
RELLEDCWDADPEARLTAECVQQRLAALAHPQESHPFPESCPRGCPPLCPEDCTSIPAPT
ILPCRPQRSACHFSVQQGPCSRNPQPACTLSPV
>sp|Q01433|AMPD2_HUMAN AMP deaminase 2 OS=Homo sapiens GN=AMPD2 PE=1 SV=2
MRNRGQGLFRLRSRCFLHQSLPLGAGRRKGLDVAEPGPSRCRSDSPAVAAVVPAMASYPS
GSGKPKAKYPFKKRASLQASTAAPEARGGLGAPPLQSARSLPGPAPCLKHFPLDLRTSMD
GKCKEIAEELFTRSLAESELRSAPYEFPEESPIEQLEERRQRLERQISQDVKLEPDILLR
AKQDFLKTDSDSDLQLYKEQGEGQGDRSLRERDVLEREFQRVTISGEEKCGVPFTDLLDA
AKSVVRALFIREKYMALSLQSFCPTTRRYLQQLAEKPLETRTYEQGPDTPVSADAPVHPP
ALEQHPYEHCEPSTMPGDLGLGLRMVRGVVHVYTRREPDEHCSEVELPYPDLQEFVADVN
VLMALIINGPIKSFCYRRLQYLSSKFQMHVLLNEMKELAAQKKVPHRDFYNIRKVDTHIH
ASSCMNQKHLLRFIKRAMKRHLEEIVHVEQGREQTLREVFESMNLTAYDLSVDTLDVHAD
RNTFHRFDKFNAKYNPIGESVLREIFIKTDNRVSGKYFAHIIKEVMSDLEESKYQNAELR
LSIYGRSRDEWDKLARWAVMHRVHSPNVRWLVQVPRLFDVYRTKGQLANFQEMLENIFLP
LFEATVHPASHPELHLFLEHVDGFDSVDDESKPENHVFNLESPLPEAWVEEDNPPYAYYL
YYTFANMAMLNHLRRQRGFHTFVLRPHCGEAGPIHHLVSAFMLAENISHGLLLRKAPVLQ
YLYYLAQIGIAMSPLSNNSLFLSYHRNPLPEYLSRGLMVSLSTDDPLQFHFTKEPLMEEY
SIATQVWKLSSCDMCELARNSVLMSGFSHKVKSHWLGPNYTKEGPEGNDIRRTNVPDIRV
GYRYETLCQELALITQAVQSEMLETIPEEAGITMSPGPQ
>sp|P49418|AMPH_HUMAN Amphiphysin OS=Homo sapiens GN=AMPH PE=1 SV=1
MADIKTGIFAKNVQKRLNRAQEKVLQKLGKADETKDEQFEEYVQNFKRQEAEGTRLQREL
RGYLAAIKGMQEASMKLTESLHEVYEPDWYGREDVKMVGEKCDVLWEDFHQKLVDGSLLT
LDTYLGQFPDIKNRIAKRSRKLVDYDSARHHLEALQSSKRKDESRISKAEEEFQKAQKVF
EEFNVDLQEELPSLWSRRVGFYVNTFKNVSSLEAKFHKEIAVLCHKLYEVMTKLGDQHAD
KAFTIQGAPSDSGPLRIAKTPSPPEEPSPLPSPTASPNHTLAPASPAPARPRSPSQTRKG
PPVPPLPKVTPTKELQQENIISFFEDNFVPEISVTTPSQNEVPEVKKEETLLDLDFDPFK
PEVTPAGSAGVTHSPMSQTLPWDLWTTSTDLVQPASGGSFNGFTQPQDTSLFTMQTDQSM
ICNLAESEQAPPTEPKAEEPLAAVTPAVGLDLGMDTRAEEPVEEAVIIPGADADAAVGTL
VSAAEGAPGEEAEAEKATVPAGEGVSLEEAKIGTETTEGAESAQPEAEELEATVPQEKVI
PSVVIEPASNHEEEGENEITIGAEPKETTEDAAPPGPTSETPELATEQKPIQDPQPTPSA
PAMGAADQLASAREASQELPPGFLYKVETLHDFEAANSDELTLQRGDVVLVVPSDSEADQ
DAGWLVGVKESDWLQYRDLATYKGLFPENFTRRLD
>sp|Q9C0C7|AMRA1_HUMAN Activating molecule in BECN1-regulated autophagy protein 1 OS=Homo sapiens GN=AMBRA1 PE=1 SV=2
MKVVPEKNAVRILWGRERGARAMGAQRLLQELVEDKTRWMKWEGKRVELPDSPRSTFLLA

FSPDRTLLASTHVNHNIYITEVKTGKCVHSLIGHRRTPWCVTFHPTISGLIASGCLDGEV
RIWDLHGGSESWFTDSNNAIASLAFHPTAQLLLIATANEIHFWDWSRREPFAVVKTASEM
ERVRLVRFDPLGHYLLTAIVNPSNQQGDDEPEIPIDGTELSHYRQRALLQSQPVRRTPLL
HNFLHMLSSRSSGIQVGEQSTVQDSATPSPPPPPPQPSTERPRTSAYIRLRQRVSYPTAE
CCQHLGILCLCSRCSGTRVPSLLPHQDSVPPASARATTPSFSFVQTEPFHPPEQASSTQQ
DQGLLNRPSAFSTVQSSTAGNTLRNLSLGPTRRSLGGPLSSHPSRYHREIAPGLTGSEWT
RTVLSLNSRSEAESMPPPRTSASSVSLLSVLRQQEGGSQASVYTSATEGRGFPASGLATE
SDGGNGSSQNNSGSIRHELQCDLRRFFLEYDRLQELDQSLSGEAPQTQQAQEMLNNNIES
ERPGPSHQPTPHSSENNSNLSRGHLNRCRACHNLLTFNNDTLRWERTTPNYSSGEASSSW
QVPSSFESVPSSGSQLPPLERTEGQTPSSSRLELSSSASPQEERTVGVAFNQETGHWERI
YTQSSRSGTVSQEALHQDMPEESSEEDSLRRRLLESSLISLSRYDGAGSREHPIYPDPAR
LSPAAYYAQRMIQYLSRRDSIRQRSMRYQQNRLRSSTSSSSSDNQGPSVEGTDLEFEDFE
DNGDRSRHRAPRNARMSAPSLGRFVPRRFLLPEYLPYAGIFHERGQPGLATHSSVNRVLA
GAVIGDGQSAVASNIANTTYRLQWWDFTKFDLPEISNASVNVLVQNCKIYNDASCDISAD
GQLLAAFIPSSQRGFPDEGILAVYSLAPHNLGEMLYTKRFGPNAISVSLSPMGRYVMVGL
ASRRILLHPSTEHMVAQVFRLQQAHGGETSMRRVFNVLYPMPADQRRHVSINSARWLPEP
GLGLAYGTNKGDLVICRPEALNSGVEYYWDQLNETVFTVHSNSRSSERPGTSRATWRTDR
DMGLMNAIGLQPRNPATSVTSQGTQTLALQLQNAETQTEREVPEPGTAASGPGEGEGSEY
GASGEDALSRIQRLMAEGGMTAVVQREQSTTMASMGGFGNNIIVSHRIHRSSQTGTEPGA
AHTSSPQPSTSRGLLPEAGQLAERGLSPRTASWDQPGTPGREPTQPTLPSSSPVPIPVSL
PSAEGPTLHCELTNNNHLLDGGSSRGDAAGPRGEPRNR
>sp|P04746|AMYP_HUMAN Pancreatic alpha-amylase OS=Homo sapiens GN=AMY2A PE=1 SV=2
MKFFLLLFTIGFCWAQYSPNTQQGRTSIVHLFEWRWVDIALECERYLAPKGFGGVQVSPP
NENVAIYNPFRPWWERYQPVSYKLCTRSGNEDEFRNMVTRCNNVGVRIYVDAVINHMCGN
AVSAGTSSTCGSYFNPGSRDFPAVPYSGWDFNDGKCKTGSGDIENYNDATQVRDCRLTGL
LDLALEKDYVRSKIAEYMNHLIDIGVAGFRLDASKHMWPGDIKAILDKLHNLNSNWFPAG
SKPFIYQEVIDLGGEPIKSSDYFGNGRVTEFKYGAKLGTVIRKWNGEKMSYLKNWGEGWG
FVPSDRALVFVDNHDNQRGHGAGGASILTFWDARLYKMAVGFMLAHPYGFTRVMSSYRWP
RQFQNGNDVNDWVGPPNNNGVIKEVTINPDTTCGNDWVCEHRWRQIRNMVIFRNVVDGQP
FTNWYDNGSNQVAFGRGNRGFIVFNNDDWSFSLTLQTGLPAGTYCDVISGDKINGNCTGI
KIYVSDDGKAHFSISNSAEDPFIAIHAESKL
>sp|Q6ZTN6|AN13D_HUMAN Ankyrin repeat domain-containing protein 13D OS=Homo sapiens GN=ANKRD13D PE=1 SV=2
MVQLVLQYRDYQRATQRLAGIPELLNKLRQAPDFYVEMKWEFTSWVPLVSKMCPSDVYRV
WKRGESLRVDTSLLGFEHMTWQRGRRSFIFKGQEAGALVMEVDHDRQVVHVETLGLTLQE
PETLLAAMRPSEEHVASRLTSPIVSTHLDTRNVAFERNKCGIWGWRSEKMETVSGYEAKV
YSATNVELVTRTRTEHLSDQDKSRSKAGKTPFQSFLGMAQQHSSHTGAPVQQAASPTNPT
AISPEEYFDPNFSLESRNIGRPIEMSSKVQRFKATLWLSEEHPLSLGDQVTPIIDLMAIS
NAHFAKLRDFITLRLPPGFPVKIEIPLFHVLNARITFSNLCGCDEPLSSVWVPAPSSAVA
ASGNPFPCEVDPTVFEVPNGYSVLGMERNEPLRDEDDDLLQFAIQQSLLEAGTEAEQVTV
WEALTNTRPGARPPPQATVYEEQLQLERALQESLQLSTEPRGPGSPPRTPPAPGPPSFEE
QLRLALELSSREQEERERRGQQEEEDLQRILQLSLTEH
>sp|Q9BXX2|AN30B_HUMAN Ankyrin repeat domain-containing protein 30B OS=Homo sapiens GN=ANKRD30B PE=2 SV=3

MKRLLAAAGKGVRGPEPPNPFSERVYTEKDYGTIYFGDLGKIHTAASRGQVQKLEKMTVG
KKPVNLNKRDMKKRTALHWACVNGHAEVVTFLVDRKCQLNVLDGEGRTPLMKALQCEREA
CANILIDAGADLNYVDVYGNTALHYAVYSENLLMVATLLSYGAVIEVQNKASLTPLLLAI
QKRSKQTVEFLLTKNANANAFNESKCTALMLAICEGSSEIVGMLLQQNVDVFAEDIHGIT
AERYAACGVNYIHQQLLEHIRKLPKNPQNTNPEGTSTGTPDEAAPLAERTPDTAESLLE
KTPDEAARLVEGTSAKIQCLGKATSGKFEQSTEETPRKILRPTKETSEKFSWPAKERSRK
ITWEEKETSVKTECVAGVTPNKTEVLEKGTSNMIACPTKETSTKASTNVDVSSVEPIFSL
FGTRTIENSQCTKVEEDFNLATKIISKSAAQNYTCLPDATYQKDIKTINHKIEDQMFPSE
SKREEDEEYSWDSGSLFESSAKTQVCIPESMYQKVMEINREVEELPEKPSAFKPAVEMQK
TVPNKAFELKNEQTLRAAQMFPSESKQKDDEENSWDSESPCETVSQKDVYLPKATHQKEF
DTLSGKLEESPVKDGLLKPTCGRKVSLPNKALELKDRETFKAESPDKDGLLKPTCGRKVS
LPNKALELKDRETLKAESPDNDGLLKPTCGRKVSLPNKALELKDRETFKAAQMFPSESKQ
KDDEENSWDFESFLETLLQNDVCLPKATHQKEFDTLSGKLEESPDKDGLLKPTCGMKISL
PNKALELKDRETFKAEDVSSVESTFSLFGKPTTENSQSTKVEEDFNLTTKEGATKTVTGQ
QERDIGIIERAPQDQTNKMPTSELGRKEDTKSTSDSEIISVSDTQNYECLPEATYQKEIK
TTNGKIEESPEKPSHFEPATEMQNSVPNKGLEWKNKQTLRADSTTLSKILDALPSCERGR
ELKKDNCEQITAKMEQTKNKFCVLQKELSEAKEIKSQLENQKAKWEQELCSVRLTLNQEE
EKRRNVDILKEKIRPEEQLRKKLEVKQQLEQTLRIQDIELKSVTSNLNQVSHTHESENDL
FHENCMLKKEIAMLKLEVATLKHQHQVKENKYFEDIKILQEKNAELQMTLKLKQKTVTKR
ASQYREQLKVLTAENTMLTSKLKEKQDKEILETEIESHHPRLASALQDHDQSVTSRKNQE
LAFHSAGDAPLQGIMNVDVSNTIYNNEVLHQPLYEAQRKSKSPKINLNYAGDDLRENALV
SEHAQRDRCETQCQMKKAEHMYQNEQDNVDKHTEQQESLEQKLFQLESKNRWLRQQLVYA
HKKVNKSKVTINIQFPEMKMQRHLNEKNEEVFNYGNHLKERIDQYEKEKAEREVSIKKYK
YFSNFLKESGLG
>sp|O95626|AN32D_HUMAN Acidic leucine-rich nuclear phosphoprotein 32 family member D OS=Homo sapiens GN=ANP32D PE=2 SV=2
MEMGKWIHLELRNRTPSDVKELFLDNSQSNEGKLEGLTDEFEELELLNTINIGLTSIANL
PKLNKLKKLELSSNRASVGLEVLAEKCPNLIHLNLSGNKIKDLSTIEPLKKLENLESLDL
FTCEVTNLNNY
>sp|Q8N2N9|AN36B_HUMAN Ankyrin repeat domain-containing protein 36B OS=Homo sapiens GN=ANKRD36B PE=1 SV=4
MERLCSDGFAFPHYYIKPYHLKRIHRAVLRGNLEKLKYLLLTYYDANKRDRKERTALHLA
CATGQPEMVHLLVSRRCELNLCDREDRTPLIKAVQLRQEACATLLLQNGADPNITDVFGR
TALHYAVYNEDTSMIEKLLSHGTNIEECSKNEYQPLLLAVSRRKVKMVEFLLKKKANVNA
IDYLGRSALILAVTLGEKDIVILLLQHNIDVFSRDVYGKLAEDYASEAENRVIFDLIYEY
KRKRYEDLPINSNPVSPQKQRAEKATSDDKDSVSNIATEIKEGPISGTVSSQKQPAEKAT
SDEKDSVSNIATEIKEGQQSGTVSPQKQSAQKVIFKKKVSLLNIATRIMGGGKSGTVSSQ
KQPASKTASDKTDSALNTATEIKDGLQCGTVSSQKQQALKATTDEEGSVSNIATEIKDGE
KSGTVSSQKKPALKATSDEKDSFSNITREKKDGEISRTVSSQKPPALKATSVKEDSVLNI
AREKKDGEKSRTVSFEQPPGLKATRDEKDSLLNIARGKKDGEKTRRVSSHKQPSLKATSD
KEDSVPNMATETKDEQISGTVSCQKQPALKATSDKKDSVSNIPTEIKDGQQSGTVSSQKQ
PAWKATSVKKDSVSNIATEIKDGQIRGTVSSQRRPALKTTGDEKDSVSNIAREIKDGEKS
GTVSPQKQSAQKVIFKKKVSLLNIATRITGGGKSGTEYPENLRTLKATIENKDSVLNTAT
KMKEVQTSTPAEQDLEMASEGEQKRLEEYENNQPQVKNQIHSRDDLDDIIQSSQTVSEDG

DSLCCNCKNVILLIDQHEMKCKDCVHLLKIKNTFCLWKRLIKLKDNHCEQLRVKIRKLKN
KASVLQKRISEKEEIKSQLKHEILELEKELCSLRFAIQQEKKKRRNVEELHQKVREKLRI
TEEQYRIEADVTKPIKPALKSAEVELKTGGNNSNQVSETDEKEDLLHENRLMQDEIARLR
LEKDTIKNQNLEKKYLKDFEIVKRKHEDLQKALKRNGETLAKTIACYSGQLAALTDENTT
LRSKLEKQRESRQRLETEMQSYRCRLNAARCDHDQSHSSKRDQELAFQGTVDKCRHLQEN
LNSHVLILSLQLSKAESKSRVLKTELHYTGEALKEKALVFEHVQSELKQKQSQMKDIEKM
YKSGYNTMEKCIEKQERFCQLKKQNMLLQQQLDDARNKADNQEKAILNIQARCDARVQNL
QAECRKHRLLLEEDNKMLVNELNHSKEKECQYEKEKAEREVAVRQLQQKRDDVLNKGSAT
KALLDASSRHCTYLENGMQDSRKKLDQMRSQFQEIQDQLTATIRCTKEMEGDTQKLEVEH
VMMRKIIKKQDDQIERLEKILQHSSLMLQVFES
>sp|Q9NU02|ANKE1_HUMAN Ankyrin repeat and EF-hand domain-containing protein 1 OS=Homo sapiens GN=ANKEF1 PE=2 SV=2
MALADKRLENLQIYKVLQCVRNKDKKQIEKLTKLGYPELINYTEPINGLSALHLASVSND
IDMVSFLLDLGAHPDVQDRMGCTPTMRAAELGHELSMEILAKAKADMTIVDNEGKGVLFY
CILPTKRHYRCALIALEHGADVNNSTYEGKPIFLRACEDAHDVKDVCLTFLEKGANPNAI
NSSTGRTALMEASREGVVEIVRGILERGGEVNAFDNDRHHAAHFAAKGGFFDILKLLFAY
NGDVGLISINGNTPLHYAAMGGFADCCKYIAQRGCDLKWKNLDHKTPRAVAKEGGFKAAS
KEIRRAERIANKLARPGAKNPNPLWALRLHDWSVEREAFLREAFAVLDRGDGSISKNDFV
MVLEERQDYASSEQLAAIAHLHEKTRGGGVNINEFFKGTRYLNKSFVLGSYGPKKKEKGM
GKKGKKGKFVLPLPICVIPEYAFPRRQDGGPPYYMIETYKNVTDSSRFNRDHPPEHPIQD
DSVWYIDDSEKVFSNINIITKAGDLASLKKAFESGIPVDMKDNYYKTPLMTACASGNIDV
VKFLLEKGANVNATDNFLWTPLHFACHAGQQDIVELLVESGALIDAASINNSTPLNRAIE
SCRLDTVKYLLDIGAKFQLENRKGHSAMDVAKAYADYRIIDLIKEKLDNLPKPAENQKLK
GKTPPILKTEGPEIKKEEELLSSIYGVPTTSEGKKVQKGNVVHLNSLITSGYTKKVDITF
IPRRIWSPEATTAELIRKRELRRERFTHEVDFDDFMMPFQKNITEKARALEAALKT
>sp|Q9NW15|ANO10_HUMAN Anoctamin-10 OS=Homo sapiens GN=ANO10 PE=1 SV=2
MKVTLSALDTSESSFTPLVVIELAQDVKEETKEWLKNRIIAKKKDGGAQLLFRPLLNKYE
QETLENQNLYLVGASKIRMLLGAEAVGLVKECNDNTMRAFTYRTRQNFKGFDDNNDDFLT
MAECQFIIKHELENLRAKDEKMIPGYPQAKLYPGKSLLRRLLTSGIVIQVFPLHDSEALK
KLEDTWYTRFALKYQPIDSIRGYFGETIALYFGFLEYFTFALIPMAVIGLPYYLFVWEDY
DKYVIFASFNLIWSTVILELWKRGCANMTYRWGTLLMKRKFEEPRPGFHGVLGINSITGK
EEPLYPSYKRQLRIYLVSLPFVCLCLYFSLYVMMIYFDMEVWALGLHENSGSEWTSVLLY
VPSIIYAIVIEIMNRLYRYAAEFLTSWENHRLESAYQNHLILKVLVFNFLNCFASLFYIA
FVLKDMKLLRQSLATLLITSQILNQIMESFLPYWLQRKHGVRVKRKVQALKADIDATLYE
QVILEKEMGTYLGTFDDYLELFLQFGYVSLFSCVYPLAAAFAVLNNFTEVNSDALKMCRV
FKRPFSEPSANIGVWQLAFETMSVISVVTNCALIGMSPQVNAVFPESKADLILIVVAVEH
ALLALKFILAFAIPDKPRHIQMKLARLEFESLEALKQQQMKLVTENLKEEPMESGKEKAT
>sp|Q32M45|ANO4_HUMAN Anoctamin-4 OS=Homo sapiens GN=ANO4 PE=2 SV=1
MEASSSGITNGKTKVFHPEGGVDLQGYQLDMQILPDGPKSDVDFSEILNAIQEMAKDVNI
LFDELEAVSSPCKDDDSLLHPGNLTSTSDDASRLEAGGETVPERNKSNGLYFRDGKCRID
YILVYRKSNPQTEKREVFERNIRAEGLQMEKESSLINSDIIFVKLHAPWEVLGRYAEQMN
VRMPFRRKIYYLPRRYKFMSRIDKQISRFRRWLPKKPMRLDKETLPDLEENDCYTAPFSQ
QRIHHFIIHNKETFFNNATRSRIVHHILQRIKYEEGKNKIGLNRLLTNGSYEAAFPLHEG
SYRSKNSIRTHGAENHRHLLYECWASWGVWYKYQPLDLVRRYFGEKIGLYFAWLGWYTGM

LFPAAFIGLFVFLYGVTTLDHSQVSKEVCQATDIIMCPVCDKYCPFMRLSDSCVYAKVTH
LFDNGATVFFAVFMAVWATVFLEFWKRRRAVIAYDWDLIDWEEEEEEIRPQFEAKYSKKE
RMNPISGKPEPYQAFTDKCSRLIVSASGIFFMICVVIAAVFGIVIYRVVTVSTFAAFKWA
LIRNNSQVATTGTAVCINFCIIMLLNVLYEKVALLLTNLEQPRTESEWENSFTLKMFLFQ
FVNLNSSTFYIAFFLGRFTGHPGAYLRLINRWRLEECHPSGCLIDLCMQMGIIMVLKQTW
NNFMELGYPLIQNWWTRRKVRQEHGPERKISFPQWEKDYNLQPMNAYGLFDEYLEMILQF
GFTTIFVAAFPLAPLLALLNNIIEIRLDAYKFVTQWRRPLASRAKDIGIWYGILEGIGIL
SVITNAFVIAITSDFIPRLVYAYKYGPCAGQGEAGQKCMVGYVNASLSVFRISDFENRSE
PESDGSEFSGTPLKYCRYRDYRDPPHSLVPYGYTLQFWHVLAARLAFIIVFEHLVFCIKH
LISYLIPDLPKDLRDRMRREKYLIQEMMYEAELERLQKERKERKKNGKAHHNEWP
>sp|Q4KMQ2|ANO6_HUMAN Anoctamin-6 OS=Homo sapiens GN=ANO6 PE=1 SV=2
MKKMSRNVLLQMEEEEDDDDGDIVLENLGQTIVPDLGSLESQHDFRTPEFEEFNGKPDSL
FFNDGQRRIDFVLVYEDESRKETNKKGTNEKQRRKRQAYESNLICHGLQLEATRSVLDDK
LVFVKVHAPWEVLCTYAEIMHIKLPLKPNDLKNRSSAFGTLNWFTKVLSVDESIIKPEQE
FFTAPFEKNRMNDFYIVDRDAFFNPATRSRIVYFILSRVKYQVINNVSKFGINRLVNSGI
YKAAFPLHDCKFRRQSEDPSCPNERYLLYREWAHPRSIYKKQPLDLIRKYYGEKIGIYFA
WLGYYTQMLLLAAVVGVACFLYGYLNQDNCTWSKEVCHPDIGGKIIMCPQCDRLCPFWKL
NITCESSKKLCIFDSFGTLVFAVFMGVWVTLFLEFWKRRQAELEYEWDTVELQQEEQARP
EYEARCTHVVINEITQEEERIPFTAWGKCIRITLCASAVFFWILLIIASVIGIIVYRLSV
FIVFSAKLPKNINGTDPIQKYLTPQTATSITASIISFIIIMILNTIYEKVAIMITNFELP
RTQTDYENSLTMKMFLFQFVNYYSSCFYIAFFKGKFVGYPGDPVYWLGKYRNEECDPGGC
LLELTTQLTIIMGGKAIWNNIQEVLLPWIMNLIGRFHRVSGSEKITPRWEQDYHLQPMGK
LGLFYEYLEMIIQFGFVTLFVASFPLAPLLALVNNILEIRVDAWKLTTQFRRLVPEKAQD
IGAWQPIMQGIAILAVVTNAMIIAFTSDMIPRLVYYWSFSVPPYGDHTSYTMEGYINNTL
SIFKVADFKNKSKGNPYSDLGNHTTCRYRDFRYPPGHPQEYKHNIYYWHVIAAKLAFIIV
MEHVIYSVKFFISYAIPDVSKRTKSKIQREKYLTQKLLHENHLKDMTKNMGVIAERMIEA
VDNNLRPKSE
>sp|Q5VYY1|ANR22_HUMAN Ankyrin repeat domain-containing protein 22 OS=Homo sapiens GN=ANKRD22 PE=2 SV=1
MGILYSEPICQAAYQNDFGQVWRWVKEDSSYANVQDGFNGDTPLICACRRGHVRIVSFLL
RRNANVNLKNQKERTCLHYAVKKKFTFIDYLLIILLMPVLLIGYFLMVSKTKQNEALVRM
LLDAGVEVNATDCYGCTALHYACEMKNQSLIPLLLEARADPTIKNKHGESSLDIARRLKF
SQIELMLRKAL
>sp|Q8N8V4|ANS4B_HUMAN Ankyrin repeat and SAM domain-containing protein 4B OS=Homo sapiens GN=ANKS4B PE=1 SV=2
MSTRYHQAASDSYLELLKEATKRDLNLSDEDGMTPTLLAAYHGNLEALEIICSRGGDPDR
CDIWGNTPLHFAASNGHAHCVSFLVNFGANIFALDNDLQTPLDAAASREQNECVALLDKA
ATAQNIMNPKKVTRLKEQAQKNARRQIKECERLQEKHQNKMAHTYSKEESGTLSSSKGTF
SRSSPSNASAPGTFGSLSKGIKDTFKIKFKKNKDTAEQVGKEGRSGQRNVMEVFREEEED
SFSGDFKEKLQLSAEEDGSVHHESILNRPGLGSIVFRRNRISSPEDISDSKREFGFKLPS
ELLQRQGASEADEGAADEEGEENGLKDDLPWDDDEVEWEEDVVDATPLEVFLLSQHLEEF
LPIFKREQIDLEALLLCSDEDLQSIQMQLGPRKKVLNAINRRKQVLQQPGQLVDTSL
>sp|Q9BXS5|AP1M1_HUMAN AP-1 complex subunit mu-1 OS=Homo sapiens GN=AP1M1 PE=1 SV=3
MSASAVYVLDLKGKVLICRNYRGDVDMSEVEHFMPILMEKEEEGMLSPILAHGGVRFMWI

KHNNLYLVATSKKNACVSLVFSFLYKVVQVFSEYFKELEEESIRDNFVIIYELLDELMDF
GYPQTTDSKILQEYITQEGHKLETGAPRPPATVTNAVSWRSEGIKYRKNEVFLDVIESVN
LLVSANGNVLRSEIVGSIKMRVFLSGMPELRLGLNDKVLFDNTGRGKSKSVELEDVKFHQ
CVRLSRFENDRTISFIPPDGEFELMSYRLNTHVKPLIWIESVIEKHSHSRIEYMIKAKSQ
FKRRSTANNVEIHIPVPNDADSPKFKTTVGSVKWVPENSEIVWSIKSFPGGKEYLMRAHF
GLPSVEAEDKEGKPPISVKFEIPYFTTSGIQVRYLKIIEKSGYQALPWVRYITQNGDYQL
RTQ
>sp|Q92754|AP2C_HUMAN Transcription factor AP-2 gamma OS=Homo sapiens GN=TFAP2C PE=1 SV=1
MLWKITDNVKYEEDCEDRHDGSSNGNPRVPHLSSAGQHLYSPAPPLSHTGVAEYQPPPYF
PPPYQQLAYSQSADPYSHLGEAYAAAINPLHQPAPTGSQQQAWPGRQSQEGAGLPSHHGR
PAGLLPHLSGLEAGAVSARRDAYRRSDLLLPHAHALDAAGLAENLGLHDMPHQMDEVQNV
DDQHLLLHDQTVIRKGPISMTKNPLNLPCQKELVGAVMNPTEVFCSVPGRLSLLSSTSKY
KVTVAEVQRRLSPPECLNASLLGGVLRRAKSKNGGRSLREKLDKIGLNLPAGRRKAAHVT
LLTSLVEGEAVHLARDFAYVCEAEFPSKPVAEYLTRPHLGGRNEMAARKNMLLAAQQLCK
EFTELLSQDRTPHGTSRLAPVLETNIQNCLSHFSLITHGFGSQAICAAVSALQNYIKEAL
IVIDKSYMNPGDQSPADSNKTLEKMEKHRK
>sp|Q96CW1|AP2M1_HUMAN AP-2 complex subunit mu OS=Homo sapiens GN=AP2M1 PE=1 SV=2
MIGGLFIYNHKGEVLISRVYRDDIGRNAVDAFRVNVIHARQQVRSPVTNIARTSFFHVKR
SNIWLAAVTKQNVNAAMVFEFLYKMCDVMAAYFGKISEENIKNNFVLIYELLDEILDFGY
PQNSETGALKTFITQQGIKSQHQTKEEQSQITSQVTGQIGWRREGIKYRRNELFLDVLES
VNLLMSPQGQVLSAHVSGRVVMKSYLSGMPECKFGMNDKIVIEKQGKGTADETSKSGKQS
IAIDDCTFHQCVRLSKFDSERSISFIPPDGEFELMRYRTTKDIILPFRVIPLVREVGRTK
LEVKVVIKSNFKPSLLAQKIEVRIPTPLNTSGVQVICMKGKAKYKASENAIVWKIKRMAG
MKESQISAEIELLPTNDKKKWARPPISMNFEVPFAPSGLKVRYLKVFEPKLNYSDHDVIK
WVRYIGRSGIYETRC
>sp|P53680|AP2S1_HUMAN AP-2 complex subunit sigma OS=Homo sapiens GN=AP2S1 PE=1 SV=2
MIRFILIQNRAGKTRLAKWYMQFDDDEKQKLIEEVHAVVTVRDAKHTNFVEFRNFKIIYR
RYAGLYFCICVDVNDNNLAYLEAIHNFVEVLNEYFHNVCELDLVFNFYKVYTVVDEMFLA
GEIRETSQTKVLKQLLMLQSLE
>sp|O43299|AP5Z1_HUMAN AP-5 complex subunit zeta-1 OS=Homo sapiens GN=AP5Z1 PE=1 SV=2
MFSAGAESLLHQAREIQDEELKKFCSRICKLLQAEDLGPDTLDSLQRLFLIISATKYSRR
LEKTCVDLLQATLGLPACPEQLQVLCAAILREMSPSDSLSLAWDHTQNSRQLSLVASVLL
AQGDRNEEVRAVGQGVLRALESRQPEGPSLRHLLPVMAKVVVLSPGTLQEDQATLLSKRL
VDWLRYASLQQGLPHSGGFFSTPRARQPGPVTEVDGAVATDFFTVLSSGHRFTDDQWLNV
QAFSMLRAWLLHSGPEGPGTLDTDDRSEQEGSTLSVISATSSAGRLLPPRERLREVAFEY
CQRLIEQSNRRALRKGDSDLQKACLVEAVLVLDVLCRQDPSFLYRSLSCLKALHGRVRGD
PASVRVLLPLAHFFLSHGEAAAVDSEAVYQHLFTRIPVEQFHSPMLAFEFIQFCRDNLHL
FSGHLSTLRLSFPNLFKFLAWNSPPLTSEFVALLPALVDAGTALEMLHALLDLPCLTAVL
DLQLRSAPAASERPLWDTSLRAPSCLEAFRDPQFQGLFQYLLRPKASGATERLAPLHQLL
QPMAGCARVAQCAQAVPTLLQAFFSAVTQVADGSLINQLALLLLGRSDSLYPAPGYAAGV
HSVLSSQFLALCTLKPSLVVELARDLLEFLGSVNGLCSRASLVTSVVWAIGEYLSVTYDR
RCTVEQINKFFEALEALLFEVTQCRPSAALPRCPPQVVTVLMTTLTKLASRSQDLIPRAS
LLLSKMRTLAHSPATSSTHSEEGAEAIRTRATELLTLLKMPSVAQFVLTPSTEVCSPRYH
RDANTALPLALRTVSRLVEREAGLMPG

>sp|Q9BS18|APC13_HUMAN Anaphase-promoting complex subunit 13 OS=Homo sapiens GN=ANAPC13 PE=1 SV=1
MDSEVQRDGRILDLIDDAWREDKLPYEDVAIPLNELPEPEQDNGGTTESVKEQEMKWTDL
ALQYLHENVPPIGN
>sp|Q9UJX4|APC5_HUMAN Anaphase-promoting complex subunit 5 OS=Homo sapiens GN=ANAPC5 PE=1 SV=2
MASVHESLYFNPMMTNGVVHANVFGIKDWVTPYKIAVLVLLNEMSRTGEGAVSLMERRRL
NQLLLPLLQGPDITLSKLYKLIEESCPQLANSVQIRIKLMAEGELKDMEQFFDDLSDSFS
GTEPEVHKTSVVGLFLRHMILAYSKLSFSQVFKLYTALQQYFQNGEKKTVEDADMELTSR
DEGERKMEKEELDVSVREEEVSCSGPLSQKQAEFFLSQQASLLKNDETKALTPASLQKEL
NNLLKFNPDFAEAHYLSYLNNLRVQDVFSSTHSLLHYFDRLILTGAESKSNGEEGYGRSL
RYAALNLAALHCRFGHYQQAELALQEAIRIAQESNDHVCLQHCLSWLYVLGQKRSDSYVL
LEHSVKKAVHFGLPYLASLGIQSLVQQRAFAGKTANKLMDALKDSDLLHWKHSLSELIDI
SIAQKTAIWRLYGRSTMALQQAQMLLSMNSLEAVNAGVQQNNTESFAVALCHLAELHAEQ
GCFAAASEVLKHLKERFPPNSQHAQLWMLCDQKIQFDRAMNDGKYHLADSLVTGITALNS
IEGVYRKAVVLQAQNQMSEAHKLLQKLLVHCQKLKNTEMVISVLLSVAELYWRSSSPTIA
LPMLLQALALSKEYRLQYLASETVLNLAFAQLILGIPEQALSLLHMAIEPILADGAILDK
GRAMFLVAKCQVASAASYDQPKKAEALEAAIENLNEAKNYFAKVDCKERIRDVVYFQARL
YHTLGKTQERNRCAMLFRQLHQELPSHGVPLINHL
>sp|Q9ULZ1|APEL_HUMAN Apelin OS=Homo sapiens GN=APLN PE=2 SV=1
MNLRLCVQALLLLWLSLTAVCGGSLMPLPDGNGLEDGNVRHLVQPRGSRNGPGPWQGGRR
KFRRQRPRLSHKGPMPF
>sp|P27695|APEX1_HUMAN DNA-(apurinic or apyrimidinic site) lyase OS=Homo sapiens GN=APEX1 PE=1 SV=2
MPKRGKKGAVAEDGDELRTEPEAKKSKTAAKKNDKEAAGEGPALYEDPPDQKTSPSGKPA
TLKICSWNVDGLRAWIKKKGLDWVKEEAPDILCLQETKCSENKLPAELQELPGLSHQYWS
APSDKEGYSGVGLLSRQCPLKVSYGIGDEEHDQEGRVIVAEFDSFVLVTAYVPNAGRGLV
RLEYRQRWDEAFRKFLKGLASRKPLVLCGDLNVAHEEIDLRNPKGNKKNAGFTPQERQGF
GELLQAVPLADSFRHLYPNTPYAYTFWTYMMNARSKNVGWRLDYFLLSHSLLPALCDSKI
RSKALGSDHCPITLYLAL
>sp|P35414|APJ_HUMAN Apelin receptor OS=Homo sapiens GN=APLNR PE=1 SV=1
MEEGGDFDNYYGADNQSECEYTDWKSSGALIPAIYMLVFLLGTTGNGLVLWTVFRSSREK
RRSADIFIASLAVADLTFVVTLPLWATYTYRDYDWPFGTFFCKLSSYLIFVNMYASVFCL
TGLSFDRYLAIVRPVANARLRLRVSGAVATAVLWVLAALLAMPVMVLRTTGDLENTTKVQ
CYMDYSMVATVSSEWAWEVGLGVSSTTVGFVVPFTIMLTCYFFIAQTIAGHFRKERIEGL
RKRRRLLSIIVVLVVTFALCWMPYHLVKTLYMLGSLLHWPCDFDLFLMNIFPYCTCISYV
NSCLNPFLYAFFDPRFRQACTSMLCCGQSRCAGTSHSSSGEKSASYSSGHSQGPGPNMGK
GGEQMHEKSIPYSQETLVVD
>sp|P04745|AMY1_HUMAN Alpha-amylase 1 OS=Homo sapiens GN=AMY1A PE=1 SV=2
MKLFWLLFTIGFCWAQYSSNTQQGRTSIVHLFEWRWVDIALECERYLAPKGFGGVQVSPP
NENVAIHNPFRPWWERYQPVSYKLCTRSGNEDEFRNMVTRCNNVGVRIYVDAVINHMCGN
AVSAGTSSTCGSYFNPGSRDFPAVPYSGWDFNDGKCKTGSGDIENYNDATQVRDCRLSGL
LDLALGKDYVRSKIAEYMNHLIDIGVAGFRIDASKHMWPGDIKAILDKLHNLNSNWFPEG
SKPFIYQEVIDLGGEPIKSSDYFGNGRVTEFKYGAKLGTVIRKWNGEKMSYLKNWGEGWG

FMPSDRALVFVDNHDNQRGHGAGGASILTFWDARLYKMAVGFMLAHPYGFTRVMSSYRWP
RYFENGKDVNDWVGPPNDNGVTKEVTINPDTTCGNDWVCEHRWRQIRNMVNFRNVVDGQP
FTNWYDNGSNQVAFGRGNRGFIVFNNDDWTFSLTLQTGLPAGTYCDVISGDKINGNCTGI
KIYVSDDGKAHFSISNSAEDPFIAIHAESKL
>sp|P19961|AMY2B_HUMAN Alpha-amylase 2B OS=Homo sapiens GN=AMY2B PE=1 SV=1
MKFFLLLFTIGFCWAQYSPNTQQGRTSIVHLFEWRWVDIALECERYLAPKGFGGVQVSPP
NENVAIHNPFRPWWERYQPVSYKLCTRSGNEDEFRNMVTRCNNVGVRIYVDAVINHMSGN
AVSAGTSSTCGSYFNPGSRDFPAVPYSGWDFNDGKCKTGSGDIENYNDATQVRDCRLVGL
LDLALEKDYVRSKIAEYMNHLIDIGVAGFRLDASKHMWPGDIKAILDKLHNLNSNWFPAG
SKPFIYQEVIDLGGEPIKSSDYFGNGRVTEFKYGAKLGTVIRKWNGEKMSYLKNWGEGWG
FMPSDRALVFVDNHDNQRGHGAGGASILTFWDARLYKMAVGFMLAHPYGFTRVMSSYRWP
RQFQNGNDVNDWVGPPNNNGVIKEVTINPDTTCGNDWVCEHRWRQIRNMVNFRNVVDGQP
FTNWYDNGSNQVAFGRGNRGFIVFNNDDWTFSLTLQTGLPAGTYCDVISGDKINGNCTGI
KIYVSDDGKAHFSISNSAEDPFIAIHAESKL
>sp|Q86YJ7|AN13B_HUMAN Ankyrin repeat domain-containing protein 13B OS=Homo sapiens GN=ANKRD13B PE=1 SV=4
MIPANASARKGPEGKYPLHYLVWHNRHRELEKEVRAGQVDIEQLDPRGRTPLHLATTLGH
LECARVLLAHGADVGRENRSGWTVLQEAVSTRDLELVQLVLRYRDYQRVVKRLAGIPVLL
EKLRKAQDFYVEMKWEFTSWVPLVSKICPSDTYKVWKSGQNLRVDTTLLGFDHMTWQRGN
RSFVFRGQDTSAVVMEIDHDRRVVYTETLALAGQDRELLLAAAQPTEEQVLSRLTAPVVT
TQLDTKNISFERNKTGILGWRSEKTEMVNGYEAKVYGASNVELITRTRTEHLSEQHKGKV
KGCKTPLQSFLGIAEQHGGPQNGTLITQTLSQANPTAITAEEYFNPNFELGNRDMGRPME
LTTKTQKFKAKLWLCEEHPLSLCEQVAPIIDLMAVSNALFAKLRDFITLRLPPGFPVKIE
IPIFHILNARITFGNLNGCDEPVPSVRGSPSSETPSPGSDSSSVSSSSSTTSCRGCEISP
ALFEAPRGYSMMGGQREAATRDDDDDLLQFAIQQSLLEAGSEYDQVTIWEALTNSKPGTH
PMSYEGRRQDRSAPPTPQRQPAPPASVPSPRPSSGPGSGGHVFRSYDEQLRLAMELSAQE
QEERRRRARQEEEELERILRLSLTEQ
>sp|Q8IVF6|AN18A_HUMAN Ankyrin repeat domain-containing protein 18A OS=Homo sapiens GN=ANKRD18A PE=2 SV=3
MRKLFSFGRRLGQALLSSMDQEYAGPGYDIRDWELRKIHRAAIKGDAAEVERCLTRRFRD
LDARDRKDRTVLHLACAHGRVQVVTLLLHRRCQIDICDRLNRTPLMKAVHSQEEACAIVL
LECGANPNIEDIYGNTALHYAVYNKGTSLAERLLSHHANIEALNKEGNTPLLFAINSRRQ
HMVEFLLKNQANIHAVDNFKRTALILAVQHNLSSIVTLLLQQNIRISSQDMFGQTAEDYA
LCSDLRSIRQQILEHKNKMLKNHLRNDNQETAAMKPANLKKRKERAKAEHNLKVASEEKQ
ERLQRSENKQPQDSQSYGKKKDAMYGNFMLKKDIAMLKEELYAIKNDSLRKEKKYIQEIK
SITEINANFEKSVRLNEKMITKTVARYSQQLNDLKAENARLNSELEKEKHNKERLEAEVE
SLHSSLATAINEYNEIVERKDLELVLWRADDVSRHEKMGSNISQLTDKNELLTEQVHKAR
VKFNTLKGKLRETRDALREKTLALGSVQLDLRQAQHRIKEMKQMHPNGEAKESQSIGKQN
SLEERIRQQELENLLLERQLEDARKEGDNKEIVINIHRDCLENGKEDLLEERNKELMKEY
NYLKEKLLQCEKEKAEREVIVREFQEELVDHLKTFSISESPLEGTSHCHINLNETWTSKK
KLFQVEIQPEEKHEEFRKLFELISLLNYTADQIRKKNRELEEEATGYKKCLEMTINMLNA
FANEDFSCHGDLNTDQLKMDILFKKLKQKFNDLVAEKEAVSSECVNLAKDNEVLHQELLS
MRNVQEKCEKLEKDKKMLEEEVLNLKTHMEKDMVELGKLQEYKSELDERAVQEIEKLEEI
HLQKQAEYEKQLEQLNKDNTASLKKKELTLKDVECKFSKMKTAYEEVTTELEEFKEAFAG

AVKANNSMSKKLMKSDKKIAVISTKLFTEKQRMKYFLSTLPTRPEPELPCVENLNSIELN
RKYIPKTAIRIPTSNPQTSNNCKNFLTEVLLC
>sp|P39687|AN32A_HUMAN Acidic leucine-rich nuclear phosphoprotein 32 family member A OS=Homo sapiens GN=ANP32A PE=1 SV=1
MEMGRRIHLELRNRTPSDVKELVLDNSRSNEGKLEGLTDEFEELEFLSTINVGLTSIANL
PKLNKLKKLELSDNRVSGGLEVLAEKCPNLTHLNLSGNKIKDLSTIEPLKKLENLKSLDL
FNCEVTNLNDYRENVFKLLPQLTYLDGYDRDDKEAPDSDAEGYVEGLDDEEEDEDEEEYD
EDAQVVEDEEDEDEEEEGEEEDVSGEEEEDEEGYNDGEVDDEEDEEELGEEERGQKRKRE
PEDEGEDDD
>sp|O43423|AN32C_HUMAN Acidic leucine-rich nuclear phosphoprotein 32 family member C OS=Homo sapiens GN=ANP32C PE=2 SV=1
MEMGRRIHSELRNRAPSDVKELALDNSRSNEGKLEALTDEFEELEFLSKINGGLTSISDL
PKLKLRKLELRVSGGLEVLAEKCPNLTHLYLSGNKIKDLSTIEPLKQLENLKSLDLFNCE
VTNLNDYGENVFKLLLQLTYLDSCYWDHKEAPYSDIEDHVEGLDDEEEGEHEEEYDEDAQ
VVEDEEGEEEEEEGEEEDVSGGDEEDEEGYNDGEVDGEEDEEELGEEERGQKRK
>sp|Q5VTE6|ANGE2_HUMAN Protein angel homolog 2 OS=Homo sapiens GN=ANGEL2 PE=2 SV=1
MEAWRCVRKGYGHCVVGRGRYPMFPHHSRSLGRDWTTPWENLQRCCWNRHISSCMRWPGH
YSRAPYPYFSSRHFSLNWRPPCLFESRTQFQYCNWRPDNLSQTSLIHLSSYVMNAEGDEP
SSKRRKHQGVIKRNWEYICSHDKEKTKILGDKNVDPKCEDSENKFDFSVMSYNILSQDLL
EDNSHLYRHCRRPVLHWSFRFPNILKEIKHFDADVLCLQEVQEDHYGAEIRPSLESLGYH
CEYKMRTGRKPDGCAICFKHSKFSLLSVNPVEFFRPDISLLDRDNVGLVLLLQPKIPYAA
CPAICVANTHLLYNPRRGDIKLTQLAMLLAEISSVAHQKDGSFCPIVMCGDFNSVPGSPL
YSFIKEGKLNYEGLPIGKVSGQEQSSRGQRILSIPIWPPNLGISQNCVYEVQQVPKVEKT
DSDLTQTQLKQTEVLVTAEKLSSNLQHHFSLSSVYSHYFPDTGIPEVTTCHSRSAITVDY
IFYSAEKEDVAGHPGAEVALVGGLKLLARLSLLTEQDLWTVNGLPNENNSSDHLPLLAKF
RLEL
>sp|Q86XS5|ANGL5_HUMAN Angiopoietin-related protein 5 OS=Homo sapiens GN=ANGPTL5 PE=2 SV=3
MMSPSQASLLFLNVCIFICGEAVQGNCVHHSTDSSVVNIVEDGSNAKDESKSNDTVCKED
CEESCDVKTKITREEKHFMCRNLQNSIVSYTRSTKKLLRNMMDEQQASLDYLSNQVNELM
NRVLLLTTEVFRKQLDPFPHRPVQSHGLDCTDIKDTIGSVTKTPSGLYIIHPEGSSYPFE
VMCDMDYRGGGRTVIQKRIDGIIDFQRLWCDYLDGFGDLLGEFWLGLKKIFYIVNQKNTS
FMLYVALESEDDTLAYASYDNFWLEDETRFFKMHLGRYSGNAGDAFRGLKKEDNQNAMPF
STSDVDNDGCRPACLVNGQSVKSCSHLHNKTGWWFNECGLANLNGIHHFSGKLLATGIQW
GTWTKNNSPVKIKSVSMKIRRMYNPYFK
>sp|O43827|ANGL7_HUMAN Angiopoietin-related protein 7 OS=Homo sapiens GN=ANGPTL7 PE=1 SV=1
MLKKPLSAVTWLCIFIVAFVSHPAWLQKLSKHKTPAQPQLKAANCCEEVKELKAQVANLS
SLLSELNKKQERDWVSVVMQVMELESNSKRMESRLTDAESKYSEMNNQIDIMQLQAAQTV
TQTSADAIYDCSSLYQKNYRISGVYKLPPDDFLGSPELEVFCDMETSGGGWTIIQRRKSG
LVSFYRDWKQYKQGFGSIRGDFWLGNEHIHRLSRQPTRLRVEMEDWEGNLRYAEYSHFVL
GNELNSYRLFLGNYTGNVGNDALQYHNNTAFSTKDKDNDNCLDKCAQLRKGGYWYNCCTD
SNLNGVYYRLGEHNKHLDGITWYGWHGSTYSLKRVEMKIRPEDFKP
>sp|O15123|ANGP2_HUMAN Angiopoietin-2 OS=Homo sapiens GN=ANGPT2 PE=1 SV=1

MWQIVFFTLSCDLVLAAAYNNFRKSMDSIGKKQYQVQHGSCSYTFLLPEMDNCRSSSSPY
VSNAVQRDAPLEYDDSVQRLQVLENIMENNTQWLMKLENYIQDNMKKEMVEIQQNAVQNQ
TAVMIEIGTNLLNQTAEQTRKLTDVEAQVLNQTTRLELQLLEHSLSTNKLEKQILDQTSE
INKLQDKNSFLEKKVLAMEDKHIIQLQSIKEEKDQLQVLVSKQNSIIEELEKKIVTATVN
NSVLQKQQHDLMETVNNLLTMMSTSNSAKDPTVAKEEQISFRDCAEVFKSGHTTNGIYTL
TFPNSTEEIKAYCDMEAGGGGWTIIQRREDGSVDFQRTWKEYKVGFGNPSGEYWLGNEFV
SQLTNQQRYVLKIHLKDWEGNEAYSLYEHFYLSSEELNYRIHLKGLTGTAGKISSISQPG
NDFSTKDGDNDKCICKCSQMLTGGWWFDACGPSNLNGMYYPQRQNTNKFNGIKWYYWKGS
GYSLKATTMMIRPADF
>sp|E9PGG2|ANHX_HUMAN Anomalous homeobox protein OS=Homo sapiens GN=ANHX PE=2 SV=1
MQSFLTLLKEHEDTCAPPAELVTLAGRLCRDFQDDLAQLQPLVTAILDSQLRLHLLDNAD
VALACARVLDQQEQQQAACRLLEGCQVPGGSQELVQLWNDIHYRLVMRRLGVAALTPVQK
FRCRKRNPPPPSLCPEGLKSRNFPREVREKLHNFAVGVNTNPSKAERENLALETSLTPEQ
VYNWFANYRRRQRALPQHMKPAQQATAEDPGARERGPDLLQPSGNPRVDSGFVDRPQWSE
EREEKGPPQSPQTTQGPWEPLALAPDFPADETVSKPLDVSGHPQSVQLEEGLGTSSGRTE
LRVGSFLVTQPPLQAPEFILTQSPPELAPAPSAFPGPVSAMELSQALPSSQVQCSDSQAS
GDAFWGARMLLEFSGSSLG
>sp|Q7Z5J8|ANKAR_HUMAN Ankyrin and armadillo repeat-containing protein OS=Homo sapiens GN=ANKAR PE=2 SV=3
MLRLPKKGLPRFEQVQDEDTYLENLAIQRNASAFFEKYDRSEIQELLTTALVSWLSAKED
VRSQVDLPCGIMSQMNNVGFSTAILLTPVDPTALLDYREVHQMIRELAIGIYCLNQIPSI
SLEANYDQSSSCQLPPAYYDTRIGQILINIDYMLKALWHGIYMPKEKRARFSELWRAIMD
IDPDGKPQTNKDIFSEFSSAGLTDITKDPDFNEIYDEDVNEDPTYDPNSPEETAVFMKYA
ENIMLKLTFSTTQIQQYENVFIFETGYWLTNAIKYNQDYLDICTYQRLQQRLYLQKKIIQ
KHFEKKKDIRRGIGYLKLICFLIPFLLSLKKKMKVPYLSSLLQPFSDDKVKTERELPPFI
YGRDFKCQNFHYKENQYFHVHGGIEFDISTPSIENALEDFQKNLEKIRDCAANTFIEDSG
YKEYYSIPVMEFHGKSYYVIYFELETFYQQLYKTQWWGAINEIVNNLRLKRLPLTDAQLH
EQFKKKLGFKRAMKCKSIPFGMKSAVERGLSAVFHTFSRKTSSSTINVSDEAGYTIFHHA
ALHNRVSIICQLCNANFKVNQRRFVTFSQGPTPLHLAAQACSLETTVCLLCSKADYTLSE
KRGWMPIHFAAFYDNVCIIIALCRKDPSLLEAEATAENQCTPLLLAATSGALDTIQYLFS
IGANWRKTDIKGNNIIHLSVLTFHTEVLKYIIKLNIPELPVWKTLVEMLQCESYKRRMMA
VMSLEVICLANDQYWRCILDAGTIPALINLLKSSKIKLQCKTVGLLSNISTHKSAVHALV
EAGGIPSLINLLVCDEPEVHSRCAVILYDIAQCENKDVIAKYNGIPSLINLLNLNIENVL
VNVMNCIRVLCIGNENNQRAVREHKGLPYLIRFLSSDSDVLKAVSSAAIAEVGRDNKEIQ
DAIAMEGAIPPLVALFKGKQISVQMKGAMAVESLASHNALIQKAFLEKSLTKYLLKLLKA
FQIDVKEQGAVALWALAGQTLKQQKYMAEQIGYSFIINMLLSPSAKMQYVGGEAVIALSK
DSRMHQNQICEGNGIAPLVRLLRISTIAEGTLLSVIRAVGSICIGVAHTSNPVSQQLVVD
ENAFPVLIQLLRNHPSPNIKVEVAFSLACIVLGNDVLQKDLHENEGFEYADVLYLLHSTE
KDICLRAGYALTLFAFNNRFQQYLILESGIMTISIFERFLESTVETEKAMAAFQIVVLAK
VIRDMDHITLSARGVTILVDSLYSVQTSTIVLTGNLIASLAHSRAGIPEAFTTLGTIQRL
CYHLYSGIEEVRAACSSALGYLTYNANAFRILLKECRNKPNQFIRIKNNISRDASINPAF
LKEFQMQQTLVGLPSLSLEKNGGPSIIPIFKRGKEHRRKLKPKIQPKDSLTLLPPVTNFM
GLFKATKKTKDSHNIFSFSSTITSDITNVSRPRIVCLNQLGKHVQKANPEPAEG

>sp|Q8N957|ANKF1_HUMAN Ankyrin repeat and fibronectin type-III domain-containing protein 1 OS=Homo sapiens GN=ANKFN1 PE=2 SV=2
MEASLTRRLLFKDRHFTCSKIIGRRFACFAQRLSHRRKQSQCDLLNESTGQLPTTCSSAA
SNSINWNCRVKMTQQMQNLHLCQSKKHSAPSSPNAAKRLYRNLSEKLKGSHSSFDEAYFR
TRTDRLSLRKTSVNFQGNEAMFEAVEQQDMDAVQILLYQYTPEELDLNTPNSEGLTPLDI
AIMTNNVPIARILLRTGARESPHFVSLESRAMHLNTLVQEAQERVSELSAQVENEGFTLD
NTEKEKQLKAWEWRYRLYRRMKTGFEHARAPEMPTNVCLMVTSSTSLTVSFQEPLSVNAA
VVTRYKVEWSMSEDFSPLAGEIIMDNLQTLRCTITGLTMGQQYFVQVSAYNMKGWGPAQT
TTPACASPSNWKDYDDREPRHKGQSEVLEGLLQQVRALHQHYSCRESTKLQTTGRKQSVS
RSLKHLFHSSNKFVKTLKRGLYIAVIFYYKDNILVTNEDQVPIVEIDDSHTSSITQDFLW
FTKLSCMWEDIRWLRQSIPISSSSSTVLQTRQKMLAATAQLQNLLGTHNLGRVYYEPIKD
RHGNILIVTIREVEMLYSFFNGKWMQISKLQSQRKSLSTPEEPTALDILLITIQDILSYH
KRSHQRLFPGLYLGYLKLCSSVDQIKVLVTQKLPNILCHVKIRENNNISREEWEWIQKLS
GSESMESVDHTSDCPMQLFFYELQMAVKALLQQINIPLHQARNFRLYTQEVLEMGHNVSF
LLLLPASDDVCTAPGQNNPYTPHSGFLNLPLQMFELGIVACFT
>sp|P55345|ANM2_HUMAN Protein arginine N-methyltransferase 2 OS=Homo sapiens GN=PRMT2 PE=1 SV=1
MATSGDCPRSESQGEEPAECSEAGLLQEGVQPEEFVAIADYAATDETQLSFLRGEKILIL
RQTTADWWWGERAGCCGYIPANHVGKHVDEYDPEDTWQDEEYFGSYGTLKLHLEMLADQP
RTTKYHSVILQNKESLTDKVILDVGCGTGIISLFCAHYARPRAVYAVEASEMAQHTGQLV
LQNGFADIITVYQQKVEDVVLPEKVDVLVSEWMGTCLLFEFMIESILYARDAWLKEDGVI
WPTMAALHLVPCSADKDYRSKVLFWDNAYEFNLSALKSLAVKEFFSKPKYNHILKPEDCL
SEPCTILQLDMRTVQISDLETLRGELRFDIRKAGTLHGFTAWFSVHFQSLQEGQPPQVLS
TGPFHPTTHWKQTLFMMDDPVPVHTGDVVTGSVVLQRNPVWRRHMSVALSWAVTSRQDPT
SQKVGEKVFPIWR
>sp|O60678|ANM3_HUMAN Protein arginine N-methyltransferase 3 OS=Homo sapiens GN=PRMT3 PE=1 SV=3
MCSLASGATGGRGAVENEEDLPELSDSGDEAAWEDEDDADLPHGKQQTPCLFCNRLFTSA
EETFSHCKSEHQFNIDSMVHKHGLEFYGYIKLINFIRLKNPTVEYMNSIYNPVPWEKEEY
LKPVLEDDLLLQFDVEDLYEPVSVPFSYPNGLSENTSVVEKLKHMEARALSAEAALARAR
EDLQKMKQFAQDFVMHTDVRTCSSSTSVIADLQEDEDGVYFSSYGHYGIHEEMLKDKIRT
ESYRDFIYQNPHIFKDKVVLDVGCGTGILSMFAAKAGAKKVLGVDQSEILYQAMDIIRLN
KLEDTITLIKGKIEEVHLPVEKVDVIISEWMGYFLLFESMLDSVLYAKNKYLAKGGSVYP
DICTISLVAVSDVNKHADRIAFWDDVYGFKMSCMKKAVIPEAVVEVLDPKTLISEPCGIK
HIDCHTTSISDLEFSSDFTLKITRTSMCTAIAGYFDIYFEKNCHNRVVFSTGPQSTKTHW
KQTVFLLEKPFSVKAGEALKGKVTVHKSKKDPRSLTVTLTLNNSTQTYGLQ
>sp|Q6IWH7|ANO7_HUMAN Anoctamin-7 OS=Homo sapiens GN=ANO7 PE=1 SV=2
MRMAATAWAGLQGPPLPTLCPAVRTGLYCRDQAHAERWAMTSETSSGSHCARSRMLRRRA
QEEDSTVLIDVSPPEAEKRGSYGSTAHASEPGGQQAAACRAGSPAKPRIADFVLVWEEDL
KLDRQQDSAARDRTDMHRTWRETFLDNLRAAGLCVDQQDVQDGNTTVHYALLSASWAVLC
YYAEDLRLKLPLQELPNQASNWSAGLLAWLGIPNVLLEVVPDVPPEYYSCRFRVNKLPRF
LGSDNQDTFFTSTKRHQILFEILAKTPYGHEKKNLLGIHQLLAEGVLSAAFPLHDGPFKT
PPEGPQAPRLNQRQVLFQHWARWGKWNKYQPLDHVRRYFGEKVALYFAWLGFYTGWLLPA
AVVGTLVFLVGCFLVFSDIPTQELCGSKDSFEMCPLCLDCPFWLLSSACALAQAGRLFDH

GGTVFFSLFMALWAVLLLEYWKRKSATLAYRWDCSDYEDTEERPRPQFAASAPMTAPNPI
TGEDEPYFPERSRARRMLAGSVVIVVMVAVVVMCLVSIILYRAIMAIVVSRSGNTLLAAW
ASRIASLTGSVVNLVFILILSKIYVSLAHVLTRWEMHRTQTKFEDAFTLKVFIFQFVNFY
SSPVYIAFFKGRFVGYPGNYHTLFGVRNEECAAGGCLIELAQELLVIMVGKQVINNMQEV
LIPKLKGWWQKFRLRSKKRKAGASAGASQGPWEDDYELVPCEGLFDEYLEMVLQFGFVTI
FVAACPLAPLFALLNNWVEIRLDARKFVCEYRRPVAERAQDIGIWFHILAGLTHLAVISN
AFLLAFSSDFLPRAYYRWTRAHDLRGFLNFTLARAPSSFAAAHNRTCRYRAFRDDDGHYS
QTYWNLLAIRLAFVIVFEHVVFSVGRLLDLLVPDIPESVEIKVKREYYLAKQALAENEVL
FGTNGTKDEQPEGSELSSHWTPFTVPKASQLQQ
>sp|O75179|ANR17_HUMAN Ankyrin repeat domain-containing protein 17 OS=Homo sapiens GN=ANKRD17 PE=1 SV=3
MEKATVPVAAATAAEGEGSPPAVAAVAGPPAAAEVGGGVGGSSRARSASSPRGMVRVCDL
LLKKKPPQQQHHKAKRNRTCRPPSSSESSSDSDNSGGGGGGGGGGGGGGGTSSNNSEEEE
DDDDEEEEVSEVESFILDQDDLENPMLETASKLLLSGTADGADLRTVDPETQARLEALLE
AAGIGKLSTADGKAFADPEVLRRLTSSVSCALDEAAAALTRMRAESTANAGQSDNRSLAE
ACSEGDVNAVRKLLIEGRSVNEHTEEGESLLCLACSAGYYELAQVLLAMHANVEDRGIKG
DITPLMAAANGGHVKIVKLLLAHKADVNAQSSTGNTALTYACAGGYVDVVKVLLESGASI
EDHNENGHTPLMEAGSAGHVEVARLLLENGAGINTHSNEFKESALTLACYKGHLEMVRFL
LEAGADQEHKTDEMHTALMEACMDGHVEVARLLLDSGAQVNMPADSFESPLTLAACGGHV
ELAALLIERGASLEEVNDEGYTPLMEAAREGHEEMVALLLGQGANINAQTEETQETALTL
ACCGGFLEVADFLIKAGADIELGCSTPLMEAAQEGHLELVKYLLAAGANVHATTATGDTA
LTYACENGHTDVADVLLQAGADLEHESEGGRTPLMKAARAGHVCTVQFLISKGANVNRTT
ANNDHTVLSLACAGGHLAVVELLLAHGADPTHRLKDGSTMLIEAAKGGHTSVVCYLLDYP
NNLLSAPPPDVTQLTPPSHDLNRAPRVPVQALPMVVPPQEPDKPPANVATTLPIRNKAAS
KQKSSSHLPANSQDVQGYITNQSPESIVEEAQGKLTELEQRIKEAIEKNAQLQSLELAHA
DQLTKEKIEELNKTREEQIQKKQKILEELQKVERELQLKTQQQLKKQYLEVKAQRIQLQQ
QQQQSCQHLGLLTPVGVGEQLSEGDYARLQQVDPVLLKDEPQQTAAQMGFAPIQPLAMPQ
ALPLAAGPLPPGSIANLTELQGVIVGQPVLGQAQLAGLGQGILTETQQGLMVASPAQTLN
DTLDDIMAAVSGRASAMSNTPTHSIAASISQPQTPTPSPIISPSAMLPIYPAIDIDAQTE
SNHDTALTLACAGGHEELVQTLLERGASIEHRDKKGFTPLILAATAGHVGVVEILLDNGA
DIEAQSERTKDTPLSLACSGGRQEVVELLLARGANKEHRNVSDYTPLSLAASGGYVNIIK
ILLNAGAEINSRTGSKLGISPLMLAAMNGHTAAVKLLLDMGSDINAQIETNRNTALTLAC
FQGRTEVVSLLLDRKANVEHRAKTGLTPLMEAASGGYAEVGRVLLDKGADVNAPPVPSSR
DTALTIAADKGHYKFCELLIGRGAHIDVRNKKGNTPLWLAANGGHLDVVQLLVQAGADVD
AADNRKITPLMAAFRKGHVKVVRYLVKEVNQFPSDSECMRYIATITDKEMLKKCHLCMES
IVQAKDRQAAEANKNASILLEELDLEKLREESRRLALAAKREKRKEKRRKKKEEQRRKLE
EIEAKNKENFELQAAQEKEKLKVEDEPEVLTEPPSATTTTTIGISATWTTLAGSHGKRNN
TITTTSSKRKNRKNKITPENVQIIFDDPLPISYSQPEKVNGESKSSSTSESGDSDNMRIS
SCSDESSNSNSSRKSDNHSPAVVTTTVSSKKQPSVLVTFPKEERKSVSGKASIKLSETIS
EGTSNSLSTCTKSGPSPLSSPNGKLTVASPKRGQKREEGWKEVVRRSKKVSVPSTVISRV
IGRGGCNINAIREFTGAHIDIDKQKDKTGDRIITIRGGTESTRQATQLINALIKDPDKEI
DELIPKNRLKSSSANSKIGSSAPTTTAANTSLMGIKMTTVALSSTSQTATALTVPAISSA
STHKTIKNPVNNVRPGFPVSLPLAYPPPQFAHALLAAQTFQQIRPPRLPMTHFGGTFPPA
QSTWGPFPVRPLSPARATNSPKPHMVPRHSNQNSSGSQVNSAGSLTSSPTTTTSSSASTV

PGTSTNGSPSSPSVRRQLFVTVVKTSNATTTTVTTTASNNNTAPTNATYPMPTAKEHYPV
SSPSSPSPPAQPGGVSRNSPLDCGSASPNKVASSSEQEAGSPPVVETTNTRPPNSSSSSG
SSSAHSNQQQPPGSVSQEPRPPLQQSQVPPPEVRMTVPPLATSSAPVAVPSTAPVTYPMP
QTPMGCPQPTPKMETPAIRPPPHGTTAPHKNSASVQNSSVAVLSVNHIKRPHSVPSSVQL
PSTLSTQSACQNSVHPANKPIAPNFSAPLPFGPFSTLFENSPTSAHAFWGGSVVSSQSTP
ESMLSGKSSYLPNSDPLHQSDTSKAPGFRPPLQRPAPSPSGIVNMDSPYGSVTPSSTHLG
NFASNISGGQMYGPGAPLGGAPAAANFNRQHFSPLSLLTPCSSASNDSSAQSVSSGVRAP
SPAPSSVPLGSEKPSNVSQDRKVPVPIGTERSARIRQTGTSAPSVIGSNLSTSVGHSGIW
SFEGIGGNQDKVDWCNPGMGNPMIHRPMSDPGVFSQHQAMERDSTGIVTPSGTFHQHVPA
GYMDFPKVGGMPFSVYGNAMIPPVAPIPDGAGGPIFNGPHAADPSWNSLIKMVSSSTENN
GPQTVWTGPWAPHMNSVHMNQLG
>sp|Q9H560|ANR19_HUMAN Putative ankyrin repeat domain-containing protein 19 OS=Homo sapiens GN=ANKRD19P PE=5 SV=1
MRKLFSFGRRLGQALLDSMDQEYAGRGYHIRDWELRKIHRAAIKGDAAEVEHCLTRRFRD
LDARDRKDRTVLHLTCAHGRVEVVTLLLSRRCQINIYDRLNRTPLMKAVHCQEEACAIIL
LEHGANPNIKDIYSNTALHYAVYNKGTSLAEKLLSHHANIEALNEEGNTPLLFAINSRRQ
QIVEFLLKNQANLHAIDNFRRTALMLAVQHNSSSIVSLLLQQNINIFSQDLFGQTAEDYA
VCYNFRSIQQQILEHKNKILKSHL
>sp|Q96NW4|ANR27_HUMAN Ankyrin repeat domain-containing protein 27 OS=Homo sapiens GN=ANKRD27 PE=1 SV=2
MALYDEDLLKNPFYLALQKCRPDLCSKVAQIHGIVLVPCKGSLSSSIQSTCQFESYILIP
VEEHFQTLNGKDVFIQGNRIKLGAGFACLLSVPILFEETFYNEKEESFSILCIAHPLEKR
ESSEEPLAPSDPFSLKTIEDVREFLGRHSERFDRNIASFHRTFRECERKSLRHHIDSANA
LYTKCLQQLLRDSHLKMLAKQEAQMNLMKQAVEIYVHHEIYNLIFKYVGTMEASEDAAFN
KITRSLQDLQQKDIGVKPEFSFNIPRAKRELAQLNKCTSPQQKLVCLRKVVQLITQSPSQ
RVNLETMCADDLLSVLLYLLVKTEIPNWMANLSYIKNFRFSSLAKDELGYCLTSFEAAIE
YIRQGSLSAKPPESEGFGDRLFLKQRMSLLSQMTSSPTDCLFKHIASGNQKEVERLLSQE
DHDKDTVQKMCHPLCFCDDCEKLVSGRLNDPSVVTPFSRDDRGHTPLHVAAVCGQASLID
LLVSKGAMVNATDYHGATPLHLACQKGYQSVTLLLLHYKASAEVQDNNGNTPLHLACTYG
HEDCVKALVYYDVESCRLDIGNEKGDTPLHIAARWGYQGVIETLLQNGASTEIQNRLKET
PLKCALNSKILSVMEAYHLSFERRQKSSEAPVQSPQRSVDSISQESSTSSFSSMSASSRQ
EETKKDYREVEKLLRAVADGDLEMVRYLLEWTEEDLEDAEDTVSAADPEFCHPLCQCPKC
APAQKRLAKVPASGLGVNVTSQDGSSPLHVAALHGRADLIPLLLKHGANAGARNADQAVP
LHLACQQGHFQVVKCLLDSNAKPNKKDLSGNTPLIYACSGGHHELVALLLQHGASINASN
NKGNTALHEAVIEKHVFVVELLLLHGASVQVLNKRQRTAVDCAEQNSKIMELLQVVPSCV
ASLDDVAETDRKEYVTVKIRKKWNSKLYDLPDEPFTRQFYFVHSAGQFKGKTSREIMARD
RSVPNLTEGSLHEPGRQSVTLRQNNLPAQSGSHAAEKGNSDWPERPGLTQTGPGHRRMLR
RHTVEDAVVSQGPEAAGPLSTPQEVSASRS
>sp|Q7Z6G8|ANS1B_HUMAN Ankyrin repeat and sterile alpha motif domain-containing protein 1B OS=Homo sapiens GN=ANKS1B PE=1 SV=2
MGKDQELLEAARTGNVALVEKLLSGRKGGILGGGSGPLPLSNLLSIWRGPNVNCTDSSGY
TALHHAALNGHKDIVLKLLQYEASTNVADNKGYFPIHLAAWKGDVEIVKILIHHGPSHSR
VNEQNNENETALHCAAQYGHSEVVAVLLEELTDPTIRNSKLETPLDLAALYGRLRVVKMI
ISAHPNLMSCNTRKHTPLHLAARNGHKAVVQVLLEAGMDVSCQTEKGSALHEAALFGKVD

VVRVLLETGIDANIKDSLGRTVLDILKEHPSQKSLQIATLLQEYLEGVGRSTVLEEPVQE
DATQETHISSPVESPSQKTKSETVTGELSKLLDEIKLCQEKDYSFEDLCHTISDHYLDNL
SKISEEELGKNGSQSVRTSSTINLSPGEVEEEDDDENTCGPSGLWEALTPCNGCRNLGFP
MLAQESYPKKRNYTMEIVPSASLDTFPSENENFLCDLMDTAVTKKPCSLEIARAPSPRTD
NASEVAVTTPGTSNHRNSSTGPTPDCSPPSPDTALKNIVKVIRPQPKQRTSIVSSLDFHR
MNHNQEYFEINTSTGCTSFTASPPASPPTSSVGTTEVKNEGTNHTDDLSRQDDNDPPKEY
DPGQFAGLLHGSSPACESPENPFHLYGKREQCEKGQDEVSLANSPLPFKQSPIENNSEPL
VKKIKPKVVSRTIFHKKSNQLENHTIVGTRSTRSGSRNGDQWVMNAGGFVERACTLGRIR
SLPKALIDMHLSKSVSKSDSDLIAYPSNEKTSRVNWSESSTAEHSSKGNSERTPSFTSEW
EEIDKIMSSIDVGINNELKEMNGETTRPRCPVQTVGQWLESIGLPQYENHLMANGFDNVQ
FMGSNVMEDQDLLEIGILNSGHRQRILQAIQLLPKMRPIGHDGYHPTSVAEWLDSIELGD
YTKAFLINGYTSMDLLKKIWEVELINVLKINLIGHRKRILASLGDRLHDDPPQKPPRSIT
LREPSGNHTPPQLSPSLSQSTYTTGGSLDVPHIIMQGDARRRRNENYFDDIPRSKLERQM
AQTGDWGEPSITLRPPNEATASTPVQYWQHHPEKLIFQSCDYKAFYLGSMLIKELRGTES
TQDACAKMRANCQKSTEQMKKVPTIILSVSYKGVKFIDATNKNIIAEHEIRNISCAAQDP
EDLSTFAYITKDLKSNHHYCHVFTAFDVNLAYEIILTLGQAFEVAYQLALQARKGGHSST
LPESFENKPSKPIPKPRVSIRKSVDLLHASHTGQEPSERHTEEALRKF
>sp|Q9H6X2|ANTR1_HUMAN Anthrax toxin receptor 1 OS=Homo sapiens GN=ANTXR1 PE=1 SV=2
MATAERRALGIGFQWLSLATLVLICAGQGGRREDGGPACYGGFDLYFILDKSGSVLHHWN
EIYYFVEQLAHKFISPQLRMSFIVFSTRGTTLMKLTEDREQIRQGLEELQKVLPGGDTYM
HEGFERASEQIYYENRQGYRTASVIIALTDGELHEDLFFYSEREANRSRDLGAIVYCVGV
KDFNETQLARIADSKDHVFPVNDGFQALQGIIHSILKKSCIEILAAEPSTICAGESFQVV
VRGNGFRHARNVDRVLCSFKINDSVTLNEKPFSVEDTYLLCPAPILKEVGMKAALQVSMN
DGLSFISSSVIITTTHCSDGSILAIALLILFLLLALALLWWFWPLCCTVIIKEVPPPPAE
ESEEEDDDGLPKKKWPTVDASYYGGRGVGGIKRMEVRWGEKGSTEEGAKLEKAKNARVKM
PEQEYEFPEPRNLNNNMRRPSSPRKWYSPIKGKLDALWVLLRKGYDRVSVMRPQPGDTGR
CINFTRVKNNQPAKYPLNNAYHTSSPPPAPIYTPPPPAPHCPPPPPSAPTPPIPSPPSTL
PPPPQAPPPNRAPPPSRPPPRPSV
>sp|A6NF34|ANTRL_HUMAN Anthrax toxin receptor-like OS=Homo sapiens GN=ANTXRL PE=3 SV=3
MGSHESLGPYFLVFLLLLLLPPPLFRAGSLRYHGPDWRIFHRLALGSRRAHHHHGPGWRQ
HWRQGQAGHRCQGSFDLYFILDKSGSVNNNWIDLYMWVEETVARFQSPNIRMCFITYSTD
GQTVLPLTSDKNRIKNGLDQLQKIVPDGHTFMQAGFRKAIQQIESFNSGNKVPSMIIAMT
DGELVAHAFQDTLREAQKARKLGANVYTLGVADYNLDQITAIADSPGHVFAVENGFKALR
STIDALTSKVCLDVTSVEPSSECVGEPYHVVIHGNGFQNLKKRDEVICRFIFNESTIIDE
KPTSIDNNSMNCPGPKLEKPGEEYSIEVSLNKGKTFFKSNVSITSTTCGIFRNWLYFVPL
LLLVPLLLCCVWRLCRKQTVKEPPPVQKPEKEPEQEKPPSPPPPPPPPPPPLPPPPPAPV
NTCPTVIICCCGCQGVGGMRRIEGNLDTFCDLSHASCHQVPWMCCQSRDQGRYLSLALAQ
SQYAQAPCCPRICFPHSQECLSLPQAPCSPRMCLRHSRECLALKQARCSPNICLRHSQHS
RECLARKQAPCSPRICLRHSPEYFSQAQTLCNPKSCLQPSRECLPLTCSSRCRLPPARCL
RPPSRMLPLLSPLLRHTAEPPLSLPPSEPNF
>sp|P20073|ANXA7_HUMAN Annexin A7 OS=Homo sapiens GN=ANXA7 PE=1 SV=3
MSYPGYPPTGYPPFPGYPPAGQESSFPPSGQYPYPSGFPPMGGGAYPQVPSSGYPGAGGY
PAPGGYPAPGGYPGAPQPGGAPSYPGVPPGQGFGVPPGGAGFSGYPQPPSQSYGGGPAQV
PLPGGFPGGQMPSQYPGGQPTYPSQINTDSFSSYPVFSPVSLDYSSEPATVTQVTQGTIR

PAANFDAIRDAEILRKAMKGFGTDEQAIVDVVANRSNDQRQKIKAAFKTSYGKDLIKDLK
SELSGNMEELILALFMPPTYYDAWSLRKAMQGAGTQERVLIEILCTRTNQEIREIVRCYQ
SEFGRDLEKDIRSDTSGHFERLLVSMCQGNRDENQSINHQMAQEDAQRLYQAGEGRLGTD
ESCFNMILATRSFPQLRATMEAYSRMANRDLLSSVSREFSGYVESGLKTILQCALNRPAF
FAERLYYAMKGAGTDDSTLVRIVVTRSEIDLVQIKQMFAQMYQKTLGTMIAGDTSGDYRR
LLLAIVGQ
>sp|P28039|AOAH_HUMAN Acyloxyacyl hydrolase OS=Homo sapiens GN=AOAH PE=1 SV=1
MQSPWKILTVAPLFLLLSLQSSASPANDDQSRPSLSNGHTCVGCVLVVSVIEQLAQVHNS
TVQASMERLCSYLPEKLFLKTTCYLVIDKFGSDIIKLLSADMNADVVCHTLEFCKQNTGQ
PLCHLYPLPKETWKFTLQKARQIVKKSPILKYSRSGSDICSLPVLAKICQKIKLAMEQSV
PFKDVDSDKYSVFPTLRGYHWRGRDCNDSDESVYPGRRPNNWDVHQDSNCNGIWGVDPKD
GVPYEKKFCEGSQPRGIILLGDSAGAHFHISPEWITASQMSLNSFINLPTALTNELDWPQ
LSGATGFLDSTVGIKEKSIYLRLWKRNHCNHRDYQNISRNGASSRNLKKFIESLSRNKVL
DYPAIVIYAMIGNDVCSGKSDPVPAMTTPEKLYSNVMQTLKHLNSHLPNGSHVILYGLPD
GTFLWDNLHNRYHPLGQLNKDMTYAQLYSFLNCLQVSPCHGWMSSNKTLRTLTSERAEQL
SNTLKKIAASEKFTNFNLFYMDFAFHEIIQEWQKRGGQPWQLIEPVDGFHPNEVALLLLA
DHFWKKVQLQWPQILGKENPFNPQIKQVFGDQGGH
>sp|O60641|AP180_HUMAN Clathrin coat assembly protein AP180 OS=Homo sapiens GN=SNAP91 PE=1 SV=2
MSGQTLTDRIAAAQYSVTGSAVARAVCKATTHEVMGPKKKHLDYLIQATNETNVNIPQMA
DTLFERATNSSWVVVFKALVTTHHLMVHGNERFIQYLASRNTLFNLSNFLDKSGSHGYDM
STFIRRYSRYLNEKAFSYRQMAFDFARVKKGADGVMRTMAPEKLLKSMPILQGQIDALLE
FDVHPNELTNGVINAAFMLLFKDLIKLFACYNDGVINLLEKFFEMKKGQCKDALEIYKRF
LTRMTRVSEFLKVAEQVGIDKGDIPDLTQAPSSLMETLEQHLNTLEGKKPGNNEGSGAPS
PLSKSSPATTVTSPNSTPAKTIDTSPPVDLFATASAAVPVSTSKPSSDLLDLQPDFSSGG
AAAAAAPAPPPPAGGATAWGDLLGEDSLAALSSVPSEAQISDPFAPEPTPPTTTAEIATA
SASASTTTTVTAVTAEVDLFGDAFAASPGEAPAASEGAAAPATPTPVAAALDACSGNDPF
APSEGSAEAAPELDLFAMKPPETSVPVVTPTASTAPPVPATAPSPAPAVAAAAAATTAAT
AAATTTTTTSAATATTAPPALDIFGDLFESTPEVAAAPKPDAAPSIDLFSTDAFSSPPQG
ASPVPESSLTADLLSVDAFAAPSPATTASPAKVDSSGVIDLFGDAFGSSASEPQPASQAA
SSSSASADLLAGFGGSFMAPSPSPVTPAQNNLLQPNFEAAFGTTPSTSSSSSFDPSVFDG
LGDLLMPTMAPAGQPAPVSMVPPSPAMAASKALGSDLDSSLASLVGNLGISGTTTKKGDL
QWNAGEKKLTGGANWQPKVAPATWSAGVPPSAPLQGAVPPTSSVPPVAGAPSVGQPGAGF
GMPPAGTGMPMMPQQPVMFAQPMMRPPFGAAAVPGTQLSPSPTPASQSPKKPPAKDPLAD
LNIKDFL
>sp|Q63HQ0|AP1AR_HUMAN AP-1 complex-associated regulatory protein OS=Homo sapiens GN=AP1AR PE=1 SV=1
MGNCCWTQCFGLLRKEAGRLQRVGGGGGSKYFRTCSRGEHLTIEFENLVESDEGESPGSS
HRPLTEEEIVDLRERHYDSIAEKQKDLDKKIQKELALQEEKLRLEEEALYAAQREAARAA
KQRKLLEQERQRIVQQYHPSNNGEYQSSGPEDDFESCLRNMKSQYEVFRSSRLSSDATVL
TPNTESSCDLMTKTKSTSGNDDSTSLDLEWEDEEGMNRMLPMRERSKTEEDILRAALKYS
NKKTGSNPTSASDDSNGLEWENDFVSAEMDDNGNSEYSGFVNPVLELSDSGIRHSDTDQQ
TR
>sp|O43747|AP1G1_HUMAN AP-1 complex subunit gamma-1 OS=Homo sapiens GN=AP1G1 PE=1 SV=5

MPAPIRLRELIRTIRTARTQAEEREMIQKECAAIRSSFREEDNTYRCRNVAKLLYMHMLG
YPAHFGQLECLKLIASQKFTDKRIGYLGAMLLLDERQDVHLLMTNCIKNDLNHSTQFVQG
LALCTLGCMGSSEMCRDLAGEVEKLLKTSNSYLRKKAALCAVHVIRKVPELMEMFLPATK
NLLNEKNHGVLHTSVVLLTEMCERSPDMLAHFRKLVPQLVRILKNLIMSGYSPEHDVSGI
SDPFLQVRILRLLRILGRNDDDSSEAMNDILAQVATNTETSKNVGNAILYETVLTIMDIK
SESGLRVLAINILGRFLLNNDKNIRYVALTSLLKTVQTDHNAVQRHRSTIVDCLKDLDVS
IKRRAMELSFALVNGNNIRGMMKELLYFLDSCEPEFKADCASGIFLAAEKYAPSKRWHID
TIMRVLTTAGSYVRDDAVPNLIQLITNSVEMHAYTVQRLYKAILGDYSQQPLVQVAAWCI
GEYGDLLVSGQCEEEEPIQVTEDEVLDILESVLISNMSTSVTRGYALTAIMKLSTRFTCT
VNRIKKVVSIYGSSIDVELQQRAVEYNALFKKYDHMRSALLERMPVMEKVTTNGPTEIVQ
TNGETEPAPLETKPPPSGPQPTSQANDLLDLLGGNDITPVIPTAPTSKPSSAGGELLDLL
GDINLTGAPAAAPAPASVPQISQPPFLLDGLSSQPLFNDIAAGIPSITAYSKNGLKIEFT
FERSNTNPSVTVITIQASNSTELDMTDFVFQAAVPKTFQLQLLSPSSSIVPAFNTGTITQ
VIKVLNPQKQQLRMRIKLTYNHKGSAMQDLAEVNNFPPQSWQ
>sp|Q7Z6R9|AP2D_HUMAN Transcription factor AP-2-delta OS=Homo sapiens GN=TFAP2D PE=2 SV=1
MSTTFPGLVHDAEIRHDGSNSYRLMQLGCLESVANSTVAYSSSSPLTYSTTGTEFASPYF
STNHQYTPLHHQSFHYEFQHSHPAVTPDAYSLNSLHHSQQYYQQIHHGEPTDFINLHNAR
ALKSSCLDEQRRELGCLDAYRRHDLSLMSHGSQYGMHPDQRLLPGPSLGLAAAGADDLQG
SVEAQCGLVLNGQGGVIRRGGTCVVNPTDLFCSVPGRLSLLSSTSKYKVTIAEVKRRLSP
PECLNASLLGGILRRAKSKNGGRCLREKLDRLGLNLPAGRRKAANVTLLTSLVEGEALHL
ARDFGYTCETEFPAKAVGEHLARQHMEQKEQTARKKMILATKQICKEFQDLLSQDRSPLG
SSRPTPILDLDIQRHLTHFSLITHGFGTPAICAALSTFQTVLSEMLNYLEKHTTHKNGGA
ADSGQGHANSEKAPLRKTSEAAVKEGKTEKTD
>sp|Q9Y6B7|AP4B1_HUMAN AP-4 complex subunit beta-1 OS=Homo sapiens GN=AP4B1 PE=1 SV=2
MPYLGSEDVVKELKKALCNPHIQADRLRYRNVIQRVIRYMTQGLDMSGVFMEMVKASATV
DIVQKKLVYLYMCTYAPLKPDLALLAINTLCKDCSDPNPMVRGLALRSMCSLRMPGVQEY
IQQPILNGLRDKASYVRRVAVLGCAKMHNLHGDSEVDGALVNELYSLLRDQDPIVVVNCL
RSLEEILKQEGGVVINKPIAHHLLNRMSKLDQWGQAEVLNFLLRYQPRSEEELFDILNLL
DSFLKSSSPGVVMGATKLFLILAKMFPHVQTDVLVRVKGPLLAACSSESRELCFVALCHV
RQILHSLPGHFSSHYKKFFCSYSEPHYIKLQKVEVLCELVNDENVQQVLEELRGYCTDVS
ADFAQAAIFAIGGIARTYTDQCVQILTELLGLRQEHITTVVVQTFRDLVWLCPQCTEAVC
QALPGCEENIQDSEGKQALIWLLGVHGERIPNAPYVLEDFVENVKSETFPAVKMELLTAL
LRLFLSRPAECQDMLGRLLYYCIEEEKDMAVRDRGLFYYRLLLVGIDEVKRILCSPKSDP
TLGLLEDPAERPVNSWASDFNTLVPVYGKAHWATISKCQGAERCDPELPKTSSFAASGPL
IPEENKERVQELPDSGALMLVPNRQLTADYFEKTWLSLKVAHQQVLPWRGEFHPDTLQMA
LQVVNIQTIAMSRAGSRPWKAYLSAQDDTGCLFLTELLLEPGNSEMQISVKQNEARTETL
NSFISVLETVIGTIEEIKS
>sp|Q8N6S5|AR6P6_HUMAN ADP-ribosylation factor-like protein 6-interacting protein 6 OS=Homo sapiens GN=ARL6IP6 PE=1 SV=1
MSFAESGWRSALRRRGPGTPGPVARPSYSSFTQGDSWGEGEVDEEEGCDQVARDLRAEFS
AGAWSEPRKRSVLPPDGNGSPVLPDKRNGIFPAAAGSRAQPRRWPVQVLSILCSLLFAIL
LAFLLAIAYLIVKELHAENLKNEDDVDTGLLGFWTLLIISLTAGFSCCSFSWTVTYFDSF
EPGMFPPTPLSPARFKKLTGHSFHMGYSMAILNGIVAALTVAWCLM
>sp|P84077|ARF1_HUMAN ADP-ribosylation factor 1 OS=Homo sapiens GN=ARF1 PE=1 SV=2

MGNIFANLFKGLFGKKEMRILMVGLDAAGKTTILYKLKLGEIVTTIPTIGFNVETVEYKN
ISFTVWDVGGQDKIRPLWRHYFQNTQGLIFVVDSNDRERVNEAREELMRMLAEDELRDAV
LLVFANKQDLPNAMNAAEITDKLGLHSLRHRNWYIQATCATSGDGLYEGLDWLSNQLRNQ
K
>sp|Q8N6T3|ARFG1_HUMAN ADP-ribosylation factor GTPase-activating protein 1 OS=Homo sapiens GN=ARFGAP1 PE=1 SV=2
MASPRTRKVLKEVRVQDENNVCFECGAFNPQWVSVTYGIWICLECSGRHRGLGVHLSFVR
SVTMDKWKDIELEKMKAGGNAKFREFLESQEDYDPCWSLQEKYNSRAAALFRDKVVALAE
GREWSLESSPAQNWTPPQPRTLPSMVHRVSGQPQSVTASSDKAFEDWLNDDLGSYQGAQG
NRYVGFGNTPPPQKKEDDFLNNAMSSLYSGWSSFTTGASRFASAAKEGATKFGSQASQKA
SELGHSLNENVLKPAQEKVKEGKIFDDVSSGVSQLASKVQGVGSKGWRDVTTFFSGKAEG
PLDSPSEGHSYQNSGLDHFQNSNIDQSFWETFGSAEPTKTRKSPSSDSWTCADTSTERRS
SDSWEVWGSASTNRNSNSDGGEGGEGTKKAVPPAVPTDDGWDNQNW
>sp|P53365|ARFP2_HUMAN Arfaptin-2 OS=Homo sapiens GN=ARFIP2 PE=1 SV=1
MTDGILGKAATMEIPIHGNGEARQLPEDDGLEQDLQQVMVSGPNLNETSIVSGGYGGSGD
GLIPTGSGRHPSHSTTPSGPGDEVARGIAGEKFDIVKKWGINTYKCTKQLLSERFGRGSR
TVDLELELQIELLRETKRKYESVLQLGRALTAHLYSLLQTQHALGDAFADLSQKSPELQE
EFGYNAETQKLLCKNGETLLGAVNFFVSSINTLVTKTMEDTLMTVKQYEAARLEYDAYRT
DLEELSLGPRDAGTRGRLESAQATFQAHRDKYEKLRGDVAIKLKFLEENKIKVMHKQLLL
FHNAVSAYFAGNQKQLEQTLQQFNIKLRPPGAEKPSWLEEQ
>sp|Q8NDY3|ARHL1_HUMAN [Protein ADP-ribosylarginine] hydrolase-like protein 1 OS=Homo sapiens GN=ADPRHL1 PE=2 SV=1
MEKFKAAMLLGSVGDALGYRNVCKENSTVGMKIQEELQRSGGLDHLVLSPGEWPVSDNTI
MHIATAEALTTDYWCLDDLYREMVRCYVEIVEKLPERRPDPATIEGCAQLKPNNYLLAWH
TPFNEKGSGFGAATKAMCIGLRYWKPERLETLIEVSVECGRMTHNHPTGFLGSLCTALFV
SFAAQGKPLVQWGRDMLRAVPLAEEYCRKTIRHTAEYQEHWFYFEAKWQFYLEERKISKD
SENKAIFPDNYDAEEREKTYRKWSSEGRGGRRGHDAPMIAYDALLAAGNSWTELCHRAMF
HGGESAATGTIAGCLFGLLYGLDLVPKGLYQDLEDKEKLEDLGAALYRLSTEEK
>sp|O14497|ARI1A_HUMAN AT-rich interactive domain-containing protein 1A OS=Homo sapiens GN=ARID1A PE=1 SV=3
MAAQVAPAAASSLGNPPPPPPSELKKAEQQQREEAGGEAAAAAAAERGEMKAAAGQESEG
PAVGPPQPLGKELQDGAESNGGGGGGGAGSGGGPGAEPDLKNSNGNAGPRPALNNNLTEP
PGGGGGGSSDGVGAPPHSAAAALPPPAYGFGQPYGRSPSAVAAAAAAVFHQQHGGQQSPG
LAALQSGGGGGLEPYAGPQQNSHDHGFPNHQYNSYYPNRSAYPPPAPAYALSSPRGGTPG
SGAAAAAGSKPPPSSSASASSSSSSFAQQRFGAMGGGGPSAAGGGTPQPTATPTLNQLLT
SPSSARGYQGYPGGDYSGGPQDGGAGKGPADMASQCWGAAAAAAAAAAASGGAQQRSHHA
PMSPGSSGGGGQPLARTPQPSSPMDQMGKMRPQPYGGTNPYSQQQGPPSGPQQGHGYPGQ
PYGSQTPQRYPMTMQGRAQSAMGGLSYTQQIPPYGQQGPSGYGQQGQTPYYNQQSPHPQQ
QQPPYSQQPPSQTPHAQPSYQQQPQSQPPQLQSSQPPYSQQPSQPPHQQSPAPYPSQQST
TQQHPQSQPPYSQPQAQSPYQQQQPQQPAPSTLSQQAAYPQPQSQQSQQTAYSQQRFPPP
QELSQDSFGSQASSAPSMTSSKGGQEDMNLSLQSRPSSLPDLSGSIDDLPMGTEGALSPG
VSTSGISSSQGEQSNPAQSPFSPHTSPHLPGIRGPSPSPVGSPASVAQSRSGPLSPAAVP
GNQMPPRPPSGQSDSIMHPSMNQSSIAQDRGYMQRNPQMPQYSSPQPGSALSPRQPSGGQ
IHTGMGSYQQNSMGSYGPQGGQYGPQGGYPRQPNYNALPNANYPSAGMAGGINPMGAGGQ

MHGQPGIPPYGTLPPGRMSHASMGNRPYGPNMANMPPQVGSGMCPPPGGMNRKTQETAVA
MHVAANSIQNRPPGYPNMNQGGMMGTGPPYGQGINSMAGMINPQGPPYSMGGTMANNSAG
MAASPEMMGLGDVKLTPATKMNNKADGTPKTESKSKKSSSSTTTNEKITKLYELGGEPER
KMWVDRYLAFTEEKAMGMTNLPAVGRKPLDLYRLYVSVKEIGGLTQVNKNKKWRELATNL
NVGTSSSAASSLKKQYIQCLYAFECKIERGEDPPPDIFAAADSKKSQPKIQPPSPAGSGS
MQGPQTPQSTSSSMAEGGDLKPPTPASTPHSQIPPLPGMSRSNSVGIQDAFNDGSDSTFQ
KRNSMTPNPGYQPSMNTSDMMGRMSYEPNKDPYGSMRKAPGSDPFMSSGQGPNGGMGDPY
SRAAGPGLGNVAMGPRQHYPYGGPYDRVRTEPGIGPEGNMSTGAPQPNLMPSNPDSGMYS
PSRYPPQQQQQQQQRHDSYGNQFSTQGTPSGSPFPSQQTTMYQQQQQNYKRPMDGTYGPP
AKRHEGEMYSVPYSTGQGQPQQQQLPPAQPQPASQQQAAQPSPQQDVYNQYGNAYPATAT
AATERRPAGGPQNQFPFQFGRDRVSAPPGTNAQQNMPPQMMGGPIQASAEVAQQGTMWQG
RNDMTYNYANRQSTGSAPQGPAYHGVNRTDEMLHTDQRANHEGSWPSHGTRQPPYGPSAP
VPPMTRPPPSNYQPPPSMQNHIPQVSSPAPLPRPMENRTSPSKSPFLHSGMKMQKAGPPV
PASHIAPAPVQPPMIRRDITFPPGSVEATQPVLKQRRRLTMKDIGTPEAWRVMMSLKSGL
LAESTWALDTINILLYDDNSIMTFNLSQLPGLLELLVEYFRRCLIEIFGILKEYEVGDPG
QRTLLDPGRFSKVSSPAPMEGGEEEEELLGPKLEEEEEEEVVENDEEIAFSGKDKPASEN
SEEKLISKFDKLPVKIVQKNDPFVVDCSDKLGRVQEFDSGLLHWRIGGGDTTEHIQTHFE
SKTELLPSRPHAPCPPAPRKHVTTAEGTPGTTDQEGPPPDGPPEKRITATMDDMLSTRSS
TLTEDGAKSSEAIKESSKFPFGISPAQSHRNIKILEDEPHSKDETPLCTLLDWQDSLAKR
CVCVSNTIRSLSFVPGNDFEMSKHPGLLLILGKLILLHHKHPERKQAPLTYEKEEEQDQG
VSCNKVEWWWDCLEMLRENTLVTLANISGQLDLSPYPESICLPVLDGLLHWAVCPSAEAQ
DPFSTLGPNAVLSPQRLVLETLSKLSIQDNNVDLILATPPFSRLEKLYSTMVRFLSDRKN
PVCREMAVVLLANLAQGDSLAARAIAVQKGSIGNLLGFLEDSLAATQFQQSQASLLHMQN
PPFEPTSVDMMRRAARALLALAKVDENHSEFTLYESRLLDISVSPLMNSLVSQVICDVLF
LIGQS
>sp|Q9Y4X5|ARI1_HUMAN E3 ubiquitin-protein ligase ARIH1 OS=Homo sapiens GN=ARIH1 PE=1 SV=2
MDSDEGYNYEFDEDEECSEEDSGAEEEEDEDDDEPDDDTLDLGEVELVEPGLGVGGERDG
LLCGETGGGGGSALGPGGGGGGGGGGGGGGPGHEQEEDYRYEVLTAEQILQHMVECIREV
NEVIQNPATITRILLSHFNWDKEKLMERYFDGNLEKLFAECHVINPSKKSRTRQMNTRSS
AQDMPCQICYLNYPNSYFTGLECGHKFCMQCWSEYLTTKIMEEGMGQTISCPAHGCDILV
DDNTVMRLITDSKVKLKYQHLITNSFVECNRLLKWCPAPDCHHVVKVQYPDAKPVRCKCG
RQFCFNCGENWHDPVKCKWLKKWIKKCDDDSETSNWIAANTKECPKCHVTIEKDGGCNHM
VCRNQNCKAEFCWVCLGPWEPHGSAWYNCNRYNEDDAKAARDAQERSRAALQRYLFYCNR
YMNHMQSLRFEHKLYAQVKQKMEEMQQHNMSWIEVQFLKKAVDVLCQCRATLMYTYVFAF
YLKKNNQSIIFENNQADLENATEVLSGYLERDISQDSLQDIKQKVQDKYRYCESRRRVLL
QHVHEGYEKDLWEYIED
>sp|Q14865|ARI5B_HUMAN AT-rich interactive domain-containing protein 5B OS=Homo sapiens GN=ARID5B PE=1 SV=3
MEPNSLQWVGSPCGLHGPYIFYKAFQFHLEGKPRILSLGDFFFVRCTPKDPICIAELQLL
WEERTSRQLLSSSKLYFLPEDTPQGRNSDHGEDEVIAVSEKVIVKLEDLVKWVHSDFSKW
RCGFHAGPVKTEALGRNGQKEALLKYRQSTLNSGLNFKDVLKEKADLGEDEEETNVIVLS
YPQYCRYRSMLKRIQDKPSSILTDQFALALGGIAVVSRNPQILYCRDTFDHPTLIENESI
CDEFAPNLKGRPRKKKPCPQRRDSFSGVKDSNNNSDGKAVAKVKCEARSALTKPKNNHNC

KKVSNEEKPKVAIGEECRADEQAFLVALYKYMKERKTPIERIPYLGFKQINLWTMFQAAQ
KLGGYETITARRQWKHIYDELGGNPGSTSAATCTRRHYERLILPYERFIKGEEDKPLPPI
KPRKQENSSQENENKTKVSGTKRIKHEIPKSKKEKENAPKPQDAAEVSSEQEKEQETLIS
QKSIPEPLPAADMKKKIEGYQEFSAKPLASRVDPEKDNETDQGSNSEKVAEEAGEKGPTP
PLPSAPLAPEKDSALVPGASKQPLTSPSALVDSKQESKLCCFTESPESEPQEASFPSFPT
TQPPLANQNETEDDKLPAMADYIANCTVKVDQLGSDDIHNALKQTPKVLVVQSFDMFKDK
DLTGPMNENHGLNYTPLLYSRGNPGIMSPLAKKKLLSQVSGASLSSSYPYGSPPPLISKK
KLIARDDLCSSLSQTHHGQSTDHMAVSRPSVIQHVQSFRSKPSEERKTINDIFKHEKLSR
SDPHRCSFSKHHLNPLADSYVLKQEIQEGKDKLLEKRALPHSHMPSFLADFYSSPHLHSL
YRHTEHHLHNEQTSKYPSRDMYRESENSSFPSHRHQEKLHVNYLTSLHLQDKKSAAAEAP
TDDQPTDLSLPKNPHKPTGKVLGLAHSTTGPQESKGISQFQVLGSQSRDCHPKACRVSPM
TMSGPKKYPESLSRSGKPHHVRLENFRKMEGMVHPILHRKMSPQNIGAARPIKRSLEDLD
LVIAGKKARAVSPLDPSKEVSGKEKASEQESEGSKAAHGGHSGGGSEGHKLPLSSPIFPG
LYSGSLCNSGLNSRLPAGYSHSLQYLKNQTVLSPLMQPLAFHSLVMQRGIFTSPTNSQQL
YRHLAAATPVGSSYGDLLHNSIYPLAAINPQAAFPSSQLSSVHPSTKL
>sp|Q9Y4B4|ARIP4_HUMAN Helicase ARIP4 OS=Homo sapiens GN=RAD54L2 PE=1 SV=4
MSDESASGSDPDLDPDVELEDAEEEEEEEEVAVEECDRDDEEDLLDDPSLEGMCGTEHAQ
LGEDGQQPPRCTSTTSSQSEPSEQLRRHQGKNLASEDPKKKRAQKPSHMRRNIRKLLRED
QLEPVTKAAQQEELERRKRLEQQRKDYAAPIPTVPLEFLPEEIALRASDGPQLPPRVLAQ
EVICLDSSSGSEDEKSSRDEVIELSSGEEDTLHIVDSSESVSEDDEEEEKGGTHVNDVLN
QRDALGRVLVNLNHPPEEENVFLAPQLARAVKPHQIGGIRFLYDNLVESLERFKTSSGFG
CILAHSMGLGKTLQVISFIDVLFRHTPAKTVLAIVPVNTLQNWLAEFNMWLPPPEALPAD
NKPEEVQPRFFKVHILNDEHKTMASRAKVMADWVSEGGVLLMGYEMYRLLTLKKSFATGR
PKKTKKRSHPVIIDLDEEDRQQEFRREFEKALCRPGPDVVICDEGHRIKNCQASTSQALK
NIRSRRRVVLTGYPLQNNLIEYWCMVDFVRPDFLGTRQEFSNMFERPILNGQCIDSTPQD
VRLMRYRSHVLHSLLEGFVQRRGHTVLKIHLPAKEENVILVRLSKIQRDLYTQFMDRFRD
CGSSGWLGLNPLKAFCVCCKIWNHPDVLYEALQKESLANEQDLDVEELGSAGTSARCPPQ
GTKGKGEDSTLASSMGEATNSKFLQGVGFNPFQERGNNIVTYEWAKDLLTNYQTGVLENS
PKMVLLFHLIEESVKLGDKILVFSQSLSTLALIEEFLGKREVPCPPGTEGQGAQKWVRNI
SYFRLDGSTPAFERERLINQFNDPSNLTTWLFLLSTRAGCLGVNLIGANRVVVFDASWNP
CHDAQAVCRVYRYGQKKPCYIYRLVADYTLEKKIYDRQISKQGMSDRVVDDLNPMLNFTR
KEVENLLHFVEKEPAPQVSLNVKGIKESVLQLACLKYPHLITKEPFEHESLLLNRKDHKL
TKAEKKAAKKSYEEDKRTSVPYTRPSYAQYYPASDQSLTSIPAFSQRNWQPTLKGDEKPV
ASVRPVQSTPIPMMPRHVPLGGSVSSASSTNPSMNFPINYLQRAGVLVQKVVTTTDIVIP
GLNSSTDVQARINAGESIHIIRGTKGTYIRTSDGRIFAVRATGKPKVPEDGRMAASGSQG
PSCESTSNGRHSASSPKAPDPEGLARPVSPDSPEIISELQQYADVAAARESRQSSPSTNA
ALPGPPAQLMDSSAVPGTALGTEPRLGGHCLNSSLLVTGQPCGDRHPVLDLRGHKRKLAT
PPAAQESSRRRSRKGHLPAPVQPYEHGYPVSGGFAMPPVSLNHNLTTPFTSQAGENSLFM
GSTPSYYQLSNLLADARLVFPVTTDPLVPAGPVSSSSTATSVTASNPSFMLNPSVPGILP
SYSLPFSQPLLSEPRMFAPFPSPVLPSNLSRGMSIYPGYMSPHAGYPAGGLLRSQVPPFD
SHEVAEVGFSSNDDEDKDDDVIEVTGK
>sp|O43488|ARK72_HUMAN Aflatoxin B1 aldehyde reductase member 2 OS=Homo sapiens GN=AKR7A2
PE=1 SV=3
MLSAASRVVSRAAVHCALRSPPPEARALAMSRPPPPRVASVLGTMEMGRRMDAPASAAAV

RAFLERGHTELDTAFMYSDGQSETILGGLGLGLGGGDCRVKIATKANPWDGKSLKPDSVR
SQLETSLKRLQCPQVDLFYLHAPDHGTPVEETLHACQRLHQEGKFVELGLSNYASWEVAE
ICTLCKSNGWILPTVYQGMYNATTRQVETELFPCLRHFGLRFYAYNPLAGGLLTGKYKYE
DKDGKQPVGRFFGNSWAETYRNRFWKEHHFEAIALVEKALQAAYGASAPSVTSAALRWMY
HHSQLQGAHGDAVILGMSSLEQLEQNLAATEEGPLEPAVVDAFNQAWHLVAHECPNYFR
>sp|Q96BM9|ARL8A_HUMAN ADP-ribosylation factor-like protein 8A OS=Homo sapiens GN=ARL8A PE=1 SV=1
MIALFNKLLDWFKALFWKEEMELTLVGLQYSGKTTFVNVIASGQFNEDMIPTVGFNMRKI
TKGNVTIKLWDIGGQPRFRSMWERYCRGVSAIVYMVDAADQEKIEASKNELHNLLDKPQL
QGIPVLVLGNKRDLPGALDEKELIEKMNLSAIQDREICCYSISCKEKDNIDITLQWLIQH
SKSRRS
>sp|Q8N1P7|AIM1L_HUMAN Absent in melanoma 1-like protein OS=Homo sapiens GN=AIM1L PE=2 SV=1
MELRTPGTKWSPQGIGSLRRVVWDYSTPEISLFSEEGLKGEQVKLTEALKNSQGLEKPLQ
VASATVSAGLWLLYPKPLFEDTPYILEPGEYPTSEAWGTSDPSVGSLKPMRLGCPSVEKP
GEPRAVVYEAPGFQGRSWEVSRDIYNLQQPEDSQSPHLASVGSLRVLGGCWVGYEKEGFR
GHQYLLEEGEYPDWSHWGGYDELLTSLRVIRTDFGDPAVVLFEAMDFEGHGVEVSKALPD
VELVQHGPSTQAIHVLSGVWVAYQEVGFSGEQYVLEKGVYRNCEDWGAGNSTLASLQPVL
QVGEHDLHFVSKIQLFSRPDFLGDHFSFEDDQAALPASFRPQSCRVHGGSWILFDETNFE
GDQHILSEGEFPTLTAMGCLASTVLGSLQKVSLHFSEPSIFLYGLECFEGKEIELSREVR
SLQAEGFNNHVLSVRIKGGIWVLCEHSDFRGRQWLVGSCEITNWLTYSGTQRVGSLYPIK
QRRVYFRLWNAALGGFLAVPDHVEDMKAGRVVVADPQAGGSCIWYYEDGLLKNQMAPTMS
LQVIGPPSPGSKVVLWAESRLPRQTWSISESGHICSQMFEGQILDVKGGRGYDRDHVVLW
EPDEDRASQIWTIHVL
>sp|Q12904|AIMP1_HUMAN Aminoacyl tRNA synthase complex-interacting multifunctional protein 1 OS=Homo sapiens GN=AIMP1 PE=1 SV=2
MANNDAVLKRLEQKGAEADQIIEYLKQQVSLLKEKAILQATLREEKKLRVENAKLKKEIE
ELKQELIQAEIQNGVKQIPFPSGTPLHANSMVSENVIQSTAVTTVSSGTKEQIKGGTGDE
KKAKEKIEKKGEKKEKKQQSIAGSADSKPIDVSRLDLRIGCIITARKHPDADSLYVEEVD
VGEIAPRTVVSGLVNHVPLEQMQNRMVILLCNLKPAKMRGVLSQAMVMCASSPEKIEILA
PPNGSVPGDRITFDAFPGEPDKELNPKKKIWEQIQPDLHTNDECVATYKGVPFEVKGKGV
CRAQTMSNSGIK
>sp|Q04828|AK1C1_HUMAN Aldo-keto reductase family 1 member C1 OS=Homo sapiens GN=AKR1C1 PE=1 SV=1
MDSKYQCVKLNDGHFMPVLGFGTYAPAEVPKSKALEATKLAIEAGFRHIDSAHLYNNEEQ
VGLAIRSKIADGSVKREDIFYTSKLWCNSHRPELVRPALERSLKNLQLDYVDLYLIHFPV
SVKPGEEVIPKDENGKILFDTVDLCATWEAVEKCKDAGLAKSIGVSNFNRRQLEMILNKP
GLKYKPVCNQVECHPYFNQRKLLDFCKSKDIVLVAYSALGSHREEPWVDPNSPVLLEDPV
LCALAKKHKRTPALIALRYQLQRGVVVLAKSYNEQRIRQNVQVFEFQLTSEEMKAIDGLN
RNVRYLTLDIFAGPPNYPFSDEY
>sp|P52895|AK1C2_HUMAN Aldo-keto reductase family 1 member C2 OS=Homo sapiens GN=AKR1C2 PE=1 SV=3
MDSKYQCVKLNDGHFMPVLGFGTYAPAEVPKSKALEAVKLAIEAGFHHIDSAHVYNNEEQ
VGLAIRSKIADGSVKREDIFYTSKLWSNSHRPELVRPALERSLKNLQLDYVDLYLIHFPV

SVKPGEEVIPKDENGKILFDTVDLCATWEAMEKCKDAGLAKSIGVSNFNHRLLEMILNKP
GLKYKPVCNQVECHPYFNQRKLLDFCKSKDIVLVAYSALGSHREEPWVDPNSPVLLEDPV
LCALAKKHKRTPALIALRYQLQRGVVVLAKSYNEQRIRQNVQVFEFQLTSEEMKAIDGLN
RNVRYLTLDIFAGPPNYPFSDEY
>sp|P0CW23|AKAI1_HUMAN A-kinase anchor protein inhibitor 1 OS=Homo sapiens GN=AKAIN1 PE=1 SV=1
MVFAPGEKPGNEPEEVKLQNASKQIVQNAILQAVQQVSQESQRREERISDNRDHIQLGVG
ELTKKHEKK
>sp|Q99996|AKAP9_HUMAN A-kinase anchor protein 9 OS=Homo sapiens GN=AKAP9 PE=1 SV=3
MEDEERQKKLEAGKAKIEELSLAFLVRQLAQFRQRKAQSDGQSPSKKQKKKRKTSSSKHD
VSAHHDLNIDQSQCNEMYINSSQRVESTVIPESTIMRTLHSGEITSHEQGFSVELESEIS
TTADDCSSEVNGCSFVMRTGKPTNLLREEEFGVDDSYSEQGAQDSPTHLEMMESELAGKQ
HEIEELNRELEEMRVTYGTEGLQQLQEFEAAIKQRDGIITQLTANLQQARREKDETMREF
LELTEQSQKLQIQFQQLQASETLRNSTHSSTAADLLQAKQQILTHQQQLEEQDHLLEDYQ
KKKEDFTMQISFLQEKIKVYEMEQDKKVENSNKEEIQEKETIIEELNTKIIEEEKKTLEL
KDKLTTADKLLGELQEQIVQKNQEIKNMKLELTNSKQKERQSSEEIKQLMGTVEELQKRN
HKDSQFETDIVQRMEQETQRKLEQLRAELDEMYGQQIVQMKQELIRQHMAQMEEMKTRHK
GEMENALRSYSNITVNEDQIKLMNVAINELNIKLQDTNSQKEKLKEELGLILEEKCALQR
QLEDLVEELSFSREQIQRARQTIAEQESKLNEAHKSLSTVEDLKAEIVSASESRKELELK
HEAEVTNYKIKLEMLEKEKNAVLDRMAESQEAELERLRTQLLFSHEEELSKLKEDLEIEH
RINIEKLKDNLGIHYKQQIDGLQNEMSQKIETMQFEKDNLITKQNQLILEISKLKDLQQS
LVNSKSEEMTLQINELQKEIEILRQEEKEKGTLEQEVQELQLKTELLEKQMKEKENDLQE
KFAQLEAENSILKDEKKTLEDMLKIHTPVSQEERLIFLDSIKSKSKDSVWEKEIEILIEE
NEDLKQQCIQLNEEIEKQRNTFSFAEKNFEVNYQELQEEYACLLKVKDDLEDSKNKQELE
YKSKLKALNEELHLQRINPTTVKMKSSVFDEDKTFVAETLEMGEVVEKDTTELMEKLEVT
KREKLELSQRLSDLSEQLKQKHGEISFLNEEVKSLKQEKEQVSLRCRELEIIINHNRAEN
VQSCDTQVSSLLDGVVTMTSRGAEGSVSKVNKSFGEESKIMVEDKVSFENMTVGEESKQE
QLILDHLPSVTKESSLRATQPSENDKLQKELNVLKSEQNDLRLQMEAQRICLSLVYSTHV
DQVREYMENEKDKALCSLKEELIFAQEEKIKELQKIHQLELQTMKTQETGDEGKPLHLLI
GKLQKAVSEECSYFLQTLCSVLGEYYTPALKCEVNAEDKENSGDYISENEDPELQDYRYE
VQDFQENMHTLLNKVTEEYNKLLVLQTRLSKIWGQQTDGMKLEFGEENLPKEETEFLSIH
SQMTNLEDIDVNHKSKLSSLQDLEKTKLEEQVQELESLISSLQQQLKETEQNYEAEIHCL
QKRLQAVSESTVPPSLPVDSVVITESDAQRTMYPGSCVKKNIDGTIEFSGEFGVKEETNI
VKLLEKQYQEQLEEEVAKVIVSMSIAFAQQTELSRISGGKENTASSKQAHAVCQQEQHYF
NEMKLSQDQIGFQTFETVDVKFKEEFKPLSKELGEHGKEILLSNSDPHDIPESKDCVLTI
SEEMFSKDKTFIVRQSIHDEISVSSMDASRQLMLNEEQLEDMRQELVRQYQEHQQATELL
RQAHMRQMERQREDQEQLQEEIKRLNRQLAQRSSIDNENLVSERERVLLEELEALKQLSL
AGREKLCCELRNSSTQTQNGNENQGEVEEQTFKEKELDRKPEDVPPEILSNERYALQKAN
NRLLKILLEVVKTTAAVEETIGRHVLGILDRSSKSQSSASLIWRSEAEASVKSCVHEEHT
RVTDESIPSYSGSDMPRNDINMWSKVTEEGTELSQRLVRSGFAGTEIDPENEELMLNISS
RLQAAVEKLLEAISETSSQLEHAKVTQTELMRESFRQKQEATESLKCQEELRERLHEESR
AREQLAVELSKAEGVIDGYADEKTLFERQIQEKTDIIDRLEQELLCASNRLQELEAEQQQ
IQEERELLSRQKEAMKAEAGPVEQQLLQETEKLMKEKLEVQCQAEKVRDDLQKQVKALEI
DVEEQVSRFIELEQEKNTELMDLRQQNQALEKQLEKMRKFLDEQAIDREHERDVFQQEIQ

KLEQQLKVVPRFQPISEHQTREVEQLANHLKEKTDKCSELLLSKEQLQRDIQERNEEIEK
LEFRVRELEQALLVSADTFQKVEDRKHFGAVEAKPELSLEVQLQAERDAIDRKEKEITNL
EEQLEQFREELENKNEEVQQLHMQLEIQKKESTTRLQELEQENKLFKDDMEKLGLAIKES
DAMSTQDQHVLFGKFAQIIQEKEVEIDQLNEQVTKLQQQLKITTDNKVIEEKNELIRDLE
TQIECLMSDQECVKRNREEEIEQLNEVIEKLQQELANIGQKTSMNAHSLSEEADSLKHQL
DVVIAEKLALEQQVETANEEMTFMKNVLKETNFKMNQLTQELFSLKRERESVEKIQSIPE
NSVNVAIDHLSKDKPELEVVLTEDALKSLENQTYFKSFEENGKGSIINLETRLLQLESTV
SAKDLELTQCYKQIKDMQEQGQFETEMLQKKIVNLQKIVEEKVAAALVSQIQLEAVQEYA
KFCQDNQTISSEPERTNIQNLNQLREDELGSDISALTLRISELESQVVEMHTSLILEKEQ
VEIAEKNVLEKEKKLLELQKLLEGNEKKQREKEKKRSPQDVEVLKTTTELFHSNEESGFF
NELEALRAESVATKAELASYKEKAEKLQEELLVKETNMTSLQKDLSQVRDHLAEAKEKLS
ILEKEDETEVQESKKACMFEPLPIKLSKSIASQTDGTLKISSSNQTPQILVKNAGIQINL
QSECSSEEVTEIISQFTEKIEKMQELHAAEILDMESRHISETETLKREHYVAVQLLKEEC
GTLKAVIQCLRSKEVFGFYNMCFSTLCDSGSDWGQGIYLTHSQGFDIASEGRGEESESAT
DSFPKKIKGLLRAVHNEGMQVLSLTESPYSDGEDHSIQQVSEPWLEERKAYINTISSLKD
LITKMQLQREAEVYDSSQSHESFSDWRGELLLALQQVFLEERSVLLAAFRTELTALGTTD
AVGLLNCLEQRIQEQGVEYQAAMECLQKADRRSLLSEIQALHAQMNGRKITLKREQESEK
PSQELLEYNIQQKQSQMLEMQVELSSMKDRATELQEQLSSEKMVVAELKSELAQTKLELE
TTLKAQHKHLKELEAFRLEVKDKTDEVHLLNDTLASEQKKSRELQWALEKEKAKLGRSEE
RDKEELEDLKFSLESQKQRNLQLNLLLEQQKQLLNESQQKIESQRMLYDAQLSEEQGRNL
ELQVLLESEKVRIREMSSTLDRERELHAQLQSSDGTGQSRPPLPSEDLLKELQKQLEEKH
SRIVELLNETEKYKLDSLQTRQQMEKDRQVHRKTLQTEQEANTEGQKKMHELQSKVEDLQ
RQLEEKRQQVYKLDLEGQRLQGIMQEFQKQELEREEKRESRRILYQNLNEPTTWSLTSDR
TRNWVLQQKIEGETKESNYAKLIEMNGGGTGCNHELEMIRQKLQCVASKLQVLPQKASER
LQFETADDEDFIWVQENIDEIILQLQKLTGQQGEEPSLVSPSTSCGSLTERLLRQNAELT
GHISQLTEEKNDLRNMVMKLEEQIRWYRQTGAGRDNSSRFSLNGGANIEAIIASEKEVWN
REKLTLQKSLKRAEAEVYKLKAELRNDSLLQTLSPDSEHVTLKRIYGKYLRAESFRKALI
YQKKYLLLLLGGFQECEDATLALLARMGGQPAFTDLEVITNRPKGFTRFRSAVRVSIAIS
RMKFLVRRWHRVTGSVSININRDGFGLNQGAEKTDSFYHSSGGLELYGEPRHTTYRSRSD
LDYIRSPLPFQNRYPGTPADFNPGSLACSQLQNYDPDRALTDYITRLEALQRRLGTIQSG
STTQFHAGMRR
>sp|Q9P2G1|AKIB1_HUMAN Ankyrin repeat and IBR domain-containing protein 1 OS=Homo sapiens
GN=ANKIB1 PE=1 SV=3
MGNTTTKFRKALINGDENLACQIYENNPQLKESLDPNTSYGEPYQHNTPLHYAARHGMNK
ILGTFLGRDGNPNKRNVHNETSMHLLCMGPQIMISEGALHPRLARPTEDDFRRADCLQMI
LKWKGAKLDQGEYERAAIDAVDNKKNTPLHYAAASGMKACVELLVKHGGDLFAENENKDT
PCDCAEKQHHKDLALNLESQMVFSRDPEAEEIEAEYAALDKREPYEGLRPQDLRRLKDML
IVETADMLQAPLFTAEALLRAHDWDREKLLEAWMSNPENCCQRSGVQMPTPPPSGYNAWD
TLPSPRTPRTTRSSVTSPDEISLSPGDLDTSLCDICMCSISVFEDPVDMPCGHDFCRGCW
ESFLNLKIQEGEAHNIFCPAYDCFQLVPVDIIESVVSKEMDKRYLQFDIKAFVENNPAIK
WCPTPGCDRAVRLTKQGSNTSGSDTLSFPLLRAPAVDCGKGHLFCWECLGEAHEPCDCQT
WKNWLQKITEMKPEELVGVSEAYEDAANCLWLLTNSKPCANCKSPIQKNEGCNHMQCAKC
KYDFCWICLEEWKKHSSSTGGYYRCTRYEVIQHVEEQSKEMTVEAEKKHKRFQELDRFMH
YYTRFKNHEHSYQLEQRLLKTAKEKMEQLSRALKETEGGCPDTTFIEDAVHVLLKTRRIL

KCSYPYGFFLEPKSTKKEIFELMQTDLEMVTEDLAQKVNRPYLRTPRHKIIKAACLVQQK
RQEFLASVARGVAPADSPEAPRRSFAGGTWDWEYLGFASPEEYAEFQYRRRHRQRRRGDV
HSLLSNPPDPDEPSESTLDIPEGGSSSRRPGTSVVSSASMSVLHSSSLRDYTPASRSENQ
DSLQALSSLDEDDPNILLAIQLSLQESGLALDEETRDFLSNEASLGAIGTSLPSRLDSVP
RNTDSPRAALSSSELLELGDSLMRLGAENDPFSTDTLSSHPLSEARSDFCPSSSDPDSAG
QDPNINDNLLGNIMAWFHDMNPQSIALIPPATTEISADSQLPCIKDGSEGVKDVELVLPE
DSMFEDASVSEGRGTQIEENPLEENILAGEAASQAGDSGNEAANRGDGSDVSSQTPQTSS
DWLEQVHLV
>sp|Q53H80|AKIR2_HUMAN Akirin-2 OS=Homo sapiens GN=AKIRIN2 PE=1 SV=2
MACGATLKRTLDFDPLLSPASPKRRRCAPLSAPTSAAASPLSAAAATAASFSAAAASPQK
YLRMEPSPFGDVSSRLTTEQILYNIKQEYKRMQKRRHLETSFQQTDPCCTSDAQPHAFLL
SGPASPGTSSAASSPLKKEQPLFTLRQVGMICERLLKEREEKVREEYEEILNTKLAEQYD
AFVKFTHDQIMRRYGEQPASYVS
>sp|Q6GMV1|ALG1L_HUMAN Putative glycosyltransferase ALG1-like OS=Homo sapiens GN=ALG1L PE=2 SV=2
MERSAFMELDAGSRLVMHLREWPALLVSSTGWTEFEQLTLDGHNLPSLVCVITGSVDLGV
CLHMSSSGLDLPMKVVDMFGCCLPVCAVNFKCLHELVKHEENGLVFEDSEELAALQMLFS
NFPDPAGKLNQFWKNLRESQQLRWDESWVQTVLPLVMDIQLLGQRLKPRDPCCPSRSFFS
ESQGKPF
>sp|Q9BVK2|ALG8_HUMAN Probable dolichyl pyrophosphate Glc1Man9GlcNAc2 alpha-1,3-glucosyltransferase OS=Homo sapiens GN=ALG8 PE=1 SV=2
MAALTIATGTGNWFSALALGVTLLKCLLIPTYHSTDFEVHRNWLAITHSLPISQWYYEAT
SEWTLDYPPFFAWFEYILSHVAKYFDQEMLNVHNLNYSSSRTLLFQRFSVIFMDVLFVYA
VRECCKCIDGKKVGKELTEKPKFILSVLLLWNFGLLIVDHIHFQYNGFLFGLMLLSIARL
FQKRHMEGAFLFAVLLHFKHIYLYVAPAYGVYLLRSYCFTANKPDGSIRWKSFSFVRVIS
LGLVVFLVSALSLGPFLALNQLPQVFSRLFPFKRGLCHAYWAPNFWALYNALDKVLSVIG
LKLKFLDPNNIPKASMTSGLVQQFQHTVLPSVTPLATLICTLIAILPSIFCLWFKPQGPR
GFLRCLTLCALSSFMFGWHVHEKAILLAILPMSLLSVGKAGDASIFLILTTTGHYSLFPL
LFTAPELPIKILLMLLFTIYSISSLKTLFRKEKPLFNWMETFYLLGLGPLEVCCEFVFPF
TSWKVKYPFIPLLLTSVYCAVGITYAWFKLYVSVLIDSAIGKTKKQ
>sp|Q6ZNB7|ALKMO_HUMAN Alkylglycerol monooxygenase OS=Homo sapiens GN=AGMO PE=1 SV=1
MKNPEAQQDVSVSQGFRMLFYTMKPSETSFQTLEEVPDYVKKATPFFISLMLLELVVSWI
LKGKPPGRLDDALTSISAGVLSRLPSLFFRSIELTSYIYIWENYRLFNLPWDSPWTWYSA
FLGVDFGYYWFHRMAHEVNIMWAGHQTHHSSEDYNLSTALRQSVLQIYTSWIFYSPLALF
IPPSVYAVHLQFNLLYQFWIHTEVINNLGPLELILNTPSHHRVHHGRNRYCIDKNYAGVL
IIWDKIFGTFEAENEKVVYGLTHPINTFEPIKVQFHHLFSIWTTFWATPGFFNKFSVIFK
GPGWGPGKPRLGLSEEIPEVTGKEVPFSSSSSQLLKIYTVVQFALMLAFYEETFADTAAL
SQVTLLLRVCFIILTLTSIGFLLDQRPKAAIMETLRCLMFLMLYRFGHLKPLVPSLSSAF
EIVFSICIAFWGVRSMKQLTSHPWK
>sp|Q8N944|AMER3_HUMAN APC membrane recruitment protein 3 OS=Homo sapiens GN=AMER3 PE=1 SV=2
MELKRGKTFIKSSLQVSHEKPPDPAAVAAAREGTGPWSVLPGGQQRPHSEKGPQASPSAQ
EYDRCPNKGAQLDPKGGPAALCGATFKPVRKCKTHDSMSGAGRATAATGQLVGSASFPGS
PGSRRMIDYRHFVPQMPFVPAVAKSIPRKRISLKRPKKCFRNLFHIRRNKTEDLASLAAE

GKSLPSPGDPSDPGGRRSKAFLPPGEGPGLDGLCQDLLDSELLADASFGLCRALCEDVAS
LQSFDSLTGCGEVFADESSVPSLELNEGPESPTQAAQGLESKVPRGPLQGSVEQLASPAQ
NEASDFTRFWDSVNRSVRQQQRALLGPWLSGPQGTDRDQSRLDTAGLAELPLCPCRDPRS
GSKASSIDTGTPKSEQPESVSTSDEGYYDSFSPGLEEDKKEAESPGTPAATFPRDSYSGD
ALYELFHDPSEGPLGPSPDDDLCVSESLSGPALGTPLSICSFRVGAEENLAPAPGPDLLS
QGFLQSSWKGKECLLKLCDTELAITMGIVSWLRRGPTPRAPPTPGQPAAPPGSQGAPRAP
TEKLGGREGLASDAGGATVCSAPSRQELWAHPGTTGLLAGESKALGGATQGTGTLSRDAS
REEETRGHSEGLFSSMESAATSTTDTSGKNKAPVPSTWPCSQKEPGPPGVLGCFRGPWRP
GHGGDTLDAEPMLAGCVARVAALKISSNEQPPAAWPPRQDMGSGLFGQRWARGPDMLEQK
QSSSSPSMTTIHGLPYSASTQDQRCRDRVQDLSWLRVEPTGLGVQAWASVEDQPLQLSTE
AVEQVAHGSQLDSEPRSAPAARWSSQGHHPESLGLTLNSQQEGGVSASAPECRCSLLARE
GLLCGQPEVGASGPAMAEPHL
>sp|Q86WK6|AMGO1_HUMAN Amphoterin-induced protein 1 OS=Homo sapiens GN=AMIGO1 PE=1 SV=1
MHPHRDPRGLWLLLPSLSLLLFEVARAGRAVVSCPAACLCASNILSCSKQQLPNVPHSLP
SYTALLDLSHNNLSRLRAEWTPTRLTQLHSLLLSHNHLNFISSEAFSPVPNLRYLDLSSN
QLRTLDEFLFSDLQVLEVLLLYNNHIMAVDRCAFDDMAQLQKLYLSQNQISRFPLELVKE
GAKLPKLTLLDLSSNKLKNLPLPDLQKLPAWIKNGLYLHNNPLNCDCELYQLFSHWQYRQ
LSSVMDFQEDLYCMNSKKLHNVFNLSFLNCGEYKERAWEAHLGDTLIIKCDTKQQGMTKV
WVTPSNERVLDEVTNGTVSVSKDGSLLFQQVQVEDGGVYTCYAMGETFNETLSVELKVHN
FTLHGHHDTLNTAYTTLVGCILSVVLVLIYLYLTPCRCWCRGVEKPSSHQGDSLSSSMLS
TTPNHDPMAGGDKDDGFDRRVAFLEPAGPGQGQNGKLKPGNTLPVPEATGKGQRRMSDPE
SVSSVFSDTPIVV
>sp|Q86WK7|AMGO3_HUMAN Amphoterin-induced protein 3 OS=Homo sapiens GN=AMIGO3 PE=2 SV=1
MTWLVLLGTLLCMLRVGLGTPDSEGFPPRALHNCPYKCICAADLLSCTGLGLQDVPAELP
AATADLDLSHNALQRLRPGWLAPLFQLRALHLDHNELDALGRGVFVNASGLRLLDLSSNT
LRALGRHDLDGLGALEKLLLFNNRLVHLDEHAFHGLRALSHLYLGCNELASFSFDHLHGL
SATHLLTLDLSSNRLGHISVPELAALPAFLKNGLYLHNNPLPCDCRLYHLLQRWHQRGLS
AVRDFAREYVCLAFKVPASRVRFFQHSRVFENCSSAPALGLERPEEHLYALVGRSLRLYC
NTSVPAMRIAWVSPQQELLRAPGSRDGSIAVLADGSLAIGNVQEQHAGLFVCLATGPRLH
HNQTHEYNVSVHFPRPEPEAFNTGFTTLLGCAVGLVLVLLYLFAPPCRCCRRACRCRRWP
QTPSPLQELSAQSSVLSTTPPDAPSRKASVHKHVVFLEPGRRGLNGRVQLAVAEEFDLYN
PGGLQLKAGSESASSIGSEGPMTT
>sp|Q9Y4X0|AMMR1_HUMAN AMME syndrome candidate gene 1 protein OS=Homo sapiens GN=AMMECR1 PE=1 SV=1
MAAGCCGVKKQKLSSSPPSGSGGGGGASSSSHCSGESQCRAGELGLGGAGTRLNGLGGLT
GGGSGSGCTLSPPQGCGGGGGGIALSPPPSCGVGTLLSTPAAATSSSPSSSSAASSSSPG
SRKMVVSAEMCCFCFDVLYCHLYGYQQPRTPRFTNEPYPLFVTWKIGRDKRLRGCIGTFS
AMNLHSGLREYTLTSALKDSRFPPMTRDELPRLFCSVSLLTNFEDVCDYLDWEVGVHGIR
IEFINEKGSKRTATYLPEVAKEQGWDHIQTIDSLLRKGGYKAPITNEFRKTIKLTRYRSE
KMTLSYAEYLAHRQHHHFQNGIGHPLPPYNHYS
>sp|Q8IY63|AMOL1_HUMAN Angiomotin-like protein 1 OS=Homo sapiens GN=AMOTL1 PE=1 SV=1
MWRAKLRRGTCEPAVKGSPSACYSPSSPVQVLEDSTYFSPDFQLYSGRHETSALTVEATS
SIREKVVEDPLCNFHSPNFLRISEVEMRGSEDAAAGTVLQRLIQEQLRYGTPTENMNLLA
IQHQATGSAGPAHPTNNFSSTENLTQEDPQMVYQSARQEPQGQEHQVDNTVMEKQVRSTQ

PQQNNEELPTYEEAKAQSQFFRGQQQQQQQQGAVGHGYYMAGGTSQKSRTEGRPTVNRAN
SGQAHKDEALKELKQGHVRSLSERIMQLSLERNGAKQHLPGSGNGKGFKVGGGPSPAQPA
GKVLDPRGPPPEYPFKTKQMMSPVSKTQEHGLFYGDQHPGMLHEMVKPYPAPQPVRTDVA
VLRYQPPPEYGVTSRPCQLPFPSTMQQHSPMSSQTSSASGPLHSVSLPLPLPMALGAPQP
PPAASPSQQLGPDAFAIVERAQQMVEILTEENRVLHQELQGYYDNADKLHKFEKELQRIS
EAYESLVKSTTKRESLDKAMRNKLEGEIRRLHDFNRDLRDRLETANRQLSSREYEGHEDK
AAEGHYASQNKEFLKEKEKLEMELAAVRTASEDHRRHIEILDQALSNAQARVIKLEEELR
EKQAYVEKVEKLQQALTQLQSACEKREQMERRLRTWLERELDALRTQQKHGNGQPANMPE
YNAPALLELVREKEERILALEADMTKWEQKYLEESTIRHFAMNAAATAAAERDTTIINHS
RNGSYGESSLEAHIWQEEEEVVQANRRCQDMEYTIKNLHAKIIEKDAMIKVLQQRSRKDA
GKTDSSSLRPARSVPSIAAATGTHSRQTSLTSSQLAEEKKEEKTWKGSIGLLLGKEHHEH
ASAPLLPPPPTSALSSIASTTAASSAHAKTGSKDSSTQTDKSAELFWPSMASLPSRGRLS
TTPAHSPVLKHPAAKGTAEKLENSPGHGKSPDHRGRVSSLLHKPEFPDGEMMEVLI
>sp|Q4VCS5|AMOT_HUMAN Angiomotin OS=Homo sapiens GN=AMOT PE=1 SV=1
MRNSEEQPSGGTTVLQRLLQEQLRYGNPSENRSLLAIHQQATGNGPPFPSGSGNPGPQSD
VLSPQDHHQQLVAHAARQEPQGQEIQSENLIMEKQLSPRMQNNEELPTYEEAKVQSQYFR
GQQHASVGAAFYVTGVTNQKMRTEGRPSVQRLNPGKMHQDEGLRDLKQGHVRSLSERLMQ
MSLATSGVKAHPPVTSAPLSPPQPNDLYKNPTSSSEFYKAQGPLPNQHSLKGMEHRGPPP
EYPFKGMPPQSVVCKPQEPGHFYSEHRLNQPGRTEGQLMRYQHPPEYGAARPAQDISLPL
SARNSQPHSPTSSLTSGGSLPLLQSPPSTRLSPARHPLVPNQGDHSAHLPRPQQHFLPNQ
AHQGDHYRLSQPGLSQQQQQQQQQHHHHHHQQQQQQQPQQQPGEAYSAMPRAQPSSASY
QPVPADPFAIVSRAQQMVEILSDENRNLRQELEGCYEKVARLQKVETEIQRVSEAYENLV
KSSSKREALEKAMRNKLEGEIRRMHDFNRDLRERLETANKQLAEKEYEGSEDTRKTISQL
FAKNKESQREKEKLEAELATARSTNEDQRRHIEIRDQALSNAQAKVVKLEEELKKKQVYV
DKVEKMQQALVQLQAACEKREQLEHRLRTRLERELESLRIQQRQGNCQPTNVSEYNAAAL
MELLREKEERILALEADMTKWEQKYLEENVMRHFALDAAATVAAQRDTTVISHSPNTSYD
TALEARIQKEEEEILMANKRCLDMEGRIKTLHAQIIEKDAMIKVLQQRSRKEPSKTEQLS
CMRPAKSLMSISNAGSGLLSHSSTLTGSPIMEEKRDDKSWKGSLGILLGGDYRAEYVPST
PSPVPPSTPLLSAHSKTGSRDCSTQTERGTESNKTAAVAPISVPAPVAAAATAAAITATA
ATITTTMVAAAPVAVAAAAAPAAAAAPSPATAAATAAAVSPAAAGQIPAAASVASAAAVA
PSAAAAAAVQVAPAAPAPVPAPALVPVPAPAAAQASAPAQTQAPTSAPAVAPTPAPTPTP
AVAQAEVPASPATGPGPHRLSIPSLTCNPDKTDGPVFHSNTLERKTPIQILGQEPDAEMV
EYLI
>sp|P23109|AMPD1_HUMAN AMP deaminase 1 OS=Homo sapiens GN=AMPD1 PE=1 SV=2
MNVRIFYSVSQSPHSLLSLLFYCAILESRISATMPLFKLPAEEKQIDDAMRNFAEKVFAS
EVKDEGGRQEISPFDVDEICPISHHEMQAHIFHLETLSTSTEARRKKRFQGRKTVNLSIP
LSETSSTKLSHIDEYISSSPTYQTVPDFQRVQITGDYASGVTVEDFEIVCKGLYRALCIR
EKYMQKSFQRFPKTPSKYLRNIDGEAWVANESFYPVFTPPVKKGEDPFRTDNLPENLGYH
LKMKDGVVYVYPNEAAVSKDEPKPLPYPNLDTFLDDMNFLLALIAQGPVKTYTHRRLKFL
SSKFQVHQMLNEMDELKELKNNPHRDFYNCRKVDTHIHAACMNQKHLLRFIKKSYQIDA
DRVVYSTKEKNLTLKELFAKLKMHPYDLTVDSLDVHAGRQTFQRFDKFNDKYNPVGASEL
RDLYLKTDNYINGEYFATIIKEVGADLVEAKYQHAEPRLSIYGRSPDEWSKLSSWFVCNR
IHCPNMTWMIQVPRIYDVFRSKNFLPHFGKMLENIFMPVFEATINPQADPELSVFLKHIT
GFDSVDDESKHSGHMFSSKSPKPQEWTLEKNPSYTYYAYYMYANIMVLNSLRKERGMNTF

LFRPHCGEAGALTHLMTAFMIADDISHGLNLKKSPVLQYLFFLAQIPIAMSPLSNNSLFL
EYAKNPFLDFLQKGLMISLSTDDPMQFHFTKEPLMEEYAIAAQVFKLSTCDMCEVARNSV
LQCGISHEEKVKFLGDNYLEEGPAGNDIRRTNVAQIRMAYRYETWCYELNLIAEGLKSTE
>sp|Q07075|AMPE_HUMAN Glutamyl aminopeptidase OS=Homo sapiens GN=ENPEP PE=1 SV=3
MNFAEREGSKRYCIQTKHVAILCAVVVGVGLIVGLAVGLTRSCDSSGDGGPGTAPAPSHL
PSSTASPSGPPAQDQDICPASEDESGQWKNFRLPDFVNPVHYDLHVKPLLEEDTYTGTVS
ISINLSAPTRYLWLHLRETRITRLPELKRPSGDQVQVRRCFEYKKQEYVVVEAEEELTPS
SGDGLYLLTMEFAGWLNGSLVGFYRTTYTENGQVKSIVATDHEPTDARKSFPCFDEPNKK
ATYTISITHPKEYGALSNMPVAKEESVDDKWTRTTFEKSVPMSTYLVCFAVHQFDSVKRI
SNSGKPLTIYVQPEQKHTAEYAANITKSVFDYFEEYFAMNYSLPKLDKIAIPDFGTGAME
NWGLITYRETNLLYDPKESASSNQQRVATVVAHELVHQWFGNIVTMDWWEDLWLNEGFAS
FFEFLGVNHAETDWQMRDQMLLEDVLPVQEDDSLMSSHPIIVTVTTPDEITSVFDGISYS
KGSSILRMLEDWIKPENFQKGCQMYLEKYQFKNAKTSDFWAALEEASRLPVKEVMDTWTR
QMGYPVLNVNGVKNITQKRFLLDPRANPSQPPSDLGYTWNIPVKWTEDNITSSVLFNRSE
KEGITLNSSNPSGNAFLKINPDHIGFYRVNYEVATWDSIATALSLNHKTFSSADRASLID
DAFALARAQLLDYKVALNLTKYLKREENFLPWQRVISAVTYIISMFEDDKELYPMIEEYF
QGQVKPIADSLGWNDAGDHVTKLLRSSVLGFACKMGDREALNNASSLFEQWLNGTVSLPV
NLRLLVYRYGMQNSGNEISWNYTLEQYQKTSLAQEKEKLLYGLASVKNVTLLSRYLDLLK
DTNLIKTQDVFTVIRYISYNSYGKNMAWNWIQLNWDYLVNRYTLNNRNLGRIVTIAEPFN
TELQLWQMESFFAKYPQAGAGEKPREQVLETVKNNIEWLKQHRNTIREWFFNLLESG
>sp|Q8N6M6|AMPO_HUMAN Aminopeptidase O OS=Homo sapiens GN=AOPEP PE=1 SV=2
MDIQLDPARDDLPLMANTSHILVKHYVLDLDVDFESQVIEGTIVLFLEDGNRFKKQNSSI
EEACQSESNKACKFGMPEPCHIPVTNARTFSSEMEYNDFAICSKGEKDTSDKDGNHDNQE
HASGISSSKYCCDTGNHGSEDFLLVLDCCDLSVLKVEEVDVAAVPGLEKFTRSPELTVVS
EEFRNQIVRELVTLPANRWREQLDYYARCSQAPGCGELLFDTDTWSLQIRKTGAQTATDF
PHAIRIWYKTKPEGRSVTWTSDQSGRPCVYTVGSPINNRALFPCQEPPVAMSTWQATVRA
AASFVVLMSGENSAKPTQLWEECSSWYYYVTMPMPASTFTIAVGCWTEMKMETWSSNDLA
TERPFSPSEANFRHVGVCSHMEYPCRFQNASATTQEIIPHRVFAPVCLTGACQETLLRLI
PPCLSAAHSVLGAHPFSRLDVLIVPANFPSLGMASPHIMFLSQSILTGGNHLCGTRLCHE
IAHAWFGLAIGARDWTEEWLSEGFATHLEDVFWATAQQLAPYEAREQQELRACLRWRRLQ
DEMQCSPEEMQVLRPSKDKTGHTSDSGASVIKHGLNPEKIFMQVHYLKGYFLLRFLAKRL
GDETYFSFLRKFVHTFHGQLILSQDFLQMLLENIPEEKRLELSVENIYQDWLESSGIPKP
LQRERRAGAECGLARQVRAEVTKWIGVNRRPRKRKRREKEEVFEKLLPDQLVLLLEHLLE
QKTLSPRTLQSLQRTYHLQDQDAEVRHRWCELIVKHKFTKAYKSVERFLQEDQAMGVYLY
GELMVSEDARQQQLARRCFERTKEQMDRSSAQVVAEMLF
>sp|Q400G9|AMZ1_HUMAN Archaemetzincin-1 OS=Homo sapiens GN=AMZ1 PE=1 SV=1
MLQCRPAQEFSFGPRALKDALVSTDAALQQLYVSAFSPAERLFLAEAYNPQRTLFCTLLI
RTGFDWLLSRPEAPEDFQTFHASLQHRKPRLARKHIYLQPIDLSEEPVGSSLLHQLCSCT
EAFFLGLRVKCLPSVAAASIRCSSRPSRDSDRLQLHTDGILSFLKNNKPGDALCVLGLTL
SDLYPHEAWSFTFSKFLPGHEVGVCSFARFSGEFPKSGPSAPDLALVEAAADGPEAPLQD
RGWALCFSALGMVQCCKVTCHELCHLLGLGNCRWLRCLMQGALSLDEALRRPLDLCPICL
RKLQHVLGFRLIERYQRLYTWTQAVVGTWPSQEAGEPSVWEDTPPASADSGMCCESDSEP
GTSVSEPLTPDAGSHTFASGPEEGLSYLAASEAPLPPGGPAEAIKEHERWLAMCIQALQR
EVAEEDLVQVDRAVDALDRWEMFTGQLPATRQDPPSSRDSVGLRKVLGDKFSSLRRKLSA

RKLARAESAPRPWDGEES
>sp|A2A2Z9|AN18B_HUMAN Ankyrin repeat domain-containing protein 18B OS=Homo sapiens GN=ANKRD18B PE=1 SV=1
MRKLLSFGRRLGQALLSSMDQEYAGRGYHIRDWELRKIHRAAIKGDAAEVEHCLTRRFRD
LDVRDRKDRTVLHLACAHGRVQVVTLLLDRKCQINICDRLNRTPLMKAVHCQEEACAIIL
LKRGANPNIKDIYGNTALHYAVYNEGTSLAERLLSHHANIEALNKEGNTPLLFAINSRRQ
HMVEFLLKNQANIHAVDNFKRTALILAVQHNLSSIVTLLLQQNIHISSQDMFGQTAEDYA
FCCDLRSIQQQILEHKNKMLKNHLRNDNQETAAMKPENLKKRKKRKKLKKRKEGAKAEHN
LKVASEEKQERLERSENKQPQDSQSYGKKKDEMFGNFMLKRDIAMLKEELYAIKNDSLRK
EKKYIQEIKSITEINANFEKSVRLNEEMITKKVAQYSQQLNDLKAENARLNSKLEKEKHN
KERLEAEVESLHSNLATAINEYNEILERKDLELVLWRADDVSRHETMGSNISQLTDKNEL
LTEQVHKARVKFNTLKGKLRETRDALREKTLALESVQLDLKQAQHRIKEMKQMHPNGEAK
ESQSIGKQNSSEERIRQRELENLLLERQLEDARKEGDNKEIVINIHRDCLENGKEDLLEE
RNKELMNEYNYLKEKLLQYEKEKAEREVIVREFQEELVDHLKKFSMSESPLEGTSHCHIN
LDETWTSKKKLFQVEIQPEEKHEEFRKVFELISLLNYTADQIRKKNRELEEEATGYKKCL
EMTINMLNAFANEDFSCHGDLNTDQLKMDILFKKLKQKFDDLMAEKEAVSSKCVNLAKDN
EVLHQELLSMGKVQEKCEKLEKDKKMLEEKVLNLKTHMEKDMVELGKVQEYKSELDERAM
QAIEKLEEIHLQKQAEYEKQLEQLNKDNTASLKKKELTLKDVECKFSKMKTAYEDVTTEL
EEYKEAFAVALKANSSMSEKITKSDKKIAVISTKLFMEKERMEYFLSTLPMRPDPELPCV
ENLNSIELNRKYIPKMAIRIPTSNPQTSNNCKNSLTELLLRWALAPIYFLL
>sp|Q9BXX3|AN30A_HUMAN Ankyrin repeat domain-containing protein 30A OS=Homo sapiens GN=ANKRD30A PE=2 SV=3
MEEISAAAVKVVPGPERPSPFSQLVYTSNDSYIVHSGDLRKIHKAASRGQVRKLEKMTKR
KKTINLNIQDAQKRTALHWACVNGHEEVVTFLVDRKCQLDVLDGEHRTPLMKALQCHQEA
CANILIDSGADINLVDVYGNTALHYAVYSEILSVVAKLLSHGAVIEVHNKASLTPLLLSI
TKRSEQIVEFLLIKNANANAVNKYKCTALMLAVCHGSSEIVGMLLQQNVDVFAADICGVT
AEHYAVTCGFHHIHEQIMEYIRKLSKNHQNTNPEGTSAGTPDEAAPLAERTPDTAESLVE
KTPDEAAPLVERTPDTAESLVEKTPDEAASLVEGTSDKIQCLEKATSGKFEQSAEETPRE
ITSPAKETSEKFTWPAKGRPRKIAWEKKEDTPREIMSPAKETSEKFTWAAKGRPRKIAWE
KKETPVKTGCVARVTSNKTKVLEKGRSKMIACPTKESSTKASANDQRFPSESKQEEDEEY
SCDSRSLFESSAKIQVCIPESIYQKVMEINREVEEPPKKPSAFKPAIEMQNSVPNKAFEL
KNEQTLRADPMFPPESKQKDYEENSWDSESLCETVSQKDVCLPKATHQKEIDKINGKLEE
SPNKDGLLKATCGMKVSIPTKALELKDMQTFKAEPPGKPSAFEPATEMQKSVPNKALELK
NEQTLRADEILPSESKQKDYEENSWDTESLCETVSQKDVCLPKAAHQKEIDKINGKLEGS
PVKDGLLKANCGMKVSIPTKALELMDMQTFKAEPPEKPSAFEPAIEMQKSVPNKALELKN
EQTLRADEILPSESKQKDYEESSWDSESLCETVSQKDVCLPKATHQKEIDKINGKLEESP
DNDGFLKAPCRMKVSIPTKALELMDMQTFKAEPPEKPSAFEPAIEMQKSVPNKALELKNE
QTLRADQMFPSESKQKKVEENSWDSESLRETVSQKDVCVPKATHQKEMDKISGKLEDSTS
LSKILDTVHSCERARELQKDHCEQRTGKMEQMKKKFCVLKKKLSEAKEIKSQLENQKVKW
EQELCSVRLTLNQEEEKRRNADILNEKIREELGRIEEQHRKELEVKQQLEQALRIQDIEL
KSVESNLNQVSHTHENENYLLHENCMLKKEIAMLKLEIATLKHQYQEKENKYFEDIKILK
EKNAELQMTLKLKEESLTKRASQYSGQLKVLIAENTMLTSKLKEKQDKEILEAEIESHHP
RLASAVQDHDQIVTSRKSQEPAFHIAGDACLQRKMNVDVSSTIYNNEVLHQPLSEAQRKS
KSLKINLNYAGDALRENTLVSEHAQRDQRETQCQMKEAEHMYQNEQDNVNKHTEQQESLD

QKLFQLQSKNMWLQQQLVHAHKKADNKSKITIDIHFLERKMQHHLLKEKNEEIFNYNNHL
KNRIYQYEKEKAETENS
>sp|Q92688|AN32B_HUMAN Acidic leucine-rich nuclear phosphoprotein 32 family member B OS=Homo sapiens GN=ANP32B PE=1 SV=1
MDMKRRIHLELRNRTPAAVRELVLDNCKSNDGKIEGLTAEFVNLEFLSLINVGLISVSNL
PKLPKLKKLELSENRIFGGLDMLAEKLPNLTHLNLSGNKLKDISTLEPLKKLECLKSLDL
FNCEVTNLNDYRESVFKLLPQLTYLDGYDREDQEAPDSDAEVDGVDEEEEDEEGEDEEDE
DDEDGEEEEFDEEDDEDEDVEGDEDDDEVSEEEEEFGLDEEDEDEDEDEEEEEGGKGEKR
KRETDDEGEDD
>sp|Q9BTT0|AN32E_HUMAN Acidic leucine-rich nuclear phosphoprotein 32 family member E OS=Homo sapiens GN=ANP32E PE=1 SV=1
MEMKKKINLELRNRSPEEVTELVLDNCLCVNGEIEGLNDTFKELEFLSMANVELSSLARL
PSLNKLRKLELSDNIISGGLEVLAEKCPNLTYLNLSGNKIKDLSTVEALQNLKNLKSLDL
FNCEITNLEDYRESIFELLQQITYLDGFDQEDNEAPDSEEEDDEDGDEDDEEEEENEAGP
PEGYEEEEEEEEEEDEDEDEDEDEAGSELGEGEEEVGLSYLMKEEIQDEEDDDDYVEEGE
EEEEEEEGGLRGEKRKRDAEDDGEEEDD
>sp|A6NCL7|AN33B_HUMAN Ankyrin repeat domain-containing protein 33B OS=Homo sapiens GN=ANKRD33B PE=3 SV=1
MVLLAGTGPEGGGARCMTPPPPSPPRGAQVEEDPADYEEFEDFSSLPDTRSIASDDSFYP
FEDEEEHGVESAESVPEGVPESVPETATLLRAACANNVGLLRTLVRRGVSVEEAQETDRN
GRTGLIVACYHGFVDTVVALAECPHVDVNWQDSEGNTALITAAQAGHAIITNYLLNYFPG
LDLERRNAFGFTALMKAAMQGRTDCIRALMLAGADVHARDPRRGMSPQEWATYTGRVDAV
RLMQRLLERPCPEQFWEKYRPELPPPPEAARKPAGSKNCLQRLTDCVLSVLTPRSVRGPE
DGGVLDHMVRMTTSLYSPAVAIVCQTVCPESPPSVGKRRLAVQEILAARAARGPQAQEED
EVGGAGQRGRTGQEDADSREGSPRAGLPPALGSRGPAAPAPRKASLLPLQRLRRRSVRPG
VVVPRVRVSKAPAPTFQPERPARKGSTKDSGHLQIPKWRYKEAKEEKRKAEEAEKKRQAE
AQKERRTAPWKKRT
>sp|P0C6C1|AN34C_HUMAN Ankyrin repeat domain-containing protein 34C OS=Homo sapiens GN=ANKRD34C PE=3 SV=2
MMDDDTELRTDGNSLLKAVWLGRLRLTRLLLEGGAYINESNDKGETALMVACITKHVDQQ
SISKSKMVKYLLDNRADPNIQDKSGKTALIHACIRRAGGEVVSLLLENGADPSLEDRTGA
SALVYAINADDKDALKHLLDACKAKGKEVIIITTDKSSSGTKTTKQYLNVPPSPKVEDRH
SPPLCASPSDIELKALGLDSPLTEKEDDFFSLQAGHPSSCNTSKAVNEPGSPTRKVSNLK
RARLPQLKRLQSEPWGLIAPSVLAASTRQDETHGASTDNEVIKSISDISFPKRGPLSRTN
SIDSKDPTLFHTVTEQVLKIPVSSAPASWKAAYEKGQAPHPRLARRGTLPVDQEKCGMGP
SGPSALKEPASLKWLENDLYDLDIQPGPDPPNSISLESGKGPLDRKKLNSSHLSLFHGSR
ESLDTVPSTSPSSARRRPPHLLERRGSGTLLLDRISHTRPGFLPPLNVNLNPPIPDIRSS
SKPSCSLASGLKSMVPVAPSSPKRVDLRSKKKLLRRHSMQIEQMKQLSDFEEIMT
>sp|Q9UNK9|ANGE1_HUMAN Protein angel homolog 1 OS=Homo sapiens GN=ANGEL1 PE=1 SV=1
MIASCLCYLLLPATRLFRALSDAFFTCRKNVLLANSSSPQVEGDFAMAPRGPEQEECEGL
LQQWREEGLSQVLSTASEGPLIDKGLAQSSLALLMDNPGEENAASEDRWSSRQLSDLRAA
ENLDEPFPEMLGEEPLLEVEGVEGSMWAAIPMQSEPQYADCAALPVGALATEQWEEDPAV
LAWSIAPEPVPQEEASIWPFEGLGQLQPPAVEIPYHEILWREWEDFSTQPDAQGLKAGDG
PQFQFTLMSYNILAQDLMQQSSELYLHCHPDILNWNYRFVNLMQEFQHWDPDILCLQEVQ

EDHYWEQLEPSLRMMGFTCFYKRRTGCKTDGCAVCYKPTRFRLLCASPVEYFRPGLELLN
RDNVGLVLLLQPLVPEGLGQVSVAPLCVANTHILYNPRRGDVKLAQMAILLAEVDKVARL
SDGSHCPIILCGDLNSVPDSPLYNFIRDGELQYHGMPAWKVSGQEDFSHQLYQRKLQAPL
WPSSLGITDCCQYVTSCHPKRSERRKYGRDFLLRFRFCSIACQRPVGLVLMEGVTDTKPE
RPAGWAESVLEEDASELEPAFSRTVGTIQHCLHLTSVYTHFLPQRGRPEVTTMPLGLGMT
VDYIFFSAESCENGNRTDHRLYRDGTLKLLGRLSLLSEEILWAANGLPNPFCSSDHLCLL
ASFGMEVTAP
>sp|Q9UKU9|ANGL2_HUMAN Angiopoietin-related protein 2 OS=Homo sapiens GN=ANGPTL2 PE=2 SV=1
MRPLCVTCWWLGLLAAMGAVAGQEDGFEGTEEGSPREFIYLNRYKRAGESQDKCTYTFIV
PQQRVTGAICVNSKEPEVLLENRVHKQELELLNNELLKQKRQIETLQQLVEVDGGIVSEV
KLLRKESRNMNSRVTQLYMQLLHEIIRKRDNALELSQLENRILNQTADMLQLASKYKDLE
HKYQHLATLAHNQSEIIAQLEEHCQRVPSARPVPQPPPAAPPRVYQPPTYNRIINQISTN
EIQSDQNLKVLPPPLPTMPTLTSLPSSTDKPSGPWRDCLQALEDGHDTSSIYLVKPENTN
RLMQVWCDQRHDPGGWTVIQRRLDGSVNFFRNWETYKQGFGNIDGEYWLGLENIYWLTNQ
GNYKLLVTMEDWSGRKVFAEYASFRLEPESEYYKLRLGRYHGNAGDSFTWHNGKQFTTLD
RDHDVYTGNCAHYQKGGWWYNACAHSNLNGVWYRGGHYRSRYQDGVYWAEFRGGSYSLKK
VVMMIRPNPNTFH
>sp|Q9Y5C1|ANGL3_HUMAN Angiopoietin-related protein 3 OS=Homo sapiens GN=ANGPTL3 PE=1 SV=1
MFTIKLLLFIVPLVISSRIDQDNSSFDSLSPEPKSRFAMLDDVKILANGLLQLGHGLKDF
VHKTKGQINDIFQKLNIFDQSFYDLSLQTSEIKEEEKELRRTTYKLQVKNEEVKNMSLEL
NSKLESLLEEKILLQQKVKYLEEQLTNLIQNQPETPEHPEVTSLKTFVEKQDNSIKDLLQ
TVEDQYKQLNQQHSQIKEIENQLRRTSIQEPTEISLSSKPRAPRTTPFLQLNEIRNVKHD
GIPAECTTIYNRGEHTSGMYAIRPSNSQVFHVYCDVISGSPWTLIQHRIDGSQNFNETWE
NYKYGFGRLDGEFWLGLEKIYSIVKQSNYVLRIELEDWKDNKHYIEYSFYLGNHETNYTL
HLVAITGNVPNAIPENKDLVFSTWDHKAKGHFNCPEGYSGGWWWHDECGENNLNGKYNKP
RAKSKPERRRGLSWKSQNGRLYSIKSTKMLIHPTDSESFE
>sp|Q12955|ANK3_HUMAN Ankyrin-3 OS=Homo sapiens GN=ANK3 PE=1 SV=3
MAHAASQLKKNRDLEINAEEEPEKKRKHRKRSRDRKKKSDANASYLRAARAGHLEKALDY
IKNGVDINICNQNGLNALHLASKEGHVEVVSELLQREANVDAATKKGNTALHIASLAGQA
EVVKVLVTNGANVNAQSQNGFTPLYMAAQENHLEVVKFLLDNGASQSLATEDGFTPLAVA
LQQGHDQVVSLLLENDTKGKVRLPALHIAARKDDTKAAALLLQNDNNADVESKSGFTPLH
IAAHYGNINVATLLLNRAAAVDFTARNDITPLHVASKRGNANMVKLLLDRGAKIDAKTRD
GLTPLHCGARSGHEQVVEMLLDRAAPILSKTKNGLSPLHMATQGDHLNCVQLLLQHNVPV
DDVTNDYLTALHVAAHCGHYKVAKVLLDKKANPNAKALNGFTPLHIACKKNRIKVMELLL
KHGASIQAVTESGLTPIHVAAFMGHVNIVSQLMHHGASPNTTNVRGETALHMAARSGQAE
VVRYLVQDGAQVEAKAKDDQTPLHISARLGKADIVQQLLQQGASPNAATTSGYTPLHLSA
REGHEDVAAFLLDHGASLSITTKKGFTPLHVAAKYGKLEVANLLLQKSASPDAAGKSGLT
PLHVAAHYDNQKVALLLLDQGASPHAAAKNGYTPLHIAAKKNQMDIATTLLEYGADANAV
TRQGIASVHLAAQEGHVDMVSLLLGRNANVNLSNKSGLTPLHLAAQEDRVNVAEVLVNQG
AHVDAQTKMGYTPLHVGCHYGNIKIVNFLLQHSAKVNAKTKNGYTPLHQAAQQGHTHIIN
VLLQNNASPNELTVNGNTALGIARRLGYISVVDTLKIVTEETMTTTTVTEKHKMNVPETM
NEVLDMSDDEVRKANAPEMLSDGEYISDVEEGEDAMTGDTDKYLGPQDLKELGDDSLPAE

GYMGFSLGARSASLRSFSSDRSYTLNRSSYARDSMMIEELLVPSKEQHLTFTREFDSDSL
RHYSWAADTLDNVNLVSSPIHSGFLVSFMVDARGGSMRGSRHHGMRIIIPPRKCTAPTRI
TCRLVKRHKLANPPPMVEGEGLASRLVEMGPAGAQFLGPVIVEIPHFGSMRGKERELIVL
RSENGETWKEHQFDSKNEDLTELLNGMDEELDSPEELGKKRICRIITKDFPQYFAVVSRI
KQESNQIGPEGGILSSTTVPLVQASFPEGALTKRIRVGLQAQPVPDEIVKKILGNKATFS
PIVTVEPRRRKFHKPITMTIPVPPPSGEGVSNGYKGDTTPNLRLLCSITGGTSPAQWEDI
TGTTPLTFIKDCVSFTTNVSARFWLADCHQVLETVGLATQLYRELICVPYMAKFVVFAKM
NDPVESSLRCFCMTDDKVDKTLEQQENFEEVARSKDIEVLEGKPIYVDCYGNLAPLTKGG
QQLVFNFYSFKENRLPFSIKIRDTSQEPCGRLSFLKEPKTTKGLPQTAVCNLNITLPAHK
KETESDQDDEIEKTDRRQSFASLALRKRYSYLTEPGMIERSTGATRSLPTTYSYKPFFST
RPYQSWTTAPITVPGPAKSGFTSLSSSSSNTPSASPLKSIWSVSTPSPIKSTLGASTTSS
VKSISDVASPIRSFRTMSSPIKTVVSQSPYNIQVSSGTLARAPAVTEATPLKGLASNSTF
SSRTSPVTTAGSLLERSSITMTPPASPKSNINMYSSSLPFKSIITSAAPLISSPLKSVVS
PVKSAVDVISSAKITMASSLSSPVKQMPGHAEVALVNGSISPLKYPSSSTLINGCKATAT
LQEKISSATNSVSSVVSAATDTVEKVFSTTTAMPFSPLRSYVSAAPSAFQSLRTPSASAL
YTSLGSSISATTSSVTSSIITVPVYSVVNVLPEPALKKLPDSNSFTKSAAALLSPIKTLT
TETHPQPHFSRTSSPVKSSLFLAPSALKLSTPSSLSSSQEILKDVAEMKEDLMRMTAILQ
TDVPEEKPFQPELPKEGRIDDEEPFKIVEKVKEDLVKVSEILKKDVCVDNKGSPKSPKSD
KGHSPEDDWIEFSSEEIREARQQAAASQSPSLPERVQVKAKAASEKDYNLTKVIDYLTND
IGSSSLTNLKYKFEDAKKDGEERQKRVLKPAIALQEHKLKMPPASMRTSTSEKELCKMAD
SFFGTDTILESPDDFSQHDQDKSPLSDSGFETRSEKTPSAPQSAESTGPKPLFHEVPIPP
VITETRTEVVHVIRSYDPSAGDVPQTQPEEPVSPKPSPTFMELEPKPTTSSIKEKVKAFQ
MKASSEEDDHNRVLSKGMRVKEETHITTTTRMVYHSPPGGEGASERIEETMSVHDIMKAF
QSGRDPSKELAGLFEHKSAVSPDVHKSAAETSAQHAEKDNQMKPKLERIIEVHIEKGNQA
EPTEVIIRETKKHPEKEMYVYQKDLSRGDINLKDFLPEKHDAFPCSEEQGQQEEEELTAE
ESLPSYLESSRVNTPVSQEEDSRPSSAQLISDDSYKTLKLLSQHSIEYHDDELSELRGES
YRFAEKMLLSEKLDVSHSDTEESVTDHAGPPSSELQGSDKRSREKIATAPKKEILSKIYK
DVSENGVGKVSKDEHFDKVTVLHYSGNVSSPKHAMWMRFTEDRLDRGREKLIYEDRVDRT
VKEAEEKLTEVSQFFRDKTEKLNDELQSPEKKARPKNGKEYSSQSPTSSSPEKVLLTELL
ASNDEWVKARQHGPDGQGFPKAEEKAPSLPSSPEKMVLSQQTEDSKSTVEAKGSISQSKA
PDGPQSGFQLKQSKLSSIRLKFEQGTHAKSKDMSQEDRKSDGQSRIPVKKIQESKLPVYQ
VFAREKQQKAIDLPDESVSVQKDFMVLKTKDEHAQSNEIVVNDSGSDNVKKQRTEMSSKA
MPDSFSEQQAKDLACHITSDLATRGPWDKKVFRTWESSGATNNKSQKEKLSHVLVHDVRE
NHIGHPESKSVDQKNEFMSVTERERKLLTNGSLSEIKEMTVKSPSKKVLYREYVVKEGDH
PGGLLDQPSRRSESSAVSHIPVRVADERRMLSSNIPDGFCEQSAFPKHELSQKLSQSSMS
KETVETQHFNSIEDEKVTYSEISKVSKHQSYVGLCPPLEETETSPTKSPDSLEFSPGKES
PSSDVFDHSPIDGLEKLAPLAQTEGGKEIKTLPVYVSFVQVGKQYEKEIQQGGVKKIISQ
ECKTVQETRGTFYTTRQQKQPPSPQGSPEDDTLEQVSFLDSSGKSPLTPETPSSEEVSYE
FTSKTPDSLIAYIPGKPSPIPEVSEESEEEEQAKSTSLKQTTVEETAVEREMPNDVSKDS
NQRPKNNRVAYIEFPPPPPLDADQIESDKKHHYLPEKEVDMIEVNLQDEHDKYQLAEPVI
RVQPPSPVPPGADVSDSSDDESIYQPVPVKKYTFKLKEVDDEQKEKPKASAEKASNQKEL
ESNGSGKDNEFGLGLDSPQNEIAQNGNNDQSITECSIATTAEFSHDTDATEIDSLDGYDL
QDEDDGLTESDSKLPIQAMEIKKDIWNTEGILKPADRSFSQSKLEVIEEEGKVGPDEDKP
PSKSSSSEKTPDKTDQKSGAQFFTLEGRHPDRSVFPDTYFSYKVDEEFATPFKTVATKGL

DFDPWSNNRGDDEVFDSKSREDETKPFGLAVEDRSPATTPDTTPARTPTDESTPTSEPNP
FPFHEGKMFEMTRSGAIDMSKRDFVEERLQFFQIGEHTSEGKSGDQGEGDKSMVTATPQP
QSGDTTVETNLERNVETPTVEPNPSIPTSGECQEGTSSSGSLEKSAAATNTSKVDPKLRT
PIKMGISASTMTMKKEGPGEITDKIEAVMTSCQGLENETITMISNTANSQMGVRPHEKHD
FQKDNFNNNNNLDSSTIQTDNIMSNIVLTEHSAPTCTTEKDNPVKVSSGKKTGVLQGHCV
RDKQKVLGEQQKTKELIGIRQKSKLPIKATSPKDTFPPNHMSNTKASKMKQVSQSEKTKA
LTTSSCVDVKSRIPVKNTHRDNIIAVRKACATQKQGQPEKGKAKQLPSKLPVKVRSTCVT
TTTTTATTTTTTTTTTTSCTVKVRKSQLKEVCKHSIEYFKGISGETLKLVDRLSEEEKK
MQSELSDEEESTSRNTSLSETSRGGQPSVTTKSARDKKTEAAPLKSKSEKAGSEKRSSRR
TGPQSPCERTDIRMAIVADHLGLSWTELARELNFSVDEINQIRVENPNSLISQSFMLLKK
WVTRDGKNATTDALTSVLTKINRIDIVTLLEGPIFDYGNISGTRSFADENNVFHDPVDGW
QNETSSGNLESCAQARRVTGGLLDRLDDSPDQCRDSITSYLKGEAGKFEANGSHTEITPE
AKTKSYFPESQNDVGKQSTKETLKPKIHGSGHVEEPASPLAAYQKSLEETSKLIIEETKP
CVPVSMKKMSRTSPADGKPRLSLHEEEGSSGSEQKQGEGFKVKTKKEIRHVEKKSHS
>sp|Q9NQW6|ANLN_HUMAN Anillin OS=Homo sapiens GN=ANLN PE=1 SV=2
MDPFTEKLLERTRARRENLQRKMAERPTAAPRSMTHAKRARQPLSEASNQQPLSGGEEKS
CTKPSPSKKRCSDNTEVEVSNLENKQPVESTSAKSCSPSPVSPQVQPQAADTISDSVAVP
ASLLGMRRGLNSRLEATAASSVKTRMQKLAEQRRRWDNDDMTDDIPESSLFSPMPSEEKA
ASPPRPLLSNASATPVGRRGRLANLAATICSWEDDVNHSFAKQNSVQEQPGTACLSKFSS
ASGASARINSSSVKQEATFCSQRDGDASLNKALSSSADDASLVNASISSSVKATSPVKST
TSITDAKSCEGQNPELLPKTPISPLKTGVSKPIVKSTLSQTVPSKGELSREICLQSQSKD
KSTTPGGTGIKPFLERFGERCQEHSKESPARSTPHRTPIITPNTKAIQERLFKQDTSSST
THLAQQLKQERQKELACLRGRFDKGNIWSAEKGGNSKSKQLETKQETHCQSTPLKKHQGV
SKTQSLPVTEKVTENQIPAKNSSTEPKGFTECEMTKSSPLKITLFLEEDKSLKVTSDPKV
EQKIEVIREIEMSVDDDDINSSKVINDLFSDVLEEGELDMEKSQEEMDQALAESSEEQED
ALNISSMSLLAPLAQTVGVVSPESLVSTPRLELKDTSRSDESPKPGKFQRTRVPRAESGD
SLGSEDRDLLYSIDAYRSQRFKETERPSIKQVIVRKEDVTSKLDEKNNAFPCQVNIKQKM
QELNNEINMQQTVIYQASQALNCCVDEEHGKGSLEEAEAERLLLIATGKRTLLIDELNKL
KNEGPQRKNKASPQSEFMPSKGSVTLSEIRLPLKADFVCSTVQKPDAANYYYLIILKAGA
ENMVATPLASTSNSLNGDALTFTTTFTLQDVSNDFEINIEVYSLVQKKDPSGLDKKKKTS
KSKAITPKRLLTSITTKSNIHSSVMASPGGLSAVRTSNFALVGSYTLSLSSVGNTKFVLD
KVPFLSSLEGHIYLKIKCQVNSSVEERGFLTIFEDVSGFGAWHRRWCVLSGNCISYWTYP
DDEKRKNPIGRINLANCTSRQIEPANREFCARRNTFELITVRPQREDDRETLVSQCRDTL
CVTKNWLSADTKEERDLWMQKLNQVLVDIRLWQPDACYKPIGKP
>sp|Q99873|ANM1_HUMAN Protein arginine N-methyltransferase 1 OS=Homo sapiens GN=PRMT1 PE=1 SV=2
MENFVATLANGMSLQPPLEEVSCGQAESSEKPNAEDMTSKDYYFDSYAHFGIHEEMLKDE
VRTLTYRNSMFHNRHLFKDKVVLDVGSGTGILCMFAAKAGARKVIGIECSSISDYAVKIV
KANKLDHVVTIIKGKVEEVELPVEKVDIIISEWMGYCLFYESMLNTVLYARDKWLAPDGL
IFPDRATLYVTAIEDRQYKDYKIHWWENVYGFDMSCIKDVAIKEPLVDVVDPKQLVTNAC
LIKEVDIYTVKVEDLTFTSPFCLQVKRNDYVHALVAYFNIEFTRCHKRTGFSTSPESPYT
HWKQTVFYMEDYLTVKTGEEIFGTIGMRPNAKNNRDLDFTIDLDFKGQLCELSCSTDYRM
R

>sp|Q9NR22|ANM8_HUMAN Protein arginine N-methyltransferase 8 OS=Homo sapiens GN=PRMT8 PE=1 SV=2
MGMKHSSRCLLLRRKMAENAAESTEVNSPPSQPPQPVVPAKPVQCVHHVSTQPSCPGRGK
MSKLLNPEEMTSRDYYFDSYAHFGIHEEMLKDEVRTLTYRNSMYHNKHVFKDKVVLDVGS
GTGILSMFAAKAGAKKVFGIECSSISDYSEKIIKANHLDNIITIFKGKVEEVELPVEKVD
IIISEWMGYCLFYESMLNTVIFARDKWLKPGGLMFPDRAALYVVAIEDRQYKDFKIHWWE
NVYGFDMTCIRDVAMKEPLVDIVDPKQVVTNACLIKEVDIYTVKTEELSFTSAFCLQIQR
NDYVHALVTYFNIEFTKCHKKMGFSTAPDAPYTHWKQTVFYLEDYLTVRRGEEIYGTISM
KPNAKNVRDLDFTVDLDFKGQLCETSVSNDYKMR
>sp|Q75V66|ANO5_HUMAN Anoctamin-5 OS=Homo sapiens GN=ANO5 PE=1 SV=1
MGDPDLLEVLAEEGEKVNKHIDYSFQMSEQSLSSRETSFLINEETMPAKRFNLFLRRRLM
FQKNQQSKDSIFFRDGIRQIDFVLSYVDDVKKDAELKAERRKEFETNLRKTGLELEIEDK
RDSEDGRTYFVKIHAPWEVLVTYAEVLGIKMPIKESDIPRPKHTPISYVLGPVRLPLSVK
YPHPEYFTAQFSRHRQELFLIEDQATFFPSSSRNRIVYYILSRCPFGIEDGKKRFGIERL
LNSNTYSSAYPLHDGQYWKPSEPPNPTNERYTLHQNWARFSYFYKEQPLDLIKNYYGEKI
GIYFVFLGFYTEMLFFAAVVGLACFIYGLLSMEHNTSSTEICDPEIGGQMIMCPLCDQVC
DYWRLNSTCLASKFSHLFDNESTVFFAIFMGIWVTLFLEFWKQRQARLEYEWDLVDFEEE
QQQLQLRPEFEAMCKHRKLNAVTKEMEPYMPLYTRIPWYFLSGATVTLWMSLVVTSMVAV
IVYRLSVFATFASFMESDASLKQVKSFLTPQITTSLTGSCLNFIVILILNFFYEKISAWI
TKMEIPRTYQEYESSLTLKMFLFQFVNFYSSCFYVAFFKGKFVGYPGKYTYLFNEWRSEE
CDPGGCLIELTTQLTIIMTGKQIFGNIKEAIYPLALNWWRRRKARTNSEKLYSRWEQDHD
LESFGPLGLFYEYLETVTQFGFVTLFVASFPLAPLLALINNIVEIRVDAWKLTTQYRRTV
ASKAHSIGVWQDILYGMAVLSVATNAFIVAFTSDIIPRLVYYYAYSTNATQPMTGYVNNS
LSVFLIADFPNHTAPSEKRDFITCRYRDYRYPPDDENKYFHNMQFWHVLAAKMTFIIVME
HVVFLVKFLLAWMIPDVPKDVVERIKREKLMTIKILHDFELNKLKENLGINSNEFAKHVM
IEENKAQLAKSTL
>sp|P17342|ANPRC_HUMAN Atrial natriuretic peptide receptor 3 OS=Homo sapiens GN=NPR3 PE=1 SV=2
MPSLLVLTFSPCVLLGWALLAGGTGGGGVGGGGGGAGIGGGRQEREALPPQKIEVLVLLP
QDDSYLFSLTRVRPAIEYALRSVEGNGTGRRLLPPGTRFQVAYEDSDCGNRALFSLVDRV
AAARGAKPDLILGPVCEYAAAPVARLASHWDLPMLSAGALAAGFQHKDSEYSHLTRVAPA
YAKMGEMMLALFRHHHWSRAALVYSDDKLERNCYFTLEGVHEVFQEEGLHTSIYSFDETK
DLDLEDIVRNIQASERVVIMCASSDTIRSIMLVAHRHGMTSGDYAFFNIELFNSSSYGDG
SWKRGDKHDFEAKQAYSSLQTVTLLRTVKPEFEKFSMEVKSSVEKQGLNMEDYVNMFVEG
FHDAILLYVLALHEVLRAGYSKKDGGKIIQQTWNRTFEGIAGQVSIDANGDRYGDFSVIA
MTDVEAGTQEVIGDYFGKEGRFEMRPNVKYPWGPLKLRIDENRIVEHTNSSPCKSSGGLE
ESAVTGIVVGALLGAGLLMAFYFFRKKYRITIERRTQQEESNLGKHRELREDSIRSHFSV
A
>sp|Q8N6D5|ANR29_HUMAN Ankyrin repeat domain-containing protein 29 OS=Homo sapiens GN=ANKRD29 PE=2 SV=2
MCRMSFKKETPLANAAFWAARRGNLALLKLLLNSGRVDVDCRDSHGTTLLMVAAYAGHID
CVRELVLQGADINLQRESGTTALFFAAQQGHNDVVRFLFGFGASTEFRTKDGGTALLAAS
QYGHMQVVETLLKHGANIHDQLYDGATALFLAAQGGYLDVIRLLLASGAKVNQPRQDGTA
PLWIASQMGHSEVVRVMLLRGADRDAARNDGTTALLKAANKGYNDVIKELLKFSPTLGIL

KNGTSALHAAVLSGNIKTVALLLEAGADPSLRNKANELPAELTKNERILRLLRSKEGPRK
S
>sp|Q3KP44|ANR55_HUMAN Ankyrin repeat domain-containing protein 55 OS=Homo sapiens GN=ANKRD55 PE=1 SV=3
MMRQATMDFSTPSVFDQQRGDSSEEVDLTMVYQAASNGDVNALTAVIREDPSILECCDSE
GCTPLMHAVSGRQADTVKLLLKMGANINMQDAYGRTSLCLATYLGWLEGCVSLLRNGAKH
NIPDKNGRLPLHAATAEPDMRLLTVLLQQSNISEINHQDNEGMTPLHWAAFHNQPQHTQM
LLKKGADPTLVDKDFKTALHWAVQSGNRILCSIILSHHQGPSIINYDDESGKTCVHIAAA
AGFSDIIHELARVPECNLQALDVDDRTPLHWAAAAGKAECVQSLLELGMDSNLRDINEST
PLAYALYCGHTACVKLLSQESRTEPTRPPPSQSSRPQKKERRFNVLNQIFCKNKKEEQRA
HQKDPSRDRYREEDTSEVNDIITTFDSIVGTNCQEQPGDQVAMVEFKKKTSDNSKYLLPE
KKPLARKGLPPIRTQSLPPITLGNNFLTASHRATSHAGLSSAPHHMAQRSQKSRSEQDLL
NNRTGCQMLLDNPWKSDSNQVFSYKVWTVSSSDKLLDRLLSVRPGHQEVSVPPHLRHLHN
PSSGQNFQHLSPNRHKIRDLPFTRNNLAPLPDQKFLSGEPLRTNRVLPAIPSQRRHSTAA
EESEHSANPTSDEN
>sp|P58335|ANTR2_HUMAN Anthrax toxin receptor 2 OS=Homo sapiens GN=ANTXR2 PE=1 SV=5
MVAERSPARSPGSWLFPGLWLLVLSGPGGLLRAQEQPSCRRAFDLYFVLDKSGSVANNWI
EIYNFVQQLAERFVSPEMRLSFIVFSSQATIILPLTGDRGKISKGLEDLKRVSPVGETYI
HEGLKLANEQIQKAGGLKTSSIIIALTDGKLDGLVPSYAEKEAKISRSLGASVYCVGVLD
FEQAQLERIADSKEQVFPVKGGFQALKGIINSILAQSCTEILELQPSSVCVGEEFQIVLS
GRGFMLGSRNGSVLCTYTVNETYTTSVKPVSVQLNSMLCPAPILNKAGETLDVSVSFNGG
KSVISGSLIVTATECSNGIAAIIVILVLLLLLGIGLMWWFWPLCCKVVIKDPPPPPAPAP
KEEEEEPLPTKKWPTVDASYYGGRGVGGIKRMEVRWGDKGSTEEGARLEKAKNAVVKIPE
ETEEPIRPRPPRPKPTHQPPQTKWYTPIKGRLDALWALLRRQYDRVSLMRPQEGDEVCIW
ECIEKELTA
>sp|P61966|AP1S1_HUMAN AP-1 complex subunit sigma-1A OS=Homo sapiens GN=AP1S1 PE=1 SV=1
MMRFMLLFSRQGKLRLQKWYLATSDKERKKMVRELMQVVLARKPKMCSFLEWRDLKVVYK
RYASLYFCCAIEGQDNELITLELIHRYVELLDKYFGSVCELDIIFNFEKAYFILDEFLMG
GDVQDTSKKSVLKAIEQADLLQEEDESPRSVLEEMGLA
>sp|O00203|AP3B1_HUMAN AP-3 complex subunit beta-1 OS=Homo sapiens GN=AP3B1 PE=1 SV=3
MSSNSFPYNEQSGGGEATELGQEATSTISPSGAFGLFSSDLKKNEDLKQMLESNKDSAKL
DAMKRIVGMIAKGKNASELFPAVVKNVASKNIEIKKLVYVYLVRYAEEQQDLALLSISTF
QRALKDPNQLIRASALRVLSSIRVPIIVPIMMLAIKEASADLSPYVRKNAAHAIQKLYSL
DPEQKEMLIEVIEKLLKDKSTLVAGSVVMAFEEVCPDRIDLIHKNYRKLCNLLVDVEEWG
QVVIIHMLTRYARTQFVSPWKEGDELEDNGKNFYESDDDQKEKTDKKKKPYTMDPDHRLL
IRNTKPLLQSRNAAVVMAVAQLYWHISPKSEAGIISKSLVRLLRSNREVQYIVLQNIATM
SIQRKGMFEPYLKSFYVRSTDPTMIKTLKLEILTNLANEANISTLLREFQTYVKSQDKQF
AAATIQTIGRCATNILEVTDTCLNGLVCLLSNRDEIVVAESVVVIKKLLQMQPAQHGEII
KHMAKLLDSITVPVARASILWLIGENCERVPKIAPDVLRKMAKSFTSEDDLVKLQILNLG
AKLYLTNSKQTKLLTQYILNLGKYDQNYDIRDRTRFIRQLIVPNVKSGALSKYAKKIFLA
QKPAPLLESPFKDRDHFQLGTLSHTLNIKATGYLELSNWPEVAPDPSVRNVEVIELAKEW
TPAGKAKQENSAKKFYSESEEEEDSSDSSSDSESESGSESGEQGESGEEGDSNEDSSEDS
SSEQDSESGRESGLENKRTAKRNSKAKGKSDSEDGEKENEKSKTSDSSNDESSSIEDSSS
DSESESEPESESESRRVTKEKEKKTKQDRTPLTKDVSLLDLDDFNPVSTPVALPTPALSP

SLMADLEGLHLSTSSSVISVSTPAFVPTKTHVLLHRMSGKGLAAHYFFPRQPCIFGDKMV
SIQITLNNTTDRKIENIHIGEKKLPIGMKMHVFNPIDSLEPEGSITVSMGIDFCDSTQTA
SFQLCTKDDCFNVNIQPPVGELLLPVAMSEKDFKKEQGVLTGMNETSAVIIAAPQNFTPS
VIFQKVVNVANVGAVPSGQDNIHRFAAKTVHSGSLMLVTVELKEGSTAQLIINTEKTVIG
SVLLRELKPVLSQG
>sp|Q9Y2T2|AP3M1_HUMAN AP-3 complex subunit mu-1 OS=Homo sapiens GN=AP3M1 PE=1 SV=1
MIHSLFLINCSGDIFLEKHWKSVVSQSVCDYFFEAQEKAADVENVPPVISTPHHYLISIY
RDKLFFVSVIQTEVPPLFVIEFLHRVADTFQDYFGECSEAAIKDNVVIVYELLEEMLDNG
FPLATESNILKELIKPPTILRSVVNSITGSSNVGDTLPTGQLSNIPWRRAGVKYTNNEAY
FDVVEEIDAIIDKSGSTVFAEIQGVIDACIKLSGMPDLSLSFMNPRLLDDVSFHPCIRFK
RWESERVLSFIPPDGNFRLISYRVSSQNLVAIPVYVKHSISFKENSSCGRFDITIGPKQN
MGKTIEGITVTVHMPKVVLNMNLTPTQGSYTFDPVTKVLTWDVGKITPQKLPSLKGLVNL
QSGAPKPEENPSLNIQFKIQQLAISGLKVNRLDMYGEKYKPFKGVKYVTKAGKFQVRT
>sp|Q92572|AP3S1_HUMAN AP-3 complex subunit sigma-1 OS=Homo sapiens GN=AP3S1 PE=1 SV=1
MIKAILIFNNHGKPRLSKFYQPYSEDTQQQIIRETFHLVSKRDENVCNFLEGGLLIGGSD
NKLIYRHYATLYFVFCVDSSESELGILDLIQVFVETLDKCFENVCELDLIFHVDKVHNIL
AEMVMGGMVLETNMNEIVTQIDAQNKLEKSEAGLAGAPARAVSAVKNMNLPEIPRNINIG
DISIKVPNLPSFK
>sp|P59780|AP3S2_HUMAN AP-3 complex subunit sigma-2 OS=Homo sapiens GN=AP3S2 PE=2 SV=1
MIQAILVFNNHGKPRLVRFYQRFPEEIQQQIVRETFHLVLKRDDNICNFLEGGSLIGGSD
YKLIYRHYATLYFVFCVDSSESELGILDLIQVFVETLDKCFENVCELDLIFHMDKVHYIL
QEVVMGGMVLETNMNEIVAQIEAQNRLEKSEGGLSAAPARAVSAVKNINLPEIPRNINIG
DLNIKVPNLSQFV
>sp|Q2VPB7|AP5B1_HUMAN AP-5 complex subunit beta-1 OS=Homo sapiens GN=AP5B1 PE=1 SV=4
MGPLSRDAWAQRLGAFRASPSAFMAGPEGEDLGRDLLSDLRSEKLSEQTKVSLLALSMEY
PAQLWPDASAAEVAATSLLDTLVLLPPRPSALRRPLLLAATTALAAGGALGPTSGASCRL
LPLLLGLAAGSDLGRGFVPASEQRPLQATACECLRELESCKPGLLGGSLGLLRGLLGQEG
PVQPLSLLLALALRNTLVLQSRVGAGLGGLLTDKVSPTGGGPWDWTLVEEGDGRLQPQAP
SWPAAEEGEGERSLTAREHSPEEARELRAAVIQLLDTSYLLTPVAQAQLLWLLGWALRGL
QGQPPALFKPQLVRLLGTAQLTLLHAMLALKAAFGEALFTAQDEALLLRRLTLAAQHPAL
PPPTHLFYLHCVLSFPENWPLGPEGEEAAPLLLGPQLCRGLLPSLLHDPMALLARLHLLC
LLCAEEEEEEKGQLPSPRHYLEELLAGLRQRAALDGGPRALATLCFQASYLVACCLAGQP
TVLTPLIHGLAQLYQARPMLAPHFVDLLDQVDSELREPLKVVLRQVVVSRPGRDEALCWH
LQMLAKVADGDAQSATLNFLQAAAAHCTNWDLQQGLLRVCRALLRAGVRGGLVDLLQVLA
RQLEDPDGRDHARLYYILLAHLAAPKLGVALGPSLAAPALASSLVAENQGFVAALMVQEA
PALVRLSLGSHRVKGPLPVLKLQPEALEPIYSLELRFRVEGQLYAPLEAVHVPCLCPGRP
ARPLLLPLQPRCPAPARLDVHALYTTSTGLTCHAHLPPLFVNFADLFLPFPQPPEGAGLG
FFEELWDSCLPEGAESRVWCPLGPQGLEGLVSRHLEPFVVVAQPPTSYCVAIHLPPDSKL
LLRLEAALADGVPVALRTDDWAVLPLAGDYLRGLAAAV
>sp|Q9P2R3|ANFY1_HUMAN Rabankyrin-5 OS=Homo sapiens GN=ANKFY1 PE=1 SV=2
MAEEEVAKLEKHLMLLRQEYVKLQKKLAETEKRCALLAAQANKESSSESFISRLLAIVAD
LYEQEQYSDLKIKVGDRHISAHKFVLAARSDSWSLANLSSTKELDLSDANPEVTMTMLRW
IYTDELEFREDDVFLTELMKLANRFQLQLLRERCEKGVMSLVNVRNCIRFYQTAEELNAS
TLMNYCAEIIASHWDDLRKEDFSSMSAQLLYKMIKSKTEYPLHKAIKVEREDVVFLYLIE

MDSQLPGKLNEADHNGDLALDLALSRRLESIATTLVSHKADVDMVDKSGWSLLHKGIQRG
DLFAATFLIKNGAFVNAATLGAQETPLHLVALYSSKKHSADVMSEMAQIAEALLQAGANP
NMQDSKGRTPLHVSIMAGNEYVFSQLLQCKQLDLELKDHEGSTALWLAVQHITVSSDQSV
NPFEDVPVVNGTSFDENSFAARLIQRGSHTDAPDTATGNCLLQRAAGAGNEAAALFLATN
GAHVNHRNKWGETPLHTACRHGLANLTAELLQQGANPNLQTEEALPLPKEAASLTSLADS
VHLQTPLHMAIAYNHPDVVSVILEQKANALHATNNLQIIPDFSLKDSRDQTVLGLALWTG
MHTIAAQLLGSGAAINDTMSDGQTLLHMAIQRQDSKSALFLLEHQADINVRTQDGETALQ
LAIRNQLPLVVDAICTRGADMSVPDEKGNPPLWLALANNLEDIASTLVRHGCDATCWGPG
PGGCLQTLLHRAIDENNEPTACFLIRSGCDVNSPRQPGANGEGEEEARDGQTPLHLAASW
GLEETVQCLLEFGANVNAQDAEGRTPIHVAISSQHGVIIQLLVSHPDIHLNVRDRQGLTP
FACAMTFKNNKSAEAILKRESGAAEQVDNKGRNFLHVAVQNSDIESVLFLISVHANVNSR
VQDASKLTPLHLAVQAGSEIIVRNLLLAGAKVNELTKHRQTALHLAAQQDLPTICSVLLE
NGVDFAAVDENGNNALHLAVMHGRLNNIRVLLTECTVDAEAFNLRGQSPLHILGQYGKEN
AAAIFDLFLECMPGYPLDKPDADGSTVLLLAYMKGNANLCRAIVRSGARLGVNNNQGVNI
FNYQVATKQLLFRLLDMLSKEPPWCDGSYCYECTARFGVTTRKHHCRHCGRLLCHKCSTK
EIPIIKFDLNKPVRVCNICFDVLTLGGVS
>sp|P03950|ANGI_HUMAN Angiogenin OS=Homo sapiens GN=ANG PE=1 SV=1
MVMGLGVLLLVFVLGLGLTPPTLAQDNSRYTHFLTQHYDAKPQGRDDRYCESIMRRRGLT
SPCKDINTFIHGNKRSIKAICENKNGNPHRENLRISKSSFQVTTCKLHGGSPWPPCQYRA
TAGFRNVVVACENGLPVHLDQSIFRRP
>sp|Q8NI99|ANGL6_HUMAN Angiopoietin-related protein 6 OS=Homo sapiens GN=ANGPTL6 PE=1 SV=1
MGKPWLRALQLLLLLGASWARAGAPRCTYTFVLPPQKFTGAVCWSGPASTRATPEAANAS
ELAALRMRVGRHEELLRELQRLAAADGAVAGEVRALRKESRGLSARLGQLRAQLQHEAGP
GAGPGADLGAEPAAALALLGERVLNASAEAQRAAARFHQLDVKFRELAQLVTQQSSLIAR
LERLCPGGAGGQQQVLPPPPLVPVVPVRLVGSTSDTSRMLDPAPEPQRDQTQRQQEPMAS
PMPAGHPAVPTKPVGPWQDCAEARQAGHEQSGVYELRVGRHVVSVWCEQQLEGGGWTVIQ
RRQDGSVNFFTTWQHYKAGFGRPDGEYWLGLEPVYQLTSRGDHELLVLLEDWGGRGARAH
YDGFSLEPESDHYRLRLGQYHGDAGDSLSWHNDKPFSTVDRDRDSYSGNCALYQRGGWWY
HACAHSNLNGVWHHGGHYRSRYQDGVYWAEFRGGAYSLRKAAMLIRPLKL
>sp|Q9NQ90|ANO2_HUMAN Anoctamin-2 OS=Homo sapiens GN=ANO2 PE=1 SV=2
MATPGPRDIPLLPGSPRRLSPQAGSRGGQGPKHGQQCLKMPGPRAPGLQGGSNRDPGQPC
GGESTRSSSVINNYLDANEPVSLEARLSRMHFHDSQRKVDYVLAYHYRKRGVHLAQGFPG
HSLAIVSNGETGKEPHAGGPGDIELGPLDALEEERKEQREEFEHNLMEAGLELEKDLENK
SQGSIFVRIHAPWQVLAREAEFLKIKVPTKKEMYEIKAGGSIAKKFSAALQKLSSHLQPR
VPEHSNNKMKNLSYPFSREKMYLYNIQEKDTFFDNATRSRIVHEILKRTACSRANNTMGI
NSLIANNIYEAAYPLHDGEYDSPEDDMNDRKLLYQEWARYGVFYKFQPIDLIRKYFGEKI
GLYFAWLGLYTSFLIPSSVIGVIVFLYGCATIEEDIPSREMCDQQNAFTMCPLCDKSCDY
WNLSSACGTAQASHLFDNPATVFFSIFMALWATMFLENWKRLQMRLGYFWDLTGIEEEEE
RAQEHSRPEYETKVREKMLKESNQSAVQKLETNTTECGDEDDEDKLTWKDRFPGYLMNFA
SILFMIALTFSIVFGVIVYRITTAAALSLNKATRSNVRVTVTATAVIINLVVILILDEIY
GAVAKWLTKIEVPKTEQTFEERLILKAFLLKFVNAYSPIFYVAFFKGRFVGRPGSYVYVF
DGYRMEECAPGGCLMELCIQLSIIMLGKQLIQNNIFEIGVPKLKKLFRKLKDETEAGETD
SAHSKHPEQWDLDYSLEPYTGLTPEYMEMIIQFGFVTLFVASFPLAPVFALLNNVIEVRL

DAKKFVTELRRPDAVRTKDIGIWFDILSGIGKFSVISNAFVIAITSDFIPRLVYQYSYSH
NGTLHGFVNHTLSFFNVSQLKEGTQPENSQFDQEVQFCRFKDYREPPWAPNPYEFSKQYW
FILSARLAFVIIFQNLVMFLSVLVDWMIPDIPTDISDQIKKEKSLLVDFFLKEEHEKLKL
MDEPALRSPGGGDRSRSRAASSAPSGQSQLGSMMSSGSQHTNV
>sp|Q9BYT9|ANO3_HUMAN Anoctamin-3 OS=Homo sapiens GN=ANO3 PE=1 SV=2
MVHHSGSIQSFKQQKGMNISKSEITKETSLKPSRRSLPCLAQSYAYSKSLSQSTSLFQST
ESESQAPTSITLISTDKAEQVNTEENKNDSVLRCSFADLSDFCLALGKDKDYTDESEHAT
YDRSRLINDFVIKDKSEFKTKLSKNDMNYIASSGPLFKDGKKRIDYILVYRKTNIQYDKR
NTFEKNLRAEGLMLEKEPAIASPDIMFIKIHIPWDTLCKYAERLNIRMPFRKKCYYTDGR
SKSMGRMQTYFRRIKNWMAQNPMVLDKSAFPDLEESDCYTGPFSRARIHHFIINNKDTFF
SNATRSRIVYHMLERTKYENGISKVGIRKLINNGSYIAAFPPHEGAYKSSQPIKTHGPQN
NRHLLYERWARWGMWYKHQPLDLIRLYFGEKIGLYFAWLGWYTGMLIPAAIVGLCVFFYG
LFTMNNSQVSQEICKATEVFMCPLCDKNCSLQRLNDSCIYAKVTYLFDNGGTVFFAIFMA
IWATVFLEFWKRRRSILTYTWDLIEWEEEEETLRPQFEAKYYKMEIVNPITGKPEPHQPS
SDKVTRLLVSVSGIFFMISLVITAVFGVVVYRLVVMEQFASFKWNFIKQYWQFATSAAAV
CINFIIIMLLNLAYEKIAYLLTNLEYPRTESEWENSFALKMFLFQFVNLNSSIFYIAFFL
GRFVGHPGKYNKLFDRWRLEECHPSGCLIDLCLQMGVIMFLKQIWNNFMELGYPLIQNWW
SRHKIKRGIHDASIPQWENDWNLQPMNLHGLMDEYLEMVLQFGFTTIFVAAFPLAPLLAL
LNNIIEIRLDAYKFVTQWRRPLPARATDIGIWLGILEGIGILAVITNAFVIAITSDYIPR
FVYEYKYGPCANHVEPSENCLKGYVNNSLSFFDLSELGMGKSGYCRYRDYRGPPWSSKPY
EFTLQYWHILAARLAFIIVFEHLVFGIKSFIAYLIPDVPKGLHDRIRREKYLVQEMMYEA
ELEHLQQQRRKSGQPVHHEWP
>sp|A1A5B4|ANO9_HUMAN Anoctamin-9 OS=Homo sapiens GN=ANO9 PE=2 SV=3
MQGEESLRILVEPEGDSFPLMEISTCETEASEQWDYVLVAQRHTQRDPRQARQQQFLEEL
RRKGFHIKVIRDQKQVFFGIRADNSVFGLYRTLLLEPEGPAPHAELAAPTTIPVTTSLRI
RIVNFVVMNNKTSAGETFEDLMKDGVFEARFPLHKGEGRLKKTWARWRHMFREQPVDEIR
NYFGEKVALYFVWLGWYTYMLVPAALTGLLVFLSGFSLFEASQISKEICEAHDILMCPLG
DHSRRYQRLSETCTFAKLTHLFDNDGTVVFAIFMALWATVFLEIWKRQRARVVLHWDLYV
WDEEQEEMALQLINCPDYKLRPYQHSYLRSTVILVLTLLMICLMIGMAHVLVVYRVLASA
LFSSSAVPFLEEQVTTAVVVTGALVHYVTIIIMTKINRCVALKLCDFEMPRTFSERESRF
TIRFFTLQFFTHFSSLIYIAFILGRINGHPGKSTRLAGLWKLEECHASGCMMDLFVQMAI
IMGLKQTLSNCVEYLVPWVTHKCRSLRASESGHLPRDPELRDWRRNYLLNPVNTFSLFDE
FMEMMIQYGFTTIFVAAFPLAPLLALFSNLVEIRLDAIKMVWLQRRLVPRKAKDIGTWLQ
VLETIGVLAVIANGMVIAFTSEFIPRVVYKYRYSPCLKEGNSTVDCLKGYVNHSLSVFHT
KDFQDPDGIEGSENVTLCRYRDYRNPPDYNFSEQFWFLLAIRLAFVILFEHVALCIKLIA
AWFVPDIPQSVKNKVLEVKYQRLREKMWHGRQRLGGVGAGSRPPMPAHPTPASIFSARST
DV
>sp|Q6UB99|ANR11_HUMAN Ankyrin repeat domain-containing protein 11 OS=Homo sapiens GN=ANKRD11 PE=1 SV=3
MPKGGCPKAPQQEELPLSSDMVEKQTGKKDKDKVSLTKTPKLERGDGGKEVRERASKRKL
PFTAGANGEQKDSDTEKQGPERKRIKKEPVTRKAGLLFGMGLSGIRAGYPLSERQQVALL
MQMTAEESANSPVDTTPKHPSQSTVCQKGTPNSASKTKDKVNKRNERGETRLHRAAIRGD
ARRIKELISEGADVNVKDFAGWTALHEACNRGYYDVAKQLLAAGAEVNTKGLDDDTPLHD
AANNGHYKVVKLLLRYGGNPQQSNRKGETPLKVANSPTMVNLLLGKGTYTSSEESSTESS

EEEDAPSFAPSSSVDGNNTDSEFEKGLKHKAKNPEPQKATAPVKDEYEFDEDDEQDRVPP
VDDKHLLKKDYRKETKSNSFISIPKMEVKSYTKNNTIAPKKASHRILSDTSDEEDASVTV
GTGEKLRLSAHTILPGSKTREPSNAKQQKEKNKVKKKRKKETKGREVRFGKRSDKFCSSE
SESESSESGEDDRDSLGSSGCLKGSPLVLKDPSLFSSLSASSTSSHGSSAAQKQNPSHTD
QHTKHWRTDNWKTISSPAWSEVSSLSDSTRTRLTSESDYSSEGSSVESLKPVRKRQEHRK
RASLSEKKSPFLSSAEGAVPKLDKEGKVVKKHKTKHKHKNKEKGQCSISQELKLKSFTYE
YEDSKQKSDKAILLENDLSTENKLKVLKHDRDHFKKEEKLSKMKLEEKEWLFKDEKSLKR
IKDTNKDISRSFREEKDRSNKAEKERSLKEKSPKEEKLRLYKEERKKKSKDRPSKLEKKN
DLKEDKISKEKEKIFKEDKEKLKKEKVYREDSAFDEYCNKNQFLENEDTKFSLSDDQRDR
WFSDLSDSSFDFKGEDSWDSPVTDYRDMKSDSVAKLILETVKEDSKERRRDSRAREKRDY
REPFFRKKDRDYLDKNSEKRKEQTEKHKSVPGYLSEKDKKRRESAEAGRDRKDALESCKE
RRDGRAKPEEAHREELKECGCESGFKDKSDGDFGKGLEPWERHHPAREKEKKDGPDKERK
EKTKPERYKEKSSDKDKSEKSILEKCQKDKEFDKCFKEKKDTKEKHKDTHGKDKERKASL
DQGKEKKEKAFPGIISEDFSEKKDDKKGKEKSWYIADIFTDESEDDRDSCMGSGFKMGEA
SDLPRTDGLQEKEEGREAYASDRHRKSSDKQHPERQKDKEPRDRRKDRGAADAGRDKKEK
VFEKHKEKKDKESTEKYKDRKDRASVDSTQDKKNKQKLPEKAEKKHAAEDKAKSKHKEKS
DKEHSKERKSSRSADAEKSLLEKLEEEALHEYREDSNDKISEVSSDSFTDRGQEPGLTAF
LEVSFTEPPGDDKPRESACLPEKLKEKERHRHSSSSSKKSHDRERAKKEKAEKKEKGEDY
KEGGSRKDSGQYEKDFLEADAYGVSYNMKADIEDELDKTIELFSTEKKDKNDSEREPSKK
IEKELKPYGSSAINILKEKKKREKHREKWRDEKERHRDRHADGLLRHHRDELLRHHRDEQ
KPATRDKDSPPRVLKDKSRDEGPRLGDAKLKEKFKDGAEKEKGDPVKMSNGNDKVAPSKD
PGKKDARPREKLLGDGDLMMTSFERMLSQKDLEIEERHKRHKERMKQMEKLRHRSGDPKL
KEKAKPADDGRKKGLDIPAKKPPGLDPPFKDKKLKESTPIPPAAENKLHPASGADSKDWL
AGPHMKEVLPASPRPDQSRPTGVPTPTSVLSCPSYEEVMHTPRTPSCSADDYADLVFDCA
DSQHSTPVPTAPTSACSPSFFDRFSVASSGLSENASQAPARPLSTNLYRSVSVDIRRTPE
EEFSVGDKLFRQQSVPAASSYDSPMPPSMEDRAPLPPVPAEKFACLSPGYYSPDYGLPSP
KVDALHCPPAAVVTVTPSPEGVFSSLQAKPSPSPRAELLVPSLEGALPPDLDTSEDQQAT
AAIIPPEPSYLEPLDEGPFSAVITEEPVEWAHPSEQALASSLIGGTSENPVSWPVGSDLL
LKSPQRFPESPKRFCPADPLHSAAPGPFSASEAPYPAPPASPAPYALPVAEPGLEDVKDG
VDAVPAAISTSEAAPYAPPSGLESFFSNCKSLPEAPLDVAPEPACVAAVAQVEALGPLEN
SFLDGSRGLSHLGQVEPVPWADAFAGPEDDLDLGPFSLPELPLQTKDAADGEAEPVEESL
APPEEMPPGAPGVINGGDVSTVVAEEPPALPPDQASTRLPAELEPEPSGEPKLDVALEAA
VEAETVPEERARGDPDSSVEPAPVPPEQRPLGSGDQGAEAEGPPAASLCAPDGPAPNTVA
QAQAADGAGPEDDTEASRAAAPAEGPPGGIQPEAAEPKPTAEAPKAPRVEEIPQRMTRNR
AQMLANQSKQGPPPSEKECAPTPAPVTRAKARGSEDDDAQAQHPRKRRFQRSTQQLQQQL
NTSTQQTREVIQQTLAAIVDAIKLDAIEPYHSDRANPYFEYLQIRKKIEEKRKILCCITP
QAPQCYAEYVTYTGSYLLDGKPLSKLHIPVIAPPPSLAEPLKELFRQQEAVRGKLRLQHS
IEREKLIVSCEQEILRVHCRAARTIANQAVPFSACTMLLDSEVYNMPLESQGDENKSVRD
RFNARQFISWLQDVDDKYDRMKTCLLMRQQHEAAALNAVQRMEWQLKVQELDPAGHKSLC
VNEVPSFYVPMVDVNDDFVLLPA
>sp|Q6UB98|ANR12_HUMAN Ankyrin repeat domain-containing protein 12 OS=Homo sapiens GN=ANKRD12 PE=1 SV=3
MPKSGFTKPIQSENSDSDSNMVEKPYGRKSKDKIASYSKTPKIERSDVSKEMKEKSSMKR
KLPFTISPSRNEERDSDTDSDPGHTSENWGERLISSYRTYSEKEGPEKKKTKKEAGNKKS

TPVSILFGYPLSERKQMALLMQMTARDNSPDSTPNHPSQTTPAQKKTPSSSSRQKDKVNK
RNERGETPLHMAAIRGDVKQVKELISLGANVNVKDFAGWTPLHEACNVGYYDVAKILIAA
GADVNTQGLDDDTPLHDSASSGHRDIVKLLLRHGGNPFQANKHGERPVDVAETEELELLL
KREVPLSDDDESYTDSEEAQSVNPSSVDENIDSETEKDSLICESKQILPSKTPLPSALDE
YEFKDDDDEEINKMIDDRHILRKEQRKENEPEAEKTHLFAKQEKAFYPKSFKSKKQKPSR
VLYSSTESSDEEALQNKKISTSCSVIPETSNSDMQTKKEYVVSGEHKQKGKVKRKLKNQN
KNKENQELKQEKEGKENTRITNLTVNTGLDCSEKTREEGNFRKSFSPKDDTSLHLFHIST
GKSPKHSCGLSEKQSTPLKQEHTKTCLSPGSSEMSLQPDLVRYDNTESEFLPESSSVKSC
KHKEKSKHQKDFHLEFGEKSNAKIKDEDHSPTFENSDCTLKKMDKEGKTLKKHKLKHKER
EKEKHKKEIEGEKEKYKTKDSAKELQRSVEFDREFWKENFFKSDETEDLFLNMEHESLTL
EKKSKLEKNIKDDKSTKEKHVSKERNFKEERDKIKKESEKSFREEKIKDLKEERENIPTD
KDSEFTSLGMSAIEESIGLHLVEKEIDIEKQEKHIKESKEKPEKRSQIKEKDIEKMERKT
FEKEKKIKHEHKSEKDKLDLSECVDKIKEKDKLYSHHTEKCHKEGEKSKNTAAIKKTDDR
EKSREKMDRKHDKEKPEKERHLAESKEKHLMEKKNKQSDNSEYSKSEKGKNKEKDRELDK
KEKSRDKESINITNSKHIQEEKKSSIVDGNKAQHEKPLSLKEKTKDEPLKTPDGKEKDKK
DKDIDRYKERDKHKDKIQINSLLKLKSEADKPKPKSSPASKDTRPKEKRLVNDDLMQTSF
ERMLSLKDLEIEQWHKKHKEKIKQKEKERLRNRNCLELKIKDKEKTKHTPTESKNKELTR
SKSSEVTDAYTKEKQPKDAVSNRSQSVDTKNVMTLGKSSFVSDNSLNRSPRSENEKPGLS
SRSVSMISVASSEDSCHTTVTTPRPPVEYDSDFMLESSESQMSFSQSPFLSIAKSPALHE
RELDSLADLPERIKPPYANRLSTSHLRSSSVEDVKLIISEGRPTIEVRRCSMPSVICEHT
KQFQTISEESNQGSLLTVPGDTSPSPKPEVFSNVPERDLSNVSNIHSSFATSPTGASNSK
YVSADRNLIKNTAPVNTVMDSPVHLEPSSQVGVIQNKSWEMPVDRLETLSTRDFICPNSN
IPDQESSLQSFCNSENKVLKENADFLSLRQTELPGNSCAQDPASFMPPQQPCSFPSQSLS
DAESISKHMSLSYVANQEPGILQQKNAVQIISSALDTDNESTKDTENTFVLGDVQKTDAF
VPVYSDSTIQEASPNFEKAYTLPVLPSEKDFNGSDASTQLNTHYAFSKLTYKSSSGHEVE
NSTTDTQVISHEKENKLESLVLTHLSRCDSDLCEMNAGMPKGNLNEQDPKHCPESEKCLL
SIEDEESQQSILSSLENHSQQSTQPEMHKYGQLVKVELEENAEDDKTENQIPQRMTRNKA
NTMANQSKQILASCTLLSEKDSESSSPRGRIRLTEDDDPQIHHPRKRKVSRVPQPVQVSP
SLLQAKEKTQQSLAAIVDSLKLDEIQPYSSERANPYFEYLHIRKKIEEKRKLLCSVIPQA
PQYYDEYVTFNGSYLLDGNPLSKICIPTITPPPSLSDPLKELFRQQEVVRMKLRLQHSIE
REKLIVSNEQEVLRVHYRAARTLANQTLPFSACTVLLDAEVYNVPLDSQSDDSKTSVRDR
FNARQFMSWLQDVDDKFDKLKTCLLMRQQHEAAALNAVQRLEWQLKLQELDPATYKSISI
YEIQEFYVPLVDVNDDFELTPI
>sp|Q5TZF3|ANR45_HUMAN Ankyrin repeat domain-containing protein 45 OS=Homo sapiens GN=ANKRD45 PE=1 SV=1
MVTEEAGARVWVFLELMESEGPPESESSEFFSQQEEENEEEEAQEPEETGPKNPLLQPAL
TGDVEGLQKIFEDPENPHHEQAMQLLLEEDIVGRNLLYAACMAGQSDVIRALAKYGVNLN
EKTTRGYTLLHCAAAWGRLETLKALVELDVDIEALNFREERARDVAARYSQTECVEFLDW
ADARLTLKKYIAKVSLAVTDTEKGSGKLLKEDKNTILSACRAKNEWLETHTEASINELFE
QRQQLEDIVTPIFTKMTTPCQVKSAKSVTSHDQKRSQDDTSN
>sp|Q8WVL7|ANR49_HUMAN Ankyrin repeat domain-containing protein 49 OS=Homo sapiens GN=ANKRD49 PE=1 SV=1
MEKEKGNDDGIPDQENSLDFSEHFNQLELLETHGHLIPTGTQSLWVGNSDEDEEQDDKNE
EWYRLQEKKMEKDPSRLLLWAAEKNRLTTVRRLLSEKATHVNTRDEDEYTPLHRAAYSGH

LDIVQELIAQGADVHAVTVDGWTPLHSACKWNNTRVASFLLQHDADINAQTKGLLTPLHL
AAGNRDSKDTLELLLMNRYVKPGLKNNLEETAFDIARRTSIYHYLFEIVEGCTNSSPQS
>sp|Q9BZ19|ANR60_HUMAN Ankyrin repeat domain-containing protein 60 OS=Homo sapiens GN=ANKRD60 PE=3 SV=3
MTRGRAWGMRRAAAGAGGARAAGPTGGASRLHPNAGRRSGARAGAQGCGGPRVGSADSRA
LPAQPLACARGRSQRLVCDPKAASALPDLAPDVFVLRVRLEETGEMFRVANCRGDMTVRE
LKEELDLMVGIPFNLQRLQYLDEGVLMDDTTLKFHDVVPGGIISLCIWHHDGWTELVLAA
VEGDPSKLSCLGLTEDSFYRTANSEHFEGEKWKHWTSQRAFVALYVASHRGHFDAVQYLL
EHGASCLSRSPLGRTPLHVAAAMGRSDCIILLLQHGASIHDRDAKGETPISIAHRLNHTL
SERQMVLLHRIAKSGIRDLNDLVMKNALQRVKSGFRSEKMTMTPH
>sp|Q9H9E1|ANRA2_HUMAN Ankyrin repeat family A protein 2 OS=Homo sapiens GN=ANKRA2 PE=1 SV=1
MDTSTNLDIGAQLIVEECPSTYSLTGMPDIKIEHPLDPNSEEGSAQGVAMGMKFILPNRF
DMNVCSRFVKSLNEEDSKNIQDQVNSDLEVASVLFKAECNIHTSPSPGIQVRHVYTPSTT
KHFSPIKQSTTLTNKHRGNEVSTTPLLANSLSVHQLAAQGEMLYLATRIEQENVINHTDE
EGFTPLMWAAAHGQIAVVEFLLQNGADPQLLGKGRESALSLACSKGYTDIVKMLLDCGVD
VNEYDWNGGTPLLYAVHGNHVKCVKMLLESGADPTIETDSGYNSMDLAVALGYRSVQQVI
ESHLLKLLQNIKE
>sp|P07355|ANXA2_HUMAN Annexin A2 OS=Homo sapiens GN=ANXA2 PE=1 SV=2
MSTVHEILCKLSLEGDHSTPPSAYGSVKAYTNFDAERDALNIETAIKTKGVDEVTIVNIL
TNRSNAQRQDIAFAYQRRTKKELASALKSALSGHLETVILGLLKTPAQYDASELKASMKG
LGTDEDSLIEIICSRTNQELQEINRVYKEMYKTDLEKDIISDTSGDFRKLMVALAKGRRA
EDGSVIDYELIDQDARDLYDAGVKRKGTDVPKWISIMTERSVPHLQKVFDRYKSYSPYDM
LESIRKEVKGDLENAFLNLVQCIQNKPLYFADRLYDSMKGKGTRDKVLIRIMVSRSEVDM
LKIRSEFKRKYGKSLYYYIQQDTKGDYQKALLYLCGGDD
>sp|P09525|ANXA4_HUMAN Annexin A4 OS=Homo sapiens GN=ANXA4 PE=1 SV=4
MATKGGTVKAASGFNAMEDAQTLRKAMKGLGTDEDAIISVLAYRNTAQRQEIRTAYKSTI
GRDLIDDLKSELSGNFEQVIVGMMTPTVLYDVQELRRAMKGAGTDEGCLIEILASRTPEE
IRRISQTYQQQYGRSLEDDIRSDTSFMFQRVLVSLSAGGRDEGNYLDDALVRQDAQDLYE
AGEKKWGTDEVKFLTVLCSRNRNHLLHVFDEYKRISQKDIEQSIKSETSGSFEDALLAIV
KCMRNKSAYFAEKLYKSMKGLGTDDNTLIRVMVSRAEIDMLDIRAHFKRLYGKSLYSFIK
GDTSGDYRKVLLVLCGGDD
>sp|P13928|ANXA8_HUMAN Annexin A8 OS=Homo sapiens GN=ANXA8 PE=1 SV=3
MAWWKSWIEQEGVTVKSSSHFNPDPDAETLYKAMKGIGTNEQAIIDVLTKRSNTQRQQIA
KSFKAQFGKDLTETLKSELSGKFERLIVALMYPPYRYEAKELHDAMKGLGTKEGVIIEIL
ASRTKNQLREIMKAYEEDYGSSLEEDIQADTSGYLERILVCLLQGSRDDVSSFVDPGLAL
QDAQDLYAAGEKIRGTDEMKFITILCTRSATHLLRVFEEYEKIANKSIEDSIKSETHGSL
EEAMLTVVKCTQNLHSYFAERLYYAMKGAGTRDGTLIRNIVSRSEIDLNLIKCHFKKMYG
KTLSSMIMEDTSGDYKNALLSLVGSDP
>sp|P21397|AOFA_HUMAN Amine oxidase [flavin-containing] A OS=Homo sapiens GN=MAOA PE=1 SV=1
MENQEKASIAGHMFDVVVIGGGISGLSAAKLLTEYGVSVLVLEARDRVGGRTYTIRNEHV
DYVDVGGAYVGPTQNRILRLSKELGIETYKVNVSERLVQYVKGKTYPFRGAFPPVWNPIA
YLDYNNLWRTIDNMGKEIPTDAPWEAQHADKWDKMTMKELIDKICWTKTARRFAYLFVNI

NVTSEPHEVSALWFLWYVKQCGGTTRIFSVTNGGQERKFVGGSGQVSERIMDLLGDQVKL
NHPVTHVDQSSDNIIIETLNHEHYECKYVINAIPPTLTAKIHFRPELPAERNQLIQRLPM
GAVIKCMMYYKEAFWKKKDYCGCMIIEDEDAPISITLDDTKPDGSLPAIMGFILARKADR
LAKLHKEIRKKKICELYAKVLGSQEALHPVHYEEKNWCEEQYSGGCYTAYFPPGIMTQYG
RVIRQPVGRIFFAGTETATKWSGYMEGAVEAGERAAREVLNGLGKVTEKDIWVQEPESKD
VPAVEITHTFWERNLPSVSGLLKIIGFSTSVTALGFVLYKYKLLPRS
>sp|P27338|AOFB_HUMAN Amine oxidase [flavin-containing] B OS=Homo sapiens GN=MAOB PE=1 SV=3
MSNKCDVVVVGGGISGMAAAKLLHDSGLNVVVLEARDRVGGRTYTLRNQKVKYVDLGGSY
VGPTQNRILRLAKELGLETYKVNEVERLIHHVKGKSYPFRGPFPPVWNPITYLDHNNFWR
TMDDMGREIPSDAPWKAPLAEEWDNMTMKELLDKLCWTESAKQLATLFVNLCVTAETHEV
SALWFLWYVKQCGGTTRIISTTNGGQERKFVGGSGQVSERIMDLLGDRVKLERPVIYIDQ
TRENVLVETLNHEMYEAKYVISAIPPTLGMKIHFNPPLPMMRNQMITRVPLGSVIKCIVY
YKEPFWRKKDYCGTMIIDGEEAPVAYTLDDTKPEGNYAAIMGFILAHKARKLARLTKEER
LKKLCELYAKVLGSLEALEPVHYEEKNWCEEQYSGGCYTTYFPPGILTQYGRVLRQPVDR
IYFAGTETATHWSGYMEGAVEAGERAAREILHAMGKIPEDEIWQSEPESVDVPAQPITTT
FLERHLPSVPGLLRLIGLTTIFSATALGFLAHKRGLLVRV
>sp|Q9Y6Q5|AP1M2_HUMAN AP-1 complex subunit mu-2 OS=Homo sapiens GN=AP1M2 PE=1 SV=4
MSASAVFILDVKGKPLISRNYKGDVAMSKIEHFMPLLVQREEEGALAPLLSHGQVHFLWI
KHSNLYLVATTSKNANASLVYSFLYKTIEVFCEYFKELEEESIRDNFVIVYELLDELMDF
GFPQTTDSKILQEYITQQSNKLETGKSRVPPTVTNAVSWRSEGIKYKKNEVFIDVIESVN
LLVNANGSVLLSEIVGTIKLKVFLSGMPELRLGLNDRVLFELTGRSKNKSVELEDVKFHQ
CVRLSRFDNDRTISFIPPDGDFELMSYRLSTQVKPLIWIESVIEKFSHSRVEIMVKAKGQ
FKKQSVANGVEISVPVPSDADSPRFKTSVGSAKYVPERNVVIWSIKSFPGGKEYLMRAHF
GLPSVEKEEVEGRPPIGVKFEIPYFTVSGIQVRYMKIIEKSGYQALPWVRYITQSGDYQL
RTS
>sp|P05549|AP2A_HUMAN Transcription factor AP-2-alpha OS=Homo sapiens GN=TFAP2A PE=1 SV=1
MLWKLTDNIKYEDCEDRHDGTSNGTARLPQLGTVGQSPYTSAPPLSHTPNADFQPPYFPP
PYQPIYPQSQDPYSHVNDPYSLNPLHAQPQPQHPGWPGQRQSQESGLLHTHRGLPHQLSG
LDPRRDYRRHEDLLHGPHALSSGLGDLSIHSLPHAIEEVPHVEDPGINIPDQTVIKKGPV
SLSKSNSNAVSAIPINKDNLFGGVVNPNEVFCSVPGRLSLLSSTSKYKVTVAEVQRRLSP
PECLNASLLGGVLRRAKSKNGGRSLREKLDKIGLNLPAGRRKAANVTLLTSLVEGEAVHL
ARDFGYVCETEFPAKAVAEFLNRQHSDPNEQVTRKNMLLATKQICKEFTDLLAQDRSPLG
NSRPNPILEPGIQSCLTHFNLISHGFGSPAVCAAVTALQNYLTEALKAMDKMYLSNNPNS
HTDNNAKSSDKEEKHRK
>sp|Q92481|AP2B_HUMAN Transcription factor AP-2-beta OS=Homo sapiens GN=TFAP2B PE=1 SV=2
MHSPPRDQAAIMLWKLVENVKYEDIYEDRHDGVPSHSSRLSQLGSVSQGPYSSAPPLSHT
PSSDFQPPYFPPPYQPLPYHQSQDPYSHVNDPYSLNPLHQPQQHPWGQRQRQEVGSEAGS
LLPQPRAALPQLSGLDPRRDYHSVRRPDVLLHSAHHGLDAGMGDSLSLHGLGHPGMEDVQ
SVEDANNSGMNLLDQSVIKKVPVPPKSVTSLMMNKDGFLGGMSVNTGEVFCSVPGRLSLL
SSTSKYKVTVGEVQRRLSPPECLNASLLGGVLRRAKSKNGGRSLRERLEKIGLNLPAGRR
KAANVTLLTSLVEGEAVHLARDFGYICETEFPAKAVSEYLNRQHTDPSDLHSRKNMLLAT
KQLCKEFTDLLAQDRTPIGNSRPSPILEPGIQSCLTHFSLITHGFGAPAICAALTALQNY
LTEALKGMDKMFLNNTTTNRHTSGEGPGSKTGDKEEKHRK

>sp|Q9UJX3|APC7_HUMAN Anaphase-promoting complex subunit 7 OS=Homo sapiens GN=ANAPC7 PE=1 SV=4
MDPGDAAILESSLRILYRLFESVLPPLPAALQSRMNVIDHVRDMAAAGLHSNVRLLSSLL
LTMSNNNPELFSPPQKYQLLVYHADSLFHDKEYRNAVSKYTMALQQKKALSKTSKVRPST
GNSASTPQSQCLPSEIEVKYKMAECYTMLKQDKDAIAILDGIPSRQRTPKINMMLANLYK
KAGQERPSVTSYKEVLRQCPLALDAILGLLSLSVKGAEVASMTMNVIQTVPNLDWLSVWI
KAYAFVHTGDNSRAISTICSLEKKSLLRDNVDLLGSLADLYFRAGDNKNSVLKFEQAQML
DPYLIKGMDVYGYLLAREGRLEDVENLGCRLFNISDQHAEPWVVSGCHSFYSKRYSRALY
LGAKAIQLNSNSVQALLLKGAALRNMGRVQEAIIHFREAIRLAPCRLDCYEGLIECYLAS
NSIREAMVMANNVYKTLGANAQTLTLLATVCLEDPVTQEKAKTLLDKALTQRPDYIKAVV
KKAELLSREQKYEDGIALLRNALANQSDCVLHRILGDFLVAVNEYQEAMDQYSIALSLDP
NDQKSLEGMQKMEKEESPTDATQEEDVDDMEGSGEEGDLEGSDSEAAQWADQEQWFGMQ
>sp|Q9UBZ4|APEX2_HUMAN DNA-(apurinic or apyrimidinic site) lyase 2 OS=Homo sapiens GN=APEX2 PE=1 SV=1
MLRVVSWNINGIRRPLQGVANQEPSNCAAVAVGRILDELDADIVCLQETKVTRDALTEPL
AIVEGYNSYFSFSRNRSGYSGVATFCKDNATPVAAEEGLSGLFATQNGDVGCYGNMDEFT
QEELRALDSEGRALLTQHKIRTWEGKEKTLTLINVYCPHADPGRPERLVFKMRFYRLLQI
RAEALLAAGSHVIILGDLNTAHRPIDHWDAVNLECFEEDPGRKWMDSLLSNLGCQSASHV
GPFIDSYRCFQPKQEGAFTCWSAVTGARHLNYGSRLDYVLGDRTLVIDTFQASFLLPEVM
GSDHCPVGAVLSVSSVPAKQCPPLCTRFLPEFAGTQLKILRFLVPLEQSPVLEQSTLQHN
NQTRVQTCQNKAQVRSTRPQPSQVGSSRGQKNLKSYFQPSPSCPQASPDIELPSLPLMSA
LMTPKTPEEKAVAKVVKGQAKTSEAKDEKELRTSFWKSVLAGPLRTPLCGGHREPCVMRT
VKKPGPNLGRRFYMCARPRGPPTDPSSRCNFFLWSRPS
>sp|A6NM10|AQ12B_HUMAN Aquaporin-12B OS=Homo sapiens GN=AQP12B PE=2 SV=1
MAGLNVSLSFFFATFTLCEAARRASKALLPVGAYEVFAREAMRTLVELGPWAGDFGPDLL
LTLLFLLFLAHGVTLDGASANPTVSLQEFLMAEESLPGTLLKLAAQGLGMQAACTLTRLC
WAWELSDLHLLQSLMAQSCSSALRTSVPHGALVEAACAFCFHLTLLHLRHSPPAYSGPAV
ALLVTVTAYTAGPFTSAFFNPALAASVTFACSGHTLLEYVQVYWLGPLTGMVLAVLLHQG
RLPHLFQRNLFYGQKNKYRAPRGKPAPASGDTQTPAKGSSVREPGRSGVEGPHSS
>sp|Q8NBQ7|AQP11_HUMAN Aquaporin-11 OS=Homo sapiens GN=AQP11 PE=2 SV=1
MSPLLGLRSELQDTCTSLGLMLSVVLLMGLARVVARQQLHRPVAHAFVLEFLATFQLCCC
THELQLLSEQHPAHPTWTLTLVYFFSLVHGLTLVGTSSNPCGVMMQMMLGGMSPETGAVR
LLAQLVSALCSRYCTSALWSLGLTQYHVSERSFACKNPIRVDLLKAVITEAVCSFLFHSA
LLHFQEVRTKLRIHLLAALITFLVYAGGSLTGAVFNPALALSLHFMCFDEAFPQFFIVYW
LAPSLGILLMILMFSFFLPWLHNNHTINKKE
>sp|P29972|AQP1_HUMAN Aquaporin-1 OS=Homo sapiens GN=AQP1 PE=1 SV=3
MASEFKKKLFWRAVVAEFLATTLFVFISIGSALGFKYPVGNNQTAVQDNVKVSLAFGLSI
ATLAQSVGHISGAHLNPAVTLGLLLSCQISIFRALMYIIAQCVGAIVATAILSGITSSLT
GNSLGRNDLADGVNSGQGLGIEIIGTLQLVLCVLATTDRRRRDLGGSAPLAIGLSVALGH
LLAIDYTGCGINPARSFGSAVITHNFSNHWIFWVGPFIGGALAVLIYDFILAPRSSDLTD
RVKVWTSGQVEEYDLDADDINSRVEMKPK
>sp|O14520|AQP7_HUMAN Aquaporin-7 OS=Homo sapiens GN=AQP7 PE=1 SV=1
MVQASGHRRSTRGSKMVSWSVIAKIQEILQRKMVREFLAEFMSTYVMMVFGLGSVAHMVL
NKKYGSYLGVNLGFGFGVTMGVHVAGRISGAHMNAAVTFANCALGRVPWRKFPVYVLGQF

LGSFLAAATIYSLFYTAILHFSGGQLMVTGPVATAGIFATYLPDHMTLWRGFLNEAWLTG
MLQLCLFAITDQENNPALPGTEALVIGILVVIIGVSLGMNTGYAINPSRDLPPRIFTFIA
GWGKQVFSNGENWWWVPVVAPLLGAYLGGIIYLVFIGSTIPREPLKLEDSVAYEDHGITV
LPKMGSHEPTISPLTPVSVSPANRSSVHPAPPLHESMALEHF
>sp|O94778|AQP8_HUMAN Aquaporin-8 OS=Homo sapiens GN=AQP8 PE=2 SV=2
MSGEIAMCEPEFGNDKAREPSVGGRWRVSWYERFVQPCLVELLGSALFIFIGCLSVIENG
TDTGLLQPALAHGLALGLVIATLGNISGGHFNPAVSLAAMLIGGLNLVMLLPYWVSQLLG
GMLGAALAKAVSPEERFWNASGAAFVTVQEQGQVAGALVAEIILTTLLALAVCMGAINEK
TKGPLAPFSIGFAVTVDILAGGPVSGGCMNPARAFGPAVVANHWNFHWIYWLGPLLAGLL
VGLLIRCFIGDGKTRLILKAR
>sp|O43315|AQP9_HUMAN Aquaporin-9 OS=Homo sapiens GN=AQP9 PE=2 SV=2
MQPEGAEKGKSFKQRLVLKSSLAKETLSEFLGTFILIVLGCGCVAQAILSRGRFGGVITI
NVGFSMAVAMAIYVAGGVSGGHINPAVSLAMCLFGRMKWFKLPFYVGAQFLGAFVGAATV
FGIYYDGLMSFAGGKLLIVGENATAHIFATYPAPYLSLANAFADQVVATMILLIIVFAIF
DSRNLGAPRGLEPIAIGLLIIVIASSLGLNSGCAMNPARDLSPRLFTALAGWGFEVFRAG
NNFWWIPVVGPLVGAVIGGLIYVLVIEIHHPEPDSVFKTEQSEDKPEKYELSVIM
>sp|Q5H913|AR13A_HUMAN ADP-ribosylation factor-like protein 13A OS=Homo sapiens GN=ARL13A PE=2 SV=2
MFRLLSSCCSCLRTTEETRRNVTIPIIGLNNSGKTVLVEAFQKLLPSKTDHCMKSELTTL
LLDEYELSIYDLNGDLKGREAWPNYYAQAHGLVFVLDSSDIRRMQEVKIILTHLLSDKRV
AGKPILILANKQDKKKALMPCDIIDYLLLKKLVKENKCPCRVEPCSAIRNLERRNHQPIV
EGLRWLLAVIDTCQLPPTSSISISKNNTGSGERCSSHSFSTRTGMSKEKRQHLEQCSIEA
KPLKSILQKEGTRLWSKKNMSVTFALDEPMKEGECSRRMRAQNTTKLCYN
>sp|P61204|ARF3_HUMAN ADP-ribosylation factor 3 OS=Homo sapiens GN=ARF3 PE=1 SV=2
MGNIFGNLLKSLIGKKEMRILMVGLDAAGKTTILYKLKLGEIVTTIPTIGFNVETVEYKN
ISFTVWDVGGQDKIRPLWRHYFQNTQGLIFVVDSNDRERVNEAREELMRMLAEDELRDAV
LLVFANKQDLPNAMNAAEITDKLGLHSLRHRNWYIQATCATSGDGLYEGLDWLANQLKNK
K
>sp|Q13795|ARFRP_HUMAN ADP-ribosylation factor-related protein 1 OS=Homo sapiens GN=ARFRP1 PE=1 SV=1
MYTLLSGLYKYMFQKDEYCILILGLDNAGKTTFLEQSKTRFNKNYKGMSLSKITTTVGLN
IGTVDVGKARLMFWDLGGQEELQSLWDKYYAECHGVIYVIDSTDEERLAESKQAFEKVVT
SEALCGVPVLVLANKQDVETCLSIPDIKTAFSDCTSKIGRRDCLTQACSALTGKGVREGI
EWMVKCVVRNVHRPPRQRDIT
>sp|Q8N1W1|ARG28_HUMAN Rho guanine nucleotide exchange factor 28 OS=Homo sapiens GN=ARHGEF28 PE=1 SV=3
MELSCSEAPLYGQMMIYAKFDKNVYLPEDAEFYFTYDGSHQRHVMIAERIEDNVLQSSVP
GHGLQETVTVSVCLCSEGYSPVTMGSGSVTYYVDNMACRLARLLVTQANRLTACSHQTLLT
PFALTAGALPALDEELVLALTHLELPLEWTVLGSSSLEVSSHRESLLHLAMRWGLAKLSQ
FFLCLPGGVQALALPNEEGATPLDLALREGHSKLVEDVTNFQGRWSPSFSRVQLSEEASL
HYIHSSETLTLTLNHTAEHLLEADIKLFRKYFWDRAFLVKAFEPEARPEERTAMPSSGAE
TEEEIKNSVSSRSAAEKEDIKRVKSLVVQHNEHEDQHSLDLDRSFDILKKSKPPSTLLAA
GRLSDMLNGGDEVYANCMVIDQVGDLDISYINIEGITATTSPESRGCTLWPQSSKHTLPT
ETSPSVYPLSENVEGTAHTEAQQSFMSPSSSCASNLNLSFGWHGFEKEQSHLKKRSSSLD

ALDADSEGEGHSEPSHICYTPGSQSSSRTGIPSGDELDSFETNTEPDFNISRAESLPLSS
NLQSKESLLSGVRSRSYSCSSPKISLGKTRLVRELTVCSSSEEQRAYSLSEPPRENRIQE
EEWDKYIIPAKSESEKYKVSRTFSFLMNRMTSPRNKSKTKSKDAKDKEKLNRHQFAPGTF
SGVLQCLVCDKTLLGKESLQCSNCNANVHKGCKDAAPACTKKFQEKYNKNKPQTILGNSS
FRDIPQPGLSLHPSSSVPVGLPTGRRETVGQVHPLSRSVPGTTLESFRRSATSLESESDH
NSCRSRSHSDELLQSMGSSPSTESFIMEDVVDSSLWSDLSSDAQEFEAESWSLVVDPSFC
NRQEKDVIKRQDVIFELMQTEMHHIQTLFIMSEIFRKGMKEELQLDHSTVDKIFPCLDEL
LEIHRHFFYSMKERRQESCAGSDRNFVIDRIGDILVQQFSEENASKMKKIYGEFCCHHKE
AVNLFKELQQNKKFQNFIKLRNSNLLARRRGIPECILLVTQRITKYPVLVERILQYTKER
TEEHKDLRKALCLIKDMIATVDLKVNEYEKNQKWLEILNKIENKTYTKLKNGHVFRKQAL
MSEERTLLYDGLVYWKTATGRFKDILALLLTDVLLFLQEKDQKYIFAAVDQKPSVISLQK
LIAREVANEERGMFLISASSAGPEMYEIHTNSKEERNNWMRRIQQAVESCPEEKGGRTSE
SDEDKRKAEARVAKIQQCQEILTNQDQQICAYLEEKLHIYAELGELSGFEDVHLEPHLLI
KPDPGEPPQAASLLAAALKEAESLQVAVKASQMGAVSQSCEDSCGDSVLADTLSSHDVPG
SPTASLVTGGREGRGCSDVDPGIQGVVTDLAVSDAGEKVECRNFPGSSQSEIIQAIQNLT
RLLYSLQAALTIQDSHIEIHRLVLQQQEGLSLGHSILRGGPLQDQKSRDADRQHEELANV
HQLQHQLQQEQRRWLRRCEQQQRAQATRESWLQERERECQSQEELLLRSRGELDLQLQEY
QHSLERLREGQRLVEREQARMRAQQSLLGHWKHGRQRSLPAVLLPGGPEVMELNRSESLC
HENSFFINEALVQMSFNTFNKLNPSVIHQDATYPTTQSHSDLVRTSEHQVDLKVDPSQPS
NVSHKLWTAAGSGHQILPFHESSKDSCKNDLDTSHTESPTPHDSNSHRPQLQAFITEAKL
NLPTRTMTRQDGETGDGAKENIVYL
>sp|Q96CM8|ACSF2_HUMAN Acyl-CoA synthetase family member 2, mitochondrial OS=Homo sapiens GN=ACSF2 PE=1 SV=2
MAVYVGMLRLGRLCAGSSGVLGARAALSRSWQEARLQGVRFLSSREVDRMVSTPIGGLSY
VQGCTKKHLNSKTVGQCLETTAQRVPEREALVVLHEDVRLTFAQLKEEVDKAASGLLSIG
LCKGDRLGMWGPNSYAWVLMQLATAQAGIILVSVNPAYQAMELEYVLKKVGCKALVFPKQ
FKTQQYYNVLKQICPEVENAQPGALKSQRLPDLTTVISVDAPLPGTLLLDEVVAAGSTRQ
HLDQLQYNQQFLSCHDPINIQFTSGTTGSPKGATLSHYNIVNNSNILGERLKLHEKTPEQ
LRMILPNPLYHCLGSVAGTMMCLMYGATLILASPIFNGKKALEAISRERGTFLYGTPTMF
VDILNQPDFSSYDISTMCGGVIAGSPAPPELIRAIINKINMKDLVVAYGTTENSPVTFAH
FPEDTVEQKAESVGRIMPHTEARIMNMEAGTLAKLNTPGELCIRGYCVMLGYWGEPQKTE
EAVDQDKWYWTGDVATMNEQGFCKIVGRSKDMIIRGGENIYPAELEDFFHTHPKVQEVQV
VGVKDDRMGEEICACIRLKDGEETTVEEIKAFCKGKISHFKIPKYIVFVTNYPLTISGKI
QKFKLREQMERHLNL
>sp|Q9UKU0|ACSL6_HUMAN Long-chain-fatty-acid--CoA ligase 6 OS=Homo sapiens GN=ACSL6 PE=2 SV=4
MQTQEILRILRLPELGDLGQFFRSLSATTLVSMGALAAILAYWFTHRPKALQPPCNLLMQ
SEEVEDSGGARRSVIGSGPQLLTHYYDDARTMYQVFRRGLSISGNGPCLGFRKPKQPYQW
LSYQEVADRAEFLGSGLLQHNCKACTDQFIGVFAQNRPEWIIVELACYTYSMVVVPLYDT
LGPGAIRYIINTADISTVIVDKPQKAVLLLEHVERKETPGLKLIILMDPFEEALKERGQK
CGVVIKSMQAVEDCGQENHQAPVPPQPDDLSIVCFTSGTTGNPKGAMLTHGNVVADFSGF
LKVTEKVIFPRQDDVLISFLPLAHMFERVIQSVVYCHGGRVGFFQGDIRLLSDDMKALCP
TIFPVVPRLLNRMYDKIFSQANTPLKRWLLEFAAKRKQAEVRSGIIRNDSIWDELFFNKI
QASLGGCVRMIVTGAAPASPTVLGFLRAALGCQVYEGYGQTECTAGCTFTTPGDWTSGHV

GAPLPCNHIKLVDVEELNYWACKGEGEICVRGPNVFKGYLKDPDRTKEALDSDGWLHTGD
IGKWLPAGTLKIIDRKKHIFKLAQGEYVAPEKIENIYIRSQPVAQIYVHGDSLKAFLVGI
VVPDPEVMPSWAQKRGIEGTYADLCTNKDLKKAILEDMVRLGKESGLHSFEQVKAIHIHS
DMFSVQNGLLTPTLKAKRPELREYFKKQIEELYSISM
>sp|Q08AH1|ACSM1_HUMAN Acyl-coenzyme A synthetase ACSM1, mitochondrial OS=Homo sapiens GN=ACSM1 PE=1 SV=1
MQWLMRFRTLWGIHKSFHNIHPAPSQLRCRSLSEFGAPRWNDYEVPEEFNFASYVLDYWA
QKEKEGKRGPNPAFWWVNGQGDEVKWSFREMGDLTRRVANVFTQTCGLQQGDHLALMLPR
VPEWWLVAVGCMRTGIIFIPATILLKAKDILYRLQLSKAKGIVTIDALASEVDSIASQCP
SLKTKLLVSDHSREGWLDFRSLVKSASPEHTCVKSKTLDPMVIFFTSGTTGFPKMAKHSH
GLALQPSFPGSRKLRSLKTSDVSWCLSDSGWIVATIWTLVEPWTAGCTVFIHHLPQFDTK
VIIQTLLKYPINHFWGVSSIYRMILQQDFTSIRFPALEHCYTGGEVVLPKDQEEWKRRTG
LLLYENYGQSETGLICATYWGMKIKPGFMGKATPPYDVQVIDDKGSILPPNTEGNIGIRI
KPVRPVSLFMCYEGDPEKTAKVECGDFYNTGDRGKMDEEGYICFLGRSDDIINASGYRIG
PAEVESALVEHPAVAESAVVGSPDPIRGEVVKAFIVLTPQFLSHDKDQLTKELQQHVKSV
TAPYKYPRKVEFVSELPKTITGKIERKELRKKETGQM
>sp|Q03154|ACY1_HUMAN Aminoacylase-1 OS=Homo sapiens GN=ACY1 PE=1 SV=1
MTSKGPEEEHPSVTLFRQYLRIRTVQPKPDYGAAVAFFEETARQLGLGCQKVEVAPGYVV
TVLTWPGTNPTLSSILLNSHTDVVPVFKEHWSHDPFEAFKDSEGYIYARGAQDMKCVSIQ
YLEAVRRLKVEGHRFPRTIHMTFVPDEEVGGHQGMELFVQRPEFHALRAGFALDEGIANP
TDAFTVFYSERSPWWVRVTSTGRPGHASRFMEDTAAEKLHKVVNSILAFREKEWQRLQSN
PHLKEGSVTSVNLTKLEGGVAYNVIPATMSASFDFRVAPDVDFKAFEEQLQSWCQAAGEG
VTLEFAQKWMHPQVTPTDDSNPWWAAFSRVCKDMNLTLEPEIMPAATDNRYIRAVGVPAL
GFSPMNRTPVLLHDHDERLHEAVFLRGVDIYTRLLPALASVPALPSDS
>sp|P78536|ADA17_HUMAN Disintegrin and metalloproteinase domain-containing protein 17 OS=Homo sapiens GN=ADAM17 PE=1 SV=1
MRQSLLFLTSVVPFVLAPRPPDDPGFGPHQRLEKLDSLLSDYDILSLSNIQQHSVRKRDL
QTSTHVETLLTFSALKRHFKLYLTSSTERFSQNFKVVVVDGKNESEYTVKWQDFFTGHVV
GEPDSRVLAHIRDDDVIIRINTDGAEYNIEPLWRFVNDTKDKRMLVYKSEDIKNVSRLQS
PKVCGYLKVDNEELLPKGLVDREPPEELVHRVKRRADPDPMKNTCKLLVVADHRFYRYMG
RGEESTTTNYLIELIDRVDDIYRNTSWDNAGFKGYGIQIEQIRILKSPQEVKPGEKHYNM
AKSYPNEEKDAWDVKMLLEQFSFDIAEEASKVCLAHLFTYQDFDMGTLGLAYVGSPRANS
HGGVCPKAYYSPVGKKNIYLNSGLTSTKNYGKTILTKEADLVTTHELGHNFGAEHDPDGL
AECAPNEDQGGKYVMYPIAVSGDHENNKMFSNCSKQSIYKTIESKAQECFQERSNKVCGN
SRVDEGEECDPGIMYLNNDTCCNSDCTLKEGVQCSDRNSPCCKNCQFETAQKKCQEAINA
TCKGVSYCTGNSSECPPPGNAEDDTVCLDLGKCKDGKCIPFCEREQQLESCACNETDNSC
KVCCRDLSGRCVPYVDAEQKNLFLRKGKPCTVGFCDMNGKCEKRVQDVIERFWDFIDQLS
INTFGKFLADNIVGSVLVFSLIFWIPFSILVHCVDKKLDKQYESLSLFHPSNVEMLSSMD
SASVRIIKPFPAPQTPGRLQPAPVIPSAPAAPKLDHQRMDTIQEDPSTDSHMDEDGFEKD
PFPNSSTAAKSFEDLTDHPVTRSEKAASFKLQRQNRVDSKETEC
>sp|P18825|ADA2C_HUMAN Alpha-2C adrenergic receptor OS=Homo sapiens GN=ADRA2C PE=2 SV=2
MASPALAAALAVAAAAGPNASGAGERGSGGVANASGASWGPPRGQYSAGAVAGLAAVVGF
LIVFTVVGNVLVVIAVLTSRALRAPQNLFLVSLASADILVATLVMPFSLANELMAYWYFG
QVWCGVYLALDVLFCTSSIVHLCAISLDRYWSVTQAVEYNLKRTPRRVKATIVAVWLISA

VISFPPLVSLYRQPDGAAYPQCGLNDETWYILSSCIGSFFAPCLIMGLVYARIYRVAKLR
TRTLSEKRAPVGPDGASPTTENGLGAAAGAGENGHCAPPPADVEPDESSAAAERRRRRGA
LRRGGRRRAGAEGGAGGADGQGAGPGAAESGALTASRSPGPGGRLSRASSRSVEFFLSRR
RRARSSVCRRKVAQAREKRFTFVLAVVMGVFVLCWFPFFFSYSLYGICREACQVPGPLFK
FFFWIGYCNSSLNPVIYTVFNQDFRRSFKHILFRRRRRGFRQ
>sp|Q99965|ADAM2_HUMAN Disintegrin and metalloproteinase domain-containing protein 2 OS=Homo sapiens GN=ADAM2 PE=2 SV=2
MWRVLFLLSGLGGLRMDSNFDSLPVQITVPEKIRSIIKEGIESQASYKIVIEGKPYTVNL
MQKNFLPHNFRVYSYSGTGIMKPLDQDFQNFCHYQGYIEGYPKSVVMVSTCTGLRGVLQF
ENVSYGIEPLESSVGFEHVIYQVKHKKADVSLYNEKDIESRDLSFKLQSVEPQQDFAKYI
EMHVIVEKQLYNHMGSDTTVVAQKVFQLIGLTNAIFVSFNITIILSSLELWIDENKIATT
GEANELLHTFLRWKTSYLVLRPHDVAFLLVYREKSNYVGATFQGKMCDANYAGGVVLHPR
TISLESLAVILAQLLSLSMGITYDDINKCQCSGAVCIMNPEAIHFSGVKIFSNCSFEDFA
HFISKQKSQCLHNQPRLDPFFKQQAVCGNAKLEAGEECDCGTEQDCALIGETCCDIATCR
FKAGSNCAEGPCCENCLFMSKERMCRPSFEECDLPEYCNGSSASCPENHYVQTGHPCGLN
QWICIDGVCMSGDKQCTDTFGKEVEFGPSECYSHLNSKTDVSGNCGISDSGYTQCEADNL
QCGKLICKYVGKFLLQIPRATIIYANISGHLCIAVEFASDHADSQKMWIKDGTSCGSNKV
CRNQRCVSSSYLGYDCTTDKCNDRGVCNNKKHCHCSASYLPPDCSVQSDLWPGGSIDSGN
FPPVAIPARLPERRYIENIYHSKPMRWPFFLFIPFFIIFCVLIAIMVKVNFQRKKWRTED
YSSDEQPESESEPKG
>sp|Q9UEY8|ADDG_HUMAN Gamma-adducin OS=Homo sapiens GN=ADD3 PE=1 SV=1
MSSDASQGVITTPPPPSMPHKERYFDRINENDPEYIRERNMSPDLRQDFNMMEQRKRVTQ
ILQSPAFREDLECLIQEQMKKGHNPTGLLALQQIADYIMANSFSGFSSPPLSLGMVTPIN
DLPGADTSSYVKGEKLTRCKLASLYRLVDLFGWAHLANTYISVRISKEQDHIIIIPRGLS
FSEATASNLVKVNIIGEVVDQGSTNLKIDHTGFSPHAAIYSTRPDVKCVIHIHTLATAAV
SSMKCGILPISQESLLLGDVAYYDYQGSLEEQEERIQLQKVLGPSCKVLVLRNHGVVALG
ETLEEAFHYIFNVQLACEIQVQALAGAGGVDNLHVLDFQKYKAFTYTVAASGGGGVNMGS
HQKWKVGEIEFEGLMRTLDNLGYRTGYAYRHPLIREKPRHKSDVEIPATVTAFSFEDDTV
PLSPLKYMAQRQQREKTRWLNSPNTYMKVNVPEESRNGETSPRTKITWMKAEDSSKVSGG
TPIKIEDPNQFVPLNTNPNEVLEKRNKIREQNRYDLKTAGPQSQLLAGIVVDKPPSTMQF
EDDDHGPPAPPNPFSHLTEGELEEYKRTIERKQQGLEDAEQELLSDDASSVSQIQSQTQS
PQNVPEKLEENHELFSKSFISMEVPVMVVNGKDDMHDVEDELAKRVSRLSTSTTIENIEI
TIKSPEKIEEVLSPEGSPSKSPSKKKKKFRTPSFLKKNKKKEKVEA
>sp|P08588|ADRB1_HUMAN Beta-1 adrenergic receptor OS=Homo sapiens GN=ADRB1 PE=1 SV=2
MGAGVLVLGASEPGNLSSAAPLPDGAATAARLLVPASPPASLLPPASESPEPLSQQWTAG
MGLLMALIVLLIVAGNVLVIVAIAKTPRLQTLTNLFIMSLASADLVMGLLVVPFGATIVV
WGRWEYGSFFCELWTSVDVLCVTASIETLCVIALDRYLAITSPFRYQSLLTRARARGLVC
TVWAISALVSFLPILMHWWRAESDEARRCYNDPKCCDFVTNRAYAIASSVVSFYVPLCIM
AFVYLRVFREAQKQVKKIDSCERRFLGGPARPPSPSPSPVPAPAPPPGPPRPAAAAATAP
LANGRAGKRRPSRLVALREQKALKTLGIIMGVFTLCWLPFFLANVVKAFHRELVPDRLFV
FFNWLGYANSAFNPIIYCRSPDFRKAFQRLLCCARRAARRRHATHGDRPRASGCLARPGP
PPSPGAASDDDDDDVVGATPPARLLEPWAGCNGGAAADSDSSLDEPCRPGFASESKV
>sp|P13945|ADRB3_HUMAN Beta-3 adrenergic receptor OS=Homo sapiens GN=ADRB3 PE=1 SV=3
MAPWPHENSSLAPWPDLPTLAPNTANTSGLPGVPWEAALAGALLALAVLATVGGNLLVIV

AIAWTPRLQTMTNVFVTSLAAADLVMGLLVVPPAATLALTGHWPLGATGCELWTSVDVLC
VTASIETLCALAVDRYLAVTNPLRYGALVTKRCARTAVVLVWVVSAAVSFAPIMSQWWRV
GADAEAQRCHSNPRCCAFASNMPYVLLSSSVSFYLPLLVMLFVYARVFVVATRQLRLLRG
ELGRFPPEESPPAPSRSLAPAPVGTCAPPEGVPACGRRPARLLPLREHRALCTLGLIMGT
FTLCWLPFFLANVLRALGGPSLVPGPAFLALNWLGYANSAFNPLIYCRSPDFRSAFRRLL
CRCGRRLPPEPCAAARPALFPSGVPAARSSPAQPRLCQRLDGASWGVS
>sp|Q16186|ADRM1_HUMAN Proteasomal ubiquitin receptor ADRM1 OS=Homo sapiens GN=ADRM1 PE=1 SV=2
MTTSGALFPSLVPGSRGASNKYLVEFRAGKMSLKGTTVTPDKRKGLVYIQQTDDSLIHFC
WKDRTSGNVEDDLIIFPDDCEFKRVPQCPSGRVYVLKFKAGSKRLFFWMQEPKTDQDEEH
CRKVNEYLNNPPMPGALGASGSSGHELSALGGEGGLQSLLGNMSHSQLMQLIGPAGLGGL
GGLGALTGPGLASLLGSSGPPGSSSSSSSSRSQSAAVTPSSTTSSTRATPAPSAPAAASAT
SPSPAPSSGNGASTAASPTQPIQLSDLQSILATMNVPAGPAGGQQVDLASVLTPEIMAPI
LANADVQERLLPYLPSGESLPQTADEIQNTLTSPQFQQALGMFSAALASGQLGPLMCQFG
LPAEAVEAANKGDVEAFAKAMQNNAKPEQKEGDTKDKKDEEEDMSLD
>sp|Q5I7T1|AG10B_HUMAN Putative Dol-P-Glc:Glc(2)Man(9)GlcNAc(2)-PP-Dol alpha-1,2-glucosyltransferase OS=Homo sapiens GN=ALG10B PE=1 SV=2
MAQLEGYCFSAALSCTFLVSCLLFSAFSRALREPYMDEIFHLPQAQRYCEGHFSLSQWDP
MITTLPGLYLVSVGVVKPAIWIFAWSEHVVCSIGMLRFVNLLFSVGNFYLLYLLFHKVQP
RNKAASSIQRVLSTLTLAVFPTLYFFNFLYYTEAGSMFFTLFAYLMCLYGNHKTSAFLGF
CGFMFRQTNIIWAVFCAGNVIAQKLTEAWKTELQKKEDRLPPIKGPFAEFRKILQFLLAY
SMSFKNLSMLFCLTWPYILLGFLFCAFVVVNGGIVIGDRSSHEACLHFPQLFYFFSFTLF
FSFPHLLSPSKIKTFLSLVWKHGILFLVVTLVSVFLVWKFTYAHKYLLADNRHYTFYVWK
RVFQRYAILKYLLVPAYIFAGWSIADSLKSKPIFWNLMFFICLFIVIVPQKLLEFRYFIL
PYVIYRLNITLPPTSRLVCELSCYAIVNFITFYIFLNKTFQWPNSQDIQRFMW
>sp|Q99490|AGAP2_HUMAN Arf-GAP with GTPase, ANK repeat and PH domain-containing protein 2 OS=Homo sapiens GN=AGAP2 PE=1 SV=2
MSRGAGALQRRTTTYLISLTLVKLESVPPPPPSPSAAAVGAPGARGSEPRDPGSPRGAEE
PGKKRHERLFHRQDALWISTSSAGAGGAEPPALSPAPASPARPVSPAPGRRLSLWAAPPG
PPLSGGLSPDSKPGGAPSSSRRPLLSSPSWGGPEPEGRTGGGVPGSSSPHPGTGSRRLKV
APPPPAPKPCKTVTTSGAKAGGGKGAGSRLSWPESEGKPRVKGSKSSAGTGASVSAAATA
AAAGGGGSTASTSGGVGAGAGARGKLSPRKGKSKTLDNSDLHPGPPAGSPPPLTLPPTPS
PATAVTAASAQPPGPAPPITLEPPAPGLKRGREGGRASTRDRKMLKFISGIFTKSTGGPP
GSGPLPGPPSLSSGSGSRELLGAELRASPKAVINSQEWTLSRSIPELRLGVLGDARSGKS
SLIHRFLTGSYQVLEKTESEQYKKEMLVDGQTHLVLIREEAGAPDAKFSGWADAVIFVFS
LEDENSFQAVSRLHGQLSSLRGEGRGGLALALVGTQDRISASSPRVVGDARARALCADMK
RCSYYETCATYGLNVDRVFQEVAQKVVTLRKQQQLLAACKSLPSSPSHSAASTPVAGQAS
NGGHTSDYSSSLPSSPNVGHRELRAEAAAVAGLSTPGSLHRAAKRRTSLFANRRGSDSEK
RSLDSRGETTGSGRAIPIKQSFLLKRSGNSLNKEWKKKYVTLSSNGFLLYHPSINDYIHS
THGKEMDLLRTTVKVPGKRPPRAISAFGPSASINGLVKDMSTVQMGEGLEATTPMPSPSP
SPSSLQPPPDQTSKHLLKPDRNLARALSTDCTPSGDLSPLSREPPPSPMVKKQRRKKLTT
PSKTEGSAGQAEAKRKMWKLKSFGSLRNIYKAEENFEFLIVSSTGQTWHFEAASFEERDA
WVQAIESQILASLQCCESSKVKLRTDSQSEAVAIQAIRNAKGNSICVDCGAPNPTWASLN
LGALICIECSGIHRNLGTHLSRVRSLDLDDWPRELTLVLTAIGNDTANRVWESDTRGRAK

PSRDSSREERESWIRAKYEQLLFLAPLSTSEEPLGRQLWAAVQAQDVATVLLLLAHARHG
PLDTSVEDPQLRSPLHLAAELAHVVITQLLLWYGADVAARDAQGRTALFYARQAGSQLCA
DILLQHGCPGEGGSAATTPSAATTPSITATPSPRRRSSAASVGRADAPVALV
>sp|A6NIR3|AGAP5_HUMAN Arf-GAP with GTPase, ANK repeat and PH domain-containing protein 5 OS=Homo sapiens GN=AGAP5 PE=2 SV=2
MGNILTCCVHPSVSLEFDQQQGSVCPSESEIYEAGAGDRMAGAPMAAAVQPAEVTVEVGE
DLHMHHIRDQEMPEALEFSLSANPEASTIFQRNSQTDALEFNPSANPEASTIFQRNSQTD
VVEIRRSNCTNHVSTERFSQQYSSCSTIFLDDSTASQHYLTMTIISVTLEIPHHITQRDA
DRSLSIPDEQLHSFAVSTVHITKNRNGGGSLNNYSSSIPSTPSTSQEDPQFSVPPTANTP
TPVCKRSMRWSNLFTSEKGSHPDKERKAPENHADTIGSGRAIPIKQGMLLKRSGKWLKTW
KKKYVTLCSNGVLTYYSSLGDYMKNIHKKEIDLRTSTIKVPGKWPSLATSACAPISSSKS
NGLSKDMDTGLGDSICFSPSISSTTSPKLNPPPSPHANKKKHLKKKSTNNFMIVSATGQT
WHFEATTYEERDAWVQAIQSQILASLQSCESSKSKSQLTSQSEAMALQSIQNMRGNAHCV
DYETQNPKWASLNLGVLMCIECSGIHRSLGTRLSRVRSLELDDWPVELRKVMSSIGNDLA
NSIWEGSSQGQTKPSVKSTREEKERWIRSKYEEKLFLAPLPCTELSLGQHLLRATADEDL
QTAILLLAHGSREEVNETCGEGDGCTALHLACRKGNVVLAQLLIWYGVDVMARDAHGNTA
LTYARQASSQECINVLLQYGCPDECV
>sp|Q5VTM2|AGAP9_HUMAN Arf-GAP with GTPase, ANK repeat and PH domain-containing protein 9 OS=Homo sapiens GN=AGAP9 PE=3 SV=2
MFEDVFSDSGNTGNFDRGKKRRLTIIECGCDINMMIDLAKVADLVLMLIDASFGFEMEMF
EFLNICQAHGFPKILGVLTHLDSFKHNKQLKKTKKRLKHRFWTEVYQDKVGLTHELVQSL
ISTYSTIDAKMASSRVTLLSNSKPLGSEAIDNQGVSLEFDQQQGSVCPSESEIYEAGAED
RMAGAPMAAAVQPAEVTVEVGEDLHMHQVRDREMPEVVEIRRSNCTNHCDLGDTSSYHTK
VSTVHIMKKRNGGGSLNNYSSSIPPTPSTSQEDPQFSVPPTANTPTPVCKRSMRWSNLFT
SEKGSDPDKERKAPENHADTIGSGRAIPIKQGMLLKRSGKWLKTWKKKYVTLCSNGVLTY
YSSLGDYMKNIHKKEIDLRTSTIKVPGKWPSLATSACAPISSSKSNGLSKDMDTGLGDSI
CFSPGISSTTSPKLNPPPSPHANKKKHLKKKSTNNFMIVSATGQTWHFEATTYEERDAWV
QAIQSQILASLQSCKSSKSKSQLTSQSEAMALQSIQNMRGNAHCVDCETQNPKWASLNLG
VLMCIECSGIHRSFGTRLSRVRSLELDDWPVELRKVMSSIGNELANSIWEGSSQGQTKPS
IKSTREEKEWWIRSKYEEKLFLAPLPCTELSLGQQLLRATTDEDLQTAILLLAHGSREEV
NETCGEGDGCTALHLACRKGNVVLEQLLTGWTSWPEMPTGTQR
>sp|O75969|AKAP3_HUMAN A-kinase anchor protein 3 OS=Homo sapiens GN=AKAP3 PE=1 SV=2
MSEKVDWLQSQNGVCKVDVYSPGDNQAQDWKMDTSTDPVRVLSWLRRDLEKSTAEFQDVR
FKPGESFGGETSNSGDPHKGFSVDYYNTTTKGTPERLHFEMTHKEIPCQGPRAQLGNGSS
VDEVSFYANRLTNLVIAMARKEINEKIDGSENKCVYQSLYMGNEPTPTKSLSKIASELVN
ETVSACSRNAAPDKAPGSGDRVSGSSQSPPNLKYKSTLKIKESTKERQGPDDKPPSKKSF
FYKEVFESRNGDYAREGGRFFPRERKRFRGQERPDDFTASVSEGIMTYANSVVSDMMVSI
MKTLKIQVKDTTIATILLKKVLLKHAKEVVSDLIDSFLRNLHSVTGTLMTDTQFVSAVKR
TVFSHGSQKATDIMDAMLRKLYNVMFAKKVPEHVRKAQDKAESYSLISMKGMGDPKNRNV
NFAMKSETKLREKMYSEPKSEEETCAKTLGEHIIKEGLTLWHKTQQKECKSLGFQHAAFE
APNTQRKPASDISFEYPEDIGNLSLPPYPPEKPENFMYDSDSWAEDLIVSALLLIQYHLA
QGGRRDARSFVEAAGTTNFPANEPPVAPDESCLKSAPIVGDQEQAEKKDLRSVFFNFIRN
LLSETIFKRDQSPEPKVPEQPVKEDRKLCERPLASSPPRLYEDDETPGALSGLTKMAVSQ
IDGHMSGQMVEHLMNSVMKLCVIIAKSCDASLAELGDDKSGDASRLTSAFPDSLYECLPA

KGTGSAEAVLQNAYQAIHNEMRGTSGQPPEGCAAPTVIVSNHNLTDTVQNKQLQAVLQWV
AASELNVPILYFAGDDEGIQEKLLQLSAAAVDKGCSVGEVLQSVLRYEKERQLNEAVGNV
TPLQLLDWLMVNL
>sp|Q92667|AKAP1_HUMAN A-kinase anchor protein 1, mitochondrial OS=Homo sapiens GN=AKAP1 PE=1 SV=1
MAIQFRSLFPLALPGMLALLGWWWFFSRKKGHVSSHDEQQVEAGAVQLRADPAIKEPLPV
EDVCPKVVSTPPSVTEPPEKELSTVSKLPAEPPALLQTHPPCRRSESSGILPNTTDMRLR
PGTRRDDSTKLELALTGGEAKSIPLECPLSSPKGVLFSSKSAEVCKQDSPFSRVPRKVQP
GYPVVPAEKRSSGERARETGGAEGTGDAVLGEKVLEEALLSREHVLELENSKGPSLASLE
GEEDKGKSSSSQVVGPVQEEEYVAEKLPSRFIESAHTELAKDDAAPAPPVADAKAQDRGV
EGELGNEESLDRNEEGLDRNEEGLDRNEESLDRNEEGLDRNEEIKRAAFQIISQVISEAT
EQVLATTVGKVAGRVCQASQLQGQKEESCVPVHQKTVLGPDTAEPATAEAAVAPPDAGLP
LPGLPAEGSPPPKTYVSCLKSLLSSPTKDSKPNISAHHISLASCLALTTPSEELPDRAGI
LVEDATCVTCMSDSSQSVPLVASPGHCSDSFSTSGLEDSCTETSSSPRDKAITPPLPEST
VPFSNGVLKGELSDLGAEDGWTMDAEADHSGGSDRNSMDSVDSCCSLKKTESFQNAQAGS
NPKKVDLIIWEIEVPKHLVGRLIGKQGRYVSFLKQTSGAKIYISTLPYTQSVQICHIEGS
QHHVDKALNLIGKKFKELNLTNIYAPPLPSLALPSLPMTSWLMLPDGITVEVIVVNQVNA
GHLFVQQHTHPTFHALRSLDQQMYLCYSQPGIPTLPTPVEITVICAAPGADGAWWRAQVV
ASYEETNEVEIRYVDYGGYKRVKVDVLRQIRSDFVTLPFQGAEVLLDSVMPLSDDDQFSP
EADAAMSEMTGNTALLAQVTSYSPTGLPLIQLWSVVGDEVVLINRSLVERGLAQWVDSYY
TSL
>sp|Q5T2L2|AKCL1_HUMAN Putative aldo-keto reductase family 1 member C8 OS=Homo sapiens GN=AKR1C8P PE=5 SV=2
MMTDLKQSHSVRLNDGPFMPVLGFGTYAPDHTPKSQAAEATKVAIDVGFRHIDSAYLYQN
EEEVGQAIWEKIADGTVKREEIFYTIKLWATFFRAELVHPALERSLKKLGPDYVDLFIIH
VPFAMKGSS
>sp|P24298|ALAT1_HUMAN Alanine aminotransferase 1 OS=Homo sapiens GN=GPT PE=1 SV=3
MASSTGDRSQAVRHGLRAKVLTLDGMNPRVRRVEYAVRGPIVQRALELEQELRQGVKKPF
TEVIRANIGDAQAMGQRPITFLRQVLALCVNPDLLSSPNFPDDAKKRAERILQACGGHSL
GAYSVSSGIQLIREDVARYIERRDGGIPADPNNVFLSTGASDAIVTVLKLLVAGEGHTRT
GVLIPIPQYPLYSATLAELGAVQVDYYLDEERAWALDVAELHRALGQARDHCRPRALCVI
NPGNPTGQVQTRECIEAVIRFAFEERLFLLADEVYQDNVYAAGSQFHSFKKVLMEMGPPY
AGQQELASFHSTSKGYMGECGFRGGYVEVVNMDAAVQQQMLKLMSVRLCPPVPGQALLDL
VVSPPAPTDPSFAQFQAEKQAVLAELAAKAKLTEQVFNEAPGISCNPVQGAMYSFPRVQL
PPRAVERAQELGLAPDMFFCLRLLEETGICVVPGSGFGQREGTYHFRMTILPPLEKLRLL
LEKLSRFHAKFTLEYS
>sp|Q8TD30|ALAT2_HUMAN Alanine aminotransferase 2 OS=Homo sapiens GN=GPT2 PE=1 SV=1
MQRAAALVRRGCGPRTPSSWGRSQSSAAAEASAVLKVRPERSRRERILTLESMNPQVKAV
EYAVRGPIVLKAGEIELELQRGIKKPFTEVIRANIGDAQAMGQQPITFLRQVMALCTYPN
LLDSPSFPEDAKKRARRILQACGGNSLGSYSASQGVNCIREDVAAYITRRDGGVPADPDN
IYLTTGASDGISTILKILVSGGGKSRTGVMIPIPQYPLYSAVISELDAIQVNYYLDEENC
WALNVNELRRAVQEAKDHCDPKVLCIINPGNPTGQVQSRKCIEDVIHFAWEEKLFLLADE
VYQDNVYSPDCRFHSFKKVLYEMGPEYSSNVELASFHSTSKGYMGECGYRGGYMEVINLH
PEIKGQLVKLLSVRLCPPVSGQAAMDIVVNPPVAGEESFEQFSREKESVLGNLAKKAKLT

EDLFNQVPGIHCNPLQGAMYAFPRIFIPAKAVEAAQAHQMAPDMFYCMKLLEETGICVVP
GSGFGQREGTYHFRMTILPPVEKLKTVLQKVKDFHINFLEKYA
>sp|Q9Y673|ALG5_HUMAN Dolichyl-phosphate beta-glucosyltransferase OS=Homo sapiens GN=ALG5 PE=1 SV=1
MAPLLLQLAVLGAALAAAALVLISIVAFTTATKMPALHRHEEEKFFLNAKGQKETLPSIW
DSPTKQLSVVVPSYNEEKRLPVMMDEALSYLEKRQKRDPAFTYEVIVVDDGSKDQTSKVA
FKYCQKYGSDKVRVITLVKNRGKGGAIRMGIFSSRGEKILMADADGATKFPDVEKLEKGL
NDLQPWPNQMAIACGSRAHLEKESIAQRSYFRTLLMYGFHFLVWFLCVKGIRDTQCGFKL
FTREAASRTFSSLHVERWAFDVELLYIAQFFKIPIAEIAVNWTEIEGSKLVPFWSWLQMG
KDLLFIRLRYLTGAWRLEQTRKMN
>sp|O14744|ANM5_HUMAN Protein arginine N-methyltransferase 5 OS=Homo sapiens GN=PRMT5 PE=1 SV=4
MAAMAVGGAGGSRVSSGRDLNCVPEIADTLGAVAKQGFDFLCMPVFHPRFKREFIQEPAK
NRPGPQTRSDLLLSGRDWNTLIVGKLSPWIRPDSKVEKIRRNSEAAMLQELNFGAYLGLP
AFLLPLNQEDNTNLARVLTNHIHTGHHSSMFWMRVPLVAPEDLRDDIIENAPTTHTEEYS
GEEKTWMWWHNFRTLCDYSKRIAVALEIGADLPSNHVIDRWLGEPIKAAILPTSIFLTNK
KGFPVLSKMHQRLIFRLLKLEVQFIITGTNHHSEKEFCSYLQYLEYLSQNRPPPNAYELF
AKGYEDYLQSPLQPLMDNLESQTYEVFEKDPIKYSQYQQAIYKCLLDRVPEEEKDTNVQV
LMVLGAGRGPLVNASLRAAKQADRRIKLYAVEKNPNAVVTLENWQFEEWGSQVTVVSSDM
REWVAPEKADIIVSELLGSFADNELSPECLDGAQHFLKDDGVSIPGEYTSFLAPISSSKL
YNEVRACREKDRDPEAQFEMPYVVRLHNFHQLSAPQPCFTFSHPNRDPMIDNNRYCTLEF
PVEVNTVLHGFAGYFETVLYQDITLSIRPETHSPGMFSWFPILFPIKQPITVREGQTICV
RFWRCSNSKKVWYEWAVTAPVCSAIHNPTGRSYTIGL
>sp|Q5XXA6|ANO1_HUMAN Anoctamin-1 OS=Homo sapiens GN=ANO1 PE=1 SV=1
MRVNEKYSTLPAEDRSVHIINICAIEDIGYLPSEGTLLNSLSVDPDAECKYGLYFRDGRR
KVDYILVYHHKRPSGNRTLVRRVQHSDTPSGARSVKQDHPLPGKGASLDAGSGEPPMDYH
EDDKRFRREEYEGNLLEAGLELERDEDTKIHGVGFVKIHAPWNVLCREAEFLKLKMPTKK
MYHINETRGLLKKINSVLQKITDPIQPKVAEHRPQTMKRLSYPFSREKQHLFDLSDKDSF
FDSKTRSTIVYEILKRTTCTKAKYSMGITSLLANGVYAAAYPLHDGDYNGENVEFNDRKL
LYEEWARYGVFYKYQPIDLVRKYFGEKIGLYFAWLGVYTQMLIPASIVGIIVFLYGCATM
DENIPSMEMCDQRHNITMCPLCDKTCSYWKMSSACATARASHLFDNPATVFFSVFMALWA
ATFMEHWKRKQMRLNYRWDLTGFEEEEEAVKDHPRAEYEARVLEKSLKKESRNKEKRRHI
PEESTNKWKQRVKTAMAGVKLTDKVKLTWRDRFPAYLTNLVSIIFMIAVTFAIVLGVIIY
RISMAAALAMNSSPSVRSNIRVTVTATAVIINLVVIILLDEVYGCIARWLTKIEVPKTEK
SFEERLIFKAFLLKFVNSYTPIFYVAFFKGRFVGRPGDYVYIFRSFRMEECAPGGCLMEL
CIQLSIIMLGKQLIQNNLFEIGIPKMKKLIRYLKLKQQSPPDHEECVKRKQRYEVDYNLE
PFAGLTPEYMEMIIQFGFVTLFVASFPLAPLFALLNNIIEIRLDAKKFVTELRRPVAVRA
KDIGIWYNILRGIGKLAVIINAFVISFTSDFIPRLVYLYMYSKNGTMHGFVNHTLSSFNV
SDFQNGTAPNDPLDLGYEVQICRYKDYREPPWSENKYDISKDFWAVLAARLAFVIVFQNL
VMFMSDFVDWVIPDIPKDISQQIHKEKVLMVELFMREEQDKQQLLETWMEKERQKDEPPC
NHHNTKACPDSLGSPAPSHAYHGGVL
>sp|Q9HCE9|ANO8_HUMAN Anoctamin-8 OS=Homo sapiens GN=ANO8 PE=1 SV=3
MAEAASGAGGTSLEGERGKRPPPEGEPAAPASGVLDKLFGKRLLQAGRYLVSHKAWMKTV
PTENCDVLMTFPDTTDDHTLLWLLNHIRVGIPELIVQVRHHRHTRAYAFFVTATYESLLR

GADELGLRKAVKAEFGGGTRGFSCEEDFIYENVESELRFFTSQERQSIIRFWLQNLRAKQ
GEALHNVRFLEDQPIIPELAARGIIQQVFPVHEQRILNRLMKSWVQAVCENQPLDDICDY
FGVKIAMYFAWLGFYTSAMVYPAVFGSVLYTFTEADQTSRDVSCVVFALFNVIWSTLFLE
EWKRRGAELAYKWGTLDSPGEAVEEPRPQFRGVRRISPITRAEEFYYPPWKRLLFQLLVS
LPLCLACLVCVFLLMLGCFQLQELVLSVKGLPRLARFLPKVMLALLVSVSAEGYKKLAIW
LNDMENYRLESAYEKHLIIKVVLFQFVNSYLSLFYIGFYLKDMERLKEMLATLLITRQFL
QNVREVLQPHLYRRLGRGELGLRAVWELARALLGLLSLRRPAPRRLEPQADEGGGGGSGG
GGRRCLSGGCGAPEEEEEAALVERRRAGEGGEEGDGPPGGKEEDEDDEEEEDEEEEEDEE
EGEEGGLLDCGLRLKKVSFAERGAGRRRPGPSPEALLEEGSPTMVEKGLEPGVFTLAEED
DEAEGAPGSPEREPPAILFRRAGGEGRDQGPDGGPDPEPGSNSDSTRRQRRQNRSSWIDP
PEEEHSPQLTQAELESCMKKYEDTFQDYQEMFVQFGYVVLFSSAFPLAALCALVNNLIEI
RSDAFKLCTGLQRPFGQRVESIGQWQKVMEAMGVLAIVVNCYLIGQCGQLQRLFPWLSPE
AAIVSVVVLEHFALLLKYLIHVAIPDIPGWVAEEMAKLEYQRREAFKRHERQAQHRYQQQ
QRRRREEEERQRHAEHHARREHDSGGREEARAEGSGLDPATSSEKASAKAKGSTAGGHGP
ERPKRPGSLLAPNNVMKLKQIIPLQGKFLSSGATSSLAAAGAGATTRPPPAQSPTGSDTR
LPAFLSFKFLKSPETRRDSERSHSPPKAFHAGKLFPFGGTRAEPGSNGAGGQARPDGTPS
SGSSRVQRSGPVDEALAEELEAPRPEEEGSGTALAPVGAPALRTRRSRSPAPPPPMPLPR
PPTPPAGCWQWDGPWGCGGEGAAPRQALAAAECPPCAMAGPPPAPQPLPGDASFYSLPPP
PLPPTSDPLETPAPSPSPSPSPQAVCWPSGWH
>sp|P16066|ANPRA_HUMAN Atrial natriuretic peptide receptor 1 OS=Homo sapiens GN=NPR1 PE=1 SV=1
MPGPRRPAGSRLRLLLLLLLPPLLLLLRGSHAGNLTVAVVLPLANTSYPWSWARVGPAVE
LALAQVKARPDLLPGWTVRTVLGSSENALGVCSDTAAPLAAVDLKWEHNPAVFLGPGCVY
AAAPVGRFTAHWRVPLLTAGAPALGFGVKDEYALTTRAGPSYAKLGDFVAALHRRLGWER
QALMLYAYRPGDEEHCFFLVEGLFMRVRDRLNITVDHLEFAEDDLSHYTRLLRTMPRKGR
VIYICSSPDAFRTLMLLALEAGLCGEDYVFFHLDIFGQSLQGGQGPAPRRPWERGDGQDV
SARQAFQAAKIITYKDPDNPEYLEFLKQLKHLAYEQFNFTMEDGLVNTIPASFHDGLLLY
IQAVTETLAHGGTVTDGENITQRMWNRSFQGVTGYLKIDSSGDRETDFSLWDMDPENGAF
RVVLNYNGTSQELVAVSGRKLNWPLGYPPPDIPKCGFDNEDPACNQDHLSTLEVLALVGS
LSLLGILIVSFFIYRKMQLEKELASELWRVRWEDVEPSSLERHLRSAGSRLTLSGRGSNY
GSLLTTEGQFQVFAKTAYYKGNLVAVKRVNRKRIELTRKVLFELKHMRDVQNEHLTRFVG
ACTDPPNICILTEYCPRGSLQDILENESITLDWMFRYSLTNDIVKGMLFLHNGAICSHGN
LKSSNCVVDGRFVLKITDYGLESFRDLDPEQGHTVYAKKLWTAPELLRMASPPVRGSQAG
DVYSFGIILQEIALRSGVFHVEGLDLSPKEIIERVTRGEQPPFRPSLALQSHLEELGLLM
QRCWAEDPQERPPFQQIRLTLRKFNRENSSNILDNLLSRMEQYANNLEELVEERTQAYLE
EKRKAEALLYQILPHSVAEQLKRGETVQAEAFDSVTIYFSDIVGFTALSAESTPMQVVTL
LNDLYTCFDAVIDNFDVYKVETIGDAYMVVSGLPVRNGRLHACEVARMALALLDAVRSFR
IRHRPQEQLRLRIGIHTGPVCAGVVGLKMPRYCLFGDTVNTASRMESNGEALKIHLSSET
KAVLEEFGGFELELRGDVEMKGKGKVRTYWLLGERGSSTRG
>sp|A6NGH8|ANR61_HUMAN Ankyrin repeat domain-containing protein 61 OS=Homo sapiens GN=ANKRD61 PE=3 SV=2
MGNITRKGSRDLVVDSAKSLEDGPSAALHSKLYEAIMREDCTTIEVLLRNHPVNQPITIL
PNSASNRLLLTQPTESIIPIHLAAKYHKAQSLLCLLRHGADPEVRDTTGLTTLNLMLLHW
PVTSTTWAKPGNRTHRILTDIQNSSITCLRILCAHGAQVNTQGEISNKRSPLHLAIAYGC

YPVLSILTQNGADVNAINEASMTPLHMAANMLNKEMMETLIAYGANVNCAVSSTGNTPLK
LAVCTASSKAGRLLGAGVSCIRLLLTHGAKVNAQDYKGQTAIHEACFGGREAIINLLLEF
EANVNILTRNGESPIYMYLQRSCNVRDTALLARLLYHTYPLRMTNNQGILPAGIMLPEFR
LLRDTLIKQSQKPLSLQGICKRNIRNIYGEKYKQHLKQFLPVTIWNSVYCCYDLAYTS
>sp|E5RJM6|ANR65_HUMAN Ankyrin repeat domain-containing protein 65 OS=Homo sapiens GN=ANKRD65 PE=2 SV=2
MDSQRPEPREEEEEEQELRWMELDSEEALGTRTEGPSVVQGWGHLLQAVWRGPAGLVTQL
LRQGASVEERDHAGRTPLHLAVLRGHAPLVRLLLQRGAPVGAVDRAGRTALHEAAWHGHS
RVAELLLQRGASAAARSGTGLTPLHWAAALGHTLLAARLLEAPGPGPAAAEAEDARGWTA
AHWAAAGGRLAVLELLAAGGAGLDGALLVAAAAGRGAALRFLLARGARVDARDGAGATAL
GLAAALGRSQDIEVLLGHGADPGIRDRHGRSALHRAAARGHLLAVQLLVTQGAEVDARDT
LGLTPLHHASREGHVEVAGCLLDRGAQVDATGWLRKTPLHLAAERGHGPTVGLLLSRGAS
PTLRTQWAEVAQMPEGDLPQALPELGGGEKECEGIESTG
>sp|Q92625|ANS1A_HUMAN Ankyrin repeat and SAM domain-containing protein 1A OS=Homo sapiens GN=ANKS1A PE=1 SV=4
MGKEQELLEAARTGHLPAVEKLLSGKRLSSGFGGGGGGGSGGGGGGSGGGGGGLGSSSHP
LSSLLSMWRGPNVNCVDSTGYTPLHHAALNGHKDVVEVLLRNDALTNVADSKGCYPLHLA
AWKGDAQIVRLLIHQGPSHTRVNEQNNDNETALHCAAQYGHTEVVKVLLEELTDPTMRNN
KFETPLDLAALYGRLEVVKMLLNAHPNLLSCNTKKHTPLHLAARNGHKAVVQVLLDAGMD
SNYQTEMGSALHEAALFGKTDVVQILLAAGTDVNIKDNHGLTALDTVRELPSQKSQQIAA
LIEDHMTGKRSTKEVDKTPPPQPPLISSMDSISQKSQGDVEKAVTELIIDFDANAEEEGP
YEALYNAISCHSLDSMASGRSSDQDSTNKEAEAAGVKPAGVRPRERPPPPAKPPPDEEEE
DHIDKKYFPLTASEVLSMRPRIHGSAAREEDEHPYELLLTAETKKVVLVDGKTKDHRRSS
SSRSQDSAEGQDGQVPEQFSGLLHGSSPVCEVGQDPFQLLCTAGQSHPDGSPQQGACHKA
SMQLEETGVHAPGASQPSALDQSKRVGYLTGLPTTNSRSHPETLTHTASPHPGGAEEGDR
SGARSRAPPTSKPKAELKLSRSLSKSDSDLLTCSPTEDATMGSRSESLSNCSIGKKRLEK
SPSFASEWDEIEKIMSSIGEGIDFSQERQKISGLRTLEQSVGEWLESIGLQQYESKLLLN
GFDDVHFLGSNVMEEQDLRDIGISDPQHRRKLLQAARSLPKVKALGYDGNSPPSVPSWLD
SLGLQDYVHSFLSSGYSSIDTVKNLWELELVNVLKVQLLGHRKRIIASLADRPYEEPPQK
PPRFSQLRCQDLLSQTSSPLSQNDSCTGRSADLLLPPGDTGRRRHDSLHDPAAPSRAERF
RIQEEHREAKLTLRPPSLAAPYAPVQSWQHQPEKLIFESCGYEANYLGSMLIKDLRGTES
TQDACAKMRKSTEHMKKIPTIILSITYKGVKFIDASNKNVIAEHEIRNISCAAQDPEDLC
TFAYITKDLQTSHHYCHVFSTVDVNLTYEIILTLGQAFEVAYQLALQAQKSRATGASAAE
MIETKSSKPVPKPRVGVRKSALEPPDMDQDAQSHASVSWVVDPKPDSKRSLSTN
>sp|Q6Q4G3|AMPQ_HUMAN Aminopeptidase Q OS=Homo sapiens GN=LVRN PE=1 SV=4
MGPPSSSGFYVSRAVALLLAGLVAALLLALAVLAALYGHCERVPPSELPGLRDLEAESSP
PLRQKPTPTPKPSSARELAVTTTPSNWRPPGPWDQLRLPPWLVPLHYDLELWPQLRPDEL
PAGSLPFTGRVNITVRCTVATSRLLLHSLFQDCERAEVRGPLSPGTGNATVGRVPVDDVW
FALDTEYMVLELSEPLKPGSSYELQLSFSGLVKEDLREGLFLNVYTDQGERRALLASQLE
PTFARYVFPCFDEPALKATFNITMIHHPSYVALSNMPKLGQSEKEDVNGSKWTVTTFSTT
PHMPTYLVAFVICDYDHVNRTERGKEIRIWARKDAIANGSADFALNITGPIFSFLEDLFN
ISYSLPKTDIIALPSFDNHAMENWGLMIFDESGLLLEPKDQLTEKKTLISYVVSHEIGHQ
WFGNLVTMNWWNNIWLNEGFASYFEFEVINYFNPKLPRNEIFFSNILHNILREDHALVTR
AVAMKVENFKTSEIQELFDIFTYSKGASMARMLSCFLNEHLFVSALKSYLKTFSYSNAEQ

DDLWRHFQMAIDDQSTVILPATIKNIMDSWTHQSGFPVITLNVSTGVMKQEPFYLENIKN
RTLLTSNDTWIVPILWIKNGTTQPLVWLDQSSKVFPEMQVSDSDHDWVILNLNMTGYYRV
NYDKLGWKKLNQQLEKDPKAIPVIHRLQLIDDAFSLSKNNYIEIETALELTKYLAEEDEI
IVWHTVLVNLVTRDLVSEVNIYDIYSLLKRYLLKRLNLIWNIYSTIIRENVLALQDDYLA
LISLEKLFVTACWLGLEDCLQLSKELFAKWVDHPENEIPYPIKDVVLCYGIALGSDKEWD
ILLNTYTNTTNKEEKIQLAYAMSCSKDPWILNRYMEYAISTSPFTSNETNIIEVVASSEV
GRYVAKDFLVNNWQAVSKRYGTQSLINLIYTIGRTVTTDLQIVELQQFFSNMLEEHQRIR
VHANLQTIKNENLKNKKLSARIAAWLRRNT
>sp|Q8IZ07|AN13A_HUMAN Ankyrin repeat domain-containing protein 13A OS=Homo sapiens GN=ANKRD13A PE=1 SV=3
MSSACDAGDHYPLHLLVWKNDYRQLEKELQGQNVEAVDPRGRTLLHLAVSLGHLESARVL
LRHKADVTKENRQGWTVLHEAVSTGDPEMVYTVLQHRDYHNTSMALEGVPELLQKILEAP
DFYVQMKWEFTSWVPLVSRICPNDVCRIWKSGAKLRVDITLLGFENMSWIRGRRSFIFKG
EDNWAELMEVNHDDKVVTTERFDLSQEMERLTLDLMKPKSREVERRLTSPVINTSLDTKN
IAFERTKSGFWGWRTDKAEVVNGYEAKVYTVNNVNVITKIRTEHLTEEEKKRYKADRNPL
ESLLGTVEHQFGAQGDLTTECATANNPTAITPDEYFNEEFDLKDRDIGRPKELTIRTQKF
KAMLWMCEEFPLSLVEQVIPIIDLMARTSAHFARLRDFIKLEFPPGFPVKIEIPLFHVLN
ARITFGNVNGCSTAEESVSQNVEGTQADSASHITNFEVDQSVFEIPESYYVQDNGRNVHL
QDEDYEIMQFAIQQSLLESSRSQELSGPASNGGISQTNTYDAQYERAIQESLLTSTEGLC
PSALSETSRFDNDLQLAMELSAKELEEWELRLQEEEAELQQVLQLSLTDK
>sp|Q8N6S4|AN13C_HUMAN Ankyrin repeat domain-containing protein 13C OS=Homo sapiens GN=ANKRD13C PE=2 SV=2
MTGEKIRSLRRDHKPSKEEGDLLEPGDEEAAAALGGTFTRSRIGKGGKACHKIFSNHHHR
LQLKAAPASSNPPGAPALPLHNSSVTANSQSPALLAGTNPVAVVADGGSCPAHYPVHECV
FKGDVRRLSSLIRTHNIGQKDNHGNTPLHLAVMLGNKECAHLLLAHNAPVKVKNAQGWSP
LAEAISYGDRQMITALLRKLKQQSRESVEEKRPRLLKALKELGDFYLELHWDFQSWVPLL
SRILPSDACKIYKQGINIRLDTTLIDFTDMKCQRGDLSFIFNGDAAPSESFVVLDNEQKV
YQRIHHEESEMETEEEVDILMSSDIYSATLSTKSISFTRAQTGWLFREDKTERVGNFLAD
FYLVNGLVLESRKRREHLSEEDILRNKAIMESLSKGGNIMEQNFEPIRRQSLTPPPQNTI
TWEEYISAENGKAPHLGRELVCKESKKTFKATIAMSQEFPLGIELLLNVLEVVAPFKHFN
KLREFVQMKLPPGFPVKLDIPVFPTITATVTFQEFRYDEFDGSIFTIPDDYKEDPSRFPD
L
>sp|Q5CZ79|AN20B_HUMAN Ankyrin repeat domain-containing protein 20B OS=Homo sapiens GN=ANKRD20A8P PE=2 SV=2
MKLFGFGSRRGQTAEGSIDHVYTGSGYRIRDSELQKIHRAAVKGDAAEVERCLARRSGDL
DARDKQHRTALHLACASGHVQVVTLLVNRKCQIDICDKENRTPLIQAVHCQEEACAVILL
KHGANPNLKDIYGNTALHYAVYSESTSLAEKLLSHGAHIEALDKDSNTPLLFAIICKKEK
MVEFLLKKKASTHAVDRLRRSALMLAVYYDSPGIVSILLKQNIDVFAQDMCGRDAEDYAI
SHHLTKIQQQILEHKQKILKKEKSDVGSSDESAVSIFHELRVDSLPASDDKDLSVATKQC
VPEKVSEPLPGPSHEKGNRIVNGQGEGPPAKHPSLKPTTGVEDPAVKGAVQRKNVQTLRA
EQALPVASEEEQERHERSEKKQPQVKKGNNTNKSEKIQLSENICDSTSSAAAGRLTQQRK
IGKTYPQQFPKKLKEEHDKCTLKQENEEKTNVNMLYKKNREELERKEKQYKKEVEAKQLE
PTVQSLEMKSKTARNTPNRDFHNHEETKGLMDENCILKADIAILRQEICTMKNDNLEKEN
KYLKDIKIVKETNAALEKYIKLNEEMITETAFRYQQELNDLKAENTRLNAELLKEKESKK

RLEADIESYQSRLAAAIGKHSESVKTERNVKLALERTQDVSVQVEMSSAISKVKDENEFL
TEQLSETQIKFNALKDKFRKTRDSLRKKSLALETVQNDLRKTQQQTQEMKEMYQNAEAKV
NNSTGKWNCVEERICHLQRENAWLVQQLDDVHQKEDHKEIVTNIQRGFIESGKKDLVLEE
KSKKLMNECDHLKESLFQYEREKAEGVVSIKEDKYFQTSRKKI
>sp|Q5JPF3|AN36C_HUMAN Ankyrin repeat domain-containing protein 36C OS=Homo sapiens GN=ANKRD36C PE=1 SV=3
MDDKEPKRWPTLRDRLCSDGFLFPQYPIKPYHLKGIHRAVFYRDLEELKFVLLTRYDINK
RDRKERTALHLACATGQPEMVHLLVSRRCELNLCDREDRTPLIKAVQLRQEACATLLLQN
GADPNITDVFGRTALHYAVYNEDTSMIEKLLSYGANIEECSEDEYPPLFLAVSQRKVKMV
EFLLKKKANINAVDYLGRSALIHAVTLGEKDIVILLLQHNIDVFSRDVYGKLAEDYASEA
KNRVIFELIYEYERKKHEELSINSNPVSSQKQPALKATSGKEDSISNIATEIKDGQKSGT
VSSQKQPALKGTSDKNDSVSNTATEIKDEQKSGTVSSQKQPALKDTSDKNDSVSNTATEI
KDEQKSGTVLPAVEQCLNRSLYRPDAVAQPVTEDEFALESEIISKLYIPKRKIISPRSIE
DVLPPVEEAVDRCLYLLDRFAQAVTKDKFALESENISEPYFTNRRTISQQSAEKLDAACG
IDKTENGTLFEDQNVDKEGKALPATGQKANVSPEQPPLFTHTVKDSDHISTRFLGSMDSL
TSSEESSERPPLSTLTLKEADPSSKAAMRRKDSPPPGKVSSQKQPAEKATSDDKDSVSNI
ATEIKEGPISGTVSSQKQPAEKATSDEKDSVSNIATEIKEGQQSGTVSPQKQSAWKVIFK
KKVSLLNIATRITGGGKSGTVSSQKQPPSKATSDKTDSALNIATEIKDGLQCGTVSSQKQ
PALKATTDEEDSVSNIATEIKDGEKSGTVSSQKQPALKATTDEEDSVSIIATEIKDGEKS
GTVSSRKKPALKATSDEKDSFSNITREKKDGEISRTVTSEKPAGLKATSDEEDSVLNIAR
GKEDGEKTRRVSSRKKPALKATSDEKDSFSNITREKKDGETSRTVSSQKPPALKATSDEE
DSVLNIAREKKDGEKSRTVSSEKPSGLKATSDEKDSVLNIARGKKHGEKTRRVSSHKQPA
LKATSDKENSVPNMATETKDEQISGTVSSQKQPALKATSDKKDSVSNIPTEIKDGQQSGT
VSSQKQLAWKATSVKKDSVSNIATEIKDGQIRGTVSSQRQPALKATGDEKDSVSNIAREI
KDGEKSGTVSPQKQSAQKVIFKKKVSLLNIATRITGGWKSGTEYPENLPTLKATIENKNS
VLNTATKMKDVQTSTPEQDLEMASEGEQKRLEEYENNQPQVKNQIHSRDDLDDIIQSSQT
VSEDGDSLCCNCKNVILLIDQHEMKCKDCVHLLKIKKTFCLCKRLTELKDNHCEQLRVKI
RKLKNKASVLQKRLSEKEEIKSQLKHETLELEKELCSLRFAIQQEKKKRRNVEELHQKVR
EKLRITEEQYRIEADVTKPIKPALKSAEVELKTGGNNSNQVSETDEKEDLLHENRLMQDE
IARLRLEKDTIKNQNLEKKYLKDFEIVKRKHEDLQKALKRNEETLAETIACYSGQLAALT
DENTTLRSKLEKQRESGQRLETEMQSYRCRLNAALCDHDQSHSSKRDQELAFQGTVDKCC
HLQENLNSHVLILSLQLSKAESKFRVLETELHYTGEALKEKALVFEHVQSELKQKQSQMK
DIEKMYKSGYNTMEKCIEKQERFCQLKKQNMLLQQQLDDARNKADNQEKAILNIQARCDA
RVENLQAECRKHRLLLEEDNKMLVNELNHSKEKKCQYEKEKAEREVAVRQLQQKQDDVLN
KRSATKALLDASSRHCIYLENGMQDSRKKLDQMRSQFQEIQDQLTATIRCTKEMEGDAQK
LEVENVMMRKIIKKQDEQIERFEKILQHSSLMLQVFES
>sp|Q9BY76|ANGL4_HUMAN Angiopoietin-related protein 4 OS=Homo sapiens GN=ANGPTL4 PE=1 SV=2
MSGAPTAGAALMLCAATAVLLSAQGGPVQSKSPRFASWDEMNVLAHGLLQLGQGLREHAE
RTRSQLSALERRLSACGSACQGTEGSTDLPLAPESRVDPEVLHSLQTQLKAQNSRIQQLF
HKVAQQQRHLEKQHLRIQHLQSQFGLLDHKHLDHEVAKPARRKRLPEMAQPVDPAHNVSR
LHRLPRDCQELFQVGERQSGLFEIQPQGSPPFLVNCKMTSDGGWTVIQRRHDGSVDFNRP
WEAYKAGFGDPHGEFWLGLEKVHSITGDRNSRLAVQLRDWDGNAELLQFSVHLGGEDTAY
SLQLTAPVAGQLGATTVPPSGLSVPFSTWDQDHDLRRDKNCAKSLSGGWWFGTCSHSNLN

GQYFRSIPQQRQKLKKGIFWKTWRGRYYPLQATTMLIQPMAAEAAS
>sp|Q9HCJ1|ANKH_HUMAN Progressive ankylosis protein homolog OS=Homo sapiens GN=ANKH PE=1 SV=2
MVKFPALTHYWPLIRFLVPLGITNIAIDFGEQALNRGIAAVKEDAVEMLASYGLAYSLMK
FFTGPMSDFKNVGLVFVNSKRDRTKAVLCMVVAGAIAAVFHTLIAYSDLGYYIINKLHHV
DESVGSKTRRAFLYLAAFPFMDAMAWTHAGILLKHKYSFLVGCASISDVIAQVVFVAILL
HSHLECREPLLIPILSLYMGALVRCTTLCLGYYKNIHDIIPDRSGPELGGDATIRKMLSF
WWPLALILATQRISRPIVNLFVSRDLGGSSAATEAVAILTATYPVGHMPYGWLTEIRAVY
PAFDKNNPSNKLVSTSNTVTAAHIKKFTFVCMALSLTLCFVMFWTPNVSEKILIDIIGVD
FAFAELCVVPLRIFSFFPVPVTVRAHLTGWLMTLKKTFVLAPSSVLRIIVLIASLVVLPY
LGVHGATLGVGSLLAGFVGESTMVAIAACYVYRKQKKKMENESATEGEDSAMTDMPPTEE
VTDIVEMREENE
>sp|Q8NFD2|ANKK1_HUMAN Ankyrin repeat and protein kinase domain-containing protein 1 OS=Homo sapiens GN=ANKK1 PE=2 SV=1
MAADPTELRLGSLPVFTRDDFEGDWRLVASGGFSQVFQARHRRWRTEYAIKCAPCLPPDA
ASSDVNYLIEEAAKMKKIKFQHIVSIYGVCKQPLGIVMEFMANGSLEKVLSTHSLCWKLR
FRIIHETSLAMNFLHSIKPPLLHLDLKPGNILLDSNMHVKISDFGLSKWMEQSTRMQYIE
RSALRGMLSYIPPEMFLESNKAPGPKYDVYSFAIVIWELLTQKKPYSGFNMMMIIIRVAA
GMRPSLQPVSDQWPSEAQQMVDLMKRCWDQDPKKRPCFLDITIETDILLSLLQSRVAVPE
SKALARKVSCKLSLRQPGEVNEDISQELMDSDSGNYLKRALQLSDRKNLVPRDEELCIYE
NKVTPLHFLVAQGSVEQVRLLLAHEVDVDCQTASGYTPLLIAAQDQQPDLCALLLAHGAD
ANRVDEDGWAPLHFAAQNGDDGTARLLLDHGACVDAQEREGWTPLHLAAQNNFENVARLL
VSRQADPNLHEAEGKTPLHVAAYFGHVSLVKLLTSQGAELDAQQRNLRTPLHLAVERGKV
RAIQHLLKSGAVPDALDQSGYGPLHTAAARGKYLICKMLLRYGASLELPTHQGWTPLHLA
AYKGHLEIIHLLAESHANMGALGAVNWTPLHLAARHGEEAVVSALLQCGADPNAAEQSGW
TPLHLAVQRSTFLSVINLLEHHANVHARNKVGWTPAHLAALKGNTAILKVLVEAGAQLDV
QDGVSCTPLQLALRSRKQGIMSFLEGKEPSVATLGGSKPGAEMEI
>sp|Q8NAG6|ANKL1_HUMAN Ankyrin repeat and LEM domain-containing protein 1 OS=Homo sapiens GN=ANKLE1 PE=1 SV=2
MCSEARLARRLRDALREEEPWAVEELLRCGADPNLVLEDGAAAVHLAAGARHPRGLRCLG
ALLRQGGDPNARSVEALTPLHVAAAWGCRRGLELLLSQGADPALRDQDGLRPLDLALQQG
HLECARVLQDLDTRTRTRTRIGAETQEPEPAPGTPGLSGPTDETLDSIALQKQPCRGDNR
DIGLEADPGPPSLPVPLETVDKHGSSASPPGHWDYSSDASFVTAVEVSGAEDPASDTPPW
AGSLPPTRQGLLHVVHANQRVPRSQGTEAELNARLQALTLTPPNAAGFQSSPSSMPLLDR
SPAHSPPRTPTPGASDCHCLWEHQTSIDSDMATLWLTEDEASSTGGREPVGPCRHLPVST
VSDLELLKGLRALGENPHPITPFTRQLYHQQLEEAQIAPGPEFSGHSLELAAALRTGCIP
DVQADEDALAQQFEQPDPARRWREGVVKSSFTYLLLDPRETQDLPARAFSLTPAERLQTF
IRAIFYVGKGTRARPYVHLWEALGHHGRSRKQPHQACPKVRQILDIWASGCGVVSLHCFQ
HVVAVEAYTREACIVEALGIQTLTNQKQGHCYGVVAGWPPARRRRLGVHLLHRALLVFLA
EGERQLHPQDIQARG
>sp|Q15327|ANKR1_HUMAN Ankyrin repeat domain-containing protein 1 OS=Homo sapiens GN=ANKRD1 PE=1 SV=2
MMVLKVEELVTGKKNGNGEAGEFLPEDFRDGEYEAAVTLEKQEDLKTLLAHPVTLGEQQW
KSEKQREAELKKKKLEQRSKLENLEDLEIIIQLKKRKKYRKTKVPVVKEPEPEIITEPVD

VPTFLKAALENKLPVVEKFLSDKNNPDVCDEYKRTALHRACLEGHLAIVEKLMEAGAQIE
FRDMLESTAIHWASRGGNLDVLKLLLNKGAKISARDKLLSTALHVAVRTGHYECAEHLIA
CEADLNAKDREGDTPLHDAVRLNRYKMIRLLIMYGADLNIKNCAGKTPMDLVLHWQNGTK
AIFDSLRENSYKTSRIATF
>sp|Q6ZW76|ANKS3_HUMAN Ankyrin repeat and SAM domain-containing protein 3 OS=Homo sapiens GN=ANKS3 PE=1 SV=1
MSELSDEASEPELLNRSLSMWHGLGTQVSGEELDVPLDLHTAASIGQYEVVKECVQRREL
DLNKKNGGGWTPLMYASYIGHDTIVHLLLEAGVSVNVPTPEGQTPLMLASSCGNESIAYF
LLQQGAELEMKDIQGWTALFHCTSAGHQHMVRFLLDSGANANVREPICGFTPLMEAAAAG
HEIIVQYFLNHGVKVDARDHSGATARMLAKQYGHMKIVALMDTYSPSLPKSLYRSPEKYE
DLSSSDESCPAPQRQRPCRKKGVSIHEGPRALARITGIGLGGRAPRPRYEQAPPRGYVTF
NSSGENPLEEEGLCCRDVTSPINERDVESSSSSSSREEHAFCANLGPVQSSSSSEGLARA
QGLSSEASVESNEDSDHACKSSARKQAKSYMKTKNPDSQWPPRAATDREGFLAESSPQTQ
RAPYSGPQDLAALLEQIGCLKYLQVFEEQDVDLRIFLTLTESDLKEIGITLFGPKRKMTS
AIARWHSSARPPGDALELAYADRLEAEMQELAIQLHKRCEEVEATRGQVCQEQELRAVVE
SCLLEQDRAREDLQARLRETWALARDAALVLDQLRACQAELSSRVRQDQPPGAATLGLAV
PPADSKGWQASLQAMSLPELSGALEDRVREMGQALCLVTQSLEKLQVLNGKKWRET
>sp|A6NFN9|ANKUB_HUMAN Protein ANKUB1 OS=Homo sapiens GN=ANKUB1 PE=2 SV=2
MRIFIAFEGSFEPFDVSADETVEVVKLMIKDYFHIPLSEDKQGRRYLELMYAGAALKDSW
SLADVGISFCSTLKCFVKEEDKPTLYVFNAVTQDTMPVMESISLLDKTVSDLRTLVTLRC
GLPVSVYCLRTPRGLEMYDCNTLKDYQTDIGTTLRLDVWDGWKEFLMGCLLGQKLKVQRY
LSKEGPVLKYQKRVALYIAAFCGYIELTEWALKQGARPHEAVGVHPYRAWCHEALHADVS
KCPIHAAAEAGQLLILKAFVNYSVLCLECKNAAGQTPLTIVFKHKHKDCVLYLLSKMWST
VSFPKISVPMRIYIKIKQWILRAQSHSLHKSQFCGARVFGAKVGDTVMVDGFTKPKMTSK
SWHKAGNSDSQSIVLKLPSLSKQTASSKPVNPLAISQPDTRKQALKFHPLKGESPKADKS
SGIM
>sp|Q9P2S6|ANKY1_HUMAN Ankyrin repeat and MYND domain-containing protein 1 OS=Homo sapiens GN=ANKMY1 PE=2 SV=2
MYQGEFGLNMKLGYGKFSWPTGESYHGQFYRDHCHGLGTYMWPDGSSFTGTFYLSHREGY
GTMYMKTRLFQGLYKADQRFGPGVETYPDGSQDVGLWFREQLIKLCTQIPSGFSLLRYPE
FSSFITHSPARISLSEEEKTEWGLQEGQDPFFYDYKRFLLNDNLTLPPEMYVYSTNSDHL
PMTSSFRKELDARIFLNEIPPFVEDGEPWFIINETPLLVKIQKQTYKFRNKPAHTSWNMG
AILEGKRSGFAPCGPKEQLSMEMILKAEEGNHEWICRILKDNFASADVADAKGYTVLAAA
ATHCHNDIVNLLLDCGADVNKCSDEGLTALSMCFLLHYPAQSFKPNVAERTIPEPQEPPK
FPVVPILSSSFMDTNLESLYYEVNVPSQGSYELRPPPAPLLLPRVSGSHEGGHFQDTGQC
GGSIDHRSSSLKGDSPLVKGSLGHVESGLEDVLGNTDRGSLCSAETKFESNVCVCDFSIE
LSQAMLERSAQSHSLLKMASPSPCTSSFDKGTMRRMALSMIERRKRWRTIKLLLRRGADP
NLCCVPMQVLFLAVKAGDVDGVRLLLEHGARTDICFPPQLSTLTPLHIAAALPGEEGVQI
VELLLHAITDVDAKASDEDDTYKPGKLDLLPSSLKLSNEPGPPQAYYSTDTALPEEGGRT
ALHMACEREDDNKCARDIVRLLLSHGANPNLLWSGHSPLSLSIASGNELVVKELLTQGAD
PNLPLTKGLGSALCVACDLTYEHQRNMDSKLALIDRLISHGADILKPVMLRQGEKEAVGT
AVDYGYFRFFQDRRIARCPFHTLMPAERETFLARKRLLEYMGLQLRQAVFAKESQWDPTW
LYLCKRAELIPSHRMKKKGPSLPRGLDVKEQGQIPFFKFCYQCGRSIGVRLLPCPRCYGI
LTCSKYCKTKAWTEFHKKDCGDLVAIVTQLEQVSRRREEFQ

>sp|Q9H8Y5|ANKZ1_HUMAN Ankyrin repeat and zinc finger domain-containing protein 1 OS=Homo sapiens GN=ANKZF1 PE=1 SV=1
MSPAPDAAPAPASISLFDLSADAPVFQGLSLVSHAPGEALARAPRTSCSGSGERESPERK
LLQGPMDISEKLFCSTCDQTFQNHQEQREHYKLDWHRFNLKQRLKDKPLLSALDFEKQSS
TGDLSSISGSEDSDSASEEDLQTLDRERATFEKLSRPPGFYPHRVLFQNAQGQFLYAYRC
VLGPHQDPPEEAELLLQNLQSRGPRDCVVLMAAAGHFAGAIFQGREVVTHKTFHRYTVRA
KRGTAQGLRDARGGPSHSAGANLRRYNEATLYKDVRDLLAGPSWAKALEEAGTILLRAPR
SGRSLFFGGKGAPLQRGDPRLWDIPLATRRPTFQELQRVLHKLTTLHVYEEDPREAVRLH
SPQTHWKTVREERKKPTEEEIRKICRDEKEALGQNEESPKQGSGSEGEDGFQVELELVEL
TVGTLDLCESEVLPKRRRRKRNKKEKSRDQEAGAHRTLLQQTQEEEPSTQSSQAVAAPLG
PLLDEAKAPGQPELWNALLAACRAGDVGVLKLQLAPSPADPRVLSLLSAPLGSGGFTLLH
AAAAAGRGSVVRLLLEAGADPTVQDSRARPPYTVAADKSTRNEFRRFMEKNPDAYDYNKA
QVPGPLTPEMEARQATRKREQKAARRQREEQQQRQQEQEEREREEQRRFAALSDREKRAL
AAERRLAAQLGAPTSPIPDSAIVNTRRCWSCGASLQGLTPFHYLDFSFCSTRCLQDHRRQ
AGRPSS
>sp|P20594|ANPRB_HUMAN Atrial natriuretic peptide receptor 2 OS=Homo sapiens GN=NPR2 PE=1 SV=1
MALPSLLLLVAALAGGVRPPGARNLTLAVVLPEHNLSYAWAWPRVGPAVALAVEALGRAL
PVDLRFVSSELEGACSEYLAPLSAVDLKLYHDPDLLLGPGCVYPAASVARFASHWRLPLL
TAGAVASGFSAKNDHYRTLVRTGPSAPKLGEFVVTLHGHFNWTARAALLYLDARTDDRPH
YFTIEGVFEALQGSNLSVQHQVYAREPGGPEQATHFIRANGRIVYICGPLEMLHEILLQA
QRENLTNGDYVFFYLDVFGESLRAGPTRATGRPWQDNRTREQAQALREAFQTVLVITYRE
PPNPEYQEFQNRLLIRAREDFGVELGPSLMNLIAGCFYDGILLYAEVLNETIQEGGTRED
GLRIVEKMQGRRYHGVTGLVVMDKNNDRETDFVLWAMGDLDSGDFQPAAHYSGAEKQIWW
TGRPIPWVKGAPPSDNPPCAFDLDDPSCDKTPLSTLAIVALGTGITFIMFGVSSFLIFRK
LMLEKELASMLWRIRWEELQFGNSERYHKGAGSRLTLSLRGSSYGSLMTAHGKYQIFANT
GHFKGNVVAIKHVNKKRIELTRQVLFELKHMRDVQFNHLTRFIGACIDPPNICIVTEYCP
RGSLQDILENDSINLDWMFRYSLINDLVKGMAFLHNSIISSHGSLKSSNCVVDSRFVLKI
TDYGLASFRSTAEPDDSHALYAKKLWTAPELLSGNPLPTTGMQKADVYSFGIILQEIALR
SGPFYLEGLDLSPKEIVQKVRNGQRPYFRPSIDRTQLNEELVLLMERCWAQDPAERPDFG
QIKGFIRRFNKEGGTSILDNLLLRMEQYANNLEKLVEERTQAYLEEKRKAEALLYQILPH
SVAEQLKRGETVQAEAFDSVTIYFSDIVGFTALSAESTPMQVVTLLNDLYTCFDAIIDNF
DVYKVETIGDAYMVVSGLPGRNGQRHAPEIARMALALLDAVSSFRIRHRPHDQLRLRIGV
HTGPVCAGVVGLKMPRYCLFGDTVNTASRMESNGQALKIHVSSTTKDALDELGCFQLELR
GDVEMKGKGKMRTYWLLGERKGPPGLL
>sp|Q6P6B7|ANR16_HUMAN Ankyrin repeat domain-containing protein 16 OS=Homo sapiens GN=ANKRD16 PE=1 SV=1
MAQPGDPRRLCRLVQEGRLRALKEELQAAGGCPGPAGDTLLHCAARHGHRDVLAYLAEAW
GMDIEATNRDYKRPLHEAASMGHRDCVRYLLGRGAAVDCLKKADWTPLMMACTRKNLGVI
QELVEHGANPLLKNKDGWNSFHIASREGDPLILQYLLTVCPGAWKTESKIRRTPLHTAAM
HGHLEAVKVLLKRCQYEPDYRDNCGVTALMDAIQCGHIDVARLLLDEHGACLSAEDSLGA
QALHRAAVTGQDEAIRFLVSELGVDVDVRATSTHLTALHYAAKEGHTSTIQTLLSLGADI
NSKDEKNRSALHLACAGQHLACAKFLLQSGLKDSEDITGTLAQQLPRRADVLQGSGHSAM
T

>sp|Q8TF21|ANR24_HUMAN Ankyrin repeat domain-containing protein 24 OS=Homo sapiens GN=ANKRD24 PE=2 SV=2
MKTLRARFKKTELRLSPTDLGSCPPCGPCPIPKPAARGRRQSQDWGKSDERLLQAVENND
APRVAALIARKGLVPTKLDPEGKSAFHLAAMRGAASCLEVMIAHGSNVMSADGAGYNALH
LAAKYGHPQCLKQLLQASCVVDVVDSSGWTALHHAAAGGCLSCSEVLCSFKAHLNPQDRS
GATPLIIAAQMCHTDLCRLLLQQGAAANDQDLQGRTALMLACEGASPETVEVLLQGGAQP
GITDALGQDAAHYGALAGDKLILHLLQEAAQRPSPPSALTEDDSGEASSQNSMSSHGKQG
APKKRKAPPPPASIPMPDDRDAYEEIVRLRQERGRLLQKIRGLEQHKERRQQESPEASSL
HILERQVQELQQLLVERQEEKESLGREVESLQSRLSLLENERENTSYDVTTLQDEEGELP
DLPGAEVLLSRQLSPSAQEHLASLQEQVAVLTRQNQELMEKVQILENFEKDETQMEVEAL
AEVIPLALYDSLRAEFDQLRRQHAEALQALRQQETREVPREEGAACGESEVAGATATKNG
PTHMELNGSVAPETKVNGAETIDEEAAGDETMEARTMEAEATGAEATGAEATGAKVTETK
PTGAEVREMETTEEEANMETKPTGAQATDTETTGVEAMGVEATKTKAEEAEMQAYGVGAG
QAEPPVTGTTNMEATGSRATGMESTGVSATGVENPGVEATVPGISAGPILHPGAAEASEK
LQVELETRIRGLEEALRQREREAAAELEAALGKCEAAEAEAGRLRERVREAEGSGASGGG
GGDTTQLRAALEQAREDLRDRDSRLRELEAASACLDEARASRLLAEEEARGLRAELAQRE
EARLEQSRELEVLREQLATARATGEQQRTAAAELGRARDAAEARVAELPAACEEARQGLA
ELREASEALRQSVVPASEHRRLQEEALELRGRAASLEQEVVATGKEAARLRAELERERVC
SVALSEHERIVGTLQANVAQLEGQLEELGRRHEKTSAEVFQVQREALFMKSERHAAEAQL
ATAEQQLRGLRTEAERARQAQSRAQEALDKAKEKDKKITELSKEVFNLKEALKEQPAALA
TPEVEALRDQVKDLQQQLQEAARDHSSVVALYRSHLLYAIQGQMDEDVQRILSQILQMQR
LQAQGR
>sp|O15084|ANR28_HUMAN Serine/threonine-protein phosphatase 6 regulatory ankyrin repeat subunit A OS=Homo sapiens GN=ANKRD28 PE=1 SV=5
MAFLKLRDQPSLVQAIFNGDPDEVRALIFKKEDVNFQDNEKRTPLHAAAYLGDAEIIELL
ILSGARVNAKDSKWLTPLHRAVASCSEEAVQVLLKHSADVNARDKNWQTPLHIAAANKAV
KCAEALVPLLSNVNVSDRAGRTALHHAAFSGHGEMVKLLLSRGANINAFDKKDRRAIHWA
AYMGHIEVVKLLVSHGAEVTCKDKKSYTPLHAAASSGMISVVKYLLDLGVDMNEPNAYGN
TPLHVACYNGQDVVVNELIDCGAIVNQKNEKGFTPLHFAAASTHGALCLELLVGNGADVN
MKSKDGKTPLHMTALHGRFSRSQTIIQSGAVIDCEDKNGNTPLHIAARYGHELLINTLIT
SGADTAKRGIHGMFPLHLAALSGFSDCCRKLLSSGFDIDTPDDFGRTCLHAAAAGGNLEC
LNLLLNTGADFNKKDKFGRSPLHYAAANCNYQCLFALVGSGASVNDLDERGCTPLHYAAT
SDTDGKCLEYLLRNDANPGIRDKQGYNAVHYSAAYGHRLCLQLIASETPLDVLMETSGTD
MLSDSDNRATISPLHLAAYHGHHQALEVLVQSLLDLDVRNSSGRTPLDLAAFKGHVECVD
VLINQGASILVKDYILKRTPIHAAATNGHSECLRLLIGNAEPQNAVDIQDGNGQTPLMLS
VLNGHTDCVYSLLNKGANVDAKDKWGRTALHRGAVTGHEECVDALLQHGAKCLLRDSRGR
TPIHLSAACGHIGVLGALLQSAASMDANPATADNHGYTALHWACYNGHETCVELLLEQEV
FQKTEGNAFSPLHCAVINDNEGAAEMLIDTLGASIVNATDSKGRTPLHAAAFTDHVECLQ
LLLSHNAQVNSVDSTGKTPLMMAAENGQTNTVEMLVSSASAELTLQDNSKNTALHLACSK
GHETSALLILEKITDRNLINATNAALQTPLHVAARNGLTMVVQELLGKGASVLAVDENGY
TPALACAPNKDVADCLALILATMMPVSSSSPLSSLTFNAINRYTNTSKTVSFEALPIMRN
EPSSYCSFNNIGGEQEYLYTDVDELNDSDSETY
>sp|Q9ULJ7|ANR50_HUMAN Ankyrin repeat domain-containing protein 50 OS=Homo sapiens GN=ANKRD50 PE=1 SV=4

MTNPWEEKVCKMAQTSLLQGKQFYCREWVFHKLQHCLQEKSNCCNSAVNAPSLVMNSGNN
ASGVSGKGAAWGVLLVGGPGSGKTALCTELLWPSSPASLQRGLHRQALAFHFCKAQDSDT
LCVGGFIRGLVAQICRSGLLQGYEDKLRDPAVQSLLQPGECERNPAEAFKRCVLLPLLGM
KPPQQSLYLLVDSVDEGCNITEGEQTSTSLSGTVAALLAGHHEFFPPWLLLLCSARKQSK
AVTKMFTGFRKISLDDLRKAYIVKDVQQYILHRLDQEEALRQHLTKETAEMLNQLHIKSS
GCFLYLERVLDGVVENFIMLREIRDIPGTLNGLYLWLCQRLFVRKQFAKVQPILNVILAA
CRPLTITELYHAVWTKNMSLTLEDFQRKLDILSKLLVDGLGNTKILFHYSFAEWLLDVKH
CTQKYLCNAAEGHRMLAMSYTCQAKNLTPLEAQEFALHLINSNLQLETAELALWMIWNGT
PVRDSLSTLIPKEQEVLQLLVKAGAHVNSEDDRTSCIVRQALEREDSIRTLLDNGASVNQ
CDSNGRTLLANAAYSGSLDVVNLLVSRGADLEIEDAHGHTPLTLAARQGHTKVVNCLIGC
GANINHTDQDGWTALRSAAWGGHTEVVSALLYAGVKVDCADADSRTALRAAAWGGHEDIV
LNLLQHGAEVNKADNEGRTALIAAAYMGHREIVEHLLDHGAEVNHEDVDGRTALSVAALC
VPASKGHASVVSLLIDRGAEVDHCDKDGMTPLLVAAYEGHVDVVDLLLEGGADVDHTDNN
GRTPLLAAASMGHASVVNTLLFWGAAVDSIDSEGRTVLSIASAQGNVEVVRTLLDRGLDE
NHRDDAGWTPLHMAAFEGHRLICEALIEQGARTNEIDNDGRIPFILASQEGHYDCVQILL
ENKSNIDQRGYDGRNALRVAALEGHRDIVELLFSHGADVNCKDADGRPTLYILALENQLT
MAEYFLENGANVEASDAEGRTALHVSCWQGHMEMVQVLIAYHADVNAADNEKRSALQSAA
WQGHVKVVQLLIEHGAVVDHTCNQGATALCIAAQEGHIDVVQVLLEHGADPNHADQFGRT
AMRVAAKNGHSQIIKLLEKYGASSLNGCSPSPVHTMEQKPLQSLSSKVQSLTIKSNSSGS
TGGGDMQPSLRGLPNGPTHAFSSPSESPDSTVDRQKSSLSNNSLKSSKNSSLRTTSSTAT
AQTVPIDSFHNLSFTEQIQQHSLPRSRSRQSIVSPSSTTQSLGQSHNSPSSEFEWSQVKP
SLKSTKASKGGKSENSAKSGSAGKKAKQSNSSQPKVLEYEMTQFDRRGPIAKSGTAAPPK
QMPAESQCKIMIPSAQQEIGRSQQQFLIHQQSGEQKKRNGIMTNPNYHLQSNQVFLGRVS
VPRTMQDRGHQEVLEGYPSSETELSLKQALKLQIEGSDPSFNYKKETPL
>sp|B4E2M5|ANR66_HUMAN Ankyrin repeat domain-containing protein 66 OS=Homo sapiens GN=ANKRD66 PE=2 SV=2
MDTTLRMVRTACQHRAPQISHKTGCSHISMHSPGGLTTTKMAGPLPRVSDSLFSAMELAK
MSDMTKLHQAVAAGDYSLVKKILKKGLCDPNYKDVDWNDRTPLHWAAIKGQMEVIRLLIE
YGARPCLVTSVGWTPAHFAAEAGHLNILKTLHALHAAIDAPDFFGDTPKRIAQIYGQKAC
VAFLEKAEPECQDHRCAAQQKGLPLDERDEDWDAKKRELELSLPSLNQNMNKKNKKSRGP
TRPSNTKGRRV
>sp|O76027|ANXA9_HUMAN Annexin A9 OS=Homo sapiens GN=ANXA9 PE=1 SV=3
MSVTGGKMAPSLTQEILSHLGLASKTAAWGTLGTLRTFLNFSVDKDAQRLLRAITGQGVD
RSAIVDVLTNRSREQRQLISRNFQERTQQDLMKSLQAALSGNLERIVMALLQPTAQFDAQ
ELRTALKASDSAVDVAIEILATRTPPQLQECLAVYKHNFQVEAVDDITSETSGILQDLLL
ALAKGGRDSYSGIIDYNLAEQDVQALQRAEGPSREETWVPVFTQRNPEHLIRVFDQYQRS
TGQELEEAVQNRFHGDAQVALLGLASVIKNTPLYFADKLHQALQETEPNYQVLIRILISR
CETDLLSIRAEFRKKFGKSLYSSLQDAVKGDCQSALLALCRAEDM
>sp|P19801|AOC1_HUMAN Amiloride-sensitive amine oxidase [copper-containing] OS=Homo sapiens GN=AOC1 PE=1 SV=4
MPALGWAVAAILMLQTAMAEPSPGTLPRKAGVFSDLSNQELKAVHSFLWSKKELRLQPSS
TTTMAKNTVFLIEMLLPKKYHVLRFLDKGERHPVREARAVIFFGDQEHPNVTEFAVGPLP
GPCYMRALSPRPGYQSSWASRPISTAEYALLYHTLQEATKPLHQFFLNTTGFSFQDCHDR
CLAFTDVAPRGVASGQRRSWLIIQRYVEGYFLHPTGLELLVDHGSTDAGHWAVEQVWYNG

KFYGSPEELARKYADGEVDVVVLEDPLPGGKGHDSTEEPPLFSSHKPRGDFPSPIHVSGP
RLVQPHGPRFRLEGNAVLYGGWSFAFRLRSSSGLQVLNVHFGGERIAYEVSVQEAVALYG
GHTPAGMQTKYLDVGWGLGSVTHELAPGIDCPETATFLDTFHYYDADDPVHYPRALCLFE
MPTGVPLRRHFNSNFKGGFNFYAGLKGQVLVLRTTSTVYNYDYIWDFIFYPNGVMEAKMH
ATGYVHATFYTPEGLRHGTRLHTHLIGNIHTHLVHYRVDLDVAGTKNSFQTLQMKLENIT
NPWSPRHRVVQPTLEQTQYSWERQAAFRFKRKLPKYLLFTSPQENPWGHKRTYRLQIHSM
ADQVLPPGWQEEQAITWARYPLAVTKYRESELCSSSIYHQNDPWHPPVVFEQFLHNNENI
ENEDLVAWVTVGFLHIPHSEDIPNTATPGNSVGFLLRPFNFFPEDPSLASRDTVIVWPRD
NGPNYVQRWIPEDRDCSMPPPFSYNGTYRPV
>sp|O75106|AOC2_HUMAN Retina-specific copper amine oxidase OS=Homo sapiens GN=AOC2 PE=1 SV=2
MHLKIVLAFLALSLITIFALAYVLLTSPGGSSQPPHCPSVSHRAQPWPHPGQSQLFADLS
REELTAVMRFLTQRLGPGLVDAAQAQPSDNCIFSVELQLPPKAAALAHLDRGSPPPAREA
LAIVLFGGQPQPNVSELVVGPLPHPSYMRDVTVERHGGPLPYHRRPVLRAEFTQMWRHLK
EVELPKAPIFLSSTFNYNGSTLAAVHATPRGLRSGDRATWMALYHNISGVGLFLHPVGLE
LLLDHRALDPAHWTVQQVFYLGHYYADLGQLEREFKSGRLEVVRVPLPPPNGASSLRSRN
SPGPLPPLQFSPQGSQYSVQGNLVVSSLWSFTFGHGVFSGLRIFDVRFQGERIAYEVSVQ
ECVSIYGADSPKTMLTRYLDSSFGLGRNSRGLVRGVDCPYQATMVDIHILVGKGAVQLLP
GAVCVFEEAQGLPLRRHHNYLQNHFYGGLASSALVVRSVSSVGNYDYIWDFVLYPNGALE
GRVHATGYINTAFLKGGEEGLLFGNRVGERVLGTVHTHAFHFKLDLDVAGLKNWVVAEDV
VFKPVAAPWNPEHWLQRPQLTRQVLGKEDLTAFSLGSPLPRYLYLASNQTNAWGHQRGYR
IQIHSPLGIHIPLESDMERALSWGRYQLVVTQRKEEESQSSSIYHQNDIWTPTVTFADFI
NNETLLGEDLVAWVTASFLHIPHAEDIPNTVTLGNRVGFLLRPYNFFDEDPSIFSPGSVY
FEKGQDAGLCSINPVACLPDLAACVPDLPPFSYHGF
>sp|Q16853|AOC3_HUMAN Membrane primary amine oxidase OS=Homo sapiens GN=AOC3 PE=1 SV=3
MNQKTILVLLILAVITIFALVCVLLVGRGGDGGEPSQLPHCPSVSPSAQPWTHPGQSQLF
ADLSREELTAVMRFLTQRLGPGLVDAAQARPSDNCVFSVELQLPPKAAALAHLDRGSPPP
AREALAIVFFGRQPQPNVSELVVGPLPHPSYMRDVTVERHGGPLPYHRRPVLFQEYLDID
QMIFNRELPQASGLLHHCCFYKHRGRNLVTMTTAPRGLQSGDRATWFGLYYNISGAGFFL
HHVGLELLVNHKALDPARWTIQKVFYQGRYYDSLAQLEAQFEAGLVNVVLIPDNGTGGSW
SLKSPVPPGPAPPLQFYPQGPRFSVQGSRVASSLWTFSFGLGAFSGPRIFDVRFQGERLV
YEISLQEALAIYGGNSPAAMTTRYVDGGFGMGKYTTPLTRGVDCPYLATYVDWHFLLESQ
APKTIRDAFCVFEQNQGLPLRRHHSDLYSHYFGGLAETVLVVRSMSTLLNYDYVWDTVFH
PSGAIEIRFYATGYISSAFLFGATGKYGNQVSEHTLGTVHTHSAHFKVDLDVAGLENWVW
AEDMVFVPMAVPWSPEHQLQRLQVTRKLLEMEEQAAFLVGSATPRYLYLASNHSNKWGHP
RGYRIQMLSFAGEPLPQNSSMARGFSWERYQLAVTQRKEEEPSSSSVFNQNDPWAPTVDF
SDFINNETIAGKDLVAWVTAGFLHIPHAEDIPNTVTVGNGVGFFLRPYNFFDEDPSFYSA
DSIYFRGDQDAGACEVNPLACLPQAAACAPDLPAFSHGGFSHN
>sp|Q06278|AOXA_HUMAN Aldehyde oxidase OS=Homo sapiens GN=AOX1 PE=1 SV=2
MDRASELLFYVNGRKVIEKNVDPETMLLPYLRKKLRLTGTKYGCGGGGCGACTVMISRYN
PITKRIRHHPANACLIPICSLYGAAVTTVEGIGSTHTRIHPVQERIAKCHGTQCGFCTPG
MVMSIYTLLRNHPEPTLDQLTDALGGNLCRCTGYRPIIDACKTFCKTSGCCQSKENGVCC
LDQGINGLPEFEEGSKTSPKLFAEEEFLPLDPTQELIFPPELMIMAEKQSQRTRVFGSER
MMWFSPVTLKELLEFKFKYPQAPVIMGNTSVGPEVKFKGVFHPVIISPDRIEELSVVNHA

YNGLTLGAGLSLAQVKDILADVVQKLPEEKTQMYHALLKHLGTLAGSQIRNMASLGGHII
SRHPDSDLNPILAVGNCTLNLLSKEGKRQIPLNEQFLSKCPNADLKPQEILVSVNIPYSR
KWEFVSAFRQAQRQENALAIVNSGMRVFFGEGDGIIRELCISYGGVGPATICAKNSCQKL
IGRHWNEQMLDIACRLILNEVSLLGSAPGGKVEFKRTLIISFLFKFYLEVSQILKKMDPV
HYPSLADKYESALEDLHSKHHCSTLKYQNIGPKQHPEDPIGHPIMHLSGVKHATGEAIYC
DDMPLVDQELFLTFVTSSRAHAKIVSIDLSEALSMPGVVDIMTAEHLSDVNSFCFFTEAE
KFLATDKVFCVGQLVCAVLADSEVQAKRAAKRVKIVYQDLEPLILTIEESIQHNSSFKPE
RKLEYGNVDEAFKVVDQILEGEIHMGGQEHFYMETQSMLVVPKGEDQEMDVYVSTQFPKY
IQDIVASTLKLPANKVMCHVRRVGGAFGGKVLKTGIIAAVTAFAANKHGRAVRCVLERGE
DMLITGGRHPYLGKYKAGFMNDGRILALDMEHYSNAGASLDESLFVIEMGLLKMDNAYKF
PNLRCRGWACRTNLPSNTAFRGFGFPQAALITESCITEVAAKCGLSPEKVRIINMYKEID
QTPYKQEINAKNLIQCWRECMAMSSYSLRKVAVEKFNAENYWKKKGLAMVPLKFPVGLGS
RAAGQAAALVHIYLDGSVLVTHGGIEMGQGVHTKMIQVVSRELRMPMSNVHLRGTSTETV
PNANISGGSVVADLNGLAVKDACQTLLKRLEPIISKNPKGTWKDWAQTAFDESINLSAVG
YFRGYESDMNWEKGEGQPFEYFVYGAACSEVEIDCLTGDHKNIRTDIVMDVGCSINPAID
IGQIEGAFIQGMGLYTIEELNYSPQGILHTRGPDQYKIPAICDMPTELHIALLPPSQNSN
TLYSSKGLGESGVFLGCSVFFAIHDAVSAARQERGLHGPLTLNSPLTPEKIRMACEDKFT
KMIPRDEPGSYVPWNVPI
>sp|Q96PC3|AP1S3_HUMAN AP-1 complex subunit sigma-3 OS=Homo sapiens GN=AP1S3 PE=1 SV=1
MIHFILLFSRQGKLRLQKWYITLPDKERKKITREIVQIILSRGHRTSSFVDWKELKLVYK
RYASLYFCCAIENQDNELLTLEIVHRYVELLDKYFGNVCELDIIFNFEKAYFILDEFIIG
GEIQETSKKIAVKAIEDSDMLQEVSTVSQTMGER
>sp|P02649|APOE_HUMAN Apolipoprotein E OS=Homo sapiens GN=APOE PE=1 SV=1
MKVLWAALLVTFLAGCQAKVEQAVETEPEPELRQQTEWQSGQRWELALGRFWDYLRWVQT
LSEQVQEELLSSQVTQELRALMDETMKELKAYKSELEEQLTPVAEETRARLSKELQAAQA
RLGADMEDVCGRLVQYRGEVQAMLGQSTEELRVRLASHLRKLRKRLLRDADDLQKRLAVY
QAGAREGAERGLSAIRERLGPLVEQGRVRAATVGSLAGQPLQERAQAWGERLRARMEEMG
SRTRDRLDEVKEQVAEVRAKLEEQAQQIRLQAEAFQARLKSWFEPLVEDMQRQWAGLVEK
VQAAVGTSAAPVPSDNH
>sp|Q9BQE5|APOL2_HUMAN Apolipoprotein L2 OS=Homo sapiens GN=APOL2 PE=1 SV=1
MNPESSIFIEDYLKYFQDQVSRENLLQLLTDDEAWNGFVAAAELPRDEADELRKALNKLA
SHMVMKDKNRHDKDQQHRQWFLKEFPRLKRELEDHIRKLRALAEEVEQVHRGTTIANVVS
NSVGTTSGILTLLGLGLAPFTEGISFVLLDTGMGLGAAAAVAGITCSVVELVNKLRARAQ
ARNLDQSGTNVAKVMKEFVGGNTPNVLTLVDNWYQVTQGIGRNIRAIRRARANPQLGAYA
PPPHIIGRISAEGGEQVERVVEGPAQAMSRGTMIVGAATGGILLLLDVVSLAYESKHLLE
GAKSESAEELKKRAQELEGKLNFLTKIHEMLQPGQDQ
>sp|O95445|APOM_HUMAN Apolipoprotein M OS=Homo sapiens GN=APOM PE=1 SV=2
MFHQIWAALLYFYGIILNSIYQCPEHSQLTTLGVDGKEFPEVHLGQWYFIAGAAPTKEEL
ATFDPVDNIVFNMAAGSAPMQLHLRATIRMKDGLCVPRKWIYHLTEGSTDLRTEGRPDMK
TELFSSSCPGGIMLNETGQGYQRFLLYNRSPHPPEKCVEEFKSLTSCLDSKAFLLTPRNQ
EACELSNN
>sp|F7VJQ1|APRIO_HUMAN Alternative prion protein OS=Homo sapiens GN=PRNP PE=1 SV=1
MEHWGQPIPGAGQPWRQPLPTSGRWWLGAASWWWLGAASWWWLGAAPWWWLGTASWWWLG
SRRWHPQSVEQAE

>sp|Q53RT3|APRV1_HUMAN Retroviral-like aspartic protease 1 OS=Homo sapiens GN=ASPRV1 PE=1 SV=1
MGSPGASLGIKKALQSEQATALPASAPAVSQPTAPAPSCLPKAGQVIPTLLREAPFSSVI
APTLLCGFLFLAWVAAEVPEESSRMAGSGARSEEGRRQHAFVPEPFDGANVVPNLWLHSF
EVINDLNHWDHITKLRFLKESLRGEALGVYNRLSPQDQGDYGTVKEALLKAFGVPGAAPS
HLPKEIVFANSMGKGYYLKGKIGKVPVRFLVDSGAQVSVVHPNLWEEVTDGDLDTLQPFE
NVVKVANGAEMKILGVWDTAVSLGKLKLKAQFLVANASAEEAIIGTDVLQDHNAILDFEH
RTCTLKGKKFRLLPVGGSLEDEFDLELIEEDPSSEEGRQELSH
>sp|O60306|AQR_HUMAN Intron-binding protein aquarius OS=Homo sapiens GN=AQR PE=1 SV=4
MAAPAQPKKIVAPTVSQINAEFVTQLACKYWAPHIKKKSPFDIKVIEDIYEKEIVKSRFA
IRKIMLLEFSQYLENYLWMNYSPEVSSKAYLMSICCMVNEKFRENVPAWEIFKKKPDHFP
FFFKHILKAALAETDGEFSLHEQTVLLLFLDHCFNSLEVDLIRSQVQQLISLPMWMGLQL
ARLELELKKTPKLRKFWNLIKKNDEKMDPEAREQAYQERRFLSQLIQKFISVLKSVPLSE
PVTMDKVHYCERFIELMIDLEALLPTRRWFNTILDDSHLLVHCYLSNLVRREEDGHLFSQ
LLDMLKFYTGFEINDQTGNALTENEMTTIHYDRITSLQRAAFAHFPELYDFALSNVAEVD
TRESLVKFFGPLSSNTLHQVASYLCLLPTLPKNEDTTFDKEFLLELLVSRHERRISQIQQ
LNQMPLYPTEKIIWDENIVPTEYYSGEGCLALPKLNLQFLTLHDYLLRNFNLFRLESTYE
IRQDIEDSVSRMKPWQSEYGGVVFGGWARMAQPIVAFTVVEVAKPNIGENWPTRVRADVT
INLNVRDHIKDEWEGLRKHDVCFLITVRPTKPYGTKFDRRRPFIEQVGLVYVRGCEIQGM
LDDKGRVIEDGPEPRPNLRGESRTFRVFLDPNQYQQDMTNTIQNGAEDVYETFNIIMRRK
PKENNFKAVLETIRNLMNTDCVVPDWLHDIILGYGDPSSAHYSKMPNQIATLDFNDTFLS
IEHLKASFPGHNVKVTVEDPALQIPPFRITFPVRSGKGKKRKDADVEDEDTEEAKTLIVE
PHVIPNRGPYPYNQPKRNTIQFTHTQIEAIRAGMQPGLTMVVGPPGTGKTDVAVQIISNI
YHNFPEQRTLIVTHSNQALNQLFEKIMALDIDERHLLRLGHGEEELETEKDFSRYGRVNY
VLARRIELLEEVKRLQKSLGVPGDASYTCETAGYFFLYQVMSRWEEYISKVKNKGSTLPD
VTEVSTFFPFHEYFANAPQPIFKGRSYEEDMEIAEGCFRHIKKIFTQLEEFRASELLRSG
LDRSKYLLVKEAKIIAMTCTHAALKRHDLVKLGFKYDNILMEEAAQILEIETFIPLLLQN
PQDGFSRLKRWIMIGDHHQLPPVIKNMAFQKYSNMEQSLFTRFVRVGVPTVDLDAQGRAR
ASLCNLYNWRYKNLGNLPHVQLLPEFSTANAGLLYDFQLINVEDFQGVGESEPNPYFYQN
LGEAEYVVALFMYMCLLGYPADKISILTTYNGQKHLIRDIINRRCGNNPLIGRPNKVTTV
DRFQGQQNDYILLSLVRTRAVGHLRDVRRLVVAMSRARLGLYIFARVSLFQNCFELTPAF
SQLTARPLHLHIIPTEPFPTTRKNGERPSHEVQIIKNMPQMANFVYNMYMHLIQTTHHYH
QTLLQLPPAMVEEGEEVQNQETELETEEEAMTVQADIIPSPTDTSCRQETPAFQTDTTPS
ETGATSTPEAIPALSETTPTVVGAVSAPAEANTPQDATSAPEETK
>sp|A6NJG6|ARGFX_HUMAN Arginine-fifty homeobox OS=Homo sapiens GN=ARGFX PE=2 SV=1
MRNRMAPENPQPDPFINRNYSNMKVIPPQDPASPSFTLLSKLECSGTVSAYCSLNLPGST
DPPTSASRVAATTAIRRRHKERTSFTHQQYEELEALFSQTMFPDRNLQEKLALRLDLPES
TVKVWFRNRRFKLKKQQQQQSAKQRNQILPSKKNVPTSPRTSPSPYAFSPVISDFYSSLP
SQPLDPSNWAWNSTFTESSTSDFQMQDTQWERLVASVPALYSDAYDIFQIIELYNLPDEN
EISSSSFHCLYQYLSPTKYQVGGQGSSLSIFAGPAVGLSPAQTWPNMTSQAFEAYSLTDS
LEFQKTSNMVDLGFL
>sp|P05089|ARGI1_HUMAN Arginase-1 OS=Homo sapiens GN=ARG1 PE=1 SV=2
MSAKSRTIGIIGAPFSKGQPRGGVEEGPTVLRKAGLLEKLKEQECDVKDYGDLPFADIPN
DSPFQIVKNPRSVGKASEQLAGKVAEVKKNGRISLVLGGDHSLAIGSISGHARVHPDLGV

IWVDAHTDINTPLTTTSGNLHGQPVSFLLKELKGKIPDVPGFSWVTPCISAKDIVYIGLR
DVDPGEHYILKTLGIKYFSMTEVDRLGIGKVMEETLSYLLGRKKRPIHLSFDVDGLDPSF
TPATGTPVVGGLTYREGLYITEEIYKTGLLSGLDIMEVNPSLGKTPEEVTRTVNTAVAIT
LACFGLAREGNHKPIDYLNPPK
>sp|Q02040|AK17A_HUMAN A-kinase anchor protein 17A OS=Homo sapiens GN=AKAP17A PE=1 SV=2
MAAATIVHDTSEAVELCPAYGLYLKPITKMTISVALPQLKQPGKSISNWEVMERLKGMVQ
NHQFSTLRISKSTMDFIRFEGEVENKSLVKSFLACLDGKTIKLSGFSDILKVRAAEFKID
FPTRHDWDSFFRDAKDMNETLPGERPDTIHLEGLPCKWFALKESGSEKPSEDVLVKVFEK
FGEIRNVDIPMLDPYREEMTGRNFHTFSFGGHLNFEAYVVQYREYMGFIQAMSALRGMKLM
YKGEDGKAVACNIKVSFDSTKHLSDASIKKRQLERQKLQELEQQREEQKRREKEAEERQR
AEERKQKELEELERERKREEKLRKREQKQRDRELRRNQKKLEKLQAEEQKQLQEKIKLEE
RKLLLAQRNLQSIRLIAELLSRAKAVKLREQEQKEEKLRLQQQEERRRLQEAELRRVEEE
KERALGLQRKERELRERLLSILLSKKPDDSHTHDELGVAHADLLQPVLDILQTVSSGCVS
ATTLHPLGGQPPAGAPKESPAHPEADGAPKSVNGSVAEEAPCKEVQSSCRVVPEDGSPEK
RCPGGVLSCIPDNNQQPKGIPACEQNVSRKDTRSEQDKCNREPSKGRGRATGDGLADRHK
RERSRARRASSREDGRPRKERRPHKKHAYKDDSPRRRSTSPDHTRSRRSHSKDRHRRERS
RERRGSASRKHSRHRRRSERSRSRSPSRHRSTWNR
>sp|O60218|AK1BA_HUMAN Aldo-keto reductase family 1 member B10 OS=Homo sapiens GN=AKR1B10 PE=1 SV=2
MATFVELSTKAKMPIVGLGTWKSPLGKVKEAVKVAIDAGYRHIDCAYVYQNEHEVGEAIQ
EKIQEKAVKREDLFIVSKLWPTFFERPLVRKAFEKTLKDLKLSYLDVYLIHWPQGFKSGD
DLFPKDDKGNAIGGKATFLDAWEAMEELVDEGLVKALGVSNFSHFQIEKLLNKPGLKYKP
VTNQVECHPYLTQEKLIQYCHSKGITVTAYSPLGSPDRPWAKPEDPSLLEDPKIKEIAAK
HKKTAAQVLIRFHIQRNVIVIPKSVTPARIVENIQVFDFKLSDEEMATILSFNRNWRACN
VLQSSHLEDYPFNAEY
>sp|P17516|AK1C4_HUMAN Aldo-keto reductase family 1 member C4 OS=Homo sapiens GN=AKR1C4 PE=1 SV=3
MDPKYQRVELNDGHFMPVLGFGTYAPPEVPRNRAVEVTKLAIEAGFRHIDSAYLYNNEEQ
VGLAIRSKIADGSVKREDIFYTSKLWCTFFQPQMVQPALESSLKKLQLDYVDLYLLHFPM
ALKPGETPLPKDENGKVIFDTVDLSATWEVMEKCKDAGLAKSIGVSNFNCRQLEMILNKP
GLKYKPVCNQVECHPYLNQSKLLDFCKSKDIVLVAHSALGTQRHKLWVDPNSPVLLEDPV
LCALAKKHKQTPALIALRYQLQRGVVVLAKSYNEQRIRENIQVFEFQLTSEDMKVLDGLN
RNYRYVVMDFLMDHPDYPFSDEY
>sp|Q5T1N1|AKND1_HUMAN Protein AKNAD1 OS=Homo sapiens GN=AKNAD1 PE=2 SV=3
MDEADFSEHTTYKQEDLPYDGDLSQIKIGNDYSFTSKKDGLEVLNQIIFIADDPQEKAMH
SETCGNTAVTIPLGKITENAANKKDEKEKQCTAALHIPANEGDASKSSISDILLHHLSKE
PFLRGQGIDCETLPEISNADSFEEEAIIKSIISCYNKNSWPKEQTPELTDQLNPKRDGEN
SNKPGSATTTEENTSDLEGPVAAGDSSHQENVNVLTKTKGPGDKQKSYQGQSPQKQQTEK
ANSGNTFKYGQGQVHYQLPDFSKIAPKVKIPKNKIINKPLAIAKQASFSSKSRDKPTLVQ
DSLETTPESNCVEKQHQEQKGKITEPSQQIQMEPIVHIHQELLTGIESEASLSKLSPTSQ
KGTSSSSSYIFQKISQGKQMCQKLKEQTDQLKTKVQEFSKRIKQDSPYHLQDKKLVLEKL
QGHLELLEQNFLATKDKHLTLQQQVHKHESTIVGDFDPERKVEGEIFKLEMLLEDVKEKM
DESKYTSAPSLPVSSPVTLDDLASTFSSLSNEIPKEHPGHPSGPRGSGGSEVTGTPQGGP
QEAPNEELCELAPQTYLNGHYGDAAAQNKPDQVAMRLSSNSGEDPNGTPRRQDCAEMTAP

SPSCAFCRRLLEWKQNVEKKGHGRINCGRFSIVLHEKAPHSDSTPNSDTGHSFCSDSGTE
MQSNKCQDCGTKIPTSRRACRKEPTKEFHYRYNTPGQNYSNHSKRGAFVQPHSLDESKNS
SPSFLKPKRICSQRVNSKSFKGEHEPTPGKKKLQAFMTYSSDPATPSPHFYSCRISGSKS
LCDFDSTEEIKSEILNSALDHALRTATILKETTDQMIKTIAEDLAKAQRWRNRLKY
>sp|Q01432|AMPD3_HUMAN AMP deaminase 3 OS=Homo sapiens GN=AMPD3 PE=1 SV=1
MPRQFPKLNISEVDEQVRLLAEKVFAKVLREEDSKDALSLFTVPEDCPIGQKEAKERELQ
KELAEQKSVETAKRKKSFKMIRSQSLSLQMPPQQDWKGPPAASPAMSPTTPVVTGATSLP
TPAPYAMPEFQRVTISGDYCAGITLEDYEQAAKSLAKALMIREKYARLAYHRFPRITSQY
LGHPRADTAPPEEGLPDFHPPPLPQEDPYCLDDAPPNLDYLVHMQGGILFVYDNKKMLEH
QEPHSLPYPDLETYTVDMSHILALITDGPTKTYCHRRLNFLESKFSLHEMLNEMSEFKEL
KSNPHRDFYNVRKVDTHIHAAACMNQKHLLRFIKHTYQTEPDRTVAEKRGRKITLRQVFD
GLHMDPYDLTVDSLDVHAGRQTFHRFDKFNSKYNPVGASELRDLYLKTENYLGGEYFARM
VKEVARELEESKYQYSEPRLSIYGRSPEEWPNLAYWFIQHKVYSPNMRWIIQVPRIYDIF
RSKKLLPNFGKMLENIFLPLFKATINPQDHRELHLFLKYVTGFDSVDDESKHSDHMFSDK
SPNPDVWTSEQNPPYSYYLYYMYANIMVLNNLRRERGLSTFLFRPHCGEAGSITHLVSAF
LTADNISHGLLLKKSPVLQYLYYLAQIPIAMSPLSNNSLFLEYSKNPLREFLHKGLHVSL
STDDPMQFHYTKEALMEEYAIAAQVWKLSTCDLCEIARNSVLQSGLSHQEKQKFLGQNYY
KEGPEGNDIRKTNVAQIRMAFRYETLCNELSFLSDAMKSEEITALTN
>sp|Q6UX39|AMTN_HUMAN Amelotin OS=Homo sapiens GN=AMTN PE=1 SV=1
MRSTILLFCLLGSTRSLPQLKPALGLPPTKLAPDQGTLPNQQQSNQVFPSLSLIPLTQML
TLGPDLHLLNPAAGMTPGTQTHPLTLGGLNVQQQLHPHVLPIFVTQLGAQGTILSSEELP
QIFTSLIIHSLFPGGILPTSQAGANPDVQDGSLPAGGAGVNPATQGTPAGRLPTPSGTDD
DFAVTTPAGIQRSTHAIEEATTESANGIQ
>sp|Q86W34|AMZ2_HUMAN Archaemetzincin-2 OS=Homo sapiens GN=AMZ2 PE=1 SV=2
MQIIRHSEQTLKTALISKNPVLVSQYEKLNAGEQRLMNEAFQPASDLFGPITLHSPSDWI
TSHPEAPQDFEQFFSDPYRKTPSPNKRSIYIQSIGSLGNTRIISEEYIKWLTGYCKAYFY
GLRVKLLEPVPVSVTRCSFRVNENTHNLQIHAGDILKFLKKKKPEDAFCVVGITMIDLYP
RDSWNFVFGQASLTDGVGIFSFARYGSDFYSMHYKGKVKKLKKTSSSDYSIFDNYYIPEI
TSVLLLRSCKTLTHEIGHIFGLRHCQWLACLMQGSNHLEEADRRPLNLCPICLHKLQCAV
GFSIVERYKALVRWIDDESSDTPGATPEHSHEDNGNLPKPVEAFKEWKEWIIKCLAVLQK
>sp|A5PLL1|AN34B_HUMAN Ankyrin repeat domain-containing protein 34B OS=Homo sapiens GN=ANKRD34B PE=2 SV=3
MDEGMEISSEGNSLIKAVHQSRLRLTRLLLEGGAYINESNDRGETPLMIACKTKHVDHQS
VSKAKMVKYLLENNADPNIQDKSGKTALMHACLEKAGPEVVSLLLKSGADLSLQDHSSYS
ALVYAINSEDTETLKVLLSACKAKGKEVIIITTAKLPCGKHTTKQYLNMPPVDIDGCHSP
ATCTTPSEIDIKTASSPLSHSSETELTLFGFKDLELAGSNDDTWDPGSPVRKPALAPKGP
KLPHAPPWVKSPPLLMHQNRVASLQEELQDITPEEELSYKTNGLALSKRFITRHQSIDVK
DTAHLLRAFDQASSRKMSYDEINCQSYLSEGNQQCIEVPVDQDPDSNQTIFASTLRSIVQ
KRNLGANHYSSDSQLSAGLTPPTSEDGKALIGKKKILSPSPSQLSESKELLENIPPGPLS
RRNHAVLERRGSGAFPLDHSVTQTRQGFLPPLNVNSHPPISDINVNNKICSLLSCGQKVL
MPTVPIFPKEFKSKKMLLRRQSLQTEQIKQLVNF
>sp|P10275|ANDR_HUMAN Androgen receptor OS=Homo sapiens GN=AR PE=1 SV=3
MEVQLGLGRVYPRPPSKTYRGAFQNLFQSVREVIQNPGPRHPEAASAAPPGASLLLLQQQ
QQQQQQQQQQQQQQQQQQQQETSPRQQQQQQGEDGSPQAHRRGPTGYLVLDEEQQPSQPQ

SALECHPERGCVPEPGAAVAASKGLPQQLPAPPDEDDSAAPSTLSLLGPTFPGLSSCSAD
LKDILSEASTMQLLQQQQQEAVSEGSSSGRAREASGAPTSSKDNYLGGTSTISDNAKELC
KAVSVSMGLGVEALEHLSPGEQLRGDCMYAPLLGVPPAVRPTPCAPLAECKGSLLDDSAG
KSTEDTAEYSPFKGGYTKGLEGESLGCSGSAAAGSSGTLELPSTLSLYKSGALDEAAAYQ
SRDYYNFPLALAGPPPPPPPPHPHARIKLENPLDYGSAWAAAAAQCRYGDLASLHGAGAA
GPGSGSPSAAASSSWHTLFTAEEGQLYGPCGGGGGGGGGGGGGGGGGGGGGGGGEAGAVAP
YGYTRPPQGLAGQESDFTAPDVWYPGGMVSRVPYPSPTCVKSEMGPWMDSYSGPYGDMRL
ETARDHVLPIDYYFPPQKTCLICGDEASGCHYGALTCGSCKVFFKRAAEGKQKYLCASRN
DCTIDKFRRKNCPSCRLRKCYEAGMTLGARKLKKLGNLKLQEEGEASSTTSPTEETTQKL
TVSHIEGYECQPIFLNVLEAIEPGVVCAGHDNNQPDSFAALLSSLNELGERQLVHVVKWA
KALPGFRNLHVDDQMAVIQYSWMGLMVFAMGWRSFTNVNSRMLYFAPDLVFNEYRMHKSR
MYSQCVRMRHLSQEFGWLQITPQEFLCMKALLLFSIIPVDGLKNQKFFDELRMNYIKELD
RIIACKRKNPTSCSRRFYQLTKLLDSVQPIARELHQFTFDLLIKSHMVSVDFPEMMAEII
SVQVPKILSGKVKPIYFHTQ
>sp|Q9NUS5|AP5S1_HUMAN AP-5 complex subunit sigma-1 OS=Homo sapiens GN=AP5S1 PE=1 SV=1
MVHAFLIHTLRAPNTEDTGLCRVLYSCVFGAEKSPDDPRPHGAERDRLLRKEQILAVARQ
VESMCRLQQQASGRPPMDLQPQSSDEQVPLHEAPRGAFRLAAENPFQEPRTVVWLGVLSL
GFALVLDAHENLLLAEGTLRLLTRLLLDHLRLLAPSTSLLLRADRIEGILTRFLPHGQLL
FLNDQFVQGLEKEFSAAWPR
>sp|O96018|APBA3_HUMAN Amyloid beta A4 precursor protein-binding family A member 3 OS=Homo sapiens GN=APBA3 PE=1 SV=1
MDFPTISRSPSGPPAMDLEGPRDILVPSEDLTPDSQWDPMPGGPGSLSRMELDESSLQEL
VQQFEALPGDLVGPSPGGAPCPLHIATGHGLASQEIADAHGLLSAEAGRDDLLGLLHCEE
CPPSQTGPEEPLEPAPRLLQPPEDPDEDSDSPEWVEGASAEQEGSRSSSSSPEPWLETVP
LVTPEEPPAGAQSPETLASYPAPQEVPGPCDHEDLLDGVIFGARYLGSTQLVSERNPPTS
TRMAQAREAMDRVKAPDGETQPMTEVDLFVSTKRIKVLTADSQEAMMDHALHTISYTADI
GCVLVLMARRRLARRPAPQDHGRRLYKMLCHVFYAEDAQLIAQAIGQAFAAAYSQFLRES
GIDPSQVGVHPSPGACHLHNGDLDHFSNSDNCREVHLEKRRGEGLGVALVESGWGSLLPT
AVIANLLHGGPAERSGALSIGDRLTAINGTSLVGLPLAACQAAVRETKSQTSVTLSIVHC
PPVTTAIIHRPHAREQLGFCVEDGIICSLLRGGIAERGGIRVGHRIIEINGQSVVATPHA
RIIELLTEAYGEVHIKTMPAATYRLLTGQEQPVYL
>sp|O00213|APBB1_HUMAN Amyloid beta A4 precursor protein-binding family B member 1 OS=Homo sapiens GN=APBB1 PE=1 SV=2
MSVPSSLSQSAINANSHGGPALSLPLPLHAAHNQLLNAKLQATAVGPKDLRSAMGEGGGP
EPGPANAKWLKEGQNQLRRAATAHRDQNRNVTLTLAEEASQEPEMAPLGPKGLIHLYSEL
ELSAHNAANRGLRGPGLIISTQEQGPDEGEEKAAGEAEEEEEDDDDEEEEEDLSSPPGLP
EPLESVEAPPRPQALTDGPREHSKSASLLFGMRNSAASDEDSSWATLSQGSPSYGSPEDT
DSFWNPNAFETDSDLPAGWMRVQDTSGTYYWHIPTGTTQWEPPGRASPSQGSSPQEESQL
TWTGFAHGEGFEDGEFWKDEPSDEAPMELGLKEPEEGTLTFPAQSLSPEPLPQEEEKLPP
RNTNPGIKCFAVRSLGWVEMTEEELAPGRSSVAVNNCIRQLSYHKNNLHDPMSGGWGEGK
DLLLQLEDETLKLVEPQSQALLHAQPIISIRVWGVGRDSGRERDFAYVARDKLTQMLKCH
VFRCEAPAKNIATSLHEICSKIMAERRNARCLVNGLSLDHSKLVDVPFQVEFPAPKNELV
QKFQVYYLGNVPVAKPVGVDVINGALESVLSSSSREQWTPSHVSVAPATLTILHQQTEAV
LGECRVRFLSFLAVGRDVHTFAFIMAAGPASFCCHMFWCEPNAASLSEAVQAACMLRYQK

CLDARSQASTSCLPAPPAESVARRVGWTVRRGVQSLWGSLKPKRLGAHTP
>sp|Q92870|APBB2_HUMAN Amyloid beta A4 precursor protein-binding family B member 2 OS=Homo sapiens GN=APBB2 PE=1 SV=3
MSEVLPADSGVDTLAVFMASSGTTDVTNRNSPATPPNTLNLRSSHNELLNAEIKHTETKN
STPPKCRKKYALTNIQAAMGLSDPAAQPLLGNGSANIKLVKNGENQLRKAAEQGQQDPNK
NLSPTAVINITSEKLEGKEPHPQDSSSCEILPSQPRRTKSFLNYYADLETSARELEQNRG
NHHGTAEEKSQPVQGQASTIIGNGDLLLQKPNRPQSSPEDGQVATVSSSPETKKDHPKTG
AKTDCALHRIQNLAPSDEESSWTTLSQDSASPSSPDETDIWSDHSFQTDPDLPPGWKRVS
DIAGTYYWHIPTGTTQWERPVSIPADLQGSRKGSLSSVTPSPTPENEKQPWSDFAVLNGG
KINSDIWKDLHAATVNPDPSLKEFEGATLRYASLKLRNAPHPDDDDSCSINSDPEAKCFA
VRSLGWVEMAEEDLAPGKSSVAVNNCIRQLSYCKNDIRDTVGIWGEGKDMYLILENDMLS
LVDPMDRSVLHSQPIVSIRVWGVGRDNGRDFAYVARDKDTRILKCHVFRCDTPAKAIATS
LHEICSKIMAERKNAKALACSSLQERANVNLDVPLQVDFPTPKTELVQKFHVQYLGMLPV
DKPVGMDILNSAIENLMTSSNKEDWLSVNMNVADATVTVISEKNEEEVLVECRVRFLSFM
GVGKDVHTFAFIMDTGNQRFECHVFWCEPNAGNVSEAVQAACMLRYQKCLVARPPSQKVR
PPPPPADSVTRRVTTNVKRGVLSLIDTLKQKRPVTEMP
>sp|O95236|APOL3_HUMAN Apolipoprotein L3 OS=Homo sapiens GN=APOL3 PE=1 SV=3
MGLGQGWGWEASCFACLIRSCCQVVTFTFPFGFQGISQSLENVSGYYADARLEVGSTQLR
TAGSCSHSFKRSFLEKKRFTEEATKYFRERVSPVHLQILLTNNEAWKRFVTAAELPRDEA
DALYEALKKLRTYAAIEDEYVQQKDEQFREWFLKEFPQVKRKIQESIEKLRALANGIEEV
HRGCTISNVVSSSTGAASGIMSLAGLVLAPFTAGTSLALTAAGVGLGAASAVTGITTSIV
EHSYTSSAEAEASRLTATSIDRLKVFKEVMRDITPNLLSLLNNYYEATQTIGSEIRAIRQ
ARARARLPVTTWRISAGSGGQAERTIAGTTRAVSRGARILSATTSGIFLALDVVNLVYES
KHLHEGAKSASAEELRRQAQELEENLMELTQIYQRLNPCHTH
>sp|Q96P48|ARAP1_HUMAN Arf-GAP with Rho-GAP domain, ANK repeat and PH domain-containing protein 1 OS=Homo sapiens GN=ARAP1 PE=1 SV=3
MAEAGDAALSVAEWLRALHLEQYTGLFEQHGLVWATECQGLSDTRLMDMGMLLPGHRRRI
LAGLLRAHTSPAPAPRPTPRPVPMKRHIFRSPPVPATPPEPLPTTTEDEGLPAAPPIPPR
RSCLPPTCFTTPSTAAPDPVLPPLPAKRHLAELSVPPVPPRTGPPRLLVSLPTKEEESLL
PSLSSPPQPQSEEPLSTLPQGPPQPPSPPPCPPEIPPKPVRLFPEFDDSDYDEVPEEGPG
APARVMTKKEEPPPSRVPRAVRVASLLSEGEELSGDDQGDEEEDDHAYEGVPNGGWHTSS
LSLSLPSTIAAPHPMDGPPGGSTPVTPVIKAGWLDKNPPQGSYIYQKRWVRLDTDHLRYF
DSNKDAYSKRFISVACISHVAAIGDQKFEVITNNRTFAFRAESDVERKEWMQALQQAMAE
QRARARLSSAYLLGVPGSEQPDRAGSLELRGFKNKLYVAVVGDKVQLYKNLEEYHLGIGI
TFIDMSVGNVKEVDRRSFDLTTPYRIFSFSADSELEKEQWLEAMQGAIAEALSTSEVAER
IWAAAPNRFCADCGAPQPDWASINLCVVICKRCAGEHRGLGAGVSKVRSLKMDRKVWTET
LIELFLQLGNGAGNRFWAANVPPSEALQPSSSPSTRRCHLEAKYREGKYRRYHPLFGNQE
ELDKALCAAVTTTDLAETQALLGCGAGINCFSGDPEAPTPLALAEQAGQTLQMEFLRNNR
TTEVPRLDSMKPLEKHYSVVLPTVSHSGFLYKTASAGKLLQDRRAREEFSRRWCVLGDGV
LSYFENERAVTPNGEIRASEIVCLAVPPPDTHGFEHTFEVYTEGERLYLFGLESAEQAHE
WVKCIAKAFVPPLAEDLLARDFERLGRLPYKAGLSLQRAQEGWFSLSGSELRAVFPEGPC
EEPLQLRKLQELSIQGDSENQVLVLVERRRTLYIQGERRLDFMGWLGAIQKAAASMGDTL
SEQQLGDSDIPVIVYRCVDYITQCGLTSEGIYRKCGQTSKTQRLLESLRQDARSVHLKEG
EQHVDDVSSALKRFLRDLPDGLFTRAQRLTWLEASEIEDEEEKVSRYRELLVRLPPVNRA

TVKALISHLYCVQCFSDTNQMNVHNLAIVFGPTLFQTDGQDYKAGRVVEDLINHYVVVFS
VDEEELRKQREEITAIVKMRVAGTASGTQHAGDFICTVYLEEKKAETEQHIKVPASMTAE
ELTLEILDRRNVGIREKDYWTCFEVNEREEAERPLHFAEKVLPILHGLGTDSHLVVKKHQ
AMEAMLLYLASRVGDTKHGMMKFREDRSLLGLGLPSGGFHDRYFILNSSCLRLYKEVRSQ
RPWSGAPETSHRPEKEWPIKSLKVYLGVKKKLRPPTCWGFTVVHETEKHEKQQWYLCCDT
QMELREWFATFLFVQHDGLVWPSEPSRVSRAVPEVRLGSVSLIPLRGSENEMRRSVAAFT
ADPLSLLRNV
>sp|Q8WZ64|ARAP2_HUMAN Arf-GAP with Rho-GAP domain, ANK repeat and PH domain-containing protein 2 OS=Homo sapiens GN=ARAP2 PE=1 SV=3
MSSVSEVNVDIKDFLMSINLEQYLLHFHESGFTTVKDCAAINDSLLQKIGISPTGHRRRI
LKQLQIILSKMQDIPIYANVHKTKKNDDPSKDYHVPSSDQNICIELSNSGSVQTSSPPQL
ETVRKNLEDSDASVERSQYPQSDDKLSPPKRDFPTAEEPHLNLGSLNDSLFGSDNIKIES
LITKKTVDHTVEEQQTEKVKLITENLSKLPNADSECLSFVGCSTSGTNSGNGTNGLLEGS
PPSPFFKFQGEMIVNDLYVPSSPILAPVRSRSKLVSRPSRSFLLRHRPVPEIPGSTKGVS
GSYFRERRNVATSTEKSVAWQNSNEENSSSIFPYGETFLFQRLENSKKRSIKNEFLTQGE
ALKGEAATATNSFIIKSSIYDNRKEKISEDKVEDIWIPREDKNNFLIDTASESEYSTVEE
CFQSLRRKNSKASKSRTQKALILDSVNRHSYPLSSTSGNADSSAVSSQAISPYACFYGAS
AKKVKSGWLDKLSPQGKRMFQKRWVKFDGLSISYYNNEKEMYSKGIIPLSAISTVRVQGD
NKFEVVTTQRTFVFRVEKEEERNDWISILLNALKSQSLTSQSQAVVTPEKCGYLELRGYK
AKIFTVLSGNSVWLCKNEQDFKSGLGITIIPMNVANVKQVDRTVKQSFEIITPYRSFSFT
AETEKEKQDWIEAVQQSIAETLSDYEVAEKIWFNESNRSCADCKAPDPDWASINLCVVIC
KKCAGQHRSLGPKDSKVRSLKMDASIWSNELIELFIVIGNKRANDFWAGNLQKDEELHMD
SPVEKRKNFITQKYKEGKFRKTLLASLTKEELNKALCAAVVKPDVLETMALLFSGADVMC
ATGDPVHSTPYLLAKKAGQSLQMEFLYHNKFSDFPQHDIHSEGVLSQESSQSTFLCDFLY
QAPSAASKLSSEKKLLEETNKKWCVLEGGFLSYYENDKSTTPNGTININEVICLAIHKED
FYLNTGPIFIFEIYLPSERVFLFGAETSQAQRKWTEAIAKHFVPLFAENLTEADYDLIGQ
LFYKDCHALDQWRKGWFAMDKSSLHFCLQMQEVQGDRMHLRRLQELTISTMVQNGEKLDV
LLLVEKGRTLYIHGHTKLDFTVWHTAIEKAAGTDGNALQDQQLSKNDVPIIVNSCIAFVT
QYGLGCKYIYQKNGDPLHISELLESFKKDARSFKLRAGKHQLEDVTAVLKSFLSDIDDAL
LTKELYPYWISALDTQDDKERIKKYGAFIRSLPGVNRATLAAIIEHLYRVQKCSEINHMN
AHNLALVFSSCLFQTKGQTSEEVNVIEDLINNYVEIFEVKEDQVKQMDIENSFITKWKDT
QVSQAGDLLIEVYVERKEPDCSIIIRISPVMEAEELTNDILAIKNIIPTKGDIWATFEVI
ENEELERPLHYKENVLEQVLRWSSLAEPGSAYLVVKRFLTADTIKHCSDRSTLGSIKEGI
LKIKEEPSKILSGNKFQDRYFVLRDGFLFLYKDVKSSKHDKMFSLSSMKFYRGVKKKMKP
PTSWGLTAYSEKHHWHLCCDSSRTQTEWMTSIFIAQHEYDIWPPAGKERKRSITKNPKIG
GLPLIPIQHEGNATLARKNIESARAELERLRLSEKCDKESVDSSLKERASMVAHCLEHKD
DKLRNRPRKHRSFNCLEDTEPEAPLGQPKGHKGLKTLRKTEDRNSKATLDSDHKLPSRVI
EELNVVLQRSRTLPKELQDEQILK
>sp|Q96IT6|ARAS1_HUMAN Putative uncharacterized protein ARHGAP5-AS1 OS=Homo sapiens GN=ARHGAP5-AS1 PE=5 SV=1
MQAPDSVRSVKVEREAKTWIEKPRGAGLRVAQKTPVHATTSLTLGTVVHLAFIILP
>sp|P35626|ARBK2_HUMAN Beta-adrenergic receptor kinase 2 OS=Homo sapiens GN=GRK3 PE=1 SV=2
MADLEAVLADVSYLMAMEKSKATPAARASKRIVLPEPSIRSVMQKYLAERNEITFDKIFN

QKIGFLLFKDFCLNEINEAVPQVKFYEEIKEYEKLDNEEDRLCRSRQIYDAYIMKELLSC
SHPFSKQAVEHVQSHLSKKQVTSTLFQPYIEEICESLRGDIFQKFMESDKFTRFCQWKNV
ELNIHLTMNEFSVHRIIGRGGFGEVYGCRKADTGKMYAMKCLDKKRIKMKQGETLALNER
IMLSLVSTGDCPFIVCMTYAFHTPDKLCFILDLMNGGDLHYHLSQHGVFSEKEMRFYATE
IILGLEHMHNRFVVYRDLKPANILLDEHGHARISDLGLACDFSKKKPHASVGTHGYMAPE
VLQKGTAYDSSADWFSLGCMLFKLLRGHSPFRQHKTKDKHEIDRMTLTVNVELPDTFSPE
LKSLLEGLLQRDVSKRLGCHGGGSQEVKEHSFFKGVDWQHVYLQKYPPPLIPPRGEVNAA
DAFDIGSFDEEDTKGIKLLDCDQELYKNFPLVISERWQQEVTETVYEAVNADTDKIEARK
RAKNKQLGHEEDYALGKDCIMHGYMLKLGNPFLTQWQRRYFYLFPNRLEWRGEGESRQNL
LTMEQILSVEETQIKDKKCILFRIKGGKQFVLQCESDPEFVQWKKELNETFKEAQRLLRR
APKFLNKPRSGTVELPKPSLCHRNSNGL
>sp|Q92747|ARC1A_HUMAN Actin-related protein 2/3 complex subunit 1A OS=Homo sapiens GN=ARPC1A PE=1 SV=2
MSLHQFLLEPITCHAWNRDRTQIALSPNNHEVHIYKKNGSQWVKAHELKEHNGHITGIDW
APKSDRIVTCGADRNAYVWSQKDGVWKPTLVILRINRAATFVKWSPLENKFAVGSGARLI
SVCYFESENDWWVSKHIKKPIRSTVLSLDWHPNNVLLAAGSCDFKCRVFSAYIKEVDEKP
ASTPWGSKMPFGQLMSEFGGSGTGGWVHGVSFSASGSRLAWVSHDSTVSVADASKSVQVS
TLKTEFLPLLSVSFVSENSVVAAGHDCCPMLFNYDDRGCLTFVSKLDIPKQSIQRNMSAM
ERFRNMDKRATTEDRNTALETLHQNSITQVSIYEVDKQDCRKFCTTGIDGAMTIWDFKTL
ESSIQGLRIM
>sp|Q7LC44|ARC_HUMAN Activity-regulated cytoskeleton-associated protein OS=Homo sapiens GN=ARC PE=2 SV=1
MELDHRTSGGLHAYPGPRGGQVAKPNVILQIGKCRAEMLEHVRRTHRHLLAEVSKQVERE
LKGLHRSVGKLESNLDGYVPTSDSQRWKKSIKACLCRCQETIANLERWVKREMHVWREVF
YRLERWADRLESTGGKYPVGSESARHTVSVGVGGPESYCHEADGYDYTVSPYAITPPPAA
GELPGQEPAEAQQYQPWVPGEDGQPSPGVDTQIFEDPREFLSHLEEYLRQVGGSEEYWLS
QIQNHMNGPAKKWWEFKQGSVKNWVEFKKEFLQYSEGTLSREAIQRELDLPQKQGEPLDQ
FLWRKRDLYQTLYVDADEEEIIQYVVGTLQPKLKRFLRHPLPKTLEQLIQRGMEVQDDLE
QAAEPAGPHLPVEDEAETLTPAPNSESVASDRTQPE
>sp|P84085|ARF5_HUMAN ADP-ribosylation factor 5 OS=Homo sapiens GN=ARF5 PE=1 SV=2
MGLTVSALFSRIFGKKQMRILMVGLDAAGKTTILYKLKLGEIVTTIPTIGFNVETVEYKN
ICFTVWDVGGQDKIRPLWRHYFQNTQGLIFVVDSNDRERVQESADELQKMLQEDELRDAV
LLVFANKQDMPNAMPVSELTDKLGLQHLRSRTWYVQATCATQGTGLYDGLDWLSHELSKR
>sp|P62330|ARF6_HUMAN ADP-ribosylation factor 6 OS=Homo sapiens GN=ARF6 PE=1 SV=2
MGKVLSKIFGNKEMRILMLGLDAAGKTTILYKLKLGQSVTTIPTVGFNVETVTYKNVKFN
VWDVGGQDKIRPLWRHYYTGTQGLIFVVDCADRDRIDEARQELHRIINDREMRDAIILIF
ANKQDLPDAMKPHEIQEKLGLTRIRDRNWYVQPSCATSGDGLYEGLTWLTSNYKS
>sp|Q69YU3|AN34A_HUMAN Ankyrin repeat domain-containing protein 34A OS=Homo sapiens GN=ANKRD34A PE=2 SV=2
MLHTEGHALLRAVGQGKLRLARLLLEGGAYVNEGDAQGETALMAACRARYDDPQNKARMV
RYLLEQGADPNIADRLGRTALMHACAGGGGAAVASLLLAHGADPSVRDHAGASALVHALD
RGDRETLATLLDACKAKGTEVIIITTDTSPSGTKKTRQYLNSPPSPGVEDPAPASPSPGF
CTSPSEIQLQTAGGGGRGMLSPRAQEEEEKRDVFEFPLPKPPDDPSPSEPLPKPPRHPPK
PLKRLNSEPWGLVAPPQPVPPTEGRPGIERLTAEFNGLTLTGRPRLSRRHSTEGPEDPPP

WAEKVTSGGPLSRRNTAPEAQESGPPSGLRQKLSRMEPVELDTPGHLCPDSPESSRLSLE
RRRYSASPLTLPPAGSAPSPRQSQESLPGAVSPLSGRRRSPGLLERRGSGTLLLDHISQT
RPGFLPPLNVSPHPPIPDIRPQPGGRAPSLPAPPYAGAPGSPRTKRKLVRRHSMQTEQIR
LLGGFQSLGGPGEPGR
>sp|A6QL64|AN36A_HUMAN Ankyrin repeat domain-containing protein 36A OS=Homo sapiens GN=ANKRD36 PE=2 SV=3
MEDGKRERWPTLMERLCSDGFAFPQYPIKPYHLKRIHRAVLHGNLEKLKYLLLTYYDANK
RDRKERTALHLACATGQPEMVHLLVSRRCELNLCDREDRTPLIKAVQLRQEACATLLLQN
GANPNITDFFGRTALHYAVYNEDTSMIEKLLSHGTNIEECSKCEYQPLLFAVSRRKVKMV
EFLLKKKANVNAIDYLGRSALIHAVTLGEKDIVILLLQHNIDVLSRDAFRKIAGDYAIEA
KNRVIFDLIYEYERKRYEDLPINSNPVSSQKQPALKATSGKEDSISNIATEIKDGQKSGT
VSSQKQPALKDTSDKDDSVSNTATEIKDEQKSGTVLPAVEQCLNRSLYRPDAVAQPVTEN
EFSLESEIISKLYIPKRKIISPRSIKDVLPPVEEAVDRCLYLLDRFAQPVTKDKFALESE
NISEPYFTNRRTISQQSAENLDAACGIDKTENGNMFEDQNVDKEGKALPATGQKANVSPE
QPPLFTHTVKDRDHISTRFLGGMDSLTSSEESSERPPLSTLTLKEADPSSKAAMRRKDSP
PPGKVSSQKQPAEKATSDDKDSVSNIATEIKEGPISGTVSSQKQPAEKATSDEKDSVSNI
ATEIKKGQQSGTVSPQKQSAWKVIFKKKVSLLNIATRIMGGGKSGTVSSQKQPASKATSD
KTDSALNIATEIKDGLQCGTVSSQKQPALKATTDEEDSVSNIATEIKDGEKSGTVSSQKQ
PALKATTDEEDSVSNIATEIKDGEKSGTVSSQKQPALKATTDEKDSVSNIATEIKDGEKS
GTVSSQKPPALTATSDEEGSVLSIARENKDGEKSRTVSSRKKPALKATSDEKDSFSNITR
GKKDGEISRKVSSQKPPTLKGTSDEEDSVLGIARENKDGEKSRTVSSEKPPGLKASSAEK
DSVLNIARGKKDGEKTKRVSSRKKPSLEATSDEKDSFSNITREKKDGEISRKVSSQKPPA
LKGTSDEEDSVLGIARENKDGEKSRTVSSEKPPGLKATSDEKDSVLNIARGKKDGEKTRT
VSSQKPPTLKATSDEEDSVLSIARENKDGEKSRTVSSEKPSGLKATSAEKDSVLNIARGK
KYGEKTKRVSSRKKPALKATSDEKDSVLYIAREKKDGEKSRTVSSPKQPALKAICDKEDS
VPNMATEKKDEQISGTVSCQKQPALKATSDKKDSVSNIPTEIKDGQQSGTVSSQKQPAWK
ATSVKKDSVSNIATEIKDGQIRGTGILEYTFNVMFDQIEEKFTSLNKSAGVSPQKQSAQK
VIFKKKVSLLNIATRITGGWKSGTEYPENLPTLKATIENKNSVLNTATKMKDVQTSTPAE
QDLEMASEGEQKRLEEYENNQPQVKNQIHSRDDLDDIIQSSQTVSEDGDSLCCNCKNVIL
LIDQHEMKCKDCVHLLKIKNTFCLWKRLIKLKDNHCEQLRVKIRKLKNKASVLQKRISEK
EEIKSQLKHEILELEKELCSLRFAIQQEKKKRRNVEEVHQKVREKLRITEEQYRIEADVT
KPIKPALKSAEVELKTGGNNSNQVSETDEKEDLLHENRLMQDEIARLRLEKDTIKNQNLE
KKYLKDFEIVKRKHEDLQKALKRNGETLAKTIACYSGQLAALTDENTTLRSKLEKQRESR
QRLETEMQSYHCRLNAARCDHDQSHSSKRDQELAFQGTVDKCRHLQENLNSHVLILSLQL
SKAESKSRVLKTELHYTGEALKEKALVFEHVQSELKQKQSQMKDIEKMYKSGYNTMEKCI
EKQERFCQLKKQNMLLQQQLDDARNKADNQEKAILNIQARCDARVQNLQAECRKHRLLLE
EDNKMLVNELNHSKEKECQYEKEKAEREVAVRQLQQKRDDVLNKGSATKALLDASSRHCT
YLENGMQDSRKKLDQMRSQFQEIQDQLTATIRCTKEMEGDTQKLEVEHVMMRKIIKKQDD
QIERLEKILQHSSLMLQVFES
>sp|P54802|ANAG_HUMAN Alpha-N-acetylglucosaminidase OS=Homo sapiens GN=NAGLU PE=1 SV=2
MEAVAVAAAVGVLLLAGAGGAAGDEAREAAAVRALVARLLGPGPAADFSVSVERALAAKP
GLDTYSLGGGGAARVRVRGSTGVAAAAGLHRYLRDFCGCHVAWSGSQLRLPRPLPAVPGE
LTEATPNRYRYYQNVCTQSYSFVWWDWARWEREIDWMALNGINLALAWSGQEAIWQRVYL
ALGLTQAEINEFFTGPAFLAWGRMGNLHTWDGPLPPSWHIKQLYLQHRVLDQMRSFGMTP

VLPAFAGHVPEAVTRVFPQVNVTKMGSWGHFNCSYSCSFLLAPEDPIFPIIGSLFLRELI
KEFGTDHIYGADTFNEMQPPSSEPSYLAAATTAVYEAMTAVDTEAVWLLQGWLFQHQPQF
WGPAQIRAVLGAVPRGRLLVLDLFAESQPVYTRTASFQGQPFIWCMLHNFGGNHGLFGAL
EAVNGGPEAARLFPNSTMVGTGMAPEGISQNEVVYSLMAELGWRKDPVPDLAAWVTSFAA
RRYGVSHPDAGAAWRLLLRSVYNCSGEACRGHNRSPLVRRPSLQMNTSIWYNRSDVFEAW
RLLLTSAPSLATSPAFRYDLLDLTRQAVQELVSLYYEEARSAYLSKELASLLRAGGVLAY
ELLPALDEVLASDSRFLLGSWLEQARAAAVSEAEADFYEQNSRYQLTLWGPEGNILDYAN
KQLAGLVANYYTPRWRLFLEALVDSVAQGIPFQQHQFDKNVFQLEQAFVLSKQRYPSQPR
GDTVDLAKKIFLKYYPRWVAGSW
>sp|Q96K21|ANCHR_HUMAN Abscission/NoCut checkpoint regulator OS=Homo sapiens GN=ZFYVE19 PE=1 SV=3
MNYDSQQPPLPPLPYAGCRRASGFPALGRGGTVPVGVWGGAGQGREGRSWGEGPRGPGLG
RRDLSSADPAVLGATMESRCYGCAVKFTLFKKEYGCKNCGRAFCSGCLSFSAAVPRTGNT
QQKVCKQCHEVLTRGSSANASKWSPPQNYKKRVAALEAKQKPSTSQSQGLTRQDQMIAER
LARLRQENKPKLVPSQAEIEARLAALKDERQGSIPSTQEMEARLAALQGRVLPSQTPQPA
HHTPDTRTQAQQTQDLLTQLAAEVAIDESWKGGGPAASLQNDLNQGGPGSTNSKRQANWS
LEEEKSRLLAEAALELREENTRQERILALAKRLAMLRGQDPERVTLQDYRLPDSDDDEDE
ETAIQRVLQQLTEEASLDEASGFNIPAEQASRPWTQPRGAEPEAQDVDPRPEAEEEELPW
CCICNEDATLRCAGCDGDLFCARCFREGHDAFELKEHQTSAYSPPRAGQEH
>sp|Q9Y264|ANGP4_HUMAN Angiopoietin-4 OS=Homo sapiens GN=ANGPT4 PE=1 SV=1
MLSQLAMLQGSLLLVVATMSVAQQTRQEADRGCETLVVQHGHCSYTFLLPKSEPCPPGPE
VSRDSNTLQRESLANPLHLGKLPTQQVKQLEQALQNNTQWLKKLERAIKTILRSKLEQVQ
QQMAQNQTAPMLELGTSLLNQTTAQIRKLTDMEAQLLNQTSRMDAQMPETFLSTNKLENQ
LLLQRQKLQQLQGQNSALEKRLQALETKQQEELASILSKKAKLLNTLSRQSAALTNIERG
LRGVRHNSSLLQDQQHSLRQLLVLLRHLVQERANASAPAFIMAGEQVFQDCAEIQRSGAS
ASGVYTIQVSNATKPRKVFCDLQSSGGRWTLIQRRENGTVNFQRNWKDYKQGFGDPAGEH
WLGNEVVHQLTRRAAYSLRVELQDWEGHEAYAQYEHFHLGSENQLYRLSVVGYSGSAGRQ
SSLVLQNTSFSTLDSDNDHCLCKCAQVMSGGWWFDACGLSNLNGVYYHAPDNKYKMDGIR
WHYFKGPSYSLRASRMMIRPLDI
>sp|Q96BM1|ANKR9_HUMAN Ankyrin repeat domain-containing protein 9 OS=Homo sapiens GN=ANKRD9 PE=2 SV=1
MPWDARRPGGGADGGPEASGAARSRAQKQCRKSSFAFYQAVRDLLPVWLLEDMRASEAFH
WDERGRAAAYSPSEALLYALVHDHQAYAHYLLATFPRRALAPPSAGFRCCAAPGPHVALA
VRYNRVGILRRILRTLRDFPAEERARVLDRRGCSRVEGGGTSLHVACELARPECLFLLLG
HGASPGLRDGGGLTPLELLLRQLGRDAGATPSAAGAPASAPGEPRQRRLLLLDLLALYTP
VGAAGSARQELLGDRPRWQRLLGEDKFQWLAGLAPPSLFARAMQVLVTAISPGRFPEALD
ELPLPPFLQPLDLTGKG
>sp|Q68DC2|ANKS6_HUMAN Ankyrin repeat and SAM domain-containing protein 6 OS=Homo sapiens GN=ANKS6 PE=1 SV=2
MGEGGLPPAFQLLLRACDQGDTETARRLLEPGAAEPAERGAEPEAGAEPAGAEVAGPGAA
AAGAVGAPVPVDCSDEAGNTALQFAAAGGHEPLVRFLLRRGASVNSRNHYGWSALMQAAR
FGHVSVAHLLLDHGADVNAQNRLGASVLTVASRGGHLGVVKLLLEAGAFVDHHHPSGEQL
GLGGSRDEPLDITALMAAIQHGHEAVVRLLMEWGADPNHAARTVGWSPLMLAALTGRLGV
AQQLVEKGANPDHLSVLEKTAFEVALDCKHRDLVDYLDPLTTVRPKTDEEKRRPDIFHAL

KMGNFQLVKEIADEDPSHVNLVNGDGATPLMLAAVTGQLALVQLLVERHADVDKQDSVHG
WTALMQATYHGNKEIVKYLLNQGADVTLRAKNGYTAFDLVMLLNDPDTELVRLLASVCMQ
VNKDKGRPSHQPPLPHSKVRQPWSIPVLPDDKGGLKSWWNRMSNRFRKLKLMQTLPRGLS
SNQPLPFSDEPEPALDSTMRAAPQDKTSRSALPDAAPVTKDNGPGSTRGEKEDTLLTTML
RNGAPLTRLPSDKLKAVIPPFLPPSSFELWSSDRSRTRHNGKADPMKTALPQRASRGHPV
GGGGTDTTPVRPVKFPSLPRSPASSANSGNFNHSPHSSGGSSGVGVSRHGGELLNRSGGS
IDNVLSQIAAQRKKAAGLLEQKPSHRSSPVGPAPGSSPSELPASPAGGSAPVGKKLETSK
RPPSGTSTTSKSTSPTLTPSPSPKGHTAESSVSSSSSHRQSKSSGGSSSGTITDEDELTG
ILKKLSLEKYQPIFEEQEVDMEAFLTLTDGDLKELGIKTDGSRQQILAAISELNAGKGRE
RQILQETIHNFHSSFESSASNTRAPGNSPCA
>sp|Q8IV38|ANKY2_HUMAN Ankyrin repeat and MYND domain-containing protein 2 OS=Homo sapiens GN=ANKMY2 PE=1 SV=1
MVHIKKGELTQEEKELLEVIGKGTVQEAGTLLSSKNVRVNCLDENGMTPLMHAAYKGKLD
MCKLLLRHGADVNCHQHEHGYTALMFAALSGNKDITWVMLEAGAETDVVNSVGRTAAQMA
AFVGQHDCVTIINNFFPRERLDYYTKPQGLDKEPKLPPKLAGPLHKIITTTNLHPVKIVM
LVNENPLLTEEAALNKCYRVMDLICEKCMKQRDMNEVLAMKMHYISCIFQKCINFLKDGE
NKLDTLIKSLLKGRASDGFPVYQEKIIRESIRKFPYCEATLLQQLVRSIAPVEIGSDPTA
FSVLTQAITGQVGFVDVEFCTTCGEKGASKRCSVCKMVIYCDQTCQKTHWFTHKKICKNL
KDIYEKQQLEAAKEKRQEENHGKLDVNSNCVNEEQPEAEVGISQKDSNPEDSGEGKKESL
ESEAELEGLQDAPAGPQVSEE
>sp|Q9NXR5|ANR10_HUMAN Ankyrin repeat domain-containing protein 10 OS=Homo sapiens GN=ANKRD10 PE=1 SV=2
MSAAGAGAGVEAGFSSEELLSLRFPLHRACRDGDLATLCSLLQQTPHAHLASEDSFYGWT
PVHWAAHFGKLECLVQLVRAGATLNVSTTRYAQTPAHIAAFGGHPQCLVWLIQAGANINK
PDCEGETPIHKAARSGSLECISALVANGAHVDLRNASGLTAADIAQTQGFQECAQFLLNL
QNCHLNHFYNNGILNGGHQNVFPNHISVGTNRKRCLEDSEDFGVKKARTEAQSLDSAVPL
TNGDTEDDADKMHVDREFAVVTDMKNSSSVSNTLTNGCVINGHLDFPSTTPLSGMESRNG
QCLTGTNGISSGLAPGQPFPSSQGSLCISGTEEPEKTLRANPELCGSLHLNGSPSSCIAS
RPSWVEDIGDNLYYGHYHGFGDTAESIPELNSVVEHSKSVKVQERYDSAVLGTMHLHHGS
>sp|Q86SG2|ANR23_HUMAN Ankyrin repeat domain-containing protein 23 OS=Homo sapiens GN=ANKRD23 PE=1 SV=1
MDFISIQQLVSGERVEGKVLGFGHGVPDPGAWPSDWRRGPQEAVAREKLKLEEEKKKKLE
RFNSTRFNLDNLADLENLVQRRKKRLRHRVPPRKPEPLVKPQSQAQVEPVGLEMFLKAAA
ENQEYLIDKYLTDGGDPNAHDKLHRTALHWACLKGHSQLVNKLLVAGATVDARDLLDRTP
VFWACRGGHLVILKQLLNQGARVNARDKIGSTPLHVAVRTRHPDCLEHLIECGAHLNAQD
KEGDTALHEAVRHGSYKAMKLLLLYGAELGVRNAASVTPVQLARDWQRGIREALQAHVAH
PRTRC
>sp|Q9UPS8|ANR26_HUMAN Ankyrin repeat domain-containing protein 26 OS=Homo sapiens GN=ANKRD26 PE=1 SV=3
MKKIFSKKGESPLGSFARRQRSSAGGGGEPGEGAYSQPGYHVRDRDLGKIHKAASAGNVA
KVQQILLLRKNGLNDRDKMNRTALHLACANGHPEVVTLLVDRKCQLNVCDNENRTALMKA
VQCQEEKCATILLEHGADPNLADVHGNTALHYAVYNEDISVATKLLLYDANIEAKNKDDL
TPLLLAVSGKKQQMVEFLIKKKANVNAVDKLESSHQLISEYKEERIPKHSSQNSNSVDES
SEDSLSRLSGKPGVDDSWPTSDDEDLNFDTKNVPKPSLAKLMTASQQSRKNLEATYGTVR

TGNRTLFEDRDSDSQDEVVVESLPTTSIKVQCFSHPTYQSPDLLPKPSHKSLANPGLMKE
EPTKPGIAKKENGIDIIESAPLEQTNNDNLTYVDEVHKNNRSDMMSALGLGQEEDIESPW
DSESISENFPQKYVDPLAGAADGKEKNIGNEQAEDVFYIPSCMSGSRNFKMAKLEDTRNV
GMPVAHMESPERYLHLKPTIEMKDSVPNKAGGMKDVQTSKAAEHDLEVASEEEQEREGSE
NNQPQVEEERKKHRNNEMEVSANIHDGATDDAEDDDDDDGLIQKRKSGETDHQQFPRKEN
KEYASGPALQMKEVKSTEKEKRTSKESVNSPVFGKASLLTGGLLQVDDDSSLSEIDEDEG
RPTKKTSNEKNKVKNQIQSMDDVDDLTQSSETASEDCELPHSSYKNFMLLIEQLGMECKD
SVSLLKIQDAALSCERLLELKKNHCELLTVKIKKMEDKVNVLQRELSETKEIKSQLEHQK
VEWERELCSLRFSLNQEEEKRRNADTLYEKIREQLRRKEEQYRKEVEVKQQLELSLQTLE
MELRTVKSNLNQVVQERNDAQRQLSREQNARMLQDGILTNHLSKQKEIEMAQKKMNSENS
HSHEEEKDLSHKNSMLQEEIAMLRLEIDTIKNQNQEKEKKCFEDLKIVKEKNEDLQKTIK
QNEETLTQTISQYNGRLSVLTAENAMLNSKLENEKQSKERLEAEVESYHSRLAAAIHDRD
QSETSKRELELAFQRARDECSRLQDKMNFDVSNLKDNNEILSQQLFKTESKLNSLEIEFH
HTRDALREKTLGLERVQKDLSQTQCQMKEMEQKYQNEQVKVNKYIGKQESVEERLSQLQS
ENMLLRQQLDDAHNKADNKEKTVINIQDQFHAIVQKLQAESEKQSLLLEERNKELISECN
HLKERQYQYENEKAEREVVVRQLQQELADTLKKQSMSEASLEVTSRYRINLEDETQDLKK
KLGQIRNQLQEAQDRHTEAVRCAEKMQDHKQKLEKDNAKLKVTVKKQMDKIEELQKNLLN
ANLSEDEKEQLKKLMELKQSLECNLDQEMKKNVELEREITGFKNLLKMTRKKLNEYENGE
FSFHGDLKTSQFEMDIQINKLKHKIDDLTAELETAGSKCLHLDTKNQILQEELLSMKTVQ
KKCEKLQKNKKKLEQEVINLRSHIERNMVELGQVKQYKQEIEERARQEIAEKLKEVNLFL
QAQAASQENLEQFRENNFASMKSQMELRIKDLESELSKIKTSQEDFNKTELEKYKQLYLE
ELKVRKSLSSKLTKTNERLAEVNTKLLVEKQQSRSLFTTLTTRPVMEPPCVGNLNNSLDL
NRKLIPRENLVISTSNPRASNNSMENYLSKMQQELEKNITRELKEAAAELESGSIASPLG
STDESNLNQDLVWKASREYVQVLKKNYMI
>sp|Q7Z3H0|ANR33_HUMAN Ankyrin repeat domain-containing protein 33 OS=Homo sapiens GN=ANKRD33 PE=2 SV=3
MVACYHGFQSVVALLSHCPFLDVNQQDKGGDTALMLAAQAGHVPLVSLLLNYYVGLDLER
RDQRGLTALMKAAMRNRCECVATLLMAGADLTAVDPVRGKTALEWAVLTDSFDTVWRIRQ
LLRRPQVEQLSQHYKPEWPALSGLVAQAQAQAQVAPSLLERLQATLSLPFAPSPQEGGVL
DHLVTATTSLASPFVTTACHTLCPDHPPSLGTRSKSVPELLVPAEAQSFRTPKSGPSSLA
IPGAQDREEETGGGGQNGTEVGEDGIGQAGNR
>sp|Q53RE8|ANR39_HUMAN Ankyrin repeat domain-containing protein 39 OS=Homo sapiens GN=ANKRD39 PE=1 SV=1
MATPRPCADGPCCSHPSAVLGVQQTLEEMDFERGIWSAALNGDLGRVKHLIQKAEDPSQP
DSAGYTALHYASRNGHYAVCQFLLESGAKCDAQTHGGATALHRASYCGHTEIARLLLSHG
SNPRVVDDDGMTSLHKAAERGHGDICSLLLQHSPALKAIRDRKARLACDLLPCNSDLRDL
LSS
>sp|Q7Z713|ANR37_HUMAN Ankyrin repeat domain-containing protein 37 OS=Homo sapiens GN=ANKRD37 PE=2 SV=1
MLLLDCNPEVDGLKHLLETGASVNAPPDPCKQSPVHLAAGSGLACFLLWQLQTGADLNQQ
DVLGEAPLHKAAKVGSLECLSLLVASDAQIDLCNKNGQTAEDLAWSCGFPDCAKFLTTIK
CMQTIKASEHPDRNDCVAVLRQKRSLGSVENTSGKRKC
>sp|Q86W74|ANR46_HUMAN Ankyrin repeat domain-containing protein 46 OS=Homo sapiens GN=ANKRD46 PE=1 SV=1

MSYVFVNDSSQTNVPLLQACIDGDFNYSKRLLESGFDPNIRDSRGRTGLHLAAARGNVDI
CQLLHKFGADLLATDYQGNTALHLCGHVDTIQFLVSNGLKIDICNHQGATPLVLAKRRGV
NKDVIRLLESLEEQEVKGFNRGTHSKLETMQTAESESAMESHSLLNPNLQQGEGVLSSFR
TTWQEFVEDLGFWRVLLLIFVIALLSLGIAYYRRTLRLGSFARQDRSRIQAI
>sp|Q8NB46|ANR52_HUMAN Serine/threonine-protein phosphatase 6 regulatory ankyrin repeat subunit C OS=Homo sapiens GN=ANKRD52 PE=1 SV=3
MGILSITDQPPLVQAIFSRDVEEVRSLLSQKENINVLDQERRTPLHAAAYVGDVPILQLL
LMSGANVNAKDTLWLTPLHRAAASRNEKVLGLLLAHSADVNARDKLWQTPLHVAAANRAT
KCAEALAPLLSSLNVADRSGRSALHHAVHSGHLETVNLLLNKGASLNVCDKKERQPLHWA
AFLGHLEVLKLLVARGADLGCKDRKGYGLLHTAAASGQIEVVKYLLRMGAEIDEPNAFGN
TALHIACYLGQDAVAIELVNAGANVNQPNDKGFTPLHVAAVSTNGALCLELLVNNGADVN
YQSKEGKSPLHMAAIHGRFTRSQILIQNGSEIDCADKFGNTPLHVAARYGHELLISTLMT
NGADTARRGIHDMFPLHLAVLFGFSDCCRKLLSSGQLYSIVSSLSNEHVLSAGFDINTPD
NLGRTCLHAAASGGNVECLNLLLSSGADLRRRDKFGRTPLHYAAANGSYQCAVTLVTAGA
GVNEADCKGCSPLHYAAASDTYRRAEPHTPSSHDAEEDEPLKESRRKEAFFCLEFLLDNG
ADPSLRDRQGYTAVHYAAAYGNRQNLELLLEMSFNCLEDVESTIPVSPLHLAAYNGHCEA
LKTLAETLVNLDVRDHKGRTALFLATERGSTECVEVLTAHGASALIKERKRKWTPLHAAA
ASGHTDSLHLLIDSGERADITDVMDAYGQTPLMLAIMNGHVDCVHLLLEKGSTADAADLR
GRTALHRGAVTGCEDCLAALLDHDAFVLCRDFKGRTPIHLASACGHTAVLRTLLQAALST
DPLDAGVDYSGYSPMHWASYTGHEDCLELLLEHSPFSYLEGNPFTPLHCAVINNQDSTTE
MLLGALGAKIVNSRDAKGRTPLHAAAFADNVSGLRMLLQHQAEVNATDHTGRTALMTAAE
NGQTAAVEFLLYRGKADLTVLDENKNTALHLACSKGHEKCALMILAETQDLGLINATNSA
LQMPLHIAARNGLASVVQALLSHGATVLAVDEEGHTPALACAPNKDVADCLALILSTMKP
FPPKDAVSPFSFSLLKNCSIAAAKTVGGCGALPHGASCPYSQERPGAIGLDGCYSE
>sp|A6NC57|ANR62_HUMAN Ankyrin repeat domain-containing protein 62 OS=Homo sapiens GN=ANKRD62 PE=2 SV=4
MEVRGSFLAACRRRMATWRKNRDKDGFSNPGYRVRQKDLGMIHKAAIAGDVNKVMESILL
RLNDLNDRDKKNRTALLLACAHGRPGVVADLVARKCQLNLTDSENRTALIKAVQCQEEVC
ASILLEHGANPNVRDMYGNTALHYAIDNENISMARKLLAYGADIEARSQDGHTSLLLAVN
RKKEQMVAFLLKKKPDLTAIDNFGRTALILAARNGSTSVVYQLLQHNIDVFCQDISGWTA
EDYAVASKFQAIRGMISEYKANKRCKSLQNSNSEQDLEMTSEGEQERLEGCESSQPQVEE
KMKKCRNKKMEVSRNVHADDSDNYNDDVDELIHKIKNRKPDNHQSPGKENGEFDRLARKT
SNEKSKVKSQIYFTDDLNDISGSSEKTSEDDELPYSDDENFMLLIEQSGMECKDFVSLSK
SKNATAACGRSIEDQKCYCERLKVKFQKMKNNISVLQKVLSETDKTKSQSEHQNLQGKKK
LCNLRFILQQQEEERIKAEELYEKDIEELKIMEEQYRTQTEVKKQSKLTLKSLEVELKTV
RSNSNQNFHTHERERDLWQENHLMRDEIARLRLEIDTIKHQNQETENKYFKDIEIIKENN
EDLEKTLKRNEEALTKTITRYSKELNVLMDENTMLNSELQKEKQSMSRLETEMESYRCRL
AAALCDHDQRQSSKRDLQLAFQSTVNEWCHLQEDTNSHIQILSQQLSKAESTSSGLETEL
HYEREALKEKTLHIEHMQGVLSRTQRRLEDIEHMYQNDQPILEKYVRKQQSVEDGLFQLQ
SQNLLYQQQCNDARKKADNQEKTIINIQVKCEDTVEKLQAECRKLEENNKGLMKECTLLK
ERQCQYEKEKEEREVVRRQLQREVDDALNKQLLLEAMLEISSERRINLEDEAQSLKKKLG
QMRSQVCMKLSMSTVTL
>sp|P01008|ANT3_HUMAN Antithrombin-III OS=Homo sapiens GN=SERPINC1 PE=1 SV=1
MYSNVIGTVTSGKRKVYLLSLLLIGFWDCVTCHGSPVDICTAKPRDIPMNPMCIYRSPEK

KATEDEGSEQKIPEATNRRVWELSKANSRFATTFYQHLADSKNDNDNIFLSPLSISTAFA
MTKLGACNDTLQQLMEVFKFDTISEKTSDQIHFFFAKLNCRLYRKANKSSKLVSANRLFG
DKSLTFNETYQDISELVYGAKLQPLDFKENAEQSRAAINKWVSNKTEGRITDVIPSEAIN
ELTVLVLVNTIYFKGLWKSKFSPENTRKELFYKADGESCSASMMYQEGKFRYRRVAEGTQ
VLELPFKGDDITMVLILPKPEKSLAKVEKELTPEVLQEWLDELEEMMLVVHMPRFRIEDG
FSLKEQLQDMGLVDLFSPEKSKLPGIVAEGRDDLYVSDAFHKAFLEVNEEGSEAAASTAV
VIAGRSLNPNRVTFKANRPFLVFIREVPLNTIIFMGRVANPCVK
>sp|P27216|ANX13_HUMAN Annexin A13 OS=Homo sapiens GN=ANXA13 PE=1 SV=3
MGNRHAKASSPQGFDVDRDAKKLNKACKGMGTNEAAIIEILSGRTSDERQQIKQKYKATY
GKELEEVLKSELSGNFEKTALALLDRPSEYAARQLQKAMKGLGTDESVLIEVLCTRTNKE
IIAIKEAYQRLFDRSLESDVKGDTSGNLKKILVSLLQANRNEGDDVDKDLAGQDAKDLYD
AGEGRWGTDELAFNEVLAKRSYKQLRATFQAYQILIGKDIEEAIEEETSGDLQKAYLTLV
RCAQDCEDYFAERLYKSMKGAGTDEETLIRIVVTRAEVDLQGIKAKFQEKYQKSLSDMVR
SDTSGDFRKLLVALLH
>sp|P56377|AP1S2_HUMAN AP-1 complex subunit sigma-2 OS=Homo sapiens GN=AP1S2 PE=1 SV=1
MQFMLLFSRQGKLRLQKWYVPLSDKEKKKITRELVQTVLARKPKMCSFLEWRDLKIVYKR
YASLYFCCAIEDQDNELITLEIIHRYVELLDKYFGSVCELDIIFNFEKAYFILDEFLLGG
EVQETSKKNVLKAIEQADLLQEEAETPRSVLEEIGLT
>sp|O94973|AP2A2_HUMAN AP-2 complex subunit alpha-2 OS=Homo sapiens GN=AP2A2 PE=1 SV=2
MPAVSKGDGMRGLAVFISDIRNCKSKEAEIKRINKELANIRSKFKGDKALDGYSKKKYVC
KLLFIFLLGHDIDFGHMEAVNLLSSNRYTEKQIGYLFISVLVNSNSELIRLINNAIKNDL
ASRNPTFMGLALHCIASVGSREMAEAFAGEIPKVLVAGDTMDSVKQSAALCLLRLYRTSP
DLVPMGDWTSRVVHLLNDQHLGVVTAATSLITTLAQKNPEEFKTSVSLAVSRLSRIVTSA
STDLQDYTYYFVPAPWLSVKLLRLLQCYPPPDPAVRGRLTECLETILNKAQEPPKSKKVQ
HSNAKNAVLFEAISLIIHHDSEPNLLVRACNQLGQFLQHRETNLRYLALESMCTLASSEF
SHEAVKTHIETVINALKTERDVSVRQRAVDLLYAMCDRSNAPQIVAEMLSYLETADYSIR
EEIVLKVAILAEKYAVDYTWYVDTILNLIRIAGDYVSEEVWYRVIQIVINRDDVQGYAAK
TVFEALQAPACHENLVKVGGYILGEFGNLIAGDPRSSPLIQFHLLHSKFHLCSVPTRALL
LSTYIKFVNLFPEVKPTIQDVLRSDSQLRNADVELQQRAVEYLRLSTVASTDILATVLEE
MPPFPERESSILAKLKKKKGPSTVTDLEDTKRDRSVDVNGGPEPAPASTSAVSTPSPSAD
LLGLGAAPPAPAGPPPSSGGSGLLVDVFSDSASVVAPLAPGSEDNFARFVCKNNGVLFEN
QLLQIGLKSEFRQNLGRMFIFYGNKTSTQFLNFTPTLICSDDLQPNLNLQTKPVDPTVEG
GAQVQQVVNIECVSDFTEAPVLNIQFRYGGTFQNVSVQLPITLNKFFQPTEMASQDFFQR
WKQLSNPQQEVQNIFKAKHPMDTEVTKAKIIGFGSALLEEVDPNPANFVGAGIIHTKTTQ
IGCLLRLEPNLQAQMYRLTLRTSKEAVSQRLCELLSAQF
>sp|P63010|AP2B1_HUMAN AP-2 complex subunit beta OS=Homo sapiens GN=AP2B1 PE=1 SV=1
MTDSKYFTTNKKGEIFELKAELNNEKKEKRKEAVKKVIAAMTVGKDVSSLFPDVVNCMQT
DNLELKKLVYLYLMNYAKSQPDMAIMAVNSFVKDCEDPNPLIRALAVRTMGCIRVDKITE
YLCEPLRKCLKDEDPYVRKTAAVCVAKLHDINAQMVEDQGFLDSLRDLIADSNPMVVANA
VAALSEISESHPNSNLLDLNPQNINKLLTALNECTEWGQIFILDCLSNYNPKDDREAQSI
CERVTPRLSHANSAVVLSAVKVLMKFLELLPKDSDYYNMLLKKLAPPLVTLLSGEPEVQY
VALRNINLIVQKRPEILKQEIKVFFVKYNDPIYVKLEKLDIMIRLASQANIAQVLAELKE
YATEVDVDFVRKAVRAIGRCAIKVEQSAERCVSTLLDLIQTKVNYVVQEAIVVIRDIFRK
YPNKYESIIATLCENLDSLDEPDARAAMIWIVGEYAERIDNADELLESFLEGFHDESTQV

QLTLLTAIVKLFLKKPSETQELVQQVLSLATQDSDNPDLRDRGYIYWRLLSTDPVTAKEV
VLSEKPLISEETDLIEPTLLDELICHIGSLASVYHKPPNAFVEGSHGIHRKHLPIHHGST
DAGDSPVGTTTATNLEQPQVIPSQGDLLGDLLNLDLGPPVNVPQVSSMQMGAVDLLGGGL
DSLVGQSFIPSSVPATFAPSPTPAVVSSGLNDLFELSTGIGMAPGGYVAPKAVWLPAVKA
KGLEISGTFTHRQGHIYMEMNFTNKALQHMTDFAIQFNKNSFGVIPSTPLAIHTPLMPNQ
SIDVSLPLNTLGPVMKMEPLNNLQVAVKNNIDVFYFSCLIPLNVLFVEDGKMERQVFLAT
WKDIPNENELQFQIKECHLNADTVSSKLQNNNVYTIAKRNVEGQDMLYQSLKLTNGIWIL
AELRIQPGNPNYTLSLKCRAPEVSQYIYQVYDSILKN
>sp|Q96N21|AP4AT_HUMAN AP-4 complex accessory subunit Tepsin OS=Homo sapiens GN=TEPSIN PE=1 SV=1
MAAAPPLRDRLSFLHRLPILLKGTSDDDVPCPGYLFEEIAKISHESPGSSQCLLEYLLSR
LHSSSGHGKLKVLKILLYLCSHGSSFFLLILKRNSAFIQEAAAFAGPPDPLHGNSLYQKV
RAAAQDLGSTLFSDTVLPLAPSQPLGTPPATGMGSQARPHSTLQGFGYSKEHGRTAVRHQ
PGQAGGGWDELDSGPSSQNSSQNSDLSRVSDSGSHSGSDSHSGASREPGDLAERVEVVAL
SDCQQELSLVRTVTRGPRAFLSREEAQHFIKACGLLNCEAVLQLLTCHLRGTSECTQLRA
LCAIASLGSSDLLPQEHILLRTRPWLQELSMGSPGPVTNKATKILRHFEASCGQLSPARG
TSAEPGPTAALPGPSDLLTDAVPLPGSQVFLQPLSSTPVSSRSPAPSSGMPSSPVPTPPP
DASPIPAPGDPSEAEARLAESRRWRPERIPGGTDSPKRGPSSCAWSRDSLFAGMELVACP
RLVGAGAAAGESCPDAPRAPQTSSQRTAAKEPPGSEPSAFAFLNA
>sp|P60006|APC15_HUMAN Anaphase-promoting complex subunit 15 OS=Homo sapiens GN=ANAPC15 PE=1 SV=1
MSTLFPSLFPRVTETLWFNLDRPCVEETELQQQEQQHQAWLQSIAEKDNNLVPIGKPASE
HYDDEEEEDDEDDEDSEEDSEDDEDMQDMDEMNDYNESPDDGEVNEVDMEGNEQDQDQWM
I
>sp|Q9H1A4|APC1_HUMAN Anaphase-promoting complex subunit 1 OS=Homo sapiens GN=ANAPC1 PE=1 SV=1
MSNFYEERTTMIAARDLQEFVPFGRDHCKHHPNALNLQLRQLQPASELWSSDGAAGLVGS
LQEVTIHEKQKESWQLRKGVSEIGEDVDYDEELYVAGNMVIWSKGSKSQALAVYKAFTVD
SPVQQALWCDFIISQDKSEKAYSSNEVEKCICILQSSCINMHSIEGKDYIASLPFQVANV
WPTKYGLLFERSASSHEVPPGSPREPLPTMFSMLHPLDEITPLVCKSGSLFGSSRVQYVV
DHAMKIVFLNTDPSIVMTYDAVQNVHSVWTLRRVKSEEENVVLKFSEQGGTPQNVATSSS
LTAHLRSLSKGDSPVTSPFQNYSSIHSQSRSTSSPSLHSRSPSISNMAALSRAHSPALGV
HSFSGVQRFNISSHNQSPKRHSISHSPNSNSNGSFLAPETEPIVPELCIDHLWTETITNI
REKNSQASKVFITSDLCGQKFLCFLVESQLQLRCVKFQESNDKTQLIFGSVTNIPAKDAA
PVEKIDTMLVLEGSGNLVLYTGVVRVGKVFIPGLPAPSLTMSNTMPRPSTPLDGVSTPKP
LSKLLGSLDEVVLLSPVPELRDSSKLHDSLYNEDCTFQQLGTYIHSIRDPVHNRVTLELS
NGSMVRITIPEIATSELVQTCLQAIKFILPKEIAVQMLVKWYNVHSAPGGPSYHSEWNLF
VTCLMNMMGYNTDRLAWTRNFDFEGSLSPVIAPKKARPSETGSDDDWEYLLNSDYHQNVE
SHLLNRSLCLSPSEASQMKDEDFSQNLSLDSSTLLFTHIPAIFFVLHLVYEELKLNTLMG
EGICSLVELLVQLARDLKLGPYVDHYYRDYPTLVRTTGQVCTIDPGQTGFMHHPSFFTSE
PPSIYQWVSSCLKGEGMPPYPYLPGICERSRLVVLSIALYILGDESLVSDESSQYLTRIT
IAPQKLQVEQEENRFSFRHSTSVSSLAERLVVWMTNVGFTLRDLETLPFGIALPIRDAIY
HCREQPASDWPEAVCLLIGRQDLSKQACEGNLPKGKSVLSSDVPSGTETEEEDDGMNDMN
HEVMSLIWSEDLRVQDVRRLLQSAHPVRVNVVQYPELSDHEFIEEKENRLLQLCQRTMAL

PVGRGMFTLFSYHPVPTEPLPIPKLNLTGRAPPRNTTVDLNSGNIDVPPNMTSWASFHNG
VAAGLKIAPASQIDSAWIVYNKPKHAELANEYAGFLMALGLNGHLTKLATLNIHDYLTKG
HEMTSIGLLLGVSAAKLGTMDMSITRLLSIHIPALLPPTSTELDVPHNVQVAAVVGIGLV
YQGTAHRHTAEVLLAEIGRPPGPEMEYCTDRESYSLAAGLALGMVCLGHGSNLIGMSDLN
VPEQLYQYMVGGHRRFQTGMHREKHKSPSYQIKEGDTINVDVTCPGATLALAMIYLKTNN
RSIADWLRAPDTMYLLDFVKPEFLLLRTLARCLILWDDILPNSKWVDSNVPQIIRENSIS
LSEIELPCSEDLNLETLSQAHVYIIAGACLSLGFRFAGSENLSAFNCLHKFAKDFMTYLS
APNASVTGPHNLETCLSVVLLSLAMVMAGSGNLKVLQLCRFLHMKTGGEMNYGFHLAHHM
ALGLLFLGGGRYSLSTSNSSIAALLCALYPHFPAHSTDNRYHLQALRHLYVLAAEPRLLV
PVDVDTNTPCYALLEVTYKGTQWYEQTKEELMAPTLLPELHLLKQIKVKGPRYWELLIDL
SKGTQHLKSILSKDGVLYVKLRAGQLSYKEDPMGWQSLLAQTVANRNSEARAFKPETISA
FTSDPALLSFAEYFCKPTVNMGQKQEILDLFSSVLYECVTQETPEMLPAYIAMDQAIRRL
GRREMSETSELWQIKLVLEFFSSRSHQERLQNHPKRGLFMNSEFLPVVKCTIDNTLDQWL
QVGGDMCVHAYLSGQPLEESQLSMLACFLVYHSVPAPQHLPPIGLEGSTSFAELLFKFKQ
LKMPVRALLRLAPLLLGNPQPMVM
>sp|O95996|APC2_HUMAN Adenomatous polyposis coli protein 2 OS=Homo sapiens GN=APC2 PE=1 SV=1
MASSVAPYEQLVRQVEALKAENSHLRQELRDNSSHLSKLETETSGMKEVLKHLQGKLEQE
ARVLVSSGQTEVLEQLKALQMDITSLYNLKFQPPTLGPEPAARTPEGSPVHGSGPSKDSF
GELSRATIRLLEELDRERCFLLNEIEKEEKEKLWYYSQLQGLSKRLDELPHVETQFSMQM
DLIRQQLEFEAQHIRSLMEERFGTSDEMVQRAQIRASRLEQIDKELLEAQDRVQQTEPQA
LLAVKSVPVDEDPETEVPTHPEDGTPQPGNSKVEVVFWLLSMLATRDQEDTARTLLAMSS
SPESCVAMRRSGCLPLLLQILHGTEAAAGGRAGAPGAPGAKDARMRANAALHNIVFSQPD
QGLARKEMRVLHVLEQIRAYCETCWDWLQARDGGPEGGGAGSAPIPIEPQICQATCAVMK
LSFDEEYRRAMNELGGLQAVAELLQVDYEMHKMTRDPLNLALRRYAGMTLTNLTFGDVAN
KATLCARRGCMEAIVAQLASDSEELHQVVSSILRNLSWRADINSKKVLREAGSVTALVQC
VLRATKESTLKSVLSALWNLSAHSTENKAAICQVDGALGFLVSTLTYKCQSNSLAIIESG
GGILRNVSSLVATREDYRQVLRDHNCLQTLLQHLTSHSLTIVSNACGTLWNLSARSARDQ
ELLWDLGAVGMLRNLVHSKHKMIAMGSAAALRNLLAHRPAKHQAAATAVSPGSCVPSLYV
RKQRALEAELDARHLAQALEHLEKQGPPAAEAATKKPLPPLRHLDGLAQDYASDSGCFDD
DDAPSSLAAAAATGEPASPAALSLFLGSPFLQGQALARTPPTRRGGKEAEKDTSGEAAVA
AKAKAKLALAVARIDQLVEDISALHTSSDDSFSLSSGDPGQEAPREGRAQSCSPCRGPEG
GRREAGSRAHPLLRLKAAHASLSNDSLNSGSASDGYCPREHMLPCPLAALASRREDPRCG
QPRPSRLDLDLPGCQAEPPAREATSADARVRTIKLSPTYQHVPLLEGASRAGAEPLAGPG
ISPGARKQAWLPADHLSKVPEKLAAAPLSVASKALQKLAAQEGPLSLSRCSSLSSLSSAG
RPGPSEGGDLDDSDSSLEGLEEAGPSEAELDSTWRAPGATSLPVAIPAPRRNRGRGLGVE
DATPSSSSENYVQETPLVLSRCSSVSSLGSFESPSIASSIPSEPCSGQGSGTISPSELPD
SPGQTMPPSRSKTPPLAPAPQGPPEATQFSLQWESYVKRFLDIADCRERCRLPSELDAGS
VRFTVEKPDENFSCASSLSALALHEHYVQQDVELRLLPSACPERGGGAGGAGLHFAGHRR
REEGPAPTGSRPRGAADQELELLRECLGAAVPARLRKVASALVPGRRALPVPVYMLVPAP
APAQEDDSCTDSAEGTPVNFSSAASLSDETLQGPPRDQPGGPAGRQRPTGRPTSARQAMG
HRHKAGGAGRSAEQSRGAGKNRAGLELPLGRPPSAPADKDGSKPGRTRGDGALQSLCLTT
PTEEAVYCFYGNDSDEEPPAAAPTPTHRRTSAIPRAFTRERPQGRKEAPAPSKAAPAAPP
PARTQPSLIADETPPCYSLSSSASSLSEPEPSEPPAVHPRGREPAVTKDPGPGGGRDSSP

SPRAAEELLQRCISSALPRRRPPVSGLRRRKPRATRLDERPAEGSRERGEEAAGSDRASD
LDSVEWRAIQEGANSIVTWLHQAAAATREASSESDSILSFVSGLSVGSTLQPPKHRKGRQ
AEGEMGSARRPEKRGAASVKTSGSPRSPAGPEKPRGTQKTTPGVPAVLRGRTVIYVPSPA
PRAQPKGTPGPRATPRKVAPPCLAQPAAPAKVPSPGQQRSRSLHRPAKTSELATLSQPPR
SATPPARLAKTPSSSSSQTSPASQPLPRKRPPVTQAAGALPGPGASPVPKTPARTLLAKQ
HKTQRSPVRIPFMQRPARRGPPPLARAVPEPGPRGRAGTEAGPGARGGRLGLVRVASALS
SGSESSDRSGFRRQLTFIKESPGLRRRRSELSSAESAASAPQGASPRRGRPALPAVFLCS
SRCEELRAAPRQGPAPARQRPPAARPSPGERPARRTTSESPSRLPVRAPAARPETVKRYA
SLPHISVARRPDGAVPAAPASADAARRSSDGEPRPLPRVAAPGTTWRRIRDEDVPHILRS
TLPATALPLRGSTPEDAPAGPPPRKTSDAVVQTEEVAAPKTNSSTSPSLETREPPGAPAG
GQLSLLGSDVDGPSLAKAPISAPFVHEGLGVAVGGFPASRHGSPSRSARVPPFNYVPSPM
VVAATTDSAAEKAPATASATLLE
>sp|Q8NCL9|APCDL_HUMAN Protein APCDD1-like OS=Homo sapiens GN=APCDD1L PE=2 SV=1
MPAAMLPYACVLVLLGAHTAPAAGEAGGSCLRWEPHCQQPLPDRVPSTAILPPRLNGPWI
STGCEVRPGPEFLTRAYTFYPSRLFRAHQFYYEDPFCGEPAHSLLVKGKVRLRRASWVTR
GATEADYHLHKVGIVFHSRRALVDVTGRLNQTRAGRDCARRLPPARAWLPGALYELRSAR
AQGDCLEALGLTMHELSLVRVQRRLQPQPRASPRLVEELYLGDIHTDPAERRHYRPTGYQ
RPLQSALHHVQPCPACGLIARSDVHHPPVLPPPLALPLHLGGWWVSSGCEVRPAVLFLTR
LFTFHGHSRSWEGYYHHFSDPACRQPTFTVVYAAGRYTRGTPSTRVRGGTELVFEVTRAHV
TPMDQVTTAMLNFSEPSSCGGAGAWSMGTERDVTATNGCLPLGIRLPHVEYELFKMEQDP
LGQSLLFIGQRPTDGSSPDTPEKRPTSYQAPLVLCHGEAPDFSRPPQHRPSLQKHPSTGG
LHIAPFPLLPLVLGLAFLHWL
>sp|Q06481|APLP2_HUMAN Amyloid-like protein 2 OS=Homo sapiens GN=APLP2 PE=1 SV=2
MAATGTAAAAATGRLLLLLLVGLTAPALALAGYIEALAANAGTGFAVAEPQIAMFCGKLN
MHVNIQTGKWEPDPTGTKSCFETKEEVLQYCQEMYPELQITNVMEANQRVSIDNWCRRDK
KQCKSRFVTPFKCLVGEFVSDVLLVPEKCQFFHKERMEVCENHQHWHTVVKEACLTQGMT
LYSYGMLLPCGVDQFHGTEYVCCPQTKIIGSVSKEEEEEDEEEEEEEDEEEDYDVYKSEF
PTEADLEDFTEAAVDEDDEDEEEGEEVVEDRDYYYDTFKGDDYNEENPTEPGSDGTMSDK
EITHDVKAVCSQEAMTGPCRAVMPRWYFDLSKGKCVRFIYGGCGGNRNNFESEDYCMAVC
KAMIPPTPLPTNDVDVYFETSADDNEHARFQKAKEQLEIRHRNRMDRVKKEWEEAELQAK
NLPKAERQTLIQHFQAMVKALEKEAASEKQQLVETHLARVEAMLNDRRRMALENYLAALQ
SDPPRPHRILQALRRYVRAENKDRLHTIRHYQHVLAVDPEKAAQMKSQVMTHLHVIEERR
NQSLSLLYKVPYVAQEIQEEIDELLQEQRADMDQFTASISETPVDVRVSSEESEEIPPFH
PFHPFPALPENEDTQPELYHPMKKGSGVGEQDGGLIGAEEKVINSKNKVDENMVIDETLD
VKEMIFNAERVGGLEEERESVGPLREDFSLSSSALIGLLVIAVAIATVIVISLVMLRKRQ
YGTISHGIVEVDPMLTPEERHLNKMQNHGYENPTYKYLEQMQI
>sp|P02655|APOC2_HUMAN Apolipoprotein C-II OS=Homo sapiens GN=APOC2 PE=1 SV=1
MGTRLLPALFLVLLVLGFEVQGTQQPQQDEMPSPTFLTQVKESLSSYWESAKTAAQNLYE
KTYLPAVDEKLRDLYSKSTAAMSTYTGIFTDQVLSVLKGEE
>sp|Q13790|APOF_HUMAN Apolipoprotein F OS=Homo sapiens GN=APOF PE=1 SV=2
MTGLCGYSAPDMRGLRLIMIPVELLLCYLLLHPVDATSYGKQTNVLMHFPLSLESQTPSS
DPLSCQFLHPKSLPGFSHMAPLPKFLVSLALRNALEEAGCQADVWALQLQLYRQGGVNAT
QVLIQHLRGLQKGRSTERNVSVEALASALQLLAREQQSTGRVGRSLPTEDCENEKEQAVH
NVVQLLPGVGTFYNLGTALYYATQNCLGKARERGRDGAIDLGYDLLMTMAGMSGGPMGLA

ISAALKPALRSGVQQLIQYYQDQKDANISQPETTKEGLRAISDVSDLEETTTLASFISEV
VSSAPYWGWAIIKSYDLDPGAGSLEI
>sp|Q9BPW4|APOL4_HUMAN Apolipoprotein L4 OS=Homo sapiens GN=APOL4 PE=2 SV=3
MEGAALLKIFVVCIWVQQNHPGWTVAGQFQEKKRFTEEVIEYFQKKVSPVHLKILLTSDE
AWKRFVRVAELPREEADALYEALKNLTPYVAIEDKDMQQKEQQFREWFLKEFPQIRWKIQ
ESIERLRVIANEIEKVHRGCVIANVVSGSTGILSVIGVMLAPFTAGLSLSITAAGVGLGI
ASATAGIASSIVENTYTRSAELTASRLTATSTDQLEALRDILRDITPNVLSFALDFDEAT
KMIANDVHTLRRSKATVGRPLIAWRYVPINVVETLRTRGAPTRIVRKVARNLGKATSGVL
VVLDVVNLVQDSLDLHKGAKSESAESLRQWAQELEENLNELTHIHQSLKAG
>sp|Q9BWW8|APOL6_HUMAN Apolipoprotein L6 OS=Homo sapiens GN=APOL6 PE=2 SV=1
MDNQAERESEAGVGLQRDEDDAPLCEDVELQDGDLSPEEKIFLREFPRLKEDLKGNIDKL
RALADDIDKTHKKFTKANMVATSTAVISGVMSLLGLALAPATGGGSLLLSTAGQGLATAA
GVTSIVSGTLERSKNKEAQARAEDILPTYDQEDREDEEEKADYVTAAGKIIYNLRNTLKY
AKKNVRAFWKLRANPRLANATKRLLTTGQVSSRSRVQVQKAFAGTTLAMTKNARVLGGVM
SAFSLGYDLATLSKEWKHLKEGARTKFAEELRAKALELERKLTELTQLYKSLQQKVRSRA
RGVGKDLTGTCETEAYWKELREHVWMWLWLCVCLCVCVYVQFT
>sp|Q9NXU5|ARL15_HUMAN ADP-ribosylation factor-like protein 15 OS=Homo sapiens GN=ARL15 PE=1 SV=1
MSDLRITEAFLYMDYLCFRALCCKGPPPARPEYDLVCIGLTGSGKTSLLSKLCSESPDNV
VSTTGFSIKAVPFQNAILNVKELGGADNIRKYWSRYYQGSQGVIFVLDSASSEDDLEAAR
NELHSALQHPQLCTLPFLILANHQDKPAARSVQEIKKYFELEPLARGKRWILQPCSLDDM
DALKDSFSQLINLLEEKDHEAVRM
>sp|P36404|ARL2_HUMAN ADP-ribosylation factor-like protein 2 OS=Homo sapiens GN=ARL2 PE=1 SV=4
MGLLTILKKMKQKERELRLLMLGLDNAGKTTILKKFNGEDIDTISPTLGFNIKTLEHRGF
KLNIWDVGGQKSLRSYWRNYFESTDGLIWVVDSADRQRMQDCQRELQSLLVEERLAGATL
LIFANKQDLPGALSSNAIREVLELDSIRSHHWCIQGCSAVTGENLLPGIDWLLDDISSRI
FTAD
>sp|Q9NVT9|ARMC1_HUMAN Armadillo repeat-containing protein 1 OS=Homo sapiens GN=ARMC1 PE=1 SV=1
MNSSTSTMSEEPDALSVVNQLRDLAADPLNRRAIVQDQGCLPGLILFMDHPNPPVVHSAL
LALRYLAECRANREKMKGELGMMLSLQNVIQKTTTPGETKLLASEIYDILQSSNMADGDS
FNEMNSRRRKAQFFLGTTNKRAKTVVLHIDGLDDTSRRNLCEEALLKIKGVISFTFQMAV
QRCVVRIRSDLKAEALASAIASTKVMKAQQVVKSESGEEMLVPFQDTPVEVEQNTELPDY
LPEDESPTKEQDKAVSRVGSHPEGGASWLSTAANFLSRSFYW
>sp|Q5T9G4|ARM12_HUMAN Armadillo repeat-containing protein 12 OS=Homo sapiens GN=ARMC12 PE=2 SV=1
MGKSIPQYLGQLDIRKSVVSLATGAGAIYLLYKAIKAGIKCKPPLCSNSPICIARLAVER
ERHGRDSGELRRLLNSLECKQDEYAKSMILHSITRCVYLLEAEASACTTDDIVLLGYMLD
DKDNSVKTQALNTLKAFSGIRKFRLKIQEHSIKVLELISTIWDTELHIAGLRLLNNLPLP
DYVHPQLRRVMPALMEILQSDYILAQVQAVRLLSYLAQKNDLLYDILNCQVHSNFLNLFQ
PTQSGSLLYEVLVFAERLSEGRNAPHYHVVKWHYNEQSLHESLFGEESRLADRLLALVIH
PEEDVQIQACKVIVSLQYPQDLRARPSSCQPSRSYFKNTE

>sp|Q5T2S8|ARMC4_HUMAN Armadillo repeat-containing protein 4 OS=Homo sapiens GN=ARMC4 PE=1 SV=1
MGVALRKLTQWTAAGHGTGILEITPLNEAILKEIIVFVESFIYKHPQEAKFVFVEPLEWN
TSLAPSAFESGYVVSETTVKSEEVDKNGQPLLFLSVPQIKIRSFGQLSRLLLIAKTGKLK
EAQACVEANRDPIVKILGSDYNTMKENSIALNILGKITRDDDPESEIKMKIAMLLKQLDL
HLLNHSLKHISLEISLSPMTVKKDIELLKRFSGKGNQTVLESIEYTSDYEFSNGCRAPPW
RQIRGEICYVLVKPHDGETLCITCSAGGVFLNGGKTDDEGDVNYERKGSIYKNLVTFLRE
KSPKFSENMSKLGISFSEDQQKEKDQLGKAPKKEEAAALRKDISGSDKRSLEKNQINFWR
NQMTKRWEPSLNWKTTVNYKGKGSAKEIQEDKHTGKLEKPRPSVSHGRAQLLRKSAEKIE
ETVSDSSSESEEDEEPPDHRQEASADLPSEYWQIQKLVKYLKGGNQTATVIALCSMRDFS
LAQETCQLAIRDVGGLEVLINLLETDEVKCKIGSLKILKEISHNPQIRQNIVDLGGLPIM
VNILDSPHKSLKCLAAETIANVAKFKRARRVVRQHGGITKLVALLDCAHDSTKPAQSSLY
EARDVEVARCGALALWSCSKSHTNKEAIRKAGGIPLLARLLKTSHENMLIPVVGTLQECA
SEENYRAAIKAERIIENLVKNLNSENEQLQEHCAMAIYQCAEDKETRDLVRLHGGLKPLA
SLLNNTDNKERLAAVTGAIWKCSISKENVTKFREYKAIETLVGLLTDQPEEVLVNVVGAL
GECCQERENRVIVRKCGGIQPLVNLLVGINQALLVNVTKAVGACAVEPESMMIIDRLDGV
RLLWSLLKNPHPDVKASAAWALCPCIKNAKDAGEMVRSFVGGLELIVNLLKSDNKEVLAS
VCAAITNIAKDQENLAVITDHGVVPLLSKLANTNNNKLRHHLAEAISRCCMWGRNRVAFG
EHKAVAPLVRYLKSNDTNVHRATAQALYQLSEDADNCITMHENGAVKLLLDMVGSPDQDL
QEAAAGCISNIRRLALATEKARYT
>sp|P48448|AL3B2_HUMAN Aldehyde dehydrogenase family 3 member B2 OS=Homo sapiens GN=ALDH3B2 PE=2 SV=3
MKDEPRSTNLFMKLDSVFIWKEPFGLVLIIAPWNYPLNLTLVLLVGALAAGSCVVLKPSE
ISQGTEKVLAEVLPQYLDQSCFAVVLGGPQETGQLLEHKLDYIFFTGSPRVGKIVMTAAT
KHLTPVTLELGGKNPCYVDDNCDPQTVANRVAWFCYFNAGQTCVAPDYVLCSPEMQERLL
PALQSTITRFYGDDPQSSPNLGHIINQKQFQRLRALLGCSRVAIGGQSNESDRYIAPTVL
VDVQETEPVMQEEIFGPILPIVNVQSVDEAIKFINRQEKPLALYAFSNSSQVVNQMLERT
SSGSFGGNEGFTYISLLSVPFGGVGHSGMGRYHGKFTFDTFSHHRTCLLAPSGLEKLKEI
HYPPYTDWNQQLLRWGMGSQSCTLL
>sp|P20292|AL5AP_HUMAN Arachidonate 5-lipoxygenase-activating protein OS=Homo sapiens GN=ALOX5AP PE=1 SV=2
MDQETVGNVVLLAIVTLISVVQNGFFAHKVEHESRTQNGRSFQRTGTLAFERVYTANQNC
VDAYPTFLAVLWSAGLLCSQVPAAFAGLMYLFVRQKYFVGYLGERTQSTPGYIFGKRIIL
FLFLMSVAGIFNYYLIFFFGSDFENYIKTISTTISPLLLIP
>sp|P49419|AL7A1_HUMAN Alpha-aminoadipic semialdehyde dehydrogenase OS=Homo sapiens GN=ALDH7A1 PE=1 SV=5
MWRLPRALCVHAAKTSKLSGPWSRPAAFMSTLLINQPQYAWLKELGLREENEGVYNGSWG
GRGEVITTYCPANNEPIARVRQASVADYEETVKKAREAWKIWADIPAPKRGEIVRQIGDA
LREKIQVLGSLVSLEMGKILVEGVGEVQEYVDICDYAVGLSRMIGGPILPSERSGHALIE
QWNPVGLVGIITAFNFPVAVYGWNNAIAMICGNVCLWKGAPTTSLISVAVTKIIAKVLED
NKLPGAICSLTCGGADIGTAMAKDERVNLLSFTGSTQVGKQVGLMVQERFGRSLLELGGN
NAIIAFEDADLSLVVPSALFAAVGTAGQRCTTARRLFIHESIHDEVVNRLKKAYAQIRVG
NPWDPNVLYGPLHTKQAVSMFLGAVEEAKKEGGTVVYGGKVMDRPGNYVEPTIVTGLGHD
ASIAHTETFAPILYVFKFKNEEEVFAWNNEVKQGLSSSIFTKDLGRIFRWLGPKGSDCGI

VNVNIPTSGAEIGGAFGGEKHTGGGRESGSDAWKQYMRRSTCTINYSKDLPLAQGIKFQ
>sp|P02768|ALBU_HUMAN Serum albumin OS=Homo sapiens GN=ALB PE=1 SV=2
MKWVTFISLLFLFSSAYSRGVFRRDAHKSEVAHRFKDLGEENFKALVLIAFAQYLQQCPF
EDHVKLVNEVTEFAKTCVADESAENCDKSLHTLFGDKLCTVATLRETYGEMADCCAKQEP
ERNECFLQHKDDNPNLPRLVRPEVDVMCTAFHDNEETFLKKYLYEIARRHPYFYAPELLF
FAKRYKAAFTECCQAADKAACLLPKLDELRDEGKASSAKQRLKCASLQKFGERAFKAWAV
ARLSQRFPKAEFAEVSKLVTDLTKVHTECCHGDLLECADDRADLAKYICENQDSISSKLK
ECCEKPLLEKSHCIAEVENDEMPADLPSLAADFVESKDVCKNYAEAKDVFLGMFLYEYAR
RHPDYSVVLLLRLAKTYETTLEKCCAAADPHECYAKVFDEFKPLVEEPQNLIKQNCELFE
QLGEYKFQNALLVRYTKKVPQVSTPTLVEVSRNLGKVGSKCCKHPEAKRMPCAEDYLSVV
LNQLCVLHEKTPVSDRVTKCCTESLVNRRPCFSALEVDETYVPKEFNAETFTFHADICTL
SEKERQIKKQTALVELVKHKPKATKEQLKAVMDDFAAFVEKCCKADDKETCFAEEGKKLV
AASQAALGL
>sp|P05091|ALDH2_HUMAN Aldehyde dehydrogenase, mitochondrial OS=Homo sapiens GN=ALDH2 PE=1 SV=2
MLRAAARFGPRLGRRLLSAAATQAVPAPNQQPEVFCNQIFINNEWHDAVSRKTFPTVNPS
TGEVICQVAEGDKEDVDKAVKAARAAFQLGSPWRRMDASHRGRLLNRLADLIERDRTYLA
ALETLDNGKPYVISYLVDLDMVLKCLRYYAGWADKYHGKTIPIDGDFFSYTRHEPVGVCG
QIIPWNFPLLMQAWKLGPALATGNVVVMKVAEQTPLTALYVANLIKEAGFPPGVVNIVPG
FGPTAGAAIASHEDVDKVAFTGSTEIGRVIQVAAGSSNLKRVTLELGGKSPNIIMSDADM
DWAVEQAHFALFFNQGQCCCAGSRTFVQEDIYDEFVERSVARAKSRVVGNPFDSKTEQGP
QVDETQFKKILGYINTGKQEGAKLLCGGGIAADRGYFIQPTVFGDVQDGMTIAKEEIFGP
VMQILKFKTIEEVVGRANNSTYGLAAAVFTKDLDKANYLSQALQAGTVWVNCYDVFGAQS
PFGGYKMSGSGRELGEYGLQAYTEVKTVTVKVPQKNS
>sp|P05062|ALDOB_HUMAN Fructose-bisphosphate aldolase B OS=Homo sapiens GN=ALDOB PE=1 SV=2
MAHRFPALTQEQKKELSEIAQSIVANGKGILAADESVGTMGNRLQRIKVENTEENRRQFR
EILFSVDSSINQSIGGVILFHETLYQKDSQGKLFRNILKEKGIVVGIKLDQGGAPLAGTN
KETTIQGLDGLSERCAQYKKDGVDFGKWRAVLRIADQCPSSLAIQENANALARYASICQQ
NGLVPIVEPEVIPDGDHDLEHCQYVTEKVLAAVYKALNDHHVYLEGTLLKPNMVTAGHAC
TKKYTPEQVAMATVTALHRTVPAAVPGICFLSGGMSEEDATLNLNAINLCPLPKPWKLSF
SYGRALQASALAAWGGKAANKEATQEAFMKRAMANCQAAKGQYVHTGSSGAASTQSLFTA
CYTY
>sp|Q2TAA5|ALG11_HUMAN GDP-Man:Man(3)GlcNAc(2)-PP-Dol alpha-1,2-mannosyltransferase OS=Homo sapiens GN=ALG11 PE=1 SV=2
MAAGERSWCLCKLLRFFYSLFFPGLIVCGTLCVCLVIVLWGIRLLLQRKKKLVSTSKNGK
NQMVIAFFHPYCNAGGGGERVLWCALRALQKKYPEAVYVVYTGDVNVNGQQILEGAFRRF
NIRLIHPVQFVFLRKRYLVEDSLYPHFTLLGQSLGSIFLGWEALMQCVPDVYIDSMGYAF
TLPLFKYIGGCQVGSYVHYPTISTDMLSVVKNQNIGFNNAAFITRNPFLSKVKLIYYYLF
AFIYGLVGSCSDVVMVNSSWTLNHILSLWKVGNCTNIVYPPCDVQTFLDIPLHEKKMTPG
HLLVSVGQFRPEKNHPLQIRAFAKLLNKKMVESPPSLKLVLIGGCRNKDDELRVNQLRRL
SEDLGVQEYVEFKINIPFDELKNYLSEATIGLHTMWNEHFGIGVVECMAAGTIILAHNSG
GPKLDIVVPHEGDITGFLAESEEDYAETIAHILSMSAEKRLQIRKSARASVSRFSDQEFE
VTFLSSVEKLFK

>sp|Q92685|ALG3_HUMAN Dol-P-Man:Man(5)GlcNAc(2)-PP-Dol alpha-1,3-mannosyltransferase OS=Homo sapiens GN=ALG3 PE=1 SV=1
MAAGLRKRGRSGSAAQAEGLCKQWLQRAWQERRLLLREPRYTLLVAACLCLAEVGITFWV
IHRVAYTEIDWKAYMAEVEGVINGTYDYTQLQGDTGPLVYPAGFVYIFMGLYYATSRGTD
IRMAQNIFAVLYLATLLLVFLIYHQTCKVPPFVFFFMCCASYRVHSIFVLRLFNDPVAMV
LLFLSINLLLAQRWGWGCCFFSLAVSVKMNVLLFAPGLLFLLLTQFGFRGALPKLGICAG
LQVVLGLPFLLENPSGYLSRSFDLGRQFLFHWTVNWRFLPEALFLHRAFHLALLTAHLTL
LLLFALCRWHRTGESILSLLRDPSKRKVPPQPLTPNQIVSTLFTSNFIGICFSRSLHYQF
YVWYFHTLPYLLWAMPARWLTHLLRLLVLGLIELSWNTYPSTSCSSAALHICHAVILLQL
WLGPQPFPKSTQHSKKAH
>sp|Q9Y672|ALG6_HUMAN Dolichyl pyrophosphate Man9GlcNAc2 alpha-1,3-glucosyltransferase OS=Homo sapiens GN=ALG6 PE=1 SV=1
MEKWYLMTVVVLIGLTVRWTVSLNSYSGAGKPPMFGDYEAQRHWQEITFNLPVKQWYFNS
SDNNLQYWGLDYPPLTAYHSLLCAYVAKFINPDWIALHTSRGYESQAHKLFMRTTVLIAD
LLIYIPAVVLYCCCLKEISTKKKIANALCILLYPGLILIDYGHFQYNSVSLGFALWGVLG
ISCDCDLLGSLAFCLAINYKQMELYHALPFFCFLLGKCFKKGLKGKGFVLLVKLACIVVA
SFVLCWLPFFTEREQTLQVLRRLFPVDRGLFEDKVANIWCSFNVFLKIKDILPRHIQLIM
SFCFTFLSLLPACIKLILQPSSKGFKFTLVSCALSFFLFSFQVHEKSILLVSLPVCLVLS
EIPFMSTWFLLVSTFSMLPLLLKDELLMPSVVTTMAFFIACVTSFSIFEKTSEEELQLKS
FSISVRKYLPCFTFLSRIIQYLFLISVITMVLLTLMTVTLDPPQKLPDLFSVLVCFVSCL
NFLFFLVYFNIIIMWDSKSGRNQKKIS
>sp|Q6NS38|ALKB2_HUMAN DNA oxidative demethylase ALKBH2 OS=Homo sapiens GN=ALKBH2 PE=1 SV=1
MDRFLVKGAQGGLLRKQEEQEPTGEEPAVLGGDKESTRKRPRREAPGNGGHSAGPSWRHI
RAEGLDCSYTVLFGKAEADEIFQELEKEVEYFTGALARVQVFGKWHSVPRKQATYGDAGL
TYTFSGLTLSPKPWIPVLERIRDHVSGVTGQTFNFVLINRYKDGCDHIGEHRDDERELAP
GSPIASVSFGACRDFVFRHKDSRGKSPSRRVAVVRLPLAHGSLLMMNHPTNTHWYHSLPV
RKKVLAPRVNLTFRKILLTKK
>sp|Q9NXW9|ALKB4_HUMAN Alpha-ketoglutarate-dependent dioxygenase alkB homolog 4 OS=Homo sapiens GN=ALKBH4 PE=1 SV=1
MAAAAAETPEVLRECGCKGIRTCLICERQRGSDPPWELPPAKTYRFIYCSDTGWAVGTEE
SDFEGWAFPFPGVMLIEDFVTREEEAELVRLMDRDPWKLSQSGRRKQDYGPKVNFRKQKL
KTEGFCGLPSFSREVVRRMGLYPGLEGFRPVEQCNLDYCPERGSAIDPHLDDAWLWGERL
VSLNLLSPTVLSMCREAPGSLLLCSAPSAAPEALVDSVIAPSRSVLCQEVEVAIPLPARS
LLVLTGAARHQWKHAIHRRHIEARRVCVTFRELSAEFGPGGRQQELGQELLRIALSFQGR
PV
>sp|Q96BT7|ALKB8_HUMAN Alkylated DNA repair protein alkB homolog 8 OS=Homo sapiens GN=ALKBH8 PE=1 SV=2
MDSNHQSNYKLSKTEKKFLRKQIKAKHTLLRHEGIETVSYATQSLVVANGGLGNGVSRNQ
LLPVLEKCGLVDALLMPPNKPYSFARYRTTEESKRAYVTLNGKEVVDDLGQKITLYLNFV
EKVQWKELRPQALPPGLMVVEEIISSEEEKMLLESVDWTEDTDNQNSQKSLKHRRVKHFG
YEFHYENNNVDKDKPLSGGLPDICESFLEKWLRKGYIKHKPDQMTINQYEPGQGIPAHID
THSAFEDEIVSLSLGSEIVMDFKHPDGIAVPVMLPRRSLLVMTGESRYLWTHGITCRKFD
TVQASESLKSGIITSDVGDLTLSKRGLRTSFTFRKVRQTPCNCSYPLVCDSQRKETPPSF

PESDKEASRLEQEYVHQVYEEIAGHFSSTRHTPWPHIVEFLKALPSGSIVADIGCGNGKY
LGINKELYMIGCDRSQNLVDICRERQFQAFVCDALAVPVRSGSCDACISIAVIHHFATAE
RRVAALQEIVRLLRPGGKALIYVWAMEQEYNKQKSKYLRGNRNSQGKKEEMNSDTSVQRS
LVEQMRDMGSRDSASSVPRINDSQEGGCNSRQVSNSKLPVHVNRTSFYSQDVLVPWHLKG
NPDKGKPVEPFGPIGSQDPSPVFHRYYHVFREGELEGACRTVSDVRILQSYYDQGNWCVI
LQKA
>sp|P30533|AMRP_HUMAN Alpha-2-macroglobulin receptor-associated protein OS=Homo sapiens GN=LRPAP1 PE=1 SV=1
MAPRRVRSFLRGLPALLLLLLFLGPWPAASHGGKYSREKNQPKPSPKRESGEEFRMEKLN
QLWEKAQRLHLPPVRLAELHADLKIQERDELAWKKLKLDGLDEDGEKEARLIRNLNVILA
KYGLDGKKDARQVTSNSLSGTQEDGLDDPRLEKLWHKAKTSGKFSGEELDKLWREFLHHK
EKVHEYNVLLETLSRTEEIHENVISPSDLSDIKGSVLHSRHTELKEKLRSINQGLDRLRR
VSHQGYSTEAEFEEPRVIDLWDLAQSANLTDKELEAFREELKHFEAKIEKHNHYQKQLEI
AHEKLRHAESVGDGERVSRSREKHALLEGRTKELGYTVKKHLQDLSGRISRARHNEL
>sp|Q9UJX6|ANC2_HUMAN Anaphase-promoting complex subunit 2 OS=Homo sapiens GN=ANAPC2 PE=1 SV=1
MAAAVVVAEGDSDSRPGQELLVAWNTVSTGLVPPAALGLVSSRTSGAVPPKEEELRAAVE
VLRGHGLHSVLEEWFVEVLQNDLQANISPEFWNAISQCENSADEPQCLLLLLDAFGLLES
RLDPYLRSLELLEKWTRLGLLMGTGAQGLREEVHTMLRGVLFFSTPRTFQEMIQRLYGCF
LRVYMQSKRKGEGGTDPELEGELDSRYARRRYYRLLQSPLCAGCSSDKQQCWCRQALEQF
HQLSQVLHRLSLLERVSAEAVTTTLHQVTRERMEDRCRGEYERSFLREFHKWIERVVGWL
GKVFLQDGPARPASPEAGNTLRRWRCHVQRFFYRIYASLRIEELFSIVRDFPDSRPAIED
LKYCLERTDQRQQLLVSLKAALETRLLHPGVNTCDIITLYISAIKALRVLDPSMVILEVA
CEPIRRYLRTREDTVRQIVAGLTGDSDGTGDLAVELSKTDPASLETGQDSEDDSGEPEDW
VPDPVDADPGKSSSKRRSSDIISLLVSIYGSKDLFINEYRSLLADRLLHQFSFSPEREIR
NVELLKLRFGEAPMHFCEVMLKDMADSRRINANIREEDEKRPAEEQPPFGVYAVILSSEF
WPPFKDEKLEVPEDIRAALEAYCKKYEQLKAMRTLSWKHTLGLVTMDVELADRTLSVAVT
PVQAVILLYFQDQASWTLEELSKAVKMPVALLRRRMSVWLQQGVLREEPPGTFSVIEEER
PQDRDNMVLIDSDDESDSGMASQADQKEEELLLFWTYIQAMLTNLESLSLDRIYNMLRMF
VVTGPALAEIDLQELQGYLQKKVRDQQLVYSAGVYRLPKNCS
>sp|P16860|ANFB_HUMAN Natriuretic peptides B OS=Homo sapiens GN=NPPB PE=1 SV=1
MDPQTAPSRALLLLLFLHLAFLGGRSHPLGSPGSASDLETSGLQEQRNHLQGKLSELQVE
QTSLEPLQESPRPTGVWKSREVATEGIRGHRKMVLYTLRAPRSPKMVQGSGCFGRKMDRI
SSSSGLGCKVLRRH
>sp|P53677|AP3M2_HUMAN AP-3 complex subunit mu-2 OS=Homo sapiens GN=AP3M2 PE=2 SV=1
MIHSLFLINSSGDIFLEKHWKSVVSRSVCDYFFEAQERATEAENVPPVIPTPHHYLLSVY
RHKIFFVAVIQTEVPPLFVIEFLHRVVDTFQDYFGVCSEPVIKDNVVVVYEVLEEMLDNG
FPLATESNILKELIKPPTILRTVVNTITGSTNVGDQLPTGQLSVVPWRRTGVKYTNNEAY
FDVIEEIDAIIDKSGSTITAEIQGVIDACVKLTGMPDLTLSFMNPRLLDDVSFHPCVRFK
RWESERILSFIPPDGNFRLLSYHVSAQNLVAIPVYVKHNISFRDSSSLGRFEITVGPKQT
MGKTIEGVTVTSQMPKGVLNMSLTPSQGTHTFDPVTKMLSWDVGKINPQKLPSLKGTMSL
QAGASKPDENPTINLQFKIQQLAISGLKVNRLDMYGEKYKPFKGIKYMTKAGKFQVRT
>sp|P50583|AP4A_HUMAN Bis(5'-nucleosyl)-tetraphosphatase [asymmetrical] OS=Homo sapiens GN=NUDT2 PE=1 SV=3

MALRACGLIIFRRCLIPKVDNNAIEFLLLQASDGIHHWTPPKGHVEPGEDDLETALRETQ
EEAGIEAGQLTIIEGFKRELNYVARNKPKTVIYWLAEVKDYDVEIRLSHEHQAYRWLGLE
EACQLAQFKEMKAALQEGHQFLCSIEA
>sp|Q9UPM8|AP4E1_HUMAN AP-4 complex subunit epsilon-1 OS=Homo sapiens GN=AP4E1 PE=1 SV=2
MSDIVEKTLTALPGLFLQNQPGGGPAAAKASFSSRLGSLVRGITALTSKHEEEKLIQQEL
SSLKATVSAPTTTLKMMKECMVRLIYCEMLGYDASFGYIHAIKLAQQGNLLEKRVGYLAV
SLFLHESHELLLLLVNTVVKDLQSTNLVEVCMALTVVSQIFPCEMIPAVLPLIEDKLQHS
KEIVRRKAVLALYKFHLIAPNQVQHIHIKFRKALCDRDVGVMAASLHIYLRMIKENSSGY
KDLTGSFVTILKQVVGGKLPVEFNYHSVPAPWLQIQLLRILGLLGKDDQRTSELMYDVLD
ESLRRAELNHNVTYAILFECVHTVYSIYPKSELLEKAAKCIGKFVLSPKINLKYLGLKAL
TYVIQQDPTLALQHQMTIIECLDHPDPIIKRETLELLYRITNAQNITVIVQKMLEYLHQS
KEEYVIVNLVGKIAELAEKYAPDNAWFIQTMNAVFSVGGDVMHPDIPNNFLRLLAEGFDD
ETEDQQLRLYAVQSYLTLLDMENVFYPQRFLQVMSWVLGEYSYLLDKETPEEVIAKLYKL
LMNDSVSSETKAWLIAAVTKLTSQAHSSNTVERLIHEFTISLDTCMRQHAFELKHLHENV
ELMKSLLPVDRSCEDLVVDASLSFLDGFVAEGLSQGAAPYKPPHQRQEEKLSQEKVLNFE
PYGLSFSSSGFTGRQSPAGISLGSDVSGNSAETGLKETNSLKLEGIKKLWGKEGYLPKKE
SKTGDESGALPVPQESIMENVDQAITKKDQSQVLTQSKEEKEKQLLASSLFVGLGSESTI
NLLGKADTVSHKFRRKSKVKEAKSGETTSTHNMTCSSFSSLSNVAYEDDYYSNTLHDTGD
KELKKFSLTSELLDSESLTELPLVEKFSYCSLSTPSLFANNNMEIFHPPQSTAASVAKES
SLASSFLEETTEYIHSNAMEVCNNETISVSSYKIWKDDCLLMVWSVTNKSGLELKSADLE
IFPAENFKVTEQPGCCLPVMEAESTKSFQYSVQIEKPFTEGNLTGFISYHMMDTHSAQLE
FSVNLSLLDFIRPLKISSDDFGKLWLSFANDVKQNVKMSESQAALPSALKTLQQKLRLHI
IEIIGNEGLLACQLLPSIPCLLHCRVHADVLALWFRSSCSTLPDYLLYQCQKVMEGS
>sp|O00189|AP4M1_HUMAN AP-4 complex subunit mu-1 OS=Homo sapiens GN=AP4M1 PE=1 SV=2
MISQFFILSSKGDPLIYKDFRGDSGGRDVAELFYRKLTGLPGDESPVVMHHHGRHFIHIR
HSGLYLVVTTSENVSPFSLLELLSRLATLLGDYCGSLGEGTISRNVALVYELLDEVLDYG
YVQTTSTEMLRNFIQTEAVVSKPFSLFDLSSVGLFGAETQQSKVAPSSAASRPVLSSRSD
QSQKNEVFLDVVERLSVLIASNGSLLKVDVQGEIRLKSFLPSGSEMRIGLTEEFCVGKSE
LRGYGPGIRVDEVSFHSSVNLDEFESHRILRLQPPQGELTVMRYQLSDDLPSPLPFRLFP
SVQWDRGSGRLQVYLKLRCDLLSKSQALNVRLHLPLPRGVVSLSQELSSPEQKAELAEGA
LRWDLPRVQGGSQLSGLFQMDVPGPPGPPSHGLSTSASPLGLGPASLSFELPRHTCSGLQ
VRFLRLAFRPCGNANPHKWVRHLSHSDAYVIRI
>sp|Q9H0R1|AP5M1_HUMAN AP-5 complex subunit mu-1 OS=Homo sapiens GN=AP5M1 PE=1 SV=2
MAQRAVWLISHEPGTPLCGTVRFSRRYPTVEKRARVFNGASYVPVPEDGPFLKALLFELR
LLDDDKDFVESRDSCSRINKTSIYGLLIGGEELWPVVAFLKNDMIYACVPLVEQTLSPRP
PLISVSGVSQGFEFLFGIQDFLYSGQKNDSELNTKLSQLPDLLLQACPFGTLLDANLQNS
LDNTNFASVTQPQKQPAWKTGTYKGKPQVSISITEKVKSMQYDKQGIADTWQVVGTVTCK
CDLEGIMPNVTISLSLPTNGSPLQDILVHPCVTSLDSAILTSSSIDAMDDSAFSGPYKFP
FTPPLESFNLCFYTSQVPVPPILGFYQMKEEEVQLRITINLKLHESVKNNFEFCEAHIPF
YNRGPITHLEYKTSFGQLEVFREKSLLIWIIGQKFPKSMEISLSGTVTFGAKSHEKQPFD
PICTGETAYLKLHFRILDYTLTGCYADQHSVQVFASGKPKISAHRKLISSDYYIWNSKAP
APVTYGSLLL
>sp|O14727|APAF_HUMAN Apoptotic protease-activating factor 1 OS=Homo sapiens GN=APAF1 PE=1 SV=2

MDAKARNCLLQHREALEKDIKTSYIMDHMISDGFLTISEEEKVRNEPTQQQRAAMLIKMI
LKKDNDSYVSFYNALLHEGYKDLAALLHDGIPVVSSSSGKDSVSGITSYVRTVLCEGGVP
QRPVVFVTRKKLVNAIQQKLSKLKGEPGWVTIHGMAGCGKSVLAAEAVRDHSLLEGCFPG
GVHWVSVGKQDKSGLLMKLQNLCTRLDQDESFSQRLPLNIEEAKDRLRILMLRKHPRSLL
ILDDVWDSWVLKAFDSQCQILLTTRDKSVTDSVMGPKYVVPVESSLGKEKGLEILSLFVN
MKKADLPEQAHSIIKECKGSPLVVSLIGALLRDFPNRWEYYLKQLQNKQFKRIRKSSSYD
YEALDEAMSISVEMLREDIKDYYTDLSILQKDVKVPTKVLCILWDMETEEVEDILQEFVN
KSLLFCDRNGKSFRYYLHDLQVDFLTEKNCSQLQDLHKKIITQFQRYHQPHTLSPDQEDC
MYWYNFLAYHMASAKMHKELCALMFSLDWIKAKTELVGPAHLIHEFVEYRHILDEKDCAV
SENFQEFLSLNGHLLGRQPFPNIVQLGLCEPETSEVYQQAKLQAKQEVDNGMLYLEWINK
KNITNLSRLVVRPHTDAVYHACFSEDGQRIASCGADKTLQVFKAETGEKLLEIKAHEDEV
LCCAFSTDDRFIATCSVDKKVKIWNSMTGELVHTYDEHSEQVNCCHFTNSSHHLLLATGS
SDCFLKLWDLNQKECRNTMFGHTNSVNHCRFSPDDKLLASCSADGTLKLWDATSANERKS
INVKQFFLNLEDPQEDMEVIVKCCSWSADGARIMVAAKNKIFLFDIHTSGLLGEIHTGHH
STIQYCDFSPQNHLAVVALSQYCVELWNTDSRSKVADCRGHLSWVHGVMFSPDGSSFLTS
SDDQTIRLWETKKVCKNSAVMLKQEVDVVFQENEVMVLAVDHIRRLQLINGRTGQIDYLT
EAQVSCCCLSPHLQYIAFGDENGAIEILELVNNRIFQSRFQHKKTVWHIQFTADEKTLIS
SSDDAEIQVWNWQLDKCIFLRGHQETVKDFRLLKNSRLLSWSFDGTVKVWNIITGNKEKD
FVCHQGTVLSCDISHDATKFSSTSADKTAKIWSFDLLLPLHELRGHNGCVRCSAFSVDST
LLATGDDNGEIRIWNVSNGELLHLCAPLSEEGAATHGGWVTDLCFSPDGKMLISAGGYIK
WWNVVTGESSQTFYTNGTNLKKIHVSPDFKTYVTVDNLGILYILQTLE
>sp|Q13794|APR_HUMAN Phorbol-12-myristate-13-acetate-induced protein 1 OS=Homo sapiens GN=PMAIP1 PE=1 SV=1
MPGKKARKNAQPSPARAPAELEVECATQLRRFGDKLNFRQKLLNLISKLFCSGT
>sp|P07741|APT_HUMAN Adenine phosphoribosyltransferase OS=Homo sapiens GN=APRT PE=1 SV=2
MADSELQLVEQRIRSFPDFPTPGVVFRDISPVLKDPASFRAAIGLLARHLKATHGGRIDY
IAGLDSRGFLFGPSLAQELGLGCVLIRKRGKLPGPTLWASYSLEYGKAELEIQKDALEPG
QRVVVVDDLLATGGTMNAACELLGRLQAEVLECVSLVELTSLKGREKLAPVPFFSLLQYE
>sp|Q8IXF9|AQ12A_HUMAN Aquaporin-12A OS=Homo sapiens GN=AQP12A PE=2 SV=1
MAGLNVSLSFFFATFALCEAARRASKALLPVGAYEVFAREAMRTLVELGPWAGDFGPDLL
LTLLFLLFLAHGVTLDGASANPTVSLQEFLMAEQSLPGTLLKLAAQGLGMQAACTLMRLC
WAWELSDLHLLQSLMAQSCSSALRTSVPHGALVEAACAFCFHLTLLHLRHSPPAYSGPAV
ALLVTVTAYTAGPFTSAFFNPALAASVTFACSGHTLLEYVQVYWLGPLTGMVLAVLLHQG
RLPHLFQRNLFYGQKNKYRAPRGKPAPASGDTQTPAKGSSVREPGRSGVEGPHSS
>sp|P41181|AQP2_HUMAN Aquaporin-2 OS=Homo sapiens GN=AQP2 PE=1 SV=1
MWELRSIAFSRAVFAEFLATLLFVFFGLGSALNWPQALPSVLQIAMAFGLGIGTLVQALG
HISGAHINPAVTVACLVGCHVSVLRAAFYVAAQLLGAVAGAALLHEITPADIRGDLAVNA
LSNSTTAGQAVTVELFLTLQLVLCIFASTDERRGENPGTPALSIGFSVALGHLLGIHYTG
CSMNPARSLAPAVVTGKFDDHWVFWIGPLVGAILGSLLYNYVLFPPAKSLSERLAVLKGL
EPDTDWEEREVRRRQSVELHSPQSLPRGTKA
>sp|Q13520|AQP6_HUMAN Aquaporin-6 OS=Homo sapiens GN=AQP6 PE=2 SV=2
MDAVEPGGRGWASMLACRLWKAISRALFAEFLATGLYVFFGVGSVMRWPTALPSVLQIAI
TFNLVTAMAVQVTWKASGAHANPAVTLAFLVGSHISLPRAVAYVAAQLVGATVGAALLYG
VMPGDIRETLGINVVRNSVSTGQAVAVELLLTLQLVLCVFASTDSRQTSGSPATMIGISV

ALGHLIGIHFTGCSMNPARSFGPAIIIGKFTVHWVFWVGPLMGALLASLIYNFVLFPDTK
TLAQRLAILTGTVEVGTGAGAGAEPLKKESQPGSGAVEMESV
>sp|Q66PJ3|AR6P4_HUMAN ADP-ribosylation factor-like protein 6-interacting protein 4 OS=Homo sapiens GN=ARL6IP4 PE=1 SV=2
MPRCTYQLEQNPGFLPDGPGVHARAHCQDLSGPYGHEFATSESLGGRVGKTRAPQSGARS
RMERAGPAGEEGGAREGRLLPRAPGAWVLRACAERAALEVGAASADTGVRGCGARGPAPL
LASAGGGRARDGTWGVRTKGSGAALPSRPASRAAPRPEASSPPLPLEKARGGLSGPQGGR
ARGAMAHVGSRKRSRSRSRSRGRGSEKRKKKSRKDTSRNCSASTSQGRKASTAPGAEASP
SPCITERSKQKARRRTRSSSSSSSSSSSSSSSSSSSSSSSSDGRKKRGKYKDKRRKKKK
KRKKLKKKGKEKAEAQQVEALPGPSLDQWHRSAGEEEDGPVLTDEQKSRIQAMKPMTKEE
WDARQSIIRKVVDPETGRTRLIKGDGEVLEEIVTKERHREINKQATRGDCLAFQMRAGLL
P
>sp|O15143|ARC1B_HUMAN Actin-related protein 2/3 complex subunit 1B OS=Homo sapiens GN=ARPC1B PE=1 SV=3
MAYHSFLVEPISCHAWNKDRTQIAICPNNHEVHIYEKSGAKWTKVHELKEHNGQVTGIDW
APESNRIVTCGTDRNAYVWTLKGRTWKPTLVILRINRAARCVRWAPNENKFAVGSGSRVI
SICYFEQENDWWVCKHIKKPIRSTVLSLDWHPNNVLLAAGSCDFKCRIFSAYIKEVEERP
APTPWGSKMPFGELMFESSSSCGWVHGVCFSASGSRVAWVSHDSTVCLADADKKMAVATL
ASETLPLLALTFITDNSLVAAGHDCFPVLFTYDAAAGMLSFGGRLDVPKQSSQRGLTARE
RFQNLDKKASSEGGTAAGAGLDSLHKNSVSQISVLSGGKAKCSQFCTTGMDGGMSIWDVK
SLESALKDLKIK
>sp|Q9NR81|ARHG3_HUMAN Rho guanine nucleotide exchange factor 3 OS=Homo sapiens GN=ARHGEF3 PE=1 SV=1
MVAKDYPFYLTVKRANCSLELPPASGPAKDAEEPSNKRVKPLSRVTSLANLIPPVKATPL
KRFSQTLQRSISFRSESRPDILAPRPWSRNAAPSSTKRRDSKLWSETFDVCVNQMLTSKE
IKRQEAIFELSQGEEDLIEDLKLAKKAYHDPMLKLSIMTEQELNQIFGTLDSLIPLHEEL
LSQLRDVRKPDGSTEHVGPILVGWLPCLSSYDSYCSNQVAAKALLDHKKQDHRVQDFLQR
CLESPFSRKLDLWNFLDIPRSRLVKYPLLLREILRHTPNDNPDQQHLEEAINIIQGIVAE
INTKTGESECRYYKERLLYLEEGQKDSLIDSSRVLCCHGELKNNRGVKLHVFLFQEVLVI
TRAVTHNEQLCYQLYRQPIPVKDLLLEDLQDGEVRLGGSLRGAFSNNERIKNFFRVSFKN
GSQSQTHSLQANDTFNKQQWLNCIRQAKETVLCAAGQAGVLDSEGSFLNPTTGSRELQGE
TKLEQMDQSDSESDCSMDTSEVSLDCERMEQTDSSCGNSRHGESNV
>sp|O15013|ARHGA_HUMAN Rho guanine nucleotide exchange factor 10 OS=Homo sapiens GN=ARHGEF10 PE=1 SV=4
MRPPGFLSRAPSLNRAERGIWSCSMDQREPLPPAPAENEMKYDTNNNEEEEGEQFDFDSG
DEIPEADRQAPSAPETGGAGASEAPAPTGGEDGAGAETTPVAEPTKLVLPMKVNPYSVID
ITPFQEDQPPTPVPSAEEENVGLHVPCGYLVPVPCGYAVPSNLPLLLPAYSSPVIICATS
LDEEAETPEVTEDRQPNSLSSEEPPTSEDQVGREDSALARWAADPANTAWMENPEEAIYD
DVPRENSDSEPDEMIYDDVENGDEGGNSSLEYGWSSSEFESYEEQSDSECKNGIPRSFLR
SNHKKQLSHDLTRLKEHYEKKMRDLMASTVGVVEIQQLRQKHELKMQKLVKAAKDGTKDG
LERTRAAVKRGRSFIRTKSLIAQDHRSSLEEEQNLFIDVDCKHPEAILTPMPEGLSQQQV
VRRYILGSVVDSEKNYVDALKRILEQYEKPLSEMEPKVLSERKLKTVFYRVKEILQCHSL
FQIALASRVSEWDSVEMIGDVFVASFSKSMVLDAYSEYVNNFSTAVAVLKKTCATKPAFL
EFLKQEQEASPDRTTLYSLMMKPIQRFPQFILLLQDMLKNTSKGHPDRLPLQMALTELET

LAEKLNERKRDADQRCEVKQIAKAINERYLNKLLSSGSRYLIRSDDMIETVYNDRGEIVK
TKERRVFMLNDVLMCATVSSRPSHDSRVMSSQRYLLKWSVPLGHVDAIEYGSSAGTGEHS
RHLAVHPPESLAVVANAKPNKVYMGPGQLYQDLQNLLHDLNVIGQITQLIGNLKGNYQNL
NQSVAHDWTSGLQRLILKKEDEIRAADCCRIQLQLPGKQDKSGRPTFFTAVFNTFTPAIK
ESWVNSLQMAKLALEEENHMGWFCVEDDGNHIKKEKHPLLVGHMPVMVAKQQEFKIECAA
YNPEPYLNNESQPDSFSTAHGFLWIGSCTHQMGQIAIVSFQNSTPKVIECFNVESRILCM
LYVPVEEKRREPGAPPDPETPAVRASDVPTICVGTEEGSISIYKSSQGSKKVRLQHFFTP
EKSTVMSLACTSQSLYAGLVNGAVASYARAPDGSWDSEPQKVIKLGVLPVRSLLMMEDTL
WAASGGQVFIISVETHAVEGQLEAHQEEGMVISHMAVSGVGIWIAFTSGSTLRLFHTETL
KHLQDINIATPVHNMLPGHQRLSVTSLLVCHGLLMVGTSLGVLVALPVPRLQGIPKVTGR
GMVSYHAHNSPVKFIVLATALHEKDKDKSRDSLAPGPEPQDEDQKDALPSGGAGSSLSQG
DPDAAIWLGDSLGSMTQKSDLSSSSGSLSLSHGSSSLEHRSEDSTIYDLLKDPVSLRSKA
RRAKKAKASSALVVCGGQGHRRVHRKARQPHQEELAPTVMVWQIPLLNI
>sp|O15085|ARHGB_HUMAN Rho guanine nucleotide exchange factor 11 OS=Homo sapiens GN=ARHGEF11 PE=1 SV=1
MSVRLPQSIDRLSSLSSLGDSAPERKSPSHHRQPSDASETTGLVQRCVIIQKDQHGFGFT
VSGDRIVLVQSVRPGGAAMKAGVKEGDRIIKVNGTMVTNSSHLEVVKLIKSGAYVALTLL
GSSPSSMGISGLQQDPSPAGAPRITSVIPSPPPPPPLPPPQRITGPKPLQDPEVQKHATQ
ILRNMLRQEEKELQDILPLYGDTSQRPSEGRLSLDSQEGDSGLDSGTERFPSLSESLMNR
NSVLSDPGLDSPRTSPVIMARVAQHHRRQGSDAAVPSTGDQGVDQSPKPLIIGPEEDYDP
GYFNNESDIIFQDLEKLKSRPAHLGVFLRYIFSQADPSPLLFYLCAEVYQQASPKDSRSL
GKDIWNIFLEKNAPLRVKIPEMLQAEIDSRLRNSEDARGVLCEAQEAAMPEIQEQIHDYR
TKRTLGLGSLYGENDLLDLDGDPLRERQVAEKQLAALGDILSKYEEDRSAPMDFALNTYM
SHAGIRLREARPSNTAEKAQSAPDKDKWLPFFPKTKKSSNSKKEKDALEDKKRNPILKYI
GKPKSSSQSTFHIPLSPVEVKPGNVRNIIQHFENNQQYDAPEPGTQRLSTGSFPEDLLES
DSSRSEIRLGRSESLKGREEMKRSRKAENVPRSRSDVDMDAAAEATRLHQSASSSTSSLS
TRSLENPTPPFTPKMGRRSIESPSLGFCTDTLLPHLLEDDLGQLSDLEPEPDAQNWQHTV
GKDVVAGLTQREIDRQEVINELFVTEASHLRTLRVLDLIFYQRMKKENLMPREELARLFP
NLPELIEIHNSWCEAMKKLREEGPIIKEISDLMLARFDGPAREELQQVAAQFCSYQSIAL
ELIKTKQRKESRFQLFMQEAESHPQCRRLQLRDLIISEMQRLTKYPLLLESIIKHTEGGT
SEHEKLCRARDQCREILKYVNEAVKQTENRHRLEGYQKRLDATALERASNPLAAEFKSLD
LTTRKMIHEGPLTWRISKDKTLDLHVLLLEDLLVLLQKQDEKLLLKCHSKTAVGSSDSKQ
TFSPVLKLNAVLIRSVATDKRAFFIICTSKLGPPQIYELVALTSSDKNTWMELLEEAVRN
ATRHPGAAPMPVHPPPPGPREPAQQGPTPSRVELDDSDVFHGEPEPEELPGGTGSQQRVQ
GKHQVLLEDPEQEGSAEEEELGVLPCPSTSLDGENRGIRTRNPIHLAFPGPLFMEGLADS
ALEDVENLRHLILWSLLPGHTMETQAAQEPEDDLTPTPSVISVTSHPWDPGSPGQAPPGG
EGDNTQLAGLEGERPEQEDMGLCSLEHLPPRTRNSGIWESPELDRNLAEDASSTEAAGGY
KVVRKAEVAGSKVVPALPESGQSEPGPPEVEGGTKATGNCFYVSMPSGPPDSSTDHSEAP
MSPPQPDSLPAGQTEPQPQLQGGNDDPRRPSRSPPSLALRDVGMIFHTIEQLTLKLNRLK
DMELAHRELLKSLGGESSGGTTPVGSFHTEAARWTDGSLSPPAKEPLASDSRNSHELGPC
PEDGSDAPLEDSTADAAASPGP
>sp|Q96PE2|ARHGH_HUMAN Rho guanine nucleotide exchange factor 17 OS=Homo sapiens GN=ARHGEF17 PE=1 SV=1
MADGAPRPQLYRSVSFKLLERWSGGPGLREEDTDTPGLRRRASCRPTTAARGQPSRRVSK

LASGPLAAPAQPRPLRSLSPSVRQLSRRFDAPRLDDGSAGTRDGGVLPAAAEEAAEGPAR
GAWPSVTEMRKLFGGPGSRRPSADSESPGTPSPDGAAWEPPARESRQPPTPPPRTCFPLA
GLRSARPLTGPETEGRLRRPQQQQERAQRPADGLHSWHIFSQPQAGARASCSSSSIAASY
PVSRSRAASSSEEEEEGPPQLPGAQSPAYHGGHSSGSDDDRDGEGGHRWGGRPGLRPGSS
LLDQDCRPDSDGLNLSSMNSAGVSGSPEPPTSPRAPREEGLREWGSGSPPCVPGPQEGLR
PMSDSVGGAFRVAKVSFPSYLASPAGSRGSSRYSSTETLKDDDLWSSRGSGGWGVYRSPS
FGAGEGLLRSQARTRAKGPGGTSRALRDGGFEPEKSRQRKSLSNPDIASETLTLLSFLRS
DLSELRVRKPGGSSGDRGSNPLDGRDSPSAGGPVGQLEPIPIPAPASPGTRPTLKDLTAT
LRRAKSFTCSEKPMARRLPRTSALKSSSSELLLTGPGAEEDPLPLIVQDQYVQEARQVFE
KIQRMGAQQDDGSDAPPGSPDWAGDVTRGQRSQEELSGPESSLTDEGIGADPEPPVAAFC
GLGTTGMWRPLSSSSAQTNHHGPGTEDSLGGWALVSPETPPTPGALRRRRKVPPSGSGGS
ELSNGEAGEAYRSLSDPIPQRHRAATSEEPTGFSVDSNLLGSLSPKTGLPATSAMDEGLT
SGHSDWSVGSEESKGYQEVIQSIVQGPGTLGRVVDDRIAGKAPKKKSLSDPSRRGELAGP
GFEGPGGEPIREVEPMLPPSSSEPILVEQRAEPEEPGATRSRAQSERALPEALPPPATAH
RNFHLDPKLADILSPRLIRRGSKKRPARSSHQELRRDEGSQDQTGSLSRARPSSRHVRHA
SVPATFMPIVVPEPPTSVGPPVAVPEPIGFPTRAHPTLQAPSLEDVTKQYMLNLHSGEVP
APVPVDMPCLPLAAPPSAEAKPPEAARPADEPTPASKCCSKPQVDMRKHVAMTLLDTEQS
YVESLRTLMQGYMQPLKQPENSVLCDPSLVDEIFDQIPELLEHHEQFLEQVRHCMQTWHA
QQKVGALLVQSFSKDVLVNIYSAYIDNFLNAKDAVRVAKEARPAFLKFLEQSMRENKEKQ
ALSDLMIKPVQRIPRYELLVKDLLKHTPEDHPDHPLLLEAQRNIKQVAERINKGVRSAEE
AERHARVLQEIEAHIEGMEDLQAPLRRFLRQEMVIEVKAIGGKKDRSLFLFTDLIVCTTL
KRKSGSLRRSSMSLYTAASVIDTASKYKMLWKLPLEDADIIKGASQATNRENIQKAISRL
DEDLTTLGQMSKLSESLGFPHQSLDDALRDLSAAMHRDLSEKQALCYALSFPPTKLELCA
TRPEGTDSYIFEFPHPDARLGFEQAFDEAKRKLASSKSCLDPEFLKAIPIMKTRSGMQFS
CAAPTLNSCPEPSPEVWVCNSDGYVGQVCLLSLRAEPDVEACIAVCSARILCIGAVPGLQ
PRCHREPPPSLRSPPETAPEPAGPELDVEAAADEEAATLAEPGPQPCLHISIAGSGLEMT
PGLGEGDPRPELVPFDSDSDDESSPSPSGTLQSQASRSTISSSFGNEETPSSKEATAETT
SSEEEQEPGFLPLSGSFGPGGPCGTSPMDGRALRRSSHGSFTRGSLEDLLSVDPEAYQSS
VWLGTEDGCVHVYQSSDSIRDRRNSMKLQHAASVTCILYLNNQVFVSLANGELVVYQREA
GHFWDPQNFKSVTLGTQGSPITKMVSVGGRLWCGCQNRVLVLSPDTLQLEHMFYVGQDSS
RCVACMVDSSLGVWVTLKGSAHVCLYHPDTFEQLAEVDVTPPVHRMLAGSDAIIRQHKAA
CLRITALLVCEELLWVGTSAGVVLTMPTSPGTVSCPRAPLSPTGLGQGHTGHVRFLAAVQ
LPDGFNLLCPTPPPPPDTGPEKLPSLEHRDSPWHRGPAPARPKMLVISGGDGYEDFRLSS
GGGSSSETVGRDDSTNHLLLWRV
>sp|Q8IW93|ARHGJ_HUMAN Rho guanine nucleotide exchange factor 19 OS=Homo sapiens GN=ARHGEF19 PE=1 SV=1
MDCGPPATLQPHLTGPPGTAHHPVAVCQQESLSFAELPALKPPSPVCLDLFPVAPEELRA
PGSRWSLGTPAPLQGLLWPLSPGGSDTEITSGGMRPSRAGSWPHCPGAQPPALEGPWSPR
HTQPQRRASHGSEKKSAWRKMRVYQREEVPGCPEAHAVFLEPGQVVQEQALSTEEPRVEL
SGSTRVSLEGPERRRFSASELMTRLHSSLRLGRNSAARALISGSGTGAAREGKASGMEAR
SVEMSGDRVSRPAPGDSREGDWSEPRLDTQEEPPLGSRSTNERRQSRFLLNSVLYQEYSD
VASARELRRQQREEEGPGDEAEGAEEGPGPPRANLSPSSSFRAQRSARGSTFSLWQDIPD
VRGSGVLATLSLRDCKLQEAKFELITSEASYIHSLSVAVGHFLGSAELSECLGAQDKQWL
FSKLPEVKSTSERFLQDLEQRLEADVLRFSVCDVVLDHCPAFRRVYLPYVTNQAYQERTY

QRLLLENPRFPGILARLEESPVCQRLPLTSFLILPFQRITRLKMLVENILKRTAQGSEDE
DMATKAFNALKELVQECNASVQSMKRTEELIHLSKKIHFEGKIFPLISQARWLVRHGELV
ELAPLPAAPPAKLKLSSKAVYLHLFNDCLLLSRRKELGKFAVFVHAKMAELQVRDLSLKL
QGIPGHVFLLQLLHGQHMKHQFLLRARTESEKQRWISALCPSSPQEDKEVISEGEDCPQV
QCVRTYKALHPDELTLEKTDILSVRTWTSDGWLEGVRLADGEKGWVPQAYVEEISSLSAR
LRNLRENKRVTSATSKLGEAPV
>sp|Q99856|ARI3A_HUMAN AT-rich interactive domain-containing protein 3A OS=Homo sapiens GN=ARID3A PE=1 SV=2
MKLQAVMETLLQRQQRARQELEARQQLPPDPPAAPPGRARAAPDEDREPESARMQRAQMA
ALAAMRAAAAGLGHPASPGGSEDGPPGSEEEDAAREGTPGSPGRGREGPGEEHFEDMASD
EDMKPKWEEEEMEEDLGEDEEEEEEDYEDEEEEEDEEGLGPPGPASLGTTALFPRKAQPP
QAFRGDGVPRVLGGQERPGPGPAHPGGAAHVAPQLQPPDHGDWTYEEQFKQLYELDGDPK
RKEFLDDLFSFMQKRGTPVNRIPIMAKQVLDLFMLYVLVTEKGGLVEVINKKLWREITKG
LNLPTSITSAAFTLRTQYMKYLYPYECEKRGLSNPNELQAAIDSNRREGRRQSFGGSLFA
YSPGGAHGMLSSPKLPVSSLGLAASTNGSSITPAPKIKKEEDSAIPITVPGRLPVSLAGH
PVVAAQAAAVQAAAAQAAVAAQAAALEQLREKLESAEPPEKKMALVADEQQRLMQRALQQ
NFLAMAAQLPMSIRINSQASESRQDSAVNLTGTNGSNSISMSVEINGIMYTGVLFAQPPA
PTPTSAPNKGGGGGGGSSSNAGGRGGNTGTSGGQAGPAGLSTPSTSTSNNSLP
>sp|Q4LE39|ARI4B_HUMAN AT-rich interactive domain-containing protein 4B OS=Homo sapiens GN=ARID4B PE=1 SV=2
MKALDEPPYLTVGTDVSAKYRGAFCEAKIKTAKRLVKVKVTFRHDSSTVEVQDDHIKGPL
KVGAIVEVKNLDGAYQEAVINKLTDASWYTVVFDDGDEKTLRRSSLCLKGERHFAESETL
DQLPLTNPEHFGTPVIGKKTNRGRRSNHIPEEESSSSSSDEDEDDRKQIDELLGKVVCVD
YISLDKKKALWFPALVVCPDCSDEIAVKKDNILVRSFKDGKFTSVPRKDVHEITSDTAPK
PDAVLKQAFEQALEFHKSRTIPANWKTELKEDSSSSEAEEEEEEEDDEKEKEDNSSEEEE
EIEPFPEERENFLQQLYKFMEDRGTPINKRPVLGYRNLNLFKLFRLVHKLGGFDNIESGA
VWKQVYQDLGIPVLNSAAGYNVKCAYKKYLYGFEEYCRSANIEFQMALPEKVVNKQCKEC
ENVKEIKVKEENETEIKEIKMEEERNIIPREEKPIEDEIERKENIKPSLGSKKNLLESIP
THSDQEKEVNIKKPEDNENLDDKDDDTTRVDESLNIKVEAEEEKAKSGDETNKEEDEDDE
EAEEEEEEEEEEEDEDDDDNNEEEEFECYPPGMKVQVRYGRGKNQKMYEASIKDSDVEGG
EVLYLVHYCGWNVRYDEWIKADKIVRPADKNVPKIKHRKKIKNKLDKEKDKDEKYSPKNC
KLRRLSKPPFQTNPSPEMVSKLDLTDAKNSDTAHIKSIEITSILNGLQASESSAEDSEQE
DERGAQDMDNNGKEESKIDHLTNNRNDLISKEEQNSSSLLEENKVHADLVISKPVSKSPE
RLRKDIEVLSEDTDYEEDEVTKKRKDVKKDTTDKSSKPQIKRGKRRYCNTEECLKTGSPG
KKEEKAKNKESLCMENSSNSSSDEDEEETKAKMTPTKKYNGLEEKRKSLRTTGFYSGFSE
VAEKRIKLLNNSDERLQNSRAKDRKDVWSSIQGQWPKKTLKELFSDSDTEAAASPPHPAP
EEGVAEESLQTVAEEESCSPSVELEKPPPVNVDSKPIEEKTVEVNDRKAEFPSSGSNSVL
NTPPTTPESPSSVTVTEGSRQQSSVTVSEPLAPNQEEVRSIKSETDSTIEVDSVAGELQD
LQSEGNSSPAGFDASVSSSSSNQPEPEHPEKACTGQKRVKDAQGGGSSSKKQKRSHKATV
VNNKKKGKGTNSSDSEELSAGESITKSQPVKSVSTGMKSHSTKSPARTQSPGKCGKNGDK
DPDLKEPSNRLPKVYKWSFQMSDLENMTSAERITILQEKLQEIRKHYLSLKSEVASIDRR
RKRLKKKERESAATSSSSSSPSSSSITAAVMLTLAEPSMSSASQNGMSVECR
>sp|Q7L311|ARMX2_HUMAN Armadillo repeat-containing X-linked protein 2 OS=Homo sapiens GN=ARMCX2 PE=2 SV=1

MSRVRDAGCVAAGIVIGAGAWYCVYKYTRGRDQTKKRMAKPKNRAVAGTGARARAGLRAG
FTIDLGSGFSPPTPVRAEAEDRAQDEASALDTVGAEAVAPAASSAEAQSGAGSQAQEADG
AGVGPKAESVVGAAMASAIAPPPGVTEALGAAEAPAMAGAPKVAEAPREAETSRAAVPPG
TVVPTEAAAPTEVTEGPGVAAPTKVAEAPGVASPTEAAAEAPVPATPTGAAAPTGAAESPG
TSGSPRTAVVPGTSAAKKATPGAHTGAIPKATSATGAVPKGGGKGVTRSRNGGKGKGKKS
KVEVDELGMGFRPGDGAAAAAAASANGGQAFLAEVPDSEEGESGWTDTESDSDSEPETQR
RGRGRRPVAMQKRPFPYEIDEILGVRDLRKVLALLQKSDDPFIQQVALLTLSNNANYSCN
QETIRKLGGLPIIANMINKTDPHIKEKALMAMNNLSENYENQGRLQVYMNKVMDDIMASN
LNSAVQVVGLKFLTNMTITNDYQHLLVNSIANFFRLLSQGGGKIKVEILKILSNFAENPD
MLKKLLSTQVPASFSSLYNSYVESEILINALTLFEIIYDNLRAEVFNYREFNKGSLFYLC
TTSGVCVKKIRALANHHDLLVKVKVIKLVNKF
>sp|Q6P1M9|ARMX5_HUMAN Armadillo repeat-containing X-linked protein 5 OS=Homo sapiens GN=ARMCX5 PE=1 SV=1
MVDSGTEARARGKAEAGLQDGISGPATARVNGKTQAEAVAEAELKTESVTQAKAGDGAMT
RTHTVTYREAMAVTREVIKVEDTTKTRVMVETKTKPLAERSIVPQTKSKAMPMSRVSTVT
KSEVKVVAVIEANIRSYAKSHDKANTGSRPDRREETSIGMKSSDEDEENICSWFWTGEEP
SVGSWFWPEEETSLQVYKPLPKIQEKPKPTHKPTLTIKQKVIAWSRARYIVLVPVEGGEQ
SLPPEGNWTLVETLIETPLGIRPLTKIPPYHGPYYQTLAEIKKQIRQREKYGPNPKACHC
KSRGFSLEPKEFDKLVALLKLTKDPFIHEIATMIMGISPAYPFTQDIIHDVGITVMIENL
VNNPNVKEHPGALSMVDDSSESSEEPKSGESYIHQVCKGIISCPLNSPVQLAGLKLLGHL
SIKFEDHYVITSYIPDFLTLLNKGSVKTKFYVLKVFSCLSKNHANTRELISAKVLSSLVA
PFNKNESKANILNIIEIFENINFQFKTKAKLFTKEKFTKSELISIFQEAKQFGQKLQDLA
EHSDPEVRDKVIRLILKL
>sp|P55087|AQP4_HUMAN Aquaporin-4 OS=Homo sapiens GN=AQP4 PE=1 SV=2
MSDRPTARRWGKCGPLCTRENIMVAFKGVWTQAFWKAVTAEFLAMLIFVLLSLGSTINWG
GTEKPLPVDMVLISLCFGLSIATMVQCFGHISGGHINPAVTVAMVCTRKISIAKSVFYIA
AQCLGAIIGAGILYLVTPPSVVGGLGVTMVHGNLTAGHGLLVELIITFQLVFTIFASCDS
KRTDVTGSIALAIGFSVAIGHLFAINYTGASMNPARSFGPAVIMGNWENHWIYWVGPIIG
AVLAGGLYEYVFCPDVEFKRRFKEAFSKAAQQTKGSYMEVEDNRSQVETDDLILKPGVVH
VIDVDRGEEKKGKDQSGEVLSSV
>sp|P55064|AQP5_HUMAN Aquaporin-5 OS=Homo sapiens GN=AQP5 PE=1 SV=1
MKKEVCSVAFLKAVFAEFLATLIFVFFGLGSALKWPSALPTILQIALAFGLAIGTLAQAL
GPVSGGHINPAITLALLVGNQISLLRAFFYVAAQLVGAIAGAGILYGVAPLNARGNLAVN
ALNNNTTQGQAMVVELILTFQLALCIFASTDSRRTSPVGSPALSIGLSVTLGHLVGIYFT
GCSMNPARSFGPAVVMNRFSPAHWVFWVGPIVGAVLAAILYFYLLFPNSLSLSERVAIIK
GTYEPDEDWEEQREERKKTMELTTR
>sp|Q6NSI1|AR26L_HUMAN Putative ankyrin repeat domain-containing protein 26-like protein OS=Homo sapiens GN=ANKRD26P1 PE=5 SV=2
MRRSSGFGGQKGQGPSCSFTGCWCCRGDDVAESDDSPFAQCGYNIQEKHLGKLHRAASRG
EVSKVECILSSGSADLDERDKKKRTALHLACANGHPEVVALLVDRGCQLDVFDNKNRTAL
LKAVQCQEEECATILLEHGADPDLPDVYGNTTLHYAIYNEDIPMTKKLLLHHANIESANK
DELTPFLLAVHEQKQQMEDFLRKQKENLTAVKLESIHQVMSEYKENETPRNPQNSNPEGT
SNKMACLGEGAAGAKVDEIPGNPVKRLFNKPSIDDSRPMSANEDFDFDTEEKATEPANGK
RQNGMGIIESAPQEHTNNENI

>sp|Q9Y2Y0|AR2BP_HUMAN ADP-ribosylation factor-like protein 2-binding protein OS=Homo sapiens GN=ARL2BP PE=1 SV=1
MDALEGESFALSFSSASDAEFDAVVGYLEDIIMDDEFQLLQRNFMDKYYLEFEDTEENKL
IYTPIFNEYISLVEKYIEEQLLQRIPEFNMAAFTTTLQHHKDEVAGDIFDMLLTFTDFLA
FKEMFLDYRAEKEGRGLDLSSGLVVTSLCKSSSLPASQNNLRH
>sp|P56211|ARP19_HUMAN cAMP-regulated phosphoprotein 19 OS=Homo sapiens GN=ARPP19 PE=1 SV=2
MSAEVPEAASAEEQKEMEDKVTSPEKAEEAKLKARYPHLGQKPGGSDFLRKRLQKGQKYF
DSGDYNMAKAKMKNKQLPTAAPDKTEVTGDHIPTPQDLPQRKPSLVASKLAG
>sp|Q9H981|ARP8_HUMAN Actin-related protein 8 OS=Homo sapiens GN=ACTR8 PE=1 SV=2
MTQAEKGDTENGKEKGGEKEKEQRGVKRPIVPALVPESLQEQIQSNFIIVIHPGSTTLRI
GRATDTLPASIPHVIARRHKQQGQPLYKDSWLLREGLNKPESNEQRQNGLKMVDQAIWSK
KMSNGTRRIPVSPEQARSYNKQMRPAILDHCSGNKWTNTSHHPEYLVGEEALYVNPLDCY
NIHWPIRRGQLNIHPGPGGSLTAVLADIEVIWSHAIQKYLEIPLKDLKYYRCILLIPDIY
NKQHVKELVNMILMKMGFSGIVVHQESVCATYGSGLSSTCIVDVGDQKTSVCCVEDGVSH
RNTRLCLAYGGSDVSRCFYWLMQRAGFPYRECQLTNKMDCLLLQHLKETFCHLDQDISGL
QDHEFQIRHPDSPALLYQFRLGDEKLQAPMALFYPATFGIVGQKMTTLQHRSQGDPEDPH
DEHYLLATQSKQEQSAKATADRKSASKPIGFEGDLRGQSSDLPERLHSQEVDLGSAQGDG
LMAGNDSEEALTALMSRKTAISLFEGKALGLDKAILHSIDCCSSDDTKKKMYSSILVVGG
GLMFHKAQEFLQHRILNKMPPSFRRIIENVDVITRPKDMDPRLIAWKGGAVLACLDTTQE
LWIYQREWQRFGVRMLRERAAFVW
>sp|O15144|ARPC2_HUMAN Actin-related protein 2/3 complex subunit 2 OS=Homo sapiens GN=ARPC2 PE=1 SV=1
MILLEVNNRIIEETLALKFENAAAGNKPEAVEVTFADFDGVLYHISNPNGDKTKVMVSIS
LKFYKELQAHGADELLKRVYGSFLVNPESGYNVSLLYDLENLPASKDSIVHQAGMLKRNC
FASVFEKYFQFQEEGKEGENRAVIHYRDDETMYVESKKDRVTVVFSTVFKDDDDVVIGKV
FMQEFKEGRRASHTAPQVLFSHREPPLELKDTDAAVGDNIGYITFVLFPRHTNASARDNT
INLIHTFRDYLHYHIKCSKAYIHTRMRAKTSDFLKVLNRARPDAEKKEMKTITGKTFSSR
>sp|Q96B67|ARRD3_HUMAN Arrestin domain-containing protein 3 OS=Homo sapiens GN=ARRDC3 PE=1 SV=1
MVLGKVKSLTISFDCLNDSNVPVYSSGDTVSGRVNLEVTGEIRVKSLKIHARGHAKVRWT
ESRNAGSNTAYTQNYTEEVEYFNHKDILIGHERDDDNSEEGFHTIHSGRHEYAFSFELPQ
TPLATSFEGRHGSVRYWVKAELHRPWLLPVKLKKEFTVFEHIDINTPSLLSPQAGTKEKT
LCCWFCTSGPISLSAKIERKGYTPGESIQIFAEIENCSSRMVVPKAAIYQTQAFYAKGKM
KEVKQLVANLRGESLSSGKTETWNGKLLKIPPVSPSILDCSIIRVEYSLMVYVDIPGAMD
LFLNLPLVIGTIPLHPFGSRTSSVSSQCSMNMNWLSLSLPERPEAPPSYAEVVTEEQRRN
NLAPVSACDDFERALQGPLFAYIQEFRFLPPPLYSEIDPNPDQSADDRPSCPSR
>sp|P15289|ARSA_HUMAN Arylsulfatase A OS=Homo sapiens GN=ARSA PE=1 SV=3
MGAPRSLLLALAAGLAVARPPNIVLIFADDLGYGDLGCYGHPSSTTPNLDQLAAGGLRFT
DFYVPVSLCTPSRAALLTGRLPVRMGMYPGVLVPSSRGGLPLEEVTVAEVLAARGYLTGM
AGKWHLGVGPEGAFLPPHQGFHRFLGIPYSHDQGPCQNLTCFPPATPCDGGCDQGLVPIP
LLANLSVEAQPPWLPGLEARYMAFAHDLMADAQRQDRPFFLYYASHHTHYPQFSGQSFAE
RSGRGPFGDSLMELDAAVGTLMTAIGDLGLLEETLVIFTADNGPETMRMSRGGCSGLLRC
GKGTTYEGGVREPALAFWPGHIAPGVTHELASSLDLLPTLAALAGAPLPNVTLDGFDLSP

LLLGTGKSPRQSLFFYPSYPDEVRGVFAVRTGKYKAHFFTQGSAHSDTTADPACHASSSL
TAHEPPLLYDLSKDPGENYNLLGGVAGATPEVLQALKQLQLLKAQLDAAVTFGPSQVARG
EDPALQICCHPGCTPRPACCHCPDPHA
>sp|P18440|ARY1_HUMAN Arylamine N-acetyltransferase 1 OS=Homo sapiens GN=NAT1 PE=1 SV=2
MDIEAYLERIGYKKSRNKLDLETLTDILQHQIRAVPFENLNIHCGDAMDLGLEAIFDQVV
RRNRGGWCLQVNHLLYWALTTIGFETTMLGGYVYSTPAKKYSTGMIHLLLQVTIDGRNYI
VDAGFGRSYQMWQPLELISGKDQPQVPCVFRLTEENGFWYLDQIRREQYIPNEEFLHSDL
LEDSKYRKIYSFTLKPRTIEDFESMNTYLQTSPSSVFTSKSFCSLQTPDGVHCLVGFTLT
HRRFNYKDNTDLIEFKTLSEEEIEKVLKNIFNISLQRKLVPKHGDRFFTI
>sp|P11245|ARY2_HUMAN Arylamine N-acetyltransferase 2 OS=Homo sapiens GN=NAT2 PE=1 SV=1
MDIEAYFERIGYKNSRNKLDLETLTDILEHQIRAVPFENLNMHCGQAMELGLEAIFDHIV
RRNRGGWCLQVNQLLYWALTTIGFQTTMLGGYFYIPPVNKYSTGMVHLLLQVTIDGRNYI
VDAGSGSSSQMWQPLELISGKDQPQVPCIFCLTEERGIWYLDQIRREQYITNKEFLNSHL
LPKKKHQKIYLFTLEPRTIEDFESMNTYLQTSPTSSFITTSFCSLQTPEGVYCLVGFILT
YRKFNYKDNTDLVEFKTLTEEEVEEVLKNIFKISLGRNLVPKPGDGSLTI
>sp|Q9NR71|ASAH2_HUMAN Neutral ceramidase OS=Homo sapiens GN=ASAH2 PE=1 SV=2
MAKRTFSNLETFLIFLLVMMSAITVALLSLLFITSGTIENHKDLGGHFFSTTQSPPATQG
STAAQRSTATQHSTATQSSTATQTSPVPLTPESPLFQNFSGYHIGVGRADCTGQVADINL
MGYGKSGQNAQGILTRLYSRAFIMAEPDGSNRTVFVSIDIGMVSQRLRLEVLNRLQSKYG
SLYRRDNVILSGTHTHSGPAGYFQYTVFVIASEGFSNQTFQHMVTGILKSIDIAHTNMKP
GKIFINKGNVDGVQINRSPYSYLQNPQSERARYSSNTDKEMIVLKMVDLNGDDLGLISWF
AIHPVSMNNSNHLVNSDNVGYASYLLEQEKNKGYLPGQGPFVAAFASSNLGDVSPNILGP
RCINTGESCDNANSTCPIGGPSMCIAKGPGQDMFDSTQIIGRAMYQRAKELYASASQEVT
GPLASAHQWVDMTDVTVWLNSTHASKTCKPALGYSFAAGTIDGVGGLNFTQGKTEGDPFW
DTIRDQILGKPSEEIKECHKPKPILLHTGELSKPHPWHPDIVDVQIITLGSLAITAIPGE
FTTMSGRRLREAVQAEFASHGMQNMTVVISGLCNVYTHYITTYEEYQAQRYEAASTIYGP
HTLSAYIQLFRNLAKAIATDTVANLSRGPEPPFFKQLIVPLIPSIVDRAPKGRTFGDVLQ
PAKPEYRVGEVAEVIFVGANPKNSVQNQTHQTFLTVEKYEATSTSWQIVCNDASWETRFY
WHKGLLGLSNATVEWHIPDTAQPGIYRIRYFGHNRKQDILKPAVILSFEGTSPAFEVVTI
>sp|Q495Z4|ASAS1_HUMAN Putative uncharacterized protein ASB16-AS1 OS=Homo sapiens GN=ASB16-AS1 PE=5 SV=2
MSGPPSAPQGALAAPRSPAVRRKGLQAPSWGSPGRPAAHSPWACGPPHWGPQRGPRNAAA
ARPGPRSRWHKRCAAACGACARPPGHQLQPPGAGAPQPGVACSYLGPRPQRTPCSAQSRP
GWCAGPRRRHAPGTEPHVAPGRAPPPRAGASPGSRLLPGSPSLLLPAATWRTWGQESKVL
RKILKAWDPFSLL
>sp|Q9Y294|ASF1A_HUMAN Histone chaperone ASF1A OS=Homo sapiens GN=ASF1A PE=1 SV=1
MAKVQVNNVVVLDNPSPFYNPFQFEITFECIEDLSEDLEWKIIYVGSAESEEYDQVLDSV
LVGPVPAGRHMFVFQADAPNPGLIPDADAVGVTVVLITCTYRGQEFIRVGYYVNNEYTET
ELRENPPVKPDFSKLQRNILASNPRVTRFHINWEDNTEKLEDAESSNPNLQSLLSTDALP
SASKGWSTSENSLNVMLESHMDCM
>sp|P08243|ASNS_HUMAN Asparagine synthetase [glutamine-hydrolyzing] OS=Homo sapiens GN=ASNS PE=1 SV=4
MCGIWALFGSDDCLSVQCLSAMKIAHRGPDAFRFENVNGYTNCCFGFHRLAVVDPLFGMQ
PIRVKKYPYLWLCYNGEIYNHKKMQQHFEFEYQTKVDGEIILHLYDKGGIEQTICMLDGV

FAFVLLDTANKKVFLGRDTYGVRPLFKAMTEDGFLAVCSEAKGLVTLKHSATPFLKVEPF
LPGHYEVLDLKPNGKVASVEMVKYHHCRDVPLHALYDNVEKLFPGFEIETVKNNLRILFN
NAVKKRLMTDRRIGCLLSGGLDSSLVAATLLKQLKEAQVQYPLQTFAIGMEDSPDLLAAR
KVADHIGSEHYEVLFNSEEGIQALDEVIFSLETYDITTVRASVGMYLISKYIRKNTDSVV
IFSGEGSDELTQGYIYFHKAPSPEKAEEESERLLRELYLFDVLRADRTTAAHGLELRVPF
LDHRFSSYYLSLPPEMRIPKNGIEKHLLRETFEDSNLIPKEILWRPKEAFSDGITSVKNS
WFKILQEYVEHQVDDAMMANAAQKFPFNTPKTKEGYYYRQVFERHYPGRADWLSHYWMPK
WINATDPSARTLTHYKSAVKA
>sp|Q5U4P2|ASPH1_HUMAN Aspartate beta-hydroxylase domain-containing protein 1 OS=Homo sapiens GN=ASPHD1 PE=1 SV=3
MKEGRGSFSVERGPRKERETAQSGMWKGNSPAGSQGAAMEGTGGELGGQGNWGPEDAPGL
LARASLIMLPWPLPLASSALTLLFGALTSLFLWYCYRLGSQDMQALGAGSRAGGVRGGPV
GCSEAGGPSPGGPGDPGEGPRTEGLVSRRLRAYARRYSWAGMGRVRRAAQGGPGPGRGPG
VLGIQRPGLLFLPDLPSAPFVPRDAQRHDVELLESSFPAILRDFGAVSWDFSGTTPPPRG
WSPPLAPGCYQLLLYQAGRCQPSNCRRCPGAYRALRGLRSFMSANTFGNAGFSVLLPGAR
LEGRCGPTNARVRCHLGLKIPPGCELVVGGEPQCWAEGHCLLVDDSFLHTVAHNGSPEDG
PRVVFIVDLWHPNVAGAERQALDFVFAPDP
>sp|Q8IZT6|ASPM_HUMAN Abnormal spindle-like microcephaly-associated protein OS=Homo sapiens GN=ASPM PE=1 SV=2
MANRRVGRGCWEVSPTERRPPAGLRGPAAEEEASSPPVLSLSHFCRSPFLCFGDVLLGAS
RTLSLALDNPNEEVAEVKISHFPAADLGFSVSQRCFVLQPKEKIVISVNWTPLKEGRVRE
IMTFLVNDVLKHQAILLGNAEEQKKKKRSLWDTIKKKKISASTSHNRRVSNIQNVNKTFS
VSQKVDRVRSPLQACENLAMNEGGPPTENNSLILEENKIPISPISPAFNECHGATCLPLS
VRRSTTYSSLHASENRELLNVHSANVSKVSFNEKAVTETSFNSVNVNGQRGENSKLSLTP
NCSSTLNITQSQIHFLSPDSFVNNSHGANNELELVTCLSSDMFMKDNSQPVHLESTIAHE
IYQKILSPDSFIKDNYGLNQDLESESVNPILSPNQFLKDNMAYMCTSQQTCKVPLSNENS
QVPQSPEDWRKSEVSPRIPECQGSKSPKAIFEELVEMKSNYYSFIKQNNPKFSAVQDISS
HSHNKQPKRRPILSATVTKRKATCTRENQTEINKPKAKRCLNSAVGEHEKVINNQKEKED
FHSYLPIIDPILSKSKSYKNEVTPSSTTASVARKRKSDGSMEDANVRVAITEHTEVREIK
RIHFSPSEPKTSAVKKTKNVTTPISKRISNREKLNLKKKTDLSIFRTPISKTNKRTKPII
AVAQSSLTFIKPLKTDIPRHPMPFAAKNMFYDERWKEKQEQGFTWWLNFILTPDDFTVKT
NISEVNAATLLLGIENQHKISVPRAPTKEEMSLRAYTARCRLNRLRRAACRLFTSEKMVK
AIKKLEIEIEARRLIVRKDRHLWKDVGERQKVLNWLLSYNPLWLRIGLETTYGELISLED
NSDVTGLAMFILNRLLWNPDIAAEYRHPTVPHLYRDGHEEALSKFTLKKLLLLVCFLDYA
KISRLIDHDPCLFCKDAEFKASKEILLAFSRDFLSGEGDLSRHLGLLGLPVNHVQTPFDE
FDFAVTNLAVDLQCGVRLVRTMELLTQNWDLSKKLRIPAISRLQKMHNVDIVLQVLKSRG
IELSDEHGNTILSKDIVDRHREKTLRLLWKIAFAFQVDISLNLDQLKEEIAFLKHTKSIK
KTISLLSCHSDDLINKKKGKRDSGSFEQYSENIKLLMDWVNAVCAFYNKKVENFTVSFSD
GRVLCYLIHHYHPCYVPFDAICQRTTQTVECTQTGSVVLNSSSESDDSSLDMSLKAFDHE
NTSELYKELLENEKKNFHLVRSAVRDLGGIPAMINHSDMSNTIPDEKVVITYLSFLCARL
LDLRKEIRAARLIQTTWRKYKLKTDLKRHQEREKAARIIQLAVINFLAKQRLRKRVNAAL
VIQKYWRRVLAQRKLLMLKKEKLEKVQNKAASLIQGYWRRYSTRQRFLKLKYYSIILQSR
IRMIIAVTSYKRYLWATVTIQRHWRAYLRRKQDQQRYEMLKSSTLIIQSMFRKWKQRKMQ
SQVKATVILQRAFREWHLRKQAKEENSAIIIQSWYRMHKELRKYIYIRSCVVIIQKRFRC

FQAQKLYKRRKESILTIQKYYKAYLKGKIERTNYLQKRAAAIQLQAAFRRLKAHNLCRQI
RAACVIQSYWRMRQDRVRFLNLKKTIIKFQAHVRKHQQRQKYKKMKKAAVIIQTHFRAYI
FAMKVLASYQKTRSAVIVLQSAYRGMQARKMYIHILTSVIKIQSYYRAYVSKKEFLSLKN
ATIKLQSTVKMKQTRKQYLHLRAAALFIQQCYRSKKIAAQKREEYMQMRESCIKLQAFVR
GYLVRKQMRLQRKAVISLQSYFRMRKARQYYLKMYKAIIVIQNYYHAYKAQVNQRKNFLQ
VKKAATCLQAAYRGYKVRQLIKQQSIAALKIQSAFRGYNKRVKYQSVLQSIIKIQRWYRA
YKTLHDTRTHFLKTKAAVISLQSAYRGWKVRKQIRREHQAALKIQSAFRMAKAQKQFRLF
KTAALVIQQNFRAWTAGRKQCMEYIELRHAVLVLQSMWKGKTLRRQLQRQHKCAIIIQSY
YRMHVQQKKWKIMKKAALLIQKYYRAYSIGREQNHLYLKTKAAVVTLQSAYRGMKVRKRI
KDCNKAAVTIQSKYRAYKTKKKYATYRASAIIIQRWYRGIKITNHQHKEYLNLKKTAIKI
QSVYRGIRVRRHIQHMHRAATFIKAMFKMHQSRISYHTMRKAAIVIQVRCRAYYQGKMQR
EKYLTILKAVKVLQASFRGVRVRRTLRKMQTAATLIQSNYRRYRQQTYFNKLKKITKTVQ
QRYWAMKERNIQFQRYNKLRHSVIYIQAIFRGKKARRHLKMMHIAATLIQRRFRTLMMRR
RFLSLKKTAILIQRKYRAHLCTKHHLQFLQVQNAVIKIQSSYRRWMIRKRMREMHRAATF
IQSTFRMHRLHMRYQALKQASVVIQQQYQANRAAKLQRQHYLRQRHSAVILQAAFRGMKT
RRHLKSMHSSATLIQSRFRSLLVRRRFISLKKATIFVQRKYRATICAKHKLYQFLHLRKA
AITIQSSYRRLMVKKKLQEMQRAAVLIQATFRMYRTYITFQTWKHASILIQQHYRTYRAA
KLQRENYIRQWHSAVVIQAAYKGMKARQLLREKHKASIVIQSTYRMYRQYCFYQKLQWAT
KIIQEKYRANKKKQKVFQHNELKKETCVQAGFQDMNIKKQIQEQHQAAIIIQKHCKAFKI
RKHYLHLRATVVSIQRRYRKLTAVRTQAVICIQSYYRGFKVRKDIQNMHRAATLIQSFYR
MHRAKVDYETKKTAIVVIQNYYRLYVRVKTERKNFLAVQKSVRTIQAAFRGMKVRQKLKN
VSEEKMAAIVNQSALCCYRSKTQYEAVQSEGVMIQEWYKASGLACSQEAEYHSQSRAAVT
IQKAFCRMVTRKLETQKCAALRIQFFLQMAVYRRRFVQQKRAAITLQHYFRTWQTRKQFL
LYRKAAVVLQNHYRAFLSAKHQRQVYLQIRSSVIIIQARSKGFIQKRKFQEIKNSTIKIQ
AMWRRYRAKKYLCKVKAACKIQAWYRCWRAHKEYLAILKAVKIIQGCFYTKLERTRFLNV
RASAIIIQRKWRAILPAKIAHEHFLMIKRHRAACLIQAHYRGYKGRQVFLRQKSAALIIQ
KYIRAREAGKHERIKYIEFKKSTVILQALVRGWLVRKRFLEQRAKIRLLHFTAAAYYHLN
AVRIQRAYKLYLAVKNANKQVNSVICIQRWFRARLQEKRFIQKYHSIKKIEHEGQECLSQ
RNRAASVIQKAVRHFLLRKKQEKFTSGIIKIQALWRGYSWRKKNDCTKIKAIRLSLQVVN
REIREENKLYKRTALALHYLLTYKHLSAILEALKHLEVVTRLSPLCCENMAQSGAISKIF
VLIRSCNRSIPCMEVIRYAVQVLLNVSKYEKTTSAVYDVENCIDILLELLQIYREKPGNK
VADKGGSIFTKTCCLLAILLKTTNRASDVRSRSKVVDRIYSLYKLTAHKHKMNTERILYK
QKKNSSISIPFIPETPVRTRIVSRLKPDWVLRRDNMEEITNPLQAIQMVMDTLGIPY
>sp|Q9BXN1|ASPN_HUMAN Asporin OS=Homo sapiens GN=ASPN PE=1 SV=2
MKEYVLLLFLALCSAKPFFSPSHIALKNMMLKDMEDTDDDDDDDDDDDDDDDEDNSLFPTR
EPRSHFFPFDLFPMCPFGCQCYSRVVHCSDLGLTSVPTNIPFDTRMLDLQNNKIKEIKEN
DFKGLTSLYGLILNNNKLTKIHPKAFLTTKKLRRLYLSHNQLSEIPLNLPKSLAELRIHE
NKVKKIQKDTFKGMNALHVLEMSANPLDNNGIEPGAFEGVTVFHIRIAEAKLTSVPKGLP
PTLLELHLDYNKISTVELEDFKRYKELQRLGLGNNKITDIENGSLANIPRVREIHLENNK
LKKIPSGLPELKYLQIIFLHSNSIARVGVNDFCPTVPKMKKSLYSAISLFNNPVKYWEMQ
PATFRCVLSRMSVQLGNFGM
>sp|Q13625|ASPP2_HUMAN Apoptosis-stimulating of p53 protein 2 OS=Homo sapiens GN=TP53BP2 PE=1 SV=2
MMPMFLTVYLSNNEQHFTEVPVTPETICRDVVDLCKEPGESDCHLAEVWCGSERPVADNE

RMFDVLQRFGSQRNEVRFFLRHERPPGRDIVSGPRSQDPSLKRNGVKVPGEYRRKENGVN
SPRMDLTLAELQEMASRQQQQIEAQQQLLATKEQRLKFLKQQDQRQQQQVAEQEKLKRLK
EIAENQEAKLKKVRALKGHVEQKRLSNGKLVEEIEQMNNLFQQKQRELVLAVSKVEELTR
QLEMLKNGRIDSHHDNQSAVAELDRLYKELQLRNKLNQEQNAKLQQQRECLNKRNSEVAV
MDKRVNELRDRLWKKKAALQQKENLPVSSDGNLPQQAASAPSRVAAVGPYIQSSTMPRMP
SRPELLVKPALPDGSLVIQASEGPMKIQTLPNMRSGAASQTKGSKIHPVGPDWSPSNADL
FPSQGSASVPQSTGNALDQVDDGEVPLREKEKKVRPFSMFDAVDQSNAPPSFGTLRKNQS
SEDILRDAQVANKNVAKVPPPVPTKPKQINLPYFGQTNQPPSDIKPDGSSQQLSTVVPSM
GTKPKPAGQQPRVLLSPSIPSVGQDQTLSPGSKQESPPAAAVRPFTPQPSKDTLLPPFRK
PQTVAASSIYSMYTQQQAPGKNFQQAVQSALTKTHTRGPHFSSVYGKPVIAAAQNQQQHP
ENIYSNSQGKPGSPEPETEPVSSVQENHENERIPRPLSPTKLLPFLSNPYRNQSDADLEA
LRKKLSNAPRPLKKRSSITEPEGPNGPNIQKLLYQRTTIAAMETISVPSYPSKSASVTAS
SESPVEIQNPYLHVEPEKEVVSLVPESLSPEDVGNASTENSDMPAPSPGLDYEPEGVPDN
SPNLQNNPEEPNPEAPHVLDVYLEEYPPYPPPPYPSGEPEGPGEDSVSMRPPEITGQVSL
PPGKRTNLRKTGSERIAHGMRVKFNPLALLLDSSLEGEFDLVQRIIYEVDDPSLPNDEGI
TALHNAVCAGHTEIVKFLVQFGVNVNAADSDGWTPLHCAASCNNVQVCKFLVESGAAVFA
MTYSDMQTAADKCEEMEEGYTQCSQFLYGVQEKMGIMNKGVIYALWDYEPQNDDELPMKE
GDCMTIIHREDEDEIEWWWARLNDKEGYVPRNLLGLYPRIKPRQRSLA
>sp|P00966|ASSY_HUMAN Argininosuccinate synthase OS=Homo sapiens GN=ASS1 PE=1 SV=2
MSSKGSVVLAYSGGLDTSCILVWLKEQGYDVIAYLANIGQKEDFEEARKKALKLGAKKVF
IEDVSREFVEEFIWPAIQSSALYEDRYLLGTSLARPCIARKQVEIAQREGAKYVSHGATG
KGNDQVRFELSCYSLAPQIKVIAPWRMPEFYNRFKGRNDLMEYAKQHGIPIPVTPKNPWS
MDENLMHISYEAGILENPKNQAPPGLYTKTQDPAKAPNTPDILEIEFKKGVPVKVTNVKD
GTTHQTSLELFMYLNEVAGKHGVGRIDIVENRFIGMKSRGIYETPAGTILYHAHLDIEAF
TMDREVRKIKQGLGLKFAELVYTGFWHSPECEFVRHCIAKSQERVEGKVQVSVLKGQVYI
LGRESPLSLYNEELVSMNVQGDYEPTDATGFININSLRLKEYHRLQSKVTAK
>sp|O60312|AT10A_HUMAN Probable phospholipid-transporting ATPase VA OS=Homo sapiens GN=ATP10A PE=2 SV=2
MEREPAGTEEPGPPGRRRRREGRTRTVRSNLLPPPGAEDPAAGAAKGERRRRRGCAQHLA
DNRLKTTKYTLLSFLPKNLFEQFHRPANVYFVFIALLNFVPAVNAFQPGLALAPVLFILA
ITAFRDLWEDYSRHRSDHKINHLGCLVFSREEKKYVNRFWKEIHVGDFVRLRCNEIFPAD
ILLLSSSDPDGLCHIETANLDGETNLKRRQVVRGFSELVSEFNPLTFTSVIECEKPNNDL
SRFRGCIIHDNGKKAGLYKENLLLRGCTLRNTDAVVGIVIYAGHETKALLNNSGPRYKRS
KLERQMNCDVLWCVLLLVCMSLFSAVGHGLWIWRYQEKKSLFYVPKSDGSSLSPVTAAVY
SFLTMIIVLQVLIPISLYVSIEIVKACQVYFINQDMQLYDEETDSQLQCRALNITEDLGQ
IQYIFSDKTGTLTENKMVFRRCTVSGVEYSHDANAQRLARYQEADSEEEEVVPRGGSVSQ
RGSIGSHQSVRVVHRTQSTKSHRRTGSRAEAKRASMLSKHTAFSSPMEKDITPDPKLLEK
VSECDKSLAVARHQEHLLAHLSPELSDVFDFFIALTICNTVVVTSPDQPRTKVRVRFELK
SPVKTIEDFLRRFTPSCLTSGCSSIGSLAANKSSHKLGSSFPSTPSSDGMLLRLEERLGQ
PTSAIASNGYSSQADNWASELAQEQESERELRYEAESPDEAALVYAARAYNCVLVERLHD
QVSVELPHLGRLTFELLHTLGFDSVRKRMSVVIRHPLTDEINVYTKGADSVVMDLLQPCS
SVDARGRHQKKIRSKTQNYLNVYAAEGLRTLCIAKRVLSKEEYACWLQSHLEAESSLENS
EELLFQSAIRLETNLHLLGATGIEDRLQDGVPETISKLRQAGLQIWVLTGDKQETAVNIA
YACKLLDHDEEVITLNATSQEACAALLDQCLCYVQSRGLQRAPEKTKGKVSMRFSSLCPP

STSTASGRRPSLVIDGRSLAYALEKNLEDKFLFLAKQCRSVLCCRSTPLQKSMVVKLVRS
KLKAMTLAIGDGANDVSMIQVADVGVGISGQEGMQAVMASDFAVPKFRYLERLLILHGHW
CYSRLANMVLYFFYKNTMFVGLLFWFQFFCGFSASTMIDQWYLIFFNLLFSSLPPLVTGV
LDRDVPANVLLTNPQLYKSGQNMEEYRPRTFWFNMADAAFQSLVCFSIPYLAYYDSNVDL
FTWGTPIVTIALLTFLLHLGIETKTWTWLNWITCGFSVLLFFTVALIYNASCATCYPPSN
PYWTMQALLGDPVFYLTCLMTPVAALLPRLFFRSLQGRVFPTQLQLARQLTRKSPRRCSA
PKETFAQGRLPKDSGTEHSSGRTVKTSVPLSQPSWHTQQPVCSLEASGEPSTVDMSMPVR
EHTLLEGLSAPAPMSSAPGEAVLRSPGGCPEESKVRAASTGRVTPLSSLFSLPTFSLLNW
ISSWSLVSRLGSVLQFSRTEQLADGQAGRGLPVQPHSGRSGLQGPDHRLLIGASSRRSQ
>sp|Q9P241|AT10D_HUMAN Probable phospholipid-transporting ATPase VD OS=Homo sapiens GN=ATP10D PE=2 SV=3
MTEALQWARYHWRRLIRGATRDDDSGPYNYSSLLACGRKSSQTPKLSGRHRIVVPHIQPF
KDEYEKFSGAYVNNRIRTTKYTLLNFVPRNLFEQFHRAANLYFLFLVVLNWVPLVEAFQK
EITMLPLVVVLTIIAIKDGLEDYRKYKIDKQINNLITKVYSRKEKKYIDRCWKDVTVGDF
IRLSCNEVIPADMVLLFSTDPDGICHIETSGLDGESNLKQRQVVRGYAEQDSEVDPEKFS
SRIECESPNNDLSRFRGFLEHSNKERVGLSKENLLLRGCTIRNTEAVVGIVVYAGHETKA
MLNNSGPRYKRSKLERRANTDVLWCVMLLVIMCLTGAVGHGIWLSRYEKMHFFNVPEPDG
HIISPLLAGFYMFWTMIILLQVLIPISLYVSIEIVKLGQIYFIQSDVDFYNEKMDSIVQC
RALNIAEDLGQIQYLFSDKTGTLTENKMVFRRCSVAGFDYCHEENARRLESYQEAVSEDE
DFIDTVSGSLSNMAKPRAPSCRTVHNGPLGNKPSNHLAGSSFTLGSGEGASEVPHSRQAA
FSSPIETDVVPDTRLLDKFSQITPRLFMPLDETIQNPPMETLYIIDFFIALAICNTVVVS
APNQPRQKIRHPSLGGLPIKSLEEIKSLFQRWSVRRSSSPSLNSGKEPSSGVPNAFVSRL
PLFSRMKPASPVEEEVSQVCESPQCSSSSACCTETEKQHGDAGLLNGKAESLPGQPLACN
LCYEAESPDEAALVYAARAYQCTLRSRTPEQVMVDFAALGPLTFQLLHILPFDSVRKRMS
VVVRHPLSNQVVVYTKGADSVIMELLSVASPDGASLEKQQMIVREKTQKHLDDYAKQGLR
TLCIAKKVMSDTEYAEWLRNHFLAETSIDNREELLLESAMRLENKLTLLGATGIEDRLQE
GVPESIEALHKAGIKIWMLTGDKQETAVNIAYACKLLEPDDKLFILNTQSKDACGMLMST
ILKELQKKTQALPEQVSLSEDLLQPPVPRDSGLRAGLIITGKTLEFALQESLQKQFLELT
SWCQAVVCCRATPLQKSEVVKLVRSHLQVMTLAIGDGANDVSMIQVADIGIGVSGQEGMQ
AVMASDFAVSQFKHLSKLLLVHGHWCYTRLSNMILYFFYKNVAYVNLLFWYQFFCGFSGT
SMTDYWVLIFFNLLFTSAPPVIYGVLEKDVSAETLMQLPELYRSGQKSEAYLPHTFWITL
LDAFYQSLVCFFVPYFTYQGSDTDIFAFGNPLNTAALFIVLLHLVIESKSLTWIHLLVII
GSILSYFLFAIVFGAMCVTCNPPSNPYWIMQEHMLDPVFYLVCILTTSIALLPRFVYRVL
QGSLFPSPILRAKHFDRLTPEERTKALKKWRGAGKMNQVTSKYANQSAGKSGRRPMPGPS
AVFAMKSASSCAIEQGNLSLCETALDQGYSETKAFEMAGPSKGKES
>sp|P14415|AT1B2_HUMAN Sodium/potassium-transporting ATPase subunit beta-2 OS=Homo sapiens GN=ATP1B2 PE=1 SV=3
MVIQKEKKSCGQVVEEWKEFVWNPRTHQFMGRTGTSWAFILLFYLVFYGFLTAMFTLTMW
VMLQTVSDHTPKYQDRLATPGLMIRPKTENLDVIVNVSDTESWDQHVQKLNKFLEPYNDS
IQAQKNDVCRPGRYYEQPDNGVLNYPKRACQFNRTQLGNCSGIGDSTHYGYSTGQPCVFI
KMNRVINFYAGANQSMNVTCAGKRDEDAENLGNFVMFPANGNIDLMYFPYYGKKFHVNYT
QPLVAVKFLNVTPNVEVNVECRINAANIATDDERDKFAGRVAFKLRINKT
>sp|P54709|AT1B3_HUMAN Sodium/potassium-transporting ATPase subunit beta-3 OS=Homo sapiens GN=ATP1B3 PE=1 SV=1

MTKNEKKSLNQSLAEWKLFIYNPTTGEFLGRTAKSWGLILLFYLVFYGFLAALFSFTMWV
MLQTLNDEVPKYRDQIPSPGLMVFPKPVTALEYTFSRSDPTSYAGYIEDLKKFLKPYTLE
EQKNLTVCPDGALFEQKGPVYVACQFPISLLQACSGMNDPDFGYSQGNPCILVKMNRIIG
LKPEGVPRIDCVSKNEDIPNVAVYPHNGMIDLKYFPYYGKKLHVGYLQPLVAVQVSFAPN
NTGKEVTVECKIDGSANLKSQDDRDKFLGRVMFKITARA
>sp|Q9NXL2|ARH38_HUMAN Rho guanine nucleotide exchange factor 38 OS=Homo sapiens GN=ARHGEF38 PE=2 SV=2
MEPKEATGKENMVTKKKNLAFLRSRLYMLERRKTDTVVESSVSGDHSGTLRRSQSDRTEY
NQKLQEKMTPQGECSVAETLTPEEEHHMKRMMAKREKIIKELIQTEKDYLNDLELCVREV
VQPLRNKKTDRLDVDSLFSNIESVHQISAKLLSLLEEATTDVEPAMQVIGEVFLQIKGPL
EDIYKIYCYHHDEAHSILESYEKEEELKEHLSHCIQSLKKIYMQEGKPNLLDMGSLMIKP
IQRVMKYPLLLCELRNSTPPSHPDYRALDDAFAAVKDINVNINELKRRKDLVLKYKKNDE
DESLKDKLSKLNIHSISKKSKRVTNHLKILTRGESQVKDNTFNREEKLFRALEKTVRLCV
KNISLCLQHIQDAMPLALQSVMDLQEISYNKDDEMDYSETLSNALNSCHDFASHLQRLIL
TPLSALLSLFPGPHKLIQKRYDKLLDCNSYLQRSTGEESDLAKKEYEALNAQLVEELQAF
NQAARKILLNCLCSFITLLRDLMLVAQQAYSTLVPMPLLVSSISEIQNQVLEEIQNLNCV
KENSATFIERKLSFEKKKPVQILPEMPHQTDIHRSKLLSTYSAEELYQAKRKCNATQEYD
INLLEGDLVAVIEQKDPLGSTSRWLVDTGNVKGYVYSSFLKPYNPAKMQKVDAENRFCDD
DFENISLFVSSRPASDSVTGTSESSIGDSSSSLSGTCGKFETNGTDVDSFQEVDEQIFYA
VHAFQARSDHELSLQEYQRVHILRFCDLSGNKEWWLAEAQGQKGYVPANYLGKMTYA
>sp|Q8TER5|ARH40_HUMAN Rho guanine nucleotide exchange factor 40 OS=Homo sapiens GN=ARHGEF40 PE=1 SV=3
MEPEPVEDCVQSTLAALYPPFEATAPTLLGQVFQVVERTYREDALRYTLDFLVPAKHLLA
KVQQEACAQYSGFLFFHEGWPLCLHEQVVVQLAALPWQLLRPGDFYLQVVPSAAQAPRLA
LKCLAPGGGRVQEVPVPNEACAYLFTPEWLQGINKDRPTGRLSTCLLSAPSGIQRLPWAE
LICPRFVHKEGLMVGHQPSTLPPELPSGPPGLPSPPLPEEALGTRSPGDGHNAPVEGPEG
EYVELLEVTLPVRGSPTDAEGSPGLSRVRTVPTRKGAGGKGRHRRHRAWMHQKGLGPRGQ
DGARPPGEGSSTGASPESPPGAEAVPEAAVLEVSEPPAEAVGEASGSCPLRPGELRGGGG
GGQGAEGPPGTPRRTGKGNRRKKRAAGRGALSRGGDSAPLSPGDKEDASHQEALGNLPSP
SEHKLPECHLVKEEYEGSGKPESEPKELKTAGEKEPQLSEACGPTEEGAGERELEGPGLL
CMAGHTGPEGPLSDTPTPPLETVQEGKGDNIPEEALAVSVSDHPDVAWDLMASGFLILTG
GVDQSGRALLTITPPCPPEEPPPSRDTLNTTLHYLHSLLRPDLQTLGLSVLLDLRQAPPL
PPALIPALSQLQDSGDPPLVQRLLILIHDDLPTELCGFQGAEVLSENDLKRVAKPEELQW
ELGGHRDPSPSHWVEIHQEVVRLCRLCQGVLGSVRQAIEELEGAAEPEEEEAVGMPKPLQ
KVLADPRLTALQRDGGAILMRLRSTPSSKLEGQGPATLYQEVDEAIHQLVRLSNLHVQQQ
EQRQCLRRLQQVLQWLSGPGEEQLASFAMPGDTLSALQETELRFRAFSAEVQERLAQARE
ALALEENATSQKVLDIFEQRLEQVESGLHRALRLQRFFQQAHEWVDEGFARLAGAGPGRE
AVLAALALRRAPEPSAGTFQEMRALALDLGSPAALREWGRCQARCQELERRIQQHVGEEA
SPRGYRRRRADGASSGGAQWGPRSPSPSLSSLLLPSSPGPRPAPSHCSLAPCGEDYEEEG
PELAPEAEGRPPRAVLIRGLEVTSTEVVDRTCSPREHVLLGRARGPDGPWGVGTPRMERK
RSISAQQRLVSELIACEQDYVATLSEPVPPPGPELTPELRGTWAAALSARERLRSFHRTH
FLRELQGCATHPLRIGACFLRHGDQFSLYAQYVKHRHKLENGLAALSPLSKGSMEAGPYL
PRALQQPLEQLTRYGRLLEELLREAGPELSSECRALGAAVQLLREQEARGRDLLAVEAVR
GCEIDLKEQGQLLHRDPFTVICGRKKCLRHVFLFEHLLLFSKLKGPEGGSEMFVYKQAFK

TADMGLTENIGDSGLCFELWFRRRRAREAYTLQATSPEIKLKWTSSIAQLLWRQAAHNKE
LRVQQMVSMGIGNKPFLDIKALGERTLSALLTGRAARTRASVAVSSFEHAGPSLPGLSPG
ACSLPARVEEEAWDLDVKQISLAPETLDSSGDVSPGPRNSPSLQPPHPGSSTPTLASRGI
LGLSRQSHARALSDPTTPL
>sp|Q14155|ARHG7_HUMAN Rho guanine nucleotide exchange factor 7 OS=Homo sapiens GN=ARHGEF7 PE=1 SV=2
MNSAEQTVTWLITLGVLESPKKTISDPEGFLQASLKDGVVLCRLLERLLPGTIEKVYPEP
RSESECLSNIREFLRGCGASLRLELLFPPSQPPQHLVTTILLSASTFDANDLYQGQNFNK
VLSSLVTLNKVTADIGLGSDSVCARPSSHRIKSFDSLGSQSLHTRTSKLFQGQYRSLDMT
DNSNNQLVVRAKFNFQQTNEDELSFSKGDVIHVTRVEEGGWWEGTLNGRTGWFPSNYVRE
VKASEKPVSPKSGTLKSPPKGFDTTAINKSYYNVVLQNILETENEYSKELQTVLSTYLRP
LQTSEKLSSANISYLMGNLEEICSFQQMLVQSLEECTKLPEAQQRVGGCFLNLMPQMKTL
YLTYCANHPSAVNVLTEHSEELGEFMETKGASSPGILVLTTGLSKPFMRLDKYPTLLKEL
ERHMEDYHTDRQDIQKSMAAFKNLSAQCQEVRKRKELELQILTEAIRNWEGDDIKTLGNV
TYMSQVLIQCAGSEEKNERYLLLFPNVLLMLSASPRMSGFIYQGKLPTTGMTITKLEDSE
NHRNAFEISGSMIERILVSCNNQQDLQEWVEHLQKQTKVTSVGNPTIKPHSVPSHTLPSH
PVTPSSKHADSKPAPLTPAYHTLPHPSHHGTPHTTINWGPLEPPKTPKPWSLSCLRPAPP
LRPSAALCYKEDLSKSPKTMKKLLPKRKPERKPSDEEFASRKSTAALEEDAQILKVIEAY
CTSAKTRQTLNSTWQGTDLMHNHVLADDDQPSLDSLGRRSSLSRLEPSDLSEDSDYDSIW
TAHSYRMGSTSRKSCCSYISHQN
>sp|Q7Z628|ARHG8_HUMAN Neuroepithelial cell-transforming gene 1 protein OS=Homo sapiens GN=NET1 PE=1 SV=1
MEPELAAQKQPRPRRRSRRASGLSTEGATGPSADTSGSELDGRCSLRRGSSFTFLTPGPN
WDFTLKRKRREKDDDVVSLSSLDLKEPSNKRVRPLARVTSLANLISPVRNGAVRRFGQTI
QSFTLRGDHRSPASAQKFSSRSTVPTPAKRRSSALWSEMLDITMKESLTTREIRRQEAIY
EMSRGEQDLIEDLKLARKAYHDPMLKLSIMSEEELTHIFGDLDSYIPLHEDLLTRIGEAT
KPDGTVEQIGHILVSWLPRLNAYRGYCSNQLAAKALLDQKKQDPRVQDFLQRCLESPFSR
KLDLWSFLDIPRSRLVKYPLLLKEILKHTPKEHPDVQLLEDAILIIQGVLSDINLKKGES
ECQYYIDKLEYLDEKQRDPRIEASKVLLCHGELRSKSGHKLYIFLFQDILVLTRPVTRNE
RHSYQVYRQPIPVQELVLEDLQDGDVRMGGSFRGAFSNSEKAKNIFRIRFHDPSPAQSHT
LQANDVFHKQQWFNCIRAAIAPFQSAGSPPELQGLPELHEECEGNHPSARKLTAQRRAST
VSSVTQVEVDENAYRCGSGMQMAEDSKSLKTHQTQPGIRRARDKALSGGKRKETLV
>sp|Q86VW2|ARHGP_HUMAN Rho guanine nucleotide exchange factor 25 OS=Homo sapiens GN=ARHGEF25 PE=1 SV=2
MRGGHKGGRCACPRVIRKVLAKCGCCFARGGRESYSIAGSEGSISASAASGLAAPSGPSS
GLSSGPCSPGPPGPVSGLRRWLDHSKHCLSVETEADSGQAGPYENWMLEPALATGEELPE
LTLLTTLLEGPGDKTQPPEEETLSQAPESEEEQKKKALERSMYVLSELVETEKMYVDDLG
QIVEGYMATMAAQGVPESLRGRDRIVFGNIQQIYEWHRDYFLQELQRCLKDPDWLAQLFI
KHERRLHMYVVYCQNKPKSEHVVSEFGDSYFEELRQQLGHRLQLNDLLIKPVQRIMKYQL
LLKDFLKYYNRAGMDTADLEQAVEVMCFVPKRCNDMMTLGRLRGFEGKLTAQGKLLGQDT
FWVTEPEAGGLLSSRGRERRVFLFEQIIIFSEALGGGVRGGTQPGYVYKNSIKVSCLGLE
GNLQGDPCRFALTSRGPEGGIQRYVLQAADPAISQAWIKHVAQILESQRDFLNALQSPIE
YQRRESQTNSLGRPRGPGVGSPGRIQLGDQAQGSTHTPINGSLPSLLLSPKGEVARALLP
LDKQALGDIPQAPHDSPPVSPTPKTPPCQARLAKLDEDEL

>sp|Q8IVW6|ARI3B_HUMAN AT-rich interactive domain-containing protein 3B OS=Homo sapiens GN=ARID3B PE=1 SV=2
MEPLQQQQQQQQQQQKQPHLAPLQMDAREKQGQQMREAQFLYAQKLVTQPTLLSATAGRP
SGSTPLGPLARVPPTAAVAQVFERGNMNSEPEEEDGGLEDEDGDDEVAEVAEKETQAASK
YFHVQKVARQDPRVAPMSNLLPAPGLPPHGQQAKEDHTKDASKASPSVSTAGQPNWNLDE
QLKQNGGLAWSDDADGGRGREISRDFAKLYELDGDPERKEFLDDLFVFMQKRGTPINRIP
IMAKQILDLYMLYKLVTEKGGLVEIINKKIWREITKGLNLPTSITSAAFTLRTQYMKYLY
AYECEKKALSSPAELQAAIDGNRREGRRPSYSSSLFGYSPAAATAAAAAGAPALLSPPKI
RFPILGLGSSSGTNTSSPRISPATTLRKGDGAPVTTVPVPNRLAVPVTLASQQAGTRTAA
LEQLRERLESGEPAEKKASRLSEEEQRLVQQAFQRNFFSMARQLPMKIRINGRAEDRAEA
SAAALNLTTSSIGSINMSVDIDGTTYAGVLFAQKPVVHLITGSAPQSLGSSASSSSSSHC
SPSPTSSRGTPSAEPSTSWSL
>sp|A6NKF2|ARI3C_HUMAN AT-rich interactive domain-containing protein 3C OS=Homo sapiens GN=ARID3C PE=3 SV=1
MEALQKQQAARLAQGVGPLAPACPLLPPQPPLPDHRTLQAPEGALGNVGAEEEEDAEEDE
EKREEAGAEEEAAEESRPGAQGPSSPSSQPPGLHPHEWTYEEQFKQLYELDADPKRKEFL
DDLFSFMQKRGTPVNRVPIMAKQVLDLYALFRLVTAKGGLVEVINRKVWREVTRGLSLPT
TITSAAFTLRTQYMKYLYPYECETRALSSPGELQAAIDSNRREGRRQAYTATPLFGLAGP
PPRGAQDPALGPGPAPPATQSSPGPAQGSTSGLPAHACAQLSPSPIKKEESGIPNPCLAL
PVGLALGPTREKLAPEEPPEKRAVLMGPMDPPRPCMPPSFLPRGKVPLREERLDGPLNLA
GSGISSINMALEINGVVYTGVLFARRQPVPASQGPTNPAPPPSTGPPSSILP
>sp|Q68CP9|ARID2_HUMAN AT-rich interactive domain-containing protein 2 OS=Homo sapiens GN=ARID2 PE=1 SV=2
MANSTGKAPPDERRKGLAFLDELRQFHHSRGSPFKKIPAVGGKELDLHGLYTRVTTLGGF
AKVSEKNQWGEIVEEFNFPRSCSNAAFALKQYYLRYLEKYEKVHHFGEDDDEVPPGNPKP
QLPIGAIPSSYNYQQHSVSDYLRQSYGLSMDFNSPNDYNKLVLSLLSGLPNEVDFAINVC
TLLSNESKHVMQLEKDPKIITLLLANAGVFDDTLGSFSTVFGEEWKEKTDRDFVKFWKDI
VDDNEVRDLISDRNKSHEGTSGEWIWESLFHPPRKLGINDIEGQRVLQIAVILRNLSFEE
GNVKLLAANRTCLRFLLLSAHSHFISLRQLGLDTLGNIAAELLLDPVDFKTTHLMFHTVT
KCLMSRDRFLKMRGMEILGNLCKAEDNGVLICEYVDQDSYREIICHLTLPDVLLVISTLE
VLYMLTEMGDVACTKIAKVEKSIDMLVCLVSMDIQMFGPDALAAVKLIEHPSSSHQMLSE
IRPQAIEQVQTQTHVASAPASRAVVAQHVAPPPGIVEIDSEKFACQWLNAHFEVNPDCSV
SRAEMYSEYLSTCSKLARGGILTSTGFYKCLRTVFPNHTVKRVEDSSSNGQAHIHVVGVK
RRAIPLPIQMYYQQQPVSTSVVRVDSVPDVSPAPSPAGIPHGSQTIGNHFQRTPVANQSS
NLTATQMSFPVQGVHTVAQTVSRIPQNPSPHTHQQQNAPVTVIQSKAPIPCEVVKATVIQ
NSIPQTGVPVSIAVGGGPPQSSVVQNHSTGPQPVTVVNSQTLLHHPSVIPQQSPLHTVVP
GQIPSGTPVTVIQQAVPQSHMFGRVQNIPACTSTVSQGQQLITTSPQPVQTSSQQTSAGS
QSQDTVIIAPPQYVTTSASNIVSATSVQNFQVATGQMVTIAGVPSPQASRVGFQNIAPKP
LPSQQVSSTVVQQPIQQPQQPTQQSVVIVSQPAQQGQTYAPAIHQIVLANPAALPAGQTV
QLTGQPNITPSSSPSPVPATNNQVPTAMSSSSTPQSQGPPPTVSQMLSVKRQQQQQHSPA
PPPQQVQVQVQQPQQVQMQVQPQQSNAGVGQPASGESSLIKQLLLPKRGPSTPGGKLILP
APQIPPPNNARAPSPQVVYQVASNQAAGFGVQGQTPAQQLLVGQQNVQLVPSAMPPSGGV
QTVPISNLQILPGPLISNSPATIFQGTSGNQVTITVVPNTSFAPATVSQGNATQLIAPAG
ITMSGTQTGVGLPVQTLPATQASPAGQSSCTTATPPFKGDKIICQKEEEAKEATGLHVHE

RKIEVMENPSCRRGATNTSNGDTKENEMHVGSLLNGRKYSDSSLPPSNSGKIQSETNQCS
LISNGPSLELGENGASGKQNSEQIDMQDIKSDLRKPLVNGICDFDKGDGSHLSKNIPNHK
TSNHVGNGEISPMEPQGTLDITQQDTAKGDQLERISNGPVLTLGGSSVSSIQEASNAATQ
QFSGTDLLNGPLASSLNSDVPQQRPSVVVSPHSTTSVIQGHQIIAVPDSGSKVSHSPALS
SDVRSTNGTAECKTVKRPAEDTDRETVAGIPNKVGVRIVTISDPNNAGCSATMVAVPAGA
DPSTVAKVAIESAVQQKQQHPPTYVQNVVPQNTPMPPSPAVQVQGQPNSSQPSPFSGSSQ
PGDPMRKPGQNFMCLWQSCKKWFQTPSQVFYHAATEHGGKDVYPGQCLWEGCEPFQRQRF
SFITHLQDKHCSKDALLAGLKQDEPGQAGSQKSSTKQPTVGGTSSTPRAQKAIVNHPSAA
LMALRRGSRNLVFRDFTDEKEGPITKHIRLTAALILKNIGKYSECGRRLLKRHENNLSVL
AISNMEASSTLAKCLYELNFTVQSKEQEKDSEMLQ
>sp|Q969Q4|ARL11_HUMAN ADP-ribosylation factor-like protein 11 OS=Homo sapiens GN=ARL11 PE=1 SV=1
MGSVNSRGHKAEAQVVMMGLDSAGKTTLLYKLKGHQLVETLPTVGFNVEPLKAPGHVSLT
LWDVGGQAPLRASWKDYLEGTDILVYVLDSTDEARLPESAAELTEVLNDPNMAGVPFLVL
ANKQEAPDALPLLKIRNRLSLERFQDHCWELRGCSALTGEGLPEALQSLWSLLKSRSCMC
LQARAHGAERGDSKRS
>sp|Q8IVW1|ARL17_HUMAN ADP-ribosylation factor-like protein 17 OS=Homo sapiens GN=ARL17A PE=2 SV=2
MGNIFEKLFKSLLGKKKMRILILSLDTAGKTTILYKLKLGETVPAVPTVGFCVETVEYKN
NTFAVWDVGSHFKIRPLWQHFFQNTKGARSPGSTHQGSLASGVLPIKCSHVEFGMWKGGR
SHPFLPHSSRCAGSGGQLDSILPHQSPAWGPWGCKDLSSGFPSFLTSSILWKSAVVK
>sp|P56559|ARL4C_HUMAN ADP-ribosylation factor-like protein 4C OS=Homo sapiens GN=ARL4C PE=1 SV=1
MGNISSNISAFQSLHIVMLGLDSAGKTTVLYRLKFNEFVNTVPTIGFNTEKIKLSNGTAK
GISCHFWDVGGQEKLRPLWKSYSRCTDGIIYVVDSVDVDRLEEAKTELHKVTKFAENQGT
PLLVIANKQDLPKSLPVAEIEKQLALHELIPATTYHVQPACAIIGEGLTEGMDKLYEMIL
KRRKSLKQKKKR
>sp|P49703|ARL4D_HUMAN ADP-ribosylation factor-like protein 4D OS=Homo sapiens GN=ARL4D PE=1 SV=2
MGNHLTEMAPTASSFLPHFQALHVVVIGLDSAGKTSLLYRLKFKEFVQSVPTKGFNTEKI
RVPLGGSRGITFQVWDVGGQEKLRPLWRSYTRRTDGLVFVVDAAEAERLEEAKVELHRIS
RASDNQGVPVLVLANKQDQPGALSAAEVEKRLAVRELAAATLTHVQGCSAVDGLGLQQGL
ERLYEMILKRKKAARGGKKRR
>sp|Q96KC2|ARL5B_HUMAN ADP-ribosylation factor-like protein 5B OS=Homo sapiens GN=ARL5B PE=1 SV=1
MGLIFAKLWSLFCNQEHKVIIVGLDNAGKTTILYQFLMNEVVHTSPTIGSNVEEIVVKNT
HFLMWDIGGQESLRSSWNTYYSNTEFIILVVDSIDRERLAITKEELYRMLAHEDLRKAAV
LIFANKQDMKGCMTAAEISKYLTLSSIKDHPWHIQSCCALTGEGLCQGLEWMTSRIGVR
>sp|Q8NEN0|ARMC2_HUMAN Armadillo repeat-containing protein 2 OS=Homo sapiens GN=ARMC2 PE=2 SV=4
MLSPNDKMLGKLDPFYQPSVSKQKTSAEIISEARNALRTVRTQRPFTPQEAQRKLFGPAS
SRTSENRPPSSFSLHASSFESSDSRPISGTRLSPLELKPKVPASPTREEDSCFSFPKPPV
DPAKIRRVSNARARLFRAASQRALLPDRSLPPSDSKKTVESKETVMMGDSMVKINGIYLT
KSNAICHLKSHPLQLTDDGGFSEIKEQEMFKGTTSLPSHLKNGGDQGKRHARASSCPSSS

DLSRLQTKAVPKADLQEEDAEIEVDEVFWNTRIVPILRELEKEENIETVCAACTQLHHAL
EEGNMLGNKFKGRSILLKTLCKLVDVGSDSLSLKLAKIILALKVSRKNLLNVCKLIFKIS
RNEKNDSLIQNDSILESLLEVLRSEDLQTNMEAFLYCMGSIKFISGNLGFLNEMISKGAV
EILINLIKQINENIKKCGTFLPNSGHLLVQVTATLRNLVDSSLVRSKFLNISALPQLCTA
MEQYKGDKDVCTNIARIFSKLTSYRDCCTALASYSRCYALFLNLINKYQKKQDLVVRVVF
ILGNLTAKNNQAREQFSKEKGSIQTLLSLFQTFHQLDLHSQKPVGQRGEQHRAQRPPSEA
EDVLIKLTRVLANIAIHPGVGPVLAANPGIVGLLLTTLEYKSLDDCEELVINATATINNL
SYYQVKNSIIQDKKLYIAELLLKLLVSNNMDGILEAVRVFGNLSQDHDVCDFIVQNNVHR
FMMALLDAQHQDICFSACGVLLNLTVDKDKRVILKEGGGIKKLVDCLRDLGPTDWQLACL
VCKTLWNFSENITNASSCFGNEDTNTLLLLLSSFLDEELALDGSFDPDLKNYHKLHWETE
FKPVAQQLLNRIQRHHTFLEPLPIPSF
>sp|P27540|ARNT_HUMAN Aryl hydrocarbon receptor nuclear translocator OS=Homo sapiens GN=ARNT PE=1 SV=1
MAATTANPEMTSDVPSLGPAIASGNSGPGIQGGGAIVQRAIKRRPGLDFDDDGEGNSKFL
RCDDDQMSNDKERFARSDDEQSSADKERLARENHSEIERRRRNKMTAYITELSDMVPTCS
ALARKPDKLTILRMAVSHMKSLRGTGNTSTDGSYKPSFLTDQELKHLILEAADGFLFIVS
CETGRVVYVSDSVTPVLNQPQSEWFGSTLYDQVHPDDVDKLREQLSTSENALTGRILDLK
TGTVKKEGQQSSMRMCMGSRRSFICRMRCGSSSVDPVSVNRLSFVRNRCRNGLGSVKDGE
PHFVVVHCTGYIKAWPPAGVSLPDDDPEAGQGSKFCLVAIGRLQVTSSPNCTDMSNVCQP
TEFISRHNIEGIFTFVDHRCVATVGYQPQELLGKNIVEFCHPEDQQLLRDSFQQVVKLKG
QVLSVMFRFRSKNQEWLWMRTSSFTFQNPYSDEIEYIICTNTNVKNSSQEPRPTLSNTIQ
RPQLGPTANLPLEMGSGQLAPRQQQQQTELDMVPGRDGLASYNHSQVVQPVTTTGPEHSK
PLEKSDGLFAQDRDPRFSEIYHNINADQSKGISSSTVPATQQLFSQGNTFPPTPRPAENF
RNSGLAPPVTIVQPSASAGQMLAQISRHSNPTQGATPTWTPTTRSGFSAQQVATQATAKT
RTSQFGVGSFQTPSSFSSMSLPGAPTASPGAAAYPSLTNRGSNFAPETGQTAGQFQTRTA
EGVGVWPQWQGQQPHHRSSSSEQHVQQPPAQQPGQPEVFQEMLSMLGDQSNSYNNEEFPD
LTMFPPFSE
>sp|Q8TBH0|ARRD2_HUMAN Arrestin domain-containing protein 2 OS=Homo sapiens GN=ARRDC2 PE=2 SV=2
MLFDKVKAFSVQLDGATAGVEPVFSGGQAVAGRVLLELSSAARVGALRLRARGRAHVHWT
ESRSAGSSTAYTQSYSERVEVVSHRATLLAPDTGETTTLPPGRHEFLFSFQLPPTLVTSF
EGKHGSVRYCIKATLHRPWVPARRARKVFTVIEPVDINTPALLAPQAGAREKVARSWYCN
RGLVSLSAKIDRKGYTPGEVIPVFAEIDNGSTRPVLPRAAVVQTQTFMARGARKQKRAVV
ASLAGEPVGPGQRALWQGRALRIPPVGPSILHCRVLHVDYALKVCVDIPGTSKLLLELPL
VIGTIPLHPFGSRSSSVGSHASFLLDWRLGALPERPEAPPEYSEVVADTEEAALGQSPFP
LPQDPDMSLEGPFFAYIQEFRYRPPPLYSEEDPNPLLGDMRPRCMTC
>sp|Q5FYB0|ARSJ_HUMAN Arylsulfatase J OS=Homo sapiens GN=ARSJ PE=2 SV=1
MAPRGCAGHPPPPSPQACVCPGKMLAMGALAGFWILCLLTYGYLSWGQALEEEEEGALLA
QAGEKLEPSTTSTSQPHLIFILADDQGFRDVGYHGSEIKTPTLDKLAAEGVKLENYYVQP
ICTPSRSQFITGKYQIHTGLQHSIIRPTQPNCLPLDNATLPQKLKEVGYSTHMVGKWHLG
FYRKECMPTRRGFDTFFGSLLGSGDYYTHYKCDSPGMCGYDLYENDNAAWDYDNGIYSTQ
MYTQRVQQILASHNPTKPIFLYIAYQAVHSPLQAPGRYFEHYRSIININRRRYAAMLSCL
DEAINNVTLALKTYGFYNNSIIIYSSDNGGQPTAGGSNWPLRGSKGTYWEGGIRAVGFVH
SPLLKNKGTVCKELVHITDWYPTLISLAEGQIDEDIQLDGYDIWETISEGLRSPRVDILH

NIDPIYTKAKNGSWAAGYGIWNTAIQSAIRVQHWKLLTGNPGYSDWVPPQSFSNLGPNRW
HNERITLSTGKSVWLFNITADPYERVDLSNRYPGIVKKLLRRLSQFNKTAVPVRYPPKDP
RSNPRLNGGVWGPWYKEETKKKKPSKNQAEKKQKKSKKKKKKQQKAVSGSTCHSGVTCG
>sp|Q96QS3|ARX_HUMAN Homeobox protein ARX OS=Homo sapiens GN=ARX PE=1 SV=1
MSNQYQEEGCSERPECKSKSPTLLSSYCIDSILGRRSPCKMRLLGAAQSLPAPLTSRADP
EKAVQGSPKSSSAPFEAELHLPPKLRRLYGPGGGRLLQGAAAAAAAAAAAAAAATATAG
PRGEAPPPPPPTARPGERPDGAGAAAAAAAAAAAAAWDTLKISQAPQVSISRSKSYRENGA
PFVPPPPALDELGGPGGVTHPEERLGVAGGPGSAPAAGGGTGTEDDEEELLEDEEDEDEE
EELLEDDEEELLEDDARALLKEPRRCPVAATGAVAAAAAAAVATEGGELSPKEELLLHPE
DAEGKDGEDSVCLSAGSDSEEGLLKRKQRRYRTTFTSYQLEELERAFQKTHYPDVFTREE
LAMRLDLTEARVQVWFQNRRAKWRKREKAGAQTHPPGLPFPGPLSATHPLSPYLDASPFP
PHHPALDSAWTAAAAAAAAAFPSLPPPPGSASLPPSGAPLGLSTFLGAAVFRHPAFISPA
FGRLFSTMAPLTSASTAAALLRQPTPAVEGAVASGALADPATAAADRRASSIAALRLKAK
EHAAQLTQLNILPGTSTGKEVC
>sp|Q9HBK9|AS3MT_HUMAN Arsenite methyltransferase OS=Homo sapiens GN=AS3MT PE=1 SV=3
MAALRDAEIQKDVQTYYGQVLKRSADLQTNGCVTTARPVPKHIREALQNVHEEVALRYYG
CGLVIPEHLENCWILDLGSGSGRDCYVLSQLVGEKGHVTGIDMTKGQVEVAEKYLDYHME
KYGFQASNVTFIHGYIEKLGEAGIKNESHDIVVSNCVINLVPDKQQVLQEAYRVLKHGGE
LYFSDVYTSLELPEEIRTHKVLWGECLGGALYWKELAVLAQKIGFCPPRLVTANLITIQN
KELERVIGDCRFVSATFRLFKHSKTGPTKRCQVIYNGGITGHEKELMFDANFTFKEGEIV
EVDEETAAILKNSRFAQDFLIRPIGEKLPTSGGCSALELKDIITDPFKLAEESDSMKSRC
VPDAAGGCCGTKKSC
>sp|Q8WXK4|ASB12_HUMAN Ankyrin repeat and SOCS box protein 12 OS=Homo sapiens GN=ASB12 PE=1 SV=2
MNLMDITKIFSLLQPDKEEEDTDTEEKQALNQAVYDNDSYTLDQLLRQERYKRFINSRSG
WGVPGTPLRLAASYGHLSCLQVLLAHGADVDSLDVKAQTPLFTAVSHGHLDCVRVLLEAG
ASPGGSIYNNCSPVLTAARDGAVAILQELLDHGAEANVKAKLPVWASNIASCSGPLYLAA
VYGHLDCFRLLLLHGADPDYNCTDQGLLARVPRPRTLLEICLHHNCEPEYIQLLIDFGAN
IYLPSLSLDLTSQDDKGIALLLQARATPRSLLSQVRLVVRRALCQAGQPQAINQLDIPPM
LISYLKHQL
>sp|Q02410|APBA1_HUMAN Amyloid beta A4 precursor protein-binding family A member 1 OS=Homo sapiens GN=APBA1 PE=1 SV=3
MNHLEGSAEVEVTDEAAGGEVNESVEADLEHPEVEEEQQQPPQQQHYVGRHQRGRALEDL
RAQLGQEEEERGECLARSASTESGFHNHTDTAEGDVIAAARDGYDAERAQDPEDESAYAV
QYRPEAEEYTEQAEAEHAEATHRRALPNHLHFHSLEHEEAMNAAYSGYVYTHRLFHRGED
EPYSEPYADYGGLQEHVYEEIGDAPELDARDGLRLYEQERDEAAAYRQEALGARLHHYDE
RSDGESDSPEKEAEFAPYPRMDSYEQEEDIDQIVAEVKQSMSSQSLDKAAEDMPEAEQDL
ERPPTPAGGRPDSPGLQAPAGQQRAVGPAGGGEAGQRYSKEKRDAISLAIKDIKEAIEEV
KTRTIRSPYTPDEPKEPIWVMRQDISPTRDCDDQRPMDGDSPSPGSSSPLGAESSSTSLH
PSDPVEASTNKESRKSLASFPTYVEVPGPCDPEDLIDGIIFAANYLGSTQLLSDKTPSKN
VRMMQAQEAVSRIKMAQKLAKSRKKAPEGESQPMTEVDLFISTQRIKVLNADTQETMMDH
PLRTISYIADIGNIVVLMARRRMPRSNSQENVEASHPSQDGKRQYKMICHVFESEDAQLI
AQSIGQAFSVAYQEFLRANGINPEDLSQKEYSDLLNTQDMYNDDLIHFSKSENCKDVFIE
KQKGEILGVVIVESGWGSILPTVIIANMMHGGPAEKSGKLNIGDQIMSINGTSLVGLPLS

TCQSIIKGLKNQSRVKLNIVRCPPVTTVLIRRPDLRYQLGFSVQNGIICSLMRGGIAERG
GVRVGHRIIEINGQSVVATPHEKIVHILSNAVGEIHMKTMPAAMYRLLTAQEQPVYI
>sp|Q99767|APBA2_HUMAN Amyloid beta A4 precursor protein-binding family A member 2 OS=Homo sapiens GN=APBA2 PE=1 SV=3
MAHRKLESVGSGMLDHRVRPGPVPHSQEPESEDMELPLEGYVPEGLELAALRPESPAPEE
QECHNHSPDGDSSSDYVNNTSEEEDYDEGLPEEEEGITYYIRYCPEDDSYLEGMDCNGEE
YLAHSAHPVDTDECQEAVEEWTDSAGPHPHGHEAEGSQDYPDGQLPIPEDEPSVLEAHDQ
EEDGHYCASKEGYQDYYPEEANGNTGASPYRLRRGDGDLEDQEEDIDQIVAEIKMSLSMT
SITSASEASPEHGPEPGPEDSVEACPPIKASCSPSRHEARPKSLNLLPEAKHPGDPQRGF
KPKTRTPEERLKWPHEQVCNGLEQPRKQQRSDLNGPVDNNNIPETKKVASFPSFVAVPGP
CEPEDLIDGIIFAANYLGSTQLLSERNPSKNIRMMQAQEAVSRVKRMQKAAKIKKKANSE
GDAQTLTEVDLFISTQRIKVLNADTQETMMDHALRTISYIADIGNIVVLMARRRMPRSAS
QDCIETTPGAQEGKKQYKMICHVFESEDAQLIAQSIGQAFSVAYQEFLRANGINPEDLSQ
KEYSDIINTQEMYNDDLIHFSNSENCKELQLEKHKGEILGVVVVESGWGSILPTVILANM
MNGGPAARSGKLSIGDQIMSINGTSLVGLPLATCQGIIKGLKNQTQVKLNIVSCPPVTTV
LIKRPDLKYQLGFSVQNGIICSLMRGGIAERGGVRVGHRIIEINGQSVVATAHEKIVQAL
SNSVGEIHMKTMPAAMFRLLTGQETPLYI
>sp|O95704|APBB3_HUMAN Amyloid beta A4 precursor protein-binding family B member 3 OS=Homo sapiens GN=APBB3 PE=1 SV=2
MLGKDYMLAIILVNCDDDLWGDHSLEVEAGLPPGWRKIHDAAGTYYWHVPSGSTQWQRPT
WELGDAEDPGTGTEGIWGLRPPKGRSFSSLESSLDRSNSLSWYGGESYIQSMEPGAKCFA
VRSLGWVEVPEEDLAPGKSSIAVNNCIQQLAQTRSRSQPPDGAWGEGQNMLMILKKDAMS
LVNPLDHSLIHCQPLVHIRVWGVGSSKGRDRDFAFVASDKDSCMLKCHVFCCDVPAKAIA
SALHGLCAQILSERVEVSGDASCCSPDPISPEDLPRQVELLDAVSQAAQKYEALYMGTLP
VTKAMGMDVLNEAIGTLTARGDRNAWVPTMLSVSDSLMTAHPIQAEASTEEEPLWQCPVR
LVTFIGVGRDPHTFGLIADLGRQSFQCAAFWCQPHAGGLSEAVQAACMVQYQKCLVASAA
RGKAWGAQARARLRLKRTSSMDSPGGPLPLPLLKGGVGGAGATPRKRGVFSFLDAFRLKP
SLLHMP
>sp|Q92624|APBP2_HUMAN Amyloid protein-binding protein 2 OS=Homo sapiens GN=APPBP2 PE=1 SV=2
MAAVELEWIPETLYNTAISAVVDNYIRSRRDIRSLPENIQFDVYYKLYQQGRLCQLGSEF
CELEVFAKVLRALDKRHLLHHCFQALMDHGVKVASVLAYSFSRRCSYIAESDAAVKEKAI
QVGFVLGGFLSDAGWYSDAEKVFLSCLQLCTLHDEMLHWFRAVECCVRLLHVRNGNCKYH
LGEETFKLAQTYMDKLSKHGQQANKAALYGELCALLFAKSHYDEAYKWCIEAMKEITAGL
PVKVVVDVLRQASKACVVKREFKKAEQLIKHAVYLARDHFGSKHPKYSDTLLDYGFYLLN
VDNICQSVAIYQAALDIRQSVFGGKNIHVATAHEDLAYSSYVHQYSSGKFDNALFHAERA
IGIITHILPEDHLLLASSKRVKALILEEIAIDCHNKETEQRLLQEAHDLHLSSLQLAKKA
FGEFNVQTAKHYGNLGRLYQSMRKFKEAEEMHIKAIQIKEQLLGQEDYEVALSVGHLASL
YNYDMNQYENAEKLYLRSIAIGKKLFGEGYSGLEYDYRGLIKLYNSIGNYEKVFEYHNVL
SNWNRLRDRQYSVTDALEDVSTSPQSTEEVVQSFLISQNVEGPSC
>sp|Q9UM13|APC10_HUMAN Anaphase-promoting complex subunit 10 OS=Homo sapiens GN=ANAPC10 PE=1 SV=1
MTTPNKTPPGADPKQLERTGTVREIGSQAVWSLSSCKPGFGVDQLRDDNLETYWQSDGSQ
PHLVNIQFRRKTTVKTLCIYADYKSDESYTPSKISVRVGNNFHNLQEIRQLELVEPSGWI

HVPLTDNHKKPTRTFMIQIAVLANHQNGRDTHMRQIKIYTPVEESSIGKFPRCTTIDFMM
YRSIR
>sp|Q9NYG5|APC11_HUMAN Anaphase-promoting complex subunit 11 OS=Homo sapiens GN=ANAPC11 PE=1 SV=1
MKVKIKCWNGVATWLWVANDENCGICRMAFNGCCPDCKVPGDDCPLVWGQCSHCFHMHCI
LKWLHAQQVQQHCPMCRQEWKFKE
>sp|Q8WW43|APH1B_HUMAN Gamma-secretase subunit APH-1B OS=Homo sapiens GN=APH1B PE=1 SV=3
MTAAVFFGCAFIAFGPALALYVFTIATEPLRIIFLIAGAFFWLVSLLISSLVWFMARVII
DNKDGPTQKYLLIFGAFVSVYIQEMFRFAYYKLLKKASEGLKSINPGETAPSMRLLAYVS
GLGFGIMSGVFSFVNTLSDSLGPGTVGIHGDSPQFFLYSAFMTLVIILLHVFWGIVFFDG
CEKKKWGILLIVLLTHLLVSAQTFISSYYGINLASAFIILVLMGTWAFLAAGGSCRSLKL
CLLCQDKNFLLYNQRSR
>sp|P05090|APOD_HUMAN Apolipoprotein D OS=Homo sapiens GN=APOD PE=1 SV=1
MVMLLLLLSALAGLFGAAEGQAFHLGKCPNPPVQENFDVNKYLGRWYEIEKIPTTFENGR
CIQANYSLMENGKIKVLNQELRADGTVNQIEGEATPVNLTEPAKLEVKFSWFMPSAPYWI
LATDYENYALVYSCTCIIQLFHVDFAWILARNPNLPPETVDSLKNILTSNNIDVKKMTVT
DQVNCPKLS
>sp|P10398|ARAF_HUMAN Serine/threonine-protein kinase A-Raf OS=Homo sapiens GN=ARAF PE=1 SV=2
MEPPRGPPANGAEPSRAVGTVKVYLPNKQRTVVTVRDGMSVYDSLDKALKVRGLNQDCCV
VYRLIKGRKTVTAWDTAIAPLDGEELIVEVLEDVPLTMHNFVRKTFFSLAFCDFCLKFLF
HGFRCQTCGYKFHQHCSSKVPTVCVDMSTNRQQFYHSVQDLSGGSRQHEAPSNRPLNELL
TPQGPSPRTQHCDPEHFPFPAPANAPLQRIRSTSTPNVHMVSTTAPMDSNLIQLTGQSFS
TDAAGSRGGSDGTPRGSPSPASVSSGRKSPHSKSPAEQRERKSLADDKKKVKNLGYRDSG
YYWEVPPSEVQLLKRIGTGSFGTVFRGRWHGDVAVKVLKVSQPTAEQAQAFKNEMQVLRK
TRHVNILLFMGFMTRPGFAIITQWCEGSSLYHHLHVADTRFDMVQLIDVARQTAQGMDYL
HAKNIIHRDLKSNNIFLHEGLTVKIGDFGLATVKTRWSGAQPLEQPSGSVLWMAAEVIRM
QDPNPYSFQSDVYAYGVVLYELMTGSLPYSHIGCRDQIIFMVGRGYLSPDLSKISSNCPK
AMRRLLSDCLKFQREERPLFPQILATIELLQRSLPKIERSASEPSLHRTQADELPACLLS
AARLVP
>sp|Q6UW56|ARAID_HUMAN All-trans retinoic acid-induced differentiation factor OS=Homo sapiens GN=ATRAID PE=1 SV=2
MAPHDPGSLTTLVPWAAALLLALGVERALALPEICTQCPGSVQNLSKVAFYCKTTRELML
HARCCLNQKGTILGLDLQNCSLEDPGPNFHQAHTTVIIDLQANPLKGDLANTFRGFTQLQ
TLILPQHVNCPGGINAWNTITSYIDNQICQGQKNLCNNTGDPEMCPENGSCVPDGPGLLQ
CVCADGFHGYKCMRQGSFSLLMFFGILGATTLSVSILLWATQRRKAKTS
>sp|Q8WWN8|ARAP3_HUMAN Arf-GAP with Rho-GAP domain, ANK repeat and PH domain-containing protein 3 OS=Homo sapiens GN=ARAP3 PE=1 SV=1
MAAPQDLDIAVWLATVHLEQYADTFRRHGLATAGAARGLGHEELKQLGISATGHRKRILR
LLQTGTEEGSLDPKSDSAMEPSPSPAPQAQPPKPVPKPRTVFGGLSGPATTQRPGLSPAL
GGPGVSRSPEPSPRPPPLPTSSSEQSSALNTVEMMPNSIYFGLDSRGRAQAAQDKAPDSS
QISAPTPALRPTTGTVHIMDPGCLYYGVQPVGTPGAPDRRESRGVCQGRAEHRLSRQDLE
AREDAGYASLELPGDSTLLSPTLETEETSDDLISPYASFSFTADRLTPLLSGWLDKLSPQ
GNYVFQRRFVQFNGRSLMYFGSDKDPFPKGVIPLTAIEMTRSSKDNKFQVITGQRVFVFR

TESEAQRDMWCSTLQSCLKEQRLLGHPRPPQPPRPLRTGMLELRGHKAKVFAALSPGELA
LYKSEQAFSLGIGICFIELQGCSVRETKSRSFDLLTPHRCFSFTAESGGARQSWAAALQE
AVTETLSDYEVAEKIWSNRANRQCADCGSSRPDWAAVNLGVVICKQCAGQHRALGSGISK
VQSLKLDTSVWSNEIVQLFIVLGNDRANRFWAGTLPPGEGLHPDATPGPRGEFISRKYRL
GLFRKPHPQYPDHSQLLQALCAAVARPNLLKNMTQLLCVEAFEGEEPWFPPAPDGSCPGL
LPSDPSPGVYNEVVVRATYSGFLYCSPVSNKAGPSPPRRGRDAPPRLWCVLGAALEMFAS
ENSPEPLSLIQPQDIVCLGVSPPPTDPGDRFPFSFELILAGGRIQHFGTDGADSLEAWTS
AVGKWFSPLSCHQLLGPGLLRLGRLWLRSPSHTAPAPGLWLSGFGLLRGDHLFLCSAPGP
GPPAPEDMVHLRRLQEISVVSAADTPDKKEHLVLVETGRTLYLQGEGRLDFTAWNAAIGG
AAGGGGTGLQEQQMSRGDIPIIVDACISFVTQHGLRLEGVYRKGGARARSLRLLAEFRRD
ARSVKLRPGEHFVEDVTDTLKRFFRELDDPVTSARLLPRWREAAELPQKNQRLEKYKDVI
GCLPRVNRRTLATLIGHLYRVQKCAALNQMCTRNLALLFAPSVFQTDGRGEHEVRVLQEL
IDGYISVFDIDSDQVAQIDLEVSLITTWKDVQLSQAGDLIMEVYIEQQLPDNCVTLKVSP
TLTAEELTNQVLEMRGTAAGMDLWVTFEIREHGELERPLHPKEKVLEQALQWCQLPEPCS
ASLLLKKVPLAQAGCLFTGIRRESPRVGLLRCREEPPRLLGSRFQERFFLLRGRCLLLLK
EKKSSKPEREWPLEGAKVYLGIRKKLKPPTPWGFTLILEKMHLYLSCTDEDEMWDWTTSI
LKAQHDDQQPVVLRRHSSSDLARQKFGTMPLLPIRGDDSGATLLSANQTLRRLHNRRTLS
MFFPMKSSQGSVEEQEELEEPVYEEPVYEEVGAFPELIQDTSTSFSTTREWTVKPENPLT
SQKSLDQPFLSKSSTLGQEERPPEPPPGPPSKSSPQARGSLEEQLLQELSSLILRKGETT
AGLGSPSQPSSPQSPSPTGLPTQTPGFPTQPPCTSSPPSSQPLT
>sp|Q8N726|ARF_HUMAN Tumor suppressor ARF OS=Homo sapiens GN=CDKN2A PE=1 SV=2
MVRRFLVTLRIRRACGPPRVRVFVVHIPRLTGEWAAPGAPAAVALVLMLLRSQRLGQQPL
PRRPGHDDGQRPSGGAAAAPRRGAQLRRPRHSHPTRARRCPGGLPGHAGGAAPGRGAAGR
ARCLGPSARGPG
>sp|P78540|ARGI2_HUMAN Arginase-2, mitochondrial OS=Homo sapiens GN=ARG2 PE=1 SV=1
MSLRGSLSRLLQTRVHSILKKSVHSVAVIGAPFSQGQKRKGVEHGPAAIREAGLMKRLSS
LGCHLKDFGDLSFTPVPKDDLYNNLIVNPRSVGLANQELAEVVSRAVSDGYSCVTLGGDH
SLAIGTISGHARHCPDLCVVWVDAHADINTPLTTSSGNLHGQPVSFLLRELQDKVPQLPG
FSWIKPCISSASIVYIGLRDVDPPEHFILKNYDIQYFSMRDIDRLGIQKVMERTFDLLIG
KRQRPIHLSFDIDAFDPTLAPATGTPVVGGLTYREGMYIAEEIHNTGLLSALDLVEVNPQ
LATSEEEAKTTANLAVDVIASSFGQTREGGHIVYDQLPTPSSPDESENQARVRI
>sp|Q9NWB6|ARGL1_HUMAN Arginine and glutamate-rich protein 1 OS=Homo sapiens GN=ARGLU1 PE=1 SV=1
MGRSRSRSSSRSKHTKSSKHNKKRSRSRSRSRDKERVRKRSKSRESKRNRRRESRSRSRS
TNTAVSRRERDRERASSPPDRIDIFGRTVSKRSSLDEKQKREEEEKKAEFERQRKIRQQE
IEEKLIEEETARRVEELVAKRVEEELEKRKDEIEREVLRRVEEAKRIMEKQLLEELERQR
QAELAAQKAREEEERAKREELERILEENNRKIAEAQAKLAEEQLRIVEEQRKIHEERMKL
EQERQRQQKEEQKIILGKGKSRPKLSFSLKTQD
>sp|Q96PS8|AQP10_HUMAN Aquaporin-10 OS=Homo sapiens GN=AQP10 PE=2 SV=2
MVFTQAPAEIMGHLRIRSLLARQCLAEFLGVFVLMLLTQGAVAQAVTSGETKGNFFTMFL
AGSLAVTIAIYVGGNVSGAHLNPAFSLAMCIVGRLPWVKLPIYILVQLLSAFCASGATYV
LYHDALQNYTGGNLTVTGPKETASIFATYPAPYLSLNNGFLDQVLGTGMLIVGLLAILDR
RNKGVPAGLEPVVVGMLILALGLSMGANCGIPLNPARDLGPRLFTYVAGWGPEVFSAGNG
WWWVPVVAPLVGATVGTATYQLLVALHHPEGPEPAQDLVSAQHKASELETPASAQMLECK

L
>sp|A6NL99|AQP73_HUMAN Putative aquaporin-7-like protein 3 OS=Homo sapiens GN=AQP7P3 PE=5 SV=2
MVQASGHRRSTRGSKMVSWSVIAKIQEICERKMAREFLAEFMSTYVMMVFGLGSVAHMLL
NKTFGSYLGVNLGFGFGVTMGVHMAGRTSGAHMNAAVSLTNCALGRVPWRKFPVYVLGQF
LGSFLAAATIYSLFYTAILHFSGGELMVTGPVATVGIFATYLPDHMTLWRGFLNEEWLTG
MLQLCLFAIVDQENNPALPGTHALVIGILVVIIRVYHGMNTGYAINPSRDLPPRIFTFIA
GWGKLVFSDGENLWWVPVVAPLLGASLGGIIYLVFIGSTIPREPLKLEDSVAYEDHGITV
LPKMGSHEPMISPLTLISVSPANRSSVHPAPPLHESMALEHF
>sp|Q3SXY8|AR13B_HUMAN ADP-ribosylation factor-like protein 13B OS=Homo sapiens GN=ARL13B PE=1 SV=1
MFSLMASCCGWFKRWREPVRKVTLLMVGLDNAGKTATAKGIQGEYPEDVAPTVGFSKINL
RQGKFEVTIFDLGGGIRIRGIWKNYYAESYGVIFVVDSSDEERMEETKEAMSEMLRHPRI
SGKPILVLANKQDKEGALGEADVIECLSLEKLVNEHKCLCQIEPCSAISGYGKKIDKSIK
KGLYWLLHVIARDFDALNERIQKETTEQRALEEQEKQERAERVRKLREERKQNEQEQAEL
DGTSGLAELDPEPTNPFQPIASVIIENEGKLEREKKNQKMEKDSDGCHLKHKMEHEQIET
QGQVNHNGQKNNEFGLVENYKEALTQQLKNEDETDRPSLESANGKKKTKKLRMKRNHRVE
PLNIDDCAPESPTPPPPPPPVGWGTPKVTRLPKLEPLGETHHNDFYRKPLPPLAVPQRPN
SDAHDVIS
>sp|Q8IWT0|ARCH_HUMAN Protein archease OS=Homo sapiens GN=ZBTB8OS PE=1 SV=2
MAQEEEDVRDYNLTEEQKAIKAKYPPVNRKYEYLDHTADVQLHAWGDTLEEAFEQCAMAM
FGYMTDTGTVEPLQTVEVETQGDDLQSLLFHFLDEWLYKFSADEFFIPREVKVLSIDQRN
FKLRSIGWGEEFSLSKHPQGTEVKAITYSAMQVYNEENPEVFVIIDI
>sp|O15033|AREL1_HUMAN Apoptosis-resistant E3 ubiquitin protein ligase 1 OS=Homo sapiens GN=AREL1 PE=1 SV=3
MFYVIGGITVSVVAFFFTIKFLFELAARVVSFLQNEDRERRGDRTIYDYVRGNYLDPRSC
KVSWDWKDPYEVGHSMAFRVHLFYKNGQPFPAHRPVGLRVHISHVELAVEIPVTQEVLQE
PNSNVVKVAFTVRKAGRYEITVKLGGLNVAYSPYYKIFQPGMVVPSKTKIVCHFSTLVLT
CGQPHTLQIVPRDEYDNPTNNSMSLRDEHNYTLSIHELGPQEEESTGVSFEKSVTSNRQT
FQVFLRLTLHSRGCFHACISYQNQPINNGEFDIIVLSEDEKNIVERNVSTSGVSIYFEAY
LYNATNCSSTPWHLPPMHMTSSQRRPSTAVDEEDEDSPSECHTPEKVKKPKKVYCYVSPK
QFSVKEFYLKIIPWRLYTFRVCPGTKFSYLGPDPVHKLLTLVVDDGIQPPVELSCKERNI
LAATFIRSLHKNIGGSETFQDKVNFFQRELRQVHMKRPHSKVTLKVSRHALLESSLKATR
NFSISDWSKNFEVVFQDEEALDWGGPRREWFELICKALFDTTNQLFTRFSDNNQALVHPN
PNRPAHLRLKMYEFAGRLVGKCLYESSLGGAYKQLVRARFTRSFLAQIIGLRMHYKYFET
DDPEFYKSKVCFILNNDMSEMELVFAEEKYNKSGQLDKVVELMTGGAQTPVTNANKIFYL
NLLAQYRLASQVKEEVEHFLKGLNELVPENLLAIFDENELELLMCGTGDISVSDFKAHAV
VVGGSWHFREKVMRWFWTVVSSLTQEELARLLQFTTGSSQLPPGGFAALCPSFQIIAAPT
HSTLPTAHTCFNQLCLPTYDSYEEVHRMLQLAISEGCEGFGML
>sp|Q9NP61|ARFG3_HUMAN ADP-ribosylation factor GTPase-activating protein 3 OS=Homo sapiens GN=ARFGAP3 PE=1 SV=1
MGDPSKQDILTIFKRLRSVPTNKVCFDCGAKNPSWASITYGVFLCIDCSGSHRSLGVHLS
FIRSTELDSNWSWFQLRCMQVGGNASASSFFHQHGCSTNDTNAKYNSRAAQLYREKIKSL
ASQATRKHGTDLWLDSCVVPPLSPPPKEEDFFASHVSPEVSDTAWASAIAEPSSLTSRPV

ETTLENNEGGQEQGPSVEGLNVPTKATLEVSSIIKKKPNQAKKGLGAKKGSLGAQKLANT
CFNEIEKQAQAADKMKEQEDLAKVVSKEESIVSSLRLAYKDLEIQMKKDEKMNISGKKNV
DSDRLGMGFGNCRSVISHSVTSDMQTIEQESPIMAKPRKKYNDDSDDSYFTSSSSYFDEP
VELRSSSFSSWDDSSDSYWKKETSKDTETVLKTTGYSDRPTARRKPDYEPVENTDEAQKK
FGNVKAISSDMYFGRQSQADYETRARLERLSASSSISSADLFEEPRKQPAGNYSLSSVLP
NAPDMAQFKQGVRSVAGKLSVFANGVVTSIQDRYGS
>sp|Q9HCE6|ARGAL_HUMAN Rho guanine nucleotide exchange factor 10-like protein OS=Homo sapiens GN=ARHGEF10L PE=1 SV=3
MASSNPPPQPAIGDQLVPGVPGPSSEAEDDPGEAFEFDDSDDEEDTSAALGVPSLAPERD
TDPPLIHLDSIPVTDPDPAAAPPGTGVPAWVSNGDAADAAFSGARHSSWKRKSSRRIDRF
TFPALEEDVIYDDVPCESPDAHQPGAERNLLYEDAHRAGAPRQAEDLGWSSSEFESYSED
SGEEAKPEVEVEPAKHRVSFQPKLSPDLTRLKERYARTKRDILALRVGGRDMQELKHKYD
CKMTQLMKAAKSGTKDGLEKTRMAVMRKVSFLHRKDVLGDSEEEDMGLLEVSVSDIKPPA
PELGPMPEGLSPQQVVRRHILGSIVQSEGSYVESLKRILQDYRNPLMEMEPKALSARKCQ
VVFFRVKEILHCHSMFQIALSSRVAEWDSTEKIGDLFVASFSKSMVLDVYSDYVNNFTSA
MSIIKKACLTKPAFLEFLKRRQVCSPDRVTLYGLMVKPIQRFPQFILLLQDMLKNTPRGH
PDRLSLQLALTELETLAEKLNEQKRLADQVAEIQQLTKSVSDRSSLNKLLTSGQRQLLLC
ETLTETVYGDRGQLIKSKERRVFLLNDMLVCANINFKPANHRGQLEISSLVPLGPKYVVK
WNTALPQVQVVEVGQDGGTYDKDNVLIQHSGAKKASASGQAQNKVYLGPPRLFQELQDLQ
KDLAVVEQITLLISTLHGTYQNLNMTVAQDWCLALQRLMRVKEEEIHSANKCRLRLLLPG
KPDKSGRPISFMVVFITPNPLSKISWVNRLHLAKIGLREENQPGWLCPDEDKKSKAPFWC
PILACCIPAFSSRALSLQLGALVHSPVNCPLLGFSAVSTSLPQGYLWVGGGQEGAGGQVE
IFSLNRPSPRTVKSFPLAAPVLCMEYIPELEEEAESRDESPTVADPSATVHPTICLGLQD
GSILLYSSVDTGTQCLVSCRSPGLQPVLCLRHSPFHLLAGLQDGTLAAYPRTSGGVLWDL
ESPPVCLTVGPGPVRTLLSLEDAVWASCGPWVTVLEATTLQPQQSFEAHQDEAVSVTHMV
KAGSGVWMAFSSGTSIRLFHTETLEHLQEINIATRTTFLLPGQKHLCVTSLLICQGLLWV
GTDQGVIVLLPVPRLEGIPKITGKGMVSLNGHCGPVAFLAVATSILAPDILRSDQEEAEG
PRAEEDKPDGQAHEPMPDSHVGRELTRKKGILLQYRLRSTAHLPGPLLSMREPAPADGAA
LEHSEEDGSIYEMADDPDIWVRSRPCARDAHRKEICSVAIISGGQGYRNFGSALGSSGRQ
APCGETDSTLLIWQVPLML
>sp|Q92888|ARHG1_HUMAN Rho guanine nucleotide exchange factor 1 OS=Homo sapiens GN=ARHGEF1 PE=1 SV=2
MEDFARGAASPGPSRPGLVPVSIIGAEDEDFENELETNSEEQNSQFQSLEQVKRRPAHLM
ALLQHVALQFEPGPLLCCLHADMLGSLGPKEAKKAFLDFYHSFLEKTAVLRVPVPPNVAF
ELDRTRADLISEDVQRRFVQEVVQSQQVAVGRQLEDFRSKRLMGMTPWEQELAQLEAWVG
RDRASYEARERHVAERLLMHLEEMQHTISTDEEKSAAVVNAIGLYMRHLGVRTKSGDKKS
GRNFFRKKVMGNRRSDEPAKTKKGLSSILDAARWNRGEPQVPDFRHLKAEVDAEKPGATD
RKGGVGMPSRDRNIGAPGQDTPGVSLHPLSLDSPDREPGADAPLELGDSSPQGPMSLESL
APPESTDEGAETESPEPGDEGEPGRSGLELEPEEPPGWRELVPPDTLHSLPKSQVKRQEV
ISELLVTEAAHVRMLRVLHDLFFQPMAECLFFPLEELQNIFPSLDELIEVHSLFLDRLMK
RRQESGYLIEEIGDVLLARFDGAEGSWFQKISSRFCSRQSFALEQLKAKQRKDPRFCAFV
QEAESRPRCRRLQLKDMIPTEMQRLTKYPLLLQSIGQNTEEPTEREKVELAAECCREILH
HVNQAVRDMEDLLRLKDYQRRLDLSHLRQSSDPMLSEFKNLDITKKKLVHEGPLTWRVTK
DKAVEVHVLLLDDLLLLLQRQDERLLLKSHSRTLTPTPDGKTMLRPVLRLTSAMTREVAT

DHKAFYVLFTWDQEAQIYELVAQTVSERKNWCALITETAGSLKVPAPASRPKPRPSPSST
REPLLSSSENGNGGRETSPADARTERILSDLLPFCRPGPEGQLAATALRKVLSLKQLLFP
AEEDNGAGPPRDGDGVPGGGPLSPARTQEIQENLLSLEETMKQLEELEEEFCRLRPLLSQ
LGGNSVPQPGCT
>sp|Q6ZSZ5|ARHGI_HUMAN Rho guanine nucleotide exchange factor 18 OS=Homo sapiens GN=ARHGEF18 PE=1 SV=3
MVTVGTNILPSRPAASANTAREDAALFSRRIPPRHKNGAAQPGAAPGPGAPGANMGNAHS
KSGDRHSALPGRPELSFYGSFPRKWSENVFLDNELLTSKILSVLRPQSERGFRAGDLRYP
THFLSTNSVLASVTASLKEHPRGTLLSDGSPALSRNVGMTVSQKGGPQPTPSPAGPGTQL
GPITGEMDEADSAFLKFKQTADDSLSLTSPNTESIFVEDPYTASLRSEIESDGHEFEAES
WSLAVDAAYAKKQKREVVKRQDVLYELMQTEVHHVRTLKIMLKVYSRALQEELQFSSKAI
GRLFPCADDLLETHSHFLARLKERRQESLEEGSDRNYVIQKIGDLLVQQFSGENGERMKE
KYGVFCSGHNEAVSHYKLLLQQNKKFQNLIKKIGNFSIVRRLGVQECILLVTQRITKYPV
LVERIIQNTEAGTEDYEDLTQALNLIKDIISQVDAKVSECEKGQRLREIAGKMDLKSSSK
LKNGLTFRKEDMLQRQLHLEGMLCWKTTSGRLKDILAILLTDVLLLLQEKDQKYVFASVD
SKPPVISLQKLIVREVANEEKAMFLISASLQGPEMYEIYTSSKEDRNAWMAHIQRAVESC
PDEEEGPFSLPEEERKVVEARATRLRDFQERLSMKDQLIAQSLLEKQQIYLEMAEMGGLE
DLPQPRGLFRGGDPSETLQGELILKSAMSEIEGIQSLICRQLGSANGQAEDGGSSTGPPR
RAETFAGYDCTNSPTKNGSFKKKVSSTDPRPRDWRGPPNSPDLKLSDSDIPGSSEESPQV
VEAPGTESDPRLPTVLESELVQRIQTLSQLLLNLQAVIAHQDSYVETQRAAIQEREKQFR
LQSTRGNLLLEQERQRNFEKQREERAALEKLQSQLRHEQQRWERERQWQHQELERAGARL
QEREGEARQLRERLEQERAELERQRQAYQHDLERLREAQRAVERERERLELLRRLKKQNT
APGALPPDTLAEAQPPSHPPSFNGEGLEGPRVSMLPSGVGPEYAERPEVARRDSAPTENR
LAKSDVPIQLLSATNQFQRQAAVQQQIPTKLAASTKGGKDKGGKSRGSQRWESSASFDLK
QQLLLNKLMGKDESTSRNRRSLSPILPGRHSPAPPPDPGFPAPSPPPADSPSEGFSLKAG
GTALLPGPPAPSPLPATPLSAKEDASKEDVIFF
>sp|Q8N7S6|ARI2O_HUMAN Uncharacterized protein ARIH2OS OS=Homo sapiens GN=ARIH2OS PE=2 SV=1
MLGQRAGDGERPGLPGDGEGGVPARPGRRAERPPQRPAKVNKAVTCAAHLPGAAASRPLS
PNKPDRVRPGQRDRIGAKRQRRRRADAGQARAASSRRVVPTAPEVLGAVASLPDRGRPTV
ARVATGSRLEGLFSAASLKLSALTQSLTRVRQAPTASGATIRLPASPVEMFLTSAFLTGF
SFHCLYSGIGHGEDILASVEQITIVSRPLSGQRGAGPGNSAYTPRRSQGGPRAATTPGFR
FPCRGLVRRAVLRLTVTVQDCILTALLAVSFHSIGVVIMTSSYLLGPVVK
>sp|O95376|ARI2_HUMAN E3 ubiquitin-protein ligase ARIH2 OS=Homo sapiens GN=ARIH2 PE=1 SV=1
MSVDMNSQGSDSNEEDYDPNCEEEEEEEEEDDPGDIEDYYVGVASDVEQQGADAFDPEEYQ
FTCLTYKESEGALNEHMTSLASVLKVSHSVAKLILVNFHWQVSEILDRYKSNSAQLLVEA
RVQPNPSKHVPTSHPPHHCAVCMQFVRKENLLSLACQHQFCRSCWEQHCSVLVKDGVGVG
VSCMAQDCPLRTPEDFVFPLLPNEELREKYRRYLFRDYVESHYQLQLCPGADCPMVIRVQ
EPRARRVQCNRCNEVFCFKCRQMYHAPTDCATIRKWLTKCADDSETANYISAHTKDCPKC
NICIEKNGGCNHMQCSKCKHDFCWMCLGDWKTHGSEYYECSRYKENPDIVNQSQQAQARE
ALKKYLFYFERWENHNKSLQLEAQTYQRIHEKIQERVMNNLGTWIDWQYLQNAAKLLAKC
RYTLQYTYPYAYYMESGPRKKLFEYQQAQLEAEIENLSWKVERADSYDRGDLENQMHIAE
QRRRTLLKDFHDT

>sp|P29374|ARI4A_HUMAN AT-rich interactive domain-containing protein 4A OS=Homo sapiens GN=ARID4A PE=1 SV=3
MKAADEPAYLTVGTDVSAKYRGAFCEAKIKTVKRLVKVKVLLKQDNTTQLVQDDQVKGPL
RVGAIVETRTSDGSFQEAIISKLTDASWYTVVFDDGDERTLRRTSLCLKGERHFAESETL
DQLPLTNPEHFGTPVIAKKTNRGRRSSLPVTEDEKEEESSEEEDEDKRRLNDELLGKVVS
VVSATERTEWYPALVISPSCNDDITVKKDQCLVRSFIDSKFYSIARKDIKEVDILNLPES
ELSTKPGLQKASIFLKTRVVPDNWKMDISEILESSSSDDEDGPAEENDEEKEKEAKKTEE
EVPEEELDPEERDNFLQQLYKFMEDRGTPINKPPVLGYKDLNLFKLFRLVYHQGGCDNID
SGAVWKQIYMDLGIPILNSAASYNVKTAYRKYLYGFEEYCRSANIQFRTVHHHEPKVKEE
KKDLEESMEEALKLDQEMPLTEVKSEPEENIDSNSESEREEIELKSPRGRRRIARDVNSI
KKEIEEEKTEDKLKDNDTENKDVDDDYETAEKKENELLLGRKNTPKQKEKKIKKQEDSDK
DSDEEEEKSQEREETESKCDSEGEEDEEDMEPCLTGTKVKVKYGRGKTQKIYEASIKSTE
IDDGEVLYLVHYYGWNVRYDEWVKADRIIWPLDKGGPKKKQKKKAKNKEDSEKDEKRDEE
RQKSKRGRPPLKSTLSSNMPYGLSKTANSEGKSDSCSSDSETEDALEKNLINEELSLKDE
LEKNENLNDDKLDEENPKISAHILKENDRTQMQPLETLKLEVGENEQIVQIFGNKMEKTE
EVKKEAEKSPKGKGRRSKTKDLSLEIIKISSFGQNEAGSEPHIEAHSLELSSLDNKNFSS
ATEDEIDQCVKEKKLKRKILGQSSPEKKIRIENGMEMTNTVSQERTSDCIGSEGMKNLNF
EQHFERENEGMPSLIAESNQCIQQLTSERFDSPAEETVNIPLKEDEDAMPLIGPETLVCH
EVDLDDLDEKDKTSIEDVAVESSESNSLVSIPPALPPVVQHNFSVASPLTLSQDESRSVK
SESDITIEVDSIAEESQEGLCERESANGFETNVASGTCSIIVQERESREKGQKRPSDGNS
GLMAKKQKRTPKRTSAAAKNEKNGTGQSSDSEDLPVLDNSSKCTPVKHLNVSKPQKLARS
PARISPHIKDGEKDKHREKHPNSSPRTYKWSFQLNELDNMNSTERISFLQEKLQEIRKYY
MSLKSEVATIDRRRKRLKKKDREVSHAGASMSSASSDTGMSPSSSSPPQNVLAVECR
>sp|Q03989|ARI5A_HUMAN AT-rich interactive domain-containing protein 5A OS=Homo sapiens GN=ARID5A PE=1 SV=2
MAAPVKGNRKQSTEGDALDPPASPKPAGKQNGIQNPISLEDSPEAGGEREEEQEREEEQA
FLVSLYKFMKERHTPIERVPHLGFKQINLWKIYKAVEKLGAYELVTGRRLWKNVYDELGG
SPGSTSAATCTRRHYERLVLPYVRHLKGEDDKPLPTSKPRKQYKMAKENRGDDGATERPK
KAKEERRMDQMMPGKTKADAADPAPLPSQEPPRNSTEQQGLASGSSVSFVGASGCPEAYK
RLLSSFYCKGTHGIMSPLAKKKLLAQVSKVEALQCQEEGCRHGAEPQASPAVHLPESPQS
PKGLTENSRHRLTPQEGLQAPGGSLREEAQAGPCPAAPIFKGCFYTHPTEVLKPVSQHPR
DFFSRLKDGVLLGPPGKEGLSVKEPQLVWGGDANRPSAFHKGGSRKGILYPKPKACWVSP
MAKVPAESPTLPPTFPSSPGLGSKRSLEEEGAAHSGKRLRAVSPFLKEADAKKCGAKPAG
SGLVSCLLGPALGPVPPEAYRGTMLHCPLNFTGTPGPLKGQAALPFSPLVIPAFPAHFLA
TAGPSPMAAGLMHFPPTSFDSALRHRLCPASSAWHAPPVTTYAAPHFFHLNTKL
>sp|P04424|ARLY_HUMAN Argininosuccinate lyase OS=Homo sapiens GN=ASL PE=1 SV=4
MASESGKLWGGRFVGAVDPIMEKFNASIAYDRHLWEVDVQGSKAYSRGLEKAGLLTKAEM
DQILHGLDKVAEEWAQGTFKLNSNDEDIHTANERRLKELIGATAGKLHTGRSRNDQVVTD
LRLWMRQTCSTLSGLLWELIRTMVDRAEAERDVLFPGYTHLQRAQPIRWSHWILSHAVAL
TRDSERLLEVRKRINVLPLGSGAIAGNPLGVDRELLRAELNFGAITLNSMDATSERDFVA
EFLFWASLCMTHLSRMAEDLILYCTKEFSFVQLSDAYSTGSSLMPQKKNPDSLELIRSKA
GRVFGRCAGLLMTLKGLPSTYNKDLQEDKEAVFEVSDTMSAVLQVATGVISTLQIHQENM
GQALSPDMLATDLAYYLVRKGMPFRQAHEASGKAVFMAETKGVALNQLSLQELQTISPLF
SGDVICVWDYGHSVEQYGALGGTARSSVDWQIRQVRALLQAQQA

>sp|Q7Z3E5|ARMC9_HUMAN LisH domain-containing protein ARMC9 OS=Homo sapiens GN=ARMC9 PE=1 SV=2
MGDILAHESELLGLVKEYLDFAEFEDTLKTFSKECKIKGKPLCKTVGGSFRDSKSLTIQK
DLVAAFDNGDQKVFFDLWEEHISSSIRDGDSFAQKLEFYLHIHFAIYLLKYSVGRPDKEE
LDEKISYFKTYLETKGAALSQTTEFLPFYALPFVPNPMVHPSFKELFQDSWTPELKLKLI
KFLALISKASNTPKLLTIYKENGQSNKEILQQLHQQLVEAERRSVTYLKRYNKIQADYHN
LIGVTAELVDSLEATVSGKMITPEYLQSVCVRLFSNQMRQSLAHSVDFTRPGTASTMLRA
SLAPVKLKDVPLLPSLDYEKLKKDLILGSDRLKAFLLQALRWRLTTSHPGEQRETVLQAY
ISNDLLDCYSHNQRSVLQLLHSTSDVVRQYMARLINAFASLAEGRLYLAQNTKVLQMLEG
RLKEEDKDIITRENVLGALQKFSLRRPLQTAMIQDGLIFWLVDVLKDPDCLSDYTLEYSV
ALLMNLCLRSTGKNMCAKVAGLVLKVLSDLLGHENHEIQPYVNGALYSILSVPSIREEAR
AMGMEDILRCFIKEGNAEMIRQIEFIIKQLNSEELPDGVLESDDDEDEDDEEDHDIMEAD
LDKDELIQPQLGELSGEKLLTTEYLGIMTNTGKTRRKGLANVQWSGDEPLQRPVTPGGHR
NGYPVVEDQHTPPQTAQHARNGHPQALPAAHEAVYREGKPSTPESCVSSSSAIIAKPGEW
LPRGRQEEPRPAPTTPRQPREAPQDPGNGVTTRECASAFTCKPRAPCTPEMLDWNPPKAK
ASVLAPLFSSCGPQQASRPGSTASSTRGLPSSQSHRK
>sp|Q9H993|ARMT1_HUMAN Protein-glutamate O-methyltransferase OS=Homo sapiens GN=ARMT1 PE=1 SV=1
MAVVPASLSGQDVGSFAYLTIKDRIPQILTKVIDTLHRHKSEFFEKHGEEGVEAEKKAIS
LLSKLRNELQTDKPFIPLVEKFVDTDIWNQYLEYQQSLLNESDGKSRWFYSPWLLVECYM
YRRIHEAIIQSPPIDYFDVFKESKEQNFYGSQESIIALCTHLQQLIRTIEDLDENQLKDE
FFKLLQISLWGNKCDLSLSGGESSSQNTNVLNSLEDLKPFILLNDMEHLWSLLSNCKKTR
EKASATRVYIVLDNSGFELVTDLILADFLLSSELATEVHFYGKTIPWFVSDTTIHDFNWL
IEQVKHSNHKWMSKCGADWEEYIKMGKWVYHNHIFWTLPHEYCAMPQVAPDLYAELQKAH
LILFKGDLNYRKLTGDRKWEFSVPFHQALNGFHPAPLCTIRTLKAEIQVGLQPGQGEQLL
ASEPSWWTTGKYGIFQYDGPL
>sp|P23582|ANFC_HUMAN C-type natriuretic peptide OS=Homo sapiens GN=NPPC PE=1 SV=1
MHLSQLLACALLLTLLSLRPSEAKPGAPPKVPRTPPAEELAEPQAAGGGQKKGDKAPGGG
GANLKGDRSRLLRDLRVDTKSRAAWARLLQEHPNARKYKGANKKGLSKGCFGLKLDRIGS
MSGLGC
>sp|Q86XL3|ANKL2_HUMAN Ankyrin repeat and LEM domain-containing protein 2 OS=Homo sapiens GN=ANKLE2 PE=1 SV=4
MLWPRLAAAEWAALAWELLGASVLLIAVRWLVRRLGPRPGGLGRSGTPVPPPSAAAAPAS
GEMTMDALLARLKLLNPDDLREEIVKAGLKCGPITSTTRFIFEKKLAQALLEQGGRLSSF
YHHEAGVTALSQDPQRILKPAEGNPTDQAGFSEDRDFGYSVGLNPPEEEAVTSKTCSVPP
SDTDTYRAGATASKEPPLYYGVCPVYEDVPARNERIYVYENKKEALQAVKMIKGSRFKAF
STREDAEKFARGICDYFPSPSKTSLPLSPVKTAPLFSNDRLKDGLCLSESETVNKERANS
YKNPRTQDLTAKLRKAVEKGEEDTFSDLIWSNPRYLIGSGDNPTIVQEGCRYNVMHVAAK
ENQASICQLTLDVLENPDFMRLMYPDDDEAMLQKRIRYVVDLYLNTPDKMGYDTPLHFAC
KFGNADVVNVLSSHHLIVKNSRNKYDKTPEDVICERSKNKSVELKERIREYLKGHYYVPL
LRAEETSSPVIGELWSPDQTAEASHVSRYGGSPRDPVLTLRAFAGPLSPAKAEDFRKLWK
TPPREKAGFLHHVKKSDPERGFERVGRELAHELGYPWVEYWEFLGCFVDLSSQEGLQRLE
EYLTQQEIGKKAQQETGEREASCRDKATTSGSNSISVRAFLDEDDMSLEEIKNRQNAARN
NSPPTVGAFGHTRCSAFPLEQEADLIEAAEPGGPHSSRNGLCHPLNHSRTLAGKRPKAPR

GEEAHLPPVSDLTVEFDKLNLQNIGRSVSKTPDESTKTKDQILTSRINAVERDLLEPSPA
DQLGNGHRRTESEMSARIAKMSLSPSSPRHEDQLEVTREPARRLFLFGEEPSKLDQDVLA
ALECADVDPHQFPAVHRWKSAVLCYSPSDRQSWPSPAVKGRFKSQLPDLSGPHSYSPGRN
SVAGSNPAKPGLGSPGRYSPVHGSQLRRMARLAELAAL
>sp|Q9GZV1|ANKR2_HUMAN Ankyrin repeat domain-containing protein 2 OS=Homo sapiens GN=ANKRD2 PE=1 SV=3
MAKAPSWAGVGALAYKAPEALWPAEAVMDGTMEDSEAVQRATALIEQRLAQEEENEKLRG
DARQKLPMDLLVLEDEKHHGAQSAALQKVKGQERVRKTSLDLRREIIDVGGIQNLIELRK
KRKQKKRDALAASHEPPPEPEEITGPVDEETFLKAAVEGKMKVIEKFLADGGSADTCDQF
RRTALHRASLEGHMEILEKLLDNGATVDFQDRLDCTAMHWACRGGHLEVVKLLQSHGADT
NVRDKLLSTPLHVAVRTGQVEIVEHFLSLGLEINARDREGDTALHDAVRLNRYKIIKLLL
LHGADMMTKNLAGKTPTDLVQLWQADTRHALEHPEPGAEHNGLEGPNDSGRETPQPVPAQ
>sp|Q6P2P2|ANM9_HUMAN Putative protein arginine N-methyltransferase 9 OS=Homo sapiens GN=PRMT9 PE=2 SV=1
MSNSRPRSRRDAGGGAGAAGRDELVSRSLQSAEHCLGVQDFGTAYAHYLLVLSLAPELKH
DVKETFQYTLFRWAEELDALSRIQDLLGCYEQALELFPDDEVICNSMGEHLFRMGFRDEA
AGYFHKAVKLNPDFSDAKENFYRVANWLVERWHFIMLNDTKRNTIYNAAIQKAVCLGSKS
VLDIGAGTGILSMFAKKAGAHSVYACELSKTMYELACDVVAANKMEAGIKLLHTKSLDIE
IPKHIPERVSLVVTETVDAGLFGEGIVESLIHAWEHLLLQPKTKGESANCEKYGKVIPAS
AVIFGMAVECAEIRRHHRVGIKDIAGIHLPTNVKFQSPAYSSVDTEETIEPYTTEKMSRV
PGGYLALTECFEIMTVDFNNLQELKSLATKKPDKIGIPVIKEGILDAIMVWFVLQLDDEH
SLSTSPSEETCWEQAVYPVQDLADYWIKPGDHVMMEVSCQDCYLRIQSISVLGLECEMDV
AKSFTQNKDLLSLGNEAELCSALANLQTSKPDAVEQTCILESTEIALLNNIPYHEGFKMA
MSKVLSSLTPEKLYQTMDTHCQNEMSSGTGQSNTVQNILEPFYVLDVSEGFSVLPVIAGT
LGQVKPYSSVEKDQHRIALDLISEANHFPKETLEFWLRHVEDESAMLQRPKSDKLWSIII
LDVIEPSGLIQQEIMEKAAISRCLLQSGGKIFPQYVLMFGLLVESQTLLEENAVQGTERT
LGLNIAPFINQFQVPIRVFLDLSSLPCIPLSKPVELLRLDLMTPYLNTSNREVKVYVCKS
GRLTAIPFWYHMYLDEEIRLDTSSEASHWKQAAVVLDNPIQVEMGEELVLSIQHHKSNVS
ITVKQ
>sp|Q8N7Z5|ANR31_HUMAN Putative ankyrin repeat domain-containing protein 31 OS=Homo sapiens GN=ANKRD31 PE=5 SV=2
MEEGVQAPDWDSDETVIEGSVTESDLEEKELPWRRLLFDQDASLKSEFSLHPDTRGMCKG
MPSPEIQLGFKLREDLQEQMNKNKMMPVLSEDTILQSQDETERNQALLQTRKNCSMFIGS
FRQSGLSLNHQNIEGPEAESPEVLPHIEKELSEGRDSPEVSLLSGTAITVSDTVAVKETS
LVEPEKILAAPNTFFEPRKEVTMTMTSEETKDEESSLETFVSALESLLTSPESTQEERLF
ELVSDFDRKELMNPLSDSLSSISIPLNSWSACHRDLLEDAKDDALPAELLEALNTLSEAK
VETICHRKEGGSSLIARNECLEVEFNTSQTNEDCTQIAETLQDPNPSGLQTLAHQNITSC
EPLSNKRNSNSVTNSSDQETACVLRRSSRLEKLKVSRDAKYSDHMYKMPEKILPKILGCE
DLTNNNSSAQNFRMQDPALMIDGKEKNMHSARFKNGKQIRKNEQFSGKKEKMKVNKISLH
SINRRNIFGENLVYKAALHDDADLVHHCIKKGGNVNQPSYAGWTALHEASVGGFYRTASE
LLKGGADVNIKGLYQITPLHDAVMNGHYKVAELLLLNGADPLFRNDDGKCALDEAKDLCM
KRLLERYIPKHQKCLTSAQRSSIDPLDIEDVYQHKKPKFSSKSHIWHVYNENSNRQKLEH
VKVNKGSKASLFINKEDVYEYYQKDPKNTKFGKSKHKQSTLDQIYSTGLRKGNLHNVKDP
NTNVPKGIGRRKTQHKRTQVDDVDCNPRKILAVSPSRRINRLVTYQQHIPETHNDLPEEL

CEPSSLTLSSLRNGLDSSTEACSVSKEKHIQNLDLSDSQEVQCLELESVDQTEAVSFPGL
LLHKEIKLPVVTTDKQPHTLQEQHHVLYKSHENSNLVPKDERFNKWENSFLSFVKENSDN
DDDDDCSTSEKAITSKKVLCSTGGKKHYNFKENLTNKKEMGFQQFLLSEDHLSQENELKA
VSLTTLPEQEAVNFSYSDNAVISEHVANYEQCIFGPSFDHSNGNPEQNSLACMRTLLTHE
ASKLTNHVELFKKPQDYIPRAPTFLMNQTDTHIVEKMAKNCDTERNYIDRDQKIIYSNEP
LSIVAHSQVIETTKVEKRRQNHLESETIHNIDSHSTDNMSKELANISKLSQREKKEISHK
PGMKAGRINKRNARGESQLHLAVRRGNLPLVKALIESGADVNLNDNAGWTPLHEASNEGS
IDIIVELLKAGAKVNCENIDGILPLHDAVANNHLKAAEILLQNGANPNQKDQKQKSALDE
ADDEKMKELLRSYGAIETVNRDESDAIVNEKIPAVRSKRHKQCFCDDGKTIDSSSLSHQE
RSRESLSVHQTLSAILQDIEEKQEYLLEFEIRNPEDAEQYIEKMLKIKKIMDNVLAKQKA
ERDDLAKKYRVSIESFKHGALREQLANLAARQKSLLVVAKKQKKISLKIQNCRNVTSLPC
LSLRKLPPRSEISSEKDSQELTSLENLEHPQSGSLSPVSGSMQETQLSLETWNYSQNTNI
CLNSEAVRRGEFSGNDMNSKQNGSDCTLDGFPKSRHSDGTEKNKLPSQPVAFIGQTEYSQ
KENDLTEATDKDHEFYVSSPVIGKLNISETASVLAENAAHPSNIICDQDLSNYDPKRGNR
KTSSQQSPTGASESLAHQGIAVLGSDTVHQMKPYLKKSVSVVPCADDSQISSSSGSGQQD
TIKKALNYSTAPKKKCIQIKDLILLGRINPGNNILEFKTQETTHKASILLNGKLKVESGQ
IYKNPVTWLKDLLGGNSYVTWNYAWSKVTYLGKELLRYVSEDAPILPEPNSVPQQYQPCL
PEVACLDDPVQEPNKSMFEKTKFGQGTSRESMQSSPRYLQINEILLISDQEFLPCHIMDQ
HWKFCVECEELTP
>sp|Q8N283|ANR35_HUMAN Ankyrin repeat domain-containing protein 35 OS=Homo sapiens GN=ANKRD35 PE=2 SV=2
MKRIFSCSSTQVAVERWNRHDQKLLEAVHRGDVGRVAALASRKSARPTKLDSNGQSPFHL
AASKGLTECLTILLANGADINSKNEDGSTALHLATISCQPQCVKVLLQHGANEDAVDAEN
RSPLHWAASSGCASSVLLLCDHEAFLDVLDNDGRTPLMIASLGGHAAICSQLLQRGARVN
VTDKNDKSALILACEKGSAEVAELLLSHGADAGAVDSTGHDALHYALHTQDKALWRHLQQ
ALSRRRRGGQRLVQHPDLASQASPSEPQAGSPPKSSWRAEPEEEQEEKEDEDPCSEEWRW
KYEEERRKVVRLEQELVQKTEECKTQAAAYLDLENQIREQAQELGVLLSWEPRASGKQGS
SLRPGGDGMEQGCPKDLLAESTQELKKQQQAAATVNPVLAPKKAEDSAPGKIQYEVHGRS
QPEEQGPPQSPASETIRKATGQQLTTNGAQTFGPDHADQLPAGQKESSQVLGVEPGGTVA
EPVGPAAMNQLLLQLREELAAVWREKDAARGALSRPVMEGALGTPRAEAAAAAWEKMEAR
LERVLARLEWAKAGLQVKPEVPSQESREGALKAAPGSIKQDEEKEKRVPGARGEPLGALG
GEKALGGLAKGQLEKEMSVLRLSNSNLLEELGELGRERQRLQRELQSLSQRLQREFVPKP
EAQVQLQQLRQSVGLLTNELAMEKEATEKLRKLLASQSSGLRGLWDCLPADLVGERSAQS
KAAESLEELRACISTLVDRHREAQQVLARLQEENQQLRGSLSPCREPGTSLKAPASPQVA
ALEQDLGKLEEELRAVQATMSGKSQEIGKLKQLLYQATEEVAELRAREAASLRQHEKTRG
SLVAQAQAWGQELKALLEKYNTACREVGRLREAVAEERRRSGDLAAQAAEQERQASEMRG
RSEQFEKTAELLKEKMEHLIGACRDKEAKIKELLKKLEQLSEEVLAIRGENARLALQLQD
SQKNHEEIISTYRNHLLNAARGYMEHEVYNILLQILSMEEE
>sp|Q6AI12|ANR40_HUMAN Ankyrin repeat domain-containing protein 40 OS=Homo sapiens GN=ANKRD40 PE=1 SV=2
MNALLEQKEQQERLREAAALGDIREVQKLVESGVDVNSQNEVNGWTCLHWACKRNHGQVV
SYLLKSGADKEILTTKGEMPVQLTSRREIRKIMGVEEEDDDDDDDDNLPQLKKESELPFV
PNYLANPAFPFIYTPTAEDSAQMQNGGPSTPPASPPADGSPPLLPPGEPPLLGTFPRDHT
SLALVQNGDVSAPSAILRTPESTKPGPVCQPPVSQSRSLFSSVPSKPPMSLEPQNGTYAG

PAPAFQPFFFTGAFPFNMQELVLKVRIQNPSLRENDFIEIELDRQELTYQELLRVCCCEL
GVNPDQVEKIRKLPNTLLRKDKDVARLQDFQELELVLMISENNFLFRNAASTLTERPCYN
RRASKLTY
>sp|Q8N9B4|ANR42_HUMAN Ankyrin repeat domain-containing protein 42 OS=Homo sapiens GN=ANKRD42 PE=2 SV=2
MPGVANSGPSTSSRETANPCSRKKVHFGSIHDAVRAGDVKQLSEIVCLHWLLWHGADITH
VTTRGWTASHIAAIRGQDACVQALIMNGANLTAQDDRGCTPLHLAATHGHSFTLQIMLRS
GVDPSVTDKREWRPVHYAAFHGRLGCLQLLVKWGCSIEDVDYNGNLPVHLAAMEGHLHCF
KFLVSRMSSATQVLKAFNDNGENVLDLAQRFFKQNILQFIQGAEYEGKDLEDQETLAFPG
HVAAFKGDLGMLKKLVEDGVININERADNGSTPMHKAAGQGHIECLQWLIKMGADSNITN
KAGERPSDVAKRFAHLAAVKLLEELQKYDIDDENEIDENDVKYFIRHGVEGSTDAKDDLC
LSDLDKTDARRPSKNCRASWSMNDYVEKN
>sp|Q8N8A2|ANR44_HUMAN Serine/threonine-protein phosphatase 6 regulatory ankyrin repeat subunit B OS=Homo sapiens GN=ANKRD44 PE=1 SV=3
MAVLKLTDQPPLVQAIFSGDPEEIRMLIHKTEDVNTLDSEKRTPLHVAAFLGDAEIIELL
ILSGARVNAKDNMWLTPLHRAVASRSEEAVQVLIKHSADVNARDKNWQTPLHVAAANKAV
KCAEVIIPLLSSVNVSDRGGRTALHHAALNGHVEMVNLLLAKGANINAFDKKDRRALHWA
AYMGHLDVVALLINHGAEVTCKDKKGYTPLHAAASNGQINVVKHLLNLGVEIDEINVYGN
TALHIACYNGQDAVVNELIDYGANVNQPNNNGFTPLHFAAASTHGALCLELLVNNGADVN
IQSKDGKSPLHMTAVHGRFTRSQTLIQNGGEIDCVDKDGNTPLHVAARYGHELLINTLIT
SGADTAKCGIHSMFPLHLAALNAHSDCCRKLLSSGFEIDTPDKFGRTCLHAAAAGGNVEC
IKLLQSSGADFHKKDKCGRTPLHYAAANCHFHCIETLVTTGANVNETDDWGRTALHYAAA
SDMDRNKTILGNAHDNSEELERARELKEKEATLCLEFLLQNDANPSIRDKEGYNSIHYAA
AYGHRQCLELLLERTNSGFEESDSGATKSPLHLAAYNGHHQALEVLLQSLVDLDIRDEKG
RTALDLAAFKGHTECVEALINQGASIFVKDNVTKRTPLHASVINGHTLCLRLLLEIADNP
EAVDVKDAKGQTPLMLAVAYGHIDAVSLLLEKEANVDTVDILGCTALHRGIMTGHEECVQ
MLLEQEVSILCKDSRGRTPLHYAAARGHATWLSELLQMALSEEDCCFKDNQGYTPLHWAC
YNGNENCIEVLLEQKCFRKFIGNPFTPLHCAIINDHGNCASLLLGAIDSSIVSCRDDKGR
TPLHAAAFADHVECLQLLLRHSAPVNAVDNSGKTALMMAAENGQAGAVDILVNSAQADLT
VKDKDLNTPLHLACSKGHEKCALLILDKIQDESLINEKNNALQTPLHVAARNGLKVVVEE
LLAKGACVLAVDENASRSNGPRSTPGTAVQKEE
>sp|Q8N9V6|ANR53_HUMAN Ankyrin repeat domain-containing protein 53 OS=Homo sapiens GN=ANKRD53 PE=1 SV=3
MASAGSTARRAGSGSWHSERGEGRGARPQPTPSGSMQQANKVSLKATWTDAESKQPSQPL
PDLADHLSAQATALARPRRPASLTPPRADPSPSKESDQTAIDQTAIGSYYQLFAAAVGNV
EWLRFCLNQSLREIPTDDKGFTAIHFAAQWGKLACLQVLVEEYKFPVDLLTNNSQTPLHL
VIHRDNTTVALPCIYYLLEKGADLNAQTCNGSTPLHLAARDGLLDCVKVLVQSGANVHAQ
DAMGYKPIDFCKIWNHRACARFLKDAMWKKDKKDFAREMTKMKMFKSQLTLMEHNYLIEY
QKEHKILREAAIRKWLHGKLHPGHSLVSNTKQARATALSKTPEQRESQRSRSFHPSVDAR
LQCIPQPTEMPKPIYRKPTVKRPTMWNVSNNPARPPTTQISHSQGIRLGVHPDPTPEHDF
SSFLEVRPDGHGGARLHTVDGHWVAPVPRLPFEVLLRMLYPRVWPYRMKVPQGFYPISMR
EVPRKRHLGDNTFWTDTLAMNLRDTFDEAFLAAVRSHQGLPTLPSPQTNP
>sp|Q6NXT1|ANR54_HUMAN Ankyrin repeat domain-containing protein 54 OS=Homo sapiens GN=ANKRD54 PE=1 SV=2

MAAAAGDADDEPRSGHSSSEGECAVAPEPLTDAEGLFSFADFGSALGGGGAGLSGRASGG
AQSPLRYLHVLWQQDAEPRDELRCKIPAGRLRRAARPHRRLGPTGKEVHALKRLRDSANA
NDVETVQQLLEDGADPCAADDKGRTALHFASCNGNDQIVQLLLDHGADPNQRDGLGNTPL
HLAACTNHVPVITTLLRGGARVDALDRAGRTPLHLAKSKLNILQEGHAQCLEAVRLEVKQ
IIHMLREYLERLGQHEQRERLDDLCTRLQMTSTKEQVDEVTDLLASFTSLSLQMQSMEKR
>sp|C9JTQ0|ANR63_HUMAN Ankyrin repeat domain-containing protein 63 OS=Homo sapiens GN=ANKRD63 PE=3 SV=1
MLKPKDLCPRAGTRTFLEAMQAGKVHLARFVLDALDRSIIDCRAEQGRTPLMVAVGLPDP
ALRARFVRLLLEQGAAVNLRDERGRTALSLACERGHLDAVQLLVQFSGDPEAADSAGNSP
VMWAAACGHGAVLEFLVRSFRRLGLRLDRTNRAGLTALQLAAARGHGTCVQALTGPWGRA
AAAAAARGSNSDSPPGRPAPAASPEHRRPSPRRLPRPLLARFARAAGGHGGEAGSAGKNS
GRHRAQGSERPELGRSMSLALGAVTEEEAARLRAGALMALPNSPQSSGTGRWRSQEVLEG
APPTLAQAPIGLSPHPEGGPGSGRLGLRRRSTAPDIPSLVGEAPGPESGPELEANALSVS
VPGPNPWQAGTEAVVLRAQR
>sp|P04083|ANXA1_HUMAN Annexin A1 OS=Homo sapiens GN=ANXA1 PE=1 SV=2
MAMVSEFLKQAWFIENEEQEYVQTVKSSKGGPGSAVSPYPTFNPSSDVAALHKAIMVKGV
DEATIIDILTKRNNAQRQQIKAAYLQETGKPLDETLKKALTGHLEEVVLALLKTPAQFDA
DELRAAMKGLGTDEDTLIEILASRTNKEIRDINRVYREELKRDLAKDITSDTSGDFRNAL
LSLAKGDRSEDFGVNEDLADSDARALYEAGERRKGTDVNVFNTILTTRSYPQLRRVFQKY
TKYSKHDMNKVLDLELKGDIEKCLTAIVKCATSKPAFFAEKLHQAMKGVGTRHKALIRIM
VSRSEIDMNDIKAFYQKMYGISLCQAILDETKGDYEKILVALCGGN
>sp|P12429|ANXA3_HUMAN Annexin A3 OS=Homo sapiens GN=ANXA3 PE=1 SV=3
MASIWVGHRGTVRDYPDFSPSVDAEAIQKAIRGIGTDEKMLISILTERSNAQRQLIVKEY
QAAYGKELKDDLKGDLSGHFEHLMVALVTPPAVFDAKQLKKSMKGAGTNEDALIEILTTR
TSRQMKDISQAYYTVYKKSLGDDISSETSGDFRKALLTLADGRRDESLKVDEHLAKQDAQ
ILYKAGENRWGTDEDKFTEILCLRSFPQLKLTFDEYRNISQKDIVDSIKGELSGHFEDLL
LAIVNCVRNTPAFLAERLHRALKGIGTDEFTLNRIMVSRSEIDLLDIRTEFKKHYGYSLY
SAIKSDTSGDYEITLLKICGGDD
>sp|P08758|ANXA5_HUMAN Annexin A5 OS=Homo sapiens GN=ANXA5 PE=1 SV=2
MAQVLRGTVTDFPGFDERADAETLRKAMKGLGTDEESILTLLTSRSNAQRQEISAAFKTL
FGRDLLDDLKSELTGKFEKLIVALMKPSRLYDAYELKHALKGAGTNEKVLTEIIASRTPE
ELRAIKQVYEEEYGSSLEDDVVGDTSGYYQRMLVVLLQANRDPDAGIDEAQVEQDAQALF
QAGELKWGTDEEKFITIFGTRSVSHLRKVFDKYMTISGFQIEETIDRETSGNLEQLLLAV
VKSIRSIPAYLAETLYYAMKGAGTDDHTLIRVMVSRSEIDLFNIRKEFRKNFATSLYSMI
KGDTSGDYKKALLLLCGEDD
>sp|P08133|ANXA6_HUMAN Annexin A6 OS=Homo sapiens GN=ANXA6 PE=1 SV=3
MAKPAQGAKYRGSIHDFPGFDPNQDAEALYTAMKGFGSDKEAILDIITSRSNRQRQEVCQ
SYKSLYGKDLIADLKYELTGKFERLIVGLMRPPAYCDAKEIKDAISGIGTDEKCLIEILA
SRTNEQMHQLVAAYKDAYERDLEADIIGDTSGHFQKMLVVLLQGTREEDDVVSEDLVQQD
VQDLYEAGELKWGTDEAQFIYILGNRSKQHLRLVFDEYLKTTGKPIEASIRGELSGDFEK
LMLAVVKCIRSTPEYFAERLFKAMKGLGTRDNTLIRIMVSRSELDMLDIREIFRTKYEKS
LYSMIKNDTSGEYKKTLLKLSGGDDDAAGQFFPEAAQVAYQMWELSAVARVELKGTVRPA
NDFNPDADAKALRKAMKGLGTDEDTIIDIITHRSNVQRQQIRQTFKSHFGRDLMTDLKSE
ISGDLARLILGLMMPPAHYDAKQLKKAMEGAGTDEKALIEILATRTNAEIRAINEAYKED

YHKSLEDALSSDTSGHFRRILISLATGHREEGGENLDQAREDAQVAAEILEIADTPSGDK
TSLETRFMTILCTRSYPHLRRVFQEFIKMTNYDVEHTIKKEMSGDVRDAFVAIVQSVKNK
PLFFADKLYKSMKGAGTDEKTLTRIMVSRSEIDLLNIRREFIEKYDKSLHQAIEGDTSGD
FLKALLALCGGED
>sp|O95782|AP2A1_HUMAN AP-2 complex subunit alpha-1 OS=Homo sapiens GN=AP2A1 PE=1 SV=3
MPAVSKGDGMRGLAVFISDIRNCKSKEAEIKRINKELANIRSKFKGDKALDGYSKKKYVC
KLLFIFLLGHDIDFGHMEAVNLLSSNKYTEKQIGYLFISVLVNSNSELIRLINNAIKNDL
ASRNPTFMCLALHCIANVGSREMGEAFAADIPRILVAGDSMDSVKQSAALCLLRLYKASP
DLVPMGEWTARVVHLLNDQHMGVVTAAVSLITCLCKKNPDDFKTCVSLAVSRLSRIVSSA
STDLQDYTYYFVPAPWLSVKLLRLLQCYPPPEDAAVKGRLVECLETVLNKAQEPPKSKKV
QHSNAKNAILFETISLIIHYDSEPNLLVRACNQLGQFLQHRETNLRYLALESMCTLASSE
FSHEAVKTHIDTVINALKTERDVSVRQRAADLLYAMCDRSNAKQIVSEMLRYLETADYAI
REEIVLKVAILAEKYAVDYSWYVDTILNLIRIAGDYVSEEVWYRVLQIVTNRDDVQGYAA
KTVFEALQAPACHENMVKVGGYILGEFGNLIAGDPRSSPPVQFSLLHSKFHLCSVATRAL
LLSTYIKFINLFPETKATIQGVLRAGSQLRNADVELQQRAVEYLTLSSVASTDVLATVLE
EMPPFPERESSILAKLKRKKGPGAGSALDDGRRDPSSNDINGGMEPTPSTVSTPSPSADL
LGLRAAPPPAAPPASAGAGNLLVDVFDGPAAQPSLGPTPEEAFLSELEPPAPESPMALLA
DPAPAADPGPEDIGPPIPEADELLNKFVCKNNGVLFENQLLQIGVKSEFRQNLGRMYLFY
GNKTSVQFQNFSPTVVHPGDLQTQLAVQTKRVAAQVDGGAQVQQVLNIECLRDFLTPPLL
SVRFRYGGAPQALTLKLPVTINKFFQPTEMAAQDFFQRWKQLSLPQQEAQKIFKANHPMD
AEVTKAKLLGFGSALLDNVDPNPENFVGAGIIQTKALQVGCLLRLEPNAQAQMYRLTLRT
SKEPVSRHLCELLAQQF
>sp|Q96DE5|APC16_HUMAN Anaphase-promoting complex subunit 16 OS=Homo sapiens GN=ANAPC16 PE=1 SV=1
MAASSSSSSAGGVSGSSVTGSGFSVSDLAPPRKALFTYPKGAGEMLEDGSERFLCESVFS
YQVASTLKQVKHDQQVARMEKLAGLVEELEADEWRFKPIEQLLGFTPSSG
>sp|Q8J025|APCD1_HUMAN Protein APCDD1 OS=Homo sapiens GN=APCDD1 PE=1 SV=1
MSWPRRLLLRYLFPALLLHGLGEGSALLHPDSRSHPRSLEKSAWRAFKESQCHHMLKHLH
NGARITVQMPPTIEGHWVSTGCEVRSGPEFITRSYRFYHNNTFKAYQFYYGSNRCTNPTY
TLIIRGKIRLRQASWIIRGGTEADYQLHNVQVICHTEAVAEKLGQQVNRTCPGFLADGGP
WVQDVAYDLWREENGCECTKAVNFAMHELQLIRVEKQYLHHNLDHLVEELFLGDIHTDAT
QRMFYRPSSYQPPLQNAKNHDHACIACRIIYRSDEHHPPILPPKADLTIGLHGEWVSQRC
EVRPEVLFLTRHFIFHDNNNTWEGHYYHYSDPVCKHPTFSIYARGRYSRGVLSSRVMGGT
EFVFKVNHMKVTPMDAATASLLNVFNGNECGAEGSWQVGIQQDVTHTNGCVALGIKLPHT
EYEIFKMEQDARGRYLLFNGQRPSDGSSPDRPEKRATSYQMPLVQCASSSPRAEDLAEDS
GSSLYGRAPGRHTWSLLLAALACLVPLLHWNIRR
>sp|Q96BI3|APH1A_HUMAN Gamma-secretase subunit APH-1A OS=Homo sapiens GN=APH1A PE=1 SV=1
MGAAVFFGCTFVAFGPAFALFLITVAGDPLRVIILVAGAFFWLVSLLLASVVWFILVHVT
DRSDARLQYGLLIFGAAVSVLLQEVFRFAYYKLLKKADEGLASLSEDGRSPISIRQMAYV
SGLSFGIISGVFSVINILADALGPGVVGIHGDSPYYFLTSAFLTAAIILLHTFWGVVFFD
ACERRRYWALGLVVGSHLLTSGLTFLNPWYEASLLPIYAVTVSMGLWAFITAGGSLRSIQ
RSLLCRRQEDSRVMVYSALRIPPED
>sp|Q8IW19|APLF_HUMAN Aprataxin and PNK-like factor OS=Homo sapiens GN=APLF PE=1 SV=1
MSGGFELQPRDGGPRVALAPGETVIGRGPLLGITDKRVSRRHAILEVAGGQLRIKPIHTN

PCFYQSSEKSQLLPLKPNLWCYLNPGDSFSLLVDKYIFRILSIPSEVEMQCTLRNSQVLD
EDNILNETPKSPVINLPHETTGASQLEGSTEIAKTQMTPTNSVSFLGENRDCNKQQPILA
ERKRILPTWMLAEHLSDQNLSVPAISGGNVIQGSGKEEICKDKSQLNTTQQGRRQLISSG
SSENTSAEQDTGEECKNTDQEESTISSKEMPQSFSAITLSNTEMNNIKTNAQRNKLPIEE
LGKVSKHKIATKRTPHKEDEAMSCSENCSSAQGDSLQDESQGSHSESSSNPSNPETLHAK
ATDSVLQGSEGNKVKRTSCMYGANCYRKNPVHFQHFSHPGDSDYGGVQIVGQDETDDRPE
CPYGPSCYRKNPQHKIEYRHNTLPVRNVLDEDNDNVGQPNEYDLNDSFLDDEEEDYEPTD
EDSDWEPGKEDEEKEDVEELLKEAKRFMKRK
>sp|P02656|APOC3_HUMAN Apolipoprotein C-III OS=Homo sapiens GN=APOC3 PE=1 SV=1
MQPRVLLVVALLALLASARASEAEDASLLSFMQGYMKHATKTAKDALSSVQESQVAQQAR
GWVTDGFSSLKDYWSTVKDKFSEFWDLDPEVRPTSAVAA
>sp|P02749|APOH_HUMAN Beta-2-glycoprotein 1 OS=Homo sapiens GN=APOH PE=1 SV=3
MISPVLILFSSFLCHVAIAGRTCPKPDDLPFSTVVPLKTFYEPGEEITYSCKPGYVSRGG
MRKFICPLTGLWPINTLKCTPRVCPFAGILENGAVRYTTFEYPNTISFSCNTGFYLNGAD
SAKCTEEGKWSPELPVCAPIICPPPSIPTFATLRVYKPSAGNNSLYRDTAVFECLPQHAM
FGNDTITCTTHGNWTKLPECREVKCPFPSRPDNGFVNYPAKPTLYYKDKATFGCHDGYSL
DGPEEIECTKLGNWSAMPSCKASCKVPVKKATVVYQGERVKIQEKFKNGMLHGDKVSFFC
KNKEKKCSYTEDAQCIDGTIEVPKCFKEHSSLAFWKTDASDVKPC
>sp|O14791|APOL1_HUMAN Apolipoprotein L1 OS=Homo sapiens GN=APOL1 PE=1 SV=5
MEGAALLRVSVLCIWMSALFLGVGVRAEEAGARVQQNVPSGTDTGDPQSKPLGDWAAGTM
DPESSIFIEDAIKYFKEKVSTQNLLLLLTDNEAWNGFVAAAELPRNEADELRKALDNLAR
QMIMKDKNWHDKGQQYRNWFLKEFPRLKSELEDNIRRLRALADGVQKVHKGTTIANVVSG
SLSISSGILTLVGMGLAPFTEGGSLVLLEPGMELGITAALTGITSSTMDYGKKWWTQAQA
HDLVIKSLDKLKEVREFLGENISNFLSLAGNTYQLTRGIGKDIRALRRARANLQSVPHAS
ASRPRVTEPISAESGEQVERVNEPSILEMSRGVKLTDVAPVSFFLVLDVVYLVYESKHLH
EGAKSETAEELKKVAQELEEKLNILNNNYKILQADQEL
>sp|Q8IVJ8|APRG1_HUMAN AP20 region protein 1 OS=Homo sapiens GN=APRG1 PE=2 SV=2
MKTMATRKRCKLSRTGPEFENVIKRLLCARTFHTRIGGDLTHGIINRGRRANAEQMGLQG
SAQHFNIFPLDLWTQGKKTEVQKREGTDSIPAAGRSGTANQPSIAPHRCLFSRGITALDG
LKRGRGCNGAAHLVRGDAWKTKLGEPWVSIALALAGPGAILILELSWFLG
>sp|Q8N6H7|ARFG2_HUMAN ADP-ribosylation factor GTPase-activating protein 2 OS=Homo sapiens GN=ARFGAP2 PE=1 SV=1
MAAEPNKTEIQTLFKRLRAVPTNKACFDCGAKNPSWASITYGVFLCIDCSGVHRSLGVHL
SFIRSTELDSNWNWFQLRCMQVGGNANATAFFRQHGCTANDANTKYNSRAAQMYREKIRQ
LGSAALARHGTDLWIDNMSSAVPNHSPEKKDSDFFTEHTQPPAWDAPATEPSGTQQPAPS
TESSGLAQPEHGPNTDLLGTSPKASLELKSSIIGKKKPAAAKKGLGAKKGLGAQKVSSQS
FSEIERQAQVAEKLREQQAADAKKQAEESMVASMRLAYQELQIDRKKEEKKLQNLEGKKR
EQAERLGMGLVSRSSVSHSVLSEMQVIEQETPVSAKSSRSQLDLFDDVGTFASGPPKYKD
NPFSLGESFGSRWDTDAAWGMDRVEEKEPEVTISSIRPISERATNRREVESRSSGLESSE
ARQKFAGAKAISSDMFFGREVDAEYEARSRLQQLSGSSAISSSDLFGDMDGAHGAGSVSL
GNVLPTADIAQFKQGVKSVAGKMAVLANGVMNSLQDRYGSY
>sp|A8MVX0|ARG33_HUMAN Rho guanine nucleotide exchange factor 33 OS=Homo sapiens GN=ARHGEF33 PE=2 SV=2
MEKTKTKQGENEHMPVNNPSTQIYQLQALASELKTGFTEAMQELSRIQHGEYALEEKVKS

CRCSMEEKVTEMKNSLNYFKEELSNAMSMIQAITSKQEEMQQKIEQLQQEKRRESRKVKA
KKTQKEEHSSQAGPAQAQGSPFRSINIPEPVLPSEDFTNLLPSQAYEKAQESRSVHVGDS
NVKGMMGPGVNPTTPEAEENLKSCLSADIQSKGHLPSGMWRQPKDGKEWGEEYVTKDHPD
KLKEAGQGRHSSLENVLCETSLAAKRQTVALELLESERKYVINISLILKIKATFQGSDGK
RNSKERSLFPGSLRYLVQQHLDLLHALQERVLKWPRQGVLGDLFLKLTNDENNFLDYYVA
YLRDLPECISLVHVVVLKEGDEEIKSDIYTLFFHIVQRIPEYLIHLQNVLKFTEQEHPDY
YLLLVCVQRLRVFISHYTLLFQCNEDLLIQKRKKLKKSSMAKLYKGLASQCANAGQDASP
TAGPEAVRDTGIHSEELLQPYPSAPSSGPAITHLMPPVKKSQQQQSLMESMQPGKPSDWE
LEGRKHERPESLLAPTQFCAAEQDVKALAGPLQAIPEMDFESSPAEPLGNVERSLRAPAE
LLPDARGFVPAAYEEFEYGGEIFALPAPYDEEPFQAPALFENCSPASSESSLDICFLRPV
SFAMEAERPEHPLQPLPKSATSPAGSSSAYKLEAAAQAHGKAKPLSRSLKEFPRAPPADG
VAPRLYSTRSSSGGRAPIKAERAAQAHGPAAAAVAARGASRTFFPQQRSQSEKQTYLEVR
REMHLEDTTRFCPKEERESEQTSFSDQNPRQDQKGGFRSSFRKLFKKKNGNATGEDFCGP
WGWW
>sp|Q8N4T4|ARG39_HUMAN Rho guanine nucleotide exchange factor 39 OS=Homo sapiens GN=ARHGEF39 PE=1 SV=1
MELSCPGSRCPVQEQRARWERKRACTARELLETERRYQEQLGLVATYFLGILKAKGTLRP
PERQALFGSWELIYGASQELLPYLEGGCWGQGLEGFCRHLELYNQFAANSERSQTTLQEQ
LKKNKGFRRFVRLQEGRPEFGGLQLQDLLPLPLQRLQQYENLVVALAENTGPNSPDHQQL
TRAARLISETAQRVHTIGQKQKNDQHLRRVQALLSGRQAKGLTSGRWFLRQGWLLVVPPH
GEPRPRMFFLFTDVLLMAKPRPPLHLLRSGTFACKALYPMAQCHLSRVFGHSGGPCGGLL
SLSFPHEKLLLMSTDQEELSRWYHSLTWAISSQKN
>sp|A1IGU5|ARH37_HUMAN Rho guanine nucleotide exchange factor 37 OS=Homo sapiens GN=ARHGEF37 PE=2 SV=2
MAKHGADEPSSRSGSPDREGRASEDRSLLHQRLAVRELIDTEVSYLHMLQLCASDIRSRL
QQLPQGDLDVLFSNIDDIIKVNSRFLHDLQETASKEEEQVQLVGNIFLEFQEELEQVYKV
YCASYDQALLLVDTYRKEPELQRHIQGIVEAVVPQAGSSGLSFLLVIPLQRITRYPLLLQ
KILENTVPDASAYPVLQRAVSALQDVNTNINEYKMRKEVASKYTKVEQLTLRERLARINT
HTLSKKTTRLSQLLKQEAGLIPRTEDKEFDDLEERFQWVSLCVTELKNNVAAYLDNLQAF
LYFRPHEYNLDIPEGPAVQYCNLARDLHLEAFLKFKQRLEGLVWQPLCSLAKALLGPQNL
IKKRLDKLLDFERVEEKLLEVGSVTYQEEAARHTYQALNSLLVAELPQFNQLVMQWLGQI
MCTFVTLQRDLAKQVLQRAEGSMAQLPHHHVPEPAFRKLVEDALGRTSNQLRSFQETFEK
VQPPPTTQPLLPGSERQVQALLSRYGPGKLYQVTSNISGTGTLDLTLPRGQIVAILQNKD
TKGNSGRWLVDTGGHRGYVPAGKLQLYHVVPSAEELRRQAGLNKDPRCLTPEPSPALVPS
IPTMNQVIAAYPFVARSSHEVSLQAGQPVTILEAQDKKGNPEWSLVEVNGQRGYVPSGFL
ARARSPVLWGWSLPS
>sp|Q92974|ARHG2_HUMAN Rho guanine nucleotide exchange factor 2 OS=Homo sapiens GN=ARHGEF2 PE=1 SV=4
MSRIESLTRARIDRSRELASKTREKEKMKEAKDARYTNGHLFTTISVSGMTMCYACNKSI
TAKEALICPTCNVTIHNRCKDTLANCTKVKQKQQKAALLKNNTALQSVSLRSKTTIRERP
SSAIYPSDSFRQSLLGSRRGRSSLSLAKSVSTTNIAGHFNDESPLGLRRILSQSTDSLNM
RNRTLSVESLIDEAEVIYSELMSDFEMDEKDFAADSWSLAVDSSFLQQHKKEVMKQQDVI
YELIQTELHHVRTLKIMTRLFRTGMLEELHLEPGVVQGLFPCVDELSDIHTRFLSQLLER
RRQALCPGSTRNFVIHRLGDLLISQFSGPSAEQMCKTYSEFCSRHSKALKLYKELYARDK

RFQQFIRKVTRPAVLKRHGVQECILLVTQRITKYPLLISRILQHSHGIEEERQDLTTALG
LVKELLSNVDEGIYQLEKGARLQEIYNRMDPRAQTPVPGKGPFGREELLRRKLIHDGCLL
WKTATGRFKDVLVLLMTDVLVFLQEKDQKYIFPTLDKPSVVSLQNLIVRDIANQEKGMFL
ISAAPPEMYEVHTASRDDRSTWIRVIQQSVRTCPSREDFPLIETEDEAYLRRIKMELQQK
DRALVELLREKVGLFAEMTHFQAEEDGGSGMALPTLPRGLFRSESLESPRGERLLQDAIR
EVEGLKDLLVGPGVELLLTPREPALPLEPDSGGNTSPGVTANGEARTFNGSIELCRADSD
SSQRDRNGNQLRSPQEEALQRLVNLYGLLHGLQAAVAQQDTLMEARFPEGPERREKLCRA
NSRDGEAGRAGAAPVAPEKQATELALLQRQHALLQEELRRCRRLGEERATEAGSLEARLR
ESEQARALLEREAEEARRQLAALGQTEPLPAEAPWARRPVDPRRRSLPAGDALYLSFNPP
QPSRGTDRLDLPVTTRSVHRNFEDRERQELGSPEERLQDSSDPDTGSEEEGSSRLSPPHS
PRDFTRMQDIPEETESRDGEAVASES
>sp|Q8NFD5|ARI1B_HUMAN AT-rich interactive domain-containing protein 1B OS=Homo sapiens GN=ARID1B PE=1 SV=2
MAHNAGAAAAAGTHSAKSGGSEAALKEGGSAAALSSSSSSSAAAAAASSSSSSGPGSAME
TGLLPNHKLKTVGEAPAAPPHQQHHHHHHAHHHHHHAHHLHHHHALQQQLNQFQQQQQQQ
QQQQQQQQQQQHPISNNNSLGGAGGGAPQPGPDMEQPQHGGAKDSAAGGQADPPGPPLLS
KPGDEDDAPPKMGEPAGGRYEHPGLGALGTQQPPVAVPGGGGGPAAVPEFNNYYGSAAPA
SGGPGGRAGPCFDQHGGQQSPGMGMMHSASAAAAGAPGSMDPLQNSHEGYPNSQCNHYPG
YSRPGAGGGGGGGGGGGGGSGGGGGGGGAGAGGAGAGAVAAAAAAAAAAAGGGGGGGYGG
SSAGYGVLSSPRQQGGGMMMGPGGGGAASLSKAAAGSAAGGFQRFAGQNQHPSGATPTLN
QLLTSPSPMMRSYGGSYPEYSSPSAPPPPPSQPQSQAAAAGAAAGGQQAAAGMGLGKDMG
AQYAAASPAWAAAQQRSHPAMSPGTPGPTMGRSQGSPMDPMVMKRPQLYGMGSNPHSQPQ
QSSPYPGGSYGPPGPQRYPIGIQGRTPGAMAGMQYPQQQMPPQYGQQGVSGYCQQGQQPY
YSQQPQPPHLPPQAQYLPSQSQQRYQPQQDMSQEGYGTRSQPPLAPGKPNHEDLNLIQQE
RPSSLPDLSGSIDDLPTGTEATLSSAVSASGSTSSQGDQSNPAQSPFSPHASPHLSSIPG
GPSPSPVGSPVGSNQSRSGPISPASIPGSQMPPQPPGSQSESSSHPALSQSPMPQERGFM
AGTQRNPQMAQYGPQQTGPSMSPHPSPGGQMHAGISSFQQSNSSGTYGPQMSQYGPQGNY
SRPPAYSGVPSASYSGPGPGMGISANNQMHGQGPSQPCGAVPLGRMPSAGMQNRPFPGNM
SSMTPSSPGMSQQGGPGMGPPMPTVNRKAQEAAAAVMQAAANSAQSRQGSFPGMNQSGLM
ASSSPYSQPMNNSSSLMNTQAPPYSMAPAMVNSSAASVGLADMMSPGESKLPLPLKADGK
EEGTPQPESKSKKSSSSTTTGEKITKVYELGNEPERKLWVDRYLTFMEERGSPVSSLPAV
GKKPLDLFRLYVCVKEIGGLAQVNKNKKWRELATNLNVGTSSSAASSLKKQYIQYLFAFE
CKIERGEEPPPEVFSTGDTKKQPKLQPPSPANSGSLQGPQTPQSTGSNSMAEVPGDLKPP
TPASTPHGQMTPMQGGRSSTISVHDPFSDVSDSSFPKRNSMTPNAPYQQGMSMPDVMGRM
PYEPNKDPFGGMRKVPGSSEPFMTQGQMPNSSMQDMYNQSPSGAMSNLGMGQRQQFPYGA
SYDRRHEPYGQQYPGQGPPSGQPPYGGHQPGLYPQQPNYKRHMDGMYGPPAKRHEGDMYN
MQYSSQQQEMYNQYGGSYSGPDRRPIQGQYPYPYSRERMQGPGQIQTHGIPPQMMGGPLQ
SSSSEGPQQNMWAARNDMPYPYQNRQGPGGPTQAPPYPGMNRTDDMMVPDQRINHESQWP
SHVSQRQPYMSSSASMQPITRPPQPSYQTPPSLPNHISRAPSPASFQRSLENRMSPSKSP
FLPSMKMQKVMPTVPTSQVTGPPPQPPPIRREITFPPGSVEASQPVLKQRRKITSKDIVT
PEAWRVMMSLKSGLLAESTWALDTINILLYDDSTVATFNLSQLSGFLELLVEYFRKCLID
IFGILMEYEVGDPSQKALDHNAARKDDSQSLADDSGKEEEDAECIDDDEEDEEDEEEDSE
KTESDEKSSIALTAPDAAADPKEKPKQASKFDKLPIKIVKKNNLFVVDRSDKLGRVQEFN
SGLLHWQLGGGDTTEHIQTHFESKMEIPPRRRPPPPLSSAGRKKEQEGKGDSEEQQEKSI

IATIDDVLSARPGALPEDANPGPQTESSKFPFGIQQAKSHRNIKLLEDEPRSRDETPLCT
IAHWQDSLAKRCICVSNIVRSLSFVPGNDAEMSKHPGLVLILGKLILLHHEHPERKRAPQ
TYEKEEDEDKGVACSKDEWWWDCLEVLRDNTLVTLANISGQLDLSAYTESICLPILDGLL
HWMVCPSAEAQDPFPTVGPNSVLSPQRLVLETLCKLSIQDNNVDLILATPPFSRQEKFYA
TLVRYVGDRKNPVCREMSMALLSNLAQGDALAARAIAVQKGSIGNLISFLEDGVTMAQYQ
QSQHNLMHMQPPPLEPPSVDMMCRAAKALLAMARVDENRSEFLLHEGRLLDISISAVLNS
LVASVICDVLFQIGQL
>sp|Q8N4G2|ARL14_HUMAN ADP-ribosylation factor-like protein 14 OS=Homo sapiens GN=ARL14 PE=1 SV=2
MGSLGSKNPQTKQAQVLLLGLDSAGKSTLLYKLKLAKDITTIPTIGFNVEMIELERNLSL
TVWDVGGQEKMRTVWGCYCENTDGLVYVVDSTDKQRLEESQRQFEHILKNEHIKNVPVVL
LANKQDMPGALTAEDITRMFKVKKLCSDRNWYVQPCCALTGEGLAQGFRKLTGFVKSHMK
SRGDTLAFFKQN
>sp|P40617|ARL4A_HUMAN ADP-ribosylation factor-like protein 4A OS=Homo sapiens GN=ARL4A PE=1 SV=2
MGNGLSDQTSILSNLPSFQSFHIVILGLDCAGKTTVLYRLQFNEFVNTVPTKGFNTEKIK
VTLGNSKTVTFHFWDVGGQEKLRPLWKSYTRCTDGIVFVVDSVDVERMEEAKTELHKITR
ISENQGVPVLIVANKQDLRNSLSLSEIEKLLAMGELSSSTPWHLQPTCAIIGDGLKEGLE
KLHDMIIKRRKMLRQQKKKR
>sp|Q9H0F7|ARL6_HUMAN ADP-ribosylation factor-like protein 6 OS=Homo sapiens GN=ARL6 PE=1 SV=1
MGLLDRLSVLLGLKKKEVHVLCLGLDNSGKTTIINKLKPSNAQSQNILPTIGFSIEKFKS
SSLSFTVFDMSGQGRYRNLWEHYYKEGQAIIFVIDSSDRLRMVVAKEELDTLLNHPDIKH
RRIPILFFANKMDLRDAVTSVKVSQLLCLENIKDKPWHICASDAIKGEGLQEGVDWLQDQ
IQTVKT
>sp|Q6NXE6|ARMC6_HUMAN Armadillo repeat-containing protein 6 OS=Homo sapiens GN=ARMC6 PE=1 SV=2
MSERCCSRYSSGASIGCTPTSTQAKMVSKRIAQETFDAAVRENIEEFAMGPEEAVKEAVE
QFESQGVDLSNIVKTAPKVSADGSQEPTHDILQMLSDLQESVASSRPQEVSAYLTRFCDQ
CKQDKACRFLAAQKGAYPIIFTAWKLATAGDQGLLLQSLNALSVLTDGQPDLLDAQGLQL
LVATLTQNADEADLTCSGIRCVRHACLKHEQNRQDLVKAGVLPLLTGAITHHGHHTDVVR
EACWALRVMTFDDDIRVPFGHAHNHAKMIVQENKGLKVLIEATKAFLDNPGILSELCGTL
SRLAIRNEFCQEVVDLGGLSILVSLLADCNDHQMRDQSGVQELVKQVLSTLRAIAGNDDV
KDAIVRAGGTESIVAAMTQHLTSPQVCEQSCAALCFLALRKPDNSRIIVEGGGAVAALQA
MKAHPQKAGVQKQACMLIRNLVAHGQAFSKPILDLGAEALIMQARSAHRDCEDVAKAALR
DLGCHVELRELWTGQRGNLAP
>sp|Q5H9R4|ARMX4_HUMAN Armadillo repeat-containing X-linked protein 4 OS=Homo sapiens GN=ARMCX4 PE=2 SV=2
MSAAGLKITGSKETKRRLLLISIDWSRDLMNLCIYFRVYCQEKQEERRELPRIITGPPPE
AAVVAFEWLKTSTLTGLHPQLPLSLPQPECALPYLVRAFSRGDYMGRIQEVGWVTAGLVI
WAGTCYYIYKFTKGRAQSVRTLARNGSTVKMETVVGVQSQTLAINEAEIKTKPQVEIGAE
TGARSGPRAEVETKATAIAIHRANSQAKAMVGAEPETQSESKVVAGTLVMTEAVTLTEVK
AKAREVAMKEAVTQTDAEAGKIVKKEAVTQTKAKAWALVAKTEAKREAMTQTKAETHILA
EKETEINRVMVTQSETLAVPREVAKMGATNKTGIVDETKTRALEETVLIPRAFPSKNASC

>sp|Q9NZ32|ARP10_HUMAN Actin-related protein 10 OS=Homo sapiens GN=ACTR10 PE=1 SV=1
MPLYEGLGSGGEKTAVVIDLGEAFTKCGFAGETGPRCIIPSVIKRAGMPKPVRVVQYNIN
TEELYSYLKEFIHILYFRHLLVNPRDRRVVIIESVLCPSHFRETLTRVLFKYFEVPSVLL
APSHLMALLTLGINSAMVLDCGYRESLVLPIYEGIPVLNCWGALPLGGKALHKELETQLL
EQCTVDTSVAKEQSLPSVMGSVPEGVLEDIKARTCFVSDLKRGLKIQAAKFNIDGNNERP
SPPPNVDYPLDGEKILHILGSIRDSVVEILFEQDNEEQSVATLILDSLIQCPIDTRKQLA
ENLVVIGGTSMLPGFLHRLLAEIRYLVEKPKYKKALGTKTFRIHTPPAKANCVAWLGGAI
FGALQDILGSRSVSKEYYNQTGRIPDWCSLNNPPLEMMFDVGKTQPPLMKRAFSTEK
>sp|Q9Y575|ASB3_HUMAN Ankyrin repeat and SOCS box protein 3 OS=Homo sapiens GN=ASB3 PE=1 SV=1
MDFTEAYADTCSTVGLAAREGNVKVLRKLLKKGRSVDVADNRGWMPIHEAAYHNSVECLQ
MLINADSSENYIKMKTFEGFCALHLAASQGHWKIVQILLEAGADPNATTLEETTPLFLAV
ENGQIDVLRLLLQHGANVNGSHSMCGWNSLHQASFQENAEIIKLLLRKGANKECQDDFGI
TPLFVAAQYGKLESLSILISSGANVNCQALDKATPLFIAAQEGHTKCVELLLSSGADPDL
YCNEDSWQLPIHAAAQMGHTKILDLLIPLTNRACDTGLNKVSPVYSAVFGGHEDCLEILL
RNGYSPDAQACLVFGFSSPVCMAFQKDCEFFGIVNILLKYGAQINELHLAYCLKYEKFSI
FRYFLRKGCSLGPWNHIYEFVNHAIKAQAKYKEWLPHLLVAGFDPLILLCNSWIDSVSID
TLIFTLEFTNWKTLAPAVERMLSARASNAWILQQHIATVPSLTHLCRLEIRSSLKSERLR
SDSYISQLPLPRSLHNYLLYEDVLRMYEVPELAAIQDG
>sp|P15514|AREG_HUMAN Amphiregulin OS=Homo sapiens GN=AREG PE=1 SV=2
MRAPLLPPAPVVLSLLILGSGHYAAGLDLNDTYSGKREPFSGDHSADGFEVTSRSEMSSG
SEISPVSEMPSSSEPSSGADYDYSEEYDNEPQIPGYIVDDSVRVEQVVKPPQNKTESENT
SDKPKRKKKGGKNGKNRRNRKKKNPCNAEFQNFCIHGECKYIEHLEAVTCKCQQEYFGER
CGEKSMKTHSMIDSSLSKIALAAIAAFMSAVILTAVAVITVQLRRQYVRKYEGEAEERKK
LRQENGNVHAIA
>sp|P18085|ARF4_HUMAN ADP-ribosylation factor 4 OS=Homo sapiens GN=ARF4 PE=1 SV=3
MGLTISSLFSRLFGKKQMRILMVGLDAAGKTTILYKLKLGEIVTTIPTIGFNVETVEYKN
ICFTVWDVGGQDRIRPLWKHYFQNTQGLIFVVDSNDRERIQEVADELQKMLLVDELRDAV
LLLFANKQDLPNAMAISEMTDKLGLQSLRNRTWYVQATCATQGTGLYEGLDWLSNELSKR
>sp|A5YM69|ARG35_HUMAN Rho guanine nucleotide exchange factor 35 OS=Homo sapiens GN=ARHGEF35 PE=1 SV=1
MEAEEAQHGASPPISAIEEFSIIPEAPMRSSQVSALGLEAQEDEDPSYKWREEHRLSATQ
QSELRDVCDYAIETMPSFPKEGSADVEPNQESLVAEACDTPEHWEAVPQSLAGRQARTLA
PPELWACPIQSEHLDMAPFSSDLGSEEEEVEFWPGLTSLTLGSGQAEEEEETSSDNSGQT
RYYSPCEEHPAETNQNEGAESGTIRQGEELPSEELQESQGLLHPQEVQVLEEQGQQEAGF
RGEGTLREDVCADGLLGEEQMIEQVNDEKGEQKQKQEQVQDVMLGRQGERMGLTGEPEGL
NDGEWEQEDMERKAQGQGGPEQGEERKRELQVPEENRADSQDEKSQTFLGKSEEVTGKQE
DHGIKEKGVPVSGQEAKEPESWDGGRLGAVGRARSREEENEHHGPSMPALIAPEDSPHCD
LFPGASYLVTQIPGTQTESRAEELSPAALSPLLEPIRCSHQPISLLGSFLTEESPDKEKL
LSVL
>sp|Q15052|ARHG6_HUMAN Rho guanine nucleotide exchange factor 6 OS=Homo sapiens GN=ARHGEF6 PE=1 SV=2
MNPEEQIVTWLISLGVLESPKKTICDPEEFLKSSLKNGVVLCKLINRLMPGSVEKFCLDP
QTEADCINNINDFLKGCATLQVEIFDPDDLYSGVNFSKVLSTLLAVNKATEDQLSERPCG

RSSSLSAANTSQTNPQGAVSSTVSGLQRQSKTVEMTENGSHQLIVKARFNFKQTNEDELS
VCKGDIIYVTRVEEGGWWEGTLNGRTGWFPSNYVREIKSSERPLSPKAVKGFETAPLTKN
YYTVVLQNILDTEKEYAKELQSLLVTYLRPLQSNNNLSTVEVTSLLGNFEEVCTFQQTLC
QALEECSKFPENQHKVGGCLLSLMPHFKSMYLAYCANHPSAVNVLTQHSDELEQFMENQG
ASSPGILILTTNLSKPFMRLEKYVTLLQELERHMEDTHPDHQDILKAIVAFKTLMGQCQD
LRKRKQLELQILSEPIQAWEGEDIKNLGNVIFMSQVMVQYGACEEKEERYLMLFSNVLIM
LSASPRMSGFIYQGKIPIAGTVVTRLDEIEGNDCTFEITGNTVERIVVHCNNNQDFQEWL
EQLNRLIRGPASCSSLSKTSSSSCSAHSSFSSTGQPRGPLEPPQIIKPWSLSCLRPAPPL
RPSAALGYKERMSYILKESSKSPKTMKKFLHKRKTERKPSEEEYVIRKSTAALEEDAQIL
KVIEAYCTSANFQQGHGSSTRKDSIPQVLLPEEEKLIIEETRSNGQTIMEEKSLVDTVYA
LKDEVRELKQENKRMKQCLEEELKSRRDLEKLVRRLLKQTDECIRGESSSKTSILP
>sp|Q5VV41|ARHGG_HUMAN Rho guanine nucleotide exchange factor 16 OS=Homo sapiens GN=ARHGEF16 PE=1 SV=1
MAQRHSDSSLEEKLLGHRFHSELRLDAGGNPASGLPMVRGSPRVRDDAAFQPQVPAPPQP
RPPGHEEPWPIVLSTESPAALKLGTQQLIPKSLAVASKAKTPARHQSFGAAVLSREAARR
DPKLLPAPSFSLDDMDVDKDPGGMLRRNLRNQSYRAAMKGLGKPGGQGDAIQLSPKLQAL
AEEPSQPHTRSPAKNKKTLGRKRGHKGSFKDDPQLYQEIQERGLNTSQESDDDILDESSS
PEGTQKVDATIVVKSYRPAQVTWSQLPEVVELGILDQLSTEERKRQEAMFEILTSEFSYQ
HSLSILVEEFLQSKELRATVTQMEHHHLFSNILDVLGASQRFFEDLEQRHKAQVLVEDIS
DILEEHAEKHFHPYIAYCSNEVYQQRTLQKLISSNAAFREALREIERRPACGGLPMLSFL
ILPMQRVTRLPLLMDTLCLKTQGHSERYKAASRALKAISKLVRQCNEGAHRMERMEQMYT
LHTQLDFSKVKSLPLISASRWLLKRGELFLVEETGLFRKIASRPTCYLFLFNDVLVVTKK
KSEESYMVQDYAQMNHIQVEKIEPSELPLPGGGNRSSSVPHPFQVTLLRNSEGRQEQLLL
SSDSASDRARWIVALTHSERQWQGLSSKGDLPQVEITKAFFAKQADEVTLQQADVVLVLQ
QEDGWLYGERLRDGETGWFPEDFARFITSRVAVEGNVRRMERLRVETDV
>sp|Q96DR7|ARHGQ_HUMAN Rho guanine nucleotide exchange factor 26 OS=Homo sapiens GN=ARHGEF26 PE=1 SV=4
MDGESEVDFSSNSITPLWRRRSIPQPHQVLGRSKPRPQSYQSPNGLLITDFPVEDGGTLL
AAQIPAQVPTASDSRTVHRSPLLLGAQRRAVANGGTASPEYRAASPRLRRPKSPKLPKAV
PGGSPKSPANGAVTLPAPPPPPVLRPPRTPNAPAPCTPEEDLTGLTASPVPSPTANGLAA
NNDSPGSGSQSGRKAKDPERGLFPGPQKSSSEQKLPLQRLPSQENELLENPSVVLSTNSP
AALKVGKQQIIPKSLASEIKISKSNNQNVEPHKRLLKVRSMVEGLGGPLGHAGEESEVDN
DVDSPGSLRRGLRSTSYRRAVVSGFDFDSPTSSKKKNRMSQPVLKVVMEDKEKFSSLGRI
KKKMLKGQGTFDGEENAVLYQNYKEKALDIDSDEESEPKEQKSDEKIVIHHKPLRSTWSQ
LSAVKRKGLSQTVSQEERKRQEAIFEVISSEHSYLLSLEILIRMFKNSKELSDTMTKTER
HHLFSNITDVCEASKKFFIELEARHQNNIFIDDISDIVEKHTASTFDPYVKYCTNEVYQQ
RTLQKLLATNPSFKEVLSRIESHEDCRNLPMISFLILPMQRVTRLPLLMDTICQKTPKDS
PKYEVCKRALKEVSKLVRLCNEGARKMERTEMMYTINSQLEFKIKPFPLVSSSRWLVKRG
ELTAYVEDTVLFSRRTSKQQVYFFLFNDVLIITKKKSEESYNVNDYSLRDQLLVESCDNE
ELNSSPGKNSSTMLYSRQSSASHLFTLTVLSNHANEKVEMLLGAETQSERARWITALGHS
SGKPPADRTSLTQVEIVRSFTAKQPDELSLQVADVVLIYQRVSDGWYEGERLRDGERGWF
PMECAKEITCQATIDKNVERMGRLLGLETNV
>sp|Q9NX46|ARHL2_HUMAN Poly(ADP-ribose) glycohydrolase ARH3 OS=Homo sapiens GN=ADPRHL2 PE=1 SV=1

MAAAAMAAAAGGGAGAARSLSRFRGCLAGALLGDCVGSFYEAHDTVDLTSVLRHVQSLEP
DPGTPGSERTEALYYTDDTAMARALVQSLLAKEAFDEVDMAHRFAQEYKKDPDRGYGAGV
VTVFKKLLNPKCRDVFEPARAQFNGKGSYGNGGAMRVAGISLAYSSVQDVQKFARLSAQL
THASSLGYNGAILQALAVHLALQGESSSEHFLKQLLGHMEDLEGDAQSVLDARELGMEER
PYSSRLKKIGELLDQASVTREEVVSELGNGIAAFESVPTAIYCFLRCMEPDPEIPSAFNS
LQRTLIYSISLGGDTDTIATMAGAIAGAYYGMDQVPESWQQSCEGYEETDILAQSLHRVF
QKS
>sp|Q5SW96|ARH_HUMAN Low density lipoprotein receptor adapter protein 1 OS=Homo sapiens GN=LDLRAP1 PE=1 SV=3
MDALKSAGRALIRSPSLAKQSWGGGGRHRKLPENWTDTRETLLEGMLFSLKYLGMTLVEQ
PKGEELSAAAIKRIVATAKASGKKLQKVTLKVSPRGIILTDNLTNQLIENVSIYRISYCT
ADKMHDKVFAYIAQSQHNQSLECHAFLCTKRKMAQAVTLTVAQAFKVAFEFWQVSKEEKE
KRDKASQEGGDVLGARQDCTPSLKSLVATGNLLDLEETAKAPLSTVSANTTNMDEVPRPQ
ALSGSSVVWELDDGLDEAFSRLAQSRTNPQVLDTGLTAQDMHYAQCLSPVDWDKPDSSGT
EQDDLFSF
>sp|Q8NHP1|ARK74_HUMAN Aflatoxin B1 aldehyde reductase member 4 OS=Homo sapiens GN=AKR7L PE=2 SV=6
MSRQLSRARPATVLGAMEMGRRMDAPTSAAVTRAFLERGHTEIDTAFLYSDGQSETILGG
LGLRMGSSDCRVKIATKANPWIGNSLKPDSVRSQLETSLKRLQCPUVDLFYLHAPDHSAP
VEETLRACHQLHQEGKFVELGLSNYAAWEVAEICTLCKSNGWILPTVYQGMYSATTRQVE
TELFPCLRHFGLRFYAYNPLAGGLLTGKYKYEDKDGKQPVGRFFGTQWAEIYRNHFWKEH
HFEGIALVEKALQAAYGASAPSMTSAALRWMYHHSQLQGAHGDAVILGMSSLEQLEQNLA
AAEEGPLEPAVVDAFNQAWHLFAHECPNYFI
>sp|Q0P5N6|ARL16_HUMAN ADP-ribosylation factor-like protein 16 OS=Homo sapiens GN=ARL16 PE=1 SV=1
MRVAGGRALSRGAELRVPGGAKHGMCLLLGATGVGKTLLVKRLQEVSSRDGKGDLGEPPP
TRPTVGTNLTDIVAQRKITIRELGGCMGPIWSSYYGNCRSLLFVMDASDPTQLSASCVQL
LGLLSAEQLAEASVLILFNKIDLPCYMSTEEMKSLIRLPDIIACAKQNITTAEISAREGT
GLAGVLAWLQATHRAND
>sp|P40616|ARL1_HUMAN ADP-ribosylation factor-like protein 1 OS=Homo sapiens GN=ARL1 PE=1 SV=1
MGGFFSSIFSSLFGTREMRILILGLDGAGKTTILYRLQVGEVVTTIPTIGFNVETVTYKN
LKFQVWDLGGQTSIRPYWRCYYSNTDAVIYVVDSCDRDRIGISKSELVAMLEEEELRKAI
LVVFANKQDMEQAMTSSEMANSLGLPALKDRKWQIFKTSATKGTGLDEAMEWLVETLKSR
Q
>sp|P36405|ARL3_HUMAN ADP-ribosylation factor-like protein 3 OS=Homo sapiens GN=ARL3 PE=1 SV=2
MGLLSILRKLKSAPDQEVRILLLGLDNAGKTTLLKQLASEDISHITPTQGFNIKSVQSQG
FKLNVWDIGGQRKIRPYWKNYFENTDILIYVIDSADRKRFEETGQELAELLEEEKLSCVP
VLIFANKQDLLTAAPASEIAEGLNLHTIRDRVWQIQSCSALTGEGVQDGMNWVCKNVNAK
KK
>sp|Q9Y689|ARL5A_HUMAN ADP-ribosylation factor-like protein 5A OS=Homo sapiens GN=ARL5A PE=1 SV=1
MGILFTRIWRLFNHQEHKVIIVGLDNAGKTTILYQFSMNEVVHTSPTIGSNVEEIVINNT

RFLMWDIGGQESLRSSWNTYYTNTEFVIVVVDSTDRERISVTREELYKMLAHEDLRKAGL
LIFANKQDVKECMTVAEISQFLKLTSIKDHQWHIQACCALTGEGLCQGLEWMMSRLKIR
>sp|A6NH57|ARL5C_HUMAN Putative ADP-ribosylation factor-like protein 5C OS=Homo sapiens GN=ARL5C PE=3 SV=4
MGQLIAKLMSIFGNQEHTVIIVGLDNEGKTTILYRFLTNEVVHMCPTIGSNVEEIILPKT
HFFMWDIVRPEALSFIWNTYYSNTEFIILVIDSTDRDRLLTTREELYKMLAHEALQDASV
LIFANKQDVKDSMRMVEISHFLTLSTIKDHSWHIQGCCALTREGLPARLQWMESQAAAN
>sp|Q9NVJ2|ARL8B_HUMAN ADP-ribosylation factor-like protein 8B OS=Homo sapiens GN=ARL8B PE=1 SV=1
MLALISRLLDWFRSLFWKEEMELTLVGLQYSGKTTFVNVIASGQFSEDMIPTVGFNMRKV
TKGNVTIKIWDIGGQPRFRSMWERYCRGVNAIVYMIDAADREKIEASRNELHNLLDKPQL
QGIPVLVLGNKRDLPNALDEKQLIEKMNLSAIQDREICCYSISCKEKDNIDITLQWLIQH
SKSRRS
>sp|Q9UBL0|ARP21_HUMAN cAMP-regulated phosphoprotein 21 OS=Homo sapiens GN=ARPP21 PE=1 SV=2
MSEQGDLNQAIAEEGGTEQETATPENGIVKSESLDEEEKLELQRRLEAQNQERRKSKSGA
GKGKLTRSLAVCEESSARPGGESLQDQESIHLQLSSFSSLQEEDKSRKDDSEREKEKDKN
KDKTSEKPKIRMLSKDCSQEYTDSTGIDLHEFLINTLKNNSRDRMILLKMEQEIIDFIAD
NNNHYKKFPQMSSYQRMLVHRVAAYFGLDHNVDQTGKSVIINKTSSTRIPEQRFCEHLKD
EKGEESQKRFILKRDNSSIDKEDNQQNRMHPFRDDRRSKSIEEREEEYQRVRERIFAHDS
VCSQESLFVENSRLLEDSNICNETYKKRQLFRGNRDGSGRTSGSRQSSSENELKWSDHQR
AWSSTDSDSSNRNLKPAMTKTASFGGITVLTRGDSTSSTRSTGKLSKAGSESSSSAGSSG
SLSRTHPPLQSTPLVSGVAAGSPGCVPYPENGIGGQVAPSSTSYILLPLEAATGIPPGSI
LLNPHTGQPFVNPDGTPAIYNPPTSQQPLRSAMVGQSQQQPPQQQPSPQPQQQVQPPQPQ
MAGPLVTQRDDVATQFGQMTLSRQSSGETPEPPSGPVYPSSLMPQPAQQPSYVIASTGQQ
LPTGGFSGSGPPISQQVLQPPPSPQGFVQQPPPAQMPVYYYPSGQYPTSTTQQYRPMAPV
QYNAQRSQQMPQAAQQAGYQPVLSGQQGFQGLIGVQQPPQSQNVINNQQGTPVQSVMVSY
PTMSSYQVPMTQGSQGLPQQSYQQPIMLPNQAGQGSLPATGMPVYCNVTPPTPQNNLRLI
GPHCPSSTVPVMSASCRTNCASMSNAGWQVKF
>sp|P61160|ARP2_HUMAN Actin-related protein 2 OS=Homo sapiens GN=ACTR2 PE=1 SV=1
MDSQGRKVVVCDNGTGFVKCGYAGSNFPEHIFPALVGRPIIRSTTKVGNIEIKDLMVGDE
ASELRSMLEVNYPMENGIVRNWDDMKHLWDYTFGPEKLNIDTRNCKILLTEPPMNPTKNR
EKIVEVMFETYQFSGVYVAIQAVLTLYAQGLLTGVVVDSGDGVTHICPVYEGFSLPHLTR
RLDIAGRDITRYLIKLLLLRGYAFNHSADFETVRMIKEKLCYVGYNIEQEQKLALETTVL
VESYTLPDGRIIKVGGERFEAPEALFQPHLINVEGVGVAELLFNTIQAADIDTRSEFYKH
IVLSGGSTMYPGLPSRLERELKQLYLERVLKGDVEKLSKFKIRIEDPPRRKHMVFLGGAV
LADIMKDKDNFWMTRQEYQEKGVRVLEKLGVTVR
>sp|Q9C0K3|ARP3C_HUMAN Actin-related protein 3C OS=Homo sapiens GN=ACTR3C PE=2 SV=1
MFESFNVPGLYIAVQAVLALAASWTSRQVGERTLTGIVIDSGDGVTHVIPVAEGYVIGSC
IKHIPIAGRDITYFIQQLLREREVGIPPEQSLETAKAIKEKYCYICPDIVKEFAKYDVDP
QKWIKQYTGINAINQKKFVIDVGYERFLGPEIFFHPEFANPDSMESISDVVDEVIQNCPI
DVRRPLYKMEQIPLSYPQGHGFHPLSPPFH
>sp|Q9BPX5|ARP5L_HUMAN Actin-related protein 2/3 complex subunit 5-like protein OS=Homo sapiens GN=ARPC5L PE=1 SV=1

MARNTLSSRFRRVDIDEFDENKFVDEQEEAAAAAAEPGPDPSEVDGLLRQGDMLRAFHAA
LRNSPVNTKNQAVKERAQGVVLKVLTNFKSSEIEQAVQSLDRNGVDLLMKYIYKGFEKPT
ENSSAVLLQWHEKALAVGGLGSIIRVLTARKTV
>sp|Q9NVP2|ASF1B_HUMAN Histone chaperone ASF1B OS=Homo sapiens GN=ASF1B PE=1 SV=1
MAKVSVLNVAVLENPSPFHSPFRFEISFECSEALADDLEWKIIYVGSAESEEFDQILDSV
LVGPVPAGRHMFVFQADAPNPSLIPETDAVGVTVVLITCTYHGQEFIRVGYYVNNEYLNP
ELRENPPMKPDFSQLQRNILASNPRVTRFHINWDNNMDRLEAIETQDPSLGCGLPLNCTP
IKGLGLPGCIPGLLPENSMDCI
>sp|Q9NR48|ASH1L_HUMAN Histone-lysine N-methyltransferase ASH1L OS=Homo sapiens GN=ASH1L
PE=1 SV=2
MDPRNTAMLGLGSDSEGFSRKSPSAISTGTLVSKREVELEKNTKEEEDLRKRNRERNIEA
GKDDGLTDAQQQFSVKETNFSEGNLKLKIGLQAKRTKKPPKNLENYVCRPAIKTTIKHPR
KALKSGKMTDEKNEHCPSKRDPSKLYKKADDVAAIECQSEEVIRLHSQGENNPLSKKLSP
VHSEMADYINATPSTLLGSRDPDLKDRALLNGGTSVTEKLAQLIATCPPSKSSKTKPKKL
GTGTTAGLVSKDLIRKAGVGSVAGIIHKDLIKKPTISTAVGLVTKDPGKKPVFNAAVGLV
NKDSVKKLGTGTTAVFINKNLGKKPGTITTVGLLSKDSGKKLGIGIVPGLVHKESGKKLG
LGTVVGLVNKDLGKKLGSTVGLVAKDCAKKIVASSAMGLVNKDIGKKLMSCPLAGLISKD
AINLKAEALLPTQEPLKASCSTNINNQESQELSESLKDSATSKTFEKNVVRQNKESILEK
FSVRKEIINLEKEMFNEGTCIQQDSFSSSEKGSYETSKHEKQPPVYCTSPDFKMGGASDV
STAKSPFSAVGESNLPSPSPTVSVNPLTRSPPETSSQLAPNPLLLSSTTELIEEISESVG
KNQFTSESTHLNVGHRSVGHSISIECKGIDKEVNDSKTTHIDIPRISSSLGKKPSLTSES
SIHTITPSVVNFTSLFSNKPFLKLGAVSASDKHCQVAESLSTSLQSKPLKKRKGRKPRWT
KVVARSTCRSPKGLELERSELFKNVSCSSLSNSNSEPAKFMKNIGPPSFVDHDFLKRRLP
KLSKSTAPSLALLADSEKPSHKSFATHKLSSSMCVSSDLLSDIYKPKRGRPKSKEMPQLE
GPPKRTLKIPASKVFSLQSKEEQEPPILQPEIEIPSFKQGLSVSPFPKKRGRPKRQMRSP
VKMKPPVLSVAPFVATESPSKLESESDNHRSSSDFFESEDQLQDPDDLDDSHRPSVCSMS
DLEMEPDKKITKRNNGQLMKTIIRKINKMKTLKRKKLLNQILSSSVESSNKGKVQSKLHN
TVSSLAATFGSKLGQQINVSKKGTIYIGKRRGRKPKTVLNGILSGSPTSLAVLEQTAQQA
AGSALGQILPPLLPSSASSSEILPSPICSQSSGTSGGQSPVSSDAGFVEPSSVPYLHLHS
RQGSMIQTLAMKKASKGRRRLSPPTLLPNSPSHLSELTSLKEATPSPISESHSDETIPSD
SGIGTDNNSTSDRAEKFCGQKKRRHSFEHVSLIPPETSTVLSSLKEKHKHKCKRRNHDYL
SYDKMKRQKRKRKKKYPQLRNRQDPDFIAELEELISRLSEIRITHRSHHFIPRDLLPTIF
RINFNSFYTHPSFPLDPLHYIRKPDLKKKRGRPPKMREAMAEMPFMHSLSFPLSSTGFYP
SYGMPYSPSPLTAAPIGLGYYGRYPPTLYPPPPSPSFTTPLPPPSYMHAGHLLLNPAKYH
KKKHKLLRQEAFLTTSRTPLLSMSTYPSVPPEMAYGWMVEHKHRHRHKHREHRSSEQPQV
SMDTGSSRSVLESLKRYRFGKDAVGERYKHKEKHRCHMSCPHLSPSKSLINREEQWVHRE
PSESSPLALGLQTPLQIDCSESSPSLSLGGFTPNSEPASSDEHTNLFTSAIGSCRVSNPN
SSGRKKLTDSPGLFSAQDTSLNRLHRKESLPSNERAVQTLAGSQPTSDKPSQRPSESTNC
SPTRKRSSSESTSSTVNGVPSRSPRLVASGDDSVDSLLQRMVQNEDQEPMEKSIDAVIAT
ASAPPSSSPGRSHSKDRTLGKPDSLLVPAVTSDSCNNSISLLSEKLTSSCSPHHIKRSVV
EAMQRQARKMCNYDKILATKKNLDHVNKILKAKKLQRQARTGNNFVKRRPGRPRKCPLQA
VVSMQAFQAAQFVNPELNRDEEGAALHLSPDTVTDVIEAVVQSVNLNPEHKKGLKRKGWL
LEEQTRKKQKPLPEEEEQENNKSFNEAPVEIPSPSETPAKPSEPESTLQPVLSLIPREKK
PPRPPKKKYQKAGLYSDVYKTTDPKSRLIQLKKEKLEYTPGEHEYGLFPAPIHVVFFVSG

KYLRQKRIDFQLPYDILWQWKHNQLYKKPDVPLYKKIRSNVYVDVKPLSGYEATTCNCKK
PDDDTRKGCVDDCLNRMIFAECSPNTCPCGEQCCNQRIQRHEWVQCLERFRAEEKGWGIR
TKEPLKAGQFIIEYLGEVVSEQEFRNRMIEQYHNHSDHYCLNLDSGMVIDSYRMGNEARF
INHSCDPNCEMQKWSVNGVYRIGLYALKDMPAGTELTYDYNFHSFNVEKQQLCKCGFEKC
RGIIGGKSQRVNGLTSSKNSQPMATHKKSGRSKEKRKSKHKLKKRRGHLSEEPSENINTP
TRLTPQLQMKPMSNRERNFVLKHHVFLVRNWEKIRQKQEEVKHTSDNIHSASLYTRWNGI
CRDDGNIKSDVFMTQFSALQTARSVRTRRLAAAEENIEVARAARLAQIFKEICDGIISYK
DSSRQALAAPLLNLPPKKKNADYYEKISDPLDLITIEKQILTGYYKTVEAFDADMLKVFR
NAEKYYGRKSPVGRDVCRLRKAYYNARHEASAQIDEIVGETASEADSSETSVSEKENGHE
KDDDVIRCICGLYKDEGLMIQCDKCMVWQHCDCMGVNSDVEHYLCEQCDPRPVDREVPMI
PRPHYAQPGCVYFICLLRDDLLLRQGDCVYLMRDSRRTPDGHPVRQSYRLLSHINRDKLD
IFRIEKLWKNEKEERFAFGHHYFRPHETHHSPSRRFYHNELFRVPLYEIIPLEAVVGTCC
VLDLYTYCKGRPKGVKEQDVYICDYRLDKSAHLFYKIHRNRYPVCTKPYAFDHFPKKLTP
KKDFSPHYVPDNYKRNGGRSSWKSERSKPPLKDLGQEDDALPLIEEVLASQEQAANEIPS
LEEPEREGATANVSEGEKKTEESSQEPQSTCTPEERRHNQRERLNQILLNLLEKIPGKNA
IDVTYLLEEGSGRKLRRRTLFIPENSFRK
>sp|P17405|ASM_HUMAN Sphingomyelin phosphodiesterase OS=Homo sapiens GN=SMPD1 PE=1 SV=4
MPRYGASLRQSCPRSGREQGQDGTAGAPGLLWMGLVLALALALALALSDSRVLWAPAEAH
PLSPQGHPARLHRIVPRLRDVFGWGNLTCPICKGLFTAINLGLKKEPNVARVGSVAIKLC
NLLKIAPPAVCQSIVHLFEDDMVEVWRRSVLSPSEACGLLLGSTCGHWDIFSSWNISLPT
VPKPPPKPPSPPAPGAPVSRILFLTDLHWDHDYLEGTDPDCADPLCCRRGSGLPPASRPG
AGYWGEYSKCDLPLRTLESLLSGLGPAGPFDMVYWTGDIPAHDVWHQTRQDQLRALTTVT
ALVRKFLGPVPVYPAVGNHESTPVNSFPPPFIEGNHSSRWLYEAMAKAWEPWLPAEALRT
LRIGGFYALSPYPGLRLISLNMNFCSRENFWLLINSTDPAGQLQWLVGELQAAEDRGDKV
HIIGHIPPGHCLKSWSWNYYRIVARYENTLAAQFFGHTHVDEFEVFYDEETLSRPLAVAF
LAPSATTYIGLNPGYRVYQIDGNYSGSSHVVLDHETYILNLTQANIPGAIPHWQLLYRAR
ETYGLPNTLPTAWHNLVYRMRGDMQLFQTFWFLYHKGHPPSEPCGTPCRLATLCAQLSAR
ADSPALCRHLMPDGSLPEAQSLWPRPLFC
>sp|P20933|ASPG_HUMAN N(4)-(beta-N-acetylglucosaminyl)-L-asparaginase OS=Homo sapiens GN=AGA PE=1 SV=2
MARKSNLPVLLVPFLLCQALVRCSSPLPLVVNTWPFKNATEAAWRALASGGSALDAVESG
CAMCEREQCDGSVGFGGSPDELGETTLDAMIMDGTTMDVGAVGDLRRIKNAIGVARKVLE
HTTHTLLVGESATTFAQSMGFINEDLSTTASQALHSDWLARNCQPNYWRNVIPDPSKYCG
PYKPPGILKQDIPIHKETEDDRGHDTIGMVVIHKTGHIAAGTSTNGIKFKIHGRVGDSPI
PGAGAYADDTAGAAAATGNGDILMRFLPSYQAVEYMRRGEDPTIACQKVISRIQKHFPEF
FGAVICANVTGSYGAACNKLSTFTQFSFMVYNSEKNQPTEEKVDCI
>sp|O75110|ATP9A_HUMAN Probable phospholipid-transporting ATPase IIA OS=Homo sapiens GN=ATP9A PE=1 SV=3
MTDNIPLQPVRQKKRMDSRPRAGCCEWLRCCGGGEARPRTVWLGHPEKRDQRYPRNVINN
QKYNFFTFLPGVLFNQFKYFFNLYFLLLACSQFVPEMRLGALYTYWVPLGFVLAVTVIRE
AVEEIRCYVRDKEVNSQVYSRLTARGTVKVKSSNIQVGDLIIVEKNQRVPADMIFLRTSE
KNGSCFLRTDQLDGETDWKLRLPVACTQRLPTAADLLQIRSYVYAEEPNIDIHNFVGTFT
REDSDPPISESLSIENTLWAGTVVASGTVVGVVLYTGRELRSVMNTSNPRSKIGLFDLEV
NCLTKILFGALVVVSLVMVALQHFAGRWYLQIIRFLLLFSNIIPISLRVNLDMGKIVYSW

VIRRDSKIPGTVVRSSTIPEQLGRISYLLTDKTGTLTQNEMIFKRLHLGTVAYGLDSMDE
VQSHIFSIYTQQSQDPPAQKGPTLTTKVRRTMSSRVHEAVKAIALCHNVTPVYESNGVTD
QAEAEKQYEDSCRVYQASSPDEVALVQWTESVGLTLVGRDQSSMQLRTPGDQILNFTILQ
IFPFTYESKRMGIIVRDESTGEITFYMKGADVVMAGIVQYNDWLEEECGNMAREGLRVLV
VAKKSLAEEQYQDFEARYVQAKLSVHDRSLKVATVIESLEMEMELLCLTGVEDQLQADVR
PTLETLRNAGIKVWMLTGDKLETATCTAKNAHLVTRNQDIHVFRLVTNRGEAHLELNAFR
RKHDCALVISGDSLEVCLKYYEYEFMELACQCPAVVCCRCAPTQKAQIVRLLQERTGKLT
CAVGDGGNDVSMIQESDCGVGVEGKEGKQASLAADFSITQFKHLGRLLMVHGRNSYKRSA
ALSQFVIHRSLCISTMQAVFSSVFYFASVPLYQGFLIIGYSTIYTMFPVFSLVLDKDVKS
EVAMLYPELYKDLLKGRPLSYKTFLIWVLISIYQGSTIMYGALLLFESEFVHIVAISFTS
LILTELLMVALTIQTWHWLMTVAELLSLACYIASLVFLHEFIDVYFIATLSFLWKVSVIT
LVSCLPLYVLKYLRRRFSPPSYSKLTS
>sp|P25705|ATPA_HUMAN ATP synthase subunit alpha, mitochondrial OS=Homo sapiens GN=ATP5A1 PE=1 SV=1
MLSVRVAAAVVRALPRRAGLVSRNALGSSFIAARNFHASNTHLQKTGTAEMSSILEERIL
GADTSVDLEETGRVLSIGDGIARVHGLRNVQAEEMVEFSSGLKGMSLNLEPDNVGVVVFG
NDKLIKEGDIVKRTGAIVDVPVGEELLGRVVDALGNAIDGKGPIGSKTRRRVGLKAPGII
PRISVREPMQTGIKAVDSLVPIGRGQRELIIGDRQTGKTSIAIDTIINQKRFNDGSDEKK
KLYCIYVAIGQKRSTVAQLVKRLTDADAMKYTIVVSATASDAAPLQYLAPYSGCSMGEYF
RDNGKHALIIYDDLSKQAVAYRQMSLLLRRPPGREAYPGDVFYLHSRLLERAAKMNDAFG
GGSLTALPVIETQAGDVSAYIPTNVISITDGQIFLETELFYKGIRPAINVGLSVSRVGSA
AQTRAMKQVAGTMKLELAQYREVAAFAQFGSDLDAATQQLLSRGVRLTELLKQGQYSPMA
IEEQVAVIYAGVRGYLDKLEPSKITKFENAFLSHVVSQHQALLGTIRADGKISEQSDAKL
KEIVTNFLAGFEA
>sp|Q8N5M1|ATPF2_HUMAN ATP synthase mitochondrial F1 complex assembly factor 2 OS=Homo sapiens GN=ATPAF2 PE=1 SV=1
MWRSCLRLRDGGRRLLNRPAGGPSASMSPGPTIPSPARAYAPPTERKRFYQNVSITQGEG
GFEINLDHRKLKTPQAKLFTVPSEALAIAVATEWDSQQDTIKYYTMHLTTLCNTSLDNPT
QRNKDQLIRAAVKFLDTDTICYRVEEPETLVELQRNEWDPIIEWAEKRYGVEISSSTSIM
GPSIPAKTREVLVSHLASYNTWALQGIEFVAAQLKSMVLTLGLIDLRLTVEQAVLLSRLE
EEYQIQKWGNIEWAHDYELQELRARTAAGTLFIHLCSESTTVKHKLLKE
>sp|Q7Z4Y8|AT5L2_HUMAN ATP synthase subunit g 2, mitochondrial OS=Homo sapiens GN=ATP5L2 PE=5 SV=1
MAPFVRNLVEKTPALVNAAVTYLKPRLAAFWYYTTVELVPPTPAEIPRAIQSLKKIVSSA
QTGSFKQLTVKEALLNGLVATEVSTWFYVREITGKRGIIG
>sp|Q9Y2Q0|AT8A1_HUMAN Phospholipid-transporting ATPase IA OS=Homo sapiens GN=ATP8A1 PE=1 SV=1
MPTMRRTVSEIRSRAEGYEKTDDVSEKTSLADQEEVRTIFINQPQLTKFCNNHVSTAKYN
IITFLPRFLYSQFRRAANSFFLFIALLQQIPDVSPTGRYTTLVPLLFILAVAAIKEIIED
IKRHKADNAVNKKQTQVLRNGAWEIVHWEKVAVGEIVKVTNGEHLPADLISLSSSEPQAM
CYIETSNLDGETNLKIRQGLPATSDIKDVDSLMRISGRIECESPNRHLYDFVGNIRLDGH
GTVPLGADQILLRGAQLRNTQWVHGIVVYTGHDTKLMQNSTSPPLKLSNVERITNVQILI
LFCILIAMSLVCSVGSAIWNRRHSGKDWYLNLNYGGASNFGLNFLTFIILFNNLIPISLL
VTLEVVKFTQAYFINWDLDMHYEPTDTAAMARTSNLNEELGQVKYIFSDKTGTLTCNVMQ

FKKCTIAGVAYGHVPEPEDYGCSPDEWQNSQFGDEKTFSDSSLLENLQNNHPTAPIICEF
LTMMAVCHTAVPEREGDKIIYQAASPDEGALVRAAKQLNFVFTGRTPDSVIIDSLGQEER
YELLNVLEFTSARKRMSVIVRTPSGKLRLYCKGADTVIYDRLAETSKYKEITLKHLEQFA
TEGLRTLCFAVAEISESDFQEWRAVYQRASTSVQNRLLKLEESYELIEKNLQLLGATAIE
DKLQDQVPETIETLMKADIKIWILTGDKQETAINIGHSCKLLKKNMGMIVINEGSLDGTR
ETLSRHCTTLGDALRKENDFALIIDGKTLKYALTFGVRQYFLDLALSCKAVICCRVSPLQ
KSEVVEMVKKQVKVVTLAIGDGANDVSMIQTAHVGVGISGNEGLQAANSSDYSIAQFKYL
KNLLMIHGAWNYNRVSKCILYCFYKNIVLYIIEIWFAFVNGFSGQILFERWCIGLYNVMF
TAMPPLTLGIFERSCRKENMLKYPELYKTSQNALDFNTKVFWVHCLNGLFHSVILFWFPL
KALQYGTAFGNGKTSDYLLLGNFVYTFVVITVCLKAGLETSYWTWFSHIAIWGSIALWVV
FFGIYSSLWPAIPMAPDMSGEAAMLFSSGVFWMGLLFIPVASLLLDVVYKVIKRTAFKTL
VDEVQELEAKSQDPGAVVLGKSLTERAQLLKNVFKKNHVNLYRSESLQQNLLHGYAFSQD
ENGIVSQSEVIRAYDTTKQRPDEW
>sp|Q8NBU5|ATAD1_HUMAN ATPase family AAA domain-containing protein 1 OS=Homo sapiens GN=ATAD1 PE=1 SV=1
MVHAEAFSRPLSRNEVVGLIFRLTIFGAVTYFTIKWMVDAIDPTRKQKVEAQKQAEKLMK
QIGVKNVKLSEYEMSIAAHLVDPLNMHVTWSDIAGLDDVITDLKDTVILPIKKKHLFENS
RLLQPPKGVLLYGPPGCGKTLIAKATAKEAGCRFINLQPSTLTDKWYGESQKLAAAVFSL
AIKLQPSIIFIDEIDSFLRNRSSSDHEATAMMKAQFMSLWDGLDTDHSCQVIVMGATNRP
QDLDSAIMRRMPTRFHINQPALKQREAILKLILKNENVDRHVDLLEVAQETDGFSGSDLK
EMCRDAALLCVREYVNSTSEESHDEDEIRPVQQQDLHRAIEKMKKSKDAAFQNVLTHVCL
D
>sp|P18847|ATF3_HUMAN Cyclic AMP-dependent transcription factor ATF-3 OS=Homo sapiens GN=ATF3 PE=1 SV=2
MMLQHPGQVSASEVSASAIVPCLSPPGSLVFEDFANLTPFVKEELRFAIQNKHLCHRMSS
ALESVTVSDRPLGVSITKAEVAPEEDERKKRRRERNKIAAAKCRNKKKEKTECLQKESEK
LESVNAELKAQIEELKNEKQHLIYMLNLHRPTCIVRAQNGRTPEDERNLFIQQIKEGTLQ
S
>sp|Q9Y2D1|ATF5_HUMAN Cyclic AMP-dependent transcription factor ATF-5 OS=Homo sapiens GN=ATF5 PE=1 SV=4
MSLLATLGLELDRALLPASGLGWLVDYGKLPPAPAPLAPYEVLGGALEGGLPVGGEPLAG
DGFSDWMTERVDFTALLPLEPPLPPGTLPQPSPTPPDLEAMASLLKKELEQMEDFFLDAP
PLPPPSPPPLPPPPLPPAPSLPLSLPSFDLPQPPVLDTLDLLAIYCRNEAGQEEVGMPPL
PPPQQPPPPSPPQPSRLAPYPHPATTRGDRKQKKRDQNKSAALRYRQRKRAEGEALEGEC
QGLEARNRELKERAESVEREIQYVKDLLIEVYKARSQRTRSC
>sp|P18850|ATF6A_HUMAN Cyclic AMP-dependent transcription factor ATF-6 alpha OS=Homo sapiens GN=ATF6 PE=1 SV=3
MGEPAGVAGTMESPFSPGLFHRLDEDWDSALFAELGYFTDTDELQLEAANETYENNFDNL
DFDLDLMPWESDIWDINNQICTVKDIKAEPQPLSPASSSYSVSSPRSVDSYSSTQHVPEE
LDLSSSSQMSPLSLYGENSNSLSSAEPLKEDKPVTGPRNKTENGLTPKKKIQVNSKPSIQ
PKPLLLPAAPKTQTNSSVPAKTIIIQTVPTLMPLAKQQPIISLQPAPTKGQTVLLSQPTV
VQLQAPGVLPSAQPVLAVAGGVTQLPNHVVNVVPAPSANSPVNGKLSVTKPVLQSTMRNV
GSDIAVLRRQQRMIKNRESACQSRKKKKEYMLGLEARLKAALSENEQLKKENGTLKRQLD
EVVSENQRLKVPSPKRRVVCVMIVLAFIILNYGPMSMLEQDSRRMNPSVSPANQRRHLLG

FSAKEAQDTSDGIIQKNSYRYDHSVSNDKALMVLTEEPLLYIPPPPCQPLINTTESLRLN
HELRGWVHRHEVERTKSRRMTNNQQKTRILQGALEQGSNSQLMAVQYTETTSSISRNSGS
ELQVYYASPRSYQDFFEAIRRRGDTFYVVSFRRDHLLLPATTHNKTTRPKMSIVLPAINI
NENVINGQDYEVMMQIDCQVMDTRILHIKSSSVPPYLRDQQRNQTNTFFGSPPAATEATH
VVSTIPESLQ
>sp|Q96DT6|ATG4C_HUMAN Cysteine protease ATG4C OS=Homo sapiens GN=ATG4C PE=1 SV=1
MEATGTDEVDKLKTKFISAWNNMKYSWVLKTKTYFSRNSPVLLLGKCYHFKYEDEDKTLP
AESGCTIEDHVIAGNVEEFRKDFISRIWLTYREEFPQIEGSALTTDCGWGCTLRTGQMLL
AQGLILHFLGRAWTWPDALNIENSDSESWTSHTVKKFTASFEASLSGEREFKTPTISLKE
TIGKYSDDHEMRNEVYHRKIISWFGDSPLALFGLHQLIEYGKKSGKKAGDWYGPAVVAHI
LRKAVEEARHPDLQGITIYVAQDCTVYNSDVIDKQSASMTSDNADDKAVIILVPVRLGGE
RTNTDYLEFVKGILSLEYCVGIIGGKPKQSYYFAGFQDDSLIYMDPHYCQSFVDVSIKDF
PLETFHCPSPKKMSFRKMDPSCTIGFYCRNVQDFKRASEEITKMLKFSSKEKYPLFTFVN
GHSRDYDFTSTTTNEEDLFSEDEKKQLKRFSTEEFVLL
>sp|O95352|ATG7_HUMAN Ubiquitin-like modifier-activating enzyme ATG7 OS=Homo sapiens GN=ATG7 PE=1 SV=1
MAAATGDPGLSKLQFAPFSSALDVGFWHELTQKKLNEYRLDEAPKDIKGYYYNGDSAGLP
ARLTLEFSAFDMSAPTPARCCPAIGTLYNTNTLESFKTADKKLLLEQAANEIWESIKSGT
ALENPVLLNKFLLLTFADLKKYHFYYWFCYPALCLPESLPLIQGPVGLDQRFSLKQIEAL
ECAYDNLCQTEGVTALPYFLIKYDENMVLVSLLKHYSDFFQGQRTKITIGVYDPCNLAQY
PGWPLRNFLVLAAHRWSSSFQSVEVVCFRDRTMQGARDVAHSIIFEVKLPEMAFSPDCPK
AVGWEKNQKGGMGPRMVNLSECMDPKRLAESSVDLNLKLMCWRLVPTLDLDKVVSVKCLL
LGAGTLGCNVARTLMGWGVRHITFVDNAKISYSNPVRQPLYEFEDCLGGGKPKALAAADR
LQKIFPGVNARGFNMSIPMPGHPVNFSSVTLEQARRDVEQLEQLIESHDVVFLLMDTRES
RWLPAVIAASKRKLVINAALGFDTFVVMRHGLKKPKQQGAGDLCPNHPVASADLLGSSLF
ANIPGYKLGCYFCNDVVAPGDSTRDRTLDQQCTVSRPGLAVIAGALAVELMVSVLQHPEG
GYAIASSSDDRMNEPPTSLGLVPHQIRGFLSRFDNVLPVSLAFDKCTACSSKVLDQYERE
GFNFLAKVFNSSHSFLEDLTGLTLLHQETQAAEIWDMSDDETI
>sp|Q674R7|ATG9B_HUMAN Autophagy-related protein 9B OS=Homo sapiens GN=ATG9B PE=2 SV=1
MVSRMGWGGRRRRLGRWGDLGPGSVPLLPMPLPPPPPPSCRGPGGGRISIFSLSPAPHTR
SSPSSFSPPTAGPPCSVLQGTGASQSCHSALPIPATPPTQAQPAMTPASASPSWGSHSTP
PLAPATPTPSQQCPQDSPGLRVGPLIPEQDYERLEDCDPEGSQDSPIHGEEQQPLLHVPE
GLRGSWHHIQNLDSFFTKIYSYHQRNGFACILLEDVFQLGQFIFIVTFTTFLLRCVDYNV
LFANQPSNHTRPGPFHSKVTLSDAILPSAQCAERIRSSPLLVLLLVLAAGFWLVQLLRSV
CNLFSYWDIQVFYREALHIPPEELSSVPWAEVQSRLLALQRSGGLCVQPRPLTELDIHHR
ILRYTNYQVALANKGLLPARCPLPWGGSAAFLSRGLALNVDLLLFRGPFSLFRGGWELPH
AYKRSDQRGALAARWGRTVLLLAALNLALSPLVLAWQVLHVFYSHVELLRREPGALGARG
WSRLARLQLRHFNELPHELRARLARAYRPAAAFLRTAAPPAPLRTLLARQLVFFAGALFA
ALLVLTVYDEDVLAVEHVLTAMTALGVTATVARSFIPEEQCQGRAPQLLLQTALAHMHYL
PEEPGPGGRDRAYRQMAQLLQYRAVSLLEELLSPLLTPLFLLFWFRPRALEIIDFFHHFT
VDVAGVGDICSFALMDVKRHGHPQWLSAGQTEASLSQRAEDGKTELSLMRFSLAHPLWRP
PGHSSKFLGHLWGRVQQDAAAWGATSARGPSTPGVLSNCTSPLPEAFLANLFVHPLLPPR
DLSPTAPCPAAATASLLASISRIAQDPSSVSPGGTGGQKLAQLPELASAEMSLHVIYLHQ
LHQQQQQQEPWGEAAASILSRPCSSPSQPPSPDEEKPSWSSDGSSPASSPRQQWGTQKAR

NLFPGGFQVTTDTQKEPDRASCTD
>sp|Q8N100|ATOH7_HUMAN Protein atonal homolog 7 OS=Homo sapiens GN=ATOH7 PE=1 SV=1
MKSCKPSGPPAGARVAPPCAGGTECAGTCAGAGRLESAARRRLAANARERRRMQGLNTAF
DRLRRVVPQWGQDKKLSKYETLQMALSYIMALTRILAEAERFGSERDWVGLHCEHFGRDH
YLPFPGAKLPGESELYSQRLFGFQPEPFQMAT
>sp|P51164|ATP4B_HUMAN Potassium-transporting ATPase subunit beta OS=Homo sapiens GN=ATP4B PE=1 SV=1
MAALQEKKTCGQRMEEFQRYCWNPDTGQMLGRTLSRWVWISLYYVAFYVVMTGLFALCLY
VLMQTVDPYTPDYQDQLRSPGVTLRPDVYGEKGLEIVYNVSDNRTWADLTQTLHAFLAGY
SPAAQEDSINCTSEQYFFQESFRAPNHTKFSCKFTADMLQNCSGLADPNFGFEEGKPCFI
IKMNRIVKFLPSNGSAPRVDCAFLDQPRELGQPLQVKYYPPNGTFSLHYFPYYGKKAQPH
YSNPLVAAKLLNIPRNAEVAIVCKVMAEHVTFNNPHDPYEGKVEFKLKIEK
>sp|Q86WX3|AROS_HUMAN Active regulator of SIRT1 OS=Homo sapiens GN=RPS19BP1 PE=1 SV=1
MSAALLRRGLELLAASEAPRDPPGQAKPRGAPVKRPRKTKAIQAQKLRNSAKGKVPKSAL
DEYRKRECRDHLRVNLKFLTRTRSTVAESVSQQILRQNRGRKACDRPVAKTKKKKAEGTV
FTEEDFQKFQQEYFGS
>sp|Q9P1U1|ARP3B_HUMAN Actin-related protein 3B OS=Homo sapiens GN=ACTR3B PE=2 SV=1
MAGSLPPCVVDCGTGYTKLGYAGNTEPQFIIPSCIAIRESAKVVDQAQRRVLRGVDDLDF
FIGDEAIDKPTYATKWPIRHGIIEDWDLMERFMEQVVFKYLRAEPEDHYFLMTEPPLNTP
ENREYLAEIMFESFNVPGLYIAVQAVLALAASWTSRQVGERTLTGIVIDSGDGVTHVIPV
AEGYVIGSCIKHIPIAGRDITYFIQQLLREREVGIPPEQSLETAKAIKEKYCYICPDIVK
EFAKYDVDPRKWIKQYTGINAINQKKFVIDVGYERFLGPEIFFHPEFANPDFMESISDVV
DEVIQNCPIDVRRPLYKNVVLSGGSTMFRDFGRRLQRDLKRVVDARLRLSEELSGGRIKP
KPVEVQVVTHHMQRYAVWFGGSMLASTPEFFQVCHTKKDYEEYGPSICRHNPVFGVMS
>sp|P61158|ARP3_HUMAN Actin-related protein 3 OS=Homo sapiens GN=ACTR3 PE=1 SV=3
MAGRLPACVVDCGTGYTKLGYAGNTEPQFIIPSCIAIKESAKVGDQAQRRVMKGVDDLDF
FIGDEAIEKPTYATKWPIRHGIVEDWDLMERFMEQVIFKYLRAEPEDHYFLLTEPPLNTP
ENREYTAEIMFESFNVPGLYIAVQAVLALAASWTSRQVGERTLTGTVIDSGDGVTHVIPV
AEGYVIGSCIKHIPIAGRDITYFIQQLLRDREVGIPPEQSLETAKAVKERYSYVCPDLVK
EFNKYDTDGSKWIKQYTGINAISKKEFSIDVGYERFLGPEIFFHPEFANPDFTQPISEVV
DEVIQNCPIDVRRPLYKNIVLSGGSTMFRDFGRRLQRDLKRTVDARLKLSEELSGGRLKP
KPIDVQVITHHMQRYAVWFGGSMLASTPEFYQVCHTKKDYEEIGPSICRHNPVFGVMS
>sp|P59998|ARPC4_HUMAN Actin-related protein 2/3 complex subunit 4 OS=Homo sapiens GN=ARPC4 PE=1 SV=3
MTATLRPYLSAVRATLQAALCLENFSSQVVERHNKPEVEVRSSKELLLQPVTISRNEKEK
VLIEGSINSVRVSIAVKQADEIEKILCHKFMRFMMMRAENFFILRRKPVEGYDISFLITN
FHTEQMYKHKLVDFVIHFMEEIDKEISEMKLSVNARARIVAEEFLKNF
>sp|O15511|ARPC5_HUMAN Actin-related protein 2/3 complex subunit 5 OS=Homo sapiens GN=ARPC5 PE=1 SV=3
MSKNTVSSARFRKVDVDEYDENKFVDEEDGGDGQAGPDEGEVDSCLRQGNMTAALQAALK
NPPINTKSQAVKDRAGSIVLKVLISFKANDIEKAVQSLDKNGVDLLMKYIYKGFESPSDN
SSAMLLQWHEKALAAGGVGSIVRVLTARKTV
>sp|P10523|ARRS_HUMAN S-arrestin OS=Homo sapiens GN=SAG PE=1 SV=3
MAASGKTSKSEPNHVIFKKISRDKSVTIYLGNRDYIDHVSQVQPVDGVVLVDPDLVKGKK

VYVTLTCAFRYGQEDIDVIGLTFRRDLYFSRVQVYPPVGAASTPTKLQESLLKKLGSNTY
PFLLTFPDYLPCSVMLQPAPQDSGKSCGVDFEVKAFATDSTDAEEDKIPKKSSVRLLIRK
VQHAPLEMGPQPRAEAAWQFFMSDKPLHLAVSLNKEIYFHGEPIPVTVTVTNNTEKTVKK
IKAFVEQVANVVLYSSDYYVKPVAMEEAQEKVPPNSTLTKTLTLLPLLANNRERRGIALD
GKIKHEDTNLASSTIIKEGIDRTVLGILVSYQIKVKLTVSGFLGELTSSEVATEVPFRLM
HPQPEDPAKESYQDANLVFEEFARHNLKDAGEAEEGKRDKNDVDE
>sp|P54793|ARSF_HUMAN Arylsulfatase F OS=Homo sapiens GN=ARSF PE=1 SV=4
MRPRRPLVFMSLVCALLNTCQAHRVHDDKPNIVLIMVDDLGIGDLGCYGNDTMRTPHIDR
LAREGVRLTQHISAASLCSPSRSAFLTGRYPIRSGMVSSGNRRVIQNLAVPAGLPLNETT
LAALLKKQGYSTGLIGKWHQGLNCDSRSDQCHHPYNYGFDYYYGMPFTLVDSCWPDPSRN
TELAFESQLWLCVQLVAIAILTLTFGKLSGWVSVPWLLIFSMILFIFLLGYAWFSSHTSP
LYWDCLLMRGHEITEQPMKAERAGSIMVKEAISFLERHSKETFLLFFSFLHVHTPLPTTD
DFTGTSKHGLYGDNVEEMDSMVGKILDAIDDFGLRNNTLVYFTSDHGGHLEARRGHAQLG
GWNGIYKGGKGMGGWEGGIRVPGIVRWPGKVPAGRLIKEPTSLMDILPTVASVSGGSLPQ
DRVIDGRDLMPLLQGNVRHSEHEFLFHYCGSYLHAVRWIPKDDSGSVWKAHYVTPVFQPP
ASGGCYVTSLCRCFGEQVTYHNPPLLFDLSRDPSESTPLTPATEPLHDFVIKKVANALKE
HQETIVPVTYQLSELNQGRTWLKPCCGVFPFCLCDKEEEVSQPRGPNEKR
>sp|Q9H2C2|ARV1_HUMAN Protein ARV1 OS=Homo sapiens GN=ARV1 PE=2 SV=1
MGNGGRSGLQQGKGNVDGVAATPTAASASCQYRCIECNQEAKELYRDYNHGVLKITICKS
CQKPVDKYIEYDPVIILINAILCKAQAYRHILFNTQINIHGKLCIFCLLCEAYLRWWQLQ
DSNQNTAPDDLIRYAKEWDFYRMFAIAALEQTAYFIGIFTFLWVERPMTAKKKPNFILLL
KALLLSSYGKLLLIPAVIWEHDYTSVCLKLIKVFVLTSNFQAIRVTLNINRKLSFLAVLS
GLLLESIMVYFFQSMEWDVGSDYAIFKSQDF
>sp|P0C7U1|ASA2B_HUMAN Putative inactive neutral ceramidase B OS=Homo sapiens GN=ASAH2B PE=2 SV=1
MRQHRQFMDRTHYLLTFSSSETLLRLLLRIVDRAPKGRTFGDVLQPAKPEYRVGEVAEVI
FVGANPKNSVQNQTHQTFLTVEKYEATSTSWQIVCNDASWETRFYWHKGLLGLSNATVEW
HIPDTAQPGIYRIRYFGHNRKQDILKPAVILSFEGTSPAFEVVTI
>sp|Q13510|ASAH1_HUMAN Acid ceramidase OS=Homo sapiens GN=ASAH1 PE=1 SV=5
MPGRSCVALVLLAAAVSCAVAQHAPPWTEDCRKSTYPPSGPTYRGAVPWYTINLDLPPYK
RWHELMLDKAPVLKVIVNSLKNMINTFVPSGKIMQVVDEKLPGLLGNFPGPFEEEMKGIA
AVTDIPLGEIISFNIFYELFTICTSIVAEDKKGHLIHGRNMDFGVFLGWNINNDTWVITE
QLKPLTVNLDFQRNNKTVFKASSFAGYVGMLTGFKPGLFSLTLNERFSINGGYLGILEWI
LGKKDVMWIGFLTRTVLENSTSYEEAKNLLTKTKILAPAYFILGGNQSGEGCVITRDRKE
SLDVYELDAKQGRWYVVQTNYDRWKHPFFLDDRRTPAKMCLNRTSQENISFETMYDVLST
KPVLNKLTVYTTLIDVTKGQFETYLRDCPDPCIGW
>sp|O43150|ASAP2_HUMAN Arf-GAP with SH3 domain, ANK repeat and PH domain-containing protein 2 OS=Homo sapiens GN=ASAP2 PE=1 SV=3
MPDQISVSEFVAETHEDYKAPTASSFTTRTAQCRNTVAAIEEALDVDRMVLYKMKKSVKA
INSSGLAHVENEEQYTQALEKFGGNCVCRDDPDLGSAFLKFSVFTKELTALFKNLIQNMN
NIISFPLDSLLKGDLKGVKGDLKKPFDKAWKDYETKITKIEKEKKEHAKLHGMIRTEISG
AEIAEEMEKERRFFQLQMCEYLLKVNEIKIKKGVDLLQNLIKYFHAQCNFFQDGLKAVES
LKPSIETLSTDLHTIKQAQDEERRQLIQLRDILKSALQVEQKEDSQIRQSTAYSLHQPQG
NKEHGTERNGSLYKKSDGIRKVWQKRKCSVKNGFLTISHGTANRPPAKLNLLTCQVKTNP

EEKKCFDLISHDRTYHFQAEDEQECQIWMSVLQNSKEEALNNAFKGDDNTGENNIVQELT
KEIISEVQRMTGNDVCCDCGAPDPTWLSTNLGILTCIECSGIHRELGVHYSRMQSLTLDV
LGTSELLLAKNIGNAGFNEIMECCLPAEDSVKPNPGSDMNARKDYITAKYIERRYARKKH
ADNAAKLHSLCEAVKTRDIFGLLQAYADGVDLTEKIPLANGHEPDETALHLAVRSVDRTS
LHIVDFLVQNSGNLDKQTGKGSTALHYCCLTDNAECLKLLLRGKASIEIANESGETPLDI
AKRLKHEHCEELLTQALSGRFNSHVHVEYEWRLLHEDLDESDDDMDEKLQPSPNRREDRP
ISFYQLGSNQLQSNAVSLARDAANLAKEKQRAFMPSILQNETYGALLSGSPPPAQPAAPS
TTSAPPLPPRNVGKVQTASSANTLWKTNSVSVDGGSRQRSSSDPPAVHPPLPPLRVTSTN
PLTPTPPPPVAKTPSVMEALSQPSKPAPPGISQIRPPPLPPQPPSRLPQKKPAPGADKST
PLTNKGQPRGPVDLSATEALGPLSNAMVLQPPAPMPRKSQATKLKPKRVKALYNCVADNP
DELTFSEGDVIIVDGEEDQEWWIGHIDGDPGRKGAFPVSFVHFIAD
>sp|Q8TDY4|ASAP3_HUMAN Arf-GAP with SH3 domain, ANK repeat and PH domain-containing protein 3 OS=Homo sapiens GN=ASAP3 PE=1 SV=1
MPEQFSVAEFLAVTAEDLSSPAGAAAFAAKMPRYRGAALAREEILEGDQAILQRIKKAVR
AIHSSGLGHVENEEQYREAVESLGNSHLSQNSHELSTGFLNLAVFTREVAALFKNLIQNL
NNIVSFPLDSLMKGQLRDGRQDSKKQLEKAWKDYEAKMAKLEKERDRARVTGGIPGEVAQ
DMQRERRIFQLHMCEYLLKAGESQMKQGPDFLQSLIKFFHAQHNFFQDGWKAAQSLFPFI
EKLAASVHALHQAQEDELQKLTQLRDSLRGTLQLESREEHLSRKNSGCGYSIHQHQGNKQ
FGTEKVGFLYKKSDGIRRVWQKRKCGVKYGCLTISHSTINRPPVKLTLLTCQVRPNPEEK
KCFDLVTHNRTYHFQAEDEHECEAWVSVLQNSKDEALSSAFLGEPSAGPGSWGSAGHDGE
PHDLTKLLIAEVKSRPGNSQCCDCGAADPTWLSTNLGVLTCIQCSGVHRELGVRFSRMQS
LTLDLLGPSELLLALNMGNTSFNEVMEAQLPSHGGPKPSAESDMGTRRDYIMAKYVEHRF
ARRCTPEPQRLWTAICNRDLLSVLEAFANGQDFGQPLPGPDAQAPEELVLHLAVKVANQA
SLPLVDFIIQNGGHLDAKAADGNTALHYAALYNQPDCLKLLLKGRALVGTVNEAGETALD
IARKKHHKECEELLEQAQAGTFAFPLHVDYSWVISTEPGSDSEEDEEEKRCLLKLPAQAH
WASGRLDISNKTYETVASLGAATPQGESEDCPPPLPVKNSSRTLVQGCARHASGDRSEVS
SLSSEAPETPESLGSPASSSSLMSPLEPGDPSQAPPNSEEGLREPPGTSRPSLTSGTTPS
EMYLPVRFSSESTRSYRRGARSPEDGPSARQPLPRRNVPVGITEGDGSRTGSLPASSVQL
LQD
>sp|P42127|ASIP_HUMAN Agouti-signaling protein OS=Homo sapiens GN=ASIP PE=1 SV=1
MDVTRLLLATLLVFLCFFTANSHLPPEEKLRDDRSLRSNSSVNLLDVPSVSIVALNKKSK
QIGRKAAEKKRSSKKEASMKKVVRPRTPLSAPCVATRNSCKPPAPACCDPCASCQCRFFR
SACSCRVLSLNC
>sp|A6ND91|ASPD_HUMAN Putative L-aspartate dehydrogenase OS=Homo sapiens GN=ASPDH PE=1 SV=2
MADRGPWRVGVVGYGRLGQSLVSRLLAQGPELGLELVFVWNRDPGRMAGSVPPSLQLQNL
AALGERRPDLVVEVAHPKIIHESGAQILRHANLLVGSPSALSDQTTERQLLEASQHWDHA
VFVARGALWGAEDIRRLDAAGGLRSLRVTMATHPDGFRLEGPLAAAHSPGPCTVLYEGPV
RGLCPFAPRNSNTMAAAALAAPSLGFDGVIGVLVADTSLTDMHVVDVELSGPRGPTGRSF
AVHTRRENPAEPGAVTGSATVTAFWQSLLACCQLPSRPGIHLC
>sp|Q9Y284|ASTER_HUMAN Protein Asterix OS=Homo sapiens GN=WDR83OS PE=1 SV=1
MSTNNMSDPRRPNKVLRYKPPPSECNPALDDPTPDYMNLLGMIFSMCGLMLKLKWCAWVA
VYCSFISFANSRSSEDTKQMMSSFMLSISAVVMSYLQNPQPMTPPW

>sp|P98196|AT11A_HUMAN Probable phospholipid-transporting ATPase IH OS=Homo sapiens GN=ATP11A PE=1 SV=3
MDCSLVRTLVHRYCAGEENWVDSRTIYVGHREPPPGAEAYIPQRYPDNRIVSSKYTFWNF
IPKNLFEQFRRVANFYFLIIFLVQLIIDTPTSPVTSGLPLFFVITVTAIKQGYEDWLRHK
ADNAMNQCPVHFIQHGKLVRKQSRKLRVGDIVMVKEDETFPCDLIFLSSNRGDGTCHVTT
ASLDGESSHKTHYAVQDTKGFHTEEDIGGLHATIECEQPQPDLYKFVGRINVYSDLNDPV
VRPLGSENLLLRGATLKNTEKIFGVAIYTGMETKMALNYQSKSQKRSAVEKSMNAFLIVY
LCILISKALINTVLKYMWQSEPFRDEPWYNQKTESERQRNLFLKAFTDFLAFMVLFNYII
PVSMYVTVEMQKFLGSYFITWDEDMFDEETGEGPLVNTSDLNEELGQVEYIFTDKTGTLT
ENNMEFKECCIEGHVYVPHVICNGQVLPESSGIDMIDSSPSVNGREREELFFRALCLCHT
VQVKDDDSVDGPRKSPDGGKSCVYISSSPDEVALVEGVQRLGFTYLRLKDNYMEILNREN
HIERFELLEILSFDSVRRRMSVIVKSATGEIYLFCKGADSSIFPRVIEGKVDQIRARVER
NAVEGLRTLCVAYKRLIQEEYEGICKLLQAAKVALQDREKKLAEAYEQIEKDLTLLGATA
VEDRLQEKAADTIEALQKAGIKVWVLTGDKMETAAATCYACKLFRRNTQLLELTTKRIEE
QSLHDVLFELSKTVLRHSGSLTRDNLSGLSADMQDYGLIIDGAALSLIMKPREDGSSGNY
RELFLEICRSCSAVLCCRMAPLQKAQIVKLIKFSKEHPITLAIGDGANDVSMILEAHVGI
GVIGKEGRQAARNSDYAIPKFKHLKKMLLVHGHFYYIRISELVQYFFYKNVCFIFPQFLY
QFFCGFSQQTLYDTAYLTLYNISFTSLPILLYSLMEQHVGIDVLKRDPTLYRDVAKNALL
RWRVFIYWTLLGLFDALVFFFGAYFVFENTTVTSNGQIFGNWTFGTLVFTVMVFTVTLKL
ALDTHYWTWINHFVIWGSLLFYVVFSLLWGGVIWPFLNYQRMYYVFIQMLSSGPAWLAIV
LLVTISLLPDVLKKVLCRQLWPTATERVQTKSQCLSVEQSTIFMLSQTSSSLSF
>sp|Q8NB49|AT11C_HUMAN Phospholipid-transporting ATPase IG OS=Homo sapiens GN=ATP11C PE=1 SV=3
MQMVPSLPPASECAGEEKRVGTRTVFVGNHPVSETEAYIAQRFCDNRIVSSKYTLWNFLP
KNLFEQFRRIANFYFLIIFLVQVTVDTPTSPVTSGLPLFFVITVTAIKQGYEDCLRHRAD
NEVNKSTVYIIENAKRVRKESEKIKVGDVVEVQADETFPCDLILLSSCTTDGTCYVTTAS
LDGESNCKTHYAVRDTIALCTAESIDTLRAAIECEQPQPDLYKFVGRINIYSNSLEAVAR
SLGPENLLLKGATLKNTEKIYGVAVYTGMETKMALNYQGKSQKRSAVEKSINAFLIVYLF
ILLTKAAVCTTLKYVWQSTPYNDEPWYNQKTQKERETLKVLKMFTDFLSFMVLFNFIIPV
SMYVTVEMQKFLGSFFISWDKDFYDEEINEGALVNTSDLNEELGQVDYVFTDKTGTLTEN
SMEFIECCIDGHKYKGVTQEVDGLSQTDGTLTYFDKVDKNREELFLRALCLCHTVEIKTN
DAVDGATESAELTYISSSPDEIALVKGAKRYGFTFLGNRNGYMRVENQRKEIEEYELLHT
LNFDAVRRRMSVIVKTQEGDILLFCKGADSAVFPRVQNHEIELTKVHVERNAMDGYRTLC
VAFKEIAPDDYERINRQLIEAKMALQDREEKMEKVFDDIETNMNLIGATAVEDKLQDQAA
ETIEALHAAGLKVWVLTGDKMETAKSTCYACRLFQTNTELLELTTKTIEESERKEDRLHE
LLIEYRKKLLHEFPKSTRSFKKAWTEHQEYGLIIDGSTLSLILNSSQDSSSNNYKSIFLQ
ICMKCTAVLCCRMAPLQKAQIVRMVKNLKGSPITLSIGDGANDVSMILESHVGIGIKGKE
GRQAARNSDYSVPKFKHLKKLLLAHGHLYYVRIAHLVQYFFYKNLCFILPQFLYQFFCGF
SQQPLYDAAYLTMYNICFTSLPILAYSLLEQHINIDTLTSDPRLYMKISGNAMLQLGPFL
YWTFLAAFEGTVFFFGTYFLFQTASLEENGKVYGNWTFGTIVFTVLVFTVTLKLALDTRF
WTWINHFVIWGSLAFYVFFSFFWGGIIWPFLKQQRMYFVFAQMLSSVSTWLAIILLIFIS
LFPEILLIVLKNVRRRSARRNLSCRRASDSLSARPSVRPLLLRTFSDESNVL
>sp|Q9NQ11|AT132_HUMAN Probable cation-transporting ATPase 13A2 OS=Homo sapiens GN=ATP13A2 PE=1 SV=2

MSADSSPLVGSTPTGYGTLTIGTSIDPLSSSVSSVRLSGYCGSPWRVIGYHVVVWMMAGI
PLLLFRWKPLWGVRLRLRPCNLAHAETLVIEIRDKEDSSWQLFTVQVQTEAIGEGSLEPS
PQSQAEDGRSQAAVGAVPEGAWKDTAQLHKSEEAVSVGQKRVLRYYLFQGQRYIWIETQQ
AFYQVSLLDHGRSCDDVHRSRHGLSLQDQMVRKAIYGPNVISIPVKSYPQLLVDEALNPY
YGFQAFSIALWLADHYYWYALCIFLISSISICLSLYKTRKQSQTLRDMVKLSMRVCVCRP
GGEEEWVDSSELVPGDCLVLPQEGGLMPCDAALVAGECMVNESSLTGESIPVLKTALPEG
LGPYCAETHRRHTLFCGTLILQARAYVGPHVLAVVTRTGFCTAKGGLVSSILHPRPINFK
FYKHSMKFVAALSVLALLGTIYSIFILYRNRVPLNEIVIRALDLVTVVVPPALPAAMTVC
TLYAQSRLRRQGIFCIHPLRINLGGKLQLVCFDKTGTLTEDGLDVMGVVPLKGQAFLPLV
PEPRRLPVGPLLRALATCHALSRLQDTPVGDPMDLKMVESTGWVLEEEPAADSAFGTQVL
AVMRPPLWEPQLQAMEEPPVPVSVLHRFPFSSALQRMSVVVAWPGATQPEAYVKGSPELV
AGLCNPETVPTDFAQMLQSYTAAGYRVVALASKPLPTVPSLEAAQQLTRDTVEGDLSLLG
LLVMRNLLKPQTTPVIQALRRTRIRAVMVTGDNLQTAVTVARGCGMVAPQEHLIIVHATH
PERGQPASLEFLPMESPTAVNGVKDPDQAASYTVEPDPRSRHLALSGPTFGIIVKHFPKL
LPKVLVQGTVFARMAPEQKTELVCELQKLQYCVGMCGDGANDCGALKAADVGISLSQAEA
SVVSPFTSSMASIECVPMVIREGRCSLDTSFSVFKYMALYSLTQFISVLILYTINTNLGD
LQFLAIDLVITTTVAVLMSRTGPALVLGRVRPPGALLSVPVLSSLLLQMVLVTGVQLGGY
FLTLAQPWFVPLNRTVAAPDNLPNYENTVVFSLSSFQYLILAAAVSKGAPFRRPLYTNVP
FLVALALLSSVLVGLVLVPGLLQGPLALRNITDTGFKLLLLGLVTLNFVGAFMLESVLDQ
CLPACLRRLRPKRASKKRFKQLERELAEQPWPPLPAGPLR
>sp|Q93084|AT2A3_HUMAN Sarcoplasmic/endoplasmic reticulum calcium ATPase 3 OS=Homo sapiens GN=ATP2A3 PE=1 SV=2
MEAAHLLPAADVLRHFSVTAEGGLSPAQVTGARERYGPNELPSEEGKSLWELVLEQFEDL
LVRILLLAALVSFVLAWFEEGEETTTAFVEPLVIMLILVANAIVGVWQERNAESAIEALK
EYEPEMGKVIRSDRKGVQRIRARDIVPGDIVEVAVGDKVPADLRLIEIKSTTLRVDQSIL
TGESVSVTKHTEAIPDPRAVNQDKKNMLFSGTNITSGKAVGVAVATGLHTELGKIRSQMA
AVEPERTPLQRKLDEFGRQLSHAISVICVAVWVINIGHFADPAHGGSWLRGAVYYFKIAV
ALAVAAIPEGLPAVITTCLALGTRRMARKNAIVRSLPSVETLGCTSVICSDKTGTLTTNQ
MSVCRMFVVAEADAGSCLLHEFTISGTTYTPEGEVRQGDQPVRCGQFDGLVELATICALC
NDSALDYNEAKGVYEKVGEATETALTCLVEKMNVFDTDLQALSRVERAGACNTVIKQLMR
KEFTLEFSRDRKSMSVYCTPTRPHPTGQGSKMFVKGAPESVIERCSSVRVGSRTAPLTPT
SREQILAKIRDWGSGSDTLRCLALATRDAPPRKEDMELDDCSKFVQYETDLTFVGCVGML
DPPRPEVAACITRCYQAGIRVVMITGDNKGTAVAICRRLGIFGDTEDVAGKAYTGREFDD
LSPEQQRQACRTARCFARVEPAHKSRIVENLQSFNEITAMTGDGVNDAPALKKAEIGIAM
GSGTAVAKSAAEMVLSDDNFASIVAAVEEGRAIYSNMKQFIRYLISSNVGEVVCIFLTAI
LGLPEALIPVQLLWVNLVTDGLPATALGFNPPDLDIMEKLPRSPREALISGWLFFRYLAI
GVYVGLATVAAATWWFVYDAEGPHINFYQLRNFLKCSEDNPLFAGIDCEVFESRFPTTMA
LSVLVTIEMCNALNSVSENQSLLRMPPWMNPWLLVAVAMSMALHFLILLVPPLPLIFQVT
PLSGRQWVVVLQISLPVILLDEALKYLSRNHMHACLYPGLLRTVSQAWSRQPLTTSWTPD
HTGRNEPEVSAGNRVESPVCTSD
>sp|Q16720|AT2B3_HUMAN Plasma membrane calcium-transporting ATPase 3 OS=Homo sapiens GN=ATP2B3 PE=1 SV=3
MGDMANSSIEFHPKPQQQRDVPQAGGFGCTLAELRTLMELRGAEALQKIEEAYGDVSGLC
RRLKTSPTEGLADNTNDLEKRRQIYGQNFIPPKQPKTFLQLVWEALQDVTLIILEVAAIV

SLGLSFYAPPGEESEACGNVSGGAEDEGEAEAGWIEGAAILLSVICVVLVTAFNDWSKEK
QFRGLQSRIEQEQKFTVIRNGQLLQVPVAALVVGDIAQVKYGDLLPADGVLIQANDLKID
ESSLTGESDHVRKSADKDPMLLSGTHVMEGSGRMVVTAVGVNSQTGIIFTLLGAGGEEEE
KKDKKGKQQDGAMESSQTKAKKQDGAVAMEMQPLKSAEGGEMEEREKKKANAPKKEKSVL
QGKLTKLAVQIGKAGLVMSAITVIILVLYFVIETFVVEGRTWLAECTPVYVQYFVKFFII
GVTVLVVAVPEGLPLAVTISLAYSVKKMMKDNNLVRHLDACETMGNATAICSDKTGTLTT
NRMTVVQSYLGDTHYKEIPAPSALTPKILDLLVHAISINSAYTTKILPPEKEGALPRQVG
NKTECALLGFVLDLKRDFQPVREQIPEDKLYKVYTFNSVRKSMSTVIRMPDGGFRLFSKG
ASEILLKKCTNILNSNGELRGFRPRDRDDMVRKIIEPMACDGLRTICIAYRDFSAGQEPD
WDNENEVVGDLTCIAVVGIEDPVRPEVPEAIRKCQRAGITVRMVTGDNINTARAIAAKCG
IIQPGEDFLCLEGKEFNRRIRNEKGEIEQERLDKVWPKLRVLARSSPTDKHTLVKGIIDS
TTGEQRQVVAVTGDGTNDGPALKKADVGFAMGIAGTDVAKEASDIILTDDNFTSIVKAVM
WGRNVYDSISKFLQFQLTVNVVAVIVAFTGACITQDSPLKAVQMLWVNLIMDTFASLALA
TEPPTESLLLRKPYGRDKPLISRTMMKNILGHAVYQLAIIFTLLFVGELFFDIDSGRNAP
LHSPPSEHYTIIFNTFVMMQLFNEINARKIHGERNVFDGIFSNPIFCTIVLGTFGIQIVI
VQFGGKPFSCSPLSTEQWLWCLFVGVGELVWGQVIATIPTSQLKCLKEAGHGPGKDEMTD
EELAEGEEEIDHAERELRRGQILWFRGLNRIQTQIRVVKAFRSSLYEGLEKPESKTSIHN
FMATPEFLINDYTHNIPLIDDTDVDENEERLRAPPPPSPNQNNNAIDSGIYLTTHVTKSA
TSSVFSSSPGSPLHSVETSL
>sp|Q9NW81|AT5SL_HUMAN ATP synthase subunit s-like protein OS=Homo sapiens GN=ATP5SL PE=1 SV=3
MAAPWASLRLVAPMWNGRIRGIHRLGAAVAPEGNQKKKRTILQFLTNYFYDVEALRDYLL
QREMYKVHEKNRSYTWLEKQHGPYGAGAFFILKQGGAVKFRDKEWIRPDKYGHFSQEFWN
FCEVPVEAVDAGDCDINYEGLDNLLRLKELQSLSLQRCCHVDDWCLSRLYPLADSLQELS
LAGCPRISERGLACLHHLQNLRRLDISDLPAVSNPGLTQILVEEMLPNCEVVGVDWAEGL
KSGPEEQPRDTASPVPA
>sp|Q9Y6H3|ATP23_HUMAN Mitochondrial inner membrane protease ATP23 homolog OS=Homo sapiens GN=ATP23 PE=1 SV=3
MAGAPDERRRGPAAGEQLQQQHVSCQVFPERLAQGNPQQGFFSSFFTSNQKCQLRLLKTL
ETNPYVKLLLDAMKHSGCAVNKDRHFSCEDCNGNVSGGFDASTSQIVLCQNNIHNQAHMN
RVVTHELIHAFDHCRAHVDWFTNIRHLACSEVRAANLSGDCSLVNEIFRLHFGLKQHHQT
CVRDRATLSILAVRNISKEVAKKAVDEVFESCFNDHEPFGRIPHNKTYARYAHRDFENRD
RYYSNI
>sp|Q99766|ATP5S_HUMAN ATP synthase subunit s, mitochondrial OS=Homo sapiens GN=ATP5S PE=1 SV=3
MCCAVSEQRLTCADQMMPFGKISQQLCGVKKLPWSCDSRYFWGWLNAVFNKVDYDRIRDV
GPDRAASEWLLRCGAMVRYHGQERWQKDYNHLPTGPLDKYKIQAIDATDSCIMSIGFDHM
EGLEHVEKIRLCKCHYIEDDCLLRLSQLENLQKTILEMEIISCGNITDKGIIALRHLRNL
KYLLLSDLPGVREKENLVQAFKTALPSLELKLQLK
>sp|P06576|ATPB_HUMAN ATP synthase subunit beta, mitochondrial OS=Homo sapiens GN=ATP5B PE=1 SV=3
MLGFVGRVAAAPASGALRRLTPSASLPPAQLLLRAAPTAVHPVRDYAAQTSPSPKAGAAT
GRIVAVIGAVVDVQFDEGLPPILNALEVQGRETRLVLEVAQHLGESTVRTIAMDGTEGLV
RGQKVLDSGAPIKIPVGPETLGRIMNVIGEPIDERGPIKTKQFAPIHAEAPEFMEMSVEQ

EILVTGIKVVDLLAPYAKGGKIGLFGGAGVGKTVLIMELINNVAKAHGGYSVFAGVGERT
REGNDLYHEMIESGVINLKDATSKVALVYGQMNEPPGARARVALTGLTVAEYFRDQEGQD
VLLFIDNIFRFTQAGSEVSALLGRIPSAVGYQPTLATDMGTMQERITTTKKGSITSVQAI
YVPADDLTDPAPATTFAHLDATTVLSRAIAELGIYPAVDPLDSTSRIMDPNIVGSEHYDV
ARGVQKILQDYKSLQDIIAILGMDELSEEDKLTVSRARKIQRFLSQPFQVAEVFTGHMGK
LVPLKETIKGFQQILAGEYDHLPEQAFYMVGPIEEAVAKADKLAEEHSS
>sp|Q9UBL3|ASH2L_HUMAN Set1/Ash2 histone methyltransferase complex subunit ASH2 OS=Homo sapiens GN=ASH2L PE=1 SV=1
MAAAGAGPGQEAGAGPGPGAVANATGAEEGEMKPVAAGAAAPPGEGISAAPTVEPSSGEA
EGGEANLVDVSGGLETESSNGKDTLEGAGDTSEVMDTQAGSVDEENGRQLGEVELQCGIC
TKWFTADTFGIDTSSCLPFMTNYSFHCNVCHHSGNTYFLRKQANLKEMCLSALANLTWQS
RTQDEHPKTMFSKDKDIIPFIDKYWECMTTRQRPGKMTWPNNIVKTMSKERDVFLVKEHP
DPGSKDPEEDYPKFGLLDQDLSNIGPAYDNQKQSSAVSTSGNLNGGIAAGSSGKGRGAKR
KQQDGGTTGTTKKARSDPLFSAQRLPPHGYPLEHPFNKDGYRYILAEPDPHAPDPEKLEL
DCWAGKPIPGDLYRACLYERVLLALHDRAPQLKISDDRLTVVGEKGYSMVRASHGVRKGA
WYFEITVDEMPPDTAARLGWSQPLGNLQAPLGYDKFSYSWRSKKGTKFHQSIGKHYSSGY
GQGDVLGFYINLPEDTETAKSLPDTYKDKALIKFKSYLYFEEKDFVDKAEKSLKQTPHSE
IIFYKNGVNQGVAYKDIFEGVYFPAISLYKSCTVSINFGPCFKYPPKDLTYRPMSDMGWG
AVVEHTLADVLYHVETEVDGRRSPPWEP
>sp|Q92485|ASM3B_HUMAN Acid sphingomyelinase-like phosphodiesterase 3b OS=Homo sapiens GN=SMPDL3B PE=1 SV=2
MRLLAWLIFLANWGGARAEPGKFWHIADLHLDPDYKVSKDPFQVCPSAGSQPVPDAGPWG
DYLCDSPWALINSSIYAMKEIEPEPDFILWTGDDTPHVPDEKLGEAAVLEIVERLTKLIR
EVFPDTKVYAALGNHDFHPKNQFPAGSNNIYNQIAELWKPWLSNESIALFKKGAFYCEKL
PGPSGAGRIVVLNTNLYYTSNALTADMADPGQQFQWLEDVLTDASKAGDMVYIVGHVPPG
FFEKTQNKAWFREGFNEKYLKVVRKHHRVIAGQFFGHHHTDSFRMLYDDAGVPISAMFIT
PGVTPWKTTLPGVVNGANNPAIRVFEYDRATLSLKDMVTYFMNLSQANAQGTPRWELEYQ
LTEAYGVPDASAHSMHTVLDRIAGDQSTLQRYYVYNSVSYSAGVCDEACSMQHVCAMRQV
DIDAYTTCLYASGTTPVPQLPLLLMALLGLCTLVL
>sp|O95671|ASML_HUMAN N-acetylserotonin O-methyltransferase-like protein OS=Homo sapiens GN=ASMTL PE=1 SV=3
MVLCPVIGKLLHKRVVLASASPRRQEILSNAGLRFEVVPSKFKEKLDKASFATPYGYAME
TAKQKALEVANRLYQKDLRAPDVVIGADTIVTVGGLILEKPVDKQDAYRMLSRLSGREHS
VFTGVAIVHCSSKDHQLDTRVSEFYEETKVKFSELSEELLWEYVHSGEPMDKAGGYGIQA
LGGMLVESVHGDFLNVVGFPLNHFCKQLVKLYYPPRPEDLRRSVKHDSIPAADTFEDLSD
VEGGGSEPTQRDAGSRDEKAEAGEAGQATAEAECHRTRETLPPFPTRLLELIEGFMLSKG
LLTACKLKVFDLLKDEAPQKAADIASKVDASACGMERLLDICAAMGLLEKTEQGYSNTET
ANVYLASDGEYSLHGFIMHNNDLTWNLFTYLEFAIREGTNQHHRALGKKAEDLFQDAYYQ
SPETRLRFMRAMHGMTKLTACQVATAFNLSRFSSACDVGGCTGALARELAREYPRMQVTV
FDLPDIIELAAHFQPPGPQAVQIHFAAGDFFRDPLPSAELYVLCRILHDWPDDKVHKLLS
RVAESCKPGAGLLLVETLLDEEKRVAQRALMQSLNMLVQTEGKERSLGEYQCLLELHGFH
QVQVVHLGGVLDAILATKVAP
>sp|P46597|ASMT_HUMAN Acetylserotonin O-methyltransferase OS=Homo sapiens GN=ASMT PE=1 SV=1

MGSSEDQAYRLLNDYANGFMVSQVLFAACELGVFDLLAEAPGPLDVAAVAAGVRASAHGT
ELLLDICVSLKLLKVETRGGKAFYRNTELSSDYLTTVSPTSQCSMLKYMGRTSYRCWGHL
ADAVREGRNQYLETFGVPAEELFTAIYRSEGERLQFMQALQEVWSVNGRSVLTAFDLSVF
PLMCDLGGGAGALAKECMSLYPGCKITVFDIPEVVWTAKQHFSFQEEEQIDFQEGDFFKD
PLPEADLYILARVLHDWADGKCSHLLERIYHTCKPGGGILVIESLLDEDRRGPLLTQLYS
LNMLVQTEGQERTPTHYHMLLSSAGFRDFQFKKTGAIYDAILARK
>sp|Q2TB18|ASTE1_HUMAN Protein asteroid homolog 1 OS=Homo sapiens GN=ASTE1 PE=1 SV=1
MGIRGLMSFVEDHSNEFFTDLKLRDTKIVIDGYALFHRLCFSSNLDLRYGGDYDSFADVV
QKFFESLFACNICPYVVLDGGCDISDKKLTTLKDRAREKIQMAHSLSVGGSGYVCPLLIR
EVFIQVLIKLRVCFVQCFSEADRDIMTLANHWNCPVLSSDSDFCIFDLKTGFCPLNSFQW
RNMNTIKGTQNYIPAKCFSLDAFCHHFSNMNKALLPLFAVLCGNDHVNLPIMETFLSKAR
LPLGATSSKGRRHHRILGLLNWLSHFANPTEALDNVLKYLPKKDRENVKELLCCSMEEYQ
QSQVKLQDFFQCGTYVCPDALNLGLPEWVLVALAKGQLSPFISDALVLRRTILPTQVENM
QQPNAHRISQPIRQIIYGLLLNASPHLDKTSWNALPPQPLAFSEVERINKNIRTSIIDAV
ELAKDHSDLSRLTELSLRRRQMLLLETLKVKQTILEPIPTSLKLPIAVSCYWLQHTETKA
KLHHLQSLLLTMLVGPLIAIINSPGKEELQEDGAKMLYAEFQRVKAQTRLGTRLDLDTAH
IFCQWQSCLQMGMYLNQLLSTPLPEPDLTRLYSGSLVHGLCQQLLASTSVESLLSICPEA
KQLYEYLFNATRSYAPAEIFLPKGRSNSKKKRQKKQNTSCSKNRGRTTAHTKCWYEGNNR
FGLLMVENLEEHSEASNIE
>sp|Q9C0F0|ASXL3_HUMAN Putative Polycomb group protein ASXL3 OS=Homo sapiens GN=ASXL3 PE=2 SV=3
MKDKRKKKDRTWAEAARLALEKHPNSPMTAKQILEVIQKEGLKETSGTSPLACLNAMLHT
NTRIGDGTFFKIPGKSGLYALKKEESSCPADGTLDLVCESELDGTDMAEANAHGEENGVC
SKQVTDEASSTRDSSLTNTAVQSKLVSSFQQHTKKALKQALRQQQKRRNGVSMMVNKTVP
RVVLTPLKVSDEQSDSPSGSESKNGEADSSDKEMKHGQKSPTGKQTSQHLKRLKKSGLGH
LKWTKAEDIDIETPGSILVNTNLRALINKHTFASLPQHFQQYLLLLLPEVDRQMGSDGIL
RLSTSALNNEFFAYAAQGWKQRLAEGEFTPEMQLRIRQEIEKEKKTEPWKEKFFERFYGE
KLGMSREESVKLTTGPNNAGAQSSSSCGTSGLPVSAQTALAEQQPKSMKSPASPEPGFCA
TLCPMVEIPPKDIMAELESEDILIPEESVIQEEIAEEVETSICECQDENHKTIPEFSEEA
ESLTNSHEEPQIAPPEDNLESCVMMNDVLETLPHIEVKIEGKSESPQEEMTVVIDQLEVC
DSLIPSTSSMTHVSDTEHKESETAVETSTPKIKTGSSSLEGQFPNEGIAIDMELQSDPEE
QLSENACISETSFSSESPEGACTSLPSPGGETQSTSEESCTPASLETTFCSEVSSTENTD
KYNQRNSTDENFHASLMSEISPISTSPEISEASLMSNLPLTSEASPVSNLPLTSETSPMS
DLPLTSETSSVSSMLLTSETTFVSSLPLPSETSPISNSSINERMAHQQRKSPSVSEEPLS
PQKDESSATAKPLGENLTSQQKNLSNTPEPIIMSSSSIAPEAFPSEDLHNKTLSQQTCKS
HVDTEKPYPASIPELASTEMIKVKNHSVLQRTEKKVLPSPLELSVFSEGTDNKGNELPSA
KLQDKQYISSVDKAPFSEGSRNKTHKQGSTQSRLETSHTSKSSEPSKSPDGIRNESRDSE
ISKRKTAEQHSFGICKEKRARIEDDQSTRNISSSSPPEKEQPPREEPRVPPLKIQLSKIG
PPFIIKSQPVSKPESRASTSTSVSGGRNTGARTLADIKARAQQARAQREAAAAAAVAAAA
SIVSGAMGSPGEGGKTRTLAHIKEQTKAKLFAKHQARAHLFQTSKETRLPPPLSSKEGPP
NLEVSSTPETKMEGSTGVIIVNPNCRSPSNKSAHLRETTTVLQQSLNPSKLPETATDLSV
HSSDENIPVSHLSEKIVSSTSSENSSVPMLFNKNSVPVSVCSTAISGAIKEHPFVSSVDK
SSVLMSVDSANTTISACNISMLKTIQGTDTPCIAIIPKCIESTPISATTEGSSISSSMDD
KQLLISSSSASNLVSTQYTSVPTPSIGNNLPNLSTSSVLIPPMGINNRFPSEKIAIPGSE

EQATVSMGTTVRAALSCSDSVAVTDSLVAHPTVAMFTGNMLTINSYDSPPKLSAESLDKN
SGPRNRADNSGKPQQPPGGFAPAAINRSIPCKVIVDHSTTLTSSLSLTVSVESSEASLDL
QGRPVRTEASVQPVACPQVSVISRPEPVANEGIDHSSTFIAASAAKQDSKTLPATCTSLR
ELPLVPDKLNEPTAPSHNFAEQARGPAPFKSEADTTCSNQYNPSNRICWNDDGMRSTGQP
LVTHSGSSKQKEYLEQSCPKAIKTEHANYLNVSELHPRNLVTNVALPVKSELHEADKGFR
MDTEDFPGPELPPPAAEGASSVQQTQNMKASTSSPMEEAISLATDALKRVPGAGSSGCRL
SSVEANNPLVTQLLQGNLPLEKVLPQPRLGAKLEINRLPLPLQTTSVGKTAPERNVEIPP
SSPNPDGKGYLAGTLAPLQMRKRENHPKKRVARTVGEHTQVKCEPGKLLVEPDVKGVPCV
ISSGISQLGHSQPFKQEWLNKHSMQNRIVHSPEVKQQKRLLPSCSFQQNLFHVDKNGGFH
TDAGTSHRQQFYQMPVAARGPIPTAALLQASSKTPVGCNAFAFNRHLEQKGLGEVSLSSA
PHQLRLANMLSPNMPMKEGDEVGGTAHTMPNKALVHPPPPPPPPPPPPLALPPPPPPPPP
LPPPLPNAEVPSDQKQPPVTMETTKRLSWPQSTGICSNIKSEPLSFEEGLSSSCELGMKQ
VSYDQNEMKEQLKAFALKSADFSSYLLSEPQKPFTQLAAQKMQVQQQQQLCGNYPTIHFG
STSFKRAASAIEKSIGILGSGSNPATGLSGGQNAQMPVQNFADSSNADELELKCSCRLKAM
IVCKGCGAFCHDDCIGPSKLCVACLVVR
>sp|Q5T6C5|AT7L2_HUMAN Ataxin-7-like protein 2 OS=Homo sapiens GN=ATXN7L2 PE=1 SV=1
MAVRERAAAAMAALERRVPSLDDFAGQSWSSWVERADLPAADGAELEESSKNTKKLDAMT
LIKEDMSIFGHCPAHDDFYLVVCNHCSQVVKPQAFQKHCERRHGPLSKLYGRAPPPPPAP
ASSQKCHVVNGQGPACRAPGSTKTSSREKGQGSRSRGHQPPEKTQKDNLCQPGGLTKDSP
GKPPMAPPSKEPPGRENIEIIPSEGSSHWAEGSPPEKEPSGTRLPPKTHRKMARKECDLN
RQCGVINPETKKICTRLLTCKIHSVHQRREVQGRAKDFDVLVAELKANSRKGESPKEKSP
GRKEQVLERPSQELPSSVQVVAAVAAPSSTFSVRAKQTYPYCALPRSRASSESELDDEGP
CGGDGDPGLFPFPMPRGGTQASSEESEEEGTSDDLHPPPDCHYATRPPRPQAFCTFGSRL
VSPGCYVFSRRLDRFCSALSSMLERHLSTHMWKKIPPAAEPPAHLVNSPLSAPLSPSSTG
TCPRLPGPTLRPACPASMPPTKDNLVPSYPAGSPSVAAACSQAECMGGSQAITSPLPANT
PSPSFSKLPPSKASKSSKGKDGVEVEAPSRKRKLSPGPTTLKRTCILEPTGKGKPSGCRG
LSAKTKTALSMGLNGTMGPRVKRAGPLDCRGSPHQLPTPVKASQLENRGAAGHPAKALPT
NCLSEEEVAKKRKNLATYCRPVKAKHCQAGAPADVACSVRRKKPGPALAFEEKCSTLKSK
AH
>sp|Q06055|AT5G2_HUMAN ATP synthase F(0) complex subunit C2, mitochondrial OS=Homo sapiens GN=ATP5G2 PE=2 SV=1
MFACSKFVSTPSLVKSTSQLLSRPLSAVVLKRPEILTDESLSSLAVSCPLTSLVSSRSFQ
TSAISRDIDTAAKFIGAGAATVGVAGSGAGIGTVFGSLIIGYARNPSLKQQLFSYAILGF
ALSEAMGLFCLMVAFLILFAM
>sp|Q8TF62|AT8B4_HUMAN Probable phospholipid-transporting ATPase IM OS=Homo sapiens GN=ATP8B4 PE=1 SV=3
MFCSEKKLREVERIVKANDREYNEKFQYADNRIHTSKYNILTFLPINLFEQFQRVANAYF
LCLLILQLIPEISSLTWFTTIVPLVLVITMTAVKDATDDYFRHKSDNQVNNRQSEVLINS
KLQNEKWMNVKVGDIIKLENNQFVAADLLLLSSSEPHGLCYVETAELDGETNLKVRHALS
VTSELGADISRLAGFDGIVVCEVPNNKLDKFMGILSWKDSKHSLNNEKIILRGCILRNTS
WCFGMVIFAGPDTKLMQNSGKTKFKRTSIDRLMNTLVLWIFGFLICLGIILAIGNSIWES
QTGDQFRTFLFWNEGEKSSVFSGFLTFWSYIIILNTVVPISLYVSVEVIRLGHSYFINWD
RKMYYSRKAIPAVARTTTLNEELGQIEYIFSDKTGTLTQNIMTFKRCSINGRIYGEVHDD
LDQKTEITQEKEPVDFSVKSQADREFQFFDHHLMESIKMGDPKVHEFLRLLALCHTVMSE

ENSAGELIYQVQSPDEGALVTAARNFGFIFKSRTPETITIEELGTLVTYQLLAFLDFNNT
RKRMSVIVRNPEGQIKLYSKGADTILFEKLHPSNEVLLSLTSDHLSEFAGEGLRTLAIAY
RDLDDKYFKEWHKMLEDANAATEERDERIAGLYEEIERDLMLLGATAVEDKLQEGVIETV
TSLSLANIKIWVLTGDKQETAINIGYACNMLTDDMNDVFVIAGNNAVEVREELRKAKQNL
FGQNRNFSNGHVVCEKKQQLELDSIVEETITGDYALIINGHSLAHALESDVKNDLLELAC
MCKTVICCRVTPLQKAQVVELVKKYRNAVTLAIGDGANDVSMIKSAHIGVGISGQEGLQA
VLASDYSFAQFRYLQRLLLVHGRWSYFRMCKFLCYFFYKNFAFTLVHFWFGFFCGFSAQT
VYDQWFITLFNIVYTSLPVLAMGIFDQDVSDQNSVDCPQLYKPGQLNLLFNKRKFFICVL
HGIYTSLVLFFIPYGAFYNVAGEDGQHIADYQSFAVTMATSLVIVVSVQIALDTSYWTFI
NHVFIWGSIAIYFSILFTMHSNGIFGIFPNQFPFVGNARHSLTQKCIWLVILLTTVASVM
PVVAFRFLKVDLYPTLSDQIRRWQKAQKKARPPSSRRPRTRRSSSRRSGYAFAHQEGYGE
LITSGKNMRAKNPPPTSGLEKTHYNSTSWIENLCKKTTDTVSSFSQDKTVKL
>sp|Q5T9A4|ATD3B_HUMAN ATPase family AAA domain-containing protein 3B OS=Homo sapiens GN=ATAD3B PE=1 SV=1
MSWLFGVNKGPKGEGAGPPPPLPPAQPGAEGGGDRGLGDRPAPKDKWSNFDPTGLERAAK
AARELEHSRYAKEALNLAQMQEQTLQLEQQSKLKEYEAAVEQLKSEQIRAQAEERRKTLS
EETRQHQARAQYQDKLARQRYEDQLKQQQLLNEENLRKQEESVQKQEAMRRATVEREMEL
RHKNEMLRVETEARARAKAERENADIIREQIRLKASEHRQTVLESIRTAGTLFGEGFRAF
VTDRDKVTATVAGLTLLAVGVYSAKNATAVTGRFIEARLGKPSLVRETSRITVLEALRHP
IQVSRRLLSRPQDVLEGVVLSPSLEARVRDIAIATRNTKKNRGLYRHILLYGPPGTGKTL
FAKKLALHSGMDYAIMTGGDVAPMGREGVTAMHKLFDWANTSRRGLLLFMDEADAFLRKR
ATEEISKDLRATLNAFLYHMGQHSNKFMLVLASNLPEQFDCAINSRIDVMVHFDLPQQEE
RERLVRLHFDNCVLKPATEGKRRLKLAQFDYGRKCSEVARLTEGMSGREIAQLAVSWQAT
AYASKDGVLTEAMMDACVQDAVQQYRQKMRWLKAEGPGRGVEHPLSGVQGETLTSWSLAT
DPSYPCLAGPCTFRICSWMGTGLCPGPLSPRMSCGGGRPFCPPGHPLL
>sp|P18846|ATF1_HUMAN Cyclic AMP-dependent transcription factor ATF-1 OS=Homo sapiens GN=ATF1 PE=1 SV=2
MEDSHKSTTSETAPQPGSAVQGAHISHIAQQVSSLSESEESQDSSDSIGSSQKAHGILAR
RPSYRKILKDLSSEDTRGRKGDGENSGVSAAVTSMSVPTPIYQTSSGQYIAIAPNGALQL
ASPGTDGVQGLQTLTMTNSGSTQQGTTILQYAQTSDGQQILVPSNQVVVQTASGDMQTYQ
IRTTPSATSLPQTVVMTSPVTLTSQTTKTDDPQLKREIRLMKNREAARECRRKKKEYVKC
LENRVAVLENQNKTLIEELKTLKDLYSNKSV
>sp|Q8N5I2|ARRD1_HUMAN Arrestin domain-containing protein 1 OS=Homo sapiens GN=ARRDC1 PE=1 SV=1
MGRVQLFEISLSHGRVVYSPGEPLAGTVRVRLGAPLPFRAIRVTCIGSCGVSNKANDTAW
VVEEGYFNSSLSLADKGSLPAGEHSFPFQFLLPATAPTSFEGPFGKIVHQVRAAIHTPRF
SKDHKCSLVFYILSPLNLNSIPDIEQPNVASATKKFSYKLVKTGSVVLTASTDLRGYVVG
QALQLHADVENQSGKDTSPVVASLLQKVSYKAKRWIHDVRTIAEVEGAGVKAWRRAQWHE
QILVPALPQSALPGCSLIHIDYYLQVSLKAPEATVTLPVFIGNIAVNHAPVSPRPGLGLP
PGAPPLVVPSAPPQEEAEAEAAAGGPHFLDPVFLSTKSHSQRQPLLATLSSVPGAPEPCP
QDGSPASHPLHPPLCISTGATVPYFAEGSGGPVPTTSTLILPPEYSSWGYPYEAPPSYEQ
SCGGVEPSLTPES
>sp|Q8WXJ9|ASB17_HUMAN Ankyrin repeat and SOCS box protein 17 OS=Homo sapiens GN=ASB17 PE=2 SV=2

MSKSTKLCGKTSCPRSNIFCNLLDKIVKRPSLQFLGQWGYHCYEPRIYRSLAKILRYVDL
DGFDALLTDYIAFVEKSGYRFEVSFNLDFTEICVNTILYWVFARKGNPDFVELLLKKTKD
YVQDRSCNLALIWRTFTPVYCPSPLSGITPLFYVAQTRQSNIFKILLQYGILEREKNPIN
IVLTIVLYPSRVRVMVDRELADIHEDAKTCLVLCSRVLSVISVKEIKTQLSLGRHPIISN
WFDYIPSTRYKDPCELLHLCRLTIRNQLLTNNMLPDGIFSLLIPARLQNYLNLEI
>sp|Q9NWX5|ASB6_HUMAN Ankyrin repeat and SOCS box protein 6 OS=Homo sapiens GN=ASB6 PE=1 SV=1
MPFLHGFRRIIFEYQPLVDAILGSLGIQDPERQESLDRPSYVASEESRILVLTELLERKA
HSPFYQEGVSNALLKMAELGLTRAADVLLRHGANLNFEDPVTYYTALHIAVLRNQPDMVE
LLVHHGADVNRRDRIHESSPLDLASEEPERLPCLQRLLDLGADVNAADKHGKTALLHALA
SSDGVQIHNTENIRLLLEGGADVKATTKDGDTVFTCIIFLLGETVGGDKEEAQMINRFCF
QVTRLLLAHGADPSECPAHESLTHICLKSFKLHFPLLRFLLESGAAYNCSLHGASCWSGF
HIIFERLCSHPGCTEDESHADLLRKAETVLDLMVTNSQKLQLPENFDIHPVGSLAEKIQA
LHFSLRQLESYPPPLKHLCRVAIRLYLQPWPVDVKVKALPLPDRLKWYLLSEHSGSVEDD
I
>sp|Q8N9N2|ASCC1_HUMAN Activating signal cointegrator 1 complex subunit 1 OS=Homo sapiens GN=ASCC1 PE=1 SV=1
MEVLRPQLIRIDGRNYRKNPVQEQTYQHEEDEEDFYQGSMECADEPCDAYEVEQTPQGFR
STLRAPSLLYNLIHLNTSNDCGFQKITLDCQNIYTWKSRHIVGKRGDTRKKIEMETKTSI
SIPKPGQDGEIVITGQHRNGVISARTRIDVLLDTFRRKQPFTHFLAFFLNEVEVQEGFLR
FQEEVLAKCSMDHGVDSSIFQNPKKLHLTIGMLVLLSEEEIQQTCEMLQQCKEEFINDIS
GGKPLEVEMAGIEYMNDDPGMVDVLYAKVHMKDGSNRLQELVDRVLERFQASGLIVKEWN
SVKLHATVMNTLFRKDPNAEGRYNLYTAEGKYIFKERESFDGRNILKSFALLPRLEYNDA
ISAHCNLCLPGSSDSPASASQVAGITGVSDAYSQSLPGKS
>sp|Q9H1I8|ASCC2_HUMAN Activating signal cointegrator 1 complex subunit 2 OS=Homo sapiens GN=ASCC2 PE=1 SV=3
MPALPLDQLQITHKDPKTGKLRTSPALHPEQKADRYFVLYKPPPKDNIPALVEEYLERAT
FVANDLDWLLALPHDKFWCQVIFDETLQKCLDSYLRYVPRKFDEGVASAPEVVDMQKRLH
RSVFLTFLRMSTHKESKDHFISPSAFGEILYNNFLFDIPKILDLCVLFGKGNSPLLQKMI
GNIFTQQPSYYSDLDETLPTILQVFSNILQHCGLQGDGANTTPQKLEERGRLTPSDMPLL
ELKDIVLYLCDTCTTLWAFLDIFPLACQTFQKHDFCYRLASFYEAAIPEMESAIKKRRLE
DSKLLGDLWQRLSHSRKKLMEIFHIILNQICLLPILESSCDNIQGFIEEFLQIFSSLLQE
KRFLRDYDALFPVAEDISLLQQASSVLDETRTAYILQAVESAWEGVDRRKATDAKDPSVI
EEPNGEPNGVTVTAEAVSQASSHPENSEEEECMGAAAAVGPAMCGVELDSLISQVKDLLP
DLGEGFILACLEYYHYDPEQVINNILEERLAPTLSQLDRNLDREMKPDPTPLLTSRHNVF
QNDEFDVFSRDSVDLSRVHKGKSTRKEENTRSLLNDKRAVAAQRQRYEQYSVVVEEVPLQ
PGESLPYHSVYYEDEYDDTYDGNQVGANDADSDDELISRRPFTIPQVLRTKVPREGQEED
DDDEEDDADEEAPKPDHFVQDPAVLREKAEARRMAFLAKKGYRHDSSTAVAGSPRGHGQS
RETTQERRKKEANKATRANHNRRTMADRKRSKGMIPS
>sp|Q9NQ33|ASCL3_HUMAN Achaete-scute homolog 3 OS=Homo sapiens GN=ASCL3 PE=2 SV=2
MDNRGNSSLPDKLPIFPDSARLPLTRSFYLEPMVTFHVHPEAPVSSPYSEELPRLPFPSD
SLILGNYSEPCPFSFPMPYPNYRGCEYSYGPAFTRKRNERERQRVKCVNEGYAQLRHHLP
EEYLEKRLSKVETLRAAIKYINYLQSLLYPDKAETKNNPGKVSSMIATTSHHADPMFRIV
>sp|P78348|ASIC1_HUMAN Acid-sensing ion channel 1 OS=Homo sapiens GN=ASIC1 PE=1 SV=3

MELKAEEEEVGGVQPVSIQAFASSSTLHGLAHIFSYERLSLKRALWALCFLGSLAVLLCV
CTERVQYYFHYHHVTKLDEVAASQLTFPAVTLCNLNEFRFSQVSKNDLYHAGELLALLNN
RYEIPDTQMADEKQLEILQDKANFRSFKPKPFNMREFYDRAGHDIRDMLLSCHFRGEVCS
AEDFKVVFTRYGKCYTFNSGRDGRPRLKTMKGGTGNGLEIMLDIQQDEYLPVWGETDETS
FEAGIKVQIHSQDEPPFIDQLGFGVAPGFQTFVACQEQRLIYLPPPWGTCKAVTMDSDLD
FFDSYSITACRIDCETRYLVENCNCRMVHMPGDAPYCTPEQYKECADPALDFLVEKDQEY
CVCEMPCNLTRYGKELSMVKIPSKASAKYLAKKFNKSEQYIGENILVLDIFFEVLNYETI
EQKKAYEIAGLLGDIGGQMGLFIGASILTVLELFDYAYEVIKHKLCRRGKCQKEAKRSSA
DKGVALSLDDVKRHNPCESLRGHPAGMTYAANILPHHPARGTFEDFTC
>sp|Q96FT7|ASIC4_HUMAN Acid-sensing ion channel 4 OS=Homo sapiens GN=ASIC4 PE=1 SV=2
MLSGAAGAARRGGAALAPSLTRSLAGTHAGADSCAGADKGSHKETIEERDKRQQRQQRQR
QHQGCGAAGSGSDSPTSGPHPVPVLFPLALSLEEQPLPPLPLGRAPGLLAREGQGREALA
SPSSRGQMPIEIVCKIKFAEEDAKPKEKEAGDEQSLLGAVAPGAAPRDLATFASTSTLHG
LGRACGPGPHGLRRTLWALALLTSLAAFLYQAAGLARGYLTRPHLVAMDPAAPAPVAGFP
AVTLCNINRFRHSALSDADIFHLANLTGLPPKDRDGHRAAGLRYPEPDMVDILNRTGHQL
ADMLKSCNFSGHHCSASNFSVVYTRYGKCYTFNADPRSSLPSRAGGMGSGLEIMLDIQQE
EYLPIWRETNETSFEAGIRVQIHSQEEPPYIHQLGFGVSPGFQTFVSCQEQRLTYLPQPW
GNCRAESELREPELQGYSAYSVSACRLRCEKEAVLQRCHCRMVHMPDSLGGGPEGPCFCP
TPCNLTRYGKEISMVRIPNRGSARYLARKYNRNETYIRENFLVLDVFFEALTSEAMEQRA
AYGLSALLGDLGGQMGLFIGASILTLLEILDYIYEVSWDRLKRVWRRPKTPLRTSTGGIS
TLGLQELKEQSPCPSRGRVEGGGVSSLLPNHHHPHGPPGGLFEDFAC
>sp|O43681|ASNA_HUMAN ATPase ASNA1 OS=Homo sapiens GN=ASNA1 PE=1 SV=2
MAAGVAGWGVEAEEFEDAPDVEPLEPTLSNIIEQRSLKWIFVGGKGGVGKTTCSCSLAVQ
LSKGRESVLIISTDPAHNISDAFDQKFSKVPTKVKGYDNLFAMEIDPSLGVAELPDEFFE
EDNMLSMGKKMMQEAMSAFPGIDEAMSYAEVMRLVKGMNFSVVVFDTAPTGHTLRLLNFP
TIVERGLGRLMQIKNQISPFISQMCNMLGLGDMNADQLASKLEETLPVIRSVSEQFKDPE
QTTFICVCIAEFLSLYETERLIQELAKCKIDTHNIIVNQLVFPDPEKPCKMCEARHKIQA
KYLDQMEDLYEDFHIVKLPLLPHEVRGADKVNTFSALLLEPYKPPSAQ
>sp|Q6ICH7|ASPH2_HUMAN Aspartate beta-hydroxylase domain-containing protein 2 OS=Homo sapiens GN=ASPHD2 PE=2 SV=1
MVWAPLGPPRTDCLTLLHTPSKDSPKMSLEWLVAWSWSLDGLRDCIATGIQSVRDCDTTA
VITVACLLVLFVWYCYHVGREQPRPYVSVNSLMQAADANGLQNGYVYCQSPECVRCTHNE
GLNQKLYHNLQEYAKRYSWSGMGRIHKGIREQGRYLNSRPSIQKPEVFFLPDLPTTPYFS
RDAQKHDVEVLERNFQTILCEFETLYKAFSNCSLPQGWKMNSTPSGEWFTFYLVNQGVCV
PRNCRKCPRTYRLLGSLRTCIGNNVFGNACISVLSPGTVITEHYGPTNIRIRCHLGLKTP
NGCELVVGGEPQCWAEGRCLLFDDSFLHAAFHEGSAEDGPRVVFMVDLWHPNVAAAERQA
LDFIFAPGR
>sp|Q96KQ4|ASPP1_HUMAN Apoptosis-stimulating of p53 protein 1 OS=Homo sapiens GN=PPP1R13B PE=1 SV=3
MMPMILTVFLSNNEQILTEVPITPETTCRDVVEFCKEPGEGSCHLAEVWRGNERPIPFDH
MMYEHLQKWGPRREEVKFFLRHEDSPTENSEQGGRQTQEQRTQRNVINVPGEKRTENGVG
NPRVELTLSELQDMAARQQQQIENQQQMLVAKEQRLHFLKQQERRQQQSISENEKLQKLK
ERVEAQENKLKKIRAMRGQVDYSKIMNGNLSAEIERFSAMFQEKKQEVQTAILRVDQLSQ
QLEDLKKGKLNGFQSYNGKLTGPAAVELKRLYQELQIRNQLNQEQNSKLQQQKELLNKRN

MEVAMMDKRISELRERLYGKKIQLNRVNGTSSPQSPLSTSGRVAAVGPYIQVPSAGSFPV
LGDPIKPQSLSIASNAAHGRSKSANDGNWPTLKQNSSSSVKPVQVAGADWKDPSVEGSVK
QGTVSSQPVPFSALGPTEKPGIEIGKVPPPIPGVGKQLPPSYGTYPSPTPLGPGSTSSLE
RRKEGSLPRPSAGLPSRQRPTLLPATGSTPQPGSSQQIQQRISVPPSPTYPPAGPPAFPA
GDSKPELPLTVAIRPFLADKGSRPQSPRKGPQTVNSSSIYSMYLQQATPPKNYQPAAHSA
LNKSVKAVYGKPVLPSGSTSPSPLPFLHGSLSTGTPQPQPPSESTEKEPEQDGPAAPADG
STVESLPRPLSPTKLTPIVHSPLRYQSDADLEALRRKLANAPRPLKKRSSITEPEGPGGP
NIQKLLYQRFNTLAGGMEGTPFYQPSPSQDFMGTLADVDNGNTNANGNLEELPPAQPTAP
LPAEPAPSSDANDNELPSPEPEELICPQTTHQTAEPAEDNNNNVATVPTTEQIPSPVAEA
PSPGEEQVPPAPLPPASHPPATSTNKRTNLKKPNSERTGHGLRVRFNPLALLLDASLEGE
FDLVQRIIYEVEDPSKPNDEGITPLHNAVCAGHHHIVKFLLDFGVNVNAADSDGWTPLHC
AASCNSVHLCKQLVESGAAIFASTISDIETAADKCEEMEEGYIQCSQFLYGVQEKLGVMN
KGVAYALWDYEAQNSDELSFHEGDALTILRRKDESETEWWWARLGDREGYVPKNLLGLYP
RIKPRQRTLA
>sp|O14525|ASTN1_HUMAN Astrotactin-1 OS=Homo sapiens GN=ASTN1 PE=2 SV=3
MALAGLCALLACCWGPAAVLATAAGDVDPSKELECKLKSITVSALPFLRENDLSIMHSPS
ASEPKLLFSVRNDFPGEMVVVDDLENTELPYFVLEISGNTEDIPLVRWRQQWLENGTLLF
HIHHQDGAPSLPGQDPTEEPQHESAEEELRILHISVMGGMIALLLSILCLVMILYTRRRW
CKRRRVPQPQKSASAEAANEIHYIPSVLIGGHGRESLRNARVQGHNSSGTLSIRETPILD
GYEYDITDLRHHLQRECMNGGEDFASQVTRTLDSLQGCNEKSGMDLTPGSDNAKLSLMNK
YKDNIIATSPVDSNHQQATLLSHTSSSQRKRINNKARAGSAFLNPEGDSGTEAENDPQLT
FYTDPSRSRRRSRVGSPRSPVNKTTLTLISITSCVIGLVCSSHVNCPLVVKITLHVPEHL
IADGSRFILLEGSQLDASDWLNPAQVVLFSQQNSSGPWAMDLCARRLLDPCEHQCDPETG
RREHRAAGECLCYEGYMKDPVHKHLCIRNEWGTNQGPWPYTIFQRGFDLVLGEQPSDKIF
RFTYTLGEGMWLPLSKSFVIPPAELAINPSAKCKTDMTVMEDAVEVREELMTSSSFDSLE
VLLDSFGPVRDCSKDNGGCSKNFRCISDRKLDSTGCVCPSGLSPMKDSSGCYDRHIGVDC
SDGFNGGCEQLCLQQMAPFPDDPTLYNILMFCGCIEDYKLGVDGRSCQLITETCPEGSDC
GESRELPMNQTLFGEMFFGYNNHSKEVAAGQVLKGTFRQNNFARGLDQQLPDGLVVATVP
LENQCLEEISEPTPDPDFLTGMVNFSEVSGYPVLQHWKVRSVMYHIKLNQVAISQALSNA
LHSLDGATSRADFVALLDQFGNHYIQEAIYGFEESCSIWYPNKQVQRRLWLEYEDISKGN
SPSDESEERERDPKVLTFPEYITSLSDSGTKHMAAGVRMECHSKGRCPSSCPLCHVTSSP
DTPAEPVLLEVTKAAPIYELVTNNQTQRLLQEATMSSLWCSGTGDVIEDWCRCDSTAFGA
DGLPTCAPLPQPVLRLSTVHEPSSTLVVLEWEHSEPPIGVQIVDYLLRQEKVTDRMDHSK
VETETVLSFVDDIISGAKSPCAMPSQVPDKQLTTISLIIRCLEPDTIYMFTLWGVDNTGR
RSRPSDVIVKTPCPVVDDVKAQEIADKIYNLFNGYTSGKEQQTAYNTLLDLGSPTLHRVL
YHYNQHYESFGEFTWRCEDELGPRKAGLILSQLGDLSSWCNGLLQEPKISLRRSSLKYLG
CRYSEIKPYGLDWAELSRDLRKTCEEQTLSIPYNDYGDSKEI
>sp|Q9NVM9|ASUN_HUMAN Protein asunder homolog OS=Homo sapiens GN=ASUN PE=1 SV=2
MKIFSESHKTVFVVDHCPYMAESCRQHVEFDMLVKNRTQGIIPLAPISKSLWTCSVESSM
EYCRIMYDIFPFKKLVNFIVSDSGAHVLNSWTQEDQNLQELMAALAAVGPPNPRADPECC
SILHGLVAAVETLCKITEYQHEARTLLMENAERVGNRGRIICITNAKSDSHVRMLEDCVQ
ETIHEHNKLAANSDHLMQIQKCELVLIHTYPVGEDSLVSDRSKKELSPVLTSEVHSVRAG
RHLATKLNILVQQHFDLASTTITNIPMKEEQHANTSANYDVELLHHKDAHVDFLKSGDSH
LGGGSREGSFKETITLKWCTPRTNNIELHYCTGAYRISPVDVNSRPSSCLTNFLLNGRSV

LLEQPRKSGSKVISHMLSSHGGEIFLHVLSSSRSILEDPPSISEGCGGRVTDYRITDFGE
FMRENRLTPFLDPRYKIDGSLEVPLERAKDQLEKHTRYWPMIISQTTIFNMQAVVPLASV
IVKESLTEEDVLNCQKTIYNLVDMERKNDPLPISTVGTRGKGPKRDEQYRIMWNELETLV
RAHINNSEKHQRVLECLMACRSKPPEEEERKKRGRKREDKEDKSEKAVKDYEQEKSWQDS
ERLKGILERGKEELAEAEIIKDSPDSPEPPNKKPLVEMDETPQVEKSKGPVSLLSLWSNR
INTANSRKHQEFAGRLNSVNNRAELYQHLKEENGMETTENGKASRQ
>sp|Q8IXJ9|ASXL1_HUMAN Putative Polycomb group protein ASXL1 OS=Homo sapiens GN=ASXL1 PE=1 SV=3
MKDKQKKKKERTWAEAARLVLENYSDAPMTPKQILQVIEAEGLKEMRSGTSPLACLNAML
HSNSRGGEGLFYKLPGRISLFTLKKDALQWSRHPATVEGEEPEDTADVESCGSNEASTVS
GENDVSLDETSSNASCSTESQSRPLSNPRDSYRASSQANKQKKKTGVMLPRVVLTPLKVN
GAHVESASGFSGCHADGESGSPSSSSSGSLALGSAAIRGQAEVTQDPAPLLRGFRKPATG
QMKRNRGEEIDFETPGSILVNTNLRALINSRTFHALPSHFQQQLLFLLPEVDRQVGTDGL
LRLSSSALNNEFFTHAAQSWRERLADGEFTHEMQVRIRQEMEKEKKVEQWKEKFFEDYYG
QKLGLTKEESLQQNVGQEEAEIKSGLCVPGESVRIQRGPATRQRDGHFKKRSRPDLRTRA
RRNLYKKQESEQAGVAKDAKSVASDVPLYKDGEAKTDPAGLSSPHLPGTSSAAPDLEGPE
FPVESVASRIQAEPDNLARASASPDRIPSLPQETVDQEPKDQKRKSFEQAASASFPEKKP
RLEDRQSFRNTIESVHTEKPQPTKEEPKVPPIRIQLSRIKPPWVVKGQPTYQICPRIIPT
TESSCRGWTGARTLADIKARALQVRGARGHHCHREAATTAIGGGGGPGGGGGGATDEGGG
RGSSSGDGGEACGHPEPRGGPSTPGKCTSDLQRTQLLPPYPLNGEHTQAGTAMSRARRED
LPSLRKEESCLLQRATVGLTDGLGDASQLPVAPTGDQPCQALPLLSSQTSVAERLVEQPQ
LHPDVRTECESGTTSWESDDEEQGPTVPADNGPILSLVGDDTLEKGTGQALDSHPTMKDP
VNVTPSSTPESSPTDCLQNRAFDDELGLGGSCPPMRESDTRQENLKTKALVSNSSLHWIP
IPSNDEVVKQPKPESREHIPSVEPQVGEEWEKAAPTPPALPGDLTAEEGLDPLDSLTSLW
TVPSRGGSDSNGSYCQQVDIEKLKINGDSEALSPHGESTDTASDFEGHLTEDSSEADTRE
AAVTKGSSVDKDEKPNWNQSAPLSKVNGDMRLVTRTDGMVAPQSWVSRVCAVRQKIPDSL
LLASTEYQPRAVCLSMPGSSVEATNPLVMQLLQGSLPLEKVLPPAHDDSMSESPQVPLTK
DQSHGSLRMGSLHGLGKNSGMVDGSSPSSLRALKEPLLPDSCETGTGLARIEATQAPGAP
QKNCKAVPSFDSLHPVTNPITSSRKLEEMDSKEQFSSFSCEDQKEVRAMSQDSNSNAAPG
KSPGDLTTSRTPRFSSPNVISFGPEQTGRALGDQSNVTGQGKKLFGSGNVAATLQRPRPA
DPMPLPAEIPPVFPSGKLGPSTNSMSGGVQTPREDWAPKPHAFVGSVKNEKTFVGGPLKA
NAENRKATGHSPLELVGHLEGMPFVMDLPFWKLPREPGKGLSEPLEPSSLPSQLSIKQAF
YGKLSKLQLSSTSFNYSSSSPTFPKGLAGSVVQLSHKANFGASHSASLSLQMFTDSSTVE
SISLQCACSLKAMIMCQGCGAFCHDDCIGPSKLCVLCLVVR
>sp|P54707|AT12A_HUMAN Potassium-transporting ATPase alpha chain 2 OS=Homo sapiens GN=ATP12A PE=1 SV=3
MHQKTPEIYSVELSGTKDIVKTDKGDGKEKYRGLKNNCLELKKKNHKEEFQKELHLDDHK
LSNRELEEKYGTDIIMGLSSTRAAELLARDGPNSLTPPKQTPEIVKFLKQMVGGFSILLW
VGAFLCWIAYGIQYSSDKSASLNNVYLGCVLGLVVILTGIFAYYQEAKSTNIMSSFNKMI
PQQALVIRDSEKKTIPSEQLVVGDIVEVKGGDQIPADIRVLSSQGCRVDNSSLTGESEPQ
PRSSEFTHENPLETKNICFYSTTCLEGTVTGMVINTGDRTIIGHIASLASGVGNEKTPIA
IEIEHFVHIVAGVAVSIGILFFIIAVSLKYQVLDSIIFLIGIIVANVPEGLLATVTVTLS
LTAKRMAKKNCLVKNLEAVETLGSTSIICSDKTGTLTQNRMTVAHLWFDNQIFVADTSED
HSNQVFDQSSRTWASLSKIITLCNRAEFKPGQENVPIMKKAVIGDASETALLKFSEVILG

DVMEIRKRNRKVAEIPFNSTNKFQLSIHEMDDPHGKRFLMVMKGAPERILEKCSTIMING
EEHPLDKSTAKTFHTAYMELGGLGERVLGFCHLYLPADEFPETYSFDIDAMNFPTSNLCF
VGLLSMIDPPRSTVPDAVTKCRSAGIKVIMVTGDHPITAKAIAKSVGIISANSETVEDIA
HRLNIAVEQVNKRDAKAAVVTGMELKDMSSEQLDEILANYQEIVFARTSPQQKLIIVEGC
QRQDAVVAVTGDGVNDSPALKKADIGIAMGIAGSDAAKNAADMVLLDDNFASIVTGVEEG
RLIFDNLKKTIAYSLTKNIAELCPFLIYIIVGLPLPIGTITILFIDLGTDIIPSIALAYE
KAESDIMNRKPRHKNKDRLVNQPLAVYSYLHIGLMQALGAFLVYFTVYAQEGFLPRTLIN
LRVEWEKDYVNDLKDSYGQEWTRYQREYLEWTGYTAFFVGILVQQIADLIIRKTRRNSIF
QQGLFRNKVIWVGITSQIIIGLILSYGLGSVTALSFTMLRAQYWFVAVPHAILIWVYDEV
RKLFIRLYPGSWWDKNMYY
>sp|Q13733|AT1A4_HUMAN Sodium/potassium-transporting ATPase subunit alpha-4 OS=Homo sapiens GN=ATP1A4 PE=1 SV=3
MGLWGKKGTVAPHDQSPRRRPKKGLIKKKMVKREKQKRNMEELKKEVVMDDHKLTLEELS
TKYSVDLTKGHSHQRAKEILTRGGPNTVTPPPTTPEWVKFCKQLFGGFSLLLWTGAILCF
VAYSIQIYFNEEPTKDNLYLSIVLSVVVIVTGCFSYYQEAKSSKIMESFKNMVPQQALVI
RGGEKMQINVQEVVLGDLVEIKGGDRVPADLRLISAQGCKVDNSSLTGESEPQSRSPDFT
HENPLETRNICFFSTNCVEGTARGIVIATGDSTVMGRIASLTSGLAVGQTPIAAEIEHFI
HLITVVAVFLGVTFFALSLLLGYGWLEAIIFLIGIIVANVPEGLLATVTVCLTLTAKRMA
RKNCLVKNLEAVETLGSTSTICSDKTGTLTQNRMTVAHMWFDMTVYEADTTEEQTGKTFT
KSSDTWFMLARIAGLCNRADFKANQEILPIAKRATTGDASESALLKFIEQSYSSVAEMRE
KNPKVAEIPFNSTNKYQMSIHLREDSSQTHVLMMKGAPERILEFCSTFLLNGQEYSMNDE
MKEAFQNAYLELGGLGERVLGFCFLNLPSSFSKGFPFNTDEINFPMDNLCFVGLISMIDP
PRAAVPDAVSKCRSAGIKVIMVTGDHPITAKAIAKGVGIISEGTETAEEVAARLKIPISK
VDASAAKAIVVHGAELKDIQSKQLDQILQNHPEIVFARTSPQQKLIIVEGCQRLGAVVAV
TGDGVNDSPALKKADIGIAMGISGSDVSKQAADMILLDDNFASIVTGVEEGRLIFDNLKK
SIMYTLTSNIPEITPFLMFIILGIPLPLGTITILCIDLGTDMVPAISLAYESAESDIMKR
LPRNPKTDNLVNHRLIGMAYGQIGMIQALAGFFTYFVILAENGFRPVDLLGIRLHWEDKY
LNDLEDSYGQQWTYEQRKVVEFTCQTAFFVTIVVVQWADLIISKTRRNSLFQQGMRNKVL
IFGILEETLLAAFLSYTPGMDVALRMYPLKITWWLCAIPYSILIFVYDEIRKLLIRQHPD
GWVERETYY
>sp|P20020|AT2B1_HUMAN Plasma membrane calcium-transporting ATPase 1 OS=Homo sapiens GN=ATP2B1 PE=1 SV=3
MGDMANNSVAYSGVKNSLKEANHDGDFGITLAELRALMELRSTDALRKIQESYGDVYGIC
TKLKTSPNEGLSGNPADLERREAVFGKNFIPPKKPKTFLQLVWEALQDVTLIILEIAAIV
SLGLSFYQPPEGDNALCGEVSVGEEEGEGETGWIEGAAILLSVVCVVLVTAFNDWSKEKQ
FRGLQSRIEQEQKFTVIRGGQVIQIPVADITVGDIAQVKYGDLLPADGILIQGNDLKIDE
SSLTGESDHVKKSLDKDPLLLSGTHVMEGSGRMVVTAVGVNSQTGIIFTLLGAGGEEEEK
KDEKKKEKKNKKQDGAIENRNKAKAQDGAAMEMQPLKSEEGGDGDEKDKKKANLPKKEKS
VLQGKLTKLAVQIGKAGLLMSAITVIILVLYFVIDTFWVQKRPWLAECTPIYIQYFVKFF
IIGVTVLVVAVPEGLPLAVTISLAYSVKKMMKDNNLVRHLDACETMGNATAICSDKTGTL
TMNRMTVVQAYINEKHYKKVPEPEAIPPNILSYLVTGISVNCAYTSKILPPEKEGGLPRH
VGNKTECALLGLLLDLKRDYQDVRNEIPEEALYKVYTFNSVRKSMSTVLKNSDGSYRIFS
KGASEIILKKCFKILSANGEAKVFRPRDRDDIVKTVIEPMASEGLRTICLAFRDFPAGEP
EPEWDNENDIVTGLTCIAVVGIEDPVRPEVPDAIKKCQRAGITVRMVTGDNINTARAIAT

KCGILHPGEDFLCLEGKDFNRRIRNEKGEIEQERIDKIWPKLRVLARSSPTDKHTLVKGI
IDSTVSDQRQVVAVTGDGTNDGPALKKADVGFAMGIAGTDVAKEASDIILTDDNFTSIVK
AVMWGRNVYDSISKFLQFQLTVNVVAVIVAFTGACITQDSPLKAVQMLWVNLIMDTLASL
ALATEPPTESLLLRKPYGRNKPLISRTMMKNILGHAFYQLVVVFTLLFAGEKFFDIDSGR
NAPLHAPPSEHYTIVFNTFVLMQLFNEINARKIHGERNVFEGIFNNAIFCTIVLGTFVVQ
IIIVQFGGKPFSCSELSIEQWLWSIFLGMGTLLWGQLISTIPTSRLKFLKEAGHGTQKEE
IPEEELAEDVEEIDHAERELRRGQILWFRGLNRIQTQMDVVNAFQSGSSIQGALRRQPSI
ASQHHDVTNISTPTHIRVVNAFRSSLYEGLEKPESRSSIHNFMTHPEFRIEDSEPHIPLI
DDTDAEDDAPTKRNSSPPPSPNKNNNAVDSGIHLTIEMNKSATSSSPGSPLHSLETSL
>sp|O75185|AT2C2_HUMAN Calcium-transporting ATPase type 2C member 2 OS=Homo sapiens GN=ATP2C2 PE=1 SV=2
MVEGRVSEFLKKLGFSGGGRQYQALEKDEEEALIDEQSELKAIEKEKKVTALPPKEACKC
QKEDLARAFCVDLHTGLSEFSVTQRRLAHGWNEFVADNSEPVWKKYLDQFKNPLILLLLG
SALVSVLTKEYEDAVSIATAVLVVVTVAFIQEYRSEKSLEELTKMVPPECNCLREGKLQH
LLARELVPGDVVSLSIGDRIPADIRLTEVTDLLVDESSFTGEAEPCSKTDSPLTGGGDLT
TLSNIVFMGTLVQYGRGQGVVIGTGESSQFGEVFKMMQAEETPKTPLQKSMDRLGKQLTL
FSFGIIGLIMLIGWSQGKQLLSMFTIGVSLAVAAIPEGLPIVVMVTLVLGVLRMAKKRVI
VKKLPIVETLGCCSVLCSDKTGTLTANEMTVTQLVTSDGLRAEVSGVGYDGQGTVCLLPS
KEVIKEFSNVSVGKLVEAGCVANNAVIRKNAVMGQPTEGALMALAMKMDLSDIKNSYIRK
KEIPFSSEQKWMAVKCSLKTEDQEDIYFMKGALEEVIRYCTMYNNGGIPLPLTPQQRSFC
LQEEKRMGSLGLRVLALASGPELGRLTFLGLVGIIDPPRVGVKEAVQVLSESGVSVKMIT
GDALETALAIGRNIGLCNGKLQAMSGEEVDSVEKGELADRVGKVSVFFRTSPKHKLKIIK
ALQESGAIVAMTGDGVNDAVALKSADIGIAMGQTGTDVSKEAANMILVDDDFSAIMNAVE
EGKGIFYNIKNFVRFQLSTSISALSLITLSTVFNLPSPLNAMQILWINIIMDGPPAQSLG
VEPVDKDAFRQPPRSVRDTILSRALILKILMSAAIIISGTLFIFWKEMPEDRASTPRTTT
MTFTCFVFFDLFNALTCRSQTKLIFEIGFLRNHMFLYSVLGSILGQLAVIYIPPLQRVFQ
TENLGALDLLFLTGLASSVFILSELLKLCEKYCCSPKRVQMHPEDV
>sp|Q5TC04|ATAS1_HUMAN Putative uncharacterized protein ATP1A1-AS1 OS=Homo sapiens GN=ATP1A1-AS1 PE=5 SV=1
MAHFKDDLQTNVEIIPGESAPRKESPRPPAPPSSAAGVGGCSNHSPSVQESPLSPPALAQ
LGSAQQPSMRTELSFSEKKDTMIIWQITITVWCQR
>sp|Q9ULI0|ATD2B_HUMAN ATPase family AAA domain-containing protein 2B OS=Homo sapiens GN=ATAD2B PE=1 SV=3
MVNTRKSSLRLLGSKSPGPGPGPGAGAEPGATGGSSHFISSRTRSSKTRAASCPAAKAGG
SGGAGVTLDEARKVEVDGSLSDSHVSPPAKRTLKQPDSVCKDKSKSRSTGQREEWNLSTG
QARLTSQPGATLPNGHSGLSLRSHPLRGEKKGDGDLSCINGDMEVRKSCRSRKNRFESVN
QSLLFDQLVNSTAEAVLQEMDNINIRQNRRSGEVERLRMWTDTEFENMDMYSRVKRRRKS
LRRNSYGIQNHHEVSTEGEEEESQEEDGDIEVEEAEGEENDRPYNLRQRKTVDRYQAPPI
VPAHQKKRENTLFDIHRSPARRSHIRRKKHAIHSSDTTSSDEERFERRKSKSMARARNRC
LPMNFRAEDLASGILRERVKVGASLADVDPMNIDKSVRFDSIGGLSHHIHALKEMVVFPL
LYPEIFEKFKIQPPRGCLFYGPPGTGKTLVARALANECSQGDKKVAFFMRKGADCLSKWV
GESERQLRLLFDQAYLMRPSIIFFDEIDGLAPVRSSRQDQIHSSIVSTLLALMDGLDNRG
EIVVIGATNRLDSIDPALRRPGRFDREFLFNLPDQKARKHILQIHTRDWNPKLSDAFLGE
LAEKCVGYCGADIKALCTEAALIALRRRYPQIYASSHKLQLDVSSIVLSAQDFYHAMQNI

VPASQRAVMSSGHALSPIIRPLLERSFNNILAVLQKVFPHAEISQSDKKEDIETLILEDS
EDENALSIFETNCHSGSPKKQSSSAAIHKPYLHFTMSPYHQPTSYRPRLLLSGERGSGQT
SHLAPALLHTLERFSVHRLDLPALYSVSAKTPEESCAQIFREARRTVPSIVYMPHIGDWW
EAVSETVRATFLTLLQDIPSFSPIFLLSTSETMYSELPEEVKCIFRIQYEEVLYIQRPIE
EDRRKFFQELILNQASMAPPRRKHAALCAMEVLPLALPSPPRQLSESEKSRMEDQEENTL
RELRLFLRDVTKRLATDKRFNIFSKPVDIEEVSDYLEVIKEPMDLSTVITKIDKHNYLTA
KDFLKDIDLICSNALEYNPDKDPGDKIIRHRACTLKDTAHAIIAAELDPEFNKLCEEIKE
ARIKRGLSVTSEQINPHSTGARKTETRVEEAFRHKQRNPMDVWHNSANKCAFRVRRKSRR
RSQWGKGIIKKRKVNNLKKDEEDTKFADYENHTEDRKLLENGEFEVSTDCHEENGEETGD
LSMTNDESSCDIMDLDQGQRLNNGAGTKENFASTEEESSNESLLVNSSSSLNPEQTSRKE
TFLKGNCLNGEASTDSFEGIPVLECQNGKLEVVSFCDSGDKCSSEQKILLEDQSKEKPET
STENHGDDLEKLEALECSNNEKLEPGSDVEVKDAELDKEGASKVKKYRKLILEQAKTTSL
ELVPEEPSEPVPPLIVDRERLKKLLDLLVDKSNNLAVDQLERLYSLLSQCIYRHRKDYDK
SQLVEEMERTVHMFETFL
>sp|P15336|ATF2_HUMAN Cyclic AMP-dependent transcription factor ATF-2 OS=Homo sapiens GN=ATF2 PE=1 SV=4
MKFKLHVNSARQYKDLWNMSDDKPFLCTAPGCGQRFTNEDHLAVHKHKHEMTLKFGPARN
DSVIVADQTPTPTRFLKNCEEVGLFNELASPFENEFKKASEDDIKKMPLDLSPLATPIIR
SKIEEPSVVETTHQDSPLPHPESTTSDEKEVPLAQTAQPTSAIVRPASLQVPNVLLTSSD
SSVIIQQAVPSPTSSTVITQAPSSNRPIVPVPGPFPLLLHLPNGQTMPVAIPASITSSNV
HVPAAVPLVRPVTMVPSVPGIPGPSSPQPVQSEAKMRLKAALTQQHPPVTNGDTVKGHGS
GLVRTQSEESRPQSLQQPATSTTETPASPAHTTPQTQSTSGRRRRAANEDPDEKRRKFLE
RNRAAASRCRQKRKVWVQSLEKKAEDLSSLNGQLQSEVTLLRNEVAQLKQLLLAHKDCPV
TAMQKKSGYHTADKDDSSEDISVPSSPHTEAIQHSSVSTSNGVSSTSKAEAVATSVLTQM
ADQSTEPALSQIVMAPSSQSQPSGS
>sp|Q86TH1|ATL2_HUMAN ADAMTS-like protein 2 OS=Homo sapiens GN=ADAMTSL2 PE=1 SV=1
MDGRWQCSCWAWFLLVLAVVAGDTVSTGSTDNSPTSNSLEGGTDATAFWWGEWTKWTACS
RSCGGGVTSQERHCLQQRRKSVPGPGNRTCTGTSKRYQLCRVQECPPDGRSFREEQCVSF
NSHVYNGRTHQWKPLYPDDYVHISSKPCDLHCTTVDGQRQLMVPARDGTSCKLTDLRGVC
VSGKCEPIGCDGVLFSTHTLDKCGICQGDGSSCTHVTGNYRKGNAHLGYSLVTHIPAGAR
DIQIVERKKSADVLALADEAGYYFFNGNYKVDSPKNFNIAGTVVKYRRPMDVYETGIEYI
VAQGPTNQGLNVMVWNQNGKSPSITFEYTLLQPPHESRPQPIYYGFSESAESQGLDGAGL
MGFVPHNGSLYGQASSERLGLDNRLFGHPGLDMELGPSQGQETNEVCEQAGGGACEGPPR
GKGFRDRNVTGTPLTGDKDDEEVDTHFASQEFFSANAISDQLLGAGSDLKDFTLNETVNS
IFAQGAPRSSLAESFFVDYEENEGAGPYLLNGSYLELSSDRVANSSSEAPFPNVSTSLLT
SAGNRTHKARTRPKARKQGVSPADMYRWKLSSHEPCSATCTTGVMSAYAMCVRYDGVEVD
DSYCDALTRPEPVHEFCAGRECQPRWETSSWSECSRTCGEGYQFRVVRCWKMLSPGFDSS
VYSDLCEAAEAVRPEERKTCRNPACGPQWEMSEWSECTAKCGERSVVTRDIRCSEDEKLC
DPNTRPVGEKNCTGPPCDRQWTVSDWGPCSGSCGQGRTIRHVYCKTSDGRVVPESQCQME
TKPLAIHPCGDKNCPAHWLAQDWERCNTTCGRGVKKRLVLCMELANGKPQTRSGPECGLA
KKPPEESTCFERPCFKWYTSPWSECTKTCGVGVRMRDVKCYQGTDIVRGCDPLVKPVGRQ
ACDLQPCPTEPPDDSCQDQPGTNCALAIKVNLCGHWYYSKACCRSCRPPHS
>sp|P51689|ARSD_HUMAN Arylsulfatase D OS=Homo sapiens GN=ARSD PE=1 SV=2
MRSAARRGRAAPAARDSLPVLLFLCLLLKTCEPKTANAFKPNILLIMADDLGTGDLGCYG

NNTLRTPNIDQLAEEGVRLTQHLAAAPLCTPSRAAFLTGRHSFRSGMDASNGYRALQWNA
GSGGLPENETTFARILQQHGYATGLIGKWHQGVNCASRGDHCHHPLNHGFDYFYGMPFTL
TNDCDPGRPPEVDAALRAQLWGYTQFLALGILTLAAGQTCGFFSVSARAVTGMAGVGCLF
FISWYSSFGFVRRWNCILMRNHDVTEQPMVLEKTASLMLKEAVSYIERHKHGPFLLFLSL
LHVHIPLVTTSAFLGKSQHGLYGDNVEEMDWLIGKVLNAIEDNGLKNSTFTYFTSDHGGH
LEARDGHSQLGGWNGIYKGGKGMGGWEGGIRVPGIFHWPGVLPAGRVIGEPTSLMDVFPT
VVQLVGGEVPQDRVIDGHSLVPLLQGAEARSAHEFLFHYCGQHLHAARWHQKDSGSVWKV
HYTTPQFHPEGAGACYGRGVCPCSGEGVTHHRPPLLFDLSRDPSEARPLTPDSEPLYHAV
IARVGAAVSEHRQTLSPVPQQFSMSNILWKPWLQPCCGHFPFCSCHEDGDGTP
>sp|Q5FYA8|ARSH_HUMAN Arylsulfatase H OS=Homo sapiens GN=ARSH PE=2 SV=1
MTRNARPNIVLLMADDLGVGDLCCYGNNSVSTPNIDRLASEGVRLTQHLAAASMCTPSRA
AFLTGRYPIRSGMVSAYNLNRAFTWLGGSGGLPTNETTFAKLLQHRGYRTGLIGKWHLGL
SCASRNDHCYHPLNHGFHYFYGVPFGLLSDCQASKTPELHRWLRIKLWISTVALALVPFL
LLIPKFARWFSVPWKVIFVFALLAFLFFTSWYSSYGFTRRWNCILMRNHEIIQQPMKEEK
VASLMLKEALAFIERYKREPFLLFFSFLHVHTPLISKKKFVGRSKYGRYGDNVEEMDWMV
GKILDALDQERLANHTLVYFTSDNGGHLEPLDGAVQLGGWNGIYKGGKGMGGWEGGIRVP
GIFRWPSVLEAGRVINEPTSLMDIYPTLSYIGGGILSQDRVIDGQNLMPLLEGRASHSDH
EFLFHYCGVYLHTVRWHQKDCATVWKAHYVTPKFYPEGTGACYGSGICSCSGDVTYHDPP
LLFDISRDPSEALPLNPDNEPLFDSVIKKMEAAIREHRRTLTPVPQQFSVFNTIWKPWLQ
PCCGTFPFCGCDKEDDILPMAP
>sp|Q5FYB1|ARSI_HUMAN Arylsulfatase I OS=Homo sapiens GN=ARSI PE=1 SV=1
MHTLTGFSLVSLLSFGYLSWDWAKPSFVADGPGEAGEQPSAAPPQPPHIIFILTDDQGYH
DVGYHGSDIETPTLDRLAAKGVKLENYYIQPICTPSRSQLLTGRYQIHTGLQHSIIRPQQ
PNCLPLDQVTLPQKLQEAGYSTHMVGKWHLGFYRKECLPTRRGFDTFLGSLTGNVDYYTY
DNCDGPGVCGFDLHEGENVAWGLSGQYSTMLYAQRASHILASHSPQRPLFLYVAFQAVHT
PLQSPREYLYRYRTMGNVARRKYAAMVTCMDEAVRNITWALKRYGFYNNSVIIFSSDNGG
QTFSGGSNWPLRGRKGTYWEGGVRGLGFVHSPLLKRKQRTSRALMHITDWYPTLVGLAGG
TTSAADGLDGYDVWPAISEGRASPRTEILHNIDPLYNHAQHGSLEGGFGIWNTAVQAAIR
VGEWKLLTGDPGYGDWIPPQTLATFPGSWWNLERMASVRQAVWLFNISADPYEREDLAGQ
RPDVVRTLLARLAEYNRTAIPVRYPAENPRAHPDFNGGAWGPWASDEEEEEEEGRARSFS
RGRRKKKCKICKLRSFFRKLNTRLMSQRI
>sp|Q9ULH1|ASAP1_HUMAN Arf-GAP with SH3 domain, ANK repeat and PH domain-containing protein 1 OS=Homo sapiens GN=ASAP1 PE=1 SV=4
MRSSASRLSSFSSRDSLWNRMPDQISVSEFIAETTEDYNSPTTSSFTTRLHNCRNTVTLL
EEALDQDRTALQKVKKSVKAIYNSGQDHVQNEENYAQVLDKFGSNFLSRDNPDLGTAFVK
FSTLTKELSTLLKNLLQGLSHNVIFTLDSLLKGDLKGVKGDLKKPFDKAWKDYETKFTKI
EKEKREHAKQHGMIRTEITGAEIAEEMEKERRLFQLQMCEYLIKVNEIKTKKGVDLLQNL
IKYYHAQCNFFQDGLKTADKLKQYIEKLAADLYNIKQTQDEEKKQLTALRDLIKSSLQLD
QKEDSQSRQGGYSMHQLQGNKEYGSEKKGYLLKKSDGIRKVWQRRKCSVKNGILTISHAT
SNRQPAKLNLLTCQVKPNAEDKKSFDLISHNRTYHFQAEDEQDYVAWISVLTNSKEEALT
MAFRGEQSAGENSLEDLTKAIIEDVQRLPGNDICCDCGSSEPTWLSTNLGILTCIECSGI
HREMGVHISRIQSLELDKLGTSELLLAKNVGNNSFNDIMEANLPSPSPKPTPSSDMTVRK
EYITAKYVDHRFSRKTCSTSSAKLNELLEAIKSRDLLALIQVYAEGVELMEPLLEPGQEL
GETALHLAVRTADQTSLHLVDFLVQNCGNLDKQTALGNTVLHYCSMYSKPECLKLLLRSK

PTVDIVNQAGETALDIAKRLKATQCEDLLSQAKSGKFNPHVHVEYEWNLRQEEIDESDDD
LDDKPSPIKKERSPRPQSFCHSSSISPQDKLALPGFSTPRDKQRLSYGAFTNQIFVSTST
DSPTSPTTEAPPLPPRNAGKGPTGPPSTLPLSTQTSSGSSTLSKKRPPPPPPGHKRTLSD
PPSPLPHGPPNKGAVPWGNDGGPSSSSKTTNKFEGLSQQSSTSSAKTALGPRVLPKLPQK
VALRKTDHLSLDKATIPPEIFQKSSQLAELPQKPPPGDLPPKPTELAPKPQIGDLPPKPG
ELPPKPQLGDLPPKPQLSDLPPKPQMKDLPPKPQLGDLLAKSQTGDVSPKAQQPSEVTLK
SHPLDLSPNVQSRDAIQKQASEDSNDLTPTLPETPVPLPRKINTGKNKVRRVKTIYDCQA
DNDDELTFIEGEVIIVTGEEDQEWWIGHIEGQPERKGVFPVSFVHILSD
>sp|Q8WXI3|ASB10_HUMAN Ankyrin repeat and SOCS box protein 10 OS=Homo sapiens GN=ASB10 PE=1 SV=2
MLMSWSPEECKGQGEPLDDRHPLCARLVEKPSRGSEEHLKSGPGPIVTRTASGPALAFWQ
AVLAGDVGCVSRILADSSTGLAPDSVFDTSDPERWRDFRFNIRALRLWSLTYEEELTTPL
HVAASRGHTEVLRLLLRRRARPDSAPGGRTALHEACAAGHTACVHVLLVAGADPNIADQD
GKRPLHLCRGPGTLECAELLLRFGARVDGRSEEEEETPLHVAARLGHVELADLLLRRGAC
PDARNAEGWTPLLAACDVRCQSITDAEATTARCLQLCSLLLSAGADADAADQDKQRPLHL
ACRRGHAAVVELLLSCGVSANTMDYGGHTPLHCALQGPAAALAQSPEHVVRALLNHGAVR
VWPGALPKVLERWSTCPRTIEVLMNTYSVVQLPEEAVGLVTPETLQKHQRFYSSLFALVR
QPRSLQHLSRCALRSHLEGSLPQALPRLPLPPRLLRYLQLDFEGVLY
>sp|Q96NS5|ASB16_HUMAN Ankyrin repeat and SOCS box protein 16 OS=Homo sapiens GN=ASB16 PE=1 SV=2
MARETFPFTSSMLRSLRLQQEWLEWEDRRRAAAQQCRSRRCPSSPRARLTRPHRSCRDPA
VHQALFSGNLQQVQALFQDEEAANMIVETVSNQLAWSAEQGFWVLTPKTKQTAPLAIATA
RGYTDCARHLIRQGAELDARVGGRAALHEACARAQFDCVRLLLTFGAKANVLTEEGTTPL
HLCTIPESLQCAKLLLEAGATVNLAAGESQETPLHVAAARGLEQHVALYLEHGADVGLRT
SQGETALNTACAGAEGPGSCRRHQAAARRLLEAGADARAAGRKRHTPLHNACANGCGGLA
ELLLRYGARAEVPNGAGHTPMDCALQAVQDSPNWEPEVLFAALLDYGAQPVRPEMLKHCA
NFPRALEVLLNAYPCVPSCETWVEAVLPELWKEHEAFYSSALCMVNQPRQLQHLARLAVR
ARLGSRCRQGATRLPLPPLLRDYLLLRVEGCIQ
>sp|Q6ZVZ8|ASB18_HUMAN Ankyrin repeat and SOCS box protein 18 OS=Homo sapiens GN=ASB18 PE=2 SV=2
MSNSDYLPDYPLNSDLVKRLKSALDAKDEERVRDLICTEITPVDAVIELANDDWMKDPSA
QLPTGMLLGDLDHLKPLMDQFFQDANVVFEINKDEMEWQVKSPATFGLSGLWTLEYKREL
TTPLCIAAAHGHTACVRHLLGRGADPDASPGGRGALHEACLGGHTACVRLLLQHRADPDL
LSAEGLAPLHLCRTAASLGCAQALLEHGASVQRVGGTGRDTPLHVAAQRGLDEHARLYLG
RGAHVDARNGRGETALSAACGAARRPDEHGRCLRLCALLLRRGAEADARDEDERSPLHKA
CGHASHSLARLLLRHGADAGALDYGGASPLGRVLQTASCALQASPQRTVQALLNHGSPTV
WPDAFPKVLKTCASVPAVIEVLFNSYPQLCLSESWKEVIPEEVFQMHKPFYQSLFALALT
PRCLQHLCRCALRRLFGKRCFDLIPLLPLPKPLQNYLLLEPQGVLH
>sp|Q96Q27|ASB2_HUMAN Ankyrin repeat and SOCS box protein 2 OS=Homo sapiens GN=ASB2 PE=1 SV=1
MTRFSYAEYFSLFHSCSAPSRSTAPPESSPARAPMGLFQGVMQKYSSSLFKTSQLAPADP
LIKAIKDGDEEALKTMIKEGKNLAEPNKEGWLPLHEAAYYGQVGCLKVLQRAYPGTIDQR
TLQEETAVYLATCRGHLDCLLSLLQAGAEPDISNKSRETPLYKACERKNAEAVKILVQHN
ADTNHRCNRGWTALHESVSRNDLEVMQILVSGGAKVESKNAYGITPLFVAAQSGQLEALR

FLAKYGADINTQASDNASALYEACKNEHEEVVEFLLSQGADANKTNKDGLLPLHIASKKG
NYRIVQMLLPVTSRTRIRRSGVSPLHLAAERNHDEVLEALLSARFDVNTPLAPERARLYE
DRRSSALYFAVVNNNVYATELLLQHGADPNRDVISPLLVAIRHGCLRTMQLLLDHGANID
AYIATHPTAFPATIMFAMKCLSLLKFLMDLGCDGEPCFSCLYGNGPHPPAPQPSSRFNDA
PAADKEPSVVQFCEFVSAPEVSRWAGPIIDVLLDYVGNVQLCSRLKEHIDSFEDWAVIKE
KAEPPRPLAHLCRLRVRKAIGKYRIKLLDTLPLPGRLIRYLKYENTQ
>sp|P50553|ASCL1_HUMAN Achaete-scute homolog 1 OS=Homo sapiens GN=ASCL1 PE=1 SV=2
MESSAKMESGGAGQQPQPQPQQPFLPPAACFFATAAAAAAAAAAAAQSAQQQQQQQQQQ
QQAPQLRPAADGQPSGGGHKSAPKQVKRQRSSSPELMRCKRRLNFSGFGYSLPQQQPAAV
ARRNERERNRVKLVNLGFATLREHVPNGAANKKMSKVETLRSAVEYIRALQQLLDEHDAV
SAAFQAGVLSPTISPNYSNDLNSMAGSPVSSYSSDEGSYDPLSPEEQELLDFTNWF
>sp|Q7RTU5|ASCL5_HUMAN Achaete-scute homolog 5 OS=Homo sapiens GN=ASCL5 PE=3 SV=2
MPMGAAERGAGPQSSAAPWAGSEKAAKRGPSKSWYPRAAASDVTCPTGGDGADPKPGPFG
GGLALGPAPRGTMNNNFCRALVDRRPLGPPSCMQLGVMPPPRQAPLPPAEPLGNVPFLLY
PGPAEPPYYDAYAGVFPYVPFPGAFGVYEYPFEPAFIQKRNERERQRVKCVNEGYARLRG
HLPGALAEKRLSKVETLRAAIRYIKYLQELLSSAPDGSTPPASRGLPGTGPCPAPPATPR
PDRPGDGEARAPSSLVPESSESSCFSPSPFLESEESWH
>sp|Q9ULZ3|ASC_HUMAN Apoptosis-associated speck-like protein containing a CARD OS=Homo sapiens GN=PYCARD PE=1 SV=2
MGRARDAILDALENLTAEELKKFKLKLLSVPLREGYGRIPRGALLSMDALDLTDKLVSFY
LETYGAELTANVLRDMGLQEMAGQLQAATHQGSGAAPAGIQAPPQSAAKPGLHFIDQHRA
ALIARVTNVEWLLDALYGKVLTDEQYQAVRAEPTNPSKMRKLFSFTPAWNWTCKDLLLQA
LRESQSYLVEDLERS
>sp|Q9NWL6|ASND1_HUMAN Asparagine synthetase domain-containing protein 1 OS=Homo sapiens GN=ASNSD1 PE=2 SV=2
MCGICCSVNFSAEHFSQDLKEDLLYNLKQRGPNSSKQLLKSDVNYQCLFSAHVLHLRGVL
TTQPVEDERGNVFLWNGEIFSGIKVEAEENDTQILFNYLSSCKNESEILSLFSEVQGPWS
FIYYQASSHYLWFGRDFFGRRSLLWHFSNLGKSFCLSSVGTQTSGLANQWQEVPASGLFR
IDLKSTVISGCIILQLYPWKYISRENIIEENVNSLSQISADLPAFVSVVANEAKLYLEKP
VVPLNMMLPQAALETHCSNISNVPPTREILQVFLTDVHMKEVIQQFIDVLSVAVKKRVLC
LPRDENLTANEVLKTCDRKANVAILFSGGIDSMVIATLADRHIPLDEPIDLLNVAFIAEE
KTMPTTFNREGNKQKNKCEIPSEEFSKDVAAAAADSPNKHVSVPDRITGRAGLKELQAVS
PSRIWNFVEINVSMEELQKLRRTRICHLIRPLDTVLDDSIGCAVWFASRGIGWLVAQEGV
KSYQSNAKVVLTGIGADEQLAGYSRHRVRFQSHGLEGLNKEIMMELGRISSRNLGRDDRV
IGDHGKEARFPFLDENVVSFLNSLPIWEKANLTLPRGIGEKLLLRLAAVELGLTASALLP
KRAMQFGSRIAKMEKINEKASDKCGRLQIMSLENLSIEKETKL
>sp|Q9BZE9|ASPC1_HUMAN Tether containing UBX domain for GLUT4 OS=Homo sapiens GN=ASPSCR1 PE=1 SV=1
MAAPAGGGGSAVSVLAPNGRRHTVKVTPSTVLLQVLEDTCRRQDFNPCEYDLKFQRSVLD
LSLQWRFANLPNNAKLEMVPASRSREGPENMVRIALQLDDGSRLQDSFCSGQTLWELLSH
FPQIRECLQHPGGATPVCVYTRDEVTGEAALRGTTLQSLGLTGGSATIRFVMKCYDPVGK
TPGSLGSSASAGQAAASAPLPLESGELSRGDLSRPEDADTSGPCCEHTQEKQSTRAPAAA
PFVPFSGGGQRLGGPPGPTRPLTSSSAKLPKSLSSPGGPSKPKKSKSGQDPQQEQEQERE
RDPQQEQERERPVDREPVDREPVVCHPDLEERLQAWPAELPDEFFELTVDDVRRRLAQLK

SERKRLEEAPLVTKAFREAQIKEKLERYPKVALRVLFPDRYVLQGFFRPSETVGDLRDFV
RSHLGNPELSFYLFITPPKTVLDDHTQTLFQANLFPAALVHLGAEEPAGVYLEPGLLEHA
ISPSAADVLVARYMSRAAGSPSPLPAPDPAPKSEPAAEEGALVPPEPIPGTAQPVKRSLG
KVPKWLKLPASKR
>sp|P26436|ASPX_HUMAN Acrosomal protein SP-10 OS=Homo sapiens GN=ACRV1 PE=1 SV=2
MNRFLLLMSLYLLGSARGTSSQPNELSGSIDHQTSVQQLPGEFFSLENPSDAEALYETSS
GLNTLSEHGSSEHGSSKHTVAEHTSGEHAESEHASGEPAATEHAEGEHTVGEQPSGEQPS
GEHLSGEQPLSELESGEQPSDEQPSGEHGSGEQPSGEQASGEQPSGEHASGEQASGAPIS
STSTGTILNCYTCAYMNDQGKCLRGEGTCITQNSQQCMLKKIFEGGKLQFMVQGCENMCP
SMNLFSHGTRMQIICCRNQSFCNKI
>sp|P05496|AT5G1_HUMAN ATP synthase F(0) complex subunit C1, mitochondrial OS=Homo sapiens GN=ATP5G1 PE=1 SV=2
MQTAGALFISPALIRCCTRGLIRPVSASFLNSPVNSSKQPSYSNFPLQVARREFQTSVVS
RDIDTAAKFIGAGAATVGVAGSGAGIGTVFGSLIIGYARNPSLKQQLFSYAILGFALSEA
MGLFCLMVAFLILFAM
>sp|Q5SQI0|ATAT_HUMAN Alpha-tubulin N-acetyltransferase 1 OS=Homo sapiens GN=ATAT1 PE=1 SV=1
MEFPFDVDALFPERITVLDQHLRPPARRPGTTTPARVDLQQQIMTIIDELGKASAKAQNL
SAPITSASRMQSNRHVVYILKDSSARPAGKGAIIGFIKVGYKKLFVLDDREAHNEVEPLC
ILDFYIHESVQRHGHGRELFQYMLQKERVEPHQLAIDRPSQKLLKFLNKHYNLETTVPQV
NNFVIFEGFFAHQHRPPAPSLRATRHSRAAAVDPTPAAPARKLPPKRAEGDIKPYSSSDR
EFLKVAVEPPWPLNRAPRRATPPAHPPPRSSSLGNSPERGPLRPFVPEQELLRSLRLCPP
HPTARLLLAADPGGSPAQRRRTRGTPPGLVAQSCCYSRHGGVNSSSPNTGNQDSKQGEQE
TKNRSASEEQALSQDGSGEKPMHTAPPQAPAPPAQSWTVGGDILNARFIRNLQERRSTRP
W
>sp|P18848|ATF4_HUMAN Cyclic AMP-dependent transcription factor ATF-4 OS=Homo sapiens GN=ATF4 PE=1 SV=3
MTEMSFLSSEVLVGDLMSPFDQSGLGAEESLGLLDDYLEVAKHFKPHGFSSDKAKAGSSE
WLAVDGLVSPSNNSKEDAFSGTDWMLEKMDLKEFDLDALLGIDDLETMPDDLLTTLDDTC
DLFAPLVQETNKQPPQTVNPIGHLPESLTKPDQVAPFTFLQPLPLSPGVLSSTPDHSFSL
ELGSEVDITEGDRKPDYTAYVAMIPQCIKEEDTPSDNDSGICMSPESYLGSPQHSPSTRG
SPNRSLPSPGVLCGSARPKPYDPPGEKMVAAKVKGEKLDKKLKKMEQNKTAATRYRQKKR
AEQEALTGECKELEKKNEALKERADSLAKEIQYLKDLIEEVRKARGKKRVP
>sp|Q9NT62|ATG3_HUMAN Ubiquitin-like-conjugating enzyme ATG3 OS=Homo sapiens GN=ATG3 PE=1 SV=1
MQNVINTVKGKALEVAEYLTPVLKESKFKETGVITPEEFVAAGDHLVHHCPTWQWATGEE
LKVKAYLPTGKQFLVTKNVPCYKRCKQMEYSDELEAIIEEDDGDGGWVDTYHNTGITGIT
EAVKEITLENKDNIRLQDCSALCEEEEDEDEGEAADMEEYEESGLLETDEATLDTRKIVE
ACKAKTDAGGEDAILQTRTYDLYITYDKYYQTPRLWLFGYDEQRQPLTVEHMYEDISQDH
VKKTVTIENHPHLPPPPMCSVHPCRHAEVMKKIIETVAEGGGELGVHMYLLIFLKFVQAV
IPTIEYDYTRHFTM
>sp|Q9H1Y0|ATG5_HUMAN Autophagy protein 5 OS=Homo sapiens GN=ATG5 PE=1 SV=2
MTDDKDVLRDVWFGRIPTCFTLYQDEITEREAEPYYLLLPRVSYLTLVTDKVKKHFQKVM
RQEDISEIWFEYEGTPLKWHYPIGLLFDLLASSSALPWNITVHFKSFPEKDLLHCPSKDA

IEAHFMSCMKEADALKHKSQVINEMQKKDHKQLWMGLQNDRFDQFWAINRKLMEYPAEEN
GFRYIPFRIYQTTTERPFIQKLFRPVAADGQLHTLGDLLKEVCPSAIDPEDGEKKNQVMI
HGIEPMLETPLQWLSEHLSYPDNFLHISIIPQPTD
>sp|Q6DD88|ATLA3_HUMAN Atlastin-3 OS=Homo sapiens GN=ATL3 PE=1 SV=1
MLSPQRVAAAASRGADDAMESSKPGPVQVVLVQKDQHSFELDEKALASILLQDHIRDLDV
VVVSVAGAFRKGKSFILDFMLRYLYSQKESGHSNWLGDPEEPLTGFSWRGGSDPETTGIQ
IWSEVFTVEKPGGKKVAVVLMDTQGAFDSQSTVKDCATIFALSTMTSSVQIYNLSQNIQE
DDLQQLQLFTEYGRLAMDEIFQKPFQTLMFLVRDWSFPYEYSYGLQGGMAFLDKRLQVKE
HQHEEIQNVRNHIHSCFSDVTCFLLPHPGLQVATSPDFDGKLKDIAGEFKEQLQALIPYV
LNPSKLMEKEINGSKVTCRGLLEYFKAYIKIYQGEDLPHPKSMLQATAEANNLAAAASAK
DIYYNNMEEVCGGEKPYLSPDILEEKHCEFKQLALDHFKKTKKMGGKDFSFRYQQELEEE
IKELYENFCKHNGSKNVFSTFRTPAVLFTGIVALYIASGLTGFIGLEVVAQLFNCMVGLL
LIALLTWGYIRYSGQYRELGGAIDFGAAYVLEQASSHIGNSTQATVRDAVVGRPSMDKKA
Q
>sp|O43313|ATMIN_HUMAN ATM interactor OS=Homo sapiens GN=ATMIN PE=1 SV=2
MAASEAAAAAGSAALAAGARAVPAATTGAAAAASGPWVPPGPRLRGSRPRPAGATQQPAV
PAPPAGELIQPSVSELSRAVRTNILCTVRGCGKILPNSPALNMHLVKSHRLQDGIVNPTI
RKDLKTGPKFYCCPIEGCPRGPERPFSQFSLVKQHFMKMHAEKKHKCSKCSNSYGTEWDL
KRHAEDCGKTFRCTCGCPYASRTALQSHIYRTGHEIPAEHRDPPSKKRKMENCAQNQKLS
NKTIESLNNQPIPRPDTQELEASEIKLEPSFEDSCGSNTDKQTLTTPPRYPQKLLLPKPK
VALVKLPVMQFSVMPVFVPTADSSAQPVVLGVDQGSATGAVHLMPLSVGTLILGLDSEAC
SLKESLPLFKIANPIAGEPISTGVQVNFGKSPSNPLQELGNTCQKNSISSINVQTDLSYA
SQNFIPSAQWATADSSVSSCSQTDLSFDSQVSLPISVHTQTFLPSSKVTSSIAAQTDAFM
DTCFQSGGVSRETQTSGIESPTDDHVQMDQAGMCGDIFESVHSSYNVATGNIISNSLVAE
TVTHSLLPQNEPKTLNQDIEKSAPIINFSAQNSMLPSQNMTDNQTQTIDLLSDLENILSS
NLPAQTLDHRSLLSDTNPGPDTQLPSGPAQNPGIDFDIEEFFSASNIQTQTEESELSTMT
TEPVLESLDIETQTDFLLADTSAQSYGCRGNSNFLGLEMFDTQTQTDLNFFLDSSPHLPL
GSILKHSSFSVSTDSSDTETQTEGVSTAKNIPALESKVQLNSTETQTMSSGFETLGSLFF
TSNETQTAMDDFLLADLAWNTMESQFSSVETQTSAEPHTVSNF
>sp|Q13315|ATM_HUMAN Serine-protein kinase ATM OS=Homo sapiens GN=ATM PE=1 SV=4
MSLVLNDLLICCRQLEHDRATERKKEVEKFKRLIRDPETIKHLDRHSDSKQGKYLNWDAV
FRFLQKYIQKETECLRIAKPNVSASTQASRQKKMQEISSLVKYFIKCANRRAPRLKCQEL
LNYIMDTVKDSSNGAIYGADCSNILLKDILSVRKYWCEISQQQWLELFSVYFRLYLKPSQ
DVHRVLVARIIHAVTKGCCSQTDGLNSKFLDFFSKAIQCARQEKSSSGLNHILAALTIFL
KTLAVNFRIRVCELGDEILPTLLYIWTQHRLNDSLKEVIIELFQLQIYIHHPKGAKTQEK
GAYESTKWRSILYNLYDLLVNEISHIGSRGKYSSGFRNIAVKENLIELMADICHQVFNED
TRSLEISQSYTTTQRESSDYSVPCKRKKIELGWEVIKDHLQKSQNDFDLVPWLQIATQLI
SKYPASLPNCELSPLLMILSQLLPQQRHGERTPYVLRCLTEVALCQDKRSNLESSQKSDL
LKLWNKIWCITFRGISSEQIQAENFGLLGAIIQGSLVEVDREFWKLFTGSACRPSCPAVC
CLTLALTTSIVPGTVKMGIEQNMCEVNRSFSLKESIMKWLLFYQLEGDLENSTEVPPILH
SNFPHLVLEKILVSLTMKNCKAAMNFFQSVPECEHHQKDKEELSFSEVEELFLQTTFDKM
DFLTIVRECGIEKHQSSIGFSVHQNLKESLDRCLLGLSEQLLNNYSSEITNSETLVRCSR
LLVGVLGCYCYMGVIAEEEAYKSELFQKAKSLMQCAGESITLFKNKTNEEFRIGSLRNMM
QLCTRCLSNCTKKSPNKIASGFFLRLLTSKLMNDIADICKSLASFIKKPFDRGEVESMED

DTNGNLMEVEDQSSMNLFNDYPDSSVSDANEPGESQSTIGAINPLAEEYLSKQDLLFLDM
LKFLCLCVTTAQTNTVSFRAADIRRKLLMLIDSSTLEPTKSLHLHMYLMLLKELPGEEYP
LPMEDVLELLKPLSNVCSLYRRDQDVCKTILNHVLHVVKNLGQSNMDSENTRDAQGQFLT
VIGAFWHLTKERKYIFSVRMALVNCLKTLLEADPYSKWAILNVMGKDFPVNEVFTQFLAD
NHHQVRMLAAESINRLFQDTKGDSSRLLKALPLKLQQTAFENAYLKAQEGMREMSHSAEN
PETLDEIYNRKSVLLTLIAVVLSCSPICEKQALFALCKSVKENGLEPHLVKKVLEKVSET
FGYRRLEDFMASHLDYLVLEWLNLQDTEYNLSSFPFILLNYTNIEDFYRSCYKVLIPHLV
IRSHFDEVKSIANQIQEDWKSLLTDCFPKILVNILPYFAYEGTRDSGMAQQRETATKVYD
MLKSENLLGKQIDHLFISNLPEIVVELLMTLHEPANSSASQSTDLCDFSGDLDPAPNPPH
FPSHVIKATFAYISNCHKTKLKSILEILSKSPDSYQKILLAICEQAAETNNVYKKHRILK
IYHLFVSLLLKDIKSGLGGAWAFVLRDVIYTLIHYINQRPSCIMDVSLRSFSLCCDLLSQ
VCQTAVTYCKDALENHLHVIVGTLIPLVYEQVEVQKQVLDLLKYLVIDNKDNENLYITIK
LLDPFPDHVVFKDLRITQQKIKYSRGPFSLLEEINHFLSVSVYDALPLTRLEGLKDLRRQ
LELHKDQMVDIMRASQDNPQDGIMVKLVVNLLQLSKMAINHTGEKEVLEAVGSCLGEVGP
IDFSTIAIQHSKDASYTKALKLFEDKELQWTFIMLTYLNNTLVEDCVKVRSAAVTCLKNI
LATKTGHSFWEIYKMTTDPMLAYLQPFRTSRKKFLEVPRFDKENPFEGLDDINLWIPLSE
NHDIWIKTLTCAFLDSGGTKCEILQLLKPMCEVKTDFCQTVLPYLIHDILLQDTNESWRN
LLSTHVQGFFTSCLRHFSQTSRSTTPANLDSESEHFFRCCLDKKSQRTMLAVVDYMRRQK
RPSSGTIFNDAFWLDLNYLEVAKVAQSCAAHFTALLYAEIYADKKSMDDQEKRSLAFEEG
SQSTTISSLSEKSKEETGISLQDLLLEIYRSIGEPDSLYGCGGGKMLQPITRLRTYEHEA
MWGKALVTYDLETAIPSSTRQAGIIQALQNLGLCHILSVYLKGLDYENKDWCPELEELHY
QAAWRNMQWDHCTSVSKEVEGTSYHESLYNALQSLRDREFSTFYESLKYARVKEVEEMCK
RSLESVYSLYPTLSRLQAIGELESIGELFSRSVTHRQLSEVYIKWQKHSQLLKDSDFSFQ
EPIMALRTVILEILMEKEMDNSQRECIKDILTKHLVELSILARTFKNTQLPERAIFQIKQ
YNSVSCGVSEWQLEEAQVFWAKKEQSLALSILKQMIKKLDASCAANNPSLKLTYTECLRV
CGNWLAETCLENPAVIMQTYLEKAVEVAGNYDGESSDELRNGKMKAFLSLARFSDTQYQR
IENYMKSSEFENKQALLKRAKEEVGLLREHKIQTNRYTVKVQRELELDELALRALKEDRK
RFLCKAVENYINCLLSGEEHDMWVFRLCSLWLENSGVSEVNGMMKRDGMKIPTYKFLPLM
YQLAARMGTKMMGGLGFHEVLNNLISRISMDHPHHTLFIILALANANRDEFLTKPEVARR
SRITKNVPKQSSQLDEDRTEAANRIICTIRSRRPQMVRSVEALCDAYIILANLDATQWKT
QRKGINIPADQPITKLKNLEDVVVPTMEIKVDHTGEYGNLVTIQSFKAEFRLAGGVNLPK
IIDCVGSDGKERRQLVKGRDDLRQDAVMQQVFQMCNTLLQRNTETRKRKLTICTYKVVPL
SQRSGVLEWCTGTVPIGEFLVNNEDGAHKRYRPNDFSAFQCQKKMMEVQKKSFEEKYEVF
MDVCQNFQPVFRYFCMEKFLDPAIWFEKRLAYTRSVATSSIVGYILGLGDRHVQNILINE
QSAELVHIDLGVAFEQGKILPTPETVPFRLTRDIVDGMGITGVEGVFRRCCEKTMEVMRN
SQETLLTIVEVLLYDPLFDWTMNPLKALYLQQRPEDETELHPTLNADDQECKRNLSDIDQ
SFNKVAERVLMRLQEKLKGVEEGTVLSVGGQVNLLIQQAIDPKNLSRLFPGWKAWV
>sp|Q92858|ATOH1_HUMAN Protein atonal homolog 1 OS=Homo sapiens GN=ATOH1 PE=2 SV=1
MSRLLHAEEWAEVKELGDHHRQPQPHHLPQPPPPPQPPATLQAREHPVYPPELSLLDSTD
PRAWLAPTLQGICTARAAQYLLHSPELGASEAAAPRDEVDGRGELVRRSSGGASSSKSPG
PVKVREQLCKLKGGVVVDELGCSRQRAPSSKQVNGVQKQRRLAANARERRRMHGLNHAFD
QLRNVIPSFNNDKKLSKYETLQMAQIYINALSELLQTPSGGEQPPPPPASCKSDHHHLRT
AASYEGGAGNATAAGAQQASGGSQRPTPPGSCRTRFSAPASAGGYSVQLDALHFSTFEDS
ALTAMMAQKNLSPSLPGSILQPVQEENSKTSPRSHRSDGEFSPHSHYSDSDEAS

>sp|O43861|ATP9B_HUMAN Probable phospholipid-transporting ATPase IIB OS=Homo sapiens GN=ATP9B PE=2 SV=4
MADQIPLYPVRSAAAAAANRKRAAYYSAAGPRPGADRHSRYQLEDESAHLDEMPLMMSEE
GFENEESDYHTLPRARIMQRKRGLEWFVCDGWKFLCTSCCGWLINICRRKKELKARTVWL
GCPEKCEEKHPRNSIKNQKYNVFTFIPGVLYEQFKFFLNLYFLVISCSQFVPALKIGYLY
TYWAPLGFVLAVTMTREAIDEFRRFQRDKEVNSQLYSKLTVRGKVQVKSSDIQVGDLIIV
EKNQRIPSDMVFLRTSEKAGSCFIRTDQLDGETDWKLKVAVSCTQQLPALGDLFSISAYV
YAQKPQMDIHSFEGTFTREDSDPPIHESLSIENTLWASTIVASGTVIGVVIYTGKETRSV
MNTSNPKNKVGLLDLELNRLTKALFLALVALSIVMVTLQGFVGPWYRNLFRFLLLFSYII
PISLRVNLDMGKAVYGWMMMKDENIPGTVVRTSTIPEELGRLVYLLTDKTGTLTQNEMIF
KRLHLGTVSYGADTMDEIQSHVRDSYSQMQSQAGGNNTGSTPLRKAQSSAPKVRKSVSSR
IHEAVKAIVLCHNVTPVYESRAGVTEETEFAEADQDFSDENRTYQASSPDEVALVQWTES
VGLTLVSRDLTSMQLKTPSGQVLSFCILQLFPFTSESKRMGVIVRDESTAEITFYMKGAD
VAMSPIVQYNDWLEEECGNMAREGLRTLVVAKKALTEEQYQDFESRYTQAKLSMHDRSLK
VAAVVESLEREMELLCLTGVEDQLQADVRPTLEMLRNAGIKIWMLTGDKLETATCIAKSS
HLVSRTQDIHIFRQVTSRGEAHLELNAFRRKHDCALVISGDSLEVCLKYYEHEFVELACQ
CPAVVCCRCSPTQKARIVTLLQQHTGRRTCAIGDGGNDVSMIQAADCGIGIEGKEGKQAS
LAADFSITQFRHIGRLLMVHGRNSYKRSAALGQFVMHRGLIISTMQAVFSSVFYFASVPL
YQGFLMVGYATIYTMFPVFSLVLDQDVKPEMAMLYPELYKDLTKGRSLSFKTFLIWVLIS
IYQGGILMYGALVLFESEFVHVVAISFTALILTELLMVALTVRTWHWLMVVAEFLSLGCY
VSSLAFLNEYFGIGRVSFGAFLDVAFITTVTFLWKVSAITVVSCLPLYVLKYLRRKLSPP
SYCKLAS
>sp|Q92482|AQP3_HUMAN Aquaporin-3 OS=Homo sapiens GN=AQP3 PE=2 SV=2
MGRQKELVSRCGEMLHIRYRLLRQALAECLGTLILVMFGCGSVAQVVLSRGTHGGFLTIN
LAFGFAVTLGILIAGQVSGAHLNPAVTFAMCFLAREPWIKLPIYTLAQTLGAFLGAGIVF
GLYYDAIWHFADNQLFVSGPNGTAGIFATYPSGHLDMINGFFDQFIGTASLIVCVLAIVD
PYNNPVPRGLEAFTVGLVVLVIGTSMGFNSGYAVNPARDFGPRLFTALAGWGSAVFTTGQ
HWWWVPIVSPLLGSIAGVFVYQLMIGCHLEQPPPSNEEENVKLAHVKHKEQI
>sp|Q15041|AR6P1_HUMAN ADP-ribosylation factor-like protein 6-interacting protein 1 OS=Homo sapiens GN=ARL6IP1 PE=1 SV=2
MAEGDNRSTNLLAAETASLEEQLQGWGEVMLMADKVLRWERAWFPPAIMGVVSLVFLIIY
YLDPSVLSGVSCFVMFLCLADYLVPILAPRIFGSNKWTTEQQQRFHEICSNLVKTRRRAV
GWWKRLFTLKEEKPKMYFMTMIVSLAAVAWVGQQVHNLLLTYLIVTSLLLLPGLNQHGII
LKYIGMAKREINKLLKQKEKKNE
>sp|P25098|ARBK1_HUMAN Beta-adrenergic receptor kinase 1 OS=Homo sapiens GN=GRK2 PE=1 SV=2
MADLEAVLADVSYLMAMEKSKATPAARASKKILLPEPSIRSVMQKYLEDRGEVTFEKIFS
QKLGYLLFRDFCLNHLEEARPLVEFYEEIKKYEKLETEEERVARSREIFDSYIMKELLAC
SHPFSKSATEHVQGHLGKKQVPPDLFQPYIEEICQNLRGDVFQKFIESDKFTRFCQWKNV
ELNIHLTMNDFSVHRIIGRGGFGEVYGCRKADTGKMYAMKCLDKKRIKMKQGETLALNER
IMLSLVSTGDCPFIVCMSYAFHTPDKLSFILDLMNGGDLHYHLSQHGVFSEADMRFYAAE
IILGLEHMHNRFVVYRDLKPANILLDEHGHVRISDLGLACDFSKKKPHASVGTHGYMAPE
VLQKGVAYDSSADWFSLGCMLFKLLRGHSPFRQHKTKDKHEIDRMTLTMAVELPDSFSPE
LRSLLEGLLQRDVNRRLGCLGRGAQEVKESPFFRSLDWQMVFLQKYPPPLIPPRGEVNAA

DAFDIGSFDEEDTKGIKLLDSDQELYRNFPLTISERWQQEVAETVFDTINAETDRLEARK
KAKNKQLGHEEDYALGKDCIMHGYMSKMGNPFLTQWQRRYFYLFPNRLEWRGEGEAPQSL
LTMEEIQSVEETQIKERKCLLLKIRGGKQFILQCDSDPELVQWKKELRDAYREAQQLVQR
VPKMKNKPRSPVVELSKVPLVQRGSANGL
>sp|P53367|ARFP1_HUMAN Arfaptin-1 OS=Homo sapiens GN=ARFIP1 PE=1 SV=2
MAQESPKNSAAEIPVTSNGEVDDSREHSFNRDLKHSLPSGLGLSETQITSHGFDNTKEGV
IEAGAFQGSPAPPLPSVMSPSRVAASRLAQQGSDLIVPAGGQRTQTKSGPVILADEIKNP
AMEKLELVRKWSLNTYKCTRQIISEKLGRGSRTVDLELEAQIDILRDNKKKYENILKLAQ
TLSTQLFQMVHTQRQLGDAFADLSLKSLELHEEFGYNADTQKLLAKNGETLLGAINFFIA
SVNTLVNKTIEDTLMTVKQYESARIEYDAYRTDLEELNLGPRDANTLPKIEQSQHLFQAH
KEKYDKMRNDVSVKLKFLEENKVKVLHNQLVLFHNAIAAYFAGNQKQLEQTLKQFHIKLK
TPGVDAPSWLEEQ
>sp|Q9GZN1|ARP6_HUMAN Actin-related protein 6 OS=Homo sapiens GN=ACTR6 PE=1 SV=1
MTTLVLDNGAYNAKIGYSHENVSVIPNCQFRSKTARLKTFTANQIDEIKDPSGLFYILPF
QKGYLVNWDVQRQVWDYLFGKEMYQVDFLDTNIIITEPYFNFTSIQESMNEILFEEYQFQ
AVLRVNAGALSAHRYFRDNPSELCCIIVDSGYSFTHIVPYCRSKKKKEAIIRINVGGKLL
TNHLKEIISYRQLHVMDETHVINQVKEDVCYVSQDFYRDMDIAKLKGEENTVMIDYVLPD
FSTIKKGFCKPREEMVLSGKYKSGEQILRLANERFAVPEILFNPSDIGIQEMGIPEAIVY
SIQNLPEEMQPHFFKNIVLTGGNSLFPGFRDRVYSEVRCLTPTDYDVSVVLPENPITYAW
EGGKLISENDDFEDMVVTREDYEENGHSVCEEKFDI
>sp|Q96EG1|ARSG_HUMAN Arylsulfatase G OS=Homo sapiens GN=ARSG PE=1 SV=1
MGWLFLKVLLAGVSFSGFLYPLVDFCISGKTRGQKPNFVIILADDMGWGDLGANWAETKD
TANLDKMASEGMRFVDFHAAASTCSPSRASLLTGRLGLRNGVTRNFAVTSVGGLPLNETT
LAEVLQQAGYVTGIIGKWHLGHHGSYHPNFRGFDYYFGIPYSHDMGCTDTPGYNHPPCPA
CPQGDGPSRNLQRDCYTDVALPLYENLNIVEQPVNLSSLAQKYAEKATQFIQRASTSGRP
FLLYVALAHMHVPLPVTQLPAAPRGRSLYGAGLWEMDSLVGQIKDKVDHTVKENTFLWFT
GDNGPWAQKCELAGSVGPFTGFWQTRQGGSPAKQTTWEGGHRVPALAYWPGRVPVNVTST
ALLSVLDIFPTVVALAQASLPQGRRFDGVDVSEVLFGRSQPGHRVLFHPNSGAAGEFGAL
QTVRLERYKAFYITGGARACDGSTGPELQHKFPLIFNLEDDTAEAVPLERGGAEYQAVLP
EVRKVLADVLQDIANDNISSADYTQDPSVTPCCNPYQIACRCQAA
>sp|Q8WXH4|ASB11_HUMAN Ankyrin repeat and SOCS box protein 11 OS=Homo sapiens GN=ASB11 PE=1 SV=1
MEDGPVFYGFKNIFITMFATFFFFKLLIKVFLALLTHFYIVKGNRKEAARIAEEIYGGIS
DCWADRSPLHEAAAQGRLLALKTLIAQGVNVNLVTINRVSSLHEACLGGHVACAKALLEN
GAHVNGVTVHGATPLFNACCSGSAACVNVLLEFGAKAQLEVHLASPIHEAVKRGHRECME
ILLANNVNIDHEVPQLGTPLYVACTYQRVDCVKKLLELGASVDHGQWLDTPLHAAARQSN
VEVIHLLTDYGANLKRRNAQGKSALDLAAPKSSVEQALLLREGPPALSQLCRLCVRKCLG
RACHQAIHKLHLPEPLERFLLYQ
>sp|Q8WXK3|ASB13_HUMAN Ankyrin repeat and SOCS box protein 13 OS=Homo sapiens GN=ASB13 PE=1 SV=2
MEPRAADGCFLGDVGFWVERTPVHEAAQRGESLQLQQLIESGACVNQVTVDSITPLHAAS
LQGQARCVQLLLAAGAQVDARNIDGSTPLCDACASGSIECVKLLLSYGAKVNPPLYTASP
LHEACMSGSSECVRLLIDVGANLEAHDCHFGTPLHVACAREHLDCVKVLLNAGANVNAAK
LHETALHHAAKVKNVDLIEMLIEFGGNIYARDNRGKKPSDYTWSSSAPAKCFEYYEKTPL

TLSQLCRVNLRKATGVRGLEKIAKLNIPPRLIDYLSYN
>sp|Q9Y576|ASB1_HUMAN Ankyrin repeat and SOCS box protein 1 OS=Homo sapiens GN=ASB1 PE=1 SV=1
MAEGGSPDGRAGPGSAGRNLKEWLREQFCDHPLEHCEDTRLHDAAYVGDLQTLRSLLQEE
SYRSRINEKSVWCCGWLPCTPLRIAATAGHGSCVDFLIRKGAEVDLVDVKGQTALYVAVV
NGHLESTQILLEAGADPNGSRHHRSTPVYHASRVGRADILKALIRYGADVDVNHHLTPDV
QPRFSRRLTSLVVCPLYISAAYHNLQCFRLLLLAGANPDFNCNGPVNTQGFYRGSPGCVM
DAVLRHGCEAAFVSLLVEFGANLNLVKWESLGPESRGRRKVDPEALQVFKEARSVPRTLL
CLCRVAVRRALGKHRLHLIPSLPLPDPIKKFLLHE
>sp|Q9Y574|ASB4_HUMAN Ankyrin repeat and SOCS box protein 4 OS=Homo sapiens GN=ASB4 PE=2 SV=1
MDGTTAPVTKSGAAKLVKRNFLEALKSNDFGKLKAILIQRQIDVDTVFEVEDENMVLASY
KQGYWLPSYKLKSSWATGLHLSVLFGHVECLLVLLDHNATINCRPNGKTPLHVACEMANV
DCVKILCDRGAKLNCYSLSGHTALHFCTTPSSILCAKQLVWRGANVNMKTNNQDEETPLH
TAAHFGLSELVAFYVEHGAIVDSVNAHMETPLAIAAYWALRFKEQEYSTEHHLVCRMLLD
YKAEVNARDDDFKSPLHKAAWNCDHVLMHMMLEAGAEANLMDINGCAAIQYVLKVTSVRP
AAQPEICYQLLLNHGAARIYPPQFHKVIQACHSCPKAIEVVVNAYEHIRWNTKWRRAIPD
DDLEKYWDFYHSLFTVCCNSPRTLMHLSRCAIRRTLHNRCHRAIPLLSLPLSLKKYLLLE
PEGIIY
>sp|Q8WWX0|ASB5_HUMAN Ankyrin repeat and SOCS box protein 5 OS=Homo sapiens GN=ASB5 PE=2 SV=1
MSVLEENRPFAQQLSNVYFTILSLFCFKLFVKISLAILSHFYIVKGNRKEAARIAAEFYG
VTQGQGSWADRSPLHEAASQGRLLALRTLLSQGYNVNAVTLDHVTPLHEACLGDHVACAR
TLLEAGANVNAITIDGVTPLFNACSQGSPSCAELLLEYGAKAQLESCLPSPTHEAASKGH
HECLDILISWGIDVDQEIPHLGTPLYVACMSQQFHCIWKLLYAGADVQKGKYWDTPLHAA
AQQSSTEIVNLLLEFGADINAKNTELLRPIDVATSSSMVERILLQHEATPSSLYQLCRLC
IRSYIGKPRLHLIPQLQLPTLLKNFLQYR
>sp|Q9H765|ASB8_HUMAN Ankyrin repeat and SOCS box protein 8 OS=Homo sapiens GN=ASB8 PE=2 SV=1
MSSSMWYIMQSIQSKYSLSERLIRTIAAIRSFPHDNVEDLIRGGADVNCTHGTLKPLHCA
CMVSDADCVELLLEKGAEVNALDGYNRTALHYAAEKDEACVEVLLEYGANPNALDGNRDT
PLHWAAFKNNAECVRALLESGASVNALDYNNDTPLSWAAMKGNLESVSILLDYGAEVRVI
NLIGQTPISRLVALLVRGLGTEKEDSCFELLHRAVGHFELRKNGTMPREVARDPQLCEKL
TVLCSAPGTLKTLARYAVRRSLGLQYLPDAVKGLPLPASLKEYLLLLE
>sp|Q8N3C0|ASCC3_HUMAN Activating signal cointegrator 1 complex subunit 3 OS=Homo sapiens GN=ASCC3 PE=1 SV=3
MALPRLTGALRSFSNVTKQDNYNEEVADLKIKRSKLHEQVLDLGLTWKKIIKFLNEKLEK
SKMQSINEDLKDILHAAKQIVGTDNGREAIESGAAFLFMTFHLKDSVGHKETKAIKQMFG
PFPSSSATAACNATNRIISHFSQDDLTALVQMTEKEHGDRVFFGKNLAFSFDMHDLDHFD
ELPINGETQKTISLDYKKFLNEHLQEACTPELKPVEKTNGSFLWCEVEKYLNSTLKEMTE
VPRVEDLCCTLYDMLASIKSGDELQDELFELLGPEGLELIEKLLQNRITIVDRFLNSSND
HRFQALQDNCKKILGENAKPNYGCQVTIQSEQEKQLMKQYRREEKRIARREKKAGEDLEV
SEGLMCFDPKELRIQREQALLNARSVPILSRQRDADVEKIHYPHVYDSQAEAMKTSAFIA
GAKMILPEGIQRENNKLYEEVRIPYSEPMPLSFEEKPVYIQDLDEIGQLAFKGMKRLNRI

QSIVFETAYNTNENMLICAPTGAGKTNIAMLTVLHEIRQHFQQGVIKKNEFKIVYVAPMK
ALAAEMTDYFSRRLEPLGIIVKELTGDMQLSKSEILRTQMLVTTPEKWDVVTRKSVGDVA
LSQIVRLLILDEVHLLHEDRGPVLESIVARTLRQVESTQSMIRILGLSATLPNYLDVATF
LHVNPYIGLFFFDGRFRPVPLGQTFLGIKCANKMQQLNNMDEVCYENVLKQVKAGHQVMV
FVHARNATVRTAMSLIERAKNCGHIPFFFPTQGHDYVLAEKQVQRSRNKQVRELFPDGFS
IHHAGMLRQDRNLVENLFSNGHIKVLVCTATLAWGVNLPAHAVIIKGTQIYAAKRGSFVD
LGILDVMQIFGRAGRPQFDKFGEGIIITTHDKLSHYLTLLTQRNPIESQFLESLADNLNA
EIALGTVTNVEEAVKWISYTYLYVRMRANPLAYGISHKAYQIDPTLRKHREQLVIEVGRK
LDKAQMIRFEERTGYFSSTDLGRTASHYYIKYNTIETFNELFDAHKTEGDIFAIVSKAEE
FDQIKVREEEIEELDTLLSNFCELSTPGGVENSYGKINILLQTYISRGEMDSFSLISDSA
YVAQNAARIVRALFEIALRKRWPTMTYRLLNLSKVIDKRLWGWASPLRQFSILPPHILTR
LEEKKLTVDKLKDMRKDEIGHILHHVNIGLKVKQCVHQIPSVMMEASIQPITRTVLRVTL
SIYADFTWNDQVHGTVGEPWWIWVEDPTNDHIYHSEYFLALKKQVISKEAQLLVFTIPIF
EPLPSQYYIRAVSDRWLGAEAVCIINFQHLILPERHPPHTELLDLQPLPITALGCKAYEA
LYNFSHFNPVQTQIFHTLYHTDCNVLLGAPTGSGKTVAAELAIFRVFNKYPTSKAVYIAP
LKALVRERMDDWKVRIEEKLGKKVIELTGDVTPDMKSIAKADLIVTTPEKWDGVSRSWQN
RNYVQQVTILIIDEIHLLGEERGPVLEVIVSRTNFISSHTEKPVRIVGLSTALANARDLA
DWLNIKQMGLFNFRPSVRPVPLEVHIQGFPGQHYCPRMASMNKPAFQAIRSHSPAKPVLI
FVSSRRQTRLTALELIAFLATEEDPKQWLNMDEREMENIIATVRDSNLKLTLAFGIGMHH
AGLHERDRKTVEELFVNCKVQVLIATSTLAWGVNFPAHLVIIKGTEYYDGKTRRYVDFPI
TDVLQMMGRAGRPQFDDQGKAVILVHDIKKDFYKKFLYEPFPVESSLLGVLSDHLNAEIA
GGTITSKQDALDYITWTYFFRRLIMNPSYYNLGDVSHDSVNKFLSHLIEKSLIELELSYC
IEIGEDNRSIEPLTYGRIASYYYLKHQTVKMFKDRLKPECSTEELLSILSDAEEYTDLPV
RHNEDHMNSELAKCLPIESNPHSFDSPHTKAHLLLQAHLSRAMLPCPDYDTDTKTVLDQA
LRVCQAMLDVAANQGWLVTVLNITNLIQMVIQGRWLKDSSLLTLPNIENHHLHLFKKWKP
IMKGPHARGRTSIESLPELIHACGGKDHVFSSMVESELHAAKTKQAWNFLSHLPVINVGI
SVKGSWDDLVEGHNELSVSTLTADKRDDNKWIKLHADQEYVLQVSLQRVHFGFHKGKPES
CAVTPRFPKSKDEGWFLILGEVDKRELIALKRVGYIRNHHVASLSFYTPEIPGRYIYTLY
FMSDCYLGLDQQYDIYLNVTQASLSAQVNTKVSDSLTDLALK
>sp|Q6XD76|ASCL4_HUMAN Achaete-scute homolog 4 OS=Homo sapiens GN=ASCL4 PE=1 SV=1
METRKPAERLALPYSLRTAPLGVPGTLPGLPRRDPLRVALRLDAACWEWARSGCARGWQY
LPVPLDSAFEPAFLRKRNERERQRVRCVNEGYARLRDHLPRELADKRLSKVETLRAAIDY
IKHLQELLERQAWGLEGAAGAVPQRRAECNSDGESKASSAPSPSSEPEEGGS
>sp|P24539|AT5F1_HUMAN ATP synthase F(0) complex subunit B1, mitochondrial OS=Homo sapiens GN=ATP5F1 PE=1 SV=2
MLSRVVLSAAATAAPSLKNAAFLGPGVLQATRTFHTGQPHLVPVPPLPEYGGKVRYGLIP
EEFFQFLYPKTGVTGPYVLGTGLILYALSKEIYVISAETFTALSVLGVMVYGIKKYGPFV
ADFADKLNEQKLAQLEEAKQASIQHIQNAIDTEKSQQALVQKRHYLFDVQRNNIAMALEV
TYRERLYRVYKEVKNRLDYHISVQNMMRRKEQEHMINWVEKHVVQSISTQQEKETIAKCI
ADLKLLAKKAQAQPVM
>sp|Q9ULK2|AT7L1_HUMAN Ataxin-7-like protein 1 OS=Homo sapiens GN=ATXN7L1 PE=1 SV=3
MTSERSRIPCLSAAAAEGTGKKQQEGRAMATLDRKVPSPEAFLGKPWSSWIDAAKLHCSD
NVDLEEAGKEGGKSREVMRLNKEDMHLFGHYPAHDDFYLVVCSACNQVVKPQVFQSHCER
RHGSMCRPSPSPVSPASNPRTSLVQVKTKACLSGHHSASSTSKPFKTPKDNLLTSSSKQH

TVFPAKGSRDKPCVPVPVVSLEKIPNLVKADGANVKMNSTTTTAVSASSTSSSAVSTPPL
IKPVLMSKSVPPSPEKILNGKGILPTTIDKKHQNGTKNSNKPYRRLSEREFDPNKHCGVL
DPETKKPCTRSLTCKTHSLSHRRAVPGRKKQFDLLLAEHKAKSREKEVKDKEHLLTSTRE
ILPSQSGPAQDSLLGSSGSSGPEPKVASPAKSRPPNSVLPRPSSANSISSSTSSNHSGHT
PEPPLPPVGGDLASRLSSDEGEMDGADESEKLDCQFSTHHPRPLAFCSFGSRLMGRGYYV
FDRRWDRFRFALNSMVEKHLNSQMWKKIPPAADSPLPSPAAHITTPVPASVLQPFSNPSA
VYLPSAPISSRLTSSYIMTSAMLSNAAFVTSPDPSALMSHTTAFPHVAATLSIMDSTFKA
PSAVSPIPAVIPSPSHKPSKTKTSKSSKVKDLSTRSDESPSNKKRKPQSSTSSSSSSSSS
SLQTSLSSPLSGPHKKNCVLNASSALNSYQAAPPYNSLSVHNSNNGVSPLSAKLEPSGRT
SLPGGPADIVRQVGAVGGSSDSCPLSVPSLALHAGDLSLASHNAVSSLPLSFDKSEGKKR
KNSSSSSKACKITKMPGMNSVHKKNPPSLLAPVPDPVNSTSSRQVGKNSSLALSQSSPSS
ISSPGHSRQNTNRTGRIRTLP
>sp|Q14CW9|AT7L3_HUMAN Ataxin-7-like protein 3 OS=Homo sapiens GN=ATXN7L3 PE=1 SV=1
MKMEEMSLSGLDNSKLEAIAQEIYADLVEDSCLGFCFEVHRAVKCGYFFLDDTDPDSMKD
FEIVDQPGLDIFGQVFNQWKSKECVCPNCSRSIAASRFAPHLEKCLGMGRNSSRIANRRI
ANSNNMNKSESDQEDNDDINDNDWSYGSEKKAKKRKSDKNPNSPRRSKSLKHKNGELSNS
DPFKYNNSTGISYETLGPEELRSLLTTQCGVISEHTKKMCTRSLRCPQHTDEQRRTVRIY
FLGPSAVLPEVESSLDNDSFDMTDSQALISRLQWDGSSDLSPSDSGSSKTSENQGWGLGT
NSSESRKTKKKKSHLSLVGTASGLGSNKKKKPKPPAPPTPSIYDDIN
>sp|Q96QE3|ATAD5_HUMAN ATPase family AAA domain-containing protein 5 OS=Homo sapiens GN=ATAD5 PE=1 SV=4
MVGVLAMAAAAAPPPVKDCEIEPCKKRKKDDDTSTCKTITKYLSPLGKTRDRVFAPPKPS
NILDYFRKTSPTNEKTQLGKECKIKSPESVPVDSNKDCTTPLEMFSNVEFKKKRKRVNLS
HQLNNIKTENEAPIEISSDDSKEDYSLNNDFVESSTSVLRYKKQVEVLAENIQDTKSQPN
TMTSLQNSKKVNPKQGTTKNDFKKLRKRKCRDVVDLSESLPLAEELNLLKKDGKDTKQME
NTTSHANSRDNVTEAAQLNDSIITVSYEEFLKSHKENKVEEIPDSTMSICVPSETVDEIV
KSGYISESENSEISQQVRFKTVTVLAQVHPIPPKKTGKIPRIFLKQKQFEMENSLSDPEN
EQTVQKRKSNVVIQEEELELAVLEAGSSEAVKPKCTLEERQQFMKAFRQPASDALKNGVK
KSSDKQKDLNEKCLYEVGRDDNSKKIMENSGIQMVSKNGNLQLHTDKGSFLKEKNKKLKK
KNKKTLDTGAIPGKNREGNTQKKETTFFLKEKQYQNRMSLRQRKTEFFKSSTLFNNESLV
YEDIANDDLLKVSSLCNNNKLSRKTSIPVKDIKLTQSKAESEASLLNVSTPKSTRRSGRI
SSTPTTETIRGIDSDDVQDNSQLKASTQKAANLSEKHSLYTAELITVPFDSESPIRMKFT
RISTPKKSKKKSNKRSEKSEATDGGFTSQIRKASNTSKNISKAKQLIEKAKALHISRSKV
TEEIAIPLRRSSRHQTLPERKKLSETEDSVIIIDSSPTALKHPEKNQKKLQCLNDVLGKK
LNTSTKNVPGKMKVAPLFLVRKAQKAADPVPSFDESSQDTSEKSQDCDVQCKAKRDFLMS
GLPDLLKRQIAKKAAALDVYNAVSTSFQRVVHVQQKDDGCCLWHLKPPSCPLLTKFKELN
TKVIDLSKCGIALGEFSTLNSKLKSGNSAAVFMRTRKEFTEEVRNLLLEEIRWSNPEFSL
KKYFPLLLKKQIEHQVLSSECHSKQELEADVSHKETKRKLVEAENSKSKRKKPNEYSKNL
EKTNRKSEELSKRNNSSGIKLDSSKDSGTEDMLWTEKYQPQTASELIGNELAIKKLHSWL
KDWKRRAELEERQNLKGKRDEKHEDFSGGIDFKGSSDDEEESRLCNTVLITGPTGVGKTA
AVYACAQELGFKIFEVNASSQRSGRQILSQLKEATQSHQVDKQGVNSQKPCFFNSYYIGK
SPKKISSPKKVVTSPRKVPPPSPKSSGPKRALPPKTLANYFKVSPKPKNNEEIGMLLENN
KGIKNSFEQKQITQTKSTNATNSNVKDVGAEEPSRKNATSLILFEEVDVIFDEDAGFLNA
IKTFMATTKRPVILTTSDPTFSLMFDGCFEEIKFSTPSLLNVASYLQMICLTENFRTDVK

DFVTLLTANTCDIRKSILYLQFWIRSGGGVLEERPLTLYRGNSRNVQLVCSEHGLDNKIY
PKNTKKKRVDLPKCDSGCAETLFGLKNIFSPSEDLFSFLKHKITMKEEWHKFIQLLTEFQ
MRNVDFLYSNLEFILPLPVDTIPETKNFCGPSVTVDASAATKSMNCLARKHSEREQPLKK
SQKKKQKKTLVILDDSDLFDTDLDFPDQSISLSSVSSSSNAEESKTGDEESKARDKGNNP
ETKKSIPCPPKTTAGKKCSALVSHCLNSLSEFMDNMSFLDALLTDVREQNKYGRNDFSWT
NGKVTSGLCDEFSLESNDGWTSQSSGELKAAAEALSFTKCSSAISKALETLNSCKKLGRD
PTNDLTFYVSQKRNNVYFSQSAANLDNAWKRISVIKSVFSSRSLLYVGNRQASIIEYLPT
LRNICKTEKLKEQGKSKRRFLHYFEGIHLDIPKETVNTLAADFP
>sp|Q9NVI7|ATD3A_HUMAN ATPase family AAA domain-containing protein 3A OS=Homo sapiens GN=ATAD3A PE=1 SV=2
MSWLFGINKGPKGEGAGPPPPLPPAQPGAEGGGDRGLGDRPAPKDKWSNFDPTGLERAAK
AARELEHSRYAKDALNLAQMQEQTLQLEQQSKLKMRLEALSLLHTLVWAWSLCRAGAVQT
QERLSGSASPEQVPAGECCALQEYEAAVEQLKSEQIRAQAEERRKTLSEETRQHQARAQY
QDKLARQRYEDQLKQQQLLNEENLRKQEESVQKQEAMRRATVEREMELRHKNEMLRVEAE
ARARAKAERENADIIREQIRLKAAEHRQTVLESIRTAGTLFGEGFRAFVTDWDKVTATVA
GLTLLAVGVYSAKNATLVAGRFIEARLGKPSLVRETSRITVLEALRHPIQVSRRLLSRPQ
DALEGVVLSPSLEARVRDIAIATRNTKKNRSLYRNILMYGPPGTGKTLFAKKLALHSGMD
YAIMTGGDVAPMGREGVTAMHKLFDWANTSRRGLLLFVDEADAFLRKRATEKISEDLRAT
LNAFLYRTGQHSNKFMLVLASNQPEQFDWAINDRINEMVHFDLPGQEERERLVRMYFDKY
VLKPATEGKQRLKLAQFDYGRKCSEVARLTEGMSGREIAQLAVSWQATAYASEDGVLTEA
MMDTRVQDAVQQHQQKMCWLKAEGPGRGDEPSPS
>sp|O95260|ATE1_HUMAN Arginyl-tRNA--protein transferase 1 OS=Homo sapiens GN=ATE1 PE=1 SV=2
MAFWAGGSPSVVDYFPSEDFYRCGYCKNESGSRSNGMWAHSMTVQDYQDLIDRGWRRSGK
YVYKPVMNQTCCPQYTIRCRPLQFQPSKSHKKVLKKMLKFLAKGEVPKGSCEDEPMDSTM
DDAVAGDFALINKLDIQCDLKTLSDDIKESLESEGKNSKKEEPQELLQSQDFVGEKLGSG
EPSHSVKVHTVPKPGKGADLSKPPCRKAKEIRKERKRLKLMQQNPAGELEGFQAQGHPPS
LFPPKAKSNQPKSLEDLIFESLPENASHKLEVRVVRSSPPSSQFKATLLESYQVYKRYQM
VIHKNPPDTPTESQFTRFLCSSPLEAETPPNGPDCGYGSFHQQYWLDGKIIAVGVIDILP
NCVSSVYLYYDPDYSFLSLGVYSALREIAFTRQLHEKTSQLSYYYMGFYIHSCPKMKYKG
QYRPSDLLCPETYVWVPIEQCLPSLENSKYCRFNQDPEAVDEDRSTEPDRLQVFHKRAIM
PYGVYKKQQKDPSEEAAVLQYASLVGGQKCSERMLLFRN
>sp|Q9H0Y0|ATG10_HUMAN Ubiquitin-like-conjugating enzyme ATG10 OS=Homo sapiens GN=ATG10 PE=1 SV=1
MEEDEFIGEKTFQRYCAEFIKHSQQIGDSWEWRPSKDCSDGYMCKIHFQIKNGSVMSHLG
ASTHGQTCLPMEEAFELPLDDCEVIETAAASEVIKYEYHVLYSCSYQVPVLYFRASFLDG
RPLTLKDIWEGVHECYKMRLLQGPWDTITQQEHPILGQPFFVLHPCKTNEFMTPVLKNSQ
KINKNVNYITSWLSIVGPVVGLNLPLSYAKATSQDERNVP
>sp|Q9NR80|ARHG4_HUMAN Rho guanine nucleotide exchange factor 4 OS=Homo sapiens GN=ARHGEF4 PE=1 SV=3
MPWEEPAGEKPSCSHSQKAFHMEPAQKPCFTTDMVTWALLCISAETVRGEAPSQPRGIPH
RSPVSVDDLWLEKTQRKKLQKQAHVERRLHIGAVHKDGVKCWRKTIITSPESLNLPRRSH
PLSQSAPTGLNHMGWPEHTPGTAMPDGALDTAVCADEVGSEEDLYDDLHSSSHHYSHPGG
GGEQLAINELISDGSVVCAEALWDHVTMDDQELGFKAGDVIEVMDATNREWWWGRVADGE

GWFPASFVRLRVNQDEPADDDAPLAGNSGAEDGGAEAQSSKDQMRTNVINEILSTERDYI
KHLRDICEGYVRQCRKRADMFSEEQLRTIFGNIEDIYRCQKAFVKALEQRFNRERPHLSE
LGACFLEHQADFQIYSEYCNNHPNACVELSRLTKLSKYVYFFEACRLLQKMIDISLDGFL
LTPVQKICKYPLQLAELLKYTHPQHRDFKDVEAALHAMKNVAQLINERKRRLENIDKIAQ
WQSSIEDWEGEDLLVRSSELIYSGELTRVTQPQAKSQQRMFFLFDHQLIYCKKDLLRRDV
LYYKGRLDMDGLEVVDLEDGKDRDLHVSIKNAFRLHRGATGDSHLLCTRKPEQKQRWLKA
FAREREQVQLDQETGFSITELQRKQAMLNASKQQVTGKPKAVGRPCYLTRQKHPALPSNR
PQQQVLVLAEPRRKPSTFWHSISRLAPFRK
>sp|Q12774|ARHG5_HUMAN Rho guanine nucleotide exchange factor 5 OS=Homo sapiens GN=ARHGEF5 PE=1 SV=3
MEAEEAQRGASPPISAIEEFSIIPEAPMRSSQVSALGLEAQEDEDPSYKWREEHRLSATQ
QSELRDVCDYAIETMPSFPKEGSADVEPNQESLVAEACDTPEHWEAVPQSLAGRQARTLA
PPELWACPIQSEHLDMAPFSSDLGSEEEEVEFWPGLTSLTLGSGQAEEEEETSSDNSGQT
RYYSPCEEHPAETNQNEGSESGTIRQGEELPPEELQESQGLLHPQEVQVLEEQGQQEAGF
RGEGTLREDVCADGLLGEEQMIEQVNDEKGEQKQKQEQVQDVMLGRQGERMGLTGEPEGL
NDGEWEQEDMERKAQGQGGPEQGEERKRELQVPEENRADSQDEKSQTFLGKSEEVTGKQE
DHGIKEKGVPVSGQEAKEPESWDGGRLGAVGRARSREEENEHHGPSMPALIAPEDSPHCD
LFPGASYLMTQIPGTQTESRAEELSPAALSPSLEPIRCSHQPISLLGSFLTEESPDKEID
QNSQQEESRLRKGTVSSQGTEVVFASASVTPPRTPDSAPPSPAEAYPITPASVSARPPVA
FPRRETSCAARAPETASAPLSMDDPSPCGTSEMCPAALYGFPSTGTSPPRPPANSTGTVQ
HLRSDSFPGSHRTEQTPDLVGMLLSYSHSELPQRRPPKPAIYSSVTPRRDRRSGRDYSTVS
ASPTALSTLKQDSQESISNLERPSSPPSIQPWVSPHNPAFATESPAYGSSPSFVSMEDVR
IHEPLPPPPPQRRDTHPSVVETDGHARVVVPTLKQHSHPPPLALGSGLHAPHKGPLPQAS
DPAVARQHRPLPSTPDSSHHAQATPRWRYNKPLPPTPDLPQPHLPPISAPGSSRIYRPLP
PLPIIDPPTEPPPLPPKSRGRSRSTRGGHMNSGGHAKTRPACQDWTVPLPASAGRTSWPP
ATARSTESFTSTSRSKSEVSPGMAFSNMTNFLCPSSPTTPWTPELQGPTSKDEAGVSEHP
EAPAREPLRRTTPQQGASGPGRSPVGQARQPEKPSHLHLEKASSWPHRRDSGRPPGDSSG
QAVAPSEGANKHKGWSRQGLRRPSILPEGSSDSRGPAVEKHPGPSDTVVFREKKPKEVMG
GFSRRCSKLINSSQLLYQEYSDVVLNKEIQSQQRLESLSETPGPSSPRQPRKALVSSESY
LQRLSMASSGSLWQEIPVVRNSTVLLSMTHEDQKLQEVKFELIVSEASYLRSLNIAVDHF
QLSTSLRATLSNQEHQWLFSRLQDVRDVSATFLSDLEENFENNIFSFQVCDVVLNHAPDF
RRVYLPYVTNQTYQERTFQSLMNSNSNFREVLEKLESDPVCQRLSLKSFLILPFQRITRL
KLLLQNILKRTQPGSSEEAEATKAHHALEQLIRDCNNNVQSMRRTEELIYLSQKIEFECK
IFPLISQSRWLVKSGELTALEFSASPGLRRKLNTRPVHLHLFNDCLLLSRPREGSRFLVF
DHAPFSSIRGEKCEMKLHGPHKNLFRLFLRQNTQGAQAEFLFRTETQSEKLRWISALAMP
REELDLLECYNSPQVQCLRAYKPRENDELALEKADVVMVTQQSSDGWLEGVRLSDGERGW
FPVQQVEFISNPEVRAQNLKEAHRVKTAKLQLVEQQA
>sp|O43307|ARHG9_HUMAN Rho guanine nucleotide exchange factor 9 OS=Homo sapiens GN=ARHGEF9 PE=1 SV=3
MTLLITGDSIVSAEAVWDHVTMANRELAFKAGDVIKVLDASNKDWWWGQIDDEEGWFPAS
FVRLWVNQEDEVEEGPSDVQNGHLDPNSDCLCLGRPLQNRDQMRANVINEIMSTERHYIK
HLKDICEGYLKQCRKRRDMFSDEQLKVIFGNIEDIYRFQMGFVRDLEKQYNNDDPHLSEI
GPCFLEHQDGFWIYSEYCNNHLDACMELSKLMKDSRYQHFFEACRLLQQMIDIAIDGFLL
TPVQKICKYPLQLAELLKYTAQDHSDYRYVAAALAVMRNVTQQINERKRRLENIDKIAQW

QASVLDWEGEDILDRSSELIYTGEMAWIYQPYGRNQQRVFFLFDHQMVLCKKDLIRRDIL
YYKGRIDMDKYEVVDIEDGRDDDFNVSMKNAFKLHNKETEEIHLFFAKKLEEKIRWLRAF
REERKMVQEDEKIGFEISENQKRQAAMTVRKVPKQKGVNSARSVPPSYPPPQDPLNHGQY
LVPDGIAQSQVFEFTEPKRSQSPFWQNFSRLTPFKK
>sp|Q9NZN5|ARHGC_HUMAN Rho guanine nucleotide exchange factor 12 OS=Homo sapiens GN=ARHGEF12 PE=1 SV=1
MSGTQSTITDRFPLKKPIRHGSILNRESPTDKKQKVERIASHDFDPTDSSSKKTKSSSEE
SRSEIYGLVQRCVIIQKDDNGFGLTVSGDNPVFVQSVKEDGAAMRAGVQTGDRIIKVNGT
LVTHSNHLEVVKLIKSGSYVALTVQGRPPGSPQIPLADSEVEPSVIGHMSPIMTSPHSPG
ASGNMERITSPVLMGEENNVVHNQKVEILRKMLQKEQERLQLLQEDYNRTPAQRLLKEIQ
EAKKHIPQLQEQLSKATGSAQDGAVVTPSRPLGDTLTVSEAETDPGDVLGRTDCSSGDAS
RPSSDNADSPKSGPKERIYLEENPEKSETIQDTDTQSLVGSPSTRIAPHIIGAEDDDFGT
EHEQINGQCSCFQSIELLKSRPAHLAVFLHHVVSQFDPATLLCYLYSDLYKHTNSKETRR
IFLEFHQFFLDRSAHLKVSVPDEMSADLEKRRPELIPEDLHRHYIQTMQERVHPEVQRHL
EDFRQKRSMGLTLAESELTKLDAERDKDRLTLEKERTCAEQIVAKIEEVLMTAQAVEEDK
SSTMQYVILMYMKHLGVKVKEPRNLEHKRGRIGFLPKIKQSMKKDKEGEEKGKRRGFPSI
LGPPRRPSRHDNSAIGRAMELQKARHPKHLSTPSSVSPEPQDSAKLRQSGLANEGTDAGY
LPANSMSSVASGASFSQEGGKENDTGSKQVGETSAPGDTLDGTPRTLNTVFDFPPPPLDQ
VQEEECEVERVTEHGTPKPFRKFDSVAFGESQSEDEQFENDLETDPPNWQQLVSREVLLG
LKPCEIKRQEVINELFYTERAHVRTLKVLDQVFYQRVSREGILSPSELRKIFSNLEDILQ
LHIGLNEQMKAVRKRNETSVIDQIGEDLLTWFSGPGEEKLKHAAATFCSNQPFALEMIKS
RQKKDSRFQTFVQDAESNPLCRRLQLKDIIPTQMQRLTKYPLLLDNIAKYTEWPTEREKV
KKAADHCRQILNYVNQAVKEAENKQRLEDYQRRLDTSSLKLSEYPNVEELRNLDLTKRKM
IHEGPLVWKVNRDKTIDLYTLLLEDILVLLQKQDDRLVLRCHSKILASTADSKHTFSPVI
KLSTVLVRQVATDNKALFVISMSDNGAQIYELVAQTVSEKTVWQDLICRMAASVKEQSTK
PIPLPQSTPGEGDNDEEDPSKLKEEQHGISVTGLQSPDRDLGLESTLISSKPQSHSLSTS
GKSEVRDLFVAERQFAKEQHTDGTLKEVGEDYQIAIPDSHLPVSEERWALDALRNLGLLK
QLLVQQLGLTEKSVQEDWQHFPRYRTASQGPQTDSVIQNSENIKAYHSGEGHMPFRTGTG
DIATCYSPRTSTESFAPRDSVGLAPQDSQASNILVMDHMIMTPEMPTMEPEGGLDDSGEH
FFDAREAHSDENPSEGDGAVNKEEKDVNLRISGNYLILDGYDPVQESSTDEEVASSLTLQ
PMTGIPAVESTHQQQHSPQNTHSDGAISPFTPEFLVQQRWGAMEYSCFEIQSPSSCADSQ
SQIMEYIHKIEADLEHLKKVEESYTILCQRLAGSALTDKHSDKS
>sp|O94989|ARHGF_HUMAN Rho guanine nucleotide exchange factor 15 OS=Homo sapiens GN=ARHGEF15 PE=1 SV=4
MSAQSLPAATPPTQKPPRIIRPRPPSRSRAAQSPGPPHNGSSPQELPRNSNDAPTPMCTP
IFWEPPAASLKPPALLPPSASRASLDSQTSPDSPSSTPTPSPVSRRSASPEPAPRSPVPP
PKPSGSPCTPLLPMAGVLAQNGSASAPGTVRRLAGRFEGGAEGRAQDADAPEPGLQARAD
VNGEREAPLTGSGSQENGAPDAGLACPPCCPCVCHTTRPGLELRWVPVGGYEEVPRVPRR
ASPLRTSRSRPHPPSIGHPAVVLTSYRSTAERKLLPLLKPPKPTRVRQDATIFGDPPQPD
LDLLSEDGIQTGDSPDEAPQNTPPATVEGREEEGLEVLKEQNWELPLQDEPLYQTYRAAV
LSEELWGVGEDGSPSPANAGDAPTFPRPPGPRNTLWQELPAVQASGLLDTLSPQERRMQE
SLFEVVTSEASYLRSLRLLTDTFVLSQALRDTLTPRDHHTLFSNVQRVQGVSERFLATLL
SRVRSSPHISDLCDVVHAHAVGPFSVYVDYVRNQQYQEETYSRLMDTNVRFSAELRRLQS
LPKCERLPLPSFLLLPFQRITRLRMLLQNILRQTEEGSSRQENAQKALGAVSKIIERCSA

EVGRMKQTEELIRLTQRLRFHKVKALPLVSWSRRLEFQGELTELGCRRGGVLFASRPRFT
PLCLLLFSDLLLITQPKSGQRLQVLDYAHRSLVQAQQVPDPSGPPTFRLSLLSNHQGRPT
HRLLQASSLSDMQRWLGAFPTPGPLPCSPDTIYEDCDCSQELCSESSAPAKTEGRSLESR
AAPKHLHKTPEGWLKGLPGAFPAQLVCEVTGEHERRRHLRQNQRLLEAVGSSSGTPNAPP
P
>sp|O95154|ARK73_HUMAN Aflatoxin B1 aldehyde reductase member 3 OS=Homo sapiens GN=AKR7A3 PE=1 SV=2
MSRQLSRARPATVLGAMEMGRRMDAPTSAAVTRAFLERGHTEIDTAFVYSEGQSETILGG
LGLRLGGSDCRVKIDTKAIPLFGNSLKPDSLRFQLETSLKRLQCPRVDLFYLHMPDHSTP
VEETLRACHQLHQEGKFVELGLSNYAAWEVAEICTLCKSNGWILPTVYQGMYNAITRQVE
TELFPCLRHFGLRFYAFNPLAGGLLTGKYKYEDKNGKQPVGRFFGNTWAEMYRNRYWKEH
HFEGIALVEKALQAAYGASAPSMTSATLRWMYHHSQLQGAHGDAVILGMSSLEQLEQNLA
AAEEGPLEPAVVDAFNQAWHLVTHECPNYFR
>sp|Q8N8L6|ARL10_HUMAN ADP-ribosylation factor-like protein 10 OS=Homo sapiens GN=ARL10 PE=2 SV=1
MAPRPLGPLVLALGGAAAVLGSVLFILWKTYFGRGRERRWDRGEAWWGAEAARLPEWDEW
DPEDEEDEEPALEELEQREVLVLGLDGAGKSTFLRVLSGKPPLEGHIPTWGFNSVRLPTK
DFEVDLLEIGGSQNLRFYWKEFVSEVDVLVFVVDSADRLRLPWARQELHKLLDKDPDLPV
VVVANKQDLSEAMSMGELQRELGLQAIDNQREVFLLAASIAPAGPTFEEPGTVHIWKLLL
ELLS
>sp|Q6T311|ARL9_HUMAN ADP-ribosylation factor-like protein 9 OS=Homo sapiens GN=ARL9 PE=2 SV=1
MRPTWKALSHPAWPEEKNKQILVLGLDGAGKTSVLHSLASNRVQHSVAPTQGFHAVCINT
EDSQMEFLEIGGSKPFRSYWEMYLSKGLLLIFVVDSADHSRLPEAKKYLHQLIAANPVLP
LVVFANKQDLEAAYHITDIHEALALSEVGNDRKMFLFGTYLTKNGSEIPSTMQDAKDLIA
QLAADVQ
>sp|Q8N2F6|ARM10_HUMAN Armadillo repeat-containing protein 10 OS=Homo sapiens GN=ARMC10 PE=1 SV=1
MGGPRGAGWVAAGLLLGAGACYCIYRLTRGRRRGDRELGIRSSKSAGALEEGTSEGQLCG
RSARPQTGGTWESQWSKTSQPEDLTDGSYDDVLNAEQLQKLLYLLESTEDPVIIERALIT
LGNNAAFSVNQAIIRELGGIPIVANKINHSNQSIKEKALNALNNLSVNVENQIKIKIYIS
QVCEDVFSGPLNSAVQLAGLTLLTNMTVTNDHQHMLHSYITDLFQVLLTGNGNTKVQVLK
LLLNLSENPAMTEGLLRAQVDSSFLSLYDSHVAKEILLRVLTLFQNIKNCLKIEGHLAVQ
PTFTEGSLFFLLHGEECAQKIRALVDHHDAEVKEKVVTIIPKI
>sp|Q5W041|ARMC3_HUMAN Armadillo repeat-containing protein 3 OS=Homo sapiens GN=ARMC3 PE=2 SV=2
MGKKIKKEVEPPPKDVFDPLMIESKKAATVVLMLNSPEEEILAKACEAIYKFALKGEENK
TTLLELGAVEPLTKLLTHEDKIVRRNATMIFGILASNNDVKKLLRELDVMNSVIAQLAPE
EEVVIHEFASLCLANMSAEYTSKVQIFEHGGLEPLIRLLSSPDPDVKKNSMECIYNLVQD
FQCRAKLQELNAIPPILDLLKSEYPVIQLLALKTLGVIANDKESRTMLRDNQGLDHLIKI
LETKELNDLHIEALAVIANCLEDMDTMVQIQQTGGLKKLLSFAENSTIPDIQKNAAKAIT
KAAYDPENRKLFHEQEVEKCLVALLGSENDGTKIAASQAISAMCENSGSKDFFNNQGIPQ
LIQLLKSDNEEVREAAALALANLTTCNPANANAAAEADGIDPLINLLSSKRDGAIANAAT
VLTNMAMQEPLRLNIQNHDIMHAIISPLRSANTVVQSKAALAVTATACDVEARTELRNSG

GLEPLVELLRSKNDEVRKHASWAVMVCAGDELTANELCRLGALDILEEVNVSGTRKNKFS
EAAYNKLLNNNLSLKYSQTGYLSSSNIINDGFYDYGRINPGTKLLPLKELCLQEPSDLRA
VLLINSKSYVSPPSSMEDKSDVGYGRSISSSSSLRRSSKEKNKKNSYHFSAGFGSPIEDK
SEPASGRNTVLSKSATKEKGWRKSKGKKEEEKVKEEEEVMVVPKFVGEGSSDKEWCPPSD
PDFSMYVYEVTKSILPITNIKEQIEDLAKYVAEKMGGKIPKEKLPDFSWELHISELKFQL
KSNVIPIGHVKKGIFYHRALLFKALADRIGIGCSLVRGEYGRAWNEVMLQNDSRKGVIGG
LPAPEMYVIDLMFHPGGLMKLRSREADLYRFI
>sp|Q96C12|ARMC5_HUMAN Armadillo repeat-containing protein 5 OS=Homo sapiens GN=ARMC5 PE=1 SV=2
MAAAKPTLTDSLSFCLAQLAAAAGEALGGEKDPATNETPLSRALLALRTRHIKAAGGIER
FRARGGLRPLLALLRRAAAAGSAPSQAGPGSAPSSAASGASSPAPASGPAPSAVSSSSPT
PPVRLRKTLDLALSILADCCTEGACRTEVRRLGGILPLVTILQCMKTDSIQNRTARALGN
LAMEPESCGDIHCAGAVPLLVESLTACQDSQCLQSVVRALRNLADSPQHRLALAQQGAVR
PLAELLATAPDAALTLALVRALLELSRGCSRACAEQLSLGGGLGPLVSLASHPKRAVREG
TILILANLCAQGLIRPALGNAGGVEVLVDELRQRRDPNGASPTSQQPLVRAVCLLCREAI
NRARLRDAGGLDLLMGLLRDPRASAWHPRIVAALVGFLYDTGALGRLQALGLVPLLAGQL
CGEAGEEEEEGREAASWDFPEERTPERAQGGSFRSLRSWLISEGYATGPDDISPDWSPEQ
CPPEPMEPASPAPTPTSLRAPRTQRTPGRSPAAAIEEPWGREGPALLLLSRFSQAPDPSG
ALVTGPALYGLLTYVTGAPGPPSPRALRILSRLTCNPACLEAFVRSYGAALLRAWLVLGV
APDDWPAPRARPTLHSRHRELGERLLQNLTVQAESPFGVGALTHLLLSGSPEDRVACALT
LPFICRKPSLWRRLLLEQGGLRLLLAALTRPAPHPLFLFFAADSLSCLQDLVSPTVSPAV
PQAVPMDLDSPSPCLYEPLLGPAPVPAPDLHFLLDSGLQLPAQRAASATASPFFRALLSG
SFAEAQMDLVPLRGLSPGAAWPVLHHLHGCRGCGAALGPVPPPGQPLLGSEAEEALEAAG
RFLLPGLEEELEEAVGRIHLGPQGGPESVGEVFRLGRPRLAAHCARWTLGSEQCPRKRGL
ALVGLVEAAGEEAGPLTEALLAVVMGIELGARVPA
>sp|Q9H6L4|ARMC7_HUMAN Armadillo repeat-containing protein 7 OS=Homo sapiens GN=ARMC7 PE=1 SV=1
MAQKPKVDPHVGRLGYLQALVTEFQETQSQDAKEQVLANLANFAYDPSNYEYLRQLQVLD
LFLDSLSEENETLVEFAIGGLCNLCPDRANKEHILHAGGVPLIINCLSSPNEETVLSAIT
TLMHLSPPGRSFLPELTATPVVQCMLRFSLSASARLRNLAQIFLEDFCSPRQVAEARSRQ
AHSALGIPLPRSVAPRQR
>sp|Q8IUR7|ARMC8_HUMAN Armadillo repeat-containing protein 8 OS=Homo sapiens GN=ARMC8 PE=1 SV=2
MACLLETPIRMSVLSEVTASSRHYVDRLFDPDPQKVLQGVIDMKNAVIGNNKQKANLIVL
GAVPRLLYLLQQETSSTELKTECAVVLGSLAMGTENNVKSLLDCHIIPALLQGLLSPDLK
FIEACLRCLRTIFTSPVTPEELLYTDATVIPHLMALLSRSRYTQEYICQIFSHCCKGPDH
QTILFNHGAVQNIAHLLTSLSYKVRMQALKCFSVLAFENPQVSMTLVNVLVDGELLPQIF
VKMLQRDKPIEMQLTSAKCLTYMCRAGAIRTDDNCIVLKTLPCLVRMCSKERLLEERVEG
AETLAYLIEPDVELQRIASITDHLIAMLADYFKYPSSVSAITDIKRLDHDLKHAHELRQA
AFKLYASLGANDEDIRKKIIETENMMDRIVTGLSESSVKVRLAAVRCLHSLSRSVQQLRT
SFQDHAVWKPLMKVLQNAPDEILVVASSMLCNLLLEFSPSKEPILESGAVELLCGLTQSE
NPALRVNGIWALMNMAFQAEQKIKADILRSLSTEQLFRLLSDSDLNVLMKTLGLLRNLLS
TRPHIDKIMSTHGKQIMQAVTLILEGEHNIEVKEQTLCILANIADGTTAKDLIMTNDDIL
QKIKYYMGHSHVKLQLAAMFCISNLIWNEEEGSQERQDKLRDMGIVDILHKLSQSPDSNL

CDKAKMALQQYLA
>sp|P0C7Q2|ARMS2_HUMAN Age-related maculopathy susceptibility protein 2 OS=Homo sapiens GN=ARMS2 PE=2 SV=1
MLRLYPGPMVTEAEGKGGPEMASLSSSVVPVSFISTLRESVLDPGVGGEGASDKQRSKLS
LSHSMIPAAKIHTELCLPAFFSPAGTQRRFQQPQHHLTLSIIHTAAR
>sp|Q9P291|ARMX1_HUMAN Armadillo repeat-containing X-linked protein 1 OS=Homo sapiens GN=ARMCX1 PE=1 SV=1
MGRTREAGCVAAGVVIGAGACYCVYRLAWGRDENEKIWDEDEESTDTSEIGVETVKGAKT
NAGAGSGAKLQGDSEVKPEVSLGLEDCPGVKEKAHSGSHSGGGLEAKAKALFNTLKEQAS
AKAGKGARVGTISGNRTLAPSLPCPGGRGGGCHPTRSGSRAGGRASGKSKGKARSKSTRA
PATTWPVRRGKFNFPYKIDDILSAPDLQKVLNILERTNDPFIQEVALVTLGNNAAYSFNQ
NAIRELGGVPIIAKLIKTKDPIIREKTYNALNNLSVNAENQGKIKTYISQVCDDTMVCRL
DSAVQMAGLRLLTNMTVTNHYQHLLSYSFPDFFALLFLGNHFTKIQIMKLIINFTENPAM
TRELVSCKVPSELISLFNKEWDREILLNILTLFENINDNIKNEGLASSRKEFSRSSLFFL
FKESGVCVKKIKALANHNDLVVKVKVLKVLTKL
>sp|Q9UH62|ARMX3_HUMAN Armadillo repeat-containing X-linked protein 3 OS=Homo sapiens GN=ARMCX3 PE=1 SV=1
MGYARKVGWVTAGLVIGAGACYCIYRLTRGRKQNKEKMAEGGSGDVDDAGDCSGARYNDW
SDDDDDSNESKSIVWYPPWARIGTEAGTRARARARARATRARRAVQKRASPNSDDTVLSP
QELQKVLCLVEMSEKPYILEAALIALGNNAAYAFNRDIIRDLGGLPIVAKILNTRDPIVK
EKALIVLNNLSVNAENQRRLKVYMNQVCDDTITSRLNSSVQLAGLRLLTNMTVTNEYQHM
LANSISDFFRLFSAGNEETKLQVLKLLLNLAENPAMTRELLRAQVPSSLGSLFNKKENKE
VILKLLVIFENINDNFKWEENEPTQNQFGEGSLFFFLKEFQVCADKVLGIESHHDFLVKV
KVGKFMAKLAEHMFPKSQE
>sp|Q7L4S7|ARMX6_HUMAN Protein ARMCX6 OS=Homo sapiens GN=ARMCX6 PE=2 SV=1
MGRAREVGWMAAGLMIGAGACYCVYKLTIGRDDSEKLEEEGEEEWDDDQELDEEEPDIWF
DFETMARPWTEDGDWTEPGAPGGTEDRPSGGGKANRAHPIKQRPFPYEHKNTWSAQNCKN
GSCVLDLSKCLFIQGKLLFAEPKDAGFPFSQDINSHLASLSMARNTSPTPDPTVREALCA
PDNLNASIESQGQIKMYINEVCRETVSRCCNSFLQQAGLNLLISMTVINNMLAKSASDLK
FPLISEGSGCAKVQVLKPLMGLSEKPVLAGELVGAQMLFSFMSLFIRNGNREILLETPAP
>sp|Q9HBZ2|ARNT2_HUMAN Aryl hydrocarbon receptor nuclear translocator 2 OS=Homo sapiens GN=ARNT2 PE=1 SV=2
MATPAAVNPPEMASDIPGSVTLPVAPMAATGQVRMAGAMPARGGKRRSGMDFDDEDGEGP
SKFSRENHSEIERRRRNKMTQYITELSDMVPTCSALARKPDKLTILRMAVSHMKSMRGTG
NKSTDGAYKPSFLTEQELKHLILEAADGFLFVVAAETGRVIYVSDSVTPVLNQPQSEWFG
STLYEQVHPDDVEKLREQLCTSENSMTGRILDLKTGTVKKEGQQSSMRMCMGSRRSFICR
MRCGNAPLDHLPLNRITTMRKRFRNGLGPVKEGEAQYAVVHCTGYIKAWPPAGMTIPEED
ADVGQGSKYCLVAIGRLQVTSSPVCMDMNGMSVPTEFLSRHNSDGIITFVDPRCISVIGY
QPQDLLGKDILEFCHPEDQSHLRESFQQVVKLKGQVLSVMYRFRTKNREWMLIRTSSFTF
QNPYSDEIEYIICTNTNVKQLQQQQAELEVHQRDGLSSYDLSQVPVPNLPAGVHEAGKSV
EKADAIFSQERDPRFAEMFAGISASEKKMMSSASAAGTQQIYSQGSPFPSGHSGKAFSSS
VVHVPGVNDIQSSSSTGQNMSQISRQLNQSQVAWTGSRPPFPGQQIPSQSSKTQSSPFGI
GTSHTYPADPSSYSPLSSPATSSPSGNAYSSLANRTPGFAESGQSSGQFQGRPSEVWSQW
QSQHHGQQSGEQHSHQQPGQTEVFQDMLPMPGDPTQGTGNYNIEDFADLGMFPPFSE

>sp|Q8IUZ5|AT2L2_HUMAN 5-phosphohydroxy-L-lysine phospho-lyase OS=Homo sapiens GN=PHYKPL PE=1 SV=1
MAADQRPKADTLALRQRLISSSCRLFFPEDPVKIVRAQGQYMYDEQGAEYIDCISNVAHV
GHCHPLVVQAAHEQNQVLNTNSRYLHDNIVDYAQRLSETLPEQLCVFYFLNSGSEANDLA
LRLARHYTGHQDVVVLDHAYHGHLSSLIDISPYKFRNLDGQKEWVHVAPLPDTYRGPYRE
DHPNPAMAYANEVKRVVSSAQEKGRKIAAFFAESLPSVGGQIIPPAGYFSQVAEHIRKAG
GVFVADEIQVGFGRVGKHFWAFQLQGKDFVPDIVTMGKSIGNGHPVACVAATQPVARAFE
ATGVEYFNTFGGSPVSCAVGLAVLNVLEKEQLQDHATSVGSFLMQLLGQQKIKHPIVGDV
RGVGLFIGVDLIKDEATRTPATEEAAYLVSRLKENYVLLSTDGPGRNILKFKPPMCFSLD
NARQVVAKLDAILTDMEEKVRSCETLRLQP
>sp|Q5VTU8|AT5EL_HUMAN ATP synthase subunit epsilon-like protein, mitochondrial OS=Homo sapiens GN=ATP5EP2 PE=3 SV=1
MVAYWRQAGLSYIRYSQICAKVVRDALKTEFKANAKKTSGNSVKIVKVKKE
>sp|O43520|AT8B1_HUMAN Phospholipid-transporting ATPase IC OS=Homo sapiens GN=ATP8B1 PE=1 SV=3
MSTERDSETTFDEDSQPNDEVVPYSDDETEDELDDQGSAVEPEQNRVNREAEENREPFRK
ECTWQVKANDRKYHEQPHFMNTKFLCIKESKYANNAIKTYKYNAFTFIPMNLFEQFKRAA
NLYFLALLILQAVPQISTLAWYTTLVPLLVVLGVTAIKDLVDDVARHKMDKEINNRTCEV
IKDGRFKVAKWKEIQVGDVIRLKKNDFVPADILLLSSSEPNSLCYVETAELDGETNLKFK
MSLEITDQYLQREDTLATFDGFIECEEPNNRLDKFTGTLFWRNTSFPLDADKILLRGCVI
RNTDFCHGLVIFAGADTKIMKNSGKTRFKRTKIDYLMNYMVYTIFVVLILLSAGLAIGHA
YWEAQVGNSSWYLYDGEDDTPSYRGFLIFWGYIIVLNTMVPISLYVSVEVIRLGQSHFIN
WDLQMYYAEKDTPAKARTTTLNEQLGQIHYIFSDKTGTLTQNIMTFKKCCINGQIYGDHR
DASQHNHNKIEQVDFSWNTYADGKLAFYDHYLIEQIQSGKEPEVRQFFFLLAVCHTVMVD
RTDGQLNYQAASPDEGALVNAARNFGFAFLARTQNTITISELGTERTYNVLAILDFNSDR
KRMSIIVRTPEGNIKLYCKGADTVIYERLHRMNPTKQETQDALDIFANETLRTLCLCYKE
IEEKEFTEWNKKFMAASVASTNRDEALDKVYEEIEKDLILLGATAIEDKLQDGVPETISK
LAKADIKIWVLTGDKKETAENIGFACELLTEDTTICYGEDINSLLHARMENQRNRGGVYA
KFAPPVQESFFPPGGNRALIITGSWLNEILLEKKTKRNKILKLKFPRTEEERRMRTQSKR
RLEAKKEQRQKNFVDLACECSAVICCRVTPKQKAMVVDLVKRYKKAITLAIGDGANDVNM
IKTAHIGVGISGQEGMQAVMSSDYSFAQFRYLQRLLLVHGRWSYIRMCKFLRYFFYKNFA
FTLVHFWYSFFNGYSAQTAYEDWFITLYNVLYTSLPVLLMGLLDQDVSDKLSLRFPGLYI
VGQRDLLFNYKRFFVSLLHGVLTSMILFFIPLGAYLQTVGQDGEAPSDYQSFAVTIASAL
VITVNFQIGLDTSYWTFVNAFSIFGSIALYFGIMFDFHSAGIHVLFPSAFQFTGTASNAL
RQPYIWLTIILAVAVCLLPVVAIRFLSMTIWPSESDKIQKHRKRLKAEEQWQRRQQVFRR
GVSTRRSAYAFSHQRGYADLISSGRSIRKKRSPLDAIVADGTAEYRRTGDS
>sp|P17544|ATF7_HUMAN Cyclic AMP-dependent transcription factor ATF-7 OS=Homo sapiens GN=ATF7 PE=1 SV=2
MGDDRPFVCNAPGCGQRFTNEDHLAVHKHKHEMTLKFGPARTDSVIIADQTPTPTRFLKN
CEEVGLFNELASSFEHEFKKAADEDEKKARSRTVAKKLVAAAGPLDMSLPSTPDIKIKEE
EPVEVDSSPPDSPASSPCSPPLKEKEVTPKPVLISTPTPTIVRPGSLPLHLGYDPLHPTL
PSPTSVITQAPPSNRQMGSPTGSLPLVMHLANGQTMPVLPGPPVQMPSVISLARPVSMVP
NIPGIPGPPVNSSGSISPSGHPIPSEAKMRLKATLTHQVSSINGGCGMVVGTASTMVTAR
PEQSQILIQHPDAPSPAQPQVSPAQPTPSTGGRRRRTVDEDPDERRQRFLERNRAAASRC

RQKRKLWVSSLEKKAEELTSQNIQLSNEVTLLRNEVAQLKQLLLAHKDCPVTALQKKTQG
YLESPKESSEPTGSPAPVIQHSSATAPSNGLSVRSAAEAVATSVLTQMASQRTELSMPIQ
SHVIMTPQSQSAGR
>sp|Q2TAZ0|ATG2A_HUMAN Autophagy-related protein 2 homolog A OS=Homo sapiens GN=ATG2A PE=1 SV=3
MSRWLWPWSNCVKERVCRYLLHHYLGHFFQEHLSLDQLSLDLYKGSVALRDIHLEIWSVN
EVLESMESPLELVEGFVGSIEVAVPWAALLTDHCTVRVSGLQLTLQPRRGPAPGAADSQS
WASCMTTSLQLAQECLRDGLPEPSEPPQPLEGLEMFAQTIETVLRRIKVTFLDTVVRVEH
SPGDGERGVAVEVRVQRLEYCDEAVRDPSQAPPVDVHQPPAFLHKLLQLAGVRLHYEELP
AQEEPPEPPLQIGSCSGYMELMVKLKQNEAFPGPKLEVAGQLGSLHLLLTPRQLQQLQEL
LSAVSLTDHEGLADKLNKSRPLGAEDLWLIEQDLNQQLQAGAVAEPLSPDPLTNPLLNLD
NTDLFFSMAGLTSSVASALSELSLSDVDLASSVRSDMASRRLSAQAHPAGKMAPNPLLDT
MRPDSLLKMTLGGVTLTLLQTSAPSSGPPDLATHFFTEFDATKDGPFGSRDFHHLRPRFQ
RACPCSHVRLTGTAVQLSWELRTGSRGRRTTSMEVHFGQLEVLECLWPRGTSEPEYTEIL
TFPGTLGSQASARPCAHLRHTQILRRVPKSRPRRSVACHCHSELALDLANFQADVELGAL
DRLAALLRLATVPAEPPAGLLTEPLPAMEQQTVFRLSAPRATLRLRFPIADLRPEPDPWA
GQAVRAEQLRLELSEPQFRSELSSGPGPPVPTHLELTCSDLHGIYEDGGKPPVPCLRVSK
ALDPKSTGRKYFLPQVVVTVNPQSSSTQWEVAPEKGEELELSVESPCELREPEPSPFSSK
RTMYETEEMVIPGDPEEMRTFQSRTLALSRCSLEVILPSVHIFLPSKEVYESIYNRINND
LLMWEPADLLPTPDPAAQPSGFPGPSGFWHDSFKMCKSAFKLANCFDLTPDSDSDDEDAH
FFSVGASGGPQAAAPEAPSLHLQSTFSTLVTVLKGRITALCETKDEGGKRLEAVHGELVL
DMEHGTLFSVSQYCGQPGLGYFCLEAEKATLYHRAAVDDYPLPSHLDLPSFAPPAQLAPT
IYPSEEGVTERGASGRKGQGRGPHMLSTAVRIHLDPHKNVKEFLVTLRLHKATLRHYMAL
PEQSWHSQLLEFLDVLDDPVLGYLPPTVITILHTHLFSCSVDYRPLYLPVRVLITAETFT
LSSNIIMDTSTFLLRFILDDSALYLSDKCEVETLDLRRDYVCVLDVDLLELVIKTWKGST
EGKLSQPLFELRCSNNVVHVHSCADSCALLVNLLQYVMSTGDLHPPPRPPSPTEIAGQKL
SESPASLPSCPPVETALINQRDLADALLDTERSLRELAQPSGGHLPQASPISVYLFPGER
SGAPPPSPPVGGPAGSLGSCSEEKEDEREEEGDGDTLDSDEFCILDAPGLGIPPRDGEPV
VTQLHPGPIVVRDGYFSRPIGSTDLLRAPAHFPVPSTRVVLREVSLVWHLYGGRDFGPHP
GHRARTGLSGPRSSPSRCSGPNRPQNSWRTQGGSGRQHHVLMEIQLSKVSFQHEVYPAEP
ATGPAAPSQELEERPLSRQVFIVQELEVRDRLASSQINKFLYLHTSERMPRRAHSNMLTI
KALHVAPTTNLGGPECCLRVSLMPLRLNVDQDALFFLKDFFTSLVAGINPVVPGETSAEA
RPETRAQPSSPLEGQAEGVETTGSQEAPGGGHSPSPPDQQPIYFREFRFTSEVPIWLDYH
GKHVTMDQVGTFAGLLIGLAQLNCSELKLKRLCCRHGLLGVDKVLGYALNEWLQDIRKNQ
LPGLLGGVGPMHSVVQLFQGFRDLLWLPIEQYRKDGRLMRGLQRGAASFGSSTASAALEL
SNRLVQAIQATAETVYDILSPAAPVSRSLQDKRSARRLRRGQQPADLREGVAKAYDTVRE
GILDTAQTICDVASRGHEQKGLTGAVGGVIRQLPPTVVKPLILATEATSSLLGGMRNQIV
PDAHKDHALKWRSDSAQD
>sp|Q9Y4P1|ATG4B_HUMAN Cysteine protease ATG4B OS=Homo sapiens GN=ATG4B PE=1 SV=2
MDAATLTYDTLRFAEFEDFPETSEPVWILGRKYSIFTEKDEILSDVASRLWFTYRKNFPA
IGGTGPTSDTGWGCMLRCGQMIFAQALVCRHLGRDWRWTQRKRQPDSYFSVLNAFIDRKD
SYYSIHQIAQMGVGEGKSIGQWYGPNTVAQVLKKLAVFDTWSSLAVHIAMDNTVVMEEIR
RLCRTSVPCAGATAFPADSDRHCNGFPAGAEVTNRPSPWRPLVLLIPLRLGLTDINEAYV
ETLKHCFMMPQSLGVIGGKPNSAHYFIGYVGEELIYLDPHTTQPAVEPTDGCFIPDESFH

CQHPPCRMSIAELDPSIAVGFFCKTEDDFNDWCQQVKKLSLLGGALPMFELVELQPSHLA
CPDVLNLSLDSSDVERLERFFDSEDEDFEILSL
>sp|Q8NHH9|ATLA2_HUMAN Atlastin-2 OS=Homo sapiens GN=ATL2 PE=1 SV=2
MAEGDEAARGQQPHQGLWRRRRTSDPSAAVNHVSSTTSLGENYEDDDLVNSDEVMKKPCP
VQIVLAHEDDHNFELDEEALEQILLQEHIRDLNIVVVSVAGAFRKGKSFLLDFMLRYMYN
KDSQSWIGGNNEPLTGFTWRGGCERETTGIQVWNEVFVIDRPNGTKVAVLLMDTQGAFDS
QSTIKDCATVFALSTMTSSVQVYNLSQNIQEDDLQHLQLFTEYGRLAMEEIYQKPFQTLM
FLIRDWSYPYEHSYGLEGGKQFLEKRLQVKQNQHEELQNVRKHIHNCFSNLGCFLLPHPG
LKVATNPSFDGRLKDIDEDFKRELRNLVPLLLAPENLVEKEISGSKVTCRDLVEYFKAYI
KIYQGEELPHPKSMLQATAEANNLAAVAGARDTYCKSMEQVCGGDKPYIAPSDLERKHLD
LKEVAIKQFRSVKKMGGDEFCRRYQDQLEAEIEETYANFIKHNDGKNIFYAARTPATLFA
VMFAMYIISGLTGFIGLNSIAVLCNLVMGLALIFLCTWAYVKYSGEFREIGTVIDQIAET
LWEQVLKPLGDNLMEENIRQSVTNSIKAGLTDQVSHHARLKTD
>sp|Q9H9F9|ARP5_HUMAN Actin-related protein 5 OS=Homo sapiens GN=ACTR5 PE=1 SV=2
MAANVFPFRDARAAPDPVLEAGPVAHGPLPVPLVLDNGSFQVRAGWACPGQDPGPEPRLQ
FRAVCARGRGGARGASGPQVGNALGSLEPLRWMLRSPFDRNVPVNLELQELLLDYSFQHL
GVSSQGCVDHPIVLTEAVCNPLYSRQMMSELLFECYGIPKVAYGIDSLFSFYHNKPKNSM
CSGLIISSGYQCTHVLPILEGRLDAKNCKRINLGGSQAAGYLQRLLQLKYPGHLAAITLS
RMEEILHEHSYIAEDYVEELHKWRCPDYYENNVHKMQLPFSSKLLGSTLTSEEKQERRQQ
QLRRLQELNARRREEKLQLDQERLDRLLYVQELLEDGQMDQFHKALIELNMDSPEELQSY
IQKLSIAVEQAKQKILQAEVNLEVDVVDSKPETPDLEQLEPSLEDVESMNDFDPLFSEET
PGVEKPVTTVQPVFNLAAYHQLFVGTERIRAPEIIFQPSLIGEEQAGIAETLQYILDRYP
KDIQEMLVQNVFLTGGNTMYPGMKARMEKELLEMRPFRSSFQVQLASNPVLDAWYGARDW
ALNHLDDNEVWITRKEYEEKGGEYLKEHCASNIYVPIRLPKQASRSSDAQASSKGSAAGG
GGAGEQA
>sp|P49407|ARRB1_HUMAN Beta-arrestin-1 OS=Homo sapiens GN=ARRB1 PE=1 SV=2
MGDKGTRVFKKASPNGKLTVYLGKRDFVDHIDLVDPVDGVVLVDPEYLKERRVYVTLTCA
FRYGREDLDVLGLTFRKDLFVANVQSFPPAPEDKKPLTRLQERLIKKLGEHAYPFTFEIP
PNLPCSVTLQPGPEDTGKACGVDYEVKAFCAENLEEKIHKRNSVRLVIRKVQYAPERPGP
QPTAETTRQFLMSDKPLHLEASLDKEIYYHGEPISVNVHVTNNTNKTVKKIKISVRQYAD
ICLFNTAQYKCPVAMEEADDTVAPSSTFCKVYTLTPFLANNREKRGLALDGKLKHEDTNL
ASSTLLREGANREILGIIVSYKVKVKLVVSRGGLLGDLASSDVAVELPFTLMHPKPKEEP
PHREVPENETPVDTNLIELDTNDDDIVFEDFARQRLKGMKDDKEEEEDGTGSPQLNNR
>sp|P32121|ARRB2_HUMAN Beta-arrestin-2 OS=Homo sapiens GN=ARRB2 PE=1 SV=2
MGEKPGTRVFKKSSPNCKLTVYLGKRDFVDHLDKVDPVDGVVLVDPDYLKDRKVFVTLTC
AFRYGREDLDVLGLSFRKDLFIATYQAFPPVPNPPRPPTRLQDRLLRKLGQHAHPFFFTI
PQNLPCSVTLQPGPEDTGKACGVDFEIRAFCAKSLEEKSHKRNSVRLVIRKVQFAPEKPG
PQPSAETTRHFLMSDRSLHLEASLDKELYYHGEPLNVNVHVTNNSTKTVKKIKVSVRQYA
DICLFSTAQYKCPVAQLEQDDQVSPSSTFCKVYTITPLLSDNREKRGLALDGKLKHEDTN
LASSTIVKEGANKEVLGILVSYRVKVKLVVSRGGDVSVELPFVLMHPKPHDHIPLPRPQS
AAPETDVPVDTNLIEFDTNYATDDDIVFEDFARLRLKGMKDDDYDDQLC
>sp|Q8NCT1|ARRD4_HUMAN Arrestin domain-containing protein 4 OS=Homo sapiens GN=ARRDC4 PE=1 SV=3
MGGEAGCAAAVGAEGRVKSLGLVFEDERKGCYSSGETVAGHVLLEASEPVALRALRLEAQ

GRATAAWGPSTCPRASASTAALAVFSEVEYLNVRLSLREPPAGEGIILLQPGKHEFPFRF
QLPSEPLVTSFTGKYGSIQYCVRAVLERPKVPDQSVKRELQVVSHVDVNTPALLTPVLKT
QEKMVGCWFFTSGPVSLSAKIERKGYCNGEAIPIYAEIENCSSRLIVPKAAIFQTQTYLA
SGKTKTIRHMVANVRGNHIASGSTDTWNGKTLKIPPVTPSILDCCIIRVDYSLAVYIHIP
GAKKLMLELPLVIGTIPYNGFGSRNSSIASQFSMDMSWLTLTLPEQPEAPPNYADVVSEE
EFSRHIPPYPQPPNCEGEVCCPVFACIQEFRFQPPPLYSEVDPHPSDVEESQPVSFIL
>sp|A6NEK1|ARRD5_HUMAN Arrestin domain-containing protein 5 OS=Homo sapiens GN=ARRDC5 PE=3 SV=2
MGDREECLSTPQPPMSVVKSIELVLPEDRIYLAGSSIKGQVILTLNSTLVDPIVKVELVG
RGYVEWSEEAGASCDYSRNVICNNKADYVHKTKTFPVEDNWLSAGSHTFDFHFNLPPRLP
STFTSKFGHVFYFVQASCMGREHILAKKRMYLLVQGTSTFHKETPFQNPLFVEAEEKVSY
NCCRQGTVCLQIQMERNTFTPGEKVVFTTEINNQTSKCIKTVVFALYAHIQYEGFTPSAE
RRSRLDSSELLRQEANTPVTRFNTTKVVSTFNLPLLLSVSSSTQDGEIMHTRYELVTTVH
LPWSLTSLKAKVPIIITSASVDSAICQLSEDGVLPVNPDHQN
>sp|P15848|ARSB_HUMAN Arylsulfatase B OS=Homo sapiens GN=ARSB PE=1 SV=1
MGPRGAASLPRGPGPRRLLLPVVLPLLLLLLLAPPGSGAGASRPPHLVFLLADDLGWNDV
GFHGSRIRTPHLDALAAGGVLLDNYYTQPLCTPSRSQLLTGRYQIRTGLQHQIIWPCQPS
CVPLDEKLLPQLLKEAGYTTHMVGKWHLGMYRKECLPTRRGFDTYFGYLLGSEDYYSHER
CTLIDALNVTRCALDFRDGEEVATGYKNMYSTNIFTKRAIALITNHPPEKPLFLYLALQS
VHEPLQVPEEYLKPYDFIQDKNRHHYAGMVSLMDEAVGNVTAALKSSGLWNNTVFIFSTD
NGGQTLAGGNNWPLRGRKWSLWEGGVRGVGFVASPLLKQKGVKNRELIHISDWLPTLVKL
ARGHTNGTKPLDGFDVWKTISEGSPSPRIELLHNIDPNFVDSSPCPRNSMAPAKDDSSLP
EYSAFNTSVHAAIRHGNWKLLTGYPGCGYWFPPPSQYNVSEIPSSDPPTKTLWLFDIDRD
PEERHDLSREYPHIVTKLLSRLQFYHKHSVPVYFPAQDPRCDPKATGVWGPWM
>sp|Q8WXK1|ASB15_HUMAN Ankyrin repeat and SOCS box protein 15 OS=Homo sapiens GN=ASB15 PE=2 SV=3
MDTNDDPDEDHLTSYDIQLSIQESIEASKTALCPERFVPLSAQNRKLVEAIKQGHIPELQ
EYVKYKYAMDEADEKGWFPLHEAVVQPIQQILEIVLDASYKTLWEFKTCDGETPLTLAVK
AGLVENVRTLLEKGVWPNTKNDKGETPLLIAVKKGSYDMVSTLIKHNTSLDQPCVKRWSA
MHEAAKQGRKDIVALLLKHGGNVHLRDGFGVTPLGVAAEYGHCDVLEHLIHKGGDVLALA
DDGASVLFEAAGGGNPDCISLLLEYGGSGNVPNRAGHLPIHRAAYEGHYLALKYLIPVTS
KNAIRKSGLTPIHSAADGQNAQCLELLIENGFDVNTLLADHISQSYDDERKTALYFGVSN
NDVHCTEVLLAAGADPNLDPLNCLLVAVRANNYEIVRLLLSHGANVNCYFMHVNDTRFPS
VIQYALNDEVMLRLLLNNGYQVEMCFDCMHGDIFGNSFVWSEIQEEVLPGWTSCVIKDNP
FCEFITVPWMKHLVGRVTRVLIDYMDYVPLCAKLKSALEVQREWPEIRQILENPCSLKHL
CRLKIRRLMGLQKLCQPASVEKLPLPPAIQRYILFKEYDLYGQELKLT
>sp|Q9H672|ASB7_HUMAN Ankyrin repeat and SOCS box protein 7 OS=Homo sapiens GN=ASB7 PE=1 SV=2
MLHHHCRRNPELQEELQIQAAVAAGDVHTVRKMLEQGYSPNGRDANGWTLLHFSAARGKE
RCVRVFLEHGADPTVKDLIGGFTALHYAAMHGRARIARLMLESEYRSDIINAKSNDGWTP
LHVAAHYGRDSFVRLLLEFKAEVDPLSDKGTTPLQLAIIRERSSCVKILLDHNANIDIQN
GFLLRYAVIKSNHSYCRMFLQRGADTNLGRLEDGQTPLHLSALRDDVLCARMLYNYGADT
NTRNYEGQTPLAVSISISGSSRPCLDFLQEVTRQPRNLQDLCRIKIRQCIGLQNLKLLDE
LPIAKVMKDYLKHKFDDI

>sp|Q96DX5|ASB9_HUMAN Ankyrin repeat and SOCS box protein 9 OS=Homo sapiens GN=ASB9 PE=1 SV=1
MDGKQGGMDGSKPAGPRDFPGIRLLSNPLMGDAVSDWSPMHEAAIHGHQLSLRNLISQGW
AVNIITADHVSPLHEACLGGHLSCVKILLKHGAQVNGVTADWHTPLFNACVSGSWDCVNL
LLQHGASVQPESDLASPIHEAARRGHVECVNSLIAYGGNIDHKISHLGTPLYLACENQQR
ACVKKLLESGADVNQGKGQDSPLHAVARTASEELACLLMDFGADTQAKNAEGKRPVELVP
PESPLAQLFLEREGPPSLMQLCRLRIRKCFGIQQHHKITKLVLPEDLKQFLLHL
>sp|Q99929|ASCL2_HUMAN Achaete-scute homolog 2 OS=Homo sapiens GN=ASCL2 PE=1 SV=2
MDGGTLPRSAPPAPPVPVGCAARRRPASPELLRCSRRRRPATAETGGGAAAVARRNERER
NRVKLVNLGFQALRQHVPHGGASKKLSKVETLRSAVEYIRALQRLLAEHDAVRNALAGGL
RPQAVRPSAPRGPPGTTPVAASPSRASSSPGRGGSSEPGSPRSAYSSDDSGCEGALSPAE
RELLDFSSWLGGY
>sp|P07306|ASGR1_HUMAN Asialoglycoprotein receptor 1 OS=Homo sapiens GN=ASGR1 PE=1 SV=2
MTKEYQDLQHLDNEESDHHQLRKGPPPPQPLLQRLCSGPRLLLLSLGLSLLLLVVVCVIG
SQNSQLQEELRGLRETFSNFTASTEAQVKGLSTQGGNVGRKMKSLESQLEKQQKDLSEDH
SSLLLHVKQFVSDLRSLSCQMAALQGNGSERTCCPVNWVEHERSCYWFSRSGKAWADADN
YCRLEDAHLVVVTSWEEQKFVQHHIGPVNTWMGLHDQNGPWKWVDGTDYETGFKNWRPEQ
PDDWYGHGLGGGEDCAHFTDDGRWNDDVCQRPYRWVCETELDKASQEPPLL
>sp|Q9NY37|ASIC5_HUMAN Acid-sensing ion channel 5 OS=Homo sapiens GN=ASIC5 PE=1 SV=1
MEQTEKSKVYAENGLLEKIKLCLSKKPLPSPTERKKFDHDFAISTSFHGIHNIVQNRSKI
RRVLWLVVVLGSVSLVTWQIYIRLLNYFTWPTTTSIEVQYVEKMEFPAVTFCNLNRFQTD
AVAKFGVIFFLWHIVSKVLHLQEITANSTGSREATDFAASHQNFSIVEFIRNKGFYLNNS
TLLDCEFFGKPCSPKDFAHVFTEYGNCFTFNHGETLQAKRKVSVSGRGLSLLFNVNQEAF
TDNPALGFVDAGIIFVIHSPKKVPQFDGLGLLSPVGMHARVTIRQVKTVHQEYPWGECNP
NIKLQNFSSYSTSGCLKECKAQHIKKQCGCVPFLLPGYGIECDLQKYFSCVSPVLDHIEF
KDLCTVGTHNSSCPVSCEEIEYPATISYSSFPSQKALKYLSKKLNQSRKYIRENLVKIEI
NYSDLNYKITQQQKAVSVSELLADLGGQLGLFCGASLITIIEIIEYLFTNFYWICIFFLL
KISEMTQWTPPPQNHLGNKNRIEEC
>sp|Q92484|ASM3A_HUMAN Acid sphingomyelinase-like phosphodiesterase 3a OS=Homo sapiens GN=SMPDL3A PE=1 SV=2
MALVRALVCCLLTAWHCRSGLGLPVAPAGGRNPPPAIGQFWHVTDLHLDPTYHITDDHTK
VCASSKGANASNPGPFGDVLCDSPYQLILSAFDFIKNSGQEASFMIWTGDSPPHVPVPEL
STDTVINVITNMTTTIQSLFPNLQVFPALGNHDYWPQDQLPVVTSKVYNAVANLWKPWLD
EEAISTLRKGGFYSQKVTTNPNLRIISLNTNLYYGPNIMTLNKTDPANQFEWLESTLNNS
QQNKEKVYIIAHVPVGYLPSSQNITAMREYYNEKLIDIFQKYSDVIAGQFYGHTHRDSIM
VLSDKKGSPVNSLFVAPAVTPVKSVLEKQTNNPGIRLFQYDPRDYKLLDMLQYYLNLTEA
NLKGESIWKLEYILTQTYDIEDLQPESLYGLAKQFTILDSKQFIKYYNYFFVSYDSSVTC
DKTCKAFQICAIMNLDNISYADCLKQLYIKHNY
>sp|Q12797|ASPH_HUMAN Aspartyl/asparaginyl beta-hydroxylase OS=Homo sapiens GN=ASPH PE=1 SV=3
MAQRKNAKSSGNSSSSGSGSGSTSAGSSSPGARRETKHGGHKNGRKGGLSGTSFFTWFMV
IALLGVWTSVAVVWFDLVDYEEVLGKLGIYDADGDGDFDVDDAKVLLGLKERSTSEPAVP
PEEAEPHTEPEEQVPVEAEPQNIEDEAKEQIQSLLHEMVHAEHVEGEDLQQEDGPTGEPQ
QEDDEFLMATDVDDRFETLEPEVSHEETEHSYHVEETVSQDCNQDMEEMMSEQENPDSSE

PVVEDERLHHDTDDVTYQVYEEQAVYEPLENEGIEITEVTAPPEDNPVEDSQVIVEEVSI
FPVEEQQEVPPETNRKTDDPEQKAKVKKKKPKLLNKFDKTIKAELDAAEKLRKRGKIEEA
VNAFKELVRKYPQSPRARYGKAQCEDDLAEKRRSNEVLRGAIETYQEVASLPDVPADLLK
LSLKRRSDRQQFLGHMRGSLLTLQRLVQLFPNDTSLKNDLGVGYLLIGDNDNAKKVYEEV
LSVTPNDGFAKVHYGFILKAQNKIAESIPYLKEGIESGDPGTDDGRFYFHLGDAMQRVGN
KEAYKWYELGHKRGHFASVWQRSLYNVNGLKAQPWWTPKETGYTELVKSLERNWKLIRDE
GLAVMDKAKGLFLPEDENLREKGDWSQFTLWQQGRRNENACKGAPKTCTLLEKFPETTGC
RRGQIKYSIMHPGTHVWPHTGPTNCRLRMHLGLVIPKEGCKIRCANETKTWEEGKVLIFD
DSFEHEVWQDASSFRLIFIVDVWHPELTPQQRRSLPAI
>sp|Q76L83|ASXL2_HUMAN Putative Polycomb group protein ASXL2 OS=Homo sapiens GN=ASXL2 PE=1 SV=1
MREKGRRKKGRTWAEAAKTVLEKYPNTPMSHKEILQVIQREGLKEIRSGTSPLACLNAML
HTNSRGEEGIFYKVPGRMGVYTLKKDVPDGVKELSEGSEESSDGQSDSQSSENSSSSSDG
GSNKEGKKSRWKRKVSSSSPQSGCPSPTIPAGKVISPSQKHSKKALKQALKQQQQKKQQQ
QCRPSISISSNQHLSLKTVKAASDSVPAKPATWEGKQSDGQTGSPQNSNSSFSSSVKVEN
TLLGLGKKSFQRSERLHTRQMKRTKCADIDVETPDSILVNTNLRALINKHTFSVLPGDCQ
QRLLLLLPEVDRQVGPDGLMKLNGSALNNEFFTSAAQGWKERLSEGEFTPEMQVRIRQEI
EKEKKVEPWKEQFFESYYGQSSGLSLEDSKKLTASPSDPKVKKTPAEQPKSMPVSEASLI
RIVPVVSQSECKEEALQMSSPGRKEECESQGEVQPNFSTSSEPLLSSALNTHELSSILPI
KCPKDEDLLEQKPVTSAEQESEKNHLTTASNYNKSESQESLVTSPSKPKSPGVEKPIVKP
TAGAGPQETNMKEPLATLVDQSPESLKRKSSLTQEEAPVSWEKRPRVTENRQHQQPFQVS
PQPFLNRGDRIQVRKVPPLKIPVSRISPMPFHPSQVSPRARFPVSITSPNRTGARTLADI
KAKAQLVKAQRAAAAAAAAAAAAASVGGTIPGPGPGGGQGPGEGGEGQTARGGSPGSDRV
SETGKGPTLELAGTGSRGGTRELLPCGPETQPQSETKTTPSQAQPHSVSGAQLQQTPPVP
PTPAVSGACTSVPSPAHIEKLDNEKLNPTRATATVASVSHPQGPSSCRQEKAPSPTGPAL
ISGASPVHCAADGTVELKAGPSKNIPNPSASSKTDASVPVAVTPSPLTSLLTTATLEKLP
VPQVSATTAPAGSAPPSSTLPAASSLKTPGTSLNMNGPTLRPTSSIPANNPLVTQLLQGK
DVPMEQILPKPLTKVEMKTVPLTAKEERGMGALIATNTTENSTREEVNERQSHPATQQQL
GKTLQSKQLPQVPRPLQLFSAKELRDSSIDTHQYHEGLSKATQDQILQTLIQRVRRQNLL
SVVPPSQFNFAHSGFQLEDISTSQRFMLGFAGRRTSKPAMAGHYLLNISTYGRGSESFRR
THSVNPEDRFCLSSPTEALKMGYTDCKNATGESSSSKEDDTDEESTGDEQESVTVKEEPQ
VSQSAGKGDTSSGPHSRETLSTSDCLASKNVKAEIPLNEQTTLSKENYLFTRGQTFDEKT
LARDLIQAAQKQMAHAVRGKAIRSSPELFSSTVLPLPADSPTHQPLLLPPLQTPKLYGSP
TQIGPSYRGMINVSTSSDMDHNSAVPGSQVSSNVGDVMSFSVTVTTIPASQAMNPSSHGQ
TIPVQAFSEENSIEGTPSKCYCRLKAMIMCKGCGAFCHDDCIGPSKLCVSCLVVR
>sp|O94823|AT10B_HUMAN Probable phospholipid-transporting ATPase VB OS=Homo sapiens GN=ATP10B PE=2 SV=2
MALSVDSSWHRWQWRVRDGFPHCPSETTPLLSPEKGRQSYNLTQQRVVFPNNSIFHQDWE
EVSRRYPGNRTCTTKYTLFTFLPRNLFEQFHRWANLYFLFLVILNWMPSMEVFHREITML
PLAIVLFVIMIKDGMEDFKRHRFDKAINCSNIRIYERKEQTYVQKCWKDVRVGDFIQMKC
NEIVPADILLLFSSDPNGICHLETASLDGETNLKQRCVVKGFSQQEVQFEPELFHNTIVC
EKPNNHLNKFKGYMEHPDQTRTGFGCESLLLRGCTIRNTEMAVGIVIYAGHETKAMLNNS
GPRYKRSKIERRMNIDIFFCIGILILMCLIGAVGHSIWNGTFEEHPPFDVPDANGSFLPS
ALGGFYMFLTMIILLQVLIPISLYVSIELVKLGQVFFLSNDLDLYDEETDLSIQCRALNI

AEDLGQIQYIFSDKTGTLTENKMVFRRCTIMGSEYSHQENAKRLETPKELDSDGEEWTQY
QCLSFSARWAQDPATMRSQKGAQPLRRSQSARVPIQGHYRQRSMGHRESSQPPVAFSSSI
EKDVTPDKNLLTKVRDAALWLETLSDSRPAKASLSTTSSIADFFLALTICNSVMVSTTTE
PRQRVTIKPSSKALGTSLEKIQQLFQKLKLLSLSQSFSSTAPSDTDLGESLGANVATTDS
DERDDASVCSGGDSTDDGGYRSSMWDQGDILESGSGTSLEEALEAPATDLARPEFCYEAE
SPDEAALVHAAHAYSFTLVSRTPEQVTVRLPQGTCLTFSLLCTLGFDSVRKRMSVVVRHP
LTGEIVVYTKGADSVIMDLLEDPACVPDINMEKKLRKIRARTQKHLDLYARDGLRTLCIA
KKVVSEEDFRRWASFRREAEASLDNRDELLMETAQHLENQLTLLGATGIEDRLQEGVPDT
IATLREAGIQLWVLTGDKQETAVNIAHSCRLLNQTDTVYTINTENQETCESILNCALEEL
KQFRELQKPDRKLFGFRLPSKTPSITSEAVVPEAGLVIDGKTLNAIFQGKLEKKFLELTQ
YCRSVLCCRSTPLQKSMIVKLVRDKLRVMTLSIGDGANDVSMIQAADIGIGISGQEGMQA
VMSSDFAITRFKHLKKLLLVHGHWCYSRLARMVVYYLYKNVCYVNLLFWYQFFCGFSSST
MIDYWQMIFFNLFFTSLPPLVFGVLDKDISAETLLALPELYKSGQNSECYNLSTFWISMV
DAFYQSLICFFIPYLAYKGSDIDVFTFGTPINTISLTTILLHQAMEMKTWTIFHGVVLLG
SFLMYFLVSLLYNATCVICNSPTNPYWVMEGQLSNPTFYLVCFLTPVVALLPRYFFLSLQ
GTCGKSLISKAQKIDKLPPDKRNLEIQSWRSRQRPAPVPEVARPTHHPVSSITGQDFSAS
TPKSSNPPKRKHVEESVLHEQRCGTECMRDDSCSGDSSAQLSSGEHLLGPNRIMAYSRGQ
TDMCRCSKRSSHRRSQSSLTI
>sp|Q9HD20|AT131_HUMAN Manganese-transporting ATPase 13A1 OS=Homo sapiens GN=ATP13A1 PE=1 SV=2
MAAAAAVGNAVPCGARPCGVRPDGQPKPGPQPRALLAAGPALIANGDELVAAVWPYRRLA
LLRRLTVLPFAGLLYPAWLGAAAAGCWGWGSSWVQIPEAALLVLATICLAHALTVLSGHW
SVHAHCALTCTPEYDPSKATFVKVVPTPNNGSTELVALHRNEGEDGLEVLSFEFQKIKYS
YDALEKKQFLPVAFPVGNAFSYYQSNRGFQEDSEIRAAEKKFGSNKAEMVVPDFSELFKE
RATAPFFVFQVFCVGLWCLDEYWYYSVFTLSMLVAFEASLVQQQMRNMSEIRKMGNKPHM
IQVYRSRKWRPIASDEIVPGDIVSIGRSPQENLVPCDVLLLRGRCIVDEAMLTGESVPQM
KEPIEDLSPDRVLDLQADSRLHVIFGGTKVVQHIPPQKATTGLKPVDSGCVAYVLRTGFN
TSQGKLLRTILFGVKRVTANNLETFIFILFLLVFAIAAAAYVWIEGTKDPSRNRYKLFLE
CTLILTSVVPPELPIELSLAVNTSLIALAKLYMYCTEPFRIPFAGKVEVCCFDKTGTLTS
DSLVVRGVAGLRDGKEVTPVSSIPVETHRALASCHSLMQLDDGTLVGDPLEKAMLTAVDW
TLTKDEKVFPRSIKTQGLKIHQRFHFASALKRMSVLASYEKLGSTDLCYIAAVKGAPETL
HSMFSQCPPDYHHIHTEISREGARVLALGYKELGHLTHQQAREVKREALECSLKFVGFIV
VSCPLKADSKAVIREIQNASHRVVMITGDNPLTACHVAQELHFIEKAHTLILQPPSEKGR
QCEWRSIDGSIVLPLARGSPKALALEYALCLTGDGLAHLQATDPQQLLRLIPHVQVFARV
APKQKEFVITSLKELGYVTLMCGDGTNDVGALKHADVGVALLANAPERVVERRRRPRDSP
TLSNSGIRATSRTAKQRSGLPPSEEQPTSQRDRLSQVLRDLEDESTPIVKLGDASIAAPF
TSKLSSIQCICHVIKQGRCTLVTTLQMFKILALNALILAYSQSVLYLEGVKFSDFQATLQ
GLLLAGCFLFISRSKPLKTLSRERPLPNIFNLYTILTVMLQFFVHFLSLVYLYREAQARS
PEKQEQFVDLYKEFEPSLVNSTVYIMAMAMQMATFAINYKGPPFMESLPENKPLVWSLAV
SLLAIIGLLLGSSPDFNSQFGLVDIPVEFKLVIAQVLLLDFCLALLADRVLQFFLGTPKL
KVPS
>sp|Q4VNC0|AT135_HUMAN Probable cation-transporting ATPase 13A5 OS=Homo sapiens GN=ATP13A5 PE=2 SV=1
MEENSKKDHRALLNQGEEDELEVFGYRDHNVRKAFCLVASVLTCGGLLLVFYWRPQWRVW

ANCIPCPLQEADTVLLRTTDEFQRYMRKKVFCLYLSTLKFPVSKKWEESLVADRHSVINQ
ALIKPELKLRCMEVQKIRYVWNDLEKRFQKVGLLEDSNSCSDIHQTFGLGLTSEEQEVRR
LVCGPNAIEVEIQPIWKLLVKQVLNPFYVFQAFTLTLWLSQGYIEYSVAIIILTVISIVL
SVYDLRQQSVKLHNLVEDHNKVQVTIIVKDKGLEELESRLLVPGDILILPGKFSLPCDAV
LIDGSCVVNEGMLTGESIPVTKTPLPQMENTMPWKCHSLEDYRKHVLFCGTEVIQVKPSG
QGPVRAVVLQTGYNTAKGDLVRSILYPRPLNFKLYSDAFKFIVFLACLGVMGFFYALGVY
MYHGVPPKDTVTMALILLTVTVPPVLPAALTIGNVYAQKRLKKKKIFCISPQRINMCGQI
NLVCFDKTGTLTEDGLDLWGTVPTADNCFQEAHSFASGQAVPWSPLCAAMASCHSLILLN
GTIQGDPLDLKMFEGTAWKMEDCIVDSCKFGTSVSNIIKPGPKASKSPVEAIITLCQFPF
SSSLQRMSVIAQLAGENHFHVYMKGAPEMVARFCRSETVPKNFPQELRSYTVQGFRVIAL
AHKTLKMGNLSEVEHLAREKVESELTFLGLLIMENRLKKETKLVLKELSEARIRTVMITG
DNLQTAITVAKNSEMIPPGSQVIIVEADEPEEFVPASVTWQLVENQETGPGKKEIYMHTG
NSSTPRGEGGSCYHFAMSGKSYQVIFQHFNSLLPKILVNGTVFARMSPGQKSSLIEEFQK
LNYYVGMCGDGANDCGALKAAHAGISLSEQEASVASPFTSKTTNIQCVPHLIREGRAALV
SSFGVFKYLTMYGIIQFISALLLYWQLQLFGNYQYLMQDVAITLMVCLTMSSTHAYPKLA
PYRPAGQLLSPPLLLSIFLNSCFSCIVQISAFLYVKQQPWYCEVYQYSECFLANQSNFST
NVSLERNWTGNATLIPGSILSFETTTLWPITTINYITVAFIFSKGKPFRKPIYTNYIFSF
LLLAALGLTIFILFSDFQVIYRGMELIPTITSWRVLILVVALTQFCVAFFVEDSILQNHE
LWLLIKREFGFYSKSQYRTWQKKLAEDSTWPPINRTDYSGDGKNGFYINGGYESHEQIPK
RKLKLGGQPTEQHFWARL
>sp|P98194|AT2C1_HUMAN Calcium-transporting ATPase type 2C member 1 OS=Homo sapiens GN=ATP2C1 PE=1 SV=3
MKVARFQKIPNGENETMIPVLTSKKASELPVSEVASILQADLQNGLNKCEVSHRRAFHGW
NEFDISEDEPLWKKYISQFKNPLIMLLLASAVISVLMHQFDDAVSITVAILIVVTVAFVQ
EYRSEKSLEELSKLVPPECHCVREGKLEHTLARDLVPGDTVCLSVGDRVPADLRLFEAVD
LSIDESSLTGETTPCSKVTAPQPAATNGDLASRSNIAFMGTLVRCGKAKGVVIGTGENSE
FGEVFKMMQAEEAPKTPLQKSMDLLGKQLSFYSFGIIGIIMLVGWLLGKDILEMFTISVS
LAVAAIPEGLPIVVTVTLALGVMRMVKKRAIVKKLPIVETLGCCNVICSDKTGTLTKNEM
TVTHIFTSDGLHAEVTGVGYNQFGEVIVDGDVVHGFYNPAVSRIVEAGCVCNDAVIRNNT
LMGKPTEGALIALAMKMGLDGLQQDYIRKAEYPFSSEQKWMAVKCVHRTQQDRPEICFMK
GAYEQVIKYCTTYQSKGQTLTLTQQQRDVYQQEKARMGSAGLRVLALASGPELGQLTFLG
LVGIIDPPRTGVKEAVTTLIASGVSIKMITGDSQETAVAIASRLGLYSKTSQSVSGEEID
AMDVQQLSQIVPKVAVFYRASPRHKMKIIKSLQKNGSVVAMTGDGVNDAVALKAADIGVA
MGQTGTDVCKEAADMILVDDDFQTIMSAIEEGKGIYNNIKNFVRFQLSTSIAALTLISLA
TLMNFPNPLNAMQILWINIIMDGPPAQSLGVEPVDKDVIRKPPRNWKDSILTKNLILKIL
VSSIIIVCGTLFVFWRELRDNVITPRDTTMTFTCFVFFDMFNALSSRSQTKSVFEIGLCS
NRMFCYAVLGSIMGQLLVIYFPPLQKVFQTESLSILDLLFLLGLTSSVCIVAEIIKKVER
SREKIQKHVSSTSSSFLEV
>sp|O15145|ARPC3_HUMAN Actin-related protein 2/3 complex subunit 3 OS=Homo sapiens GN=ARPC3 PE=1 SV=3
MPAYHSSLMDPDTKLIGNMALLPIRSQFKGPAPRETKDTDIVDEAIYYFKANVFFKNYEI
KNEADRTLIYITLYISECLKKLQKCNSKSQGEKEMYTLGITNFPIPGEPGFPLNAIYAKP
ANKQEDEVMRAYLQQLRQETGLRLCEKVFDPQNDKPSKWWTCFVKRQFMNKSLSGPGQ
>sp|Q7Z6K5|ARPIN_HUMAN Arpin OS=Homo sapiens GN=ARPIN PE=1 SV=1

MSRIYHDGALRNKAVQSVRLPGAWDPAAHQGGNGVLLEGELIDVSRHSILDTHGRKERYY
VLYIRPSHIHRRKFDAKGNEIEPNFSATRKVNTGFLMSSYKVEAKGDTDRLTPEALKGLV
NKPELLALTESLTPDHTVAFWMPESEMEVMELELGAGVRLKTRGDGPFLDSLAKLEAGTV
TKCNFTGDGKTGASWTDNIMAQKCSKGAAAEIREQGDGAEDEEWDD
>sp|P36575|ARRC_HUMAN Arrestin-C OS=Homo sapiens GN=ARR3 PE=1 SV=2
MSKVFKKTSSNGKLSIYLGKRDFVDHVDTVEPIDGVVLVDPEYLKCRKLFVMLTCAFRYG
RDDLEVIGLTFRKDLYVQTLQVVPAESSSPQGPLTVLQERLLHKLGDNAYPFTLQMVTNL
PCSVTLQPGPEDAGKPCGIDFEVKSFCAENPEETVSKRDYVRLVVRKVQFAPPEAGPGPS
AQTIRRFLLSAQPLQLQAWMDREVHYHGEPISVNVSINNCTNKVIKKIKISVDQITDVVL
YSLDKYTKTVFIQEFTETVAANSSFSQSFAVTPILAASCQKRGLALDGKLKHEDTNLASS
TIIRPGMDKELLGILVSYKVRVNLMVSCGGILGDLTASDVGVELPLVLIHPKPSHEAASS
EDIVIEEFTRKGEEESQKAVEAEGDEGS
>sp|P51690|ARSE_HUMAN Arylsulfatase E OS=Homo sapiens GN=ARSE PE=1 SV=2
MLHLHHSCLCFRSWLPAMLAVLLSLAPSASSDISASRPNILLLMADDLGIGDIGCYGNNT
MRTPNIDRLAEDGVKLTQHISAASLCTPSRAAFLTGRYPVRSGMVSSIGYRVLQWTGASG
GLPTNETTFAKILKEKGYATGLIGKWHLGLNCESASDHCHHPLHHGFDHFYGMPFSLMGD
CARWELSEKRVNLEQKLNFLFQVLALVALTLVAGKLTHLIPVSWMPVIWSALSAVLLLAS
SYFVGALIVHADCFLMRNHTITEQPMCFQRTTPLILQEVASFLKRNKHGPFLLFVSFLHV
HIPLITMENFLGKSLHGLYGDNVEEMDWMVGRILDTLDVEGLSNSTLIYFTSDHGGSLEN
QLGNTQYGGWNGIYKGGKGMGGWEGGIRVPGIFRWPGVLPAGRVIGEPTSLMDVFPTVVR
LAGGEVPQDRVIDGQDLLPLLLGTAQHSDHEFLMHYCERFLHAARWHQRDRGTMWKVHFV
TPVFQPEGAGACYGRKVCPCFGEKVVHHDPPLLFDLSRDPSETHILTPASEPVFYQVMER
VQQAVWEHQRTLSPVPLQLDRLGNIWRPWLQPCCGPFPLCWCLREDDPQ
>sp|Q6UWY0|ARSK_HUMAN Arylsulfatase K OS=Homo sapiens GN=ARSK PE=1 SV=1
MLLLWVSVVAALALAVLAPGAGEQRRRAAKAPNVVLVVSDSFDGRLTFHPGSQVVKLPFI
NFMKTRGTSFLNAYTNSPICCPSRAAMWSGLFTHLTESWNNFKGLDPNYTTWMDVMERHG
YRTQKFGKLDYTSGHHSISNRVEAWTRDVAFLLRQEGRPMVNLIRNRTKVRVMERDWQNT
DKAVNWLRKEAINYTEPFVIYLGLNLPHPYPSPSSGENFGSSTFHTSLYWLEKVSHDAIK
IPKWSPLSEMHPVDYYSSYTKNCTGRFTKKEIKNIRAFYYAMCAETDAMLGEIILALHQL
DLLQKTIVIYSSDHGELAMEHRQFYKMSMYEASAHVPLLMMGPGIKAGLQVSNVVSLVDI
YPTMLDIAGIPLPQNLSGYSLLPLSSETFKNEHKVKNLHPPWILSEFHGCNVNASTYMLR
TNHWKYIAYSDGASILPQLFDLSSDPDELTNVAVKFPEITYSLDQKLHSIINYPKVSASV
HQYNKEQFIKWKQSIGQNYSNVIANLRWHQDWQKEPRKYENAIDQWLKTHMNPRAV
>sp|Q5T4W7|ARTN_HUMAN Artemin OS=Homo sapiens GN=ARTN PE=1 SV=1
MELGLGGLSTLSHCPWPRQQPALWPTLAALALLSSVAEASLGSAPRSPAPREGPPPVLAS
PAGHLPGGRTARWCSGRARRPPPQPSRPAPPPPAPPSALPRGGRAARAGGPGSRARAAGA
RGCRLRSQLVPVRALGLGHRSDELVRFRFCSGSCRRARSPHDLSLASLLGAGALRPPPGS
RPVSQPCCRPTRYEAVSFMDVNSTWRTVDRLSATACGCLG
>sp|O00192|ARVC_HUMAN Armadillo repeat protein deleted in velo-cardio-facial syndrome OS=Homo sapiens GN=ARVCF PE=1 SV=1
MEDCNVHSAASILASVKEQEARFERLTRALEQERRHVALQLERAQQPGMVSGGMGSGQPL
PMAWQQLVLQEQSPGSQASLATMPEAPDVLEETVTVEEDPGTPTSHVSIVTSEDGTTRRT
ETKVTKTVKTVTTRTVRQVPVGPDGLPLLDGGPPLGPFADGALDRHFLLRGGGPVATLSR
AYLSSGGGFPEGPEPRDSPSYGSLSRGLGMRPPRAGPLGPGPGDGCFTLPGHREAFPVGP

EPGPPGGRSLPERFQAEPYGLEDDTRSLAADDEGGPELEPDYGTATRRRPECGRGLHTRA
YEDTADDGGELADERPAFPMVTAPLAQPERGSMGSLDRLVRRSPSVDSARKEPRWRDPEL
PEVLAMLRHPVDPVKANAAAYLQHLCFENEGVKRRVRQLRGLPLLVALLDHPRAEVRRRA
CGALRNLSYGRDTDNKAAIRDCGGVPALVRLLRAARDNEVRELVTGTLWNLSSYEPLKMV
IIDHGLQTLTHEVIVPHSGWEREPNEDSKPRDAEWTTVFKNTSGCLRNVSSDGAEARRRL
RECEGLVDALLHALQSAVGRKDTDNKSVENCVCIMRNLSYHVHKEVPGADRYQEAEPGPL
GSAVGSQRRRRDDASCFGGKKAKEEWFHQGKKDGEMDRNFDTLDLPKRTEAAKGFELLYQ
PEVVRLYLSLLTESRNFNTLEAAAGALQNLSAGNWMWATYIRATVRKERGLPVLVELLQS
ETDKVVRAVAIALRNLSLDRRNKDLIGSYAMAELVRNVRNAQAPPRPGACLEEDTVVAVL
NTIHEIVSDSLDNARSLLQARGVPALVALVASSQSVREAKAASHVLQTVWSYKELRGTLQ
KDGWTKARFQSAAATAKGPKGALSPGGFDDSTLPLVDKSLEGEKTGSRDVIPMDALGPDG
YSTVDRRERRPRGASSAGEASEKEPLKLDPSRKAPPPGPSRPAVRLVDAVGDAKPQPVDS
WV
>sp|A6NK59|ASB14_HUMAN Ankyrin repeat and SOCS box protein 14 OS=Homo sapiens GN=ASB14 PE=2 SV=2
MDNYTSDEDIDEDFDTQLIIQQSLQDIYKPGTAQHAPKDESLHSFLSADYKKIVETIEKV
GKEDALSHLTKYHSAFGEADEIGWIPLHKAAVQLNRKILEITLSASDPSLWEQTTHNGET
PLFLAVSSCLLENATFLLLNGCNPNAKNFEGNSPLLAAVLRDCYDMAALLINYGADVNLR
CANERTALHEAAKLGREDMVKLMLVSGAHPDPQSTYGFTPLALAAQSGHTEIMEMLLRKG
KIFCLASDSSSILLEAASGGNPDAVALLLEYGADANIPKNSGHLPIHVAADRGHLLALKI
LIPVTDLAAIKQSGISPVHCAAAGAHPQCLELLIQAGFDVNFMLDQRINKHYDDHRKSAL
YFAVSNSDLSSVKLLLSAGALPNQDPVNCLQIALRMGNYELISLLLRHGANVNYFCRVNP
LHFPSALQYTLKDEVMLRMLLNYGYDTERCFDCPHGDKVHPSYTVEGWTSTVIKDTKFCE
VITLSWLQHLSGKVVRVMLDYVDQVRICSKLKAVLQKQGIWSEIHFILTNPRSLKHLCRL
KIRKCMGRLHLRCPVFMSFLPLPNRLKAYVLYKEYDLYGQGIFTGTW
>sp|Q7L266|ASGL1_HUMAN Isoaspartyl peptidase/L-asparaginase OS=Homo sapiens GN=ASRGL1 PE=1 SV=2
MNPIVVVHGGGAGPISKDRKERVHQGMVRAATVGYGILREGGSAVDAVEGAVVALEDDPE
FNAGCGSVLNTNGEVEMDASIMDGKDLSAGAVSAVQCIANPIKLARLVMEKTPHCFLTDQ
GAAQFAAAMGVPEIPGEKLVTERNKKRLEKEKHEKGAQKTDCQKNLGTVGAVALDCKGNV
AYATSTGGIVNKMVGRVGDSPCLGAGGYADNDIGAVSTTGHGESILKVNLARLTLFHIEQ
GKTVEEAADLSLGYMKSRVKGLGGLIVVSKTGDWVAKWTSTSMPWAAAKDGKLHFGIDPD
DTTITDLP
>sp|P07307|ASGR2_HUMAN Asialoglycoprotein receptor 2 OS=Homo sapiens GN=ASGR2 PE=1 SV=2
MAKDFQDIQQLSSEENDHPFHQGEGPGTRRLNPRRGNPFLKGPPPAQPLAQRLCSMVCFS
LLALSFNILLLVVICVTGSQSEGHGGAQLQAELRSLKEAFSNFSSSTLTEVQAISTHGGS
VGDKITSLGAKLEKQQQDLKADHDALLFHLKHFPVDLRFVACQMELLHSNGSQRTCCPVN
WVEHQGSCYWFSHSGKAWAEAEKYCQLENAHLVVINSWEEQKFIVQHTNPFNTWIGLTDS
DGSWKWVDGTDYRHNYKNWAVTQPDNWHGHELGGSEDCVEVQPDGRWNDDFCLQVYRWVC
EKRRNATGEVA
>sp|Q9BVC5|ASHWN_HUMAN Ashwin OS=Homo sapiens GN=C2orf49 PE=1 SV=1
MAGDVGGRSCTDSELLLHPELLSQEFLLLTLEQKNIAVETDVRVNKDSLTDLYVQHAIPL
PQRDLPKNRWGKMMEKKREQHEIKNETKRSSTVDGLRKRPLIVFDGSSTSTSIKVKKTEN
GDNDRLKPPPQASFTSNAFRKLSNSSSSVSPLILSSNLPVNNKTEHNNNDAKQNHDLTHR

KSPSGPVKSPPLSPVGTTPVKLKRAAPKEEAEAMNNLKPPQAKRKIQHVTWP
>sp|Q16515|ASIC2_HUMAN Acid-sensing ion channel 2 OS=Homo sapiens GN=ASIC2 PE=1 SV=1
MDLKESPSEGSLQPSSIQIFANTSTLHGIRHIFVYGPLTIRRVLWAVAFVGSLGLLLVES
SERVSYYFSYQHVTKVDEVVAQSLVFPAVTLCNLNGFRFSRLTTNDLYHAGELLALLDVN
LQIPDPHLADPSVLEALRQKANFKHYKPKQFSMLEFLHRVGHDLKDMMLYCKFKGQECGH
QDFTTVFTKYGKCYMFNSGEDGKPLLTTVKGGTGNGLEIMLDIQQDEYLPIWGETEETTF
EAGVKVQIHSQSEPPFIQELGFGVAPGFQTFVATQEQRLTYLPPPWGECRSSEMGLDFFP
VYSITACRIDCETRYIVENCNCRMVHMPGDAPFCTPEQHKECAEPALGLLAEKDSNYCLC
RTPCNLTRYNKELSMVKIPSKTSAKYLEKKFNKSEKYISENILVLDIFFEALNYETIEQK
KAYEVAALLGDIGGQMGLFIGASILTILELFDYIYELIKEKLLDLLGKEEDEGSHDENVS
TCDTMPNHSETISHTVNVPLQTTLGTLEEIAC
>sp|Q9UHC3|ASIC3_HUMAN Acid-sensing ion channel 3 OS=Homo sapiens GN=ASIC3 PE=1 SV=2
MKPTSGPEEARRPASDIRVFASNCSMHGLGHVFGPGSLSLRRGMWAAAVVLSVATFLYQV
AERVRYYREFHHQTALDERESHRLIFPAVTLCNINPLRRSRLTPNDLHWAGSALLGLDPA
EHAAFLRALGRPPAPPGFMPSPTFDMAQLYARAGHSLDDMLLDCRFRGQPCGPENFTTIF
TRMGKCYTFNSGADGAELLTTTRGGMGNGLDIMLDVQQEEYLPVWRDNEETPFEVGIRVQ
IHSQEEPPIIDQLGLGVSPGYQTFVSCQQQQLSFLPPPWGDCSSASLNPNYEPEPSDPLG
SPSPSPSPPYTLMGCRLACETRYVARKCGCRMVYMPGDVPVCSPQQYKNCAHPAIDAMLR
KDSCACPNPCASTRYAKELSMVRIPSRAAARFLARKLNRSEAYIAENVLALDIFFEALNY
ETVEQKKAYEMSELLGDIGGQMGLFIGASLLTILEILDYLCEVFRDKVLGYFWNRQHSQR
HSSTNLLQEGLGSHRTQVPHLSLGPRPPTPPCAVTKTLSASHRTCYLVTQL
>sp|P05023|AT1A1_HUMAN Sodium/potassium-transporting ATPase subunit alpha-1 OS=Homo sapiens GN=ATP1A1 PE=1 SV=1
MGKGVGRDKYEPAAVSEQGDKKGKKGKKDRDMDELKKEVSMDDHKLSLDELHRKYGTDLS
RGLTSARAAEILARDGPNALTPPPTTPEWIKFCRQLFGGFSMLLWIGAILCFLAYSIQAA
TEEEPQNDNLYLGVVLSAVVIITGCFSYYQEAKSSKIMESFKNMVPQQALVIRNGEKMSI
NAEEVVVGDLVEVKGGDRIPADLRIISANGCKVDNSSLTGESEPQTRSPDFTNENPLETR
NIAFFSTNCVEGTARGIVVYTGDRTVMGRIATLASGLEGGQTPIAAEIEHFIHIITGVAV
FLGVSFFILSLILEYTWLEAVIFLIGIIVANVPEGLLATVTVCLTLTAKRMARKNCLVKN
LEAVETLGSTSTICSDKTGTLTQNRMTVAHMWFDNQIHEADTTENQSGVSFDKTSATWLA
LSRIAGLCNRAVFQANQENLPILKRAVAGDASESALLKCIELCCGSVKEMRERYAKIVEI
PFNSTNKYQLSIHKNPNTSEPQHLLVMKGAPERILDRCSSILLHGKEQPLDEELKDAFQN
AYLELGGLGERVLGFCHLFLPDEQFPEGFQFDTDDVNFPIDNLCFVGLISMIDPPRAAVP
DAVGKCRSAGIKVIMVTGDHPITAKAIAKGVGIISEGNETVEDIAARLNIPVSQVNPRDA
KACVVHGSDLKDMTSEQLDDILKYHTEIVFARTSPQQKLIIVEGCQRQGAIVAVTGDGVN
DSPALKKADIGVAMGIAGSDVSKQAADMILLDDNFASIVTGVEEGRLIFDNLKKSIAYTL
TSNIPEITPFLIFIIANIPLPLGTVTILCIDLGTDMVPAISLAYEQAESDIMKRQPRNPK
TDKLVNERLISMAYGQIGMIQALGGFFTYFVILAENGFLPIHLLGLRVDWDDRWINDVED
SYGQQWTYEQRKIVEFTCHTAFFVSIVVVQWADLVICKTRRNSVFQQGMKNKILIFGLFE
ETALAAFLSYCPGMGVALRMYPLKPTWWFCAFPYSLLIFVYDEVRKLIIRRRPGGWVEKE
TYY
>sp|P50993|AT1A2_HUMAN Sodium/potassium-transporting ATPase subunit alpha-2 OS=Homo sapiens GN=ATP1A2 PE=1 SV=1
MGRGAGREYSPAATTAENGGGKKKQKEKELDELKKEVAMDDHKLSLDELGRKYQVDLSKG

LTNQRAQDVLARDGPNALTPPPTTPEWVKFCRQLFGGFSILLWIGAILCFLAYGIQAAME
DEPSNDNLYLGVVLAAVVIVTGCFSYYQEAKSSKIMDSFKNMVPQQALVIREGEKMQINA
EEVVVGDLVEVKGGDRVPADLRIISSHGCKVDNSSLTGESEPQTRSPEFTHENPLETRNI
CFFSTNCVEGTARGIVIATGDRTVMGRIATLASGLEVGRTPIAMEIEHFIQLITGVAVFL
GVSFFVLSLILGYSWLEAVIFLIGIIVANVPEGLLATVTVCLTLTAKRMARKNCLVKNLE
AVETLGSTSTICSDKTGTLTQNRMTVAHMWFDNQIHEADTTEDQSGATFDKRSPTWTALS
RIAGLCNRAVFKAGQENISVSKRDTAGDASESALLKCIELSCGSVRKMRDRNPKVAEIPF
NSTNKYQLSIHEREDSPQSHVLVMKGAPERILDRCSTILVQGKEIPLDKEMQDAFQNAYM
ELGGLGERVLGFCQLNLPSGKFPRGFKFDTDELNFPTEKLCFVGLMSMIDPPRAAVPDAV
GKCRSAGIKVIMVTGDHPITAKAIAKGVGIISEGNETVEDIAARLNIPMSQVNPREAKAC
VVHGSDLKDMTSEQLDEILKNHTEIVFARTSPQQKLIIVEGCQRQGAIVAVTGDGVNDSP
ALKKADIGIAMGISGSDVSKQAADMILLDDNFASIVTGVEEGRLIFDNLKKSIAYTLTSN
IPEITPFLLFIIANIPLPLGTVTILCIDLGTDMVPAISLAYEAAESDIMKRQPRNSQTDK
LVNERLISMAYGQIGMIQALGGFFTYFVILAENGFLPSRLLGIRLDWDDRTMNDLEDSYG
QEWTYEQRKVVEFTCHTAFFASIVVVQWADLIICKTRRNSVFQQGMKNKILIFGLLEETA
LAAFLSYCPGMGVALRMYPLKVTWWFCAFPYSLLIFIYDEVRKLILRRYPGGWVEKETYY
>sp|P13637|AT1A3_HUMAN Sodium/potassium-transporting ATPase subunit alpha-3 OS=Homo sapiens GN=ATP1A3 PE=1 SV=3
MGDKKDDKDSPKKNKGKERRDLDDLKKEVAMTEHKMSVEEVCRKYNTDCVQGLTHSKAQE
ILARDGPNALTPPPTTPEWVKFCRQLFGGFSILLWIGAILCFLAYGIQAGTEDDPSGDNL
YLGIVLAAVVIITGCFSYYQEAKSSKIMESFKNMVPQQALVIREGEKMQVNAEEVVVGDL
VEIKGGDRVPADLRIISAHGCKVDNSSLTGESEPQTRSPDCTHDNPLETRNITFFSTNCV
EGTARGVVVATGDRTVMGRIATLASGLEVGKTPIAIEIEHFIQLITGVAVFLGVSFFILS
LILGYTWLEAVIFLIGIIVANVPEGLLATVTVCLTLTAKRMARKNCLVKNLEAVETLGST
STICSDKTGTLTQNRMTVAHMWFDNQIHEADTTEDQSGTSFDKSSHTWVALSHIAGLCNR
AVFKGGQDNIPVLKRDVAGDASESALLKCIELSSGSVKLMRERNKKVAEIPFNSTNKYQL
SIHETEDPNDNRYLLVMKGAPERILDRCSTILLQGKEQPLDEEMKEAFQNAYLELGGLGE
RVLGFCHYYLPEEQFPKGFAFDCDDVNFTTDNLCFVGLMSMIDPPRAAVPDAVGKCRSAG
IKVIMVTGDHPITAKAIAKGVGIISEGNETVEDIAARLNIPVSQVNPRDAKACVIHGTDL
KDFTSEQIDEILQNHTEIVFARTSPQQKLIIVEGCQRQGAIVAVTGDGVNDSPALKKADI
GVAMGIAGSDVSKQAADMILLDDNFASIVTGVEEGRLIFDNLKKSIAYTLTSNIPEITPF
LLFIMANIPLPLGTITILCIDLGTDMVPAISLAYEAAESDIMKRQPRNPRTDKLVNERLI
SMAYGQIGMIQALGGFFSYFVILAENGFLPGNLVGIRLNWDDRTVNDLEDSYGQQWTYEQ
RKVVEFTCHTAFFVSIVVVQWADLIICKTRRNSVFQQGMKNKILIFGLFEETALAAFLSY
CPGMDVALRMYPLKPSWWFCAFPYSFLIFVYDEIRKLILRRNPGGWVEKETYY
>sp|O14983|AT2A1_HUMAN Sarcoplasmic/endoplasmic reticulum calcium ATPase 1 OS=Homo sapiens GN=ATP2A1 PE=1 SV=1
MEAAHAKTTEECLAYFGVSETTGLTPDQVKRNLEKYGLNELPAEEGKTLWELVIEQFEDL
LVRILLLAACISFVLAWFEEGEETITAFVEPFVILLILIANAIVGVWQERNAENAIEALK
EYEPEMGKVYRADRKSVQRIKARDIVPGDIVEVAVGDKVPADIRILAIKSTTLRVDQSIL
TGESVSVIKHTEPVPDPRAVNQDKKNMLFSGTNIAAGKALGIVATTGVGTEIGKIRDQMA
ATEQDKTPLQQKLDEFGEQLSKVISLICVAVWLINIGHFNDPVHGGSWFRGAIYYFKIAV
ALAVAAIPEGLPAVITTCLALGTRRMAKKNAIVRSLPSVETLGCTSVICSDKTGTLTTNQ
MSVCKMFIIDKVDGDICLLNEFSITGSTYAPEGEVLKNDKPVRPGQYDGLVELATICALC

NDSSLDFNEAKGVYEKVGEATETALTTLVEKMNVFNTDVRSLSKVERANACNSVIRQLMK
KEFTLEFSRDRKSMSVYCSPAKSSRAAVGNKMFVKGAPEGVIDRCNYVRVGTTRVPLTGP
VKEKIMAVIKEWGTGRDTLRCLALATRDTPPKREEMVLDDSARFLEYETDLTFVGVVGML
DPPRKEVTGSIQLCRDAGIRVIMITGDNKGTAIAICRRIGIFGENEEVADRAYTGREFDD
LPLAEQREACRRACCFARVEPSHKSKIVEYLQSYDEITAMTGDGVNDAPALKKAEIGIAM
GSGTAVAKTASEMVLADDNFSTIVAAVEEGRAIYNNMKQFIRYLISSNVGEVVCIFLTAA
LGLPEALIPVQLLWVNLVTDGLPATALGFNPPDLDIMDRPPRSPKEPLISGWLFFRYMAI
GGYVGAATVGAAAWWFLYAEDGPHVNYSQLTHFMQCTEDNTHFEGIDCEVFEAPEPMTMA
LSVLVTIEMCNALNSLSENQSLLRMPPWVNIWLLGSICLSMSLHFLILYVDPLPMIFKLR
ALDLTQWLMVLKISLPVIGLDEILKFVARNYLEDPEDERRK
>sp|P16615|AT2A2_HUMAN Sarcoplasmic/endoplasmic reticulum calcium ATPase 2 OS=Homo sapiens GN=ATP2A2 PE=1 SV=1
MENAHTKTVEEVLGHFGVNESTGLSLEQVKKLKERWGSNELPAEEGKTLLELVIEQFEDL
LVRILLLAACISFVLAWFEEGEETITAFVEPFVILLILVANAIVGVWQERNAENAIEALK
EYEPEMGKVYRQDRKSVQRIKAKDIVPGDIVEIAVGDKVPADIRLTSIKSTTLRVDQSIL
TGESVSVIKHTDPVPDPRAVNQDKKNMLFSGTNIAAGKAMGVVVATGVNTEIGKIRDEMV
ATEQERTPLQQKLDEFGEQLSKVISLICIAVWIINIGHFNDPVHGGSWIRGAIYYFKIAV
ALAVAAIPEGLPAVITTCLALGTRRMAKKNAIVRSLPSVETLGCTSVICSDKTGTLTTNQ
MSVCRMFILDRVEGDTCSLNEFTITGSTYAPIGEVHKDDKPVNCHQYDGLVELATICALC
NDSALDYNEAKGVYEKVGEATETALTCLVEKMNVFDTELKGLSKIERANACNSVIKQLMK
KEFTLEFSRDRKSMSVYCTPNKPSRTSMSKMFVKGAPEGVIDRCTHIRVGSTKVPMTSGV
KQKIMSVIREWGSGSDTLRCLALATHDNPLRREEMHLEDSANFIKYETNLTFVGCVGMLD
PPRIEVASSVKLCRQAGIRVIMITGDNKGTAVAICRRIGIFGQDEDVTSKAFTGREFDEL
NPSAQRDACLNARCFARVEPSHKSKIVEFLQSFDEITAMTGDGVNDAPALKKAEIGIAMG
SGTAVAKTASEMVLADDNFSTIVAAVEEGRAIYNNMKQFIRYLISSNVGEVVCIFLTAAL
GFPEALIPVQLLWVNLVTDGLPATALGFNPPDLDIMNKPPRNPKEPLISGWLFFRYLAIG
CYVGAATVGAAAWWFIAADGGPRVSFYQLSHFLQCKEDNPDFEGVDCAIFESPYPMTMAL
SVLVTIEMCNALNSLSENQSLLRMPPWENIWLVGSICLSMSLHFLILYVEPLPLIFQITP
LNVTQWLMVLKISLPVILMDETLKFVARNYLEPGKECVQPATKSCSFSACTDGISWPFVL
LIMPLVIWVYSTDTNFSDMFWS
>sp|Q01814|AT2B2_HUMAN Plasma membrane calcium-transporting ATPase 2 OS=Homo sapiens GN=ATP2B2 PE=1 SV=2
MGDMTNSDFYSKNQRNESSHGGEFGCTMEELRSLMELRGTEAVVKIKETYGDTEAICRRL
KTSPVEGLPGTAPDLEKRKQIFGQNFIPPKKPKTFLQLVWEALQDVTLIILEIAAIISLG
LSFYHPPGEGNEGCATAQGGAEDEGEAEAGWIEGAAILLSVICVVLVTAFNDWSKEKQFR
GLQSRIEQEQKFTVVRAGQVVQIPVAEIVVGDIAQVKYGDLLPADGLFIQGNDLKIDESS
LTGESDQVRKSVDKDPMLLSGTHVMEGSGRMLVTAVGVNSQTGIIFTLLGAGGEEEEKKD
KKGVKKGDGLQLPAADGAAASNAADSANASLVNGKMQDGNVDASQSKAKQQDGAAAMEMQ
PLKSAEGGDADDRKKASMHKKEKSVLQGKLTKLAVQIGKAGLVMSAITVIILVLYFTVDT
FVVNKKPWLPECTPVYVQYFVKFFIIGVTVLVVAVPEGLPLAVTISLAYSVKKMMKDNNL
VRHLDACETMGNATAICSDKTGTLTTNRMTVVQAYVGDVHYKEIPDPSSINTKTMELLIN
AIAINSAYTTKILPPEKEGALPRQVGNKTECGLLGFVLDLKQDYEPVRSQMPEEKLYKVY
TFNSVRKSMSTVIKLPDESFRMYSKGASEIVLKKCCKILNGAGEPRVFRPRDRDEMVKKV
IEPMACDGLRTICVAYRDFPSSPEPDWDNENDILNELTCICVVGIEDPVRPEVPEAIRKC

QRAGITVRMVTGDNINTARAIAIKCGIIHPGEDFLCLEGKEFNRRIRNEKGEIEQERIDK
IWPKLRVLARSSPTDKHTLVKGIIDSTHTEQRQVVAVTGDGTNDGPALKKADVGFAMGIA
GTDVAKEASDIILTDDNFSSIVKAVMWGRNVYDSISKFLQFQLTVNVVAVIVAFTGACIT
QDSPLKAVQMLWVNLIMDTFASLALATEPPTETLLLRKPYGRNKPLISRTMMKNILGHAV
YQLALIFTLLFVGEKMFQIDSGRNAPLHSPPSEHYTIIFNTFVMMQLFNEINARKIHGER
NVFDGIFRNPIFCTIVLGTFAIQIVIVQFGGKPFSCSPLQLDQWMWCIFIGLGELVWGQV
IATIPTSRLKFLKEAGRLTQKEEIPEEELNEDVEEIDHAERELRRGQILWFRGLNRIQTQ
IRVVKAFRSSLYEGLEKPESRTSIHNFMAHPEFRIEDSQPHIPLIDDTDLEEDAALKQNS
SPPSSLNKNNSAIDSGINLTTDTSKSATSSSPGSPIHSLETSL
>sp|P23634|AT2B4_HUMAN Plasma membrane calcium-transporting ATPase 4 OS=Homo sapiens GN=ATP2B4 PE=1 SV=2
MTNPSDRVLPANSMAESREGDFGCTVMELRKLMELRSRDALTQINVHYGGVQNLCSRLKT
SPVEGLSGNPADLEKRRQVFGHNVIPPKKPKTFLELVWEALQDVTLIILEIAAIISLVLS
FYRPAGEENELCGQVATTPEDENEAQAGWIEGAAILFSVIIVVLVTAFNDWSKEKQFRGL
QCRIEQEQKFSIIRNGQLIQLPVAEIVVGDIAQVKYGDLLPADGILIQGNDLKIDESSLT
GESDHVKKSLDKDPMLLSGTHVMEGSGRMVVTAVGVNSQTGIILTLLGVNEDDEGEKKKK
GKKQGVPENRNKAKTQDGVALEIQPLNSQEGIDNEEKDKKAVKVPKKEKSVLQGKLTRLA
VQIGKAGLLMSALTVFILILYFVIDNFVINRRPWLPECTPIYIQYFVKFFIIGITVLVVA
VPEGLPLAVTISLAYSVKKMMKDNNLVRHLDACETMGNATAICSDKTGTLTMNRMTVVQA
YIGGIHYRQIPSPDVFLPKVLDLIVNGISINSAYTSKILPPEKEGGLPRQVGNKTECALL
GFVTDLKQDYQAVRNEVPEEKLYKVYTFNSVRKSMSTVIRNPNGGFRMYSKGASEIILRK
CNRILDRKGEAVPFKNKDRDDMVRTVIEPMACDGLRTICIAYRDFDDTEPSWDNENEILT
ELTCIAVVGIEDPVRPEVPDAIAKCKQAGITVRMVTGDNINTARAIATKCGILTPGDDFL
CLEGKEFNRLIRNEKGEVEQEKLDKIWPKLRVLARSSPTDKHTLVKGIIDSTVGEHRQVV
AVTGDGTNDGPALKKADVGFAMGIAGTDVAKEASDIILTDDNFTSIVKAVMWGRNVYDSI
SKFLQFQLTVNVVAVIVAFTGACITQDSPLKAVQMLWVNLIMDTFASLALATEPPTESLL
KRRPYGRNKPLISRTMMKNILGHAFYQLIVIFILVFAGEKFFDIDSGRKAPLHSPPSQHY
TIVFNTFVLMQLFNEINSRKIHGEKNVFSGIYRNIIFCSVVLGTFICQIFIVEFGGKPFS
CTSLSLSQWLWCLFIGIGELLWGQFISAIPTRSLKFLKEAGHGTTKEEITKDAEGLDEID
HAEMELRRGQILWFRGLNRIQTQIDVINTFQTGASFKGVLRRQNMGQHLDVKLVPSSSYI
KVVKAFHSSLHESIQKPYNQKSIHSFMTHPEFAIEEELPRTPLLDEEEEENPDKASKFGT
RVLLLDGEVTPYANTNNNAVDCNQVQLPQSDSSLQSLETSV
>sp|P18859|ATP5J_HUMAN ATP synthase-coupling factor 6, mitochondrial OS=Homo sapiens GN=ATP5J PE=1 SV=1
MILQRLFRFSSVIRSAVSVHLRRNIGVTAVAFNKELDPIQKLFVDKIREYKSKRQTSGGP
VDASSEYQQELERELFKLKQMFGNADMNTFPTFKFEDPKFEVIEKPQA
>sp|Q9NQS1|AVEN_HUMAN Cell death regulator Aven OS=Homo sapiens GN=AVEN PE=1 SV=1
MQAERGARGGRGRRPGRGRPGGDRHSERPGAAAAVARGGGGGGGGDGGGRRGRGRGRGFR
GARGGRGGGGAPRGSRREPGGWGAGASAPVEDDSDAETYGEENDEQGNYSKRKIVSNWDR
YQDIEKEVNNESGESQRGTDFSVLLSSAGDSFSQFRFAEEKEWDSEASCPKQNSAFYVDS
ELLVRALQELPLCLRLNVAAELVQGTVPLEVPQVKPKRTDDGKGLGMQLKGPLGPGGRGP
IFELKSVAAGCPVLLGKDNPSPGPSRDSQKPTSPLQSAGDHLEEELDLLLNLDAPIKEGD
NILPDQTSQDLKSKEDGEVVQEEEVCAKPSVTEEKNMEPEQPSTSKNVTEEELEDWLDSM
IS

>sp|Q8NBF6|AVL9_HUMAN Late secretory pathway protein AVL9 homolog OS=Homo sapiens GN=AVL9 PE=1 SV=1
MEKARRGGDGVPRGPVLHIVVVGFHHKKGCQVEFSYPPLIPGDGHDSHTLPEEWKYLPFL
ALPDGAHNYQEDTVFFHLPPRNGNGATVFGISCYRQIEAKALKVRQADITRETVQKSVCV
LSKLPLYGLLQAKLQLITHAYFEEKDFSQISILKELYEHMNSSLGGASLEGSQVYLGLSP
RDLVLHFRHKVLILFKLILLEKKVLFYISPVNKLVGALMTVLSLFPGMIEHGLSDCSQYR
PRKSMSEDGGLQESNPCADDFVSASTADVSHTNLGTIRKVMAGNHGEDAAMKTEEPLFQV
EDSSKGQEPNDTNQYLKPPSRPSPDSSESDWETLDPSVLEDPNLKEREQLGSDQTNLFPK
DSVPSESLPITVQPQANTGQVVLIPGLISGLEEDQYGMPLAIFTKGYLCLPYMALQQHHL
LSDVTVRGFVAGATNILFRQQKHLSDAIVEVEEALIQIHDPELRKLLNPTTADLRFADYL
VRHVTENRDDVFLDGTGWEGGDEWIRAQFAVYIHALLAATLQLDNEKILSDYGTTFVTAW
KNTHNYRVWNSNKHPALAEINPNHPFQGQYSVSDMKLRFSHSVQNSERGKKIGNVMVTTS
RNVVQTGKAVGQSVGGAFSSAKTAMSSWLSTFTTSTSQSLTEPPDEKP
>sp|Q9Y2C3|B3GT5_HUMAN Beta-1,3-galactosyltransferase 5 OS=Homo sapiens GN=B3GALT5 PE=2 SV=1
MAFPKMRLMYICLLVLGALCLYFSMYSLNPFKEQSFVYKKDGNFLKLPDTDCRQTPPFLV
LLVTSSHKQLAERMAIRQTWGKERMVKGKQLKTFFLLGTTSSAAETKEVDQESQRHGDII
QKDFLDVYYNLTLKTMMGIEWVHRFCPQAAFVMKTDSDMFINVDYLTELLLKKNRTTRFF
TGFLKLNEFPIRQPFSKWFVSKSEYPWDRYPPFCSGTGYVFSGDVASQVYNVSKSVPYIK
LEDVFVGLCLERLNIRLEELHSQPTFFPGGLRFSVCLFRRIVACHFIKPRTLLDYWQALE
NSRGEDCPPV
>sp|Q96L58|B3GT6_HUMAN Beta-1,3-galactosyltransferase 6 OS=Homo sapiens GN=B3GALT6 PE=1 SV=2
MKLLRRAWRRRAALGLGTLALCGAALLYLARCAAEPGDPRAMSGRSPPPPAPARAAAFLA
VLVASAPRAAERRSVIRSTWLARRGAPGDVWARFAVGTAGLGAEERRALEREQARHGDLL
LLPALRDAYENLTAKVLAMLAWLDEHVAFEFVLKADDDSFARLDALLAELRAREPARRRR
LYWGFFSGRGRVKPGGRWREAAWQLCDYYLPYALGGGYVLSADLVHYLRLSRDYLRAWHS
EDVSLGAWLAPVDVQREHDPRFDTEYRSRGCSNQYLVTHKQSLEDMLEKHATLAREGRLC
KREVQLRLSYVYDWSAPPSQCCQRREGIP
>sp|Q00973|B4GN1_HUMAN Beta-1,4 N-acetylgalactosaminyltransferase 1 OS=Homo sapiens GN=B4GALNT1 PE=1 SV=2
MWLGRRALCALVLLLACASLGLLYASTRDAPGLRLPLAPWAPPQSPRRPELPDLAPEPRY
AHIPVRIKEQVVGLLAWNNCSCESSGGGLPLPFQKQVRAIDLTKAFDPAELRAASATREQ
EFQAFLSRSQSPADQLLIAPANSPLQYPLQGVEVQPLRSILVPGLSLQAASGQEVYQVNL
TASLGTWDVAGEVTGVTLTGEGQADLTLVSPGLDQLNRQLQLVTYSSRSYQTNTADTVRF
STEGHEAAFTIRIRHPPNPRLYPPGSLPQGAQYNISALVTIATKTFLRYDRLRALITSIR
RFYPTVTVVIADDSDKPERVSGPYVEHYLMPFGKGWFAGRNLAVSQVTTKYVLWVDDDFV
FTARTRLERLVDVLERTPLDLVGGAVREISGFATTYRQLLSVEPGAPGLGNCLRQRRGFH
HELVGFPGCVVTDGVVNFFLARTDKVREVGFDPRLSRVAHLEFFLDGLGSLRVGSCSDVV
VDHASKLKLPWTSRDAGAETYARYRYPGSLDESQMAKHRLLFFKHRLQCMTSQ
>sp|O95429|BAG4_HUMAN BAG family molecular chaperone regulator 4 OS=Homo sapiens GN=BAG4 PE=1 SV=1
MSALRRSGYGPSDGPSYGRYYGPGGGDVPVHPPPPLYPLRPEPPQPPISWRVRGGGPAET
TWLGEGGGGDGYYPSGGAWPEPGRAGGSHQEQPPYPSYNSNYWNSTARSRAPYPSTYPVR

PELQGQSLNSYTNGAYGPTYPPGPGANTASYSGAYYAPGYTQTSYSTEVPSTYRSSGNSP
TPVSRWIYPQQDCQTEAPPLRGQVPGYPPSQNPGMTLPHYPYGDGNRSVPQSGPTVRPQE
DAWASPGAYGMGGRYPWPSSAPSAPPGNLYMTESTSPWPSSGSPQSPPSPPVQQPKDSSY
PYSQSDQSMNRHNFPCSVHQYESSGTVNNDDSDLLDSQVQYSAEPQLYGNATSDHPNNQD
QSSSLPEECVPSDESTPPSIKKIIHVLEKVQYLEQEVEEFVGKKTDKAYWLLEEMLTKEL
LELDSVETGGQDSVRQARKEAVCKIQAILEKLEKKGL
>sp|Q13072|BAGE1_HUMAN B melanoma antigen 1 OS=Homo sapiens GN=BAGE PE=2 SV=1
MAARAVFLALSAQLLQARLMKEESPVVSWRLEPEDGTALCFIF
>sp|Q8N9N5|BANP_HUMAN Protein BANP OS=Homo sapiens GN=BANP PE=1 SV=3
MMSEHDLADVVQIAVEDLSPDHPVVLENHVVTDEDEPALKRQRLEINCQDPSIKTICLRL
DSIEAKLQALEATCKSLEEKLDLVTNKQHSPIQVPMVAGSPLGATQTCNKVRCVVPQTTV
ILNNDRQNAIVAKMEDPLSNRAPDSLENVISNAVPGRRQNTIVVKVPGQEDSHHEDGESG
SEASDSVSSCGQAGSQSIGSNVTLITLNSEEDYPNGTWLGDENNPEMRVRCAIIPSDMLH
ISTNCRTAEKMALTLLDYLFHREVQAVSNLSGQGKHGKKQLDPLTIYGIRCHLFYKFGIT
ESDWYRIKQSIDSKCRTAWRRKQRGQSLAVKSFSRRTPNSSSYCPSEPMMSTPPPASELP
QPQPQPQALHYALANAQQVQIHQIGEDGQVQVGHLHIAQVPQGEQVQITQDSEGNLQIHH
VGQDGQLLEATRIPCLLAPSVFKASSGQVLQGAQLIAVASSDPAAAGVDGSPLQGSDIQV
QYVQLAPVSDHTAGAQTAEALQPTLQPEMQLEHGAIQIQ
>sp|Q92560|BAP1_HUMAN Ubiquitin carboxyl-terminal hydrolase BAP1 OS=Homo sapiens GN=BAP1 PE=1 SV=2
MNKGWLELESDPGLFTLLVEDFGVKGVQVEEIYDLQSKCQGPVYGFIFLFKWIEERRSRR
KVSTLVDDTSVIDDDIVNNMFFAHQLIPNSCATHALLSVLLNCSSVDLGPTLSRMKDFTK
GFSPESKGYAIGNAPELAKAHNSHARPEPRHLPEKQNGLSAVRTMEAFHFVSYVPITGRL
FELDGLKVYPIDHGPWGEDEEWTDKARRVIMERIGLATAGEPYHDIRFNLMAVVPDRRIK
YEARLHVLKVNRQTVLEALQQLIRVTQPELIQTHKSQESQLPEESKSASNKSPLVLEANR
APAASEGNHTDGAEEAAGSCAQAPSHSPPNKPKLVVKPPGSSLNGVHPNPTPIVQRLPAF
LDNHNYAKSPMQEEEDLAAGVGRSRVPVRPPQQYSDDEDDYEDDEEDDVQNTNSALRYKG
KGTGKPGALSGSADGQLSVLQPNTINVLAEKLKESQKDLSIPLSIKTSSGAGSPAVAVPT
HSQPSPTPSNESTDTASEIGSAFNSPLRSPIRSANPTRPSSPVTSHISKVLFGEDDSLLR
VDCIRYNRAVRDLGPVISTGLLHLAEDGVLSPLALTEGGKGSSPSIRPIQGSQGSSSPVE
KEVVEATDSREKTGMVRPGEPLSGEKYSPKELLALLKCVEAEIANYEACLKEEVEKRKKF
KIDDQRRTHNYDEFICTFISMLAQEGMLANLVEQNISVRRRQGVSIGRLHKQRKPDRRKR
SRPYKAKRQ
>sp|P51572|BAP31_HUMAN B-cell receptor-associated protein 31 OS=Homo sapiens GN=BCAP31 PE=1 SV=3
MSLQWTAVATFLYAEVFVVLLLCIPFISPKRWQKIFKSRLVELLVSYGNTFFVVLIVILV
LLVIDAVREIRKYDDVTEKVNLQNNPGAMEHFHMKLFRAQRNLYIAGFSLLLSFLLRRLV
TLISQQATLLASNEAFKKQAESASEAAKKYMEENDQLKKGAAVDGGKLDVGNAEVKLEEE
NRSLKADLQKLKDELASTKQKLEKAENQVLAMRKQSEGLTKEYDRLLEEHAKLQAAVDGP
MDKKEE
>sp|Q16520|BATF_HUMAN Basic leucine zipper transcriptional factor ATF-like OS=Homo sapiens GN=BATF PE=1 SV=1
MPHSSDSSDSSFSRSPPPGKQDSSDDVRRVQRREKNRIAAQKSRQRQTQKADTLHLESED
LEKQNAALRKEIKQLTEELKYFTSVLNSHEPLCSVLAASTPSPPEVVYSAHAFHQPHVSS

PRFQP
>sp|Q9UIG0|BAZ1B_HUMAN Tyrosine-protein kinase BAZ1B OS=Homo sapiens GN=BAZ1B PE=1 SV=2
MAPLLGRKPFPLVKPLPGEEPLFTIPHTQEAFRTREEYEARLERYSERIWTCKSTGSSQL
THKEAWEEEQEVAELLKEEFPAWYEKLVLEMVHHNTASLEKLVDTAWLEIMTKYAVGEEC
DFEVGKEKMLKVKIVKIHPLEKVDEEATEKKSDGACDSPSSDKENSSQIAQDHQKKETVV
KEDEGRRESINDRARRSPRKLPTSLKKGERKWAPPKFLPHKYDVKLQNEDKIISNVPADS
LIRTERPPNKEIVRYFIRHNALRAGTGENAPWVVEDELVKKYSLPSKFSDFLLDPYKYMT
LNPSTKRKNTGSPDRKPSKKSKTDNSSLSSPLNPKLWCHVHLKKSLSGSPLKVKNSKNSK
SPEEHLEEMMKMMSPNKLHTNFHIPKKGPPAKKPGKHSDKPLKAKGRSKGILNGQKSTGN
SKSPKKGLKTPKTKMKQMTLLDMAKGTQKMTRAPRNSGGTPRTSSKPHKHLPPAALHLIA
YYKENKDREDKRSALSCVISKTARLLSSEDRARLPEELRSLVQKRYELLEHKKRWASMSE
EQRKEYLKKKREELKKKLKEKAKERREKEMLERLEKQKRYEDQELTGKNLPAFRLVDTPE
GLPNTLFGDVAMVVEFLSCYSGLLLPDAQYPITAVSLMEALSADKGGFLYLNRVLVILLQ
TLLQDEIAEDYGELGMKLSEIPLTLHSVSELVRLCLRRSDVQEESEGSDTDDNKDSAAFE
DNEVQDEFLEKLETSEFFELTSEEKLQILTALCHRILMTYSVQDHMETRQQMSAELWKER
LAVLKEENDKKRAEKQKRKEMEAKNKENGKVENGLGKTDRKKEIVKFEPQVDTEAEDMIS
AVKSRRLLAIQAKKEREIQEREMKVKLERQAEEERIRKHKAAAEKAFQEGIAKAKLVMRR
TPIGTDRNHNRYWLFSDEVPGLFIEKGWVHDSIDYRFNHHCKDHTVSGDEDYCPRSKKAN
LGKNASMNTQHGTATEVAVETTTPKQGQNLWFLCDSQKELDELLNCLHPQGIRESQLKER
LEKRYQDIIHSIHLARKPNLGLKSCDGNQELLNFLRSDLIEVATRLQKGGLGYVEETSEF
EARVISLEKLKDFGECVIALQASVIKKFLQGFMAPKQKRRKLQSEDSAKTEEVDEEKKMV
EEAKVASALEKWKTAIREAQTFSRMHVLLGMLDACIKWDMSAENARCKVCRKKGEDDKLI
LCDECNKAFHLFCLRPALYEVPDGEWQCPACQPATARRNSRGRNYTEESASEDSEDDESD
EEEEEEEEEEEEDYEVAGLRLRPRKTIRGKHSVIPPAARSGRRPGKKPHSTRRSQPKAP
PVDDAEVDELVLQTKRSSRRQSLELQKCEEILHKIVKYRFSWPFREPVTRDEAEDYYDVI
THPMDFQTVQNKCSCGSYRSVQEFLTDMKQVFTNAEVYNCRGSHVLSCMVKTEQCLVALL
HKHLPGHPYVRRKRKKFPDRLAEDEGDSEPEAVGQSRGRRQKK
>sp|Q9BXH1|BBC3_HUMAN Bcl-2-binding component 3 OS=Homo sapiens GN=BBC3 PE=1 SV=1
MARARQEGSSPEPVEGLARDGPRPFPLGRLVPSAVSCGLCEPGLAAAPAAPTLLPAAYLC
APTAPPAVTAALGGSRWPGGPRSRPRGPRPDGPQPSLSLAEQHLESPVPSAPGALAGGPT
QAAPGVRGEEEQWAREIGAQLRRMADDLNAQYERRRQEEQQRHRPSPWRVLYNLIMGLLP
LPRGHRAPEMEPN
>sp|Q6ZW61|BBS12_HUMAN Bardet-Biedl syndrome 12 protein OS=Homo sapiens GN=BBS12 PE=1 SV=2
MVMACRVVNKRRHMGLQQLSSFAETGRTFLGPLKSSKFIIDEECHESVLISSTVRLLESL
DLTSAVGQLLNEAVQAQNNTYRTGISTLLFLVGAWSSAVEECLHLGVPISIIVSVMSEGL
NFCSEEVVSLHVPVHNIFDCMDSTKTFSQLETFSVSLCPFLQVPSDTDLIEELHGLKDVA
SQTLTISNLSGRPLKSYELFKPQTKVEADNNTSRTLKNSLLADTCCRQSILIHSRHFNRT
DNTEGVSKPDGFQEHVTATHKTYRCNDLVELAVGLSHGDHSSMKLVEEAVQLQYQNACVQ
QGNCTKPFMFDISRIFTCCLPGLPETSSCVCPGYITVVSVSNNPVIKELQNQPVRIVLIE
GDLTENYRHLGFNKSANIKTVLDSMRLQEDSSEELWANHVLQVLIQFKVNLVLVQGNVSE
RLIEKCINSKRLVIGSVNGSVMQAFAEAAGAVQVAYITQVNEDCVGDGVCVTFWRSSPLD
VVDRNNRIAILLKTEGINLVTAVLTNPVTAQMQIKEDRFWTCAYRLYYALKEEKVFLGGG
AVEFLCLSCLHILAEQSLKKENHACSGWLHNTSSWLASSLAIYRPTVLKFLANGWQKYLS

TLLYNTANYSSEFEASTYIQHHLQNATDSGSPSSYILNEYSKLNSRIFNSDISNKLEQIP
RVYDVVTPKIEAWRRALDLVLLVLQTDSEIITGHGHTQINSQELTGFLFL
>sp|Q96RK4|BBS4_HUMAN Bardet-Biedl syndrome 4 protein OS=Homo sapiens GN=BBS4 PE=1 SV=2
MAEERVATRTQFPVSTESQKPRQKKAPEFPILEKQNWLIHLHYIRKDYEACKAVIKEQLQ
ETQGLCEYAIYVQALIFRLEGNIQESLELFQTCAVLSPQSADNLKQVARSLFLLGKHKAA
IEVYNEAAKLNQKDWEISHNLGVCYIYLKQFNKAQDQLHNALNLNRHDLTYIMLGKIHLL
EGDLDKAIEVYKKAVEFSPENTELLTTLGLLYLQLGIYQKAFEHLGNALTYDPTNYKAIL
AAGSMMQTHGDFDVALTKYRVVACAVPESPPLWNNIGMCFFGKKKYVAAISCLKRANYLA
PFDWKILYNLGLVHLTMQQYASAFHFLSAAINFQPKMGELYMLLAVALTNLEDIENAKRA
YAEAVHLDKCNPLVNLNYAVLLYNQGEKKNALAQYQEMEKKVSLLKDNSSLEFDSEMVEM
AQKLGAALQVGEALVWTKPVKDPKSKHQTTSTSKPASFQQPLGSNQALGQAMSSAAAYRT
LPSGAGGTSQFTKPPSLPLEPEPAVESSPTETSEQIREK
>sp|Q9H165|BC11A_HUMAN B-cell lymphoma/leukemia 11A OS=Homo sapiens GN=BCL11A PE=1 SV=2
MSRRKQGKPQHLSKREFSPEPLEAILTDDEPDHGPLGAPEGDHDLLTCGQCQMNFPLGDI
LIFIEHKRKQCNGSLCLEKAVDKPPSPSPIEMKKASNPVEVGIQVTPEDDDCLSTSSRGI
CPKQEHIADKLLHWRGLSSPRSAHGALIPTPGMSAEYAPQGICKDEPSSYTCTTCKQPFT
SAWFLLQHAQNTHGLRIYLESEHGSPLTPRVGIPSGLGAECPSQPPLHGIHIADNNPFNL
LRIPGSVSREASGLAEGRFPPTPPLFSPPPRHHLDPHRIERLGAEEMALATHHPSAFDRV
LRLNPMAMEPPAMDFSRRLRELAGNTSSPPLSPGRPSPMQRLLQPFQPGSKPPFLATPPL
PPLQSAPPPSQPPVKSKSCEFCGKTFKFQSNLVVHRRSHTGEKPYKCNLCDHACTQASKL
KRHMKTHMHKSSPMTVKSDDGLSTASSPEPGTSDLVGSASSALKSVVAKFKSENDPNLIP
ENGDEEEEEDDEEEEEEEEEEEEELTESERVDYGFGLSLEAARHHENSSRGAVVGVGDES
RALPDVMQGMVLSSMQHFSEAFHQVLGEKHKRGHLAEAEGHRDTCDEDSVAGESDRIDDG
TVNGRGCSPGESASGGLSKKLLLGSPSSLSPFSKRIKLEKEFDLPPAAMPNTENVYSQWL
AGYAASRQLKDPFLSFGDSRQSPFASSSEHSSENGSLRFSTPPGELDGGISGRSGTGSGG
STPHISGPGPGRPSSKEGRRSDTCEYCGKVFKNCSNLTVHRRSHTGERPYKCELCNYACA
QSSKLTRHMKTHGQVGKDVYKCEICKMPFSVYSTLEKHMKKWHSDRVLNNDIKTE
>sp|Q9BYV7|BCDO2_HUMAN Beta,beta-carotene 9',10'-oxygenase OS=Homo sapiens GN=BCO2 PE=1 SV=5
MFFRVFLHFIRSHSATAVDFLPVMVHRLPVFKRYMGNTPQKKAVFGQCRGLPCVAPLLTT
VEEAPRGISARVWGHFPKWLNGSLLRIGPGKFEFGKDKYNHWFDGMALLHQFRMAKGTVT
YRSKFLQSDTYKANSAKNRIVISEFGTLALPDPCKNVFERFMSRFELPGKAAAMTDNTNV
NYVRYKGDYYLCTETNFMNKVDIETLEKTEKVDWSKFIAVNGATAHPHYDLDGTAYNMGN
SFGPYGFSYKVIRVPPEKVDLGETIHGVQVICSIASTEKGKPSYYHSFGMTRNYIIFIEQ
PLKMNLWKIATSKIRGKAFSDGISWEPQCNTRFHVVEKRTGQLLPGRYYSKPFVTFHQIN
AFEDQGCVIIDLCCQDNGRTLEVYQLQNLRKAGEGLDQVHNSAAKSFPRRFVLPLNVSLN
APEGDNLSPLSYTSASAVKQADGTIWCSHENLHQEDLEKEGGIEFPQIYYDRFSGKKYHF
FYGCGFRHLVGDSLIKVDVVNKTLKVWREDGFYPSEPVFVPAPGTNEEDGGVILSVVITP
NQNESNFILVLDAKNFEELGRAEVPVQMPYGFHGTFIPI
>sp|Q8N888|BCOR2_HUMAN Putative BCoR-like protein 2 OS=Homo sapiens GN=BCORP1 PE=5 SV=1
MKEKLSKKRAEVKGNRSWLEEFLKPSDNEEGPPPKNKVLSNNASSQKPTHSSCIPLLRLP
DKQQKVNESIKTDMLCTDKEEECPAASLLQKYTNNSEKPSGKRQCKTKHLISQDLRQGFL
LTGKCYVENADGKIPLGTCFLLGLI
>sp|Q6W2J9|BCOR_HUMAN BCL-6 corepressor OS=Homo sapiens GN=BCOR PE=1 SV=1

MLSATPLYGNVHSWMNSERVRMCGASEDRKILVNDGDASKARLELREENPLNHNVVDAST
AHRIDGLAALSMDRTGLIREGLRVPGNIVYSSLCGLGSEKGREAATSTLGGLGFSSERNP
EMQFKPNTPETVEASAVSGKPPNGFSAIYKTPPGIQKSAVATAEALGLDRPASDKQSPLN
INGASYLRLPWVNPYMEGATPAIYPFLDSPNKYSLNMYKALLPQQSYSLAQPLYSPVCTN
GERFLYLPPPHYVGPHIPSSLASPMRLSTPSASPAIPPLVHCADKSLPWKMGVSPGNPVD
SHAYPHIQNSKQPRVPSAKAVTSGLPGDTALLLPPSPRPSPRVHLPTQPAADTYSEFHKH
YARISTSPSVALSKPYMTVSSEFPAARLSNGKYPKAPEGGEGAQPVPGHARKTAVQDRKD
GSSPPLLEKQTVTKDVTDKPLDLSSKVVDVDASKADHMKKMAPTVLVHSRAGSGLVLSGS
EIPKETLSPPGNGCAIYRSEIISTAPSSWVVPGPSPNEENNGKSMSLKNKALDWAIPQQR
SSSCPRMGGTDAVITNVSGSVSSAGRPASASPAPNANADGTKTSRSSVETTPSVIQHVGQ
PPATPAKHSSSTSSKGAKASNPEPSFKANENGLPPSSIFLSPNEAFRSPPIPYPRSYLPY
PAPEGIAVSPLSLHGKGPVYPHPVLLPNGSLFPGHLAPKPGLPYGLPTGRPEFVTYQDAL
GLGMVHPMLIPHTPIEITKEEKPERRSRSHERARYEDPTLRNRFSEILETSSTKLHPDVP
TDKNLKPNPNWNQGKTVVKSDKLVYVDLLREEPDAKTDTNVSKPSFAAESVGQSAEPPKP
SVEPALQQHRDFIALREELGRISDFHETYTFKQPVFTVSKDSVLAGTNKENLGLPVSTPF
LEPPLGSDGPAVTFGKTQEDPKPFCVGSAPPSVDVTPTYTKDGADEAESNDGKVLKPKPS
KLAKRIANSAGYVGDRFKCVTTELYADSSQLSREQRALQMEGLQEDSILCLPAAYCERAM
MRFSELEMKEREGGHPATKDSEMCKFSPADWERLKGNQDKKPKSVTLEEAIAEQNESERC
EYSVGNKHRDPFEAPEDKDLPVEKYFVERQPVSEPPADQVASDMPHSPTLRVDRKRKVSG
DSSHTETTAEEVPEDPLLKAKRRRVSKDDWPEREMTNSSSNHLEDPHYSELTNLKVCIEL
TGLHPKKQRHLLHLRERWEQQVSAADGKPGRQSRKEVTQATQPEAIPQGTNITEEKPGRK
RAEAKGNRSWSEESLKPSDNEQGLPVFSGSPPMKSLSSTSAGGKKQAQPSCAPASRPPAK
QQKIKENQKTDVLCADEEEDCQAASLLQKYTDNSEKPSGKRLCKTKHLIPQESRRGLPLT
GEYYVENADGKVTVRRFRKRPEPSSDYDLSPAKQEPKPFDRLQQLLPASQSTQLPCSSSP
QETTQSRPMPPEARRLIVNKNAGETLLQRAARLGYEEVVLYCLENKICDVNHRDNAGYCA
LHEACARGWLNIVRHLLEYGADVNCSAQDGTRPLHDAVENDHLEIVRLLLSYGADPTLAT
YSGRTIMKMTHSELMEKFLTDYLNDLQGRNDDDASGTWDFYGSSVCEPDDESGYDVLANP
PGPEDQDDDDDAYSDVFEFEFSETPLLPCYNIQVSVAQGPRNWLLLSDVLKKLKMSSRIF
RCNFPNVEIVTIAEAEFYRQVSASLLFSCSKDLEAFNPESKELLDLVEFTNEIQTLLGSS
VEWLHPSDLASDNYW
>sp|Q02338|BDH_HUMAN D-beta-hydroxybutyrate dehydrogenase, mitochondrial OS=Homo sapiens GN=BDH1 PE=1 SV=3
MLATRLSRPLSRLPGKTLSACDRENGARRPLLLGSTSFIPIGRRTYASAAEPVGSKAVLV
TGCDSGFGFSLAKHLHSKGFLVFAGCLMKDKGHDGVKELDSLNSDRLRTVQLNVCSSEEV
EKVVEIVRSSLKDPEKGMWGLVNNAGISTFGEVEFTSLETYKQVAEVNLWGTVRMTKSFL
PLIRRAKGRVVNISSMLGRMANPARSPYCITKFGVEAFSDCLRYEMYPLGVKVSVVEPGN
FIAATSLYSPESIQAIAKKMWEELPEVVRKDYGKKYFDEKIAKMETYCSSGSTDTSPVID
AVTHALTATTPYTRYHPMDYYWWLRMQIMTHLPGAISDMIYIR
>sp|Q7Z3C6|ATG9A_HUMAN Autophagy-related protein 9A OS=Homo sapiens GN=ATG9A PE=1 SV=3
MAQFDTEYQRLEASYSDSPPGEEDLLVHVAEGSKSPWHHIENLDLFFSRVYNLHQKNGFT
CMLIGEIFELMQFLFVVAFTTFLVSCVDYDILFANKMVNHSLHPTEPVKVTLPDAFLPAQ
VCSARIQENGSLITILVIAGVFWIHRLIKFIYNICCYWEIHSFYLHALRIPMSALPYCTW
QEVQARIVQTQKEHQICIHKRELTELDIYHRILRFQNYMVALVNKSLLPLRFRLPGLGEA
VFFTRGLKYNFELILFWGPGSLFLNEWSLKAEYKRGGQRLELAQRLSNRILWIGIANFLL

CPLILIWQILYAFFSYAEVLKREPGALGARCWSLYGRCYLRHFNELEHELQSRLNRGYKP
ASKYMNCFLSPLLTLLAKNGAFFAGSILAVLIALTIYDEDVLAVEHVLTTVTLLGVTVTV
CRSFIPDQHMVFCPEQLLRVILAHIHYMPDHWQGNAHRSQTRDEFAQLFQYKAVFILEEL
LSPIVTPLILIFCLRPRALEIIDFFRNFTVEVVGVGDTCSFAQMDVRQHGHPQWLSAGQT
EASVYQQAEDGKTELSLMHFAITNPGWQPPRESTAFLGFLKEQVQRDGAAASLAQGGLLP
ENALFTSIQSLQSESEPLSLIANVVAGSSCRGPPLPRDLQGSRHRAEVASALRSFSPLQP
GQAPTGRAHSTMTGSGVDARTASSGSSVWEGQLQSLVLSEYASTEMSLHALYMHQLHKQQ
AQAEPERHVWHRRESDESGESAPDEGGEGARAPQSIPRSASYPCAAPRPGAPETTALHGG
FQRRYGGITDPGTVPRVPSHFSRLPLGGWAEDGQSASRHPEPVPEEGSEDELPPQVHKV
>sp|Q8N6G6|ATL1_HUMAN ADAMTS-like protein 1 OS=Homo sapiens GN=ADAMTSL1 PE=1 SV=4
MECCRRATPGTLLLFLAFLLLSSRTARSEEDRDGLWDAWGPWSECSRTCGGGASYSLRRC
LSSKSCEGRNIRYRTCSNVDCPPEAGDFRAQQCSAHNDVKHHGQFYEWLPVSNDPDNPCS
LKCQAKGTTLVVELAPKVLDGTRCYTESLDMCISGLCQIVGCDHQLGSTVKEDNCGVCNG
DGSTCRLVRGQYKSQLSATKSDDTVVAIPYGSRHIRLVLKGPDHLYLETKTLQGTKGENS
LSSTGTFLVDNSSVDFQKFPDKEILRMAGPLTADFIVKIRNSGSADSTVQFIFYQPIIHR
WRETDFFPCSATCGGGYQLTSAECYDLRSNRVVADQYCHYYPENIKPKPKLQECNLDPCP
ASDGYKQIMPYDLYHPLPRWEATPWTACSSSCGGGIQSRAVSCVEEDIQGHVTSVEEWKC
MYTPKMPIAQPCNIFDCPKWLAQEWSPCTVTCGQGLRYRVVLCIDHRGMHTGGCSPKTKP
HIKEECIVPTPCYKPKEKLPVEAKLPWFKQAQELEEGAAVSEEPSFIPEAWSACTVTCGV
GTQVRIVRCQVLLSFSQSVADLPIDECEGPKPASQRACYAGPCSGEIPEFNPDETDGLFG
GLQDFDELYDWEYEGFTKCSESCGGGVQEAVVSCLNKQTREPAEENLCVTSRRPPQLLKS
CNLDPCPARWEIGKWSPCSLTCGVGLQTRDVFCSHLLSREMNETVILADELCRQPKPSTV
QACNRFNCPPAWYPAQWQPCSRTCGGGVQKREVLCKQRMADGSFLELPETFCSASKPACQ
QACKKDDCPSEWLLSDWTECSTSCGEGTQTRSAICRKMLKTGLSTVVNSTLCPPLPFSSS
IRPCMLATCARPGRPSTKHSPHIAAARKVYIQTRRQRKLHFVVGGFAYLLPKTAVVLRCP
ARRVRKPLITWEKDGQHLISSTHVTVAPFGYLKIHRLKPSDAGVYTCSAGPAREHFVIKL
IGGNRKLVARPLSPRSEEEVLAGRKGGPKEALQTHKHQNGIFSNGSKAEKRGLAANPGSR
YDDLVSRLLEQGGWPGELLASWEAQDSAERNTTSEEDPGAEQVLLHLPFTMVTEQRRLDD
ILGNLSQQPEELRDLYSKHLVAQLAQEIFRSHLEHQDTLLKPSERRTSPVTLSPHKHVSG
FSSSLRTSSTGDAGGGSRRPHRKPTILRKISAAQQLSASEVVTHLGQTVALASGTLSVLL
HCEAIGHPRPTISWARNGEEVQFSDRILLQPDDSLQILAPVEADVGFYTCNATNALGYDS
VSIAVTLAGKPLVKTSRMTVINTEKPAVTVDIGSTIKTVQGVNVTINCQVAGVPEAEVTW
FRNKSKLGSPHHLHEGSLLLTNVSSSDQGLYSCRAANLHGELTESTQLLILDPPQVPTQL
EDIRALLAATGPNLPSVLTSPLGTQLVLDPGNSALLGCPIKGHPVPNITWFHGGQPIVTA
TGLTHHILAAGQILQVANLSGGSQGEFSCLAQNEAGVLMQKASLVIQDYWWSVDRLATCS
ASCGNRGVQQPRLRCLLNSTEVNPAHCAGKVRPAVQPIACNRRDCPSRWMVTSWSACTRS
CGGGVQTRRVTCQKLKASGISTPVSNDMCTQVAKRPVDTQACNQQLCVEWAFSSWGQCNG
PCIGPHLAVQHRQVFCQTRDGITLPSEQCSALPRPVSTQNCWSEACSVHWRVSLWTLCTA
TCGNYGFQSRRVECVHARTNKAVPEHLCSWGPRPANWQRCNITPCENMECRDTTRYCEKV
KQLKLCQLSQFKSRCCGTCGKA
>sp|Q6ZMM2|ATL5_HUMAN ADAMTS-like protein 5 OS=Homo sapiens GN=ADAMTSL5 PE=1 SV=3
MGKLRPGRVEWLASGHTERPHLFQNLLLFLWALLNCGLGVSAQGPGEWTPWVSWTRCSSS
CGRGVSVRSRRCLRLPGEEPCWGDSHEYRLCQLPDCPPGAVPFRDLQCALYNGRPVLGTQ
KTYQWVPFHGAPNQCDLNCLAEGHAFYHSFGRVLDGTACSPGAQGVCVAGRCLSAGCDGL

LGSGALEDRCGRCGGANDSCLFVQRVFRDAGAFAGYWNVTLIPEGARHIRVEHRSRNHLA
LMGGDGRYVLNGHWVVSPPGTYEAAGTHVVYTRDTGPQETLQAAGPTSHDLLLQVLLQEP
NPGIEFEFWLPRERYSPFQARVQALGWPLRQPQPRGVEPQPPAAPAVTPAQTPTLAPDPC
PPCPDTRGRAHRLLHYCGSDFVFQARVLGHHHQAQETRYEVRIQLVYKNRSPLRAREYVW
APGHCPCPMLAPHRDYLMAVQRLVSPDGTQDQLLLPHAGYARPWSPAEDSRIRLTARRCP
G
>sp|Q96SQ7|ATOH8_HUMAN Protein atonal homolog 8 OS=Homo sapiens GN=ATOH8 PE=2 SV=2
MKHIPVLEDGPWKTVCVKELNGLKKLKRKGKEPARRANGYKTFRLDLEAPEPRAVATNGL
RDRTHRLQPVPVPVPVPVPVAPAVPPRGGTDTAGERGGSRAPEVSDARKRCFALGAVGPG
LPTPPPPPPPAPQSQAPGGPEAQPFREPGLRPRILLCAPPARPAPSAPPAPPAPPESTVR
PAPPTRPGESSYSSISHVIYNNHQDSSASPRKRPGEATAASSEIKALQQTRRLLANARER
TRVHTISAAFEALRKQVPCYSYGQKLSKLAILRIACNYILSLARLADLDYSADHSNLSFS
ECVQRCTRTLQAEGRAKKRKE
>sp|P20648|ATP4A_HUMAN Potassium-transporting ATPase alpha chain 1 OS=Homo sapiens GN=ATP4A PE=2 SV=5
MGKAENYELYSVELGPGPGGDMAAKMSKKKKAGGGGGKRKEKLENMKKEMEINDHQLSVA
ELEQKYQTSATKGLSASLAAELLLRDGPNALRPPRGTPEYVKFARQLAGGLQCLMWVAAA
ICLIAFAIQASEGDLTTDDNLYLAIALIAVVVVTGCFGYYQEFKSTNIIASFKNLVPQQA
TVIRDGDKFQINADQLVVGDLVEMKGGDRVPADIRILAAQGCKVDNSSLTGESEPQTRSP
ECTHESPLETRNIAFFSTMCLEGTVQGLVVNTGDRTIIGRIASLASGVENEKTPIAIEIE
HFVDIIAGLAILFGATFFIVAMCIGYTFLRAMVFFMAIVVAYVPEGLLATVTVCLSLTAK
RLASKNCVVKNLEAVETLGSTSVICSDKTGTLTQNRMTVSHLWFDNHIHTADTTEDQSGQ
TFDQSSETWRALCRVLTLCNRAAFKSGQDAVPVPKRIVIGDASETALLKFSELTLGNAMG
YRDRFPKVCEIPFNSTNKFQLSIHTLEDPRDPRHLLVMKGAPERVLERCSSILIKGQELP
LDEQWREAFQTAYLSLGGLGERVLGFCQLYLNEKDYPPGYAFDVEAMNFPSSGLCFAGLV
SMIDPPRATVPDAVLKCRTAGIRVIMVTGDHPITAKAIAASVGIISEGSETVEDIAARLR
VPVDQVNRKDARACVINGMQLKDMDPSELVEALRTHPEMVFARTSPQQKLVIVESCQRLG
AIVAVTGDGVNDSPALKKADIGVAMGIAGSDAAKNAADMILLDDNFASIVTGVEQGRLIF
DNLKKSIAYTLTKNIPELTPYLIYITVSVPLPLGCITILFIELCTDIFPSVSLAYEKAES
DIMHLRPRNPKRDRLVNEPLAAYSYFQIGAIQSFAGFTDYFTAMAQEGWFPLLCVGLRAQ
WEDHHLQDLQDSYGQEWTFGQRLYQQYTCYTVFFISIEVCQIADVLIRKTRRLSAFQQGF
FRNKILVIAIVFQVCIGCFLCYCPGMPNIFNFMPIRFQWWLVPLPYGILIFVYDEIRKLG
VRCCPGSWWDQELYY
>sp|P56381|ATP5E_HUMAN ATP synthase subunit epsilon, mitochondrial OS=Homo sapiens GN=ATP5E PE=1 SV=2
MVAYWRQAGLSYIRYSQICAKAVRDALKTEFKANAEKTSGSNVKIVKVKKE
>sp|Q6E213|AWAT2_HUMAN Acyl-CoA wax alcohol acyltransferase 2 OS=Homo sapiens GN=AWAT2 PE=1 SV=1
MLLPSKKDLKTALDVFAVFQWSFSALLITTTVIAVNLYLVVFTPYWPVTVLILTWLAFDW
KTPQRGGRRFTCVRHWRLWKHYSDYFPLKLLKTHDICPSRNYILVCHPHGLFAHGWFGHF
ATEASGFSKIFPGITPYILTLGAFFWMPFLREYVMSTGACSVSRSSIDFLLTHKGTGNMV
IVVIGGLAECRYSLPGSSTLVLKNRSGFVRMALQHGVPLIPAYAFGETDLYDQHIFTPGG
FVNRFQKWFQSMVHIYPCAFYGRGFTKNSWGLLPYSRPVTTIVGEPLPMPKIENPSQEIV
AKYHTLYIDALRKLFDQHKTKFGISETQELEII

>sp|Q9Y2T1|AXIN2_HUMAN Axin-2 OS=Homo sapiens GN=AXIN2 PE=1 SV=1
MSSAMLVTCLPDPSSSFREDAPRPPVPGEEGETPPCQPGVGKGQVTKPMSVSSNTRRNED
GLGEPEGRASPDSPLTRWTKSLHSLLGDQDGAYLFRTFLEREKCVDTLDFWFACNGFRQM
NLKDTKTLRVAKAIYKRYIENNSIVSKQLKPATKTYIRDGIKKQQIDSIMFDQAQTEIQS
VMEENAYQMFLTSDIYLEYVRSGGENTAYMSNGGLGSLKVVCGYLPTLNEEEEWTCADFK
CKLSPTVVGLSSKTLRATASVRSTETVDSGYRSFKRSDPVNPYHIGSGYVFAPATSANDS
EISSDALTDDSMSMTDSSVDGIPPYRVGSKKQLQREMHRSVKANGQVSLPHFPRTHRLPK
EMTPVEPATFAAELISRLEKLKLELESRHSLEERLQQIREDEEREGSELTLNSREGAPTQ
HPLSLLPSGSYEEDPQTILDDHLSRVLKTPGCQSPGVGRYSPRSRSPDHHHHHHSQYHSL
LPPGGKLPPAAASPGACPLLGGKGFVTKQTTKHVHHHYIHHHAVPKTKEEIEAEATQRVH
CFCPGGSEYYCYSKCKSHSKAPETMPSEQFGGSRGSTLPKRNGKGTEPGLALPAREGGAP
GGAGALQLPREEGDRSQDVWQWMLESERQSKPKPHSAQSTKKAYPLESARSSPGERASRH
HLWGGNSGHPRTTPRAHLFTQDPAMPPLTPPNTLAQLEEACRRLAEVSKPPKQRCCVASQ
QRDRNHSATVQTGATPFSNPSLAPEDHKEPKKLAGVHALQASELVVTYFFCGEEIPYRRM
LKAQSLTLGHFKEQLSKKGNYRYYFKKASDEFACGAVFEEIWEDETVLPMYEGRILGKVE
RID
>sp|Q96BY7|ATG2B_HUMAN Autophagy-related protein 2 homolog B OS=Homo sapiens GN=ATG2B PE=1 SV=5
MPWPFSESIKKRACRYLLQRYLGHFLQEKLSLEQLSLDLYQGTGSLAQVPLDKWCLNEIL
ESADAPLEVTEGFIQSISLSVPWGSLLQDNCALEVRGLEMVFRPRPRPATGSEPMYWSSF
MTSSMQLAKECLSQKLTDEQGEGSQPFEGLEKFAETIETVLRRVKVTFIDTVLRIEHVPE
NSKTGTALEIRIERTVYCDETADESSGINVHQPTAFAHKLLQLSGVSLFWDEFSASAKSS
PVCSTAPVETEPKLSPSWNPKIIYEPHPQLTRNLPEIAPSDPVQIGRLIGRLELSLTLKQ
NEVLPGAKLDVDGQIDSIHLLLSPRQVHLLLDMLAAIAGPENSSKIGLANKDRKNRPMQQ
EDEYRIQMELNRYYLRKDSLSVGVSSEQSFYETETARTPSSREEEVFFSMADMDMSHSLS
SLPPLGDPPNMDLELSLTSTYTNTPAGSPLSATVLQPTWGEFLDHHKEQPVRGSTFPSNL
VHPTPLQKTSLPSRSVSVDESRPELIFRLAVGTFSISVLHIDPLSPPETSQNLNPLTPMA
VAFFTCIEKIDPARFSTEDFKSFRAVFAEACSHDHLRFIGTGIKVSYEQRQRSASRYFST
DMSIGQMEFLECLFPTDFHSVPPHYTELLTFHSKEETGSHSPVCLQLHYKHSENRGPQGN
QARLSSVPHKAELQIKLNPVCCELDISIVDRLNSLLQPQKLATVEMMASHMYTSYNKHIS
LHKAFTEVFLDDSHSPANCRISVQVATPALNLSVRFPIPDLRSDQERGPWFKKSLQKEIL
YLAFTDLEFKTEFIGGSTPEQIKLELTFRELIGSFQEEKGDPSIKFFHVSSGVDGDTTSS
DDFDWPRIVLKINPPAMHSILERIAAEEEEENDGHYQEEEEGGAHSLKDVCDLRRPAPSP
FSSRRVMFENEQMVMPGDPVEMTEFQDKAISNSHYVLELTLPNIYVTLPNKSFYEKLYNR
IFNDLLLWEPTAPSPVETFENISYGIGLSVASQLINTFNKDSFSAFKSAVHYDEESGSEE
ETLQYFSTVDPNYRSRRKKKLDSQNKNSQSFLSVLLNINHGLIAVFTDVKQDNGDLLENK
HGEFWLEFNSGSLFCVTKYEGFDDKHYICLHSSSFSLYHKGIVNGVILPTETRLPSSTRP
HWLEPTIYSSEEDGLSKTSSDGVGGDSLNMLSVAVKILSDKSESNTKEFLIAVGLKGATL
QHRMLPSGLSWHEQILYFLNIADEPVLGYNPPTSFTTFHVHLWSCALDYRPLYLPIRSLL
TVETFSVSSSVALDKSSSTLRIILDEAALHLSDKCNTVTINLSRDYVRVMDMGLLELTIT
AVKSDSDGEQTEPRFELHCSSDVVHIRTCSDSCAALMNLIQYIASYGDLQTPNKADMKPG
AFQRRSKVDSSGRSSSRGPVLPEADQQMLRDLMSDAMEEIDMQQGTSSVKPQANGVLDEK
SQIQEPCCSDLFLFPDESGNVSQESGPTYASFSHHFISDAMTGVPTENDDFCILFAPKAA
MQEKEEEPVIKIMVDDAIVIRDNYFSLPVNKTDTSKAPLHFPIPVIRYVVKEVSLVWHLY

GGKDFGIVPPTSPAKSYISPHSSPSHTPTRHGRNTVCGGKGRNHDFLMEIQLSKVKFQHE
VYPPCKPDCDSSLSEHPVSRQVFIVQDLEIRDRLATSQMNKFLYLYCSKEMPRKAHSNML
TVKALHVCPESGRSPQECCLRVSLMPLRLNIDQDALFFLKDFFTSLSAEVELQMTPDPEV
KKSPGADVTCSLPRHLSTSKEPNLVISFSGPKQPSQNDSANSVEVVNGMEEKNFSAEEAS
FRDQPVFFREFRFTSEVPIRLDYHGKHVSMDQGTLAGILIGLAQLNCSELKLKRLSYRHG
LLGVDKLFSYAITEWLNDIKKNQLPGILGGVGPMHSLVQLVQGLKDLVWLPIEQYRKDGR
IVRGFQRGAASFGTSTAMAALELTNRMVQTIQAAAETAYDMVSPGTLSIEPKKTKRFPHH
RLAHQPVDLREGVAKAYSVVKEGITDTAQTIYETAAREHESRGVTGAVGEVLRQIPPAVV
KPLIVATEATSNVLGGMRNQIRPDVRQDESQKWRHGDD
>sp|Q8WYN0|ATG4A_HUMAN Cysteine protease ATG4A OS=Homo sapiens GN=ATG4A PE=1 SV=1
MESVLSKYEDQITIFTDYLEEYPDTDELVWILGKQHLLKTEKSKLLSDISARLWFTYRRK
FSPIGGTGPSSDAGWGCMLRCGQMMLAQALICRHLGRDWSWEKQKEQPKEYQRILQCFLD
RKDCCYSIHQMAQMGVGEGKSIGEWFGPNTVAQVLKKLALFDEWNSLAVYVSMDNTVVIE
DIKKMCRVLPLSADTAGDRPPDSLTASNQSKGTSAYCSAWKPLLLIVPLRLGINQINPVY
VDAFKECFKMPQSLGALGGKPNNAYYFIGFLGDELIFLDPHTTQTFVDTEENGTVNDQTF
HCLQSPQRMNILNLDPSVALGFFCKEEKDFDNWCSLVQKEILKENLRMFELVQKHPSHWP
PFVPPAKPEVTTTGAEFIDSTEQLEEFDLEEDFEILSV
>sp|Q86TL0|ATG4D_HUMAN Cysteine protease ATG4D OS=Homo sapiens GN=ATG4D PE=1 SV=1
MNSVSPAAAQYRSSSPEDARRRPEARRPRGPRGPDPNGLGPSGASGPALGSPGAGPSEPD
EVDKFKAKFLTAWNNVKYGWVVKSRTSFSKISSIHLCGRRYRFEGEGDIQRFQRDFVSRL
WLTYRRDFPPLPGGCLTSDCGWGCMLRSGQMMLAQGLLLHFLPRDWTWAEGMGLGPPELS
GSASPSRYHGPARWMPPRWAQGAPELEQERRHRQIVSWFADHPRAPFGLHRLVELGQSSG
KKAGDWYGPSLVAHILRKAVESCSDVTRLVVYVSQDCTVYKADVARLVARPDPTAEWKSV
VILVPVRLGGETLNPVYVPCVKELLRCELCLGIMGGKPRHSLYFIGYQDDFLLYLDPHYC
QPTVDVSQADFPLESFHCTSPRKMAFAKMDPSCTVGFYAGDRKEFETLCSELTRVLSSSS
ATERYPMFTLAEGHAQDHSLDDLCSQLAQPTLRLPRTGRLLRAKRPSSEDFVFL
>sp|Q8WXF7|ATLA1_HUMAN Atlastin-1 OS=Homo sapiens GN=ATL1 PE=1 SV=1
MAKNRRDRNSWGGFSEKTYEWSSEEEEPVKKAGPVQVLIVKDDHSFELDETALNRILLSE
AVRDKEVVAVSVAGAFRKGKSFLMDFMLRYMYNQESVDWVGDYNEPLTGFSWRGGSERET
TGIQIWSEIFLINKPDGKKVAVLLMDTQGTFDSQSTLRDSATVFALSTMISSIQVYNLSQ
NVQEDDLQHLQLFTEYGRLAMEETFLKPFQSLIFLVRDWSFPYEFSYGADGGAKFLEKRL
KVSGNQHEELQNVRKHIHSCFTNISCFLLPHPGLKVATNPNFDGKLKEIDDEFIKNLKIL
IPWLLSPESLDIKEINGNKITCRGLVEYFKAYIKIYQGEELPHPKSMLQATAEANNLAAV
ATAKDTYNKKMEEICGGDKPFLAPNDLQTKHLQLKEESVKLFRGVKKMGGEEFSRRYLQQ
LESEIDELYIQYIKHNDSKNIFHAARTPATLFVVIFITYVIAGVTGFIGLDIIASLCNMI
MGLTLITLCTWAYIRYSGEYRELGAVIDQVAAALWDQGSTNEALYKLYSAAATHRHLYHQ
AFPTPKSESTEQSEKKKM
>sp|P54259|ATN1_HUMAN Atrophin-1 OS=Homo sapiens GN=ATN1 PE=1 SV=3
MKTRQNKDSMSMRSGRKKEAPGPREELRSRGRASPGGVSTSSSDGKAEKSRQTAKKARVE
EASTPKVNKQGRSEEISESESEETNAPKKTKTEQELPRPQSPSDLDSLDGRSLNDDGSSD
PRDIDQDNRSTSPSIYSPGSVENDSDSSSGLSQGPARPYHPPPLFPPSPQPPDSTPRQPE
ASFEPHPSVTPTGYHAPMEPPTSRMFQAPPGAPPPHPQLYPGGTGGVLSGPPMGPKGGGA
ASSVGGPNGGKQHPPPTTPISVSSSGASGAPPTKPPTTPVGGGNLPSAPPPANFPHVTPN
LPPPPALRPLNNASASPPGLGAQPLPGHLPSPHAMGQGMGGLPPGPEKGPTLAPSPHSLP

PASSSAPAPPMRFPYSSSSSSSAAASSSSSSSSSSSASPFPASQALPSYPHSFPPPTSLSV
SNQPPKYTQPSLPSQAVWSQGPPPPPPYGRLLANSNAHPGPFPPSTGAQSTAHPPVSTHH
HHHQQQQQQQQQQQQQQQQQQQHHGNSGPPPPGAFPHPLEGGSSHHAHPYAMSPSLGSLR
PYPPGPAHLPPPHSQVSYSQAGPNGPPVSSSSNSSSSTSQGSYPCSHPSPSQGPQGAPYP
FPPVPTVTTSSATLSTVIATVASSPAGYKTASPPGPPPYGKRAPSPGAYKTATPPGYKPG
SPPSFRTGTPPGYRGTSPPAGPGTFKPGSPTVGPGPLPPAGPSGLPSLPPPPAAPASGPP
LSATQIKQEPAEEYETPESPVPPARSPSPPPKVVDVPSHASQSARFNKHLDRGFNSCARS
DLYFVPLEGSKLAKKRADLVEKVRREAEQRAREEKEREREREREKEREREKERELERSVK
LAQEGRAPVECPSLGPVPHRPPFEPGSAVATVPPYLGPDTPALRTLSEYARPHVMSPGNR
NHPFYVPLGAVDPGLLGYNVPALYSSDPAAREREREARERDLRDRLKPGFEVKPSELEPL
HGVPGPGLDPFPRHGGLALQPGPPGLHPFPFHPSLGPLERERLALAAGPALRPDMSYAER
LAAERQHAERVAALGNDPLARLQMLNVTPHHHQHSHIHSHLHLHQQDAIHAASASVHPLI
DPLASGSHLTRIPYPAGTLPNPLLPHPLHENEVLRHQLFAAPYRDLPASLSAPMSAAHQL
QAMHAQSAELQRLALEQQQWLHAHHPLHSVPLPAQEDYYSHLKKESDKPL
>sp|Q6RW13|ATRAP_HUMAN Type-1 angiotensin II receptor-associated protein OS=Homo sapiens GN=AGTRAP PE=1 SV=1
MELPAVNLKVILLGHWLLTTWGCIVFSGSYAWANFTILALGVWAVAQRDSIDAISMFLGG
LLATIFLDIVHISIFYPRVSLTDTGRFGVGMAILSLLLKPLSCCFVYHMYRERGGELLVH
TGFLGSSQDRSAYQTIDSAEAPADPFAVPEGRSQDARGY
>sp|Q9H324|ATS10_HUMAN A disintegrin and metalloproteinase with thrombospondin motifs 10 OS=Homo sapiens GN=ADAMTS10 PE=1 SV=2
MAPACQILRWALALGLGLMFEVTHAFRSQDEFLSSLESYEIAFPTRVDHNGALLAFSPPP
PRRQRRGTGATAESRLFYKVASPSTHFLLNLTRSSRLLAGHVSVEYWTREGLAWQRAARP
HCLYAGHLQGQASTSHVAISTCGGLHGLIVADEEEYLIEPLHGGPKGSRSPEESGPHVVY
KRSSLRHPHLDTACGVRDEKPWKGRPWWLRTLKPPPARPLGNETERGQPGLKRSVSRERY
VETLVVADKMMVAYHGRRDVEQYVLAIMNIVAKLFQDSSLGSTVNILVTRLILLTEDQPT
LEITHHAGKSLDSFCKWQKSIVNHSGHGNAIPENGVANHDTAVLITRYDICIYKNKPCGT
LGLAPVGGMCERERSCSVNEDIGLATAFTIAHEIGHTFGMNHDGVGNSCGARGQDPAKLM
AAHITMKTNPFVWSSCSRDYITSFLDSGLGLCLNNRPPRQDFVYPTVAPGQAYDADEQCR
FQHGVKSRQCKYGEVCSELWCLSKSNRCITNSIPAAEGTLCQTHTIDKGWCYKRVCVPFG
SRPEGVDGAWGPWTPWGDCSRTCGGGVSSSSRHCDSPRPTIGGKYCLGERRRHRSCNTDD
CPPGSQDFREVQCSEFDSIPFRGKFYKWKTYRGGGVKACSLTCLAEGFNFYTERAAAVVD
GTPCRPDTVDICVSGECKHVGCDRVLGSDLREDKCRVCGGDGSACETIEGVFSPASPGAG
YEDVVWIPKGSVHIFIQDLNLSLSHLALKGDQESLLLEGLPGTPQPHRLPLAGTTFQLRQ
GPDQVQSLEALGPINASLIVMVLARTELPALRYRFNAPIARDSLPPYSWHYAPWTKCSAQ
CAGGSQVQAVECRNQLDSSAVAPHYCSAHSKLPKRQRACNTEPCPPDWVVGNWSLCSRSC
DAGVRSRSVVCQRRVSAAEEKALDDSACPQPRPPVLEACHGPTCPPEWAALDWSECTPSC
GPGLRHRVVLCKSADHRATLPPAHCSPAAKPPATMRCNLRRCPPARWVAGEWGECSAQCG
VGQRQRSVRCTSHTGQASHECTEALRPPTTQQCEAKCDSPTPGDGPEECKDVNKVAYCPL
VLKFQFCSRAYFRQMCCKTCHGH
>sp|Q6HA08|ASTL_HUMAN Astacin-like metalloendopeptidase OS=Homo sapiens GN=ASTL PE=1 SV=4
MEGVGGLWPWVLGLLSLPGVILGAPLASSCAGACGTSFPDGLTPEGTQASGDKDIPAINQ
GLILEETPESSFLIEGDIIRPSPFRLLSATSNKWPMGGSGVVEVPFLLSSKYDEPSRQVI
LEALAEFERSTCIRFVTYQDQRDFISIIPMYGCFSSVGRSGGMQVVSLAPTCLQKGRGIV

LHELMHVLGFWHEHTRADRDRYIRVNWNEILPGFEINFIKSQSSNMLTPYDYSSVMHYGR
LAFSRRGLPTITPLWAPSVHIGQRWNLSASDITRVLKLYGCSPSGPRPRGRGSHAHSTGR
SPAPASLSLQRLLEALSAESRSPDPSGSSAGGQPVPAGPGESPHGWESPALKKLSAEASA
RQPQTLASSPRSRPGAGAPGVAQEQSWLAGVSTKPTVPSSEAGIQPVPVQGSPALPGGCV
PRNHFKGMSED
>sp|O75129|ASTN2_HUMAN Astrotactin-2 OS=Homo sapiens GN=ASTN2 PE=1 SV=2
MAAAGARLSPGPGSGLRGRPRLCFHPGPPPLLPLLLLFLLLLPPPPLLAGATAAASREPD
SPCRLKTVTVSTLPALRESDIGWSGARAGAGAGTGAGAAAAAASPGSPGSAGTAAESRLL
LFVRNELPGRIAVQDDLDNTELPFFTLEMSGTAADISLVHWRQQWLENGTLYFHVSMSSS
GQLAQATAPTLQEPSEIVEEQMHILHISVMGGLIALLLLLLVFTVALYAQRRWQKRRRIP
QKSASTEATHEIHYIPSVLLGPQARESFRSSRLQTHNSVIGVPIRETPILDDYDCEEDEE
PPRRANHVSREDEFGSQVTHTLDSLGHPGEEKVDFEKKAAAEATQETVESLMQKFKESFR
ANTPIEIGQLQPPLRSTSAGKRKRRSKSRGGISFGRAKGTSGSEADDETQLTFYTEQYRS
RRRSKGLLKSPVNKTALTLIAVSSCILAMVCGSQMSCPLTVKVTLHVPEHFIADGSSFVV
SEGSYLDISDWLNPAKLSLYYQINATSPWVRDLCGQRTTDACEQLCDPETGECSCHEGYA
PDPVHRHLCVRSDWGQSEGPWPYTTLERGYDLVTGEQAPEKILRSTFSLGQGLWLPVSKS
FVVPPVELSINPLASCKTDVLVTEDPADVREEAMLSTYFETINDLLSSFGPVRDCSRNNG
GCTRNFKCVSDRQVDSSGCVCPEELKPMKDGSGCYDHSKGIDCSDGFNGGCEQLCLQQTL
PLPYDATSSTIFMFCGCVEEYKLAPDGKSCLMLSDVCEGPKCLKPDSKFNDTLFGEMLHG
YNNRTQHVNQGQVFQMTFRENNFIKDFPQLADGLLVIPLPVEEQCRGVLSEPLPDLQLLT
GDIRYDEAMGYPMVQQWRVRSNLYRVKLSTITLAAGFTNVLKILTKESSREELLSFIQHY
GSHYIAEALYGSELTCIIHFPSKKVQQQLWLQYQKETTELGSKKELKSMPFITYLSGLLT
AQMLSDDQLISGVEIRCEEKGRCPSTCHLCRRPGKEQLSPTPVLLEINRVVPLYTLIQDN
GTKEAFKSALMSSYWCSGKGDVIDDWCRCDLSAFDANGLPNCSPLLQPVLRLSPTVEPSS
TVVSLEWVDVQPAIGTKVSDYILQHKKVDEYTDTDLYTGEFLSFADDLLSGLGTSCVAAG
RSHGEVPEVSIYSVIFKCLEPDGLYKFTLYAVDTRGRHSELSTVTLRTACPLVDDNKAEE
IADKIYNLYNGYTSGKEQQMAYNTLMEVSASMLFRVQHHYNSHYEKFGDFVWRSEDELGP
RKAHLILRRLERVSSHCSSLLRSAYIQSRVETVPYLFCRSEEVRPAGMVWYSILKDTKIT
CEEKMVSMARNTYGESKGR
>sp|Q8WWH4|ASZ1_HUMAN Ankyrin repeat, SAM and basic leucine zipper domain-containing protein 1 OS=Homo sapiens GN=ASZ1 PE=2 SV=1
MAASALRGLPVAGGGESSESEDDGWEIGYLDRTSQKLKRLLPIEEKKEKFKKAMTIGDVS
LVQELLDSGISVDSNFQYGWTPLMYAASVANAELVRVLLDRGANASFEKDKQSILITACS
AHGSEEQILKCVELLLSRNADPNVACRRLMTPIMYAARDGHTQVVALLVAHGAEVNTQDE
NGYTALTWAARQGHKNIVLKLLELGANKMLQTKDGKMPSEIAKRNKHHEIFNLLSFTLNP
LEGKLQQLTKEDTICKILTTDSDREKDHIFSSYTAFGDLEVFLHGLGLEHMTDLLKERDI
TLRHLLTMREDEFTKNGITSKDQQKILAALKELQVEEIQFGELSEETKLEISGDEFLNFL
LKLNKQCGHLITAVQNVITELPVNSQKITLEWASPQNFTSVCEELVNNVEDLSEKVCKLK
DLIQKLQNERENDPTHIQLREEVSTWNSRILKRTAITICGFGFLLFICKLTFQRK
>sp|Q9Y2G3|AT11B_HUMAN Probable phospholipid-transporting ATPase IF OS=Homo sapiens GN=ATP11B PE=1 SV=2
MWRWIRQQLGFDPPHQSDTRTIYVANRFPQNGLYTPQKFIDNRIISSKYTVWNFVPKNLF
EQFRRVANFYFLIIFLVQLMIDTPTSPVTSGLPLFFVITVTAIKQGYEDWLRHNSDNEVN
GAPVYVVRSGGLVKTRSKNIRVGDIVRIAKDEIFPADLVLLSSDRLDGSCHVTTASLDGE

TNLKTHVAVPETALLQTVANLDTLVAVIECQQPEADLYRFMGRMIITQQMEEIVRPLGPE
SLLLRGARLKNTKEIFGVAVYTGMETKMALNYKSKSQKRSAVEKSMNTFLIIYLVILISE
AVISTILKYTWQAEEKWDEPWYNQKTEHQRNSSKILRFISDFLAFLVLYNFIIPISLYVT
VEMQKFLGSFFIGWDLDLYHEESDQKAQVNTSDLNEELGQVEYVFTDKTGTLTENEMQFR
ECSINGMKYQEINGRLVPEGPTPDSSEGNLSYLSSLSHLNNLSHLTTSSSFRTSPENETE
LIKEHDLFFKAVSLCHTVQISNVQTDCTGDGPWQSNLAPSQLEYYASSPDEKALVEAAAR
IGIVFIGNSEETMEVKTLGKLERYKLLHILEFDSDRRRMSVIVQAPSGEKLLFAKGAESS
ILPKCIGGEIEKTRIHVDEFALKGLRTLCIAYRKFTSKEYEEIDKRIFEARTALQQREEK
LAAVFQFIEKDLILLGATAVEDRLQDKVRETIEALRMAGIKVWVLTGDKHETAVSVSLSC
GHFHRTMNILELINQKSDSECAEQLRQLARRITEDHVIQHGLVVDGTSLSLALREHEKLF
MEVCRNCSAVLCCRMAPLQKAKVIRLIKISPEKPITLAVGDGANDVSMIQEAHVGIGIMG
KEGRQAARNSDYAIARFKFLSKLLFVHGHFYYIRIATLVQYFFYKNVCFITPQFLYQFYC
LFSQQTLYDSVYLTLYNICFTSLPILIYSLLEQHVDPHVLQNKPTLYRDISKNRLLSIKT
FLYWTILGFSHAFIFFFGSYLLIGKDTSLLGNGQMFGNWTFGTLVFTVMVITVTVKMALE
THFWTWINHLVTWGSIIFYFVFSLFYGGILWPFLGSQNMYFVFIQLLSSGSAWFAIILMV
VTCLFLDIIKKVFDRHLHPTSTEKAQLTETNAGIKCLDSMCCFPEGEAACASVGRMLERV
IGRCSPTHISRSWSASDPFYTNDRSILTLSTMDSSTC
>sp|Q9H7F0|AT133_HUMAN Probable cation-transporting ATPase 13A3 OS=Homo sapiens GN=ATP13A3 PE=1 SV=4
MDREERKTINQGQEDEMEIYGYNLSRWKLAIVSLGVICSGGFLLLLLYWMPEWRVKATCV
RAAIKDCEVVLLRTTDEFKMWFCAKIRVLSLETYPVSSPKSMSNKLSNGHAVCLIENPTE
ENRHRISKYSQTESQQIRYFTHHSVKYFWNDTIHNFDFLKGLDEGVSCTSIYEKHSAGLT
KGMHAYRKLLYGVNEIAVKVPSVFKLLIKEVLNPFYIFQLFSVILWSTDEYYYYALAIVV
MSIVSIVSSLYSIRKQYVMLHDMVATHSTVRVSVCRVNEEIEEIFSTDLVPGDVMVIPLN
GTIMPCDAVLINGTCIVNESMLTGESVPVTKTNLPNPSVDVKGIGDELYNPETHKRHTLF
CGTTVIQTRFYTGELVKAIVVRTGFSTSKGQLVRSILYPKPTDFKLYRDAYLFLLCLVAV
AGIGFIYTIINSILNEVQVGVIIIESLDIITITVPPALPAAMTAGIVYAQRRLKKIGIFC
ISPQRINICGQLNLVCFDKTGTLTEDGLDLWGIQRVENARFLSPEENVCNEMLVKSQFVA
CMATCHSLTKIEGVLSGDPLDLKMFEAIGWILEEATEEETALHNRIMPTVVRPPKQLLPE
STPAGNQEMELFELPATYEIGIVRQFPFSSALQRMSVVARVLGDRKMDAYMKGAPEAIAG
LCKPETVPVDFQNVLEDFTKQGFRVIALAHRKLESKLTWHKVQNISRDAIENNMDFMGLI
IMQNKLKQETPAVLEDLHKANIRTVMVTGDSMLTAVSVARDCGMILPQDKVIIAEALPPK
DGKVAKINWHYADSLTQCSHPSAIDPEAIPVKLVHDSLEDLQMTRYHFAMNGKSFSVILE
HFQDLVPKLMLHGTVFARMAPDQKTQLIEALQNVDYFVGMCGDGANDCGALKRAHGGISL
SELEASVASPFTSKTPSISCVPNLIREGRAALITSFCVFKFMALYSIIQYFSVTLLYSIL
SNLGDFQFLFIDLAIILVVVFTMSLNPAWKELVAQRPPSGLISGALLFSVLSQIIICIGF
QSLGFFWVKQQPWYEVWHPKSDACNTTGSGFWNSSHVDNETELDEHNIQNYENTTVFFIS
SFQYLIVAIAFSKGKPFRQPCYKNYFFVFSVIFLYIFILFIMLYPVASVDQVLQIVCVPY
QWRVTMLIIVLVNAFVSITVEESVDRWGKCCLPWALGCRKKTPKAKYMYLAQELLVDPEW
PPKPQTTTEAKALVKENGSCQIITIT
>sp|Q4VNC1|AT134_HUMAN Probable cation-transporting ATPase 13A4 OS=Homo sapiens GN=ATP13A4 PE=2 SV=3
MGHFEKGQHALLNEGEENEMEIFGYRTQGCRKSLCLAGSIFSFGILPLVFYWRPAWHVWA
HCVPCSLQEADTVLLRTTDEFQIYSWKKVIWIYLSALNSAFGLTPDHPLMTDEEYIINRA

IRKPDLKVRCIKVQKIRYVWNYLEGQFQKIGSLEDWLSSAKIHQKFGSGLTREEQEIRRL
ICGPNTIDVEVTPIWKLLIKEVLNPFYIFQLFSVCLWFSEDYKEYAFAIIIMSIISISLT
VYDLREQSVKLHHLVESHNSITVSVCGRKAGVQELESRVLVPGDLLILTGNKVLMPCDAV
LIEGSCVVDEGMLTGESIPVTKTPLPKMDSSVPWKTQSEADYKRHVLFCGTEVIQAKAAC
SGTVRAVVLQTGFNTAKGDLVRSILYPKPVNFQLYRDAIRFLLCLVGTATIGMIYTLCVY
VLSGEPPEEVVRKALDVITIAVPPALPAALTTGIIYAQRRLKKRGIFCISPQRINVCGQL
NLVCFDKTGTLTRDGLDLWGVVSCDRNGFQEVHSFASGQALPWGPLCAAMASCHSLILLD
GTIQGDPLDLKMFEATTWEMAFSGDDFHIKGVPAHAMVVKPCRTASQVPVEGIAILHQFP
FSSALQRMTVIVQEMGGDRLAFMKGAPERVASFCQPETVPTSFVSELQIYTTQGFRVIAL
AYKKLENDHHATTLTRETVESDLIFLGLLILENRLKEETKPVLEELISARIRTVMITGDN
LQTAITVARKSGMVSESQKVILIEANETTGSSSASISWTLVEEKKHIMYGNQDNYINIRD
EVSDKGREGSYHFALTGKSFHVISQHFSSLLPKILINGTIFARMSPGQKSSLVEEFQKLD
YFVGMCGDGANDCGALKMAHVGISLSEQEASVASPFTSKTPNIECVPHLIKEGRAALVTS
FCMFKYMALYSMIQYVGVLLLYWETNSLSNYQFLFQDLAITTLIGVTMNLNGAYPKLVPF
RPAGRLISPPLLLSVIFNILLSLAMHIAGFILVQRQPWYSVEIHSACTVQNESISELTMS
PTAPEKMESNSTFTSFENTTVWFLGTINCITVALVFSKGKPFRQPTYTNYIFVLVLIIQL
GVCLFILFADIPELYRRLDLLCTPVLWRASIVIMLSLNFIVSLVAEEAVIENRALWMMIK
RCFGYQSKSQYRIWQRDLANDPSWPPLNQTSHSDMPECGRGVSYSNPVFESNEEQL
>sp|P05026|AT1B1_HUMAN Sodium/potassium-transporting ATPase subunit beta-1 OS=Homo sapiens GN=ATP1B1 PE=1 SV=1
MARGKAKEEGSWKKFIWNSEKKEFLGRTGGSWFKILLFYVIFYGCLAGIFIGTIQVMLLT
ISEFKPTYQDRVAPPGLTQIPQIQKTEISFRPNDPKSYEAYVLNIVRFLEKYKDSAQRDD
MIFEDCGDVPSEPKERGDFNHERGERKVCRFKLEWLGNCSGLNDETYGYKEGKPCIIIKL
NRVLGFKPKPPKNESLETYPVMKYNPNVLPVQCTGKRDEDKDKVGNVEYFGLGNSPGFPL
QYYPYYGKLLQPKYLQPLLAVQFTNLTMDTEIRIECKAYGENIGYSEKDRFQGRFDVKIE
VKS
>sp|Q9UN42|AT1B4_HUMAN Protein ATP1B4 OS=Homo sapiens GN=ATP1B4 PE=2 SV=1
MRRQLRSRRAPSFPYSYRYRLDDPDEANQNYLADEEEEAEEEARVTVVPKSEEEEEEEEK
EEEEEEEKEEEEGQGQPTGNAWWQKLQIMSEYLWDPERRMFLARTGQSWSLILLIYFFFY
ASLAAVITLCMYTLFLTISPYIPTFTERVKPPGVMIRPFAHSLNFNFNVSEPDTWQHYVI
SLNGFLQGYNDSLQEEMNVDCPPGQYFIQDGNEDEDKKACQFKRSFLKNCSGLEDPTFGY
STGQPCILLKMNRIVGFRPELGDPVKVSCKVQRGDENDIRSISYYPESASFDLRYYPYYG
KLTHVNYTSPLVAMHFTDVVKNQAVPVQCQLKGKGVINDVINDRFVGRVIFTLNIET
>sp|Q8TBG4|AT2L1_HUMAN Ethanolamine-phosphate phospho-lyase OS=Homo sapiens GN=ETNPPL PE=1 SV=1
MCELYSKRDTLGLRKKHIGPSCKVFFASDPIKIVRAQRQYMFDENGEQYLDCINNVAHVG
HCHPGVVKAALKQMELLNTNSRFLHDNIVEYAKRLSATLPEKLSVCYFTNSGSEANDLAL
RLARQFRGHQDVITLDHAYHGHLSSLIEISPYKFQKGKDVKKEFVHVAPTPDTYRGKYRE
DHADSASAYADEVKKIIEDAHNSGRKIAAFIAESMQSCGGQIIPPAGYFQKVAEYVHGAG
GVFIADEVQVGFGRVGKHFWSFQMYGEDFVPDIVTMGKPMGNGHPVACVVTTKEIAEAFS
SSGMEYFNTYGGNPVSCAVGLAVLDIIENEDLQGNAKRVGNYLTELLKKQKAKHTLIGDI
RGIGLFIGIDLVKDHLKRTPATAEAQHIIYKMKEKRVLLSADGPHRNVLKIKPPMCFTEE
DAKFMVDQLDRILTVLEEAMGTKTESVTSENTPCKTKMLKEAHIELLRDSTTDSKENPSR
KRNGMCTDTHSLLSKRLKT

>sp|O75964|ATP5L_HUMAN ATP synthase subunit g, mitochondrial OS=Homo sapiens GN=ATP5L PE=1 SV=3
MAQFVRNLVEKTPALVNAAVTYSKPRLATFWYYAKVELVPPTPAEIPRAIQSLKKIVNSA
QTGSFKQLTVKEAVLNGLVATEVLMWFYVGEIIGKRGIIGYDV
>sp|P30049|ATPD_HUMAN ATP synthase subunit delta, mitochondrial OS=Homo sapiens GN=ATP5D PE=1 SV=2
MLPAALLRRPGLGRLVRHARAYAEAAAAPAAASGPNQMSFTFASPTQVFFNGANVRQVDV
PTLTGAFGILAAHVPTLQVLRPGLVVVHAEDGTTSKYFVSSGSIAVNADSSVQLLAEEAV
TLDMLDLGAAKANLEKAQAELVGTADEATRAEIQIRIEANEALVKALE
>sp|P58397|ATS12_HUMAN A disintegrin and metalloproteinase with thrombospondin motifs 12 OS=Homo sapiens GN=ADAMTS12 PE=1 SV=2
MPCAQRSWLANLSVVAQLLNFGALCYGRQPQPGPVRFPDRRQEHFIKGLPEYHVVGPVRV
DASGHFLSYGLHYPITSSRRKRDLDGSEDWVYYRISHEEKDLFFNLTVNQGFLSNSYIME
KRYGNLSHVKMMASSAPLCHLSGTVLQQGTRVGTAALSACHGLTGFFQLPHGDFFIEPVK
KHPLVEGGYHPHIVYRRQKVPETKEPTCGLKDSVNISQKQELWREKWERHNLPSRSLSRR
SISKERWVETLVVADTKMIEYHGSENVESYILTIMNMVTGLFHNPSIGNAIHIVVVRLIL
LEEEEQGLKIVHHAEKTLSSFCKWQKSINPKSDLNPVHHDVAVLLTRKDICAGFNRPCET
LGLSHLSGMCQPHRSCNINEDSGLPLAFTIAHELGHSFGIQHDGKENDCEPVGRHPYIMS
RQLQYDPTPLTWSKCSEEYITRFLDRGWGFCLDDIPKKKGLKSKVIAPGVIYDVHHQCQL
QYGPNATFCQEVENVCQTLWCSVKGFCRSKLDAAADGTQCGEKKWCMAGKCITVGKKPES
IPGGWGRWSPWSHCSRTCGAGVQSAERLCNNPEPKFGGKYCTGERKRYRLCNVHPCRSEA
PTFRQMQCSEFDTVPYKNELYHWFPIFNPAHPCELYCRPIDGQFSEKMLDAVIDGTPCFE
GGNSRNVCINGICKMVGCDYEIDSNATEDRCGVCLGDGSSCQTVRKMFKQKEGSGYVDIG
LIPKGARDIRVMEIEGAGNFLAIRSEDPEKYYLNGGFIIQWNGNYKLAGTVFQYDRKGDL
EKLMATGPTNESVWIQLLFQVTNPGIKYEYTIQKDGLDNDVEQQMYFWQYGHWTECSVTC
GTGIRRQTAHCIKKGRGMVKATFCDPETQPNGRQKKCHEKACPPRWWAGEWEACSATCGP
HGEKKRTVLCIQTMVSDEQALPPTDCQHLLKPKTLLSCNRDILCPSDWTVGNWSECSVSC
GGGVRIRSVTCAKNHDEPCDVTRKPNSRALCGLQQCPSSRRVLKPNKGTISNGKNPPTLK
PVPPPTSRPRMLTTPTGPESMSTSTPAISSPSPTTASKEGDLGGKQWQDSSTQPELSSRY
LISTGSTSQPILTSQSLSIQPSEENVSSSDTGPTSEGGLVATTTSGSGLSSSRNPITWPV
TPFYNTLTKGPEMEIHSGSGEEREQPEDKDESNPVIWTKIRVPGNDAPVESTEMPLAPPL
TPDLSRESWWPPFSTVMEGLLPSQRPTTSETGTPRVEGMVTEKPANTLLPLGGDHQPEPS
GKTANRNHLKLPNNMNQTKSSEPVLTEEDATSLITEGFLLNASNYKQLTNGHGSAHWIVG
NWSECSTTCGLGAYWRRVECSTQMDSDCAAIQRPDPAKRCHLRPCAGWKVGNWSKCSRNC
SGGFKIREIQCVDSRDHRNLRPFHCQFLAGIPPPLSMSCNPEPCEAWQVEPWSQCSRSCG
GGVQERGVFCPGGLCDWTKRPTSTMSCNEHLCCHWATGNWDLCSTSCGGGFQKRTVQCVP
SEGNKTEDQDQCLCDHKPRPPEFKKCNQQACKKSADLLCTKDKLSASFCQTLKAMKKCSV
PTVRAECCFSCPQTHITHTQRQRRQRLLQKSKEL
>sp|Q8TE58|ATS15_HUMAN A disintegrin and metalloproteinase with thrombospondin motifs 15 OS=Homo sapiens GN=ADAMTS15 PE=2 SV=1
MLLLGILTLAFAGRTAGGSEPEREVVVPIRLDPDINGRRYYWRGPEDSGDQGLIFQITAF
QEDFYLHLTPDAQFLAPAFSTEHLGVPLQGLTGGSSDLRRCFYSGDVNAEPDSFAAVSLC
GGLRGAFGYRGAEYVISPLPNASAPAAQRNSQGAHLLQRRGVPGGPSGDPTSRCGVASGW
NPAILRALDPYKPRRAGFGESRSRRRSGRAKRFVSIPRYVETLVVADESMVKFHGADLEH

YLLTLLATAARLYRHPSILNPINIVVVKVLLLRDRDSGPKVTGNAALTLRNFCAWQKKLN
KVSDKHPEYWDTAILFTRQDLCGATTCDTLGMADVGTMCDPKRSCSVIEDDGLPSAFTTA
HELGHVFNMPHDNVKVCEEVFGKLRANHMMSPTLIQIDRANPWSACSAAIITDFLDSGHG
DCLLDQPSKPISLPEDLPGASYTLSQQCELAFGVGSKPCPYMQYCTKLWCTGKAKGQMVC
QTRHFPWADGTSCGEGKLCLKGACVERHNLNKHRVDGSWAKWDPYGPCSRTCGGGVQLAR
RQCTNPTPANGGKYCEGVRVKYRSCNLEPCPSSASGKSFREEQCEAFNGYNHSTNRLTLA
VAWVPKYSGVSPRDKCKLICRANGTGYFYVLAPKVVDGTLCSPDSTSVCVQGKCIKAGCD
GNLGSKKRFDKCGVCGGDNKSCKKVTGLFTKPMHGYNFVVAIPAGASSIDIRQRGYKGLI
GDDNYLALKNSQGKYLLNGHFVVSAVERDLVVKGSLLRYSGTGTAVESLQASRPILEPLT
VEVLSVGKMTPPRVRYSFYLPKEPREDKSSHPKDPRGPSVLHNSVLSLSNQVEQPDDRPP
ARWVAGSWGPCSASCGSGLQKRAVDCRGSAGQRTVPACDAAHRPVETQACGEPCPTWELS
AWSPCSKSCGRGFQRRSLKCVGHGGRLLARDQCNLHRKPQELDFCVLRPC
>sp|Q8TE60|ATS18_HUMAN A disintegrin and metalloproteinase with thrombospondin motifs 18 OS=Homo sapiens GN=ADAMTS18 PE=1 SV=3
MECALLLACAFPAAGSGPPRGLAGLGRVAKALQLCCLCCASVAAALASDSSSGASGLNDD
YVFVTPVEVDSAGSYISHDILHNGRKKRSAQNARSSLHYRFSAFGQELHLELKPSAILSS
HFIVQVLGKDGASETQKPEVQQCFYQGFIRNDSSSSVAVSTCAGLSGLIRTRKNEFLISP
LPQLLAQEHNYSSPAGHHPHVLYKRTAEEKIQRYRGYPGSGRNYPGYSPSHIPHASQSRE
TEYHHRRLQKQHFCGRRKKYAPKPPTEDTYLRFDEYGSSGRPRRSAGKSQKGLNVETLVV
ADKKMVEKHGKGNVTTYILTVMNMVSGLFKDGTIGSDINVVVVSLILLEQEPGGLLINHH
ADQSLNSFCQWQSALIGKNGKRHDHAILLTGFDICSWKNEPCDTLGFAPISGMCSKYRSC
TINEDTGLGLAFTIAHESGHNFGMIHDGEGNPCRKAEGNIMSPTLTGNNGVFSWSSCSRQ
YLKKFLSTPQAGCLVDEPKQAGQYKYPDKLPGQIYDADTQCKWQFGAKAKLCSLGFVKDI
CKSLWCHRVGHRCETKFMPAAEGTVCGLSMWCRQGQCVKFGELGPRPIHGQWSAWSKWSE
CSRTCGGGVKFQERHCNNPKPQYGGLFCPGSSRIYQLCNINPCNENSLDFRAQQCAEYNS
KPFRGWFYQWKPYTKVEEEDRCKLYCKAENFEFFFAMSGKVKDGTPCSPNKNDVCIDGVC
ELVGCDHELGSKAVSDACGVCKGDNSTCKFYKGLYLNQHKANEYYPVVLIPAGARSIEIQ
ELQVSSSYLAVRSLSQKYYLTGGWSIDWPGEFPFAGTTFEYQRSFNRPERLYAPGPTNET
LVFEILMQGKNPGIAWKYALPKVMNGTPPATKRPAYTWSIVQSECSVSCGGGYINVKAIC
LRDQNTQVNSSFCSAKTKPVTEPKICNAFSCPAYWMPGEWSTCSKACAGGQQSRKIQCVQ
KKPFQKEEAVLHSLCPVSTPTQVQACNSHACPPQWSLGPWSQCSKTCGRGVRKRELLCKG
SAAETLPESQCTSLPRPELQEGCVLGRCPKNSRLQWVASSWSECSATCGLGVRKREMKCS
EKGFQGKLITFPERRCRNIKKPNLDLEETCNRRACPAHPVYNMVAGWYSLPWQQCTVTCG
GGVQTRSVHCVQQGRPSSSCLLHQKPPVLRACNTNFCPAPEKREDPSCVDFFNWCHLVPQ
HGVCNHKFYGKQCCKSCTRKI
>sp|O15072|ATS3_HUMAN A disintegrin and metalloproteinase with thrombospondin motifs 3 OS=Homo sapiens GN=ADAMTS3 PE=2 SV=4
MVLLSLWLIAAALVEVRTSADGQAGNEEMVQIDLPIKRYREYELVTPVSTNLEGRYLSHT
LSASHKKRSARDVSSNPEQLFFNITAFGKDFHLRLKPNTQLVAPGAVVEWHETSLVPGNI
TDPINNHQPGSATYRIRRTEPLQTNCAYVGDIVDIPGTSVAISNCDGLAGMIKSDNEEYF
IEPLERGKQMEEEKGRIHVVYKRSAVEQAPIDMSKDFHYRESDLEGLDDLGTVYGNIHQQ
LNETMRRRRHAGENDYNIEVLLGVDDSVVRFHGKEHVQNYLLTLMNIVNEIYHDESLGVH
INVVLVRMIMLGYAKSISLIERGNPSRSLENVCRWASQQQRSDLNHSEHHDHAIFLTRQD
FGPAGMQGYAPVTGMCHPVRSCTLNHEDGFSSAFVVAHETGHVLGMEHDGQGNRCGDETA

MGSVMAPLVQAAFHRYHWSRCSGQELKRYIHSYDCLLDDPFDHDWPKLPELPGINYSMDE
QCRFDFGVGYKMCTAFRTFDPCKQLWCSHPDNPYFCKTKKGPPLDGTECAAGKWCYKGHC
MWKNANQQKQDGNWGSWTKFGSCSRTCGTGVRFRTRQCNNPMPINGGQDCPGVNFEYQLC
NTEECQKHFEDFRAQQCQQRNSHFEYQNTKHHWLPYEHPDPKKRCHLYCQSKETGDVAYM
KQLVHDGTHCSYKDPYSICVRGECVKVGCDKEIGSNKVEDKCGVCGGDNSHCRTVKGTFT
RTPRKLGYLKMFDIPPGARHVLIQEDEASPHILAIKNQATGHYILNGKGEEAKSRTFIDL
GVEWDYNIEDDIESLHTDGPLHDPVIVLIIPQENDTRSSLTYKYIIHEDSVPTINSNNVI
QEELDTFEWALKSWSQCSKPCGGGFQYTKYGCRRKSDNKMVHRSFCEANKKPKPIRRMCN
IQECTHPLWVAEEWEHCTKTCGSSGYQLRTVRCLQPLLDGTNRSVHSKYCMGDRPESRRP
CNRVPCPAQWKTGPWSECSVTCGEGTEVRQVLCRAGDHCDGEKPESVRACQLPPCNDEPC
LGDKSIFCQMEVLARYCSIPGYNKLCCESCSKRSSTLPPPYLLEAAETHDDVISNPSDLP
RSLVMPTSLVPYHSETPAKKMSLSSISSVGGPNAYAAFRPNSKPDGANLRQRSAQQAGSK
TVRLVTVPSSPPTKRVHLSSASQMAAASFFAASDSIGASSQARTSKKDGKIIDNRRPTRS
STLER
>sp|O75173|ATS4_HUMAN A disintegrin and metalloproteinase with thrombospondin motifs 4
OS=Homo sapiens GN=ADAMTS4 PE=1 SV=3
MSQTGSHPGRGLAGRWLWGAQPCLLLPIVPLSWLVWLLLLLLASLLPSARLASPLPREEE
IVFPEKLNGSVLPGSGAPARLLCRLQAFGETLLLELEQDSGVQVEGLTVQYLGQAPELLG
GAEPGTYLTGTINGDPESVASLHWDGGALLGVLQYRGAELHLQPLEGGTPNSAGGPGAHI
LRRKSPASGQGPMCNVKAPLGSPSPRPRRAKRFASLSRFVETLVVADDKMAAFHGAGLKR
YLLTVMAAAAKAFKHPSIRNPVSLVVTRLVILGSGEEGPQVGPSAAQTLRSFCAWQRGLN
TPEDSDPDHFDTAILFTRQDLCGVSTCDTLGMADVGTVCDPARSCAIVEDDGLQSAFTAA
HELGHVFNMLHDNSKPCISLNGPLSTSRHVMAPVMAHVDPEEPWSPCSARFITDFLDNGY
GHCLLDKPEAPLHLPVTFPGKDYDADRQCQLTFGPDSRHCPQLPPPCAALWCSGHLNGHA
MCQTKHSPWADGTPCGPAQACMGGRCLHMDQLQDFNIPQAGGWGPWGPWGDCSRTCGGGV
QFSSRDCTRPVPRNGGKYCEGRRTRFRSCNTEDCPTGSALTFREEQCAAYNHRTDLFKSF
PGPMDWVPRYTGVAPQDQCKLTCQAQALGYYYVLEPRVVDGTPCSPDSSSVCVQGRCIHA
GCDRIIGSKKKFDKCMVCGGDGSGCSKQSGSFRKFRYGYNNVVTIPAGATHILVRQQGNP
GHRSIYLALKLPDGSYALNGEYTLMPSPTDVVLPGAVSLRYSGATAASETLSGHGPLAQP
LTLQVLVAGNPQDTRLRYSFFVPRPTPSTPRPTPQDWLHRRAQILEILRRRPWAGRK
>sp|Q9UKP5|ATS6_HUMAN A disintegrin and metalloproteinase with thrombospondin motifs 6
OS=Homo sapiens GN=ADAMTS6 PE=2 SV=2
MEILWKTLTWILSLIMASSEFHSDHRLSYSSQEEFLTYLEHYQLTIPIRVDQNGAFLSFT
VKNDKHSRRRRSMDPIDPQQAVSKLFFKLSAYGKHFHLNLTLNTDFVSKHFTVEYWGKDG
PQWKHDFLDNCHYTGYLQDQRSTTKVALSNCVGLHGVIATEDEEYFIEPLKNTTEDSKHF
SYENGHPHVIYKKSALQQRHLYDHSHCGVSDFTRSGKPWWLNDTSTVSYSLPINNTHIHH
RQKRSVSIERFVETLVVADKMMVGYHGRKDIEHYILSVMNIVAKLYRDSSLGNVVNIIVA
RLIVLTEDQPNLEINHHADKSLDSFCKWQKSILSHQSDGNTIPENGIAHHDNAVLITRYD
ICTYKNKPCGTLGLASVAGMCEPERSCSINEDIGLGSAFTIAHEIGHNFGMNHDGIGNSC
GTKGHEAAKLMAAHITANTNPFSWSACSRDYITSFLDSGRGTCLDNEPPKRDFLYPAVAP
GQVYDADEQCRFQYGATSRQCKYGEVCRELWCLSKSNRCVTNSIPAAEGTLCQTGNIEKG
WCYQGDCVPFGTWPQSIDGGWGPWSLWGECSRTCGGGVSSSLRHCDSPAPSGGGKYCLGE
RKRYRSCNTDPCPLGSRDFREKQCADFDNMPFRGKYYNWKPYTGGGVKPCALNCLAEGYN
FYTERAPAVIDGTQCNADSLDICINGECKHVGCDNILGSDAREDRCRVCGGDGSTCDAIE

GFFNDSLPRGGYMEVVQIPRGSVHIEVREVAMSKNYIALKSEGDDYYINGAWTIDWPRKF
DVAGTAFHYKRPTDEPESLEALGPTSENLIVMVLLQEQNLGIRYKFNVPITRTGSGDNEV
GFTWNHQPWSECSATCAGGVQRQEVVCKRLDDNSIVQNNYCDPDSKPPENQRACNTEPCP
PEWFIGDWLECSKTCDGGMRTRAVLCIRKIGPSEEETLDYSGCLTHRPVEKEPCNNQSCP
PQWVALDWSECTPKCGPGFKHRIVLCKSSDLSKTFPAAQCPEESKPPVRIRCSLGRCPPP
RWVTGDWGQCSAQCGLGQQMRTVQCLSYTGQASSDCLETVRPPSMQQCESKCDSTPISNT
EECKDVNKVAYCPLVLKFKFCSRAYFRQMCCKTCQGH
>sp|P0C7T5|ATX1L_HUMAN Ataxin-1-like OS=Homo sapiens GN=ATXN1L PE=1 SV=1
MKPVHERSQECLPPKKRDLPVTSEDMGRTTSCSTNHTPSSDASEWSRGVVVAGQSQAGAR
VSLGGDGAEAITGLTVDQYGMLYKVAVPPATFSPTGLPSVVNMSPLPPTFNVASSLIQHP
GIHYPPLHYAQLPSTSLQFIGSPYSLPYAVPPNFLPSPLLSPSANLATSHLPHFVPYASL
LAEGATPPPQAPSPAHSFNKAPSATSPSGQLPHHSSTQPLDLAPGRMPIYYQMSRLPAGY
TLHETPPAGASPVLTPQESQSALEAAAANGGQRPRERNLVRRESEALDSPNSKGEGQGLV
PVVECVVDGQLFSGSQTPRVEVAAPAHRGTPDTDLEVQRVVGALASQDYRVVAAQRKEEP
SPLNLSHHTPDHQGEGRGSARNPAELAEKSQARGFYPQSHQEPVKHRPLPKAMVVANGNL
VPTGTDSGLLPVGSEILVASSLDVQARATFPDKEPTPPPITSSHLPSHFMKGAIIQLATG
ELKRVEDLQTQDFVRSAEVSGGLKIDSSTVVDIQESQWPGFVMLHFVVGEQQSKVSIEVP
PEHPFFVYGQGWSSCSPGRTTQLFSLPCHRLQVGDVCISISLQSLNSNSVSQASCAPPSQ
LGPPRERPERTVLGSRELCDSEGKSQPAGEGSRVVEPSQPESGAQACWPAPSFQRYSMQG
EEARAALLRPSFIPQEVKLSIEGRSNAGK
>sp|Q8WWM7|ATX2L_HUMAN Ataxin-2-like protein OS=Homo sapiens GN=ATXN2L PE=1 SV=2
MLKPQPLQQPSQPQQPPPTQQAVARRPPGGTSPPNGGLPGPLATSAAPPGPPAAASPCLG
PVAAAGSGLRRGAEGILAPQPPPPQQHQERPGAAAIGSARGQSTGKGPPQSPVFEGVYNN
SRMLHFLTAVVGSTCDVKVKNGTTYEGIFKTLSSKFELAVDAVHRKASEPAGGPRREDIV
DTMVFKPSDVMLVHFRNVDFNYATKDKFTDSAIAMNSKVNGEHKEKVLQRWEGGDSNSDD
YDLESDMSNGWDPNEMFKFNEENYGVKTTYDSSLSSYTVPLEKDNSEEFRQRELRAAQLA
REIESSPQYRLRIAMENDDGRTEEEKHSAVQRQGSGRESPSLASREGKYIPLPQRVREGP
RGGVRCSSSRGGRPGLSSLPPRGPHHLDNSSPGPGSEARGINGGPSRMSPKAQRPLRGAK
TLSSPSNRPSGETSVPPPPAVGRMYPPRSPKSAAPAPISASCPEPPIGSAVPTSSASIPV
TSSVSDPGVGSISPASPKISLAPTDVKELSTKEPGRTLEPQELARIAGKVPGLQNEQKRF
QLEELRKFGAQFKLQPSSSPENSLDPFPPRILKEEPKGKEKEVDGLLTSEPMGSPVSSKT
ESVSDKEDKPPLAPSGGTEGPEQPPPPCPSQTGSPPVGLIKGEDKDEGPVAEQVKKSTLN
PNAKEFNPTKPLLSVNKSTSTPTSPGPRTHSTPSIPVLTAGQSGLYSPQYISYIPQIHMG
PAVQAPQMYPYPVSNSVPGQQGKYRGAKGSLPPQRSDQHQPASAPPMMQAAAAAGPPLVA
ATPYSSYIPYNPQQFPGQPAMMQPMAHYPSQPVFAPMLQSNPRMLTSGSHPQAIVSSSTP
QYPSAEQPTPQALYATVHQSYPHHATQLHAHQPQPATTPTGSQPQSQHAAPSPVQHQAGQ
APHLGSGQPQQNLYHPGALTGTPPSLPPGPSAQSPQSSFPQPAAVYAIHHQQLPHGFTNM
AHVTQAHVQTGITAAPPPHPGAPHPPQVMLLHPPQSHGGPPQGAVPQSGVPALSASTPSP
YPYIGHPQGEQPGQAPGFPGGADDRIREFSLAGGIWHGRAEGLQVGQDARVLGGE
>sp|Q9H1Z8|AUGN_HUMAN Augurin OS=Homo sapiens GN=C2orf40 PE=2 SV=1
MAASPARPAVLALTGLALLLLLCWGPGGISGNKLKLMLQKREAPVPTKTKVAVDENKAKE
FLGSLKRQKRQLWDRTRPEVQQWYQQFLYMGFDEAKFEDDITYWLNRDRNGHEYYGDYYQ
RHYDEDSAIGPRSPYGFRHGASVNYDDY
>sp|Q96GD4|AURKB_HUMAN Aurora kinase B OS=Homo sapiens GN=AURKB PE=1 SV=3

MAQKENSYPWPYGRQTAPSGLSTLPQRVLRKEPVTPSALVLMSRSNVQPTAAPGQKVMEN
SSGTPDILTRHFTIDDFEIGRPLGKGKFGNVYLAREKKSHFIVALKVLFKSQIEKEGVEH
QLRREIEIQAHLHHPNILRLYNYFYDRRRIYLILEYAPRGELYKELQKSCTFDEQRTATI
MEELADALMYCHGKKVIHRDIKPENLLLGLKGELKIADFGWSVHAPSLRRKTMCGTLDYL
PPEMIEGRMHNEKVDLWCIGVLCYELLVGNPPFESASHNETYRRIVKVDLKFPASVPMGA
QDLISKLLRHNPSERLPLAQVSAHPWVRANSRRVLPPSALQSVA
>sp|Q9BYG0|B3GN5_HUMAN Lactosylceramide 1,3-N-acetyl-beta-D-glucosaminyltransferase OS=Homo sapiens GN=B3GNT5 PE=1 SV=1
MRMLVSGRRVKKWQLIIQLFATCFLASLMFFWEPIDNHIVSHMKSYSYRYLINSYDFVND
TLSLKHTSAGPRYQYLINHKEKCQAQDVLLLLFVKTAPENYDRRSGIRRTWGNENYVRSQ
LNANIKTLFALGTPNPLEGEELQRKLAWEDQRYNDIIQQDFVDSFYNLTLKLLMQFSWAN
TYCPHAKFLMTADDDIFIHMPNLIEYLQSLEQIGVQDFWIGRVHRGAPPIRDKSSKYYVS
YEMYQWPAYPDYTAGAAYVISGDVAAKVYEASQTLNSSLYIDDVFMGLCANKIGIVPQDH
VFFSGEGKTPYHPCIYEKMMTSHGHLEDLQDLWKNATDPKVKTISKGFFGQIYCRLMKII
LLCKISYVDTYPCRAAFI
>sp|Q67FW5|B3GNL_HUMAN UDP-GlcNAc:betaGal beta-1,3-N-acetylglucosaminyltransferase-like protein 1 OS=Homo sapiens GN=B3GNTL1 PE=2 SV=2
MSGAGVGGASEESQAMQAHVSIILPVHNAEPWLDECLRSVLQQDFEGTMELSVFNDASKD
KSGAIIEKWRVKLEDSGVHVIIGGHDSPSPRGVGYAKNQAVAQSSGSYLCFLDSDDVMMP
QRVRLQHEAAVQHPSSIIGCRVRRDPPNSTERYTRWINQLTPEQLLTQVFTSNGPTVIMP
TWFCSRAWFSHVGPFNEGGQGVPEDLLFFYEHLRKGGGVIRVDQSLLLYRHHPQAATHCV
LETTIWTHRVRFLEEQALPRWAAFTIWNAGKQGRRLYRSLTAGSQRKVVAFCDVDENKIR
KGFYCHEDSQERPKPRIPILHFRAARPPFVICVKLDLTGGAFEDNLRSLHLQEGQDFLHF
S
>sp|Q9BSB4|ATGA1_HUMAN Autophagy-related protein 101 OS=Homo sapiens GN=ATG101 PE=1 SV=1
MNCRSEVLEVSVEGRQVEEAMLAVLHTVLLHRSTGKFHYKKEGTYSIGTVGTQDVDCDFI
DFTYVRVSSEELDRALRKVVGEFKDALRNSGGDGLGQMSLEFYQKKKSRWPFSDECIPWE
VWTVKVHVVALATEQERQICREKVGEKLCEKIINIVEVMNRHEYLPKMPTQSEVDNVFDT
GLRDVQPYLYKISFQITDALGTSVTTTMRRLIKDTLAL
>sp|Q32M88|ATHL1_HUMAN Acid trehalase-like protein 1 OS=Homo sapiens GN=ATHL1 PE=1 SV=2
MEDAGEDPTTFAAHSLPSDPRLLATVTNAYLGTRVFHDTLHVSGVYNGAGGDTHRAMLPS
PLNVRLEAPAGMGEQLTETFALDTNTGSFLHTLEGPRFRASQCIYAHRTLPHVLAFRVSI
ARLAPGSGPITLLLRSAFSPESPDLDLHQGPDFQGARYLYGHTLTPEQPGGPQQEVHMLW
TPAPPDLTLGEGEEARTWDFLTAVGGSQAEAQACLTEALQLQARGALYTAHAQAWAQLWV
ECGLDVVGPLQLRQALRGSLYYLLSALPQPKAPGYICHGLSPGGLSNGSREECYWGHVFW
DQDLWMFPSILMFHPEAARAILEYRIRTLDGALENAQNLGYQGAKFAWESADSGLEVCPE
DIYGVQEVHVNGAVVLAFELYYHTTQDLQLFREAGGWDVVRAVAEFWCSRVEWSPREEKY
HLRGVMSPDEYHSGVNNSVYTNVLVQNSLRFAAALAQDLGLPIPSQWLAVADKIKVPFDV
EQNFHPEFDGYEPGEVVKQADVVLLGYPVPFSLSPDVRRKNLEIYEAVTSPQGPAMTWSM
FAVGWMELKDAVRARGLLDRSFANMAEPFKVWTENADGSGAVNFLTGMGGFLQAVVFGCT
GFRVTRAGVTFDPVCLSGISRVSVSGIFYQGNKLNFSFSEDSVTVEVTARAGPWAPHLEA
ELWPSQSRLSLLPGHKVSFPRSAGRIQMSPPKLPGSSSSEFPGRTFSDVRDPLQSPLWVT
LGSSSPTESLTVDPASE

>sp|Q9UII2|ATIF1_HUMAN ATPase inhibitor, mitochondrial OS=Homo sapiens GN=ATPIF1 PE=1 SV=1
MAVTALAARTWLGVWGVRTMQARGFGSDQSENVDRGAGSIREAGGAFGKREQAEEERYFR
AQSREQLAALKKHHEEEIVHHKKEIERLQKEIERHKQKIKMLKHDD
>sp|P82987|ATL3_HUMAN ADAMTS-like protein 3 OS=Homo sapiens GN=ADAMTSL3 PE=1 SV=4
MASWTSPWWVLIGMVFMHSPLPQTTAEKSPGAYFLPEFALSPQGSFLEDTTGEQFLTYRY
DDQTSRNTRSDEDKDGNWDAWGDWSDCSRTCGGGASYSLRRCLTGRNCEGQNIRYKTCSN
HDCPPDAEDFRAQQCSAYNDVQYQGHYYEWLPRYNDPAAPCALKCHAQGQNLVVELAPKV
LDGTRCNTDSLDMCISGICQAVGCDRQLGSNAKEDNCGVCAGDGSTCRLVRGQSKSHVSP
EKREENVIAVPLGSRSVRITVKGPAHLFIESKTLQGSKGEHSFNSPGVFLVENTTVEFQR
GSERQTFKIPGPLMADFIFKTRYTAAKDSVVQFFFYQPISHQWRQTDFFPCTVTCGGGYQ
LNSAECVDIRLKRVVPDHYCHYYPENVKPKPKLKECSMDPCPSSDGFKEIMPYDHFQPLP
RWEHNPWTACSVSCGGGIQRRSFVCVEESMHGEILQVEEWKCMYAPKPKVMQTCNLFDCP
KWIAMEWSQCTVTCGRGLRYRVVLCINHRGEHVGGCNPQLKLHIKEECVIPIPCYKPKEK
SPVEAKLPWLKQAQELEETRIATEEPTFIPEPWSACSTTCGPGVQVREVKCRVLLTFTQT
ETELPEEECEGPKLPTERPCLLEACDESPASRELDIPLPEDSETTYDWEYAGFTPCTATC
VGGHQEAIAVCLHIQTQQTVNDSLCDMVHRPPAMSQACNTEPCPPRWHVGSWGPCSATCG
VGIQTRDVYCLHPGETPAPPEECRDEKPHALQACNQFDCPPGWHIEEWQQCSRTCGGGTQ
NRRVTCRQLLTDGSFLNLSDELCQGPKASSHKSCARTDCPPHLAVGDWSKCSVSCGVGIQ
RRKQVCQRLAAKGRRIPLSEMMCRDLPGLPLVRSCQMPECSKIKSEMKTKLGEQGPQILS
VQRVYIQTREEKRINLTIGSRAYLLPNTSVIIKCPVRRFQKSLIQWEKDGRCLQNSKRLG
ITKSGSLKIHGLAAPDIGVYRCIAGSAQETVVLKLIGTDNRLIARPALREPMREYPGMDH
SEANSLGVTWHKMRQMWNNKNDLYLDDDHISNQPFLRALLGHCSNSAGSTNSWELKNKQF
EAAVKQGAYSMDTAQFDELIRNMSQLMETGEVSDDLASQLIYQLVAELAKAQPTHMQWRG
IQEETPPAAQLRGETGSVSQSSHAKNSGKLTFKPKGPVLMRQSQPPSISFNKTINSRIGN
TVYITKRTEVINILCDLITPSEATYTWTKDGTLLQPSVKIILDGTGKIQIQNPTRKEQGI
YECSVANHLGSDVESSSVLYAEAPVILSVERNITKPEHNHLSVVVGGIVEAALGANVTIR
CPVKGVPQPNITWLKRGGSLSGNVSLLFNGSLLLQNVSLENEGTYVCIATNALGKAVATS
VLHLLERRWPESRIVFLQGHKKYILQATNTRTNSNDPTGEPPPQEPFWEPGNWSHCSATC
GHLGARIQRPQCVMANGQEVSEALCDHLQKPLAGFEPCNIRDCPARWFTSVWSQCSVSCG
EGYHSRQVTCKRTKANGTVQVVSPRACAPKDRPLGRKPCFGHPCVQWEPGNRCPGRCMGR
AVRMQQRHTACQHNSSDSNCDDRKRPTLRRNCTSGACDVCWHTGPWKPCTAACGRGFQSR
KVDCIHTRSCKPVAKRHCVQKKKPISWRHCLGPSCDRDCTDTTHYCMFVKHLNLCSLDRY
KQRCCQSCQEG
>sp|Q6UY14|ATL4_HUMAN ADAMTS-like protein 4 OS=Homo sapiens GN=ADAMTSL4 PE=1 SV=2
MENWTGRPWLYLLLLLSLPQLCLDQEVLSGHSLQTPTEEGQGPEGVWGPWVQWASCSQPC
GVGVQRRSRTCQLPTVQLHPSLPLPPRPPRHPEALLPRGQGPRPQTSPETLPLYRTQSRG
RGGPLRGPASHLGREETQEIRAARRSRLRDPIKPGMFGYGRVPFALPLHRNRRHPRSPPR
SELSLISSRGEEAIPSPTPRAEPFSANGSPQTELPPTELSVHTPSPQAEPLSPETAQTEV
APRTRPAPLRHHPRAQASGTEPPSPTHSLGEGGFFRASPQPRRPSSQGWASPQVAGRRPD
PFPSVPRGRGQQGQGPWGTGGTPHGPRLEPDPQHPGAWLPLLSNGPHASSLWSLFAPSSP
IPRCSGESEQLRACSQAPCPPEQPDPRALQCAAFNSQEFMGQLYQWEPFTEVQGSQRCEL
NCRPRGFRFYVRHTEKVQDGTLCQPGAPDICVAGRCLSPGCDGILGSGRRPDGCGVCGGD
DSTCRLVSGNLTDRGGPLGYQKILWIPAGALRLQIAQLRPSSNYLALRGPGGRSIINGNW

AVDPPGSYRAGGTVFRYNRPPREEGKGESLSAEGPTTQPVDVYMIFQEENPGVFYQYVIS
SPPPILENPTPEPPVPQLQPEILRVEPPLAPAPRPARTPGTLQRQVRIPQMPAPPHPRTP
LGSPAAYWKRVGHSACSASCGKGVWRPIFLCISRESGEELDERSCAAGARPPASPEPCHG
TPCPPYWEAGEWTSCSRSCGPGTQHRQLQCRQEFGGGGSSVPPERCGHLPRPNITQSCQL
RLCGHWEVGSPWSQCSVRCGRGQRSRQVRCVGNNGDEVSEQECASGPPQPPSREACDMGP
CTTAWFHSDWSSKCSAECGTGIQRRSVVCLGSGAALGPGQGEAGAGTGQSCPTGSRPPDM
RACSLGPCERTWRWYTGPWGECSSECGSGTQRRDIICVSKLGTEFNVTSPSNCSHLPRPP
ALQPCQGQACQDRWFSTPWSPCSRSCQGGTQTREVQCLSTNQTLSTRCPPQLRPSRKRPC
NSQPCSQRPDDQCKDSSPHCPLVVQARLCVYPYYTATCCRSCAHVLERSPQDPS
>sp|O75947|ATP5H_HUMAN ATP synthase subunit d, mitochondrial OS=Homo sapiens GN=ATP5H PE=1 SV=3
MAGRKLALKTIDWVAFAEIIPQNQKAIASSLKSWNETLTSRLAALPENPPAIDWAYYKAN
VAKAGLVDDFEKKFNALKVPVPEDKYTAQVDAEEKEDVKSCAEWVSLSKARIVEYEKEME
KMKNLIPFDQMTIEDLNEAFPETKLDKKKYPYWPHQPIENL
>sp|P56385|ATP5I_HUMAN ATP synthase subunit e, mitochondrial OS=Homo sapiens GN=ATP5I PE=1 SV=2
MVPPVQVSPLIKLGRYSALFLGVAYGATRYNYLKPRAEEERRIAAEEKKKQDELKRIARE
LAEDDSILK
>sp|P03928|ATP8_HUMAN ATP synthase protein 8 OS=Homo sapiens GN=MT-ATP8 PE=1 SV=1
MPQLNTTVWPTMITPMLLTLFLITQLKMLNTNYHLPPSPKPMKMKNYNKPWEPKWTKICS
LHSLPPQS
>sp|O75882|ATRN_HUMAN Attractin OS=Homo sapiens GN=ATRN PE=1 SV=2
MVAAAAATEARLRRRTAATAALAGRSGGPHWDWDVTRAGRPGLGAGLRLPRLLSPPLRPR
LLLLLLLLSPPLLLLLLPCEAEAAAAAAAVSGSAAAEAKECDRPCVNGGRCNPGTGQCVC
PAGWVGEQCQHCGGRFRLTGSSGFVTDGPGNYKYKTKCTWLIEGQPNRIMRLRFNHFATE
CSWDHLYVYDGDSIYAPLVAAFSGLIVPERDGNETVPEVVATSGYALLHFFSDAAYNLTG
FNITYSFDMCPNNCSGRGECKISNSSDTVECECSENWKGEACDIPHCTDNCGFPHRGICN
SSDVRGCSCFSDWQGPGCSVPVPANQSFWTREEYSNLKLPRASHKAVVNGNIMWVVGGYM
FNHSDYNMVLAYDLASREWLPLNRSVNNVVVRYGHSLALYKDKIYMYGGKIDSTGNVTNE
LRVFHIHNESWVLLTPKAKEQYAVVGHSAHIVTLKNGRVVMLVIFGHCPLYGYISNVQEY
DLDKNTWSILHTQGALVQGGYGHSSVYDHRTRALYVHGGYKAFSANKYRLADDLYRYDVD
TQMWTILKDSRFFRYLHTAVIVSGTMLVFGGNTHNDTSMSHGAKCFSSDFMAYDIACDRW
SVLPRPDLHHDVNRFGHSAVLHNSTMYVFGGFNSLLLSDILVFTSEQCDAHRSEAACLAA
GPGIRCVWNTGSSQCISWALATDEQEEKLKSECFSKRTLDHDRCDQHTDCYSCTANTNDC
HWCNDHCVPRNHSCSEGQISIFRYENCPKDNPMYYCNKKTSCRSCALDQNCQWEPRNQEC
IALPENICGIGWHLVGNSCLKITTAKENYDNAKLFCRNHNALLASLTTQKKVEFVLKQLR
IMQSSQSMSKLTLTPWVGLRKINVSYWCWEDMSPFTNSLLQWMPSEPSDAGFCGILSEPS
TRGLKAATCINPLNGSVCERPANHSAKQCRTPCALRTACGDCTSGSSECMWCSNMKQCVD
SNAYVASFPFGQCMEWYTMSTCPPENCSGYCTCSHCLEQPGCGWCTDPSNTGKGKCIEGS
YKGPVKMPSQAPTGNFYPQPLLNSSMCLEDSRYNWSFIHCPACQCNGHSKCINQSICEKC
ENLTTGKHCETCISGFYGDPTNGGKCQPCKCNGHASLCNTNTGKCFCTTKGVKGDECQLC
EVENRYQGNPLRGTCYYTLLIDYQFTFSLSQEDDRYYTAINFVATPDEQNRDLDMFINAS
KNFNLNITWAASFSAGTQAGEEMPVVSKTNIKEYKDSFSNEKFDFRNHPNITFFVYVSNF
TWPIKIQIAFSQHSNFMDLVQFFVTFFSCFLSLLLVAAVVWKIKQSCWASRRREQLLREM

QQMASRPFASVNVALETDEEPPDLIGGSIKTVPKPIALEPCFGNKAAVLSVFVRLPRGLG
GIPPPGQSGLAVASALVDISQQMPIVYKEKSGAVRNRKQQPPAQPGTCI
>sp|Q13535|ATR_HUMAN Serine/threonine-protein kinase ATR OS=Homo sapiens GN=ATR PE=1 SV=3
MGEHGLELASMIPALRELGSATPEEYNTVVQKPRQILCQFIDRILTDVNVVAVELVKKTD
SQPTSVMLLDFIQHIMKSSPLMFVNVSGSHEAKGSCIEFSNWIITRLLRIAATPSCHLLH
KKICEVICSLLFLFKSKSPAIFGVLTKELLQLFEDLVYLHRRNVMGHAVEWPVVMSRFLS
QLDEHMGYLQSAPLQLMSMQNLEFIEVTLLMVLTRIIAIVFFRRQELLLWQIGCVLLEYG
SPKIKSLAISFLTELFQLGGLPAQPASTFFSSFLELLKHLVEMDTDQLKLYEEPLSKLIK
TLFPFEAEAYRNIEPVYLNMLLEKLCVMFEDGVLMRLKSDLLKAALCHLLQYFLKFVPAG
YESALQVRKVYVRNICKALLDVLGIEVDAEYLLGPLYAALKMESMEIIEEIQCQTQQENL
SSNSDGISPKRRRLSSSLNPSKRAPKQTEEIKHVDMNQKSILWSALKQKAESLQISLEYS
GLKNPVIEMLEGIAVVLQLTALCTVHCSHQNMNCRTFKDCQHKSKKKPSVVITWMSLDFY
TKVLKSCRSLLESVQKLDLEATIDKVVKIYDALIYMQVNSSFEDHILEDLCGMLSLPWIY
SHSDDGCLKLTTFAANLLTLSCRISDSYSPQAQSRCVFLLTLFPRRIFLEWRTAVYNWAL
QSSHEVIRASCVSGFFILLQQQNSCNRVPKILIDKVKDDSDIVKKEFASILGQLVCTLHG
MFYLTSSLTEPFSEHGHVDLFCRNLKATSQHECSSSQLKASVCKPFLFLLKKKIPSPVKL
AFIDNLHHLCKHLDFREDETDVKAVLGTLLNLMEDPDKDVRVAFSGNIKHILESLDSEDG
FIKELFVLRMKEAYTHAQISRNNELKDTLILTTGDIGRAAKGDLVPFALLHLLHCLLSKS
ASVSGAAYTEIRALVAAKSVKLQSFFSQYKKPICQFLVESLHSSQMTALPNTPCQNADVR
KQDVAHQREMALNTLSEIANVFDFPDLNRFLTRTLQVLLPDLAAKASPAASALIRTLGKQ
LNVNRREILINNFKYIFSHLVCSCSKDELERALHYLKNETEIELGSLLRQDFQGLHNELL
LRIGEHYQQVFNGLSILASFASSDDPYQGPRDIISPELMADYLQPKLLGILAFFNMQLLS
SSVGIEDKKMALNSLMSLMKLMGPKHVSSVRVKMMTTLRTGLRFKDDFPELCCRAWDCFV
RCLDHACLGSLLSHVIVALLPLIHIQPKETAAIFHYLIIENRDAVQDFLHEIYFLPDHPE
LKKIKAVLQEYRKETSESTDLQTTLQLSMKAIQHENVDVRIHALTSLKETLYKNQEKLIK
YATDSETVEPIISQLVTVLLKGCQDANSQARLLCGECLGELGAIDPGRLDFSTTETQGKD
FTFVTGVEDSSFAYGLLMELTRAYLAYADNSRAQDSAAYAIQELLSIYDCREMETNGPGH
QLWRRFPEHVREILEPHLNTRYKSSQKSTDWSGVKKPIYLSKLGSNFAEWSASWAGYLIT
KVRHDLASKIFTCCSIMMKHDFKVTIYLLPHILVYVLLGCNQEDQQEVYAEIMAVLKHDD
QHTINTQDIASDLCQLSTQTVFSMLDHLTQWARHKFQALKAEKCPHSKSNRNKVDSMVST
VDYEDYQSVTRFLDLIPQDTLAVASFRSKAYTRAVMHFESFITEKKQNIQEHLGFLQKLY
AAMHEPDGVAGVSAIRKAEPSLKEQILEHESLGLLRDATACYDRAIQLEPDQIIHYHGVV
KSMLGLGQLSTVITQVNGVHANRSEWTDELNTYRVEAAWKLSQWDLVENYLAADGKSTTW
SVRLGQLLLSAKKRDITAFYDSLKLVRAEQIVPLSAASFERGSYQRGYEYIVRLHMLCEL
EHSIKPLFQHSPGDSSQEDSLNWVARLEMTQNSYRAKEPILALRRALLSLNKRPDYNEMV
GECWLQSARVARKAGHHQTAYNALLNAGESRLAELYVERAKWLWSKGDVHQALIVLQKGV
ELCFPENETPPEGKNMLIHGRAMLLVGRFMEETANFESNAIMKKYKDVTACLPEWEDGHF
YLAKYYDKLMPMVTDNKMEKQGDLIRYIVLHFGRSLQYGNQFIYQSMPRMLTLWLDYGTK
AYEWEKAGRSDRVQMRNDLGKINKVITEHTNYLAPYQFLTAFSQLISRICHSHDEVFVVL
MEIIAKVFLAYPQQAMWMMTAVSKSSYPMRVNRCKEILNKAIHMKKSLEKFVGDATRLTD
KLLELCNKPVDGSSSTLSMSTHFKMLKKLVEEATFSEILIPLQSVMIPTLPSILGTHANH
ASHEPFPGHWAYIAGFDDMVEILASLQKPKKISLKGSDGKFYIMMCKPKDDLRKDCRLME
FNSLINKCLRKDAESRRRELHIRTYAVIPLNDECGIIEWVNNTAGLRPILTKLYKEKGVY
MTGKELRQCMLPKSAALSEKLKVFREFLLPRHPPIFHEWFLRTFPDPTSWYSSRSAYCRS

TAVMSMVGYILGLGDRHGENILFDSLTGECVHVDFNCLFNKGETFEVPEIVPFRLTHNMV
NGMGPMGTEGLFRRACEVTMRLMRDQREPLMSVLKTFLHDPLVEWSKPVKGHSKAPLNET
GEVVNEKAKTHVLDIEQRLQGVIKTRNRVTGLPLSIEGHVHYLIQEATDENLLCQMYLGW
TPYM
>sp|Q76LX8|ATS13_HUMAN A disintegrin and metalloproteinase with thrombospondin motifs 13 OS=Homo sapiens GN=ADAMTS13 PE=1 SV=1
MHQRHPRARCPPLCVAGILACGFLLGCWGPSHFQQSCLQALEPQAVSSYLSPGAPLKGRP
PSPGFQRQRQRQRRAAGGILHLELLVAVGPDVFQAHQEDTERYVLTNLNIGAELLRDPSL
GAQFRVHLVKMVILTEPEGAPNITANLTSSLLSVCGWSQTINPEDDTDPGHADLVLYITR
FDLELPDGNRQVRGVTQLGGACSPTWSCLITEDTGFDLGVTIAHEIGHSFGLEHDGAPGS
GCGPSGHVMASDGAAPRAGLAWSPCSRRQLLSLLSAGRARCVWDPPRPQPGSAGHPPDAQ
PGLYYSANEQCRVAFGPKAVACTFAREHLDMCQALSCHTDPLDQSSCSRLLVPLLDGTEC
GVEKWCSKGRCRSLVELTPIAAVHGRWSSWGPRSPCSRSCGGGVVTRRRQCNNPRPAFGG
RACVGADLQAEMCNTQACEKTQLEFMSQQCARTDGQPLRSSPGGASFYHWGAAVPHSQGD
ALCRHMCRAIGESFIMKRGDSFLDGTRCMPSGPREDGTLSLCVSGSCRTFGCDGRMDSQQ
VWDRCQVCGGDNSTCSPRKGSFTAGRAREYVTFLTVTPNLTSVYIANHRPLFTHLAVRIG
GRYVVAGKMSISPNTTYPSLLEDGRVEYRVALTEDRLPRLEEIRIWGPLQEDADIQVYRR
YGEEYGNLTRPDITFTYFQPKPRQAWVWAAVRGPCSVSCGAGLRWVNYSCLDQARKELVE
TVQCQGSQQPPAWPEACVLEPCPPYWAVGDFGPCSASCGGGLRERPVRCVEAQGSLLKTL
PPARCRAGAQQPAVALETCNPQPCPARWEVSEPSSCTSAGGAGLALENETCVPGADGLEA
PVTEGPGSVDEKLPAPEPCVGMSCPPGWGHLDATSAGEKAPSPWGSIRTGAQAAHVWTPA
AGSCSVSCGRGLMELRFLCMDSALRVPVQEELCGLASKPGSRREVCQAVPCPARWQYKLA
ACSVSCGRGVVRRILYCARAHGEDDGEEILLDTQCQGLPRPEPQEACSLEPCPPRWKVMS
LGPCSASCGLGTARRSVACVQLDQGQDVEVDEAACAALVRPEASVPCLIADCTYRWHVGT
WMECSVSCGDGIQRRRDTCLGPQAQAPVPADFCQHLPKPVTVRGCWAGPCVGQGTPSLVP
HEEAAAPGRTTATPAGASLEWSQARGLLFSPAPQPRRLLPGPQENSVQSSACGRQHLEPT
GTIDMRGPGQADCAVAIGRPLGEVVTLRVLESSLNCSAGDMLLLWGRLTWRKMCRKLLDM
TFSSKTNTLVVRQRCGRPGGGVLLRYGSQLAPETFYRECDMQLFGPWGEIVSPSLSPATS
NAGGCRLFINVAPHARIAIHALATNMGAGTEGANASYILIRDTHSLRTTAFHGQQVLYWE
SESSQAEMEFSEGFLKAQASLRGQYWTLQSWVPEMQDPQSWKGKEGT
>sp|Q8TE56|ATS17_HUMAN A disintegrin and metalloproteinase with thrombospondin motifs 17 OS=Homo sapiens GN=ADAMTS17 PE=2 SV=2
MCDGALLPPLVLPVLLLLVWGLDPGTAVGDAAADVEVVLPWRVRPDDVHLPPLPAAPGPR
RRRRPRTPPAAPRARPGERALLLHLPAFGRDLYLQLRRDLRFLSRGFEVEEAGAARRRGR
PAELCFYSGRVLGHPGSLVSLSACGAAGGLVGLIQLGQEQVLIQPLNNSQGPFSGREHLI
RRKWSLTPSPSAEAQRPEQLCKVLTEKKKPTWGRPSRDWRERRNAIRLTSEHTVETLVVA
DADMVQYHGAEAAQRFILTVMNMVYNMFQHQSLGIKINIQVTKLVLLRQRPAKLSIGHHG
ERSLESFCHWQNEEYGGARYLGNNQVPGGKDDPPLVDAAVFVTRTDFCVHKDEPCDTVGI
AYLGGVCSAKRKCVLAEDNGLNLAFTIAHELGHNLGMNHDDDHSSCAGRSHIMSGEWVKG
RNPSDLSWSSCSRDDLENFLKSKVSTCLLVTDPRSQHTVRLPHKLPGMHYSANEQCQILF
GMNATFCRNMEHLMCAGLWCLVEGDTSCKTKLDPPLDGTECGADKWCRAGECVSKTPIPE
HVDGDWSPWGAWSMCSRTCGTGARFRQRKCDNPPPGPGGTHCPGASVEHAVCENLPCPKG
LPSFRDQQCQAHDRLSPKKKGLLTAVVVDDKPCELYCSPLGKESPLLVADRVLDGTPCGP
YETDLCVHGKCQKIGCDGIIGSAAKEDRCGVCSGDGKTCHLVKGDFSHARGTALKDSGKG

SINSDWKIELPGEFQIAGTTVRYVRRGLWEKISAKGPTKLPLHLMVLLFHDQDYGIHYEY
TVPVNRTAENQSEPEKPQDSLFIWTHSGWEGCSVQCGGGERRTIVSCTRIVNKTTTLVND
SDCPQASRPEPQVRRCNLHPCQSRWVAGPWSPCSATCEKGFQHREVTCVYQLQNGTHVAT
RPLYCPGPRPAAVQSCEGQDCLSIWEASEWSQCSASCGKGVWKRTVACTNSQGKCDASTR
PRAEEACEDYSGCYEWKTGDWSTCSSTCGKGLQSRVVQCMHKVTGRHGSECPALSKPAPY
RQCYQEVCNDRINANTITSPRLAALTYKCTRDQWTVYCRVIREKNLCQDMRWYQRCCQTC
RDFYANKMRQPPPNS
>sp|Q8TE59|ATS19_HUMAN A disintegrin and metalloproteinase with thrombospondin motifs 19 OS=Homo sapiens GN=ADAMTS19 PE=2 SV=2
MRLTHICCCCLLYQLGFLSNGIVSELQFAPDREEWEVVFPALWRREPVDPAGGSGGSADP
GWVRGVGGGGSARAQAAGSSREVRSVAPVPLEEPVEGRSESRLRPPPPSEGEEDEELESQ
ELPRGSSGAAALSPGAPASWQPPPPPQPPPSPPPAQHAEPDGDEVLLRIPAFSRDLYLLL
RRDGRFLAPRFAVEQRPNPGPGPTGAASAPQPPAPPDAGCFYTGAVLRHPGSLASFSTCG
GGLMGFIQLNEDFIFIEPLNDTMAITGHPHRVYRQKRSMEEKVTEKSALHSHYCGIISDK
GRPRSRKIAESGRGKRYSYKLPQEYNIETVVVADPAMVSYHGADAARRFILTILNMVFNL
FQHKSLSVQVNLRVIKLILLHETPPELYIGHHGEKMLESFCKWQHEEFGKKNDIHLEMST
NWGEDMTSVDAAILITRKDFCVHKDEPCDTVGIAYLSGMCSEKRKCIIAEDNGLNLAFTI
AHEMGHNMGINHDNDHPSCADGLHIMSGEWIKGQNLGDVSWSRCSKEDLERFLRSKASNC
LLQTNPQSVNSVMVPSKLPGMTYTADEQCQILFGPLASFCQEMQHVICTGLWCKVEGEKE
CRTKLDPPMDGTDCDLGKWCKAGECTSRTSAPEHLAGEWSLWSPCSRTCSAGISSRERKC
PGLDSEARDCNGPRKQYRICENPPCPAGLPGFRDWQCQAYSVRTSSPKHILQWQAVLDEE
KPCALFCSPVGKEQPILLSEKVMDGTSCGYQGLDICANGRCQKVGCDGLLGSLAREDHCG
VCNGNGKSCKIIKGDFNHTRGAGYVEVLVIPAGARRIKVVEEKPAHSYLALRDAGKQSIN
SDWKIEHSGAFNLAGTTVHYVRRGLWEKISAKGPTTAPLHLLVLLFQDQNYGLHYEYTIP
SDPLPENQSSKAPEPLFMWTHTSWEDCDATCGGGERKTTVSCTKIMSKNISIVDNEKCKY
LTKPEPQIRKCNEQPCQTRWMMTEWTPCSRTCGKGMQSRQVACTQQLSNGTLIRARERDC
IGPKPASAQRCEGQDCMTVWEAGVWSECSVKCGKGIRHRTVRCTNPRKKCVLSTRPREAE
DCEDYSKCYVWRMGDWSKCSITCGKGMQSRVIQCMHKITGRHGNECFSSEKPAAYRPCHL
QPCNEKINVNTITSPRLAALTFKCLGDQWPVYCRVIREKNLCQDMRWYQRCCETCRDFYA
QKLQQKS
>sp|O95450|ATS2_HUMAN A disintegrin and metalloproteinase with thrombospondin motifs 2 OS=Homo sapiens GN=ADAMTS2 PE=2 SV=2
MDPPAGAARRLLCPALLLLLLLLPPPLLPPPPPPANARLAAAADPPGGPLGHGAERILAV
PVRTDAQGRLVSHVVSAATSRAGVRARRAAPVRTPSFPGGNEEEPGSHLFYNVTVFGRDL
HLRLRPNARLVAPGATMEWQGEKGTTRVEPLLGSCLYVGDVAGLAEASSVALSNCDGLAG
LIRMEEEEFFIEPLEKGLAAQEAEQGRVHVVYRRPPTSPPLGGPQALDTGASLDSLDSLS
RALGVLEEHANSSRRRARRHAADDDYNIEVLLGVDDSVVQFHGKEHVQKYLLTLMNIVNE
IYHDESLGAHINVVLVRIILLSYGKSMSLIEIGNPSQSLENVCRWAYLQQKPDTGHDEYH
DHAIFLTRQDFGPSGMQGYAPVTGMCHPVRSCTLNHEDGFSSAFVVAHETGHVLGMEHDG
QGNRCGDEVRLGSIMAPLVQAAFHRFHWSRCSQQELSRYLHSYDCLLDDPFAHDWPALPQ
LPGLHYSMNEQCRFDFGLGYMMCTAFRTFDPCKQLWCSHPDNPYFCKTKKGPPLDGTMCA
PGKHCFKGHCIWLTPDILKRDGSWGAWSPFGSCSRTCGTGVKFRTRQCDNPHPANGGRTC
SGLAYDFQLCSRQDCPDSLADFREEQCRQWDLYFEHGDAQHHWLPHEHRDAKERCHLYCE
SRETGEVVSMKRMVHDGTRCSYKDAFSLCVRGDCRKVGCDGVIGSSKQEDKCGVCGGDNS

HCKVVKGTFTRSPKKHGYIKMFEIPAGARHLLIQEVDATSHHLAVKNLETGKFILNEEND
VDASSKTFIAMGVEWEYRDEDGRETLQTMGPLHGTITVLVIPVGDTRVSLTYKYMIHEDS
LNVDDNNVLEEDSVVYEWALKKWSPCSKPCGGGSQFTKYGCRRRLDHKMVHRGFCAALSK
PKAIRRACNPQECSQPVWVTGEWEPCSQTCGRTGMQVRSVRCIQPLHDNTTRSVHAKHCN
DARPESRRACSRELCPGRWRAGPWSQCSVTCGNGTQERPVLCRTADDSFGICQEERPETA
RTCRLGPCPRNISDPSKKSYVVQWLSRPDPDSPIRKISSKGHCQGDKSIFCRMEVLSRYC
SIPGYNKLCCKSCNLYNNLTNVEGRIEPPPGKHNDIDVFMPTLPVPTVAMEVRPSPSTPL
EVPLNASSTNATEDHPETNAVDEPYKIHGLEDEVQPPNLIPRRPSPYEKTRNQRIQELID
EMRKKEMLGKF
>sp|Q9H3M9|ATX3L_HUMAN Ataxin-3-like protein OS=Homo sapiens GN=ATXN3L PE=1 SV=2
MDFIFHEKQEGFLCAQHCLNNLLQGEYFSPVELASIAHQLDEEERMRMAEGGVTSEEYLA
FLQQPSENMDDTGFFSIQVISNALKFWGLEIIHFNNPEYQKLGIDPINERSFICNYKQHW
FTIRKFGKHWFNLNSLLAGPELISDTCLANFLARLQQQAYSVFVVKGDLPDCEADQLLQI
ISVEEMDTPKLNGKKLVKQKEHRVYKTVLEKVSEESDESGTSDQDEEDFQRALELSRQET
NREDEHLRSTIELSMQGSSGNTSQDLPKTSCVTPASEQPKKIKEDYFEKHQQEQKQQQQQ
SDLPGHSSYLHERPTTSSRAIESDLSDDISEGTVQAAVDTILEIMRKNLKIKGEK
>sp|P54252|ATX3_HUMAN Ataxin-3 OS=Homo sapiens GN=ATXN3 PE=1 SV=4
MESIFHEKQEGSLCAQHCLNNLLQGEYFSPVELSSIAHQLDEEERMRMAEGGVTSEDYRT
FLQQPSGNMDDSGFFSIQVISNALKVWGLELILFNSPEYQRLRIDPINERSFICNYKEHW
FTVRKLGKQWFNLNSLLTGPELISDTYLALFLAQLQQEGYSIFVVKGDLPDCEADQLLQM
IRVQQMHRPKLIGEELAQLKEQRVHKTDLERVLEANDGSGMLDEDEEDLQRALALSRQEI
DMEDEEADLRRAIQLSMQGSSRNISQDMTQTSGTNLTSEELRKRREAYFEKQQQKQQQQQ
QQQQQGDLSGQSSHPCERPATSSGALGSDLGKACSPFIMFATFTLYLTYELHVIFALHYS
SFPL
>sp|Q13825|AUHM_HUMAN Methylglutaconyl-CoA hydratase, mitochondrial OS=Homo sapiens GN=AUH PE=1 SV=1
MAAAVAAAPGALGSLHAGGARLVAACSAWLCPGLRLPGSLAGRRAGPAIWAQGWVPAAGG
PAPKRGYSSEMKTEDELRVRHLEEENRGIVVLGINRAYGKNSLSKNLIKMLSKAVDALKS
DKKVRTIIIRSEVPGIFCAGADLKERAKMSSSEVGPFVSKIRAVINDIANLPVPTIAAID
GLALGGGLELALACDIRVAASSAKMGLVETKLAIIPGGGGTQRLPRAIGMSLAKELIFSA
RVLDGKEAKAVGLISHVLEQNQEGDAAYRKALDLAREFLPQGPVAMRVAKLAINQGMEVD
LVTGLAIEEACYAQTIPTKDRLEGLLAFKEKRPPRYKGE
>sp|Q6ZP68|ATPUN_HUMAN Putative protein ATP11AUN OS=Homo sapiens GN=ATP11AUN PE=2 SV=1
MNSPEARLCVAQCRDSYPGCQPLKDTRAWASSLKMDPAGLEGGPRDESRDEPPIRAQAAS
WDQPQGCLTYKGRRSASGTQKQLQLPDTLSSLLCWRGAIMVYIKVTVQTDDSNKLLSLLY
R
>sp|Q8TE57|ATS16_HUMAN A disintegrin and metalloproteinase with thrombospondin motifs 16 OS=Homo sapiens GN=ADAMTS16 PE=2 SV=3
MKPRARGWRGLAALWMLLAQVAEQAPACAMGPAAAAPGSPSVPRPPPPAERPGWMEKGEY
DLVSAYEVDHRGDYVSHEIMHHQRRRRAVPVSEVESLHLRLKGSRHDFHMDLRTSSSLVA
PGFIVQTLGKTGTKSVQTLPPEDFCFYQGSLRSHRNSSVALSTCQGLSGMIRTEEADYFL
RPLPSHLSWKLGRAAQGSSPSHVLYKRSTEPHAPGASEVLVTSRTWELAHQPLHSSDLRL
GLPQKQHFCGRRKKYMPQPPKEDLFILPDEYKSCLRHKRSLLRSHRNEELNVETLVVVDK
KMMQNHGHENITTYVLTILNMVSALFKDGTIGGNINIAIVGLILLEDEQPGLVISHHADH

TLSSFCQWQSGLMGKDGTRHDHAILLTGLDICSWKNEPCDTLGFAPISGMCSKYRSCTIN
EDTGLGLAFTIAHESGHNFGMIHDGEGNMCKKSEGNIMSPTLAGRNGVFSWSPCSRQYLH
KFLSTAQAICLADQPKPVKEYKYPEKLPGELYDANTQCKWQFGEKAKLCMLDFKKDICKA
LWCHRIGRKCETKFMPAAEGTICGHDMWCRGGQCVKYGDEGPKPTHGHWSDWSSWSPCSR
TCGGGVSHRSRLCTNPKPSHGGKFCEGSTRTLKLCNSQKCPRDSVDFRAAQCAEHNSRRF
RGRHYKWKPYTQVEDQDLCKLYCIAEGFDFFFSLSNKVKDGTPCSEDSRNVCIDGICERV
GCDNVLGSDAVEDVCGVCNGNNSACTIHRGLYTKHHHTNQYYHMVTIPSGARSIRIYEMN
VSTSYISVRNALRRYYLNGHWTVDWPGRYKFSGTTFDYRRSYNEPENLIATGPTNETLIV
ELLFQGRNPGVAWEYSMPRLGTEKQPPAQPSYTWAIVRSECSVSCGGGQMTVREGCYRDL
KFQVNMSFCNPKTRPVTGLVPCKVSACPPSWSVGNWSACSRTCGGGAQSRPVQCTRRVHY
DSEPVPASLCPQPAPSSRQACNSQSCPPAWSAGPWAECSHTCGKGWRKRAVACKSTNPSA
RAQLLPDAVCTSEPKPRMHEACLLQRCHKPKKLQWLVSAWSQCSVTCERGTQKRFLKCAE
KYVSGKYRELASKKCSHLPKPSLELERACAPLPCPRHPPFAAAGPSRGSWFASPWSQCTA
SCGGGVQTRSVQCLAGGRPASGCLLHQKPSASLACNTHFCPIAEKKDAFCKDYFHWCYLV
PQHGMCSHKFYGKQCCKTCSKSNL
>sp|Q9UHI8|ATS1_HUMAN A disintegrin and metalloproteinase with thrombospondin motifs 1 OS=Homo sapiens GN=ADAMTS1 PE=1 SV=4
MQRAVPEGFGRRKLGSDMGNAERAPGSRSFGPVPTLLLLAAALLAVSDALGRPSEEDEEL
VVPELERAPGHGTTRLRLHAFDQQLDLELRPDSSFLAPGFTLQNVGRKSGSETPLPETDL
AHCFYSGTVNGDPSSAAALSLCEGVRGAFYLLGEAYFIQPLPAASERLATAAPGEKPPAP
LQFHLLRRNRQGDVGGTCGVVDDEPRPTGKAETEDEDEGTEGEDEGAQWSPQDPALQGVG
QPTGTGSIRKKRFVSSHRYVETMLVADQSMAEFHGSGLKHYLLTLFSVAARLYKHPSIRN
SVSLVVVKILVIHDEQKGPEVTSNAALTLRNFCNWQKQHNPPSDRDAEHYDTAILFTRQD
LCGSQTCDTLGMADVGTVCDPSRSCSVIEDDGLQAAFTTAHELGHVFNMPHDDAKQCASL
NGVNQDSHMMASMLSNLDHSQPWSPCSAYMITSFLDNGHGECLMDKPQNPIQLPGDLPGT
SYDANRQCQFTFGEDSKHCPDAASTCSTLWCTGTSGGVLVCQTKHFPWADGTSCGEGKWC
INGKCVNKTDRKHFDTPFHGSWGMWGPWGDCSRTCGGGVQYTMRECDNPVPKNGGKYCEG
KRVRYRSCNLEDCPDNNGKTFREEQCEAHNEFSKASFGSGPAVEWIPKYAGVSPKDRCKL
ICQAKGIGYFFVLQPKVVDGTPCSPDSTSVCVQGQCVKAGCDRIIDSKKKFDKCGVCGGN
GSTCKKISGSVTSAKPGYHDIITIPTGATNIEVKQRNQRGSRNNGSFLAIKAADGTYILN
GDYTLSTLEQDIMYKGVVLRYSGSSAALERIRSFSPLKEPLTIQVLTVGNALRPKIKYTY
FVKKKKESFNAIPTFSAWVIEEWGECSKSCELGWQRRLVECRDINGQPASECAKEVKPAS
TRPCADHPCPQWQLGEWSSCSKTCGKGYKKRSLKCLSHDGGVLSHESCDPLKKPKHFIDF
CTMAECS
>sp|Q9UNA0|ATS5_HUMAN A disintegrin and metalloproteinase with thrombospondin motifs 5 OS=Homo sapiens GN=ADAMTS5 PE=1 SV=2
MLLGWASLLLCAFRLPLAAVGPAATPAQDKAGQPPTAAAAAQPRRRQGEEVQERAEPPGH
PHPLAQRRRSKGLVQNIDQLYSGGGKVGYLVYAGGRRFLLDLERDGSVGIAGFVPAGGGT
SAPWRHRSHCFYRGTVDGSPRSLAVFDLCGGLDGFFAVKHARYTLKPLLRGPWAEEEKGR
VYGDGSARILHVYTREGFSFEALPPRASCETPASTPEAHEHAPAHSNPSGRAALASQLLD
QSALSPAGGSGPQTWWRRRRRSISRARQVELLLVADASMARLYGRGLQHYLLTLASIANR
LYSHASIENHIRLAVVKVVVLGDKDKSLEVSKNAATTLKNFCKWQHQHNQLGDDHEEHYD
AAILFTREDLCGHHSCDTLGMADVGTICSPERSCAVIEDDGLHAAFTVAHEIGHLLGLSH
DDSKFCEETFGSTEDKRLMSSILTSIDASKPWSKCTSATITEFLDDGHGNCLLDLPRKQI

LGPEELPGQTYDATQQCNLTFGPEYSVCPGMDVCARLWCAVVRQGQMVCLTKKLPAVEGT
PCGKGRICLQGKCVDKTKKKYYSTSSHGNWGSWGSWGQCSRSCGGGVQFAYRHCNNPAPR
NNGRYCTGKRAIYRSCSLMPCPPNGKSFRHEQCEAKNGYQSDAKGVKTFVEWVPKYAGVL
PADVCKLTCRAKGTGYYVVFSPKVTDGTECRLYSNSVCVRGKCVRTGCDGIIGSKLQYDK
CGVCGGDNSSCTKIVGTFNKKSKGYTDVVRIPEGATHIKVRQFKAKDQTRFTAYLALKKK
NGEYLINGKYMISTSETIIDINGTVMNYSGWSHRDDFLHGMGYSATKEILIVQILATDPT
KPLDVRYSFFVPKKSTPKVNSVTSHGSNKVGSHTSQPQWVTGPWLACSRTCDTGWHTRTV
QCQDGNRKLAKGCPLSQRPSAFKQCLLKKC
>sp|Q9UP79|ATS8_HUMAN A disintegrin and metalloproteinase with thrombospondin motifs 8 OS=Homo sapiens GN=ADAMTS8 PE=2 SV=2
MLPAPAAPRWPPLLLLLLLLLPLARGAPARPAAGGQASELVVPTRLPGSAGELALHLSAF
GKGFVLRLAPDDSFLAPEFKIERLGGSGRATGGERGLRGCFFSGTVNGEPESLAAVSLCR
GLSGSFLLDGEEFTIQPQGAGGSLAQPHRLQRWGPAGARPLPRGPEWEVETGEGQRQERG
DHQEDSEEESQEEEAEGASEPPPPLGATSRTKRFVSEARFVETLLVADASMAAFYGADLQ
NHILTLMSVAARIYKHPSIKNSINLMVVKVLIVEDEKWGPEVSDNGGLTLRNFCNWQRRF
NQPSDRHPEHYDTAILLTRQNFCGQEGLCDTLGVADIGTICDPNKSCSVIEDEGLQAAHT
LAHELGHVLSMPHDDSKPCTRLFGPMGKHHVMAPLFVHLNQTLPWSPCSAMYLTELLDGG
HGDCLLDAPAAALPLPTGLPGRMALYQLDQQCRQIFGPDFRHCPNTSAQDVCAQLWCHTD
GAEPLCHTKNGSLPWADGTPCGPGHLCSEGSCLPEEEVERPKPVADGGWAPWGPWGECSR
TCGGGVQFSHRECKDPEPQNGGRYCLGRRAKYQSCHTEECPPDGKSFREQQCEKYNAYNY
TDMDGNLLQWVPKYAGVSPRDRCKLFCRARGRSEFKVFEAKVIDGTLCGPETLAICVRGQ
CVKAGCDHVVDSPRKLDKCGVCGGKGNSCRKVSGSLTPTNYGYNDIVTIPAGATNIDVKQ
RSHPGVQNDGNYLALKTADGQYLLNGNLAISAIEQDILVKGTILKYSGSIATLERLQSFR
PLPEPLTVQLLTVPGEVFPPKVKYTFFVPNDVDFSMQSSKERATTNIIQPLLHAQWVLGD
WSECSSTCGAGWQRRTVECRDPSGGQASATCNKALKPEDAKPCESQLCPL
>sp|Q9P2N4|ATS9_HUMAN A disintegrin and metalloproteinase with thrombospondin motifs 9 OS=Homo sapiens GN=ADAMTS9 PE=1 SV=4
MQFVSWATLLTLLVRDLAEMGSPDAAAAVRKDRLHPRQVKLLETLSEYEIVSPIRVNALG
EPFPTNVHFKRTRRSINSATDPWPAFASSSSSSTSSQAHYRLSAFGQQFLFNLTANAGFI
APLFTVTLLGTPGVNQTKFYSEEEAELKHCFYKGYVNTNSEHTAVISLCSGMLGTFRSHD
GDYFIEPLQSMDEQEDEEEQNKPHIIYRRSAPQREPSTGRHACDTSEHKNRHSKDKKKTR
ARKWGERINLAGDVAALNSGLATEAFSAYGNKTDNTREKRTHRRTKRFLSYPRFVEVLVV
ADNRMVSYHGENLQHYILTLMSIVASIYKDPSIGNLINIVIVNLIVIHNEQDGPSISFNA
QTTLKNFCQWQHSKNSPGGIHHDTAVLLTRQDICRAHDKCDTLGLAELGTICDPYRSCSI
SEDSGLSTAFTIAHELGHVFNMPHDDNNKCKEEGVKSPQHVMAPTLNFYTNPWMWSKCSR
KYITEFLDTGYGECLLNEPESRPYPLPVQLPGILYNVNKQCELIFGPGSQVCPYMMQCRR
LWCNNVNGVHKGCRTQHTPWADGTECEPGKHCKYGFCVPKEMDVPVTDGSWGSWSPFGTC
SRTCGGGIKTAIRECNRPEPKNGGKYCVGRRMKFKSCNTEPCLKQKRDFRDEQCAHFDGK
HFNINGLLPNVRWVPKYSGILMKDRCKLFCRVAGNTAYYQLRDRVIDGTPCGQDTNDICV
QGLCRQAGCDHVLNSKARRDKCGVCGGDNSSCKTVAGTFNTVHYGYNTVVRIPAGATNID
VRQHSFSGETDDDNYLALSSSKGEFLLNGNFVVTMAKREIRIGNAVVEYSGSETAVERIN
STDRIEQELLLQVLSVGKLYNPDVRYSFNIPIEDKPQQFYWNSHGPWQACSKPCQGERKR
KLVCTRESDQLTVSDQRCDRLPQPGHITEPCGTDCDLRWHVASRSECSAQCGLGYRTLDI
YCAKYSRLDGKTEKVDDGFCSSHPKPSNREKCSGECNTGGWRYSAWTECSKSCDGGTQRR

RAICVNTRNDVLDDSKCTHQEKVTIQRCSEFPCPQWKSGDWSECLVTCGKGHKHRQVWCQ
FGEDRLNDRMCDPETKPTSMQTCQQPECASWQAGPWGQCSVTCGQGYQLRAVKCIIGTYM
SVVDDNDCNAATRPTDTQDCELPSCHPPPAAPETRRSTYSAPRTQWRFGSWTPCSATCGK
GTRMRYVSCRDENGSVADESACATLPRPVAKEECSVTPCGQWKALDWSSCSVTCGQGRAT
RQVMCVNYSDHVIDRSECDQDYIPETDQDCSMSPCPQRTPDSGLAQHPFQNEDYRPRSAS
PSRTHVLGGNQWRTGPWGACSSTCAGGSQRRVVVCQDENGYTANDCVERIKPDEQRACES
GPCPQWAYGNWGECTKLCGGGIRTRLVVCQRSNGERFPDLSCEILDKPPDREQCNTHACP
HDAAWSTGPWSSCSVSCGRGHKQRNVYCMAKDGSHLESDYCKHLAKPHGHRKCRGGRCPK
WKAGAWSQCSVSCGRGVQQRHVGCQIGTHKIARETECNPYTRPESERDCQGPRCPLYTWR
AEEWQECTKTCGEGSRYRKVVCVDDNKNEVHGARCDVSKRPVDRESCSLQPCEYVWITGE
WSECSVTCGKGYKQRLVSCSEIYTGKENYEYSYQTTINCPGTQPPSVHPCYLRDCPVSAT
WRVGNWGSCSVSCGVGVMQRSVQCLTNEDQPSHLCHTDLKPEERKTCRNVYNCELPQNCK
EVKRLKGASEDGEYFLMIRGKLLKIFCAGMHSDHPKEYVTLVHGDSENFSEVYGHRLHNP
TECPYNGSRRDDCQCRKDYTAAGFSSFQKIRIDLTSMQIITTDLQFARTSEGHPVPFATA
GDCYSAAKCPQGRFSINLYGTGLSLTESARWISQGNYAVSDIKKSPDGTRVVGKCGGYCG
KCTPSSGTGLEVRVL
>sp|Q9UQB9|AURKC_HUMAN Aurora kinase C OS=Homo sapiens GN=AURKC PE=1 SV=1
MSSPRAVVQLGKAQPAGEELATANQTAQQPSSPAMRRLTVDDFEIGRPLGKGKFGNVYLA
RLKESHFIVALKVLFKSQIEKEGLEHQLRREIEIQAHLQHPNILRLYNYFHDARRVYLIL
EYAPRGELYKELQKSEKLDEQRTATIIEELADALTYCHDKKVIHRDIKPENLLLGFRGEV
KIADFGWSVHTPSLRRKTMCGTLDYLPPEMIEGRTYDEKVDLWCIGVLCYELLVGYPPFE
SASHSETYRRILKVDVRFPLSMPLGARDLISRLLRYQPLERLPLAQILKHPWVQAHSRRV
LPPCAQMAS
>sp|O75061|AUXI_HUMAN Putative tyrosine-protein phosphatase auxilin OS=Homo sapiens GN=DNAJC6 PE=1 SV=3
MKDSENKGASSPDMEPSYGGGLFDMVKGGAGRLFSNLKDNLKDTLKDTSSRVIQSVTSYT
KGDLDFTYVTSRIIVMSFPLDNVDIGFRNQVDDIRSFLDSRHLDHYTVYNLSPKSYRTAK
FHSRVSECSWPIRQAPSLHNLFAVCRNMYNWLLQNPKNVCVVHCLDGRAASSILVGAMFI
FCNLYSTPGPAIRLLYAKRPGIGLSPSHRRYLGYMCDLLADKPYRPHFKPLTIKSITVSP
IPFFNKQRNGCRPYCDVLIGETKIYSTCTDFERMKEYRVQDGKIFIPLNITVQGDVVVSM
YHLRSTIGSRLQAKVTNTQIFQLQFHTGFIPLDTTVLKFTKPELDACDVPEKYPQLFQVT
LDVELQPHDKVIDLTPPWEHYCTKDVNPSILFSSHQEHQDTLALGGQAPIDIPPDNPRHY
GQSGFFASLCWQDQKSEKSFCEEDHAALVNQESEQSDDELLTLSSPHGNANGDKPHGVKK
PSKKQQEPAAPPPPEDVDLLGLEGSAMSNSFSPPAAPPTNSELLSDLFGGGGAAGPTQAG
QSGVEDVFHPSGPASTQSTPRRSATSTSASPTLRVGEGATFDPFGAPSKPSGQDLLGSFL
NTSSASSDPFLQPTRSPSPTVHASSTPAVNIQPDVSGGWDWHAKPGGFGMGSKSAATSPT
GSSHGTPTHQSKPQTLDPFADLGTLGSSSFASKPTTPTGLGGGFPPLSSPQKASPQPMGG
GWQQGGAYNWQQPQPKPQPSMPHSSPQNRPNYNVSFSAMPGGQNERGKGSSNLEGKQKAA
DFEDLLSGQGFNAHKDKKGPRTIAEMRKEEMAKEMDPEKLKILEWIEGKERNIRALLSTM
HTVLWAGETKWKPVGMADLVTPEQVKKVYRKAVLVVHPDKATGQPYEQYAKMIFMELNDA
WSEFENQGQKPLY
>sp|Q13705|AVR2B_HUMAN Activin receptor type-2B OS=Homo sapiens GN=ACVR2B PE=1 SV=3
MTAPWVALALLWGSLCAGSGRGEAETRECIYYNANWELERTNQSGLERCEGEQDKRLHCY
ASWRNSSGTIELVKKGCWLDDFNCYDRQECVATEENPQVYFCCCEGNFCNERFTHLPEAG

GPEVTYEPPPTAPTLLTVLAYSLLPIGGLSLIVLLAFWMYRHRKPPYGHVDIHEDPGPPP
PSPLVGLKPLQLLEIKARGRFGCVWKAQLMNDFVAVKIFPLQDKQSWQSEREIFSTPGMK
HENLLQFIAAEKRGSNLEVELWLITAFHDKGSLTDYLKGNIITWNELCHVAETMSRGLSY
LHEDVPWCRGEGHKPSIAHRDFKSKNVLLKSDLTAVLADFGLAVRFEPGKPPGDTHGQVG
TRRYMAPEVLEGAINFQRDAFLRIDMYAMGLVLWELVSRCKAADGPVDEYMLPFEEEIGQ
HPSLEELQEVVVHKKMRPTIKDHWLKHPGLAQLCVTIEECWDHDAEARLSAGCVEERVSL
IRRSVNGTTSDCLVSLVTSVTNVDLPPKESSI
>sp|O15169|AXIN1_HUMAN Axin-1 OS=Homo sapiens GN=AXIN1 PE=1 SV=2
MNIQEQGFPLDLGASFTEDAPRPPVPGEEGELVSTDPRPASYSFCSGKGVGIKGETSTAT
PRRSDLDLGYEPEGSASPTPPYLKWAESLHSLLDDQDGISLFRTFLKQEGCADLLDFWFA
CTGFRKLEPCDSNEEKRLKLARAIYRKYILDNNGIVSRQTKPATKSFIKGCIMKQLIDPA
MFDQAQTEIQATMEENTYPSFLKSDIYLEYTRTGSESPKVCSDQSSGSGTGKGISGYLPT
LNEDEEWKCDQDMDEDDGRDAAPPGRLPQKLLLETAAPRVSSSRRYSEGREFRYGSWREP
VNPYYVNAGYALAPATSANDSEQQSLSSDADTLSLTDSSVDGIPPYRIRKQHRREMQESV
QVNGRVPLPHIPRTYRVPKEVRVEPQKFAEELIHRLEAVQRTREAEEKLEERLKRVRMEE
EGEDGDPSSGPPGPCHKLPPAPAWHHFPPRCVDMGCAGLRDAHEENPESILDEHVQRVLR
TPGRQSPGPGHRSPDSGHVAKMPVALGGAASGHGKHVPKSGAKLDAAGLHHHRHVHHHVH
HSTARPKEQVEAEATRRAQSSFAWGLEPHSHGARSRGYSESVGAAPNASDGLAHSGKVGV
ACKRNAKKAESGKSASTEVPGASEDAEKNQKIMQWIIEGEKEISRHRRTGHGSSGTRKPQ
PHENSRPLSLEHPWAGPQLRTSVQPSHLFIQDPTMPPHPAPNPLTQLEEARRRLEEEEKR
ASRAPSKQRYVQEVMRRGRACVRPACAPVLHVVPAVSDMELSETETRSQRKVGGGSAQPC
DSIVVAYYFCGEPIPYRTLVRGRAVTLGQFKELLTKKGSYRYYFKKVSDEFDCGVVFEEV
REDEAVLPVFEEKIIGKVEKVD
>sp|Q96A70|AZIN2_HUMAN Antizyme inhibitor 2 OS=Homo sapiens GN=AZIN2 PE=1 SV=1
MAGYLSESDFVMVEEGFSTRDLLKELTLGASQATTDEVAAFFVADLGAIVRKHFCFLKCL
PRVRPFYAVKCNSSPGVLKVLAQLGLGFSCANKAEMELVQHIGIPASKIICANPCKQIAQ
IKYAAKHGIQLLSFDNEMELAKVVKSHPSAKMVLCIATDDSHSLSCLSLKFGVSLKSCRH
LLENAKKHHVEVVGVSFHIGSGCPDPQAYAQSIADARLVFEMGTELGHKMHVLDLGGGFP
GTEGAKVRFEEIASVINSALDLYFPEGCGVDIFAELGRYYVTSAFTVAVSIIAKKEVLLD
QPGREEENGSTSKTIVYHLDEGVYGIFNSVLFDNICPTPILQKKPSTEQPLYSSSLWGPA
VDGCDCVAEGLWLPQLHVGDWLVFDNMGAYTVGMGSPFWGTQACHITYAMSRVAWEALRR
QLMAAEQEDDVEGVCKPLSCGWEITDTLCVGPVFTPASIM
>sp|Q92843|B2CL2_HUMAN Bcl-2-like protein 2 OS=Homo sapiens GN=BCL2L2 PE=1 SV=2
MATPASAPDTRALVADFVGYKLRQKGYVCGAGPGEGPAADPLHQAMRAAGDEFETRFRRT
FSDLAAQLHVTPGSAQQRFTQVSDELFQGGPNWGRLVAFFVFGAALCAESVNKEMEPLVG
QVQEWMVAYLETQLADWIHSSGGWAEFTALYGDGALEEARRLREGNWASVRTVLTGAVAL
GALVTVGAFFASK
>sp|Q9HD36|B2L10_HUMAN Bcl-2-like protein 10 OS=Homo sapiens GN=BCL2L10 PE=1 SV=2
MADPLRERTELLLADYLGYCAREPGTPEPAPSTPEAAVLRSAAARLRQIHRSFFSAYLGY
PGNRFELVALMADSVLSDSPGPTWGRVVTLVTFAGTLLERGPLVTARWKKWGFQPRLKEQ
EGDVARDCQRLVALLSSRLMGQHRAWLQAQGGWDGFCHFFRTPFPLAFWRKQLVQAFLSC
LLTTAFIYLWTRLL
>sp|O43521|B2L11_HUMAN Bcl-2-like protein 11 OS=Homo sapiens GN=BCL2L11 PE=1 SV=1
MAKQPSDVSSECDREGRQLQPAERPPQLRPGAPTSLQTEPQGNPEGNHGGEGDSCPHGSP

QGPLAPPASPGPFATRSPLFIFMRRSSLLSRSSSGYFSFDTDRSPAPMSCDKSTQTPSPP
CQAFNHYLSAMASMRQAEPADMRPEIWIAQELRRIGDEFNAYYARRVFLNNYQAAEDHPR
MVILRLLRYIVRLVWRMH
>sp|O94766|B3GA3_HUMAN Galactosylgalactosylxylosylprotein 3-beta-glucuronosyltransferase 3 OS=Homo sapiens GN=B3GAT3 PE=1 SV=2
MKLKLKNVFLAYFLVSIAGLLYALVQLGQPCDCLPPLRAAAEQLRQKDLRISQLQAELRR
PPPAPAQPPEPEALPTIYVVTPTYARLVQKAELVRLSQTLSLVPRLHWLLVEDAEGPTPL
VSGLLAASGLLFTHLVVLTPKAQRLREGEPGWVHPRGVEQRNKALDWLRGRGGAVGGEKD
PPPPGTQGVVYFADDDNTYSRELFEEMRWTRGVSVWPVGLVGGLRFEGPQVQDGRVVGFH
TAWEPSRPFPVDMAGFAVALPLLLDKPNAQFDSTAPRGHLESSLLSHLVDPKDLEPRAAN
CTRVLVWHTRTEKPKMKQEEQLQRQGRGSDPAIEV
>sp|Q8NFL0|B3GN7_HUMAN UDP-GlcNAc:betaGal beta-1,3-N-acetylglucosaminyltransferase 7 OS=Homo sapiens GN=B3GNT7 PE=2 SV=1
MSLWKKTVYRSLCLALALLVAVTVFQRSLTPGQFLQEPPPPTLEPQKAQKPNGQLVNPNN
FWKNPKDVAAPTPMASQGPQAWDVTTTNCSANINLTHQPWFQVLEPQFRQFLFYRHCRYF
PMLLNHPEKCRGDVYLLVVVKSVITQHDRREAIRQTWGRERQSAGGGRGAVRTLFLLGTA
SKQEERTHYQQLLAYEDRLYGDILQWGFLDTFFNLTLKEIHFLKWLDIYCPHVPFIFKGD
DDVFVNPTNLLEFLADRQPQENLFVGDVLQHARPIRRKDNKYYIPGALYGKASYPPYAGG
GGFLMAGSLARRLHHACDTLELYPIDDVFLGMCLEVLGVQPTAHEGFKTFGISRNRNSRM
NKEPCFFRAMLVVHKLLPPELLAMWGLVHSNLTCSRKLQVL
>sp|Q9H503|BAFL_HUMAN Barrier-to-autointegration factor-like protein OS=Homo sapiens GN=BANF2 PE=1 SV=1
MDNMSPRLRAFLSEPIGEKDVCWVDGISHELAINLVTKGINKAYILLGQFLLMHKNEAEF
QRWLICCFGATECEAQQTSHCLKEWCACFL
>sp|Q9UL15|BAG5_HUMAN BAG family molecular chaperone regulator 5 OS=Homo sapiens GN=BAG5 PE=1 SV=1
MDMGNQHPSISRLQEIQKEVKSVEQQVIGFSGLSDDKNYKKLERILTKQLFEIDSVDTEG
KGDIQQARKRAAQETERLLKELEQNANHPHRIEIQNIFEEAQSLVREKIVPFYNGGNCVT
DEFEEGIQDIILRLTHVKTGGKISLRKARYHTLTKICAVQEIIEDCMKKQPSLPLSEDAH
PSVAKINFVMCEVNKARGVLIALLMGVNNNETCRHLSCVLSGLIADLDALDVCGRTEIRN
YRREVVEDINKLLKYLDLEEEADTTKAFDLRQNHSILKIEKVLKRMREIKNELLQAQNPS
ELYLSSKTELQGLIGQLDEVSLEKNPCIREARRRAVIEVQTLITYIDLKEALEKRKLFAC
EEHPSHKAVWNVLGNLSEIQGEVLSFDGNRTDKNYIRLEELLTKQLLALDAVDPQGEEKC
KAARKQAVRLAQNILSYLDLKSDEWEY
>sp|P46379|BAG6_HUMAN Large proline-rich protein BAG6 OS=Homo sapiens GN=BAG6 PE=1 SV=2
MEPNDSTSTAVEEPDSLEVLVKTLDSQTRTFIVGAQMNVKEFKEHIAASVSIPSEKQRLI
YQGRVLQDDKKLQEYNVGGKVIHLVERAPPQTHLPSGASSGTGSASATHGGGSPPGTRGP
GASVHDRNANSYVMVGTFNLPSDGSAVDVHINMEQAPIQSEPRVRLVMAQHMIRDIQTLL
SRMETLPYLQCRGGPQPQHSQPPPQPPAVTPEPVALSSQTSEPVESEAPPREPMEAEEVE
ERAPAQNPELTPGPAPAGPTPAPETNAPNHPSPAEYVEVLQELQRLESRLQPFLQRYYEV
LGAAATTDYNNNHEGREEDQRLINLVGESLRLLGNTFVALSDLRCNLACTPPRHLHVVRP
MSHYTTPMVLQQAAIPIQINVGTTVTMTGNGTRPPPTPNAEAPPPGPGQASSVAPSSTNV
ESSAEGAPPPGPAPPPATSHPRVIRISHQSVEPVVMMHMNIQDSGTQPGGVPSAPTGPLG
PPGHGQTLGQQVPGFPTAPTRVVIARPTPPQARPSHPGGPPVSGTLQGAGLGTNASLAQM

VSGLVGQLLMQPVLVAQGTPGMAPPPAPATASASAGTTNTATTAGPAPGGPAQPPPTPQP
SMADLQFSQLLGNLLGPAGPGAGGSGVASPTITVAMPGVPAFLQGMTDFLQATQTAPPPP
PPPPPPPPAPEQQTMPPPGSPSGGAGSPGGLGLESLSPEFFTSVVQGVLSSLLGSLGARA
GSSESIAAFIQRLSGSSNIFEPGADGALGFFGALLSLLCQNFSMVDVVMLLHGHFQPLQR
LQPQLRSFFHQHYLGGQEPTPSNIRMATHTLITGLEEYVRESFSLVQVQPGVDIIRTNLE
FLQEQFNSIAAHVLHCTDSGFGARLLELCNQGLFECLALNLHCLGGQQMELAAVINGRIR
RMSRGVNPSLVSWLTTMMGLRLQVVLEHMPVGPDAILRYVRRVGDPPQPLPEEPMEVQGA
ERASPEPQRENASPAPGTTAEEAMSRGPPPAPEGGSRDEQDGASAETEPWAAAVPPEWVP
IIQQDIQSQRKVKPQPPLSDAYLSGMPAKRRKTMQGEGPQLLLSEAVSRAAKAAGARPLT
SPESLSRDLEAPEVQESYRQQLRSDIQKRLQEDPNYSPQRFPNAQRAFADDP
>sp|Q9P281|BAHC1_HUMAN BAH and coiled-coil domain-containing protein 1 OS=Homo sapiens GN=BAHCC1 PE=1 SV=3
MDGRDFAPPPHLLSERGSLGHRSAAAAARLAPAGPAAQPPAHFQPGKYFPSPLPMASHTA
SSRLMGSSPASSFMGSFLTSSLGSAASTHPSGPSSSPPEQAYRGSHPTTSQIWFSHSHEA
PGYPRFSGSLASTFLPVSHLDHHGNSNVLYGQHRFYGTQKDNFYLRNLPPQPTLLPANHN
FPSVARAAPAHPMGSCSRDRDRGEAGSLQKGPKDFDRFLVGKELGREKAGKAAEGKERPA
AEEDGGKERHKLVLPVPADGHCREGGPAPRGACEGRPKHLTSCLLNTKVLNGEMGRAALA
SCAGGMLGRPGTGVVTSGRCAKEAAGPPEPGPAFSECLERRQMLHHTASYAGPPPPLSTA
AGSFPCLQLHGGPDGLCPLQDKAPRDLKASGPTFVPSVGHLADKGRPFQAAEACAVAGEG
KDRHLEGTMAPDHAAPYGVSYAHLKAEGKGERRPGGFEAALNPRLKGLDYLSSAGPEASF
PGLPKSGLDKSGYFELPTSSQDCARPGHQDPLGGKAPQACCTLDKTVGKEAPAGPPGAQK
VARIRHQQHLMAAEVEQGGIGAEAKRKSLELASLGYSGPHLPPWGVQAGQGTAMAISEER
KAGAYLDPFGSGLQQAALLPQELPAPPDEVSAMKNLLKYSSQALVVGQKAPLVGLGGLKA
SCIQQEAKFLSSKGPGQSERPDCARSREHDTTHGDGEVRQPPVGIAVALARQKDTVSRSE
AAYGTNTARQGRAAPAFKGGGGPRSTHALDLEAEEERTRLCDDRLGLASRELLLQDSKDR
VEFARIHPPSSCPGDLAPHLMMQSGQLGGDPAPHTHPHPPWLPRTRSPSLWMGGHSYGLG
HPALHQNLPPGFPASVAGPVPSVFPLPQDAPTQLVILPSEPTPHSAPHALADVMDQASLW
PPMYGGRGPASHMQHPGQLPVYSRPQLLRQQELYALQQQRAAQFQRKPEDQHLDLEEPAQ
EKAPKSTHKPVALTPTAPGAPSPAAGPTKLPPCCHPPDPKPPASSPTPPPRPSAPCTLNV
CPASSPGPGSRVRSAEEKNGEGQQSTADIITSEPDLPPGYLRPMAGLGFSLPSDVHSSNL
EDPETMQTTAPGAQPEPTRTFLPGEPPPCSPRSLEEPGLLSGAREATQDLAATPYPTERG
PQGKAADPSPLEGLQELQCAALLEAGGPEATGQAHSTQGGAREERSREEGEQGPSSGASS
QVLEQRAGSPGALEDEGEQPAPEEDELEEDELGQQSMEDSEEDCGGAPDNSHPPRALPGL
DALVAATINLGDLPSDSPPDPQPPAASGPPSTVPLPHSSGIHGIALLSELADLAIQRQRS
ERTVPEEEEDVLAFNLQHLATLATAWSLVEAAGLDSSTAPAQPPTANPCSGPRLTPRMQI
LQRKDTWTPKTKPVCPLKAAIDRLDTQEVGMRVRLAELQRRYKEKQRELARLQRKHDHER
DESSRSPARRGPGRPRKRKHSSSLPAPRPTGPLPRSDGKKVKAVRTSLGLLCAELRGGSG
GEPAKKRSKLERSVYAGLQTASVEKAQCKKSSCQGGLAPSVAHRVAQLKPKVKSKGLPTG
LSSFQQKEATPGGRIREKLSRAKSAKVSGATRHPQPKGHGSRETPRCPAQPSVAASQEAG
NGYDSEDCEGLLGTEAPPREAGLLLHTGASVAVLGPSPSSVVKMEANQKAKKKKERQGLL
GACRLSSPESEVKIKRRSVKAKVGTTLERAPGQRPPGALGKKKAKGKAKGSLRAEPGATP
SRDALFNPSRAFACREEGSQLASERLKRATRKGTVLQPVLRRKNGALSITLATRNAKAIL
GKGRKLSKVKHKAGKQGKGRAVSRLLESFAVEEDFEFDDNSSFSEEEEDEEEEEEDSGPL
SAEQSAALARSCAIHKEDLRDGLPVLIPKEDSLLYAGSVRTLQPPDIYSIVIEGERGNRQ

RIYSLEQLLQEAVLDVRPQSSRYLPPGTRVCAYWSQKSRCLYPGNVVRGASGDEDEDLDS
VVVEFDDGDTGHIAVSNVRLLPPDFKIQCTEPSPALLVSSSCRRTKKVSSEAPPPSEAAT
PSLSPKAQDGPEALKTPGKKSISKDKAGKAELLTSGAKSPTGASDHFLGRRGSPLLSWSA
VAQTKRKAVAAASKGPGVLQNLFQLNGSSKKLRAREALFPVHSVATPIFGNGFRADSFSS
LASSYAPFVGGTGPGLPRGAHKLLRAKKAERVEAEKGGRRRAGGEFLVKLDHEGVTSPKN
KTCKALLMGDKDFSPKLGRPLPSPSYVHPALVGKDKKGRAPIPPLPMGLALRKYAGQAEF
PLPYDSDCHSSFSDEDEDGPGLAAGVPSRFLARLSVSSSSSGSSTSSSSGSVSTSSLCSS
DNEDSSYSSDDEDPALLLQTCLTHPVPTLLAQPEALRSKGSGPHAHAQRCFLSRATVAGT
GAGSGPSSSSKSKLKRKEALSFSKAKELSRRQRPPSVENRPKISAFLPARQLWKWSGNPT
QRRGMKGKARKLFYKAIVRGEETLRVGDCAVFLSAGRPNLPYIGRIESMWESWGSNMVVK
VKWFYHPEETKLGKRQCDGKNALYQSCHEDENDVQTISHKCQVVAREQYEQMARSRKCQD
RQDLYYLAGTYDPTTGRLVTADGVPILC
>sp|Q9UQB8|BAIP2_HUMAN Brain-specific angiogenesis inhibitor 1-associated protein 2 OS=Homo sapiens GN=BAIAP2 PE=1 SV=1
MSLSRSEEMHRLTENVYKTIMEQFNPSLRNFIAMGKNYEKALAGVTYAAKGYFDALVKMG
ELASESQGSKELGDVLFQMAEVHRQIQNQLEEMLKSFHNELLTQLEQKVELDSRYLSAAL
KKYQTEQRSKGDALDKCQAELKKLRKKSQGSKNPQKYSDKELQYIDAISNKQGELENYVS
DGYKTALTEERRRFCFLVEKQCAVAKNSAAYHSKGKELLAQKLPLWQQACADPSKIPERA
VQLMQQVASNGATLPSALSASKSNLVISDPIPGAKPLPVPPELAPFVGRMSAQESTPIMN
GVTGPDGEDYSPWADRKAAQPKSLSPPQSQSKLSDSYSNTLPVRKSVTPKNSYATTENKT
LPRSSSMAAGLERNGRMRVKAIFSHAAGDNSTLLSFKEGDLITLLVPEARDGWHYGESEK
TKMRGWFPFSYTRVLDSDGSDRLHMSLQQGKSSSTGNLLDKDDLAIPPPDYGAASRAFPA
QTASGFKQRPYSVAVPAFSQGLDDYGARSMSRNPFAHVQLKPTVTNDRCDLSAQGPEGRE
HGDGSARTLAGR
>sp|P35613|BASI_HUMAN Basigin OS=Homo sapiens GN=BSG PE=1 SV=2
MAAALFVLLGFALLGTHGASGAAGFVQAPLSQQRWVGGSVELHCEAVGSPVPEIQWWFEG
QGPNDTCSQLWDGARLDRVHIHATYHQHAASTISIDTLVEEDTGTYECRASNDPDRNHLT
RAPRVKWVRAQAVVLVLEPGTVFTTVEDLGSKILLTCSLNDSATEVTGHRWLKGGVVLKE
DALPGQKTEFKVDSDDQWGEYSCVFLPEPMGTANIQLHGPPRVKAVKSSEHINEGETAML
VCKSESVPPVTDWAWYKITDSEDKALMNGSESRFFVSSSQGRSELHIENLNMEADPGQYR
CNGTSSKGSDQAIITLRVRSHLAALWPFLGIVAEVLVLVTIIFIYEKRRKPEDVLDDDDA
GSAPLKSSGQHQNDKGKNVRQRNSS
>sp|Q8N1L9|BATF2_HUMAN Basic leucine zipper transcriptional factor ATF-like 2 OS=Homo sapiens GN=BATF2 PE=1 SV=1
MHLCGGNGLLTQTDPKEQQRQLKKQKNRAAAQRSRQKHTDKADALHQQHESLEKDNLALR
KEIQSLQAELAWWSRTLHVHERLCPMDCASCSAPGLLGCWDQAEGLLGPGPQGQHGCREQ
LELFQTPGSCYPAQPLSPGPQPHDSPSLLQCPLPSLSLGPAVVAEPPVQLSPSPLLFASH
TGSSLQGSSSKLSALQPSLTAQTAPPQPLELEHPTRGKLGSSPDNPSSALGLARLQSREH
KPALSAATWQGLVVDPSPHPLLAFPLLSSAQVHF
>sp|Q9UIF9|BAZ2A_HUMAN Bromodomain adjacent to zinc finger domain protein 2A OS=Homo sapiens GN=BAZ2A PE=1 SV=4
MEMEANDHFNFTGLPPAPAASGLKPSPSSGEGLYTNGSPMNFPQQGKSLNGDVNVNGLST
VSHTTTSGILNSAPHSSSTSHLHHPSVAYDCLWNYSQYPSANPGSNLKDPPLLSQFSGGQ
YPLNGILGGSRQPSSPSHNTNLRAGSQEFWANGTQSPMGLNFDSQELYDSFPDQNFEVMP

NGPPSFFTSPQTSPMLGSSIQTFAPSQEVGSGIHPDEAAEKEMTSVVAENGTGLVGSLEL
EEEQPELKMCGYNGSVPSVESLHQEVSVLVPDPTVSCLDDPSHLPDQLEDTPILSEDSLE
PFNSLAPEPVSGGLYGIDDTELMGAEDKLPLEDSPVISALDCPSLNNATAFSLLADDSQT
STSIFASPTSPPVLGESVLQDNSFDLNNGSDAEQEEMETQSSDFPPSLTQPAPDQSSTIQ
LHPATSPAVSPTTSPAVSLVVSPAASPEISPEVCPAASTVVSPAVFSVVSPASSAVLPAV
SLEVPLTASVTSPKASPVTSPAAAFPTASPANKDVSSFLETTADVEEITGEGLTASGSGD
VMRRRIATPEEVRLPLQHGWRREVRIKKGSHRWQGETWYYGPCGKRMKQFPEVIKYLSRN
VVHSVRREHFSFSPRMPVGDFFEERDTPEGLQWVQLSAEEIPSRIQAITGKRGRPRNTEK
AKTKEVPKVKRGRGRPPKVKITELLNKTDNRPLKKLEAQETLNEEDKAKIAKSKKKMRQK
VQRGECQTTIQGQARNKRKQETKSLKQKEAKKKSKAEKEKGKTKQEKLKEKVKREKKEKV
KMKEKEEVTKAKPACKADKTLATQRRLEERQRQQMILEEMKKPTEDMCLTDHQPLPDFSR
VPGLTLPSGAFSDCLTIVEFLHSFGKVLGFDPAKDVPSLGVLQEGLLCQGDSLGEVQDLL
VRLLKAALHDPGFPSYCQSLKILGEKVSEIPLTRDNVSEILRCFLMAYGVEPALCDRLRT
QPFQAQPPQQKAAVLAFLVHELNGSTLIINEIDKTLESMSSYRKNKWIVEGRLRRLKTVL
AKRTGRSEVEMEGPEECLGRRRSSRIMEETSGMEEEEEEESIAAVPGRRGRRDGEVDATA
SSIPELERQIEKLSKRQLFFRKKLLHSSQMLRAVSLGQDRYRRRYWVLPYLAGIFVEGTE
GNLVPEEVIKKETDSLKVAAHASLNPALFSMKMELAGSNTTASSPARARGRPRKTKPGSM
QPRHLKSPVRGQDSEQPQAQLQPEAQLHAPAQPQPQLQLQLQSHKGFLEQEGSPLSLGQS
QHDLSQSAFLSWLSQTQSHSSLLSSSVLTPDSSPGKLDPAPSQPPEEPEPDEAESSPDPQ
ALWFNISAQMPCNAAPTPPPAVSEDQPTPSPQQLASSKPMNRPSAANPCSPVQFSSTPLA
GLAPKRRAGDPGEMPQSPTGLGQPKRRGRPPSKFFKQMEQRYLTQLTAQPVPPEMCSGWW
WIRDPEMLDAMLKALHPRGIREKALHKHLNKHRDFLQEVCLRPSADPIFEPRQLPAFQEG
IMSWSPKEKTYETDLAVLQWVEELEQRVIMSDLQIRGWTCPSPDSTREDLAYCEHLSDSQ
EDITWRGRGREGLAPQRKTTNPLDLAVMRLAALEQNVERRYLREPLWPTHEVVLEKALLS
TPNGAPEGTTTEISYEITPRIRVWRQTLERCRSAAQVCLCLGQLERSIAWEKSVNKVTCL
VCRKGDNDEFLLLCDGCDRGCHIYCHRPKMEAVPEGDWFCTVCLAQQVEGEFTQKPGFPK
RGQKRKSGYSLNFSEGDGRRRRVLLRGRESPAAGPRYSEEGLSPSKRRRLSMRNHHSDLT
FCEIILMEMESHDAAWPFLEPVNPRLVSGYRRIIKNPMDFSTMRERLLRGGYTSSEEFAA
DALLVFDNCQTFNEDDSEVGKAGHIMRRFFESRWEEFYQGKQANL
>sp|Q8TAM1|BBS10_HUMAN Bardet-Biedl syndrome 10 protein OS=Homo sapiens GN=BBS10 PE=1 SV=2
MLSSMAAAGSVKAALQVAEVLEAIVSCCVGPEGRQVLCTKPTGEVLLSRNGGRLLEALHL
EHPIARMIVDCVSSHLKKTGDGAKTFIIFLCHLLRGLHAITDREKDPLMCENIQTHGRHW
KNCSRWKFISQALLTFQTQILDGIMDQYLSRHFLSIFSSAKERTLCRSSLELLLEAYFCG
RVGRNNHKFISQLMCDYFFKCMTCKSGIGVFELVDDHFVELNVGVTGLPVSDSRIIAGLV
LQKDFSVYRPADGDMRMVIVTETIQPLFSTSGSEFILNSEAQFQTSQFWIMEKTKAIMKH
LHSQNVKLLISSVKQPDLVSYYAGVNGISVVECLSSEEVSLIRRIIGLSPFVPPQAFSQC
EIPNTALVKFCKPLILRSKRYVHLGLISTCAFIPHSIVLCGPVHGLIEQHEDALHGALKM
LRQLFKDLDLNYMTQTNDQNGTSSLFIYKNSGESYQAPDPGNGSIQRPYQDTVAENKDAL
EKTQTYLKVHSNLVIPDVELETYIPYSTPTLTPTDTFQTVETLTCLSLERNRLTDYYEPL
LKNNSTAYSTRGNRIEISYENLQVTNITRKGSMLPVSCKLPNMGTSQSYLSSSMPAGCVL
PVGGNFEILLHYYLLNYAKKCHQSEETMVSMIIANALLGIPKVLYKSKTGKYSFPHTYIR
AVHALQTNQPLVSSQTGLESVMGKYQLLTSVLQCLTKILTIDMVITVKRHPQKVHNQDSE
DEL

>sp|Q8NFJ9|BBS1_HUMAN Bardet-Biedl syndrome 1 protein OS=Homo sapiens GN=BBS1 PE=1 SV=1
MAAASSSDSDACGAESNEANSKWLDAHYDPMANIHTFSACLALADLHGDGEYKLVVGDLG
PGGQQPRLKVLKGPLVMTESPLPALPAAAATFLMEQHEPRTPALALASGPCVYVYKNLRP
YFKFSLPQLPPNPLEQDLWNQAKEDRIDPLTLKEMLESIRETAEEPLSIQSLRFLQLELS
EMEAFVNQHKSNSIKRQTVITTMTTLKKNLADEDAVSCLVLGTENKELLVLDPEAFTILA
KMSLPSVPVFLEVSGQFDVEFRLAAACRNGNIYILRRDSKHPKYCIELSAQPVGLIRVHK
VLVVGSTQDSLHGFTHKGKKLWTVQMPAAILTMNLLEQHSRGLQAVMAGLANGEVRIYRD
KALLNVIHTPDAVTSLCFGRYGREDNTLIMTTRGGGLIIKILKRTAVFVEGGSEVGPPPA
QAMKLNVPRKTRLYVDQTLREREAGTAMHRAFQTDLYLLRLRAARAYLQALESSLSPLST
TAREPLKLHAVVQGLGPTFKLTLHLQNTSTTRPVLGLLVCFLYNEALYSLPRAFFKVPLL
VPGLNYPLETFVESLSNKGISDIIKVLVLREGQSAPLLSAHVNMPGSEGLAAA
>sp|Q9BXC9|BBS2_HUMAN Bardet-Biedl syndrome 2 protein OS=Homo sapiens GN=BBS2 PE=1 SV=1
MLLPVFTLKLRHKISPRMVAIGRYDGTHPCLAAATQTGKVFIHNPHTRNQHVSASRVFQS
PLESDVSLLNINQAVSCLTAGVLNPELGYDALLVGTQTNLLAYDVYNNSDLFYREVADGA
NAIVLGTLGDISSPLAIIGGNCALQGFNHEGSDLFWTVTGDNVNSLALCDFDGDGKKELL
VGSEDFDIRVFKEDEIVAEMTETEIVTSLCPMYGSRFGYALSNGTVGVYDKTSRYWRIKS
KNHAMSIHAFDLNSDGVNELITGWSNGKVDARSDRTGEVIFKDNFSSAIAGVVEGDYRMD
GHIQLICCSVDGEIRGYLPGTAEMRGNLMDTSAEQDLIRELSQKKQNLLLELRNYEENAK
AELASPLNEADGHRGIIPANTRLHTTLSVSLGNETQTAHTELRISTSNDTIIRAVLIFAE
GIFTGESHVVHPSIHNLSSSICIPIVPPKDVPVDLHLKAFVGYRSSTQFHVFESTRQLPR
FSMYALTSLDPASEPISYVNFTIAERAQRVVVWLGQNFLLPEDTHIQNAPFQVCFTSLRN
GGHLHIKIKLSGEITINTDDIDLAGDIIQSMASFFAIEDLQVEADFPVYFEELRKVLVKV
DEYHSVHQKLSADMADHSNLIRSLLVGAEDARLMRDMKTMKSRYMELYDLNRDLLNGYKI
RCNNHTELLGNLKAVNQAIQRAGRLRVGKPKNQVITACRDAIRSNNINTLFKIMRVGTAS
S
>sp|P50895|BCAM_HUMAN Basal cell adhesion molecule OS=Homo sapiens GN=BCAM PE=1 SV=2
MEPPDAPAQARGAPRLLLLAVLLAAHPDAQAEVRLSVPPLVEVMRGKSVILDCTPTGTHD
HYMLEWFLTDRSGARPRLASAEMQGSELQVTMHDTRGRSPPYQLDSQGRLVLAEAQVGDE
RDYVCVVRAGAAGTAEATARLNVFAKPEATEVSPNKGTLSVMEDSAQEIATCNSRNGNPA
PKITWYRNGQRLEVPVEMNPEGYMTSRTVREASGLLSLTSTLYLRLRKDDRDASFHCAAH
YSLPEGRHGRLDSPTFHLTLHYPTEHVQFWVGSPSTPAGWVREGDTVQLLCRGDGSPSPE
YTLFRLQDEQEEVLNVNLEGNLTLEGVTRGQSGTYGCRVEDYDAADDVQLSKTLELRVAY
LDPLELSEGKVLSLPLNSSAVVNCSVHGLPTPALRWTKDSTPLGDGPMLSLSSITFDSNG
TYVCEASLPTVPVLSRTQNFTLLVQGSPELKTAEIEPKADGSWREGDEVTLICSARGHPD
PKLSWSQLGGSPAEPIPGRQGWVSSSLTLKVTSALSRDGISCEASNPHGNKRHVFHFGTV
SPQTSQAGVAVMAVAVSVGLLLLVVAVFYCVRRKGGPCCRQRREKGAPPPGEPGLSHSGS
EQPEQTGLLMGGASGGARGGSGGFGDEC
>sp|Q9NTI2|AT8A2_HUMAN Phospholipid-transporting ATPase IB OS=Homo sapiens GN=ATP8A2 PE=1 SV=2
MSRATSVGDQLEAPARTIYLNQPHLNKFRDNQISTAKYSVLTFLPRFLYEQIRRAANAFF
LFIALLQQIPDVSPTGRYTTLVPLIIILTIAGIKEIVEDFKRHKADNAVNKKKTIVLRNG
MWHTIMWKEVAVGDIVKVVNGQYLPADVVLLSSSEPQAMCYVETANLDGETNLKIRQGLS
HTADMQTREVLMKLSGTIECEGPNRHLYDFTGNLNLDGKSLVALGPDQILLRGTQLRNTQ
WVFGIVVYTGHDTKLMQNSTKAPLKRSNVEKVTNVQILVLFGILLVMALVSSAGALYWNR

SHGEKNWYIKKMDTTSDNFGYNLLTFIILYNNLIPISLLVTLEVVKYTQALFINWDTDMY
YIGNDTPAMARTSNLNEELGQVKYLFSDKTGTLTCNIMNFKKCSIAGVTYGHFPELAREP
SSDDFCRMPPPCSDSCDFDDPRLLKNIEDRHPTAPCIQEFLTLLAVCHTVVPEKDGDNII
YQASSPDEAALVKGAKKLGFVFTARTPFSVIIEAMGQEQTFGILNVLEFSSDRKRMSVIV
RTPSGRLRLYCKGADNVIFERLSKDSKYMEETLCHLEYFATEGLRTLCVAYADLSENEYE
EWLKVYQEASTILKDRAQRLEECYEIIEKNLLLLGATAIEDRLQAGVPETIATLLKAEIK
IWVLTGDKQETAINIGYSCRLVSQNMALILLKEDSLDATRAAITQHCTDLGNLLGKENDV
ALIIDGHTLKYALSFEVRRSFLDLALSCKAVICCRVSPLQKSEIVDVVKKRVKAITLAIG
DGANDVGMIQTAHVGVGISGNEGMQATNNSDYAIAQFSYLEKLLLVHGAWSYNRVTKCIL
YCFYKNVVLYIIELWFAFVNGFSGQILFERWCIGLYNVIFTALPPFTLGIFERSCTQESM
LRFPQLYKITQNGEGFNTKVFWGHCINALVHSLILFWFPMKALEHDTVLTSGHATDYLFV
GNIVYTYVVVTVCLKAGLETTAWTKFSHLAVWGSMLTWLVFFGIYSTIWPTIPIAPDMRG
QATMVLSSAHFWLGLFLVPTACLIEDVAWRAAKHTCKKTLLEEVQELETKSRVLGKAVLR
DSNGKRLNERDRLIKRLGRKTPPTLFRGSSLQQGVPHGYAFSQEEHGAVSQEEVIRAYDT
TKKKSRKK
>sp|P0DMR3|AT8OS_HUMAN Putative protein ATXN8OS OS=Homo sapiens GN=ATXN8OS PE=5 SV=1
MPCPGAPCCSLVATGSRVPFSGLKEEEEEDGEDDEEEEEEGFFQKVLTPLLSWLLSRRLW
LGPQCSKLPLPSCCRQPPPAGPPVEGDGWLKSFQRSRRMCFTSKSFRPEPDMLYAQKAKG
WQLTQDSGGWEVQDQCTRIWSKENLLALNTHSRRQKGKRENKVCVSTWQKSRGDRTYSSM
ATTPSMTKILEGCMYRKLKC
>sp|O60423|AT8B3_HUMAN Phospholipid-transporting ATPase IK OS=Homo sapiens GN=ATP8B3 PE=2 SV=4
MGTGPAQTPRSTRAGPEPSPAPPGPGDTGDSDVTQEGSGPAGIRGGETVIRAGMGDSPGR
GAPERRHKAQPGRARKYEWRPEGPTSMGSLGQREDLQDEDRNSAFTWKVQANNRAYNGQF
KEKVILCWQRKKYKTNVIRTAKYNFYSFLPLNLYEQFHRVSNLFFLIIIILQSIPDISTL
PWFSLSTPMVCLLFIRATRDLVDDMGRHKSDRAINNRPCQILMGKSFKQKKWQDLCVGDV
VCLRKDNIVPADMLLLASTEPSSLCYVETVDIDGETNLKFRQALMVTHKELATIKKMASF
QGTVTCEAPNSRMHHFVGCLEWNDKKYSLDIGNLLLRGCRIRNTDTCYGLVIYAGFDTKI
MKNCGKIHLKRTKLDLLMNKLVVVIFISVVLVCLVLAFGFGFSVKEFKDHHYYLSGVHGS
SVAAESFFVFWSFLILLSVTIPMSMFILSEFIYLGNSVFIDWDVQMYYKPQDVPAKARST
SLNDHLGQVEYIFSDKTGTLTQNILTFNKCCISGRVYGPDSEATTRPKENPYLWNKFADG
KLLFHNAALLHLVRTNGDEAVREFWRLLAICHTVMVRESPRERPDQLLYQAASPDEGALV
TAARNFGYVFLSRTQDTVTIMELGEERVYQVLAIMDFNSTRKRMSVLVRKPEGAICLYTK
GADTVIFERLHRRGAMEFATEEALAAFAQETLRTLCLAYREVAEDIYEDWQQRHQEASLL
LQNRAQALQQLLGATAIEDRLQDGVPETIKCLKKSNIKIWVLTGDKQETAVNIGFACELL
SENMLILEEKEISRILETYWENSNNLLTRESLSQVKLALVINGDFLDKLLVSLRKEPRAL
AQNVNMDEAWQELGQSRRDFLYARRLSLLCRRFGLPLAAPPAQDSRARRSSEVLQERAFV
DLASKCQAVICCRVTPKQKALIVALVKKYHQVVTLAIGDGANDINMIKTADVGVGLAGQE
GMQAVQNSDFVLGQFCFLQRLLLVHGRWSYVRICKFLRYFFYKSMASMMVQVWFACYNGF
TGQPLYEGWFLALFNLLYSTLPVLYIGLFEQDVSAEQSLEKPELYVVGQKDELFNYWVFV
QAIAHGVTTSLVNFFMTLWISRDTAGPASFSDHQSFAVVVALSCLLSITMEVILIIKYWT
ALCVATILLSLGFYAIMTTTTQSFWLFRVSPTTFPFLYADLSVMSSPSILLVVLLSVSIN
TFPVLALRVIFPALKELRAKEEKVEEGPSEEIFTMEPLPHVHRESRARRSSYAFSHREGY
ANLITQGTILRRGPGVSSDIASESLDPSDEEAASSPKESQ

>sp|Q6PL18|ATAD2_HUMAN ATPase family AAA domain-containing protein 2 OS=Homo sapiens GN=ATAD2 PE=1 SV=1
MVVLRSSLELHNHSAASATGSLDLSSDFLSLEHIGRRRLRSAGAAQKKPAATTAKAGDGS
SVKEVETYHRTRALRSLRKDAQNSSDSSFEKNVEITEQLANGRHFTRQLARQQADKKKEE
HREDKVIPVTRSLRARNIVQSTEHLHEDNGDVEVRRSCRIRSRYSGVNQSMLFDKLITNT
AEAVLQKMDDMKKMRRQRMRELEDLGVFNETEESNLNMYTRGKQKDIQRTDEETTDNQEG
SVESSEEGEDQEHEDDGEDEDDEDDDDDDDDDDDDDEDDEDEEDGEEENQKRYYLRQRK
ATVYYQAPLEKPRHQRKPNIFYSGPASPARPRYRLSSAGPRSPYCKRMNRRRHAIHSSDS
TSSSSSEDEQHFERRRKRSRNRAINRCLPLNFRKDELKGIYKDRMKIGASLADVDPMQLD
SSVRFDSVGGLSNHIAALKEMVVFPLLYPEVFEKFKIQPPRGCLFYGPPGTGKTLVARAL
ANECSQGDKRVAFFMRKGADCLSKWVGESERQLRLLFDQAYQMRPSIIFFDEIDGLAPVR
SSRQDQIHSSIVSTLLALMDGLDSRGEIVVIGATNRLDSIDPALRRPGRFDREFLFSLPD
KEARKEILKIHTRDWNPKPLDTFLEELAENCVGYCGADIKSICAEAALCALRRRYPQIYT
TSEKLQLDLSSINISAKDFEVAMQKMIPASQRAVTSPGQALSTVVKPLLQNTVDKILEAL
QRVFPHAEFRTNKTLDSDISCPLLESDLAYSDDDVPSVYENGLSQKSSHKAKDNFNFLHL
NRNACYQPMSFRPRILIVGEPGFGQGSHLAPAVIHALEKFTVYTLDIPVLFGVSTTSPEE
TCAQVIREAKRTAPSIVYVPHIHVWWEIVGPTLKATFTTLLQNIPSFAPVLLLATSDKPH
SALPEEVQELFIRDYGEIFNVQLPDKEERTKFFEDLILKQAAKPPISKKKAVLQALEVLP
VAPPPEPRSLTAEEVKRLEEQEEDTFRELRIFLRNVTHRLAIDKRFRVFTKPVDPDEVPD
YVTVIKQPMDLSSVISKIDLHKYLTVKDYLRDIDLICSNALEYNPDRDPGDRLIRHRACA
LRDTAYAIIKEELDEDFEQLCEEIQESRKKRGCSSSKYAPSYYHVMPKQNSTLVGDKRSD
PEQNEKLKTPSTPVACSTPAQLKRKIRKKSNWYLGTIKKRRKISQAKDDSQNAIDHKIES
DTEETQDTSVDHNETGNTGESSVEENEKQQNASESKLELRNNSNTCNIENELEDSRKTTA
CTELRDKIACNGDASSSQIIHISDENEGKEMCVLRMTRARRSQVEQQQLITVEKALAILS
QPTPSLVVDHERLKNLLKTVVKKSQNYNIFQLENLYAVISQCIYRHRKDHDKTSLIQKME
QEVENFSCSR
>sp|Q86WG3|ATCAY_HUMAN Caytaxin OS=Homo sapiens GN=ATCAY PE=1 SV=2
MGTTEATLRMENVDVKEEWQDEDLPRPLPEETGVELLGSPVEDTSSPPNTLNFNGAHRKR
KTLVAPEINISLDQSEGSLLSDDFLDTPDDLDINVDDIETPDETDSLEFLGNGNELEWED
DTPVATAKNMPGDSADLFGDGTTEDGSAANGRLWRTVIIGEQEHRIDLHMIRPYMKVVTH
GGYYGEGLNAIIVFAACFLPDSSLPDYHYIMENLFLYVISSLELLVAEDYMIVYLNGATP
RRRMPGIGWLKKCYQMIDRRLRKNLKSLIIVHPSWFIRTVLAISRPFISVKFINKIQYVH
SLEDLEQLIPMEHVQIPDCVLQYEEERLKARRESARPQPEFVLPRSEEKPEVAPVENRSA
LVSEDQETSMS
>sp|Q5T2N8|ATD3C_HUMAN ATPase family AAA domain-containing protein 3C OS=Homo sapiens GN=ATAD3C PE=2 SV=2
MSKDALNLAQMQEQTLQLEQQSKLKQLVNEDLRKQEESVQKHHQTFLESIRAAGTLFGEG
FRAFVTDRDKVTATVAGLTLLAVGVYSAKNATAVTGRYIEARLGKPSLVRETSRITVLEA
LRHPIQQVSRRLLSRPQDVLEGVVLSPSLEARVRDIAIMTRNIKKNRGLYRHILLYGPPG
TGKTLFAKKLALHSGMDYAIMTGGDVAPMGREGVTAMHKLFDWANTSRRGLLLFVDEADA
FLRKRATEKISEDLRATLNAFLYRTGQHSNKFMLILASCHPEQFDWAINACIDVMVHFDL
PGQEERARLVRMYLNEYVLKPATEGKRRLKLAQFDYGRKCLEIARLTEGMSCRKIAQLAV
SWQATAYASKDGVLTEAMMDACVQDFVQQHQQMMRWLKGERPGPEDEQPSS
>sp|P00846|ATP6_HUMAN ATP synthase subunit a OS=Homo sapiens GN=MT-ATP6 PE=1 SV=1

MNENLFASFIAPTILGLPAAVLIILFPPLLIPTSKYLINNRLITTQQWLIKLTSKQMMTM
HNTKGRTWSLMLVSLIIFIATTNLLGLLPHSFTPTTQLSMNLAMAIPLWAGTVIMGFRSK
IKNALAHFLPQGTPTPLIPMLVIIETISLLIQPMALAVRLTANITAGHLLMHLIGSATLA
MSTINLPSTLIIFTILILLTILEIAVALIQAYVFTLLVSLYLHDNT
>sp|Q5VV63|ATRN1_HUMAN Attractin-like protein 1 OS=Homo sapiens GN=ATRNL1 PE=2 SV=2
METGGRARTGTPQPAAPGVWRARPAGGGGGGASSWLLDGNSWLLCYGFLYLALYAQVSQS
KPCERTGSCFSGRCVNSTCLCDPGWVGDQCQHCQGRFKLTEPSGYLTDGPINYKYKTKCT
WLIEGYPNAVLRLRFNHFATECSWDHMYVYDGDSIYAPLIAVLSGLIVPEIRGNETVPEV
VTTSGYALLHFFSDAAYNLTGFNIFYSINSCPNNCSGHGKCTTSVSVPSQVYCECDKYWK
GEACDIPYCKANCGSPDHGYCDLTGEKLCVCNDSWQGPDCSLNVPSTESYWILPNVKPFS
PSVGRASHKAVLHGKFMWVIGGYTFNYSSFQMVLNYNLESSIWNVGTPSRGPLQRYGHSL
ALYQENIFMYGGRIETNDGNVTDELWVFNIHSQSWSTKTPTVLGHGQQYAVEGHSAHIME
LDSRDVVMIIIFGYSAIYGYTSSIQEYHISSNTWLVPETKGAIVQGGYGHTSVYDEITKS
IYVHGGYKALPGNKYGLVDDLYKYEVNTKTWTILKESGFARYLHSAVLINGAMLIFGGNT
HNDTSLSNGAKCFSADFLAYDIACDEWKILPKPNLHRDVNRFGHSAVVINGSMYIFGGFS
SVLLNDILVYKPPNCKAFRDEELCKNAGPGIKCVWNKNHCESWESGNTNNILRAKCPPKT
AASDDRCYRYADCASCTANTNGCQWCDDKKCISANSNCSMSVKNYTKCHVRNEQICNKLT
SCKSCSLNLNCQWDQRQQECQALPAHLCGEGWSHIGDACLRVNSSRENYDNAKLYCYNLS
GNLASLTTSKEVEFVLDEIQKYTQQKVSPWVGLRKINISYWGWEDMSPFTNTTLQWLPGE
PNDSGFCAYLERAAVAGLKANPCTSMANGLVCEKPVVSPNQNARPCKKPCSLRTSCSNCT
SNGMECMWCSSTKRCVDSNAYIISFPYGQCLEWQTATCSPQNCSGLRTCGQCLEQPGCGW
CNDPSNTGRGHCIEGSSRGPMKLIGMHHSEMVLDTNLCPKEKNYEWSFIQCPACQCNGHS
TCINNNVCEQCKNLTTGKQCQDCMPGYYGDPTNGGQCTACTCSGHANICHLHTGKCFCTT
KGIKGDQCQLCDSENRYVGNPLRGTCYYSLLIDYQFTFSLLQEDDRHHTAINFIANPEQS
NKNLDISINASNNFNLNITWSVGSTAGTISGEETSIVSKNNIKEYRDSFSYEKFNFRSNP
NITFYVYVSNFSWPIKIQIAFSQHNTIMDLVQFFVTFFSCFLSLLLVAAVVWKIKQTCWA
SRRREQLLRERQQMASRPFASVDVALEVGAEQTEFLRGPLEGAPKPIAIEPCAGNRAAVL
TVFLCLPRGSSGAPPPGQSGLAIASALIDISQQKASDSKDKTSGVRNRKHLSTRQGTCV
>sp|Q9H7T9|AUNIP_HUMAN Aurora kinase A and ninein-interacting protein OS=Homo sapiens GN=AUNIP PE=1 SV=1
MRRTGPEEEACGVWLDAAALKRRKVQTHLIKPGTKMLTLLPGERKANIYFTQRRAPSTGI
HQRSIASFFTLQPGKTNGSDQKSVSSHTESQINKESKKNATQLDHLIPGLAHDCMASPLA
TSTTADIQEAGLSPQSLQTSGHHRMKTPFSTELSLLQPDTPDCAGDSHTPLAFSFTEDLE
SSCLLDRKEEKGDSARKWEWLHESKKNYQSMEKHTKLPGDKCCQPLGKTKLERKVSAKEN
RQAPVLLQTYRESWNGENIESVKQSRSPVSVFSWDNEKNDKDSWSQLFTEDSQGQRVIAH
NTRAPFQDVTNNWNWDLGPFPNSPWAQCQEDGPTQNLKPDLLFTQDSEGNQVIRHQF
>sp|Q5T686|AVPI1_HUMAN Arginine vasopressin-induced protein 1 OS=Homo sapiens GN=AVPI1 PE=1 SV=3
MGTPASVVSEPPPWQAPIEARGRKQASANIFQDAELLQIQALFQRSGDQLAEERAQIIWE
CAGDHRVAEALKRLRRKRPPRQKPLGHSLHHCSRLRILEPHSALANPQSATETASSEQYL
HSRKKSARIRRNWRKSGPTSYLHQIRH
>sp|Q07817|B2CL1_HUMAN Bcl-2-like protein 1 OS=Homo sapiens GN=BCL2L1 PE=1 SV=1
MSQSNRELVVDFLSYKLSQKGYSWSQFSDVEENRTEAPEGTESEMETPSAINGNPSWHLA
DSPAVNGATGHSSSLDAREVIPMAAVKQALREAGDEFELRYRRAFSDLTSQLHITPGTAY

QSFEQVVNELFRDGVNWGRIVAFFSFGGALCVESVDKEMQVLVSRIAAWMATYLNDHLEP
WIQENGGWDTFVELYGNNAAAESRKGQERFNRWFLTGMTVAGVVLLGSLFSRK
>sp|Q16548|B2LA1_HUMAN Bcl-2-related protein A1 OS=Homo sapiens GN=BCL2A1 PE=1 SV=1
MTDCEFGYIYRLAQDYLQCVLQIPQPGSGPSKTSRVLQNVAFSVQKEVEKNLKSCLDNVN
VVSVDTARTLFNQVMEKEFEDGIINWGRIVTIFAFEGILIKKLLRQQIAPDVDTYKEISY
FVAEFIMNNTGEWIRQNGGWENGFVKKFEPKSGWMTFLEVTGKICEMLSLLKQYC
>sp|P61769|B2MG_HUMAN Beta-2-microglobulin OS=Homo sapiens GN=B2M PE=1 SV=1
MSRSVALAVLALLSLSGLEAIQRTPKIQVYSRHPAENGKSNFLNCYVSGFHPSDIEVDLL
KNGERIEKVEHSDLSFSKDWSFYLLYYTEFTPTEKDEYACRVNHVTLSQPKIVKWDRDM
>sp|P04920|B3A2_HUMAN Anion exchange protein 2 OS=Homo sapiens GN=SLC4A2 PE=1 SV=4
MSSAPRRPAKGADSFCTPEPESLGPGTPGFPEQEEDELHRTLGVERFEEILQEAGSRGGE
EPGRSYGEEDFEYHRQSSHHIHHPLSTHLPPDARRRKTPQGPGRKPRRRPGASPTGETPT
IEEGEEDEDEASEAEGARALTQPSPVSTPSSVQFFLQEDDSADRKAERTSPSSPAPLPHQ
EATPRASKGAQAGTQVEEAEAEAVAVASGTAGGDDGGASGRPLPKAQPGHRSYNLQERRR
IGSMTGAEQALLPRVPTDEIEAQTLATADLDLMKSHRFEDVPGVRRHLVRKNAKGSTQSG
REGREPGPTPRARPRAPHKPHEVFVELNELLLDKNQEPQWRETARWIKFEEDVEEETERW
GKPHVASLSFRSLLELRRTLAHGAVLLDLDQQTLPGVAHQVVEQMVISDQIKAEDRANVL
RALLLKHSHPSDEKDFSFPRNISAGSLGSLLGHHHGQGAESDPHVTEPLMGGVPETRLEV
ERERELPPPAPPAGITRSKSKHELKLLEKIPENAEATVVLVGCVEFLSRPTMAFVRLREA
VELDAVLEVPVPVRFLFLLLGPSSANMDYHEIGRSISTLMSDKQFHEAAYLADEREDLLT
AINAFLDCSVVLPPSEVQGEELLRSVAHFQRQMLKKREEQGRLLPTGAGLEPKSAQDKAL
LQMVEAAGAAEDDPLRRTGRPFGGLIRDVRRRYPHYLSDFRDALDPQCLAAVIFIYFAAL
SPAITFGGLLGEKTQDLIGVSELIMSTALQGVVFCLLGAQPLLVIGFSGPLLVFEEAFFS
FCSSNHLEYLVGRVWIGFWLVFLALLMVALEGSFLVRFVSRFTQEIFAFLISLIFIYETF
YKLVKIFQEHPLHGCSASNSSEVDGGENMTWAGARPTLGPGNRSLAGQSGQGKPRGQPNT
ALLSLVLMAGTFFIAFFLRKFKNSRFFPGRIRRVIGDFGVPIAILIMVLVDYSIEDTYTQ
KLSVPSGFSVTAPEKRGWVINPLGEKSPFPVWMMVASLLPAILVFILIFMETQITTLIIS
KKERMLQKGSGFHLDLLLIVAMGGICALFGLPWLAAATVRSVTHANALTVMSKAVAPGDK
PKIQEVKEQRVTGLLVALLVGLSIVIGDLLRQIPLAVLFGIFLYMGVTSLNGIQFYERLH
LLLMPPKHHPDVTYVKKVRTLRMHLFTALQLLCLALLWAVMSTAASLAFPFILILTVPLR
MVVLTRIFTDREMKCLDANEAEPVFDEREGVDEYNEMPMPV
>sp|Q9C0J1|B3GN4_HUMAN N-acetyllactosaminide beta-1,3-N-acetylglucosaminyltransferase 4 OS=Homo sapiens GN=B3GNT4 PE=1 SV=1
MLPPQPSAAHQGRGGRSGLLPKGPAMLCRLCWLVSYSLAVLLLGCLLFLRKAAKPAGDPT
AHQPFWAPPTPRHSRCPPNHTVSSASLSLPSRHRLFLTYRHCRNFSILLEPSGCSKDTFL
LLAIKSQPGHVERRAAIRSTWGRVGGWARGRQLKLVFLLGVAGSAPPAQLLAYESREFDD
ILQWDFTEDFFNLTLKELHLQRWVVAACPQAHFMLKGDDDVFVHVPNVLEFLDGWDPAQD
LLVGDVIRQALPNRNTKVKYFIPPSMYRATHYPPYAGGGGYVMSRATVRRLQAIMEDAEL
FPIDDVFVGMCLRRLGLSPMHHAGFKTFGIRRPLDPLDPCLYRGLLLVHRLSPLEMWTMW
ALVTDEGLKCAAGPIPQR
>sp|Q7Z7M8|B3GN8_HUMAN UDP-GlcNAc:betaGal beta-1,3-N-acetylglucosaminyltransferase 8 OS=Homo sapiens GN=B3GNT8 PE=1 SV=1
MRCPKCLLCLSALLTLLGLKVYIEWTSESRLSKAYPSPRGTPPSPTPANPEPTLPANLST
RLGQTIPLPFAYWNQQQWRLGSLPSGDSTETGGCQAWGAAAATEIPDFASYPKDLRRFLL

SAACRSFPQWLPGGGGSQVSSCSDTDVPYLLLAVKSEPGRFAERQAVRETWGSPAPGIRL
LFLLGSPVGEAGPDLDSLVAWESRRYSDLLLWDFLDVPFNQTLKDLLLLAWLGRHCPTVS
FVLRAQDDAFVHTPALLAHLRALPPASARSLYLGEVFTQAMPLRKPGGPFYVPESFFEGG
YPAYASGGGYVIAGRLAPWLLRAAARVAPFPFEDVYTGLCIRALGLVPQAHPGFLTAWPA
DRTADHCAFRNLLLVRPLGPQASIRLWKQLQDPRLQC
>sp|Q8NHY0|B4GN2_HUMAN Beta-1,4 N-acetylgalactosaminyltransferase 2 OS=Homo sapiens GN=B4GALNT2 PE=1 SV=2
MGSAGFSVGKFHVEVASRGRECVSGTPECGNRLGSAGFGALCLELRGADPAWGPFAAHGR
SRRQGSRFLWLLKILVIILVLGIVGFMFGSMFLQAVFSSPKPELPSPAPGVQKLKLLPEE
RLRNLFSYDGIWLFPKNQCKCEANKEQGGYNFQDAYGQSDLPAVKARRQAEFEHFQRREG
LPRPLPLLVQPNLPFGYPVHGVEVMPLHTVPIPGLQFEGPDAPVYEVTLTASLGTLNTLA
DVPDSVVQGRGQKQLIISTSDRKLLKFILQHVTYTSTGYQHQKVDIVSLESRSSVAKFPV
TIRHPVIPKLYDPGPERKLRNLVTIATKTFLRPHKLMIMLRSIREYYPDLTVIVADDSQK
PLEIKDNHVEYYTMPFGKGWFAGRNLAISQVTTKYVLWVDDDFLFNEETKIEVLVDVLEK
TELDVVGGSVLGNVFQFKLLLEQSENGACLHKRMGFFQPLDGFPSCVVTSGVVNFFLAHT
ERLQRVGFDPRLQRVAHSEFFIDGLGTLLVGSCPEVIIGHQSRSPVVDSELAALEKTYNT
YRSNTLTRVQFKLALHYFKNHLQCAA
>sp|Q76KP1|B4GN4_HUMAN N-acetyl-beta-glucosaminyl-glycoprotein 4-beta-N-acetylgalactosaminyltransferase 1 OS=Homo sapiens GN=B4GALNT4 PE=1 SV=1
MPRLPVKKIRKQMKLLLLLLLLLSCAAWLTYVHLGLVRQGRALRQRLGYGRDGEKLTSETD
GRGVHAAPSTQRAEDSSESREEEQAPEGRDLDMLFPGGAGRLPLNFTHQTPPWREEYKGQ
VNLHVFEDWCGGAVGHLRRNLHFPLFPHTRTTVKKLAVSPKWKNYGLRIFGFIHPARDGD
VQFSVASDDNSEFWLSLDESPAAAQLVAFVGKTGSEWTAPGEFTKFSSQVSKPRRLMASR
RYYFELLHKQDDRGSDHVEVGWRAFLPGLKFEVISSAHISLYTDESALKMDHVAHVPQSP
ASHVGGRPPQEETSADMLRPDPRDTFFLTPRMESSSLENVLEPCAYAPTYVVKDFPIARY
QGLQFVYLSFVYPNDYTRLTHMETDNKCFYRESPLYLERFGFYKYMKMDKEEGDEDEEDE
VQRRAFLFLNPDDFLDDEDEGELLDSLEPTEAAPPRSGPQSPAPAAPAQPGATLAPPTPP
RPRDGGTPRHSRALSWAARAARPLPLFLGRAPPPRPAVEQPPPKVYVTRVRPGQRASPRA
PAPRAPWPPFPGVFLHPRPLPRVQLRAPPRPPRPHGRRTGGPQATQPRPPARAQATQGGR
EGQARTLGPAAPTVDSNLSSEARPVTSFLSLSQVSGPQLPGEGEEEEEGEDDGAPGDEAA
SEDSEEAAGPALGRWREDAIDWQRTFSVGAVDFELLRSDWNDLRCNVSGNLQLPEAEAVD
VTAQYMERLNARHGGRFALLRIVNVEKRRDSARGSRFLLELELQERGGGRLRLSEYVFLR
LPGARVGDADGESPEPAPAASVRPDGRPELCRPLRLAWRQDVMVHFIVPVKNQARWVAQF
LADMAALHARTGDSRFSVVLVDFESEDMDVERALRAARLPRYQYLRRTGNFERSAGLQAG
VDAVEDASSIVFLCDLHIHFPPNILDGIRKHCVEGRLAFAPVVMRLSCGSSPRDPHGYWE
VNGFGLFGIYKSDFDRVGGMNTEEFRDQWGGEDWELLDRVLQAGLEVERLRLRNFYHHYH
SKRGMWSVRSRKGSRTGAS
>sp|P15291|B4GT1_HUMAN Beta-1,4-galactosyltransferase 1 OS=Homo sapiens GN=B4GALT1 PE=1 SV=5
MRLREPLLSGSAAMPGASLQRACRLLVAVCALHLGVTLVYYLAGRDLSRLPQLVGVSTPL
QGGSNSAAAIGQSSGELRTGGARPPPPLGASSQPRPGGDSSPVVDSGPGPASNLTSVPVP
HTTALSLPACPEESPLLVGPMLIEFNMPVDLELVAKQNPNVKMGGRYAPRDCVSPHKVAI
IIPFRNRQEHLKYWLYYLHPVLQRQQLDYGIYVINQAGDTIFNRAKLLNVGFQEALKDYD
YTCFVFSDVDLIPMNDHNAYRCFSQPRHISVAMDKFGFSLPYVQYFGGVSALSKQQFLTI

NGFPNNYWGWGGEDDDIFNRLVFRGMSISRPNAVVGRCRMIRHSRDKKNEPNPQRFDRIA
HTKETMLSDGLNSLTYQVLDVQRYPLYTQITVDIGTPS
>sp|O60512|B4GT3_HUMAN Beta-1,4-galactosyltransferase 3 OS=Homo sapiens GN=B4GALT3 PE=1 SV=2
MLRRLLERPCTLALLVGSQLAVMMYLSLGGFRSLSALFGRDQGPTFDYSHPRDVYSNLSH
LPGAPGGPPAPQGLPYCPERSPLLVGPVSVSFSPVPSLAEIVERNPRVEPGGRYRPAGCE
PRSRTAIIVPHRAREHHLRLLLYHLHPFLQRQQLAYGIYVIHQAGNGTFNRAKLLNVGVR
EALRDEEWDCLFLHDVDLLPENDHNLYVCDPRGPRHVAVAMNKFGYSLPYPQYFGGVSAL
TPDQYLKMNGFPNEYWGWGGEDDDIATRVRLAGMKISRPPTSVGHYKMVKHRGDKGNEEN
PHRFDLLVRTQNSWTQDGMNSLTYQLLARELGPLYTNITADIGTDPRGPRAPSGPRYPPG
SSQAFRQEMLQRRPPARPGPLSTANHTALRGSH
>sp|O60513|B4GT4_HUMAN Beta-1,4-galactosyltransferase 4 OS=Homo sapiens GN=B4GALT4 PE=1 SV=1
MGFNLTFHLSYKFRLLLLLTLCLTVVGWATSNYFVGAIQEIPKAKEFMANFHKTLILGKG
KTLTNEASTKKVELDNCPSVSPYLRGQSKLIFKPDLTLEEVQAENPKVSRGRYRPQECKA
LQRVAILVPHRNREKHLMYLLEHLHPFLQRQQLDYGIYVIHQAEGKKFNRAKLLNVGYLE
ALKEENWDCFIFHDVDLVPENDFNLYKCEEHPKHLVVGRNSTGYRLRYSGYFGGVTALSR
EQFFKVNGFSNNYWGWGGEDDDLRLRVELQRMKISRPLPEVGKYTMVFHTRDKGNEVNAE
RMKLLHQVSRVWRTDGLSSCSYKLVSVEHNPLYINITVDFWFGA
>sp|Q9UBX8|B4GT6_HUMAN Beta-1,4-galactosyltransferase 6 OS=Homo sapiens GN=B4GALT6 PE=1 SV=1
MSVLRRMMRVSNRSLLAFIFFFSLSSSCLYFIYVAPGIANTYLFMVQARGIMLRENVKTI
GHMIRLYTNKNSTLNGTDYPEGNNSSDYLVQTTTYLPENFTYSPYLPCPEKLPYMRGFLN
VNVSEVSFDEIHQLFSKDLDIEPGGHWRPKDCKPRWKVAVLIPFRNRHEHLPIFFLHLIP
MLQKQRLEFAFYVIEQTGTQPFNRAMLFNVGFKEAMKDSVWDCVIFHDVDHLPENDRNYY
GCGEMPRHFAAKLDKYMYILPYKEFFGGVSGLTVEQFRKINGFPNAFWGWGGEDDDLWNR
VHYAGYNVTRPEGDLGKYKSIPHHHRGEVQFLGRYKLLRYSKERQYIDGLNNLIYRPKIL
VDRLYTNISVNLMPELAPIEDY
>sp|Q9UPM9|B9D1_HUMAN B9 domain-containing protein 1 OS=Homo sapiens GN=B9D1 PE=2 SV=1
MATASPSVFLLMVNGQVESAQFPEYDDLYCKYCFVYGQDWAPTAGLEEGISQITSKSQDV
RQALVWNFPIDVTFKSTNPYGWPQIVLSVYGPDVFGNDVVRGYGAVHVPFSPGRHKRTIP
MFVPESTSKLQKFTSWFMGRRPEYTDPKVVAQGEGREVTRVRSQGFVTLLFNVVTKDMRK
LGYDTGPSDTQGVLGPSPPQSFPQ
>sp|Q8WXS3|BAALC_HUMAN Brain and acute leukemia cytoplasmic protein OS=Homo sapiens GN=BAALC PE=2 SV=3
MGCGGSRADAIEPRYYESWTRETESTWLTYTDSDAPPSAAAPDSGPEAGGLHSVLEAEKS
KIKAPTDSVSDEGLFSASKMAPLAVFSHGMLEDGLPSNGVPRSTAPGGIPNPEKKTNCET
QCPNPQSLSSGPLTQKQNGLQTTEAKRDAKRMPAKEVTINVTDSIQQMDRSRRITKNCVN
>sp|Q8WZ19|BACD1_HUMAN BTB/POZ domain-containing adapter for CUL3-mediated RhoA degradation protein 1 OS=Homo sapiens GN=KCTD13 PE=1 SV=1
MSAEASGPAAAAAPSLEAPKPSGLEPGPAAYGLKPLTPNSKYVKLNVGGSLHYTTLRTLT
GQDTMLKAMFSGRVEVLTDAGGWVLIDRSGRHFGTILNYLRDGSVPLPESTRELGELLGE
ARYYLVQGLIEDCQLALQQKRETLSPLCLIPMVTSPREEQQLLASTSKPVVKLLHNRSNN
KYSYTSTSDDNLLKNIELFDKLALRFHGRLLFLKDVLGDEICCWSFYGQGRKIAEVCCTS

IVYATEKKQTKVEFPEARIFEETLNILIYETPRGPDPALLEATGGAAGAGGAGRGEDEEN
REHRVRRIHVRRHITHDERPHGQQIVFKD
>sp|Q9H3F6|BACD3_HUMAN BTB/POZ domain-containing adapter for CUL3-mediated RhoA degradation protein 3 OS=Homo sapiens GN=KCTD10 PE=1 SV=1
MEEMSGESVVSSAVPAAATRTTSFKGTSPSSKYVKLNVGGALYYTTMQTLTKQDTMLKAM
FSGRMEVLTDSEGWILIDRCGKHFGTILNYLRDGAVPLPESRREIEELLAEAKYYLVQGL
VEECQAALQNKDTYEPFCKVPVITSSKEEQKLIATSNKPAVKLLYNRSNNKYSYTSNSDD
NMLKNIELFDKLSLRFNGRVLFIKDVIGDEICCWSFYGQGRKIAEVCCTSIVYATEKKQT
KVEFPEARIYEETLNILLYEAQDGRGPDNALLEATGGAAGRSHHLDEDEERERIERVRRI
HIKRPDDRAHLHQ
>sp|Q9BYV9|BACH2_HUMAN Transcription regulator protein BACH2 OS=Homo sapiens GN=BACH2 PE=1 SV=1
MSVDEKPDSPMYVYESTVHCTNILLGLNDQRKKDILCDVTLIVERKEFRAHRAVLAACSE
YFWQALVGQTKNDLVVSLPEEVTARGFGPLLQFAYTAKLLLSRENIREVIRCAEFLRMHN
LEDSCFSFLQTQLLNSEDGLFVCRKDAACQRPHEDCENSAGEEEDEEEETMDSETAKMAC
PRDQMLPEPISFEAAAIPVAEKEEALLPEPDVPTDTKESSEKDALTQYPRYKKYQLACTK
NVYNASSHSTSGFASTFREDNSSNSLKPGLARGQIKSEPPSEENEEESITLCLSGDEPDA
KDRAGDVEMDRKQPSPAPTPTAPAGAACLERSRSVASPSCLRSLFSITKSVELSGLPSTS
QQHFARSPACPFDKGITQGDLKTDYTPFTGNYGQPHVGQKEVSNFTMGSPLRGPGLEALC
KQEGELDRRSVIFSSSACDQVSTSVHSYSGVSSLDKDLSEPVPKGLWVGAGQSLPSSQAY
SHGGLMADHLPGRMRPNTSCPVPIKVCPRSPPLETRTRTSSSCSSYSYAEDGSGGSPCSL
PLCEFSSSPCSQGARFLATEHQEPGLMGDGMYNQVRPQIKCEQSYGTNSSDESGSFSEAD
SESCPVQDRGQEVKLPFPVDQITDLPRNDFQMMIKMHKLTSEQLEFIHDVRRRSKNRIAA
QRCRKRKLDCIQNLECEIRKLVCEKEKLLSERNQLKACMGELLDNFSCLSQEVCRDIQSP
EQIQALHRYCPVLRPMDLPTASSINPAPLGAEQNIAASQCAVGENVPCCLEPGAAPPGPP
WAPSNTSENCTSGRRLEGTDPGTFSERGPPLEPRSQTVTVDFCQEMTDKCTTDEQPRKDY
T
>sp|Q13145|BAMBI_HUMAN BMP and activin membrane-bound inhibitor homolog OS=Homo sapiens GN=BAMBI PE=1 SV=1
MDRHSSYIFIWLQLELCAMAVLLTKGEIRCYCDAAHCVATGYMCKSELSACFSRLLDPQN
SNSPLTHGCLDSLASTTDICQAKQARNHSGTTIPTLECCHEDMCNYRGLHDVLSPPRGEA
SGQGNRYQHDGSRNLITKVQELTSSKELWFRAAVIAVPIAGGLILVLLIMLALRMLRSEN
KRLQDQRQQMLSRLHYSFHGHHSKKGQVAKLDLECMVPVSGHENCCLTCDKMRQADLSND
KILSLVHWGMYSGHGKLEFV
>sp|Q8IXM2|BAP18_HUMAN Chromatin complexes subunit BAP18 OS=Homo sapiens GN=BAP18 PE=1 SV=1
MTSASTKVGEIFSAAGAAFTKLGELTMQLHPVADSSPAGAKWTETEIEMLRAAVKRFGDD
LNHISCVIKERTVAQIKATVKRKVYEDSGIPLPAESPKKGPKKVASGVLSPPPAAPPPSS
SSVPEAGGPPIKKQKADVTLSALNDSDANSDVVDIEGLGETPPAKKLNFDQA
>sp|Q5TC12|ATPF1_HUMAN ATP synthase mitochondrial F1 complex assembly factor 1 OS=Homo sapiens GN=ATPAF1 PE=1 SV=1
MAAVVVAAAGGAGPAVLQVAGLYRGLCAVRSRALGLGLVSPAQLRVFPVRPGSGRPEGGA
DSSGVGAEAELQANPFYDRYRDKIQLLRRSDPAAFESRLEKRSEFRKQPVGHSRQGDFIK
CVEQKTDALGKQSVNRGFTKDKTLSSIFNIEMVKEKTAEEIKQIWQQYFAAKDTVYAVIP

AEKFDLIWNRAQSCPTFLCALPRREGYEFFVGQWTGTELHFTALINIQTRGEAAASQLIL
YHYPELKEEKGIVLMTAEMDSTFLNVAEAQCIANQVQLFYATDRKETYGLVETFNLRPNE
FKYMSVIAELEQSGLGAELKCAQNQNKT
>sp|Q9Y679|AUP1_HUMAN Ancient ubiquitous protein 1 OS=Homo sapiens GN=AUP1 PE=1 SV=1
MELPSGPGPERLFDSHRLPGDCFLLLVLLLYAPVGFCLLVLRLFLGIHVFLVSCALPDSV
LRRFVVRTMCAVLGLVARQEDSGLRDHSVRVLISNHVTPFDHNIVNLLTTCSTVSESEAE
SATGRFPGAQLKAPLSPLAFRMEDTEALPLTPILYPTCQFFFFIFLNIFLLAFSSPGSQP
LLNSPPSFVCWSRGFMEMNGRGELVESLKRFCASTRLPPTPLLLFPEEEATNGREGLLRF
SSWPFSIQDVVQPLTLQVQRPLVSVTVSDASWVSELLWSLFVPFTVYQVRWLRPVHRQLG
EANEEFALRVQQLVAKELGQTGTRLTPADKAEHMKRQRHPRLRPQSAQSSFPPSPGPSPD
VQLATLAQRVKEVLPHVPLGVIQRDLAKTGCVDLTITNLLEGAVAFMPEDITKGTQSLPT
ASASKFPSSGPVTPQPTALTFAKSSWARQESLQERKQALYEYARRRFTERRAQEAD
>sp|Q9HB09|B2L12_HUMAN Bcl-2-like protein 12 OS=Homo sapiens GN=BCL2L12 PE=1 SV=1
MGRPAGLFPPLCPFLGFRPEACWERHMQIERAPSVPPFLRWAGYRPGPVRRRGKVELIKF
VRVQWRRPQVEWRRRRWGPGPGASMAGSEELGLREDTLRVLAAFLRRGEAAGSPVPTPPR
SPAQEEPTDFLSRLRRCLPCSLGRGAAPSESPRPCSLPIRPCYGLEPGPATPDFYALVAQ
RLEQLVQEQLKSPPSPELQGPPSTEKEAILRRLVALLEEEAEVINQKLASDPALRSKLVR
LSSDSFARLVELFCSRDDSSRPSRACPGPPPPSPEPLARLALAMELSRRVAGLGGTLAGL
SVEHVHSFTPWIQAHGGWEGILAVSPVDLNLPLD
>sp|Q9P2W7|B3GA1_HUMAN Galactosylgalactosylxylosylprotein 3-beta-glucuronosyltransferase 1 OS=Homo sapiens GN=B3GAT1 PE=1 SV=2
MPKRRDILAIVLIVLPWTLLITVWHQSTLAPLLAVHKDEGSDPRRETPPGADPREYCTSD
RDIVEVVRTEYVYTRPPPWSDTLPTIHVVTPTYSRPVQKAELTRMANTLLHVPNLHWLVV
EDAPRRTPLTARLLRDTGLNYTHLHVETPRNYKLRGDARDPRIPRGTMQRNLALRWLRET
FPRNSSQPGVVYFADDDNTYSLELFEEMRSTRRVSVWPVAFVGGLRYEAPRVNGAGKVVG
WKTVFDPHRPFAIDMAGFAVNLRLILQRSQAYFKLRGVKGGYQESSLLRELVTLNDLEPK
AANCTKILVWHTRTEKPVLVNEGKKGFTDPSVEI
>sp|Q9NPZ5|B3GA2_HUMAN Galactosylgalactosylxylosylprotein 3-beta-glucuronosyltransferase 2 OS=Homo sapiens GN=B3GAT2 PE=1 SV=2
MKSALFTRFFILLPWILIVIIMLDVDTRRPVPPLTPRPYFSPYAVGRGGARLPLRRGGPA
HGTQKRNQSRPQPQPEPQLPTIYAITPTYSRPVQKAELTRLANTFRQVAQLHWILVEDAA
ARSELVSRFLARAGLPSTHLHVPTPRRYKRPGLPRATEQRNAGLAWLRQRHQHQRAQPGV
LFFADDDNTYSLELFQEMRTTRKVSVWPVGLVGGRRYERPLVENGKVVGWYTGWRADRPF
AIDMAGFAVSLQVILSNPKAVFKRRGSQPGMQESDFLKQITTVEELEPKANNCTKVLVWH
TRTEKVNLANEPKYHLDTVKIEV
>sp|Q8NCR0|B3GL2_HUMAN UDP-GalNAc:beta-1,3-N-acetylgalactosaminyltransferase 2 OS=Homo sapiens GN=B3GALNT2 PE=1 SV=1
MRNWLVLLCPCVLGAALHLWLRLRSPPPACASGAGPADQLALFPQWKSTHYDVVVGVLSA
RNNHELRNVIRSTWMRHLLQHPTLSQRVLVKFIIGAHGCEVPVEDREDPYSCKLLNITNP
VLNQEIEAFSLSEDTSSGLPEDRVVSVSFRVLYPIVITSLGVFYDANDVGFQRNITVKLY
QAEQEEALFIARFSPPSCGVQVNKLWYKPVEQFILPESFEGTIVWESQDLHGLVSRNLHK
VTVNDGGGVLRVITAGEGALPHEFLEGVEGVAGGFIYTIQEGDALLHNLHSRPQRLIDHI
RNLHEEDALLKEESSIYDDIVFVDVVDTYRNVPAKLLNFYRWTVETTSFNLLLKTDDDCY
IDLEAVFNRIVQKNLDGPNFWWGNFRLNWAVDRTGKWQELEYPSPAYPAFACGSGYVISK

DIVKWLASNSGRLKTYQGEDVSMGIWMAAIGPKRYQDSLWLCEKTCETGMLSSPQYSPWE
LTELWKLKERCGDPCRCQAR
>sp|Q9Y5Z6|B3GT1_HUMAN Beta-1,3-galactosyltransferase 1 OS=Homo sapiens GN=B3GALT1 PE=1 SV=1
MASKVSCLYVLTVVCWASALWYLSITRPTSSYTGSKPFSHLTVARKNFTFGNIRTRPINP
HSFEFLINEPNKCEKNIPFLVILISTTHKEFDARQAIRETWGDENNFKGIKIATLFLLGK
NADPVLNQMVEQESQIFHDIIVEDFIDSYHNLTLKTLMGMRWVATFCSKAKYVMKTDSDI
FVNMDNLIYKLLKPSTKPRRRYFTGYVINGGPIRDVRSKWYMPRDLYPDSNYPPFCSGTG
YIFSADVAELIYKTSLHTRLLHLEDVYVGLCLRKLGIHPFQNSGFNHWKMAYSLCRYRRV
ITVHQISPEEMHRIWNDMSSKKHLRC
>sp|O43825|B3GT2_HUMAN Beta-1,3-galactosyltransferase 2 OS=Homo sapiens GN=B3GALT2 PE=1 SV=1
MLQWRRRHCCFAKMTWNAKRSLFRTHLIGVLSLVFLFAMFLFFNHHDWLPGRAGFKENPV
TYTFRGFRSTKSETNHSSLRNIWKETVPQTLRPQTATNSNNTDLSPQGVTGLENTLSANG
SIYNEKGTGHPNSYHFKYIINEPEKCQEKSPFLILLIAAEPGQIEARRAIRQTWGNESLA
PGIQITRIFLLGLSIKLNGYLQRAILEESRQYHDIIQQEYLDTYYNLTIKTLMGMNWVAT
YCPHIPYVMKTDSDMFVNTEYLINKLLKPDLPPRHNYFTGYLMRGYAPNRNKDSKWYMPP
DLYPSERYPVFCSGTGYVFSGDLAEKIFKVSLGIRRLHLEDVYVGICLAKLRIDPVPPPN
EFVFNHWRVSYSSCKYSHLITSHQFQPSELIKYWNHLQQNKHNACANAAKEKAGRYRHRK
LH
>sp|Q6L9W6|B4GN3_HUMAN Beta-1,4-N-acetylgalactosaminyltransferase 3 OS=Homo sapiens GN=B4GALNT3 PE=1 SV=2
MGSPRAARPPLLLRPVKLLRRRFRLLLALAVVSVGLWTLYLELVASAQVGGNPLNRRYGS
WRELAKALASRNIPAVDPHLQFYHPQRLSLEDHDIDQGVSSNSSYLKWNKPVPWLSEFRG
RANLHVFEDWCGSSIQQLRRNLHFPLYPHIRTTLRKLAVSPKWTNYGLRIFGYLHPFTDG
KIQFAIAADDNAEFWLSLDDQVSGLQLLASVGKTGKEWTAPGEFGKFRSQISKPVSLSAS
HRYYFEVLHKQNEEGTDHVEVAWRRNDPGAKFTIIDSLSLSLFTNETFLQMDEVGHIPQT
AASHVDSSNALPRDEQPPADMLRPDPRDTLYRVPLIPKSHLRHVLPDCPYKPSYLVDGLP
LQRYQGLRFVHLSFVYPNDYTRLSHMETHNKCFYQENAYYQDRFSFQEYIKIDQPEKQGL
EQPGFEENLLEESQYGEVAEETPASNNQNARMLEGRQTPASTLEQDATDYRLRSLRKLLA
QPREGLLAPFSKRNSTASFPGRTSHIPVQQPEKRKQKPSPEPSQDSPHSDKWPPGHPVKN
LPQMRGPRPRPAGDSPRKTQWLNQVESYIAEQRRGDRMRPQAPGRGWHGEEEVVAAAGQE
GQVEGEEEGEEEEEEEDMSEVFEYVPVFDPVVNWDQTFSARNLDFQALRTDWIDLSCNTS
GNLLLPEQEALEVTRVFLKKLNQRSRGRYQLQRIVNVEKRQDQLRGGRYLLELELLEQGQ
RVVRLSEYVSARGWQGIDPAGGEEVEARNLQGLVWDPHNRRRQVLNTRAQEPKLCWPQGF
SWSHRAVVHFVVPVKNQARWVQQFIKDMENLFQVTGDPHFNIVITDYSSEDMDVEMALKR
SKLRSYQYVKLSGNFERSAGLQAGIDLVKDPHSIIFLCDLHIHFPAGVIDAIRKHCVEGK
MAFAPMVMRLHCGATPQWPEGYWEVNGFGLLGIYKSDLDRIGGMNTKEFRDRWGGEDWEL
LDRILQAGLDVERLSLRNFFHHFHSKRGMWSRRQMKTL
>sp|O60909|B4GT2_HUMAN Beta-1,4-galactosyltransferase 2 OS=Homo sapiens GN=B4GALT2 PE=1 SV=1
MSRLLGGTLERVCKAVLLLCLLHFLVAVILYFDVYAQHLAFFSRFSARGPAHALHPAASS
SSSSSNCSRPNATASSSGLPEVPSALPGPTAPTLPPCPDSPPGLVGRLLIEFTSPMPLER
VQRENPGVLMGGRYTPPDCTPAQTVAVIIPFRHREHHLRYWLHYLHPILRRQRLRYGVYV

INQHGEDTFNRAKLLNVGFLEALKEDAAYDCFIFSDVDLVPMDDRNLYRCGDQPRHFAIA
MDKFGFRLPYAGYFGGVSGLSKAQFLRINGFPNEYWGWGGEDDDIFNRISLTGMKISRPD
IRIGRYRMIKHDRDKHNEPNPQRFTKIQNTKLTMKRDGIGSVRYQVLEVSRQPLFTNITV
DIGRPPSWPPRG
>sp|Q6ZUV0|BACHL_HUMAN Putative cytosolic acyl coenzyme A thioester hydrolase-like OS=Homo sapiens GN=ACOT7L PE=5 SV=1
MIKEAGAIISTRHCNPQNGDRCVAALARVECTHFLWPMCIGEVAHVSAEITYTSKHSVEV
QVNMMSENILTGAKKLTNKATLWYAPLSLTNVDKVLEEPPVVYFRQEQEEEGQKRYKTQK
LERMETNWRNGDIVQPVLNPEPNTVSYSQSSLIHLVGPSDCTLHSFVHEGVTMKVMDEVA
GILAARHCKTNLVTASMEAINFDNKIRKGCIKTISGRMTFTSNKSVEIEVLVDADCVVDS
SQKRYRAASVFT
>sp|Q86Y30|BAGE2_HUMAN B melanoma antigen 2 OS=Homo sapiens GN=BAGE2 PE=2 SV=1
MAAGVVFLALSAQLLQARLMKEESPVVSWRLEPEDGTALDVHFVSTLEPLSNAVKRNVPR
CIIILVLQEPTAFRISVTSSCFVQNTLTKLLKDRRKMQTVQCATARETS
>sp|Q99728|BARD1_HUMAN BRCA1-associated RING domain protein 1 OS=Homo sapiens GN=BARD1 PE=1 SV=2
MPDNRQPRNRQPRIRSGNEPRSAPAMEPDGRGAWAHSRAALDRLEKLLRCSRCTNILREP
VCLGGCEHIFCSNCVSDCIGTGCPVCYTPAWIQDLKINRQLDSMIQLCSKLRNLLHDNEL
SDLKEDKPRKSLFNDAGNKKNSIKMWFSPRSKKVRYVVSKASVQTQPAIKKDASAQQDSY
EFVSPSPPADVSERAKKASARSGKKQKKKTLAEINQKWNLEAEKEDGEFDSKEESKQKLV
SFCSQPSVISSPQINGEIDLLASGSLTESECFGSLTEVSLPLAEQIESPDTKSRNEVVTP
EKVCKNYLTSKKSLPLENNGKRGHHNRLSSPISKRCRTSILSTSGDFVKQTVPSENIPLP
ECSSPPSCKRKVGGTSGRKNSNMSDEFISLSPGTPPSTLSSSSYRRVMSSPSAMKLLPNM
AVKRNHRGETLLHIASIKGDIPSVEYLLQNGSDPNVKDHAGWTPLHEACNHGHLKVVELL
LQHKALVNTTGYQNDSPLHDAAKNGHVDIVKLLLSYGASRNAVNIFGLRPVDYTDDESMK
SLLLLPEKNESSSASHCSVMNTGQRRDGPLVLIGSGLSSEQQKMLSELAVILKAKKYTEF
DSTVTHVVVPGDAVQSTLKCMLGILNGCWILKFEWVKACLRRKVCEQEEKYEIPEGPRRS
RLNREQLLPKLFDGCYFYLWGTFKHHPKDNLIKLVTAGGGQILSRKPKPDSDVTQTINTV
AYHARPDSDQRFCTQYIIYEDLCNYHPERVRQGKVWKAPSSWFIDCVMSFELLPLDS
>sp|Q9BZE3|BARH1_HUMAN BarH-like 1 homeobox protein OS=Homo sapiens GN=BARHL1 PE=2 SV=1
MEGSNGFGIDSILSHRAGSPALPKGDPLLGDCRSPLELSPRSESSSDCSSPASPGRDCLE
TGTPRPGGASGPGLDSHLQPGQLSAPAQSRTVTSSFLIRDILADCKPLAACAPYSSSGQP
AAPEPGGRLAAKAAEDFRDKLDKSGSNASSDSEYKVKEEGDREISSSRDSPPVRLKKPRK
ARTAFTDHQLAQLERSFERQKYLSVQDRMELAASLNLTDTQVKTWYQNRRTKWKRQTAVG
LELLAEAGNYSALQRMFPSPYFYPQSLVSNLDPGAALYLYRGPSAPPPALQRPLVPRILI
HGLQGASEPPPPLPPLAGVLPRAAQPR
>sp|Q9NRL2|BAZ1A_HUMAN Bromodomain adjacent to zinc finger domain protein 1A OS=Homo sapiens GN=BAZ1A PE=1 SV=2
MPLLHRKPFVRQKPPADLRPDEEVFYCKVTNEIFRHYDDFFERTILCNSLVWSCAVTGRP
GLTYQEALESEKKARQNLQSFPEPLIIPVLYLTSLTHRSRLHEICDDIFAYVKDRYFVEE
TVEVIRNNGARLQCRILEVLPPSHQNGFANGHVNSVDGETIIISDSDDSETQSCSFQNGK
KKDAIDPLLFKYKVQPTKKELHESAIVKATQISRRKHLFSRDKLKLFLKQHCEPQDGVIK
IKASSLSTYKIAEQDFSYFFPDDPPTFIFSPANRRRGRPPKRIHISQEDNVANKQTLASY
RSKATKERDKLLKQEEMKSLAFEKAKLKREKADALEAKKKEKEDKEKKREELKKIVEEER

LKKKEEKERLKVEREKEREKLREEKRKYVEYLKQWSKPREDMECDDLKELPEPTPVKTRL
PPEIFGDALMVLEFLNAFGELFDLQDEFPDGVTLEVLEEALVGNDSEGPLCELLFFFLTA
IFQAIAEEEEEVAKEQLTDADTKDLTEALDEDADPTKSALSAVASLAAAWPQLHQGCSLK
SLDLDSCTLSEILRLHILASGADVTSANAKYRYQKRGGFDATDDACMELRLSNPSLVKKL
SSTSVYDLTPGEKMKILHALCGKLLTLVSTRDFIEDYVDILRQAKQEFRELKAEQHRKER
EEAAARIRKRKEEKLKEQEQKMKEKQEKLKEDEQRNSTADISIGEEEREDFDTSIESKDT
EQKELDQDMVTEDEDDPGSHKRGRRGKRGQNGFKEFTRQEQINCVTREPLTADEEEALKQ
EHQRKEKELLEKIQSAIACTNIFPLGRDRMYRRYWIFPSIPGLFIEEDYSGLTEDMLLPR
PSSFQNNVQSQDPQVSTKTGEPLMSESTSNIDQGPRDHSVQLPKPVHKPNRWCFYSSCEQ
LDQLIEALNSRGHRESALKETLLQEKSRICAQLARFSEEKFHFSDKPQPDSKPTYSRGRS
SNAYDPSQMCAEKQLELRLRDFLLDIEDRIYQGTLGAIKVTDRHIWRSALESGRYELLSE
ENKENGIIKTVNEDVEEMEIDEQTKVIVKDRLLGIKTETPSTVSTNASTPQSVSSVVHYL
AMALFQIEQGIERRFLKAPLDASDSGRSYKTVLDRWRESLLSSASLSQVFLHLSTLDRSV
IWSKSILNARCKICRKKGDAENMVLCDGCDRGHHTYCVRPKLKTVPEGDWFCPECRPKQR
SRRLSSRQRPSLESDEDVEDSMGGEDDEVDGDEEEGQSEEEEYEVEQDEDDSQEEEEVSL
PKRGRPQVRLPVKTRGKLSSSFSSRGQQQEPGRYPSRSQQSTPKTTVSSKTGRSLRKINS
APPTETKSLRIASRSTRHSHGPLQADVFVELLSPRRKRRGRKSANNTPENSPNFPNFRVI
ATKSSEQSRSVNIASKLSLQESESKRRCRKRQSPEPSPVTLGRRSSGRQGGVHELSAFEQ
LVVELVRHDDSWPFLKLVSKIQVPDYYDIIKKPIALNIIREKVNKCEYKLASEFIDDIEL
MFSNCFEYNPRNTSEAKAGTRLQAFFHIQAQKLGLHVTPSNVDQVSTPPAAKKSRI
>sp|Q6ZUJ8|BCAP_HUMAN Phosphoinositide 3-kinase adapter protein 1 OS=Homo sapiens GN=PIK3AP1 PE=1 SV=2
MAASGVPRGCDILIVYSPDAEEWCQYLQTLFLSSRQVRSQKILTHRLGPEASFSAEDLSL
FLSTRCVVVLLSAELVQHFHKPALLPLLQRAFHPPHRVVRLLCGVRDSEEFLDFFPDWAH
WQELTCDDEPETYVAAVKKAISEDSGCDSVTDTEPEDEKVVSYSKQQNLPTVTSPGNLMV
VQPDRIRCGAETTVYVIVRCKLDDRVATEAEFSPEDSPSVRMEAKVENEYTISVKAPNLS
SGNVSLKIYSGDLVVCETVISYYTDMEEIGNLLSNAANPVEFMCQAFKIVPYNTETLDKL
LTESLKNNIPASGLHLFGINQLEEEDMMTNQRDEELPTLLHFAAKYGLKNLTALLLTCPG
ALQAYSVANKHGHYPNTIAEKHGFRDLRQFIDEYVETVDMLKSHIKEELMHGEEADAVYE
SMAHLSTDLLMKCSLNPGCDEDLYESMAAFVPAATEDLYVEMLQASTSNPIPGDGFSRAT
KDSMIRKFLEGNSMGMTNLERDQCHLGQEEDVYHTVDDDEAFSVDLASRPPVPVPRPETT
APGAHQLPDNEPYIFKVFAEKSQERPGNFYVSSESIRKGPPVRPWRDRPQSSIYDPFAGM
KTPGQRQLITLQEQVKLGIVNVDEAVLHFKEWQLNQKKRSESFRFQQENLKRLRDSITRR
QREKQKSGKQTDLEITVPIRHSQHLPAKVEFGVYESGPRKSVIPPRTELRRGDWKTDSTS
STASSTSNRSSTRSLLSVSSGMEGDNEDNEVPEVTRSRSPGPPQVDGTPTMSLERPPRVP
PRAASQRPPTRETFHPPPPVPPRGR
>sp|P56945|BCAR1_HUMAN Breast cancer anti-estrogen resistance protein 1 OS=Homo sapiens GN=BCAR1 PE=1 SV=2
MNHLNVLAKALYDNVAESPDELSFRKGDIMTVLEQDTQGLDGWWLCSLHGRQGIVPGNRL
KILVGMYDKKPAGPGPGPPATPAQPQPGLHAPAPPASQYTPMLPNTYQPQPDSVYLVPTP
SKAQQGLYQVPGPSPQFQSPPAKQTSTFSKQTPHHPFPSPATDLYQVPPGPGGPAQDIYQ
VPPSAGMGHDIYQVPPSMDTRSWEGTKPPAKVVVPTRVGQGYVYEAAQPEQDEYDIPRHL
LAPGPQDIYDVPPVRGLLPSQYGQEVYDTPPMAVKGPNGRDPLLEVYDVPPSVEKGLPPS
NHHAVYDVPPSVSKDVPDGPLLREETYDVPPAFAKAKPFDPARTPLVLAAPPPDSPPAED

VYDVPPPAPDLYDVPPGLRRPGPGTLYDVPRERVLPPEVADGGVVDSGVYAVPPPAEREA
PAEGKRLSASSTGSTRSSQSASSLEVAGPGREPLELEVAVEALARLQQGVSATVAHLLDL
AGSAGATGSWRSPSEPQEPLVQDLQAAVAAVQSAVHELLEFARSAVGNAAHTSDRALHAK
LSRQLQKMEDVHQTLVAHGQALDAGRGGSGATLEDLDRLVACSRAVPEDAKQLASFLHGN
ASLLFRRTKATAPGPEGGGTLHPNPTDKTSSIQSRPLPSPPKFTSQDSPDGQYENSEGGW
MEDYDYVHLQGKEEFEKTQKELLEKGSITRQGKSQLELQQLKQFERLEQEVSRPIDHDLA
NWTPAQPLAPGRTGGLGPSDRQLLLFYLEQCEANLTTLTNAVDAFFTAVATNQPPKIFVA
HSKFVILSAHKLVFIGDTLSRQAKAADVRSQVTHYSNLLCDLLRGIVATTKAAALQYPSP
SAAQDMVERVKELGHSTQQFRRVLGQLAAA
>sp|Q8TDM0|BCAS4_HUMAN Breast carcinoma-amplified sequence 4 OS=Homo sapiens GN=BCAS4 PE=1 SV=1
MQRTGGGAPRPGRNHGLPGSLRQPDPVALLMLLVDADQPEPMRSGARELALFLTPEPGAE
AKEVEETIEGMLLRLEEFCSLADLIRSDTSQILEENIPVLKAKLTEMRGIYAKVDRLEAF
VKMVGHHVAFLEADVLQAERDHGAFPQALRRWLGSAGLPSFRNVECSGTIPARCNLRLPG
SSDSPASASQVAGITEVTCTGARDVRAAHTV
>sp|Q9NWK9|BCD1_HUMAN Box C/D snoRNA protein 1 OS=Homo sapiens GN=ZNHIT6 PE=1 SV=1
MEFAAENEGKSGGGLHSVAEGVRLSPEPGREGVRDLAGAEEFGGGEEGTGLTGIKEIGDG
EEGSGQRPEEIPMDLTVVKQEIIDWPGTEGRLAGQWVEQEVEDRPEVKDENAGVLEVKQE
TDSSLVVKEAKVGEPEVKEEKVKEEVMDWSEVKEEKDNLEIKQEEKFVGQCIKEELMHGE
CVKEEKDFLKKEIVDDTKVKEEPPINHPVGCKRKLAMSRCETCGTEEAKYRCPRCMRYSC
SLPCVKKHKAELTCNGVRDKTAYISIQQFTEMNLLSDYRFLEDVARTADHISRDAFLKRP
ISNKYMYFMKNRARRQGINLKLLPNGFTKRKENSTFFDKKKQQFCWHVKLQFPQSQAEYI
EKRVPDDKTINEILKPYIDPEKSDPVIRQRLKAYIRSQTGVQILMKIEYMQQNLVRYYEL
DPYKSLLDNLRNKVIIEYPTLHVVLKGSNNDMKVLHQVKSESTKNVGNEN
>sp|O60756|BCE1_HUMAN Putative protein BCE-1 OS=Homo sapiens GN=BCE1 PE=5 SV=1
MGRTPTAVQVKSFTKQGQQRRVCRDLPLKNTKNGLSPGMRTCFLYLRFFPCLSWMSLKWT
QAVHCARNIVLSFMLLLLLLNYNM
>sp|O14874|BCKD_HUMAN [3-methyl-2-oxobutanoate dehydrogenase [lipoamide]] kinase, mitochondrial OS=Homo sapiens GN=BCKDK PE=1 SV=2
MILASVLRSGPGGGLPLRPLLGPALALRARSTSATDTHHVEMARERSKTVTSFYNQSAID
AAAEKPSVRLTPTMMLYAGRSQDGSHLLKSARYLQQELPVRIAHRIKGFRCLPFIIGCNP
TILHVHELYIRAFQKLTDFPPIKDQADEAQYCQLVRQLLDDHKDVVTLLAEGLRESRKHI
EDEKLVRYFLDKTLTSRLGIRMLATHHLALHEDKPDFVGIICTRLSPKKIIEKWVDFARR
LCEHKYGNAPRVRINGHVAARFPFIPMPLDYILPELLKNAMRATMESHLDTPYNVPDVVI
TIANNDVDLIIRISDRGGGIAHKDLDRVMDYHFTTAEASTQDPRISPLFGHLDMHSGAQS
GPMHGFGFGLPTSRAYAEYLGGSLQLQSLQGIGTDVYLRLRHIDGREESFRI
>sp|Q8N143|BCL6B_HUMAN B-cell CLL/lymphoma 6 member B protein OS=Homo sapiens GN=BCL6B PE=2 SV=2
MGSPAAPEGALGYVREFTRHSSDVLGNLNELRLRGILTDVTLLVGGQPLRAHKAVLIACS
GFFYSIFRGRAGVGVDVLSLPGGPEARGFAPLLDFMYTSRLRLSPATAPAVLAAATYLQM
EHVVQACHRFIQASYEPLGISLRPLEAEPPTPPTAPPPGSPRRSEGHPDPPTESRSCSQG
PPSPASPDPKACNWKKYKYIVLNSQASQAGSLVGERSSGQPCPQARLPSGDEASSSSSSS
SSSSEEGPIPGPQSRLSPTAATVQFKCGAPASTPYLLTSQAQDTSGSPSERARPLPGSEF
FSCQNCEAVAGCSSGLDSLVPGDEDKPYKCQLCRSSFRYKGNLASHRTVHTGEKPYHCSI

CGARFNRPANLKTHSRIHSGEKPYKCETCGSRFVQVAHLRAHVLIHTGEKPYPCPTCGTR
FRHLQTLKSHVRIHTGEKPYHCDPCGLHFRHKSQLRLHLRQKHGAATNTKVHYHILGGP
>sp|Q9BQE9|BCL7B_HUMAN B-cell CLL/lymphoma 7 protein family member B OS=Homo sapiens GN=BCL7B PE=1 SV=1
MSGRSVRAETRSRAKDDIKKVMAAIEKVRKWEKKWVTVGDTSLRIFKWVPVTDSKEKEKS
KSNSSAAREPNGFPSDASANSSLLLEFQDENSNQSSVSDVYQLKVDSSTNSSPSPQQSES
LSPAHTSDFRTDDSQPPTLGQEILEEPSLPSSEVADEPPTLTKEEPVPLETQVVEEEEDS
GAPPLKRFCVDQPTVPQTASES
>sp|O00512|BCL9_HUMAN B-cell CLL/lymphoma 9 protein OS=Homo sapiens GN=BCL9 PE=1 SV=4
MHSSNPKVRSSPSGNTQSSPKSKQEVMVRPPTVMSPSGNPQLDSKFSNQGKQGGSASQSQ
PSPCDSKSGGHTPKALPGPGGSMGLKNGAGNGAKGKGKRERSISADSFDQRDPGTPNDDS
DIKECNSADHIKSQDSQHTPHSMTPSNATAPRSSTPSHGQTTATEPTPAQKTPAKVVYVF
STEMANKAAEAVLKGQVETIVSFHIQNISNNKTERSTAPLNTQISALRNDPKPLPQQPPA
PANQDQNSSQNTRLQPTPPIPAPAPKPAAPPRPLDRESPGVENKLIPSVGSPASSTPLPP
DGTGPNSTPNNRAVTPVSQGSNSSSADPKAPPPPPVSSGEPPTLGENPDGLSQEQLEHRE
RSLQTLRDIQRMLFPDEKEFTGAQSGGPQQNPGVLDGPQKKPEGPIQAMMAQSQSLGKGP
GPRTDVGAPFGPQGHRDVPFSPDEMVPPSMNSQSGTIGPDHLDHMTPEQIAWLKLQQEFY
EEKRRKQEQVVVQQCSLQDMMVHQHGPRGVVRGPPPPYQMTPSEGWAPGGTEPFSDGINM
PHSLPPRGMAPHPNMPGSQMRLPGFAGMINSEMEGPNVPNPASRPGLSGVSWPDDVPKIP
DGRNFPPGQGIFSGPGRGERFPNPQGLSEEMFQQQLAEKQLGLPPGMAMEGIRPSMEMNR
MIPGSQRHMEPGNNPIFPRIPVEGPLSPSRGDFPKGIPPQMGPGRELEFGMVPSGMKGDV
NLNVNMGSNSQMIPQKMREAGAGPEEMLKLRPGGSDMLPAQQKMVPLPFGEHPQQEYGMG
PRPFLPMSQGPGSNSGLRNLREPIGPDQRTNSRLSHMPPLPLNPSSNPTSLNTAPPVQRG
LGRKPLDISVAGSQVHSPGINPLKSPTMHQVQSPMLGSPSGNLKSPQTPSQLAGMLAGPA
AAASIKSPPVLGSAAASPVHLKSPSLPAPSPGWTSSPKPPLQSPGIPPNHKAPLTMASPA
MLGNVESGGPPPPTASQPASVNIPGSLPSSTPYTMPPEPTLSQNPLSIMMSRMSKFAMPS
STPLYHDAIKTVASSDDDSPPARSPNLPSMNNMPGMGINTQNPRISGPNPVVPMPTLSPM
GMTQPLSHSNQMPSPNAVGPNIPPHGVPMGPGLMSHNPIMGHGSQEPPMVPQGRMGFPQG
FPPVQSPPQQVPFPHNGPSGGQGSFPGGMGFPGEGPLGRPSNLPQSSADAALCKPGGPGG
PDSFTVLGNSMPSVFTDPDLQEVIRPGATGIPEFDLSRIIPSEKPSQTLQYFPRGEVPGR
KQPQGPGPGFSHMQGMMGEQAPRMGLALPGMGGPGPVGTPDIPLGTAPSMPGHNPMRPPA
FLQQGMMGPHHRMMSPAQSTMPGQPTLMSNPAAAVGMIPGKDRGPAGLYTHPGPVGSPGM
MMSMQGMMGPQQNIMIPPQMRPRGMAADVGMGGFSQGPGNPGNMMF
>sp|Q9NYF8|BCLF1_HUMAN Bcl-2-associated transcription factor 1 OS=Homo sapiens GN=BCLAF1 PE=1 SV=2
MGRSNSRSHSSRSKSRSQSSSRSRSRSHSRKKRYSSRSRSRTYSRSRSRDRMYSRDYRRD
YRNNRGMRRPYGYRGRGRGYYQGGGGRYHRGGYRPVWNRRHSRSPRRGRSRSRSPKRRSV
SSQRSRSRSRRSYRSSRSPRSSSSRSSSPYSKSPVSKRRGSQEKQTKKAEGEPQEESPLK
SKSQEEPKDTFEHDPSESIDEFNKSSATSGDIWPGLSAYDNSPRSPHSPSPIATPPSQSS
SCSDAPMLSTVHSAKNTPSQHSHSIQHSPERSGSGSVGNGSSRYSPSQNSPIHHIPSRRS
PAKTIAPQNAPRDESRGRSSFYPDGGDQETAKTGKFLKRFTDEESRVFLLDRGNTRDKEA
SKEKGSEKGRAEGEWEDQEALDYFSDKESGKQKFNDSEGDDTEETEDYRQFRKSVLADQG
KSFATASHRNTEEEGLKYKSKVSLKGNRESDGFREEKNYKLKETGYVVERPSTTKDKHKE
EDKNSERITVKKETQSPEQVKSEKLKDLFDYSPPLHKNLDAREKSTFREESPLRIKMIAS

DSHRPEVKLKMAPVPLDDSNRPASLTKDRLLASTLVHSVKKEQEFRSIFDHIKLPQASKS
TSESFIQHIVSLVHHVKEQYFKSAAMTLNERFTSYQKATEEHSTRQKSPEIHRRIDISPS
TLRKHTRLAGEERVFKEENQKGDKKLRCDSADLRHDIDRRRKERSKERGDSKGSRESSGS
RKQEKTPKDYKEYKSYKDDSKHKREQDHSRSSSSSASPSSPSSREEKESKKEREEEFKTH
HEMKEYSGFAGVSRPRGTFFRIRGRGRARGVFAGTNTGPNNSNTTFQKRPKEEEWDPEYT
PKSKKYFLHDDRDDGVDYWAKRGRGRGTFQRGRGRFNFKKSGSSPKWTHDKYQGDGIVED
EEETMENNEEKKDRRKEEKE
>sp|Q8NFC6|BD1L1_HUMAN Biorientation of chromosomes in cell division protein 1-like 1 OS=Homo sapiens GN=BOD1L1 PE=1 SV=2
MATNPQPQPPPPAPPPPPPQPQPQPPPPPPGPGAGPGAGGAGGAGAGAGDPQLVAMIVNH
LKSQGLFDQFRRDCLADVDTKPAYQNLRQRVDNFVANHLATHTWSPHLNKNQLRNNIRQQ
VLKSGMLESGIDRIISQVVDPKINHTFRPQVEKAVHEFLATLNHKEEGSGNTAPDDEKPD
TSLITQGVPTPGPSANVANDAMSILETITSLNQEASAARASTETSNAKTSERASKKLPSQ
PTTDTSTDKERTSEDMADKEKSTADSGGEGLETAPKSEEFSDLPCPVEEIKNYTKEHNNL
ILLNKDVQQESSEQKNKSTDKGEKKPDSNEKGERKKEKKEKTEKKFDHSKKSEDTQKVKD
EKQAKEKEVESLKLPSEKNSNKAKTVEGTKEDFSLIDSDVDGLTDITVSSVHTSDLSSFE
EDTEEEVVTSDSMEEGEITSDDEEKNKQNKTKTQTSDSSEGKTKSVRHAYVHKPYLYSKY
YSDSDDELTVEQRRQSIAKEKEERLLRRQINREKLEEKRKQKAEKTKSSKTKGQGRSSVD
LEESSTKSLEPKAARIKEVLKERKVLEKKVALSKKRKKDSRNVEENSKKKQQYEEDSKET
LKTSEHCEKEKISSSKELKHVHAKSEPSKPARRLSESLHVVDENKNESKLEREHKRRTST
PVIMEGVQEETDTRDVKRQVERSEICTEEPQKQKSTLKNEKHLKKDDSETPHLKSLLKKE
VKSSKEKPEREKTPSEDKLSVKHKYKGDCMHKTGDETELHSSEKGLKVEENIQKQSQQTK
LSSDDKTERKSKHRNERKLSVLGKDGKPVSEYIIKTDENVRKENNKKERRLSAEKTKAEH
KSRRSSDSKIQKDSLGSKQHGITLQRRSESYSEDKCDMDSTNMDSNLKPEEVVHKEKRRT
KSLLEEKLVLKSKSKTQGKQVKVVETELQEGATKQATTPKPDKEKNTEENDSEKQRKSKV
EDKPFEETGVEPVLETASSSAHSTQKDSSHRAKLPLAKEKYKSDKDSTSTRLERKLSDGH
KSRSLKHSSKDIKKKDENKSDDKDGKEVDSSHEKARGNSSLMEKKLSRRLCENRRGSLSQ
EMAKGEEKLAANTLSTPSGSSLQRPKKSGDMTLIPEQEPMEIDSEPGVENVFEVSKTQDN
RNNNSQQDIDSENMKQKTSATVQKDELRTCTADSKATAPAYKPGRGTGVNSNSEKHADHR
STLTKKMHIQSAVSKMNPGEKEPIHRGTTEVNIDSETVHRMLLSAPSENDRVQKNLKNTA
AEEHVAQGDATLEHSTNLDSSPSLSSVTVVPLRESYDPDVIPLFDKRTVLEGSTASTSPA
DHSALPNQSLTVRESEVLKTSDSKEGGEGFTVDTPAKASITSKRHIPEAHQATLLDGKQG
KVIMPLGSKLTGVIVENENITKEGGLVDMAKKENDLNAEPNLKQTIKATVENGKKDGIAV
DHVVGLNTEKYAETVKLKHKRSPGKVKDISIDVERRNENSEVDTSAGSGSAPSVLHQRNG
QTEDVATGPRRAEKTSVATSTEGKDKDVTLSPVKAGPATTTSSETRQSEVALPCTSIEAD
EGLIIGTHSRNNPLHVGAEASECTVFAAAEEGGAVVTEGFAESETFLTSTKEGESGECAV
AESEDRAADLLAVHAVKIEANVNSVVTEEKDDAVTSAGSEEKCDGSLSRDSEIVEGTITF
ISEVESDGAVTSAGTEIRAGSISSEEVDGSQGNMMRMGPKKETEGTVTCTGAEGRSDNFV
ICSVTGAGPREERMVTGAGVVLGDNDAPPGTSASQEGDGSVNDGTEGESAVTSTGITEDG
EGPASCTGSEDSSEGFAISSESEENGESAMDSTVAKEGTNVPLVAAGPCDDEGIVTSTGA
KEEDEEGEDVVTSTGRGNEIGHASTCTGLGEESEGVLICESAEGDSQIGTVVEHVEAEAG
AAIMNANENNVDSMSGTEKGSKDTDICSSAKGIVESSVTSAVSGKDEVTPVPGGCEGPMT
SAASDQSDSQLEKVEDTTISTGLVGGSYDVLVSGEVPECEVAHTSPSEKEDEDIITSVEN
EECDGLMATTASGDITNQNSLAGGKNQGKVLIISTSTTNDYTPQVSAITDVEGGLSDALR

TEENMEGTRVTTEEFEAPMPSAVSGDDSQLTASRSEEKDECAMISTSIGEEFELPISSAT
TIKCAESLQPVAAAVEERATGPVLISTADFEGPMPSAPPEAESPLASTSKEEKDECALIS
TSIAEECEASVSGVVVESENERAGTVMEEKDGSGIISTSSVEDCEGPVSSAVPQEEGDPS
VTPAEEMGDTAMISTSTSEGCEAVMIGAVLQDEDRLTITRVEDLSDAAIISTSTAECMPI
SASIDRHEENQLTADNPEGNGDLSATEVSKHKVPMPSLIAENNCRCPGPVRGGKEPGPVL
AVSTEEGHNGPSVHKPSAGQGHPSAVCAEKEEKHGKECPEIGPFAGRGQKESTLHLINAE
EKNVLLNSLQKEDKSPETGTAGGSSTASYSAGRGLEGNANSPAHLRGPEQTSGQTAKDPS
VSIRYLAAVNTGAIKADDMPPVQGTVAEHSFLPAEQQGSEDNLKTSTTKCITGQESKIAP
SHTMIPPATYSVALLAPKCEQDLTIKNDYSGKWTDQASAEKTGDDNSTRKSFPEEGDIMV
TVSSEENVCDIGNEESPLNVLGGLKLKANLKMEAYVPSEEEKNGEILAPPESLCGGKPSG
IAELQREPLLVNESLNVENSGFRTNEEIHSESYNKGEISSGRKDNAEAISGHSVEADPKE
VEEEERHMPKRKRKQHYLSSEDEPDDNPDVLDSRIETAQRQCPETEPHDTKEENSRDLEE
LPKTSSETNSTTSRVMEEKDEYSSSETTGEKPEQNDDDTIKSQEEDQPIIIKRKRGRPRK
YPVETTLKMKDDSKTDTGIVTVEQSPSSSKLKVMQTDESNKETANLQERSISNDDGEEKI
VTSVRRRGRKPKRSLTVSDDAESSEPERKRQKSVSDPVEDKKEQESDEEEEEEEEDEPSG
ATTRSTTRSEAQRSKTQLSPSIKRKREVSPPGARTRGQQRVEEAPVKKAKR
>sp|Q14457|BECN1_HUMAN Beclin-1 OS=Homo sapiens GN=BECN1 PE=1 SV=2
MEGSKTSNNSTMQVSFVCQRCSQPLKLDTSFKILDRVTIQELTAPLLTTAQAKPGETQEE
ETNSGEEPFIETPRQDGVSRRFIPPARMMSTESANSFTLIGEASDGGTMENLSRRLKVTG
DLFDIMSGQTDVDHPLCEECTDTLLDQLDTQLNVTENECQNYKRCLEILEQMNEDDSEQL
QMELKELALEEERLIQELEDVEKNRKIVAENLEKVQAEAERLDQEEAQYQREYSEFKRQQ
LELDDELKSVENQMRYAQTQLDKLKKTNVFNATFHIWHSGQFGTINNFRLGRLPSVPVEW
NEINAAWGQTVLLLHALANKMGLKFQRYRLVPYGNHSYLESLTDKSKELPLYCSGGLRFF
WDNKFDHAMVAFLDCVQQFKEEVEKGETRFCLPYRMDVEKGKIEDTGGSGGSYSIKTQFN
SEEQWTKALKFMLTNLKWGLAWVSSQFYNK
>sp|Q9NYM9|BET1L_HUMAN BET1-like protein OS=Homo sapiens GN=BET1L PE=1 SV=1
MADWARAQSPGAVEEILDRENKRMADSLASKVTRLKSLALDIDRDAEDQNRYLDGMDSDF
TSMTSLLTGSVKRFSTMARSGQDNRKLLCGMAVGLIVAFFILSYFLSRART
>sp|O15155|BET1_HUMAN BET1 homolog OS=Homo sapiens GN=BET1 PE=1 SV=1
MRRAGLGEGVPPGNYGNYGYANSGYSACEEENERLTESLRSKVTAIKSLSIEIGHEVKTQ
NKLLAEMDSQFDSTTGFLGKTMGKLKILSRGSQTKLLCYMMLFSLFVFFIIYWIIKLR
>sp|Q5H9J7|BEX5_HUMAN Protein BEX5 OS=Homo sapiens GN=BEX5 PE=1 SV=1
MENVPKENKVVEKAPVQNEAPALGGGEYQEPGGNVKGVWAPPAPGFGEDVPNRLVDNIDM
IDGDGDDMERFMEEMRELRRKIRELQLRYSLRILIGDPPHHDHHDEFCLMP
>sp|P16278|BGAL_HUMAN Beta-galactosidase OS=Homo sapiens GN=GLB1 PE=1 SV=2
MPGFLVRILPLLLVLLLLGPTRGLRNATQRMFEIDYSRDSFLKDGQPFRYISGSIHYSRV
PRFYWKDRLLKMKMAGLNAIQTYVPWNFHEPWPGQYQFSEDHDVEYFLRLAHELGLLVIL
RPGPYICAEWEMGGLPAWLLEKESILLRSSDPDYLAAVDKWLGVLLPKMKPLLYQNGGPV
ITVQVENEYGSYFACDFDYLRFLQKRFRHHLGDDVVLFTTDGAHKTFLKCGALQGLYTTV
DFGTGSNITDAFLSQRKCEPKGPLINSEFYTGWLDHWGQPHSTIKTEAVASSLYDILARG
ASVNLYMFIGGTNFAYWNGANSPYAAQPTSYDYDAPLSEAGDLTEKYFALRNIIQKFEKV
PEGPIPPSTPKFAYGKVTLEKLKTVGAALDILCPSGPIKSLYPLTFIQVKQHYGFVLYRT
TLPQDCSNPAPLSSPLNGVHDRAYVAVDGIPQGVLERNNVITLNITGKAGATLDLLVENM
GRVNYGAYINDFKGLVSNLTLSSNILTDWTIFPLDTEDAVRSHLGGWGHRDSGHHDEAWA

HNSSNYTLPAFYMGNFSIPSGIPDLPQDTFIQFPGWTKGQVWINGFNLGRYWPARGPQLT
LFVPQHILMTSAPNTITVLELEWAPCSSDDPELCAVTFVDRPVIGSSVTYDHPSKPVEKR
LMPPPPQKNKDSWLDHV
>sp|Q04656|ATP7A_HUMAN Copper-transporting ATPase 1 OS=Homo sapiens GN=ATP7A PE=1 SV=3
MDPSMGVNSVTISVEGMTCNSCVWTIEQQIGKVNGVHHIKVSLEEKNATIIYDPKLQTPK
TLQEAIDDMGFDAVIHNPDPLPVLTDTLFLTVTASLTLPWDHIQSTLLKTKGVTDIKIYP
QKRTVAVTIIPSIVNANQIKELVPELSLDTGTLEKKSGACEDHSMAQAGEVVLKMKVEGM
TCHSCTSTIEGKIGKLQGVQRIKVSLDNQEATIVYQPHLISVEEMKKQIEAMGFPAFVKK
QPKYLKLGAIDVERLKNTPVKSSEGSQQRSPSYTNDSTATFIIDGMHCKSCVSNIESTLS
ALQYVSSIVVSLENRSAIVKYNASSVTPESLRKAIEAVSPGLYRVSITSEVESTSNSPSS
SSLQKIPLNVVSQPLTQETVINIDGMTCNSCVQSIEGVISKKPGVKSIRVSLANSNGTVE
YDPLLTSPETLRGAIEDMGFDATLSDTNEPLVVIAQPSSEMPLLTSTNEFYTKGMTPVQD
KEEGKNSSKCYIQVTGMTCASCVANIERNLRREEGIYSILVALMAGKAEVRYNPAVIQPP
MIAEFIRELGFGATVIENADEGDGVLELVVRGMTCASCVHKIESSLTKHRGILYCSVALA
TNKAHIKYDPEIIGPRDIIHTIESLGFEASLVKKDRSASHLDHKREIRQWRRSFLVSLFF
CIPVMGLMIYMMVMDHHFATLHHNQNMSKEEMINLHSSMFLERQILPGLSVMNLLSFLLC
VPVQFFGGWYFYIQAYKALKHKTANMDVLIVLATTIAFAYSLIILLVAMYERAKVNPITF
FDTPPMLFVFIALGRWLEHIAKGKTSEALAKLISLQATEATIVTLDSDNILLSEEQVDVE
LVQRGDIIKVVPGGKFPVDGRVIEGHSMVDESLITGEAMPVAKKPGSTVIAGSINQNGSL
LICATHVGADTTLSQIVKLVEEAQTSKAPIQQFADKLSGYFVPFIVFVSIATLLVWIVIG
FLNFEIVETYFPGYNRSISRTETIIRFAFQASITVLCIACPCSLGLATPTAVMVGTGVGA
QNGILIKGGEPLEMAHKVKVVVFDKTGTITHGTPVVNQVKVLTESNRISHHKILAIVGTA
ESNSEHPLGTAITKYCKQELDTETLGTCIDFQVVPGCGISCKVTNIEGLLHKNNWNIEDN
NIKNASLVQIDASNEQSSTSSSMIIDAQISNALNAQQYKVLIGNREWMIRNGLVINNDVN
DFMTEHERKGRTAVLVAVDDELCGLIAIADTVKPEAELAIHILKSMGLEVVLMTGDNSKT
ARSIASQVGITKVFAEVLPSHKVAKVKQLQEEGKRVAMVGDGINDSPALAMANVGIAIGT
GTDVAIEAADVVLIRNDLLDVVASIDLSRETVKRIRINFVFALIYNLVGIPIAAGVFMPI
GLVLQPWMGSAAMAASSVSVVLSSLFLKLYRKPTYESYELPARSQIGQKSPSEISVHVGI
DDTSRNSPKLGLLDRIVNYSRASINSLLSDKRSLNSVVTSEPDKHSLLVGDFREDDDTAL
>sp|P35670|ATP7B_HUMAN Copper-transporting ATPase 2 OS=Homo sapiens GN=ATP7B PE=1 SV=4
MPEQERQITAREGASRKILSKLSLPTRAWEPAMKKSFAFDNVGYEGGLDGLGPSSQVATS
TVRILGMTCQSCVKSIEDRISNLKGIISMKVSLEQGSATVKYVPSVVCLQQVCHQIGDMG
FEASIAEGKAASWPSRSLPAQEAVVKLRVEGMTCQSCVSSIEGKVRKLQGVVRVKVSLSN
QEAVITYQPYLIQPEDLRDHVNDMGFEAAIKSKVAPLSLGPIDIERLQSTNPKRPLSSAN
QNFNNSETLGHQGSHVVTLQLRIDGMHCKSCVLNIEENIGQLLGVQSIQVSLENKTAQVK
YDPSCTSPVALQRAIEALPPGNFKVSLPDGAEGSGTDHRSSSSHSPGSPPRNQVQGTCST
TLIAIAGMTCASCVHSIEGMISQLEGVQQISVSLAEGTATVLYNPSVISPEELRAAIEDM
GFEASVVSESCSTNPLGNHSAGNSMVQTTDGTPTSVQEVAPHTGRLPANHAPDILAKSPQ
STRAVAPQKCFLQIKGMTCASCVSNIERNLQKEAGVLSVLVALMAGKAEIKYDPEVIQPL
EIAQFIQDLGFEAAVMEDYAGSDGNIELTITGMTCASCVHNIESKLTRTNGITYASVALA
TSKALVKFDPEIIGPRDIIKIIEEIGFHASLAQRNPNAHHLDHKMEIKQWKKSFLCSLVF
GIPVMALMIYMLIPSNEPHQSMVLDHNIIPGLSILNLIFFILCTFVQLLGGWYFYVQAYK
SLRHRSANMDVLIVLATSIAYVYSLVILVVAVAEKAERSPVTFFDTPPMLFVFIALGRWL
EHLAKSKTSEALAKLMSLQATEATVVTLGEDNLIIREEQVPMELVQRGDIVKVVPGGKFP

VDGKVLEGNTMADESLITGEAMPVTKKPGSTVIAGSINAHGSVLIKATHVGNDTTLAQIV
KLVEEAQMSKAPIQQLADRFSGYFVPFIIIMSTLTLVVWIVIGFIDFGVVQRYFPNPNKH
ISQTEVIIRFAFQTSITVLCIACPCSLGLATPTAVMVGTGVAAQNGILIKGGKPLEMAHK
IKTVMFDKTGTITHGVPRVMRVLLLGDVATLPLRKVLAVVGTAEASSEHPLGVAVTKYCK
EELGTETLGYCTDFQAVPGCGIGCKVSNVEGILAHSERPLSAPASHLNEAGSLPAEKDAV
PQTFSVLIGNREWLRRNGLTISSDVSDAMTDHEMKGQTAILVAIDGVLCGMIAIADAVKQ
EAALAVHTLQSMGVDVVLITGDNRKTARAIATQVGINKVFAEVLPSHKVAKVQELQNKGK
KVAMVGDGVNDSPALAQADMGVAIGTGTDVAIEAADVVLIRNDLLDVVASIHLSKRTVRR
IRINLVLALIYNLVGIPIAAGVFMPIGIVLQPWMGSAAMAASSVSVVLSSLQLKCYKKPD
LERYEAQAHGHMKPLTASQVSVHIGMDDRWRDSPRATPWDQVSYVSQVSLSSLTSDKPSR
HSAAADDDGDKWSLLLNGRDEEQYI
>sp|P48047|ATPO_HUMAN ATP synthase subunit O, mitochondrial OS=Homo sapiens GN=ATP5O PE=1 SV=1
MAAPAVSGLSRQVRCFSTSVVRPFAKLVRPPVQVYGIEGRYATALYSAASKQNKLEQVEK
ELLRVAQILKEPKVAASVLNPYVKRSIKVKSLNDITAKERFSPLTTNLINLLAENGRLSN
TQGVVSAFSTMMSVHRGEVPCTVTSASPLEEATLSELKTVLKSFLSQGQVLKLEAKTDPS
ILGGMIVRIGEKYVDMSVKTKIQKLGRAMREIV
>sp|Q8WXX7|AUTS2_HUMAN Autism susceptibility gene 2 protein OS=Homo sapiens GN=AUTS2 PE=1 SV=1
MDGPTRGHGLRKKRRSRSQRDRERRSRGGLGAGAAGGGGAGRTRALSLASSSGSDKEDNG
KPPSSAPSRPRPPRRKRRESTSAEEDIIDGFAMTSFVTFEALEKDVALKPQERVEKRQTP
LTKKKREALTNGLSFHSKKSRLSHPHHYSSDRENDRNLCQHLGKRKKMPKALRQLKPGQN
SCRDSDSESASGESKGFHRSSSRERLSDSSAPSSLGTGYFCDSDSDQEEKASDASSEKLF
NTVIVNKDPELGVGTLPEHDSQDAGPIVPKISGLERSQEKSQDCCKEPIFEPVVLKDPCP
QVAQPIPQPQTEPQLRAPSPDPDLVQRTEAPPQPPPLSTQPPQGPPEAQLQPAPQPQVQR
PPRPQSPTQLLHQNLPPVQAHPSAQSLSQPLSAYNSSSLSLNSLSSSRSSTPAKTQPAPP
HISHHPSASPFPLSLPNHSPLHSFTPTLQPPAHSHHPNMFAPPTALPPPPPLTSGSLQVA
GHPAGSTYSEQDILRQELNTRFLASQSADRGASLGPPPYLRTEFHQHQHQHQHTHQHTHQ
HTFTPFPHAIPPTAIMPTPAPPMFDKYPTKVDPFYRHSLFHSYPPAVSGIPPMIPPTGPF
GSLQGAFQPKTSNPIDVAARPGTVPHTLLQKDPRLTDPFRPMLRKPGKWCAMHVHIAWQI
YHHQQKVKKQMQSDPHKLDFGLKPEFLSRPPGPSLFGAIHHPHDLARPSTLFSAAGAAHP
TGTPFGPPPHHSNFLNPAAHLEPFNRPSTFTGLAAVGGNAFGGLGNPSVTPNSMFGHKDG
PSVQNFSNPHEPWNRLHRTPPSFPTPPPWLKPGELERSASAAAHDRDRDVDKRDSSVSKD
DKERESVEKRHSSHPSPAPVLPVNALGHTRSSTEQIRAHLNTEAREKDKPKERERDHSES
RKDLAADEHKAKEGHLPEKDGHGHEGRAAGEEAKQLARVPSPYVRTPVVESARPNSTSSR
EAEPRKGEPAYENPKKSSEVKVKEERKEDHDLPPEAPQTHRASEPPPPNSSSSVHPGPLA
SMPMTVGVTGIHPMNSISSLDRTRMMTPFMGISPLPGGERFPYPSFHWDPIRDPLRDPYR
ELDIHRRDPLGRDFLLRNDPLHRLSTPRLYEADRSFRDREPHDYSHHHHHHHHPLSVDPR
REHERGGHLDERERLHMLREDYEHTRLHSVHPASLDGHLPHPSLITPGLPSMHYPRISPT
AGNQNGLLNKTPPTAALSAPPPLISTLGGRPVSPRRTTPLSAEIRERPPSHTLKDIEAR
>sp|O75366|AVIL_HUMAN Advillin OS=Homo sapiens GN=AVIL PE=1 SV=3
MPLTSAFRAVDNDPGIIVWRIEKMELALVPVSAHGNFYEGDCYVILSTRRVASLLSQDIH
FWIGKDSSQDEQSCAAIYTTQLDDYLGGSPVQHREVQYHESDTFRGYFKQGIIYKQGGVA
SGMKHVETNTYDVKRLLHVKGKRNIRATEVEMSWDSFNRGDVFLLDLGKVIIQWNGPESN

SGERLKAMLLAKDIRDRERGGRAKIGVIEGDKEAASPELMKVLQDTLGRRSIIKPTVPDE
IIDQKQKSTIMLYHISDSAGQLAVTEVATRPLVQDLLNHDDCYILDQSGTKIYVWKGKGA
TKAEKQAAMSKALGFIKMKSYPSSTNVETVNDGAESAMFKQLFQKWSVKDQTMGLGKTFS
IGKIAKVFQDKFDVTLLHTKPEVAAQERMVDDGNGKVEVWRIENLELVPVEYQWYGFFYG
GDCYLVLYTYEVNGKPHHILYIWQGRHASQDELAASAYQAVEVDRQFDGAAVQVRVRMGT
EPRHFMAIFKGKLVIFEGGTSRKGNAEPDPPVRLFQIHGNDKSNTKAVEVPAFASSLNSN
DVFLLRTQAEHYLWYGKGSSGDERAMAKELASLLCDGSENTVAEGQEPAEFWDLLGGKTP
YANDKRLQQEILDVQSRLFECSNKTGQFVVTEITDFTQDDLNPTDVMLLDTWDQVFLWIG
AEANATEKESALATAQQYLHTHPSGRDPDTPILIIKQGFEPPIFTGWFLAWDPNIWSAGK
TYEQLKEELGDAAAIMRITADMKNATLSLNSNDSEPKYYPIAVLLKNQNQELPEDVNPAK
KENYLSEQDFVSVFGITRGQFAALPGWKQLQMKKEKGLF
>sp|P0C853|BAAS2_HUMAN Putative uncharacterized protein BAALC-AS2 OS=Homo sapiens GN=BAALC-AS2 PE=5 SV=1
MSLKSWHPQSKTKRVGASEGNPQWGSGSMEAPLLSSFLPPLASEAELTGNTWFLHRCSCI
LNLEESMDSDWGAWWGVSLPRRAPFLIYGSDGPWCTQAGFPGWGH
>sp|Q14032|BAAT_HUMAN Bile acid-CoA:amino acid N-acyltransferase OS=Homo sapiens GN=BAAT PE=1 SV=1
MIQLTATPVSALVDEPVHIRATGLIPFQMVSFQASLEDENGDMFYSQAHYRANEFGEVDL
NHASSLGGDYMGVHPMGLFWSLKPEKLLTRLLKRDVMNRPFQVQVKLYDLELIVNNKVAS
APKASLTLERWYVAPGVTRIKVREGRLRGALFLPPGEGLFPGVIDLFGGLGGLLEFRASL
LASRGFASLALAYHNYEDLPRKPEVTDLEYFEEAANFLLRHPKVFGSGVGVVSVCQGVQI
GLSMAIYLKQVTATVLINGTNFPFGIPQVYHGQIHQPLPHSAQLISTNALGLLELYRTFE
TTQVGASQYLFPIEEAQGQFLFIVGEGDKTINSKAHAEQAIGQLKRHGKNNWTLLSYPGA
GHLIEPPYSPLCCASTTHDLRLHWGGEVIPHAAAQEHAWKEIQRFLRKHLIPDVTSQL
>sp|Q99933|BAG1_HUMAN BAG family molecular chaperone regulator 1 OS=Homo sapiens GN=BAG1 PE=1 SV=4
MAQRGGARRPRGDRERLGSRLRALRPGREPRQSEPPAQRGPPPSGRPPARSTASGHDRPT
RGAAAGARRPRMKKKTRRRSTRSEELTRSEELTLSEEATWSEEATQSEEATQGEEMNRSQ
EVTRDEESTRSEEVTREEMAAAGLTVTVTHSNEKHDLHVTSQQGSSEPVVQDLAQVVEEV
IGVPQSFQKLIFKGKSLKEMETPLSALGIQDGCRVMLIGKKNSPQEEVELKKLKHLEKSV
EKIADQLEELNKELTGIQQGFLPKDLQAEALCKLDRRVKATIEQFMKILEEIDTLILPEN
FKDSRLKRKGLVKKVQAFLAECDTVEQNICQETERLQSTNFALAE
>sp|Q9UMQ3|BARX2_HUMAN Homeobox protein BarH-like 2 OS=Homo sapiens GN=BARX2 PE=2 SV=3
MHCHAELRLSSPGQLKAARRRYKTFMIDEILSKETCDYFEKLSLYSVCPSLVVRPKPLHS
CTGSPSLRAYPLLSVITRQPTVISHLVPATPGIAQALSCHQVTEAVSAEAPGGEALASSE
SETEQPTPRQKKPRRSRTIFTELQLMGLEKKFQKQKYLSTPDRLDLAQSLGLTQLQVKTW
YQNRRMKWKKMVLKGGQEAPTKPKGRPKKNSIPTSEEIEAEEKMNSQAQGQEQLEPSQGQ
EELCEAQEPKARDVPLEMAEPPDPPQELPIPSSEPPPLS
>sp|Q6ZU67|BEND4_HUMAN BEN domain-containing protein 4 OS=Homo sapiens GN=BEND4 PE=2 SV=3
MEEEMQPAEEGPSVPKIYKQRSPYSVLKTFPSKRPALAKRYERPTLVELPHVRAPPPPPP
PFAPHAAVSISSSEPPPQQFQAQSSYPPGPGRAAAAASSSSPSCTPATSQGHLRTPAQPP
PASPAASSSSSFAAVVRYGPGAAAAAGTGGTGSDSASLELSAESRMILDAFAQQCSRVLS
LLNCGGKLLDSNHSQSMISCVKQEGSSYNERQEHCHIGKGVHSQTSDNVDIEMQYMQRKQ
QTSAFLRVFTDSLQNYLLSGSFPTPNPSSASEYGHLADVDPLSTSPVHTLGGWTSPATSE

SHGHPSSSTLPEEEEEEDEEGYCPRCQELEQEVISLQQENEELRRKLESIPVPCQTVLDY
LKMVLQHHNQLLIPQPADQPTEGSKQLLNNYPVYITSKQWDEAVNSSKKDGRRLLRYLIR
FVFTTDELKYSCGLGKRKRSVQSGETGPERRPLDPVKVTCLREFIRMHCTSNPDWWMPSE
EQINKVFSDAVGHARQGRAVGTFLHNGGSFYEGIDHQASQDEVFNKSSQDGSGD
>sp|Q8N7W2|BEND7_HUMAN BEN domain-containing protein 7 OS=Homo sapiens GN=BEND7 PE=1 SV=2
MEFSERKRSRKSQSFKLVSRDYHHEVYKIPEFSNDVNGEAKETQPIFLGDESMEIKKQIT
GMRRLLNDSTGRIYQRVGKEGEKLKEEPQDLDLVWPPRLNSSAEAPQSLHPSSRGVWNEL
PPQSGQFSGQYGTRSRTFQSQPHPTTSSNGELPVVNSSAGSNCCTCNCQSTLQAILQELK
TMRKLMQIQAVGTQNRQQPPISLICSQRTAVSRKRNKKKKVPPKTVEPLTVKQKPSGSEM
EKKSVVASELSALQAAEHTSPEESRVLGFGIVLESPSSDPEVQLAEGFDVFMPKSQLDSI
LSNYTRSGSLLFRKLVCAFFDDKTLANSLPNGKRKRGLNDNRKGLDQNIVGAIKVFTEKY
CTANHVDKLPGPRDWVQILQDQIKLARRRLKRGSEIADSDERLDGIALPPTGACGGPCTV
LPGGSAAVTLVLQSSPQTMSQEKGQMAEPWEEQHLVLLNNLTRDRAETGALSQTSQDFKH
HSFLITQVSATLHHQRGIRNFPTPGSAKSLTLHISCLSL
>sp|O76090|BEST1_HUMAN Bestrophin-1 OS=Homo sapiens GN=BEST1 PE=1 SV=1
MTITYTSQVANARLGSFSRLLLCWRGSIYKLLYGEFLIFLLCYYIIRFIYRLALTEEQQL
MFEKLTLYCDSYIQLIPISFVLGFYVTLVVTRWWNQYENLPWPDRLMSLVSGFVEGKDEQ
GRLLRRTLIRYANLGNVLILRSVSTAVYKRFPSAQHLVQAGFMTPAEHKQLEKLSLPHNM
FWVPWVWFANLSMKAWLGGRIRDPILLQSLLNEMNTLRTQCGHLYAYDWISIPLVYTQVV
TVAVYSFFLTCLVGRQFLNPAKAYPGHELDLVVPVFTFLQFFFYVGWLKVAEQLINPFGE
DDDDFETNWIVDRNLQVSLLAVDEMHQDLPRMEPDMYWNKPEPQPPYTAASAQFRRASFM
GSTFNISLNKEEMEFQPNQEDEEDAHAGIIGRFLGLQSHDHHPPRANSRTKLLWPKRESL
LHEGLPKNHKAAKQNVRGQEDNKAWKLKAVDAFKSAPLYQRPGYYSAPQTPLSPTPMFFP
LEPSAPSKLHSVTGIDTKDKSLKTVSSGAKKSFELLSESDGALMEHPEVSQVRRKTVEFN
LTDMPEIPENHLKEPLEQSPTNIHTTLKDHMDPYWALENRDEAHS
>sp|Q00994|BEX3_HUMAN Protein BEX3 OS=Homo sapiens GN=BEX3 PE=1 SV=1
MANIHQENEEMEQPMQNGEEDRPLGGGEGHQPAGNRRGQARRLAPNFRWAIPNRQINDGM
GGDGDDMEIFMEEMREIRRKLRELQLRNCLRILMGELSNHHDHHDEFCLMP
>sp|Q9NZS9|BFAR_HUMAN Bifunctional apoptosis regulator OS=Homo sapiens GN=BFAR PE=1 SV=1
MEEPQKSYVNTMDLERDEPLKSTGPQISVSEFSCHCCYDILVNPTTLNCGHSFCRHCLAL
WWASSKKTECPECREKWEGFPKVSILLRDAIEKLFPDAIRLRFEDIQQNNDIVQSLAAFQ
KYGNDQIPLAPNTGRANQQMGGGFFSGVLTALTGVAVVLLVYHWSSRESEHDLLVHKAVA
KWTAEEVVLWLEQLGPWASLYRERFLSERVNGRLLLTLTEEEFSKTPYTIENSSHRRAIL
MELERVKALGVKPPQNLWEYKAVNPGRSLFLLYALKSSPRLSLLYLYLFDYTDTFLPFIH
TICPLQEDSSGEDIVTKLLDLKEPTWKQWREFLVKYSFLPYQLIAEFAWDWLEVHYWTSR
FLIINAMLLSVLELFSFWRIWSRSELKTVPQRMWSHFWKVSTQGLFVAMFWPLIPQFVCN
CLFYWALYFNPIINIDLVVKELRRLETQVL
>sp|Q12934|BFSP1_HUMAN Filensin OS=Homo sapiens GN=BFSP1 PE=1 SV=3
MYRRSYVFQTRKEQYEHADEASRAAEPERPADEGWAGATSLAALQGLGERVAAHVQRARA
LEQRHAGLRRQLDAFQRLGELAGPEDALARQVESNRQRVRDLEAERARLERQGTEAQRAL
DEFRSKYENECECQLLLKEMLERLNKEADEALLHNLRLQLEAQFLQDDISAAKDRHKKNL
LEVQTYISILQQIIHTTPPASIVTSGMREEKLLTEREVAALRSQLEEGREVLSHLQAQRV
ELQAQTTTLEQAIKSAHECYDDEIQLYNEQIETLRKEIEETERVLEKSSYDCRQLAVAQQ
TLKNELDRYHRIIEIEGNRLTSAFIETPIPLFTQSHGVSLSTGSGGKDLTRALQDITAAK

PRQKALPKNVPRRKEIITKDKTNGALEDAPLKGLEDTKLVQVVLKEESESKFESESKEVS
PLTQEGAPEDVPDGGQISKGFGKLYRKVKEKVRSPKEPETPTELYTKERHVLVTGDANYV
DPRFYVSSITAKGGVAVSVAEDSVLYDGQVEPSPESPKPPLENGQVGLQEKEDGQPIDQQ
PIDKEIEPDGAELEGPEEKREGEERDEESRRPCAMVTPGAEEPSIPEPPKPAADQDGAEV
LGTRSRSLPEKGPPKALAYKTVEVVESIEKISTESIQTYEETAVIVETMIGKTKSDKKKS
GEKSS
>sp|Q86UB2|BIVM_HUMAN Basic immunoglobulin-like variable motif-containing protein OS=Homo sapiens GN=BIVM PE=2 SV=3
MPNVAETERSNDSGNGEHKSERKSPEENLQGAVKSFCTSASGAPLGPKGDGHYPWSCPVT
HTREKIYAICSDYAFLNQATSIYKTPNPSRSPCLPDSTSLSAGNNSSRYIGIPTSTSEII
YNEENSLENLSNSLGKLPLAWEIDKSEFDGVTTNSKHKSGNAKKQVSKRKTSDKKGRYQK
ECPQHSPLEDIKQRKVLDLRRWYCISRPQYKTSCGISSLISCWNFLYSTMGAGNLPPITQ
EEALHILGFQPPFEDIRFGPFTGNTTLMRWFRQINDHFHVKGCSYVLYKPHGKNKTAGET
ASGALSKLTRGLKDESLAYIYHCQNHYFCPIGFEATPVKANKAFSRGPLSPQEVEYWILI
GESSRKHPAIHCKKWADIVTDLNTQNPEYLDIRHLERGLQYRKTKKVGGNLHCIIAFQRL
NWQRFGLWNFPFGTIRQESQPPTHAQGIAKSESEDNISKKQHGRLGRSFSASFHQDSAWK
KMSSIHERRNSGYQGYSDYDGND
>sp|P46663|BKRB1_HUMAN B1 bradykinin receptor OS=Homo sapiens GN=BDKRB1 PE=1 SV=3
MASSWPPLELQSSNQSQLFPQNATACDNAPEAWDLLHRVLPTFIISICFFGLLGNLFVLL
VFLLPRRQLNVAEIYLANLAASDLVFVLGLPFWAENIWNQFNWPFGALLCRVINGVIKAN
LFISIFLVVAISQDRYRVLVHPMASRRQQRRRQARVTCVLIWVVGGLLSIPTFLLRSIQA
VPDLNITACILLLPHEAWHFARIVELNILGFLLPLAAIVFFNYHILASLRTREEVSRTRC
GGRKDSKTTALILTLVVAFLVCWAPYHFFAFLEFLFQVQAVRGCFWEDFIDLGLQLANFF
AFTNSSLNPVIYVFVGRLFRTKVWELYKQCTPKSLAPISSSHRKEIFQLFWRN
>sp|Q6QNY1|BL1S2_HUMAN Biogenesis of lysosome-related organelles complex 1 subunit 2 OS=Homo sapiens GN=BLOC1S2 PE=1 SV=1
MAAAAEGVLATRSDEPARDDAAVETAEEAKEPAEADITELCRDMFSKMATYLTGELTATS
EDYKLLENMNKLTSLKYLEMKDIAINISRNLKDLNQKYAGLQPYLDQINVIEEQVAALEQ
AAYKLDAYSKKLEAKYKKLEKR
>sp|O00327|BMAL1_HUMAN Aryl hydrocarbon receptor nuclear translocator-like protein 1 OS=Homo sapiens GN=ARNTL PE=1 SV=2
MADQRMDISSTISDFMSPGPTDLLSSSLGTSGVDCNRKRKGSSTDYQESMDTDKDDPHGR
LEYTEHQGRIKNAREAHSQIEKRRRDKMNSFIDELASLVPTCNAMSRKLDKLTVLRMAVQ
HMKTLRGATNPYTEANYKPTFLSDDELKHLILRAADGFLFVVGCDRGKILFVSESVFKIL
NYSQNDLIGQSLFDYLHPKDIAKVKEQLSSSDTAPRERLIDAKTGLPVKTDITPGPSRLC
SGARRSFFCRMKCNRPSVKVEDKDFPSTCSKKKADRKSFCTIHSTGYLKSWPPTKMGLDE
DNEPDNEGCNLSCLVAIGRLHSHVVPQPVNGEIRVKSMEYVSRHAIDGKFVFVDQRATAI
LAYLPQELLGTSCYEYFHQDDIGHLAECHRQVLQTREKITTNCYKFKIKDGSFITLRSRW
FSFMNPWTKEVEYIVSTNTVVLANVLEGGDPTFPQLTASPHSMDSMLPSGEGGPKRTHPT
VPGIPGGTRAGAGKIGRMIAEEIMEIHRIRGSSPSSCGSSPLNITSTPPPDASSPGGKKI
LNGGTPDIPSSGLLSGQAQENPGYPYSDSSSILGENPHIGIDMIDNDQGSSSPSNDEAAM
AVIMSLLEADAGLGGPVDFSDLPWPL
>sp|Q8WYA1|BMAL2_HUMAN Aryl hydrocarbon receptor nuclear translocator-like protein 2 OS=Homo sapiens GN=ARNTL2 PE=1 SV=2

MAAEEEAAAGGKVLREENQCIAPVVSSRVSPGTRPTAMGSFSSHMTEFPRKRKGSDSDPS
QSGIMTEKVVEKLSQNPLTYLLSTRIEISASSGSRVEDGEHQVKMKAFREAHSQTEKRRR
DKMNNLIEELSAMIPQCNPMARKLDKLTVLRMAVQHLRSLKGLTNSYVGSNYRPSFLQDN
ELRHLILKTAEGFLFVVGCERGKILFVSKSVSKILNYDQASLTGQSLFDFLHPKDVAKVK
EQLSSFDISPREKLIDAKTGLQVHSNLHAGRTRVYSGSRRSFFCRIKSCKISVKEEHGCL
PNSKKKEHRKFYTIHCTGYLRSWPPNIVGMEEERNSKKDNSNFTCLVAIGRLQPYIVPQN
SGEINVKPTEFITRFAVNGKFVYVDQRATAILGYLPQELLGTSCYEYFHQDDHNNLTDKH
KAVLQSKEKILTDSYKFRAKDGSFVTLKSQWFSFTNPWTKELEYIVSVNTLVLGHSEPGE
ASFLPCSSQSSEESSRQSCMSVPGMSTGTVLGAGSIGTDIANEILDLQRLQSSSYLDDSS
PTGLMKDTHTVNCRSMSNKELFPPSPSEMGELEATRQNQSTVAVHSHEPLLSDGAQLDFD
ALCDNDDTAMAAFMNYLEAEGGLGDPGDFSDIQWTL
>sp|P18075|BMP7_HUMAN Bone morphogenetic protein 7 OS=Homo sapiens GN=BMP7 PE=1 SV=1
MHVRSLRAAAPHSFVALWAPLFLLRSALADFSLDNEVHSSFIHRRLRSQERREMQREILS
ILGLPHRPRPHLQGKHNSAPMFMLDLYNAMAVEEGGGPGGQGFSYPYKAVFSTQGPPLAS
LQDSHFLTDADMVMSFVNLVEHDKEFFHPRYHHREFRFDLSKIPEGEAVTAAEFRIYKDY
IRERFDNETFRISVYQVLQEHLGRESDLFLLDSRTLWASEEGWLVFDITATSNHWVVNPR
HNLGLQLSVETLDGQSINPKLAGLIGRHGPQNKQPFMVAFFKATEVHFRSIRSTGSKQRS
QNRSKTPKNQEALRMANVAENSSSDQRQACKKHELYVSFRDLGWQDWIIAPEGYAAYYCE
GECAFPLNSYMNATNHAIVQTLVHFINPETVPKPCCAPTQLNAISVLYFDDSSNVILKKY
RNMVVRACGCH
>sp|O60238|BNI3L_HUMAN BCL2/adenovirus E1B 19 kDa protein-interacting protein 3-like OS=Homo sapiens GN=BNIP3L PE=1 SV=1
MSSHLVEPPPPLHNNNNNCEENEQSLPPPAGLNSSWVELPMNSSNGNDNGNGKNGGLEHV
PSSSSIHNGDMEKILLDAQHESGQSSSRGSSHCDSPSPQEDGQIMFDVEMHTSRDHSSQS
EEEVVEGEKEVEALKKSADWVSDWSSRPENIPPKEFHFRHPKRSVSLSMRKSGAMKKGGI
FSAEFLKVFIPSLFLSHVLALGLGIYIGKRLSTPSASTY
>sp|Q12982|BNIP2_HUMAN BCL2/adenovirus E1B 19 kDa protein-interacting protein 2 OS=Homo sapiens GN=BNIP2 PE=1 SV=1
MEGVELKEEWQDEDFPIPLPEDDSIEADILAITGPEDQPGSLEVNGNKVRKKLMAPDISL
TLDPSDGSVLSDDLDESGEIDLDGLDTPSENSNEFEWEDDLPKPKTTEVIRKGSITEYTA
AEEKEDGRRWRMFRIGEQDHRVDMKAIEPYKKVISHGGYYGDGLNAIVVFAVCFMPESSQ
PNYRYLMDNLFKYVIGTLELLVAENYMIVYLNGATTRRKMPSLGWLRKCYQQIDRRLRKN
LKSLIIVHPSWFIRTLLAVTRPFISSKFSQKIRYVFNLAELAELVPMEYVGIPECIKQVD
QELNGKQDEPKNEQ
>sp|Q7Z465|BNIPL_HUMAN Bcl-2/adenovirus E1B 19 kDa-interacting protein 2-like protein OS=Homo sapiens GN=BNIPL PE=1 SV=1
MGTIQEAGKKTDVGVREIAEAPELGAALRHGELELKEEWQDEEFPRLLPEEAGTSEDPED
PKGDSQAAAGTPSTLALCGQRPMRKRLSAPELRLSLTKGPGNDGASPTQSAPSSPDGSSD
LEIDELETPSDSEQLDSGHEFEWEDELPRAEGLGTSETAERLGRGCMWDVTGEDGHHWRV
FRMGPREQRVDMTVIEPYKKVLSHGGYHGDGLNAVILFASCYLPRSSIPNYTYVMEHLFR
YMVGTLELLVAENYLLVHLSGGTSRAQVPPLSWIRQCYRTLDRRLRKNLRALVVVHATWY
VKAFLALLRPFISSKFTRKIRFLDSLGELAQLISLDQVHIPEAVRQLDRDLHGSGGT
>sp|P48201|AT5G3_HUMAN ATP synthase F(0) complex subunit C3, mitochondrial OS=Homo sapiens GN=ATP5G3 PE=2 SV=1

MFACAKLACTPSLIRAGSRVAYRPISASVLSRPEASRTGEGSTVFNGAQNGVSQLIQREF
QTSAISRDIDTAAKFIGAGAATVGVAGSGAGIGTVFGSLIIGYARNPSLKQQLFSYAILG
FALSEAMGLFCLMVAFLILFAM
>sp|P98198|AT8B2_HUMAN Phospholipid-transporting ATPase ID OS=Homo sapiens GN=ATP8B2 PE=1 SV=2
MTVPKEMPEKWARAQAPPSWSRKKPSWGTEEERRARANDREYNEKFQYASNCIKTSKYNI
LTFLPVNLFEQFQEVANTYFLFLLILQLIPQISSLSWFTTIVPLVLVLTITAVKDATDDY
FRHKSDNQVNNRQSQVLINGILQQEQWMNVCVGDIIKLENNQFVAADLLLLSSSEPHGLC
YIETAELDGETNMKVRQAIPVTSELGDISKLAKFDGEVICEPPNNKLDKFSGTLYWKENK
FPLSNQNMLLRGCVLRNTEWCFGLVIFAGPDTKLMQNSGRTKFKRTSIDRLMNTLVLWIF
GFLVCMGVILAIGNAIWEHEVGMRFQVYLPWDEAVDSAFFSGFLSFWSYIIILNTVVPIS
LYVSVEVIRLGHSYFINWDKKMFCMKKRTPAEARTTTLNEELGQVEYIFSDKTGTLTQNI
MVFNKCSINGHSYGDVFDVLGHKAELGERPEPVDFSFNPLADKKFLFWDPSLLEAVKIGD
PHTHEFFRLLSLCHTVMSEEKNEGELYYKAQSPDEGALVTAARNFGFVFRSRTPKTITVH
EMGTAITYQLLAILDFNNIRKRMSVIVRNPEGKIRLYCKGADTILLDRLHHSTQELLNTT
MDHLNEYAGEGLRTLVLAYKDLDEEYYEEWAERRLQASLAQDSREDRLASIYEEVENNMM
LLGATAIEDKLQQGVPETIALLTLANIKIWVLTGDKQETAVNIGYSCKMLTDDMTEVFIV
TGHTVLEVREELRKAREKMMDSSRSVGNGFTYQDKLSSSKLTSVLEAVAGEYALVINGHS
LAHALEADMELEFLETACACKAVICCRVTPLQKAQVVELVKKYKKAVTLAIGDGANDVSM
IKTAHIGVGISGQEGIQAVLASDYSFSQFKFLQRLLLVHGRWSYLRMCKFLCYFFYKNFA
FTMVHFWFGFFCGFSAQTVYDQYFITLYNIVYTSLPVLAMGVFDQDVPEQRSMEYPKLYE
PGQLNLLFNKREFFICIAQGIYTSVLMFFIPYGVFADATRDDGTQLADYQSFAVTVATSL
VIVVSVQIGLDTGYWTAINHFFIWGSLAVYFAILFAMHSNGLFDMFPNQFRFVGNAQNTL
AQPTVWLTIVLTTVVCIMPVVAFRFLRLNLKPDLSDTVRYTQLVRKKQKAQHRCMRRVGR
TGSRRSGYAFSHQEGFGELIMSGKNMRLSSLALSSFTTRSSSSWIESLRRKKSDSASSPS
GGADKPLKG
>sp|Q99941|ATF6B_HUMAN Cyclic AMP-dependent transcription factor ATF-6 beta OS=Homo sapiens GN=ATF6B PE=1 SV=2
MAELMLLSEIADPTRFFTDNLLSPEDWGLQNSTLYSGLDEVAEEQTQLFRCPEQDVPFDG
SSLDVGMDVSPSEPPWELLPIFPDLQVKSEPSSPCSSSSLSSESSRLSTEPSSEALGVGE
VLHVKTESLAPPLCLLGDDPTSSFETVQINVIPTSDDSSDVQTKIEPVSPCSSVNSEASL
LSADSSSQAFIGEEVLEVKTESLSPSGCLLWDVPAPSLGAVQISMGPSLDGSSGKALPTR
KPPLQPKPVVLTTVPMPSRAVPPSTTVLLQSLVQPPPVSPVVLIQGAIRVQPEGPAPSLP
RPERKSIVPAPMPGNSCPPEVDAKLLKRQQRMIKNRESACQSRRKKKEYLQGLEARLQAV
LADNQQLRRENAALRRRLEALLAENSELKLGSGNRKVVCIMVFLLFIAFNFGPVSISEPP
SAPISPRMNKGEPQPRRHLLGFSEQEPVQGVEPLQGSSQGPKEPQPSPTDQPSFSNLTAF
PGGAKELLLRDLDQLFLSSDCRHFNRTESLRLADELSGWVQRHQRGRRKIPQRAQERQKS
QPRKKSPPVKAVPIQPPGPPERDSVGQLQLYRHPDRSQPAFLDAIDRREDTFYVVSFRRD
HLLLPAISHNKTSRPKMSLVMPAMAPNETLSGRGAPGDYEEMMQIECEVMDTRVIHIKTS
TVPPSLRKQPSPTPGNATGGPLPVSAASQAHQASHQPLYLNHP
>sp|O94817|ATG12_HUMAN Ubiquitin-like protein ATG12 OS=Homo sapiens GN=ATG12 PE=1 SV=1
MAEEPQSVLQLPTSIAAGGEGLTDVSPETTTPEPPSSAAVSPGTEEPAGDTKKKIDILLK
AVGDTPIMKTKKWAVERTRTIQGLIDFIKKFLKLVASEQLFIYVNQSFAPSPDQEVGTLY
ECFGSDGKLVLHYCKSQAWG

>sp|O75143|ATG13_HUMAN Autophagy-related protein 13 OS=Homo sapiens GN=ATG13 PE=1 SV=1
METDLNSQDRKDLDKFIKFFALKTVQVIVQARLGEKICTRSSSSPTGSDWFNLAIKDIPE
VTHEAKKALAGQLPAVGRSMCVEISLKTSEGDSMELEIWCLEMNEKCDKEIKVSYTVYNR
LSLLLKSLLAITRVTPAYRLSRKQGHEYVILYRIYFGEVQLSGLGEGFQTVRVGTVGTPV
GTITLSCAYRINLAFMSTRQFERTPPIMGIIIDHFVDRPYPSSSPMHPCNYRTAGEDTGV
IYPSVEDSQEVCTTSFSTSPPSQLSSSRLSYQPAALGVGSADLAYPVVFAAGLNATHPHQ
LMVPGKEGGVPLAPNQPVHGTQADQERLATCTPSDRTHCAATPSSSEDTETVSNSSEGRA
SPHDVLETIFVRKVGAFVNKPINQVTLTSLDIPFAMFAPKNLELEDTDPMVNPPDSPETE
SPLQGSLHSDGSSGGSSGNTHDDFVMIDFKPAFSKDDILPMDLGTFYREFQNPPQLSSLS
IDIGAQSMAEDLDSLPEKLAVHEKNVREFDAFVETLQ
>sp|P54710|ATNG_HUMAN Sodium/potassium-transporting ATPase subunit gamma OS=Homo sapiens GN=FXYD2 PE=1 SV=3
MTGLSMDGGGSPKGDVDPFYYDYETVRNGGLIFAGLAFIVGLLILLSRRFRCGGNKKRRQ
INEDEP
>sp|O00244|ATOX1_HUMAN Copper transport protein ATOX1 OS=Homo sapiens GN=ATOX1 PE=1 SV=1
MPKHEFSVDMTCGGCAEAVSRVLNKLGGVKYDIDLPNKKVCIESEHSMDTLLATLKKTGK
TVSYLGLE
>sp|P56134|ATPK_HUMAN ATP synthase subunit f, mitochondrial OS=Homo sapiens GN=ATP5J2 PE=1 SV=3
MASVGECPAPVPVKDKKLLEVKLGELPSWILMRDFSPSGIFGAFQRGYYRYYNKYINVKK
GSISGITMVLACYVLFSYSFSYKHLKHERLRKYH
>sp|Q6ZMB0|B3GN6_HUMAN Acetylgalactosaminyl-O-glycosyl-glycoprotein beta-1,3-N-acetylglucosaminyltransferase OS=Homo sapiens GN=B3GNT6 PE=1 SV=2
MAFPCRRSLTAKTLACLLVGVSFLALQQWFLQAPRSPREERSPQEETPEGPTDAPAADEP
PSELVPGPPCVANASANATADFEQLPARIQDFLRYRHCRHFPLLWDAPAKCAGGRGVFLL
LAVKSAPEHYERRELIRRTWGQERSYGGRPVRRLFLLGTPGPEDEARAERLAELVALEAR
EHGDVLQWAFADTFLNLTLKHLHLLDWLAARCPHARFLLSGDDDVFVHTANVVRFLQAQP
PGRHLFSGQLMEGSVPIRDSWSKYFVPPQLFPGSAYPVYCSGGGFLLSGPTARALRAAAR
HTPLFPIDDAYMGMCLERAGLAPSGHEGIRPFGVQLPGAQQSSFDPCMYRELLLVHRFAP
YEMLLMWKALHSPALSCDRGHRVS
>sp|O43505|B4GA1_HUMAN Beta-1,4-glucuronyltransferase 1 OS=Homo sapiens GN=B4GAT1 PE=1 SV=1
MQMSYAIRCAFYQLLLAALMLVAMLQLLYLSLLSGLHGQEEQDQYFEFFPPSPRSVDQVK
AQLRTALASGGVLDASGDYRVYRGLLKTTMDPNDVILATHASVDNLLHLSGLLERWEGPL
SVSVFAATKEEAQLATVLAYALSSHCPDMRARVAMHLVCPSRYEAAVPDPREPGEFALLR
SCQEVFDKLARVAQPGINYALGTNVSYPNNLLRNLAREGANYALVIDVDMVPSEGLWRGL
REMLDQSNQWGGTALVVPAFEIRRARRMPMNKNELVQLYQVGEVRPFYYGLCTPCQAPTN
YSRWVNLPEESLLRPAYVVPWQDPWEPFYVAGGKVPTFDERFRQYGFNRISQACELHVAG
FDFEVLNEGFLVHKGFKEALKFHPQKEAENQHNKILYRQFKQELKAKYPNSPRRC
>sp|O43286|B4GT5_HUMAN Beta-1,4-galactosyltransferase 5 OS=Homo sapiens GN=B4GALT5 PE=2 SV=1
MRARRGLLRLPRRSLLAALFFFSLSSSLLYFVYVAPGIVNTYLFMMQAQGILIRDNVRTI
GAQVYEQVLRSAYAKRNSSVNDSDYPLDLNHSETFLQTTTFLPEDFTYFANHTCPERLPS
MKGPIDINMSEIGMDYIHELFSKDPTIKLGGHWKPSDCMPRWKVAILIPFRNRHEHLPVL

FRHLLPMLQRQRLQFAFYVVEQVGTQPFNRAMLFNVGFQEAMKDLDWDCLIFHDVDHIPE
SDRNYYGCGQMPRHFATKLDKYMYLLPYTEFFGGVSGLTVEQFRKINGFPNAFWGWGGED
DDLWNRVQNAGYSVSRPEGDTGKYKSIPHHHRGEVQFLGRYALLRKSKERQGLDGLNNLN
YFANITYDALYKNITVNLTPELAQVNEY
>sp|O00154|BACH_HUMAN Cytosolic acyl coenzyme A thioester hydrolase OS=Homo sapiens GN=ACOT7 PE=1 SV=3
MKLLARALRLCEFGRQASSRRLVAGQGCVGPRRGCCAPVQVVGPRADLPPCGACITGRIM
RPDDANVAGNVHGGTILKMIEEAGAIISTRHCNSQNGERCVAALARVERTDFLSPMCIGE
VAHVSAEITYTSKHSVEVQVNVMSENILTGAKKLTNKATLWYVPLSLKNVDKVLEVPPVV
YSRQEQEEEGRKRYEAQKLERMETKWRNGDIVQPVLNPEPNTVSYSQSSLIHLVGPSDCT
LHGFVHGGVTMKLMDEVAGIVAARHCKTNIVTASVDAINFHDKIRKGCVITISGRMTFTS
NKSMEIEVLVDADPVVDSSQKRYRAASAFFTYVSLSQEGRSLPVPQLVPETEDEKKRFEE
GKGRYLQMKAKRQGHAEPQP
>sp|Q6P575|BGP11_HUMAN Putative inactive beta-glucuronidase protein GUSBP11 OS=Homo sapiens GN=GUSBP11 PE=5 SV=2
MTAAETGRGKPRLGGGSGLGGSPAAVVWLHVGATGRDAVSPREPVVAAQAAGRPLKLPRR
LLRQPTPGLRGAESGPTVDMPVPSSFNDIGQGWRLRHFVSWLWYEREVTLLERWIQDLCT
RVVLRIGSAHFYAIVWVNGVDTVEHEGGYLPFEADISSLFQVEPLPSHLCITIAINNTLT
PQPCHQGPSVHDRHLQVGTILPPLHAPTFPPHPVVFLPGTGYPKGYFVQNTDFDFFSYAG
LLWSLLLYTTPPTYIDDVTVTTGVKRDSGEGFW
>sp|P30411|BKRB2_HUMAN B2 bradykinin receptor OS=Homo sapiens GN=BDKRB2 PE=1 SV=2
MFSPWKISMFLSVREDSVPTTASFSADMLNVTLQGPTLNGTFAQSKCPQVEWLGWLNTIQ
PPFLWVLFVLATLENIFVLSVFCLHKSSCTVAEIYLGNLAAADLILACGLPFWAITISNN
FDWLFGETLCRVVNAIISMNLYSSICFLMLVSIDRYLALVKTMSMGRMRGVRWAKLYSLV
IWGCTLLLSSPMLVFRTMKEYSDEGHNVTACVISYPSLIWEVFTNMLLNVVGFLLPLSVI
TFCTMQIMQVLRNNEMQKFKEIQTERRATVLVLVVLLLFIICWLPFQISTFLDTLHRLGI
LSSCQDERIIDVITQIASFMAYSNSCLNPLVYVIVGKRFRKKSWEVYQGVCQKGGCRSEP
IQMENSMGTLRTSISVERQIHKLQDWAGSRQ
>sp|Q8IZY5|BLID_HUMAN BH3-like motif-containing cell death inducer OS=Homo sapiens GN=BLID PE=1 SV=2
MVTLLPIEGQEIHFFEILESECVLYTGWIERASGSSIYPEAKARLPLEALLGSNKEPMLP
KETVLSLKRYNLGSSAMKRNVPGHVLQRPSYLTRIQVTLLCNSSAEAL
>sp|P51451|BLK_HUMAN Tyrosine-protein kinase Blk OS=Homo sapiens GN=BLK PE=1 SV=3
MGLVSSKKPDKEKPIKEKDKGQWSPLKVSAQDKDAPPLPPLVVFNHLTPPPPDEHLDEDK
HFVVALYDYTAMNDRDLQMLKGEKLQVLKGTGDWWLARSLVTGREGYVPSNFVARVESLE
MERWFFRSQGRKEAERQLLAPINKAGSFLIRESETNKGAFSLSVKDVTTQGELIKHYKIR
CLDEGGYYISPRITFPSLQALVQHYSKKGDGLCQRLTLPCVRPAPQNPWAQDEWEIPRQS
LRLVRKLGSGQFGEVWMGYYKNNMKVAIKTLKEGTMSPEAFLGEANVMKALQHERLVRLY
AVVTKEPIYIVTEYMARGCLLDFLKTDEGSRLSLPRLIDMSAQIAEGMAYIERMNSIHRD
LRAANILVSEALCCKIADFGLARIIDSEYTAQEGAKFPIKWTAPEAIHFGVFTIKADVWS
FGVLLMEVVTYGRVPYPGMSNPEVIRNLERGYRMPRPDTCPPELYRGVIAECWRSRPEER
PTFEFLQSVLEDFYTATERQYELQP
>sp|P30043|BLVRB_HUMAN Flavin reductase (NADPH) OS=Homo sapiens GN=BLVRB PE=1 SV=3
MAVKKIAIFGATGQTGLTTLAQAVQAGYEVTVLVRDSSRLPSEGPRPAHVVVGDVLQAAD

VDKTVAGQDAVIVLLGTRNDLSPTTVMSEGARNIVAAMKAHGVDKVVACTSAFLLWDPTK
VPPRLQAVTDDHIRMHKVLRESGLKYVAVMPPHIGDQPLTGAYTVTLDGRGPSRVISKHD
LGHFMLRCLTTDEYDGHSTYPSHQYQ
>sp|P22003|BMP5_HUMAN Bone morphogenetic protein 5 OS=Homo sapiens GN=BMP5 PE=2 SV=1
MHLTVFLLKGIVGFLWSCWVLVGYAKGGLGDNHVHSSFIYRRLRNHERREIQREILSILG
LPHRPRPFSPGKQASSAPLFMLDLYNAMTNEENPEESEYSVRASLAEETRGARKGYPASP
NGYPRRIQLSRTTPLTTQSPPLASLHDTNFLNDADMVMSFVNLVERDKDFSHQRRHYKEF
RFDLTQIPHGEAVTAAEFRIYKDRSNNRFENETIKISIYQIIKEYTNRDADLFLLDTRKA
QALDVGWLVFDITVTSNHWVINPQNNLGLQLCAETGDGRSINVKSAGLVGRQGPQSKQPF
MVAFFKASEVLLRSVRAANKRKNQNRNKSSSHQDSSRMSSVGDYNTSEQKQACKKHELYV
SFRDLGWQDWIIAPEGYAAFYCDGECSFPLNAHMNATNHAIVQTLVHLMFPDHVPKPCCA
PTKLNAISVLYFDDSSNVILKKYRNMVVRSCGCH
>sp|P36894|BMR1A_HUMAN Bone morphogenetic protein receptor type-1A OS=Homo sapiens GN=BMPR1A PE=1 SV=2
MPQLYIYIRLLGAYLFIISRVQGQNLDSMLHGTGMKSDSDQKKSENGVTLAPEDTLPFLK
CYCSGHCPDDAINNTCITNGHCFAIIEEDDQGETTLASGCMKYEGSDFQCKDSPKAQLRR
TIECCRTNLCNQYLQPTLPPVVIGPFFDGSIRWLVLLISMAVCIIAMIIFSSCFCYKHYC
KSISSRRRYNRDLEQDEAFIPVGESLKDLIDQSQSSGSGSGLPLLVQRTIAKQIQMVRQV
GKGRYGEVWMGKWRGEKVAVKVFFTTEEASWFRETEIYQTVLMRHENILGFIAADIKGTG
SWTQLYLITDYHENGSLYDFLKCATLDTRALLKLAYSAACGLCHLHTEIYGTQGKPAIAH
RDLKSKNILIKKNGSCCIADLGLAVKFNSDTNEVDVPLNTRVGTKRYMAPEVLDESLNKN
HFQPYIMADIYSFGLIIWEMARRCITGGIVEEYQLPYYNMVPSDPSYEDMREVVCVKRLR
PIVSNRWNSDECLRAVLKLMSECWAHNPASRLTALRIKKTLAKMVESQDVKI
>sp|Q9BZR8|B2L14_HUMAN Apoptosis facilitator Bcl-2-like protein 14 OS=Homo sapiens GN=BCL2L14 PE=1 SV=1
MCSTSGCDLEEIPLDDDDLNTIEFKILAYYTRHHVFKSTPALFSPKLLRTRSLSQRGLGN
CSANESWTEVSWPCRNSQSSEKAINLGKKKSSWKAFFGVVEKEDSQSTPAKVSAQGQRTL
EYQDSHSQQWSRCLSNVEQCLEHEAVDPKVISIANRVAEIVYSWPPPQATQAGGFKSKEI
FVTEGLSFQLQGHVPVASSSKKDEEEQILAKIVELLKYSGDQLERKLKKDKALMGHFQDG
LSYSVFKTITDQVLMGVDPRGESEVKAQGFKAALVIDVTAKLTAIDNHPMNRVLGFGTKY
LKENFSPWIQQHGGWEKILGISHEEVD
>sp|Q9UBV7|B4GT7_HUMAN Beta-1,4-galactosyltransferase 7 OS=Homo sapiens GN=B4GALT7 PE=1 SV=1
MFPSRRKAAQLPWEDGRSGLLSGGLPRKCSVFHLFVACLSLGFFSLLWLQLSCSGDVARA
VRGQGQETSGPPRACPPEPPPEHWEEDASWGPHRLAVLVPFRERFEELLVFVPHMRRFLS
RKKIRHHIYVLNQVDHFRFNRAALINVGFLESSNSTDYIAMHDVDLLPLNEELDYGFPEA
GPFHVASPELHPLYHYKTYVGGILLLSKQHYRLCNGMSNRFWGWGREDDEFYRRIKGAGL
QLFRPSGITTGYKTFRHLHDPAWRKRDQKRIAAQKQEQFKVDREGGLNTVKYHVASRTAL
SVGGAPCTVLNIMLDCDKTATPWCTFS
>sp|Q13829|BACD2_HUMAN BTB/POZ domain-containing adapter for CUL3-mediated RhoA degradation protein 2 OS=Homo sapiens GN=TNFAIP1 PE=1 SV=1
MSGDTCLCPASGAKPKLSGFKGGGLGNKYVQLNVGGSLYYTTVRALTRHDTMLKAMFSGR
MEVLTDKEGWILIDRCGKHFGTILNYLRDDTITLPQNRQEIKELMAEAKYYLIQGLVNMC
QSALQDKKDSYQPVCNIPIITSLKEEERLIESSTKPVVKLLYNRSNNKYSYTSNSDDHLL

KNIELFDKLSLRFNGRVLFIKDVIGDEICCWSFYGQGRKLAEVCCTSIVYATEKKQTKVE
FPEARIYEETLNVLLYETPRVPDNSLLEATSRSRSQASPSEDEETFELRDRVRRIHVKRY
STYDDRQLGHQSTHRD
>sp|P56817|BACE1_HUMAN Beta-secretase 1 OS=Homo sapiens GN=BACE1 PE=1 SV=2
MAQALPWLLLWMGAGVLPAHGTQHGIRLPLRSGLGGAPLGLRLPRETDEEPEEPGRRGSF
VEMVDNLRGKSGQGYYVEMTVGSPPQTLNILVDTGSSNFAVGAAPHPFLHRYYQRQLSST
YRDLRKGVYVPYTQGKWEGELGTDLVSIPHGPNVTVRANIAAITESDKFFINGSNWEGIL
GLAYAEIARPDDSLEPFFDSLVKQTHVPNLFSLQLCGAGFPLNQSEVLASVGGSMIIGGI
DHSLYTGSLWYTPIRREWYYEVIIVRVEINGQDLKMDCKEYNYDKSIVDSGTTNLRLPKK
VFEAAVKSIKAASSTEKFPDGFWLGEQLVCWQAGTTPWNIFPVISLYLMGEVTNQSFRIT
ILPQQYLRPVEDVATSQDDCYKFAISQSSTGTVMGAVIMEGFYVVFDRARKRIGFAVSAC
HVHDEFRTAAVEGPFVTLDMEDCGYNIPQTDESTLMTIAYVMAAICALFMLPLCLMVCQW
CCLRCLRQQHDDFADDISLLK
>sp|O14867|BACH1_HUMAN Transcription regulator protein BACH1 OS=Homo sapiens GN=BACH1 PE=1 SV=2
MSLSENSVFAYESSVHSTNVLLSLNDQRKKDVLCDVTIFVEGQRFRAHRSVLAACSSYFH
SRIVGQADGELNITLPEEVTVKGFEPLIQFAYTAKLILSKENVDEVCKCVEFLSVHNIEE
SCFQFLKFKFLDSTADQQECPRKKCFSSHCQKTDLKLSLLDQRDLETDEVEEFLENKNVQ
TPQCKLRRYQGNAKASPPLQDSASQTYESMCLEKDAALALPSLCPKYRKFQKAFGTDRVR
TGESSVKDIHASVQPNERSENECLGGVPECRDLQVMLKCDESKLAMEPEETKKDPASQCP
TEKSEVTPFPHNSSIDPHGLYSLSLLHTYDQYGDLNFAGMQNTTVLTEKPLSGTDVQEKT
FGESQDLPLKSDLGTREDSSVASSDRSSVEREVAEHLAKGFWSDICSTDTPCQMQLSPAV
AKDGSEQISQKRSECPWLGIRISESPEPGQRTFTTLSSVNCPFISTLSTEGCSSNLEIGN
DDYVSEPQQEPCPYACVISLGDDSETDTEGDSESCSAREQECEVKLPFNAQRIISLSRND
FQSLLKMHKLTPEQLDCIHDIRRRSKNRIAAQRCRKRKLDCIQNLESEIEKLQSEKESLL
KERDHILSTLGETKQNLTGLCQKVCKEAALSQEQIQILAKYSAADCPLSFLISEKDKSTP
DGELALPSIFSLSDRPPAVLPPCARGNSEPGYARGQESQQMSTATSEQAGPAEQCRQSGG
ISDFCQQMTDKCTTDE
>sp|O95816|BAG2_HUMAN BAG family molecular chaperone regulator 2 OS=Homo sapiens GN=BAG2 PE=1 SV=1
MAQAKINAKANEGRFCRSSSMADRSSRLLESLDQLELRVEALREAATAVEQEKEILLEMI
HSIQNSQDMRQISDGEREELNLTANRLMGRTLTVEVSVETIRNPQQQESLKHATRIIDEV
VNKFLDDLGNAKSHLMSLYSACSSEVPHGPVDQKFQSIVIGCALEDQKKIKRRLETLLRN
IENSDKAIKLLEHSKGAGSKTLQQNAESRFN
>sp|O95817|BAG3_HUMAN BAG family molecular chaperone regulator 3 OS=Homo sapiens GN=BAG3 PE=1 SV=3
MSAATHSPMMQVASGNGDRDPLPPGWEIKIDPQTGWPFFVDHNSRTTTWNDPRVPSEGPK
ETPSSANGPSREGSRLPPAREGHPVYPQLRPGYIPIPVLHEGAENRQVHPFHVYPQPGMQ
RFRTEAAAAAPQRSQSPLRGMPETTQPDKQCGQVAAAAAAQPPASHGPERSQSPAASDCS
SSSSSASLPSSGRSSLGSHQLPRGYISIPVIHEQNVTRPAAQPSFHQAQKTHYPAQQGEY
QTHQPVYHKIQGDDWEPRPLRAASPFRSSVQGASSREGSPARSSTPLHSPSPIRVHTVVD
RPQQPMTHRETAPVSQPENKPESKPGPVGPELPPGHIPIQVIRKEVDSKPVSQKPPPPSE
KVEVKVPPAPVPCPPPSPGPSAVPSSPKSVATEERAAPSTAPAEATPPKPGEAEAPPKHP
GVLKVEAILEKVQGLEQAVDNFEGKKTDKKYLMIEEYLTKELLALDSVDPEGRADVRQAR

RDGVRKVQTILEKLEQKAIDVPGQVQVYELQPSNLEADQPLQAIMEMGAVAADKGKKNAG
NAEDPHTETQQPEATAAATSNPSSMTDTPGNPAAP
>sp|Q86Y27|BAGE5_HUMAN B melanoma antigen 5 OS=Homo sapiens GN=BAGE5 PE=2 SV=1
MAAGAVFLALSAQLLQARLMKEESPVVSWRLEPEDGTALCFIF
>sp|Q8TBE0|BAHD1_HUMAN Bromo adjacent homology domain-containing 1 protein OS=Homo sapiens GN=BAHD1 PE=1 SV=2
MTHTRRKSLPMLSSGLTGRREPLQMEDSNMEQGVEGVEPGMPESPGHLTGRRKNYPLRKR
PLVPEKPKACKVLLTRLENVAGPRSADEADELPPDLPKPPSPAPSSEDPGLAQPRKRRLA
SLNAEALNNLLLEREDTSSLAGTRRSRAGDPHRSRDRDRATGGWSSSKKRPRLGDLGGGS
RDLSPEPAPDEGPRRDGDPAPKRLASLNAAAFLKLSQERELPLRLPRAHAEVDGRSTEPP
APKAPRPKWPKVNGKNYPKAWQGASSGEAAGPPGWQGCPDEPWPSATPCGPSVQPSHQPL
SKALESPLGLRPHLPLLMGGQAALKPEPGRPGEESPAPKQELHQPSFPTPQLSPLPMPGN
PADYNGLCVGPELTALGSFYLYCGQEGLQCGGYSPCPMLPEGKLSPVAAPHEEGLLLAPS
SVPSGTPFQHPPWGSSRYCSSEDTGVNGYSICGVLPLSVTHAGTTCGGCPYKMPFAAEGC
RSLGQLEFPLPEAGHPASPAHPLLGCPVPSVPPAAEPVPHLQTPTSEPQTVARACPQSAK
PPSGSKSGLRTGSSCRHTARSKAARRPSHPKQPRVQRPRPRRRRRRRTNGWVPVGAACEK
AVYVLDEPEPAIRKSYQAVERHGETIRVRDTVLLKSGPRKTSTPYVAKISALWENPESGE
LMMSLLWYYRPEHLQGGRSPSMHEPLQNEVFASRHQDQNSVACIEEKCYVLTFAEYCRFC
AMAKRRGEGLPSRKTALVPPSADYSTPPHRTVPEDTDPELVFLCRHVYDFRHGRILKNPQ
>sp|O14514|BAI1_HUMAN Brain-specific angiogenesis inhibitor 1 OS=Homo sapiens GN=BAI1 PE=1 SV=2
MRGQAAAPGPVWILAPLLLLLLLLGRRARAAAGADAGPGPEPCATLVQGKFFGYFSAAAV
FPANASRCSWTLRNPDPRRYTLYMKVAKAPVPCSGPGRVRTYQFDSFLESTRTYLGVESF
DEVLRLCDPSAPLAFLQASKQFLQMRRQQPPQHDGLRPRAGPPGPTDDFSVEYLVVGNRN
PSRAACQMLCRWLDACLAGSRSSHPCGIMQTPCACLGGEAGGPAAGPLAPRGDVCLRDAV
AGGPENCLTSLTQDRGGHGATGGWKLWSLWGECTRDCGGGLQTRTRTCLPAPGVEGGGCE
GVLEEGRQCNREACGPAGRTSSRSQSLRSTDARRREELGDELQQFGFPAPQTGDPAAEEW
SPWSVCSSTCGEGWQTRTRFCVSSSYSTQCSGPLREQRLCNNSAVCPVHGAWDEWSPWSL
CSSTCGRGFRDRTRTCRPPQFGGNPCEGPEKQTKFCNIALCPGRAVDGNWNEWSSWSACS
ASCSQGRQQRTRECNGPSYGGAECQGHWVETRDCFLQQCPVDGKWQAWASWGSCSVTCGA
GSQRRERVCSGPFFGGAACQGPQDEYRQCGTQRCPEPHEICDEDNFGAVIWKETPAGEVA
AVRCPRNATGLILRRCELDEEGIAYWEPPTYIRCVSIDYRNIQMMTREHLAKAQRGLPGE
GVSEVIQTLVEISQDGTSYSGDLLSTIDVLRNMTEIFRRAYYSPTPGDVQNFVQILSNLL
AEENRDKWEEAQLAGPNAKELFRLVEDFVDVIGFRMKDLRDAYQVTDNLVLSIHKLPASG
ATDISFPMKGWRATGDWAKVPEDRVTVSKSVFSTGLTEADEASVFVVGTVLYRNLGSFLA
LQRNTTVLNSKVISVTVKPPPRSLRTPLEIEFAHMYNGTTNQTCILWDETDVPSSSAPPQ
LGPWSWRGCRTVPLDALRTRCLCDRLSTFAILAQLSADANMEKATLPSVTLIVGCGVSSL
TLLMLVIIYVSVWRYIRSERSVILINFCLSIISSNALILIGQTQTRNKVVCTLVAAFLHF
FFLSSFCWVLTEAWQSYMAVTGHLRNRLIRKRFLCLGWGLPALVVAISVGFTKAKGYSTM
NYCWLSLEGGLLYAFVGPAAAVVLVNMVIGILVFNKLVSKDGITDKKLKERAGASLWSSC
VVLPLLALTWMSAVLAVTDRRSALFQILFAVFDSLEGFVIVMVHCILRREVQDAVKCRVV
DRQEEGNGDSGGSFQNGHAQLMTDFEKDVDLACRSVLNKDIAACRTATITGTLKRPSLPE
EEKLKLAHAKGPPTNFNSLPANVSKLHLHGSPRYPGGPLPDFPNHSLTLKRDKAPKSSFV
GDGDIFKKLDSELSRAQEKALDTSYVILPTATATLRPKPKEEPKYSIHIDQMPQTRLIHL

STAPEASLPARSPPSRQPPSGGPPEAPPAQPPPPPPPPPPPPQQPLPPPPNLEPAPPSLG
DPGEPAAHPGPSTGPSTKNENVATLSVSSLERRKSRYAELDFEKIMHTRKRHQDMFQDLN
RKLQHAAEKDKEVLGPDSKPEKQQTPNKRPWESLRKAHGTPTWVKKELEPLQPSPLELRS
VEWERSGATIPLVGQDIIDLQTEV
>sp|O94812|BAIP3_HUMAN BAI1-associated protein 3 OS=Homo sapiens GN=BAIAP3 PE=1 SV=2
MRPRGAAFAAGPPGDLHLGTAIGFAGAIWRSRSPAMSTLLDIKSSVLRQVQVCPSFRRRT
EQDPGSASADPQEPATGAWKPGDGVEFFAHMRLMLKKGEGRQGLPCLEVPLRSGSPAPPE
PVDPSLGLRALAPEEVEMLYEEALYTVLYRAGTMGPDQVDDEEALLSYLQQVFGTSLEEH
TEAIERVRKAKAPTYALKVSVMRAKNLLAKDPNGFSDPYCMLGILPASDATREPRAQKEQ
RFGFRKGSKRGGPLPAKCIQVTEVKSSTLNPVWKEHFLFEIEDVSTDQLHLDIWDHDDDV
SLVEACRKLNEVIGLKGMGRYFKQIVKSARANGTAGPTEDHTDDFLGCLNIPVREVPVAG
VDRWFKLEPRSSASRVQGHCHLVLKLITTQRDTAMSQRGRSGFLSHLLLLSHLLRLEHSA
EEPNSSSWRGELSTPAATILCLHGAQSNLSPLQLAVLHWQVSSRHHQTCTLDYSYLLGLL
EDMQAHWEEAPSLPQEQEESLADSLSAFSEFGLQLLRQLRDYFPATNSTAVHRLELLLKC
LGKLQLFQPSFEICPFESELNMDIAAALKRGNREWYDRILNDKSPREQPGPQRLPGLVVL
ADAVYDDLQFCYSVYASLFHSILNVDVFTLTFRQLERLVAEEAWVLTEELSPKMTLEVAS
GLFELYLTLADLQRFWDSIPGRDSRSLALAGIHAPFLPAVKLWFQVLRDQAKWRLQGAVD
MDTLEPVDASSRHSSSAATAGLCLSHIQELWVRLAWPDPAQAQGLGTQLGQDVCEATLFY
TELLRKKVDTQPGAAGEAVSEALCVVLNNVELVRKAAGQALKGLAWPEGATGPEGVLPRP
LLSCTQALDDDLQREAHTVTAHLTSKMVGDIRKYVQHISLSPDSIQNDEAVAPLMKYLDE
KLALLNASLVKGNLSRVLEALWELLLQAILQALGANRDVSADFYSRFHFTLEALVSFFHA
EGQGLPLESLRDGSYKRLKEELRLHKCSTRECIEQFYLDKLKQRTLEQNRFGRLSVRCHY
EAAEQRLAVEVLHAADLLPLDANGLSDPFVIVELGPPHLFPLVRSQRTQVKTRTLHPVYD
ELFYFSVPAEACRRRAACVLFTVMDHDWLSTNDFAGEAALGLGGVTGVARPQVGGGARAG
QPVTLHLCRPRAQVRSALRRLEGRTSKEAQEFVKKLKELEKCMEADP
>sp|Q16611|BAK_HUMAN Bcl-2 homologous antagonist/killer OS=Homo sapiens GN=BAK1 PE=1 SV=1
MASGQGPGPPRQECGEPALPSASEEQVAQDTEEVFRSYVFYRHQQEQEAEGVAAPADPEM
VTLPLQPSSTMGQVGRQLAIIGDDINRRYDSEFQTMLQHLQPTAENAYEYFTKIATSLFE
SGINWGRVVALLGFGYRLALHVYQHGLTGFLGQVTRFVVDFMLHHCIARWIAQRGGWVAA
LNLGNGPILNVLVVLGVVLLGQFVVRRFFKS
>sp|P82251|BAT1_HUMAN b(0,+)-type amino acid transporter 1 OS=Homo sapiens GN=SLC7A9 PE=1
SV=1
MGDTGLRKRREDEKSIQSQEPKTTSLQKELGLISGISIIVGTIIGSGIFVSPKSVLSNTE
AVGPCLIIWAACGVLATLGALCFAELGTMITKSGGEYPYLMEAYGPIPAYLFSWASLIVI
KPTSFAIICLSFSEYVCAPFYVGCKPPQIVVKCLAAAAILFISTVNSLSVRLGSYVQNIF
TAAKLVIVAIIIISGLVLLAQGNTKNFDNSFEGAQLSVGAISLAFYNGLWAYDGWNQLNY
ITEELRNPYRNLPLAIIIGIPLVTACYILMNVSYFTVMTATELLQSQAVAVTFGDRVLYP
ASWIVPLFVAFSTIGAANGTCFTAGRLIYVAGREGHMLKVLSYISVRRLTPAPAIIFYGI
IATIYIIPGDINSLVNYFSFAAWLFYGLTILGLIVMRFTRKELERPIKVPVVIPVLMTLI
SVFLVLAPIISKPTWEYLYCVLFILSGLLFYFLFVHYKFGWAQKISKPITMHLQMLMEVV
PPEEDPE
>sp|Q07812|BAX_HUMAN Apoptosis regulator BAX OS=Homo sapiens GN=BAX PE=1 SV=1
MDGSGEQPRGGGPTSSEQIMKTGALLLQGFIQDRAGRMGGEAPELALDPVPQDASTKKLS
ECLKRIGDELDSNMELQRMIAAVDTDSPREVFFRVAADMFSDGNFNWGRVVALFYFASKL

VLKALCTKVPELIRTIMGWTLDFLRERLLGWIQDQGGWDGLLSYFGTPTWQTVTIFVAGV
LTASLTIWKKMG
>sp|A8MTZ0|BBIP1_HUMAN BBSome-interacting protein 1 OS=Homo sapiens GN=BBIP1 PE=1 SV=2
MLKAAAKRPELSGKNTISNNSDMAEVKSMFREVLPKQGPLFVEDIMTMVLCKPKLLPLKS
LTLEKLEKMHQAAQNTIRQQEMAEKDQRQITH
>sp|Q8IWZ6|BBS7_HUMAN Bardet-Biedl syndrome 7 protein OS=Homo sapiens GN=BBS7 PE=1 SV=2
MDLILNRMDYLQVGVTSQKTMKLIPASRHRATQKVVIGDHDGVVMCFGMKKGEAAAVFKT
LPGPKIARLELGGVINTPQEKIFIAAASEIRGFTKRGKQFLSFETNLTESIKAMHISGSD
LFLSASYIYNHYCDCKDQHYYLSGDKINDVICLPVERLSRITPVLACQDRVLRVLQGSDV
MYAVEVPGPPTVLALHNGNGGDSGEDLLFGTSDGKLALIQITTSKPVRKWEIQNEKKRGG
ILCIDSFDIVGDGVKDLLVGRDDGMVEVYSFDNANEPVLRFDQMLSESVTSIQGGCVGKD
SYDEIVVSTYSGWVTGLTTEPIHKESGPGEELKINQEMQNKISSLRNELEHLQYKVLQER
ENYQQSSQSSKAKSAVPSFGINDKFTLNKDDASYSLILEVQTAIDNVLIQSDVPIDLLDV
DKNSAVVSFSSCDSESNDNFLLATYRCQADTTRLELKIRSIEGQYGTLQAYVTPRIQPKT
CQVRQYHIKPLSLHQRTHFIDHDRPMNTLTLTGQFSFAEVHSWVVFCLPEVPEKPPAGEC
VTFYFQNTFLDTQLESTYRKGEGVFKSDNISTISILKDVLSKEATKRKINLNISYEINEV
SVKHTLKLIHPKLEYQLLLAKKVQLIDALKELQIHEGNTNFLIPEYHCILEEADHLQEEY
KKQPAHLERLYGMITDLFIDKFKFKGTNVKTKVPLLLEILDSYDQNALISFFDAA
>sp|Q9C0K0|BC11B_HUMAN B-cell lymphoma/leukemia 11B OS=Homo sapiens GN=BCL11B PE=1 SV=1
MSRRKQGNPQHLSQRELITPEADHVEAAILEEDEGLEIEEPSGLGLMVGGPDPDLLTCGQ
CQMNFPLGDILVFIEHKRKQCGGSLGACYDKALDKDSPPPSSRSELRKVSEPVEIGIQVT
PDEDDHLLSPTKGICPKQENIAGPCRPAQLPAVAPIAASSHPHSSVITSPLRALGALPPC
LPLPCCSARPVSGDGTQGEGQTEAPFGCQCQLSGKDEPSSYICTTCKQPFNSAWFLLQHA
QNTHGFRIYLEPGPASSSLTPRLTIPPPLGPEAVAQSPLMNFLGDSNPFNLLRMTGPILR
DHPGFGEGRLPGTPPLFSPPPRHHLDPHRLSAEEMGLVAQHPSAFDRVMRLNPMAIDSPA
MDFSRRLRELAGNSSTPPPVSPGRGNPMHRLLNPFQPSPKSPFLSTPPLPPMPPGGTPPP
QPPAKSKSCEFCGKTFKFQSNLIVHRRSHTGEKPYKCQLCDHACSQASKLKRHMKTHMHK
AGSLAGRSDDGLSAASSPEPGTSELAGEGLKAADGDFRHHESDPSLGHEPEEEDEEEEEE
EEELLLENESRPESSFSMDSELSRNRENGGGGVPGVPGAGGGAAKALADEKALVLGKVME
NVGLGALPQYGELLADKQKRGAFLKRAAGGGDAGDDDDAGGCGDAGAGGAVNGRGGGFAP
GTEPFPGLFPRKPAPLPSPGLNSAAKRIKVEKDLELPPAALIPSENVYSQWLVGYAASRH
FMKDPFLGFTDARQSPFATSSEHSSENGSLRFSTPPGDLLDGGLSGRSGTASGGSTPHLG
GPGPGRPSSKEGRRSDTCEYCGKVFKNCSNLTVHRRSHTGERPYKCELCNYACAQSSKLT
RHMKTHGQIGKEVYRCDICQMPFSVYSTLEKHMKKWHGEHLLTNDVKIEQAERS
>sp|P41182|BCL6_HUMAN B-cell lymphoma 6 protein OS=Homo sapiens GN=BCL6 PE=1 SV=1
MASPADSCIQFTRHASDVLLNLNRLRSRDILTDVVIVVSREQFRAHKTVLMACSGLFYSI
FTDQLKCNLSVINLDPEINPEGFCILLDFMYTSRLNLREGNIMAVMATAMYLQMEHVVDT
CRKFIKASEAEMVSAIKPPREEFLNSRMLMPQDIMAYRGREVVENNLPLRSAPGCESRAF
APSLYSGLSTPPASYSMYSHLPVSSLLFSDEEFRDVRMPVANPFPKERALPCDSARPVPG
EYSRPTLEVSPNVCHSNIYSPKETIPEEARSDMHYSVAEGLKPAAPSARNAPYFPCDKAS
KEEERPSSEDEIALHFEPPNAPLNRKGLVSPQSPQKSDCQPNSPTESCSSKNACILQASG
SPPAKSPTDPKACNWKKYKFIVLNSLNQNAKPEGPEQAELGRLSPRAYTAPPACQPPMEP
ENLDLQSPTKLSASGEDSTIPQASRLNNIVNRSMTGSPRSSSESHSPLYMHPPKCTSCGS
QSPQHAEMCLHTAGPTFPEEMGETQSEYSDSSCENGAFFCNECDCRFSEEASLKRHTLQT

HSDKPYKCDRCQASFRYKGNLASHKTVHTGEKPYRCNICGAQFNRPANLKTHTRIHSGEK
PYKCETCGARFVQVAHLRAHVLIHTGEKPYPCEICGTRFRHLQTLKSHLRIHTGEKPYHC
EKCNLHFRHKSQLRLHLRQKHGAITNTKVQYRVSATDLPPELPKAC
>sp|Q9Y276|BCS1_HUMAN Mitochondrial chaperone BCS1 OS=Homo sapiens GN=BCS1L PE=1 SV=1
MPLSDFILALKDNPYFGAGFGLVGVGTALALARKGVQLGLVAFRRHYMITLEVPARDRSY
AWLLSWLTRHSTRTQHLSVETSYLQHESGRISTKFEFVPSPGNHFIWYRGKWIRVERSRE
MQMIDLQTGTPWESVTFTALGTDRKVFFNILEEARELALQQEEGKTVMYTAVGSEWRPFG
YPRRRRPLNSVVLQQGLADRIVRDVQEFIDNPKWYTDRGIPYRRGYLLYGPPGCGKSSFI
TALAGELEHSICLLSLTDSSLSDDRLNHLLSVAPQQSLVLLEDVDAAFLSRDLAVENPVK
YQGLGRLTFSGLLNALDGVASTEARIVFMTTNHVDRLDPALIRPGRVDLKEYVGYCSHWQ
LTQMFQRFYPGQAPSLAENFAEHVLRATNQISPAQVQGYFMLYKNDPVGAIHNAESLRR
>sp|Q8IYS8|BD1L2_HUMAN Biorientation of chromosomes in cell division protein 1-like 2 OS=Homo sapiens GN=BOD1L2 PE=2 SV=2
MADGGGGGSGGAGPASTRASGGGGPINPASLPPGDPQLIAIIVGQLKSRGLFDSFRRDCK
ADVDTKPAYQNLSQKADNFVSTHLDKQEWNPPANDNQLHDGLRQSVVQSGRSEAGVDRIS
SQVVDPKLNHIFRPQIEQIIHEFLVAQKEAAVPALPPEPEGQDPPAPSQDTS
>sp|P23560|BDNF_HUMAN Brain-derived neurotrophic factor OS=Homo sapiens GN=BDNF PE=1 SV=1
MTILFLTMVISYFGCMKAAPMKEANIRGQGGLAYPGVRTHGTLESVNGPKAGSRGLTSLA
DTFEHVIEELLDEDQKVRPNEENNKDADLYTSRVMLSSQVPLEPPLLFLLEEYKNYLDAA
NMSMRVRRHSDPARRGELSVCDSISEWVTAADKKTAVDMSGGTVTVLEKVPVSKGQLKQY
FYETKCNPMGYTKEGCRGIDKRHWNSQCRTTQSYVRALTMDSKKRIGWRFIRIDTSCVCT
LTIKRGR
>sp|A8MW95|BECN2_HUMAN Beclin-2 OS=Homo sapiens GN=BECN2 PE=1 SV=2
MSSIRFLCQRCHQALKLSGSSESRSLPAAPAPTSGQAEPGDTREPGVTTREVTDAEEQQD
GASSRSPPGDGSVSKGHANIFTLLGELGAMHMLSSIQKAAGDIFDIVSGQAVVDHPLCEE
CTDSLLEQLDIQLALTEADSQNYQRCLETGELATSEDEAAALRAELRDLELEEARLVQEL
EDVDRNNARAAADLQAAQAEAAELDQQERQHYRDYSALKRQQLELLDQLGNVENQLQYAR
VQRDRLKEINCFTATFEIWVEGPLGVINNFRLGRLPTVRVGWNEINTAWGQAALLLLTLA
NTIGLQFQRYRLIPCGNHSYLKSLTDDRTELPLFCYGGQDVFLNNKYDRAMVAFLDCMQQ
FKEEAEKGELGLSLPYGIQVETGLMEDVGGRGECYSIRTHLNTQELWTKALKFMLINFKW
SLIWVASRYQK
>sp|Q8NFU1|BEST2_HUMAN Bestrophin-2 OS=Homo sapiens GN=BEST2 PE=2 SV=1
MTVTYTARVANARFGGFSQLLLLWRGSIYKLLWRELLCFLGFYMALSAAYRFVLTEGQKR
YFEKLVIYCDQYASLIPVSFVLGFYVTLVVNRWWSQYLCMPLPDALMCVVAGTVHGRDDR
GRLYRRTLMRYAGLSAVLILRSVSTAVFKRFPTIDHVVEAGFMTREERKKFENLNSSYNK
YWVPCVWFSNLAAQARREGRIRDNSALKLLLEELNVFRGKCGMLFHYDWISVPLVYTQVV
TIALYSYFLACLIGRQFLDPAQGYKDHDLDLCVPIFTLLQFFFYAGWLKVAEQLINPFGE
DDDDFETNFLIDRNFQVSMLAVDEMYDDLAVLEKDLYWDAAEARAPYTAATVFQLRQPSF
QGSTFDITLAKEDMQFQRLDGLDGPMGEAPGDFLQRLLPAGAGMVAGGPLGRRLSFLLRK
NSCVSEASTGASCSCAVVPEGAAPECSCGDPLLDPGLPEPEAPPPAGPEPLTLIPGPVEP
FSIVTMPGPRGPAPPWLPSPIGEEEENLA
>sp|Q8N1M1|BEST3_HUMAN Bestrophin-3 OS=Homo sapiens GN=BEST3 PE=2 SV=1
MTVTYSSKVANATFFGFHRLLLKWRGSIYKLLYREFIVFAVLYTAISLVYRLLLTGVQKR
YFEKLSIYCDRYAEQIPVTFVLGFYVTLVVNRWWNQFVNLPWPDRLMFLISSSVHGSDEH

GRLLRRTLMRYVNLTSLLIFRSVSTAVYKRFPTMDHVVEAGFMTTDERKLFNHLKSPHLK
YWVPFIWFGNLATKARNEGRIRDSVDLQSLMTEMNRYRSWCSLLFGYDWVGIPLVYTQVV
TLAVYTFFFACLIGRQFLDPTKGYAGHDLDLYIPIFTLLQFFFYAGWLKVAEQLINPFGE
DDDDFETNWCIDRNLQVSLLAVDEMHMSLPKMKKDIYWDDSAARPPYTLAAADYCIPSFL
GSTVQMGLSGSDFPDEEWLWDYEKHGHRHSMIRRVKRFLSAHEHPSSPRRRSYRRQTSDS
SMFLPRDDLSPARDLLDVPSRNPPRASPTWKKSCFPEGSPTLHFSMGELSTIRETSQTST
LQSLTPQSSVRTSPIKMPLVPEVLITAAEAPVPTSGGYHHDSATSILSSEFTGVQPSKTE
QQQGPMGSILSPSEKETPPGGPSPQTVSASAEENIFNCEEDPGDTFLKRWSLPGFLGSSH
TSLGNLSPDPMSSQPALLIDTETSSEISGINIVAGSRVSSDMLYLMENLDTKETDIIELN
KETEESPK
>sp|Q8NFU0|BEST4_HUMAN Bestrophin-4 OS=Homo sapiens GN=BEST4 PE=2 SV=1
MTVSYTLKVAEARFGGFSGLLLRWRGSIYKLLYKEFLLFGALYAVLSITYRLLLTQEQRY
VYAQVARYCNRSADLIPLSFVLGFYVTLVVNRWWSQYTSIPLPDQLMCVISASVHGVDQR
GRLLRRTLIRYANLASVLVLRSVSTRVLKRFPTMEHVVDAGFMSQEERKKFESLKSDFNK
YWVPCVWFTNLAAQARRDGRIRDDIALCLLLEELNKYRAKCSMLFHYDWISIPLVYTQVV
TIAVYSFFALSLVGRQFVEPEAGAAKPQKLLKPGGQEPAPALGDPDMYVPLTTLLQFFFYA
GWLKVAEQIINPFGEDDDDFETNQLIDRNLQVSLLSVDEMYQNLPPAEKDQYWDEDQPQP
PYTVATAAESLRPSFLGSTFNLRMSDDPEQSLQVEASPGSGRPAPAAQTPLLGRFLGVGA
PSPAISLRNFGRVRGTPRPPHLLRFRAEEGGDPEAAARIEEESAESGDEALEP
>sp|Q7RTU4|BHA09_HUMAN Class A basic helix-loop-helix protein 9 OS=Homo sapiens GN=BHLHA9 PE=1 SV=2
MLRGAPGLGLTARKGAEDSAEDLGGPCPEPGGDSGVLGANGASCSRGEAEEPAGRRRARP
VRSKARRMAANVRERKRILDYNEAFNALRRALRHDLGGKRLSKIATLRRAIHRIAALSLV
LRASPAPRGPCGHLECHGPAARGDTGDTGASPPPPAGPSLARPDAARPSVPSAPRCASCP
PHAPLARPSAVAEGPGLAQASGGSWRRCPGASSAGPPPWPRGYLRSAPGMGHPRS
>sp|Q8NFJ8|BHE22_HUMAN Class E basic helix-loop-helix protein 22 OS=Homo sapiens GN=BHLHE22 PE=2 SV=1
MERGMHLGAAAAGEDDLFLHKSLSASTSKRLEAAFRSTPPGMDLSLAPPPRERPASSSSS
PLGCFEPADPEGAGLLLPPPGGGGGGSAGSGGGGGGGVGVPGLLVGSAGVGGDPSLSSLP
AGAALCLKYGESASRGSVAESSGGEQSPDDDSDGRCELVLRAGVADPRASPGAGGGGAKA
AEGCSNAHLHGGASVPPGGLGGGGGGGSSSGSSGGGGGSGSGSGGSSSSSSSSSKKSKEQ
KALRLNINARERRRMHDLNDALDELRAVIPYAHSPSVRKLSKIATLLLAKNYILMQAQAL
EEMRRLVAYLNQGQAISAASLPSSAAAAAAAAALHPALGAYEQAAGYPFSAGLPPAASCP
EKCALFNSVSSSLCKQCTEKP
>sp|Q9H6S1|AZI2_HUMAN 5-azacytidine-induced protein 2 OS=Homo sapiens GN=AZI2 PE=1 SV=1
MDALVEDDICILNHEKAHKRDTVTPVSIYSGDESVASHFALVTAYEDIKKRLKDSEKENS
LLKKRIRFLEEKLIARFEEETSSVGREQVNKAYHAYREVCIDRDNLKSKLDKMNKDNSES
LKVLNEQLQSKEVELLQLRTEVETQQVMRNLNPPSSNWEVEKLSCDLKIHGLEQELELMR
KECSDLKIELQKAKQTDPYQEDNLKSRDLQKLSISSDNMQHAYWELKREMSNLHLVTQVQ
AELLRKLKTSTAIKKACAPVGCSEDLGRDSTKLHLMNFTATYTRHPPLLPNGKALCHTTS
SPLPGDVKVLSEKAILQSWTDNERSIPNDGTCFQEHSSYGRNSLEDNSWVFPSPPKSSET
AFGETKTKTLPLPNLPPLHYLDQHNQNCLYKN
>sp|Q9BXK5|B2L13_HUMAN Bcl-2-like protein 13 OS=Homo sapiens GN=BCL2L13 PE=1 SV=1
MASSSTVPLGFHYETKYVVLSYLGLLSQEKLQEQHLSSPQGVQLDIASQSLDQEILLKVK

TEIEEELKSLDKEISEAFTSTGFDRHTSPVFSPANPESSMEDCLAHLGEKVSQELKEPLH
KALQMLLSQPVTYQAFRECTLETTVHASGWNKILVPLVLLRQMLLELTRRGQEPLSALLQ
FGVTYLEDYSAEYIIQQGGWGTVFSLESEEEEYPGITAEDSNDIYILPSDNSGQVSPPES
PTVTTSWQSESLPVSLSASQSWHTESLPVSLGPESWQQIAMDPEEVKSLDSNGAGEKSEN
NSSNSDIVHVEKEEVPEGMEEAAVASVVLPARELQEALPEAPAPLLPHITATSLLGTREP
DTEVITVEKSSPATSLFVELDEEEVKAATTEPTEVEEVVPALEPTETLLSEKEINAREES
LVEELSPASEKKPVPPSEGKSRLSPAGEMKPMPLSEGKSILLFGGAAAVAILAVAIGVAL
ALRKK
>sp|P48751|B3A3_HUMAN Anion exchange protein 3 OS=Homo sapiens GN=SLC4A3 PE=2 SV=2
MANGVIPPPGGASPLPQVRVPLEEPPLSPDVEEEDDDLGKTLAVSRFGDLISKPPAWDPE
KPSRSYSERDFEFHRHTSHHTHHPLSARLPPPHKLRRLPPTSARHTRRKRKKEKTSAPPS
EGTPPIQEEGGAGVDEEEEEEEEEEEGESEAEPVEPPHSGTPQKAKFSIGSDEDDSPGLPG
RAAVTKPLPSVGPHTDKSPQHSSSSPSPRARASRLAGEKSRPWSPSASYDLRERLCPGSA
LGNPGGPEQQVPTDEAEAQMLGSADLDDMKSHRLEDNPGVRRHLVKKPSRTQGGRGSPSG
LAPILRRKKKKKKLDRRPHEVFVELNELMLDRSQEPHWRETARWIKFEEDVEEETERWGK
PHVASLSFRSLLELRRTIAHGAALLDLEQTTLPGIAHLVVETMIVSDQIRPEDRASVLRT
LLLKHSHPNDDKDSGFFPRNPSSSSMNSVLGNHHPTPSHGPDGAVPTMADDLGEPAPLWP
HDPDAKEKPLHMPGGDGHRGKSLKLLEKIPEDAEATVVLVGCVPFLEQPAAAFVRLNEAV
LLESVLEVPVPVRFLFVMLGPSHTSTDYHELGRSIATLMSDKLFHEAAYQADDRQDLLSA
ISEFLDGSIVIPPSEVEGRDLLRSVAAFQRELLRKRREREQTKVEMTTRGGYTAPGKELS
LELGGSEATPEDDPLLRTGSVFGGLVRDVRRRYPHYPSDLRDALHSQCVAAVLFIYFAAL
SPAITFGGLLGEKTEGLMGVSELIVSTAVLGVLFSLLGAQPLLVVGFSGPLLVFEEAFFK
FCRAQDLEYLTGRVWVGLWLVVFVLALVAAEGSFLVRYISPFTQEIFAFLISLIFIYETF
YKLYKVFTEHPLLPFYPPEGALEGSLDAGLEPNGSALPPTEGPPSPRNQPNTALLSLILM
LGTFFIAFFLRKFRNSRFLGGKARRIIGDFGIPISILVMVLVDYSITDTYTQKLTVPTGL
SVTSPDKRSWFIPPLGSARPFPPWMMVAAAVPALLVLILIFMETQITALIVSQKARRLLK
GSGFHLDLLLIGSLGGLCGLFGLPWLTAATVRSVTHVNALTVMRTAIAPGDKPQIQEVRE
QRVTGVLIASLVGLSIVMGAVLRRIPLAVLFGIFLYMGVTSLSGIQLSQRLLLILMPAKH
HPEQPYVTKVKTWRMHLFTCIQLGCIALLWVVKSTAASLAFPFLLLLTVPLRHCLLPRLF
QDRELQALDSEDAEPNFDEDGQDEYNELHMPV
>sp|O75752|B3GL1_HUMAN UDP-GalNAc:beta-1,3-N-acetylgalactosaminyltransferase 1 OS=Homo sapiens GN=B3GALNT1 PE=1 SV=1
MASALWTVLPSRMSLRSLKWSLLLLSLLSFFVMWYLSLPHYNVIERVNWMYFYEYEPIYR
QDFHFTLREHSNCSHQNPFLVILVTSHPSDVKARQAIRVTWGEKKSWWGYEVLTFFLLGQ
EAEKEDKMLALSLEDEHLLYGDIIRQDFLDTYNNLTLKTIMAFRWVTEFCPNAKYVMKTD
TDVFINTGNLVKYLLNLNHSEKFFTGYPLIDNYSYRGFYQKTHISYQEYPFKVFPPYCSG
LGYIMSRDLVPRIYEMMGHVKPIKFEDVYVGICLNLLKVNIHIPEDTNLFFLYRIHLDVC
QLRRVIAAHGFSSKEIITFWQVMLRNTTCHY
>sp|Q9Y2A9|B3GN3_HUMAN N-acetyllactosaminide beta-1,3-N-acetylglucosaminyltransferase 3 OS=Homo sapiens GN=B3GNT3 PE=1 SV=2
MKYLRHRRPNATLILAIGAFTLLLFSLLVSPPTCKVQEQPPAIPEALAWPTPPTRPAPAP
CHANTSMVTHPDFATQPQHVQNFLLYRHCRHFPLLQDVPPSKCAQPVFLLLVIKSSPSNY
VRRELLRRTWGRERKVRGLQLRLLFLVGTASNPHEARKVNRLLELEAQTHGDILQWDFHD
SFFNLTLKQVLFLQWQETRCANASFVLNGDDDVFAHTDNMVFYLQDHDPGRHLFVGQLIQ

NVGPIRAFWSKYYVPEVVTQNERYPPYCGGGGFLLSRFTAAALRRAAHVLDIFPIDDVFL
GMCLELEGLKPASHSGIRTSGVRAPSQRLSSFDPCFYRDLLLVHRFLPYEMLLMWDALNQ
PNLTCGNQTQIY
>sp|Q6UX72|B3GN9_HUMAN UDP-GlcNAc:betaGal beta-1,3-N-acetylglucosaminyltransferase 9 OS=Homo sapiens GN=B3GNT9 PE=2 SV=1
MRRRLRLRRDALLTLLLGASLGLLLYAQRDGAAPTASAPRGRGRAAPRPTPGPRAFQLPD
AGAAPPAYEGDTPAPPTPTGPFDFARYLRAKDQRRFPLLINQPHKCRGDGAPGGRPDLLI
AVKSVAEDFERRQAVRQTWGAEGRVQGALVRRVFLLGVPRGAGSGGADEVGEGARTHWRA
LLRAESLAYADILLWAFDDTFFNLTLKEIHFLAWASAFCPDVRFVFKGDADVFVNVGNLL
EFLAPRDPAQDLLAGDVIVHARPIRTRASKYYIPEAVYGLPAYPAYAGGGGFVLSGATLH
RLAGACAQVELFPIDDVFLGMCLQRLRLTPEPHPAFRTFGIPQPSAAPHLSTFDPCFYRE
LVVVHGLSAADIWLMWRLLHGPHGPACAHPQPVAAGPFQWDS
>sp|Q9BPU9|B9D2_HUMAN B9 domain-containing protein 2 OS=Homo sapiens GN=B9D2 PE=1 SV=2
MAEVHVIGQIIGASGFSESSLFCKWGIHTGAAWKLLSGVREGQTQVDTPQIGDMAYWSHP
IDLHFATKGLQGWPRLHFQVWSQDSFGRCQLAGYGFCHVPSSPGTHQLACPTWRPLGSWR
EQLARAFVGGGPQLLHGDTIYSGADRYRLHTAAGGTVHLEIGLLLRNFDRYGVEC
>sp|Q9NWV8|BABA1_HUMAN BRISC and BRCA1-A complex member 1 OS=Homo sapiens GN=BABAM1 PE=1 SV=1
MEVAEPSSPTEEEEEEEEHSAEPRPRTRSNPEGAEDRAVGAQASVGSRSEGEGEAASADD
GSLNTSGAGPKSWQVPPPAPEVQIRTPRVNCPEKVIICLDLSEEMSLPKLESFNGSKTNA
LNVSQKMIEMFVRTKHKIDKSHEFALVVVNDDTAWLSGLTSDPRELCSCLYDLETASCST
FNLEGLFSLIQQKTELPVTENVQTIPPPYVVRTILVYSRPPCQPQFSLTEPMKKMFQCPY
FFFDVVYIHNGTEEKEEEMSWKDMFAFMGSLDTKGTSYKYEVALAGPALELHNCMAKLLA
HPLQRPCQSHASYSLLEEEDEAIEVEATV
>sp|Q9Y5Z0|BACE2_HUMAN Beta-secretase 2 OS=Homo sapiens GN=BACE2 PE=1 SV=1
MGALARALLLPLLAQWLLRAAPELAPAPFTLPLRVAAATNRVVAPTPGPGTPAERHADGL
ALALEPALASPAGAANFLAMVDNLQGDSGRGYYLEMLIGTPPQKLQILVDTGSSNFAVAG
TPHSYIDTYFDTERSSTYRSKGFDVTVKYTQGSWTGFVGEDLVTIPKGFNTSFLVNIATI
FESENFFLPGIKWNGILGLAYATLAKPSSSLETFFDSLVTQANIPNVFSMQMCGAGLPVA
GSGTNGGSLVLGGIEPSLYKGDIWYTPIKEEWYYQIEILKLEIGGQSLNLDCREYNADKA
IVDSGTTLLRLPQKVFDAVVEAVARASLIPEFSDGFWTGSQLACWTNSETPWSYFPKISI
YLRDENSSRSFRITILPQLYIQPMMGAGLNYECYRFGISPSTNALVIGATVMEGFYVIFD
RAQKRVGFAASPCAEIAGAAVSEISGPFSTEDVASNCVPAQSLSEPILWIVSYALMSVCG
AILLVLIVLLLLPFRCQRRPRDPEVVNDESSLVRHRWK
>sp|Q92934|BAD_HUMAN Bcl2-associated agonist of cell death OS=Homo sapiens GN=BAD PE=1 SV=3
MFQIPEFEPSEQEDSSSAERGLGPSPAGDGPSGSGKHHRQAPGLLWDASHQQEQPTSSSH
HGGAGAVEIRSRHSSYPAGTEDDEGMGEEPSPFRGRSRSAPPNLWAAQRYGRELRRMSDE
FVDSFKKGLPRPKSAGTATQMRQSSSWTRVFQSWWDRNLGRGSSAPSQ
>sp|O75531|BAF_HUMAN Barrier-to-autointegration factor OS=Homo sapiens GN=BANF1 PE=1 SV=1
MTTSQKHRDFVAEPMGEKPVGSLAGIGEVLGKKLEERGFDKAYVVLGQFLVLKKDEDLFR
EWLKDTCGANAKQSRDCFGCLREWCDAFL
>sp|Q86Y28|BAGE4_HUMAN B melanoma antigen 4 OS=Homo sapiens GN=BAGE4 PE=2 SV=1
MAAGAVFLALSAQLLQARLMKEESPVVSWWLEPEDGTAL

>sp|Q6ZNE5|BAKOR_HUMAN Beclin 1-associated autophagy-related key regulator OS=Homo sapiens GN=ATG14 PE=1 SV=1
MASPSGKGARALEAPGCGPRPLARDLVDSVDDAEGLYVAVERCPLCNTTRRRLTCAKCVQ
SGDFVYFDGRDRERFIDKKERLSRLKSKQEEFQKEVLKAMEGKWITDQLRWKIMSCKMRI
EQLKQTICKGNEEMEKNSEGLLKTKEKNQKLYSRAQRHQEKKEKIQRHNRKLGDLVEKKT
IDLRSHYERLANLRRSHILELTSVIFPIEEVKTGVRDPADVSSESDSAMTSSTVSKLAEA
RRTTYLSGRWVCDDHNGDTSISITGPWISLPNNGDYSAYYSWVEEKKTTQGPDMEQSNPA
YTISAALCYATQLVNILSHILDVNLPKKLCNSEFCGENLSKQKFTRAVKKLNANILYLCF
SQHVNLDQLQPLHTLRNLMYLVSPSSEHLGRSGPFEVRADLEESMEFVDPGVAGESDESG
DERVSDEETDLGTDWENLPSPRFCDIPSQSVEVSQSQSTQASPPIASSSAGGMISSAAAS
VTSWFKAYTGHR
>sp|Q8NDB2|BANK1_HUMAN B-cell scaffold protein with ankyrin repeats OS=Homo sapiens GN=BANK1 PE=1 SV=3
MLPAAPGKGLGSPDPAPCGPAPPGNTKDIIMIYEEDAEEWALYLTEVFLHVVKREAILLY
RLENFSFRHLELLNLTSYKCKLLILSNSLLRDLTPKKCQFLEKILHSPKSVVTLLCGVKS
SDQLYELLNISQSRWEISTEQEPEDYISVIQSIIFKDSEDYFEVNIPTDLRAKHSGEISE
RKEIEELSEASRNTIPLAVVLPTEIPCENPGEIFIILRDEVIGDTVEVEFTSSNKRIRTR
PALWNKKVWCMKALEFPAGSVHVNVYCDGIVKATTKIKYYPTAKAKECLFRMADSGESLC
QNSIEELDGVLTSIFKHEIPYYEFQSLQTEICSQNKYTHFKELPTLLHCAAKFGLKNLAI
HLLQCSGATWASKMKNMEGSDPAHIAERHGHKELKKIFEDFSIQEIDINNEQENDYEEDI
ASFSTYIPSTQNPAFHHESRKTYGQSADGAEANEMEGEGKQNGSGMETKHSPLEVGSESS
EDQYDDLYVFIPGADPENNSQEPLMSSRPPLPPPRPVANAFQLERPHFTLPGTMVEGQME
RSQNWGHPGVRQETGDEPKGEKEKKEEEKEQEEEEDPYTFAEIDDSEYDMILANLSIKKK
TGSRSFIINRPPAPTPRPTSIPPKEETTPYIAQVFQQKTARRQSDDDKFCGLPKKQDRAR
IESPAFSTLRGCLTDGQEELILLQEKVKNGKMSMDEALEKFKHWQMGKSGLEMIQQEKLR
QLRDCIIGKRPEEENVYNKLTIVHHPGGKETAHNENKFYNVHFSNKLPARPQVEKEFGFC
CKKDH
>sp|Q9UHQ4|BAP29_HUMAN B-cell receptor-associated protein 29 OS=Homo sapiens GN=BCAP29 PE=1 SV=2
MTLQWAAVATFLYAEIGLILIFCLPFIPPQRWQKIFSFNVWGKIATFWNKAFLTIIILLI
VLFLDAVREVRKYSSVHTIEKSSTSRPDAYEHTQMKLFRSQRNLYISGFSLFFWLVLRRL
VTLITQLAKELSNKGVLKTQAENTNKAAKKFMEENEKLKRILKSHGKDEECVLEAENKKL
VEDQEKLKTELRKTSDALSKAQNDVMEMKMQSERLSKEYDQLLKEHSELQDRLERGNKKR
L
>sp|Q8N3I7|BBS5_HUMAN Bardet-Biedl syndrome 5 protein OS=Homo sapiens GN=BBS5 PE=1 SV=1
MSVLDALWEDRDVRFDLSAQQMKTRPGEVLIDCLDSIEDTKGNNGDRGRLLVTNLRILWH
SLALSRVNVSVGYNCILNITTRTANSKLRGQTEALYILTKCNSTRFEFIFTNLVPGSPRL
FTSVMAVHRAYETSKMYRDFKLRSALIQNKQLRLLPQEHVYDKINGVWNLSSDQGNLGTF
FITNVRIVWHANMNDSFNVSIPYLQIRSIKIRDSKFGLALVIESSQQSGGYVLGFKIDPV
EKLQESVKEINSLHKVYSASPIFGVDYEMEEKPQPLEALTVEQIQDDVEIDSDGHTDAFV
AYFADGNKQQDREPVFSEELGLAIEKLKDGFTLQGLWEVMS
>sp|O75363|BCAS1_HUMAN Breast carcinoma-amplified sequence 1 OS=Homo sapiens GN=BCAS1 PE=1 SV=2
MGNQMSVPQRVEDQENEPEAETYQDNASALNGVPVVVSTHTVQHLEEVDLGISVKTDNVA

TSSPETTEISAVADANGKNLGKEAKPEAPAAKSRFFLMLSRPVPGRTGDQAADSSLGSVK
LDVSSNKAPANKDPSESWTLPVAAGPGQDTDKTPGHAPAQDKVLSAARDPTLLPPETGGA
GGEAPSKPKDSSFFDKFFKLDKGQEKVPGDSQQEAKRAEHQDKVDEVPGLSGQSDDVPAG
KDIVDGKEKEGQELGTADCSVPGDPEGLETAKDDSQAAAIAENNNSIMSFFKTLVSPNKA
ETKKDPEDTGAEKSPTTSADLKSDKANFTSQETQGAGKNSKGCNPSGHTQSVTTPEPAKE
GTKEKSGPTSLPLGKLFWKKSVKEDSVPTGAEENVVCESPVEIIKSKEVESALQTVDLNE
GDAAPEPTEAKLKREESKPRTSLMAFLRQMSVKGDGGITHSEEINGKDSSCQTSDSTEKT
ITPPEPEPTGAPQKGKEGSSKDKKSAAEMNKQKSNKQEAKEPAQCTEQATVDTNSLQNGD
KLQKRPEKRQQSLGGFFKGLGPKRMLDAQVQTDPVSIGPVGKSK
>sp|Q9H6U6|BCAS3_HUMAN Breast carcinoma-amplified sequence 3 OS=Homo sapiens GN=BCAS3 PE=1 SV=3
MNEAMATDSPRRPSRCTGGVVVRPQAVTEQSYMESVVTFLQDVVPQAYSGTPLTEEKEKI
VWVRFENADLNDTSRNLEFHEIHSTGNEPPLLIMIGYSDGMQVWSIPISGEAQELFSVRH
GPIRAARILPAPQFGAQKCDNFAEKRPLLGVCKSIGSSGTSPPYCCVDLYSLRTGEMVKS
IQFKTPIYDLHCNKRILVVVLQEKIAAFDSCTFTKKFFVTSCYPCPGPNMNPIALGSRWL
AYAENKLIRCHQSRGGACGDNIQSYTATVISAAKTLKSGLTMVGKVVTQLTGTLPSGVTE
DDVAIHSNSRRSPLVPGIITVIDTETVGEGQVLVSEDSDSDGIVAHFPAHEKPVCCMAFN
TSGMLLVTTDTLGHDFHVFQILTHPWSSSQCAVHHLYTLHRGETEAKVQDICFSHDCRWV
VVSTLRGTSHVFPINPYGGQPCVRTHMSPRVVNRMSRFQKSAGLEEIEQELTSKQGGRCS
PVPGLSSSPSGSPLHGKLNSQDSYNNFTNNNPGNPRLSPLPSLMVVMPLAQIKQPMTLGT
ITKRTGPYLFGAGCFSIKAPCKVKPPPQISPSKSMGGEFCVAAIFGTSRSWFANNAGLKR
EKDQSKQVVVESLYIISCYGTLVEHMMEPRPLSTAPKISDDTPLEMMTSPRASWTLVRTP
QWNELQPPFNANHPLLLAADAVQYYQFLLAGLVPPGSPGPITRHGSYDSLASDHSGQEDE
EWLSQVEIVTHTGPHRRLWMGPQFQFKTIHPSGQTTVISSSSSVLQSHGPSDTPQPLLDF
DTDDLDLNSLRIQPVRSDPVSMPGSSRPVSDRRGVSTVIDAASGTFDRSVTLLEVCGSWP
EGFGLRHMSSMEHTEEGLRERLADAMAESPSRDVVGSGTELQREGSIETLSNSSGSTSGS
IPRNFDGYRSPLPTNESQPLSLFPTGFP
>sp|Q4VC05|BCL7A_HUMAN B-cell CLL/lymphoma 7 protein family member A OS=Homo sapiens GN=BCL7A PE=1 SV=1
MSGRSVRAETRSRAKDDIKRVMAAIEKVRKWEKKWVTVGDTSLRIYKWVPVTEPKVDDKN
KNKKKGKDEKCGSEVTTPENSSSPGMMDMHDDNSNQSSIADASPIKQENSSNSSPAPEPN
SAVPSDGTEAKVDEAQADGKEHPGAEDASDEQNSQSSMEHSMNSSEKVDRQPSGDSGLAA
ETSAISQDLEGVPPSKKMKLEASQQNSEEM
>sp|Q86UU0|BCL9L_HUMAN B-cell CLL/lymphoma 9-like protein OS=Homo sapiens GN=BCL9L PE=1 SV=1
MRILANKTRLPHPRRREAPGSPPLSPRGHCPPAPAKPMHPENKLTNHGKTGNGGAQSQHQ
NVNQGPTCNVGSKGVGAGNHGAKANQISPSNSSLKNPQAGVPPFSSLKGKVKRDRSVSVD
SGEQREAGTPSLDSEAKEVAPRSKRRCVLERKQPYSGDEWCSGPDSEEDDKPIGATHNCN
VADPAMAAPQLGPGQTTQLPLSESSVPGAPHGPPPGLRPDAPGGGGGGGGVPGKPPSQFV
YVFTTHLANTAAEAVLQGRADSILAYHQQNVPRAKLDQAPKVPPTPEPLPLSTPSAGTPQ
SQPPPLPPPPPPAPGSAPPALPPEGPPEDSSQDLAPNSVGAASTGGGTGGTHPNTPTATT
ANNPLPPGGDPSSAPGPALLGEAAAPGNGQRSLVGSEGLSKEQLEHRERSLQTLRDIERL
LLRSGETEPFLKGPPGGAGEGGPPAQAPPPPQQPPTAPPSGLKKYEEPLQSMISQTQSLG
GPPLEHEVPGHPPGGDMGQQMNMMIQRLGQDSLTPEQVAWRKLQEEYYEEKRRKEEQIGL

HGSRPLQDMMGMGGMMVRGPPPPYHSKPGDQWPPGMGAQLRGPMDVQDPMQLRGGPPFPG
PRFPGNQIQRVPGFGGMQSMPMEVPMNAMQRPVRPGMGWTEDLPPMGGPSNFAQNTMPYP
GGQGEAERFMTPRVREELLRHQLLEKRSMGMQRPLGMAGSGMGQSMEMERMMQAHRQMDP
AMFPGQMAGGEGLAGTPMGMEFGGGRGLLSPPMGQSGLREVDPPMGPGNLNMNMNVNMNM
NMNLNVQMTPQQQMLMSQKMRGPGDLMGPQGLSPEEMARVRAQNSSGVMGGPQKMLMPSQ
FPNQGQQGFSGGQGPYQAMSQDMGNTQDMFSPDQSSMPMSNVGTTRLSHMPLPPASNPPG
TVHSAPNRGLGRRPSDLTISINQMGSPGMGHLKSPTLSQVHSPLVTSPSANLKSPQTPSQ
MVPLPSANPPGPLKSPQVLGSSLSVRSPTGSPSRLKSPSMAVPSPGWVASPKTAMPSPGV
SQNKQPPLNMNSSTTLSNMEQGTLPPSGPRSSSSAPPANPPSGLMNPSLPFTSSPDPTPS
QNPLSLMMTQMSKYAMPSSTPLYHNAIKTIATSDDELLPDRPLLPPPPPPQGSGPGISNS
QPSQMHLNSAAAQSPMGMNLPGQQPLSHEPPPAMLPSPTPLGSNIPLHPNAQGTGGPPQN
SMMMAPGGPDSLNAPCGPVPSSSQMMPFPPRLQQPHGAMAPTGGGGGGPGLQQHYPSGMA
LPPEDLPNQPPGPMPPQQHLMGKAMAGRMGDAYPPGVLPGVASVLNDPELSEVIRPTPTG
IPEFDLSRIIPSEKPSSTLQYFPKSENQPPKAQPPNLHLMNLQNMMAEQTPSRPPNLPGQ
QGVQRGLNMSMCHPGQMSLLGRTGVPPQQGMVPHGLHQGVMSPPQGLMTQQNFMLMKQRG
VGGEVYSQPPHMLSPQGSLMGPPPQQNLMVSHPLRQRSVSLDSQMGYLPAPGGMANLPF
>sp|Q5H9F3|BCORL_HUMAN BCL-6 corepressor-like protein 1 OS=Homo sapiens GN=BCORL1 PE=1 SV=1
MISTAPLYSGVHNWTSSDRIRMCGINEERRAPLSDEESTTGDCQHFGSQEFCVSSSFSKV
ELTAVGSGSNARGADPDGSATEKLGHKSEDKPDDPQPKMDYAGNVAEAEGFLVPLSSPGD
GLKLPASDSAEASNSRADCSWTPLNTQMSKQVDCSPAGVKALDSRQGVGEKNTFILATLG
TGVPVEGTLPLVTTNFSPLPAPICPPAPGSASVPHSVPDAFQVPLSVPAPVPHSGLVPVQ
VATSVPAPSPPLAPVPALAPAPPSVPTLISDSNPLSVSASVLVPVPASAPPSGPVPLSAP
APAPLSVPVSAPPLALIQAPVPPSAPTLVLAPVPTPVLAPMPASTPPAAPAPPSVPMPTP
TPSSGPPSTPTLIPAFAPTPVPAPTPAPIFTPAPTPMPAATPAAIPTSAPIPASFSLSRV
CFPAAQAPAMQKVPLSFQPGTVLTPSQPLVYIPPPSCGQPLSVATLPTTLGVSSTLTLPV
LPSYLQDRCLPGVLASPELRSYPYAFSVARPLTSDSKLVSLEVNRLPCTSPSGSTTTQPA
PDGVPGPLADTSLVTASAKVLPTPQPLLPAPSGSSAPPHPAKMPSGTEQQTEGTSVTFSP
LKSPPQLEREMASPPECSEMPLDLSSKSNRQKLPLPNQRKTPPMPVLTPVHTSSKALLST
VLSRSQRTTQAAGGNVTSCLGSTSSPFVIFPEIVRNGDPSTWVKNSTALISTIPGTYVGV
ANPVPASLLLNKDPNLGLNRDPRHLPKQEPISIIDQGEPKGTGATCGKKGSQAGAEGQPS
TVKRYTPARIAPGLPGCQTKELSLWKPTGPANIYPRCSVNGKPTSTQVLPVGWSPYHQAS
LLSIGISSAGQLTPSQGAPIRPTSVVSEFSGVPSLSSSEAVHGLPEGQPRPGGSFVPEQD
PVTKNKTCRIAAKPYEEQVNPVLLTLSPQTGTLALSVQPSGGDIRMNQGPEESESHLCSD
STPKMEGPQGACGLKLAGDTKPKNQVLATYMSHELVLATPQNLPKMPELPLLPHDSHPKE
LILDVVPSSRRGSSTERPQLGSQVDLGRVKMEKVDGDVVFNLATCFRADGLPVAPQRGQA
EVRAKAGQARVKQESVGVFACKNKWQPDDVTESLPPKKMKCGKEKDSEEQQLQPQAKAVV
RSSHRPKCRKLPSDPQESTKKSPRGASDSGKEHNGVRGKHKHRKPTKPESQSPGKRADSH
EEGSLEKKAKSSFRDFIPVVLSTRTRSQSGSICSSFAGMADSDMGSQEVFPTEEEEEVTP
TPAKRRKVRKTQRDTQYRSHHAQDKSLLSQGRRHLWRAREMPWRTEAARQMWDTNEEEEE
EEEEGLLKRKKRRRQKSRKYQTGEYLTEQEDEQRRKGRADLKARKQKTSSSQSLEHRLRN
RNLLLPNKVQGISDSPNGFLPNNLEEPACLENSEKPSGKRKCKTKHMATVSEEAKDVVLY
CLQKDSEDVNHRDNAGYTALHEACSRGWTDILNILLEHGANVNCSAQDGTRPVHDAVVND
NLETIWLLLSYGADPTLATYSGQTAMKLASSDTMKRFLSDHLSDLQGRAEGDPGVSWDFY

SSSVLEEKDGFACDLLHNPPGSSDQEGDDPMEEDDFMFELSDKPLLPCYNLQVSVSRGPC
NWFLFSDVLKRLKLSSRIFQARFPHFEITTMPKAEFYRQVASSQLLTPAERPGGLDDRSP
PGSSETVELVRYEPDLLRLLGSEVEFQSCNS
>sp|Q9BUH8|BEGIN_HUMAN Brain-enriched guanylate kinase-associated protein OS=Homo sapiens GN=BEGAIN PE=1 SV=1
MEKLSALQEQKGELRKRLSYTTHKLEKLETEFDSTRHYLEIELRRAQEELEKVTEKLRRI
QSNYMALQRINQELEDKLYRMGQHYEEEKRALSHEIVALNSHLLEAKVTIDKLSEDNELY
RKDCNLAAQLLQCSQTYGRVHKVSELPSDFQERVSLHMEKHGCSLPSPLCHPAYADSVPT
CVIAKVLEKPDPASLSSRLSDASARDLAFCDGVEKPGPRPPYKGDIYCSDTALYCPEERR
RDRRPSVDAPVTDVGFLRAQNSTDSAAEEEEEAEAAAFPAGFQHEAFPSYAGSLPTSSSY
SSFSATSEEKEHAQASTLTASQQAIYLNSRDELFDRKPPATTYEGSPRFAKATAAVAAPL
EAEVAPGFGRTMSPYPAETFRFPASPGPQQALMPPNLWSLRAKPGTARLPGEDMRGQWRP
LSVEDIGAYSYPVSAAGRASPCSFSERYYGGAGGSPGKKADGRASPLYASYKADSFSEGD
DLSQGHLAEPCFLRAGGDLSLSPGRSADPLPGYAPSEGGDGDRLGVQLCGTASSPEPEQG
SRDSLEPSSMEASPEMHPAARLSPQQAFPRTGGSGLSRKDSLTKAQLYGTLLN
>sp|Q5SZJ8|BEND6_HUMAN BEN domain-containing protein 6 OS=Homo sapiens GN=BEND6 PE=1 SV=2
MQKIVQTDEITNTQAFRKGKRKRTETMDSENANSDMDKGQRDPYSGNAFLPGESSSEDEE
PLAELSKEELCAKIKSLKEKLTNTRKENSRLRQSLVMLQVLPQAVTQFEELVGMAEALLK
GGGTMSTSASTLWRATNNSSPDSFASTCSNSNSNSSSPVSLKPEEEHQTDEKQFQIEKWQ
IARCNKSKPQKFINDLMQVLYTNEYMATHSLTGAKSSTSRDKAVKPAMNQNEVQEIIGVT
KQLFPNTDDVSIRRMIGQKLNNCTKKPNLSKNLNSQDIK
>sp|Q9HBH7|BEX1_HUMAN Protein BEX1 OS=Homo sapiens GN=BEX1 PE=1 SV=2
MESKEKRAVNSLSMENANQENEEKEQVANKGEPLALPLDAGEYCVPRGNRRRFRVRQPIL
QYRWDMMHRLGEPQARMREENMERIGEEVRQLMEKLREKQLSHSLRAVSTDPPHHDHHDE
FCLMP
>sp|P16442|BGAT_HUMAN Histo-blood group ABO system transferase OS=Homo sapiens GN=ABO PE=1 SV=2
MAEVLRTLAGKPKCHALRPMILFLIMLVLVLFGYGVLSPRSLMPGSLERGFCMAVREPDH
LQRVSLPRMVYPQPKVLTPCRKDVLVVTPWLAPIVWEGTFNIDILNEQFRLQNTTIGLTV
FAIKKYVAFLKLFLETAEKHFMVGHRVHYYVFTDQPAAVPRVTLGTGRQLSVLEVRAYKR
WQDVSMRRMEMISDFCERRFLSEVDYLVCVDVDMEFRDHVGVEILTPLFGTLHPGFYGSS
REAFTYERRPQSQAYIPKDEGDFYYLGGFFGGSVQEVQRLTRACHQAMMVDQANGIEAVW
HDESHLNKYLLRHKPTKVLSPEYLWDQQLLGWPAVLRKLRFTAVPKNHQAVRNP
>sp|Q7RTS1|BHA15_HUMAN Class A basic helix-loop-helix protein 15 OS=Homo sapiens GN=BHLHA15 PE=1 SV=1
MKTKNRPPRRRAPVQDTEATPGEGTPDGSLPNPGPEPAKGLRSRPARAAARAPGEGRRRR
PGPSGPGGRRDSSIQRRLESNERERQRMHKLNNAFQALREVIPHVRADKKLSKIETLTLA
KNYIKSLTATILTMSSSRLPGLEGPGPKLYQHYQQQQQVAGGALGATEAQPQGHLQRYST
QIHSFREGT
>sp|Q9C0J9|BHE41_HUMAN Class E basic helix-loop-helix protein 41 OS=Homo sapiens GN=BHLHE41 PE=1 SV=1
MDEGIPHLQERQLLEHRDFIGLDYSSLYMCKPKRSMKRDDTKDTYKLPHRLIEKKRRDRI
NECIAQLKDLLPEHLKLTTLGHLEKAVVLELTLKHLKALTALTEQQHQKIIALQNGERSL
KSPIQSDLDAFHSGFQTCAKEVLQYLSRFESWTPREPRCVQLINHLHAVATQFLPTPQLL

TQQVPLSKGTGAPSAAGSAAAPCLERAGQKLEPLAYCVPVIQRTQPSAELAAENDTDTDS
GYGGEAEARPDREKGKGAGASRVTIKQEPPGEDSPAPKRMKLDSRGGGSGGGPGGGAAAA
AAALLGPDPAAAAALLRPDAALLSSLVAFGGGGGAPFPQPAAAAAPFCLPFCFLSPSAAA
AYVQPFLDKSGLEKYLYPAAAAAPFPLLYPGIPAPAAAAAAAAAAAAAAAFPCLSSVLS
PPPEKAGAAAATLLPHEVAPLGAPHPQHPHGRTHLPFAGPREPGNPESSAQEDPSQPGKE
AP
>sp|Q9H2M3|BHMT2_HUMAN S-methylmethionine--homocysteine S-methyltransferase BHMT2 OS=Homo sapiens GN=BHMT2 PE=1 SV=1
MAPAGRPGAKKGILERLESGEVVIGDGSFLITLEKRGYVKAGLWTPEAVIEHPDAVRQLH
MEFLRAGSNVMQTFTFSASEDNMESKWEDVNAAACDLAREVAGKGDALVAGGICQTSIYK
YQKDEARIKKLFRQQLEVFAWKNVDFLIAEYFEHVEEAVWAVEVLKESDRPVAVTMCIGP
EGDMHDITPGECAVRLVKAGASIVGVNCRFGPDTSLKTMELMKEGLEWAGLKAHLMVQPL
GFHAPDCGKEGFVDLPEYPFGLESRVATRWDIQKYAREAYNLGVRYIGGCCGFEPYHIRA
IAEELAPERGFLPPASEKHGSWGSGLDMHTKPWIRARARREYWENLLPASGRPFCPSLSK
PDF
>sp|A1A5D9|BICL2_HUMAN BICD family-like cargo adapter 2 OS=Homo sapiens GN=BICDL2 PE=1 SV=2
MSSPDGPSFPSGPLSGGASPSGDEGFFPFVLERRDSFLGGGPGPEEPEDLALQLQQKEKD
LLLAAELGKMLLERNEELRRQLETLSAQHLEREERLQQENHELRRGLAARGAEWEARAVE
LEGDVEALRAQLGEQRSEQQDSGRERARALSELSEQNLRLSQQLAQASQTEQELQRELDA
LRGQCQAQALAGAELRTRLESLQGENQMLQSRRQDLEAQIRGLREEVEKGEGRLQTTHEE
LLLLRRERREHSLELERARSEAGEALSALRRLQRRVSELEEESRLQDADVSAASLQSELA
HSLDDGDQGQGADAPGDTPTTRSPKTRKASSPQPSPPEEILEPPKKRTSLSPAEILEEKE
VEVAKLQDEISLQQAELQSLREELQRQKELRAQEDPGEALHSALSDRDEAVNKALELSLQ
LNRVSLERDSLSRELLRAIRQKVALTQELEAWQDDMQVVIGQQLRSQRQKELSASASSST
PRRAAPRFSLRLGPGPAGGFLSNLFRRT
>sp|Q8TDH9|BL1S5_HUMAN Biogenesis of lysosome-related organelles complex 1 subunit 5 OS=Homo sapiens GN=BLOC1S5 PE=1 SV=1
MSGGGTETPVGCEAAPGGGSKKRDSLGTAGSAHLIIKDLGEIHSRLLDHRPVIQGETRYF
VKEFEEKRGLREMRVLENLKNMIHETNEHTLPKCRDTMRDSLSQVLQRLQAANDSVCRLQ
QREQERKKIHSDHLVASEKQHMLQWDNFMKEQPNKRAEVDEEHRKAMERLKEQYAEMEKD
LAKFSTF
>sp|P12644|BMP4_HUMAN Bone morphogenetic protein 4 OS=Homo sapiens GN=BMP4 PE=1 SV=1
MIPGNRMLMVVLLCQVLLGGASHASLIPETGKKKVAEIQGHAGGRRSGQSHELLRDFEAT
LLQMFGLRRRPQPSKSAVIPDYMRDLYRLQSGEEEEEQIHSTGLEYPERPASRANTVRSF
HHEEHLENIPGTSENSAFRFLFNLSSIPENEVISSAELRLFREQVDQGPDWERGFHRINI
YEVMKPPAEVVPGHLITRLLDTRLVHHNVTRWETFDVSPAVLRWTREKQPNYGLAIEVTH
LHQTRTHQGQHVRISRSLPQGSGNWAQLRPLLVTFGHDGRGHALTRRRRAKRSPKHHSQR
ARKKNKNCRRHSLYVDFSDVGWNDWIVAPPGYQAFYCHGDCPFPLADHLNSTNHAIVQTL
VNSVNSSIPKACCVPTELSAISMLYLDEYDKVVLKNYQEMVVEGCGCR
>sp|Q1RMZ1|BMT2_HUMAN Probable methyltransferase BMT2 homolog OS=Homo sapiens GN=BMT2 PE=2 SV=1
MEPGAGGRNTARAQRAGSPNTPPPREQERKLEQEKLSGVVKSVHRRLRKKYREVGDFDKI
WREHCEDEETLCEYAVAMKNLADNHWAKTCEGEGRIEWCCSVCREYFQNGGKRKALEKDE

KRAVLATKTTPALNMHESSQLEGHLTNLSFTNPEFITELLQASGKIRLLDVGSCFNPFLK
FEEFLTVGIDIVPAVESVYKCDFLNLQLQQPLQLAQDAIDAFLKQLKNPIDSLPGELFHV
VVFSLLLSYFPSPYQRWICCKKAHELLVLNGLLLIITPDSSHQNRHAMMMKSWKIAIESL
GFKRFKYSKFSHMHLMAFRKISLKTTSDLVSRNYPGMLYIPQDFNSIEDEEYSNPSCYVR
SDIEDEQLAYGFTELPDAPYDSDSGESQASSIPFYELEDPILLLS
>sp|P51813|BMX_HUMAN Cytoplasmic tyrosine-protein kinase BMX OS=Homo sapiens GN=BMX PE=1 SV=1
MDTKSILEELLLKRSQQKKKMSPNNYKERLFVLTKTNLSYYEYDKMKRGSRKGSIEIKKI
RCVEKVNLEEQTPVERQYPFQIVYKDGLLYVYASNEESRSQWLKALQKEIRGNPHLLVKY
HSGFFVDGKFLCCQQSCKAAPGCTLWEAYANLHTAVNEEKHRVPTFPDRVLKIPRAVPVL
KMDAPSSSTTLAQYDNESKKNYGSQPPSSSTSLAQYDSNSKKIYGSQPNFNMQYIPREDF
PDWWQVRKLKSSSSSEDVASSNQKERNVNHTTSKISWEFPESSSSEEEENLDDYDWFAGN
ISRSQSEQLLRQKGKEGAFMVRNSSQVGMYTVSLFSKAVNDKKGTVKHYHVHTNAENKLY
LAENYCFDSIPKLIHYHQHNSAGMITRLRHPVSTKANKVPDSVSLGNGIWELKREEITLL
KELGSGQFGVVQLGKWKGQYDVAVKMIKEGSMSEDEFFQEAQTMMKLSHPKLVKFYGVCS
KEYPIYIVTEYISNGCLLNYLRSHGKGLEPSQLLEMCYDVCEGMAFLESHQFIHRDLAAR
NCLVDRDLCVKVSDFGMTRYVLDDQYVSSVGTKFPVKWSAPEVFHYFKYSSKSDVWAFGI
LMWEVFSLGKQPYDLYDNSQVVLKVSQGHRLYRPHLASDTIYQIMYSCWHELPEKRPTFQ
QLLSSIEPLREKDKH
>sp|Q9BWV1|BOC_HUMAN Brother of CDO OS=Homo sapiens GN=BOC PE=1 SV=1
MLRGTMTAWRGMRPEVTLACLLLATAGCFADLNEVPQVTVQPASTVQKPGGTVILGCVVE
PPRMNVTWRLNGKELNGSDDALGVLITHGTLVITALNNHTVGRYQCVARMPAGAVASVPA
TVTLANLQDFKLDVQHVIEVDEGNTAVIACHLPESHPKAQVRYSVKQEWLEASRGNYLIM
PSGNLQIVNASQEDEGMYKCAAYNPVTQEVKTSGSSDRLRVRRSTAEAARIIYPPEAQTI
IVTKGQSLILECVASGIPPPRVTWAKDGSSVTGYNKTRFLLSNLLIDTTSEEDSGTYRCM
ADNGVGQPGAAVILYNVQVFEPPEVTMELSQLVIPWGQSAKLTCEVRGNPPPSVLWLRNA
VPLISSQRLRLSRRALRVLSMGPEDEGVYQCMAENEVGSAHAVVQLRTSRPSITPRLWQD
AELATGTPPVSPSKLGNPEQMLRGQPALPRPPTSVGPASPQCPGEKGQGAPAEAPIILSS
PRTSKTDSYELVWRPRHEGSGRAPILYYVVKHRKVTNSSDDWTISGIPANQHRLTLTRLD
PGSLYEVEMAAYNCAGEGQTAMVTFRTGRRPKPEIMASKEQQIQRDDPGASPQSSSQPDH
GRLSPPEAPDRPTISTASETSVYVTWIPRGNGGFPIQSFRVEYKKLKKVGDWILATSAIP
PSRLSVEITGLEKGTSYKFRVRALNMLGESEPSAPSRPYVVSGYSGRVYERPVAGPYITF
TDAVNETTIMLKWMYIPASNNNTPIHGFYIYYRPTDSDNDSDYKKDMVEGDKYWHSISHL
QPETSYDIKMQCFNEGGESEFSNVMICETKARKSSGQPGRLPPPTLAPPQPPLPETIERP
VGTGAMVARSSDLPYLIVGVVLGSIVLIIVTFIPFCLWRAWSKQKHTTDLGFPRSALPPS
CPYTMVPLGGLPGHQASGQPYLSGISGRACANGIHMNRGCPSAAVGYPGMKPQQHCPGEL
QQQSDTSSLLRQTHLGNGYDPQSHQITRGPKSSPDEGSFLYTLPDDSTHQLLQPHHDCCQ
RQEQPAAVGQSGVRRAPDSPVLEAVWDPPFHSGPPCCLGLVPVEEVDSPDSCQVSGGDWC
PQHPVGAYVGQEPGMQLSPGPLVRVSFETPPLTI
>sp|Q96IK1|BOD1_HUMAN Biorientation of chromosomes in cell division protein 1 OS=Homo sapiens GN=BOD1 PE=1 SV=2
MADGGGGGGTGAVGGGGTSQASAGAATGATGASGGGGPINPASLPPGDPQLIALIVEQLK
SRGLFDSFRRDCLADVDTKPAYQNLRQKVDNFVSTHLDKQEWNPTMNKNQLRNGLRQSVV
QSGMLEAGVDRIISQVVDPKLNHIFRPQIERAIHEFLAAQKKAAVPAPPPEPEGQDPPAP

SQDTS
>sp|Q9BQP9|BPIA3_HUMAN BPI fold-containing family A member 3 OS=Homo sapiens GN=BPIFA3 PE=2 SV=3
MMCPLWRLLIFLGLLALPLAPHKQPWPGLAQAHRDNKSTLARIIAQGLIKHNAESRIQNI
HFGDRLNASAQVAPGLVGWLISGRKHQQQQESSINITNIQLDCGGIQISFHKEWFSANIS
LEFDLELRPSFDNNIVKMCAHMSIVVEFWLEKDEFGRRDLVIGKCDAEPSSVHVAILTEA
IPPKMNQFLYNLKENLQKVLPHMVESQVCPLIGEILGQLDVKLLKSLIEQEAAHEPTHHE
TSQPSACQAGESPS
>sp|P50747|BPL1_HUMAN Biotin--protein ligase OS=Homo sapiens GN=HLCS PE=1 SV=1
MEDRLHMDNGLVPQKIVSVHLQDSTLKEVKDQVSNKQAQILEPKPEPSLEIKPEQDGMEH
VGRDDPKALGEEPKQRRGSASGSEPAGDSDRGGGPVEHYHLHLSSCHECLELENSTIESV
KFASAENIPDLPYDYSSSLESVADETSPEREGRRVNLTGKAPNILLYVGSDSQEALGRFH
EVRSVLADCVDIDSYILYHLLEDSALRDPWTDNCLLLVIATRESIPEDLYQKFMAYLSQG
GKVLGLSSSFTFGGFQVTSKGALHKTVQNLVFSKADQSEVKLSVLSSGCRYQEGPVRLSP
GRLQGHLENEDKDRMIVHVPFGTRGGEAVLCQVHLELPPSSNIVQTPEDFNLLKSSNFRR
YEVLREILTTLGLSCDMKQVPALTPLYLLSAAEEIRDPLMQWLGKHVDSEGEIKSGQLSL
RFVSSYVSEVEITPSCIPVVTNMEAFSSEHFNLEIYRQNLQTKQLGKVILFAEVTPTTMR
LLDGLMFQTPQEMGLIVIAARQTEGKGRGGNVWLSPVGCALSTLLISIPLRSQLGQRIPF
VQHLMSVAVVEAVRSIPEYQDINLRVKWPNDIYYSDLMKIGGVLVNSTLMGETFYILIGC
GFNVTNSNPTICINDLITEYNKQHKAELKPLRADYLIARVVTVLEKLIKEFQDKGPNSVL
PLYYRYWVHSGQQVHLGSAEGPKVSIVGLDDSGFLQVHQEGGEVVTVHPDGNSFDMLRNL
ILPKRR
>sp|P46736|BRCC3_HUMAN Lys-63-specific deubiquitinase BRCC36 OS=Homo sapiens GN=BRCC3 PE=1 SV=2
MAVQVVQAVQAVHLESDAFLVCLNHALSTEKEEVMGLCIGELNDDTRSDSKFAYTGTEMR
TVAEKVDAVRIVHIHSVIILRRSDKRKDRVEISPEQLSAASTEAERLAELTGRPMRVVGW
YHSHPHITVWPSHVDVRTQAMYQMMDQGFVGLIFSCFIEDKNTKTGRVLYTCFQSIQAQK
SSESLHGPRDFWSSSQHISIEGQKEEERYERIEIPIHIVPHVTIGKVCLESAVELPKILC
QEEQDAYRRIHSLTHLDSVTKIHNGSVFTKNLCSQMSAVSGPLLQWLEDRLEQNQQHLQE
LQQEKEELMQELSSLE
>sp|P25440|BRD2_HUMAN Bromodomain-containing protein 2 OS=Homo sapiens GN=BRD2 PE=1 SV=2
MLQNVTPHNKLPGEGNAGLLGLGPEAAAPGKRIRKPSLLYEGFESPTMASVPALQLTPAN
PPPPEVSNPKKPGRVTNQLQYLHKVVMKALWKHQFAWPFRQPVDAVKLGLPDYHKIIKQP
MDMGTIKRRLENNYYWAASECMQDFNTMFTNCYIYNKPTDDIVLMAQTLEKIFLQKVASM
PQEEQELVVTIPKNSHKKGAKLAALQGSVTSAHQVPAVSSVSHTALYTPPPEIPTTVLNI
PHPSVISSPLLKSLHSAGPPLLAVTAAPPAQPLAKKKGVKRKADTTTPTPTAILAPGSPA
SPPGSLEPKAARLPPMRRESGRPIKPPRKDLPDSQQQHQSSKKGKLSEQLKHCNGILKEL
LSKKHAAYAWPFYKPVDASALGLHDYHDIIKHPMDLSTVKRKMENRDYRDAQEFAADVRL
MFSNCYKYNPPDHDVVAMARKLQDVFEFRYAKMPDEPLEPGPLPVSTAMPPGLAKSSSES
SSEESSSESSSEEEEEEDEEDEEEEESESSDSEEERAHRLAELQEQLRAVHEQLAALSQG
PISKPKRKREKKEKKKKRKAEKHRGRAGADEDDKGPRAPRPPQPKKSKKASGSGGGSAAL
GPSGFGPSGGSGTKLPKKATKTAPPALPTGYDSEEEEESRPMSYDEKRQLSLDINKLPGE
KLGRVVHIIQAREPSLRDSNPEEIEIDFETLKPSTLRELERYVLSCLRKKPRKPYTIKKP
VGKTKEELALEKKRELEKRLQDVSGQLNSTKKPPKKANEKTESSSAQQVAVSRLSASSSS

SDSSSSSSSSSSSSDTSDSDSG
>sp|Q9H8M2|BRD9_HUMAN Bromodomain-containing protein 9 OS=Homo sapiens GN=BRD9 PE=1 SV=2
MGKKHKKHKAEWRSSYEDYADKPLEKPLKLVLKVGGSEVTELSGSGHDSSYYDDRSDHER
ERHKEKKKKKKKKSEKEKHLDDEERRKRKEEKKRKREREHCDTEGEADDFDPGKKVEVEP
PPDRPVRACRTQPAENESTPIQQLLEHFLRQLQRKDPHGFFAFPVTDAIAPGYSMIIKHP
MDFGTMKDKIVANEYKSVTEFKADFKLMCDNAMTYNRPDTVYYKLAKKILHAGFKMMSKQ
AALLGNEDTAVEEPVPEVVPVQVETAKKSKKPSREVISCMFEPEGNACSLTDSTAEEHVL
ALVEHAADEARDRINRFLPGGKMGYLKRNGDGSLLYSVVNTAEPDADEEETHPVDLSSLS
SKLLPGFTTLGFKDERRNKVTFLSSATTALSMQNNSVFGDLKSDEMELLYSAYGDETGVQ
CALSLQEFVKDAGSYSKKVVDDLLDQITGGDHSRTLFQLKQRRNVPMKPPDEAKVGDTLG
DSSSSVLEFMSMKSYPDVSVDISMLSSLGKVKKELDPDDSHLNLDETTKLLQDLHEAQAE
RGGSRPSSNLSSLSNASERDQHHLGSPSRLSVGEQPDVTHDPYEFLQSPEPAASAKT
>sp|Q58F21|BRDT_HUMAN Bromodomain testis-specific protein OS=Homo sapiens GN=BRDT PE=1 SV=4
MSLPSRQTAIIVNPPPPEYINTKKNGRLTNQLQYLQKVVLKDLWKHSFSWPFQRPVDAVK
LQLPDYYTIIKNPMDLNTIKKRLENKYYAKASECIEDFNTMFSNCYLYNKPGDDIVLMAQ
ALEKLFMQKLSQMPQEEQVVGVKERIKKGTQQNIAVSSAKEKSSPSATEKVFKQQEIPSV
FPKTSISPLNVVQGASVNSSSQTAAQVTKGVKRKADTTTPATSAVKASSEFSPTFTEKSV
ALPPIKENMPKNVLPDSQQQYNVVKTVKVTEQLRHCSEILKEMLAKKHFSYAWPFYNPVD
VNALGLHNYYDVVKNPMDLGTIKEKMDNQEYKDAYKFAADVRLMFMNCYKYNPPDHEVVT
MARMLQDVFETHFSKIPIEPVESMPLCYIKTDITETTGRENTNEASSEGNSSDDSEDERV
KRLAKLQEQLKAVHQQLQVLSQVPFRKLNKKKEKSKKEKKKEKVNNSNENPRKMCEQMRL
KEKSKRNQPKKRKQQFIGLKSEDEDNAKPMNYDEKRQLSLNINKLPGDKLGRVVHIIQSR
EPSLSNSNPDEIEIDFETLKASTLRELEKYVSACLRKRPLKPPAKKIMMSKEELHSQKKQ
ELEKRLLDVNNQLNSRKRQTKSDKTQPSKAVENVSRLSESSSSSSSSESESSSSDLSSS
DSSDSESEMFPKFTEVKPNDSPSKENVKKMKNECIPPEGRTGVTQIGYCVQDTTSANTTL
VHQTTPSHVMPPNHHQLAFNYQELEHLQTVKNISPLQILPPSGDSEQLSNGITVMHPSGD
SDTTMLESECQAPVQKDIKIKNADSWKSLGKPVKPSGVMKSSDELFNQFRKAAIEKEVKA
RTQELIRKHLEQNTKELKASQENQRDLGNGLTVESFSNKIQNKCSGEEQKEHQQSSEAQD
KSKLWLLKDRDLARQKEQERRRREAMVGTIDMTLQSDIMTMFENNFD
>sp|Q96KV6|BT2A3_HUMAN Putative butyrophilin subfamily 2 member A3 OS=Homo sapiens GN=BTN2A3P PE=5 SV=2
MEPAAALHFSRPASLLLLLSLCALVSAQVTVVGPTDPILAMVGENTTLRCCLSPEENAED
MEVRWFQSQFSPAVFVYKGGRERTEEQKEEYRGRTTFVSKDSRGSVALIIHNVTAEDNGI
YQCYFQEGRSCNEAILHLVVAGLDSEPVIEMRDHEDGGIQLECISGGWYPKPLTVWRDPY
GEVVPALKEVSTPDADSLFMVTTAVIIRDKSVRNVSCSINDTLLGQKKESVIFIPESFMP
SRSPCVVILPVIMIILMIPIAICIYWINNLQKEKKDSHLMTFNLCLSLAGWRRTFLHAAN
VVLDQDTGHPYLFVSEDKRSVTLDPSRESIPGNPERFDSQLCVLGQESFASGKHYLEVDV
ENVIEWTVGICRDNVERKWEVPLLPQNGFWTLEMHKRKYWALTSLKWILSLEEPLCQVGI
FLDYEAGDVSFYNMRDRSHIYTFPHSAFSVPVRPFFSLGSYDSQILICSAFTGASGVTVP
EEGWTLHRAGTHHSPQNQFPSLTAMETSPGHLSSHCTMPLVEDTPSSPLVTQENIFQLPL
SHPLQTSAPVHLLIRCGFSSSFGCNYGMESRHRELVVPQLPARKKV
>sp|O00481|BT3A1_HUMAN Butyrophilin subfamily 3 member A1 OS=Homo sapiens GN=BTN3A1 PE=1 SV=3

MKMASFLAFLLLNFRVCLLLLQLLMPHSAQFSVLGPSGPILAMVGEDADLPCHLFPTMSA
ETMELKWVSSSLRQVVNVYADGKEVEDRQSAPYRGRTSILRDGITAGKAALRIHNVTASD
SGKYLCYFQDGDFYEKALVELKVAALGSDLHVDVKGYKDGGIHLECRSTGWYPQPQIQWS
NNKGENIPTVEAPVVADGVGLYAVAASVIMRGSSGEGVSCTIRSSLLGLEKTASISIADP
FFRSAQRWIAALAGTLPVLLLLLGGAGYFLWQQQEEKKTQFRKKKREQELREMAWSTMKQ
EQSTRVKLLEELRWRSIQYASRGERHSAYNEWKKALFKPADVILDPKTANPILLVSEDQR
SVQRAKEPQDLPDNPERFNWHYCVLGCESFISGRHYWEVEVGDRKEWHIGVCSKNVQRKG
WVKMTPENGFWTMGLTDGNKYRTLTEPRTNLKLPKPPKKVGVFLDYETGDISFYNAVDGS
HIHTFLDVSFSEALYPVFRILTLEPTALTICPA
>sp|O00478|BT3A3_HUMAN Butyrophilin subfamily 3 member A3 OS=Homo sapiens GN=BTN3A3 PE=1 SV=1
MKMASSLAFLLLNFHVSLFLVQLLTPCSAQFSVLGPSGPILAMVGEDADLPCHLFPTMSA
ETMELRWVSSSLRQVVNVYADGKEVEDRQSAPYRGRTSILRDGITAGKAALRIHNVTASD
SGKYLCYFQDGDFYEKALVELKVAALGSDLHIEVKGYEDGGIHLECRSTGWYPQPQIKWS
DTKGENIPAVEAPVVADGVGLYAVAASVIMRGSSGGGVSCIIRNSLLGLEKTASISIADP
FFRSAQPWIAALAGTLPISLLLLAGASYFLWRQQKEKIALSRETERERERMKEMGYAATEQ
EISLREKLQEELKWRKIQYMARGEKSLAYHEWKMALFKPADVILDPDTANAILLVSEDQR
SVQRAEEPRDLPDNPERFEWRYCVLGCENFTSGRHYWEVEVGDRKEWHIGVCSKNVERKK
GWVKMTPENGYWTMGLTDGNKYRALTEPRTNLKLPEPPRKVGIFLDYETGEISFYNATDG
SHIYTFPHASFSEPLYPVFRILTLEPTALTICPIPKEVESSPDPDLVPDHSLETPLTPGL
ANESGEPQAEVTSLLLPAHPGAEVSPSATTNQNHKLQARTEALY
>sp|Q8WXE1|ATRIP_HUMAN ATR-interacting protein OS=Homo sapiens GN=ATRIP PE=1 SV=1
MAGTSAPGSKRRSEPPAPRPGPPPGTGHPPSKRARGFSAAAAPDPDDPFGAHGDFTADDL
EELDTLASQALSQCPAAARDVSSDHKVHRLLDGMSKNPSGKNRETVPIKDNFELEVLQAQ
YKELKEKMKVMEEEVLIKNGEIKILRDSLHQTESVLEEQRRSHFLLEQEKTQALSDKEKE
FSKKLQSLQSELQFKDAEMNELRTKLQTSERANKLAAPSVSHVSPRKNPSVVIKPEACSP
QFGKTSFPTKESFSANMSLPHPCQTESGYKPLVGREDSKPHSLRGDSIKQEEAQKSFVDS
WRQRSNTQGSILINLLLKQPLIPGSSLSLCHLLSSSSESPAGTPLQPPGFGSTLAGMSGL
RTTGSYDGSFSLSALREAQNLAFTGLNLVARNECSRDGDPAEGGRRAFPLCQLPGAVHFL
PLVQFFIGLHCQALQDLAAAKRSGAPGDSPTHSSCVSSGVETNPEDSVCILEGFSVTALS
ILQHLVCHSGAVVSLLLSGVGADSAAGEGNRSLVHRLSDGDMTSALRGVADDQGQHPLLK
MLLHLLAFSSAATGHLQASVLTQCLKVLVKLAENTSCDFLPRFQCVFQVLPKCLSPETPL
PSVLLAVELLSLLADHDQLAPQLCSHSEGCLLLLLYMYITSRPDRVALETQWLQLEQEVV
WLLAKLGVQSPLPPVTGSNCQCNVEVVRALTVMLHRQWLTVRRAGGPPRTDQQRRTVRCL
RDTVLLLHGLSQKDKLFMMHCVEVLHQFDQVMPGVSMLIRGLPDVTDCEEAALDDLCAAE
TDVEDPEVECG
>sp|P46100|ATRX_HUMAN Transcriptional regulator ATRX OS=Homo sapiens GN=ATRX PE=1 SV=5
MTAEPMSESKLNTLVQKLHDFLAHSSEESEETSSPPRLAMNQNTDKISGSGSNSDMMENS
KEEGTSSSEKSKSSGSSRSKRKPSIVTKYVESDDEKPLDDETVNEDASNENSENDITMQS
LPKGTVIVQPEPVLNEDKDDFKGPEFRSRSKMKTENLKKRGEDGLHGIVSCTACGQQVNH
FQKDSIYRHPSLQVLICKNCFKYYMSDDISRDSDGMDEQCRWCAEGGNLICCDFCHNAFC
KKCILRNLGRKELSTIMDENNQWYCYICHPEPLLDLVTACNSVFENLEQLLQQNKKKIKV
DSEKSNKVYEHTSRFSPKKTSSNCNGEEKKLDDSCSGSVTYSYSALIVPKEMIKKAKKLI
ETTANMNSSYVKFLKQATDNSEISSATKLRQLKAFKSVLADIKKAHLALEEDLNSEFRAM

DAVNKEKNTKEHKVIDAKFETKARKGEKPCALEKKDISKSEAKLSRKQVDSEHMHQNVPT
EEQRTNKSTGGEHKKSDRKEEPQYEPANTSEDLDMDIVSVPSSVPEDIFENLETAMEVQS
SVDHQGDGSSGTEQEVESSSVKLNISSKDNRGGIKSKTTAKVTKELYVKLTPVSLSNSPI
KGADCQEVPQDKDGYKSCGLNPKLEKCGLGQENSDNEHLVENEVSLLLEESDLRRSPRVK
TTPLRRPTETNPVTSNSDEECNETVKEKQKLSVPVRKKDKRNSSDSAIDNPKPNKLPKSK
QSETVDQNSDSDEMLAILKEVSRMSHSSSSDTDINEIHTNHKTLYDLKTQAGKDDKGKRK
RKSSTSGSDFDTKKGKSAKSSIISKKKRQTQSESSNYDSELEKEIKSMSKIGAARTTKKR
IPNTKDFDSSEDEKHSKKGMDNQGHKNLKTSQEGSSDDAERKQERETFSSAEGTVDKDTT
IMELRDRLPKKQQASASTDGVDKLSGKEQSFTSLEVRKVAETKEKSKHLKTKTCKKVQDG
LSDIAEKFLKKDQSDETSEDDKKQSKKGTEEKKKPSDFKKKVIKMEQQYESSSDGTEKLP
EREEICHFPKGIKQIKNGTTDGEKKSKKIRDKTSKKKDELSDYAEKSTGKGDSCDSSEDK
KSKNGAYGREKKRCKLLGKSSRKRQDCSSSDTEKYSMKEDGCNSSDKRLKRIELRERRNL
SSKRNTKEIQSGSSSSDAEESSEDNKKKKQRTSSKKKAVIVKEKKRNSLRTSTKRKQADI
TSSSSSDIEDDDQNSIGEGSSDEQKIKPVTENLVLSSHTGFCQSSGDEALSKSVPVTVDD
DDDDNDPENRIAKKMLLEEIKANLSSDEDGSSDDEPEEGKKRTGKQNEENPGDEEAKNQV
NSESDSDSEESKKPRYRHRLLRHKLTVSDGESGEEKKTKPKEHKEVKGRNRRKVSSEDSE
DSDFQESGVSEEVSESEDEQRPRTRSAKKAELEENQRSYKQKKKRRRIKVQEDSSSENKS
NSEEEEEEKEEEEEEEEEEEEEEEDENDDSKSPGKGRKKIRKILKDDKLRTETQNALKEE
EERRKRIAERERERERKLREVIEIEDASPTKCPITTKLVLDEDEETKEPLVQVHRNMVIKL
KPHQVDGVQFMWDCCCESVKKTKKSPGSGCILAHCMGLGKTLQVVSFLHTVLLCDKLDFS
TALVVCPLNTALNWMNEFEKWQEGLKDDEKLEVSELATVKRPQERSYMLQRWQEDGGVMI
IGYEMYRNLAQGRNVKSRKLKEIFNKALVDPGPDFVVCDEGHILKNEASAVSKAMNSIRS
RRRIILTGTPLQNNLIEYHCMVNFIKENLLGSIKEFRNRFINPIQNGQCADSTMVDVRVM
KKRAHILYEMLAGCVQRKDYTALTKFLPPKHEYVLAVRMTSIQCKLYQYYLDHLTGVGNN
SEGGRGKAGAKLFQDFQMLSRIWTHPWCLQLDYISKENKGYFDEDSMDEFIASDSDETSM
SLSSDDYTKKKKKGKKGKKDSSSSGSGSDNDVEVIKVWNSRSRGGGEGNVDETGNNPSVS
LKLEESKATSSSNPSSPAPDWYKDFVTDADAEVLEHSGKMVLLFEILRMAEEIGDKVLVF
SQSLISLDLIEDFLELASREKTEDKDKPLIYKGEGKWLRNIDYYRLDGSTTAQSRKKWAE
EFNDETNVRGRLFIISTKAGSLGINLVAANRVIIFDASWNPSYDIQSIFRVYRFGQTKPV
YVYRFLAQGTMEDKIYDRQVTKQSLSFRVVDQQQVERHFTMNELTELYTFEPDLLDDPNS
EKKKKRDTPMLPKDTILAELLQIHKEHIVGYHEHDSLLDHKEEEELTEEERKAAWAEYEA
EKKGLTMRFNIPTGTNLPPVSFNSQTPYIPFNLGALSAMSNQQLEDLINQGREKVVEATN
SVTAVRIQPLEDIISAVWKENMNLSEAQVQALALSRQASQELDVKRREAIYNDVLTKQQM
LISCVQRILMNRRLQQQYNQQQQQMTYQQATLGHLMMPKPPNLIMNPSNYQQIDMRGMY
QPVAGGMQPPPLQRAPPPMRSKNPGPSQGKSM
>sp|Q8WXS8|ATS14_HUMAN A disintegrin and metalloproteinase with thrombospondin motifs 14 OS=Homo sapiens GN=ADAMTS14 PE=2 SV=2
MAPLRALLSYLLPLHCALCAAAGSRTPELHLSGKLSDYGVTVPCSTDFRGRFLSHVVSGP
AAASAGSMVVDTPPTLPRHSSHLRVARSPLHPGGTLWPGRVGRHSLYFNVTVFGKELHLR
LRPNRRLVVPGSSVEWQEDFRELFRQPLRQECVYTGGVTGMPGAAVAISNCDGLAGLIRT
DSTDFFIEPLERGQQEKEASGRTHVVYRREAVQQEWAEPDGDLHNEAFGLGDLPNLLGLV
GDQLGDTERKRRHAKPGSYSIEVLLVVDDSVVRFHGKEHVQNYVLTLMNIVDEIYHDESL
GVHINIALVRLIMVGYRQSLSLIERGNPSRSLEQVCRWAHSQQRQDPSHAEHHDHVVFLT
RQDFGPSGYAPVTGMCHPLRSCALNHEDGFSSAFVIAHETGHVLGMEHDGQGNGCADETS

LGSVMAPLVQAAFHRFHWSRCSKLELSRYLPSYDCLLDDPFDPAWPQPPELPGINYSMDE
QCRFDFGSGYQTCLAFRTFEPCKQLWCSHPDNPYFCKTKKGPPLDGTECAPGKWCFKGHC
IWKSPEQTYGQDGGWSSWTKFGSCSRSCGGGVRSRSRSCNNPSPAYGGRLCLGPMFEYQV
CNSEECPGTYEDFRAQQCAKRNSYYVHQNAKHSWVPYEPDDDAQKCELICQSADTGDVVF
MNQVVHDGTRCSYRDPYSVCARGECVPVGCDKEVGSMKADDKCGVCGGDNSHCRTVKGTL
GKASKQAGALKLVQIPAGARHIQIEALEKSPHRIVVKNQVTGSFILNPKGKEATSRTFTA
MGLEWEDAVEDAKESLKTSGPLPEAIAILALPPTEGGPRSSLAYKYVIHEDLLPLIGSNN
VLLEEMDTYEWALKSWAPCSKACGGGIQFTKYGCRRRRDHHMVQRHLCDHKKRPKPIRRR
CNQHPCSQPVWVTEEWGACSRSCGKLGVQTRGIQCLLPLSNGTHKVMPAKACAGDRPEAR
RPCLRVPCPAQWRLGAWSQCSATCGEGIQQRQVVCRTNANSLGHCEGDRPDTVQVCSLPA
CGGNHQNSTVRADVWELGTPEGQWVPQSEPLHPINKISSTEPCTGDRSVFCQMEVLDRYC
SIPGYHRLCCVSCIKKASGPNPGPDPGPTSLPPFSTPGSPLPGPQDPADAAEPPGKPTGS
EDHQHGRATQLPGALDTSSPGTQHPFAPETPIPGASWSISPTTPGGLPWGWTQTPTPVPE
DKGQPGEDLRHPGTSLPAASPVT
>sp|Q9UKP4|ATS7_HUMAN A disintegrin and metalloproteinase with thrombospondin motifs 7 OS=Homo sapiens GN=ADAMTS7 PE=1 SV=2
MPGGPSPRSPAPLLRPLLLLLCALAPGAPGPAPGRATEGRAALDIVHPVRVDAGGSFLSY
ELWPRALRKRDVSVRRDAPAFYELQYRGRELRFNLTANQHLLAPGFVSETRRRGGLGRAH
IRAHTPACHLLGEVQDPELEGGLAAISACDGLKGVFQLSNEDYFIEPLDSAPARPGHAQP
HVVYKRQAPERLAQRGDSSAPSTCGVQVYPELESRRERWEQRQQWRRPRLRRLHQRSVSK
EKWVETLVVADAKMVEYHGQPQVESYVLTIMNMVAGLFHDPSIGNPIHITIVRLVLLEDE
EEDLKITHHADNTLKSFCKWQKSINMKGDAHPLHHDTAILLTRKDLCAAMNRPCETLGLS
HVAGMCQPHRSCSINEDTGLPLAFTVAHELGHSFGIQHDGSGNDCEPVGKRPFIMSPQLL
YDAAPLTWSRCSRQYITRFLDRGWGLCLDDPPAKDIIDFPSVPPGVLYDVSHQCRLQYGA
YSAFCEDMDNVCHTLWCSVGTTCHSKLDAAVDGTRCGENKWCLSGECVPVGFRPEAVDGG
WSGWSAWSICSRSCGMGVQSAERQCTQPTPKYKGRYCVGERKRFRLCNLQACPAGRPSFR
HVQCSHFDAMLYKGQLHTWVPVVNDVNPCELHCRPANEYFAEKLRDAVVDGTPCYQVRAS
RDLCINGICKNVGCDFEIDSGAMEDRCGVCHGNGSTCHTVSGTFEEAEGLGYVDVGLIPA
GAREIRIQEVAEAANFLALRSEDPEKYFLNGGWTIQWNGDYQVAGTTFTYARRGNWENLT
SPGPTKEPVWIQLLFQESNPGVHYEYTIHREAGGHDEVPPPVFSWHYGPWTKCTVTCGRG
VQRQNVYCLERQAGPVDEEHCDPLGRPDDQQRKCSEQPCPARWWAGEWQLCSSSCGPGGL
SRRAVLCIRSVGLDEQSALEPPACEHLPRPPTETPCNRHVPCPATWAVGNWSQCSVTCGE
GTQRRNVLCTNDTGVPCDEAQQPASEVTCSLPLCRWPLGTLGPEGSGSGSSSHELFNEAD
FIPHHLAPRPSPASSPKPGTMGNAIEEEAPELDLPGPVFVDDFYYDYNFINFHEDLSYGP
SEEPDLDLAGTGDRTPPPHSHPAAPSTGSPVPATEPPAAKEEGVLGPWSPSPWPSQAGRS
PPPPSEQTPGNPLINFLPEEDTPIGAPDLGLPSLSWPRVSTDGLQTPATPESQNDFPVGK
DSQSQLPPPWRDRTNEVFKDDEEPKGRGAPHLPPRPSSTLPPLSPVGSTHSSPSPDVAEL
WTGGTVAWEPALEGGLGPVDSELWPTVGVASLLPPPIAPLPEMKVRDSSLEPGTPSFPTP
GPGSWDLQTVAVWGTFLPTTLTGLGHMPEPALNPGPKGQPESLSPEVPLSSRLLSTPAWD
SPANSHRVPETQPLAPSLAEAGPPADPLVVRNAGWQAGNWSECSTTCGLGAVWRPVRCSS
GRDEDCAPAGRPQPARRCHLRPCATWHSGNWSKCSRSCGGGSSVRDVQCVDTRDLRPLRP
FHCQPGPAKPPAHRPCGAQPCLSWYTSSWRECSEACGGGEQQRLVTCPEPGLCEEALRPN
TTRPCNTHPCTQWVVGPWGQCSGPCGGGVQRRLVKCVNTQTGLPEEDSDQCGHEAWPESS
RPCGTEDCEPVEPPRCERDRLSFGFCETLRLLGRCQLPTIRTQCCRSCSPPSHGAPSRGH

QRVARR
>sp|P17735|ATTY_HUMAN Tyrosine aminotransferase OS=Homo sapiens GN=TAT PE=1 SV=1
MDPYMIQMSSKGNLPSILDVHVNVGGRSSVPGKMKGRKARWSVRPSDMAKKTFNPIRAIV
DNMKVKPNPNKTMISLSIGDPTVFGNLPTDPEVTQAMKDALDSGKYNGYAPSIGFLSSRE
EIASYYHCPEAPLEAKDVILTSGCSQAIDLCLAVLANPGQNILVPRPGFSLYKTLAESMG
IEVKLYNLLPEKSWEIDLKQLEYLIDEKTACLIVNNPSNPCGSVFSKRHLQKILAVAARQ
CVPILADEIYGDMVFSDCKYEPLATLSTDVPILSCGGLAKRWLVPGWRLGWILIHDRRDI
FGNEIRDGLVKLSQRILGPCTIVQGALKSILCRTPGEFYHNTLSFLKSNADLCYGALAAI
PGLRPVRPSGAMYLMVGIEMEHFPEFENDVEFTERLVAEQSVHCLPATCFEYPNFIRVVI
TVPEVMMLEACSRIQEFCEQHYHCAEGSQEECDK
>sp|Q9UBB4|ATX10_HUMAN Ataxin-10 OS=Homo sapiens GN=ATXN10 PE=1 SV=1
MAAPRPPPARLSGVMVPAPIQDLEALRALTALFKEQRNRETAPRTIFQRVLDILKKSSHA
VELACRDPSQVENLASSLQLITECFRCLRNACIECSVNQNSIRNLDTIGVAVDLILLFRE
LRVEQESLLTAFRCGLQFLGNIASRNEDSQSIVWVHAFPELFLSCLNHPDKKIVAYSSMI
LFTSLNHERMKELEENLNIAIDVIDAYQKHPESEWPFLIITDLFLKSPELVQAMFPKLNN
QERVTLLDLMIAKITSDEPLTKDDIPVFLRHAELIASTFVDQCKTVLKLASEEPPDDEEA
LATIRLLDVLCEMTVNTELLGYLQVFPGLLERVIDLLRVIHVAGKETTNIFSNCGCVRAE
GDISNVANGFKSHLIRLIGNLCYKNKDNQDKVNELDGIPLILDNCNISDSNPFLTQWVIY
AIRNLTEDNSQNQDLIAKMEEQGLADASLLKKVGFEVEKKGEKLILKSTRDTPKP
>sp|Q99700|ATX2_HUMAN Ataxin-2 OS=Homo sapiens GN=ATXN2 PE=1 SV=2
MRSAAAAPRSPAVATESRRFAAARWPGWRSLQRPARRSGRGGGGAAPGPYPSAAPPPPGP
GPPPSRQSSPPSASDCFGSNGNGGGAFRPGSRRLLGLGGPPRPFVVLLLPLASPGAPPAA
PTRASPLGARASPPRSGVSLARPAPGCPRPACEPVYGPLTMSLKPQQQQQQQQQQQQQQQ
QQQQQQQQPPPAAANVRKPGGSGLLASPAAAPSPSSSSVSSSSATAPSSVVAATSGGGRP
GLGRGRNSNKGLPQSTISFDGIYANMRMVHILTSVVGSKCEVQVKNGGIYEGVFKTYSPK
CDLVLDAAHEKSTESSSGPKREEIMESILFKCSDFVVVQFKDMDSSYAKRDAFTDSAISA
KVNGEHKEKDLEPWDAGELTANEELEALENDVSNGWDPNDMFRYNEENYGVVSTYDSSLS
SYTVPLERDNSEEFLKREARANQLAEEIESSAQYKARVALENDDRSEEEKYTAVQRNSSE
REGHSINTRENKYIPPGQRNREVISWGSGRQNSPRMGQPGSGSMPSRSTSHTSDFNPNSG
SDQRVVNGGVPWPSPCPSPSSRPPSRYQSGPNSLPPRAATPTRPPSRPPSRPSRPPSHPS
AHGSPAPVSTMPKRMSSEGPPRMSPKAQRHPRNHRVSAGRGSISSGLEFVSHNPPSEAAT
PPVARTSPSGGTWSSVVSGVPRLSPKTHRPRSPRQNSIGNTPSGPVLASPQAGIIPTEAV
AMPIPAASPTPASPASNRAVTPSSEAKDSRLQDQRQNSPAGNKENIKPNETSPSFSKAEN
KGISPVVSEHRKQIDDLKKFKNDFRLQPSSTSESMDQLLNKNREGEKSRDLIKDKIEPSA
KDSFIENSSSNCTSGSSKPNSPSISPSILSNTEHKRGPEVTSQGVQTSSPACKQEKDDKE
EKKDAAEQVRKSTLNPNAKEFNPRSFSQPKPSTTPTSPRPQAQPSPSMVGHQQPTPVYTQ
PVCFAPNMMYPVPVSPGVQPLYPIPMTPMPVNQAKTYRAVPNMPQQRQDQHHQSAMMHPA
SAAGPPIAATPPAYSTQYVAYSPQQFPNQPLVQHVPHYQSQHPHVYSPVIQGNARMMAPP
THAQPGLVSSSATQYGAHEQTHAMYACPKLPYNKETSPSFYFAISTGSLAQQYAHPNATL
HPHTPHPQPSATPTGQQQSQHGGSHPAPSPVQHHQHQAAQALHLASPQQQSAIYHAGLAP
TPPSMTPASNTQSPQNSFPAAQQTVFTIHPSHVQPAYTNPPHMAHVPQAHVQSGMVPSHP
TAHAPMMLMTTQPPGGPQAALAQSALQPIPVSTTAHFPYMTHPSVQAHHQQQL
>sp|Q58HT5|AWAT1_HUMAN Acyl-CoA wax alcohol acyltransferase 1 OS=Homo sapiens GN=AWAT1 PE=1 SV=1

MAHSKQPSHFQSLMLLQWPLSYLAIFWILQPLFVYLLFTSLWPLPVLYFAWLFLDWKTPE
RGGRRSAWVRNWCVWTHIRDYFPITILKTKDLSPEHNYLMGVHPHGLLTFGAFCNFCTEA
TGFSKTFPGITPHLATLSWFFKIPFVREYLMAKGVCSVSQPAINYLLSHGTGNLVGIVVG
GVGEALQSVPNTTTLILQKRKGFVRTALQHGAHLVPTFTFGETEVYDQVLFHKDSRMYKF
QSCFRRIFGFYCCVFYGQSFCQGSTGLLPYSRPIVTVVGEPLPLPQIEKPSQEMVDKYHA
LYMDALHKLFDQHKTHYGCSETQKLFFL
>sp|Q3ZCQ2|AX2R_HUMAN Annexin-2 receptor OS=Homo sapiens GN=ANXA2R PE=1 SV=2
MEQHFLGCVKRAWDSAEVAPEPQPPPIVSSEDRGPWPLPLYPVLGEYSLDSCDLGLLSSP
CWRLPGVYWQNGLSPGVQSTLEPSTAKPTEFSWPGTQKQQEAPVEEVGQAEEPDRLRLQQ
LPWSSPLHPWDRQQDTEVCDSGCLLERRHPPALQPWRHLPGFSDCLEWILRVGFAAFSVL
WACCSRICGAKQP
>sp|A6NMY6|AXA2L_HUMAN Putative annexin A2-like protein OS=Homo sapiens GN=ANXA2P2 PE=5 SV=2
MSTVHEILCKLSLEGDHSTPPSAYGSVKAYTNFDAERDALNIETAIKTKGVDEVTIVNIV
TNRDNAQRQDIVFSYQRRTKKELASALKSALSGHLETVILGLLKTPAQYDASELKASMKG
LGTDEDSLIEIICSRTNQELQEINRVYKEMYKTDLEKDIISDTSGDFRKLMVALAKGRRA
EDGSVIDYELIDQDAQDLYDAGVKRKGTDVPKWISIMTERSVPHLQKVFDRYKSYSPYDM
LESIRKEVKGDLENAFLNLVQRIQNKPLYFADQLYDSMKGKGTRDKVLIRIMVSRSEVDM
LKIRSEFKRKYGKSLYYYIQQDTKGDYQKALLYLCGGDD
>sp|Q5VT79|AXA81_HUMAN Annexin A8-like protein 1 OS=Homo sapiens GN=ANXA8L1 PE=2 SV=2
MAWWKAWIEQEGVTVKSSSHFNPDPDAETLYKAMKGIGTNEQAIIDVLTKRSNTQRQQIA
KSFKAQFGKDLTETLKSELSGKFERLIVALMYPPYRYEAKELHDAMKGLGTKEGVIIEIL
ASRTKNQLREIMKAYEEDYGSSLEEDIQADTSGYLERILVCLLQGSRDDVSSFVDPALAL
QDAQDLYAAGEKIRGTDEMKFITILCTRSATHLLRVFEEYEKIANKSIEDSIKSETHGSL
EEAMLTVVKCTQNLHSYFAERLYYAMKGAGTRDGTLIRNIVSRSEIDLNLIKCHFKKMYG
KTLSSMIMEDTSGDYKNALLSLVGSDP
>sp|Q5T1B0|AXDN1_HUMAN Axonemal dynein light chain domain-containing protein 1 OS=Homo sapiens GN=AXDND1 PE=2 SV=1
MSLPKTPSTPLNSTSTSESKKLKVSVAKEGTRGLPELKEKKNMVDRSKLLPTSLQNEFIP
KEVLLSLTYAANAGPCPENLLPPKKIKTPKGTLPRLVDHVWHHPVRRNKFKYLIDHPVSL
TGAGRDISFLYDVTYAKGQTREKAVCPPHLARSLQSHDGVIVPHKPKTLTDTLIPEEFHI
VSSTGVSGLECYDDKYTTLLTDSENRLLLFPSMKPNKRVEVAQLNDVMDTMLERAGVENQ
EYTGPTKMHKLLHILKKEQTIYNMIFHELIRQVSVDCADRGELLSKVRERYVQMLDQIAR
QMIDFYKDLVTQRVMDQRILEELYNFKHVIEELTRELCLVRAHDVKLTKETEKAHKDLAQ
ALLNAEKNAKIVEEYHDLYTLQRERMENDMKKLVAERDIWSSATYELALKVIERNRVILA
RRLYLNEKGWNKYTKHFIILLSNKDTEDLALLQKLTQKWRNLVNKLKQEVEQMEESTSET
LKIVKDGLIKWQEFFNEKDILSPNKGNIFNSVLLDFKQWQKILNEKKEEFTGDVLLSKYD
TLKIIKHLQENWADIGLGIFNRHKSLEGEMPSERQYMEEIIKNIQKLYKEYEIRINGDNG
YSKILPSLISSLDFCSFKLENLEFPDTPLEEWQEIDEKINEMKSHLDILLNLTGIVPQHI
DVDSVSVLQAYIFNMIQQWLLKIGNEINNGNIELQHHMDELHISMIQWMVNLLILMIPNF
TDQDCLLKLEEESAEKHDIGVARLELDAIELTRKLYQYSSYLSSCCKGMVTAMALSKSTN
SHKNATEDLYEVDKLKKECYEWINTCSCLLSNIKGRKITLLTYEEIERLLEEEAVKEFIE
PEIDESFKEDEEESKEDRKLQEENKERAEEQPSTSTEKEKLIRFIGEDENVHSKPLFETD
VLSSWRESAKQGTLAQKYLEAMAVIEHMQEKLLEVENRARQAEEKFEDAYEKLHHTLIKN

KDLEELVMTSRKESKEEKENQDEREVKEEEEQQEEEEVRSAENSSKSPKKGH
>sp|O14977|AZIN1_HUMAN Antizyme inhibitor 1 OS=Homo sapiens GN=AZIN1 PE=1 SV=2
MKGFIDDANYSVGLLDEGTNLGNVIDNYVYEHTLTGKNAFFVGDLGKIVKKHSQWQNVVA
QIKPFYTVKCNSAPAVLEILAALGTGFACSSKNEMALVQELGVPPENIIYISPCKQVSQI
KYAAKVGVNILTCDNEIELKKIARNHPNAKVLLHIATEDNIGGEEGNMKFGTTLKNCRHL
LECAKELDVQIIGVKFHVSSACKESQVYVHALSDARCVFDMAGEIGFTMNMLDIGGGFTG
TEFQLEEVNHVISPLLDIYFPEGSGVKIISEPGSYYVSSAFTLAVNIIAKKVVENDKFPS
GVEKTGSDEPAFMYYMNDGVYGSFASKLSEDLNTIPEVHKKYKEDEPLFTSSLWGPSCDE
LDQIVESCLLPELNVGDWLIFDNMGADSFHEPSAFNDFQRPAIYYMMSFSDWYEMQDAGI
TSDSMMKNFFFVPSCIQLSQEDSFSAEA
>sp|Q9P287|BCCIP_HUMAN BRCA2 and CDKN1A-interacting protein OS=Homo sapiens GN=BCCIP PE=1 SV=1
MASRSKRRAVESGVPQPPDPPVQRDEEEEKEVENEDEDDDDSDKEKDEEDEVIDEEVNIE
FEAYSLSDNDYDGIKKLLQQLFLKAPVNTAELTDLLIQQNHIGSVIKQTDVSEDSNDDMD
EDEVFGFISLLNLTERKGTQCVEQIQELVLRFCEKNCEKSMVEQLDKFLNDTTKPVGLLL
SERFINVPPQIALPMYQQLQKELAGAHRTNKPCGKCYFYLLISKTFVEAGKNNSKKKPSN
KKKAALMFANAEEEFFYEKAILKFNYSVQEESDTCLGGKWSFDDVPMTPLRTVMLIPGDK
MNEIMDKLKEYLSV
>sp|P0C6P0|BCL8_HUMAN Putative protein BCL8 OS=Homo sapiens GN=NBEAP1 PE=5 SV=1
MSCCLSSRVHITRPVLEQFLSFAKYLDGLSHGVPLLKQLCDHILFINPAIWIHTPAKVQL
SLYTYLSAEFIGTATIYTTICRIGTVIKDNAHLKILLLGY
>sp|Q8NDZ0|BEND2_HUMAN BEN domain-containing protein 2 OS=Homo sapiens GN=BEND2 PE=2 SV=2
MSERTQEQDFVIITVDDSDDNNDCSIEMVEVSETADNSTNDIADDSTYVTADNPTDDTAT
QPNFPGGNDGHHRPLQMSYGSGSVTQAGVQWHDHSSLQPQPLGLKQFFHLSLPSSWDDRR
TPPCPVAHGDQIVSQINHPVHLRRYSYNSEEVDFPKRGRFYTPEVQSSISPPAERQETHA
WASPAVTSLESAACHELQEADLSESLSYPRIVSSSSLQQYVAQGGSFPCFGMPWNFISGG
AESTNAVISFANATTAVPMAVLSRRESSLANNPGVVNYSALPENENVGPGRALSSFCFHP
NLEMPERPANSSKNSTETANYPTLMGNYNGQNTASLSVFIPPYFAEKIILTEMPGTTETN
VENNSQTVYYPALSGNTSAPYPASSYLPITSNFESGPQMSYGTMSYSTEMKNNCDQDDAS
ASACLTPDFALLPLNILVKVDTNTENSVNTMNRSTLLDSDSGQDSSSSSVCIPPKYGYLG
DPKRNVRVLKIHLLAVQNMAKPKQAACYLVRILFSKEILISSSVDIHLKDSQSLDPNKMA
ALREYLATTFPTCDLHEHGKDWQDCISGINSMIYCLCSEGKSTPKTVRKNKKRTNRVASA
SADRNDQRGRDGGEGCSWMFQPMNNSKMREKRNLQPNSNAIPEGMREPSTDNPEEPGEAW
SYFGRPWRNIRMPCSVLTLAKTKSCASLSARYLIQKLFTKDVLVQSNVYGNLKHGLCALD
PNKISALREFLQENYPICDLSENGRDWKSCVTSINSGIRSLRHDVRRAEARSQSLPAVTP
PELEQESKPGDPDATDPST
>sp|Q5T5X7|BEND3_HUMAN BEN domain-containing protein 3 OS=Homo sapiens GN=BEND3 PE=1 SV=1
MNSTEFTEDVEEVLKSITVKVETEAEDAALDCSVNSRTSEKHSVDSVLTALQDSSKRKQL
VSDGLLDSVPGVKRRRLIPEALLAGMRNRENSSPCQGNGEQAGRGRSLGNVWPGEEEPCN
DATTPSYKKPLYGISHKIMEKKNPPSGDLLNVYELFEKANASNSPSSLRLLNEPQKRDCG
STGAGTDNDPNIYFLIQKMFYMLNTLTSNMSQLHSKVDLLSLEVSRIKKQVSPTEMVAKF
QPPPEYQLTAAELKQIVDQSLSGGDLACRLLVQLFPELFSDVDFSRGCSACGFAAKRKLE
SLHLQLIRNYVEVYYPSVKDTAVWQAECLPQLNDFFSRFWAQREMEDSQPSGQVASFFEA
EQVDPGHFLDNKDQEEALSLDRSSTIASDHVVDTQDLTEFLDEASSPGEFAVFLLHRLFP

ELFDHRKLGEQYSCYGDGGKQELDPQRLQIIRNYTEIYFPDMQEEEAWLQQCAQRINDEL
EGLGLDAGSEGDPPRDDCYDSSSLPDDISVVKVEDSFEGERPGRRSKKIWLVPIDFDKLE
IPQPDFEVPGADCLLSKEQLRSIYESSLSIGNFASRLLVHLFPELFTHENLRKQYNCSGS
LGKKQLDPSRIKLIRHYVQLLYPRAKNDRVWTLEFVGKLDERCRRRDTEQRRSYQQQRKV
HVPGPECRDLTSYAINPERFREEFEGPPLPPERSSKDFCKIPLDELVVPSPDFPVPSPYL
LSDKEVREIVQQSLSVGNFAARLLVRLFPELFTAENLRLQYNHSGACNKKQLDPTRLRLI
RHYVEAVYPVEKMEEVWHYECIPSIDERCRRPNRKKCDILKKAKKVEK
>sp|Q9NWD9|BEX4_HUMAN Protein BEX4 OS=Homo sapiens GN=BEX4 PE=2 SV=1
MESKEELAANNLNGENAQQENEGGEQAPTQNEEESRHLGGGEGQKPGGNIRRGRVRRLVP
NFRWAIPNRHIEHNEARDDVERFVGQMMEIKRKTREQQMRHYMRFQTPEPDNHYDFCLIP
>sp|Q13515|BFSP2_HUMAN Phakinin OS=Homo sapiens GN=BFSP2 PE=1 SV=1
MSERRVVVDLPTSASSSMPLQRRRASFRGPRSSSSLESPPASRTNAMSGLVRAPGVYVGT
APSGCIGGLGARVTRRALGISSVFLQGLRSSGLATVPAPGLERDHGAVEDLGGCLVEYMA
KVHALEQVSQELETQLRMHLESKATRSGNWGALRASWASSCQQVGEAVLENARLMLQTET
IQAGADDFKERYENEQPFRKAAEEEINSLYKVIDEANLTKMDLESQIESLKEELGSLSRN
YEEDVKLLHKQLAGCELEQMDAPIGTGLDDILETIRIQWERDVEKNRVEAGALLQAKQQA
EVAHMSQTQEEKLAAALRVELHNTSCQVQSLQAETESLRALKRGLENTLHDAKHWHDMEL
QNLGAVVGRLEAELREIRAEAEQQQQERAHLLARKCQLQKDVASYHALLDREESG
>sp|C9JSJ3|BHMG1_HUMAN Basic helix-loop-helix and HMG box domain-containing protein 1 OS=Homo sapiens GN=BHMG1 PE=3 SV=2
MFGSSRYLGSSEQPRANSLGPSDRTLVLCSLVEGEDKVNPSEPHGLRMEEKWLLKGKLRN
QRNQNKLLSPNKKQRKNHTSKLQELALLLPIALKTGTKKLTKKEILVHVLQYIQYLQRNI
DAAKALFKCHITTGEGGLAGLGQKPAWGPARRRRHSTPSSSPSSQKSCLQGACQKPRKKK
LTQASESQTRTPKPRRSLALNKPEKLVAPSPDQKGSGTGGTTTPPRCPDSCGHPRPASSS
PPGDRKGGQSQLTLLDLAEDTIHCDISSCWCQGSVQDDAPFPALLAQEDVARIHFLNKTQ
PHPRQKLVFYDSSEDVDKGSLDADPWLPAWTPENSPQGSPLFLGPPQIDVWSGTGHPSEI
LGLSPSLFSSPGKLLPDEILEDDMEYLTQAAFFEEVCLDLESSPSAYTQEAPQEKDTASK
APKDPPESHSLHRSSVSLDHCYLSLSGNSKAPSSSSSSSSSSSSSEDSDSEPLWKQREDM
QANPVGTPGSSEEDEDTTWTPTRLASPLLAAEKKATKGQVARAPVKPKEKKKGPCPPQMK
KKCVNGFIMFCRMNRKQYIRSCPGTASTAATKELAQLWRVMTQQERRPYCTKARRFSRQH
NRIVKQDGSSSEAEDWETPKPFYQLLAEKALPLPPHLQ
>sp|P55061|BI1_HUMAN Bax inhibitor 1 OS=Homo sapiens GN=TMBIM6 PE=1 SV=2
MNIFDRKINFDALLKFSHITPSTQQHLKKVYASFALCMFVAAAGAYVHMVTHFIQAGLLS
ALGSLILMIWLMATPHSHETEQKRLGLLAGFAFLTGVGLGPALEFCIAVNPSILPTAFMG
TAMIFTCFTLSALYARRRSYLFLGGILMSALSLLLLSSLGNVFFGSIWLFQANLYVGLVV
MCGFVLFDTQLIIEKAEHGDQDYIWHCIDLFLDFITVFRKLMMILAMNEKDKKKEKK
>sp|Q9UHR4|BI2L1_HUMAN Brain-specific angiogenesis inhibitor 1-associated protein 2-like protein 1 OS=Homo sapiens GN=BAIAP2L1 PE=1 SV=2
MSRGPEEVNRLTESTYRNVMEQFNPGLRNLINLGKNYEKAVNAMILAGKAYYDGVAKIGE
IATGSPVSTELGHVLIEISSTHKKLNESLDENFKKFHKEIIHELEKKIELDVKYMNATLK
RYQTEHKNKLESLEKSQAELKKIRRKSQGSRNALKYEHKEIEYVETVTSRQSEIQKFIAD
GCKEALLEEKRRFCFLVDKHCGFANHIHYYHLQSAELLNSKLPRWQETCVDAIKVPEKIM
NMIEEIKTPASTPVSGTPQASPMIERSNVVRKDYDTLSKCSPKMPPAPSGRAYTSPLIDM
FNNPATAAPNSQRVNNSTGTSEDPSLQRSVSVATGLNMMKKQKVKTIFPHTAGSNKTLLS

FAQGDVITLLIPEEKDGWLYGEHDVSKARGWFPSSYTKLLEENETEAVTVPTPSPTPVRS
ISTVNLSENSSVVIPPPDYLECLSMGAAADRRADSARTTSTFKAPASKPETAAPNDANGT
AKPPFLSGENPFATVKLRPTVTNDRSAPIIR
>sp|Q96G01|BICD1_HUMAN Protein bicaudal D homolog 1 OS=Homo sapiens GN=BICD1 PE=1 SV=3
MAAEEVLQTVDHYKTEIERLTKELTETTHEKIQAAEYGLVVLEEKLTLKQQYDELEAEYD
SLKQELEQLKEAFGQSFSIHRKVAEDGETREETLLQESASKEAYYLGKILEMQNELKQSR
AVVTNVQAENERLTAVVQDLKENNEMVELQRIRMKDEIREYKFREARLLQDYTELEEENI
TLQKLVSTLKQNQVEYEGLKHEIKRFEEETVLLNSQLEDAIRLKEIAEHQLEEALETLKN
EREQKNNLRKELSQYISLNDNHISISVDGLKFAEDGSEPNNDDKMNGHIHGPLVKLNGDY
RTPTLRKGESLNPVSDLFSELNISEIQKLKQQLMQVEREKAILLANLQESQTQLEHTKGA
LTEQHERVHRLTEHVNAMRGLQSSKELKAELDGEKGRDSGEEAHDYEVDINGLEILECKY
RVAVTEVIDLKAEIKALKEKYNKSVENYTDEKAKYESKIQMYDEQVTSLEKTTKESGEKM
AHMEKELQKMTSIANENHSTLNTAQDELVTFSEELAQLYHHVCLCNNETPNRVMLDYYRQ
SRVTRSGSLKGPDDPRGLLSPRLARRGVSSPVETRTSSEPVAKESTEASKEPSPTKTPTI
SPVITAPPSSPVLDTSDIRKEPMNIYNLNAIIRDQIKHLQKAVDRSLQLSRQRAAARELA
PMIDKDKEALMEEILKLKSLLSTKREQIATLRAVLKANKQTAEVALANLKNKYENEKAMV
TETMTKLRNELKALKEDAATFSSLRAMFATRCDEYVTQLDEMQRQLAAAEDEKKTLNTLL
RMAIQQKLALTQRLEDLEFDHEQSRRSKGKLGKSKIGSPKVSGEASVTVPTIDTYLLHSQ
GPQTPNIRVSSGTQRKRQFSPSLCDQSRPRTSGASYLQNLLRVPPDPTSTESFLLKGPPS
MSEFIQGHRLSKEKRLTVAPPDCQQPAASVPPQCSQLAGRQDCPTVSPDTALPEEQPHSS
SQCAPLHCLSKPPHP
>sp|Q9UBW5|BIN2_HUMAN Bridging integrator 2 OS=Homo sapiens GN=BIN2 PE=1 SV=3
MAEGKAGGAAGLFAKQVQKKFSRAQEKVLQKLGKAVETKDERFEQSASNFYQQQAEGHKL
YKDLKNFLSAVKVMHESSKRVSETLQEIYSSEWDGHEELKAIVWNNDLLWEDYEEKLADQ
AVRTMEIYVAQFSEIKERIAKRGRKLVDYDSARHHLEAVQNAKKKDEAKTAKAEEEFNKA
QTVFEDLNQELLEELPILYNSRIGCYVTIFQNISNLRDVFYREMSKLNHNLYEVMSKLEK
QHSNKVFVVKGLSSSSRRSLVISPPVRTATVSSPLTSPTSPSTLSLKSESESVSATEDLA
PDAAQGEDNSEIKELLEEEEIEKEGSEASSSEEDEPLPACNGPAQAQPSPTTERAKSQEE
VLPSSTTPSPGGALSPSGQPSSSATEVVLRTRTASEGSEQPKKRASIQRTSAPPSRPPPP
RATASPRPSSGNIPSSPTASGGGSPTSPRASLGTGTASPRTSLEVSPNPEPPEKPVRTPE
AKENENIHNQNPEELCTSPTLMTSQVASEPGEAKKMEDKEKDNKLISANSSEGQDQLQVS
MVPENNNLTAPEPQEEVSTSENPQL
>sp|Q156A1|ATX8_HUMAN Ataxin-8 OS=Homo sapiens GN=ATXN8 PE=1 SV=1
MQQQQQQQQQQQQQQQQQQQQQQQQQQQQQQQQQQQQQQQQQQQQQQQQQQQQQQQQQQQ
QQQQQQQQQQQQQQQQQQQQ
>sp|O14965|AURKA_HUMAN Aurora kinase A OS=Homo sapiens GN=AURKA PE=1 SV=2
MDRSKENCISGPVKATAPVGGPKRVLVTQQFPCQNPLPVNSGQAQRVLCPSNSSQRVPLQ
AQKLVSSHKPVQNQKQKQLQATSVPHPVSRPLNNTQKSKQPLPSAPENNPEEELASKQKN
EESKKRQWALEDFEIGRPLGKGKFGNVYLAREKQSKFILALKVLFKAQLEKAGVEHQLRR
EVEIQSHLRHPNILRLYGYFHDATRVYLILEYAPLGTVYRELQKLSKFDEQRTATYITEL
ANALSYCHSKRVIHRDIKPENLLLGSAGELKIADFGWSVHAPSSRRTTLCGTLDYLPPEM
IEGRMHDEKVDLWSLGVLCYEFLVGKPPFEANTYQETYKRISRVEFTFPDFVTEGARDLI
SRLLKHNPSQRPMLREVLEHPWITANSSKPSNCQNKESASKQS
>sp|Q5TBC7|B2L15_HUMAN Bcl-2-like protein 15 OS=Homo sapiens GN=BCL2L15 PE=1 SV=1

MKSSQTFEEQTECIVNTLLMDFLSPTLQVASRNLCCVDEVDSGEPCSFDVAIIAGRLRML
GDQFNGELEASAKNVIAETIKGQTGAILQDTVESLSKTWCAQDSSLAYERAFLAVSVKLL
EYMAHIAPEVVGQVAIPMTGMINGNQAIREFIQGQGGWENLES
>sp|P59052|B3AS1_HUMAN Putative uncharacterized protein B3GALT5-AS1 OS=Homo sapiens GN=B3GALT5-AS1 PE=5 SV=2
MRRLRHREVRGPVLGHTATGGPQNGTSGCTTAPQQRPPPGTQGMLEQYLNRGGQKSHGLC
WLLCFVSQGQNQDVISAELWCRIHVQAHWGCWQNSAVWGCRNEVLVSLLAVGQGLPSASG
GRLPSLVHGPSHPDSQHPREVPLAL
>sp|Q96Q91|B3A4_HUMAN Anion exchange protein 4 OS=Homo sapiens GN=SLC4A9 PE=2 SV=2
MEMKLPGQEGFEASSAPRNIPSGELDSNPDPGTGPSPDGPSDTESKELGVPKDPLLFIQL
NELLGWPQALEWRETGSSSASLLLDMGEMPSITLSTHLHHRWVLFEEKLEVAAGRWSAPH
VPTLALPSLQKLRSLLAEGLVLLDCPAQSLLELVEQVTRVESLSPELRGQLQALLLQRPQ
HYNQTTGTRPCWGSTHPRKASDNEEAPLREQCQNPLRQKLPPGAEAGTVLAGELGFLAQP
LGAFVRLRNPVVLGSLTEVSLPSRFFCLLLGPCMLGKGYHEMGRAAAVLLSDPQFQWSVR
RASNLHDLLAALDAFLEEVTVLPPGRWDPTARIPPPKCLPSQHKRLPSQQREIRGPAVPR
LTSAEDRHRHGPHAHSPELQRTGRLFGGLIQDVRRKVPWYPSDFLDALHLQCFSAVLYIY
LATVTNAITFGGLLGDATDGAQGVLESFLGTAVAGAAFCLMAGQPLTILSSTGPVLVFER
LLFSFSRDYSLDYLPFRLWVGIWVATFCLVLVATEASVLVRYFTRFTEEGFCALISLIFI
YDAVGKMLNLTHTYPIQKPGSSAYGCLCQYPGPGGNESQWIRTRPKDRDDIVSMDLGLIN
ASLLPPPECTRQGGHPRGPGCHTVPDIAFFSLLLFLTSFFFAMALKCVKTSRFFPSVVRK
GLSDFSSVLAILLGCGLDAFLGLATPKLMVPREFKPTLPGRGWLVSPFGANPWWWSVAAA
LPALLLSILIFMDQQITAVILNRMEYRLQKGAGFHLDLFCVAVLMLLTSALGLPWYVSAT
VISLAHMDSLRRESRACAPGERPNFLGIREQRLTGLVVFILTGASIFLAPVLKFIPMPVL
YGIFLYMGVAALSSIQFTNRVKLLLMPAKHQPDLLLLRHVPLTRVHLFTAIQLACLGLLW
IIKSTPAAIIFPLMLLGLVGVRKALERVFSPQELLWLDELMPEEERSIPEKGLEPEHSFS
GSDSEDSELMYQPKAPEINISVN
>sp|P02730|B3AT_HUMAN Band 3 anion transport protein OS=Homo sapiens GN=SLC4A1 PE=1 SV=3
MEELQDDYEDMMEENLEQEEYEDPDIPESQMEEPAAHDTEATATDYHTTSHPGTHKVYVE
LQELVMDEKNQELRWMEAARWVQLEENLGENGAWGRPHLSHLTFWSLLELRRVFTKGTVL
LDLQETSLAGVANQLLDRFIFEDQIRPQDREELLRALLLKHSHAGELEALGGVKPAVLTR
SGDPSQPLLPQHSSLETQLFCEQGDGGTEGHSPSGILEKIPPDSEATLVLVGRADFLEQP
VLGFVRLQEAAELEAVELPVPIRFLFVLLGPEAPHIDYTQLGRAAATLMSERVFRIDAYM
AQSRGELLHSLEGFLDCSLVLPPTDAPSEQALLSLVPVQRELLRRRYQSSPAKPDSSFYK
GLDLNGGPDDPLQQTGQLFGGLVRDIRRRYPYYLSDITDAFSPQVLAAVIFIYFAALSPA
ITFGGLLGEKTRNQMGVSELLISTAVQGILFALLGAQPLLVVGFSGPLLVFEEAFFSFCE
TNGLEYIVGRVWIGFWLILLVVLVVAFEGSFLVRFISRYTQEIFSFLISLIFIYETFSKL
IKIFQDHPLQKTYNYNVLMVPKPQGPLPNTALLSLVLMAGTFFFAMMLRKFKNSSYFPGK
LRRVIGDFGVPISILIMVLVDFFIQDTYTQKLSVPDGFKVSNSSARGWVIHPLGLRSEFP
IWMMFASALPALLVFILIFLESQITTLIVSKPERKMVKGSGFHLDLLLVVGMGGVAALFG
MPWLSATTVRSVTHANALTVMGKASTPGAAAQIQEVKEQRISGLLVAVLVGLSILMEPIL
SRIPLAVLFGIFLYMGVTSLSGIQLFDRILLLFKPPKYHPDVPYVKRVKTWRMHLFTGIQ
IICLAVLWVVKSTPASLALPFVLILTVPLRRVLLPLIFRNVELQCLDADDAKATFDEEEG
RDEYDEVAMPV
>sp|Q6Y288|B3GLT_HUMAN Beta-1,3-glucosyltransferase OS=Homo sapiens GN=B3GLCT PE=1 SV=2

MRPPACWWLLAPPALLALLTCSLAFGLASEDTKKEVKQSQDLEKSGISRKNDIDLKGIVF
VIQSQSNSFHAKRAEQLKKSILKQAADLTQELPSVLLLHQLAKQEGAWTILPLLPHFSVT
YSRNSSWIFFCEEETRIQIPKLLETLRRYDPSKEWFLGKALHDEEATIIHHYAFSENPTV
FKYPDFAAGWALSIPLVNKLTKRLKSESLKSDFTIDLKHEIALYIWDKGGGPPLTPVPEF
CTNDVDFYCATTFHSFLPLCRKPVKKKDIFVAVKTCKKFHGDRIPIVKQTWESQASLIEY
YSDYTENSIPTVDLGIPNTDRGHCGKTFAILERFLNRSQDKTAWLVIVDDDTLISISRLQ
HLLSCYDSGEPVFLGERYGYGLGTGGYSYITGGGGMVFSREAVRRLLASKCRCYSNDAPD
DMVLGMCFSGLGIPVTHSPLFHQARPVDYPKDYLSHQVPISFHKHWNIDPVKVYFTWLAP
SDEDKARQETQKGFREEL
>sp|Q9NY97|B3GN2_HUMAN N-acetyllactosaminide beta-1,3-N-acetylglucosaminyltransferase 2 OS=Homo sapiens GN=B3GNT2 PE=1 SV=2
MSVGRRRIKLLGILMMANVFIYFIMEVSKSSSQEKNGKGEVIIPKEKFWKISTPPEAYWN
REQEKLNRQYNPILSMLTNQTGEAGRLSNISHLNYCEPDLRVTSVVTGFNNLPDRFKDFL
LYLRCRNYSLLIDQPDKCAKKPFLLLAIKSLTPHFARRQAIRESWGQESNAGNQTVVRVF
LLGQTPPEDNHPDLSDMLKFESEKHQDILMWNYRDTFFNLSLKEVLFLRWVSTSCPDTEF
VFKGDDDVFVNTHHILNYLNSLSKTKAKDLFIGDVIHNAGPHRDKKLKYYIPEVVYSGLY
PPYAGGGGFLYSGHLALRLYHITDQVHLYPIDDVYTGMCLQKLGLVPEKHKGFRTFDIEE
KNKNNICSYVDLMLVHSRKPQEMIDIWSQLQSAHLKC
>sp|O96024|B3GT4_HUMAN Beta-1,3-galactosyltransferase 4 OS=Homo sapiens GN=B3GALT4 PE=1 SV=1
MQLRLFRRLLLAALLLVIVWTLFGPSGLGEELLSLSLASLLPAPASPGPPLALPRLLIPN
QEACSGPGAPPFLLILVCTAPENLNQRNAIRASWGGLREARGLRVQTLFLLGEPNAQHPV
WGSQGSDLASESAAQGDILQAAFQDSYRNLTLKTLSGLNWAEKHCPMARYVLKTDDDVYV
NVPELVSELVLRGGRWGQWERSTEPQREAEQEGGQVLHSEEVPLLYLGRVHWRVNPSRTP
GGRHRVSEEQWPHTWGPFPPYASGTGYVLSASAVQLILKVASRAPLLPLEDVFVGVSARR
GGLAPTQCVKLAGATHYPLDRCCYGKFLLTSHRLDPWKMQEAWKLVGGSDGERTAPFCSW
FQGVLGILRCRAIAWLQS
>sp|Q86Y29|BAGE3_HUMAN B melanoma antigen 3 OS=Homo sapiens GN=BAGE3 PE=2 SV=1
MAAGVVFLALSAQLLQARLMKEESPVVSWRLEPEDGTALDVHFVSTLEPLSNAVKRNVPR
CIIILVLQEPTPFRISVTSSCFVQNTLTKLLKDRRKMQTVQCATAQETS
>sp|Q9NY43|BARH2_HUMAN BarH-like 2 homeobox protein OS=Homo sapiens GN=BARHL2 PE=1 SV=2
MTMEGASGSSFGIDTILSSASSGSPGMMNGDFRPLGEARTADFRSQATPSPCSEIDTVGT
APSSPISVTMEPPEPHLVADATQHHHHLHHSQQPPPPAAAPTQSLQPLPQQQQPLPPQQP
PPPPPQQLGSAASAPRTSTSSFLIKDILGDSKPLAACAPYSTSVSSPHHTPKQESNAVHE
SFRPKLEQEDSKTKLDKREDSQSDIKCHGTKEEGDREITSSRESPPVRAKKPRKARTAFS
DHQLNQLERSFERQKYLSVQDRMDLAAALNLTDTQVKTWYQNRRTKWKRQTAVGLELLAE
AGNYSALQRMFPSPYFYHPSLLGSMDSTTAAAAAAAMYSSMYRTPPAPHPQLQRPLVPRV
LIHGLGPGGQPALNPLSSPIPGTPHPR
>sp|Q9HBU1|BARX1_HUMAN Homeobox protein BarH-like 1 OS=Homo sapiens GN=BARX1 PE=1 SV=2
MQRPGEPGAARFGPPEGCADHRPHRYRSFMIEEILTEPPGPKGAAPAAAAAAAGELLKFG
VQALLAARPFHSHLAVLKAEQAAVFKFPLAPLGCSGLSSALLAAGPGLPGAAGAPHLPLE
LQLRGKLEAAGPGEPGTKAKKGRRSRTVFTELQLMGLEKRFEKQKYLSTPDRIDLAESLG
LSQLQVKTWYQNRRMKWKKIVLQGGGLESPTKPKGRPKKNSIPTSEQLTEQERAKDAEKP
AEVPGEPSDRSRED

>sp|Q8WY36|BBX_HUMAN HMG box transcription factor BBX OS=Homo sapiens GN=BBX PE=1 SV=1
MKGSNRNKDHSAEGEGVGKRPKRKCLQWHPLLAKKLLDFSEEEEEEDEEEDIDKVQLLGA
DGLEQDVGETEDDESPEQRARRPMNAFLLFCKRHRSLVRQEHPRLDNRGATKILADWWAV
LDPKEKQKYTDMAKEYKDAFMKANPGYKWCPTTNKPVKSPTPTVNPRKKLWAFPSDSSRD
LPSPKKAKTEEMPQLNFGMADPTQMGGLSMLLLAGEHALGTPEVSSGTCRPDVSESPELR
QKSPLFQFAEISSSTSHSDASTKQCQTSALFQFAEISSNTSQLGGAEPVKRCGKSALFQL
AEMCLASEGMKMEESKLIKAKESDGGRIKELEKGKEEKEIKMEKTDETRLQKEAEFEKSA
KENLRDSKELRNFEALQIDDIMAIKMEDPKEIRKEELEEDHKCSHFPDFSYSASSKIIIS
DVPSRKDHMCHPHGIMIIEDPAALNKPEKLKKKKKSKMDRHGNDKSTPKKTCKKRQSSE
SDIESVIYTIEAVAKGDWGIEKLGDTPRKKVRTSSSGKGSILDAKPPKKKVKSREKKMSK
EKSSDTTKESRPPDFISISASKNISGETPEGIKAEPLTPMEDALPPSLSGQAKPEDSDCH
RKIETCGSRKSERSCKGALYKTLVSEGMLTSLRANVDRGKRSSGKGNSSDHEGCWNEESW
TFSQSGTSGSKKFKKTKPKEDCLLGSAKLDEEFEKKFNSLPQYSPVTFDRKCVPVPRKKK
KTGNVSSEPTKTSKGPFQSQKKNLFHKIVSKYKHKKEKPNVPEKGSGDKWSNKQLFLDAI
HPTEAIFSEDRNTMEPVHKVKNIPSIFNTPEPTTTQEPLVGSQKRKARKTKITHLVRTAD
GRVSPAGGTLDDKPKEQLQRSLPKATETDCNDKCSHNTEVGETRSSTPEMPAVSAFFSLA
ALAEVAAMENVHRGQRSTPLTHDGQPKEMPQAPVLISCADQ
>sp|O95999|BCL10_HUMAN B-cell lymphoma/leukemia 10 OS=Homo sapiens GN=BCL10 PE=1 SV=1
MEPTAPSLTEEDLTEVKKDALENLRVYLCEKIIAERHFDHLRAKKILSREDTEEISCRTS
SRKRAGKLLDYLQENPKGLDTLVESIRREKTQNFLIQKITDEVLKLRNIKLEHLKGLKCS
SCEPFPDGATNNLSRSNSDESNFSEKLRASTVMYHPEGESSTTPFFSTNSSLNLPVLEVG
RTENTIFSSTTLPRPGDPGAPPLPPDLQLEEEGTCANSSEMFLPLRSRTVSRQ
>sp|P10415|BCL2_HUMAN Apoptosis regulator Bcl-2 OS=Homo sapiens GN=BCL2 PE=1 SV=2
MAHAGRTGYDNREIVMKYIHYKLSQRGYEWDAGDVGAAPPGAAPAPGIFSSQPGHTPHPA
ASRDPVARTSPLQTPAAPGAAAGPALSPVPPVVHLTLRQAGDDFSRRYRRDFAEMSSQLH
LTPFTARGRFATVVEELFRDGVNWGRIVAFFEFGGVMCVESVNREMSPLVDNIALWMTEY
LNRHLHTWIQDNGGWDAFVELYGPSMRPLFDFSWLSLKTLLSLALVGACITLGAYLGHK
>sp|Q9BUT1|BDH2_HUMAN 3-hydroxybutyrate dehydrogenase type 2 OS=Homo sapiens GN=BDH2 PE=1 SV=2
MGRLDGKVIILTAAAQGIGQAAALAFAREGAKVIATDINESKLQELEKYPGIQTRVLDVT
KKKQIDQFANEVERLDVLFNVAGFVHHGTVLDCEEKDWDFSMNLNVRSMYLMIKAFLPKM
LAQKSGNIINMSSVASSVKGVVNRCVYSTTKAAVIGLTKSVAADFIQQGIRCNCVCPGTV
DTPSLQERIQARGNPEEARNDFLKRQKTGRFATAEEIAMLCVYLASDESAYVTGNPVIID
GGWSL
>sp|A6H8Y1|BDP1_HUMAN Transcription factor TFIIIB component B'' homolog OS=Homo sapiens GN=BDP1 PE=1 SV=3
MFRRARLSVKPNVRPGVGARGSTASNPQRGRESPRPPDPATDSASKPAEPTDVPTVDFGG
AEPQEKAPRSSTEKTGGDNDVEESSRSSSTVSQRRKRISSTSSLVKSSVSVPSESHPLST
INQEAPQPTATSTKEKQPCSDRYRIYKAQKLREMLKEELRKEKKQWKNKYAINESQRPPD
RSKMTMRDFIYYLPDNNPMTSSLEQEKKTEKPSTPVQTREQEGKSTPNAEDNEMEEETDD
GPLLVPRVKVAEDGSIILDEESLTVEVLRTKGPCVVEENDPIFERGSTTTYSSFRKNYYS
KPWSNKETDMFFLAISMVGTDFSMIGQLFPHRARIEIKNKFKREEKTNGWRIDKAFQEKR
PFDFDFFAHLLQKVLAEEEKRKQKSVKNHSLKEKKSTKPRKNVKVKKVACEGVNNDPDES
MSSRISDTERSQKDAQTVEEESLTLSREDAEQVALEVDLNQKKRRRKKQDGANELGVNNL

LENATVQAGPSKGEKHKNKCQAIRPELKEGECSKEQMLSCTQNIDGIVGFASTEKVEKRT
DPILSLSNQQDATSVATESSESSTSDLPSFEVGIRALCEVNNAEGSCIEERNVDLKNNSL
EIDQTENVKPMLRGRFQRPKPNLSRAGKKSVLSQGKTESESKNSHSKTSVEKNHVEKDKM
NTLDILRMETTERENPEAETVSVLGEKNCLQEGSQLKALRPVQVRGRLQKPKPNAGKAAE
RKEILISQEEIGANVEKNENESCADRDTPQHMEDQSRKDFEEEDVILQPEKNDSFQNVQP
DEPKVLNECLSVQENNKANKLNQVPILRTRFQKPKPNIGRGTGRREISSKEEVLEKILVS
GEMAAALRETVRLDTSPKEMVPAEINTKEMQSDLKETGRRAISPREKILDVIDDTIEMET
GLKAMGREICLREKTPEVIDATEEIDKDLEEAGRREISPQKNGPEEVKPLGEVETDLKAT
GNESSPREKTPEVTDATEEIDKNLEETGRRKISPRENGPEEVKPVDEMETDLNATGRESS
PREKTPEVIDATEEIDLEETEREVSPQENGLEEVKPLGEMETDLKATGRDSFPRGKTPEV
IDAIEEIEIDLEETEREISPQENGLEEVKPLGEMQTDLKATGREISPREKTPEVIDATEE
IDKDLEETGRREISPEENGPEEVKPVDEMETDLKTTGREGSSREKTREVIDAAEVIETDL
EETEREISPQENGPEEVKPVGKMETDLKEIREEISQREKVLAEFSAIREKEIDLKETGKR
DIPIMEKVSGKMAVVEEMEADLKETGKENFRERGSEEICVTEEKVAELKQTGKTDISPRE
NELEETSTSRQTDTHLMQSGSNDFSAVPSLDIQNISSEVLSMMHTPVEEKRNSEKEVSSH
FSHFKISSQTHESDKTEVQGIQSPDVPEQFSDINLSKSLPQEQKPLEIKPAPFVRSRFKR
PKPNLARAALKRETTESEKYIYEKKSETKKMETIVMQENNEQTDTLPSQHDEASLMISRE
KDTLGHRNEEAVILPCTQTERNLSPSNSCEPKEESQSAPVQKNDSVVSVGTNNVNTFQQE
MKESVIQTARQVRGRLQRPRPNIRKTGQRQIVDKGEAKGIIKEGRTILPKDETEKKVLTV
SNSQIETEIEVPSSAVPEHRMYENQSQVVLVENLHVNKTNETIRHENKPYVPSSAQMTRR
KFQKAKPNLGRAHSKKEEPVLEKVTTDQSKEGKPEDHLLQKGASNTQLLLKEKAELLTSL
EVSARKDCVGSKESALAKIDAELEEVGPSRRVGEETVGDNSPSSVVEEQYLNKLTSCPQP
LNETSYSKIALDGKTTISSTSEYERNRGERRSHKKFKPNVTRGRGSKRVRGKTSKKEPRA
SKAMLVTLRASQEEDDDADDFESDYEEESYHLAPEEVNKAPVFVPVGLRSPEPVSAQIEE
TMEELEITVNVPDVGCIAVVEHELPNTDVTTEEMKQEENLSVPFEMTTSEHIQDEPGTND
GSTEAAITLLTMGDLVLQSEISSEQGDVGVCIIPHVHSKDKSHIPSSLDNVNHKIVHECQ
ELSSPVITTSPASFEENKIVLEEQSSREEISLMEKVKENATPTRNTISKVTSNLRIRSRL
AKPKPNLEKTLGTNRLDDYQEVSSLCVTKGAEMETQRETEKNASKATELENKNLGPVTTA
ENKDQSKLACVHGIKGTSISSEVNLTERNENQEESSQEVHMLSVAPVASSETGPCTLGLD
RGLGENSVEEPQIKDSKGDSVLTLPVPEYTPTSIPEVQQENIINPQDLTVNLVANVPQDG
EDEQAFILTLVEIPANAVEEFTDATAQFMPNPLLPAPILVKSVNTEERGDMSICLPATSV
GQDAMGLSISGRDNSKKPPDNLDLVSRKRFQCRLDKNDHIPPAKKRSLTLRDDCQEYTTE
VHSKELTNVFEETGESHKGQDIFLTSGSTLTTPEPQRQQVEAAFQSRGSRSPDACMDKNV
PQLPQDEMIVSDKEERTDAAPKSQQMDSRTSSSKASLSRPGRRPLGFLSLICSKNSLESD
EPMQVHSKKRLKPLIPGLRKKLKRSNPFNESQEKNRESSDLLPSPSVITTQSENISSSAT
QVSCDQPLLKEGYKSAQKRAPQGEATTVSEYFFNDIFIEVDETE
>sp|Q7L4P6|BEND5_HUMAN BEN domain-containing protein 5 OS=Homo sapiens GN=BEND5 PE=1 SV=1
MYAFVRFLEDNVCYALPVSCVRDFSPRSRLDFDNQKVYAVYRGPEELGAGPESPPRAPRD
WGALLLHKAQILALAEDKSDLENSVMQKKIKIPKLSLNHVEEDGEVKDYGEEDLQLRHIK
RPEGRKPSEVAHKSIEAVVARLEKQNGLSLGHSTCPEEVFVEASPGTEDMDSLEDAVVPR
ALYEELLRNYQQQQEEMRHLQQELERTRRQLVQQAKKLKEYGALVSEMKELRDLNRRLQD
VLLLRLGSGPAIDLEKVKSECLEPEPELRSTFSEEANTSSYYPAPAPVMDKYILDNGKVH
LGSGIWVDEEKWHQLQVTQGDSKYTKNLAVMIWGTDVLKNRSVTGVATKKKKDAVPKPPL
SPHKLSIVRECLYDRIAQETVDETEIAQRLSKVNKYICEKIMDINKSCKNEERREAKYNL

Q
>sp|Q9BXY8|BEX2_HUMAN Protein BEX2 OS=Homo sapiens GN=BEX2 PE=1 SV=1
MESKEERALNNLIVENVNQENDEKDEKEQVANKGEPLALPLNVSEYCVPRGNRRRFRVRQ
PILQYRWDIMHRLGEPQARMREENMERIGEEVRQLMEKLREKQLSHSLRAVSTDPPHHDH
HDEFCLMP
>sp|P08236|BGLR_HUMAN Beta-glucuronidase OS=Homo sapiens GN=GUSB PE=1 SV=2
MARGSAVAWAALGPLLWGCALGLQGGMLYPQESPSRECKELDGLWSFRADFSDNRRRGFE
EQWYRRPLWESGPTVDMPVPSSFNDISQDWRLRHFVGWVWYEREVILPERWTQDLRTRVV
LRIGSAHSYAIVWVNGVDTLEHEGGYLPFEADISNLVQVGPLPSRLRITIAINNTLTPTT
LPPGTIQYLTDTSKYPKGYFVQNTYFDFFNYAGLQRSVLLYTTPTTYIDDITVTTSVEQD
SGLVNYQISVKGSNLFKLEVRLLDAENKVVANGTGTQGQLKVPGVSLWWPYLMHERPAYL
YSLEVQLTAQTSLGPVSDFYTLPVGIRTVAVTKSQFLINGKPFYFHGVNKHEDADIRGKG
FDWPLLVKDFNLLRWLGANAFRTSHYPYAEEVMQMCDRYGIVVIDECPGVGLALPQFFNN
VSLHHHMQVMEEVVRRDKNHPAVVMWSVANEPASHLESAGYYLKMVIAHTKSLDPSRPVT
FVSNSNYAADKGAPYVDVICLNSYYSWYHDYGHLELIQLQLATQFENWYKKYQKPIIQSE
YGAETIAGFHQDPPLMFTEEYQKSLLEQYHLGLDQKRRKYVVGELIWNFADFMTEQSPTR
VLGNKKGIFTRQRQPKSAAFLLRERYWKIANETRYPHSVAKSQCLENSLFT
>sp|Q8NDY6|BHE23_HUMAN Class E basic helix-loop-helix protein 23 OS=Homo sapiens GN=BHLHE23 PE=2 SV=1
MAELKSLSGDAYLALSHGYAAAAAGLAYGAAREPEAARGYGTPGPGGDLPAAPAPRAPAQ
AAESSGEQSGDEDDAFEQRRRRRGPGSAADGRRRPREQRSLRLSINARERRRMHDLNDAL
DGLRAVIPYAHSPSVRKLSKIATLLLAKNYILMQAQALDEMRRLVAFLNQGQGLAAPVNA
APLTPFGQATVCPFSAGAALGPCPDKCAAFSGTPSALCKHCHEKP
>sp|P53004|BIEA_HUMAN Biliverdin reductase A OS=Homo sapiens GN=BLVRA PE=1 SV=2
MNAEPERKFGVVVVGVGRAGSVRMRDLRNPHPSSAFLNLIGFVSRRELGSIDGVQQISLE
DALSSQEVEVAYICSESSSHEDYIRQFLNAGKHVLVEYPMTLSLAAAQELWELAEQKGKV
LHEEHVELLMEEFAFLKKEVVGKDLLKGSLLFTAGPLEEERFGFPAFSGISRLTWLVSLF
GELSLVSATLEERKEDQYMKMTVCLETEKKSPLSWIEEKGPGLKRNRYLSFHFKSGSLEN
VPNVGVNKNIFLKDQNIFVQKLLGQFSEKELAAEKKRILHCLGLAEEIQKYCCSRK
>sp|O00499|BIN1_HUMAN Myc box-dependent-interacting protein 1 OS=Homo sapiens GN=BIN1 PE=1 SV=1
MAEMGSKGVTAGKIASNVQKKLTRAQEKVLQKLGKADETKDEQFEQCVQNFNKQLTEGTR
LQKDLRTYLASVKAMHEASKKLNECLQEVYEPDWPGRDEANKIAENNDLLWMDYHQKLVD
QALLTMDTYLGQFPDIKSRIAKRGRKLVDYDSARHHYESLQTAKKKDEAKIAKPVSLLEK
AAPQWCQGKLQAHLVAQTNLLRNQAEEELIKAQKVFEEMNVDLQEELPSLWNSRVGFYVN
TFQSIAGLEENFHKEMSKLNQNLNDVLVGLEKQHGSNTFTVKAQPSDNAPAKGNKSPSPP
DGSPAATPEIRVNHEPEPAGGATPGATLPKSPSQLRKGPPVPPPPKHTPSKEVKQEQILS
LFEDTFVPEISVTTPSQFEAPGPFSEQASLLDLDFDPLPPVTSPVKAPTPSGQSIPWDLW
EPTESPAGSLPSGEPSAAEGTFAVSWPSQTAEPGPAQPAEASEVAGGTQPAAGAQEPGET
AASEAASSSLPAVVVETFPATVNGTVEGGSGAGRLDLPPGFMFKVQAQHDYTATDTDELQ
LKAGDVVLVIPFQNPEEQDEGWLMGVKESDWNQHKELEKCRGVFPENFTERVP
>sp|P36542|ATPG_HUMAN ATP synthase subunit gamma, mitochondrial OS=Homo sapiens GN=ATP5C1 PE=1 SV=1
MFSRAGVAGLSAWTLQPQWIQVRNMATLKDITRRLKSIKNIQKITKSMKMVAAAKYARAE

RELKPARIYGLGSLALYEKADIKGPEDKKKHLLIGVSSDRGLCGAIHSSIAKQMKSEVAT
LTAAGKEVMLVGIGDKIRGILYRTHSDQFLVAFKEVGRKPPTFGDASVIALELLNSGYEF
DEGSIIFNKFRSVISYKTEEKPIFSLNTVASADSMSIYDDIDADVLQNYQEYNLANIIYY
SLKESTTSEQSARMTAMDNASKNASEMIDKLTLTFNRTRQAVITKELIEIISGAAALD
>sp|P59510|ATS20_HUMAN A disintegrin and metalloproteinase with thrombospondin motifs 20 OS=Homo sapiens GN=ADAMTS20 PE=2 SV=2
MWVAKWLTGLLYHLSLFITRSWEVDFHPRQEALVRTLTSYEVVIPERVNEFGEVFPQSHH
FSRQKRSSEALEPMPFRTHYRFTAYGQLFQLNLTADASFLAAGYTEVHLGTPERGAWESD
AGPSDLRHCFYRGQVNSQEDYKAVVSLCGGLTGTFKGQNGEYFLEPIMKADGNEYEDGHN
KPHLIYRQDLNNSFLQTLKYCSVSESQIKETSLPFHTYSNMNEDLNVMKERVLGHTSKNV
PLKDERRHSRKKRLISYPRYIEIMVTADAKVVSAHGSNLQNYILTLMSIVATIYKDPSIG
NLIHIVVVKLVMIHREEEGPVINFDGATTLKNFCSWQQTQNDLDDVHPSHHDTAVLITRE
DICSSKEKCNMLGLSYLGTICDPLQSCFINEEKGLISAFTIAHELGHTLGVQHDDNPRCK
EMKVTKYHVMAPALSFHMSPWSWSNCSRKYVTEFLDTGYGECLLDKPDEEIYNLPSELPG
SRYDGNKQCELAFGPGSQMCPHINICMHLWCTSTEKLHKGCFTQHVPPADGTDCGPGMHC
RHGLCVNKETETRPVNGEWGPWEPYSSCSRTCGGGIESATRRCNRPEPRNGGNYCVGRRM
KFRSCNTDSCPKGTQDFREKQCSDFNGKHLDISGIPSNVRWLPRYSGIGTKDRCKLYCQV
AGTNYFYLLKDMVEDGTPCGTETHDICVQGQCMAAGCDHVLNSSAKIDKCGVCGGDNSSC
KTITGVFNSSHYGYNVVVKIPAGATNVDIRQYSYSGQPDDSYLALSDAEGNFLFNGNFLL
STSKKEINVQGTRTVIEYSGSNNAVERINSTNRQEKEILIEVLCVGNLYNPDVHYSFNIP
LEERSDMFTWDPYGPWEGCTKMCQGLQRRNITCIHKSDHSVVSDKECDHLPLPSFVTQSC
NTDCELRWHVIGKSECSSQCGQGYRTLDIHCMKYSIHEGQTVQVDDHYCGDQLKPPTQEL
CHGNCVFTRWHYSEWSQCSRSCGGGERSRESYCMNNFGHRLADNECQELSRVTRENCNEF
SCPSWAASEWSECLVTCGKGTKQRQVWCQLNVDHLSDGFCNSSTKPESLSPCELHTCASW
QVGPWGPCTTTCGHGYQMRDVKCVNELASAVLEDTECHEASRPSDRQSCVLTPCSFISKL
ETALLPTVLIKKMAQWRHGSWTPCSVSCGRGTQARYVSCRDALDRIADESYCAHLPRPAE
IWDCFTPCGEWQAGDWSPCSASCGHGKTTRQVLCMNYHQPIDENYCDPEVRPLMEQECSL
AACPPAHSHFPSSPVQPSYYLSTNLPLTQKLEDNENQVVHPSVRGNQWRTGPWGSCSSSC
SGGLQHRAVVCQDENGQSASYCDAASKPPELQQCGPGPCPQWNYGNWGECSQTCGGGIKS
RLVICQFPNGQILEDHNCEIVNKPPSVIQCHMHACPADVSWHQEPWTSCSASCGKGRKYR
EVFCIDQFQRKLEDTNCSQVQKPPTHKACRSVRCPSWKANSWNECSVTCGSGVQQRDVYC
RLKGVGQVVEEMCDQSTRPCSQRRCWSQDCVQHKGMERGRLNCSTSCERKDSHQRMECTD
NQIRQVNEIVYNSSTISLTSKNCRNPPCNYIVVTADSSQCANNCGFSYRQRITYCTEIPS
TKKHKLHRLRPIVYQECPVVPSSQVYQCINSCLHLATWKVGKWSKCSVTCGIGIMKRQVK
CITKHGLSSDLCLNHLKPGAQKKCYANDCKSFTTCKEIQVKNHIRKDGDYYLNIKGRIIK
IYCADMYLENPKEYLTLVQGEENFSEVYGFRLKNPYQCPFNGSRREDCECDNGHLAAGYT
VFSKIRIDLTSMQIKTTDLLFSKTIFGNAVPFATAGDCYSAFRCPQGQFSINLSGTGMKI
SSTAKWLTQGSYTSVSIRRSEDGTRFFGKCGGYCGKCLPHMTTGLPIQVI
>sp|P54253|ATX1_HUMAN Ataxin-1 OS=Homo sapiens GN=ATXN1 PE=1 SV=2
MKSNQERSNECLPPKKREIPATSRSSEEKAPTLPSDNHRVEGTAWLPGNPGGRGHGGGRH
GPAGTSVELGLQQGIGLHKALSTGLDYSPPSAPRSVPVATTLPAAYATPQPGTPVSPVQY
AHLPHTFQFIGSSQYSGTYASFIPSQLIPPTANPVTSAVASAAGATTPSQRSQLEAYSTL
LANMGSLSQTPGHKAEQQQQQQQQQQQQHQHQQQQQQQQQQQQQQHLSRAPGLITPGSPP
PAQQNQYVHISSSPQNTGRTASPPAIPVHLHPHQTMIPHTLTLGPPSQVVMQYADSGSHF

VPREATKKAESSRLQQAIQAKEVLNGEMEKSRRYGAPSSADLGLGKAGGKSVPHPYESRH
VVVHPSPSDYSSRDPSGVRASVMVLPNSNTPAADLEVQQATHREASPSTLNDKSGLHLGK
PGHRSYALSPHTVIQTTHSASEPLPVGLPATAFYAGTQPPVIGYLSGQQQAITYAGSLPQ
HLVIPGTQPLLIPVGSTDMEASGAAPAIVTSSPQFAAVPHTFVTTALPKSENFNPEALVT
QAAYPAMVQAQIHLPVVQSVASPAAAPPTLPPYFMKGSIIQLANGELKKVEDLKTEDFIQ
SAEISNDLKIDSSTVERIEDSHSPGVAVIQFAVGEHRAQVSVEVLVEYPFFVFGQGWSSC
CPERTSQLFDLPCSKLSVGDVCISLTLKNLKNGSVKKGQPVDPASVLLKHSKADGLAGSR
HRYAEQENGINQGSAQMLSENGELKFPEKMGLPAAPFLTKIEPSKPAATRKRRWSAPESR
KLEKSEDEPPLTLPKPSLIPQEVKICIEGRSNVGK
>sp|O15265|ATX7_HUMAN Ataxin-7 OS=Homo sapiens GN=ATXN7 PE=1 SV=1
MSERAADDVRGEPRRAAAAAGGAAAAAARQQQQQQQQQQPPPPQPQRQQHPPPPPRRTRP
EDGGPGAASTSAAAMATVGERRPLPSPEVMLGQSWNLWVEASKLPGKDGTELDESFKEFG
KNREVMGLCREDMPIFGFCPAHDDFYLVVCNDCNQVVKPQAFQSHYERRHSSSSKPPLAV
PPTSVFSFFPSLSKSKGGSASGSNRSSSGGVLSASSSSSKLLKSPKEKLQLRGNTRPMHP
IQQSRVPHGRIMTPSVKVEKIHPKMDGTLLKSAVGPTCPATVSSLVKPGLNCPSIPKPTL
PSPGQILNGKGLPAPPTLEKKPEDNSNNRKFLNKRLSEREFDPDIHCGVIDLDTKKPCTR
SLTCKTHSLTQRRAVQGRRKRFDVLLAEHKNKTREKELIRHPDSQQPPQPLRDPHPAPPR
TSQEPHQNPHGVIPSESKPFVASKPKPHTPSLPRPPGCPAQQGGSAPIDPPPVHESPHPP
LPATEPASRLSSEEGEGDDKEESVEKLDCHYSGHHPQPASFCTFGSRQIGRGYYVFDSRW
NRLRCALNLMVEKHLNAQLWKKIPPVPSTTSPISTRIPHRTNSVPTSQCGVSYLAAATVS
TSPVLLSSTCISPNSKSVPAHGTTLNAQPAASGAMDPVCSMQSRQVSSSSSSPSTPSGLS
SVPSSPMSRKPQKLKSSKSLRPKESSGNSTNCQNASSSTSGGSGKKRKNSSPLLVHSSSS
SSSSSSSSHSMESFRKNCVAHSGPPYPSTVTSSHSIGLNCVTNKANAVNVRHDQSGRGPP
TGSPAESIKRMSVMVNSSDSTLSLGPFIHQSNELPVNSHGSFSHSHTPLDKLIGKKRKCS
PSSSSINNSSSKPTKVAKVPAVNNVHMKHTGTIPGAQGLMNSSLLHQPKARP
>sp|P27037|AVR2A_HUMAN Activin receptor type-2A OS=Homo sapiens GN=ACVR2A PE=1 SV=1
MGAAAKLAFAVFLISCSSGAILGRSETQECLFFNANWEKDRTNQTGVEPCYGDKDKRRHC
FATWKNISGSIEIVKQGCWLDDINCYDRTDCVEKKDSPEVYFCCCEGNMCNEKFSYFPEM
EVTQPTSNPVTPKPPYYNILLYSLVPLMLIAGIVICAFWVYRHHKMAYPPVLVPTQDPGP
PPPSPLLGLKPLQLLEVKARGRFGCVWKAQLLNEYVAVKIFPIQDKQSWQNEYEVYSLPG
MKHENILQFIGAEKRGTSVDVDLWLITAFHEKGSLSDFLKANVVSWNELCHIAETMARGL
AYLHEDIPGLKDGHKPAISHRDIKSKNVLLKNNLTACIADFGLALKFEAGKSAGDTHGQV
GTRRYMAPEVLEGAINFQRDAFLRIDMYAMGLVLWELASRCTAADGPVDEYMLPFEEEIG
QHPSLEDMQEVVVHKKKRPVLRDYWQKHAGMAMLCETIEECWDHDAEARLSAGCVGERIT
QMQRLTNIITTEDIVTVVTMVTNVDFPPKESSL
>sp|Q8ND07|BBOF1_HUMAN Basal body-orientation factor 1 OS=Homo sapiens GN=BBOF1 PE=2 SV=3
MPSKGKDKKKGKSKGKDTKKLIKTDESVVDRAKANASLWEARLEVTELSRIKYRDTSRIL
AKSNEDLKKKQCKMEKDIMSVLSYLKKQDQEKDNMIEKLKQQLNETKEKAQEEKDKLEQK
YTRQINELEGQFHQKAKEIGMIHTELKAVRQFQKRKIQVERELDDLKENLRNTERIHQET
LRRLESRFFEEKHRLEQEAEKKIIMLAERAHHEAIVQLNDAGRNVFKENDYLQKALAYHL
KETDALQKNSQKLQESHTLLLHQKEINDLLVKEKIMQLVQQRSQIQTLQKKVVNLETALS
YMTKEFESEVLKLQQHAMIENQAGQVEIDKLQHLLQMKDREMNRVKKLAKNILDERTEVE
RFFLDALHQVKQQILISRKHYKQIAQAAFNLKMRAACTGRTEYPKIRTFDGREHSTNSVN
QDLLEAEKWTHIEGNVDIGDLTWEQKEKVLRLLFAKMNGCPSRKYNQSSRPPVPDYVVSD

SGETKEFGDESKLQDKIFITQQIAISDSSGEVVLPTIPKEPQESDTGTF
>sp|O75815|BCAR3_HUMAN Breast cancer anti-estrogen resistance protein 3 OS=Homo sapiens GN=BCAR3 PE=1 SV=1
MAAGKFASLPRNMPVNHQFPLASSMDLLSSRSPLAEHRPDAYQDVSIHGTLPRKKKGPPP
IRSCDDFSHMGTLPHSKSPRQNSPVTQDGIQESPWQDRHGETFTFRDPHLLDPTVEYVKF
SKERHIMDRTPEKLKKELEEELLLSSEDLRSHAWYHGRIPRQVSENLVQRDGDFLVRDSL
SSPGNFVLTCQWKNLAQHFKINRTVLRLSEAYSRVQYQFEMESFDSIPGLVRCYVGNRRP
ISQQSGAIIFQPINRTVPLRCLEEHYGTSPGQAREGSLTKGRPDVAKRLSLTMGGVQARE
QNLPRGNLLRNKEKSGSQPACLDHMQDRRALSLKAHQSESYLPIGCKLPPQSSGVDTSPC
PNSPVFRTGSEPALSPAVVRRVSSDARAGEALRGSDSQLCPKPPPKPCKVPFLKVPSSPS
AWLNSEANYCELNPAFATGCGRGAKLPSCAQGSHTELLTAKQNEAPGPRNSGVNYLILDD
DDRERPWEPAAAQMEKGQWDKGEFVTPLLETVSSFRPNEFESKFLPPENKPLETAMLKRA
KELFTNNDPKVIAQHVLSMDCRVARILGVSEEMRRNMGVSSGLELITLPHGHQLRLDIIE
RHNTMAIGIAVDILGCTGTLEDRAATLSKIIQVAVELKDSMGDLYSFSALMKALEMPQIT
RLEKTWTALRHQYTQTAILYEKQLKPFSKLLHEGRESTCVPPNNVSVPLLMPLVTLMERQ
AVTFEGTDMWEKNDQSCEIMLNHLATARFMAEAADSYRMNAERILAGFQPDEEMNEICKT
EFQMRLLWGSKGAQVNQTERYEKFNQILTALSRKLEPPPVKQAEL
>sp|P54687|BCAT1_HUMAN Branched-chain-amino-acid aminotransferase, cytosolic OS=Homo sapiens GN=BCAT1 PE=1 SV=3
MKDCSNGCSAECTGEGGSKEVVGTFKAKDLIVTPATILKEKPDPNNLVFGTVFTDHMLTV
EWSSEFGWEKPHIKPLQNLSLHPGSSALHYAVELFEGLKAFRGVDNKIRLFQPNLNMDRM
YRSAVRATLPVFDKEELLECIQQLVKLDQEWVPYSTSASLYIRPTFIGTEPSLGVKKPTK
ALLFVLLSPVGPYFSSGTFNPVSLWANPKYVRAWKGGTGDCKMGGNYGSSLFAQCEAVDN
GCQQVLWLYGEDHQITEVGTMNLFLYWINEDGEEELATPPLDGIILPGVTRRCILDLAHQ
WGEFKVSERYLTMDDLTTALEGNRVREMFGSGTACVVCPVSDILYKGETIHIPTMENGPK
LASRILSKLTDIQYGREESDWTIVLS
>sp|O15382|BCAT2_HUMAN Branched-chain-amino-acid aminotransferase, mitochondrial OS=Homo sapiens GN=BCAT2 PE=1 SV=2
MAAAALGQIWARKLLSVPWLLCGPRRYASSSFKAADLQLEMTQKPHKKPGPGEPLVFGKT
FTDHMLMVEWNDKGWGQPRIQPFQNLTLHPASSSLHYSLQLFEGMKAFKGKDQQVRLFRP
WLNMDRMLRSAMRLCLPSFDKLELLECIRRLIEVDKDWVPDAAGTSLYVRPVLIGNEPSL
GVSQPTRALLFVILCPVGAYFPGGSVTPVSLLADPAFIRAWVGGVGNYKLGGNYGPTVLV
QQEALKRGCEQVLWLYGPDHQLTEVGTMNIFVYWTHEDGVLELVTPPLNGVILPGVVRQS
LLDMAQTWGEFRVVERTITMKQLLRALEEGRVREVFGSGTACQVCPVHRILYKDRNLHIP
TMENGPELILRFQKELKEIQYGIRAHEWMFPV
>sp|Q9HAY6|BCDO1_HUMAN Beta,beta-carotene 15,15'-dioxygenase OS=Homo sapiens GN=BCO1 PE=1 SV=1
MDIIFGRNRKEQLEPVRAKVTGKIPAWLQGTLLRNGPGMHTVGESRYNHWFDGLALLHSF
TIRDGEVYYRSKYLRSDTYNTNIEANRIVVSEFGTMAYPDPCKNIFSKAFSYLSHTIPDF
TDNCLINIMKCGEDFYATSETNYIRKINPQTLETLEKVDYRKYVAVNLATSHPHYDEAGN
VLNMGTSIVEKGKTKYVIFKIPATVPEGKKQGKSPWKHTEVFCSIPSRSLLSPSYYHSFG
VTENYVIFLEQPFRLDILKMATAYIRRMSWASCLAFHREEKTYIHIIDQRTRQPVQTKFY
TDAMVVFHHVNAYEEDGCIVFDVIAYEDNSLYQLFYLANLNQDFKENSRLTSVPTLRRFA
VPLHVDKNAEVGTNLIKVASTTATALKEEDGQVYCQPEFLYEGLELPRVNYAHNGKQYRY

VFATGVQWSPIPTKIIKYDILTKSSLKWREDDCWPAEPLFVPAPGAKDEDDGVILSAIVS
TDPQKLPFLLILDAKSFTELARASVDVDMHMDLHGLFITDMDWDTKKQAASEEQRDRASD
CHGAPLT
>sp|P20749|BCL3_HUMAN B-cell lymphoma 3 protein OS=Homo sapiens GN=BCL3 PE=1 SV=2
MPRCPAGAMDEGPVDLRTRPKAAGLPGAALPLRKRPLRAPSPEPAAPRGAAGLVVPLDPL
RGGCDLPAVPGPPHGLARPEALYYPGALLPLYPTRAMGSPFPLVNLPTPLYPMMCPMEHP
LSADIAMATRADEDGDTPLHIAVVQGNLPAVHRLVNLFQQGGRELDIYNNLRQTPLHLAV
ITTLPSVVRLLVTAGASPMALDRHGQTAAHLACEHRSPTCLRALLDSAAPGTLDLEARNY
DGLTALHVAVNTECQETVQLLLERGADIDAVDIKSGRSPLIHAVENNSLSMVQLLLQHGA
NVNAQMYSGSSALHSASGRGLLPLVRTLVRSGADSSLKNCHNDTPLMVARSRRVIDILRG
KATRPASTSQPDPSPDRSANTSPESSSRLSSNGLLSASPSSSPSQSPPRDPPGFPMAPPN
FFLPSPSPPAFLPFAGVLRGPGRPVPPSPAPGGS
>sp|Q8WUZ0|BCL7C_HUMAN B-cell CLL/lymphoma 7 protein family member C OS=Homo sapiens GN=BCL7C PE=1 SV=3
MAGRTVRAETRSRAKDDIKKVMATIEKVRRWEKRWVTVGDTSLRIFKWVPVVDPQEEERR
RAGGGAERSRGRERRGRGASPRGGGPLILLDLNDENSNQSFHSEGSLQKGTEPSPGGTPQ
PSRPVSPAGPPEGVPEEAQPPRLGQERDPGGITAGSTDEPPMLTKEEPVPELLEAEAPEA
YPVFEPVPPVPEAAQGDTEDSEGAPPLKRICPNAPDP
>sp|P11274|BCR_HUMAN Breakpoint cluster region protein OS=Homo sapiens GN=BCR PE=1 SV=2
MVDPVGFAEAWKAQFPDSEPPRMELRSVGDIEQELERCKASIRRLEQEVNQERFRMIYLQ
TLLAKEKKSYDRQRWGFRRAAQAPDGASEPRASASRPQPAPADGADPPPAEEPEARPDGE
GSPGKARPGTARRPGAAASGERDDRGPPASVAALRSNFERIRKGHGQPGADAEKPFYVNV
EFHHERGLVKVNDKEVSDRISSLGSQAMQMERKKSQHGAGSSVGDASRPPYRGRSSESSC
GVDGDYEDAELNPRFLKDNLIDANGGSRPPWPPLEYQPYQSIYVGGMMEGEGKGPLLRSQ
STSEQEKRLTWPRRSYSPRSFEDCGGGYTPDCSSNENLTSSEEDFSSGQSSRVSPSPTTY
RMFRDKSRSPSQNSQQSFDSSSPPTPQCHKRHRHCPVVVSEATIVGVRKTGQIWPNDGEG
AFHGDADGSFGTPPGYGCAADRAEEQRRHQDGLPYIDDSPSSSPHLSSKGRGSRDALVSG
ALESTKASELDLEKGLEMRKWVLSGILASEETYLSHLEALLLPMKPLKAAATTSQPVLTS
QQIETIFFKVPELYEIHKEFYDGLFPRVQQWSHQQRVGDLFQKLASQLGVYRAFVDNYGV
AMEMAEKCCQANAQFAEISENLRARSNKDAKDPTTKNSLETLLYKPVDRVTRSTLVLHDL
LKHTPASHPDHPLLQDALRISQNFLSSINEEITPRRQSMTVKKGEHRQLLKDSFMVELVE
GARKLRHVFLFTDLLLCTKLKKQSGGKTQQYDCKWYIPLTDLSFQMVDELEAVPNIPLVP
DEELDALKIKISQIKNDIQREKRANKGSKATERLKKKLSEQESLLLLMSPSMAFRVHSRN
GKSYTFLISSDYERAEWRENIREQQKKCFRSFSLTSVELQMLTNSCVKLQTVHSIPLTIN
KEDDESPGLYGFLNVIVHSATGFKQSSNLYCTLEVDSFGYFVNKAKTRVYRDTAEPNWNE
EFEIELEGSQTLRILCYEKCYNKTKIPKEDGESTDRLMGKGQVQLDPQALQDRDWQRTVI
AMNGIEVKLSVKFNSREFSLKRMPSRKQTGVFGVKIAVVTKRERSKVPYIVRQCVEEIER
RGMEEVGIYRVSGVATDIQALKAAFDVNNKDVSVMMSEMDVNAIAGTLKLYFRELPEPLF
TDEFYPNFAEGIALSDPVAKESCMLNLLLSLPEANLLTFLFLLDHLKRVAEKEAVNKMSL
HNLATVFGPTLLRPSEKESKLPANPSQPITMTDSWSLEVMSQVQVLLYFLQLEAIPAPDS
KRQSILFSTEV
>sp|Q93088|BHMT1_HUMAN Betaine--homocysteine S-methyltransferase 1 OS=Homo sapiens GN=BHMT PE=1 SV=2
MPPVGGKKAKKGILERLNAGEIVIGDGGFVFALEKRGYVKAGPWTPEAAVEHPEAVRQLH

REFLRAGSNVMQTFTFYASEDKLENRGNYVLEKISGQEVNEAACDIARQVADEGDALVAG
GVSQTPSYLSCKSETEVKKVFLQQLEVFMKKNVDFLIAEYFEHVEEAVWAVETLIASGKP
VAATMCIGPEGDLHGVPPGECAVRLVKAGASIIGVNCHFDPTISLKTVKLMKEGLEAARL
KAHLMSQPLAYHTPDCNKQGFIDLPEFPFGLEPRVATRWDIQKYAREAYNLGVRYIGGCC
GFEPYHIRAIAEELAPERGFLPPASEKHGSWGSGLDMHTKPWVRARARKEYWENLRIASG
RPYNPSMSKPDGWGVTKGTAELMQQKEATTEQQLKELFEKQKFKSQ
>sp|P55957|BID_HUMAN BH3-interacting domain death agonist OS=Homo sapiens GN=BID PE=1 SV=1
MDCEVNNGSSLRDECITNLLVFGFLQSCSDNSFRRELDALGHELPVLAPQWEGYDELQTD
GNRSSHSRLGRIEADSESQEDIIRNIARHLAQVGDSMDRSIPPGLVNGLALQLRNTSRSE
EDRNRDLATALEQLLQAYPRDMEKEKTMLVLALLLAKKVASHTPSLLRDVFHTTVNFINQ
NLRTYVRSLARNGMD
>sp|Q9Y6D6|BIG1_HUMAN Brefeldin A-inhibited guanine nucleotide-exchange protein 1 OS=Homo sapiens GN=ARFGEF1 PE=1 SV=2
MYEGKKTKNMFLTRALEKILADKEVKKAHHSQLRKACEVALEEIKAETEKQSPPHGEAKA
GSSTLPPVKSKTNFIEADKYFLPFELACQSKCPRIVSTSLDCLQKLIAYGHLTGNAPDST
TPGKKLIDRIIETICGCFQGPQTDEGVQLQIIKALLTAVTSQHIEIHEGTVLQAVRTCYN
IYLASKNLINQTTAKATLTQMLNVIFARMENQALQEAKQMEKERHRQHHHLLQSPVSHHE
PESPQLRYLPPQTVDHISQEHEGDLDLHTNDVDKSLQDDTEPENGSDISSAENEQTEADQ
ATAAETLSKNEVLYDGENHDCEEKPQDIVQNIVEEMVNIVVGDMGEGTTINASADGNIGT
IEDGSDSENIQANGIPGTPISVAYTPSLPDDRLSVSSNDTQESGNSSGPSPGAKFSHILQ
KDAFLVFRSLCKLSMKPLSDGPPDPKSHELRSKILSLQLLLSILQNAGPIFRTNEMFINA
IKQYLCVALSKNGVSSVPEVFELSLSIFLTLLSNFKTHLKMQIEVFFKEIFLYILETSTS
SFDHKWMVIQTLTRICADAQSVVDIYVNYDCDLNAANIFERLVNDLSKIAQGRGSQELGM
SNVQELSLRKKGLECLVSILKCMVEWSKDQYVNPNSQTTLGQEKPSEQEMSEIKHPETIN
RYGSLNSLESTSSSGIGSYSTQMSGTDNPEQFEVLKQQKEIIEQGIDLFNKKPKRGIQYL
QEQGMLGTTPEDIAQFLHQEERLDSTQVGEFLGDNDKFNKEVMYAYVDQHDFSGKDFVSA
LRMFLEGFRLPGEAQKIDRLMEKFAARYLECNQGQTLFASADTAYVLAYSIIMLTTDLHS
PQVKNKMTKEQYIKMNRGINDSKDLPEEYLSAIYNEIAGKKISMKETKELTIPTKSSKQN
VASEKQRRLLYNLEMEQMAKTAKALMEAVSHVQAPFTSATHLEHVRPMFKLAWTPFLAAF
SVGLQDCDDTEVASLCLEGIRCAIRIACIFSIQLERDAYVQALARFTLLTVSSGITEMKQ
KNIDTIKTLITVAHTDGNYLGNSWHEILKCISQLELAQLIGTGVKPRYISGTVRGREGSL
TGTKDQAPDEFVGLGLVGGNVDWKQIASIQESIGETSSQSVVVAVDRIFTGSTRLDGNAI
VDFVRWLCAVSMDELLSTTHPRMFSLQKIVEISYYNMGRIRLQWSRIWEVIGDHFNKVGC
NPNEDVAIFAVDSLRQLSMKFLEKGELANFRFQKDFLRPFEHIMKRNRSPTIRDMVVRCI
AQMVNSQAANIRSGWKNIFSVFHLAASDQDESIVELAFQTTGHIVTLVFEKHFPATIDSF
QDAVKCLSEFACNAAFPDTSMEAIRLIRHCAKYVSDRPQAFKEYTSDDMNVAPEDRVWVR
GWFPILFELSCIINRCKLDVRTRGLTVMFEIMKTYGHTYEKHWWQDLFRIVFRIFDNMKL
PEQQTEKAEWMTTTCNHALYAICDVFTQYLEVLSDVLLDDIFAQLYWCVQQDNEQLARSG
TNCLENVVILNGEKFTLEIWDKTCNCTLDIFKTTIPHALLTWRPNSGETAPPPPSPVSEK
PLDTISQKSVDIHDSIQPRSVDNRPQAPLVSASAVNEEVSKIKSTAKFPEQKLFAALLIK
CVVQLELIQTIDNIVFFPATSKKEDAENLAAAQRDAVDFDVRVDTQDQGMYRFLTSQQLF
KLLDCLLESHRFAKAFNSNNEQRTALWKAGFKGKSKPNLLKQETSSLACGLRILFRMYMD
ESRVSAWEEVQQRLLNVCSEALSYFLTLTSESHREAWTNLLLLFLTKVLKISDNRFKAHA

SFYYPLLCEIMQFDLIPELRAVLRRFFLRIGVVFQISQPPEQELGINKQ
>sp|Q9Y6D5|BIG2_HUMAN Brefeldin A-inhibited guanine nucleotide-exchange protein 2 OS=Homo sapiens GN=ARFGEF2 PE=1 SV=3
MQESQTKSMFVSRALEKILADKEVKRPQHSQLRRACQVALDEIKAEIEKQRLGTAAPPKA
NFIEADKYFLPFELACQSKSPRVVSTSLDCLQKLIAYGHITGNAPDSGAPGKRLIDRIVE
TICSCFQGPQTDEGVQLQIIKALLTAVTSPHIEIHEGTILQTVRTCYNIYLASKNLINQT
TAKATLTQMLNVIFTRMENQVLQEARELEKPIQSKPQSPVIQAAAVSPKFVRLKHSQAQS
KPTTPEKTDLTNGEHARSDSGKVSTENGDAPRERGSSLSGTDDGAQEVVKDILEDVVTSA
IKEAAEKHGLTEPERVLGELECQECAIPPGVDENSQTNGIADDRQSLSSADNLESDAQGH
QVAARFSHVLQKDAFLVFRSLCKLSMKPLGEGPPDPKSHELRSKVVSLQLLLSVLQNAGP
VFRTHEMFINAIKQYLCVALSKNGVSSVPDVFELSLAIFLTLLSNFKMHLKMQIEVFFKE
IFLNILETSTSSFEHRWMVIQTLTRICADAQCVVDIYVNYDCDLNAANIFERLVNDLSKI
AQGRSGHELGMTPLQELSLRKKGLECLVSILKCMVEWSKDLYVNPNHQTSLGQERLTDQE
IGDGKGLDMARRCSVTSMESTVSSGTQTTVQDDPEQFEVIKQQKEIIEHGIELFNKKPKR
GIQFLQEQGMLGTSVEDIAQFLHQEERLDSTQVGDFLGDSARFNKEVMYAYVDQLDFCEK
EFVSALRTFLEGFRLPGEAQKIDRLMEKFAARYIECNQGQTLFASADTAYVLAYSIIMLT
TDLHSPQVKNKMTKEQYIKMNRGINDSKDLPEEYLSSIYEEIEGKKIAMKETKELTIATK
STKQNVASEKQRRLLYNLEMEQMAKTAKALMEAVSHAKAPFTSATHLDHVRPMFKLVWTP
LLAAYSIGLQNCDDTEVASLCLEGIRCAIRIACIFGMQLERDAYVQALARFSLLTASSSI
TEMKQKNIDTIKTLITVAHTDGNYLGNSWHEILKCISQLELAQLIGTGVKTRYLSGSGRE
REGSLKGHTLAGEEFMGLGLGNLVSGGVDKRQMASFQESVGETSSQSVVVAVDRIFTGST
RLDGNAIVDFVRWLCAVSMDELASPHHPRMFSLQKIVEISYYNMNRIRLQWSRIWHVIGD
HFNKVGCNPNEDVAIFAVDSLRQLSMKFLEKGELANFRFQKDFLRPFEHIMKKNRSPTIR
DMAIRCIAQMVNSQAANIRSGWKNIFAVFHQAASDHDGNIVELAFQTTCHIVTTIFQHHF
PAAIDSFQDAVKCLSEFACNAAFPDTSMEAIRLIRFCGKYVSERPRVLQEYTSDDMNVAP
GDRVWVRGWFPILFELSCIINRCKLDVRTRGLTVMFEIMKSYGHTFEKHWWQDLFRIVFR
IFDNMKLPEQLSEKSEWMTTTCNHALYAICDVFTQFYEALNEVLLSDVFAQLQWCVKQDN
EQLARSGTNCLENLVISNGEKFSPEVWDETCNCMLDIFKTTIPHVLLTWRPVGMEEDSSE
KHLDVDLDRQSLSSIDKNPSERGQSQLSNPTDDSWKGRPYANQKLFASLLIKCVVQLELI
QTIDNIVFYPATSKKEDAEHMVAAQQDTLDADIHIETEDQGMYKYMSSQHLFKLLDCLQE
SHSFSKAFNSNYEQRTVLWRAGFKGKSKPNLLKQETSSLACCLRILFRMYVDENRRDSWE
EIQQRLLTVCSEALAYFITVNSESHREAWTSLLLLLLTKTLKINDEKFKAHASMYYPYLC
EIMQFDLIPELRAVLRKFFLRIGVVYKIWIPEEPSQVPAALSPVW
>sp|Q5TH69|BIG3_HUMAN Brefeldin A-inhibited guanine nucleotide-exchange protein 3 OS=Homo sapiens GN=ARFGEF3 PE=1 SV=3
MEEILRKLQKEASGSKYKAIKESCTWALETLGGLDTIVKIPPHVLREKCLLPLQLALESK
NVKLAQHALAGMQKLLSEERFVSMETDSDEKQLLNQILNAVKVTPSLNEDLQVEVMKVLL
CITYTPTFDLNGSAVLKIAEVCIETYISSCHQRSINTAVRATLSQMLSDLTLQLRQRQEN
TIIENPDVPQDFGNQGSTVESLCDDVVSVLTVLCEKLQAAINDSQQLQLLYLECILSVLS
SSSSSMHLHRRFTDLIWKNLCPALIVILGNPIHDKTITSAHTSSTSTSLESDSASPGVSD
HGRGSGCSCTAPALSGPVARTIYYIAAELVRLVGSVDSMKPVLQSLYHRVLLYPPPQHRV
EAIKIMKEILGSPQRLCDLAGPSSTESESRKRSISKRKSHLDLLKLIMDGMTEACIKGGI
EACYAAVSCVCTLLGALDELSQGKGLSEGQVQLLLLRLEELKDGAEWSRDSMEINEADFR
WQRRVLSSEHTPWESGNERSLDISISVTTDTGQTTLEGELGQTTPEDHSGNHKNSLKSPA

IPEGKETLSKVLETEAVDQPDVVQRSHTVPYPDITNFLSVDCRTRSYGSRYSESNFSVDD
QDLSRTEFDSCDQYSMAAEKDSGRSDVSDIGSDNCSLADEEQTPRDCLGHRSLRTAALSL
KLLKNQEADQHSARLFIQSLEGLLPRLLSLSNVEEVDTALQNFASTFCSGMMHSPGFDGN
SSLSFQMLMNADSLYTAAHCALLLNLKLSHGDYYRKRPTLAPGVMKDFMKQVQTSGVLMV
FSQAWIEELYHQVLDRNMLGEAGYWGSPEDNSLPLITMLTDIDGLESSAIGGQLMASAAT
ESPFAQSRRIDDSTVAGVAFARYILVGCWKNLIDTLSTPLTGRMAGSSKGLAFILGAEGI
KEQNQKERDAICMSLDGLRKAARLSCALGVAANCASALAQMAAASCVQEEKEEREAQEPS
DAITQVKLKVEQKLEQIGKVQGVWLHTAHVLCMEAILSVGLEMGSHNPDCWPHVFRVCEY
VGTLEHNHFSDGASQPPLTISQPQKATGSAGLLGDPECEGSPPEHSPEQGRSLSTAPVVQ
PLSIQDLVREGSRGRASDFRGGSLMSGSSAAKVVLTLSTQADRLFEDATDKLNLMALGGF
LYQLKKASQSQLFHSVTDTVDYSLAMPGEVKSTQDRKSALHLFRLGNAMLRIVRSKARPL
LHVMRCWSLVAPHLVEAACHKERHVSQKAVSFIHDILTEVLTDWNEPPHFHFNEALFRPF
ERIMQLELCDEDVQDQVVTSIGELVEVCSTQIQSGWRPLFSALETVHGGNKSEMKEYLVG
DYSMGKGQAPVFDVFEAFLNTDNIQVFANAATSYIMCLMKFVKGLGEVDCKEIGDCAPAP
GAPSTDLCLPALDYLRRCSQLLAKIYKMPLKPIFLSGRLAGLPRRLQEQSASSEDGIESV
LSDFDDDTGLIEVWIILLEQLTAAVSNCPRQHQPPTLDLLFELLRDVTKTPGPGFGIYAV
VHLLLPVMSVWLRRSHKDHSYWDMASANFKHAIGLSCELVVEHIQSFLHSDIRYESMINT
MLKDLFELLVACVAKPTETISRVGCSCIRYVLVTAGPVFTEEMWRLACCALQDAFSATLK
PVKDLLGCFHSGTESFSGEGCQVRVAAPSSSPSAEAEYWRIRAMAQQVFMLDTQCSPKTP
NNFDHAQSCQLIIELPPDEKPNGHTKKSVSFREIVVSLLSHQVLLQNLYDILLEEFVKGP
SPGEEKTIQVPEAKLAGFLRYISMQNLAVIFDLLLDSYRTAREFDTSPGLKCLLKKVSGI
GGAANLYRQSAMSFNIYFHALVCAVLTNQETITAEQVKKVLFEDDERSTDSSQQCSSEDE
DIFEETAQVSPPRGKEKRQWRARMPLLSVQPVSNADWVWLVKRLHKLCMELCNNYIQMHL
DLENCMEEPPIFKGDPFFILPSFQSESSTPSTGGFSGKETPSEDDRSQSREHMGESLSLK
AGGGDLLLPPSPKVEKKDPSRKKEWWENAGNKIYTMAADKTISKLMTEYKKRKQQHNLSA
FPKEVKVEKKGEPLGPRGQDSPLLQRPQHLMDQGQMRHSFSAGPELLRQDKRPRSGSTGS
SLSVSVRDAEAQIQAWTNMVLTVLNQIQILPDQTFTALQPAVFPCISQLTCHVTDIRVRQ
AVREWLGRVGRVYDIIV
>sp|O95393|BMP10_HUMAN Bone morphogenetic protein 10 OS=Homo sapiens GN=BMP10 PE=1 SV=1
MGSLVLTLCALFCLAAYLVSGSPIMNLEQSPLEEDMSLFGDVFSEQDGVDFNTLLQSMKD
EFLKTLNLSDIPTQDSAKVDPPEYMLELYNKFATDRTSMPSANIIRSFKNEDLFSQPVSF
NGLRKYPLLFNVSIPHHEEVIMAELRLYTLVQRDRMIYDGVDRKITIFEVLESKGDNEGE
RNMLVLVSGEIYGTNSEWETFDVTDAIRRWQKSGSSTHQLEVHIESKHDEAEDASSGRLE
IDTSAQNKHNPLLIVFSDDQSSDKERKEELNEMISHEQLPELDNLGLDSFSSGPGEEALL
QMRSNIIYDSTARIRRNAKGNYCKRTPLYIDFKEIGWDSWIIAPPGYEAYECRGVCNYPL
AEHLTPTKHAIIQALVHLKNSQKASKACCVPTKLEPISILYLDKGVVTYKFKYEGMAVSE
CGCR
>sp|O95972|BMP15_HUMAN Bone morphogenetic protein 15 OS=Homo sapiens GN=BMP15 PE=1 SV=2
MVLLSILRILFLCELVLFMEHRAQMAEGGQSSIALLAEAPTLPLIEELLEESPGEQPRKP
RLLGHSLRYMLELYRRSADSHGHPRENRTIGATMVRLVKPLTNVARPHRGTWHIQILGFP
LRPNRGLYQLVRATVVYRHHLQLTRFNLSCHVEPWVQKNPTNHFPSSEGDSSKPSLMSNA
WKEMDITQLVQQRFWNNKGHRILRLRFMCQQQKDSGGLELWHGTSSLDIAFLLLYFNDTH
KSIRKAKFLPRGMEEFMERESLLRRTRQADGISAEVTASSSKHSGPENNQCSLHPFQISF
RQLGWDHWIIAPPFYTPNYCKGTCLRVLRDGLNSPNHAIIQNLINQLVDQSVPRPSCVPY

KYVPISVLMIEANGSILYKEYEGMIAESCTCR
>sp|Q9NSY1|BMP2K_HUMAN BMP-2-inducible protein kinase OS=Homo sapiens GN=BMP2K PE=1 SV=2
MKKFSRMPKSEGGSGGGAAGGGAGGAGAGAGCGSGGSSVGVRVFAVGRHQVTLEESLAEG
GFSTVFLVRTHGGIRCALKRMYVNNMPDLNVCKREITIMKELSGHKNIVGYLDCAVNSIS
DNVWEVLILMEYCRAGQVVNQMNKKLQTGFTEPEVLQIFCDTCEAVARLHQCKTPIIHRD
LKVENILLNDGGNYVLCDFGSATNKFLNPQKDGVNVVEEEIKKYTTLSYRAPEMINLYGG
KPITTKADIWALGCLLYKLCFFTLPFGESQVAICDGNFTIPDNSRYSRNIHCLIRFMLEP
DPEHRPDIFQVSYFAFKFAKKDCPVSNINNSSIPSALPEPMTASEAAARKSQIKARITDT
IGPTETSIAPRQRPKANSATTATPSVLTIQSSATPVKVLAPGEFGNHRPKGALRPGNGPE
ILLGQGPPQQPPQQHRVLQQLQQGDWRLQQLHLQHRHPHQQQQQQQQQQQQQQQQQQQQQ
QQQQQQHHHHHHHHLLQDAYMQQYQHATQQQQMLQQQFLMHSVYQPQPSASQYPTMMPQY
QQAFFQQQMLAQHQPSQQQASPEYLTSPQEFSPALVSYTSSLPAQVGTIMDSSYSANRSV
ADKEAIANFTNQKNISNPPDMSGWNPFGEDNFSKLTEEELLDREFDLLRSNRLEERASSD
KNVDSLSAPHNHPPEDPFGSVPFISHSGSPEKKAEHSSINQENGTANPIKNGKTSPASKD
QRTGKKTSVQGQVQKGNDESESDFESDPPSPKSSEEEEQDDEEVLQGEQGDFNDDDTEPE
NLGHRPLLMDSEDEEEEEKHSSDSDYEQAKAKYSDMSSVYRDRSGSGPTQDLNTILLTSA
QLSSDVAVETPKQEFDVFGAVPFFAVRAQQPQQEKNEKNLPQHRFPAAGLEQEEFDVFTK
APFSKKVNVQECHAVGPEAHTIPGYPKSVDVFGSTPFQPFLTSTSKSESNEDLFGLVPFD
EITGSQQQKVKQRSLQKLSSRQRRTKQDMSKSNGKRHHGTPTSTKKTLKPTYRTPERARR
HKKVGRRDSQSSNEFLTISDSKENISVALTDGKDRGNVLQPEESLLDPFGAKPFHSPDLS
WHPPHQGLSDIRADHNTVLPGRPRQNSLHGSFHSADVLKMDDFGAVPFTELVVQSITPHQ
SQQSQPVELDPFGAAPFPSKQ
>sp|P12645|BMP3_HUMAN Bone morphogenetic protein 3 OS=Homo sapiens GN=BMP3 PE=1 SV=2
MAGASRLLFLWLGCFCVSLAQGERPKPPFPELRKAVPGDRTAGGGPDSELQPQDKVSEHM
LRLYDRYSTVQAARTPGSLEGGSQPWRPRLLREGNTVRSFRAAAAETLERKGLYIFNLTS
LTKSENILSATLYFCIGELGNISLSCPVSGGCSHHAQRKHIQIDLSAWTLKFSRNQSQLL
GHLSVDMAKSHRDIMSWLSKDITQLLRKAKENEEFLIGFNITSKGRQLPKRRLPFPEPYI
LVYANDAAISEPESVVSSLQGHRNFPTGTVPKWDSHIRAALSIERRKKRSTGVLLPLQNN
ELPGAEYQYKKDEVWEERKPYKTLQAQAPEKSKNKKKQRKGPHRKSQTLQFDEQTLKKAR
RKQWIEPRNCARRYLKVDFADIGWSEWIISPKSFDAYYCSGACQFPMPKSLKPSNHATIQ
SIVRAVGVVPGIPEPCCVPEKMSSLSILFFDENKNVVLKVYPNMTVESCACR
>sp|Q7Z5Y6|BMP8A_HUMAN Bone morphogenetic protein 8A OS=Homo sapiens GN=BMP8A PE=2 SV=2
MAARPGPLWLLGLTLCALGGGGPGLRPPPGCPQRRLGARERRDVQREILAVLGLPGRPRP
RAPPAASRLPASAPLFMLDLYHAMAGDDDEDGAPAEQRLGRADLVMSFVNMVERDRALGH
QEPHWKEFRFDLTQIPAGEAVTAAEFRIYKVPSIHLLNRTLHVSMFQVVQEQSNRESDLF
FLDLQTLRAGDEGWLVLDVTAASDCWLLKRHKDLGLRLYVETEDGHSVDPGLAGLLGQRA
PRSQQPFVVTFFRASPSPIRTPRAVRPLRRRQPKKSNELPQANRLPGIFDDVRGSHGRQV
CRRHELYVSFQDLGWLDWVIAPQGYSAYYCEGECSFPLDSCMNATNHAILQSLVHLMKPN
AVPKACCAPTKLSATSVLYYDSSNNVILRKHRNMVVKACGCH
>sp|Q53S33|BOLA3_HUMAN BolA-like protein 3 OS=Homo sapiens GN=BOLA3 PE=1 SV=1
MAAWSPAAAAPLLRGIRGLPLHHRMFATQTEGELRVTQILKEKFPRATAIKVTDISGGCG
AMYEIKIESEEFKEKRTVQQHQMVNQALKEEIKEMHGLRIFTSVPKR
>sp|Q96FH0|BORC8_HUMAN BLOC-1-related complex subunit 8 OS=Homo sapiens GN=BORCS8 PE=1 SV=1

MEEPEMQLKGKKVTDKFTESVYVLANEPSVALYRLQEHVRRSLPELAQHKADMQRWEEQS
QGAIYTVEYACSAVKNLVDSSVYFRSVEGLLKQAISIRDHMNASAQGHSPEEPPPPSSA
>sp|Q9GZL8|BPEC1_HUMAN Putative BPES syndrome breakpoint region protein OS=Homo sapiens GN=BPESC1 PE=2 SV=1
MGGRDQITNLGSEGETHPWGYSSPPYPLHTFFIPFLPSGFGGSGLGIPSDNEKHDLQDCV
EVSRPEGPAPELPSSLCGWNKISSLCGLGFPSRDPKTWDLAMLLSPWVDFFELQLL
>sp|Q9NP55|BPIA1_HUMAN BPI fold-containing family A member 1 OS=Homo sapiens GN=BPIFA1 PE=1 SV=1
MFQTGGLIVFYGLLAQTMAQFGGLPVPLDQTLPLNVNPALPLSPTGLAGSLTNALSNGLL
SGGLLGILENLPLLDILKPGGGTSGGLLGGLLGKVTSVIPGLNNIIDIKVTDPQLLELGL
VQSPDGHRLYVTIPLGIKLQVNTPLVGASLLRLAVKLDITAEILAVRDKQERIHLVLGDC
THSPGSLQISLLDGLGPLPIQGLLDSLTGILNKVLPELVQGNVCPLVNEVLRGLDITLVH
DIVNMLIHGLQFVIKV
>sp|Q8N4F0|BPIB2_HUMAN BPI fold-containing family B member 2 OS=Homo sapiens GN=BPIFB2 PE=1 SV=2
MAWASRLGLLLALLLPVVGASTPGTVVRLNKAALSYVSEIGKAPLQRALQVTVPHFLDWS
GEALQPTRIRILNVHVPRLHLKFIAGFGVRLLAAANFTFKVFRAPEPLELTLPVELLADT
RVTQSSIRTPVVSISACSLFSGHANEFDGSNSTSHALLVLVQKHIKAVLSNKLCLSISNL
VQGVNVHLGTLIGLNPVGPESQIRYSMVSVPTVTSDYISLEVNAVLFLLGKPIILPTDAT
PFVLPRHVGTEGSMATVGLSQQLFDSALLLLQKAGALNLDITGQLRSDDNLLNTSALGRL
IPEVARQFPEPMPVVLKVRLGATPVAMLHTNNATLRLQPFVEVLATASNSAFQSLFSLDV
VVNLRLQLSVSKVKLQGTTSVLGDVQLTVASSNVGFIDTDQVRTLMGTVFEKPLLDHLNA
LLAMGIALPGVVNLHYVAPEIFVYEGYVVISSGLFYQS
>sp|Q8NFQ5|BPIB6_HUMAN BPI fold-containing family B member 6 OS=Homo sapiens GN=BPIFB6 PE=1 SV=1
MLRILCLALCSLLTGTRADPGALLRLGMDIMNQVQSAMDESHILEKMAAEAGKKQPGMKP
IKGITNLKVKDVQLPVITLNFVPGVGIFQCVSTGMTVTGKSFMGGNMEIIVALNITATNR
LLRDEETGLPVFKSEGCEVILVNVKTNLPSNMLPKMVNKFLDSTLHKVLPGLMCPAIDAV
LVYVNRKWTNLSDPMPVGQMGTVKYVLMSAPATTASYIQLDFSPVVQQQKGKTIKLADAG
EALTFPEGYAKGSSQLLLPATFLSAELALLQKSFHVNIQDTMIGELPPQTTKTLARFIPE
VAVAYPKSKPLTTQIKIKKPPKVTMKTGKSLLHLHSTLEMFAARWRSKAPMSLFLLEVHF
NLKVQYSVHENQLQMATSLDRLLSLSRKSSSIGNFNERELTGFITSYLEEAYIPVVNDVL
QVGLPLPDFLAMNYNLAELDIVENALMLDLKLG
>sp|P59051|BRAS2_HUMAN Putative uncharacterized protein encoded by BRWD1-AS2 OS=Homo sapiens GN=BRWD1-AS2 PE=5 SV=1
MLGWIQPSRQPQLRAAPPTRTPSAKRCILCNFLPGCWLVGDVAGSRQPSAPQTLRQRQHT
RPPPQERGSGRRSPLREARRANPHFKSFPVLEARGLPCGARRTGPRRPVREMTLPSDPER
ATLPNPRLGAPAVPRRGPRSHGGRR
>sp|Q9UPA5|BSN_HUMAN Protein bassoon OS=Homo sapiens GN=BSN PE=1 SV=4
MGNEVSLEGGAGDGPLPPGGAGPGPGPGPGPGAGKPPSAPAGGGQLPAAGAARSTAVPPV
PGPGPGPGPGPGPGSTSRRLDPKEPLGNQRAASPTPKQASATTPGHESPRETRAQGPAGQ
EADGPRRTLQVDSRTQRSGRSPSVSPDRGSTPTSPYSVPQIAPLPSSTLCPICKTSDLTS
TPSQPNFNTCTQCHNKVCNQCGFNPNPHLTQVKEWLCLNCQMQRALGMDMTTAPRSKSQQ
QLHSPALSPAHSPAKQPLGKPDQERSRGPGGPQPGSRQAETARATSVPGPAQAAAPPEVG

RVSPQPPQPTKPSTAEPRPPAGEAPAKSATAVPAGLGATEQTQEGLTGKLFGLGASLLTQ
ASTLMSVQPEADTQGQPAPSKGTPKIVFNDASKEAGPKPLGSGPGPGPAPGAKTEPGARM
GPGSGPGALPKTGGTTSPKHGRAEHQAASKAAAKPKTMPKERAICPLCQAELNVGSKSPA
NYNTCTTCRLQVCNLCGFNPTPHLVEKTEWLCLNCQTKRLLEGSLGEPTPLPPPTSQQPP
VGAPHRASGTSPLKQKGPQGLGQPSGPLPAKASPLSTKASPLPSKASPQAKPLRASEPSK
TPSSVQEKKTRVPTKAEPMPKPPPETTPTPATPKVKSGVRRAEPATPVVKAVPEAPKGGE
AEDLVGKPYSQDASRSPQSLSDTGYSSDGISSSQSEITGVVQQEVEQLDSAGVTGPHPPS
PSEIHKVGSSMRPLLQAQGLAPSERSKPLSSGTGEEQKQRPHSLSITPEAFDSDEELEDI
LEEDEDSAEWRRRREQQDTAESSDDFGSQLRHDYVEDSSEGGLSPLPPQPPARAAELTDE
DFMRRQILEMSAEEDNLEEDDTATSGRGLAKHGTQKGGPRPRPEPSQEPAALPKRRLPHN
ATTGYEELLPEGGSAEATDGSGTLQGGLRRFKTIELNSTGSYGHELDLGQGPDPSLDREP
ELEMESLTGSPEDRSRGEHSSTLPASTPSYTSGTSPTSLSSLEEDSDSSPSRRQRLEEAK
QQRKARHRSHGPLLPTIEDSSEEEELREEEELLREQEKMREVEQQRIRSTARKTRRDKEE
LRAQRRRERSKTPPSNLSPIEDASPTEELRQAAEMEELHRSSCSEYSPSPSLDSEAEALD
GGPSRLYKSGSEYNLPTFMSLYSPTETPSGSSTTPSSGRPLKSAEEAYEEMMRKAELLQR
QQGQAAGARGPHGGPSQPTGPRGLGSFEYQDTTDREYGQAAQPAAEGTPASLGAAVYEEI
LQTSQSIVRMRQASSRDLAFAEDKKKEKQFLNAESAYMDPMKQNGGPLTPGTSPTQLAAP
VSFSTPTSSDSSGGRVIPDVRVTQHFAKETQDPLKLHSSPASPSSASKEIGMPFSQGPGT
PATTAVAPCPAGLPRGYMTPASPAGSERSPSPSSTAHSYGHSPTTANYGSQTEDLPQAPS
GLAAAGRAAREKPLSASDGEGGTPQPSRAYSYFASSSPPLSPSSPSESPTFSPGKMGPRA
TAEFSTQTPSPAPASDMPRSPGAPTPSPMVAQGTQTPHRPSTPRLVWQESSQEAPFMVIT
LASDASSQTRMVHASASTSPLCSPTETQPTTHGYSQTTPPSVSQLPPEPPGPPGFPRVPS
AGADGPLALYGWGALPAENISLCRISSVPGTSRVEPGPRTPGTAVVDLRTAVKPTPIILT
DQGMDLTSLAVEARKYGLALDPIPGRQSTAVQPLVINLNAQEHTFLATATTVSITMASSV
FMAQQKQPVVYGDPYQSRLDFGQGGGSPVCLAQVKQVEQAVQTAPYRSGPRGRPREAKFA
RYNLPNQVAPLARRDVLITQMGTAQSIGLKPGPVPEPGAEPHRATPAELRSHALPGARKP
HTVVVQMGEGTAGTVTTLLPEEPAGALDLTGMRPESQLACCDMVYKLPFGSSCTGTFHPA
PSVPEKSMADAAPPGQSSSPFYGPRDPEPPEPPTYRAQGVVGPGPHEEQRPYPQGLPGRL
YSSMSDTNLAEAGLNYHAQRIGQLFQGPGRDSAMDLSSLKHSYSLGFADGRYLGQGLQYG
SVTDLRHPTDLLAHPLPMRRYSSVSNIYSDHRYGPRGDAVGFQEASLAQYSATTAREISR
MCAALNSMDQYGGRHGSGGGGPDLVQYQPQHGPGLSAPQSLVPLRPGLLGNPTFPEGHPS
PGNLAQYGPAAGQGTAVRQLLPSTATVRAADGMIYSTINTPIAATLPITTQPASVLRPMV
RGGMYRPYASGGITAVPLTSLTRVPMIAPRVPLGPTGLYRYPAPSRFPIASSVPPAEGPV
YLGKPAAAKAPGAGGPSRPEMPVGAAREEPLPTTTPAAIKEAAGAPAPAPLAGQKPPADA
APGGGSGALSRPGFEKEEASQEERQRKQQEQLLQLERERVELEKLRQLRLQEELERERVE
LQRHREEEQLLVQRELQELQTIKHHVLQQQQEERQAQFALQREQLAQQRLQLEQIQQLQQ
QLQQQLEEQKQRQKAPFPAACEAPGRGPPLAAAELAQNGQYWPPLTHAAFIAMAGPEGLG
QPREPVLHRGLPSSASDMSLQTEEQWEASRSGIKKRHSMPRLRDACELESGTEPCVVRRI
ADSSVQTDDEDGESRYLLSRRRRARRSADCSVQTDDEDSAEWEQPVRRRRSRLPRHSDSG
SDSKHDATASSSSAAATVRAMSSVGIQTISDCSVQTEPDQLPRVSPAIHITAATDPKVEI
VRYISAPEKTGRGESLACQTEPDGQAQGVAGPQLVGPTAISPYLPGIQIVTPGPLGRFEK
KKPDPLEIGYQAHLPPESLSQLVSRQPPKSPQVLYSPVSPLSPHRLLDTSFASSERLNKA
HVSPQKHFTADSALRQQTLPRPMKTLQRSLSDPKPLSPTAEESAKERFSLYQHQGGLGSQ
VSALPPNSLVRKVKRTLPSPPPEEAHLPLAGQASPQLYAASLLQRGLTGPTTVPATKASL

LRELDRDLRLVEHESTKLRKKQAELDEEEKEIDAKLKYLELGITQRKESLAKDRGGRDYP
PLRGLGEHRDYLSDSELNQLRLQGCTTPAGQFVDFPATAAAPATPSGPTAFQQPRFQPPA
PQYSAGSGGPTQNGFPAHQAPTYPGPSTYPAPAFPPGASYPAEPGLPNQQAFRPTGHYAG
QTPMPTTQSTLFPVPADSRAPLQKPRQTSLADLEQKVPTNYEVIASPVVPMSSAPSETSY
SGPAVSSGYEQGKVPEVPRAGDRGSVSQSPAPTYPSDSHYTSLEQNVPRNYVMIDDISEL
TKDSTSTAPDSQRLEPLGPGSSGRPGKEPGEPGVLDGPTLPCCYARGEEESEEDSYDPRG
KGGHLRSMESNGRPASTHYYGDSDYRHGARVEKYGPGPMGPKHPSKSLAPAAISSKRSKH
RKQGMEQKISKFSPIEEAKDVESDLASYPPPAVSSSLVSRGRKFQDEITYGLKKNVYEQQ
KYYGMSSRDAVEDDRIYGGSSRSRAPSAYSGEKLSSHDFSGWGKGYEREREAVERLQKAG
PKPSSLSMAHSRVRPPMRSQASEEESPVSPLGRPRPAGGPLPPGGDTCPQFCSSHSMPDV
QEHVKDGPRAHAYKREEGYILDDSHCVVSDSEAYHLGQEETDWFDKPRDARSDRFRHHGG
HAVSSSSQKRGPARHSYHDYDEPPEEGLWPHDEGGPGRHASAKEHRHGDHGRHSGRHTGE
EPGRRAAKPHARDLGRHEARPHSQPSSAPAMPKKGQPGYPSSAEYSQPSRASSAYHHASD
SKKGSRQAHSGPAALQSKAEPQAQPQLQGRQAAPGPQQSQSPSSRQIPSGAASRQPQTQQ
QQQGLGLQPPQQALTQARLQQQSQPTTRGSAPAASQPAGKPQPGPSTATGPQPAGPPRAE
QTNGSKGTAKAPQQGRAPQAQPAPGPGPAGVKAGARPGGTPGAPAGQPGADGESVFSKIL
PGGAAEQAGKLTEAVSAFGKKFSSFW
>sp|Q13410|BT1A1_HUMAN Butyrophilin subfamily 1 member A1 OS=Homo sapiens GN=BTN1A1 PE=1 SV=3
MAVFPSSGLPRCLLTLILLQLPKLDSAPFDVIGPPEPILAVVGEDAELPCRLSPNASAEH
LELRWFRKKVSPAVLVHRDGREQEAEQMPEYRGRATLVQDGIAKGRVALRIRGVRVSDDG
EYTCFFREDGSYEEALVHLKVAALGSDPHISMQVQENGEICLECTSVGWYPEPQVQWRTS
KGEKFPSTSESRNPDEEGLFTVAASVIIRDTSAKNVSCYIQNLLLGQEKKVEISIPASSL
PRLTPWIVAVAVILMVLGLLTIGSIFFTWRLYNERPRERRNEFSSKERLLEELKWKKATL
HAVDVTLDPDTAHPHLFLYEDSKSVRLEDSRQKLPEKTERFDSWPCVLGRETFTSGRHYW
EVEVGDRTDWAIGVCRENVMKKGFDPMTPENGFWAVELYGNGYWALTPLRTPLPLAGPPR
RVGIFLDYESGDISFYNMNDGSDIYTFSNVTFSGPLRPFFCLWSSGKKPLTICPIADGPE
RVTVIANAQDLSKEIPLSPMGEDSAPRDADTLHSKLIPTQPSQGAP
>sp|O14981|BTAF1_HUMAN TATA-binding protein-associated factor 172 OS=Homo sapiens GN=BTAF1 PE=1 SV=2
MAVSRLDRLFILLDTGTTPVTRKAAAQQLGEVVKLHPHELNNLLSKVLIYLRSANWDTRI
AAGQAVEAIVKNVPEWNPVPRTRQEPTSESSMEDSPTTERLNFDRFDICRLLQHGASLLG
SAGAEFEVQDEKSGEVDPKERIARQRKLLQKKLGLNMGEAIGMSTEELFNDEDLDYTPTS
ASFVNKQPTLQAAELIDSEFRAGMSNRQKNKAKRMAKLFAKQRSRDAVETNEKSNDSTDG
EPEEKRRKIANVVINQSANDSKVLIDNIPDSSSLIEETNEWPLESFCEELCNDLFNPSWE
VRHGAGTGLREILKAHGKSGGKMGDSTLEEMIQQHQEWLEDLVIRLLCVFALDRFGDFVS
DEVVAPVRETCAQTLGVVLKHMNETGVHKTVDVLLKLLTQEQWEVRHGGLLGIKYALAVR
QDVINTLLPKVLTRIIEGLQDLDDDVRAVAAASLVPVVESLVYLQTQKVPFIINTLWDAL
LELDDLTASTNSIMTLLSSLLTYPQVQQCSIQQSLTVLVPRVWPFLHHTISSVRRAALET
LFTLLSTQDQNSSSWLIPILPDMLRHIFQFCVLESSQEILDLIHKVWMELLSKASVQYVV
AAACPWMGAWLCLMMQPSHLPIDLNMLLEVKARAKEKTGGKVRQGQSQNKEVLQEYIAGA
DTIMEDPATRDFVVMRARMMAAKLLGALCCCICDPGVNVVTQEIKPAESLGQLLLFHLNS
KSALQRISVALVICEWAALQKECKAVTLAVQPRLLDILSEHLYYDEIAVPFTRMQNECKQ
LISSLADVHIEVGNRVNNNVLTIDQASDLVTTVFNEATSSFDLNPQVLQQLDSKRQQVQM

TVTETNQEWQVLQLRVHTFAACAVVSLQQLPEKLNPIIKPLMETIKKEENTLVQNYAAQC
IAKLLQQCTTRTPCPNSKIIKNLCSSLCVDPYLTPCVTCPVPTQSGQENSKGSTSEKDGM
HHTVTKHRGIITLYRHQKAAFAITSRRGPTPKAVKAQIADLPAGSSGNILVELDEAQKPY
LVQRRGAEFALTTIVKHFGGEMAVKLPHLWDAMVGPLRNTIDINNFDGKSLLDKGDSPAQ
ELVNSLQVFETAAASMDSELHPLLVQHLPHLYMCLQYPSTAVRHMAARCVGVMSKIATME
TMNIFLEKVLPWLGAIDDSVKQEGAIEALACVMEQLDVGIVPYIVLLVVPVLGRMSDQTD
SVRFMATQCFATLIRLMPLEAGIPDPPNMSAELIQLKAKERHFLEQLLDGKKLENYKIPV
PINAELRKYQQDGVNWLAFLNKYKLHGILCDDMGLGKTLQSICILAGDHCHRAQEYARSK
LAECMPLPSLVVCPPTLTGHWVDEVGKFCSREYLNPLHYTGPPTERIRLQHQVKRHNLIV
ASYDVVRNDIDFFRNIKFNYCILDEGHVIKNGKTKLSKAVKQLTANYRIILSGTPIQNNV
LELWSLFDFLMPGFLGTERQFAARYGKPILASRDARSSSREQEAGVLAMDALHRQVLPFL
LRRMKEDVLQDLPPKIIQDYYCTLSPLQVQLYEDFAKSRAKCDVDETVSSATLSEETEKP
KLKATGHVFQALQYLRKLCNHPALVLTPQHPEFKTTAEKLAVQNSSLHDIQHAPKLSALK
QLLLDCGLGNGSTSESGTESVVAQHRILIFCQLKSMLDIVEHDLLKPHLPSVTYLRLDGS
IPPGQRHSIVSRFNNDPSIDVLLLTTHVGGLGLNLTGADTVVFVEHDWNPMRDLQAMDRA
HRIGQKRVVNVYRLITRGTLEEKIMGLQKFKMNIANTVISQENSSLQSMGTDQLLDLFTL
DKDGKAEKADTSTSGKASMKSILENLSDLWDQEQYDSEYSLENFMHSLK
>sp|Q9H0C5|BTBD1_HUMAN BTB/POZ domain-containing protein 1 OS=Homo sapiens GN=BTBD1 PE=1 SV=1
MASLGPAAAGEQASGAEAEPGPAGPPPPPSPSSLGPLLPLQREPLYNWQATKASLKERFA
FLFNSELLSDVRFVLGKGRGAAAAGGPQRIPAHRFVLAAGSAVFDAMFNGGMATTSAEIE
LPDVEPAAFLALLRFLYSDEVQIGPETVMTTLYTAKKYAVPALEAHCVEFLTKHLRADNA
FMLLTQARLFDEPQLASLCLDTIDKSTMDAISAEGFTDIDIDTLCAVLERDTLSIRESRL
FGAVVRWAEAECQRQQLPVTFGNKQKVLGKALSLIRFPLMTIEEFAAGPAQSGILSDREV
VNLFLHFTVNPKPRVEYIDRPRCCLRGKECCINRFQQVESRWGYSGTSDRIRFTVNRRIS
IVGFGLYGSIHGPTDYQVNIQIIEYEKKQTLGQNDTGFSCDGTANTFRVMFKEPIEILPN
VCYTACATLKGPDSHYGTKGLKKVVHETPAASKTVFFFFSSPGNNNGTSIEDGQIPEIIF
YT
>sp|Q53HL2|BOREA_HUMAN Borealin OS=Homo sapiens GN=CDCA8 PE=1 SV=2
MAPRKGSSRVAKTNSLRRRKLASFLKDFDREVEIRIKQIESDRQNLLKEVDNLYNIEILR
LPKALREMNWLDYFALGGNKQALEEAATADLDITEINKLTAEAIQTPLKSAKTRKVIQVD
EMIVEEEEEEENERKNLQTARVKRCPPSKKRTQSIQGKGKGKRSSRANTVTPAVGRLEVS
MVKPTPGLTPRFDSRVFKTPGLRTPAAGERIYNISGNGSPLADSKEIFLTVPVGGGESLR
LLASDLQRHSIAQLDPEALGNIKKLSNRLAQICSSIRTHK
>sp|Q86WA6|BPHL_HUMAN Valacyclovir hydrolase OS=Homo sapiens GN=BPHL PE=1 SV=1
MVAVLGGRGVLRLRLLLSALKPGIHVPRAGPAAAFGTSVTSAKVAVNGVQLHYQQTGEGD
HAVLLLPGMLGSGETDFGPQLKNLNKKLFTVVAWDPRGYGHSRPPDRDFPADFFERDAKD
AVDLMKALKFKKVSLLGWSDGGITALIAAAKYPSYIHKMVIWGANAYVTDEDSMIYEGIR
DVSKWSERTRKPLEALYGYDYFARTCEKWVDGIRQFKHLPDGNICRHLLPRVQCPALIVH
GEKDPLVPRFHADFIHKHVKGSRLHLMPEGKHNLHLRFADEFNKLAEDFLQ
>sp|Q96DR5|BPIA2_HUMAN BPI fold-containing family A member 2 OS=Homo sapiens GN=BPIFA2 PE=1 SV=2
MLQLWKLVLLCGVLTGTSESLLDNLGNDLSNVVDKLEPVLHEGLETVDNTLKGILEKLKV
DLGVLQKSSAWQLAKQKAQEAEKLLNNVISKLLPTNTDIFGLKISNSLILDVKAEPIDDG

KGLNLSFPVTANVTVAGPIIGQIINLKASLDLLTAVTIETDPQTHQPVAVLGECASDPTS
ISLSLLDKHSQIINKFVNSVINTLKSTVSSLLQKEICPLIRIFIHSLDVNVIQQVVDNPQ
HKTQLQTLI
>sp|O95861|BPNT1_HUMAN 3'(2'),5'-bisphosphate nucleotidase 1 OS=Homo sapiens GN=BPNT1 PE=1 SV=1
MASSNTVLMRLVASAYSIAQKAGMIVRRVIAEGDLGIVEKTCATDLQTKADRLAQMSICS
SLARKFPKLTIIGEEDLPSEEVDQELIEDSQWEEILKQPCPSQYSAIKEEDLVVWVDPLD
GTKEYTEGLLDNVTVLIGIAYEGKAIAGVINQPYYNYEAGPDAVLGRTIWGVLGLGAFGF
QLKEVPAGKHIITTTRSHSNKLVTDCVAAMNPDAVLRVGGAGNKIIQLIEGKASAYVFAS
PGCKKWDTCAPEVILHAVGGKLTDIHGNVLQYHKDVKHMNSAGVLATLRNYDYYASRVPE
SIKNALVP
>sp|Q12830|BPTF_HUMAN Nucleosome-remodeling factor subunit BPTF OS=Homo sapiens GN=BPTF PE=1 SV=3
MRGRRGRPPKQPAAPAAERCAPAPPPPPPPPTSGPIGGLRSRHRGSSRGRWAAAQAEVAP
KTRLSSPRGGSSSRRKPPPPPPAPPSTSAPGRGGRGGGGGRTGGGGGGGHLARTTAARRA
VNKVVYDDHESEEEEEEEDMVSEEEEEEDGDAEETQDSEDDEEDEMEEDDDDSDYPEEME
DDDDDASYCTESSFRSHSTYSSTPGRRKPRVHRPRSPILEEKDIPPLEFPKSSEDLMVPN
EHIMNVIAIYEVLRNFGTVLRLSPFRFEDFCAALVSQEQCTLMAEMHVVLLKAVLREEDT
SNTTFGPADLKDSVNSTLYFIDGMTWPEVLRVYCESDKEYHHVLPYQEAEDYPYGPVENK
IKVLQFLVDQFLTTNIAREELMSEGVIQYDDHCRVCHKLGDLLCCETCSAVYHLECVKPP
LEEVPEDEWQCEVCVAHKVPGVTDCVAEIQKNKPYIRHEPIGYDRSRRKYWFLNRRLIIE
EDTENENEKKIWYYSTKVQLAELIDCLDKDYWEAELCKILEEMREEIHRHMDITEDLTNK
ARGSNKSFLAAANEEILESIRAKKGDIDNVKSPEETEKDKNETENDSKDAEKNREEFEDQ
SLEKDSDDKTPDDDPEQGKSEEPTEVGDKGNSVSANLGDNTTNATSEETSPSEGRSPVGC
LSETPDSSNMAEKKVASELPQDVPEEPNKTCESSNTSATTTSIQPNLENSNSSSELNSSQ
SESAKAADDPENGERESHTPVSIQEEIVGDFKSEKSNGELSESPGAGKGASGSTRIITRL
RNPDSKLSQLKSQQVAAAAHEANKLFKEGKEVLVVNSQGEISRLSTKKEVIMKGNINNYF
KLGQEGKYRVYHNQYSTNSFALNKHQHREDHDKRRHLAHKFCLTPAGEFKWNGSVHGSKV
LTISTLRLTITQLENNIPSSFLHPNWASHRANWIKAVQMCSKPREFALALAILECAVKPV
VMLPIWRESLGHTRLHRMTSIEREEKEKVKKKEKKQEEEETMQQATWVKYTFPVKHQVWK
QKGEEYRVTGYGGWSWISKTHVYRFVPKLPGNTNVNYRKSLEGTKNNMDENMDESDKRKC
SRSPKKIKIEPDSEKDEVKGSDAAKGADQNEMDISKITEKKDQDVKELLDSDSDKPCKEE
PMEVDDDMKTESHVNCQESSQVDVVNVSEGFHLRTSYKKKTKSSKLDGLLERRIKQFTLE
EKQRLEKIKLEGGIKGIGKTSTNSSKNLSESPVITKAKEGCQSDSMRQEQSPNANNDQPE
DLIQGCSESDSSVLRMSDPSHTTNKLYPKDRVLDDVSIRSPETKCPKQNSIENDIEEKVS
DLASRGQEPSKSKTKGNDFFIDDSKLASADDIGTLICKNKKPLIQEESDTIVSSSKSALH
SSVPKSTNDRDATPLSRAMDFEGKLGCDSESNSTLENSSDTVSIQDSSEEDMIVQNSNES
ISEQFRTREQDVEVLEPLKCELVSGESTGNCEDRLPVKGTEANGKKPSQQKKLEERPVNK
CSDQIKLKNTTDKKNNENRESEKKGQRTSTFQINGKDNKPKIYLKGECLKEISESRVVSG
NVEPKVNNINKIIPENDIKSLTVKESAIRPFINGDVIMEDFNERNSSETKSHLLSSSDAE
GNYRDSLETLPSTKESDSTQTTTPSASCPESNSVNQVEDMEIETSEVKKVTSSPITSEEE
SNLSNDFIDENGLPINKNENVNGESKRKTVITEVTTMTSTVATESKTVIKVEKGDKQTVV
SSTENCAKSTVTTTTTTVTKLSTPSTGGSVDIISVKEQSKTVVTTTVTDSLTTTGGTLVT
SMTVSKEYSTRDKVKLMKFSRPKKTRSGTALPSYRKFVTKSSKKSIFVLPNDDLKKLARK

GGIREVPYFNYNAKPALDIWPYPSPRPTFGITWRYRLQTVKSLAGVSLMLRLLWASLRWD
DMAAKAPPGGGTTRTETSETEITTTEIIKRRDVGPYGIRSEYCIRKIICPIGVPETPKET
PTPQRKGLRSSALRPKRPETPKQTGPVIIETWVAEEELELWEIRAFAERVEKEKAQAVEQ
QAKKRLEQQKPTVIATSTTSPTSSTTSTISPAQKVMVAPISGSVTTGTKMVLTTKVGSPA
TVTFQQNKNFHQTFATWVKQGQSNSGVVQVQQKVLGIIPSSTGTSQQTFTSFQPRTATVT
IRPNTSGSGGTTSNSQVITGPQIRPGMTVIRTPLQQSTLGKAIIRTPVMVQPGAPQQVMT
QIIRGQPVSTAVSAPNTVSSTPGQKSLTSATSTSNIQSSASQPPRPQQGQVKLTMAQLTQ
LTQGHGGNQGLTVVIQGQGQTTGQLQLIPQGVTVLPGPGQQLMQAAMPNGTVQRFLFTPL
ATTATTASTTTTTVSTTAAGTGEQRQSKLSPQMQVHQDKTLPPAQSSSVGPAEAQPQTAQ
PSAQPQPQTQPQSPAQPEVQTQPEVQTQTTVSSHVPSEAQPTHAQSSKPQVAAQSQPQSN
VQGQSPVRVQSPSQTRIRPSTPSQLSPGQQSQVQTTTSQPIPIQPHTSLQIPSQGQPQSQ
PQVQSSTQTLSSGQTLNQVTVSSPSRPQLQIQQPQPQVIAVPQLQQQVQVLSQIQSQVVA
QIQAQQSGVPQQIKLQLPIQIQQSSAVQTHQIQNVVTVQAASVQEQLQRVQQLRDQQQKK
KQQQIEIKREHTLQASNQSEIIQKQVVMKHNAVIEHLKQKKSMTPAEREENQRMIVCNQV
MKYILDKIDKEEKQAAKKRKREESVEQKRSKQNATKLSALLFKHKEQLRAEILKKRALLD
KDLQIEVQEELKRDLKIKKEKDLMQLAQATAVAAPCPPVTPAPPAPPAPPPSPPPPPAVQ
HTGLLSTPTLPAASQKRKREEEKDSSSKSKKKKMISTTSKETKKDTKLYCICKTPYDESK
FYIGCDRCQNWYHGRCVGILQSEAELIDEYVCPQCQSTEDAMTVLTPLTEKDYEGLKRVL
RSLQAHKMAWPFLEPVDPNDAPDYYGVIKEPMDLATMEERVQRRYYEKLTEFVADMTKIF
DNCRYYNPSDSPFYQCAEVLESFFVQKLKGFKASRSHNNKLQSTAS
>sp|Q6PJG6|BRAT1_HUMAN BRCA1-associated ATM activator 1 OS=Homo sapiens GN=BRAT1 PE=1 SV=2
MDPECAQLLPALCAVLVDPRQPVADDTCLEKLLDWFKTVTEGESSVVLLQEHPCLVELLS
HVLKVQDLSSGVLSFSLRLAGTFAAQENCFQYLQQGELLPGLFGEPGPLGRATWAVPTVR
SGWIQGLRSLAQHPSALRFLADHGAVDTIFSLQGDSSLFVASAASQLLVHVLALSMRGGA
EGQPCLPGGDWPACAQKIMDHVEESLCSAATPKVTQALNVLTTTFGRCQSPWTEALWVRL
SPRVACLLERDPIPAAHSFVDLLLCVARSPVFSSSDGSLWETVARALSCLGPTHMGPLAL
GILKLEHCPQALRTQAFQVLLQPLACVLKATVQAPGPPGLLDGTADDATTVDTLLASKSS
CAGLLCRTLAHLEELQPLPQRPSPWPQASLLGATVTVLRLCDGSAAPASSVGGHLCGTLA
GCVRVQRAALDFLGTLSQGTGPQELVTQALAVLLECLESPGSSPTVLKKAFQATLRWLLS
SPKTPGCSDLGPLIPQFLRELFPVLQKRLCHPCWEVRDSALEFLTQLSRHWGGQADFRCA
LLASEVPQLALQLLQDPESYVRASAVTAMGQLSSQGLHAPTSPEHAEARQSLFLELLHIL
SVDSEGFPRRAVMQVFTEWLRDGHADAAQDTEQFVATVLQAASRDLDWEVRAQGLELALV
FLGQTLGPPRTHCPYAVALPEVAPAQPLTEALRALCHVGLFDFAFCALFDCDRPVAQKSC
DLLLFLRDKIASYSSLREARGSPNTASAEATLPRWRAGEQAQPPGDQEPEAVLAMLRSLD
LEGLRSTLAESSDHVEKSPQSLLQDMLATGGFLQGDEADCY
>sp|Q8WUW1|BRK1_HUMAN Protein BRICK1 OS=Homo sapiens GN=BRK1 PE=1 SV=1
MAGQEDPVQREIHQDWANREYIEIITSSIKKIADFLNSFDMSCRSRLATLNEKLTALERR
IEYIEARVTKGETLT
>sp|Q9HCU9|BRMS1_HUMAN Breast cancer metastasis-suppressor 1 OS=Homo sapiens GN=BRMS1 PE=1 SV=1
MPVQPPSKDTEEMEAEGDSAAEMNGEEEESEEERSGSQTESEEESSEMDDEDYERRRSEC
VSEMLDLEKQFSELKEKLFRERLSQLRLRLEEVGAERAPEYTEPLGGLQRSLKIRIQVAG
IYKGFCLDVIRNKYECELQGAKQHLESEKLLLYDTLQGELQERIQRLEEDRQSLDLSSEW

WDDKLHARGSSRSWDSLPPSKRKKAPLVSGPYIVYMLQEIDILEDWTAIKKARAAVSPQK
RKSDGP
>sp|P32247|BRS3_HUMAN Bombesin receptor subtype-3 OS=Homo sapiens GN=BRS3 PE=1 SV=1
MAQRQPHSPNQTLISITNDTESSSSVVSNDNTNKGWSGDNSPGIEALCAIYITYAVIISV
GILGNAILIKVFFKTKSMQTVPNIFITSLAFGDLLLLLTCVPVDATHYLAEGWLFGRIGC
KVLSFIRLTSVGVSVFTLTILSADRYKAVVKPLERQPSNAILKTCVKAGCVWIVSMIFAL
PEAIFSNVYTFRDPNKNMTFESCTSYPVSKKLLQEIHSLLCFLVFYIIPLSIISVYYSLI
ARTLYKSTLNIPTEEQSHARKQIESRKRIARTVLVLVALFALCWLPNHLLYLYHSFTSQT
YVDPSAMHFIFTIFSRVLAFSNSCVNPFALYWLSKSFQKHFKAQLFCCKAERPEPPVADT
SLTTLAVMGTVPGTGSIQMSEISVTSFTGCSVKQAEDRF
>sp|Q9NSI6|BRWD1_HUMAN Bromodomain and WD repeat-containing protein 1 OS=Homo sapiens GN=BRWD1 PE=1 SV=4
MAEPSSARRPVPLIESELYFLIARYLSAGPCRRAAQVLVQELEQYQLLPKRLDWEGNEHN
RSYEELVLSNKHVAPDHLLQICQRIGPMLDKEIPPSISRVTSLLGAGRQSLLRTAKDCRH
TVWKGSAFAALHRGRPPEMPVNYGSPPNLVEIHRGKQLTGCSTFSTAFPGTMYQHIKMHR
RILGHLSAVYCVAFDRTGHRIFTGSDDCLVKIWSTHNGRLLSTLRGHSAEISDMAVNYEN
TMIAAGSCDKIIRVWCLRTCAPVAVLQGHTGSITSLQFSPMAKGSQRYMVSTGADGTVCF
WQWDLESLKFSPRPLKFTEKPRPGVQMLCSSFSVGGMFLATGSTDHVIRMYFLGFEAPEK
IAELESHTDKVDSIQFCNNGDRFLSGSRDGTARIWRFEQLEWRSILLDMATRISGDLSSE
EERFMKPKVTMIAWNQNDSIVVTAVNDHVLKVWNSYTGQLLHNLMGHADEVFVLETHPFD
SRIMLSAGHDGSIFIWDITKGTKMKHYFNMIEGQGHGAVFDCKFSQDGQHFACTDSHGHL
LIFGFGCSKPYEKIPDQMFFHTDYRPLIRDSNNYVLDEQTQQAPHLMPPPFLVDVDGNPH
PTKYQRLVPGRENSADEHLIPQLGYVATSDGEVIEQIISLQTNDNDERSPESSILDGMIR
QLQQQQDQRMGADQDTIPRGLSNGEETPRRGFRRLSLDIQSPPNIGLRRSGQVEGVRQMH
QNAPRSQIATERDLQAWKRRVVVPEVPLGIFRKLEDFRLEKGEEERNLYIIGRKRKTLQL
SHKSDSVVLVSQSRQRTCRRKYPNYGRRNRSWRELSSGNESSSSVRHETSCDQSEGSGSS
EEDEWRSDRKSESYSESSSDSSSRYSDWTADAGINLQPPLRTSCRRRITRFCSSSEDEIS
TENLSPPKRRRKRKKENKPKKENLRRMTPAELANMEHLYEFHPPVWITDTTLRKSPFVPQ
MGDEVIYFRQGHEAYIEAVRRNNIYELNPNKEPWRKMDLRDQELVKIVGIRYEVGPPTLC
CLKLAFIDPATGKLMDKSFSIRYHDMPDVIDFLVLRQFYDEARQRNWQSCDRFRSIIDDA
WWFGTVLSQEPYQPQYPDSHFQCYIVRWDNTEIEKLSPWDMEPIPDNVDPPEELGASISV
TTDELEKLLYKPQAGEWGQKSRDEECDRIISGIDQLLNLDIAAAFAGPVDLCTYPKYCTV
VAYPTDLYTIRMRLVNRFYRRLSALVWEVRYIEHNARTFNEPESVIARSAKKITDQLLKF
IKNQHCTNISELSNTSENDEQNAEDLDDSDLPKTSSGRRRVHDGKKSIRATNYVESNWKK
QCKELVNLIFQCEDSEPFRQPVDLVEYPDYRDIIDTPMDFGTVRETLDAGNYDSPLEFCK
DIRLIFSNAKAYTPNKRSKIYSMTLRLSALFEEKMKKISSDFKIGQKFNEKLRRSQRFKQ
RQNCKGDSQPNKSIRNLKPKRLKSQTKIIPELVGSPTQSTSSRTAYLGTHKTSAGISSGV
TSGDSSDSAESSERRKRNRPITNGSTLSESEVEDSLATSLSSSASSSSEESKESSRARES
SSRSGLSRSSNLRVTRTRAAQRKTGPVSLANGCGRKATRKRVYLSDSDNNSLETGEILKA
RAGNNRKVLRKCAAVAANKIKLMSDVEENSSSESVCSGRKLPHRNASAVARKKLLHNSED
EQSLKSEIEEEELKDENQLLPVSSSHTAQSNVDESENRDSESESDLRVARKNWHANGYKS
HTPAPSKTKFLKIESSEEDSKSHDSDHACNRTAGPSTSVQKLKAESISEEADSEPGRSGG
RKYNTFHKNASFFKKTKILSDSEDSESEEQDREDGKCHKMEMNPISGNLNCDPIAMSQCS
SDHGCETDLDSDDDKIEKPNNFMKDSASQDNGLSRKISRKRVCSSDSDSSLQVVKKSSKA

RTGLLRITRRCAATAANKIKLMSDVEDVSLENVHTRSKNGRKKPLHLACTTAKKKLSDCE
GSVHCEVPSEQYACEGKPPDPDSEGSTKVLSQALNGDSDSEDMLNSEHKHRHTNIHKIDA
PSKRKSSSVTSSGEDSKSHIPGSETDRTFSSESTLAQKATAENNFEVELNYGLRRWNGRR
LRTYGKAPFSKTKVIHDSQETAEKEVKRKRSHPELENVKISETTGNSKFRPDTSSKSSDL
GSVTESDIDCTDNTKTKRRKTKGKAKVVRKEFVPRDREPNTKVRTCMHNQKDAVQMPSET
LKAKMVPEKVPRRCATVAANKIKIMSNLKETISGPENVWIRKSSRKLPHRNASAAAKKKL
LNVYKEDDTTINSESEKELEDINRKMLFLRGFRSWKENAQ
>sp|Q96G97|BSCL2_HUMAN Seipin OS=Homo sapiens GN=BSCL2 PE=1 SV=3
MVNDPPVPALLWAQEVGQVLAGRARRLLLQFGVLFCTILLLLWVSVFLYGSFYYSYMPTV
SHLSPVHFYYRTDCDSSTTSLCSFPVANVSLTKGGRDRVLMYGQPYRVTLELELPESPVN
QDLGMFLVTISCYTRGGRIISTSSRSVMLHYRSDLLQMLDTLVFSSLLLFGFAEQKQLLE
VELYADYRENSYVPTTGAIIEIHSKRIQLYGAYLRIHAHFTGLRYLLYNFPMTCAFIGVA
SNFTFLSVIVLFSYMQWVWGGIWPRHRFSLQVNIRKRDNSRKEVQRRISAHQPGPEGQEE
STPQSDVTEDGESPEDPSGTEGQLSEEEKPDQQPLSGEEELEPEASDGSGSWEDAALLTE
ANLPAPAPASASAPVLETLGSSEPAGGALRQRPTCSSS
>sp|Q7KYR7|BT2A1_HUMAN Butyrophilin subfamily 2 member A1 OS=Homo sapiens GN=BTN2A1 PE=1 SV=3
MESAAALHFSRPASLLLLLLSLCALVSAQFIVVGPTDPILATVGENTTLRCHLSPEKNAE
DMEVRWFRSQFSPAVFVYKGGRERTEEQMEEYRGRTTFVSKDISRGSVALVIHNITAQEN
GTYRCYFQEGRSYDEAILHLVVAGLGSKPLISMRGHEDGGIRLECISRGWYPKPLTVWRD
PYGGVAPALKEVSMPDADGLFMVTTAVIIRDKSVRNMSCSINNTLLGQKKESVIFIPESF
MPSVSPCAVALPIIVVILMIPIAVCIYWINKLQKEKKILSGEKEFERETREIALKELEKE
RVQKEEELQVKEKLQEELRWRRTFLHAVDVVLDPDTAHPDLFLSEDRRSVRRCPFRHLGE
SVPDNPERFDSQPCVLGRESFASGKHYWEVEVENVIEWTVGVCRDSVERKGEVLLIPQNG
FWTLEMHKGQYRAVSSPDRILPLKESLCRVGVFLDYEAGDVSFYNMRDRSHIYTCPRSAF
SVPVRPFFRLGCEDSPIFICPALTGANGVTVPEEGLTLHRVGTHQSL
>sp|Q5XKL5|BTBD8_HUMAN BTB/POZ domain-containing protein 8 OS=Homo sapiens GN=BTBD8 PE=2 SV=2
MARCGEGSAAPMVLLGSAGVCSKGLQRKGPCERRRLKATVSEQLSQDLLRLLREEFHTDV
TFSVGCTLFKAHKAVLLARVPDFYFHTIGQTSNSLTNQEPIAVENVEALEFRTFLQIIYS
SNRNIKNYEEEILRKKIMEIGISQKQLDISFPKCENSSDCSLQKHEIPEDISDRDDDFIS
NDNYDLEPASELGEDLLKLYVKPCCPDIDIFVDGKRFKAHRAILSARSSYFAAMLSGCWA
ESSQEYVTLQGISHVELNVMMHFIYGGTLDIPDKTNVGQILNMADMYGLEGLKEVAIYIL
RRDYCNFFQKPVPRTLTSILECLIIAHSVGVESLFADCMKWIVKHFARFWSERSFANIPP
EIQKSCLNMLIQSLVSIT
>sp|Q96Q07|BTBD9_HUMAN BTB/POZ domain-containing protein 9 OS=Homo sapiens GN=BTBD9 PE=1 SV=2
MSNSHPLRPFTAVGEIDHVHILSEHIGALLIGEEYGDVTFVVEKKRFPAHRVILAARCQY
FRALLYGGMRESQPEAEIPLQDTTAEAFTMLLKYIYTGRATLTDEKEEVLLDFLSLAHKY
GFPELEDSTSEYLCTILNIQNVCMTFDVASLYSLPKLTCMCCMFMDRNAQEVLSSEGFLS
LSKTALLNIVLRDSFAAPEKDIFLALLNWCKHNSKENHAEIMQAVRLPLMSLTELLNVVR
PSGLLSPDAILDAIKVRSESRDMDLNYRGMLIPEENIATMKYGAQVVKGELKSALLDGDT
QNYDLDHGFSRHPIDDDCRSGIEIKLGQPSIINHIRILLWDRDSRSYSYFIEVSMDELDW
VRVIDHSQYLCRSWQKLYFPARVCRYIRIVGTHNTVNKIFHIVAFECMFTNKTFTLEKGL

IVPMENVATIADCASVIEGVSRSRNALLNGDTKNYDWDSGYTCHQLGSGAIVVQLAQPYM
IGSIRLLLWDCDDRSYSYYVEVSTNQQQWTMVADRTKVSCKSWQSVTFERQPASFIRIVG
THNTANEVFHCVHFECPEQQSSQKEENSEESGTGDTSLAGQQLDSHALRAPSGSSLPSSP
GSNSRSPNRQHQ
>sp|Q32M84|BTBDG_HUMAN BTB/POZ domain-containing protein 16 OS=Homo sapiens GN=BTBD16 PE=2 SV=2
MIMSNTHKARLERRVTGSTNRWRLPKQPFSGDLLSLSQMCKALSIDFEEALRNPDRLCIS
QIQKFFFENFKNKDIQSGEADVILECLGFKWELHQPQLFQSETLAKLYLKALAQGTTHPL
RELEELLRAQSPKKTKEKSPAKRIIISLKINDPLVTKVAFATALKNLYMSEVEINLEDLL
GVLASAHILQFSGLFQRCVDVMIARLKPSTIKKFYEAGCKYKEEQLTTGCEKWLEMNLVP
LGGTQIHLHKIPQDLLHKVLKSPRLFTFSEFHLLKTMLLWVFLQLNYKIQAIPTYETVMT
FFKSFPENCCFLDRDIGRSLRPLFLCLRLHGITKGKDLEVLRHLNFFPESWLDQVTVNHY
HALENGGDMVHLKDLNTQAVRFGLLFNQENTTYSKTIALYGFFFKIKGLKHDTTSYSFYM
QRIKHTDLESPSAVYEHNHVSLRAARLVKYEIRAEALVDGKWQEFRTNQIKQKFGLTTSS
CKSHTLKIQTVGIPIYVSFAFIFPAS
>sp|C9JJ37|BTBDJ_HUMAN BTB/POZ domain-containing protein 19 OS=Homo sapiens GN=BTBD19 PE=2 SV=1
MEPLGLVVHGKAEPFSAALRSLVNNPRYSDVCFVVGQERQEVFAHRCLLACRCNFFQRLL
GTEPGPGVPSPVVLSTVPTEAFLAVLEFLYTNSVKLYRHSVLEVLTAAVEYGLEELRELC
LQFVVKVLDVDLVCEALQVAVTFGLGQLQERCVAFIEAHSQEALRTRGFLELSAAALLPL
LRSDKLCVDEAELVRAARSWARVGAAVLERPVAEVAAPVVKELRLALLAPAELSALEEQN
RQEPLIPVEQIVEAWKCHALRRGDEARGAPCRRRRGTLPREHHRFLDLSFK
>sp|Q7Z6A9|BTLA_HUMAN B- and T-lymphocyte attenuator OS=Homo sapiens GN=BTLA PE=1 SV=3
MKTLPAMLGTGKLFWVFFLIPYLDIWNIHGKESCDVQLYIKRQSEHSILAGDPFELECPV
KYCANRPHVTWCKLNGTTCVKLEDRQTSWKEEKNISFFILHFEPVLPNDNGSYRCSANFQ
SNLIESHSTTLYVTDVKSASERPSKDEMASRPWLLYRLLPLGGLPLLITTCFCLFCCLRR
HQGKQNELSDTAGREINLVDAHLKSEQTEASTRQNSQVLLSETGIYDNDPDLCFRMQEGS
EVYSNPCLEENKPGIVYASLNHSVIGPNSRLARNVKEAPTEYASICVRS
>sp|O60566|BUB1B_HUMAN Mitotic checkpoint serine/threonine-protein kinase BUB1 beta OS=Homo sapiens GN=BUB1B PE=1 SV=3
MAAVKKEGGALSEAMSLEGDEWELSKENVQPLRQGRIMSTLQGALAQESACNNTLQQQKR
AFEYEIRFYTGNDPLDVWDRYISWTEQNYPQGGKESNMSTLLERAVEALQGEKRYYSDPR
FLNLWLKLGRLCNEPLDMYSYLHNQGIGVSLAQFYISWAEEYEARENFRKADAIFQEGIQ
QKAEPLERLQSQHRQFQARVSRQTLLALEKEEEEEVFESSVPQRSTLAELKSKGKKTARA
PIIRVGGALKAPSQNRGLQNPFPQQMQNNSRITVFDENADEASTAELSKPTVQPWIAPPM
PRAKENELQAGPWNTGRSLEHRPRGNTASLIAVPAVLPSFTPYVEETARQPVMTPCKIEP
SINHILSTRKPGKEEGDPLQRVQSHQQASEEKKEKMMYCKEKIYAGVGEFSFEEIRAEVF
RKKLKEQREAELLTSAEKRAEMQKQIEEMEKKLKEIQTTQQERTGDQQEETMPTKETTKL
QIASESQKIPGMTLSSSVCQVNCCARETSLAENIWQEQPHSKGPSVPFSIFDEFLLSEKK
NKSPPADPPRVLAQRRPLAVLKTSESITSNEDVSPDVCDEFTGIEPLSEDAIITGFRNVT
ICPNPEDTCDFARAARFVSTPFHEIMSLKDLPSDPERLLPEEDLDVKTSEDQQTACGTIY
SQTLSIKKLSPIIEDSREATHSSGFSGSSASVASTSSIKCLQIPEKLELTNETSENPTQS
PWCSQYRRQLLKSLPELSASAELCIEDRPMPKLEIEKEIELGNEDYCIKREYLICEDYKL
FWVAPRNSAELTVIKVSSQPVPWDFYINLKLKERLNEDFDHFCSCYQYQDGCIVWHQYIN

CFTLQDLLQHSEYITHEITVLIIYNLLTIVEMLHKAEIVHGDLSPRCLILRNRIHDPYDC
NKNNQALKIVDFSYSVDLRVQLDVFTLSGFRTVQILEGQKILANCSSPYQVDLFGIADLA
HLLLFKEHLQVFWDGSFWKLSQNISELKDGELWNKFFVRILNANDEATVSVLGELAAEMN
GVFDTTFQSHLNKALWKVGKLTSPGALLFQ
>sp|Q5T3Y7|BVAS1_HUMAN Putative uncharacterized protein BVES-AS1 OS=Homo sapiens GN=BVES-AS1 PE=5 SV=1
MLRSTFTVHWTTILGICRPIKPIYKSQACPLSEKRNADNHLVSEDNTFKEPERYISSSCV
LTSILYLGEKTFSFRTLISPRGMKLVEILQQPSLTMVA
>sp|Q8N1D0|BWR1B_HUMAN Beckwith-Wiedemann syndrome chromosomal region 1 candidate gene B protein OS=Homo sapiens GN=SLC22A18AS PE=2 SV=3
MGELPGSEGMWENCPLGWVKKKASGTLAPLDFLLQRKRLWLWASEPVRPQPQGIHRFREA
RRQFCRMRGSRLTGGRKGFGSSGLRFGRGGFSEEVMPQPVLKAMRCAEGAWWFSPDGPAG
SAASIWPAEGAEGLPGQLGRDRLEVVYSVPDNVPGQNGSRRPLVCKITGKCLSVCSEENA
KAGGCSAFPLLLSQLGARMTGREHAHKGPELTTPDSGLPRPPNPALAGFRALAQHSPPLG
TSTPSAVLLSAAT
>sp|Q07021|C1QBP_HUMAN Complement component 1 Q subcomponent-binding protein, mitochondrial OS=Homo sapiens GN=C1QBP PE=1 SV=1
MLPLLRCVPRVLGSSVAGLRAAAPASPFRQLLQPAPRLCTRPFGLLSVRAGSERRPGLLR
PRGPCACGCGCGSLHTDGDKAFVDFLSDEIKEERKIQKHKTLPKMSGGWELELNGTEAKL
VRKVAGEKITVTFNINNSIPPTFDGEEEPSQGQKVEEQEPELTSTPNFVVEVIKNDDGKK
ALVLDCHYPEDEVGQEDEAESDIFSIREVSFQSTGESEWKDTNYTLNTDSLDWALYDHLM
DFLADRGVDNTFADELVELSTALEHQEYITFLEDLKSFVKSQ
>sp|Q86Z23|C1QL4_HUMAN Complement C1q-like protein 4 OS=Homo sapiens GN=C1QL4 PE=2 SV=1
MVLLLLVAIPLLVHSSRGPAHYEMLGRCRMVCDPHGPRGPGPDGAPASVPPFPPGAKGEV
GRRGKAGLRGPPGPPGPRGPPGEPGRPGPPGPPGPGPGGVAPAAGYVPRIAFYAGLRRPH
EGYEVLRFDDVVTNVGNAYEAASGKFTCPMPGVYFFAYHVLMRGGDGTSMWADLMKNGQV
RASAIAQDADQNYDYASNSVILHLDVGDEVFIKLDGGKVHGGNTNKYSTFSGFIIYPD
>sp|Q9BXJ1|C1QT1_HUMAN Complement C1q tumor necrosis factor-related protein 1 OS=Homo sapiens GN=C1QTNF1 PE=1 SV=1
MGSRGQGLLLAYCLLLAFASGLVLSRVPHVQGEQQEWEGTEELPSPPDHAERAEEQHEKY
RPSQDQGLPASRCLRCCDPGTSMYPATAVPQINITILKGEKGDRGDRGLQGKYGKTGSAG
ARGHTGPKGQKGSMGAPGERCKSHYAAFSVGRKKPMHSNHYYQTVIFDTEFVNLYDHFNM
FTGKFYCYVPGLYFFSLNVHTWNQKETYLHIMKNEEEVVILFAQVGDRSIMQSQSLMLEL
REQDQVWVRLYKGERENAIFSEELDTYITFSGYLVKHATEP
>sp|Q9BXJ3|C1QT4_HUMAN Complement C1q tumor necrosis factor-related protein 4 OS=Homo sapiens GN=C1QTNF4 PE=1 SV=2
MLPLLLGLLGPAACWALGPTPGPGSSELRSAFSAARTTPLEGTSEMAVTFDKVYVNIGGD
FDVATGQFRCRVPGAYFFSFTAGKAPHKSLSVMLVRNRDEVQALAFDEQRRPGARRAASQ
SAMLQLDYGDTVWLRLHGAPQYALGAPGATFSGYLVYADADADAPARGPPAPPEPRSAFS
AARTRSLVGSDAGPGPRHQPLAFDTEFVNIGGDFDAAAGVFRCRLPGAYFFSFTLGKLPR
KTLSVKLMKNRDEVQAMIYDDGASRRREMQSQSVMLALRRGDAVWLLSHDHDGYGAYSNH
GKYITFSGFLVYPDLAPAAPPGLGASELL
>sp|P09871|C1S_HUMAN Complement C1s subcomponent OS=Homo sapiens GN=C1S PE=1 SV=1
MWCIVLFSLLAWVYAEPTMYGEILSPNYPQAYPSEVEKSWDIEVPEGYGIHLYFTHLDIE

LSENCAYDSVQIISGDTEEGRLCGQRSSNNPHSPIVEEFQVPYNKLQVIFKSDFSNEERF
TGFAAYYVATDINECTDFVDVPCSHFCNNFIGGYFCSCPPEYFLHDDMKNCGVNCSGDVF
TALIGEIASPNYPKPYPENSRCEYQIRLEKGFQVVVTLRREDFDVEAADSAGNCLDSLVF
VAGDRQFGPYCGHGFPGPLNIETKSNALDIIFQTDLTGQKKGWKLRYHGDPMPCPKEDTP
NSVWEPAKAKYVFRDVVQITCLDGFEVVEGRVGATSFYSTCQSNGKWSNSKLKCQPVDCG
IPESIENGKVEDPESTLFGSVIRYTCEEPYYYMENGGGGEYHCAGNGSWVNEVLGPELPK
CVPVCGVPREPFEEKQRIIGGSDADIKNFPWQVFFDNPWAGGALINEYWVLTAAHVVEGN
REPTMYVGSTSVQTSRLAKSKMLTPEHVFIHPGWKLLEVPEGRTNFDNDIALVRLKDPVK
MGPTVSPICLPGTSSDYNLMDGDLGLISGWGRTEKRDRAVRLKAARLPVAPLRKCKEVKV
EKPTADAEAYVFTPNMICAGGEKGMDSCKGDSGGAFAVQDPNDKTKFYAAGLVSWGPQCG
TYGLYTRVKNYVDWIMKTMQENSTPRED
>sp|Q96NR2|C2AS1_HUMAN Putative uncharacterized protein C20orf166-AS1 OS=Homo sapiens GN=C20orf166-AS1 PE=2 SV=3
MQTLATGHLAHEGSSPHKLSGGRTQESSGFSEAKGGHGAPRHSASHGATPSGLRRGLHCR
EGPSDSGFQPHPGPPLSVPSGPLAPRARRHPNPLGMSVLRMWHTRPGRAILLLLHPPQPW
IRGLYRGQKFTQSR
>sp|Q86YS7|C2CD5_HUMAN C2 domain-containing protein 5 OS=Homo sapiens GN=C2CD5 PE=1 SV=1
MPGKLKVKIVAGRHLPVMDRASDLTDAFVEVKFGNTTFKTDVYLKSLNPQWNSEWFKFEV
DDEDLQDEPLQITVLDHDTYSANDAIGKVYIDIDPLLYSEAATVISGWFPIYDTIHGIRG
EINVVVKVDLFNDLNRFRQSSCGVKFFCTTSIPKCYRAVIIHGFVEELVVNEDPEYQWID
RIRTPRASNEARQRLISLMSGELQRKIGLKVLEMRGNAVVGYLQCFDLEGESGLVVRAIG
TACTLDKLSSPAAFLPACNSPSKEMKEIPFNEDPNPNTHSSGPSTPLKNQTYSFSPSKSY
SRQSSSSDTDLSLTPKTGMGSGSAGKEGGPFKALLRQQTQSALEQREFPFFTLTAFPPGF
LVHVGGVVSARSVKLLDRIHNPDEPETRDAWWAEIRQEIKSHAKALGCHAVVGYSESTSI
CEEVCILSASGTAAVLNPRFLQDGTVEGCLEQRLEENLPTRCGFCHIPYDELNMPFPAHL
TYCYNCRKQKVPDVLFTTIDLPTDATVIGKGCLIQARLCRLKKKAQAEANATAISNLLPF
MEYEVHTQLMNKLKLKGMNALFGLRIQITVGENMLMGLASATGVYLAALPTPGGIQIAGK
TPNDGSYEQHISHMQKKINDTIAKNKELYEINPPEISEEIIGSPIPEPRQRSRLLRSQSE
SSDEVTELDLSHGKKDAFVLEIDDTDAMEDVHSLLTDVPPPSGFYSCNTEIMPGINNWTS
EIQMFTSVRVIRLSSLNLTNQALNKNFNDLCENLLKSLYFKLRSMIPCCLCHVNFTVSLP
EDELIQVTVTAVAITFDKNQALQTTKTPVEKSLQRASTDNEELLQFPLELCSDSLPSHPF
PPAKAMTVEKASPVGDGNFRNRSAPPCANSTVGVVKMTPLSFIPGAKITKYLGIINMFFI
RETTSLREEGGVSGFLHAFIAEVFAMVRAHVAALGGNAVVSYIMKQCVFMENPNKNQAQC
LINVSGDAVVFVRESDLEVVSSQQPTTNCQSSCTEGEVTT
>sp|Q9P2K1|C2D2A_HUMAN Coiled-coil and C2 domain-containing protein 2A OS=Homo sapiens GN=CC2D2A PE=1 SV=3
MNPREEKVKIITEEFIENDEDADMGRQNKNSKVRRQPRKKQPPTAVPKEMVSEKSHLGNP
QEPVQEEPKTRLLSMTVRRGPRSLPPIPSTSRTGFAEFSMRGRMREKLQAARSKAESALL
QEIPTPRPRRLRSPSKKELETEFGTEPGKEVERTQQEVDSQSYSRVKFHDSARKIKPKPQ
VPPGFPSAEEAYNFFTFNFDPEPEGSEEKPKARHRAGTNQEEEEGEEEEPPAQGGGKEMD
EEELLNGDDAEDFLLGLDHVADDFVAVRPADYESIHDRLQMEREMLFIPSRQTVPTYKKL
PENVQPRFLEDEGLYTGVRPEVARTNQNIMENRLLMQDPERRWFGDDGRILALPNPIKPF
PSRPPVLTQEQSIKAELETLYKKAVKYVHSSQHVIRSGDPPGNFQLDIDISGLIFTHHPC
FSREHVLAAKLAQLYDQYLARHQRNKAKFLTDKLQALRNAVQTGLDPEKPHQSLDTIQKT

INEYKSEIRQTRKFRDAEQEKDRTLLKTIIKVWKEMKSLREFQRFTNTPLKLVLRKEKAD
QKADEEAYEAEIQAEISELLEEHTEEYAQKMEEYRTSLQQWKAWRKVQRAKKKKRKQAAE
EHPGDEIAEPYPEEDLVKPSPPEPTDRAVIEQEVRERAAQSRRRPWEPTLVPELSLAGSV
TPNDQCPRAEVSRREDVKKRSVYLKVLFNNKEVSRTVSRPLGADFRVHFGQIFNLQIVNW
PESLTLQVYETVGHSSPTLLAEVFLPIPETTVVTGRAPTEEVEFSSNQHVTLDHEGVGSG
VPFSFEADGSNQLTLMTSGKVSHSVAWAIGENGIPLIPPLSQQNIGFRSALKKADAISSI
GTSGLTDMKKLAKWAAESKLDPNDPNNAPLMQLISVATSGESYVPDFFRLEQLQQEFNFV
SDQELNRSKRFRLLHLRSQEVPEFRNYKQVPVYDREIMEKVFQDYEKRLRDRNVIETKEH
IDTHRAIVAKYLQQVRESVINRFLIAKQYFLLADMIVEEEVPNISILGLSLFKLAEQKRP
LRPRRKGRKKVTAQNLSDGDIKLLVNIVRAYDIPVRKPAVSKFQQPSRSSRMFSEKHAAS
PSTYSPTHNADYPLGQVLVRPFVEVSFQRTVCHTTTAEGPNPSWNEELELPFRAPNGDYS
TASLQSVKDVVFINIFDEVLHDVLEDDRERGSGIHTRIERHWLGCVKMPFSTIYFQARID
GTFKIDIPPVLLGYSKERNMILERGFDSVRSLSEGSYITLFITIEPQLVPGESIREKFES
QEDEKLLQATEKFQAECALKFPNRQCLTTVIDISGKTVFITRYLKPLNPPQELLNVYPNN
LQATAELVARYVSLIPFLPDTVSFGGICDLWSTSDQFLDLLAGDEEEHAVLLCNYFLSLG
KKAWLLMGNAIPEGPTAYVLTWEQGRYLIWNPCSGHFYGQFDTFCPLKNVGCLIGPDNIW
FNIQRYESPLRINFDVTRPKLWKSFFSRSLPYPGLSSVQPEELIYQRSDKAAAAELQDRI
EKILKEKIMDWRPRHLTRWNRYCTSTLRHFLPLLEKSQGEDVEDDHRAELLKQLGDYRFS
GFPLHMPYSEVKPLIDAVYSTGVHNIDVPNVEFALAVYIHPYPKNVLSVWIYVASLIRNR
>sp|P04003|C4BPA_HUMAN C4b-binding protein alpha chain OS=Homo sapiens GN=C4BPA PE=1 SV=2
MHPPKTPSGALHRKRKMAAWPFSRLWKVSDPILFQMTLIAALLPAVLGNCGPPPTLSFAA
PMDITLTETRFKTGTTLKYTCLPGYVRSHSTQTLTCNSDGEWVYNTFCIYKRCRHPGELR
NGQVEIKTDLSFGSQIEFSCSEGFFLIGSTTSRCEVQDRGVGWSHPLPQCEIVKCKPPPD
IRNGRHSGEENFYAYGFSVTYSCDPRFSLLGHASISCTVENETIGVWRPSPPTCEKITCR
KPDVSHGEMVSGFGPIYNYKDTIVFKCQKGFVLRGSSVIHCDADSKWNPSPPACEPNSCI
NLPDIPHASWETYPRPTKEDVYVVGTVLRYRCHPGYKPTTDEPTTVICQKNLRWTPYQGC
EALCCPEPKLNNGEITQHRKSRPANHCVYFYGDEISFSCHETSRFSAICQGDGTWSPRTP
SCGDICNFPPKIAHGHYKQSSSYSFFKEEIIYECDKGYILVGQAKLSCSYSHWSAPAPQC
KALCRKPELVNGRLSVDKDQYVEPENVTIQCDSGYGVVGPQSITCSGNRTWYPEVPKCEW
ETPEGCEQVLTGKRLMQCLPNPEDVKMALEVYKLSLEIEQLELQRDSARQSTLDKEL
>sp|P21730|C5AR1_HUMAN C5a anaphylatoxin chemotactic receptor 1 OS=Homo sapiens GN=C5AR1 PE=1 SV=2
MDSFNYTTPDYGHYDDKDTLDLNTPVDKTSNTLRVPDILALVIFAVVFLVGVLGNALVVW
VTAFEAKRTINAIWFLNLAVADFLSCLALPILFTSIVQHHHWPFGGAACSILPSLILLNM
YASILLLATISADRFLLVFKPIWCQNFRGAGLAWIACAVAWGLALLLTIPSFLYRVVREE
YFPPKVLCGVDYSHDKRRERAVAIVRLVLGFLWPLLTLTICYTFILLRTWSRRATRSTKT
LKVVVAVVASFFIFWLPYQVTGIMMSFLEPSSPTFLLLKKLDSLCVSFAYINCCINPIIY
VVAGQGFQGRLRKSLPSLLRNVLTEESVVRESKSFTRSTVDTMAQKTQAV
>sp|Q9NSG2|CA112_HUMAN Uncharacterized protein C1orf112 OS=Homo sapiens GN=C1orf112 PE=1 SV=1
MFLPHMNHLTLEQTFFSQVLPKTVKLFDDMMYELTSQARGLSSQNLEIQTTLRNILQTMV
QLLGALTGCVQHICATQESIILENIQSLPSSVLHIIKSTFVHCKNSESVYSGCLHLVSDL
LQALFKEAYSLQKQLMELLDMVCMDPLVDDNDDILNMVIVIHSLLDICSVISSMDHAFHA
NTWKFIIKQSLKHQSIIKSQLKHKDIITSLCEDILFSFHSCLQLAEQMTQSDAQDNADYR

LFQKTLKLCRFFANSLLHYAKEFLPFLSDSCCTLHQLYLQIHSKFPPSLYATRISKAHQE
EIAGAFLVTLDPLISQLLTFQPFMQVVLDSKLDLPCELQFPQCLLLVVVMDKLPSQPKEV
QTLWCTDSQVSETTTRISLLKAVFYSFEQCSGELSLPVHLQGLKSKGKAEVAVTLYQHVC
VHLCTFITSFHPSLFAELDAALLNAVLSANMITSLLAMDAWCFLARYGTAELCAHHVTIV
AHLIKSCPGECYQLINLSILLKRLFFFMAPPHQLEFIQKFSPKEAENLPLWQHISFQALP
PELREQTVHEVTTVGTAECRKWLSRSRTLGELESLNTVLSALLAVCNSAGEALDTGKQTA
IIEVVSQLWAFLNIKQVADQPYVQQTFSLLLPLLGFFIQTLDPKLILQAVTLQTSLLKLE
LPDYVRLAMLDFVSSLGKLFIPEAIQDRILPNLSCMFALLLADRSWLLEQHTLEAFTQFA
EGTNHEEIVPQCLSSEETKNKVVSFLEKTGFVDETEAAKVERVKQEKGIFWEPFANVTVE
EAKRSSLQPYAKRARQEFPWEEEYRSALHTIAGALEATESLLQKGPAPAWLSMEMEALQE
RMDKLKRYIHTLG
>sp|Q8N9H9|CA127_HUMAN Uncharacterized protein C1orf127 OS=Homo sapiens GN=C1orf127 PE=2 SV=2
MKCPMLRSRLGQESVHCGPMFIQVSRPLPLWRDNRQTPWLLSLRGELVASLEDASLMGLY
VDMNATTVTVQSPRQGLLQRWEVSGGQQALPGVSFQPESEVLVHIPKQRLGLVKRGSYIE
ETLSLRFLRVHQSNIFMVTENKDFVVVSIPAAGVLQVQRCQEVGGTPGTQAFYRVDLSLE
FAEMAAPVLWTVESFFQCVGSGTESPASTAALRTTPSPPSPGPETPPAGVPPAASSQVWA
AGPAAQEWLSRDLLHRPSDALAKKGLGPFLQTAKPARRGQTSASILPRVVQAQRGPQPPP
GEAGIPGHPTPPATLPSEPVEGVQASPWRPRPVLPTHPALTLPVSSDASSPSPPAPRPER
PESLLVSGPSVTLTEGLGTVRPEQDPAKSPGSPLLLRGLSSGDVAAPEPIMGEPGQASEE
FQPLARPWRATLAAEELVSHRSPGEPQETCSGTEVERPRQTGPGLPREGARGHMDLSSSE
PSQDIEGPGLSILPARDATFSTPSVRQPDPSAWLSSGPELTGMPRVRLAAPLAVLPMEPL
PPEPVRPAALLTPEASSVGGPDQARYLESAPGWPVGQEEWGVAHTSSPPSTQTLSLWAPT
GVLLPSLVELEYPFQAGRGASLQQELTEPTLALSAESHRPPELQDSVEGLSERPSR
>sp|Q5TEV5|CA134_HUMAN Putative uncharacterized protein C1orf134 OS=Homo sapiens GN=C1orf134 PE=3 SV=1
MLCCCPLADALLIFLETGSCFQPGQQSETLSQKQNKTNNKKWAGGAGCRPFRLWWVGRSR
GAGVQLYSTKKGWILRGKGGTEA
>sp|Q96MR7|CA145_HUMAN Putative uncharacterized protein C1orf145 OS=Homo sapiens GN=C1orf145 PE=5 SV=1
MYTASSSAETLRTVRRRSVPSSSMPYLALAHNRVSSLNHAASVDGWGTSHRNVADSFSRT
SRSCSRFLKGTAGSARREDWNGHLQPWIPRPDRRGWETADRKGERTQVHGLRRSLGPRAP
HPGAHRALRPAQSCRSGPRGWTPCRCRGRGPTACRRGS
>sp|Q8NAE3|CA180_HUMAN Putative uncharacterized protein encoded by LINC01555 OS=Homo sapiens GN=LINC01555 PE=2 SV=1
MLGPNAQVTVVVAQPGPDHLDHLLLDTQHCQHSSCGSAACTQLTWVPCLGGSHKASIKMS
AQAVVSTEAPWERPCFQAHLGCLQNSVLCSCRMEGFSLFLAMLWGGVGELPSDPRGHLQF
LAT
>sp|Q5TG92|CA195_HUMAN Putative uncharacterized protein C1orf195 OS=Homo sapiens GN=C1orf195 PE=2 SV=1
MPSSTLTCKPFDLKQRSSRRGPKSTASASPRSCLKPSCGKQVCLLLSVRRSQSLAHPGRD
STRVLSYQQTSSDAVSQYKHTGKVEELYGLRLSWLSQAQFLLAQDKTAYAVKSVVLPASN
HKTKGQ

>sp|Q8IVY1|CA210_HUMAN Type III endosome membrane protein TEMP OS=Homo sapiens GN=C1orf210 PE=3 SV=1
MNETNKTLVGPSELPTASAVAPGPGTGARAWPVLVGFVLGAVVLSLLIALAAKCHLCRRY
HASYRHRPLPETGRGGRPQVAEDEDDDGFIEDNYIQPGTGELGTEGSRDHFSL
>sp|Q6PIY5|CA228_HUMAN Uncharacterized protein C1orf228 OS=Homo sapiens GN=C1orf228 PE=2 SV=2
MTSIKEQAAISRLLSFLQEWDNAGKVARSHILDKFIETNQGKTAPELEQEFSQGASLFLV
RLTTSLRITYMTDSCLEKLLRSIGIFLSAVSSNRYLIEFLEVGGVLTLLEILGLEKIKEE
AKKESVKLLQVIANSGRTYKELICESYGVRSIAEFLAKSKSEETQEEVQVLLDSLVHGNP
KYQNQVYKGLIALLPCESPKAQQLSLQTLRTAQPIIGTTHPSIVDCVLKVLGTMHLEVQY
EAIELIKDLVGYDVRQALLKGLVALLIPSVKEISKLQAKILSDPSVLQLTPSLPMFLQQA
AAAKAIGVLARNDMSIAEELLYLRVVRGLMAAMGNTDHSNSQRLASLTLECFVQMFPLVA
EHVRKCMGEELYQLFLSNAEDLYMKIDSIQADILAANTVNVTKALCLHGSSYSMNTLYGS
RDSAQMAYLTHFEEDVESKE
>sp|Q9NPB3|CABP2_HUMAN Calcium-binding protein 2 OS=Homo sapiens GN=CABP2 PE=1 SV=4
MGNCAKRPWRRGPKDPLQWLGSPPRGSCPSPSSSPKEQGDPAPGVQGYSVLNSLVGPACI
FLRPSIAATQLDRELRPEEIEELQVAFQEFDRDRDGYIGCRELGACMRTLGYMPTEMELI
EISQQISGGKVDFEDFVELMGPKLLAETADMIGVRELRDAFREFDTNGDGRISVGELRAA
LKALLGERLSQREVDEILQDVDLNGDGLVDFEEFVRMMSR
>sp|P57796|CABP4_HUMAN Calcium-binding protein 4 OS=Homo sapiens GN=CABP4 PE=1 SV=2
MTTEQARGQQGPNLAIGRQKPPAGVVTPKSDAEEPPLTRKRSKKERGLRGSRKRTGSSGE
QTGPEAPGSSNNPPSTGEGPAGAPPASPGPASSRQSHRHRPDSLHDAAQRTYGPLLNRVF
GKDRELGPEELDELQAAFEEFDTDRDGYISHRELGDCMRTLGYMPTEMELLEVSQHIKMR
MGGRVDFEEFVELIGPKLREETAHMLGVRELRIAFREFDRDRDGRITVAELREAVPALLG
EPLAGPELDEMLREVDLNGDGTVDFDEFVMMLSRH
>sp|Q01668|CAC1D_HUMAN Voltage-dependent L-type calcium channel subunit alpha-1D OS=Homo sapiens GN=CACNA1D PE=1 SV=2
MMMMMMMKKMQHQRQQQADHANEANYARGTRLPLSGEGPTSQPNSSKQTVLSWQAAIDAA
RQAKAAQTMSTSAPPPVGSLSQRKRQQYAKSKKQGNSSNSRPARALFCLSLNNPIRRACI
SIVEWKPFDIFILLAIFANCVALAIYIPFPEDDSNSTNHNLEKVEYAFLIIFTVETFLKI
IAYGLLLHPNAYVRNGWNLLDFVIVIVGLFSVILEQLTKETEGGNHSSGKSGGFDVKALR
AFRVLRPLRLVSGVPSLQVVLNSIIKAMVPLLHIALLVLFVIIIYAIIGLELFIGKMHKT
CFFADSDIVAEEDPAPCAFSGNGRQCTANGTECRSGWVGPNGGITNFDNFAFAMLTVFQC
ITMEGWTDVLYWMNDAMGFELPWVYFVSLVIFGSFFVLNLVLGVLSGEFSKEREKAKARG
DFQKLREKQQLEEDLKGYLDWITQAEDIDPENEEEGGEEGKRNTSMPTSETESVNTENVS
GEGENRGCCGSLCQAISKSKLSRRWRRWNRFNRRRCRAAVKSVTFYWLVIVLVFLNTLTI
SSEHYNQPDWLTQIQDIANKVLLALFTCEMLVKMYSLGLQAYFVSLFNRFDCFVVCGGIT
ETILVELEIMSPLGISVFRCVRLLRIFKVTRHWTSLSNLVASLLNSMKSIASLLLLLFLF
IIIFSLLGMQLFGGKFNFDETQTKRSTFDNFPQALLTVFQILTGEDWNAVMYDGIMAYGG
PSSSGMIVCIYFIILFICGNYILLNVFLAIAVDNLADAESLNTAQKEEAEEKERKKIARK
ESLENKKNNKPEVNQIANSDNKVTIDDYREEDEDKDPYPPCDVPVGEEEEEEEEDEPEVP
AGPRPRRISELNMKEKIAPIPEGSAFFILSKTNPIRVGCHKLINHHIFTNLILVFIMLSS
AALAAEDPIRSHSFRNTILGYFDYAFTAIFTVEILLKMTTFGAFLHKGAFCRNYFNLLDM
LVVGVSLVSFGIQSSAISVVKILRVLRVLRPLRAINRAKGLKHVVQCVFVAIRTIGNIMI

VTTLLQFMFACIGVQLFKGKFYRCTDEAKSNPEECRGLFILYKDGDVDSPVVRERIWQNS
DFNFDNVLSAMMALFTVSTFEGWPALLYKAIDSNGENIGPIYNHRVEISIFFIIYIIIVA
FFMMNIFVGFVIVTFQEQGEKEYKNCELDKNQRQCVEYALKARPLRRYIPKNPYQYKFWY
VVNSSPFEYMMFVLIMLNTLCLAMQHYEQSKMFNDAMDILNMVFTGVFTVEMVLKVIAFK
PKGYFSDAWNTFDSLIVIGSIIDVALSEADPTESENVPVPTATPGNSEESNRISITFFRL
FRVMRLVKLLSRGEGIRTLLWTFIKSFQALPYVALLIAMLFFIYAVIGMQMFGKVAMRDN
NQINRNNNFQTFPQAVLLLFRCATGEAWQEIMLACLPGKLCDPESDYNPGEEYTCGSNFA
IVYFISFYMLCAFLIINLFVAVIMDNFDYLTRDWSILGPHHLDEFKRIWSEYDPEAKGRI
KHLDVVTLLRRIQPPLGFGKLCPHRVACKRLVAMNMPLNSDGTVMFNATLFALVRTALKI
KTEGNLEQANEELRAVIKKIWKKTSMKLLDQVVPPAGDDEVTVGKFYATFLIQDYFRKFK
KRKEQGLVGKYPAKNTTIALQAGLRTLHDIGPEIRRAISCDLQDDEPEETKREEEDDVFK
RNGALLGNHVNHVNSDRRDSLQQTNTTHRPLHVQRPSIPPASDTEKPLFPPAGNSVCHNH
HNHNSIGKQVPTSTNANLNNANMSKAAHGKRPSIGNLEHVSENGHHSSHKHDREPQRRSS
VKRTRYYETYIRSDSGDEQLPTICREDPEIHGYFRDPHCLGEQEYFSSEECYEDDSSPTW
SRQNYGYYSRYPGRNIDSERPRGYHHPQGFLEDDDSPVCYDSRRSPRRRLLPPTPASHRR
SSFNFECLRRQSSQEEVPSSPIFPHRTALPLHLMQQQIMAVAGLDSSKAQKYSPSHSTRS
WATPPATPPYRDWTPCYTPLIQVEQSEALDQVNGSLPSLHRSSWYTDEPDISYRTFTPAS
LTVPSSFRNKNSDKQRSADSLVEAVLISEGLGRYARDPKFVSATKHEIADACDLTIDEME
SAASTLLNGNVRPRANGDVGPLSHRQDYELQDFGPGYSDEEPDPGRDEEDLADEMICITT
L
>sp|Q15878|CAC1E_HUMAN Voltage-dependent R-type calcium channel subunit alpha-1E OS=Homo sapiens GN=CACNA1E PE=1 SV=3
MARFGEAVVARPGSGDGDSDQSRNRQGTPVPASGQAAAYKQTKAQRARTMALYNPIPVRQ
NCFTVNRSLFIFGEDNIVRKYAKKLIDWPPFEYMILATIIANCIVLALEQHLPEDDKTPM
SRRLEKTEPYFIGIFCFEAGIKIVALGFIFHKGSYLRNGWNVMDFIVVLSGILATAGTHF
NTHVDLRTLRAVRVLRPLKLVSGIPSLQIVLKSIMKAMVPLLQIGLLLFFAILMFAIIGL
EFYSGKLHRACFMNNSGILEGFDPPHPCGVQGCPAGYECKDWIGPNDGITQFDNILFAVL
TVFQCITMEGWTTVLYNTNDALGATWNWLYFIPLIIIGSFFVLNLVLGVLSGEFAKERER
VENRRAFMKLRRQQQIERELNGYRAWIDKAEEVMLAEENKNAGTSALEVLRRATIKRSRT
EAMTRDSSDEHCVDISSVGTPLARASIKSAKVDGVSYFRHKERLLRISIRHMVKSQVFYW
IVLSLVALNTACVAIVHHNQPQWLTHLLYYAEFLFLGLFLLEMSLKMYGMGPRLYFHSSF
NCFDFGVTVGSIFEVVWAIFRPGTSFGISVLRALRLLRIFKITKYWASLRNLVVSLMSSM
KSIISLLFLLFLFIVVFALLGMQLFGGRFNFNDGTPSANFDTFPAAIMTVFQILTGEDWN
EVMYNGIRSQGGVSSGMWSAIYFIVLTLFGNYTLLNVFLAIAVDNLANAQELTKDEQEEE
EAFNQKHALQKAKEVSPMSAPNMPSIERDRRRRHHMSMWEPRSSHLRERRRRHHMSVWEQ
RTSQLRKHMQMSSQEALNREEAPTMNPLNPLNPLSSLNPLNAHPSLYRRPRAIEGLALGL
ALEKFEEERISRGGSLKGDGGDRSSALDNQRTPLSLGQREPPWLARPCHGNCDPTQQEAG
GGEAVVTFEDRARHRQSQRRSRHRRVRTEGKESSSASRSRSASQERSLDEAMPTEGEKDH
ELRGNHGAKEPTIQEERAQDLRRTNSLMVSRGSGLAGGLDEADTPLVLPHPELEVGKHVV
LTEQEPEGSSEQALLGNVQLDMGRVISQSEPDLSCITANTDKATTESTSVTVAIPDVDPL
VDSTVVHISNKTDGEASPLKEAEIREDEEEVEKKKQKKEKRETGKAMVPHSSMFIFSTTN
PIRRACHYIVNLRYFEMCILLVIAASSIALAAEDPVLTNSERNKVLRYFDYVFTGVFTFE
MVIKMIDQGLILQDGSYFRDLWNILDFVVVVGALVAFALANALGTNKGRDIKTIKSLRVL
RVLRPLKTIKRLPKLKAVFDCVVTSLKNVFNILIVYKLFMFIFAVIAVQLFKGKFFYCTD

SSKDTEKECIGNYVDHEKNKMEVKGREWKRHEFHYDNIIWALLTLFTVSTGEGWPQVLQH
SVDVTEEDRGPSRSNRMEMSIFYVVYFVVFPFFFVNIFVALIIITFQEQGDKMMEECSLE
KNERACIDFAISAKPLTRYMPQNRHTFQYRVWHFVVSPSFEYTIMAMIALNTVVLMMKYY
SAPCTYELALKYLNIAFTMVFSLECVLKVIAFGFLNYFRDTWNIFDFITVIGSITEIILT
DSKLVNTSGFNMSFLKLFRAARLIKLLRQGYTIRILLWTFVQSFKALPYVCLLIAMLFFI
YAIIGMQVFGNIKLDEESHINRHNNFRSFFGSLMLLFRSATGEAWQEIMLSCLGEKGCEP
DTTAPSGQNENERCGTDLAYVYFVSFIFFCSFLMLNLFVAVIMDNFEYLTRDSSILGPHH
LDEFVRVWAEYDRAACGRIHYTEMYEMLTLMSPPLGLGKRCPSKVAYKRLVLMNMPVAED
MTVHFTSTLMALIRTALDIKIAKGGADRQQLDSELQKETLAIWPHLSQKMLDLLVPMPKA
SDLTVGKIYAAMMIMDYYKQSKVKKQRQQLEEQKNAPMFQRMEPSSLPQEIIANAKALPY
LQQDPVSGLSGRSGYPSMSPLSPQDIFQLACMDPADDGQFQERQSLEPEVSELKSVQPSN
HGIYLPSDTQEHAGSGRASSMPRLTVDPQVVTDPSSMRRSFSTIRDKRSNSSWLEEFSME
RSSENTYKSRRRSYHSSLRLSAHRLNSDSGHKSDTHRSGGRERGRSKERKHLLSPDVSRC
NSEERGTQADWESPERRQSRSPSEGRSQTPNRQGTGSLSESSIPSVSDTSTPRRSRRQLP
PVPPKPRPLLSYSSLIRHAGSISPPADGSEEGSPLTSQALESNNACLTESSNSPHPQQSQ
HASPQRYISEPYLALHEDSHASDCGEEETLTFEAAVATSLGRSNTIGSAPPLRHSWQMPN
GHYRRRRRGGPGPGMMCGAVNNLLSDTEEDDKC
>sp|Q9P0X4|CAC1I_HUMAN Voltage-dependent T-type calcium channel subunit alpha-1I OS=Homo sapiens GN=CACNA1I PE=1 SV=1
MAESASPPSSSAAAPAAEPGVTTEQPGPRSPPSSPPGLEEPLDGADPHVPHPDLAPIAFF
CLRQTTSPRNWCIKMVCNPWFECVSMLVILLNCVTLGMYQPCDDMDCLSDRCKILQVFDD
FIFIFFAMEMVLKMVALGIFGKKCYLGDTWNRLDFFIVMAGMVEYSLDLQNINLSAIRTV
RVLRPLKAINRVPSMRILVNLLLDTLPMLGNVLLLCFFVFFIFGIIGVQLWAGLLRNRCF
LEENFTIQGDVALPPYYQPEEDDEMPFICSLSGDNGIMGCHEIPPLKEQGRECCLSKDDV
YDFGAGRQDLNASGLCVNWNRYYNVCRTGSANPHKGAINFDNIGYAWIVIFQVITLEGWV
EIMYYVMDAHSFYNFIYFILLIIVGSFFMINLCLVVIATQFSETKQREHRLMLEQRQRYL
SSSTVASYAEPGDCYEEIFQYVCHILRKAKRRALGLYQALQSRRQALGPEAPAPAKPGPH
AKEPRHYHGKTKGQGDEGRHLGSRHCQTLHGPASPGNDHSGRELCPQHSPLDATPHTLVQ
PIPATLASDPASCPCCQHEDGRRPSGLGSTDSGQEGSGSGSSAGGEDEADGDGARSSEDG
ASSELGKEEEEEEQADGAVWLCGDVWRETRAKLRGIVDSKYFNRGIMMAILVNTVSMGIE
HHEQPEELTNILEICNVVFTSMFALEMILKLAAFGLFDYLRNPYNIFDSIIVIISIWEIV
GQADGGLSVLRTFRLLRVLKLVRFMPALRRQLVVLMKTMDNVATFCMLLMLFIFIFSILG
MHIFGCKFSLRTDTGDTVPDRKNFDSLLWAIVTVFQILTQEDWNVVLYNGMASTSPWASL
YFVALMTFGNYVLFNLLVAILVEGFQAEGDANRSYSDEDQSSSNIEEFDKLQEGLDSSGD
PKLCPIPMTPNGHLDPSLPLGGHLGPAGAAGPAPRLSLQPDPMLVALGSRKSSVMSLGRM
SYDQRSLSSSRSSYYGPWGRSAAWASRRSSWNSLKHKPPSAEHESLLSAERGGGARVCEV
AADEGPPRAAPLHTPHAHHIHHGPHLAHRHRHHRRTLSLDNRDSVDLAELVPAVGAHPRA
AWRAAGPAPGHEDCNGRMPSIAKDVFTKMGDRGDRGEDEEEIDYTLCFRVRKMIDVYKPD
WCEVREDWSVYLFSPENRFRVLCQTIIAHKLFDYVVLAFIFLNCITIALERPQIEAGSTE
RIFLTVSNYIFTAIFVGEMTLKVVSLGLYFGEQAYLRSSWNVLDGFLVFVSIIDIVVSLA
SAGGAKILGVLRVLRLLRTLRPLRVISRAPGLKLVVETLISSLKPIGNIVLICCAFFIIF
GILGVQLFKGKFYHCLGVDTRNITNRSDCMAANYRWVHHKYNFDNLGQALMSLFVLASKD
GWVNIMYNGLDAVAVDQQPVTNHNPWMLLYFISFLLIVSFFVLNMFVGVVVENFHKCRQH
QEAEEARRREEKRLRRLEKKRRKAQRLPYYATYCHTRLLIHSMCTSHYLDIFITFIICLN

VVTMSLEHYNQPTSLETALKYCNYMFTTVFVLEAVLKLVAFGLRRFFKDRWNQLDLAIVL
LSVMGITLEEIEINAALPINPTIIRIMRVLRIARVLKLLKMATGMRALLDTVVQALPQVG
NLGLLFMLLFFIYAALGVELFGKLVCNDENPCEGMSRHATFENFGMAFLTLFQVSTGDNW
NGIMKDTLRDCTHDERSCLSSLQFVSPLYFVSFVLTAQFVLINVVVAVLMKHLDDSNKEA
QEDAEMDAELELEMAHGLGPGPRLPTGSPGAPGRGPGGAGGGGDTEGGLCRRCYSPAQEN
LWLDSVSLIIKDSLEGELTIIDNLSGSIFHHYSSPAGCKKCHHDKQEVQLAETEAFSLNS
DRSSSILLGDDLSLEDPTACPPGRKDSKGELDPPEPMRVGDLGECFFPLSSTAVSPDPEN
FLCEMEEIPFNPVRSWLKHDSSQAPPSPFSPDASSPLLPMPAEFFHPAVSASQKGPEKGT
GTGTLPKIALQGSWASLRSPRVNCTLLRQATGSDTSLDASPSSSAGSLQTTLEDSLTLSD
SPRRALGPPAPAPGPRAGLSPAARRRLSLRGRGLFSLRGLRAHQRSHSSGGSTSPGCTHH
DSMDPSDEEGRGGAGGGGAGSEHSETLSSLSLTSLFCPPPPPPAPGLTPARKFSSTSSLA
APGRPHAAALAHGLARSPSWAADRSKDPPGRAPLPMGLGPLAPPPQPLPGELEPGDAASK
RKR
>sp|O00305|CACB4_HUMAN Voltage-dependent L-type calcium channel subunit beta-4 OS=Homo sapiens GN=CACNB4 PE=1 SV=2
MSSSSYAKNGTADGPHSPTSQVARGTTTRRSRLKRSDGSTTSTSFILRQGSADSYTSRPS
DSDVSLEEDREAIRQEREQQAAIQLERAKSKPVAFAVKTNVSYCGALDEDVPVPSTAISF
DAKDFLHIKEKYNNDWWIGRLVKEGCEIGFIPSPLRLENIRIQQEQKRGRFHGGKSSGNS
SSSLGEMVSGTFRATPTSTAKQKQKVTEHIPPYDVVPSMRPVVLVGPSLKGYEVTDMMQK
ALFDFLKHRFDGRISITRVTADISLAKRSVLNNPSKRAIIERSNTRSSLAEVQSEIERIF
ELARSLQLVVLDADTINHPAQLIKTSLAPIIVHVKVSSPKVLQRLIKSRGKSQSKHLNVQ
LVAADKLAQCPPEMFDVILDENQLEDACEHLGEYLEAYWRATHTTSSTPMTPLLGRNLGS
TALSPYPTAISGLQSQRMRHSNHSTENSPIERRSLMTSDENYHNERARKSRNRLSSSSQH
SRDHYPLVEEDYPDSYQDTYKPHRNRGSPGGYSHDSRHRL
>sp|Q9P1Z2|CACO1_HUMAN Calcium-binding and coiled-coil domain-containing protein 1 OS=Homo sapiens GN=CALCOCO1 PE=1 SV=2
MEESPLSRAPSRGGVNFLNVARTYIPNTKVECHYTLPPGTMPSASDWIGIFKVEAACVRD
YHTFVWSSVPESTTDGSPIHTSVQFQASYLPKPGAQLYQFRYVNRQGQVCGQSPPFQFRE
PRPMDELVTLEEADGGSDILLVVPKATVLQNQLDESQQERNDLMQLKLQLEGQVTELRSR
VQELERALATARQEHTELMEQYKGISRSHGEITEERDILSRQQGDHVARILELEDDIQTI
SEKVLTKEVELDRLRDTVKALTREQEKLLGQLKEVQADKEQSEAELQVAQQENHHLNLDL
KEAKSWQEEQSAQAQRLKDKVAQMKDTLGQAQQRVAELEPLKEQLRGAQELAASSQQKAT
LLGEELASAAAARDRTIAELHRSRLEVAEVNGRLAELGLHLKEEKCQWSKERAGLLQSVE
AEKDKILKLSAEILRLEKAVQEERTQNQVFKTELAREKDSSLVQLSESKRELTELRSALR
VLQKEKEQLQEEKQELLEYMRKLEARLEKVADEKWNEDATTEDEEAAVGLSCPAALTDSE
DESPEDMRLPPYGLCERGDPGSSPAGPREASPLVVISQPAPISPHLSGPAEDSSSDSEAE
DEKSVLMAAVQSGGEEANLLLPELGSAFYDMASGFTVGTLSETSTGGPATPTWKECPICK
ERFPAESDKDALEDHMDGHFFFSTQDPFTFE
>sp|P55289|CAD12_HUMAN Cadherin-12 OS=Homo sapiens GN=CDH12 PE=2 SV=2
MLTRNCLSLLLWVLFDGGLLTPLQPQPQQTLATEPRENVIHLPGQRSHFQRVKRGWVWNQ
FFVLEEYVGSEPQYVGKLHSDLDKGEGTVKYTLSGDGAGTVFTIDETTGDIHAIRSLDRE
EKPFYTLRAQAVDIETRKPLEPESEFIIKVQDINDNEPKFLDGPYVATVPEMSPVGAYVL
QVKATDADDPTYGNSARVVYSILQGQPYFSIDPKTGVIRTALPNMDREVKEQYQVLIQAK
DMGGQLGGLAGTTIVNITLTDVNDNPPRFPKSIFHLKVPESSPIGSAIGRIRAVDPDFGQ

NAEIEYNIVPGDGGNLFDIVTDEDTQEGVIKLKKPLDFETKKAYTFKVEASNLHLDHRFH
SAGPFKDTATVKISVLDVDEPPVFSKPLYTMEVYEDTPVGTIIGAVTAQDLDVGSSAVRY
FIDWKSDGDSYFTIDGNEGTIATNELLDRESTAQYNFSIIASKVSNPLLTSKVNILINVL
DVNEFPPEISVPYETAVCENAKPGQIIQIVSAADRDLSPAGQQFSFRLSPEAAIKPNFTV
RDFRNNTAGIETRRNGYSRRQQELYFLPVVIEDSSYPVQSSTNTMTIRVCRCDSDGTILS
CNVEAIFLPVGLSTGALIAILLCIVILLAIVVLYVALRRQKKKDTLMTSKEDIRDNVIHY
DDEGGGEEDTQAFDIGALRNPKVIEENKIRRDIKPDSLCLPRQRPPMEDNTDIRDFIHQR
LQENDVDPTAPPYDSLATYAYEGSGSVAESLSSIDSLTTEADQDYDYLTDWGPRFKVLAD
MFGEEESYNPDKVT
>sp|P55290|CAD13_HUMAN Cadherin-13 OS=Homo sapiens GN=CDH13 PE=1 SV=1
MQPRTPLVLCVLLSQVLLLTSAEDLDCTPGFQQKVFHINQPAEFIEDQSILNLTFSDCKG
NDKLRYEVSSPYFKVNSDGGLVALRNITAVGKTLFVHARTPHAEDMAELVIVGGKDIQGS
LQDIFKFARTSPVPRQKRSIVVSPILIPENQRQPFPRDVGKVVDSDRPERSKFRLTGKGV
DQEPKGIFRINENTGSVSVTRTLDREVIAVYQLFVETTDVNGKTLEGPVPLEVIVIDQND
NRPIFREGPYIGHVMEGSPTGTTVMRMTAFDADDPATDNALLRYNIRQQTPDKPSPNMFY
IDPEKGDIVTVVSPALLDRETLENPKYELIIEAQDMAGLDVGLTGTATATIMIDDKNDHS
PKFTKKEFQATVEEGAVGVIVNLTVEDKDDPTTGAWRAAYTIINGNPGQSFEIHTNPQTN
EGMLSVVKPLDYEISAFHTLLIKVENEDPLVPDVSYGPSSTATVHITVLDVNEGPVFYPD
PMMVTRQEDLSVGSVLLTVNATDPDSLQHQTIRYSVYKDPAGWLNINPINGTVDTTAVLD
RESPFVDNSVYTALFLAIDSGNPPATGTGTLLITLEDVNDNAPFIYPTVAEVCDDAKNLS
VVILGASDKDLHPNTDPFKFEIHKQAVPDKVWKISKINNTHALVSLLQNLNKANYNLPIM
VTDSGKPPMTNITDLRVQVCSCRNSKVDCNAAGALRFSLPSVLLLSLFSLACL
>sp|P22223|CADH3_HUMAN Cadherin-3 OS=Homo sapiens GN=CDH3 PE=1 SV=2
MGLPRGPLASLLLLQVCWLQCAASEPCRAVFREAEVTLEAGGAEQEPGQALGKVFMGCPG
QEPALFSTDNDDFTVRNGETVQERRSLKERNPLKIFPSKRILRRHKRDWVVAPISVPENG
KGPFPQRLNQLKSNKDRDTKIFYSITGPGADSPPEGVFAVEKETGWLLLNKPLDREEIAK
YELFGHAVSENGASVEDPMNISIIVTDQNDHKPKFTQDTFRGSVLEGVLPGTSVMQVTAT
DEDDAIYTYNGVVAYSIHSQEPKDPHDLMFTIHRSTGTISVISSGLDREKVPEYTLTIQA
TDMDGDGSTTTAVAVVEILDANDNAPMFDPQKYEAHVPENAVGHEVQRLTVTDLDAPNSP
AWRATYLIMGGDDGDHFTITTHPESNQGILTTRKGLDFEAKNQHTLYVEVTNEAPFVLKL
PTSTATIVVHVEDVNEAPVFVPPSKVVEVQEGIPTGEPVCVYTAEDPDKENQKISYRILR
DPAGWLAMDPDSGQVTAVGTLDREDEQFVRNNIYEVMVLAMDNGSPPTTGTGTLLLTLID
VNDHGPVPEPRQITICNQSPVRQVLNITDKDLSPHTSPFQAQLTDDSDIYWTAEVNEEGD
TVVLSLKKFLKQDTYDVHLSLSDHGNKEQLTVIRATVCDCHGHVETCPGPWKGGFILPVL
GAVLALLFLLLVLLLLVRKKRKIKEPLLLPEDDTRDNVFYYGEEGGGEEDQDYDITQLHR
GLEARPEVVLRNDVAPTIIPTPMYRPRPANPDEIGNFIIENLKAANTDPTAPPYDTLLVF
DYEGSGSDAASLSSLTSSASDQDQDYDYLNEWGSRFKKLADMYGGGEDD
>sp|P55283|CADH4_HUMAN Cadherin-4 OS=Homo sapiens GN=CDH4 PE=2 SV=2
MTAGAGVLLLLLSLSGALRAHNEDLTTRETCKAGFSEDDYTALISQNILEGEKLLQVKFS
SCVGTKGTQYETNSMDFKVGADGTVFATRELQVPSEQVAFTVTAWDSQTAEKWDAVVRLL
VAQTSSPHSGHKPQKGKKVVALDPSPPPKDTLLPWPQHQNANGLRRRKRDWVIPPINVPE
NSRGPFPQQLVRIRSDKDNDIPIRYSITGVGADQPPMEVFSIDSMSGRMYVTRPMDREEH
ASYHLRAHAVDMNGNKVENPIDLYIYVIDMNDNRPEFINQVYNGSVDEGSKPGTYVMTVT
ANDADDSTTANGMVRYRIVTQTPQSPSQNMFTINSETGDIVTVAAGLDREKVQQYTVIVQ

ATDMEGNLNYGLSNTATAIITVTDVNDNPPEFTASTFAGEVPENRVETVVANLTVMDRDQ
PHSPNWNAVYRIISGDPSGHFSVRTDPVTNEGMVTVVKAVDYELNRAFMLTVMVSNQAPL
ASGIQMSFQSTAGVTISIMDINEAPYFPSNHKLIRLEEGVPPGTVLTTFSAVDPDRFMQQ
AVRYSKLSDPASWLHINATNGQITTAAVLDRESLYTKNNVYEATFLAADNGIPPASGTGT
LQIYLIDINDNAPELLPKEAQICEKPNLNAINITAADADVDPNIGPYVFELPFVPAAVRK
NWTITRLNGDYAQLSLRILYLEAGMYDVPIIVTDSGNPPLSNTSIIKVKVCPCDDNGDCT
TIGAVAAAGLGTGAIVAILICILILLTMVLLFVMWMKRREKERHTKQLLIDPEDDVRDNI
LKYDEEGGGEEDQDYDLSQLQQPEAMGHVPSKAPGVRRVDERPVGAEPQYPIRPMVPHPG
DIGDFINEGLRAADNDPTAPPYDSLLVFDYEGSGSTAGSVSSLNSSSSGDQDYDYLNDWG
PRFKKLADMYGGGEED
>sp|P55285|CADH6_HUMAN Cadherin-6 OS=Homo sapiens GN=CDH6 PE=1 SV=1
MRTYRYFLLLFWVGQPYPTLSTPLSKRTSGFPAKKRALELSGNSKNELNRSKRSWMWNQF
FLLEEYTGSDYQYVGKLHSDQDRGDGSLKYILSGDGAGDLFIINENTGDIQATKRLDREE
KPVYILRAQAINRRTGRPVEPESEFIIKIHDINDNEPIFTKEVYTATVPEMSDVGTFVVQ
VTATDADDPTYGNSAKVVYSILQGQPYFSVESETGIIKTALLNMDRENREQYQVVIQAKD
MGGQMGGLSGTTTVNITLTDVNDNPPRFPQSTYQFKTPESSPPGTPIGRIKASDADVGEN
AEIEYSITDGEGLDMFDVITDQETQEGIITVKKLLDFEKKKVYTLKVEASNPYVEPRFLY
LGPFKDSATVRIVVEDVDEPPVFSKLAYILQIREDAQINTTIGSVTAQDPDAARNPVKYS
VDRHTDMDRIFNIDSGNGSIFTSKLLDRETLLWHNITVIATEINNPKQSSRVPLYIKVLD
VNDNAPEFAEFYETFVCEKAKADQLIQTLHAVDKDDPYSGHQFSFSLAPEAASGSNFTIQ
DNKDNTAGILTRKNGYNRHEMSTYLLPVVISDNDYPVQSSTGTVTVRVCACDHHGNMQSC
HAEALIHPTGLSTGALVAILLCIVILLVTVVLFAALRRQRKKEPLIISKEDIRDNIVSYN
DEGGGEEDTQAFDIGTLRNPEAIEDNKLRRDIVPEALFLPRRTPTARDNTDVRDFINQRL
KENDTDPTAPPYDSLATYAYEGTGSVADSLSSLESVTTDADQDYDYLSDWGPRFKKLADM
YGGVDSDKDS
>sp|Q9ULB4|CADH9_HUMAN Cadherin-9 OS=Homo sapiens GN=CDH9 PE=2 SV=2
MRTYHYIPLFIWTYMFHTVDTILLQEKPNSYLSSKKIAGLTKDDGKMLRRTKRGWMWNQF
FLLEEYTGTDTQYVGKLHTDQDKGDGNLKYILTGDGAGSLFVIDENTGDIHAAKKLDREE
KSLYILRAKAIDRKTGRQVEPESEFIIKIHDINDNEPKFTKDLYTASVPEMSGVGTSVIQ
VTATDADDANYGNSAKVVYSILQGQPYFSVDPESGIIKTALPDMSRENREQYQVVIQAKD
MGGQMGGLSGTTTVNITLTDVNNNPPRFPQSTYQFNSPESVPLGTHLGRIKANDPDVGEN
AEMEYSIAEGDGADMFDVITDKDTQEGIITVKQNLDFENQMLYTLRVDASNTHPDPRFLH
LGPFKDTAVVKISVEDIDEPPVFTKVSYLIEVDEDVKEGSIIGQVTAYDPDARNNLIKYS
VDRHTDMDRIFGIHSENGSIFTLKALDRESSPWHNITVTATEINNPKQSSHIPVFIRILD
INDHAPEFAMYYETFVCENAKPGQLIQTVSVMDKDDPPRGHKFFFEPVPEFTLNPNFTIV
DNKDNTAGIMTRKDGYSRNKMSTYLLPILIFDNDYPIQSSTGTLTIRVCACDNQGNMQSC
TAEALILSAGLSTGALVAILLCVLILLILVVLFAALKRQRKKEPLIISKDDVRDNIVTYN
DEGGGEEDTQAFDIGTLRNPEAREDSKLRRDVMPETIFQIRRTVPLWENIDVQDFIHRRL
KENDADPSAPPYDSLATYAYEGNDSIADSLSSLESLTADCNQDYDYLSDWGPRFKKLADM
YGGDDSDRD
>sp|Q9BY67|CADM1_HUMAN Cell adhesion molecule 1 OS=Homo sapiens GN=CADM1 PE=1 SV=2
MASVVLPSGSQCAAAAAAAAPPGLRLRLLLLLFSAALIPTGDGQNLFTKDVTVIEGEVA
TISCQVNKSDDSVIQLLNPNRQTIYFRDFRPLKDSRFQLLNFSSSELKVSLTNVSISDEG
RYFCQLYTDPPQESYTTITVLVPPRNLMIDIQKDTAVEGEEIEVNCTAMASKPATTIRWF

KGNTELKGKSEVEEWSDMYTVTSQLMLKVHKEDDGVPVICQVEHPAVTGNLQTQRYLEVQ
YKPQVHIQMTYPLQGLTREGDALELTCEAIGKPQPVMVTWVRVDDEMPQHAVLSGPNLFI
NNLNKTDNGTYRCEASNIVGKAHSDYMLYVYDPPTTIPPPTTTTTTTTTTTTILTIITD
SRAGEEGSIRAVDHAVIGGVVAVVVFAMLCLLIILGRYFARHKGTYFTHEAKGADDAADA
DTAIINAEGGQNNSEEKKEYFI
>sp|P22748|CAH4_HUMAN Carbonic anhydrase 4 OS=Homo sapiens GN=CA4 PE=1 SV=2
MRMLLALLALSAARPSASAESHWCYEVQAESSNYPCLVPVKWGGNCQKDRQSPINIVTTK
AKVDKKLGRFFFSGYDKKQTWTVQNNGHSVMMLLENKASISGGGLPAPYQAKQLHLHWSD
LPYKGSEHSLDGEHFAMEMHIVHEKEKGTSRNVKEAQDPEDEIAVLAFLVEAGTQVNEGF
QPLVEALSNIPKPEMSTTMAESSLLDLLPKEEKLRHYFRYLGSLTTPTCDEKVVWTVFRE
PIQLHREQILAFSQKLYYDKEQTVSMKDNVRPLQQLGQRTVIKSGAPGRPLPWALPALLG
PMLACLLAGFLR
>sp|Q9Y2D0|CAH5B_HUMAN Carbonic anhydrase 5B, mitochondrial OS=Homo sapiens GN=CA5B PE=1 SV=1
MVVMNSLRVILQASPGKLLWRKFQIPRFMPARPCSLYTCTYKTRNRALHPLWESVDLVPG
GDRQSPINIRWRDSVYDPGLKPLTISYDPATCLHVWNNGYSFLVEFEDSTDKSVIKGGPL
EHNYRLKQFHFHWGAIDAWGSEHTVDSKCFPAELHLVHWNAVRFENFEDAALEENGLAVI
GVFLKLGKHHKELQKLVDTLPSIKHKDALVEFGSFDPSCLMPTCPDYWTYSGSLTTPPLS
ESVTWIIKKQPVEVDHDQLEQFRTLLFTSEGEKEKRMVDNFRPLQPLMNRTVRSSFRHDY
VLNVQAKPKPATSQATP
>sp|P23280|CAH6_HUMAN Carbonic anhydrase 6 OS=Homo sapiens GN=CA6 PE=1 SV=3
MRALVLLLSLFLLGGQAQHVSDWTYSEGALDEAHWPQHYPACGGQRQSPINLQRTKVRYN
PSLKGLNMTGYETQAGEFPMVNNGHTVQISLPSTMRMTVADGTVYIAQQMHFHWGGASSE
ISGSEHTVDGIRHVIEIHIVHYNSKYKSYDIAQDAPDGLAVLAAFVEVKNYPENTYYSNF
ISHLANIKYPGQRTTLTGLDVQDMLPRNLQHYYTYHGSLTTPPCTENVHWFVLADFVKLS
RTQVWKLENSLLDHRNKTIHNDYRRTQPLNHRVVESNFPNQEYTLGSEFQFYLHKIEEIL
DYLRRALN
>sp|P43166|CAH7_HUMAN Carbonic anhydrase 7 OS=Homo sapiens GN=CA7 PE=1 SV=1
MTGHHGWGYGQDDGPSHWHKLYPIAQGDRQSPINIISSQAVYSPSLQPLELSYEACMSLS
ITNNGHSVQVDFNDSDDRTVVTGGPLEGPYRLKQFHFHWGKKHDVGSEHTVDGKSFPSEL
HLVHWNAKKYSTFGEAASAPDGLAVVGVFLETGDEHPSMNRLTDALYMVRFKGTKAQFSC
FNPKCLLPASRHYWTYPGSLTTPPLSESVTWIVLREPICISERQMGKFRSLLFTSEDDER
IHMVNNFRPPQPLKGRVVKASFRA
>sp|Q86XJ0|CAHM3_HUMAN Calcium homeostasis modulator protein 3 OS=Homo sapiens GN=CALHM3 PE=2 SV=1
MDKFRMLFQHFQSSSESVMNGICLLLAAVTVKLYSSFDFNCPCLVHYNALYGLGLLLTPP
LALFLCGLLANRQSVVMVEEWRRPAGHRRKDPGIIRYMCSSVLQRALAAPLVWILLALLD
GKCFVCAFSSSVDPEKFLDFANMTPSQVQLFLAKVPCKEDELVRDSPARKAVSRYLRCLS
QAIGWSVTLLLIIAAFLARCLRPCFDQTVFLQRRYWSNYVDLEQKLFDETCCEHARDFAH
RCVLHFFASMRSELQARGLRRGNAGRRLELPAVPEPPAVPEPPEGLDSGSGKAHLRAISS
REQVDRLLSTWYSSKPPLDLAASPGLCGGGLSHRAPTLALGTRLSQHTDV
>sp|P80723|BASP1_HUMAN Brain acid soluble protein 1 OS=Homo sapiens GN=BASP1 PE=1 SV=2
MGGKLSKKKKGYNVNDEKAKEKDKKAEGAATEEEGTPKESEPQAAAEPAEAKEGKEKPDQ
DAEGKAEEKEGEKDAAAAKEEAPKAEPEKTEGAAEAKAEPPKAPEQEQAAPGPAAGGEAP

KAAEAAAAPAESAAPAAGEEPSKEEGEPKKTEAPAAPAAQETKSDGAPASDSKPGSSEAA
PSSKETPAATEAPSSTPKAQGPAASAEEPKPVEAPAANSDQTVTVKE
>sp|Q9NR55|BATF3_HUMAN Basic leucine zipper transcriptional factor ATF-like 3 OS=Homo sapiens GN=BATF3 PE=1 SV=1
MSQGLPAAGSVLQRSVAAPGNQPQPQPQQQSPEDDDRKVRRREKNRVAAQRSRKKQTQKA
DKLHEEYESLEQENTMLRREIGKLTEELKHLTEALKEHEKMCPLLLCPMNFVPVPPRPDP
VAGCLPR
>sp|Q9UIF8|BAZ2B_HUMAN Bromodomain adjacent to zinc finger domain protein 2B OS=Homo sapiens GN=BAZ2B PE=1 SV=3
MESGERLPSSAASSTTPTSSSTPSVASVVSKGGLSTGVASLSSTINPCGHLFRTAGDQPF
NLSTVSSAFPMVSHPVFGLHSASSGHSEFGGLGTLGTPTALAAHPQLASFPGAEWWRTTD
AHTRTGATFFPPLLGIPPLFAPPAQNHDSSSFHSRTSGKSNRNGPEKGVNGSINGSNTSS
VIGINTSVLSTTASSSMGQTKSTSSGGGNRKCNQEQSKNQPLDARVDKIKDKKPRKKAME
SSSNSDSDSGTSSDTSSEGISSSDSDDLEEDEEEEDQSIEESEDDDSDSESEAQHKSNNQ
VLLHGISDPKADGQKATEKAQEKRIHQPLPLASESQTHSFQSQQKQPQVLSQQLPFIFQS
SQAKEESVNKHTSVIQSTGLVSNVKPLSLVNQAKKETYMKLIVPSPDVLKAGNKNTSEES
SLLTSELRSKREQYKQAFPSQLKKQESSKSLKKVIAALSNPKATSSSPAHPKQTLENNHP
NPFLTNALLGNHQPNGVIQSVIQEAPLALTTKTKMQSKINENIAAASSTPFSSPVNLSTS
GRRTPGNQTPVMPSASPILHSQGKEKAVSNNVNPVKTQHHSHPAKSLVEQFRGTDSDIPS
SKDSEDSNEDEEEDDEEEDEEDDEDDESDDSQSESDSNSESDTEGSEEEDDDDKDQDESD
SDTEGEKTSMKLNKTTSSVKSPSMSLTGHSTPRNLHIAKAPGSAPAALCSESQSPAFLGT
SSSTLTSSPHSGTSKRRRVTDERELRIPLEYGWQRETRIRNFGGRLQGEVAYYAPCGKKL
RQYPEVIKYLSRNGIMDISRDNFSFSAKIRVGDFYEARDGPQGMQWCLLKEEDVIPRIRA
MEGRRGRPPNPDRQRAREESRMRRRKGRPPNVGNAEFLDNADAKLLRKLQAQEIARQAAQ
IKLLRKLQKQEQARVAKEAKKQQAIMAAEEKRKQKEQIKIMKQQEKIKRIQQIRMEKELR
AQQILEAKKKKKEEAANAKLLEAEKRIKEKEMRRQQAVLLKHQERERRRQHMMLMKAMEA
RKKAEEKERLKQEKRDEKRLNKERKLEQRRLELEMAKELKKPNEDMCLADQKPLPELPRI
PGLVLSGSTFSDCLMVVQFLRNFGKVLGFDVNIDVPNLSVLQEGLLNIGDSMGEVQDLLV
RLLSAAVCDPGLITGYKAKTALGEHLLNVGVNRDNVSEILQIFMEAHCGQTELTESLKTK
AFQAHTPAQKASVLAFLINELACSKSVVSEIDKNIDYMSNLRRDKWVVEGKLRKLRIIHA
KKTGKRDTSGGIDLGEEQHPLGTPTPGRKRRRKGGDSDYDDDDDDDSDDQGDEDDEDEED
KEDKKGKKTDICEDEDEGDQAASVEELEKQIEKLSKQQSQYRRKLFDASHSLRSVMFGQD
RYRRRYWILPQCGGIFVEGMESGEGLEEIAKEREKLKKAESVQIKEEMFETSGDSLNCSN
TDHCEQKEDLKEKDNTNLFLQKPGSFSKLSKLLEVAKMPPESEVMTPKPNAGANGCTLSY
QNSGKHSLGSVQSTATQSNVEKADSNNLFNTGSSGPGKFYSPLPNDQLLKTLTEKNRQWF
SLLPRTPCDDTSLTHADMSTASLVTPQSQPPSKSPSPTPAPLGSSAQNPVGLNPFALSPL
QVKGGVSMMGLQFCGWPTGVVTSNIPFTSSVPSLGSGLGLSEGNGNSFLTSNVASSKSES
PVPQNEKATSAQPAAVEVAKPVDFPSPKPIPEEMQFGWWRIIDPEDLKALLKVLHLRGIR
EKALQKQIQKHLDYITQACLKNKDVAIIELNENEENQVTRDIVENWSVEEQAMEMDLSVL
QQVEDLERRVASASLQVKGWMCPEPASEREDLVYFEHKSFTKLCKEHDGEFTGEDESSAH
ALERKSDNPLDIAVTRLADLERNIERRIEEDIAPGLRVWRRALSEARSAAQVALCIQQLQ
KSIAWEKSIMKVYCQICRKGDNEELLLLCDGCDKGCHTYCHRPKITTIPDGDWFCPACIA
KASGQTLKIKKLHVKGKKTNESKKGKKVTLTGDTEDEDSASTSSSLKRGNKDLKKRKMEE
NTSINLSKQESFTSVKKPKRDDSKDLALCSMILTEMETHEDAWPFLLPVNLKLVPGYKKV

IKKPMDFSTIREKLSSGQYPNLETFALDVRLVFDNCETFNEDDSDIGRAGHNMRKYFEKK
WTDTFKVS
>sp|Q96PG8|BBC3B_HUMAN Bcl-2-binding component 3 OS=Homo sapiens GN=BBC3 PE=2 SV=2
MKFGMGSAQACPCQVPRAASTTWVPCQICGPRERHGPRTPGGQLPGARRGPGPRRPAPLP
ARPPGALGSVLRPLRARPGCRPRRPHPAARCLPLRPHRPTRRHRRPGGFPLAWGSPQPAP
RPAPGRSSALALAGGAAPGVARAQRPGGSGGRSHPGGPGSPRGGGTVGPGDRGPAAADGG
RPQRTVRAAETRGAAAAPPLTLEGPVQSHHGTPALTQGPQSPRDGAQLGACTRPVDVRDS
GGRPLPPPDTLASAGDFLCTM
>sp|Q3B7T3|BEAN1_HUMAN Protein BEAN1 OS=Homo sapiens GN=BEAN1 PE=2 SV=2
MSFKRPCPLARYNRTSYFYPTFSESSEHSHLLVSPVLVASAVIGVVIILSCITIIVGSIR
RDRQARLQRHRHRHHRHHHHHHHHRRRRHREYEHGYVSDEHTYSRSSRRMRYACSSSEDW
PPPLDISSDGDVDATVLRELYPDSPPGYEECVGPGATQLYVPTDAPPPYSLTDSCPTLDG
TSDSGSGHSPGRHQQEQRTPAQGGLHTVSMDTLPPYEAVCGAGPPSGLLPLPGPDPGPRG
SQGSPTPTRAPASGPERIV
>sp|Q15582|BGH3_HUMAN Transforming growth factor-beta-induced protein ig-h3 OS=Homo sapiens GN=TGFBI PE=1 SV=1
MALFVRLLALALALALGPAATLAGPAKSPYQLVLQHSRLRGRQHGPNVCAVQKVIGTNRK
YFTNCKQWYQRKICGKSTVISYECCPGYEKVPGEKGCPAALPLSNLYETLGVVGSTTTQL
YTDRTEKLRPEMEGPGSFTIFAPSNEAWASLPAEVLDSLVSNVNIELLNALRYHMVGRRV
LTDELKHGMTLTSMYQNSNIQIHHYPNGIVTVNCARLLKADHHATNGVVHLIDKVISTIT
NNIQQIIEIEDTFETLRAAVAASGLNTMLEGNGQYTLLAPTNEAFEKIPSETLNRILGDP
EALRDLLNNHILKSAMCAEAIVAGLSVETLEGTTLEVGCSGDMLTINGKAIISNKDILAT
NGVIHYIDELLIPDSAKTLFELAAESDVSTAIDLFRQAGLGNHLSGSERLTLLAPLNSVF
KDGTPPIDAHTRNLLRNHIIKDQLASKYLYHGQTLETLGGKKLRVFVYRNSLCIENSCIA
AHDKRGRYGTLFTMDRVLTPPMGTVMDVLKGDNRFSMLVAAIQSAGLTETLNREGVYTVF
APTNEAFRALPPRERSRLLGDAKELANILKYHIGDEILVSGGIGALVRLKSLQGDKLEVS
LKNNVVSVNKEPVAEPDIMATNGVVHVITNVLQPPANRPQERGDELADSALEIFKQASAF
SRASQRSVRLAPVYQKLLERMKH
>sp|Q6UXY1|BI2L2_HUMAN Brain-specific angiogenesis inhibitor 1-associated protein 2-like protein 2 OS=Homo sapiens GN=BAIAP2L2 PE=1 SV=1
MAPEMDQFYRSTMAIYKSIMEQFNPALENLVYLGNNYLRAFHALSEAAEVYFSAIQKIGE
RALQSPTSQILGEILVQMSDTQRHLNSDLEVVVQTFHGGLLQHMEKNTKLDMQFIKDSRQ
HYELEYRHRAANLEKCMSELWRMERKRDKNVREMKESVNRLHAQMQAFVSESQRAAELEE
KRRYRFLAEKHLLLSNTFLQFFGRARGMLQNRVLLWKEQSEASRSPSRAHSPGLLGPALG
PPYPSGRLTPTCLDMPPRPLGEFSSPRSRHGSGSYGTEPDARPASQLEPDRRSLPRTPSA
SSLYSGSAQSSRSNSFGERPGGGGGARRVRALVSHSEGANHTLLRFSAGDVVEVLVPEAQ
NGWLYGKLEGSSASGWFPEAYVKALEEGPVNPMTPVTPMTSMTSMSPMTPMNPGNELPSR
SYPLRGSHSLDDLLDRPGNSIAPSEYWDGQSRSRTPSRVPSRAPSPAPPPLPSSRRSSMG
STAVATDVKKLMSSEQYPPQELFPRGTNPFATVKLRPTITNDRSAPLIR
>sp|Q9H694|BICC1_HUMAN Protein bicaudal C homolog 1 OS=Homo sapiens GN=BICC1 PE=1 SV=2
MAAQGEPGYLAAQSDPGSNSERSTDSPVPGSEDDLVAGATLHSPEWSEERFRVDRKKLEA
MLQAAAEGKGRSGEDFFQKIMEETNTQIAWPSKLKIGAKSKKDPHIKVSGKKEDVKEAKE
MIMSVLDTKSNRVTLKMDVSHTEHSHVIGKGGNNIKKVMEETGCHIHFPDSNRNNQAEKS
NQVSIAGQPAGVESARVRIRELLPLVLMFELPIAGILQPVPDPNSPSIQHISQTYNISVS

FKQRSRMYGATVIVRGSQNNTSAVKEGTAMLLEHLAGSLASAIPVSTQLDIAAQHHLFMM
GRNGSNIKHIMQRTGAQIHFPDPSNPQKKSTVYLQGTIESVCLARQYLMGCLPLVLMFDM
KEEIEVDPQFIAQLMEQLDVFISIKPKPKQPSKSVIVKSVERNALNMYEARKCLLGLESS
GVTIATSPSPASCPAGLACPSLDILASAGLGLTGLGLLGPTTLSLNTSTTPNSLLNALNS
SVSPLQSPSSGTPSPTLWAPPLANTSSATGFSAIPHLMIPSTAQATLTNILLSGVPTYGH
TAPSPPPGLTPVDVHINSMQTEGKKISAALNGHAQSPDIKYGAISTSSLGEKVLSANHGD
PSIQTSGSEQTSPKSSPTEGCNDAFVEVGMPRSPSHSGNAGDLKQMMCPSKVSCAKRQTV
ELLQGTKNSHLHSTDRLLSDPELSATESPLADKKAPGSERAAERAAAAQQNSERAHLAPR
SSYVNMQAFDYEQKKLLATKAMLKKPVVTEVRTPTNTWSGLGFSKSMPAETIKELRRANH
VSYKPTMTTTYEGSSMSLSRSNSREHLGGGSESDNWRDRNGIGPGSHSEFAASIGSPKRK
QNKSTEHYLSSSNYMDCISSLTGSNGCNLNSSFKGSDLPELFSKLGLGKYTDVFQQQEID
LQTFLTLTDQDLKELGITTFGARRKMLLAISELNKNRRKLFESPNARTSFLEGGASGRLP
RQYHSDIASVSGRW
>sp|Q13075|BIRC1_HUMAN Baculoviral IAP repeat-containing protein 1 OS=Homo sapiens GN=NAIP PE=1 SV=3
MATQQKASDERISQFDHNLLPELSALLGLDAVQLAKELEEEEQKERAKMQKGYNSQMRSE
AKRLKTFVTYEPYSSWIPQEMAAAGFYFTGVKSGIQCFCCSLILFGAGLTRLPIEDHKRF
HPDCGFLLNKDVGNIAKYDIRVKNLKSRLRGGKMRYQEEEARLASFRNWPFYVQGISPCV
LSEAGFVFTGKQDTVQCFSCGGCLGNWEEGDDPWKEHAKWFPKCEFLRSKKSSEEITQYI
QSYKGFVDITGEHFVNSWVQRELPMASAYCNDSIFAYEELRLDSFKDWPRESAVGVAALA
KAGLFYTGIKDIVQCFSCGGCLEKWQEGDDPLDDHTRCFPNCPFLQNMKSSAEVTPDLQS
RGELCELLETTSESNLEDSIAVGPIVPEMAQGEAQWFQEAKNLNEQLRAAYTSASFRHMS
LLDISSDLATDHLLGCDLSIASKHISKPVQEPLVLPEVFGNLNSVMCVEGEAGSGKTVLL
KKIAFLWASGCCPLLNRFQLVFYLSLSSTRPDEGLASIICDQLLEKEGSVTEMCVRNIIQ
QLKNQVLFLLDDYKEICSIPQVIGKLIQKNHLSRTCLLIAVRTNRARDIRRYLETILEIK
AFPFYNTVCILRKLFSHNMTRLRKFMVYFGKNQSLQKIQKTPLFVAAICAHWFQYPFDPS
FDDVAVFKSYMERLSLRNKATAEILKATVSSCGELALKGFFSCCFEFNDDDLAEAGVDED
EDLTMCLMSKFTAQRLRPFYRFLSPAFQEFLAGMRLIELLDSDRQEHQDLGLYHLKQINS
PMMTVSAYNNFLNYVSSLPSTKAGPKIVSHLLHLVDNKESLENISENDDYLKHQPEISLQ
MQLLRGLWQICPQAYFSMVSEHLLVLALKTAYQSNTVAACSPFVLQFLQGRTLTLGALNL
QYFFDHPESLSLLRSIHFPIRGNKTSPRAHFSVLETCFDKSQVPTIDQDYASAFEPMNEW
ERNLAEKEDNVKSYMDMQRRASPDLSTGYWKLSPKQYKIPCLEVDVNDIDVVGQDMLEIL
MTVFSASQRIELHLNHSRGFIESIRPALELSKASVTKCSISKLELSAAEQELLLTLPSLE
SLEVSGTIQSQDQIFPNLDKFLCLKELSVDLEGNINVFSVIPEEFPNFHHMEKLLIQISA
EYDPSKLVKLIQNSPNLHVFHLKCNFFSDFGSLMTMLVSCKKLTEIKFSDSFFQAVPFVA
SLPNFISLKILNLEGQQFPDEETSEKFAYILGSLSNLEELILPTGDGIYRVAKLIIQQCQ
QLHCLRVLSFFKTLNDDSVVEIAKVAISGGFQKLENLKLSINHKITEEGYRNFFQALDNM
PNLQELDISRHFTECIKAQATTVKSLSQCVLRLPRLIRLNMLSWLLDADDIALLNVMKER
HPQSKYLTILQKWILPFSPIIQK
>sp|Q96P09|BIRC8_HUMAN Baculoviral IAP repeat-containing protein 8 OS=Homo sapiens GN=BIRC8 PE=1 SV=2
MTGYEARLITFGTWMYSVNKEQLARAGFYAIGQEDKVQCFHCGGGLANWKPKEDPWEQHA
KWYPGCKYLLEEKGHEYINNIHLTRSLEGALVQTTKKTPSLTKRISDTIFPNPMLQEAIR
MGFDFKDVKKIMEERIQTSGSNYKTLEVLVADLVSAQKDTTENELNQTSLQREISPEEPL

RRLQEEKLCKICMDRHIAVVFIPCGHLVTCKQCAEAVDRCPMCSAVIDFKQRVFMS
>sp|Q3C1V8|BSH_HUMAN Brain-specific homeobox protein homolog OS=Homo sapiens GN=BSX PE=2 SV=2
MNLNFTSPLHPASSQRPTSFFIEDILLHKPKPLREVAPDHFASSLASRVPLLDYGYPLMP
TPTLLAPHAHHPLHKGDHHHPYFLTTSGMPVPALFPHPQHAELPGKHCRRRKARTVFSDS
QLSGLEKRFEIQRYLSTPERVELATALSLSETQVKTWFQNRRMKHKKQLRKSQDEPKAPD
GPESPEGSPRGSEAATAAEARLSLPAGPFVLTEPEDEVDIGDEGELGSGPHVL
>sp|Q075Z2|BSPH1_HUMAN Binder of sperm protein homolog 1 OS=Homo sapiens GN=BSPH1 PE=2 SV=1
MGSLMLLFVETTRNSSACIFPVILNELSSTVETITHFPEVTDGECVFPFHYKNGTYYDCI
KSKARHKWCSLNKTYEGYWKFCSAEDFANCVFPFWYRRLIYWECTDDGEAFGKKWCSLTK
NFNKDRIWKYCE
>sp|Q10588|BST1_HUMAN ADP-ribosyl cyclase/cyclic ADP-ribose hydrolase 2 OS=Homo sapiens GN=BST1 PE=1 SV=2
MAAQGCAASRLLQLLLQLLLLLLLLAAGGARARWRGEGTSAHLRDIFLGRCAEYRALLSP
EQRNKNCTAIWEAFKVALDKDPCSVLPSDYDLFINLSRHSIPRDKSLFWENSHLLVNSFA
DNTRRFMPLSDVLYGRVADFLSWCRQKNDSGLDYQSCPTSEDCENNPVDSFWKRASIQYS
KDSSGVIHVMLNGSEPTGAYPIKGFFADYEIPNLQKEKITRIEIWVMHEIGGPNVESCGE
GSMKVLEKRLKDMGFQYSCINDYRPVKLLQCVDHSTHPDCALKSAAAATQRKAPSLYTEQ
RAGLIIPLFLVLASRTQL
>sp|Q10589|BST2_HUMAN Bone marrow stromal antigen 2 OS=Homo sapiens GN=BST2 PE=1 SV=1
MASTSYDYCRVPMEDGDKRCKLLLGIGILVLLIIVILGVPLIIFTIKANSEACRDGLRAV
MECRNVTHLLQQELTEAQKGFQDVEAQAATCNHTVMALMASLDAEKAQGQKKVEELEGEI
TTLNHKLQDASAEVERLRRENQVLSVRIADKKYYPSSQDSSSAAAPQLLIVLLGLSALLQ
>sp|P78410|BT3A2_HUMAN Butyrophilin subfamily 3 member A2 OS=Homo sapiens GN=BTN3A2 PE=1 SV=2
MKMASSLAFLLLNFHVSLLLVQLLTPCSAQFSVLGPSGPILAMVGEDADLPCHLFPTMSA
ETMELKWVSSSLRQVVNVYADGKEVEDRQSAPYRGRTSILRDGITAGKAALRIHNVTASD
SGKYLCYFQDGDFYEKALVELKVAALGSNLHVEVKGYEDGGIHLECRSTGWYPQPQIQWS
NAKGENIPAVEAPVVADGVGLYEVAASVIMRGGSGEGVSCIIRNSLLGLEKTASISIADP
FFRSAQPWIAALAGTLPILLLLLAGASYFLWRQQKEITALSSEIESEQEMKEMGYAATER
EISLRESLQEELKRKKIQYLTRGEESSSDTNKSA
>sp|A8MVZ5|BTNLA_HUMAN Butyrophilin-like protein 10 OS=Homo sapiens GN=BTNL10 PE=5 SV=2
MAVTCDPEAFLSICFVTLVFLQLPLASIWKADFDVTGPHAPILAMAGGHVELQCQLFPNI
SAEDMELRWYRCQPSLAVHMHERGMDMDGEQKWQYRGRTTFMSDHVARGKAMVRSHRVTT
FDNRTYCCRFKDGVKFGEATVQVQVAGLGREPRIQVTDQQDGVRAECTSAGCFPKSWVER
RDFRGQARPAVTNLSASATTRLWAVASSLTLWDRAVEGLSCSISSPLLPERRKVAESHLP
ATFSRSSQFTAWKAALPLILVAMGLVIAGGICIFWKRQREKNKASLEEERE
>sp|O43683|BUB1_HUMAN Mitotic checkpoint serine/threonine-protein kinase BUB1 OS=Homo sapiens GN=BUB1 PE=1 SV=1
MDTPENVLQMLEAHMQSYKGNDPLGEWERYIQWVEENFPENKEYLITLLEHLMKEFLDKK
KYHNDPRFISYCLKFAEYNSDLHQFFEFLYNHGIGTLSSPLYIAWAGHLEAQGELQHASA
VLQRGIQNQAEPREFLQQQYRLFQTRLTETHLPAQARTSEPLHNVQVLNQMITSKSNPGN
NMACISKNQGSELSGVISSACDKESNMERRVITISKSEYSVHSSLASKVDVEQVVMYCKE

KLIRGESEFSFEELRAQKYNQRRKHEQWVNEDRHYMKRKEANAFEEQLLKQKMDELHKKL
HQVVETSHEDLPASQERSEVNPARMGPSVGSQQELRAPCLPVTYQQTPVNMEKNPREAPP
VVPPLANAISAALVSPATSQSIAPPVPLKAQTVTDSMFAVASKDAGCVNKSTHEFKPQSG
AEIKEGCETHKVANTSSFHTTPNTSLGMVQATPSKVQPSPTVHTKEALGFIMNMFQAPTL
PDISDDKDEWQSLDQNEDAFEAQFQKNVRSSGAWGVNKIISSLSSAFHVFEDGNKENYGL
PQPKNKPTGARTFGERSVSRLPSKPKEEVPHAEEFLDDSTVWGIRCNKTLAPSPKSPGDF
TSAAQLASTPFHKLPVESVHILEDKENVVAKQCTQATLDSCEENMVVPSRDGKFSPIQEK
SPKQALSSHMYSASLLRLSQPAAGGVLTCEAELGVEACRLTDTDAAIAEDPPDAIAGLQA
EWMQMSSLGTVDAPNFIVGNPWDDKLIFKLLSGLSKPVSSYPNTFEWQCKLPAIKPKTEF
QLGSKLVYVHHLLGEGAFAQVYEATQGDLNDAKNKQKFVLKVQKPANPWEFYIGTQLMER
LKPSMQHMFMKFYSAHLFQNGSVLVGELYSYGTLLNAINLYKNTPEKVMPQGLVISFAMR
MLYMIEQVHDCEIIHGDIKPDNFILGNGFLEQDDEDDLSAGLALIDLGQSIDMKLFPKGT
IFTAKCETSGFQCVEMLSNKPWNYQIDYFGVAATVYCMLFGTYMKVKNEGGECKPEGLFR
RLPHLDMWNEFFHVMLNIPDCHHLPSLDLLRQKLKKVFQQHYTNKIRALRNRLIVLLLEC
KRSRK
>sp|Q2TBE0|C19L2_HUMAN CWF19-like protein 2 OS=Homo sapiens GN=CWF19L2 PE=1 SV=4
MATSMAAASGRFESAKSIEERKEQTRNARAEVLRQAKANFEKEERRKELKRLRGEDTWML
PDVNERIEQFSQEHSVKKKKKKDKHSKKAKKEKKKKSKKQKYEKNNESSDSSSSSEDEWV
EAVPSQTPDKEKAWKVKDEKSGKDDTQIIKRDEWMTVDFMSVKTVSSSSLKAEKETMRKI
EQEKNQALEQSKLMERELNPYWKDGGTGLPPEDCSVSSITKVSVVEDGGLSWLRKSYLRM
KEQAEKQSRNFEDIVAERYGSMEIFQSKLEDAEKAASTKEDYRRERWRKPTYSDKAQNCQ
ESRESDLVKYGNSSRDRYATTDTAKNSNNEKFIGDEKDKRPGSLETCRRESNPRQNQEFS
FGNLRAKFLRPSDDEELSFHSKGRKFEPLSSSSALVAQGSLCSGFRKPTKNSEERLTSWS
RSDGRGDKKHSNQKPSETSTDEHQHVPEDPREKSQDEVLRDDPPKKEHLRDTKSTFAGSP
ERESIHILSVDEKNKLGAKIIKAEMMGNMELAEQLKVQLEKANKFKETITQIPKKSGVEN
EDQQEVILVRTDQSGRVWPVNTPGKSLESQGGRRKRQMVSTHEERERVRYFHDDDNLSLN
DLVKNEKMGTAENQNKLFMRMASKFMGKTDGDYYTLDDMFVSKAAERERLGEEEENQRKK
AIAEHRSLAAQMEKCLYCFDSSQFPKHLIVAIGVKVYLCLPNVRSLTEGHCLIVPLQHHR
AATLLDEDIWEEIQMFRKSLVKMFEDKGLDCIFLETNMSMKKQYHMVYECIPLPKEVGDM
APIYFKKAIMESDEEWSMNKKLIDLSSKDIRKSVPRGLPYFSVDFGLHGGFAHVIEDQHK
FPHYFGKEIIGGMLDIEPRLWRKGIRESFEDQRKKALQFAQWWKPYDFTKSKNY
>sp|P02746|C1QB_HUMAN Complement C1q subcomponent subunit B OS=Homo sapiens GN=C1QB PE=1 SV=3
MMMKIPWGSIPVLMLLLLLGLIDISQAQLSCTGPPAIPGIPGIPGTPGPDGQPGTPGIKG
EKGLPGLAGDHGEFGEKGDPGIPGNPGKVGPKGPMGPKGGPGAPGAPGPKGESGDYKATQ
KIAFSATRTINVPLRRDQTIRFDHVITNMNNNYEPRSGKFTCKVPGLYYFTYHASSRGNL
CVNLMRGRERAQKVVTFCDYAYNTFQVTTGGMVLKLEQGENVFLQATDKNSLLGMEGANS
IFSGFLLFPDMEA
>sp|Q6P1N0|C2D1A_HUMAN Coiled-coil and C2 domain-containing protein 1A OS=Homo sapiens GN=CC2D1A PE=1 SV=1
MHKRKGPPGPPGRGAAAARQLGLLVDLSPDGLMIPEDGANDEELEAEFLALVGGQPPALE
KLKGKGPLPMEAIEKMASLCMRDPDEDEEEGTDEDDLEADDDLLAELNEVLGEEQKASET
PPPVAQPKPEAPHPGLETTLQERLALYQTAIESARQAGDSAKMRRYDRGLKTLENLLASI
RKGNAIDEADIPPPVAIGKGPASTPTYSPAPTQPAPRIASAPEPRVTLEGPSATAPASSP

GLAKPQMPPGPCSPGPLAQLQSRQRDYKLAALHAKQQGDTTAAARHFRVAKSFDAVLEAL
SRGEPVDLSCLPPPPDQLPPDPPSPPSQPPTPATAPSTTEVPPPPRTLLEALEQRMERYQ
VAAAQAKSKGDQRKARMHERIVKQYQDAIRAHKAGRAVDVAELPVPPGFPPIQGLEATKP
TQQSLVGVLETAMKLANQDEGPEDEEDEVPKKQNSPVAPTAQPKAPPSRTPQSGSAPTAK
APPKATSTRAQQQLAFLEGRKKQLLQAALRAKQKNDVEGAKMHLRQAKGLEPMLEASRNG
LPVDITKVPPAPVNKDDFALVQRPGPGLSQEAARRYGELTKLIRQQHEMCLNHSNQFTQL
GNITETTKFEKLAEDCKRSMDILKQAFVRGLPTPTARFEQRTFSVIKIFPDLSSNDMLLF
IVKGINLPTPPGLSPGDLDVFVRFDFPYPNVEEAQKDKTSVIKNTDSPEFKEQFKLCINR
SHRGFRRAIQTKGIKFEVVHKGGLFKTDRVLGTAQLKLDALEIACEVREILEVLDGRRPT
GGRLEVMVRIREPLTAQQLETTTERWLVIDPVPAAVPTQVAGPKGKAPPVPAPARESGNR
SARPLHSLSVLAFDQERLERKILALRQARRPVPPEVAQQYQDIMQRSQWQRAQLEQGGVG
IRREYAAQLERQLQFYTEAARRLGNDGSRDAAKEALYRRNLVESELQRLRR
>sp|Q16581|C3AR_HUMAN C3a anaphylatoxin chemotactic receptor OS=Homo sapiens GN=C3AR1 PE=1 SV=2
MASFSAETNSTDLLSQPWNEPPVILSMVILSLTFLLGLPGNGLVLWVAGLKMQRTVNTIW
FLHLTLADLLCCLSLPFSLAHLALQGQWPYGRFLCKLIPSIIVLNMFASVFLLTAISLDR
CLVVFKPIWCQNHRNVGMACSICGCIWVVAFVMCIPVFVYREIFTTDNHNRCGYKFGLSS
SLDYPDFYGDPLENRSLENIVQPPGEMNDRLDPSSFQTNDHPWTVPTVFQPQTFQRPSAD
SLPRGSARLTSQNLYSNVFKPADVVSPKIPSGFPIEDHETSPLDNSDAFLSTHLKLFPSA
SSNSFYESELPQGFQDYYNLGQFTDDDQVPTPLVAITITRLVVGFLLPSVIMIACYSFIV
FRMQRGRFAKSQSKTFRVAVVVVAVFLVCWTPYHIFGVLSLLTDPETPLGKTLMSWDHVC
IALASANSCFNPFLYALLGKDFRKKARQSIQGILEAAFSEELTRSTHCPSNNVISERNST
TV
>sp|Q9NRR8|C42S1_HUMAN CDC42 small effector protein 1 OS=Homo sapiens GN=CDC42SE1 PE=1 SV=1
MSEFWHKLGCCVVEKPQPKKKRRRIDRTMIGEPMNFVHLTHIGSGEMGAGDGLAMTGAVQ
EQMRSKGNRDRPWSNSRGL
>sp|Q99643|C560_HUMAN Succinate dehydrogenase cytochrome b560 subunit, mitochondrial OS=Homo sapiens GN=SDHC PE=1 SV=1
MAALLLRHVGRHCLRAHFSPQLCIRNAVPLGTTAKEEMERFWNKNIGSNRPLSPHITIYS
WSLPMAMSICHRGTGIALSAGVSLFGMSALLLPGNFESYLELVKSLCLGPALIHTAKFAL
VFPLMYHTWNGIRHLMWDLGKGLKIPQLYQSGVVVLVLTVLSSMGLAAM
>sp|O14503|BHE40_HUMAN Class E basic helix-loop-helix protein 40 OS=Homo sapiens GN=BHLHE40 PE=1 SV=1
MERIPSAQPPPACLPKAPGLEHGDLPGMYPAHMYQVYKSRRGIKRSEDSKETYKLPHRLI
EKKRRDRINECIAQLKDLLPEHLKLTTLGHLEKAVVLELTLKHVKALTNLIDQQQQKIIA
LQSGLQAGELSGRNVETGQEMFCSGFQTCAREVLQYLAKHENTRDLKSSQLVTHLHRVVS
ELLQGGTSRKPSDPAPKVMDFKEKPSSPAKGSEGPGKNCVPVIQRTFAHSSGEQSGSDTD
TDSGYGGESEKGDLRSEQPCFKSDHGRRFTMGERIGAIKQESEEPPTKKNRMQLSDDEGH
FTSSDLISSPFLGPHPHQPPFCLPFYLIPPSATAYLPMLEKCWYPTSVPVLYPGLNASAA
ALSSFMNPDKISAPLLMPQRLPSPLPAHPSVDSSVLLQALKPIPPLNLETKD
>sp|Q6PI77|BHLH9_HUMAN Protein BHLHb9 OS=Homo sapiens GN=BHLHB9 PE=2 SV=1
MAGTKNKTRAQAKTEKKAAIQAKAGAEREATGVVRPVAKTRAKAKAKTGSKTDAVAEMKA
VSKNKVVAETKEGALSEPKTLGKAMGDFTPKAGNESTSSTCKNEAGTDAWFWAGEEATIN

SWFWNGEEAGNSFSTKNDKPEIGAQVCAEELEPAAGADCKPRSGAEEEEEEENVIGNWFWE
GDDTSFDPNPKPVSRIVKPQPVYEINEKNRPKDWSEVTIWPNAPAVTPAVLGFRSQAPSE
ASPPSYIVLASAEENACSLPVATACRPSRNTRSCSQPIPECRFDSDPCIQTIDEIRRQIR
IREVNGIKPFACPCKMECYMDSEEFEKLVSLLKSTTDPLIHKIARIAMGVHNVHPFAQEF
INEVGVVTLIESLLSFPSPEMRKKTVITLNPPSGDERQRKIELHVKHMCKETMSFPLNSP
GQQSGLKILGQLTTDFVHHYIVANYFSELFHLLSSGNCKTRNLVLKLLLNMSENPTAARD
MINMKALAALKLIFNQKEAKANLVSGVAIFINIKEHIRKGSIVVVDHLSYNTLMAIFREV
KEIIETM
>sp|Q8TD16|BICD2_HUMAN Protein bicaudal D homolog 2 OS=Homo sapiens GN=BICD2 PE=1 SV=1
MSAPSEEEEYARLVMEAQPEWLRAEVKRLSHELAETTREKIQAAEYGLAVLEEKHQLKLQ
FEELEVDYEAIRSEMEQLKEAFGQAHTNHKKVAADGESREESLIQESASKEQYYVRKVLE
LQTELKQLRNVLTNTQSENERLASVAQELKEINQNVEIQRGRLRDDIKEYKFREARLLQD
YSELEEENISLQKQVSVLRQNQVEFEGLKHEIKRLEEETEYLNSQLEDAIRLKEISERQL
EEALETLKTEREQKNSLRKELSHYMSINDSFYTSHLHVSLDGLKFSDDAAEPNNDAEALV
NGFEHGGLAKLPLDNKTSTPKKEGLAPPSPSLVSDLLSELNISEIQKLKQQLMQMEREKA
GLLATLQDTQKQLEHTRGSLSEQQEKVTRLTENLSALRRLQASKERQTALDNEKDRDSHE
DGDYYEVDINGPEILACKYHVAVAEAGELREQLKALRSTHEAREAQHAEEKGRYEAEGQA
LTEKVSLLEKASRQDRELLARLEKELKKVSDVAGETQGSLSVAQDELVTFSEELANLYHH
VCMCNNETPNRVMLDYYREGQGGAGRTSPGGRTSPEARGRRSPILLPKGLLAPEAGRADG
GTGDSSPSPGSSLPSPLSDPRREPMNIYNLIAIIRDQIKHLQAAVDRTTELSRQRIASQE
LGPAVDKDKEALMEEILKLKSLLSTKREQITTLRTVLKANKQTAEVALANLKSKYENEKA
MVTETMMKLRNELKALKEDAATFSSLRAMFATRCDEYITQLDEMQRQLAAAEDEKKTLNS
LLRMAIQQKLALTQRLELLELDHEQTRRGRAKAAPKTKPATPSL
>sp|Q6ZP65|BICL1_HUMAN BICD family-like cargo adapter 1 OS=Homo sapiens GN=BICDL1 PE=2 SV=2
MSAFCLGLVGRASAPAEPDSACCMELPAAAGDAVRSPAAAAALIFPGGSGELELALEEEL
ALLAAGERPSDPGEHPQAEPGSLAEGAGPQPPPSQDPELLSVIRQKEKDLVLAARLGKAL
LERNQDMSRQYEQMHKELTDKLEHLEQEKHELRRRFENREGEWEGRVSELESDVKQLQDE
LERQQIHLREADREKSRAVQELSEQNQRLLDQLSRASEVERQLSMQVHALREDFREKNSS
TNQHIIRLESLQAEIKMLSDRKRELEHRLSATLEENDLLQGTVEELQDRVLILERQGHDK
DLQLHQSQLELQEVRLSCRQLQVKVEELTEERSLQSSAATSTSLLSEIEQSMEAEELEQE
REQLRLQLWEAYCQVRYLCSHLRGNDSADSAVSTDSSMDESSETSSAKDVPAGSLRTALN
ELKRLIQSIVDGMEPTVTLLSVEMTALKEERDRLRVTSEDKEPKEQLQKAIRDRDEAIAK
KNAVELELAKCRMDMMSLNSQLLDAIQQKLNLSQQLEAWQDDMHRVIDRQLMDTHLKERS
QPAAALCRGHSAGRGDEPSIAEGKRLFSFFRKI
>sp|Q13323|BIK_HUMAN Bcl-2-interacting killer OS=Homo sapiens GN=BIK PE=1 SV=2
MSEVRPLSRDILMETLLYEQLLEPPTMEVLGMTDSEEDLDPMEDFDSLECMEGSDALALR
LACIGDEMDVSLRAPRLAQLSEVAMHSLGLAFIYDQTEDIRDVLRSFMDGFTTLKENIMR
FWRSPNPGSWVSCEQVLLALLLLLALLLPLLSGGLHLLLK
>sp|Q9NQY0|BIN3_HUMAN Bridging integrator 3 OS=Homo sapiens GN=BIN3 PE=1 SV=1
MSWIPFKIGQPKKQIVPKTVERDFEREYGKLQQLEEQTRRLQKDMKKSTDADLAMSKSAV
KISLDLLSNPLCEQDQDLLNMVTALDTAMKRMDAFNQEKVNQIQKTVIEPLKKFGSVFPS
LNMAVKRREQALQDYRRLQAKVEKYEEKEKTGPVLAKLHQAREELRPVREDFEAKNRQLL
EEMPRFYGSRLDYFQPSFESLIRAQVVYYSEMHKIFGDLSHQLDQPGHSDEQRERENEAK

LSELRALSIVADD
>sp|Q13867|BLMH_HUMAN Bleomycin hydrolase OS=Homo sapiens GN=BLMH PE=1 SV=1
MSSSGLNSEKVAALIQKLNSDPQFVLAQNVGTTHDLLDICLKRATVQRAQHVFQHAVPQE
GKPITNQKSSGRCWIFSCLNVMRLPFMKKLNIEEFEFSQSYLFFWDKVERCYFFLSAFVD
TAQRKEPEDGRLVQFLLMNPANDGGQWDMLVNIVEKYGVIPKKCFPESYTTEATRRMNDI
LNHKMREFCIRLRNLVHSGATKGEISATQDVMMEEIFRVVCICLGNPPETFTWEYRDKDK
NYQKIGPITPLEFYREHVKPLFNMEDKICLVNDPRPQHKYNKLYTVEYLSNMVGGRKTLY
NNQPIDFLKKMVAASIKDGEAVWFGCDVGKHFNSKLGLSDMNLYDHELVFGVSLKNMNKA
ERLTFGESLMTHAMTFTAVSEKDDQDGAFTKWRVENSWGEDHGHKGYLCMTDEWFSEYVY
EVVVDRKHVPEEVLAVLEQEPIILPAWDPMGALAE
>sp|Q8WV28|BLNK_HUMAN B-cell linker protein OS=Homo sapiens GN=BLNK PE=1 SV=2
MDKLNKITVPASQKLRQLQKMVHDIKNNEGGIMNKIKKLKVKAPPSVPRRDYASESPADE
EEQWSDDFDSDYENPDEHSDSEMYVMPAEENADDSYEPPPVEQETRPVHPALPFARGEYI
DNRSSQRHSPPFSKTLPSKPSWPSEKARLTSTLPALTALQKPQVPPKPKGLLEDEADYVV
PVEDNDENYIHPTESSSPPPEKAPMVNRSTKPNSSTPASPPGTASGRNSGAWETKSPPPA
APSPLPRAGKKPTTPLKTTPVASQQNASSVCEEKPIPAERHRGSSHRQEAVQSPVFPPAQ
KQIHQKPIPLPRFTEGGNPTVDGPLPSFSSNSTISEQEAGVLCKPWYAGACDRKSAEEAL
HRSNKDGSFLIRKSSGHDSKQPYTLVVFFNKRVYNIPVRFIEATKQYALGRKKNGEEYFG
SVAEIIRNHQHSPLVLIDSQNNTKDSTRLKYAVKVS
>sp|Q8N8U9|BMPER_HUMAN BMP-binding endothelial regulator protein OS=Homo sapiens GN=BMPER PE=1 SV=3
MLWFSGVGALAERYCRRSPGITCCVLLLLNCSGVPMSLASSFLTGSVAKCENEGEVLQIP
FITDNPCIMCVCLNKEVTCKREKCPVLSRDCALAIKQRGACCEQCKGCTYEGNTYNSSFK
WQSPAEPCVLRQCQEGVVTESGVRCVVHCKNPLEHLGMCCPTCPGCVFEGVQYQEGEEFQ
PEGSKCTKCSCTGGRTQCVREVCPILSCPQHLSHIPPGQCCPKCLGQRKVFDLPFGSCLF
RSDVYDNGSSFLYDNCTACTCRDSTVVCKRKCSHPGGCDQGQEGCCEECLLRVPPEDIKV
CKFGNKIFQDGEMWSSINCTICACVKGRTECRNKQCIPISSCPQGKILNRKGCCPICTEK
PGVCTVFGDPHYNTFDGRTFNFQGTCQYVLTKDCSSPASPFQVLVKNDARRTRSFSWTKS
VELVLGESRVSLQQHLTVRWNGSRIALPCRAPHFHIDLDGYLLKVTTKAGLEISWDGDSF
VEVMAAPHLKGKLCGLCGNYNGHKRDDLIGGDGNFKFDVDDFAESWRVESNEFCNRPQRK
PVPELCQGTVKVKLRAHRECQKLKSWEFQTCHSTVDYATFYRSCVTDMCECPVHKNCYCE
SFLAYTRACQREGIKVHWEPQQNCAATQCKHGAVYDTCGPGCIKTCDNWNEIGPCNKPCV
AGCHCPANLVLHKGRCIKPVLCPQR
>sp|O00238|BMR1B_HUMAN Bone morphogenetic protein receptor type-1B OS=Homo sapiens GN=BMPR1B PE=1 SV=1
MLLRSAGKLNVGTKKEDGESTAPTPRPKVLRCKCHHHCPEDSVNNICSTDGYCFTMIEED
DSGLPVVTSGCLGLEGSDFQCRDTPIPHQRRSIECCTERNECNKDLHPTLPPLKNRDFVD
GPIHHRALLISVTVCSLLLVLIILFCYFRYKRQETRPRYSIGLEQDETYIPPGESLRDLI
EQSQSSGSGSGLPLLVQRTIAKQIQMVKQIGKGRYGEVWMGKWRGEKVAVKVFFTTEEAS
WFRETEIYQTVLMRHENILGFIAADIKGTGSWTQLYLITDYHENGSLYDYLKSTTLDAKS
MLKLAYSSVSGLCHLHTEIFSTQGKPAIAHRDLKSKNILVKKNGTCCIADLGLAVKFISD
TNEVDIPPNTRVGTKRYMPPEVLDESLNRNHFQSYIMADMYSFGLILWEVARRCVSGGIV
EEYQLPYHDLVPSDPSYEDMREIVCIKKLRPSFPNRWSSDECLRQMGKLMTECWAHNPAS
RLTALRVKKTLAKMSESQDIKL

>sp|Q01954|BNC1_HUMAN Zinc finger protein basonuclin-1 OS=Homo sapiens GN=BNC1 PE=1 SV=2
MRRRPPSRGGRGAARARETRRQPRHRSGRRMAEAISCTLNCSCQSFKPGKINHRQCDQCK
HGWVAHALSKLRIPPMYPTSQVEIVQSNVVFDISSLMLYGTQAIPVRLKILLDRLFSVLK
QDEVLQILHALDWTLQDYIRGYVLQDASGKVLDHWSIMTSEEEVATLQQFLRFGETKSIV
ELMAIQEKEEQSIIIPPSTANVDIRAFIESCSHRSSSLPTPVDKGNPSSIHPFENLISNM
TFMLPFQFFNPLPPALIGSLPEQYMLEQGHDQSQDPKQEVHGPFPDSSFLTSSSTPFQVE
KDQCLNCPDAITKKEDSTHLSDSSSYNIVTKFERTQLSPEAKVKPERNSLGTKKGRVFCT
ACEKTFYDKGTLKIHYNAVHLKIKHKCTIEGCNMVFSSLRSRNRHSANPNPRLHMPMNRN
NRDKDLRNSLNLASSENYKCPGFTVTSPDCRPPPSYPGSGEDSKGQPAFPNIGQNGVLFP
NLKTVQPVLPFYRSPATPAEVANTPGILPSLPLLSSSIPEQLISNEMPFDALPKKKSRKS
SMPIKIEKEAVEIANEKRHNLSSDEDMPLQVVSEDEQEACSPQSHRVSEEQHVQSGGLGK
PFPEGERPCHRESVIESSGAISQTPEQATHNSERETEQTPALIMVPREVEDGGHEHYFTP
GMEPQVPFSDYMELQQRLLAGGLFSALSNRGMAFPCLEDSKELEHVGQHALARQIEENRF
QCDICKKTFKNACSVKIHHKNMHVKEMHTCTVEGCNATFPSRRSRDRHSSNLNLHQKALS
QEALESSEDHFRAAYLLKDVAKEAYQDVAFTQQASQTSVIFKGTSRMGSLVYPITQVHSA
SLESYNSGPLSEGTILDLSTTSSMKSESSSHSSWDSDGVSEEGTVLMEDSDGNCEGSSLV
PGEDEYPICVLMEKADQSLASLPSGLPITCHLCQKTYSNKGTFRAHYKTVHLRQLHKCKV
PGCNTMFSSVRSRNRHSQNPNLHKSLASSPSHLQ
>sp|O75936|BODG_HUMAN Gamma-butyrobetaine dioxygenase OS=Homo sapiens GN=BBOX1 PE=1 SV=1
MACTIQKAEALDGAHLMQILWYDEEESLYPAVWLRDNCPCSDCYLDSAKARKLLVEALDV
NIGIKGLIFDRKKVYITWPDEHYSEFQADWLKKRCFSKQARAKLQRELFFPECQYWGSEL
QLPTLDFEDVLRYDEHAYKWLSTLKKVGIVRLTGASDKPGEVSKLGKRMGFLYLTFYGHT
WQVQDKIDANNVAYTTGKLSFHTDYPALHHPPGVQLLHCIKQTVTGGDSEIVDGFNVCQK
LKKNNPQAFQILSSTFVDFTDIGVDYCDFSVQSKHKIIELDDKGQVVRINFNNATRDTIF
DVPVERVQPFYAALKEFVDLMNSKESKFTFKMNPGDVITFDNWRLLHGRRSYEAGTEISR
HLEGAYADWDVVMSRLRILRQRVENGN
>sp|Q9UMX3|BOK_HUMAN Bcl-2-related ovarian killer protein OS=Homo sapiens GN=BOK PE=1 SV=1
MEVLRRSSVFAAEIMDAFDRSPTDKELVAQAKALGREYVHARLLRAGLSWSAPERAAPVP
GRLAEVCAVLLRLGDELEMIRPSVYRNVARQLHISLQSEPVVTDAFLAVAGHIFSAGITW
GKVVSLYAVAAGLAVDCVRQAQPAMVHALVDCLGEFVRKTLATWLRRRGGWTDVLKCVVS
TDPGLRSHWLVAALCSFGRFLKAAFFVLLPER
>sp|Q9H3K6|BOLA2_HUMAN BolA-like protein 2 OS=Homo sapiens GN=BOLA2 PE=1 SV=1
MELSAEYLREKLQRDLEAEHVEVEDTTLNRCSCSFRVLVVSAKFEGKPLLQRHRLVNACL
AEELPHIHAFEQKTLTPDQWARERQK
>sp|Q8N9W6|BOLL_HUMAN Protein boule-like OS=Homo sapiens GN=BOLL PE=1 SV=2
MQTDSLSPSPNPVSPVPLNNPTSAPRYGTVIPNRIFVGGIDFKTNESDLRKFFSQYGSVK
EVKIVNDRAGVSKGYGFVTFETQEDAQKILQEAEKLNYKDKKLNIGPAIRKQQVGIPRSS
IMPAAGTMYLTTSTGYPYTYHNGVAYFHTPEVTSVPPPWPSRSVCSSPVMVAQPIYQQPA
YHYQATTQYLPGQWQWSVPQPSASSAPFLYLQPSEVIYQPVEIAQDGGCVPPPLSLMETS
VPEPYSDHGVQATYHQVYAPSAITMPAPVMQPEPIKTVWSIHY
>sp|Q14137|BOP1_HUMAN Ribosome biogenesis protein BOP1 OS=Homo sapiens GN=BOP1 PE=1 SV=2
MAGSRGAGRTAAPSVRPEKRRSEPELEPEPEPEPPLLCTSPLSHSTGSDSGVSDSEESVF
SGLEDSGSDSSEDDDEGDEEGEDGALDDEGHSGIKKTTEEQVQASTPCPRTEMASARIGD

EYAEDSSDEEDIRNTVGNVPLEWYDDFPHVGYDLDGRRIYKPLRTRDELDQFLDKMDDPD
YWRTVQDPMTGRDLRLTDEQVALVRRLQSGQFGDVGFNPYEPAVDFFSGDVMIHPVTNRP
ADKRSFIPSLVEKEKVSRMVHAIKMGWIQPRRPRDPTPSFYDLWAQEDPNAVLGRHKMHV
PAPKLALPGHAESYNPPPEYLLSEEERLAWEQQEPGERKLSFLPRKFPSLRAVPAYGRFI
QERFERCLDLYLCPRQRKMRVNVDPEDLIPKLPRPRDLQPFPTCQALVYRGHSDLVRCLS
VSPGGQWLVSGSDDGSLRLWEVATARCVRTVPVGGVVKSVAWNPSPAVCLVAAAVEDSVL
LLNPALGDRLVAGSTDQLLSAFVPPEEPPLQPARWLEASEEERQVGLRLRICHGKPVTQV
TWHGRGDYLAVVLATQGHTQVLIHQLSRRRSQSPFRRSHGQVQRVAFHPARPFLLVASQR
SVRLYHLLRQELTKKLMPNCKWVSSLAVHPAGDNVICGSYDSKLVWFDLDLSTKPYRMLR
HHKKALRAVAFHPRYPLFASGSDDGSVIVCHGMVYNDLLQNPLLVPVKVLKGHVLTRDLG
VLDVIFHPTQPWVFSSGADGTVRLFT
>sp|Q8NFQ6|BPIFC_HUMAN BPI fold-containing family C protein OS=Homo sapiens GN=BPIFC PE=2 SV=1
MCTKTIPVLWGCFLLWNLYVSSSQTIYPGIKARITQRALDYGVQAGMKMIEQMLKEKKLP
DLSGSESLEFLKVDYVNYNFSNIKISAFSFPNTSLAFVPGVGIKALTNHGTANISTDWGF
ESPLFQDTGGADLFLSGVYFTGIIILTRNDFGHPTLKLQDCYAQLSHAHVSFSGELSVLY
NSFAEPMEKPILKNLNEMLCPIIASEVKALNANLSTLEVLTKIDNYTLLDYSLISSPEIT
ENYLDLNLKGVFYPLENLTDPPFSPVPFVLPERSNSMLYIGIAEYFFKSASFAHFTAGVF
NVTLSTEEISNHFVQNSQGLGNVLSRIAEIYILSQPFMVRIMATEPPIINLQPGNFTLDI
PASIMMLTQPKNSTVETIVSMDFVASTSVGLVILGQRLVCSLSLNRFRLALPESNRSNIE
VLRFENILSSILHFGVLPLANAKLQQGFPLSNPHKFLFVNSDIEVLEGFLLISTDLKYET
SSKQQPSFHVWEGLNLISRQWRGKSAP
>sp|O60477|BRNP1_HUMAN BMP/retinoic acid-inducible neural-specific protein 1 OS=Homo sapiens GN=BRINP1 PE=1 SV=2
MNWRFVELLYFLFIWGRISVQPSHQEPAGTDQHVSKEFDWLISDRGPFHHSRSYLSFVER
HRQGFTTRYKIYREFARWKVRNTAIERRDLVRHPVPLMPEFQRSIRLLGRRPTTQQFIDT
IIKKYGTHLLISATLGGEEALTMYMDKSRLDRKSGNATQSVEALHQLASSYFVDRDGTMR
RLHEIQISTGAIKVTETRTGPLGCNSYDNLDSVSSVLLQSTESKLHLQGLQIIFPQYLQE
KFVQSALSYIMCNGEGEYLCQNSQCRCQCAEEFPQCNCPITDIQIMEYTLANMAKSWAEA
YKDLENSDEFKSFMKRLPSNHFLTIGSIHQHWGNDWDLQNRYKLLQSATEAQRQKIQRTA
RKLFGLSVRCRHNPNHQLPRERTIQQWLARVQSLLYCNENGFWGTFLESQRSCVCHGSTT
LCQRPIPCVIGGNNSCAMCSLANISLCGSCNKGYKLYRGRCEPQNVDSERSEQFISFETD
LDFQDLELKYLLQKMDSRLYVHTTFISNEIRLDTFFDPRWRKRMSLTLKSNKNRMDFIHM
VIGMSMRICQMRNSSLDPMFFVYVNPFSGSHSEGWNMPFGEFGYPRWEKIRLQNSQCYNW
TLLLGNRWKTFFETVHIYLRSRTRLPTLLRNETGQGPVDLSDPSKRQFYIKISDVQVFGY
SLRFNADLLRSAVQQVNQSYTQGGQFYSSSSVMLLLLDIRDRINRLAPPVAPGKPQLDLF
SCMLKHRLKLTNSEIIRVNHALDLYNTEILKQSDQMTAKLC
>sp|P55201|BRPF1_HUMAN Peregrin OS=Homo sapiens GN=BRPF1 PE=1 SV=2
MGVDFDVKTFCHNLRATKPPYECPVETCRKVYKSYSGIEYHLYHYDHDNPPPPQQTPLRK
HKKKGRQSRPANKQSPSPSEVSQSPGREVMSYAQAQRMVEVDLHGRVHRISIFDNLDVVS
EDEEAPEEAPENGSNKENTETPAATPKSGKHKNKEKRKDSNHHHHHNVSASTTPKLPEVV
YRELEQDTPDAPPRPTSYYRYIEKSAEELDEEVEYDMDEEDYIWLDIMNERRKTEGVSPI
PQEIFEYLMDRLEKESYFESHNKGDPNALVDEDAVCCICNDGECQNSNVILFCDMCNLAV
HQECYGVPYIPEGQWLCRRCLQSPSRAVDCALCPNKGGAFKQTDDGRWAHVVCALWIPEV

CFANTVFLEPIDSIEHIPPARWKLTCYICKQRGSGACIQCHKANCYTAFHVTCAQQAGLY
MKMEPVRETGANGTSFSVRKTAYCDIHTPPGSARRLPALSHSEGEEDEDEEEDEGKGWSS
EKVKKAKAKSRIKMKKARKILAEKRAAAPVVSVPCIPPHRLSKITNRLTIQRKSQFMQRL
HSYWTLKRQSRNGVPLLRRLQTHLQSQRNCDQVGRDSEDKNWALKEQLKSWQRLRHDLER
ARLLVELIRKREKLKRETIKVQQIAMEMQLTPFLILLRKTLEQLQEKDTGNIFSEPVPLS
EVPDYLDHIKKPMDFFTMKQNLEAYRYLNFDDFEEDFNLIVSNCLKYNAKDTIFYRAAVR
LREQGGAVLRQARRQAEKMGIDFETGMHIPHSLAGDEATHHTEDAAEEERLVLLENQKHL
PVEEQLKLLLERLDEVNASKQSVGRSRRAKMIKKEMTALRRKLAHQRETGRDGPERHGPS
SRGSLTPHPAACDKDGQTDSAAEESSSQETSKGLGPNMSSTPAHEVGRRTSVLFSKKNPK
TAGPPKRPGRPPKNRESQMTPSHGGSPVGPPQLPIMSSLRQRKRGRSPRPSSSSDSDSDK
STEDPPMDLPANGFSGGNQPVKKSFLVYRNDCSLPRSSSDSESSSSSSSSAASDRTSTTP
SKQGRGKPSFSRGTFPEDSSEDTSGTENEAYSVGTGRGVGHSMVRKSLGRGAGWLSEDED
SPLDALDLVWAKCRGYPSYPALIIDPKMPREGMFHHGVPIPVPPLEVLKLGEQMTQEARE
HLYLVLFFDNKRTWQWLPRTKLVPLGVNQDLDKEKMLEGRKSNIRKSVQIAYHRALQHRS
KVQGEQSSETSDSD
>sp|Q8TDN6|BRX1_HUMAN Ribosome biogenesis protein BRX1 homolog OS=Homo sapiens GN=BRIX1 PE=1 SV=2
MAATKRKRRGGFAVQAKKPKRNEIDAEPPAKRHATAEEVEEEERDRIPGPVCKGKWKNKE
RILIFSSRGINFRTRHLMQDLRMLMPHSKADTKMDRKDKLFVINEVCEMKNCNKCIYFEA
KKKQDLYMWLSNSPHGPSAKFLVQNIHTLAELKMTGNCLKGSRPLLSFDPAFDELPHYAL
LKELLIQIFSTPRYHPKSQPFVDHVFTFTILDNRIWFRNFQIIEEDAALVEIGPRFVLNL
IKIFQGSFGGPTLYENPHYQSPNMHRRVIRSITAAKYREKQQVKDVQKLRKKEPKTLLPH
DPTADVFVTPAEEKPIEIQWVKPEPKVDLKARKKRIYKRQRKMKQRMDSGKTK
>sp|P19099|C11B2_HUMAN Cytochrome P450 11B2, mitochondrial OS=Homo sapiens GN=CYP11B2 PE=1 SV=3
MALRAKAEVCVAAPWLSLQRARALGTRAARAPRTVLPFEAMPQHPGNRWLRLLQIWREQG
YEHLHLEMHQTFQELGPIFRYNLGGPRMVCVMLPEDVEKLQQVDSLHPCRMILEPWVAYR
QHRGHKCGVFLLNGPEWRFNRLRLNPDVLSPKAVQRFLPMVDAVARDFSQALKKKVLQNA
RGSLTLDVQPSIFHYTIEASNLALFGERLGLVGHSPSSASLNFLHALEVMFKSTVQLMFM
PRSLSRWISPKVWKEHFEAWDCIFQYGDNCIQKIYQELAFNRPQHYTGIVAELLLKAELS
LEAIKANSMELTAGSVDTTAFPLLMTLFELARNPDVQQILRQESLAAAASISEHPQKATT
ELPLLRAALKETLRLYPVGLFLERVVSSDLVLQNYHIPAGTLVQVFLYSLGRNAALFPRP
ERYNPQRWLDIRGSGRNFHHVPFGFGMRQCLGRRLAEAEMLLLLHHVLKHFLVETLTQED
IKMVYSFILRPGTSPLLTFRAIN
>sp|Q6UB35|C1TM_HUMAN Monofunctional C1-tetrahydrofolate synthase, mitochondrial OS=Homo sapiens GN=MTHFD1L PE=1 SV=1
MGTRLPLVLRQLRRPPQPPGPPRRLRVPCRASSGGGGGGGGGREGLLGQRRPQDGQARSS
CSPGGRTPAARDSIVREVIQNSKEVLSLLQEKNPAFKPVLAIIQAGDDNLMQEINQNLAE
EAGLNITHICLPPDSSEAEIIDEILKINEDTRVHGLALQISENLFSNKVLNALKPEKDVD
GVTDINLGKLVRGDAHECFVSPVAKAVIELLEKSGVNLDGKKILVVGAHGSLEAALQCLF
QRKGSMTMSIQWKTRQLQSKLHEADIVVLGSPKPEEIPLTWIQPGTTVLNCSHDFLSGKV
GCGSPRIHFGGLIEEDDVILLAAALRIQNMVSSGRRWLREQQHRRWRLHCLKLQPLSPVP
SDIEISRGQTPKAVDVLAKEIGLLADEIEIYGKSKAKVRLSVLERLKDQADGKYVLVAGI
TPTPLGEGKSTVTIGLVQALTAHLNVNSFACLRQPSQGPTFGVKGGAAGGGYAQVIPMEE

FNLHLTGDIHAITAANNLLAAAIDTRILHENTQTDKALYNRLVPLVNGVREFSEIQLARL
KKLGINKTDPSTLTEEEVSKFARLDIDPSTITWQRVLDTNDRFLRKITIGQGNTEKGHYR
QAQFDIAVASEIMAVLALTDSLADMKARLGRMVVASDKSGQPVTADDLGVTGALTVLMKD
AIKPNLMQTLEGTPVFVHAGPFANIAHGNSSVLADKIALKLVGEEGFVVTEAGFGADIGM
EKFFNIKCRASGLVPNVVVLVATVRALKMHGGGPSVTAGVPLKKEYTEENIQLVADGCCN
LQKQIQITQLFGVPVVVALNVFKTDTRAEIDLVCELAKRAGAFDAVPCYHWSVGGKGSVD
LARAVREAASKRSRFQFLYDVQVPIVDKIRTIAQAVYGAKDIELSPEAQAKIDRYTQQGF
GNLPICMAKTHLSLSHQPDKKGVPRDFILPISDVRASIGAGFIYPLVGTMSTMPGLPTRP
CFYDIDLDTETEQVKGLF
>sp|Q6ZSU1|C2G1P_HUMAN Putative inactive cytochrome P450 2G1 OS=Homo sapiens GN=CYP2G1P PE=5 SV=1
MPYTDVVIHEIQRLVDIVPMGVPHNIIQDTQFRGYLLPKGTDVFPLLGSVLKDPKYFRYP
DAFYPQHFLDEQGRFKKNEAFVPFSSGKRICLGEAMARMELFLYFTSTLQNFSLCSLVPL
VDIDITPKLSGFGNITPTYELCLVAR
>sp|Q6ZMU1|C3P1_HUMAN Putative protein C3P1 OS=Homo sapiens GN=C3P1 PE=5 SV=3
MPVRTLFPESWLWRKFTLPKSKSGISHYPISVKVPDSITTWQFVVVSLKAGQGLCVSDPF
ELTVMKSFFVDLKLPSSVIRNEQVQIQAMLYNFRDRQAKVRVEFPHKETLCSASKPGAPS
HQAGVQIQQTSYSIVLEPQGGQTQTKLVPRQEFLNMVPDTEAEVFISVQGYTQMLTHRSSD
GTYHTSKGNPGSTWLTSYVFRVFALAYSMMTTQVLSLSSLCDMANWIIIDRQAEDGHFLE
KGPVVMTSMQDGYQGSEEDVSLTALVLIALNEGKELCRQKNLMASIERARGFLERKLPDI
QTTFAVAIASYALALANSSQANDCLDSFASPSGCGMLLNQPQSWSGEGVISNPAMCYSSL
SVS
>sp|P20851|C4BPB_HUMAN C4b-binding protein beta chain OS=Homo sapiens GN=C4BPB PE=1 SV=1
MFFWCACCLMVAWRVSASDAEHCPELPPVDNSIFVAKEVEGQILGTYVCIKGYHLVGKKT
LFCNASKEWDNTTTECRLGHCPDPVLVNGEFSSSGPVNVSDKITFMCNDHYILKGSNRSQ
CLEDHTWAPPFPICKSRDCDPPGNPVHGYFEGNNFTLGSTISYYCEDRYYLVGVQEQQCV
DGEWSSALPVCKLIQEAPKPECEKALLAFQESKNLCEAMENFMQQLKESGMTMEELKYSL
ELKKAELKAKLL
>sp|Q8TCZ2|C99L2_HUMAN CD99 antigen-like protein 2 OS=Homo sapiens GN=CD99L2 PE=1 SV=1
MVAWRSAFLVCLAFSLATLVQRGSGDFDDFNLEDAVKETSSVKQPWDHTTTTTTNRPGTT
RAPAKPPGSGLDLADALDDQDDGRRKPGIGGRERWNHVTTTTKRPVTTRAPANTLGNDFD
LADALDDRNDRDDGRRKPIAGGGGFSDKDLEDIVGGGEYKPDKGKGDGRYGSNDDPGSGM
VAEPGTIAGVASALAMALIGAVSSYISYQQKKFCFSIQQGLNADYVKGENLEAVVCEEPQ
VKYSTLHTQSAEPPPPPEPARI
>sp|Q8N6N3|CA052_HUMAN UPF0690 protein C1orf52 OS=Homo sapiens GN=C1orf52 PE=1 SV=1
MAAEEKDPLSYFAAYGSSSSGSSDEEDNIEPEETSRRTPDPAKSAGGCRNKAEKRLPGPD
ELFRSVTRPAFLYNPLNKQIDWERHVVKAPEEPPKEFKIWKSNYVPPPETYTTEKKPPPP
ELDMAIKWSNIYEDNGDDAPQNAKKARLLPEGEETLESDDEKDEHTSKKRKVEPGEPAKK
KK
>sp|Q9NX04|CA109_HUMAN Uncharacterized protein C1orf109 OS=Homo sapiens GN=C1orf109 PE=1 SV=1
MTQDRPLLAVQEALKKCFPVVEEQQGLWQSALRDCQPLLSSLSNLAEQLQAAQNLRFEDV
PALRAFPDLKERLRRKQLVAGDIVLDKLGERLAILLKVRDMVSSHVERVFQIYEQHADTV
GIDAVLQPSAVSPSVADMLEWLQDIERHYRKSYLKRKYLLSSIQWGDLANIQALPKAWDR

ISKDEHQDLVQDILLNVSFFLEE
>sp|Q5JT78|CA137_HUMAN Putative uncharacterized protein C1orf137 OS=Homo sapiens GN=C1orf137 PE=4 SV=1
MALKTSLGSVQFMEQFCWPHLRSFCETCVGSWSIQMRQHHHQSSFPGSLPQETNLTLKVC
KPQAPQKLDPTLSSCFFIESCGAQVPVEGSRRETSITV
>sp|Q9NUP1|BL1S4_HUMAN Biogenesis of lysosome-related organelles complex 1 subunit 4 OS=Homo sapiens GN=BLOC1S4 PE=1 SV=1
MEGSFSDGGALPEGLAEEAEPQGAAWSGDSGTVSQSHSSASGPWEDEGAEDGAPGRDLPL
LRRAAAGYAACLLPGAGARPEVEALDASLEDLLTRVDEFVGMLDMLRGDSSHVVSEGVPR
IHAKAAEMRRIYSRIDRLEAFVRMVGGRVARMEEQVTKAEAELGTFPRAFKKLLHTMNVP
SLFSKSAPSRPQQAGYEAPVLFRTEDYFPCCSERPQL
>sp|Q9Y3E2|BOLA1_HUMAN BolA-like protein 1 OS=Homo sapiens GN=BOLA1 PE=1 SV=1
MLSGRLVLGLVSMAGRVCLCQGSAGSGAIGPVEAAIRTKLEEALSPEVLELRNESGGHAV
PPGSETHFRVAVVSSRFEGLSPLQRHRLVHAALAEELGGPVHALAIQARTPAQWRENSQL
DTSPPCLGGNKKTLGTP
>sp|Q9UKI2|BORG2_HUMAN Cdc42 effector protein 3 OS=Homo sapiens GN=CDC42EP3 PE=1 SV=1
MPAKTPIYLKAANNKKGKKFKLRDILSPDMISPPLGDFRHTIHIGKEGQHDVFGDISFLQ
GNYELLPGNQEKAHLGQFPGHNEFFRANSTSDSVFTETPSPVLKNAISLPTIGGSQALML
PLLSPVTFNSKQESFGPAKLPRLSCEPVMEEKAQEKSSLLENGTVHQGDTSWGSSGSASQ
SSQGRDSHSSSLSEQYPDWPAEDMFDHPTPCELIKGKTKSEESLSDLTGSLLSLQLDLGP
SLLDEVLNVMDKNK
>sp|P15056|BRAF_HUMAN Serine/threonine-protein kinase B-raf OS=Homo sapiens GN=BRAF PE=1 SV=4
MAALSGGGGGGAEPGQALFNGDMEPEAGAGAGAAASSAADPAIPEEVWNIKQMIKLTQEH
IEALLDKFGGEHNPPSIYLEAYEEYTSKLDALQQREQQLLESLGNGTDFSVSSSASMDTV
TSSSSSSLSVLPSSLSVFQNPTDVARSNPKSPQKPIVRVFLPNKQRTVVPARCGVTVRDS
LKKALMMRGLIPECCAVYRIQDGEKKPIGWDTDISWLTGEELHVEVLENVPLTTHNFVRK
TFFTLAFCDFCRKLLFQGFRCQTCGYKFHQRCSTEVPLMCVNYDQLDLLFVSKFFEHHPI
PQEEASLAETALTSGSSPSAPASDSIGPQILTSPSPSKSIPIPQPFRPADEDHRNQFGQR
DRSSSAPNVHINTIEPVNIDDLIRDQGFRGDGGSTTGLSATPPASLPGSLTNVKALQKSP
GPQRERKSSSSSEDRNRMKTLGRRDSSDDWEIPDGQITVGQRIGSGSFGTVYKGKWHGDV
AVKMLNVTAPTPQQLQAFKNEVGVLRKTRHVNILLFMGYSTKPQLAIVTQWCEGSSLYHH
LHIIETKFEMIKLIDIARQTAQGMDYLHAKSIIHRDLKSNNIFLHEDLTVKIGDFGLATV
KSRWSGSHQFEQLSGSILWMAPEVIRMQDKNPYSFQSDVYAFGIVLYELMTGQLPYSNIN
NRDQIIFMVGRGYLSPDLSKVRSNCPKAMKRLMAECLKKKRDERPLFPQILASIELLARS
LPKIHRSASEPSLNRAGFQTEDFSLYACASPKTPIQAGGYGAFPVH
>sp|P38398|BRCA1_HUMAN Breast cancer type 1 susceptibility protein OS=Homo sapiens GN=BRCA1 PE=1 SV=2
MDLSALRVEEVQNVINAMQKILECPICLELIKEPVSTKCDHIFCKFCMLKLLNQKKGPSQ
CPLCKNDITKRSLQESTRFSQLVEELLKIICAFQLDTGLEYANSYNFAKKENNSPEHLKD
EVSIIQSMGYRNRAKRLLQSEPENPSLQETSLSVQLSNLGTVRTLRTKQRIQPQKTSVYI
ELGSDSSEDTVNKATYCSVGDQELLQITPQGTRDEISLDSAKKAACEFSETDVTNTEHHQ
PSNNDLNTTEKRAAERHPEKYQGSSVSNLHVEPCGTNTHASSLQHENSSLLLTKDRMNVE
KAEFCNKSKQPGLARSQHNRWAGSKETCNDRRTPSTEKKVDLNADPLCERKEWNKQKLPC

SENPRDTEDVPWITLNSSIQKVNEWFSRSDELLGSDDSHDGESESNAKVADVLDVLNEVD
EYSGSSEKIDLLASDPHEALICKSERVHSKSVESNIEDKIFGKTYRKKASLPNLSHVTEN
LIIGAFVTEPQIIQERPLTNKLKRKRRPTSGLHPEDFIKKADLAVQKTPEMINQGTNQTE
QNGQVMNITNSGHENKTKGDSIQNEKNPNPIESLEKESAFKTKAEPISSSISNMELELNI
HNSKAPKKNRLRRKSSTRHIHALELVVSRNLSPPNCTELQIDSCSSSEEIKKKKYNQMPV
RHSRNLQLMEGKEPATGAKKSNKPNEQTSKRHDSDTFPELKLTNAPGSFTKCSNTSELKE
FVNPSLPREEKEEKLETVKVSNNAEDPKDLMLSGERVLQTERSVESSSISLVPGTDYGTQ
ESISLLEVSTLGKAKTEPNKCVSQCAAFENPKGLIHGCSKDNRNDTEGFKYPLGHEVNHS
RETSIEMEESELDAQYLQNTFKVSKRQSFAPFSNPGNAEEECATFSAHSGSLKKQSPKVT
FECEQKEENQGKNESNIKPVQTVNITAGFPVVGQKDKPVDNAKCSIKGGSRFCLSSQFRG
NETGLITPNKHGLLQNPYRIPPLFPIKSFVKTKCKKNLLEENFEEHSMSPEREMGNENIP
STVSTISRNNIRENVFKEASSSNINEVGSSTNEVGSSINEIGSSDENIQAELGRNRGPKL
NAMLRLGVLQPEVYKQSLPGSNCKHPEIKKQEYEEVVQTVNTDFSPYLISDNLEQPMGSS
HASQVCSETPDDLLDDGEIKEDTSFAENDIKESSAVFSKSVQKGELSRSPSPFTHTHLAQ
GYRRGAKKLESSEENLSSEDEELPCFQHLLFGKVNNIPSQSTRHSTVATECLSKNTEENL
LSLKNSLNDCSNQVILAKASQEHHLSEETKCSASLFSSQCSELEDLTANTNTQDPFLIGS
SKQMRHQSESQGVGLSDKELVSDDEERGTGLEENNQEEQSMDSNLGEAASGCESETSVSE
DCSGLSSQSDILTTQQRDTMQHNLIKLQQEMAELEAVLEQHGSQPSNSYPSIISDSSALE
DLRNPEQSTSEKAVLTSQKSSEYPISQNPEGLSADKFEVSADSSTSKNKEPGVERSSPSK
CPSLDDRWYMHSCSGSLQNRNYPSQEELIKVVDVEEQQLEESGPHDLTETSYLPRQDLEG
TPYLESGISLFSDDPESDPSEDRAPESARVGNIPSSTSALKVPQLKVAESAQSPAAAHTT
DTAGYNAMEESVSREKPELTASTERVNKRMSMVVSGLTPEEFMLVYKFARKHHITLTNLI
TEETTHVVMKTDAEFVCERTLKYFLGIAGGKWVVSYFWVTQSIKERKMLNEHDFEVRGDV
VNGRNHQGPKRARESQDRKIFRGLEICCYGPFTNMPTDQLEWMVQLCGASVVKELSSFTL
GTGVHPIVVVQPDAWTEDNGFHAIGQMCEAPVVTREWVLDSVALYQCQELDTYLIPQIPH
SHY
>sp|Q9C0B6|BRNP2_HUMAN BMP/retinoic acid-inducible neural-specific protein 2 OS=Homo sapiens GN=BRINP2 PE=2 SV=2
MRWQCGTRFRGLRPAVAPWTALLALGLPGWVLAVSATAAAVVPEQHASVAGQHPLDWLLT
DRGPFHRAQEYADFMERYRQGFTTRYRIYREFARWKVNNLALERKDFFSLPLPLAPEFIR
NIRLLGRRPNLQQVTENLIKKYGTHFLLSATLGGEESLTIFVDKQKLGRKTETTGGASII
GGSGNSTAVSLETLHQLAASYFIDRESTLRRLHHIQIATGAIKVTETRTGPLGCSNYDNL
DSVSSVLVQSPENKVQLLGLQVLLPEYLRERFVAAALSYITCSSEGELVCKENDCWCKCS
PTFPECNCPDADIQAMEDSLLQIQDSWATHNRQFEESEEFQALLKRLPDDRFLNSTAISQ
FWAMDTSLQHRYQQLGAGLKVLFKKTHRILRRLFNLCKRCHRQPRFRLPKERSLSYWWNR
IQSLLYCGESTFPGTFLEQSHSCTCPYDQSSCQGPIPCALGEGPACAHCAPDNSTRCGSC
NPGYVLAQGLCRPEVAESLENFLGLETDLQDLELKYLLQKQDSRIEVHSIFISNDMRLGS
WFDPSWRKRMLLTLKSNKYKPGLVHVMLALSLQICLTKNSTLEPVMAIYVNPFGGSHSES
WFMPVNEGSFPDWERTNVDAAAQCQNWTITLGNRWKTFFETVHVYLRSRIKSLDDSSNET
IYYEPLEMTDPSKNLGYMKINTLQVFGYSLPFDPDAIRDLILQLDYPYTQGSQDSALLQL
IELRDRVNQLSPPGKVRLDLFSCLLRHRLKLANNEVGRIQSSLRAFNSKLPNPVEYETGK
LCS
>sp|Q76B58|BRNP3_HUMAN BMP/retinoic acid-inducible neural-specific protein 3 OS=Homo sapiens GN=BRINP3 PE=1 SV=1

MIWRSRAGAELFSLMALWEWIALSLHCWVLAVAAVSDQHATSPFDWLLSDKGPFHRSQEY
TDFVDRSRQGFSTRYKIYREFGRWKVNNLAVERRNFLGSPLPLAPEFFRNIRLLGRRPTL
QQITENLIKKYGTHFLLSATLGGEESLTIFVDKRKLSKRAEGSDSTTNSSSVTLETLHQL
AASYFIDRDSTLRRLHHIQIASTAIKVTETRTGPLGCSNYDNLDSVSSVLVQSPENKIQL
QGLQVLLPDYLQERFVQAALSYIACNSEGEFICKENDCWCHCGPKFPECNCPSMDIQAME
ENLLRITETWKAYNSDFEESDEFKLFMKRLPMNYFLNTSTIMHLWTMDSNFQRRYEQLEN
SMKQLFLKAQKIVHKLFSLSKRCHKQPLISLPRQRTSTYWLTRIQSFLYCNENGLLGSFS
EETHSCTCPNDQVVCTAFLPCTVGDASACLTCAPDNRTRCGTCNTGYMLSQGLCKPEVAE
STDHYIGFETDLQDLEMKYLLQKTDRRIEVHAIFISNDMRLNSWFDPSWRKRMLLTLKSN
KYKSSLVHMILGLSLQICLTKNSTLEPVLAVYVNPFGGSHSESWFMPVNENSFPDWERTK
LDLPLQCYNWTLTLGNKWKTFFETVHIYLRSRIKSNGPNGNESIYYEPLEFIDPSRNLGY
MKINNIQVFGYSMHFDPEAIRDLILQLDYPYTQGSQDSALLQLLEIRDRVNKLSPPGQRR
LDLFSCLLRHRLKLSTSEVVRIQSALQAFNAKLPNTMDYDTTKLCS
>sp|Q9ULD4|BRPF3_HUMAN Bromodomain and PHD finger-containing protein 3 OS=Homo sapiens GN=BRPF3 PE=1 SV=2
MRKPRRKSRQNAEGRRSPSPYSLKCSPTRETLTYAQAQRIVEVDIDGRLHRISIYDPLKI
ITEDELTAQDITECNSNKENSEQPQFPGKSKKPSSKGKKKESCSKHASGTSFHLPQPSFR
MVDSGIQPEAPPLPAAYYRYIEKPPEDLDAEVEYDMDEEDLAWLDMVNEKRRVDGHSLVS
ADTFELLVDRLEKESYLESRSSGAQQSLIDEDAFCCVCLDDECHNSNVILFCDICNLAVH
QECYGVPYIPEGQWLCRCCLQSPSRPVDCILCPNKGGAFKQTSDGHWAHVVCAIWIPEVC
FANTVFLEPIEGIDNIPPARWKLTCYICKQKGLGAAIQCHKVNCYTAFHVTCAQRAGLFM
KIEPMRETSLNGTIFTVRKTAYCEAHSPPGAATARRKGDSPRSISETGDEEGLKEGDGEE
EEEEEVEEEEQEAQGGVSGSLKGVPKKSKMSLKQKIKKEPEEAGQDTPSTLPMLAVPQIP
SYRLNKICSGLSFQRKNQFMQRLHNYWLLKRQARNGVPLIRRLHSHLQSQRNAEQREQDE
KTSAVKEELKYWQKLRHDLERARLLIELIRKREKLKREQVKVQQAAMELELMPFNVLLRT
TLDLLQEKDPAHIFAEPVNLSEVPDYLEFISKPMDFSTMRRKLESHLYRTLEEFEEDFNL
IVTNCMKYNAKDTIFHRAAVRLRDLGGAILRHARRQAENIGYDPERGTHLPESPKLEDFY
RFSWEDVDNILIPENRAHLSPEVQLKELLEKLDLVSAMRSSGARTRRVRLLRREINALRQ
KLAQPPPPQPPSLNKTVSNGELPAGPQGDAAVLEQALQEEPEDDGDRDDSKLPPPPTLEP
TGPAPSLSEQESPPEPPTLKPINDSKPPSRFLKPRKVEEDELLEKSPLQLGNEPLQRLLS
DNGINRLSLMAPDTPAGTPLSGVGRRTSVLFKKAKNGVKLQRSPDRVLENGEDHGVAGSP
ASPASIEEERHSRKRPRSRSCSESEGERSPQQEEETGMTNGFGKHTESGSDSECSLGLSG
GLAFEACSGLTPPKRSRGKPALSRVPFLEGVNGDSDYNGSGRSLLLPFEDRGDLEPLELV
WAKCRGYPSYPALIIDPKMPREGLLHNGVPIPVPPLDVLKLGEQKQAEAGEKLFLVLFFD
NKRTWQWLPRDKVLPLGVEDTVDKLKMLEGRKTSIRKSVQVAYDRAMIHLSRVRGPHSFV
TSSYL
>sp|P43251|BTD_HUMAN Biotinidase OS=Homo sapiens GN=BTD PE=1 SV=2
MAHAHIQGGRRAKSRFVVCIMSGARSKLALFLCGCYVVALGAHTGEESVADHHEAEYYVA
AVYEHPSILSLNPLALISRQEALELMNQNLDIYEQQVMTAAQKDVQIIVFPEDGIHGFNF
TRTSIYPFLDFMPSPQVVRWNPCLEPHRFNDTEVLQRLSCMAIRGDMFLVANLGTKEPCH
SSDPRCPKDGRYQFNTNVVFSNNGTLVDRYRKHNLYFEAAFDVPLKVDLITFDTPFAGRF
GIFTCFDILFFDPAIRVLRDYKVKHVVYPTAWMNQLPLLAAIEIQKAFAVAFGINVLAAN
VHHPVLGMTGSGIHTPLESFWYHDMENPKSHLIIAQVAKNPVGLIGAENATGETDPSHSK
FLKILSGDPYCEKDAQEVHCDEATKWNVNAPPTFHSEMMYDNFTLVPVWGKEGYLHVCSN

GLCCYLLYERPTLSKELYALGVFDGLHTVHGTYYIQVCALVRCGGLGFDTCGQEITEATG
IFEFHLWGNFSTSYIFPLFLTSGMTLEVPDQLGWENDHYFLRKSRLSSGLVTAALYGRLY
ERD
>sp|Q14201|BTG3_HUMAN Protein BTG3 OS=Homo sapiens GN=BTG3 PE=1 SV=3
MKNEIAAVVFFFTRLVRKHDKLKKEAVERFAEKLTLILQEKYKNHWYPEKPSKGQAYRCI
RVNKFQRVDPDVLKACENSCILYSDLGLPKELTLWVDPCEVCCRYGEKNNAFIVASFENK
DENKDEISRKVTRALDKVTSDYHSGSSSSDEETSKEMEVKPSSVTAAASPVYQISELIFP
PLPMWHPLPRKKPGMYRGNGHQNHYPPPVPFGYPNQGRKNKPYRPIPVTWVPPPGMHCDR
NHWINPHMLAPH
>sp|A2RUR9|C144A_HUMAN Coiled-coil domain-containing protein 144A OS=Homo sapiens GN=CCDC144A PE=2 SV=1
MASWGGEKRGGAEGSPKPAVYATRKTPSVGSQGDQWYLGYPGDQWSSGFPYSWWKNSVGS
ESKHGEGALDQPQHDVRLEDLGELHRAARSGDVPGVEHILAPGDTGVDKRDRKKSIQQLV
PEYKEKQTPESLPQNNNPDWHPTNLTLSDETCQRSKNLKVDDKCPSVSPSMPENQSATKE
LGQMNLTEREKMDTGVVLLSGNDTLHDLCQSQLPENKESKEAEQDSELTSEEEQERLKGC
ENKQPQKTSQEPEMAKDCDREDIPIYPVLPHVQKSEEMWIEQGKLEWKNQLKLVINELKQ
RFGEIYEKYKIPACPEEEPLLDNSTRGTDVKDIPFNLTNNIPGCEEEDASEISVSVVFET
FPEQKEPSLKNIIHPYYHPYSGSQEHVCQSSSKFHLHENKLDCDNDNKPGIGHIFSTDKN
FHNDASTKKARNPEVVMVEMKEDQEFDLQMTKNMNQNSDSGSTNNYKSLKPKLENLSSLP
PDSDRTSEVYLHEELQQDMQKFKNEVNTLEEEFLALKKEDVQLHKDVEEEMEKHRSNSTE
LSGTLTDGTTVGNDDDGLNQQIPRKENGEHDRPADKTSNEKNEVKNQIYPEADFADSMEP
SEIASEDCELSHSVYENFMLLIEQLRMEYKDSASLPRIQDTFCLCEHLLKLKNNHCDQLT
VKLKQMENMVSVLQNELSETKKTKLQLELQKIEWEKELYDLRLALKQENEEKRNADMLYN
KDSEQLRIKEEECGKVVETKQQLKWNLRRLVKELRTVRNNLDLVVQERNDAQKQLSEEQD
ARILQDQILTSKQKELEMARKKMNSEISHRHQKEKDLFHEDCMLQEEIALLRLEIDTIKN
QNKQKEKKYFEDIEAVKEKNDNLQKIIKLNEETLTETILQYSGQLNNLTAENKILNSELE
NGKQNQERLEIEMESYRCRLAAAVRDCDQSQTARDLKLDFQRTRQEWVRLHDKMKVDMSG
LQAKNEILSEKLSNAESKINSLQIQLHNTRDALGRESLILERVQRDLSQTQCQKKETEQM
YQIEQSKLKKYIAKQESVEERLSQLQSENMLLRQQLDDAHKKANSQEKTSSTIQDQFHSA
AKNLQAESEKQILSLQEKNKELMDEYNHLKERMDQCEKEKAGRKIDLTEAQETVPSRCLH
LDAENEVLQLQQTLFSMKAIQKQCETLQKNKKQLKQEVVNLKSYMERNMLERGKAEWHKL
LIEERARKEIEEKLNEAILTLQKQAAVSHEQLVQLREDNTTSIKTQMELTIKDLESEISR
IKTSQADFNKTELERYKELYLEEVKVRESLSNELSRTNEMIAEVSTQLTVEKEQTRSRSL
FTAYATRPVLESPCVGNLNDSEGLNRKHIPRKKRSALKDMESYLLKMQQKLQNDLTAEVA
GSSQTGLHRIPQCSSFSSSSLHLLLCSICQPFFLILQLLLNMNLDPI
>sp|Q9BXJ0|C1QT5_HUMAN Complement C1q tumor necrosis factor-related protein 5 OS=Homo sapiens GN=C1QTNF5 PE=1 SV=1
MRPLLVLLLLGLAAGSPPLDDNKIPSLCPGHPGLPGTPGHHGSQGLPGRDGRDGRDGAPG
APGEKGEGGRPGLPGPRGDPGPRGEAGPAGPTGPAGECSVPPRSAFSAKRSESRVPPPSD
APLPFDRVLVNEQGHYDAVTGKFTCQVPGVYYFAVHATVYRASLQFDLVKNGESIASFFQ
FFGGWPKPASLSGGAMVRLEPEDQVWVQVGVGDYIGIYASIKTDSTFSGFLVYSDWHSSP
VFA
>sp|Q5T0F9|C2D1B_HUMAN Coiled-coil and C2 domain-containing protein 1B OS=Homo sapiens GN=CC2D1B PE=1 SV=1

MMPGPRPRKGPQARGQGVAAAKQMGLFMEFGPEDMLLGMDEAEDDEDLEAELLALTGEAQ
TTGKKPAPKGQAPLPMAHIEKLAADCMRDVEEEEEEEGLEEDAELLTELQEVLGVDEETE
PLDGDEVADPGGSEEENGLEDTEPPVQTAVLTASAPAAQAGASQGLHALLEERIHNYREA
AASAKEAGEAAKARRCERGLKTLESQLASVRRGRKINEDEIPPPVALGKRPLAPQEPANR
SPETDPPAPPALESDNPSQPETSLPGISAQPVSDLDPDPRALLSSRQREYKVAALSAKRA
GELDRARELMRIGKRFGAVLEALEKGQPVDLSAMPPAPEDLKPQQASQAPTAPSVIPPAV
ERVQPVMAPDVPATPVAPTESQTVLDALQQRLNKYREAGIQARSGGDERKARMHERIAKQ
YQDAIRAHRAGRKVNFAELPVPPGFPPIPGLESTMGVEEDAVAATLAAAEKLASAEDSAP
ADKDEDEPPGHLQGEPPAQAPVAKKPARPTVPSSQRLPEPRASSSKESPSPSVREQLALL
EARKLQYQRAALQAKRSQDLEQAKAYLRVAKWLEAQIIQARSGRPVDLSKVPSPLTDEEG
DFILIHHEDLRLSQKAEEVYAQLQKMLLEQQEKCLLFSKQFMHQGNVAETTRFEKLAQDR
KKQLEILQLAQAQGLDPPTHHFELKTFQTVRIFSELNSTEMHLIIVRGMNLPAPPGVTPD
DLDAFVRFEFHYPNSDQAQKSKTAVVKNTNSPEFDQLFKLNINRNHRGFKRVIQSKGIKF
EIFHKGSFFRSDKLVGTAHLKLERLENECEIREIVEVLDGRKPTGGKLEVKVRLREPLSG
QDVQMVTENWLVLEPRGL
>sp|Q9BWL3|CA043_HUMAN Uncharacterized protein C1orf43 OS=Homo sapiens GN=C1orf43 PE=2 SV=1
MASGSNWLSGVNVVLVMAYGSLVFVLLFIFVKRQIMRFAMKSRRGPHVPVGHNAPKDLKE
EIDIRLSRVQDIKYEPQLLADDDARLLQLETQGNQSCYNYLYRMKALDAIRTSEIPFHSE
GRHPRSLMGKNFRSYLLDLRNTSTPFKGVRKALIDTLLDGYETARYGTGVFGQNEYLRYQ
EALSELATAVKARIGSSQRHHQSAAKDLTQSPEVSPTTIQVTYLPSSQKSKRAKHFLELK
SFKDNYNTLESTL
>sp|Q8WWF1|CA054_HUMAN Uncharacterized protein C1orf54 OS=Homo sapiens GN=C1orf54 PE=2 SV=1
MDVLFVAIFAVPLILGQEYEDEERLGEDEYYQVVYYYTVTPSYDDFSADFTIDYSIFESE
DRLNRLDKDITEAIETTISLETARADHPKPVTVKPVTTEPSPDLNDAVSSLRSPIPLLLS
CAFVQVGMYFM
>sp|Q8TC20|CAGE1_HUMAN Cancer-associated gene 1 protein OS=Homo sapiens GN=CAGE1 PE=1 SV=2
MNKDYQKFWSSPSDPVHFEVDTSHEKVESMSESDTMNVSNLSQGVMLSHSPICMETTGTT
CDLPQNEIKNFERENEYESTLCEDAYGTLDNLLNDNNIENYSTNALIQPVDTISISSLRQ
FETVCKFHWVEAFDDEMTEKPEFQSQVYNYAKDNNIKQDSFKEENPMETSVSANTDQLGN
EYFRQPPPRSPPLIHCSGEMLKFTEKSLAKSIAKESALNPSQPPSFLCKTAVPSKEIQNY
GEIPEMSVSYEKEVTAEGVERPEIVSTWSSAGISWRSEACRENCEMPDWEQSAESLQPVQ
EDMALNEVLQKLKHTNRKQEVRIQELQCSNLYLEKRVKELQMKITKQQVFIDVINKLKEN
VEELIEDKYKIILEKNDTKKTLQNLEEVLANTQKHLQESRNDKEMLQLQFKKIKANYVCL
QERYMTEMQQKNKSVSQYLEMDKTLSKKEEEVERLQQLKKELEKATASALDLLKREKEAQ
EQEFLSLQEEFQKLEKENLEERQKLKSRLEKLLTQVRNLQFMSENERTKNIKLQQQINEV
KNENAKLKQQVARSEEQNYVPKFETAQLKDQLEEVLKSDITKDTKTTHSNLLPDCSPCEE
RLNPADIKRASQLASKMHSLLALMVGLLTCQDIINSDAEHFKESEKVSDIMLQKLKSLHL
KKKTLDKEVIDCDSDEAKSIRDVPTLLGAKLDKYHSLNEELDFLVTSYEEIIECADQRLA
ISHSQIAHLEERNKHLEDLIRKPREKARKPRSKSLENHPKSMTMMPALFKENRNDLD
>sp|Q9ULX7|CAH14_HUMAN Carbonic anhydrase 14 OS=Homo sapiens GN=CA14 PE=1 SV=1
MLFSALLLEVIWILAADGGQHWTYEGPHGQDHWPASYPECGNNAQSPIDIQTDSVTFDPD

LPALQPHGYDQPGTEPLDLHNNGHTVQLSLPSTLYLGGLPRKYVAAQLHLHWGQKGSPGG
SEHQINSEATFAELHIVHYDSDSYDSLSEAAERPQGLAVLGILIEVGETKNIAYEHILSH
LHEVRHKDQKTSVPPFNLRELLPKQLGQYFRYNGSLTTPPCYQSVLWTVFYRRSQISMEQ
LEKLQGTLFSTEEEPSKLLVQNYRALQPLNQRMVFASFIQAGSSYTTGEMLSLGVGILVG
CLCLLLAVYFIARKIRKKRLENRKSVVFTSAQATTEA
>sp|P00915|CAH1_HUMAN Carbonic anhydrase 1 OS=Homo sapiens GN=CA1 PE=1 SV=2
MASPDWGYDDKNGPEQWSKLYPIANGNNQSPVDIKTSETKHDTSLKPISVSYNPATAKEI
INVGHSFHVNFEDNDNRSVLKGGPFSDSYRLFQFHFHWGSTNEHGSEHTVDGVKYSAELH
VAHWNSAKYSSLAEAASKADGLAVIGVLMKVGEANPKLQKVLDALQAIKTKGKRAPFTNF
DPSTLLPSSLDFWTYPGSLTHPPLYESVTWIICKESISVSSEQLAQFRSLLSNVEGDNAV
PMQHNNRPTQPLKGRTVRASF
>sp|P00918|CAH2_HUMAN Carbonic anhydrase 2 OS=Homo sapiens GN=CA2 PE=1 SV=2
MSHHWGYGKHNGPEHWHKDFPIAKGERQSPVDIDTHTAKYDPSLKPLSVSYDQATSLRIL
NNGHAFNVEFDDSQDKAVLKGGPLDGTYRLIQFHFHWGSLDGQGSEHTVDKKKYAAELHL
VHWNTKYGDFGKAVQQPDGLAVLGIFLKVGSAKPGLQKVVDVLDSIKTKGKSADFTNFDP
RGLLPESLDYWTYPGSLTTPPLLECVTWIVLKEPISVSSEQVLKFRKLNFNGEGEPEELM
VDNWRPAQPLKNRQIKASFK
>sp|P35219|CAH8_HUMAN Carbonic anhydrase-related protein OS=Homo sapiens GN=CA8 PE=1 SV=3
MADLSFIEDTVAFPEKEEDEEEEEEGVEWGYEEGVEWGLVFPDANGEYQSPINLNSREAR
YDPSLLDVRLSPNYVVCRDCEVTNDGHTIQVILKSKSVLSGGPLPQGHEFELYEVRFHWG
RENQRGSEHTVNFKAFPMELHLIHWNSTLFGSIDEAVGKPHGIAIIALFVQIGKEHVGLK
AVTEILQDIQYKGKSKTIPCFNPNTLLPDPLLRDYWVYEGSLTIPPCSEGVTWILFRYPL
TISQLQIEEFRRLRTHVKGAELVEGCDGILGDNFRPTQPLSDRVIRAAFQ
>sp|P22676|CALB2_HUMAN Calretinin OS=Homo sapiens GN=CALB2 PE=2 SV=2
MAGPQQQPPYLHLAELTASQFLEIWKHFDADGNGYIEGKELENFFQELEKARKGSGMMSK
SDNFGEKMKEFMQKYDKNSDGKIEMAELAQILPTEENFLLCFRQHVGSSAEFMEAWRKYD
TDRSGYIEANELKGFLSDLLKKANRPYDEPKLQEYTQTILRMFDLNGDGKLGLSEMSRLL
PVQENFLLKFQGMKLTSEEFNAIFTFYDKDRSGYIDEHELDALLKDLYEKNKKEMNIQQL
TNYRKSVMSLAEAGKLYRKDLEIVLCSEPPM
>sp|Q13939|CALI_HUMAN Calicin OS=Homo sapiens GN=CCIN PE=2 SV=3
MKLEFTEKNYNSFVLQNLNRQRKRKEYWDMALSVDNHVFFAHRNVLAAVSPLVRSLISSN
DMKTADELFITIDTSYLSPVTVDQLLDYFYSGKVVISEQNVEELLRGAQYFNTPRLRVHC
NDFLIKSICRANCLRYLFLAELFELKEVSDVAYSGIRDNFHYWASPEGSMHFMRCPPVIF
GRLLRDENLHVLNEDQALSALINWVYFRKEDREKYFKKFFNYINLNAVSNKTLVFASNKL
VGMENTSSHTTLIESVLMDRKQERPCSLLVYQRKGALLDSVVILGGQKAHGQFNDGVFAY
IIQENLWMKLSDMPYRAAALSATSAGRYIYISGGTTEQISGLKTAWRYDMDDNSWTKLPD
LPIGLVFHTMVTCGGTVYSVGGSIAPRRYVSNIYRYDERKEVWCLAGKMSIPMDGTAVIT
KGDRHLYIVTGRCLVKGYISRVGVVDCFDTSTGDVVQCITFPIEFNHRPLLSFQQDNILC
VHSHRQSVEINLQKVKASKTTTSVPVLPNSCPLDVSHAICSIGDSKVFVCGGVTTASDVQ
TKDYTINPNAFLLDQKTGKWKTLAPPPEALDCPACCLAKLPCKILQRI
>sp|Q96GE6|CALL4_HUMAN Calmodulin-like protein 4 OS=Homo sapiens GN=CALML4 PE=2 SV=3
MAAEHLLPGPPPSLADFRLEAGGKGTERGSGSSKPTGSSRGPRMAKFLSQDQINEYKECF
SLYDKQQRGKIKATDLMVAMRCLGASPTPGEVQRHLQTHGIDGNGELDFSTFLTIMHMQI
KQEDPKKEILLAMLMVDKEKKGYVMASDLRSKLTSLGEKLTHKEVDDLFREADIEPNGKV

KYDEFIHKITLPGRDY
>sp|Q8TD86|CALL6_HUMAN Calmodulin-like protein 6 OS=Homo sapiens GN=CALML6 PE=2 SV=2
MGLQQEISLQPWCHHPAESCQTTTDMTERLSAEQIKEYKGVFEMFDEEGNGEVKTGELEW
LMSLLGINPTKSELASMAKDVDRDNKGFFNCDGFLALMGVYHEKAQNQESELRAAFRVFD
KEGKGYIDWNTLKYVLMNAGEPLNEVEAEQMMKEADKDGDRTIDYEEFVAMMTGESFKLI
Q
>sp|P27797|CALR_HUMAN Calreticulin OS=Homo sapiens GN=CALR PE=1 SV=1
MLLSVPLLLGLLGLAVAEPAVYFKEQFLDGDGWTSRWIESKHKSDFGKFVLSSGKFYGDE
EKDKGLQTSQDARFYALSASFEPFSNKGQTLVVQFTVKHEQNIDCGGGYVKLFPNSLDQT
DMHGDSEYNIMFGPDICGPGTKKVHVIFNYKGKNVLINKDIRCKDDEFTHLYTLIVRPDN
TYEVKIDNSQVESGSLEDDWDFLPPKKIKDPDASKPEDWDERAKIDDPTDSKPEDWDKPE
HIPDPDAKKPEDWDEEMDGEWEPPVIQNPEYKGEWKPRQIDNPDYKGTWIHPEIDNPEYS
PDPSIYAYDNFGVLGLDLWQVKSGTIFDNFLITNDEAYAEEFGNETWGVTKAAEKQMKDK
QDEEQRLKEEEEDKKRKEEEEAEDKEDDEDKDEDEEDEEDKEEDEEEDVPGQAKDEL
>sp|Q9P1Y5|CAMP3_HUMAN Calmodulin-regulated spectrin-associated protein 3 OS=Homo sapiens GN=CAMSAP3 PE=1 SV=2
MVEAAPPGPGPLRRTFLVPEIKSLDQYDFSRAKAAASLAWVLRAAFGGAEHVPPELWEPF
YTDQYAQEHVKPPVTRLLLSAELYCRAWRQALPQLETPPNPSALLALLARRGTVPALPER
PVREADLRHQPILMGAHLAVIDALMAAFAFEWTKTLPGPLALTSLEHKLLFWVDTTVRRL
QEKTEQEAAQRASPAAPADGAAPAQPSIRYRKDRVVARRAPCFPTVTSLQDLASGAALAA
TIHCYCPQLLRLEEVCLKDPMSVADSLYNLQLVQDFCASRLPRGCPLSLEDLLYVPPPLK
VNLVVMLAELFMCFEVLKPDFVQVKDLPDGHAASPRGTEASPPQNNSGSSSPVFTFRHPL
LSSGGPQSPLRGSTGSLKSSPSMSHMEALGKAWNRQLSRPLSQAVSFSTPFGLDSDVDVV
MGDPVLLRSVSSDSLGPPRPAPARTPTQPPPEPGDLPTIEEALQIIHSAEPRLLPDGAAD
GSFYLHSPEGPSKPSLASPYLPEGTSKPLSDRPTKAPVYMPHPETPSKPSPCLVGEASKP
PAPSEGSPKAVASSPAATNSEVKMTSFAERKKQLVKAEAEAGAGSPTSTPAPPEALSSEM
SELSARLEEKRRAIEAQKRRIEAIFAKHRQRLGKSAFLQVQPREASGEAEAEAEEADSGP
VPGGERPAGEGQGEPTSRPKAVTFSPDLGPVPHEGLGEYNRAVSKLSAALSSLQRDMQRL
TDQQQRLLAPPEAPGSAPPPAAWVIPGPTTGPKAASPSPARRVPATRRSPGPGPSQSPRS
PKHTRPAELRLAPLTRVLTPPHDVDSLPHLRKFSPSQVPVQTRSSILLAEETPPEEPAAR
PGLIEIPLGSLADPAAEDEGDGSPAGAEDSLEEEASSEGEPRVGLGFFYKDEDKPEDEMA
QKRASLLERQQRRAEEARRRKQWQEVEKEQRREEAARLAQEEAPGPAPLVSAVPMATPAP
AARAPAEEEVGPRKGDFTRQEYERRAQLKLMDDLDKVLRPRAAGSGGPGRGGRRATRPRS
GCCDDSALARSPARGLLGSRLSKIYSQSTLSLSTVANEAHNNLGVKRPTSRAPSPSGLMS
PSRLPGSRERDWENGSNASSPASVPEYTGPRLYKEPSAKSNKFIIHNALSHCCLAGKVNE
PQKNRILEEIEKSKANHFLILFRDSSCQFRALYTLSGETEELSRLAGYGPRTVTPAMVEG
IYKYNSDRKRFTQIPAKTMSMSVDAFTIQGHLWQGKKPTTPKKGGGTPK
>sp|P17655|CAN2_HUMAN Calpain-2 catalytic subunit OS=Homo sapiens GN=CAPN2 PE=1 SV=6
MAGIAAKLAKDREAAEGLGSHDRAIKYLNQDYEALRNECLEAGTLFQDPSFPAIPSALGF
KELGPYSSKTRGIEWKRPTEICADPQFIIGGATRTDICQGALGDCWLLAAIASLTLNEEI
LARVVPLNQSFQENYAGIFHFQFWQYGEWVEVVVDDRLPTKDGELLFVHSAEGSEFWSAL
LEKAYAKINGCYEALSGGATTEGFEDFTGGIAEWYELKKPPPNLFKIIQKALQKGSLLGC
SIDITSAADSEAITFQKLVKGHAYSVTGAEEVESNGSLQKLIRIRNPWGEVEWTGRWNDN
CPSWNTIDPEERERLTRRHEDGEFWMSFSDFLRHYSRLEICNLTPDTLTSDTYKKWKLTK

MDGNWRRGSTAGGCRNYPNTFWMNPQYLIKLEEEDEDEEDGESGCTFLVGLIQKHRRRQR
KMGEDMHTIGFGIYEVPEELSGQTNIHLSKNFFLTNRARERSDTFINLREVLNRFKLPPG
EYILVPSTFEPNKDGDFCIRVFSEKKADYQAVDDEIEANLEEFDISEDDIDDGFRRLFAQ
LAGEDAEISAFELQTILRRVLAKRQDIKSDGFSIETCKIMVDMLDSDGSGKLGLKEFYIL
WTKIQKYQKIYREIDVDRSGTMNSYEMRKALEEAGFKMPCQLHQVIVARFADDQLIIDFD
NFVRCLVRLETLFKIFKQLDPENTGTIELDLISWLCFSVL
>sp|O15484|CAN5_HUMAN Calpain-5 OS=Homo sapiens GN=CAPN5 PE=1 SV=2
MFSCVKPYEDQNYSALRRDCRRRKVLFEDPLFPATDDSLYYKGTPGPAVRWKRPKGICED
PRLFVDGISSHDLHQGQVGNCWFVAACSSLASRESLWQKVIPDWKEQEWDPEKPNAYAGI
FHFHFWRFGEWVDVVIDDRLPTVNNQLIYCHSNSRNEFWCALVEKAYAKLAGCYQALDGG
NTADALVDFTGGVSEPIDLTEGDFANDETKRNQLFERMLKVHSRGGLISASIKAVTAADM
EARLACGLVKGHAYAVTDVRKVRLGHGLLAFFKSEKLDMIRLRNPWGEREWNGPWSDTSE
EWQKVSKSEREKMGVTVQDDGEFWMTFEDVCRYFTDIIKCRVINTSHLSIHKTWEEARLH
GAWTLHEDPRQNRGGGCINHKDTFFQNPQYIFEVKKPEDEVLICIQQRPKRSTRREGKGE
NLAIGFDIYKVEENRQYRMHSLQHKAASSIYINSRSVFLRTDQPEGRYVIIPTTFEPGHT
GEFLLRVFTDVPSNCRELRLDEPPHTCWSSLCGYPQLVTQVHVLGAAGLKDSPTGANSYV
IIKCEGDKVRSAVQKGTSTPEYNVKGIFYRKKLSQPITVQVWNHRVLKDEFLGQVHLKAD
PDNLQALHTLHLRDRNSRQPSNLPGTVAVHILSSTSLMAV
>sp|Q6IMN6|CAPR2_HUMAN Caprin-2 OS=Homo sapiens GN=CAPRIN2 PE=1 SV=1
MEVQVSQASLGFELTSVEKSLREWSRLSREVIAWLCPSSPNFILNFPPPPSASSVSMVQL
FSSPFGYQSPSGHSEEEREGNMKSAKPQVNHSQHGESQRALSPLQSTLSSAASPSQAYET
YIENGLICLKHKIRNIEKKKLKLEDYKDRLKSGEHLNPDQLEAVEKYEEVLHNLEFAKEL
QKTFSGLSLDLLKAQKKAQRREHMLKLEAEKKKLRTILQVQYVLQNLTQEHVQKDFKGGL
NGAVYLPSKELDYLIKFSKLTCPERNESLSVEDQMEQSSLYFWDLLEGSEKAVVGTTYKH
LKDLLSKLLNSGYFESIPVPKNAKEKEVPLEEEMLIQSEKKTQLSKTESVKESESLMEFA
QPEIQPQEFLNRRYMTEVDYSNKQGEEQPWEADYARKPNLPKRWDMLTEPDGQEKKQESF
KSWEASGKHQEVSKPAVSLEQRKQDTSKLRSTLPEEQKKQEISKSKPSPSQWKQDTPKSK
AGYVQEEQKKQETPKLWPVQLQKEQDPKKQTPKSWTPSMQSEQNTTKSWTTPMCEEQDSK
QPETPKSWENNVESQKHSLTSQSQISPKSWGVATASLIPNDQLLPRKLNTEPKDVPKPVH
QPVGSSSTLPKDPVLRKEKLQDLMTQIQGTCNFMQESVLDFDKPSSAIPTSQPPSATPGS
PVASKEQNLSSQSDFLQEPLQATSSPVTCSSNACLVTTDQASSGSETEFMTSETPEAAIP
PGKQPSSLASPNPPMAKGSEQGFQSPPASSSSVTINTAPFQAMQTVFNVNAPLPPRKEQE
IKESPYSPGYNQSFTTASTQTPPQCQLPSIHVEQTVHSQETAANYHPDGTIQVSNGSLAF
YPAQTNVFPRPTQPFVNSRGSVRGCTRGGRLITNSYRSPGGYKGFDTYRGLPSISNGNYS
QLQFQAREYSGAPYSQRDNFQQCYKRGGTSGGPRANSRAGWSDSSQVSSPERDNETFNSG
DSGQGDSRSMTPVDVPVTNPAATILPVHVYPLPQQMRVAFSAARTSNLAPGTLDQPIVFD
LLLNNLGETFDLQLGRFNCPVNGTYVFIFHMLKLAVNVPLYVNLMKNEEVLVSAYANDGA
PDHETASNHAILQLFQGDQIWLRLHRGAIYGSSWKYSTFSGYLLYQD
>sp|Q86UW7|CAPS2_HUMAN Calcium-dependent secretion activator 2 OS=Homo sapiens GN=CADPS2 PE=1 SV=2
MLDPSSSEEESDEGLEEESRDVLVAAGSSQRAPPAPTREGRRDAPGRAGGGGAARSVSPS
PSVLSEGRDEPQRQLDDEQERRIRLQLYVFVVRCIAYPFNAKQPTDMARRQQKLNKQQLQ
LLKERFQAFLNGETQIVADEAFCNAVRSYYEVFLKSDRVARMVQSGGCSANDFREVFKKN
IEKRVRSLPEIDGLSKETVLSSWIAKYDAIYRGEEDLCKQPNRMALSAVSELILSKEQLY

EMFQQILGIKKLEHQLLYNACQLDNADEQAAQIRRELDGRLQLADKMAKERKFPKFIAKD
MENMYIEELRSSVNLLMANLESLPVSKGGPEFKLQKLKRSQNSAFLDIGDENEIQLSKSD
VVLSFTLEIVIMEVQGLKSVAPNRIVYCTMEVEGEKLQTDQAEASRPQWGTQGDFTTTHP
RPVVKVKLFTESTGVLALEDKELGRVILYPTSNSSKSAELHRMVVPKNSQDSDLKIKLAV
RMDKPAHMKHSGYLYALGQKVWKRWKKRYFVLVQVSQYTFAMCSYREKKSEPQELMQLEG
YTVDYTDPHPGLQGGCMFFNAVKEGDTVIFASDDEQDRILWVQAMYRATGQSYKPVPAIQ
TQKLNPKGGTLHADAQLSGKDADRFQKHGMDEFISANPCKLDHAFLFRILQRQTLDHRLN
DSYSCLGWFSPGQVFVLDEYCARYGVRGCHRHLCYLAELMEHSENGAVIDPTLLHYSFAF
CASHVHGNRPDGIGTVSVEEKERFEEIKERLSSLLENQISHFRYCFPFGRPEGALKATLS
LLERVLMKDIATPIPAEEVKKVVRKCLEKAALINYTRLTEYAKIEETMNQASPARKLEEI
LHLAELCIEVLQQNEEHHAEGREAFAWWPDLLAEHAEKFWALFTVDMDTALEAQPQDSWD
SFPLFQLLNNFLRNDTLLCNGKFHKHLQEIFVPLVVRYVDLMESSIAQSIHRGFEQETWQ
PVKNIANSLPNVALPKVPSLPLNLPQIPNISTASWMPSLYESTNGSATSEDLFWKLDALQ
MFVFDLHWPEQEFAHHLEQRLKLMASDMLEACVKRTRTAFELKLQKASKTTDLRIPASVC
TMFNVLVDAKKQSTKLCALDGGQEQQYHSKIDDLIDNSVKEIISLLVSKFVSVLEGVLSK
LSRYDEGTFFSSILSFTVKAAAKYVDVPKPGMDLADTYIMFVRQNQDILREKVNEEMYIE
KLFDQWYSSSMKVICVWLTDRLDLQLHIYQLKTLIKIVKKTYRDFRLQGVLEGTLNSKTY
DTVHRRLTVEEATASVSEGGGLQGITMKDSDEEEEG
>sp|Q8WWF8|CAPSL_HUMAN Calcyphosin-like protein OS=Homo sapiens GN=CAPSL PE=2 SV=4
MAGTARHDREMAIQAKKKLTTATDPIERLRLQCLARGSAGIKGLGRVFRIMDDDNNRTLD
FKEFMKGLNDYAVVMEKEEVEELFRRFDKDGNGTIDFNEFLLTLRPPMSRARKEVIMQAF
RKLDKTGDGVITIEDLREVYNAKHHPKYQNGEWSEEQVFRKFLDNFDSPYDKDGLVTPEE
FMNYYAGVSASIDTDVYFIIMMRTAWKL
>sp|Q9BXL6|CAR14_HUMAN Caspase recruitment domain-containing protein 14 OS=Homo sapiens GN=CARD14 PE=1 SV=2
MGELCRRDSALTALDEETLWEMMESHRHRIVRCICPSRLTPYLRQAKVLCQLDEEEVLHS
PRLTNSAMRAGHLLDLLKTRGKNGAIAFLESLKFHNPDVYTLVTGLQPDVDFSNFSGLME
TSKLTECLAGAIGSLQEELNQEKGQKEVLLRRCQQLQEHLGLAETRAEGLHQLEADHSRM
KREVSAHFHEVLRLKDEMLSLSLHYSNALQEKELAASRCRSLQEELYLLKQELQRANMVS
SCELELQEQSLRTASDQESGDEELNRLKEENEKLRSLTFSLAEKDILEQSLDEARGSRQE
LVERIHSLRERAVAAERQREQYWEEKEQTLLQFQKSKMACQLYREKVNALQAQVCELQKE
RDQAYSARDSAQREISQSLVEKDSLRRQVFELTDQVCELRTQLRQLQAEPPGVLKQEART
REPCPREKQRLVRMHAICPRDDSDCSLVSSTESQLLSDLSATSSRELVDSFRSSSPAPPS
QQSLYKRVAEDFGEEPWSFSSCLEIPEGDPGALPGAKAGDPHLDYELLDTADLPQLESSL
QPVSPGRLDVSESGVLMRRRPARRILSQVTMLAFQGDALLEQISVIGGNLTGIFIHRVTP
GSAADQMALRPGTQIVMVDYEASEPLFKAVLEDTTLEEAVGLLRRVDGFCCLSVKVNTDG
YKRLLQDLEAKVATSGDSFYIRVNLAMEGRAKGELQVHCNEVLHVTDTMFQGCGCWHAHR
VNSYTMKDTAAHGTIPNYSRAQQQLIALIQDMTQQCTVTRKPSSGGPQKLVRIVSMDKAK
ASPLRLSFDRGQLDPSRMEGSSTCFWAESCLTLVPYTLVRPHRPARPRPVLLVPRAVGKI
LSEKLCLLQGFKKCLAEYLSQEEYEAWSQRGDIIQEGEVSGGRCWVTRHAVESLMEKNTH
ALLDVQLDSVCTLHRMDIFPIVIHVSVNEKMAKKLKKGLQRLGTSEEQLLEAARQEEGDL
DRAPCLYSSLAPDGWSDLDGLLSCVRQAIADEQKKVVWTEQSPR
>sp|Q9BX69|CARD6_HUMAN Caspase recruitment domain-containing protein 6 OS=Homo sapiens GN=CARD6 PE=1 SV=2

MATESTPSEIIERERKKLLEILQHDPDSILDTLTSRRLISEEEYETLENVTDLLKKSRKL
LILVQKKGEATCQHFLKCLFSTFPQSAAICGLRHEVLKHENTVPPQSMGASSNSEDAFSP
GIKQPEAPEITVFFSEKEHLDLETSEFFRDKKTSYRETALSARKNEKEYDTPEVTLSYSV
EKVGCEVPATITYIKDGQRYEELDDSLYLGKEEYLGSVDTPEDAEATVEEEVYDDPEHVG
YDGEEDFENSETTEFSGEEPSYEGSETSLSLEEEQEKSIEERKKVFKDVLLCLNMDRSRK
VLPDFVKQFSLDRGCKWTPESPGDLAWNFLMKVQARDVTARDSILSHKVLDEDSKEDLLA
GVENLEIRDIQTINPLDVLCATMLCSDSSLQRQVMSNMYQCQFALPLLLPDAENNKSILM
LGAMKDIVKKQSTQFSGGPTEDTEKFLTLMKMPVISFVRLGYCSFSKSRILNTLLSPAQL
KLHKIFLHQDLPLLVLPRQISDGLVEITWCFPDSDDRKENPFFQKPVALANLRGNLESFW
TQFGFLMEVSSAVFFFTDCLGEKEWDLLMFLGEAAIERCYFVLSSQARESEEAQIFQRIL
NLKPAQLLFWERGDAGDRRKNMEGLQAALQEVMFSSCLRCVSVEDMAALARELGIQVDED
FENTQRIQVSSGENMAGTAEGEGQQRHSQLKSSSKSQALMPIQEPGTQCELSQNLQNLYG
TPVFRPVLENSWLFPTRIGGNFNHVSLKASWVMGRPFGSEQRPKWFHPLPFQNAGAQGRG
KSFGIQSFHPQIFYSGERFMKFSRVARGCHSNGTFGRLPRPICQHVQACPERPQMMGTLE
RSRAVASKIGHSYSLDSQPARAVGKPWPQQACTRVTELTEATGKLIRTSHIGKPHPQSFQ
PAAATQKLRPASQQGVQMKTQGGASNPALQIGSHPMCKSSQFKSDQSNPSTVKHSQPKPF
HSVPSQPKSSQTKSCQSQPSQTKPSPCKSTQPKPSQPWPPQSKPSQPRPPQPKSSSTNPS
QAKAHHSKAGQKRGGKH
>sp|Q9NXV6|CARF_HUMAN CDKN2A-interacting protein OS=Homo sapiens GN=CDKN2AIP PE=1 SV=3
MAQEVSEYLSQNPRVAAWVEALRCDGETDKHWRHRRDFLLRNAGDLAPAGGAASASTDEA
ADAESGTRNRQLQQLISFSMAWANHVFLGCRYPQKVMDKILSMAEGIKVTDAPTYTTRDE
LVAKVKKRGISSSNEGVEEPSKKRVIEGKNSSAVEQDHAKTSAKTERASAQQENSSTCIG
SAIKSESGNSARSSGISSQNSSTSDGDRSVSSQSSSSVSSQVTTAGSGKASEAEAPDKHG
SASFVSLLKSSVNSHMTQSTDSRQQSGSPKKSALEGSSASASQSSSEIEVPLLGSSGSSE
VELPLLSSKPSSETASSGLTSKTSSEASVSSSVAKNSSSSGTSLLTPKSSSSTNTSLLTS
KSTSQVAASLLASKSSSQTSGSLVSKSTSLASVSQLASKSSSQTSTSQLPSKSTSQSSES
SVKFSCKLTNEDVKQKQPFFNRLYKTVAWKLVAVGGFSPNVNHGELLNAAIEALKATLDV
FFVPLKELADLPQNKSSQESIVCELRCKSVYLGTGCGKSKENAKAVASREALKLFLKKKV
VVKICKRKYRGSEIEDLVLLDEESRPVNLPPALKHPQELL
>sp|Q5VZK9|CARL1_HUMAN F-actin-uncapping protein LRRC16A OS=Homo sapiens GN=CARMIL1 PE=1
SV=1
MTEESSDVPRELIESIKDVIGRKIKISVKKKVKLEVKGDKVENKVLVLTSCRAFLVTARI
PTKLELTFSYLEIHGVVCSKSAQMIVETEKCSISMKMASPEDVSEVLAHIGTCLRKIFPG
LSPVRIMKKVSMEPSERLASLQALWDSQTVAEQGPCGGFSQMYACVCDWLGFSYREEVQW
DVDTIYLTQDTRELNLQDFSHLDHRDLIPIIAALEYNQWFTKLSSKDLKLSTDVCEQILR
VVSRSNRLEELVLENAGLRTDFAQKLASALAHNPNSGLHTINLAGNPLEDRGVSSLSIQF
AKLPKGLKHLNLSKTSLSPKGVNSLSQSLSANPLTASTLVHLDLSGNVLRGDDLSHMYNF
LAQPNAIVHLDLSNTECSLDMVCGALLRGCLQYLAVLNLSRTVFSHRKGKEVPPSFKQFF
SSSLALMHINLSGTKLSPEPLKALLLGLACNHNLKGVSLDLSNCELRSGGAQVLEGCIAE
IHNITSLDISDNGLESDLSTLIVWLSKNRSIQHLALGKNFNNMKSKNLTPVLDNLVQMIQ
DEESPLQSLSLADSKLKTEVTIIINALGSNTSLTKVDISGNGMGDMGAKMLAKALQINTK
LRTVIWDKNNITAQGFQDIAVAMEKNYTLRFMPIPMYDASQALKTNPEKTEDALQKIENY
LLRNHETRKYLQEQAYRLQQGIVTSTTQQMIDRICVKVQDHLNSLRNCGGDAIQEDLKSA
ERLMRDAKNSKTLLPNLYHVGGASWAGASGLLSSPIQETLESMAGEVTRVVDEQLKALLE

SMVDAAENLCPNVMKKAHIRQDLIHASTEKISIPRTFVKNVLLEQSGIDILNKISEVKLT
VASFLSDRIVDEILDALSHCHHKLADHFSRRGKTLPQQESLEIELAEEKPVKRSIITVEE
LTEIERLEDLDTCMMTPKSKRKSIHSRMLRPVSRAFEMEFDLDKALEEVPIHIEDPPFPS
LRQEKRSSGFISELPSEEGKKLEHFTKLRPKRNKKQQPTQAAVCAANIVSQDGEQNGLMG
RVDEGVDEFFTKKVTKMDSKKWSTRGSESHELNEGGDEKKKRDSRKSSGFLNLIKSRSKS
ERPPTILMTEEPSSPKGAVRSPPVDCPRKDTKAAEHNGNSERIEEIKTPDSFEESQGEEI
GKVERSDSKSSPQAGRRYGVQVMGSGLLAEMKAKQEKRAACAQKKLGNDAVSQDSSSPAL
SGVERSDGGGAVPKLHPGLPENRFGLGTPEKNTKAEPKAEAGSRSRSSSSTPTSPKPLLQ
SPKPSLAARPVIPQKPRTASRPDDIPDSPSSPKVALLPPVLKKVPSDKERDGQSSPQPSP
RTFSQEVSRRSWGQQAQEYQEQKQRSSSKDGHQGSKSNDSGEEAEKEFIFV
>sp|Q6F5E8|CARL2_HUMAN Capping protein, Arp2/3 and myosin-I linker protein 2 OS=Homo sapiens GN=CARMIL2 PE=1 SV=2
MAQTPDGISCELRGEITRFLWPKEVELLLKTWLPGEGAVQNHVLALLRWRAYLLHTTCLP
LRVDCTFSYLEVQAMALQETPPQVTFELESLRELVLEFPGVAALEQLAQHVAAAIKKVFP
RSTLGKLFRRPTPASMLARLERSSPSESTDPCSPCGGFLETYEALCDYNGFPFREEIQWD
VDTIYHRQGCRHFSLGDFSHLGSRDLALSVAALSYNLWFRCLSCVDMKLSLEVSEQILHM
MSQSSHLEELVLETCSLRGDFVRRLAQALAGHSSSGLRELSLAGNLLDDRGMTALSRHLE
RCPGALRRLSLAQTGLTPRGMRALGRALATNAAFDSTLTHLDLSGNPGALGASEDSGGLY
SFLSRPNVLSFLNLAGTDTALDTVRGCSVGGWMTGRADWRAGRGGLGPPAGVANSLPPQL
FAAVSRGCCTSLTHLDASRNVFSRTKSRAAPAALQLFLSRARTLRHLGLAGCKLPPDALR
ALLDGLALNTHLRDLHLDLSACELRSAGAQVIQDLVCDAGAVSSLDLADNGFGSDMVTLV
LAIGRSRSLRHVALGRNFNVRCKETLDDVLHRIVQLMQDDDCPLQSLSVAESRLKLGASV
LLRALATNPNLTALDISGNAMGDAGAKLLAKALRVNSRLRSVVWDRNHTSALGLLDVAQA
LEQNHSLKAMPLPLNDVAQAQRSRPELTARAVHQIQACLLRNNRADPASSDHTTRLQPLG
LVSDPSEQEVNELCQSVQEHVELLGCGAGPQGEAAVRQAEDAIQNANFSLSILPILYEAG
SSPSHHWQLGQKLEGLLRQVGEVCRQDIQDFTQATLDTARSLCPQMLQGSSWREQLEGVL
AGSRGLPELLPEQLLQDAFTRLRDMRLSITGTLAESIVAQALAGLSAARDQLVESLAQQA
TVTMPPALPAPDGGEPSLLEPGELEGLFFPEEKEEEKEKDDSPPQKWPELSHGLHLVPFI
HSAAEEAEPEPELAAPGEDAEPQAGPSARGSPSPAAPGPPAGPLPRMDLPLAGQPLRHPT
RARPRPRRQHHHRPPPGGPQVPPALPQEGNGLSARVDEGVEEFFSKRLIQQDRLWAPEED
PATEGGATPVPRTLRKKLGTLFAFKKPRSTRGPRTDLETSPGAAPRTRKTTFGDLLRPPT
RPSRGEELGGAEGDTSSPDPAGRSRPRYTRDSKAYSMILLPAEEEATLGARPDKRRPLER
GETELAPSFEQRVQVMLQRIGVSRGSGGAEGKRKQSKDGEIKKAGSDGDIMDSSTEAPPI
SIKSRTHSVSADPSCRPGPGSQGPESATWKTLGQQLNAELRSRGWGQQDGPGPPSPGQSP
SPCRTSPSPDSLGLPEDPCLGPRNEDGQLRPRPLSAGRRAVSVHEDQLQAPAERPLRLQR
SPVLKRRPKLEAPPSPSLGSGLGTEPLPPQPTEPSSPERSPPSPATDQRGGGPNP
>sp|Q8ND23|CARL3_HUMAN Capping protein, Arp2/3 and myosin-I linker protein 3 OS=Homo sapiens GN=CARMIL3 PE=2 SV=2
MAKPSVELTRELQDSIRRCLSQGAVLQQHHVKLETKPKKFEDRVLALTSWRLHLFLLKVP
AKVESSFNVLEIRAFNTLSQNQILVETERGMVSMRLPSAESVDQVTRHVSSALSKVCPGP
GCLIRRGNADTPEGPRDTSPNSETSTSTTHSVCGGFSETYAALCDYNGLHCREEVQWDVD
TIYHAEDNREFNLLDFSHLESRDLALMVAALAYNQWFTKLYCKDLRLGSEVLEQVLHTLS
KSGSLEELVLDNAGLKTDFVQKLAGVFGENGSCVLHALTLSHNPIEDKGFLSLSQQLLCF
PSGLTKLCLAKTAISPRGLQALGQTFGANPAFASSLRYLDLSKNPGLLATDEANALYSFL

AQPNALVHLDLSGTDCVIDLLLGALLHGCCSHLTYLNLARNSCSHRKGREAPPAFKQFFS
SAYTLSHVNLSATKLPLEALRALLQGLSLNSHLSDLHLDLSSCELRSAGAQALQEQLGAV
TCVGSLDLSDNGFDSDLLTLVPALGKNKSLKHLFLGKNFNVKAKTLEEILHKLVQLIQEE
DCSLQSLSVADSRLKLRTSILINALGSNTCLAKVDLSGNGMEDIGAKMLSKALQINSSLR
TILWDRNNTSALGFLDIARALESNHTLRFMSFPVSDISQAYRSAPERTEDVWQKIQWCLV
RNNHSQTCPQEQAFRLQQGLVTSSAEQMLQRLCGRVQEEVRALRLCPLEPVQDELLYARD
LIKDAKNSRALFPSLYELGHVLANDGPVRQRLESVASEVSKAVDKELQVILESMVSLTQE
LCPVAMRVAEGHNKMLSNVAERVTVPRNFIRGALLEQAGQDIQNKLDEVKLSVVTYLTSS
IVDEILQELYHSHKSLARHLTQLRTLSDPPGCPGQGQDLSSRGRGRNHDHEETTDDELGT
NIDTMAIKKQKRCRKIRPVSAFISGSPQDMESQLGNLGIPPGWFSGLGGSQPTASGSWEG
LSELPTHGYKLRHQTQGRPRPPRTTPPGPGRPSMPAPGTRQENGMATRLDEGLEDFFSRR
VLEESSSYPRTLRTVRPGLSEAPLPPLQKKRRRGLFHFRRPRSFKGDRGPGSPTTGLLLP
PPPPPPPTQESPPSPDPPSLGNNSSPCWSPEEESSLLPGFGGGRGPSFRRKMGTEGSEPG
EGGPAPGTAQQPRVHGVALPGLERAKGWSFDGKREGPGPDQEGSTQAWQKRRSSDDAGPG
SWKPPPPPQSTKPSFSAMRRAEATWHIAEESAPNHSCQSPSPASQDGEEEKEGTLFPERT
LPARNAKLQDPALAPWPPKPVAVPRGRQPPQEPGVREEAEAGDAAPGVNKPRLRLSSQQD
QEEPEVQGPPDPGRRTAPLKPKRTRRAQSCDKLEPDRRRPPDPTGTSEPGTD
>sp|Q13490|BIRC2_HUMAN Baculoviral IAP repeat-containing protein 2 OS=Homo sapiens GN=BIRC2 PE=1 SV=2
MHKTASQRLFPGPSYQNIKSIMEDSTILSDWTNSNKQKMKYDFSCELYRMSTYSTFPAGV
PVSERSLARAGFYYTGVNDKVKCFCCGLMLDNWKLGDSPIQKHKQLYPSCSFIQNLVSAS
LGSTSKNTSPMRNSFAHSLSPTLEHSSLFSGSYSSLSPNPLNSRAVEDISSSRTNPYSYA
MSTEEARFLTYHMWPLTFLSPSELARAGFYYIGPGDRVACFACGGKLSNWEPKDDAMSEH
RRHFPNCPFLENSLETLRFSISNLSMQTHAARMRTFMYWPSSVPVQPEQLASAGFYYVGR
NDDVKCFCCDGGLRCWESGDDPWVEHAKWFPRCEFLIRMKGQEFVDEIQGRYPHLLEQLL
STSDTTGEENADPPIIHFGPGESSSEDAVMMNTPVVKSALEMGFNRDLVKQTVQSKILTT
GENYKTVNDIVSALLNAEDEKREEEKEKQAEEMASDDLSLIRKNRMALFQQLTCVLPILD
NLLKANVINKQEHDIIKQKTQIPLQARELIDTILVKGNAAANIFKNCLKEIDSTLYKNLF
VDKNMKYIPTEDVSGLSLEEQLRRLQEERTCKVCMDKEVSVVFIPCGHLVVCQECAPSLR
KCPICRGIIKGTVRTFLS
>sp|P78537|BL1S1_HUMAN Biogenesis of lysosome-related organelles complex 1 subunit 1 OS=Homo sapiens GN=BLOC1S1 PE=1 SV=2
MAPGSRGERSSFRSRRGPGVPSPQPDVTMLSRLLKEHQAKQNERKELQEKRRREAITAAT
CLTEALVDHLNVGVAQAYMNQRKLDHEVKTLQVQAAQFAKQTGQWIGMVENFNQALKEIG
DVENWARSIELDMRTIATALEYVYKGQLQSAPS
>sp|Q6QNY0|BL1S3_HUMAN Biogenesis of lysosome-related organelles complex 1 subunit 3 OS=Homo sapiens GN=BLOC1S3 PE=1 SV=1
MASQGRRRRPLRRPETVVPGEATETDSERSASSSEEEELYLGPSGPTRGRPTGLRVAGEA
AETDSEPEPEPEPTAAPRDLPPLVVQRESAEEAWGTEEAPAPAPARSLLQLRLAESQARL
DHDVAAAVSGVYRRAGRDVAALASRLAAAQAAGLAAAHSVRLARGDLCALAERLDIVAGC
RLLPDIRGVPGTEPEKDPGPRA
>sp|O95696|BRD1_HUMAN Bromodomain-containing protein 1 OS=Homo sapiens GN=BRD1 PE=1 SV=1
MRRKGRCHRGSAARHPSSPCSVKHSPTRETLTYAQAQRMVEIEIEGRLHRISIFDPLEII
LEDDLTAQEMSECNSNKENSERPPVCLRTKRHKNNRVKKKNEALPSAHGTPASASALPEP

KVRIVEYSPPSAPRRPPVYYKFIEKSAEELDNEVEYDMDEEDYAWLEIVNEKRKGDCVPA
VSQSMFEFLMDRFEKESHCENQKQGEQQSLIDEDAVCCICMDGECQNSNVILFCDMCNLA
VHQECYGVPYIPEGQWLCRHCLQSRARPADCVLCPNKGGAFKKTDDDRWGHVVCALWIPE
VGFANTVFIEPIDGVRNIPPARWKLTCYLCKQKGVGACIQCHKANCYTAFHVTCAQKAGL
YMKMEPVKELTGGGTTFSVRKTAYCDVHTPPGCTRRPLNIYGDVEMKNGVCRKESSVKTV
RSTSKVRKKAKKAKKALAEPCAVLPTVCAPYIPPQRLNRIANQVAIQRKKQFVERAHSYW
LLKRLSRNGAPLLRRLQSSLQSQRSSQQRENDEEMKAAKEKLKYWQRLRHDLERARLLIE
LLRKREKLKREQVKVEQVAMELRLTPLTVLLRSVLDQLQDKDPARIFAQPVSLKEVPDYL
DHIKHPMDFATMRKRLEAQGYKNLHEFEEDFDLIIDNCMKYNARDTVFYRAAVRLRDQGG
VVLRQARREVDSIGLEEASGMHLPERPAAAPRRPFSWEDVDRLLDPANRAHLGLEEQLRE
LLDMLDLTCAMKSSGSRSKRAKLLKKEIALLRNKLSQQHSQPLPTGPGLEGFEEDGAALG
PEAGEEVLPRLETLLQPRKRSRSTCGDSEVEEESPGKRLDAGLTNGFGGARSEQEPGGGL
GRKATPRRRCASESSISSSNSPLCDSSFNAPKCGRGKPALVRRHTLEDRSELISCIENGN
YAKAARIAAEVGQSSMWISTDAAASVLEPLKVVWAKCSGYPSYPALIIDPKMPRVPGHHN
GVTIPAPPLDVLKIGEHMQTKSDEKLFLVLFFDNKRSWQWLPKSKMVPLGIDETIDKLKM
MEGRNSSIRKAVRIAFDRAMNHLSRVHGEPTSDLSDID
>sp|O75150|BRE1B_HUMAN E3 ubiquitin-protein ligase BRE1B OS=Homo sapiens GN=RNF40 PE=1 SV=4
MSGPGNKRAAGDGGSGPPEKKLSREEKTTTTLIEPIRLGGISSTEEMDLKVLQFKNKKLA
ERLEQRQACEDELRERIEKLEKRQATDDATLLIVNRYWAQLDETVEALLRCHESQGELSS
APEAPGTQEGPTCDGTPLPEPGTSELRDPLLMQLRPPLSEPALAFVVALGASSSEEVELE
LQGRMEFSKAAVSRVVEASDRLQRRVEELCQRVYSRGDSEPLSEAAQAHTRELGRENRRL
QDLATQLQEKHHRISLEYSELQDKVTSAETKVLEMETTVEDLQWDIEKLRKREQKLNKHL
AEALEQLNSGYYVSGSSSGFQGGQITLSMQKFEMLNAELEENQELANSRMAELEKLQAEL
QGAVRTNERLKVALRSLPEEVVRETGEYRMLQAQFSLLYNESLQVKTQLDEARGLLLATK
NSHLRHIEHMESDELGLQKKLRTEVIQLEDTLAQVRKEYEMLRIEFEQNLAANEQAGPIN
REMRHLISSLQNHNHQLKGDAQRYKRKLREVQAEIGKLRAQASGSAHSTPNLGHPEDSGV
SAPAPGKEEGGPGPVSTPDNRKEMAPVPGTTTTTTSVKKEELVPSEEDFQGITPGAQGPS
SRGREPEARPKRELQEREGPSLGPPPVASALSRADREKAKVEETKRKESELLKGLRAELK
KAQESQKEMKLLLDMYKSAPKEQRDKVQLMAAERKAKAEVDELRSRIRELEERDRRESKK
IADEDALRRIRQAEEQIEHLQRKLGATKQEEEALLSEMDVTGQAFEDMQEQNGRLLQQLR
EKDDANFKLMSERIKANQIHKLLREEKDELGEQVLGLKSQVDAQLLTVQKLEEKERALQG
SLGGVEKELTLRSQALELNKRKAVEAAQLAEDLKVQLEHVQTRLREIQPCLAESRAAREK
ESFNLKRAQEDISRLRRKLEKQRKVEVYADADEILQEEIKEYKARLTCPCCNTRKKDAVL
TKCFHVFCFECVRGRYEARQRKCPKCNAAFGAHDFHRIYIS
>sp|Q5PSV4|BRM1L_HUMAN Breast cancer metastasis-suppressor 1-like protein OS=Homo sapiens GN=BRMS1L PE=1 SV=2
MPVHSRGDKKETNHHDEMEVDYAENEGSSSEDEDTESSSVSEDGDSSEMDDEDCERRRME
CLDEMSNLEKQFTDLKDQLYKERLSQVDAKLQEVIAGKAPEYLEPLATLQENMQIRTKVA
GIYRELCLESVKNKYECEIQASRQHCESEKLLLYDTVQSELEEKIRRLEEDRHSIDITSE
LWNDELQSRKKRKDPFSPDKKKPVVVSGPYIVYMLQDLDILEDWTTIRKAMATLGPHRVK
TEPPVKLEKHLHSARSEEGRLYYDGEWYIRGQTICIDKKDECPTSAVITTINHDEVWFKR
PDGSKSKLYISQLQKGKYSIKHS
>sp|Q96NH3|BROMI_HUMAN Protein broad-minded OS=Homo sapiens GN=TBC1D32 PE=2 SV=4

MAHFSSEDQAMLQAMLRRLFQSVKEKITGAPSLECAEEILLHLEETDENFHNYEFVKYLR
QHIGNTLGSMIEEEMEKCTSDRNQGEECGYDTVVQQVTKRTQESKEYKEMMHYLKNIMIA
VVESMINKFEEDETRNQERQKKIQKEKSHSYRTDNCSDSDSSLNQSYKFCQGKLQLILDQ
LDPGQPKEVRYEALQTLCSAPPSDVLNCENWTTLCEKLTVSLSDPDPVFSDRILKFCAQT
FLLSPLHMTKEIYTSLAKYLESYFLSRENHIPTLSAGVDITNPNMTRLLKKVRLLNEYQK
EAPSFWIRHPEKYMEEIVESTLSLLTVKHNQSHVVSQKILDPIYFFALVDTKAVWFKKWM
HAHYSRTTVLRLLETKYKSLVTTAIQQCVQYFEMCKTRKADETLGHSKHCRNKQKTFYYL
GQELQYIYFIHSLCLLGRLLIYKQGRKLFPIKLKNKKGLVSLIDLLVLFTQLIYYSPSCP
KMTSAAHSENYSPASMVTEVLWILSDQKECAVECLYNNIVIETLLQPIHNLMKGNEASPN
CSETALIHIAGILARIASVEEGLILLLYGANMNSSEESPTGAHIIAQFSKKLLDEDISIF
SGSEMLPVVKGAFISVCRHIYSTCEGLQVLITYNLHESIAKAWKKTSLLSERIPTPVEGS
DSVSSVSQESQNIMAWEDNLLDDLLHFAATPKGLLLLQRTGAINECVTFIFNRYAKKLQV
SRHKKFGYGVLVTRVASTAAGGIALKKSGFINELITELWSNLEYGRDDVRVTHPRTTPVD
PIDRSCQKSFLALVNLLSYPAIYELVRNQDLPNKTEYSLREVPTCVIDIIDRLIILNSEA
KIRSLFNYEQSHIFGLRDFIIDGLSVERNHVLVRINLVGGPLERILPPRLLEKSDNPYPW
PMFSSYPLPNCYLSDITRNAGIKQDNDLDKLLLCLKISDKQTEWIENCQRQFCKMMKAKP
DIISGEALIELLEKFVLHLTESPSECYFPSVEYTATDANVKNESLSSVQQLGIKMTVRYG
KFLSLLKDGAENDLTWVLKHCERFLKQQQTSIKSSLLCLQGNYAGHDWFVSSLFMIMLGD
KEKTFQFLHQFSRLLTSAFLWLPRLHISSYLPNDTVESGIHPVYFCSTHYIEMLLKAELP
LVFSAFHMSGFAPSQICLQWITQCFWNYLDWIEICHYIATCVFLGPDYQVYICIAVFKHL
QQDILQHTQTQDLQVFLKEEALHGFRVSDYFEYMEILEQNYRTVLLRDMRNIRLQST
>sp|Q8TDC3|BRSK1_HUMAN Serine/threonine-protein kinase BRSK1 OS=Homo sapiens GN=BRSK1 PE=1 SV=2
MSSGAKEGGGGSPAYHLPHPHPHPPQHAQYVGPYRLEKTLGKGQTGLVKLGVHCITGQKV
AIKIVNREKLSESVLMKVEREIAILKLIEHPHVLKLHDVYENKKYLYLVLEHVSGGELFD
YLVKKGRLTPKEARKFFRQIVSALDFCHSYSICHRDLKPENLLLDEKNNIRIADFGMASL
QVGDSLLETSCGSPHYACPEVIKGEKYDGRRADMWSCGVILFALLVGALPFDDDNLRQLL
EKVKRGVFHMPHFIPPDCQSLLRGMIEVEPEKRLSLEQIQKHPWYLGGKHEPDPCLEPAP
GRRVAMRSLPSNGELDPDVLESMASLGCFRDRERLHRELRSEEENQEKMIYYLLLDRKER
YPSCEDQDLPPRNDVDPPRKRVDSPMLSRHGKRRPERKSMEVLSITDAGGGGSPVPTRRA
LEMAQHSQRSRSVSGASTGLSSSPLSSPRSPVFSFSPEPGAGDEARGGGSPTSKTQTLPS
RGPRGGGAGEQPPPPSARSTPLPGPPGSPRSSGGTPLHSPLHTPRASPTGTPGTTPPPSP
GGGVGGAAWRSRLNSIRNSFLGSPRFHRRKMQVPTAEEMSSLTPESSPELAKRSWFGNFI
SLDKEEQIFLVLKDKPLSSIKADIVHAFLSIPSLSHSVLSQTSFRAEYKASGGPSVFQKP
VRFQVDISSSEGPEPSPRRDGSGGGGIYSVTFTLISGPSRRFKRVVETIQAQLLSTHDQP
SVQALADEKNGAQTRPAGAPPRSLQPPPGRPDPELSSSPRRGPPKDKKLLATNGTPLP
>sp|Q6UXE8|BTNL3_HUMAN Butyrophilin-like protein 3 OS=Homo sapiens GN=BTNL3 PE=2 SV=2
MAFVLILVLSFYELVSGQWQVTGPGKFVQALVGEDAVFSCSLFPETSAEAMEVRFFRNQF
HAVVHLYRDGEDWESKQMPQYRGRTEFVKDSIAGGRVSLRLKNITPSDIGLYGCWFSSQI
YDEEATWELRVAALGSLPLISIVGYVDGGIQLLCLSSGWFPQPTAKWKGPQGQDLSSDSR
ANADGYSLYDVEISIIVQENAGSILCSIHLAEQSHEVESKVLIGETFFQPSPWRLASILL
GLLCGALCGVVMGMIIVFFKSKGKIQAELDWRRKHGQAELRDARKHAVEVTLDPETAHPK
LCVSDLKTVTHRKAPQEVPHSEKRFTRKSVVASQGFQAGKHYWEVDVGQNVGWYVGVCRD
DVDRGKNNVTLSPNNGYWVLRLTTEHLYFTFNPHFISLPPSTPPTRVGVFLDYEGGTISF

FNTNDQSLIYTLLTCQFEGLLRPYIQHAMYDEEKGTPIFICPVSWG
>sp|O95971|BY55_HUMAN CD160 antigen OS=Homo sapiens GN=CD160 PE=1 SV=1
MLLEPGRGCCALAILLAIVDIQSGGCINITSSASQEGTRLNLICTVWHKKEEAEGFVVFL
CKDRSGDCSPETSLKQLRLKRDPGIDGVGEISSQLMFTISQVTPLHSGTYQCCARSQKSG
IRLQGHFFSILFTETGNYTVTGLKQRQHLEFSHNEGTLSSGFLQEKVWVMLVTSLVALQA
L
>sp|Q13895|BYST_HUMAN Bystin OS=Homo sapiens GN=BYSL PE=1 SV=3
MPKFKAARGVGGQEKHAPLADQILAGNAVRAGVREKRRGRGTGEAEEEYVGPRLSRRILQ
QARQQQEELEAEHGTGDKPAAPRERTTRLGPRMPQDGSDDEDEEWPTLEKAATMTAAGHH
AEVVVDPEDERAIEMFMNKNPPARRTLADIIMEKLTEKQTEVETVMSEVSGFPMPQLDPR
VLEVYRGVREVLSKYRSGKLPKAFKIIPALSNWEQILYVTEPEAWTAAAMYQATRIFASN
LKERMAQRFYNLVLLPRVRDDVAEYKRLNFHLYMALKKALFKPGAWFKGILIPLCESGTC
TLREAIIVGSIITKCSIPVLHSSAAMLKIAEMEYSGANSIFLRLLLDKKYALPYRVLDAL
VFHFLGFRTEKRELPVLWHQCLLTLVQRYKADLATDQKEALLELLRLQPHPQLSPEIRRE
LQSAVPRDVEDVPITVE
>sp|Q96A19|C102A_HUMAN Coiled-coil domain-containing protein 102A OS=Homo sapiens GN=CCDC102A PE=1 SV=2
MSHGPSPRLAESPQLSKGSLLTILGSPSPERMGPADSLPPTPPSGTPSPGPPPALPLPPA
PALLADGDWESREELRLRELEEARARAAQMEKTMRRWSDCTANWREKWSKVRAERNRARE
EVRQLRQRLDALTKELAGARRERQEAQGECEARGRELARLRGARGVADQTRDGPEPEAER
EPVRDVGSERPPGSQELELVESLLKSMPEESEDCWEARSLGAGGPRGSSGRQERSRLPWE
DTAATEEEASKLTALRLRLDESQKVLLKEREDKLALSRNIEKLEGELSQWKIKYEELSKT
KQEMLKQLSILKEAHQDELGRMSEDLEDELGARSSMDRKMAELRGEMERLQAENAAEWGR
RERLETEKLGLERENKKLRAQVGDLEEALARRRRQTASALDCDLRASQAALFEKNKELAD
LKHVHGKLKKQFQEKVAELAHANRRVEQHEAEVKKLRLRVEELKKELAQAEDELDEAHNQ
ARKLQRSLDEQTEQSENLQVQLEHLQSRLRRQQQNAPLFGKIRSARFGTEEAEDGTSDLD
EDEDLQIQVA
>sp|Q99622|C10_HUMAN Protein C10 OS=Homo sapiens GN=C12orf57 PE=1 SV=1
MASASTQPAALSAEQAKVVLAEVIQAFSAPENAVRMDEARDNACNDMGKMLQFVLPVATQ
IQQEVIKAYGFSCDGEGVLKFARLVKSYEAQDPEIASLSGKLKALFLPPMTLPPHGPAAG
GSVAAS
>sp|Q3MJ40|C144B_HUMAN Coiled-coil domain-containing protein 144B OS=Homo sapiens GN=CCDC144B PE=2 SV=1
MASWGGEKRGGAEGSPKLAVYATRKTRSVRSQEDQWYLGYPGDQWSSGFSYSWWKNSVGS
ESKHGEGALDQPQHDVRLEDLGELHRAARSGDVPGVEHVLAPGDTGVDKRDRKKSIQQLV
PEYKEKQTPESLPQNNNPDWHPTNLTLSDETCQRSKNLKVGDKSPSVSPSMPENQSATKE
LGQMNLTEREKMDTGVVLLSGNDTLHDLCQSQLPENKESKEAEQDLELTSEEEQERLKGC
ENKQPQKTSQEPEMAKDCDREDIPIYPVLPHVQKSEEMWIEQGKLEWKNQLKLVINELKQ
RFGEIYEKYKIPACPEEEPLLDNSTRGTDVKDIPFNLTNNIPGCEEEDASEISVSVVFET
FPEQKEPSLKNIIHPYYHPYSGSQEHVCQSSSKLHLHENKLDCDNDNKLGIGHIFSTDNN
FHNDASTKKARNPEVVTVEMKEDQEFDLQMTKNMNQNSDSGSTNNYKSLKPKLENLSSLP
PDSDRTSEVYLHEELQQDMQKFKNEVNTLEEEFLALKKENVQLHKEVEEEMEKHRSNSTE
LSGTLTDGTTVGNDDDGLNQQIPRKENEEHDRPADKTANEKNKVKNQIYPEADFADSMEP
SEIASEDCELSHSVYENFMLLIEQLRMEYKDSASLPRIQDTFCLCEHLLKLKNNHCDQLT

VKLKQMENMVSVLQNELSETKKTKLQLELQKIEWEKELYDLRLALKQENGRKEMPICCII
KIVNS
>sp|Q6NUI1|C144L_HUMAN Putative coiled-coil domain-containing protein 144 N-terminal-like OS=Homo sapiens GN=CCDC144NL PE=5 SV=1
MASWGGEKRGGAGGSPKPAVYATRKTPSVGSQEDQWYLDYPGDQWSLGFSYSWWKNSVGS
ESKHGEGALDQLQHDVRLEDLGELHRAARSGDVPGVEHVLAPGDTGVDKRDRKKSIQQLV
PEYKEKQTPESLPQNNNPAAPSQAEGGEGGVACGTVEQMTWLCSLPHAVGGGDGDHSSTG
AVGGHPRGPGEYCHLHEQRVHHHIFARGKRKGKNHVSNVVR
>sp|Q9Y4F5|C170B_HUMAN Centrosomal protein of 170 kDa protein B OS=Homo sapiens GN=CEP170B PE=1 SV=4
MSATSWFLVSSSGARHRLPRELIFVGREECELMLQSRSVDKQHAVINYDQDRDEHWVKDL
GSLNGTFVNDMRIPDQKYVTLKLNDVIRFGYDSNMYVLERVQHRVPEEALKHEKYTSQLQ
VSVKGLAPKRSEALPEHTPYCEASNPRPEKGDRRPGTEAASYRTPLYGQPSWWGEDDGST
LPDAQRQGEPYPERPKGPVQQDGELHGFRAPAEPQGCSFRREPSYFEIPTKETPQPSQPP
EVPAHEMPTKDAEAGGGGAAPVVQSHASFTIEFDDCSPGKMKIKDHITKFSLRQRRPPGK
EATPGEMVSAETKVADWLVQNDPSLLHRVGPGDDRHSTKSDLPVHTRTLKGHKHEDGTQS
DSEDPLAKAASAAGVPLEASGEQVRLQRQIKRDPQELLHNQQAFVIEFFDEDTPRKKRSQ
SFTHSPSGDPKADKRRGPTPADRDRPSVPAPVQAGGRSSGPQRAGSLKREKTEERLGSPS
PASRTPARPFGSVGRRSRLAQDFMAQCLRESSPAARPSPEKVPPVLPAPLTPHGTSPVGP
PTPPPAPTDPQLTKARKQEEDDSLSDAGTYTIETEAQDTEVEEARKMIDQVFGVLESPEL
SRASSATFRPVIRGDRDESDDGGVAQRMALLQEFASRPLGAAPQAEHQGLPVPGSPGGQK
WVSRWASLADSYSDPGLTEDGLGRRGGEPEGSLPVRMRRRLPQLPSERADSPAGPESSRR
SGPGPPELDSEQPSRLFGQEELDPDSLSDASGSDGGRGPEPGVEPQDSRRRSPQEGPTWS
RGRRSPRAPGEPTPASFFIGDQNGDAVLSRKPLAAPGDGEGLGQTAQPSPPARDGVYVSA
NGRMVIQLRPGRSPEPDGPAPAFLRQESFTKEPASGPPAPGKPPHISSHPLLQDLAATRA
ARMDFHSQDTHLILKETETALAALEARLLSNSVDAECEGGSTPRPPEDALSGDSDVDTAS
TVSLRSGKSGPSPTTPQPLRAQKEMSPSPPAAQDPGGTALVSAREQSSERQHHPLGPTDM
GRGEPVRRSAIRRGHRPRGSLDWPSEERGPVLAHLPSSDVMASNHETPEATGAGRLGSRR
KPAAPPPSPAAREEQSRSSASSQKGPQALTRSNSLSTPRPTRASRLRRARLGDASDTEAA
DGERGSLGNPEPVGRPAAEQAKKLSRLDILAMPRKRAGSFTGTSDPEAAPARTSFSGRSV
ELCCASRKPTMAEARAVSRKAANTATTTGPRQPFSRARSGSARYTSNTRRRQQGSDYTST
SEEEYGSRHGSPKHTRSHTSTATQTPRAGSSSRARSRAPGPRDTDDDEEEPDPYGFIVQT
AEIAEIARLSQTLVKDVAILAQEIHDVAGDGDTLGSSEPAHSASLSNMPSTPASTISARE
ELVQRIPEASLNFQKVPPGSLNSRDFDQNMNDSCEDALANKTRPRNREEVIFDNLMLNPV
SQLSQAIRENTEHLAEKMKILFQNTGRAWEDLEARINAENEVPILKTSNKEISSILKELR
RVQKQLEVINAIVDPSGSLDLLTGNRSLASSAQPGLGKGRVAAQSPPSPASAEALLPALP
LRNFPQRASCGPPSLPDPTFLPDAERFLI
>sp|Q96L14|C170L_HUMAN Cep170-like protein OS=Homo sapiens GN=CEP170P1 PE=5 SV=2
MPTSSSFKHRIKEQEDYIRDWTAHREEIARISQDLALIAREINDVAGEIDSVTSSGTAPS
TTLVDRVFDESLNFQKIPPLVHSKTPEGNNGRSGDPRPQAAEPPDHLTITRRRTWSRDEV
MGDNLLLSSVFQFSKKIRQSIDKTAGKIRILFKDKDRNWDDIESKLRAESEVPIVKTSSM
EISSILQELKRVEKQLQAINAMIDPDGTLEALNNMGFPSAMLPSPPKQKSSPVNNHHSPG
QTPTLGQPEARALHPAAVSAAAEFENAESEADFSIHFNRVNPDGEEEDVTVHK

>sp|Q8N1F1|C1AS1_HUMAN Putative uncharacterized protein LRRC75A-AS1, mitochondrial OS=Homo sapiens GN=LRRC75A-AS1 PE=5 SV=1
MFPGSLSRGRRAAVEMAWLPGSCARVAFAAGAAARYWTAWQGSAGPNPAAVAEAHGSLFC
GRATSARAWSLRRPGPGSPAHSGGVQTRENWIAYPLQSAEDGVATRLQIREESASCLAAE
YWSQEPAMRF
>sp|Q96EU7|C1GLC_HUMAN C1GALT1-specific chaperone 1 OS=Homo sapiens GN=C1GALT1C1 PE=1 SV=1
MLSESSSFLKGVMLGSIFCALITMLGHIRIGHGNRMHHHEHHHLQAPNKEDILKISEDER
MELSKSFRVYCIILVKPKDVSLWAAVKETWTKHCDKAEFFSSENVKVFESINMDTNDMWL
MMRKAYKYAFDKYRDQYNWFFLARPTTFAIIENLKYFLLKKDPSQPFYLGHTIKSGDLEY
VGMEGGIVLSVESMKRLNSLLNIPEKCPEQGGMIWKISEDKQLAVCLKYAGVFAENAEDA
DGKDVFNTKSVGLSIKEAMTYHPNQVVEGCCSDMAVTFNGLTPNQMHVMMYGVYRLRAFG
HIFNDALVFLPPNGSDND
>sp|P02747|C1QC_HUMAN Complement C1q subcomponent subunit C OS=Homo sapiens GN=C1QC PE=1 SV=3
MDVGPSSLPHLGLKLLLLLLLLPLRGQANTGCYGIPGMPGLPGAPGKDGYDGLPGPKGEP
GIPAIPGIRGPKGQKGEPGLPGHPGKNGPMGPPGMPGVPGPMGIPGEPGEEGRYKQKFQS
VFTVTRQTHQPPAPNSLIRFNAVLTNPQGDYDTSTGKFTCKVPGLYYFVYHASHTANLCV
LLYRSGVKVVTFCGHTSKTNQVNSGGVLLRLQVGEEVWLAVNDYYDMVGIQGSDSVFSGF
LLFPD
>sp|Q9NPY3|C1QR1_HUMAN Complement component C1q receptor OS=Homo sapiens GN=CD93 PE=1 SV=3
MATSMGLLLLLLLLLTQPGAGTGADTEAVVCVGTACYTAHSGKLSAAEAQNHCNQNGGNL
ATVKSKEEAQHVQRVLAQLLRREAALTARMSKFWIGLQREKGKCLDPSLPLKGFSWVGGG
EDTPYSNWHKELRNSCISKRCVSLLLDLSQPLLPSRLPKWSEGPCGSPGSPGSNIEGFVC
KFSFKGMCRPLALGGPGQVTYTTPFQTTSSSLEAVPFASAANVACGEGDKDETQSHYFLC
KEKAPDVFDWGSSGPLCVSPKYGCNFNNGGCHQDCFEGGDGSFLCGCRPGFRLLDDLVTC
ASRNPCSSSPCRGGATCVLGPHGKNYTCRCPQGYQLDSSQLDCVDVDECQDSPCAQECVN
TPGGFRCECWVGYEPGGPGEGACQDVDECALGRSPCAQGCTNTDGSFHCSCEEGYVLAGE
DGTQCQDVDECVGPGGPLCDSLCFNTQGSFHCGCLPGWVLAPNGVSCTMGPVSLGPPSGP
PDEEDKGEKEGSTVPRAATASPTRGPEGTPKATPTTSRPSLSSDAPITSAPLKMLAPSGS
PGVWREPSIHHATAASGPQEPAGGDSSVATQNNDGTDGQKLLLFYILGTVVAILLLLALA
LGLLVYRKRRAKREEKKEKKPQNAADSYSWVPERAESRAMENQYSPTPGTDC
>sp|O75973|C1QRF_HUMAN C1q-related factor OS=Homo sapiens GN=C1QL1 PE=1 SV=1
MLLVLVVLIPVLVSSGGPEGHYEMLGTCRMVCDPYPARGPGAGARTDGGDALSEQSGAPP
PSTLVQGPQGKPGRTGKPGPPGPPGDPGPPGPVGPPGEKGEPGKPGPPGLPGAGGSGAIS
TATYTTVPRVAFYAGLKNPHEGYEVLKFDDVVTNLGNNYDAASGKFTCNIPGTYFFTYHV
LMRGGDGTSMWADLCKNGQVRASAIAQDADQNYDYASNSVILHLDAGDEVFIKLDGGKAH
GGNSNKYSTFSGFIIYSD
>sp|B2RNN3|C1T9B_HUMAN Complement C1q and tumor necrosis factor-related protein 9B OS=Homo sapiens GN=C1QTNF9B PE=1 SV=1
MRIWWLLLAIEICTGNINSQDTCRQGHPGIPGNPGHNGLPGRDGRDGAKGDKGDAGEPGC
PGSPGKDGTSGEKGERGADGKVEAKGIKGDQGSRGSPGKHGPKGLAGPMGEKGLRGETGP
QGQKGNKGDVGPTGPEGPRGNIGPLGPTGLPGPMGPIGKPGPKGEAGPTGPQGEPGVRGI

RGWKGDRGEKGKIGETLVLPKSAFTVGLTVLSKFPSSDVPIKFDKILYNEFNHYDTAVGK
FTCHIAGVYYFTYHITVFSRNVQVSLVKNGVKILHTRDAYVSSEDQASGSIVLQLKLGDE
MWLQVTGGERFNGLFADEDDDTTFTGFLLFSSQ
>sp|Q4G0S4|C27C1_HUMAN Cytochrome P450 27C1 OS=Homo sapiens GN=CYP27C1 PE=2 SV=2
MRSVLRQRILKPKDVAIYSGEVNQVIADLIKRIYLLRSQAEDGETVTNVNDLFFKYSMEG
VATILYESRLGCLENSIPQLTVEYIEALELMFSMFKTSMYAGAIPRWLRPFIPKPWREFC
RSWDGLFKFSQIHVDNKLRDIQYQMDRGRRVSGGLLTYLFLSQALTLQEIYANVTEMLLA
GVDTTSFTLSWTVYLLARHPEVQQTVYREIVKNLGERHVPTAADVPKVPLVRALLKETLR
LFPVLPGNGRVTQEDLVIGGYLIPKGTQLALCHYATSYQDENFPRAKEFRPERWLRKGDL
DRVDNFGSIPFGHGVRSCIGRRIAELEIHLVVIQLLQHFEIKTSSQTNAVHAKTHGLLTP
GGPIHVRFVNRK
>sp|A6NLJ0|C2C4B_HUMAN C2 calcium-dependent domain-containing protein 4B OS=Homo sapiens GN=C2CD4B PE=2 SV=1
MRLLEKLCSSAAGSSAPKPAFAKVLTPNRIPEFCIPPRLPAPCTLESPIRAAAVPRRCAA
ESDLWPRAADEDAGRTDWDPRSQAALSLPHLPRVRTTYGFCALLESPHTRRKESLLLGGP
PAPRPRAHSCGGGGGPDAPLGTLCGPRGPGPATPAAPGGPRLPQDALAAGPRRCRLLRVP
DGLLSRALRAGRSRRLARVRSVSSGNEDEERRAGSESPARAPSSSPLSSRAPLPERLEAK
GTVALGRAGDALRLAAEYCPGTRRLRLRLLRAESLFGGAPGPRAVRCRLSLVLRPPGTAR
WQCSAVVGRSRKASFDQDFCFDGLSEDEVRRLAVRVKARDEGRGRDRGRLLGQGELSLGA
LLLL
>sp|Q9BRT7|CA097_HUMAN Putative uncharacterized protein encoded by LINC00467 OS=Homo sapiens GN=LINC00467 PE=5 SV=1
MDKKSTHRNPEDARAGKYEGKHKRKKRRKQNQNQHRSRHRSVTSFSSDDPMFPSSSSSSS
GSQTDSSIEDAAKGKIKKKRREKTNKWEKRKDKI
>sp|Q3KP66|CA106_HUMAN Uncharacterized protein C1orf106 OS=Homo sapiens GN=C1orf106 PE=2 SV=2
MLQMPKLNEIPPGRAGRREARGEGRWPGQTGPEAARLEWRAQGQAGGARAPWDSWGSSRL
PTQPGPGWSRCPPSLLCALSFQKSTMESKDEVSDTDSGIILQSGPDSPVSPMKELTHAVH
KQQRALEARLEACLEELRRLCLREAELTGTLPAEYPLKPGEKAPKVRRRIGAAYKLDDWA
LHREDPLSSLERQLALQLQITEAARRLCLEENLSRQARRQRKHSMLQEEKKLQELQRCLV
ERRRNSEPPPAAALPLGRELSASDDSSLSDGLLLEEEESQVPKPPPESPAPPSRPLPPQT
LEGLQPTGPEAGSPERAPVQNSPWKETSLDHPYEKPRKSSEPWSESSSPATTPQDGPSAS
SLWLLEPASYHVVPIRGVPGQWQGRTSAPATPEIQGRRGQSQSLRVDSFRAGPEGRGRSA
FPRRRPTHYTVTVPDSCFPATKPPLPHAACHSCSEDSGSDVSSISHPTSPGSSSPDISFL
QPLSPPKTHRHRGAWVPAGSRELVAHHPKLLLPPGYFPAGRYVVVAESPLPPGEWELRRA
APGPAYEEEGTPLRYQRLVPSRSRIVRTPSLKDSPAGRGLSKAAVSEELKWWHERARLRS
TRPHSLDRQGAFRVRSLPLGREGFGRALGPRAQVPTVCVLRRSPDGAPVQVFVPEKGEII
SQV
>sp|Q5T0L3|CA111_HUMAN Uncharacterized protein C1orf111 OS=Homo sapiens GN=C1orf111 PE=1 SV=3
MENFSLLSISGPPISSSALSAFPDIMFSRATSLPDIAKTAVPTEASSPAQALPPQYQSII
VRQGIQNTALSPDCSLGDTQHGEKLRRNCTIYRPWFSPYSYFVCADKESQLEAYDFPEVQ
QDEGKWDNCLSEDMAENICSSSSSPENTCPREATKKSRHGLDSITSQDILMASRWHPAQQ
NGYKCVACCRMYPTLDFLKSHIKRGFREGFSCKVYYRKLKALWSKEQKARLGDRLSSGSC

QAFNSPAEHLRQIGGEAYLCL
>sp|Q6ZSJ8|CA122_HUMAN Uncharacterized protein C1orf122 OS=Homo sapiens GN=C1orf122 PE=4 SV=2
MEWGPGSDWSRGEAAGVDRGKAGLGLGGRPPPQPPREERAQQLLDAVEQRQRQLLDTIAA
CEEMLRQLGRRRPEPAGGGNVSAKPGAPPQPAVSARGGFPKDAGDGAAEP
>sp|Q5JVX7|CA141_HUMAN Uncharacterized protein C1orf141 OS=Homo sapiens GN=C1orf141 PE=2 SV=1
MAEKILEKLDVLDKQAEIILARRTKINRLQSEGRKTTMAIPLTFDFQLEFEEALATSASK
AISKIKEDKSCSITKSKMHVSFKCEPEPRKSNFEKSNLRPFFIQTNVKNKESESTAQIEK
KPRKPLDSVGLLEGDRNKRKKSPQMNDFNIKENKSVRNYQLSKYRSVRKKSLLPLCFEDE
LKNPHAKIVNVSPTKTVTSHMEQKDTNPIIFHDTEYVRMLLLTKNRFSSHPLENENIYPH
KRTNFILERNCEILKSIIGNQSISLFKPQKTMPTVQRKDIQIPMSFKAGHTTVDDKLKKK
TNKQTLENRSWNTLYNFSQNFSSLTKQFVGYLDKAVIHEMSAQTGKFERMFSAGKPTSIP
TSSALPVKCYSKPFKYIYELNNVTPLDNLLNLSNEILNAS
>sp|Q5VVC0|CA146_HUMAN Uncharacterized protein C1orf146 OS=Homo sapiens GN=C1orf146 PE=2 SV=1
MAESGKEKIKWTTTIIISSSLKSYEVATALENRSHKVRYSDSVENGSIIFSLSGVAFLLM
DTKECLLSTEEIFLAKIEKFINIHQNSFLVLSAALHGPEEWKLMFRIQQRFLGCNLRILP
VHNTVNAINLMCTIAKTTSKPYIDSICYRMITAKAYIIEQSPVWKTLQKIKLNSDSVNPN
>sp|Q5SNV9|CA167_HUMAN Uncharacterized protein C1orf167 OS=Homo sapiens GN=C1orf167 PE=2 SV=2
MTRMSCPWGSYQWEPGACPAAPRGIGGGDMAGGIPDVRGLQEAALGAGRSQEEARLVEEA
QTPVMLPQDSGQRVEEVPGDLMAKRMSLILHVQKLPWDHVPCLRRTRQNLYQDVGGHAHG
SGLGGAKRGAARSALRRPLPPATCRPAGIVSGPSPRLDSNPTGAHLIKQTRPLTVEWTKD
TPVPEPMELRSDASHKENVSPKPAALPKPGKRLKQRRFRRSLGIGLSGRHDQWVPGCQVE
RGGPAATPSPGAVLDQEPCRVQTNLASPGPRLGLALKDTTGQLVNSSFWQQSNLQSLARR
RQGKAREFAIQQSNLSINETSSPHLCPEPGGSSGPHKLPWGPLLSQEPLARPSSCLRQSG
LPAPGTPSGDFRPTEAFAPLDGHTQPGLRSWGGLGSWRSRLVGEPLTLEDLAVPSQNQTQ
APSRAAVHQLLASVHCLAQEAARLRCQAPQEPPAWGVSPKQKGEEGAPRERVHREEERTA
FHLSDTVPASSASKNKAQNITAPESEAICWQLLSRCFRSWRHLVKRQREPAAAAVALGRW
QLLRKCLQALWLREAQLEAAWGQYTKVLLVRSFREVSGLQVGPGGRVKQCPGSLREEEIA
QRLLSHPRQRTDSRHERVQILQALQLAVFFLWCQQKKRARQERETLRKATRATQRTGSFP
QAWHSTAAGVAWVAPLSPQHQRAWLCRCFGAWQQFVQRGSRYRDHLADRRTGTLRKCLEQ
WVRMKQLRESDGAKVTQLSLCRQKAGREAVYTAGPGACGLGAVGQAQGQQEQGRGSLQDA
CWTLALCWALLLWKMRLFQRQWANSFFQGLQQRMLQRSLRWWHLRALGPDATSSCTKTPS
ALEPLSSSTLQDSLEKVPRAPTLPDTLQGSLLWAAGQRQQGQCLLLWQARAQQFQGTARW
YQHTRQRRIFLSWSRWATAQWAWRELASHRAWDRTCRAVLGLWRQRLLQSRLVEWWAQER
GWRLARDALCHWHSCWQGQQFLHEKCQTWVQVHLQGLQKVVFRSWQQAAAHQRCTVTRPE
QLLLQSYFQAWCEVVRDTGVLRAQHQAFQDGLRRRALGAVFATWREAQEVAAGAQEQRVA
QASLARWRSCGQQGQEDGQQKKARAPQAFPAWPVAPGMHHEAQQQAGESAGAQAAQCWTW
CWALWVHESCRGQVSRAHASWKPRAWVLEASVQSAVRGGVQRAILTQLRPAELRRFLRTV
QLRVRLGLPGAGKTRSCWTQATELVPPAPSLQCSLGGRRKPRGTAWAQRCREHSLCPAFQ
LWPQWPGQSSWVPGLPLWTRDQGPRAHSSPEPRACKAQSKAHKRRLRILEKQAQAHGSAL
LLALKGHDALGHQEEVPAAPVPRGTASRAAGFPAGQVPGSGMAALGGCPRGRAAGADPAQ

GVAPEMGLADVVAADPATASGSAVTAAGRWAFKKWHQRLAARSPRRGAASSPRPWSKPGP
KGPESGQEAARAPRGWGLGAEHGAQLQL
>sp|Q9NZU7|CABP1_HUMAN Calcium-binding protein 1 OS=Homo sapiens GN=CABP1 PE=1 SV=5
MGGGDGAAFKRPGDGARLQRVLGLGSRREPRSLPAGGPAPRRTAPPPPGHASAGPAAMSS
HIAKSESKTSLLKAAAAAASGGSRAPRHGPARDPGLPSRRLPGSCPATPQSSGDPSSRRP
LCRPAPREEGARGSQRVLPQAHCRPREALPAAASRPSPSSPLPPARGRDGEERGLSPALG
LRGSLRARGRGDSVPAAASEADPFLHRLRPMLSSAFGQDRSLRPEEIEELREAFREFDKD
KDGYINCRDLGNCMRTMGYMPTEMELIELSQQINMNLGGHVDFDDFVELMGPKLLAETAD
MIGVKELRDAFREFDTNGDGEISTSELREAMRKLLGHQVGHRDIEEIIRDVDLNGDGRVD
FEEFVRMMSR
>sp|Q9BXU9|CABP8_HUMAN Calcium-binding protein 8 OS=Homo sapiens GN=CALN1 PE=2 SV=1
MPFHHVTAGLLYKGNYLNRSLSAGSDSEQLANISVEELDEIREAFRVLDRDGNGFISKQE
LGMAMRSLGYMPSEVELAIIMQRLDMDGDGQVDFDEFMTILGPKLVSSEGRDGFLGNTID
SIFWQFDMQRITLEELKHILYHAFRDHLTMKDIENIIINEEESLNETSGNCQTEFEGVHS
QKQNRQTCVRKSLICAFAMAFIISVMLIAANQILRSGME
>sp|Q96KC9|CABS1_HUMAN Calcium-binding and spermatid-specific protein 1 OS=Homo sapiens GN=CABS1 PE=2 SV=3
MAEDGLPKIYSHPPTESSKTPTAATIFFGADNAIPKSETTITSEGDHVTSVNEYMLESDF
STTTDNKLTAKKEKLKSEDDMGTDFIKSTTHLQKEITSLTGTTNSITRDSITEHFMPVKI
GNISSPVTTVSLIDFSTDIAKEDILLATIDTGDAEISITSEVSGTLKDSSAGVADAPAFP
RKKDEADMSNYNSSIKSNVPADEAVQVTDSTIPEAEIPPAPEESFTTIPDITALEEEKIT
EIDLSVLEDDTSAVATLTDSDEKFITVFELTTSAEKDKDKREDTLLTDEETTEGASIWME
RDTANEAETHSVLLTAVESRYDFVVPASIATNLVEESSTEEDLSETDNTETVPKITEPFS
GTTSVLDTPDYKEDTSTTETDIFELLKEEPDEFMI
>sp|O43497|CAC1G_HUMAN Voltage-dependent T-type calcium channel subunit alpha-1G OS=Homo sapiens GN=CACNA1G PE=1 SV=3
MDEEEDGAGAEESGQPRSFMRLNDLSGAGGRPGPGSAEKDPGSADSEAEGLPYPALAPVV
FFYLSQDSRPRSWCLRTVCNPWFERISMLVILLNCVTLGMFRPCEDIACDSQRCRILQAF
DDFIFAFFAVEMVVKMVALGIFGKKCYLGDTWNRLDFFIVIAGMLEYSLDLQNVSFSAVR
TVRVLRPLRAINRVPSMRILVTLLLDTLPMLGNVLLLCFFVFFIFGIVGVQLWAGLLRNR
CFLPENFSLPLSVDLERYYQTENEDESPFICSQPRENGMRSCRSVPTLRGDGGGGPPCGL
DYEAYNSSSNTTCVNWNQYYTNCSAGEHNPFKGAINFDNIGYAWIAIFQVITLEGWVDIM
YFVMDAHSFYNFIYFILLIIVGSFFMINLCLVVIATQFSETKQRESQLMREQRVRFLSNA
STLASFSEPGSCYEELLKYLVYILRKAARRLAQVSRAAGVRVGLLSSPAPLGGQETQPSS
SCSRSHRRLSVHHLVHHHHHHHHHYHLGNGTLRAPRASPEIQDRDANGSRRLMLPPPSTP
ALSGAPPGGAESVHSFYHADCHLEPVRCQAPPPRSPSEASGRTVGSGKVYPTVHTSPPPE
TLKEKALVEVAASSGPPTLTSLNIPPGPYSSMHKLLETQSTGACQSSCKISSPCLKADSG
ACGPDSCPYCARAGAGEVELADREMPDSDSEAVYEFTQDAQHSDLRDPHSRRQRSLGPDA
EPSSVLAFWRLICDTFRKIVDSKYFGRGIMIAILVNTLSMGIEYHEQPEELTNALEISNI
VFTSLFALEMLLKLLVYGPFGYIKNPYNIFDGVIVVISVWEIVGQQGGGLSVLRTFRLMR
VLKLVRFLPALQRQLVVLMKTMDNVATFCMLLMLFIFIFSILGMHLFGCKFASERDGDTL
PDRKNFDSLLWAIVTVFQILTQEDWNKVLYNGMASTSSWAALYFIALMTFGNYVLFNLLV
AILVEGFQAEEISKREDASGQLSCIQLPVDSQGGDANKSESEPDFFSPSLDGDGDRKKCL
ALVSLGEHPELRKSLLPPLIIHTAATPMSLPKSTSTGLGEALGPASRRTSSSGSAEPGAA

HEMKSPPSARSSPHSPWSAASSWTSRRSSRNSLGRAPSLKRRSPSGERRSLLSGEGQESQ
DEEESSEEERASPAGSDHRHRGSLEREAKSSFDLPDTLQVPGLHRTASGRGSASEHQDCN
GKSASGRLARALRPDDPPLDGDDADDEGNLSKGERVRAWIRARLPACCLERDSWSAYIFP
PQSRFRLLCHRIITHKMFDHVVLVIIFLNCITIAMERPKIDPHSAERIFLTLSNYIFTAV
FLAEMTVKVVALGWCFGEQAYLRSSWNVLDGLLVLISVIDILVSMVSDSGTKILGMLRVL
RLLRTLRPLRVISRAQGLKLVVETLMSSLKPIGNIVVICCAFFIIFGILGVQLFKGKFFV
CQGEDTRNITNKSDCAEASYRWVRHKYNFDNLGQALMSLFVLASKDGWVDIMYDGLDAVG
VDQQPIMNHNPWMLLYFISFLLIVAFFVLNMFVGVVVENFHKCRQHQEEEEARRREEKRL
RRLEKKRRNLMLDDVIASGSSASAASEAQCKPYYSDYSRFRLLVHHLCTSHYLDLFITGV
IGLNVVTMAMEHYQQPQILDEALKICNYIFTVIFVLESVFKLVAFGFRRFFQDRWNQLDL
AIVLLSIMGITLEEIEVNASLPINPTIIRIMRVLRIARVLKLLKMAVGMRALLDTVMQAL
PQVGNLGLLFMLLFFIFAALGVELFGDLECDETHPCEGLGRHATFRNFGMAFLTLFRVST
GDNWNGIMKDTLRDCDQESTCYNTVISPIYFVSFVLTAQFVLVNVVIAVLMKHLEESNKE
AKEEAELEAELELEMKTLSPQPHSPLGSPFLWPGVEGPDSPDSPKPGALHPAAHARSASH
FSLEHPTDRQLFDTISLLIQGSLEWELKLMDELAGPGGQPSAFPSAPSLGGSDPQIPLAE
MEALSLTSEIVSEPSCSLALTDDSLPDDMHTLLLSALESNMQPHPTELPGPDLLTVRKSG
VSRTHSLPNDSYMCRHGSTAEGPLGHRGWGLPKAQSGSVLSVHSQPADTSYILQLPKDAP
HLLQPHSAPTWGTIPKLPPPGRSPLAQRPLRRQAAIRTDSLDVQGLGSREDLLAEVSGPS
PPLARAYSFWGQSSTQAQQHSRSHSKISKHMTPPAPCPGPEPNWGKGPPETRSSLELDTE
LSWISGDLLPPGGQEEPPSPRDLKKCYSVEAQSCQRRPTSWLDEQRRHSIAVSCLDSGSQ
PHLGTDPSNLGGQPLGGPGSRPKKKLSPPSITIDPPESQGPRTPPSPGICLRRRAPSSDS
KDPLASGPPDSMAASPSPKKDVLSLSGLSSDPADLDP
>sp|Q08289|CACB2_HUMAN Voltage-dependent L-type calcium channel subunit beta-2 OS=Homo sapiens GN=CACNB2 PE=1 SV=3
MVQRDMSKSPPTAAAAVAQEIQMELLENVAPAGALGAAAQSYGKGARRKNRFKGSDGSTS
SDTTSNSFVRQGSADSYTSRPSDSDVSLEEDREAVRREAERQAQAQLEKAKTKPVAFAVR
TNVSYSAAHEDDVPVPGMAISFEAKDFLHVKEKFNNDWWIGRLVKEGCEIGFIPSPVKLE
NMRLQHEQRAKQGKFYSSKSGGNSSSSLGDIVPSSRKSTPPSSAIDIDATGLDAEENDIP
ANHRSPKPSANSVTSPHSKEKRMPFFKKTEHTPPYDVVPSMRPVVLVGPSLKGYEVTDMM
QKALFDFLKHRFEGRISITRVTADISLAKRSVLNNPSKHAIIERSNTRSSLAEVQSEIER
IFELARTLQLVVLDADTINHPAQLSKTSLAPIIVYVKISSPKVLQRLIKSRGKSQAKHLN
VQMVAADKLAQCPPELFDVILDENQLEDACEHLADYLEAYWKATHPPSSSLPNPLLSRTL
ATSSLPLSPTLASNSQGSQGDQRTDRSAPIRSASQAEEEPSVEPVKKSQHRSSSSAPHHN
HRSGTSRGLSRQETFDSETQESRDSAYVEPKEDYSHDHVDHYASHRDHNHRDETHGSSDH
RHRESRHRSRDVDREQDHNECNKQRSRHKSKDRYCEKDGEVISKKRNEAGEWNRDVYIRQ
>sp|O75493|CAH11_HUMAN Carbonic anhydrase-related protein 11 OS=Homo sapiens GN=CA11 PE=1 SV=2
MGAAARLSAPRALVLWAALGAAAHIGPAPDPEDWWSYKDNLQGNFVPGPPFWGLVNAAWS
LCAVGKRQSPVDVELKRVLYDPFLPPLRLSTGGEKLRGTLYNTGRHVSFLPAPRPVVNVS
GGPLLYSHRLSELRLLFGARDGAGSEHQINHQGFSAEVQLIHFNQELYGNFSAASRGPNG
LAILSLFVNVASTSNPFLSRLLNRDTITRISYKNDAYFLQDLSLELLFPESFGFITYQGS
LSTPPCSETVTWILIDRALNITSLQMHSLRLLSQNPPSQIFQSLSGNSRPLQPLAHRALR
GNRDPRHPERRCRGPNYRLHVDGVPHGR
>sp|O43570|CAH12_HUMAN Carbonic anhydrase 12 OS=Homo sapiens GN=CA12 PE=1 SV=1

MPRRSLHAAAVLLLVILKEQPSSPAPVNGSKWTYFGPDGENSWSKKYPSCGGLLQSPIDL
HSDILQYDASLTPLEFQGYNLSANKQFLLTNNGHSVKLNLPSDMHIQGLQSRYSATQLHL
HWGNPNDPHGSEHTVSGQHFAAELHIVHYNSDLYPDASTASNKSEGLAVLAVLIEMGSFN
PSYDKIFSHLQHVKYKGQEAFVPGFNIEELLPERTAEYYRYRGSLTTPPCNPTVLWTVFR
NPVQISQEQLLALETALYCTHMDDPSPREMINNFRQVQKFDERLVYTSFSQVQVCTAAGL
SLGIILSLALAGILGICIVVVVSIWLFRRKSIKKGDNKGVIYKPATKMETEAHA
>sp|P07451|CAH3_HUMAN Carbonic anhydrase 3 OS=Homo sapiens GN=CA3 PE=1 SV=3
MAKEWGYASHNGPDHWHELFPNAKGENQSPVELHTKDIRHDPSLQPWSVSYDGGSAKTIL
NNGKTCRVVFDDTYDRSMLRGGPLPGPYRLRQFHLHWGSSDDHGSEHTVDGVKYAAELHL
VHWNPKYNTFKEALKQRDGIAVIGIFLKIGHENGEFQIFLDALDKIKTKGKEAPFTKFDP
SCLFPACRDYWTYQGSFTTPPCEECIVWLLLKEPMTVSSDQMAKLRSLLSSAENEPPVPL
VSNWRPPQPINNRVVRASFK
>sp|Q16790|CAH9_HUMAN Carbonic anhydrase 9 OS=Homo sapiens GN=CA9 PE=1 SV=2
MAPLCPSPWLPLLIPAPAPGLTVQLLLSLLLLVPVHPQRLPRMQEDSPLGGGSSGEDDPL
GEEDLPSEEDSPREEDPPGEEDLPGEEDLPGEEDLPEVKPKSEEEGSLKLEDLPTVEAPG
DPQEPQNNAHRDKEGDDQSHWRYGGDPPWPRVSPACAGRFQSPVDIRPQLAAFCPALRPL
ELLGFQLPPLPELRLRNNGHSVQLTLPPGLEMALGPGREYRALQLHLHWGAAGRPGSEHT
VEGHRFPAEIHVVHLSTAFARVDEALGRPGGLAVLAAFLEEGPEENSAYEQLLSRLEEIA
EEGSETQVPGLDISALLPSDFSRYFQYEGSLTTPPCAQGVIWTVFNQTVMLSAKQLHTLS
DTLWGPGDSRLQLNFRATQPLNGRVIEASFPAGVDSSPRAAEPVQLNSCLAAGDILALVF
GLLFAVTSVAFLVQMRRQHRRGTKGGVSYRPAEVAETGA
>sp|Q8IU99|CAHM1_HUMAN Calcium homeostasis modulator protein 1 OS=Homo sapiens GN=CALHM1 PE=1 SV=2
MMDKFRMIFQFLQSNQESFMNGICGIMALASAQMYSAFDFNCPCLPGYNAAYSAGILLAP
PLVLFLLGLVMNNNVSMLAEEWKRPLGRRAKDPAVLRYMFCSMAQRALIAPVVWVAVTLL
DGKCFLCAFCTAVPVSALGNGSLAPGLPAPELARLLARVPCPEIYDGDWLLAREVAVRYL
RCISQALGWSFVLLTTLLAFVVRSVRPCFTQAAFLKSKYWSHYIDIERKLFDETCTEHAK
AFAKVCIQQFFEAMNHDLELGHTHGTLATAPASAAAPTTPDGAEEEREKLRGITDQGTMN
RLLTSWHKCKPPLRLGQEEPPLMGNGWAGGGPRPPRKEVATYFSKV
>sp|P05937|CALB1_HUMAN Calbindin OS=Homo sapiens GN=CALB1 PE=1 SV=2
MAESHLQSSLITASQFFEIWLHFDADGSGYLEGKELQNLIQELQQARKKAGLELSPEMKT
FVDQYGQRDDGKIGIVELAHVLPTEENFLLLFRCQQLKSCEEFMKTWRKYDTDHSGFIET
EELKNFLKDLLEKANKTVDDTKLAEYTDLMLKLFDSNNDGKLELTEMARLLPVQENFLLK
FQGIKMCGKEFNKAFELYDQDGNGYIDENELDALLKDLCEKNKQDLDINNITTYKKNIMA
LSDGGKLYRTDLALILCAGDN
>sp|P06881|CALCA_HUMAN Calcitonin gene-related peptide 1 OS=Homo sapiens GN=CALCA PE=1 SV=3
MGFQKFSPFLALSILVLLQAGSLHAAPFRSALESSPADPATLSEDEARLLLAALVQNYVQ
MKASELEQEQEREGSRIIAQKRACDTATCVTHRLAGLLSRSGGVVKNNFVPTNVGSKAFG
RRRRDLQA
>sp|P62158|CALM_HUMAN Calmodulin OS=Homo sapiens GN=CALM1 PE=1 SV=2
MADQLTEEQIAEFKEAFSLFDKDGDGTITTKELGTVMRSLGQNPTEAELQDMINEVDADG
NGTIDFPEFLTMMARKMKDTDSEEEIREAFRVFDKDGNGYISAAELRHVMTNLGEKLTDE
EVDEMIREADIDGDGQVNYEEFVQMMTAK

>sp|Q8NCB2|CAMKV_HUMAN CaM kinase-like vesicle-associated protein OS=Homo sapiens GN=CAMKV PE=2 SV=2
MPFGCVTLGDKKNYNQPSEVTDRYDLGQVIKTEEFCEIFRAKDKTTGKLHTCKKFQKRDG
RKVRKAAKNEIGILKMVKHPNILQLVDVFVTRKEYFIFLELATGREVFDWILDQGYYSER
DTSNVVRQVLEAVAYLHSLKIVHRNLKLENLVYYNRLKNSKIVISDFHLAKLENGLIKEP
CGTPEYLAPEVVGRQRYGRPVDCWAIGVIMYILLSGNPPFYEEVEEDDYENHDKNLFRKI
LAGDYEFDSPYWDDISQAAKDLVTRLMEVEQDQRITAEEAISHEWISGNAASDKNIKDGV
CAQIEKNFARAKWKKAVRVTTLMKRLRAPEQSSTAAAQSASATDTATPGAAGGATAAAAS
GATSAPEGDAARAAKSDNVAPADRSATPATDGSATPATDGSVTPATDGSITPATDGSVTP
ATDRSATPATDGRATPATEESTVPTTQSSAMLATKAAATPEPAMAQPDSTAPEGATGQAP
PSSKGEEAAGYAQESQREEAS
>sp|A6NHC0|CAN8_HUMAN Calpain-8 OS=Homo sapiens GN=CAPN8 PE=2 SV=3
MAAQAAGVSRQRAATQGLGSNQNALKYLGQDFKTLRQQCLDSGVLFKDPEFPACPSALGY
KDLGPGSPQTQGIIWKRPTELCPSPQFIVGGATRTDICQGGLGDCWLLAAIASLTLNEEL
LYRVVPRDQDFQENYAGIFHFQFWQYGEWVEVVIDDRLPTKNGQLLFLHSEQGNEFWSAL
LEKAYAKLNGCYEALAGGSTVEGFEDFTGGISEFYDLKKPPANLYQIIRKALCAGSLLGC
SIDVSSAAEAEAITSQKLVKSHAYSVTGVEEVNFQGHPEKLIRLRNPWGEVEWSGAWSDD
APEWNHIDPRRKEELDKKVEDGEFWMSLSDFVRQFSRLEICNLSPDSLSSEEVHKWNLVL
FNGHWTRGSTAGGCQNYPATYWTNPQFKIRLDEVDEDQEESIGEPCCTVLLGLMQKNRRW
RKRIGQGMLSIGYAVYQVPKELESHTDAHLGRDFFLAYQPSARTSTYVNLREVSGRARLP
PGEYLVVPSTFEPFKDGEFCLRVFSEKKAQALEIGDVVAGNPYEPHPSEVDQEDDQFRRL
FEKLAGKDSEITANALKILLNEAFSKRTDIKFDGFNINTCREMISLLDSNGTGTLGAVEF
KTLWLKIQKYLEIYWETDYNHSGTIDAHEMRTALRKAGFTLNSQVQQTIALRYACSKLGI
NFDSFVACMIRLETLFKLFSLLDEDKDGMVQLSLAEWLCCVLV
>sp|Q9BXL7|CAR11_HUMAN Caspase recruitment domain-containing protein 11 OS=Homo sapiens GN=CARD11 PE=1 SV=3
MPGGGPEMDDYMETLKDEEDALWENVECNRHMLSRYINPAKLTPYLRQCKVIDEQDEDEV
LNAPMLPSKINRAGRLLDILHTKGQRGYVVFLESLEFYYPELYKLVTGKEPTRRFSTIVV
EEGHEGLTHFLMNEVIKLQQQMKAKDLQRCELLARLRQLEDEKKQMTLTRVELLTFQERY
YKMKEERDSYNDELVKVKDDNYNLAMRYAQLSEEKNMAVMRSRDLQLEIDQLKHRLNKME
EECKLERNQSLKLKNDIENRPKKEQVLELERENEMLKTKNQELQSIIQAGKRSLPDSDKA
ILDILEHDRKEALEDRQELVNRIYNLQEEARQAEELRDKYLEEKEDLELKCSTLGKDCEM
YKHRMNTVMLQLEEVERERDQAFHSRDEAQTQYSQCLIEKDKYRKQIRELEEKNDEMRIE
MVRREACIVNLESKLRRLSKDSNNLDQSLPRNLPVTIISQDFGDASPRTNGQEADDSSTS
EESPEDSKYFLPYHPPQRRMNLKGIQLQRAKSPISLKRTSDFQAKGHEEEGTDASPSSCG
SLPITNSFTKMQPPRSRSSIMSITAEPPGNDSIVRRYKEDAPHRSTVEEDNDSGGFDALD
LDDDSHERYSFGPSSIHSSSSSHQSEGLDAYDLEQVNLMFRKFSLERPFRPSVTSVGHVR
GPGPSVQHTTLNGDSLTSQLTLLGGNARGSFVHSVKPGSLAEKAGLREGHQLLLLEGCIR
GERQSVPLDTCTKEEAHWTIQRCSGPVTLHYKVNHEGYRKLVKDMEDGLITSGDSFYIRL
NLNISSQLDACTMSLKCDDVVHVRDTMYQDRHEWLCARVDPFTDHDLDMGTIPSYSRAQQ
LLLVKLQRLMHRGSREEVDGTHHTLRALRNTLQPEEALSTSDPRVSPRLSRASFLFGQLL
QFVSRSENKYKRMNSNERVRIISGSPLGSLARSSLDATKLLTEKQEELDPESELGKNLSL
IPYSLVRAFYCERRRPVLFTPTVLAKTLVQRLLNSGGAMEFTICKSDIVTRDEFLRRQKT
ETIIYSREKNPNAFECIAPANIEAVAAKNKHCLLEAGIGCTRDLIKSNIYPIVLFIRVCE

KNIKRFRKLLPRPETEEEFLRVCRLKEKELEALPCLYATVEPDMWGSVEELLRVVKDKIG
EEQRKTIWVDEDQL
>sp|P05814|CASB_HUMAN Beta-casein OS=Homo sapiens GN=CSN2 PE=1 SV=4
MKVLILACLVALALARETIESLSSSEESITEYKQKVEKVKHEDQQQGEDEHQDKIYPSFQ
PQPLIYPFVEPIPYGFLPQNILPLAQPAVVLPVPQPEIMEVPKAKDTVYTKGRVMPVLKS
PTIPFFDPQIPKLTDLENLHLPLPLLQPLMQQVPQPIPQTLALPPQPLWSVPQPKVLPIP
QQVVPYPQRAVPVQALLLNQELLLNPTHQIYPVTQPLAPVHNPISV
>sp|P31944|CASPE_HUMAN Caspase-14 OS=Homo sapiens GN=CASP14 PE=1 SV=2
MSNPRSLEEEKYDMSGARLALILCVTKAREGSEEDLDALEHMFRQLRFESTMKRDPTAEQ
FQEELEKFQQAIDSREDPVSCAFVVLMAHGREGFLKGEDGEMVKLENLFEALNNKNCQAL
RAKPKVYIIQACRGEQRDPGETVGGDEIVMVIKDSPQTIPTYTDALHVYSTVEGYIAYRH
DQKGSCFIQTLVDVFTKRKGHILELLTEVTRRMAEAELVQEGKARKTNPEIQSTLRKRLY
LQ
>sp|Q13948|CASP_HUMAN Protein CASP OS=Homo sapiens GN=CUX1 PE=1 SV=2
MAANVGSMFQYWKRFDLQQLQRELDATATVLANRQDESEQSRKRLIEQSREFKKNTPEDL
RKQVAPLLKSFQGEIDALSKRSKEAEAAFLNVYKRLIDVPDPVPALDLGQQLQLKVQRLH
DIETENQKLRETLEEYNKEFAEVKNQEVTIKALKEKIREYEQTLKNQAETIALEKEQKLQ
NDFAEKERKLQETQMSTTSKLEEAEHKVQSLQTALEKTRTELFDLKTKYDEETTAKADEI
EMIMTDLERANQRAEVAQREAETLREQLSSANHSLQLASQIQKAPDVEQAIEVLTRSSLE
VELAAKEREIAQLVEDVQRLQASLTKLRENSASQISQLEQQLSAKNSTLKQLEEKLKGQA
DYEEVKKELNILKSMEFAPSEGAGTQDAAKPLEVLLLEKNRSLQSENAALRISNSDLSGR
CAELQVRITEAVATATEQRELIARLEQDLSIIQSIQRPDAEGAAEHRLEKIPEPIKEATA
LFYGPAAPASGALPEGQVDSLLSIISSQRERFRARNQELEAENRLAQHTLQALQSELDSL
RADNIKLFEKIKFLQSYPGRGSGSDDTELRYSSQYEERLDPFSSFSKRERQRKYLSLSPW
DKATLSMGRLVLSNKMARTIGFFYTLFLHCLVFLVLYKLAWSESMERDCATFCAKKFADH
LHKFHENDNGAAAGDLWQ
>sp|Q9Y6E2|BZW2_HUMAN Basic leucine zipper and W2 domain-containing protein 2 OS=Homo sapiens GN=BZW2 PE=1 SV=1
MNKHQKPVLTGQRFKTRKRDEKEKFEPTVFRDTLVQGLNEAGDDLEAVAKFLDSTGSRLD
YRRYADTLFDILVAGSMLAPGGTRIDDGDKTKMTNHCVFSANEDHETIRNYAQVFNKLIR
RYKYLEKAFEDEMKKLLLFLKAFSETEQTKLAMLSGILLGNGTLPATILTSLFTDSLVKE
GIAASFAVKLFKAWMAEKDANSVTSSLRKANLDKRLLELFPVNRQSVDHFAKYFTDAGLK
ELSDFLRVQQSLGTRKELQKELQERLSQECPIKEVVLYVKEEMKRNDLPETAVIGLLWTC
IMNAVEWNKKEELVAEQALKHLKQYAPLLAVFSSQGQSELILLQKVQEYCYDNIHFMKAF
QKIVVLFYKADVLSEEAILKWYKEAHVAKGKSVFLDQMKKFVEWLQNAEEESESEGEEN
>sp|Q8IYA2|C144C_HUMAN Putative coiled-coil domain-containing protein 144C OS=Homo sapiens GN=CCDC144CP PE=5 SV=3
MVSWGGEKRGGAEGSPKPAVYATRKTGSVRSQEDQWYLGYPGDQWSSGFSYSWWKNSVGS
ESKHGEGALDQPQHDVRLEDLGELHRAARSGDVPGVEHVLVPGDTGVDKRDRKKSIQQLV
PEYKEKQTPESLPQNNNPDWHPTNLTLSDETCQRSKNLKVDDKCPSVSPSMPENQSATKE
LGQMNLTEREKMDTGVKTSQEPEMAKDCDREDIPIYPVLPHVQKSEEMRIEQGKLEWKNQ
LKLVINELKQRFGEIYEKYKIPACPEEEPLLDNSTRGTDVKDIPFNLTNNIPGCEEEDAS
EISVSVVFETFPEQKEPSLKNIIHSYYHPYSGSQEHVCQSSSKLHLHENKLDCDNDNKPG
IGHIFSTDKNFHNDASTKKARNPEVVTVEMKEDQEFDLQMTKNMNQNSDSGSTNNYKSLK

PKLENLSSLPPDSDRTSEVYLHEELQQDMQKFKNEVNTLEEEFLALKKENVQLHKEVEEE
MEKHRSNSTELSGTLTDGTTVGNDDDGLNQQIPRKENGEHDRLALKQENEEKRNADMLYN
KDSEQLRIKEEECGKVVETKQQLKWNLRRLVKELRTVVQERNDAQKQLSEEQDARILQDQ
ILTSKQKELEMAQKKRNPEISHRHQKEKDLFHENCMLQEEIALLRLEIDTIKNQNKQKEK
KYFEDIEVVKEKNDNLQKIIKRNEETLTETILQYSGQLNNLTAENKMLNSELENGKENQE
RLEIEMESYRCRLAAAVHDCDQSQTARDLKLDFQRTRQEWVRLHDKMKVDMSGLQAKNEI
LSEKLSNAESKINSLQIQLHNTRDALGRESLILERVQRDLSQTQCQKKETEQMYQSKLKK
YIAKQESVEERLSQLQSENMLLRQQLDDVHKKANSQEKTISTIQDQFHSAAKNLQAESEK
QILSLQEKNKELMDEYNHLKERMDQCEKEKAGRKIDLTEAQETVPSRCLHLDAENEVLQL
QQTLFSMKAIQKQCETLQKNKKQLKQEVVNLKSYMERNMLERGEAEWHKLLIEERARKEI
EEKLNEAILTLQKQAAVSHEQLAQLREDNTTSIKTQMELTVIDLESEISRIKTSQADFNK
TKLERYKELYLEEVKVRESLSNELSRTNEMIAEVSTQLTVEKEQTRSRSLFTAYATRPVL
ESPCVGNLNDSEGLNRKHIPRKKRSALKDMESYLLKMQQKLQNDLTAEVAGSSQTGLHRI
PQCSSFSSSSLHLLLCSICQPFFLILQLLLNMNLDPI
>sp|Q86VB7|C163A_HUMAN Scavenger receptor cysteine-rich type 1 protein M130 OS=Homo sapiens GN=CD163 PE=1 SV=2
MSKLRMVLLEDSGSADFRRHFVNLSPFTITVVLLLSACFVTSSLGGTDKELRLVDGENKC
SGRVEVKVQEEWGTVCNNGWSMEAVSVICNQLGCPTAIKAPGWANSSAGSGRIWMDHVSC
RGNESALWDCKHDGWGKHSNCTHQQDAGVTCSDGSNLEMRLTRGGNMCSGRIEIKFQGRW
GTVCDDNFNIDHASVICRQLECGSAVSFSGSSNFGEGSGPIWFDDLICNGNESALWNCKH
QGWGKHNCDHAEDAGVICSKGADLSLRLVDGVTECSGRLEVRFQGEWGTICDDGWDSYDA
AVACKQLGCPTAVTAIGRVNASKGFGHIWLDSVSCQGHEPAIWQCKHHEWGKHYCNHNED
AGVTCSDGSDLELRLRGGGSRCAGTVEVEIQRLLGKVCDRGWGLKEADVVCRQLGCGSAL
KTSYQVYSKIQATNTWLFLSSCNGNETSLWDCKNWQWGGLTCDHYEEAKITCSAHREPRL
VGGDIPCSGRVEVKHGDTWGSICDSDFSLEAASVLCRELQCGTVVSILGGAHFGEGNGQI
WAEEFQCEGHESHLSLCPVAPRPEGTCSHSRDVGVVCSRYTEIRLVNGKTPCEGRVELKT
LGAWGSLCNSHWDIEDAHVLCQQLKCGVALSTPGGARFGKGNGQIWRHMFHCTGTEQHMG
DCPVTALGASLCPSEQVASVICSGNQSQTLSSCNSSSLGPTRPTIPEESAVACIESGQLR
LVNGGGRCAGRVEIYHEGSWGTICDDSWDLSDAHVVCRQLGCGEAINATGSAHFGEGTGP
IWLDEMKCNGKESRIWQCHSHGWGQQNCRHKEDAGVICSEFMSLRLTSEASREACAGRLE
VFYNGAWGTVGKSSMSETTVGVVCRQLGCADKGKINPASLDKAMSIPMWVDNVQCPKGPD
TLWQCPSSPWEKRLASPSEETWITCDNKIRLQEGPTSCSGRVEIWHGGSWGTVCDDSWDL
DDAQVVCQQLGCGPALKAFKEAEFGQGTGPIWLNEVKCKGNESSLWDCPARRWGHSECGH
KEDAAVNCTDISVQKTPQKATTGRSSRQSSFIAVGILGVVLLAIFVALFFLTKKRRQRQR
LAVSSRGENLVHQIQYREMNSCLNADDLDLMNSSENSHESADFSAAELISVSKFLPISGM
EKEAILSHTEKENGNL
>sp|Q9NR16|C163B_HUMAN Scavenger receptor cysteine-rich type 1 protein M160 OS=Homo sapiens GN=CD163L1 PE=1 SV=2
MMLPQNSWHIDFGRCCCHQNLFSAVVTCILLLNSCFLISSFNGTDLELRLVNGDGPCSGT
VEVKFQGQWGTVCDDGWNTTASTVVCKQLGCPFSFAMFRFGQAVTRHGKIWLDDVSCYGN
ESALWECQHREWGSHNCYHGEDVGVNCYGEANLGLRLVDGNNSCSGRVEVKFQERWGTIC
DDGWNLNTAAVVCRQLGCPSSFISSGVVNSPAVLRPIWLDDILCQGNELALWNCRHRGWG
NHDCSHNEDVTLTCYDSSDLELRLVGGTNRCMGRVELKIQGRWGTVCHHKWNNAAADVVC
KQLGCGTALHFAGLPHLQSGSDVVWLDGVSCSGNESFLWDCRHSGTVNFDCLHQNDVSVI

CSDGADLELRLADGSNNCSGRVEVRIHEQWWTICDQNWKNEQALVVCKQLGCPFSVFGSR
RAKPSNEARDIWINSISCTGNESALWDCTYDGKAKRTCFRRSDAGVICSDKADLDLRLVG
AHSPCYGRLEVKYQGEWGTVCHDRWSTRNAAVVCKQLGCGKPLHVFGMTYFKEASGPIWL
DDVSCIGNESNIWDCEHSGWGKHNCVHREDVIVTCSGDATWGLRLVGGSNRCSGRLEVYF
QGRWGTVCDDGWNSKAAAVVCSQLDCPSSIIGMGLGNASTGYGKIWLDDVSCDGDESDLW
SCRNSGWGNNDCSHSEDVGVICSDASDMELRLVGGSSRCAGKVEVNVQGAVGILCANGWG
MNIAEVVCRQLECGSAIRVSREPHFTERTLHILMSNSGCTGGEASLWDCIRWEWKQTACH
LNMEASLICSAHRQPRLVGADMPCSGRVEVKHADTWRSVCDSDFSLHAANVLCRELNCGD
AISLSVGDHFGKGNGLTWAEKFQCEGSETHLALCPIVQHPEDTCIHSREVGVVCSRYTDV
RLVNGKSQCDGQVEINVLGHWGSLCDTHWDPEDARVLCRQLSCGTALSTTGGKYIGERSV
RVWGHRFHCLGNESLLDNCQMTVLGAPPCIHGNTVSVICTGSLTQPLFPCLANVSDPYLS
AVPEGSALICLEDKRLRLVDGDSRCAGRVEIYHDGFWGTICDDGWDLSDAHVVCQKLGCG
VAFNATVSAHFGEGSGPIWLDDLNCTGMESHLWQCPSRGWGQHDCRHKEDAGVICSEFTA
LRLYSETETESCAGRLEVFYNGTWGSVGRRNITTAIAGIVCRQLGCGENGVVSLAPLSKT
GSGFMWVDDIQCPKTHISIWQCLSAPWERRISSPAEETWITCEDRIRVRGGDTECSGRVE
IWHAGSWGTVCDDSWDLAEAEVVCQQLGCGSALAALRDASFGQGTGTIWLDDMRCKGNES
FLWDCHAKPWGQSDCGHKEDAGVRCSGQSLKSLNASSGHLALILSSIFGLLLLVLFILFL
TWCRVQKQKHLPLRVSTRRRGSLEENLFHEMETCLKREDPHGTRTSDDTPNHGCEDASDT
SLLGVLPASEATK
>sp|Q6UWJ8|C16L2_HUMAN CD164 sialomucin-like 2 protein OS=Homo sapiens GN=CD164L2 PE=1 SV=2
MEAPGPRALRTALCGGCCCLLLCAQLAVAGKGARGFGRGALIRLNIWPAVQGACKQLEVC
EHCVEGDRARNLSSCMWEQCRPEEPGHCVAQSEVVKEGCSIYNRSEACPAAHHHPTYEPK
TVTTGSPPVPEAHSPGFDGASFIGGVVLVLSLQAVAFFVLHFLKAKDSTYQTLI
>sp|P0DN25|C1C1L_HUMAN C1GALT1-specific chaperone 1-like protein OS=Homo sapiens GN=C1GALT1C1L PE=3 SV=1
MVSASGTSFFKGMLLGSISWVLITMFGQIHIRHRGQTQDHEHHHLRPPNRNDFLNTSKVI
LLELSKSIRVFCIIFGESEDESYWAVLKETWTKHCDKAELYDTKNDNLFNIESNDRWVQM
RTAYKYVFEKYGDNYNWFFLALPTTFAVIENLKYLLFTRDASQPFYLGHTVIFGDLEYVT
VEGGIVLSRELMKRLNRLLDNSETCADQSVIWKLSEDKQLAICLKYAGVHAENAEDYEGR
DVFNTKPIAQLIEEALSNNPQQVVEGCCSDMAITFNGLTPQKMEVMMYGLYRLRAFGHYF
NDTLVFLPPVGSEND
>sp|Q9BXI9|C1QT6_HUMAN Complement C1q tumor necrosis factor-related protein 6 OS=Homo sapiens GN=C1QTNF6 PE=1 SV=3
MGTAALGPVWAALLLFLLMCEIPMVELTFDRAVASGCQRCCDSEDPLDPAHVSSASSSGR
PHALPEIRPYINITILKGDKGDPGPMGLPGYMGREGPQGEPGPQGSKGDKGEMGSPGAPC
QKRFFAFSVGRKTALHSGEDFQTLLFERVFVNLDGCFDMATGQFAAPLRGIYFFSLNVHS
WNYKETYVHIMHNQKEAVILYAQPSERSIMQSQSVMLDLAYGDRVWVRLFKRQRENAIYS
NDFDTYITFSGHLIKAEDD
>sp|P60827|C1QT8_HUMAN Complement C1q tumor necrosis factor-related protein 8 OS=Homo sapiens GN=C1QTNF8 PE=1 SV=2
MAAPALLLLALLLPVGAWPGLPRRPCVHCCRPAWPPGPYARVSDRDLWRGDLWRGLPRVR
PTIDIEILKGEKGEAGVRGRAGRSGKEGPPGARGLQGRRGQKGQVGPPGAACRRAYAAFS
VGRREGLHSSDHFQAVPFDTELVNLDGAFDLAAGRFLCTVPGVYFLSLNVHTWNYKETYL

HIMLNRRPAAVLYAQPSERSVMQAQSLMLLLAAGDAVWVRMFQRDRDNAIYGEHGDLYIT
FSGHLVKPAAEL
>sp|P00736|C1R_HUMAN Complement C1r subcomponent OS=Homo sapiens GN=C1R PE=1 SV=2
MWLLYLLVPALFCRAGGSIPIPQKLFGEVTSPLFPKPYPNNFETTTVITVPTGYRVKLVF
QQFDLEPSEGCFYDYVKISADKKSLGRFCGQLGSPLGNPPGKKEFMSQGNKMLLTFHTDF
SNEENGTIMFYKGFLAYYQAVDLDECASRSKSGEEDPQPQCQHLCHNYVGGYFCSCRPGY
ELQEDTHSCQAECSSELYTEASGYISSLEYPRSYPPDLRCNYSIRVERGLTLHLKFLEPF
DIDDHQQVHCPYDQLQIYANGKNIGEFCGKQRPPDLDTSSNAVDLLFFTDESGDSRGWKL
RYTTEIIKCPQPKTLDEFTIIQNLQPQYQFRDYFIATCKQGYQLIEGNQVLHSFTAVCQD
DGTWHRAMPRCKIKDCGQPRNLPNGDFRYTTTMGVNTYKARIQYYCHEPYYKMQTRAGSR
ESEQGVYTCTAQGIWKNEQKGEKIPRCLPVCGKPVNPVEQRQRIIGGQKAKMGNFPWQVF
TNIHGRGGGALLGDRWILTAAHTLYPKEHEAQSNASLDVFLGHTNVEELMKLGNHPIRRV
SVHPDYRQDESYNFEGDIALLELENSVTLGPNLLPICLPDNDTFYDLGLMGYVSGFGVME
EKIAHDLRFVRLPVANPQACENWLRGKNRMDVFSQNMFCAGHPSLKQDACQGDSGGVFAV
RDPNTDRWVATGIVSWGIGCSRGYGFYTKVLNYVDWIKKEMEEED
>sp|P0C862|C1T9A_HUMAN Complement C1q and tumor necrosis factor-related protein 9A OS=Homo sapiens GN=C1QTNF9 PE=1 SV=1
MRIWWLLLAIEICTGNINSQDTCRQGHPGIPGNPGHNGLPGRDGRDGAKGDKGDAGEPGR
PGSPGKDGTSGEKGERGADGKVEAKGIKGDQGSRGSPGKHGPKGLAGPMGEKGLRGETGP
QGQKGNKGDVGPTGPEGPRGNIGPLGPTGLPGPMGPIGKPGPKGEAGPTGPQGEPGVRGI
RGWKGDRGEKGKIGETLVLPKSAFTVGLTVLSKFPSSDMPIKFDKILYNEFNHYDTAAGK
FTCHIAGVYYFTYHITVFSRNVQVSLVKNGVKILHTKDAYMSSEDQASGGIVLQLKLGDE
VWLQVTGGERFNGLFADEDDDTTFTGFLLFSSP
>sp|P11586|C1TC_HUMAN C-1-tetrahydrofolate synthase, cytoplasmic OS=Homo sapiens GN=MTHFD1 PE=1 SV=3
MAPAEILNGKEISAQIRARLKNQVTQLKEQVPGFTPRLAILQVGNRDDSNLYINVKLKAA
EEIGIKATHIKLPRTTTESEVMKYITSLNEDSTVHGFLVQLPLDSENSINTEEVINAIAP
EKDVDGLTSINAGRLARGDLNDCFIPCTPKGCLELIKETGVPIAGRHAVVVGRSKIVGAP
MHDLLLWNNATVTTCHSKTAHLDEEVNKGDILVVATGQPEMVKGEWIKPGAIVIDCGINY
VPDDKKPNGRKVVGDVAYDEAKERASFITPVPGGVGPMTVAMLMQSTVESAKRFLEKFKP
GKWMIQYNNLNLKTPVPSDIDISRSCKPKPIGKLAREIGLLSEEVELYGETKAKVLLSAL
ERLKHRPDGKYVVVTGITPTPLGEGKSTTTIGLVQALGAHLYQNVFACVRQPSQGPTFGI
KGGAAGGGYSQVIPMEEFNLHLTGDIHAITAANNLVAAAIDARIFHELTQTDKALFNRLV
PSVNGVRRFSDIQIRRLKRLGIEKTDPTTLTDEEINRFARLDIDPETITWQRVLDTNDRF
LRKITIGQAPTEKGHTRTAQFDISVASEIMAVLALTTSLEDMRERLGKMVVASSKKGEPV
SAEDLGVSGALTVLMKDAIKPNLMQTLEGTPVFVHAGPFANIAHGNSSIIADRIALKLVG
PEGFVVTEAGFGADIGMEKFFNIKCRYSGLCPHVVVLVATVRALKMHGGGPTVTAGLPLP
KAYIQENLELVEKGFSNLKKQIENARMFGIPVVVAVNAFKTDTESELDLISRLSREHGAF
DAVKCTHWAEGGKGALALAQAVQRAAQAPSSFQLLYDLKLPVEDKIRIIAQKIYGADDIE
LLPEAQHKAEVYTKQGFGNLPICMAKTHLSLSHNPEQKGVPTGFILPIRDIRASVGAGFL
YPLVGTMSTMPGLPTRPCFYDIDLDPETEQVNGLF
>sp|Q96HQ2|C2AIL_HUMAN CDKN2AIP N-terminal-like protein OS=Homo sapiens GN=CDKN2AIPNL PE=1 SV=1
MVGGEAAAAVEELVSGVRQAADFAEQFRSYSESEKQWKARMEFILRHLPDYRDPPDGSGR

LDQLLSLSMVWANHLFLGCSYNKDLLDKVMEMADGIEVEDLPQFTTRSELMKKHQS
>sp|Q6DHV5|C2D2B_HUMAN Protein CC2D2B OS=Homo sapiens GN=CC2D2B PE=2 SV=2
MMTEKHEDHCLKSCSGHSYIRKNWLGCIVFPFSALLQQSEFLDQTEVLQRAQIFKKNCKA
MFPNRRIVTTVFNDEGIQFLVTRYIKALNPPQQLLDIFLHNSNATFDLIARFVSLIPFVP
NTPDENDGSDIWMTSEHCISLAIGNKEEHAILLCNFFLYFGKKALVLLGTSVLEGHVAYV
VTQETNEYLLWNPSTGQCYKQFDPFCPLKSVDCLFDDRNVWFNIQQNNTPMAVFFDYSKE
SFWKQLLPKNVQGTKIQSIQVTGFPIQMPYIDVQSIIDAVYQTGIHSAEFPQTEFALAVY
IHPYPNNILSVWVYLASLVQHQ
>sp|P33076|C2TA_HUMAN MHC class II transactivator OS=Homo sapiens GN=CIITA PE=1 SV=3
MRCLAPRPAGSYLSEPQGSSQCATMELGPLEGGYLELLNSDADPLCLYHFYDQMDLAGEE
EIELYSEPDTDTINCDQFSRLLCDMEGDEETREAYANIAELDQYVFQDSQLEGLSKDIFK
HIGPDEVIGESMEMPAEVGQKSQKRPFPEELPADLKHWKPAEPPTVVTGSLLVRPVSDCS
TLPCLPLPALFNQEPASGQMRLEKTDQIPMPFSSSSLSCLNLPEGPIQFVPTISTLPHGL
WQISEAGTGVSSIFIYHGEVPQASQVPPPSGFTVHGLPTSPDRPGSTSPFAPSATDLPSM
PEPALTSRANMTEHKTSPTQCPAAGEVSNKLPKWPEPVEQFYRSLQDTYGAEPAGPDGIL
VEVDLVQARLERSSSKSLERELATPDWAERQLAQGGLAEVLLAAKEHRRPRETRVIAVLG
KAGQGKSYWAGAVSRAWACGRLPQYDFVFSVPCHCLNRPGDAYGLQDLLFSLGPQPLVAA
DEVFSHILKRPDRVLLILDGFEELEAQDGFLHSTCGPAPAEPCSLRGLLAGLFQKKLLRG
CTLLLTARPRGRLVQSLSKADALFELSGFSMEQAQAYVMRYFESSGMTEHQDRALTLLRD
RPLLLSHSHSPTLCRAVCQLSEALLELGEDAKLPSTLTGLYVGLLGRAALDSPPGALAEL
AKLAWELGRRHQSTLQEDQFPSADVRTWAMAKGLVQHPPRAAESELAFPSFLLQCFLGAL
WLALSGEIKDKELPQYLALTPRKKRPYDNWLEGVPRFLAGLIFQPPARCLGALLGPSAAA
SVDRKQKVLARYLKRLQPGTLRARQLLELLHCAHEAEEAGIWQHVVQELPGRLSFLGTRL
TPPDAHVLGKALEAAGQDFSLDLRSTGICPSGLGSLVGLSCVTRFRAALSDTVALWESLQ
QHGETKLLQAAEEKFTIEPFKAKSLKDVEDLGKLVQTQRTRSSSEDTAGELPAVRDLKKL
EFALGPVSGPQAFPKLVRILTAFSSLQHLDLDALSENKIGDEGVSQLSATFPQLKSLETL
NLSQNNITDLGAYKLAEALPSLAASLLRLSLYNNCICDVGAESLARVLPDMVSLRVMDVQ
YNKFTAAGAQQLAASLRRCPHVETLAMWTPTIPFSVQEHLQQQDSRISLR
>sp|Q8NEQ6|CA064_HUMAN Uncharacterized protein C1orf64 OS=Homo sapiens GN=C1orf64 PE=2 SV=1
MAPSEDPRDWRANLKGTIRETGLETSSGGKLAGHQKTVPTAHLTFVIDCTHGKQLSLAAT
ASPPQAPSPNRGLVTPPMKTYIVFCGENWPHLTRVTPMGGGCLAQARATLPLCRGSVASA
SFPVSPLCPQEVPEAKGKPVKAAPVRSSTWGTVKDSLKALSSCVCGQAD
>sp|O95561|CA105_HUMAN Uncharacterized protein C1orf105 OS=Homo sapiens GN=C1orf105 PE=1 SV=1
MEKRELKASVPKFDKIPWLSEASLVNKPLVLSLPRRYPHTSATFLTSSKKNMNLPILFQV
PDVLSKARRNQCDSMLLRNQQLCSTCQEMKMVQPRTMKIPDDPKASFENCMSYRMSLHQP
KFQTTPEPFHDDIPTESIHYRLPILGPRTAVFHGLLTEAYKTLKERQRSSLPRKEPIGKT
TRQ
>sp|Q5VVS0|CA140_HUMAN Putative uncharacterized protein C1orf140 OS=Homo sapiens GN=C1orf140 PE=5 SV=1
MTQNSSSLGDRIITPYTPPRSQPMNKHLFVSLSTLFQTTELDFANKKSCFLCLPLRADGF
PEMPHSPAQTERMQKLAKDPPHARSEDVPDHLFFVDTAGLREIVCFCGVLSLRTSEGLMR
KETA

>sp|Q8N6G1|CA211_HUMAN Putative uncharacterized protein encoded by LINC00337 OS=Homo sapiens GN=LINC00337 PE=5 SV=1
MGIEKRFNVLKIKVTQLQCHMGRRPGCECSWELRAPVTVASFLWSPTDSGHLPLLQDTSG
PPEGTHRTFTPGRELVLGPKPTVPGKPFLASALLNV
>sp|Q8TAB5|CA216_HUMAN UPF0500 protein C1orf216 OS=Homo sapiens GN=C1orf216 PE=1 SV=1
MFAIQPGLAEGGQFLGDPPPGLCQPELQPDSNSNFMASAKDANENWHGMPGRVEPILRRS
SSESPSDNQAFQAPGSPEEGVRSPPEGAEIPGAEPEKMGGAGTVCSPLEDNGYASSSLSI
DSRSSSPEPACGTPRGPGPPDPLLPSVAQAVQHLQVQERYKEQEKEKHHVHLVMYRRLAL
LQWIRGLQHQLIDQQARLQESFDTILDNRKELIRCLQQRAAPSRPQDQA
>sp|H3BTG2|CA234_HUMAN Uncharacterized protein C1orf234 OS=Homo sapiens GN=C1orf234 PE=2 SV=1
MSLHGILASAGTIGAVAAWLMSYKPALFGFLFLLLLLSNWLVKYEHKLTLPEPQQDEILQ
RLLFSEMKMKVLENQMFIIWNKMNHHGRSSRHRNFPMKKHRMRRHESICPTLSDCTSSSP
S
>sp|Q9NY47|CA2D2_HUMAN Voltage-dependent calcium channel subunit alpha-2/delta-2 OS=Homo sapiens GN=CACNA2D2 PE=1 SV=2
MAVPARTCGASRPGPARTARPWPGCGPHPGPGTRRPTSGPPRPLWLLLPLLPLLAAPGAS
AYSFPQQHTMQHWARRLEQEVDGVMRIFGGVQQLREIYKDNRNLFEVQENEPQKLVEKVA
GDIESLLDRKVQALKRLADAAENFQKAHRWQDNIKEEDIVYYDAKADAELDDPESEDVER
GSKASTLRLDFIEDPNFKNKVNYSYAAVQIPTDIYKGSTVILNELNWTEALENVFMENRR
QDPTLLWQVFGSATGVTRYYPATPWRAPKKIDLYDVRRRPWYIQGASSPKDMVIIVDVSG
SVSGLTLKLMKTSVCEMLDTLSDDDYVNVASFNEKAQPVSCFTHLVQANVRNKKVFKEAV
QGMVAKGTTGYKAGFEYAFDQLQNSNITRANCNKMIMMFTDGGEDRVQDVFEKYNWPNRT
VRVFTFSVGQHNYDVTPLQWMACANKGYYFEIPSIGAIRINTQEYLDVLGRPMVLAGKEA
KQVQWTNVYEDALGLGLVVTGTLPVFNLTQDGPGEKKNQLILGVMGIDVALNDIKRLTPN
YTLGANGYVFAIDLNGYVLLHPNLKPQTTNFREPVTLDFLDAELEDENKEEIRRSMIDGN
KGHKQIRTLVKSLDERYIDEVTRNYTWVPIRSTNYSLGLVLPPYSTFYLQANLSDQILQV
KLPISKLKDFEFLLPSSFESEGHVFIAPREYCKDLNASDNNTEFLKNFIELMEKVTPDSK
QCNNFLLHNLILDTGITQQLVERVWRDQDLNTYSLLAVFAATDGGITRVFPNKAAEDWTE
NPEPFNASFYRRSLDNHGYVFKPPHQDALLRPLELENDTVGILVSTAVELSLGRRTLRPA
VVGVKLDLEAWAEKFKVLASNRTHQDQPQKCGPNSHCEMDCEVNNEDLLCVLIDDGGFLV
LSNQNHQWDQVGRFFSEVDANLMLALYNNSFYTRKESYDYQAACAPQPPGNLGAAPRGVF
VPTVADFLNLAWWTSAAAWSLFQQLLYGLIYHSWFQADPAEAEGSPETRESSCVMKQTQY
YFGSVNASYNAIIDCGNCSRLFHAQRLTNTNLLFVVAEKPLCSQCEAGRLLQKETHSDGP
EQCELVQRPRYRRGPHICFDYNATEDTSDCGRGASFPPSLGVLVSLQLLLLLGLPPRPQP
QVLVHASRRL
>sp|Q8N1I8|CAAS1_HUMAN Putative uncharacterized protein encoded by CACTIN-AS1 OS=Homo sapiens GN=CACTIN-AS1 PE=2 SV=1
MRPEVGREPAALQPRQRPRSDHQLHRSPFTVPPRTPACRSPGPSPPIAVALEVKPQLEDA
IGKLAAEAVAVRVFPLAVDDLESDVLVGRPRVEAQDGEILVVGAGLQEVLGRGALVDEVG
VEDVELVSLHDLGRWVVKVVVRLVVLVPLEARVHAVEEARLAWPVLVGPQDGGCPGSART
AAGKSFHHLWPQGQWASKPRMNEGTPQCACR
>sp|Q9Y6J0|CABIN_HUMAN Calcineurin-binding protein cabin-1 OS=Homo sapiens GN=CABIN1 PE=1 SV=1

MIRIAALNASSTIEDDHEGSFKSHKTQTKEAQEAEAFALYHKALDLQKHDRFEESAKAYH
ELLEASLLREAVSSGDEKEGLKHPGLILKYSTYKNLAQLAAQREDLETAMEFYLEAVMLD
STDVNLWYKIGHVALRLIRIPLARHAFEEGLRCNPDHWPCLDNLITVLYTLSDYTTCLYF
ICKALEKDCRYSKGLVLKEKIFEEQPCLRKDSLRMFLKCDMSIHDVSVSAAETQAIVDEA
LGLRKKRQALIVREKEPDLKLVQPIPFFTWKCLGESLLAMYNHLTTCEPPRPSLGKRIDL
SDYQDPSQPLESSMVVTPVNVIQPSTVSTNPAVAVAEPVVSYTSVATTSFPLHSPGLLET
GAPVGDISGGDKSKKGVKRKKISEESGETAKRRSARVRNTKCKKEEKVDFQELLMKFLPS
RLRKLDPEEEDDSFNNYEVQSEAKLESFPSIGPQRLSFDSATFMESEKQDVHEFLLENLT
NGGILELMMRYLKAMGHKFLVRWPPGLAEVVLSVYHSWRRHSTSLPNPLLRDCSNKHIKD
MMLMSLSCMELQLDQWLLTKGRSSAVSPRNCPAGMVNGRFGPDFPGTHCLGDLLQLSFAS
SQRDLFEDGWLEFVVRVYWLKARFLALQGDMEQALENYDICTEMLQSSTAIQVEAGAERR
DIVIRLPNLHNDSVVSLEEIDKNLKSLERCQSLEEIQRLYEAGDYKAVVHLLRPTLCTSG
FDRAKHLEFMTSIPERPAQLLLLQDSLLRLKDYRQCFECSDVALNEAVQQMVNSGEAAAK
EEWVATVTQLLMGIEQALSADSSGSILKVSSSTTGLVRLTNNLIQVIDCSMAVQEEAKEP
HVSSVLPWIILHRIIWQEEDTFHSLCHQQQLQNPAEEGMSETPMLPSSLMLLNTAHEYLG
RRSWCCNSDGALLRFYVRVLQKELAASTSEDTHPYKEELETALEQCFYCLYSFPSKKSKA
RYLEEHSAQQVDLIWEDALFMFEYFKPKTLPEFDSYKTSTVSADLANLLKRIATIVPRTE
RPALSLDKVSAYIEGTSTEVPCLPEGADPSPPVVNELYYLLADYHFKNKEQSKAIKFYMH
DICICPNRFDSWAGMALARASRIQDKLNSNELKSDGPIWKHATPVLNCFRRALEIDSSNL
SLWIEYGTMSYALHSFASRQLKQWRGELPPELVQQMEGRRDSMLETAKHCFTSAARCEGD
GDEEEWLIHYMLGKVAEKQQQPPTVYLLHYRQAGHYLHEEAARYPKKIHYHNPPELAMEA
LEVYFRLHASILKLLGKPDSGVGAEVLVNFMKEAAEGPFARGEEKNTPKASEKEKACLVD
EDSHSSAGTLPGPGASLPSSSGPGLTSPPYTATPIDHDYVKCKKPHQQATPDDRSQDSTA
VALSDSSSTQDFFNEPTSLLEGSRKSYTEKRLPILSSQAGATGKDLQGATEERGKNEESL
ESTEGFRAAEQGVQKPAAETPASACIPGKPSASTPTLWDGKKRGDLPGEPVAFPQGLPAG
AEEQRQFLTEQCIASFRLCLSRFPQHYKSLYRLAFLYTYSKTHRNLQWARDVLLGSSIPW
QQLQHMPAQGLFCERNKTNFFNGIWRIPVDEIDRPGSFAWHMNRSIVLLLKVLAQLRDHS
TLLKVSSMLQRTPDQGKKYLRDADRQVLAQRAFILTVKVLEDTLSELAEGSERPGPKVCG
LPGARMTTDVSHKASPEDGQEGLPQPKKPPLADGSGPGPEPGGKVGLLNHRPVAMDAGDS
ADQSGERKDKESPRAGPTEPMDTSEATVCHSDLERTPPLLPGRPARDRGPESRPTELSLE
ELSISARQQPTPLTPAQPAPAPAPATTTGTRAGGHPEEPLSRLSRKRKLLEDTESGKTLL
LDAYRVWQQGQKGVAYDLGRVERIMSETYMLIKQVDEEAALEQAVKFCQVHLGAAAQRQA
SGDTPTTPKHPKDSRENFFPVTVVPTAPDPVPADSVQRPSDAHTKPRPALAAATTIITCP
PSASASTLDQSKDPGPPRPHRPEATPSMASLGPEGEELARVAEGTSFPPQEPRHSPQVKM
APTSSPAEPHCWPAEAALGTGAEPTCSQEGKLRPEPRRDGEAQEAASETQPLSSPPTAAS
SKAPSSGSAQPPEGHPGKPEPSRAKSRPLPNMPKLVIPSAATKFPPEITVTPPTPTLLSP
KGSISEETKQKLKSAILSAQSAANVRKESLCQPALEVLETSSQESSLESETDEDDDYMDI
>sp|Q9BTV7|CABL2_HUMAN CDK5 and ABL1 enzyme substrate 2 OS=Homo sapiens GN=CABLES2 PE=1 SV=3
MAAAAAGGAPGPAPGPAGPPPPAAPTSAARAPPQALRRRGDSRRRQAALFFLNNISLDGR
PPSLGPGGEKPPPPPAEAREPPAPPPPEPPTGLPARTPAPQGLLSPTQVPTGLGLDGQRQ
RKRVTSQRCSLEFLEDAVGCAPAQRTKHTSGSPRHKGLKKTHFIKNMRQYDTRNSRIVLI
CAKRSLCAAFSVLPYGEGLRISDLRVDSQKQRHPSGGVSVSSEMVFELEGVELGADGKVV
SYAKFLYPTNALVTHKSDSHGLLPTPRPSVPRTLPGSRHKPAPTKSAPASTELGSDVGDT

LEYNPNLLDDPQWPCGKHKRVLIFASYMTTVIEYVKPSDLKKDMNETFREKFPHVKLTLS
KIRSLKREMRSLSEECSLEPVTVAMAYVVYFEKLVLQGKLSKQNRKLCAGACVLLAAKISS
DLRKSGVTQLIDKLEERFRFNRRDLIGFEFTVLVALELALYLPENQVLPHYRRLTQQF
>sp|O75952|CABYR_HUMAN Calcium-binding tyrosine phosphorylation-regulated protein OS=Homo sapiens GN=CABYR PE=1 SV=2
MISSKPRLVVPYGLKTLLEGISRAVLKTNPSNINQFAAAYFQELTMYRGNTTMDIKDLVK
QFHQIKVEKWSEGTTPQKKLECLKEPGKTSVESKVPTQMEKSTDTDEDNVTRTEYSDKTT
QFPSVYAVPGTEQTEAVGGLSSKPATPKTTTPPSSPPPTAVSPEFAYVPADPAQLAAQML
GKVSSIHSDQSDVLMVDVATSMPVVIKEVPSSEAAEDVMVAAPLVCSGKVLEVQVVNQTS
VHVDLGSQPKENEAEPSTASSVPLQDEQEPPAYDQAPEVTLQADIEVMSTVHISSVYNDV
PVTEGVVYIEQLPEQIVIPFTDQVACLKENEQSKENEQSPRVSPKSVVEKTTSGMSKKSV
ESVKLAQLEENAKYSSVYMEAEATALLSDTSLKGQPEVPAQLLDAEGAIKIGSEKSLHLE
VEITSIVSDNTGQEESGENSVPQEMEGKPVLSGEAAEAVHSGTSVKSSSGPFPPAPEGLT
APEIEPEGESTAE
>sp|O00555|CAC1A_HUMAN Voltage-dependent P/Q-type calcium channel subunit alpha-1A OS=Homo sapiens GN=CACNA1A PE=1 SV=2
MARFGDEMPARYGGGGSGAAAGVVVGSGGGRGAGGSRQGGQPGAQRMYKQSMAQRARTMA
LYNPIPVRQNCLTVNRSLFLFSEDNVVRKYAKKITEWPPFEYMILATIIANCIVLALEQH
LPDDDKTPMSERLDDTEPYFIGIFCFEAGIKIIALGFAFHKGSYLRNGWNVMDFVVVLTG
ILATVGTEFDLRTLRAVRVLRPLKLVSGIPSLQVVLKSIMKAMIPLLQIGLLLFFAILIF
AIIGLEFYMGKFHTTCFEEGTDDIQGESPAPCGTEEPARTCPNGTKCQPYWEGPNNGITQ
FDNILFAVLTVFQCITMEGWTDLLYNSNDASGNTWNWLYFIPLIIIGSFFMLNLVLGVLS
GEFAKERERVENRRAFLKLRRQQQIERELNGYMEWISKAEEVILAEDETDGEQRHPFDGA
LRRTTIKKSKTDLLNPEEAEDQLADIASVGSPFARASIKSAKLENSTFFHKKERRMRFYI
RRMVKTQAFYWTVLSLVALNTLCVAIVHYNQPEWLSDFLYYAEFIFLGLFMSEMFIKMYG
LGTRPYFHSSFNCFDCGVIIGSIFEVIWAVIKPGTSFGISVLRALRLLRIFKVTKYWASL
RNLVVSLLNSMKSIISLLFLLFLFIVVFALLGMQLFGGQFNFDEGTPPTNFDTFPAAIMT
VFQILTGEDWNEVMYDGIKSQGGVQGGMVFSIYFIVLTLFGNYTLLNVFLAIAVDNLANA
QELTKDEQEEEEAANQKLALQKAKEVAEVSPLSAANMSIAVKEQQKNQKPAKSVWEQRTS
EMRKQNLLASREALYNEMDPDERWKAAYTRHLRPDMKTHLDRPLVVDPQENRNNNTNKSR
AAEPTVDQRLGQQRAEDFLRKQARYHDRARDPSGSAGLDARRPWAGSQEAELSREGPYGR
ESDHHAREGSLEQPGFWEGEAERGKAGDPHRRHVHRQGGSRESRSGSPRTGADGEHRRHR
AHRRPGEEGPEDKAERRARHREGSRPARGGEGEGEGPDGGERRRRHRHGAPATYEGDARR
EDKERRHRRRKENQGSGVPVSGPNLSTTRPIQQDLGRQDPPLAEDIDNMKNNKLATAESA
APHGSLGHAGLPQSPAKMGNSTDPGPMLAIPAMATNPQNAASRRTPNNPGNPSNPGPPKT
PENSLIVTNPSGTQTNSAKTARKPDHTTVDIPPACPPPLNHTVVQVNKNANPDPLPKKEE
EKKEEEEDDRGEDGPKPMPPYSSMFILSTTNPLRRLCHYILNLRYFEMCILMVIAMSSIA
LAAEDPVQPNAPRNNVLRYFDYVFTGVFTFEMVIKMIDLGLVLHQGAYFRDLWNILDFIV
VSGALVAFAFTGNSKGKDINTIKSLRVLRVLRPLKTIKRLPKLKAVFDCVVNSLKNVFNI
LIVYMLFMFIFAVVAVQLFKGKFFHCTDESKEFEKDCRGKYLLYEKNEVKARDREWKKYE
FHYDNVLWALLTLFTVSTGEGWPQVLKHSVDATFENQGPSPGYRMEMSIFYVVYFVVFPF
FFVNIFVALIIITFQEQGDKMMEEYSLEKNERACIDFAISAKPLTRHMPQNKQSFQYRMW
QFVVSPPFEYTIMAMIALNTIVLMMKFYGASVAYENALRVFNIVFTSLFSLECVLKVMAF
GILNYFRDAWNIFDFVTVLGSITDILVTEFGNNFINLSFLRLFRAARLIKLLRQGYTIRI

LLWTFVQSFKALPYVCLLIAMLFFIYAIIGMQVFGNIGIDVEDEDSDEDEFQITEHNNFR
TFFQALMLLFRSATGEAWHNIMLSCLSGKPCDKNSGILTRECGNEFAYFYFVSFIFLCSF
LMLNLFVAVIMDNFEYLTRDSSILGPHHLDEYVRVWAEYDPAAWGRMPYLDMYQMLRHMS
PPLGLGKKCPARVAYKRLLRMDLPVADDNTVHFNSTLMALIRTALDIKIAKGGADKQQMD
AELRKEMMAIWPNLSQKTLDLLVTPHKSTDLTVGKIYAAMMIMEYYRQSKAKKLQAMREE
QDRTPLMFQRMEPPSPTQEGGPGQNALPSTQLDPGGALMAHESGLKESPSWVTQRAQEMF
QKTGTWSPEQGPPTDMPNSQPNSQSVEMREMGRDGYSDSEHYLPMEGQGRAASMPRLPAE
NQRRRGRPRGNNLSTISDTSPMKRSASVLGPKARRLDDYSLERVPPEENQRHHQRRRDRS
HRASERSLGRYTDVDTGLGTDLSMTTQSGDLPSKERDQERGRPKDRKHRQHHHHHHHHHH
PPPPDKDRYAQERPDHGRARARDQRWSRSPSEGREHMAHRQGSSSVSGSPAPSTSGTSTP
RRGRRQLPQTPSTPRPHVSYSPVIRKAGGSGPPQQQQQQQQQQQAVARPGRAATSGPRRY
PGPTAEPLAGDRPPTGGHSSGRSPRMERRVPGPARSESPRACRHGGARWPASGPHVSEGP
PGPRHHGYYRGSDYDEADGPGSGGGEEAMAGAYDAPPPVRHASSGATGRSPRTPRASGPA
CASPSRHGRRLPNGYYPAHGLARPRGPGSRKGLHEPYSESDDDWC
>sp|Q00975|CAC1B_HUMAN Voltage-dependent N-type calcium channel subunit alpha-1B OS=Homo sapiens GN=CACNA1B PE=1 SV=1
MVRFGDELGGRYGGPGGGERARGGGAGGAGGPGPGGLQPGQRVLYKQSIAQRARTMALYN
PIPVKQNCFTVNRSLFVFSEDNVVRKYAKRITEWPPFEYMILATIIANCIVLALEQHLPD
GDKTPMSERLDDTEPYFIGIFCFEAGIKIIALGFVFHKGSYLRNGWNVMDFVVVLTGILA
TAGTDFDLRTLRAVRVLRPLKLVSGIPSLQVVLKSIMKAMVPLLQIGLLLFFAILMFAII
GLEFYMGKFHKACFPNSTDAEPVGDFPCGKEAPARLCEGDTECREYWPGPNFGITNFDNI
LFAILTVFQCITMEGWTDILYNTNDAAGNTWNWLYFIPLIIIGSFFMLNLVLGVLSGEFA
KERERVENRRAFLKLRRQQQIERELNGYLEWIFKAEEVMLAEEDRNAEEKSPLDVLKRAA
TKKSRNDLIHAEEGEDRFADLCAVGSPFARASLKSGKTESSSYFRRKEKMFRFFIRRMVK
AQSFYWVVLCVVALNTLCVAMVHYNQPRRLTTTLYFAEFVFLGLFLTEMSLKMYGLGPRS
YFRSSFNCFDFGVIVGSVFEVVWAAIKPGSSFGISVLRALRLLRIFKVTKYWSSLRNLVV
SLLNSMKSIISLLFLLFLFIVVFALLGMQLFGGQFNFQDETPTTNFDTFPAAILTVFQIL
TGEDWNAVMYHGIESQGGVSKGMFSSFYFIVLTLFGNYTLLNVFLAIAVDNLANAQELTK
DEEEMEEAANQKLALQKAKEVAEVSPMSAANISIAARQQNSAKARSVWEQRASQLRLQNL
RASCEALYSEMDPEERLRFATTRHLRPDMKTHLDRPLVVELGRDGARGPVGGKARPEAAE
APEGVDPPRRHHRHRDKDKTPAAGDQDRAEAPKAESGEPGAREERPRPHRSHSKEAAGPP
EARSERGRGPGPEGGRRHHRRGSPEEAAEREPRRHRAHRHQDPSKECAGAKGERRARHRG
GPRAGPREAESGEEPARRHRARHKAQPAHEAVEKETTEKEATEKEAEIVEADKEKELRNH
QPREPHCDLETSGTVTVGPMHTLPSTCLQKVEEQPEDADNQRNVTRMGSQPPDPNTIVHI
PVMLTGPLGEATVVPSGNVDLESQAEGKKEVEADDVMRSGPRPIVPYSSMFCLSPTNLLR
RFCHYIVTMRYFEVVILVVIALSSIALAAEDPVRTDSPRNNALKYLDYIFTGVFTFEMVI
KMIDLGLLLHPGAYFRDLWNILDFIVVSGALVAFAFSGSKGKDINTIKSLRVLRVLRPLK
TIKRLPKLKAVFDCVVNSLKNVLNILIVYMLFMFIFAVIAVQLFKGKFFYCTDESKELER
DCRGQYLDYEKEEVEAQPRQWKKYDFHYDNVLWALLTLFTVSTGEGWPMVLKHSVDATYE
EQGPSPGYRMELSIFYVVYFVVFPFFFVNIFVALIIITFQEQGDKVMSECSLEKNERACI
DFAISAKPLTRYMPQNRQSFQYKTWTFVVSPPFEYFIMAMIALNTVVLMMKFYDAPYEYE
LMLKCLNIVFTSMFSMECVLKIIAFGVLNYFRDAWNVFDFVTVLGSITDILVTEIAETNN
FINLSFLRLFRAARLIKLLRQGYTIRILLWTFVQSFKALPYVCLLIAMLFFIYAIIGMQV
FGNIALDDDTSINRHNNFRTFLQALMLLFRSATGEAWHEIMLSCLSNQACDEQANATECG

SDFAYFYFVSFIFLCSFLMLNLFVAVIMDNFEYLTRDSSILGPHHLDEFIRVWAEYDPAA
CGRISYNDMFEMLKHMSPPLGLGKKCPARVAYKRLVRMNMPISNEDMTVHFTSTLMALIR
TALEIKLAPAGTKQHQCDAELRKEISVVWANLPQKTLDLLVPPHKPDEMTVGKVYAALMI
FDFYKQNKTTRDQMQQAPGGLSQMGPVSLFHPLKATLEQTQPAVLRGARVFLRQKSSTSL
SNGGAIQNQESGIKESVSWGTQRTQDAPHEARPPLERGHSTEIPVGRSGALAVDVQMQSI
TRRGPDGEPQPGLESQGRAASMPRLAAETQPVTDASPMKRSISTLAQRPRGTHLCSTTPD
RPPPSQASSHHHHHRCHRRRDRKQRSLEKGPSLSADMDGAPSSAVGPGLPPGEGPTGCRR
ERERRQERGRSQERRQPSSSSSEKQRFYSCDRFGGREPPKPKPSLSSHPTSPTAGQEPGP
HPQGSGSVNGSPLLSTSGASTPGRGGRRQLPQTPLTPRPSITYKTANSSPIHFAGAQTSL
PAFSPGRLSRGLSEHNALLQRDPLSQPLAPGSRIGSDPYLGQRLDSEASVHALPEDTLTF
EEAVATNSGRSSRTSYVSSLTSQSHPLRRVPNGYHCTLGLSSGGRARHSYHHPDQDHWC
>sp|Q13936|CAC1C_HUMAN Voltage-dependent L-type calcium channel subunit alpha-1C OS=Homo sapiens GN=CACNA1C PE=1 SV=4
MVNENTRMYIPEENHQGSNYGSPRPAHANMNANAAAGLAPEHIPTPGAALSWQAAIDAAR
QAKLMGSAGNATISTVSSTQRKRQQYGKPKKQGSTTATRPPRALLCLTLKNPIRRACISI
VEWKPFEIIILLTIFANCVALAIYIPFPEDDSNATNSNLERVEYLFLIIFTVEAFLKVIA
YGLLFHPNAYLRNGWNLLDFIIVVVGLFSAILEQATKADGANALGGKGAGFDVKALRAFR
VLRPLRLVSGVPSLQVVLNSIIKAMVPLLHIALLVLFVIIIYAIIGLELFMGKMHKTCYN
QEGIADVPAEDDPSPCALETGHGRQCQNGTVCKPGWDGPKHGITNFDNFAFAMLTVFQCI
TMEGWTDVLYWVNDAVGRDWPWIYFVTLIIIGSFFVLNLVLGVLSGEFSKEREKAKARGD
FQKLREKQQLEEDLKGYLDWITQAEDIDPENEDEGMDEEKPRNMSMPTSETESVNTENVA
GGDIEGENCGARLAHRISKSKFSRYWRRWNRFCRRKCRAAVKSNVFYWLVIFLVFLNTLT
IASEHYNQPNWLTEVQDTANKALLALFTAEMLLKMYSLGLQAYFVSLFNRFDCFVVCGGI
LETILVETKIMSPLGISVLRCVRLLRIFKITRYWNSLSNLVASLLNSVRSIASLLLLLFL
FIIIFSLLGMQLFGGKFNFDEMQTRRSTFDNFPQSLLTVFQILTGEDWNSVMYDGIMAYG
GPSFPGMLVCIYFIILFICGNYILLNVFLAIAVDNLADAESLTSAQKEEEEEKERKKLAR
TASPEKKQELVEKPAVGESKEEKIELKSITADGESPPATKINMDDLQPNENEDKSPYPNP
ETTGEEDEEEPEMPVGPRPRPLSELHLKEKAVPMPEASAFFIFSSNNRFRLQCHRIVNDT
IFTNLILFFILLSSISLAAEDPVQHTSFRNHILFYFDIVFTTIFTIEIALKILGNADYVF
TSIFTLEIILKMTAYGAFLHKGSFCRNYFNILDLLVVSVSLISFGIQSSAINVVKILRVL
RVLRPLRAINRAKGLKHVVQCVFVAIRTIGNIVIVTTLLQFMFACIGVQLFKGKLYTCSD
SSKQTEAECKGNYITYKDGEVDHPIIQPRSWENSKFDFDNVLAAMMALFTVSTFEGWPEL
LYRSIDSHTEDKGPIYNYRVEISIFFIIYIIIIAFFMMNIFVGFVIVTFQEQGEQEYKNC
ELDKNQRQCVEYALKARPLRRYIPKNQHQYKVWYVVNSTYFEYLMFVLILLNTICLAMQH
YGQSCLFKIAMNILNMLFTGLFTVEMILKLIAFKPKGYFSDPWNVFDFLIVIGSIIDVIL
SETNHYFCDAWNTFDALIVVGSIVDIAITEVNPAEHTQCSPSMNAEENSRISITFFRLFR
VMRLVKLLSRGEGIRTLLWTFIKSFQALPYVALLIVMLFFIYAVIGMQVFGKIALNDTTE
INRNNNFQTFPQAVLLLFRCATGEAWQDIMLACMPGKKCAPESEPSNSTEGETPCGSSFA
VFYFISFYMLCAFLIINLFVAVIMDNFDYLTRDWSILGPHHLDEFKRIWAEYDPEAKGRI
KHLDVVTLLRRIQPPLGFGKLCPHRVACKRLVSMNMPLNSDGTVMFNATLFALVRTALRI
KTEGNLEQANEELRAIIKKIWKRTSMKLLDQVVPPAGDDEVTVGKFYATFLIQEYFRKFK
KRKEQGLVGKPSQRNALSLQAGLRTLHDIGPEIRRAISGDLTAEEELDKAMKEAVSAASE
DDIFRRAGGLFGNHVSYYQSDGRSAFPQTFTTQRPLHINKAGSSQGDTESPSHEKLVDST
FTPSSYSSTGSNANINNANNTALGRLPRPAGYPSTVSTVEGHGPPLSPAIRVQEVAWKLS

SNRERHVPMCEDLELRRDSGSAGTQAHCLLLRKANPSRCHSRESQAAMAGQEETSQDETY
EVKMNHDTEACSEPSLLSTEMLSYQDDENRQLTLPEEDKRDIRQSPKRGFLRSASLGRRA
SFHLECLKRQKDRGGDISQKTVLPLHLVHHQALAVAGLSPLLQRSHSPASFPRPFATPPA
TPGSRGWPPQPVPTLRLEGVESSEKLNSSFPSIHCGSWAETTPGGGGSSAARRVRPVSLM
VPSQAGAPGRQFHGSASSLVEAVLISEGLGQFAQDPKFIEVTTQELADACDMTIEEMESA
ADNILSGGAPQSPNGALLPFVNCRDAGQDRAGGEEDAGCVRARGRPSEEELQDSRVYVSS
L
>sp|O60840|CAC1F_HUMAN Voltage-dependent L-type calcium channel subunit alpha-1F OS=Homo sapiens GN=CACNA1F PE=1 SV=2
MSESEGGKDTTPEPSPANGAGPGPEWGLCPGPPAVEGESSGASGLGTPKRRNQHSKHKTV
AVASAQRSPRALFCLTLANPLRRSCISIVEWKPFDILILLTIFANCVALGVYIPFPEDDS
NTANHNLEQVEYVFLVIFTVETVLKIVAYGLVLHPSAYIRNGWNLLDFIIVVVGLFSVLL
EQGPGRPGDAPHTGGKPGGFDVKALRAFRVLRPLRLVSGVPSLHIVLNSIMKALVPLLHI
ALLVLFVIIIYAIIGLELFLGRMHKTCYFLGSDMEAEEDPSPCASSGSGRACTLNQTECR
GRWPGPNGGITNFDNFFFAMLTVFQCVTMEGWTDVLYWMQDAMGYELPWVYFVSLVIFGS
FFVLNLVLGVLSGEFSKEREKAKARGDFQKQREKQQMEEDLRGYLDWITQAEELDMEDPS
ADDNLGSMAEEGRAGHRPQLAELTNRRRGRLRWFSHSTRSTHSTSSHASLPASDTGSMTE
TQGDEDEEEGALASCTRCLNKIMKTRVCRRLRRANRVLRARCRRAVKSNACYWAVLLLVF
LNTLTIASEHHGQPVWLTQIQEYANKVLLCLFTVEMLLKLYGLGPSAYVSSFFNRFDCFV
VCGGILETTLVEVGAMQPLGISVLRCVRLLRIFKVTRHWASLSNLVASLLNSMKSIASLL
LLLFLFIIIFSLLGMQLFGGKFNFDQTHTKRSTFDTFPQALLTVFQILTGEDWNVVMYDG
IMAYGGPFFPGMLVCIYFIILFICGNYILLNVFLAIAVDNLASGDAGTAKDKGGEKSNEK
DLPQENEGLVPGVEKEEEEGARREGADMEEEEEEEEEEEEEEEEEEGAGGVELLQEVVPKE
KVVPIPEGSAFFCLSQTNPLRKGCHTLIHHHVFTNLILVFIILSSVSLAAEDPIRAHSFR
NHILGYFDYAFTSIFTVEILLKMTVFGAFLHRGSFCRSWFNMLDLLVVSVSLISFGIHSS
AISVVKILRVLRVLRPLRAINRAKGLKHVVQCVFVAIRTIGNIMIVTTLLQFMFACIGVQ
LFKGKFYTCTDEAKHTPQECKGSFLVYPDGDVSRPLVRERLWVNSDFNFDNVLSAMMALF
TVSTFEGWPALLYKAIDAYAEDHGPIYNYRVEISVFFIVYIIIIAFFMMNIFVGFVIITF
RAQGEQEYQNCELDKNQRQCVEYALKAQPLRRYIPKNPHQYRVWATVNSAAFEYLMFLLI
LLNTVALAMQHYEQTAPFNYAMDILNMVFTGLFTIEMVLKIIAFKPKHYFTDAWNTFDAL
IVVGSIVDIAVTEVNNGGHLGESSEDSSRISITFFRLFRVMRLVKLLSKGEGIRTLLWTF
IKSFQALPYVALLIAMIFFIYAVIGMQMFGKVALQDGTQINRNNNFQTFPQAVLLLFRCA
TGEAWQEIMLASLPGNRCDPESDFGPGEEFTCGSNFAIAYFISFFMLCAFLIINLFVAVI
MDNFDYLTRDWSILGPHHLDEFKRIWSEYDPGAKGRIKHLDVVALLRRIQPPLGFGKLCP
HRVACKRLVAMNMPLNSDGTVTFNATLFALVRTSLKIKTEGNLEQANQELRIVIKKIWKR
MKQKLLDEVIPPPDEEEVTVGKFYATFLIQDYFRKFRRRKEKGLLGNDAAPSTSSALQAG
LRSLQDLGPEMRQALTCDTEEEEEEGQEGVEEEDEKDLETNKATMVSQPSARRGSGISVS
LPVGDRLPDSLSFGPSDDDRGTPTSSQPSVPQAGSNTHRRGSGALIFTIPEEGNSQPKGT
KGQNKQDEDEEVPDRLSYLDEQAGTPPCSVLLPPHRAQRYMDGHLVPRRRLLPPTPAGRK
PSFTIQCLQRQGSCEDLPIPGTYHRGRNSGPNRAQGSWATPPQRGRLLYAPLLLVEEGAA
GEGYLGRSSGPLRTFTCLHVPGTHSDPSHGKRGSADSLVEAVLISEGLGLFARDPRFVAL
AKQEIADACRLTLDEMDNAASDLLAQGTSSLYSDEESILSRFDEEDLGDEMACVHAL
>sp|O95180|CAC1H_HUMAN Voltage-dependent T-type calcium channel subunit alpha-1H OS=Homo sapiens GN=CACNA1H PE=1 SV=4

MTEGARAADEVRVPLGAPPPGPAALVGASPESPGAPGREAERGSELGVSPSESPAAERGA
ELGADEEQRVPYPALAATVFFCLGQTTRPRSWCLRLVCNPWFEHVSMLVIMLNCVTLGMF
RPCEDVECGSERCNILEAFDAFIFAFFAVEMVIKMVALGLFGQKCYLGDTWNRLDFFIVV
AGMMEYSLDGHNVSLSAIRTVRVLRPLRAINRVPSMRILVTLLLDTLPMLGNVLLLCFFV
FFIFGIVGVQLWAGLLRNRCFLDSAFVRNNNLTFLRPYYQTEEGEENPFICSSRRDNGMQ
KCSHIPGRRELRMPCTLGWEAYTQPQAEGVGAARNACINWNQYYNVCRSGDSNPHNGAIN
FDNIGYAWIAIFQVITLEGWVDIMYYVMDAHSFYNFIYFILLIIVGSFFMINLCLVVIAT
QFSETKQRESQLMREQRARHLSNDSTLASFSEPGSCYEELLKYVGHIFRKVKRRSLRLYA
RWQSRWRKKVDPSAVQGQGPGHRQRRAGRHTASVHHLVYHHHHHHHHHYHFSHGSPRRPG
PEPGACDTRLVRAGAPPSPPSPGRGPPDAESVHSIYHADCHIEGPQERARVAHAAATAAA
SLRLATGLGTMNYPTILPSGVGSGKGSTSPGPKGKWAGGPPGTGGHGPLSLNSPDPYEKI
PHVVGEHGLGQAPGHLSGLSVPCPLPSPPAGTLTCELKSCPYCTRALEDPEGELSGSESG
DSDGRGVYEFTQDVRHGDRWDPTRPPRATDTPGPGPGSPQRRAQQRAAPGEPGWMGRLWV
TFSGKLRRIVDSKYFSRGIMMAILVNTLSMGVEYHEQPEELTNALEISNIVFTSMFALEM
LLKLLACGPLGYIRNPYNIFDGIIVVISVWEIVGQADGGLSVLRTFRLLRVLKLVRFLPA
LRRQLVVLVKTMDNVATFCTLLMLFIFIFSILGMHLFGCKFSLKTDTGDTVPDRKNFDSL
LWAIVTVFQILTQEDWNVVLYNGMASTSSWAALYFVALMTFGNYVLFNLLVAILVEGFQA
EGDANRSDTDEDKTSVHFEEDFHKLRELQTTELKMCSLAVTPNGHLEGRGSLSPPLIMCT
AATPMPTPKSSPFLDAAPSLPDSRRGSSSSGDPPLGDQKPPASLRSSPCAPWGPSGAWSS
RRSSWSSLGRAPSLKRRGQCGERESLLSGEGKGSTDDEAEDGRAAPGPRATPLRRAESLD
PRPLRPAALPPTKCRDRDGQVVALPSDFFLRIDSHREDAAELDDDSEDSCCLRLHKVLEP
YKPQWCRSREAWALYLFSPQNRFRVSCQKVITHKMFDHVVLVFIFLNCVTIALERPDIDP
GSTERVFLSVSNYIFTAIFVAEMMVKVVALGLLSGEHAYLQSSWNLLDGLLVLVSLVDIV
VAMASAGGAKILGVLRVLRLLRTLRPLRVISRAPGLKLVVETLISSLRPIGNIVLICCAF
FIIFGILGVQLFKGKFYYCEGPDTRNISTKAQCRAAHYRWVRRKYNFDNLGQALMSLFVL
SSKDGWVNIMYDGLDAVGVDQQPVQNHNPWMLLYFISFLLIVSFFVLNMFVGVVVENFHK
CRQHQEAEEARRREEKRLRRLERRRRSTFPSPEAQRRPYYADYSPTRRSIHSLCTSHYLD
LFITFIICVNVITMSMEHYNQPKSLDEALKYCNYVFTIVFVFEAALKLVAFGFRRFFKDR
WNQLDLAIVLLSLMGITLEEIEMSAALPINPTIIRIMRVLRIARVLKLLKMATGMRALLD
TVVQALPQVGNLGLLFMLLFFIYAALGVELFGRLECSEDNPCEGLSRHATFSNFGMAFLT
LFRVSTGDNWNGIMKDTLRECSREDKHCLSYLPALSPVYFVTFVLVAQFVLVNVVVAVLM
KHLEESNKEAREDAELDAEIELEMAQGPGSARRVDADRPPLPQESPGARDAPNLVARKVS
VSRMLSLPNDSYMFRPVVPASAPHPRPLQEVEMETYGAGTPLGSVASVHSPPAESCASLQ
IPLAVSSPARSGEPLHALSPRGTARSPSLSRLLCRQEAVHTDSLEGKIDSPRDTLDPAEP
GEKTPVRPVTQGGSLQSPPRSPRPASVRTRKHTFGQRCVSSRPAAPGGEEAEASDPADEE
VSHITSSACPWQPTAEPHGPEASPVAGGERDLRRLYSVDAQGFLDKPGRADEQWRPSAEL
GSGEPGEAKAWGPEAEPALGARRKKKMSPPCISVEPPAEDEGSARPSAAEGGSTTLRRRT
PSCEATPHRDSLEPTEGSGAGGDPAAKGERWGQASCRAEHLTVPSFAFEPLDLGVPSGDP
FLDGSHSVTPESRASSSGAIVPLEPPESEPPMPVGDPPEKRRGLYLTVPQCPLEKPGSPS
ATPAPGGGADDPV
>sp|P55291|CAD15_HUMAN Cadherin-15 OS=Homo sapiens GN=CDH15 PE=1 SV=1
MDAAFLLVLGLLAQSLCLSLGVPGWRRPTTLYPWRRAPALSRVRRAWVIPPISVSENHKR
LPYPLVQIKSDKQQLGSVIYSIQGPGVDEEPRGVFSIDKFTGKVFLNAMLDREKTDRFRL
RAFALDLGGSTLEDPTDLEIVVVDQNDNRPAFLQEAFTGRVLEGAVPGTYVTRAEATDAD

DPETDNAALRFSILQQGSPELFSIDELTGEIRTVQVGLDREVVAVYNLTLQVADMSGDGL
TATASAIITLDDINDNAPEFTRDEFFMEAIEAVSGVDVGRLEVEDRDLPGSPNWVARFTI
LEGDPDGQFTIRTDPKTNEGVLSIVKALDYESCEHYELKVSVQNEAPLQAAALRAERGQA
KVRVHVQDTNEPPVFQENPLRTSLAEGAPPGTLVATFSARDPDTEQLQRLSYSKDYDPED
WLQVDAATGRIQTQHVLSPASPFLKGGWYRAIVLAQDDASQPRTATGTLSIEILEVNDHA
PVLAPPPPGSLCSEPHQGPGLLLGATDEDLPPHGAPFHFQLSPRLPELGRNWSLSQVNVS
HARLRPRHQVPEGLHRLSLLLRDSGQPPQQREQPLNVTVCRCGKDGVCLPGAAALLAGGT
GLSLGALVIVLASALLLLVLVLLVALRARFWKQSRGKGLLHGPQDDLRDNVLNYDEQGGG
EEDQDAYDISQLRHPTALSLPLGPPPLRRDAPQGRLHPQPPRVLPTSPLDIADFINDGLE
AADSDPSVPPYDTALIYDYEGDGSVAGTLSSILSSQGDEDQDYDYLRDWGPRFARLADMY
GHPCGLEYGARWDHQAREGLSPGALLPRHRGRTA
>sp|Q12864|CAD17_HUMAN Cadherin-17 OS=Homo sapiens GN=CDH17 PE=2 SV=3
MILQAHLHSLCLLMLYLATGYGQEGKFSGPLKPMTFSIYEGQEPSQIIFQFKANPPAVTF
ELTGETDNIFVIEREGLLYYNRALDRETRSTHNLQVAALDANGIIVEGPVPITIKVKDIN
DNRPTFLQSKYEGSVRQNSRPGKPFLYVNATDLDDPATPNGQLYYQIVIQLPMINNVMYF
QINNKTGAISLTREGSQELNPAKNPSYNLVISVKDMGGQSENSFSDTTSVDIIVTENIWK
APKPVEMVENSTDPHPIKITQVRWNDPGAQYSLVDKEKLPRFPFSIDQEGDIYVTQPLDR
EEKDAYVFYAVAKDEYGKPLSYPLEIHVKVKDINDNPPTCPSPVTVFEVQENERLGNSIG
TLTAHDRDEENTANSFLNYRIVEQTPKLPMDGLFLIQTYAGMLQLAKQSLKKQDTPQYNL
TIEVSDKDFKTLCFVQINVIDINDQIPIFEKSDYGNLTLAEDTNIGSTILTIQATDADEP
FTGSSKILYHIIKGDSEGRLGVDTDPHTNTGYVIIKKPLDFETAAVSNIVFKAENPEPLV
FGVKYNASSFAKFTLIVTDVNEAPQFSQHVFQAKVSEDVAIGTKVGNVTAKDPEGLDISY
SLRGDTRGWLKIDHVTGEIFSVAPLDREAGSPYRVQVVATEVGGSSLSSVSEFHLILMDV
NDNPPRLAKDYTGLFFCHPLSAPGSLIFEATDDDQHLFRGPHFTFSLGSGSLQNDWEVSK
INGTHARLSTRHTEFEEREYVVLIRINDGGRPPLEGIVSLPVTFCSCVEGSCFRPAGHQT
GIPTVGMAVGILLTTLLVIGIILAVVFIRIKKDKGKDNVESAQASEVKPLRS
>sp|Q8N3J6|CADM2_HUMAN Cell adhesion molecule 2 OS=Homo sapiens GN=CADM2 PE=2 SV=1
MIWKRSAVLRFYSVCGLLLQGSQGQFPLTQNVTVVEGGTAILTCRVDQNDNTSLQWSNPA
QQTLYFDDKKALRDNRIELVRASWHELSISVSDVSLSDEGQYTCSLFTMPVKTSKAYLTV
LGVPEKPQISGFSSPVMEGDLMQLTCKTSGSKPAADIRWFKNDKEIKDVKYLKEEDANRK
TFTVSSTLDFRVDRSDDGVAVICRVDHESLNATPQVAMQVLEIHYTPSVKIIPSTPFPQE
GQPLILTCESKGKPLPEPVLWTKDGGELPDPDRMVVSGRELNILFLNKTDNGTYRCEATN
TIGQSSAEYVLIVHDVPNTLLPTTIIPSLTTATVTTTVAITTSPTTSATTSSIRDPNALA
GQNGPDHALIGGIVAVVVFVTLCSIFLLGRYLARHKGTYLTNEAKGAEDAPDADTAIINA
EGSQVNAEEKKEYFI
>sp|Q9NS85|CAH10_HUMAN Carbonic anhydrase-related protein 10 OS=Homo sapiens GN=CA10 PE=2
SV=1
MEIVWEVLFLLQANFIVCISAQQNSPKIHEGWWAYKEVVQGSFVPVPSFWGLVNSAWNLC
SVGKRQSPVNIETSHMIFDPFLTPLRINTGGRKVSGTMYNTGRHVSLRLDKEHLVNISGG
PMTYSHRLEEIRLHFGSEDSQGSEHLLNGQAFSGEVQLIHYNHELYTNVTEAAKSPNGLV
VVSIFIKVSDSSNPFLNRMLNRDTITRITYKNDAYLLQGLNIEELYPETSSFITYDGSMT
IPPCYETASWIIMNKPVYITRMQMHSLRLLSQNQPSQIFLSMSDNFRPVQPLNNRCIRTN
INFSLQGKDCPNNRAQKLQYRVNEWLLK
>sp|Q8N1Q1|CAH13_HUMAN Carbonic anhydrase 13 OS=Homo sapiens GN=CA13 PE=1 SV=1

MSRLSWGYREHNGPIHWKEFFPIADGDQQSPIEIKTKEVKYDSSLRPLSIKYDPSSAKII
SNSGHSFNVDFDDTENKSVLRGGPLTGSYRLRQVHLHWGSADDHGSEHIVDGVSYAAELH
VVHWNSDKYPSFVEAAHEPDGLAVLGVFLQIGEPNSQLQKITDTLDSIKEKGKQTRFTNF
DLLSLLPPSWDYWTYPGSLTVPPLLESVTWIVLKQPINISSQQLAKFRSLLCTAEGEAAA
FLVSNHRPPQPLKGRKVRASFH
>sp|P10092|CALCB_HUMAN Calcitonin gene-related peptide 2 OS=Homo sapiens GN=CALCB PE=1 SV=1
MGFRKFSPFLALSILVLYQAGSLQAAPFRSALESSPDPATLSKEDARLLLAALVQDYVQM
KASELKQEQETQGSSSAAQKRACNTATCVTHRLAGLLSRSGGMVKSNFVPTNVGSKAFGR
RRRDLQA
>sp|Q96L12|CALR3_HUMAN Calreticulin-3 OS=Homo sapiens GN=CALR3 PE=1 SV=2
MARALVQLWAICMLRVALATVYFQEEFLDGEHWRNRWLQSTNDSRFGHFRLSSGKFYGHK
EKDKGLQTTQNGRFYAISARFKPFSNKGKTLVIQYTVKHEQKMDCGGGYIKVFPADIDQK
NLNGKSQYYIMFGPDICGFDIKKVHVILHFKNKYHENKKLIRCKVDGFTHLYTLILRPDL
SYDVKIDGQSIESGSIEYDWNLTSLKKETSPAESKDWEQTKDNKAQDWEKHFLDASTSKQ
SDWNGDLDGDWPAPMLQKPPYQDGLKPEGIHKDVWLHRKMKNTDYLTQYDLSEFENIGAI
GLELWQVRSGTIFDNFLITDDEEYADNFGKATWGETKGPEREMDAIQAKEEMKKAREEEE
EELLSGKINRHEHYFNQFHRRNEL
>sp|Q16602|CALRL_HUMAN Calcitonin gene-related peptide type 1 receptor OS=Homo sapiens GN=CALCRL PE=1 SV=2
MEKKCTLNFLVLLPFFMILVTAELEESPEDSIQLGVTRNKIMTAQYECYQKIMQDPIQQA
EGVYCNRTWDGWLCWNDVAAGTESMQLCPDYFQDFDPSEKVTKICDQDGNWFRHPASNRT
WTNYTQCNVNTHEKVKTALNLFYLTIIGHGLSIASLLISLGIFFYFKSLSCQRITLHKNL
FFSFVCNSVVTIIHLTAVANNQALVATNPVSCKVSQFIHLYLMGCNYFWMLCEGIYLHTL
IVVAVFAEKQHLMWYYFLGWGFPLIPACIHAIARSLYYNDNCWISSDTHLLYIIHGPICA
ALLVNLFFLLNIVRVLITKLKVTHQAESNLYMKAVRATLILVPLLGIEFVLIPWRPEGKI
AEEVYDYIMHILMHFQGLLVSTIFCFFNGEVQAILRRNWNQYKIQFGNSFSNSEALRSAS
YTVSTISDGPGYSHDCPSEHLNGKSIHDIENVLLKPENLYN
>sp|P49069|CAMLG_HUMAN Calcium signal-modulating cyclophilin ligand OS=Homo sapiens GN=CAMLG PE=1 SV=1
MESMAVATDGGERPGVPAGSGLSASQRRAELRRRKLLMNSEQRINRIMGFHRPGSGAEEE
SQTKSKQQDSDKLNSLSVPSVSKRVVLGDSVSTGTTDQQGGVAEVKGTQLGDKLDSFIKP
PECSSDVNLELRQRNRGDLTADSVQRGSRHGLEQYLSRFEEAMKLRKQLISEKPSQEDGN
TTEEFDSFRIFRLVGCALLALGVRAFVCKYLSIFAPFLTLQLAYMGLYKYFPKSEKKIKT
TVLTAALLLSGIPAEVINRSMDTYSKMGEVFTDLCVYFFTFIFCHELLDYWGSEVP
>sp|Q08AD1|CAMP2_HUMAN Calmodulin-regulated spectrin-associated protein 2 OS=Homo sapiens GN=CAMSAP2 PE=1 SV=3
MGDAADPREMRKTFIVPAIKPFDHYDFSRAKIACNLAWLVAKAFGTENVPEELQEPFYTD
QYDQEHIKPPVVNLLLSAELYCRAGSLILKSDAAKPLLGHDAVIQALAQKGLYVTDQEKL
VTERDLHKKPIQMSAHLAMIDTLMMAYTVEMVSIEKVIACAQQYSAFFQATDLPYDIEDA
VMYWINKVNEHLKDIMEQEQKLKEHHTVEAPGGQKSPSKWFWKLVPARYRKEQTLLKQLP
CIPLVENLLKDGTDGCALAALIHFYCPDVVRLEDICLKETMSLADSLYNLQLIQEFCQEY
LNQCCHFTLEDMLYAASSIKSNYLVFMAELFWWFEVVKPSFVQPRVVRPQGAEPVKDMPS
IPVLNAAKRNVLDSSSDFPSSGEGATFTQSHHHLPSRYSRPQAHSSASGGIRRSSSMSYV

DGFIGTWPKEKRSSVHGVSFDISFDKEDSVQRSTPNRGITRSISNEGLTLNNSHVSKHIR
KNLSFKPINGEEEAESIEEELNIDSHSDLKSCVPLNTNELNSNENIHYKLPNGALQNRIL
LDEFGNQIETPSIEEALQIIHDTEKSPHTPQPDQIANGFFLHSQEMSILNSNIKLNQSSP
DNVTDTKGALSPITDNTEVDTGIHVPSEDIPETMDEDSSLRDYTVSLDSDMDDASKFLQD
YDIRTGNTREALSPCPSTVSTKSQPGSSASSSSGVKMTSFAEQKFRKLNHTDGKSSGSSS
QKTTPEGSELNIPHVVAWAQIPEETGLPQGRDTTQLLASEMVHLRMKLEEKRRAIEAQKK
KMEAAFTKQRQKMGRTAFLTVVKKKGDGISPLREEAAGAEDEKVYTDRAKEKESQKTDGQ
RSKSLADIKESMENPQAKWLKSPTTPIDPEKQWNLASPSEETLNEGEILEYTKSIEKLNS
SLHFLQQEMQRLSLQQEMLMQMREQQSWVISPPQPSPQKQIRDFKPSKQAGLSSAIAPFS
SDSPRPTHPSPQSSNRKSASFSVKSQRTPRPNELKITPLNRTLTPPRSVDSLPRLRRFSP
SQVPIQTRSFVCFGDDGEPQLKESKPKEEVKKEELESKGTLEQRGHNPEEKEIKPFESTV
SEVLSLPVTETVCLTPNEDQLNQPTEPPPKPVFPPTAPKNVNLIEVSLSDLKPPEKADVP
VEKYDGESDKEQFDDDQKVCCGFFFKDDQKAENDMAMKRAALLEKRLRREKETQLRKQQL
EAEMEHKKEETRRKTEEERQKKEDERARREFIRQEYMRRKQLKLMEDMDTVIKPRPQVVK
QKKQRPKSIHRDHIESPKTPIKGPPVSSLSLASLNTGDNESVHSGKRTPRSESVEGFLSP
SRCGSRNGEKDWENASTTSSVASGTEYTGPKLYKEPSAKSNKHIIQNALAHCCLAGKVNE
GQKKKILEEMEKSDANNFLILFRDSGCQFRSLYTYCPETEEINKLTGIGPKSITKKMIEG
LYKYNSDRKQFSHIPAKTLSASVDAITIHSHLWQTKRPVTPKKLLPTKA
>sp|P49913|CAMP_HUMAN Cathelicidin antimicrobial peptide OS=Homo sapiens GN=CAMP PE=1 SV=1
MKTQRDGHSLGRWSLVLLLLGLVMPLAIIAQVLSYKEAVLRAIDGINQRSSDANLYRLLD
LDPRPTMDGDPDTPKPVSFTVKETVCPRTTQQSPEDCDFKKDGLVKRCMGTVTLNQARGS
FDISCDKDNKRFALLGDFFRKSKEKIGKEFKRIVQRIKDFLRNLVPRTES
>sp|Q9UMQ6|CAN11_HUMAN Calpain-11 OS=Homo sapiens GN=CAPN11 PE=2 SV=2
MLYSPGPSLPESAESLDGSQEDKPRGSCAEPTFTDTGMVAHINNSRLKAKGVGQHDNAQN
FGNQSFEELRAACLRKGELFEDPLFPAEPSSLGFKDLGPNSKNVQNISWQRPKDIINNPL
FIMDGISPTDICQGILGDCWLLAAIGSLTTCPKLLYRVVPRGQSFKKNYAGIFHFQIWQF
GQWVNVVVDDRLPTKNDKLVFVHSTERSEFWSALLEKAYAKLSGSYEALSGGSTMEGLED
FTGGVAQSFQLQRPPQNLLRLLRKAVERSSLMGCSIEVTSDSELESMTDKMLVRGHAYSV
TGLQDVHYRGKMETLIRVRNPWGRIEWNGAWSDSAREWEEVASDIQMQLLHKTEDGEFWM
SYQDFLNNFTLLEICNLTPDTLSGDYKSYWHTTFYEGSWRRGSSAGGCRNHPGTFWTNPQ
FKISLPEGDDPEDDAEGNVVVCTCLVALMQKNWRHARQQGAQLQTIGFVLYAVPKEFQNI
QDVHLKKEFFTKYQDHGFSEIFTNSREVSSQLRLPPGEYIIIPSTFEPHRDADFLLRVFT
EKHSESWELDEVNYAEQLQEEKVSEDDMDQDFLHLFKIVAGEGKEIGVYELQRLLNRMAI
KFKSFKTKGFGLDACRCMINLMDKDGSGKLGLLEFKILWKKLKKWMDIFRECDQDHSGTL
NSYEMRLVIEKAGIKLNNKVMQVLVARYADDDLIIDFDSFISCFLRLKTMFTFFLTMDPK
NTGHICLSLEQWLQMTMWG
>sp|O75808|CAN15_HUMAN Calpain-15 OS=Homo sapiens GN=CAPN15 PE=1 SV=1
MATVGEWSCVRCTFLNPAGQRQCSICEAPRHKPDLNHILRLSVEEQKWPCARCTFRNFLG
KEACEVCGFTPEPAPGAAFLPVLNGVLPKPPAILGEPKGSCQEEAGPVRTAGLVATEPAR
GQCEDKDEEEKEEQEEEEGAAEPRGGWACPRCTLHNTPVASSCSVCGGPRRLSLPRIPPE
ALVVPEVVAPAGFHVVPAAPPPGLPGEGAEANPPATSQGPAAEPEPPRVPPFSPFSSTLQ
NNPVPRSRREVPPQLQPPVPEAAQPSPSAGCRGAPQGSGWAGASRLAELLSGKRLSVLEE
EATEGGTSRVEAGSSTSGSDIIDLAGDTVRYTPASPSSPDFTTWSCAKCTLRNPTVAPRC

SACGCSKLHGFQEHGEPPTHCPDCGADKPSPCGRSCGRVSSAQKAARVLPERPGQWACPA
CTLLNALRAKHCAACHTPQLLVAQRRGAAPLRRRESMHVEQRRQTDEGEAKALWENIVAF
CRENNVSFVDDSFPPGPESVGFPAGDSVQQRVRQWLRPQEINCSVFRDHRATWSVFHTLR
PSDILQGLLGNCWFLSALAVLAERPDLVERVMVTRSLCAEGAYQVRLCKDGTWTTVLVDD
MLPCDEAGCLLFSQAQRKQLWVALIEKALAKLHGSYFALQAGRAIEGLATLTGAPCESLA
LQLSSTNPREEPVDTDLIWAKMLSSKEAGFLMGASCGGGNMKVDDSAYESLGLRPRHAYS
ILDVRDVQGTRLLRLRNPWGRFSWNGSWSDEWPHWPGHLRGELMPHGSSEGVFWMEYGDF
VRYFDSVDICKVHSDWQEARVQGCFPSSASAPVGVTALTVLERASLEFALFQEGSRRSDA
VDSHLLDLCILVFRATFGSGGHLSLGRLLAHSKRAVKKFVSCDVMLEPGEYAVVCCAFNH
WGPPLPGTPAPQASSPSAGVPRASPEPPGHVLAVYSSRLVMVEPVEAQPTTLADAIILLT
ESRGERHEGREGMTCYYLTHGWAGLIVVVENRHPKAYLHVQCDCTDSFNVVSTRGSLRTQ
DSVPPLHRQVLVILSQLEGNAGFSITHRLAHRKAAQAFLSDWTASKGTHSPPLTPEVAGL
HGPRPL
>sp|Q9Y6Q1|CAN6_HUMAN Calpain-6 OS=Homo sapiens GN=CAPN6 PE=1 SV=2
MGPPLKLFKNQKYQELKQECIKDSRLFCDPTFLPENDSLFYNRLLPGKVVWKRPQDICDD
PHLIVGNISNHQLTQGRLGHKPMVSAFSCLAVQESHWTKTIPNHKEQEWDPQKTEKYAGI
FHFRFWHFGEWTEVVIDDLLPTINGDLVFSFSTSMNEFWNALLEKAYAKLLGCYEALDGL
TITDIIVDFTGTLAETVDMQKGRYTELVEEKYKLFGELYKTFTKGGLICCSIESPNQEEQ
EVETDWGLLKGHTYTMTDIRKIRLGERLVEVFSAEKVYMVRLRNPLGRQEWSGPWSEISE
EWQQLTASDRKNLGLVMSDDGEFWMSLEDFCRNFHKLNVCRNVNNPIFGRKELESVLGCW
TVDDDPLMNRSGGCYNNRDTFLQNPQYIFTVPEDGHKVIMSLQQKDLRTYRRMGRPDNYI
IGFELFKVEMNRKFRLHHLYIQERAGTSTYIDTRTVFLSKYLKKGNYVLVPTMFQHGRTS
EFLLRIFSEVPVQLRELTLDMPKMSCWNLARGYPKVVTQITVHSAEDLEKKYANETVNPY
LVIKCGKEEVRSPVQKNTVHAIFDTQAIFYRRTTDIPIIVQVWNSRKFCDQFLGQVTLDA
DPSDCRDLKSLYLRKKGGPTAKVKQGHISFKVISSDDLTEL
>sp|O14815|CAN9_HUMAN Calpain-9 OS=Homo sapiens GN=CAPN9 PE=1 SV=1
MPYLYRAPGPQAHPVPKDARITHSSGQSFEQMRQECLQRGTLFEDADFPASNSSLFYSER
PQIPFVWKRPGEIVKNPEFILGGATRTDICQGELGDCWLLAAIASLTLNQKALARVIPQD
QSFGPGYAGIFHFQFWQHSEWLDVVIDDRLPTFRDRLVFLHSADHNEFWSALLEKAYAKL
NGSYEALKGGSAIEAMEDFTGGVAETFQTKEAPENFYEILEKALKRGSLLGCFIDTRSAA
ESEARTPFGLIKGHAYSVTGIDQVSFRGQRIELIRIRNPWGQVEWNGSWSDSSPEWRSVG
PAEQKRLCHTALDDGEFWMAFKDFKAHFDKVEICNLTPDALEEDAIHKWEVTVHQGSWVR
GSTAGGCRNFLDTFWTNPQIKLSLTEKDEGQEECSFLVALMQKDRRKLKRFGANVLTIGY
AIYECPDKDEHLNKDFFRYHASRARSKTFINLREVSDRFKLPPGEYILIPSTFEPHQEAD
FCLRIFSEKKAITRDMDGNVDIDLPEPPKPTPPDQETEEEQRFRALFEQVAGEDMEVTAE
ELEYVLNAVLQKKKDIKFKKLSLISCKNIISLMDTSGNGKLEFDEFKVFWDKLKQWINLF
LRFDADKSGTMSTYELRTALKAAGFQLSSHLLQLIVLRYADEELQLDFDDFLNCLVRLEN
ASRVFQALSTKNKEFIHLNINEFIHLTMNI
>sp|Q5XLA6|CAR17_HUMAN Caspase recruitment domain-containing protein 17 OS=Homo sapiens GN=CARD17 PE=1 SV=1
MADKVLKEKRKQFIRSVGEGTINGLLGELLETRVLSQEEIEIVKCENATVMDKARALLDS
VIRKGAPACQICITYICEEDSHLAGTLGLSAGPTSGNHLTTQDSQIVLPS
>sp|Q96LW7|CAR19_HUMAN Caspase recruitment domain-containing protein 19 OS=Homo sapiens GN=CARD19 PE=1 SV=1

MTDQTYCDRLVQDTPFLTGHGRLSEQQVDRIILQLNRYYPQILTNKEAEKFRNPKASLRV
RLCDLLSHLQRSGERDCQEFYRALYIHAQPLHSRLPSRHALRKFHITNHACLVLARGGHP
SLPLMAWMSSMTTQVCCSPGLASPLASAPPQRPPSGPEGRVWQAQAVQMLVSVSHFLPLP
PSLSHGSFHTAWGILYVHSCPSFSNLIPRGSLHVCVDSNLVPTAAWRS
>sp|Q9Y2G2|CARD8_HUMAN Caspase recruitment domain-containing protein 8 OS=Homo sapiens GN=CARD8 PE=1 SV=1
MMRQRQSHYCSVLFLSVNYLGGTFPGDICSEENQIVSSYASKVCFEIEEDYKNRQFLGPE
GNVDVELIDKSTNRYSVWFPTAGWYLWSATGLGFLVRDEVTVTIAFGSWSQHLALDLQHH
EQWLVGGPLFDVTAEPEEAVAEIHLPHFISLQGEVDVSWFLVAHFKNEGMVLEHPARVEP
FYAVLESPSFSLMGILLRIASGTRLSIPITSNTLIYYHPHPEDIKFHLYLVPSDALLTKA
IDDEEDRFHGVRLQTSPPMEPLNFGSSYIVSNSANLKVMPKELKLSYRSPGEIQHFSKFY
AGQMKEPIQLEITEKRHGTLVWDTEVKPVDLQLVAASAPPPFSGAAFVKENHRQLQARMG
DLKGVLDDLQDNEVLTENEKELVEQEKTRQSKNEALLSMVEKKGDLALDVLFRSISERDP
YLVSYLRQQNL
>sp|Q9H257|CARD9_HUMAN Caspase recruitment domain-containing protein 9 OS=Homo sapiens GN=CARD9 PE=1 SV=2
MSDYENDDECWSVLEGFRVTLTSVIDPSRITPYLRQCKVLNPDDEEQVLSDPNLVIRKRK
VGVLLDILQRTGHKGYVAFLESLELYYPQLYKKVTGKEPARVFSMIIDASGESGLTQLLM
TEVMKLQKKVQDLTALLSSKDDFIKELRVKDSLLRKHQERVQRLKEECEAGSRELKRCKE
ENYDLAMRLAHQSEEKGAALMRNRDLQLEIDQLKHSLMKAEDDCKVERKHTLKLRHAMEQ
RPSQELLWELQQEKALLQARVQELEASVQEGKLDRSSPYIQVLEEDWRQALRDHQEQANT
IFSLRKDLRQGEARRLRCMEEKEMFELQCLALRKDSKMYKDRIEAILLQMEEVAIERDQA
IATREELHAQHARGLQEKDALRKQVRELGEKADELQLQVFQCEAQLLAVEGRLRRQQLET
LVLSSDLEDGSPRRSQELSLPQDLEDTQLSDKGCLAGGGSPKQPFAALHQEQVLRNPHDA
GLSSGEPPEKERRRLKESFENYRRKRALRKMQKGWRQGEEDRENTTGSDNTDTEGS
>sp|Q13938|CAYP1_HUMAN Calcyphosin OS=Homo sapiens GN=CAPS PE=1 SV=1
MDAVDATMEKLRAQCLSRGASGIQGLARFFRQLDRDGSRSLDADEFRQGLAKLGLVLDQA
EAEGVCRKWDRNGSGTLDLEEFLRALRPPMSQAREAVIAAAFAKLDRSGDGVVTVDDLRG
VYSGRAHPKVRSGEWTEDEVLRRFLDNFDSSEKDGQVTLAEFQDYYSGVSASMNTDEEFV
AMMTSAWQL
>sp|P52907|CAZA1_HUMAN F-actin-capping protein subunit alpha-1 OS=Homo sapiens GN=CAPZA1 PE=1 SV=3
MADFDDRVSDEEKVRIAAKFITHAPPGEFNEVFNDVRLLLNNDNLLREGAAHAFAQYNMD
QFTPVKIEGYEDQVLITEHGDLGNSRFLDPRNKISFKFDHLRKEASDPQPEEADGGLKSW
RESCDSALRAYVKDHYSNGFCTVYAKTIDGQQTIIACIESHQFQPKNFWNGRWRSEWKFT
ITPPTAQVVGVLKIQVHYYEDGNVQLVSHKDVQDSLTVSNEAQTAKEFIKIIENAENEYQ
TAISENYQTMSDTTFKALRRQLPVTRTKIDWNKILSYKIGKEMQNA
>sp|P0DN79|CBSL_HUMAN Cystathionine beta-synthase-like protein OS=Homo sapiens GN=CBSL PE=1 SV=1
MPSETPQAEVGPTGCPHRSGPHSAKGSLEKGSPEDKEAKEPLWIRPDAPSRCTWQLGRPA
SESPHHHTAPAKSPKILPDILKKIGDTPMVRINKIGKKFGLKCELLAKCEFFNAGGSVKD
RISLRMIEDAERDGTLKPGDTIIEPTSGNTGIGLALAAAVRGYRCIIVMPEKMSSEKVDV
LRALGAEIVRTPTNARFDSPESHVGVAWRLKNEIPNSHILDQYRNASNPLAHYDTTADEI
LQQCDGKLDMLVASVGTGGTITGIARKLKEKCPGCRIIGVDPEGSILAEPEELNQTEQTT

YEVEGIGYDFIPTVLDRTVVDKWFKSNDEEAFTFARMLIAQEGLLCGGSAGSTVAVAVKA
AQELQEGQRCVVILPDSVRNYMTKFLSDRWMLQKGFLKEEDLTEKKPWWWHLRVQELGLS
APLTVLPTITCGHTIEILREKGFDQAPVVDEAGVILGMVTLGNMLSSLLAGKVQPSDQVG
KVIYKQFKQIRLTDTLGRLSHILEMDHFALVVHEQIQYHSTGKSSQRQMVFGVVTAIDLL
NFVAAQERDQK
>sp|Q8IUF1|CBWD2_HUMAN COBW domain-containing protein 2 OS=Homo sapiens GN=CBWD2 PE=2 SV=1
MLPAVGSADEEEDPAEEDCPELVPMETTQSEEEEKSGLGAKIPVTIITGYLGAGKTTLLN
YILTEQHSKRVAVILNEFGEGSALEKSLAVSQGGELYEEWLELRNGCLCCSVKDNGLRAI
ENLMQKKGKFDYILLETTGLADPGAVASMFWVDAELGSDIYLDGIITIVDSKYGLKHLAE
EKPDGLINEATRQVALADAILINKTDLVPEEDVKKLRATIRSINGLGQILETQRSRVDLS
NVLDLHAFDSLSGISLQKKLQHVPGTQPHLDQSIVTITFEVPGNAKEEHLNMFIQNLLWE
KNVRNKDNHCMEVIRLKGLVSIKDKSQQVIVQGVHELYDLEETPVSWKDDTERTNRLVLL
GRNLDKDILKQLFIATVTETEKQWTTHFKEDQVCT
>sp|P13497|BMP1_HUMAN Bone morphogenetic protein 1 OS=Homo sapiens GN=BMP1 PE=1 SV=2
MPGVARLPLLLGLLLLPRPGRPLDLADYTYDLAEEDDSEPLNYKDPCKAAAFLGDIALDE
EDLRAFQVQQAVDLRRHTARKSSIKAAVPGNTSTPSCQSTNGQPQRGACGRWRGRSRSRR
AATSRPERVWPDGVIPFVIGGNFTGSQRAVFRQAMRHWEKHTCVTFLERTDEDSYIVFTY
RPCGCCSYVGRRGGGPQAISIGKNCDKFGIVVHELGHVVGFWHEHTRPDRDRHVSIVREN
IQPGQEYNFLKMEPQEVESLGETYDFDSIMHYARNTFSRGIFLDTIVPKYEVNGVKPPIG
QRTRLSKGDIAQARKLYKCPACGETLQDSTGNFSSPEYPNGYSAHMHCVWRISVTPGEKI
ILNFTSLDLYRSRLCWYDYVEVRDGFWRKAPLRGRFCGSKLPEPIVSTDSRLWVEFRSSS
NWVGKGFFAVYEAICGGDVKKDYGHIQSPNYPDDYRPSKVCIWRIQVSEGFHVGLTFQSF
EIERHDSCAYDYLEVRDGHSESSTLIGRYCGYEKPDDIKSTSSRLWLKFVSDGSINKAGF
AVNFFKEVDECSRPNRGGCEQRCLNTLGSYKCSCDPGYELAPDKRRCEAACGGFLTKLNG
SITSPGWPKEYPPNKNCIWQLVAPTQYRISLQFDFFETEGNDVCKYDFVEVRSGLTADSK
LHGKFCGSEKPEVITSQYNNMRVEFKSDNTVSKKGFKAHFFSDKDECSKDNGGCQQDCVN
TFGSYECQCRSGFVLHDNKHDCKEAGCDHKVTSTSGTITSPNWPDKYPSKKECTWAISST
PGHRVKLTFMEMDIESQPECAYDHLEVFDGRDAKAPVLGRFCGSKKPEPVLATGSRMFLR
FYSDNSVQRKGFQASHATECGGQVRADVKTKDLYSHAQFGDNNYPGGVDCEWVIVAEEGY
GVELVFQTFEVEEETDCGYDYMELFDGYDSTAPRLGRYCGSGPPEEVYSAGDSVLVKFHS
DDTITKKGFHLRYTSTKFQDTLHSRK
>sp|P22004|BMP6_HUMAN Bone morphogenetic protein 6 OS=Homo sapiens GN=BMP6 PE=1 SV=1
MPGLGRRAQWLCWWWGLLCSCCGPPPLRPPLPAAAAAAAGGQLLGDGGSPGRTEQPPPSP
QSSSGFLYRRLKTQEKREMQKEILSVLGLPHRPRPLHGLQQPQPPALRQQEEQQQQQQLP
RGEPPPGRLKSAPLFMLDLYNALSADNDEDGASEGERQQSWPHEAASSSQRRQPPPGAAH
PLNRKSLLAPGSGSGGASPLTSAQDSAFLNDADMVMSFVNLVEYDKEFSPRQRHHKEFKF
NLSQIPEGEVVTAAEFRIYKDCVMGSFKNQTFLISIYQVLQEHQHRDSDLFLLDTRVVWA
SEEGWLEFDITATSNLWVVTPQHNMGLQLSVVTRDGVHVHPRAAGLVGRDGPYDKQPFMV
AFFKVSEVHVRTTRSASSRRRQQSRNRSTQSQDVARVSSASDYNSSELKTACRKHELYVS
FQDLGWQDWIIAPKGYAANYCDGECSFPLNAHMNATNHAIVQTLVHLMNPEYVPKPCCAP
TKLNAISVLYFDDNSNVILKKYRNMVVRACGCH
>sp|Q13873|BMPR2_HUMAN Bone morphogenetic protein receptor type-2 OS=Homo sapiens GN=BMPR2 PE=1 SV=2

MTSSLQRPWRVPWLPWTILLVSTAAASQNQERLCAFKDPYQQDLGIGESRISHENGTILC
SKGSTCYGLWEKSKGDINLVKQGCWSHIGDPQECHYEECVVTTTPPSIQNGTYRFCCCST
DLCNVNFTENFPPPDTTPLSPPHSFNRDETIIIALASVSVLAVLIVALCFGYRMLTGDRK
QGLHSMNMMEAAASEPSLDLDNLKLLELIGRGRYGAVYKGSLDERPVAVKVFSFANRQNF
INEKNIYRVPLMEHDNIARFIVGDERVTADGRMEYLLVMEYYPNGSLCKYLSLHTSDWVS
SCRLAHSVTRGLAYLHTELPRGDHYKPAISHRDLNSRNVLVKNDGTCVISDFGLSMRLTG
NRLVRPGEEDNAAISEVGTIRYMAPEVLEGAVNLRDCESALKQVDMYALGLIYWEIFMRC
TDLFPGESVPEYQMAFQTEVGNHPTFEDMQVLVSREKQRPKFPEAWKENSLAVRSLKETI
EDCWDQDAEARLTAQCAEERMAELMMIWERNKSVSPTVNPMSTAMQNERNLSHNRRVPKI
GPYPDYSSSSYIEDSIHHTDSIVKNISSEHSMSSTPLTIGEKNRNSINYERQQAQARIPS
PETSVTSLSTNTTTTNTTGLTPSTGMTTISEMPYPDETNLHTTNVAQSIGPTPVCLQLTE
EDLETNKLDPKEVDKNLKESSDENLMEHSLKQFSGPDPLSSTSSSLLYPLIKLAVEATGQ
QDFTQTANGQACLIPDVLPTQIYPLPKQQNLPKRPTSLPLNTKNSTKEPRLKFGSKHKSN
LKQVETGVAKMNTINAAEPHVVTVTMNGVAGRNHSVNSHAATTQYANGTVLSGQTTNIVT
HRAQEMLQNQFIGEDTRLNINSSPDEHEPLLRREQQAGHDEGVLDRLVDRRERPLEGGRT
NSNNNNSNPCSEQDVLAQGVPSTAADPGPSKPRRAQRPNSLDLSATNVLDGSSIQIGEST
QDGKSGSGEKIKKRVKTPYSLKRWRPSTWVISTESLDCEVNNNGSNRAVHSKSSTAVYLA
EGGTATTMVSKDIGMNCL
>sp|Q14692|BMS1_HUMAN Ribosome biogenesis protein BMS1 homolog OS=Homo sapiens GN=BMS1 PE=1 SV=1
MEAKDQKKHRKKNSGPKAAKKKKRLLQDLQLGDEEDARKRNPKAFAVQSAVRMARSFHRT
QDLKTKKHHIPVVDRTPLEPPPIVVVVMGPPKVGKSTLIQCLIRNFTRQKLTEIRGPVTI
VSGKKRRLTIIECGCDINMMIDLAKVADLVLMLIDASFGFEMETFEFLNICQVHGFPKIM
GVLTHLDSFKHNKQLKKTKKRLKHRFWTEVYPGAKLFYLSGMVHGEYQNQEIHNLGRFIT
VMKFRPLTWQTSHPYILADRMEDLTNPEDIRTNIKCDRKVSLYGYLRGAHLKNKSQIHMP
GVGDFAVSDISFLPDPCALPEQQKKRCLNEKEKLVYAPLSGVGGVLYDKDAVYVDLGGSH
VFQDEVGPTHELVQSLISTHSTIDAKMASSRVTLFSDSKPLGSEDIDNQGLMMPKEEKQM
DLNTGRMRRKAIFGDEDESGDSDDEEDDEMSEDDGLENGSSDEEAEEEENAEMTDQYMAV
KGIKRRKLELEEDSEMDLPAFADSDDDLERSSAEEGEAEEADESSEEEDCTAGEKGISGS
KAAGEGSKAGLSPANCQSDRVNLEKSLLMKKAALPTFDSGHCTAEEVFASEDESEESSSL
SAEEEDSENEEAIRKKLSKPSQVSSGQKLGPQNFIDETSDIENLLKEEEDYKEENNDSKE
TSGALKWKEDLSRKAAEAFLRQQQAAPNLRKLIYGTVTEDNEEEDDDTLEELGGLFRVNQ
PDRECKHKADSLDCSRFLVEAPHDWDLEEVMNSIRDCFVTGKWEDDKDAAKVLAEDEELY
GDFEDLETGDVHKGKSGPNTQNEDIEKEVKEEIDPDEEESAKKKHLDKKRKLKEMFDAEY
DEGESTYFDDLKGEMQKQAQLNRAEFEDQDDEARVQYEGFRPGMYVRIEIENVPCEFVQN
FDPHYPIILGGLGNSEGNVGYVQMRLKKHRWYKKILKSRDPIIFSVGWRRFQTIPLYYIE
DHNGRQRLLKYTPQHMHCGAAFWGPITPQGTGFLAIQSVSGIMPDFRIAATGVVLDLDKS
IKIVKKLKLTGFPYKIFKNTSFIKGMFNSALEVAKFEGAVIRTVSGIRGQIKKALRAPEG
AFRASFEDKLLMSDIVFMRTWYPVSIPAFYNPVTSLLKPVGEKDTWSGMRTTGQLRLAHG
VRLKANKDSLYKPILRQKKHFNSLHIPKALQKALPFKNKPKTQAKAGKVPKDRRRPAVIR
EPHERKILALLDALSTVHSQKMKKAKEQRHLHNKEHFRAKQKEEEEKLKRQKDLRKKLFR
IQGQKERRNQKSSLKGAEGQLQ
>sp|Q6ZN30|BNC2_HUMAN Zinc finger protein basonuclin-2 OS=Homo sapiens GN=BNC2 PE=1 SV=1
MAHLGPTPPPHSLNYKSEDRLSEQDWPAYFKVPCCGVDTSQIESEEAEVDVRERETQRDR

EPKRARDLTLRDSCTDNSMQFGTRTTTAEPGFMGTWQNADTNLLFRMSQQAIRCTLVNCT
CECFQPGKINLRTCDQCKHGWVAHALDKLSTQHLYHPTQVEIVQSNVVFDISSLMLYGTQ
AVPVRLKILLDRLFSVLKQEEVLHILHGLGWTLRDYVRGYILQDAAGKVLDRWAIMSREE
EIITLQQFLRFGETKSIVELMAIQEKEGQAVAVPSSKTDSDIRTFIESNNRTRSPSLLAH
LENSNPSSIHHFENIPNSLAFLLPFQYINPVSAPLLGLPPNGLLLEQPGLRLREPSLSTQ
NEYNESSESEVSPTPYKNDQTPNRNALTSITNVEPKTEPACVSPIQNSAPVSDLTKTEHP
KSSFRIHRMRRMGSASRKGRVFCNACGKTFYDKGTLKIHYNAVHLKIKHRCTIEGCNMVF
SSLRSRNRHSANPNPRLHMPMLRNNRDKDLIRATSGAATPVIASTKSNLALTSPGRPPMG
FTTPPLDPVLQNPLPSQLVFSGLKTVQPVPPFYRSLLTPGEMVSPPTSLPTSPIIPTSGT
IEQHPPPPSEPVVPAVMMATHEPSADLAPKKKPRKSSMPVKIEKEIIDTADEFDDEDDDP
NDGGAVVNDMSHDNHCHSQEEMSPGMSVKDFSKHNRTRCISRTEIRRADSMTSEDQEPER
DYENESESSEPKLGEESMEGDEHIHSEVSEKVLMNSERPDENHSEPSHQDVIKVKEEFTD
PTYDMFYMSQYGLYNGGGASMAALHESFTSSLNYGSPQKFSPEGDLCSSPDPKICYVCKK
SFKSSYSVKLHYRNVHLKEMHVCTVAGCNAAFPSRRSRDRHSANINLHRKLLTKELDDMG
LDSSQPSLSKDLRDEFLVKIYGAQHPMGLDVREDASSPAGTEDSHLNGYGRGMAEDYMVL
DLSTTSSLQSSSSIHSSRESDAGSDEGILLDDIDGASDSGESAHKAEAPALPGSLGAEVS
GSLMFSSLSGSNGGIMCNICHKMYSNKGTLRVHYKTVHLREMHKCKVPGCNMMFSSVRSR
NRHSQNPNLHKNIPFTSVD
>sp|Q6PGQ7|BORA_HUMAN Protein aurora borealis OS=Homo sapiens GN=BORA PE=1 SV=2
MGDVKESKMQITPETPGRIPVLNPFESPSDYSNLHEQTLASPSVFKSTKLPTPGKFRWSI
DQLAVINPVEIDPEDIHRQALYLSHSRIDKDVEDKRQKAIEEFFTKDVIVPSPWTDHEGK
QLSQCHSSKCTNINSDSPVGKKLTIHSEKSDAACQTLLSLPVDFNLENILGDYFRADEFA
DQSPGNLSSSSLRRKLFLDGNGSISDSLPSASPGSPHSGVQTSLEMFYSIDLSPVKCRSP
LQTPSSGQFSSSPIQASAKKYSLGSITSPSPISSPTFSPIEFQIGETPLSEQRKFTVHSP
DASSGTNSNGITNPCIRSPYIDGCSPIKNWSPMRLQMYSGGTQYRTSVIQIPFTLETQGE
DEEDKENIPSTDVSSPAMDAAGIHLRQFSNEASTHGTHLVVTAMSVTQNQSSASEKELAL
LQDVEREKDNNTVDMVDPIEIADETTWIKEPVDNGSLPMTDFVSGIAFSIENSHMCMSPL
AESSVIPCESSNIQMDSGYNTQNCGSNIMDTVGAESYCKESDAQTCEVESKSQAFNMKQD
HTTQRCWMKTASPFQCSSP
>sp|Q96GS4|BORC6_HUMAN BLOC-1-related complex subunit 6 OS=Homo sapiens GN=BORCS6 PE=1 SV=2
MESSRGRPGPETDLLAVAEHQALVFGGGPGRTSSEPPAGLRVSGEEETENVGGANRHPRT
SPKTSSCGVVHRPEREALENEPGPQGTLSGAGSRRGAPGAEHEPSLSSRHKNPAPPEGKP
SSGRDCRRGGPGGGMDVEQQEEEDNDEEAAAGSRAGRSFSSRLQDSRSLDGLSEACGGAG
SSGSAESGAGGGRRATISSPLELEGTVSRHGDLTHFVANNLQLKIRLSGAPPPPPSAPAR
PCPAPAPTPTPAIPPIDPEVLRDLERLSRELGGRVDRLLRGLGGAVQELTALSVGCIQTY
RDAVDSLGEAVDMSIKGMYTLLARCEELERALQPVQGLARQVRDIRRTLEVLEALCK
>sp|O14613|BORG1_HUMAN Cdc42 effector protein 2 OS=Homo sapiens GN=CDC42EP2 PE=1 SV=1
MSTKVPIYLKRGSRKGKKEKLRDLLSSDMISPPLGDFRHTIHIGSGGGSDMFGDISFLQG
KFHLLPGTMVEGPEEDGTFDLPFQFTRTATVCGRELPDGPSPLLKNAISLPVIGGPQALT
LPTAQAPPKPPRLHLETPQPSPQEGGSVDIWRIPETGSPNSGLTPESGAEEPFLSNASSL
LSLHVDLGPSILDDVLQIMDQDLDSMQIPT
>sp|P17213|BPI_HUMAN Bactericidal permeability-increasing protein OS=Homo sapiens GN=BPI PE=1 SV=4

MRENMARGPCNAPRWASLMVLVAIGTAVTAAVNPGVVVRISQKGLDYASQQGTAALQKEL
KRIKIPDYSDSFKIKHLGKGHYSFYSMDIREFQLPSSQISMVPNVGLKFSISNANIKISG
KWKAQKRFLKMSGNFDLSIEGMSISADLKLGSNPTSGKPTITCSSCSSHINSVHVHISKS
KVGWLIQLFHKKIESALRNKMNSQVCEKVTNSVSSELQPYFQTLPVMTKIDSVAGINYGL
VAPPATTAETLDVQMKGEFYSENHHNPPPFAPPVMEFPAAHDRMVYLGLSDYFFNTAGLV
YQEAGVLKMTLRDDMIPKESKFRLTTKFFGTFLPEVAKKFPNMKIQIHVSASTPPHLSVQ
PTGLTFYPAVDVQAFAVLPNSSLASLFLIGMHTTGSMEVSAESNRLVGELKLDRLLLELK
HSNIGPFPVELLQDIMNYIVPILVLPRVNEKLQKGFPLPTPARVQLYNVVLQPHQNFLLF
GADVVYK
>sp|O15178|BRAC_HUMAN Brachyury protein OS=Homo sapiens GN=T PE=1 SV=1
MSSPGTESAGKSLQYRVDHLLSAVENELQAGSEKGDPTERELRVGLEESELWLRFKELTN
EMIVTKNGRRMFPVLKVNVSGLDPNAMYSFLLDFVAADNHRWKYVNGEWVPGGKPEPQAP
SCVYIHPDSPNFGAHWMKAPVSFSKVKLTNKLNGGGQIMLNSLHKYEPRIHIVRVGGPQR
MITSHCFPETQFIAVTAYQNEEITALKIKYNPFAKAFLDAKERSDHKEMMEEPGDSQQPG
YSQWGWLLPGTSTLCPPANPHPQFGGALSLPSTHSCDRYPTLRSHRSSPYPSPYAHRNNS
PTYSDNSPACLSMLQSHDNWSSLGMPAHPSMLPVSHNASPPTSSSQYPSLWSVSNGAVTP
GSQAAAVSNGLGAQFFRGSPAHYTPLTHPVSAPSSSGSPLYEGAAAATDIVDSQYDAAAQ
GRLIASWTPVSPPSM
>sp|Q9HAW0|BRF2_HUMAN Transcription factor IIIB 50 kDa subunit OS=Homo sapiens GN=BRF2 PE=1 SV=1
MPGRGRCPDCGSTELVEDSHYSQSQLVCSDCGCVVTEGVLTTTFSDEGNLREVTYSRSTG
ENEQVSRSQQRGLRRVRDLCRVLQLPPTFEDTAVAYYQQAYRHSGIRAARLQKKEVLVGC
CVLITCRQHNWPLTMGAICTLLYADLDVFSSTYMQIVKLLGLDVPSLCLAELVKTYCSSF
KLFQASPSVPAKYVEDKEKMLSRTMQLVELANETWLVTGRHPLPVITAATFLAWQSLQPA
DRLSCSLARFCKLANVDLPYPASSRLQELLAVLLRMAEQLAWLRVLRLDKRSVVKHIGDL
LQHRQSLVRSAFRDGTAEVETREKEPPGWGQGQGEGEVGNNSLGLPQGKRPASPALLLPP
CMLKSPKRICPVPPVSTVTGDENISDSEIEQYLRTPQEVRDFQRAQAARQAATSVPNPP
>sp|Q8WY22|BRI3B_HUMAN BRI3-binding protein OS=Homo sapiens GN=BRI3BP PE=1 SV=1
MGARASGGPLARAGLLLLLLLLLLLGLLAPGAQGARGRGGAEKNSYRRTVNTFSQSVSSL
FGEDNVRAAQKFLARLTERFVLGVDMFVETLWKVWTELLDVLGLDVSNLSQYFSPASVSS
SPARALLLVGVVLLAYWFLSLTLGFTFSVLHVVFGRFFWIVRVVLFSMSCVYILHKYEGE
PENAVLPLCFVVAVYFMTGPMGFYWRSSPSGPSNPSNPSVEEKLEHLEKQVRLLNIRLNR
VLESLDRSKDK
>sp|Q8IWQ3|BRSK2_HUMAN Serine/threonine-protein kinase BRSK2 OS=Homo sapiens GN=BRSK2 PE=1 SV=3
MTSTGKDGGAQHAQYVGPYRLEKTLGKGQTGLVKLGVHCVTCQKVAIKIVNREKLSESVL
MKVEREIAILKLIEHPHVLKLHDVYENKKYLYLVLEHVSGGELFDYLVKKGRLTPKEARK
FFRQIISALDFCHSHSICHRDLKPENLLLDEKNNIRIADFGMASLQVGDSLLETSCGSPH
YACPEVIRGEKYDGRKADVWSCGVILFALLVGALPFDDDNLRQLLEKVKRGVFHMPHFIP
PDCQSLLRGMIEVDAARRLTLEHIQKHIWYIGGKNEPEPEQPIPRKVQIRSLPSLEDIDP
DVLDSMHSLGCFRDRNKLLQDLLSEEENQEKMIYFLLLDRKERYPSQEDEDLPPRNEIDP
PRKRVDSPMLNRHGKRRPERKSMEVLSVTDGGSPVPARRAIEMAQHGQRSRSISGASSGL
STSPLSSPRVTPHPSPRGSPLPTPKGTPVHTPKESPAGTPNPTPPSSPSVGGVPWRARLN
SIKNSFLGSPRFHRRKLQVPTPEEMSNLTPESSPELAKKSWFGNFISLEKEEQIFVVIKD

KPLSSIKADIVHAFLSIPSLSHSVISQTSFRAEYKATGGPAVFQKPVKFQVDITYTEGGE
AQKENGIYSVTFTLLSGPSRRFKRVVETIQAQLLSTHDPPAAQHLSDTTNCMEMMTGRLS
KCGSPLSNFFDVIKQLFSDEKNGQAAQAPSTPAKRSAHGPLGDSAAAGPGPGGDAEYPTG
KDTAKMGPPTARREQP
>sp|Q6RI45|BRWD3_HUMAN Bromodomain and WD repeat-containing protein 3 OS=Homo sapiens GN=BRWD3 PE=1 SV=2
MAAAPTQIEAELYYLIARFLQSGPCNKSAQVLVQELEEHQLIPRRLDWEGKEHRRSFEDL
VAANAHIPPDYLLKICERIGPLLDKEIPQSVPGVQTLLGVGRQSLLRDAKDCKSTLWNGS
AFAALHRGRPPELPVNYVKPPNVVNITSARQLTGCSRFGHIFPSSAYQHIKMHKRILGHL
SSVYCVAFDRSGRRIFTGSDDCLVKIWATDDGRLLATLRGHSAEISDMAVNYENTLIAAG
SCDKVVRVWCLRTCAPVAVLQGHSASITSIQFCPSTKGTNRYLTSTGADGTICFWQWHVK
TMKFRDRPVKFTERSRPGVQISCSSFSSGGMFITTGSTDHVIRIYYLGSEVPEKIAELES
HTDKVVAVQFCNNGDSLRFVSGSRDGTARIWQYQQQEWKSIVLDMATKMTGNNLPSGEDK
ITKLKVTMVAWDRYDTTVITAVNNFLLKVWNSITGQLLHTLSGHDDEVFVLEAHPFDQRI
ILSAGHDGNIFIWDLDRGTKIRNYFNMIEGQGHGAVFDCKFSPDGNHFACTDSHGHLLLF
GFGCSKYYEKIPDQMFFHTDYRPLIRDANNYVLDEQTQQAPHLMPPPFLVDVDGNPHPTK
FQRLVPGRENCKDEQLIPQLGYVANGDGEVVEQVIGQQTNDQDESILDGIIRELQREQDL
RLINEGDVPHLPVNRAYSVNGALRSPNMDISSSPNIRLRRHSSQIEGVRQMHNNAPRSQM
ATERDLMAWSRRVVVNELNNGVSRVQEECRTAKGDIEISLYTVEKKKKPSYTTQRNDYEP
SCGRSLRRTQRKRQHTYQTRSNIEHNSQASCQNSGVQEDSDSSSEEDETVGTSDASVEDP
VVEWQSESSSSDSSSEYSDWTADAGINLQPPKRQTRQTTRKICSSSDEENLKSLEERQKK
PKQTRKKKGGLVSIAGEPNEEWFAPQWILDTIPRRSPFVPQMGDELIYFRQGHEAYVRAV
RKSKIYSVNLQKQPWNKMDLREQEFVKIVGIKYEVGPPTLCCLKLAFLDPISGKMTGESF
SIKYHDMPDVIDFLVLHQFYNEAKERNWQIGDRFRSIIDDAWWFGTVESQQPFQPEYPDS
SFQCYSVHWDNNEREKMSPWDMEPIPEGTAFPDEVGAGVPVSQEELTALLYKPQEGEWGA
HSRDEECERVIQGINHLLSLDFASPFAVPVDLSAYPLYCTVVAYPTDLNTIRRRLENRFY
RRISALMWEVRYIEHNARTFNEPDSPIVKAAKIVTDVLLRFIGDQSCTDILDTYNKIKAE
ERNSTDAEEDTEIVDLDSDGPGTSSGRKVKCRGRRQSLKCNPDAWKKQCKELLSLIYERE
DSEPFRQPADLLSYPGHQEQEGESSESVVPERQQDSSLSEDYQDVIDTPVDFSTVKETLE
AGNYGSPLEFYKDVRQIFNNSKAYTSNKKSRIYSMMLRLSALFESHIKNIISEYKSAIQS
QKRRRPRYRKRLRSSSSSLSSSGAPSPKGKQKQMKLQPKNDQNTSVSHARTSSPFSSPVS
DAAEGLSLYLLDDEPDGPFSSSSFGGYSRSGNSHDPGKAKSFRNRVLPVKQDHSLDGPLT
NGDGREPRTGIKRKLLSASEEDENMGGEDKEKKETKEKSHLSTSESGELGSSLSSESTCG
SDSDSESTSRTDQDYVDGDHDYSKFIQTRPKRKLRKQHGNGKRNWKTRGTGGRGRWGRWG
RWSRGGRGRGGRGRGSRGRGGGGTRGRGRGRGGRGASRGATRAKRARIADDEFDTMFSGR
FSRLPRIKTRNQGRRTVLYNDDSDNDNFVSTEDPLNLGTSRSGRVRKMTEKARVSHLMGW
NY
>sp|Q9BX70|BTBD2_HUMAN BTB/POZ domain-containing protein 2 OS=Homo sapiens GN=BTBD2 PE=1 SV=1
MAAGGSGGRASCPPGVGVGPGTGGSPGPSANAAATPAPGNAAAAAAAAAAAAAAPGPTPP
APPGPGTDAQAAGAERAEEAAGPGAAALQREAAYNWQASKPTVQERFAFLFNNEVLCDVH
FLVGKGLSSQRIPAHRFVLAVGSAVFDAMFNGGMATTSTEIELPDVEPAAFLALLKFLYS
DEVQIGPETVMTTLYTAKKYAVPALEAHCVEFLKKNLRADNAFMLLTQARLFDEPQLASL
CLENIDKNTADAITAEGFTDIDLDTLVAVLERDTLGIREVRLFNAVVRWSEAECQRQQLQ

VTPENRRKVLGKALGLIRFPLMTIEEFAAGPAQSGILVDREVVSLFLHFTVNPKPRVEFI
DRPRCCLRGKECSINRFQQVESRWGYSGTSDRIRFSVNKRIFVVGFGLYGSIHGPTDYQV
NIQIIHTDSNTVLGQNDTGFSCDGSASTFRVMFKEPVEVLPNVNYTACATLKGPDSHYGT
KGLRKVTHESPTTGAKTCFTFCYAAGNNNGTSVEDGQIPEVIFYT
>sp|Q9BSF8|BTBDA_HUMAN BTB/POZ domain-containing protein 10 OS=Homo sapiens GN=BTBD10 PE=1 SV=2
MAGRPHPYDGNSSDPENWDRKLHSRPRKLYKHSSTSSRIAKGGVDHTKMSLHGASGGHER
SRDRRRSSDRSRDSSHERTESQLTPCIRNVTSPTRQHHVEREKDHSSSRPSSPRPQKASP
NGSISSAGNSSRNSSQSSSDGSCKTAGEMVFVYENAKEGARNIRTSERVTLIVDNTRFVV
DPSIFTAQPNTMLGRMFGSGREHNFTRPNEKGEYEVAEGIGSTVFRAILDYYKTGIIRCP
DGISIPELREACDYLCISFEYSTIKCRDLSALMHELSNDGARRQFEFYLEEMILPLMVAS
AQSGERECHIVVLTDDDVVDWDEEYPPQMGEEYSQIIYSTKLYRFFKYIENRDVAKSVLK
ERGLKKIRLGIEGYPTYKEKVKKRPGGRPEVIYNYVQRPFIRMSWEKEEGKSRHVDFQCV
KSKSITNLAAAAADIPQDQLVVMHPTPQVDELDILPIHPPSGNSDLDPDAQNPML
>sp|Q9BRD0|BUD13_HUMAN BUD13 homolog OS=Homo sapiens GN=BUD13 PE=1 SV=1
MAAAPPLSKAEYLKRYLSGADAGVDRGSESGRKRRKKRPKPGGAGGKGMRIVDDDVSWTA
ISTTKLEKEEEEDDGDLPVVAEFVDERPEEVKQMEAFRSSAKWKLLGGHNEDLPSNRHFR
HDTPDSSPRRVRHGTPDPSPRKDRHDTPDPSPRRARHDTPDPSPLRGARHDSDTSPPRRI
RHDSSDTSPPRRARHDSPDPSPPRRPQHNSSGASPRRVRHDSPDPSPPRRARHGSSDISS
PRRVHNNSPDTSRRTLGSSDTQQLRRARHDSPDLAPNVTYSLPRTKSGKAPERASSKTSP
HWKESGASHLSFPKNSKYEYDPDISPPRKKQAKSHFGDKKQLDSKGDCQKATDSDLSSPR
HKQSPGHQDSDSDLSPPRNRPRHRSSDSDLSPPRRRQRTKSSDSDLSPPRRSQPPGKKAA
HMYSGAKTGLVLTDIQREQQELKEQDQETMAFEAEFQYAETVFRDKSGRKRNLKLERLEQ
RRKAEKDSERDELYAQWGKGLAQSRQQQQNVEDAMKEMQKPLARYIDDEDLDRMLREQER
EGDPMANFIKKNKAKENKNKKVRPRYSGPAPPPNRFNIWPGYRWDGVDRSNGFEQKRFAR
LASKKAVEELAYKWSVEDM
>sp|Q68D86|C102B_HUMAN Coiled-coil domain-containing protein 102B OS=Homo sapiens GN=CCDC102B PE=1 SV=4
MNLDSIHRLIEETQIFQMQQSSIKSRGDMVAPASPPRDTCNTCFPLHGLQSHAAHNFCAH
SYNTNKWDICEELRLRELEEVKARAAQMEKTMRWWSDCTANWREKWSKVRAERNSAREEG
RQLRIKLEMAMKELSTLKKKQSLPPQKEALEAKVTQDLKLPGFVEESCEHTDQFQLSSQM
HESIREYLVKRQFSTKEDTNNKEQGVVIDSLKLSEEMKPNLDGVDLFNNGGSGNGETKTG
LRLKAINLPLENEVTEISALQVHLDEFQKILWKEREMRTALEKEIERLESALSLWKWKYE
ELKESKPKNVKEFDILLGQHNDEMQELSGNIKEESKSQNSKDRVICELRAELERLQAENT
SEWDKREILEREKQGLERENRRLKIQVKEMEELLDKKNRLSANSQSPDFKMSQIDLQEKN
QELLNLQHAYYKLNRQYQANIAELTHANNRVDQNEAEVKKLRLRVEELKQGLNQKEDELD
DSLNQIRKLQRSLDEEKERNENLETELRHLQNW
>sp|Q5VWW1|C1QL3_HUMAN Complement C1q-like protein 3 OS=Homo sapiens GN=C1QL3 PE=2 SV=1
MVLLLVILIPVLVSSAGTSAHYEMLGTCRMVCDPYGGTKAPSTAATPDRGLMQSLPTFIQ
GPKGEAGRPGKAGPRGPPGEPGPPGPMGPPGEKGEPGRQGLPGPPGAPGLNAAGAISAAT
YSTVPKIAFYAGLKRQHEGYEVLKFDDVVTNLGNHYDPTTGKFTCSIPGIYFFTYHVLMR
GGDGTSMWADLCKNNQVRASAIAQDADQNYDYASNSVVLHLEPGDEVYIKLDGGKAHGGN
NNKYSTFSGFIIYAD

>sp|Q9BXJ2|C1QT7_HUMAN Complement C1q tumor necrosis factor-related protein 7 OS=Homo sapiens GN=C1QTNF7 PE=2 SV=1
MFVLLYVTSFAICASGQPRGNQLKGENYSPRYICSIPGLPGPPGPPGANGSPGPHGRIGL
PGRDGRDGRKGEKGEKGTAGLRGKTGPLGLAGEKGDQGETGKKGPIGPEGEKGEVGPIGP
PGPKGDRGEQGDPGLPGVCRCGSIVLKSAFSVGITTSYPEERLPIIFNKVLFNEGEHYNP
ATGKFICAFPGIYYFSYDITLANKHLAIGLVHNGQYRIKTFDANTGNHDVASGSTVIYLQ
PEDEVWLEIFFTDQNGLFSDPGWADSLFSGFLLYVDTDYLDSISEDDEL
>sp|Q9NZP8|C1RL_HUMAN Complement C1r subcomponent-like protein OS=Homo sapiens GN=C1RL PE=1 SV=2
MPGPRVWGKYLWRSPHSKGCPGAMWWLLLWGVLQACPTRGSVLLAQELPQQLTSPGYPEP
YGKGQESSTDIKAPEGFAVRLVFQDFDLEPSQDCAGDSVTISFVGSDPSQFCGQQGSPLG
RPPGQREFVSSGRSLRLTFRTQPSSENKTAHLHKGFLALYQTVAVNYSQPISEASRGSEA
INAPGDNPAKVQNHCQEPYYQAAAAGALTCATPGTWKDRQDGEEVLQCMPVCGRPVTPIA
QNQTTLGSSRAKLGNFPWQAFTSIHGRGGGALLGDRWILTAAHTIYPKDSVSLRKNQSVN
VFLGHTAIDEMLKLGNHPVHRVVVHPDYRQNESHNFSGDIALLELQHSIPLGPNVLPVCL
PDNETLYRSGLLGYVSGFGMEMGWLTTELKYSRLPVAPREACNAWLQKRQRPEVFSDNMF
CVGDETQRHSVCQGDSGSVYVVWDNHAHHWVATGIVSWGIGCGEGYDFYTKVLSYVDWIK
GVMNGKN
>sp|O14523|C2C2L_HUMAN C2 domain-containing protein 2-like OS=Homo sapiens GN=C2CD2L PE=1 SV=3
MDPGWGQRDVGWAALLILFAASLLTVFAWLLQYARGLWLARARGDRGPGPALAGEPAGSL
RELGVWRSLLRLRATRAGAAEEPGVRGLLASLFAFKSFRENWQRAWVRALNEQACRNGSS
IQIAFEEVPQLPPRASISHVTCVDQSEHTMVLRCQLSAEEVRFPVSVTQQSPAAVSMETY
HVTLTLPPTQLEVNLEEIPGEGLLISWAFTDRPDLSLTVLPKLQARERGEEQVELSTIEE
LIKDAIVSTQPAMMVNLRACSAPGGLVPSEKPPMMPQAQPAIPRPNRLFLRQLRASHLGN
ELEGTEELCCVAELDNPMQQKWTKPARAGSEVEWTEDLALDLGPQSRELTLKVLRSSSCG
DTELLGQATLPVGSPSRPLSRRQLCPLTPGPGKALGPAATMAVELHYEEGSPRNLGTPTS
STPRPSITPTKKIELDRTIMPDGTIVTTVTTVQSRPRIDGKLDSPSRSPSKVEVTEKTTT
VLSESSGPSNTSHSSSRDSHLSNGLDPVAETAIRQLTEPSGRVAKKTPTKRSTLIISGVS
KVPIAQDELALSLGYAASLEASVQDDAGTSGGPSSPPSDPPAMSPGPLDALSSPTSVQEA
DETTRSDISERPSVDDIESETGSTGALETRSLKDHKVSFLRSGTKLIFRRRPRQKEAGLS
QSHDDLSNATATPSVRKKAGSFSRRLIKRFSFKSKPKANGNPSPQL
>sp|Q8NCU7|C2C4A_HUMAN C2 calcium-dependent domain-containing protein 4A OS=Homo sapiens GN=C2CD4A PE=2 SV=2
MWCLERLRLGPECLRRSGDWLLPGRARGAKSRTTAACANVLTPDRIPEFCIPPRLMPRLA
LAALRNSWVEEAGMDEGAGRTDWDPRSQAALSLPHLPRVRTAYGFCALLESPHTRRKESL
LLGGPPAPRPRAHTYGGGGGPDALLGTLRVPRAPGPATPAAPGCPRPPQDALARRPRGCR
LLRVPDGLLSRALRAGRSRRLTRVRSVSSGNEDKERRAGSQSPARAPSTSPPSSRVPFPE
RLEAEGTVALGRAGDALRLAAEYCPGTGRLRLRLLRAESPAGGAPGPRAVSCRLSLVLRP
PGTALRQCSTVVGRSRKASFDQDFCFDGLSEDEVRRLAVRVKARDEGRGRERGRLLGQGE
LSLGALLLL
>sp|Q9NRR3|C42S2_HUMAN CDC42 small effector protein 2 OS=Homo sapiens GN=CDC42SE2 PE=1 SV=1
MSEFWLCFNCCIAEQPQPKRRRRIDRSMIGEPTNFVHTAHVGSGDLFSGMNSVSSIQNQM

QSKGGYGGGMPANVQMQLVDTKAG
>sp|Q9Y5P4|C43BP_HUMAN Collagen type IV alpha-3-binding protein OS=Homo sapiens GN=COL4A3BP PE=1 SV=1
MSDNQSWNSSGSEEDPETESGPPVERCGVLSKWTNYIHGWQDRWVVLKNNALSYYKSEDE
TEYGCRGSICLSKAVITPHDFDECRFDISVNDSVWYLRAQDPDHRQQWIDAIEQHKTESG
YGSESSLRRHGSMVSLVSGASGYSATSTSSFKKGHSLREKLAEMETFRDILCRQVDTLQK
YFDACADAVSKDELQRDKVVEDDEDDFPTTRSDGDFLHSTNGNKEKLFPHVTPKGINGID
FKGEAITFKATTAGILATLSHCIELMVKREDSWQKRLDKETEKKRRTEEAYKNAMTELKK
KSHFGGPDYEEGPNSLINEEEFFDAVEAALDRQDKIEEQSQSEKVRLHWPTSLPSGDAFS
SVGTHRFVQKPYSRSSSMSSIDLVSASDDVHRFSSQVEEMVQNHMTYSLQDVGGDANWQL
VVEEGEMKVYRREVEENGIVLDPLKATHAVKGVTGHEVCNYFWNVDVRNDWETTIENFHV
VETLADNAIIIYQTHKRVWPASQRDVLYLSVIRKIPALTENDPETWIVCNFSVDHDSAPL
NNRCVRAKINVAMICQTLVSPPEGNQEISRDNILCKITYVANVNPGGWAPASVLRAVAKR
EYPKFLKRFTSYVQEKTAGKPILF
>sp|O14569|C56D2_HUMAN Cytochrome b561 domain-containing protein 2 OS=Homo sapiens GN=CYB561D2 PE=1 SV=1
MALSAETESHIYRALRTASGAAAHLVALGFTIFVAVLARPGSSLFSWHPVLMSLAFSFLM
TEALLVFSPESSLLHSLSRKGRARCHWVLQLLALLCALLGLGLVILHKEQLGKAHLVTRH
GQAGLLAVLWAGLQCSGGVGLLYPKLLPRWPLAKLKLYHATSGLVGYLLGSASLLLGMCS
LWFTASVTGAAWYLAVLCPVLTSLVIMNQVSNAYLYRKRIQP
>sp|Q6P1W5|CA094_HUMAN Uncharacterized protein C1orf94 OS=Homo sapiens GN=C1orf94 PE=1 SV=2
MRGGGGCVLALGGQRGFQKERRRMASGNGLPSSSALVAKGPCALGPFPRYIWIHQDTPQD
SLDKTCHEIWKRVQGLPEASQPWTSMEQLSVPVVGTLRGNELSFQEEALELSSGKDEISL
LVEQEFLSLTKEHSILVEESSGELEVPGSSPEGTRELAPCILAPPLVAGSNERPRASIIV
GDKLLKQKVAMPVISSRQDCDSATSTVTDILCAAEVKSSKGTEDRGRILGDSNLQVSKLL
SQFPLKSTETSKVPDNKNVLDKTRVTKDFLQDNLFSGPGPKEPTGLSPFLLLPPRPPPAR
PDKLPELPAQKRQLPVFAKICSKPKADPAVERHHLMEWSPGTKEPKKGQGSLFLSQWPQS
QKDACGEEGCCDAVGTASLTLPPKKPTCPAEKNLLYEFLGATKNPSGQPRLRNKVEVDGP
ELKFNAPVTVADKNNPKYTGNVFTPHFPTAMTSATLNQPLWLNLNYPPPPVFTNHSTFLQ
YQGLYPQQAARMPYQQALHPQLGCYSQQVMPYNPQQMGQQIFRSSYTPLLSYIPFVQPNY
PYPQRTPPKMSANPRDPPLMAGDGPQYLFPQGYGFGSTSGGPLMHSPYFSSSGNGINF
>sp|Q9H7X2|CA115_HUMAN Uncharacterized protein C1orf115 OS=Homo sapiens GN=C1orf115 PE=2 SV=1
MTVGARLRSKAESSLLRRGPRGRGRTEGDEEAAAILEHLEYADEAEAAAESGTSAADERG
PGTRGARRVHFALLPERYEPLEEPAPSEQPRKRYRRKLKKYGKNVGKVIIKGCRYVVIGL
QGFAAAYSAPFAVATSVVSFVR
>sp|Q9NWV4|CA123_HUMAN UPF0587 protein C1orf123 OS=Homo sapiens GN=C1orf123 PE=1 SV=1
MGKIALQLKATLENITNLRPVGEDFRWYLKMKCGNCGEISDKWQYIRLMDSVALKGGRGS
ASMVQKCKLCARENSIEILSSTIKPYNAEDNENFKTIVEFECRGLEPVDFQPQAGFAAEG
VESGTAFSDINLQEKDWTDYDEKAQESVGIYEVTHQFVKC
>sp|Q8NDD1|CA131_HUMAN Uncharacterized protein C1orf131 OS=Homo sapiens GN=C1orf131 PE=1 SV=3
MRVDSSADPTMSQEQGPGSSTPPSSPTLLDALLQNLYDFGGTEGETEQKKIIKKRENKKR

DVMASAALAAEPSPLPGSLIRGQRKSASSFFKELREERHCAPSGTPTGPEILAAAVPPSS
LKNNREQVEVVEFHSNKKRKLTPDHNKNTKQANPSVLERDVDTQEFNLEKARLEVHRFGI
TGYGKGKERILEQERAIMLGAKPPKKSYVNYKVLQEQIKEKKAAKEEEKRLAQETDIFKK
KKRKGQEDRRKSKKKSAPSILSNGRIGQVGKFKNGTLILSPVDIKKINSSRVAK
>sp|Q5T7R7|CA185_HUMAN Uncharacterized protein C1orf185 OS=Homo sapiens GN=C1orf185 PE=2 SV=1
MASPKGFFNYLTYFLAAGAVTLGIGFFALASALWFLICKRREIFQNSKFKAIDERCRQRP
SMAKIKSHSQCVFISRNFHTGRFQLQEEQRKKEAAHIKAIKDHSKDEPQLATKNIICDPS
ETSSTTNRSSVTLSLSTLPSDSYYSQSIEAADDWFSDDSLVKRNSPMPSLGEPLMEKVFS
YLSTISLEEGTESVLNDTL
>sp|Q9H425|CA198_HUMAN Uncharacterized protein C1orf198 OS=Homo sapiens GN=C1orf198 PE=1 SV=1
MASMAAAIAASRSAVMSGNRPLDDRERKRFTYFSSLSPMARKIMQDKEKIREKYGPEWAR
LPPAQQDEIIDRCLVGPRAPAPRDPGDSEELTRFPGLRGPTGQKVVRFGDEDLTWQDEHS
APFSWETKSQMEFSISALSIQEPSNGTAASEPRPLSKASQGSQALKSSQGSRSSSLDALG
PTRKEEEASFWKINAERSRGEGPEAEFQSLTPSQIKSMEKGEKVLPPCYRQEPAPKDREA
KVERPSTLRQEQRPLPNVSTERERPQPVQAFSSALHEAAPSQLEGKLPSPDVRQDDGEDT
LFSEPKFAQVSSSNVVLKTGFDFLDNW
>sp|Q5SR53|CA200_HUMAN Putative uncharacterized protein PIK3CD-AS1 OS=Homo sapiens GN=PIK3CD-AS1 PE=2 SV=1
MPSQSACPVLSTAPGTPCDLRKHLLNMVSEEKRSPQLSAKTWRRGLRLQKRRNALFLPEG
DICVVGSTSGARALIPETSKLERSGTVIAYCNLELLASSDPPVWASQSTGMTGMSYRSQP
QLGFKSTPPAHSSVFHHSVKAPKEDQAQEAASRPLTSQDGWNPNIKK
>sp|O75309|CAD16_HUMAN Cadherin-16 OS=Homo sapiens GN=CDH16 PE=2 SV=1
MVPAWLWLLCVSVPQALPKAQPAELSVEVPENYGGNFPLYLTKLPLPREGAEGQIVLSGD
SGKATEGPFAMDPDSGFLLVTRALDREEQAEYQLQVTLEMQDGHVLWGPQPVLVHVKDEN
DQVPHFSQAIYRARLSRGTRPGIPFLFLEASDRDEPGTANSDLRFHILSQAPAQPSPDMF
QLEPRLGALALSPKGSTSLDHALERTYQLLVQVKDMGDQASGHQATATVEVSIIESTWVS
LEPIHLAENLKVLYPHHMAQVHWSGGDVHYHLESHPPGPFEVNAEGNLYVTRELDREAQA
EYLLQVRAQNSHGEDYAAPLELHVLVMDENDNVPICPPRDPTVSIPELSPPGTEVTRLSA
EDADAPGSPNSHVVYQLLSPEPEDGVEGRAFQVDPTSGSVTLGVLPLRAGQNILLLVLAM
DLAGAEGGFSSTCEVEVAVTDINDHAPEFITSQIGPISLPEDVEPGTLVAMLTAIDADLE
PAFRLMDFAIERGDTEGTFGLDWEPDSGHVRLRLCKNLSYEAAPSHEVVVVVQSVAKLVG
PGPGPGATATVTVLVERVMPPPKLDQESYEASVPISAPAGSFLLTIQPSDPISRTLRFSL
VNDSEGWLCIEKFSGEVHTAQSLQGAQPGDTYTVLVEAQDTDEPRLSASAPLVIHFLKAP
PAPALTLAPVPSQYLCTPRQDHGLIVSGPSKDPDLASGHGPYSFTLGPNPTVQRDWRLQT
LNGSHAYLTLALHWVEPREHIIPVVVSHNAQMWQLLVRVIVCRCNVEGQCMRKVGRMKGM
PTKLSAVGILVGTLVAIGIFLILIFTHWTMSRKKDPDQPADSVPLKATV
>sp|Q9H159|CAD19_HUMAN Cadherin-19 OS=Homo sapiens GN=CDH19 PE=2 SV=1
MNCYLLLRFMLGIPLLWPCLGATENSQTKKVKQPVRSHLRVKRGWVWNQFFVPEEMNTTS
HHIGQLRSDLDNGNNSFQYKLLGAGAGSTFIIDERTGDIYAIQKLDREERSLYILRAQVI
DIATGRAVEPESEFVIKVSDINDNEPKFLDEPYEAIVPEMSPEGTLVIQVTASDADDPSS
GNNARLLYSLLQGQPYFSVEPTTGVIRISSKMDRELQDEYWVIIQAKDMIGQPGALSGTT
SVLIKLSDVNDNKPIFKESLYRLTVSESAPTGTSIGTIMAYDNDIGENAEMDYSIEEDDS

QTFDIITNHETQEGIVILKKKVDFEHQNHYGIRAKVKNHHVPEQLMKYHTEASTTFIKIQ
VEDVDEPPLFLLPYYVFEVFEETPQGSFVGVVSATDPDNRKSPIRYSITRSKVFNINDNG
TITTSNSLDREISAWYNLSITATEKYNIEQISSIPLYVQVLNINDHAPEFSQYYETYVCE
NAGSGQVIQTISAVDRDESIEEHHFYFNLSVEDTNNSSFTIIDNQDNTAVILTNRTGFNL
QEEPVFYISILIADNGIPSLTSTNTLTIHVCDCGDSGSTQTCQYQELVLSMGFKTEVIIA
ILICIMIIFGFIFLTLGLKQRRKQILFPEKSEDFRENIFQYDDEGGGEEDTEAFDIAELR
SSTIMRERKTRKTTSAEIRSLYRQSLQVGPDSAIFRKFILEKLEEANTDPCAPPFDSLQT
YAFEGTGSLAGSLSSLESAVSDQDESYDYLNELGPRFKRLACMFGSAVQSNN
>sp|Q9HBT6|CAD20_HUMAN Cadherin-20 OS=Homo sapiens GN=CDH20 PE=2 SV=2
MWTSGRMSNAKNWLGLGMSLYFWGLMDLTTTVLSDTPTPQGELEALLSDKPQSHQRTKRS
WVWNQFFVLEEYTGTDPLYVGKLHSDMDRGDGSIKYILSGEGAGIVFTIDDTTGDIHAIQ
RLDREERAQYTLRAQALDRRTGRPMEPESEFIIKIQDINDNEPKFLDGPYVATVPEMSPV
GTSVIQVTATDADDPTYGNSARVVYSILQGQPYFSVDSKTGVIRTALMNMDREAKEYYEV
IIQAKDMGGQLGGLAGTTTVNITLSDVNDNPPRFPQKHYQMSVLESAPISSTVGRVFAKD
LDEGINAEMKYTIVDGDGADAFDISTDPNFQVGIITVKKPLSFESKKSYTLKVEGANPHL
EMRFLNLGPFQDTTTVHISVEDVDEPPVFEPGFYFVEVPEDVAIGTTIQIISAKDPDVTN
NSIRYSIDRSSDPGRFFYVDITTGALMTARPLDREEFSWHNITVLAMEMNNPSQVGSVPV
TIKVLDVNDNAPEFPRFYEAFVCENAKAGQLIQTVSAVDQDDPRNGQHFYYSLAPEAANN
PNFTIRDNQDNTARILTRRSGFRQQEQSVFHLPILIADSGQPVLSSTGTLTIQVCSCDDD
GHVMSCSPEAYMLPVSLSRGALIAILACIFVLLVLVLLILSMRRHRKQPYIIDDEENIHE
NIVRYDDEGGGEEDTEAFDIAAMWNPREAQAGAAPKTRQDMLPEIESLSRYVPQTCAVNS
TVHSYVLAKLYEADMDLWAPPFDSLQTYMFEGDGSVAGSLSSLQSATSDSEQSFDFLTDW
GPRFRKLAELYGASEGPAPLW
>sp|Q9UJ99|CAD22_HUMAN Cadherin-22 OS=Homo sapiens GN=CDH22 PE=2 SV=2
MRPRPEGRGLRAGVALSPALLLLLLLPPPPTLLGRLWAAGTPSPSAPGARQDGALGAGRV
KRGWVWNQFFVVEEYTGTEPLYVGKIHSDSDEGDGAIKYTISGEGAGTIFLIDELTGDIH
AMERLDREQKTFYTLRAQARDRATNRLLEPESEFIIKVQDINDSEPRFLHGPYIGSVAEL
SPTGTSVMQVMASDADDPTYGSSARLVYSVLDGEHHFTVDPKTGVIRTAVPDLDRESQER
YEVVIQATDMAGQLGGLSGSTTVTIVVTDVNDNPPRFPQKMYQFSIQESAPIGTAVGRVK
AEDSDVGENTDMTYHLKDESSSGGDVFKVTTDSDTQEAIIVVQKRLDFESQPVHTVILEA
LNKFVDPRFADLGTFRDQAIVRVAVTDVDEPPEFRPPSGLLEVQEDAQVGSLVGVVTARD
PDAANRPVRYAIDRESDLDQIFDIDADTGAIVTGKGLDRETAGWHNITVLAMEADNHAQL
SRASLRIRILDVNDNPPELATPYEAAVCEDAKPGQLIQTISVVDRDEPQGGHRFYFRLVP
EAPSNPHFSLLDIQDNTAAVHTQHVGFNRQEQDVFFLPILVVDSGPPTLSSTGTLTIRIC
GCDSSGTIQSCNTTAFVMAASLSPGALIALLVCVLILVVLVLLILTLRRHHKSHLSSDED
EDMRDNVIKYNDEGGGEQDTEAYDMSALRSLYDFGELKGGDGGGSAGGGAGGGSGGGAGS
PPQAHLPSERHSLPQGPPSPEPDFSVFRDFISRKVALADGDLSVPPYDAFQTYAFEGADS
PAASLSSLHSGSSGSEQDFAYLSSWGPRFRPLAALYAGHRGDDEAQAS
>sp|P19022|CADH2_HUMAN Cadherin-2 OS=Homo sapiens GN=CDH2 PE=1 SV=4
MCRIAGALRTLLPLLAALLQASVEASGEIALCKTGFPEDVYSAVLSKDVHEGQPLLNVKF
SNCNGKRKVQYESSEPADFKVDEDGMVYAVRSFPLSSEHAKFLIYAQDKETQEKWQVAVK
LSLKPTLTEESVKESAEVEEIVFPRQFSKHSGHLQRQKRDWVIPPINLPENSRGPFPQEL
VRIRSDRDKNLSLRYSVTGPGADQPPTGIFIINPISGQLSVTKPLDREQIARFHLRAHAV
DINGNQVENPIDIVINVIDMNDNRPEFLHQVWNGTVPEGSKPGTYVMTVTAIDADDPNAL

NGMLRYRIVSQAPSTPSPNMFTINNETGDIITVAAGLDREKVQQYTLIIQATDMEGNPTY
GLSNTATAVITVTDVNDNPPEFTAMTFYGEVPENRVDIIVANLTVTDKDQPHTPAWNAVY
RISGGDPTGRFAIQTDPNSNDGLVTVVKPIDFETNRMFVLTVAAENQVPLAKGIQHPPQS
TATVSVTVIDVNENPYFAPNPKIIRQEEGLHAGTMLTTFTAQDPDRYMQQNIRYTKLSDP
ANWLKIDPVNGQITTIAVLDRESPNVKNNIYNATFLASDNGIPPMSGTGTLQIYLLDIND
NAPQVLPQEAETCETPDPNSINITALDYDIDPNAGPFAFDLPLSPVTIKRNWTITRLNGD
FAQLNLKIKFLEAGIYEVPIIITDSGNPPKSNISILRVKVCQCDSNGDCTDVDRIVGAGL
GTGAIIAILLCIIILLILVLMFVVWMKRRDKERQAKQLLIDPEDDVRDNILKYDEEGGGE
EDQDYDLSQLQQPDTVEPDAIKPVGIRRMDERPIHAEPQYPVRSAAPHPGDIGDFINEGL
KAADNDPTAPPYDSLLVFDYEGSGSTAGSLSSLNSSSSGGEQDYDYLNDWGPRFKKLADM
YGGGDD
>sp|Q8NFZ8|CADM4_HUMAN Cell adhesion molecule 4 OS=Homo sapiens GN=CADM4 PE=1 SV=1
MGRARRFQWPLLLLWAAAAGPGAGQEVQTENVTVAEGGVAEITCRLHQYDGSIVVIQNPA
RQTLFFNGTRALKDERFQLEEFSPRRVRIRLSDARLEDEGGYFCQLYTEDTHHQIATLTV
LVAPENPVVEVREQAVEGGEVELSCLVPRSRPAATLRWYRDRKELKGVSSSQENGKVWSV
ASTVRFRVDRKDDGGIIICEAQNQALPSGHSKQTQYVLDVQYSPTARIHASQAVVREGDT
LVLTCAVTGNPRPNQIRWNRGNESLPERAEAVGETLTLPGLVSADNGTYTCEASNKHGHA
RALYVLVVYDPGAVVEAQTSVPYAIVGGILALLVFLIICVLVGMVWCSVRQKGSYLTHEA
SGLDEQGEAREAFLNGSDGHKRKEEFFI
>sp|Q13111|CAF1A_HUMAN Chromatin assembly factor 1 subunit A OS=Homo sapiens GN=CHAF1A PE=1 SV=2
MLEELECGAPGARGAATAMDCKDRPAFPVKKLIQARLPFKRLNLVPKGKADDMSDDQGTS
VQSKSPDLEASLDTLENNCHVGSDIDFRPKLVNGKGPLDNFLRNRIETSIGQSTVIIDLT
EDSNEQPDSLVDHNKLNSEASPSREAINGQREDTGDQQGLLKAIQNDKLAFPGETLSDIP
CKTEEEGVGCGGAGRRGDSQECSPRSCPELTSGPRMCPRKEQDSWSEAGGILFKGKVPMV
VLQDILAVRPPQIKSLPATPQGKNMTPESEVLESFPEEDSVLSHSSLSSPSSTSSPEGPP
APPKQHSSTSPFPTSTPLRRITKKFVKGSTEKNKLRLQRDQERLGKQLKLRAEREEKEKL
KEEAKRAKEEAKKKKEEEKELKEKERREKREKDEKEKAEKQRLKEERRKERQEALEAKLE
EKRKKEEEKRLREEEKRIKAEKAEITRFFQKPKTPQAPKTLAGSCGKFAPFEIKEHMVLA
PRRRTAFHPDLCSQLDQLLQQQSGEFSFLKDLKGRQPLRSGPTHVSTRNADIFNSDVVIV
ERGKGDGVPERRKFGRMKLLQFCENHRPAYWGTWNKKTALIRARDPWAQDTKLLDYEVDS
DEEWEEEEPGESLSHSEGDDDDDMGEDEDEDDGFFVPHGYLSEDEGVTEECADPENHKVR
QKLKAKEWDEFLAKGKRFRVLQPVKIGCVWAADRDCAGDDLKVLQQFAACFLETLPAQEE
QTPKASKRERRDEQILAQLLPLLHGNVNGSKVIIREFQEHCRRGLLSNHTGSPRSPSTTY
LHTPTPSEDAAIPSKSRLKRLISENSVYEKRPDFRMCWYVHPQVLQSFQQEHLPVPCQWS
YVTSVPSAPKEDSGSVPSTGPSQGTPISLKRKSAGSMCITQFMKKRRHDGQIGAEDMDGF
QADTEEEEEEEEGDCMIVDVPDAAEVQAPCGAASGAGGGVGVDTGKATLTSSPLGAS
>sp|Q13112|CAF1B_HUMAN Chromatin assembly factor 1 subunit B OS=Homo sapiens GN=CHAF1B PE=1 SV=1
MKVITCEIAWHNKEPVYSLDFQHGTAGRIHRLASAGVDTNVRIWKVEKGPDGKAIVEFLS
NLARHTKAVNVVRFSPTGEILASGGDDAVILLWKVNDNKEPEQIAFQDEDEAQLNKENWT
VVKTLRGHLEDVYDICWATDGNLMASASVDNTAIIWDVSKGQKISIFNEHKSYVQGVTWD
PLGQYVATLSCDRVLRVYSIQKKRVAFNVSKMLSGIGAEGEARSYRMFHDDSMKSFFRRL
SFTPDGSLLLTPAGCVESGENVMNTTYVFSRKNLKRPIAHLPCPGKATLAVRCCPVYFEL

RPVVETGVELMSLPYRLVFAVASEDSVLLYDTQQSFPFGYVSNIHYHTLSDISWSSDGAF
LAISSTDGYCSFVTFEKDELGIPLKEKPVLNMRTPDTAKKTKSQTHRGSSPGPRPVEGTP
ASRTQDPSSPGTTPPQARQAPAPTVIRDPPSITPAVKSPLPGPSEEKTLQPSSQNTKAHP
SRRVTLNTLQAWSKTTPRRINLTPLKTDTPPSSVPTSVISTPSTEEIQSETPGDAQGSPP
ELKRPRLDENKGGTESLDP
>sp|Q5VU97|CAHD1_HUMAN VWFA and cache domain-containing protein 1 OS=Homo sapiens GN=CACHD1 PE=2 SV=2
MARQPEEEETAVARARRPPLWLLCLVACWLLGAGAEADFSILDEAQVLASQMRRLAAEEL
GVVTMQRIFNSFVYTEKISNGESEVQQLAKKIREKFNRYLDVVNRNKQVVEASYTAHLTS
PLTAIQDCCTIPPSMMEFDGNFNTNVSRTISCDRLSTTVNSRAFNPGRDLNSVLADNLKS
NPGIKWQYFSSEEGIFTVFPAHKFRCKGSYEHRSRPIYVSTVRPQSKHIVVILDHGASVT
DTQLQIAKDAAQVILSAIDEHDKISVLTVADTVRTCSLDQCYKTFLSPATSETKRKMSTF
VSSVKSSDSPTQHAVGFQKAFQLIRSTNNNTKFQANTDMVIIYLSAGITSKDSSEEDKKA
TLQVINEENSFLNNSVMILTYALMNDGVTGLKELAFLRDLAEQNSGKYGVPDRMALPVIK
GSMMVLNQLSNLETTVGRFYTNLPNRMIDEAVFSLPFSDEMGDGLIMTVSKPCYFGNLLL
GIVGVDVNLAYILEDVTYYQDSLASYTFLIDDKGYTLMHPSLTRPYLLSEPPLHTDIIHY
ENIPKFELVRQNILSLPLGSQIIAVPVNSSLSWHINKLRETGKEAYNVSYAWKMVQDTSF
ILCIVVIQPEIPVKQLKNLNTVPSSKLLYHRLDLLGQPSACLHFKQLATLESPTIMLSAG
SFSSPYEHLSQPETKRMVEHYTAYLSDNTRLIANPGLKFSVRNEVMATSHVTDEWMTQME
MSSLNTYIVRRYIATPNGVLRIYPGSLMDKAFDPTRRQWYLHAVANPGLISLTGPYLDVG
GAGYVVTISHTIHSSSTQLSSGHTVAVMGIDFTLRYFYKVLMDLLPVCNQDGGNKIRCFI
MEDRGYLVAHPTLIDPKGHAPVEQQHITHKEPLVANDILNHPNFVKKNLCNSFSDRTVQR
FYKFNTSLAGDLTNLVHGSHCSKYRLARIPGTNAFVGIVNETCDSLAFCACSMVDRLCLN
CHRMEQNECECPCECPLEVNECTGNLTNAENRNPSCEVHQEPVTYTAIDPGLQDALHQCV
NSRCSQRLESGDCFGVLDCEWCMVDSDGKTHLDKPYCAPQKECFGGIVGAKSPYVDDMGA
IGDEVITLNMIKSAPVGPVAGGIMGCIMVLVLAVYAYRHQIHRRSHQHMSPLAAQEMSVR
MSNLENDRDERDDDSHEDRGIISNTRFIAAVIERHAHSPERRRRYWGRSGTESDHGYSTM
SPQEDSENPPCNNDPLSAGVDVGNHDEDLDLDTPPQTAALLSHKFHHYRSHHPTLHHSHH
LQAAVTVHTVDAEC
>sp|P27482|CALL3_HUMAN Calmodulin-like protein 3 OS=Homo sapiens GN=CALML3 PE=1 SV=2
MADQLTEEQVTEFKEAFSLFDKDGDGCITTRELGTVMRSLGQNPTEAELRDMMSEIDRDG
NGTVDFPEFLGMMARKMKDTDNEEEIREAFRVFDKDGNGFVSAAELRHVMTRLGEKLSDE
EVDEMIRAADTDGDGQVNYEEFVRVLVSK
>sp|Q9NZT1|CALL5_HUMAN Calmodulin-like protein 5 OS=Homo sapiens GN=CALML5 PE=1 SV=2
MAGELTPEEEAQYKKAFSAVDTDGNGTINAQELGAALKATGKNLSEAQLRKLISEVDSDG
DGEISFQEFLTAAKKARAGLEDLQVAFRAFDQDGDGHITVDELRRAMAGLGQPLPQEELD
AMIREADVDQDGRVNYEEFARMLAQE
>sp|P07384|CAN1_HUMAN Calpain-1 catalytic subunit OS=Homo sapiens GN=CAPN1 PE=1 SV=1
MSEEIITPVYCTGVSAQVQKQRARELGLGRHENAIKYLGQDYEQLRVRCLQSGTLFRDEA
FPPVPQSLGYKDLGPNSSKTYGIKWKRPTELLSNPQFIVDGATRTDICQGALGDCWLLAA
IASLTLNDTLLHRVVPHGQSFQNGYAGIFHFQLWQFGEWVDVVVDDLLPIKDGKLVFVHS
AEGNEFWSALLEKAYAKVNGSYEALSGGSTSEGFEDFTGGVTEWYELRKAPSDLYQIILK
ALERGSLLGCSIDISSVLDMEAITFKKLVKGHAYSVTGAKQVNYRGQVVSLIRMRNPWGE
VEWTGAWSDSSSEWNNVDPYERDQLRVKMEDGEFWMSFRDFMREFTRLEICNLTPDALKS

RTIRKWNTTLYEGTWRRGSTAGGCRNYPATFWVNPQFKIRLDETDDPDDYGDRESGCSFV
LALMQKHRRRERRFGRDMETIGFAVYEVPPELVGQPAVHLKRDFFLANASRARSEQFINL
REVSTRFRLPPGEYVVVPSTFEPNKEGDFVLRFFSEKSAGTVELDDQIQANLPDEQVLSE
EEIDENFKALFRQLAGEDMEISVKELRTILNRIISKHKDLRTKGFSLESCRSMVNLMDRD
GNGKLGLVEFNILWNRIRNYLSIFRKFDLDKSGSMSAYEMRMAIESAGFKLNKKLYELII
TRYSEPDLAVDFDNFVCCLVRLETMFRFFKTLDTDLDGVVTFDLFKWLQLTMFA
>sp|P20807|CAN3_HUMAN Calpain-3 OS=Homo sapiens GN=CAPN3 PE=1 SV=2
MPTVISASVAPRTAAEPRSPGPVPHPAQSKATEAGGGNPSGIYSAIISRNFPIIGVKEKT
FEQLHKKCLEKKVLYVDPEFPPDETSLFYSQKFPIQFVWKRPPEICENPRFIIDGANRTD
ICQGELGDCWFLAAIACLTLNQHLLFRVIPHDQSFIENYAGIFHFQFWRYGEWVDVVIDD
CLPTYNNQLVFTKSNHRNEFWSALLEKAYAKLHGSYEALKGGNTTEAMEDFTGGVAEFFE
IRDAPSDMYKIMKKAIERGSLMGCSIDDGTNMTYGTSPSGLNMGELIARMVRNMDNSLLQ
DSDLDPRGSDERPTRTIIPVQYETRMACGLVRGHAYSVTGLDEVPFKGEKVKLVRLRNPW
GQVEWNGSWSDRWKDWSFVDKDEKARLQHQVTEDGEFWMSYEDFIYHFTKLEICNLTADA
LQSDKLQTWTVSVNEGRWVRGCSAGGCRNFPDTFWTNPQYRLKLLEEDDDPDDSEVICSF
LVALMQKNRRKDRKLGASLFTIGFAIYEVPKEMHGNKQHLQKDFFLYNASKARSKTYINM
REVSQRFRLPPSEYVIVPSTYEPHQEGEFILRVFSEKRNLSEEVENTISVDRPVKKKKTK
PIIFVSDRANSNKELGVDQESEEGKGKTSPDKQKQSPQPQPGSSDQESEEQQQFRNIFKQ
IAGDDMEICADELKKVLNTVVNKHKDLKTHGFTLESCRSMIALMDTDGSGKLNLQEFHHL
WNKIKAWQKIFKHYDTDQSGTINSYEMRNAVNDAGFHLNNQLYDIITMRYADKHMNIDFD
SFICCFVRLEGMFRAFHAFDKDGDGIIKLNVLEWLQLTMYA
>sp|Q96LZ3|CANB2_HUMAN Calcineurin subunit B type 2 OS=Homo sapiens GN=PPP3R2 PE=1 SV=3
MGNEASYPAEMCSHFDNDEIKRLGRRFKKLDLDKSGSLSVEEFMSLPELRHNPLVRRVID
VFDTDGDGEVDFKEFILGTSQFSVKGDEEQKLRFAFSIYDMDKDGYISNGELFQVLKMMV
GNNLTDWQLQQLVDKTIIILDKDGDGKISFEEFSAVVRDLEIHKKLVLIV
>sp|O75052|CAPON_HUMAN Carboxyl-terminal PDZ ligand of neuronal nitric oxide synthase protein OS=Homo sapiens GN=NOS1AP PE=1 SV=3
MPSKTKYNLVDDGHDLRIPLHNEDAFQHGICFEAKYVGSLDVPRPNSRVEIVAAMRRIRY
EFKAKNIKKKKVSIMVSVDGVKVILKKKKKLLLLQKKEWTWDESKMLVMQDPIYRIFYVS
HDSQDLKIFSYIARDGASNIFRCNVFKSKKKSQAMRIVRTVGQAFEVCHKLSLQHTQQNA
DGQEDGESERNSNSSGDPGRQLTGAERASTATAEETDIDAVEVPLPGNDVLEFSRGVTDL
DAVGKEGGSHTGSKVSHPQEPMLTASPRMLLPSSSSKPPGLGTETPLSTHHQMQLLQQLL
QQQQQQTQVAVAQVHLLKDQLAAEAAARLEAQARVHQLLLQNKDMLQHISLLVKQVQELE
LKLSGQNAMGSQDSLLEITFRSGALPVLCDPTTPKPEDLHSPPLGAGLADFAHPAGSPLG
RRDCLVKLECFRFLPPEDTPPPAQGEALLGGLELIKFRESGIASEYESNTDESEERDSWS
QEELPRLLNVLQRQELGDGLDDEIAV
>sp|Q9H246|CA021_HUMAN Uncharacterized protein C1orf21 OS=Homo sapiens GN=C1orf21 PE=1 SV=1
MGCASAKHVATVQNEEEAQKGKNYQNGDVFGDEYRIKPVEEVKYMKNGAEEEQKIAARNQ
ENLEKSASSNVRLKTNKEVPGLVHQPRANMHISESQQEFFRMLDEKIEKGRDYCSEEEDI
T
>sp|Q5VUE5|CA053_HUMAN Uncharacterized protein C1orf53 OS=Homo sapiens GN=C1orf53 PE=1 SV=1
MAARQIWARTGAALCRQPSAAPPPAPLWVRAGFRQQLSLTLCPANEGNCGGSAPSTPGRP

ERAARPSVSEELTAAERQIAELHAAACAAGQLNYVDPATGYVVLTQIAHLQRGECCGSAC
RHCPYGQVNVKDPSKKKQFNSYFYV
>sp|Q8N0U7|CA087_HUMAN Uncharacterized protein C1orf87 OS=Homo sapiens GN=C1orf87 PE=2 SV=1
MSSAWKTPRGSDAMPEIMVKIIGSKHFQYLVEKPKIKENDSLKTETQTMHQKPMTDNARQ
MSRDTPVPINFTDQQTTDNPDDVKEKKHPENNQKSENNQKLLTGANSSRFLDGNIPSQAN
VHCSSVPTGDQSLSYVHGIPRRKLRDWSLEQMVRGSSDQPEDIGQSPSGTTNEDAFLLAL
VRRELKSRPLSSNLLEKLQKELKILDPISSGFLLQSQLSRLFLKHEVPLQLPTVKILCQR
FSKRGSPEMVNYEKLLWFLNSAASDYPQQNKAAADLRKTESHGTHSQSTPPQHSSSQPEV
NRSLLEILKMALRTTNGRLNIDNLNLSFRKEDRSFSGCLPLPKVRAICGKHGLYLTLSLL
ETLLNHQDLGYQNEIKWQNFVEMLTRASSDLLSDLPTGKNEKKAPAPPMEPEVPEMSQSK
TEHMKTPEEELQPESSPAETSACKDPLKPLKIRPVSQPFVNPAVKNKAEECETWIDRFRK
LENALYLCDLSNTGVLEKERARRLIHNYNLIYNLSLSPQKIDQALRRFRSGENMLLEPAL
RYLKEL
>sp|Q96HA4|CA159_HUMAN Uncharacterized protein C1orf159 OS=Homo sapiens GN=C1orf159 PE=2 SV=2
MALRHLALLAGLLVGVASKSMENTVTRNSTAVINTQAEGTLSPPGLSSLPVVREWALTHT
AQLPECCVDVVGVNASCPGASLCGPGCYRRWNADGSASCVRCGNGTLPAYNGSECRSFAG
PGAPFPMNRSSGTPGRPHPGAPRVAASLFLGTFFISSGLILSVAGFFYLKRSSKLPRACY
RRNKAPALQPGEAAAMIPPPQSSGNSSCRIPLWGFPSLGQSQGALWVCPQTGLPGSGSRP
PLPGSPGDPPTRQGQGRIWLVPPALDLSWIWPAPPARPPLIPVTSMLFPVPETWGLQERR
THHDRADPQYLLLLEVQLHPRTDAAGLRQALLSSHRFSGAGSGGPKSQPVRKPRYVRRER
PLDRATDPAAFPGEARISNV
>sp|Q5VWT5|CA168_HUMAN Uncharacterized protein C1orf168 OS=Homo sapiens GN=C1orf168 PE=2 SV=1
MEGEGVRNFKELRAKFQNLDAPPLPGPIKFPAGVSPKGDIGGTQSTQILANGKPLSSNHK
QRTPYCSSSESQPLQPQKIKLAQKSEIPKCSNSPGPLGKSTVCSATSSQKASLLLEVTQS
NVEIITKEKVMVANSFRNKLWNWEKVSSQKSEMSSALLLANYGSKAIHLEGQKGMGLTPE
EPRKKLETKGAQTLPSQKHVVAPKILHNVSEDPSFVISQHIRKSWENPPPERSPASSPCQ
PIYECELASQAPEKQPDVRHHHLPKTKPLPSIDSLGPPPPKPSRPPIVNLQAFQRQPAAV
PKTQGEVTVEEGSLSPERLFNAEFEEPHNYEATISYLRHSGNSINLCTAKEIADPTYEVG
IEELQKPGKNFPYPEPSAKHEDKKMKEKQPCELKPKNTEKEPYSNHVFKVDACEGTPEKI
QMTNVHTGRRNMLAGKQEAMIDIIQTNPCPEGPKLARHSQGHCGHLEVLESTKETPDLGV
SKTSSISEEIYDDVEYSRKEVPKLNYSSSLASSSEENRELYEDVYKTKNNYPKIDLDGKE
ALKRLQQFFKKEKDRFKIKKTKSKENLSAFSILLPDLELKSQEVIIYDDVDLSEKESKDE
DKLKMWKPKFLTPKEKKEKNGAEESESFSPRNFFKTKKQNLEKNRMKREEKLFRERFKYD
KEIIVINTAVACSNNSRNGIFDLPISPGEELEVIDTTEQNLVICRNSKGKYGYVLIEHLD
FKHQSWSP
>sp|B1AJZ1|CA196_HUMAN Putative uncharacterized protein C1orf196 OS=Homo sapiens GN=C1orf196 PE=5 SV=2
MTNTKDPKRAMESTLATSNSATGPVTFSHVFGQQCQLMQAAVQSLHTLNDQISHFIVTKS
KALEEDKDPFLPTEKETLKSSMILMRHLLMDAQVQRSIQKFSMEKVESFFLFGISCPKMF
NYHTY

>sp|Q8NC38|CA213_HUMAN Putative uncharacterized protein ZNF436-AS1 OS=Homo sapiens GN=ZNF436-AS1 PE=2 SV=2
MLAVPVRLKVGSRKPEWGTNRLTSCPAKDPLDRRLQNLRDRERVPEPQRSLRPGVQEDSR
EHGQVPEVSDPQVDLEFVDLQAKPRYRRLILKTQIPEASDSQAAQKPQAHRQIPETTEAG
RETTSN
>sp|Q6ZS94|CA229_HUMAN Putative uncharacterized protein C1orf229 OS=Homo sapiens GN=C1orf229 PE=2 SV=1
MGLCTLQPLGPPRKSSTSCGTWTASGLPSLGHLPRRLRLAFDFLEPRARGQRGGSGGCQS
TRAAERTRPPHPPNAVLLLQPEPSLTWAQGRGCRGHFRSLPAAASRSGSRTLRCASSDRS
LREQKQRRAGPDPTPSPAPPPAGPRPSPGSLGPSAPAAPRTARGAYELQGGASQDGPGQA
AVGATPTTGPGTGGEGALLGCGSGRTPPTSATWRRRLLPAEVPPGAAAANFPERERL
>sp|Q9H8G2|CAAP1_HUMAN Caspase activity and apoptosis inhibitor 1 OS=Homo sapiens GN=CAAP1 PE=1 SV=2
MTGKKSSREKRRKRSSQEAAAALAAPDIVPALASGSSGSTSGCGSAGGCGSVSCCGNANF
SGSVTGGGSGGSCWGGSSVERSERRKRRSTDSSSVSGSLQQETKYILPTLEKELFLAEHS
DLEEGGLDLTVSLKPVSFYISDKKEMLQQCFCIIGEKKLQKMLPDVLKNCSIEEIKKLCQ
EQLELLSEKKILKILEGDNGMDSDMEEEADDGSKMGSDLVSQQDICIDSASSVRENKQPE
GLELKQGKGEDSDVLSINADAYDSDIEGPCNEEAAAPEAPENTVQSEAGQIDDLEKDIEK
SVNEILGLAESSPNEPKAATLAVPPPEDVQPSAQQLELLELEMRARAIKALMKAGDIKKP
A
>sp|Q9Y376|CAB39_HUMAN Calcium-binding protein 39 OS=Homo sapiens GN=CAB39 PE=1 SV=1
MPFPFGKSHKSPADIVKNLKESMAVLEKQDISDKKAEKATEEVSKNLVAMKEILYGTNEK
EPQTEAVAQLAQELYNSGLLSTLVADLQLIDFEGKKDVAQIFNNILRRQIGTRTPTVEYI
CTQQNILFMLLKGYESPEIALNCGIMLRECIRHEPLAKIILWSEQFYDFFRYVEMSTFDI
ASDAFATFKDLLTRHKLLSAEFLEQHYDRFFSEYEKLLHSENYVTKRQSLKLLGELLLDR
HNFTIMTKYISKPENLKLMMNLLRDKSRNIQFEAFHVFKVFVANPNKTQPILDILLKNQA
KLIEFLSKFQNDRTEDEQFNDEKTYLVKQIRDLKRPAQQEA
>sp|Q9BRK5|CAB45_HUMAN 45 kDa calcium-binding protein OS=Homo sapiens GN=SDF4 PE=1 SV=1
MVWPWVAMASRWGPLIGLAPCCLWLLGAVLLMDASARPANHSSTRERVANREENEILPPD
HLNGVKLEMDGHLNRGFHQEVFLGKDLGGFDEDAEPRRSRRKLMVIFSKVDVNTDRKISA
KEMQRWIMEKTAEHFQEAMEESKTHFRAVDPDGDGHVSWDEYKVKFLASKGHSEKEVADA
IRLNEELKVDEETQEVLENLKDRWYQADSPPADLLLTEEEFLSFLHPEHSRGMLRFMVKE
IVRDLDQDGDKQLSVPEFISLPVGTVENQQGQDIDDNWVKDRKKEFEELIDSNHDGIVTA
EELESYMDPMNEYNALNEAKQMIAVADENQNHHLEPEEVLKYSEFFTGSKLVDYARSVHE
EF
>sp|Q8TDN4|CABL1_HUMAN CDK5 and ABL1 enzyme substrate 1 OS=Homo sapiens GN=CABLES1 PE=1 SV=2
MAAAAAAATTAACSSGSAGTDAAGASGLQQPPPQPQPQPAAAAPAQPPPEPPRKPRMDPR
RRQAALSFLTNISLDGRLPPQDAEWGGGEEGGAAKPGAGGACGARTRFSLLAAAERGGCI
ALAAPGTPAAGLAAGSGPCLPQPSSLPPLIPGGHATVSGPGVARGFASPLGAGRASGEQW
QPPRPAPLAACAQLQLLDGSGAAGQEELEEDDAFISVQVPAAAFLGSGTPGSGSGSRGRL
NSFTQGILPIAFSRPTSQNYCSLEQPGQGGGSTSAFEQLQRSRRRLISQRSSLETLEDIEE
NAPLRRCRTLSGSPRPKNFKKIHFIKNMRQHDTRNGRIVLISGRRSFCSIFSVLPYRDST
QVGDLKLDGGRQSTGAVSLKEIIGLEGVELGADGKTVSYTQFLLPTNAFGARRNTIDSTS

SFSQFRNLSHRSLSIGRASGTQGSLDTGSDLGDFMDYDPNLLDDPQWPCGKHKRVLIFPS
YMTTVIDYVKPSDLKKDMNETFKEKFPHIKLTLSKIRSLKREMRKLAQEDCGLEEPTVAM
AFVYFEKLALKGKLNKQNRKLCAGACVLLAAKIGSDLKKHEVKHLIDKLEEKFRLNRREL
IAFEFPVLVALEFALHLPEHEVMPHYRRLVQSS
>sp|Q86V35|CABP7_HUMAN Calcium-binding protein 7 OS=Homo sapiens GN=CABP7 PE=1 SV=1
MPFHPVTAALMYRGIYTVPNLLSEQRPVDIPEDELEEIREAFKVFDRDGNGFISKQELGT
AMRSLGYMPNEVELEVIIQRLDMDGDGQVDFEEFVTLLGPKLSTSGIPEKFHGTDFDTVF
WKCDMQKLTVDELKRLLYDTFCEHLSMKDIENIIMTEEESHLGTAEECPVDVETCSNQQI
RQTCVRKSLICAFAIAFIISVMLIAANQVLRSGMK
>sp|P43155|CACP_HUMAN Carnitine O-acetyltransferase OS=Homo sapiens GN=CRAT PE=1 SV=5
MLAFAARTVVKPLGFLKPFSLMKASSRFKAHQDALPRLPVPPLQQSLDHYLKALQPIVSE
EEWAHTKQLVDEFQASGGVGERLQKGLERRARKTENWLSEWWLKTAYLQYRQPVVIYSSP
GVMLPKQDFVDLQGQLRFAAKLIEGVLDFKVMIDNETLPVEYLGGKPLCMNQYYQILSSC
RVPGPKQDTVSNFSKTKKPPTHITVVHNYQFFELDVYHSDGTPLTADQIFVQLEKIWNSS
LQTNKEPVGILTSNHRNSWAKAYNTLIKDKVNRDSVRSIQKSIFTVCLDATMPRVSEDVY
RSHVAGQMLHGGGSRLNSGNRWFDKTLQFIVAEDGSCGLVYEHAAAEGPPIVTLLDYVIE
YTKKPELVRSPLVPLPMPKKLRFNITPEIKSDIEKAKQNLSIMIQDLDITVMVFHHFGKD
FPKSEKLSPDAFIQMALQLAYYRIYGQACATYESASLRMFHLGRTDTIRSASMDSLTFVK
AMDDSSVTEHQKVELLRKAVQAHRGYTDRAIRGEAFDRHLLGLKLQAIEDLVSMPDIFMD
TSYAIAMHFHLSTSQVPAKTDCVMFFGPVVPDGYGVCYNPMEAHINFSLSAYNSCAETNA
ARLAHYLEKALLDMRALLQSHPRAKL
>sp|Q9Y6N8|CAD10_HUMAN Cadherin-10 OS=Homo sapiens GN=CDH10 PE=1 SV=2
MTIHQFLLLFLFWVCLPHFCSPEIMFRRTPVPQQRILSSRVPRSDGKILHRQKRGWMWNQ
FFLLEEYTGSDYQYVGKLHSDQDKGDGSLKYILSGDGAGTLFIIDEKTGDIHATRRIDRE
EKAFYTLRAQAINRRTLRPVEPESEFVIKIHDINDNEPTFPEEIYTASVPEMSVVGTSVV
QVTATDADDPSYGNSARVIYSILQGQPYFSVEPETGIIRTALPNMNRENREQYQVVIQAK
DMGGQMGGLSGTTTVNITLTDVNDNPPRFPQNTIHLRVLESSPVGTAIGSVKATDADTGK
NAEVEYRIIDGDGTDMFDIVTEKDTQEGIITVKKPLDYESRRLYTLKVEAENTHVDPRFY
YLGPFKDTTIVKISIEDVDEPPVFSRSSYLFEVHEDIEVGTIIGTVMARDPDSISSPIRF
SLDRHTDLDRIFNIHSGNGSLYTSKPLDRELSQWHNLTVIAAEINNPKETTRVAVFVRIL
DVNDNAPQFAVFYDTFVCENARPGQLIQTISAVDKDDPLGGQKFFFSLAAVNPNFTVQDN
EDNTARILTRKNGFNRHEISTYLLPVVISDNDYPIQSSTGTLTIRVCACDSQGNMQSCSA
EALLLPAGLSTGALIAILLCIIILLVIVVLFAALKRQRKKEPLILSKEDIRDNIVSYNDE
GGGEEDTQAFDIGTLRNPAAIEEKKLRRDIIPETLFIPRRTPTAPDNTDVRDFINERLKE
HDLDPTAPPYDSLATYAYEGNDSIAESLSSLESGTTEGDQNYDYLREWGPRFNKLAEMYG
GGESDKDS
>sp|P12830|CADH1_HUMAN Cadherin-1 OS=Homo sapiens GN=CDH1 PE=1 SV=3
MGPWSRSLSALLLLLQVSSWLCQEPEPCHPGFDAESYTFTVPRRHLERGRVLGRVNFEDC
TGRQRTAYFSLDTRFKVGTDGVITVKRPLRFHNPQIHFLVYAWDSTYRKFSTKVTLNTVG
HHHRPPPHQASVSGIQAELLTFPNSSPGLRRQKRDWVIPPISCPENEKGPFPKNLVQIKS
NKDKEGKVFYSITGQGADTPPVGVFIIERETGWLKVTEPLDRERIATYTLFSHAVSSNGN
AVEDPMEILITVTDQNDNKPEFTQEVFKGSVMEGALPGTSVMEVTATDADDDVNTYNAAI
AYTILSQDPELPDKNMFTINRNTGVISVVTTGLDRESFPTYTLVVQAADLQGEGLSTTAT
AVITVTDTNDNPPIFNPTTYKGQVPENEANVVITTLKVTDADAPNTPAWEAVYTILNDDG

GQFVVTTNPVNNDGILKTAKGLDFEAKQQYILHVAVTNVVPFEVSLTTSTATVTVDVLDV
NEAPIFVPPEKRVEVSEDFGVGQEITSYTAQEPDTFMEQKITYRIWRDTANWLEINPDTG
AISTRAELDREDFEHVKNSTYTALIIATDNGSPVATGTGTLLLILSDVNDNAPIPEPRTI
FFCERNPKPQVINIIDADLPPNTSPFTAELTHGASANWTIQYNDPTQESIILKPKMALEV
GDYKINLKLMDNQNKDQVTTLEVSVCDCEGAAGVCRKAQPVEAGLQIPAILGILGGILAL
LILILLLLLFLRRRAVVKEPLLPPEDDTRDNVYYYDEEGGGEEDQDFDLSQLHRGLDARP
EVTRNDVAPTLMSVPRYLPRPANPDEIGNFIDENLKAADTDPTAPPYDSLLVFDYEGSGS
EAASLSSLNSSESDKDQDYDYLNEWGNRFKKLADMYGGGEDD
>sp|P33151|CADH5_HUMAN Cadherin-5 OS=Homo sapiens GN=CDH5 PE=1 SV=5
MQRLMMLLATSGACLGLLAVAAVAAAGANPAQRDTHSLLPTHRRQKRDWIWNQMHIDEEK
NTSLPHHVGKIKSSVSRKNAKYLLKGEYVGKVFRVDAETGDVFAIERLDRENISEYHLTA
VIVDKDTGENLETPSSFTIKVHDVNDNWPVFTHRLFNASVPESSAVGTSVISVTAVDADD
PTVGDHASVMYQILKGKEYFAIDNSGRIITITKSLDREKQARYEIVVEARDAQGLRGDSG
TATVLVTLQDINDNFPFFTQTKYTFVVPEDTRVGTSVGSLFVEDPDEPQNRMTKYSILRG
DYQDAFTIETNPAHNEGIIKPMKPLDYEYIQQYSFIVEATDPTIDLRYMSPPAGNRAQVI
INITDVDEPPIFQQPFYHFQLKENQKKPLIGTVLAMDPDAARHSIGYSIRRTSDKGQFFR
VTKKGDIYNEKELDREVYPWYNLTVEAKELDSTGTPTGKESIVQVHIEVLDENDNAPEFA
KPYQPKVCENAVHGQLVLQISAIDKDITPRNVKFKFILNTENNFTLTDNHDNTANITVKY
GQFDREHTKVHFLPVVISDNGMPSRTGTSTLTVAVCKCNEQGEFTFCEDMAAQVGVSIQA
VVAILLCILTITVITLLIFLRRRLRKQARAHGKSVPEIHEQLVTYDEEGGGEMDTTSYDV
SVLNSVRRGGAKPPRPALDARPSLYAQVQKPPRHAPGAHGGPGEMAAMIEVKKDEADHDG
DGPPYDTLHIYGYEGSESIAESLSSLGTDSSDSDVDYDFLNDWGPRFKMLAELYGSDPRE
ELLY
>sp|Q9ULB5|CADH7_HUMAN Cadherin-7 OS=Homo sapiens GN=CDH7 PE=2 SV=2
MKLGKVEFCHFLQLIALFLCFSGMSQAELSRSRSKPYFQSGRSRTKRSWVWNQFFVLEEY
MGSDPLYVGKLHSDVDKGDGSIKYILSGEGASSIFIIDENTGDIHATKRLDREEQAYYTL
RAQALDRLTNKPVEPESEFVIKIQDINDNEPKFLDGPYTAGVPEMSPVGTSVVQVTATDA
DDPTYGNSARVVYSILQGQPYFSVEPKTGVIKTALPNMDREAKDQYLLVIQAKDMVGQNG
GLSGTTSVTVTLTDVNDNPPRFPRRSYQYNVPESLPVASVVARIKAADADIGANAEMEYK
IVDGDGLGIFKISVDKETQEGIITIQKELDFEAKTSYTLRIEAANKDADPRFLSLGPFSD
TTTVKIIVEDVDEPPVFSSPLYPMEVSEATQVGNIIGTVAAHDPDSSNSPVRYSIDRNTD
LERYFNIDANSGVITTAKSLDRETNAIHNITVLAMESQNPSQVGRGYVAITILDINDNAP
EFAMDYETTVCENAQPGQVIQKISAVDKDEPSNGHQFYFSLTTDATNNHNFSLKDNKDNT
ASILTRRNGFRRQEQSVYYLPIFIVDSGSPSLSSTNTLTIRVCDCDADGVAQTCNAEAYV
LPAGLSTGALIAILACVLTLLVLILLIVTMRRRKKEPLIFDEERDIRENIVRYDDEGGGE
EDTEAFDMAALRNLNVIRDTKTRRDVTPEIQFLSRPAFKSIPDNVIFREFIWERLKEADV
DPGAPPYDSLQTYAFEGNGSVAESLSSLDSISSNSDQNYDYLSDWGPRFKRLADMYGTGQ
ESLYS
>sp|Q5T440|CAF17_HUMAN Putative transferase CAF17, mitochondrial OS=Homo sapiens GN=IBA57
PE=1 SV=1
MATAALLRGATPGRGGPVWRWRLRAAPRCRLAHSSCSPGGDPTAGAAWACFRLDGRTLLR
VRGPDAAPFLLGLLTNELPLPSPAAAGAPPAARAGYAHFLNVQGRTLYDVILYGLQEHSE
VSGFLLECDSSVQGALQKHLALYRIRRKVTVEPHPELRVWAVLPSSPEACGAASLQERAG
AAAILIRDPRTARMGWRLLTQDEGPALVPGGRLGDLWDYHQHRYLQGVPEGVRDLPPGVA

LPLESNLAFMNGVSFTKGCYIGQELTARTHHMGVIRKRLFPVRFLDPLPTSGITPGATVL
TASGQTVGKFRAGQGNVGLALLWSEKIKGPLHIRASEGAQVALAASVPDWWPTVSK
>sp|P35218|CAH5A_HUMAN Carbonic anhydrase 5A, mitochondrial OS=Homo sapiens GN=CA5A PE=1 SV=1
MLGRNTWKTSAFSFLVEQMWAPLWSRSMRPGRWCSQRSCAWQTSNNTLHPLWTVPVSVPG
GTRQSPINIQWRDSVYDPQLKPLRVSYEAASCLYIWNTGYLFQVEFDDATEASGISGGPL
ENHYRLKQFHFHWGAVNEGGSEHTVDGHAYPAELHLVHWNSVKYQNYKEAVVGENGLAVI
GVFLKLGAHHQTLQRLVDILPEIKHKDARAAMRPFDPSTLLPTCWDYWTYAGSLTTPPLT
ESVTWIIQKEPVEVAPSQLSAFRTLLFSALGEEEKMMVNNYRPLQPLMNRKVWASFQATN
EGTRS
>sp|Q9HA72|CAHM2_HUMAN Calcium homeostasis modulator protein 2 OS=Homo sapiens GN=CALHM2 PE=2 SV=1
MAALIAENFRFLSLFFKSKDVMIFNGLVALGTVGSQELFSVVAFHCPCSPARNYLYGLAA
IGVPALVLFIIGIILNNHTWNLVAECQHRRTKNCSAAPTFLLLSSILGRAAVAPVTWSVI
SLLRGEAYVCALSEFVDPSSLTAREEHFPSAHATEILARFPCKENPDNLSDFREEVSRRL
RYESQLFGWLLIGVVAILVFLTKCLKHYCSPLSYRQEAYWAQYRANEDQLFQRTAEVHSR
VLAANNVRRFFGFVALNKDDEELIANFPVEGTQPRPQWNAITGVYLYRENQGLPLYSRLH
KWAQGLAGNGAAPDNVEMALLPS
>sp|P30988|CALCR_HUMAN Calcitonin receptor OS=Homo sapiens GN=CALCR PE=1 SV=2
MQFSGEKISGQRDLQKSKMRFTFTSRCLALFLLLNHPTPILPAFSNQTYPTIEPKPFLYV
VGRKKMMDAQYKCYDRMQQLPAYQGEGPYCNRTWDGWLCWDDTPAGVLSYQFCPDYFPDF
DPSEKVTKYCDEKGVWFKHPENNRTWSNYTMCNAFTPEKLKNAYVLYYLAIVGHSLSIFT
LVISLGIFVFFRKLTTIFPLNWKYRKALSLGCQRVTLHKNMFLTYILNSMIIIIHLVEVV
PNGELVRRDPVSCKILHFFHQYMMACNYFWMLCEGIYLHTLIVVAVFTEKQRLRWYYLLG
WGFPLVPTTIHAITRAVYFNDNCWLSVETHLLYIIHGPVMAALVVNFFFLLNIVRVLVTK
MRETHEAESHMYLKAVKATMILVPLLGIQFVVFPWRPSNKMLGKIYDYVMHSLIHFQGFF
VATIYCFCNNEVQTTVKRQWAQFKIQWNQRWGRRPSNRSARAAAAAAEAGDIPIYICHQE
PRNEPANNQGEESAEIIPLNIIEQESSA
>sp|P01258|CALC_HUMAN Calcitonin OS=Homo sapiens GN=CALCA PE=1 SV=2
MGFQKFSPFLALSILVLLQAGSLHAAPFRSALESSPADPATLSEDEARLLLAALVQNYVQ
MKASELEQEQEREGSSLDSPRSKRCGNLSTCMLGTYTQDFNKFHTFPQTAIGVGAPGKKR
DMSSDLERDHRPHVSMPQNAN
>sp|Q05682|CALD1_HUMAN Caldesmon OS=Homo sapiens GN=CALD1 PE=1 SV=3
MDDFERRRELRRQKREEMRLEAERIAYQRNDDDEEEAARERRRRARQERLRQKQEEESLG
QVTDQVEVNAQNSVPDEEAKTTTTNTQVEGDDEAAFLERLARREERRQKRLQEALERQKE
FDPTITDASLSLPSRRMQNDTAENETTEKEEKSESRQERYEIEETETVTKSYQKNDWRDA
EENKKEDKEKEEEEEEKPKRGSIGENQVEVMVEEKTTESQEETVVMSLKNGQISSEEPKQ
EEEREQGSDEISHHEKMEEEDKERAEAERARLEAEERERIKAEQDKKIADERARIEAEEK
AAAQERERREAEERERMREEEKRAAEERQRIKEEEKRAAEERQRIKEEEKRAAEERQRIK
EEEKRAAEERQRARAEEEEKAKVEEQKRNKQLEEKKHAMQETKIKGEKVEQKIEGKWVNE
KKAQEDKLQTAVLKKQGEEKGTKVQAKREKLQEDKPTFKKEEIKDEKIKKDKEPKEEVKS
FMDRKKGFTEVKSQNGEFMTHKLKHTENTFSRPGGRASVDTKEAEGAPQVEAGKRLEELR
RRRGETESEEFEKLKQKQQEAALELEELKKKREERRKVLEEEEQRRKQEEADRKLREEEE
KRRLKEEIERRRAEAAEKRQKMPEDGLSDDKKPFKCFTPKGSSLKIEERAEFLNKSVQKS

SGVKSTHQAAIVSKIDSRLEQYTSAIEGTKSAKPTKPAASDLPVPAEGVRNIKSMWEKGN
VFSSPTAAGTPNKETAGLKVGVSSRINEWLTKTPDGNKSPAPKPSDLRPGDVSSKRNLWE
KQSVDKVTSPTKV
>sp|Q5T5Y3|CAMP1_HUMAN Calmodulin-regulated spectrin-associated protein 1 OS=Homo sapiens GN=CAMSAP1 PE=1 SV=2
MVDASGRAAAEGWRKMEAPPDGAADLVPLDRYDAARAKIAANLQWICAKAYGRDNIPEDL
RDPFYVDQYEQEHIKPPVIKLLLSSELYCRVCSLILKGDQVAALQGHQSVIQALSRKGIY
VMESDDTPVTESDLSRAPIKMSAHMAMVDALMMAYTVEMISIEKVVASVKRFSTFSASKE
LPYDLEDAMVFWINKVNLKMREITEKEVKLKQQLLESPAHQKVRYRREHLSARQSPYFPL
LEDLMRDGSDGAALLAVIHYYCPEQMKLDDICLKEVTSMADSLYNIRLLREFSNEYLNKC
FYLTLEDMLYAPLVLKPNVMVFIAELFWWFENVKPDFVQPRDVQELKDAKTVLHQKSSRP
PVPISNATKRSFLGSPAAGTLAELQPPVQLPAEGCHRHYLHPEEPEYLGKGTAAFSPSHP
LLPLRQKQQKSIQGEDIPDQRHRSNSLTRVDGQPRGAAIAWPEKKTRPASQPTPFALHHA
ASCEVDPSSGDSISLARSISKDSLASNIVNLTPQNQPHPTATKSHGKSLLSNVSIEDEEE
ELVAIVRADVVPQQADPEFPRASPRALGLTANARSPQGQLDTSESKPDSFFLEPLMPAVL
KPAKEKQVITKEDERGEGRPRSIVSRRPSEGPQPLVRRKMTGSRDLNRTFTPIPCSEFPM
GIDPTETGPLSVETAGEVCGGPLALGGFDPFPQGPSTDGFFLHVGRADEDTEGRLYVSCS
KSPNSHDSEPWTLLRQDSDSDVVDIEEAEHDFMGEAHPVVFSRYIGEEESAKLQEDMKVK
EHEDKDDASGRSSPCLSTASQMSSVSMASGSVKMTSFAERKLQRLNSCETKSSTSSSQKT
TPDASESCPAPLTTWRQKREQSPSQHGKDPASLLASELVQLHMQLEEKRRAIEAQKKKME
ALSARQRLKLGKAAFLHVVKKGKAEAAPPLRPEHFAKEYSQHNGEDCGDAVSKTEDFLVK
EEQREELLHEPQDVDKESLAFAQQHKAKDPVALHELERNKVISAALLEDTVGEVVDVNEC
DLSIEKLNETISTLQQAILKISQQQEQLLMKSPTVPVPGSKNNSQDHKVKAPVHFVEPLS
PTGVAGHRKAPRLGQGRNSRSGRPAELKVPKDRPQGSSRSKTPTPSVETLPHLRPFPASS
HPRTPTDPGLDSALEPSGDPHGKCLFDSYRLHDESNQRTLTLSSSKDANILSEQMSLKEV
LDASVKEVGSSSSDVSGKESVPVEEPLRSRASLIEVDLSDLKAPDEDGELVSLDGSADLV
SEGDQKPGVGFFFKDEQKAEDELAKKRAAFLLKQQRKAEEARVRKQQLEAEVELKRDEAR
RKAEEDRVRKEEEKARRELIKQEYLRRKQQQILEEQGLGKPKSKPKKPRPKSVHREESCS
DSGTKCSSTPDNLSRTQSGSSLSLASAATTEPESVHSGGTPSQRVESMEALPILSRNPSR
STDRDWETASAASSLASVAEYTGPKLFKEPSSKSNKPIIHNAISHCCLAGKVNEPHKNSI
LEELEKCDANHYIILFRDAGCQFRALYCYYPDTEEIYKLTGTGPKNITKKMIDKLYKYSS
DRKQFNLIPAKTMSVSVDALTIHNHLWQPKRPAVPKKAQTRK
>sp|Q6MZZ7|CAN13_HUMAN Calpain-13 OS=Homo sapiens GN=CAPN13 PE=1 SV=2
MAYYQEPSVETSIIKFKDQDFTTLRDHCLSMGRTFKDETFPAADSSIGQKLLQEKRLSNV
IWKRPQDLPGGPPHFILDDISRFDIQQGGAADCWFLAALGSLTQNPQYRQKILMVQSFSH
QYAGIFRFRFWQCGQWVEVVIDDRLPVQGDKCLFVRPRHQNQEFWPCLLEKAYAKLLGSY
SDLHYGFLEDALVDLTGGVITNIHLHSSPVDLVKAVKTATKAGSLITCATPSGPTDTAQA
MENGLVSLHAYTVTGAEQIQYRRGWEEIISLWNPWGWGEAEWRGRWSDGSQEWEETCDPR
KSQLHKKREDGEFWMSCQDFQQKFIAMFICSEIPITLDHGNTLHEGWSQIMFRKQVILGN
TAGGPRNDAQFNFSVQEPMEGTNVVVCVTVAVTPSNLKAEDAKFPLDFQVILAGSQRFRE
KFPPVFFSSFRNTVQSSNNKFRRNFTMTYHLSPGNYVVVAQTRRKSAEFLLRIFLKMPDS
DRHLSSHFNLRMKGSPSEHGSQQSIFNRYAQQRLDIDATQLQGLLNQELLTGPPGDMFSL
DECRSLVALMELKVNGRLDQEEFARLWKRLVHYQHVFQKVQTSPGVLLSSDLWKAIENTD
FLRGIFISRELLHLVTLRYSDSVGRVSFPSLVCFLMRLEAMAKTFRNLSKDGKGLYLTEM

EWMSLVMYN
>sp|A8MX76|CAN14_HUMAN Calpain-14 OS=Homo sapiens GN=CAPN14 PE=2 SV=2
MSLWPPFRCRWKLAPRYSRRASPQQPQQDFEALLAECLRNGCLFEDTSFPATLSSIGSGS
LLQKLPPRLQWKRPPELHSNPQFYFAKAKRLDLCQGIVGDCWFLAALQALALHQDILSRV
VPLNQSFTEKYAGIFRFWFWHYGNWVPVVIDDRLPVNEAGQLVFVSSTYKNLFWGALLEK
AYAKLSGSYEDLQSGQVSEALVDFTGGVTMTINLAEAHGNLWDILIEATYNRTLIGCQTH
SGEKILENGLVEGHAYTLTGIRKVTCKHRPEYLVKLRNPWGKVEWKGDWSDSSSKWELLS
PKEKILLLRKDNDGEFWMTLQDFKTHFVLLVICKLTPGLLSQEAAQKWTYTMREGRWEKR
STAGGQRQLLQDTFWKNPQFLLSVWRPEEGRRSLRPCSVLVSLLQKPRHRCRKRKPLLAI
GFYLYRMNKYHDDQRRLPPEFFQRNTPLSQPDRFLKEKEVSQELCLEPGTYLIVPCILEA
HQKSEFVLRVFSRKHIFYEIGSNSGVVFSKEIEDQNERQDEFFTKFFEKHPEINAVQLQN
LLNQMTWSSLGSRQPFFSLEACQGILALLDLNASGTMSIQEFRDLWKQLKLSQKVFHKQD
RGSGYLNWEQLHAAMREAGIMLSDDVCQLMLIRYGGPRLQMDFVSFIHLMLRVENMEDVF
QNLTQDGKGIYLQKPEWMMMALYS
>sp|Q8WVQ1|CANT1_HUMAN Soluble calcium-activated nucleotidase 1 OS=Homo sapiens GN=CANT1 PE=1 SV=1
MPVQLSEHPEWNESMHSLRISVGGLPVLASMTKAADPRFRPRWKVILTFFVGAAILWLLC
SHRPAPGRPPTHNAHNWRLGQAPANWYNDTYPLSPPQRTPAGIRYRIAVIADLDTESRAQ
EENTWFSYLKKGYLTLSDSGDKVAVEWDKDHGVLESHLAEKGRGMELSDLIVFNGKLYSV
DDRTGVVYQIEGSKAVPWVILSDGDGTVEKGFKAEWLAVKDERLYVGGLGKEWTTTTGDV
VNENPEWVKVVGYKGSVDHENWVSNYNALRAAAGIQPPGYLIHESACWSDTLQRWFFLPR
RASQERYSEKDDERKGANLLLSASPDFGDIAVSHVGAVVPTHGFSSFKFIPNTDDQIIVA
LKSEEDSGRVASYIMAFTLDGRFLLPETKIGSVKYEGIEFI
>sp|Q01518|CAP1_HUMAN Adenylyl cyclase-associated protein 1 OS=Homo sapiens GN=CAP1 PE=1 SV=5
MADMQNLVERLERAVGRLEAVSHTSDMHRGYADSPSKAGAAPYVQAFDSLLAGPVAEYLK
ISKEIGGDVQKHAEMVHTGLKLERALLVTASQCQQPAENKLSDLLAPISEQIKEVITFRE
KNRGSKLFNHLSAVSESIQALGWVAMAPKPGPYVKEMNDAAMFYTNRVLKEYKDVDKKHV
DWVKAYLSIWTELQAYIKEFHTTGLAWSKTGPVAKELSGLPSGPSAGSCPPPPPPCPPPP
PVSTISCSYESASRSSLFAQINQGESITHALKHVSDDMKTHKNPALKAQSGPVRSGPKPF
SAPKPQTSPSPKRATKKEPAVLELEGKKWRVENQENVSNLVIEDTELKQVAYIYKCVNTT
LQIKGKINSITVDNCKKLGLVFDDVVGIVEIINSKDVKVQVMGKVPTISINKTDGCHAYL
SKNSLDCEIVSAKSSEMNVLIPTEGGDFNEFPVPEQFKTLWNGQKLVTTVTEIAG
>sp|P47710|CASA1_HUMAN Alpha-S1-casein OS=Homo sapiens GN=CSN1S1 PE=1 SV=1
MRLLILTCLVAVALARPKLPLRYPERLQNPSESSEPIPLESREEYMNGMNRQRNILREKQ
TDEIKDTRNESTQNCVVAEPEKMESSISSSSEEMSLSKCAEQFCRLNEYNQLQLQAAHAQ
EQIRRMNENSHVQVPFQQLNQLAAYPYAVWYYPQIMQYVPFPPFSDISNPTAHENYEKNN
VMLQW
>sp|Q8NG31|CASC5_HUMAN Protein CASC5 OS=Homo sapiens GN=CASC5 PE=1 SV=3
MDGVSSEANEENDNIERPVRRRHSSILKPPRSPLQDLRGGNERVQESNALRNKKNSRRVS
FADTIKVFQTESHMKIVRKSEMEGCSAMVPSQLQLLPPGFKRFSCLSLPETETGENLLLI
QNKKLEDNYCEITGMNTLLSAPIHTQMQQKEFSIIEHTRERKHANDQTVIFSDENQMDLT
SSHTVMITKGLLDNPISEKSTKIDTTSFLANLKLHTEDSRMKKEVNFSVDQNTSSENKID
FNDFIKRLKTGKCSAFPDVPDKENFEIPIYSKEPNSASSTHQMHVSLKEDENNSNITRLF

REKDDGMNFTQCHTANIQTLIPTSSETNSRESKGNDITIYGNDFMDLTFNHTLQILPATG
NFSEIENQTQNAMDVTTGYGTKASGNKTVFKSKQNTAFQDLSINSADKIHITRSHIMGAE
THIVSQTCNQDARILAMTPESIYSNPSIQGCKTVFYSSCNDAMEMTKCLSNMREEKNLLK
HDSNYAKMYCNPDAMSSLTEKTIYSGEENMDITKSHTVAIDNQIFKQDQSNVQIAAAPTP
EKEMMLQNLMTTSEDGKMNVNCNSVPHVSKERIQQSLSNPLSISLTDRKTELLSGENMDL
TESHTSNLGSQVPLAAYNLAPESTSESHSQSKSSSDECEEITKSRNEPFQRSDIIAKNSL
TDTWNKDKDWVLKILPYLDKDSPQSADCNQEIATSHNIVYCGGVLDKQITNRNTVSWEQS
LFSTTKPLFSSGQFSMKNHDTAISSHTVKSVLGQNSKLAEPLRKSLSNPTPDYCHDKMII
CSEEEQNMDLTKSHTVVIGFGPSELQELGKTNLEHTTGQLTTMNRQIAVKVEKCGKSPIE
KSGVLKSNCIMDVLEDESVQKPKFPKEKQNVKIWGRKSVGGPKIDKTIVFSEDDKNDMDI
TKSYTIEINHRPLLEKRDCHLVPLAGTSETILYTCRQDDMEITRSHTTALECKTVSPDEI
TTRPMDKTVVFVDNHVELEMTESHTVFIDYQEKERTDRPNFELSQRKSLGTPTVICTPTE
ESVFFPGNGESDRLVANDSQLTPLEEWSNNRGPVEVADNMELSKSATCKNIKDVQSPGFL
NEPLSSKSQRRKSLKLKNDKTIVFSENHKNDMDITQSCMVEIDNESALEDKEDFHLAGAS
KTILYSCGQDDMEITRSHTTALECKTLLPNEIAIRPMDKTVLFTDNYSDLEVTDSHTVFI
DCQATEKILEENPKFGIGKGKNLGVSFPKDNSCVQEIAEKQALAVGNKIVLHTEQKQQLF
AATNRTTNEIIKFHSAAMDEKVIGKVVDQACTLEKAQVESCQLNNRDRRNVDFTSSHATA
VCGSSDNYSCLPNVISCTDNLEGSAMLLCDKDEEKANYCPVQNDLAYANDFASEYYLESE
GQPLSAPCPLLEKEEVIQTSTKGQLDCVITLHKDQDLIKDPRNLLANQTLVYSQDLGEMT
KLNSKRVSFKLPKDQMKVYVDDIYVIPQPHFSTDQPPLPKKGQSSINKEEVILSKAGNKS
LNIIENSSAPICENKPKILNSEEWFAAACKKELKENIQTTNYNTALDFHSNSDVTKQVIQ
THVNAGEAPDPVITSNVPCFHSIKPNLNNLNGKTGEFLAFQTVHLPPLPEQLLELGNKAH
NDMHIVQATEIHNINIISSNAKDSRDEENKKSHNGAETTSLPPKTVFKDKVRRCSLGIFL
PRLPNKRNCSVTGIDDLEQIPADTTDINHLETQPVSSKDSGIGSVAGKLNLSPSQYINEE
NLPVYPDEINSSDSINIETEEKALIETYQKEISPYENKMGKTCNSQKRTWVQEEEDIHKE
KKIRKNEIKFSDTTQDREIFDHHTEEDIDKSANSVLIKNLSRTPSSCSSSLDSIKADGTS
LDFSTYRSSQMESQFLRDTICEESLREKLQDGRITIREFFILLQVHILIQKPRQSNLPGN
FTVNTPPTPEDLMLSQYVVYRPKIQIYREDCEARRQKIEELKLSASNQDKLLVDINKNLWE
KMRHCSDKELKAFGIYLNKIKSCFTKMTKVFTHQGKVALYGKLVQSAQNEREKLQIKIDE
MDKILKKIDNCLTEMETETKNLEDEEKNNPVEEWDSEMRAAEKELEQLKTEEEELQRNLL
ELEVQKEQTLAQIDFMQKQRNRTEELLDQLSLSEWDVVEWSDDQAVFTFVYDTIQLTITF
EESVVGFPFLDKRYRKIVDVNFQSLLDEDQAPPSSLLVHKLIFQYVEEKESWKKTCTTQH
QLPKMLEEFSLVVHHCRLLGEEIEYLKRWGPNYNLMNIDINNNELRLLFSSSAAFAKFEI
TLFLSAYYPSVPLPSTIQNHVGNTSQDDIATILSKVPLENNYLKNVVKQIYQDLFQDCHF
YH
>sp|P07498|CASK_HUMAN Kappa-casein OS=Homo sapiens GN=CSN3 PE=1 SV=3
MKSFLLVVNALALTLPFLAVEVQNQKQPACHENDERPFYQKTAPYVPMYYVPNSYPYYGT
NLYQRRPAIAINNPYVPRTYYANPAVVRPHAQIPQRQYLPNSHPPTVVRRPNLHPSFIAI
PPKKIQDKIIIPTINTIATVEPTPAPATEPTVDSVVTPEAFSESIITSTPETTTVAVTPP
TA
>sp|Q92851|CASPA_HUMAN Caspase-10 OS=Homo sapiens GN=CASP10 PE=1 SV=3
MKSQGQHWYSSSDKNCKVSFREKLLIIDSNLGVQDVENLKFLCIGLVPNKKLEKSSSASD
VFEHLLAEDLLSEEDPFFLAELLYIIRQKKLLQHLNCTKEEVERLLPTRQRVSLFRNLLY
ELSEGIDSENLKDMIFLLKDSLPKTEMTSLSFLAFLEKQGKIDEDNLTCLEDLCKTVVPK

LLRNIEKYKREKAIQIVTPPVDKEAESYQGEEELVSQTDVKTFLEALPQESWQNKHAGSN
GNRATNGAPSLVSRGMQGASANTLNSETSTKRAAVYRMNRNHRGLCVIVNNHSFTSLKDR
QGTHKDAEILSHVFQWLGFTVHIHNNVTKVEMEMVLQKQKCNPAHADGDCFVFCILTHGR
FGAVYSSDEALIPIREIMSHFTALQCPRLAEKPKLFFIQACQGEEIQPSVSIEADALNPE
QAPTSLQDSIPAEADFLLGLATVPGYVSFRHVEEGSWYIQSLCNHLKKLVPRMLKFLEKT
MEIRGRKRTVWGAKQISATSLPTAISAQTPRPPMRRWSSVS
>sp|Q8WUQ7|CATIN_HUMAN Cactin OS=Homo sapiens GN=CACTIN PE=1 SV=3
MGRDTRSRSRSAGRRGRRRQSQSGSRSRSRSHGRRNRRRREDEGRRRRRRRSRERRSDSE
EERWQRSGMRSRSPPRPKWHSRDGSSQSDSGEEQSRGQWARRRRRARSWSPSSSASSSAS
PGRSQSPRAAAAAALSQQQSLQERLRLREERKQQEELMKAFETPEEKRARRLAKKEAKERK
KREKMGWGEEYMGYTNTDNPFGDNNLLGTFIWNKALEKKGISHLEEKELKERNKRIQEDN
RLELQKVKQLRLEREREKAMREQELEMLQREKEAEHFKTWEEQEDNFHLQQAKLRSKIRI
RDGRAKPIDLLAKYISAEDDDLAVEMHEPYTFLNGLTVADMEDLLEDIQVYMELEQGKNA
DFWRDMTTITEDEISKLRKLEASGKGPGERREGVNASVSSDVQSVFKGKTYNQLQVIFQG
IEGKIRAGGPNLDMGYWESLLQQLRAHMARARLRERHQDVLRQKLYKLKQEQGVESEPLF
PILKQEPQSPSRSLEPEDAAPTPPGPSSEGGPAEAEVDGATPTEGDGDGDGEGEGEGEAV
LMEEDLIQQSLDDYDAGRYSPRLLTAHELPLDAHVLEPDEDLQRLQLSRQQLQVTGDASE
SAEDIFFRRAKEGMGQDEAQFSVEMPLTGKAYLWADKYRPRKPRFFNRVHTGFEWNKYNQ
THYDFDNPPPKIVQGYKFNIFYPDLIDKRSTPEYFLEACADNKDFAILRFHAGPPYEDIA
FKIVNREWEYSHRHGFRCQFANGIFQLWFHFKRYRYRR
>sp|P07711|CATL1_HUMAN Cathepsin L1 OS=Homo sapiens GN=CTSL PE=1 SV=2
MNPTLILAAFCLGIASATLTFDHSLEAQWTKWKAMHNRLYGMNEEGWRRAVWEKNMKMIE
LHNQEYREGKHSFTMAMNAFGDMTSEEFRQVMNGFQNRKPRKGKVFQEPLFYEAPRSVDW
REKGYVTPVKNQGQCGSCWAFSATGALEGQMFRKTGRLISLSEQNLVDCSGPQGNEGCNG
GLMDYAFQYVQDNGGLDSEESYPYEATEESCKYNPKYSVANDTGFVDIPKQEKALMKAVA
TVGPISVAIDAGHESFLFYKEGIYFEPDCSSEDMDHGVLVVGYGFESTESDNNKYWLVKN
SWGEEWGMGGYVKMAKDRRNHCGIASAASYPTV
>sp|P43234|CATO_HUMAN Cathepsin O OS=Homo sapiens GN=CTSO PE=2 SV=1
MDVRALPWLPWLLWLLCRGGGDADSRAPFTPTWPRSREREAAAFRESLNRHRYLNSLFPS
ENSTAFYGINQFSYLFPEEFKAIYLRSKPSKFPRYSAEVHMSIPNVSLPLRFDWRDKQVV
TQVRNQQMCGGCWAFSVVGAVESAYAIKGKPLEDLSVQQVIDCSYNNYGCNGGSTLNALN
WLNKMQVKLVKDSEYPFKAQNGLCHYFSGSHSGFSIKGYSAYDFSDQEDEMAKALLTFGP
LVVIVDAVSWQDYLGGIIQHHCSSGEANHAVLITGFDKTGSTPYWIVRNSWGSSWGVDGY
AHVKMGSNVCGIADSVSSIFV
>sp|Q8WU43|CB015_HUMAN Uncharacterized protein C2orf15 OS=Homo sapiens GN=C2orf15 PE=2 SV=1
MSSTLGKLSNQVEETLPLLKKVPANYFHICSAILMGFSLSKSATQVSAIHMDSKVDDHLI
RGTEKSRLEPATQLFQNTKKIRLEDTNQENFTRIEGTGTGSLSGKALGSVVYVKESDGLE
MTDVE
>sp|Q68DN1|CB016_HUMAN Uncharacterized protein C2orf16 OS=Homo sapiens GN=C2orf16 PE=2 SV=3
MELTPGAQQQGINYQELTSGWQDVKSMMLVPEPTRKFPSGPLLTSVRFSNLSPESQQQDV
KSLEFTVEPKLQSVKHVKLSSVSLQQTIKSVELAPGSLPQRVKYGEQTPRTNYQIMESSE
LIPRPGHQFAKYAEMIPQPKYQIPKSANLISIPIYHATESSEMAQGLAYKGIDTVEKSVG

LTPKLTGRAKESLGMLLQPDLQVPKFVDLTPMVRDQGSKFLGLTPEKSYQILETMELLSQ
SRPRVKDVGELYMKPLQQTVEYEGITPELKHYFTEAMGLTAEARIQANEFFGMTPKPTSQ
ATGFAERSPRLCPQNLECVEVISEKRLQGEESVVLIPKSLHHVPDSASGMTPGLGHRVPE
SVELTSKSGVQVEKTLQLTPKPQHHVGSPGIISGLGHQVPESVNLTCKQWLQMEESLEVP
LKQTSQVIGHEESVELTSEARQHREVSMGLTKSKNQSMKSPGTTPGPLGRIVEFMRISPE
PLDQVTESARTQLQVAQSEEVILIDVPKVVQSVKVTPGPPFQIVKSVTIPRPTPQMVEYI
ELTPKLQYVRPSEHHTGPCLQDVKSTKLITKPKHQILETVELTGFQIVKTMLIPGPSLQI
VKSEELAPGPIPQVVEPIGVALESGIEAINCVDLLPRPHLQELIVPAELTPSPCTQVKSA
ELTSPQTSPFEEHTILTHKQGLQAVKSTVIKTEPPKVMETEDLNLGHVCQNRDCQKLTSE
ELQVGTDFSRFLQSSSTTLISSSVRTASELGGLWDSGIQEVSRALDIKNPGTDILQPEET
YIDPTMIQSLTFPLALHNQSSDKTANIVENPCPEILGVDVISKETTKRKQMEELENSLQR
HLPQSWRSRSRTFQAESGVQKGLIKSFPGRQHNVWESHAWRQRLPRKYLSTMLMLGNILG
TTMERKLCSQTSLAERATADTCQSIQNLFGIPAELMEPSQSLPEKGPVTISQPSVVKNYI
QRHTFYHGHKKRMALRIWTRGSTSSIIQQYSGTRVRIKKTNSTFNGISQEVIQHMPVSCA
GGQLPVLVKSESSLSIFYDREDLVPMEESEDSQSDSQTRISESQHSLKPNYLSQAKTDFS
EQFQLLEDLQLKIAAKLLRSQIPPDVPPPLASGLVLKYPICLQCGRCSGLNCHHKLQTTS
GPYLLIYPQLHLVRTPEGHGEVRLHLGFRLRIGKRSQISKYRERDRPVIRRSPISPSQRK
AKIYTQASKSPTSTIDLQSGPSQSPAPVQVYIRRGQRSRPDLVEKTKTRAPGHYEFTQVH
NLPESDSESTQNEKRAKVRTKKTSDSKYPMKRITKRLRKHRKFYTNSRTTIESPSRELAA
HLRRKRIGATQTSTASLKRQPKKPSQPKFMQLLFQSLKRAFQTAHRVIASVGRKPVDGTR
PDNLWASKNYYPKQNARDYCLPSSIKRDKRSADKLTPAGSTIKQEDILWGGTVQCRSAQQ
PRRAYSFQPRPLRLPKPTDSQSGIAFQTASVGQPLRTVQKDSSSRSKKNFYRNETSSQES
KNLSTPGTRVQARGRILPGSPVKRTWHRHLKDKLTHKEHNHPSFYRERTPRGPSERTRHN
PSWRNHRSPSERSQRSSLERRHHSPSQRSHCSPSRKNHSSPSERSWRSPSQRNHCSPPER
SCHSLSERGLHSPSQRSHRGPSQRRHHSPSERSHRSPSERSHRSSSERRHRSPSQRSHRG
PSERSHCSPSERRHRSPSQRSHRGPSERRHHSPSKRSHRSPARRSHRSPSERSHHSPSER
SHHSPSERRHHSPSERSHCSPSERSHCSPSERRHRSPSERRHHSPSEKSHHSPSERSHHS
PSERRRHSPLERSRHSLLERSHRSPSERRSHRSFERSHRRISERSHSPSEKSHLSPLERS
RCSPSERRGHSSSGKTCHSPSERSHRSPSGMRQGRTSERSHRSSCERTRHSPSEMRPGRP
SGRNHCSPSERSRRSPLKEGLKYSFPGERPSHSLSRDFKNQTTLLGTTHKNPKAGQVWRP
EATR
>sp|Q08AI8|CB054_HUMAN Uncharacterized protein C2orf54 OS=Homo sapiens GN=C2orf54 PE=2 SV=2
MPAPALPTSAMAVQVPLWHHYLQAIRSREAPRAQDFQRAENVLLTVLERVHALDPRFIVD
YSRGLEAFQFALRSSEDPMDMEVPLWVDAEALLIEEPEATQPEDGLELCHLGVPREGAGL
ERWTTEDTFTASSEGDAKCRGHIVPSKVLCVLKDLLVAAIVHCKHHSLIAPGSLNAASLR
EEQLHLSLLVSSGWRTISFHVVPVVRRKLGAPALEGVQQMPGFPEGSLRRILSQGVDLVP
ASAQLWRTSTDYLLTRLLGELGSLQGHRLDSLSILDRVNHESWRDSGQTDGLTFGHLKMV
LLWASVLFLAPEDWAELQGAVYRLLVVLLCCLATRKLPHFLHPQRNLLQGSGLDLGAIYQ
RVEGFASQPEAALRIHATHLGRSPPPRIGSGLKALLQLPASDPTYWATAYFDVLLDKFQV
FNIQDKDRISAMQSIFQKTRTLGGEES
>sp|A6NCS6|CB072_HUMAN Uncharacterized protein C2orf72 OS=Homo sapiens GN=C2orf72 PE=1 SV=2
MERELEALAARLARPAEPPFQALVEAAGGRGQVLLVGELWEREQSRALLRDFARAVFPPE

PGAAKPGGAAAEGAGPGAARGAQRAARAAGAAGAAAAAARAIRSPLVFVLCRASSLAARE
PRRRLREMLRDVRGRRRAGAALVGVLVAEAGPEDAVAPGLRLLEALLRAVFGRQAGGPVQ
AAAYCPGLPASCLAVQAAACRALQAAGAGQPVEGAWERPGLPGLLACFSWGPWSRRKNQD
VAACRSSAQEDFQEPEEELPLTAIFPNGDCDDLGRGSKACDGVVHTPAEPTGDSR
>sp|Q6ZV80|CB091_HUMAN Uncharacterized protein C2orf91 OS=Homo sapiens GN=C2orf91 PE=2 SV=1
MVRMSRPLFLDWAWRPLCSPSQSLPLTYGPEGWILQWKGTCRQQTALHCPFDFPQAPLRG
RHTLSQVPNKGHEKASAVQLPEKQGTDQSRRGPTSAVTKARTSYPESETFIVYLCSYFWN
SSKGVYMSGST
>sp|Q5RIA9|CBWD5_HUMAN COBW domain-containing protein 5 OS=Homo sapiens GN=CBWD5 PE=2 SV=1
MLPAVGSVDEEEDPAEEDCPELVPIETTQSEEEEKSGLGAKIPVTIITGYLGAGKTTLLN
YILTEQHSKRVAVILNESGEGSALEKSLAVSQGGELYEEWLELRNGCLCCSVKDNGLRAI
ENLMQKKGKFDDILLETTGLADPGAVTSMFWVDAELGSDIYLDGIITIVDSKYGLKHLTE
EKPDGLINEATRQVALADIILINKTDLVPEEDVKKLRTTIRSINGLGQILETQRSRVDLS
NVLDLHAFDSLSGISLQKKLQHVPGTQPHLDQSIVTITFEVPGNAKEEHLNMFIQNLLWE
KNVRNKDNHCMEVIRLKGLVSIKDKSQQVIVQGVHELYDLEETPVSWKDDTERTNRLVLI
GRNLDKDILKQLFIATVTETEKQWTTHFKEDQVCT
>sp|Q14781|CBX2_HUMAN Chromobox protein homolog 2 OS=Homo sapiens GN=CBX2 PE=1 SV=2
MEELSSVGEQVFAAECILSKRLRKGKLEYLVKWRGWSSKHNSWEPEENILDPRLLLAFQK
KEHEKEVQNRKRGKRPRGRPRKLTAMSSCSRRSKLKEPDAPSKSKSSSSSSSSTSSSSSS
DEEDDSDLDAKRGPRGRETHPVPQKKAQILVAKPELKDPIRKKRGRKPLPPEQKATRRPV
SLAKVLKTARKDLGAPASKLPPPLSAPVAGLAALKAHAKEACGGPSAMATPENLASLMKG
MASSPGRGGISWQSSIVHYMNRMTQSQAQAASRLALKAQATNKCGLGLDLKVRTQKGELG
MSPPGSKIPKAPSGGAVEQKVGNTGGPPHTHGASRVPAGCPGPQPAPTQELSLQVLDLQS
VKNGMPGVGLLARHATATKGVPATNPAPGKGTGSGLIGASGATMPTDTSKSEKLASRAVA
PPTPASKRDCVKGSATPSGQESRTAPGEARKAATLPEMSAGEESSSSDSDPDSASPPSTG
QNPSVSVQTSQDWKPTRSLIEHVFVTDVTANLITVTVKESPTSVGFFNLRHY
>sp|Q13185|CBX3_HUMAN Chromobox protein homolog 3 OS=Homo sapiens GN=CBX3 PE=1 SV=4
MASNKTTLQKMGKKQNGKSKKVEEAEPEEFVVEKVLDRRVVNGKVEYFLKWKGFTDADNT
WEPEENLDCPELIEAFLNSQKAGKEKDGTKRKSLSDSESDDSKSKKKRDAADKPRGFARG
LDPERIIGATDSSGELMFLMKWKDSDEADLVLAKEANMKCPQIVIAFYEERLTWHSCPED
EAQ
>sp|A6NI87|CBY3_HUMAN Protein chibby homolog 3 OS=Homo sapiens GN=CBY3 PE=3 SV=3
MWASRDHLPEPDLGDAAPPGSPSSFWTSGLPRQERSTSRQRSRGSPSSTCVPYKVHALAT
FECSATSHASRLWQTLQQFWADHISRPFSPRRPPLRRMPSLSTFYLLDHNTRQAELGLAY
GAPCMRLSNQAFVFRGGRWTTESQLARTRSPLLSRTAWGWKAQVQRSKSQVLLEENNYLK
LQQELLIDMLTETMARMHLLEKQRNPEVIPTAAARAGQRKMRKRAGASAGVLMIQPCALD
SQ
>sp|Q9HBI5|CC014_HUMAN Uncharacterized protein C3orf14 OS=Homo sapiens GN=C3orf14 PE=2 SV=1
MTSLFAQEIRLSKRHEEIVSQRLMLLQQMENKLGDQHTEKASQLQTVETAFKRNLSLLKD
IEAAEKSLQTRIHPLPRPEVVSLETRYWASVEEYIPKWEQFLLGRAPYPFAVENQNEAEN
TIQNEAQR

>sp|Q3SXR2|CC036_HUMAN Uncharacterized protein C3orf36 OS=Homo sapiens GN=C3orf36 PE=2 SV=1
MQAETILEGLEAGLPQAVSSGLSLVPAPGLVLTCLSAPSGPGGMALEPPPTTLRKAFLAQ
STLLESTLEGAPEWAAPHPEEQRRSPPACSQHTPPLPSTPTGPPPCSPGGNHPLCALSGR
GGGRCSIPSLSSSSTFSLFSSGCWNPRVKLRVRKSQSQGRAGQLI
>sp|Q9BWC9|CC106_HUMAN Coiled-coil domain-containing protein 106 OS=Homo sapiens GN=CCDC106 PE=1 SV=1
MNDRSSRRRTMKDDETFEISIPFDEAPHLDPQIFYSLSPSRRNFEEPPEAASSALALMNS
VKTQLHMALERNSWLQKRIEDLEEERDFLRCQLDKFISSARMEAEDHCRMKPGPRRMEGD
SRGGAGGEASDPESAASSLSGASEEGSASERRRQKQKGGASRRRFGKPKARERQRVKDAD
GVLCRYKKILGTFQKLKSMSRAFEHHRVDRNTVALTTPIAELLIVAPEKLAEVGEFDPSK
ERLLEYSRRCFLALDDETLKKVQALKKSKLLLPITYRFKR
>sp|Q8TBZ0|CC110_HUMAN Coiled-coil domain-containing protein 110 OS=Homo sapiens GN=CCDC110 PE=1 SV=1
MSPEKQHREEDEVDSVLLSASKILNSSEGVKESGCSDTEYGCIAESENQIQPQSALKVLQ
QQLESFQALRMQTLQNVSMVQSEISEILNKSIIEVENPQFSSEKNLVFGTRIEKDLPTEN
QEENLSMEKSHHFEDSKTLHSVEEKLSGDSVNSLPQSVNVPSQIHSEDTLTLRTSTDNLS
SNIIIHPSENSDILKNYNNFYRFLPTAPPNVMSQADTVILDKSKITVPFLKHGFCENLDD
ICHSIKQMKEELQKSHDGEVALTNELQTLQTDPDVHRNGKYDMSPIHQDKMNFIKEENLD
GNLNEDIKSKRISELEALVKKLLPFRETVSKFHVHFCRKCKKLSKSEMHRGKKNEKNNKE
IPITGKNITDLKFHSRVPRYTLSFLDQTKHEMKDKERQPFLVKQGSIISENEKTSKVNSV
TEQCVAKIQYLQNYLKESVQIQKKVMELESENLNLKSKMKPLIFTTQSLIQKVETYEKQL
KNLVEEKSTIQSKLSKTEEYSKECLKEFKKIISKYNVLQGQNKTLEEKNIQLSLEKQQMM
EALDQLKSKEHKTQSDMAIVNNENNRMSIEMEAMKTNILLIQDEKEMLEKKTHQLLKEKS
SLGNELKESQLEIIQLKEKERLAKTEQETLLQIIETVKDEKLNLETTLQESTAARQIMER
EIENIQTYQSTAEENFLQEIKNAKSEASIYKNSLSEIGKECEMLSKMVMETKTDNQILKE
ELKKHSQENIKFENSISRLTEDKILLENYVRSIENERDTLEFEMRHLQREYLSLSDKICN
QHNDPSKTTYISRREKFHFDNYTHEDTSSPQSRPLASDLKGYFKVKDRTLKHH
>sp|Q494R4|CC153_HUMAN Coiled-coil domain-containing protein 153 OS=Homo sapiens GN=CCDC153 PE=1 SV=2
MPPKNKEKGKKSGAQKKKKNWGADVVAESRHRLVVLEKELLRDHLALRRDEARRAKASED
QLRQRLQGVEAELEGARSEGKAIYAEMSRQCHALQEDMQTRSKQLEEEVKGLRGQLEACQ
REAAAAREEAEQALGERDQALAQLRAHMADMEAKYEEILHDSLDRLLAKLRAIKQQWDGA
ALRLHARHKEQQRQFGLTPPGSLRPPAPSL
>sp|Q569K6|CC157_HUMAN Coiled-coil domain-containing protein 157 OS=Homo sapiens GN=CCDC157 PE=2 SV=3
MAHLLGSQACMESLRTDLTDLQGAIVDVFSRAGPVRFPSWKFPDRMACDLDMVALLEHYD
HVPGDPEFTQLSHAVLLELVIDRLLLLLQSCMSYLENLGSEQMMPPAQAAGPCMSVGLTV
RRFWDSLLRLGTLHQQPLPQKGANQRETPTSKPTTKGEPARSPEYLTTKLIKPSSPVLGL
PQTCQEPESIPVRASLQFPATTFKNTRSVHSQTIETALVPCDACASVQGSLQKVGKVVIS
LCQSQNLPSSLGQFQQLVQDSMGLRPLPAATVGRWAAEQRKDLTRLSKHVEALRAQLEEA
EGQKDGLRKQAGKLEQALKQEQGARRRQAEEDEQCLSEWEHDKQQLLTETSDLKTKMATL
ERELKQQRESTQAVEAKAQQLQEEGERRAAAERQVQQLEEQVQQLEAQVQLLVGRLEGAG
QQVCWASTELDKEKARVDSMVRHQESLQAKQRALLKQLDSLDQEREELRGSLDEAEAQRA

RVEEQLQSEREQGQCQLRAQQELLQSLQREKQGLEQATTDLRLTILELERELEELKERER
LLVAFPDLHRPTETQIHGGRSSSVESQITCPTDSGNVTDHMERQVQSNDIRIRVLQEENG
RLQSMLSKIREVAQQGGLKLIPQDRLWSPSSKGTQGATPPVQAKSTSPGPLGRQHLPSSR
TGRTLLGQPCTSPPRQPCTSPPRQPCTSPPRQPCTSPSRQPCSQPSKSLLEGVTHLDTCT
QNPIKVLVRLRKRLSPGRGQASSAHQPQERPM
>sp|Q8IYT3|CC170_HUMAN Coiled-coil domain-containing protein 170 OS=Homo sapiens GN=CCDC170 PE=1 SV=3
MSLDCTSHIALGAASPAPEETYDHLSEVPVTREQLNHYRNVAQNARSELAATLVKFECAQ
SELQDLRSKMLSKEVSCQELKAEMESYKENNARKSSLLTSLRDRVQELEEESAALSTSKI
RTEITAHAAIKENQELKKKVVELNEKLQKCSKENEENKKQVSKNCRKHEEFLTQLRDCLD
PDERNDKASDEDLILKLRDLRKENEFVKGQIVILEETINVHEMEAKASRETIMRLASEVN
REQKKAASCTEEKEKLNQDLLSAVEAKEALEREVKIFQERLLAGQQVWDASKQEVSLLKK
SSSELEKSLKASQDAVTTSQSQYFSFREKIAALLRGRLSMTGSTEDTILEKIREMDSREE
SRDRMVSQLEAQISELVEQLGKESGFHQKALQRAQKAENMLETLQGQLTHLEAELVSGGV
LRDNLNFEKQKYLKFLDQLSQKMKLDQMAAELGFDMRLDVVLARTEQLVRLESNAVIENK
TIAHNLQRKLKTQKERLESKELHMSLLRQKIAQLEEEKQARTALVVERDNAHLTIRNLQK
KVERLQKELNTCRDLHTELKAKLADTNELKIKTLEQTKAIEDLNKSRDQLEKMKEKAEKK
LMSVKSELDTTEHEAKENKERARNMIEVVTSEMKTLKKSLEEAEKREKQLADFREVVSQM
LGLNVTSLALPDYEIIKCLERLVHSHQHHFVTCACLKDVTTGQERHPQGHLQLLH
>sp|Q6PII3|CC174_HUMAN Coiled-coil domain-containing protein 174 OS=Homo sapiens GN=CCDC174 PE=1 SV=3
MDRRKKPLDVTASSLVDLKAELFRKQEEFKQEKLLKDSGVFGKPKTTNKKPSIWSKQNVG
VSNRAEKDAEQKIEEQKTLDKAREKLEEKAKLYEKMTKGDFIDEEVEDMYLVDFTQKIID
KRKEMEASGAHRDSQKAGERDDDEENLPEGEIPPPQDPSEEWVDYVDSLGRSRRCMRKDL
PDLLEMDKNLQGRLFISPANEKTLLSEDMRKELQRQQWEEEEREALKRPMGPVHYEDIRE
NEARQLGVGYFAFARDKELRNKQMKTLEMLREQTTDQRTKRENIKEKRKAILEARLAKLR
QKKMKKSKEGGTEEENRDGDVIGPLPPEPEAVPTPRPAAQSSKVEVIVQERKDTKPGVPH
IREWDRGKEFSFGYWSKRQSDLRAERDPEFAPPSDYFVGQKRTGFSSSQAWSRPGPAQSD
PGQCPDQSHGPSPEHTSPTPAPDNPPQAPTVTFKTLDDMISYYKQVT
>sp|P0DO97|CC192_HUMAN Coiled-coil domain-containing protein 192 OS=Homo sapiens GN=CCDC192 PE=4 SV=1
MMPVDVCPRDRGSQWVWLEMGQCYSKKSVVPESDTSERSSMTSGSSESDIPQENKVSKAS
LDTGQMAFTLAQLESLEICLKEAEEKAKALSEQLSVSEGTKSKLLEQVSRLEEKLEAVDH
KEASGGPYEKMVLVKDQCIQKLQAEVKASQEQLIAQKLKHEKKVKKLQTDLATANAITVL
ELNEKIKTLYEGKPAPREDSLLEGFCGGLPPVEEGDRKISLIMELSTQVSLQTERITQLK
EVLEEKERKIQQLEAERSPHPPQEVKDPPGCLPEAPVFSTHDIPPVVSDENL
>sp|Q8IWP9|CC28A_HUMAN Coiled-coil domain-containing protein 28A OS=Homo sapiens GN=CCDC28A PE=1 SV=1
MPRAEPRATLGEQEKAGLPLGAWRLYLLRHFRKQTELRRSGSRDVTGALLVAAAVASEAV
GSLRVAEGGPNTLLLQVLRSWPWCNKELKTMEERKVKRRSPKSFSAHCTQVVNAKKNAIP
VSKSTGFSNPASQSTSQRPKLKRVMKEKTKPQGGEGKGAQSTPIQHSFLTDVSDVQEMER
GLLSLLNDFHSGKLQAFGNECSIEQMEHVRGMQEKLARLNLELYGELEELPEDKRKTASD
SNLDRLLSDLEELNSSIQKLHLADAQDVPNTSAS

>sp|Q9BUN5|CC28B_HUMAN Coiled-coil domain-containing protein 28B OS=Homo sapiens GN=CCDC28B PE=1 SV=2
MDDKKKKRSPKPCLAQPAQAPGTLRRVPVPTSHSGSLALGLPHLPSPKQRAKFKRVGKEK
CRPVLAGGGSGSAGTPLQHSFLTEVTDVYEMEGGLLNLLNDFHSGRLQAFGKECSFEQLE
HVREMQEKLARLHFSLDVCGEEEDDEEEEDGVTEGLPEEQKKTMADRNLDQLLSNLEDLS
NSIQKLHLAENAEPEEQSAA
>sp|Q8N9Z2|CC71L_HUMAN Coiled-coil domain-containing protein 71L OS=Homo sapiens GN=CCDC71L PE=1 SV=2
MRRSMKRRRRRRPVAPATAARGGDFRAEDGAGLEAREEKVVYSRSQLSLADSTKALGDAF
KLFMPRSTEFMSSDAELWSFLCSLKHQFSPHILRSKDVYGYSSCRALVPDPPGPPTARGQ
ARRPVPRAAARRRRRGARAAAARRRKPRPPPPPPPPPEESCPAKPVAPGPCFGGRTLEEI
WRAATPTLTTFPTIRVGSDVWGERSLAAARRRARQVLRVNLEPMVRLRRFPVPRA
>sp|Q96A33|CCD47_HUMAN Coiled-coil domain-containing protein 47 OS=Homo sapiens GN=CCDC47 PE=1 SV=1
MKAFHTFCVVLLVFGSVSEAKFDDFEDEEDIVEYDDNDFAEFEDVMEDSVTESPQRVIIT
EDDEDETTVELEGQDENQEGDFEDADTQEGDTESEPYDDEEFEGYEDKPDTSSSKNKDPI
TIVDVPAHLQNSWESYYLEILMVTGLLAYIMNYIIGKNKNSRLAQAWFNTHRELLESNFT
LVGDDGTNKEATSTGKLNQENEHIYNLWCSGRVCCEGMLIQLRFLKRQDLLNVLARMMRP
VSDQVQIKVTMNDEDMDTYVFAVGTRKALVRLQKEMQDLSEFCSDKPKSGAKYGLPDSLA
ILSEMGEVTDGMMDTKMVHFLTHYADKIESVHFSDQFSGPKIMQEEGQPLKLPDTKRTLL
FTFNVPGSGNTYPKDMEALLPLMNMVIYSIDKAKKFRLNREGKQKADKNRARVEENFLKL
THVQRQEAAQSRREEKKRAEKERIMNEEDPEKQRRLEEAALRREQKKLEKKQMKMKQIKV
KAM
>sp|Q9H2F9|CCD68_HUMAN Coiled-coil domain-containing protein 68 OS=Homo sapiens GN=CCDC68 PE=1 SV=1
MTTVTVTTEIPPRDKMEDNSALYESTSAHIIEETEYVKKIRTTLQKIRTQMFKDEIRHDS
TNHKLDAKHCGNLQQGSDSEMDPSCCSLDLLMKKIKGKDLQLLEMNKENEVLKIKLQASR
EAGAAALRNVAQRLFENYQTQSEEVRKKQEDSKQLLQVNKLEKEQKLKQHVENLNQVAEK
LEEKHSQITELENLVQRMEKEKRTLLERKLSLENKLLQLKSSATYGKSCQDLQREISILQ
EQISHLQFVIHSQHQNLRSVIQEMEGLKNNLKEQDKRIENLREKVNILEAQNKELKTQVA
LSSETPRTKVSKAVSTSELKTEGVSPYLMLIRLRK
>sp|Q6ZRK6|CCD73_HUMAN Coiled-coil domain-containing protein 73 OS=Homo sapiens GN=CCDC73 PE=2 SV=2
MESNFNTESSSTFTLQSSSETLFSIQLLDFKTSLLEALEELRMRREAEIHYEEQIGKIIV
ETQELKWQKETLQNQKETLAEQHKEAMAVFKKQLQMKMCALEEEKGKYQLATEIKEKEIE
GLKETLKALQVSKYSLQKKVSEMEQKVQLHLLAKEDYHKQLSEIEKYYATITGQFGLVKE
NHEKLEQNVREAIQSNKRLSALNKKQEAEICSLKKELKKAASDLIKSKVTCQYKMGEENI
NLTIKEQKFQELQERLNMELELNEKINEEITHIQEEKQDIIISFQHMQQLLRQQIQANTE
MEAELKVLKENNQTLERDNELQREKVKENEEKFLNLQNEHEKALGTWKRHAEELNGEINK
IKNELSSLKETHIKLQEHYNKLCNQKTFEEDKKFQNVPEVNNENSEMSTEKSENTIIQKY
NTEQEIREENMENFCSDTEYREKEEKKEGSFIEEIIIDDLQLFEKSFKNEIDTVVSQDEN
QSEISLSKTLSLDKEVISQGQTSNVTDNRKSVTTEIKDKICLEKDNGCTEFKSPNNHFVV
LDTAIETEKIHLERTRGLDVHHTDVNLEVENNKTSFNSILNETAHNTYHNNNKDVSENEP
FKQFRLLPGTREHALEKEITNSDQTKADLDSSLDIKKNPVPCQKYSLRNSSNVMLDDKQC

KIKQIQLLTKKSECSILLSKQTSDFLQVCNDTLEKSELTVPCDIVIDHHVSYAAFSANSK
LLLKNSDKNVHSMSMLVKPNSSPGGKTMCKNMSDMQNSQFNNCLGYLENTNVNISHLHLN
NENSHASQAKDVKTAVHMKTCTETEFSNKKNQIDENQVTEATKNDLFLFVSINERQHTLL
NNTEKTESLNDIVSGKMFSEGQLEESHSFHIEPSGDLVNRSGRSTFDLSTSDKKTEKTPV
YMNFSDPGPWSKVNHIESQTASSSTPCISLLLKERPLDPSENKKIISMALCKNIGVDDVG
KDIGPDTTSINRVADTLNNWSIHPDPKGEPSEEKNAMAKTFYDSSFPTEHVKTKPLISTP
LQSHLQAIKTTKNTSGDDDWQSLITNQLNKSENLLSLENDNQPKKRKAEETLEKNNRLK
>sp|Q9NR09|BIRC6_HUMAN Baculoviral IAP repeat-containing protein 6 OS=Homo sapiens GN=BIRC6 PE=1 SV=2
MVTGGGAAPPGTVTEPLPSVIVLSAGRKMAAAAAAASGPGCSSAAGAGAAGVSEWLVLRD
GCMHCDADGLHSLSYHPALNAILAVTSRGTIKVIDGTSGATLQASALSAKPGGQVKCQYI
SAVDKVIFVDDYAVGCRKDLNGILLLDTALQTPVSKQDDVVQLELPVTEAQQLLSACLEK
VDISSTEGYDLFITQLKDGLKNTSHETAANHKVAKWATVTFHLPHHVLKSIASAIVNELK
KINQNVAALPVASSVMDRLSYLLPSARPELGVGPGRSVDRSLMYSEANRRETFTSWPHVG
YRWAQPDPMAQAGFYHQPASSGDDRAMCFTCSVCLVCWEPTDEPWSEHERHSPNCPFVKG
EHTQNVPLSVTLATSPAQFPCTDGTDRISCFGSGSCPHFLAAATKRGKICIWDVSKLMKV
HLKFEINAYDPAIVQQLILSGDPSSGVDSRRPTLAWLEDSSSCSDIPKLEGDSDDLLEDS
DSEEHSRSDSVTGHTSQKEAMEVSLDITALSILQQPEKLQWEIVANVLEDTVKDLEELGA
NPCLTNSKSEKTKEKHQEQHNIPFPCLLAGGLLTYKSPATSPISSNSHRSLDGLSRTQGE
SISEQGSTDNESCTNSELNSPLVRRTLPVLLLYSIKESDEKAGKIFSQMNNIMSKSLHDD
GFTVPQIIEMELDSQEQLLLQDPPVTYIQQFADAAANLTSPDSEKWNSVFPKPGTLVQCL
RLPKFAEEENLCIDSITPCADGIHLLVGLRTCPVESLSAINQVEALNNLNKLNSALCNRR
KGELESNLAVVNGANISVIQHESPADVQTPLIIQPEQRNVSGGYLVLYKMNYATRIVTLE
EEPIKIQHIKDPQDTITSLILLPPDILDNREDDCEEPIEDMQLTSKNGFEREKTSDISTL
GHLVITTQGGYVKILDLSNFEILAKVEPPKKEGTEEQDTFVSVIYCSGTDRLCACTKGGE
LHFLQIGGTCDDIDEADILVDGSLSKGIEPSSEGSKPLSNPSSPGISGVDLLVDQPFTLE
ILTSLVELTRFETLTPRFSATVPPCWVEVQQEQQQRRHPQHLHQQHHGDAAQHTRTWKLQ
TDSNSWDEHVFELVLPKACMVGHVDFKFVLNSNITNIPQIQVTLLKNKAPGLGKVNALNI
EVEQNGKPSLVDLNEEMQHMDVEESQCLRLCPFLEDHKEDILCGPVWLASGLDLSGHAGM
LTLTSPKLVKGMAGGKYRSFLIHVKAVNERGTEEICNGGMRPVVRLPSLKHQSNKGYSLA
SLLAKVAAGKEKSSNVKNENTSGTRKSENLRGCDLLQEVSVTIRRFKKTSISKERVQRCA
MLQFSEFHEKLVNTLCRKTDDGQITEHAQSLVLDTLCWLAGVHSNGPGSSKEGNENLLSK
TRKFLSDIVRVCFFEAGRSIAHKCARFLALCISNGKCDPCQPAFGPVLLKALLDNMSFLP
AATTGGSVYWYFVLLNYVKDEDLAGCSTACASLLTAVSRQLQDRLTPMEALLQTRYGLYS
SPFDPVLFDLEMSGSSCKNVYNSSIGVQSDEIDLSDVLSGNGKVSSCTAAEGSFTSLTGL
LEVEPLHFTCVSTSDGTRIERDDAMSSFGVTPAVGGLSSGTVGEASTALSSAAQVALQSL
SHAMASAEQQLQVLQEKQQQLLKLQQQKAKLEAKLHQTTAAAAAAASAVGPVHNSVPSNP
VAAPGFFIHPSDVIPPTPKTTPLFMTPPLTPPNEAVSVVINAELAQLFPGSVIDPPAVNL
AAHNKNSNKSRMNPLGSGLALAISHASHFLQPPPHQSIIIERMHSGARRFVTLDFGRPIL
LTDVLIPTCGDLASLSIDIWTLGEEVDGRRLVVATDISTHSLILHDLIPPPVCRFMKITV
IGRYGSTNARAKIPLGFYYGHTYILPWESELKLMHDPLKGEGESANQPEIDQHLAMMVAL
QEDIQCRYNLACHRLETLLQSIDLPPLNSANNAQYFLRKPDKAVEEDSRVFSAYQDCIQL
QLQLNLAHNAVQRLKVALGASRKMLSETSNPEDLIQTSSTEQLRTIIRYLLDTLLSLLHA
SNGHSVPAVLQSTFHAQACEELFKHLCISGTPKIRLHTGLLLVQLCGGERWWGQFLSNVL

QELYNSEQLLIFPQDRVFMLLSCIGQRSLSNSGVLESLLNLLDNLLSPLQPQLPMHRRTE
GVLDIPMISWVVMLVSRLLDYVATVEDEAAAAKKPLNGNQWSFINNNLHTQSLNRSSKGS
SSLDRLYSRKIRKQLVHHKQQLNLLKAKQKALVEQMEKEKIQSNKGSSYKLLVEQAKLKQ
ATSKHFKDLIRLRRTAEWSRSNLDTEVTTAKESPEIEPLPFTLAHERCISVVQKLVLFLL
SMDFTCHADLLLFVCKVLARIANATRPTIHLCEIVNEPQLERLLLLLVGTDFNRGDISWG
GAWAQYSLTCMLQDILAGELLAPVAAEAMEEGTVGDDVGATAGDSDDSLQQSSVQLLETI
DEPLTHDITGAPPLSSLEKDKEIDLELLQDLMEVDIDPLDIDLEKDPLAAKVFKPISSTW
YDYWGADYGTYNYNPYIGGLGIPVAKPPANTEKNGSQTVSVSVSQALDARLEVGLEQQAE
LMLKMMSTLEADSILQALTNTSPTLSQSPTGTDDSLLGGLQAANQTSQLIIQLSSVPMLN
VCFNKLFSMLQVHHVQLESLLQLWLTLSLNSSSTGNKENGADIFLYNANRIPVISLNQAS
ITSFLTVLAWYPNTLLRTWCLVLHSLTLMTNMQLNSGSSSAIGTQESTAHLLVSDPNLIH
VLVKFLSGTSPHGTNQHSPQVGPTATQAMQEFLTRLQVHLSSTCPQIFSEFLLKLIHILS
TERGAFQTGQGPLDAQVKLLEFTLEQNFEVVSVSTISAVIESVTFLVHHYITCSDKVMSR
SGSDSSVGARACFGGLFANLIRPGDAKAVCGEMTRDQLMFDLLKLVNILVQLPLSGNREY
SARVSVTTNTTDSVSDEEKVSGGKDGNGSSTSVQGSPAYVADLVLANQQIMSQILSALGL
CNSSAMAMIIGASGLHLTKHENFHGGLDAISVGDGLFTILTTLSKKASTVHMMLQPILTY
MACGYMGRQGSLATCQLSEPLLWFILRVLDTSDALKAFHDMGGVQLICNNMVTSTRAIVN
TARSMVSTIMKFLDSGPNKAVDSTLKTRILASEPDNAEGIHNFAPLGTITSSSPTAQPAE
VLLQATPPHRRARSAAWSYIFLPEEAWCDLTIHLPAAVLLKEIHIQPHLASLATCPSSVS
VEVSADGVNMLPLSTPVVTSGLTYIKIQLVKAEVASAVCLRLHRPRDASTLGLSQIKLLG
LTAFGTTSSATVNNPFLPSEDQVSKTSIGWLRLLHHCLTHISDLEGMMASAAAPTANLLQ
TCAALLMSPYCGMHSPNIEVVLVKIGLQSTRIGLKLIDILLRNCAASGSDPTDLNSPLLF
GRLNGLSSDSTIDILYQLGTTQDPGTKDRIQALLKWVSDSARVAAMKRSGRMNYMCPNSS
TVEYGLLMPSPSHLHCVAAILWHSYELLVEYDLPALLDQELFELLFNWSMSLPCNMVLKK
AVDSLLCSMCHVHPNYFSLLMGWMGITPPPVQCHHRLSMTDDSKKQDLSSSLTDDSKNAQ
APLALTESHLATLASSSQSPEAIKQLLDSGLPSLLVRSLASFCFSHISSSESIAQSIDIS
QDKLRRHHVPQQCNKMPITADLVAPILRFLTEVGNSHIMKDWLGGSEVNPLWTALLFLLC
HSGSTSGSHNLGAQQTSARSASLSSAATTGLTTQQRTAIENATVAFFLQCISCHPNNQKL
MAQVLCELFQTSPQRGNLPTSGNISGFIRRLFLQLMLEDEKVTMFLQSPCPLYKGRINAT
SHVIQHPMYGAGHKFRTLHLPVSTTLSDVLDRVSDTPSITAKLISEQKDDKEKKNHEEKE
KVKAENGFQDNYSVVVASGLKSQSKRAVSATPPRPPSRRGRTIPDKIGSTSGAEAANKII
TVPVFHLFHKLLAGQPLPAEMTLAQLLTLLYDRKLPQGYRSIDLTVKLGSRVITDPSLSK
TDSYKRLHPEKDHGDLLASCPEDEALTPGDECMDGILDESLLETCPIQSPLQVFAGMGGL
ALIAERLPMLYPEVIQQVSAPVVTSTTQEKPKDSDQFEWVTIEQSGELVYEAPETVAAEP
PPIKSAVQTMSPIPAHSLAAFGLFLRLPGYAEVLLKERKHAQCLLRLVLGVTDDGEGSHI
LQSPSANVLPTLPFHVLRSLFSTTPLTTDDGVLLRRMALEIGALHLILVCLSALSHHSPR
VPNSSVNQTEPQVSSSHNPTSTEEQQLYWAKGTGFGTGSTASGWDVEQALTKQRLEEEHV
TCLLQVLASYINPVSSAVNGEAQSSHETRGQNSNALPSVLLELLSQSCLIPAMSSYLRND
SVLDMARHVPLYRALLELLRAIASCAAMVPLLLPLSTENGEEEEEQSECQTSVGTLLAKM
KTCVDTYTNRLRSKRENVKTGVKPDASDQEPEGLTLLVPDIQKTAEIVYAATTSLRQANQ
EKKLGEYSKKAAMKPKPLSVLKSLEEKYVAVMKKLQFDTFEMVSEDEDGKLGFKVNYHYM
SQVKNANDANSAARARRLAQEAVTLSTSLPLSSSSSVFVRCDEERLDIMKVLITGPADTP
YANGCFEFDVYFPQDYPSSPPLVNLETTGGHSVRFNPNLYNDGKVCLSILNTWHGRPEEK
WNPQTSSFLQVLVSVQSLILVAEPYFNEPGYERSRGTPSGTQSSREYDGNIRQATVKWAM

LEQIRNPSPCFKEVIHKHFYLKRVEIMAQCEEWIADIQQYSSDKRVGRTMSHHAAALKRH
TAQLREELLKLPCPEGLDPDTDDAPEVCRATTGAEETLMHDQVKPSSSKELPSDFQL
>sp|Q96CA5|BIRC7_HUMAN Baculoviral IAP repeat-containing protein 7 OS=Homo sapiens GN=BIRC7 PE=1 SV=2
MGPKDSAKCLHRGPQPSHWAAGDGPTQERCGPRSLGSPVLGLDTCRAWDHVDGQILGQLR
PLTEEEEEEGAGATLSRGPAFPGMGSEELRLASFYDWPLTAEVPPELLAAAGFFHTGHQD
KVRCFFCYGGLQSWKRGDDPWTEHAKWFPSCQFLLRSKGRDFVHSVQETHSQLLGSWDPW
EEPEDAAPVAPSVPASGYPELPTPRREVQSESAQEPGGVSPAEAQRAWWVLEPPGARDVE
AQLRRLQEERTCKVCLDRAVSIVFVPCGHLVCAECAPGLQLCPICRAPVRSRVRTFLS
>sp|A8MY62|BLML_HUMAN Putative beta-lactamase-like 1 OS=Homo sapiens GN=LACTBL1 PE=2 SV=2
MCPRHPEPVPLAHPLPVLKEALEKVDQILRQAMSAPGVAAMSAVVIHNDTVLWTGNFGKK
NGSDPASGAPNEYTMYRISSISKIFPVLMLYRLWEEGIVASLDDPLERYASTFTINNPLG
LASAEQQGLILRRMASQLSGLPRRLRSTSLLWKGSTQEALNLLKDDVLVVDPGTRCHYST
LAFSLLAHVLAAHTAQGDYQRWVSENVLEPLGMADTGFDLTPDVRARLAAGFYGSGRPAP
LYDLGWYRPSGQMYSTAADLAKLAVALLGGGPRRLLRPDAAKTLLAPLLACPGAYFANET
GTPWEFHAQRGYRVVRKDGDLDGYAATFSLVPPLRLGLVLLLAGPRPPGPDLVARAYDEL
LPALERALREAEPGPAPPPTAHPFAGYFTFANLTFYEVRAGPAGELRLRQFGPRVEALVP
PAFRTLALRHLHGRVFQLHVAHEFPCALPLGDAWLSLEAQHGQLVNFYPLDHHGLSPGFD
VPGLNTYRVLRLRGKPVFKT
>sp|P54132|BLM_HUMAN Bloom syndrome protein OS=Homo sapiens GN=BLM PE=1 SV=1
MAAVPQNNLQEQLERHSARTLNNKLSLSKPKFSGFTFKKKTSSDNNVSVTNVSVAKTPVL
RNKDVNVTEDFSFSEPLPNTTNQQRVKDFFKNAPAGQETQRGGSKSLLPDFLQTPKEVVC
TTQNTPTVKKSRDTALKKLEFSSSPDSLSTINDWDDMDDFDTSETSKSFVTPPQSHFVRV
STAQKSKKGKRNFFKAQLYTTNTVKTDLPPPSSESEQIDLTEEQKDDSEWLSSDVICIDD
GPIAEVHINEDAQESDSLKTHLEDERDNSEKKKNLEEAELHSTEKVPCIEFDDDDYDTDF
VPPSPEEIISASSSSSKCLSTLKDLDTSDRKEDVLSTSKDLLSKPEKMSMQELNPETSTD
CDARQISLQQQLIHVMEHICKLIDTIPDDKLKLLDCGNELLQQRNIRRKLLTEVDFNKSD
ASLLGSLWRYRPDSLDGPMEGDSCPTGNSMKELNFSHLPSNSVSPGDCLLTTTLGKTGFS
ATRKNLFERPLFNTHLQKSFVSSNWAETPRLGKKNESSYFPGNVLTSTAVKDQNKHTASI
NDLERETQPSYDIDNFDIDDFDDDDDWEDIMHNLAASKSSTAAYQPIKEGRPIKSVSERL
SSAKTDCLPVSSTAQNINFSESIQNYTDKSAQNLASRNLKHERFQSLSFPHTKEMMKIFH
KKFGLHNFRTNQLEAINAALLGEDCFILMPTGGGKSLCYQLPACVSPGVTVVISPLRSLI
VDQVQKLTSLDIPATYLTGDKTDSEATNIYLQLSKKDPIIKLLYVTPEKICASNRLISTL
ENLYERKLLARFVIDEAHCVSQWGHDFRQDYKRMNMLRQKFPSVPVMALTATANPRVQKD
ILTQLKILRPQVFSMSFNRHNLKYYVLPKKPKKVAFDCLEWIRKHHPYDSGIIYCLSRRE
CDTMADTLQRDGLAALAYHAGLSDSARDEVQQKWINQDGCQVICATIAFGMGIDKPDVRF
VIHASLPKSVEGYYQESGRAGRDGEISHCLLFYTYHDVTRLKRLIMMEKDGNHHTRETHF
NNLYSMVHYCENITECRRIQLLAYFGENGFNPDFCKKHPDVSCDNCCKTKDYKTRDVTDD
VKSIVRFVQEHSSSQGMRNIKHVGPSGRFTMNMLVDIFLGSKSAKIQSGIFGKGSAYSRH
NAERLFKKLILDKILDEDLYINANDQAIAYVMLGNKAQTVLNGNLKVDFMETENSSSVKK
QKALVAKVSQREEMVKKCLGELTEVCKSLGKVFGVHYFNIFNTVTLKKLAESLSSDPEVL
LQIDGVTEDKLEKYGAEVISVLQKYSEWTSPAEDSSPGISLSSSRGPGRSAAEELDEEIP
VSSHYFASKTRNERKRKKMPASQRSKRRKTASSGSKAKGGSATCRKISSKTKSSSIIGSS
SASHTSQATSGANSKLGIMAPPKPINRPFLKPSYAFS

>sp|Q5H9B9|BM2KL_HUMAN Putative BMP-2-inducible kinase-like protein OS=Homo sapiens GN=BMP2KL PE=5 SV=2
MIAPSPKSSEEEGQKDEEVLQGEQGDFNDNDTEPENLGHRPLLMDSEDEEEEEKRSSDSD
YEQAKAKYSDMSPVYRDKSGSGPTQDINTILLTSAQLSSDVGVETPKQEFDIFGAVPFFA
VCAQQPQQEKNEKSLPQHRFPATGLQQEEFDVFTKAPFSKKVNVQECHAVGPETHPKSID
IFDFTPFQPFLTSTSKSESNEDLFGLVPFEEIMGSQQQKVKQRSLQKLSSRQRRTKQDMS
KSNGKRHHGTPTSKKKTLKPTYRTPERARRHKKVGRRVSQTSNEFVTISDSKENIGAAVT
DGNDRGNVLQLEESLLDPFGAKPFHPPDLSWHPLHQGLNDIRADHNTVLPKQPRQNSLHG
SFHSADVLTMDDFGAMPFTELVVQSITLQQSQQSQPVELDPFGAAPFPSKQ
>sp|Q96LC9|BMF_HUMAN Bcl-2-modifying factor OS=Homo sapiens GN=BMF PE=1 SV=1
MEPSQCVEELEDDVFQPEDGEPVTQPGSLLSADLFAQSLLDCPLSRLQLFPLTHCCGPGL
RPTSQEDKATQTLSPASPSQGVMLPCGVTEEPQRLFYGNAGYRLPLPASFPAVLPIGEQP
PEGQWQHQAEVQIARKLQCIADQFHRLHVQQHQQNQNRVWWQILLFLHNLALNGEENRNG
AGPR
>sp|P35226|BMI1_HUMAN Polycomb complex protein BMI-1 OS=Homo sapiens GN=BMI1 PE=1 SV=2
MHRTTRIKITELNPHLMCVLCGGYFIDATTIIECLHSFCKTCIVRYLETSKYCPICDVQV
HKTRPLLNIRSDKTLQDIVYKLVPGLFKNEMKRRRDFYAAHPSADAANGSNEDRGEVADE
DKRIITDDEIISLSIEFFDQNRLDRKVNKDKEKSKEEVNDKRYLRCPAAMTVMHLRKFLR
SKMDIPNTFQIDVMYEEEPLKDYYTLMDIAYIYTWRRNGPLPLKYRVRPTCKRMKISHQR
DGLTNAGELESDSGSDKANSPAGGIPSTSSCLPSPSTPVQSPHPQFPHISSTMNGTSNSP
SGNHQSSFANRPRKSSVNGSSATSSG
>sp|P12643|BMP2_HUMAN Bone morphogenetic protein 2 OS=Homo sapiens GN=BMP2 PE=1 SV=1
MVAGTRCLLALLLPQVLLGGAAGLVPELGRRKFAAASSGRPSSQPSDEVLSEFELRLLSM
FGLKQRPTPSRDAVVPPYMLDLYRRHSGQPGSPAPDHRLERAASRANTVRSFHHEESLEE
LPETSGKTTRRFFFNLSSIPTEEFITSAELQVFREQMQDALGNNSSFHHRINIYEIIKPA
TANSKFPVTRLLDTRLVNQNASRWESFDVTPAVMRWTAQGHANHGFVVEVAHLEEKQGVS
KRHVRISRSLHQDEHSWSQIRPLLVTFGHDGKGHPLHKREKRQAKHKQRKRLKSSCKRHP
LYVDFSDVGWNDWIVAPPGYHAFYCHGECPFPLADHLNSTNHAIVQTLVNSVNSKIPKAC
CVPTELSAISMLYLDENEKVVLKNYQDMVVEGCGCR
>sp|Q8WZ55|BSND_HUMAN Barttin OS=Homo sapiens GN=BSND PE=1 SV=1
MADEKTFRIGFIVLGLFLLALGTFLMSHDRPQVYGTFYAMGSVMVIGGIIWSMCQCYPKI
TFVPADSDFQGILSPKAMGLLENGLAAEMKSPSPQPPYVRLWEEAAYDQSLPDFSHIQMK
VMSYSEDHRSLLAPEMGQPKLGTSDGGEGGPGDVQAWMEAAVVIHKGSDESEGERRLTQS
WPGPLACPQGPAPLASFQDDLDMDSSEGSSPNASPHDREEACSPQQEPQGCRCPLDRFQD
FALIDAPTLEDEPQEGQQWEIALPNNWQRYPRTKVEEKEASDTGGEEPEKEEEDLYYGLP
DGAGDLLPDKELGFEPDTQG
>sp|Q5W0U4|BSPRY_HUMAN B box and SPRY domain-containing protein OS=Homo sapiens GN=BSPRY PE=2 SV=1
MSAEGAEPGPGSGSGPGPGPLCPEHGQALSWFCGSERRPVCAACAGLGGRCRGHRIRRAE
ERAEELRNKIVDQCERLQLQSAAITKYVADVLPGKNQRAVSMASAARELVIQRLSLVRSL
CESEEQRLLEQVHGEEERAHQSILTQRVHWAEALQKLDTIRTGLVGMLTHLDDLQLIQKE
QEIFERTEEAEGILDPQESEMLNFNEKCTRSPLLTQLWATAVLGSLSGTEDIRIDERTVS
PFLQLSDDRKTLTFSTKKSKACADGPERFDHWPNALAATSFQNGLHAWMVNVQNSCAYKV
GVASGHLPRKGSGSDCRLGHNAFSWVFSRYDQEFRFSHNGQHEPLGLLRGPAQLGVVLDL

QVQELLFYEPASGTVLCAHHVSFPGPLFPVFAVADQTISIVR
>sp|Q96KE9|BTBD6_HUMAN BTB/POZ domain-containing protein 6 OS=Homo sapiens GN=BTBD6 PE=1 SV=3
MAAELYAPASAAAADLANSNAGAAVGRKAGPRSPPSAPAPAPPPPAPAPPTLGNNHQESP
GWRCCRPTLRERNALMFNNELMADVHFVVGPPGATRTVPAHKYVLAVGSSVFYAMFYGDL
AEVKSEIHIPDVEPAAFLILLKYMYSDEIDLEADTVLATLYAAKKYIVPALAKACVNFLE
TSLEAKNACVLLSQSRLFEEPELTQRCWEVIDAQAEMALRSEGFCEIDRQTLEIIVTREA
LNTKEAVVFEAVLNWAEAECKRQGLPITPRNKRHVLGRALYLVRIPTMTLEEFANGAAQS
DILTLEETHSIFLWYTATNKPRLDFPLTKRKGLAPQRCHRFQSSAYRSNQWRYRGRCDSI
QFAVDRRVFIAGLGLYGSSSGKAEYSVKIELKRLGVVLAQNLTKFMSDGSSNTFPVWFEH
PVQVEQDTFYTASAVLDGSELSYFGQEGMTEVQCGKVAFQFQCSSDSTNGTGVQGGQIPE
LIFYA
>sp|A6NE02|BTBDH_HUMAN BTB/POZ domain-containing protein 17 OS=Homo sapiens GN=BTBD17 PE=3 SV=1
MPRRGYSKPGSWGSFWAMLTLVGLVTHAAQRADVGGEAAGTSINHSQAVLQRLQELLRQG
NASDVVLRVQAAGTDEVRVFHAHRLLLGLHSELFLELLSNQSEAVLQEPQDCAAVFDKFI
RYLYCGELTVLLTQAIPLHRLATKYGVSSLQRGVADYMRAHLAGGAGPAVGWYHYAVGTG
DEALRESCLQFLAWNLSAVAASTEWGAVSPELLWQLLQRSDLVLQDELELFHALEAWLGR
ARPPPAVAERALRAIRYPMIPPAQLFQLQARSAALARHGPAVADLLLQAYQFHAASPLHY
AKFFDVNGSAFLPRNYLAPAWGAPWVINNPARDDRSTSFQTQLGPSGHDAGRRVTWNVLF
SPRWLPVSLRPVYADAAGTALPAARPEDGRPRLVVTPASSGGDAAGVSFQKTVLVGARQQ
GRLLVRHAYSFHQSSEEAGDFLAHADLQRRNSEYLVENALHLHLIVKPVYHTLIRTPK
>sp|P20290|BTF3_HUMAN Transcription factor BTF3 OS=Homo sapiens GN=BTF3 PE=1 SV=1
MRRTGAPAQADSRGRGRARGGCPGGEATLSQPPPRGGTRGQEPQMKETIMNQEKLAKLQA
QVRIGGKGTARRKKKVVHRTATADDKKLQFSLKKLGVNNISGIEEVNMFTNQGTVIHFNN
PKVQASLAANTFTITGHAETKQLTEMLPSILNQLGADSLTSLRRLAEALPKQSVDGKAPL
ATGEDDDDEVPDLVENFDEASKNEAN
>sp|Q06187|BTK_HUMAN Tyrosine-protein kinase BTK OS=Homo sapiens GN=BTK PE=1 SV=3
MAAVILESIFLKRSQQKKKTSPLNFKKRLFLLTVHKLSYYEYDFERGRRGSKKGSIDVEK
ITCVETVVPEKNPPPERQIPRRGEESSEMEQISIIERFPYPFQVVYDEGPLYVFSPTEEL
RKRWIHQLKNVIRYNSDLVQKYHPCFWIDGQYLCCSQTAKNAMGCQILENRNGSLKPGSS
HRKTKKPLPPTPEEDQILKKPLPPEPAAAPVSTSELKKVVALYDYMPMNANDLQLRKGDE
YFILEESNLPWWRARDKNGQEGYIPSNYVTEAEDSIEMYEWYSKHMTRSQAEQLLKQEGK
EGGFIVRDSSKAGKYTVSVFAKSTGDPQGVIRHYVVCSTPQSQYYLAEKHLFSTIPELIN
YHQHNSAGLISRLKYPVSSQNKNAPSTAGLGYGSWEIDPKDLTFLKELGTGQFGVVKYGK
WRGQYDVAIKMIKEGSMSEDEFIEEAKVMMNLSHEKLVQLYGVCTKQRPIFIITEYMANG
CLLNYLREMRHRFQTQQLLEMCKDVCEAMEYLESKQFLHRDLAARNCLVNDQGVVKVSDF
GLSRYVLDDEYTSSVGSKFPVRWSPPEVLMYSKFSSKSDIWAFGVLMWEIYSLGKMPYER
FTNSETAEHIAQGLRLYRPHLASEKVYTIMYSCWHEKADERPTFKILLSNILDVMDEES
>sp|Q6UXG8|BTNL9_HUMAN Butyrophilin-like protein 9 OS=Homo sapiens GN=BTNL9 PE=2 SV=1
MVDLSVSPDSLKPVSLTSSLVFLMHLLLLQPGEPSSEVKVLGPEYPILALVGEEVEFPCH
LWPQLDAQQMEIRWFRSQTFNVVHLYQEQQELPGRQMPAFRNRTKLVKDDIAYGSVVLQL
HSIIPSDKGTYGCRFHSDNFSGEALWELEVAGLGSDPHLSLEGFKEGGIQLRLRSSGWYP
KPKVQWRDHQGQCLPPEFEAIVWDAQDLFSLETSVVVRAGALSNVSVSIQNLLLSQKKEL

VVQIADVFVPGASAWKSAFVATLPLLLVLAALALGVLRKQRRSREKLRKQAEKRQEKLTA
ELEKLQTELDWRRAEGQAEWRAAQKYAVDVTLDPASAHPSLEVSEDGKSVSSRGAPPGPA
PGHPQRFSEQTCALSLERFSAGRHYWEVHVGRRSRWFLGACLAAVPRAGPARLSPAAGYW
VLGLWNGCEYFVLAPHRVALTLRVPPRRLGVFLDYEAGELSFFNVSDGSHIFTFHDTFSG
ALCAYFRPRAHDGGEHPDPLTICPLPVRGTGVPEENDSDTWLQPYEPADPALDWW
>sp|P41223|BUD31_HUMAN Protein BUD31 homolog OS=Homo sapiens GN=BUD31 PE=1 SV=2
MPKVKRSRKAPPDGWELIEPTLDELDQKMREAETEPHEGKRKVESLWPIFRIHHQKTRYI
FDLFYKRKAISRELYEYCIKEGYADKNLIAKWKKQGYENLCCLRCIQTRDTNFGTNCICR
VPKSKLEVGRIIECTHCGCRGCSG
>sp|Q9UBR1|BUP1_HUMAN Beta-ureidopropionase OS=Homo sapiens GN=UPB1 PE=1 SV=1
MAGAEWKSLEECLEKHLPLPDLQEVKRVLYGKELRKLDLPREAFEAASREDFELQGYAFE
AAEEQLRRPRIVHVGLVQNRIPLPANAPVAEQVSALHRRIKAIVEVAAMCGVNIICFQEA
WTMPFAFCTREKLPWTEFAESAEDGPTTRFCQKLAKNHDMVVVSPILERDSEHGDVLWNT
AVVISNSGAVLGKTRKNHIPRVGDFNESTYYMEGNLGHPVFQTQFGRIAVNICYGRHHPL
NWLMYSINGAEIIFNPSATIGALSESLWPIEARNAAIANHCFTCAINRVGTEHFPNEFTS
GDGKKAHQDFGYFYGSSYVAAPDSSRTPGLSRSRDGLLVAKLDLNLCQQVNDVWNFKMTG
RYEMYARELAEAVKSNYSPTIVKE
>sp|P27824|CALX_HUMAN Calnexin OS=Homo sapiens GN=CANX PE=1 SV=2
MEGKWLLCMLLVLGTAIVEAHDGHDDDVIDIEDDLDDVIEEVEDSKPDTTAPPSSPKVTY
KAPVPTGEVYFADSFDRGTLSGWILSKAKKDDTDDEIAKYDGKWEVEEMKESKLPGDKGL
VLMSRAKHHAISAKLNKPFLFDTKPLIVQYEVNFQNGIECGGAYVKLLSKTPELNLDQFH
DKTPYTIMFGPDKCGEDYKLHFIFRHKNPKTGIYEEKHAKRPDADLKTYFTDKKTHLYTL
ILNPDNSFEILVDQSVVNSGNLLNDMTPPVNPSREIEDPEDRKPEDWDERPKIPDPEAVK
PDDWDEDAPAKIPDEEATKPEGWLDDEPEYVPDPDAEKPEDWDEDMDGEWEAPQIANPRC
ESAPGCGVWQRPVIDNPNYKGKWKPPMIDNPSYQGIWKPRKIPNPDFFEDLEPFRMTPFS
AIGLELWSMTSDIFFDNFIICADRRIVDDWANDGWGLKKAADGAAEPGVVGQMIEAAEER
PWLWVVYILTVALPVFLVILFCCSGKKQTSGMEYKKTDAPQPDVKEEEEEKEEEKDKGDE
EEEGEEKLEEKQKSDAEEDGGTVSQEEEDRKPKAEEDEILNRSPRNRKPRRE
>sp|Q9NYX4|CALY_HUMAN Neuron-specific vesicular protein calcyon OS=Homo sapiens GN=CALY PE=1 SV=1
MVKLGCSFSGKPGKDPGDQDGAAMDSVPLISPLDISQLQPPLPDQVVIKTQTEYQLSSPD
QQNFPDLEGQRLNCSHPEEGRRLPTARMIAFAMALLGCVLIMYKAIWYDQFTCPDGFLLR
HKICTPLTLEMYYTEMDPERHRSILAAIGAYPLSRKHGTETPAAWGDGYRAAKEERKGPT
QAGAAAAATEPPGKPSAKAEKEAARKAAGSAAPPPAQ
>sp|Q86VP6|CAND1_HUMAN Cullin-associated NEDD8-dissociated protein 1 OS=Homo sapiens GN=CAND1 PE=1 SV=2
MASASYHISNLLEKMTSSDKDFRFMATNDLMTELQKDSIKLDDDSERKVVKMILKLLEDK
NGEVQNLAVKCLGPLVSKVKEYQVETIVDTLCTNMLSDKEQLRDISSIGLKTVIGELPPA
SSGSALAANVCKKITGRLTSAIAKQEDVSVQLEALDIMADMLSRQGGLLVNFHPSILTCL
LPQLTSPRLAVRKRTIIALGHLVMSCGNIVFVDLIEHLLSELSKNDSMSTTRTYIQCIAA
ISRQAGHRIGEYLEKIIPLVVKFCNVDDDELREYCIQAFESFVRRCPKEVYPHVSTIINI
CLKYLTYDPNYNYDDEDEDENAMDADGGDDDDQGSDDEYSDDDDMSWKVRRAAAKCLDAV
VSTRHEMLPEFYKTVSPALISRFKEREENVKADVFHAYLSLLKQTRPVQSWLCDPDAMEQ
GETPLTMLQSQVPNIVKALHKQMKEKSVKTRQCCFNMLTELVNVLPGALTQHIPVLVPGI

IFSLNDKSSSSNLKIDALSCLYVILCNHSPQVFHPHVQALVPPVVACVGDPFYKITSEAL
LVTQQLVKVIRPLDQPSSFDATPYIKDLFTCTIKRLKAADIDQEVKERAISCMGQIICNL
GDNLGSDLPNTLQIFLERLKNEITRLTTVKALTLIAGSPLKIDLRPVLGEGVPILASFLR
KNQRALKLGTLSALDILIKNYSDSLTAAMIDAVLDELPPLISESDMHVSQMAISFLTTLA
KVYPSSLSKISGSILNELIGLVRSPLLQGGALSAMLDFFQALVVTGTNNLGYMDLLRMLT
GPVYSQSTALTHKQSYYSIAKCVAALTRACPKEGPAVVGQFIQDVKNSRSTDSIRLLALL
SLGEVGHHIDLSGQLELKSVILEAFSSPSEEVKSAASYALGSISVGNLPEYLPFVLQEIT
SQPKRQYLLLHSLKEIISSASVVGLKPYVENIWALLLKHCECAEEGTRNVVAECLGKLTL
IDPETLLPRLKGYLISGSSYARSSVVTAVKFTISDHPQPIDPLLKNCIGDFLKTLEDPDL
NVRRVALVTFNSAAHNKPSLIRDLLDTVLPHLYNETKVRKELIREVEMGPFKHTVDDGLD
IRKAAFECMYTLLDSCLDRLDIFEFLNHVEDGLKDHYDIKMLTFLMLVRLSTLCPSAVLQ
RLDRLVEPLRATCTTKVKANSVKQEFEKQDELKRSAMRAVAALLTIPEAEKSPLMSEFQS
QISSNPELAAIFESIQKDSSSTNLESMDTS
>sp|O75155|CAND2_HUMAN Cullin-associated NEDD8-dissociated protein 2 OS=Homo sapiens GN=CAND2 PE=1 SV=3
MSTAAFHISSLLEKMTSSDKDFRFMATSDLMSELQKDSIQLDEDSERKVVKMLLRLLEDK
NGEVQNLAVKCLGPLVVKVKEYQVETIVDTLCTNMRSDKEQLRDIAGIGLKTVLSELPPA
ATGSGLATNVCRKITGQLTSAIAQQEDVAVQLEALDILSDMLSRLGVPLGAFHASLLHCL
LPQLSSPRLAVRKRAVGALGHLAAACSTDLFVELADHLLDRLPGPRVPTSPTAIRTLIQC
LGSVGRQAGHRLGAHLDRLVPLVEDFCNLDDDELRESCLQAFEAFLRKCPKEMGPHVPNV
TSLCLQYIKHDPNYNYDSDEDEEQMETEDSEFSEQESEDEYSDDDDMSWKVRRAAAKCIA
ALISSRPDLLPDFHCTLAPVLIRRFKEREENVKADVFTAYIVLLRQTQPPKGWLEAMEEP
TQTGSNLHMLRGQVPLVVKALQRQLKDRSVRARQGCFSLLTELAGVLPGSLAEHMPVLVS
GIIFSLADRSSSSTIRMDALAFLQGLLGTEPAEAFHPHLPILLPPVMACVADSFYKIAAE
ALVVLQELVRALWPLHRPRMLDPEPYVGEMSAVTLARLRATDLDQEVKERAISCMGHLVG
HLGDRLGDDLEPTLLLLLDRLRNEITRLPAIKALTLVAVSPLQLDLQPILAEALHILASF
LRKNQRALRLATLAALDALAQSQGLSLPPSAVQAVLAELPALVNESDMHVAQLAVDFLAT
VTQAQPASLVEVSGPVLSELLRLLRSPLLPAGVLAAAEGFLQALVGTRPPCVDYAKLISL
LTAPVYEQAVDGGPGLHKQVFHSLARCVAALSAACPQEAASTASRLVCDARSPHSSTGVK
VLAFLSLAEVGQVAGPGHQRELKAVLLEALGSPSEDVRAAASYALGRVGAGSLPDFLPFL
LEQIEAEPRRQYLLLHSLREALGAAQPDSLKPYAEDIWALLFQRCEGAEEGTRGVVAECI
GKLVLVNPSFLLPRLRKQLAAGRPHTRSTVITAVKFLISDQPHPIDPLLKSFIGEFMESL
QDPDLNVRRATLAFFNSAVHNKPSLVRDLLDDILPLLYQETKIRRDLIREVEMGPFKHTV
DDGLDVRKAAFECMYSLLESCLGQLDICEFLNHVEDGLKDHYDIRMLTFIMVARLATLCP
APVLQRVDRLIEPLRATCTAKVKAGSVKQEFEKQDELKRSAMRAVAALLTIPEVGKSPIM
ADFSSQIRSNPELAALFESIQKDSASAPSTDSMELS
>sp|P20160|CAP7_HUMAN Azurocidin OS=Homo sapiens GN=AZU1 PE=1 SV=3
MTRLTVLALLAGLLASSRAGSSPLLDIVGGRKARPRQFPFLASIQNQGRHFCGGALIHAR
FVMTAASCFQSQNPGVSTVVLGAYDLRRRERQSRQTFSISSMSENGYDPQQNLNDLMLLQ
LDREANLTSSVTILPLPLQNATVEAGTRCQVAGWGSQRSGGRLSRFPRFVNVTVTPEDQC
RPNNVCTGVLTRRGGICNGDGGTPLVCEGLAHGVASFSLGPCGRGPDFFTRVALFRDWID
GVLNNPGPGPA
>sp|Q86X55|CARM1_HUMAN Histone-arginine methyltransferase CARM1 OS=Homo sapiens GN=CARM1 PE=1 SV=3

MAAAAAAVGPGAGGAGSAVPGGAGPCATVSVFPGARLLTIGDANGEIQRHAEQQALRLEV
RAGPDSAGIALYSHEDVCVFKCSVSRETECSRVGKQSFIITLGCNSVLIQFATPNDFCSF
YNILKTCRGHTLERSVFSERTEESSAVQYFQFYGYLSQQQNMMQDYVRTGTYQRAILQNH
TDFKDKIVLDVGCGSGILSFFAAQAGARKIYAVEASTMAQHAEVLVKSNNLTDRIVVIPG
KVEEVSLPEQVDIIISEPMGYMLFNERMLESYLHAKKYLKPSGNMFPTIGDVHLAPFTDE
QLYMEQFTKANFWYQPSFHGVDLSALRGAAVDEYFRQPVVDTFDIRILMAKSVKYTVNFL
EAKEGDLHRIEIPFKFHMLHSGLVHGLAFWFDVAFIGSIMTVWLSTAPTEPLTHWYQVRC
LFQSPLFAKAGDTLSGTCLLIANKRQSYDISIVAQVDQTGSKSSNLLDLKNPFFRYTGTT
PSPPPGSHYTSPSENMWNTGSTYNLSSGMAVAGMPTAYDLSSVIASGSSVGHNNLIPLAN
TGIVNHTHSRMGSIMSTGIVQGSSGAQGSGGGSTSAHYAVNSQFTMGGPAISMASPMSIP
TNTMHYGS
>sp|P14091|CATE_HUMAN Cathepsin E OS=Homo sapiens GN=CTSE PE=1 SV=2
MKTLLLLLLVLLELGEAQGSLHRVPLRRHPSLKKKLRARSQLSEFWKSHNLDMIQFTESC
SMDQSAKEPLINYLDMEYFGTISIGSPPQNFTVIFDTGSSNLWVPSVYCTSPACKTHSRF
QPSQSSTYSQPGQSFSIQYGTGSLSGIIGADQVSAFATQVEGLTVVGQQFGESVTEPGQT
FVDAEFDGILGLGYPSLAVGGVTPVFDNMMAQNLVDLPMFSVYMSSNPEGGAGSELIFGG
YDHSHFSGSLNWVPVTKQAYWQIALDNIQVGGTVMFCSEGCQAIVDTGTSLITGPSDKIK
QLQNAIGAAPVDGEYAVECANLNVMPDVTFTINGVPYTLSPTAYTLLDFVDGMQFCSSGF
QGLDIHPPAGPLWILGDVFIRQFYSVFDRGNNRVGLAPAVP
>sp|P43235|CATK_HUMAN Cathepsin K OS=Homo sapiens GN=CTSK PE=1 SV=1
MWGLKVLLLPVVSFALYPEEILDTHWELWKKTHRKQYNNKVDEISRRLIWEKNLKYISIH
NLEASLGVHTYELAMNHLGDMTSEEVVQKMTGLKVPLSHSRSNDTLYIPEWEGRAPDSVD
YRKKGYVTPVKNQGQCGSCWAFSSVGALEGQLKKKTGKLLNLSPQNLVDCVSENDGCGGG
YMTNAFQYVQKNRGIDSEDAYPYVGQEESCMYNPTGKAAKCRGYREIPEGNEKALKRAVA
RVGPVSVAIDASLTSFQFYSKGVYYDESCNSDNLNHAVLAVGYGIQKGNKHWIIKNSWGE
NWGNKGYILMARNKNNACGIANLASFPKM
>sp|Q13166|CATR1_HUMAN CATR tumorigenic conversion 1 protein OS=Homo sapiens GN=CATR1 PE=2 SV=1
MVLNEEIPRHLLLTQNNDIIPKHHILILPAVDSYQKSVNDLRALTFSKFQELKHAHELRN
LCVSQSRFLAIMWFGTNTN
>sp|Q9UBR2|CATZ_HUMAN Cathepsin Z OS=Homo sapiens GN=CTSZ PE=1 SV=1
MARRGPGWRPLLLLVLLAGAAQGGLYFRRGQTCYRPLRGDGLAPLGRSTYPRPHEYLSPA
DLPKSWDWRNVDGVNYASITRNQHIPQYCGSCWAHASTSAMADRINIKRKGAWPSTLLSV
QNVIDCGNAGSCEGGNDLSVWDYAHQHGIPDETCNNYQAKDQECDKFNQCGTCNEFKECH
AIRNYTLWRVGDYGSLSGREKMMAEIYANGPISCGIMATERLANYTGGIYAEYQDTTYIN
HVVSVAGWGISDGTEYWIVRNSWGEPWGERGWLRIVTSTYKDGKGARYNLAIEEHCTFGD
PIV
>sp|Q03135|CAV1_HUMAN Caveolin-1 OS=Homo sapiens GN=CAV1 PE=1 SV=4
MSGGKYVDSEGHLYTVPIREQGNIYKPNNKAMADELSEKQVYDAHTKEIDLVNRDPKHLN
DDVVKIDFEDVIAEPEGTHSFDGIWKASFTTFTVTKYWFYRLLSALFGIPMALIWGIYFA
ILSFLHIWAVVPCIKSFLIEIQCISRVYSIYVHTVCDPLFEAVGKIFSNVRINLQKEI
>sp|Q580R0|CB027_HUMAN Uncharacterized protein C2orf27 OS=Homo sapiens GN=C2orf27A PE=2 SV=1
MTVKWKQLSAPASGAEIQRFPVPAVEPVPAPGADSPPGTALELEEAPEPSCRCPGTAQDQ

PSEELPDFMAPPVEPPASALELKVWLELEVAERGGQHSSSQQLPHCSQSWAQWKLWRQRP
GFAIWAPLPHWRGTSLIQQSSSPAAEGPAATAAGAVCLPAGGAGEQEKEPVSRGSSRSSC
SQRRPPPPGMEVCPQLGIWAICP
>sp|Q96LS8|CB048_HUMAN Uncharacterized protein C2orf48 OS=Homo sapiens GN=C2orf48 PE=2 SV=1
MESRPSGRQHASEGDGDQSPTQCAGMRSSGRSDQPYVLKGNPLLLRVRCYSGCASGSGRQ
LQLSVFQDLNQFSHCRVWRSPALIVKGEPPWCSQQDTQSPFQTGTPLERPCFRMKLSGWE
LTKNAQRALGSKLQHILSLDSTQACGAGGPTILRPPRAP
>sp|Q2NKX9|CB068_HUMAN UPF0561 protein C2orf68 OS=Homo sapiens GN=C2orf68 PE=2 SV=1
MEAGPHPRPGHCCKPGGRLDMNHGFVHHIRRNQIARDDYDKKVKQAAKEKVRRRHTPAPT
RPRKPDLQVYLPRHRDVSAHPRNPDYEESGESSSSGGSELEPSGHQLFCLEYEADSGEVT
SVIVYQGDDPGKVSEKVSAHTPLDPPMREALKLRIQEEIAKRQSQH
>sp|A6NJV1|CB070_HUMAN UPF0573 protein C2orf70 OS=Homo sapiens GN=C2orf70 PE=3 SV=1
MASRSAGTLLTEFNAAYVPPGLMPGYQGHVPTVAFSFGAPYGTTTLKYFQDHRNRAMEKS
HTPFSQGGHFPTIFSTNPNLLLMERASTRDRWLHKPSYTRFNLDSHRSTELTNFYQMVQQ
HRKYYQDKTGTVPRVPYFAMPVREPERYPLPTVLPPLCPKKKWHLLRLAPENLKTYQTFP
SGKRVSPQERKKRDCYFEFRA
>sp|Q8N5S3|CB073_HUMAN Uncharacterized protein C2orf73 OS=Homo sapiens GN=C2orf73 PE=2 SV=3
MEEKEDKHQQHKIEDAAITYVSENEEIKHEEKPGKSIHHSKSHVGRGRIYYAKFINTNAR
TYNEPFPYIDPKKGPEIQGDWWSHGKALEPVFLPPYDSKSTQRSDFQKPSCPLVLPVKHS
KMQKPSCGIVPLASPGTSAELQNNFIEYISFIHQYDARKTPNEPLQGKRHGAFVQREIKP
GSRPTVPKGAEVLLNTPGSRSSEQSKKTEKGNSAESRMISPGLCQQNSQELLEPKTHLSE
TDVRQAAKACPSTPESREKTSGATQTTVGDALFTRHKPLNPPIKKSE
>sp|A6NCI8|CB078_HUMAN Uncharacterized protein C2orf78 OS=Homo sapiens GN=C2orf78 PE=2 SV=3
MHWLASATQTSASIVSSSLLSAVDVSSSLTMSEYFQNTSLPGTANSRQFSLPVVSNAAFL
TGSISNFSRASAPAISSAWLQPSASGTSFQPLMGSAYLYQHSSTTMLSGVTGQSHICTSA
ASYPGVFEWDSTASTVKKSSSLRDFTVTVIDQNTAVSSMSMTAQYYKTSDTNTMVPLYPS
LSASLVQGTLTQIPNQQGHNLSLPCQIGSQVYYYNQGTLGPQLSCLQSYGSVSYTGYRAS
AHQPEMVMVLKEVQPTNVLPPVSTSGMYYSVSSQPITETSVQVMETSLGMDTSLGLQSPS
QTFCLPQTPEFSKSFSSRNTQTLESNPSPELGDISITPVQSPTNLLTLSPAPSQEKNENE
NLDEIKTNLSKPLDVHQILIGNQDPPLLPVEIPDIHPLLACIDPLGQEEQPGSENANLRN
KSLSLEDQGIFENGIESSSDLADITTWVEDTYLPPIFSSLQDLDQPESPSAKKAKDTSAI
KVNQVQEKSCVIKGHSDQVRKNKHKASEPIQGAPKAKIQPKNPECLLEREVVVGSATVSN
SASVNKAKHSSNKPHKAASSRISKTKSHGQEKTKGNRKNSSKKSEESKQSGKKVKVEEKQ
TIPNMKRKKNQPELSQKTLKKPRSSLGMHMLESVQVFHALGKKIDMKTGFSSSRTLGSSS
NTQNRQPFPALKPWLDIQHEGKGPEKIQVKAQKLDGSAEKECTSPSHSELPPPGKVKLIP
LPFLTLDQPQARHVSRRPNPLASRRPAVAYPARPDSTNSAQSNAVNPSRPAPTNTSLTGP
ATPAQPISAKATQPSSANPTQPTVPQSAASRPSAYKTSSCSSLQREPVSTAVTSLRSLPK
PQNQFLIQDFSLQPRPWRKPTVPEPVMSTPITEEQRPEREAMKRKAQQERENAAKYTSLG
KVQFFIERERDMEIAEYYGYTI
>sp|P08185|CBG_HUMAN Corticosteroid-binding globulin OS=Homo sapiens GN=SERPINA6 PE=1 SV=1

MPLLLYTCLLWLPTSGLWTVQAMDPNAAYVNMSNHHRGLASANVDFAFSLYKHLVALSPK
KNIFISPVSISMALAMLSLGTCGHTRAQLLQGLGFNLTERSETEIHQGFQHLHQLFAKSD
TSLEMTMGNALFLDGSLELLESFSADIKHYYESEVLAMNFQDWATASRQINSYVKNKTQG
KIVDLFSGLDSPAILVLVNYIFFKGTWTQPFDLASTREENFYVDETTVVKVPMMLQSSTI
SYLHDSELPCQLVQMNYVGNGTVFFILPDKGKMNTVIAALSRDTINRWSAGLTSSQVDLY
IPKVTISGVYDLGDVLEEMGIADLFTNQANFSRITQDAQLKSSKVVHKAVLQLNEEGVDT
AGSTGVTLNLTSKPIILRFNQPFIIMIFDHFTWSSLFLARVMNPV
>sp|Q13489|BIRC3_HUMAN Baculoviral IAP repeat-containing protein 3 OS=Homo sapiens GN=BIRC3 PE=1 SV=2
MNIVENSIFLSNLMKSANTFELKYDLSCELYRMSTYSTFPAGVPVSERSLARAGFYYTGV
NDKVKCFCCGLMLDNWKRGDSPTEKHKKLYPSCRFVQSLNSVNNLEATSQPTFPSSVTNS
THSLLPGTENSGYFRGSYSNSPSNPVNSRANQDFSALMRSSYHCAMNNENARLLTFQTWP
LTFLSPTDLAKAGFYYIGPGDRVACFACGGKLSNWEPKDNAMSEHLRHFPKCPFIENQLQ
DTSRYTVSNLSMQTHAARFKTFFNWPSSVLVNPEQLASAGFYYVGNSDDVKCFCCDGGLR
CWESGDDPWVQHAKWFPRCEYLIRIKGQEFIRQVQASYPHLLEQLLSTSDSPGDENAESS
IIHFEPGEDHSEDAIMMNTPVINAAVEMGFSRSLVKQTVQRKILATGENYRLVNDLVLDL
LNAEDEIREEERERATEEKESNDLLLIRKNRMALFQHLTCVIPILDSLLTAGIINEQEHD
VIKQKTQTSLQARELIDTILVKGNIAATVFRNSLQEAEAVLYEHLFVQQDIKYIPTEDVS
DLPVEEQLRRLQEERTCKVCMDKEVSIVFIPCGHLVVCKDCAPSLRKCPICRSTIKGTVR
TFLS
>sp|O15392|BIRC5_HUMAN Baculoviral IAP repeat-containing protein 5 OS=Homo sapiens GN=BIRC5 PE=1 SV=3
MGAPTLPPAWQPFLKDHRISTFKNWPFLEGCACTPERMAEAGFIHCPTENEPDLAQCFFC
FKELEGWEPDDDPIEEHKKHSSGCAFLSVKKQFEELTLGEFLKLDRERAKNKIAKETNNK
KKEFEETAKKVRRAIEQLAAMD
>sp|A4D250|BLACE_HUMAN B-cell acute lymphoblastic leukemia-expressed protein OS=Homo sapiens GN=BLACE PE=2 SV=2
MMKDIIPASSWASEESTDLQNGSFPLSVAPRSEFPRRRALEDWLLSVFLADQAESEGQLV
LERVRDTPPPVTSPRGDGICVSRGKAPSSPGGSTHAWLYLTRHFPWSPFPHGGWTDTSEP
CVLETLGGSSLAALRGNSLWVQSSGACAFCVYESLIEQSLPNERFEELLLGPSPGEVMK
>sp|P34820|BMP8B_HUMAN Bone morphogenetic protein 8B OS=Homo sapiens GN=BMP8B PE=2 SV=2
MTALPGPLWLLGLALCALGGGGPGLRPPPGCPQRRLGARERRDVQREILAVLGLPGRPRP
RAPPAASRLPASAPLFMLDLYHAMAGDDDEDGAPAERRLGRADLVMSFVNMVERDRALGH
QEPHWKEFRFDLTQIPAGEAVTAAEFRIYKVPSIHLLNRTLHVSMFQVVQEQSNRESDLF
FLDLQTLRAGDEGWLVLDVTAASDCWLLKRHKDLGLRLYVETEDGHSVDPGLAGLLGQRA
PRSQQPFVVTFFRASPSPIRTPRAVRPLRRRQPKKSNELPQANRLPGIFDDVHGSHGRQV
CRRHELYVSFQDLGWLDWVIAPQGYSAYYCEGECSFPLDSCMNATNHAILQSLVHLMMPD
AVPKACCAPTKLSATSVLYYDSSNNVILRKHRNMVVKACGCH
>sp|Q7Z5W3|BN3D2_HUMAN Pre-miRNA 5'-monophosphate methyltransferase OS=Homo sapiens GN=BCDIN3D PE=1 SV=1
MAVPTELDGGSVKETAAEEESRVLAPGAAPFGNFPHYSRFHPPEQRLRLLPPELLRQLFP
ESPENGPILGLDVGCNSGDLSVALYKHFLSLPDGETCSDASREFRLLCCDIDPVLVKRAE
KECPFPDALTFITLDFMNQRTRKVLLSSFLSQFGRSVFDIGFCMSITMWIHLNHGDHGLW
EFLAHLSSLCHYLLVEPQPWKCYRAAARRLRKLGLHDFDHFHSLAIRGDMPNQIVQILTQ

DHGMELICCFGNTSWDRSLLLFRAKQTIETHPIPESLIEKGKEKNRLSFQKQ
>sp|Q12983|BNIP3_HUMAN BCL2/adenovirus E1B 19 kDa protein-interacting protein 3 OS=Homo sapiens GN=BNIP3 PE=1 SV=2
MSQNGAPGMQEESLQGSWVELHFSNNGNGGSVPASVSIYNGDMEKILLDAQHESGRSSSK
SSHCDSPPRSQTPQDTNRASETDTHSIGEKNSSQSEEDDIERRKEVESILKKNSDWIWDW
SSRPENIPPKEFLFKHPKRTATLSMRNTSVMKKGGIFSAEFLKVFLPSLLLSHLLAIGLG
IYIGRRLTTSTSTF
>sp|Q96B45|BORC7_HUMAN BLOC-1-related complex subunit 7 OS=Homo sapiens GN=BORCS7 PE=3 SV=1
MATGTPESQARFGQSVKGLLTEKVTTCGTDVIALTKQVLKGSRSSELLGQAARNMVLQED
AILHSEDSLRKMAIITTHLQYQQEAIQKNVEQSSDLQDQLNHLLK
>sp|Q6NZY7|BORG3_HUMAN Cdc42 effector protein 5 OS=Homo sapiens GN=CDC42EP5 PE=2 SV=1
MPVLKQLGPAQPKKRPDRGALSISAPLGDFRHTLHVGRGGDAFGDTSFLSRHGGGPPPEP
RAPPAGAPRSPPPPAVPQSAAPSPADPLLSFHLDLGPSMLDAVLGVMDAARPEAAAAKPD
AEPRPGTQPPQARCRPNADLELNDVIGL
>sp|Q7Z569|BRAP_HUMAN BRCA1-associated protein OS=Homo sapiens GN=BRAP PE=1 SV=2
MSVSLVVIRLELAEHSPVPAGFGFSAAAGEMSDEEIKKTTLASAVACLEGKSPGEKVAII
HQHLGRREMTDVIIETMKSNPDELKTTVEERKSSEASPTAQRSKDHSKECINAAPDSPSK
QLPDQISFFSGNPSVEIVHGIMHLYKTNKMTSLKEDVRRSAMLCILTVPAAMTSHDLMKF
VAPFNEVIEQMKIIRDSTPNQYMVLIKFRAQADADSFYMTCNGRQFNSIEDDVCQLVYVE
RAEVLKSEDGASLPVMDLTELPKCTVCLERMDESVNGILTTLCNHSFHSQCLQRWDDTTC
PVCRYCQTPEPVEENKCFECGVQENLWICLICGHIGCGRYVSRHAYKHFEETQHTYAMQL
TNHRVWDYAGDNYVHRLVASKTDGKIVQYECEGDTCQEEKIDALQLEYSYLLTSQLESQR
IYWENKIVRIEKDTAEEINNMKTKFKETIEKCDNLEHKLNDLLKEKQSVERKCTQLNTKV
AKLTNELKEEQEMNKCLRANQVLLQNKLKEEERVLKETCDQKDLQITEIQEQLRDVMFYL
ETQQKINHLPAETRQEIQEGQINIAMASASSPASSGGSGKLPSRKGRSKRGK
>sp|Q15059|BRD3_HUMAN Bromodomain-containing protein 3 OS=Homo sapiens GN=BRD3 PE=1 SV=1
MSTATTVAPAGIPATPGPVNPPPPEVSNPSKPGRKTNQLQYMQNVVVKTLWKHQFAWPFY
QPVDAIKLNLPDYHKIIKNPMDMGTIKKRLENNYYWSASECMQDFNTMFTNCYIYNKPTD
DIVLMAQALEKIFLQKVAQMPQEEVELLPPAPKGKGRKPAAGAQSAGTQQVAAVSSVSPA
TPFQSVPPTVSQTPVIAATPVPTITANVTSVPVPPAAAPPPPATPIVPVVPPTPPVVKKK
GVKRKADTTTPTTSAITASRSESPPPLSDPKQAKVVARRESGGRPIKPPKKDLEDGEVPQ
HAGKKGKLSEHLRYCDSILREMLSKKHAAYAWPFYKPVDAEALELHDYHDIIKHPMDLST
VKRKMDGREYPDAQGFAADVRLMFSNCYKYNPPDHEVVAMARKLQDVFEMRFAKMPDEPV
EAPALPAPAAPMVSKGAESSRSSEESSSDSGSSDSEEERATRLAELQEQLKAVHEQLAAL
SQAPVNKPKKKKEKKEKEKKKKDKEKEKEKHKVKAEEEKKAKVAPPAKQAQQKKAPAKKA
NSTTTAGRQLKKGGKQASASYDSEEEEEGLPMSYDEKRQLSLDINRLPGEKLGRVVHIIQ
SREPSLRDSNPDEIEIDFETLKPTTLRELERYVKSCLQKKQRKPFSASGKKQAAKSKEEL
AQEKKKELEKRLQDVSGQLSSSKKPARKEKPGSAPSGGPSRLSSSSSSESGSSSSSGSSS
DSSDSE
>sp|O60885|BRD4_HUMAN Bromodomain-containing protein 4 OS=Homo sapiens GN=BRD4 PE=1 SV=2
MSAESGPGTRLRNLPVMGDGLETSQMSTTQAQAQPQPANAASTNPPPPETSNPNKPKRQT
NQLQYLLRVVLKTLWKHQFAWPFQQPVDAVKLNLPDYYKIIKTPMDMGTIKKRLENNYYW
NAQECIQDFNTMFTNCYIYNKPGDDIVLMAEALEKLFLQKINELPTEETEIMIVQAKGRG

RGRKETGTAKPGVSTVPNTTQASTPPQTQTPQPNPPPVQATPHPFPAVTPDLIVQTPVMT
VVPPQPLQTPPPVPPQPQPPPAPAPQPVQSHPPIIAATPQPVKTKKGVKRKADTTTPTTI
DPIHEPPSLPPEPKTTKLGQRRESSRPVKPPKKDVPDSQQHPAPEKSSKVSEQLKCCSGI
LKEMFAKKHAAYAWPFYKPVDVEALGLHDYCDIIKHPMDMSTIKSKLEAREYRDAQEFGA
DVRLMFSNCYKYNPPDHEVVAMARKLQDVFEMRFAKMPDEPEEPVVAVSSPAVPPPTKVV
APPSSSDSSSDSSSDSDSSTDDSEEERAQRLAELQEQLKAVHEQLAALSQPQQNKPKKKE
KDKKEKKKEKHKRKEEVEENKKSKAKEPPPKKTKKNNSSNSNVSKKEPAPMKSKPPPTYE
SEEEDKCKPMSYEEKRQLSLDINKLPGEKLGRVVHIIQSREPSLKNSNPDEIEIDFETLK
PSTLRELERYVTSCLRKKRKPQAEKVDVIAGSSKMKGFSSSESESSSESSSSDSEDSETE
MAPKSKKKGHPGREQKKHHHHHHQQMQQAPAPVPQQPPPPPQQPPPPPPPQQQQQPPPPP
PPPSMPQQAAPAMKSSPPPFIATQVPVLEPQLPGSVFDPIGHFTQPILHLPQPELPPHLP
QPPEHSTPPHLNQHAVVSPPALHNALPQQPSRPSNRAAALPPKPARPPAVSPALTQTPLL
PQPPMAQPPQVLLEDEEPPAPPLTSMQMQLYLQQLQKVQPPTPLLPSVKVQSQPPPPLPP
PPHPSVQQQLQQQPPPPPPPQPQPPPQQQHQPPPRPVHLQPMQFSTHIQQPPPPQGQQPP
HPPPGQQPPPPQPAKPQQVIQHHHSPRHHKSDPYSTGHLREAPSPLMIHSPQMSQFQSLT
HQSPPQQNVQPKKQELRAASVVQPQPLVVVKEEKIHSPIIRSEPFSPSLRPEPPKHPESI
KAPVHLPQRPEMKPVDVGRPVIRPPEQNAPPPGAPDKDKQKQEPKTPVAPKKDLKIKNMG
SWASLVQKHPTTPSSTAKSSSDSFEQFRRAAREKEEREKALKAQAEHAEKEKERLRQERM
RSREDEDALEQARRAHEEARRRQEQQQQQRQEQQQQQQQQAAAVAAAATPQAQSSQPQSM
LDQQRELARKREQERRRREAMAATIDMNFQSDLLSIFEENLF
>sp|Q9H0E9|BRD8_HUMAN Bromodomain-containing protein 8 OS=Homo sapiens GN=BRD8 PE=1 SV=2
MATGTGKHKLLSTGPTEPWSIREKLCLASSVMRSGDQNWVSVSRAIKPFAEPGRPPDWFS
QKHCASQYSELLETTETPKRKRGEKGEVVETVEDVIVRKLTAERVEELKKVIKETQERYR
RLKRDAELIQAGHMDSRLDELCNDIATKKKLEEEEAEVKRKATDAAYQARQAVKTPPRRL
PTVMVRSPIDSASPGGDYPLGDLTPTTMEEATSGVNESEMAVASGHLNSTGVLLEVGGVL
PMIHGGEIQQTPNTVAASPAASGAPTLSRLLEAGPTQFTTPLASFTTVASEPPVKLVPPP
VESVSQATIVMMPALPAPSSAPAVSTTESVAPVSQPDNCVPMEAVGDPHTVTVSMDSSEI
SMIINSIKEECFRSGVAEAPVGSKAPSIDGKEELDLAEKMDIAVSYTGEELDFETVGDII
AIIEDKVDDHPEVLDVAAVEAALSFCEENDDPQSLPGPWEHPIQQERDKPVPLPAPEMTV
KQERLDFEETENKGIHELVDIREPSAEIKVEPAEPEPVISGAEIVAGVVPATSMEPPELR
SQDLDEELGSTAAGEIVEADVAIGKGDETPLTNVKTEASPESMLSPSHGSNPIEDPLEAE
TQHKFEMSDSLKEESGTIFGSQIKDAPGEDEEEDGVSEAASLEEPKEEDQGEGYLSEMDN
EPPVSESDDGFSIHNATLQSHTLADSIPSSPASSQFSVCSEDQEAIQAQKIWKKAIMLVW
RAAANHRYANVFLQPVTDDIAPGYHSIVQRPMDLSTIKKNIENGLIRSTAEFQRDIMLMF
QNAVMYNSSDHDVYHMAVEMQRDVLEQIQQFLATQLIMQTSESGISAKSLRGRDSTRKQD
ASEKDSVPMGSPAFLLSLFMGHEWVWLDSEQDHPNDSELSNDCRSLFSSWDSSLDLDVGN
WRETEDPEAEELEESSPEREPSELLVGDGGSEESQEAARKASHQNLLHFLSEVAYLMEPL
CISSNESSEGCCPPSGTRQEGREIKASEGERELCRETEELSAKGDPLVAEKPLGENGKPE
VASAPSVICTVQGLLTESEEGEAQQESKGEDQGEVYVSEMEDQPPSGECDDAFNIKETPL
VDTLFSHATSSKLTDLSQDDPVQDHLLFKKTLLPVWKMIASHRFSSPFLKPVSERQAPGY
KDVVKRPMDLTSLKRNLSKGRIRTMAQFLRDLMLMFQNAVMYNDSDHHVYHMAVEMRQEV
LEQIQVLNIWLDKRKGSSSLEGEPANPVDDGKPVF
>sp|Q9GZN4|BSSP4_HUMAN Brain-specific serine protease 4 OS=Homo sapiens GN=PRSS22 PE=1
SV=1

MVVSGAPPALGGGCLGTFTSLLLLASTAILNAARIPVPPACGKPQQLNRVVGGEDSTDSE
WPWIVSIQKNGTHHCAGSLLTSRWVITAAHCFKDNLNKPYLFSVLLGAWQLGNPGSRSQK
VGVAWVEPHPVYSWKEGACADIALVRLERSIQFSERVLPICLPDASIHLPPNTHCWISGW
GSIQDGVPLPHPQTLQKLKVPIIDSEVCSHLYWRGAGQGPITEDMLCAGYLEGERDACLG
DSGGPLMCQVDGAWLLAGIISWGEGCAERNRPGVYISLSAHRSWVEKIVQGVQLRGRAQG
GGALRAPSQGSGAAARS
>sp|Q8WVV5|BT2A2_HUMAN Butyrophilin subfamily 2 member A2 OS=Homo sapiens GN=BTN2A2 PE=1 SV=2
MEPAAALHFSLPASLLLLLLLLLLSLCALVSAQFTVVGPANPILAMVGENTTLRCHLSPE
KNAEDMEVRWFRSQFSPAVFVYKGGRERTEEQMEEYRGRITFVSKDINRGSVALVIHNVT
AQENGIYRCYFQEGRSYDEAILRLVVAGLGSKPLIEIKAQEDGSIWLECISGGWYPEPLT
VWRDPYGEVVPALKEVSIADADGLFMVTTAVIIRDKYVRNVSCSVNNTLLGQEKETVIFI
PESFMPSASPWMVALAVILTASPWMVSMTVILAVFIIFMAVSICCIKKLQREKKILSGEK
KVEQEEKEIAQQLQEELRWRRTFLHAADVVLDPDTAHPELFLSEDRRSVRRGPYRQRVPD
NPERFDSQPCVLGWESFASGKHYWEVEVENVMVWTVGVCRHSVERKGEVLLIPQNGFWTL
EMFGNQYRALSSPERILPLKESLCRVGVFLDYEAGDVSFYNMRDRSHIYTCPRSAFTVPV
RPFFRLGSDDSPIFICPALTGASGVMVPEEGLKLHRVGTHQSL
>sp|Q96K17|BT3L4_HUMAN Transcription factor BTF3 homolog 4 OS=Homo sapiens GN=BTF3L4 PE=1 SV=1
MNQEKLAKLQAQVRIGGKGTARRKKKVVHRTATADDKKLQSSLKKLAVNNIAGIEEVNMI
KDDGTVIHFNNPKVQASLSANTFAITGHAEAKPITEMLPGILSQLGADSLTSLRKLAEQF
PRQVLDSKAPKPEDIDEEDDDVPDLVENFDEASKNEAN
>sp|Q9P203|BTBD7_HUMAN BTB/POZ domain-containing protein 7 OS=Homo sapiens GN=BTBD7 PE=1 SV=3
MGANASNYPHSCSPRVGGNSQAQQTFIGTSSYSQQGYGCESKLYSLDHGHEKPQDKKKRT
SGLATLKKKFIKRRKSNRSADHAKQMRELLSGWDVRDVNALVEEYEGTSALKELSLQASL
ARPEARTLQKDMADLYEYKYCTDVDLIFQETCFPVHRAILAARCPFFKTLLSSSPEYGAE
IIMDINTAGIDMPMFSALLHYLYTGEFGMEDSRFQNVDILVQLSEEFGTPNSLDVDMRGL
FDYMCYYDVVLSFSSDSELVEAFGGNQNCLDEELKAHKAVISARSPFFRNLLQRRIRTGE
EITDRTLRTPTRIILDESIIPKKYATVILHCMYTDVVDLSVLHCSPSVGSLSEVQALVAG
KPNMTRAEEAMELYHIALFLEFNMLAQGCEDIIAESISLDTLIAILKWSSHPYGSKWVHR
QALHFLCEEFSQVMTSDVFYELSKDHLLTAIQSDYLQASEQDILKYLIKWGEHQLMKRIA
DREPNLLSGTAHSVNKRGVKRRDLDMEELREILSSLLPFVRIEHILPINSEVLSDAMKRG
LISTPPSDMLPTTEGGKSNAWLRQKNAGIYVRPRLFSPYVEEAKSVLDEMMVEQTDLVRL
RMVRMSNVPDTLYMVNNAVPQCCHMISHQQISSNQSSPPSVVANEIPVPRLLIMKDMVRR
LQELRHTEQVQRAYALNCGEGATVSYEIQIRVLREFGLADAAAELLQNPHKFFPDERFGD
ESPLLTMRQPGRCRVNSTPPAETMFTDLDSFVAFHPPLPPPPPPYHPPATPIHNQLKAGW
KQRPPSQHPSRSFSYPCNHSLFHSRTAPKAGPPPVYLPSVKAAPPDCTSTAGLGRQTVAA
AAATTTSTATAAAAAASEKQVRTQPVLNDLMPDIAVGVSTLSLKDRRLPELAVDTELSQS
VSEAGPGPPQHLSCIPQRHTHTSRKKHTLEQKTDTRENPQEYPDFYDFSNAACRPSTPAL
SRRTPSPSQGGYFGPDLYSHNKASPSGLKSAYLPGQTSPKKQEEARREYPLSPDGHLHRQ
KNEPIHLDVVEQPPQRSDFPLAAPENASTGPAHVRGRTAVETDLTFGLTPNRPSLSACSS
EAPEERSGRRLADSESLGHGAQRNTDLEREDSISRGRRSPSKPDFLYKKSAL

>sp|B2RXH4|BTBDI_HUMAN BTB/POZ domain-containing protein 18 OS=Homo sapiens GN=BTBD18 PE=2 SV=1
MCSPASPKILYRNPRFLRLAFLQLHHQQQSDVFCDVLLQAEGEAVPAHCCILSACSPFFT
ERLERERPAQGGKVVLELGGLKISTLRKLVDFLYTSEMEVSQEEAQDVLSAARQLRVSEL
ESLQLEGGKLVKAPQGRRLNRECLQPTSAAPISARVVTPSHHPHTPLPTNQTPCPLGAIR
LKSLGKEEGPQENNRQNADNLSGTLLLKRKARACPTPQEKNSSPSSHSQEPRENKNDTAL
DPTVLSPPSLYPSVDKHLLPRKIRLSRSKPSPGICTSKPSSILSGSSSVPATPGRRLWRQ
RSVNKETPEDKPKPGRASPLQSTPNPSGLGKTGGSRKRSPEVRAPNSDSAEEGQVGRVKL
RKIVNGTCWEVVQETPLKNTQDSPQIPDPGGDFQEPSGTQPFSSNEQEMSPTRTELCQDS
PMCTKLQDILVSASHSPDHPVVKSEFESSPELVEKEPMLAIDCREPYAFDTALLEQPCEA
EEYRITSAAATSELEEILDFMLCGSDIEPPIGSLESPGAEGCRTPTYHLTETGKNWIEGE
EWCLPDMELWPRELTELEKEPAGENRGPTELLSPLVMPSEVSEVLSVGGRWTPDLEITSS
QPLDGQEDKLLHVSSLDTPQRSYGDLSPPCSNWVETGLEVSLTTDELLYPSPKAGKEVSG
HSELLGSLPASSEEEEIDVVDWTAEGRLVPTTVPSVWPDPSSESETEVDILT
>sp|P35070|BTC_HUMAN Probetacellulin OS=Homo sapiens GN=BTC PE=1 SV=1
MDRAARCSGASSLPLLLALALGLVILHCVVADGNSTRSPETNGLLCGDPEENCAATTTQS
KRKGHFSRCPKQYKHYCIKGRCRFVVAEQTPSCVCDEGYIGARCERVDLFYLRGDRGQIL
VICLIAVMVVFIILVIGVCTCCHPLRKRRKRKKKEEEMETLGKDITPINEDIEETNIA
>sp|Q9NY30|BTG4_HUMAN Protein BTG4 OS=Homo sapiens GN=BTG4 PE=2 SV=1
MRDEIATTVFFVTRLVKKHDKLSKQQIEDFAEKLMTILFETYRSHWHSDCPSKGQAFRCI
RINNNQNKDPILERACVESNVDFSHLGLPKEMTIWVDPFEVCCRYGEKNHPFTVASFKGR
WEEWELYQQISYAVSRASSDVSSGTSCDEESCSKEPRVIPKVSNPKSIYQVENLKQPFQS
WLQIPRKKNVVDGRVGLLGNTYHGSQKHPKCYRPAMHRLDRIL
>sp|O43684|BUB3_HUMAN Mitotic checkpoint protein BUB3 OS=Homo sapiens GN=BUB3 PE=1 SV=1
MTGSNEFKLNQPPEDGISSVKFSPNTSQFLLVSSWDTSVRLYDVPANSMRLKYQHTGAVL
DCAFYDPTHAWSGGLDHQLKMHDLNTDQENLVGTHDAPIRCVEYCPEVNVMVTGSWDQTV
KLWDPRTPCNAGTFSQPEKVYTLSVSGDRLIVGTAGRRVLVWDLRNMGYVQQRRESSLKY
QTRCIRAFPNKQGYVLSSIEGRVAVEYLDPSPEVQKKKYAFKCHRLKENNIEQIYPVNAI
SFHNIHNTFATGGSDGFVNIWDPFNKKRLCQFHRYPTSIASLAFSNDGTTLAIASSYMYE
MDDTEHPEDGIFIRQVTDAETKPKSPCT
>sp|Q7L1Q6|BZW1_HUMAN Basic leucine zipper and W2 domain-containing protein 1 OS=Homo sapiens GN=BZW1 PE=1 SV=1
MNNQKQQKPTLSGQRFKTRKRDEKERFDPTQFQDCIIQGLTETGTDLEAVAKFLDASGAK
LDYRRYAETLFDILVAGGMLAPGGTLADDMMRTDVCVFAAQEDLETMQAFAQVFNKLIRR
YKYLEKGFEDEVKKLLLFLKGFSESERNKLAMLTGVLLANGTLNASILNSLYNENLVKEG
VSAAFAVKLFKSWINEKDINAVAASLRKVSMDNRLMELFPANKQSVEHFTKYFTEAGLKE
LSEYVRNQQTIGARKELQKELQEQMSRGDPFKDIILYVKEEMKKNNIPEPVVIGIVWSSV
MSTVEWNKKEELVAEQAIKHLKQYSPLLAAFTTQGQSELTLLLKIQEYCYDNIHFMKAFQ
KIVVLFYKAEVLSEEPILKWYKDAHVAKGKSVFLEQMKKFVEWLKNAEEESESEAEEGD
>sp|P15538|C11B1_HUMAN Cytochrome P450 11B1, mitochondrial OS=Homo sapiens GN=CYP11B1 PE=1 SV=5
MALRAKAEVCMAVPWLSLQRAQALGTRAARVPRTVLPFEAMPRRPGNRWLRLLQIWREQG
YEDLHLEVHQTFQELGPIFRYDLGGAGMVCVMLPEDVEKLQQVDSLHPHRMSLEPWVAYR
QHRGHKCGVFLLNGPEWRFNRLRLNPEVLSPNAVQRFLPMVDAVARDFSQALKKKVLQNA

RGSLTLDVQPSIFHYTIEASNLALFGERLGLVGHSPSSASLNFLHALEVMFKSTVQLMFM
PRSLSRWTSPKVWKEHFEAWDCIFQYGDNCIQKIYQELAFSRPQQYTSIVAELLLNAELS
PDAIKANSMELTAGSVDTTVFPLLMTLFELARNPNVQQALRQESLAAAASISEHPQKATT
ELPLLRAALKETLRLYPVGLFLERVASSDLVLQNYHIPAGTLVRVFLYSLGRNPALFPRP
ERYNPQRWLDIRGSGRNFYHVPFGFGMRQCLGRRLAEAEMLLLLHHVLKHLQVETLTQED
IKMVYSFILRPSMFPLLTFRAIN
>sp|Q69YN2|C19L1_HUMAN CWF19-like protein 1 OS=Homo sapiens GN=CWF19L1 PE=1 SV=2
MAQKPLRLLACGDVEGKFDILFNRVQAIQKKSGNFDLLLCVGNFFGSTQDAEWEEYKTGI
KKAPIQTYVLGANNQETVKYFQDADGCELAENITYLGRKGIFTGSSGLQIVYLSGTESLN
EPVPGYSFSPKDVSSLRMMLCTTSQFKGVDILLTSPWPKCVGNFGNSSGEVDTKKCGSAL
VSSLATGLKPRYHFAALEKTYYERLPYRNHIILQENAQHATRFIALANVGNPEKKKYLYA
FSIVPMKLMDAAELVKQPPDVTENPYRKSGQEASIGKQILAPVEESACQFFFDLNEKQGR
KRSSTGRDSKSSPHPKQPRKPPQPPGPCWFCLASPEVEKHLVVNIGTHCYLALAKGGLSD
DHVLILPIGHYQSVVELSAEVVEEVEKYKATLRRFFKSRGKWCVVFERNYKSHHLQLQVI
PVPISCSTTDDIKDAFITQAQEQQIELLEIPEHSDIKQIAQPGAAYFYVELDTGEKLFHR
IKKNFPLQFGREVLASEAILNVPDKSDWRQCQISKEDEETLARRFRKDFEPYDFTLDD
>sp|Q13901|C1D_HUMAN Nuclear nucleic acid-binding protein C1D OS=Homo sapiens GN=C1D PE=1 SV=1
MAGEEINEDYPVEIHEYLSAFENSIGAVDEMLKTMMSVSRNELLQKLDPLEQAKVDLVSA
YTLNSMFWVYLATQGVNPKEHPVKQELERIRVYMNRVKEITDKKKAGKLDRGAASRFVKN
ALWEPKSKNASKVANKGKSKS
>sp|Q9NS00|C1GLT_HUMAN Glycoprotein-N-acetylgalactosamine 3-beta-galactosyltransferase 1 OS=Homo sapiens GN=C1GALT1 PE=1 SV=1
MASKSWLNFLTFLCGSAIGFLLCSQLFSILLGEKVDTQPNVLHNDPHARHSDDNGQNHLE
GQMNFNADSSQHKDENTDIAENLYQKVRILCWVMTGPQNLEKKAKHVKATWAQRCNKVLF
MSSEENKDFPAVGLKTKEGRDQLYWKTIKAFQYVHEHYLEDADWFLKADDDTYVILDNLR
WLLSKYDPEEPIYFGRRFKPYVKQGYMSGGAGYVLSKEALKRFVDAFKTDKCTHSSSIED
LALGRCMEIMNVEAGDSRDTIGKETFHPFVPEHHLIKGYLPRTFWYWNYNYYPPVEGPGC
CSDLAVSFHYVDSTTMYELEYLVYHLRPYGYLYRYQPTLPERILKEISQANKNEDTKVKL
GNP
>sp|P02745|C1QA_HUMAN Complement C1q subcomponent subunit A OS=Homo sapiens GN=C1QA PE=1 SV=2
MEGPRGWLVLCVLAISLASMVTEDLCRAPDGKKGEAGRPGRRGRPGLKGEQGEPGAPGIR
TGIQGLKGDQGEPGPSGNPGKVGYPGPSGPLGARGIPGIKGTKGSPGNIKDQPRPAFSAI
RRNPPMGGNVVIFDTVITNQEEPYQNHSGRFVCTVPGYYYFTFQVLSQWEICLSIVSSSR
GQVRRSLGFCDTTNKGLFQVVSGGMVLQLQQGDQVWVEKDPKKGHIYQGSEADSVFSGFL
IFPSA
>sp|Q9BXJ5|C1QT2_HUMAN Complement C1q tumor necrosis factor-related protein 2 OS=Homo sapiens GN=C1QTNF2 PE=1 SV=1
MIPWVLLACALPCAADPLLGAFARRDFRKGSPQLVCSLPGPQGPPGPPGAPGPSGMMGRM
GFPGKDGQDGHDGDRGDSGEEGPPGRTGNRGKPGPKGKAGAIGRAGPRGPKGVNGTPGKH
GTPGKKGPKGKKGEPGLPGPCSCGSGHTKSAFSVAVTKSYPRERLPIKFDKILMNEGGHY
NASSGKFVCGVPGIYYFTYDITLANKHLAIGLVHNGQYRIRTFDANTGNHDVASGSTILA
LKQGDEVWLQIFYSEQNGLFYDPYWTDSLFTGFLIYADQDDPNEV

>sp|Q9BXJ4|C1QT3_HUMAN Complement C1q tumor necrosis factor-related protein 3 OS=Homo sapiens GN=C1QTNF3 PE=1 SV=1
MLWRQLIYWQLLALFFLPFCLCQDEYMESPQTGGLPPDCSKCCHGDYSFRGYQGPPGPPG
PPGIPGNHGNNGNNGATGHEGAKGEKGDKGDLGPRGERGQHGPKGEKGYPGIPPELQIAF
MASLATHFSNQNSGIIFSSVETNIGNFFDVMTGRFGAPVSGVYFFTFSMMKHEDVEEVYV
YLMHNGNTVFSMYSYEMKGKSDTSSNHAVLKLAKGDEVWLRMGNGALHGDHQRFSTFAGF
LLFETK
>sp|Q9BV19|CA050_HUMAN Uncharacterized protein C1orf50 OS=Homo sapiens GN=C1orf50 PE=1 SV=2
MEDAAAPGRTEGVLERQGAPPAAGQGGALVELTPTPGGLALVSPYHTHRAGDPLDLVALA
EQVQKADEFIRANATNKLTVIAEQIQHLQEQARKVLEDAHRDANLHHVACNIVKKPGNIY
YLYKRESGQQYFSIISPKEWGTSCPHDFLGAYKLQHDLSWTPYEDIEKQDAKISMMDTLL
SQSVALPPCTEPNFQGLTH
>sp|Q5SVJ3|CA100_HUMAN Uncharacterized protein C1orf100 OS=Homo sapiens GN=C1orf100 PE=2 SV=1
MTAIRLREFIERRPVIPPSIFIAHQGRDVQGYYPGQLARLHFDHSAKRAPRPLIDLTIPP
KTKYHYQPQLDQQTLIRYICLRRHSKPAEPWYKETTYRRDYSLPFYEIDWNQKLATVSLN
PRPLNSLPELYCCEERSSFERNAFKLK
>sp|Q8N1D5|CA158_HUMAN Uncharacterized protein C1orf158 OS=Homo sapiens GN=C1orf158 PE=2 SV=2
MFLTAVNPQPLSTPSWQIETKYSTKVLTGNWMEERRKFTRDTDKTPQSIYRKEYIPFPDH
RPDQISRWYGKRKVEGLPYKHLITHHQEPPHRYLISTYDDHYNRHGYNPGLPPLRTWNGQ
KLLWLPEKSDFPLLAPPTNYGLYEQLKQRQLTPKAGLKQSTYTSSYPRPPLCAMSWREHA
VPVPPHRLHPFPHF
>sp|Q8IYL3|CA174_HUMAN UPF0688 protein C1orf174 OS=Homo sapiens GN=C1orf174 PE=1 SV=2
MRSRKLTGAVRSSARLKARSCSAARLASAQEVAGSTSAKTACLTSSSHKATDTRTSKKFK
CDKGHLVKSELQKLVPKNDSASLPKVTPETPCENEFAEGSALLPGSEAGVSVQQGAASLP
LGGCRVVSDSRLAKTRDGLSVPKHSAGSGAEESNSSSTVQKQNEPGLQTEDVQKPPLQMD
NSVFLDDDSNQPMPVSRFFGNVELMQDLPPASSSCPSMSRREFRKMHFRAKDDDDDDDDD
AEM
>sp|Q6ZWK4|CA186_HUMAN Uncharacterized protein C1orf186 OS=Homo sapiens GN=C1orf186 PE=2 SV=1
MLTEVMEVWHGLVIAVVSLFLQACFLTAINYLLSRHMAHKSEQILKAASLQVPRPSPGHH
HPPAVKEMKETQTERDIPMSDSLYRHDSDTPSDSLDSSCSSPPACQATEDVDYTQVVFSD
PGELKNDSPLDYENIKEITDYVNVNPERHKPSFWYFVNPALSEPAEYDQVAM
>sp|Q5T5A4|CA194_HUMAN Uncharacterized protein C1orf194 OS=Homo sapiens GN=C1orf194 PE=2 SV=1
MPPTRDPFQQPTLDNDDSYLGELRASKKLPYKNPTHLAQQQEPWSRLNSTPTITSMRRDA
YYFDPEIPKDDLDFRLAALYNHHTGTFKNKSEILLNQKTTQDTYRTKIQFPGEFLTPPTP
PITFLANIRHWINPKKESIHSIQGSIVSPHTAATNGGYSRKKDGGFFST
>sp|A1L170|CA226_HUMAN Uncharacterized protein C1orf226 OS=Homo sapiens GN=C1orf226 PE=1 SV=1
MFENLNTALTPKLQASRSFPHLSKPVAPGSAPLGSGEPGGPGLWVGSSQHLKNLGKAMGA
KVNDFLRRKEPSSLGSVGVTEINKTAGAQLASGTDAAPEAWLEDERSVLQETFPRLDPPP

PITRKRTPRALKTTQDMLISSQPVLSSLEYGTEPSPGQAQDSAPTAQPDVPADASQPEAT
MEREERGKVLPNGEVSLSVPDLIHKDSQDESKLKMTECRRASSPSLIERNGFKLSLSPIS
LAESWEDGSPPPQARTSSLDNEGPHPDLLSFE
>sp|P54289|CA2D1_HUMAN Voltage-dependent calcium channel subunit alpha-2/delta-1 OS=Homo sapiens GN=CACNA2D1 PE=1 SV=3
MAAGCLLALTLTLFQSLLIGPSSEEPFPSAVTIKSWVDKMQEDLVTLAKTASGVNQLVDI
YEKYQDLYTVEPNNARQLVEIAARDIEKLLSNRSKALVRLALEAEKVQAAHQWREDFASN
EVVYYNAKDDLDPEKNDSEPGSQRIKPVFIEDANFGRQISYQHAAVHIPTDIYEGSTIVL
NELNWTSALDEVFKKNREEDPSLLWQVFGSATGLARYYPASPWVDNSRTPNKIDLYDVRR
RPWYIQGAASPKDMLILVDVSGSVSGLTLKLIRTSVSEMLETLSDDDFVNVASFNSNAQD
VSCFQHLVQANVRNKKVLKDAVNNITAKGITDYKKGFSFAFEQLLNYNVSRANCNKIIML
FTDGGEERAQEIFNKYNKDKKVRVFTFSVGQHNYDRGPIQWMACENKGYYYEIPSIGAIR
INTQEYLDVLGRPMVLAGDKAKQVQWTNVYLDALELGLVITGTLPVFNITGQFENKTNLK
NQLILGVMGVDVSLEDIKRLTPRFTLCPNGYYFAIDPNGYVLLHPNLQPKPIGVGIPTIN
LRKRRPNIQNPKSQEPVTLDFLDAELENDIKVEIRNKMIDGESGEKTFRTLVKSQDERYI
DKGNRTYTWTPVNGTDYSLALVLPTYSFYYIKAKLEETITQARYSETLKPDNFEESGYTF
IAPRDYCNDLKISDNNTEFLLNFNEFIDRKTPNNPSCNADLINRVLLDAGFTNELVQNYW
SKQKNIKGVKARFVVTDGGITRVYPKEAGENWQENPETYEDSFYKRSLDNDNYVFTAPYF
NKSGPGAYESGIMVSKAVEIYIQGKLLKPAVVGIKIDVNSWIENFTKTSIRDPCAGPVCD
CKRNSDVMDCVILDDGGFLLMANHDDYTNQIGRFFGEIDPSLMRHLVNISVYAFNKSYDY
QSVCEPGAAPKQGAGHRSAYVPSVADILQIGWWATAAAWSILQQFLLSLTFPRLLEAVEM
EDDDFTASLSKQSCITEQTQYFFDNDSKSFSGVLDCGNCSRIFHGEKLMNTNLIFIMVES
KGTCPCDTRLLIQAEQTSDGPNPCDMVKQPRYRKGPDVCFDNNVLEDYTDCGGVSGLNPS
LWYIIGIQFLLLWLVSGSTHRLL
>sp|Q8IZS8|CA2D3_HUMAN Voltage-dependent calcium channel subunit alpha-2/delta-3 OS=Homo sapiens GN=CACNA2D3 PE=1 SV=1
MAGPGSPRRASRGASALLAAALLYAALGDVVRSEQQIPLSVVKLWASAFGGEIKSIAAKY
SGSQLLQKKYKEYEKDVAIEEIDGLQLVKKLAKNMEEMFHKKSEAVRRLVEAAEEAHLKH
EFDADLQYEYFNAVLINERDKDGNFLELGKEFILAPNDHFNNLPVNISLSDVQVPTNMYN
KDPAIVNGVYWSESLNKVFVDNFDRDPSLIWQYFGSAKGFFRQYPGIKWEPDENGVIAFD
CRNRKWYIQAATSPKDVVILVDVSGSMKGLRLTIAKQTVSSILDTLGDDDFFNIIAYNEE
LHYVEPCLNGTLVQADRTNKEHFREHLDKLFAKGIGMLDIALNEAFNILSDFNHTGQGSI
CSQAIMLITDGAVDTYDTIFAKYNWPDRKVRIFTYLIGREAAFADNLKWMACANKGFFTQ
ISTLADVQENVMEYLHVLSRPKVIDQEHDVVWTEAYIDSTLPQAQKLTDDQGPVLMTTVA
MPVFSKQNETRSKGILLGVVGTDVPVKELLKTIPKYKLGIHGYAFAITNNGYILTHPELR
LLYEEGKKRRKPNYSSVDLSEVEWEDRDDVLRNAMVNRKTGKFSMEVKKTVDKGKRVLVM
TNDYYYTDIKGTPFSLGVALSRGHGKYFFRGNVTIEEGLHDLEHPDVSLADEWSYCNTDL
HPEHRHLSQLEAIKLYLKGKEPLLQCDKELIQEVLFDAVVSAPIEAYWTSLALNKSENSD
KGVEVAFLGTRTGLSRINLFVGAEQLTNQDFLKAGDKENIFNADHFPLWYRRAAEQIPGS
FVYSIPFSTGPVNKSNVVTASTSIQLLDERKSPVVAAVGIQMKLEFFQRKFWTASRQCAS
LDGKCSISCDDETVNCYLIDNNGFILVSEDYTQTGDFFGEIEGAVMNKLLTMGSFKRITL
YDYQAMCRANKESSDGAHGLLDPYNAFLSAVKWIMTELVLFLVEFNLCSWWHSDMTAKAQ
KLKQTLEPCDTEYPAFVSERTIKETTGNIACEDCSKSFVIQQIPSSNLFMVVVDSSCLCE
SVAPITMAPIEIRYNESLKCERLKAQKIRRRPESCHGFHPEENARECGGAPSLQAQTVLL

LLPLLLMLFSR
>sp|P55286|CADH8_HUMAN Cadherin-8 OS=Homo sapiens GN=CDH8 PE=2 SV=2
MPERLAEMLLDLWTPLIILWITLPPCIYMAPMNQSQVLMSGSPLELNSLGEEQRILNRSK
RGWVWNQMFVLEEFSGPEPILVGRLHTDLDPGSKKIKYILSGDGAGTIFQINDVTGDIHA
IKRLDREEKAEYTLTAQAVDWETSKPLEPPSEFIIKVQDINDNAPEFLNGPYHATVPEMS
ILGTSVTNVTATDADDPVYGNSAKLVYSILEGQPYFSIEPETAIIKTALPNMDREAKEEY
LVVIQAKDMGGHSGGLSGTTTLTVTLTDVNDNPPKFAQSLYHFSVPEDVVLGTAIGRVKA
NDQDIGENAQSSYDIIDGDGTALFEITSDAQAQDGIIRLRKPLDFETKKSYTLKVEAANV
HIDPRFSGRGPFKDTATVKIVVEDADEPPVFSSPTYLLEVHENAALNSVIGQVTARDPDI
TSSPIRFSIDRHTDLERQFNINADDGKITLATPLDRELSVWHNITIIATEIRNHSQISRV
PVAIKVLDVNDNAPEFASEYEAFLCENGKPGQVIQTVSAMDKDDPKNGHYFLYSLLPEMV
NNPNFTIKKNEDNSLSILAKHNGFNRQKQEVYLLPIIISDSGNPPLSSTSTLTIRVCGCS
NDGVVQSCNVEAYVLPIGLSMGALIAILACIILLLVIVVLFVTLRRHKNEPLIIKDDEDV
RENIIRYDDEGGGEEDTEAFDIATLQNPDGINGFLPRKDIKPDLQFMPRQGLAPVPNGVD
VDEFINVRLHEADNDPTAPPYDSIQIYGYEGRGSVAGSLSSLESTTSDSDQNFDYLSDWG
PRFKRLGELYSVGESDKET
>sp|O43852|CALU_HUMAN Calumenin OS=Homo sapiens GN=CALU PE=1 SV=2
MDLRQFLMCLSLCTAFALSKPTEKKDRVHHEPQLSDKVHNDAQSFDYDHDAFLGAEEAKT
FDQLTPEESKERLGKIVSKIDGDKDGFVTVDELKDWIKFAQKRWIYEDVERQWKGHDLNE
DGLVSWEEYKNATYGYVLDDPDPDDGFNYKQMMVRDERRFKMADKDGDLIATKEEFTAFL
HPEEYDYMKDIVVQETMEDIDKNADGFIDLEEYIGDMYSHDGNTDEPEWVKTEREQFVEF
RDKNRDGKMDKEETKDWILPSDYDHAEAEARHLVYESDQNKDGKLTKEEIVDKYDLFVGS
QATDFGEALVRHDEF
>sp|Q9HC96|CAN10_HUMAN Calpain-10 OS=Homo sapiens GN=CAPN10 PE=1 SV=2
MRAGRGATPARELFRDAAFPAADSSLFCDLSTPLAQFREDITWRRPQEICATPRLFPDDP
REGQVKQGLLGDCWFLCACAALQKSRHLLDQVIPPGQPSWADQEYRGSFTCRIWQFGRWV
EVTTDDRLPCLAGRLCFSRCQREDVFWLPLLEKVYAKVHGSYEHLWAGQVADALVDLTGG
LAERWNLKGVAGSGGQQDRPGRWEHRTCRQLLHLKDQCLISCCVLSPRAGARELGEFHAF
IVSDLRELQGQAGQCILLLRIQNPWGRRCWQGLWREGGEGWSQVDAAVASELLSQLQEGE
FWVEEEEFLREFDELTVGYPVTEAGHLQSLYTERLLCHTRALPGAWVKGQSAGGCRNNSG
FPSNPKFWLRVSEPSEVYIAVLQRSRLHAADWAGRARALVGDSHTSWSPASIPGKHYQAV
GLHLWKVEKRRVNLPRVLSMPPVAGTACHAYDREVHLRCELSPGYYLAVPSTFLKDAPGE
FLLRVFSTGRVSLSAIRAVAKNTTPGAALPAGEWGTVQLRGSWRVGQTAGGSRNFASYPT
NPCFPFSVPEGPGPRCVRITLHQHCRPSDTEFHPIGFHIFQVPEGGRSQDAPPLLLQEPL
LSCVPHRYAQEVSRLCLLPAGTYKVVPSTYLPDTEGAFTVTIATRIDRPSIHSQEMLGQF
LQEVSIMAVMKT
>sp|Q6ZSI9|CAN12_HUMAN Calpain-12 OS=Homo sapiens GN=CAPN12 PE=2 SV=1
MASSSGRVTIQLVDEEAGVGAGRLQLFRGQSYEAIRAACLDSGILFRDPYFPAGPDALGY
DQLGPDSEKAKGVKWMRPHEFCAEPKFICEDMSRTDVCQGSLGNCWFLAAAASLTLYPRL
LRRVVPPGQDFQHGYAGVFHFQLWQFGRWMDVVVDDRLPVREGKLMFVRSEQRNEFWAPL
LEKAYAKLHGSYEVMRGGHMNEAFVDFTGGVGEVLYLRQNSMGLFSALRHALAKESLVGA
TALSDRGEYRTEEGLVKGHAYSITGTHKVFLGFTKVRLLRLRNPWGCVEWTGAWSDSCPR
WDTLPTECRDALLVKKEDGEFWMELRDFLLHFDTVQICSLSPEVLGPSPEGGGWHVHTFQ
GRWVRGFNSGGSQPNAETFWTNPQFRLTLLEPDEEDDEDEEGPWGGWGAAGARGPARGGR

TPKCTVLLSLIQRNRRRLRAKGLTYLTVGFHVFQIPEELLGLWDSPRSHALLPRLLRADR
SPLSARRDVTRRCCLRPGHYLVVPSTAHAGDEADFTLRVFSERRHTAVEIDDVISADLQS
LQGPYLPLELGLEQLFQELAGEEEELNASQLQALLSIALEPARAHTSTPREIGLRTCEQL
LQCFGHGQSLALHHFQQLWGYLLEWQAIFNKFDEDTSGTMNSYELRLALNAAGFHLNNQL
TQTLTSRYRDSRLRVDFERFVSCVAHLTCIFCHCSQHLDGGEGVICLTHRQWMEVATFS
>sp|Q9Y6W3|CAN7_HUMAN Calpain-7 OS=Homo sapiens GN=CAPN7 PE=1 SV=1
MDATALERDAVQFARLAVQRDHEGRYSEAVFYYKEAAQALIYAEMAGSSLENIQEKITEY
LERVQALHSAVQSKSADPLKSKHQLDLERAHFLVTQAFDEDEKENVEDAIELYTEAVDLC
LKTSYETADKVLQNKLKQLARQALDRAEALSEPLTKPVGKISSTSVKPKPPPVRAHFPLG
ANPFLERPQSFISPQSCDAQGQRYTAEEIEVLRTTSKINGIEYVPFMNVDLRERFAYPMP
FCDRWGKLPLSPKQKTTFSKWVRPEDLTNNPTMIYTVSSFSIKQTIVSDCSFVASLAISA
AYERRFNKKLITGIIYPQNKDGEPEYNPCGKYMVKLHLNGVPRKVIIDDQLPVDHKGELL
CSYSNNKSELWVSLIEKAYMKVMGGYDFPGSNSNIDLHALTGWIPERIAMHSDSQTFSKD
NSFRMLYQRFHKGDVLITASTGMMTEAEGEKWGLVPTHAYAVLDIREFKGLRFIQLKNPW
SHLRWKGRYSENDVKNWTPELQKYLNFDPRTAQKIDNGIFWISWDDLCQYYDVIYLSWNP
GLFKESTCIHSTWDAKQGPVKDAYSLANNPQYKLEVQCPQGGAAVWVLLSRHITDKDDFA
NNREFITMVVYKTDGKKVYYPADPPPYIDGIRINSPHYLTKIKLTTPGTHTFTLVVSQYE
KQNTIHYTVRVYSACSFTFSKIPSPYTLSKRINGKWSGQSAGGCGNFQETHKNNPIYQFH
IEKTGPLLIELRGPRQYSVGFEVVTVSTLGDPGPHGFLRKSSGDYRCGFCYLELENIPSG
IFNIIPSTFLPKQEGPFFLDFNSIIPIKITQLQ
>sp|P63098|CANB1_HUMAN Calcineurin subunit B type 1 OS=Homo sapiens GN=PPP3R1 PE=1 SV=2
MGNEASYPLEMCSHFDADEIKRLGKRFKKLDLDNSGSLSVEEFMSLPELQQNPLVQRVID
IFDTDGNGEVDFKEFIEGVSQFSVKGDKEQKLRFAFRIYDMDKDGYISNGELFQVLKMMV
GNNLKDTQLQQIVDKTIINADKDGDGRISFEEFCAVVGGLDIHKKMVVDV
>sp|P40121|CAPG_HUMAN Macrophage-capping protein OS=Homo sapiens GN=CAPG PE=1 SV=2
MYTAIPQSGSPFPGSVQDPGLHVWRVEKLKPVPVAQENQGVFFSGDSYLVLHNGPEEVSH
LHLWIGQQSSRDEQGACAVLAVHLNTLLGERPVQHREVQGNESDLFMSYFPRGLKYQEGG
VESAFHKTSTGAPAAIKKLYQVKGKKNIRATERALNWDSFNTGDCFILDLGQNIFAWCGG
KSNILERNKARDLALAIRDSERQGKAQVEIVTDGEEPAEMIQVLGPKPALKEGNPEEDLT
ADKANAQAAALYKVSDATGQMNLTKVADSSPFALELLISDDCFVLDNGLCGKIYIWKGRK
ANEKERQAALQVAEGFISRMQYAPNTQVEILPQGHESPIFKQFFKDWK
>sp|Q9UL45|BL1S6_HUMAN Biogenesis of lysosome-related organelles complex 1 subunit 6 OS=Homo sapiens GN=BLOC1S6 PE=1 SV=1
MSVPGPSSPDGALTRPPYCLEAGEPTPGLSDTSPDEGLIEDLTIEDKAVEQLAEGLLSHY
LPDLQRSKQALQELTQNQVVLLDTLEQEISKFKECHSMLDINALFAEAKHYHAKLVNIRK
EMLMLHEKTSKLKKRALKLQQKRQKEELEREQQREKEFEREKQLTARPAKRM
>sp|P62952|BLCAP_HUMAN Bladder cancer-associated protein OS=Homo sapiens GN=BLCAP PE=1 SV=1
MYCLQWLLPVLLIPKPLNPALWFSHSMFMGFYLLSFLLERKPCTICALVFLAALFLICYS
CWGNCFLYHCSDSPLPESAHDPGVVGT
>sp|Q7L3V2|BOP_HUMAN Protein Bop OS=Homo sapiens GN=BOP PE=1 SV=1
MPRGRCRQQGPRIPIWAAANYANAHPWQQMDKASPGVAYTPLVDPWIERPCCGDTVCVRT
TMEQKSTASGTCGGKPAERGPLAGHMPSSRPHRVDFCWVPGSDPGTFDGSPWLLDRFLAQ
LGDYMSFHFEHYQDNISRVCEILRRLTGRAQAWAAPYLDGDLPLPDDYELFCQDLKEVVQ

DPNSFAEYHAVVTCPLPLASSQLPVAPQLPVVRQYLARFLEGLALDMGTAPRSLPAAMAT
PAVSGSNSVSRSALFEQQLTKESTPGPKEPPVLPSSTCSSKPGPVEPASSQPEEAAPTPV
PRLSESANPPAQRPDPAHPGGPKPQKTEEEVLETEGDQEVSLGTPQEVVEAPETPGEPPL
SPGF
>sp|Q969J3|BORC5_HUMAN BLOC-1-related complex subunit 5 OS=Homo sapiens GN=BORCS5 PE=1 SV=1
MGSEQSSEAESRPNDLNSSVTPSPAKHRAKMDDIVVVAQGSQASRNVSNDPDVIKLQEIP
TFQPLLKGLLSGQTSPTNAKLEKLDSQQVLQLCLRYQDHLHQCAEAVAFDQNALVKRIKE
MDLSVETLFSFMQERQKRYAKYAEQIQKVNEMSAILRRIQMGIDQTVPLLDRLNSMLPEG
ERLEPFSMKPDRELRL
>sp|Q9H3Q1|BORG4_HUMAN Cdc42 effector protein 4 OS=Homo sapiens GN=CDC42EP4 PE=1 SV=1
MPILKQLVSSSVHSKRRSRADLTAEMISAPLGDFRHTMHVGRAGDAFGDTSFLNSKAGEP
DGESLDEQPSSSSSKRSLLSRKFRGSKRSQSVTRGEREQRDMLGSLRDSALFVKNAMSLP
QLNEKEAAEKGTSKLPKSLSSSPVKKANDGEGGDEEAGTEEAVPRRNGAAGPHSPDPLLD
EQAFGDLTDLPVVPKATYGLKHAESIMSFHIDLGPSMLGDVLSIMDKEEWDPEEGEGGYH
GDEGAAGTITQAPPYAVAAPPLARQEGKAGPDLPSLPSHALEDEGWAAAAPSPGSARSMG
SHTTRDSSSLSSCTSGILEERSPAFRGPDRARAAVSRQPDKEFSFMDEEEEDEIRV
>sp|Q00587|BORG5_HUMAN Cdc42 effector protein 1 OS=Homo sapiens GN=CDC42EP1 PE=1 SV=1
MPGPQGGRGAATMSLGKLSPVGWVSSSQGKRRLTADMISHPLGDFRHTMHVGRGGDVFGD
TSFLSNHGGSSGSTHRSPRSFLAKKLQLVRRVGAPPRRMASPPAPSPAPPAISPIIKNAI
SLPQLNQAAYDSLVVGKLSFDSSPTSSTDGHSSYGLDSGFCTISRLPRSEKPHDRDRDGS
FPSEPGLRRSDSLLSFRLDLDLGPSLLSELLGVMSLPEAPAAETPAPAANPPAPTANPTG
PAANPPATTANPPAPAANPSAPAATPTGPAANPPAPAASSTPHGHCPNGVTAGLGPVAEV
KSSPVGGGPRGPAGPALGRHWGAGWDGGHHYPEMDARQERVEVLPQARASWESLDEEWRA
PQAGSRTPVPSTVQANTFEFADAEEDDEVKV
>sp|Q8TDL5|BPIB1_HUMAN BPI fold-containing family B member 1 OS=Homo sapiens GN=BPIFB1 PE=1 SV=1
MAGPWTFTLLCGLLAATLIQATLSPTAVLILGPKVIKEKLTQELKDHNATSILQQLPLLS
AMREKPAGGIPVLGSLVNTVLKHIIWLKVITANILQLQVKPSANDQELLVKIPLDMVAGF
NTPLVKTIVEFHMTTEAQATIRMDTSASGPTRLVLSDCATSHGSLRIQLLHKLSFLVNAL
AKQVMNLLVPSLPNLVKNQLCPVIEASFNGMYADLLQLVKVPISLSIDRLEFDLLYPAIK
GDTIQLYLGAKLLDSQGKVTKWFNNSAASLTMPTLDNIPFSLIVSQDVVKAAVAAVLSPE
EFMVLLDSVLPESAHRLKSSIGLINEKAADKLGSTQIVKILTQDTPEFFIDQGHAKVAQL
IVLEVFPSSEALRPLFTLGIEASSEAQFYTKGDQLILNLNNISSDRIQLMNSGIGWFQPD
VLKNIITEIIHSILLPNQNGKLRSGVPVSLVKALGFEAAESSLTKDALVLTPASLWKPSS
PVSQ
>sp|P59826|BPIB3_HUMAN BPI fold-containing family B member 3 OS=Homo sapiens GN=BPIFB3 PE=2 SV=2
MQPVMLALWSLLLLWGLATPCQELLETVGTLARIDKDELGKAIQNSLVGEPILQNVLGSV
TAVNRGLLGSGGLLGGGGLLGHGGVFGVVEELSGLKIEELTLPKVLLKLLPGFGVQLSLH
TKVGMHCSGPLGGLLQLAAEVNVTSRVALAVSSRGTPILILKRCSTLLGHISLFSGLLPT
PLFGVVEQMLFKVLPGLLCPVVDSVLGVVNELLGAVLGLVSLGALGSVEFSLATLPLISN
QYIELDINPIVKSVAGDIIDFPKSRAPAKVPPKKDHTSQVMVPLYLFNTTFGLLQTNGAL
DMDITPELVPSDVPLTTTDLAALLPEALGKLPLHQQLLLFLRVREAPTVTLHNKKALVSL

PANIHVLFYVPKGTPESLFELNSVMTVRAQLAPSATKLHISLSLERLSVKVASSFTHAFD
GSRLEEWLSHVVGAVYAPKLNVALDVGIPLPKVLNINFSNSVLEIVENAVVLTVAS
>sp|P59827|BPIB4_HUMAN BPI fold-containing family B member 4 OS=Homo sapiens GN=BPIFB4 PE=2 SV=2
MWMAWCVAALSVVAVCGTSHETNTVLRVTKDVLSNAISGMLQQSDALHSALREVPLGVGD
IPYNDFHVRGPPPVYTNGKKLDGIYQYGHIETNDNTAQLGGKYRYGEILESEGSIRDLRN
SGYRSAENAYGGHRGLGRYRAAPVGRLHRRELQPGEIPPGVATGAVGPGGLLGTGGMLAA
DGILAGQGGLLGGGGLLGDGGLLGGGGVLGVLGEGGILSTVQGITGLRIVELTLPRVSVR
LLPGVGVYLSLYTRVAINGKSLIGFLDIAVEVNITAKVRLTMDRTGYPRLVIERCDTLLG
GIKVKLLRGLLPNLVDNLVNRVLADVLPDLLCPIVDVVLGLVNDQLGLVDSLIPLGILGS
VQYTFSSLPLVTGEFLELDLNTLVGEAGGGLIDYPLGWPAVSPKPMPELPPMGDNTKSQL
AMSANFLGSVLTLLQKQHALDLDITNGMFEELPPLTTATLGALIPKVFQQYPESCPLIIR
IQVLNPPSVMLQKDKALVKVLATAEVMVSQPKDLETTICLIDVDTEFLASFSTEGDKLMI
DAKLEKTSLNLRTSNVGNFDIGLMEVLVEKIFDLAFMPAMNAVLGSGVPLPKILNIDFSN
ADIDVLEDLLVLSA
>sp|P51587|BRCA2_HUMAN Breast cancer type 2 susceptibility protein OS=Homo sapiens GN=BRCA2 PE=1 SV=3
MPIGSKERPTFFEIFKTRCNKADLGPISLNWFEELSSEAPPYNSEPAEESEHKNNNYEPN
LFKTPQRKPSYNQLASTPIIFKEQGLTLPLYQSPVKELDKFKLDLGRNVPNSRHKSLRTV
KTKMDQADDVSCPLLNSCLSESPVVLQCTHVTPQRDKSVVCGSLFHTPKFVKGRQTPKHI
SESLGAEVDPDMSWSSSLATPPTLSSTVLIVRNEEASETVFPHDTTANVKSYFSNHDESL
KKNDRFIASVTDSENTNQREAASHGFGKTSGNSFKVNSCKDHIGKSMPNVLEDEVYETVV
DTSEEDSFSLCFSKCRTKNLQKVRTSKTRKKIFHEANADECEKSKNQVKEKYSFVSEVEP
NDTDPLDSNVANQKPFESGSDKISKEVVPSLACEWSQLTLSGLNGAQMEKIPLLHISSCD
QNISEKDLLDTENKRKKDFLTSENSLPRISSLPKSEKPLNEETVVNKRDEEQHLESHTDC
ILAVKQAISGTSPVASSFQGIKKSIFRIRESPKETFNASFSGHMTDPNFKKETEASESGL
EIHTVCSQKEDSLCPNLIDNGSWPATTTQNSVALKNAGLISTLKKKTNKFIYAIHDETSY
KGKKIPKDQKSELINCSAQFEANAFEAPLTFANADSGLLHSSVKRSCSQNDSEEPTLSLT
SSFGTILRKCSRNETCSNNTVISQDLDYKEAKCNKEKLQLFITPEADSLSCLQEGQCEND
PKSKKVSDIKEEVLAAACHPVQHSKVEYSDTDFQSQKSLLYDHENASTLILTPTSKDVLS
NLVMISRGKESYKMSDKLKGNNYESDVELTKNIPMEKNQDVCALNENYKNVELLPPEKYM
RVASPSRKVQFNQNTNLRVIQKNQEETTSISKITVNPDSEELFSDNENNFVFQVANERNN
LALGNTKELHETDLTCVNEPIFKNSTMVLYGDTGDKQATQVSIKKDLVYVLAEENKNSVK
QHIKMTLGQDLKSDISLNIDKIPEKNNDYMNKWAGLLGPISNHSFGGSFRTASNKEIKLS
EHNIKKSKMFFKDIEEQYPTSLACVEIVNTLALDNQKKLSKPQSINTVSAHLQSSVVVSD
CKNSHITPQMLFSKQDFNSNHNLTPSQKAEITELSTILEESGSQFEFTQFRKPSYILQKS
TFEVPENQMTILKTTSEECRDADLHVIMNAPSIGQVDSSKQFEGTVEIKRKFAGLLKNDC
NKSASGYLTDENEVGFRGFYSAHGTKLNVSTEALQKAVKLFSDIENISEETSAEVHPISL
SSSKCHDSVVSMFKIENHNDKTVSEKNNKCQLILQNNIEMTTGTFVEEITENYKRNTENE
DNKYTAASRNSHNLEFDGSDSSKNDTVCIHKDETDLLFTDQHNICLKLSGQFMKEGNTQI
KEDLSDLTFLEVAKAQEACHGNTSNKEQLTATKTEQNIKDFETSDTFFQTASGKNISVAK
ESFNKIVNFFDQKPEELHNFSLNSELHSDIRKNKMDILSYEETDIVKHKILKESVPVGTG
NQLVTFQGQPERDEKIKEPTLLGFHTASGKKVKIAKESLDKVKNLFDEKEQGTSEITSFS
HQWAKTLKYREACKDLELACETIEITAAPKCKEMQNSLNNDKNLVSIETVVPPKLLSDNL

CRQTENLKTSKSIFLKVKVHENVEKETAKSPATCYTNQSPYSVIENSALAFYTSCSRKTS
VSQTSLLEAKKWLREGIFDGQPERINTADYVGNYLYENNSNSTIAENDKNHLSEKQDTYL
SNSSMSNSYSYHSDEVYNDSGYLSKNKLDSGIEPVLKNVEDQKNTSFSKVISNVKDANAY
PQTVNEDICVEELVTSSSPCKNKNAAIKLSISNSNNFEVGPPAFRIASGKIVCVSHETIK
KVKDIFTDSFSKVIKENNENKSKICQTKIMAGCYEALDDSEDILHNSLDNDECSTHSHKV
FADIQSEEILQHNQNMSGLEKVSKISPCDVSLETSDICKCSIGKLHKSVSSANTCGIFST
ASGKSVQVSDASLQNARQVFSEIEDSTKQVFSKVLFKSNEHSDQLTREENTAIRTPEHLI
SQKGFSYNVVNSSAFSGFSTASGKQVSILESSLHKVKGVLEEFDLIRTEHSLHYSPTSRQ
NVSKILPRVDKRNPEHCVNSEMEKTCSKEFKLSNNLNVEGGSSENNHSIKVSPYLSQFQQ
DKQQLVLGTKVSLVENIHVLGKEQASPKNVKMEIGKTETFSDVPVKTNIEVCSTYSKDSE
NYFETEAVEIAKAFMEDDELTDSKLPSHATHSLFTCPENEEMVLSNSRIGKRRGEPLILV
GEPSIKRNLLNEFDRIIENQEKSLKASKSTPDGTIKDRRLFMHHVSLEPITCVPFRTTKE
RQEIQNPNFTAPGQEFLSKSHLYEHLTLEKSSSNLAVSGHPFYQVSATRNEKMRHLITTG
RPTKVFVPPFKTKSHFHRVEQCVRNINLEENRQKQNIDGHGSDDSKNKINDNEIHQFNKN
NSNQAAAVTFTKCEEEPLDLITSLQNARDIQDMRIKKKQRQRVFPQPGSLYLAKTSTLPR
ISLKAAVGGQVPSACSHKQLYTYGVSKHCIKINSKNAESFQFHTEDYFGKESLWTGKGIQ
LADGGWLIPSNDGKAGKEEFYRALCDTPGVDPKLISRIWVYNHYRWIIWKLAAMECAFPK
EFANRCLSPERVLLQLKYRYDTEIDRSRRSAIKKIMERDDTAAKTLVLCVSDIISLSANI
SETSSNKTSSADTQKVAIIELTDGWYAVKAQLDPPLLAVLKNGRLTVGQKIILHGAELVG
SPDACTPLEAPESLMLKISANSTRPARWYTKLGFFPDPRPFPLPLSSLFSDGGNVGCVDV
IIQRAYPIQWMEKTSSGLYIFRNEREEEKEAAKYVEAQQKRLEALFTKIQEEFEEHEENT
TKPYLPSRALTRQQVRALQDGAELYEAVKNAADPAYLEGYFSEEQLRALNNHRQMLNDKK
QAQIQLEIRKAMESAEQKEQGLSRDVTTVWKLRIVSYSKKEKDSVILSIWRPSSDLYSLL
TEGKRYRIYHLATSKSKSKSERANIQLAATKKTQYQQLPVSDEILFQIYQPREPLHFSKF
LDPDFQPSCSEVDLIGFVVSVVKKTGLAPFVYLSDECYNLLAIKFWIDLNEDIIKPHMLI
AASNLQWRPESKSGLLTLFAGDFSVFSASPKEGHFQETFNKMKNTVENIDILCNEAENKL
MHILHANDPKWSTPTKDCTSGPYTAQIIPGTGNKLLMSSPNCEIYYQSPLSLCMAKRKSV
STPVSAQMTSKSCKGEKEIDDQKNCKKRRALDFLSRLPLPPPVSPICTFVSPAAQKAFQP
PRSCGTKYETPIKKKELNSPQMTPFKKFNEISLLESNSIADEELALINTQALLSGSTGEK
QFISVSESTRTAPTSSEDYLRLKRRCTTSLIKEQESSQASTEECEKNKQDTITTKKYI
>sp|Q9NPI1|BRD7_HUMAN Bromodomain-containing protein 7 OS=Homo sapiens GN=BRD7 PE=1 SV=1
MGKKHKKHKSDKHLYEEYVEKPLKLVLKVGGNEVTELSTGSSGHDSSLFEDKNDHDKHKD
RKRKKRKKGEKQIPGEEKGRKRRRVKEDKKKRDRDRVENEAEKDLQCHAPVRLDLPPEKP
LTSSLAKQEEVEQTPLQEALNQLMRQLQRKDPSAFFSFPVTDFIAPGYSMIIKHPMDFST
MKEKIKNNDYQSIEELKDNFKLMCTNAMIYNKPETIYYKAAKKLLHSGMKILSQERIQSL
KQSIDFMADLQKTRKQKDGTDTSQSGEDGGCWQREREDSGDAEAHAFKSPSKENKKKDKD
MLEDKFKSNNLEREQEQLDRIVKESGGKLTRRLVNSQCEFERRKPDGTTTLGLLHPVDPI
VGEPGYCPVRLGMTTGRLQSGVNTLQGFKEDKRNKVTPVLYLNYGPYSSYAPHYDSTFAN
ISKDDSDLIYSTYGEDSDLPSDFSIHEFLATCQDYPYVMADSLLDVLTKGGHSRTLQEME
MSLPEDEGHTRTLDTAKEMEITEVEPPGRLDSSTQDRLIALKAVTNFGVPVEVFDSEEAE
IFQKKLDETTRLLRELQEAQNERLSTRPPPNMICLLGPSYREMHLAEQVTNNLKELAQQV
TPGDIVSTYGVRKAMGISIPSPVMENNFVDLTEDTEEPKKTDVAECGPGGS
>sp|Q5VTR2|BRE1A_HUMAN E3 ubiquitin-protein ligase BRE1A OS=Homo sapiens GN=RNF20 PE=1 SV=2

MSGIGNKRAAGEPGTSMPPEKKAAVEDSGTTVETIKLGGVSSTEELDIRTLQTKNRKLAE
MLDQRQAIEDELREHIEKLERRQATDDASLLIVNRYWSQFDENIRIILKRYDLEQGLGDL
LTERKALVVPEPEPDSDSNQERKDDRERGEGQEPAFSFLATLASSSSEEMESQLQERVES
SRRAVSQIVTVYDKLQEKVELLSRKLNSGDNLIVEEAVQELNSFLAQENMRLQELTDLLQ
EKHRTMSQEFSKLQSKVETAESRVSVLESMIDDLQWDIDKIRKREQRLNRHLAEVLERVN
SKGYKVYGAGSSLYGGTITINARKFEEMNAELEENKELAQNRLCELEKLRQDFEEVTTQN
EKLKVELRSAVEQVVKETPEYRCMQSQFSVLYNESLQLKAHLDEARTLLHGTRGTHQHQV
ELIERDEVSLHKKLRTEVIQLEDTLAQVRKEYEMLRIEFEQTLAANEQAGPINREMRHLI
SSLQNHNHQLKGEVLRYKRKLREAQSDLNKTRLRSGSALLQSQSSTEDPKDEPAELKPDS
EDLSSQSSASKASQEDANEIKSKRDEEERERERREKEREREREREKEKEREREKQKLKES
EKERDSAKDKEKGKHDDGRKKEAEIIKQLKIELKKAQESQKEMKLLLDMYRSAPKEQRDK
VQLMAAEKKSKAELEDLRQRLKDLEDKEKKENKKMADEDALRKIRAVEEQIEYLQKKLAM
AKQEEEALLSEMDVTGQAFEDMQEQNIRLMQQLREKDDANFKLMSERIKSNQIHKLLKEE
KEELADQVLTLKTQVDAQLQVVRKLEEKEHLLQSNIGTGEKELGLRTQALEMNKRKAMEA
AQLADDLKAQLELAQKKLHDFQDEIVENSVTKEKDMFNFKRAQEDISRLRRKLETTKKPD
NVPKCDEILMEEIKDYKARLTCPCCNMRKKDAVLTKCFHVFCFECVKTRYDTRQRKCPKC
NAAFGANDFHRIYIG
>sp|Q9NXR7|BRE_HUMAN BRCA1-A complex subunit BRE OS=Homo sapiens GN=BRE PE=1 SV=2
MSPEVALNRISPMLSPFISSVVRNGKVGLDATNCLRITDLKSGCTSLTPGPNCDRFKLHI
PYAGETLKWDIIFNAQYPELPPDFIFGEDAEFLPDPSALQNLASWNPSNPECLLLVVKEL
VQQYHQFQCSRLRESSRLMFEYQTLLEEPQYGENMEIYAGKKNNWTGEFSARFLLKLPVD
FSNIPTYLLKDVNEDPGEDVALLSVSFEDTEATQVYPKLYLSPRIEHALGGSSALHIPAF
PGGGCLIDYVPQVCHLLTNKVQYVIQGYHKRREYIAAFLSHFGTGVVEYDAEGFTKLTLL
LMWKDFCFLVHIDLPLFFPRDQPTLTFQSVYHFTNSGQLYSQAQKNYPYSPRWDGNEMAK
RAKAYFKTFVPQFQEAAFANGKL
>sp|O95415|BRI3_HUMAN Brain protein I3 OS=Homo sapiens GN=BRI3 PE=2 SV=1
MDHKPLLQERPPAYNLEAGQGDYACGPHGYGAIPAAPPPPPYPYLVTGIPTHHPRVYNIH
SRTVTRYPANSIVVVGGCPVCRVGVLEDCFTFLGIFLAIILFPFGFICCFALRKRRCPNC
GATFA
>sp|Q6PL45|BRID5_HUMAN BRICHOS domain-containing protein 5 OS=Homo sapiens GN=BRICD5 PE=1 SV=3
MEPASCCAERPKPGPTGVKTKPSCGGWRAVSLLLLLLLLVLAAVGVVAGGLLGSAQGPPK
PRLQTLRMTLPSPHMPRPNQTILVDVARNAATITVTPPQSNHSWAVLFDGQSGCICYRPE
EHQVCFLRLMEDSDRETLRLLVDTSKVQEAWVPSQDTHHTQELLAVQGSLEVDPAQAGAL
VQRLCMRTPIYWARRAEGESGPLWGKARPSGWFEELGAEPLEIHGTLATGPRRQRLIYLC
IDICFPSNICVSVCFYYLPD
>sp|Q5VW32|BROX_HUMAN BRO1 domain-containing protein BROX OS=Homo sapiens GN=BROX PE=1 SV=1
MTHWFHRNPLKATAPVSFNYYGVVTGPSASKICNDLRSSRARLLELFTDLSCNPEMMKNA
ADSYFSLLQGFINSLDESTQESKLRYIQNFKWTDTLQGQVPSAQQDAVFELISMGFNVAL
WYTKYASRLAGKENITEDEAKEVHRSLKIAAGIFKHLKESHLPKLITPAEKGRDLESRLI
EAYVIQCQAEAQEVTIARAIELKHAPGLIAALAYETANFYQKADHTLSSLEPAYSAKWRK
YLHLKMCFYTAYAYCYHGETLLASDKCGEAIRSLQEAEKLYAKAEALCKEYGETKGPGPT
VKPSGHLFFRKLGNLVKNTLEKCQRENGFIYFQKIPTEAPQLELKANYGLVEPIPFEFPP

TSVQWTPETLAAFDLTKRPKDDSTKPKPEEEVKPVKEPDIKPQKDTGCYIS
>sp|Q9NW68|BSDC1_HUMAN BSD domain-containing protein 1 OS=Homo sapiens GN=BSDC1 PE=1 SV=1
MAEGEDVGWWRSWLQQSYQAVKEKSSEALEFMKRDLTEFTQVVQHDTACTIAATASVVKE
KLATEGSSGATEKMKKGLSDFLGVISDTFAPSPDKTIDCDVITLMGTPSGTAEPYDGTKA
RLYSLQSDPATYCNEPDGPPELFDAWLSQFCLEEKKGEISELLVGSPSIRALYTKMVPAA
VSHSEFWHRYFYKVHQLEQEQARRDALKQRAEQSISEEPGWEEEEEELMGISPISPKEAK
VPVAKISTFPEGEPGPQSPCEENLVTSVEPPAEVTPSESSESISLVTQIANPATAPEARV
LPKDLSQKLLEASLEEQGLAVDVGETGPSPPIHSKPLTPAGHTGGPEPRPPARVETLREE
APTDLRVFELNSDSGKSTPSNNGKKGSSTDISEDWEKDFDLDMTEEEVQMALSKVDASGE
LEDVEWEDWE
>sp|Q9Y2F9|BTBD3_HUMAN BTB/POZ domain-containing protein 3 OS=Homo sapiens GN=BTBD3 PE=2 SV=1
MVDDKEKNMKCLTFFLMLPETVKNRSKKSSKKANTSSSSSNSSKLPPVCYEIITLKTKKK
KMAADIFPRKKPANSSSTSVQQYHQQNLSNNNLIPAPNWQGLYPTIRERNAMMFNNDLMA
DVHFVVGPPGGTQRLPGHKYVLAVGSSVFHAMFYGELAEDKDEIRIPDVEPAAFLAMLKY
IYCDEIDLAADTVLATLYAAKKYIVPHLARACVNFLETSLSAKNACVLLSQSCLFEEPDL
TQRCWEVIDAQAELALKSEGFCDIDFQTLESILRRETLNAKEIVVFEAALNWAEVECQRQ
DLALSIENKRKVLGKALYLIRIPTMALDDFANGAAQSGVLTLNETNDIFLWYTAAKKPEL
QFVSKARKGLVPQRCHRFQSCAYRSNQWRYRGRCDSIQFAVDKRVFIAGFGLYGSSCGSA
EYSAKIELKRQGVVLGQNLSKYFSDGSSNTFPVWFEYPVQIEPDTFYTASVILDGNELSY
FGQEGMTEVQCGKVTVQFQCSSDSTNGTGVQGGQIPELIFYA
>sp|A6QL63|BTBDB_HUMAN Ankyrin repeat and BTB/POZ domain-containing protein BTBD11 OS=Homo sapiens GN=BTBD11 PE=2 SV=3
MARRGKKPVVRTLEDLTLDSGYGGAADSVRSSNLSLCCSDSHPASPYGGSCWPPLADSMH
SRHNSFDTVNTALVEDSEGLDCAGQHCSRLLPDLDEVPWTLQELEALLLRSRDPRAGPAV
PGGLPKDALAKLSTLVSRALVRIAKEAQRLSLRFAKCTKYEIQSAMEIVLSWGLAAHCTA
AALAALSLYNMSSAGGDRLGRGKSARCGLTFSVGRVYRWMVDSRVALRIHEHAAIYLTAC
MESLFRDIYSRVVASGVPRSCSGPGSGSGSGPGPSSGPGAAPAADKEREAPGGGAASGGA
CSAASSASGGSSCCAPPAAAAAAVPPAAAANHHHHHHHALHEAPKFTVETLEHTVNNDSE
IWGLLQPYQHLICGKNASGVLCLPDSLNLHRDPQRSNKPGELPMFSQSELRTIEQSLLAT
RVGSIAELSDLVSRAMHHLQPLNAKHHGNGTPLHHKQGALYWEPEALYTLCYFMHCPQME
WENPNVEPSKVNLQVERPFLVLPPLMEWIRVAVAHAGHRRSFSMDSDDVRQAARLLLPGV
DCEPRQLRADDCFCASRKLDAVAIEAKFKQDLGFRMLNCGRTDLVKQAVSLLGPDGINTM
SEQGMTPLMYACVRGDEAMVQMLLDAGADLNVEVVSTPHKYPSVHPETRHWTALTFAVLH
GHIPVVQLLLDAGAKVEGSVEHGEENYSETPLQLAAAVGNFELVSLLLERGADPLIGTMY
RNGISTTPQGDMNSFSQAAAHGHRNVFRKLLAQPEKEKSDILSLEEILAEGTDLAETAPP
PLCASRNSKAKLRALREAMYHSAEHGYVDVTIDIRSIGVPWTLHTWLESLRIAFQQHRRP
LIQCLLKEFKTIQEEEYTEELVTQGLPLMFEILKASKNEVISQQLCVIFTHCYGPYPIPK
LTEIKRKQTSRLDPHFLNNKEMSDVTFLVEGRPFYAHKVLLFTASPRFKALLSSKPTNDG
TCIEIGYVKYSIFQLVMQYLYYGGPESLLIKNNEIMELLSAAKFFQLEALQRHCEIICAK
SINTDNCVDIYNHAKFLGVTELSAYCEGYFLKNMMVLIENEAFKQLLYDKNGEGTGQDVL
QDLQRTLAIRIQSIHLSSSKGSVV
>sp|P62324|BTG1_HUMAN Protein BTG1 OS=Homo sapiens GN=BTG1 PE=1 SV=1
MHPFYTRAATMIGEIAAAVSFISKFLRTKGLTSERQLQTFSQSLQELLAEHYKHHWFPEK

PCKGSGYRCIRINHKMDPLIGQAAQRIGLSSQELFRLLPSELTLWVDPYEVSYRIGEDGS
ICVLYEASPAGGSTQNSTNVQMVDSRISCKEELLLGRTSPSKNYNMMTVSG
>sp|P78543|BTG2_HUMAN Protein BTG2 OS=Homo sapiens GN=BTG2 PE=1 SV=1
MSHGKGTDMLPEIAAAVGFLSSLLRTRGCVSEQRLKVFSGALQEALTEHYKHHWFPEKPS
KGSGYRCIRINHKMDPIISRVASQIGLSQPQLHQLLPSELTLWVDPYEVSYRIGEDGSIC
VLYEEAPLAASCGLLTCKNQVLLGRSSPSKNYVMAVSS
>sp|Q9UIR0|BTNL2_HUMAN Butyrophilin-like protein 2 OS=Homo sapiens GN=BTNL2 PE=2 SV=1
MVDFPGYNLSGAVASFLFILLTMKQSEDFRVIGPAHPILAGVGEDALLTCQLLPKRTTMH
VEVRWYRSEPSTPVFVHRDGVEVTEMQMEEYRGWVEWIENGIAKGNVALKIHNIQPSDNG
QYWCHFQDGNYCGETSLLLKVAGLGSAPSIHMEGPGESGVQLVCTARGWFPEPQVYWEDI
RGEKLLAVSEHRIQDKDGLFYAEATLVVRNASAESVSCLVHNPVLTEEKGSVISLPEKLQ
TELASLKVNGPSQPILVRVGEDIQLTCYLSPKANAQSMEVRWDRSHRYPAVHVYMDGDHV
AGEQMAEYRGRTVLVSDAIDEGRLTLQILSARPSDDGQYRCLFEKDDVYQEASLDLKVVS
LGSSPLITVEGQEDGEMQPMCSSDGWFPQPHVPWRDMEGKTIPSSSQALTQGSHGLFHVQ
TLLRVTNISAVDVTCSISIPFLGEEKIATFSLSGW
>sp|Q6UX41|BTNL8_HUMAN Butyrophilin-like protein 8 OS=Homo sapiens GN=BTNL8 PE=1 SV=1
MALMLSLVLSLLKLGSGQWQVFGPDKPVQALVGEDAAFSCFLSPKTNAEAMEVRFFRGQF
SSVVHLYRDGKDQPFMQMPQYQGRTKLVKDSIAEGRISLRLENITVLDAGLYGCRISSQS
YYQKAIWELQVSALGSVPLISITGYVDRDIQLLCQSSGWFPRPTAKWKGPQGQDLSTDSR
TNRDMHGLFDVEISLTVQENAGSISCSMRHAHLSREVESRVQIGDTFFEPISWHLATKVL
GILCCGLFFGIVGLKIFFSKFQWKIQAELDWRRKHGQAELRDARKHAVEVTLDPETAHPK
LCVSDLKTVTHRKAPQEVPHSEKRFTRKSVVASQSFQAGKHYWEVDGGHNKRWRVGVCRD
DVDRRKEYVTLSPDHGYWVLRLNGEHLYFTLNPRFISVFPRTPPTKIGVFLDYECGTISF
FNINDQSLIYTLTCRFEGLLRPYIEYPSYNEQNGTPIVICPVTQESEKEASWQRASAIPE
TSNSESSSQATTPFLPRGEM
>sp|Q7Z5L3|C1QL2_HUMAN Complement C1q-like protein 2 OS=Homo sapiens GN=C1QL2 PE=2 SV=2
MALGLLIAVPLLLQAAPRGAAHYEMMGTCRMICDPYTAAPGGEPPGAKAQPPGPSTAALE
VMQDLSANPPPPFIQGPKGDPGRPGKPGPRGPPGEPGPPGPRGPPGEKGDSGRPGLPGLQ
LTAGTASGVGVVGGGAGVGGDSEGEVTSALSATFSGPKIAFYVGLKSPHEGYEVLKFDDV
VTNLGNHYDPTTGKFSCQVRGIYFFTYHILMRGGDGTSMWADLCKNGQVRASAIAQDADQ
NYDYASNSVVLHLDSGDEVYVKLDGGKAHGGNNNKYSTFSGFLLYPD
>sp|Q96MC4|C295L_HUMAN CEP295 N-terminal-like protein OS=Homo sapiens GN=CEP295NL PE=2 SV=1
MCSGWSSSVIWRHTQFAVERCGFCGSSGPGAPLEPSTLGSKHLPWEAVSAGFADRNRNMD
GAMWLSLCPDNEDLLWRKKHKLLQARGKGDLALQRRADAKLWKNYQLQRLAEELRRGYQE
AQHLHVGGLDRLQSARLLGWGGGRARENEPDSQGPIQRRSARPPRAKEKHRAALSEERSC
REELGQQHPRHSRPRKTAASPEKPQTTKATGRMNSHLAPPEKRKGRPEPSTKSGGGRCAI
HPRRSKGADLERSNPLVAAVGEIGLVEEKEKGTARAGRRQLGKGAVCFVPALTSRSQGQS
LEGKLRDLGQLWPADSSCRREAVSPASQCTLREKNKWQKELELAFEELFNINRKLKKHLC
LYLALKPRMDQRPGEGHAFSEMQECGAGTPRGKKMADPEMLPAGEPRSPAEEEAQQAASK
TDLKTFMGKAQNQKYQGTVKPTFRNGSQTLSPEAGIFINKEDSLLYSTESGQETPKLGTL
AEGSLQLHLQDQADRVGSTASRQRQKAEMEQRRQKQLESLEQMEHPDMSLEIHYKAELEK
ERREQRRARLAHLKSSSTRAQERERGSELSTTSPSGTSLADDDRHSQMIRDQQQQILQQN
RLHKQFLEEARKCLREFQNIC

>sp|Q8TF44|C2C4C_HUMAN C2 calcium-dependent domain-containing protein 4C OS=Homo sapiens GN=C2CD4C PE=1 SV=2
MRKTNMWFLERLRGSGENGAARGVGSEAGDKASKGPLYSNVLTPDKIPDFFIPPKLPSGP
AEGEGQAALGPSTSEQNLASAAPRQTPRSPRLPAKLAAESKSLLKAATRHVIQIESAEDW
LSEEATDADPQAQGAMSLPSVPKAQTSYGFAMLAESPHTRRKESLFHSEHGALAQVGSPG
AGRRRAAAKANGGDGGPREAGGALMSPGRYFSGGESDTGSSAESSPFGSPLLSRSVSLLK
GFAQDSQAKVSQLRHSVGRHGSLSADDSTPDASPGSRRRLTRRAPPEPGPESGQARGEHT
VHVGPRGSVRLLAEYEAGQARLRVHLLAAEGLYDRLCDARSINCCVGLCLVPGKLQKQRS
TIVKNSRRPVFNEDFFFDGLGPASVRKLALRIKVVNKGSSLKRDTLLGEKELPLTSLLPF
L
>sp|Q4AC94|C2CD3_HUMAN C2 domain-containing protein 3 OS=Homo sapiens GN=C2CD3 PE=1 SV=4
MKQRKGQGSGGSRGRKKRGLSDISPSTSLPPLVEGQLRCFLKLTVNRVIWKIAKPPTCVL
VRVRWWGETSDGTLFCPRDALQTEPKAVRTTTRYAIRCGPKQFTSYLTDMAVLVLEVITK
LDGLPIGRVQINGLAQLSPTHQINGFFTIVSSTSKKLGELQVSLALEPLSETYDSYHPLP
TTDMTENVLLSKQGFRENTEPSSTQFQVPSRPRDIHTIKIDGKELAANSSRSTTPRGKDH
VCFAENPDTIKDSSFGLQHSLNSGQSLESVTLKGRAPRKQMSLLNSSEFQPQIRTVAKSH
SDSCILSSNNLPTKDLLSALLEQGNKLRNAMVISAMKSSPETSMLLDQVHPPINEDSLRA
STQIRAFSRNRFKDHIEDHLLPSTENTFWRHDTKADTRAIQLLLGSAELSQGNFWDGLGS
PPDSPSPGSDVYCISELNDPQYDQSLLENLFYTAPKSDTSISDFLSEEDDIVPSKKISQS
TALARSSKVLESSDHKLKKRSAGKRNRNLVEQQMLSETPEDAQTMTLSVDRLALLGRTHS
VRIIIETMGVPPDSPQMTPGKKSYAGPPPKVTTAKKRTFFVEYHFPVGFSESGLGKTALI
TEVVRLASSKITDGKVKFQQRFVFPVQFGGPMIEHWWNSNLTFQIYVKKTPQKKPEVIGS
VSLSLRAVIQSELLSFSDQLPVQQENGQSPFGPLKVTMELITDNKDFTGINTKLSGNTHY
TPLCAPTSPNKALPELNQDMTCTKNPQNLNQIHEETAKKAQNLVLPNRKSPSPVAPHPST
FVATPASHNLVNQTNGTTKESALLLHVLLMVPDGKDFISGESEKQSPCNVYLNCKLFSTE
EVTRSVIAWGTTQPVFNFSQVIPVSLSSKYLERLKNNVMVIETWNKVRSPGQDKLLGLVK
LPLHQFYMSFKDAKISRLLLDAQYPVVAVDSYMPVIDVFSGHQNGSLRVFLAMGSSNQIM
ALQRLKNEEGTLPPFSPRPAHFLDQPTAASVAMAEDRGNGLMEHCFEIHIEMVKGLAPLQ
ATVWGEADCYVQYYFPVQHSQSSVLKGPEFLENGITLKPFRTATTLCVPDPIFNSEHHHS
LLLPAEVPVQRLLLSAFSAQGLVPGGGVQFEIWCRYYYPNVRDQKVAKGTLPLSRICAMV
TTQHREDVGIQTFNLPLTPRIENRKELRNQSSGLLDVGLRYRRSPRTAEGVLAARTVSIS
VQIIRACGLQAAAKALAEREPALQFSATVGVNASVTTHLSFLPQGEQRRTHPVACSFCPE
FSHHVEFTCNLVTQHCSGEACFLAELLEFAEVIFAVYHENTKSASDIISIESCKEYLLGV
VKVPTKELLIKRSGITGWYPIILPEDGGLPHGLELMQKIVGGLELSISFTHRGDRERVLE
AAEHLGWSFENSLKDFVRMDEGEPATVTISTPRLWLPIHCVLLAGHNHIHKNTYCYLRYK
FYDHEAFWTPLKKPKESVNKKQIMVTFKASKRAEVTRGPSLLWYFREERLEIQVWRAYGN
DSVERPHQTDSWIGSAYVDLARLGERSARTLTVSGVYPLFGRNASNLSGAALRVHVVLSS
LSSHLEPTHELDSMDCSSHSESEQLPRRNDEVQLSPPEVISCHQKSPASTQVPCSSTTAE
VRLTQEGPADLDGTFAVSILVERAMHLSLKGSPLTERKVSIPSCCVSFATADESSPVYTQ
VVENTDSPIWNFQQQSRLSKELLLDPQQTLVFKVWHKGDEERVIGFASVDLSPLLSGFQF
VCGWYNITDFSGECQGQIKVAVSPLESLIHFKEERQARRGVETSKSLIPIYSPFSFPASD
TYAAFSSHMARQTLDQLAHASSKELDFSSPGRSDTTRSQASRHEEHVQNIRRFHESLHLQ
GEAPLPCDDKLTTSPLSSQTSILTSLRKNLSELDQIQRYFRQKLTKPFLPLSPQTQTAIS
QHQESCRDHLGPGASSLDPGSQCILEKSSNLVLQVSSLITDLQTITRDSQAALSSHRARS

RSNKATTLPDAQDTEALQERCTMPDEPLVRAPDKGTDSPSPPPLEETSNGGRMLHESLRH
AVPITRMQSSEDTEAGPAYSDEDYEEDIIEPRTLNEITTVTDKTSPWSSVISDTSEVISP
QPDEVQREGPSCPSPGPFCREELMVKSSFLSSPERAVNPHLPRQGSPSQSLVACECEASK
ARVGGESASANPQPIPCPTLSGAQQSSTFVGWSSPQTDQNKEPKSEAPAENEAATSELGD
SADSFKKLPLNLASQSRRENHKGPPIDSSDIRQRQVTTGSETSTKQSLLLPGPIVVPNFF
LPPQQLEASLRMLSLSATLPPAATTDQDKSEATRGALSQRPCRPRPNSLPLNLPEEETLR
IARIFSSQYSQKD
>sp|B7Z1M9|C2D4D_HUMAN C2 calcium-dependent domain-containing protein 4D OS=Homo sapiens GN=C2CD4D PE=2 SV=2
MWLLEKAGYKVGAAEPAARWAPSGLFSKRRAPGPPTSACPNVLTPDRIPQFFIPPRLPDP
GGAVPAARRHVAGRGLPATCSLPHLAGREGWAFLPESPHTRRRESLFHGPPPAPAGGLPA
AQSRLHVSAPDLRLCRAPDSDTASSPDSSPFGSPRPGLGRRRVSRPHSLSPEKASSADTS
PHSPRRAGPPTPPLFHLDFLCCQLRPTRESVLRLGPRGGQLRLSTEYQAGPGRLRLRLVS
AEGLPRPRSRPGSGGGGCCVVLRLRPRVRPREQQSRVVKCSANPIFNEDFFFDGLGPPDL
AARSLRAKVLDRGAGLRRDVLLGECETPLIALLPPLGGGLGPGSSLAPTHLSL
>sp|Q9H0H9|C4F30_HUMAN Putative cytochrome P450 family member 4F30 OS=Homo sapiens GN=CYP4F30P PE=5 SV=1
MVTPAGCLGGRNQGPREIPGTAFPCSSRAGQTGQAVSGAQVSSWRERQPFGGSRGPLHIL
GTDGNVDTTGKLGLVPTPPRIQKETKQGALCGMKPPFLPEALLTVWWLPFVAVSLCLF
>sp|Q8N8Q1|C56D1_HUMAN Cytochrome b561 domain-containing protein 1 OS=Homo sapiens GN=CYB561D1 PE=2 SV=1
MQPLEVGLVPAPAGEPRLTRWLRRGSGILAHLVALGFTIFLTALSRPGTSLFSWHPVFMA
LAFCLCMAEAILLFSPEHSLFFFCSRKARIRLHWAGQTLAILCAALGLGFIISSRTRSEL
PHLVSWHSWVGALTLLATAVQALCGLCLLCPRAARVSRVARLKLYHLTCGLVVYLMATVT
VLLGMYSVWFQAQIKGAAWYLCLALPVYPALVIMHQISRSYLPRKKMEM
>sp|Q9P296|C5AR2_HUMAN C5a anaphylatoxin chemotactic receptor 2 OS=Homo sapiens GN=C5AR2 PE=1 SV=1
MGNDSVSYEYGDYSDLSDRPVDCLDGACLAIDPLRVAPLPLYAAIFLVGVPGNAMVAWVA
GKVARRRVGATWLLHLAVADLLCCLSLPILAVPIARGGHWPYGAVGCRALPSIILLTMYA
SVLLLAALSADLCFLALGPAWWSTVQRACGVQVACGAAWTLALLLTVPSAIYRRLHQEHF
PARLQCVVDYGGSSSTENAVTAIRFLFGFLGPLVAVASCHSALLCWAARRCRPLGTAIVV
GFFVCWAPYHLLGLVLTVAAPNSALLARALRAEPLIVGLALAHSCLNPMLFLYFGRAQLR
RSLPAACHWALRESQGQDESVDSKKSTSHDLVSEMEV
>sp|Q13698|CAC1S_HUMAN Voltage-dependent L-type calcium channel subunit alpha-1S OS=Homo sapiens GN=CACNA1S PE=1 SV=4
MEPSSPQDEGLRKKQPKKPVPEILPRPPRALFCLTLENPLRKACISIVEWKPFETIILLT
IFANCVALAVYLPMPEDDNNSLNLGLEKLEYFFLIVFSIEAAMKIIAYGFLFHQDAYLRS
GWNVLDFTIVFLGVFTVILEQVNVIQSHTAPMSSKGAGLDVKALRAFRVLRPLRLVSGVP
SLQVVLNSIFKAMLPLFHIALLVLFMVIIYAIIGLELFKGKMHKTCYFIGTDIVATVENE
EPSPCARTGSGRRCTINGSECRGGWPGPNHGITHFDNFGFSMLTVYQCITMEGWTDVLYW
VNDAIGNEWPWIYFVTLILLGSFFILNLVLGVLSGEFTKEREKAKSRGTFQKLREKQQLD
EDLRGYMSWITQGEVMDVEDFREGKLSLDEGGSDTESLYEIAGLNKIIQFIRHWRQWNRI
FRWKCHDIVKSKVFYWLVILIVALNTLSIASEHHNQPLWLTRLQDIANRVLLSLFTTEML
MKMYGLGLRQYFMSIFNRFDCFVVCSGILEILLVESGAMTPLGISVLRCIRLLRIFKITK

YWTSLSNLVASLLNSIRSIASLLLLLFLFIVIFALLGMQLFGGRYDFEDTEVRRSNFDNF
PQALISVFQVLTGEDWTSMMYNGIMAYGGPSYPGMLVCIYFIILFVCGNYILLNVFLAIA
VDNLAEAESLTSAQKAKAEEKKRRKMSKGLPDKSEEEKSTMAKKLEQKPKGEGIPTTAKL
KIDEFESNVNEVKDPYPSADFPGDDEEDEPEIPLSPRPRPLAELQLKEKAVPIPEASSFF
IFSPTNKIRVLCHRIVNATWFTNFILLFILLSSAALAAEDPIRADSMRNQILKHFDIGFT
SVFTVEIVLKMTTYGAFLHKGSFCRNYFNMLDLLVVAVSLISMGLESSAISVVKILRVLR
VLRPLRAINRAKGLKHVVQCMFVAISTIGNIVLVTTLLQFMFACIGVQLFKGKFFRCTDL
SKMTEEECRGYYYVYKDGDPMQIELRHREWVHSDFHFDNVLSAMMSLFTVSTFEGWPQLL
YKAIDSNAEDVGPIYNNRVEMAIFFIIYIILIAFFMMNIFVGFVIVTFQEQGETEYKNCE
LDKNQRQCVQYALKARPLRCYIPKNPYQYQVWYIVTSSYFEYLMFALIMLNTICLGMQHY
NQSEQMNHISDILNVAFTIIFTLEMILKLMAFKARGYFGDPWNVFDFLIVIGSIIDVILS
EIDTFLASSGGLYCLGGGCGNVDPDESARISSAFFRLFRVMRLIKLLSRAEGVRTLLWTF
IKSFQALPYVALLIVMLFFIYAVIGMQMFGKIALVDGTQINRNNNFQTFPQAVLLLFRCA
TGEAWQEILLACSYGKLCDPESDYAPGEEYTCGTNFAYYYFISFYMLCAFLVINLFVAVI
MDNFDYLTRDWSILGPHHLDEFKAIWAEYDPEAKGRIKHLDVVTLLRRIQPPLGFGKFCP
HRVACKRLVGMNMPLNSDGTVTFNATLFALVRTALKIKTEGNFEQANEELRAIIKKIWKR
TSMKLLDQVIPPIGDDEVTVGKFYATFLIQEHFRKFMKRQEEYYGYRPKKDIVQIQAGLR
TIEEEAAPEICRTVSGDLAAEEELERAMVEAAMEEGIFRRTGGLFGQVDNFLERTNSLPP
VMANQRPLQFAEIEMEEMESPVFLEDFPQDPRTNPLARANTNNANANVAYGNSNHSNSHV
FSSVHYEREFPEETETPATRGRALGQPCRVLGPHSKPCVEMLKGLLTQRAMPRGQAPPAP
CQCPRVESSMPEDRKSSTPGSLHEETPHSRSTRENTSRCSAPATALLIQKALVRGGLGTL
AADANFIMATGQALADACQMEPEEVEIMATELLKGREAPEGMASSLGCLNLGSSLGSLDQ
HQGSQETLIPPRL
>sp|Q02641|CACB1_HUMAN Voltage-dependent L-type calcium channel subunit beta-1 OS=Homo sapiens GN=CACNB1 PE=2 SV=3
MVQKTSMSRGPYPPSQEIPMEVFDPSPQGKYSKRKGRFKRSDGSTSSDTTSNSFVRQGSA
ESYTSRPSDSDVSLEEDREALRKEAERQALAQLEKAKTKPVAFAVRTNVGYNPSPGDEVP
VQGVAITFEPKDFLHIKEKYNNDWWIGRLVKEGCEVGFIPSPVKLDSLRLLQEQKLRQNR
LGSSKSGDNSSSSLGDVVTGTRRPTPPASAKQKQKSTEHVPPYDVVPSMRPIILVGPSLK
GYEVTDMMQKALFDFLKHRFDGRISITRVTADISLAKRSVLNNPSKHIIIERSNTRSSLA
EVQSEIERIFELARTLQLVALDADTINHPAQLSKTSLAPIIVYIKITSPKVLQRLIKSRG
KSQSKHLNVQIAASEKLAQCPPEMFDIILDENQLEDACEHLAEYLEAYWKATHPPSSTPP
NPLLNRTMATAALAASPAPVSNLQGPYLASGDQPLERATGEHASMHEYPGELGQPPGLYP
SSHPPGRAGTLRALSRQDTFDADTPGSRNSAYTELGDSCVDMETDPSEGPGLGDPAGGGT
PPARQGSWEDEEEDYEEELTDNRNRGRNKARYCAEGGGPVLGRNKNELEGWGRGVYIR
>sp|P54284|CACB3_HUMAN Voltage-dependent L-type calcium channel subunit beta-3 OS=Homo sapiens GN=CACNB3 PE=1 SV=1
MYDDSYVPGFEDSEAGSADSYTSRPSLDSDVSLEEDRESARREVESQAQQQLERAKHKPV
AFAVRTNVSYCGVLDEECPVQGSGVNFEAKDFLHIKEKYSNDWWIGRLVKEGGDIAFIPS
PQRLESIRLKQEQKARRSGNPSSLSDIGNRRSPPPSLAKQKQKQAEHVPPYDVVPSMRPV
VLVGPSLKGYEVTDMMQKALFDFLKHRFDGRISITRVTADLSLAKRSVLNNPGKRTIIER
SSARSSIAEVQSEIERIFELAKSLQLVVLDADTINHPAQLAKTSLAPIIVFVKVSSPKVL
QRLIRSRGKSQMKHLTVQMMAYDKLVQCPPESFDVILDENQLEDACEHLAEYLEVYWRAT
HHPAPGPGLLGPPSAIPGLQNQQLLGERGEEHSPLERDSLMPSDEASESSRQAWTGSSQR

SSRHLEEDYADAYQDLYQPHRQHTSGLPSANGHDPQDRLLAQDSEHNHSDRNWQRNRPWP
KDSY
>sp|P55287|CAD11_HUMAN Cadherin-11 OS=Homo sapiens GN=CDH11 PE=2 SV=2
MKENYCLQAALVCLGMLCHSHAFAPERRGHLRPSFHGHHEKGKEGQVLQRSKRGWVWNQF
FVIEEYTGPDPVLVGRLHSDIDSGDGNIKYILSGEGAGTIFVIDDKSGNIHATKTLDREE
RAQYTLMAQAVDRDTNRPLEPPSEFIVKVQDINDNPPEFLHETYHANVPERSNVGTSVIQ
VTASDADDPTYGNSAKLVYSILEGQPYFSVEAQTGIIRTALPNMDREAKEEYHVVIQAKD
MGGHMGGLSGTTKVTITLTDVNDNPPKFPQSVYQMSVSEAAVPGEEVGRVKAKDPDIGEN
GLVTYNIVDGDGMESFEITTDYETQEGVIKLKKPVDFETKRAYSLKVEAANVHIDPKFIS
NGPFKDTVTVKISVEDADEPPMFLAPSYIHEVQENAAAGTVVGRVHAKDPDAANSPIRYS
IDRHTDLDRFFTINPEDGFIKTTKPLDREETAWLNITVFAAEIHNRHQEAKVPVAIRVLD
VNDNAPKFAAPYEGFICESDQTKPLSNQPIVTISADDKDDTANGPRFIFSLPPEIIHNPN
FTVRDNRDNTAGVYARRGGFSRQKQDLYLLPIVISDGGIPPMSSTNTLTIKVCGCDVNGA
LLSCNAEAYILNAGLSTGALIAILACIVILLVIVVLFVTLRRQKKEPLIVFEEEDVRENI
ITYDDEGGGEEDTEAFDIATLQNPDGINGFIPRKDIKPEYQYMPRPGLRPAPNSVDVDDF
INTRIQEADNDPTAPPYDSIQIYGYEGRGSVAGSLSSLESATTDSDLDYDYLQNWGPRFK
KLADLYGSKDTFDDDS
>sp|Q13634|CAD18_HUMAN Cadherin-18 OS=Homo sapiens GN=CDH18 PE=2 SV=1
MKITSTSCICPVLVCLCFVQRCYGTAHHSSIKVMRNQTKHIEGETEVHHRPKRGWVWNQF
FVLEEHMGPDPQYVGKLHSNSDKGDGSVKYILTGEGAGTIFIIDDTTGDIHSTKSLDREQ
KTHYVLHAQAIDRRTNKPLEPESEFIIKVQDINDNAPKFTDGPYIVTVPEMSDMGTSVLQ
VTATDADDPTYGNSARVVYSILQGQPYFSVDPKTGVIRTALHNMDREAREHYSVVIQAKD
MAGQVGGLSGSTTVNITLTDVNDNPPRFPQKHYQLYVPESAQVGSAVGKIKANDADTGSN
ADMTYSIINGDGMGIFSISTDKETREGILSLKKPLNYEKKKSYTLNIEGANTHLDFRFSH
LGPFKDATMLKIIVGDVDEPPLFSMPSYLMEVYENAKIGTVVGTVLAQDPDSTNSLVRYF
INYNVEDDRFFNIDANTGTIRTTKVLDREETPWYNITVTASEIDNPDLLSHVTVGIRVLD
VNDNPPELAREYDIIVCENSKPGQVIHTISATDKDDFANGPRFNFFLDERLPVNPNFTLK
DNEDNTASILTRRRRFSRTVQDVYYLPIMISDGGIPSLSSSSTLTIRVCACERDGRVRTC
HAEAFLSSAGLSTGALIAILLCVLILLAIVVLFITLRRSKKEPLIISEEDVRENVVTYDD
EGGGEEDTEAFDITALRNPSAAEELKYRRDIRPEVKLTPRHQTSSTLESIDVQEFIKQRL
AEADLDPSVPPYDSLQTYAYEGQRSEAGSISSLDSATTQSDQDYHYLGDWGPEFKKLAEL
YGEIESERTT
>sp|Q9H251|CAD23_HUMAN Cadherin-23 OS=Homo sapiens GN=CDH23 PE=1 SV=2
MGRHVATSCHVAWLLVLISGCWGQVNRLPFFTNHFFDTYLLISEDTPVGSSVTQLLAQDM
DNDPLVFGVSGEEASRFFAVEPDTGVVWLRQPLDRETKSEFTVEFSVSDHQGVITRKVNI
QVGDVNDNAPTFHNQPYSVRIPENTPVGTPIFIVNATDPDLGAGGSVLYSFQPPSQFFAI
DSARGIVTVIRELDYETTQAYQLTVNATDQDKTRPLSTLANLAIIITDVQDMDPIFINLP
YSTNIYEHSPPGTTVRIITAIDQDKGRPRGIGYTIVSGNTNSIFALDYISGVLTLNGLLD
RENPLYSHGFILTVKGTELNDDRTPSDATVTTTFNILVIDINDNAPEFNSSEYSVAITEL
AQVGFALPLFIQVVDKDENLGLNSMFEVYLVGNNSHHFIISPTSVQGKADIRIRVAIPLD
YETVDRYDFDLFANESVPDHVGYAKVKITLINENDNRPIFSQPLYNISLYENVTVGTSVL
TVLATDNDAGTFGEVSYFFSDDPDRFSLDKDTGLIMLIARLDYELIQRFTLTIIARDGGG
EETTGRVRINVLDVNDNVPTFQKDAYVGALRENEPSVTQLVRLRATDEDSPPNNQITYSI
VSASAFGSYFDISLYEGYGVISVSRPLDYEQISNGLIYLTVMAMDAGNPPLNSTVPVTIE

VFDENDNPPTFSKPAYFVSVVENIMAGATVLFLNATDLDRSREYGQESIIYSLEGSTQFR
INARSGEITTTSLLDRETKSEYILIVRAVDGGVGHNQKTGIATVNITLLDINDNHPTWKD
APYYINLVEMTPPDSDVTTVVAVDPDLGENGTLVYSIQPPNKFYSLNSTTGKIRTTHAML
DRENPDPHEAELMRKIVVSVTDCGRPPLKATSSATVFVNLLDLNDNDPTFQNLPFVAEVL
EGIPAGVSIYQVVAIDLDEGLNGLVSYRMPVGMPRMDFLINSSSGVVVTTTELDRERIAE
YQLRVVASDAGTPTKSSTSTLTIHVLDVNDETPTFFPAVYNVSVSEDVPREFRVVWLNCT
DNDVGLNAELSYFITGGNVDGKFSVGYRDAVVRTVVGLDRETTAAYMLILEAIDNGPVGK
RHTGTATVFVTVLDVNDNRPIFLQSSYEASVPEDIPEGHSILQLKATDADEGEFGRVWYR
ILHGNHGNNFRIHVSNGLLMRGPRPLDRERNSSHVLIVEAYNHDLGPMRSSVRVIVYVED
INDEAPVFTQQQYSRLGLRETAGIGTSVIVVQATDRDSGDGGLVNYRILSGAEGKFEIDE
STGLIITVNYLDYETKTSYMMNVSATDQAPPFNQGFCSVYITLLNELDEAVQFSNASYEA
AILENLALGTEIVRVQAYSIDNLNQITYRFNAYTSTQAKALFKIDAITGVITVQGLVDRE
KGDFYTLTVVADDGGPKVDSTVKVYITVLDENDNSPRFDFTSDSAVSIPEDCPVGQRVAT
VKAWDPDAGSNGQVVFSLASGNIAGAFEIVTTNDSIGEVFVARPLDREELDHYILQVVAS
DRGTPPRKKDHILQVTILDINDNPPVIESPFGYNVSVNENVGGGTAVVQVRATDRDIGIN
SVLSYYITEGNKDMAFRMDRISGEIATRPAPPDRERQSFYHLVATVEDEGTPTLSATTHV
YVTIVDENDNAPMFQQPHYEVLLDEGPDTLNTSLITIQALDLDEGPNGTVTYAIVAGNIV
NTFRIDRHMGVITAAKELDYEISHGRYTLIVTATDQCPILSHRLTSTTTVLVNVNDINDN
VPTFPRDYEGPFEVTEGQPGPRVWTFLAHDRDSGPNGQVEYSIMDGDPLGEFVISPVEGV
LRVRKDVELDRETIAFYNLTICARDRGMPPLSSTMLVGIRVLDINDNDPVLLNLPMNITI
SENSPVSSFVAHVLASDADSGCNARLTFNITAGNRERAFFINATTGIVTVNRPLDRERIP
EYKLTISVKDNPENPRIARRDYDLLLIFLSDENDNHPLFTKSTYQAEVMENSPAGTPLTV
LNGPILALDADQDIYAVVTYQLLGAQSGLFDINSSTGVVTVRSGVIIDREAFSPPILELL
LLAEDIGLLNSTAHLLITILDDNDNRPTFSPATLTVHLLENCPPGFSVLQVTATDEDSGL
NGELVYRIEAGAQDRFLIHLVTGVIRVGNATIDREEQESYRLTVVATDRGTVPLSGTAIV
TILIDDINDSRPEFLNPIQTVSVLESAEPGTVIANITAIDHDLNPKLEYHIVGIVAKDDT
DRLVPNQEDAFAVNINTGSVMVKSPMNRELVATYEVTLSVIDNASDLPERSVSVPNAKLT
VNVLDVNDNTPQFKPFGITYYMERILEGATPGTTLIAVAAVDPDKGLNGLVTYTLLDLVP
PGYVQLEDSSAGKVIANRTVDYEEVHWLNFTVRASDNGSPPRAAEIPVYLEIVDINDNNP
IFDQPSYQEAVFEDVPVGTIILTVTATDADSGNFALIEYSLGDGESKFAINPTTGDIYVL
SSLDREKKDHYILTALAKDNPGDVASNRRENSVQVVIQVLDVNDCRPQFSKPQFSTSVYE
NEPAGTSVITMMATDQDEGPNGELTYSLEGPGVEAFHVDMDSGLVTTQRPLQSYEKFSLT
VVATDGGEPPLWGTTMLLVEVIDVNDNRPVFVRPPNGTILHIREEIPLRSNVYEVYATDK
DEGLNGAVRYSFLKTAGNRDWEFFIIDPISGLIQTAQRLDRESQAVYSLILVASDLGQPV
PYETMQPLQVALEDIDDNEPLFVRPPKGSPQYQLLTVPEHSPRGTLVGNVTGAVDADEGP
NAIVYYFIAAGNEEKNFHLQPDGCLLVLRDLDREREAIFSFIVKASSNRSWTPPRGPSPT
LDLVADLTLQEVRVVLEDINDQPPRFTKAEYTAGVATDAKVGSELIQVLALDADIGNNSL
VFYSILAIHYFRALANDSEDVGQVFTMGSMDGILRTFDLFMAYSPGYFVVDIVARDLAGH
NDTAIIGIYILRDDQRVKIVINEIPDRVRGFEEEFIHLLSNITGAIVNTDNVQFHVDKKG
RVNFAQTELLIHVVNRDTNRILDVDRVIQMIDENKEQLRNLFRNYNVLDVQPAISVRLPD
DMSALQMAIIVLAILLFLAAMLFVLMNWYYRTVHKRKLKAIVAGSAGNRGFIDIMDMPNT
NKYSFDGANPVWLDPFCRNLELAAQAEHEDDLPENLSEIADLWNSPTRTHGTFGREPAAV
KPDDDRYLRAAIQEYDNIAKLGQIIREGPIKGSLLKVVLEDYLRLKKLFAQRMVQKASSC
HSSISELIQTELDEEPGDHSPGQGSLRFRHKPPVELKGPDGIHVVHGSTGTLLATDLNSL

PEEDQKGLGRSLETLTAAEATAFERNARTESAKSTPLHKLRDVIMETPLEITEL
>sp|Q86UP0|CAD24_HUMAN Cadherin-24 OS=Homo sapiens GN=CDH24 PE=1 SV=1
MWGLVRLLLAWLGGWGCMGRLAAPARAWAGSREHPGPALLRTRRSWVWNQFFVIEEYAGP
EPVLIGKLHSDVDRGEGRTKYLLTGEGAGTVFVIDEATGNIHVTKSLDREEKAQYVLLAQ
AVDRASNRPLEPPSEFIIKVQDINDNPPIFPLGPYHATVPEMSNVGTSVIQVTAHDADDP
SYGNSAKLVYTVLDGLPFFSVDPQTGVVRTAIPNMDRETQEEFLVVIQAKDMGGHMGGLS
GSTTVTVTLSDVNDNPPKFPQSLYQFSVVETAGPGTLVGRLRAQDPDLGDNALMAYSILD
GEGSEAFSISTDLQGRDGLLTVRKPLDFESQRSYSFRVEATNTLIDPAYLRRGPFKDVAS
VRVAVQDAPEPPAFTQAAYHLTVPENKAPGTLVGQISAADLDSPASPIRYSILPHSDPER
CFSIQPEEGTIHTAAPLDREARAWHNLTVLATELGWSWGPERGWVPLLVAEWSAPAAPPQ
RSPVGSAVGIPQDSSAQASRVQVAIQTLDENDNAPQLAEPYDTFVCDSAAPGQLIQVIRA
LDRDEVGNSSHVSFQGPLGPDANFTVQDNRDGSASLLLPSRPAPPRHAPYLVPIELWDWG
QPALSSTATVTVSVCRCQPDGSVASCWPEAHLSAAGLSTGALLAIITCVGALLALVVLFV
ALRRQKQEALMVLEEEDVRENIITYDDEGGGEEDTEAFDITALQNPDGAAPPAPGPPARR
DVLPRARVSRQPRPPGPADVAQLLALRLREADEDPGVPPYDSVQVYGYEGRGSSCGSLSS
LGSGSEAGGAPGPAEPLDDWGPLFRTLAELYGAKEPPAP
>sp|Q8IXH8|CAD26_HUMAN Cadherin-like protein 26 OS=Homo sapiens GN=CDH26 PE=2 SV=3
MAMRSGRHPSLLLLLVLLLWLLQVSIIDSVQQETDDLTKQTKEKIYQPLRRSKRRWVITT
LELEEEDPGPFPKLIGELFNNMSYNMSLMYLISGPGVDEYPEIGLFSLEDHENGRIYVHR
PVDREMTPSFTVYFDVVERSTGKIVDTSLIFNIRISDVNDHAPQFPEKEFNITVQENQSA
GQPIFQMLAVDLDEENTPNSQVLYFLISQTPLLKESGFRVDRLSGEIRLSGCLDYETAPQ
FTLLIRARDCGEPSLSSTTTVHVDVQEGNNHRPAFTQENYKVQIPEGRASQGVLRLLVQD
RDSPFTSAWRAKFNILHGNEEGHFDISTDPETNEGILNVIKPLDYETRPAQSLIIVVENE
ERLVFCERGKLQPPRKAAASATVSVQVTDANDPPAFHPQSFIVNKEEGARPGTLLGTFNA
MDPDSQIRYELVHDPANWVSVDKNSGVVITVEPIDRESPHVNNSFYVIIIHAVDDGFPPQ
TATGTLMLFLSDINDNVPTLRPRSRYMEVCESAVHEPLHIEAEDPDLEPFSDPFTFELDN
TWGNAEDTWKLGRNWGQSVELLTLRSLPRGNYLVPLFIGDKQGLSQKQTVHVRICPCASG
LTCVELADAEVGLHVGALFPVCAAFVALAVALLFLLRCYFVLEPKRHGCSVSNDEGHQTL
VMYNAESKGTSAQTWSDVEGQRPALLICTAAAGPTQGVKAYPDATMHRQLLAPVEGRMAE
TLNQSKERNRFSLSRGCIIPQGRATAGRGLPQDIYKEMMPRRLTQTGKRKHGALARTPSF
KKVVYDHKEDEENKAGRKQRSHLFKVMQLRNEQGGVRVQSAHSPSPLNKKACFPGDYRGE
SAGGHNCRAVSG
>sp|Q8N126|CADM3_HUMAN Cell adhesion molecule 3 OS=Homo sapiens GN=CADM3 PE=1 SV=1
MGAPAASLLLLLLLFACCWAPGGANLSQDDSQPWTSDETVVAGGTVVLKCQVKDHEDSSL
QWSNPAQQTLYFGEKRALRDNRIQLVTSTPHELSISISNVALADEGEYTCSIFTMPVRTA
KSLVTVLGIPQKPIITGYKSSLREKDTATLNCQSSGSKPAARLTWRKGDQELHGEPTRIQ
EDPNGKTFTVSSSVTFQVTREDDGASIVCSVNHESLKGADRSTSQRIEVLYTPTAMIRPD
PPHPREGQKLLLHCEGRGNPVPQQYLWEKEGSVPPLKMTQESALIFPFLNKSDSGTYGCT
ATSNMGSYKAYYTLNVNDPSPVPSSSSTYHAIIGGIVAFIVFLLLIMLIFLGHYLIRHKG
TYLTHEAKGSDDAPDADTAIINAEGGQSGGDDKKEYFI
>sp|Q5NE16|CATL3_HUMAN Putative inactive cathepsin L-like protein CTSL3P OS=Homo sapiens GN=CTSL3P PE=5 SV=1
MKMIEQHNQEYREGKHSFTMAMNAFGEMTSEEFRQVVNGFQNQKHRKGKVLQEPLLHDIR
KSVDWREKGYVTPVKDQCNWGSVRTDVRKTEKLVSLSVQTWWTALGFKAMLAAFLENHYF

ASSMLPTMEAWTLRKPFHMKKSSGDWKVQGHRGASGESLLASGESQQSPEVAQYSGKHQV
QCHLIEEALQMLSGGDEDHDEDKWPHDMRNHLAGEAQV
>sp|A6NM15|CBWD7_HUMAN Putative COBW domain-containing protein 7 OS=Homo sapiens GN=CBWD7 PE=5 SV=3
MFWVDAELGSDIYLDGIITIVDSKYGLKHLTEEKPDGLINEATRQVALADIILINKTDLV
PEEDVKKLRTTIRSINGLGQILETQRSRVDLSNVLDLHAFDSLSGISLQKKLQHVPGTQP
HLDQSIVTITFEVPGNAKEEHLNMFIQNLLWEKNVRNKDNHCMEVIRLKGLVSIKDKSQQ
VIVQGVHELYDLEETPVSWKDDTERTNRLVLIGRNLDKDILKQLFIATVTETEKQWTTHF
KEDQVCT
>sp|P83916|CBX1_HUMAN Chromobox protein homolog 1 OS=Homo sapiens GN=CBX1 PE=1 SV=1
MGKKQNKKKVEEVLEEEEEEYVVEKVLDRRVVKGKVEYLLKWKGFSDEDNTWEPEENLDC
PDLIAEFLQSQKTAHETDKSEGGKRKADSDSEDKGEESKPKKKKEESEKPRGFARGLEPE
RIIGATDSSGELMFLMKWKNSDEADLVPAKEANVKCPQVVISFYEERLTWHSYPSEDDDK
KDDKN
>sp|P45973|CBX5_HUMAN Chromobox protein homolog 5 OS=Homo sapiens GN=CBX5 PE=1 SV=1
MGKKTKRTADSSSSEDEEEYVVEKVLDRRVVKGQVEYLLKWKGFSEEHNTWEPEKNLDCP
ELISEFMKKYKKMKEGENNKPREKSESNKRKSNFSNSADDIKSKKKREQSNDIARGFERG
LEPEKIIGATDSCGDLMFLMKWKDTDEADLVLAKEANVKCPQIVIAFYEERLTWHAYPED
AENKEKETAKS
>sp|Q6TFL3|CC171_HUMAN Coiled-coil domain-containing protein 171 OS=Homo sapiens GN=CCDC171 PE=2 SV=1
MNLNTSSNTGDTQRLKIASLDVKQILKNETELDITDNLRKKLHWAKKEKLEITTKHNAEL
ASYESQIAKLRSEVEKGEALRQSLEYDLAVARKEAGLGRRAAEERLAEAHRIQEKLCAQN
SELQAKTNETEKAFQTSQQKWKEECRRFEHDLEERDNMIQNCNREYDLLMKEKSRLEKTL
QEALEKHQREKNEMESHIRETALEEFRLQEEQWEAERRELQFIVQEQDTAVQNMHKKVEK
LETEHMDCSDLLRRQTSELEFSTQREERLRKEFEATTLRVRKLEENIEAERAAHLESKFN
SEIIQLRIRDLEGALQVEKASQAEAVADLEIIKNEFKEVESAYEREKHNAQESFAKLNLL
EKEYFSKNKKLNEDIEEQKKVIIDLSKRLQYNEKSCSELQEELVMAKKHQAFLVETCENN
VKELESILDSFTVSGQWTSGIHKDKDKPPSFSVVLERLRRTLTDYQNKLEDASNEEKACN
ELDSTKQKIDSHTKNIKELQDKLADVNKELSHLHTKCADREALISTLKVELQNVLHCWEK
EKAQAAQSESELQKLSQAFHKDAEEKLTFLHTLYQHLVAGCVLIKQPEGMLDKFSWSELC
AVLQENVDALIADLNRANEKIRHLEYICKNKSDTMRELQQTQEDTFTKVAEQIKAQESCW
HRQKKELELQYSELFLEVQKRAQKFQEIAEKNMEKLNHIEKSHEQLVLENSHFKKLLSQT
QREQMSLLAACALMAGALYPLYSRSCALSTQRDFLQEQVNTFELFKLEIRTLAQALSTVE
EKKQEEAKMKKKTFKGLIRIFRKGVIAVLAANRLKILGQSCASLFTWMESFKEGIGMLVC
TGEPQDKHKFPKHQKEQLRCLQALSWLTSSDLLAAIISSMAELQDVIGKADPNSRICGHL
LIGAAKNSFAKLMDKISLVMECIPLHSSRSITYVEKDSLVQRLAHGLHKVNTLALKYGLR
GHVPITKSTASLQKQILGFTQRLHAAEVERRSLRLEVTEFKRSVNEMKKELDKAQGLQMQ
LNEFKQSKLITHEKFESACEELNNALLREEQAQMLLNEQAQQLQELNYKLELHSSEEADK
NQTLGEAVKSLSEAKMELRRKDQSLRQLNRHLTQLEQDKRRLEENIHDAESALRMAAKDK
ECVANHMRAVENTLHKVRDQISLSWSAASRNDFTLQLPKLHLETFAMEGLKGGPEVVACQ
AMIKSFMDVYQLASTRIMTLEKEMTSHRSHIAALKSELHTACLRENASLQSIGSRDHSNL
SIPSRAPLPADTTGIGDFLPLKAELDTTYTFLKETFINTVPHALTSSHSSPVTMSANANR
PTQIGL

>sp|P0C221|CC175_HUMAN Coiled-coil domain-containing protein 175 OS=Homo sapiens GN=CCDC175 PE=4 SV=2
MALSPWTPGLGAGEKLVQAAAVSTGPSLELCTLPSTLGSSVAVEALEQLFVVEQSLQSDY
FKCNEEAKIFLKDIAVAVKKLEEMRKATIDLLEIESMELNKLYYLLETLPNSIKRELEEC
VRDARRLNLFEINTIKMRITRTENEIELLKKKITDLTKYNEALGEKQEELARKHARFVLS
LNQTMEKKATTTVYINETYTKINLKREDIALQKKCIQEAEELMEKERAEYLIRKQELTAQ
INEFENTREVKRMETYQKKKELDKLQTKMSKIKETVTVSAAVLSDHNLEIARLHESIRYW
EQEVSELKKDLAILEAKLCFFTDNKEKLDDISNDEKNEFLNKIKQLVETLHAARMEYKDL
REKMKTLARQYKIVLSEEEKAFLQKQKIHDENQKQLTFISQKEYFLSQKRVDIKNMEEGL
ITLQELQQATKTVYQQQIKILSANLERESQRCVITQWKMACLRKKHARWTAKIKAEIQAI
TEKIQNAEVRRIELLNETSFRQQEISGFVAQIEKLTTELKEEEKAFVNKEKMLMKELSKY
EEIFVKETQINKEKEEELVEYLPQLQVAEQEYKEKRRKLEELSNIITAQRQEEDLLNNHI
FLFTRDFSRYISNMEDVKQELQQLRDQESKKNKDHFETLKNLENGFYINDQKADLLLLEN
KKLKEYILYLKNNIEKYREGQEALMHTSSDLSRQLIAQEAQYKDLWAEFQTTVKILVDNG
EETLQDINNLTDKLRERDEKMQHVSTWLRGSLEGLRLLVEQESPMDLLKKKKHIRTRVHF
PVVKCTEKNTLTK
>sp|Q5BJE1|CC178_HUMAN Coiled-coil domain-containing protein 178 OS=Homo sapiens GN=CCDC178 PE=2 SV=3
MTENKTVSSSSTRDDQTNIGLTCQEVKALREKAWSRTNEGNAMSQSLVLYGASKENSEGF
HESKMTNTEGVNKGIYFSYPCRRHSCAVVNIPAPCVNKMISHIQDVESKIQEHLKRFETS
FEEWSRTSSTKDLKEDWSVTTPVKEVKPGEKRDEKCPELKQEMETLLSEAIRLIKSLETD
RADAEEALKQQRSRKNMINMKIDSWSVWKLQELPLAVQKEHEAYLSDVIELQWHLEDKAN
QLQHFEKQKTELEEANAKIQADIDYMNEHGPLLDSKQNQELQDLKNHYKKKMEVMDLHRK
VNEELEEALEACENARLKAQQIKEEIDKDIYQDEKTIEAYKREIYQLNSLFDHYSSSVIN
VNTNIEEKEEEVTEAIRETKSSKNELHSLSKMLEDLRRVYDQLTWKQKSHENQYLEAVND
FYAAKKTWDIELSDVAKDFSAISLACTKLTEDNKKLEIDINKITVKTNESIRKKSKYESE
IKYLTIMKLKNDKHLKNIYKEAYRIGTLFHLTKHKTDEMEDKIAEVRRKFKGREEFLKKL
TQGEVAAGMVLQKKLYSIYEVQALERKELIKNRAICAMSLAELQEPLLQLEDEAERIRSL
DKEHSVMLNNIIDQKDLIRRKVGKVKKKLRKKGKKTLDALIETESKRSAIFKDLEATKSK
TMIFYAKINELNEELKAKEEEKKSFDQTLEILKNKFITMRFKREHAQTVFDHYMQEKKDC
EERIFEEDQRFRVLLAVRQKTLQDTQKIIADSLEENLRLAQEYQQLQITFLKEKDNYFNI
YDKQLSLDTSIRDKKQLCQLQRRMHTLWQEHFKLVVLFSQMRLANFQTDSQESIQKILAV
QEESSNLMQHILGFFQTLTDGTCENDG
>sp|Q5TID7|CC181_HUMAN Coiled-coil domain-containing protein 181 OS=Homo sapiens GN=CCDC181 PE=2 SV=1
MNENKDTDSKKSEEYEDDFEKDLEWLINENEKSDASIIEMACEKEENINQDLKENETVME
HTKRHSDPDKSLQDEVSPRRNDIISVPGIQPLDPISDSDSENSFQESKLESQKDLEEEED
EEVRRYIMEKIVQANKLLQNQEPVNDKRERKLKFKDQLVDLEVPPLEDTTTFKNYFENER
NMFGKLSQLCISNDFGQEDVLLSLTNGSCEENKDRTILVERDGKFELLNLQDIASQGFLP
PINNANSTENDPQQLLPRSSNSSVSGTKKEDSTAKIHAVTHSSTGEPLAYIAQPPLNRKT
CPSSAVNSDRSKGNGKSNHRTQSAHISPVTSTYCLSPRQKELQKQLEEKREKLKREEERR
KIEEEKEKKRENDIVFKAWLQKKREQVLEMRRIQRAKEIEDMNSRQENRDPQQAFRLWLK
KKHEEQMKERQTEELRKQEECLFFLKGTEGRERAFKQWLRRKRMEKMAEQQAVRERTRQL
RLEAKRSKQLQHHLYMSEAKPFRFTDHYN

>sp|Q8N715|CC185_HUMAN Coiled-coil domain-containing protein 185 OS=Homo sapiens GN=CCDC185 PE=1 SV=2
MAGFSHFSQPPYRDLWEPPRPGGERESTQRLGGQRSGADSTACSRAGTPGAESEAGACWL
HPHCSFTPRPRRRGCSDSLRGSRSLSDVARRPLERSRKHRPRSRRLEDAWGETGTKPRPA
WQPQTQLPPQRPQPCPHYPLAQGDSPPPCPGGAGTPLSGTFRVEKAQGGDQWAVPLGRHL
GRWSPSSVPSERSSVPSQKFKRHSACVCAQKRDSSDQVESLASRDSQPLASSKEMRSPHT
QVLKSKLEEVVVSSQDQQIVALVLTRLKKAQRIRELQQQAAKAWEELKRSDQKVQMTLER
ERRLLLRQSQEQWQEKEQRKTLQSPEQRGLRRDSQRKNVPPGESRWKEQPEDQESPRQEK
LEKARAQAEHRKQCQVRRLREQEKMLRNLREQHSLQLQRRLVEACRKRHLHAVEGQKKVQ
DTNLSSLINYQARKVLMDCQAKAEELLRQLSLEQSFQRSQEIHQGLRKERQRELREKAQK
EEEQLQQARWRAGESEEQRKMRKRILVELADEKIRQARSHVHKTTRDKVQHLRELNHLRE
KNHHILKLKAEKEEKCHIEGIKEAIKKKEQRVQHISQGKDPNFQEFQKLPQASRREERAP
PNSSLDQMVLEAQLRACQQNRGY
>sp|A1A4V9|CC189_HUMAN Coiled-coil domain-containing protein 189 OS=Homo sapiens GN=CCDC189 PE=2 SV=1
MLNRKTSHFLGMRVQSELEHLSELRREAGKDRSSVHGSAARTRASVRTQWTTAAAAKADE
DPGANLFPPPLPRPRICMWKYLDVHSMHQLEKTTNAEMREVLAELLELGCPEQSLRDAIT
LDLFCHALIFCRQQGFSLEQTSAACALLQDLHKACIATPLGNVEECYRYFTSVLFCHGVR
RPPFSIDLFKEEQLLALEDYVVNTYFRHFKLYKYVFTPQVRLDLSLTYMGLQPPKLWPES
ETEKEESKEMEEQAVTPQKEELETVAPPEPEPSHIHVLRAYIKTQVNKELEQLQGLVEER
LKASEERLSSKLTALERPFQLPPGKGKSKTK
>sp|Q6ZRV3|CC074_HUMAN Putative uncharacterized protein encoded by LINC00696 OS=Homo sapiens GN=LINC00696 PE=5 SV=1
MQSVDSMLGTVGGCGGGEAASTFSKDPSGCCVGNDCRDGGRGLERRHGRWSRGEGGESGL
CSGGQMASEIEYWDGLAISVMEVEKAFGSTNVLWKTEPFSLWACTAACPPSLSPTLLALG
LPRDGKELAEQGSLWTVLEPGGDWSHSQSQLGTPGRGKGALGF
>sp|Q8NCX0|CC150_HUMAN Coiled-coil domain-containing protein 150 OS=Homo sapiens GN=CCDC150 PE=1 SV=2
MDCKVHMETTVSRPVLSPTHINATASETFTVLQQRMRIVEEQTSSLRDDLIMLDFGEKRG
YLEAPDCLEDLDSQKVISPIQNEAICAGKTDILWKNCEFLVNRMCRLESLMQSLKMNIFR
LQTEKDLNPQKTAFLKDRLNAIQEEHSKDLKLLHLEVMNLRQQLRAVKEEEDKAQDEVQR
LTATLKIASQTKKNAAIIEEELKTTKRKMNLKIQELRRQLAQEKYLRESLEKSASAMLLK
IQEMGSTVEVERKQVHILQQNCIALRDSIQSAQELLAQEQKKKEELEIATSQLKSDLTSR
DDLISKLVEENKNLQISFNKEHEENAYLRSEIMSLHEASEKAQVLNDQLTKKCSELSCML
QTVTMEKARIIADHQAILQVEQKMMTQTFQEQNLLLDAAHASITNELQTVQNEKTQLQAH
LDHLILEHNQCIQKAQDAEKRTAVQKELLESTIARLRGELEASMQEKKSLLEEKERFQRE
VNKTEKEIVQERCNLEKELAKNKVDINTLTHNLQTLEEENKHLADQMASLELQQVTSDYH
GLAQQKVEKITESKNKLAYENGKLQIKVKQLEEQVQSFTDTSLQNDHLRKMNKYLQTKYA
QANSELSAKRVHLQQADAHLKEVKSILERSKEELSRTVKCRNAALKESQKLKEDLEAVED
RENKKVGNFQRQLAEAKEDNCKVTIMLENVLASHSKMQGALEKVQIELGRRDSEIAGLKK
ERDLNQQRVQKLEAEVDQWQARMLVMEDQHNSEIESLQKALGVAREDNRKLAMSLEQALQ
TNNHLQTKLDHIQEQLESKELERQNLETFKDRMTEESKVEAELHAERIEALRKQFQTERE
TTKKVAQREVAELKKALDEANFRSVEVSRTNRELRQKLAELEKILESNKEKIKNQKTQIK
LHLSAKANNAQNIERMKQIEKELKQMELIKDQYQKKNYEQSLSIQRFVCEMTNLQKEMQM

LAKSQYDASVRNKQQELHLEAERKIRQELENRCQELEETVRHLKKCKEATENTLKEASVE
SEQITANLEEAHRWFKHRFDGLQLELTKNRLQRPSGEDRWQEKDQDVKHDVMSNQSVLHR
WERKQNLRPMPKKYHSEVQRK
>sp|Q9NQR7|CC177_HUMAN Coiled-coil domain-containing protein 177 OS=Homo sapiens GN=CCDC177 PE=2 SV=3
MVDPVPEEEKAGAEPGDSGGDEAVASVPPDSQGAQEPAASSASASASAAVPRKAEVPCAA
AEGGRREQSPLLHLDLFNFDCPEAEGSRYVLTSPRSLEACARCAVKPVELLPRALADLVR
EAPGRSMRVATGLYEAYEAERRAKLQQCRAERERIMREEKRRLFTPLSPAAAAAAAAAAA
SAPSAGSSSSCSSASLPASPAPRAARKASPSPSSARTQPPPAGSRTGRKSHSLDSLSRRR
EGALSSESGASSSSYSGESLRELRWPPRASARNSCPAGSASSTTNAPGRPSALTLVPITG
RSFSLGDLSHSPQTAQHVERIVRQVRAERGLRGVPERDRKIAALMLARHQEELLLLEQRA
AAHGQWELQRVHAKQRREREEREKQRALEQGRRAWAAQVEERRGRRGREEREAARRRQRQ
YERSEERRRELAERQGLLRRERAERAAREDRLRKLQQEQNLKQREEGLQEGRERAEQIRR
ERAQRAARAKQRQEGQLQREKRELSRAERARHEALLQGRTRQQRQEREGLRSSLEASLGR
AQENYEHLVEQRTRELRERARREELQGRRAKEAAERKEREHQAHLEALARAGERRLQHAT
QVAEEAVQQKARRVGQSRLEKERAQRANKEKVERDEDCRRRELLQAIGRKLERSEQLTRE
RRSALESARSTARASFHVREKVREETNTRSFDRMVREAQLHASLDRK
>sp|Q9P1Z9|CC180_HUMAN Coiled-coil domain-containing protein 180 OS=Homo sapiens GN=CCDC180 PE=2 SV=2
MWHGNHVQPGATHRPNQGLEMLQGLGIGMKAFHNFNYFLFFYNVLLGLGACLSRLLISCL
LGMWLIARIDRTIMQSGYEGADMGFSAWIGMLYMDHYHINPVLVSFCHILITNHREKKLQ
QSTKYWCLNQSAESLRICAMRGGENRPPARVQSSSEELELRHQSLDAFPGRRLPGRGIQP
AAKMSSVGKVTQVPNGKAYQQIFQAEVQLVHSLAATRKRAAERSVTLKSGRIPMMKKVET
PEGEVMSPRQQKWMHSLPNDWIMENPVLHREKERAKREKARESENTIAAREVRGLMDTIV
PEKISTSTFQRQAEHKRKSYESALASFQEEIAQVGKEMEPLIVDTGGLFLKKLTESDEEM
NRLFLKVENDTNLEDYTIQALLELWDKVAGRLLLRKQEIKELDEALHSLEFSRTDKLKSV
LKKYAEVIEKTSYLMRPEVYRLINEEAMVMNYALLGNRKALAQLFVNLMESTLQQELDSR
HRWQGLVDTWKALKKEALLQSFSEFMASESIHTPPAVTKELEVMLKTQNVLQQRRLKHLC
TICDLLPPSYSKTQLTEWHSSLNSLNKELDTYHVDCMMRIRLLYEKTWQECLMHVQNCKK
QLLDWKAFTEEEAETLVNQFFFQMVGALQGKVEEDLELLDKSFETLADQTEWQSSHLFKY
FQEVVQLWEAHQSELLVQELELEKRMEQHRQKHSLESQVQEAHLDRLLDQLRQQSDKETL
AFHLEKVKDYLKNMKSRYECFHTLLTKEVMEYPAIMLKELNSYSSALSQYFFVREIFEQN
LAGEVIFKFRQPEAHEKPSQKRVKKLRKKQGSKEDMTRSEESISSGTSTARSVEEVEEEN
DQEMESFITEEVLGQQKKSPLHAKMDESKEGSIQGLEEMQVEREGSLNPSLNEENVKGQG
EKKEESEEEDEKEEEEEEEKLEEEKEEKEAQEEQESLSVGEEEDKEEGLEEIYYEDMESF
TISSGNTYFVFVPLEEEHCRKSHSTFSAMFINDTSSAKFIEQVTIPSRLILEIKKQLFSE
GGNFSPKEINSLCSRLEKEAARIELVESVIMLNMEKLENEYLDQANDVINKFESKFHNLS
VDLIFIEKIQRLLTNLQVKIKCQVAKSNSQTNGLNFSLQQLQNKIKTCQESRGEKTTVTT
EELLSFVQTWKEKLSQRIQYLNCSLDRVSMTELVFTNTILKDQEEDSDILTSSEALEEEA
KLDVVTPESFTQLSRVGKPLIEDPAVDVIRKLLQLPNTKWPTHHCDKDPSQTGFKRHRCQ
PENSGKKAVPSASATSAGSLQTTHPPLSHSFTPHPKPNKMERKYRVLGDKPPPAAEDFKG
IILTLLWESSENLLTVAEEFYRKEKRPVTRPDCMCDTFDQCAENISKKILEYQSQANKYH
NSCLIELRIQIRRFEELLPQVCWLVMENFKEHHWKKFFTSVKEIRGQFEEQQKRLEKRKD
KNAQKLHLNLGHPVHFQEMESLHLSEEERQEELDSMIRMNKEKLEECTRRNGQVFITNLA

TFTEKFLLQLDEVVTIDDVQVARMEPPKQKLSMLIRRKLAGLSLKEESEKPLIERGSRKW
PGIKPTEVTIQNKILLQPTSSISTTKTTLGHLAAVEARDAVYLKYLASFEEELKRIQDDC
TSQIKEAQRWKDSWKQSLHTIQGLYV
>sp|A6NF36|CC182_HUMAN Coiled-coil domain-containing protein 182 OS=Homo sapiens GN=CCDC182 PE=2 SV=1
MEPLYQAGSILMTVNTLQGKKMIESGLQSGDFSLSQSWPSCLPPPADLEILQQKVAGVQR
ELEDFKKEALKSIHYLEDAFCEMNGALVQQEEQAARVRQRLREEEDRGIVRNKVLTFLLP
REKQLREHCKRLEDLLLDRGRDALRATKKSQAD
>sp|Q7Z3E2|CC186_HUMAN Coiled-coil domain-containing protein 186 OS=Homo sapiens GN=CCDC186 PE=1 SV=2
MSETDHIASTSSDKNVGKTPELKEDSCNLFSGNESSKLENESKLLSLNTDKTLCQPNEHN
NRIEAQENYIPDHGGGEDSCAKTDTGSENSEQIANFPSGNFAKHISKTNETEQKVTQILV
ELRSSTFPESANEKTYSESPYDTDCTKKFISKIKSVSASEDLLEEIESELLSTEFAEHRV
PNGMNKGEHALVLFEKCVQDKYLQQEHIIKKLIKENKKHQELFVDICSEKDNLREELKKR
TETEKQHMNTIKQLESRIEELNKEVKASRDQLIAQDVTAKNAVQQLHKEMAQRMEQANKK
CEEARQEKEAMVMKYVRGEKESLDLRKEKETLEKKLRDANKELEKNTNKIKQLSQEKGRL
HQLYETKEGETTRLIREIDKLKEDINSHVIKVKWAQNKLKAEMDSHKETKDKLKETTTKL
TQAKEEADQIRKNCQDMIKTYQESEEIKSNELDAKLRVTKGELEKQMQEKSDQLEMHHAK
IKELEDLKRTFKEGMDELRTLRTKVKCLEDERLRTEDELSKYKEIINRQKAEIQNLLDKV
KTADQLQEQLQRGKQEIENLKEEVESLNSLINDLQKDIEGSRKRESELLLFTERLTSKNA
QLQSESNSLQSQFDKVSCSESQLQSQCEQMKQTNINLESRLLKEEELRKEEVQTLQAELA
CRQTEVKALSTQVEELKDELVTQRRKHASSIKDLTKQLQQARRKLDQVESGSYDKEVSSM
GSRSSSSGSLNARSSAEDRSPENTGSSVAVDNFPQVDKAMLIERIVRLQKAHARKNEKIE
FMEDHIKQLVEEIRKKTKIIQSYILREESGTLSSEASDFNKVHLSRRGGIMASLYTSHPA
DNGLTLELSLEINRKLQAVLEDTLLKNITLKENLQTLGTEIERLIKHQHELEQRTKKT
>sp|Q9GZT6|CC90B_HUMAN Coiled-coil domain-containing protein 90B, mitochondrial OS=Homo sapiens GN=CCDC90B PE=1 SV=2
MNSRQAWRLFLSQGRGDRWVSRPRGHFSPALRREFFTTTTKEGYDRRPVDITPLEQRKLT
FDTHALVQDLETHGFDKTQAETIVSALTALSNVSLDTIYKEMVTQAQQEITVQQLMAHLD
AIRKDMVILEKSEFANLRAENEKMKIELDQVKQQLMHETSRIRADNKLDINLERSRVTDM
FTDQEKQLMETTTEFTKKDTQTKSIISETSNKIDAEIASLKTLMESNKLETIRYLAASVF
TCLAIALGFYRFWK
>sp|Q8IYE1|CCD13_HUMAN Coiled-coil domain-containing protein 13 OS=Homo sapiens GN=CCDC13 PE=1 SV=2
MAADESSQNTLRLQFKAMQEMQHKRLQKQMEKKREKELSLKSRADDQEEPLEVSDGLSLL
HAGEPNSKNSFEKRVLEDEIEHLRNELRETVDENGRLYKLLKERDFEIKHLKKKIEEDRF
AFTGTAGVAGDVVATKIVELSKKNRLLMAESEGAKTRVKQLTNRIQELERELQTALTRLS
AKGATDAGAKPPRAQMGDRALLETPEVKALQDRLVATNLKMSDLRNQIQSVKQELRMAQK
VLAREVGEDINVQQLLSSPGTWRGRAQQILVLQSKVQELEKQLGQARSQSAGTASDELSV
YPDPRKLSAQEKNLLRIRSLEREKQEGLEKLASERDVLQRELEELKKKFEGMRSRNKLLS
SEMKTLKSQMGTLVEKGRHDDELIDALMDQLKQLQEILGSLSLQEEKTRVSQHHLDQQLN
SEAQRSNSLVAQLQAMVAEREAKVRQLEMEIGQLNVHYLRNKGVGEGSSGREVSPAYTQF
LEDPGLTKSPASAGDHVGRLGSSRSVTSLGHTLVESALTRPSLPSPHRTSPRFSDSPEQK
GWQAQVSEIKALWQAAEVERDRLTEFVTVLQKRVEESNSKLLESERKLQEERHRTVVLEQ

HLEKIRLEPGKASASQRAAPRTKTGLPTSNNRHNPTGSEKKDPSFAQLSDVPVESQMEEL
TTRLAIQVEENEMLKAALGSALRGKEEDFRMYHEILGQVKSVFLQALRQQKTGKQ
>sp|Q8N4L8|CCD24_HUMAN Coiled-coil domain-containing protein 24 OS=Homo sapiens GN=CCDC24 PE=1 SV=1
MLRHSPSLWELVEEHVPLRERREVKRILGEAAVDLSLELRAEVAMLRALLQEARSSQAPS
SRPISDPSSLLAPPPLLKDLLRQELRQLLQGLRHKAICEGRDQAQAWVQYSPRVLHFALE
EPRCDLPEQEIFQMRGGGPSSGHRDLSIIKDQLNVSNIDQVARHLRGLLEEECHTLEREI
LILQRCLEEEYLRPCHPSEAALEPTLAELKEQKKAMEQELQASVGPSCVSPNHRQRPLGS
STQGLRPPLPLCGVAPLQCCLPAPPLEPYLRPRGQSATHRWGRQLQCSPREGPASTPMSS
AAPQAPA
>sp|Q8TAB7|CCD26_HUMAN Putative coiled-coil domain-containing protein 26 OS=Homo sapiens GN=CCDC26 PE=5 SV=1
MERLCLQPGLLPTAVYLPHWERSSRDREEKEAPFFRLLSRRLMFCVHARQRTQNVPINKY
QRLVVKMKEEEALREKLNMQNITHKENQNAGSLEMIDNMLKQEERRELK
>sp|Q8IYA8|CCD36_HUMAN Coiled-coil domain-containing protein 36 OS=Homo sapiens GN=CCDC36 PE=1 SV=2
MNFNVWNIKEMLSIPSGSGNKKSSNWNNNQNDYSSLSDSQFLFGSQFCPENSETLSAPLD
FGAHLRHSKQSQQNYLEGEPSIFTKYQTKPQLFGGDIKDGGLFPPPLSVGKSKGLLEQFE
EKKKRAKDKCDSETLYNFVSNVRESILRLQTSVEKSEDHLSSRSQSILDSLETVAKTLQE
TIQAQNDLVFEAVQDKGNMEQAILEMKKRFEARQGEFIEMKSNLKHLEVLVAQQSQEFQQ
LCEQLGQLNVPSVLAELKRLISVPPVKDSASQTSPPLAQSLNLTRQEKYTSEKPVLWQAQ
ALPAAWNPGMGSLQPGEFDVWGEGAKNDDLQEEAALPAFGSHERNRHVKDKVVQTNCKNW
AVTKTGAKNHGSSVPGHKIPSDRDLVSQGASQLTSLEINFSTSIKNACQKYQAQSMFLCD
PREHLVIKQKDGTVEMRGKDKKQQPRKAHRAHRGRLIASKQKQIPIQTCKFNSKYQSPQP
AISVPQSPFLGQQEPRAQPLHLQCPRSPRKPVCPILGGTVMPNKTVRAVQGRLLQLSRCS
SQDNWLLSSSSQGDHQMSWFSDLNLGCSETPLCKEAGKNLLYDLGFDSSDDDGF
>sp|Q96MW1|CCD43_HUMAN Coiled-coil domain-containing protein 43 OS=Homo sapiens GN=CCDC43 PE=1 SV=2
MAAPSEVAAIAPGEGDGGGGGFGSWLDGRLEALGVDRAVYGAYILGILQEEEEEEKLDAL
QGILSAFLEEDSLLNICKEIVERWSETQNVVTKVKKEDEVQAIATLIEKQAQIVVKPRMV
SEEEKQRKAALLAQYADVTDEEDEADEKDDSGATTMNIGSDKLLFRNTNVEDVLNARKLE
RDSLRDESQRKKEQDKLQRERDKLAKQERKEKEKKRTQRGERKR
>sp|Q96ER9|CCD51_HUMAN Coiled-coil domain-containing protein 51 OS=Homo sapiens GN=CCDC51 PE=1 SV=2
MMGRSPGFAMQHIVGVPHVLVRRGLLGRDLFMTRTLCSPGPSQPGEKRPEEVALGLHHRL
PALGRALGHSIQQRATSTAKTWWDRYEEFVGLNEVREAQGKVTEAEKVFMVARGLVREAR
EDLEVHQAKLKEVRDRLDRVSREDSQYLELATLEHRMLQEEKRLRTAYLRAEDSEREKFS
LFSAAVRESHEKERTRAERTKNWSLIGSVLGALIGVAGSTYVNRVRLQELKALLLEAQKG
PVSLQEAIREQASSYSRQQRDLHNLMVDLRGLVHAAGPGQDSGSQAGSPPTRDRDVDVLS
AALKEQLSHSRQVHSCLEGLREQLDGLEKTCSQMAGVVQLVKSAAHPGLVEPADGAMPSF
LLEQGSMILALSDTEQRLEAQVNRNTIYSTLVTCVTFVATLPVLYMLFKAS
>sp|Q8NEL0|CCD54_HUMAN Coiled-coil domain-containing protein 54 OS=Homo sapiens GN=CCDC54 PE=1 SV=2
MYTLHTKRVKAAARQMWTSNLSKVRQSLKNVYHKCKIRHQDSTGYPTVTSDDCNQDDDSY

DGKMNLPVVLQDVKTAQVELFSQMTDIVHMIPKVQEKTDLYQKQMEVLETRMNVNEDKQC
TTTKDILSMKEDIKALKKKVTELEIQNSCSTIHCLEILEGERGKEITELLYKLIQPATLK
NTLASTDMEISSAEPEKVPSYPKSTDHLEKKTISPQMKTLKKRNHQNASRSFEKAKPNIY
IYPDFSTWIKLTFVHGGKWTFFLSATKLEEFIQWLLSRPTILPEEPQVITQRYCPFTGPI
LSLTTICLSIFNNIYGFICSLKEEVTRL
>sp|A6NI79|CCD69_HUMAN Coiled-coil domain-containing protein 69 OS=Homo sapiens GN=CCDC69 PE=1 SV=1
MGCRHSRLSSCKPPKKKRQEPEPEQPPRPEPHELGPLNGDTAITVQLCASEEAERHQKDI
TRILQQHEEEKKKWAQQVEKERELELRDRLDEQQRVLEGKNEEALQVLRASYEQEKEALT
HSFREASSTQQETIDRLTSQLEAFQAKMKRVEESILSRNYKKHIQDYGSPSQFWEQELES
LHFVIEMKNERIHELDRRLILMETVKEKNLILEEKITTLQQENEDLHVRSRNQVVLSRQL
SEDLLLTREALEKEVQLRRQLQQEKEELLYRVLGANASPAFPLAPVTPTEVSFLAT
>sp|Q86UT8|CCD84_HUMAN Coiled-coil domain-containing protein 84 OS=Homo sapiens GN=CCDC84 PE=1 SV=1
MAPAQRCPLCRQTFFCGRGHVYSRKHQRQLKEALERLLPQVEAARKAIRAAQVERYVPEH
ERCCWCLCCGCEVREHLSHGNLTVLYGGLLEHLASPEHKKATNKFWWENKAEVQMKEKFL
VTPQDYARFKKSMVKGLDSYEEKEDKVIKEMAAQIREVEQSRQEVVRSVLEPQAVPDPEE
GSSAPRSWKGMNSQVASSLQQPSNLDLPPAPELDWMETGPSLTFIGHQDIPGVGNIHSGA
TPPWMIQDEEYIAGNQEIGPSYEEFLKEKEKQKLKKLPPDRVGANFDHSSRTSAGWLPSF
GRVWNNGRRWQSRHQFKTEAAAMKKQSHTEKS
>sp|Q9NVE4|CCD87_HUMAN Coiled-coil domain-containing protein 87 OS=Homo sapiens GN=CCDC87 PE=1 SV=2
MMEPPKPEPELQRFYHRLLRPLSLFPTRTTSPEPQKRPPQEGRILQSFPLAKLTVASLCS
QVAKLLAGSGIAAGVPPEARLRLIKVILDELKCSWREPPAELSLSHKNNQKLRKRLEAYV
LLSSEQLFLRYLHLLVTMSTPRGVFTESATLTRLAASLARDCTLFLTSPNVYRGLLADFQ
ALLRAEQASGDVDKLHPVCPAGTFKLCPIPWPHSTGFAQVQCSNLNLNYLIQLSRPPEFL
NEPGRMDPVKELKSIPRLKRKKPFHWLPSIGKKREIDISSSQMVSLPSYPVAPTSRASPS
PFCPELRRGQSMPSLREGWRLADELGLPPLPSRPLTPLVLATESKPELTGLIVAEDLKQL
IKKMKLEGTRYPPLDSGLPPLLGVVTRHPAAGHRLEELEKMLRNLQEEEASGQWDPQPPK
SFPLHPQPVTITLKLRNEVVVQAAAVRVSDRNFLDSFHIEGAGALYNHLAGELDPKAIEK
MDIDNFVGSTTREVYKELMSHVSSDHLHFDQGPLVEPAADKDWSTFLSSAFLRQEKQPQI
INPELVGLYSQRANTLQSNTKKMPSLPSLQATKSWEKWSNKASLMNSWKTTLSVDDYFKY
LTNHETDFLHVIFQMHEEEVPVEIVAPARESLEIQHPPPLLEDEEPDFVPGEWDWNTVLE
HRLGAGKTPHLGEPHKILSLQKHLEQLWSVLEVPDKDQVDMTIKYSSKARLRQLPSLVNA
WERALKPIQLREALLARLEWFEGQASNPNRFFKKTNLSSSHFLEENQVRSHLHRKLNLME
SSLVSLLEEIELIFGEPVIFKGRPYLDKMKSDKVEMLYWLQQQRRVRHLVSALKDPHQST
LFRSSAASL
>sp|Q53HC0|CCD92_HUMAN Coiled-coil domain-containing protein 92 OS=Homo sapiens GN=CCDC92 PE=1 SV=2
MTSPHFSSYDEGPLDVSMAATNLENQLHSAQKNLLFLQREHASTLKGLHSEIRRLQQHCT
DLTYELTVKSSEQTGDGTSKSSELKKRCEELEAQLKVKENENAELLKELEQKNAMITVLE
NTIKEREKKYLEELKAKSHKLTLLSSELEQRASTIAYLTSQLHAAKKKLMSSSGTSDASP
SGSPVLASYKPAPPKDKLPETPRRRMKKSLSAPLHPEFEEVYRFGAESRKLLLREPVDAM
PDPTPFLLARESAEVHLIKERPLVIPPIASDRSGEQHSPAREKPHKAHVGVAHRIHHATP

PQAQPEVKTLAVDQVNGGKVVRKHSGTDRTV
>sp|Q8WXS5|CCG8_HUMAN Voltage-dependent calcium channel gamma-8 subunit OS=Homo sapiens GN=CACNG8 PE=2 SV=3
MESLKRWNEERGLWCEKGVQVLLTTVGAFAAFGLMTIAISTDYWLYTRALICNTTNLTAG
GDDGTPHRGGGGASEKKDPGGLTHSGLWRICCLEGLKRGVCVKINHFPEDTDYDHDSAEY
LLRVVRASSIFPILSAILLLLGGVCVAASRVYKSKRNIILGAGILFVAAGLSNIIGVIVY
ISANAGEPGPKRDEEKKNHYSYGWSFYFGGLSFILAEVIGVLAVNIYIERSREAHCQSRS
DLLKAGGGAGGSGGSGPSAILRLPSYRFRYRRRSRSSSRSSEPSPSRDASPGGPGGPGFA
STDISMYTLSRDPSKGSVAAGLAGAGGGGGGAVGAFGGAAGGAGGGGGGGGGAGAERDRG
GASGFLTLHNAFPKEAGGGVTVTVTGPPAPPAPAPPAPSAPAPGTLAKEAAASNTNTLNR
KTTPV
>sp|P32238|CCKAR_HUMAN Cholecystokinin receptor type A OS=Homo sapiens GN=CCKAR PE=1 SV=1
MDVVDSLLVNGSNITPPCELGLENETLFCLDQPRPSKEWQPAVQILLYSLIFLLSVLGNT
LVITVLIRNKRMRTVTNIFLLSLAVSDLMLCLFCMPFNLIPNLLKDFIFGSAVCKTTTYF
MGTSVSVSTFNLVAISLERYGAICKPLQSRVWQTKSHALKVIAATWCLSFTIMTPYPIYS
NLVPFTKNNNQTANMCRFLLPNDVMQQSWHTFLLLILFLIPGIVMMVAYGLISLELYQGI
KFEASQKKSAKERKPSTTSSGKYEDSDGCYLQKTRPPRKLELRQLSTGSSSRANRIRSNS
SAANLMAKKRVIRMLIVIVVLFFLCWMPIFSANAWRAYDTASAERRLSGTPISFILLLSY
TSSCVNPIIYCFMNKRFRLGFMATFPCCPNPGPPGARGEVGEEEEGGTTGASLSRFSYSH
MSASVPPQ
>sp|P80098|CCL7_HUMAN C-C motif chemokine 7 OS=Homo sapiens GN=CCL7 PE=1 SV=3
MKASAALLCLLLTAAAFSPQGLAQPVGINTSTTCCYRFINKKIPKQRLESYRRTTSSHCP
REAVIFKTKLDKEICADPTQKWVQDFMKHLDKKTQTPKL
>sp|Q14094|CCNI_HUMAN Cyclin-I OS=Homo sapiens GN=CCNI PE=2 SV=1
MKFPGPLENQRLSFLLEKAITREAQMWKVNVRKMPSNQNVSPSQRDEVIQWLAKLKYQFN
LYPETFALASSLLDRFLATVKAHPKYLSCIAISCFFLAAKTVEEDERIPVLKVLARDSFC
GCSSSEILRMERIILDKLNWDLHTATPLDFLHIFHAIAVSTRPQLLFSLPKLSPSQHLAV
LTKQLLHCMACNQLLQFRGSMLALAMVSLEMEKLIPDWLSLTIELLQKAQMDSSQLIHCR
ELVAHHLSTLQSSLPLNSVYVYRPLKHTLVTCDKGVFRLHPSSVPGPDFSKDNSKPEVPV
RGTAAFYHHLPAASGCKQTSTKRKVEEMEVDDFYDGIKRLYNEDNVSENVGSVCGTDLSR
QEGHASPCPPLQPVSVM
>sp|P22674|CCNO_HUMAN Cyclin-O OS=Homo sapiens GN=CCNO PE=1 SV=2
MVTPCPTSPSSPAARAGRRDNDQNLRAPVKKSRRPRLRRKQPLHPLNPCPLPGDSGICDL
FESPSSGSDGAESPSAARGGSPLPGPAQPVAQLDLQTFRDYGQSCYAFRKAQESHFHPRE
ALARQPQVTAESRCKLLSWLIPVHRQFGLSFESLCLTVNTLDRFLTTTPVAADCFQLLGV
TSLLIACKQVEVHPPRVKQLLALCCGAFSRQQLCNLECIVLHKLHFTLGAPTISFFLEHF
THARVEAGQAEASEALEAQALARGVAELSLADYAFTSYSPSLLAICCLALADRMLRVSRP
VDLRLGDHPEAALEDCMGKLQLLVAINSTSLTHMLPVQICEKCSLPPSSK
>sp|O60583|CCNT2_HUMAN Cyclin-T2 OS=Homo sapiens GN=CCNT2 PE=1 SV=2
MASGRGASSRWFFTREQLENTPSRRCGVEADKELSCRQQAANLIQEMGQRLNVSQLTINT
AIVYMHRFYMHHSFTKFNKNIISSTALFLAAKVEEQARKLEHVIKVAHACLHPLEPLLDT
KCDAYLQQTQELVILETIMLQTLGFEITIEHPHTDVVKCTQLVRASKDLAQTSYFMATNS
LHLTTFCLQYKPTVIACVCIHLACKWSNWEIPVSTDGKHWWEYVDPTVTLELLDELTHEF
LQILEKTPNRLKKIRNWRANQAARKPKVDGQVSETPLLGSSLVQNSILVDSVTGVPTNPS

FQKPSTSAFPAPVPLNSGNISVQDSHTSDNLSMLATGMPSTSYGLSSHQEWPQHQDSART
EQLYSQKQETSLSGSQYNINFQQGPSISLHSGLHHRPDKISDHSSVKQEYTHKAGSSKHH
GPISTTPGIIPQKMSLDKYREKRKLETLDLDVRDHYIAAQVEQQHKQGQSQAASSSSVTS
PIKMKIPIANTEKYMADKKEKSGSLKLRIPIPPTDKSASKEELKMKIKVSSSERHSSSDE
GSGKSKHSSPHISRDHKEKHKEHPSSRHHTSSHKHSHSHSGSSSGGSKHSADGIPPTVLR
SPVGLSSDGISSSSSSSRKRLHVNDASHNHHSKMSKSSKSSGSSSSSSSSVKQYISSHNS
VFNHPLPPPPPVTYQVGYGHLSTLVKLDKKPVETNGPDANHEYSTSSQHMDYKDTFDMLD
SLLSAQGMNM
>sp|Q9ULG6|CCPG1_HUMAN Cell cycle progression protein 1 OS=Homo sapiens GN=CCPG1 PE=1 SV=3
MSENSSDSDSSCGWTVISHEGSDIEMLNSVTPTDSCEPAPECSSLEQEELQALQIEQGES
SQNGTVLMEETAYPALEETSSTIEAEEQKIPEDSIYIGTASDDSDIVTLEPPKLEEIGNQ
EVVIVEEAQSSEDFNMGSSSSSQYTFCQPETVFSSQPSDDESSSDETSNQPSPAFRRRRA
RKKTVSASESEDRLVAEQETEPSKELSKRQFSSGLNKCVILALVIAISMGFGHFYGTIQI
QKRQQLVRKIHEDELNDMKDYLSQCQQEQESFIDYKSLKENLARCWTLTEAEKMSFETQK
TNLATENQYLRVSLEKEEKALSSLQEELNKLREQIRILEDKGTSTELVKENQKLKQHLEE
EKQKKHSFLSQRETLLTEAKMLKRELERERLVTTALRGELQQLSGSQLHGKSDSPNVYTE
KKEIAILRERLTELERKLTFEQQRSDLWERLYVEAKDQNGKQGTDGKKKGGRGSHRAKNK
SKETFLGSVKETFDAMKNSTKEFVRHHKEKIKQAKEAVKENLKKFSDSVKSTFRHFKDTT
KNIFDEKGNKRFGATKEAAEKPRTVFSDYLHPQYKAPTENHHNRGPTMQNDGRKEKPVHF
KEFRKNTNSKKCSPGHDCRENSHSFRKACSGVFDCAQQESMSLFNTVVNPIRMDEFRQII
QRYMLKELDTFCHWNELDQFINKFFLNGVFIHDQKLFTDFVNDVKDYLRNMKEYEVDNDG
VFEKLDEYIYRHFFGHTFSPPYGPRSVYIKPCHYSSL
>sp|P46092|CCR10_HUMAN C-C chemokine receptor type 10 OS=Homo sapiens GN=CCR10 PE=1 SV=3
MGTEATEQVSWGHYSGDEEDAYSAEPLPELCYKADVQAFSRAFQPSVSLTVAALGLAGNG
LVLATHLAARRAARSPTSAHLLQLALADLLLALTLPFAAAGALQGWSLGSATCRTISGLY
SASFHAGFLFLACISADRYVAIARALPAGPRPSTPGRAHLVSVIVWLLSLLLALPALLFS
QDGQREGQRRCRLIFPEGLTQTVKGASAVAQVALGFALPLGVMVACYALLGRTLLAARGP
ERRRALRVVVALVAAFVVLQLPYSLALLLDTADLLAARERSCPASKRKDVALLVTSGLAL
ARCGLNPVLYAFLGLRFRQDLRRLLRGGSCPSGPQPRRGCPRRPRLSSCSAPTETHSLSW
DN
>sp|P51677|CCR3_HUMAN C-C chemokine receptor type 3 OS=Homo sapiens GN=CCR3 PE=1 SV=1
MTTSLDTVETFGTTSYYDDVGLLCEKADTRALMAQFVPPLYSLVFTVGLLGNVVVVMILI
KYRRLRIMTNIYLLNLAISDLLFLVTLPFWIHYVRGHNWVFGHGMCKLLSGFYHTGLYSE
IFFIILLTIDRYLAIVHAVFALRARTVTFGVITSIVTWGLAVLAALPEFIFYETEELFEE
TLCSALYPEDTVYSWRHFHTLRMTIFCLVLPLLVMAICYTGIIKTLLRCPSKKKYKAIRL
IFVIMAVFFIFWTPYNVAILLSSYQSILFGNDCERSKHLDLVMLVTEVIAYSHCCMNPVI
YAFVGERFRKYLRHFFHRHLLMHLGRYIPFLPSEKLERTSSVSPSTAEPELSIVF
>sp|P51679|CCR4_HUMAN C-C chemokine receptor type 4 OS=Homo sapiens GN=CCR4 PE=1 SV=1
MNPTDIADTTLDESIYSNYYLYESIPKPCTKEGIKAFGELFLPPLYSLVFVFGLLGNSVV
VLVLFKYKRLRSMTDVYLLNLAISDLLFVFSLPFWGYYAADQWVFGLGLCKMISWMYLVG
FYSGIFFVMLMSIDRYLAIVHAVFSLRARTLTYGVITSLATWSVAVFASLPGFLFSTCYT
ERNHTYCKTKYSLNSTTWKVLSSLEINILGLVIPLGIMLFCYSMIIRTLQHCKNEKKNKA
VKMIFAVVVLFLGFWTPYNIVLFLETLVELEVLQDCTFERYLDYAIQATETLAFVHCCLN

PIIYFFLGEKFRKYILQLFKTCRGLFVLCQYCGLLQIYSADTPSSSYTQSTMDHDLHDAL
>sp|P86791|CCZ1_HUMAN Vacuolar fusion protein CCZ1 homolog OS=Homo sapiens GN=CCZ1 PE=1 SV=1
MAAAAAGAGSGPWAAQEKQFPPALLSFFIYNPRFGPREGQEENKILFYHPNEVEKNEKIR
NVGLCEAIVQFTRTFSPSKPAKSLHTQKNRQFFNEPEENFWMVMVVRNPIIEKQSKDGKP
VIEYQEEELLDKVYSSVLRQCYSMYKLFNGTFLKAMEDGGVKLLKERLEKFFHRYLQTLH
LQSCDLLDIFGGISFFPLDKMTYLKIQSFINRMEESLNIVKYTAFLYNDQLIWSGLEQDD
MRILYKYLTTSLFPRHIEPELAGRDSPIRAEMPGNLQHYGRFLTGPLNLNDPDAKCRFPK
IFVNTDDTYEELHLIVYKAMSAAVCFMIDASVHPTLDFCRRLDSIVGPQLTVLASDICEQ
FNINKRMSGSEKEPQFKFIYFNHMNLAEKSTVHMRKTPSVSLTSVHPDLMKILGDINSDF
TRVDEDEEIIVKAMSDYWVVGKKSDRRELYVILNQKNANLIEVNEEVKKLCATQFNNIFF
LD
>sp|Q53FE4|CD017_HUMAN Uncharacterized protein C4orf17 OS=Homo sapiens GN=C4orf17 PE=2 SV=3
MNLNPPTSALQIEGKGSHIMARNVSCFLVRHTPHPRRVCHIKGLNNIPICTVNDDENAFG
TLWGVGQSNYLEKNRIPFANCSYPSSTAVQESPVRGMSPAPNGAKVPPRPHSEPSRKIKE
CFKTSSENPLVIKKEEIKAKRPPSPPKACSTPGSCSSGMTSTKNDVKANTICIPNYLDQE
IKILAKLCSILHTDSLAEVLQWLLHATSKEKEWVSALIHSELAEINLLTHHRRNTSMEPA
AETGKPPTVKSPPTVKLPPNFTAKSKVLTRDTEGDQPTRVSSQGSEENKEVPKEAEHKPP
LLIRRNNMKIPVAEYFSKPNSPPRPNTQESGSAKPVSARSIQEYNLCPQRACYPSTHRR
>sp|Q6ZRC1|CD050_HUMAN Uncharacterized protein C4orf50 OS=Homo sapiens GN=C4orf50 PE=2 SV=1
MLRDSEAEVTEEDPRLRAQQLHHRVLTLQCQLRDQGAAHQASLDEATRLQEELQAKLEEL
QKKQHEAKLAVTPLKAKIASLVRKCRERNRLITHLLQELHRHGLGNLLLSELAQNMLNDV
ALAEYTATFLAPGVPETSHHLDVKSEMTAALRAQTYLLNPEMDSVLQSSLSSESWPIPEP
EWPAQTAQLDSLKLPLSLVSTLDPGTCLAAVTVEPGLPAQRLQEKGGMPCPALQVDNVPA
PSELLSPARILAFHQELRQSICSNSQVHKSPLELEM
>sp|P08571|CD14_HUMAN Monocyte differentiation antigen CD14 OS=Homo sapiens GN=CD14 PE=1 SV=2
MERASCLLLLLLPLVHVSATTPEPCELDDEDFRCVCNFSEPQPDWSEAFQCVSAVEVEIH
AGGLNLEPFLKRVDADADPRQYADTVKALRVRRLTVGAAQVPAQLLVGALRVLAYSRLKE
LTLEDLKITGTMPPLPLEATGLALSSLRLRNVSWATGRSWLAELQQWLKPGLKVLSIAQA
HSPAFSCEQVRAFPALTSLDLSDNPGLGERGLMAALCPHKFPAIQNLALRNTGMETPTGV
CAALAAAGVQPHSLDLSHNSLRATVNPSAPRCMWSSALNSLNLSFAGLEQVPKGLPAKLR
VLDLSCNRLNRAPQPDELPEVDNLTLDGNPFLVPGTALPHEGSMNSGVVPACARSTLSVG
VSGTLVLLQGARGFA
>sp|A8MXV6|CD15L_HUMAN CMT1A duplicated region transcript 15 protein-like protein OS=Homo sapiens GN=CDRT15L2 PE=2 SV=1
MFSCCFPTSRGCCFRNGGSESLFRQCRRRLIPHPRRLWPFVRRRTQVPQDSPGQALAGQA
TPEIPSGLPLHIVLVQEEIREPMEAQTHAPGPYADIAALAAPAVEPKPAWEEPPPERALE
VEGAPAKDQPSQELPEIMAPTVATGLNAGAENVAGERSGREGVTSTAPASRSHAAPSPGH
GGKHGGGDQGIQTGLLYLAGERLLSFAGTTALLLQGLFIVLILVGYISVKVMLKSIKTRL
GRRVPAAPPALRRNLLLQAWKCVCNWASRLFAPNVLPRTGS
>sp|Q13740|CD166_HUMAN CD166 antigen OS=Homo sapiens GN=ALCAM PE=1 SV=2

MESKGASSCRLLFCLLISATVFRPGLGWYTVNSAYGDTIIIPCRLDVPQNLMFGKWKYEK
PDGSPVFIAFRSSTKKSVQYDDVPEYKDRLNLSENYTLSISNARISDEKRFVCMLVTEDN
VFEAPTIVKVFKQPSKPEIVSKALFLETEQLKKLGDCISEDSYPDGNITWYRNGKVLHPL
EGAVVIIFKKEMDPVTQLYTMTSTLEYKTTKADIQMPFTCSVTYYGPSGQKTIHSEQAVF
DIYYPTEQVTIQVLPPKNAIKEGDNITLKCLGNGNPPPEEFLFYLPGQPEGIRSSNTYTL
TDVRRNATGDYKCSLIDKKSMIASTAITVHYLDLSLNPSGEVTRQIGDALPVSCTISASR
NATVVWMKDNIRLRSSPSFSSLHYQDAGNYVCETALQEVEGLKKRESLTLIVEGKPQIKM
TKKTDPSGLSKTIICHVEGFPKPAIQWTITGSGSVINQTEESPYINGRYYSKIIISPEEN
VTLTCTAENQLERTVNSLNVSAISIPEHDEADEISDENREKVNDQAKLIVGIVVGLLLAA
LVAGVVYWLYMKKSKTASKHVNKDLGNMEENKKLEENNHKTEA
>sp|P15391|CD19_HUMAN B-lymphocyte antigen CD19 OS=Homo sapiens GN=CD19 PE=1 SV=6
MPPPRLLFFLLFLTPMEVRPEEPLVVKVEEGDNAVLQCLKGTSDGPTQQLTWSRESPLKP
FLKLSLGLPGLGIHMRPLAIWLFIFNVSQQMGGFYLCQPGPPSEKAWQPGWTVNVEGSGE
LFRWNVSDLGGLGCGLKNRSSEGPSSPSGKLMSPKLYVWAKDRPEIWEGEPPCLPPRDSL
NQSLSQDLTMAPGSTLWLSCGVPPDSVSRGPLSWTHVHPKGPKSLLSLELKDDRPARDMW
VMETGLLLPRATAQDAGKYYCHRGNLTMSFHLEITARPVLWHWLLRTGGWKVSAVTLAYL
IFCLCSLVGILHLQRALVLRRKRKRMTDPTRRFFKVTPPPGSGPQNQYGNVLSLPTPTSG
LGRAQRWAAGLGGTAPSYGNPSSDVQADGALGSRSPPGVGPEEEEGEGYEEPDSEEDSEF
YENDSNLGQDQLSQDGSGYENPEDEPLGPEDEDSFSNAESYENEDEELTQPVARTMDFLS
PHGSAWDPSREATSLGSQSYEDMRGILYAAPQLRSIRGQPGPNHEEDADSYENMDNPDGP
DPAWGGGGRMGTWSTR
>sp|P06126|CD1A_HUMAN T-cell surface glycoprotein CD1a OS=Homo sapiens GN=CD1A PE=1 SV=4
MLFLLLPLLAVLPGDGNADGLKEPLSFHVTWIASFYNHSWKQNLVSGWLSDLQTHTWDSN
SSTIVFLCPWSRGNFSNEEWKELETLFRIRTIRSFEGIRRYAHELQFEYPFEIQVTGGCE
LHSGKVSGSFLQLAYQGSDFVSFQNNSWLPYPVAGNMAKHFCKVLNQNQHENDITHNLLS
DTCPRFILGLLDAGKAHLQRQVKPEAWLSHGPSPGPGHLQLVCHVSGFYPKPVWVMWMRG
EQEQQGTQRGDILPSADGTWYLRATLEVAAGEAADLSCRVKHSSLEGQDIVLYWEHHSSV
GFIILAVIVPLLLLIGLALWFRKRCFC
>sp|P15812|CD1E_HUMAN T-cell surface glycoprotein CD1e, membrane-associated OS=Homo sapiens GN=CD1E PE=1 SV=2
MLLLFLLFEGLCCPGENTAAPQALQSYHLAAEEQLSFRMLQTSSFANHSWAHSEGSGWLG
DLQTHGWDTVLGTIRFLKPWSHGNFSKQELKNLQSLFQLYFHSFIQIVQASAGQFQLEYP
FEIQILAGCRMNAPQIFLNMAYQGSDFLSFQGISWEPSPGAGIRAQNICKVLNRYLDIKE
ILQSLLGHTCPRFLAGLMEAGESELKRKVKPEAWLSCGPSPGPGRLQLVCHVSGFYPKPV
WVMWMRGEQEQRGTQRGDVLPNADETWYLRATLDVAAGEAAGLSCRVKHSSLGGHDLIIH
WGGYSIFLILICLTVIVTLVILVVVDSRLKKQSSNKNILSPHTPSPVFLMGANTQDTKNS
RHQFCLAQVSWIKNRVLKKWKTRLNQLW
>sp|P20273|CD22_HUMAN B-cell receptor CD22 OS=Homo sapiens GN=CD22 PE=1 SV=2
MHLLGPWLLLLVLEYLAFSDSSKWVFEHPETLYAWEGACVWIPCTYRALDGDLESFILFH
NPEYNKNTSKFDGTRLYESTKDGKVPSEQKRVQFLGDKNKNCTLSIHPVHLNDSGQLGLR
MESKTEKWMERIHLNVSERPFPPHIQLPPEIQESQEVTLTCLLNFSCYGYPIQLQWLLEG
VPMRQAAVTSTSLTIKSVFTRSELKFSPQWSHHGKIVTCQLQDADGKFLSNDTVQLNVKH
TPKLEIKVTPSDAIVREGDSVTMTCEVSSSNPEYTTVSWLKDGTSLKKQNTFTLNLREVT
KDQSGKYCCQVSNDVGPGRSEEVFLQVQYAPEPSTVQILHSPAVEGSQVEFLCMSLANPL

PTNYTWYHNGKEMQGRTEEKVHIPKILPWHAGTYSCVAENILGTGQRGPGAELDVQYPPK
KVTTVIQNPMPIREGDTVTLSCNYNSSNPSVTRYEWKPHGAWEEPSLGVLKIQNVGWDNT
TIACAACNSWCSWASPVALNVQYAPRDVRVRKIKPLSEIHSGNSVSLQCDFSSSHPKEVQ
FFWEKNGRLLGKESQLNFDSISPEDAGSYSCWVNNSIGQTASKAWTLEVLYAPRRLRVSM
SPGDQVMEGKSATLTCESDANPPVSHYTWFDWNNQSLPYHSQKLRLEPVKVQHSGAYWCQ
GTNSVGKGRSPLSTLTVYYSPETIGRRVAVGLGSCLAILILAICGLKLQRRWKRTQSQQG
LQENSSGQSFFVRNKKVRRAPLSEGPHSLGCYNPMMEDGISYTTLRFPEMNIPRTGDAES
SEMQRPPPDCDDTVTYSALHKRQVGDYENVIPDFPEDEGIHYSELIQFGVGERPQAQENV
DYVILKH
>sp|Q9BZW8|CD244_HUMAN Natural killer cell receptor 2B4 OS=Homo sapiens GN=CD244 PE=1 SV=2
MLGQVVTLILLLLLKVYQGKGCQGSADHVVSISGVPLQLQPNSIQTKVDSIAWKKLLPSQ
NGFHHILKWENGSLPSNTSNDRFSFIVKNLSLLIKAAQQQDSGLYCLEVTSISGKVQTAT
FQVFVFESLLPDKVEKPRLQGQGKILDRGRCQVALSCLVSRDGNVSYAWYRGSKLIQTAG
NLTYLDEEVDINGTHTYTCNVSNPVSWESHTLNLTQDCQNAHQEFRFWPFLVIIVILSAL
FLGTLACFCVWRRKRKEKQSETSPKEFLTIYEDVKDLKTRRNHEQEQTFPGGGSTIYSMI
QSQSSAPTSQEPAYTLYSLIQPSRKSGSRKRNHSPSFNSTIYEVIGKSQPKAQNPARLSR
KELENFDVYS
>sp|P16070|CD44_HUMAN CD44 antigen OS=Homo sapiens GN=CD44 PE=1 SV=3
MDKFWWHAAWGLCLVPLSLAQIDLNITCRFAGVFHVEKNGRYSISRTEAADLCKAFNSTL
PTMAQMEKALSIGFETCRYGFIEGHVVIPRIHPNSICAANNTGVYILTSNTSQYDTYCFN
ASAPPEEDCTSVTDLPNAFDGPITITIVNRDGTRYVQKGEYRTNPEDIYPSNPTDDDVSS
GSSSERSSTSGGYIFYTFSTVHPIPDEDSPWITDSTDRIPATTLMSTSATATETATKRQE
TWDWFSWLFLPSESKNHLHTTTQMAGTSSNTISAGWEPNEENEDERDRHLSFSGSGIDDD
EDFISSTISTTPRAFDHTKQNQDWTQWNPSHSNPEVLLQTTTRMTDVDRNGTTAYEGNWN
PEAHPPLIHHEHHEEEETPHSTSTIQATPSSTTEETATQKEQWFGNRWHEGYRQTPKEDS
HSTTGTAAASAHTSHPMQGRTTPSPEDSSWTDFFNPISHPMGRGHQAGRRMDMDSSHSIT
LQPTANPNTGLVEDLDRTGPLSMTTQQSNSQSFSTSHEGLEEDKDHPTTSTLTSSNRNDV
TGGRRDPNHSEGSTTLLEGYTSHYPHTKESRTFIPVTSAKTGSFGVTAVTVGDSNSNVNR
SLSGDQDTFHPSGGSHTTHGSESDGHSHGSQEGGANTTSGPIRTPQIPEWLIILASLLAL
ALILAVCIAVNSRRRCGQKKKLVINSGNGAVEDRKPSGLNGEASKSQEMVHLVNKESSET
PDQFMTADETRNLQNVDMKIGV
>sp|P30203|CD6_HUMAN T-cell differentiation antigen CD6 OS=Homo sapiens GN=CD6 PE=1 SV=3
MWLFFGITGLLTAALSGHPSPAPPDQLNTSSAESELWEPGERLPVRLTNGSSSCSGTVEV
RLEASWEPACGALWDSRAAEAVCRALGCGGAEAASQLAPPTPELPPPPAAGNTSVAANAT
LAGAPALLCSGAEWRLCEVVEHACRSDGRRARVTCAENRALRLVDGGGACAGRVEMLEHG
EWGSVCDDTWDLEDAHVVCRQLGCGWAVQALPGLHFTPGRGPIHRDQVNCSGAEAYLWDC
PGLPGQHYCGHKEDAGAVCSEHQSWRLTGGADRCEGQVEVHFRGVWNTVCDSEWYPSEAK
VLCQSLGCGTAVERPKGLPHSLSGRMYYSCNGEELTLSNCSWRFNNSNLCSQSLAARVLC
SASRSLHNLSTPEVPASVQTVTIESSVTVKIENKESRELMLLIPSIVLGILLLGSLIFIA
FILLRIKGKYALPVMVNHQHLPTTIPAGSNSYQPVPITIPKEVFMLPIQVQAPPPEDSDS
GSDSDYEHYDFSAQPPVALTTFYNSQRHRVTDEEVQQSRFQMPPLEEGLEELHASHIPTA
NPGHCITDPPSLGPQYHPRSNSESSTSSGEDYCNSPKSKLPPWNPQVFSSERSSFLEQPP
NLELAGTQPAFSAGPPADDSSSTSSGEWYQNFQPPPQPPSEEQFGCPGSPSPQPDSTDND

DYDDISAA
>sp|P21854|CD72_HUMAN B-cell differentiation antigen CD72 OS=Homo sapiens GN=CD72 PE=2 SV=1
MAEAITYADLRFVKAPLKKSISSRLGQDPGADDDGEITYENVQVPAVLGVPSSLASSVLG
DKAAVKSEQPTASWRAVTSPAVGRILPCRTTCLRYLLLGLLLTCLLLGVTAICLGVRYLQ
VSQQLQQTNRVLEVTNSSLRQQLRLKITQLGQSAEDLQGSRRELAQSQEALQVEQRAHQA
AEGQLQACQADRQKTKETLQSEEQQRRALEQKLSNMENRLKPFFTCGSADTCCPSGWIMH
QKSCFYISLTSKNWQESQKQCETLSSKLATFSEIYPQSHSYYFLNSLLPNGGSGNSYWTG
LSSNKDWKLTDDTQRTRTYAQSSKCNKVHKTWSWWTLESESCRSSLPYICEMTAFRFPD
>sp|P11912|CD79A_HUMAN B-cell antigen receptor complex-associated protein alpha chain OS=Homo sapiens GN=CD79A PE=1 SV=2
MPGGPGVLQALPATIFLLFLLSAVYLGPGCQALWMHKVPASLMVSLGEDAHFQCPHNSSN
NANVTWWRVLHGNYTWPPEFLGPGEDPNGTLIIQNVNKSHGGIYVCRVQEGNESYQQSCG
TYLRVRQPPPRPFLDMGEGTKNRIITAEGIILLFCAVVPGTLLLFRKRWQNEKLGLDAGD
EYEDENLYEGLNLDDCSMYEDISRGLQGTYQDVGSLNIGDVQLEKP
>sp|P40259|CD79B_HUMAN B-cell antigen receptor complex-associated protein beta chain OS=Homo sapiens GN=CD79B PE=1 SV=1
MARLALSPVPSHWMVALLLLLSAEPVPAARSEDRYRNPKGSACSRIWQSPRFIARKRGFT
VKMHCYMNSASGNVSWLWKQEMDENPQQLKLEKGRMEESQNESLATLTIQGIRFEDNGIY
FCQQKCNNTSEVYQGCGTELRVMGFSTLAQLKQRNTLKDGIIMIQTLLIILFIIVPIFLL
LDKDDSKAGMEEDHTYEGLDIDQTATYEDIVTLRTGEVKWSVGEHPGQE
>sp|P09564|CD7_HUMAN T-cell antigen CD7 OS=Homo sapiens GN=CD7 PE=1 SV=1
MAGPPRLLLLPLLLALARGLPGALAAQEVQQSPHCTTVPVGASVNITCSTSGGLRGIYLR
QLGPQPQDIIYYEDGVVPTTDRRFRGRIDFSGSQDNLTITMHRLQLSDTGTYTCQAITEV
NVYGSGTLVLVTEEQSQGWHRCSDAPPRASALPAPPTGSALPDPQTASALPDPPAASALP
AALAVISFLLGLGLGVACVLARTQIKKLCSWRDKNSAACVVYEDMSHSRCNTLSSPNQYQ
>sp|Q01151|CD83_HUMAN CD83 antigen OS=Homo sapiens GN=CD83 PE=1 SV=1
MSRGLQLLLLSCAYSLAPATPEVKVACSEDVDLPCTAPWDPQVPYTVSWVKLLEGGEERM
ETPQEDHLRGQHYHQKGQNGSFDAPNERPYSLKIRNTTSCNSGTYRCTLQDPDGQRNLSG
KVILRVTGCPAQRKEETFKKYRAEIVLLLALVIFYLTLIIFTCKFARLQSIFPDFSKAGM
ERAFLPVTSPNKHLGLVTPHKTELV
>sp|A6NJW9|CD8BL_HUMAN Putative T-cell surface glycoprotein CD8 beta-2 chain OS=Homo sapiens GN=CD8BP PE=5 SV=2
MRPRLWLLLAAQLTVLHGNSVLQQTPAYIKVQTNKMVMLSCEAKISLSNMCIYWLRQRQA
PSSDSHHEFLTLWDSAKGTIHGEEVEQEKIAVFRDASRFILNLTSVKPEDSGIYFCMIVG
SPELTFGKGTQLSVVVDFLPTTAQPTKKSTLKKRVCRLPRPETQKGPLCSPVTLGLLVAG
VLVLLVSLGVAMHLCCRRRRARLRFMKQLYK
>sp|Q9BWV3|CDAC1_HUMAN Cytidine and dCMP deaminase domain-containing protein 1 OS=Homo sapiens GN=CDADC1 PE=2 SV=1
MKEAGQMQNLESARAGRSVSTQTGSMTGGQIPRLSKVNLFTLLSLWMELFPAEAQRQKSQK
NEEGKHGPLGDNEERTRVSTDKRQVKRTGLVVVKNMKIVGLHCSSEDLHAGQIALIKHGS
RLKNCDLYFSRKPCSACLKMIVNAGVNRISYWPADPEISLLTEASSSEDAKLDAKAVERL
KSNSRAHVCVLLQPLVCYMVQFVEETSYKCDFIQKITKTLPDANTDFYYECKQERIKEYE
MLFLVSNEEMHKQILMTIGLENLCENPYFSNLRQNMKDLILLLATVASSVPNFKHFGFYR

SNPEQINEIHNQSLPQEIARHCMVQARLLAYRTEDHKTGVGAVIWAEGKSRSCDGTGAMY
FVGCGYNAFPVGSEYADFPHMDDKQKDREIRKFRYIIHAEQNALTFRCQEIKPEERSMIF
VTKCPCDECVPLIKGAGIKQIYAGDVDVGKKKADISYMRFGELEGVSKFTWQLNPSGAYG
LEQNEPERRENGVLRPVPQKEEQHQDKKLRLGIH
>sp|Q9UJX2|CDC23_HUMAN Cell division cycle protein 23 homolog OS=Homo sapiens GN=CDC23 PE=1 SV=3
MAASTSMVPVAVTAAVAPVLSINSDFSDLREIKKQLLLIAGLTRERGLLHSSKWSAELAF
SLPALPLAELQPPPPITEEDAQDMDAYTLAKAYFDVKEYDRAAHFLHGCNSKKAYFLYMY
SRYLSGEKKKDDETVDSLGPLEKGQVKNEALRELRVELSKKHQARELDGFGLYLYGVVLR
KLDLVKEAIDVFVEATHVLPLHWGAWLELCNLITDKEMLKFLSLPDTWMKEFFLAHIYTE
LQLIEEALQKYQNLIDVGFSKSSYIVSQIAVAYHNIRDIDKALSIFNELRKQDPYRIENM
DTFSNLLYVRSMKSELSYLAHNLCEIDKYRVETCCVIGNYYSLRSQHEKAALYFQRALKL
NPRYLGAWTLMGHEYMEMKNTSAAIQAYRHAIEVNKRDYRAWYGLGQTYEILKMPFYCLY
YYRRAHQLRPNDSRMLVALGECYEKLNQLVEAKKCYWRAYAVGDVEKMALVKLAKLHEQL
TESEQAAQCYIKYIQDIYSCGEIVEHLEESTAFRYLAQYYFKCKLWDEASTCAQKCCAFN
DTREEGKALLRQILQLRNQGETPTTEVPAPFFLPASLSANNTPTRRVSPLNLSSVTP
>sp|Q9NYV4|CDK12_HUMAN Cyclin-dependent kinase 12 OS=Homo sapiens GN=CDK12 PE=1 SV=2
MPNSERHGGKKDGSGGASGTLQPSSGGGSSNSRERHRLVSKHKRHKSKHSKDMGLVTPEA
ASLGTVIKPLVEYDDISSDSDTFSDDMAFKLDRRENDERRGSDRSDRLHKHRHHQHRRSR
DLLKAKQTEKEKSQEVSSKSGSMKDRISGSSKRSNEETDDYGKAQVAKSSSKESRSSKLH
KEKTRKERELKSGHKDRSKSHRKRETPKSYKTVDSPKRRSRSPHRKWSDSSKQDDSPSGA
SYGQDYDLSPSRSHTSSNYDSYKKSPGSTSRRQSVSPPYKEPSAYQSSTRSPSPYSRRQR
SVSPYSRRRSSSYERSGSYSGRSPSPYGRRRSSSPFLSKRSLSRSPLPSRKSMKSRSRSP
AYSRHSSSHSKKKRSSSRSRHSSISPVRLPLNSSLGAELSRKKKERAAAAAAAKMDGKES
KGSPVFLPRKENSSVEAKDSGLESKKLPRSVKLEKSAPDTELVNVTHLNTEVKNSSDTGK
VKLDENSEKHLVKDLKAQGTRDSKPIALKEEIVTPKETETSEKETPPPLPTIASPPPPLP
TTTPPPQTPPLPPLPPIPALPQQPPLPPSQPAFSQVPASSTSTLPPSTHSKTSAVSSQAN
SQPPVQVSVKTQVSVTAAIPHLKTSTLPPLPLPPLLPGDDDMDSPKETLPSKPVKKEKEQ
RTRHLLTDLPLPPELPGGDLSPPDSPEPKAITPPQQPYKKRPKICCPRYGERRQTESDWG
KRCVDKFDIIGIIGEGTYGQVYKAKDKDTGELVALKKVRLDNEKEGFPITAIREIKILRQ
LIHRSVVNMKEIVTDKQDALDFKKDKGAFYLVFEYMDHDLMGLLESGLVHFSEDHIKSFM
KQLMEGLEYCHKKNFLHRDIKCSNILLNNSGQIKLADFGLARLYNSEESRPYTNKVITLW
YRPPELLLGEERYTPAIDVWSCGCILGELFTKKPIFQANLELAQLELISRLCGSPCPAVW
PDVIKLPYFNTMKPKKQYRRRLREEFSFIPSAALDLLDHMLTLDPSKRCTAEQTLQSDFL
KDVELSKMAPPDLPHWQDCHELWSKKRRRQRQSGVVVEEPPPSKTSRKETTSGTSTEPVK
NSSPAPPQPAPGKVESGAGDAIGLADITQQLNQSELAVLLNLLQSQTDLSIPQMAQLLNI
HSNPEMQQQLEALNQSISALTEATSQQQDSETMAPEESLKEAPSAPVILPSAEQTTLEAS
STPADMQNILAVLLSQLMKTQEPAGSLEENNSDKNSGPQGPRRTPTMPQEEAAACPPHIL
PPEKRPPEPPGPPPPPPPPPLVEGDLSSAPQELNPAVTAALLQLLSQPEAEPPGHLPHEH
QALRPMEYSTRPRPNRTYGNTDGPETGFSAIDTDERNSGPALTESLVQTLVKNRTFSGSL
SHLGESSSYQGTGSVQFPGDQDLRFARVPLALHPVVGQPFLKAEGSSNSVVHAETKLQNY
GELGPGTTGASSSGAGLHWGGPTQSSAYGKLYRGPTRVPPRGGRGRGVPY
>sp|Q9BWU1|CDK19_HUMAN Cyclin-dependent kinase 19 OS=Homo sapiens GN=CDK19 PE=1 SV=1
MDYDFKAKLAAERERVEDLFEYEGCKVGRGTYGHVYKARRKDGKDEKEYALKQIEGTGIS

MSACREIALLRELKHPNVIALQKVFLSHSDRKVWLLFDYAEHDLWHIIKFHRASKANKKP
MQLPRSMVKSLLYQILDGIHYLHANWVLHRDLKPANILVMGEGPERGRVKIADMGFARLF
NSPLKPLADLDPVVVTFWYRAPELLLGARHYTKAIDIWAIGCIFAELLTSEPIFHCRQED
IKTSNPFHHDQLDRIFSVMGFPADKDWEDIRKMPEYPTLQKDFRRTTYANSSLIKYMEKH
KVKPDSKVFLLLQKLLTMDPTKRITSEQALQDPYFQEDPLPTLDVFAGCQIPYPKREFLN
EDDPEEKGDKNQQQQQNQHQQPTAPPQQAAAPPQAPPPQQNSTQTNGTAGGAGAGVGGTG
AGLQHSQDSSLNQVPPNKKPRLGPSGANSGGPVMPSDYQHSSSRLNYQSSVQGSSQSQST
LGYSSSSQQSSQYHPSHQAHRY
>sp|P11802|CDK4_HUMAN Cyclin-dependent kinase 4 OS=Homo sapiens GN=CDK4 PE=1 SV=2
MATSRYEPVAEIGVGAYGTVYKARDPHSGHFVALKSVRVPNGGGGGGGLPISTVREVALL
RRLEAFEHPNVVRLMDVCATSRTDREIKVTLVFEHVDQDLRTYLDKAPPPGLPAETIKDL
MRQFLRGLDFLHANCIVHRDLKPENILVTSGGTVKLADFGLARIYSYQMALTPVVVTLWY
RAPEVLLQSTYATPVDMWSVGCIFAEMFRRKPLFCGNSEADQLGKIFDLIGLPPEDDWPR
DVSLPRGAFPPRGPRPVQSVVPEMEESGAQLLLEMLTFNPHKRISAFRALQHSYLHKDEG
NPE
>sp|Q00534|CDK6_HUMAN Cyclin-dependent kinase 6 OS=Homo sapiens GN=CDK6 PE=1 SV=1
MEKDGLCRADQQYECVAEIGEGAYGKVFKARDLKNGGRFVALKRVRVQTGEEGMPLSTIR
EVAVLRHLETFEHPNVVRLFDVCTVSRTDRETKLTLVFEHVDQDLTTYLDKVPEPGVPTE
TIKDMMFQLLRGLDFLHSHRVVHRDLKPQNILVTSSGQIKLADFGLARIYSFQMALTSVV
VTLWYRAPEVLLQSSYATPVDLWSVGCIFAEMFRRKPLFRGSSDVDQLGKILDVIGLPGE
EDWPRDVALPRQAFHSKSAQPIEKFVTDIDELGKDLLLKCLTFNPAKRISAYSALSHPYF
QDLERCKENLDSHLPPSQNTSELNTA
>sp|P50750|CDK9_HUMAN Cyclin-dependent kinase 9 OS=Homo sapiens GN=CDK9 PE=1 SV=3
MAKQYDSVECPFCDEVSKYEKLAKIGQGTFGEVFKARHRKTGQKVALKKVLMENEKEGFP
ITALREIKILQLLKHENVVNLIEICRTKASPYNRCKGSIYLVFDFCEHDLAGLLSNVLVK
FTLSEIKRVMQMLLNGLYYIHRNKILHRDMKAANVLITRDGVLKLADFGLARAFSLAKNS
QPNRYTNRVVTLWYRPPELLLGERDYGPPIDLWGAGCIMAEMWTRSPIMQGNTEQHQLAL
ISQLCGSITPEVWPNVDNYELYEKLELVKGQKRKVKDRLKAYVRDPYALDLIDKLLVLDP
AQRIDSDDALNHDFFWSDPMPSDLKGMLSTHLTSMFEYLAPPRRKGSQITQQSTNQSRNP
ATTNQTEFERVF
>sp|P55273|CDN2D_HUMAN Cyclin-dependent kinase 4 inhibitor D OS=Homo sapiens GN=CDKN2D PE=1 SV=1
MLLEEVRAGDRLSGAAARGDVQEVRRLLHRELVHPDALNRFGKTALQVMMFGSTAIALEL
LKQGASPNVQDTSGTSPVHDAARTGFLDTLKVLVEHGADVNVPDGTGALPIHLAVQEGHT
AVVSFLAAESDLHRRDARGLTPLELALQRGAQDLVDILQGHMVAPL
>sp|Q9Y6F8|CDY1_HUMAN Testis-specific chromodomain protein Y 1 OS=Homo sapiens GN=CDY1 PE=1 SV=1
MASQEFEVEAIVDKRQDKNGNTQYLVRWKGYDKQDDTWEPEQHLMNCEKCVHDFNRRQTE
KQKKLTWTTTSRIFSNNARRRTSRSTKANYSKNSPKTPVTDKHHRSKNRKLFAASKNVRR
KAASILSDTKNMEIINSTIETLAPDSPFDHKTVSGFQKLEKLDPIAADQQDTVVFKVTEG
KLLRDPLSRPGAEQTGIQNKTQIHPLMSQMSGSVTASMATGSATRKGIVVLIDPLAANGT
TDMHTSVPRVKGGQRNITDDSRDQPFIKKMHFTIRLTESASTYRDIVVKKEDGFTQIVLS
TRSTEKNALNTEVIKEIVNALNSAAADDSKLVLFSAAGSVFCCGLDFGYFVKHLRNNRNT
ASLEMVDTIKNFVNTFIQFKKPIVVSVNGPAIGLGASILPLCDLVWANEKAWFQTPYTTF

GQSPDGCSSITFPKMMGKASANEMLIAGRKLTAREACAKGLVSQVFLTGTFTQEVMIQIK
ELASYNPIVLEECKALVRCNIKLELEQANERECEVLRKIWSSAQGIESMLKYVENKIDEF
>sp|Q49AR2|CE022_HUMAN UPF0489 protein C5orf22 OS=Homo sapiens GN=C5orf22 PE=1 SV=2
MSDSAGGRAGLRRYPKLPVWVVEDHQEVLPFIYRAIGSKHLPASNVSFLHFDSHPDLLIP
VNMPADTVFDKETLFGELSIENWIMPAVYAGHFSHVIWFHPTWAQQIREGRHHFLVGKDT
STTTIRVTSTDHYFLSDGLYVPEDQLENQKPLQLDVIMVKPYKLCNNQEENDAVSSAKKP
KLALEDSENTASTNCDSSSEGLEKDTATQRSDQTCLEPSCSCSSENQECQTAASTGEILE
ILKKGKAFVLDIDLDFFSVKNPFKEMFTQEEYKILQELYQFKKPGTNLTEEDLVDIVDTR
IHQLEDLEATFADLCDGDDEETVQRWASNPGMESLVPLVQSLKKRMEVPDYEMVHQAGLT
CDYSELPHHISTEQEIECLIQSVHYLLKNLPNPTLVTIARSSLDDYCPSDQVDTIQEKVL
NMLRALYGNLDLQVYAAESPPS
>sp|Q8N8E3|CE112_HUMAN Centrosomal protein of 112 kDa OS=Homo sapiens GN=CEP112 PE=1 SV=2
MEVGSEEEKWEKLDAEFDHFVVDMKPFVLKLPHRTERQRCALWIRKLCEPSGTGAGIMGR
KNRNLYAKLLLHMLKRGALEGPFTHRPEPGTLKILPSYMSIYFDEPNPARAKGSSPEGLP
AWVLGELETSEHKLNESWKLSSGEDNTLVQSPTDVYSREQYTGKLRVRSHSLSPTHREDG
QNITPKICEVYSKKSPVSLDDSDIEARLNSWNLGIENPRYLRQKPIPVSLMTPKFSLRKS
SSFHDDHFLSRIREKELDMKTKMMEAKFHEEKLKLQQKHDADVQKILERKNNEIEELKTL
YRSKQHETEETIRKLEKKVQTLIRDCQVIRETKEDQIAELKKICEQSTESLNNDWEKKLH
NAVAEMEQEKFDLQKQHTENIQELLEDTNVRLNKMESEYMAQTQSTNHMIKELEARVQQL
TGEAENSNLQRQKLIQEKAELERCYQITCSELQEVKARRNTLHKEKDHLVNDYEQNMKLL
QTKYDADINLLKQEHALSASKASSMIEELEQNVCQLKQQLQESELQRKQQLRDQENKFQM
EKSHLKHIYEKKAHDLQSELDKGKEDTQKKIHKFEEALKEKEEQLTRVTEVQRLQAQQAD
AALEEFKRQVELNSEKVYAEMKEQMEKVEADLTRSKSLREKQSKEFLWQLEDIRQRYEQQ
IVELKLEHEQEKTHLLQQHNAEKDSLVRDHEREIENLEKQLRAANMEHENQIQEFKKRDA
QVIADMEAQVHKLREELINVNSQRKQQLVELGLLREEEKQRATREHEIVVNKLKAESEKM
KIELKKTHAAETEMTLEKANSKLKQIEKEYTQKLAKSSQIIAELQTTISSLKEENSQQQL
AAERRLQDVRQKFEDEKKQLIRDNDQAIKVLQDELENRSNQVRCAEKKLQHKELESQEQI
TYIRQEYETKLKGLMPASLRQELEDTISSLKSQVNFLQKRASILQEELTTYQGRR
>sp|Q03701|CEBPZ_HUMAN CCAAT/enhancer-binding protein zeta OS=Homo sapiens GN=CEBPZ PE=1 SV=3
MAAVKEPLEFHAKRPWRPEEAVEDPDEEDEDNTSEAENGFSLEEVLRLGGTKQDYLMLAT
LDENEEVIDGGKKGAIDDLQQGELEAFIQNLNLAKYTKASLVEEDEPAEKENSSKKEVKI
PKINNKNTAESQRTSVNKVKNKNRPEPHSDENGSTTPKVKKDKQNIFEFFERQTLLLRPG
GKWYDLEYSNEYSLKPQPQDVVSKYKTLAQKLYQHEINLFKSKTNSQKGASSTWMKAIVS
SGTLGDRMAAMILLIQDDAVHTLQFVETLVNLVKKKGSKQQCLMALDTFKELLITDLLPD
NRKLRIFSQRPFDKLEQLSSGNKDSRDRRLILWYFEHQLKHLVAEFVQVLETLSHDTLVT
TKTRALTVAHELLCNKPEEEKALLVQVVNKLGDPQNRIATKASHLLETLLCKHPNMKGVV
SGEVERLLFRSNISSKAQYYAICFLNQMALSHEESELANKLITVYFCFFRTCVKKKDVES
KMLSALLTGVNRAYPYSQTGDDKVREQIDTLFKVLHIVNFNTSVQALMLLFQVMNSQQTI
SDRYYTALYRKMLDPGLMTCSKQAMFLNLVYKSLKADIVLRRVKAFVKRLLQVTCQQMPP
FICGALYLVSEILKAKPGLRSQLDDHPESDDEENFIDANDDEDMEKFTDADKETEIVKKL
ETEETVPETDVETKKPEVASWVHFDNLKGGKQLNKYDPFSRNPLFCGAENTSLWELKKLS
VHFHPSVALFAKTILQGNYIQYSGDPLQDFTLMRFLDRFVYRNPKPHKGKENTDSVVMQP
KRKHFIKDIRHLPVNSKEFLAKEESQIPVDEVFFHRYYKKVAVKEKQKRDADEESIEDVD

DEEFEELIDTFEDDNCFSSGKDDMDFAGNVKKRTKGAKDNTLDEDSEGSDDELGNLDDDE
VSLGSMDDEEFAEVDEDGGTFMDVLDDESESVPELEVHSKVSTKKSKRKGTDDFDFAGSF
QGPRKKKRNLNDSSLFVSAEEFGHLLDENMGSKFDNIGMNAMANKDNASLKQLRWEAERD
DWLHNRDAKSIIKKKKHFKKKRIKTTQKTKKQRK
>sp|Q9BXF3|CECR2_HUMAN Cat eye syndrome critical region protein 2 OS=Homo sapiens GN=CECR2 PE=1 SV=2
MCPEEGGAAGLGELRSWWEVPAIAHFCSLFRTAFRLPDFEIEELEAALHRDDVEFISDLI
ACLLQGCYQRRDITPQTFHSYLEDIINYRWELEEGKPNPLREASFQDLPLRTRVEILHRL
CDYRLDADDVFDLLKGLDADSLRVEPLGEDNSGALYWYFYGTRMYKEDPVQGKSNGELSL
SRESEGQKNVSSIPGKTGKRRGRPPKRKKLQEEILLSEKQEENSLASEPQTRHGSQGPGQ
GTWWLLCQTEEEWRQVTESFRERTSLRERQLYKLLSEDFLPEICNMIAQKGKRPQRTKAE
LHPRWMSDHLSIKPVKQEETPVLTRIEKQKRKEEEEERQILLAVQKKEQEQMLKEERKRE
LEEKVKAVEGMCSVRVVWRGACLSTSRPVDRAKRRKLREERAWLLAQGKELPPELSHLDP
NSPMREEKKTKDLFELDDDFTAMYKVLDVVKAHKDSWPFLEPVDESYAPNYYQIIKAPMD
ISSMEKKLNGGLYCTKEEFVNDMKTMFRNCRKYNGESSEYTKMSDNLERCFHRAMMKHFP
GEDGDTDEEFWIREDEKREKRRSRAGRSGGSHVWTRSRDPEGSSRKQQPMENGGKSLPPT
RRAPSSGDDQSSSSTQPPREVGTSNGRGFSHPLHCGGTPSQAPFLNQMRPAVPGTFGPLR
GSDPATLYGSSGVPEPHPGEPVQQRQPFTMQPPVGINSLRGPRLGTPEEKQMCGGLTHLS
NMGPHPGSLQLGQISGPSQDGSMYAPAQFQPGFIPPRHGGAPARPPDFPESSEIPPSHMY
RSYKYLNRVHSAVWNGNHGATNQGPLGPDEKPHLGPGPSHQPRTLGHVMDSRVMRPPVPP
NQWTEQSGFLPHGVPSSGYMRPPCKSAGHRLQPPPVPAPSSLFGAPAQALRGVQGGDSMM
DSPEMIAMQQLSSRVCPPGVPYHPHQPAHPRLPGPFPQVAHPMSVTVSAPKPALGNPGRA
PENSEAQEPENDQAEPLPGLEEKPPGVGTSEGVYLTQLPHPTPPLQTDCTRQSSPQERET
VGPELKSSSSESADNCKAMKGKNPWPSDSSYPGPAAQGCVRDLSTVADRGALSENGVIGE
ASPCGSEGKGLGSSGSEKLLCPRGRTLQETMPCTGQNAATPPSTDPGLTGGTVSQFPPLY
MPGLEYPNSAAHYHISPGLQGVGPVMGGKSPASHPQHFPPRGFQSNHPHSGGFPRYRPPQ
GMRYSYHPPPQPSYHHYQRTPYYACPQSFSDWQRPLHPQGSPSGPPASQPPPPRSLFSDK
NAMASLQGCETLNAALTSPTRMDAVAAKVPNDGQNPGPEEEKLDESMERPESPKEFLDLD
NHNAATKRQSSLSASEYLYGTPPPLSSGMGFGSSAFPPHSVMLQTGPPYTPQRPASHFQP
RAYSSPVAALPPHHPGATQPNGLSQEGPIYRCQEEGLGHFQAVMMEQIGTRSGIRGPFQE
MYRPSGMQMHPVQSQASFPKTPTAATSQEEVPPHKPPTLPLDQS
>sp|Q96J87|CELF6_HUMAN CUGBP Elav-like family member 6 OS=Homo sapiens GN=CELF6 PE=1 SV=1
MAAAPGGSAQPAGPGPRLGFSTADSGVGMSGLNPGPAVPMKDHDAIKLFVGQIPRGLDEQ
DLKPLFEEFGRIYELTVLKDRLTGLHKGCAFLTYCARDSALKAQSALHEQKTLPGMNRPI
QVKPAASEGRGEDRKLFVGMLGKQQGEEDVRRLFQPFGHIEECTVLRSPDGTSKGCAFVK
FGSQGEAQAAIRGLHGSRTMAGASSSLVVKLADTDRERALRRMQQMAGHLGAFHPAPLPL
GACGAYTTAILQHQAALLAAAQGPGLGPVAAVAAQMQHVAAFSLVAAPLLPAAAANSPPG
SGPGTLPGLPAPIGVNGFGPLTPQTNGQPGSDTLYNNGLSPYPAQSPGVADPLQQAYAGM
HHYAAAYPSAYAPVSTAFPQQPSALPQQQREGPEGCNLFIYHLPQEFGDAELIQTFLPFG
AVVSAKVFVDRATNQSKCFGFVSFDNPTSAQTAIQAMNGFQIGMKRLKVQLKRPKDANRP
Y
>sp|Q9NYQ7|CELR3_HUMAN Cadherin EGF LAG seven-pass G-type receptor 3 OS=Homo sapiens GN=CELSR3 PE=1 SV=2
MMARRPPWRGLGGRSTPILLLLLLLSLFPLSQEELGGGGHQGWDPGLAATTGPRAHIGGGA

LALCPESSGVREDGGPGLGVREPIFVGLRGRRQSARNSRGPPEQPNEELGIEHGVQPLGS
RERETGQGPGSVLYWRPEVSSCGRTGPLQRGSLSPGALSSGVPGSGNSSPLPSDFLIRHH
GPKPVSSQRNAGTGSRKRVGTARCCGELWATGSKGQGERATTSGAERTAPRRNCLPGASG
SGPELDSAPRTARTAPASGSAPRESRTAPEPAPKRMRSRGLFRCRFLPQRPGPRPPGLPA
RPEARKVTSANRARFRRAANRHPQFPQYNYQTLVPENEAAGTAVLRVVAQDPDAGEAGRL
VYSLAALMNSRSLELFSIDPQSGLIRTAAALDRESMERHYLRVTAQDHGSPRLSATTMVA
VTVADRNDHSPVFEQAQYRETLRENVEEGYPILQLRATDGDAPPNANLRYRFVGPPAARA
AAAAAFEIDPRSGLISTSGRVDREHMESYELVVEASDQGQEPGPRSATVRVHITVLDEND
NAPQFSEKRYVAQVREDVRPHTVVLRVTATDRDKDANGLVHYNIISGNSRGHFAIDSLTG
EIQVVAPLDFEAEREYALRIRAQDAGRPPLSNNTGLASIQVVDINDHIPIFVSTPFQVSV
LENAPLGHSVIHIQAVDADHGENARLEYSLTGVAPDTPFVINSATGWVSVSGPLDRESVE
HYFFGVEARDHGSPPLSASASVTVTVLDVNDNRPEFTMKEYHLRLNEDAAVGTSVVSVTA
VDRDANSAISYQITGGNTRNRFAISTQGGVGLVTLALPLDYKQERYFKLVLTASDRALHD
HCYVHINITDANTHRPVFQSAHYSVSVNEDRPMGSTIVVISASDDDVGENARITYLLEDN
LPQFRIDADSGAITLQAPLDYEDQVTYTLAITARDNGIPQKADTTYVEVMVNDVNDNAPQ
FVASHYTGLVSEDAPPFTSVLQISATDRDAHANGRVQYTFQNGEDGDGDFTIEPTSGIVR
TVRRLDREAVSVYELTAYAVDRGVPPLRTPVSIQVMVQDVNDNAPVFPAEEFEVRVKENS
IVGSVVAQITAVDPDEGPNAHIMYQIVEGNIPELFQMDIFSGELTALIDLDYEARQEYVI
VVQATSAPLVSRATVHVRLVDQNDNSPVLNNFQILFNNYVSNRSDTFPSGIIGRIPAYDP
DVSDHLFYSFERGNELQLLVVNQTSGELRLSRKLDNNRPLVASMLVTVTDGLHSVTAQCV
LRVVIITEELLANSLTVRLENMWQERFLSPLLGRFLEGVAAVLATPAEDVFIFNIQNDTD
VGGTVLNVSFSALAPRGAGAGAAGPWFSSEELQEQLYVRRAALAARSLLDVLPFDDNVCL
REPCENYMKCVSVLRFDSSAPFLASASTLFRPIQPIAGLRCRCPPGFTGDFCETELDLCY
SNPCRNGGACARREGGYTCVCRPRFTGEDCELDTEAGRCVPGVCRNGGTCTDAPNGGFRC
QCPAGGAFEGPRCEVAARSFPPSSFVMFRGLRQRFHLTLSLSFATVQQSGLLFYNGRLNE
KHDFLALELVAGQVRLTYSTGESNTVVSPTVPGGLSDGQWHTVHLRYYNKPRTDALGGAQ
GPSKDKVAVLSVDDCDVAVALQFGAEIGNYSCAAAGVQTSSKKSLDLTGPLLLGGVPNLP
ENFPVSHKDFIGCMRDLHIDGRRVDMAAFVANNGTMAGCQAKLHFCDSGPCKNSGFCSER
WGSFSCDCPVGFGGKDCQLTMAHPHHFRGNGTLSWNFGSDMAVSVPWYLGLAFRTRATQG
VLMQVQAGPHSTLLCQLDRGLLSVTVTRGSGRASHLLLDQVTVSDGRWHDLRLELQEEPG
GRRGHHVLMVSLDFSLFQDTMAVGSELQGLKVKQLHVGGLPPGSAEEAPQGLVGCIQGVW
LGSTPSGSPALLPPSHRVNAEPGCVVTNACASGPCPPHADCRDLWQTFSCTCQPGYYGPG
CVDACLLNPCQNQGSCRHLPGAPHGYTCDCVGGYFGHHCEHRMDQQCPRGWWGSPTCGPC
NCDVHKGFDPNCNKTNGQCHCKEFHYRPRGSDSCLPCDCYPVGSTSRSCAPHSGQCPCRP
GALGRQCNSCDSPFAEVTASGCRVLYDACPKSLRSGVWWPQTKFGVLATVPCPRGALGAA
VRLCDEAQGWLEPDLFNCTSPAFRELSLLLDGLELNKTALDTMEAKKLAQRLREVTGHTD
HYFSQDVRVTARLLAHLLAFESHQQGFGLTATQDAHFNENLLWAGSALLAPETGDLWAAL
GQRAPGGSPGSAGLVRHLEEYAATLARNMELTYLNPMGLVTPNIMLSIDRMEHPSSPRGA
RRYPRYHSNLFRGQDAWDPHTHVLLPSQSPRPSPSEVLPTSSSIENSTTSSVVPPPAPPE
PEPGISIIILLVYRTLGGLLPAQFQAERRGARLPQNPVMNSPVVSVAVFHGRNFLRGILE
SPISLEFRLLQTANRSKAICVQWDPPGLAEQHGVWTARDCELVHRNGSHARCRCSRTGTF
GVLMDASPRERLEGDLELLAVFTHVVVAVSVAALVLTAAILLSLRSLKSNVRGIHANVAA
ALGVAELLFLLGIHRTHNQLVCTAVAILLHYFFLSTFAWLFVQGLHLYRMQVEPRNVDRG
AMRFYHALGWGVPAVLLGLAVGLDPEGYGNPDFCWISVHEPLIWSFAGPVVLVIVMNGTM

FLLAARTSCSTGQREAKKTSALTLRSSFLLLLLVSASWLFGLLAVNHSILAFHYLHAGLC
GLQGLAVLLLFCVLNADARAAWMPACLGRKAAPEEARPAPGLGPGAYNNTALFEESGLIR
ITLGASTVSSVSSARSGRTQDQDSQRGRSYLRDNVLVRHGSAADHTDHSLQAHAGPTDLD
VAMFHRDAGADSDSDSDLSLEEERSLSIPSSESEDNGRTRGRFQRPLCRAAQSERLLTHP
KDVDGNDLLSYWPALGECEAAPCALQTWGSERRLGLDTSKDAANNNQPDPALTSGDETSL
GRAQRQRKGILKNRLQYPLVPQTRGAPELSWCRAATLGHRAVPAASYGRIYAGGGTGSLS
QPASRYSSREQLDLLLRRQLSRERLEEAPAPVLRPLSRPGSQECMDAAPGRLEPKDRGST
LPRRQPPRDYPGAMAGRFGSRDALDLGAPREWLSTLPPPRRTRDLDPQPPPLPLSPQRQL
SRDPLLPSRPLDSLSRSSNSREQLDQVPSRHPSREALGPLPQLLRAREDSVSGPSHGPST
EQLDILSSILASFNSSALSSVQSSSTPLGPHTTATPSATASVLGPSTPRSATSHSISELS
PDSEVPRSEGHS
>sp|Q9BS16|CENPK_HUMAN Centromere protein K OS=Homo sapiens GN=CENPK PE=1 SV=1
MNQEDLDPDSTTDVGDVTNTEEELIRECEEMWKDMEECQNKLSLIGTETLTDSNAQLSLL
IMQVKCLTAELSQWQKKTPETIPLTEDVLITLGKEEFQKLRQDLEMVLSTKESKNEKLKE
DLEREQRWLDEQQQIMESLNVLHSELKNKVETFSESRIFNELKTKMLNIKEYKEKLLSTL
GEFLEDHFPLPDRSVKKKKKNIQESSVNLITLHEMLEILINRLFDVPHDPYVKISDSFWP
PYVELLLRNGIALRHPEDPTRIRLEAFHQ
>sp|Q9BU64|CENPO_HUMAN Centromere protein O OS=Homo sapiens GN=CENPO PE=1 SV=1
MEQANPLRPDGESKGGVLAHLERLETQVSRSRKQSEELQSVQAQEGALGTKIHKLRRLRD
ELRAVVRHRRASVKACIANVEPNQTVEINEQEALEEKLENVKAILQAYHFTGLSGKLTSR
GVCVCISTAFEGNLLDSYFVDLVIQKPLRIHHHSVPVFIPLEEIAAKYLQTNIQHFLFSL
CEYLNAYSGRKYQADRLQSDFAALLTGPLQRNPLCNLLSFTYKLDPGGQSFPFCARLLYK
DLTATLPTDVTVTCQGVEVLSTSWEEQRASHETLFCTKPLHQVFASFTRKGEKLDMSLVS
>sp|Q8N2Z9|CENPS_HUMAN Centromere protein S OS=Homo sapiens GN=APITD1 PE=1 SV=1
MEEEAETEEQQRFSYQQRLKAAVHYTVGCLCEEVALDKEMQFSKQTIAAISELTFRQCEN
FAKDLEMFARHAKRTTINTEDVKLLARRSNSLLKYITDKSEEIAQINLERKAQKKKKSED
GSKNSRQPAEAGVVESEN
>sp|Q71F23|CENPU_HUMAN Centromere protein U OS=Homo sapiens GN=CENPU PE=1 SV=1
MAPRGRRRPRPHRSEGARRSKNTLERTHSMKDKAGQKCKPIDVFDFPDNSDVSSIGRLGE
NEKDEETYETFDPPLHSTAIYADEEEFSKHCGLSLSSTPPGKEAKRSSDTSGNEASEIES
VKISAKKPGRKLRPISDDSESIEESDTRRKVKSAEKISTQRHEVIRTTASSELSEKPAES
VTSKKTGPLSAQPSVEKENLAIESQSKTQKKGKISHDKRKKSRSKAIGSDTSDIVHIWCP
EGMKTSDIKELNIVLPEFEKTHLEHQQRIESKVCKAAIATFYVNVKEQFIKMLKESQMLT
NLKRKNAKMISDIEKKRQRMIEVQDELLRLEPQLKQLQTKYDELKERKSSLRNAAYFLSN
LKQLYQDYSDVQAQEPNVKETYDSSSLPALLFKARTLLGAESHLRNINHQLEKLLDQG
>sp|Q9H6K1|CF106_HUMAN Uncharacterized protein C6orf106 OS=Homo sapiens GN=C6orf106 PE=1 SV=2
MEGMDVDLDPELMQKFSCLGTTDKDVLISEFQRLLGFQLNPAGCAFFLDMTNWNLQAAIG
AYYDFESPNISVPSMSFVEDVTIGEGESIPPDTQFVKTWRIQNSGAEAWPPGVCLKYVGG
DQFGHVNMVMVRSLEPQEIADVSVQMCSPSRAGMYQGQWRMCTATGLYYGDVIWVILSVE
VGGLLGVTQQLSSFETEFNTQPHRKVEGNFNPFASPQKNRQSDENNLKDPGGSEFDSISK
NTWAPAPDTWAPAPDQTEQDQNRLSQNSVNLSPSSHANNLSVVTYSKGLHGPYPFGQS
>sp|A8K010|CF176_HUMAN Putative transcriptional regulator encoded by LINC00473 OS=Homo sapiens GN=LINC00473 PE=5 SV=2

MELSAAAGRRRQAPWREFTGRHRTERSQERGSTPRKERSMGSRQPRKREREPRDCTPTCT
NAASVSRSGARRAPGCAGRCITMKNCARAWRELSERACCFQAVPPSNMKQSWLIFLDTTL
CSAWLLASSCPRLYAPLISFHCMCFNTVLLFPRLFGPTLSAKIQFNTDLSLRAIAWKCTK
DTTTYC
>sp|Q96MR6|CFA57_HUMAN Cilia- and flagella-associated protein 57 OS=Homo sapiens GN=CFAP57 PE=2 SV=3
MSAVVAQTLHVFGLRSHVANNIFYFDEQIIIFPSGNHCVKYNVDQKWQKFIPGSEKSQGM
LALSISPNRRYLAISETVQEKPAITIYELSSIPCRKRKVLNNFDFQVQKFISMAFSPDSK
YLLAQTSPPESNLVYWLWEKQKVMAIVRIDTQNNPVYQVSFSPQDNTQVCVTGNGMFKLL
RFAEGTLKQTSFQRGEPQNYLAHTWVADDKIVVGTDTGKLFLFESGDQRWETSIMVKEPT
NGSKSLDVIQESESLIEFPPVSSPLPSYEQMVAASSHSQMSMPQVFAIAAYSKGFACSAG
PGRVLLFEKMEEKDFYRESREIRIPVDPQSNDPSQSDKQDVLCLCFSPSEETLVASTSKN
QLYSITMSLTEISKGEPAHFEYLMYPLHSAPITGLATCIRKPLIATCSLDRSIRLWNYET
NTLELFKEYQEEAYSISLHPSGHFIVVGFADKLRLMNLLIDDIRSFKEYSVRGCGECSFS
NGGHLFAAVNGNVIHVYTTTSLENISSLKGHTGKIRSIVWNADDSKLISGGTDGAVYEWN
LSTGKRETECVLKSCSYNCVTVSPDAKIIFAVGSDHTLKEIADSLILREISAFDVTYTAI
VISHSGRMMFVGTSVGTIRAMKYPLPLQKEFNEYQAHAGPITKMLLTFDDQFLLTAAEDG
CLFTWKVFDKDGRGIKREREVGFAEEVLVTKTDMEEKAQVMLELKTRVEELKMENEYQLR
LKDMNYSEKIKELTDKFIQEMESLKTKNQVLRTEKEKQDVYHHEHIEDLLDKQSRELQDM
ECCNNQKLLLEYEKYQELQLKSQRMQEEYEKQLRDNDETKSQALEELTEFYEAKLQEKTT
LLEEAQEDVRQQLREFEETKKQIEEDEDREIQDIKTKYEKKLRDEKESNLRLKGETGIMR
KKFSSLQKEIEERTNDIETLKGEQMKLQGVIKSLEKDIQGLKREIQERDETIQDKEKRIY
DLKKKNQELGKFKFVLDYKIKELKKQIEPRENEIRVMKEQIQEMEAELENFHKQNTQLEL
NITELWQKLRATDQEMRRERQKERDLEALVKRFKTDLHNCVAYIQEPRLLKEKVRGLFEK
YVQRADMVEIAGLNTDLQQEYTRQREHLERNLATLKKKVVKEGELHRTDYVRIMQENVSL
IKEINELRRELKFTRSQVYDLEAALKLTKKVRPQEVSETEPSRDMLSTAPTARLNEQEET
GRIIEMQRLEIQRLRDQIQEQEQVTGFHTLAGVRLPSLSNSEVDLEVKTN
>sp|Q8NHU2|CFA61_HUMAN Cilia- and flagella-associated protein 61 OS=Homo sapiens GN=CFAP61 PE=2 SV=3
MSVLTSPRGKVEVVHCRRTESQDVYCIKSLIRKFTCKLFGKLNIIYLLEKANLAVTLCND
KEEIMAQATFLDYPNWNVAKQDDWVSVFRELDSDIPCTPLNTLFMHLFVAVDEYSVGCCK
EILRTVYKAVPELHFIFLIVPSYMSLGSTLITVFDQVGNIPCLTYEEDFAVHICHRHSHY
PQLHVRKARVEDHDDLMPIFMRYDTILKETYGEYFLAELIEAQDEENHAVVCEVEGTAVG
FMSVCSRVNMQLLHECFDLGPFHGLCFPHPDDVLESPQDLSVRRSQDAELRSSSQGSQKI
VEELQEPVSPDTMENIQGNIAREAASEEALTAVQSGNVSEPEDIEKLSDISTGYAQYHHV
SSRSLASLVLPEEPVHFRPIYRGASAAFCIQLFCIDEKYEARSLDFMNFVFSLFSDKNFC
VISLPHLTPEFFLIQNFVKMVPFNTCTLEQDLYVFHRAGLLKSINIRFATLLDTPGVENL
VSTLMLNKSILEDLDRYNKARKDPDGTLLQAFVAEVAEQIVGIAVIRNEMDIEYIRSHYN
IEDFIYFSHHQREEHGHMHHFALNPIFRHYTKFFLKEILRLGFKSCLYYRVYPKSREGKF
QNPYAHSLTSALHYLVPVRPRRQIVYPLEKLGINAPSKAVSKDPMSYALNHTNRKLTLEP
KITVNAKIIVVGASSVGISFLETLVFCSHMKFNNLTLISTHGLPGKKLLDTEQRKFLASD
HCFNDKDYALMSLCSWVNVVVGRMTGIDRAAKHVVLSTDEIVPYDHLILCTGQQYQVPCP
TEADISQHLTNREVPNSSQRRYTGKVPCNHFTLNEEEDCFKALIWIRNNSITTEGNIIVY
GNTIDTYTTVETLLNLGVSGSRIHLVQPPPASTITCINNYSVESAVADALGAAGVTMYRD

AILAQWNDGLHPDPIYSASFTTPTKPFRLQCSMFFSFCEKNVDYETFKALNDACLVYDSR
LVIDTNFHTNDIAIRAAGSLTKFSNRYYSNEWTHSNFSSKEIGFQLAAAMLHLFDPTLEP
VTEPPANLDRLIPMYKGAKIQGGILPGSYHYLHIAKPAIPTPLEVQMAQPNYGLELVTGS
AKNGTYFRIHINKYKMVETITCLSREPFPASNYIRLFGQHEQLLNNLCARYDENLITDLY
SYFTEPWCLALFHDRFIDLRKELRQILASKEEEDLPSIEQLAHQIEDEEINPTEKPRQYL
KRVFEESIYKTLVERSTLDYLHYNRYHLPMYAWPGIV
>sp|Q9C0B2|CFA74_HUMAN Cilia- and flagella-associated protein 74 OS=Homo sapiens GN=CFAP74 PE=2 SV=3
MEDDGSLLPEDELLADALLLEDERDELEDPEFDIKCLLQEAEDDVDPGHSSSVKELDTDA
DKLKKKTAEDRTQAFHLRQNLSALDKMHEEQELFTEKMRGELRACRQRRDLIDKQQEAVA
AEIATEEEAGNMAAVGRLQAVSRRLFAELENERDLQSRTEAVLKESENTMWHIEIQEGRL
EAFRTADREEVEATGRRLQVRAAEQLCREQEALGKVERNRLLRIRKSLNTQKELGLRHQK
LLEDARKNHKVAVRFLKASLGRIREQEKKEEMECHEYMRRRMDAVVALKGSISANRDTLR
KFQAWDRAKAELAEQRVQAEKKAILAQGRDAFRHLVHQRRRQELEAQKRAFEEEQKLRKQ
EIISRILKEEAEEEKRKKQHPPTSARHRLTLRDKTWNYISDFCKKTTVPTNTYTLDYEAA
AGPGPSRLLEVVSSELIQGDPGASSEEETLAEPEISGLWNEDYKPYQVPKEDVDRKPVGG
TKMDKDILERTVERLRSRVVHKQVVWGREFQGRPFNSKPELLHFQDFDIGKVYKKKITLV
NTTYTINYCKLVGVEEHLRDFIHVDFDPPGPLSAGMSCEVLVTFKPMINKDLEGNISFLA
QTGEFSVPLKCSTKKCSLSLDKELIDFGSYVVGETTSRTITLTNVGGLGTTFKFLPASEP
CEMDDSQSALKLSSLLTYEDKSLYDKAATSFSEQQLEGTESSQADMQSRKELEKLDKEQE
EEQPAEPERLTTVIPPSEEQTEITLGEVTEGEIGPFSSIKVPIVFTPVVPGDVQARFKVT
FKNPQCPTLHFRVVGVAIDVPVWVPKPSVDLKICMYDRLYQDSVLVHTRSKAALRLKFEV
CKELRAHLELLPKTGYIQAQSSYSVQLKFLPRHSLPEDAGRYFDKETRVLEAPMTIWVAD
QNKPVGFTVHAIVTTSDLELSPSEVDFGYCTIYEAIRTEISLHNHSLLPQEFGFVRLPKF
VDVQPNDGFGTILPLETLQFCVIFQPTKAEEHRFQLTCKSEINRCFKLSCRAVGVHPPLE
LSHYQIKFAATALYDTSVATVYVINSHLSMSSPTHSKPRIGSEDASPMGPTSFEFLLPPD
SPITISPSVGTVWPGKRCLVQVAFRPVLPEKLIRQEALPLLNKEMETKSFRKNMAPQRKD
LHGLSFSVLRAQNRDKLFKVSVPHVLEMRKRELRPSSDEYQAARATLLRAFQAKFDTFVV
PCVVASGDIKDRKGSEPLSFSPHNTLYLELWCPTVAPSVVVTSHKGKTIFNFGDVAVGHR
SIKKISIQNVSPEDLALDFSLLNPNGPFVLLNHSSLLRAGGTQVLVLSFSPHESILAQET
LDIITKRGTLTLTLMGTGVASMITCSIEGSVLNMGYVIAGESVSSGFKLQNNSLLPIKFS
MHLDSLSSTRGRGQQQLPQFLSSPSQRTEVVGTQNLNGQSVFSVAPVKGVMDPGKTQDFT
VTFSPDHESLYFSDKLQVVLFEKKISHQILLKGAACQHMMFVEGGDPLDVPVESLTAIPV
FDPRHREEAEELRPILVTLDYIQFDTDTPAPPATRELQVGCIRTTQPSPKKPDHPLMVSA
LLQLRGDVKETYKVIFVAQVLTGP
>sp|Q9P2B7|CFA97_HUMAN Cilia- and flagella-associated protein 97 OS=Homo sapiens GN=CFAP97 PE=1 SV=2
MDQFGDILEGEVDHSFFDSDFEEGKKCETNSVFDKQNDDPKERIDKDTKNVNSNTGMQTT
ENYLTEKGNERNVKFPPEHPVENDVTQTVSSFSLPASSRSKKLCDVTTGLKIHVSIPNRI
PKIVKEGEDDYYTDGEESSDDGKKYHVKSKSAKPSTNVKKSIRKKYCKVSSSSSSSLSSS
SSGSGTDCLDAGSDSHLSDSSPSSKSSKKHVSGITLLSPKHKYKSGIKSTETQPSSTTPK
CGHYPEESEDTVTDVSPLSTPDISPLQSFELGIANDQKVKIKKQENVSQEIYEDVEDLKN
NSKYLKAAKKGKEKHEPDVSSKSSSVLDSSLDHRHKQKVLHDTMDLNHLLKAFLQLDKKG
PQKHHFDQPSVAPGKNYSFTREEVRQIDRENQRLLKELSRQAEKPGSKSTIPRSADHPPK

LYHSALNRQKEQQRIERENLALLKRLEAVKPTVGMKRSEQLMDYHRNMGYLNSSPLSRRA
RSTLGQYSPLRASRTSSATSGLSCRSERSAVDPSSGHPRRRPKPPNVRTAWL
>sp|Q8NEG2|CG057_HUMAN Uncharacterized protein C7orf57 OS=Homo sapiens GN=C7orf57 PE=2 SV=3
MRNTSKELQGATHRYAPCDWYYHVPVKRSEKAVDAPPASQIPGLSNLGDSHSENLPGTRR
YWIKETDSEYVKLAKQGGRPDLLKHFAPGTRKGSPVAYSLPDWYIHHSKPPTASQQEVRA
VSMPDYMVHEEFNPDQANGSYASRRGPFDFDMKTVWQREAEELEKEKKKLRLPAIDSKYL
SKAGTPLGPKNPAGSRLSFPPVPGQKNSSPTNFSKLISNGYKDEWLQQQQRADSDKRTPK
TSRASVLSQSPRDLEGPQDAARLQDAEASEGPEDTPESSQSPEESVSASTPAELK
>sp|A4D263|CG072_HUMAN Uncharacterized protein C7orf72 OS=Homo sapiens GN=C7orf72 PE=4 SV=2
MDVEIQDTPGKISISKRSILSGTVENIDYPHYCDLLRKMNMPFVKGLENRHNYGRFEKKC
NPAFLKFHPYPPSVLPDYHLHDPYPPPYGPHYPLFPLRDDVTLGDSCSGFMSPGGDADLN
PGIGRTIPTLVDFSDVKPQHRVPRPDTGFQTTIKRQKILSEELQQNRRWNSREVPDISIR
ARLGGWTSPLKVTPLQPHHEGRSLSHIFTFDEEATCTDEGEPLVQTNKKCNAKDSFYKSS
TQKAYEDVPWDKMLPPKLVPEETTLEKTADPISQCFTLKRYKGVPAITQMVGELWDRFQT
RSFLAPVKPINFVSSSSRSKYIPLYTGHVQSTNADDVDNPLGDIASLAKQRYSKPLYTNT
SRAANIPGYTGKVHFTATHPANSNIPSTTPSPDSELHRVFQKEMAVDLFRHQAPLSRLVT
TVRPYNPFNKKDKETIDY
>sp|A4D0Y5|CG077_HUMAN Uncharacterized protein C7orf77 OS=Homo sapiens GN=C7orf77 PE=4 SV=1
MGAERVCTKAPEITQDEAEIYSLTNMEGNIGIKGCEFKSWLFKFYQARSQVLLCGEVKNP
YLLTSNKTTVKEQNACLTHPDRSAMAGLLL
>sp|Q8N884|CGAS_HUMAN Cyclic GMP-AMP synthase OS=Homo sapiens GN=MB21D1 PE=1 SV=2
MQPWHGKAMQRASEAGATAPKASARNARGAPMDPTESPAAPEAALPKAGKFGPARKSGSR
QKKSAPDTQERPPVRATGARAKKAPQRAQDTQPSDATSAPGAEGLEPPAAREPALSRAGS
CRQRGARCSTKPRPPPGPWDVPSPGLPVSAPILVRRDAAPGASKLRAVLEKLKLSRDDIS
TAAGMVKGVVDHLLLRLKCDSAFRGVGLLNTGSYYEHVKISAPNEFDVMFKLEVPRIQLE
EYSNTRAYYFVKFKRNPKENPLSQFLEGEILSASKMLSKFRKIIKEEINDIKDTDVIMKR
KRGGSPAVTLLISEKISVDITLALESKSSWPASTQEGLRIQNWLSAKVRKQLRLKPFYLV
PKHAKEGNGFQEETWRLSFSHIEKEILNNHGKSKTCCENKEEKCCRKDCLKLMKYLLEQL
KERFKDKKHLDKFSSYHVKTAFFHVCTQNPQDSQWDRKDLGLCFDNCVTYFLQCLRTEKL
ENYFIPEFNLFSSNLIDKRSKEFLTKQIEYERNNEFPVFDEF
>sp|Q8TDX6|CGAT1_HUMAN Chondroitin sulfate N-acetylgalactosaminyltransferase 1 OS=Homo sapiens GN=CSGALNACT1 PE=1 SV=2
MMMVRRGLLAWISRVVVLLVLLCCAISVLYMLACTPKGDEEQLALPRANSPTGKEGYQAV
LQEWEEQHRNYVSSLKRQIAQLKEELQERSEQLRNGQYQASDAAGLGLDRSPPEKTQADL
LAFLHSQVDKAEVNAGVKLATEYAAVPFDSFTLQKVYQLETGLTRHPEEKPVRKDKRDEL
VEAIESALETLNSPAENSPNHRPYTASDFIEGIYRTERDKGTLYELTFKGDHKHEFKRLI
LFRPFGPIMKVKNEKLNMANTLINVIVPLAKRVDKFRQFMQNFREMCIEQDGRVHLTVVY
FGKEEINEVKGILENTSKAANFRNFTFIQLNGEFSRGKGLDVGARFWKGSNVLLFFCDVD
IYFTSEFLNTCRLNTQPGKKVFYPVLFSQYNPGIIYGHHDAVPPLEQQLVIKKETGFWRD
FGFGMTCQYRSDFINIGGFDLDIKGWGGEDVHLYRKYLHSNLIVVRTPVRGLFHLWHEKR
CMDELTPEQYKMCMQSKAMNEASHGQLGMLVFRHEIEAHLRKQKQKTSSKKT

>sp|Q99674|CGRE1_HUMAN Cell growth regulator with EF hand domain protein 1 OS=Homo sapiens GN=CGREF1 PE=2 SV=2
MLPLTMTVLILLLLPTGQAAPKDGVTRPDSEVQHQLLPNPFQPGQEQLGLLQSYLKGLGR
TEVQLEHLSREQVLLYLFALHDYDQSGQLDGLELLSMLTAALAPGAANSPTTNPVILIVD
KVLETQDLNGDGLMTPAELINFPGVALRHVEPGEPLAPSPQEPQAVGRQSLLAKSPLRQE
TQEAPGPREEAKGQVEARRESLDPVQEPGGQAEADGDVPGPRGEAEGQAEAKGDAPGPRG
EAGGQAEAEGDAPGPRGEAGGQAEARENGEEAKELPGETLESKNTQNDFEVHIVQVENDE
I
>sp|Q99675|CGRF1_HUMAN Cell growth regulator with RING finger domain protein 1 OS=Homo sapiens GN=CGRRF1 PE=1 SV=1
MAAVFLVTLYEYSPLFYIAVVFTCFIVTTGLVLGWFGWDVPVILRNSEETQFSTRVFKKQ
MRQVKNPFGLEITNPSSASITTGITLTTDCLEDSLLTCYWGCSVQKLYEALQKHVYCFRI
STPQALEDALYSEYLYQEQYFIKKDSKEEIYCQLPRDTKIEDFGTVPRSRYPLVALLTLA
DEDDREIYDIISMVSVIHIPDRTYKLSCRILYQYLLLAQGQFHDLKQLFMSANNNFTPSN
NSSSEEKNTDRSLLEKVGLSESEVEPSEENSKDCVVCQNGTVNWVLLPCRHTCLCDGCVK
YFQQCPMCRQFVQESFALCSQKEQDKDKPKTL
>sp|Q9H7E9|CH033_HUMAN UPF0488 protein C8orf33 OS=Homo sapiens GN=C8orf33 PE=1 SV=1
MAALGHLAGEAAAAPGPGTPCASRGARLPGPVSSARNPSTVCLCPEQPTCSNADSRAHPL
GDEGGTASKKQKNKKKTRNRASVANGGEKASEKLAPEEVPLSAEAQAQQLAQELAWCVEQ
LELGLKRQKPTPKQKEQAIGAIRTLRSKRTPLPRKRQLMHSLFGDYRAQMEAEWREALRA
LRAAAYSAQVQPVDGATRKKSQRVCRPRSIWRAKATLDMPDEEFRFNFF
>sp|Q8TAG6|CH046_HUMAN Uncharacterized protein C8orf46 OS=Homo sapiens GN=C8orf46 PE=2 SV=2
MMHQIYSCSDENIEVFTTVIPSKVSSPARRRAKSSQHLLTKNVVIESDLYTHQPLELLPH
RGDRRDPGDRRRFGRLQTARPPTAHPAKASARPVGISEPKTSNLCGNRAYGKSLIPPVPR
ISVKTSASASLEATAMGTEKGAVLMRGSRHLKKMTEEYPALPQGAEASLPLTGSASCGVP
GILRKMWTRHKKKSEYVGATNSAFEAD
>sp|Q8NAV2|CH058_HUMAN Uncharacterized protein C8orf58 OS=Homo sapiens GN=C8orf58 PE=2 SV=2
MMGRRRAFAVDGRDGAGEGLARGCIVPGVTSTYRRIPDAAHGCSSWERGDKFRGVGREAL
FLKLASRDSGVEMAVGDSPLAALPGLSQDSLDFESSGSSEPPAQVGRLLASQKLGEVLER
SRRLPTAPTSLSGQHRSLRLASKPEREVPLGAGQQESMEADTDLEAGLEEEAVGGLGPGA
WACLPGQGLRYLEHLCLVLEQMARLQQLYLQLRIQRPPGDPGEEESTRAPLPSPLHTPGN
RGQGPWELLSQTEHTGAKAASPPKVEVPSANPPRLPETPVEPTYHLPSSQGHKRDISHWD
KVKVLLNRICRRSHHHPEPPAPPDGSDPRIESRDLPERPQCRPHRKTFMPSLVVKKQRAK
NLSVG
>sp|Q8N0T1|CH059_HUMAN Uncharacterized protein C8orf59 OS=Homo sapiens GN=C8orf59 PE=1 SV=4
MAKNKLRGPKSRNVFHIASQKNFKAKNKAKPVTTNLKKINIMNEEKVNRVNKAFVNVQKE
LAHFAKSISLEPLQKELIPQQRHESKPVNVDEATRLMALL
>sp|P0DMB2|CH088_HUMAN Uncharacterized protein C8orf88 OS=Homo sapiens GN=C8orf88 PE=4 SV=1
METKKLIGKPLQPARPVRHLTSPPGAVFPFNFQNEYPCNTQCIQSGVSRCKTNGMQAFSQ
GLNEQQQQQSPVKKERIKYSRDFLLKLSSVSICRKKPDFLPDHPIVLQKPENNQSFK

>sp|P61604|CH10_HUMAN 10 kDa heat shock protein, mitochondrial OS=Homo sapiens GN=HSPE1 PE=1 SV=2
MAGQAFRKFLPLFDRVLVERSAAETVTKGGIMLPEKSQGKVLQATVVAVGSGSKGKGGEI
QPVSVKVGDKVLLPEYGGTKVVLDDKDYFLFRDGDILGKYVD
>sp|Q96KX2|CAZA3_HUMAN F-actin-capping protein subunit alpha-3 OS=Homo sapiens GN=CAPZA3 PE=1 SV=2
MTLSVLSRKDKERVIRRLLLQAPPGEFVNAFDDLCLLIRDEKLMHHQGECAGHQHCQKYS
VPLCIDGNPVLLSHHNVMGDYRFFDHQSKLSFKYDLLQNQLKDIQSHGIIQNEAEYLRVV
LLCALKLYVNDHYPKGNCNMLRKTVKSKEYLIACIEDHNYETGECWNGLWKSKWIFQVNP
FLTQVTGRIFVQAHFFRCVNLHIEISKDLKESLEIVNQAQLALSFARLVEEQENKFQAAV
LEELQELSNEALRKILRRDLPVTRTLIDWHRILSDLNLVMYPKLGYVIYSRSVLCNWII
>sp|Q6P9G0|CB5D1_HUMAN Cytochrome b5 domain-containing protein 1 OS=Homo sapiens GN=CYB5D1 PE=2 SV=1
MPRRGLVAGPDLEYFQRRYFTPAEVAQHNRPEDLWVSYLGRVYDLTSLAQEYKGNLLLKP
IVEVAGQDISHWFDPKTRDIRKHIDPLTGCLRYCTPRGRFVHVPPQLPCSDWANDFGKPW
WQGSYYEVGRLSAKTRSIRIINTLTSQEHTLEVGVLESIWEILHRYLPYNSHAASYTWKY
EGKNLNMDFTLEENGIRDEEEEFDYLSMDGTLHTPAILLYFNDDLTEL
>sp|Q9UI42|CBPA4_HUMAN Carboxypeptidase A4 OS=Homo sapiens GN=CPA4 PE=1 SV=2
MRWILFIGALIGSSICGQEKFFGDQVLRINVRNGDEISKLSQLVNSNNLKLNFWKSPSSF
NRPVDVLVPSVSLQAFKSFLRSQGLEYAVTIEDLQALLDNEDDEMQHNEGQERSSNNFNY
GAYHSLEAIYHEMDNIAADFPDLARRVKIGHSFENRPMYVLKFSTGKGVRRPAVWLNAGI
HSREWISQATAIWTARKIVSDYQRDPAITSILEKMDIFLLPVANPDGYVYTQTQNRLWRK
TRSRNPGSSCIGADPNRNWNASFAGKGASDNPCSEVYHGPHANSEVEVKSVVDFIQKHGN
FKGFIDLHSYSQLLMYPYGYSVKKAPDAEELDKVARLAAKALASVSGTEYQVGPTCTTVY
PASGSSIDWAYDNGIKFAFTFELRDTGTYGFLLPANQIIPTAEETWLGLKTIMEHVRDNL
Y
>sp|P16152|CBR1_HUMAN Carbonyl reductase [NADPH] 1 OS=Homo sapiens GN=CBR1 PE=1 SV=3
MSSGIHVALVTGGNKGIGLAIVRDLCRLFSGDVVLTARDVTRGQAAVQQLQAEGLSPRFH
QLDIDDLQSIRALRDFLRKEYGGLDVLVNNAGIAFKVADPTPFHIQAEVTMKTNFFGTRD
VCTELLPLIKPQGRVVNVSSIMSVRALKSCSPELQQKFRSETITEEELVGLMNKFVEDTK
KGVHQKEGWPSSAYGVTKIGVTVLSRIHARKLSEQRKGDKILLNACCPGWVRTDMAGPKA
TKSPEEGAETPVYLALLPPDAEGPHGQFVSEKRVEQW
>sp|Q5JTY5|CBWD3_HUMAN COBW domain-containing protein 3 OS=Homo sapiens GN=CBWD3 PE=2 SV=1
MLPAVGSVDEEEDPAEEDCPELVPIETTQSEEEEKSGLGAKIPVTIITGYLGAGKTTLLN
YILTEQHSKRVAVILNESGEGSALEKSLAVSQGGELYEEWLELRNGCLCCSVKDNGLRAI
ENLMQKKGKFDDILLETTGLADPGAVASMFWVDAELGSDIYLDGIITIVDSKYGLKHLTE
EKPDGLINEATRQVALADIILINKTDLVPEEDVKKLRTTIRSINGLGQILETQRSRVDLS
NVLDLHAFDSLSGISLQKKLQHVPGTQPHLDQSIVTITFEVPGNAKEEHLNMFIQNLLWE
KNVRNKDNHCMEVIRLKGLVSIKDKSQQVIVQGVHELYDLEETPVSWKDDTERTNRLVLI
GRNLDKDILKQLFIATVTETEKQWTTHFKEDQVCT
>sp|O00257|CBX4_HUMAN E3 SUMO-protein ligase CBX4 OS=Homo sapiens GN=CBX4 PE=1 SV=3
MELPAVGEHVFAVESIEKKRIRKGRVEYLVKWRGWSPKYNTWEPEENILDPRLLIAFQNR
ERQEQLMGYRKRGPKPKPLVVQVPTFARRSNVLTGLQDSSTDNRAKLDLGAQGKGQGHQY

ELNSKKHHQYQPHSKERAGKPPPPGKSGKYYYQLNSKKHHPYQPDPKMYDLQYQGGHKEA
PSPTCPDLGAKSHPPDKWAQGAGAKGYLGAVKPLAGAAGAPGKGSEKGPPNGMMPAPKEA
VTGNGIGGKMKIVKNKNKNGRIVIVMSKYMENGMQAVKIKSGEVAEGEARSPSHKKRAAD
ERHPPADRTFKKAAGAEEKKVEAPPKRREEEVSGVSDPQPQDAGSRKLSPTKEAFGEQPL
QLTTKPDLLAWDPARNTHPPSHHPHPHPHHHHHHHHHHHHAVGLNLSHVRKRCLSETHGE
REPCKKRLTARSISTPTCLGGSPAAERPADLPPAAALPQPEVILLDSDLDEPIDLRCVKT
RSEAGEPPSSLQVKPETPASAAVAVAAAAAPTTTAEKPPAEAQDEPAESLSEFKPFFGNI
IITDVTANCLTVTFKEYVTV
>sp|O95503|CBX6_HUMAN Chromobox protein homolog 6 OS=Homo sapiens GN=CBX6 PE=1 SV=1
MELSAVGERVFAAESIIKRRIRKGRIEYLVKWKGWAIKYSTWEPEENILDSRLIAAFEQK
ERERELYGPKKRGPKPKTFLLKARAQAEALRISDVHFSVKPSASASSPKLHSSAAVHRLK
KDIRRCHRMSRRPLPRPDPQGGSPGLRPPISPFSETVRIINRKVKPREPKRNRIILNLKV
IDKGAGGGGAGQGAGALARPKVPSRNRVIGKSKKFSESVLRTQIRHMKFGAFALYKPPPA
PLVAPSPGKAEASAPGPGLLLAAPAAPYDARSSGSSGCPSPTPQSSDPDDTPPKLLPETV
SPSAPSWREPEVLDLSLPPESAATSKRAPPEVTAAAGPAPPTAPEPAGASSEPEAGDWRP
EMSPCSNVVVTDVTSNLLTVTIKEFCNPEDFEKVAAGVAGAAGGGGSIGASK
>sp|A1A4F0|CC055_HUMAN Putative uncharacterized protein PQLC2L OS=Homo sapiens GN=PQLC2L PE=2 SV=2
MKVVGNYRVNTANSSTDTSGEHLTCLRSQLFVAYRNGRVDEAVSLGFLDCWIGGDLTNFK
GCYLTNQLPIQIFTAIFDMNTDVIILSQFMYYRLKNQKKKMIFQPQLFKDSITREKVRLS
LWGVLCPVYIPYSFR
>sp|F5H4A9|CC080_HUMAN Uncharacterized membrane protein C3orf80 OS=Homo sapiens GN=C3orf80 PE=2 SV=1
MWGPGVTAEGLSVAPAPPPLLPLLLLLALALVAPSRGGGGCAELACGERERCCDATNATA
VRCCKLPLHAFLDNVGWFVRKLSGLLILLVLFAIGYFLQRIICPSPRRYPRGQARPGQRP
GPPGGAGPLGGAGPPDDDDDSPALLRDEAAAGSQDSLLDSGGGGRGRGGGGRSDPSCASE
HEMRVVSPVFLQLPSYEEVKYLPTYEESMRLQQLSPGEVVLPVSVLGRPRGGVAAEPDGG
EGRYPLI
>sp|Q96HB5|CC120_HUMAN Coiled-coil domain-containing protein 120 OS=Homo sapiens GN=CCDC120 PE=1 SV=1
MEVKGQLISSPTFNAPAALFGEAAPQVKSERLRGLLDRQRTLQEALSLKLQELRKVCLQE
AELTGQLPPECPLEPGERPQLVRRRPPTARAYPPPHPNQAHHSLCPAEELALEALEREVS
VQQQIAAAARRLALAPDLSTEQRRRRRQVQADALRRLHELEEQLRDVRARLGLPVLPLPQ
PLPLSTGSVITTQGVCLGMRLAQLSQEDVVLHSESSSLSESGASHDNEEPHGCFSLAERP
SPPKAWDQLRAVSGGSPERRTPWKPPPSDLYGDLKSRRNSVASPTSPTRSLPRSASSFEG
RSVPATPVLTRGAGPQLCKPEGLHSRQWSGSQDSQMGFPRADPASDRASLFVARTRRSNS
SEALLVDRAAGGGAGSPPAPLAPSASGPPVCKSSEVLYERPQPTPAFSSRTAGPPDPPRA
ARPSSAAPASRGAPRLPPVCGDFLLDYSLDRGLPRSGGGTGWGELPPAAEVPGPLSRRDG
LLTMLPGPPPVYAADSNSPLLRTKDPHTRATRTKPCGLPPEAAEGPEVHPNPLLWMPPPT
RIPSAGERSGHKNLALEGLRDWYIRNSGLAAGPQRRPVLPSVGPPHPPFLHARCYEVGQA
LYGAPSQAPLPHSRSFTAPPVSGRYGGCFY
>sp|Q86Z20|CC125_HUMAN Coiled-coil domain-containing protein 125 OS=Homo sapiens GN=CCDC125 PE=1 SV=2
MSKVARSSSESDVQLWETEEDDMTEGDLGYGLGRKPGGIYEIEFSHRSRKRSDGKNFSPP

PFPRKGEERNEASFQYSKHKSQQDTFPQVSRISNYRRQSSTVDSNSELSNEELRQCLNET
LEEVEMLKTELEASQRQLRGKEEALKILQSMAILGKATSHTQAVLQKTMEQNRSLEKEIN
ALQWEIEFDHNRFKNIEESWIQKYDRLNCENAVLKENLKVKTEEIKMLKSDNAVLNQRYL
EALAMLDIKQQKMAQENMCCDKSGFAEASGLELAVLGACLCHGPGGNPCSCARMAASTRK
LLLQLKQELEILQKSKEEAYVMADAFRIAFEQQLMRKNDQALQLTQMDKMHKKATKWMNW
KHLKEDGFPSPRSKKTFGQRLLGMLPSENSSKRMEDQDSPQEVLKMLIDLLNDKEEALAH
QRKVSYMLARALEDKDTASNENKEKNPIKENFPFNNPWRKTSEFSVLGDPIHSSVCILNS
VGCICSIQHSQIDPNYRTLKRSHSLPSSIIF
>sp|Q96EE4|CC126_HUMAN Coiled-coil domain-containing protein 126 OS=Homo sapiens GN=CCDC126 PE=2 SV=2
MFFTISRKNMSQKLSLLLLVFGLIWGLMLLHYTFQQPRHQSSVKLREQILDLSKRYVKAL
AEENKNTVDVENGASMAGYADLKRTIAVLLDDILQRLVKLENKVDYIVVNGSAANTTNGT
SGNLVPVTTNKRTNVSGSIR
>sp|Q96MF4|CC140_HUMAN Coiled-coil domain-containing protein 140 OS=Homo sapiens GN=CCDC140 PE=2 SV=1
MGDECSNPDLLAEPGSSPPWDHGNQRQEAANESNTRVPRVLKAHLGPETAQPTKRSKRNR
WRRQSCQGPSPARSGQFLGSADLGLQRGVLKSAARTCLSEISNSTRASPESAQSTDPGRA
ARPRTRTLPTPHSFKIGEEAAEEMKKKKERKRRKERKKERNFKK
>sp|A5D8V7|CC151_HUMAN Coiled-coil domain-containing protein 151 OS=Homo sapiens GN=CCDC151 PE=1 SV=1
MTSPLCRAASANALPPQDQASTPSSRVKGREASGKPSHLRGKGTAQAWTPGRSKGGSFHR
GAGKPSVHSQVAELHKKIQLLEGDRKAFFESSQWNIKKNQETISQLRKETKALELKLLDL
LKGDEKVVQAVIREWKWEKPYLKNRTGQALEHLDHRLREKVKQQNALRHQVVLRQRRLEE
LQLQHSLRLLEMAEAQNRHTEVAKTMRNLENRLEKAQMKAQEAEHITSVYLQLKAYLMDE
SLNLENRLDSMEAEVVRTKHELEALHVVNQEALNARDIAKNQLQYLEETLVRERKKRERY
ISECKKRAEEKKLENERMERKTHREHLLLQSDDTIQDSLHAKEEELRQRWSMYQMEVIFG
KVKDATGTDETHSLVRRFLAQGDTFAQLETLKSENEQTLVRLKQEKQQLQRELEDLKYSG
EATLVSQQKLQAEAQERLKKEERRHAEAKDQLERALRAMQVAKDSLEHLASKLIHITVED
GRFAGKELDPQADNYVPNLLGLVEEKLLKLQAQLQGHDVQEMLCHIANREFLASLEGRLP
EYNTRIALPLATSKDKFFDEESEEEDNEVVTRASLKIRSQKLIESHKKHRRSRRS
>sp|Q9P0B6|CC167_HUMAN Coiled-coil domain-containing protein 167 OS=Homo sapiens GN=CCDC167 PE=1 SV=2
MTKKKRENLGVALEIDGLEEKLSQCRRDLEAVNSRLHSRELSPEARRSLEKEKNSLMNKA
SNYEKELKFLRQENRKNMLLSVAIFILLTLVYAYWTM
>sp|Q8WUD4|CCD12_HUMAN Coiled-coil domain-containing protein 12 OS=Homo sapiens GN=CCDC12 PE=1 SV=1
MEATTAGVGRLEEEALRRKERLKALREKTGRKDKEDGEPKTKHLREEEEEGEKHRELRLR
NYVPEDEDLKKRRVPQAKPVAVEEKVKEQLEAAKPEPVIEEVDLANLAPRKPDWDLKRDV
AKKLEKLKKRTQRAIAELIRERLKGQEDSLASAVDAATEQKTCDSD
>sp|Q502W7|CCD38_HUMAN Coiled-coil domain-containing protein 38 OS=Homo sapiens GN=CCDC38 PE=2 SV=1
MSSNLLPTLNSGGKVKDGSTKEDRPYKIFFRDLFLVKENEMAAKETEKFMNRNMKVYQKT
TFSSRMKSHSYLSQLAFYPKRSGRSFEKFGPGPAPIPRLIEGSDTKRTVHEFINDQRDRF
LLEYALSTKRNTIKKFEKDIAMRERQLKKAEKKLQDDALAFEEFLRENDQRSVDALKMAA

QETINKLQMTAELKKASMEVQAVKSEIAKTEFLLREYMKYGFFLLQMSPKHWQIQQALKR
AQASKSKANIILPKILAKLSLHSSNKEGILEESGRTAVLSEDASQGRDSQGKPSRSLTRT
PEKKKSNLAESFGSEDSLEFLLDDEMDVDLEPALYFKEPEELLQVLRELEEQNLTLFQYS
QDVDENLEEVNKREKVIQDKTNSNIEFLLEQEKMLKANCVREEEKAAELQLKSKLFSFGE
FNSDAQEILIDSLSKKITQVYKVCIGDAEDDGLNPIQKLVKVESRLVELCDLIESIPKEN
VEAIERMKQKEWRQKFRDEKMKEKQRHQQERLKAALEKAVAQPKKKLGRRLVFHSKPPSG
NKQQLPLVNETKTKSQEEEYFFT
>sp|Q92583|CCL17_HUMAN C-C motif chemokine 17 OS=Homo sapiens GN=CCL17 PE=1 SV=1
MAPLKMLALVTLLLGASLQHIHAARGTNVGRECCLEYFKGAIPLRKLKTWYQTSEDCSRD
AIVFVTVQGRAICSDPNNKRVKNAVKYLQSLERS
>sp|P22362|CCL1_HUMAN C-C motif chemokine 1 OS=Homo sapiens GN=CCL1 PE=1 SV=1
MQIITTALVCLLLAGMWPEDVDSKSMQVPFSRCCFSFAEQEIPLRAILCYRNTSSICSNE
GLIFKLKRGKEACALDTVGWVQRHRKMLRHCPSKRK
>sp|O00626|CCL22_HUMAN C-C motif chemokine 22 OS=Homo sapiens GN=CCL22 PE=1 SV=2
MDRLQTALLVVLVLLAVALQATEAGPYGANMEDSVCCRDYVRYRLPLRVVKHFYWTSDSC
PRPGVVLLTFRDKEICADPRVPWVKMILNKLSQ
>sp|P80075|CCL8_HUMAN C-C motif chemokine 8 OS=Homo sapiens GN=CCL8 PE=1 SV=2
MKVSAALLCLLLMAATFSPQGLAQPDSVSIPITCCFNVINRKIPIQRLESYTRITNIQCP
KEAVIFKTKRGKEVCADPKERWVRDSMKHLDQIFQNLKP
>sp|P14635|CCNB1_HUMAN G2/mitotic-specific cyclin-B1 OS=Homo sapiens GN=CCNB1 PE=1 SV=1
MALRVTRNSKINAENKAKINMAGAKRVPTAPAATSKPGLRPRTALGDIGNKVSEQLQAKM
PMKKEAKPSATGKVIDKKLPKPLEKVPMLVPVPVSEPVPEPEPEPEPEPVKEEKLSPEPI
LVDTASPSPMETSGCAPAEEDLCQAFSDVILAVNDVDAEDGADPNLCSEYVKDIYAYLRQ
LEEEQAVRPKYLLGREVTGNMRAILIDWLVQVQMKFRLLQETMYMTVSIIDRFMQNNCVP
KKMLQLVGVTAMFIASKYEEMYPPEIGDFAFVTDNTYTKHQIRQMEMKILRALNFGLGRP
LPLHFLRRASKIGEVDVEQHTLAKYLMELTMLDYDMVHFPPSQIAAGAFCLALKILDNGE
WTPTLQHYLSYTEESLLPVMQHLAKNVVMVNQGLTKHMTVKNKYATSKHAKISTLPQLNS
ALVQDLAKAVAKV
>sp|P24385|CCND1_HUMAN G1/S-specific cyclin-D1 OS=Homo sapiens GN=CCND1 PE=1 SV=1
MEHQLLCCEVETIRRAYPDANLLNDRVLRAMLKAEETCAPSVSYFKCVQKEVLPSMRKIV
ATWMLEVCEEQKCEEEVFPLAMNYLDRFLSLEPVKKSRLQLLGATCMFVASKMKETIPLT
AEKLCIYTDNSIRPEELLQMELLLVNKLKWNLAAMTPHDFIEHFLSKMPEAEENKQIIRK
HAQTFVALCATDVKFISNPPSMVAAGSVVAAVQGLNLRSPNNFLSYYRLTRFLSRVIKCD
PDCLRACQEQIEALLESSLRQAQQNMDPKAAEEEEEEEEEVDLACTPTDVRDVDI
>sp|P32246|CCR1_HUMAN C-C chemokine receptor type 1 OS=Homo sapiens GN=CCR1 PE=1 SV=1
METPNTTEDYDTTTEFDYGDATPCQKVNERAFGAQLLPPLYSLVFVIGLVGNILVVLVLV
QYKRLKNMTSIYLLNLAISDLLFLFTLPFWIDYKLKDDWVFGDAMCKILSGFYYTGLYSE
IFFIILLTIDRYLAIVHAVFALRARTVTFGVITSIIIWALAILASMPGLYFSKTQWEFTH
HTCSLHFPHESLREWKLFQALKLNLFGLVLPLLVMIICYTGIIKILLRRPNEKKSKAVRL
IFVIMIIFFLFWTPYNLTILISVFQDFLFTHECEQSRHLDLAVQVTEVIAYTHCCVNPVI
YAFVGERFRKYLRQLFHRRVAVHLVKWLPFLSVDRLERVSSTSPSTGEHELSAGF
>sp|P41597|CCR2_HUMAN C-C chemokine receptor type 2 OS=Homo sapiens GN=CCR2 PE=1 SV=1
MLSTSRSRFIRNTNESGEEVTTFFDYDYGAPCHKFDVKQIGAQLLPPLYSLVFIFGFVGN
MLVVLILINCKKLKCLTDIYLLNLAISDLLFLITLPLWAHSAANEWVFGNAMCKLFTGLY

HIGYFGGIFFIILLTIDRYLAIVHAVFALKARTVTFGVVTSVITWLVAVFASVPGIIFTK
CQKEDSVYVCGPYFPRGWNNFHTIMRNILGLVLPLLIMVICYSGILKTLLRCRNEKKRHR
AVRVIFTIMIVYFLFWTPYNIVILLNTFQEFFGLSNCESTSQLDQATQVTETLGMTHCCI
NPIIYAFVGEKFRSLFHIALGCRIAPLQKPVCGGPGVRPGKNVKVTTQGLLDGRGKGKSI
GRAPEASLQDKEGA
>sp|Q6IQ19|CCSAP_HUMAN Centriole, cilia and spindle-associated protein OS=Homo sapiens GN=CCSAP PE=1 SV=2
MSPGSGVKSEYMKRYQEPRWEEYGPCYRELLHYRLGRRLLEQAHAPWLWDDWGPAGSSED
SASSESSGAGGPAPRCAPPSPPPPVEPATQEEAERRARGAPEEQDAEAGDAEAEDAEDAA
LPALPVKDVEDKPEQQTRTRETDKSPTSTEPRQQPSALFARGNRKAVKSPQRSSSKIKEN
KHPFALYGWGEKQTDTGSQKTHNVCASAPVHEIHESALRAKNRRQVEKRKLVAQRQRAHS
VDVEKNRKMKASSSENPWMTEYMRCYSARA
>sp|Q96FF9|CDCA5_HUMAN Sororin OS=Homo sapiens GN=CDCA5 PE=1 SV=1
MSGRRTRSGGAAQRSGPRAPSPTKPLRRSQRKSGSELPSILPEIWPKTPSAAAVRKPIVL
KRIVAHAVEVPAVQSPRRSPRISFFLEKENEPPGRELTKEDLFKTHSVPATPTSTPVPNP
EAESSSKEGELDARDLEMSKKVRRSYSRLETLGSASTSTPGRRSCFGFEGLLGAEDLSGV
SPVVCSKLTEVPRVCAKPWAPDMTLPGISPPPEKQKRKKKKMPEILKTELDEWAAAMNAE
FEAAEQFDLLVE
>sp|Q9H5V8|CDCP1_HUMAN CUB domain-containing protein 1 OS=Homo sapiens GN=CDCP1 PE=1 SV=3
MAGLNCGVSIALLGVLLLGAARLPRGAEAFEIALPRESNITVLIKLGTPTLLAKPCYIVI
SKRHITMLSIKSGERIVFTFSCQSPENHFVIEIQKNIDCMSGPCPFGEVQLQPSTSLLPT
LNRTFIWDVKAHKSIGLELQFSIPRLRQIGPGESCPDGVTHSISGRIDATVVRIGTFCSN
GTVSRIKMQEGVKMALHLPWFHPRNVSGFSIANRSSIKRLCIIESVFEGEGSATLMSANY
PEGFPEDELMTWQFVVPAHLRASVSFLNFNLSNCERKEERVEYYIPGSTTNPEVFKLEDK
QPGNMAGNFNLSLQGCDQDAQSPGILRLQFQVLVQHPQNESNKIYVVDLSNERAMSLTIE
PRPVKQSRKFVPGCFVCLESRTCSSNLTLTSGSKHKISFLCDDLTRLWMNVEKTISCTDH
RYCQRKSYSLQVPSDILHLPVELHDFSWKLLVPKDRLSLVLVPAQKLQQHTHEKPCNTSF
SYLVASAIPSQDLYFGSFCPGGSIKQIQVKQNISVTLRTFAPSFQQEASRQGLTVSFIPY
FKEEGVFTVTPDTKSKVYLRTPNWDRGLPSLTSVSWNISVPRDQVACLTFFKERSGVVCQ
TGRAFMIIQEQRTRAEEIFSLDEDVLPKPSFHHHSFWVNISNCSPTSGKQLDLLFSVTLT
PRTVDLTVILIAAVGGGVLLLSALGLIICCVKKKKKKTNKGPAVGIYNDNINTEMPRQPK
KFQKGRKDNDSHVYAVIEDTMVYGHLLQDSSGSFLQPEVDTYRPFQGTMGVCPPSPPTIC
SRAPTAKLATEEPPPRSPPESESEPYTFSHPNNGDVSSKDTDIPLLNTQEPMEPAE
>sp|Q9BYE9|CDHR2_HUMAN Cadherin-related family member 2 OS=Homo sapiens GN=CDHR2 PE=1 SV=2
MAQLWLSCFLLPALVVSVAANVAPKFLANMTSVILPEDLPVGAQAFWLVAEDQDNDPLTY
GMSGPNAYFFAVTPKTGEVKLASALDYETLYTFKVTISVSDPYIQVQREMLVIVEDRNDN
APVFQNTAFSTSINETLPVGSVVFSVLAVDKDMGSAGMVVYSIEKVIPSTGDSEHLFRIL
ANGSIVLNGSLSYNNKSAFYQLELKACDLGGMYHNTFTIQCSLPVFLSISVVDQPDLDPQ
FVREFYSASVAEDAAKGTSVLTVEAVDGDKGINDPVIYSISYSTRPGWFDIGADGVIRVN
GSLDREQLLEADEEVQLQVTATETHLNIYGQEAKVSIWVTVRVMDVNDHKPEFYNCSLPA
CTFTPEEAQVNFTGYVDEHASPRIPIDDLTMVVYDPDKGSNGTFLLSLGGPDAEAFSVSP
ERAVGSASVQVLVRVSALVDYERQTAMAVQVVATDSVSQNFSVAMVTIHLRDINDHRPTF
PQSLYVLTVPEHSATGSVVTDSIHATDPDTGAWGQITYSLLPGNGADLFQVDPVSGTVTV

RNGELLDRESQAVYYLTLQATDGGNLSSSTTLQIHLLDINDNAPVVSGSYNIFVQEEEGN
VSVTIQAHDNDEPGTNNSRLLFNLLPGPYSHNFSLDPDTGLLRNLGPLDREAIDPALEGR
IVLTVLVSDCGEPVLGTKVNVTITVEDINDNLPIFNQSSYNFTVKEEDPGVLVGVVKAWD
ADQTEANNRISFSLSGSGANYFMIRGLVLGAGWAEGYLRLPPDVSLDYETQPVFNLTVSA
ENPDPQGGETIVDVCVNVKDVNDNPPTLDVASLRGIRVAENGSQHGQVAVVVASDVDTSA
QLEIQLVNILCTKAGVDVGSLCWGWFSVAANGSVYINQSKAIDYEACDLVTLVVRACDLA
TDPGFQAYSNNGSLLITIEDVNDNAPYFLPENKTFVIIPELVLPNREVASVRARDDDSGN
NGVILFSILRVDFISKDGATIPFQGVFSIFTSSEADVFAGSIQPVTSLDSTLQGTYQVTV
QARDRPSLGPFLEATTTLNLFTVDQSYRSRLQFSTPKEEVGANRQAINAALTQATRTTVY
IVDIQDIDSAARARPHSYLDAYFVFPNGSALTLDELSVMIRNDQDSLTQLLQLGLVVLGS
QESQESDLSKQLISVIIGLGVALLLVLVIMTMAFVCVRKSYNRKLQAMKAAKEARKTAAG
VMPSAPAIPGTNMYNTERANPMLNLPNKDLGLEYLSPSNDLDSVSVNSLDDNSVDVDKNS
QEIKEHRPPHTPPEPDPEPLSVVLLGRQAGASGQLEGPSYTNAGLDTTDL
>sp|Q6ZTQ4|CDHR3_HUMAN Cadherin-related family member 3 OS=Homo sapiens GN=CDHR3 PE=1 SV=1
MQEAIILLALLGAMSGGEALHLILLPATGNVAENSPPGTSVHKFSVKLSASLSPVIPGFP
QIVNSNPLTEAFRVNWLSGTYFEVVTTGMEQLDFETGPNIFDLQIYVKDEVGVTDLQVLT
VQVTDVNEPPQFQGNLAEGLHLYIVERANPGFIYQVEAFDPEDTSRNIPLSYFLISPPKS
FRMSANGTLFSTTELDFEAGHRSFHLIVEVRDSGGLKASTELQVNIVNLNDEVPRFTSPT
RVYTVLEELSPGTIVANITAEDPDDEGFPSHLLYSITTVSKYFMINQLTGTIQVAQRIDR
DAGELRQNPTISLEVLVKDRPYGGQENRIQITFIVEDVNDNPATCQKFTFSIMVPERTAK
GTLLLDLNKFCFDDDSEAPNNRFNFTMPSGVGSGSRFLQDPAGSGKIVLIGDLDYENPSN
LAAGNKYTVIIQVQDVAPPYYKNNVYVYILTSPENEFPLIFDRPSYVFDVSERRPARTRV
GQVRATDKDLPQSSLLYSISTGGASLQYPNVFWINPKTGELQLVTKVDCETTPIYILRIQ
ATNNEDTSSVTVTVNILEENDEKPICTPNSYFLALPVDLKVGTNIQNFKLTCTDLDSSPR
SFRYSIGPGNVNNHFTFSPNAGSNVTRLLLTSRFDYAGGFDKIWDYKLLVYVTDDNLMSD
RKKAEALVETGTVTLSIKVIPHPTTIITTTPRPRVTYQVLRKNVYSPSAWYVPFVITLGS
ILLLGLLVYLVVLLAKAIHRHCPCKTGKNKEPLTKKGETKTAERDVVVETIQMNTIFDGE
AIDPVTGETYEFNSKTGARKWKDPLTQMPKWKESSHQGAAPRRVTAGEGMGSLRSANWEE
DELSGKAWAEDAGLGSRNEGGKLGNPKNRNPAFMNRAYPKPHPGK
>sp|Q00537|CDK17_HUMAN Cyclin-dependent kinase 17 OS=Homo sapiens GN=CDK17 PE=1 SV=2
MKKFKRRLSLTLRGSQTIDESLSELAEQMTIEENSSKDNEPIVKNGRPPTSHSMHSFLHQ
YTGSFKKPPLRRPHSVIGGSLGSFMAMPRNGSRLDIVHENLKMGSDGESDQASGTSSDEV
QSPTGVCLRNRIHRRISMEDLNKRLSLPADIRIPDGYLEKLQINSPPFDQPMSRRSRRAS
LSEIGFGKMETYIKLEKLGEGTYATVYKGRSKLTENLVALKEIRLEHEEGAPCTAIREVS
LLKDLKHANIVTLHDIVHTDKSLTLVFEYLDKDLKQYMDDCGNIMSMHNVKLFLYQILRG
LAYCHRRKVLHRDLKPQNLLINEKGELKLADFGLARAKSVPTKTYSNEVVTLWYRPPDVL
LGSSEYSTQIDMWGVGCIFFEMASGRPLFPGSTVEDELHLIFRLLGTPSQETWPGISSNE
EFKNYNFPKYKPQPLINHAPRLDSEGIELITKFLQYESKKRVSAEEAMKHVYFRSLGPRI
HALPESVSIFSLKEIQLQKDPGFRNSSYPETGHGKNRRQSMLF
>sp|Q16667|CDKN3_HUMAN Cyclin-dependent kinase inhibitor 3 OS=Homo sapiens GN=CDKN3 PE=1 SV=1
MKPPSSIQTSEFDSSDEEPIEDEQTPIHISWLSLSRVNCSQFLGLCALPGCKFKDVRRNV
QKDTEELKSCGIQDIFVFCTRGELSKYRVPNLLDLYQQCGIITHHHPIADGGTPDIASCC

EIMEELTTCLKNYRKTLIHCYGGLGRSCLVAACLLLYLSDTISPEQAIDSLRDLRGSGAI
QTIKQYNYLHEFRDKLAAHLSSRDSQSRSVSR
>sp|Q8N8D9|CE056_HUMAN Uncharacterized protein C5orf56 OS=Homo sapiens GN=C5orf56 PE=2 SV=2
MDSLAAGELNASHQPWVPEFVAYWRKTHQGNLQTLLLHWLLLCSCLHHLYNASCHPAFPV
EYSHAVCCLQPRFWKEMSPFSSSSTTHLFSKCFFYTCLCWAWQQCSSEQNHQPCSHAAYN
QVERQT
>sp|Q9BXQ6|CECR6_HUMAN Cat eye syndrome critical region protein 6 OS=Homo sapiens GN=CECR6 PE=2 SV=1
MRPALGHPRSVSSASGSFPPPPAAARLQPLFLRGGSFRGRRGSGDSSTSTSTSRGGGGGR
RGGGGGSPSSSTGAEREDDDESLSVSKPLVPNAALLGPPAQVGAPAGPAPVAFSSSAATS
SSTSTPTSSCSMTAADFGGGAAAGAVGGPGSRSAGGAGGTGTGSGASCCPCCCCCGCPDR
PGRRGRRRGCAPSPRCRWGYQALSVVLLLAQGGLLDLYLIAVTDLYWCSWIATDLVVVVG
WAIFFAKNSRGRRGGAASGAHNHHLHHHHAAPPLHLPAPSAATAGAKARGARGGAGGAGG
GLGAAAAAGEFAFAYLAWLIYSIAFTPKVVLILGTSILDLIELRAPFGTTGFRLTMALSV
PLLYSLVRAISEAGAPPGSAGPLLLQPQRHRAAGCFLGTCLDLLDSFTLVELMLEGRVPL
PAHLRYLLIAVYFLTLASPVLWLYELNAAAAAAASWGQASGPGSCSRLLRLLGGCLVDVP
LLALRCLLVVSYQQPLSIFMLKNLFFLGCRGLEALEGCWDRGNRASPSRARGGYGAPPSA
PPPPPPPPQGGSQLGHCISENEGGAHGYVNTLAVASQN
>sp|Q92879|CELF1_HUMAN CUGBP Elav-like family member 1 OS=Homo sapiens GN=CELF1 PE=1 SV=2
MNGTLDHPDQPDLDAIKMFVGQVPRTWSEKDLRELFEQYGAVYEINVLRDRSQNPPQSKG
CCFVTFYTRKAALEAQNALHNMKVLPGMHHPIQMKPADSEKNNAVEDRKLFIGMISKKCT
ENDIRVMFSSFGQIEECRILRGPDGLSRGCAFVTFTTRAMAQTAIKAMHQAQTMEGCSSP
MVVKFADTQKDKEQKRMAQQLQQQMQQISAASVWGNLAGLNTLGPQYLALYLQLLQQTAS
SGNLNTLSSLHPMGGLNAMQLQNLAALAAAASAAQNTPSGTNALTTSSSPLSVLTSSGSS
PSSSSSNSVNPIASLGALQTLAGATAGLNVGSLAGMAALNGGLGSSGLSNGTGSTMEALT
QAYSGIQQYAAAALPTLYNQNLLTQQSIGAAGSQKEGPEGANLFIYHLPQEFGDQDLLQM
FMPFGNVVSAKVFIDKQTNLSKCFGFVSYDNPVSAQAAIQSMNGFQIGMKRLKVQLKRSK
NDSKPY
>sp|Q9BZC1|CELF4_HUMAN CUGBP Elav-like family member 4 OS=Homo sapiens GN=CELF4 PE=1 SV=1
MYIKMATLANGQADNASLSTNGLGSSPGSAGHMNGLSHSPGNPSTIPMKDHDAIKLFIGQ
IPRNLDEKDLKPLFEEFGKIYELTVLKDRFTGMHKGCAFLTYCERESALKAQSALHEQKT
LPGMNRPIQVKPADSESRGGSSCLRQPPSQDRKLFVGMLNKQQSEDDVRRLFEAFGNIEE
CTILRGPDGNSKGCAFVKYSSHAEAQAAINALHGSQTMPGASSSLVVKFADTDKERTMRR
MQQMAGQMGMFNPMAIPFGAYGAYAQALMQQQAALMASVAQGGYLNPMAAFAAAQMQQMA
ALNMNGLAAAPMTPTSGGSTPPGITAPAVPSIPSPIGVNGFTGLPPQANGQPAAEAVFAN
GIHPYPAQSPTAADPLQQAYAGVQQYAGPAAYPAAYGQISQAFPQPPPMIPQQQREGPEG
CNLFIYHLPQEFGDAELMQMFLPFGFVSFDNPASAQTAIQAMNGFQIGMKRLKVQLKRPK
DANRPY
>sp|Q9NYQ6|CELR1_HUMAN Cadherin EGF LAG seven-pass G-type receptor 1 OS=Homo sapiens GN=CELSR1 PE=1 SV=1
MAPPPPPVLPVLLLLAAAAALPAMGLRAAAWEPRVPGGTRAFALRPGCTYAVGAACTPRA
PRELLDVGRDGRLAGRRRVSGAGRPLPLQVRLVARSAPTALSRRLRARTHLPGCGARARL
CGTGARLCGALCFPVPGGCAAAQHSALAAPTTLPACRCPPRPRPRCPGRPICLPPGGSVR

LRLLCALRRAAGAVRVGLALEAATAGTPSASPSPSPPLPPNLPEARAGPARRARRGTSGR
GSLKFPMPNYQVALFENEPAGTLILQLHAHYTIEGEEERVSYYMEGLFDERSRGYFRIDS
ATGAVSTDSVLDRETKETHVLRVKAVDYSTPPRSATTYITVLVKDTNDHSPVFEQSEYRE
RVRENLEVGYEVLTIRASDRDSPINANLRYRVLGGAWDVFQLNESSGVVSTRAVLDREEA
AEYQLLVEANDQGRNPGPLSATATVYIEVEDENDNYPQFSEQNYVVQVPEDVGLNTAVLR
VQATDRDQGQNAAIHYSILSGNVAGQFYLHSLSGILDVINPLDFEDVQKYSLSIKAQDGG
RPPLINSSGVVSVQVLDVNDNEPIFVSSPFQATVLENVPLGYPVVHIQAVDADSGENARL
HYRLVDTASTFLGGGSAGPKNPAPTPDFPFQIHNSSGWITVCAELDREEVEHYSFGVEAV
DHGSPPMSSSTSVSITVLDVNDNDPVFTQPTYELRLNEDAAVGSSVLTLQARDRDANSVI
TYQLTGGNTRNRFALSSQRGGGLITLALPLDYKQEQQYVLAVTASDGTRSHTAHVLINVT
DANTHRPVFQSSHYTVSVSEDRPVGTSIATLSANDEDTGENARITYVIQDPVPQFRIDPD
SGTMYTMMELDYENQVAYTLTIMAQDNGIPQKSDTTTLEILILDANDNAPQFLWDFYQGS
IFEDAPPSTSILQVSATDRDSGPNGRLLYTFQGGDDGDGDFYIEPTSGVIRTQRRLDREN
VAVYNLWALAVDRGSPTPLSASVEIQVTILDINDNAPMFEKDELELFVEENNPVGSVVAK
IRANDPDEGPNAQIMYQIVEGDMRHFFQLDLLNGDLRAMVELDFEVRREYVLVVQATSAP
LVSRATVHILLVDQNDNPPVLPDFQILFNNYVTNKSNSFPTGVIGCIPAHDPDVSDSLNY
TFVQGNELRLLLLDPATGELQLSRDLDNNRPLEALMEVSVSDGIHSVTAFCTLRVTIITD
DMLTNSITVRLENMSQEKFLSPLLALFVEGVAAVLSTTKDDVFVFNVQNDTDVSSNILNV
TFSALLPGGVRGQFFPSEDLQEQIYLNRTLLTTISTQRVLPFDDNICLREPCENYMKCVS
VLRFDSSAPFLSSTTVLFRPIHPINGLRCRCPPGFTGDYCETEIDLCYSDPCGANGRCRS
REGGYTCECFEDFTGEHCEVDARSGRCANGVCKNGGTCVNLLIGGFHCVCPPGEYERPYC
EVTTRSFPPQSFVTFRGLRQRFHFTISLTFATQERNGLLLYNGRFNEKHDFIALEIVDEQ
VQLTFSAGETTTTVAPKVPSGVSDGRWHSVQVQYYNKPNIGHLGLPHGPSGEKMAVVTVD
DCDTTMAVRFGKDIGNYSCAAQGTQTGSKKSLDLTGPLLLGGVPNLPEDFPVHNRQFVGC
MRNLSVDGKNVDMAGFIANNGTREGCAARRNFCDGRRCQNGGTCVNRWNMYLCECPLRFG
GKNCEQAMPHPQLFSGESVVSWSDLNIIISVPWYLGLMFRTRKEDSVLMEATSGGPTSFR
LQILNNYLQFEVSHGPSDVESVMLSGLRVTDGEWHHLLIELKNVKEDSEMKHLVTMTLDY
GMDQNKADIGGMLPGLTVRSVVVGGASEDKVSVRRGFRGCMQGVRMGGTPTNVATLNMNN
ALKVRVKDGCDVDDPCTSSPCPPNSRCHDAWEDYSCVCDKGYLGINCVDACHLNPCENMG
ACVRSPGSPQGYVCECGPSHYGPYCENKLDLPCPRGWWGNPVCGPCHCAVSKGFDPDCNK
TNGQCQCKENYYKLLAQDTCLPCDCFPHGSHSRTCDMATGQCACKPGVIGRQCNRCDNPF
AEVTTLGCEVIYNGCPKAFEAGIWWPQTKFGQPAAVPCPKGSVGNAVRHCSGEKGWLPPE
LFNCTTISFVDLRAMNEKLSRNETQVDGARALQLVRALRSATQHTGTLFGNDVRTAYQLL
GHVLQHESWQQGFDLAATQDADFHEDVIHSGSALLAPATRAAWEQIQRSEGGTAQLLRRL
EGYFSNVARNVRRTYLRPFVIVTANMILAVDIFDKFNFTGARVPRFDTIHEEFPRELESS
VSFPADFFRPPEEKEGPLLRPAGRRTTPQTTRPGPGTEREAPISRRRRHPDDAGQFAVAL
VIIYRTLGQLLPERYDPDRRSLRLPHRPIINTPMVSTLVYSEGAPLPRPLERPVLVEFAL
LEVEERTKPVCVFWNHSLAVGGTGGWSARGCELLSRNRTHVACQCSHTASFAVLMDISRR
ENGEVLPLKIVTYAAVSLSLAALLVAFVLLSLVRMLRSNLHSIHKHLAVALFLSQLVFVI
GINQTENPFLCTVVAILLHYIYMSTFAWTLVESLHVYRMLTEVRNIDTGPMRFYYVVGWG
IPAIVTGLAVGLDPQGYGNPDFCWLSLQDTLIWSFAGPIGAVIIINTVTSVLSAKVSCQR
KHHYYGKKGIVSLLRTAFLLLLLISATWLLGLLAVNRDALSFHYLFAIFSGLQGPFVLLF
HCVLNQEVRKHLKGVLGGRKLHLEDSATTRATLLTRSLNCNTTFGDGPDMLRTDLGESTA
SLDSIVRDEGIQKLGVSSGLVRGSHGEPDASLMPRSCKDPPGHDSDSDSELSLDEQSSSY

ASSHSSDSEDDGVGAEEKWDPARGAVHSTPKGDAVANHVPAGWPDQSLAESDSEDPSGKP
RLKVETKVSVELHREEQGSHRGEYPPDQESGGAARLASSQPPEQRKGILKNKVTYPPPLT
LTEQTLKGRLREKLADCEQSPTSSRTSSLGSGGPDCAITVKSPGREPGRDHLNGVAMNVR
TGSAQADGSDSEKP
>sp|P19835|CEL_HUMAN Bile salt-activated lipase OS=Homo sapiens GN=CEL PE=1 SV=3
MGRLQLVVLGLTCCWAVASAAKLGAVYTEGGFVEGVNKKLGLLGDSVDIFKGIPFAAPTK
ALENPQPHPGWQGTLKAKNFKKRCLQATITQDSTYGDEDCLYLNIWVPQGRKQVSRDLPV
MIWIYGGAFLMGSGHGANFLNNYLYDGEEIATRGNVIVVTFNYRVGPLGFLSTGDANLPG
NYGLRDQHMAIAWVKRNIAAFGGDPNNITLFGESAGGASVSLQTLSPYNKGLIRRAISQS
GVALSPWVIQKNPLFWAKKVAEKVGCPVGDAARMAQCLKVTDPRALTLAYKVPLAGLEYP
MLHYVGFVPVIDGDFIPADPINLYANAADIDYIAGTNNMDGHIFASIDMPAINKGNKKVT
EEDFYKLVSEFTITKGLRGAKTTFDVYTESWAQDPSQENKKKTVVDFETDVLFLVPTEIA
LAQHRANAKSAKTYAYLFSHPSRMPVYPKWVGADHADDIQYVFGKPFATPTGYRPQDRTV
SKAMIAYWTNFAKTGDPNMGDSAVPTHWEPYTTENSGYLEITKKMGSSSMKRSLRTNFLR
YWTLTYLALPTVTDQEATPVPPTGDSEATPVPPTGDSETAPVPPTGDSGAPPVPPTGDSG
APPVPPTGDSGAPPVPPTGDSGAPPVPPTGDSGAPPVPPTGDSGAPPVPPTGDSGAPPVP
PTGDSGAPPVPPTGDAGPPPVPPTGDSGAPPVPPTGDSGAPPVTPTGDSETAPVPPTGDS
GAPPVPPTGDSEAAPVPPTDDSKEAQMPAVIRF
>sp|B2RD01|CENP1_HUMAN CENPB DNA-binding domain-containing protein 1 OS=Homo sapiens GN=CENPBD1 PE=2 SV=1
MPGERPTDATVIPSAKRERKAITLDLKLEVLRRFEAGEKLSQIAKALDLAISTVATIRDS
KEKIKASSQIATPLRASRLTRHRSAVMESMEQLLSLWLEDQSQPNATLSAAIVQEKAEFD
DLQREHGEGSQTERFHASQGWLVRFKECHCLPHFKMNSAAPSNKDMYTEMLKSIIEEGEY
TPQVSLT
>sp|Q96H22|CENPN_HUMAN Centromere protein N OS=Homo sapiens GN=CENPN PE=1 SV=2
MDETVAEFIKRTILKIPMNELTTILKAWDFLSENQLQTVNFRQRKESVVQHLIHLCEEKR
ASISDAALLDIIYMQFHQHQKVWEVFQMSKGPGEDVDLFDMKQFKNSFKKILQRALKNVT
VSFRETEENAVWIRIAWGTQYTKPNQYKPTYVVYYSQTPYAFTSSSMLRRNTPLLGQALT
IASKHHQIVKMDLRSRYLDSLKAIVFKQYNQTFETHNSTTPLQERSLGLDINMDSRIIHE
NIVEKERVQRITQETFGDYPQPQLEFAQYKLETKFKSGLNGSILAEREEPLRCLIKFSSP
HLLEALKSLAPAGIADAPLSPLLTCIPNKRMNYFKIRDK
>sp|Q7L2Z9|CENPQ_HUMAN Centromere protein Q OS=Homo sapiens GN=CENPQ PE=1 SV=1
MSGKANASKKNAQQLKRNPKRKKDNEEVVLSENKVRNTVKKNKNHLKDLSSEGQTKHTNL
KHGKTAASKRKTWQPLSKSTRDHLQTMMESVIMTILSNSIKEKEEIQYHLNFLKKRLLQQ
CETLKVPPKKMEDLTNVSSLLNMERARDKANEEGLALLQEEIDKMVETTELMTGNIQSLK
NKIQILASEVEEEEERVKQMHQINSSGVLSLPELSQKTLKAPTLQKEILALIPNQNALLK
DLDILHNSSQMKSMSTFIEEAYKKLDAS
>sp|Q96BT3|CENPT_HUMAN Centromere protein T OS=Homo sapiens GN=CENPT PE=1 SV=2
MADHNPDSDSTPRTLLRRVLDTADPRTPRRPRSARAGARRALLETASPRKLSGQTRTIAR
GRSHGARSVGRSAHIQASGHLEEQTPRTLLKNILLTAPESSILMPESVVKPVPAPQAVQP
SRQESSCGSLELQLPELEPPTTLAPGLLAPGRRKQRLRLSVFQQGVDQGLSLSQEPQGNA
DASSLTRSLNLTFATPLQPQSVQRPGLARRPPARRAVDVGAFLRDLRDTSLAPPNIVLED
TQPFSQPMVGSPNVYHSLPCTPHTGAEDAEQAAGRKTQSSGPGLQKNSPGKPAQFLAGEA
EEVNAFALGFLSTSSGVSGEDEVEPLHDGVEEAEKKMEEEGVSVSEMEATGAQGPSRVEE

AEGHTEVTEAEGSQGTAEADGPGASSGDEDASGRAASPESASSTPESLQARRHHQFLEPA
PAPGAAVLSSEPAEPLLVRHPPRPRTTGPRPRQDPHKAGLSHYVKLFSFYAKMPMERKAL
EMVEKCLDKYFQHLCDDLEVFAAHAGRKTVKPEDLELLMRRQGLVTDQVSLHVLVERHLP
LEYRQLLIPCAYSGNSVFPAQ
>sp|Q9BYV8|CEP41_HUMAN Centrosomal protein of 41 kDa OS=Homo sapiens GN=CEP41 PE=1 SV=1
MSLRRHIGNPEYLMKRIPQNPRYQHIKSRLDTGNSMTKYTEKLEEIKKNYRYKKDELFKR
LKVTTFAQLIIQVASLSDQTLEVTAEEIQRLEDNDSAASDPDAETTARTNGKGNPGEQSP
SPEQFINNAGAGDSSRSTLQSVISGVGELDLDKGPVKKAEPHTKDKPYPDCPFLLLDVRD
RDSYQQCHIVGAYSYPIATLSRTMNPYSNDILEYKNAHGKIIILYDDDERLASQAATTMC
ERGFENLFMLSGGLKVLAQKFPEGLITGSLPASCQQALPPGSARKRSSPKGPPLPAENKW
RFTPEDLKKIEYYLEEEQGPADHPSRLNQANSSGRESKVPGARSAQNLPGGGPASHSNPR
SLSSGHLQGKPWK
>sp|Q53EZ4|CEP55_HUMAN Centrosomal protein of 55 kDa OS=Homo sapiens GN=CEP55 PE=1 SV=3
MSSRSTKDLIKSKWGSKPSNSKSETTLEKLKGEIAHLKTSVDEITSGKGKLTDKERHRLL
EKIRVLEAEKEKNAYQLTEKDKEIQRLRDQLKARYSTTTLLEQLEETTREGERREQVLKA
LSEEKDVLKQQLSAATSRIAELESKTNTLRLSQTVAPNCFNSSINNIHEMEIQLKDALEK
NQQWLVYDQQREVYVKGLLAKIFELEKKTETAAHSLPQQTKKPESEGYLQEEKQKCYNDL
LASAKKDLEVERQTITQLSFELSEFRRKYEETQKEVHNLNQLLYSQRRADVQHLEDDRHK
TEKIQKLREENDIARGKLEEEKKRSEELLSQVQFLYTSLLKQQEEQTRVALLEQQMQACT
LDFENEKLDRQHVQHQLHVILKELRKARNQITQLESLKQLHEFAITEPLVTFQGETENRE
KVAASPKSPTAALNESLVECPKCNIQYPATEHRDLLVHVEYCSK
>sp|Q5JTW2|CEP78_HUMAN Centrosomal protein of 78 kDa OS=Homo sapiens GN=CEP78 PE=1 SV=1
MIDSVKLRRDSAADFFSHYEYLCALQNSVPLPAVRACLREGVLDFNADRLRGVDWAPLLS
TLKINKDLPLVSIKSFFQPWLGDTGSDMNKFCRSRVPAIRYKDVTFQLCKALKGCLSISS
VLKNLELNGLILRERDLTILAKGLNKSASLVHLSLANCPIGDGGLEIICQGIKSSITLKT
VNFTGCNLTWQGADHMAKILKYQTMRRHEETWAESLRYRRPDLDCMAGLRRITLNCNTLI
GDLGACAFADSLSEDLWLRALDLQQCGLTNEGAKALLEALETNTTLVVLDIRKNPLIDHS
MMKAVIKKVLQNGRSAKSEYQWITSPSVKEPSKTAKQKRRTIILGSGHKGKATIRIGLAT
KKPVSSGRKHSLGKEYYAPAPLPPGVSGFLPWRTAERAKRHRGFPLIKTRDICNQLQQPG
FPVTVTVESPSSSEVEEVDDSSESVHEVPEKTSIEQEALQEKLEECLKQLKEERVIRLKV
DKRVSELEHENAQLRNINFSLSEALHAQSLTNMILDDEGVLGSIENSFQKFHAFLDLLKD
AGLGQLATMAGIDQSDFQLLGHPQMTSTVSNPPKEEKKALEDEKPEPKQNALGQMQNIQF
QKITGDARIPLPLDSFPVPVSTPEGLGTSSNNLGVPATEQRQESFEGFIARMCSPSPDAT
SGTGSQRKEEELSRNSRSSSEKKTKTESH
>sp|O95813|CER1_HUMAN Cerberus OS=Homo sapiens GN=CER1 PE=1 SV=1
MHLLLFQLLVLLPLGKTTRHQDGRQNQSSLSPVLLPRNQRELPTGNHEEAEEKPDLFVAV
PHLVATSPAGEGQRQREKMLSRFGRFWKKPEREMHPSRDSDSEPFPPGTQSLIQPIDGMK
MEKSPLREEAKKFWHHFMFRKTPASQGVILPIKSHEVHWETCRTVPFSQTITHEGCEKVV
VQNNLCFGKCGSVHFPGAAQHSHTSCSHCLPAKFTTMHLPLNCTELSSVIKVVMLVEECQ
CKVKTEHEDGHILHAGSQDSFIPGVSA
>sp|Q12798|CETN1_HUMAN Centrin-1 OS=Homo sapiens GN=CETN1 PE=1 SV=1
MASGFKKPSAASTGQKRKVAPKPELTEDQKQEVREAFDLFDVDGSGTIDAKELKVAMRAL
GFEPRKEEMKKMISEVDREGTGKISFNDFLAVMTQKMSEKDTKEEILKAFRLFDDDETGK
ISFKNLKRVANELGENLTDEELQEMIDEADRDGDGEVNEEEFLRIMKKTSLY

>sp|P41208|CETN2_HUMAN Centrin-2 OS=Homo sapiens GN=CETN2 PE=1 SV=1
MASNFKKANMASSSQRKRMSPKPELTEEQKQEIREAFDLFDADGTGTIDVKELKVAMRAL
GFEPKKEEIKKMISEIDKEGTGKMNFGDFLTVMTQKMSEKDTKEEILKAFKLFDDDETGK
ISFKNLKRVAKELGENLTDEELQEMIDEADRDGDGEVSEQEFLRIMKKTSLY
>sp|Q5JQF7|CF100_HUMAN Putative uncharacterized protein encoded by LINC01556 OS=Homo sapiens GN=LINC01556 PE=4 SV=2
MLQVVQEGNPAPFIINTVKRGRRDRERQRTPWAPHPLGFQGRRYIYESPNHRGKDSSFLA
QK
>sp|Q9Y6Z2|CF123_HUMAN Uncharacterized protein encoded by LINC01558 OS=Homo sapiens GN=LINC01558 PE=4 SV=1
MGTAVGPHHSPAPHDSALPARLLTSDFPYGRSCQIEQVKYSVPDTGLFQHWEGSIPT
>sp|Q5T0Z8|CF132_HUMAN Uncharacterized protein C6orf132 OS=Homo sapiens GN=C6orf132 PE=1 SV=4
MKKKQTVQGTFSKLFGKKHTTTPSTSLYATNPPWIFTQEAPEEGTGGFDGIYYGDNRFNT
VSESGTATLKARPRVRPLLTFLPLNAQENHGLAVPTPSVPDDFADKEVTGTSSLVNGNLR
LYSSVGDLRPGQYGQDLLIPPPPPGPAPGPPQDISEPPGGSPLPSPPSTAPPPPPLLLEP
PPPPSMAPPPPPVLEALSPPHTLSSPSIPTPPDFIPPAPPLAFLAPPPPPVPAPAPPAPA
SPHTVGTRLFPPGGVTKWKSDVALNGRQAEATRASPPRSPAEPKGSALGPNPEPHLTFPR
SFKVPPPTPVRTSSIPVQEAQEAPRKEEGATKKAPSRLPLPPSFHIRPASQVYPDRAPEP
DCPGELKATAPASPRLGQSQSQADERAGTPPPAPPLPPPAPPLPPPAPPLPPAAPPLPCA
QKAAHPPAGFTKTPKSSSPALKPKPNPPSPENTASSAPVDWRDPSQMEKLRNELAAYLCG
SRREDRFLSHRPGPTVAPQSKEGKKGPRLPEKETLLSLPAKDTPPGVPEKSLGGSSLTET
EAAPSLTLPSVDYIPQDSPTPSVRQIRNELEARLSSAAEKEAKPSIGSLPPKPRLEGGRI
CENGADDDKLSKPVAKNLPPQSTTLLPTTSLQPKAMLGPAIPPKATPEPAIPPKATLWPA
TPPKATLGPATPLKATSGPTTPLKATSGPAIASTATTLPTTTSQLMAEKDSGPAGQPEKP
ASQEVSTPSQARGEGSPSEATRLPTQGARSSAAFPPKTSPGGGEVPCLYKPHCHQSSLSR
EVAVVMPTLARGGAAGPGEPVEVKEPPGLPAKPPASAQPTDELLRHPVTGEVVERGSPMA
LLLAARQRAQKGRSVGAALGRSSLPGSLRDHSHQAEASSDSIFHSQGTPNSFTVVPKLPK
EAEKDSPLTTEIPNKWGPRLGRDAEGTELSRRHNWTKPEPQAPVAWERVAPSNLPQGHPL
PKSFSSPPSPSNKREEEEEEFNFEVIPPPPEFSNDPEPPAPALQYLGRQSSPPRNNYSDL
RQLPNAGPGAPPALGFSRFPAGARYAGAGGLERFSGGGRSLIKKRLYVGEPHRGPGLPHG
GTGRSLSSPNCFGPQPGGPEMRRVNSAGRAPPGGLHAPRLSLEGAARGAAEAKHKAPGSA
DYGFAPAAGRSPYTTTRYGSPINTFTVRPGTRHPISYVCSGAHRKATS
>sp|Q9H8W2|CF155_HUMAN Putative uncharacterized protein encoded by LINC00472 OS=Homo sapiens GN=LINC00472 PE=5 SV=2
MRPGSSPRAPECGAPALPRPQLDRLPARPAPSRGRGAPSLRWPAKEVGPRPQIPATCEPG
KVCGASAGRRDAARPSRPRSSRVTFSTRRQPGPQRGRWGLRGGPESVRGLPHLGLRISGT
PLGIFSSWSL
>sp|Q6P656|CF161_HUMAN Cilia- and flagella-associated protein 161 OS=Homo sapiens GN=CFAP161 PE=2 SV=1
MAQNVYGPGVRIGNWNEDVYLEEELMKDFLEKRDKGKLLIQRSRRLKQNLLRPMQLSVTE
DGYIHYGDKVMLVNPDDPDTEADVFLRGDLSLCMTPDEIQSHLKDELEVPCGLSAVQAKT
PIGRNTFIILSVHRDATGQVLRYGQDFCLGITGGFDNKMLYLSSDHRTLLKSSKRSWLQE
VYLTDEVSHVNCWQAAFPDPQLRLEYEGFPVPANAKILINHCHTNRGLAAHRHLFLSTYF

GKEAEVVAHTYLDSHRVEKPRNHWMLVTGNPRDASSSMLDLPKPPTEDTRAMEQAMGLDT
Q
>sp|Q96MT7|CFA44_HUMAN Cilia- and flagella-associated protein 44 OS=Homo sapiens GN=CFAP44 PE=1 SV=1
MKEPDDQDTDGEKSVTSKSDGKKSLRSSKSESRSPVQEDNTFLEDDTDETFTKGEGSYLE
EDSDEERLEGSLSSFQYGDLQSTTVPQQTPAPAVEEAEEEVKKKISESFFYDYMELASMP
FVTLDSNIPLDLLTLVHSFGYDCRKRANLQLLDDSIAIYIAGNQLIFLNLKTKEQIYLRS
SSGEGIGVIGVHPHKTYFTVAEKGSFPDIIIYEYPSLRPYRVLRDGTEKGYAYVDFNYSG
NLLASVGSNPDYTLTIWNWKEEQPILRTKAFSQEVFKVTFNPDKEEQLTTSGSGHIKFWE
MAFTFTGLKLQGSLGRFGKTITTDIEGYMELPDGKVLSGSEWGNMLLWEGGLIKVELCRG
TSKSCHNGPINQIMLYEGEVITVGSDGYVRIWDFETIDTADVIDETGLLEIEPINELQVD
KNVNLFSMIKMNETGNNFWLAQDANGAIWKLDLSFSNITQDPECLFSFHSGAIEAVAVSP
LTYLMATTALDCSVRIYDFASKTPLAQMKFKQGGTALVWVPRMVNFTGAQIIVGFEDGVV
RILELYDPKGLTIFAGRKKILDADIQLKQVFKPHTACVTALAYERDGEILATGSKDQTVF
FFEVERDYKPIGYINTPGPVCQLMWSPMSHPESTLLIICENGYILEAPLPTIKQEEDDHD
VVSYEIKDMCIKCFHFSSVKSKILRLIEIEKRERQRELKEKIREERRNKLAAEMGEDGEK
EFQEEEEEKEEEEEEEEPLPEIFIPSTPSPILCGFYSEPGKFWVSLGGYDSGFLYHCEFP
PCDESSDFKEQKDEPIDVRYLADTEDNPIQTITFNINKVMMFCGMKNGAIRVYVLNQNDP
SLTSLVDYWHFNMHDNNYGCIKSIANSFDDRFLVTAGADGNIFVFNIFSEFMLRKDMKAK
VPSPRFGIETEPIPEDIEDPKAYSIENARRKREHDKLMKEVGEIKARKREQIKALRSEFC
NLLEMNEKLPKHMQFKRTVSLI
>sp|Q8IYW2|CFA46_HUMAN Cilia- and flagella-associated protein 46 OS=Homo sapiens GN=CFAP46 PE=2 SV=3
MDLVITQELARAESQQDAASLKKAYELIKSANLGKSEFDPSESFSPDLFVLCAEQALKMR
QPEVSEDCIQMYFKVKAPITQFLGRAHLCRAQMCAPKSAENLEEFENCVTEYMKAINFAK
GEPRYYFLVYNASVLYWQMVRPFLKPGYRHHLIPSLSQIINVLSQTEEEDKEWRAELMLE
LLECYLQAGRKEEAARFCSTAAPFIKSHVPQKYRQIFSVMVRHELMDELQLKEEKKNSIS
LSVTFYINMLKAKAEQNDLPGDISVILRKAYRHLGHYNHQRFPSISEEKMLLLFELARFS
LTLKCMEISSACLSDLKKMESKDPGKLIEMECLECESEALRLESKMKVYNRAAVEAQLDI
IQRLDVALQRAVRLGDPRVIHVVCATQWNTCLPLLQHNLRHHLRKPLAGVADVLEKLDSL
MTLLRCQVHMEMAQIEEDEDRLEPATEHLRKAARLDSLGLYRDRIQMASTRLRLCTTLYQ
APERAEDKAIMAVEQAKKATPKDSVRKKRALLVNAGLALAPDAFQIVLDSENEAKVSTGK
NRGRFTYLCAKAWHHTVSVDKAAGHLRRLGNENDKERIQIWAELAKVARKQGVWDVCRTA
SRFCLLYDNVKVKKLRLRRGKKKRGRDGSVQDTWSQPEVVLQRQVCPDLLRKFAEVGFIH
AEATVHLLRSEGVELNDRAIPPEDLSQHPAGYVPEPPEVNAEWITYRTWIESLSRCAMNN
WLRSAEIGQEIQEAWIVQNAVVYVLNHNHHLILAGRQKELVDALYHLLSIVKATGHSGDP
VMLVTLCNTLARGLIISWIPVQAAEKSRKFMRPNAFHSPLDAGATSEIKTAVEVCEFALN
LTNGSAPEETVPTGTRQQLIATWVKAKQLLQQQIGPRLGTEEQGTNEDVSSVTRVLVALE
MYSCNGLGLMDFTVPSLAQLVKMASECNWSDPLVELQTLTRLTHFAHAARDHETTMACAH
RALEMGIKYLKKFGPEESRLVAEMLCTATAIQGRSIMENLKGRKQLRLVAAKAFTESARF
GGIAGSSALVMLAARHYWNAWLPLLSSAVYRKKAKGALKRLIGIINKTEARKQEKGKTLL
LHQWPTADFQGGGTTEGYFLPGAEDDLALRAALYGLLFHSHADQDDWEGGLKVLDEAVQV
LPRTAHRLLIFKHMVIVKAKLGQNFSMEIQKFKAESEDYLARMWHRLALNSPSVSGELAC
YNNAIQALQKPEMEWQKVEYLMEFGQWLHHRHFPLEDVVFHLRWAVEILLAMKPPGDVPE

PQPTPDGEYVAVEMPPRSPVSEAEEAVSLEQLRSVRQLEALARVHILLALVLSPGAEGYE
DCCLAAYAFFRHIWQVSLMTAGKSVLENRPLAATSSHLLLPKKEKENERSKEKEKERSKE
KENERSKEKDKEKGKEEKVKEPKQSQSPAPIKQLEDLPMSIEEWASYSCPEEVLSVLKQD
RSDSTVNPSSIQKPTYSLYFLDHLVKALQKMCLHELTVPVLQLGVLISDSVVGSKGLSDL
YHLRLAHACSELKLREAAARHEEAVGQVCVSELEQASCRKEIALKKEKNKEPLLEESLPA
LNEQTLPVQPGEIKPLDAKDKILKMNGETGRDLDGTSFPHLWMLKAEVLLEMNLYQPARL
LLSEAYLAFQELDEPCAEAQCLLLLAQLANKEKNYGQAKKMIAQAQHLGGSEEFWYNSTL
TLAEALLSMEHSGREATVCHIFQKLINAFKILKKERPNRLPLLEFMITDLEARCLSLRVR
VAQHSAVTEPTECSLLLKEMDDGLLEIERKFIDCGCKENCVDVKLERAKIKRLRAQNEKD
EEQKTAYYLEAYGLAQGAVAEEEGRLHSIQGLYGLAQGAMAEEEGRLHSVQGLLSLQDLQ
NVNTPLMRKLARLKLGLVEMALDMLQFIWEEAHGQQSEQGSLEKLLADYLQNTSDYTSVG
LQWFTLKRTLAHGALAQLGSLQPLSVGCVEIRARLLGLAGRALHLLAMQADPVHPTCYWE
AGPSVGAKLSGLKSLELEVEEEGATKSSRDPPASRAAPEEHCRRGEDLKRRMVLAQQYLA
QASEVLLQCLQVALGSGLLDVAAAASLEMVECVGTLDPATTCQFLALSQSCSASETMRDV
LLAATANTSSSQLAALLQLQHQLRCQDRTTTSLGARVEQRLAAVSKAWQNLCVTEQHFNL
LNEMPPTFWILFLHLSGDRSRLYGAAYEKPKFITAAKGKVQAVGGSCKVMRLAISPTAFS
HLLACAQQFRKQTQAQVYSEDMALNIGSEPEGLQVEEKERPVQRLSSVLGPLEELLQPLF
PLLSLSKARVQTPAVVADSGKSKGKDKERKTSTGQHSTVQPEVADKIVLVADRHLLELPL
EGLSVFDEGTISSVSREFSLQMLWNRLHKEETEGGVKKEGRSRDPKKRSLAKKGRKGSIP
RTIPPDCIIVDSDNFKFVVDPYEEAQGPEMLTPVSITQDILERFQDTFTSRWAGHLGSKH
FPSQAQWEQALGSCSGFFFYGMESFLSHILVERLVAMNLQECQVAVLLDLARSYQSLKRH
MESVEHRRSVGRWEANWRNSASPSEDEWRRGGEPRRGFSDLEGQAAAAPKLRAPSHHAQL
GPVWAAAPSHRVVQAWTCLPSAAGAPALASALGSAPLPTHPHLPAPIPSSQLALPFLGLS
PALGAASARDPPPATSRKAAAWTSSSACLCAPWGLRRGWSCVSSRGQDKGGLPLAALVLS
CLDQKTIQTVSLFLI
>sp|Q96N23|CFA54_HUMAN Cilia- and flagella-associated protein 54 OS=Homo sapiens GN=CFAP54 PE=2 SV=3
MAAQGSPSSSPSDDSTTSGSLPELPPTSTATSRSPPESKGSSRSSLLQWTCPEDSLPLAV
FYGPLDAKNPLLASCEKEIQELLGFMRKKKALATTEEEKHEFRRRCATSLFNIWTKYAPR
LPADYYNEKLLKVGDSLCQMKEYKLALLQCYGRYLQQFNTNFDENKVDVTQFKATFFPKG
FKDKTAGLTFHALSGKNMCNYQLVCDSDENLKNKESVVQCLHILSSLRLIMQVALPQEHL
CWIIFNGTIYIYTICRKLMVIGQSSKALEYLLWASMCMESLVPLLSLRYLTWRATLYTAV
CQCCYDCHAGIHGEAFARRALAKIDELRQLELMSSSKSQEESRRYFREATMKMAVMIFKR
GVFESRRKNKAVFRPKIRINLREVQTLSWPRTVTERLLDEMFDSTASQFLAVLEALSDSN
RRILQTGPIVTDEVEIHDVVSELFMAGKELLIMSNIGADGMLDFPKTSLLELMIGRKDVI
SVDAAVKFIKLAFTYEEWSLFESSAVHLIYFLQRQDDPESKKAEKDLTLLIAMEPLINVK
RNKGLIFPLENYKEGQSTQIYLKKIAVHDTCLKTCGYSEDIFHLAATLYVCVCTAPQDVQ
PDKEIVVDTIMFLWQKCKLGIQRLNISRNDYAKFTQKISTNKWIYLLWQINEVIHCYKME
DIDIVVVAEVTLRLSEILESLGSPGRKFKQSLDVPLREGTNKFPGAPKGITEILPILQKN
PVEQLLFAYKLLDRAIGGINLNCMLTSLPNGSSVIDHCYAKRTHHIDGDTYKPLASNSFM
MDLHLELIQAQHRIAVVLLDKLQVLQTPTVSKDISTKGPEKLKQSGSTDCFTELNIMNKI
KKNTLSKAIYLMQKALLIFEKDATSTSSWELLMEAYSLIQRIEAEQNALYSYQKYLESSK
RKKSRVPPPPILLSRTHCSVTLKPAPFTSEVKVSWYCILGCKAEGSYGKVRLNNNHLPNS
GEAIPADGKSVFEVKGLETNEKYVFAVAAYSNNGKLVGGAIGETTKPILVYPPLSTITAR

MFLTQVAYQVGNYELAKKVFSPVWDYFVASPLQDEQSVICLSNIITITQRRLHSDILAET
SSILLYLFLRNIFVTSDIKIKEENLFCDNIKGNEIFPSQQIARLIECERVLVALELSNFL
NDSSYALQAVTQCYGLLAPIIYHNIVLVPVVQILIKCIVVLQGLPSIVCSKKHTASFESI
QHMIACCIFYITKILRSWREYDLAVMIINYGKKMLDITPGCKSLFDGSNEQEEMPEEDSS
KKSLKTKKPQQILLPEKINEQLALLETHLLKLTKQYVTSELSGGEDPIFLYPVVLNWSVK
GAVKEVMKFKQKPRFLEFFTQVMLKCMNEEKFHLMVEVTTPVHDFLKRRNESLLGLIKVK
YKDSALNKKANKSLKFKAAVMEIGRSAEMQQRIRSKKKETLRDFIFKNPAISEMVAHERN
RRTSVRKAAQRYLMDYLNPLILSYVKRKRFHRLSLEEMPWRAQMNLYLAGAHFNLVLQKL
WECTKMKFGTSHMMVSFRSCDPNMFSLYNSGTVLPTRKLTVENYKAMLDFLLTAKKRKAN
LPSDAEEFSTFINSIMSDENMSKTQTVYDSDSQSGSSAKEKDRGANLCVMDHFMKIFLYC
RRAMVLAHRGGYWTLLQNCCRALWNFTQELQILLKQAVDLDKTFPISQDGFLCTSVLPFY
LGAELLIDMLIQLQNTSSIKPIEDKGEFSVPSCYGNIKNDNGGSSLTFEHPLDDVNVVDL
KWIHDFVLKSLEVLYQVEKWETLVSLAIQFNTVSHERYTEQVTPLLVYAQRQLLLRIQKF
KGPDITQQPCARYEAEYGEKITCRNFIGKQLKINSSTIEATSNCTDLLKMLISSEYSRAK
ALVCVPVDVTDTLRCFRETLEKSKYHNRSIRHSRKLLSLFLAQTQDVLQASNQRSLKVQA
LHSLGSLLIFAEKKRAAFKCWCQALDDIFRKPDVLHTWKEFGPSLTNVTNSHSPPGFKDY
SEEFLSRVGIWGCLQGAVISAKIAQFIKSLNVEKKTDCCILSALLFQGLLRTTLPHPKAE
RCYAQYEITQLLPGIELFSDRYRADICSVIASLYYIIRELHFVRQNLIVLPLLALYQYFV
SGICQDITRNLEARILKIEVLIDLRFFSEAFYEISQIFYGKNMPCPIPAGYKATGKMKIF
QSFDSGKLLTSKENIQAIDELRNKGLPAVLVTIGQPHLLNKFNFVKAYFFLSVAATINCV
PENKFKTVITNKSKPNLPNLEEIYSKDDGSSFYNLTKLKDEITLSMLKSMLLMEAEDRLN
FLLSEVEQKTLSQCSAGELEIVVEARLQLAAVALQRHRAAYSAAIVFSTLTLLQDSKLFE
KKVVQDDTENPVSPGTSVTENKDDSEFLDPISLNAREYFNIHLWLRCRLALVTAFVAQIH
GIGIVKEDDMTDCLSLINEVCMEAKSAGDTELQAEFLTQAVILGLQEKHLKADIMTNLQD
IIHLLEGNEFISPQSRLTLARSLVLLDDLTKAEKFKESPSSKTGKLNLLTRAHSILTEQM
LAFGETIEFRSSNTKYANPLQPLKNIYLPHVMLLAKIKMRIGHTVAKQVYYKNKRKDPSK
WLPALHLFDVALKLCRTTAVEEHEVEAEILFQKGKIERQILMEEKSPSFQLESLYEAIQL
SLKNDQNSGLIRDSYLEMALLYFHLKKPKIKISGSPLTLKPPLRRSSSVKETSANKFEMY
SSLAWIAIRAAAQVSEAVLAINLLIGKKNTRMHKVNQVALPNIPEFAALDLLSSYTDYLL
DNYQVLFQTSCTFLYQNDDVCDSADGRKKTQTKVDITWILLLRYYIHLQRINNLSKLLAS
ATPVSGISLPDDTLLTSLYNSELILRQKEVHFFLKKFLQLYSSSCIDEFPKELLCQLENP
PLSEKDLRESSAKLYRDSSVQSILSFKPVSGSSCVDITPIEMVTQASNKELCFQWYIPPL
DRPPKETEPMVLLLYAYNLKPLKISDVRHSTYNSTCVGSLWIPLNRVIAIHEKLSNLAQI
AELSLPAAPEITSNENIYEVEVEEESVDNEMEDMIIQCCSEIASLFLNDKEPTPLSEVPF
DISLPSIFNLERLFDLANGCILSGGSLFNWIVSIIP
>sp|Q5T0N1|CFA70_HUMAN Cilia- and flagella-associated protein 70 OS=Homo sapiens GN=CFAP70 PE=2 SV=3
MEQVPSAGRLVQITVTEGYDLKGFKGDTPVTFIRAEFNQVVLGDSAKITVSPEGSAKYNF
TSSFEFNPEGGITSDDLAHKPVFLTVTEVLPKEKKQKEEKTLILGQAVVDLLPLLEGQSS
FQTTVPLHPVQGSPLETPRSSAKQCSLEVKVLVAEPLLTTAQISGGNLLKVTLEAAYSVP
ESFIPTGPGQNYMVGLQVPSLGEKDYPILFKNGTLKLGGEREPVPRPKKWPIANILAPGA
NNIPDAFIVGGPYEEEEGELNHPEDSEFRNQAECIKKRIIWDLESRCYLDPSAVVSFQKR
IADCRLWPVEITRVPLVTIPKGKAGKTEKTDEEAQLSFHGVAYVNMVPLLYPGVKRIRGA
FHVYPYLDSVVHEKTKCLLSLFRDIGHHLIHNNKIGGINSLLSKQAVSKNLKEDKPVKEK

DIDGRPRPGDVQAPSIKSQSSDTPLEGEPPLSHNPEGQQYVEAGTYIVLEIQLDKALVPK
RMPEELARRVKEMIPPRPPLTRRTGGAQKAMSDYHIQIKNISRAILDEYYRMFGKQVAKL
ESDMDSETLEEQKCQLSYELNCSGKYFAFKEQLKHAVVKIVRDKYLKTTSFESQEELQTF
ISELYVFLVDQMHVALNQTMPDDVQGTVATIYTSSEQLQLFAFEAEVNENFEMAAAYYKE
RLVREPQNLDHWLDYGAFCLLTEDNIKAQECFQKALSLNQSHIHSLLLCGVLAVLLENYE
QAEIFFEDATCLEPTNVVAWTLLGLYYEIQNNDIRMEMAFHEASKQLQARMLQAQVTKQK
STGVEDTEERGKRESSLGPWGITNGSATAIKVEAPAGPGAALSILDKFLEESSKLQSDSQ
EPILTTQTWDPSISQKPSNTFIKEIPTKKEASKCQDSSALLHPGLHYGVSQTTTIFMETI
HFLMKVKAVQYVHRVLAHELLCPQGGPSCEYYLVLAQTHILKKNFAKAEEYLQQAAQMDY
LNPNVWGLKGHLYFLSGNHSEAKACYERTISFVVDASEMHFIFLRLGLIYLEEKEYEKAK
KTYMQACKRSPSCLTWLGLGIACYRLEELTEAEDALSEANALNNYNAEVWAYLALVCLKV
GRQLEAEQAYKYMIKLKLKDEALLAEIHTLQETVGFGNPSF
>sp|P0CG37|CFC1_HUMAN Cryptic protein OS=Homo sapiens GN=CFC1 PE=1 SV=1
MTWRHHVRLLFTVSLALQIINLGNSYQREKHNGGREEVTKVATQKHRQSPLNWTSSHFGE
VTGSAEGWGPEEPLPYSRAFGEGASARPRCCRNGGTCVLGSFCVCPAHFTGRYCEHDQRR
SECGALEHGAWTLRACHLCRCIFGALHCLPLQTPDRCDPKDFLASHAHGPSAGGAPSLLL
LLPCALLHRLLRPDAPAHPRSLVPSVLQRERRPCGRPGLGHRL
>sp|P13569|CFTR_HUMAN Cystic fibrosis transmembrane conductance regulator OS=Homo sapiens GN=CFTR PE=1 SV=3
MQRSPLEKASVVSKLFFSWTRPILRKGYRQRLELSDIYQIPSVDSADNLSEKLEREWDRE
LASKKNPKLINALRRCFFWRFMFYGIFLYLGEVTKAVQPLLLGRIIASYDPDNKEERSIA
IYLGIGLCLLFIVRTLLLHPAIFGLHHIGMQMRIAMFSLIYKKTLKLSSRVLDKISIGQL
VSLLSNNLNKFDEGLALAHFVWIAPLQVALLMGLIWELLQASAFCGLGFLIVLALFQAGL
GRMMMKYRDQRAGKISERLVITSEMIENIQSVKAYCWEEAMEKMIENLRQTELKLTRKAA
YVRYFNSSAFFFSGFFVVFLSVLPYALIKGIILRKIFTTISFCIVLRMAVTRQFPWAVQT
WYDSLGAINKIQDFLQKQEYKTLEYNLTTTEVVMENVTAFWEEGFGELFEKAKQNNNNRK
TSNGDDSLFFSNFSLLGTPVLKDINFKIERGQLLAVAGSTGAGKTSLLMVIMGELEPSEG
KIKHSGRISFCSQFSWIMPGTIKENIIFGVSYDEYRYRSVIKACQLEEDISKFAEKDNIV
LGEGGITLSGGQRARISLARAVYKDADLYLLDSPFGYLDVLTEKEIFESCVCKLMANKTR
ILVTSKMEHLKKADKILILHEGSSYFYGTFSELQNLQPDFSSKLMGCDSFDQFSAERRNS
ILTETLHRFSLEGDAPVSWTETKKQSFKQTGEFGEKRKNSILNPINSIRKFSIVQKTPLQ
MNGIEEDSDEPLERRLSLVPDSEQGEAILPRISVISTGPTLQARRRQSVLNLMTHSVNQG
QNIHRKTTASTRKVSLAPQANLTELDIYSRRLSQETGLEISEEINEEDLKECFFDDMESI
PAVTTWNTYLRYITVHKSLIFVLIWCLVIFLAEVAASLVVLWLLGNTPLQDKGNSTHSRN
NSYAVIITSTSSYYVFYIYVGVADTLLAMGFFRGLPLVHTLITVSKILHHKMLHSVLQAP
MSTLNTLKAGGILNRFSKDIAILDDLLPLTIFDFIQLLLIVIGAIAVVAVLQPYIFVATV
PVIVAFIMLRAYFLQTSQQLKQLESEGRSPIFTHLVTSLKGLWTLRAFGRQPYFETLFHK
ALNLHTANWFLYLSTLRWFQMRIEMIFVIFFIAVTFISILTTGEGEGRVGIILTLAMNIM
STLQWAVNSSIDVDSLMRSVSRVFKFIDMPTEGKPTKSTKPYKNGQLSKVMIIENSHVKK
DDIWPSGGQMTVKDLTAKYTEGGNAILENISFSISPGQRVGLLGRTGSGKSTLLSAFLRL
LNTEGEIQIDGVSWDSITLQQWRKAFGVIPQKVFIFSGTFRKNLDPYEQWSDQEIWKVAD
EVGLRSVIEQFPGKLDFVLVDGGCVLSHGHKQLMCLARSVLSKAKILLLDEPSAHLDPVT
YQIIRRTLKQAFADCTVILCEHRIEAMLECQQFLVIEENKVRQYDSIQKLLNERSLFRQA
ISPSDRVKLFPHRNSSKCKSKPQIAALKEETEEEVQDTRL

>sp|Q8WU49|CG033_HUMAN Uncharacterized protein C7orf33 OS=Homo sapiens GN=C7orf33 PE=2 SV=1
MQVEVQSLSLEECPWRLPGPQCECEALLPSGARRRIDLRLSGRAVAVWVHVRGGPGQFNL
SYATGRHKKPNPHQNMNRGMEFIAPVSAPTKSGAPWHFLSQGPTDAQRAVRIRPGTRMGL
SSDPVVGTLSSSYLDLLTLSYKPGRTVTSSYLNVRGHEVRKLQNSVEATRISRTDSS
>sp|Q96HJ9|CG055_HUMAN UPF0562 protein C7orf55 OS=Homo sapiens GN=C7orf55 PE=1 SV=2
MAALGSPSHTFRGLLRELRYLSAATGRPYRDTAAYRYLVKAFRAHRVTSEKLCRAQHELH
FQAATYLCLLRSIRKHVALHQEFHGKGERSVEESAGLVGLKLPHQPGGKGWEP
>sp|Q8TBZ9|CG062_HUMAN Uncharacterized protein C7orf62 OS=Homo sapiens GN=C7orf62 PE=2 SV=1
MSFSVHNQKGSKRPLPLEPLLFLQVPRSNYLHFQEEKQRLHLKKFLLDRMFLVAKIQANV
ERKDVADYYEQMFQSVLKHHLGEAVTGLLLIYPTSILHILESSSDTLYKVLLDYIGHVKD
ETVFFIQQMKIIVISHNIPMRLFMQWHVSVIKVPVMYLDDVTQSQSLKEVITDFLTQTHK
LSLYLCQTMKVGTKGPGDNLHQVAPDLLLPEQIIKYLCKSEEFMDPATFINMYNRPIHIT
LDSEVVWPAPSRF
>sp|Q9H7B7|CG069_HUMAN Uncharacterized protein C7orf69 OS=Homo sapiens GN=C7orf69 PE=2 SV=3
MGFHFCIWIIFLLPPPCKKCLSPPTMNLRPPKSCGNVFYWVLVLNSGLLYKFCQTIKCRA
NWRPARAPRGWNEATERHQERRTQMETEMGGISTTYWHRLCTCTDRRAEKLVMDGNNCWF
HK
>sp|A6NKQ9|CGB1_HUMAN Choriogonadotropin subunit beta variant 1 OS=Homo sapiens GN=CGB1 PE=2 SV=3
MSTFPVLAEDIPLRERHVKGRVDPHFRAPKMEMFQRLLLLLLLSMGGTWASKEPLRPRCR
PINATLAVEKEGCPVCITVNTTICAGYCPTMTRVLQGVLPALPQVVCNYRDVRFESIRLP
GCPRGVNPVVSYAVALSCQCALCRRSTTDCGGPKDHPLTCDDPRFQDSSSSKAPPPSLPS
PSRLPGP
>sp|Q9NR00|CH004_HUMAN Protein C8orf4 OS=Homo sapiens GN=C8orf4 PE=1 SV=2
MKAKRSHQAVIMSTSLRVSPSIHGYHFDTASRKKAVGNIFENTDQESLERLFRNSGDKKA
EERAKIIFAIDQDVEEKTRALMALKKRTKDKLFQFLKLRKYSIKVH
>sp|Q49A92|CH034_HUMAN Uncharacterized protein C8orf34 OS=Homo sapiens GN=C8orf34 PE=2 SV=2
MASHPQTRIQAYLEKNKIGPLFEELMTKLITETPDQPIPFLIDHLQSKQGNRGQLQRTLS
GSAALWAESEKSESKGTRRDFRSYDKPWQLNAKKPKKSKSDLAVSNISPPSPDSKSLPRS
VEHPKWNWRTKPQSRDFDELNHILQESKKLGKALENLSRSIAISDELDKETVTFNSSLLR
PRVIGEWIGREENDADPLAAEMLQPPIPRSKNDQWESEDSGSSPAGSLKMEPKNKGLKQQ
QQQHKKLLAAMLSQDSFESIHSPTPSVTEEDIDNEDDAMELLEDLNDLRMEGVTTLVPSG
SKFNQGRPTYPAEPQAKVTLNICSRCARLQGDNLEERTEESLPILHSPDEKIPDSFDSLP
GTEEALMEEGDEFEKASKLTGPGEASSGVGHSLKNYMEEDESLKQLQVVHQPWILPSDTE
SEGVEAEQEKRSADLLLCVPCSSCPTLVYSGL
>sp|Q6P047|CH074_HUMAN Uncharacterized protein C8orf74 OS=Homo sapiens GN=C8orf74 PE=1 SV=3
MALLTPQGVKEVFQLQRPQGRERLRRLLNWEEFDEQRDSRRSILLDTLYESIIFAVGKGF
PWVEVAQVVKFTEELLRETKGCSITEAVTILGNKLRDYRGHFNTTHLLALCDYFHHTFIR
HYKLYQYVLGQDQQVDLTVAHLEVCMPPHPLPLAEGMDRDLWIHEQQVATLTEAEAQKRA

DVLLLKEALRLERENSLQKAFAAAAPAQPGQVLERQELESLICQAVHTQMELLQELLQRQ
IQNTFAILDLKLQKKTLNLNAPTPIPPPITSHAGQEEALKPQRASKGKKAKARK
>sp|E5RJ46|CH087_HUMAN Uncharacterized protein C8orf87 OS=Homo sapiens GN=C8orf87 PE=4 SV=1
MRTPKRTRSPKTKVSLRGETLTLQLTTVSLDTRHMVKRCDERHGRPLPHSQESQHGSATS
KKAVRGTADTAPLERISAARGWALPMEATVSVFRAHQWQWN
>sp|Q8N4J0|CARME_HUMAN Carnosine N-methyltransferase OS=Homo sapiens GN=CARNMT1 PE=1 SV=1
MQRRRRPPPPTSRLPEGCGGGGGGSEEVEVQFSAGRWGSAAAVSAAAAAATRSTEEEEER
LEREHFWKIINAFRYYGTSMHERVNRTERQFRSLPANQQKLLPQFLLHLDKIRKCIDHNQ
EILLTIVNDCIHMFENKEYGEDGNGKIMPASTFDMDKLKSTLKQFVRDWSETGKAERDAC
YQPIIKEILKNFPKERWDPSKVNILVPGAGLGRLAWEIAMLGYACQGNEWSFFMLFSSNF
VLNRCSEINKYKLYPWIHQFSNNRRSADQIRPIFFPDVDPHSLPPGSNFSMTAGDFQEIY
SECNTWDCIATCFFIDTAHNVIDYIDTIWKILKPGGIWINLGPLLYHFENLANELSIELS
YEDIKNVVLQYGFKVEVEKESVLSTYTVNDLSMMKYYYECVLFVVRKPQ
>sp|Q6TDU7|CASC1_HUMAN Protein CASC1 OS=Homo sapiens GN=CASC1 PE=2 SV=2
MSGSKKKKVTKAERLKLLQEEEERRLKEEEEARLKYEKEEMERLEIQRIEKEKWHRLEAK
DLERRNEELEELYLLERCFPEAEKLKQETKLLSQWKHYIQCDGSPDPSVAQEMNTFISLW
KEKTNETFEEVIEKSKVVLNLIEKLKFILLETPPCDLQDKNIIQYQESILQLQELLHLKF
GVATEILLKQASTLADLDSGNMEKVIKDENVTLYVWANLKKNPRHRSVRFSETQIGFEIP
RILATSDIAVRLLHTHYDHVSALHPVSTPSKEYTSAVTELVKDDVKNVEKAISKEVEEES
KQQERGSHLIQEEEIKVEEEQGDIEVKMSSAEEESEAIKCEREMKVLSETVSAAQLLLVE
NSSEKPDFFEDNVVDLCQFTTLGGVYHLDILELPPQCKPVKGWMIVEILKEGLQKYTYPP
ETTEEFETENAFPPIEVTLEVHENVIFFEDPVVVRWDAEGKHWRTDGISNVSYKPKERLV
TFSLDTFGPVTLIQDAHINMPYQSWELRPLDVNKVLLTVTTVFTEIQIQIKENLCMLSSI
KLKDKKHISILEGTWMTPIPFIIALKEAGLNIFPTRHSHFYVIINNKVPLVEVKAYRQMA
LLSSAFAFGWSKWNLLCNSTKVVFKVREHLTEACTENPNWALLMFSGDRAQRLKIKEESE
AFSEALKEETEFHSTLYHMVKDFASEEAMEKVRSSNCQFVNSVCHMLLSTRLLSYS
>sp|Q14511|CASL_HUMAN Enhancer of filamentation 1 OS=Homo sapiens GN=NEDD9 PE=1 SV=1
MKYKNLMARALYDNVPECAEELAFRKGDILTVIEQNTGGLEGWWLCSLHGRQGIVPGNRV
KLLIGPMQETASSHEQPASGLMQQTFGQQKLYQVPNPQAAPRDTIYQVPPSYQNQGIYQV
PTGHGTQEQEVYQVPPSVQRSIGGTSGPHVGKKVITPVRTGHGYVYEYPSRYQKDVYDIP
PSHTTQGVYDIPPSSAKGPVFSVPVGEIKPQGVYDIPPTKGVYAIPPSACRDEAGLREKD
YDFPPPMRQAGRPDLRPEGVYDIPPTCTKPAGKDLHVKYNCDIPGAAEPVARRHQSLSPN
HPPPQLGQSVGSQNDAYDVPRGVQFLEPPAETSEKANPQERDGVYDVPLHNPPDAKGSRD
LVDGINRLSFSSTGSTRSNMSTSSTSSKESSLSASPAQDKRLFLDPDTAIERLQRLQQAL
EMGVSSLMALVTTDWRCYGYMERHINEIRTAVDKVELFLKEYLHFVKGAVANAACLPELI
LHNKMKRELQRVEDSHQILSQTSHDLNECSWSLNILAINKPQNKCDDLDRFVMVAKTVPD
DAKQLTTTINTNAEALFRPGPGSLHLKNGPESIMNSTEYPHGGSQGQLLHPGDHKAQAHN
KALPPGLSKEQAPDCSSSDGSERSWMDDYDYVHLQGKEEFERQQKELLEKENIMKQNKMQ
LEHHQLSQFQLLEQEITKPVENDISKWKPSQSLPTTNSGVSAQDRQLLCFYYDQCETHFI
SLLNAIDALFSCVSSAQPPRIFVAHSKFVILSAHKLVFIGDTLTRQVTAQDIRNKVMNSS
NQLCEQLKTIVMATKMAALHYPSTTALQEMVHQVTDLSRNAQLFKRSLLEMATF
>sp|P51878|CASP5_HUMAN Caspase-5 OS=Homo sapiens GN=CASP5 PE=1 SV=3
MAEDSGKKKRRKNFEAMFKGILQSGLDNFVINHMLKNNVAGQTSIQTLVPNTDQKSTSVK

KDNHKKKTVKMLEYLGKDVLHGVFNYLAKHDVLTLKEEEKKKYYDTKIEDKALILVDSLR
KNRVAHQMFTQTLLNMDQKITSVKPLLQIEAGPPESAESTNILKLCPREEFLRLCKKNHD
EIYPIKKREDRRRLALIICNTKFDHLPARNGAHYDIVGMKRLLQGLGYTVVDEKNLTARD
MESVLRAFAARPEHKSSDSTFLVLMSHGILEGICGTAHKKKKPDVLLYDTIFQIFNNRNC
LSLKDKPKVIIVQACRGEKHGELWVRDSPASLALISSQSSENLEADSVCKIHEEKDFIAF
CSSTPHNVSWRDRTRGSIFITELITCFQKYSCCCHLMEIFRKVQKSFEVPQAKAQMPTIE
RATLTRDFYLFPGN
>sp|P55211|CASP9_HUMAN Caspase-9 OS=Homo sapiens GN=CASP9 PE=1 SV=3
MDEADRRLLRRCRLRLVEELQVDQLWDALLSRELFRPHMIEDIQRAGSGSRRDQARQLII
DLETRGSQALPLFISCLEDTGQDMLASFLRTNRQAAKLSKPTLENLTPVVLRPEIRKPEV
LRPETPRPVDIGSGGFGDVGALESLRGNADLAYILSMEPCGHCLIINNVNFCRESGLRTR
TGSNIDCEKLRRRFSSLHFMVEVKGDLTAKKMVLALLELAQQDHGALDCCVVVILSHGCQ
ASHLQFPGAVYGTDGCPVSVEKIVNIFNGTSCPSLGGKPKLFFIQACGGEQKDHGFEVAS
TSPEDESPGSNPEPDATPFQEGLRTFDQLDAISSLPTPSDIFVSYSTFPGFVSWRDPKSG
SWYVETLDDIFEQWAHSEDLQSLLLRVANAVSVKGIYKQMPGCFNFLRKKLFFKTS
>sp|Q6UXS9|CASPC_HUMAN Inactive caspase-12 OS=Homo sapiens GN=CASP12 PE=2 SV=2
MADEKPSNGVLVHMVKLLIKTFLDGIFDDLMENNVLNTDEIHLIGKCLKFVVSNAENLVD
DITETAQTAGKIFREHLWNSKKQLSSDISSDGEREANMPGLNIRNKEFNYLHNRNGSELD
LLGMRDLLENLGYSVVIKENLTAQEMETALRQFAAHPEHQSSDSTFLVFMSHSILNGICG
TKHWDQEPDVLHDDTIFEIFNNRNCQSLKDKPKVIIMQACRGNGAGIVWFTTDSGKAGAD
THGRLLQGNICNDAVTKAHVEKDFIAFKSSTPHNVSWRHETNGSVFISQIIYYFREYSWS
HHLEEIFQKVQHSFETPNILTQLPTIERLSMTRYFYLFPGN
>sp|Q9NWW7|CB042_HUMAN Uncharacterized protein C2orf42 OS=Homo sapiens GN=C2orf42 PE=1 SV=1
MEPNSLRTKVPAFLSDLGKATLRGIRKCPRCGTYNGTRGLSCKNKTCGTIFRYGARKQPS
VEAVKIITGSDLQVYSVRQRDRGPDYRCFVELGVSETTIQTVDGTIITQLSSGRCYVPSC
LKAATQGVVENQCQHIKLAVNCQAEATPLTLKSSVLNAMQASPETKQTIWQLATEPTGPL
VQRITKNILVVKCKASQKHSLGYLHTSFVQKVSGKSLPERRFFCSCQTLKSHKSNASKDE
TAQRCIHFFACICAFASDETLAQEFSDFLNFDSSGLKEIIVPQLGCHSESTVSACESTAS
KSKKRRKDEVSGAQMNSSLLPQDAVSSNLRKSGLKKPVVASSLKRQACGQLLDEAQVTLS
FQDWLASVTERIHQTMHYQFDGKPEPLVFHIPQSFFDALQQRISIGSAKKRLPNSTTAFV
RKDALPLGTFSKYTWHITNILQVKQILDTPEMPLEITRSFIQNRDGTYELFKCPKVEVES
IAETYGRIEKQPVLRPLELKTFLKVGNTSPDQKEPTPFIIEWIPDILPQSKIGELRIKFE
YGHHRNGHVAEYQDQRPPLDQPLELAPLTTITFP
>sp|Q96LR7|CB050_HUMAN Uncharacterized protein C2orf50 OS=Homo sapiens GN=C2orf50 PE=1 SV=1
MGSHPTPGLQRTTSAGYRLPPTRPPASVSPAARGGPMASRGLAGGCQAPQALKAQRVAQG
AACDGVQQDQLWRELLEAERRGQQRWIQNWSFLKDYDPMGNKKEPEKLPDHVPLFSDTVP
SSTNQVVGSRLDTPLGQTLIRMDFFFTEGARKKKLEDQMQPI
>sp|Q6UXQ4|CB066_HUMAN Uncharacterized protein C2orf66 OS=Homo sapiens GN=C2orf66 PE=3 SV=1
MIIDSSRIPSFTQLHSTMTRAPLLLLCVALVLLGHVNGATVRNEDKWKPLNNPRNRDLFF
RRLQAYFKGRGLDLGTFPNPFPTNENPRPLSFQSELTASASADYEEQKNSFHNYLKG
>sp|Q3KRA6|CB076_HUMAN UPF0538 protein C2orf76 OS=Homo sapiens GN=C2orf76 PE=1 SV=3

MAPGEVTITVRLIRSFEHRNFKPVVYHGVNLDQTVKEFIVFLKQDIPLRTNLPPPFRNYK
YDALKIIHQAHKSKTNELVLSLEDDERLLLKEDSTLKAAGIASETEIAFFCEEDYKNYKA
NPISSW
>sp|Q0P641|CB080_HUMAN Uncharacterized protein C2orf80 OS=Homo sapiens GN=C2orf80 PE=2 SV=2
MERRLIKKEMKKLLGDYIGIRLRENEFDPKGRRQLTFLDDMAHYDLAISVALQWLDPSED
LTWLEWEELKIPLHGRPIYPNRREREAMILSSYAGILMNSIPIEEVFKIYGADSSADSGT
IKVPRVSSLCLSLHPFAMLTAPKAAAYARKQSVKSRKVTTNKNATSISAKEANATEWKSS
QRFSDTQPKHKVT
>sp|A6NN90|CB081_HUMAN Uncharacterized protein C2orf81 OS=Homo sapiens GN=C2orf81 PE=3 SV=2
MAHEGSRQVRDRGVTRSKAEKVRPPTVPVPQVDIVPGRLSEAEMALTALEEGEDVVGDIL
ADLLARVMDSAFKVYLTQQCIPFTISQAREAMLQITEWRFLARDEGESAVAEDPTWGEDE
EPSACTTDSWAQGSVPVLHASTSEGLENFQGEDPGGVDRIPLGRSWMGRGSQEQMESWEP
SPQLRVTSAPPPTSELFQEAGPGGPVEEADGQSRGLSSAGSLSASFQLSVEEAPADDADP
SLDPYLVASPQASTGRGHPLGFHLSLEDLYCCMPQLDAAGDRLELRSEGVPCIASGVLVS
YPSVGGATRPSASCQQQRAGHSDVRLSAHHHRMRRKAAVKRLDPARLPCHWVRPLAEVLV
PDSQTRPLEAYRGRQRGEKTKARAEPQALGPGTRVSPAAFFPLRPGIPFRDLDSGPALLF
PTLNLGLSSPSLESKLPLPNSRIRFLTTHPVLPDVARSRSPKLWPSVRWPSGWEGKAELL
GELWAGRTRVPPQGLELADREGQDPGRWPRTTPPVLEATSQVMWKPVLLPEALKLAPGVS
MWNRSTQVLLSSGVPEQEDKEGSTFPPVEQHPIQTGAPKPR
>sp|Q6UX34|CB082_HUMAN Uncharacterized protein C2orf82 OS=Homo sapiens GN=C2orf82 PE=2 SV=1
MASCLALRMALLLVSGVLAPAVLTDDVPQEPVPTLWNEPAELPSGEGPVESTSPGREPVD
TGPPAPTVAPGPEDSTAQERLDQGGGSLGPGAIAAIVIAALLATCVVLALVVVALRKFSA
S
>sp|Q53S99|CB083_HUMAN Folate transporter-like protein C2orf83 OS=Homo sapiens GN=C2orf83 PE=2 SV=1
MEDYALTFGINPFIALMIQPIVTMTVVDNQGLGLPVDIQHKAKPSPKASQMLPPDLGPPS
FQNLLSPSEKLEPWDPGMRKLTVQTCGLTFIHPAGHGLCHPTAEASAETLSSTALNRPSV
REGACNEKSTENKKPQDSVLWSHSRWFQGN
>sp|P23435|CBLN1_HUMAN Cerebellin-1 OS=Homo sapiens GN=CBLN1 PE=1 SV=1
MLGVLELLLLGAAWLAGPARGQNETEPIVLEGKCLVVCDSNPTSDPTGTALGISVRSGSA
KVAFSAIRSTNHEPSEMSNRTMIIYFDQVLVNIGNNFDSERSTFIAPRKGIYSFNFHVVK
VYNRQTIQVSLMLNGWPVISAFAGDQDVTREAASNGVLIQMEKGDRAYLKLERGNLMGGW
KYSTFSGFLVFPL
>sp|Q6UW01|CBLN3_HUMAN Cerebellin-3 OS=Homo sapiens GN=CBLN3 PE=1 SV=1
MLGAKPHWLPGPLHSPGLPLVLVLLALGAGWAQEGSEPVLLEGECLVVCEPGRAAAGGPG
GAALGEAPPGRVAFAAVRSHHHEPAGETGNGTSGAIYFDQVLVNEGGGFDRASGSFVAPV
RGVYSFRFHVVKVYNRQTVQVSLMLNTWPVISAFANDPDVTREAATSSVLLPLDPGDRVS
LRLRRGNLLGGWKYSSFSGFLIFPL
>sp|P15086|CBPB1_HUMAN Carboxypeptidase B OS=Homo sapiens GN=CPB1 PE=1 SV=4
MLALLVLVTVALASAHHGGEHFEGEKVFRVNVEDENHINIIRELASTTQIDFWKPDSVTQ
IKPHSTVDFRVKAEDTVTVENVLKQNELQYKVLISNLRNVVEAQFDSRVRATGHSYEKYN

KWETIEAWTQQVATENPALISRSVIGTTFEGRAIYLLKVGKAGQNKPAIFMDCGFHAREW
ISPAFCQWFVREAVRTYGREIQVTELLDKLDFYVLPVLNIDGYIYTWTKSRFWRKTRSTH
TGSSCIGTDPNRNFDAGWCEIGASRNPCDETYCGPAAESEKETKALADFIRNKLSSIKAY
LTIHSYSQMMIYPYSYAYKLGENNAELNALAKATVKELASLHGTKYTYGPGATTIYPAAG
GSDDWAYDQGIRYSFTFELRDTGRYGFLLPESQIRATCEETFLAIKYVASYVLEHLY
>sp|Q8NDL9|CBPC5_HUMAN Cytosolic carboxypeptidase-like protein 5 OS=Homo sapiens GN=AGBL5 PE=2 SV=1
MELRCGGLLFSSRFDSGNLAHVEKVESLSSDGEGVGGGASALTSGIASSPDYEFNVWTRP
DCAETEFENGNRSWFYFSVRGGMPGKLIKINIMNMNKQSKLYSQGMAPFVRTLPTRPRWE
RIRDRPTFEMTETQFVLSFVHRFVEGRGATTFFAFCYPFSYSDCQELLNQLDQRFPENHP
THSSPLDTIYYHRELLCYSLDGLRVDLLTITSCHGLREDREPRLEQLFPDTSTPRPFRFA
GKRIFFLSSRVHPGETPSSFVFNGFLDFILRPDDPRAQTLRRLFVFKLIPMLNPDGVVRG
HYRTDSRGVNLNRQYLKPDAVLHPAIYGAKAVLLYHHVHSRLNSQSSSEHQPSSCLPPDA
PVSDLEKANNLQNEAQCGHSADRHNAEAWKQTEPAEQKLNSVWIMPQQSAGLEESAPDTI
PPKESGVAYYVDLHGHASKRGCFMYGNSFSDESTQVENMLYPKLISLNSAHFDFQGCNFS
EKNMYARDRRDGQSKEGSGRVAIYKASGIIHSYTLECNYNTGRSVNSIPAACHDNGRASP
PPPPAFPSRYTVELFEQVGRAMAIAALDMAECNPWPRIVLSEHSSLTNLRAWMLKHVRNS
RGLSSTLNVGVNKKRGLRTPPKSHNGLPVSCSENTLSRARSFSTGTSAGGSSSSQQNSPQ
MKNSPSFPFHGSRPAGLPGLGSSTQKVTHRVLGPVREPRSQDRRRQQQPLNHRPAGSLAP
SPAPTSSGPASSHKLGSCLLPDSFNIPGSSCSLLSSGDKPEAVMVIGKGLLGTGARMPCI
KTRLQARPRLGRGSPPTRRGMKGSSGPTSPTPRTRESSELELGSCSATPGLPQARPPRPR
SAPAFSPISCSLSDSPSWNCYSRGPLGQPEVCFVPKSPPLTVSPRV
>sp|Q66K79|CBPZ_HUMAN Carboxypeptidase Z OS=Homo sapiens GN=CPZ PE=1 SV=2
MPPPLPLLLLTVLVVAAARPGCEFERNPAGECHRPPAADSATCVDLQLRTCSDAAYNHTT
FPNLLQHRSWEVVEASSEYILLSVLHQLLEGQCNPDLRLLGCAVLAPRCEGGWVRRPCRH
ICEGLREVCQPAFDAIDMAWPYFLDCHRYFTREDEGCYDPLEKLRGGLEADEALPSGLPP
TFIRFSHHSYAQMVRVLRRTASRCAHVARTYSIGRSFDGRELLVIEFSSRPGQHELMEPE
VKLIGNIHGNEVAGREMLIYLAQYLCSEYLLGNPRIQRLLNTTRIHLLPSMNPDGYEVAA
AEGAGYNGWTSGRQNAQNLDLNRNFPDLTSEYYRLAETRGARSDHIPIPQHYWWGKVAPE
TKAIMKWMQTIPFVLSASLHGGDLVVSYPFDFSKHPQEEKMFSPTPDEKMFKLLSRAYAD
VHPMMMDRSENRCGGNFLKRGSIINGADWYSFTGGMSDFNYLHTNCFEITVELGCVKFPP
EEALYILWQHNKESLLNFVETVHRGIKGVVTDKFGKPVKNARISVKGIRHDITTAPDGDY
WRLLPPGIHIVIAQAPGYAKVIKKVIIPARMKRAGRVDFILQPLGMGPKNFIHGLRRTGP
HDPLGGASSLGEATEPDPLRARRQPSADGSKPWWWSYFTSLSTHRPRWLLKY
>sp|Q9UK00|CC018_HUMAN Uncharacterized protein C3orf18 OS=Homo sapiens GN=C3orf18 PE=2 SV=2
MNSRTASARGWFSSRPPTSESDLEPATDGPASETTTLSPEATTFNDTRIPDAAGGTAGVG
TMLLSFGIITVIGLAVALVLYIRKKKRLEKLRHQLMPMYNFDPTEEQDELEQELLEHGRD
AASVQAATSVQAMQGKTTLPSQGPLQRPSRLVFTDVANAIHA
>sp|Q8WV48|CC107_HUMAN Coiled-coil domain-containing protein 107 OS=Homo sapiens GN=CCDC107 PE=2 SV=2
MAGAVSLLGVVGLLLVSALSGVLGDRANPDLRAHPGNAAHPGSGATEPRRRPPLKDQRER
TRAGSLPLGALYTAAVAAFVLYKCLQGKDETAVLHEEASKQQPLQSEQQLAQLTQQLAQT
EQHLNNLMAQLDPLFERVTTLAGAQQELLNMKLWTIHELLQDSKPDKDMEASEPGEGSGG

ESAGGGDKVSETGTFLISPHTEASRPLPEDFCLKEDEEEIGDSQAWEEPTNWSTETWNLA
TSWEVGRGLRRRCSQAVAKGPSHSLGWEGGTTAEGRLKQSLFS
>sp|Q5T0U0|CC122_HUMAN Coiled-coil domain-containing protein 122 OS=Homo sapiens GN=CCDC122 PE=2 SV=1
MSDNKERKSQGFPKEDNQDTSSLADAVEKVAKQQQSQASEIEKNKKVLFNLKNELHELEK
EIAAISAETKETERQIYQQDSAIENTKLHCDSLETQIKSLHSENVKLKFDIETAQEDFEE
HMIKYNAYYAKIKAHKNSLGEVESKWSFMTELHEKRDFVKKLKTMKEELMQDLQNPGGNR
ITQVQEDITNLKDKIITVKESIIEKTCFLEEEKKTHEKLRKEIEVQHKRYDAILKRLHCQ
VNKLQSNRRQWQWNIQQLEKTAAELRKCIGMQE
>sp|Q9H6E4|CC134_HUMAN Coiled-coil domain-containing protein 134 OS=Homo sapiens GN=CCDC134 PE=1 SV=1
MDLLQFLAFLFVLLLSGMGATGTLRTSLDPSLEIYKKMFEVKRREQLLALKNLAQLNDIH
QQYKILDVMLKGLFKVLEDSRTVLTAADVLPDGPFPQDEKLKDAFSHVVENTAFFGDVVL
RFPRIVHYYFDHNSNWNLLIRWGISFCNQTGVFNQGPHSPILSLMAQELGISEKDSNFQN
PFKIDRTEFIPSTDPFQKALREEEKRRKKEEKRKEIRKGPRISRSQSEL
>sp|Q17RM4|CC142_HUMAN Coiled-coil domain-containing protein 142 OS=Homo sapiens GN=CCDC142 PE=2 SV=1
MAQASRSGSLPPLVIVPPLRAQPGGTGEEQWERSRTGGLRWEVHCWPSGTSGGTPWWPTP
ADVSEDYEADAAAWRRGPAGGGPIPPALQRLRAVLLRLHREREQLLQARDCAYHLQSAVR
LMKTLSPGSPSGGPSPLPQWCRDLQLHPSQGAVLRIGPGETLEPLLLARPIGLAAQCLEA
VIEMQLRALGREPASPGLSSQLAELLFALPAYHTLQRKALSHVPGAARPFPTSRVLRLLT
GERGCQVASRLDEALQGSALRDQLRRRCQEEGDLLPGLLGLVGGVAGSASCGLGLGGAGA
LWSQYWTLLWAACAQSLDLNLGPWRDPRATAQQLSQALGQASLPQECEKELASLCHRLLH
QSLIWSWDQGFCQALGSALGGQSSLPTSSGTAELLQQLFPPLLDALREPRLRRIFCQPAD
PAPVALGLCTLQTTLLWFLGRAQQYLAAWDPASFLLLIQKDLPPLLHEAEALYSLASEES
LALEVEQQLGLEIQKLTAQIQLLPEESLSVFSQECHKQAMQGFKLYMPRGRYWRLRLCPE
PPSAPSEYAGLVVRTVLEPVLQGLQGLPPQAQAPALGQALTAIVGAWLDHILTHGIRFSL
QGALQLKQDFGVVRELLEEEQWSLSPDLRQTLLMLSIFQQLDGALLCLLQQPLPKSQVHR
RPPCCCACQEVQTTKLPSSCLNSLESLEPPLQPGTSPAQTGQLQSTLGGRGPSPEGYLVG
NQQAWLALRQHQRPRWHLPFFSCLGTSPES
>sp|A4D256|CC14C_HUMAN Dual specificity protein phosphatase CDC14C OS=Homo sapiens GN=CDC14C PE=2 SV=2
MNEVSSECGKKCEPLGCSSTNGDLQGEAGAVVSIFLRMVPRIKSNEGYGYSNRNWRKENT
MHSLDRNIVDGGQALGQWKRKSKGRSSWAAAPHCSPRCSLTSQGVKKMRSSTLQDPRRRD
PQDDVYVDITDRLRFAILYSRPKSASNVHYFSIDNELEYENFSEDFGPLNLAMVYRYCCK
INKKLKSITMLRKKIVHFTGSDQRKQANAAFLVGCYMVIYLGRTPEAAYRILIFGDTPYI
PFRDAAYGSCNFYITLLDCFHAVKKAMQYGFLNFNSFNLDEYEHYEKAENGDLNWIIPDR
FIAFCGPHSRARLESGYHQHSPETYIQYFKNHNVTTIIRLNKRMYDAKRFTDAGFDHHDL
FFADGSTPTDAIVKRFLDICENAEGAIAVHCKAGLGRTGTLIACYIMKHYRMTAAETIAW
VRICRPGLVIGPQQQFLVMKQTSLWLEGDYFRQRLKGQENGQHRAAFSKLLSGVDDISIN
GVENQDQQEPKPYSDDDEINGVTQGDRSRALKRRRQSKTNDILLPSPLAVLTFTLCSVVI
WWIVCDYILPILLF
>sp|Q4G0S7|CC152_HUMAN Coiled-coil domain-containing protein 152 OS=Homo sapiens GN=CCDC152 PE=2 SV=3

MDQSSEGCMKKISSVNLDKLINDFSQIEKKMVETNGKNNILDIQLEKSNCLLKVMQAKEV
SIKEECATLHNIIKGLQQTIEYQQNLKGENEQLKISADLIKEKLKSHEQEYKNNIAKLVS
EMKIKEEGYKKEISKLYQDMQRKVELNEEKHKELIEKKEMEISELNAKLRSQEKEKQNEI
IKLQLEFDAKLARVQTKSKSYQDSTVLPQSIYRRKLQHFQEEKNKEIAILRNTIRDLEQR
LSVGKDSHLKRRRF
>sp|Q8NDH2|CC168_HUMAN Coiled-coil domain-containing protein 168 OS=Homo sapiens GN=CCDC168 PE=2 SV=2
MAQIQKDKANISEKSVMHPEYIAVKAEKSPLSHILKTKELQVNISQQGEKAQEGEVEIVV
LLSKTCPFVTSSAFLELDSIKEEEGEPRITRSFMPHLEIQESLPSRQTAPTKPTESLVKK
EKQLLPQKEDRVQTVSMHGLMHPNGAVFKAKTSAPPQVFSITEHSPLSKRKEPQWGMKER
AGQKQDRTGRPHVILTKTHPFMPSLSHHRFSPSQPKLPISSGAGKSRLANSNEGISSHKV
ILKANQQMPYKEAKDRVKIEGREGRILPKRIHLRAEALPLALLCNGKNYSLHIEEQGEGV
QESKKEPGVVPRKSASFPPPPFYLNCDTRRNEKEGTLGKTQFSFPPLKIQDSSDSGKKAY
TESLHGYTLSNSKGPVQPTAQGEEKGGLRIDMEDKMLPKCTDLKAKQLLLSDILNTKKLQ
WKSKEQKRKIQEDKNKQVKGLPSINTSLLTPPYLKFDTTEGQENVIRIAKVSLPQSRSKE
SSDAGRIACPEATHGELSSDVKQLKAHLLQKEEKDREKVADMTSVLDPNKMYLKAKKSPV
LHTHSFSDLQWKTREQEEEKVQKVKSGPGVMLSKSPSRSSPLHLNVNTGFQEESIPILTR
PSFPLVKLQVSPDTEGGTCIRPIAGDILIYLQKGKHVSQNKEEDDVQIVSILIFPKHQEE
KVQECEGEPGVVLTKSTSLPSLSQLELDKETHLGNEMLRLKRPILRRISHIGETVHRESV
VGDIPKDVKNEKQHIPQKEERNQKKIIDMRGTDITLKSKKSPRSCMLHRTELHVNIGGQG
RKEHEGQDKPPGMIQRKMCILFSKPLPSNLKLERATHADEERLGGKTSFVLPLMPSALPD
TEKTADAEARSGDVRKGKPHRSQKENRHEVKTIDMRFRIHCQEARISPMSHILNAKELVL
NINKLEKKVHKDKDEACVVLSRTFLSIPSAPPLYLDSGNKTDKDTPGITGSSCPQRTLHV
PSNTQKITNRDSVEGVDKNVVKQAEQYVPRPEAEQQLTSNFMISVQQRNQPSRVRSEEDL
NQLVLNSRDEDIYFTGFGTIRSGKRPEWLFTGKKAQPVKYKTETLTAFLSYPTMDATKMG
GLEEDTEIMDNLNHKISPKASVSLIRKISKELYVTLGTPANSKGFSVSERYAHQQETSSK
VSPELAGSCKFDKPKEDGQSNDRISKMFSPKVLAPQTKGSLKKISIVTNWNAPQNIEEQD
IVMKKQVIRRCEHGHKTRTNTILSKFPLQSGKQKTPSETDVDKKTTAHLSLQMLPGIHMD
MTEIDPAKGGRKQALLISEQEEGVLEFLPKSLFPPWTFQFQSGDLEEKHQTDANTNINLE
QKKLEMDNDSTVNQKEGKLKIGTNRALHLQEEKTEMHKARTANLEKERGRMDTSSSAHPH
LLSLKAEESQMKTQVITHRENSRLIMQKQKKELEASNAKQSIQLQKLFQRNVLDSFYSYV
PLSPKRKDQKGRLTIRDLKRELSTKYLTMKIQNHPIPQMLNITGRGTPSNRKKLEYDVKL
KNIASWSKDVSGIFIRSLSISIMRSPHTDPKTNLEREKRICLPKFQEKSPNTSEMSKRDT
LTIVKGEQNFTNTVPQDPQPFAVDKQQMQKLPNVKSEANLRSEMNKKYLKAQTKERIVPE
HDVSRIIKKPDLRIIEQEEKILKRILTPTECPSMLEDPKLPKQRDQSEPVWDMTTQKVQQ
QKAFPGTVPIPPQVKSSEVKIVADSTNAEHLLPICEATKAISESQVKNMIQDKVSSDKLD
NIQAYKPDDLKSPPFPEGPDTISTAIYPKTQHKSLLEQFTPKEKNKLTSHLESKALEIQL
NLIPEMARKSLQMFNFYPKGTISKDNSWRFYSRHKTMNFMSLEGTDTIEPNSKHKHQKDS
PLASNMKTLIVDVSSDSEETITKLQSINKLENGTSAVTSASEMLLPHTLQNHSVKEKGKL
LMHFSVKTLEIQMKAFPRIVRESYAMTSAHERKKPLSNCIHPGFTGPKRQNRILLLSEEK
SLHQIDLDLQYKYLRFLLGLPVGSTFPKPNVLPKHSKLNTIAVCKNVNAGGQSGSLSIDT
ELLEQHISFKKQSPHENSSLIRKFPQPTLVCASDRDLHSPRKKDTQVLSESEFHVTPEKN
KQYHVWFQERNTCESVDLRTQRNATGSAVSCETQISEDFVDIQTDIESPADLDECSCLEV
SESEECVFLEANSYLSQESENILFELQTGIPLENVYKITTDLKSFYSEDSGSHCTRECRK

ETLIITPPSCKSHKSRKYRSSSKMKSPDWLCHSSSNTAEIQSRSSSVSFSEEKISWTTKS
RTSYSSAPLTESNIKSHLAKNQGKSHRHPESQERKKARSDLFRKNSSHWDHDYSCTHSKG
KRDRKKRVYDYESERLDCFQSKHKSASKPHHDDINFYSERKQNRPFFFACVPADSLEVIP
KTIRWTIPPETLRKRNFRIPLVAKISSSWNIWSSSKKLLGSLSGSLTTVFHS
>sp|A6NC98|CC88B_HUMAN Coiled-coil domain-containing protein 88B OS=Homo sapiens GN=CCDC88B PE=1 SV=1
MEGGKGPRLRDFLSGSLATWALGLAGLVGEAEDSEGEEEEEEEEPPLWLEKRFLRLSDGA
LLLRVLGIIAPSSRGGPRMLRGLDGPAAWRVWNLNHLWGRLRDFYQEELQLLILSPPPDL
QTLGFDPLSEEAVEQLEGVLRLLLGASVQCEHRELFIRHIQGLSLEVQSELAAAIQEVTQ
PGAGVVLALSGPDPGELAPAELEMLSRSLMGTLSKLARERDLGAQRLAELLLEREPLCLR
PEAPSRAPAEGPSHHLALQLANAKAQLRRLRQELEEKAELLLDSQAEVQGLEAEIRRLRQ
EAQALSGQAKRAELYREEAEALRERAGRLPRLQEELRRCRERLQAAEAYKSQLEEERVLS
GVLEASKALLEEQLEAARERCARLHETQRENLLLRTRLGEAHAELDSLRHQVDQLAEENV
ELELELQRSLEPPPGSPGEAPLAGAAPSLQDEVREAEAGRLRTLERENRELRGLLQVLQG
QPGGQHPLLEAPREDPVLPVLEEAPQTPVAFDHSPQGLVQKARDGGPQALDLAPPALDSV
LEASAECPQAPDSDPQEAESPLQAAAMDPQASDWSPQESGSPVETQESPEKAGRRSSLQS
PASVAPPQGPGTKIQAPQLLGGETEGREAPQGELVPEAWGLRQEGPEHKPGPSEPSSVQL
EEQEGPNQGLDLATGQAEAREHDQRLEGTVRDPAWQKPQQKSEGALEVQVWEGPIPGESL
ASGVAEQEALREEVAQLRRKAEALGDELEAQARKLEAQNTEAARLSKELAQARRAEAEAH
REAEAQAWEQARLREAVEAAGQELESASQEREALVEALAAAGRERRQWEREGSRLRAQSE
AAEERMQVLESEGRQHLEEAERERREKEALQAELEKAVVRGKELGDRLEHLQRELEQAAL
ERQEFLREKESQHQRYQGLEQRLEAELQAAATSKEEALMELKTRALQLEEELFQLRQGPA
GLGPKKRAEPQLVETQNVRLIEVERSNAMLVAEKAALQGQLQHLEGQLGSLQGRAQELLL
QSQRAQEHSSRLQAEKSVLEIQGQELHRKLEVLEEEVRAARQSQEETRGQQQALLRDHKA
LAQLQRRQEAELEGLLVRHRDLKANMRALELAHRELQGRHEQLQAQRASVEAQEVALLAE
RERLMQDGHRQRGLEEELRRLQSEHDRAQMLLAELSRERGELQGERGELRGRLARLELER
AQLEMQSQQLRESNQQLDLSACRLTTQCELLTQLRSAQEEENRQLLAEVQALSRENRELL
ERSLESRDHLHREQREYLDQLNALRREKQKLVEKIMDQYRVLEPVPLPRTKKGSWLADKV
KRLMRPRREGGPPGGLRLGADGAGSTESLGGPPETELPEGREADGTGSPSPAPMRRAQSS
LCLRDETLAGGQRRKLSSRFPVGRSSESFSPGDTPRQRFRQRHPGPLGAPVSHSKGPGVG
WENSAETLQEHETDANREGPEVQEPEKRPLTPSLSQ
>sp|Q49A88|CCD14_HUMAN Coiled-coil domain-containing protein 14 OS=Homo sapiens GN=CCDC14 PE=1 SV=3
MKRGIRRDPFRKRKLGGRAKKVREPTAVNSFYREASLPSVWASLRRREMVRSGARPGQVL
SSGRHTGPAKLTNGKKATYLRKIPRFNADSGYSIHSDSESQAETVHGLDGCASLLRDILR
NEDSGSETAYLENRSNSRPLESKRYGSKKKRHEKHTIPLVVQKETSSSDNKKQIPNEASA
RSERDTSDLEQNWSLQDHYRMYSPIIYQALCEHVQTQMSLMNDLTSKNIPNGIPAVPCHA
PSHSESQATPHSSYGLCTSTPVWSLQRPPCPPKVHSEVQTDGNSQFASQGKTVSATCTDV
LRNSFNTSPGVPCSLPKTDISAIPTLQQLGLVNGILPQQGIHKETDLLKCIQTYLSLFRS
HGKETHLDSQTHRSPTQSQPAFLATNEEKCAREQIREATSERKDLNIHVRDTKTVKDVQK
AKNVNKTAEKVRIIKYLLGELKALVAEQEDSEIQRLITEMEACISVLPTVSGNTDIQVEI
ALAMQPLRSENAQLRRQLRILNQQLREQQKTQKPSGAVDCNLELFSLQSLNMSLQNQLEE
SLKSQELLQSKNEELLKVIENQKDENKKFSSIFKDKDQTILENKQQYDIEITRIKIELEE
ALVNVKSSQFKLETAEKENQILGITLRQRDAEVTRLRELTRTLQTSMAKLLSDLSVDSAR

CKPGNNLTKSLLNIHDKQLQHDPAPAHTSIMSYLNKLETNYSFTHSEPLSTIKNEETIEP
DKTYENVLSSRGPQNSNTRGMEEASAPGIISALSKQDSDEGSETMALIEDEHNLDNTIYI
PFARSTPEKKSPLSKRLSPQPQIRAATTQLVSNSGLAVSGKENKLCTPVICSSSTKEAED
APEKLSRASDMKDTQLLKKIKEAIGKIPAATKEPEEQTACHGPSGCLSNSLQVKGNTVCD
GSVFTSDLMSDWSISSFSTFTSRDEQDFRNGLAALDANIARLQKSLRTGLLEK
>sp|Q0P6D6|CCD15_HUMAN Coiled-coil domain-containing protein 15 OS=Homo sapiens GN=CCDC15 PE=2 SV=2
MLGSMARKKPRNTSRLPLALNPLKSKDVLAVLAERNEAIVPVGAWVEPASPGSSEIPAYT
SAYLIEEELKEQLRKKQEALKHFQKQVKYRVNQQIRLRKKQQLQKSYERAQKEGSIAMQS
SATHLTSKRTSVFPNNLNVAIGSSRLPPSLMPGDGIEDEENQNELFQQQAQALSETMKQA
RHRLASFKTVIKKKGSVFPDDGRKSFLTREEVLSRKPASTGINTGIRGELPIKVHQGLLA
AVPYQNYMENQELDYEEPDYEESSSLVTDEKGKEDLFGRGQQDQQAIHSEDKNKPFSRVQ
KVKFKNPLFVLMEEEEQKQLHFEGLQDILPEAQDYFLEAQGDLLETQGDLTGIQSVKPDT
QAVEMKVQVTEPEGQAIEPEGQPIKTETQGIMLKAQSIELEEGSIVLKTQDFLPTNQALL
TKNQDVLLKDHCVLPKDQSILLKYQDQDFLPRDQHVLHKDQDILPKYQDQNFLPKDQNFL
SRDQHVLPKDQDILPKYQDQNFLPKDQNFLSRDQHVLPKDQNILPKYQGQDFLPKDQDFL
SRDQHVLPKDWNILPKCQDQDFLPRDQGVLPKDQNILPICQDQDFLPRDQGYLPKDQNIL
PICQDRDFLPRDLHVLSNDQNILPKCQDQDFLPKYQKVHFKEPYSDMTDEKGREDFSLAD
YQCLPPKSQDQDDIKNQQPASFMREERVREELPLDYHQYVVPKIQDQDSPREQNKHIKLP
SSFEKWEIARGNTPGVPLAYDRYQSGLSTEFQAPLAFQSDVDKEEDKKERQKQYLRHRRL
FMDIEREQVKEQQRQKEQKKKIEKIKKKREQECYAAEQRILRMNFHEDPYSGEKLSEILA
QLQLQEIKGTREKQQREKEYLRYVEALRAQIQEKMQLYNITLPPLCCCGPDFWDAHPDTC
ANNCIFYKNHRAYTRALHSFINSCDVPGGNSTLRVAIHNFASAHRRTLKNL
>sp|Q96LX7|CCD17_HUMAN Coiled-coil domain-containing protein 17 OS=Homo sapiens GN=CCDC17 PE=1 SV=2
MDSHSGEPALLPCGTCDMVFRSSALLATHTQRFCIGHPTQEMTFGAQASVATEPQRAAVV
PQEHQGVPQEPQGLPDQQASRSALKRLTEEVQWLRLSLQEMRPWITEVPRVFAGPWTRSE
ARPQSPMSEAVGSPSERLRALFRTRARRVAEMEAQSRALQLRGEELSRRLQVVACTRGGM
SRLFGLEQEIRELQAEAGRTRGALEVLGARIQELQAEPGNPLSSRREAELYSPVQKANPG
TLAAEIRALREAYIRDGGRDPGVLGQIWQLQVEASALELQRSQTRRGRAGATSGELPVVE
AENRRLEAEILALQMQRGRAPLGPQDLRLLGDASLQPKGRRDPPLLPPPVAPPLPPLPGF
SEPQLPGTMTRNLGLDSHFLLPTSDMLGPAPYDPGAGLVIFYDFLRGLEASWIWVQLRTG
LARDGRDTGRTTALPPALCLPPPPAPGPMGNCAILASRQPVPRLPPSSSVSLVCELQVWQ
GLAWARAPQPKAWVSLGLFDQDQRVLSGRWRLPLRALPLDPSLSLGQLNGIPQAGQAELF
LRLVNARDAAVQTLAEINPASVHEYQYPPPVSSTSSLEASFLTPAVGFADPPPRTEEPLS
GVKDRDEGLGPHHSSDLPPVSF
>sp|Q5T9S5|CCD18_HUMAN Coiled-coil domain-containing protein 18 OS=Homo sapiens GN=CCDC18 PE=1 SV=1
MESSSSDYYNKDNEEESLLANVASLRHELKITEWSLQSLGEELSSVSPSENSDYAPNPSR
SEKLILDVQPSHPGLLNYSPYENVCKISGSSTDFQKKPRDKMFSSSAPVDQEIKSLREKL
NKLRQQNACLVTQNHSLMTKFESIHFELTQSRAKVSMLESAQQQAASVPILEEQIINLEA
EVSAQDKVLREAENKLEQSQKMVIEKEQSLQESKEECIKLKVDLLEQTKQGKRAERQRNE
ALYNAEELSKAFQQYKKKVAEKLEKVQAEEEILERNLTNCEKENKRLQERCGLYKSELEI
LKEKLRQLKEENNNGKEKLRIMAVKNSEVMAQLTESRQSILKLESELENKDEILRDKFSL

MNENRELKVRVAAQNERLDLCQQEIESSRVELRSLEKIISQLPLKRELFGFKSYLSKYQM
SSFSNKEDRCIGCCEANKLVISELRIKLAIKEAEIQKLHANLTANQLSQSLITCNDSQES
SKLSSLETEPVKLGGHQVESVKDQNQHTMNKQYEKERQRLVTGIEELRTKLIQIEAENSD
LKVNMAHRTSQFQLIQEELLEKASNSSKLESEMTKKCSQLLTLEKQLEEKIVAYSSIAAK
NAELEQELMEKNEKIRSLETNINTEHEKICLAFEKAKKIHLEQHKEMEKQIERLEAQLEK
KDQQFKEQEKTMSMLQQDIICKQHHLESLDRLLTESKGEMKKENMKKDEALKALQNQVSE
ETIKVRQLDSALEICKEELVLHLNQLEGNKEKFEKQLKKKSEEVYCLQKELKIKNHSLQE
TSEQNVILQHTLQQQQQMLQQETIRNGELEDTQTKLEKQVSKLEQELQKQRESSAEKLRK
MEEKCESAAHEADLKRQKVIELTGTARQVKIEMDQYKEELSKMEKEIMHLKRDGENKAMH
LSQLDMILDQTKTELEKKTNAVKELEKLQHSTETELTEALQKREVLETELQNAHGELKST
LRQLQELRDVLQKAQLSLEEKYTTIKDLTAELRECKMEIEDKKQELLEMDQALKERNWEL
KQRAAQVTHLDMTIREHRGEMEQKIIKLEGTLEKSELELKECNKQIESLNDKLQNAKEQL
REKEFIMLQNEQEISQLKKEIERTQQRMKEMESVMKEQEQYIATQYKEAIDLGQELRLTR
EQVQNSHTELAEARHQQVQAQREIERLSSELEDMKQLSKEKDAHGNHLAEELGASKVREA
HLEARMQAEIKKLSAEVESLKEAYHMEMISHQENHAKWKISADSQKSSVQQLNEQLEKAK
LELEEAQDTVSNLHQQVQDRNEVIEAANEALLTKESELTRLQAKISGHEKAEDIKFLPAP
FTSPTEIMPDVQDPKFAKCFHTSFSKCTKLRRSISASDLTFKIHGDEDLSEELLQDLKKM
QLEQPSTLEESHKNLTYTQPDSFKPLTYNLEADSSENNDFNTLSGMLRYINKEVRLLKKS
SMQTGAGLNQGENV
>sp|Q9BXT2|CCG6_HUMAN Voltage-dependent calcium channel gamma-6 subunit OS=Homo sapiens GN=CACNG6 PE=2 SV=1
MMWSNFFLQEENRRRGAAGRRRAHGQGRSGLTPEREGKVKLALLLAAVGATLAVLSVGTE
FWVELNTYKANGSAVCEAAHLGLWKACTKRLWQADVPVDRDTCGPAELPGEANCTYFKFF
TTGENARIFQRTTKKEVNLAAAVIAVLGLAVMALGCLCIIMVLSKGAEFLLRVGAVCFGL
SGLLLLVSLEVFRHSVRALLQRVSPEPPPAPRLTYEYSWSLGCGVGAGLILLLGAGCFLL
LTLPSWPWGSLCPKRGHRAT
>sp|Q8TD31|CCHCR_HUMAN Coiled-coil alpha-helical rod protein 1 OS=Homo sapiens GN=CCHCR1 PE=1 SV=2
MFPPSGSTGLIPPSHFQARPLSTLPRMAPTWLSDIPLVQPPGHQDVSERRLDTQRPQVTM
WERDVSSDRQEPGRRGRSWGLEGSQALSQQAEVIVRQLQELRRLEEEVRLLRETSLQQKM
RLEAQAMELEALARAEKAGRAEAEGLRAALAGAEVVRKNLEEGSQRELEEVQRLHQEQLS
SLTQAHEEALSSLTSKAEGLEKSLSSLETRRAGEAKELAEAQREAELLRKQLSKTQEDLE
AQVTLVENLRKYVGEQVPSEVHSQTWELERQKLLETMQHLQEDRDSLHATAELLQVRVQS
LTHILALQEEELTRKVQPSDSLEPEFTRKCQSLLNRWREKVFALMVQLKAQELEHSDSVK
QLKGQVASLQEKVTSQSQEQAILQRSLQDKAAEVEVERMGAKGLQLELSRAQEARRRWQQ
QTASAEEQLRLVVNAVSSSQIWLETTMAKVEGAAAQLPSLNNRLSYAVRKVHTIRGLIAR
KLALAQLRQESCPLPPPVTDVSLELQQLREERNRLDAELQLSARLIQQEVGRAREQGEAE
RQQLSKVAQQLEQELQQTQESLASLGLQLEVARQGQQESTEEAASLRQELTQQQELYGQA
LQEKVAEVETRLREQLSDTERRLNEARREHAKAVVSLRQIQRRAAQEKERSQELRRLQEE
ARKEEGQRLARRLQELERDKNLMLATLQQEGLLSRYKQQRLLTVLPSLLDKKKSVVSSPR
PPECSASAPVAAAVPTRESIKGSLSVLLDDLQDLSEAISKEEAVCQGDNLDRCSSSNPQM
SS
>sp|P51671|CCL11_HUMAN Eotaxin OS=Homo sapiens GN=CCL11 PE=1 SV=1
MKVSAALLWLLLIAAAFSPQGLAGPASVPTTCCFNLANRKIPLQRLESYRRITSGKCPQK

AVIFKTKLAKDICADPKKKWVQDSMKYLDQKSPTPKP
>sp|O00585|CCL21_HUMAN C-C motif chemokine 21 OS=Homo sapiens GN=CCL21 PE=1 SV=1
MAQSLALSLLILVLAFGIPRTQGSDGGAQDCCLKYSQRKIPAKVVRSYRKQEPSLGCSIP
AILFLPRKRSQAELCADPKELWVQQLMQHLDKTPSPQKPAQGCRKDRGASKTGKKGKGSK
GCKRTERSQTPKGP
>sp|O15444|CCL25_HUMAN C-C motif chemokine 25 OS=Homo sapiens GN=CCL25 PE=1 SV=2
MNLWLLACLVAGFLGAWAPAVHTQGVFEDCCLAYHYPIGWAVLRRAWTYRIQEVSGSCNL
PAAIFYLPKRHRKVCGNPKSREVQRAMKLLDARNKVFAKLHHNTQTFQAGPHAVKKLSSG
NSKLSSSKFSNPISSSKRNVSLLISANSGL
>sp|P24863|CCNC_HUMAN Cyclin-C OS=Homo sapiens GN=CCNC PE=1 SV=2
MAGNFWQSSHYLQWILDKQDLLKERQKDLKFLSEEEYWKLQIFFTNVIQALGEHLKLRQQ
VIATATVYFKRFYARYSLKSIDPVLMAPTCVFLASKVEEFGVVSNTRLIAAATSVLKTRF
SYAFPKEFPYRMNHILECEFYLLELMDCCLIVYHPYRPLLQYVQDMGQEDMLLPLAWRIV
NDTYRTDLCLLYPPFMIALACLHVACVVQQKDARQWFAELSVDMEKILEIIRVILKLYEQ
WKNFDERKEMATILSKMPKPKPPPNSEGEQGPNGSQNSSYSQS
>sp|P51681|CCR5_HUMAN C-C chemokine receptor type 5 OS=Homo sapiens GN=CCR5 PE=1 SV=1
MDYQVSSPIYDINYYTSEPCQKINVKQIAARLLPPLYSLVFIFGFVGNMLVILILINCKR
LKSMTDIYLLNLAISDLFFLLTVPFWAHYAAAQWDFGNTMCQLLTGLYFIGFFSGIFFII
LLTIDRYLAVVHAVFALKARTVTFGVVTSVITWVVAVFASLPGIIFTRSQKEGLHYTCSS
HFPYSQYQFWKNFQTLKIVILGLVLPLLVMVICYSGILKTLLRCRNEKKRHRAVRLIFTI
MIVYFLFWAPYNIVLLLNTFQEFFGLNNCSSSNRLDQAMQVTETLGMTHCCINPIIYAFV
GEKFRNYLLVFFQKHIAKRFCKCCSIFQQEAPERASSVYTRSTGEQEISVGL
>sp|Q6W349|CD011_HUMAN Putative uncharacterized protein encoded by LINC00575 OS=Homo sapiens GN=LINC00575 PE=5 SV=1
MPTSETSWWPGACLCSSCAWTSDSRFFNLWTLGLAPAASQGFSGLKPQTDDCTVSFPGFE
AFGLGLSHYWHLSFPACRQSIMGLCLVIVLANSS
>sp|P11836|CD20_HUMAN B-lymphocyte antigen CD20 OS=Homo sapiens GN=MS4A1 PE=1 SV=1
MTTPRNSVNGTFPAEPMKGPIAMQSGPKPLFRRMSSLVGPTQSFFMRESKTLGAVQIMNG
LFHIALGGLLMIPAGIYAPICVTVWYPLWGGIMYIISGSLLAATEKNSRKCLVKGKMIMN
SLSLFAAISGMILSIMDILNIKISHFLKMESLNFIRAHTPYINIYNCEPANPSEKNSPST
QYCYSIQSLFLGILSVMLIFAFFQELVIAGIVENEWKRTCSRPKSNIVLLSAEEKKEQTI
EIKEEVVGLTETSSQPKNEEDIEIIPIQEEEEEETETNFPEPPQDQESSPIENDSSP
>sp|Q9HCU0|CD248_HUMAN Endosialin OS=Homo sapiens GN=CD248 PE=1 SV=1
MLLRLLLAWAAAGPTLGQDPWAAEPRAACGPSSCYALFPRRRTFLEAWRACRELGGDLAT
PRTPEEAQRVDSLVGAGPASRLLWIGLQRQARQCQLQRPLRGFTWTTGDQDTAFTNWAQP
ASGGPCPAQRCVALEASGEHRWLEGSCTLAVDGYLCQFGFEGACPALQDEAGQAGPAVYT
TPFHLVSTEFEWLPFGSVAAVQCQAGRGASLLCVKQPEGGVGWSRAGPLCLGTGCSPDNG
GCEHECVEEVDGHVSCRCTEGFRLAADGRSCEDPCAQAPCEQQCEPGGPQGYSCHCRLGF
RPAEDDPHRCVDTDECQIAGVCQQMCVNYVGGFECYCSEGHELEADGISCSPAGAMGAQA
SQDLGDELLDDGEDEEDEDEAWKAFNGGWTEMPGILWMEPTQPPDFALAYRPSFPEDREP
QIPYPEPTWPPPLSAPRVPYHSSVLSVTRPVVVSATHPTLPSAHQPPVIPATHPALSRDH
QIPVIAANYPDLPSAYQPGILSVSHSAQPPAHQPPMISTKYPELFPAHQSPMFPDTRVAG
TQTTTHLPGIPPNHAPLVTTLGAQLPPQAPDALVLRTQATQLPIIPTAQPSLTTTSRSPV
SPAHQISVPAATQPAALPTLLPSQSPTNQTSPISPTHPHSKAPQIPREDGPSPKLALWLP

SPAPTAAPTALGEAGLAEHSQRDDRWLLVALLVPTCVFLVVLLALGIVYCTRCGPHAPNK
RITDCYRWVIHAGSKSPTEPMPPRGSLTGVQTCRTSV
>sp|Q5ZPR3|CD276_HUMAN CD276 antigen OS=Homo sapiens GN=CD276 PE=1 SV=1
MLRRRGSPGMGVHVGAALGALWFCLTGALEVQVPEDPVVALVGTDATLCCSFSPEPGFSL
AQLNLIWQLTDTKQLVHSFAEGQDQGSAYANRTALFPDLLAQGNASLRLQRVRVADEGSF
TCFVSIRDFGSAAVSLQVAAPYSKPSMTLEPNKDLRPGDTVTITCSSYQGYPEAEVFWQD
GQGVPLTGNVTTSQMANEQGLFDVHSILRVVLGANGTYSCLVRNPVLQQDAHSSVTITPQ
RSPTGAVEVQVPEDPVVALVGTDATLRCSFSPEPGFSLAQLNLIWQLTDTKQLVHSFTEG
RDQGSAYANRTALFPDLLAQGNASLRLQRVRVADEGSFTCFVSIRDFGSAAVSLQVAAPY
SKPSMTLEPNKDLRPGDTVTITCSSYRGYPEAEVFWQDGQGVPLTGNVTTSQMANEQGLF
DVHSVLRVVLGANGTYSCLVRNPVLQQDAHGSVTITGQPMTFPPEALWVTVGLSVCLIAL
LVALAFVCWRKIKQSCEEENAGAEDQDGEGEGSKTALQPLKHSDSKEDDGQEIA
>sp|P10747|CD28_HUMAN T-cell-specific surface glycoprotein CD28 OS=Homo sapiens GN=CD28 PE=1 SV=1
MLRLLLALNLFPSIQVTGNKILVKQSPMLVAYDNAVNLSCKYSYNLFSREFRASLHKGLD
SAVEVCVVYGNYSQQLQVYSKTGFNCDGKLGNESVTFYLQNLYVNQTDIYFCKIEVMYPP
PYLDNEKSNGTIIHVKGKHLCPSPLFPGPSKPFWVLVVVGGVLACYSLLVTVAFIIFWVR
SKRSRLLHSDYMNMTPRRPGPTRKHYQPYAPPRDFAAYRS
>sp|Q7L3B6|CD37L_HUMAN Hsp90 co-chaperone Cdc37-like 1 OS=Homo sapiens GN=CDC37L1 PE=1 SV=1
MEQPWPPPGPWSLPRAEGEAEEESDFDVFPSSPRCPQLPGGGAQMYSHGIELACQKQKEF
VKSSVACKWNLAEAQQKLGSLALHNSESLDQEHAKAQTAVSELRQREEEWRQKEEALVQR
EKMCLWSTDAISKDVFNKSFINQDKRKDTEDEDKSESFMQKYEQKIRHFGMLSRWDDSQR
FLSDHPYLVCEETAKYLILWCFHLEAEKKGALMEQIAHQAVVMQFIMEMAKNCNVDPRGC
FRLFFQKAKAEEEGYFEAFKNELEAFKSRVRLYSQSQSFQPMTVQNHVPHSGVGSIGLLE
SLPQNPDYLQYSISTALCSLNSVVHKEDDEPKMMDTV
>sp|P07766|CD3E_HUMAN T-cell surface glycoprotein CD3 epsilon chain OS=Homo sapiens GN=CD3E PE=1 SV=2
MQSGTHWRVLGLCLLSVGVWGQDGNEEMGGITQTPYKVSISGTTVILTCPQYPGSEILWQ
HNDKNIGGDEDDKNIGSDEDHLSLKEFSELEQSGYYVCYPRGSKPEDANFYLYLRARVCE
NCMEMDVMSVATIVIVDICITGGLLLLVYYWSKNRKAKAKPVTRGAGAGGRQRGQNKERP
PPVPNPDYEPIRKGQRDLYSGLNQRRI
>sp|P31358|CD52_HUMAN CAMPATH-1 antigen OS=Homo sapiens GN=CD52 PE=1 SV=1
MKRFLFLLLTISLLVMVQIQTGLSGQNDTSQTSSPSASSNISGGIFLFFVANAIIHLFCF
S
>sp|P34810|CD68_HUMAN Macrosialin OS=Homo sapiens GN=CD68 PE=1 SV=2
MRLAVLFSGALLGLLAAQGTGNDCPHKKSATLLPSFTVTPTVTESTGTTSHRTTKSHKTT
THRTTTTGTTSHGPTTATHNPTTTSHGNVTVHPTSNSTATSQGPSTATHSPATTSHGNAT
VHPTSNSTATSPGFTSSAHPEPPPPSPSPSPTSKETIGDYTWTNGSQPCVHLQAQIQIRV
MYTTQGGGEAWGISVLNPNKTKVQGSCEGAHPHLLLSFPYGHLSFGFMQDLQQKVVYLSY
MAVEYNVSFPHAAQWTFSAQNASLRDLQAPLGQSFSCSNSSIILSPAVHLDLLSLRLQAA
QLPHTGVFGQSFSCPSDRSILLPLIIGLILLGLLALVLIAFCIIRRRPSAYQAL
>sp|P42081|CD86_HUMAN T-lymphocyte activation antigen CD86 OS=Homo sapiens GN=CD86 PE=1 SV=2

MDPQCTMGLSNILFVMAFLLSGAAPLKIQAYFNETADLPCQFANSQNQSLSELVVFWQDQ
ENLVLNEVYLGKEKFDSVHSKYMGRTSFDSDSWTLRLHNLQIKDKGLYQCIIHHKKPTGM
IRIHQMNSELSVLANFSQPEIVPISNITENVYINLTCSSIHGYPEPKKMSVLLRTKNSTI
EYDGVMQKSQDNVTELYDVSISLSVSFPDVTSNMTIFCILETDKTRLLSSPFSIELEDPQ
PPPDHIPWITAVLPTVIICVMVFCLILWKWKKKKRPRNSYKCGTNTMEREESEQTKKREK
IHIPERSDEAQRVFKSSKTSSCDKSDTCF
>sp|Q8IWY9|CDAN1_HUMAN Codanin-1 OS=Homo sapiens GN=CDAN1 PE=1 SV=4
MAAVLESLLREEVSVAAVVRWIARSTQGSEDNAGEAAALSSLRALRKEFVPFLLNFLREQ
SSRVLPQGPPTPAKTPGASAALPGRPGGPPRGSRGARSQLFPPTEAQSTAAEAPLARRGG
RRRGPGPARERGGRGLEEGVSGESLPGAGGRRLRGSGSPSRPSLTLSDPPNLSNLEEFPP
VGSVPPGPTGTKPSRRINPTPVSEERSLSKPKTCFTSPPISCVPSSQPSALDTSPWGLGL
PPGCRSLQEEREMLRKERSKQLQQSPTPTCPTPELGSPLPSRTGSLTDEPADPARVSSRQ
RLELVALVYSSCIAENLVPNLFLELFFVFQLLTARRMVTAKDSDPELSPAVLDSLESPLF
QSIHDCVFFAVQVLECHFQVLSNLDKGTLKLLAENERLLCFSPALQGRLRAAYEGSVAKV
SLVMPPSTQAVSFQPETDNRANFSSDRAFHTFKKQRDVFYEVLREWEDHHEEPGWDFEKG
LGSRIRAMMGQLSAACSHSHFVRLFQKQLLQMCQSPGGAGGTVLGEAPDVLSMLGADKLG
RLWRLQERLMAPQSSGGPCPPPTFPGCQGFFRDFILSASSFQFNQHLMDSLSLKIQELNG
LALPQHEPNDEDGESDVDWQGERKQFAVVLLSLRLLAKFLGFVAFLPYRGPEPPPTGELQ
DSILALRSQVPPVLDVRTLLQRGLQARRAVLTVPWLVEFLSFADHVVPLLEYYRDIFTLL
LRLHRSLVLSQESEGKMCFLNKLLLLAVLGWLFQIPTVPEDLFFLEEGPSYAFEVDTVAP
EHGLDNAPVVDQQLLYTCCPYIGELRKLLASWVSGSSGRSGGFMRKITPTTTTSLGAQPS
QTSQGLQAQLAQAFFHNQPPSLRRTVEFVAERIGSNCVKHIKATLVADLVRQAESLLQEQ
LVTQGEEGGDPAQLLEILCSQLCPHGAQALALGREFCQRKSPGAVRALLPEETPAAVLSS
AENIAVGLATEKACAWLSANITALIRREVKAAVSRTLRAQGPEPAARGERRGCSRACEHH
APLPSHLISEIKDVLSLAVGPRDPDEGVSPEHLEQLLGQLGQTLRCRQFLCPPAEQHLAK
CSVELASLLVADQIPILGPPAQYRLERGQARRLLHMLLSLWKEDFQGPVPLQLLLSPRNV
GLLADTRPREWDLLLFLLRELVEKGLMGRMEIEACLGSLHQAQWPGDFAEELATLSNLFL
AEPHLPEPQLRACELVQPNRGTVLAQS
>sp|Q99459|CDC5L_HUMAN Cell division cycle 5-like protein OS=Homo sapiens GN=CDC5L PE=1 SV=2
MPRIMIKGGVWRNTEDEILKAAVMKYGKNQWSRIASLLHRKSAKQCKARWYEWLDPSIKK
TEWSREEEEKLLHLAKLMPTQWRTIAPIIGRTAAQCLEHYEFLLDKAAQRDNEEETTDDP
RKLKPGEIDPNPETKPARPDPIDMDEDELEMLSEARARLANTQGKKAKRKAREKQLEEAR
RLAALQKRRELRAAGIEIQKKRKRKRGVDYNAEIPFEKKPALGFYDTSEENYQALDADFR
KLRQQDLDGELRSEKEGRDRKKDKQHLKRKKESDLPSAILQTSGVSEFTKKRSKLVLPAP
QISDAELQEVVKVGQASEIARQTAEESGITNSASSTLLSEYNVTNNSVALRTPRTPASQD
RILQEAQNLMALTNVDTPLKGGLNTPLHESDFSGVTPQRQVVQTPNTVLSTPFRTPSNGA
EGLTPRSGTTPKPVINSTPGRTPLRDKLNINPEDGMADYSDPSYVKQMERESREHLRLGL
LGLPAPKNDFEIVLPENAEKELEEREIDDTYIEDAADVDARKQAIRDAERVKEMKRMHKA
VQKDLPRPSEVNETILRPLNVEPPLTDLQKSEELIKKEMITMLHYDLLHHPYEPSGNKKG
KTVGFGTNNSEHITYLEHNPYEKFSKEELKKAQDVLVQEMEVVKQGMSHGELSSEAYNQV
WEECYSQVLYLPGQSRYTRANLASKKDRIESLEKRLEINRGHMTTEAKRAAKMEKKMKIL
LGGYQSRAMGLMKQLNDLWDQIEQAHLELRTFEELKKHEDSAIPRRLECLKEDVQRQQER
EKELQHRYADLLLEKETLKSKF

>sp|Q6P1J9|CDC73_HUMAN Parafibromin OS=Homo sapiens GN=CDC73 PE=1 SV=1
MADVLSVLRQYNIQKKEIVVKGDEVIFGEFSWPKNVKTNYVVWGTGKEGQPREYYTLDSI
LFLLNNVHLSHPVYVRRAATENIPVVRRPDRKDLLGYLNGEASTSASIDRSAPLEIGLQR
STQVKRAADEVLAEAKKPRIEDEECVRLDKERLAARLEGHKEGIVQTEQIRSLSEAMSVE
KIAAIKAKIMAKKRSTIKTDLDDDITALKQRSFVDAEVDVTRDIVSRERVWRTRTTILQS
TGKNFSKNIFAILQSVKAREEGRAPEQRPAPNAAPVDPTLRTKQPIPAAYNRYDQERFKG
KEETEGFKIDTMGTYHGMTLKSVTEGASARKTQTPAAQPVPRPVSQARPPPNQKKGSRTP
IIIIPAATTSLITMLNAKDLLQDLKFVPSDEKKKQGCQRENETLIQRRKDQMQPGGTAIS
VTVPYRVVDQPLKLMPQDWDRVVAVFVQGPAWQFKGWPWLLPDGSPVDIFAKIKAFHLKY
DEVRLDPNVQKWDVTVLELSYHKRHLDRPVFLRFWETLDRYMVKHKSHLRF
>sp|Q96Q40|CDK15_HUMAN Cyclin-dependent kinase 15 OS=Homo sapiens GN=CDK15 PE=1 SV=2
MGQELCAKTVQPGCSCYHCSEGGEAHSCRRSQPETTEAAFKLTDLKEASCSMTSFHPRGL
QAARAQKFKSKRPRSNSDCFQEEDLRQGFQWRKSLPFGAASSYLNLEKLGEGSYATVYKG
ISRINGQLVALKVISMNAEEGVPFTAIREASLLKGLKHANIVLLHDIIHTKETLTFVFEY
MHTDLAQYMSQHPGGLHPHNVRLFMFQLLRGLAYIHHQHVLHRDLKPQNLLISHLGELKL
ADFGLARAKSIPSQTYSSEVVTLWYRPPDALLGATEYSSELDIWGAGCIFIEMFQGQPLF
PGVSNILEQLEKIWEVLGVPTEDTWPGVSKLPNYNPEWFPLPTPRSLHVVWNRLGRVPEA
EDLASQMLKGFPRDRVSAQEALVHDYFSALPSQLYQLPDEESLFTVSGVRLKPEMCDLLA
SYQKGHHPAQFSKCW
>sp|P24941|CDK2_HUMAN Cyclin-dependent kinase 2 OS=Homo sapiens GN=CDK2 PE=1 SV=2
MENFQKVEKIGEGTYGVVYKARNKLTGEVVALKKIRLDTETEGVPSTAIREISLLKELNH
PNIVKLLDVIHTENKLYLVFEFLHQDLKKFMDASALTGIPLPLIKSYLFQLLQGLAFCHS
HRVLHRDLKPQNLLINTEGAIKLADFGLARAFGVPVRTYTHEVVTLWYRAPEILLGCKYY
STAVDIWSLGCIFAEMVTRRALFPGDSEIDQLFRIFRTLGTPDEVVWPGVTSMPDYKPSF
PKWARQDFSKVVPPLDEDGRSLLSQMLHYDPNKRISAKAALAHPFFQDVTKPVPHLRL
>sp|Q9UKL3|C8AP2_HUMAN CASP8-associated protein 2 OS=Homo sapiens GN=CASP8AP2 PE=1 SV=1
MAADDDNGDGTSLFDVFSASPLKNNDEGSLDIYAGLDSAVSDSASKSCVPSRNCLDLYEE
ILTEEGTAKEATYNDLQVEYGKCQLQMKELMKKFKEIQTQNFSLINENQSLKKNISALIK
TARVEINRKDEEISNLHQRLSEFPHFRNNHKTARTFDTVKTKDLKSRSPHLDDCSKTDHR
AKSDVSKDVHHSTSLPNLEKEGKPHSDKRSTSHLPTSVEKHCTNGVWSRSHYQVGEGSSN
EDSRRGRKDIRHSQFNRGTERVRKDLSTGCGDGEPRILEASQRLQGHPEKYGKGEPKTES
KSSKFKSNSDSDYKGERINSSWEKETPGERSHSRVDSQSDKKLERQSERSQNINRKEVKS
QDKEERKVDQKPKSVVKDQDHWRRSERASLPHSKNEITFSHNSSKYHLEERRGWEDCKRD
KSVNSHSFQDGRCPSSLSNSRTHKNIDSKEVDAMHQWENTPLKAERHRTEDKRKREQESK
EENRHIRNEKRVPTEHLQKTNKETKKTTTDLKKQNEPKTDKGEVLDNGVSEGADNKELAM
KAESGPNETKNKDLKLSFMKKLNLTLSPAKKQPVSQDNQHKITDIPKSSGVCDSESSMQV
KTVAYVPSISEHILGEAAVSEHTMGETKSTLLEPKVALLAVTEPRIGISETNKEDENSLL
VRSVDNTMHCEEPICGTETSFPSPMEIQQTESLFPSTGMKQTINNGRAAAPVVMDVLQTD
VSQNFGLELDTKRNDNSDYCGISEGMEMKVALSTTVSETTESILQPSIEEADILPIMLSE
DNNPKFEPSVIVTPLVESKSCHLEPCLPKETLDSSLQQTELMDHRMATGETNSVYHDDDN
SVLSIDLNHLRPIPEAISPLNSPVRPVAKVLRNESPPQVPVYNNSHKDVFLPNSAHSTSK
SQSDLNKENQKPIYKSDKCTEADTCKNSPLDELEEGEIRSDSETSKPQESFEKNSKRRVS
ADVRKSKTIPRRGKSTVCLDKDSRKTHVRIHQTNNKWNKRPDKSSRSSKTEKKDKVMSTS
SLEKIVPIIAVPSSEQEIMHMLRMIRKHVRKNYMKFKAKFSLIQFHRIIESAILSFTSLI

KHLNLHKISKSVTTLQKNLCDIIESKLKQVKKNGIVDRLFEQQLPDMKKKLWKFVDDQLD
YLFAKLKKILVCDSKSFGRDSDEGKLEKTSKQNAQYSNSQKRSVDNSNRELLKEKLSKSE
DPVHYKSLVGCKKSEENYQDQNNSSINTVKHDIKKNFNICFDNIKNSQSEERSLEVHCPS
TPKSEKNEGSSIEDAQTSQHATLKPERSFEILTEQQASSLTFNLVSDAQMGEIFKSLLQG
SDLLDSSVNCTEKSEWELKTPEKQLLETLKCESIPACTTEELVSGVASPCPKMISDDNWS
LLSSEKGPSLSSGLSLPVHPDVLDESCMFEVSTNLPLSKDNVCSVEKSKPCVSSILLEDL
AVSLTVPSPLKSDGHLSFLKPDMSSSSTPEEVISAHFSEDALLEEEDASEQDIHLALESD
NSSSKSSCSSSWTSRSVAPGFQYHPNLPMHAVIMEKSNDHFIVKIRRATPSTSSGLKQSM
MPDELLTSLPRHGKEADEGPEKEYISCQNTVFKSVEELENSNKNVDGSKSTHEEQSSMIQ
TQVPDIYEFLKDASDKMGHSDEVADECFKLHQVWETKVPESIEELPSMEEISHSVGEHLP
NTYVDLTKDPVTETKNLGEFIEVTVLHIDQLGCSGGNLNQSAQILDNSLQADTVGAFIDL
TQDASSEAKSEGNHPALAVEDLGCGVIQVDEDNCKEEKAQVANRPLKCIVEETYIDLTTE
SPSSCEVKKDELKSEPGSNCDNSELPGTLHNSHKKRRNISDLNHPHKKQRKETDLTNKEK
TKKPTQDSCENTEAHQKKASKKKAPPVTKDPSSLKATPGIKDSSAALATSTSLSAKNVIK
KKGEIIILWTRNDDREILLECQKRGPSFKTFAYLAAKLDKNPNQVSERFQQLMKLFEKSK
CR
>sp|Q96LT6|CA074_HUMAN UPF0739 protein C1orf74 OS=Homo sapiens GN=C1orf74 PE=2 SV=1
MLLLDLMSSPSPQLLVAAAQQTLGMGKRRSPPQAICLHLAGEVLAVARGLKPAVLYDCNC
AGASELQSYLEELKGLGFLTFGLHILEIGENSLIVSPEHVCQHLEQVLLGTIAFVDVSSC
QRHPSVCSLDQLQDLKALVAEIITHLQGLQRDLSLAVSYSRLHSSDWNLCTVFGILLGYP
VPYTFHLNQGDDNCLALTPLRVFTARISWLLGQPPILLYSFSVPESLFPGLRDILNTWEK
DLRTRFRTQNDFADLSISSEIVTLPAVAL
>sp|Q5SY80|CA101_HUMAN Uncharacterized protein C1orf101 OS=Homo sapiens GN=C1orf101 PE=2 SV=1
MSAREVAVLLLWLSCYGSALWRYSTNSPNYRIFSTRSTIKLEYEGTLFTEWSVPETCFVL
NKSSPTTELRCSSPGVHAIKPIVTGPDEEERYLFVESSHTCFLWYYRVRHFFNNFTQLIT
VWAYDPESADPDELLGNAEEPSINSIVLSTQMATLGQKPVIHTVLKRKVYSSNEKMRRGT
WRIVVPMTKDDALKEIRGNQVTFQDCFIADFLILLTFPLLTIPEIPGYLPISSPRGSQLM
ASWDACVVASAVLVTDMETFHTTDSFKSWTRIRVPPDILSDDERRSVAHVILSRDGIVFL
INGVLYIKSFRGFIRLGGIVNLPDGGITGISSRKWCWVNYLLKAKGRRSTFAVWTENEIY
LGSILLKFARLVTTTELKNILSLSVTATLTIDRVEYTGHPLEIAVFLNYCTVCNVTKKIF
LVIYNEDTKQWVSQDFTLDAPIDSVTMPHFTFSALPGLLLWNKHSIYYCYHNFTFTGILQ
TPAGHGNLSMLSNDSIIHEVFIDYYGDILVKMENNVIFYSKINTRDAVKLHLWTNYTTRA
FIFLSTSGQTYFLYALDDGTIQIQDYPLHLEAQSIAFTTKDKCPYMAFHNNVAHVFYFLD
KGEALTVWTQIVYPENTGLYVIVESYGPKILQESHEISFEAAFGYCTKTLTLTFYQNVDY
ERISDYFETQDKHTGLVLVQFRPSEYSKACPIAQKVFQIAVGCDDKKFIAIKGFSKKGCH
HHDFSYVIEKSYLRHQPSKNLRVRYIWGEYGCPLRLDFTEKFQPVVQLFDDNGYVKDVEA
NFIVWEIHGRDDYSFNNTMAQSGCLHEAQTWKSMIELNKHLPLEEVWGPENYKHCFSYAI
GKPGDLNQPYEIINSSNGNHIFWPMGHSGMYVFRVKILDPNYSFCNLTAMFAIETFGLIP
SPSVYLVASFLFVLMLLFFTILVLSYFRYMRIYRRYIYEPLHKPQRKRKKN
>sp|Q96MC9|CA147_HUMAN Uncharacterized protein C1orf147 OS=Homo sapiens GN=C1orf147 PE=2 SV=2
MQGNNLQPPSPRPLECWSLHREAPQRTEASRTHPSPFLALPGSHRTANQAQVVPGLVHHP
QENTQRLRIRAGLSPFPRARLAAIGSTHGHRWVPGICLPRCLCPASCALHACPPHCNNAE

AHSWLRLGGPLQSRSVEHQQHACGWGWVTHATYGERGTSQAAERQRIAHTRALSVGNCPR
SHFQCEFSSISKWSEGTGTFSPRPRAVKSGVGGQPSPHIRITWRSCTTLTSKPCSRAITP
ECVVGWSRRSRHWYSDMRTGECQGLSPITR
>sp|Q3SY05|CA157_HUMAN Putative uncharacterized protein encoded by LINC00303 OS=Homo sapiens GN=LINC00303 PE=5 SV=2
MYKSWTLGDPKEKEGVGNILEETKRTQNNTEQASRAINSPLQSPYTDSMKALAISSHWFL
PQIHTLPPITKATRHRWPPACCGRRGGQTTLPPLTPIVRACESMERSCPGNYPMQHERKV
MQRHPTLR
>sp|Q8NEQ5|CA162_HUMAN Transmembrane protein C1orf162 OS=Homo sapiens GN=C1orf162 PE=2 SV=1
MGGNGSTCKPDTERQGTLSTAAPTTSPAPCLSNHHNKKHLILAFCAGVLLTLLLIAFIFL
IIKSYRKYRRERLPISPGPLLRWVPLLSGTMADHSKPQAPDPHSDPPAKLSSIPGESLTY
ASTTFKLSEEKSNHLAENHSADFDPIVYAQIKVTN
>sp|Q5VU69|CA189_HUMAN Uncharacterized protein C1orf189 OS=Homo sapiens GN=C1orf189 PE=1 SV=1
MSVEKMTKVEESFQKAMGLKKTVDRWRNSHTHCLWQMALGQRRNPYATLRMQDTMVQELA
LAKKQLLMVRQAALHQLFEKEHQQYQQELNQMGKAFYVERF
>sp|Q5T0J3|CA220_HUMAN Putative uncharacterized protein C1orf220 OS=Homo sapiens GN=C1orf220 PE=2 SV=1
MIPVRGLWYCYLQVKKVRLREAERLGPKSQCPAECGAASWISWVLQHQQLLASEWATKDG
RGTWGSKPSCTVPALSATSQLLQLGRLNSDLQFVRQRAKKSVSSPSLLQFWCCLSRLSFH
ALNQKINSTNINIT
>sp|Q7Z3S7|CA2D4_HUMAN Voltage-dependent calcium channel subunit alpha-2/delta-4 OS=Homo sapiens GN=CACNA2D4 PE=1 SV=2
MVCGCSALLPLPNPRPTMPATPNFLANPSSSSRWIPLQPMPVAWAFVQKTSALLWLLLLG
TSLSPAWGQAKIPLETVKLWADTFGGDLYNTVTKYSGSLLLQKKYKDVESSLKIEEVDGL
ELVRKFSEDMENMLRRKVEAVQNLVEAAEEADLNHEFNESLVFDYYNSVLINERDEKGNF
VELGAEFLLESNAHFSNLPVNTSISSVQLPTNVYNKDPDILNGVYMSEALNAVFVENFQR
DPTLTWQYFGSATGFFRIYPGIKWTPDENGVITFDCRNRGWYIQAATSPKDIVILVDVSG
SMKGLRMTIAKHTITTILDTLGENDFINIIAYNDYVHYIEPCFKGILVQADRDNREHFKL
LVEELMVKGVGVVDQALREAFQILKQFQEAKQGSLCNQAIMLISDGAVEDYEPVFEKYNW
PDCKVRVFTYLIGREVSFADRMKWIACNNKGYYTQISTLADTQENVMEYLHVLSRPMVIN
HDHDIIWTEAYMDSKLLSSQAQSLTLLTTVAMPVFSKKNETRSHGILLGVVGSDVALREL
MKLAPRYKLGVHGYAFLNTNNGYILSHPDLRPLYREGKKLKPKPNYNSVDLSEVEWEDQA
ESLRTAMINRETGTLSMDVKVPMDKGKRVLFLTNDYFFTDISDTPFSLGVVLSRGHGEYI
LLGNTSVEEGLHDLLHPDLALAGDWIYCITDIDPDHRKLSQLEAMIRFLTRKDPDLECDE
ELVREVLFDAVVTAPMEAYWTALALNMSEESEHVVDMAFLGTRAGLLRSSLFVGSEKVSD
RKFLTPEDEASVFTLDRFPLWYRQASEHPAGSFVFNLRWAEGPESAGEPMVVTASTAVAV
TVDKRTAIAAAAGVQMKLEFLQRKFWAATRQCSTVDGPCTQSCEDSDLDCFVIDNNGFIL
ISKRSRETGRFLGEVDGAVLTQLLSMGVFSQVTMYDYQAMCKPSSHHHSAAQPLVSPISA
FLTATRWLLQELVLFLLEWSVWGSWYDRGAEAKSVFHHSHKHKKQDPLQPCDTEYPVFVY
QPAIREANGIVECGPCQKVFVVQQIPNSNLLLLVTDPTCDCSIFPPVLQEATEVKYNASV
KCDRMRSQKLRRRPDSCHAFHPEENAQDCGGASDTSASPPLLLLPVCAWGLLPQLLR

>sp|Q8WTZ4|CA5BL_HUMAN Putative inactive carbonic anhydrase 5B-like protein OS=Homo sapiens GN=CA5BP1 PE=5 SV=2
MTQPPASTSGIMGTLSSWNLKILQINQLHLVHWNAVKFENFEDAALEENGLAVIGVFLKI
SETSGSPVSTGRPKPLARKLRPAQKHWVLQSRPFLSSQVQENCKVTYFHRKHWVRIRPLR
TTPPSWDYTRICIQREMVPARIRVLREMVPEAWRCFPNRLPLLSNIRPDFSKAPLAYVKR
WLWTARHPHSLSAAW
>sp|Q9NP86|CABP5_HUMAN Calcium-binding protein 5 OS=Homo sapiens GN=CABP5 PE=1 SV=1
MQFPMGPACIFLRKGIAEKQRERPLGQDEIEELREAFLEFDKDRDGFISCKDLGNLMRTM
GYMPTEMELIELGQQIRMNLGGRVDFDDFVELMTPKLLAETAGMIGVQEMRDAFKEFDTN
GDGEITLVELQQAMQRLLGERLTPREISEVVREADVNGDGTVDFEEFVKMMSR
>sp|Q86Y37|CACL1_HUMAN CDK2-associated and cullin domain-containing protein 1 OS=Homo sapiens GN=CACUL1 PE=1 SV=1
MEESMEEEEGGSYEAMMDDQNHNNWEAAVDGFRQPLPPPPPPSSIPAPAREPPGGQLLAV
PAVSVDRKGPKEGLPMGPQPPPEANGVIMMLKSCDAAAAVAKAAPAPTASSTININTSTS
KFLMNVITIEDYKSTYWPKLDGAIDQLLTQSPGDYIPISYEQIYSCVYKCVCQQHSEQMY
SDLIKKITNHLERVSKELQASPPDLYIERFNIALGQYMGALQSIVPLFIYMNKFYIETKL
NRDLKDDLIKLFTEHVAEKHIYSLMPLLLEAQSTPFQVTPSTMANIVKGLYTLRPEWVQM
APTLFSKFIPNILPPAVESELSEYAAQDQKFQRELIQNGFTRGDQSRKRAGDELAYNSSS
ACASSRGYR
>sp|Q13137|CACO2_HUMAN Calcium-binding and coiled-coil domain-containing protein 2 OS=Homo sapiens GN=CALCOCO2 PE=1 SV=1
MEETIKDPPTSAVLLDHCHFSQVIFNSVEKFYIPGGDVTCHYTFTQHFIPRRKDWIGIFR
VGWKTTREYYTFMWVTLPIDLNNKSAKQQEVQFKAYYLPKDDEYYQFCYVDEDGVVRGAS
IPFQFRPENEEDILVVTTQGEVEEIEQHNKELCKENQELKDSCISLQKQNSDMQAELQKK
QEELETLQSINKKLELKVKEQKDYWETELLQLKEQNQKMSSENEKMGIRVDQLQAQLSTQ
EKEMEKLVQGDQDKTEQLEQLKKENDHLFLSLTEQRKDQKKLEQTVEQMKQNETTAMKKQ
QELMDENFDLSKRLSENEIICNALQRQKERLEGENDLLKRENSRLLSYMGLDFNSLPYQV
PTSDEGGARQNPGLAYGNPYSGIQESSSPSPLSIKKCPICKADDICDHTLEQQQMQPLCF
NCPICDKIFPATEKQIFEDHVFCHSL
>sp|P40123|CAP2_HUMAN Adenylyl cyclase-associated protein 2 OS=Homo sapiens GN=CAP2 PE=1 SV=1
MANMQGLVERLERAVSRLESLSAESHRPPGNCGEVNGVIAGVAPSVEAFDKLMDSMVAEF
LKNSRILAGDVETHAEMVHSAFQAQRAFLLMASQYQQPHENDVAALLKPISEKIQEIQTF
RERNRGSNMFNHLSAVSESIPALGWIAVSPKPGPYVKEMNDAATFYTNRVLKDYKHSDLR
HVDWVKSYLNIWSELQAYIKEHHTTGLTWSKTGPVASTVSAFSVLSSGPGLPPPPPPLPP
PGPPPLFENEGKKEESSPSRSALFAQLNQGEAITKGLRHVTDDQKTYKNPSLRAQGGQTQ
SPTKSHTPSPTSPKSYPSQKHAPVLELEGKKWRVEYQEDRNDLVISETELKQVAYIFKCE
KSTIQIKGKVNSIIIDNCKKLGLVFDNVVGIVEVINSQDIQIQVMGRVPTISINKTEGCH
IYLSEDALDCEIVSAKSSEMNILIPQDGDYREFPIPEQFKTAWDGSKLITEPAEIMA
>sp|P47756|CAPZB_HUMAN F-actin-capping protein subunit beta OS=Homo sapiens GN=CAPZB PE=1 SV=4
MSDQQLDCALDLMRRLPPQQIEKNLSDLIDLVPSLCEDLLSSVDQPLKIARDKVVGKDYL
LCDYNRDGDSYRSPWSNKYDPPLEDGAMPSARLRKLEVEANNAFDQYRDLYFEGGVSSVY
LWDLDHGFAGVILIKKAGDGSKKIKGCWDSIHVVEVQEKSSGRTAHYKLTSTVMLWLQTN

KSGSGTMNLGGSLTRQMEKDETVSDCSPHIANIGRLVEDMENKIRSTLNEIYFGKTKDIV
NGLRSIDAIPDNQKFKQLQRELSQVLTQRQIYIQPDN
>sp|Q9BWT7|CAR10_HUMAN Caspase recruitment domain-containing protein 10 OS=Homo sapiens GN=CARD10 PE=1 SV=2
MPGRAEAGEAEEEAGAGSGSEAEEDALWERIEGVRHRLARALNPAKLTPYLRQCRVIDEQ
DEEEVLSTYRFPCRVNRTGRLMDILRCRGKRGYEAFLEALEFYYPEHFTLLTGQEPAQRC
SMILDEEGPEGLTQFLMTEVRRLREARKSQLQREQQLQARGRVLEEERAGLEQRLRDQQQ
AQERCQRLREDWEAGSLELLRLKDENYMIAMRLAQLSEEKNSAVLRSRDLQLAVDQLKLK
VSRLEEECALLRRARGPPPGAEEKEKEKEKEKEPDNVDLVSELRAENQRLTASLRELQEG
LQQEASRPGAPGSERILLDILEHDWREAQDSRQELCQKLHAVQGELQWAEELRDQYLQEM
EDLRLKHRTLQKDCDLYKHRMATVLAQLEEIEKERDQAIQSRDRIQLQYSQSLIEKDQYR
KQVRGLEAERDELLTTLTSLEGTKALLEVQLQRAQGGTCLKACASSHSLCSNLSSTWSLS
EFPSPLGGPEATGEAAVMGGPEPHNSEEATDSEKEINRLSILPFPPSAGSILRRQREEDP
APPKRSFSSMSDITGSVTLKPWSPGLSSSSSSDSVWPLGKPEGLLARGCGLDFLNRSLAI
RVSGRSPPGGPEPQDKGPDGLSFYGDRWSGAVVRRVLSGPGSARMEPREQRVEAAGLEGA
CLEAEAQQRTLLWNQGSTLPSLMDSKACQSFHEALEAWAKGPGAEPFYIRANLTLPERAD
PHALCVKAQEILRLVDSAYKRRQEWFCTRVDPLTLRDLDRGTVPNYQRAQQLLEVQEKCL
PSSRHRGPRSNLKKRALDQLRLVRPKPVGAPAGDSPDQLLLEPCAEPERSLRPYSLVRPL
LVSALRPVVLLPECLAPRLIRNLLDLPSSRLDFQVCPAESLSGEELCPSSAPGAPKAQPA
TPGLGSRIRAIQESVGKKHCLLELGARGVRELVQNEIYPIVIHVEVTEKNVREVRGLLGR
PGWRDSELLRQCRGSEQVLWGLPCSWVQVPAHEWGHAEELAKVVRGRILQEQARLVWVEC
GSSRGCPSSSEA
>sp|P57730|CAR18_HUMAN Caspase recruitment domain-containing protein 18 OS=Homo sapiens GN=CARD18 PE=1 SV=1
MADQLLRKKRRIFIHSVGAGTINALLDCLLEDEVISQEDMNKVRDENDTVMDKARVLIDL
VTGKGPKSCCKFIKHLCEEDPQLASKMGLH
>sp|P29466|CASP1_HUMAN Caspase-1 OS=Homo sapiens GN=CASP1 PE=1 SV=1
MADKVLKEKRKLFIRSMGEGTINGLLDELLQTRVLNKEEMEKVKRENATVMDKTRALIDS
VIPKGAQACQICITYICEEDSYLAGTLGLSADQTSGNYLNMQDSQGVLSSFPAPQAVQDN
PAMPTSSGSEGNVKLCSLEEAQRIWKQKSAEIYPIMDKSSRTRLALIICNEEFDSIPRRT
GAEVDITGMTMLLQNLGYSVDVKKNLTASDMTTELEAFAHRPEHKTSDSTFLVFMSHGIR
EGICGKKHSEQVPDILQLNAIFNMLNTKNCPSLKDKPKVIIIQACRGDSPGVVWFKDSVG
VSGNLSLPTTEEFEDDAIKKAHIEKDFIAFCSSTPDNVSWRHPTMGSVFIGRLIEHMQEY
ACSCDVEEIFRKVRFSFEQPDGRAQMPTTERVTLTRCFYLFPGH
>sp|P42575|CASP2_HUMAN Caspase-2 OS=Homo sapiens GN=CASP2 PE=1 SV=2
MAAPSAGSWSTFQHKELMAADRGRRILGVCGMHPHHQETLKKNRVVLAKQLLLSELLEHL
LEKDIITLEMRELIQAKVGSFSQNVELLNLLPKRGPQAFDAFCEALRETKQGHLEDMLLT
TLSGLQHVLPPLSCDYDLSLPFPVCESCPLYKKLRLSTDTVEHSLDNKDGPVCLQVKPCT
PEFYQTHFQLAYRLQSRPRGLALVLSNVHFTGEKELEFRSGGDVDHSTLVTLFKLLGYDV
HVLCDQTAQEMQEKLQNFAQLPAHRVTDSCIVALLSHGVEGAIYGVDGKLLQLQEVFQLF
DNANCPSLQNKPKMFFIQACRGDETDRGVDQQDGKNHAGSPGCEESDAGKEKLPKMRLPT
RSDMICGYACLKGTAAMRNTKRGSWYIEALAQVFSERACDMHVADMLVKVNALIKDREGY
APGTEFHRCKEMSEYCSTLCRHLYLFPGHPPT
>sp|P55212|CASP6_HUMAN Caspase-6 OS=Homo sapiens GN=CASP6 PE=1 SV=2

MSSASGLRRGHPAGGEENMTETDAFYKREMFDPAEKYKMDHRRRGIALIFNHERFFWHLT
LPERRGTCADRDNLTRRFSDLGFEVKCFNDLKAEELLLKIHEVSTVSHADADCFVCVFLS
HGEGNHIYAYDAKIEIQTLTGLFKGDKCHSLVGKPKIFIIQACRGNQHDVPVIPLDVVDN
QTEKLDTNITEVDAASVYTLPAGADFLMCYSVAEGYYSHRETVNGSWYIQDLCEMLGKYG
SSLEFTELLTLVNRKVSQRRVDFCKDPSAIGKKQVPCFASMLTKKLHFFPKSN
>sp|Q86V15|CASZ1_HUMAN Zinc finger protein castor homolog 1 OS=Homo sapiens GN=CASZ1 PE=1 SV=4
MDLGTAEGTRCTDPPAGKPAMAPKRKGGLKLNAICAKLSRQVVVEKRADAGSHTEGSPSQ
PRDQERSGPESGAARAPRSEEDKRRAVIEKWVNGEYSEEPAPTPVLGRIAREGLELPPEG
VYMVQPQGCSDEEDHAEEPSKDGGALEEKDSDGAASKEDSGPSTRQASGEASSLRDYAAS
TMTEFLGMFGYDDQNTRDELARKISFEKLHAGSTPEAATSSMLPTSEDTLSKRARFSKYE
EYIRKLKAGEQLSWPAPSTKTEERVGKEVVGTLPGLRLPSSTAHLETKATILPLPSHSSV
QMQNLVARASKYDFFIQKLKTGENLRPQNGSTYKKPSKYDLENVKYLHLFKPGEGSPDMG
GAIAFKTGKVGRPSKYDVRGIQKPGPAKVPPTPSLAPAPLASVPSAPSAPGPGPEPPASL
SFNTPEYLKSTFSKTDSITTGTVSTVKNGLPTDKPAVTEDVNIYQKYIARFSGSQHCGHI
HCAYQYREHYHCLDPECNYQRFTSKQDVIRHYNMHKKRDNSLQHGFMRFSPLDDCSVYYH
GCHLNGKSTHYHCMQVGCNKVYTSTSDVMTHENFHKKNTQLINDGFQRFRATEDCGTADC
QFYGQKTTHFHCRRPGCTFTFKNKCDIEKHKSYHIKDDAYAKDGFKKFYKYEECKYEGCV
YSKATNHFHCIRAGCGFTFTSTSQMTSHKRKHERRHIRSSGALGLPPSLLGAKDTEHEES
SNDDLVDFSALSSKNSSLSASPTSQQSSASLAAATAATEAGPSATKPPNSKISGLLPQGL
PGSIPLALALSNSGLPTPTPYFPILAGRGSTSLPVGTPSLLGAVSSGSAASATPDTPTLV
ASGAGDSAPVAAASVPAPPASIMERISASKGLISPMMARLAAAALKPSATFDPGSGQQVT
PARFPPAQVKPEPGESTGAPGPHEASQDRSLDLTVKEPSNESNGHAVPANSSLLSSLMNK
MSQGNPGLGSLLNIKAEAEGSPAAEPSPFLGKAVKALVQEKLAEPWKVYLRRFGTKDFCD
GQCDFLHKAHFHCVVEECGALFSTLDGAIKHANFHFRTEGGAAKGNTEAAFPASAAETKP
PMAPSSPPVPPVTTATVSSLEGPAPSPASVPSTPTLLAWKQLASTIPQMPQIPASVPHLP
ASPLATTSLENAKPQVKPGFLQFQENDPCLATDCKYANKFHFHCLFGNCKYVCKTSGKAE
SHCLDHINPNNNLVNVRDQFAYYSLQCLCPNQHCEFRMRGHYHCLRTGCYFVTNITTKLP
WHIKKHEKAERRAANGFKYFTKREECGRLGCKYNQVNSHFHCIREGCQFSFLLKHQMTSH
ARKHMRRMLGKNFDRVPPSQGPPGLMDAETDECMDYTGCSPGAMSSESSTMDRSCSSTPV
GNESTAAGNTISMPTASGAKKRFWIIEDMSPFGKRRKTASSRKMLDEGMMLEGFRRFDLY
EDCKDAACQFSLKVTHYHCTRENCGYKFCGRTHMYKHAQHHDRVDNLVLDDFKRFKASLS
CHFADCPFSGTSTHFHCLRCRFRCTDSTKVTAHRKHHGKQDVISAAGFCQFSSSADCAVP
DCKYKLKCSHFHCTFPGCRHTVVGMSQMDSHKRKHEKQERGEPAAEGPAPGPPISLDGSL
SLGAEPGSLLFLQSAAAGLGLALGDAGDPGPPDAAAPGPREGAAAAAAAAGESSQEDEEE
ELELPEEEAEDDEDEDDDEDDDDEDDDEDDDDEDLRTDSEESLPEAAAEAAGAGARTPAL
AALAALGAPGPAPTAASSP
>sp|P04040|CATA_HUMAN Catalase OS=Homo sapiens GN=CAT PE=1 SV=3
MADSRDPASDQMQHWKEQRAAQKADVLTTGAGNPVGDKLNVITVGPRGPLLVQDVVFTDE
MAHFDRERIPERVVHAKGAGAFGYFEVTHDITKYSKAKVFEHIGKKTPIAVRFSTVAGES
GSADTVRDPRGFAVKFYTEDGNWDLVGNNTPIFFIRDPILFPSFIHSQKRNPQTHLKDPD
MVWDFWSLRPESLHQVSFLFSDRGIPDGHRHMNGYGSHTFKLVNANGEAVYCKFHYKTDQ
GIKNLSVEDAARLSQEDPDYGIRDLFNAIATGKYPSWTFYIQVMTFNQAETFPFNPFDLT
KVWPHKDYPLIPVGKLVLNRNPVNYFAEVEQIAFDPSNMPPGIEASPDKMLQGRLFAYPD

THRHRLGPNYLHIPVNCPYRARVANYQRDGPMCMQDNQGGAPNYYPNSFGAPEQQPSALE
HSIQYSGEVRRFNTANDDNVTQVRAFYVNVLNEEQRKRLCENIAGHLKDAQIFIQKKAVK
NFTEVHPDYGSHIQALLDKYNAEKPKNAIHTFVQSGSHLAAREKANL
>sp|Q7Z7H3|CATIP_HUMAN Ciliogenesis-associated TTC17-interacting protein OS=Homo sapiens GN=CATIP PE=1 SV=1
MSSKVYSTGSRAKDHQPSGPECLPLPEANAEAIDFLSSLHKEELQMLFFSETLAMVSDTG
EPQGELTIEVQRGKYQEKLGMLTYCLFVHASSRGFLDKMLCGNSLLGYLSEKLELMEQHS
QDFIKFLILPMERKMSLLKQDDQLAVTRSIKEGEEVKTGVTSFPWSSIKGFISEAANLVL
LRVMAWRRMVPSNARFLTLDTEGKLCYLTYQNLGFQTIQVDHQQAEVFIVEQTVHAEEGI
PMSCQYYLLSDGHLAKRIQVGSPGCCIITKMPILREEDEIEPRPVFEKKPLVWEEDMELY
SKFLDRKEELRLGHASYLRQHPEAHALISDFLLFLLLRQPEDVVTFAAEFFGPFDPWRPS
SPALGSSHRPNPFRSLEPEGDARSGAA
>sp|Q8IUK8|CBLN2_HUMAN Cerebellin-2 OS=Homo sapiens GN=CBLN2 PE=2 SV=1
MQAPGRGPLGLRLMMPGRRGALREPGGCGSCLGVALALLLLLLPACCPVRAQNDTEPIVL
EGKCLVVCDSSPSADGAVTSSLGISVRSGSAKVAFSATRSTNHEPSEMSNRTMTIYFDQV
LVNIGNHFDLASSIFVAPRKGIYSFSFHVVKVYNRQTIQVSLMQNGYPVISAFAGDQDVT
REAASNGVLLLMEREDKVHLKLERGNLMGGWKYSTFSGFLVFPL
>sp|Q8N5N4|CC022_HUMAN Uncharacterized protein C3orf22 OS=Homo sapiens GN=C3orf22 PE=2 SV=1
MDSSACKKSHQSKKWRIQAQENFAKKFPYRLSWLTEPDPEPLQPWEVTNDSNTVQLPLQK
RLVPTRSIPVRGLGAPDFTSPSGSCPAPLPAPSPPPLCNLWELKLLSRRFPRQLAFLLST
RHTEAACPQTSKAAGLSRGLS
>sp|O96020|CCNE2_HUMAN G1/S-specific cyclin-E2 OS=Homo sapiens GN=CCNE2 PE=1 SV=1
MSRRSSRLQAKQQPQPSQTESPQEAQIIQAKKRKTTQDVKKRREEVTKKHQYEIRNCWPP
VLSGGISPCIIIETPHKEIGTSDFSRFTNYRFKNLFINPSPLPDLSWGCSKEVWLNMLKK
ESRYVHDKHFEVLHSDLEPQMRSILLDWLLEVCEVYTLHRETFYLAQDFFDRFMLTQKDI
NKNMLQLIGITSLFIASKLEEIYAPKLQEFAYVTDGACSEEDILRMELIILKALKWELCP
VTIISWLNLFLQVDALKDAPKVLLPQYSQETFIQIAQLLDLCILAIDSLEFQYRILTAAA
LCHFTSIEVVKKASGLEWDSISECVDWMVPFVNVVKSTSPVKLKTFKKIPMEDRHNIQTH
TNYLAMLEEVNYINTFRKGGQLSPVCNGGIMTPPKSTEKPPGKH
>sp|Q96S94|CCNL2_HUMAN Cyclin-L2 OS=Homo sapiens GN=CCNL2 PE=1 SV=1
MAAAAAAAGAAGSAAPAAAAGAPGSGGAPSGSQGVLIGDRLYSGVLITLENCLLPDDKLR
FTPSMSSGLDTDTETDLRVVGCELIQAAGILLRLPQVAMATGQVLFQRFFYTKSFVKHSM
EHVSMACVHLASKIEEAPRRIRDVINVFHRLRQLRDKKKPVPLLLDQDYVNLKNQIIKAE
RRVLKELGFCVHVKHPHKIIVMYLQVLECERNQHLVQTSWNYMNDSLRTDVFVRFQPESI
ACACIYLAARTLEIPLPNRPHWFLLFGATEEEIQEICLKILQLYARKKVDLTHLEGEVEK
RKHAIEEAKAQARGLLPGGTQVLDGTSGFSPAPKLVESPKEGKGSKPSPLSVKNTKRRLE
GAKKAKADSPVNGLPKGRESRSRSRSRSREQSYSRSPSRSASPKRRKSDSGSTSGGSKSQSR
SRSRSDSPPRQAPRSAPYKGSEIRGSRKSKDCKYPQKPHKSRSRSSSRSRSRSRERADNP
GKYKKKSHYYRDQRRERSRSYERTGRRYERDHPGHSRHRR
>sp|O60563|CCNT1_HUMAN Cyclin-T1 OS=Homo sapiens GN=CCNT1 PE=1 SV=1
MEGERKNNNKRWYFTREQLENSPSRRFGVDPDKELSYRQQAANLLQDMGQRLNVSQLTIN
TAIVYMHRFYMIQSFTQFPGNSVAPAALFLAAKVEEQPKKLEHVIKVAHTCLHPQESLPD
TRSEAYLQQVQDLVILESIILQTLGFELTIDHPHTHVVKCTQLVRASKDLAQTSYFMATN

SLHLTTFSLQYTPPVVACVCIHLACKWSNWEIPVSTDGKHWWEYVDATVTLELLDELTHE
FLQILEKTPNRLKRIWNWRACEAAKKTKADDRGTDEKTSEQTILNMISQSSSDTTIAGLM
SMSTSTTSAVPSLPVSEESSSNLTSVEMLPGKRWLSSQPSFKLEPTQGHRTSENLALTGV
DHSLPQDGSNAFISQKQNSKSVPSAKVSLKEYRAKHAEELAAQKRQLENMEANVKSQYAY
AAQNLLSHHDSHSSVILKMPIEGSENPERPFLEKADKTALKMRIPVAGGDKAASSKPEEI
KMRIKVHAAADKHNSVEDSVTKSREHKEKHKTHPSNHHHHHNHHSHKHSHSQLPVGTGNK
RPGDPKHSSQTSNLAHKTYSLSSSFSSSSSTRKRGPSEETGGAVFDHPAKIAKSTKSSSL
NFSFPSLPTMGQMPGHSSDTSGLSFSQPSCKTRVPHSKLDKGPTGANGHNTTQTIDYQDT
VNMLHSLLSAQGVQPTQPTAFEFVRPYSDYLNPRSGGISSRSGNTDKPRPPPLPSEPPPP
LPPLPK
>sp|Q8ND76|CCNY_HUMAN Cyclin-Y OS=Homo sapiens GN=CCNY PE=1 SV=2
MGNTTSCCVSSSPKLRRNAHSRLESYRPDTDLSREDTGCNLQHISDRENIDDLNMEFNPS
DHPRASTIFLSKSQTDVREKRKSLFINHHPPGQIARKYSSCSTIFLDDSTVSQPNLKYTI
KCVALAIYYHIKNRDPDGRMLLDIFDENLHPLSKSEVPPDYDKHNPEQKQIYRFVRTLFS
AAQLTAECAIVTLVYLERLLTYAEIDICPANWKRIVLGAILLASKVWDDQAVWNVDYCQI
LKDITVEDMNELERQFLELLQFNINVPSSVYAKYYFDLRSLAEANNLSFPLEPLSRERAH
KLEAISRLCEDKYKDLRRSARKRSASADNLTLPRWSPAIIS
>sp|O14618|CCS_HUMAN Copper chaperone for superoxide dismutase OS=Homo sapiens GN=CCS PE=1 SV=1
MASDSGNQGTLCTLEFAVQMTCQSCVDAVRKSLQGVAGVQDVEVHLEDQMVLVHTTLPSQ
EVQALLEGTGRQAVLKGMGSGQLQNLGAAVAILGGPGTVQGVVRFLQLTPERCLIEGTID
GLEPGLHGLHVHQYGDLTNNCNSCGNHFNPDGASHGGPQDSDRHRGDLGNVRADADGRAI
FRMEDEQLKVWDVIGRSLIIDEGEDDLGRGGHPLSKITGNSGERLACGIIARSAGLFQNP
KQICSCDGLTIWEERGRPIAGKGRKESAQPPAHL
>sp|Q8N7R7|CCYL1_HUMAN Cyclin-Y-like protein 1 OS=Homo sapiens GN=CCNYL1 PE=1 SV=2
MGNTLTCCVSPNASPKLGRRAGSAELYCASDIYEAVSGDAVAVAPAVVEPAELDFGEGEG
HHLQHISDREMPEDLALESNPSDHPRASTIFLSKSQTDVREKRKSNHLNHVSPGQLTKKY
SSCSTIFLDDSTVSQPNLRTTVKCVTLAIYYHIKNRDANRSLDIFDERSHPLTREKVPEE
YFKHDPEHKFIYRFVRTLFSAAQLTAECAIVTLVYLERLLTYAEIDICPTNWKRIVLGAI
LLASKVWDDQAVWNVDYCQILKDITVEDMNEMERHFLELLQFNINVPASVYAKYYFDLRS
LADDNNLNFLFAPLSKERAQNLEAISRLCEDKDLCRAAMRRSFSADNFIGIQRSKAILS
>sp|Q8IY42|CD019_HUMAN Uncharacterized protein C4orf19 OS=Homo sapiens GN=C4orf19 PE=1 SV=1
MGCRCCKIIQSYLFDPVQVPSPGYVNEVNSCKLDEDDTDKLKGKWSSEVLVQKNDPQRQG
SKKTESSSRTADPWEPCWPHQGPLPQGDAGGEHHACGVNGIGPAATPQPTGNSSPTQDDR
GSWASTANTVPPTQPFLEGGGTRKQDCVLLASEGTQVMRNGDSRAPSEAESFALEVQDHV
FQIPAPDYLQHWGPAGDNVDHNEKDCVFKNHTEDESLEGIQPPVGEHGLNTPFSVRRSWD
SLNEDVETEVLSICFNEKGPVHAMPVVDSGNRQEDTHGSDGDGDGEIVDEDAAVAEALAA
LEAATAGEDLDETD
>sp|P06127|CD5_HUMAN T-cell surface glycoprotein CD5 OS=Homo sapiens GN=CD5 PE=1 SV=2
MPMGSLQPLATLYLLGMLVASCLGRLSWYDPDFQARLTRSNSKCQGQLEVYLKDGWHMVC
SQSWGRSSKQWEDPSQASKVCQRLNCGVPLSLGPFLVTYTPQSSIICYGQLGSFSNCSHS
RNDMCHSLGLTCLEPQKTTPPTTRPPPTTTPEPTAPPRLQLVAQSGGQHCAGVVEFYSGS
LGGTISYEAQDKTQDLENFLCNNLQCGSFLKHLPETEAGRAQDPGEPREHQPLPIQWKIQ

NSSCTSLEHCFRKIKPQKSGRVLALLCSGFQPKVQSRLVGGSSICEGTVEVRQGAQWAAL
CDSSSARSSLRWEEVCREQQCGSVNSYRVLDAGDPTSRGLFCPHQKLSQCHELWERNSYC
KKVFVTCQDPNPAGLAAGTVASIILALVLLVVLLVVCGPLAYKKLVKKFRQKKQRQWIGP
TGMNQNMSFHRNHTATVRSHAENPTASHVDNEYSQPPRNSHLSAYPALEGALHRSSMQPD
NSSDSDYDLHGAQRL
>sp|Q99618|CDCA3_HUMAN Cell division cycle-associated protein 3 OS=Homo sapiens GN=CDCA3 PE=1 SV=1
MGSAKSVPVTPARPPPHNKHLARVADPRSPSAGILRTPIQVESSPQPGLPAGEQLEGLKH
AQDSDPRSPTLGIARTPMKTSSGDPPSPLVKQLSEVFETEDSKSNLPPEPVLPPEAPLSS
ELDLPLGTQLSVEEQMPPWNQTEFPSKQVFSKEEARQPTETPVASQSSDKPSRDPETPRS
SGSMRNRWKPNSSKVLGRSPLTILQDDNSPGTLTLRQGKRPSPLSENVSELKEGAILGTG
RLLKTGGRAWEQGQDHDKENQHFPLVES
>sp|Q9BWT1|CDCA7_HUMAN Cell division cycle-associated protein 7 OS=Homo sapiens GN=CDCA7 PE=1 SV=1
MDARRVPQKDLRVKKNLKKFRYVKLISMETSSSSDDSCDSFASDNFANTRLQSVREGCRT
RSQCRHSGPLRVAMKFPARSTRGATNKKAESRQPSENSVTDSNSDSEDESGMNFLEKRAL
NIKQNKAMLAKLMSELESFPGSFRGRHPLPGSDSQSRRPRRRTFPGVASRRNPERRARPL
TRSRSRILGSLDALPMEEEEEEDKYMLVRKRKTVDGYMNEDDLPRSRRSRSSVTLPHIIR
PVEEITEEELENVCSNSREKIYNRSLGSTCHQCRQKTIDTKTNCRNPDCWGVRGQFCGPC
LRNRYGEEVRDALLDPNWHCPPCRGICNCSFCRQRDGRCATGVLVYLAKYHGFGNVHAYL
KSLKQEFEMQA
>sp|Q15131|CDK10_HUMAN Cyclin-dependent kinase 10 OS=Homo sapiens GN=CDK10 PE=1 SV=1
MAEPDLECEQIRLKCIRKEGFFTVPPEHRLGRCRSVKEFEKLNRIGEGTYGIVYRARDTQ
TDEIVALKKVRMDKEKDGIPISSLREITLLLRLRHPNIVELKEVVVGNHLESIFLVMGYC
EQDLASLLENMPTPFSEAQVKCIVLQVLRGLQYLHRNFIIHRDLKVSNLLMTDKGCVKTA
DFGLARAYGVPVKPMTPKVVTLWYRAPELLLGTTTQTTSIDMWAVGCILAELLAHRPLLP
GTSEIHQIDLIVQLLGTPSENIWPGFSKLPLVGQYSLRKQPYNNLKHKFPWLSEAGLRLL
HFLFMYDPKKRATAGDCLESSYFKEKPLPCEPELMPTFPHHRNKRAAPATSEGQSKRCKP
>sp|Q14004|CDK13_HUMAN Cyclin-dependent kinase 13 OS=Homo sapiens GN=CDK13 PE=1 SV=2
MPSSSDTALGGGGGLSWAEKKLEERRKRRRFLSPQQPPLLLPLLQPQLLQPPPPPPPLLF
LAAPGTAAAAAAAAAASSSCFSPGPPLEVKRLARGKRRAGGRQKRRRGPRAGQEAEKRRV
FSLPQPQQDGGGGASSGGGVTPLVEYEDVSSQSEQGLLLGGASAATAATAAGGTGGSGGS
PASSSGTQRRGEGSERRPRRDRRSSSGRSKERHREHRRRDGQRGGSEASKSRSRHSHSGE
ERAEVAKSGSSSSSGGRRKSASATSSSSSSRKDRDSKAHRSRTKSSKEPPSAYKEPPKAY
REDKTEPKAYRRRRSLSPLGGRDDSPVSHRASQSLRSRKSPSPAGGGSSPYSRRLPRSPS
PYSRRRSPSYSRHSSYERGGDVSPSPYSSSSWRRSRSPYSPVLRRSGKSRSRSPYSSRHS
RSRSRHRLSRSRSRHSSISPSTLTLKSSLAAELNKNKKARAAEAARAAEAAKAAEATKAA
EAAAKAAKASNTSTPTKGNTETSASASQTNHVKDVKKIKIEHAPSPSSGGTLKNDKAKTK
PPLQVTKVENNLIVDKATKKAVIVGKESKSAATKEESVSLKEKTKPLTPSIGAKEKEQHV
ALVTSTLPPLPLPPMLPEDKEADSLRGNISVKAVKKEVEKKLRCLLADLPLPPELPGGDD
LSKSPEEKKTATQLHSKRRPKICGPRYGETKEKDIDWGKRCVDKFDIIGIIGEGTYGQVY
KARDKDTGEMVALKKVRLDNEKEGFPITAIREIKILRQLTHQSIINMKEIVTDKEDALDF
KKDKGAFYLVFEYMDHDLMGLLESGLVHFNENHIKSFMRQLMEGLDYCHKKNFLHRDIKC
SNILLNNRGQIKLADFGLARLYSSEESRPYTNKVITLWYRPPELLLGEERYTPAIDVWSC

GCILGELFTKKPIFQANQELAQLELISRICGSPCPAVWPDVIKLPYFNTMKPKKQYRRKL
REEFVFIPAAALDLFDYMLALDPSKRCTAEQALQCEFLRDVEPSKMPPPDLPLWQDCHEL
WSKKRRRQKQMGMTDDVSTIKAPRKDLSLGLDDSRTNTPQGVLPSSQLKSQGSSNVAPVK
TGPGQHLNHSELAILLNLLQSKTSVNMADFVQVLNIKVNSETQQQLNKINLPAGILATGE
KQTDPSTPQQESSKPLGGIQPSSQTIQPKVETDAAQAAVQSAFAVLLTQLIKAQQSKQKD
VLLEERENGSGHEASLQLRPPPEPSTPVSGQDDLIQHQDMRILELTPEPDRPRILPPDQR
PPEPPEPPPVTEEDLDYRTENQHVPTTSSSLTDPHAGVKAALLQLLAQHQPQDDPKREGG
IDYQAGDTYVSTSDYKDNFGSSSFSSAPYVSNDGLGSSSAPPLERRSFIGNSDIQSLDNY
STASSHSGGPPQPSAFSESFPSSVAGYGDIYLNAGPMLFSGDKDHRFEYSHGPIAVLANS
SDPSTGPESTHPLPAKMHNYNYGGNLQENPSGPSLMHGQTWTSPAQGPGYSQGYRGHIST
STGRGRGRGLPY
>sp|Q07002|CDK18_HUMAN Cyclin-dependent kinase 18 OS=Homo sapiens GN=CDK18 PE=1 SV=3
MNKMKNFKRRFSLSVPRTETIEESLAEFTEQFNQLHNRRNENLQLGPLGRDPPQECSTFS
PTDSGEEPGQLSPGVQFQRRQNQRRFSMEDVSKRLSLPMDIRLPQEFLQKLQMESPDLPK
PLSRMSRRASLSDIGFGKLETYVKLDKLGEGTYATVFKGRSKLTENLVALKEIRLEHEEG
APCTAIREVSLLKNLKHANIVTLHDLIHTDRSLTLVFEYLDSDLKQYLDHCGNLMSMHNV
KIFMFQLLRGLAYCHHRKILHRDLKPQNLLINERGELKLADFGLARAKSVPTKTYSNEVV
TLWYRPPDVLLGSTEYSTPIDMWGVGCIHYEMATGRPLFPGSTVKEELHLIFRLLGTPTE
ETWPGVTAFSEFRTYSFPCYLPQPLINHAPRLDTDGIHLLSSLLLYESKSRMSAEAALSH
SYFRSLGERVHQLEDTASIFSLKEIQLQKDPGYRGLAFQQPGRGKNRRQSIF
>sp|Q5VV42|CDKAL_HUMAN Threonylcarbamoyladenosine tRNA methylthiotransferase OS=Homo sapiens GN=CDKAL1 PE=1 SV=1
MPSASCDTLLDDIEDIVSQEDSKPQDRHFVRKDVVPKVRRRNTQKYLQEEENSPPSDSTI
PGIQKIWIRTWGCSHNNSDGEYMAGQLAAYGYKITENASDADLWLLNSCTVKNPAEDHFR
NSIKKAQEENKKIVLAGCVPQAQPRQDYLKGLSIIGVQQIDRVVEVVEETIKGHSVRLLG
QKKDNGRRLGGARLDLPKIRKNPLIEIISINTGCLNACTYCKTKHARGNLASYPIDELVD
RAKQSFQEGVCEIWLTSEDTGAYGRDIGTNLPTLLWKLVEVIPEGAMLRLGMTNPPYILE
HLEEMAKILNHPRVYAFLHIPVQSASDSVLMEMKREYCVADFKRVVDFLKEKVPGITIAT
DIICGFPGETDQDFQETVKLVEEYKFPSLFINQFYPRPGTPAAKMEQVPAQVKKQRTKDL
SRVFHSYSPYDHKIGERQQVLVTEESFDSKFYVAHNQFYEQVLVPKNPAFMGKMVEVDIY
ESGKHFMKGQPVSDAKVYTPSISKPLAKGEVSGLTKDFRNGLGNQLSSGSHTSAASQCDS
ASSRMVLPMPRLHQDCALRMSVGLALLGLLFAFFVKVYN
>sp|O76039|CDKL5_HUMAN Cyclin-dependent kinase-like 5 OS=Homo sapiens GN=CDKL5 PE=1 SV=1
MKIPNIGNVMNKFEILGVVGEGAYGVVLKCRHKETHEIVAIKKFKDSEENEEVKETTLRE
LKMLRTLKQENIVELKEAFRRRGKLYLVFEYVEKNMLELLEEMPNGVPPEKVKSYIYQLI
KAIHWCHKNDIVHRDIKPENLLISHNDVLKLCDFGFARNLSEGNNANYTEYVATRWYRSP
ELLLGAPYGKSVDMWSVGCILGELSDGQPLFPGESEIDQLFTIQKVLGPLPSEQMKLFYS
NPRFHGLRFPAVNHPQSLERRYLGILNSVLLDLMKNLLKLDPADRYLTEQCLNHPTFQTQ
RLLDRSPSRSAKRKPYHVESSTLSNRNQAGKSTALQSHHRSNSKDIQNLSVGLPRADEGL
PANESFLNGNLAGASLSPLHTKTYQASSQPGSTSKDLTNNNIPHLLSPKEAKSKTEFDFN
IDPKPSEGPGTKYLKSNSRSQQNRHSFMESSQSKAGTLQPNEKQSRHSYIDTIPQSSRSP
SYRTKAKSHGALSDSKSVSNLSEARAQIAEPSTSRYFPSSCLDLNSPTSPTPTRHSDTRT
LLSPSGRNNRNEGTLDSRRTTTRHSKTMEELKLPEHMDSSHSHSLSAPHESFSYGLGYTS
PFSSQQRPHRHSMYVTRDKVRAKGLDGSLSIGQGMAARANSLQLLSPQPGEQLPPEMTVA

RSSVKETSREGTSSFHTRQKSEGGVYHDPHSDDGTAPKENRHLYNDPVPRRVGSFYRVPS
PRPDNSFHENNVSTRVSSLPSESSSGTNHSKRQPAFDPWKSPENISHSEQLKEKEKQGFF
RSMKKKKKKSQTVPNSDSPDLLTLQKSIHSASTPSSRPKEWRPEKISDLQTQSQPLKSLR
KLLHLSSASNHPASSDPRFQPLTAQQTKNSFSEIRIHPLSQASGGSSNIRQEPAPKGRPA
LQLPDGGCDGRRQRHHSGPQDRRFMLRTTEQQGEYFCCGDPKKPHTPCVPNRALHRPISS
PAPYPVLQVRGTSMCPTLQVRGTDAFSCPTQQSGFSFFVRHVMREALIHRAQVNQAALLT
YHENAALTGK
>sp|P46527|CDN1B_HUMAN Cyclin-dependent kinase inhibitor 1B OS=Homo sapiens GN=CDKN1B PE=1 SV=1
MSNVRVSNGSPSLERMDARQAEHPKPSACRNLFGPVDHEELTRDLEKHCRDMEEASQRKW
NFDFQNHKPLEGKYEWQEVEKGSLPEFYYRPPRPPKGACKVPAQESQDVSGSRPAAPLIG
APANSEDTHLVDPKTDPSDSQTGLAEQCAGIRKRPATDDSSTQNKRANRTEENVSDGSPN
AGSVEQTPKKPGLRRRQT
>sp|P42773|CDN2C_HUMAN Cyclin-dependent kinase 4 inhibitor C OS=Homo sapiens GN=CDKN2C PE=1 SV=1
MAEPWGNELASAAARGDLEQLTSLLQNNVNVNAQNGFGRTALQVMKLGNPEIARRLLLRG
ANPDLKDRTGFAVIHDAARAGFLDTLQTLLEFQADVNIEDNEGNLPLHLAAKEGHLRVVE
FLVKHTASNVGHRNHKGDTACDLARLYGRNEVVSLMQANGAGGATNLQ
>sp|Q8IU53|CASC2_HUMAN Protein CASC2, isoforms 1/2 OS=Homo sapiens GN=CASC2 PE=4 SV=1
MAGTRGLMLLGPGPVAGPRDVGTCRGRQMEIQKHKDNKKLPQGIIIVFRLQTHTTPQIYT
QLKGKLRKFFKEPYSE
>sp|O15234|CASC3_HUMAN Protein CASC3 OS=Homo sapiens GN=CASC3 PE=1 SV=2
MADRRRQRASQDTEDEESGASGSDSGGSPLRGGGSCSGSAGGGGSGSLPSQRGGRTGALH
LRRVESGGAKSAEESECESEDGIEGDAVLSDYESAEDSEGEEGEYSEEENSKVELKSEAN
DAVNSSTKEEKGEEKPDTKSTVTGERQSGDGQESTEPVENKVGKKGPKHLDDDEDRKNPA
YIPRKGLFFEHDLRGQTQEEEVRPKGRQRKLWKDEGRWEHDKFREDEQAPKSRQELIALY
GYDIRSAHNPDDIKPRRIRKPRYGSPPQRDPNWNGERLNKSHRHQGLGGTLPPRTFINRN
AAGTGRMSAPRNYSRSGGFKEGRAGFRPVEAGGQHGGRSGETVKHEISYRSRRLEQTSVR
DPSPEADAPVLGSPEKEEAASEPPAAAPDAAPPPPDRPIEKKSYSRARRTRTKVGDAVKL
AEEVPPPPEGLIPAPPVPETTPTPPTKTGTWEAPVDSSTSGLEQDVAQLNIAEQNWSPGQ
PSFLQPRELRGMPNHIHMGAGPPPQFNRMEEMGVQGGRAKRYSSQRQRPVPEPPAPPVHI
SIMEGHYYDPLQFQGPIYTHGDSPAPLPPQGMLVQPGMNLPHPGLHPHQTPAPLPNPGLY
PPPVSMSPGQPPPQQLLAPTYFSAPGVMNFGNPSYPYAPGALPPPPPPHLYPNTQAPSQV
YGGVTYYNPAQQQVQPKPSPPRRTPQPVTIKPPPPEVVSRGSS
>sp|P42574|CASP3_HUMAN Caspase-3 OS=Homo sapiens GN=CASP3 PE=1 SV=2
MENTENSVDSKSIKNLEPKIIHGSESMDSGISLDNSYKMDYPEMGLCIIINNKNFHKSTG
MTSRSGTDVDAANLRETFRNLKYEVRNKNDLTREEIVELMRDVSKEDHSKRSSFVCVLLS
HGEEGIIFGTNGPVDLKKITNFFRGDRCRSLTGKPKLFIIQACRGTELDCGIETDSGVDD
DMACHKIPVEADFLYAYSTAPGYYSWRNSKDGSWFIQSLCAMLKQYADKLEFMHILTRVN
RKVATEFESFSFDATFHAKKQIPCIVSMLTKELYFYH
>sp|P49662|CASP4_HUMAN Caspase-4 OS=Homo sapiens GN=CASP4 PE=1 SV=1
MAEGNHRKKPLKVLESLGKDFLTGVLDNLVEQNVLNWKEEEKKKYYDAKTEDKVRVMADS
MQEKQRMAGQMLLQTFFNIDQISPNKKAHPNMEAGPPESGESTDALKLCPHEEFLRLCKE
RAEEIYPIKERNNRTRLALIICNTEFDHLPPRNGADFDITGMKELLEGLDYSVDVEENLT

ARDMESALRAFATRPEHKSSDSTFLVLMSHGILEGICGTVHDEKKPDVLLYDTIFQIFNN
RNCLSLKDKPKVIIVQACRGANRGELWVRDSPASLEVASSQSSENLEEDAVYKTHVEKDF
IAFCSSTPHNVSWRDSTMGSIFITQLITCFQKYSWCCHLEEVFRKVQQSFETPRAKAQMP
TIERLSMTRYFYLFPGN
>sp|P55210|CASP7_HUMAN Caspase-7 OS=Homo sapiens GN=CASP7 PE=1 SV=1
MADDQGCIEEQGVEDSANEDSVDAKPDRSSFVPSLFSKKKKNVTMRSIKTTRDRVPTYQY
NMNFEKLGKCIIINNKNFDKVTGMGVRNGTDKDAEALFKCFRSLGFDVIVYNDCSCAKMQ
DLLKKASEEDHTNAACFACILLSHGEENVIYGKDGVTPIKDLTAHFRGDRCKTLLEKPKL
FFIQACRGTELDDGIQADSGPINDTDANPRYKIPVEADFLFAYSTVPGYYSWRSPGRGSW
FVQALCSILEEHGKDLEIMQILTRVNDRVARHFESQSDDPHFHEKKQIPCVVSMLTKELY
FSQ
>sp|Q14790|CASP8_HUMAN Caspase-8 OS=Homo sapiens GN=CASP8 PE=1 SV=1
MDFSRNLYDIGEQLDSEDLASLKFLSLDYIPQRKQEPIKDALMLFQRLQEKRMLEESNLS
FLKELLFRINRLDLLITYLNTRKEEMERELQTPGRAQISAYRVMLYQISEEVSRSELRSF
KFLLQEEISKCKLDDDMNLLDIFIEMEKRVILGEGKLDILKRVCAQINKSLLKIINDYEE
FSKERSSSLEGSPDEFSNGEELCGVMTISDSPREQDSESQTLDKVYQMKSKPRGYCLIIN
NHNFAKAREKVPKLHSIRDRNGTHLDAGALTTTFEELHFEIKPHDDCTVEQIYEILKIYQ
LMDHSNMDCFICCILSHGDKGIIYGTDGQEAPIYELTSQFTGLKCPSLAGKPKVFFIQAC
QGDNYQKGIPVETDSEEQPYLEMDLSSPQTRYIPDEADFLLGMATVNNCVSYRNPAEGTW
YIQSLCQSLRERCPRGDDILTILTEVNYEVSNKDDKKNMGKQMPQPTFTLRKKLVFPSD
>sp|P41180|CASR_HUMAN Extracellular calcium-sensing receptor OS=Homo sapiens GN=CASR PE=1
SV=2
MAFYSCCWVLLALTWHTSAYGPDQRAQKKGDIILGGLFPIHFGVAAKDQDLKSRPESVEC
IRYNFRGFRWLQAMIFAIEEINSSPALLPNLTLGYRIFDTCNTVSKALEATLSFVAQNKI
DSLNLDEFCNCSEHIPSTIAVVGATGSGVSTAVANLLGLFYIPQVSYASSSRLLSNKNQF
KSFLRTIPNDEHQATAMADIIEYFRWNWVGTIAADDDYGRPGIEKFREEAEERDICIDFS
ELISQYSDEEEIQHVVEVIQNSTAKVIVVFSSGPDLEPLIKEIVRRNITGKIWLASEAWA
SSSLIAMPQYFHVVGGTIGFALKAGQIPGFREFLKKVHPRKSVHNGFAKEFWEETFNCHL
QEGAKGPLPVDTFLRGHEESGDRFSNSSTAFRPLCTGDENISSVETPYIDYTHLRISYNV
YLAVYSIAHALQDIYTCLPGRGLFTNGSCADIKKVEAWQVLKHLRHLNFTNNMGEQVTFD
ECGDLVGNYSIINWHLSPEDGSIVFKEVGYYNVYAKKGERLFINEEKILWSGFSREVPFS
NCSRDCLAGTRKGIIEGEPTCCFECVECPDGEYSDETDASACNKCPDDFWSNENHTSCIA
KEIEFLSWTEPFGIALTLFAVLGIFLTAFVLGVFIKFRNTPIVKATNRELSYLLLFSLLC
CFSSSLFFIGEPQDWTCRLRQPAFGISFVLCISCILVKTNRVLLVFEAKIPTSFHRKWWG
LNLQFLLVFLCTFMQIVICVIWLYTAPPSSYRNQELEDEIIFITCHEGSLMALGFLIGYT
CLLAAICFFFAFKSRKLPENFNEAKFITFSMLIFFIVWISFIPAYASTYGKFVSAVEVIA
ILAASFGLLACIFFNKIYIILFKPSRNTIEEVRCSTAAHAFKVAARATLRRSNVSRKRSS
SLGGSTGSTPSSSISSKSNSEDPFPQPERQKQQQPLALTQQEQQQQPLTLPQQQRSQQQP
RCKQKVIFGSGTVTFSLSFDEPQKNAMAHRNSTHQNSLEAQKSSDTLTRHQPLLPLQCGE
TDLDLTVQETGLQGPVGGDQRPEVEDPEELSPALVVSSSQSFVISGGGSTVTENVVNS
>sp|P07339|CATD_HUMAN Cathepsin D OS=Homo sapiens GN=CTSD PE=1 SV=1
MQPSSLLPLALCLLAAPASALVRIPLHKFTSIRRTMSEVGGSVEDLIAKGPVSKYSQAVP
AVTEGPIPEVLKNYMDAQYYGEIGIGTPPQCFTVVFDTGSSNLWVPSIHCKLLDIACWIH
HKYNSDKSSTYVKNGTSFDIHYGSGSLSGYLSQDTVSVPCQSASSASALGGVKVERQVFG

EATKQPGITFIAAKFDGILGMAYPRISVNNVLPVFDNLMQQKLVDQNIFSFYLSRDPDAQ
PGGELMLGGTDSKYYKGSLSYLNVTRKAYWQVHLDQVEVASGLTLCKEGCEAIVDTGTSL
MVGPVDEVRELQKAIGAVPLIQGEYMIPCEKVSTLPAITLKLGGKGYKLSPEDYTLKVSQ
AGKTLCLSGFMGMDIPPPSGPLWILGDVFIGRYYTVFDRDNNRVGFAEAARL
>sp|P08311|CATG_HUMAN Cathepsin G OS=Homo sapiens GN=CTSG PE=1 SV=2
MQPLLLLLAFLLPTGAEAGEIIGGRESRPHSRPYMAYLQIQSPAGQSRCGGFLVREDFVL
TAAHCWGSNINVTLGAHNIQRRENTQQHITARRAIRHPQYNQRTIQNDIMLLQLSRRVRR
NRNVNPVALPRAQEGLRPGTLCTVAGWGRVSMRRGTDTLREVQLRVQRDRQCLRIFGSYD
PRRQICVGDRRERKAAFKGDSGGPLLCNNVAHGIVSYGKSSGVPPEVFTRVSSFLPWIRT
TMRSFKLLDQMETPL
>sp|P51636|CAV2_HUMAN Caveolin-2 OS=Homo sapiens GN=CAV2 PE=1 SV=2
MGLETEKADVQLFMDDDSYSHHSGLEYADPEKFADSDQDRDPHRLNSHLKLGFEDVIAEP
VTTHSFDKVWICSHALFEISKYVMYKFLTVFLAIPLAFIAGILFATLSCLHIWILMPFVK
TCLMVLPSVQTIWKSVTDVIIAPLCTSVGRCFSSVSLQLSQD
>sp|Q9H9S4|CB39L_HUMAN Calcium-binding protein 39-like OS=Homo sapiens GN=CAB39L PE=1 SV=3
MKKMPLFSKSHKNPAEIVKILKDNLAILEKQDKKTDKASEEVSKSLQAMKEILCGTNEKE
PPTEAVAQLAQELYSSGLLVTLIADLQLIDFEGKKDVTQIFNNILRRQIGTRSPTVEYIS
AHPHILFMLLKGYEAPQIALRCGIMLRECIRHEPLAKIILFSNQFRDFFKYVELSTFDIA
SDAFATFKDLLTRHKVLVADFLEQNYDTIFEDYEKLLQSENYVTKRQSLKLLGELILDRH
NFAIMTKYISKPENLKLMMNLLRDKSPNIQFEAFHVFKVFVASPHKTQPIVEILLKNQPK
LIEFLSSFQKERTDDEQFADEKNYLIKQIRDLKKTAP
>sp|P15085|CBPA1_HUMAN Carboxypeptidase A1 OS=Homo sapiens GN=CPA1 PE=1 SV=2
MRGLLVLSVLLGAVFGKEDFVGHQVLRISVADEAQVQKVKELEDLEHLQLDFWRGPAHPG
SPIDVRVPFPSIQAVKIFLESHGISYETMIEDVQSLLDEEQEQMFAFRSRARSTDTFNYA
TYHTLEEIYDFLDLLVAENPHLVSKIQIGNTYEGRPIYVLKFSTGGSKRPAIWIDTGIHS
REWVTQASGVWFAKKITQDYGQDAAFTAILDTLDIFLEIVTNPDGFAFTHSTNRMWRKTR
SHTAGSLCIGVDPNRNWDAGFGLSGASSNPCSETYHGKFANSEVEVKSIVDFVKDHGNIK
AFISIHSYSQLLMYPYGYKTEPVPDQDELDQLSKAAVTALASLYGTKFNYGSIIKAIYQA
SGSTIDWTYSQGIKYSFTFELRDTGRYGFLLPASQIIPTAKETWLALLTIMEHTLNHPY
>sp|Q96IY4|CBPB2_HUMAN Carboxypeptidase B2 OS=Homo sapiens GN=CPB2 PE=1 SV=2
MKLCSLAVLVPIVLFCEQHVFAFQSGQVLAALPRTSRQVQVLQNLTTTYEIVLWQPVTAD
LIVKKKQVHFFVNASDVDNVKAHLNVSGIPCSVLLADVEDLIQQQISNDTVSPRASASYY
EQYHSLNEIYSWIEFITERHPDMLTKIHIGSSFEKYPLYVLKVSGKEQAAKNAIWIDCGI
HAREWISPAFCLWFIGHITQFYGIIGQYTNLLRLVDFYVMPVVNVDGYDYSWKKNRMWRK
NRSFYANNHCIGTDLNRNFASKHWCEEGASSSSCSETYCGLYPESEPEVKAVASFLRRNI
NQIKAYISMHSYSQHIVFPYSYTRSKSKDHEELSLVASEAVRAIEKISKNTRYTHGHGSE
TLYLAPGGGDDWIYDLGIKYSFTIELRDTGTYGFLLPERYIKPTCREAFAAVSKIAWHVI
RNV
>sp|Q5U5Z8|CBPC2_HUMAN Cytosolic carboxypeptidase 2 OS=Homo sapiens GN=AGBL2 PE=1 SV=2
MFPALETHLKQTIPDPYEDFMYRHLQYYGYFKAQRGSLPNSATHQHVRKNNPQCLLNGSL
GEKDDLIPDTLQKEKLLWPISLSSAVHRQIEAINRDFHSCLGWMQWRGLSSLQPPPPRFK
DSPASAFRVAGITDSHMLSLPHLRSRQLLYDELDEVNPRLREPQELFSILSTKRPLQAPR
WPIECEVIKENIHHIEWAPPQPEYFYQPKGNEKVPEIVGEKKGTVVYQLDSVPIEGSYFT

SSRVGGKRGIVKELAVTLQGPEDNTLLFESRFESGNLQKAVRVDTYEYELTLRTDLYTNK
HTQWFYFRVQNTRKDATYRFTIVNLLKPKSLYTVGMKPLLYSQLDANTRNIGWRREGNEI
KYYKNNTDDGQQPFYCLTWTIQFPYDQDTCFFAHFYPYTYTDLQCYLLSVANNPIQSQFC
KLQTLCRSLAGNTVYLLTITNPSQTPQEAAAKKAVVLSARVHPGESNGSWVMKGFLDFIL
SNSPDAQLLRDIFVFKVLPMLNPDGVIVGNYRCSLAGRDLNRHYKTILKESFPCIWYTRN
MIKRLLEEREVLLYCDFHGHSRKNNIFLYGCNNNNRKYWLHERVFPLMLCKNAPDKFSFH
SCNFKVQKCKEGTGRVVMWRMGILNSYTMESTFGGSTLGNKRDTHFTIEDLKSLGYHVCD
TLLDFCDPDQMKFTQCLAELKELLRQEIHKKFHELGQDVDLEGSWSDISLSDIESSTSGS
DSSLSDGLPVHLANIADELTQKKKMFKKKKKKSLQTRKQRNEQYQKKNLMQKLKLTEDTS
EKAGFASTLQKQPTFFKNSENSSFLPMKNENPRLNETNLNRRDKDTPLDPSMATLILPKN
KGRMQNKKPGFTVSCSPKRTINSSQEPAPGMKPNWPRSRYPATKRGCAAMAAYPSLHIYT
YP
>sp|O75976|CBPD_HUMAN Carboxypeptidase D OS=Homo sapiens GN=CPD PE=1 SV=2
MASGRDERPPWRLGRLLLLMCLLLLGSSARAAHIKKAEATTTTTSAGAEAAEGQFDRYYH
EEELESALREAAAAGLPGLARLFSIGRSVEGRPLWVLRLTAGLGSLIPEGDAGPDAAGPD
AAGPLLPGRPQVKLVGNMHGDETVSRQVLIYLARELAAGYRRGDPRLVRLLNTTDVYLLP
SLNPDGFERAREGDCGFGDGGPSGASGRDNSRGRDLNRSFPDQFSTGEPPALDEVPEVRA
LIEWIRRNKFVLSGNLHGGSVVASYPFDDSPEHKATGIYSKTSDDEVFKYLAKAYASNHP
IMKTGEPHCPGDEDETFKDGITNGAHWYDVEGGMQDYNYVWANCFEITLELSCCKYPPAS
QLRQEWENNRESLITLIEKVHIGVKGFVKDSITGSGLENATISVAGINHNITTGRFGDFY
RLLVPGTYNLTVVLTGYMPLTVTNVVVKEGPATEVDFSLRPTVTSVIPDTTEAVSTASTV
AIPNILSGTSSSYQPIQPKDFHHHHFPDMEIFLRRFANEYPNITRLYSLGKSVESRELYV
MEISDNPGVHEPGEPEFKYIGNMHGNEVVGRELLLNLIEYLCKNFGTDPEVTDLVHNTRI
HLMPSMNPDGYEKSQEGDSISVIGRNNSNNFDLNRNFPDQFVQITDPTQPETIAVMSWMK
SYPFVLSANLHGGSLVVNYPFDDDEQGLATYSKSPDDAVFQQIALSYSKENSQMFQGRPC
KNMYPNEYFPHGITNGASWYNVPGGMQDWNYLQTNCFEVTIELGCVKYPLEKELPNFWEQ
NRRSLIQFMKQVHQGVRGFVLDATDGRGILNATISVAEINHPVTTYKTGDYWRLLVPGTY
KITASARGYNPVTKNVTVKSEGAIQVNFTLVRSSTDSNNESKKGKGASSSTNDASDPTTK
EFETLIKDLSAENGLESLMLRSSSNLALALYRYHSYKDLSEFLRGLVMNYPHITNLTNLG
QSTEYRHIWSLEISNKPNVSEPEEPKIRFVAGIHGNAPVGTELLLALAEFLCLNYKKNPA
VTQLVDRTRIVIVPSLNPDGRERAQEKDCTSKIGQTNARGKDLDTDFTNNASQPETKAII
ENLIQKQDFSLSVALDGGSMLVTYPYDKPVQTVENKETLKHLASLYANNHPSMHMGQPSC
PNKSDENIPGGVMRGAEWHSHLGSMKDYSVTYGHCPEITVYTSCCYFPSAARLPSLWADN
KRSLLSMLVEVHKGVHGFVKDKTGKPISKAVIVLNEGIKVQTKEGGYFHVLLAPGVHNII
AIADGYQQQHSQVFVHHDAASSVVIVFDTDNRIFGLPRELVVTVSGATMSALILTACIIW
CICSIKSNRHKDGFHRLRQHHDEYEDEIRMMSTGSKKSLLSHEFQDETDTEEETLYSSKH
>sp|P16870|CBPE_HUMAN Carboxypeptidase E OS=Homo sapiens GN=CPE PE=1 SV=1
MAGRGGSALLALCGALAACGWLLGAEAQEPGAPAAGMRRRRRLQQEDGISFEYHRYPELR
EALVSVWLQCTAISRIYTVGRSFEGRELLVIELSDNPGVHEPGEPEFKYIGNMHGNEAVG
RELLIFLAQYLCNEYQKGNETIVNLIHSTRIHIMPSLNPDGFEKAASQPGELKDWFVGRS
NAQGIDLNRNFPDLDRIVYVNEKEGGPNNHLLKNMKKIVDQNTKLAPETKAVIHWIMDIP
FVLSANLHGGDLVANYPYDETRSGSAHEYSSSPDDAIFQSLARAYSSFNPAMSDPNRPPC
RKNDDDSSFVDGTTNGGAWYSVPGGMQDFNYLSSNCFEITVELSCEKFPPEETLKTYWED
NKNSLISYLEQIHRGVKGFVRDLQGNPIANATISVEGIDHDVTSAKDGDYWRLLIPGNYK

LTASAPGYLAITKKVAVPYSPAAGVDFELESFSERKEEEKEELMEWWKMMSETLNF
>sp|Q9Y646|CBPQ_HUMAN Carboxypeptidase Q OS=Homo sapiens GN=CPQ PE=1 SV=1
MKFLIFAFFGGVHLLSLCSGKAICKNGISKRTFEEIKEEIASCGDVAKAIINLAVYGKAQ
NRSYERLALLVDTVGPRLSGSKNLEKAIQIMYQNLQQDGLEKVHLEPVRIPHWERGEESA
VMLEPRIHKIAILGLGSSIGTPPEGITAEVLVVTSFDELQRRASEARGKIVVYNQPYINY
SRTVQYRTQGAVEAAKVGALASLIRSVASFSIYSPHTGIQEYQDGVPKIPTACITVEDAE
MMSRMASHGIKIVIQLKMGAKTYPDTDSFNTVAEITGSKYPEQVVLVSGHLDSWDVGQGA
MDDGGGAFISWEALSLIKDLGLRPKRTLRLVLWTAEEQGGVGAFQYYQLHKVNISNYSLV
MESDAGTFLPTGLQFTGSEKARAIMEEVMSLLQPLNITQVLSHGEGTDINFWIQAGVPGA
SLLDDLYKYFFFHHSHGDTMTVMDPKQMNVAAAVWAVVSYVVADMEEMLPRS
>sp|Q5JPI3|CC038_HUMAN Uncharacterized protein C3orf38 OS=Homo sapiens GN=C3orf38 PE=1 SV=1
MEMSGLSFSEMEGCRNLLGLLDNDEIMALCDTVTNRLVQPQDRQDAVHAILAYSQSAEEL
LRRRKVHREVIFKYLATQGIVIPPATEKHNLIQHAKDYWQKQPQLKLKETPEPVTKTEDI
HLFQQQVKEDKKAEKVDFRRLGEEFCHWFFGLLNSQNPFLGPPQDEWGPQHFWHDVKLRF
YYNTSEQNVMDYHGAEIVSLRLLSLVKEEFLFLSPNLDSHGLKCASSPHGLVMVGVAGTV
HRGNTCLGIFEQIFGLIRCPFVENTWKIKFINLKIMGESSLAPGTLPKPSVKFEQSDLEA
FYNVITVCGTNEVRHNVKQASDSGTGDQV
>sp|Q8IYK2|CC105_HUMAN Coiled-coil domain-containing protein 105 OS=Homo sapiens GN=CCDC105 PE=2 SV=3
MRVLVPPAERSQDTRVGAPAWREAAQAMARTAHILTDRCGQEAVTMWQPKDSVLDPNVAH
HLGRAAYIQPWRFRVEMIKGGGTLEKPPPGEGVTLWKGKMKPPAWYARLPLPLHRKARAL
QTTEVVHAHARGARLTAARLGRAQHQINGRVRQLLRQREVTDHRLSEVRKGLLINQQSVK
LRGYRPKSEKVPDKADSMLTWEKEELKSMKRKMERDMEKSEVLLKTLASCRDTLNFCFKE
RLQAVDLMNQPLDKVLEQARRHSWVNLSRAPTPRTQGQKTPPPDPVGTYNPACALALNEA
KRLLVESKDTLVEMAKNEVDVREQQLQISDRVCASLAQKASETLELKERLNMTLGLMRGT
ILRCTKYNQELYTTHGLIKGPLSKVHLETAEKLDRPLVRMYQRHVGTQLPEAARLAQGTD
KLQCHITYLEKNLDELLATHKNLSWGLNCKNIGHEVDGNVVRLRLRQRQPHVCYEQAQRL
VKDWDPRTPPPRSKSSADP
>sp|Q96NT0|CC115_HUMAN Coiled-coil domain-containing protein 115 OS=Homo sapiens GN=CCDC115 PE=1 SV=1
MAALDLRAELDSLVLQLLGDLEELEGKRTVLNARVEEGWLSLAKARYAMGAKSVGPLQYA
SHMEPQVCLHASEAQEGLQKFKVVRAGVHAPEEVGPREAGLRRRKGPTKTPEPESSEAPQ
DPLNWFGILVPHSLRQAQASFRDGLQLAADIASLQNRIDWGRSQLRGLQEKLKQLEPGAA
>sp|Q8IWD4|CC117_HUMAN Coiled-coil domain-containing protein 117 OS=Homo sapiens GN=CCDC117 PE=1 SV=1
MAALGRPFSGLPLSGGSDFLQPPQPAFPGRAFPPGADGAELAPRPGPRAVPSSPAGSAAR
GRVSVHCKKKHKREEEEDDDCPVRKKRITEAELCAGPNDWILCAHQDVEGHGVNPSVSGL
SIPGILDVICEEMDQTTGEPQCEVARRKLQEIEDRIIDEDEEVEADRNVNHLPSLVLSDT
MKTGLKREFDEVFTKKMIESMSRPSMELVLWKPLPELLSDKPKPSSNTKNYTGESQAKHV
AAGTAFPQRTELFSEPRPTGMSLYNSLETATSTEEEMEL
>sp|Q6ZUS5|CC121_HUMAN Coiled-coil domain-containing protein 121 OS=Homo sapiens GN=CCDC121 PE=1 SV=1
MTDLNKHIKQAQTQRKQLLEESRELHREKLLVQAENRFFLEYLTNKTEEYTEQPEKVWNS

YLQKSGEIERRRQESASRYAEQISVLKTALLQKENIQSSLKRKLQAMRDIAILKEKQEKE
IQTLQEETKKVQAETASKTREVQAQLLQEKRLLEKQLSEPDRRLLGKRKRRELNMKAQAL
KLAAKRFIFEYSCGINRENQQFKKELLQLIEQAQKLTATQSHLENRKQQLQQEQWYLESL
IQARQRLQGSHNQCLNRQDVPKTTPSLPQGTKSRINPK
>sp|Q8NFR7|CC148_HUMAN Coiled-coil domain-containing protein 148 OS=Homo sapiens GN=CCDC148 PE=1 SV=2
MCAASASPDNLVFHMKNEMRNIKYKPVDYQQLRALTEAKKLASASAKLKIRKAMLTSKLS
KEQTLIKQHKQVWWQEYQRLNEVRCKMESEIKSLLNEENIGNECLCDLTNFEQELSEQQC
TYLKNVINPIQQLRADLKYRQHHTLQHSHPHIEFNSMKVLEEVDFVKKQLKTVFERLRLE
QQRIENDLSDWSIKILDHSLEEKTNPLSELPIELESLECPYPDLKSSILSEFYKFTQKYQ
KKLQDFNLQLEDIYRNCQLSEEDHWIYQAILDQYPGDLFGRRTLYLDMLQRYFPHKSRHD
LVEHEKYCDQYRFAIEQQNILISNWNKNKKDFIQKAVLTLTEACATHEMESMLAKDKKKQ
QELCADLKAKVRQWRAHQEEVARLEMEISARRREKEEEKEKLWKKKELLQRAEKKKKIKK
YWAKKKQKWQEMEMRDLQRLEELKKLIAEQSLKDRERVKYRQELLERRLMEKKEVALQEA
HEDKERARRLEALRKQVAVVAQFDPVRMMSDTMASKARMGIEIEEEFILQKPLFTLNTYN
EQQIISDPRLRFELALREAGLHRTLYAKEILPKISPQKPPRKDMESTVFKI
>sp|P0CW27|CC166_HUMAN Coiled-coil domain-containing protein 166 OS=Homo sapiens GN=CCDC166 PE=4 SV=1
MAPKKKRGPSAGSQPGGAAAAGAEQPLSERAQYLQREHALLSEQLDTCEESVDQVLRENA
FLDREALRLREENRLYASYVSARAQRCAKAIVRLDEQNRVDLAQIHWQRAELASLYHGRE
DGVRAQLLEMEARAAQMAQQVQELQPYKVLQLEQLARIRALERELLHMRVEHTQLLHRVK
RRFLEDKAAFEREARQRVQSLARRAEREAVRALVAHTQAIKADNGRLRQELLLLLRRTQL
LHHTRRQLLEQREQLHREHEDTRDLARVHGWLRRGPGGPPLWERPAFSQPTSRPGSLAAP
ISPSRAASQTPSVVPSRAAPRASSVVPSREASRVPSLVLSSMDSRVPSLATSKVGSRMPS
LTASRAGSRALSLVQSLEGSGISSGSSPRVSSQDTLRSTKSGPKLLSGLSRDRDPALLPP
QSEDSVNAEAAAEASPGRA
>sp|Q8IXS2|CCD65_HUMAN Coiled-coil domain-containing protein 65 OS=Homo sapiens GN=CCDC65 PE=1 SV=2
MPKKEKMAKTPLSDEKQLLLFQQKLLAEEEMAKKKERLLSQFLKDKLAKEEHNSALNLNK
INTQWRTVLREVKTRELHKDIEILSQTFERVVDCKDNVIKSLAKDLSEAEEQYAHALRSH
LHNVDQLLALQRHRLSLLEESYNMELEALTKEFETERKTIIDQHEKEIHYLQDIFMAMEQ
NYIDSEYESKLEFQSMWNDLKNMNLEEKHFLRLHLENRVEDLWRKFQDVLKNYTDATEDR
KAAFETLQVKDEKSSKEIEVQMKKIQKLQDAITISKGKIMIHSRESEDENRYIRNDKELV
LVQLRKLKAQRTQARAASQKNLVRLTLESNATLKALRKIVDKGEKILKLAEICRKFETEE
EKVLPFYSSVLTPKEQEGIQKNNLEELTEELTKVMVDYIGMENFWKRYNKVKLEQLSLQH
RRAQLLDINGKLREMLKQYLDGISVSDEVLSQLNPLFIVNYQSNLLQPLSIRIAHPGDKQ
HPTT
>sp|Q8IV32|CCD71_HUMAN Coiled-coil domain-containing protein 71 OS=Homo sapiens GN=CCDC71 PE=1 SV=3
MSVVVQHVEEKAVHSWSRISTAGKKALEEALLVFNPMSQDLSATEAQLVAFLQGLRDDGF
QPTILRSGDVYGYSSCTANPPSQTKLQARAPNPTATSPPASAPRTAMRLPAGRATLLPMP
LSGRLAKASTPALAKHATTNLLLSSLKQSSASHARGAAVGFPTHLYPGVYPAMRLSVVLE
ALVPLKTPMPCLGAKHKAQSLQLSLADSPLKLRKSSGKGPGNPRPKAPRKTTSKGPKCLT
RKGPGAGPRRGSGHQSKTNRATGSPSVRRMKGGSALGTKTAQAKVARTLAKAARAQKVA

RTQAKAAKARAKAKAAQVKAKAKAKAAQVKAKAKVMAAWAKAKAKAKAVRAKAKVARTQP
RGRGRPKGSAKARTTRKGQKNRPETVGQKRKRAEEAKDLPPKKRTRLGPRSPKAWLGPGT
AKLLKFRAIKVDRRSSDDEVRQRAQRILRVNLSPVIRLQPLLPYSAV
>sp|Q9BR77|CCD77_HUMAN Coiled-coil domain-containing protein 77 OS=Homo sapiens GN=CCDC77 PE=2 SV=1
MNFTPTHTPVCRKRTVVSKRGVAVSGPTKRRGMADSLESTPLPSPEDRLAKLHPSKELLE
YYQKKMAECEAENEDLLKKLELYKEACEGQHKLECDLQQREEEIAELQKALSDMQVCLFQ
EREHVLRLYSENDRLRIRELEDKKKIQNLLALVGTDAGEVTYFCKEPPHKVTILQKTIQA
VGECEQSESSAFKADPKISKRRPSRERKESSEHYQRDIQTLILQVEALQAQLGEQTKLSR
EQIEGLIEDRRIHLEEIQVQHQRNQNKIKELTKNLHHTQELLYESTKDFLQLRSENQNKE
KSWMLEKDNLMSKIKQYRVQCKKKEDKIGKVLPVMHESHHAQSEYIKSLKDKLVQEKKLS
NMYQEQCISLEEELARIREEEGMRREIFKDRTNKMGKRLQIMTKRYEALERRRILEVEGF
KTDIKVLRQKLKDLEQMLYKATVNARANQDLALLCEVRDSNRRAHKIQGELKNLKSKVFG
LENELRLC
>sp|Q8IWF9|CCD83_HUMAN Coiled-coil domain-containing protein 83 OS=Homo sapiens GN=CCDC83 PE=2 SV=2
MENSGKANKKDTHDGPPKEIKLPTSEALLDYQCQIKEDAVEQFMFQIKTLRKKNQKYHER
NSRLKEEQIWHIRHLLKELSEEKAEGLPVVTREDVEEAMKEKWKFERDQEKNLRDMRMQI
SNAEKLFLEKLSEKEYWEEYKNVGSERHAKLITSLQNDINTVKENAEKMSEHYKITLEDT
RKKIIKETLLQLDQKKEWATQNAVKLIDKGSYLEIWENDWLKKEVAIHRKEVEELKNAIH
ELEAENLVLIDQLSNCRLVDLKIPRRLYLTQAAGLEVPPEEMSLELPETHIEEKSELQPT
EVESRDLMSSSDESTILHLSHENSIEDLQYVKIDKEENSGTEFGDTDMKYLLYEDEKDFK
DYVNLGPLGVKLMSVESKKMPIHFQEKEIPVKLYKDVRSPESHITYKMMKSFL
>sp|Q567U6|CCD93_HUMAN Coiled-coil domain-containing protein 93 OS=Homo sapiens GN=CCDC93 PE=1 SV=2
MGLPRGPEGQGLPEVETREDEEQNVKLTEILELLVAAGYFRARIKGLSPFDKVVGGMTWC
ITTCNFDVDVDLLFQENSTIGQKIALSEKIVSVLPRMKCPHQLEPHQIQGMDFIHIFPVV
QWLVKRAIETKEEMGDYIRSYSVSQFQKTYSLPEDDDFIKRKEKAIKTVVDLSEVYKPRR
KYKRHQGAEELLDEESRIHATLLEYGRRYGFSRQSKMEKAEDKKTALPAGLSATEKADAH
EEDELRAAEEQRIQSLMTKMTAMANEESRLTASSVGQIVGLCSAEIKQIVSEYAEKQSEL
SAEESPEKLGTSQLHRRKVISLNKQIAQKTKHLEELRASHTSLQARYNEAKKTLTELKTY
SEKLDKEQAALEKIESKADPSILQNLRALVAMNENLKSQEQEFKAHCREEMTRLQQEIEN
LKAERAPRGDEKTLSSGEPPGTLTSAMTHDEDLDRRYNMEKEKLYKIRLLQARRNREIAI
LHRKIDEVPSRAELIQYQKRFIELYRQISAVHKETKQFFTLYNTLDDKKVYLEKEISLLN
SIHENFSQAMASPAARDQFLRQMEQIVEGIKQSRMKMEKKKQENKMRRDQLNDQYLELLE
KQRLYFKTVKEFKEEGRKNEMLLSKVKAKAS
>sp|Q2M329|CCD96_HUMAN Coiled-coil domain-containing protein 96 OS=Homo sapiens GN=CCDC96 PE=2 SV=2
MDVSSEHTKDPGGEGGDGESLAARPSKIKASSGPPTSPEPGELESEPEEEEEEQAASQGG
TAADEQAEAPKGLTAAEAAGEEGPGEPGRPAEPQPEPEEPAEVGAEEPAQPEPGAGPEEL
EAEAGAEELEQAAEGKEVRFQASLPLTRIDEEEAAAAPEAETERVEGEEEDKEETQRDGA
ESKERDGEGRPAKSQEEGKRLYGRDEFEDLEWSEEVQKLQEQQLRSDLLDQYRSLLVERN
RSQRYNLYLQHKIFEALRRKKGLEAAEVADRGAEAEAPEKEQAYLRHLGMLEELKKQQAD
DLQWYHQELGQLKRQCQEKLTRVEKEWRRFQALKKQVVMQAMGSCRMRGGRQAALREVEQ

IQALEDKKEKEMSAVRLENIQLKQSLVHFETRMRTQEDLTQGLLLIDFEQLKIENQTFNE
KIEERNEELLKLRSKVTNSVQVITHVKEKLHFMDMENACKKTQLAEIEAQAALGRDILTK
TKQAREGLRTDNIRLNQKCGLLGKDSLLRDLEEKVDKTELLHRRLESLKRHHASLTLSCR
GVRQKIREAKAFLPS
>sp|P62955|CCG7_HUMAN Voltage-dependent calcium channel gamma-7 subunit OS=Homo sapiens GN=CACNG7 PE=2 SV=1
MSHCSSRALTLLSSVFGACGLLLVGIAVSTDYWLYMEEGTVLPQNQTTEVKMALHAGLWR
VCFFAGREKGRCVASEYFLEPEINLVTENTENILKTVRTATPFPMVSLFLVFTAFVISNI
GHIRPQRTILAFVSGIFFILSGLSLVVGLVLYISSINDEVMNRPSSSEQYFHYRYGWSFA
FAASSFLLKEGAGVMSVYLFTKRYAEEEMYRPHPAFYRPRLSDCSDYSGQFLQPEAWRRG
RSPSDISSDVSIQMTQNYPPAIKYPDHLHISTSPC
>sp|Q8WWL7|CCNB3_HUMAN G2/mitotic-specific cyclin-B3 OS=Homo sapiens GN=CCNB3 PE=1 SV=2
MLLPLPPQSSKPVPKKSQSSKIVPSHHDPSEKTGENCQTKISPSSLQESPSSLQGALKKR
SAFEDLTNASQCQPVQPKKEANKEFVKVVSKKINRNTHALGLAKKNKRNLKWHKLEVTPV
VASTTVVPNIMEKPLILDISTTSKTPNTEEASLFRKPLVLKEEPTIEDETLINKSLSLKK
CSNHEEVSLLEKLQPLQEESDSDDAFVIEPMTFKKTHKTEEAAITKKTLSLKKKMCASQR
KQSCQEESLAVQDVNMEEDSFFMESMSFKKKPKTEESIPTHKLSSLKKKCTIYGKICHFR
KPPVLQTTICGAMSSIKKPTTEKETLFQELSVLQEKHTTEHEMSILKKSLALQKTNFKED
SLVKESLAFKKKPSTEEAIMMPVILKEQCMTEGKRSRLKPLVLQEITSGEKSLIMKPLSI
KEKPSTEKESFSQEPSALQKKHTTQEEVSILKEPSSLLKSPTEESPFDEALAFTKKCTIE
EAPPTKKPLILKRKHATQGTMSHLKKPLILQTTSGEKSLIKEPLPFKEEKVSLKKKCTTQ
EMMSICPELLDFQDMIGEDKNSFFMEPMSFRKNPTTEETVLTKTSLSLQEKKITQGKMSH
LKKPLVLQKITSEEESFYKKLLPFKMKSTTEEKFLSQEPSALKEKHTTLQEVSLSKESLA
IQEKATTEEEFSQELFSLHVKHTNKSGSLFQEALVLQEKTDAEEDSLKNLLALQEKSTME
EESLINKLLALKEELSAEAATNIQTQLSLKKKSTSHGKVFFLKKQLALNETINEEEFLNK
QPLALEGYPSIAEGETLFKKLLAMQEEPSIEKEAVLKEPTIDTEAHFKEPLALQEEPSTE
KEAVLKEPSVDTEAHFKETLALQEKPSIEQEALFKRHSALWEKPSTEKETIFKESLDLQE
KPSIKKETLLKKPLALKMSTINEAVLFEDMIALNEKPTTGKELSFKEPLALQESPTYKED
TFLKTLLVPQVGTSPNVSSTAPESITSKSSIATMTSVGKSGTINEAFLFEDMITLNEKPT
TGKELSFKEPLALQESPTCKEDTFLETFLIPQIGTSPYVFSTTPESITEKSSIATMTSVG
KSRTTTESSACESASDKPVSPQAKGTPKEITPREDIDEDSSDPSFNPMYAKEIFSYMKER
EEQFILTDYMNRQIEITSDMRAILVDWLVEVQVSFEMTHETLYLAVKLVDLYLMKAVCKK
DKLQLLGATAFMIAAKFEEHNSPRVDDFVYICDDNYQRSEVLSMEINILNVLKCDINIPI
AYHFLRRYARCIHTNMKTLTLSRYICEMTLQEYHYVQEKASKLAAASLLLALYMKKLGYW
VPFLEHYSGYSISELHPLVRQLNKLLTFSSYDSLKAVYYKYSHPVFFEVAKIPALDMLKL
EEILNCDCEAQGLVL
>sp|P51959|CCNG1_HUMAN Cyclin-G1 OS=Homo sapiens GN=CCNG1 PE=1 SV=2
MIEVLTTTDSQKLLHQLNALLEQESRCQPKVCGLRLIESAHDNGLRMTARLRDFEVKDLL
SLTQFFGFDTETFSLAVNLLDRFLSKMKVQPKHLGCVGLSCFYLAVKSIEEERNVPLATD
LIRISQYRFTVSDLMRMEKIVLEKVCWKVKATTAFQFLQLYYSLLQENLPLERRNSINFE
RLEAQLKACHCRIIFSKAKPSVLALSIIALEIQAQKCVELTEGIECLQKHSKINGRDLTF
WQELVSKCLTEYSSNKCSKPNVQKLKWIVSGRTARQLKHSYYRITHLPTIPEMVP
>sp|Q9C0I3|CCSE1_HUMAN Serine-rich coiled-coil domain-containing protein 1 OS=Homo sapiens GN=CCSER1 PE=2 SV=2

MGDSGSRRSTLVSRLPIFRRSINRRHDSLPSSPSSSNTVGVHSSSPSSTNSSSGSTGKRR
SIFRTPSISFHHKKGSEPKQEPTNQNLSISNGAQPGHSNMQKLSLEEHIKTRGRHSVGFS
SSRNKKITRSLTEDFEREKEHSTNKNVFINCLSSGKSEGDDSGFTEDQTRRSVKQSTRKL
LPKSFSSHYKFSKPVLQSQSISLVQQSEFSLEVTQYQEREPVLVRASPSCSVDVTERAGS
SLQSPLLSADLTTAQTPSEFLALTEDSVSEMDAFSKSGSMASHCDNFGHNDSTSQMSLNS
AAVTKTTTELTGTVPCAIMSPGKYRLEGQCSTESNSLPETSAANQKEVLLQIAELPATSV
SHSESNLPADSEREENIGLQNGETMLGTNSPRKLGFYEQHKAIAEHVKGIHPISDSKIIP
TSGDHHIFNKTSHGYEANPAKVLASSLSPFREGRFIERRLRSSSEGTAGSSRMILKPKDG
NIEEVNSLRKQRAGSSSSKMNSLDVLNNLGSCELDEDDLMLDLEFLEEQSLHPSVCREDS
YHSVVSCAAVVLTPMEPMIEMKKREEPEFPEPSKQNLSLKLTKDVDQEARCSHISRMPNS
PSADWPLQGVEENGGIDSLPFRLMLQDCTAVKTLLLKMKRVLQESADMSPASSTTSLPVS
PLTEEPVPFKDIMKDECSMLKLQLKEKDELISQLQEELGKVRHLQKAFASRVDKSTQTEL
LCYDGLNLKRLETVQGGREATYRNRIVSQNLSTRDRKAIHTPTEDRFRYSAADQTSPYKN
KTCQLPSLCLSNFLKDKELAEVIKHSRGTYETLTSDVTQNLRATVGQSSLKPTAKTEGLS
TFLEKPKDQVATARQHSTFTGRFGQPPRGPISLHMYSRKNVFLHHNLHSTELQTLGQQDG
>sp|Q9H7U1|CCSE2_HUMAN Serine-rich coiled-coil domain-containing protein 2 OS=Homo sapiens GN=CCSER2 PE=1 SV=2
MEEKTQIKTFLGSKLPKYGTKSVRSTLQPMPNGTPVNLLGTSKNSNVKSYIKNNGSDCPS
SHSFNWRKANKYQLCAQGVEEPNNTQNSHDKIIDPEKRVPTQGMFDKNGIKGGLKSVSLF
TSKLAKPSTMFVSSTEELNQKSFSGPSNLGKFTKGTLLGRTSYSSINTPKSQLNGFYGNR
SAGSMQRPRANSCATRSSSGESLAQSPDSSKSINCEKMVRSQSFSHSIQNSFLPPSSITR
SHSFNRAVDLTKPYQNQQLSIRVPLRSSMLTRNSRQPEVLNGNEHLGYGFNRPYAAGGKK
LALPNGPGVTSTLGYRMVHPSLLKSSRSPFSGTMTVDGNKNSPADTCVEEDATVLAKDRA
ANKDQELIENESYRTKNNQTMKHDAKMRYLSDDVDDISLSSLSSSDKNDLSEDFSDDFID
IEDSNRTRITPEEMSLKEEKHENGPPQDMFDSPKENEKAFSKTDEWIDISVSDRSECTKH
TSGNNLVSPDTDYRAGSSFELSPSDSSDGTYMWDEEGLEPIGNVHPVGSYESSEMNSIDI
LNNLESCDLEDDDLMLDVDLPEDAPLENVECDNMNRFDRPDRNVRQPQEGFWKRPPQRWS
GQEHYHLSHPDHYHHHGKSDLSRGSPYRESPLGHFESYGGMPFFQAQKMFVDVPENTVIL
DEMTLRHMVQDCTAVKTQLLKLKRLLHQHDGSGSLHDIQLSLPSSPEPEDGDKVYKNEDL
LNEIKQLKDEIKKKDEKIQLLELQLATQHICHQKCKEEKCTYADKYTQTPWRRIPGGYSA
PSFSPWQGSFQGIPRTVPPHRRQTSSTTAFQQPSQTHRSHPGKTNKATTYRGPQ
>sp|Q5T2Q4|CCYL2_HUMAN Cyclin-Y-like protein 2 OS=Homo sapiens GN=CCNYL2 PE=3 SV=2
MGNILTCCVCPRASPGLHQHQGLVCHCESEIYEAAAGDLIAGVPVAAAVEPGEVTFVAGE
GLHMHHICEREMPEDIPLEPNPSDHPKASTIFLRKSQTDVQEKKKKQLCKVSTEHFTQQY
SSCSTIFLDDSIASQPHLTMTLKSCDLGTILSYQAKRDAHRSLGIFDEQLHPLTVRKEVL
EEYFKYDPEHKLIFRFVRTLFKAMRLTAEFAIVSLIYIERLVSYADIDICPTNWKRIVLG
AILLASKVWSDMAVWNEDYCKLFKNITVEEMNELERQFLKLINYNNSITNSVYSRFYFDL
RTLAHNNGLYSPVYLLDRERAWKLEAFSRMEQDKVFYSAAKNGSLSADDLIHLQRAKAIL
F
>sp|Q99440|CD006_HUMAN Uncharacterized protein encoded by LINC01587 OS=Homo sapiens GN=LINC01587 PE=2 SV=1
MDTQKQIHKTHNSKNQFFTIFFFLSVEFGKEGTRKNFYLLLSIGHYGRKSRRADLGTADT
ADKTEPECFAASWTFDPNPSVTVSGAHSTAVHQ

>sp|Q8N8J7|CD032_HUMAN Uncharacterized protein C4orf32 OS=Homo sapiens GN=C4orf32 PE=2 SV=2
MCSAGELLRGGDGGERDEDGDALAEREAAGTGWDPGASPRRRGQRPKESEQDVEDSQNHT
GEPVGDDYKKMGTLFGELNKNLINMGFTRMYFGERIVEPVIVIFFWVMLWFLGLQALGLV
AVLCLVIIYVQQ
>sp|Q96KX1|CD036_HUMAN Uncharacterized protein C4orf36 OS=Homo sapiens GN=C4orf36 PE=4 SV=2
MAYGVPRKNTVKTILRGSCYNVQEPWDIALLAKTWSTNLANIKLPFLEEISFGGSVQLTK
CTTIKDGLLPSAESIKLEREYEVKRLCKLKCQENTSKEIQLLLRERPAGLRRPLPSK
>sp|Q504U0|CD046_HUMAN Renal cancer differentiation gene 1 protein OS=Homo sapiens GN=C4orf46 PE=1 SV=1
MADPEELQVSSPPPPPPSSPSSSDASAASSPGGPVSLGWPVPSRSSGPTVDQLEEVELQI
GDAAFSLTKLLEATSAVSAQVEELAFKCTENARFLKTWRDLLKEGYDSLKPDD
>sp|O95400|CD2B2_HUMAN CD2 antigen cytoplasmic tail-binding protein 2 OS=Homo sapiens GN=CD2BP2 PE=1 SV=1
MPKRKVTFQGVGDEEDEDEIIVPKKKLVDPVAGSGGPGSRFKGKHSLDSDEEEDDDDGGS
SKYDILASEDVEGQEAATLPSEGGVRITPFNLQEEMEEGHFDADGNYFLNRDAQIRDSWL
DNIDWVKIRERPPGQRQASDSEEEDSLGQTSMSAQALLEGLLELLLPRETVAGALRRLGA
RGGGKGRKGPGQPSSPQRLDRLSGLADQMVARGNLGVYQETRERLAMRLKGLGCQTLGPH
NPTPPPSLDMFAEELAEEELETPTPTQRGEAESRGDGLVDVMWEYKWENTGDAELYGPFT
SAQMQTWVSEGYFPDGVYCRKLDPPGGQFYNSKRIDFDLYT
>sp|Q9NPF0|CD320_HUMAN CD320 antigen OS=Homo sapiens GN=CD320 PE=1 SV=1
MSGGWMAQVGAWRTGALGLALLLLLGLGLGLEAAASPLSTPTSAQAAGPSSGSCPPTKFQ
CRTSGLCVPLTWRCDRDLDCSDGSDEEECRIEPCTQKGQCPPPPGLPCPCTGVSDCSGGT
DKKLRNCSRLACLAGELRCTLSDDCIPLTWRCDGHPDCPDSSDELGCGTNEILPEGDATT
MGPPVTLESVTSLRNATTMGPPVTLESVPSVGNATSSSAGDQSGSPTAYGVIAAAAVLSA
SLVTATLLLLSWLRAQERLRPLGLLVAMKESLLLSEQKTSLP
>sp|P20138|CD33_HUMAN Myeloid cell surface antigen CD33 OS=Homo sapiens GN=CD33 PE=1 SV=2
MPLLLLLPLLWAGALAMDPNFWLQVQESVTVQEGLCVLVPCTFFHPIPYYDKNSPVHGYW
FREGAIISRDSPVATNKLDQEVQEETQGRFRLLGDPSRNNCSLSIVDARRRDNGSYFFRM
ERGSTKYSYKSPQLSVHVTDLTHRPKILIPGTLEPGHSKNLTCSVSWACEQGTPPIFSWL
SAAPTSLGPRTTHSSVLIITPRPQDHGTNLTCQVKFAGAGVTTERTIQLNVTYVPQNPTT
GIFPGDGSGKQETRAGVVHGAIGGAGVTALLALCLCLIFFIVKTHRRKAARTAVGRNDTH
PTTGSASPKHQKKSKLHGPTETSSCSGAAPTVEMDEELHYASLNFHGMNPSKDTSTEYSE
VRTQ
>sp|Q08722|CD47_HUMAN Leukocyte surface antigen CD47 OS=Homo sapiens GN=CD47 PE=1 SV=1
MWPLVAALLLGSACCGSAQLLFNKTKSVEFTFCNDTVVIPCFVTNMEAQNTTEVYVKWKF
KGRDIYTFDGALNKSTVPTDFSSAKIEVSQLLKGDASLKMDKSDAVSHTGNYTCEVTELT
REGETIIELKYRVVSWFSPNENILIVIFPIFAILLFWGQFGIKTLKYRSGGMDEKTIALL
VAGLVITVIVIVGAILFVPGEYSLKNATGLGLIVTSTGILILLHYYVFSTAIGLTSFVIA
ILVIQVIAYILAVVGLSLCIAACIPMHGPLLISGLSILALAQLLGLVYMKFVASNQKTIQ
PPRKAVEEPLNAFKESKGMMNDE
>sp|P19397|CD53_HUMAN Leukocyte surface antigen CD53 OS=Homo sapiens GN=CD53 PE=1 SV=1
MGMSSLKLLKYVLFFFNLLFWICGCCILGFGIYLLIHNNFGVLFHNLPSLTLGNVFVIVG

SIIMVVAFLGCMGSIKENKCLLMSFFILLLIILLAEVTLAILLFVYEQKLNEYVAKGLTD
SIHRYHSDNSTKAAWDSIQSFLQCCGINGTSDWTSGPPASCPSDRKVEGCYAKARLWFHS
NFLYIGIITICVCVIEVLGMSFALTLNCQIDKTSQTIGL
>sp|P13987|CD59_HUMAN CD59 glycoprotein OS=Homo sapiens GN=CD59 PE=1 SV=1
MGIQGGSVLFGLLLVLAVFCHSGHSLQCYNCPNPTADCKTAVNCSSDFDACLITKAGLQV
YNKCWKFEHCNFNDVTTRLRENELTYYCCKKDLCNFNEQLENGGTSLSEKTVLLLVTPFL
AAAWSLHP
>sp|Q15078|CD5R1_HUMAN Cyclin-dependent kinase 5 activator 1 OS=Homo sapiens GN=CDK5R1 PE=1 SV=1
MGTVLSLSPSYRKATLFEDGAATVGHYTAVQNSKNAKDKNLKRHSIISVLPWKRIVAVSA
KKKNSKKVQPNSSYQNNITHLNNENLKKSLSCANLSTFAQPPPAQPPAPPASQLSGSQTG
GSSSVKKAPHPAVTSAGTPKRVIVQASTSELLRCLGEFLCRRCYRLKHLSPTDPVLWLRS
VDRSLLLQGWQDQGFITPANVVFLYMLCRDVISSEVGSDHELQAVLLTCLYLSYSYMGNE
ISYPLKPFLVESCKEAFWDRCLSVINLMSSKMLQINADPHYFTQVFSDLKNESGQEDKKR
LLLGLDR
>sp|P08962|CD63_HUMAN CD63 antigen OS=Homo sapiens GN=CD63 PE=1 SV=2
MAVEGGMKCVKFLLYVLLLAFCACAVGLIAVGVGAQLVLSQTIIQGATPGSLLPVVIIAV
GVFLFLVAFVGCCGACKENYCLMITFAIFLSLIMLVEVAAAIAGYVFRDKVMSEFNNNFR
QQMENYPKNNHTASILDRMQADFKCCGAANYTDWEKIPSMSKNRVPDSCCINVTVGCGIN
FNEKAIHKEGCVEKIGGWLRKNVLVVAAAALGIAFVEVLGIVFACCLVKSIRSGYEVM
>sp|P27701|CD82_HUMAN CD82 antigen OS=Homo sapiens GN=CD82 PE=1 SV=1
MGSACIKVTKYFLFLFNLIFFILGAVILGFGVWILADKSSFISVLQTSSSSLRMGAYVFI
GVGAVTMLMGFLGCIGAVNEVRCLLGLYFAFLLLILIAQVTAGALFYFNMGKLKQEMGGI
VTELIRDYNSSREDSLQDAWDYVQAQVKCCGWVSFYNWTDNAELMNRPEVTYPCSCEVKG
EEDNSLSVRKGFCEAPGNRTQSGNHPEDWPVYQEGCMEKVQAWLQENLGIILGVGVGVAI
IELLGMVLSICLCRHVHSEDYSKVPKY
>sp|Q96BT1|CC049_HUMAN Putative uncharacterized protein C3orf49 OS=Homo sapiens GN=C3orf49 PE=2 SV=1
MAQPQLYLPEPFKIAYRKVGGQCRRFQQLKKKNGSFKRKGIERWHRAVSTNLLKQNVLVPK
EESSSDSDMGFHESQQNQKSNLKTKVKTAFGRMLSYKYRSKPACASQEGSTDHKEALLSN
TQSLLPRIVKEFSSPKLFTAKMRKLSENATIQLDVVEAETEEITQGNTLLRARRTTKRLS
VTSLPSGLQKGPYSPKKRPHFPALKKKKRGMENILRKSDLTVGKLQMQVDDLIETVTDKS
MKLLAQRHAELQQCEFLGDEILQSSKQFQRISKRTMRKYKLKNMTTKGPGDS
>sp|Q8IYX3|CC116_HUMAN Coiled-coil domain-containing protein 116 OS=Homo sapiens GN=CCDC116 PE=1 SV=2
MARCRHHSGYLADDEASHSMCSARVQLPKKPLVPEMRPACKPGRVPHPPSTCGSSALQGQ
RRNKRHPQPFGHFLDFLTESQVLDSLETVVEKATERMAAMKTEAGVPLVEVQDPVEVPSG
GRRAHARPSLSTVHRHRVRPTLCTGHPNNYPSSSSSMSNCHSSLMAGCLGSHSRDSDLGA
QGSLPPVRDKLLLEKNLKRLLQLEREGKGLSQSCSQRDSLLWDSLGSQTSFQWTQEQPLS
WFSGLLGSSSGVPEASEPRPGEQEPIFRKREFNKEIKSLLSQLESLDLPGYCPLREPHRT
LNFLADHRLFPALQSVVSQAVDKLRGAHCRDGRPLFPTSLEPTSDLPPLGSEPAKPTNGG
QPYASPRPTVSSPKMLQRKRKDRGGSPSMSSAQVATRFKLKSPCSSSRFTKKKPLPSISS
KSSMSHFSNRLYEELADFLTQQAASLVIRKYEFEKDLSKQLGFFSFPITHVLRDLSLGLK
KVKGSRIHLSSETHRSCLLRKLEESKRAAAVSLEA

>sp|Q96BQ5|CC127_HUMAN Coiled-coil domain-containing protein 127 OS=Homo sapiens GN=CCDC127 PE=1 SV=1
MNNLNDPPNWNIRPNSRADGGDGSRWNYALLVPMLGLAAFRWIWSRESQKEVEKEREAYR
RRTAAFQQDLEAKYHAMISENRRAVAQLSLELEKEQNRTASYREALISQGRKLVEEKKLL
EQERAQVMQEKRQVQPLRSAYLSCLQREENWQRRARLLLKEFEAVLTERQNIYCSLFLPR
SKRLEIEKSLLVRASVDPVAADLEMAAGLTDIFQHDTYCGDVWNTNKRQNGRLMWLYLKY
WELVVELKKFKRVEEAILEK
>sp|Q6ZRS4|CC129_HUMAN Coiled-coil domain-containing protein 129 OS=Homo sapiens GN=CCDC129 PE=2 SV=2
MMAQKSQGSDNLQEGQEKSKREILKCTKSAWAPLDEWLPPDPEEESQSLTIPMLEDSKQE
SIQQWLDSGFFVSANENFQQVIDRTVSLYEQGMVQMTVKDYMRSLHQFSETPILSRGTSF
NSCYSTASVPQSIPEWLEFWEIDPVEILLDLGFGADEPDICMQIPARFLGCGSAARGINI
RVFLEAQKQRMDIENPNLYGRFRQLEILDHVTNAFSSLLSDVSILPNRAEEKAGGESVQR
TSVSAAKEHRRRMGKLLRRASKQNIRRDCNPEVSESFKVKDEVFVPFTKPWDCGAELAAT
SINHKQNHLSLSVEHQSLQACDDLLPYPPHGLLSKQWPCSSMPAKQAPPSCVSEGSVKGR
TQKENLFQTNKLKSLSHLAGKGPDSFEMEEVQSFEEETGNPLDMTSGTVGARVDRANSCQ
SDSSGFLEEPLEPLPLQMPSLPNSQSPAENGGRKPRDQSHSLVSSQDCQLESDGPDSKSR
ASMSFSSQEANALEQRASVSVMEEEFLLEAMEGPPELYIPDMACAKTTTRGECPRKDSHL
WQLLPMPHAEYEVTRPTATSKYDHPLGFMVTHVTEMQDSFVRPEGAGKVQSHHNESQRSP
GNDHTQDKFLHVDSEAPREEESSGFCPHTNHSLLVPESSSQCIPKHSEITPYATDLAQTS
EKLIPHLHKLPGDPAQVKSRSGTLGQILPGTEAEMENLPLNTGSSRSVMTQMSSSLVSAA
QRAVALGTGPRGTSLECTVCDPVTATETRLGTKARQLNDASIQTSALSNKTLTHGPQPLT
KSVSLDSGFSSICPMGTCHAIPAHCCICCHHHPHCHGERQSPGPEPSVCRHCLCSLTGHQ
EAQFMTTLKALQDTTVRELCSCTVHEMEAMKTICQSFREYLEEIEQHLMGQQALFSRDMS
EEEREEAEQLQTLREALRQQVAELEFQLGDRAQQIREGILLQLEVLTAEPPEHYSNLHQY
NWIEESNGQTSCSKIHPGMAPRTVFPPDDGQEAPCSGGTQLAAFTPPTLENSTRMSPSSS
AWAKLGPTPLSNCPVGEKDADVFL
>sp|Q96JN2|CC136_HUMAN Coiled-coil domain-containing protein 136 OS=Homo sapiens GN=CCDC136 PE=1 SV=3
MQAMEGEVLLPALYEEEEEEEEEEEEVEEEEEQVQKGGSVGSLSVNKHRGLSLTETELEE
LRAQVLQLVAELEETRELAGQHEDDSLELQGLLEDERLASAQQAEVFTKQIQQLQGELRS
LREEISLLEHEKESELKEIEQELHLAQAEIQSLRQAAEDSATEHESDIASLQEDLCRMQN
ELEDMERIRGDYEMEIASLRAEMEMKSSEPSGSLGLSDYSGLQEELQELRERYHFLNEEY
RALQESNSSLTGQLADLESERTQRATERWLQSQTLSMTSAESQTSEMDFLEPDPEMQLLR
QQLRDAEEQMHGMKNKCQELCCELEELQHHRQVSEEEQRRLQRELKCAQNEVLRFQTSHS
VTQNEELKSRLCTLQKKYDTSQDEQNELLKMQLQLQTELRQLKVMKSTLVENQSEKELLC
RLQKLHLQHQNVTCEKEKLLERQQQLQEELQCHEAELQHLRDTVASFKESNEKDTETHAQ
LQEMKQLYQASKDELERQKHMYDQLEQDLLLCQLELKELKASHPIPEDKGKCANKCDTLL
SRLTELQEKYKASQKEMGQLQMEQCELLEDQRRMQEEQGQLQEELHRLTLPLPKSGLLLK
SQELLTKLEDLCELQLLYQGMQEEQKKLIQNQDCVLKEQLEIHEELRRFKESHFQEVLEN
PDDSKLAKSSKCNRNKQSKLLMEQMQALQVMYDAGQAKQELLQQEQGRLLEERKRLQADL
QLCLEEMQLLQVQSPSIKMSLESYGKSYGSMVPSNENCRKTYDTTVDDNESYYKSYTSTQ
TSSKSFLKSYDSSTSASEAYGKSYCTTSNSSITYKKSYGSTSSSDTCQKSFVSSCTDEEP
AEPEDMERFEEMVVKVLIKLQAVQAMYQISQEEHSQLQEQMEKLLAKQKDLKEELDACER

EFKECMECLEKPMAPQNDKNEIKELQTKLRELQLQYQASMDEQGRLLVVQEQLEGQLQCC
QEELRQLREKRPSVVKEARGKNANKNMNKNANGVKMKKVTKPCSDTSESDLETRKSLEVV
LYYKASQRKLDGLAKEEEKKEEMEEEKKQVKEEAKEQCGDELVAEPADPEEAKSTEDQEE
NEEDKEEEEKEEDSEEEEDDADSSLESPEENNPLRLSESKKSSPTPNPPIFSLPLVGLVV
ISALLWCWWAETSS
>sp|Q96M89|CC138_HUMAN Coiled-coil domain-containing protein 138 OS=Homo sapiens GN=CCDC138 PE=1 SV=1
MEPRVVKPPGQDLVVESLKSRYGLGGSCPDEYDFSNFYQSKYKRRTLTSPGDLDIYSGDK
VGSSLKYSDESKHCRTPLGSLFKHVNVNCLDDELDSFHDLKKQETEEELIENDYRVSTSK
ITKQSFKEIEKVALPTNTTSSRPRTECCSDAGDSPLKPVSCPKSKASDKRSLLPHQISQI
YDELFQIHLKLQCETAAQQKFAEELQKRERFLLEREQLLFRHENALSKIKGVEEEVLTRF
QIIKEQHDAEVEHLTEVLKEKNKETKRLRSSFDALKELNDTLKKQLNEASEENRKIDIQA
KRVQARLDNLQRKYEFMTIQRLKGSSHAVHEMKSLKQEKAPVSKTYKVPLNGQVYELLTV
FMDWISDHHLSKVKHEESGMDGKKPQLKFASQRNDIQEKCVKLLPLMTEQLQWMPFVNIK
LHEPFVKFIYWSLRQLDAGAQHSTMTSTLRRLGEDIFKGVVTKGIQDNSPQHSVENKPKT
AAFFKSSNLPLRFLSTLIVLKTVTQADYLAQAFDSLCLDLKTEEGKTLFLEYQAVPVILS
HLRISSKGLLSNVIDSLLQMTVESKSLQPFLEACSNSLFFRTCSVLLRAPKLDLQILEKL
SIILQKLSKIKSNKKLFELFTIHLMLQEIQRTTNPEHAFLCINLNSTLFNLGLTKCNSLV
SSASP
>sp|Q6ZP82|CC141_HUMAN Coiled-coil domain-containing protein 141 OS=Homo sapiens GN=CCDC141 PE=1 SV=2
MSSQGSPSVALSTTTVSSVAVQAGDSKIVIAVIKCGKWVQLQLAESQPNLLEIGSSQDET
KKLLHDHELLLAKLKALEDRVWELLQEADKTAEENKDQSQVYDAMAETLGEAWAALVSML
ERRTELLRLTSEFFENALEFAIKIDQAEDFLQNTHEFESAESLKSLLQLHEHHTKELLER
SLALLNKSQQLTDFIEKFKCEGPNVNPELTQGAHSSCLKVDRLLELLQDRRRQLDKYLKQ
QWQELSQVLQICQWDQQENQVTCWFQKTIRNLQEQSLGSSLSDNEDRIHKQEELIIKAKE
WNSAVEKLKSEALRILLSKDYVEKEHLQLSHQKLSQLQEEFGQLMVERNTWLKKANEFFN
SANKAFDVLGRVEAYLKLLKSEGLSLAVLAVRHEELHRKIKDCTTDALQKGQTLISQVDS
CSSQVSGIHEMMGCIKRRVDHLTEQCSAHKEYALKKQQLTASVEGYLRKVEMSIQKISPV
LSNAMDVGSTRSESEKILNKYLELDIQAKETSHELEAAAKTMMEKNEFVSDEMVSLSSKA
RWLAEELNLFGQSIDYRSQVLQTYVAFLKSSEEVEMQFQSLKEFYETEIPQKEQDDAKAK
HCSDSAEKQWQLFLKKSFITQDLGLEFLNLINMAKENEILDVKNEVYLMKNTMENQKAER
EELSLLRLAWQLKATESKPGKQQWAAFKEQLKKTSHNLKLLQEALMPVSALDLGGSLQFI
LDLRQKWNDMKPQFQQLNDEVQYIMKESEELTGRGAPVKEKSQQLKDLIHFHQKQKERIQ
DYEDILYKVVQFHQVKEELGRLIKSRELEFVEQPKELGDAHDVQIHLRCSQEKQARVDHL
HRLALSLGVDIISSVQRPHCSNVSAKNLQQQLELLEEDSMKWRAKAEEYGRTLSRSVEYC
AMRDEINELKDSFKDIKKKFNNLKFNYTKKNEKSRNLKALKYQIQQVDMYAEKMQALKRK
MEKVSNKTSDSFLNYPSDKVNVLLEVMKDLQKHVDDFDKVVTDYKKNLDLTEHFQEVIEE
CHFWYEDASATVVRVGKYSTECKTKEAVKILHQQFNKFIAPSVPQQEERIQEATDLAQHL
YGLEEGQKYIEKIVTKHKEVLESVTELCESLTELEEKLKQGDVLKMNPNLEDFHYDYIDL
LKEPAKNKQTIFNEERNKGQVQVADLLGINGTGEERLPQDLKVSTDKEGGVQDLLLPEDM
LSGEEYECVSPDDISLPPLPGSPESPLAPSDMEVEEPVSSSLSLHISSYGVQAGTSSPGD
AQESVLPPPVAFADACNDKRETFSSHFERPYLQFKAEPPLTSRGFVEKSTALHRISAEHP
ESMMSEVHERALQQHPQAQGGLLETREKMHADNNFTKTQDRLHASSDAFSGLRFQSGTSR

GYQRQMVPREEIKSTSAKSSVVSLADQAPNFSRLLSNVTVMEGSPVTLEVEVTGFPEPTL
TWWVAYNDKP
>sp|A6NI56|CC154_HUMAN Coiled-coil domain-containing protein 154 OS=Homo sapiens GN=CCDC154 PE=2 SV=4
MSELADSGPSGASAPSQLRAVTLEDLGLLLAGGLASPEPLSLEELSERYESSHPTSTASV
PEQDTAKHWNQLEQWVVELQAEVACLREHKQRCERATRSLLRELLQVRARVQLQGSELRQ
LQQEARPAAQAPEKEAPEFSGLQNQMQALDKRLVEVREALTRLRRRQVQQEAERRGAEQE
AGLRLAKLTDLLQQEEQGREVACGALQKNQEDSSRRVDLEVARMQAQVTKLGEEVSLRFL
KREAKLCGFLQKSFLALEKRMKASESSRLKLEGSLRGELESRWEKLRGLMEERLRALQGQ
HEVGPGGRRAEESHLLEQCQGLDAAVAQLTKFVQQNQASLNRVLLAEEKAWDAKGRLEES
RAGELAAYVQENLEAAQLAGELARQEMHGELVLLREKSRALEASVAQLAGQLKELSGHLP
ALSSRLDLQEQMLGLRLSEAKTEWEGAERKSLEDLARWRKEVTEHLRGVREKVDGLPQQI
ESVSDKCLLHKSDSDLRISAEGKAREFKVGALRQELATLLSSVQLLKEDNPGRKIAEMQG
KLATNQIMKLENCVQANKTIQNLRFNTEARLRTQEMATLWESVLRLWSEEGPRTPLGSWK
ALPSLVRPRVFIKDMAPGKVVPMNCWGVYQAVRWLRWKASLIKLRALRRPGGVLEKPHSQ
EQVQQLTPSLFIQK
>sp|P0C7W6|CC172_HUMAN Coiled-coil domain-containing protein 172 OS=Homo sapiens GN=CCDC172 PE=1 SV=1
MSLESLFQHIIFTEHQAEESRRLMREVRSEITRCREKIKKATEELNEEKIKLESKVQQFF
EKSFFLQLLKAHENALEKQYSEITNHRNMLLQTFEAIKKQMIEEEDKFIKEITDFNNDYE
ITKKRELLMKENVKIEISDLENQANMLKSEMKSMEHDSSQLNELQKQKSELIQELFTLQR
KLKVFEDEENESICTTKYLEAEKIKISEKPQNDTECLRLKKELELYKEDDMESVYEALQT
EIEFLELTLAQKDLQESK
>sp|Q52MB2|CC184_HUMAN Coiled-coil domain-containing protein 184 OS=Homo sapiens GN=CCDC184 PE=1 SV=2
MEDGLLEIMTKDGGDMPAPLEVSTVPAVGDVISGEYNGGMKELMEHLKAQLQALFEDVRA
MRGALDEQASHIQVLSDDVCANQRAIVSMCQIMTTAPRQGGLGVVGGKGSFQSDPQEPET
PSPGIGDSGLLGRDPEDEEEEEEEKEMPSPATPSSHCERPESPCAGLLGGDGPLVEPLDM
PDITLLQLEGEASL
>sp|A0A096LP49|CC187_HUMAN Coiled-coil domain-containing protein 187 OS=Homo sapiens GN=CCDC187 PE=2 SV=1
MPTLVVGTPPTCLGDTPQPCHKNSQRQGPFSHGAPGRAADWKAVAKPRLCAPAAEDDVAA
LRWPGPSQQPDPPWAAPHVVGSDDLKEPGPWGKACSLPMWSTGPEARDGDSSVSSGRLSC
SSGGHDVCVSWKERPPQVLGPQQRPRKSDARLEQLRDKIRAQAWQQGSCASLGTSAPSSA
SRLHKASTLMLRRKGQEAKNPPPAPECSGFSILSAAERRVEAKASHGQGRELSRVSQHQV
PVLREKPKRVKSSSCKREKTPKLPSPRRAAKDKHKDEDSELVGVYAWRKGQALVRSLLGP
PPVLRRHHSKDPSRDPALTVDLGDSEKVIAAKCSPVCAQLPDATSAYSDQQVSGNTPSLA
SFDQPATIQTAMAILQDLRQQIQAGLELAQARKGGQELGPSKRRLQDVAGRGCCRDPNAQ
SSFSKSPWAMTERKHSSLERARSVHTWEPWSSSTARESCPQRAWGAQGQDRSFQRPESPH
ERLGHFSQRPWSALAGQACSPQRAWGAQRQGPSSQRPGSPPEKRSPFPQQPWSAVATQPC
PRRAWTACETWEDPGPRLRNPLERPSPPAQRPWSSSGVQRAGPQGKGRGIGSPVSAAKHA
LPRPTGSFPQNPLGKEKDTLRPCPRSRGLLGPSHSSESLREFMRQKAQARRRQALEEKAS
ALRTRELRSRRLQEVYRQQREAVLGRAVPVVSRTTPGIVTFVPSSAQSGGLEASGSLESP
VLEWSKVTSGMVLGGQEAPGSFCLCLNRAWNHAETLDPPGMGGPQDGRDAPVLLSASPSL

GSLELQDLTTRYLPRGMCIYLDPKEAEHLGTSSSLHLRHKQAQLQALETTAKVLKQRVDS
LTAKLQGAEALDTVRDPAVGLLRSCPHSLPAAPTLATPTLATPACPGALGPNWGRGAPGE
WVSMQPQPLLPPTYFLDGETLSWGPSWEQQQSVSPRAHCESKPRGFPEEGHVDVKPDKRL
QRGVAPFQALSPSAGSSYAGPATLHPIWGSLGLEETPSVGGADSVAPCTPRSCGKGDPAD
RPWAGWSGGRGIHREHLPLPSTRAWPYGVEKSFSTWRASTPRY
>sp|Q96PX6|CC85A_HUMAN Coiled-coil domain-containing protein 85A OS=Homo sapiens GN=CCDC85A PE=2 SV=3
MSKAAGGAAAAAAAAAESCSPAPAGSSAAPPAPVEDLSKVSDEELLQWSKEELIRSLRRAE
AEKVSAMLDHSNLIREVNRRLQLHLGEIRGLKDINQKLQEDNQELRDLCCFLDDDRQKGK
RVSREWQRLGRYTAGVMHKEVALYLQKLKDLEVKQEEVVKENMELKELCVLLDEEKGAGC
AGSRCSIDSQASLCQLTASTAPYVRDVGDGSSTSSTGSTDSPDHHKHHASSGSPEHLQKP
RSEGSPEHSKHRSASPEHPQKPRACGTPDRPKALKGPSPEHHKPLCKGSPEQQRHPHPGS
SPETLPKHVLSGSPEHFQKHRSGSSPEHARHSGGSPEHLQKHALGGSLEHLPRARGTSPE
HLKQHYGGSPDHKHGGGSGGSGGSGGGSREGTLRRQAQEDGSPHHRNVYSGMNESTLSYV
RQLEARVRQLEEENRMLPQASQNRRQPPTRNSSNMEKGWGSRARRVLQWWQGCRGIGRCL
PTLPGSFRLSSGADGSNSSPNSAASFSGHATPSQQPEPVVHSLKVVWRKLGDAAGSCPGI
RQHLSGNQYKGPM
>sp|A6NKD9|CC85C_HUMAN Coiled-coil domain-containing protein 85C OS=Homo sapiens GN=CCDC85C PE=1 SV=1
MAKPAATAAAASEELSQVPDEELLRWSKEELARRLRRAEGEKVGLMLEHGGLMRDVNRRL
QQHLLEIRGLKDVNQRLQDDNQELRELCCFLDDDRQKGRKLAREWQRFGRHAAGAVWHEV
ARSQQKLRELEARQEALLRENLELKELVLLLDEERAALAATGAASGGGGGGGGAGSRSSI
DSQASLSGPLSGGAPGAGARDVGDGSSTSSAGSGGSPDHHHHVPPPLLPPGPHKAPDGKA
GATRRSLDDLSAPPHHRSIPNGLHDPSSTYIRQLESKVRLLEGDKLLAQQAGSGEFRTLR
KGFSPYHSESQLASLPPSYQDSLQNGPACPAPELPSPPSAGYSPAGQKPEAVVHAMKVLE
VHENLDRQLQDSCEEDLSEKEKAIVREMCNVVWRKLGDAASSKPSIRQHLSGNQFKGPL
>sp|Q8IX12|CCAR1_HUMAN Cell division cycle and apoptosis regulator protein 1 OS=Homo sapiens GN=CCAR1 PE=1 SV=2
MAQFGGQKNPPWATQFTATAVSQPAALGVQQPSLLGASPTIYTQQTALAAAGLTTQTPAN
YQLTQTAALQQQAAAAAAALQQQYSQPQQALYSVQQQLQQPQQTLLTQPAVALPTSLSLS
TPQPTAQITVSYPTPRSSQQQTQPQKQRVFTGVVTKLHDTFGFVDEDVFFQLSAVKGKTP
QVGDRVLVEATYNPNMPFKWNAQRIQTLPNQNQSQTQPLLKTPPAVLQPIAPQTTFGVQT
QPQPQSLLQAQISAASITPLLQTQPQPLLQQPQQKAGLLQPPVRIVSQPQPARRLDPPSR
FSGRNDRGDQVPNRKDDRSRERERERRRSRERSPQRKRSRERSPRRERERSPRRVRRVVP
RYTVQFSKFSLDCPSCDMMELRRRYQNLYIPSDFFDAQFTWVDAFPLSRPFQLGNYCNFY
VMHREVESLEKNMAILDPPDADHLYSAKVMLMASPSMEDLYHKSCALAEDPQELRDGFQH
PARLVKFLVGMKGKDEAMAIGGHWSPSLDGPDPEKDPSVLIKTAIRCCKALTGIDLSVCT
QWYRFAEIRYHRPEETHKGRTVPAHVETVVLFFPDVWHCLPTRSEWETLSRGYKQQLVEK
LQGERKEADGEQDEEEKDDGEAKEISTPTHWSKLDPKTMKVNDLRKELESRALSSKGLKS
QLIARLTKQLKVEEQKEEQKELEKSEKEEDEDDDRKSEDDKEEEERKRQEEIERQRRERR
YILPDEPAIIVHPNWAAKSGKFDCSIMSLSVLLDYRLEDNKEHSFEVSLFAELFNEMLQR
DFGVRIYKSLLSLPEKEDKKEKDKKSKKDERKDKKEERDDETDEPKPKRRKSGDDKDKKE
DRDERKKEDKRKDDSKDDDETEEDNNQDEYDPMEAEEAEDEEDDRDEEEMTKRDDKRDIN
RYCKERPSKDKEKEKTQMITINRDLLMAFVYFDQSHCGYLLEKDLEEILYTLGLHLSRAQ

VKKLLNKVVLRESCFYRKLTDTSKDEENHEESESLQEDMLGNRLLLPTPTVKQESKDVEE
NVGLIVYNGAMVDVGSLLQKLEKSEKVRAEVEQKLQLLEEKTDEDEKTILNLENSNKSLS
GELREVKKDLSQLQENLKISENMNLQFENQMNKTIRNLSTVMDEIHTVLKKDNVKNEDKD
QKSKENGASV
>sp|Q8N163|CCAR2_HUMAN Cell cycle and apoptosis regulator protein 2 OS=Homo sapiens GN=CCAR2 PE=1 SV=2
MSQFKRQRINPLPGGRNFSGTASTSLLGPPPGLLTPPVATELSQNARHLQGGEKQRVFTG
IVTSLHDYFGVVDEEVFFQLSVVKGRLPQLGEKVLVKAAYNPGQAVPWNAVKVQTLSNQP
LLKSPAPPLLHVAALGQKQGILGAQPQLIFQPHRIPPLFPQKPLSLFQTSHTLHLSHLNR
FPARGPHGRLDQGRSDDYDSKKRKQRAGGEPWGAKKPRHDLPPYRVHLTPYTVDSPICDF
LELQRRYRSLLVPSDFLSVHLSWLSAFPLSQPFSLHHPSRIQVSSEKEAAPDAGAEPITA
DSDPAYSSKVLLLSSPGLEELYRCCMLFVDDMAEPRETPEHPLKQIKFLLGRKEEEAVLV
GGEWSPSLDGLDPQADPQVLVRTAIRCAQAQTGIDLSGCTKWWRFAEFQYLQPGPPRRLQ
TVVVYLPDVWTIMPTLEEWEALCQQKAAEAAPPTQEAQGETEPTEQAPDALEQAADTSRR
NAETPEATTQQETDTDLPEAPPPPLEPAVIARPGCVNLSLHGIVEDRRPKERISFEVMVL
AELFLEMLQRDFGYRVYKMLLSLPEKVVSPPEPEKEEAAKEEATKEEEAIKEEVVKEPKD
EAQNEGPATESEAPLKEDGLLPKPLSSGGEEEEKPRGEASEDLCEMALDPELLLLRDDGE
EEFAGAKLEDSEVRSVASNQSEMEFSSLQDMPKELDPSAVLPLDCLLAFVFFDANWCGYL
HRRDLERILLTLGIRLSAEQAKQLVSRVVTQNICQYRSLQYSRQEGLDGGLPEEVLFGNL
DLLPPPGKSTKPGAAPTEHKALVSHNGSLINVGSLLQRAEQQDSGRLYLENKIHTLELKL
EESHNRFSATEVTNKTLAAEMQELRVRLAEAEETARTAERQKSQLQRLLQELRRRLTPLQ
LEIQRVVEKADSWVEKEEPAPSN
>sp|Q1T7F1|CCB42_HUMAN Putative chemokine-related protein B42 OS=Homo sapiens PE=5 SV=1
MPLSDWCCGICEEAPLGRAYTQTWMETGCGPHGVTALGQQELKDCLRARSGGTASSVDWI
MEAARGSLNVHNCLIKFGRRD
>sp|Q2M243|CCD27_HUMAN Coiled-coil domain-containing protein 27 OS=Homo sapiens GN=CCDC27 PE=2 SV=2
MFEAIFPSTPQARLKRDPREKPGLSSFRSTFRQQSSLGLCIPRLMLPKEASPSQRHSSMS
SSMARALVLLQSMASRDARCPEWKPHQKPRTLSKSVQTISRYYRKTSEPKDAASLTGFMS
KMELRRVFPTHPDCPQFSTRATSMSHCGSPTEADLSGEIDNSSETWRGTQDLFLARRGSD
TNVDGYLLPFSKSICEFDYLRKRRKSQTLSPVTSSSVASQSCLRKRMPWYLSVIHEKDHC
LSELEIQVQKKDEEILLLQEEREALKMQLKCLLKGKGQETSMSPGRREQLSDASLKLGRL
SLLKAFSRHEEELQHWWQMQEESAAPERGKEPDLGGGEEDEGLEGEPDGVEDTGAWGGVS
QMGSVHEEGSEEEEEEEGDRDEDSEERELPEEEEIPRRRASSLAESFEEELLAQLEEYEQ
VILDFQFNLEATRTRYSLATGVIASLQQQVDFQETQLRKINTENETLQKELRERRQQLQA
MTDKFSNLREDKKHQEMMGLIEKDNQLLRQQVSELERKLTKRDCVISELDTKVSQLQEQV
ELDQNHLQRWKQLQEDLQSKKEMIQQAEQHTRVALESSQSRLERLRNKIIQATFSISGTK
SLANEISDNDILEALQRIISERSDYYNQLKQKGVKVPPLQQSEAFLTSKSKKGTSK
>sp|O60359|CCG3_HUMAN Voltage-dependent calcium channel gamma-3 subunit OS=Homo sapiens GN=CACNG3 PE=2 SV=1
MRMCDRGIQMLITTVGAFAAFSLMTIAVGTDYWLYSRGVCRTKSTSDNETSRKNEEVMTH
SGLWRTCCLEGAFRGVCKKIDHFPEDADYEQDTAEYLLRAVRASSVFPILSVTLLFFGGL
CVAASEFHRSRHNVILSAGIFFVSAGLSNIIGIIVYISANAGDPGQRDSKKSYSYGWSFY
FGAFSFIIAEIVGVVAVHIYIEKHQQLRAKSHSEFLKKSTFARLPPYRYRFRRRSSSRST

EPRSRDLSPISKGFHTIPSTDISMFTLSRDPSKITMGTLLNSDRDHAFLQFHNSTPKEFK
ESLHNNPANRRTTPV
>sp|Q16663|CCL15_HUMAN C-C motif chemokine 15 OS=Homo sapiens GN=CCL15 PE=1 SV=2
MKVSVAALSCLMLVAVLGSQAQFINDAETELMMSKLPLENPVVLNSFHFAADCCTSYISQ
SIPCSLMKSYFETSSECSKPGVIFLTKKGRQVCAKPSGPGVQDCMKKLKPYSI
>sp|P55773|CCL23_HUMAN C-C motif chemokine 23 OS=Homo sapiens GN=CCL23 PE=1 SV=3
MKVSVAALSCLMLVTALGSQARVTKDAETEFMMSKLPLENPVLLDRFHATSADCCISYTP
RSIPCSLLESYFETNSECSKPGVIFLTKKGRRFCANPSDKQVQVCVRMLKLDTRIKTRKN
>sp|Q99731|CCL19_HUMAN C-C motif chemokine 19 OS=Homo sapiens GN=CCL19 PE=1 SV=1
MALLLALSLLVLWTSPAPTLSGTNDAEDCCLSVTQKPIPGYIVRNFHYLLIKDGCRVPAV
VFTTLRGRQLCAPPDQPWVERIIQRLQRTSAKMKRRSS
>sp|Q9NRJ3|CCL28_HUMAN C-C motif chemokine 28 OS=Homo sapiens GN=CCL28 PE=1 SV=1
MQQRGLAIVALAVCAALHASEAILPIASSCCTEVSHHISRRLLERVNMCRIQRADGDCDL
AAVILHVKRRRICVSPHNHTVKQWMKVQAAKKNGKGNVCHRKKHHGKRNSNRAHQGKHET
YGHKTPY
>sp|P13236|CCL4_HUMAN C-C motif chemokine 4 OS=Homo sapiens GN=CCL4 PE=1 SV=1
MKLCVTVLSLLMLVAAFCSPALSAPMGSDPPTACCFSYTARKLPRNFVVDYYETSSLCSQ
PAVVFQTKRSKQVCADPSESWVQEYVYDLELN
>sp|P13501|CCL5_HUMAN C-C motif chemokine 5 OS=Homo sapiens GN=CCL5 PE=1 SV=3
MKVSAAALAVILIATALCAPASASPYSSDTTPCCFAYIARPLPRAHIKEYFYTSGKCSNP
AVVFVTRKNRQVCANPEKKWVREYINSLEMS
>sp|Q9BSQ5|CCM2_HUMAN Cerebral cavernous malformations 2 protein OS=Homo sapiens GN=CCM2 PE=1 SV=1
MEEEGKKGKKPGIVSPFKRVFLKGEKSRDKKAHEKVTERRPLHTVVLSLPERVEPDRLLS
DYIEKEVKYLGQLTSIPGYLNPSSRTEILHFIDNAKRAHQLPGHLTQEHDAVLSLSAYNV
KLAWRDGEDIILRVPIHDIAAVSYVRDDAAHLVVLKTAQDPGISPSQSLCAESSRGLSAG
SLSESAVGPVEACCLVILAAESKVAAEELCCLLGQVFQVVYTESTIDFLDRAIFDGASTP
THHLSLHSDDSSTKVDIKETYEVEASTFCFPESVDVGGASPHSKTISESELSASATELLQ
DYMLTLRTKLSSQEIQQFAALLHEYRNGASIHEFCINLRQLYGDSRKFLLLGLRPFIPEK
DSQHFENFLETIGVKDGRGIITDSFGRHRRALSTTSSSTTNGNRATGSSDDRSAPSEGDE
WDRMISDISSDIEALGCSMDQDSA
>sp|P32248|CCR7_HUMAN C-C chemokine receptor type 7 OS=Homo sapiens GN=CCR7 PE=2 SV=2
MDLGKPMKSVLVVALLVIFQVCLCQDEVTDDYIGDNTTVDYTLFESLCSKKDVRNFKAWF
LPIMYSIICFVGLLGNGLVVLTYIYFKRLKTMTDTYLLNLAVADILFLLTLPFWAYSAAK
SWVFGVHFCKLIFAIYKMSFFSGMLLLLCISIDRYVAIVQAVSAHRHRARVLLISKLSCV
GIWILATVLSIPELLYSDLQRSSSEQAMRCSLITEHVEAFITIQVAQMVIGFLVPLLAMS
FCYLVIIRTLLQARNFERNKAIKVIIAVVVVFIVFQLPYNGVVLAQTVANFNITSSTCEL
SKQLNIAYDVTYSLACVRCCVNPFLYAFIGVKFRNDLFKLFKDLGCLSQEQLRQWSSCRH
IRRSSMSVEAETTTTFSP
>sp|P51686|CCR9_HUMAN C-C chemokine receptor type 9 OS=Homo sapiens GN=CCR9 PE=1 SV=2
MTPTDFTSPIPNMADDYGSESTSSMEDYVNFNFTDFYCEKNNVRQFASHFLPPLYWLVFI
VGALGNSLVILVYWYCTRVKTMTDMFLLNLAIADLLFLVTLPFWAIAAADQWKFQTFMCK
VVNSMYKMNFYSCVLLIMCISVDRYIAIAQAMRAHTWREKRLLYSKMVCFTIWVLAAALC
IPEILYSQIKEESGIAICTMVYPSDESTKLKSAVLTLKVILGFFLPFVVMACCYTIIIHT

LIQAKKSSKHKALKVTITVLTVFVLSQFPYNCILLVQTIDAYAMFISNCAVSTNIDICFQ
VTQTIAFFHSCLNPVLYVFVGERFRRDLVKTLKNLGCISQAQWVSFTRREGSLKLSSMLL
ETTSGALSL
>sp|Q5M9N0|CD158_HUMAN Coiled-coil domain-containing protein 158 OS=Homo sapiens GN=CCDC158 PE=2 SV=2
MESKAWESNNEDLLSSSGVTSNGGSSSSFFVSSIRGTIIENTSSAGTLTQVPFFPKYEVE
LDSPRKIIPSPGKEHFERVLEEYSHQVKDLQRRLNESNELHEKQKFYLRQSVIDLQTKLQ
EMQMERDAMADIRRRESQSQEDLRNQLQNTVHELEAAKCLKEDMLKDSNTQIEQLRKMML
SHEGVLQEIRSILVDFEEASGKKICEHDSMSTLHFRSLGSAISKILRELDTEISYLKGRI
FPVEDQLEALKSESQNKIELLLQQHQDRIEQLISEHEVEITGLTEKASSARSQANSIQSQ
MEIIQEQARNQNSMYMRQLSDLESTVSQLRSELREAKRMYEDKTEELEKQLVLANSELTE
ARTERDQFSQESGNLDDQLQKLLADLHKREKELSLEKEQNKRLWDRDTGNSITIDHLRRE
LDNRNMEVQRLEALLKALKSECQGQMERQMAAIQGKNESLEKVSSLTAQLESTKEMLRKV
VEELTAKKMTLESSERTISDLTTSLQEKERAIEATNAEITKLRSRVDLKLQELQHLKNEG
DHLRNVQTECEALKLQMTEKDKVIEILRQQIENMTQLVGQHGRTAGAMQVEKAQLEKEIN
DRRMELKELKILKDKKDAKIRELEARVSDLELEKVKLVNAGSERLRAVKDIKQERDQLLN
EVKTSRSELNNLSEEYEVLKRNFRNKSEEMEMTTNKLKMQLKSAQSELEQTRNTLKSMEG
SDGHAMKVAMGMQKQITAKRGQIDALQSKIQFLEEAMTNANKEKHFLKEEKSKLSQELST
VATEKNKMAGELEVLRSQERRLKEKVTNMEVALDKASLQFAECQDIIQRQEQESVRLKLQ
HTLDIKELQGPGYTSNSSLKPRLLQPASVTRSHSNVPSSQSTASFLSHHSTKANTLKEDP
TRDLKQLLQELRSVINEEPAVSLSKTEEDGRTSLGALEDRVRDCITESSLRSDMCHRSNN
SLRDSTEGSKSSETLSREPVTLHAGDREDPSGCFTFTSAASPSVKNSASRSFNSSPKKSP
VHSLLTSSVEGSIGSTSQYRSAKPIHSSDSVKDSQSPPIETTGKTCRKLQNRLESLQTLV
EDLQLKNQAMSSMIRNQEKRIQKVKDQEKMLLK
>sp|P29017|CD1C_HUMAN T-cell surface glycoprotein CD1c OS=Homo sapiens GN=CD1C PE=1 SV=2
MLFLQFLLLALLLPGGDNADASQEHVSFHVIQIFSFVNQSWARGQGSGWLDELQTHGWDS
ESGTIIFLHNWSKGNFSNEELSDLELLFRFYLFGLTREIQDHASQDYSKYPFEVQVKAGC
ELHSGKSPEGFFQVAFNGLDLLSFQNTTWVPSPGCGSLAQSVCHLLNHQYEGVTETVYNL
IRSTCPRFLLGLLDAGKMYVHRQVRPEAWLSSRPSLGSGQLLLVCHASGFYPKPVWVTWM
RNEQEQLGTKHGDILPNADGTWYLQVILEVASEEPAGLSCRVRHSSLGGQDIILYWGHHF
SMNWIALVVIVPLVILIVLVLWFKKHCSYQDIL
>sp|Q86Y33|CD20B_HUMAN Cell division cycle protein 20 homolog B OS=Homo sapiens GN=CDC20B PE=1 SV=3
MEWKLERTAPRRVRTEEEMLWESIMRVLSKDLKQKRSQDSANVLDSVNATYSDFKSNFAK
RLSAEVPVASSPITTRWQQSQTRALSSDSFGEEQSTTYLPEASGSVLKTPPEKETLTLGS
RKEQLKTPSKGISETSNSALHFCKAPHAMDRDWKESVASKGQKCLKQLFVTQNVVQQANG
KMQLCEQSECVWKGCKDGVRDESFHLKSSGDINDSILQPEVKIHITGLRNDYYLNILDWS
FQNLVAIALGSAVYIWNGENHNGIENIDLSLTCNYISSVSWIKEGTCLAVGTSEGEVQLW
DVVTKKRLRNMLGHLSVVGALSWNHFILSSGSRLGRVYHHDVRVAQHHVGTLRHKQAVCA
LKWSPDGRLLSSGCSDGLLTIWPHDPGASAQGQPLKVITQSTAVKAMDWCPWQSGVLAIG
GGMKDGRLHILDINAGKSIQTPSTNSQICSLIWLPKTKEIATGQGTPKNDVTVWTCPTVS
RSGGFFGHRGRVLHLSLSPDQTRVFSAAADGTASVWNCY
>sp|Q15762|CD226_HUMAN CD226 antigen OS=Homo sapiens GN=CD226 PE=1 SV=2
MDYPTLLLALLHVYRALCEEVLWHTSVPFAENMSLECVYPSMGILTQVEWFKIGTQQDSI

AIFSPTHGMVIRKPYAERVYFLNSTMASNNMTLFFRNASEDDVGYYSCSLYTYPQGTWQK
VIQVVQSDSFEAAVPSNSHIVSEPGKNVTLTCQPQMTWPVQAVRWEKIQPRQIDLLTYCN
LVHGRNFTSKFPRQIVSNCSHGRWSVIVIPDVTVSDSGLYRCYLQASAGENETFVMRLTV
AEGKTDNQYTLFVAGGTVLLLLFVISITTIIVIFLNRRRRRERRDLFTESWDTQKAPNNY
RSPISTSQPTNQSMDDTREDIYVNYPTFSRRPKTRV
>sp|P25063|CD24_HUMAN Signal transducer CD24 OS=Homo sapiens GN=CD24 PE=1 SV=2
MGRAMVARLGLGLLLLALLLPTQIYSSETTTGTSSNSSQSTSNTGLAPNPTNATTKAAGG
ALQSTASLFVVSLSLLHLYS
>sp|P26842|CD27_HUMAN CD27 antigen OS=Homo sapiens GN=CD27 PE=1 SV=2
MARPHPWWLCVLGTLVGLSATPAPKSCPERHYWAQGGKLCCQMCEPGTFLVKDCDQHRKAA
QCDPCIPGVSFSPDHHTRPHCESCRHCNSGLLVRNCTITANAECACRNGWQCRDKECTEC
DPLPNPSLTARSSQALSPHPQPTHLPYVSEMLEARTAGHMQTLADFRQLPARTLSTHWPP
QRSLCSSDFIRILVIFSGMFLVFTLAGALFLHQRRKYRSNKGESPVEPAEPCHYSCPREE
EGSTIPIQEDYRKPEPACSP
>sp|Q8IX05|CD302_HUMAN CD302 antigen OS=Homo sapiens GN=CD302 PE=1 SV=1
MLRAALPALLLPLLGLAAAAVADCPSSTWIQFQDSCYIFLQEAIKVESIEDVRNQCTDHG
ADMISIHNEEENAFILDTLKKQWKGPDDILLGMFYDTDDASFKWFDNSNMTFDKWTDQDD
DEDLVDTCAFLHIKTGEWKKGNCEVSSVEGTLCKTAIPYKRKYLSDNHILISALVIASTV
ILTVLGAIIWFLYKKHSDSRFTTVFSTAPQSPYNEDCVLVVGEENEYPVQFD
>sp|P28906|CD34_HUMAN Hematopoietic progenitor cell antigen CD34 OS=Homo sapiens GN=CD34 PE=1 SV=2
MLVRRGARAGPRMPRGWTALCLLSLLPSGFMSLDNNGTATPELPTQGTFSNVSTNVSYQE
TTTPSTLGSTSLHPVSQHGNEATTNITETTVKFTSTSVITSVYGNTNSSVQSQTSVISTV
FTTPANVSTPETTLKPSLSPGNVSDLSTTSTSLATSPTKPYTSSSPILSDIKAEIKCSGI
REVKLTQGICLEQNKTSSCAEFKKDRGEGLARVLCGEEQADADAGAQVCSLLLAQSEVRP
QCLLLVLANRTEISSKLQLMKKHQSDLKKLGILDFTEQDVASHQSYSQKTLIALVTSGAL
LAVLGITGYFLMNRRSWSPTGERLGEDPYYTENGGGQGYSSGPGTSPEAQGKASVNRGAQ
ENGTGQATSRNGHSARQHVVADTEL
>sp|P29965|CD40L_HUMAN CD40 ligand OS=Homo sapiens GN=CD40LG PE=1 SV=1
MIETYNQTSPRSAATGLPISMKIFMYLLTVFLITQMIGSALFAVYLHRRLDKIEDERNLH
EDFVFMKTIQRCNTGERSLSLLNCEEIKSQFEGFVKDIMLNKEETKKENSFEMQKGDQNP
QIAAHVISEASSKTTSVLQWAEKGYYTMSNNLVTLENGKQLTVKRQGLYYIYAQVTFCSN
REASSQAPFIASLCLKSPGRFERILLRAANTHSSAKPCGQQSIHLGGVFELQPGASVFVN
VTDPSQVSHGTGFTSFGLLKL
>sp|O43866|CD5L_HUMAN CD5 antigen-like OS=Homo sapiens GN=CD5L PE=1 SV=1
MALLFSLILAICTRPGFLASPSGVRLVGGLHRCEGRVEVEQKGQWGTVCDDGWDIKDVAV
LCRELGCGAASGTPSGILYEPPAEKEQKVLIQSVSCTGTEDTLAQCEQEEVYDCSHDEDA
GASCENPESSFSPVPEGVRLADGPGHCKGRVEVKHQNQWYTVCQTGWSLRAAKVVCRQLG
CGRAVLTQKRCNKHAYGRKPIWLSQMSCSGREATLQDCPSGPWGKNTCNHDEDTWVECED
PFDLRLVGGDNLCSGRLEVLHKGVWGSVCDDNWGEKEDQVVCKQLGCGKSLSPSFRDRKC
YGPGVGRIWLDNVRCSGEEQSLEQCQHRFWGFHDCTHQEDVAVICSG
>sp|P01732|CD8A_HUMAN T-cell surface glycoprotein CD8 alpha chain OS=Homo sapiens GN=CD8A PE=1 SV=1
MALPVTALLLPLALLLHAARPSQFRVSPLDRTWNLGETVELKCQVLLSNPTSGCSWLFQP

RGAAASPTFLLYLSQNKPKAAEGLDTQRFSGKRLGDTFVLTLSDFRRENEGYYFCSALSN
SIMYFSHFVPVFLPAKPTTTPAPRPPTPAPTIASQPLSLRPEACRPAAGGAVHTRGLDFA
CDIYIWAPLAGTCGVLLLSLVITLYCNHRNRRRVCKCPRPVVKSGDKPSLSARYV
>sp|P14209|CD99_HUMAN CD99 antigen OS=Homo sapiens GN=CD99 PE=1 SV=1
MARGAALALLLFGLLGVLVAAPDGGFDLSDALPDNENKKPTAIPKKPSAGDDFDLGDAVV
DGENDDPRPPNPPKPMPNPNPNHPSSSGSFSDADLADGVSGGEGKGGSDGGGSHRKEGEE
ADAPGVIPGIVGAVVVAVAGAISSFIAYQKKKLCFKENAEQGEVDMESHRNANAEPAVQR
TLLEK
>sp|Q96GN5|CDA7L_HUMAN Cell division cycle-associated 7-like protein OS=Homo sapiens GN=CDCA7L PE=1 SV=2
MELATRYQIPKEVADIFNAPSDDEEFVGFRDDVPMETLSSEESCDSFDSLESGKQQDVRF
HSKYFTEELRRIFIEDTDSETEDFAGFTQSDLNGKTNPEVMVVESDLSDDGKASLVSEEE
EDEEEDKATPRRSRSRRSSIGLRVAFQFPTKKLANKPDKNSSSEQLFSSARLQNEKKTIL
ERKKDCRQVIQREDSTSESEDDSRDESQESSDALLKRTMNIKENKAMLAQLLAELNSMPD
FFPVRTPTSASRKKTVRRAFSEGQITRRMNPTRSARPPEKFALENFTVSAAKFAEEFYSF
RRRKTIGGKCREYRRRHRISSFRPVEDITEEDLENVAITVRDKIYDKVLGNTCHQCRQKT
IDTKTVCRNQGCCGVRGQFCGPCLRNRYGEDVRSALLDPDWVCPPCRGICNCSYCRKRDG
RCATGILIHLAKFYGYDNVKEYLESLQKELVEDN
>sp|Q12834|CDC20_HUMAN Cell division cycle protein 20 homolog OS=Homo sapiens GN=CDC20 PE=1 SV=2
MAQFAFESDLHSLLQLDAPIPNAPPARWQRKAKEAAGPAPSPMRAANRSHSAGRTPGRTP
GKSSSKVQTTPSKPGGDRYIPHRSAAQMEVASFLLSKENQPENSQTPTKKEHQKAWALNL
NGFDVEEAKILRLSGKPQNAPEGYQNRLKVLYSQKATPGSSRKTCRYIPSLPDRILDAPE
IRNDYYLNLVDWSSGNVLAVALDNSVYLWSASSGDILQLLQMEQPGEYISSVAWIKEGNY
LAVGTSSAEVQLWDVQQQKRLRNMTSHSARVGSLSWNSYILSSGSRSGHIHHHDVRVAEH
HVATLSGHSQEVCGLRWAPDGRHLASGGNDNLVNVWPSAPGEGGWVPLQTFTQHQGAVKA
VAWCPWQSNVLATGGGTSDRHIRIWNVCSGACLSAVDAHSQVCSILWSPHYKELISGHGF
AQNQLVIWKYPTMAKVAELKGHTSRVLSLTMSPDGATVASAAADETLRLWRCFELDPARR
REREKASAAKSSLIHQGIR
>sp|P60953|CDC42_HUMAN Cell division control protein 42 homolog OS=Homo sapiens GN=CDC42 PE=1 SV=2
MQTIKCVVVGDGAVGKTCLLISYTTNKFPSEYVPTVFDNYAVTVMIGGEPYTLGLFDTAG
QEDYDRLRPLSYPQTDVFLVCFSVVSPSSFENVKEKWVPEITHHCPKTPFLLVGTQIDLR
DDPSTIEKLAKNKQKPITPETAEKLARDLKAVKYVECSALTQKGLKNVFDEAILAALEPP
EPKKSRRCVLL
>sp|Q99741|CDC6_HUMAN Cell division control protein 6 homolog OS=Homo sapiens GN=CDC6 PE=1 SV=1
MPQTRSQAQATISFPKRKLSRALNKAKNSSDAKLEPTNVQTVTCSPRVKALPLSPRKRLG
DDNLCNTPHLPPCSPPKQGKKENGPPHSHTLKGRRLVFDNQLTIKSPSKRELAKVHQNKI
LSSVRKSQEITTNSEQRCPLKKESACVRLFKQEGTCYQQAKLVLNTAVPDRLPAREREMD
VIRNFLREHICGKKAGSLYLSGAPGTGKTACLSRILQDLKKELKGFKTIMLNCMSLRTAQ
AVFPAIAQEICQEEVSRPAGKDMMRKLEKHMTAEKGPMIVLVLDEMDQLDSKGQDVLYTL
FEWPWLSNSHLVLIGIANTLDLTDRILPRLQAREKCKPQLLNFPPYTRNQIVTILQDRLN
QVSRDQVLDNAAVQFCARKVSAVSGDVRKALDVCRRAIEIVESDVKSQTILKPLSECKSP

SEPLIPKRVGLIHISQVISEVDGNRMTLSQEGAQDSFPLQQKILVCSLMLLIRQLKIKEV
TLGKLYEAYSKVCRKQQVAAVDQSECLSLSGLLEARGILGLKRNKETRLTKVFFKIEEKE
IEHALKDKALIGNILATGLP
>sp|Q69YH5|CDCA2_HUMAN Cell division cycle-associated protein 2 OS=Homo sapiens GN=CDCA2 PE=1 SV=2
MDANSKDKPPETKESAMNNAGNASFILGTGKIVTPQKHAELPPNPCTPDTFKSPLNFSTV
TVEQLGITPESFVRNSAGKSSSYLKKCRRRSAVGARGSPETNHLIRFIARQQNIKNARKS
PLAQDSPSQGSPALYRNVNTLRERISAFQSAFHSIKENEKMTGCLEFSEAGKESEMTDLT
RKEGLSACQQSGFPAVLSSKRRRISYQRDSDENLTDAEGKVIGLQIFNIDTDRACAVETS
VDLSEISSKLGSTQSGFLVEESLPLSELTETSNALKVADCVVGKGSSDAVSPDTFTAEVS
SDAVPDVRSPATPACRRDLPTPKTFVLRSVLKKPSVKMCLESLQEHCNNLYDDDGTHPSL
ISNLPNCCKEKEAEDEENFEAPAFLNMRKRKRVTFGEDLSPEVFDESLPANTPLRKGGTP
VCKKDFSGLSSLLLEQSPVPEPLPQPDFDDKGENLENIEPLQVSFAVLSSPNKSSISETL
SGTDTFSSSNNHEKISSPKVGRITRTSNRRNQLVSVVEESVCNLLNTEVQPCKEKKINRR
KSQETKCTKRALPKKSQVLKSCRKKKGKGKKSVQKSLYGERDIASKKPLLSPIPELPEVP
EMTPSIPSIRRLGSGYFSSNGKLEEVKTPKNPVKRKDLLRHDPDLHMHQGYDKYDVSEFC
SYIKSSSSLGNATSDEDPNTNIMNINENKNIPKAKNKSESENEPKAGTDSPVSCASVTEE
RVASDSPKPALTLQQGQEFSAGGQNAENLCQFFKISPDLNIKCERKDDFLGAAEGKLQCN
RLMPNSQKDCHCLGDVLIENTKESKSQSEDLGRKPMESSSVVSCRDRKDRRRSMCYSDGR
SLHLEKNGNHTPSSSVGSSVEISLENSELFKDLSDAIEQTFQRRNSETKVRRSTRLQKDL
ENEGLVWISLPLPSTSQKAKRRTICTFDSSGFESMSPIKETVSSRQKPQMAPPVSDPENS
QGPAAGSSDEPGKRRKSFCISTLANTKATSQFKGYRRRSSLNGKGESSLTALERIEHNGE
RKQ
>sp|P32320|CDD_HUMAN Cytidine deaminase OS=Homo sapiens GN=CDA PE=1 SV=2
MAQKRPACTLKPECVQQLLVCSQEAKKSAYCPYSHFPVGAALLTQEGRIFKGCNIENACY
PLGICAERTAIQKAVSEGYKDFRAIAIASDMQDDFISPCGACRQVMREFGTNWPVYMTKP
DGTYIVMTVQELLPSSFGPEDLQKTQ
>sp|Q9H305|CDIP1_HUMAN Cell death-inducing p53-target protein 1 OS=Homo sapiens GN=CDIP1 PE=1 SV=1
MSSEPPPPYPGGPTAPLLEEKSGAPPTPGRSSPAVMQPPPGMPLPPADIGPPPYEPPGHP
MPQPGFIPPHMSADGTYMPPGFYPPPGPHPPMGYYPPGPYTPGPYPGPGGHTATVLVPSG
AATTVTVLQGEIFEGAPVQTVCPHCQQAITTKISYEIGLMNFVLGFFCCFMGCDLGCCLI
PCLINDFKDVTHTCPSCKAYIYTYKRLC
>sp|Q00536|CDK16_HUMAN Cyclin-dependent kinase 16 OS=Homo sapiens GN=CDK16 PE=1 SV=1
MDRMKKIKRQLSMTLRGGRGIDKTNGAPEQIGLDESGGGGGSDPGEAPTRAAPGELRSAR
GPLSSAPEIVHEDLKMGSDGESDQASATSSDEVQSPVRVRMRNHPPRKISTEDINKRLSL
PADIRLPEGYLEKLTLNSPIFDKPLSRRLRRVSLSEIGFGKLETYIKLDKLGEGTYATVY
KGKSKLTDNLVALKEIRLEHEEGAPCTAIREVSLLKDLKHANIVTLHDIIHTEKSLTLVF
EYLDKDLKQYLDDCGNIINMHNVKLFLFQLLRGLAYCHRQKVLHRDLKPQNLLINERGEL
KLADFGLARAKSIPTKTYSNEVVTLWYRPPDILLGSTDYSTQIDMWGVGCIFYEMATGRP
LFPGSTVEEQLHFIFRILGTPTEETWPGILSNEEFKTYNYPKYRAEALLSHAPRLDSDGA
DLLTKLLQFEGRNRISAEDAMKHPFFLSLGERIHKLPDTTSIFALKEIQLQKEASLRSSS
MPDSGRPAFRVVDTEF
>sp|Q8IZL9|CDK20_HUMAN Cyclin-dependent kinase 20 OS=Homo sapiens GN=CDK20 PE=1 SV=1

MDQYCILGRIGEGAHGIVFKAKHVETGEIVALKKVALRRLEDGFPNQALREIKALQEMED
NQYVVQLKAVFPHGGGFVLAFEFMLSDLAEVVRHAQRPLAQAQVKSYLQMLLKGVAFCHA
NNIVHRDLKPANLLISASGQLKIADFGLARVFSPDGSRLYTHQVATRWYRAPELLYGARQ
YDQGVDLWSVGCIMGELLNGSPLFPGKNDIEQLCYVLRILGTPNPQVWPELTELPDYNKI
SFKEQVPMPLEEVLPDVSPQALDLLGQFLLYPPHQRIAASKALLHQYFFTAPLPAHPSEL
PIPQRLGGPAPKAHPGPPHIHDFHVDRPLEESLLNPELIRPFILEG
>sp|P38936|CDN1A_HUMAN Cyclin-dependent kinase inhibitor 1 OS=Homo sapiens GN=CDKN1A PE=1 SV=3
MSEPAGDVRQNPCGSKACRRLFGPVDSEQLSRDCDALMAGCIQEARERWNFDFVTETPLE
GDFAWERVRGLGLPKLYLPTGPRRGRDELGGGRRPGTSPALLQGTAEEDHVDLSLSCTLV
PRSGEQAEGSPGGPGDSQGRKRRQTSMTDFYHSKRRLIFSKRKP
>sp|Q9Y6F7|CDY2_HUMAN Testis-specific chromodomain protein Y 2 OS=Homo sapiens GN=CDY2A PE=1 SV=1
MASQEFEVEAIVDKRQDKNGNTQYLVRWKGYDKQDDTWEPEQHLMNCEKCVHDFNRRQTE
KQKKLTWTTTSRIFSNNARRRTSRSTKANYSKNSPKTPVTDKHHRSKNCKLFAASKNVRR
KAASTLSDTKNMEIINSTIETLAPDSPFDHKKTVSGFQKLEKLDPIAADQQDTVVFKVTE
GKLLRDPLSHPGAEQTGIQNKTQMHPLMSQMSGSVTASMATGSATRKGIVVLIDPLAANG
TTDMHTSVPRVKGGQRNITDDSRGQPFIKKMHFTIRLTESAITYRDIVVKKEDGFTQIVL
STRSTEKNALNTEVIKEMVNALNSAAADDSKLVLFSAAGSVFCCGLDFGYFVRHLRNDRN
TASLEMVDTIKNFVNTFIQFKKPIVVSVNGPAIGLGASILPLCDLVWANEKAWFQTPYTT
FGQSPDGCSSITFPKMMGKASANEMLIAGRKLTAREACAKGLVSQVFLTGTFTQEVMIQI
KELASYNAIVLEECKALVRCNIKLELEQANERECEVLRKIWSSAQGIESMLKYVENKIDE
F
>sp|Q8NAS9|CE017_HUMAN Uncharacterized protein C5orf17 OS=Homo sapiens GN=C5orf17 PE=2 SV=1
MPEPPTPSVGSCAARASRMSAAPCSRAPSPIDHPRAEECGRAARDWQAAPPAAPVRDPLG
EASWAPESGEDMENLYVQLKLQSVGTCFNREELPSVLQWPQGEELGATSVTNKPGLSAMR
KETDQQVGERKLTYCGDQVTLFTDQALSQILSSEEGRIKVM
>sp|A6NDU8|CE051_HUMAN UPF0600 protein C5orf51 OS=Homo sapiens GN=C5orf51 PE=1 SV=1
MAAAVSSVVRRVEELGDLAQAHIQQLSEAAGEDDHFLIRASAALEKLKLLCGEEKECSNP
SNLLELYTQAILDMTYFEENKLVDEDFPEDSSSQKVKELISFLSEPEILVKENNMHPKHC
NLLGDELLECLSWRRGALLYMYCHSLTKRREWLLRKSSLLKKYLLDGISYLLQMLNYRCP
IQLNEGVSFQDLDTAKLLSAGIFSDIHLLAMMYSGEMCYWGSKYCADQQPENHEVDTSVS
GAGCTTYKEPLDFREVGEKILKKYVSVCEGPLKEQEWNTTNAKQILNFFHHRCN
>sp|O60308|CE104_HUMAN Centrosomal protein of 104 kDa OS=Homo sapiens GN=CEP104 PE=1 SV=1
MPHKIGFVVVSSSGHEDGFSARELMIHAPTVSGWRSPRFCQFPQEIVLQMVERCRIRKLQ
LLAHQYMISSKIEFYISESLPEYFAPYQAERFRRLGYVSLCDNEKTGCKARELKSVYVDA
VGQFLKLIFHQNHVNKYNIYNQVALVAINIIGDPADFSDESNTASREKLIDHYLGHNSED
PALEGTYARKSDYISPLDDLAFDMYQDPEVAQIIRKLDERKREAVQKERYDYAKKLKQAI
ADLQKVGERLGRYEVEKRCAVEKEDYDLAKEKKQQMEQYRAEVYEQLELHSLLDAELMRR
PFDLPLQPLARSGSPCHQKPMPSLPQLEERGTENQFAEPFLQEKPSSYSLTISPQHSAVD
PLLPATDPHPKINAESLPYDERPLPAIRKHYGEAVVEPEMSNADISDARRGGMLGEPEPL
TEKALREASSAIDVLGETLVAEAYCKTWSYREDALLALSKKLMEMPVGTPKEDLKNTLRA
SVFLVRRAIKDIVTSVFQASLKLLKMIITQYIPKHKLSKLETAHCVERTIPVLLTRTGDS

SARLRVTAANFIQEMALFKEVKSLQIIPSYLVQPLKANSSVHLAMSQMGLLARLLKDLGT
GSSGFTIDNVMKFSVSALEHRVYEVRETAVRIILDMYRQHQASILEYLPPDDSNTRRNIL
YKTIFEGFAKIDGRATDAEMRARRKAATEEAEKQKKEEIKALQGQLAALKEIQAEVQEKE
SDAVKPKNQDIQGGKAAPAEALGIPDEHYLDNLCIFCGERSESFTEEGLDLHYWKHCLML
TRCDHCKQVVEISSLTEHLLTECDKKDGFGKCYRCSEAVFKEELPRHIKHKDCNPAKPEK
LANRCPLCHENFSPGEEAWKAHLMGPAGCTMNLRKTHILQKAPALQPGKSSAVAASGPLG
SKAGSKIPTPKGGLSKSSSRTYAKR
>sp|Q9P2H0|CE126_HUMAN Centrosomal protein of 126 kDa OS=Homo sapiens GN=CEP126 PE=1 SV=3
MLAGRPGTRSAVGELGTESSDNLDRAPLGPRESGGHHRPGSYLDMKIHLEKNLEEERQIL
LQQQKICRNRARKYFVESNRRKKAFEEKRKEQEEKEHQIREQILQQRKQKFEEVTEKFQR
AHVPLSQRRKAVSRKPVPPLEEALKQIQESNLKSEVNLPFSRRPTINWRAIDSALPSALS
KNDHKHQKQLLSKINCEKEMNENMRATLATSKNVFQLKLEETQKLLEDQHLSNLQKFGDE
VNQITNSETLSSIDSLEATEHEEIYLTLNKEHSTSIQRNTISLKPANMQSTNLSCFDEDK
LAFSKTQHINNWLTNLDASNTQNVTAFSDILSKSNVLPSWEYFNSKEQNPSPLNGTVERA
TNTANNSVPFVSSPPMFVLDKKCEKTSETSTMRTTDSTSGAFKRERPLVTESPTFKFSKS
QSTSDSLTQEVATFPDQEKYSELNQENGTTSIPTSCVPVATPLVLPSNIQSARPSAKNSI
HIKEIDAVQCSDKLDELKDGKEEEIKYFNCNKEELPLFSDSFQDAYIPHNPDSKDEKQKL
AETSSLSNVTSNYDFVGQHKKMKYNIHERNGVRFLKSILKKESKYEHGYLKALIINQSFK
FGNQKAAAIRDSIELTKEKGAEIPKTIKKLRWFDETSNIENNAENSHSLKNKTGTTQQHS
QQFHIQSGAGSNIISVSTCAVNSADTKKSREDSISENVTTLGGSGADHMPLNCFIPSGYN
FAKHAWPASKKEESKIPVHDDSKTKQGKPQRGRAKIIRKPGSAKVQSGFICTNRKGAVIQ
PQSASKVNIFTQAQGKLIIPCPPPQSTSNIRSGKNIQVSQCQPVTPENPQNIITHNSFNS
KHVLPTEHSLNQWNQESSSPLSNACSDLVTVIPSLPSYCSSECQTFAKINHSNGTQAVAR
QDATLYCTQRSPVCEESYPSVTLRTAEEESVPLWKRGPNVLHQNKRATGSTVMRRKRIAE
TKRRNILEQKRQNPGSVGQKYSEQINNFGQSVLLSSSEPKQTTRGTSYIEEVSDSTSEFL
MAENLVKASVPEDEILTVLNSKQIQKSNLPLNKTQQFNICTLSAEEQKILESLNDLSERL
HYIQESICKNPSIKNTLQIIPLLEKREDRTSSCRDKR
>sp|Q5SW79|CE170_HUMAN Centrosomal protein of 170 kDa OS=Homo sapiens GN=CEP170 PE=1 SV=1
MSLTSWFLVSSGGTRHRLPREMIFVGRDDCELMLQSRSVDKQHAVINYDASTDEHLVKDL
GSLNGTFVNDVRIPEQTYITLKLEDKLRFGYDTNLFTVVQGEMRVPEEALKHEKFTIQLQ
LSQKSSESELSKSASAKSIDSKVADAATEVQHKTTEALKSEEKAMDISAMPRGTPLYGQP
SWWGDDEVDEKRAFKTNGKPEEKNHEAGTSGCGIDAKQVEEQSAAANEEVLFPFCREPSY
FEIPTKEFQQPSQITESTIHEIPTKDTPSSHITGAGHASFTIEFDDSTPGKVTIRDHVTK
FTSDQRHKSKKSSPGTQDLLGIQTGMMAPENKVADWLAQNNPPQMLWERTEEDSKSIKSD
VPVYLKRLKGNKHDDGTQSDSENAGAHRRCSKRATLEEHLRRHHSEHKKLQKVQATEKHQ
DQAVTSSAHHRGGHGVPHGKLLKQKSEEPSVSIPFLQTALLRSSGSLGHRPSQEMDKMLK
NQATSATSEKDNDDDQSDKGTYTIELENPNSEEVEARKMIDKVFGVDDNQDYNRPVINEK
HKDLIKDWALSSAAAVMEERKPLTTSGFHHSEEGTSSSGSKRWVSQWASLAANHTRHDQE
ERIMEFSAPLPLENETEISESGMTVRSTGSATSLASQGERRRRTLPQLPNEEKSLESHRA
KVVTQRSEIGEKQDTELQEKETPTQVYQKDKQDADRPLSKMNRAVNGETLKTGGDNKTLL
HLGSSAPGKEKSETDKETSLVKQTLAKLQQQEQREEAQWTPTKLSSKNVSGQTDKCREET
FKQESQPPEKNSGHSTSKGDRVAQSESKRRKAEEILKSQTPKGGDKKESSKSLVRQGSFT
IEKPSPNIPIELIPHINKQTSSTPSSLALTSASRIRERSESLDPDSSMDTTLILKDTEAV
MAFLEAKLREDNKTDEGPDTPSYNRDNSISPESDVDTASTISLVTGETERKSTQKRKSFT

SLYKDRCSTGSPSKDVTKSSSSGAREKMEKKTKSRSTDVGSRADGRKFVQSSGRIRQPSV
DLTDDDQTSSVPHSAISDIMSSDQETYSCKPHGRTPLTSADEHVHSKLEGSKVTKSKTSP
VVSGSSSKSTTLPRPRPTRTSLLRRARLGEASDSELADADKASVASEVSTTSSTSKPPTG
RRNISRIDLLAQPRRTRLGSLSARSDSEATISRSSASSRTAEAIIRSGARLVPSDKFSPR
IRANSISRLSDSKVKSMTSAHGSASVNSRWRRFPTDYASTSEDEFGSNRNSPKHTRLRTS
PALKTTRLQSAGSAMPTSSSFKHRIKEQEDYIRDWTAHREEIARISQDLALIAREINDVA
GEIDSVTSSGTAPSTTVSTAATTPGSAIDTREELVDRVFDESLNFRKIPPLVHSKTPEGN
NGRSGDPRPQAAEPPDHLTITRRRTWSRDEVMGDNLLLSSVFQFSKKIRQSIDKTAGKIR
ILFKDKDRNWDDIESKLRAESEVPIVKTSSMEISSILQELKRVEKQLQAINAMIDPDGTL
EALNNMGFPSAMLPSPPKQKSSPVNNHHSPGQTPTLGQPEARALHPAAVSAAAEFENAES
EADFSIHFNRFNPDGEEEDVTVQE
>sp|Q9C0D2|CE295_HUMAN Centrosomal protein of 295 kDa OS=Homo sapiens GN=CEP295 PE=1 SV=4
MKRKVVNTHKLRLSPNEEAFILKEDYERRRKLRLLQVREQERDIALQIREDIKQRRNQQF
TRLAEELRAEWEESQTQKIQNLEKLYLASLRSMGEGHRQAKENEPDLDALAQRAAERKRK
ADLRHKEALKVQKNQKEILLKQKTWHIKARKEALLVEKERSAKITSLPPPPPTLFENIEV
KRISAVKTNSSTYHHLHTFVNRETDTKRPDARLAAEEEAKRLEELQKQAAQERMERFEKA
HVRGFQAMKKIHLAQNQEKLMKELKQLQQEDLARRRQTVAQMPPQLVELPYKRSEMKEDW
QRELEFAFEDMYNADRKVVKGNLILHLEPEPLPTVTNQIQDEELDLSMEQENLGAAEDLPV
TEAEICSSETDVPLVMKTQQIPSKVLFKKLLNKIRSQKSLWTIKSMSEDESEMITTVSEI
ESKAPTVESGTIASKERTLSSGQEQVVESDTLTIESGPLASEDKPLSCGTNSGKEQEINE
TLPITTVAQSSVLLHPQEAAARIRMSARQKQIMEIEEQKQKQLELLEQIEQQKLRLETDC
FRAQLEEEKRKKTQPTGVGIAPASCPVISDEDSHRQMIRNYQHQLLQQNRLHRQSVETAR
KQLLEYQTMLKGRCPSVSAPSLITDSVISVPSWKSERPTAISEHWDQGQRLKLSPNKYQP
IQPIQTSKLEQDHFQVARQNHFPQRQVETTETLRASDILTNQALESQEHLRQFSQTETQQ
RDYKLVPKDSETLSRALSHDRQLISQDARKISETFGATTFQSLESQQLFSENSENISYHL
TEPSSFVPLVPQHSFSSLPVKVESGKIQEPFSAMSKSTVSTSHSIISQMHDRPLLPSENI
TAQQGNMKALQEQLDLQKKVLQATQEAQEQLLLCKQKEVEQQTGLSVFLPLVTPDSSALL
PSAKADLGRIQESSPTKNNIAVSSDHHVISQLQDKRLSLSQPILSQQNNFKFLQEQLNIQ
KDSLQARREAQEVLYVHKQSELDRRVCSEQAEPSFPFQVAQHTFTSLPSADTKSGKIQEQ
HSSKSEKGLVSCQSDIPISQDGSLSFLQQFLPLHDSLKLLQEQLTKQRDTLQARHEAQVE
LLLHRQRDLGDSKSGLVSSSSSPVVVQHSVASQASAKAEPRRIQELYLSEKENVGPSCHL
IIPTFQDKSLSFPQHSLAQQENLTILQEQSQIQRVILGAKEGTQEFVHTESELEKRISSE
QTGTSSSLSQVDESERFQECISIKSDSTIPLSHPKIPRCQERLLRVSQHMLPLQDNLEEH
QAWLDTEKEAFHFSQKTQENTSSEQTGSSSFIPQLVQLSFTSLASAESGTILEPLFTESE
SKIFSSHLQIPQLQDRLLRISQLIQPQQDNLKALQEQLATQREAIILARQEAREELLLHQ
SEWEGRISPEQVDTSSLPLVPQHSFASLPLNESERNQEPCSINSDNIVSSGHSEIPTLPD
GLLGLSHLVLPQQDNLIALEEHLHAQTDFLPSIEKTQKELVLSKPCKFEEKVSSEHFIQS
HHGDLQALQQQLDTQKKAIRSIQEVQEELLLQRLSELEKRVSSEQVCSSSFVSQVPVADS
ERTQKSFPTKSNDTLPSSHREIPRLQDRLLSLSKPILPQQDNMTAQLDAQREVMYSYEKP
QEELSLNKQRKLNKSESAEHTIPSLFLPKETEHSFIPLPFAEAKPKSTCELYSSQNEHAA
PPSNPVIPGFQDRLLSFSQSVLTQQDNLGLQKQLDLQREVLHYSQKAQEKLLVQRQTALQ
QQIQKHEETLKDFFKDSQISKPTVENDLKTQKMGQLRDWFPNTQDLAGNDQENIRHADRN
NSDDNHLASEDTSAKQSGEHLEKDLGRRSSKPPVAKVKCGLDLNQHELSAIQEVESPAIG
RTSILGKPGIYEDRDPLRVSISREQSFFGSPLAHDPFSCLQLVGQENVCGDDYDEAVKLK

ESVVENHAVLSYAVEEEHAYLGPTVKPDDKAKTLSYEPLSSATVSTGSLLSYENTDLSLT
DPESFSEHMDDSKQESTTSKEEETNIISSIVPSTQDIYQRQNSSDVHKSLLPAVDETTCG
HTHFQQMIDKYINEANLIPEKTDLQELEHIFPNLHHQLFKPLEPHPDFDLSSSSSGISPD
NRDFYQRSDSSSESHCATGLSKSTVYFTALRRTSMHSSLNTSPNQQPDTNLAHVGAHSFA
TENIIGGSEQCFEQLQPEYSSQEESQHADLPSIFSIEARDSSQGMKNQNYPSEEHTEILQ
NKKKIVHFQLSIGNLSSVYSSSDEANVFDQLNVQHSTPCGSNSSECSTKHQLESRKESMG
FEELSKRGVVTMLQSQGLIEDNKNETCRVLDINPQVEETDSRLCVRTVEMGTSIQAPYSL
TTQNEKYFENSAETDIPKITKKLSQLGESELFASSGSFSLQSSIPVWETETGHGIMEEPE
LTLISTTDTSIAEMDFANLTLEEKSENEAKCFFQVSEFLPLVSATEASDYPAVSELSIEK
PRTASTETPRRLTPVPGSLQEAFIKRKKSFMERSHQRQKEIRNKIHVSENSQIKTVKEKP
SISSSVSRLKGVNKVRASFPEDRKTTQALRHQRGLRLYNQLAEVKQQKEEKTKQEAYAQN
RARAKEFHKKTLEKLRAKNTC
>sp|P13688|CEAM1_HUMAN Carcinoembryonic antigen-related cell adhesion molecule 1 OS=Homo sapiens GN=CEACAM1 PE=1 SV=2
MGHLSAPLHRVRVPWQGLLLTASLLTFWNPPTTAQLTTESMPFNVAEGKEVLLLVHNLPQ
QLFGYSWYKGERVDGNRQIVGYAIGTQQATPGPANSGRETIYPNASLLIQNVTQNDTGFY
TLQVIKSDLVNEEATGQFHVYPELPKPSISSNNSNPVEDKDAVAFTCEPETQDTTYLWWI
NNQSLPVSPRLQLSNGNRTLTLLSVTRNDTGPYECEIQNPVSANRSDPVTLNVTYGPDTP
TISPSDTYYRPGANLSLSCYAASNPPAQYSWLINGTFQQSTQELFIPNITVNNSGSYTCH
ANNSVTGCNRTTVKTIIVTELSPVVAKPQIKASKTTVTGDKDSVNLTCSTNDTGISIRWF
FKNQSLPSSERMKLSQGNTTLSINPVKREDAGTYWCEVFNPISKNQSDPIMLNVNYNALP
QENGLSPGAIAGIVIGVVALVALIAVALACFLHFGKTGRASDQRDLTEHKPSVSNHTQDH
SNDPPNKMNEVTYSTLNFEAQQPTQPTSASPSLTATEIIYSEVKKQ
>sp|O75871|CEAM4_HUMAN Carcinoembryonic antigen-related cell adhesion molecule 4 OS=Homo sapiens GN=CEACAM4 PE=1 SV=2
MGPPSAAPRGGHRPWQGLLITASLLTFWHPPTTVQFTIEALPSSAAEGKDVLLLACNISE
TIQAYYWHKGKTAEGSPLIAGYITDIQANIPGAAYSGRETVYPNGSLLFQNITLEDAGSY
TLRTINASYDSDQATGQLHVHQNNVPGLPVGAVAGIVTGVLVGVALVAALVCFLLLSRTG
RASIQRDLREQPPPASTPGHGPSHRSTFSAPLPSPRTATPIYEELLYSDANIYCQIDHKA
DVVS
>sp|Q14002|CEAM7_HUMAN Carcinoembryonic antigen-related cell adhesion molecule 7 OS=Homo sapiens GN=CEACAM7 PE=1 SV=1
MGSPSACPYRVCIPWQGLLLTASLLTFWNLPNSAQTNIDVVPFNVAEGKEVLLVVHNESQ
NLYGYNWYKGERVHANYRIIGYVKNISQENAPGPAHNGRETIYPNGTLLIQNVTHNDAGF
YTLHVIKENLVNEEVTRQFYVFSEPPKPSITSNNFNPVENKDIVVLTCQPETQNTTYLWW
VNNQSLLVSPRLLLSTDNRTLVLLSATKNDIGPYECEIQNPVGASRSDPVTLNVRYESVQ
ASSPDLSAGTAVSIMIGVLAGMALI
>sp|P49715|CEBPA_HUMAN CCAAT/enhancer-binding protein alpha OS=Homo sapiens GN=CEBPA PE=1 SV=3
MESADFYEAEPRPPMSSHLQSPPHAPSSAAFGFPRGAGPAQPPAPPAAPEPLGGICEHET
SIDISAYIDPAAFNDEFLADLFQHSRQQEKAKAAVGPTGGGGGGDFDYPGAPAGPGGAVM
PGGAHGPPPGYGCAAAGYLDGRLEPLYERVGAPALRPLVIKQEPREEDEAKQLALAGLFP
YQPPPPPPPSHPHPHPPPAHLAAPHLQFQIAHCGQTTMHLQPGHPTPPPTPVPSPHPAPA
LGAAGLPGPGSALKGLGAAHPDLRASGGSGAGKAKKSVDKNSNEYRVRRERNNIAVRKSR

DKAKQRNVETQQKVLELTSDNDRLRKRVEQLSRELDTLRGIFRQLPESSLVKAMGNCA
>sp|Q9NZK5|CECR1_HUMAN Adenosine deaminase CECR1 OS=Homo sapiens GN=CECR1 PE=1 SV=2
MLVDGPSERPALCFLLLAVAMSFFGSALSIDETRAHLLLKEKMMRLGGRLVLNTKEELAN
ERLMTLKIAEMKEAMRTLIFPPSMHFFQAKHLIERSQVFNILRMMPKGAALHLHDIGIVT
MDWLVRNVTYRPHCHICFTPRGIMQFRFAHPTPRPSEKCSKWILLEDYRKRVQNVTEFDD
SLLRNFTLVTQHPEVIYTNQNVVWSKFETIFFTISGLIHYAPVFRDYVFRSMQEFYEDNV
LYMEIRARLLPVYELSGEHHDEEWSVKTYQEVAQKFVETHPEFIGIKIIYSDHRSKDVAV
IAESIRMAMGLRIKFPTVVAGFDLVGHEDTGHSLHDYKEALMIPAKDGVKLPYFFHAGET
DWQGTSIDRNILDALMLNTTRIGHGFALSKHPAVRTYSWKKDIPIEVCPISNQVLKLVSD
LRNHPVATLMATGHPMVISSDDPAMFGAKGLSYDFYEVFMGIGGMKADLRTLKQLAMNSI
KYSTLLESEKNTFMEIWKKRWDKFIADVATK
>sp|Q86SI9|CEI_HUMAN Protein CEI OS=Homo sapiens GN=C5orf38 PE=2 SV=1
MVAPAARVFLRAVRAALTSTVPDLLCLLARGSPRGLASGRLPLAVHSAQHGPGSGAPWLR
IARRALRFVLSKHWGDDCYLTNRLWQDLKPPSHVENGQELRLAPPVQWALQVQGNQLQTA
VLCLRMAPPEPAGSRQRI
>sp|Q9UNI1|CELA1_HUMAN Chymotrypsin-like elastase family member 1 OS=Homo sapiens GN=CELA1 PE=1 SV=2
MLVLYGHSTQDLPETNARVVGGTEAGRNSWPSQISLQYRSGGSRYHTCGGTLIRQNWVMT
AAHCVDYQKTFRVVAGDHNLSQNDGTEQYVSVQKIVVHPYWNSDNVAAGYDIALLRLAQS
VTLNSYVQLGVLPQEGAILANNSPCYITGWGKTKTNGQLAQTLQQAYLPSVDYAICSSSS
YWGSTVKNTMVCAGGDGVRSGCQGDSGGPLHCLVNGKYSVHGVTSFVSSRGCNVSRKPTV
FTQVSAYISWINNVIASN
>sp|P07199|CENPB_HUMAN Major centromere autoantigen B OS=Homo sapiens GN=CENPB PE=1 SV=2
MGPKRRQLTFREKSRIIQEVEENPDLRKGEIARRFNIPPSTLSTILKNKRAILASERKYG
VASTCRKTNKLSPYDKLEGLLIAWFQQIRAAGLPVKGIILKEKALRIAEELGMDDFTASN
GWLDRFRRRHGVVSCSGVARARARNAAPRTPAAPASPAAVPSEGSGGSTTGWRAREEQPP
SVAEGYASQDVFSATETSLWYDFLPDQAAGLCGGDGRPRQATQRLSVLLCANADGSEKLP
PLVAGKSAKPRAGQAGLPCDYTANSKGGVTTQALAKYLKALDTRMAAESRRVLLLAGRLA
AQSLDTSGLRHVQLAFFPPGTVHPLERGVVQQVKGHYRQAMLLKAMAALEGQDPSGLQLG
LTEALHFVAAAWQAVEPSDIAACFREAGFGGGPNATITTSLKSEGEEEEEEEEEEEEEEG
EGEEEEEEGEEEEEEEGGEGEELGEEEEVEEEGDVDSDEEEEEDEESSSEGLEAEDWAQGV
VEAGGSFGAYGAQEEAQCPTLHFLEGGEDSDSDSEEEDDEEEDDEDEDDDDDEEDGDEVP
VPSFGEAMAYFAMVKRYLTSFPIDDRVQSHILHLEHDLVHVTRKNHARQAGVRGLGHQS
>sp|Q02224|CENPE_HUMAN Centromere-associated protein E OS=Homo sapiens GN=CENPE PE=1 SV=2
MAEEGAVAVCVRVRPLNSREESLGETAQVYWKTDNNVIYQVDGSKSFNFDRVFHGNETTK
NVYEEIAAPIIDSAIQGYNGTIFAYGQTASGKTYTMMGSEDHLGVIPRAIHDIFQKIKKF
PDREFLLRVSYMEIYNETITDLLCGTQKMKPLIIREDVNRNVYVADLTEEVVYTSEMALK
WITKGEKSRHYGETKMNQRSSRSHTIFRMILESREKGEPSNCEGSVKVSHLNLVDLAGSE
RAAQTGAAGVRLKEGCNINRSLFILGQVIKKLSDGQVGGFINYRDSKLTRILQNSLGGNA
KTRIICTITPVSFDETLTALQFASTAKYMKNTPYVNEVSTDEALLKRYRKEIMDLKKQLE
EVSLETRAQAMEKDQLAQLLEEKDLLQKVQNEKIENLTRMLVTSSSLTLQQELKAKRKRR
VTWCLGKINKMKNSNYADQFNIPTNITTKTHKLSINLLREIDESVCSESDVFSNTLDTLS
EIEWNPATKLLNQENIESELNSLRADYDNLVLDYEQLRTEKEEMELKLKEKNDLDEFEAL
ERKTKKDQEMQLIHEISNLKNLVKHAEVYNQDLENELSSKVELLREKEDQIKKLQEYIDS

QKLENIKMDLSYSLESIEDPKQMKQTLFDAETVALDAKRESAFLRSENLELKEKMKELAT
TYKQMENDIQLYQSQLEAKKKMQVDLEKELQSAFNEITKLTSLIDGKVPKDLLCNLELEG
KITDLQKELNKEVEENEALREEVILLSELKSLPSEVERLRKEIQDKSEELHIITSEKDKL
FSEVVHKESRVQGLLEEIGKTKDDLATTQSNYKSTDQEFQNFKTLHMDFEQKYKMVLEEN
ERMNQEIVNLSKEAQKFDSSLGALKTELSYKTQELQEKTREVQERLNEMEQLKEQLENRD
STLQTVEREKTLITEKLQQTLEEVKTLTQEKDDLKQLQESLQIERDQLKSDIHDTVNMNI
DTQEQLRNALESLKQHQETINTLKSKISEEVSRNLHMEENTGETKDEFQQKMVGIDKKQD
LEAKNTQTLTADVKDNEIIEQQRKIFSLIQEKNELQQMLESVIAEKEQLKTDLKENIEMT
IENQEELRLLGDELKKQQEIVAQEKNHAIKKEGELSRTCDRLAEVEEKLKEKSQQLQEKQ
QQLLNVQEEMSEMQKKINEIENLKNELKNKELTLEHMETERLELAQKLNENYEEVKSITK
ERKVLKELQKSFETERDHLRGYIREIEATGLQTKEELKIAHIHLKEHQETIDELRRSVSE
KTAQIINTQDLEKSHTKLQEEIPVLHEEQELLPNVKEVSETQETMNELELLTEQSTTKDS
TTLARIEMERLRLNEKFQESQEEIKSLTKERDNLKTIKEALEVKHDQLKEHIRETLAKIQ
ESQSKQEQSLNMKEKDNETTKIVSEMEQFKPKDSALLRIEIEMLGLSKRLQESHDEMKSV
AKEKDDLQRLQEVLQSESDQLKENIKEIVAKHLETEEELKVAHCCLKEQEETINELRVNL
SEKETEISTIQKQLEAINDKLQNKIQEIYEKEEQFNIKQISEVQEKVNELKQFKEHRKAK
DSALQSIESKMLELTNRLQESQEEIQIMIKEKEEMKRVQEALQIERDQLKENTKEIVAKM
KESQEKEYQFLKMTAVNETQEKMCEIEHLKEQFETQKLNLENIETENIRLTQILHENLEE
MRSVTKERDDLRSVEETLKVERDQLKENLRETITRDLEKQEELKIVHMHLKEHQETIDKL
RGIVSEKTNEISNMQKDLEHSNDALKAQDLKIQEELRIAHMHLKEQQETIDKLRGIVSEK
TDKLSNMQKDLENSNAKLQEKIQELKANEHQLITLKKDVNETQKKVSEMEQLKKQIKDQS
LTLSKLEIENLNLAQKLHENLEEMKSVMKERDNLRRVEETLKLERDQLKESLQETKARDL
EIQQELKTARMLSKEHKETVDKLREKISEKTIQISDIQKDLDKSKDELQKKIQELQKKEL
QLLRVKEDVNMSHKKINEMEQLKKQFEAQNLSMQSVRMDNFQLTKKLHESLEEIRIVAKE
RDELRRIKESLKMERDQFIATLREMIARDRQNHQVKPEKRLLSDGQQHLTESLREKCSRI
KELLKRYSEMDDHYECLNRLSLDLEKEIEFQKELSMRVKANLSLPYLQTKHIEKLFTANQ
RCSMEFHRIMKKLKYVLSYVTKIKEEQHESINKFEMDFIDEVEKQKELLIKIQHLQQDCD
VPSRELRDLKLNQNMDLHIEEILKDFSESEFPSIKTEFQQVLSNRKEMTQFLEEWLNTRF
DIEKLKNGIQKENDRICQVNNFFNNRIIAIMNESTEFEERSATISKEWEQDLKSLKEKNE
KLFKNYQTLKTSLASGAQVNPTTQDNKNPHVTSRATQLTTEKIRELENSLHEAKESAMHK
ESKIIKMQKELEVTNDIIAKLQAKVHESNKCLEKTKETIQVLQDKVALGAKPYKEEIEDL
KMKLVKIDLEKMKNAKEFEKEISATKATVEYQKEVIRLLRENLRRSQQAQDTSVISEHTD
PQPSNKPLTCGGGSGIVQNTKALILKSEHIRLEKEISKLKQQNEQLIKQKNELLSNNQHL
SNEVKTWKERTLKREAHKQVTCENSPKSPKVTGTASKKKQITPSQCKERNLQDPVPKESP
KSCFFDSRSKSLPSPHPVRYFDNSSLGLCPEVQNAGAESVDSQPGPWHASSGKDVPECKT
Q
>sp|P49454|CENPF_HUMAN Centromere protein F OS=Homo sapiens GN=CENPF PE=1 SV=2
MSWALEEWKEGLPTRALQKIQELEGQLDKLKKEKQQRQFQLDSLEAALQKQKQKVENEKT
EGTNLKRENQRLMEICESLEKTKQKISHELQVKESQVNFQEGQLNSGKKQIEKLEQELKR
CKSELERSQQAAQSADVSLNPCNTPQKIFTTPLTPSQYYSGSKYEDLKEKYNKEVEERKR
LEAEVKALQAKKASQTLPQATMNHRDIARHQASSSVFSWQQEKTPSHLSSNSQRTPIRRD
FSASYFSGEQEVTPSRSTLQIGKRDANSSFFDNSSSPHLLDQLKAQNQELRNKINELELR
LQGHEKEMKGQVNKFQELQLQLEKAKVELIEKEKVLNKCRDELVRTTAQYDQASTKYTAL
EQKLKKLTEDLSCQRQNAESARCSLEQKIKEKEKEFQEELSRQQRSFQTLDQECIQMKAR

LTQELQQAKNMHNVLQAELDKLTSVKQQLENNLEEFKQKLCRAEQAFQASQIKENELRRS
MEEMKKENNLLKSHSEQKAREVCHLEAELKNIKQCLNQSQNFAEEMKAKNTSQETMLRDL
QEKINQQENSLTLEKLKLAVADLEKQRDCSQDLLKKREHHIEQLNDKLSKTEKESKALLS
ALELKKKEYEELKEEKTLFSCWKSENEKLLTQMESEKENLQSKINHLETCLKTQQIKSHE
YNERVRTLEMDRENLSVEIRNLHNVLDSKSVEVETQKLAYMELQQKAEFSDQKHQKEIEN
MCLKTSQLTGQVEDLEHKLQLLSNEIMDKDRCYQDLHAEYESLRDLLKSKDASLVTNEDH
QRSLLAFDQQPAMHHSFANIIGEQGSMPSERSECRLEADQSPKNSAILQNRVDSLEFSLE
SQKQMNSDLQKQCEELVQIKGEIEENLMKAEQMHQSFVAETSQRISKLQEDTSAHQNVVA
ETLSALENKEKELQLLNDKVETEQAEIQELKKSNHLLEDSLKELQLLSETLSLEKKEMSS
IISLNKREIEELTQENGTLKEINASLNQEKMNLIQKSESFANYIDEREKSISELSDQYKQ
EKLILLQRCEETGNAYEDLSQKYKAAQEKNSKLECLLNECTSLCENRKNELEQLKEAFAK
EHQEFLTKLAFAEERNQNLMLELETVQQALRSEMTDNQNNSKSEAGGLKQEIMTLKEEQN
KMQKEVNDLLQENEQLMKVMKTKHECQNLESEPIRNSVKERESERNQCNFKPQMDLEVKE
ISLDSYNAQLVQLEAMLRNKELKLQESEKEKECLQHELQTIRGDLETSNLQDMQSQEISG
LKDCEIDAEEKYISGPHELSTSQNDNAHLQCSLQTTMNKLNELEKICEILQAEKYELVTE
LNDSRSECITATRKMAEEVGKLLNEVKILNDDSGLLHGELVEDIPGGEFGEQPNEQHPVS
LAPLDESNSYEHLTLSDKEVQMHFAELQEKFLSLQSEHKILHDQHCQMSSKMSELQTYVD
SLKAENLVLSTNLRNFQGDLVKEMQLGLEEGLVPSLSSSCVPDSSSLSSLGDSSFYRALL
EQTGDMSLLSNLEGAVSANQCSVDEVFCSSLQTYVDSLKAENLVLSTNLRNFQGDLVKEM
QLGLEEGLVPSLSSSCVPDSSSLSSLGDSSFYRALLEQTGDMSLLSNLEGVVSANQCSVD
EVFCSSLQEENLTRKETPSAPAKGVEELESLCEVYRQSLEKLEEKMESQGIMKNKEIQEL
EQLLSSERQELDCLRKQYLSENEQWQQKLTSVTLEMESKLAAEKKQTEQLSLELEVARLQ
LQGLDLSSRSLLGIDTEDAIQGRNESCDISKEHTSETTERTPKHDVHQICDKDAQQDLNL
DIEKITETGAVKPTGECSGEQSPDTNYEPPGEDKTQGSSECISELSFSGPNALVPMDFLG
NQEDIHNLQLRVKETSNENLRLLHVIEDRDRKVESLLNEMKELDSKLHLQEVQLMTKIEA
CIELEKIVGELKKENSDLSEKLEYFSCDHQELLQRVETSEGLNSDLEMHADKSSREDIGD
NVAKVNDSWKERFLDVENELSRIRSEKASIEHEALYLEADLEVVQTEKLCLEKDNENKQK
VIVCLEEELSVVTSERNQLRGELDTMSKKTTALDQLSEKMKEKTQELESHQSECLHCIQV
AEAEVKEKTELLQTLSSDVSELLKDKTHLQEKLQSLEKDSQALSLTKCELENQIAQLNKE
KELLVKESESLQARLSESDYEKLNVSKALEAALVEKGEFALRLSSTQEEVHQLRRGIEKL
RVRIEADEKKQLHIAEKLKERERENDSLKDKVENLERELQMSEENQELVILDAENSKAEV
ETLKTQIEEMARSLKVFELDLVTLRSEKENLTKQIQEKQGQLSELDKLLSSFKSLLEEKE
QAEIQIKEESKTAVEMLQNQLKELNEAVAALCGDQEIMKATEQSLDPPIEEEHQLRNSIE
KLRARLEADEKKQLCVLQQLKESEHHADLLKGRVENLERELEIARTNQEHAALEAENSKG
EVETLKAKIEGMTQSLRGLELDVVTIRSEKENLTNELQKEQERISELEIINSSFENILQE
KEQEKVQMKEKSSTAMEMLQTQLKELNERVAALHNDQEACKAKEQNLSSQVECLELEKAQ
LLQGLDEAKNNYIVLQSSVNGLIQEVEDGKQKLEKKDEEISRLKNQIQDQEQLVSKLSQV
EGEHQLWKEQNLELRNLTVELEQKIQVLQSKNASLQDTLEVLQSSYKNLENELELTKMDK
MSFVEKVNKMTAKETELQREMHEMAQKTAELQEELSGEKNRLAGELQLLLEEIKSSKDQL
KELTLENSELKKSLDCMHKDQVEKEGKVREEIAEYQLRLHEAEKKHQALLLDTNKQYEVE
IQTYREKLTSKEECLSSQKLEIDLLKSSKEELNNSLKATTQILEELKKTKMDNLKYVNQL
KKENERAQGKMKLLIKSCKQLEEEKEILQKELSQLQAAQEKQKTGTVMDTKVDELTTEIK
ELKETLEEKTKEADEYLDKYCSLLISHEKLEKAKEMLETQVAHLCSQQSKQDSRGSPLLG
PVVPGPSPIPSVTEKRLSSGQNKASGKRQRSSGIWENGRGPTPATPESFSKKSKKAVMSG

IHPAEDTEGTEFEPEGLPEVVKKGFADIPTGKTSPYILRRTTMATRTSPRLAAQKLALSP
LSLGKENLAESSKPTAGGSRSQKVKVAQRSPVDSGTILREPTTKSVPVNNLPERSPTDSP
REGLRVKRGRLVPSPKAGLESNGSENCKVQ
>sp|Q96ST8|CEP89_HUMAN Centrosomal protein of 89 kDa OS=Homo sapiens GN=CEP89 PE=1 SV=3
MLLGFRRGRRSHFKHIIHGLLPAASVAPKAAVPRTPPPRSPNPSPERPRSALAAAILATT
LTGRTVAIPQPRQRSRSESDVSSVEQDSFIEPYATTSQLRPRPNWQSEMGRRSSLPSFET
LDYGDEEDIETQLSSSGKELGDVSAREDRGGHSDDLYAVPHRNQVPLLHEVNSEDDENIS
HQDGFPGSPPAPQRTQQKDGKHPVLNLKDEKPPLCEKPPPSPDITGRARQRYTEITREKF
EALKEENMDLNNMNQSLTLELNTMKQAMKELQLKLKGMEKEKRKLKEAEKASSQEVAAPE
LLYLRKQAQELVDENDGLKMTVHRLNVELSRYQTKFRHLSKEESLNIEGLPSKGPIPPWL
LDIKYLSPLLLAYEDMMKEKDELNATLKEEMRMFRMRVQEVVKENEELHQELNKSSAVTS
EEWRQLQTQAKLVLEENKLLLEQLEIQQRKAKDSHQERLQEVSKLTKQLMLLEAKTHGQE
KELAENREQLEILRAKCQELKTHSDGKIAVEVHKSIVNELKSQLQKEEEKERAEMEELME
KLTVLQAQKKSLLLEKNSLTEQNKALEAELERAQKINRKSQKKIEVLKKQVEKAMGNEMS
AHQYLANLVGLAENITQERDSLMCLAKCLESEKDGVLNKVIKSNIRLGKLEEKVKGYKKQ
AALKLGDISHRLLEQQEDFAGKTAQYRQEMRHLHQVLKDKQEVLDQALQQNREMEGELEV
IWESTFRENRRIRELLQDTLTRTGVQDNPRALVAPSLNGVSQADLLDGCDVCSYDLKSHA
PTC
>sp|Q49MI3|CERKL_HUMAN Ceramide kinase-like protein OS=Homo sapiens GN=CERKL PE=1 SV=1
MPWRRRRNRVSALEGGREEEAPPEAAAVPPALLTSPQQTEAAAERILLRGIFEIGRDSCD
VVLSERALRWRPIQPERPAGDSKYDLLCKEEFIELKDIFSVKLKRRCSVKQQRSGTLLGI
TLFICLKKEQNKLKNSTLDLINLSEDHCDIWFRQFKKILAGFPNRPKSLKILLNPQSHKK
EATQVYYEKVEPLLKLAGIKTDVTIMEYEGHALSLLKECELQGFDGGHRKPLFAIHWSVQ
RLFTGMQTLEPSVVCVGGDGSASEVAHALLLRAQKNAGMETDRILTPVRAQLPLGLIPAG
STNVLAHSLHGVPHVITATLHIIMGHVQLVDVCTFSTAGKLLRFGFSAMFGFGGRTLALA
EKYRWMSPNQRRDFAVVKALAKLKAEDCEISFLPFNSSDDVQERRAQGSPKSDCNDQWQM
IQGQFLNVSIMAIPCLCSVAPRGLAPNTRLNNGSMALIIARNTSRPEFIKHLKRYASVKN
QFNFPFVETYTVEEVKVHPRNNTGGYNPEEEEDETASENCFPWNVDGDLMEVASEVHIRL
HPRLISLYGGSMEEMIPK
>sp|Q96G23|CERS2_HUMAN Ceramide synthase 2 OS=Homo sapiens GN=CERS2 PE=1 SV=1
MLQTLYDYFWWERLWLPVNLTWADLEDRDGRVYAKASDLYITLPLALLFLIVRYFFELYV
ATPLAALLNIKEKTRLRAPPNATLEHFYLTSGKQPKQVEVELLSRQSGLSGRQVERWFRR
RRNQDRPSLLKKFREASWRFTFYLIAFIAGMAVIVDKPWFYDMKKVWEGYPIQSTIPSQY
WYYMIELSFYWSLLFSIASDVKRKDFKEQIIHHVATIILISFSWFANYIRAGTLIMALHD
SSDYLLESAKMFNYAGWKNTCNNIFIVFAIVFIITRLVILPFWILHCTLVYPLELYPAFF
GYYFFNSMMGVLQLLHIFWAYLILRMAHKFITGKLVEDERSDREETESSEGEEAAAGGGA
KSRPLANGHPILNNNHRKND
>sp|O95873|CF047_HUMAN Uncharacterized protein C6orf47 OS=Homo sapiens GN=C6orf47 PE=1 SV=2
MFLRRLGGWLPRPWGRRKPMRPDPPYPEPRRVDSSSENSGSDWDSAPETMEDVGHPKTKD
SGALRVSGAASEPSKEEPQVEQLGSKRMDSLKWDQPISSTQESGRLEAGGASPKLRWDHV
DSGGTRRPGVSPEGGLSVPGPGAPLEKPGRREKLLGWLRGEPGAPSRYLGGPEECLQIST
NLTLHLLELLASALLALCSRPLRAALDTLGLRGPLGLWLHGLLSFLAALHGLHAVLSLLT
AHPLHFACLFGLLQALVLAVSLREPNGDEAATDWESEGLEREGEEQRGDPGKGL

>sp|Q5T4I8|CF052_HUMAN Putative uncharacterized protein C6orf52 OS=Homo sapiens GN=C6orf52 PE=2 SV=2
MAQPESSADFGIAQQNNYYCYWQSLPSAIRVKQEFQPSQSYRYGNWYARQHGSYLLSGYS
YGCAVDGNGKDCFSAHETPEHTAGTLVMPKETTPLAENQDEDPLEDPHLHLNIEESNQEF
MVKSEELYDSLMNCHWQPLDTVHSEIPDETPK
>sp|Q8N319|CF223_HUMAN Uncharacterized protein C6orf223 OS=Homo sapiens GN=C6orf223 PE=2 SV=2
MMPLAEAGALAQGGGPSATEWACILRRKTPRHKQPTLLMVRASRRSGKTSAVLKAGRQSV
SGRKNSTSKDLVTLGASSLREERGHPLHPRHRKAVHLRTRGRTRGWVQTLARMSRRTRGP
VERAAAAAAAAAGGDAGHAPFPPPPAADGARAPRSPGQVTPRGLRLRLPRRESLLRGLCR
PLRPLLGFRESDSAKPASLRLLQHTPSARRNYRIAGARLMRSNYPPPLSSAALRGAGPTR
RN
>sp|Q5EG05|CAR16_HUMAN Caspase recruitment domain-containing protein 16 OS=Homo sapiens GN=CARD16 PE=1 SV=1
MADKVLKEKRKLFIHSMGEGTINGLLDELLQTRVLNQEEMEKVKRENATVMDKTRALIDS
VIPKGAQACQICITYICEEDSYLAETLGLSAALQAVQDNPAMPTCSSPEGRIKLCFLEDA
QRIWKQKLQRCHVQNTIIKWSERYTSGSFEMQWLFLRTNFIERFWRNILLLPLHKGSLYP
RIPGLGKELQTGTHKLS
>sp|P53634|CATC_HUMAN Dipeptidyl peptidase 1 OS=Homo sapiens GN=CTSC PE=1 SV=2
MGAGPSLLLAALLLLLSGDGAVRCDTPANCTYLDLLGTWVFQVGSSGSQRDVNCSVMGPQ
EKKVVVYLQKLDTAYDDLGNSGHFTIIYNQGFEIVLNDYKWFAFFKYKEEGSKVTTYCNE
TMTGWVHDVLGRNWACFTGKKVGTASENVYVNIAHLKNSQEKYSNRLYKYDHNFVKAINA
IQKSWTATTYMEYETLTLGDMIRRSGGHSRKIPRPKPAPLTAEIQQKILHLPTSWDWRNV
HGINFVSPVRNQASCGSCYSFASMGMLEARIRILTNNSQTPILSPQEVVSCSQYAQGCEG
GFPYLIAGKYAQDFGLVEEACFPYTGTDSPCKMKEDCFRYYSSEYHYVGGFYGGCNEALM
KLELVHHGPMAVAFEVYDDFLHYKKGIYHHTGLRDPFNPFELTNHAVLLVGYGTDSASGM
DYWIVKNSWGTGWGENGYFRIRRGTDECAIESIAVAATPIPKL
>sp|Q9UBX1|CATF_HUMAN Cathepsin F OS=Homo sapiens GN=CTSF PE=1 SV=1
MAPWLQLLSLLGLLPGAVAAPAQPRAASFQAWGPPSPELLAPTRFALEMFNRGRAAGTRA
VLGLVRGRVRRAGQGSLYSLEATLEEPPCNDPMVCRLPVSKKTLLCSFQVLDELGRHVLL
RKDCGPVDTKVPGAGEPKSAFTQGSAMISSLSQNHPDNRNETFSSVISLLNEDPLSQDLP
VKMASIFKNFVITYNRTYESKEEARWRLSVFVNNMVRAQKIQALDRGTAQYGVTKFSDLT
EEEFRTIYLNTLLRKEPGNKMKQAKSVGDLAPPEWDWRSKGAVTKVKDQGMCGSCWAFSV
TGNVEGQWFLNQGTLLSLSEQELLDCDKMDKACMGGLPSNAYSAIKNLGGLETEDDYSYQ
GHMQSCNFSAEKAKVYINDSVELSQNEQKLAAWLAKRGPISVAINAFGMQFYRHGISRPL
RPLCSPWLIDHAVLLVGYGNRSDVPFWAIKNSWGTDWGEKGYYYLHRGSGACGVNTMASS
AVVD
>sp|P25774|CATS_HUMAN Cathepsin S OS=Homo sapiens GN=CTSS PE=1 SV=3
MKRLVCVLLVCSSAVAQLHKDPTLDHHWHLWKKTYGKQYKEKNEEAVRRLIWEKNLKFVM
LHNLEHSMGMHSYDLGMNHLGDMTSEEVMSLMSSLRVPSQWQRNITYKSNPNRILPDSVD
WREKGCVTEVKYQGSCGACWAFSAVGALEAQLKLKTGKLVSLSAQNLVDCSTEKYGNKGC
NGGFMTTAFQYIIDNKGIDSDASYPYKAMDQKCQYDSKYRAATCSKYTELPYGREDVLKE
AVANKGPVSVGVDARHPSFFLYRSGVYYEPSCTQNVNHGVLVVGYGDLNGKEYWLVKNSW
GHNFGEEGYIRMARNKGNHCGIASFPSYPEI

>sp|P48052|CBPA2_HUMAN Carboxypeptidase A2 OS=Homo sapiens GN=CPA2 PE=1 SV=3
MAMRLILFFGALFGHIYCLETFVGDQVLEIVPSNEEQIKNLLQLEAQEHLQLDFWKSPTT
PGETAHVRVPFVNVQAVKVFLESQGIAYSIMIEDVQVLLDKENEEMLFNRRRERSGNFNF
GAYHTLEEISQEMDNLVAEHPGLVSKVNIGSSFENRPMNVLKFSTGGDKPAIWLDAGIHA
REWVTQATALWTANKIVSDYGKDPSITSILDALDIFLLPVTNPDGYVFSQTKNRMWRKTR
SKVSGSLCVGVDPNRNWDAGFGGPGASSNPCSDSYHGPSANSEVEVKSIVDFIKSHGKVK
AFITLHSYSQLLMFPYGYKCTKLDDFDELSEVAQKAAQSLRSLHGTKYKVGPICSVIYQA
SGGSIDWSYDYGIKYSFAFELRDTGRYGFLLPARQILPTAEETWLGLKAIMEHVRDHPY
>sp|Q8N4T0|CBPA6_HUMAN Carboxypeptidase A6 OS=Homo sapiens GN=CPA6 PE=1 SV=2
MKCLGKRRGQAAAFLPLCWLFLKILQPGHSHLYNNRYAGDKVIRFIPKTEEEAYALKKIS
YQLKVDLWQPSSISYVSEGTVTDVHIPQNGSRALLAFLQEANIQYKVLIEDLQKTLEKGS
SLHTQRNRRSLSGYNYEVYHSLEEIQNWMHHLNKTHSGLIHMFSIGRSYEGRSLFILKLG
RRSRLKRAVWIDCGIHAREWIGPAFCQWFVKEALLTYKSDPAMRKMLNHLYFYIMPVFNV
DGYHFSWTNDRFWRKTRSRNSRFRCRGVDANRNWKVKWCDEGASMHPCDDTYCGPFPESE
PEVKAVANFLRKHRKHIRAYLSFHAYAQMLLYPYSYKYATIPNFRCVESAAYKAVNALQS
VYGVRYRYGPASTTLYVSSGSSMDWAYKNGIPYAFAFELRDTGYFGFLLPEMLIKPTCTE
TMLAVKNITMHLLKKCP
>sp|Q8NEM8|CBPC3_HUMAN Cytosolic carboxypeptidase 3 OS=Homo sapiens GN=AGBL3 PE=2 SV=2
MSEDSEKEDYSDRTISDEDESDEDMFMKFVSEDLHRCALLTADSFGDPFFPRTTQILLEY
QLGRWVPRLREPRDLYGVSSSGPLSPTRWPYHCEVIDEKVQHIDWTPSCPEPVYIPTGLE
TEPLYPDSKEATVVYLAEDAYKEPCFVYSRVGGNRTPLKQPVDYRDNTLMFEARFESGNL
QKVVKVAEYEYQLTVRPDLFTNKHTQWYYFQVTNMRAGIVYRFTIVNFTKPASLYSRGMR
PLFYSEKEAKAHHIGWQRIGDQIKYYRNNPGQDGRHYFSLTWTFQFPHNKDTCYFAHCYP
YTYTNLQEYLSGINNDPVRSKFCKIRVLCHTLARNMVYILTITTPLKNSDSRKRKAVILT
ARVHPGETNSSWIMKGFLDYILGNSSDAQLLRDTFVFKVVPMLNPDGVIVGNYRCSLAGR
DLNRNYTSLLKESFPSVWYTRNMVHRLMEKREVILYCDLHGHSRKENIFMYGCDGSDRSK
TLYLQQRIFPLMLSKNCPDKFSFSACKFNVQKSKEGTGRVVMWKMGIRNSFTMEATFCGS
TLGNKRGTHFSTKDLESMGYHFCDSLLDYCDPDRTKYYRCLKELEEMERHITLEKVFEDS
DTPVIDITLDVESSSRGSDSSESIDSLTYLLKLTSQKKHLKTKKERNSTIASHQNARGQE
VYDRGHLLQRHTQSNSDVKDTRPNEPDDYMVDYFRRQLPNQGLAHCKLRLPGSRHSPASA
SRVAGTTGTRHHTWLIFVFLVEMGKKIPLKGTDLYGNCFKVTSLQSPMGKQTSTWTEKTR
IPTEDLHHNLKSKIKECISFQSKKTGINWTDDEKRSYKDKGIVQTQEILQYLLPIVHSTK
NMQTTQIKQLFNPRTNFQIQHQLNPATCRNIKKYSTSWTAPRNHPFVIQGDVMANSSEWV
QSKPHRSLESLSPLKGPKKNKHSQIWAIKNEDIKPLSSKWETASSSFGMDANVLKYKSLQ
AEETNQQSSKHTALHLTKNKDEQANKNDGQPTLYLKFQRES
>sp|Q96MI9|CBPC4_HUMAN Cytosolic carboxypeptidase 4 OS=Homo sapiens GN=AGBL1 PE=1 SV=2
MISKGGSEALLQTLVDTARTAPPDYDILLPLFRLLAKVGLRDKKIGRKALELEALDVTLI
LARKNLSHGQNLLHCLWALRVFASSVSMGAMLGINGAMELLFKVITPYTRKRTQAIRAAT
EVLAALLKSKSNGRRAVNRGYVTSLLGLHQDWHSHDTANAYVQIRRGLLLCLRHIAALRS
GREAFLAAQGMEILFSTTQNCLDDKSMEPVISVVLQILRQCYPTSPLPLVTASSAYAFPV
PGCITTEPPHDLPEEDFEDDGDDEVDKDSDTEDGKVEDDDLETDVNKLSSKPGLDRPEEE
LMQYEVMCLELSYSFEELQSKLGDDLNSEKTQYANHHHIPAAASSKQHCYSKDQSSCGQE
REYAVQTSLLCRVKTGRSTVHLGSKKNPGVNLYQNVQSNSLRRDSSESEIPDIQASPKAD
AWDVDAIFCPRMSASFSNSTRTREVVKVIDKLLQTHLKRVPFHDPYLYMAKARRTSSVVD

FKMMAFPDVWGHCPPPTTQPMLERKCGVQRIRIFEDIRRLIQPSDVINKVVFSLDEPWPL
QDNASNCLRFFSKFESGNLRKAIQVREFEYDLLVNADVNSTQHQQWFYFKVSGMQAAIPY
HFNIINCEKPNSQFNYGMQPTLYSVKEALLGKPTWIRTGHEICYYKNHYRQSTAVAGGAS
GKCYYTLTFAVTFPHSEDVCYLAYHYPYTYTALMTHLDILEKSVNLKEVYFRQDVLCQTL
GGNPCPLVTITAMPESNSDEHLEQFRHRPYQVITARVHPGESNASWVMKGTLEFLVSSDP
VARLLRENFIFKIIPMLNPDGVINGNHRCSLSGEDLNRQWLSPSAHLQPTIYHAKGLLYH
LSSIGRSPVVFCDFHGHSQKKNVFLYGCSIKETLWQAACTVGTSTILEEVNYRTLPKILD
KLAPAFTMSSCSFLVEKSRASTARVVVWREMGVSRSYTMESSYCGCNQGPYQCTQRLLER
TKNERAHPVDGLQGLQFGTRELEEMGAMFCLGLLILELKSASCSHQLLAQAATLLSAEED
ALDQHLQRLKSSNFLPKHIWFAYHFFAITNFFKMNLLLHVSPVCDT
>sp|Q5VU57|CBPC6_HUMAN Cytosolic carboxypeptidase 6 OS=Homo sapiens GN=AGBL4 PE=2 SV=3
MAEGSQSAPEAGNDMGNDDAIGGNVSKYIVLPTGYCGQPKKGHLIFDACFESGNLGRVDQ
VSEFEYDLFIRPDTCNPRFRVWFNFTVENVKESQRVIFNIVNFSKTKSLYRDGMAPMVKS
TSRPKWQRLPPKNVYYYRCPDHRKNYVMSFAFCFDREEDIYQFAYCYPYTYTRFQHYLDS
LQKRNMDYFFREQLGQSVQQRKLDLLTITSPDNLREGAEQKVVFITGRVHPGETPSSFVC
QGIIDFLVSQHPIACVLREYLVFKIAPMLNPDGVYLGNYRCSLMGFDLNRHWLDPSPWVH
PTLHGVKQLIVQMYNDPKTSLEFYIDIHAHSTMMNGFMYGNIFEDEERFQRQAIFPKLLC
QNAEDFSYSSTSFNRDAVKAGTGRRFLGGLLDHTSYCYTLEVSFYSYIISGTTAAVPYTE
EAYMKLGRNVARTFLDYYRLNPVVEKVAIPMPRLRNKEIEVQRRKEKSPPYKHPLLRGPA
SNYPNSKGDKKSSVNHKDPSTPF
>sp|Q92793|CBP_HUMAN CREB-binding protein OS=Homo sapiens GN=CREBBP PE=1 SV=3
MAENLLDGPPNPKRAKLSSPGFSANDSTDFGSLFDLENDLPDELIPNGGELGLLNSGNLV
PDAASKHKQLSELLRGGSGSSINPGIGNVSASSPVQQGLGGQAQGQPNSANMASLSAMGK
SPLSQGDSSAPSLPKQAASTSGPTPAASQALNPQAQKQVGLATSSPATSQTGPGICMNAN
FNQTHPGLLNSNSGHSLINQASQGQAQVMNGSLGAAGRGRGAGMPYPTPAMQGASSSVLA
ETLTQVSPQMTGHAGLNTAQAGGMAKMGITGNTSPFGQPFSQAGGQPMGATGVNPQLASK
QSMVNSLPTFPTDIKNTSVTNVPNMSQMQTSVGIVPTQAIATGPTADPEKRKLIQQQLVL
LLHAHKCQRREQANGEVRACSLPHCRTMKNVLNHMTHCQAGKACQVAHCASSRQIISHWK
NCTRHDCPVCLPLKNASDKRNQQTILGSPASGIQNTIGSVGTGQQNATSLSNPNPIDPSS
MQRAYAALGLPYMNQPQTQLQPQVPGQQPAQPQTHQQMRTLNPLGNNPMNIPAGGITTDQ
QPPNLISESALPTSLGATNPLMNDGSNSGNIGTLSTIPTAAPPSSTGVRKGWHEHVTQDL
RSHLVHKLVQAIFPTPDPAALKDRRMENLVAYAKKVEGDMYESANSRDEYYHLLAEKIYK
IQKELEEKRRSRLHKQGILGNQPALPAPGAQPPVIPQAQPVRPPNGPLSLPVNRMQVSQG
MNSFNPMSLGNVQLPQAPMGPRAASPMNHSVQMNSMGSVPGMAISPSRMPQPPNMMGAHT
NNMMAQAPAQSQFLPQNQFPSSSGAMSVGMGQPPAQTGVSQGQVPGAALPNPLNMLGPQA
SQLPCPPVTQSPLHPTPPPASTAAGMPSLQHTTPPGMTPPQPAAPTQPSTPVSSSGQTPT
PTPGSVPSATQTQSTPTVQAAAQAQVTPQPQTPVQPPSVATPQSSQQQPTPVHAQPPGTP
LSQAAASIDNRVPTPSSVASAETNSQQPGPDVPVLEMKTETQAEDTEPDPGESKGEPRSE
MMEEDLQGASQVKEETDIAEQKSEPMEVDEKKPEVKVEVKEEEESSSNGTASQSTSPSQP
RKKIFKPEELRQALMPTLEALYRQDPESLPFRQPVDPQLLGIPDYFDIVKNPMDLSTIKR
KLDTGQYQEPWQYVDDVWLMFNNAWLYNRKTSRVYKFCSKLAEVFEQEIDPVMQSLGYCC
GRKYEFSPQTLCCYGKQLCTIPRDAAYYSYQNRYHFCEKCFTEIQGENVTLGDDPSQPQT
TISKDQFEKKKNDTLDPEPFVDCKECGRKMHQICVLHYDIIWPSGFVCDNCLKKTGRPRK
ENKFSAKRLQTTRLGNHLEDRVNKFLRRQNHPEAGEVFVRVVASSDKTVEVKPGMKSRFV

DSGEMSESFPYRTKALFAFEEIDGVDVCFFGMHVQEYGSDCPPPNTRRVYISYLDSIHFF
RPRCLRTAVYHEILIGYLEYVKKLGYVTGHIWACPPSEGDDYIFHCHPPDQKIPKPKRLQ
EWYKKMLDKAFAERIIHDYKDIFKQATEDRLTSAKELPYFEGDFWPNVLEESIKELEQEE
EERKKEESTAASETTEGSQGDSKNAKKKNNKKTNKNKSSISRANKKKPSMPNVSNDLSQK
LYATMEKHKEVFFVIHLHAGPVINTLPPIVDPDPLLSCDLMDGRDAFLTLARDKHWEFSS
LRRSKWSTLCMLVELHTQGQDRFVYTCNECKHHVETRWHCTVCEDYDLCINCYNTKSHAH
KMVKWGLGLDDEGSSQGEPQSKSPQESRRLSIQRCIQSLVHACQCRNANCSLPSCQKMKR
VVQHTKGCKRKTNGGCPVCKQLIALCCYHAKHCQENKCPVPFCLNIKHKLRQQQIQHRLQ
QAQLMRRRMATMNTRNVPQQSLPSPTSAPPGTPTQQPSTPQTPQPPAQPQPSPVSMSPAG
FPSVARTQPPTTVSTGKPTSQVPAPPPPAQPPPAAVEAARQIEREAQQQQHLYRVNINNS
MPPGRTGMGTPGSQMAPVSLNVPRPNQVSGPVMPSMPPGQWQQAPLPQQQPMPGLPRPVI
SMQAQAAVAGPRMPSVQPPRSISPSALQDLLRTLKSPSSPQQQQQVLNILKSNPQLMAAF
IKQRTAKYVANQPGMQPQPGLQSQPGMQPQPGMHQQPSLQNLNAMQAGVPRPGVPPQQQA
MGGLNPQGQALNIMNPGHNPNMASMNPQYREMLRRQLLQQQQQQQQQQQQQQQQQQGSAG
MAGGMAGHGQFQQPQGPGGYPPAMQQQQRMQQHLPLQGSSMGQMAAQMGQLGQMGQPGLG
ADSTPNIQQALQQRILQQQQMKQQIGSPGQPNPMSPQQHMLSGQPQASHLPGQQIATSLS
NQVRSPAPVQSPRPQSQPPHSSPSPRIQPQPSPHHVSPQTGSPHPGLAVTMASSIDQGHL
GNPEQSAMLPQLNTPSRSALSSELSLVGDTTGDTLEKFVEGL
>sp|Q8N4T8|CBR4_HUMAN Carbonyl reductase family member 4 OS=Homo sapiens GN=CBR4 PE=1 SV=3
MDKVCAVFGGSRGIGRAVAQLMARKGYRLAVIARNLEGAKAAAGDLGGDHLAFSCDVAKE
HDVQNTFEELEKHLGRVNFLVNAAGINRDGLLVRTKTEDMVSQLHTNLLGSMLTCKAAMR
TMIQQQGGSIVNVGSIVGLKGNSGQSVYSASKGGLVGFSRALAKEVARKKIRVNVVAPGF
VHTDMTKDLKEEHLKKNIPLGRFGETIEVAHAVVFLLESPYITGHVLVVDGGLQLIL
>sp|P35520|CBS_HUMAN Cystathionine beta-synthase OS=Homo sapiens GN=CBS PE=1 SV=2
MPSETPQAEVGPTGCPHRSGPHSAKGSLEKGSPEDKEAKEPLWIRPDAPSRCTWQLGRPA
SESPHHHTAPAKSPKILPDILKKIGDTPMVRINKIGKKFGLKCELLAKCEFFNAGGSVKD
RISLRMIEDAERDGTLKPGDTIIEPTSGNTGIGLALAAAVRGYRCIIVMPEKMSSEKVDV
LRALGAEIVRTPTNARFDSPESHVGVAWRLKNEIPNSHILDQYRNASNPLAHYDTTADEI
LQQCDGKLDMLVASVGTGGTITGIARKLKEKCPGCRIIGVDPEGSILAEPEELNQTEQTT
YEVEGIGYDFIPTVLDRTVVDKWFKSNDEEAFTFARMLIAQEGLLCGGSAGSTVAVAVKA
AQELQEGQRCVVILPDSVRNYMTKFLSDRWMLQKGFLKEEDLTEKKPWWWHLRVQELGLS
APLTVLPTITCGHTIEILREKGFDQAPVVDEAGVILGMVTLGNMLSSLLAGKVQPSDQVG
KVIYKQFKQIRLTDTLGRLSHILEMDHFALVVHEQIQYHSTGKSSQRQMVFGVVTAIDLL
NFVAAQERDQK
>sp|O95931|CBX7_HUMAN Chromobox protein homolog 7 OS=Homo sapiens GN=CBX7 PE=1 SV=1
MELSAIGEQVFAVESIRKKRVRKGKVEYLVKWKGWPPKYSTWEPEEHILDPRLVMAYEEK
EERDRASGYRKRGPKPKRLLLQRLYSMDLRSSHKAKGKEKLCFSLTCPLGSGSPEGVVKA
GAPELVDKGPLVPTLPFPLRKPRKAHKYLRLSRKKFPPRGPNLESHSHRRELFLQEPPAP
DVLQAAGEWEPAAQPPEEEADADLAEGPPPWTPALPSSEVTVTDITANSITVTFREAQAA
EGFFRDRSGKF
>sp|Q9Y3M2|CBY1_HUMAN Protein chibby homolog 1 OS=Homo sapiens GN=CBY1 PE=1 SV=1
MPFFGNTFSPKKTPPRKSASLSNLHSLDRSTREVELGLEYGSPTMNLAGQSLKFENGQWI
AETGVSGGVDRREVQRLRRRNQQLEEENNLLRLKVDILLDMLSESTAESHLMEKELDELR

ISRKRK
>sp|Q96M83|CCDC7_HUMAN Coiled-coil domain-containing protein 7 OS=Homo sapiens GN=CCDC7 PE=2 SV=3
MKPVKHLLTTSNKSANVPALTTKKGLHNLPLSPELKEKHNAKLIHDKIEPMVLRSPPTGE
SILRYALPIPSSKTKNLLPEDEMIGKIIKHLKMVVSTLEETYGHCDQNGEEPFVKHEHEE
LSLSVGDDMNSFLTYCSQFAAQLEEALKEEQNILESLFKWFQWQVNQMEEISKDQTLLQA
EPPKPDKTVILNIAEIVRLVQRFEELKNRLKQRSKSSVKVMLSKTMDKENRPEAVKSCEA
LAQKIEEFLEAHSTDEFKDVSATEPQTAHSMTNRFNAMLKVFENQANMLERAVNDQVLLD
AEYKQMQCDFQLLSEEKLVLENELQKLKDKEKTKPTNNRTKKAVKTVKKKDKGKSEDSEK
KMSPEKEFKIKEDLDQVQKVARLEIENKVLQEQLKQALQEAEKAKHQLNYFLNQEKLLKS
EGKTETTMQVGNSQTKVKGEDSKNIPLEKETRKSLVSDSGGQRTSDKIQEYPQITAQSGR
LIEKSSEKKRSSPAISDLSQILKSQDESAFLESSNEVSVAENQSYKSPSETHDKSLTTVS
SSKEVQDSLSVGTLAQKNETVISPFILPPVLTESKKADVSEEQLQKMTEEQTYQAAEKSQ
ADSEVPDENLMVENKDSVTKVQIEQMKQRTSSMERHEETLTTPQLPEDMVLVSRIQSETK
NLKATRNESFHSHNDVPEENLMLEQDTKSKTEVEVKKQKSFQDNQLSTHNEVPNERLVVE
HQESLSKTKLQIKKQETSTEQPLTTPDKEPNENLILRHQDSMSKSEMQVKEQRTLKGQRI
ITHDEEPGKNLVLEHQDSVSKLEMQIEKTKKLPREKRHSTHDEESGENPMLKHQDSVSKI
QVQLEIQETSEGEGRSIPDKNSMFVHQDSVSKLQMQEKKKITPGRERRNTRIVVPNENVI
SVHQDSKSKLQMQEKKQINSGVERHKTFPLEIKKKDISLEHLLPEEKVLLSRSESQTKKL
QAKVTSRKIKNEAASELPDTAENLPAMYPSISDLIIQFDLNKVVETDIESLRGALGRRLL
NDEFKTQSKSFPGPDIEQLTDAFGRDILKDEFKTRSKSLPETDERLHSTTERGTINDAIK
TQLKRKSYPETVLKHLKGVNGKDIIKHLINIQSKSHGETDKEHLADDTGRGIIKGSINAQ
LKGHQKTDKNFFAYATGRGLMKESTTTQLKSHPETDKEFLADAIGRGIIIGPITTQLKSH
RETDKELLKDAIGRDIIKGPISAQLKSHQETDVEPLTNAIGSSKTIGEIKTQLRTHYDVN
LFKNKDMSVQRQEGIFTRSITPSKFPTKVINLSPFENKEETYEYSSPYVTAPSKAIYRTY
RAGPSFSKDIHLPLLNQLPSGHSKVVTLSQKTIEFTLPTVTNTVGKPTYKVLHAAARKSV
PHPYF
>sp|Q9H0W5|CCDC8_HUMAN Coiled-coil domain-containing protein 8 OS=Homo sapiens GN=CCDC8 PE=1 SV=2
MLQIGEDVDYLLIPREVRLAGGVWRVISKPATKEAEFRERLTQFLEEEGRTLEDVARIME
KSTPHPPQPPKKPKEPRVRRRVQQMVTPPPRLVVGTYDSSNASDSEFSDFETSRDKSRQG
PRRGKKVRKMPVSYLGSKFLGSDLESEDDEELVEAFLRRQEKQPSAPPARRRVNLPVPMF
EDNLGPQLSKADRWREYVSQVSWGKLKRRVKGWAPRAGPGVGEARLASTAVESAGVSSAP
EGTSPGDRLGNAGDVCVPQASPRRWRPKINWASFRRRRKEQTAPTGQGADIEADQGGEAA
DSQREEAIADQREGAAGNQRAGAPADQGAEAADNQREEAADNQRAGAPAEEGAEAADNQR
EEAADNQRAEAPADQRSQGTDNHREEAADNQRAEAPADQGSEVTDNQREEAVHDQRERAP
AVQGADNQRAQARAGQRAEAAHNQRAGAPGIQEAEVSAAQGTTGTAPGARARKQVKTVRF
QTPGRFSWFCKRRRAFWHTPRLPTLPKRVPRAGEARNLRVLRAEARAEAEQGEQEDQL
>sp|Q8WXS4|CCGL_HUMAN Voltage-dependent calcium channel gamma-like subunit OS=Homo sapiens GN=TMEM37 PE=2 SV=2
MTAVGVQAQRPLGQRQPRRSFFESFIRTLIITCVALAVVLSSVSICDGHWLLAEDRLFGL
WHFCTTTNQTICFRDLGQAHVPGLAVGMGLVRSVGALAVVAAIFGLEFLMVSQLCEDKHS
QCKWVMGSILLLVSFVLSSGGLLGFVILLRNQVTLIGFTLMFWCEFTASFLLFLNAISGL
HINSITHPWE

>sp|Q99616|CCL13_HUMAN C-C motif chemokine 13 OS=Homo sapiens GN=CCL13 PE=1 SV=1
MKVSAVLLCLLLMTAAFNPQGLAQPDALNVPSTCCFTFSSKKISLQRLKSYVITTSRCPQ
KAVIFRTKLGKEICADPKEKWVQNYMKHLGRKAHTLKT
>sp|P78556|CCL20_HUMAN C-C motif chemokine 20 OS=Homo sapiens GN=CCL20 PE=1 SV=1
MCCTKSLLLAALMSVLLLHLCGESEAASNFDCCLGYTDRILHPKFIVGFTRQLANEGCDI
NAIIFHTKKKLSVCANPKQTWVKYIVRLLSKKVKNM
>sp|O00175|CCL24_HUMAN C-C motif chemokine 24 OS=Homo sapiens GN=CCL24 PE=1 SV=2
MAGLMTIVTSLLFLGVCAHHIIPTGSVVIPSPCCMFFVSKRIPENRVVSYQLSSRSTCLK
AGVIFTTKKGQQFCGDPKQEWVQRYMKNLDAKQKKASPRARAVAVKGPVQRYPGNQTTC
>sp|P78396|CCNA1_HUMAN Cyclin-A1 OS=Homo sapiens GN=CCNA1 PE=1 SV=1
METGFPAIMYPGSFIGGWGEEYLSWEGPGLPDFVFQQQPVESEAMHCSNPKSGVVLATVA
RGPDACQILTRAPLGQDPPQRTVLGLLTANGQYRRTCGQGITRIRCYSGSENAFPPAGKK
ALPDCGVQEPPKQGFDIYMDELEQGDRDSCSVREGMAFEDVYEVDTGTLKSDLHFLLDFN
TVSPMLVDSSLLSQSEDISSLGTDVINVTEYAEEIYQYLREAEIRHRPKAHYMKKQPDIT
EGMRTILVDWLVEVGEEYKLRAETLYLAVNFLDRFLSCMSVLRGKLQLVGTAAMLLASKY
EEIYPPEVDEFVYITDDTYTKRQLLKMEHLLLKVLAFDLTVPTTNQFLLQYLRRQGVCVR
TENLAKYVAELSLLEADPFLKYLPSLIAAAAFCLANYTVNKHFWPETLAAFTGYSLSEIV
PCLSELHKAYLDIPHRPQQAIREKYKASKYLCVSLMEPPAVLLLQ
>sp|O95067|CCNB2_HUMAN G2/mitotic-specific cyclin-B2 OS=Homo sapiens GN=CCNB2 PE=1 SV=1
MALLRRPTVSSDLENIDTGVNSKVKSHVTIRRTVLEEIGNRVTTRAAQVAKKAQNTKVPV
QPTKTTNVNKQLKPTASVKPVQMEKLAPKGPSPTPEDVSMKEENLCQAFSDALLCKIEDI
DNEDWENPQLCSDYVKDIYQYLRQLEVLQSINPHFLDGRDINGRMRAILVDWLVQVHSKF
RLLQETLYMCVGIMDRFLQVQPVSRKKLQLVGITALLLASKYEEMFSPNIEDFVYITDNA
YTSSQIREMETLILKELKFELGRPLPLHFLRRASKAGEVDVEQHTLAKYLMELTLIDYDM
VHYHPSKVAAAASCLSQKVLGQGKWNLKQQYYTGYTENEVLEVMQHMAKNVVKVNENLTK
FIAIKNKYASSKLLKISMIPQLNSKAVKDLASPLIGRS
>sp|P30279|CCND2_HUMAN G1/S-specific cyclin-D2 OS=Homo sapiens GN=CCND2 PE=1 SV=1
MELLCHEVDPVRRAVRDRNLLRDDRVLQNLLTIEERYLPQCSYFKCVQKDIQPYMRRMVA
TWMLEVCEEQKCEEEVFPLAMNYLDRFLAGVPTPKSHLQLLGAVCMFLASKLKETSPLTA
EKLCIYTDNSIKPQELLEWELVVLGKLKWNLAAVTPHDFIEHILRKLPQQREKLSLIRKH
AQTFIALCATDFKFAMYPPSMIATGSVGAAICGLQQDEEVSSLTCDALTELLAKITNTDV
DCLKACQEQIEAVLLNSLQQYRQDQRDGSKSEDELDQASTPTDVRDIDL
>sp|Q6ZMN8|CCNI2_HUMAN Cyclin-I2 OS=Homo sapiens GN=CCNI2 PE=2 SV=1
MASGAQLPPQPSSSEVSAVQSPGGRPGAGLEETALGVPLPPSPGEAPLPRSNRSRCPGTR
QPGAASLHAASAAVPVRPRRGTAPAGKTADAVPAAAPEQAPRPAPQSRKPRNLEGDLDER
RLLCHLQLAQDREARLWRGGKPQDEICDAFEEVVLWLLRLQNTFYFSQSTFNLALTIFGR
LLISVKVKEKYLHCATITSLRLAAKVNEEEEFIPQVKDFTKHYGSDYSPNELLRMELAIL
DRLHWDLYIGTPLDFLTIFHALVVLSWPHVLELLPQRNPSLHVASLTRQLQHCMAGHQLL
QFKGSTLALVIITLELERLMPGWCAPISDLLKKAQVGDMQYSCCKELVMQQLRSLQSSSC
TDNFVSPAN
>sp|Q5T5M9|CCNJ_HUMAN Cyclin-J OS=Homo sapiens GN=CCNJ PE=2 SV=2
MELEGQWWRGQLAADIHQALRYKELKLPSYKGQSPQLSLRRYFADLIAIVSNRFTLCPSA
RHLAVYLLDLFMDRYDISIQQLHLVALSCLLLASKFEEKEDSVPKLEQLNSLGCMTNMNL
VLTKQNLLHMELLLLETFQWNLCLPTAAHFIEYYLSEAVHETDLHDGWPMICLEKTKLYM

AKYADYFLEVSLQVAAACVASSRIILRLSPTWPTRLHRLTAYSWDFLVQCIERLLIAHDN
DVKEANKQRGQAGPQSAQLSVFQTASQPSRPVHFQQPQYLHQTHQTSLQYRHPTSEQPSC
QQIVSTTHTSSYTLQTCPAGFQTSVQGLGHMQTGVGMSLAIPVEVKPCLSVSYNRSYQIN
EHYPCITPCFER
>sp|Q9UK58|CCNL1_HUMAN Cyclin-L1 OS=Homo sapiens GN=CCNL1 PE=1 SV=1
MASGPHSTATAAAAASSAAPSAGGSSSGTTTTTTTTTGGILIGDRLYSEVSLTIDHSLIP
EERLSPTPSMQDGLDLPSETDLRILGCELIQAAGILLRLPQVAMATGQVLFHRFFYSKSF
VKHSFEIVAMACINLASKIEEAPRRIRDVINVFHHLRQLRGKRTPSPLILDQNYINTKNQ
VIKAERRVLKELGFCVHVKHPHKIIVMYLQVLECERNQTLVQTAWNYMNDSLRTNVFVRF
QPETIACACIYLAARALQIPLPTRPHWFLLFGTTEEEIQEICIETLRLYTRKKPNYELLE
KEVEKRKVALQEAKLKAKGLNPDGTPALSTLGGFSPASKPSSPREVKAEEKSPISINVKT
VKKEPEDRQQASKSPYNGVRKDSKRSRNSRSASRSRSRTRSRSRSHTPRRHYNNRRSRSG
TYSSRSRSRSRSHSESPRRHHNHGSPHLKAKHTRDDLKSSNRHGHKRKKSRSRSQSKSRD
HSDAAKKHRHERGHHRDRRERSRSFERSHKSKHHGGSRSGHGRHRR
>sp|Q8WVX3|CD003_HUMAN Uncharacterized protein C4orf3 OS=Homo sapiens GN=C4orf3 PE=1 SV=2
MEVDAPGVDGRDGLRERRGFSEGGRQNFDVRPQSGANGLPKHSYWLDLWLFILFDVVVFL
FVYFLP
>sp|Q6V702|CD022_HUMAN Uncharacterized protein C4orf22 OS=Homo sapiens GN=C4orf22 PE=2 SV=4
MDQEEGLKALDNIVTQFNAYEDFLDSQITTVDLYYLEDETLARQLVELGYRGTGERVKRE
DFEARKAAIEIARLAERAQQKTLTSAGKDLQDNFLTALAMREEDNRSGKLSSVIFIRDRN
SHGQEISGYIDYAHRLKTEDFEVYFTGKKRLLPRPTDISFYNWDADIAVSNSSPNYQVIA
DNPEGLLFRYKRDRKILNVDPKAQPGDNSTRITILTELYVQAVIFDHISRRKT
>sp|Q96LM5|CD045_HUMAN Uncharacterized protein C4orf45 OS=Homo sapiens GN=C4orf45 PE=2 SV=3
MASVSYQKPTSTTVGKQMIFTGPDYIKDYLPKIHQHTSYVGEQHLALEKTGDLRYLWRPA
SNRSLPAKYKHEYVSEIGWRIPQYNFINKSRLGSGFHIKYEELSQASLDSITHRYQNPWQ
PKPHVLDMQGKQSRASFAWHMSAFEDTDQRNSKWAILVRQCKSSLPRASKPPKLPKLPKK
EKKRKH
>sp|A7E2U8|CD047_HUMAN UPF0602 protein C4orf47 OS=Homo sapiens GN=C4orf47 PE=2 SV=1
MPAEGGKTDMERIGLFSEMEYITVGDKYVSQFNRPFNEAASKNKQMLPGGSKEMSDLQAG
YFDPHFVRIFEGEGYINLNQVRRRDMVEAAKKNLGKAFLPSNGEKKPCGLGSYYGTIGGP
VPFFSAQSKPREKYKAPGKNLYTNPGKKGTGYGYANITIGKQFSHSADFYDAAKLKYKKA
NEEHHRLLKGAPFKLNLHPRDYFDANPYFSEESLPPIKKEEKKKTISNTFKPSSPGKKPG
GMKAGTFDPYPSHSADPYVAKLANISGKDDKIFHPPSGPKSRPVESIMTLNVRRALNSKN
YKTSSVPSY
>sp|P21127|CD11B_HUMAN Cyclin-dependent kinase 11B OS=Homo sapiens GN=CDK11B PE=1 SV=3
MGDEKDSWKVKTLDEILQEKKRRKEQEEKAEIKRLKNSDDRDSKRDSLEEGELRDHRMEI
TIRNSPYRREDSMEDRGEEDDSLAIKPPQQMSRKEKVHHRKDEKRKEKRRHRSHSAEGGK
HARVKEKEREHERRKRHREEQDKARREWERQKRREMAREHSRRERDRLEQLERKRERERK
MREQQKEQREQKERERRAEERRKEREARREVSAHHRTMREDYSDKVKASHWSRSPPRPPR
ERFELGDGRKPGEARPARAQKPAQLKEEKMEERDLLSDLQDISDSERKTSSAESSSAESG
SGSEEEEEEEEEEEEEGSTSEESEEEEEEEEEEEEETGSNSEEASEQSAEEVSEEEMSED
EERENENHLLVVPESRFDRDSGESEEAEEEVGEGTPQSSALTEGDYVPDSPALSPIELKQ

ELPKYLPALQGCRSVEEFQCLNRIEEGTYGVVYRAKDKKTDEIVALKRLKMEKEKEGFPI
TSLREINTILKAQHPNIVTVREIVVGSNMDKIYIVMNYVEHDLKSLMETMKQPFLPGEVK
TLMIQLLRGVKHLHDNWILHRDLKTSNLLLSHAGILKVGDFGLAREYGSPLKAYTPVVVT
LWYRAPELLLGAKEYSTAVDMWSVGCIFGELLTQKPLFPGKSEIDQINKVFKDLGTPSEK
IWPGYSELPAVKKMTFSEHPYNNLRKRFGALLSDQGFDLMNKFLTYFPGRRISAEDGLKH
EYFRETPLPIDPSMFPTWPAKSEQQRVKRGTSPRPPEGGLGYSQLGDDDLKETGFHLTTT
NQGASAAGPGFSLKF
>sp|Q8NHZ8|CDC26_HUMAN Anaphase-promoting complex subunit CDC26 OS=Homo sapiens GN=CDC26 PE=1 SV=1
MLRRKPTRLELKLDDIEEFENIRKDLETRKKQKEDVEVVGGSDGEGAIGLSSDPKSREQM
INDRIGYKPQPKPNNRSSQFGSLEF
>sp|P30260|CDC27_HUMAN Cell division cycle protein 27 homolog OS=Homo sapiens GN=CDC27 PE=1 SV=2
MTVLQEPVQAAIWQALNHYAYRDAVFLAERLYAEVHSEEALFLLATCYYRSGKAYKAYRL
LKGHSCTTPQCKYLLAKCCVDLSKLAEGEQILSGGVFNKQKSHDDIVTEFGDSACFTLSL
LGHVYCKTDRLAKGSECYQKSLSLNPFLWSPFESLCEIGEKPDPDQTFKFTSLQNFSNCL
PNSCTTQVPNHSLSHRQPETVLTETPQDTIELNRLNLESSNSKYSLNTDSSVSYIDSAVI
SPDTVPLGTGTSILSKQVQNKPKTGRSLLGGPAALSPLTPSFGILPLETPSPGDGSYLQN
YTNTPPVIDVPSTGAPSKKSVARIGQTGTKSVFSQSGNSREVTPILAQTQSSGPQTSTTP
QVLSPTITSPPNALPRRSSRLFTSDSSTTKENSKKLKMKFPPKIPNRKTKSKTNKGGITQ
PNINDSLEITKLDSSIISEGKISTITPQIQAFNLQKAAAEGLMSLLREMGKGYLALCSYN
CKEAINILSHLPSHHYNTGWVLCQIGRAYFELSEYMQAERIFSEVRRIENYRVEGMEIYS
TTLWHLQKDVALSVLSKDLTDMDKNSPEAWCAAGNCFSLQREHDIAIKFFQRAIQVDPNY
AYAYTLLGHEFVLTEELDKALACFRNAIRVNPRHYNAWYGLGMIYYKQEKFSLAEMHFQK
ALDINPQSSVLLCHIGVVQHALKKSEKALDTLNKAIVIDPKNPLCKFHRASVLFANEKYK
SALQELEELKQIVPKESLVYFLIGKVYKKLGQTHLALMNFSWAMDLDPKGANNQIKEAID
KRYLPDDEEPITQEEQIMGTDESQESSMTDADDTQLHAAESDEF
>sp|Q9BXL8|CDCA4_HUMAN Cell division cycle-associated protein 4 OS=Homo sapiens GN=CDCA4 PE=1 SV=1
MFARGLKRKCVGHEEDVEGALAGLKTVSSYSLQRQSLLDMSLVKLQLCHMLVEPNLCRSV
LIANTVRQIQEEMTQDGTWRTVAPQAAERAPLDRLVSTEILCRAAWGQEGAHPAPGLGDG
HTQGPVSDLCPVTSAQAPRHLQSSAWEMDGPRENRGSFHKSLDQIFETLETKNPSCMEEL
FSDVDSPYYDLDTVLTGMMGGARPGPCEGLEGLAPATPGPSSSCKSDLGELDHVVEILVE
T
>sp|A6H8M9|CDHR4_HUMAN Cadherin-related family member 4 OS=Homo sapiens GN=CDHR4 PE=2 SV=1
MVLLRLLVFLFAPVVSDLCSLPCFINVSESQGPGTVLQFLSFNCSSYTPTPTLELLNVQP
PTTFFNPPSLARWQGTYVGKLTLSSSAQLDALMVNHYKVQLKFTCGNHVMEGSLSVDVQR
DLSHIQCAGQFASPAGEMIQVPETVTPGARLYTLLLPGLELHGAQMSIISAQDLPHFPGP
FSINEQGWLQAPSQGLLGQAQKVFQLQISVSFGQRQSCQGMVIVKVLPVPSSQVSFLEQA
QNITIPENLAPGSEVVQVQARGVDLRYEILSPVPSPLFSIGRADGVVRTTTPLELARTSG
TAVSRLQVKAFEQGQLWASAKLNLTMNVQLVNLWPPRCLPALLVSQIPETAPVGTVLNTL
TCEDPDSVGATLDYKLWFRSSSNPASLCLYDRVLEVNATLDCDTPGACFQHAASILVLDG
GQPQMTTEVPVLVMVTPINEFSPACAPRTFRVQEDAAPHTLLGSVVGTDMDYPHDNIEYY

TSGGPTTFAVDRLSGEVHLLGPLDYEQQRLYRLTVLVIDHGQDQNPNHHLSGSCTITIEV
EDVNDHAPECEPPFQELTIYAPLGRSVEVTKMSCQIPQEPQRLIYSYSIVGGNSQNRFIL
QGAILVHSDLVLGPFWPEQPRTYELLICVADAGPSTPHLSTTATIIVHLVPRRASTVATS
THRTTVPSTMTPMLVTDTEAFWQPQPWFVVVLTATGALLLLALGWLLGRLLQGLAQLLQA
PSKPAQALLLNSIQGTEGSIEGFLEAPKMEMSQAPSSVMSLHFDGRAQDSRTGRDYLFNT
HTGARRWL
>sp|P06493|CDK1_HUMAN Cyclin-dependent kinase 1 OS=Homo sapiens GN=CDK1 PE=1 SV=3
MEDYTKIEKIGEGTYGVVYKGRHKTTGQVVAMKKIRLESEEEGVPSTAIREISLLKELRH
PNIVSLQDVLMQDSRLYLIFEFLSMDLKKYLDSIPPGQYMDSSLVKSYLYQILQGIVFCH
SRRVLHRDLKPQNLLIDDKGTIKLADFGLARAFGIPIRVYTHEVVTLWYRSPEVLLGSAR
YSTPVDIWSIGTIFAELATKKPLFHGDSEIDQLFRIFRALGTPNNEVWPEVESLQDYKNT
FPKWKPGSLASHVKNLDENGLDLLSKMLIYDPAKRISGKMALNHPYFNDLDNQIKKM
>sp|Q00526|CDK3_HUMAN Cyclin-dependent kinase 3 OS=Homo sapiens GN=CDK3 PE=1 SV=1
MDMFQKVEKIGEGTYGVVYKAKNRETGQLVALKKIRLDLEMEGVPSTAIREISLLKELKH
PNIVRLLDVVHNERKLYLVFEFLSQDLKKYMDSTPGSELPLHLIKSYLFQLLQGVSFCHS
HRVIHRDLKPQNLLINELGAIKLADFGLARAFGVPLRTYTHEVVTLWYRAPEILLGSKFY
TTAVDIWSIGCIFAEMVTRKALFPGDSEIDQLFRIFRMLGTPSEDTWPGVTQLPDYKGSF
PKWTRKGLEEIVPNLEPEGRDLLMQLLQYDPSQRITAKTALAHPYFSSPEPSPAARQYVL
QRFRH
>sp|P42771|CDN2A_HUMAN Cyclin-dependent kinase inhibitor 2A OS=Homo sapiens GN=CDKN2A PE=1 SV=2
MEPAAGSSMEPSADWLATAAARGRVEEVRALLEAGALPNAPNSYGRRPIQVMMMGSARVA
ELLLLHGAEPNCADPATLTRPVHDAAREGFLDTLVVLHRAGARLDVRDAWGRLPVDLAEE
LGHRDVARYLRAAAGGTRGSNHARIDAAEGPSDIPD
>sp|Q49AH0|CDNF_HUMAN Cerebral dopamine neurotrophic factor OS=Homo sapiens GN=CDNF PE=1 SV=2
MWCASPVAVVAFCAGLLVSHPVLTQGQEAGGRPGADCEVCKEFLNRFYKSLIDRGVNFSL
DTIEKELISFCLDTKGKENRLCYYLGATKDAATKILSEVTRPMSVHMPAMKICEKLKKLD
SQICELKYEKTLDLASVDLRKMRVAELKQILHSWGEECRACAEKTDYVNLIQELAPKYAA
THPKTEL
>sp|Q7Z6I8|CE024_HUMAN UPF0461 protein C5orf24 OS=Homo sapiens GN=C5orf24 PE=1 SV=1
MMHPVASSNPAFCGPGKPSCLNEDAMRAADQFDIYSSQQSKYSHTVNHKPMVCQRQDPLN
ETHLQTTSGRSIEIKDELKKKKNLNRSGKRGRPSGTTKSAGYRTSTGRPLGTTKAAGFKT
SPGRPLGTTKAAGYKVSPGRPPGSIKALSRLADLGYGCGTAAFPYPMMHGRAVHGVEETS
SEVKPPNE
>sp|O94986|CE152_HUMAN Centrosomal protein of 152 kDa OS=Homo sapiens GN=CEP152 PE=1 SV=4
MSLDFGSVALPVQNEDEEYDEEDYEREKELQQLLTDLPHDMLDDDLSSPELQYSDCSEDG
TDGQPHHPEQLEMSWNEQMLPKSQSVNGYNEIQSLYAGEKCGNVWEENRSKTEDRHPVYH
PEEGGDEGGSGYSPPSKCEQTDLYHLPENFRPYTNGQKQEFNNQATNVIKFSDPQWNHFQ
GPSCQGLEPYNKVTYKPYQSSAQNNGSPAQEITGSDTFEGLQQQFLGANENSAENMQIIQ
LQVLNKAKERQLENLIEKLNESERQIRYLNHQLVIIKDEKDGLTLSLRESQKLFQNGKER
EIQLEAQIKALETQIQALKVNEEQMIKKSRTTEMALESLKQQLVDLHHSESLQRAREQHE
SIVMGLTKKYEEQVLSLQKNLDATVTALKEQEDICSRLKDHVKQLERNQEAIKLEKTEII
NKLTRSLEESQKQCAHLLQSGSVQEVAQLQFQLQQAQKAHAMSANMNKALQEELTELKDE

ISLYESAAKLGIHPSDSEGELNIELTESYVDLGIKKVNWKKSKVTSIVQEEDPNEELSKD
EFILKLKAEVQRLLGSNSMKRHLVSQLQNDLKDCHKKIEDLHQVKKDEKSIEVETKTDTS
EKPKNQLWPESSTSDVVRDDILLLKNEIQVLQQQNQELKETEGKLRNTNQDLCNQMRQMV
QDFDHDKQEAVDRCERTYQQHHEAMKTQIRESLLAKHALEKQQLFEAYERTHLQLRSELD
KLNKEVTAVQECYLEVCREKDNLELTLRKTTEKEQQTQEKIKEKLIQQLEKEWQSKLDQT
IKAMKKKTLDCGSQTDQVTTSDVISKKEMAIMIEEQKCTIQQNLEQEKDIAIKGAMKKLE
IELELKHCENITKQVEIAVQNAHQRWLGELPELAEYQALVKAEQKKWEEQHEVSVNKRIS
FAVSEAKEKWKSELENMRKNILPGKELEEKIHSLQKELELKNEEVPVVIRAELAKARSEW
NKEKQEEIHRIQEQNEQDYRQFLDDHRNKINEVLAAAKEDFMKQKTELLLQKETELQTCL
DQSRREWTMQEAKRIQLEIYQYEEDILTVLGVLLSDTQKEHISDSEDKQLLEIMSTCSSK
WMSVQYFEKLKGCIQKAFQDTLPLLVENADPEWKKRNMAELSKDSASQGTGQGDPGPAAG
HHAQPLALQATEAEADKKKVLEIKDLCCGHCFQELEKAKQECQDLKGKLEKCCRHLQHLE
RKHKAVVEKIGEENNKVVEELIEENNDMKNKLEELQTLCKTPPRSLSAGAIENACLPCSG
GALEELRGQYIKAVKKIKCDMLRYIQESKERAAEMVKAEVLRERQETARKMRKYYLICLQ
QILQDDGKEGAEKKIMNAASKLATMAKLLETPISSKSQSKTTQSALPLTSEMLIAVKKSK
RNDVNQKIPCCIESKSNSVNTITRTLCEQAPKRRAACNLQRLLENSEHQSIKHVGSKETH
LEFQFGDGSCKHLNSLPRNVSPEFVPCEGEGGFGLHKKKDLLSDNGSESLPHSAAYPFLG
TLGNKPSPRCTPGPSESGCMHITFRDSNERLGLKVYKCNPLMESENAASEKSQGLDVQEP
PVKDGGDLSDCLGWPSSSATLSFDSREASFVHGRPQGTLEIPSESVKSKQFSPSGYLSDT
EESNMICQTMKCQRYQTPYLSEETTYLEPGKISVNCGHPSRHKADRLKSDFKKLSSTLPS
SVCQQPSRKLIVPLSSQQDSGFDSPFVNLD
>sp|Q9UPV0|CE164_HUMAN Centrosomal protein of 164 kDa OS=Homo sapiens GN=CEP164 PE=1 SV=3
MAGRPLRIGDQLVLEEDYDETYIPSEQEILEFAREIGIDPIKEPELMWLAREGIVAPLPG
EWKPCQDITGDIYYFNFANGQSMWDHPCDEHYRSLVIQERAKLSTSGAIKKKKKKKEKKD
KKDRDPPKSSLALGSSLAPVHVPLGGLAPLRGLVDTPPSALRGSQSVSLGSSVESGRQLG
ELMLPSQGLKTSAYTKGLLGSIYEDKTALSLLGLGEETNEEDEEESDNQSVHSSSEPLRN
LHLDIGALGGDFEYEESLRTSQPEEKKDVSLDSDAAGPPTPCKPSSPGADSSLSSAVGKG
RQGSGARPGLPEKEENEKSEPKICRNLVTPKADPTGSEPAKASEKEAPEDTVDAGEEGSR
REEAAKEPKKKASALEEGSSDASQELEISEHMKEPQLSDSIASDPKSFHGLDFGFRSRIS
EHLLDVDVLSPVLGGACRQAQQPLGIEDKDDSQSSQDELQSKQSKGLEERLSPPLPHEER
AQSPPRSLATEEEPPQGPEGQPEWKEAEELGEDSAASLSLQLSLQREQAPSPPAACEKGK
EQHSQAEELGPGQEEAEDPEEKVAVSPTPPVSPEVRSTEPVAPPEQLSEAALKAMEEAVA
QVLEQDQRHLLESKQEKMQQLREKLCQEEEEEILRLHQQKEQSLSSLRERLQKAIEEEEA
RMREEESQRLSWLRAQVQSSTQADEDQIRAEQEASLQKLREELESQQKAERASLEQKNRQ
MLEQLKEEIEASEKSEQAALNAAKEKALQQLREQLEGERKEAVATLEKEHSAELERLCSS
LEAKHREVVSSLQKKIQEAQQKEEAQLQKCLGQVEHRVHQKSYHVAGYEHELSSLLREKR
QEVEGEHERRLDKMKEEHQQVMAKAREQYEAEERKQRAELLGHLTGELERLQRAHERELE
TVRQEQHKRLEDLRRRHREQERKLQDLELDLETRAKDVKARLALLEVQEETARREKQQLL
DVQRQVALKSEEATATHQQLEEAQKEHTHLLQSNQQLREILDELQARKLKLESQVDLLQA
QSQQLQKHFSSLEAEAQKKQHLLREVTVEENNASPHFEPDLHIEDLRKSLGTNQTKEVSS
SLSQSKEDLYLDSLSSHNVWHLLSAEGVALRSAKEFLVQQTRSMRRRQTALKAAQQHWRH
ELASAQEVAKDPPGIKALEDMRKNLEKETRHLDEMKSAMRKGHNLLKKKEEKLNQLESSL
WEEASDEGTLGGSPTKKAVTFDLSDMDSLSSESSESFSPPHREWWRQQRIDSTPSLTSRK
IHGLSHSLRQISSQLSSVLSILDSLNPQSPPPLLASMPAQLPPRDPKSTPTPTYYGSLAR

FSALSSATPTSTQWAWDSGQGPRLPSSVAQTVDDFLLEKWRKYFPSGIPLLSNSPTPLES
RLGYMSASEQLRLLQHSHSQVPEAGSTTFQGIIEANRRWLERVKNDPRLPLFSSTPKPKA
TLSLLQLGLDEHNRVKVYRF
>sp|Q8TEP8|CE192_HUMAN Centrosomal protein of 192 kDa OS=Homo sapiens GN=CEP192 PE=1 SV=2
MKTSDLVPSFGYFIRSPEKREPIALIRKSDVSRGNLEKEMAHLNHDLYSGDLNEQSQAQL
SEGSITLQVEAVESTSQVDENDVTLTADKGKTEDTFFMSNKPQRYKDKLPDSGDSMLRIS
TIASAIAEASVNTDPSQLAAMIKALSNKTRDKTFQEDEKQKDYSHVRHFLPNDLEKSNGS
NALDMEKYLKKTEVSRYESALENFSRASMSDTWDLSLPKEQTTQDIHPVDLSATSVSVRA
PEENTAAIVYVENGESENQESFRTINSSNSVTNRENNSAVVDVKTCSIDNKLQDVGNDEK
ATSISTPSDSYSSVRNPRITSLCLLKDCEEIRDNRENQRQNECVSEISNSEKHVTFENHR
IVSPKNSDLKNTSPEHGGRGSEDEQESFRPSTSPLSHSSPSEISGTSSSGCALESFGSAA
QQQQPPCEQELSPLVCSPAGVSRLTYVSEPESSYPTTATDDALEDRKSDITSELSTTIIQ
GSPAALEERAMEKLREKVPFQNRGKGTLSSIIQNNSDTRKATETTSLSSKPEYVKPDFRW
SKDPSSKSGNLLETSEVGWTSNPEELDPIRLALLGKSGLSCQVGSATSHPVSCQEPIDED
QRISPKDKSTAGREFSGQVSHQTTSENQCTPIPSSTVHSSVADMQNMPAAVHALLTQPSL
SAAPFAQRYLGTLPSTGSTTLPQCHAGNATVCGFSGGLPYPAVAGEPVQNSVAVGICLGS
NIGSGWMGTSSLCNPYSNTLNQNLLSTTKPFPVPSVGTNCGIEPWDSGVTSGLGSVRVPE
ELKLPHACCVGIASQTLLSVLNPTDRWLQVSIGVLSISVNGEKVDLSTYRCLVFKNKAII
RPHATEEIKVLFIPSSPGVFRCTFSVASWPCSTDAETIVQAEALASTVTLTAIAESPVIE
VETEKKDVLDFGDLTYGGWKALPLKLINRTHATVPIRLIINANAVAWRCFTFSKESVRAP
VEVAPCADVVTRLAGPSVVNHMMPASYDGQDPEFLMIWVLFHSPKKQISSSDILDSAEEF
SAKVDIEVDSPNPTPVLRSVSLRARAGIARIHAPRDLQTMHFLAKVASSRKQHLPLKNAG
NIEVYLDIKVPEQGSHFSVDPKNLLLKPGEEHEVIVSFTPKDPEACEERILKIFVQPFGP
QYEVVLKGEVISSGSKPLSPGPCLDIPSILSNKQFLAWGGVPLGRTQLQKLALRNNSAST
TQHLRLLIRGQDQDCFQLQNTFGSEQRLTSNCEIRIHPKEDIFISVLFAPTRLSCMLARL
EIKQLGNRSQPGIKFTIPLSGYGGTSNLILEGVKKLSDSYMVTVNGLVPGKESKIVFSVR
NTGSRAAFVKAVGFKDSQKKVLLDPKVLRIFPDKFVLKERTQENVTLIYNPSDRGINNKT
ATELSTVYLFGGDEISRQQYRRALLHKPEMIKQILPEHSVLQNINFVEAFQDELLVTEVY
DLPQRPNDVQLFYGSMCKIILSVIGEFRDCISSREFLQPSSKASLESTSDLGASGKHGGN
VSLDVLPVKGPQGSPLLSRAARPPLDQLASEEPWTVLPEHLILVAPSPCDMAKTGRFQIV
NNSVRLLRFELCWPAHCLTVTPQHGCVAPESKLQILVSPNSSLSTKQSMFPWSGLIYIHC
DDGQKKIVKVQIREDLTQVELLTRLTSKPFGILSPVSEPSVSHLVKPMTKPPSTKVEIRN
KSITFPTTEPGETSESCLELENHGTTDVKWHLSSLAPPYVKGVDESGDVFRATYAAFRCS
PISGLLESHGIQKVSITFLPRGRGDYAQFWDVECHPLKEPHMKHTLRFQLSGQSIEAENE
PENACLSTDSLIKIDHLVKPRRQAVSEASARIPEQLDVTARGVYAPEDVYRFRPTSVGES
RTLKVNLRNNSFITHSLKFLSPREPFYVKHSKYSLRAQHYINMPVQFKPKSAGKFEALLV
IQTDEGKSIAIRLIGEALGKN
>sp|O15078|CE290_HUMAN Centrosomal protein of 290 kDa OS=Homo sapiens GN=CEP290 PE=1 SV=2
MPPNINWKEIMKVDPDDLPRQEELADNLLISLSKVEVNELKSEKQENVIHLFRITQSLMK
MKAQEVELALEEVEKAGEEQAKFENQLKTKVMKLENELEMAQQSAGGRDTRFLRNEICQL
EKQLEQKDRELEDMEKELEKEKKVNEQLALRNEEAENENSKLRRENKRLKKKNEQLCQDI
IDYQKQIDSQKETLLSRRGEDSDYRSQLSKKNYELIQYLDEIQTLTEANEKIEVQNQEMR
KNLEESVQEMEKMTDEYNRMKAIVHQTDNVIDQLKKENDHYQLQVQELTDLLKSKNEEDD
PIMVAVNAKVEEWKLILSSKDDEIIEYQQMLHNLREKLKNAQLDADKSNVMALQQGIQER

DSQIKMLTEQVEQYTKEMEKNTCIIEDLKNELQRNKGASTLSQQTHMKIQSTLDILKEKT
KEAERTAELAEADAREKDKELVEALKRLKDYESGVYGLEDAVVEIKNCKNQIKIRDREIE
ILTKEINKLELKISDFLDENEALRERVGLEPKTMIDLTEFRNSKHLKQQQYRAENQILLK
EIESLEEERLDLKKKIRQMAQERGKRSATSGLTTEDLNLTENISQGDRISERKLDLLSLK
NMSEAQSKNEFLSRELIEKERDLERSRTVIAKFQNKLKELVEENKQLEEGMKEILQAIKE
MQKDPDVKGGETSLIIPSLERLVNAIESKNAEGIFDASLHLKAQVDQLTGRNEELRQELR
ESRKEAINYSQQLAKANLKIDHLEKETSLLRQSEGSNVVFKGIDLPDGIAPSSASIINSQ
NEYLIHLLQELENKEKKLKNLEDSLEDYNRKFAVIRHQQSLLYKEYLSEKETWKTESKTI
KEEKRKLEDQVQQDAIKVKEYNNLLNALQMDSDEMKKILAENSRKITVLQVNEKSLIRQY
TTLVELERQLRKENEKQKNELLSMEAEVCEKIGCLQRFKEMAIFKIAALQKVVDNSVSLS
ELELANKQYNELTAKYRDILQKDNMLVQRTSNLEHLECENISLKEQVESINKELEITKEK
LHTIEQAWEQETKLGNESSMDKAKKSITNSDIVSISKKITMLEMKELNERQRAEHCQKMY
EHLRTSLKQMEERNFELETKFAELTKINLDAQKVEQMLRDELADSVSKAVSDADRQRILE
LEKNEMELKVEVSKLREISDIARRQVEILNAQQQSRDKEVESLRMQLLDYQAQSDEKSLI
AKLHQHNVSLQLSEATALGKLESITSKLQKMEAYNLRLEQKLDEKEQALYYARLEGRNRA
KHLRQTIQSLRRQFSGALPLAQQEKFSKTMIQLQNDKLKIMQEMKNSQQEHRNMENKTLE
MELKLKGLEELISTLKDTKGAQKVINWHMKIEELRLQELKLNRELVKDKEEIKYLNNIIS
EYERTISSLEEEIVQQNKFHEERQMAWDQREVDLERQLDIFDRQQNEILNAAQKFEEATG
SIPDPSLPLPNQLEIALRKIKENIRIILETRATCKSLEEKLKEKESALRLAEQNILSRDK
VINELRLRLPATAEREKLIAELGRKEMEPKSHHTLKIAHQTIANMQARLNQKEEVLKKYQ
RLLEKAREEQREIVKKHEEDLHILHHRLELQADSSLNKFKQTAWDLMKQSPTPVPTNKHF
IRLAEMEQTVAEQDDSLSSLLVKLKKVSQDLERQREITELKVKEFENIKLQLQENHEDEV
KKVKAEVEDLKYLLDQSQKESQCLKSELQAQKEANSRAPTTTMRNLVERLKSQLALKEKQ
QKALSRALLELRAEMTAAAEERIISATSQKEAHLNVQQIVDRHTRELKTQVEDLNENLLK
LKEALKTSKNRENSLTDNLNDLNNELQKKQKAYNKILREKEEIDQENDELKRQIKRLTSG
LQGKPLTDNKQSLIEELQRKVKKLENQLEGKVEEVDLKPMKEKNAKEELIRWEEGKKWQA
KIEGIRNKLKEKEGEVFTLTKQLNTLKDLFAKADKEKLTLQRKLKTTGMTVDQVLGIRAL
ESEKELEELKKRNLDLENDILYMRAHQALPRDSVVEDLHLQNRYLQEKLHALEKQFSKDT
YSKPSISGIESDDHCQREQELQKENLKLSSENIELKFQLEQANKDLPRLKNQVRDLKEMC
EFLKKEKAEVQRKLGHVRGSGRSGKTIPELEKTIGLMKKVVEKVQRENEQLKKASGILTS
EKMANIEQENEKLKAELEKLKAHLGHQLSMHYESKTKGTEKIIAENERLRKELKKETDAA
EKLRIAKNNLEILNEKMTVQLEETGKRLQFAESRGPQLEGADSKSWKSIVVTRMYETKLK
ELETDIAKKNQSITDLKQLVKEATEREQKVNKYNEDLEQQIKILKHVPEGAETEQGLKRE
LQVLRLANHQLDKEKAELIHQIEANKDQSGAESTIPDADQLKEKIKDLETQLKMSDLEKQ
HLKEEIKKLKKELENFDPSFFEEIEDLKYNYKEEVKKNILLEEKVKKLSEQLGVELTSPV
AASEEFEDEEESPVNFPIY
>sp|Q2WEN9|CEA16_HUMAN Carcinoembryonic antigen-related cell adhesion molecule 16 OS=Homo sapiens GN=CEACAM16 PE=1 SV=2
MALTGYSWLLLSATFLNVGAEISITLEPAQPSEGDNVTLVVHGLSGELLAYSWYAGPTLS
VSYLVASYIVSTGDETPGPAHTGREAVRPDGSLDIQGILPRHSGTYILQTFNRQLQTEVG
YGHVQVHEILAQPTVLANSTALVERRDTLRLMCSSPSPTAEVRWFFNGGALPVALRLGLS
PDGRVLARHGIRREEAGAYQCEVWNPVSVSRSEPINLTVYFGPERVAILQDSTTRTGCTI
KVDFNTSLTLWCVSRSCPEPEYVWTFNGQALKNGQDHLNISSMTAAQEGTYTCIAKNTKT
LLSGSASVVVKLSAAAVATMIVPVPTKPTEGQDVTLTVQGYPKDLLVYAWYRGPASEPNR

LLSQLPSGTWIAGPAHTGREVGFPNCSLLVQKLNLTDTGRYTLKTVTVQGKTETLEVELQ
VAPLG
>sp|Q6UY09|CEA20_HUMAN Carcinoembryonic antigen-related cell adhesion molecule 20 OS=Homo sapiens GN=CEACAM20 PE=2 SV=1
MGPADSWGHHWMGILLSASLCTVWSPPAAAQLTLNANPLDATQSEDVVLPVFGTPRTPQI
HGRSRELAKPSIAVSPGTAIEQKDMVTFYCTTKDVNITIHWVSNNLSIVFHERMQLSKDG
KILTILIVQREDSGTYQCEARDALLSQRSDPIFLDVKYGPDPVEIKLESGVASGEVVEVM
EGSSMTFLAETKSHPPCAYTWFLLDSILSHTTRTFTIHAVSREHEGLYRCLVSNSATHLS
SLGTLKVRVLETLTMPQVVPSSLNLVENARSVDLTCQTVNQSVNVQWFLSGQPLLPSEHL
QLSADNRTLIIHGLQRNDTGPYACEVWNWGSRARSEPLELTINYGPDQVHITRESASEMI
STIEAELNSSLTLQCWAESKPGAEYRWTLEHSTGEHLGEQLIIRALTWEHDGIYNCTASN
SLTGLARSTSVLVKVVGPQSSSLSSGAIAGIVIGILAVIAVASELGYFLCIRNARRPSRK
TTEDPSHETSQPIPKEEHPTEPSSESLSPEYCNISQLQGRIRVELMQPPDLPEETYETKL
PSASRRGNSFSPWKPPPKPLMPPLRLVSTVPKNMESIYEVLGMQQ
>sp|Q3KPI0|CEA21_HUMAN Carcinoembryonic antigen-related cell adhesion molecule 21 OS=Homo sapiens GN=CEACAM21 PE=2 SV=2
MGPPSACPHRECIPWQGLLLTASLLTFWNAPTTAWLFIASAPFEVAEGENVHLSVVYLPE
NLYSYGWYKGKTVEPNQLIAAYVIDTHVRTPGPAYSGRETISPSGDLHFQNVTLEDTGYY
TLQVTYRNSQIEQASHHLRVYESVAQPSIQASSTTVTEKGSVVLTCHTNNTGTSFQWIFN
NQRLQVTKRMKLSWFNHMLTIDPIRQEDAGEYQCEVSNPVSSNRSDPLKLTVKSDDNTLG
ILIGVLVGSLLVAALVCFLLLRKTGRASDQSDFREQQPPASTPGHGPSDSSIS
>sp|P40198|CEAM3_HUMAN Carcinoembryonic antigen-related cell adhesion molecule 3 OS=Homo sapiens GN=CEACAM3 PE=1 SV=2
MGPPSASPHRECIPWQGLLLTASLLNFWNPPTTAKLTIESMPLSVAEGKEVLLLVHNLPQ
HLFGYSWYKGERVDGNSLIVGYVIGTQQATPGAAYSGRETIYTNASLLIQNVTQNDIGFY
TLQVIKSDLVNEEATGQFHVYQENAPGLPVGAVAGIVTGVLVGVALVAALVCFLLLAKTG
RTSIQRDLKEQQPQALAPGRGPSHSSAFSMSPLSTAQAPLPNPRTAASIYEELLKHDTNI
YCRMDHKAEVAS
>sp|P06731|CEAM5_HUMAN Carcinoembryonic antigen-related cell adhesion molecule 5 OS=Homo sapiens GN=CEACAM5 PE=1 SV=3
MESPSAPPHRWCIPWQRLLLTASLLTFWNPPTTAKLTIESTPFNVAEGKEVLLLVHNLPQ
HLFGYSWYKGERVDGNRQIIGYVIGTQQATPGPAYSGREIIYPNASLLIQNIIQNDTGFY
TLHVIKSDLVNEEATGQFRVYPELPKPSISSNNSKPVEDKDAVAFTCEPETQDATYLWWV
NNQSLPVSPRLQLSNGNRTLTLFNVTRNDTASYKCETQNPVSARRSDSVILNVLYGPDAP
TISPLNTSYRSGENLNLSCHAASNPPAQYSWFVNGTFQQSTQELFIPNITVNNSGSYTCQ
AHNSDTGLNRTTVTTITVYAEPPKPFITSNNSNPVEDEDAVALTCEPEIQNTTYLWWVNN
QSLPVSPRLQLSNDNRTLTLLSVTRNDVGPYECGIQNKLSVDHSDPVILNVLYGPDDPTI
SPSYTYYRPGVNLSLSCHAASNPPAQYSWLIDGNIQQHTQELFISNITEKNSGLYTCQAN
NSASGHSRTTVKTITVSAELPKPSISSNNSKPVEDKDAVAFTCEPEAQNTTYLWWVNGQS
LPVSPRLQLSNGNRTLTLFNVTRNDARAYVCGIQNSVSANRSDPVTLDVLYGPDTPIISP
PDSSYLSGANLNLSCHSASNPSPQYSWRINGIPQQHTQVLFIAKITPNNNGTYACFVSNL
ATGRNNSIVKSITVSASGTSPGLSAGATVGIMIGVLVGVALI
>sp|Q14444|CAPR1_HUMAN Caprin-1 OS=Homo sapiens GN=CAPRIN1 PE=1 SV=2
MPSATSHSGSGSKSSGPPPPSGSSGSEAAAGAGAAAPASQHPATGTGAVQTEAMKQILGV

IDKKLRNLEKKKGKLDDYQERMNKGERLNQDQLDAVSKYQEVTNNLEFAKELQRSFMALS
QDIQKTIKKTARREQLMREEAEQKRLKTVLELQYVLDKLGDDEVRTDLKQGLNGVPILSE
EELSLLDEFYKLVDPERDMSLRLNEQYEHASIHLWDLLEGKEKPVCGTTYKVLKEIVERV
FQSNYFDSTHNHQNGLCEEEEAASAPAVEDQVPEAEPEPAEEYTEQSEVESTEYVNRQFM
AETQFTSGEKEQVDEWTVETVEVVNSLQQQPQAASPSVPEPHSLTPVAQADPLVRRQRVQ
DLMAQMQGPYNFIQDSMLDFENQTLDPAIVSAQPMNPTQNMDMPQLVCPPVHSESRLAQP
NQVPVQPEATQVPLVSSTSEGYTASQPLYQPSHATEQRPQKEPIDQIQATISLNTDQTTA
SSSLPAASQPQVFQAGTSKPLHSSGINVNAAPFQSMQTVFNMNAPVPPVNEPETLKQQNQ
YQASYNQSFSSQPHQVEQTELQQEQLQTVVGTYHGSPDQSHQVTGNHQQPPQQNTGFPRS
NQPYYNSRGVSRGGSRGARGLMNGYRGPANGFRGGYDGYRPSFSNTPNSGYTQSQFSAPR
DYSGYQRDGYQQNFKRGSGQSGPRGAPRGRGGPPRPNRGMPQMNTQQVN
>sp|Q9ULU8|CAPS1_HUMAN Calcium-dependent secretion activator 1 OS=Homo sapiens GN=CADPS PE=1 SV=3
MLDPSSSEEESDEIVEEESGKEVLGSAPSGARLSPSRTSEGSAGSAGLGGGGAGAGAGVG
AGGGGGSGASSGGGAGGLQPSSRAGGGRPSSPSPSVVSEKEKEELERLQKEEEERKKRLQ
LYVFVMRCIAYPFNAKQPTDMARRQQKISKQQLQTVKDRFQAFLNGETQIMADEAFMNAV
QSYYEVFLKSDRVARMVQSGGCSANDSREVFKKHIEKRVRSLPEIDGLSKETVLSSWMAK
FDAIYRGEEDPRKQQARMTASAASELILSKEQLYEMFQNILGIKKFEHQLLYNACQLDNP
DEQAAQIRRELDGRLQMADQIARERKFPKFVSKEMENMYIEELKSSVNLLMANLESMPVS
KGGEFKLQKLKRSHNASIIDMGEESENQLSKSDVVLSFSLEVVIMEVQGLKSLAPNRIVY
CTMEVEGGEKLQTDQAEASKPTWGTQGDFSTTHALPAVKVKLFTESTGVLALEDKELGRV
ILHPTPNSPKQSEWHKMTVSKNCPDQDLKIKLAVRMDKPQNMKHSGYLWAIGKNVWKRWK
KRFFVLVQVSQYTFAMCSYREKKAEPQELLQLDGYTVDYTDPQPGLEGGRAFFNAVKEGD
TVIFASDDEQDRILWVQAMYRATGQSHKPVPPTQVQKLNAKGGNVPQLDAPISQFYADRA
QKHGMDEFISSNPCNFDHASLFEMVQRLTLDHRLNDSYSCLGWFSPGQVFVLDEYCARNG
VRGCHRHLCYLRDLLERAENGAMIDPTLLHYSFAFCASHVHGNRPDGIGTVTVEEKERFE
EIKERLRVLLENQITHFRYCFPFGRPEGALKATLSLLERVLMKDIVTPVPQEEVKTVIRK
CLEQAALVNYSRLSEYAKIEENQKDAENVGRLITPAKKLEDTIRLAELVIEVLQQNEEHH
AEPHVDKGEAFAWWSDLMVEHAETFLSLFAVDMDAALEVQPPDTWDSFPLFQLLNDFLRT
DYNLCNGKFHKHLQDLFAPLVVRYVDLMESSIAQSIHRGFERESWEPVKSLTSNLPNVNL
PNVNLPKVPNLPVNIPLGIPQMPTFSAPSWMAAIYDADNGSGTSEDLFWKLDALQTFIRD
LHWPEEEFGKHLEQRLKLMASDMIESCVKRTRIAFEVKLQKTSRSTDFRVPQSICTMFNV
MVDAKAQSTKLCSMEMGQEHQYHSKIDELIEETVKEMITLLVAKFVTILEGVLAKLSRYD
EGTLFSSFLSFTVKAASKYVDVPKPGMDVADAYVTFVRHSQDVLRDKVNEEMYIERLFDQ
WYNSSMNVICTWLTDRMDLQLHIYQLKTLIRMVKKTYRDFRLQGVLDSTLNSKTYETIRN
RLTVEEATASVSEGGGLQGISMKDSDEEDEEDD
>sp|O14958|CASQ2_HUMAN Calsequestrin-2 OS=Homo sapiens GN=CASQ2 PE=1 SV=2
MKRTHLFIVGIYFLSSCRAEEGLNFPTYDGKDRVVSLSEKNFKQVLKKYDLLCLYYHEPV
SSDKVTQKQFQLKEIVLELVAQVLEHKAIGFVMVDAKKEAKLAKKLGFDEEGSLYILKGD
RTIEFDGEFAADVLVEFLLDLIEDPVEIISSKLEVQAFERIEDYIKLIGFFKSEDSEYYK
AFEEAAEHFQPYIKFFATFDKGVAKKLSLKMNEVDFYEPFMDEPIAIPNKPYTEEELVEF
VKEHQRPTLRRLRPEEMFETWEDDLNGIHIVAFAEKSDPDGYEFLEILKQVARDNTDNPD
LSILWIDPDDFPLLVAYWEKTFKIDLFRPQIGVVNVTDADSVWMEIPDDDDLPTAEELED
WIEDVLSGKINTEDDDEDDDDDDNSDEEDNDDSDDDDDE

>sp|Q9NQ75|CASS4_HUMAN Cas scaffolding protein family member 4 OS=Homo sapiens GN=CASS4 PE=1 SV=2
MKGTGIMDCAPKALLARALYDNCPDCSDELAFSRGDILTILEQHVPESEGWWKCLLHGRQ
GLAPANRLQILTEVAADRPCPPFLRGLEEAPASSEETYQVPTLPRPPTPGPVYEQMRSWA
EGPQPPTAQVYEFPDPPTSARIICEKTLSFPKQAILTLPRPVRASLPTLPSQVYDVPTQH
RGPVVLKEPEKQQLYDIPASPKKAGLHPPDSQASGQGVPLISVTTLRRGGYSTLPNPQKS
EWIYDTPVSPGKASVRNTPLTSFAEESRPHALPSSSSTFYNPPSGRSRSLTPQLNNNVPM
QKKLSLPEIPSYGFLVPRGTFPLDEDVSYKVPSSFLIPRVEQQNTKPNIYDIPKATSSVS
QAGKELEKAKEVSENSAGHNSSWFSRRTTSPSPEPDRLSGSSSDSRASIVSSCSTTSTDD
SSSSSSEESAKELSLDLDVAKETVMALQHKVVSSVAGLMLFVSRKWRFRDYLEANIDAIH
RSTDHIEESVREFLDFARGVHGTACNLTDSNLQNRIRDQMQTISNSYRILLETKESLDNR
NWPLEVLVTDSVQNSPDDLERFVMVARMLPEDIKRFASIVIANGRLLFKRNCEKEETVQL
TPNAEFKCEKYIQPPQRETESHQKSTPSTKQREDEHSSELLKKNRANICGQNPGPLIPQP
SSQQTPERKPRLSEHCRLYFGALFKAISAFHGSLSSSQPAEIITQSKLVIMVGQKLVDTL
CMETQERDVRNEILRGSSHLCSLLKDVALATKNAVLTYPSPAALGHLQAEAEKLEQHTRQ
FRGTLG
>sp|P07858|CATB_HUMAN Cathepsin B OS=Homo sapiens GN=CTSB PE=1 SV=3
MWQLWASLCCLLVLANARSRPSFHPLSDELVNYVNKRNTTWQAGHNFYNVDMSYLKRLCG
TFLGGPKPPQRVMFTEDLKLPASFDAREQWPQCPTIKEIRDQGSCGSCWAFGAVEAISDR
ICIHTNAHVSVEVSAEDLLTCCGSMCGDGCNGGYPAEAWNFWTRKGLVSGGLYESHVGCR
PYSIPPCEHHVNGSRPPCTGEGDTPKCSKICEPGYSPTYKQDKHYGYNSYSVSNSEKDIM
AEIYKNGPVEGAFSVYSDFLLYKSGVYQHVTGEMMGGHAIRILGWGVENGTPYWLVANSW
NTDWGDNGFFKILRGQDHCGIESEVVAGIPRTDQYWEKI
>sp|P56539|CAV3_HUMAN Caveolin-3 OS=Homo sapiens GN=CAV3 PE=1 SV=1
MMAEEHTDLEAQIVKDIHCKEIDLVNRDPKNINEDIVKVDFEDVIAEPVGTYSFDGVWKV
SYTTFTVSKYWCYRLLSTLLGVPLALLWGFLFACISFCHIWAVVPCIKSYLIEIQCISHI
YSLCIRTFCNPLFAALGQVCSSIKVVLRKEV
>sp|Q9BXY5|CAYP2_HUMAN Calcyphosin-2 OS=Homo sapiens GN=CAPS2 PE=2 SV=2
MGNSFCYTAVYCMINTGTQMDLEVKGVAATSRSQIQPFFGRKKPLQQRWTSESWTNQNSC
PPVVPRLDLGSLVDSDDEDNFSYIPLSTANLPNSSSTLGWVTPCQTPYTQYHLNKLDQNI
IPENLPAPTDKCKLKYQQCKTEIKEGYKQYSQRNAENTKSNVTHKQSPRNKIDEKCVQDE
EANTDDLTTLDRKAILQQGYADNSCDKQQRARKLDAEIVAAEKKKQIVAEQVMIDHLSRA
VISDPEQNLAIEQKESDHILPDSKMTPLRFRKRTLHETKIRTHSTLTENVLSHKLQFDGR
IVSRNGRDACRELIGFFFTHDQSLTIYEYRQFGKNRTNVLPFIQKSIYSHQCGRRKGKQY
RLGDFYVGATLTFLSSDHLSLPESIKENTLLKLRITNIDQIALDSLKTASMEQEDDIIIQ
ETNDRLVFKAIQDVLKEKLHKRGVRILTGLGKYFQQLDKEGNGLLDKADFKQALKVFHLE
VSEKDFESAWLILNDNGNGKVDYGEFKRGIIGEMNEYRKSYVRKAFMKLDFNKSGSVPII
NIRKCYCAKKHSQVISG
>sp|P47755|CAZA2_HUMAN F-actin-capping protein subunit alpha-2 OS=Homo sapiens GN=CAPZA2 PE=1 SV=3
MADLEEQLSDEEKVRIAAKFIIHAPPGEFNEVFNDVRLLLNNDNLLREGAAHAFAQYNLD
QFTPVKIEGYEDQVLITEHGDLGNGKFLDPKNRICFKFDHLRKEATDPRPCEVENAVESW
RTSVETALRAYVKEHYPNGVCTVYGKKIDGQQTIIACIESHQFQAKNFWNGRWRSEWKFT
ITPSTTQVVGILKIQVHYYEDGNVQLVSHKDIQDSLTVSNEVQTAKEFIKIVEAAENEYQ

TAISENYQTMSDTTFKALRRQLPVTRTKIDWNKILSYKIGKEMQNA
>sp|Q8N535|CB052_HUMAN Putative uncharacterized protein encoded by LINC00471 OS=Homo sapiens GN=LINC00471 PE=5 SV=1
MSEAKDNGSRDEVLVPHKNCRKNTTVPGKKGEEKSLAPVFAEKLISPSRRGAKLKDRESH
QENEDRNSELDQDEEDKESFCRGFPMSGCELETSCCVCHSTALGERFC
>sp|Q53QW1|CB057_HUMAN Uncharacterized protein C2orf57 OS=Homo sapiens GN=C2orf57 PE=1 SV=1
MALPGYPLGNVDDSRSKDSPAGEPQGQVPLTADVLAVSSSVASTDWQDIDQASFKTATPR
AISTSGDKDKSAVVPEHGQKTPRKITPLLPSQNPSPLQVSMSLQNPAWDRQVQDARTSQS
LVVFPSHLLGKDKMSQMASVPEREPESAPSAPSAELQSTQHMEAQPVESDADHVTAGANG
QHGPQAASTTKSAEEKAEHPKAPHPEAEALPSDESPVAMGANVVDSLGDLQTWFFPPPPA
GSVSPSPGPHEVALGRRPLDSSLYTASEENSYMRSMTSLLDRGEGSISSLADILVWSETT
MGMAIATGFLDSGHSTVADLLHSSGPSLRSVPSLVGSVSSAFSSGLVSGTSSALRTITRV
LETVEQRTVEGIRSAMRYLTSHLTPRQAQADPNYD
>sp|Q8N801|CB061_HUMAN Uncharacterized protein C2orf61 OS=Homo sapiens GN=C2orf61 PE=2 SV=2
MDQPAVATASTSIREDLVGGESFITASKPAQKTSSFEREGWWRIALTDTPIPGTYHLKTF
IEESLLNPVIATYNFKNEGRKKPPLVQRNNPVLNDLPQYMPPDFLDLLKKQVATYSFKDK
PRPSPSTLVDKDQSLQLSPGQYNVLPAPVPKYASRSCVFRSTVQRFPTTYFIPHEGPGPG
HYNVKMPPTSSVTSCFQSRVPRFLPSCSKTPGPGAYTTLRQFPKQSPTIAKMGQEHSLFF
NNNNWLLK
>sp|A8MZ97|CB074_HUMAN Uncharacterized protein C2orf74 OS=Homo sapiens GN=C2orf74 PE=4 SV=3
MSLLAKPMSFETTAITFFIILLICLICILLLLVVFLYKCFQGRKGKETKKVPCTDANGGV
DCAAAKVVTSNPEDHERILMQVMNLNVPMRPGILVQRQSKEVLATPLENRRDMEAEEENQ
INEKQEPENAGETGQEEDDGLQKIHTSVTRTPSVVESQKRPLKGVTFSREVIVVDLGNEY
PTPRSYTREHKERK
>sp|Q13191|CBLB_HUMAN E3 ubiquitin-protein ligase CBL-B OS=Homo sapiens GN=CBLB PE=1 SV=2
MANSMNGRNPGGRGGNPRKGRILGIIDAIQDAVGPPKQAAADRRTVEKTWKLMDKVVRLC
QNPKLQLKNSPPYILDILPDTYQHLRLILSKYDDNQKLAQLSENEYFKIYIDSLMKKSKR
AIRLFKEGKERMYEEQSQDRRNLTKLSLIFSHMLAEIKAIFPNGQFQGDNFRITKADAAE
FWRKFFGDKTIVPWKVFRQCLHEVHQISSGLEAMALKSTIDLTCNDYISVFEFDIFTRLF
QPWGSILRNWNFLAVTHPGYMAFLTYDEVKARLQKYSTKPGSYIFRLSCTRLGQWAIGYV
TGDGNILQTIPHNKPLFQALIDGSREGFYLYPDGRSYNPDLTGLCEPTPHDHIKVTQEQY
ELYCEMGSTFQLCKICAENDKDVKIEPCGHLMCTSCLTAWQESDGQGCPFCRCEIKGTEP
IIVDPFDPRDEGSRCCSIIDPFGMPMLDLDDDDDREESLMMNRLANVRKCTDRQNSPVTS
PGSSPLAQRRKPQPDPLQIPHLSLPPVPPRLDLIQKGIVRSPCGSPTGSPKSSPCMVRKQ
DKPLPAPPPPLRDPPPPPPERPPPIPPDNRLSRHIHHVESVPSRDPPMPLEAWCPRDVFG
TNQLVGCRLLGEGSPKPGITASSNVNGRHSRVGSDPVLMRKHRRHDLPLEGAKVFSNGHL
GSEEYDVPPRLSPPPPVTTLLPSIKCTGPLANSLSEKTRDPVEEDDDEYKIPSSHPVSLN
SQPSHCHNVKPPVRSCDNGHCMLNGTHGPSSEKKSNIPDLSIYLKGDVFDSASDPVPLPP
ARPPTRDNPKHGSSLNRTPSDYDLLIPPLGEDAFDALPPSLPPPPPPARHSLIEHSKPPG
SSSRPSSGQDLFLLPSDPFVDLASGQVPLPPARRLPGENVKTNRTSQDYDQLPSCSDGSQ
APARPPKPRPRRTAPEIHHRKPHGPEAALENVDAKIAKLMGEGYAFEEVKRALEIAQNNV

EVARSILREFAFPPPVSPRLNL
>sp|P15088|CBPA3_HUMAN Mast cell carboxypeptidase A OS=Homo sapiens GN=CPA3 PE=1 SV=2
MRLILPVGLIATTLAIAPVRFDREKVFRVKPQDEKQADIIKDLAKTNELDFWYPGATHHV
AANMMVDFRVSEKESQAIQSALDQNKMHYEILIHDLQEEIEKQFDVKEDIPGRHSYAKYN
NWEKIVAWTEKMMDKYPEMVSRIKIGSTVEDNPLYVLKIGEKNERRKAIFTDCGIHAREW
VSPAFCQWFVYQATKTYGRNKIMTKLLDRMNFYILPVFNVDGYIWSWTKNRMWRKNRSKN
QNSKCIGTDLNRNFNASWNSIPNTNDPCADNYRGSAPESEKETKAVTNFIRSHLNEIKVY
ITFHSYSQMLLFPYGYTSKLPPNHEDLAKVAKIGTDVLSTRYETRYIYGPIESTIYPISG
SSLDWAYDLGIKHTFAFELRDKGKFGFLLPESRIKPTCRETMLAVKFIAKYILKHTS
>sp|Q8WXQ8|CBPA5_HUMAN Carboxypeptidase A5 OS=Homo sapiens GN=CPA5 PE=2 SV=1
MQGTPGGGTRPGPSPVDRRTLLVFSFILAAALGQMNFTGDQVLRVLAKDEKQLSLLGDLE
GLKPQKVDFWRGPARPSLPVDMRVPFSELKDIKAYLESHGLAYSIMIKDIQVLLDEERQA
MAKSRRLERSTNSFSYSSYHTLEEIYSWIDNFVMEHSDIVSKIQIGNSFENQSILVLKFS
TGGSRHPAIWIDTGIHSREWITHATGIWTANKIVSDYGKDRVLTDILNAMDIFIELVTNP
DGFAFTHSMNRLWRKNKSIRPGIFCIGVDLNRNWKSGFGGNGSNSNPCSETYHGPSPQSE
PEVAAIVNFITAHGNFKALISIHSYSQMLMYPYGRLLEPVSNQRELYDLAKDAVEALYKV
HGIEYIFGSISTTLYVASGITVDWAYDSGIKYAFSFELRDTGQYGFLLPATQIIPTAQET
WMALRTIMEHTLNHPY
>sp|P14384|CBPM_HUMAN Carboxypeptidase M OS=Homo sapiens GN=CPM PE=1 SV=2
MDFPCLWLGLLLPLVAALDFNYHRQEGMEAFLKTVAQNYSSVTHLHSIGKSVKGRNLWVL
VVGRFPKEHRIGIPEFKYVANMHGDETVGRELLLHLIDYLVTSDGKDPEITNLINSTRIH
IMPSMNPDGFEAVKKPDCYYSIGRENYNQYDLNRNFPDAFEYNNVSRQPETVAVMKWLKT
ETFVLSANLHGGALVASYPFDNGVQATGALYSRSLTPDDDVFQYLAHTYASRNPNMKKGD
ECKNKMNFPNGVTNGYSWYPLQGGMQDYNYIWAQCFEITLELSCCKYPREEKLPSFWNNN
KASLIEYIKQVHLGVKGQVFDQNGNPLPNVIVEVQDRKHICPYRTNKYGEYYLLLLPGSY
IINVTVPGHDPHITKVIIPEKSQNFSALKKDILLPFQGQLDSIPVSNPSCPMIPLYRNLP
DHSAATKPSLFLFLVSLLHIFFK
>sp|P15169|CBPN_HUMAN Carboxypeptidase N catalytic chain OS=Homo sapiens GN=CPN1 PE=1 SV=1
MSDLLSVFLHLLLLFKLVAPVTFRHHRYDDLVRTLYKVQNECPGITRVYSIGRSVEGRHL
YVLEFSDHPGIHEPLEPEVKYVGNMHGNEALGRELMLQLSEFLCEEFRNRNQRIVQLIQD
TRIHILPSMNPDGYEVAAAQGPNKPGYLVGRNNANGVDLNRNFPDLNTYIYYNEKYGGPN
HHLPLPDNWKSQVEPETRAVIRWMHSFNFVLSANLHGGAVVANYPYDKSFEHRVRGVRRT
ASTPTPDDKLFQKLAKVYSYAHGWMFQGWNCGDYFPDGITNGASWYSLSKGMQDFNYLHT
NCFEITLELSCDKFPPEEELQREWLGNREALIQFLEQVHQGIKGMVLDENYNNLANAVIS
VSGINHDVTSGDHGDYFRLLLPGIYTVSATAPGYDPETVTVTVGPAEPTLVNFHLKRSIP
QVSPVRRAPSRRHGVRAKVQPQARKKEMEMRQLQRGPA
>sp|Q8IVL8|CBPO_HUMAN Carboxypeptidase O OS=Homo sapiens GN=CPO PE=1 SV=1
MKPLLETLYLLGMLVPGGLGYDRSLAQHRQEIVDKSVSPWSLETYSYNIYHPMGEIYEWM
REISEKYKEVVTQHFLGVTYETHPMYYLKISQPSGNPKKIIWMDCGIHAREWIAPAFCQW
FVKEILQNHKDNSSIRKLLRNLDFYVLPVLNIDGYIYTWTTDRLWRKSRSPHNNGTCFGT
DLNRNFNASWCSIGASRNCQDQTFCGTGPVSEPETKAVASFIESKKDDILCFLTMHSYGQ
LILTPYGYTKNKSSNHPEMIQVGQKAANALKAKYGTNYRVGSSADILYASSGSSRDWARD
IGIPFSYTFELRDSGTYGFVLPEAQIQPTCEETMEAVLSVLDDVYAKHWHSDSAGRVTSA

TMLLGLLVSCMSLL
>sp|Q9BRT8|CBWD1_HUMAN COBW domain-containing protein 1 OS=Homo sapiens GN=CBWD1 PE=2 SV=1
MLPAVGSADEEEDPAEEDCPELVPIETTQSEEEEKSGLGAKIPVTIITGYLGAGKTTLLN
YILTEQHSKRVAVILNEFGEGSALEKSLAVSQGGELYEEWLELRNGCLCCSVKDSGLRAI
ENLMQKKGKFDYILLETTGLADPGAVASMFWVDAELGSDIYLDGIITIVDSKYGLKHLTE
EKPDGLINEATRQVALADAILINKTDLVPEEDVKKLRTTIRSINGLGQILETQRSRVDLS
NVLDLHAFDSLSGISLQKKLQHVPGTQPHLDQSIVTITFEVPGNAKEEHLNMFIQNLLWE
KNVRNKDNHCMEVIRLKGLVSIKDKSQQVIVQGVHELYDLEETPVSWKDDTERTNRLVLL
GRNLDKDILKQLFIATVTETEKQWTTRFQEDQVCT
>sp|Q9HC52|CBX8_HUMAN Chromobox protein homolog 8 OS=Homo sapiens GN=CBX8 PE=1 SV=3
MELSAVGERVFAAEALLKRRIRKGRMEYLVKWKGWSQKYSTWEPEENILDARLLAAFEER
EREMELYGPKKRGPKPKTFLLKAQAKAKAKTYEFRSDSARGIRIPYPGRSPQDLASTSRA
REGLRNMGLSPPASSTSTSSTCRAEAPRDRDRDRDRDRERDRERERERERERERERERER
GTSRVDDKPSSPGDSSKKRGPKPRKELPDPSQRPLGEPSAGLGEYLKGRKLDDTPSGAGK
FPAGHSVIQLARRQDSDLVQCGVTSPSSAEATGKLAVDTFPARVIKHRAAFLEAKGQGAL
DPNGTRVRHGSGPPSSGGGLYRDMGAQGGRPSLIARIPVARILGDPEEESWSPSLTNLEK
VVVTDVTSNFLTVTIKESNTDQGFFKEKR
>sp|Q8N813|CC056_HUMAN Putative uncharacterized protein C3orf56 OS=Homo sapiens GN=C3orf56 PE=2 SV=1
MGTGASEKQAEQKVRRAFEASEEAHGTLAASTPWVAMGSAYGSCTCLGAQPVTDLALWPV
IYSCMGFSPQAYPAFWAYPWVLYGGYLWMGYPPPAALVPSVWLYWRGASSFDPLIGSPYL
AALAPNLFPFPMKFPPTYSLASPTLGGATSSHCPQVGCWTPASSAPRAAVEGPSRGAPYL
KTCKAPPSEWASRFGIWAPLPCCSSELRPLPPSPIEDSQLDPGCSRSSSRSPCRARRRLF
EC
>sp|Q86WR0|CCD25_HUMAN Coiled-coil domain-containing protein 25 OS=Homo sapiens GN=CCDC25 PE=1 SV=2
MVFYFTSSSVNSSAYTIYMGKDKYENEDLIKHGWPEDIWFHVDKLSSAHVYLRLHKGENI
EDIPKEVLMDCAHLVKANSIQGCKMNNVNVVYTPWSNLKKTADMDVGQIGFHRQKDVKIV
TVEKKVNEILNRLEKTKVERFPDLAAEKECRDREERNEKKAQIQEMKKREKEEMKKKREM
DELRSYSSLMKVENMSSNQDGNDSDEFM
>sp|Q9UFE4|CCD39_HUMAN Coiled-coil domain-containing protein 39 OS=Homo sapiens GN=CCDC39 PE=2 SV=3
MSSEFLAELHWEDGFAIPVANEENKLLEDQLSKLKDERASLQDELREYEERINSMTSHFK
NVKQELSITQSLCKARERETESEEHFKAIAQRELGRVKDEIQRLENEMASILEKKSDKEN
GIFKATQKLDGLKCQMNWDQQALEAWLEESAHKDSDALTLQKYAQQDDNKIRALTLQLER
LTLECNQKRKILDNELTETISAQLELDKAAQDFRKIHNERQELIKQWENTIEQMQKRDGD
IDNCALELARIKQETREKENLVKEKIKFLESEIGNNTEFEKRISVADRKLLKCRTAYQDH
ETSRIQLKGELDSLKATVNRTSSDLEALRKNISKIKKDIHEETARLQKTKNHNEIIQTKL
KEITEKTMSVEEKATNLEDMLKEEEKDVKEVDVQLNLIKGVLFKKAQELQTETMKEKAVL
SEIEGTRSSLKHLNHQLQKLDFETLKQQEIMYSQDFHIQQVERRMSRLKGEINSEEKQAL
EAKIVELRKSLEEKKSTCGLLETQIKKLHNDLYFIKKAHSKNSDEKQSLMTKINELNLFI
DRSEKELDKAKGFKQDLMIEDNLLKLEVKRTREMLHSKAEEVLSLEKRKQQLYTAMEERT
EEIKVHKTMLASQIRYVDQERENISTEFRERLSKIEKLKNRYEILTVVMLPPEGEEEKTQ

AYYVIKAAQEKEELQREGDCLDAKINKAEKEIYALENTLQVLNSCNNNYKQSFKKVTPSS
DEYELKIQLEEQKRAVDEKYRYKQRQIRELQEDIQSMENTLDVIEHLANNVKEKLSEKQA
YSFQLSKETEEQKPKLERVTKQCAKLTKEIRLLKDTKDETMEEQDIKLREMKQFHKVIDE
MLVDIIEENTEIRIILQTYFQQSGLELPTASTKGSRQSSRSPSHTSLSARSSRSTSTSTS
QSSIKVLELKFPASSSLVGSPSRPSSASSSSSNVKSKKSSK
>sp|Q4G0X9|CCD40_HUMAN Coiled-coil domain-containing protein 40 OS=Homo sapiens GN=CCDC40 PE=2 SV=2
MAEPGGAAGRSHPEDGSASEGEKEGNNESHMVSPPEKDDGQKGEEAVGSTEHPEEVTTQA
EAAIEEGEVETEGEAAVEGEEEAVSYGDAESEEEYYYTETSSPEGQISAADTTYPYFSPP
QELPGEEAYDSVSGEAGLQGFQQEATGPPESRERRVTSPEPSHGVLGPSEQMGQVTSGPA
VGRLTGSTEEPQGQVLPMGVQHRFRLSHGSDIESSDLEEFVSQEPVIPPGVPDAHPREGD
LPVFQDQIQQPSTEEGAMAERVESEGSDEEAEDEGSQLVVLDPDHPLMVRFQAALKNYLN
RQIEKLKLDLQELVVATKQSRAQRQELGVNLYEVQQHLVHLQKLLEKSHDRHAMASSERR
QKEEELQAARALYTKTCAAANEERKKLAALQTEMENLALHLFYMQNIDQDMRDDIRVMTQ
VVKKAETERIRAEIEKKKQDLYVDQLTTRAQQLEEDIALFEAQYLAQAEDTRILRKAVSE
ACTEIDAISVEKRRIMQQWASSLVGMKHRDEAHRAVLEALRGCQHQAKSTDGEIEAYKKS
IMKEEEKNEKLASILNRTETEATLLQKLTTQCLTKQVALQSQFNTYRLTLQDTEDALSQD
QLEQMILTEELQAIRQAIQGELELRRKTDAAIREKLQEHMTSNKTTKYFNQLILRLQKEK
TNMMTHLSKINGDIAQTTLDITHTSSRLDAHQKTLVELDQDVKKVNELITNSQSEISRRT
ILIERKQGLINFLNKQLERMVSELGGEEVGPLELEIKRLSKLIDEHDGKAVQAQVTWLRL
QQEMVKVTQEQEEQLASLDASKKELHIMEQKKLRVESKIEQEKKEQKEIEHHMKDLDNDL
KKLNMLMNKNRCSSEELEQNNRVTENEFVRSLKASERETIKMQDKLNQLSEEKATLLNQL
VEAEHQIMLWEKKIQLAKEMRSSVDSEIGQTEIRAMKGEIHRMKVRLGQLLKQQEKMIRA
MELAVARRETVTTQAEGQRKMDRKALTRTDFHHKQLELRRKIRDVRKATDECTKTVLELE
ETQRNVSSSLLEKQEKLSVIQADFDTLEADLTRLGALKRQNLSEIVALQTRLKHLQAVKE
GRYVFLFRSKQSLVLERQRLDKRLALIATILDRVRDEYPQFQEALHKVSQMIANKLESPG
PS
>sp|Q9Y6R9|CCD61_HUMAN Coiled-coil domain-containing protein 61 OS=Homo sapiens GN=CCDC61 PE=1 SV=3
MDQPAGLQVDYVFRGVEHAVRVMVSGQVLELEVEDRMTADQWRGEFDAGFIEDLTHKTGN
FKQFNIFCHMLESALTQSSESVTLDLLTYTDLESLRNRKMGGRPGSLAPRSAQLNSKRYL
ILIYSVEFDRIHYPLPLPYQGKPDPVVLQGIIRSLKEELGRLQGLDGQNTRDTRENEIWH
LREQVSRLASEKRELEAQLGRSREEALAGRAARQEAEALRGLVRGLELELRQERGLGHRV
AGRRGQDCRRLAKELEEAKASERSLRARLKTLTSELALYKRGRRTPPVQPPPTREDRASS
SRERSASRGRGAARSSSRESGRGSRGRGRPARPSPSPTGGRALRFDPTAFVKAKERKQRE
IQMKQQQRNRLGSGGSGDGPSVSWSRQTQPPAALTGRGDAPNRSRNRSSSVDSFRSRCSS
ASSCSDLEDFSESLSRGGHRRRGKPPSPTPWSGSNMKSPPVERSHHQKSLANSGGWVPIK
EYSSEHQAADMAEIDARLKALQEYMNRLDMRS
>sp|Q6P9F0|CCD62_HUMAN Coiled-coil domain-containing protein 62 OS=Homo sapiens GN=CCDC62 PE=1 SV=2
MNPPAAFLAGRQNIGSEVEISTIEKQRKELQLLIGELKDRDKELNDMVAVHQQQLLSWEE
DRQKVLTLEERCSKLEGELHKRTEIIRSLTKKVKALESNQMECQTALQKTQLQLQEMAQK
ATHSSLLSEDLEARNETLSNTLVELSAQVGQLQAREQALTTMIKLKDKDIIEAVNHIADC
SGKFKMLEHALRDAKMAETCIVKEKQDYKQKLKALKIEVNKLKEDLNEKTTENNEQREEI

IRLKQEKSCLHDELLFTVEREKRKDELLNIAKSKQERTNSELHNLRQIYVKQQSDLQFLN
FNVENSQELIQMYDSKMEESKALDSSRDMCLSDLENNHPKVDIKREKNQKSLFKDQKFEA
MLVQQNRSDKSSCDECKEKKQQIDTVFGEKSVITLSSIFTKDLVEKHNLPWSLGGKTQIE
PENKITLCKIHTKSPKCHGTGVQNEGKQPSETPTLSDEKQWHDVSVYLGLTNCPSSKHPE
KLDVECQDQMERSEISCCQKNEACLGESGMCDSKCCHPSNFIIEAPGHMSDVEWMSIFKP
SKMQRIVRLKSGCTCSESICGTQHDSPASELIAIQDSHSLGSSKSALREDETESSSNKKN
SPTSLLIYKDAPAFNEKASIVLPSQDDFSPTSKLQRLLAESRQMVTDLELSTLLPISHEN
LTGSATNKSEVPEESAQKNTFVSY
>sp|A2IDD5|CCD78_HUMAN Coiled-coil domain-containing protein 78 OS=Homo sapiens GN=CCDC78 PE=2 SV=1
MEHAATTGPRPGPPSRRVENVVLRAKDWLPGAPGGTAVWATSLEAEVPPDLALNKEQQLQ
ISKELVDIQITTHHLHEQHEAEIFQLKSEILRLESRVLELELRGDGTSQGCAVPVESDPR
HPRAAAQELRHKAQVPGHSDDHRFQVQPKNTMNPENEQHRLGSGLQGEVKWALEHQEARQ
QALVTRVATLGRQLQGAREEARAAGQRLATQAVVLCSCQGQLRQAEAENARLQLQLKKLK
DEYVLRLQHCAWQAVEHADGAGQAPATTALRTFLEATLEDIRAAHRSREQQLARAARSYH
KRLVDLSRRHEELLVAYRAPGNPQAIFDIASLDLEPLPVPLVTDFSHREDQHGGPGALLS
SPKKRPGGASQGGTSEPQGLDAASWAQIHQKLRDFSRSTQSWNGSGHSCWSGPRWLKSNF
LSYRSTWTSTWAGTSTKS
>sp|Q76M96|CCD80_HUMAN Coiled-coil domain-containing protein 80 OS=Homo sapiens GN=CCDC80 PE=1 SV=1
MTWRMGPRFTMLLAMWLVCGSEPHPHATIRGSHGGRKVPLVSPDSSRPARFLRHTGRSRG
IERSTLEEPNLQPLQRRRSVPVLRLARPTEPPARSDINGAAVRPEQRPAARGSPREMIRD
EGSSARSRMLRFPSGSSSPNILASFAGKNRVWVISAPHASEGYYRLMMSLLKDDVYCELA
ERHIQQIVLFHQAGEEGGKVRRITSEGQILEQPLDPSLIPKLMSFLKLEKGKFGMVLLKK
TLQVEERYPYPVRLEAMYEVIDQGPIRRIEKIRQKGFVQKCKASGVEGQVVAEGNDGGGG
AGRPSLGSEKKKEDPRRAQVPPTRESRVKVLRKLAATAPALPQPPSTPRATTLPPAPATT
VTRSTSRAVTVAARPMTTTAFPTTQRPWTPSPSHRPPTTTEVITARRPSVSENLYPPSRK
DQHRERPQTTRRPSKATSLESFTNAPPTTISEPSTRAAGPGRFRDNRMDRREHGHRDPNV
VPGPPKPAKEKPPKKKAQDKILSNEYEEKYDLSRPTASQLEDELQVGNVPLKKAKESKKH
EKLEKPEKEKKKKMKNENADKLLKSEKQMKKSEKKSKQEKEKSKKKKGGKTEQDGYQKPT
NKHFTQSPKKSVADLLGSFEGKRRLLLITAPKAENNMYVQQRDEYLESFCKMATRKISVI
TIFGPVNNSTMKIDHFQLDNEKPMRVVDDEDLVDQRLISELRKEYGMTYNDFFMVLTDVD
LRVKQYYEVPITMKSVFDLIDTFQSRIKDMEKQKKEGIVCKEDKKQSLENFLSRFRWRRR
LLVISAPNDEDWAYSQQLSALSGQACNFGLRHITILKLLGVGEEVGGVLELFPINGSSVV
EREDVPAHLVKDIRNYFQVSPEYFSMLLVGKDGNVKSWYPSPMWSMVIVYDLIDSMQLRR
QEMAIQQSLGMRCPEDEYAGYGYHSYHQGYQDGYQDDYRHHESYHHGYPY
>sp|Q8N4S0|CCD82_HUMAN Coiled-coil domain-containing protein 82 OS=Homo sapiens GN=CCDC82 PE=1 SV=2
MIHVRRHETRRNSKSHVPEQKSRVDWRRTKRSSISQLLDSDEELDSEEFDSDEELDSDES
FENDEELDSNKGPDCNKTPGSERELNLSKIQSEGNDSKCLINSGNGSTYEEETNKIKHRN
IDLQDQEKHLSQEDNDLNKQTGQIIEDDQEKHLSQEDNDLNKQTGQIIEDDLEEEDIKRG
KRKRLSSVMCDSDESDDSDILVRKVGVKRPRRVVEDEGSSVEMEQKTPEKTLAAQKREKL
QKLKELSKQRSRQRRSSGRDFEDSEKESCPSSDEVDEEEEEDNYESDEDGDDYIIDDFVV
QDEEGDEENKNQQGEKLTTSQLKLVKQNSLYSFSDHYTHFERVVKALLINALDESFLGTL

YDGTRQKSYAKDMLTSLHYLDNRFVQPRLESLVSRSRWKEQYKERVENYSNVSIHLKNPE
NCSCQACGLHRYCKYSVHLSGELYNTRTMQIDNFMSHDKQVFTVGRICASRTRIYHKLKH
FKFKLYQECCTIAMTEEVEDEQVKETVERIFRRSKENGWIKEKYGQLEEYLNFADYFQEE
KFEL
>sp|Q9UF02|CCG5_HUMAN Voltage-dependent calcium channel gamma-5 subunit OS=Homo sapiens GN=CACNG5 PE=2 SV=2
MSACGRKALTLLSSVFAVCGLGLLGIAVSTDYWLYLEEGVIVPQNQSTEIKMSLHSGLWR
VCFLAGEERGRCFTIEYVMPMNTQLTSESTVNVLKMIRSATPFPLVSLFFMFIGFILNNI
GHIRPHRTILAFVSGIFFILSGLSLVVGLVLYISSINDEMLNRTKDAETYFNYKYGWSFA
FAAISFLLTESAGVMSVYLFMKRYTAEDMYRPHPGFYRPRLSNCSDYSGQFLHPDAWVRG
RSPSDISSEASLQMNSNYPALLKCPDYDQMSSSPC
>sp|P53701|CCHL_HUMAN Cytochrome c-type heme lyase OS=Homo sapiens GN=HCCS PE=1 SV=1
MGLSPSAPAVAVQASNASASPPSGCPMHEGKMKGCPVNTEPSGPTCEKKTYSVPAHQERA
YEYVECPIRGTAAENKENLDPSNLMPPPNQTPAPDQPFALSTVREESSIPRADSEKKWVY
PSEQMFWNAMLKKGWKWKDEDISQKDMYNIIRIHNQNNEQAWKEILKWEALHAAECPCGP
SLIRFGGKAKEYSPRARIRSWMGYELPFDRHDWIINRCGTEVRYVIDYYDGGEVNKDYQF
TILDVRPALDSLSAVWDRMKVAWWRWTS
>sp|Q16627|CCL14_HUMAN C-C motif chemokine 14 OS=Homo sapiens GN=CCL14 PE=1 SV=1
MKISVAAIPFFLLITIALGTKTESSSRGPYHPSECCFTYTTYKIPRQRIMDYYETNSQCS
KPGIVFITKRGHSVCTNPSDKWVQDYIKDMKEN
>sp|P55774|CCL18_HUMAN C-C motif chemokine 18 OS=Homo sapiens GN=CCL18 PE=1 SV=1
MKGLAAALLVLVCTMALCSCAQVGTNKELCCLVYTSWQIPQKFIVDYSETSPQCPKPGVI
LLTKRGRQICADPNKKWVQKYISDLKLNA
>sp|Q9Y258|CCL26_HUMAN C-C motif chemokine 26 OS=Homo sapiens GN=CCL26 PE=1 SV=1
MMGLSLASAVLLASLLSLHLGTATRGSDISKTCCFQYSHKPLPWTWVRSYEFTSNSCSQR
AVIFTTKRGKKVCTHPRKKWVQKYISLLKTPKQL
>sp|Q9NUG4|CCM2L_HUMAN Cerebral cavernous malformations 2 protein-like OS=Homo sapiens GN=CCM2L PE=2 SV=3
MEYEVKKGKKGFVSPIRRLVFPKAGRRAACRSSVSRRPLHSMPLYPPDYLIDPQILLCDY
LEKEVKFLGHLTWVTSSLNPSSRDELLQLLDTARQLKELPLKTTAEQDSILSLSARCLLL
TWRDNEELILRIPTHEIAAASYLQDDALHLLVLKTGLGVDPVPAGVDASPGGAGRDPGPP
GGAPEKRRVGTAERRHTICSLDWRMGWGGGAAEARAGGGGGGSLERQRAGARASGSWERR
QTFSGSWERRHGGGGGGGGAGKPGGSWERRQAGSGGGGSWERRHPGPNPLDPQDPSPDAY
CNLVILAVANRDAAEESCALICQVFQIIYGDQSIECVDRAGYHYTSTPERPWLCSRSESC
HTDGTYAYDADFSCCSSFNGSQDTFEACYSGTSTPSFHGSHCSGSDHSSLGLEQLQDYMV
TLRSKLGPLEIQQFAMLLREYRLGLPIQDYCTGLLKLYGDRRKFLLLGMRPFIPDQDIGY
FEGFLEGVGIREGGILTDSFGRIKRSMSSTSASAVRSYDGAAQRPEAQAFHRLLADITHD
IEALAPDDDDDDEDEPRGSRGGSDAAEDNYL
>sp|O75909|CCNK_HUMAN Cyclin-K OS=Homo sapiens GN=CCNK PE=1 SV=2
MKENKENSSPSVTSANLDHTKPCWYWDKKDLAHTPSQLEGLDPATEARYRREGARFIFDV
GTRLGLHYDTLATGIIYFHRFYMFHSFKQFPRYVTGACCLFLAGKVEETPKKCKDIIKTA
RSLLNDVQFGQFGDDPKEEVMVLERILLQTIKFDLQVEHPYQFLLKYAKQLKGDKNKIQK
LVQMAWTFVNDSLCTTLSLQWEPEIIAVAVMYLAGRLCKFEIQEWTSKPMYRRWWEQFVQ
DVPVDVLEDICHQILDLYSQGKQQMPHHTPHQLQQPPSLQPTPQVPQVQQSQPSQSSEPS

QPQQKDPQQPAQQQQPAQQPKKPSPQPSSPRQVKRAVVVSPKEENKAAEPPPPKIPKIET
THPPLPPAHPPPDRKPPLAAALGEAEPPGPVDATDLPKVQIPPPAHPAPVHQPPPLPHRP
PPPPPSSYMTGMSTTSSYMSGEGYQSLQSMMKTEGPSYGALPPAYGPPAHLPYHPHVYPP
NPPPPPVPPPPASFPPPAIPPPTPGYPPPPPTYNPNFPPPPPRLPPTHAVPPHPPPGLGL
PPASYPPPAVPPGGQPPVPPPIPPPGMPPVGGLGRAAWMR
>sp|P51684|CCR6_HUMAN C-C chemokine receptor type 6 OS=Homo sapiens GN=CCR6 PE=1 SV=2
MSGESMNFSDVFDSSEDYFVSVNTSYYSVDSEMLLCSLQEVRQFSRLFVPIAYSLICVFG
LLGNILVVITFAFYKKARSMTDVYLLNMAIADILFVLTLPFWAVSHATGAWVFSNATCKL
LKGIYAINFNCGMLLLTCISMDRYIAIVQATKSFRLRSRTLPRSKIICLVVWGLSVIISS
STFVFNQKYNTQGSDVCEPKYQTVSEPIRWKLLMLGLELLFGFFIPLMFMIFCYTFIVKT
LVQAQNSKRHKAIRVIIAVVLVFLACQIPHNMVLLVTAANLGKMNRSCQSEKLIGYTKTV
TEVLAFLHCCLNPVLYAFIGQKFRNYFLKILKDLWCVRRKYKSSGFSCAGRYSENISRQT
SETADNDNASSFTM
>sp|P51685|CCR8_HUMAN C-C chemokine receptor type 8 OS=Homo sapiens GN=CCR8 PE=1 SV=1
MDYTLDLSVTTVTDYYYPDIFSSPCDAELIQTNGKLLLAVFYCLLFVFSLLGNSLVILVL
VVCKKLRSITDVYLLNLALSDLLFVFSFPFQTYYLLDQWVFGTVMCKVVSGFYYIGFYSS
MFFITLMSVDRYLAVVHAVYALKVRTIRMGTTLCLAVWLTAIMATIPLLVFYQVASEDGV
LQCYSFYNQQTLKWKIFTNFKMNILGLLIPFTIFMFCYIKILHQLKRCQNHNKTKAIRLV
LIVVIASLLFWVPFNVVLFLTSLHSMHILDGCSISQQLTYATHVTEIISFTHCCVNPVIY
AFVGEKFKKHLSEIFQKSCSQIFNYLGRQMPRESCEKSSSCQQHSSRSSSVDYIL
>sp|O00421|CCRL2_HUMAN C-C chemokine receptor-like 2 OS=Homo sapiens GN=CCRL2 PE=1 SV=2
MANYTLAPEDEYDVLIEGELESDEAEQCDKYDAQALSAQLVPSLCSAVFVIGVLDNLLVV
LILVKYKGLKRVENIYLLNLAVSNLCFLLTLPFWAHAGGDPMCKILIGLYFVGLYSETFF
NCLLTVQRYLVFLHKGNFFSARRRVPCGIITSVLAWVTAILATLPEFVVYKPQMEDQKYK
CAFSRTPFLPADETFWKHFLTLKMNISVLVLPLFIFTFLYVQMRKTLRFREQRYSLFKLV
FAIMVVFLLMWAPYNIAFFLSTFKEHFSLSDCKSSYNLDKSVHITKLIATTHCCINPLLY
AFLDGTFSKYLCRCFHLRSNTPLQPRGQSAQGTSREEPDHSTEV
>sp|P0C7X3|CCYL3_HUMAN Putative cyclin-Y-like protein 3 OS=Homo sapiens GN=CCNYL3 PE=3 SV=1
MACAVFQIPPWHLDRKYGSCSTILLDNSTASQPDLRHTLERYANRSLAIFEEPVHPLPQE
KLPGKSFKHDPKRNCIFRHFCTLFQVIKLTAPCAIVALVYIKRLLTSANIDLCPTNWKKI
VLGTMLLASKVWRNHGLWSVDDSQNSKDTAVENMSKMEKCFLELLEFNIHVSASVYAKYY
FDLCALANDHDLYFLFSFLHKDKAQKLEGHTSQHSLSLGPCRVGMENYATYSPLSSQPVL
GALSLAEVLPQEIKQEIHQVLMDHGVPCHYTCFLLHPEDSTLERFLVLHSIQGLQEDSVL
CKVEIHGLSPQPQTYQSHFRSPSLAELLNRISGTCRNHFAGMQK
>sp|P86790|CCZ1B_HUMAN Vacuolar fusion protein CCZ1 homolog B OS=Homo sapiens GN=CCZ1B PE=1 SV=1
MAAAAAGAGSGPWAAQEKQFPPALLSFFIYNPRFGPREGQEENKILFYHPNEVEKNEKIR
NVGLCEAIVQFTRTFSPSKPAKSLHTQKNRQFFNEPEENFWMVMVVRNPIIEKQSKDGKP
VIEYQEEELLDKVYSSVLRQCYSMYKLFNGTFLKAMEDGGVKLLKERLEKFFHRYLQTLH
LQSCDLLDIFGGISFFPLDKMTYLKIQSFINRMEESLNIVKYTAFLYNDQLIWSGLEQDD
MRILYKYLTTSLFPRHIEPELAGRDSPIRAEMPGNLQHYGRFLTGPLNLNDPDAKCRFPK
IFVNTDDTYEELHLIVYKAMSAAVCFMIDASVHPTLDFCRRLDSIVGPQLTVLASDICEQ
FNINKRMSGSEKEPQFKFIYFNHMNLAEKSTVHMRKTPSVSLTSVHPDLMKILGDINSDF

TRVDEDEEIIVKAMSDYWVVGKKSDRRELYVILNQKNANLIEVNEEVKKLCATQFNNIFF
LD
>sp|Q6ZUJ4|CC062_HUMAN Uncharacterized protein C3orf62 OS=Homo sapiens GN=C3orf62 PE=1 SV=1
MHYIKTWSLLGEMSEKLRRCRKELTAAIDRAFEGVSYSQECTGQQRLELSAAPLSFSLPV
HRLLCRRHPLAACSSAAPFAAVPCAPENENPAFATNHAPVNAKPHALCPERKPLTSKENV
LMHSSILAPERESWRTAGEGENWRKENLRKDMERDLKADSNMPLNNSSQEVTKDLLDMID
HTSIRTIEELAGKIEFENELNHMCGHCQDSPFKEEAWALLMDKSPQKATDADPGSLKQAF
DDHNIVETVLDLEEDYNVMTSFKYQIE
>sp|A6NLC5|CC070_HUMAN UPF0524 protein C3orf70 OS=Homo sapiens GN=C3orf70 PE=2 SV=1
MSAAASPASERGWKSEKLDEAQALARSCAARRPDFQPCDGLSICATHSHGKCFKLHWCCH
LGWCHCKYMYQPMTPVEQLPSTEIPARPREPTNTIQISVSLTEHFLKFASVFQPPLPPDS
PRYCMISDLFIDNYQVKCINGKMCYVQKQPAPHSHRMSPEEVSAHDALISKESNTPKIDH
CSSPSSSEDSGINAIGAHYVESCDEDTEEGAELSSEEDYSPESSWEPDECTLLSPSQSDL
EVIETIETTV
>sp|A2VCL2|CC162_HUMAN Coiled-coil domain-containing protein 162 OS=Homo sapiens GN=CCDC162P PE=2 SV=3
MFKTLPEKAAFKALKRTLQLIAPLHDIVAYLVSFAKLGNCPACFEFPRSPNPLRGDWGGT
EGIGSELQELQNMIDSLQSPQDPIRVAQALLLRREVIFLQFDAAVRHLIRRTFLAAGNVP
AYQSVTDGMCHGLPALSNSLRKSIFASQLSLPQPLDPRSLQAFELFPWRAFLEDGGPFPV
MSNSPDTLEYNMQLCLCGLSDRDRKVAHGELVGVQLLLEDVLSSYHVTMEAPQRQQATLG
KNTQPDWSKVPGFRSQFRSSPKTSELLEGLCDAVMSFALLRSFLILWKQLEVLKEHWGRL
KLQGQDINSVSLHKRFSELYETDVLYPSMKAIARQMGKEDEFEGFIVNNQSVLPPSGASE
VEIKTHQLQKLLENCEIQMIQEVLRKVNREMTLVLSEKCKEECSLPTDLWKHQVMKENFS
VSRPQIVEKFIQRLMNYQDDGVEITFRKDHLEACLLSLGCDVMARECSNFETYSMCYEHV
LHHARQRLSQKEQELDATQRGQGPPEDSAGGQIAELSHDMIMEITTLRAQLTDLEEVNLNL
KKQIRKEVQEEYEALVRALFETCLHIKEKLDDNQLNLIQKVCELIGEVRTEGIDNMKDLK
KKWCSASPDEGMKENPAKEQLWALEQDNCSLANLVCKVRSLGRWRLAVQQACFQAQLSRT
EKESIQSKKEYLRIKLMAEREVGLFRQQVLALRQALARAQADSARMWKQQDSQAQLLKEL
EHRVTQEALTQQQLHFMKTSRMEKLLEDVGQKEQQLQLLSKEAERASKLGQLQQKKMKRD
LHQMRSRLAQERSVKLDALQRAEELQGQLHDAQRSAVPMGSSGDLISQAQYSPTSASTSS
RYSQQRFLKTNLKGSKITRWIQRPQTVPIKHKKRTDDVFLPNMAENVQLTAFQVQTAPSR
FPFRADW
>sp|A6NNP5|CC169_HUMAN Coiled-coil domain-containing protein 169 OS=Homo sapiens GN=CCDC169 PE=2 SV=4
MKEERNYNFDGVSTNRLKQQLLEEVRKKDAVQLSIFELRHKITELEAKLNTDNEGSEWKT
RYETQLELNDELEKQIVYLKEKVEKIHGNSSDRLSSIRVYERMPVESLNTLLKQLEEEKK
TLESQVKYYALKLEQESKAYQKINNERRTYLAEMSQGSGLHQVSKRQQVDQLPRMQENLV
KTGRYNPAKQKTVSAKRGPVKKITRPNHLPELHP
>sp|Q5T5S1|CC183_HUMAN Coiled-coil domain-containing protein 183 OS=Homo sapiens GN=CCDC183 PE=2 SV=3
MRRHSETDVEEQTQELKTITQLQEQCRALQIQGVKENMDQNKATLALLRSNIRRGAQDWA
LAKKYDQWTISKACGKNLPLRLAHCRSTMEVVREKLRKYVFDRVNMHNLLIHLVRRRGQK
LESMQLELDSLRSQPDASKEELRLLQIIRQLENNIEKTMIKIITSQNIHLLYLDLLDYLK

TVLAGYPIELDKLQNLVVNYCSELSDMKIMSQDAMMITDEVKRNMRQREASFIEERRARE
NRLNQQKKLIDKIHTKETSEKYRRGQMDLDFPSNLMSTETLKLRRKETSTAEMEYQSGVT
AVVEKVKSAVRCSHVWDITSRFLAQRNTEENLELQMEDCEEWRVQLKALVKQLELEEAVL
KFRQKPSSISFKSVEKKMTDMLKEEEERLQLAHSNMTKGQELLLTIQMGIDNLYVRLMGI
NLPATQREVVLSNTLDLNSKLAYCEGKLTYLADRVQMVSRTEEGDTKVRDTLESSTLMEK
YNTRISFENREEDMIDTFQFPDMDHSYVPSRAEIKRQAQRLIEGKLKAAKKKKK
>sp|Q86UF4|CC190_HUMAN Coiled-coil domain-containing protein 190 OS=Homo sapiens GN=CCDC190 PE=2 SV=2
MERHMVRGQLYKHFDLERKNAKQAEARLDQRLQRLKVICLYHVKLLTWEQRQLQKELQRL
QQAETMKKKFSSYLGNGFQKRPEDVLVFSPQGRQKHRAPQAKKMRALATRMAQDTCKSKS
QVPPSHDAGLKDPMKSKKQPLSQNNRTACFIKEQPQAQEKDSVNPSKDVDPSKGISVPCQ
NQEVSTNTIEQGPSSSPASDSGMACADETRSKDVALKPDGNTGKQIPPKHMECAGSFEGE
FTKPTFLELLSKARNAHYLRHRVPPESERLLSIGEIFGHGESSSSRAGKECENRVPSKFL
PL
>sp|Q8NHW4|CC4L_HUMAN C-C motif chemokine 4-like OS=Homo sapiens GN=CCL4L1 PE=1 SV=1
MKLCVTVLSLLVLVAAFCSLALSAPMGSDPPTACCFSYTARKLPRNFVVDYYETSSLCSQ
PAVVFQTKRGKQVCADPSESWVQEYVYDLELN
>sp|Q3MIR4|CC50B_HUMAN Cell cycle control protein 50B OS=Homo sapiens GN=TMEM30B PE=1 SV=1
MTWSATARGAHQPDNTAFTQQRLPAWQPLLSASIALPLFFCAGLAFIGLGLGLYYSSNGI
KELEYDYTGDPGTGNCSVCAAAGQGRALPPPCSCAWYFSLPELFQGPVYLYYELTNFYQN
NRRYGVSRDDAQLSGLPSALRHPVNECAPYQRSAAGLPIAPCGAIANSLFNDSFSLWHQR
QPGGPYVEVPLDRSGIAWWTDYHVKFRNPPLVNGSLALAFQGTAPPPNWRRPVYELSPDP
NNTGFINQDFVVWMRTAALPTFRKLYARIRQGNYSAGLPRGAYRVNITYNYPVRAFGGHK
LLIFSSISWMGGKNPFLGIAYLVVGSLCILTGFVMLVVYIRYQDQDDDDEE
>sp|Q96AQ1|CC74A_HUMAN Coiled-coil domain-containing protein 74A OS=Homo sapiens GN=CCDC74A PE=2 SV=1
MSGAGVAAGTRPPSSPTPGSRRRRQRPSVGVQSLRPQSPQLRQSDPQKRNLDLEKSLQFL
QQQHSEMLAKLHEEIEHLKRENKDLHYKLIMNQTSQKKDGPSGNHLSRASAPLGARWVCI
NGVWVEPGGPSPARLKEGSSRTHRPGGKRGRLAGGSADTVRSPADSLSMSSFQSVKSISN
SGKARPQPGSFNKQDSKADVSQKADLEEEPLLHNSKLDKVPGVQGQARKEKAEASNAGAA
CMGNSQHQGRQMGAGAHPPMILPLPLRKPTTLRQCEVLIRELWNTNLLQTQELRHLKSLL
EGSQRPQAAPEEASFPRDQEATHFPKVSTKSLSKKCLSPPVAERAILPALKQTPKNNFAE
RQKRLQAMQKRRLHRSVL
>sp|Q15834|CC85B_HUMAN Coiled-coil domain-containing protein 85B OS=Homo sapiens GN=CCDC85B PE=1 SV=2
MEAEAGGLEELTDEEMAALGKEELVRRLRREEAARLAALVQRGRLMQEVNRQLQGHLGEI
RELKQLNRRLQAENRELRDLCCFLDSERQRGRRAARQWQLFGTQASRAVREDLGGCWQKL
AELEGRQEELLRENLALKELCLALGEEWGPRGGPSGAGGSGAGPAPELALPPCGPRDLGD
GSSSTGSVGSPDQLPLACSPDD
>sp|Q6UXH8|CCBE1_HUMAN Collagen and calcium-binding EGF domain-containing protein 1 OS=Homo sapiens GN=CCBE1 PE=1 SV=1
MVPPPPSRGGAARGQLGRSLGPLLLLLALGHTWTYREEPEDGDREICSESKIATTKYPCL
KSSGELTTCYRKKCCKGYKFVLGQCIPEDYDVCAEAPCEQQCTDNFGRVLCTCYPGYRYD

RERHRKREKPYCLDIDECASSNGTLCAHICINTLGSYRCECREGYIREDDGKTCTRGDKY
PNDTGHEKSENMVKAGTCCATCKEFYQMKQTVLQLKQKIALLPNNAADLGKYITGDKVLA
SNTYLPGPPGLPGGQGPPGSPGPKGSPGFPGMPGPPGQPGPRGSMGPMGPSPDLSHIKQG
RRGPVGPPGAPGRDGSKGERGAPGPRGSPGPPGSFDFLLLMLADIRNDITELQEKVFGHR
THSSAEEFPLPQEFPSYPEAMDLGSGDDHPRRTETRDLRAPRDFYP
>sp|Q5VVM6|CCD30_HUMAN Coiled-coil domain-containing protein 30 OS=Homo sapiens GN=CCDC30 PE=2 SV=1
MSQEKNEMFESEWSKEREREKQLASGLDTAEKALKVESEELQKSKSELICLYNEVHNLPG
ESESKDHFLIACDLLQRENSELETKVLKLSQEFAQLNHFTLGGKTAPSNLITSENTCKDP
ESNEPILETEIQSRKEETEELCPKLGERKQKEIPEESVKEGSFPREGQKEEGSQQNRDMK
DEEKEQQLTMKPEEIVRLREELSHINQSLLQSQSSGDSSDDSGAQHPSSGEKLKYNQQGE
VQQLHQNLHRLQILCNSAENELRYERGQNLDLKQHNSLLQEENIKIKIELKHAQQKLLDS
TKMCSSLTAEYKHCQQKIKELELEVLKHTQSIKSQNNLQEKLVQEKSKVADAEEKILDLQ
RKLEHAHKVCLTDTCISEKQQLEEKIKEATQNEAKVKQQYQEEQQKRKLLYQNVDELHRQ
VRTLQDKENLLEMTCSQQQSRIQQQEALLKQLENEKRKYDEHVKSNQELSEKLSKLQQEK
EALREEYLRLLKLLNVHVRNYNEKHHQQKVKLQKVKYRLTNEVELRDKRINQFEDEIGIL
QHKIEKEKAIQDQITAQNDTLLLEKRKLQEQVIEQEQLIHSNKWTISSIQSRVLYMDKEN
KQLQENSLRLTQQIGFLERIIRSIHIRRGENLKEFPVPKWWHRGKLASLPPTKKQKEIYS
TEVFTSNNAELQHEDESVPEATEKWKHSEQMETTISDILESEVVNEILPLSNSSFSGKGL
VESFASLQETEEIKSKEAMASSKSPEKSPENLVCSQNSEAGYINVASLKETHGIQEQDQK
SEL
>sp|Q8N5R6|CCD33_HUMAN Coiled-coil domain-containing protein 33 OS=Homo sapiens GN=CCDC33 PE=1 SV=3
MAFRGPEPWVSASLLRQRLKAEEKTLDLEFEVLSVGFNEAGRYALRLSAENPLQVGSGAG
VQLQVNDGDPFPACSAITDVIEQQEPGQSLTLTRSKFIFTLPKGFCKNDGQHDAQLHVEA
LRLDEPLGRAAQRVGEAIFPIYPRPDQPRMNPKAQDHEDLYRYCGNLALLRASTDPTARH
CGSLAYSVAFHVHRGPQPPVSDSPPRAGQPELMSPEEPLIASQSTEPEIGHLSPSKKETI
MVTLHGATNLPACKDGSEPWPYVVVKSTSEEKNNQSSKAVTSVTSEPTRAPIWGDTVNVE
IQAEDAGQEDVILKVVDNRKKQELLSYKIPIKYLRVFHPYHFELVKPTESGKADEATAKT
QLYATVVRKSSFIPRYIGCNHMALEIFLRGVNEPLANNPNPIVVIARVVPNYKEFKVSQA
NRDLASVGLPITPLSFPIPSMMNFDVPRVSQNGCPQLSKPGGPPEQPLWNQSFLFQGRDG
ATSFSEDTALVLEYYSSTSMKGSQPWTLNQPLGISVLPLKSRLYQKMLTGKGLDGLHVER
LPIMDTSLKTINDEAPTVALSFQLLSSERPENFLTPNNSKALPTLDPKILDKKLRTIQES
WSKDTVSSTMDLSTSTPREAEEEPLVPEMSHDTEMNNYRRAMQKMAEDILSLRRQASILE
GENRILRSRLAQQEEEEGQGKASEAQNTVSMKQKLLLSELDMKKLRDRVQHLQNELIRKN
DREKELLLLYQAQQPQAALLKQYQGKLQKMKALEETVRHQEKVIEKMERVLEDRLQDRSK
PPPLNRQQGKPYTGFPMLSASGLPLGSMGENLPVELYSVLLAENAKLRTELDKNRHQQAP
IILQQQALPDLLSGTSDKFNLLAKLEHAQSRILSLESQLEDSARRWGREKQDLATRLQEQ
EKGFRHPSNSIIIEQPSALTHSMDLKQPSELEPLLPSSDSKLNKPLSPQKETANSQQT
>sp|Q9Y3C0|CCD53_HUMAN WASH complex subunit CCDC53 OS=Homo sapiens GN=CCDC53 PE=1 SV=1
MDEDGLPLMGSGIDLTKVPAIQQKRTVAFLNQFVVHTVQFLNRFSTVCEEKLADLSLRIQ
QIETTLNILDAKLSSIPGLDDVTVEVSPLNVTSVTNGAHPEATSEQPQQNSTQDSGLQES
EVSAENILTVAKDPRYARYLKMVQVGVPVMAIRNKMISEGLDPDLLERPDAPVPDGESEK
TVEESSDSESSFSD

>sp|Q8IWA6|CCD60_HUMAN Coiled-coil domain-containing protein 60 OS=Homo sapiens GN=CCDC60 PE=1 SV=2
MTKVPATKKLQSSPNSGAVRPFYASENLRQVPDKPMKSIKYMDKEIINLKKDLIRSRFLI
QSVKIGRGYFAILREETAKKKKQQQLQKLKEEERNKFQPAEKISEIHYGDTLLSTYDDEK
LKTLGARVTRRPFTPIHSCIISPSLTEAHVEPLFRQLCALHWLLEALTIDHTHHTMKPVI
TCWNPKDPGGSKSTIKKINKDKSMGQKWEHFITAPKTKKFKIPTMRVTNRKPSRRGSTLS
LSRASGGSSPQSSMISVNPGSDEPPSVNTQVTSSKDIEDNESSSTKPDEEPLYMNLQKLL
EMVREDARRTVTIENGMQRKAPSILSVLKQNKSNSAYKEMQTTLKSSERSSSTSAESHIQ
PVQKKSKNRTNCDINIHYKSGVCNTMRAKFYSVAQEAGFCLQDKMEILMKRQEERGIQKF
RAFVLVSNFQKDIAKMRHHISVVKGDAEEIADHWYFDLLSKLPEDLKNFRPAKKILVKLQ
KFGENLDLRIRPHVLLKVLQDLRIWELCSPDIAVAIEFVREHIIHMPQEDYISWLQSRIN
IPIGPYSALR
>sp|Q8NA47|CCD63_HUMAN Coiled-coil domain-containing protein 63 OS=Homo sapiens GN=CCDC63 PE=2 SV=1
MSVLKKNRRKDSDTPQEPSEKAKEQQAEAELRKLRQQFRKMVESRKSFKFRNQQKIASQY
KEIKTLKTEQDEITLLLSLMKSSRNMNRSEKNYMELRLLLQTKEDYEALIKSLKVLLAEL
DEKILQMEKKIANQKQIFAKMQEANNPRKLQKQIHILETRLNLVTVHFDKMLTTNAKLRK
EIEDLRFEKAAYDNVYQQLQHCLLMEKKTMNLAIEQSSQAYEQRVEAMARMAAMKDRQKK
DTSQYNLEIRELERLYAHESKLKSFLLVKLNDRNEFEEQAKREEALKAKKHVKKNRGESF
ESYEVAHLRLLKLAESGNLNQLIEDFLAKEEKNFARFTYVTELNNDMEMMHKRTQRIQDE
IILLRSQQKLSHDDNHSVLRQLEDKLRKTTEEADMYESKYGEVSKTLDLLKNSVEKLFKK
INCDATKILVQLGETGKVTDINLPQYFAIIEKKTNDLLLLETYRRILEVEGAEAEIPPPF
INPFWGGSALLKPPEPIKVIPPVLGADPFSDRLDDVEQPLDHSSLRQLVLDNYILKENRS
KEVRGDSLPEKVDDFRSRKKVTM
>sp|A2RUB6|CCD66_HUMAN Coiled-coil domain-containing protein 66 OS=Homo sapiens GN=CCDC66 PE=1 SV=4
MNLGDGLKLETELLDGKTKLILSPYEHKSKISVKMGNKAKIAKCPLRTKTGHILKSTQDT
CIGSEKLLQKKPVGSETSQAKGEKNGMTFSSTKDLCKQCIDKDCLHIQKEISPATPNMQK
TRNTVNTSLVGKQKPHKKHITAENMKSSLVCLTQDQLQQILMTVNQGNRSLSLTENGKEA
KSQYSLYLNSISNQPKDENIMGLFKKTEMVSSVPAENKSVLNEHQETSKQCEQKIAIENE
WKPADIFSTLGERECDRSSLEAKKAQWRKELDEQVALKKKEKEVSEKWNDPWKKSESDKI
IWEKHQILDQSRETVLLEHPFSAVKQELQRKWIEELNKQIEDDRQRKIEEKIIYSKGEEH
DRWAMHFDSLKSYPGSQSQLFSQSTHKQPEYFCVSPDTQELADVSSVCTPTTGSQVEPSE
EEHIAKPIKDVVMANSKKTNFLRSMTALLDPAQIEERDRRRQKQLEHQKAITAQVEEKRR
KKQLEEEQRKKEEQEEELRLAQEREEMQKQYEEDILKQKQKEEIMTLKTNELFQTMQRAQ
ELAQRLKQEQRIRELAQKGHDTSRLIKNLGVDTIQMEYNASNISNSRHDSDEISGKMNTY
MNSTTSKKDTGVQTDDLNIGIFTNAESHCGSLMERDITNCSSPEISAELIGQFSTKKNKQ
ELTQDKGASLEKENNRCNDQCNQFTRIEKQTKHMKKYPKRPDWNINKPPKRYIPASEKYP
KQLQKQREEKKVRRQMELLHLVEKNNPGHLSQNRGISPEIFHSSHQETESKLRWHLVKKE
EEPLNIHSFSKERSPSSPVPVVKNRTQQTQNTLHLPLKNSSYERENLISGSNQTELSSGI
SESSHFIPYVRTNEIYYLDPDAPLSGPSTQDPQYQNSQDCGQKRQLFDSDCVRDPLLNPN
MVKNRDRQQAILKGLSELRQGLLQKQKELESSLLPLAENQEESFGSSF
>sp|Q6NSX1|CCD70_HUMAN Coiled-coil domain-containing protein 70 OS=Homo sapiens GN=CCDC70 PE=1 SV=1

MATPPFRLIRKMFSFKVSRWMGLACFRSLAASSPSIRQKKLMHKLQEEKAFREEMKIFRE
KIEDFREEMWTFRGKIHAFRGQILGFWEEERPFWEEEKTFWKEEKSFWEMEKSFREEEKT
FWKKYRTFWKEDKAFWKEDNALWERDRNLLQEDKALWEEEKALWVEERALLEGEKALWED
KTSLWEEENALWEEERAFWMENNGHIAGEQMLEDGPHNANRGQRLLAFSRGRA
>sp|Q6ZN84|CCD81_HUMAN Coiled-coil domain-containing protein 81 OS=Homo sapiens GN=CCDC81 PE=2 SV=2
MLDTIARALQDLGRQVLPTLPSLSQEEVSIIWGNVSEFVRRQLTLHKGVQIPAFGTFTFI
RQKLEVGNNKFILIQRPVFIMVEKLVQIHGLKQNKVYTPGEIPIVPLNFVMISLEGPFNR
DVVEGCVKETLLFLSRSISMKQNVEFTFKGIGVLMIRDSKVKMRFYKDFLCTMDGSGALA
KALANRPGTVDSVLSSREALRKWPSSVLAFPRIELKEMENKLPMETLVEECGENRERKCK
LKDQSDKEEGTRDISSPKRLRDRQALFPAKVTNVSLLEKFERSESGGKIMTPESLSYPSC
LKHDSEMKPQTSPACQDHNKAGGQEMCYVCLQRAQRNSLLYYSEERRREIEDERLIQQYQM
LKDQEALFRHQMKSLATREQNQKNAAYNLGVAEAIRNHKNEKPEFYKSFLFDKRPLSPAL
NALKQEEYSRSLLKQMDNRQENEIKQRQYRELMDRLEQVQLTEELAAQRAKFLKDKMEET
QCYKRALDAQIKNKPSRLPPFEPDSSEPIFGKNEGELMVEKQKREQNYMKHQLEAAANHK
RKAILHQLVDQRRDLQMLQRTQREHLADRTAELERVNRVNQCLQEDWERSAAMKKQRDLE
DKAFERASDKLFLLDQCEKYRRCKQCQRRTSNVGESNLWPLNKFLPGSRLLV
>sp|Q9Y3X0|CCDC9_HUMAN Coiled-coil domain-containing protein 9 OS=Homo sapiens GN=CCDC9 PE=1 SV=1
MAATLDLKSKEEKDAELDKRIEALRRKNEALIRRYQEIEEDRKKAELEGVAVTAPRKGRS
VEKENVAVESEKNLGPSRRSPGTPRPPGASKGGRTPPQQGGRAGMGRASRSWEGSPGEQP
RGGGAGGRGRRGRGRGSPHLSGAGDTSISDRKSKEWEERRRQNIEKMNEEMEKIAEYERN
QREGVLEPNPVRNFLDDPRRRSGPLEESERDRREESRRHGRNWGGPDFERVRCGLEHERQ
GRRAGLGSAGDMTLSMTGRERSEYLRWKQEREKIDQERLQRHRKPTGQWRREWDAEKTDG
MFKDGPVPAHEPSHRYDDQAWARPPKPPTFGEFLSQHKAEASSRRRRKSSRPQAKAAPRA
YSDHDDRWETKEGAASPAPETPQPTSPETSPKETPMQPPEIPAPAHRPPEDEGEENEGEE
DEEWEDISEDEEEEEIEVEEGDEEEPAQDHQAPEAAPTGIPCSEQAHGVPFSPEEPLLEP
QAPGTPSSPFSPPSGHQPVSDWGEEVELNSPRTTHLAGALSPGEAWPFESV
>sp|I3L3R5|CCER2_HUMAN Coiled-coil domain-containing glutamate-rich protein 2 OS=Homo sapiens GN=CCER2 PE=3 SV=1
MPPRGPASELLLLRLLLLGAATAAPLAPRPSKEELTRCLAEVVTEVLTVGQVQRGPCTAL
LHKELCGTEPHGCASTEEKGLLLGDFKKQEAGKMRSSQEVRDEEEEEVAERTHKSEVQEQ
AIRMQGHRQLHQEEDEEEEKEERKRGPMETFEDLWQRHLENGGDLQKRVAEKASDKETAQ
FQAEEKGVRVLGGDRSLWQGAERGGGERREDLPHHHHHHHQPEAEPRQEKEEASEREEKE
VEQLEHLRDELKKVTETLGEQLRREG
>sp|P20248|CCNA2_HUMAN Cyclin-A2 OS=Homo sapiens GN=CCNA2 PE=1 SV=2
MLGNSAPGPATREAGSALLALQQTALQEDQENINPEKAAPVQQPRTRAALAVLKSGNPRG
LAQQQRPKTRRVAPLKDLPVNDEHVTVPPWKANSKQPAFTIHVDEAEKEAQKKPAESQKI
EREDALAFNSAISLPGPRKPLVPLDYPMDGSFESPHTMDMSIILEDEKPVSVNEVPDYHE
DIHTYLREMEVKCKPKVGYMKKQPDITNSMRAILVDWLVEVGEEYKLQNETLHLAVNYID
RFLSSMSVLRGKLQLVGTAAMLLASKFEEIYPPEVAEFVYITDDTYTKKQVLRMEHLVLK
VLTFDLAAPTVNQFLTQYFLHQQPANCKVESLAMFLGELSLIDADPYLKYLPSVIAGAAF
HLALYTVTGQSWPESLIRKTGYTLESLKPCLMDLHQTYLKAPQHAQQSIREKYKNSKYHG
VSLLNPPETLNL

>sp|P51946|CCNH_HUMAN Cyclin-H OS=Homo sapiens GN=CCNH PE=1 SV=1
MYHNSSQKRHWTFSSEEQLARLRADANRKFRCKAVANGKVLPNDPVFLEPHEEMTLCKYY
EKRLLEFCSVFKPAMPRSVVGTACMYFKRFYLNNSVMEYHPRIIMLTCAFLACKVDEFNV
SSPQFVGNLRESPLGQEKALEQILEYELLLIQQLNFHLIVHNPYRPFEGFLIDLKTRYPI
LENPEILRKTADDFLNRIALTDAYLLYTPSQIALTAILSSASRAGITMESYLSESLMLKE
NRTCLSQLLDIMKSMRNLVKKYEPPRSEEVAVLKQKLERCHSAELALNVITKKRKGYEDD
DYVSKKSKHEEEEWTDDDLVESL
>sp|Q17RF5|CD026_HUMAN Uncharacterized protein C4orf26 OS=Homo sapiens GN=C4orf26 PE=1 SV=1
MARRHCFSYWLLVCWLVVTVAEGQEEVFTPPGDSQNNADATDCQIFTLTPPPAPRSPVTR
AQPITKTPRCPFHFFPRRPRIHFRFPNRPFVPSRCNHRFPFQPFYWPHRYLTYRYFPRRR
LQRGSSSEES
>sp|Q8N1A6|CD033_HUMAN UPF0462 protein C4orf33 OS=Homo sapiens GN=C4orf33 PE=1 SV=2
MDFKIEHTWDGFPVKHEPVFIRLNPGDRGVMMDISAPFFRDPPAPLGEPGKPFNELWDYE
VVEAFFLNDITEQYLEVELCPHGQHLVLLLSGRRNVWKQELPLSFRMSRGETKWEGKAYL
PWSYFPPNVTKFNSFAIHGSKDKRSYEALYPVPQHELQQGQKPDFHCLEYFKSFNFNTLL
GEEWKQPESDLWLIEKCDI
>sp|Q5BLP8|CD048_HUMAN Neuropeptide-like protein C4orf48 OS=Homo sapiens GN=C4orf48 PE=1 SV=3
MAPPPACRSPMSPPPPPLLLLLLSLALLGARARAEPAGSAVPAQSRPCVDCHAFEFMQRA
LQDLRKTACSLDARTETLLLQAERRALCACWPAGH
>sp|C9J302|CD051_HUMAN Uncharacterized protein C4orf51 OS=Homo sapiens GN=C4orf51 PE=4 SV=1
MSHYFYLTPQILLPFSPLTSQEFDLIRRKAGASWQDETRWSDSSVTTYTGSYRKKQLDKS
MCSQFSFRAGQHEPECKQMSLTNSSACHLLCWAGTQETTDIKGLFPDITRPFKKSFDVKH
GVAHQIWDFGDCFPTPPNYGKYCVRPKKPAQEALINYSRRGKGVLKHLHGRCDSESKVCS
SEDSEADRYSDYGWGGPSSPFN
>sp|Q9UQ88|CD11A_HUMAN Cyclin-dependent kinase 11A OS=Homo sapiens GN=CDK11A PE=1 SV=4
MGDEKDSWKVKTLDEILQEKKRRKEQEEKAEIKRLKNSDDRDSKRDSLEEGELRDHCMEI
TIRNSPYRREDSMEDRGEEDDSLAIKPPQQMSRKEKVHHRKDEKRKEKWKHARVKEREHE
RRKRHREEQDKARREWERQKRREMAREHSRRERDRLEQLERKREREREKMREQQKEQREQK
ERERRAEERRKEREARREVSAHHRTMREDYSDKVKASHWSRSPPRPPRERFELGDGRKPG
EARPAPAQKPAQLKEEKMEERDLLSDLQDISDSERKTSSAESSSAESGSGSEEEEEEEEE
EEEEGSTSEESEEEEEEEEEEEEEETGSNSEEASEQSAEEVSEEEMSEDEERENENHLLVV
PESRFDRDSGESEEAEEEVGEGTPQSSALTEGDYVPDSPALLPIELKQELPKYLPALQGC
RSVEEFQCLNRIEEGTYGVVYRAKDKKTDEIVALKRLKMEKEKEGFPITSLREINTILKA
QHPNIVTVREIVVGSNMDKIYIVMNYVEHDLKSLMETMKQPFLPGEVKTLMIQLLRGVKH
LHDNWILHRDLKTSNLLLSHAGILKVGDFGLAREYGSPLKAYTPVVVTQWYRAPELLLGA
KEYSTAVDMWSVGCIFGELLTQKPLFPGNSEIDQINKVFKELGTPSEKIWPGYSELPVVK
KMTFSEHPYNNLRKRFGALLSDQGFDLMNKFLTYFPGRRISAEDGLKHEYFRETPLPIDP
SMFPTWPAKSEQQRVKRGTSPRPPEGGLGYSQLGDDDLKETGFHLTTTNQGASAAGPGFS
LKF
>sp|Q6YHK3|CD109_HUMAN CD109 antigen OS=Homo sapiens GN=CD109 PE=1 SV=2
MQGPPLLTAAHLLCVCTAALAVAPGPRFLVTAPGIIRPGGNVTIGVELLEHCPSQVTVKA

ELLKTASNLTVSVLEAEGVFEKGSFKTLTLPSLPLNSADEIYELRVTGRTQDEILFSNST
RLSFETKRISVFIQTDKALYKPKQEVKFRIVTLFSDFKPYKTSLNILIKDPKSNLIQQWL
SQQSDLGVISKTFQLSSHPILGDWSIQVQVNDQTYYQSFQVSEYVLPKFEVTLQTPLYCS
MNSKHLNGTITAKYTYGKPVKGDVTLTFLPLSFWGKKKNITKTFKINGSANFSFNDEEMK
NVMDSSNGLSEYLDLSSPGPVEILTTVTESVTGISRNVSTNVFFKQHDYIIEFFDYTTVL
KPSLNFTATVKVTRADGNQLTLEERRNNVVITVTQRNYTEYWSGSNSGNQKMEAVQKINY
TVPQSGTFKIEFPILEDSSELQLKAYFLGSKSSMAVHSLFKSPSKTYIQLKTRDENIKVG
SPFELVVSGNKRLKELSYMVVSRGQLVAVGKQNSTMFSLTPENSWTPKACVIVYYIEDDG
EIISDVLKIPVQLVFKNKIKLYWSKVKAEPSEKVSLRISVTQPDSIVGIVAVDKSVNLMN
ASNDITMENVVHELELYNTGYYLGMFMNSFAVFQECGLWVLTDANLTKDYIDGVYDNAEY
AERFMEENEGHIVDIHDFSLGSSPHVRKHFPETWIWLDTNMGYRIYQEFEVTVPDSITSW
VATGFVISEDLGLGLTTTPVELQAFQPFFIFLNLPYSVIRGEEFALEITIFNYLKDATEV
KVIIEKSDKFDILMTSNEINATGHQQTLLVPSEDGATVLFPIRPTHLGEIPITVTALSPT
ASDAVTQMILVKAEGIEKSYSQSILLDLTDNRLQSTLKTLSFSFPPNTVTGSERVQITAI
GDVLGPSINGLASLIRMPYGCGEQNMINFAPNIYILDYLTKKKQLTDNLKEKALSFMRQG
YQRELLYQREDGSFSAFGNYDPSGSTWLSAFVLRCFLEADPYIDIDQNVLHRTYTWLKGH
QKSNGEFWDPGRVIHSELQGGNKSPVTLTAYIVTSLLGYRKYQPNIDVQESIHFLESEFS
RGISDNYTLALITYALSSVGSPKAKEALNMLTWRAEQEGGMQFWVSSESKLSDSWQPRSL
DIEVAAYALLSHFLQFQTSEGIPIMRWLSRQRNSLGGFASTQDTTVALKALSEFAALMNT
ERTNIQVTVTGPSSPSPVKFLIDTHNRLLLQTAELAVVQPTAVNISANGFGFAICQLNVV
YNVKASGSSRRRRSIQNQEAFDLDVAVKENKDDLNHVDLNVCTSFSGPGRSGMALMEVNL
LSGFMVPSEAISLSETVKKVEYDHGKLNLYLDSVNETQFCVNIPAVRNFKVSNTQDASVS
IVDYYEPRRQAVRSYNSEVKLSSCDLCSDVQGCRPCEDGASGSHHHSSVIFIFCFKLLYF
MELWL
>sp|P48509|CD151_HUMAN CD151 antigen OS=Homo sapiens GN=CD151 PE=1 SV=3
MGEFNEKKTTCGTVCLKYLLFTYNCCFWLAGLAVMAVGIWTLALKSDYISLLASGTYLAT
AYILVVAGTVVMVTGVLGCCATFKERRNLLRLYFILLLIIFLLEIIAGILAYAYYQQLNT
ELKENLKDTMTKRYHQPGHEAVTSAVDQLQQEFHCCGSNNSQDWRDSEWIRSQEAGGRVV
PDSCCKTVVALCGQRDHASNIYKVEGGCITKLETFIQEHLRVIGAVGIGIACVQVFGMIF
TCCLYRSLKLEHY
>sp|Q8N6Q3|CD177_HUMAN CD177 antigen OS=Homo sapiens GN=CD177 PE=1 SV=2
MSAVLLLALLGFILPLPGVQALLCQFGTVQHVWKVSDLPRQWTPKNTSCDSGLGCQDTLM
LIESGPQVSLVLSKGCTEAKDQEPRVTEHRMGPGLSLISYTFVCRQEDFCNNLVNSLPLW
APQPPADPGSLRCPVCLSMEGCLEGTTEEICPKGTTHCYDGLLRLRGGGIFSNLRVQGCM
PQPGCNLLNGTQEIGPVGMTENCNRKDFLTCHRGTTIMTHGNLAQEPTDWTTSNTEMCEV
GQVCQETLLLLDVGLTSTLVGTKGCSTVGAQNSQKTTIHSAPPGVLVASYTHFCSSDLCN
SASSSSVLLNSLPPQAAPVPGDRQCPTCVQPLGTCSSGSPRMTCPRGATHCYDGYIHLSG
GGLSTKMSIQGCVAQPSSFLLNHTRQIGIFSAREKRDVQPPASQHEGGGAEGLESLTWGV
GLALAPALWWGVVCPSC
>sp|Q99467|CD180_HUMAN CD180 antigen OS=Homo sapiens GN=CD180 PE=1 SV=2
MAFDVSCFFWVVLFSAGCKVITSWDQMCIEKEANKTYNCENLGLSEIPDTLPNTTEFLEF
SFNFLPTIHNRTFSRLMNLTFLDLTRCQINWIHEDTFQSHHQLSTLVLTGNPLIFMAETS
LNGPKSLKHLFLIQTGISNLEFIPVHNLENLESLYLGSNHISSIKFPKDFPARNLKVLDF
QNNAIHYISREDMRSLEQAINLSLNFNGNNVKGIELGAFDSTIFQSLNFGGTPNLSVIFN

GLQNSTTQSLWLGTFEDIDDEDISSAMLKGLCEMSVESLNLQEHRFSDISSTTFQCFTQL
QELDLTATHLKGLPSGMKGLNLLKKLVLSVNHFDQLCQISAANFPSLTHLYIRGNVKKLH
LGVGCLEKLGNLQTLDLSHNDIEASDCCSLQLKNLSHLQTLNLSHNEPLGLQSQAFKECP
QLELLDLAFTRLHINAPQSPFQNLHFLQVLNLTYCFLDTSNQHLLAGLPVLRHLNLKGNH
FQDGTITKTNLLQTVGSLEVLILSSCGLLSIDQQAFHSLGKMSHVDLSHNSLTCDSIDSL
SHLKGIYLNLAANSINIISPRLLPILSQQSTINLSHNPLDCTCSNIHFLTWYKENLHKLE
GSEETTCANPPSLRGVKLSDVKLSCGITAIGIFFLIVFLLLLAILLFFAVKYLLRWKYQH
I
>sp|P29016|CD1B_HUMAN T-cell surface glycoprotein CD1b OS=Homo sapiens GN=CD1B PE=1 SV=1
MLLLPFQLLAVLFPGGNSEHAFQGPTSFHVIQTSSFTNSTWAQTQGSGWLDDLQIHGWDS
DSGTAIFLKPWSKGNFSDKEVAELEEIFRVYIFGFAREVQDFAGDFQMKYPFEIQGIAGC
ELHSGGAIVSFLRGALGGLDFLSVKNASCVPSPEGGSRAQKFCALIIQYQGIMETVRILL
YETCPRYLLGVLNAGKADLQRQVKPEAWLSSGPSPGPGRLQLVCHVSGFYPKPVWVMWMR
GEQEQQGTQLGDILPNANWTWYLRATLDVADGEAAGLSCRVKHSSLEGQDIILYWRNPTS
IGSIVLAIIVPSLLLLLCLALWYMRRRSYQNIP
>sp|P15813|CD1D_HUMAN Antigen-presenting glycoprotein CD1d OS=Homo sapiens GN=CD1D PE=1 SV=1
MGCLLFLLLWALLQAWGSAEVPQRLFPLRCLQISSFANSSWTRTDGLAWLGELQTHSWSN
DSDTVRSLKPWSQGTFSDQQWETLQHIFRVYRSSFTRDVKEFAKMLRLSYPLELQVSAGC
EVHPGNASNNFFHVAFQGKDILSFQGTSWEPTQEAPLWVNLAIQVLNQDKWTRETVQWLL
NGTCPQFVSGLLESGKSELKKQVKPKAWLSRGPSPGPGRLLLVCHVSGFYPKPVWVKWMR
GEQEQQGTQPGDILPNADETWYLRATLDVVAGEAAGLSCRVKHSSLEGQDIVLYWGGSYT
SMGLIALAVLACLLFLLIVGFTSRFKRQTSYQGVL
>sp|Q9NNX6|CD209_HUMAN CD209 antigen OS=Homo sapiens GN=CD209 PE=1 SV=1
MSDSKEPRLQQLGLLEEEQLRGLGFRQTRGYKSLAGCLGHGPLVLQLLSFTLLAGLLVQV
SKVPSSISQEQSRQDAIYQNLTQLKAAVGELSEKSKLQEIYQELTQLKAAVGELPEKSKL
QEIYQELTRLKAAVGELPEKSKLQEIYQELTWLKAAVGELPEKSKMQEIYQELTRLKAAV
GELPEKSKQQEIYQELTRLKAAVGELPEKSKQQEIYQELTRLKAAVGELPEKSKQQEIYQ
ELTQLKAAVERLCHPCPWEWTFFQGNCYFMSNSQRNWHDSITACKEVGAQLVVIKSAEEQ
NFLQLQSSRSNRFTWMGLSDLNQEGTWQWVDGSPLLPSFKQYWNRGEPNNVGEEDCAEFS
GNGWNDDKCNLAKFWICKKSAASCSRDEEQFLSPAPATPNPPPA
>sp|Q9Y5K6|CD2AP_HUMAN CD2-associated protein OS=Homo sapiens GN=CD2AP PE=1 SV=1
MVDYIVEYDYDAVHDDELTIRVGEIIRNVKKLQEEGWLEGELNGRRGMFPDNFVKEIKRE
TEFKDDSLPIKRERHGNVASLVQRISTYGLPAGGIQPHPQTKNIKKKTKKRQCKVLFEYI
PQNEDELELKVGDIIDINEEVEEGWWSGTLNNKLGLFPSNFVKELEVTDDGETHEAQDDS
ETVLAGPTSPIPSLGNVSETASGSVTQPKKIRGIGFGDIFKEGSVKLRTRTSSSETEEKK
PEKPLILQSLGPKTQSVEITKTDTEGKIKAKEYCRTLFAYEGTNEDELTFKEGEIIHLIS
KETGEAGWWRGELNGKEGVFPDNFAVQINELDKDFPKPKKPPPPAKAPAPKPELIAAEKK
YFSLKPEEKDEKSTLEQKPSKPAAPQVPPKKPTPPTKASNLLRSSGTVYPKRPEKPVPPP
PPIAKINGEVSSISSKFETEPVSKLKLDSEQLPLRPKSVDFDSLTVRTSKETDVVNFDDI
ASSENLLHLTANRPKMPGRRLPGRFNGGHSPTHSPEKILKLPKEEDSANLKPSELKKDTC
YSPKPSVYLSTPSSASKANTTAFLTPLEIKAKVETDDVKKNSLDELRAQIIELLCIVEAL
KKDHGKELEKLRKDLEEEKTMRSNLEMEIEKLKKAVLSS

>sp|P28907|CD38_HUMAN ADP-ribosyl cyclase/cyclic ADP-ribose hydrolase 1 OS=Homo sapiens GN=CD38 PE=1 SV=2
MANCEFSPVSGDKPCCRLSRRAQLCLGVSILVLILVVVLAVVVPRWRQQWSGPGTTKRFP
ETVLARCVKYTEIHPEMRHVDCQSVWDAFKGAFISKHPCNITEEDYQPLMKLGTQTVPCN
KILLWSRIKDLAHQFTQVQRDMFTLEDTLLGYLADDLTWCGEFNTSKINYQSCPDWRKDC
SNNPVSVFWKTVSRRFAEAACDVVHVMLNGSRSKIFDKNSTFGSVEVHNLQPEKVQTLEA
WVIHGGREDSRDLCQDPTIKELESIISKRNIQFSCKNIYRPDKFLQCVKNPEDSSCTSEI
>sp|P04234|CD3D_HUMAN T-cell surface glycoprotein CD3 delta chain OS=Homo sapiens GN=CD3D PE=1 SV=1
MEHSTFLSGLVLATLLSQVSPFKIPIEELEDRVFVNCNTSITWVEGTVGTLLSDITRLDL
GKRILDPRGIYRCNGTDIYKDKESTVQVHYRMCQSCVELDPATVAGIIVTDVIATLLLAL
GVFCFAGHETGRLSGAADTQALLRNDQVYQPLRDRDDAQYSHLGGNWARNK
>sp|P09693|CD3G_HUMAN T-cell surface glycoprotein CD3 gamma chain OS=Homo sapiens GN=CD3G PE=1 SV=1
MEQGKGLAVLILAIILLQGTLAQSIKGNHLVKVYDYQEDGSVLLTCDAEAKNITWFKDGK
MIGFLTEDKKKWNLGSNAKDPRGMYQCKGSQNKSKPLQVYYRMCQNCIELNAATISGFLF
AEIVSIFVLAVGVYFIAGQDGVRQSRASDKQTLLPNDQLYQPLKDREDDQYSHLQGNQLR
RN
>sp|P20963|CD3Z_HUMAN T-cell surface glycoprotein CD3 zeta chain OS=Homo sapiens GN=CD247 PE=1 SV=2
MKWKALFTAAILQAQLPITEAQSFGLLDPKLCYLLDGILFIYGVILTALFLRVKFSRSAD
APAYQQGQNQLYNELNLGRREEYDVLDKRRGRDPEMGGKPQRRKNPQEGLYNELQKDKMA
EAYSEIGMKGERRRGKGHDGLYQGLSTATKDTYDALHMQALPPR
>sp|P09326|CD48_HUMAN CD48 antigen OS=Homo sapiens GN=CD48 PE=1 SV=2
MCSRGWDSCLALELLLLPLSLLVTSIQGHLVHMTVVSGSNVTLNISESLPENYKQLTWFY
TFDQKIVEWDSRKSKYFESKFKGRVRLDPQSGALYISKVQKEDNSTYIMRVLKKTGNEQE
WKIKLQVLDPVPKPVIKIEKIEDMDDNCYLKLSCVIPGESVNYTWYGDKRPFPKELQNSV
LETTLMPHNYSRCYTCQVSNSVSSKNGTVCLSPPCTLARSFGVEWIASWLVVTVPTILGL
LLT
>sp|P01730|CD4_HUMAN T-cell surface glycoprotein CD4 OS=Homo sapiens GN=CD4 PE=1 SV=1
MNRGVPFRHLLLVLQLALLPAATQGKKVVLGKKGDTVELTCTASQKKSIQFHWKNSNQIK
ILGNQGSFLTKGPSKLNDRADSRRSLWDQGNFPLIIKNLKIEDSDTYICEVEDQKEEVQL
LVFGLTANSDTHLLQGQSLTLTLESPPGSSPSVQCRSPRGKNIQGGKTLSVSQLELQDSG
TWTCTVLQNQKKVEFKIDIVVLAFQKASSIVYKKEGEQVEFSFPLAFTVEKLTGSGELWW
QAERASSSKSWITFDLKNKEVSVKRVTQDPKLQMGKKLPLHLTLPQALPQYAGSGNLTLA
LEAKTGKLHQEVNLVVMRATQLQKNLTCEVWGPTSPKLMLSLKLENKEAKVSKREKAVWV
LNPEAGMWQCLLSDSGQVLLESNIKVLPTWSTPVQPMALIVLGGVAGLLLFIGLGIFFCV
RCRHRRRQAERMSQIKRLLSEKKTCQCPHRFQKTCSPI
>sp|Q13319|CD5R2_HUMAN Cyclin-dependent kinase 5 activator 2 OS=Homo sapiens GN=CDK5R2 PE=1 SV=1
MGTVLSLSPASSAKGRRPGGLPEEKKKAPPAGDEALGGYGAPPVGKGGKGESRLKRPSVL
ISALTWKRLVAASAKKKKGSKKVTPKPASTGPDPLVQQRNRENLLRKGRDPPDGGGTAKP
LAVPVPTVPAAAATCEPPSGGSAAAQPPGSGGGKPPPPPPPAPQVAPPVPGGSPRRVIVQ
ASTGELLRCLGDFVCRRCYRLKELSPGELVGWFRGVDRSLLLQGWQDQAFITPANLVFVY

LLCRESLRGDELASAAELQAAFLTCLYLAYSYMGNEISYPLKPFLVEPDKERFWQRCLRL
IQRLSPQMLRLNADPHFFTQVFQDLKNEGEAAASGGGPPSGGAPAASSAARDSCAAGTKH
WTMNLDR
>sp|P32970|CD70_HUMAN CD70 antigen OS=Homo sapiens GN=CD70 PE=1 SV=2
MPEEGSGCSVRRRPYGCVLRAALVPLVAGLVICLVVCIQRFAQAQQQLPLESLGWDVAEL
QLNHTGPQQDPRLYWQGGPALGRSFLHGPELDKGQLRIHRDGIYMVHIQVTLAICSSTTA
SRHHPTTLAVGICSPASRSISLLRLSFHQGCTIASQRLTPLARGDTLCTNLTGTLLPSRN
TDETFFGVQWVRP
>sp|P33681|CD80_HUMAN T-lymphocyte activation antigen CD80 OS=Homo sapiens GN=CD80 PE=1 SV=1
MGHTRRQGTSPSKCPYLNFFQLLVLAGLSHFCSGVIHVTKEVKEVATLSCGHNVSVEELA
QTRIYWQKEKKMVLTMMSGDMNIWPEYKNRTIFDITNNLSIVILALRPSDEGTYECVVLK
YEKDAFKREHLAEVTLSVKADFPTPSISDFEIPTSNIRRIICSTSGGFPEPHLSWLENGE
ELNAINTTVSQDPETELYAVSSKLDFNMTTNHSFMCLIKYGHLRVNQTFNWNTTKQEHFP
DNLLPSWAITLISVNGIFVICCLTYCFAPRCRERRRNERLRRESVRPV
>sp|P60033|CD81_HUMAN CD81 antigen OS=Homo sapiens GN=CD81 PE=1 SV=1
MGVEGCTKCIKYLLFVFNFVFWLAGGVILGVALWLRHDPQTTNLLYLELGDKPAPNTFYV
GIYILIAVGAVMMFVGFLGCYGAIQESQCLLGTFFTCLVILFACEVAAGIWGFVNKDQIA
KDVKQFYDQALQQAVVDDDANNAKAVVKTFHETLDCCGSSTLTALTTSVLKNNLCPSGSN
IISNLFKEDCHQKIDDLFSGKLYLIGIAAIVVAVIMIFEMILSMVLCCGIRNSSVY
>sp|P10966|CD8B_HUMAN T-cell surface glycoprotein CD8 beta chain OS=Homo sapiens GN=CD8B PE=2 SV=1
MRPRLWLLLAAQLTVLHGNSVLQQTPAYIKVQTNKMVMLSCEAKISLSNMRIYWLRQRQA
PSSDSHHEFLALWDSAKGTIHGEEVEQEKIAVFRDASRFILNLTSVKPEDSGIYFCMIVG
SPELTFGKGTQLSVVDFLPTTAQPTKKSTLKKRVCRLPRPETQKGPLCSPITLGLLVAGV
LVLLVSLGVAIHLCCRRRRARLRFMKQFYK
>sp|P48960|CD97_HUMAN CD97 antigen OS=Homo sapiens GN=CD97 PE=1 SV=4
MGGRVFLAFCVWLTLPGAETQDSRGCARWCPQNSSCVNATACRCNPGFSSFSEIITTPTE
TCDDINECATPSKVSCGKFSDCWNTEGSYDCVCSPGYEPVSGAKTFKNESENTCQDVDEC
QQNPRLCKSYGTCVNTLGSYTCQCLPGFKFIPEDPKVCTDVNECTSGQNPCHSSTHCLNN
VGSYQCRCRPGWQPIPGSPNGPNNTVCEDVDECSSGQHQCDSSTVCFNTVGSYSCRCRPG
WKPRHGIPNNQKDTVCEDMTFSTWTPPPGVHSQTLSRFFDKVQDLGRDSKTSSAEVTIQN
VIKLVDELMEAPGDVEALAPPVRHLIATQLLSNLEDIMRILAKSLPKGPFTYISPSNTEL
TLMIQERGDKNVTMGQSSARMKLNWAVAAGAEDPGPAVAGILSIQNMTTLLANASLNLHS
KKQAELEEIYESSIRGVQLRRLSAVNSIFLSHNNTKELNSPILFAFSHLESSDGEAGRDP
PAKDVMPGPRQELLCAFWKSDSDRGGHWATEGCQVLGSKNGSTTCQCSHLSSFAILMAHY
DVEDWKLTLITRVGLALSLFCLLLCILTFLLVRPIQGSRTTIHLHLCICLFVGSTIFLAG
IENEGGQVGLRCRLVAGLLHYCFLAAFCWMSLEGLELYFLVVRVFQGQGLSTRWLCLIGY
GVPLLIVGVSAAIYSKGYGRPRYCWLDFEQGFLWSFLGPVTFIILCNAVIFVTTVWKLTQ
KFSEINPDMKKLKKARALTITAIAQLFLLGCTWVFGLFIFDDRSLVLTYVFTILNCLQGA
FLYLLHCLLNKKVREEYRKWACLVAGGSKYSEFTSTTSGTGHNQTRALRASESGI
>sp|Q13042|CDC16_HUMAN Cell division cycle protein 16 homolog OS=Homo sapiens GN=CDC16 PE=1 SV=2
MNLERLRKRVRQYLDQQQYQSALFWADKVASLSREEPQDIYWLAQCLYLTAQYHRAAHAL

RSRKLDKLYEACRYLAARCHYAAKEHQQALDVLDMEEPINKRLFEKYLKDESGFKDPSSD
WEMSQSSIKSSICLLRGKIYDALDNRTLATYSYKEALKLDVYCFEAFDLLTSHHMLTAQE
EKELLESLPLSKLCNEEQELLRFLFENKLKKYNKPSETVIPESVDGLQENLDVVVSLAER
HYYNCDFKMCYKLTSVVMEKDPFHASCLPVHIGTLVELNKANELFYLSHKLVDLYPSNPV
SWFAVGCYYLMVGHKNEHARRYLSKATTLEKTYGPAWIAYGHSFAVESEHDQAMAAYFTA
AQLMKGCHLPMLYIGLEYGLTNNSKLAERFFSQALSIAPEDPFVMHEVGVVAFQNGEWKT
AEKWFLDALEKIKAIGNEVTVDKWEPLLNNLGHVCRKLKKYAEALDYHRQALVLIPQNAS
TYSAIGYIHSLMGNFENAVDYFHTALGLRRDDTFSVTMLGHCIEMYIGDSEAYIGADIKD
KLKCYDFDVHTMKTLKNIISPPWDFREFEVEKQTAEETGLTPLETSRKTPDSRPSLEETF
EIEMNESDMMLETSMSDHST
>sp|Q9HBB8|CDHR5_HUMAN Cadherin-related family member 5 OS=Homo sapiens GN=CDHR5 PE=1 SV=3
MGSWALLWPPLLFTGLLVRPPGTMAQAQYCSVNKDIFEVEENTNVTEPLVDIHVPEGQEV
TLGALSTPFAFRIQGNQLFLNVTPDYEEKSLLEAQLLCQSGGTLVTQLRVFVSVLDVNDN
APEFPFKTKEIRVEEDTKVNSTVIPETQLQAEDRDKDDILFYTLQEMTAGASDYFSLVSV
NRPALRLDRPLDFYERPNMTFWLLVRDTPGENVEPSHTATATLVLNVVPADLRPPWFLPC
TFSDGYVCIQAQYHGAVPTGHILPSPLVLRPGPIYAEDGDRGINQPIIYSIFRGNVNGTF
IIHPDSGNLTVARSVPSPMTFLLLVKGQQQADLARYSVTQVTVEAVAAAGSPPRFPQRLYR
GTVARGAGAGVVVKDAAAPSQPLRIQAQDPEFSDLNSAITYRITNHSHFRMEGEVVLTTT
TLAQAGAFYAEVEAHNTVTSGTATTVIEIQVSEQEPPSTDVPPSPEAGGTTGPWTSTTSE
VPRPPEPSQGPSTTSSGGGTGPHPPSGTTLRPPTSSTPGGPPGAENSTSHQPATPGGDTA
QTPKPGTSQPMPPGVGTSTSHQPATPSGGTAQTPEPGTSQPMPPSMGTSTSHQPATPGGG
TAQTPEAGTSQPMPPGMGTSTSHQPTTPGGGTAQTPEPGTSQPMPLSKSTPSSGGGPSED
KRFSVVDMAALGGVLGALLLLALLGLAVLVHKHYGPRLKCCCGKAPEPQPQGFDNQAFLP
DHKANWAPVPSPTHDPKPAEAPMPAEPAPPGPASPGGAPEPPAAARAGGSPTAVRSILTK
ERRPEGGYKAVWFGEDIGTEADVVVLNAPTLDVDGASDSGSGDEGEGAGRGGGPYDAPGG
DDSYI
>sp|O94921|CDK14_HUMAN Cyclin-dependent kinase 14 OS=Homo sapiens GN=CDK14 PE=1 SV=3
MCDLIEPQPAEKIGKMKKLRRTLSESFSRIALKKDDTTFDEICVTKMSTRNCQGMDSVIK
PLDTIPEDKKVRVQRTQSTFDPFEKPANQVKRVHSENNACINFKTSSTGKESPKVRRHSS
PSSPTSPKFGKADSYEKLEKLGEGSYATVYKGKSKVNGKLVALKVIRLQEEEGTPFTAIR
EASLLKGLKHANIVLLHDIIHTKETLTLVFEYVHTDLCQYMDKHPGGLHPDNVKLFLFQL
LRGLSYIHQRYILHRDLKPQNLLISDTGELKLADFGLARAKSVPSHTYSNEVVTLWYRPP
DVLLGSTEYSTCLDMWGVGCIFVEMIQGVAAFPGMKDIQDQLERIFLVLGTPNEDTWPGV
HSLPHFKPERFTLYSSKNLRQAWNKLSYVNHAEDLASKLLQCSPKNRLSAQAALSHEYFS
DLPPRLWELTDMSSIFTVPNVRLQPEAGESMRAFGKNNSYGKSLSNSKH
>sp|Q00535|CDK5_HUMAN Cyclin-dependent-like kinase 5 OS=Homo sapiens GN=CDK5 PE=1 SV=3
MQKYEKLEKIGEGTYGTVFKAKNRETHEIVALKRVRLDDDDEGVPSSALREICLLKELKH
KNIVRLHDVLHSDKKLTLVFEFCDQDLKKYFDSCNGDLDPEIVKSFLFQLLKGLGFCHSR
NVLHRDLKPQNLLINRNGELKLADFGLARAFGIPVRCYSAEVVTLWYRPPDVLFGAKLYS
TSIDMWSAGCIFAELANAGRPLFPGNDVDDQLKRIFRLLGTPTEEQWPSMTKLPDYKPYP
MYPATTSLVNVVPKLNATGRDLLQNLLKCNPVQRISAEEALQHPYFSDFCPP
>sp|P50613|CDK7_HUMAN Cyclin-dependent kinase 7 OS=Homo sapiens GN=CDK7 PE=1 SV=1
MALDVKSRAKRYEKLDFLGEGQFATVYKARDKNTNQIVAIKKIKLGHRSEAKDGINRTAL

REIKLLQELSHPNIIGLLDAFGHKSNISLVFDFMETDLEVIIKDNSLVLTPSHIKAYMLM
TLQGLEYLHQHWILHRDLKPNNLLLDENGVLKLADFGLAKSFGSPNRAYTHQVVTRWYRA
PELLFGARMYGVGVDMWAVGCILAELLLRVPFLPGDSDLDQLTRIFETLGTPTEEQWPDM
CSLPDYVTFKSFPGIPLHHIFSAAGDDLLDLIQGLFLFNPCARITATQALKMKYFSNRPG
PTPGCQLPRPNCPVETLKEQSNPALAIKRKRTEALEQGGLPKKLIF
>sp|O14519|CDKA1_HUMAN Cyclin-dependent kinase 2-associated protein 1 OS=Homo sapiens GN=CDK2AP1 PE=1 SV=1
MSYKPNLAAHMPAAALNAAGSVHSPSTSMATSSQYRQLLSDYGPPSLGYTQGTGNSQVPQ
SKYAELLAIIEELGKEIRPTYAGSKSAMERLKRGIIHARGLVRECLAETERNARS
>sp|P36222|CH3L1_HUMAN Chitinase-3-like protein 1 OS=Homo sapiens GN=CHI3L1 PE=1 SV=2
MGVKASQTGFVVLVLLQCCSAYKLVCYYTSWSQYREGDGSCFPDALDRFLCTHIIYSFAN
ISNDHIDTWEWNDVTLYGMLNTLKNRNPNLKTLLSVGGWNFGSQRFSKIASNTQSRRTFI
KSVPPFLRTHGFDGLDLAWLYPGRRDKQHFTTLIKEMKAEFIKEAQPGKKQLLLSAALSA
GKVTIDSSYDIAKISQHLDFISIMTYDFHGAWRGTTGHHSPLFRGQEDASPDRFSNTDYA
VGYMLRLGAPASKLVMGIPTFGRSFTLASSETGVGAPISGPGIPGRFTKEAGTLAYYEIC
DFLRGATVHRILGQQVPYATKGNQWVGYDDQESVKSKVQYLKDRQLAGAMVWALDLDDFQ
GSFCGQDLRFPLTNAIKDALAAT
>sp|Q15782|CH3L2_HUMAN Chitinase-3-like protein 2 OS=Homo sapiens GN=CHI3L2 PE=1 SV=1
MGATTMDQKSLWAGVVVLLLLQGGSAYKLVCYFTNWSQDRQEPGKFTPENIDPFLCSHLI
YSFASIENNKVIIKDKSEVMLYQTINSLKTKNPKLKILLSIGGYLFGSKGFHPMVDSSTS
RLEFINSIILFLRNHNFDGLDVSWIYPDQKENTHFTVLIHELAEAFQKDFTKSTKERLLL
TAGVSAGRQMIDNSYQVEKLAKDLDFINLLSFDFHGSWEKPLITGHNSPLSKGWQDRGPS
SYYNVEYAVGYWIHKGMPSEKVVMGIPTYGHSFTLASAETTVGAPASGPGAAGPITESSG
FLAYYEICQFLKGAKITRLQDQQVPYAVKGNQWVGYDDVKSMETKVQFLKNLNLGGAMIW
SIDMDDFTGKSCNQGPYPLVQAVKRSLGSL
>sp|Q8N187|CARTF_HUMAN Calcium-responsive transcription factor OS=Homo sapiens GN=CARF PE=1 SV=2
MEQSNDSLRVNHNDGEESKTSAQVFEHLICMDSRDSSFGQNDSPTVLPITTREANNSLIS
QNIPGPLTQTQTLSAEQFHLVDQNGQAIQYELQSLGESNAQMMIVASPTENGQVLRVIPP
TQTGMAQVIIPQGQLVDVNSPRDVPEEKPSNRNLPTVRVDTLADNTSNYILHPQTSFPLP
KKSVTGMLEEPLLGPLQPLSSNTPIWACRLRSCEKIGDSYRGYCVSETELESVLTFHKQQ
TQSVWGTRQSPSPAKPATRLMWKSQYVPYDGIPFVNAGSRAVVMECQYGPRRKGFQLKKV
SEQESRSCQLYKATCPARIYIKKVQKFPEYRVPTDPKIDKKIIRMEQEKAFNMLKKNLVD
AGGVLRWYVQLPTQQAHQYHELETPCLTLSPSPFPVSSLEEEETAVRDENCALPSRLHPQ
VAHKIQELVSQGIEQVYAVRKQLRKFVERELFKPDEVPERHNLSFFPTVNDIKNHIHEVQ
KSLRNGDTVYNSEIIPATLQWTTDSGNILKETMTVTFAEGNSPGESITTKVETNQTRGSL
SPEPTHLLSSLSSFQPKIFTQLQGLQLQPRYTSPDESPAVVSVNNQPSSSPSGLLDTIGS
AVMNNNSLLLGQSHSLQRDTCLTQNNSTASTMGNLPEPDQNLVAMDELVEVGDVEDTGNL
EGTVHRILLGDVQTIPIQIIDNHSALIEENPESTISVSQVKQEPKEPALSMEAKKTVDYK
KLSAT
>sp|Q16568|CART_HUMAN Cocaine- and amphetamine-regulated transcript protein OS=Homo sapiens GN=CARTPT PE=1 SV=1
MESSRVRLLPLLGAALLLMLPLLGTRAQEDAELQPRALDIYSAVDDASHEKELIEALQEV
LKKLKSKRVPIYEKKYGQVPMCDAGEQCAVRKGARIGKLCDCPRGTSCNSFLLKCL

>sp|Q6P4E1|CASC4_HUMAN Protein CASC4 OS=Homo sapiens GN=CASC4 PE=1 SV=1
MVGFGANRRAGRLPSLVLVVLLVVIVVLAFNYWSISSRHVLLQEEVAELQGQVQRTEVAR
GRLEKRNSDLLLLVDTHKKQIDQKEADYGRLSSRLQAREGLGKRCEDDKVKLQNNISYQM
ADIHHLKEQLAELRQEFLRQEDQLQDYRKNNTYLVKRLEYESFQCGQQMKELRAQHEENI
KKLADQFLEEQKQETQKIQSNDGKELDINNQVVPKNIPKVAENVADKNEEPSSNHIPHGK
EQIKRGGDAGMPGIEENDLAKVDDLPPALRKPPISVSQHESHQAISHLPTGQPLSPNMPP
DSHINHNGNPGTSKQNPSSPLQRLIPGSNLDSEPRIQTDILKQATKDRVSDFHKLKQSRF
FDENESPVDPQHGSKLADYNGDDGNVGEYEADKQAELAYNEEEDGDGGEEDVQGERGPGL
HAITMKPTSKFFG
>sp|Q96PB1|CASD1_HUMAN CAS1 domain-containing protein 1 OS=Homo sapiens GN=CASD1 PE=2 SV=1
MAALAYNLGKREINHYFSVRSAKVLALVAVLLLAACHLASRRYRGNDSCEYLLSSGRFLG
EKVWQPHSCMMHKYKISEAKNCLVDKHIAFIGDSRIRQLFYSFVKIINPQFKEEGNKHEN
IPFEDKTASVKVDFLWHPEVNGSMKQCIKVWTEDSIAKPHVIVAGAATWSIKIHNGSSEA
LSQYKMNITSIAPLLEKLAKTSDVYWVLQDPVYEDLLSENRKMITNEKIDAYNEAAVSIL
NSSTRNSKSNVKMFSVSKLIAQETIMESLDGLHLPESSRETTAMILMNVYCNKILKPVDG
SCCQPRPPVTLIQKLAACFFTLSIIGYLIFYIIHRNAHRKNKPCTDLESGEEKKNIINTP
VSSLEILLQSFCKLGLIMAYFYMCDRANLFMKENKFYTHSSFFIPIIYILVLGVFYNENT
KETKVLNREQTDEWKGWMQLVILIYHISGASTFLPVYMHIRVLVAAYLFQTGYGHFSYFW
IKGDFGIYRVCQVLFRLNFLVVVLCIVMDRPYQFYYFVPLVTVWFMVIYVTLALWPQIIQ
KKANGNCFWHFGLLLKLGFLLLFICFLAYSQGAFEKIFSLWPLSKCFELKGNVYEWWFRW
RLDRYVVFHGMLFAFIYLALQKRQILSEGKGEPLFSNKISNFLLFISVVSFLTYSIWASS
CKNKAECNELHPSVSVVQILAFILIRNIPGYARSVYSSFFAWFGKISLELFICQYHIWLA
ADTRGILVLIPGNPMLNIIVSTFIFVCVAHEISQITNDLAQIIIPKDNSSLLKRLACIAA
FFCGLLILSSIQDKSKH
>sp|P31415|CASQ1_HUMAN Calsequestrin-1 OS=Homo sapiens GN=CASQ1 PE=1 SV=3
MSATDRMGPRAVPGLRLALLLLLVLGTPKSGVQGQEGLDFPEYDGVDRVINVNAKNYKNV
FKKYEVLALLYHEPPEDDKASQRQFEMEELILELAAQVLEDKGVGFGLVDSEKDAAVAKK
LGLTEVDSMYVFKGDEVIEYDGEFSADTIVEFLLDVLEDPVELIEGERELQAFENIEDEI
KLIGYFKSKDSEHYKAFEDAAEEFHPYIPFFATFDSKVAKKLTLKLNEIDFYEAFMEEPV
TIPDKPNSEEEIVNFVEEHRRSTLRKLKPESMYETWEDDMDGIHIVAFAEEADPDGFEFL
ETLKAVAQDNTENPDLSIIWIDPDDFPLLVPYWEKTFDIDLSAPQIGVVNVTDADSVWME
MDDEEDLPSAEELEDWLEDVLEGEINTEDDDDDDDD
>sp|P09668|CATH_HUMAN Pro-cathepsin H OS=Homo sapiens GN=CTSH PE=1 SV=4
MWATLPLLCAGAWLLGVPVCGAAELCVNSLEKFHFKSWMSKHRKTYSTEEYHHRLQTFAS
NWRKINAHNNGNHTFKMALNQFSDMSFAEIKHKYLWSEPQNCSATKSNYLRGTGPYPPSV
DWRKKGNFVSPVKNQGACGSCWTFSTTGALESAIAIATGKMLSLAEQQLVDCAQDFNNHG
CQGGLPSQAFEYILYNKGIMGEDTYPYQGKDGYCKFQPGKAIGFVKDVANITIYDEEAMV
EAVALYNPVSFAFEVTQDFMMYRTGIYSSTSCHKTPDKVNHAVLAVGYGEKNGIPYWIVK
NSWGPQWGMNGYFLIERGKNMCGLAACASYPIPLV
>sp|O60911|CATL2_HUMAN Cathepsin L2 OS=Homo sapiens GN=CTSV PE=1 SV=2
MNLSLVLAAFCLGIASAVPKFDQNLDTKWYQWKATHRRLYGANEEGWRRAVWEKNMKMIE
LHNGEYSQGKHGFTMAMNAFGDMTNEEFRQMMGCFRNQKFRKGKVFREPLFLDLPKSVDW
RKKGYVTPVKNQKQCGSCWAFSATGALEGQMFRKTGKLVSLSEQNLVDCSRPQGNQGCNG

GFMARAFQYVKENGGLDSEESYPYVAVDEICKYRPENSVANDTGFTVVAPGKEKALMKAV
ATVGPISVAMDAGHSSFQFYKSGIYFEPDCSSKNLDHGVLVVGYGFEGANSNNSKYWLVK
NSWGPEWGSNGYVKIAKDKNNHCGIATAASYPNV
>sp|P56202|CATW_HUMAN Cathepsin W OS=Homo sapiens GN=CTSW PE=1 SV=2
MALTAHPSCLLALLVAGLAQGIRGPLRAQDLGPQPLELKEAFKLFQIQFNRSYLSPEEHA
HRLDIFAHNLAQAQRLQEEDLGTAEFGVTPFSDLTEEEFGQLYGYRRAAGGVPSMGREIR
SEEPEESVPFSCDWRKVASAISPIKDQKNCNCCWAMAAAGNIETLWRISFWDFVDVSVQE
LLDCGRCGDGCHGGFVWDAFITVLNNSGLASEKDYPFQGKVRAHRCHPKKYQKVAWIQDF
IMLQNNEHRIAQYLATYGPITVTINMKPLQLYRKGVIKATPTTCDPQLVDHSVLLVGFGS
VKSEEGIWAETVSSQSQPQPPHPTPYWILKNSWGAQWGEKGYFRLHRGSNTCGITKFPLT
ARVQKPDMKPRVSCPP
>sp|Q6ZSB3|CB046_HUMAN Putative uncharacterized protein encoded by LINC00299 OS=Homo sapiens GN=LINC00299 PE=5 SV=1
MVQREKARDNFEGGCLAELIGSPRDWKCFLAVPDPLLGVQHWLHLWRPQTKDGNSLHRHG
DQAWGKHRRQNSLKSPALSGHSIDYHFYPRLRCGMLIGPDKQAVASGLEVLVTSSTKILG
QLFPDAAHFLEEASEFKAE
>sp|Q8N8R5|CB069_HUMAN UPF0565 protein C2orf69 OS=Homo sapiens GN=C2orf69 PE=1 SV=1
MWGFRLLRSPPLLLLLPQLGIGNASSCSQARTMNPGGSGGARCSLSAEVRRRQCLQLSTV
PGADPQRSNELLLLAAAGEGLERQDLPGDPAKEEPQPPPQHHVLYFPGDVQNYHEIMTRH
PENYQWENWSLENVATILAHRFPNSYIWVIKCSRMHLHKFSCYDNFVKSNMFGAPEHNTD
FGAFKHLYMLLVNAFNLSQNSLSKKSLNVWNKDSIASNCRSSPSHTTNGCQGEKVRTCEK
SDESAMSFYPPSLNDASFTLIGFSKGCVVLNQLLFELKEAKKDKNIDAFIKSIRTMYWLD
GGHSGGSNTWVTYPEVLKEFAQTGIIVHTHVTPYQVRDPMRSWIGKEHKKFVQILGDLGM
QVTSQIHFTKEAPSIENHFRVHEVF
>sp|A6NGG8|CB071_HUMAN Uncharacterized protein C2orf71 OS=Homo sapiens GN=C2orf71 PE=1 SV=1
MGCTPSHSDLVNSVAKSGIQFLKKPKAIRPGCQGGSERGSIPLLVKNSTCYDAGEGLAEE
QPSPRRNQTTAKGLCQLMGDPASGKRKDMEGLIPGTKTSSSQLNKSQSHMAKDIPFKTQG
SHGSQGADFSGDESEESSTQDTSKWKRTAKCHTSSTQSHCYQTIHPAHEPEGKVDFPEPL
VKAHQQAYTYLHSSLSKYEAILCIIHQATQTRELLQPMVSFLLLCFEEISQLLGEISKDG
EVLLQEVREDLAWPLKKREPQEQPNLLQQLLQYTVSKLQVLNGTVASLTGSFLEGSSSYL
HSTATHLENKLSTKRNVDERLLRALRQLESLASGCGDPGVQGLPLCSEDSGIGADNESVQ
SVDKLGKQTSWDLAPEPEEWKSVTSPHTEARQSGHTWQQSPFCLGSGRPQDCLLSGAPMA
KVQPRAQDEARSPCLSSTSPENITSPPLKLGTSTPCDSFGIGVSVEPHLSKTSRPMDASS
LSDSEDSSPEEEEEDKMSSMSLCAWQEKTPHSRPQSSPADRESPFQARTRRLRSLQAQEM
ILKMKESISERIKFVPVPCGHQDWSEEEEGRTVVPPRPSTVSGSRRAPERQTRSQSESCL
QSHVEDPTFQELRRVQRDLSQKLEAFYALGAKGQGQSQEQILQPRAAAVWPNGTCRVSPS
NTTSRLKASLTKNFSILPSQDKSILQKCNPHPEDEQGKAGKLPNAIPSGEVSEAAKATDW
NVRGCPTRTSVKKLIETFSPTESLRMLGDSKDAGASPCLRNCIMPPRFPKYTGLAPLYPK
PQISPASGRESLKMGIGWKPLAPIFPPLPKAEAAKSEELSCEMEGNLEHLPPPPMEVLMD
KSFASLESPESSKSTENSPKETQEPGPGEAGPTRRTWASPKLRASVSPLDLLPSKSTASL
TKPHSTGPGSGRSSCQPRKPALDLSSPPATSQSPEVKGGTWSQAEKATSLYRQPRKAIAW
HHSGPPSGQNRTSESSLARPRQSRERSPPVGRKASPTRTHWVPQADKRRRSLPSSYRPAQ
PSPSAVQTPPSPPVSPRVLSPPTTKRRTSPPHQPKLPNPPPESAPAQCKVPSPPTQHPEA

SPPFSIPSPSPPMSPSQEHKETRDSEDSQAVIAKVSGNTHSIFCPATSSLFEAKPPLSTA
HPLTPPSLPPEAGGPLGNPAECWKNSSGPWLRADSQRRAALCALNPLPFLRRTASDRQPG
GRPQPPTLDPTSTSYESQLGQNSSSEESPKKDTEPGSSPCSPELQGGTRRASPPEFCVLG
HGLQPEPRTGHIQDKSQPEAQPQQEEVS
>sp|Q8N350|CBARP_HUMAN Voltage-dependent calcium channel beta subunit-associated regulatory protein OS=Homo sapiens GN=CBARP PE=1 SV=3
MQPTATMATAATTTTTTTATVALTTSWDNATGRPTAEPDPILDNYVLLVVVMSLFVGGTL
VVLSGVLLLCKRCWDVHQRLNRAMEEAEKTTTTYLDNGTHPAQDPDFRGEDPECQDAETE
RFLSTSSTGRRVSFNEAALFEQSRKTQDKGRRYTLTEGDFHHLKNARLTHLHLPPLKIVT
IHECDSGEASSATTPHPATSPKATLAIFQPPGKALTGRSVGPSSALPGDPYNSAAGATDF
AEISPSASSDSGEGTSLDAGTRSTKAGGPGAAAGPGEAGPGSGAGTVLQFLTRLRRHASL
DGASPYFKVKKWKLEPSQRAASLDTRGSPKRHHFQRQRAASESTEQEEGDAPQEDFIQYI
ARAGDAVAFPHPRPFLASPPPALGRLEAAEAAGGASPDSPPERGAGSAGPEQQQPPLEPD
AERDAGPEQAQTSYRDLWSLRASLELHAAASDHSSSGNDRDSVRSGDSSGSGSGGAAPAF
PPPSPPAPRPKDGEARRLLQMDSGYASIEGRGAGDDTEPPAAPARPRSPRAWPRRPRRDY
SIDEKTDALFHEFLRHDPHFDDTPAAARHRARAHPHARKQWQRGRQHSDPGARAAPALAG
TPAPPAGAARPARAPLRRGDSVDGPPDGRTLGGAGDDPAIPVIEEEPGGGGCPGSGLCVL
PSGSVLDKLAAGLDERLFPPRLAEPVVATPALVAAAPTSPDHSPA
>sp|Q9ULV8|CBLC_HUMAN E3 ubiquitin-protein ligase CBL-C OS=Homo sapiens GN=CBLC PE=1 SV=3
MALAVAPWGRQWEEARALGRAVRMLQRLEEQCVDPRLSVSPPSLRDLLPRTAQLLREVAH
SRRAAGGGGPGGPGGSGDFLLIYLANLEAKSRQVAALLPPRGRRSANDELFRAGSRLRRQ
LAKLAIIFSHMHAELHALFPGGKYCGHMYQLTKAPAHTFWRESCGARCVLPWAEFESLLG
TCHPVEPGCTALALRTTIDLTCSGHVSIFEFDVFTRLFQPWPTLLKNWQLLAVNHPGYMA
FLTYDEVQERLQACRDKPGSYIFRPSCTRLGQWAIGYVSSDGSILQTIPANKPLSQVLLE
GQKDGFYLYPDGKTHNPDLTELGQAEPQQRIHVSEEQLQLYWAMDSTFELCKICAESNKD
VKIEPCGHLLCSCCLAAWQHSDSQTCPFCRCEIKGWEAVSIYQFHGQATAEDSGNSSDQE
GRELELGQVPLSAPPLPPRPDLPPRKPRNAQPKVRLLKGNSPPAALGPQDPAPA
>sp|Q9NTU7|CBLN4_HUMAN Cerebellin-4 OS=Homo sapiens GN=CBLN4 PE=1 SV=1
MGSGRRALSAVPAVLLVLTLPGLPVWAQNDTEPIVLEGKCLVVCDSNPATDSKGSSSSPL
GISVRAANSKVAFSAVRSTNHEPSEMSNKTRIIYFDQILVNVGNFFTLESVFVAPRKGIY
SFSFHVIKVYQSQTIQVNLMLNGKPVISAFAGDKDVTREAATNGVLLYLDKEDKVYLKLE
KGNLVGGWQYSTFSGFLVFPL
>sp|P22681|CBL_HUMAN E3 ubiquitin-protein ligase CBL OS=Homo sapiens GN=CBL PE=1 SV=2
MAGNVKKSSGAGGGSGSGGSGSGGLIGLMKDAFQPHHHHHHHLSPHPPGTVDKKMVEKCW
KLMDKVVRLCQNPKLALKNSPPYILDLLPDTYQHLRTILSRYEGKMETLGENEYFRVFME
NLMKKTKQTISLFKEGKERMYEENSQPRRNLTKLSLIFSHMLAELKGIFPSGLFQGDTFR
ITKADAAEFWRKAFGEKTIVPWKSFRQALHEVHPISSGLEAMALKSTIDLTCNDYISVFE
FDIFTRLFQPWSSLLRNWNSLAVTHPGYMAFLTYDEVKARLQKFIHKPGSYIFRLSCTRL
GQWAIGYVTADGNILQTIPHNKPLFQALIDGFREGFYLFPDGRNQNPDLTGLCEPTPQDH
IKVTQEQYELYCEMGSTFQLCKICAENDKDVKIEPCGHLMCTSCLTSWQESEGQGCPFCR
CEIKGTEPIVVDPFDPRGSGSLLRQGAEGAPSPNYDDDDDERADDTLFMMKELAGAKVER
PPSPFSMAPQASLPPVPPRLDLLPQRVCVPSSASALGTASKAASGSLHKDKPLPVPPTLR
DLPPPPPPDRPYSVGAESRPQRRPLPCTPGDCPSRDKLPPVPSSRLGDSWLPRPIPKVPV
SAPSSSDPWTGRELTNRHSLPFSLPSQMEPRPDVPRLGSTFSLDTSMSMNSSPLVGPECD

HPKIKPSSSANAIYSLAARPLPVPKLPPGEQCEGEEDTEYMTPSSRPLRPLDTSQSSRAC
DCDQQIDSCTYEAMYNIQSQAPSITESSTFGEGNLAAAHANTGPEESENEDDGYDVPKPP
VPAVLARRTLSDISNASSSFGWLSLDGDPTTNVTEGSQVPERPPKPFPRRINSERKAGSC
QQGSGPAASAATASPQLSSEIENLMSQGYSYQDIQKALVIAQNNIEMAKNILREFVSISS
PAHVAT
>sp|Q9UPW5|CBPC1_HUMAN Cytosolic carboxypeptidase 1 OS=Homo sapiens GN=AGTPBP1 PE=1 SV=3
MSKLKVIPEKSLTNNSRIVGLLAQLEKINAEPSESDTARYVTSKILHLAQSQEKTRREMT
AKGSTGMEILLSTLENTKDLQTTLNILSILVELVSAGGGRRVSFLVTKGGSQILLQLLMN
ASKESPPHEDLMVQIHSILAKIGPKDKKFGVKARINGALNITLNLVKQNLQNHRLVLPCL
QLLRVYSANSVNSVSLGKNGVVELMFKIIGPFSKKNSSLIKVALDTLAALLKSKTNARRA
VDRGYVQVLLTIYVDWHRHDNRHRNMLIRKGILQSLKSVTNIKLGRKAFIDANGMKILYN
TSQECLAVRTLDPLVNTSSLIMRKCFPKNRLPLPTIKSSFHFQLPVIPVTGPVAQLYSLP
PEVDDVVDESDDNDDIDVEAENETENEDDLDQNFKNDDIETDINKLKPQQEPGRTIEDLK
MYEHLFPELVDDFQDYDLISKEPKPFVFEGKVRGPIVVPTAGEETSGNSGNLRKVVMKEN
ISSKGDEGEKKSTFMDLAKEDIKDNDRTLQQQPGDQNRTISSVHGLNNDIVKALDRITLQ
NIPSQTAPGFTAEMKKDCSLPLTVLTCAKACPHMATCGNVLFEGRTVQLGKLCCTGVETE
DDEDTESNSSVEQASVEVPDGPTLHDPDLYIEIVKNTKSVPEYSEVAYPDYFGHIPPPFK
EPILERPYGVQRTKIAQDIERLIHQSDIIDRVVYDLDNPNYTIPEEGDILKFNSKFESGN
LRKVIQIRKNEYDLILNSDINSNHYHQWFYFEVSGMRPGVAYRFNIINCEKSNSQFNYGM
QPLMYSVQEALNARPWWIRMGTDICYYKNHFSRSSVAAGGQKGKSYYTITFTVNFPHKDD
VCYFAYHYPYTYSTLQMHLQKLESAHNPQQIYFRKDVLCETLSGNSCPLVTITAMPESNY
YEHICHFRNRPYVFLSARVHPGETNASWVMKGTLEYLMSNNPTAQSLRESYIFKIVPMLN
PDGVINGNHRCSLSGEDLNRQWQSPSPDLHPTIYHAKGLLQYLAAVKRLPLVYCDYHGHS
RKKNVFMYGCSIKETVWHTNDNATSCDVVEDTGYRTLPKILSHIAPAFCMSSCSFVVEKS
KESTARVVVWREIGVQRSYTMESTLCGCDQGKYKGLQIGTRELEEMGAKFCVGLLRLKRL
TSPLEYNLPSSLLDFENDLIESSCKVTSPTTYVLDEDEPRFLEEVDYSAESNDELDIELA
ENVGDYEPSAQEEVLSDSELSRTYLP
>sp|O75828|CBR3_HUMAN Carbonyl reductase [NADPH] 3 OS=Homo sapiens GN=CBR3 PE=1 SV=3
MSSCSRVALVTGANRGIGLAIARELCRQFSGDVVLTARDVARGQAAVQQLQAEGLSPRFH
QLDIDDLQSIRALRDFLRKEYGGLNVLVNNAAVAFKSDDPMPFDIKAEMTLKTNFFATRN
MCNELLPIMKPHGRVVNISSLQCLRAFENCSEDLQERFHSETLTEGDLVDLMKKFVEDTK
NEVHEREGWPNSPYGVSKLGVTVLSRILARRLDEKRKADRILVNACCPGPVKTDMDGKDS
IRTVEEGAETPVYLALLPPDATEPQGQLVHDKVVQNW
>sp|Q4V339|CBWD6_HUMAN COBW domain-containing protein 6 OS=Homo sapiens GN=CBWD6 PE=3 SV=1
MLPAVGSVDEEEDPAEEDCPELVPIETTQSEEEEKSGLGAKIPVTIITGYLGAGKTTLLN
YILTEQHSKRVAVILNESGEGSALEKSLAVSQGGELYEEWLELRNGCLCCSVKDNGLRAI
ENLMQKKGKFDDILLETTGLADPGAVASMFWVDAELGSDIYLDGIITIVDSKYGLKHLTE
EKPDGLINEATRQVALADIILINKTDLVPEEDVKKLRTTLRSINGLGQILETQRSRVDLS
NVLDLHAFDSLSGISLQKKLQHVPGTQPHLDQSIVTITFDVPGNAKEEHLNMFIQNLLWE
KNVRNKDNHCMEVIRLKGLVSIKDKSQQVIVQGVHELCDLEETPVSWKDDTERTNRLVLI
GRNLDKDILKQLFIATVTETEKQWTTHFKEDQVCT
>sp|Q6P1S2|CC033_HUMAN Protein C3orf33 OS=Homo sapiens GN=C3orf33 PE=1 SV=2
MAGQPAATGSPSADKDGMEPNVVARISQWADDHLRLVRNISTGMAIAGIMLLLRSIRLTS

KFTSSSDIPVEFIRRNVKLRGRLRRITENGLEIEHIPITLPIIASLRKEPRGALLVKLAG
VELAETGKAWLQKELKPSQLLWFQLLGKENSALFCYLLVSKGGYFSVNLNEEILRRGLGK
TVLVKGLKYDSKIYWTVHRNLLKAELTALKKGEGIWKEDSEKESYLEKFKDSWREIWKKD
SFLKTTGSDFSLKKESYYEKLKRTYEIWKDNMNNCSLILKFRELISRINFRRKG
>sp|P0CE67|CC079_HUMAN Putative uncharacterized protein C3orf79 OS=Homo sapiens GN=C3orf79 PE=4 SV=1
MHVCDLQKLVRIQLAFTTFPWMFSCHLLPTPELSSKRNQCLLYKTSGCLTQMPILYGHPA
TLLKDYILQAILQPGKKIQGGTEIQRGSFANQYQTDASHL
>sp|Q8IVM0|CCD50_HUMAN Coiled-coil domain-containing protein 50 OS=Homo sapiens GN=CCDC50 PE=1 SV=1
MAEVSIDQSKLPGVKEVCRDFAVLEDHTLAHSLQEQEIEHHLASNVQRNRLVQHDLQVAK
QLQEEDLKAQAQLQKRYKDLEQQDCEIAQEIQEKLAIEAERRRIQEKKDEDIARLLQEKE
LQEEKKRKKHFPEFPATRAYADSYYYEDGGMKPRVMKEAVSTPSRMAHRDQEWYDAEIAR
KLQEEELLATQVDMRAAQVAQDEEIARLLMAEEKKAYKKAKEREKSSLDKRKQDPEWKPK
TAKAANSKSKESDEPHHSKNERPARPPPPIMTDGEDADYTHFTNQQSSTRHFSKSESSHK
GFHYKH
>sp|Q4VC31|CCD58_HUMAN Coiled-coil domain-containing protein 58 OS=Homo sapiens GN=CCDC58 PE=1 SV=1
MAAPSGGVNCEEFAEFQELLKVMRTIDDRIVHELNTTVPTASFAGKIDASQTCKQLYESL
MAAHASRDRVIKNCIAQTSAVVKNLREEREKNLDDLTLLKQLRKEQTKLKWMQSELNVEE
VVNDRSWKVFNERCRIHFKPPKNE
>sp|Q8N998|CCD89_HUMAN Coiled-coil domain-containing protein 89 OS=Homo sapiens GN=CCDC89 PE=2 SV=1
MRAPMLQKQQAPRMDTPPPEERLEKQNEKLNNQEEETEFKELDGLREALANLRGLSEEER
SEKAMLRSRIEEQSQLICILKRRSDEALERCQILELLNAELEEKMMQEAEKLKAQGEYSR
KLEERFMTLAANHELMLRFKDEYKSENIKLREENEKLRLENSSLFSQALKDEEAKVLQLT
VRCEALTGELETLKERCAQDACQAQAREKELLELQSQQACTHTKETEQLRSQLQTLKQQH
QQAVEQIAKAEETHSSLSQELQARLQTVTREKEELLQLSIERGKVLQNKQAEICQLEEKL
EIANEDRKHALERFEQEAVAVDSNLRVRELQRKVDGIQKAYDELRLQSEAFKKHSLDLLS
KERELNGKLRHLSP
>sp|Q9BW85|CCD94_HUMAN Coiled-coil domain-containing protein 94 OS=Homo sapiens GN=CCDC94 PE=1 SV=1
MSERKVLNKYYPPDFDPSKIPKLKLPKDRQYVVRLMAPFNMRCKTCGEYIYKGKKFNARK
ETVQNEVYLGLPIFRFYIKCTRCLAEITFKTDPENTDYTMEHGATRNFQAEKLLEEEEKR
VQKEREDEELNNPMKVLENRTKDSKLEMEVLENLQELKDLNQRQAHVDFEAMLRQHRLSE
EERRRQQQEEDEQETAALLEEARKRRLLEDSDSEDEAAPSPLQPALRPNPTAILDEAPKP
KRKVEVWEQSVGSLGSRPPLSRLVVVKKAKADPDCSNGQPQAAPTPGAPQNRKEANPTPL
TPGASSLSQLGAYLDSDDSNGSN
>sp|Q9BQI4|CCDC3_HUMAN Coiled-coil domain-containing protein 3 OS=Homo sapiens GN=CCDC3 PE=2 SV=1
MLRQLLLAALCLAGPPAPARACQLPSEWRPLSEGCRAELAETIVYARVLALHPEAPGLYN
HLPWQYHAGQGGLFYSAEVEMLCDQAWGSMLEVPAGSRLNLTGLGYFSCHSHTVVQDYSY
FFFLRMDENYNLLPHGVNFQDAIFPDTQENRRMFSSLFQFSNCSQGQQLATFSSDWEIQE
DSRLMCSSVQKALFEEEDHVKKLQQKVATLEKRNRQLRERVKKVKRSLRQARKKGRHLEL

ANQKLSEKLAAGALPHINARGPVRPPYLRG
>sp|Q16204|CCDC6_HUMAN Coiled-coil domain-containing protein 6 OS=Homo sapiens GN=CCDC6 PE=1 SV=2
MADSASESDTDGAGGNSSSSAAMQSSCSSTSGGGGGGGGGGGGGKSGGIVISPFRLEELT
NRLASLQQENKVLKIELETYKLKCKALQEENRDLRKASVTIQARAEQEEEFISNTLFKKI
QALQKEKETLAVNYEKEEEFLTNELSRKLMQLQHEKAELEQHLEQEQEFQVNKLMKKIKK
LENDTISKQLTLEQLRREKIDLENTLEQEQEALVNRLWKRMDKLEAEKRILQEKLDQPVS
APPSPRDISMEIDSPENMMRHIRFLKNEVERLKKQLRAAQLQHSEKMAQYLEEERHMREE
NLRLQRKLQREMERREALCRQLSESESSLEMDDERYFNEMSAQGLRPRTVSSPIPYTPSP
SSSRPISPGLSYASHTVGFTPPTSLTRAGMSYYNSPGLHVQHMGTSHGITRPSPRRSNSP
DKFKRPTPPPSPNTQTPVQPPPPPPPPPMQPTVPSAATSQPTPSQHSAHPSSQP
>sp|Q06432|CCG1_HUMAN Voltage-dependent calcium channel gamma-1 subunit OS=Homo sapiens GN=CACNG1 PE=1 SV=1
MSQTKMLKVRVTLFCILAGIVLAMTAVVTDHWAVLSPHMEHHNTTCEAAHFGLWRICTKR
IPMDDSKTCGPITLPGEKNCSYFRHFNPGESSEIFEFTTQKEYSISAAAIAIFSLGFIIL
GSLCVLLSLGKKRDYLLRPASMFYAFAGLCILVSVEVMRQSVKRMIDSEDTVWIEYYYSW
SFACACAAFILLFLGGLALLLFSLPRMPRNPWESCMDAEPEH
>sp|Q9Y698|CCG2_HUMAN Voltage-dependent calcium channel gamma-2 subunit OS=Homo sapiens GN=CACNG2 PE=1 SV=1
MGLFDRGVQMLLTTVGAFAAFSLMTIAVGTDYWLYSRGVCKTKSVSENETSKKNEEVMTH
SGLWRTCCLEGNFKGLCKQIDHFPEDADYEADTAEYFLRAVRASSIFPILSVILLFMGGL
CIAASEFYKTRHNIILSAGIFFVSAGLSNIIGIIVYISANAGDPSKSDSKKNSYSYGWSF
YFGALSFIIAEMVGVLAVHMFIDRHKQLRATARATDYLQASAITRIPSYRYRYQRRSRSS
SRSTEPSHSRDASPVGIKGFNTLPSTEISMYTLSRDPLKAATTPTATYNSDRDNSFLQVH
NCIQKENKDSLHSNTANRRTTPV
>sp|Q9UBN1|CCG4_HUMAN Voltage-dependent calcium channel gamma-4 subunit OS=Homo sapiens GN=CACNG4 PE=2 SV=1
MVRCDRGLQMLLTTAGAFAAFSLMAIAIGTDYWLYSSAHICNGTNLTMDDGPPPRRARGD
LTHSGLWRVCCIEGIYKGHCFRINHFPEDNDYDHDSSEYLLRIVRASSVFPILSTILLLL
GGLCIGAGRIYSRKNNIVLSAGILFVAAGLSNIIGIIVYISSNTGDPSDKRDEDKKNHYN
YGWSFYFGALSFIVAETVGVLAVNIYIEKNKELRFKTKREFLKASSSSPYARMPSYRYRR
RRSRSSSRSTEASPSRDVSPMGLKITGAIPMGELSMYTLSREPLKVTTAASYSPDQEASF
LQVHDFFQQDLKEGFHVSMLNRRTTPV
>sp|P06307|CCKN_HUMAN Cholecystokinin OS=Homo sapiens GN=CCK PE=1 SV=1
MNSGVCLCVLMAVLAAGALTQPVPPADPAGSGLQRAEEAPRRQLRVSQRTDGESRAHLGA
LLARYIQQARKAPSGRMSIVKNLQNLDPSHRISDRDYMGWMDFGRRSAEEYEYPS
>sp|O15467|CCL16_HUMAN C-C motif chemokine 16 OS=Homo sapiens GN=CCL16 PE=1 SV=1
MKVSEAALSLLVLILIITSASRSQPKVPEWVNTPSTCCLKYYEKVLPRRLVVGYRKALNC
HLPAIIFVTKRNREVCTNPNDDWVQEYIKDPNLPLLPTRNLSTVKIITAKNGQPQLLNSQ
>sp|Q9Y4X3|CCL27_HUMAN C-C motif chemokine 27 OS=Homo sapiens GN=CCL27 PE=1 SV=1
MKGPPTFCSLLLLSLLLSPDPTAAFLLPPSTACCTQLYRKPLSDKLLRKVIQVELQEADG
DCHLQAFVLHLAQRSICIHPQNPSLSQWFEHQERKLHGTLPKLNFGMLRKMG
>sp|P13500|CCL2_HUMAN C-C motif chemokine 2 OS=Homo sapiens GN=CCL2 PE=1 SV=1
MKVSAALLCLLLIAATFIPQGLAQPDAINAPVTCCYNFTNRKISVQRLASYRRITSSKCP

KEAVIFKTIVAKEICADPKQKWVQDSMDHLDKQTQTPKT
>sp|P10147|CCL3_HUMAN C-C motif chemokine 3 OS=Homo sapiens GN=CCL3 PE=1 SV=1
MQVSTAALAVLLCTMALCNQFSASLAADTPTACCFSYTSRQIPQNFIADYFETSSQCSKP
GVIFLTKRSRQVCADPSEEWVQKYVSDLELSA
>sp|O75419|CDC45_HUMAN Cell division control protein 45 homolog OS=Homo sapiens GN=CDC45 PE=1 SV=1
MFVSDFRKEFYEVVQSQRVLLFVASDVDALCACKILQALFQCDHVQYTLVPVSGWQELET
AFLEHKEQFHYFILINCGANVDLLDILQPDEDTIFFVCDTHRPVNVVNVYNDTQIKLLIK
QDDDLEVPAYEDIFRDEEEDEEHSGNDSDGSEPSEKRTRLEEEIVEQTMRRRQRREWEAR
RRDILFDYEQYEYHGTSSAMVMFELAWMLSKDLNDMLWWAIVGLTDQWVQDKITQMKYVT
DVGVLQRHVSRHNHRNEDEENTLSVDCTRISFEYDLRLVLYQHWSLHDSLCNTSYTAARF
KLWSVHGQKRLQEFLADMGLPLKQVKQKFQAMDISLKENLREMIEESANKFGMKDMRVQT
FSIHFGFKHKFLASDVVFATMSLMESPEKDGSGTDHFIQALDSLSRSNLDKLYHGLELAK
KQLRATQQTIASCLCTNLVISQGPFLYCSLMEGTPDVMLFSRPASLSLLSKHLLKSFVCS
TKNRRCKLLPLVMAAPLSMEHGTVTVVGIPPETDSSDRKNFFGRAFEKAAESTSSRMLHN
HFDLSVIELKAEDRSKFLDALISLLS
>sp|Q96JP9|CDHR1_HUMAN Cadherin-related family member 1 OS=Homo sapiens GN=CDHR1 PE=1 SV=2
MRRCRWAALALGLLRLCLAQANFAPHFFDNGVGSTNGNMALFSLPEDTPVGSHVYTLNGT
DPEGDPISYHISFDPSTRSVFSVDPTFGNITLVEELDREREDEIEAIISISDGLNLVAEK
VVILVTDANDEAPRFIQEPYVALVPEDIPAGSIIFKVHAVDRDTGSGGSVTYFLQNLHSP
FAVDRHSGVLRLQAGATLDYERSRTHYITVVAKDGGGRLHGADVVFSATTTVTVNVEDVQ
DMAPVFVGTPYYGYVYEDTLPGSEVLKVVAMDGDRGKPNRILYSLVNGNDGAFEINETSG
AISITQSPAQLQREVYELHVQVTEMSPAGSPAAQATVPVTIRIVDLNNHPPTFYGESGPQ
NRFELSMNEHPPQGEILRGLKITVNDSDQGANAKFNLQLVGPRGIFRVVPQTVLNEAQVT
IIVENSAAIDFEKSKVLTFKLLAVEVNTPEKFSSTADVVIQLLDTNDNVPKFDSLYYVAR
IPENAPGGSSVVAVTAVDPDTGPWGEVKYSTYGTGADLFLIHPSTGLIYTQPWASLDAEA
TARYNFYVKAEDMEGKYSVAEVFITLLDVNDHPPQFGKSVQKKTMVLGTPVKIEAIDEDA
EEPNNLVDYSITHAEPANVFDINSHTGEIWLKNSIRSLDALHNITPGRDCLWSLEVQAKD
RGSPSFSTTALLKIDITDAETLSRSPMAAFLIQTKDNPMKAVGVLAGTMATVVAITVLIS
TATFWRNKKSNKVLPMRRVLRKRPSPAPRTIRIEWLKSKSTKAATKFMLKEKPPNENCNN
NSPESSLLPRAPALPPPPSVAPSTGAAQWTVPTVSGSLTPQPTQPPPKPKTMGSPVQSTL
ISELKQKFEKKSVHNKAYF
>sp|Q92772|CDKL2_HUMAN Cyclin-dependent kinase-like 2 OS=Homo sapiens GN=CDKL2 PE=1 SV=1
MEKYENLGLVGEGSYGMVMKCRNKDTGRIVAIKKFLESDDDKMVKKIAMREIKLLKQLRH
ENLVNLLEVCKKKKRWYLVFEFVDHTILDDLELFPNGLDYQVVQKYLFQIINGIGFCHSH
NIIHRDIKPENILVSQSGVVKLCDFGFARTLAAPGEVYTDYVATRWYRAPELLVGDVKYG
KAVDVWAIGCLVTEMFMGEPLFPGDSDIDQLYHIMMCLGNLIPRHQELFNKNPVFAGVRL
PEIKEREPLERRYPKLSEVVIDLAKKCLHIDPDKRPFCAELLHHDFFQMDGFAERFSQEL
QLKVQKDARNVSLSKKSQNRKKEKEKDDSLVEERKTLVVQDTNADPKIKDYKLFKIKGSK
IDGEKAEKGNRASNASCLHDSRTSHNKIVPSTSLKDCSNVSVDHTRNPSVAIPPLTHNLS
AVAPSINSGMGTETIPIQGYRVDEKTKKCSIPFVKPNRHSPSGIYNINVTTLVSGPPLSD
DSGADLPQMEHQH
>sp|Q8IVW4|CDKL3_HUMAN Cyclin-dependent kinase-like 3 OS=Homo sapiens GN=CDKL3 PE=1 SV=1

MEMYETLGKVGEGSYGTVMKCKHKNTGQIVAIKIFYERPEQSVNKIAMREIKFLKQFHHE
NLVNLIEVFRQKKKIHLVFEFIDHTVLDELQHYCHGLESKRLRKYLFQILRAIDYLHSNN
IIHRDIKPENILVSQSGITKLCDFGFARTLAAPGDIYTDYVATRWYRAPELVLKDTSYGK
PVDIWALGCMIIEMATGNPYLPSSSDLDLLHKIVLKVGNLSPHLQNIFSKSPIFAGVVLP
QVQHPKNARKKYPKLNGLLADIVHACLQIDPADRISSSDLLHHEYFTRDGFIEKFMPELK
AKLLQEAKVNSLIKPKESSKENELRKDERKTVYTNTLLSSSVLGKEIEKEKKPKEIKVRV
IKVKGGRGDISEPKKKEYEGGLGQQDANENVHPMSPDTKLVTIEPPNPINPSTNCNGLKE
NPHCGGSVTMPPINLTNSNLMAANLSSNLFHPSVRLTERAKKRRTSSQSIGQVMPNSRQE
DPGPIQSQMEKGIFNERTGHSDQMANENKRKLNFSRSDRKEFHFPELPVTIQSKDTKGME
VKQIKMLKRESKKTESSKIPTLLNVDQNQEKQEGGDGHCEGKNLKRNRFFFW
>sp|P49918|CDN1C_HUMAN Cyclin-dependent kinase inhibitor 1C OS=Homo sapiens GN=CDKN1C PE=1 SV=1
MSDASLRSTSTMERLVARGTFPVLVRTSACRSLFGPVDHEELSRELQARLAELNAEDQNR
WDYDFQQDMPLRGPGRLQWTEVDSDSVPAFYRETVQVGRCRLLLAPRPVAVAVAVSPPLE
PAAESLDGLEEAPEQLPSVPVPAPASTPPPVPVLAPAPAPAPAPVAAPVAAPVAVAVLAP
APAPAPAPAPAPAPVAAPAPAPAPAPAPAPAPAPDAAPQESAEQGANQGQRGQEPLAD
QLHSGISGRPAAGTAAASANGAAIKKLSGPLISDFFAKRKRSAPEKSSGDVPAPCPSPSA
APGVGSVEQTPRKRLR
>sp|Q52M75|CE027_HUMAN Putative uncharacterized protein encoded by LINC01554 OS=Homo sapiens GN=LINC01554 PE=4 SV=2
MTPCLESFCQRAKDCKQNIPLPFLIQDWPLSESKPQGQDFWTPQPLQAAAPSHEFQQVAL
HPARTRLASRPQGGRLMSSREVGLRAPRCSCGKRKR
>sp|Q96MH7|CE034_HUMAN Uncharacterized protein C5orf34 OS=Homo sapiens GN=C5orf34 PE=2 SV=2
MAAELRMILYEDDSVQVQYVDGSTLQLSPCGTEFLFEKSPPVSAHPLEQPERIRQRTHFV
ISTYREQLQRALDFRNSSATCPFLSETIIPSERKKHIFIDITEVRWPSLDTDGTMIYMES
GIVKITSLDGHAYLCLPRSQHEFTVHFLCKVSQKSDSSAVLSETNNKAPKDKLVEKTGKI
CIRGNLPGQRLKNKENEFHCQIMKSKETLKKMSCVNGTEGREELPSPGTKHTCVYTWVKQ
CWSVAACPEEWKYPLSLALHFHNKISNMSKIDAHITQSRFLTSDISEERGKVVSVLPRAL
SLSCPVPHLHRWNFCDSLLQRQSDEYSYPELVKMVWYKGVTYRLTHQNMNSIEIYSGDGS
VFKSEGAYFGNYFTYYSIQEGSGKREEKTYSVNNLPPDRPGSPFTVGSLIKQATRILQHC
VKMRLSLSHNYRICCWKMVPGINDSNILPLVLKESLIPSVGRFLAYSDDKVHAIFLDGIT
LTLNWNFSSPIEKRQVNQGLNLGWCKLTFPDGGQEQLIQIEHPEPYERYVTTVTSWCRRLT
QTSPREMPTHSSSSVLQENWSVASELEKIQKFNLLLENSGILNQISNKKNEQQSFDHYKP
GSSETLLGEVNENRVSIALKKTSEILHDIDCLLSNSKK
>sp|Q6NTE8|CE045_HUMAN UPF0544 protein C5orf45 OS=Homo sapiens GN=C5orf45 PE=2 SV=2
MASLQRSRVLRCCSCRLFQAHQVKKSVKWTCKACGEKQSFLQAYGEGSGADCRRHVQKLN
LLQGQVSELPLRSLEETVSASEEENVGHQQAGNVKQQEKSQPSESRWLKYLEKDSQELEL
EGTGVCFSKQPSSKMEEPGPRFSQDLPRKRKWSRSTVQPPCSRGVQDSGGSEVAWGPQKG
QAGLTWKVKQGSSPCLQENSADCSAGELRGPGKELWSPIQQVTATSSKWAQFVLPPRKSS
HVDSEQPRSLQRDPRPAGPAQAKQGTPRAQASREGLSRPTAAVQLPRATHPVTSGSERPC
GKTSWDARTPWAEGGPLVLEAQNPRPTRLCDLFITGEDFDDDV
>sp|Q569G3|CE047_HUMAN Uncharacterized protein C5orf47 OS=Homo sapiens GN=C5orf47 PE=2 SV=2

MAAAGRGREQDSARFVYVTRFGSHQCSGVLQLGGRGAQGLWGQGPGAGCRQEKPREAMAV
AGVQGGSELPLGSQLRVPTTPGVEAAASASSQLRASRVQSGTRQSARAGLIQKDAAKKYD
FPIPLNEASKIMKKKKKVLVWNRVYKVISRMLEENEKYRHRLKCQRLSSESSNYTR
>sp|Q8N960|CE120_HUMAN Centrosomal protein of 120 kDa OS=Homo sapiens GN=CEP120 PE=1 SV=2
MVSKSDQLLIVVSILEGRHFPKRPKHMLVVEAKFDGEQLATDPVDHTDQPEFATELAWEI
DRKALHQHRLQRTPIKLQCFALDPVTSAKETIGYIVLDLRTAQETKQAPKWYQLLSNKYT
KFKSEIQISIALETDTKPPVDSFKAKGAPPRDGKVPAILAGLDPRDIVAVLNEEGGYHQI
GPAEYCTDSFIMSVTIAFATQLEQLIPCTMKLPERQPEFFFYYSLLGNDVTNEPFNDLIN
PNFEPERASVRIRSSVEILRVYLALQSKLQIHLCCGDQSLGSTEIPLTGLLKKGSTEINQ
HPVTVEGAFTLDPPNRAKQKLAPIPVELAPTVGVSVALQREGIDSQSLIELKTQNEHEPE
HSKKKVLTPIKEKTLTGPKSPTVSPVPSHNQSPPTKDDATESEVESLQYDKDTKPNPKAS
SSVPASLAQLVTTSNASEVASGQKIAVPATSHHFCFSIDLRSIHALEIGFPINCILRYSY
PFFGSAAPIMTNPPVEVRKNMEVFLPQSYCAFDFATMPHQLQDTFLRIPLLVELWHKDKM
SKDLLLGIARIQLSNILSSEKTRFLGSNGEQCWRQTYSESVPVIAAQGSNNRIADLSYTV
TLEDYGLVKMREIFISDSSQGVSAVQQKPSSLPPAPCPSEIQTEPRETLEYKAALELEMW
KEMQEDIFENQLKQKELAHMQALAEEWKKRDRERESLVKKKVAEYTILEGKLQKTLIDLE
KREQQLASVESELQREKKELQSERQRNLQELQDSIRRAKEDCIHQVELERLKIKQLEEDK
HRLQQQLNDAENKYKILEKEFQQFKDQQNNKPEIRLQSEINLLTLEKVELERKLESATKS
KLHYKQQWGRALKELARLKQREQESQMARLKKQQEELEQMRLRYLAAEEKDTVKTERQEL
LDIRNELNRLRQQEQKQYQDSTEIASGKKDGPHGSVLEEGLDDYLTRLIEERDTLMRTGV
YNHEDRIISELDRQIREILAKSNASN
>sp|Q8IYX8|CE57L_HUMAN Centrosomal protein CEP57L1 OS=Homo sapiens GN=CEP57L1 PE=1 SV=1
MDSELMHSIVGSYHKPPERVFVPSFTQNEPSQNCHPANLEVTSPKILHSPNSQALILALK
TLQEKIHRLELERTQAEDNLNILSREAAQYKKALENETNERNLAHQELIKQKKDISIQLS
SAQSRCTLLEKQLEYTKRMVLNVEREKNMILEQQAQLQREKEQDQMKLYAKLEKLDVLEK
ECFRLTTTQKTAEDKIKHLEEKLKEEEHQRKLFQDKASELQTGLEISKIIMSSVSNLKHS
KEKKKSSKKTKCIKRRPPWQICSKFGALPFVAEKMRQHRDPHILQKPFNVTETRCLPKPS
RTTSWCKAIPPDSEKSISICDNLSELLMAMQDELDQMSMEHQELLKQMKETESHSVCDDI
ECELECLLKKMEIKGEQISKLKKHQDSVCKLQQKVQNSKMSEASGIQQEDSYPKGSKNIK
NSPRKCLTDTNLFQKNSSFHPIRVHNLQMKLRRDDIMWEQ
>sp|P31997|CEAM8_HUMAN Carcinoembryonic antigen-related cell adhesion molecule 8 OS=Homo sapiens GN=CEACAM8 PE=1 SV=2
MGPISAPSCRWRIPWQGLLLTASLFTFWNPPTTAQLTIEAVPSNAAEGKEVLLLVHNLPQ
DPRGYNWYKGETVDANRRIIGYVISNQQITPGPAYSNRETIYPNASLLMRNVTRNDTGSY
TLQVIKLNLMSEEVTGQFSVHPETPKPSISSNNSNPVEDKDAVAFTCEPETQNTTYLWWV
NGQSLPVSPRLQLSNGNRTLTLLSVTRNDVGPYECEIQNPASANFSDPVTLNVLYGPDAP
TISPSDTYYHAGVNLNLSCHAASNPPSQYSWSVNGTFQQYTQKLFIPNITTKNSGSYACH
TTNSATGRNRTTVRMITVSDALVQGSSPGLSARATVSIMIGVLARVALI
>sp|Q8N7Q2|CEAS1_HUMAN Putative uncharacterized protein CELF2-AS1 OS=Homo sapiens GN=CELF2-AS1 PE=5 SV=2
MFCLLHLCFYLANFASSIKRTHAVNGCCGLQMIALWAQSSGNADARVEEILAGEERRLAA
LLGSQGMRFWVCLAACRAMWGLAARRGRAEDSSSSPVDASKFPWRGGQHRTTMMPCLLRV
GVFRPCHVRPTGDPSCDVQPPRLGFSRVPDTQVAFYGSGHWDPTPFSVHSCFFNFQVRKI
IFLL

>sp|A8MTT3|CEBOS_HUMAN Protein CEBPZOS OS=Homo sapiens GN=CEBPZOS PE=1 SV=2
MARTLEPLAKKIFKGVLVAELVGVFGAYFLFSKMHTSQDFRQTMSKKYPFILEVYYKSTE
KSGMYGIRELDQKTWLNSKN
>sp|P49716|CEBPD_HUMAN CCAAT/enhancer-binding protein delta OS=Homo sapiens GN=CEBPD PE=1 SV=2
MSAALFSLDGPARGAPWPAEPAPFYEPGRAGKPGRGAEPGALGEPGAAAPAMYDDESAID
FSAYIDSMAAVPTLELCHDELFADLFNSNHKAGGAGPLELLPGGPARPLGPGPAAPRLLK
REPDWGDGDAPGSLLPAQVAACAQTVVSLAAAGQPTPPTSPEPPRSSPRQTPAPGPAREK
SAGKRGPDRGSPEYRQRRERNNIAVRKSRDKAKRRNQEMQQKLVELSAENEKLHQRVEQL
TRDLAGLRQFFKQLPSPPFLPAAGTADCR
>sp|P08218|CEL2B_HUMAN Chymotrypsin-like elastase family member 2B OS=Homo sapiens GN=CELA2B PE=2 SV=2
MIRTLLLSTLVAGALSCGVSTYAPDMSRMLGGEEARPNSWPWQVSLQYSSNGQWYHTCGG
SLIANSWVLTAAHCISSSGIYRVMLGQHNLYVAESGSLAVSVSKIVVHKDWNSDQVSKGN
DIALLKLANPVSLTDKIQLACLPPAGTILPNNYPCYVTGWGRLQTNGALPDDLKQGQLLV
VDYATCSSSGWWGSTVKTNMICAGGDGVICTCNGDSGGPLNCQASDGRWEVHGIGSLTSV
LGCNYYYKPSIFTRVSNYNDWINSVIANN
>sp|P09093|CEL3A_HUMAN Chymotrypsin-like elastase family member 3A OS=Homo sapiens GN=CELA3A PE=1 SV=3
MMLRLLSSLLLVAVASGYGPPSSHSSSRVVHGEDAVPYSWPWQVSLQYEKSGSFYHTCGG
SLIAPDWVVTAGHCISRDLTYQVVLGEYNLAVKEGPEQVIPINSEELFVHPLWNRSCVAC
GNDIALIKLSRSAQLGDAVQLASLPPAGDILPNKTPCYITGWGRLYTNGPLPDKLQQARL
PVVDYKHCSRWNWWGSTVKKTMVCAGGYIRSGCNGDSGGPLNCPTEDGGWQVHGVTSFVS
AFGCNFIWKPTVFTRVSAFIDWIEETIASH
>sp|P08861|CEL3B_HUMAN Chymotrypsin-like elastase family member 3B OS=Homo sapiens GN=CELA3B PE=1 SV=3
MMLRLLSSLLLVAVASGYGPPSSRPSSRVVNGEDAVPYSWPWQVSLQYEKSGSFYHTCGG
SLIAPDWVVTAGHCISSSRTYQVVLGEYDRAVKEGPEQVIPINSGDLFVHPLWNRSCVAC
GNDIALIKLSRSAQLGDAVQLASLPPAGDILPNETPCYITGWGRLYTNGPLPDKLQEALL
PVVDYEHCSRWNWWGSSVKKTMVCAGGDIRSGCNGDSGGPLNCPTEDGGWQVHGVTSFVS
AFGCNTRRKPTVFTRVSAFIDWIEETIASH
>sp|O95319|CELF2_HUMAN CUGBP Elav-like family member 2 OS=Homo sapiens GN=CELF2 PE=1 SV=1
MRCPKSAVTMRNEELLLSNGTANKMNGALDHSDQPDPDAIKMFVGQIPRSWSEKELKELF
EPYGAVYQINVLRDRSQNPPQSKGCCFVTFYTRKAALEAQNALHNIKTLPGMHHPIQMKP
ADSEKSNAVEDRKLFIGMVSKKCNENDIRVMFSPFGQIEECRILRGPDGLSRGCAFVTFS
TRAMAQNAIKAMHQSQTMEGCSSPIVVKFADTQKDKEQRRLQQQLAQQMQQLNTATWGNL
TGLGGLTPQYLALLQQATSSSNLGAFSGIQQMAGMNALQLQNLATLAAAAAAAQTSATST
NANPLSTTSSALGALTSPVAASTPNSTAGAAMNSLTSLGTLQGLAGATVGLNNINALAGM
AALNGGLGATGLTNGTAGTMDALTQAYSGIQQYAAAALPTLYSQSLLQQQSAAGSQKEGP
EGANLFIYHLPQEFGDQDILQMFMPFGNVISAKVFIDKQTNLSKCFGFVSYDNPVSAQAA
IQAMNGFQIGMKRLKVQLKRSKNDSKPY
>sp|Q5SZQ8|CELF3_HUMAN CUGBP Elav-like family member 3 OS=Homo sapiens GN=CELF3 PE=1 SV=1
MKEPDAIKLFVGQIPRHLEEKDLKPIFEQFGRIFELTVIKDKYTGLHKGCAFLTYCARDS
ALKAQSALHEQKTLPGMNRPIQVKPADSESRGEDRKLFVGMLGKQQTDEDVRKMFEPFGT

IDECTVLRGPDGTSKGCAFVKFQTHAEAQAAINTLHSSRTLPGASSSLVVKFADTEKERG
LRRMQQVATQLGMFSPIALQFGAYSAYTQALMQQQAALVAAHSAYLSPMATMAAVQMQHM
AAINANGLIATPITPSSGTSTPPAIAATPVSAIPAALGVNGYSPVPTQPTGQPAPDALYP
NGVHPYPAQSPAAPVDPLQQAYAGMQHYTAAYPAAYSLVAPAFPQPPALVAQQPPPPPQQ
QQQQQQQQQQQQQREGPDGCNIFIYHLPQEFTDSEILQMFVPFGHVISAKVFVDRATNQS
KCFGFVSFDNPASAQAAIQAMNGFQIGMKRLKVQLKRPKDANRPY
>sp|Q03188|CENPC_HUMAN Centromere protein C OS=Homo sapiens GN=CENPC PE=1 SV=2
MAASGLDHLKNGYRRRFCRPSRARDINTEQGQNVLEILQDCFEEKSLANDFSTNSTKSVP
NSTRKIKDTCIQSPSKECQKSHPKSVPVSSKKKEASLQFVVEPSEATNRSVQAHEVHQKI
LATDVSSKNTPDSKKISSRNINDHHSEADEEFYLSVGSPSVLLDAKTSVSQNVIPSSAQK
RETYTFENSVNMLPSSTEVSVKTKKRLNFDDKVMLKKIEIDNKVSDEEDKTSEGQERKPS
GSSQNRIRDSEYEIQRQAKKSFSTLFLETVKRKSESSPIVRHAATAPPHSCPPDDTKLIE
DEFIIDESDQSFASRSWITIPRKAGSLKQRTISPAESTALLQGRKSREKHHNILPKTLAN
DKHSHKPHPVETSQPSDKTVLDTSYALIGETVNNYRSTKYEMYSKNAEKPSRSKRTIKQK
QRRKFMAKPAEEQLDVGQSKDENIHTSHITQDEFQRNSDRNMEEHEEMGNDCVSKKQMPP
VGSKKSSTRKDKEESKKKRFSSESKNKLVPEEVTSTVTKSRRISRRPSDWWVVKSEESPV
YSNSSVRNELPMHHNSSRKSTKKTNQSSKNIRKKTIPLKRQKTATKGNQRVQKFLNAEGS
GGIVGHDEISRCSLSEPLESDEADLAKKKNLDCSRSTRSSKNEDNIMTAQNVPLKPQTSG
YTCNIPTESNLDSGEHKTSVLEESGPSRLNNNYLMSGKNDVDDEEVHGSSDDSKQSKVIP
KNRIHHKLVLPSNTPNVRRTKRTRLKPLEYWRGERIDYQGRPSGGFVISGVLSPDTISSK
RKAKENIGKVNKKSNKKRICLDNDERKTNLMVNLGIPLGDPLQPTRVKDPETREIILMDL
VRPQDTYQFFVKHGELKVYKTLDTPFFSTGKLILGPQEEKGKQHVGQDILVFYVNFGDLL
CTLHETPYILSTGDSFYVPSGNYYNIKNLRNEESVLLFTQIKR
>sp|Q9HC77|CENPJ_HUMAN Centromere protein J OS=Homo sapiens GN=CENPJ PE=1 SV=2
MFLMPTSSELNSGQNFLTQWMTNPSRAGVILNRGFPILEADKEKRAAVDISTSFPIKGTH
FSDSFSFINEEDSLLEEQKLESNNPYKPQSDKSETHTAFPCIKKGPQVAACHSAPGHQEE
NKNDFIPDLASEFKEGAYKDPLFKKLEQLKEVQQKKQEQLKRQQLEQLQRLMEEQEKLLT
MVSGQCTLPGLSLLPDDQSQKHRSPGNTTTGERATCCFPSYVYPDPTQEETYPSNILSHE
QSNFCRTAHGDFVLTSKRASPNLFSEAQYQEAPVEKNNLKEENRNHPTGESILCWEKVTE
QIQEANDKNLQKHDDSSEVANIEERPIKAAIGERKQTFEDYLEEQIQLEEQELKQKQLKE
AEGPLPIKAKPKQPFLKRGEGLARFTNAKSKFQKGKESKLVTNQSTSEDQPLFKMDRQQL
QRKTALKNKELCADNPILKKDSKARTKSGSVTLSQKPKMLKCSNRKSLSPSGLKIQTGKK
CDGQFRDQIKFENKVTSNNKENVTECPKPCDTGCTGWNKTQGKDRLPLSTGPASRLAAKS
PIRETMKESESSLDVSLQKKLETWEREKEKENLELDEFLFLEQAADEISFSSNSSFVLKI
LERDQQICKGHRMSSTPVKAVPQKTNPADPISHCNRSEDLDHTAREKESECEVAPKQLHS
LSSADELREQPCKIRKAVQKSTSENQTEWNARDDEGVPNSDSSTDSEEQLDVTIKPSTED
RERGISSREDSPQVCDDKGPFKDTRTQEDKRRDVDLDLSDKDYSSDESIMESIKHKVSEP
SRSSSLSLSKMDFDDERTWTDLEENLCNHDVVLGNESTYGTPQTCYPNNEIGILDKTIKR
KIAPVKRGEDLSKSRRSRSPPTSELMMKFFPSLKPKPKSDSHLGNELKLNISQDQPPGDN
ARSQVLREKIIELETEIEKFKAENASLAKLRIERESALEKLRKEIADFEQQKAKELARIE
EFKKEEMRKLQKERKVFEKYTTAARTFPDKKEREEIQTLKQQIADLREDLKRKETKWSST
HSRLRSQIQMLVRENTDLREEIKVMERFRLDAWKRAEAIESSLEVEKKDKLANTSVRFQN
SQISSGTQVEKYKKNYLPMQGNPPRRSKSAPPRDLGNLDKGQAASPREPLEPLNFPDPEY
KEEEEDQDIQGEISHPDGKVEKVYKNGCRVILFPNGTRKEVSADGKTITVTFFNGDVKQV

MPDQRVIYYYAAAQTTHTTYPEGLEVLHFSSGQIEKHYPDGRKEITFPDQTVKNLFPDGQ
EESIFPDGTIVRVQRDGNKLIEFNNGQRELHTAQFKRREYPDGTVKTVYANGHQETKYRS
GRIRVKDKEGNVLMDTEL
>sp|Q9NSP4|CENPM_HUMAN Centromere protein M OS=Homo sapiens GN=CENPM PE=1 SV=1
MSVLRPLDKLPGLNTATILLVGTEDALLQQLADSMLKEDCASELKVHLAKSLPLPSSVNR
PRIDLIVFVVNLHSKYSLQNTEESLRHVDASFFLGKVCFLATGAGRESHCSIHRHTVVKL
AHTYQSPLLYCDLEVEGFRATMAQRLVRVLQICAGHVPGVSALNLLSLLRSSEGPSLEDL
>sp|Q13352|CENPR_HUMAN Centromere protein R OS=Homo sapiens GN=ITGB3BP PE=1 SV=2
MPVKRSLKLDGLLEENSFDPSKITRKKSVITYSPTTGTCQMSLFASPTSSEEQKHRNGLS
NEKRKKLNHPSLTESKESTTKDNDEFMMLLSKVEKLSEEIMEIMQNLSSIQALEGSRELE
NLIGISCASHFLKREMQKTKELMTKVNKQKLFEKSTGLPHKASRHLDSYEFLKAILN
>sp|Q86XR8|CEP57_HUMAN Centrosomal protein of 57 kDa OS=Homo sapiens GN=CEP57 PE=1 SV=2
MAAASVSAASGSHLSNSFAEPSRSNGSMVRHSSSPYVVYPSDKPFLNSDLRRSPSKPTLA
YPESNSRAIFSALKNLQDKIRRLELERIQAEESVKTLSRETIEYKKVLDEQIQERENSKN
EESKHNQELTSQLLAAENKCNLLEKQLEYMRNMIKHAEMERTSVLEKQVSLERERQHDQT
HVQSQLEKLDLLEQEYNKLTTMQALAEKKMQELEAKLHEEEQERKRMQAKAAELQTGLET
NRLIFEDKATPCVPNARRIKKKKSKPPEKKSSRNYFGAQPHYRLCLGDMPFVAGKSTSPS
HAVVANVQLVLHLMKQHSKALCNDRVINSIPLAKQVSSRGGKSKKLSVTPPSSNGINEEL
SEVLQTLQDEFGQMSFDHQQLAKLIQESPTVELKDKLECELEALVGRMEAKANQITKVRK
YQAQLEKQKLEKQKKELKATKKTLDEERNSSSRSGITGTTNKKDFMKLRPGEKRRKNLQL
LKDMQSIQNSLQSSSLCWDY
>sp|Q96MT8|CEP63_HUMAN Centrosomal protein of 63 kDa OS=Homo sapiens GN=CEP63 PE=1 SV=1
MEALLEGIQNRGHGGGFLTSCEAELQELMKQIDIMVAHKKSEWEGRTHALETCLKIREQE
LKSLRSQLDVTHKEVGMLHQQVEEHEKIKQEMTMEYKQELKKLHEELCILKRSYEKLQKK
QMREFRGNTKNHREDRSEIERLTAKIEEFRQKSLDWEKQRLIYQQQVSSLEAQRKALAEQ
SEIIQAQLVNRKQKLESVELSSQSEIQHLSSKLERANDTICANELEIERLTMRVNDLVGT
SMTVLQEQQQKEEKLRESEKLLEALQEEKRELKAALQSQENLIHEARIQKEKLQEKVKAT
NTQHAVEAIRPREESLAEKKYTSQGQGDLDSVLSQLNFTHTSEDLLQAEVTCLEGSLESV
SATCKQLSQELMEKYEELKRMEAHNNEYKAEIKKLKEQILQGEQSYSSALEGMKMEISHL
TQELHQRDITIASTKGSSSDMEKRLRAEMQKAEDKAVEHKEILDQLESLKLENRHLSEMV
MKLELGLHEAKEISLADLQENYIEALNKLVSENQQLQKDLMNTKSQLEISTQMCKKQNDR
IFKPTHSRTTEFKNTEFKPTHGQHRHDGIKTEHYKTDLHSPRGQASDSINPMSRVLSPLS
PQISPCSSTRSLTSYSLCKTHSLPSALDTNEANFSDTMSESMNDQEEFISSCSLPVSPLG
SIATRFLEEEELRSHHILERLDAHIEELKRESEKTVRQFTALK
>sp|Q76N32|CEP68_HUMAN Centrosomal protein of 68 kDa OS=Homo sapiens GN=CEP68 PE=1 SV=2
MALGEEKAEAEASEDTKAQSYGRGSCRERELDIPGPMSGEQPPRLEAEGGLISPVWGAEG
IPAPTCWIGTDPGGPSRAHQPQASDANREPVAERSEPALSGLPPATMGSGDLLLSGESQV
EKTKLSSSEEFPQTLSLPRTTTICSGHDADTEDDPSLADLPQALDLSQQPHSSGLSCLSQ
WKSVLSPGSAAQPSSCSISASSTGSSLQGHQERAEPRGGSLAKVSSSLEPVVPQEPSSVV
GLGPRPQWSPQPVFSGGDASGLGRRRLSFQAEYWACVLPDSLPPSPDRHSPLWNPNKEYE
DLLDYTYPLRPGPQLPKHLDSRVPADPVLQDSGVDLDSFSVSPASTLKSPTNVSPNCPPA
EATALPFSGPREPSLKQWPSRVPQKQGGMGLASWSQLASTPRAPGSRDARWERREPALRG
AKDRLTIGKHLDMGSPQLRTRDRGWPSPRPEREKRTSQSARRPTCTESRWKSEEEVESDD
EYLALPARLTQVSSLVSYLGSISTLVTLPTGDIKGQSPLEVSDSDGPASFPSSSSQSQLP

PGAALQGSGDPEGQNPCFLRSFVRAHDSAGEGSLGSSQALGVSSGLLKTRPSLPARLDRW
PFSDPDVEGQLPRKGGEQGKESLVQCVKTFCCQLEELICWLYNVADVTDHGTAARSNLTS
LKSSLQLYRQFKKDIDEHQSLTESVLQKGEILLQCLLENTPVLEDVLGRIAKQSGELESH
ADRLYDSILASLDMLAGCTLIPDKKPMAAMEHPCEGV
>sp|Q9Y592|CEP83_HUMAN Centrosomal protein of 83 kDa OS=Homo sapiens GN=CEP83 PE=1 SV=2
MDTFPNNFPPGGDSGLTGSQSEFQKMLIDERLRCEHHKANYQTLKAEHTRLQNEHVKLQN
ELKHLFNEKQTQQEKLQLLLEELRGELVEKTKDLEEMKLQILTPQKLELLRAQIQQELET
PMRERFRNLDEEVEKYRAVYNKLRYEHTFLKSEFEHQKEEYARILDEGKIKYESEIARLE
EDKEELRNQLLNVDLTKDSKRVEQLAREKVYLCQKLKGLEAEVAELKAEKENSEAQVENA
QRIQVRQLAEMQATVRSLEAEKQSANLRAERLEKELQSSSEQNTFLINKLHKAEREINTL
SSKVKELKHSNKLEITDIKLETARAKSELERERNKIQSELDGLQSDNEILKAAVEHHKVL
LVEKDRELIRKVQAAKEEGYQKLVVLQDEKLELENRLADLEKMKVEHDVWRQSEKDQYEE
KLRASQMAEEITRKELQSVRLKLQQQIVTIENAEKEKNENSDLKQQISSLQIQVTSLAQS
ENDLLNSNQMLKEMVERLKQECRNFRSQAEKAQLEAEKTLEEKQIQWLEEKHKLHERITD
REEKYNQAKEKLQRAAIAQKKRKSLHENKLKRLQEKVEVLEAKKEELETENQVLNRQNVP
FEDYTRLQKRLKDIQRRHNEFRSLILVPNMPPTASINPVSFQSSAMVPSMELPFPPHMQE
EQHQRELSLLRKRLEELETTQRKQLEELGSSGE
>sp|Q96MT4|CF195_HUMAN Uncharacterized protein encoded by LINC01600 OS=Homo sapiens GN=LINC01600 PE=2 SV=2
MIYPLDLFRNIPWKQGKCFASLSPEGERAFDGMEPLCQPGARPALRGLQHASDCYRLLSP
PGSGLVGTNPSVPAPSPHCGCCQAWSISSFTFTGPTPFKINSDQATPCHLRSPETIFRQR
EISNEAF
>sp|Q9H8X3|CF208_HUMAN Putative uncharacterized protein LINC00574 OS=Homo sapiens GN=LINC00574 PE=5 SV=1
MSLCSACTSPASHQLLLNCQGQTVAKSHSSADAGVSLVSGRWCAWWPEHCSESMFPSQHP
VLSSNLADSSGQGRSPAGAHPALCPFHKSPWFPHFPQILPREWSWCGPERPAGCSLGLKA
EAALVGKK
>sp|Q5I0X4|CF226_HUMAN Uncharacterized protein C6orf226 OS=Homo sapiens GN=C6orf226 PE=1 SV=1
MERPRSPQCSAPASASASVTLAQLLQLVQQGQELPGLEKRHIAAIHGEPTASRLPRRPKP
WEAAALAESLPPPTLRIGTAPAEPGLVEAATAPSSWHTVGP
>sp|Q6ZU64|CFA65_HUMAN Cilia- and flagella-associated protein 65 OS=Homo sapiens GN=CFAP65 PE=1 SV=2
MFTLTGCRLVEKTQKVENPSVSFASSFPLIPLLLRGKSVQKKQAESKSQIKLHTQSAPFG
LCPKDMMLTQAPSSVVRSRNSRNHTVNSGGSCLSASTVAIPAINDSSAAMSACSTISAQP
ASSMDTQMHSPKKQERVNKRVIWGIEVAEELHWKGWELGKETTRNLVLKNRSLKLQKMKY
RPPKTKFFFTVIPQPIFLSPGITLTLPIVFRPLEAKEYMDQLWFEKAEGMFCVGLRATLP
CHRLICRPPSLQLPMCAVGDTTEAFFCLDNVGDLPTFFTWEFSSPFQMLPATGLLEPGQA
SQIKVTFQPLTAVIYEVQATCWYGAGSRQRSSIQLQAVAKCAQLLVSIKHKCPEDQDAEG
FQKLLYFGSVAVGCTSERQIRLHNPSAVNAPFRIEISPDELAEDQAFSCPTAHGIVLPGE
KKCVSVFFHPKTLDTRTVDYCSIMPSGCASKTLLKVVGFCRGPAVSLQHYCVNFSWVNLG
ERSEQPLWIENQSDCTAHFQFAIDCLESVFTIRPAFGTLVGKARMTLHCAFQPTHPIICF
RRVACLIHHQDPLFLDLMGTCHSDSTKPAILKPQHLTWYRTHLARGLTLYPPDILDAMLK
EKKLAQDQNGALMIPIQDLEDMPAPQYPYIPPMTEFFFDGTSDITIFPPPISVEPVEVDF

GACPGPEAPNPVPLCLMNHTKGKIMVVWTRRSDCPFWVTPESCDVPPLKSMAMRLHFQPP
HPNCLYTVELEAFAIYKVLQSYSNIEEDCTMCPSWCLTVRARGHSYFAGFEHHIPQYSLD
VPKLFPAVSSGEPTYRSLLLVNKDCKLLTFSLAPQRGSDVILRPTSGLVAPGAHQIILIC
TYPEGSSWKQHTFYLQCNASPQYLKEVSMYSREEPLQLKLDTHKSLYFKPTWVGCSSTSP
FTFRNPSRLPLQFEWRVSEQHRKLLAVQPSRGLIQPNERLTLTWTFSPLEETKYLFQVGM
WVWEAGLSPNANPAATTHYMLRLVGVGLTSSLSAKEKELAFGNVLVNSKQSRFLVLLNDG
NCTLYYRLYLEQGSPEAVDNHPLALQLDRTEGSMPPRSQDTICLTACPKQRSQYSWTITY
SLLSHRDNKAGEKQELCCVSLVAVYPLLSILDVSSMGSAEGITRKHLWRLFSLDLLNSYL
ERDPTPCELTYKVPTRHSMSQIPPVLTPLRLDFNFGAAPFKAPPSVVFLALKNSGVVSLD
WAFLLPSDQRIDVELWAEQAELNSTELHQMRVQDNCLFSISPKAGSLSPGQEQMVELKYS
HLFIGTDHLPVLFKVSHGREILLNFIGVTVKPEQKYVHFTSTTHQFIPIPIGDTLPPRQI
YELYNGGSVPVTYEVQTDVLSQVQEKNFDHPIFCCLNPKGEIQPGSTARVLWIFSPIEAK
TYTVDVPIHILGWNSALIHFQGVGYNPHMMGDTAPFHNISSWDNSSIHSRLVVPGQNVFL
SQSHISLGNIPVQSKCSRLLFLNNISKNEEIAFSWQPSPLDFGEVSVSPMIGVVAPEETV
PFVVTLRASVHASFYSADLVCKLYSQQLMRQYHKELQEWKDEKVRQEVEFTITDMKVKKR
TCCTACEPARKYKTLPPIKNQQSVSRPASWKLQTPKEEVSWPCPQPPSPGMLCLGLTARA
HATDYFLANFFSEFPCHFLHRELPKRKAPREESETSEEKSPNKWGPVSKQKKQLLVDILT
TIIRGLLEDKNFHEAVDQSLVEQVPYFRQFWNEQSTKFMDQKNSLYLMPILPVPSSSWED
GKGKQPKEDRPEHYPGLGKKEEGEEEKGEEEEEELEEEEEEEEETEEEELGKEEIEEKEE
ERDEKEEKVSWAGIGPTPQPESQESMQWQWQQQLNVMVKEEQEQDEKEAIRRLPAFANLQ
EALLENMIQNILVEASRGEVVLTSRPRVIALPPFCVPRSLTPDTLLPTQQAEVLHPVVPL
PTDLP
>sp|D6REC4|CFA99_HUMAN Cilia- and flagella-associated protein 99 OS=Homo sapiens GN=CFAP99 PE=3 SV=1
MPRSCRERVQGSKQQLRLQFPPRIRKTPKLTFYRPDNILVKLNTTAILREGALYQRQVEQ
ELQRVDKLVDGAGDFSEFFEWQKKMQAKDREEQLAASECRRLQGKLSHEEAVLARQSLMQ
ENKQRVEQQKEQMAKLMLQRAERRLREDRSRKELVEQVIEGQKNAKAAQTKLAKGRQQTV
QEAIEESRGLLQRRAQAAQEEQRRRCELISQLRALETQPTRKGKLVDLTQIPGYGLEGEM
SIVELRERLALLKENQRRKEEEKRDQIIQGKHTKSQELQNMVEQISLCRAAMGRSAALRW
EEKKALAAAPAAPSQDERVQQLRRRISERAAERSRQAALLHVSAPRTARPKPRVSPDWWE
EPGRLKAGAGWGWRARRAGTGVPGRGWRGDRVRSAAGRYAAAGAGGGGGVPARADAFPGL
QAQLEAQHWLELERSRERRLQALQQGGSGPGPARRLEAA
>sp|P08603|CFAH_HUMAN Complement factor H OS=Homo sapiens GN=CFH PE=1 SV=4
MRLLAKIICLMLWAICVAEDCNELPPRRNTEILTGSWSDQTYPEGTQAIYKCRPGYRSLG
NVIMVCRKGEWVALNPLRKCQKRPCGHPGDTPFGTFTLTGGNVFEYGVKAVYTCNEGYQL
LGEINYRECDTDGWTNDIPICEVVKCLPVTAPENGKIVSSAMEPDREYHFGQAVRFVCNS
GYKIEGDEEMHCSDDGFWSKEKPKCVEISCKSPDVINGSPISQKIIYKENERFQYKCNMG
YEYSERGDAVCTESGWRPLPSCEEKSCDNPYIPNGDYSPLRIKHRTGDEITYQCRNGFYP
ATRGNTAKCTSTGWIPAPRCTLKPCDYPDIKHGGLYHENMRRPYFPVAVGKYYSYYCDEH
FETPSGSYWDHIHCTQDGWSPAVPCLRKCYFPYLENGYNQNYGRKFVQGKSIDVACHPGY
ALPKAQTTVTCMENGWSPTPRCIRVKTCSKSSIDIENGFISESQYTYALKEKAKYQCKLG
YVTADGETSGSITCGKDGWSAQPTCIKSCDIPVFMNARTKNDFTWFKLNDTLDYECHDGY
ESNTGSTTGSIVCGYNGWSDLPICYERECELPKIDVHLVPDRKKDQYKVGEVLKFSCKPG
FTIVGPNSVQCYHFGLSPDLPICKEQVQSCGPPPELLNGNVKEKTKEEYGHSEVVEYYCN

PRFLMKGPNKIQCVDGEWTTLPVCIVEESTCGDIPELEHGWAQLSSPPYYYGDSVEFNCS
ESFTMIGHRSITCIHGVWTQLPQCVAIDKLKKCKSSNLIILEEHLKNKKEFDHNSNIRYR
CRGKEGWIHTVCINGRWDPEVNCSMAQIQLCPPPPQIPNSHNMTTTLNYRDGEKVSVLCQ
ENYLIQEGEEITCKDGRWQSIPLCVEKIPCSQPPQIEHGTINSSRSSQESYAHGTKLSYT
CEGGFRISEENETTCYMGKWSSPPQCEGLPCKSPPEISHGVVAHMSDSYQYGEEVTYKCF
EGFGIDGPAIAKCLGEKWSHPPSCIKTDCLSLPSFENAIPMGEKKDVYKAGEQVTYTCAT
YYKMDGASNVTCINSRWTGRPTCRDTSCVNPPTVQNAYIVSRQMSKYPSGERVRYQCRSP
YEMFGDEEVMCLNGNWTEPPQCKDSTGKCGPPPPIDNGDITSFPLSVYAPASSVEYQCQN
LYQLEGNKRITCRNGQWSEPPKCLHPCVISREIMENYNIALRWTAKQKLYSRTGESVEFV
CKRGYRLSSRSHTLRTTCWDGKLEYPTCAKR
>sp|Q9UEE9|CFDP1_HUMAN Craniofacial development protein 1 OS=Homo sapiens GN=CFDP1 PE=1 SV=1
MEEFDSEDFSTSEEDEDYVPSGGEYSEDDVNELVKEDEVDGEEQTQKTQGKKRKAQSIPA
RKRRQGGLSLEEEEEEDANSESEGSSSEEEDDAAEQEKGIGSEDARKKKEDELWASFLND
VGPKSKVPPSTQVKKGEETEETSSSKLLVKAEELEKPKETEKVKITKVFDFAGEEVRVTK
EVDATSKEAKSFFKQNEKEKPQANVPSALPSLPAGSGLKRSSGMSSLLGKIGAKKQKMST
LEKSKLDWESFKEEEGIGEELAIHNRGKEGYIERKAFLDRVDHRQFEIERDLRLSKMKP
>sp|Q96N11|CG026_HUMAN Uncharacterized protein C7orf26 OS=Homo sapiens GN=C7orf26 PE=2 SV=1
MSDIRHSLLRRDALSAAKEVLYHLDIYFSSQLQSAPLPIVDKGPVELLEEFVFQVPKERS
AQPKRLNSLQELQLLEIMCNYFQEQTKDSVRQIIFSSLFSPQGNKADDSRMSLLGKLVSM
AVAVCRIPVLECAASWLQRTPVVYCVRLAKALVDDYCCLVPGSIQTLKQIFSASPRFCCQ
FITSVTALYDLSSDDLIPPMDLLEMIVTWIFEDPRLILITFLNTPIAANLPIGFLELTPL
VGLIRWCVKAPLAYKRKKKPPLSNGHVSNKVTKDPGVGMDRDSHLLYSKLHLSVLQVLMT
LQLHLTEKNLYGRLGLILFDHMVPLVEEINRLADELNPLNASQEIELSLDRLAQALQVAM
ASGALLCTRDDLRTLCSRLPHNNLLQLVISGPVQQSPHAALPPGFYPHIHTPPLGYGAVP
AHPAAHPALPTHPGHTFISGVTFPFRPIR
>sp|P0DN87|CGB7_HUMAN Choriogonadotropin subunit beta 7 OS=Homo sapiens GN=CGB7 PE=2 SV=1
MEMFQGLLLLLLLSMGGTWASREMLRPRCRPINATLAVEKEGCPVCITVNTTICAGYCPT
MTRVLQGVLPALPQVVCNYRDVRFESIRLPGCPRGVNPVVSYAVALSCQCALCRRSTTDC
GGPKDHPLTCDDPRFQASSSSKAPPPSLPSPSRLPGPSDTPILPQ
>sp|Q8N9K7|CH079_HUMAN Uncharacterized protein KIAA1456 isoform OS=Homo sapiens GN=KIAA1456 PE=2 SV=1
MQKQLLPLAAGQRCDYSQRESCCQSPHRAESAGDRSTDNGLHRGAERSFCCALDPLPRRP
PAGRRGRDRSSSACPGARARVWPPGACASFRDSHKRFIMRRTALFHFTPTSFHLRYTLRK
L
>sp|Q8WYQ3|CHC10_HUMAN Coiled-coil-helix-coiled-coil-helix domain-containing protein 10, mitochondrial OS=Homo sapiens GN=CHCHD10 PE=1 SV=1
MPRGSRSAASRPASRPAAPSAHPPAHPPPSAAAPAPAPSGQPGLMAQMATTAAGVAVGSA
VGHVMGSALTGAFSGGSSEPSQPAVQQAPTPAAPQPLQMGPCAYEIRQFLDCSTTQSDLS
LCEGFSEALKQCKYYHGLSSLP
>sp|Q9BUK0|CHCH7_HUMAN Coiled-coil-helix-coiled-coil-helix domain-containing protein 7 OS=Homo sapiens GN=CHCHD7 PE=1 SV=1
MPSVTQRLRDPDINPCLSESDASTRCLDENNYDRERCSTYFLRYKNCRRFWNSIVMQRRK

NGVKPFMPTAAERDEILRAVGNMPY
>sp|Q14839|CHD4_HUMAN Chromodomain-helicase-DNA-binding protein 4 OS=Homo sapiens GN=CHD4 PE=1 SV=2
MASGLGSPSPCSAGSEEEDMDALLNNSLPPPHPENEEDPEEDLSETETPKLKKKKKPKKP
RDPKIPKSKRQKKERMLLCRQLGDSSGEGPEFVEEEEEVALRSDSEGSDYTPGKKKKKKL
GPKKEKKSKSKRKEEEEEEEDDDDDSKEPKSSAQLLEDWGMEDIDHVFSEEDYRTLTNYKA
FSQFVRPLIAAKNPKIAVSKMMMVLGAKWREFSTNNPFKGSSGASVAAAAAAAVAVVESM
VTATEVAPPPPPVEVPIRKAKTKEGKGPNARRKPKGSPRVPDAKKPKPKKVAPLKIKLGG
FGSKRKRSSSEDDDLDVESDFDDASINSYSVSDGSTSRSSRSRKKLRTTKKKKKGEEEVT
AVDGYETDHQDYCEVCQQGGEIILCDTCPRAYHMVCLDPDMEKAPEGKWSCPHCEKEGIQ
WEAKEDNSEGEEILEEVGGDLEEEDDHHMEFCRVCKDGGELLCCDTCPSSYHIHCLNPPL
PEIPNGEWLCPRCTCPALKGKVQKILIWKWGQPPSPTPVPRPPDADPNTPSPKPLEGRPE
RQFFVKWQGMSYWHCSWVSELQLELHCQVMFRNYQRKNDMDEPPSGDFGGDEEKSRKRKN
KDPKFAEMEERFYRYGIKPEWMMIHRILNHSVDKKGHVHYLIKWRDLPYDQASWESEDVE
IQDYDLFKQSYWNHRELMRGEEGRPGKKLKKVKLRKLERPPETPTVDPTVKYERQPEYLD
ATGGTLHPYQMEGLNWLRFSWAQGTDTILADEMGLGKTVQTAVFLYSLYKEGHSKGPFLV
SAPLSTIINWEREFEMWAPDMYVVTYVGDKDSRAIIRENEFSFEDNAIRGGKKASRMKKE
ASVKFHVLLTSYELITIDMAILGSIDWACLIVDEAHRLKNNQSKFFRVLNGYSLQHKLLL
TGTPLQNNLEELFHLLNFLTPERFHNLEGFLEEFADIAKEDQIKKLHDMLGPHMLRRLKA
DVFKNMPSKTELIVRVELSPMQKKYYKYILTRNFEALNARGGGNQVSLLNVVMDLKKCCN
HPYLFPVAAMEAPKMPNGMYDGSALIRASGKLLLLQKMLKNLKEGGHRVLIFSQMTKMLD
LLEDFLEHEGYKYERIDGGITGNMRQEAIDRFNAPGAQQFCFLLSTRAGGLGINLATADT
VIIYDSDWNPHNDIQAFSRAHRIGQNKKVMIYRFVTRASVEERITQVAKKKMMLTHLVVR
PGLGSKTGSMSKQELDDILKFGTEELFKDEATDGGGDNKEGEDSSVIHYDDKAIERLLDR
NQDETEDTELQGMNEYLSSFKVAQYVVREEEMGEEEEVEREIIKQEESVDPDYWEKLLRH
HYEQQQEDLARNLGKGKRIRKQVNYNDGSQEDRDWQDDQSDNQSDYSVASEEGDEDFDER
SEAPRRPSRKGLRNDKDKPLPPLLARVGGNIEVLGFNARQRKAFLNAIMRYGMPPQDAFT
TQWLVRDLRGKSEKEFKAYVSLFMRHLCEPGADGAETFADGVPREGLSRQHVLTRIGVMS
LIRKKVQEFEHVNGRWSMPELAEVEENKKMSQPGSPSPKTPTPSTPGDTQPNTPAPVPPA
EDGIKIEENSLKEEESIEGEKEVKSTAPETAIECTQAPAPASEDEKVVVEPPEGEEKVEK
AEVKERTEEPMETEPKGAADVEKVEEKSAIDLTPIVVEDKEEKKEEEEKKEVMLQNGETP
KDLNDEKQKKNIKQRFMFNIADGGFTELHSLWQNEERAATVTKKTYEIWHRRHDYWLLAG
IINHGYARWQDIQNDPRYAILNEPFKGEMNRGNFLEIKNKFLARRFKLLEQALVIEEQLR
RAAYLNMSEDPSHPSMALNTRFAEVECLAESHQHLSKESMAGNKPANAVLHKVLKQLEEL
LSDMKADVTRLPATIARIPPVAVRLQMSERNILSRLANRAPEPTPQQVAQQQ
>sp|Q96EP1|CHFR_HUMAN E3 ubiquitin-protein ligase CHFR OS=Homo sapiens GN=CHFR PE=1 SV=2
MERPEEGKQSPPPQPWGRLLRLGAEEGEPHVLLRKREWTIGRRRGCDLSFPSNKLVSGDH
CRIVVDEKSGQVTLEDTSTSGTVINKLKVVKKQTCPLQTGDVIYLVYRKNEPEHNVAYLY
ESLSEKQGMTQESFEANKENVFHGTKDTSGAGAGRGADPRVPPSSPATQVCFEEPQPSTS
TSDLFPTASASSTEPSPAGRERSSSCGSGGGGISPKGSGPSVASDEVSSFASALPDRKTA
SFSSLEPQDQEDLEPVKKKMRGDGDLDLNGQLLVAQPRRNAQTVHEDVRAAAGKPDKMEE
TLTCIICQDLLHDCVSLQPCMHTFCAACYSGWMERSSLCPTCRCPVERICKNHILNNLVE
AYLIQHPDKSRSEEDVQSMDARNKITQDMLQPKVRRSFSDEEGSSEDLLELSDVDSESSD
ISQPYVVCRQCPEYRRQAAQPPHCPAPEGEPGAPQALGDAPSTSVSLTTAVQDYVCPLQG

SHALCTCCFQPMPDRRAEREQDPRVAPQQCAVCLQPFCHLYWGCTRTGCYGCLAPFCELN
LGDKCLDGVLNNNSYESDILKNYLATRGLTWKNMLTESLVALQRGVFLLSDYRVTGDTVL
CYCCGLRSFRELTYQYRQNIPASELPVAVTSRPDCYWGRNCRTQVKAHHAMKFNHICEQT
RFKN
>sp|Q9BZP6|CHIA_HUMAN Acidic mammalian chitinase OS=Homo sapiens GN=CHIA PE=1 SV=1
MTKLILLTGLVLILNLQLGSAYQLTCYFTNWAQYRPGLGRFMPDNIDPCLCTHLIYAFAG
RQNNEITTIEWNDVTLYQAFNGLKNKNSQLKTLLAIGGWNFGTAPFTAMVSTPENRQTFI
TSVIKFLRQYEFDGLDFDWEYPGSRGSPPQDKHLFTVLVQEMREAFEQEAKQINKPRLMV
TAAVAAGISNIQSGYEIPQLSQYLDYIHVMTYDLHGSWEGYTGENSPLYKYPTDTGSNAY
LNVDYVMNYWKDNGAPAEKLIVGFPTYGHNFILSNPSNTGIGAPTSGAGPAGPYAKESGI
WAYYEICTFLKNGATQGWDAPQEVPYAYQGNVWVGYDNIKSFDIKAQWLKHNKFGGAMVW
AIDLDDFTGTFCNQGKFPLISTLKKALGLQSASCTAPAQPIEPITAAPSGSGNGSGSSSS
GGSSGGSGFCAVRANGLYPVANNRNAFWHCVNGVTYQQNCQAGLVFDTSCDCCNWA
>sp|Q9Y259|CHKB_HUMAN Choline/ethanolamine kinase OS=Homo sapiens GN=CHKB PE=1 SV=3
MAAEATAVAGSGAVGGCLAKDGLQQSKCPDTTPKRRRASSLSRDAERRAYQWCREYLGGA
WRRVQPEELRVYPVSGGLSNLLFRCSLPDHLPSVGEEPREVLLRLYGAILQGVDSLVLES
VMFAILAERSLGPQLYGVFPEGRLEQYIPSRPLKTQELREPVLSAAIATKMAQFHGMEMP
FTKEPHWLFGTMERYLKQIQDLPPTGLPEMNLLEMYSLKDEMGNLRKLLESTPSPVVFCH
NDIQEGNILLLSEPENADSLMLVDFEYSSYNYRGFDIGNHFCEWVYDYTHEEWPFYKARP
TDYPTQEQQLHFIRHYLAEAKKGETLSQEEQRKLEEDLLVEVSRYALASHFFWGLWSILQ
ASMSTIEFGYLDYAQSRFQFYFQQKGQLTSVHSSS
>sp|P59074|CHM4P_HUMAN Putative charged multivesicular body protein 4B-like protein CHMP4BP1 OS=Homo sapiens GN=CHMP4BP1 PE=5 SV=1
MLSKKQEFLEKKIEQRHGTKNKPAALQALKRKKRYEKQLAQIDGTLSTIEFQQQALENAN
TNTEVLKNMGSAAKAKKAAHDNMDIDKVDELMQDIADQQELGEEISTAISKPVGFGEKSD
EDELMAELEELEQEEPDKNLLEVSGPETVPLPNVPSIALPSKPAKKRKTTT
>sp|Q9NRB3|CHSTC_HUMAN Carbohydrate sulfotransferase 12 OS=Homo sapiens GN=CHST12 PE=2 SV=2
MTKARLFRLWLVLGSVFMILLIIVYWDSAGAAHFYLHTSFSRPHTGPPLPTPGPDRDREL
TADSDVDEFLDKFLSAGVKQSDLPRKETEQPPAPGSMEESVRGYDWSPRDARRSPDQGRQ
QAERRSVLRGFCANSSLAFPTKERAFDDIPNSELSHLIVDDRHGAIYCYVPKVACTNWKR
VMIVLSGSLLHRGAPYRDPLRIPREHVHNASAHLTFNKFWRRYGKLSRHLMKVKLKKYTK
FLFVRDPFVRLISAFRSKFELENEEFYRKFAVPMLRLYANHTSLPASAREAFRAGLKVSF
ANFIQYLLDPHTEKLAPFNEHWRQVYRLCHPCQIDYDFVGKLETLDEDAAQLLQLLQVDR
QLRFPPSYRNRTASSWEEDWFAKIPLAWRQQLYKLYEADFVLFGYPKPENLLRD
>sp|Q5SWW7|CJ055_HUMAN Uncharacterized protein C10orf55 OS=Homo sapiens GN=C10orf55 PE=2 SV=1
MFLHLDSHSSLERTKPTVVGVDTHMELDEVHCCPGRDSGGGFIKGPMLQGLQGEGKLAPI
PKPTLPSPSRLTLFVSSSQMEDHGFPARRNGLTQASFIYQMPAGWGSPGGLFLPCQPVPT
PVVLKPPLPPCPISWGESGPAVDGIRRTPAP
>sp|Q711Q0|CJ071_HUMAN Uncharacterized protein C10orf71 OS=Homo sapiens GN=C10orf71 PE=1 SV=2
MMQGNKKCTDAFSDSSSIGSVLDDADREVSSLTDRAFRSLCISEDTSFHDSYLAVSPDIT
RQVFGTFHQRTVGHTQRKSGIWSQLPSQGTEHSGWAATFQQLPKYVQGEEKYPKTSPPPT

PVQRRLEVPVSGLRSSNKPVSKVSTLIKSFDRTESQRCESRPTASKPPALKNPPKFAPLP
ENSVNFCFDSAFLTVRRVPAEVSNTHQNSYQPGRKHGEQESSKNPEMACHGSSSFLPAAN
DTATLCESKFPSPHHKPVTGEPGRGKGTFLHSENSAFESWNAHQPKLLERKDTAGTVPES
KAPKHYGDTTLLREPCPPERTVSPCQVQASCSQEENRLAAGALSTSIPWGCRDPGAQVFA
VEGKAPSSQPDSQEKPAQPPWRKPKTGKKGKESLQDTLEEKTQTNQRGPPLYTKHNPQEQ
FSENNALDLPVEPNEHYDPPFNISKLLTPIIPSKHALDSADSQPAERTPSPPGQLNGYQE
KEPSECQSRDSYKSKAPSLLFNLKDVRKRVKSTYSSSPLLKVLDEKTRGKVDGKQEPVSN
GVILPNGLEESPPNELSKERPADDPTASHINPQKDPTADPSEPSADSYLTLSTAPTIAKA
PFYVNGEAAERSSYENKEVEGELEMGPAGSSWCPDSREHRPRKHLSLRLCNRDPEPGGAT
EKMKTHQLENGLSRSVSQETEPEREAGLQNTHLNQKFFPGPLSPEEEDVFYSDSQSDFMP
SLKGKAKFSTSSSDQSFASFDDQQKMWFTENQREDRRKDVSAGDSQKDEKENVMRKDELQ
YCALSNGHACLENRSQGEALQRERESVSGGRTRKASAEEANFRGSWIGENKGTTFSQAKD
LTPSPSSASNRHMLFTIKDNTLRATPVIKPIMLPLLRTMSLEDSLSSGHKEEELPRPEWG
EDPGFCAPENQDILGTSTPTNTRGTRVKCMANEVMEDPGQGSSMARMEASQPAPKGNFPS
MPLVGEGDRVKAPPDAAPGLVASNCKSGSADSGKLAAPWHIPTIALPEGDIEDQPPPWQP
ENCWEEQTPGFKSHFLSTPRAGPPGRRLVPSERANSPNPGSPGESSACSPAASNIWEESS
QAPGGPELLPEEPNQASPWASSSPARVTRREDLTHALVWEGGSDPLLELSAEDLRTLSPR
GSLLDVATSPAGTSGRLELPAQLERTASKPPAVPPKTEKALRRAKKLASKRRKTDQAQEK
HGESQEGKPCPEDLEQTQQRPLCPRERPRHNFPVVRSLPPPVHRHSVSGFSEPVGRRPGG
PQSLTPLPAYPATQKVLQDPQSGEYFVFDLPLQVKIKTFYDPETGKYVKVSIPSSEGASP
EPPPPDALAAPYVLYPGFQPVPVTALMPLRCSSQLSAPTFLRQGPRASAARARTQSVHES
GLQLDPGPHGDCTPHSAGQRPHGPPQSPGEEGVEAPGLGIISTDDLEDFATEGIS
>sp|Q8WW14|CJ082_HUMAN Uncharacterized protein C10orf82 OS=Homo sapiens GN=C10orf82 PE=2 SV=2
MEPSKTFMRNLPITPGYSGFVPFLSCQGMSKEDDMNHCVKTFQEKTQRYKEQLRELCCAV
ATAPKLKPVNSEETVLQALHQYNLQYHPLILECKYVKKPLQEPPIPGWAGYLPRAKVTEF
GCGTRYTVMAKNCYKDFLEITERAKKAHLKPYEEIYGVSSTKTSAPSPKVLQHEELLPKY
PDFSIPDGSCPALGRPLREDPKTPLTCGCAQRPSIPCSGKMYLEPLSSAK
>sp|Q5VZT2|CJ113_HUMAN Putative uncharacterized protein C10orf113 OS=Homo sapiens GN=C10orf113 PE=2 SV=2
MAKSERRIYSMESMAPEISEDIGCPLAYMRESVCALLINQSFFSCVAEMYIPDMNRPGKA
ERRAEKGLRPPRKLVGETRRLQSWPTLARWLRRVSFSLYKPPIQAAPEPDPGNWAKSPRL
SLGPEGITKGKHGFKFQGIKEKFNVSKKVLKMTFL
>sp|Q8N4M7|CJ126_HUMAN Putative uncharacterized protein C10orf126 OS=Homo sapiens GN=C10orf126 PE=2 SV=2
MNHCIQFSPQSLQRWLILPCYDLKLPIWANTTEFCPHGPRRASQDPQLLAWLPDQSLEVS
LELYDWNSMTFTLFLETVEPVAVESEGSGIFSFVWQQLIFPAEARWCFSWAQDCGLDGSF
PGSAHTEPFGKAAAGQGSVAGKEAKKAGPGFHRQLLYLQFQKRCLFNYPELL
>sp|B7Z368|CJ142_HUMAN Uncharacterized protein C10orf142 OS=Homo sapiens GN=C10orf142 PE=2 SV=1
MRMYSSDAHERPPSPSLGTTPPHPLPPTGSPRPRQDSAAGNSEEREPRGLRRASGVGSSC
KRPTVCMGRQQGLPFCTVCGYRCSSPERTRGRCAVGKVRVAGGGGAPGGGAGMRCCGCRE
RNINKELELF

>sp|Q9P2W6|CK021_HUMAN Uncharacterized protein C11orf21 OS=Homo sapiens GN=C11orf21 PE=2 SV=1
MGRTWCGMWRRRRPGRRSAVPRWPHLSSQSGVEPPDRWTGTPGWPSRDQEAPGSMMPPAA
AQPSAHGALVPPATAHEPVDHPALHWLACCCCLSLPGQLPLAIRLGWDLDLEAGPSSGKL
CPRARRWQPLPS
>sp|Q96A22|CK052_HUMAN Uncharacterized protein C11orf52 OS=Homo sapiens GN=C11orf52 PE=1 SV=2
MGNRVCCGGSWSCPSTFQKKKKTGSQTRRTLKPQPQQLQQNLPKGHETTGHTYERVLQQQ
GSQERSPGLMSEDSNLHYADIQVCSRPHAREVKHVHLENATEYATLRFPQATPRYDSKNG
TLV
>sp|Q495D7|CL036_HUMAN Putative uncharacterized protein encoded by LINC01559 OS=Homo sapiens GN=LINC01559 PE=2 SV=1
MERFLNSKARRLGSCSHPAFYLLCVPDEDTSCSTIYLPLKRRADPDQLFSDLLGGTQRLW
SNRFGNEESFPGRVRALVDTFCWIARAPPLGNPLRLEERIAWRIQRLKSGQTALIEKKKQ
KIEEDVRHQCQQTTCDRC
>sp|Q86WS4|CL040_HUMAN Uncharacterized protein C12orf40 OS=Homo sapiens GN=C12orf40 PE=1 SV=3
MNWVGGSRSRVLIKQERRKQKEYFEKHRLKSKMKSLGVLSPVKNSAVSLDILNLYMVNQI
SCKKKIPETVRKPTHVNMNRDIKMPLRKHNLELTMSPHCVPSKLCLDDTETNVNCQRLSS
KEDLGPVQSQGMDSYSMLHPQFSKIENCSFTPSSFSVELPSNRHISKLNFTSGIAPTPQK
LAYEKKQNDQRSTVNCSDSLLSKLNKSQDVFSPSHKTTRFGTLFERLNSLGNRNLLTKSP
AVIMDEDCRSTDEIRQSDYITEKHSIQHIWGKNGKEVSNFLEDVNQSTPNLLSENCDSFV
SQNMINVLNIDEQRIKKTFNKCDYDSMGDTCVVTSSDKNHVTDRCIRNIFTVPELTFSNS
TLNKTSYPEKCQPNKKYQREYNKNERNDLSTSFENDYYPSSSERKEKFENDYQEKTPQKS
IQKYPANSMGNIPSEELHSKQSWDFGLDEILMEEGGIYSLKSKRISTKKISLDSAQSSRS
TSYSPRPTDSCFSSSSDLPSEDEDQISQQIEDSNRMTIKTKEKMNNFYVERMAKLSGDRI
VKNDDKIHKQNENFYQFSVKNNTDQFPQLQCNSAHILQNKTNDNCVLQAARCDAGIQTES
ESVMEEKLDVAIQCDLISKCTCRSDVSLCNLERCSGNIKADTTGGQEIHKNN
>sp|Q9H3J6|CL065_HUMAN Probable peptide chain release factor C12orf65, mitochondrial OS=Homo sapiens GN=C12orf65 PE=2 SV=1
MSTVGLFHFPTPLTRICPAPWGLRLWEKLTLLSPGIAVTPVQMAGKKDYPALLSLDENEL
EEQFVKGHGPGGQATNKTSNCVVLKHIPSGIVVKCHQTRSVDQNRKLARKILQEKVDVFY
NGENSPVHKEKREAAKKKQERKKRAKETLEKKKLLKELWESSKKVH
>sp|Q96M19|CL067_HUMAN Putative transmembrane protein encoded by LINC00477 OS=Homo sapiens GN=LINC00477 PE=5 SV=1
MLPFFSNTTSKSVSVSSFQGSPATPLSFLFFFFLCRAGSSMTGCFTFFLDFIFFFAGVLG
PSPMGMYSGASTLTGFFLLRFLGQLSMDLEGLEWLGRASPSWWIFFSSSPSHRVPWGSCA
SASAPRLPVPHPPSPLSKCPQHPRPRRTKGPGLRKLWGPGPPFFPS
>sp|Q8TDQ1|CLM1_HUMAN CMRF35-like molecule 1 OS=Homo sapiens GN=CD300LF PE=1 SV=3
MPLLTLYLLLFWLSGYSIVTQITGPTTVNGLERGSLTVQCVYRSGWETYLKWWCRGAIWR
DCKILVKTSGSEQEVKRDRVSIKDNQKNRTFTVTMEDLMKTDADTYWCGIEKTGNDLGVT
VQVTIDPAPVTQEETSSSPTLTGHHLDNRHKLLKLSVLLPLIFTILLLLLVAASLLAWRM
MKYQQKAAGMSPEQVLQPLEGDLCYADLTLQLAGTSPQKATTKLSSAQVDQVEVEYVTMA
SLPKEDISYASLTLGAEDQEPTYCNMGHLSSHLPGRGPEEPTEYSTISRP

>sp|Q92989|CLP1_HUMAN Polyribonucleotide 5'-hydroxyl-kinase Clp1 OS=Homo sapiens GN=CLP1 PE=1 SV=1
MGEEANDDKKPTTKFELERETELRFEVEASQSVQLELLTGMAEIFGTELTRNKKFTFDAG
AKVAVFTWHGCSVQLSGRTEVAYVSKDTPMLLYLNTHTALEQMRRQAEKEEERGPRVMVV
GPTDVGKSTVCRLLLNYAVRLGRRPTYVELDVGQGSVSIPGTMGALYIERPADVEEGFSI
QAPLVYHFGSTTPGTNIKLYNKITSRLADVFNQRCEVNRRASVSGCVINTCGWVKGSGYQ
ALVHAASAFEVDVVVVLDQERLYNELKRDLPHFVRTVLLPKSGGVVERSKDFRRECRDER
IREYFYGFRGCFYPHAFNVKFSDVKIYKVGAPTIPDSCLPLGMSQEDNQLKLVPVTPGRD
MVHHLLSVSTAEGTEENLSETSVAGFIVVTSVDLEHQVFTVLSPAPRPLPKNFLLIMDIR
FMDLK
>sp|Q8NCR9|CLRN3_HUMAN Clarin-3 OS=Homo sapiens GN=CLRN3 PE=2 SV=1
MPTTKKTLMFLSSFFTSLGSFIVICSILGTQAWITSTIAVRDSASNGSIFITYGLFRGES
SEELSHGLAEPKKKFAVLEILNNSSQKTLHSVTILFLVLSLITSLLSSGFTFYNSISNPY
QTFLGPTGVYTWNGLGASFVFVTMILFVANTQSNQLSEELFQMLYPATTSKGTTHSYGYS
FWLILLVILLNIVTVTIIIFYQKARYQRKQEQRKPMEYAPRDGILF
>sp|Q5K130|CLU1O_HUMAN Putative chronic lymphocytic leukemia up-regulated protein 1 opposite strand transcript protein OS=Homo sapiens GN=CLLU1OS PE=5 SV=1
MNKLGHNELKECLKTATDSLQTVQPSISQTCTSYGPALGAPLPGRNEVALLTSLPPNYEI
SEGKPRAISAYVRAGKGNVTRRRKKTHLGNDDGKKEAQEKM
>sp|Q96AJ1|CLUA1_HUMAN Clusterin-associated protein 1 OS=Homo sapiens GN=CLUAP1 PE=1 SV=4
MSFRDLRNFTEMMRALGYPRHISMENFRTPNFGLVSEVLLWLVKRYEPQTDIPPDVDTEQ
DRVFFIKAIAQFMATKAHIKLNTKKLYQADGYAVKELLKITSVLYNAMKTKGMEGSEIVE
EDVNKFKFDLGSKIADLKAARQLASEITSKGASLYDLLGMEVELREMRTEAIARPLEINE
TEKVMRIAIKEILTQVQKTKDLLNNVASDEANLEAKIEKRKLELERNRKRLETLQSVRPC
FMDEYEKTEEELQKQYDTYLEKFQNLTYLEQQLEDHHRMEQERFEEAKNTLCLIQNKLKE
EEKRLLKSGSNDDSDIDIQEDDESDSELEERRLPKPQTAMEMLMQGRPGKRIVGTMQGGD
SDDNEDSEESEIDMEDDDDEDDDLEDESISLSPTKPNRRVRKSEPLDESDNDF
>sp|Q86X52|CHSS1_HUMAN Chondroitin sulfate synthase 1 OS=Homo sapiens GN=CHSY1 PE=1 SV=3
MAARGRRAWLSVLLGLVLGFVLASRLVLPRASELKRAGPRRRASPEGCRSGQAAASQAGG
ARGDARGAQLWPPGSDPDGGPRDRNFLFVGVMTAQKYLQTRAVAAYRTWSKTIPGKVQFF
SSEGSDTSVPIPVVPLRGVDDSYPPQKKSFMMLKYMHDHYLDKYEWFMRADDDVYIKGDR
LENFLRSLNSSEPLFLGQTGLGTTEEMGKLALEPGENFCMGGPGVIMSREVLRRMVPHIG
KCLREMYTTHEDVEVGRCVRRFAGVQCVWSYEMQQLFYENYEQNKKGYIRDLHNSKIHQA
ITLHPNKNPPYQYRLHSYMLSRKISELRHRTIQLHREIVLMSKYSNTEIHKEDLQLGIPP
SFMRFQPRQREEILEWEFLTGKYLYSAVDGQPPRRGMDSAQREALDDIVMQVMEMINANA
KTRGRIIDFKEIQYGYRRVNPMYGAEYILDLLLLYKKHKGKKMTVPVRRHAYLQQTFSKI
QFVEHEELDAQELAKRINQESGSLSFLSNSLKKLVPFQLPGSKSEHKEPKDKKINILIPL
SGRFDMFVRFMGNFEKTCLIPNQNVKLVVLLFNSDSNPDKAKQVELMRDYRIKYPKADMQ
ILPVSGEFSRALALEVGSSQFNNESLLFFCDVDLVFTTEFLQRCRANTVLGQQIYFPIIF
SQYDPKIVYSGKVPSDNHFAFTQKTGFWRNYGFGITCIYKGDLVRVGGFDVSIQGWGLED
VDLFNKVVQAGLKTFRSQEVGVVHVHHPVFCDPNLDPKQYKMCLGSKASTYGSTQQLAEM
WLEKNDPSYSKSSNNNGSVRTA
>sp|O43916|CHST1_HUMAN Carbohydrate sulfotransferase 1 OS=Homo sapiens GN=CHST1 PE=1 SV=1
MQCSWKAVLLLALASIAIQYTAIRTFTAKSFHTCPGLAEAGLAERLCEESPTFAYNLSRK

THILILATTRSGSSFVGQLFNQHLDVFYLFEPLYHVQNTLIPRFTQGKSPADRRVMLGAS
RDLLRSLYDCDLYFLENYIKPPPVNHTTDRIFRRGASRVLCSRPVCDPPGPADLVLEEGD
CVRKCGLLNLTVAAEACRERSHVAIKTVRVPEVNDLRALVEDPRLNLKVIQLVRDPRGIL
ASRSETFRDTYRLWRLWYGTGRKPYNLDVTQLTTVCEDFSNSVSTGLMRPPWLKGKYMLV
RYEDLARNPMKKTEEIYGFLGIPLDSHVARWIQNNTRGDPTLGKHKYGTVRNSAATAEKW
RFRLSYDIVAFAQNACQQVLAQLGYKIAASEEELKNPSVSLVEERDFRPFS
>sp|Q9GZS9|CHST5_HUMAN Carbohydrate sulfotransferase 5 OS=Homo sapiens GN=CHST5 PE=2 SV=3
MGMRARVPKVAHSTRRPPAARMWLPRFSSKTVTVLLLAQTTCLLLFIISRPGPSSPAGGE
DRVHVLVLSSWRSGSSFLGQLFSQHPDVFYLMEPAWHVWTTLSQGSAATLHMAVRDLMRS
IFLCDMDVFDAYMPQSRNLSAFFNWATSRALCSPPACSAFPRGTISKQDVCKTLCTRQPF
SLAREACRSYSHVVLKEVRFFNLQVLYPLLSDPALNLRIVHLVRDPRAVLRSREAAGPIL
ARDNGIVLGTNGKWVEADPHLRLIREVCRSHVRIAEAATLKPPPFLRGRYRLVRFEDLAR
EPLAEIRALYAFTGLTLTPQLEAWIHNITHGSGIGKPIEAFHTSSRNARNVSQAWRHALP
FTKILRVQEVCAGALQLLGYRPVYSADQQRDLTLDLVLPRGPDHFSWASPD
>sp|Q9GZX3|CHST6_HUMAN Carbohydrate sulfotransferase 6 OS=Homo sapiens GN=CHST6 PE=1 SV=1
MWLPRVSSTAVTALLLAQTFLLLFLVSRPGPSSPAGGEARVHVLVLSSWRSGSSFVGQLF
NQHPDVFYLMEPAWHVWTTLSQGSAATLHMAVRDLVRSVFLCDMDVFDAYLPWRRNLSDL
FQWAVSRALCSPPACSAFPRGAISSEAVCKPLCARQSFTLAREACRSYSHVVLKEVRFFN
LQVLYPLLSDPALNLRIVHLVRDPRAVLRSREQTAKALARDNGIVLGTNGTWVEADPGLR
VVREVCRSHVRIAEAATLKPPPFLRGRYRLVRFEDLAREPLAEIRALYAFTGLSLTPQLE
AWIHNITHGSGPGARREAFKTSSRNALNVSQAWRHALPFAKIRRVQELCAGALQLLGYRP
VYSEDEQRNLALDLVLPRGLNGFTWASSTASHPRN
>sp|P0C843|CI014_HUMAN Putative uncharacterized protein encoded by LINC00032 OS=Homo sapiens GN=LINC00032 PE=5 SV=1
MHLHVQPLRAKGKRPKDTFNKMAHRKRHSVTSEFLSKVPDEVRQRYINLFVEKYLKVCKT
EDEAVYKAKIEKKAIYERCSRRNMYVNIAVNYLKKLRDQGA
>sp|Q9P2X8|CI027_HUMAN Putative uncharacterized protein encoded by LINC00474 OS=Homo sapiens GN=LINC00474 PE=5 SV=2
MLCVSGFTSNLYSSKKDDKMKEISRTSNWGSSFSEKSGCMQTHPSMNLDCRDVTYVMNLL
LIAHHHLLQ
>sp|Q6ZRZ4|CI047_HUMAN Uncharacterized protein C9orf47 OS=Homo sapiens GN=C9orf47 PE=2 SV=1
MVRIWTTIMIVLILLLRIGPNKPSLSGRQAPAQAQTSDLVPSLFPLGLWAPGFCTWSSPD
EDKVWRPAWEQGPKGEPDPRGLRPRKPVPGTGNRDSGTRRRLQDATEQDPRPGNDVASAE
TAGPPSPSGIRAQDRAPRHRRAPPARMPVAPAPSADGEPLQEQGGGLFHRTRSVYNGLEL
NTWMKVERLFVEKFHQSFSLDN
>sp|Q5T6V5|CI064_HUMAN UPF0553 protein C9orf64 OS=Homo sapiens GN=C9orf64 PE=1 SV=1
MDGLLNPRESSKFIAENSRDVFIDSGGVRRVAELLLAKAAGPELRVEGWKALHELNPRAA
DEAAVNWVFVTDTLNFSFWSEQDEHKCVVRYRGKTYSGYWSLCAAVNRALDEGIPITSAS
YYATVTLDQVRNILRSDTDVSMPLVEERHRILNETGKILLEKFGGSFLNCVRESENSAQK
LMHLVVESFPSYRDVTLFEGKRVSFYKRAQILVADTWSVLEGKGDGCFKDISSITMFADY
RLPQVLAHLGALKYSDDLLKKLLKGEMLSYGDRQEVEIRGCSLWCVELIRDCLLELIEQK
GEKPNGEINSILLDYYLWDYAHDHREDMKGIPFHRIRCIYY

>sp|Q8N365|CIART_HUMAN Circadian-associated transcriptional repressor OS=Homo sapiens GN=CIART PE=1 SV=1
MDSPSSVSSYSSYSLSSSFPTSPVNSDFGFPSDSEREDKGAHGPRPDTVGQRGGSRPSPG
PIRCRHRSKVSGNQHTPSHPKQRGSASPMAGSGAKRSRDGELETSLNTQGCTTEGDLLFA
QKCKELQGFIPPLTDLLNGLKMGRFERGLSSFQQSVAMDRIQRIVGVLQKPQMGERYLGT
LLQVEGMLKTWFPQIAAQKSSLGGGKHQLTKHFPSHHSDSAASSPASPMEKMDQTQLGHL
ALKPKQPWHLTQWPAMNLTWIHTTPICNPPLSSPGTISFSHGPLGTGTGIGVILFLQHGV
QPFTHSAPTTPVPPTTASPVIPGEPMKLSGEGPRCYSLPVTLPSDWSYTLSPPSLPTLAR
KMTIGHREQQRSHPPVAADAHLLNL
>sp|Q9C0C6|CIPC_HUMAN CLOCK-interacting pacemaker OS=Homo sapiens GN=CIPC PE=1 SV=2
MERKNPSRESPRRLSAKVGKGTEMKKVARQLGMAAAESDKDSGFSDGSSECLSSAEQMES
EDMLSALGWSREDRPRQNSKTAKNAFPTLSPMVVMKNVLVKQGSSSSQLQSWTVQPSFEV
ISAQPQLLFLHPPVPSPVSPCHTGEKKSDSRNYLPILNSYTKIAPHPGKRGLSLGPEEKG
TSGVQKKICTERLGPSLSSSEPTKAGAVPSSPSTPAPPSAKLAEDSALQGVPSLVAGGSP
QTLQPVSSSHVAKAPSLTFASPASPVCASDSTLHGLESNSPLSPLSANYSSPLWAAEHLC
RSPDIFSEQRQSKHRRFQNTLVVLHKSGLLEITLKTKELIRQNQATQVELDQLKEQTQLF
IEATKSRAPQAWAKLQASLTPGSSNTGSDLEAFSDHPAI
>sp|Q86X95|CIR1_HUMAN Corepressor interacting with RBPJ 1 OS=Homo sapiens GN=CIR1 PE=1 SV=1
MGKSFANFMCKKDFHPASKSNIKKVWMAEQKISYDKKKQEELMQQYLKEQESYDNRLLMG
DERVKNGLNFMYEAPPGAKKENKEKEETEGETEYKFEWQKGAPREKYAKDDMNIRDQPFG
IQVRNVRCIKCHKWGHVNTDRECPLFGLSGINASSVPTDGSGPSMHPSELIAEMRNSGFA
LKRNVLGRNLTANDPSQEYVASEGEEDPEVEFLKSLTTKQKQKLLRKLDRLEKKKKKKDR
KKKKFQKSRSKHKKHKSSSSSSSSSSSSSSSTETSESSSESESESNNKEKKIQRKKRKKNKCS
GHNNSDSEEKDKSKKRKLHEELSSSHHNREKAKEKPRFLKHESSREDSKWSHSDSDKKSR
THKHSPEKRGSERKEGSSRSHGREERSRRSRSRSPGSYKQRETRKRAQRNPGEEQSRRND
SRSHGTDLYRGEKMYREHPGGTHTKVTQRE
>sp|P0C7P0|CISD3_HUMAN CDGSH iron-sulfur domain-containing protein 3, mitochondrial OS=Homo sapiens GN=CISD3 PE=1 SV=1
MRGAGAILRPAARGARDLNPRRDISSWLAQWFPRTPARSVVALKTPIKVELVAGKTYRWC
VCGRSKKQPFCDGSHFFQRTGLSPLKFKAQETRMVALCTCKATQRPPYCDGTHRSERVQK
AEVGSPL
>sp|Q9NSE2|CISH_HUMAN Cytokine-inducible SH2-containing protein OS=Homo sapiens GN=CISH PE=1 SV=1
MVLCVQGPRPLLAVERTGQRPLWAPSLELPKPVMQPLPAGAFLEEVAEGTPAQTESEPKV
LDPEEDLLCIAKTFSYLRESGWYWGSITASEARQHLQKMPEGTFLVRDSTHPSYLFTLSV
KTTRGPTNVRIEYADSSFRLDSNCLSRPRILAFPDVVSLVQHYVASCTADTRSDSPDPAP
TPALPMPKEDAPSDPALPAPPPATAVHLKLVQPFVRRSSARSLQHLCRLVINRLVADVDC
LPLPRRMADYLRQYPFQL
>sp|Q9H0W9|CK054_HUMAN Ester hydrolase C11orf54 OS=Homo sapiens GN=C11orf54 PE=1 SV=1
MACAEFSFHVPSLEELAGVMQKGLKDNFADVQVSVVDCPDLTKEPFTFPVKGICGKTRIA
EVGGVPYLLPLVNQKKVYDLNKIAKEIKLPGAFILGAGAGPFQTLGFNSEFMPVIQTESE
HKPPVNGSYFAHVNPADGGCLLEKYSEKCHDFQCALLANLFASEGQPGKVIEVKAKRRTG
PLNFVTCMRETLEKHYGNKPIGMGGTFIIQKGKVKSHIMPAEFSSCPLNSDEEVNKWLHF

YEMKAPLVCLPVFVSRDPGFDLRLEHTHFFSRHGEGGHYHYDTTPDIVEYLGYFLPAEFL
YRIDQPKETHSIGRD
>sp|Q7Z7J9|CK2N1_HUMAN Calcium/calmodulin-dependent protein kinase II inhibitor 1 OS=Homo sapiens GN=CAMK2N1 PE=1 SV=1
MSEVLPYGDEKLSPYGDGGDVGQIFSCRLQDTNNFFGAGQNKRPPKLGQIGRSKRVVIED
DRIDDVLKNMTDKAPPGV
>sp|Q96SZ6|CK5P1_HUMAN CDK5 regulatory subunit-associated protein 1 OS=Homo sapiens GN=CDK5RAP1 PE=1 SV=2
MHPLQCVLQVQRSLGWGPLASVSWLSLRMCRAHSSLSSTMCPSPERQEDGARKDFSSRLA
AGPTFQHFLKSASAPQEKLSSEVEDPPPYLMMDELLGRQRKVYLETYGCQMNVNDTEIAW
SILQKSGYLRTSNLQEADVILLVTCSIREKAEQTIWNRLHQLKALKTRRPRSRVPLRIGI
LGCMAERLKEEILNREKMVDILAGPDAYRDLPRLLAVAESGQQAANVLLSLDETYADVMP
VQTSASATSAFVSIMRGCDNMCSYCIVPFTRGRERSRPIASILEEVKKLSEQVFLPPRPP
KVLGLQGLKEVTLLGQNVNSFRDNSEVQFNSAVPTNLSRGFTTNYKTKQGGLRFAHLLDQ
VSRVDPEMRIRFTSPHPKDFPDEVLQLIHERDNICKQIHLPAQSGSSRVLEAMRRGYSRE
AYVELVHHIRESIPGVSLSSDFIAGFCGETEEDHVQTVSLLREVQYNMGFLFAYSMRQKT
RAYHRLKDDVPEEVKLRRLEELITIFREEATKANQTSVGCTQLVLVEGLSKRSATDLCGR
NDGNLKVIFPDAEMEDVNNPGLRVRAQPGDYVLVKITSASSQTLRGHVLCRTTLRDSSAY
C
>sp|Q8IZR5|CKLF4_HUMAN CKLF-like MARVEL transmembrane domain-containing protein 4 OS=Homo sapiens GN=CMTM4 PE=1 SV=1
MRSGEELDGFEGEASSTSMISGASSPYQPTTEPVSQRRGLAGLRCDPDYLRGALGRLKVA
QVILALIAFICIETIMACSPCEGLYFFEFVSCSAFVVTGVLLIMFSLNLHMRIPQINWNL
TDLVNTGLSAFLFFIASIVLAALNHRAGAEIAAVIFGFLATAAYAVNTFLAVQKWRVSVR
QQSTNDYIRARTESRDVDSRPEIQRLDTFSYSTNVTVRKKSPTNLLSLNHWQLA
>sp|Q9NX76|CKLF6_HUMAN CKLF-like MARVEL transmembrane domain-containing protein 6 OS=Homo sapiens GN=CMTM6 PE=1 SV=1
MENGAVYSPTTEEDPGPARGPRSGLAAYFFMGRLPLLRRVLKGLQLLLSLLAFICEEVVS
QCTLCGGLYFFEFVSCSAFLLSLLILIVYCTPFYERVDTTKVKSSDFYITLGTGCVFLLA
SIIFVSTHDRTSAEIAAIVFGFIASFMFLLDFITMLYEKRQESQLRKPENTTRAEALTEP
LNA
>sp|Q9UBR5|CKLF_HUMAN Chemokine-like factor OS=Homo sapiens GN=CKLF PE=2 SV=1
MDNVQPKIKHRPFCFSVKGHVKMLRLALTVTSMTFFIIAQAPEPYIVITGFEVTVILFFI
LLYVLRLDRLMKWLFWPLLDIINSLVTTVFMLIVSVLALIPETTTLTVGGGVFALVTAVC
CLADGALIYRKLLFNPSGPYQKKPVHEKKEVL
>sp|Q8IYA6|CKP2L_HUMAN Cytoskeleton-associated protein 2-like OS=Homo sapiens GN=CKAP2L PE=1 SV=4
MVGPGPTAAAAVEERQRKLQEYLAAKGKLKSQNTKPYLKSKNNCQNQPPSKSTIRPKNDV
TNHVVLPVKPKRSISIKLQPRPPNTAGSQKPKLEPPKLLGKRLTSECVSSNPYSKPSSKS
FQQCEAGSSTTGELSRKPVGSLNIEQLKTTKQQLTDQGNGKCIDFMNNIHVENESLDNFL
KETNKENLLDILTEPERKPDPKLYTRSKPKTDSYNQTKNSLVPKQALGKSSVNSAVLKDR
VNKQFVGETQSRTFPVKSQQLSRGADLARPGVKPSRTVPSHFIRTLSKVQSSKKPVVKNI
KDIKVNRSQYERPNETKIRSYPVTEQRVKHTKPRTYPSLLQGEYNNRHPNIKQDQKSSQV
CIPQTSCVLQKSKAISQRPNLTVGRFNSAIPSTPSIRPNGTSGNKHNNNGFQQKAQTLDS

KLKKAVPQNHFLNKTAPKTQADVTTVNGTQTNPNIKKKATAEDRRKQLEEWQKSKGKTYK
RPPMELKTKRKVIKEMNISFWKSIEKEEEEKKAQLELSSKINNTLTECLNLIEGGVPSNE
ILNILSSIPEAEKFAKFWICKAKLLASKGTFDVIGLYEEAIKNGATPIQELRKVVLNILQ
DSNRTTEGITSDSLVAETSITSVEELAKKMESVKSCLSPKEREQVTATPRIAKAEQHNYP
GIKLQIGPIPRINGMPEVQDMKFITPVRRSSRIERAVSRYPEMLQEHDLVVASLDELLEV
EETKCFIFRRNEALPVTLGFQTPES
>sp|P61024|CKS1_HUMAN Cyclin-dependent kinases regulatory subunit 1 OS=Homo sapiens GN=CKS1B PE=1 SV=1
MSHKQIYYSDKYDDEEFEYRHVMLPKDIAKLVPKTHLMSESEWRNLGVQQSQGWVHYMIH
EPEPHILLFRRPLPKKPKK
>sp|C9JDV5|CL097_HUMAN Putative uncharacterized protein C12orf77 OS=Homo sapiens GN=C12orf77 PE=2 SV=1
MIPKLATPRDISLLVIKEASSRARNNIQILDPLDHIARLTNYTLTSCFSSNKVPTMPGSL
PTGSKQGNVQTLDSIRWMPATPVPAPECHQKYAISTETAFPTLSTFMNTEKSVKGSNADF
TKRNPRWRFLLGIYCSLHHAMTIFI
>sp|Q2HXU8|CL12B_HUMAN C-type lectin domain family 12 member B OS=Homo sapiens GN=CLEC12B PE=1 SV=1
MSEEVTYATLTFQDSAGARNNRDGNNLRKRGHPAPSPIWRHAALGLVTLCLMLLIGLVTL
GMMFLQISNDINSDSEKLSQLQKTIQQQQDNLSQQLGNSNNLSMEEEFLKSQISSVLKRQ
EQMAIKLCQELIIHTSDHRCNPCPKMWQWYQNSCYYFTTNEEKTWANSRKDCIDKNSTLV
KIDSLEEKDFLMSQPLLMFSFFWLGLSWDSSGRSWFWEDGSVPSPSLFSTKELDQINGSK
GCAYFQKGNIYISRCSAEIFWICEKTAAPVKTEDLD
>sp|Q2KHT3|CL16A_HUMAN Protein CLEC16A OS=Homo sapiens GN=CLEC16A PE=1 SV=2
MFGRSRSWVGGGHGKTSRNIHSLDHLKYLYHVLTKNTTVTEQNRNLLVETIRSITEILIW
GDQNDSSVFDFFLEKNMFVFFLNILRQKSGRYVCVQLLQTLNILFENISHETSLYYLLSN
NYVNSIIVHKFDFSDEEIMAYYISFLKTLSLKLNNHTVHFFYNEHTNDFALYTEAIKFFN
HPESMVRIAVRTITLNVYKVSLDNQAMLHYIRDKTAVPYFSNLVWFIGSHVIELDDCVQT
DEEHRNRGKLSDLVAEHLDHLHYLNDILIINCEFLNDVLTDHLLNRLFLPLYVYSLENQD
KGGERPKISLPVSLYLLSQVFLIIHHAPLVNSLAEVILNGDLSEMYAKTEQDIQRSSAKP
SIRCFIKPTETLERSLEMNKHKGKRRVQKRPNYKNVGEEEDEEKGPTEDAQEDAEKAKGT
EGGSKGIKTSGESEEIEMVIMERSKLSELAASTSVQEQNTTDEEKSAAATCSESTQWSRP
FLDMVYHALDSPDDDYHALFVLCLLYAMSHNKGMDPEKLERIQLPVPNAAEKTTYNHPLA
ERLIRIMNNAAQPDGKIRLATLELSCLLLKQQVLMSAGCIMKDVHLACLEGAREESVHLV
RHFYKGEDIFLDMFEDEYRSMTMKPMNVEYLMMDASILLPPTGTPLTGIDFVKRLPCGDV
EKTRRAIRVFFMLRSLSLQLRGEPETQLPLTREEDLIKTDDVLDLNNSDLIACTVITKDG
GMVQRFLAVDIYQMSLVEPDVSRLGWGVVKFAGLLQDMQVTGVEDDSRALNITIHKPASS
PHSKPFPILQATFIFSDHIRCIIAKQRLAKGRIQARRMKMQRIAALLDLPIQPTTEVLGF
GLGSSTSTQHLPFRFYDQGRRGSSDPTVQRSVFASVDKVPGFAVAQCINQHSSPSLSSQS
PPSASGSPSGSGSTSHCDSGGTSSSSTPSTAQSPADAPMSPELPKPHLPDQLVIVNETEA
DSKPSKNVARSAAVETASLSPSLVPARQPTISLLCEDTADTLSVESLTLVPPVDPHSLRS
LTGMPPLSTPAAACTEPVGEEAACAEPVGTAED
>sp|P16619|CL3L1_HUMAN C-C motif chemokine 3-like 1 OS=Homo sapiens GN=CCL3L1 PE=1 SV=1
MQVSTAALAVLLCTMALCNQVLSAPLAADTPTACCFSYTSRQIPQNFIADYFETSSQCSK
PSVIFLTKRGRQVCADPSEEWVQKYVSDLELSA

>sp|O75122|CLAP2_HUMAN CLIP-associating protein 2 OS=Homo sapiens GN=CLASP2 PE=1 SV=2
MAMGDDKSFDDEESVDGNRPSSAASAFKVPAPKTSGNPANSARKPGSAGGPKVGGASKEG
GAGAVDEDDFIKAFTDVPSIQIYSSRELEETLNKIREILSDDKHDWDQRANALKKIRSLL
VAGAAQYDCFFQHLRLLDGALKLSAKDLRSQVVREACITVAHLSTVLGNKFDHGAEAIVP
TLFNLVPNSAKVMATSGCAAIRFIIRHTHVPRLIPLITSNCTSKSVPVRRRSFEFLDLLL
QEWQTHSLERHAAVLVETIKKGIHDADAEARVEARKTYMGLRNHFPGEAETLYNSLEPSY
QKSLQTYLKSSGSVASLPQSDRSSSSSQESLNRPFSSKWSTANPSTVAGRVSAGSSKASS
LPGSLQRSRSDIDVNAAAGAKAHHAAGQSVRRGRLGAGALNAGSYASLEDTSDKLDGTAS
EDGRVRAKLSAPLAGMGNAKADSRGRSRTKMVSQSQPGSRSGSPGRVLTTTALSTVSSGV
QRVLVNSASAQKRSKIPRSQGCSREASPSRLSVARSSRIPRPSVSQGCSREASRESSRDT
SPVRSFQPLASRHHSRSTGALYAPEVYGASGPGYGISQSSRLSSSVSAMRVLNTGSDVEE
AVADALKKPARRRYESYGMHSDDDANSDASSACSERSYSSRNGSIPTYMRQTEDVAEVLN
RCASSNWSERKEGLLGLQNLLKNQRTLSRVELKRLCEIFTRMFADPHGKRVFSMFLETLV
DFIQVHKDDLQDWLFVLLTQLLKKMGADLLGSVQAKVQKALDVTRESFPNDLQFNILMRF
TVDQTQTPSLKVKVAILKYIETLAKQMDPGDFINSSETRLAVSRVITWTTEPKSSDVRKA
AQSVLISLFELNTPEFTMLLGALPKTFQDGATKLLHNHLRNTGNGTQSSMGSPLTRPTPR
SPANWSSPLTSPTNTSQNTLSPSAFDYDTENMNSEDIYSSLRGVTEAIQNFSFRSQEDMN
EPLKRDSKKDDGDSMCGGPGMSDPRAGGDATDSSQTALDNKASLLHSMPTHSSPRSRDYN
PYNYSDSISPFNKSALKEAMFDDDADQFPDDLSLDHSDLVAELLKELSNHNERVEERKIA
LYELMKLTQEESFSVWDEHFKTILLLLLETLGDKEPTIRALALKVLREILRHQPARFKNY
AELTVMKTLEAHKDPHKEVVRSAEEAASVLATSISPEQCIKVLCPIIQTADYPINLAAIK
MQTKVIERVSKETLNLLLPEIMPGLIQGYDNSESSVRKACVFCLVAVHAVIGDELKPHLS
QLTGSKMKLLNLYIKRAQTGSGGADPTTDVSGQS
>sp|Q8N2M8|CLASR_HUMAN CLK4-associating serine/arginine rich protein OS=Homo sapiens GN=CLASRP PE=1 SV=4
MWHEARKHERKLRGMMVDYKKRAERRREYYEKIKKDPAQFLQVHGRACKVHLDSAVALAA
ESPVNMMPWQGDTNNMIDRFDVRAHLDHIPDYTPPLLTTISPEQESDERKCNYERYRGLV
QNDFAGISEEQCLYQIYIDELYGGLQRPSEDEKKKLAEKKASIGYTYEDSTVAKVEKAAE
KPEEEESAAEEESNSDEDEVIPDIDVEVDVDELNQEQVADLNKQATTYGMADGDFVRMLR
KDKEEAEAIKHAKALEEEKAMYSGRRSRRQRREFREKRLRGRKISPPSYARRDSPTYDPY
KRSPSESSSESRSRSRSPTPGREEKITFITSFGGSDEEAAAAAAAAAASGVTTGKPPAPP
QPGGPAPGRNASARRRSSSSSSSSSASRTSSSRSSSRSSSRSRRGGGYYRSGRHARSRSR
SWSRSRSRSRRYSRSRSRGRRHSGGGSRDGHRYSRSPARRGGYGPRRRSRSRSHSGDRYR
RGGRGLRHHSSSRSRSSWSLSPSRSRSLTRSRSHSPSPSQSRSRSRSRSQSPSPSPAREK
LTRPAASPAVGEKLKKTEPAAGKETGAAKPKLTPQEKLKLRMQKALNRQFKADKKAAQEK
MIQQEHERQEREDELRAMARKIRMKERERREKEREEWERQYSRQSRSPSPRYSREYSSSR
RRSRSRSRSPHYRH
>sp|Q9Y240|CLC11_HUMAN C-type lectin domain family 11 member A OS=Homo sapiens GN=CLEC11A PE=1 SV=1
MQAAWLLGALVVPQLLGFGHGARGAEREWEGGWGGAQEEEREREALMLKHLQEALGLPAG
RGDENPAGTVEGKEDWEMEEDQGEEEEEEATPTPSSGPSPSPTPEDIVTYILGRLAGLDA
GLHQLHVRLHALDTRVVELTQGLRQLRNAAGDTRDAVQALQEAQGRAEREHGRLEGCLKG
LRLGHKCFLLSRDFEAQAAAQARCTARGGSLAQPADRQQMEALTRYLRAALAPYNWPVWL
GVHDRRAEGLYLFENGQRVSFFAWHRSPRPELGAQPSASPHPLSPDQPNGGTLENCVAQA

SDDGSWWDHDCQRRLYYVCEFPF
>sp|Q86T13|CLC14_HUMAN C-type lectin domain family 14 member A OS=Homo sapiens GN=CLEC14A PE=1 SV=1
MRPAFALCLLWQALWPGPGGGEHPTADRAGCSASGACYSLHHATMKRQAAEEACILRGGA
LSTVRAGAELRAVLALLRAGPGPGGGSKDLLFWVALERRRSHCTLENEPLRGFSWLSSDP
GGLESDTLQWVEEPQRSCTARRCAVLQATGGVEPAGWKEMRCHLRANGYLCKYQFEVLCP
APRPGAASNLSYRAPFQLHSAALDFSPPGTEVSALCRGQLPISVTCIADEIGARWDKLSG
DVLCPCPGRYLRAGKCAELPNCLDDLGGFACECATGFELGKDGRSCVTSGEGQPTLGGTG
VPTRRPPATATSPVPQRTWPIRVDEKLGETPLVPEQDNSVTSIPEIPRWGSQSTMSTLQM
SLQAESKATITPSGSVISKFNSTTSSATPQAFDSSSAVVFIFVSTAVVVLVILTMTVLGL
VKLCFHESPSSQPRKESMGPPGLESDPEPAALGSSSAHCTNNGVKVGDCDLRDRAEGALL
AESPLGSSDA
>sp|Q8WXI8|CLC4D_HUMAN C-type lectin domain family 4 member D OS=Homo sapiens GN=CLEC4D PE=1 SV=2
MGLEKPQSKLEGGMHPQLIPSVIAVVFILLLSVCFIASCLVTHHNFSRCKRGTGVHKLEH
HAKLKCIKEKSELKSAEGSTWNCCPIDWRAFQSNCYFPLTDNKTWAESERNCSGMGAHLM
TISTEAEQNFIIQFLDRRLSYFLGLRDENAKGQWRWVDQTPFNPRRVFWHKNEPDNSQGE
NCVVLVYNQDKWAWNDVPCNFEASRICKIPGTTLN
>sp|Q9H2X3|CLC4M_HUMAN C-type lectin domain family 4 member M OS=Homo sapiens GN=CLEC4M PE=1 SV=1
MSDSKEPRVQQLGLLEEDPTTSGIRLFPRDFQFQQIHGHKSSTGCLGHGALVLQLLSFML
LAGVLVAILVQVSKVPSSLSQEQSEQDAIYQNLTQLKAAVGELSEKSKLQEIYQELTQLK
AAVGELPEKSKLQEIYQELTRLKAAVGELPEKSKLQEIYQELTRLKAAVGELPEKSKLQE
IYQELTRLKAAVGELPEKSKLQEIYQELTELKAAVGELPEKSKLQEIYQELTQLKAAVGE
LPDQSKQQQIYQELTDLKTAFERLCRHCPKDWTFFQGNCYFMSNSQRNWHDSVTACQEVR
AQLVVIKTAEEQNFLQLQTSRSNRFSWMGLSDLNQEGTWQWVDGSPLSPSFQRYWNSGEP
NNSGNEDCAEFSGSGWNDNRCDVDNYWICKKPAACFRDE
>sp|Q6EIG7|CLC6A_HUMAN C-type lectin domain family 6 member A OS=Homo sapiens GN=CLEC6A PE=2 SV=1
MMQEQQPQSTEKRGWLSLRLWSVAGISIALLSACFIVSCVVTYHFTYGETGKRLSELHSY
HSSLTCFSEGTKVPAWGCCPASWKSFGSSCYFISSEEKVWSKSEQNCVEMGAHLVVFNTE
AEQNFIVQQLNESFSYFLGLSDPQGNNNWQWIDKTPYEKNVRFWHLGEPNHSAEQCASIV
FWKPTGWGWNDVICETRRNSICEMNKIYL
>sp|Q9BXN2|CLC7A_HUMAN C-type lectin domain family 7 member A OS=Homo sapiens GN=CLEC7A PE=1 SV=1
MEYHPDLENLDEDGYTQLHFDSQSNTRIAVVSEKGSCAASPPWRLIAVILGILCLVILVI
AVVLGTMAIWRSNSGSNTLENGYFLSRNKENHSQPTQSSLEDSVTPTKAVKTTGVLSSPC
PPNWIIYEKSCYLFSMSLNSWDGSKRQCWQLGSNLLKIDSSNELGFIVKQVSSQPDNSFW
IGLSRPQTEVPWLWEDGSTFSSNLFQIRTTATQENPSPNCVWIHVSVIYDQLCSVPSYSI
CEKKFSM
>sp|P51801|CLCKB_HUMAN Chloride channel protein ClC-Kb OS=Homo sapiens GN=CLCNKB PE=1 SV=3
MEEFVGLREGSSGNPVTLQELWGPCPRIRRGIRGGLEWLKQKLFRLGEDWYFLMTLGVLM
ALVSCAMDLAVESVVRAHQWLYREIGDSHLLRYLSWTVYPVALVSFSSGFSQSITPSSGG

SGIPEVKTMLAGVVLEDYLDIKNFGAKVVGLSCTLACGSTLFLGKVGPFVHLSVMMAAYL
GRVRTTTIGEPENKSKQNEMLVAAAAVGVATVFAAPFSGVLFSIEVMSSHFSVWDYWRGF
FAATCGAFMFRLLAVFNSEQETITSLYKTSFRVDVPFDLPEIFFFVALGGLCGILGSAYL
FCQRIFFGFIRNNRFSSKLLATSKPVYSALATLVLASITYPPSAGRFLASRLSMKQHLDS
LFDNHSWALMTQNSSPPWPEELDPQHLWWEWYHPRFTIFGTLAFFLVMKFWMLILATTIP
MPAGYFMPIFVYGAAIGRLFGETLSFIFPEGIVAGGITNPIMPGGYALAGAAAFSGAVTH
TISTALLAFEVTGQIVHALPVLMAVLAANAIAQSCQPSFYDGTVIVKKLPYLPRILGRNI
GSHRVRVEHFMNHSITTLAKDMPLEEVVKVVTSTDVAKYPLVESTESQILVGIVRRAQLV
QALKAEPPSWAPGHQQCLQDILAAGCPTEPVTLKLSPETSLHEAHNLFELLNLHSLFVTS
RGRAVGCVSWVEMKKAISNLTNPPAPK
>sp|P35523|CLCN1_HUMAN Chloride channel protein 1 OS=Homo sapiens GN=CLCN1 PE=1 SV=3
MEQSRSQQRGGEQSWWGSDPQYQYMPFEHCTSYGLPSENGGLQHRLRKDAGPRHNVHPTQ
IYGHHKEQFSDREQDIGMPKKTGSSSTVDSKDEDHYSKCQDCIHRLGQVVRRKLGEDGIF
LVLLGLLMALVSWSMDYVSAKSLQAYKWSYAQMQPSLPLQFLVWVTFPLVLILFSALFCH
LISPQAVGSGIPEMKTILRGVVLKEYLTMKAFVAKVVALTAGLGSGIPVGKEGPFVHIAS
ICAAVLSKFMSVFCGVYEQPYYYSDILTVGCAVGVGCCFGTPLGGVLFSIEVTSTYFAVR
NYWRGFFAATFSAFVFRVLAVWNKDAVTITALFRTNFRMDFPFDLKELPAFAAIGICCGL
LGAVFVYLHRQVMLGVRKHKALSQFLAKHRLLYPGIVTFVIASFTFPPGMGQFMAGELMP
REAISTLFDNNTWVKHAGDPESLGQSAVWIHPRVNVVIIIFLFFVMKFWMSIVATTMPIP
CGGFMPVFVLGAAFGRLVGEIMAMLFPDGILFDDIIYKILPGGYAVIGAAALTGAVSHTV
STAVICFELTGQIAHILPMMVAVILANMVAQSLQPSLYDSIIQVKKLPYLPDLGWNQLSK
YTIFVEDIMVRDVKFVSASYTYGELRTLLQTTTVKTLPLVDSKDSMILLGSVERSELQAL
LQRHLCPERRLRAAQEMARKLSELPYDGKARLAGEGLPGAPPGRPESFAFVDEDEDEDLS
GKSELPPSLALHPSTTAPLSPEEPNGPLPGHKQQPEAPEPAGQRPSIFQSLLHCLLGRAR
PTKKKTTQDSTDLVDNMSPEEIEAWEQEQLSQPVCFDSCCIDQSPFQLVEQTTLHKTHTL
FSLLGLHLAYVTSMGKLRGVLALEELQKAIEGHTKSGVQLRPPLASFRNTTSTRKSTGAP
PSSAENWNLPEDRPGATGTGDVIAASPETPVPSPSPEPPLSLAPGKVEGELEELELVESP
GLEEELADILQGPSLRSTDEEDEDELIL
>sp|P78369|CLD10_HUMAN Claudin-10 OS=Homo sapiens GN=CLDN10 PE=2 SV=2
MASTASEIIAFMVSISGWVLVSSTLPTDYWKVSTIDGTVITTATYWANLWKACVTDSTGV
SNCKDFPSMLALDGYIQACRGLMIAAVSLGFFGSIFALFGMKCTKVGGSDKAKAKIACLA
GIVFILSGLCSMTGCSLYANKITTEFFDPLFVEQKYELGAALFIGWAGASLCIIGGVIFC
FSISDNNKTPRYTYNGATSVMSSRTKYHGGEDFKTTNPSKQFDKNAYV
>sp|Q9Y5I7|CLD16_HUMAN Claudin-16 OS=Homo sapiens GN=CLDN16 PE=1 SV=1
MTSRTPLLVTACLYYSYCNSRHLQQGVRKSKRPVFSHCQVPETQKTDTRHLSGARAGVCP
CCHPDGLLATMRDLLQYIACFFAFFSAGFLIVATWTDCWMVNADDSLEVSTKCRGLWWEC
VTNAFDGIRTCDEYDSILAEHPLKLVVTRALMITADILAGFGFLTLLLGLDCVKFLPDEP
YIKVRICFVAGATLLIAGTPGIIGSVWYAVDVYVERSTLVLHNIFLGIQYKFGWSCWLGM
AGSLGCFLAGAVLTCCLYLFKDVGPERNYPYSLRKAYSAAGVSMAKSYSAPRTETAKMYA
VDTRV
>sp|Q8N6F1|CLD19_HUMAN Claudin-19 OS=Homo sapiens GN=CLDN19 PE=1 SV=2
MANSGLQLLGYFLALGGWVGIIASTALPQWKQSSYAGDAIITAVGLYEGLWMSCASQSTG
QVQCKLYDSLLALDGHIQSARALMVVAVLLGFVAMVLSVVGMKCTRVGDSNPIAKGRVAI
AGGALFILAGLCTLTAVSWYATLVTQEFFNPSTPVNARYEFGPALFVGWASAGLAVLGGS

FLCCTCPEPERPNSSPQPYRPGPSAAAREPVVKLPASAKGPLGV
>sp|O15551|CLD3_HUMAN Claudin-3 OS=Homo sapiens GN=CLDN3 PE=1 SV=1
MSMGLEITGTALAVLGWLGTIVCCALPMWRVSAFIGSNIITSQNIWEGLWMNCVVQSTGQ
MQCKVYDSLLALPQDLQAARALIVVAILLAAFGLLVALVGAQCTNCVQDDTAKAKITIVA
GVLFLLAALLTLVPVSWSANTIIRDFYNPVVPEAQKREMGAGLYVGWAAAALQLLGGALL
CCSCPPREKKYTATKVVYSAPRSTGPGASLGTGYDRKDYV
>sp|O95471|CLD7_HUMAN Claudin-7 OS=Homo sapiens GN=CLDN7 PE=1 SV=4
MANSGLQLLGFSMALLGWVGLVACTAIPQWQMSSYAGDNIITAQAMYKGLWMDCVTQSTG
MMSCKMYDSVLALSAALQATRALMVVSLVLGFLAMFVATMGMKCTRCGGDDKVKKARIAM
GGGIIFIVAGLAALVACSWYGHQIVTDFYNPLIPTNIKYEFGPAIFIGWAGSALVILGGA
LLSCSCPGNESKAGYRVPRSYPKSNSSKEYV
>sp|Q96NY7|CLIC6_HUMAN Chloride intracellular channel protein 6 OS=Homo sapiens GN=CLIC6 PE=2 SV=3
MAEAAEPEGVAPGPQGPPEVPAPLAERPGEPGAAGGEAEGPEGSEGAEEAPRGAAAVKEA
GGGGPDRGPEAEARGTRGAHGETEAEEGAPEGAEVPQGGEETSGAQQVEGASPGRGAQGE
PRGEAQREPEDSAAPERQEEAEQRPEVPEGSASGEAGDSVDAEGPLGDNIEAEGPAGDSV
EAEGRVGDSVDAEGPAGDSVDAEGPLGDNIQAEGPAGDSVDAEGRVGDSVDAEGPAGDSV
DAEGRVGDSVEAGDPAGDGVEAGVPAGDSVEAEGPAGDSMDAEGPAGRARRVSGEPQQSG
DGSLSPQAEAIEVAAGESAGRSPGELAWDAAEEAEVPGVKGSEEAAPGDARADAGEDRVG
DGPQQEPGEDEERRERSPEGPREEEAAGGEEESPDSSPHGEASRGAAEPEAQLSNHLAEE
GPAEGSGEAARVNGRREDGEASEPRALGQEHDITLFVKVKLTALGCSRIAIKKYLRAGYD
GESIGNCPFSQRLFMILWLKGVIFNVTTVDLKRKPADLQNLAPGTNPPFMTFDGEVKTDV
NKIEEFLEEKLAPPRYPKLGTQHPESNSAGNDVFAKFSAFIKNTKKDANEIHEKNLLKAL
RKLDNYLNSPLPDEIDAYSTEDVTVSGRKFLDGDELTLADCNLLPKLHIIKIVAKKYRDF
EFPSEMTGIWRYLNNAYARDEFTNTCPADQEIEHAYSDVAKRMK
>sp|P30622|CLIP1_HUMAN CAP-Gly domain-containing linker protein 1 OS=Homo sapiens GN=CLIP1 PE=1 SV=2
MSMLKPSGLKAPTKILKPGSTALKTPTAVVAPVEKTISSEKASSTPSSETQEEFVDDFRV
GERVWVNGNKPGFIQFLGETQFAPGQWAGIVLDEPIGKNDGSVAGVRYFQCEPLKGIFTR
PSKLTRKVQAEDEANGLQTTPASRATSPLCTSTASMVSSSPSTPSNIPQKPSQPAAKEPS
ATPPISNLTKTASESISNLSEAGSIKKGERELKIGDRVLVGGTKAGVVRFLGETDFAKGE
WCGVELDEPLGKNDGAVAGTRYFQCQPKYGLFAPVHKVTKIGFPSTTPAKAKANAVRRVM
ATTSASLKRSPSASSLSSMSSVASSVSSRPSRTGLLTETSSRYARKISGTTALQEALKEK
QQHIEQLLAERDLERAEVAKATSHVGEIEQELALARDGHDQHVLELEAKMDQLRTMVEAA
DREKVELLNQLEEEKRKVEDLQFRVEEESITKGDLEQKSQISEDPENTQTKLEHARIKEL
EQSLLFEKTKADKLQRELEDTRVATVSEKSRIMELEKDLALRVQEVAELRRRLESNKPAG
DVDMSLSLLQEISSLQEKLEVTRTDHQREITSLKEHFGAREETHQKEIKALYTATEKLSK
ENESLKSKLEHANKENSDVIALWKSKLETAIASHQQAMEELKVSFSKGLGTETAEFAELK
TQIEKMRLDYQHEIENLQNQQDSERAAHAKEMEALRAKLMKVIKEKENSLEAIRSKLDKA
EDQHLVEMEDTLNKLQEAEIKVKELEVLQAKCNEQTKVIDNFTSQLKATEEKLLDLDALR
KASSEGKSEMKKLRQQLEAAEKQIKHLEIEKNAESSKASSITRELQGRELKLTNLQENLS
EVSQVKETLEKELQILKEKFAEASEEAVSVQRSMQETVNKLHQKEEQFNMLSSDLEKLRE
NLADMEAKFREKDEREEQLIKAKEKLENDIAEIMKMSGDNSSQLTKMNDELRLKERDVEE
LQLKLTKANENASFLQKSIEDMTVKAEQSQQEAAKKHEEEKKELERKLSDLEKKMETSHN

QCQELKARYERATSETKTKHEEILQNLQKTLLDTEDKLKGAREENSGLLQELEELRKQAD
KAKAAQTAEDAMQIMEQMTKEKTETLASLEDTKQTNAKLQNELDTLKENNLKNVEELNKS
KELLTVENQKMEEFRKEIETLKQAAAQKSQQLSALQEENVKLAEELGRSRDEVTSHQKLE
EERSVLNNQLLEMKKRESKFIKDADEEKASLQKSISITSALLTEKDAELEKLRNEVTVLR
GENASAKSLHSVVQTLESDKVKLELKVKNLELQLKENKRQLSSSSGNTDTQADEDERAQE
SQIDFLNSVIVDLQRKNQDLKMKVEMMSEAALNGNGDDLNNYDSDDQEKQSKKKPRLFCD
ICDCFDLHDTEDCPTQAQMSEDPPHSTHHGSRGEERPYCEICEMFGHWATNCNDDETF
>sp|Q96DZ5|CLIP3_HUMAN CAP-Gly domain-containing linker protein 3 OS=Homo sapiens GN=CLIP3 PE=1 SV=3
MTKTDPAPMAPPPRGEEEEEEEEEDEPVPEAPSPTQERRQKPVVHPSAPAPLPKDYAFTFF
DPNDPACQEILFDPQTTIPELFAIVRQWVPQVQHKIDVIGNEILRRGCHVNDRDGLTDMT
LLHYACKAGAHGVGDPAAAVRLSQQLLALGADVTLRSRWTNMNALHYAAYFDVPDLVRVL
LKGARPRVVNSTCSDFNHGSALHIAASSLCLGAAKCLLEHGANPALRNRKGQVPAEVVPD
PMDMSLDKAEAALVAKELRTLLEEAVPLSCALPKVTLPNYDNVPGNLMLSALGLRLGDRV
LLDGQKTGTLRFCGTTEFASGQWVGVELDEPEGKNDGSVGGVRYFICPPKQGLFASVSKI
SKAVDAPPSSVTSTPRTPRMDFSRVTGKGRREHKGKKKTPSSPSLGSLQQRDGAKAEVGD
QVLVAGQKQGIVRFYGKTDFAPGYWYGIELDQPTGKHDGSVFGVRYFTCPPRHGVFAPAS
RIQRIGGSTDSPGDSVGAKKVHQVTMTQPKRTFTTVRTPKDIASENSISRLLFCCWFPWM
LRAEMQS
>sp|Q6UXG3|CLM9_HUMAN CMRF35-like molecule 9 OS=Homo sapiens GN=CD300LG PE=1 SV=2
MRLLVLLWGCLLLPGYEALEGPEEISGFEGDTVSLQCTYREELRDHRKYWCRKGGILFSR
CSGTIYAEEEGQETMKGRVSIRDSRQELSLIVTLWNLTLQDAGEYWCGVEKRGPDESLLI
SLFVFPGPCCPPSPSPTFQPLATTRLQPKAKAQQTQPPGLTSPGLYPAATTAKQGKTGAE
APPLPGTSQYGHERTSQYTGTSPHPATSPPAGSSRPPMQLDSTSAEDTSPALSSGSSKPR
VSIPMVRILAPVLVLLSLLSAAGLIAFCSHLLLWRKEAQQATETQRNEKFCLSRLTAEEK
EAPSQAPEGDVISMPPLHTSEEELGFSKFVSA
>sp|O15516|CLOCK_HUMAN Circadian locomoter output cycles protein kaput OS=Homo sapiens GN=CLOCK PE=1 SV=1
MLFTVSCSKMSSIVDRDDSSIFDGLVEEDDKDKAKRVSRNKSEKKRRDQFNVLIKELGSM
LPGNARKMDKSTVLQKSIDFLRKHKEITAQSDASEIRQDWKPTFLSNEEFTQLMLEALDG
FFLAIMTDGSIIYVSESVTSLLEHLPSDLVDQSIFNFIPEGEHSEVYKILSTHLLESDSL
TPEYLKSKNQLEFCCHMLRGTIDPKEPSTYEYVKFIGNFKSLNSVSSSAHNGFEGTIQRT
HRPSYEDRVCFVATVRLATPQFIKEMCTVEEPNEEFTSRHSLEWKFLFLDHRAPPIIGYL
PFEVLGTSGYDYYHVDDLENLAKCHEHLMQYGKGKSCYYRFLTKGQQWIWLQTHYYITYH
QWNSRPEFIVCTHTVVSYAEVRAERRRELGIEESLPETAADKSQDSGSDNRINTVSLKEA
LERFDHSPTPSASSRSSRKSSHTAVSDPSSTPTKIPTDTSTPPRQHLPAHEKMVQRRSSF
SSQSINSQSVGSSLTQPVMSQATNLPIPQGMSQFQFSAQLGAMQHLKDQLEQRTRMIEAN
IHRQQEELRKIQEQLQMVHGQGLQMFLQQSNPGLNFGSVQLSSGNSSNIQQLAPINMQGQ
VVPTNQIQSGMNTGHIGTTQHMIQQQTLQSTSTQSQQNVLSGHSQQTSLPSQTQSTLTAP
LYNTMVISQPAAGSMVQIPSSMPQNSTQSAAVTTFTQDRQIRFSQGQQLVTKLVTAPVAC
GAVMVPSTMLMGQVVTAYPTFATQQQQSQTLSVTQQQQQQSSQEQQLTSVQQPSQAQLTQ
PPQQFLQTSRLLHGNPSTQLILSAAFPLQQSTFPQSHHQQHQSQQQQQLSRHRTDSLPDP
SKVQPQ
>sp|Q8IY22|CMIP_HUMAN C-Maf-inducing protein OS=Homo sapiens GN=CMIP PE=1 SV=3

MDVTSSSGGGGDPRQIEETKPLLGGDVSAPEGTKMGAVPCRRALLLCNGMRYKLLQEGDI
QVCVIRHPRTFLSKILTSKFLRRWEPHHLTLADNSLASATPTGYMENSVSYSAIEDVQLL
SWENAPKYCLQLTIPGGTVLLQAANSYLRDQWFHSLQWKKKIYKYKKVLSNPSRWEVVLK
EIRTLVDMALTSPLQDDSINQAPLEIVSKLLSENTNLTTQEHENIIVAIAPLLENNHPPP
DLCEFFCKHCRERPRSMVVIEVFTPVVQRILKHNMDFGKCPRLRLFTQEYILALNELNAG
MEVVKKFIQSMHGPTGHCPHPRVLPNLVAVCLAAIYSCYEEFINSRDNSPSLKEIRNGCQ
QPCDRKPTLPLRLLHPSPDLVSQEATLSEARLKSVVVASSEIHVEVERTSTAKPALTASA
GNDSEPNLIDCLMVSPACSTMSIELGPQADRTLGCYVEILKLLSDYDDWRPSLASLLQPI
PFPKEALAHEKFTKELKYVIQRFAEDPRQEVHSCLLSVRAGKDGWFQLYSPGGVACDDDG
ELFASMVHILMGSCYKTKKFLLSLAENKLGPCMLLALRGNQTMVEILCLMLEYNIIDNND
TQLQIISTLESTDVGKRMYEQLCDRQRELKELQRKGGPTRLTLPSKSTDADLARLLSSGS
FGNLENLSLAFTNVTSACAEHLIKLPSLKQLNLWSTQFGDAGLRLLSEHLTMLQVLNLCE
TPVTDAGLLALSSMKSLCSLNMNSTKLSADTYEDLKAKLPNLKEVDVRYTEAW
>sp|Q9BQ75|CMS1_HUMAN Protein CMSS1 OS=Homo sapiens GN=CMSS1 PE=1 SV=2
MADDLGDEWWENQPTGAGSSPEASDGEGEGDTEVMQQETVPVPVPSEKTKQPKECFLIQP
KERKENTTKTRKRRKKKITDVLAKSEPKPGLPEDLQKLMKDYYSSRRLVIELEELNLPDS
CFLKANDLTHSLSSYLKEICPKWVKLRKNHSEKKSVLMLIICSSAVRALELIRSMTAFRG
DGKVIKLFAKHIKVQAQVKLLEKRVVHLGVGTPGRIKELVKQGGLNLSPLKFLVFDWNWR
DQKLRRMMDIPEIRKEVFELLEMGVLSLCKSESLKLGLF
>sp|Q9H761|CN139_HUMAN Putative uncharacterized protein encoded by LINC00341 OS=Homo sapiens GN=LINC00341 PE=5 SV=1
MVQQDVQGLKAQSAGLSISQHLLSGCHGNIIPNPTTLSSRETAIIRRDLGFPRGSWASFL
KRLLLSSATRNTRPAHPQHLCGDEGQSPTVPLPVLMRKGGKKTYPERLQYGHDTFKKPVL
WSHDQKPECGEGQQPASEQPARRGVVNLGLVPRHDWLRAQCSTLWNVPWASVAFLSFAGR
GLLPLWQLVS
>sp|Q7Z3D6|CN159_HUMAN UPF0317 protein C14orf159, mitochondrial OS=Homo sapiens GN=C14orf159 PE=1 SV=2
MPFTLHLRSRLPSAIRSLILQKKPNIRNTSSMAGELRPASLVVLPRSLAPAFERFCQVNT
GPLPLLGQSEPEKWMLPPQGAISETRMGHPQFWKYEFGACTGSLASLEQYSEQLKDMVAF
FLGCSFSLEEALEKAGLPRRDPAGHSQTTVPCVTHAGFCCPLVVTMRPIPKDKLEGLVRA
CCSLGGEQGQPVHMGDPELLGIKELSKPAYGDAMVCPPGEVPVFWPSPLTSLGAVSSCET
PLAFASIPGCTVMTDLKDAKAPPGCLTPERIPEVHHISQDPLHYSIASVSASQKIRELES
MIGIDPGNRGIGHLLCKDELLKASLSLSHARSVLITTGFPTHFNHEPPEETDGPPGAVAL
VAFLQALEKEVAIIVDQRAWNLHQKIVEDAVEQGVLKTQIPILTYQGGSVEAAQAFLCKN
GDPQTPRFDHLVAIERAGRAADGNYYNARKMNIKHLVDPIDDLFLAAKKIPGISSTGVGD
GGNELGMGKVKEAVRRHIRHGDVIACDVEADFAVIAGVSNWGGYALACALYILYSCAVHS
QYLRKAVGPSRAPGDQAWTQALPSVIKEEKMLGILVQHKVRSGVSGIVGMEVDGLPFHNT
HAEMIQKLVDVTTAQV
>sp|P09543|CN37_HUMAN 2',3'-cyclic-nucleotide 3'-phosphodiesterase OS=Homo sapiens GN=CNP PE=1 SV=2
MNRGFSRKSHTFLPKIFFRKMSSSGAKDKPELQFPFLQDEDTVATLLECKTLFILRGLPG
SGKSTLARVIVDKYRDGTKMVSADAYKITPGARGAFSEEYKRLDEDLAAYCRRRDIRILV
LDDTNHERERLEQLFEMADQYQYQVVLVEPKTAWRLDCAQLKEKNQWQLSADDLKKLKPG
LEKDFLPLYFGWFLTKKSSETLRKAGQVFLEELGNHKAFKKELRQFVPGDEPREKMDLVT

YFGKRPPGVLHCTTKFCDYGKAPGAEEYAQQDVLKKSYSKAFTLTISALFVTPKTTGARV
ELSEQQLQLWPSDVDKLSPTDNLPRGSRAHITLGCAADVEAVQTGLDLLEILRQEKGGSR
GEEVGELSRGKLYSLGNGRWMLTLAKNMEVRAIFTGYYGKGKPVPTQGSRKGGALQSCTI
I
>sp|Q6IBW4|CNDH2_HUMAN Condensin-2 complex subunit H2 OS=Homo sapiens GN=NCAPH2 PE=1 SV=1
MEDVEARFAHLLQPIRDLTKNWEVDVAAQLGEYLEELDQICISFDEGKTTMNFIEAALLI
QGSACVYSKKVEYLYSLVYQALDFISGKRRAKQLSSVQEDRANGVASSGVPQEAENEFLS
LDDFPDSRTNVDLKNDQTPSEVLIIPLLPMALVAPDEMEKNNNPLYSRQGEVLASRKDFR
MNTCVPHPRGAFMLEPEGMSPMEPAGVSPMPGTQKDTGRTEEQPMEVSVCRSPVPALGFS
QEPGPSPEGPMPLGGGEDEDAEEAVELPEASAPKAALEPKESRSPQQSAALPRRYMLRER
EGAPEPASCVKETPDPWQSLDPFDSLESKPFKKGRPYSVPPCVEEALGQKRKRKGAAKLQ
DFHQWYLAAYADHADSRRLRRKGPSFADMEVLYWTHVKEQLETLRKLQRREVAEQWLRPA
EEDHLEDSLEDLGAADDFLEPEEYMEPEGADPREAADLDAVPMSLSYEELVRRNVELFIA
TSQKFVQETELSQRIRDWEDTVQPLLQEQEQHVPFDIHTYGDQLVSRFPQLNEWCPFAEL
VAGQPAFEVCRSMLASLQLANDYTVEITQQPGLEMAVDTMSLRLLTHQRAHKRFQTYAAP
SMAQP
>sp|Q96KP4|CNDP2_HUMAN Cytosolic non-specific dipeptidase OS=Homo sapiens GN=CNDP2 PE=1 SV=2
MAALTTLFKYIDENQDRYIKKLAKWVAIQSVSAWPEKRGEIRRMMEVAAADVKQLGGSVE
LVDIGKQKLPDGSEIPLPPILLGRLGSDPQKKTVCIYGHLDVQPAALEDGWDSEPFTLVE
RDGKLYGRGSTDDKGPVAGWINALEAYQKTGQEIPVNVRFCLEGMEESGSEGLDELIFAR
KDTFFKDVDYVCISDNYWLGKKKPCITYGLRGICYFFIEVECSNKDLHSGVYGGSVHEAM
TDLILLMGSLVDKRGNILIPGINEAVAAVTEEEHKLYDDIDFDIEEFAKDVGAQILLHSH
KKDILMHRWRYPSLSLHGIEGAFSGSGAKTVIPRKVVGKFSIRLVPNMTPEVVGEQVTSY
LTKKFAELRSPNEFKVYMGHGGKPWVSDFSHPHYLAGRRAMKTVFGVEPDLTREGGSIPV
TLTFQEATGKNVMLLPVGSADDGAHSQNEKLNRYNYIEGTKMLAAYLYEVSQLKD
>sp|Q8IV77|CNGA4_HUMAN Cyclic nucleotide-gated cation channel alpha-4 OS=Homo sapiens GN=CNGA4 PE=1 SV=3
MSQDTKVKTTESSPPAPSKARKLLPVLDPSGDYYYWWLNTMVFPVMYNLIILVCRACFPD
LQHGYLVAWLVLDYTSDLLYLLDMVVRFHTGFLEQGILVVDKGRISSRYVRTWSFFLDLA
SLMPTDVVYVRLGPHTPTLRLNRFLRAPRLFEAFDRTETRTAYPNAFRIAKLMLYIFVVI
HWNSCLYFALSRYLGFGRDAWVYPDPAQPGFERLRRQYLYSFYFSTLILTTVGDTPPPAR
EEEYLFMVGDFLLAVMGFATIMGSMSSVIYNMNTADAAFYPDHALVKKYMKLQHVNRKLE
RRVIDWYQHLQINKKMTNEVAILQHLPERLRAEVAVSVHLSTLSRVQIFQNCEASLLEEL
VLKLQPQTYSPGEYVCRKGDIGQEMYIIREGQLAVVADDGITQYAVLGAGLYFGEISIIN
IKGNMSGNRRTANIKSLGYSDLFCLSKEDLREVLSEYPQAQTIMEEKGREILLKMNKLDV
NAEAAEIALQEATESRLRGLDQQLDDLQTKFARLLAELESSALKIAYRIERLEWQTREWP
MPEDLAEADDEGEPEEGTSKDEEGRASQEGPPGPE
>sp|Q14028|CNGB1_HUMAN Cyclic nucleotide-gated cation channel beta-1 OS=Homo sapiens GN=CNGB1 PE=1 SV=2
MLGWVQRVLPQPPGTPRKTKMQEEEEVEPEPEMEAEVEPEPNPEEAETESESMPPEESFK
EEEVAVADPSPQETKEAALTSTISLRAQGAEISEMNSPSRRVLTWLMKGVEKVIPQPVHS
ITEDPAQILGHGSTGDTGCTDEPNEALEAQDTRPGLRLLLWLEQNLERVLPQPPKSSEVW
RDEPAVATGAASDPAPPGRPQEMGPKLQARETPSLPTPIPLQPKEEPKEAPAPEPQPGSQ

AQTSSLPPTRDPARLVAWVLHRLEMALPQPVLHGKIGEQEPDSPGICDVQTISILPGGQV
EPDLVLEEVEPPWEDAHQDVSTSPQGTEVVPAYEEENKAVEKMPRELSRIEEEKEDEEEE
EEEEEEEEEEEVTEVLLDSCVVSQVGVGQSEEDGTRPQSTSDQKLWEEVGEEAKKEAEEK
AKEEAEEVAEEEAEKEPQDWAETKEEPEAEAEAASSGVPATKQHPEVQVEDTDADSCPLM
AEENPPSTVLPPPSPAKSDTLIVPSSASGTHRKKLPSEDDEAEELKALSPAESPVVAWSD
PTTPKDTDGQDRAASTASTNSAIINDRLQELVKLFKERTEKVKEKLIDPDVTSDEESPKP
SPAKKAPEPAPDTKPAEAEPVEEEHYCDMLCCKFKHRPWKKYQFPQSIDPLTNLMYVLWL
FFVVMAWNWNCWLIPVRWAFPYQTPDNIHHWLLMDYLCDLIYFLDITVFQTRLQFVRGGD
IITDKKDMRNNYLKSRRFKMDLLSLLPLDFLYLKVGVNPLLRLPRCLKYMAFFEFNSRLE
SILSKAYVYRVIRTTAYLLYSLHLNSCLYYWASAYQGLGSTHWVYDGVGNSYIRCYYFAV
KTLITIGGLPDPKTLFEIVFQLLNYFTGVFAFSVMIGQMRDVVGAATAGQTYYRSCMDST
VKYMNFYKIPKSVQNRVKTWYEYTWHSQGMLDESELMVQLPDKMRLDLAIDVNYNIVSKV
ALFQGCDRQMIFDMLKRLRSVVYLPNDYVCKKGEIGREMYIIQAGQVQVLGGPDGKSVLV
TLKAGSVFGEISLLAVGGGNRRTANVVAHGFTNLFILDKKDLNEILVHYPESQKLLRKKA
RRMLRSNNKPKEEKSVLILPPRAGTPKLFNAALAMTGKMGGKGAKGGKLAHLRARLKELA
ALEAAAKQQELVEQAKSSQDVKGEEGSAAPDQHTHPKEAATDPPAPRTPPEPPGSPPSSP
PPASLGRPEGEEEGPAEPEEHSVRICMSPGPEPGEQILSVKMPEEREEKAE
>sp|O95406|CNIH1_HUMAN Protein cornichon homolog 1 OS=Homo sapiens GN=CNIH1 PE=1 SV=1
MAFTFAAFCYMLALLLTAALIFFAIWHIIAFDELKTDYKNPIDQCNTLNPLVLPEYLIHA
FFCVMFLCAAEWLTLGLNMPLLAYHIWRYMSRPVMSGPGLYDPTTIMNADILAYCQKEGW
CKLAFYLLAFFYYLYGMIYVLVSS
>sp|P51911|CNN1_HUMAN Calponin-1 OS=Homo sapiens GN=CNN1 PE=1 SV=2
MSSAHFNRGPAYGLSAEVKNKLAQKYDHQREQELREWIEGVTGRRIGNNFMDGLKDGIIL
CEFINKLQPGSVKKINESTQNWHQLENIGNFIKAITKYGVKPHDIFEANDLFENTNHTQV
QSTLLALASMAKTKGNKVNVGVKYAEKQERKFEPGKLREGRNIIGLQMGTNKFASQQGMT
AYGTRRHLYDPKLGTDQPLDQATISLQMGTNKGASQAGMTAPGTKRQIFEPGLGMEHCDT
LNVSLQMGSNKGASQRGMTVYGLPRQVYDPKYCLTPEYPELGEPAHNHHAHNYYNSA
>sp|Q9H9A5|CNO10_HUMAN CCR4-NOT transcription complex subunit 10 OS=Homo sapiens GN=CNOT10 PE=1 SV=1
MAADKPADQGAEKHEGTGQSSGITDQEKELSTNAFQAFTSGNYDACLQHLACLQDINKDD
YKIILNTAVAEFFKSNQTTTDNLRQTLNQLKNQVHSAVEEMDGLDDVENSMLYYNQAVIL
YHLRQYTEAISVGEKLYQFIEPFEEKFAQAVCFLLVDLYILTYQAEKALHLLAVLEKMIS
QGNNNKNGKNETGNNNNKDGSNHKAESGALIEAAKSKIHQYKVRAYIQMKSLKACKREIK
SVMNTAGNSAPSLFLKSNFEYLRGNYRKAVKLLNSSNIAEHPGFMKTGECLRCMFWNNLG
CIHFAMSKHNLGIFYFKKALQENDNVCAQLSAGSTDPGKKFSGRPMCTLLTNKRYELLYN
CGIQLLHIGRPLAAFECLIEAVQVYHANPRLWLRLAECCIAANKGTSEQETKGLPSKKGI
VQSIVGQGYHRKIVLASQSIQNTVYNDGQSSAIPVASMEFAAICLRNALLLLPEEQQDPK
QENGAKNSNQLGGNTESSESSETCSSKSHDGDKFIPAPPSSPLRKQELENLKCSILACSA
YVALALGDNLMALNHADKLLQQPKLSGSLKFLGHLYAAEALISLDRISDAITHLNPENVT
DVSLGISSNEQDQGSDKGENEAMESSGKRAPQCYPSSVNSARTVMLFNLGSAYCLRSEYD
KARKCLHQAASMIHPKEVPPEAILLAVYLELQNGNTQLALQIIKRNQLLPAVKTHSEVRK
KPVFQPVHPIQPIQMPAFTTVQRK
>sp|Q9UKZ1|CNO11_HUMAN CCR4-NOT transcription complex subunit 11 OS=Homo sapiens GN=CNOT11 PE=1 SV=1

MPGGGASAASGRLLTAAEQRGSREAAGSASRSGFGGSGGGRGGASGPGSGSGGPGGPAGR
MSLTPKELSSLLSIISEEAGGGSTFEGLSTAFHHYFSKADHFRLGSVLVMLLQQPDLLPS
AAQRLTALYLLWEMYRTEPLAANPFAASFAHLLNPAPPARGGQEPDRPPLSGFLPPITPP
EKFFLSQLMLAPPRELFKKTPRQIALMDVGNMGQSVDISGLQLALAERQSELPTQSKASF
PSILSDPDPDSSNSGFDSSVASQITEALVSGPKPPIESHFRPEFIRPPPPLHICEDELAW
LNPTEPDHAIQWDKSMCVKNSTGVEIKRIMAKAFKSPLSSPQQTQLLGELEKDPKLVYHI
GLTPAKLPDLVENNPLVAIEMLLKLMQSSQITEYFSVLVNMDMSLHSMEVVNRLTTAVDL
PPEFIHLYISNCISTCEQIKDKYMQNRLVRLVCVFLQSLIRNKIINVQDLFIEVQAFCIE
FSRIREAAGLFRLLKTLDTGETPSETKMSK
>sp|A5YKK6|CNOT1_HUMAN CCR4-NOT transcription complex subunit 1 OS=Homo sapiens GN=CNOT1 PE=1 SV=2
MNLDSLSLALSQISYLVDNLTKKNYRASQQEIQHIVNRHGPEADRHLLRCLFSHVDFSGD
GKSSGKDFHQTQFLIQECALLITKPNFISTLSYAIDNPLHYQKSLKPAPHLFAQLSKVLK
LSKVQEVIFGLALLNSSSSDLRGFAAQFIKQKLPDLLRSYIDADVSGNQEGGFQDIAIEV
LHLLLSHLLFGQKGAFGVGQEQIDAFLKTLRRDFPQERCPVVLAPLLYPEKRDILMDRIL
PDSGGVAKTMMESSLADFMQEVGYGFCASIEECRNIIVQFGVREVTAAQVARVLGMMART
HSGLTDGIPLQSISAPGSGIWSDGKDKSDGAQAHTWNVEVLIDVLKELNPSLNFKEVTYE
LDHPGFQIRDSKGLHNVVYGIQRGLGMEVFPVDLIYRPWKHAEGQLSFIQHSLINPEIFC
FADYPCHTVATDILKAPPEDDNREIATWKSLDLIESLLRLAEVGQYEQVKQLFSFPIKHC
PDMLVLALLQINTSWHTLRHELISTLMPIFLGNHPNSAIILHYAWHGQGQSPSIRQLIMH
AMAEWYMRGEQYDQAKLSRILDVAQDLKALSMLLNGTPFAFVIDLAALASRREYLKLDKW
LTDKIREHGEPFIQACMTFLKRRCPSILGGLAPEKDQPKSAQLPPETLATMLACLQACAG
SVSQELSETILTMVANCSNVMNKARQPPPGVMPKGRPPSASSLDAISPVQIDPLAGMTSL
SIGGSAAPHTQSMQGFPPNLGSAFSTPQSPAKAFPPLSTPNQTTAFSGIGGLSSQLPVGG
LGTGSLTGIGTGALGLPAVNNDPFVQRKLGTSGLNQPTFQQSKMKPSDLSQVWPEANQHF
SKEIDDEANSYFQRIYNHPPHPTMSVDEVLEMLQRFKDSTIKREREVFNCMLRNLFEEYR
FFPQYPDKELHITACLFGGIIEKGLVTYMALGLALRYVLEALRKPFGSKMYYFGIAALDR
FKNRLKDYPQYCQHLASISHFMQFPHHLQEYIEYGQQSRDPPVKMQGSITTPGSIALAQA
QAQAQVPAKAPLAGQVSTMVTTSTTTTVAKTVTVTRPTGVSFKKDVPPSINTTNIDTLLV
ATDQTERIVEPPENIQEKIAFIFNNLSQSNMTQKVEELKETVKEEFMPWVSQYLVMKRVS
IEPNFHSLYSNFLDTLKNPEFNKMVLNETYRNIKVLLTSDKAAANFSDRSLLKNLGHWLG
MITLAKNKPILHTDLDVKSLLLEAYVKGQQELLYVVPFVAKVLESSIRSVVFRPPNPWTM
AIMNVLAELHQEHDLKLNLKFEIEVLCKNLALDINELKPGNLLKDKDRLKNLDEQLSAPK
KDVKQPEELPPITTTTTSTTPATNTTCTATVPPQPQYSYHDINVYSLAGLAPHITLNPTI
PLFQAHPQLKQCVRQAIERAVQELVHPVVDRSIKIAMTTCEQIVRKDFALDSEESRMRIA
AHHMMRNLTAGMAMITCREPLLMSISTNLKNSFASALRTASPQQREMMDQAAAQLAQDNC
ELACCFIQKTAVEKAGPEMDKRLATEFELRKHARQEGRRYCDPVVLTYQAERMPEQIRLK
VGGVDPKQLAVYEEFARNVPGFLPTNDLSQPTGFLAQPMKQAWATDDVAQIYDKCITELE
QHLHAIPPTLAMNPQAQALRSLLEVVVLSRNSRDAIAALGLLQKAVEGLLDATSGADADL
LLRYRECHLLVLKALQDGRAYGSPWCNKQITRCLIECRDEYKYNVEAVELLIRNHLVNMQ
QYDLHLAQSMENGLNYMAVAFAMQLVKILLVDERSVAHVTEADLFHTIETLMRINAHSRG
NAPEGLPQLMEVVRSNYEAMIDRAHGGPNFMMHSGISQASEYDDPPGLREKAEYLLREWV
NLYHSAAAGRDSTKAFSAFVGQMHQQGILKTDDLITRFFRLCTEMCVEISYRAQAEQQHN
PAANPTMIRAKCYHNLDAFVRLIALLVKHSGEATNTVTKINLLNKVLGIVVGVLLQDHDV

RQSEFQQLPYHRIFIMLLLELNAPEHVLETINFQTLTAFCNTFHILRPTKAPGFVYAWLE
LISHRIFIARMLAHTPQQKGWPMYAQLLIDLFKYLAPFLRNVELTKPMQILYKGTLRVLL
VLLHDFPEFLCDYHYGFCDVIPPNCIQLRNLILSAFPRNMRLPDPFTPNLKVDMLSEINI
APRILTNFTGVMPPQFKKDLDSYLKTRSPVTFLSDLRSNLQVSNEPGNRYNLQLINALVL
YVGTQAIAHIHNKGSTPSMSTITHSAHMDIFQNLAVDLDTEGRYLFLNAIANQLRYPNSH
THYFSCTMLYLFAEANTEAIQEQITRVLLERLIVNRPHPWGLLITFIELIKNPAFKFWNH
EFVHCAPEIEKLFQSVAQCCMGQKQAQQVMEGTGAS
>sp|Q9UIV1|CNOT7_HUMAN CCR4-NOT transcription complex subunit 7 OS=Homo sapiens GN=CNOT7 PE=1 SV=3
MPAATVDHSQRICEVWACNLDEEMKKIRQVIRKYNYVAMDTEFPGVVARPIGEFRSNADY
QYQLLRCNVDLLKIIQLGLTFMNEQGEYPPGTSTWQFNFKFNLTEDMYAQDSIELLTTSG
IQFKKHEEEGIETQYFAELLMTSGVVLCEGVKWLSFHSGYDFGYLIKILTNSNLPEEELD
FFEILRLFFPVIYDVKYLMKSCKNLKGGLQEVAEQLELERIGPQHQAGSDSLLTGMAFFK
MREMFFEDHIDDAKYCGHLYGLGSGSSYVQNGTGNAYEEEANKQS
>sp|Q9Y2B0|CNPY2_HUMAN Protein canopy homolog 2 OS=Homo sapiens GN=CNPY2 PE=1 SV=1
MKGWGWLALLLGALLGTAWARRSQDLHCGACRALVDELEWEIAQVDPKKTIQMGSFRINP
DGSQSVVEVPYARSEAHLTELLEEICDRMKEYGEQIDPSTHRKNYVRVVGRNGESSELDL
QGIRIDSDISGTLKFACESIVEEYEDELIEFFSREADNVKDKLCSKRTDLCDHALHISHD
EL
>sp|P18545|CNRG_HUMAN Retinal rod rhodopsin-sensitive cGMP 3',5'-cyclic phosphodiesterase subunit gamma OS=Homo sapiens GN=PDE6G PE=1 SV=1
MNLEPPKAEFRSATRVAGGPVTPRKGPPKFKQRQTRQFKSKPPKKGVQGFGDDIPGMEGL
GTDITVICPWEAFNHLELHELAQYGII
>sp|Q9BV29|CO057_HUMAN Uncharacterized protein C15orf57 OS=Homo sapiens GN=C15orf57 PE=1 SV=2
MKMFESADSTATRSGQDLWAEICSCLPNPEQEDGANNAFSDSFVDSCPEGEGQREVADFA
VQPAVKPWAPLQDSEVYLASLEKKLRRIKGLNQEVTSKDMLRTLAQAKKECWDRFLQEKL
ASEFFVDGLDSDESTLEHFKRWLQPDKVAVSTEEVQYLIPPESQVEKPVAEDEPAAGDKP
AAAEQ
>sp|P02452|CO1A1_HUMAN Collagen alpha-1(I) chain OS=Homo sapiens GN=COL1A1 PE=1 SV=5
MFSFVDLRLLLLLAATALLTHGQEEGQVEGQDEDIPPITCVQNGLRYHDRDVWKPEPCRI
CVCDNGKVLCDDVICDETKNCPGAEVPEGECCPVCPDGSESPTDQETTGVEGPKGDTGPR
GPRGPAGPPGRDGIPGQPGLPGPPGPPGPPGPPGLGGNFAPQLSYGYDEKSTGGISVPGP
MGPSGPRGLPGPPGAPGPQGFQGPPGEPGEPGASGPMGPRGPPGPPGKNGDDGEAGKPGR
PGERGPPGPQGARGLPGTAGLPGMKGHRGFSGLDGAKGDAGPAGPKGEPGSPGENGAPGQ
MGPRGLPGERGRPGAPGPAGARGNDGATGAAGPPGPTGPAGPPGFPGAVGAKGEAGPQGP
RGSEGPQGVRGEPGPPGPAGAAGPAGNPGADGQPGAKGANGAPGIAGAPGFPGARGPSGP
QGPGGPPGPKGNSGEPGAPGSKGDTGAKGEPGPVGVQGPPGPAGEEGKRGARGEPGPTGL
PGPPGERGGPGSRGFPGADGVAGPKGPAGERGSPGPAGPKGSPGEAGRPGEAGLPGAKGL
TGSPGSPGPDGKTGPPGPAGQDGRPGPPGPPGARGQAGVMGFPGPKGAAGEPGKAGERGV
PGPPGAVGPAGKDGEAGAQGPPGPAGPAGERGEQGPAGSPGFQGLPGPAGPPGEAGKPGE
QGVPGDLGAPGPSGARGERGFPGERGVQGPPGPAGPRGANGAPGNDGAKGDAGAPGAPGS
QGAPGLQGMPGERGAAGLPGPKGDRGDAGPKGADGSPGKDGVRGLTGPIGPPGPAGAPGD
KGESGPSGPAGPTGARGAPGDRGEPGPPGPAGFAGPPGADGQPGAKGEPGDAGAKGDAGP

PGPAGPAGPPGPIGNVGAPGAKGARGSAGPPGATGFPGAAGRVGPPGPSGNAGPPGPPGP
AGKEGGKGPRGETGPAGRPGEVGPPGPPGPAGEKGSPGADGPAGAPGTPGPQGIAGQRGV
VGLPGQRGERGFPGLPGPSGEPGKQGPSGASGERGPPGPMGPPGLAGPPGESGREGAPGA
EGSPGRDGSPGAKGDRGETGPAGPPGAPGAPGAPGPVGPAGKSGDRGETGPAGPTGPVGP
VGARGPAGPQGPRGDKGETGEQGDRGIKGHRGFSGLQGPPGPPGSPGEQGPSGASGPAGP
RGPPGSAGAPGKDGLNGLPGPIGPPGPRGRTGDAGPVGPPGPPGPPGPPGPPSAGFDFSF
LPQPPQEKAHDGGRYYRADDANVVRDRDLEVDTTLKSLSQQIENIRSPEGSRKNPARTCR
DLKMCHSDWKSGEYWIDPNQGCNLDAIKVFCNMETGETCVYPTQPSVAQKNWYISKNPKD
KRHVWFGESMTDGFQFEYGGQGSDPADVAIQLTFLRLMSTEASQNITYHCKNSVAYMDQQ
TGNLKKALLLQGSNEIEIRAEGNSRFTYSVTVDGCTSHTGAWGKTVIEYKTTKTSRLPII
DVAPLDVGAPDQEFGFDVGPVCFL
>sp|Q6ZVT6|CC067_HUMAN Uncharacterized protein C3orf67 OS=Homo sapiens GN=C3orf67 PE=2 SV=2
MIKRKIWCNLCIDLVAFTSEIFKGAVFQSLDGIVVSANCKLRKIFTLKSKPQDTADKDAV
YGVPFSTDEPTDIIPRSCQLMTDVPHVTQLLNMTKLRQTEIKFGGHPLRSAESDQFINRG
TSITRNSKNQDVCHIAFGSKVLGPPPLSGRRNNMKISSETVRSVGSKNNRSCQPSTVEKC
VNGTEMSALLIPESEEQGNKENIHQIKQTVPIHAANLHIMHPHPPQEPSADKNNNRRRLR
LKSTSRERTETPSGSSSGNNRIEDKASTILTTVSQQGAELLNSGTLGPQSPDQSDEWIFP
ENADHISYLASSRQSLLLGDDSCNPSHLWLEASKESEHDQQAEESQSVPKDIFTFSSRPR
SAPHGKTQTMSPEELSFILDLKEDNSVTSRDTQSEDDFYGGDSSEEGNHSIQGSRGPTTG
PSELTQLTLESLLGKAAKRTSKEYLRSAYTEAGATESQDSSMEQIDRNNFEMSLLPTTCL
SPTGRRCGSCQKTPEPVIKAKDLSAQQVPASLNKTSLKEISGERLSSIPEASEYDWRNYQ
PSQMSESELQMLASLRWQQNEELEDAGTSHGLSASQVDNCNVSISTSSDDTTTWNSCLPP
PVNQGRHYQKEMNPPSPSNPRDWLNMLSPPIVPPSQQPAEQRPDSCESLSVQGEEDLSVE
EDEEVLTLLYDPCLNCYFDPQTGKYYELV
>sp|O75794|CD123_HUMAN Cell division cycle protein 123 homolog OS=Homo sapiens GN=CDC123 PE=1 SV=1
MKKEHVLHCQFSAWYPFFRGVTIKSVILPLPQNVKDYLLDDGTLVVSGRDDPPTHSQPDS
DDEAEEIQWSDDENTATLTAPEFPEFATKVQEAINSLGGSVFPKLNWSAPRDAYWIAMNS
SLKCKTLSDIFLLFKSSDFITRDFTQPFIHCTDDSPDPCIEYELVLRKWCELIPGAEFRC
FVKENKLIGISQRDYTQYYDHISKQKEEIRRCIQDFFKKHIQYKFLDEDFVFDIYRDSRG
KVWLIDFNPFGEVTDSLLFTWEELISENNLNGDFSEVDAQEQDSPAFRCTNSEVTVQPSP
YLSYRLPKDFVDLSTGEDAHKLIDFLKLKRNQQEDD
>sp|P06729|CD2_HUMAN T-cell surface antigen CD2 OS=Homo sapiens GN=CD2 PE=1 SV=2
MSFPCKFVASFLLIFNVSSKGAVSKEITNALETWGALGQDINLDIPSFQMSDDIDDIKWE
KTSDKKKIAQFRKEKETFKEKDTYKLFKNGTLKIKHLKTDDQDIYKVSIYDTKGKNVLEK
IFDLKIQERVSKPKISWTCINTTLTCEVMNGTDPELNLYQDGKHLKLSQRVITHKWTTSL
SAKFKCTAGNKVSKESSVEPVSCPEKGLDIYLIIGICGGGSLLMVFVALLVFYITKRKKQ
RSRRNDEELETRAHRVATEERGRKPHQIPASTPQNPATSQHPPPPPGHRSQAPSHRPPPP
GHRVQHQPQKRPPAPSGTQVHQQKGPPLPRPRVQPKPPHGAAENSLSPSSN
>sp|P16671|CD36_HUMAN Platelet glycoprotein 4 OS=Homo sapiens GN=CD36 PE=1 SV=2
MGCDRNCGLIAGAVIGAVLAVFGGILMPVGDLLIQKTIKKQVVLEEGTIAFKNWVKTGTE
VYRQFWIFDVQNPQEVMMNSSNIQVKQRGPYTYRVRFLAKENVTQDAEDNTVSFLQPNGA
IFEPSLSVGTEADNFTVLNLAVAAASHIYQNQFVQMILNSLINKSKSSMFQVRTLRELLW

GYRDPFLSLVPYPVTTTVGLFYPYNNTADGVYKVFNGKDNISKVAIIDTYKGKRNLSYWE
SHCDMINGTDAASFPPFVEKSQVLQFFSSDICRSIYAVFESDVNLKGIPVYRFVLPSKAF
ASPVENPDNYCFCTEKIISKNCTSYGVLDISKCKEGRPVYISLPHFLYASPDVSEPIDGL
NPNEEEHRTYLDIEPITGFTLQFAKRLQVNLLVKPSEKIQVLKNLKRNYIVPILWLNETG
TIGDEKANMFRSQVTGKINLLGLIEMILLSVGVVMFVAFMISYCACRSKTIK
>sp|P11049|CD37_HUMAN Leukocyte antigen CD37 OS=Homo sapiens GN=CD37 PE=1 SV=2
MSAQESCLSLIKYFLFVFNLFFFVLGSLIFCFGIWILIDKTSFVSFVGLAFVPLQIWSKV
LAISGIFTMGIALLGCVGALKELRCLLGLYFGMLLLLFATQITLGILISTQRAQLERSLR
DVVEKTIQKYGTNPEETAAEESWDYVQFQLRCCGWHYPQDWFQVLILRGNGSEAHRVPCS
CYNLSATNDSTILDKVILPQLSRLGHLARSRHSADICAVPAESHIYREGCAQGLQKWLHN
NLISIVGICLGVGLLELGFMTLSIFLCRNLDHVYNRLARYR
>sp|Q07108|CD69_HUMAN Early activation antigen CD69 OS=Homo sapiens GN=CD69 PE=1 SV=1
MSSENCFVAENSSLHPESGQENDATSPHFSTRHEGSFQVPVLCAVMNVVFITILIIALIA
LSVGQYNCPGQYTFSMPSDSHVSSCSEDWVGYQRKCYFISTVKRSWTSAQNACSEHGATL
AVIDSEKDMNFLKRYAGREEHWVGLKKEPGHPWKWSNGKEFNNWFNVTGSDKCVFLKNTE
VSSMECEKNLYWICNKPYK
>sp|P21926|CD9_HUMAN CD9 antigen OS=Homo sapiens GN=CD9 PE=1 SV=4
MPVKGGTKCIKYLLFGFNFIFWLAGIAVLAIGLWLRFDSQTKSIFEQETNNNNSSFYTGV
YILIGAGALMMLVGFLGCCGAVQESQCMLGLFFGFLLVIFAIEIAAAIWGYSHKDEVIKE
VQEFYKDTYNKLKTKDEPQRETLKAIHYALNCCGLAGGVEQFISDICPKKDVLETFTVKS
CPDAIKEVFDNKFHIIGAVGIGIAVVMIFGMIFSMILCCAIRRNREMV
>sp|P49336|CDK8_HUMAN Cyclin-dependent kinase 8 OS=Homo sapiens GN=CDK8 PE=1 SV=1
MDYDFKVKLSSERERVEDLFEYEGCKVGRGTYGHVYKAKRKDGKDDKDYALKQIEGTGIS
MSACREIALLRELKHPNVISLQKVFLSHADRKVWLLFDYAEHDLWHIIKFHRASKANKKP
VQLPRGMVKSLLYQILDGIHYLHANWVLHRDLKPANILVMGEGPERGRVKIADMGFARLF
NSPLKPLADLDPVVVTFWYRAPELLLGARHYTKAIDIWAIGCIFAELLTSEPIFHCRQED
IKTSNPYHHDQLDRIFNVMGFPADKDWEDIKKMPEHSTLMKDFRRNTYTNCSLIKYMEKH
KVKPDSKAFHLLQKLLTMDPIKRITSEQAMQDPYFLEDPLPTSDVFAGCQIPYPKREFLT
EEEPDDKGDKKNQQQQQGNNHTNGTGHPGNQDSSHTQGPPLKKVRVVPPTTTSGGLIMTS
DYQRSNPHAAYPNPGPSTSQPQSSMGYSATSQQPPQYSHQTHRY
>sp|O14627|CDX4_HUMAN Homeobox protein CDX-4 OS=Homo sapiens GN=CDX4 PE=1 SV=1
MYGSCLLEKEAGMYPGTLMSPGGDGTAGTGGTGGGGSPMPASNFAAAPAFSHYMGYPHMP
SMDPHWPSLGVWGSPYSPPREDWSVYPGPSSTMGTVPVNDVTSSPAAFCSTDYSNLGPVG
GGTSGSSLPGQAGGSLVPTDAGAAKASSPSRSRHSPYAWMRKTVQVTGKTRTKEKYRVVY
TDHQRLELEKEFHCNRYITIQRKSELAVNLGLSERQVKIWFQNRRAKERKMIKKKISQFE
NSGGSVQSDSDSISPGELPNTFFTTPSAVRGFQPIEIQQVIVSE
>sp|Q8N9H6|CH031_HUMAN Uncharacterized protein C8orf31 OS=Homo sapiens GN=C8orf31 PE=2 SV=1
MAEKPHQNSCNSVRQLFKTKQLVTHRDRGSCTHRAQGLLAARTTALQRSPLQQEIWESTT
ALNLPSALAPQGLTAKDAHFLGDTDPIQEGARDHAAGGPFQDRQASVAAQTLSWERGQGF
SRHHGNHLLYSH
>sp|Q6P1X6|CH082_HUMAN UPF0598 protein C8orf82 OS=Homo sapiens GN=C8orf82 PE=1 SV=2
MWPPCGTLRTLALARSRGARACSGDGGVSYTQGQSPEPRTREYFYYVDHQGQLFLDDSKM
KNFITCFKDPQFLVTFFSRLRPNRSGRYEAAFPFLSPCGRERNFLRCEDRPVVFTHLLTA

DHGPPRLSYCGGGEALAVPFEPARLLPLAANGRLYHPAPERAGGVGLVRSALAFELSACF
EYGPGAPALPSHVRWQGRRLALTMDLAPLLLAARSP
>sp|P0DMQ9|CH089_HUMAN Putative uncharacterized protein C8orf89 OS=Homo sapiens GN=C8orf89 PE=2 SV=1
MSVLSPEIKCETSKFTRSSFGSCLIFESSWKKAVLETQKIKKEYTTAFGLEELKECIKMP
YLPGLQSCQKSVSSTPLEVPKRLPRADAEVSAVRLKKTKETCSVAPLWEKSKGSGFSDPL
TGAPSQYLERLSKIAILEYDTIRQETTTKSKKSKKRDLRDR
>sp|Q9HD42|CHM1A_HUMAN Charged multivesicular body protein 1a OS=Homo sapiens GN=CHMP1A PE=1 SV=1
MDDTLFQLKFTAKQLEKLAKKAEKDSKAEQAKVKKALLQKNVECARVYAENAIRKKNEGV
NWLRMASRVDAVASKVQTAVTMKGVTKNMAQVTKALDKALSTMDLQKVSSVMDRFEQQVQ
NLDVHTSVMEDSMSSATTLTTPQEQVDSLIMQIAEENGLEVLDQLSQLPEGASAVGESSV
RSQEDQLSRRLAALRN
>sp|Q9UQN3|CHM2B_HUMAN Charged multivesicular body protein 2b OS=Homo sapiens GN=CHMP2B PE=1 SV=1
MASLFKKKTVDDVIKEQNRELRGTQRAIIRDRAALEKQEKQLELEIKKMAKIGNKEACKV
LAKQLVHLRKQKTRTFAVSSKVTSMSTQTKVMNSQMKMAGAMSTTAKTMQAVNKKMDPQK
TLQTMQNFQKENMKMEMTEEMINDTLDDIFDGSDDEEESQDIVNQVLDEIGIEISGKMAK
APSAARSLPSASTSKATISDEEIERQLKALGVD
>sp|Q70JA7|CHSS3_HUMAN Chondroitin sulfate synthase 3 OS=Homo sapiens GN=CHSY3 PE=2 SV=3
MAVRSRRPWMSVALGLVLGFTAASWLIAPRVAELSERKRRGSSLCSYYGRSAAGPRAGAQ
QPLPQPQSRPRQEQSPPPARQDLQGPPLPEAAPGITSFRSSPWQQPPPLQQRRRGREPEG
ATGLPGAPAAEGEPEEEDGGAAGQRRDGRPGSSHNGSGDGGAAAPSARPRDFLYVGVMTA
QKYLGSRALAAQRTWARFIPGRVEFFSSQQPPNAGQPPPPLPVIALPGVDDSYPPQKKSF
MMIKYMHDHYLDKYEWFMRADDDVYIKGDKLEEFLRSLNSSKPLYLGQTGLGNIEELGKL
GLEPGENFCMGGPGMIFSREVLRRMVPHIGECLREMYTTHEDVEVGRCVRRFGGTQCVWS
YEMQQLFHENYEHNRKGYIQDLHNSKIHAAITLHPNKRPAYQYRLHNYMLSRKISELRYR
TIQLHRESALMSKLSNTEVSKEDQQLGVIPSFNHFQPRERNEVIEWEFLTGKLLYSAAEN
QPPRQSLSSILRTALDDTVLQVMEMINENAKSRGRLIDFKEIQYGYRRVNPMHGVEYILD
LLLLYKRHKGRKLTVPVRRHAYLQQLFSKPFFRETEELDVNSLVESINSETQSFSFISNS
LKILSSFQGAKEMGGHNEKKVHILVPLIGRYDIFLRFMENFENMCLIPKQNVKLVIILFS
RDSGQDSSKHIELIKGYQNKYPKAEMTLIPMKGEFSRGLGLEMASAQFDNDTLLLFCDVD
LIFREDFLQRCRDNTIQGQQVYYPIIFSQYDPKVTNGGNPPTDDYFIFSKKTGFWRDYGY
GITCIYKSDLLGAGGFDTSIQGWGLEDVDLYNKVILSGLRPFRSQEVGVVHIFHPVHCDP
NLDPKQYKMCLGSKASTFASTMQLAELWLEKHLGVRYNRTLS
>sp|Q7LGC8|CHST3_HUMAN Carbohydrate sulfotransferase 3 OS=Homo sapiens GN=CHST3 PE=1 SV=3
MEKGLTLPQDCRDFVHSLKMRSKYALFLVFVVIVFVFIEKENKIISRVSDKLKQIPQALA
DANSTDPALILAENASLLSLSELDSAFSQLQSRLRNLSLQLGVEPAMEAAGEEEEEQRKE
EEPPRPAVAGPRRHVLLMATTRTGSSFVGEFFNQQGNIFYLFEPLWHIERTVSFEPGGAN
AAGSALVYRDVLKQLFLCDLYVLEHFITPLPEDHLTQFMFRRGSSRSLCEDPVCTPFVKK
VFEKYHCKNRRCGPLNVTLAAEACRRKEHMALKAVRIRQLEFLQPLAEDPRLDLRVIQLV
RDPRAVLASRMVAFAGKYKTWKKWLDDEGQDGLREEEVQRLRGNCESIRLSAELGLRQPA
WLRGRYMLVRYEDVARGPLQKAREMYRFAGIPLTPQVEDWIQKNTQAAHDGSGIYSTQKN
SSEQFEKWRFSMPFKLAQVVQAACGPAMRLFGYKLARDAAALTNRSVSLLEERGTFWVT

>sp|Q9NS84|CHST7_HUMAN Carbohydrate sulfotransferase 7 OS=Homo sapiens GN=CHST7 PE=1 SV=2
MKGRRRRRREYCKFALLLVLYTLVLLLVPSVLDGGRDGDKGAEHCPGLQRSLGVWSLEAA
AAGEREQGAEARAAEEGGANQSPRFPSNLSGAVGEAVSREKQHIYVHATWRTGSSFLGEL
FNQHPDVFYLYEPMWHLWQALYPGDAESLQGALRDMLRSLFRCDFSVLRLYAPPGDPAAR
APDTANLTTAALFRWRTNKVICSPPLCPGAPRARAEVGLVEDTACERSCPPVAIRALEAE
CRKYPVVVIKDVRLLDLGVLVPLLRDPGLNLKVVQLFRDPRAVHNSRLKSRQGLLRESIQ
VLRTRQRGDRFHRVLLAHGVGARPGGQSRALPAAPRADFFLTGALEVICEAWLRDLLFAR
GAPAWLRRRYLRLRYEDLVRQPRAQLRRLLRFSGLRALAALDAFALNMTRGAAYGADRPF
HLSARDAREAVHAWRERLSREQVRQVEAACAPAMRLLAYPRSGEEGDAEQPREGETPLEM
DADGAT
>sp|Q8IXQ3|CI040_HUMAN Uncharacterized protein C9orf40 OS=Homo sapiens GN=C9orf40 PE=1 SV=1
MAKRRAAEPVTFHVPWKRLLLCDFAEQPPPPPLWIRPPGVAHAGQLLGVPEQHRKRKIDA
GTMAEPSASPSKRRDSGDNSAPSGQEREDHGLETGDPPLPPPPVLPGPGEELPGARLPGG
GGDDGAGRAGPPRGDWGVASRQHNEEFWQYNTFQYWRNPLPPIDLADIEDLSEDTLTEAT
LQGRNEGAEVDMES
>sp|Q5T280|CI114_HUMAN Putative methyltransferase C9orf114 OS=Homo sapiens GN=C9orf114 PE=1 SV=3
MAERGRKRPCGPGEHGQRIEWRKWKQQKKEEKKKWKDLKLMKKLERQRAQEEQAKRLEEE
EAAAEKEDRGRPYTLSVALPGSILDNAQSPELRTYLAGQIARACAIFCVDEIVVFDEEGQ
DAKTVEGEFTGVGKKGQACVQLARILQYLECPQYLRKAFFPKHQDLQFAGLLNPLDSPHH
MRQDEESEFREGIVVDRPTRPGHGSFVNCGMKKEVKIDKNLEPGLRVTVRLNQQQHPDCK
TYHGKVVSSQDPRTKAGLYWGYTVRLASCLSAVFAEAPFQDGYDLTIGTSERGSDVASAQ
LPNFRHALVVFGGLQGLEAGADADPNLEVAEPSVLFDLYVNTCPGQGSRTIRTEEAILIS
LAALQPGLIQAGARHT
>sp|Q8N9P6|CI163_HUMAN Uncharacterized protein C9orf163 OS=Homo sapiens GN=C9orf163 PE=2 SV=1
MPGPLTCTPAWQGQGRAAAFLCCSFQRAGAVVGVPARWHRGRLSSQQRLRSSLGGSHPCP
QLGRRLVREGVISVPRQQGRRRCRESFSPADVAPGPICSANICLSGVRFLTCLNRVREHV
VGPSPSPAAPICFFPVVEALCTLRGRRCHCLPFPKRGMQRWMLPLRRGARLLPLASSKNP
RARSPGLDPLGSSETLWSHRGGH
>sp|A0PJX0|CIB4_HUMAN Calcium and integrin-binding family member 4 OS=Homo sapiens GN=CIB4 PE=1 SV=1
MGQCLRYQMHWEDLEEYQALTFLTRNEILCIHDTFLKLCPPGKYYKEATLTMDQVSSLPA
LRVNPFRDRICRVFSHKGMFSFEDVLGMASVFSEQACPSLKIEYAFRIYDFNENGFIDEE
DLQRIILRLLNSDDMSEDLLMDLTNHVLSESDLDNDNMLSFSEFEHAMAKSPDFMNSFRI
HFWGC
>sp|Q8TCG1|CIP2A_HUMAN Protein CIP2A OS=Homo sapiens GN=KIAA1524 PE=1 SV=2
MDSTACLKSLLLTVSQYKAVKSEANATQLLRHLEVISGQKLTRLFTSNQILTSECLSCLV
ELLEDPNISASLILSIIGLLSQLAVDIETRDCLQNTYNLNSVLAGVVCRSSHTDSVFLQC
IQLLQKLTYNVKIFYSGANIDELITFLIDHIQSSEDELKMPCLGLLANLCRHNLSVQTHI
KTLSNVKSFYRTLITLLAHSSLTVVVFALSILSSLTLNEEVGEKLFHARNIHQTFQLIFN
ILINGDGTLTRKYSVDLLMDLLKNPKIADYLTRYEHFSSCLHQVLGLLNGKDPDSSSKVL
ELLLAFCSVTQLRHMLTQMMFEQSPPGSATLGSHTKCLEPTVALLRWLSQPLDGSENCSV

LALELFKEIFEDVIDAANCSSADRFVTLLLPTILDQLQFTEQNLDEALTRKKCERIAKAI
EVLLTLCGDDTLKMHIAKILTTVKCTTLIEQQFTYGKIDLGFGTKVADSELCKLAADVIL
KTLDLINKLKPLVPGMEVSFYKILQDPRLITPLAFALTSDNREQVQSGLRILLEAAPLPD
FPALVLGESIAANNAYRQQETEHIPRKMPWQSSNHSFPTSIKCLTPHLKDGVPGLNIEEL
IEKLQSGMVVKDQICDVRISDIMDVYEMKLSTLASKESRLQDLLETKALALAQADRLIAQ
HRCQRTQAETEARTLASMLREVERKNEELSVLLKAQQVESERAQSDIEHLFQHNRKLESV
AEEHEILTKSYMELLQRNESTEKKNKDLQITCDSLNKQIETVKKLNESLKEQNEKSIAQL
IEKEEQRKEVQNQLVDREHKLANLHQKTKVQEEKIKTLQKEREDKEETIDILRKELSRTE
QIRKELSIKASSLEVQKAQLEGRLEEKESLVKLQQEELNKHSHMIAMIHSLSGGKINPET
VNLSI
>sp|Q15642|CIP4_HUMAN Cdc42-interacting protein 4 OS=Homo sapiens GN=TRIP10 PE=1 SV=3
MDWGTELWDQFEVLERHTQWGLDLLDRYVKFVKERTEVEQAYAKQLRSLVKKYLPKRPAK
DDPESKFSQQQSFVQILQEVNDFAGQRELVAENLSVRVCLELTKYSQEMKQERKMHFQEG
RRAQQQLENGFKQLENSKRKFERDCREAEKAAQTAERLDQDINATKADVEKAKQQAHLRS
HMAEESKNEYAAQLQRFNRDQAHFYFSQMPQIFDKLQDMDERRATRLGAGYGLLSEAELE
VVPIIAKCLEGMKVAANAVDPKNDSHVLIELHKSGFARPGDVEFEDFSQPMNRAPSDSSL
GTPSDGRPELRGPGRSRTKRWPFGKKNKPRPPPLSPLGGPVPSALPNGPPSPRSGRDPLA
ILSEISKSVKPRLASFRSLRGSRGTVVTEDFSHLPPEQQRKRLQQQLEERSRELQKEVDQ
REALKKMKDVYEKTPQMGDPASLEPQIAETLSNIERLKLEVQKYEAWLAEAESRVLSNRG
DSLSRHARPPDPPASAPPDSSSNSASQDTKESSEEPPSEESQDTPIYTEFDEDFEEEPTS
PIGHCVAIYHFEGSSEGTISMAEGEDLSLMEEDKGDGWTRVRRKEGGEGYVPTSYLRVTL
N
>sp|Q9NZ45|CISD1_HUMAN CDGSH iron-sulfur domain-containing protein 1 OS=Homo sapiens GN=CISD1 PE=1 SV=1
MSLTSSSSVRVEWIAAVTIAAGTAAIGYLAYKRFYVKDHRNKAMINLHIQKDNPKIVHAF
DMEDLGDKAVYCRCWRSKKFPFCDGAHTKHNEETGDNVGPLIIKKKET
>sp|Q99967|CITE2_HUMAN Cbp/p300-interacting transactivator 2 OS=Homo sapiens GN=CITED2 PE=1 SV=2
MADHMMAMNHGRFPDGTNGLHHHPAHRMGMGQFPSPHHHQQQQPQHAFNALMGEHIHYGA
GNMNATSGIRHAMGPGTVNGGHPPSALAPAARFNNSQFMGPPVASQGGSLPASMQLQKLN
NQYFNHHPYPHNHYMPDLHPAAGHQMNGTNQHFRDCNPKHSGGSSTPGGSGGSSTPGGSG
SSSGGGAGSSNSGGGSGSGNMPASVAHVPAAMLPPNVIDTDFIDEEVLMSLVIEMGLDRI
KELPELWLGQNEFDFMTDFVCKQQPSRVSC
>sp|Q96RK1|CITE4_HUMAN Cbp/p300-interacting transactivator 4 OS=Homo sapiens GN=CITED4 PE=1 SV=1
MADHLMLAEGYRLVQRPPSAAAAHGPHALRTLPPYAGPGLDSGLRPRGAPLGPPPPRQPG
ALAYGAFGPPSSFQPFFAVPPPAAGIAHLQPVATPYPGRAAAPPNAPGGPPGPQPAPSAA
APPPPAHALGGMDAELIDEEALTSLELELGLHRVRELPELFLGQSEFDCFSDLGSAPPAG
SVSC
>sp|Q9ULV3|CIZ1_HUMAN Cip1-interacting zinc finger protein OS=Homo sapiens GN=CIZ1 PE=1 SV=2
MFSQQQQQQLQQQQQQLQQLQQQQLQQQQLQQQQLLQLQQLLQQSPPQAPLPMAVSRGLP
PQQPQQPLLNLQGTNSASLLNGSMLQRALLLQQLQGLDQFAMPPATYDTAGLTMPTATLG
NLRGYGMASPGLAAPSLTPPQLATPNLQQFFPQATRQSLLGPPPVGVPMNPSQFNLSGRN

PQKQARTSSSTTPNRKDSSSQTMPVEDKSDPPEGSEEAAEPRMDTPEDQDLPPCPEDIAK
EKRTPAPEPEPCEASELPAKRLRSSEEPTEKEPPGQLQVKAQPQARMTVPKQTQTPDLLP
EALEAQVLPRFQPRVLQVQAQVQSQTQPRIPSTDTQVQPKLQKQAQTQTSPEHLVLQQKQ
VQPQLQQEAEPQKQVQPQVQPQAHSQGPRQVQLQQEAEPLKQVQPQVQPQAHSQPPRQVQ
LQLQKQVQTQTYPQVHTQAQPSVQPQEHPPAQVSVQPPEQTHEQPHTQPQVSLLAPEQTP
VVVHVCGLEMPPDAVEAGGGMEKTLPEPVGTQVSMEEIQNESACGLDVGECENRAREMPG
VWGAGGSLKVTILQSSDSRAFSTVPLTPVPRPSDSVSSTPAATSTPSKQALQFFCYICKA
SCSSQQEFQDHMSEPQHQQRLGEIQHMSQACLLSLLPVPRDVLETEDEEPPPRRWCNTCQ
LYYMGDLIQHRRTQDHKIAKQSLRPFCTVCNRYFKTPRKFVEHVKSQGHKDKAKELKSLE
KEIAGQDEDHFITVDAVGCFEGDEEEEEDDEDEEEIEVEEELCKQVRSRDISREEWKGSE
TYSPNTAYGVDFLVPVMGYICRICHKFYHSNSGAQLSHCKSLGHFENLQKYKAAKNPSPT
TRPVSRRCAINARNALTALFTSSGRPPSQPNTQDKTPSKVTARPSQPPLPRRSTRLKT
>sp|Q5T742|CJ025_HUMAN Uncharacterized protein C10orf25 OS=Homo sapiens GN=C10orf25 PE=2 SV=3
MVPGPPESVVRFFLWFCFLLPPTRKASCDPRDLKSCNRPCVWSRLLKPNSSLSNLETAYF
PQILRFLRPWYFSRSHLNYHQKAPARWEWLYSIYRKGTKAQRRNVLRSPCAPPQPSWPCS
VI
>sp|A4QN01|CJ040_HUMAN Putative uncharacterized protein encoded by LINC01553 OS=Homo sapiens GN=LINC01553 PE=2 SV=1
MKHKYNTLSWYKKERSTQLRRFIVHQPEVGLFFFLRDVVYNYITEKTTCRCPLTQERPCD
TGGKFGRIWICQPEELGKMQIWICQPEELGKMQIDKNRTMVQQMWYIWYGGLDTSTNSLL
PILVRGMA
>sp|Q9H8K7|CJ088_HUMAN Uncharacterized protein C10orf88 OS=Homo sapiens GN=C10orf88 PE=1 SV=2
METRTEDGGLTRRPTLASSWDVAGGALTHSLLLTRAGLGPGDFDWEELLAPPAPGQDLVI
LKRNHNNKDENPCFLYLRCGPDGGEEIASIGILSSARNMEVYLGEEYCGTSRGKNVCTVL
DDSEHEKIILYKKNLKLESSTHACKIKLLSFGERQCVFISKVVVHMRSVFANSSTSSPAL
GSRIDLDKVQTIMESMGSKLSPGAQQLMDMVRCQQRNCIPIGEQLQSVLGNSGYKHMIGL
QSSSTLGTLNKSSSTPFPFRTGLTSGNVTENLQTYIDKSTQLPGGENSTKLDECKVMPQN
HSFLENDLKNAMASFLPKKVSDNSNIPNSELLPFLQNLCSQVNHLHVGNKTECQENITKH
GERILGVGMEEQSICSYLEKILSKNMELMEKKLMDYIDQRIHELQEHIDDKIALLLDLLQ
NPNSPPTGIPLRHYDSGERLSNGER
>sp|Q6UWK7|CJ099_HUMAN Secreted protein C10orf99 OS=Homo sapiens GN=C10orf99 PE=1 SV=1
MRLLVLSSLLCILLLCFSIFSTEGKRRPAKAWSGRRTRLCCHRVPSPNSTNLKGHHVRLC
KPCKLEPEPRLWVVPGALPQV
>sp|Q5SQS8|CJ120_HUMAN Uncharacterized protein C10orf120 OS=Homo sapiens GN=C10orf120 PE=2 SV=1
MIREWKNDCQRIEKQRASDTMVQERKNEKPVRIFNTNSSFQDQAPTCCQEDLSSASPLRI
WSKFYRSDPRIALGKYSPLEKEILRLGGIHTIAARRLLAYKQEEECRMLKELQLLSPDYK
QAMEYKKKHSSPCAICVPLEKIWTAKVIAPLEAFKMPQREQVNVSKHIERMRLARALGNH
QPLPYIERFTRSSFLSGVGLGPMAKNKARRKEDNYDTHNCDDANQDKKEEAEGKNTKRRE
IKMNVVFKSKEPKKCLTYHGNDRKSFLPAKKPERSIAGLTNRNLFCISEFPGDLMLMNQD
FISRRDHFSDLVKTYSLEEESIWKERMRKATPYHY

>sp|Q8WZ69|CK040_HUMAN Putative uncharacterized protein C11orf40 OS=Homo sapiens GN=C11orf40 PE=2 SV=1
MALVQALVPREREPKLSILQMDRGDPQHSSHWCPEREKVKLLTLKPRETSKNILINFYRA
FNLDKDVFIHQANHPLTVPSSVVMGDNGHTLAEDDKRPCFRVLPCYLERVSSGISISWIS
APLPVGAMKHQLLCDLMDLITLSFWLAGQCMSLKATNMQHCKCSIATSDWAIELDRTDYK
TLPSEYSILALLQVFAGKNCMDRVLLHVDVNYLKSLP
>sp|Q9H6J7|CK049_HUMAN UPF0705 protein C11orf49 OS=Homo sapiens GN=C11orf49 PE=1 SV=2
MLSPERLALPDYEYLAQRHVLTYMEDAVCQLLENREDISQYGIARFFTEYFNSVCQGTHI
LFREFSFVQATPHNRVSFLRAFWRCFRTVGKNGDLLTMKEYHCLLQLLCPDFPLELTQKA
ARIVLMDDAMDCLMSFSDFLFAFQIQFYYSEFLDSVAAIYEDLLSGKNPNTVIVPTSSSG
QHRQRPALGGAGTLEGVEASLFYQCLENLCDRHKYSCPPPALVKEALSNVQRLTFYGFLM
ALSKHRGINQALGALPDKGDLMHDPAMDEELERLLAQVPGLVNSVTASPEASCLPSRTPP
RVGSPWRPLHHSRKVDGESDGSTEETDESET
>sp|Q9BRQ4|CK070_HUMAN Uncharacterized protein C11orf70 OS=Homo sapiens GN=C11orf70 PE=2 SV=3
MATGELGDLGGYYFRFLPQKTFQSLSSKEITSRLRQWSMLGRIKAQAFGFDQTFQSYRKD
DFVMAFFKDPNVIPNLKLLSDSSGQWIILGTEVKKIEAINVPCTQLSMSFFHRLYDEDIV
RDSGHIVKCLDSFCDPFLISDELRRVLLVEDSEKYEIFSQPDREEFLFCLFKHLCLGGAL
CQYEDVISPYLETTKLIYKDLVSVRKNPQTKKIQITSSVFKVSAYDSAGMCYPSAKNHEQ
TFSYFIVDPIRRHLHVLYHCYGVGDMS
>sp|Q86VG3|CK074_HUMAN Uncharacterized protein C11orf74 OS=Homo sapiens GN=C11orf74 PE=1 SV=1
MSAHMSGLEIMDEDQLIKDVLDKFLNCHEQTYDEEFLNTFTHLSQEDHVSKRGVFGTDSS
ENIFTSAKVTHKNEADDYHLRNKTIFLRTSSQCLEEQVDNFLDLEDLDMDEEIKPQMSED
LLLLPGEVEQDVSTSIPSCIPFVAQPPTCEVKPKPSVKRMDKQTEEILGDEVQLFSLDEE
FDYDNVMLTSKFSPAEIENIKELCKQQKRKDTSPDLEKSCD
>sp|Q6NUJ2|CK087_HUMAN Uncharacterized protein C11orf87 OS=Homo sapiens GN=C11orf87 PE=1 SV=2
MSARAPKELRLALPPCLLNRTFASPNASGSGNTGARGPGAVGSGTCITQVGQQLFQSFSS
TLVLIVLVTLIFCLIVLSLSTFHIHKRRMKKRKMQRAQEEYERDHCSGSRGGGGLPRPGR
QAPTHAKETRLERQPRDSPFCAPSNASSLSSSSPGLPCQGPCAPPPPPPASSPQGAHAAS
SCLDTAGEGLLQTVVLS
>sp|Q3C1V1|CK091_HUMAN Uncharacterized protein C11orf91 OS=Homo sapiens GN=C11orf91 PE=2 SV=2
MPKGRRGSHSPTMSQRSAPPLYFPSLYDRGISSSPLSDFNIWKKLFVPLKAGGAPVGGAA
GARSLSQALPAPAPPPPPPPGLGPSSERPWPSPWPSGLASIPYEPLRFFYSPPPGPEVVA
SPLVPCPSTPRLASASHPEELCELEIRIKELELLTITGDGFDSQSYTFLKALKDEKLQGL
KTKQPGKKSASLS
>sp|Q96S95|CK2N2_HUMAN Calcium/calmodulin-dependent protein kinase II inhibitor 2 OS=Homo sapiens GN=CAMK2N2 PE=2 SV=1
MSEILPYSEDKMGRFGADPEGSDLSFSCRLQDTNSFFAGNQAKRPPKLGQIGRAKRVVIE
DDRIDDVLKGMGEKPPSGV
>sp|Q96JB5|CK5P3_HUMAN CDK5 regulatory subunit-associated protein 3 OS=Homo sapiens GN=CDK5RAP3 PE=1 SV=2

MEDHQHVPIDIQTSKLLDWLVDRRHCSLKWQSLVLTIREKINAAIQDMPESEEIAQLLSG
SYIHYFHCLRILDLLKGTEASTKNIFGRYSSQRMKDWQEIIALYEKDNTYLVELSSLLVR
NVNYEIPSLKKQIAKCQQLQQEYSRKEEECQAGAAEMREQFYHSCKQYGITGENVRGELL
ALVKDLPSQLAEIGAAAQQSLGEAIDVYQASVGFVCESPTEQVLPMLRFVQKRGNSTVYE
WRTGTEPSVVERPHLEELPEQVAEDAIDWGDFGVEAVSEGTDSGISAEAAGIDWGIFPES
DSKDPGGDGIDWGDDAVALQITVLEAGTQAPEGVARGPDALTLLEYTETRNQFLDELMEL
EIFLAQRAVELSEEADVLSVSQFQLAPAILQGQTKEKMVTMVSVLEDLIGKLTSLQLQHL
FMILASPRYVDRVTEFLQQKLKQSQLLALKKELMVQKQQEALEEQAALEPKLDLLLEKTK
ELQKLIEADISKRYSGRPVNLMGTSL
>sp|Q8WWK9|CKAP2_HUMAN Cytoskeleton-associated protein 2 OS=Homo sapiens GN=CKAP2 PE=1 SV=1
MSTPAVPQDLQLPPSQRAQSAFKEQRRQKLKEHLLRRKTLFAYKQENEMLSSSRDQRVVT
SEDQVQEGTKVLKLKTKMADKENMKRPAESKNNTVVGKHCIPLKPSNELTNSTVVIDTHK
PKDSNQTPHLLLTEDDPQSQHMTLSQAFHLKNNSKKKQMTTEKQKQDANMPKKPVLGSYR
GQIVQSKINSFRKPLQVKDESSAATKKLSATIPKATKPQPVNTSSVTVKSNRSSNMTATT
KFVSTTSQNTQLVRPPIRSHHSNTRDTVKQGISRTSANVTIRKGPHEKELLQSKTALSSV
KTSSSQGIIRNKTLSRSIASEVIARPASLSNDKLMEKSEPVDQRRHTAGKAIVDSRSAQP
KETSEERKARLSEWKAGKGRVLKRPPNSVVTQHEPAGQNEKPVGSFWTTMAEEDEQRLFT
EKVNNTFSECLNLINEGCPKEDILVTLNDLIKNIPDAKKLVKYWICLALIEPITSPIENI
IAIYEKAILAGAQPIEEMRHTIVDILTMKSQEKANLGENMEKSCASKEEVKEVSIEDTGV
DVDPEKLEMESKLHRNLLFQDCEKEQDNKTKDPTHDVKTPNTETRTSCLIKYNVSTTPYL
QSVKKKVQFDGTNSAFKELKFLTPVRRSRRLQEKTSKLPDMLKDHYPCVSSLEQLTELGR
ETDAFVCRPNAALCRVYYEADTT
>sp|Q14008|CKAP5_HUMAN Cytoskeleton-associated protein 5 OS=Homo sapiens GN=CKAP5 PE=1 SV=3
MGDDSEWLKLPVDQKCEHKLWKARLSGYEEALKIFQKIKDEKSPEWSKFLGLIKKFVTDS
NAVVQLKGLEAALVYVENAHVAGKTTGEVVSGVVSKVFNQPKAKAKELGIEICLMYIEIE
KGEAVQEELLKGLDNKNPKIIVACIETLRKALSEFGSKIILLKPIIKVLPKLFESREKAV
RDEAKLIAVEIYRWIRDALRPPLQNINSVQLKELEEEWVKLPTSAPRPTRFLRSQQELEA
KLEQQQSAGGDAEGGGDDGDEVPQIDAYELLEAVEILSKLPKDFYDKIEAKKWQERKEAL
ESVEVLIKNPKLEAGDYADLVKALKKVVGKDTNVMLVALAAKCLTGLAVGLRKKFGQYAG
HVVPTILEKFKEKKPQVVQALQEAIDAIFLTTTLQNISEDVLAVMDNKNPTIKQQTSLFI
ARSFRHCTASTLPKSLLKPFCAALLKHINDSAPEVRDAAFEALGTALKVVGEKAVNPFLA
DVDKLKLDKIKECSEKVELIHGKKAGLAADKKEFKPLPGRTAASGAAGDKDTKDISAPKP
GPLKKAPAAKAGGPPKKGKPAAPGGAGNTGTKNKKGLETKEIVEPELSIEVCEEKASAVL
PPTCIQLLDSSNWKERLACMEEFQKAVELMDRTEMPCQALVRMLAKKPGWKETNFQVMQM
KLHIVALIAQKGNFSKTSAQVVLDGLVDKIGDVKCGNNAKEAMTAIAEACMLPWTAEQVV
SMAFSQKNPKNQSETLNWLSNAIKEFGFSGLNVKAFISNVKTALAATNPAVRTAAITLLG
VMYLYVGPSLRMFFEDEKPALLSQIDAEFEKMQGQSPPAPTRGISKHSTSGTDEGEDGDE
PDDGSNDVVDLLPRTEISDKITSELVSKIGDKNWKIRKEGLDEVAGIINDAKFIQPNIGE
LPTALKGRLNDSNKILVQQTLNILQQLAVAMGPNIKQHVKNLGIPIITVLGDSKNNVRAA
ALATVNAWAEQTGMKEWLEGEDLSEELKKENPFLRQELLGWLAEKLPTLRSTPTDLILCV
PHLYSCLEDRNGDVRKKAQDALPFFMMHLGYEKMAKATGKLKPTSKDQVLAMLEKAKVNM
PAKPAPPTKATSKPMGGSAPAKFQPASAPAEDCISSSTEPKPDPKKAKAPGLSSKAKSAQ

GKKMPSKTSLKEDEDKSGPIFIVVPNGKEQRMKDEKGLKVLKWNFTTPRDEYIEQLKTQM
SSCVAKWLQDEMFHSDFQHHNKALAVMVDHLESEKEGVIGCLDLILKWLTLRFFDTNTSV
LMKALEYLKLLFTLLSEEEYHLTENEASSFIPYLVVKVGEPKDVIRKDVRAILNRMCLVY
PASKMFPFIMEGTKSKNSKQRAECLEELGCLVESYGMNVCQPTPGKALKEIAVHIGDRDN
AVRNAALNTIVTVYNVHGDQVFKLIGNLSEKDMSMLEERIKRSAKRPSAAPIKQVEEKPQ
RAQNISSNANMLRKGPAEDMSSKLNQARSMSGHPEAAQMVRREFQLDLDEIENDNGTVRC
EMPELVQHKLDDIFEPVLIPEPKIRAVSPHFDDMHSNTASTINFIISQVASGDINTSIQA
LTQIDEVLRQEDKAEAMSGHIDQFLIATFMQLRLIYNTHMADEKLEKDEIIKLYSCIIGN
MISLFQIESLAREASTGVLKDLMHGLITLMLDSRIEDLEEGQQVIRSVNLLVVKVLEKSD
QTNILSALLVLLQDSLLATASSPKFSELVMKCLWRMVRLLPDTINSINLDRILLDIHIFM
KVFPKEKLKQCKSEFPIRTLKTLLHTLCKLKGPKILDHLTMIDNKNESELEAHLCRMMKH
SMDQTGSKSDKETEKGASRIDEKSSKAKVNDFLAEIFKKIGSKENTKEGLAELYEYKKKY
SDADIEPFLKNSSQFFQSYVERGLRVIEMEREGKGRISTSTGISPQMEVTCVPTPTSTVS
SIGNTNGEEVGPSVYLERLKILRQRCGLDNTKQDDRPPLTSLLSKPAVPTVASSTDMLHS
KLSQLRESREQHQHSDLDSNQTHSSGTVTSSSSTANIDDLKKRLERIKSSRK
>sp|Q96FZ5|CKLF7_HUMAN CKLF-like MARVEL transmembrane domain-containing protein 7 OS=Homo sapiens GN=CMTM7 PE=2 SV=1
MSHGAGLVRTTCSSGSALGPGAGAAQPSASPLEGLLDLSYPRTHAALLKVAQMVTLLIAF
ICVRSSLWTNYSAYSYFEVVTICDLIMILAFYLVHLFRFYRVLTCISWPLSELLHYLIGT
LLLLIASIVAASKSYNQSGLVAGAIFGFMATFLCMASIWLSYKISCVTQSTDAAV
>sp|Q8NA57|CL050_HUMAN Uncharacterized protein C12orf50 OS=Homo sapiens GN=C12orf50 PE=1 SV=1
MEMQQNCSISCFWETQPLGCVKISCIFYHSKPRNINGLFLPPSSNITLQKEIQEGIPLQS
QSQEPLKPQENISRPIHHPLVLKTNFEEEEEVDEQNDASSLWTKTPEEIEEKRAIKEMCY
KSGEYYRFHTPPDILSSKSMTPTAEKQLEKPLENGSELQEGDSLTVPTKLSQYERQGEIK
TSLHGKPKTDIAAFENGGGDCYVPQRVIFLGVDESEALTEEKEITISKCSNTKDNKDSPH
PKHSLTTRLVPTTHVLNATENISMKCREDPSSMNDVQPVKKPHFKGVKKRKWIYDEPQNF
PNSGMQRAVQAPRPQNKMSYHRNNKNRNAENASYIHVQRDAVRTVALNAPSRSRPTHGSY
NKVHANREPKPNLSPDKYTSTSYNDSAWRKRIPFSKTYSKSEKIYPEPRRNGSK
>sp|G3V211|CL079_HUMAN Uncharacterized protein encoded by LINC01619 OS=Homo sapiens GN=LINC01619 PE=4 SV=1
MNVCCSSHPVNEKVWKPSSRKWSSKVWSMDEFDLQTACYWFMTRCQKEAGKFGTHRGKPM
CFVRSLLRVQLLPRTFPANSFVISFFPSLIYPLQVYQLHFESSDKQRAMQFVTEG
>sp|Q8NCF0|CL18C_HUMAN C-type lectin domain family 18 member C OS=Homo sapiens GN=CLEC18C PE=2 SV=2
MLHPETSPGRGHLLAVLLALLGTAWAEVWPPQLQEQAPMAGALNRKESFLLLSLHNRLRS
WVQPPAADMRRLDWSDSLAQLAQARAALCGIPTPSLASGLWRTLQVGWNMQLLPAGLASF
VEVVSLWFAEGQRYSHAAGECARNATCTHYTQLVWATSSQLGCGRHLCSAGQAAIEAFVC
AYSPRGNWEVNGKTIVPYKKGAWCSLCTASVSGCFKAWDHAGGLCEVPRNPCRMSCQNHG
RLNISTCHCHCPPGYTGRYCQVRCSLQCVHGRFREEECSCVCDIGYGGAQCATKVHFPFH
TCDLRIDGDCFMVSSEADTYYRARMKCQRKGGVLAQIKSQKVQDILAFYLGRLETTNEVI
DSDFETRNFWIGLTYKTAKDSFRWATGEHQAFTSFAFGQPDNHGFGNCVELQASAAFNWN
NQRCKTRNRYICQFAQEHISRWGPGS

>sp|Q6UXS0|CL19A_HUMAN C-type lectin domain family 19 member A OS=Homo sapiens GN=CLEC19A PE=2 SV=1
MQRWTLWAAAFLTLHSAQAFPQTDISISPALPELPLPSLCPLFWMEFKGHCYRFFPLNKT
WAEADLYCSEFSVGRKSAKLASIHSWEENVFVYDLVNSCVPGIPADVWTGLHDHRQVRKQ
WPLGPLGSSSQDSILI
>sp|P28329|CLAT_HUMAN Choline O-acetyltransferase OS=Homo sapiens GN=CHAT PE=1 SV=4
MGLRTAKKRGLGGGGKWKREEGGGTRGRREVRPACFLQSGGRGDPGDVGGPAGNPGCSPH
PRAATRPPPLPAHTPAHTPEWCGAASAEAAEPRRAGPHLCIPAPGLTKTPILEKVPRKMA
AKTPSSEESGLPKLPVPPLQQTLATYLQCMRHLVSEEQFRKSQAIVQQFGAPGGLGETLQ
QKLLERQEKTANWVSEYWLNDMYLNNRLALPVNSSPAVIFARQHFPGTDDQLRFAASLIS
GVLSYKALLDSHSIPTDCAKGQLSGQPLCMKQYYGLFSSYRLPGHTQDTLVAQNSSIMPE
PEHVIVACCNQFFVLDVVINFRRLSEGDLFTQLRKIVKMASNEDERLPPIGLLTSDGRSE
WAEARTVLVKDSTNRDSLDMIERCICLVCLDAPGGVELSDTHRALQLLHGGGYSKNGANR
WYDKSLQFVVGRDGTCGVVCEHSPFDGIVLVQCTEHLLKHVTQSSRKLIRADSVSELPAP
RRLRWKCSPEIQGHLASSAEKLQRIVKNLDFIVYKFDNYGKTFIKKQKCSPDAFIQVALQ
LAFYRLHRRLVPTYESASIRRFQEGRVDNIRSATPEALAFVRAVTDHKAAVPASEKLLLL
KDAIRAQTAYTVMAITGMAIDNHLLALRELARAMCKELPEMFMDETYLMSNRFVLSTSQV
PTTTEMFCCYGPVVPNGYGACYNPQPETILFCISSFHSCKETSSSKFAKAVEESLIDMRD
LCSLLPPTESKPLATKEKATRPSQGHQP
>sp|Q6UVW9|CLC2A_HUMAN C-type lectin domain family 2 member A OS=Homo sapiens GN=CLEC2A PE=1 SV=2
MINPELRDGRADGFIHRIVPKLIQNWKIGLMCFLSIIITTVCIIMIATWSKHAKPVACSG
DWLGVRDKCFYFSDDTRNWTASKIFCSLQKAELAQIDTQEDMEFLKRYAGTDMHWIGLSR
KQGDSWKWTNGTTFNGWFEIIGNGSFAFLSADGVHSSRGFIDIKWICSKPKYFL
>sp|Q9UMR7|CLC4A_HUMAN C-type lectin domain family 4 member A OS=Homo sapiens GN=CLEC4A PE=1 SV=1
MTSEITYAEVRFKNEFKSSGINTASSAASKERTAPHKSNTGFPKLLCASLLIFFLLLAIS
FFIAFVIFFQKYSQLLEKKTTKELVHTTLECVKKNMPVEETAWSCCPKNWKSFSSNCYFI
STESASWQDSEKDCARMEAHLLVINTQEEQDFIFQNLQEESAYFVGLSDPEGQRHWQWVD
QTPYNESSTFWHPREPSDPNERCVVLNFRKSPKRWGWNDVNCLGPQRSVCEMMKIHL
>sp|P56880|CLD20_HUMAN Claudin-20 OS=Homo sapiens GN=CLDN20 PE=2 SV=1
MASAGLQLLAFILALSGVSGVLTATLLPNWKVNVDVDSNIITAIVQLHGLWMDCTWYSTG
MFSCALKHSILSLPIHVQAARATMVLACVLSALGICTSTVGMKCTRLGGDRETKSHASFA
GGVCFMSAGISSLISTVWYTKEIIANFLDLTVPESNKHEPGGAIYIGFISAMLLFISGMI
FCTSCIKRNPEARLDPPTQQPISNTQLENNSTHNLKDYV
>sp|A6NM45|CLD24_HUMAN Putative claudin-24 OS=Homo sapiens GN=CLDN24 PE=5 SV=2
MALIFRTAMQSVGLLLSLLGWILSIITTYLPHWKNLNLDLNEMENWTMGLWQTCVIQEEV
GMQCKDFDSFLALPAELRVSRILMFLSNGLGFLGLLVSGFGLDCLRIGESQRDLKRRLLI
LGGILSWASGITALVPVSWVAHKTVQEFWDENVPDFVPRWEFGEALFLGWFAGLSLLLGG
CLLNCAACSSHAPLALGHYAVAQMQTQCPYLEDGTADPQV
>sp|P53675|CLH2_HUMAN Clathrin heavy chain 2 OS=Homo sapiens GN=CLTCL1 PE=1 SV=2
MAQILPVRFQEHFQLQNLGINPANIGFSTLTMESDKFICIREKVGEQAQVTIIDMSDPMA
PIRRPISAESAIMNPASKVIALKAGKTLQIFNIEMKSKMKAHTMAEEVIFWKWVSVNTVA
LVTETAVYHWSMEGDSQPMKMFDRHTSLVGCQVIHYRTDEYQKWLLLVGISAQQNRVVGA

MQLYSVDRKVSQPIEGHAAAFAEFKMEGNAKPATLFCFAVRNPTGGKLHIIEVGQPAAGN
QPFVKKAVDVFFPPEAQNDFPVAMQIGAKHGVIYLITKYGYLHLYDLESGVCICMNRISA
DTIFVTAPHKPTSGIIGVNKKGQVLSVCVEEDNIVNYATNVLQNPDLGLRLAVRSNLAGA
EKLFVRKFNTLFAQGSYAEAAKVAASAPKGILRTRETVQKFQSIPAQSGQASPLLQYFGI
LLDQGQLNKLESLELCHLVLQQGRKQLLEKWLKEDKLECSEELGDLVKTTDPMLALSVYL
RANVPSKVIQCFAETGQFQKIVLYAKKVGYTPDWIFLLRGVMKISPEQGLQFSRMLVQDE
EPLANISQIVDIFMENSLIQQCTSFLLDALKNNRPAEGLLQTWLLEMNLVHAPQVADAIL
GNKMFTHYDRAHIAQLCEKAGLLQQALEHYTDLYDIKRAVVHTHLLNPEWLVNFFGSLSV
EDSVECLHAMLSANIRQNLQLCVQVASKYHEQLGTQALVELFESFKSYKGLFYFLGSIVN
FSQDPDVHLKYIQAACKTGQIKEVERICRESSCYNPERVKNFLKEAKLTDQLPLIIVCDR
FGFVHDLVLYLYRNNLQRYIEIYVQKVNPSRTPAVIGGLLDVDCSEEVIKHLIMAVRGQF
STDELVAEVEKRNRLKLLLPWLESQIQEGCEEPATHNALAKIYIDSNNSPECFLRENAYY
DSSVVGRYCEKRDPHLACVAYERGQCDLELIKVCNENSLFKSEARYLVCRKDPELWAHVL
EETNPSRRQLIDQVVQTALSETRDPEEISVTVKAFMTADLPNELIELLEKIVLDNSVFSE
HRNLQNLLILTAIKADRTRVMEYISRLDNYDALDIASIAVSSALYEEAFTVFHKFDMNAS
AIQVLIEHIGNLDRAYEFAERCNEPAVWSQLAQAQLQKDLVKEAINSYIRGDDPSSYLEV
VQSASRSNNWEDLVKFLQMARKKGRESYIETELIFALAKTSRVSELEDFINGPNNAHIQQ
VGDRCYEEGMYEAAKLLYSNVSNFARLASTLVHLGEYQAAVDNSRKASSTRTWKEVCFAC
MDGQEFRFAQLCGLHIVIHADELEELMCYYQDRGYFEELILLLEAALGLERAHMGMFTEL
AILYSKFKPQKMLEHLELFWSRVNIPKVLRAAEQAHLWAELVFLYDKYEEYDNAVLTMMS
HPTEAWKEGQFKDIITKVANVELCYRALQFYLDYKPLLINDLLLVLSPRLDHTWTVSFFS
KAGQLPLVKPYLRSVQSHNNKSVNEALNHLLTEEEDYQGLRASIDAYDNFDNISLAQQLE
KHQLMEFRCIAAYLYKGNNWWAQSVELCKKDHLYKDAMQHAAESRDAELAQKLLQWFLEE
GKRECFAACLFTCYDLLRPDMVLELAWRHNLVDLAMPYFIQVMREYLSKVDKLDALESLR
KQEEHVTEPAPLVFDFDGHE
>sp|O00299|CLIC1_HUMAN Chloride intracellular channel protein 1 OS=Homo sapiens GN=CLIC1
PE=1 SV=4
MAEEQPQVELFVKAGSDGAKIGNCPFSQRLFMVLWLKGVTFNVTTVDTKRRTETVQKLCP
GGQLPFLLYGTEVHTDTNKIEEFLEAVLCPPRYPKLAALNPESNTAGLDIFAKFSAYIKN
SNPALNDNLEKGLLKALKVLDNYLTSPLPEEVDETSAEDEGVSQRKFLDGNELTLADCNL
LPKLHIVQVVCKKYRGFTIPEAFRGVHRYLSNAYAREEFASTCPDDEEIELAYEQVAKAL
K
>sp|O15247|CLIC2_HUMAN Chloride intracellular channel protein 2 OS=Homo sapiens GN=CLIC2
PE=1 SV=3
MSGLRPGTQVDPEIELFVKAGSDGESIGNCPFCQRLFMILWLKGVKFNVTTVDMTRKPEE
LKDLAPGTNPPFLVYNKELKTDFIKIEEFLEQTLAPPRYPHLSPKYKESFDVGCNLFAKF
SAYIKNTQKEANKNFEKSLLKEFKRLDDYLNTPLLDEIDPDSAEEPPVSRRLFLDGDQLT
LADCSLLPKLNIIKVAAKKYRDFDIPAEFSGVWRYLHNAYAREEFTHTCPEDKEIENTYA
NVAKQKS
>sp|Q9Y696|CLIC4_HUMAN Chloride intracellular channel protein 4 OS=Homo sapiens GN=CLIC4
PE=1 SV=4
MALSMPLNGLKEEDKEPLIELFVKAGSDGESIGNCPFSQRLFMILWLKGVVFSVTTVDLK
RKPADLQNLAPGTHPPFITFNSEVKTDVNKIEEFLEEVLCPPKYLKLSPKHPESNTAGMD
IFAKFSAYIKNSRPEANEALERGLLKTLQKLDEYLNSPLPDEIDENSMEDIKFSTRKFLD

GNEMTLADCNLLPKLHIVKVVAKKYRNFDIPKEMTGIWRYLTNAYSRDEFTNTCPSDKEV
EIAYSDVAKRLTK
>sp|Q7Z7G1|CLNK_HUMAN Cytokine-dependent hematopoietic cell linker OS=Homo sapiens GN=CLNK PE=1 SV=2
MNRQGNRKTTKEGSNDLKFQNFSLPKNRSWPRINSATGQYQRMNKPLLDWERNFAAVLDG
AKGHSDDDYDDPELRMEETWQSIKILPARPIKESEYADTHYFKVAMDTPLPLDTRTSISI
GQPTWNTQTRLERVDKPISKDVRSQNIKGDASVRKNKIPLPPPRPLITLPKKYQPLPPEP
ESSRPPLSQRHTFPEVQRMPSQISLRDLSEVLEAEKVPHNQRKPESTHLLENQNTQEIPL
AISSSSFTTSNHSVQNRDHRGGMQPCSPQRCQPPASCSPHENILPYKYTSWRPPFPKRSD
RKDVQHNEWYIGEYSRQAVEEAFMKENKDGSFLVRDCSTKSKEEPYVLAVFYENKVYNVK
IRFLERNQQFALGTGLRGDEKFDSVEDIIEHYKNFPIILIDGKDKTGVHRKQCHLTQPLP
LTRHLLPL
>sp|Q96KA5|CLP1L_HUMAN Cleft lip and palate transmembrane protein 1-like protein OS=Homo sapiens GN=CLPTM1L PE=1 SV=1
MWSGRSSFTSLVVGVFVVYVVHTCWVMYGIVYTRPCSGDANCIQPYLARRPKLQLSVYTT
TRSHLGAENNIDLVLNVEDFDVESKFERTVNVSVPKKTRNNGTLYAYIFLHHAGVLPWHD
GKQVHLVSPLTTYMVPKPEEINLLTGESDTQQIEAEKKPTSALDEPVSHWRPRLALNVMA
DNFVFDGSSLPADVHRYMKMIQLGKTVHYLPILFIDQLSNRVKDLMVINRSTTELPLTVS
YDKVSLGRLRFWIHMQDAVYSLQQFGFSEKDADEVKGIFVDTNLYFLALTFFVAAFHLLF
DFLAFKNDISFWKKKKSMIGMSTKAVLWRCFSTVVIFLFLLDEQTSLLVLVPAGVGAAIE
LWKVKKALKMTIFWRGLMPEFQFGTYSESERKTEEYDTQAMKYLSYLLYPLCVGGAVYSL
LNIKYKSWYSWLINSFVNGVYAFGFLFMLPQLFVNYKLKSVAHLPWKAFTYKAFNTFIDD
VFAFIITMPTSHRLACFRDDVVFLVYLYQRWLYPVDKRRVNEFGESYEEKATRAPHTD
>sp|Q16740|CLPP_HUMAN ATP-dependent Clp protease proteolytic subunit, mitochondrial OS=Homo sapiens GN=CLPP PE=1 SV=1
MWPGILVGGARVASCRYPALGPRLAAHFPAQRPPQRTLQNGLALQRCLHATATRALPLIP
IVVEQTGRGERAYDIYSRLLRERIVCVMGPIDDSVASLVIAQLLFLQSESNKKPIHMYIN
SPGGVVTAGLAIYDTMQYILNPICTWCVGQAASMGSLLLAAGTPGMRHSLPNSRIMIHQP
SGGARGQATDIAIQAEEIMKLKKQLYNIYAKHTKQSLQVIESAMERDRYMSPMEAQEFGI
LDKVLVHPPQDGEDEPTLVQKEPVEAAPAAEPVPAST
>sp|Q9Y232|CDYL1_HUMAN Chromodomain Y-like protein OS=Homo sapiens GN=CDYL PE=1 SV=2
MTFQASHRSAWGKSRKKNWQYEGPTQKLFLKRNNVSAPDGPSDPSISVSSEQSGAQQPPA
LQVERIVDKRKNKKGKTEYLVRWKGYDSEDDTWEPEQHLVNCEEYIHDFNRRHTEKQKES
TLTRTNRTSPNNARKQISRSTNSNFSKTSPKALVIGKDHESKNSQLFAASQKFRKNTAPS
LSSRKNMDLAKSGIKILVPKSPVKSRTAVDGFQSESPEKLDPVEQGQEDTVAPEVAAEKP
VGALLGPGAERARMGSRPRIHPLVPQVPGPVTAAMATGLAVNGKGTSPFMDALTANGTTN
IQTSVTGVTASKRKFIDDRRDQPFDKRLRFSVRQTESAYRYRDIVVRKQDGFTHILLSTK
SSENNSLNPEVMREVQSALSTAAADDSKLVLLSAVGSVFCCGLDFIYFIRRLTDDRKRES
TKMAEAIRNFVNTFIQFKKPIIVAVNGPAIGLGASILPLCDVVWANEKAWFQTPYTTFGQ
SPDGCSTVMFPKIMGGASANEMLLSGRKLTAQEACGKGLVSQVFWPGTFTQEVMVRIKEL
ASCNPVVLEESKALVRCNMKMELEQANERECEVLKKIWGSAQGMDSMLKYLQRKIDEF
>sp|C9J3I9|CE058_HUMAN Putative uncharacterized protein C5orf58 OS=Homo sapiens GN=C5orf58 PE=4 SV=1
MREIAEILPAQEKVEARIDLKMGKKRVTDHKLNVDKVIKNINTISSELKKIKELSQLLLC

DLILHFNHPIKTENLAEAERNNPLFEESKISDVSLVSNSFSI
>sp|F2Z3F1|CE067_HUMAN Uncharacterized protein C5orf67 OS=Homo sapiens GN=C5orf67 PE=4 SV=1
MKRIFYKHRKRRAPVFKEPEHGYQSLPELVLVPAQPLVCLGDYRTPDPGGLFPWSLRLMM
PGAWTKLPGDGGSVPEKGKHGILGAQGQEHPGLNVSSPFSSPWTCYLSGHQPQNNNSPEL
QVKEILL
>sp|A8MTB9|CEA18_HUMAN Carcinoembryonic antigen-related cell adhesion molecule 18 OS=Homo sapiens GN=CEACAM18 PE=3 SV=4
MDLSRPRWSLWRRVFLMASLLACGICQASGQIFITQTLGIKGYRTVVALDKVPEDVQEYS
WYWGANDSAGNMIISHKPPSAQQPGPMYTGRERVNREGSLLIRPTALNDTGNYTVRVVAG
NETQRATGWLEVLELGSNLGISVNASSLVENMDSVAADCLTNVTNITWYVNDVPTSSSDR
MTISPDGKTLVILRVSRYDRTIQCMIESFPEIFQRSERISLTVAYGPDYVLLRSNPDDFN
GIVTAEIGSQVEMECICYSFLDLKYHWIHNGSLLNFSDAKMNLSSLAWEQMGRYRCTVEN
PVTQLIMYMDVRIQAPHEDLTCCPQRFLHLRIHGDVPHHADSAGWRLHLWSPDPCSDQPL
LNQDKSGSMSVHPRPEDKTRRASR
>sp|P53567|CEBPG_HUMAN CCAAT/enhancer-binding protein gamma OS=Homo sapiens GN=CEBPG PE=1 SV=1
MSKISQQNSTPGVNGISVIHTQAHASGLQQVPQLVPAGPGGGGKAVAPSKQSKKSSPMDR
NSDEYRQRRERNNMAVKKSRLKSKQKAQDTLQRVNQLKEENERLEAKIKLLTKELSVLKD
LFLEHAHNLADNVQSISTENTTADGDNAGQ
>sp|Q9BUX1|CHAC1_HUMAN Glutathione-specific gamma-glutamylcyclotransferase 1 OS=Homo sapiens GN=CHAC1 PE=1 SV=2
MGGAQLELPSGARPGVCVRRSFRAHAGDQPRRPPGPIPVPGTMKQESAAPNTPPTSQSPT
PSAQFPRNDGDPQALWIFGYGSLVWRPDFAYSDSRVGFVRGYSRRFWQGDTFHRGSDKMP
GRVVTLLEDHEGCTWGVAYQVQGEQVSKALKYLNVREAVLGGYDTKEVTFYPQDAPDQPL
KALAYVATPQNPGYLGPAPEEAIATQILACRGFSGHNLEYLLRLADFMQLCGPQAQDEHL
AAIVDAVGTMLPCFCPTEQALALV
>sp|Q6NUI6|CHADL_HUMAN Chondroadherin-like protein OS=Homo sapiens GN=CHADL PE=1 SV=2
MEGPRSSTHVPLVLPLLVLLLLAPARQAAAQRCPQACICDNSRRHVACRYQNLTEVPDAI
PELTQRLDLQGNLLKVIPAAAFQGVPHLTHLDLRHCEVELVAEGAFRGLGRLLLLNLASN
HLRELPQEALDGLGSLRRLELEGNALEELRPGTFGALGALATLNLAHNALVYLPAMAFQG
LLRVRWLRLSHNALSVLAPEALAGLPALRRLSLHHNELQALPGPVLSQARGLARLELGHN
PLTYAGEEDGLALPGLRELLLDGGALQALGPRAFAHCPRLHTLDLRGNQLDTLPPLQGPG
QLRRLRLQGNPLWCGCQARPLLEWLARARVRSDGACQGPRRLRGEALDALRPWDLRCPGD
AAQEEEELEERAVAGPRAPPRGPPRGPGEERAVAPCPRACVCVPESRHSSCEGCGLQAVP
RGFPSDTQLLDLRRNHFPSVPRAAFPGLGHLVSLHLQHCGIAELEAGALAGLGRLIYLYL
SDNQLAGLSAAALEGAPRLGYLYLERNRFLQVPGAALRALPSLFSLHLQDNAVDRLAPGD
LGRTRALRWVYLSGNRITEVSLGALGPARELEKLHLDRNQLREVPTGALEGLPALLELQL
SGNPLRALRDGAFQPVGRSLQHLFLNSSGLEQICPGAFSGLGPGLQSLHLQKNQLRALPA
LPSLSQLELIDLSSNPFHCDCQLLPLHRWLTGLNLRVGATCATPPNARGQRVKAAAAVFE
DCPGWAARKAKRTPASRPSARRTPIKGRQCGADKVGKEKGRL
>sp|Q8TDI0|CHD5_HUMAN Chromodomain-helicase-DNA-binding protein 5 OS=Homo sapiens GN=CHD5 PE=1 SV=1
MRGPVGTEEELPRLFAEEMENEDEMSEEEDGGLEAFDDFFPVEPVSLPKKKKPKKLKENK

CKGKRKKKEGSNDELSENEEDLEEKSESEGSDYSPNKKKKKKLKDKKEKKAKRKKKDEDE
DDNDDGCLKEPKSSGQLMAEWGLDDVDYLFSEEDYHTLTNYKAFSQFLRPLIAKKNPKIP
MSKMMTVLGAKWREFSANNPFKGSSAAAAAAAVAAAVETVTISPPLAVSPPQVPQPVPIR
KAKTKEGKGPGVRKKIKGSKDGKKKGKGKKTAGLKFRFGGISNKRKKGSSSEEDEREESD
FDSASIHSASVRSECSAALGKKSKRRRKKKRIDDGDGYETDHQDYCEVCQQGGEIILCDT
CPRAYHLVCLDPELEKAPEGKWSCPHCEKEGIQWEPKDDDDEEEEGGCEEEEDDHMEFCR
VCKDGGELLCCDACPSSYHLHCLNPPLPEIPNGEWLCPRCTCPPLKGKVQRILHWRWTEP
PAPFMVGLPGPDVEPSLPPPKPLEGIPEREFFVKWAGLSYWHCSWVKELQLELYHTVMYR
NYQRKNDMDEPPPFDYGSGDEDGKSEKRKNKDPLYAKMEERFYRYGIKPEWMMIHRILNH
SFDKKGDVHYLIKWKDLPYDQCTWEIDDIDIPYYDNLKQAYWGHRELMLGEDTRLPKRLL
KKGKKLRDDKQEKPPDTPIVDPTVKFDKQPWYIDSTGGTLHPYQLEGLNWLRFSWAQGTD
TILADEMGLGKTVQTIVFLYSLYKEGHSKGPYLVSAPLSTIINWEREFEMWAPDFYVVTY
TGDKESRSVIRENEFSFEDNAIRSGKKVFRMKKEVQIKFHVLLTSYELITIDQAILGSIE
WACLVVDEAHRLKNNQSKFFRVLNSYKIDYKLLLTGTPLQNNLEELFHLLNFLTPERFNN
LEGFLEEFADISKEDQIKKLHDLLGPHMLRRLKADVFKNMPAKTELIVRVELSQMQKKYY
KFILTRNFEALNSKGGGNQVSLLNIMMDLKKCCNHPYLFPVAAVEAPVLPNGSYDGSSLV
KSSGKLMLLQKMLKKLRDEGHRVLIFSQMTKMLDLLEDFLEYEGYKYERIDGGITGGLRQ
EAIDRFNAPGAQQFCFLLSTRAGGLGINLATADTVIIYDSDWNPHNDIQAFSRAHRIGQN
KKVMIYRFVTRASVEERITQVAKRKMMLTHLVVRPGLGSKSGSMTKQELDDILKFGTEEL
FKDDVEGMMSQGQRPVTPIPDVQSSKGGNLAASAKKKHGSTPPGDNKDVEDSSVIHYDDA
AISKLLDRNQDATDDTELQNMNEYLSSFKVAQYVVREEDGVEEVEREIIKQEENVDPDYW
EKLLRHHYEQQQEDLARNLGKGKRIRKQVNYNDASQEDQEWQDELSDNQSEYSIGSEDED
EDFEERPEGQSGRRQSRRQLKSDRDKPLPPLLARVGGNIEVLGFNARQRKAFLNAIMRWG
MPPQDAFNSHWLVRDLRGKSEKEFRAYVSLFMRHLCEPGADGAETFADGVPREGLSRQHV
LTRIGVMSLVRKKVQEFEHVNGKYSTPDLIPEGPEGKKSGEVISSDPNTPVPASPAHLLP
APLGLPDKMEAQLGYMDEKDPGAQKPRQPLEVQALPAALDRVESEDKHESPASKERAREE
RPEETEKAPPSPEQLPREEVLPEKEKILDKLELSLIHSRGDSSELRPDDTKAEEKEPIET
QQNGDKEEDDEGKKEDKKGKFKFMFNIADGGFTELHTLWQNEERAAVSSGKIYDIWHRRH
DYWLLAGIVTHGYARWQDIQNDPRYMILNEPFKSEVHKGNYLEMKNKFLARRFKLLEQAL
VIEEQLRRAAYLNMTQDPNHPAMALNARLAEVECLAESHQHLSKESLAGNKPANAVLHKV
LNQLEELLSDMKADVTRLPSMLSRIPPVAARLQMSERSILSRLTNRAGDPTIQQGAFGSS
QMYSNNFGPNFRGPGPGGIVNYNQMPLGPYVTDI
>sp|Q9HCK8|CHD8_HUMAN Chromodomain-helicase-DNA-binding protein 8 OS=Homo sapiens GN=CHD8
PE=1 SV=5
MADPIMDLFDDPNLFGLDSLTDDSFNQVTQDPIEEALGLPSSLDSLDQMNQDGGGGDVGN
SSASELVPPPEETAPTELSKESTAPAPESITLHDYTTQPASQEQPAQPVLQTSTPTSGLL
QVSKSQEILSQGNPFMGVSATAVSSSSAGGQPPQSAPKIVILKAPPSSSVTGAHVAQIQA
QGITSTAQPLVAGTANGGKVTFTKVLTGTPLRPGVSIVSGNTVLAAKVPGNQAAVQRIVQ
PSRPVKQLVLQPVKGSAPAGNPGATGPPLKPAVTLTSTPTQGESKRITLVLQQPQSGGPQ
GHRHVVLGSLPGKIVLQGNQLAALTQAKNAQGQPAKVVTIQLQVQQPQQKIQIVPQPPSS
QPQPQQPPSTQPVTLSSVQQAQIMGPGQSPGQRLSVPVKVVLQPQAGSSQGASSGLSVVK
VLSASEVAALSSPASSAPHSGGKTGMEENRRLEHQKKQEKANRIVAEAIARARARGEQNI
PRVLNEDELPSVRPEEEGEKKRRKKSAGERLKEEKPKKSKTSGASKTKGKSKLNTITPVV
GKKRKRNTSSDNSDVEVMPAQSPREDEESSIQKRRSNRQVKRKKYTEDLDIKITDDEEEE

EVDVTGPIKPEPILPEPVQEPDGETLPSMQFFVENPSEEDAAIVDKVLSMRIVKKELPSG
QYTEAEEFFVKYKNYSYLHCEWATISQLEKDKRIHQKLKRFKTKMAQMRHFFHEDEEPFN
PDYVEVDRILDESHSIDKDNGEPVIYYLVKWCSLPYEDSTWELKEDVDEGKIREFKRIQS
RHPELKRVNRPQASAWKKLELSHEYKNRNQLREYQLEGVNWLLFNWYNRQNCILADEMGL
GKTIQSIAFLQEVYNVGIHGPFLVIAPLSTITNWEREFNTWTEMNTIVYHGSLASRQMIQ
QYEMYCKDSRGRLIPGAYKFDALITTFEMILSDCPELREIEWRCVIIDEAHRLKNRNCKL
LDSLKHMDLEHKVLLTGTPLQNTVEELFSLLHFLEPSQFPSESEFLKDFGDLKTEEQVQK
LQAILKPMMLRRLKEDVEKNLAPKQETIIEVELTNIQKKYYRAILEKNFSFLSKGAGHTN
MPNLLNTMMELRKCCNHPYLINGAEEKILTEFREACHIIPHDFHLQAMVRSAGKLVLIDK
LLPKLKAGGHKVLIFSQMVRCLDILEDYLIQRRYLYERIDGRVRGNLRQAAIDRFSKPDS
DRFVFLLCTRAGGLGINLTAADTCIIFDSDWNPQNDLQAQARCHRIGQSKAVKVYRLITR
NSYEREMFDKASLKLGLDKAVLQSMSGRDGNITGIQQFSKKEIEDLLRKGAYAAIMEEDD
EGSKFCEEDIDQILLRRTTTITIESEGKGSTFAKASFVASENRTDISLDDPNFWQKWAKK
ADLDMDLLNSKNNLVIDTPRVRKQTRHFSTLKDDDLVEFSDLESEDDERPRSRRHDRHHA
YGRTDCFRVEKHLLVYGWGRWRDILSHGRFKRRMTERDVETICRAILVYCLLHYRGDENI
KGFIWDLISPAENGKTKELQNHSGLSIPVPRGRKGKKVKSQSTFDIHKADWIRKYNPDTL
FQDESYKKHLKHQCNKVLLRVRMLYYLRQEVIGDQAEKVLGGAIASEIDIWFPVVDQLEV
PTTWWDSEADKSLLIGVFKHGYEKYNTMRADPALCFLEKAGRPDDKAIAAEHRVLDNFSD
IVEGVDFDKDCEDPEYKPLQGPPKDQDDEGDPLMMMDEEISVIDGDEAQVTQQPGHLFWP
PGSALTARLRRLVTAYQRSYKREQMKIEAAERGDRRRRRCEAAFKLKEIARREKQQRWTR
REQTDFYRVVSTFGVEYDPDTMQFHWDRFRTFARLDKKTDESLTKYFHGFVAMCRQVCRL
PPAAGDEPPDPNLFIEPITEERASRTLYRIELLRRLREQVLCHPLLEDRLALCQPPGPEL
PKWWEPVRHDGELLRGAARHGVSQTDCNIMQDPDFSFLAARMNYMQNHQAGAPAPSLSRC
STPLLHQQYTSRTASPLPLRPDAPVEKSPEETATQVPSLESLTLKLEHEVVARSRPTPQD
YEMRVSPSDTTPLVSRSVPPVKLEDEDDSDSELDLSKLSPSSSSSSSSSSSSSSTDESED
EKEEKLTDQSRSKLYDEESLLSLTMSQDGFPNEDGEQMTPELLLLQERQRASEWPKDRVL
INRIDLVCQAVLSGKWPSSRRSQEMVTGGILGPGNHLLDSPSLTPGEYGDSPVPTPRSSS
[truncated: 502,257 more chars]
