# Supplementary material for: The Amino Acid Composition of Quadruplex Binding Proteins Reveals a Shared Motif and Predicts New Potential Quadruplex Interactors
Source: Molecules. 2018 Sep 13;23(9):2341. doi: 10.3390/molecules23092341 (PMC6225207; doi:10.3390/molecules23092341)
Supplement: Supplementary file 1 [file Supplementary material 3C.pdf › molecules-355581-supplementary/Supplementary material 3C.pdf]

>ENSG00000005073|ENST00000006015
MDFDERGPCSSNMYLPSCTYYVSGPDFSSLPSFLPQTPSSRPMTYSYSSNLPQVQPVREV
TFREYAIEPATKWHPRGNLAHCYSAEELVHRDCLQAPSAAGVPGDVLAKSSANVYHHPTP
AVSSNFYSTVGRNGVLPQAFDQFFETAYGTPENLASSDYPGDKSAEKGPPAATATSAAAA
AAATGAPATSSSDSGGGGGCRETAAAAEEKERRRRPESSSSPESSSGHTEDKAGGSSGQR
TRKKRCPYTKYQIRELEREFFFSVYINKEKRLQLSRMLNLTDRQVKIWFQNRRMKEKKIN
RDRLQYYSANPLL*
>ENSG00000010072|ENST00000008440
MDDDLMLALRLQEEWNLQEAERDHAQESLSLVDASWELVDPTPDLQALFVQFNDQFFWGQ
LEAVEVKWSVRMTLCAGICSYEGKGGMCSIRLSEPLLKLRPRKDLVEVYHTFHDEVDEYR
RHWWRCNGPCQHRPPYYGYVKRATNREPSAHDYWWAEHQKTCGGTYIKIKEPENYSKKGK
GKAKLGKEPVLAAENKGTFVYILLIFM*
>ENSG00000007376|ENST00000007264
MEPGSVENLSIVYRSRDFLVVNKHWDVRIDSKAWRETLTLQKQLRYRFPELADPDTCYGF
RFCHQLDFSTSGALCVALNKAAAGSAYRCFKERRVTKAYLALLRGHIQESRVTISHAIGR
NSTEGRAHTMCIEGSQGCENPKPSLTDLVVLEHGLYAGDPVSKVLLKPLTGRTHQLRVHC
SALGHPVVGDLTYGEVSGREDRPFRMMLHAFYLRIPTDTECVEVCTPDPFLPSLDACWSP
HTLLQSLDQLVQALRATPDPDPEDRGPRPGSPSALLPGPGRPPPPPTKPPETEAQRGPCL
QWLSEWTLEPDS*
>ENSG00000008988|ENST00000009589
MAFKDTGKTPVEPEVAIHRIRITLTSRNVKSLEKVCADLIRGAKEKNLKVKGPVRMPTKT
LRITTRKTPCGEGSKTWDRFQMRIHKRLIDLHSPSEIVKQITSISIEPGVEVEVTIADA*
>ENSG00000173153|ENST00000000442
MSSQVVGIEPLYIKAEPASPDSPKGSSETETEPPVALAPGPAPTRCLPGHKEEEDGEGAG
PGEQGGGKLVLSSLPKRLCLVCGDVASGYHYGVASCEACKAFFKRTIQGSIEYSCPASNE
CEITKRRRKACQACRFTKCLRVGMLKEGVRLDRVRGGRQKYKRRPEVDPLPFPGPFPAGP
LAVAGGPRKTAAPVNALVSHLLVVEPEKLYAMPDPAGPDGHLPAVATLCDLFDREIVVTI
SWAKSIPGFSSLSLSDQMSVLQSVWMEVLVLGVAQRSLPLQDELAFAEDLVLDEEGARAA
GLGELGAALLQLVRRLQALRLEREEYVLLKALALANSDSVHIEDAEAVEQLREALHEALL
EYEAGRAGPGGGAERRRAGRLLLTLPLLRQTAGKVLAHFYGVKLEGKVPMHKLFLEMLEA
MMD*
>ENSG00000008405|ENST00000008527
MGVNAVHWFRKGLRLHDNPALKECIQGADTIRCVYILDPWFAGSSNVGINRWRFLLQCLE
DLDANLRKLNSRLFVIRGQPADVFPRLFKEWNITKLSIEYDSEPFGKERDAAIKKLATEA
GVEVIVRISHTLYDLDKIIELNGGQPPLTYKRFQTLISKMEPLEIPVETITSEVIEKCTT
PLSDDHDEKYGVPSLEELGFDTDGLSSAVWPGGETEALTRLERHLERKAWVANFERPRMN
ANSLLASPTGLSPYLRFGCLSCRLFYFKLTDLYKKVKKNSSPPLSLYGQLLWREFFYTAA
TNNPRFDKMEGNPICVQIPWDKNPEALAKWAEGRTGFPWIDAIMTQLRQEGWIHHLARHA
VACFLTRGDLWISWEEGMKVFEELLLDADWSINAGSWMWLSCSSFFQQFFHCYCPVGFGR
RTDPNGDYIRRYLPVLRGFPAKYIYDPWNAPEGIQKVAKCLIGVNYPKPMVNHAEASRLN
IERMKQIYQQLSRYRGLGLLASVPSNPNGNGGFMGYSAENIPGCSSSGSCSQGSGILHYA
HGDSQQTHLLKQGRSSMGTGLSGGKRPSQEEDTQSIGPKVQRQSTN*
>ENSG00000004478|ENST00000001008
MTAEEMKATESGAQSAPLPMEGVDISPKQDEGVLKVIKREGTGTEMPMIGDRVFVHYTGW

LLDGTKFDSSLDRKDKFSFDLGKGEVIKAWDIAIATMKVGEVCHITCKPEYAYGSAGSPP
KIPPNATLVFEVELFEFKGEDLTEEEDGGIIRRIQTRGEGYAKPNEGAIVEVALEGYYKD
KLFDQRELRFEIGEGENLDLPYGLERAIQRMEKGEHSIVYLKPSYAFGSVGKEKFQIPPN
AELKYELHLKSFEKAKESWEMNSEEKLEQSTIVKERGTVYFKEGKYKQALLQYKKIVSWL
EYESSFSNEEAQKAQALRLASHLNLAMCHLKLQAFSAAIESCNKALELDSNNEKGLFRRG
EAHLAVNDFELARADFQKVLQLYPNNKAAKTQLAVCQQRIRRQLAREKKLYANMFERLAE
EENKAKAEASSGDHPTDTEMKEEQKSNTAGSQSQVETEA*
>ENSG00000005801|ENST00000005082
MTLLTFRDVAIEFSLEEWKCLDLAQQNLYRDVMLENYRNLFSVGLTVCKPGLITCLEQRK
EPWNVKRQEAADGHPEMGFHHATQACLELLGSSDLPASASQSAGITGVNHRAQPGLNVSV
DKFTAMSSHFTQDLLPEQGIQDAFPKRILRGYGNCGLDNLYLRKDWESLDECKLQKDYNG
LNQCSSTTHSKIFQYNKYVKIFDNFSNLHRRNISNTGEKPFKCQECGKSFQMLSFLTEHQ
KIHTGKKFQKCGECGKTFIQCSHFTEPENIDTGEKPYKCQECNNVIKTCSVLTKNRIYAG
GEHYRCEEFGKVFNQCSHLTEHEHGTEEKPCKYEECSSVFISCSSLSNQQMILAGEKLSK
CETWYKGFNHSPNPSKHQRNEIGGKPFKCEECDSIFKWFSDLTKHKRIHTGEKPYKCDEC
GKAYTQSSHLSEHRRIHTGEKPYQCEECGKVFRTCSSLSNHKRTHSEEKPYTCEECGNIF
KQLSDLTKHKKTHTGEKPYKCDECGKNFTQSSNLIVHKRIHTGEKPYKCEECGRVFMWFS
DITKHKKTHTGEKPYKCDECGKNFTQSSNLIVHKRIHTGEKPYKCEKCGKAFTQFSHLTV
HESIHT*
>ENSG00000006047|ENST00000007699
MSEVEAAAGATAVPAATVPATAAGVVAVVVPVPAGEPQKGGGAGGGGGAASGPAAGTPSA
PGSRTPGNPATAVSGTPAPPARSQADKPVLAIQVLGTVKWFNVRNGYGFINRNDTKEDVF
VHQTAIKRNNPRKFLRSVGDGETVEFDVVEGEKGAEATNVTGPGGVPVKGSRYAPNRRKS
RRFIPRPPSVAPPPMVAEIPSAGTGPGSKGERAEDSGQRPRRWCPPPFFYRRRFVRGPRP
PNQQQPIEGTDRVEPKETAPLEGHQQQGDERVPPPRFRPRYRRPFRPRPRQQPTTEGGDG
ETKPSQGPADGSRPEPQRPRNRPYFQRRRQQAPGPQQAPGPRQPAAPETSAPVNSGDPTT
TILE*
>ENSG00000008197|ENST00000008391
MSTTFPGLVHDAEIRHDGSNSYRLMQLGCLESVANSTVAYSSSSPLTYSTTGTEFASPYF
STNHQYTPLHHQSFHYEFQHSHPAVTPDAYSLNSLHHSQQYYQQIHHGEPTDFINLHNAR
ALKSSCLDEQRRELGCLDAYRRHDLSLMSHGSQYGMHPDQRLLPGPSLGLAAAGADDLQG
SVEAQCGLVLNGQGGVIRRGGTCVVNPTDLFCSVPGRLSLLSSTSKYKVTIAEVKRRLSP
PECLNASLLGGILRRAKSKNGGRCLREKLDRLGLNLPAGRRKAANVTLLTSLVEGEALHL
ARDFGYTCETEFPAKAVGEHLARQHMEQKEQTARKKMILATKQICKEFQDLLSQDRSPLG
SSRPTPILDLDIQRHLTHFSLITHGFGTPAICAALSTFQTVLSEMLNYLEKHTTHKNGGA
ADSGQGHANSEKAPLRKTSEAAVKEGKTEKTD*
>ENSG00000026297|ENST00000028008
MRPAALRGALLGCLCLALLCLGGADKRLRDNHEWKKLIMVQHWPETVCEKIQNDCRDPPD
YWTIHGLWQAR*
>ENSG00000011485|ENST00000012443
MAMAEGERTECAEPPRDEPPADGALKRAEELKTQANDYFKAKDYENAIKFYSQAIELNPS
NAIYYGNRSLAYLRTECYGYALGDATRAIELDKKYIKGYYRRAASNMALGKFRAALRDYE
TVVKVKPHDKDAKMKYQECNKIVKQKAFERAIAGDEHKRSVVDSLDIESMTIEDEYSGPK
LEDGKVTISFMKELMQWYKDQKKLHRKCAYQILVQVKEVLSKLSTLVETTLKETEKITVC

GDTHGQFYDLLNIFELNGLPSETNPYIFNGDFVDRGSFSVEVILTLFGFKLLYPDHFHLL
RGNHETDNMNQIYGFEGEVKAKYTAQMYELFSEVFEWLPLAQCINGKVLIMHGGLFSEDG
VTLDDIRKIERNRQPPDSGPMCDLLWSDPQPQNGRSISKRGVSCQFGPDVTKAFLEENNL
DYIIRSHEVKAEGYEVAHGGRCVTVFSAPNYCDQMGNKASYIHLQGSDLRPQFHQFTAVP
HPNVKPMAYANTLLQLGMM*
>ENSG00000023734|ENST00000025399
MAMRQTPLTCSGHTRPVVDLAFSGITPYGYFLISACKGAGQHLPRLSGQHDGKPMLRQGD
TGDWIGTFLGHKGAVWGATLNKDATKAATAAADFTAKVWDAVSGDELMTLAHKHIVKTVD
FTQDSNYLLTGGQDKLLRIYDLNKPEAEPKEISGHTSGIKKALWCSEDKQILSADDKTVR
LWDHATMTEVKSLNFNMSVSSMEYIPEGEILVITYGRSIAFHSAVSLDPIKSFEAPATIN
SASLHPEKEFLVAGGEDFKLYKYDYNSGEELESYKGHFGPIHCVRFSPDGELYASGSEDG
TLRLWQTVVGKTYGLWKCVLPEEDSGELAKPKIGFPETTEEELEEIASENSDCIFPSAPD
VKA*
>ENSG00000037965|ENST00000040584
MSSYFVNPLFSKYKAGESLEPAYYDCRFPQSVGRSHALVYGPGGSAPGFQHASHHVQDFF
HHGTSGISNSGYQQNPCSLSCHGDASKFYGYEALPRQSLYGAQQEASVVQYPDCKSSANT
NSSEGQGHLNQNSSPSLMFPWMRPHAPGRRSGRQTYSRYQTLELEKEFLFNPYLTRKRRI
EVSHALGLTERQVKIWFQNRRMKWKKENNKDKLPGARDEEKVEEEGNEEEEKEEEEKEEN
KD*
>ENSG00000019549|ENST00000020945
MPRSFLVKKHFNASKKPNYSELDTHTVIISPYLYESYSMPVIPQPEILSSGAYSPITVWT
TAAPFHAQLPNGLSPLSGYSSSLGRVSPPPPSDTSSKDHSGSESPISDEEERLQSKLSDP
HAIEAEKFQCNLCNKTYSTFSGLAKHKQLHCDAQSRKSFSCKYCDKEYVSLGALKMHIRT
HTLPCVCKICGKAFSRPWLLQGHIRTHTGEKPFSCPHCNRAFADRSNLRAHLQTHSDVKK
YQCKNCSKTFSRMSLLHKHEESGCCVAH*
>ENSG00000010818|ENST00000012134
MDTGDTALGQKATSRSGETDKASGRWRQEQSAVIKMSTFGSHEGQRQPQIEPEQIGNTAS
AQLFGSGKLASPSEVVQQVAEKQYPPHRPSPYSCQHSLSFPQHSLPQGVMHSTKPHQSLE
GPPWLFPGPLPSVASEDLFPFPIHGHSGGYPRKKISSLNPAYSQYSQKSIEQAEEAHKKE
HKPKKPGKYICPYCSRACAKPSVLKKHIRSHTGERPYPCIPCGFSFKTKSNLYKHRKSHA
HAIKAGLVPFTESAVSKLDLEAGFIDVEAEIHSDGEQSTDTDEESSLFAEASDKMSPGPP
IPLDIASRGGYHGSLEESLGGPMKVPILIIPKSGIPLPNESSQYIGPDMLPNPSLNTKAD
DSHTVKQKLALRLSEKKGQDSEPSLNLLSPHSKGSTDSGYFSRSESAEQQISPPNTNAKS
YEEIIFGKYCRLSPRNALSVTTTSQERAAMGRKGIMEPLPHVNTRLDVKMFEDPVSQLIP
SKGDVDPSQTSMLKSTKFNSESRQPQIIPSSIRNEGKLYPANFQGSNPVLLEAPVDSSPL
IRSNSVPTSSATNLTIPPSLRGSHSFDERMTGSDDVFYPGTVGIPPQRMLRRQAAFELPS
VQEGHVEVEHHGRMLKGISSSSLKEKKLSPGDRVGYDYDVCRKPYKKWEDSETPKQNYRD
ISCLSSLKHGGEYFMDPVVPLQGVPSMFGTTCENRKRRKEKSVGDEEDTPMICSSIVSTP
VGIMASDYDPKLQMQEGVRSGFAMAGHENLSHGHTERFDPCRPQLQPGSPSLVSEESPSA
IDSDKMSDLGGRKPPGNVISVIQHTNSLSRPNSFERSESAELVACTQDKAPSPSETCDSE
ISEAPVSPEWAPPGDGAESGGKPSPSQQVQQQSYHTQPRLVRQHNIQVPEIRVTEEPDKP
EKEKEAQSKEPEKPVEEFQWPQRSETLSQLPAEKLPPKKKRLRLADMEHSSGESSFESTG
TGLSRSPSQESNLSHSSSFSMSFEREETSKLSALPKQDEFGKHSEFLTVPAGSYSLSVPG
HHHQKEMRRCSSEQMPCPHPAEVPEVRSKSFDYGNLSHAPVSGAAASTVSPSRERKKCFL

VRQASFSGSPEISQGEVGMDQSVKQEQLEHLHAGLRSGWHHGPPAVLPPLQQEDPGKQVA
GPCPPLSSGPLHLAQPQIMHMDSQESLRNPLIQPTSYMTSKHLPEQPHLFPHQETIPFSP
IQNALFQFQYPTVCMVHLPAQQPPWWQAHFPHPFAQHPQKSYGKPSFQTEIHSSYPLEHV
AEHTGKKPAEYAHTKEQTYPCYSGASGLHPKNLLPKFPSDQSSKSTETPSEQVLQEDFAS
ANAGSLQSLPGTVVPVRIQTHVPSYGSVMYTSISQILGQNSPAIVICKVDENMTQRTLVT
NAAMQGIGFNIAQVLGQHAGLEKYPIWKAPQTLPLGLESSIPLCLPSTSDSVATLGGSKR
MLSPASSLELFMETKQQKRVKEEKMYGQIVEELSAVELTNSDIKKDLSRPQKPQLVRQGC
ASEPKDGLQSGSSSFSSLSPSSSQDYPSVSPSSREPFLPSKEMLSGSRAPLPGQKSSGPS
ESKESSDELDIDETASDMSMSPQSSSLPAGDGQLEEEGKGHKRPVGMLVRMASAPSGNVA
DSTLLLTDMADFQQILQFPSLRTTTTVSWCFLNYTKPNYVQQATFKSSVYASWCISSCNP
NPSGLNTKTTLALLRSKQKITAEIYTLAAMHRPGTGKLTSSSAWKQFTQMKPDASFLFGS
KLERKLVGNILKERGKGDIHGDKDIGSKQTEPIRIKIFEGGYKSNEDYVYVRGRGRGKYI
CEECGIRCKKPSMLKKHIRTHTDVRPYVCKLCNFAFKTKGNLTKHMKSKAHMKKCLELGV
SMTSVDDTETEEAENLEDLHKAAEKHSMSSISTDHQFSDAEESDGEDGDDNDDDDEDEDD
FDDQGDLTPKTRSRSTSPQPPRFSSLPVNVGAVPHGVPSDSSLGHSSLISYLVTLPSIRV
TQLMTPSDSCEDTQMTEYQRLFQSKSTDSEPDKDRLDIPSCMDEECMLPSEPSSSPRDFS
PSSHHSSPGYDSSPCRDNSPKRYLIPKGDLSPRRHLSPRRDLSPMRHLSPRKEAALRREM
SQRDVSPRRHLSPRRPVSPGKDITARRDLSPRRERRYMTTIRAPSPRRALYHNPPLSMGQ
YLQAEPIVLGPPNLRRGLPQVPYFSLYGDQEGAYEHPGSSLFPEGPNDYVFSHLPLHSQQ
QVRAPIPMVPVGGIQMVHSMPPALSSLHPSPTLPLPMEGFEEKKGASGESFSKDPYVLSK
QHEKRGPHALQSSGPPSTPSSPRLLMKQSTSEDSLNATEREQEENIQTCTKAIASLRIAT
EEAALLGPDQPARVQEPHQNPLGSAHVSIRHFSRPEPGQPCTSATHPDLHDGEKDNFGTS
QTPLAHSTFYSKSCVDDKQLDFHSSKELSSSTEESKDPSSEKSQLH*
>ENSG00000013563|ENST00000014935
MHYPTALLFLILANGAQAFRICAFNAQRLTLAKVAREQVMDTLVRILARCDIMVLQEVVD
SSGSAIPLLLRELNRFDGSGPYSTLSSPQLGRSTYMETYVYFYRSHKTQVLSSYVYNDED
DVFAREPFVAQFSLPSNVLPSLVLVPLHTTPKAVEKELNALYDVFLEVSQHWQSKDVILL
GDFNADCASLTKKRLDKLELRTEPGFHWVIADGEDTTVRASTHCTYDRVVLHGERCRSLL
HTAAAFDFPTSFQLTEEEALNISDHYPVEVELKLSQAHSVQPLSLTVLLLLSLLSPQLCP
AA*
>ENSG00000012061|ENST00000013807
MDPGKDKEGVPQPSGPPARKKFVIPLDEDEVPPGVAKPLFRSTQSLPTVDTSAQAAPQTY
AEYAISQPLEGAGATCPTGSEPLAGETPNQALKPGAKSNSIIVSPRQRGNPVLKFVRNVP
WEFGDVIPDYVLGQSTCALFLSLRYHNLHPDYIHGRLQSLGKNFALRVLLVQVDVKDPQQ
ALKELAKMCILADCTLILAWSPEEAGRYLETYKAYEQKPADLLMEKLEQDFVSRVTECLT
TVKSVNKTDSQTLLTTFGSLEQLIAASREDLALCPGLGPQKVRALGKNPRSWGKERAPNK
HNLRPQSFKVKKEPKTRHSGFRL*
>ENSG00000239672|ENST00000013034
MVLLSTLGIVFQGEGPPISSCDTGTMANCERTFIAIKPDGVQRGLVGEIIKRFEQKGFRL
VGLKFMQASEDLLKEHYVDLKDRPFFAGLVKYMHSGPVVAMVWEGLNVVKTGRVMLGETN
PADSKPGTIRGDFCIQVGRNIIHGSDSVESAEKEIGLWFHPEELVDYTSCAQNWIYE*
>ENSG00000050344|ENST00000056233
MKHLKRWWSAGGGLLHLTLLLSLAGLRVDLDLYLLLPPPTLLQDELLFLGGPASSAYALS
PFSASGGWGRAGHLHPKGRELDPAAPPEGQLLREVRALGVPFVPRTSVDAWLVHSVAAGS

ADEAHGLLGAAAASSTGGAGASVDGGSQAVQGGGGDPRAARSGPLDAGEEEKAPAEPTAQ
VPDAGGCASEENGVLREKHEAVDHSSQHEENEERVSAQKENSLQQNDDDENKIAEKPDWE
AEKTTESRNERHLNGTDTSFSLEDLFQLLSSQPENSLEGISLGDIPLPGSISDGMNSSAH
YHVNFSQAISQDVNLHEAILLCPNNTFRRDPTARTSQSQEPFLQLNSHTTNPEQTLPGTN
LTGFLSPVDNHMRNLTSQDLLYDLDINIFDEINLMSLATEDNFDPIDVSQLFDEPDSDSG
LSLDSSHNNTSVIKSNSSHSVCDEGAIGYCTDHESSSHHDLEGAVGGYYPEPSKLCHLDQ
SDSDFHGDLTFQHVFHNHTYHLQPTAPESTSEPFPWPGKSQKIRSRYLEDTDRNLSRDEQ
RAKALHIPFSVDEIVGMPVDSFNSMLSRYYLTDLQVSLIRDIRRRGKNKVAAQNCRKRKL
DIILNLEDDVCNLQAKKETLKREQAQCNKAINIMKQKLHDLYHDIFSRLRDDQGRPVNPN
HYALQCTHDGSILIVPKELVASGHKKETQKGKRK*
>ENSG00000136960|ENST00000075322
MARRSSFQSCQIISLFTFAVGVNICLGFTAHRIKRAEGWEEGPPTVLSDSPWTNISGSCK
GRCFELQEAGPPDCRCDNLCKSYTSCCHDFDELCLKTARGWECTKDRCGEVRNEENACHC
SEDCLARGDCCTNYQVVCKGESHWVDDDCEEIKAAECPAGFVRPPLIIFSVDGFRASYMK
KGSKVMPNIEKLRSCGTHSPYMRPVYPTKTFPNLYTLATGLYPESHGIVGNSMYDPVFDA
TFHLRGREKFNHRWWGGQPLWITATKQGVKAGTFFWSVVIPHERRILTILQWLTLPDHER
PSVYAFYSEQPDFSGHKYGPFGPEMTNPLREIDKIVGQLMDGLKQLKLHRCVNVIFVGDH
GMEDVTCDRTEFLSNYLTNVDDITLVPGTLGRIRSKFSNNAKYDPKAIIANLTCKKPDQH
FKPYLKQHLPKRLHYANNRRIEDIHLLVERRWHVARKPLDVYKKPSGKCFFQGDHGFDNK
VNSMQTVFVGYGSTFKYKTKVPPFENIELYNVMCDLLGLKPAPNNGTHGSLNHLLRTNTF
RPTMPEEVTRPNYPGIMYLQSDFDLGCTCDDKVEPKNKLDELNKRLHTKGSTEERHLLYG
RPAVLYRTRYDILYHTDFESGYSEIFLMPLWTSYTVSKQAEVSSVPDHLTSCVRPDVRVS
PSFSQNCLAYKNDKQMSYGFLFPPYLSSSPEAKYDAFLVTNMVPMYPAFKRVWNYFQRVL
VKKYASERNGVNVISGPIFDYDYDGLHDTEDKIKQYVEGSSIPVPTHYYSIITSCLDFTQ
PADKCDGPLSVSSFILPHRPDNEESCNSSEDESKWVEELMKMHTARVRDIEHLTSLDFFR
KTSRSYPEILTLKTYLHTYESEI*
>ENSG00000030582|ENST00000053867
MWTLVSWVALTAGLVAGTRCPDGQFCPVACCLDPGGASYSCCRPLLDKWPTTLSRHLGGP
CQVDAHCSAGHSCIFTVSGTSSCCPFPEAVACGDGHHCCPRGFHCSADGRSCFQRSGNNS
VGAIQCPDSQFECPDFSTCCVMVDGSWGCCPMPQASCCEDRVHCCPHGAFCDLVHTRCIT
PTGTHPLAKKLPAQRTNRAVALSSSVMCPDARSRCPDGSTCCELPSGKYGCCPMPNATCC
SDHLHCCPQDTVCDLIQSKCLSKENATTDLLTKLPAHTVGDVKCDMEVSCPDGYTCCRLQ
SGAWGCCPFTQAVCCEDHIHCCPAGFTCDTQKGTCEQGPHQVPWMEKAPAHLSLPDPQAL
KRDVPCDNVSSCPSSDTCCQLTSGEWGCCPIPEAVCCSDHQHCCPQGYTCVAEGQCQRGS
EIVAGLEKMPARRASLSHPRDIGCDQHTSCPVGQTCCPSLGGSWACCQLPHAVCCEDRQH
CCPAGYTCNVKARSCEKEVVSAQPATFLARSPHVGVKDVECGEGHFCHDNQTCCRDNRQG
WACCPYRQGVCCADRRHCCPAGFRCAARGTKCLRREAPRWDAPLRDPALRQLL*
>ENSG00000063515|ENST00000086933
MAAAAGGAASRRGAGRPCPFSIEHILSSLPERSLPARAACPPQPAGRQSPAKPEEPGAPE
AAPCACCCCCGPRAAPCGPPEAAAGLGARLAWPLRLGPAVPLSLGAPAGGSGALPGAVGP
GSQRRTRRHRTIFSEEQLQALEALFVQNQYPDVSTRERLAGRIRLREERVEVWFKNRRAK
WRHQKRASASARLLPGVKKSPKGSC*
>ENSG00000049541|ENST00000055077
MEVEAVCGGAGEVEAQDSDPAPAFSKAPGSAGHYELPWVEKYRPVKLNEIVGNEDTVSRL

EVFAREGNVPNIIIAGPPGTGKTTSILCLARALLGPALKDAMLELNASNDRGIDVVRNKI
KMFAQQKVTLPKGRHKIIILDEADSMTDGAQQALRRTMEIYSKTTRFALACNASDKIIEP
IQSRCAVLRYTKLTDAQILTRLMNVIEKERVPYTDDGLEAIIFTAQGDMRQALNNLQSTF
SGFGFINSENVFKVCDEPHPLLVKEMIQHCVNANIDEAYKILAHLWHLGYSPEDIIGNIF
RVCKTFQMAEYLKLEFIKEIGYTHMKIAEGVNSLLQMAGLLARLCQKTMAPVAS*
>ENSG00000059588|ENST00000040877
MEWVLAEALLSQSRDPRALLGALCQGEASAERVETLRFLLQRLEDEEARGSGGAGALPEA
AREVAAGYLVPLLRSLRGRPAGGPDPSLQPRHRRRVLRAAGAALRSCVRLAGRPQLAAAL
AEEALRDLLAGWRAPGAEAAVEVLAAVGPCLRPREDGPLLERVAGTAVALALGGGGDGDE
AGPAEDAAALVAGRLLPVLVQCGGAALRAVWGGLAAPGASLGSGRVEEKLLVLSALAEKL
LPEPGGDRARGAREAGPDARRCWRFWRTVQAGLGQADALTRKRARYLLQRAVEVSAELGA
DCTCGPQEGNGPSLFWWSERKKDELLKFWENYILIMETLEGNQIHVIKPVLPKLNNLFEY
AVSEENGCWLFHPSWHMCIYKRMFESENKILSKEGVIHFLELYETKILPFSPEFSEFIIG
PLMDALSESSLYSRSPGQPIGSCSPLGLKLQKFLVTYISLLPEEIKSSFLLKFIRKMTSR
HWCAVPILFLSKALANVPRHKALGIDGLLALRDVIHCTMITHQILLRGAAQCYLLQTAMN
LLDVEKVSLSDVSTFLMSLRQEESLGRGTSLWTELCDWLRVNESYFKPSPTCSSIGLHKT
SLNAYVKSIVQEYVKSSAWETGENCFMPDWFEAKLVSLMVLLAVDVEGMKTQYSGKQRTE
NVLRIFLDPLLDVLMKFSTNAYMPLLKTDRCLQLLLKLLNTCRLKGSSAQDDEVSTVLQN
FFMSTTESISEFILRRLTMNELNSVSDLDRCHLYLMVLTELINLHLKVGWKRGNPIWRVI
SLLKNASIQHLQEMDSGQEPTVGSQIQRVVSMAALAMVCEAIDQKPELQLDSLHAGPLES
FLSSLQLNQTLQKPHAEEQSSYAHPLECSSVLEESSSSQGWGKIVAQYIHDQWVCLSFLL
KKYHTLIPTTGSEILEPFLPAVQMPIRTLQSALEALTVLSSDQVLPVFHCLKVLVPKLLT
SSESLCIESFDMAWKIISSLSNTQLIFWANLKAFVQFVFDNKVLTIAAKIKGQAYFKIKE
IMYKIIEMSAIKTGVFNTLISYCCQSWIVSASNVSQGSLSSAKNYSELILEACIFGTVFR
RDQRLVQDVQTFIENLGHDCAANIVMENTKREDHYVRICAVKFLCLLDGSNMSHKLFIED
LAIKLLDKDELVSKSKKRYYVNSLQHRVKNRVWQTLLVLFPRLDQNFLNGIIDRIFQAGF
TNNQASIKYFIEWIIILILHKFPQFLPKFWDCFSYGEENLKTSICTFLAVLSHLDIITQN
IPEKKLILKQALIVVLQWCFNHNFSVRLYALVALKKLWTVCKVLSVEEFDALTPVIESSL
HQVESMHGAGNAKKNWQRIQEHFFFATFHPLKDYCLETIFYILPRLSGLIEDEWITIDKF
TRFTDVPLAAGFQWYLSQTQLSKLKPGDWSQQDIGTNLVEADNQAEWTDVQKKIIPWNSR
VSDLDLELLFQDRAARLGKSISRLIVVASLIDKPTNLGGLCRTCEVFGASVLVVGSLQCI
SDKQFQHLSVSAEQWLPLVEVKPPQLIDYLQQKKTEGYTIIGVEQTAKSLDLTQYCFPEK
SLLLLGNEREGIPANLIQQLDVCVEIPQQGIIRSLNVHVSGALLIWEYTRQQLLSHGDTK
P*
>ENSG00000061794|ENST00000081029
MAAAALPAWLSLQSRARTLRAFSTAVYSATPVPTPSLPERTPGNERPPRRKALPPRTEKM
AVDQDWPSVYPVAAPFKPSAVPLPVRMGYPVKKGVPMAKEGNLELLKIPNFLHLTPVAIK
KHCEALKDFCTEWPAALDSDEKCEKHFPIEIDSTDYVSSGPSVRNPRARVVVLRVKLSSL
NLDDHAKKKLIKLVGERYCKTTDVLTIKTDRCPLRRQNYDYAVYLLTVLYHESWNTEEWE
KSKTEADMEEYIWENSSSERNILETLLQMKAAEKNMEINKEELLGTKEIEEYKKSVVSLK
NEEENENSISQYKESVKRLLNVT*
>ENSG00000068394|ENST00000156109
MADSKEGVLPLTAASTAPISFGFTRTSARRRLADSGDGAGPSPEEKDFLKTVEGRELQSV
KPQEAPKELVIPLIQNGHRRQPPARPPGPSTDTGALADGVVSQAVKELIAESKKSLEERE

NAGVDPTLAIPMIQKGCTPSGEGADSEPRAETVPEEANYEAVPVEAYGLAMLRGMGWKPG
EGIGRTFNQVVKPRVNSLRPKGLGLGANLTEAQALTPTGPSRMPRPDEEQEKDKEDQPQG
LVPGGAVVVLSGPHRGLYGKVEGLDPDNVRAMVRLAVGSRVVTVSEYYLRPVSQQEFDKN
TLDLRQQNGTASSRKTLWNQELYIQQDNSERKRKHLPDRQDGPAAKSEKAAPRSQHWLHR
DLRVRFVDNMYKGGQYYNTKMIIEDVLSPDTCVCRTDEGRVLEGLREDMLETLVPKAEGD
RVMVVLGPQTGRVGHLLSRDRARSRALVQLPRENQVVELHYDAICQYMGPSDTDDD*
>ENSG00000021776|ENST00000156471
MAAPAQPKKIVAPTVSQINAEFVTQLACKYWAPHIKKKSPFDIKVIEDIYEKEIVKSRFA
IRKIMLLEFSQYLENYLWMNYSPEVSSKAYLMSICCMVNEKFRENVPAWEIFKKKPDHFP
FFFKHILKAALAETDGEFSLHEQTVLLLFLDHCFNSLEVDLIRSQVQQLISLPMWMGLQL
ARLELELKKTPKLRKFWNLIKKNDEKMDPEAREQAYQERRFLSQLIQKFISVLKSVPLSE
PVTMDKVHYCERFIELMIDLEALLPTRRWFNTILDDSHLLVHCYLSNLVRREEDGHLFSQ
LLDMLKFYTGFEINDQTGNALTENEMTTIHYDRITSLQRAAFAHFPELYDFALSNVAEVD
TRESLVKFFGPLSSNTLHQVASYLCLLPTLPKNEDTTFDKEFLLELLVSRHERRISQIQQ
LNQMPLYPTEKIIWDENIVPTEYYSGEGCLALPKLNLQFLTLHDYLLRNFNLFRLESTYE
IRQDIEDSVSRMKPWQSEYGGVVFGGWARMAQPIVAFTVVEVAKPNIGENWPTRVRADVT
INLNVRDHIKDEWEGLRKHDVCFLITVRPTKPYGTKFDRRRPFIEQVGLVYVRGCEIQGM
LDDKGRVIEDGPEPRPNLRGESRTFRVFLDPNQYQQDMTNTIQNGAEDVYETFNIIMRRK
PKENNFKAVLETIRNLMNTDCVVPDWLHDIILGYGDPSSAHYSKMPNQIATLDFNDTFLS
IEHLKASFPGHNVKVTVEDPALQIPPFRITFPVRSGKGKKRKDADVEDEDTEEAKTLIVE
PHVIPNRGPYPYNQPKRNTIQFTHTQIEAIRAGMQPGLTMVVGPPGTGKTDVAVQIISNI
YHNFPEQRTLIVTHSNQALNQLFEKIMALDIDERHLLRLGHGEEELETEKDFSRYGRVNY
VLARRIELLEEVKRLQKSLGVPGDASYTCETAGYFFLYQVMSRWEEYISKVKNKGSTLPD
VTEVSTFFPFHEYFANAPQPIFKGRSYEEDMEIAEGCFRHIKKIFTQLEEFRASELLRSG
LDRSKYLLVKEAKIIAMTCTHAALKRHDLVKLGFKYDNILMEEAAQILEIETFIPLLLQN
PQDGFSRLKRWIMIGDHHQLPPVIKNMAFQKYSNMEQSLFTRFVRVGVPTVDLDAQGRAR
ASLCNLYNWRYKNLGNLPHVQLLPEFSTANAGLLYDFQLINVEDFQGVGESEPNPYFYQN
LGEAEYVVALFMYMCLLGYPADKISILTTYNGQKHLIRDIINRRCGNNPLIGRPNKVTTV
DRFQGQQNDYILLSLVRTRAVGHLRDVRRLVVAMSRARLGLYIFARVSLFQNCFELTPAF
SQLTARPLHLHIIPTEPFPTTRKNGERPSHEVQIIKNMPQMANFVYNMYMHLIQTTHHYH
QTLLQLPPAMVEEGEEVQNQETELETEEEAMTVQADIIPSPTDTSCRQETPAFQTDTTPS
ETGATSTPEAIPALSETTPTVVGAVSAPAEANTPQDATSAPEETK*
>ENSG00000071655|ENST00000156825
MERKSPSGKKFRSKPQLARYLGGSMDLSTFDFRTGKMLMSKMNKSRQRVRYDSSNQVKGK
PDLNTALPVRQTASIFKQPVTKITNHPSNKVKSDPQKAVDQPRQLFWEKKLSGLNAFDIA
EELVKTMDLPKGLQGVGPGCTDETLLSAIASALHTSTMPITGQLSAAVEKNPGVWLNTTQ
PLCKAFMVTDEDIRKQEELVQQVRKRLEEALMADMLAHVEELARDGEAPLDKACAEDDDE
EDEEEEEEEPDPDPEMEHV*
>ENSG00000079432|ENST00000160740
MYSAHRPLMPASSAASRGLGMFVWTNVEPRSVAVFPWHSLVPFLAPSQPDPSVQPSEAQQ
PASHPVASNQSKEPAESAAVAHERPPGGTGSADPERPPGATCPESPGPGPPHPLGVVESG
KGPPPTTEEEASGPPGEPRLDSETESDHDDAFLSIMSPEIQLPLPPGKRRTQSLSALPKE
RDSSSEKDGRSPNKREKDHIRRPMNAFMIFSKRHRALVHQRHPNQDNRTVSKILGEWWYA
LGPKEKQKYHDLAFQVKEAHFKAHPDWKWCNKDRKKSSSEAKPTSLGLAGGHKETRERSM

SETGTAAAPGVSSELLSVAAQTLLSSDTKAPGSSSCGAERLHTVGGPGSARPRAFSHSGV
HSLDGGEVDSQALQELTQMVSGPASYSGPKPSTQYGAPGPFAAPGEGGALAATGRPPLLP
TRASRSQRAASEDMTSDEERMVICEEEGDDDVIADDGFGTTDIDLKCKERVTDSESGDSS
GEDPEGNKGFGRKVFSPVIRSSFTHCRPPLDPEPPGPPDPPVAFGKGYGSAPSSSASSPA
SSSASAATSFSLGSGTFKAQESGQGSTAGPLRPPPPGAGGPATPSKATRFLPMDPATFRR
KRPESVGGLEPPGPSVIAAPPSGGGNILQTLVLPPNKEEQEGGGARVPSAPAPSLAYGAP
AAPLSRPAATMVTNVVRPVSSTPVPIASKPFPTSGRAEASPNDTAGARTEMGTGSRVPGG
SPLGVSLVYSDKKSAAATSPAPHLVAGPLLGTVGKAPATVTNLLVGTPGYGAPAPPAVQF
IAQGAPGGGTTAGSGAGAGSGPNGPVPLGILQPGALGKAGGITQVQYILPTLPQQLQVAP
APAPAPGTKAAAPSGPAPTTSIRFTLPPGTSTNGKVLAATAPTPGIPILQSVPSAPPPKA
QSVSPVQAPPPGGSAQLLPGKVLVPLAAPSMSVRGGGAGQPLPLVSPPFSVPVQNGAQPP
SKIIQLTPVPVSTPSGLVPPLSPATLPGPTSQPQKVLLPSSTRITYVQSAGGHALPLGTS
PASSQAGTVTSYGPTSSVALGFTSLGPSGPAFVQPLLSGQAPLLAPGQVGVSPVPSPQLP
PACAAPGGPVITAFYSGSPAPTSSAPLAQPSQAPPSLVYTVATSTTPPAATILPKGPPAP
ATATPAPTSPFPSATGSMTYSLVAPKAQRPSPKAPQKVKAAIASIPVGSFEAGASGRPGP
APRQPLEPGPVREPTAPESELEGQPTPPAPPPLPETWTPTARSSPPLPPPAEERTSAKGP
ETMASKFPSSSSDWRVPGQGLENRGEPPTPPSPAPAPAVAPGGSSESSSGRAAGDTPERK
EAAGTGKKVKVRPPPLKKTFDSVDNRVLSEVDFEERFAELPEFRPEEVLPSPTLQSLATS
PRAILGSYRKKRKNSTDLDSAPEDPTSPKRKMRRRSSCSSEPNTPKSAKCEGDIFTFDRT
GTEAEDVLGELEYDKVPYSSLRRTLDQRRALVMQLFQDHGFFPSAQATAAFQARYADIFP
SKVCLQLKIREVRQKIMQAATPTEQPPGAEAPLPVPPPTGTAAAPAPTPSPAGGPDPTSP
SSDSGTAQAAPPLPPPPESGPGQPGWEGAPQPSPPPPGPSTAATGR*
>ENSG00000011465|ENST00000052754
MKATIILLLLAQVSWAGPFQQRGLFDFMLEDEASGIGPEVPDDRDFEPSLGPVCPFRCQC
HLRVVQCSDLGLDKVPKDLPPDTTLLDLQNNKITEIKDGDFKNLKNLHALILVNNKISKV
SPGAFTPLVKLERLYLSKNQLKELPEKMPKTLQELRAHENEITKVRKVTFNGLNQMIVIE
LGTNPLKSSGIENGAFQGMKKLSYIRIADTNITSIPQGLPPSLTELHLDGNKISRVDAAS
LKGLNNLAKLGLSFNSISAVDNGSLANTPHLRELHLDNNKLTRVPGGLAEHKYIQVVYLH
NNNISVVGSSDFCPPGHNTKKASYSGVSLFSNPVQYWEIQPSTFRCVYVRSAIQLGNYK*
>ENSG00000041988|ENST00000054650
MPKSCAARQCCNRYSSRRKQLTFHRFPFSRPELLKEWVLNIGRGNFKPKQHTVICSEHFR
PECFSAFGNRKNLKHNAVPTVFAFQDPTQQVRENTDPASERGNASSSQKEKVLPEAGAGE
DSPGRNMDTALEELQLPPNAEGHVKQVSPRRPQATEAVGRPTGPAGLRRTPNKQPSDHSY
ALLDLDSLKKKLFLTLKENEKLRKRLQAQRLVMRRMSSRLRACKGHQGLQARLGPEQQS*
>ENSG00000060566|ENST00000078445
MNTDLAAGKMASAACSMDPIDSFELLDLLFDRQDGILRHVELGEGWGHVKDQQVLPNPDS
DDFLSSILGSGDSLPSSPLWSPEGSDSGISEDLPSDPQDTPPRSGPATSPAGCHPAQPGK
GPCLSYHPGNSCSTTTPGPVIQVPEASVTIDLEMWSPGGRICAEKPADPVDLSPRCNLTV
KDLLLSGSSGDLQQHHLGASYLLRPGAGHCQELVLTEDEKKLLAKEGITLPTQLPLTKYE
ERVLKKIRRKIRNKQSAQESRKKKKEYIDGLETRMSACTAQNQELQRKVLHLEKQNLSLL
EQLKKLQAIVVQSTSKSAQTGTCVAVLLLSFALIILPSISPFGPNKTESPGDFAPVRVFS
RTLHNDAASRVAADAVPGSEAPGPRPEADTTREESPGSPGADWGFQDTANLTNSTEELDN
ATLVLRNATEGLGQVALLDWVAPGPSTGSGRAGLEAAGDEL*
>ENSG00000067646|ENST00000155093

MDEDEFELQPQEPNSFFDGIGADATHMDGDQIVVEIQEAVFVSNIVDSDITVHNFVPDDP
DSVVIQDVVEDVVIEEDVQCSDILEEADVSENVIIPEQVLDSDVTEEVSLPHCTVPDDVL
ASDITSTSMSMPEHVLTSESMHVCDIGHVEHMVHDSVVEAEIITDPLTSDIVSEEVLVAD
CAPEAVIDASGISVDQQDNDKASCEDYLMISLDDAGKIEHDGSTGVTIDAESEMDPCKVD
STCPEVIKVYIFKADPGEDDLGGTVDIVESEPENDHGVELLDQNSSIRVPREKMVYMTVN
DSQQEDEDLNVAEIADEVYMEVIVGEEDAAVAAAAAAVHEQQIDEDEMKTFVPIAWAAAY
GNNSDGIENRNGTASALLHIDESAGLGRLAKQKPKKKRRPDSRQYQTAIIIGPDGHPLTV
YPCMICGKKFKSRGFLKRHMKNHPEHLAKKKYHCTDCDYTTNKKISLHNHLESHKLTSKA
EKAIECDECGKHFSHAGALFTHKMVHKEKGANKMHKCKFCEYETAEQGLLNRHLLAVHSK
NFPHICVECGKGFRHPSELRKHMRIHTGEKPYQCQYCEYRSADSSNLKTHIKTKHSKEMP
FKCDICLLTFSDTKEVQQHTLVHQESKTHQCLHCDHKSSNSSDLKRHVISVHTKDYPHKC
EMCEKGFHRPSELKKHVAVHKGKKMHQCRHCDFKIADPFVLSRHILSVHTKDLPFRCKRC
RKGFRQQNELKKHMKTHSGRKVYQCEYCEYSTTDASGFKRHVISIHTKDYPHRCEYCKKG
FRRPSEKNQHIMRHHKEVGLP*
>ENSG00000079616|ENST00000160827
MAAGGSTQQRRREMAAASAAAISGAGRCRLSKIGATRRPPPARVRVAVRLRPFVDGTAGA
SDPPCVRGMDSCSLEIANWRNHQETLKYQFDAFYGERSTQQDIYAGSVQPILRHLLEGQN
ASVLAYGPTGAGKTHTMLGSPEQPGVIPRALMDLLQLTREEGAEGRPWALSVTMSYLEIY
QEKVLDLLDPASGDLVIREDCRGNILIPGLSQKPISSFADFERHFLPASRNRTVGATRLN
QRSSRSHAVLLVKVDQRERLAPFRQREGKLYLIDLAGSEDNRRTGNKGLRLKESGAINTS
LFVLGKVVDALNQGLPRVPYRDSKLTRLLQDSLGGSAHSILIANIAPERRFYLDTVSALN
FAARSKEVINRPFTNESLQPHALGPVKLSQKELLGPPEAKRARGPEEEEIGSPEPMAAPA
SASQKLSPLQKLSSMDPAMLERLLSLDRLLASQGSQGAPLLSTPKRERMVLMKTVEEKDL
EIERLKTKQKELEAKMLAQKAEEKENHCPTMLRPLSHRTVTGAKPLKKAVVMPLQLIQEQ
AASPNAEIHILKNKGRKRKLESLDALEPEEKAEDCWELQISPELLAHGRQKILDLLNEGS
ARDLRSLQRIGPKKAQLIVGWRELHGPFSQVEDLERVEGITGKQMESFLKANILGLAAGQ
RCGAS*
>ENSG00000088002|ENST00000201586
MDGPAEPQIPGLWDTYEDDISEISQKLPGEYFRYKGVPFPVGLYSLESISLAENTQDVRD
DDIFIITYPKSGTTWMIEIICLILKEGDPSWIRSVPIWERAPWCETIVGAFSLPDQYSPR
LMSSHLPIQIFTKAFFSSKAKVIYMGRNPRDVVVSLYHYSKIAGQLKDPGTPDQFLRDFL
KGEVQFGSWFDHIKGWLRMKGKDNFLFITYEELQQDLQGSVERICGFLGRPLGKEALGSV
VAHSTFSAMKANTMSNYTLLPPSLLDHRRGAFLRKGVCGDWKNHFTVAQSEAFDRAYRKQ
MRGMPTFPWDEDPEEDGSPDPEPSPEPEPKPSLEPNTSLEREPRPNSSPSPSPGQASETP
HPRPS*
>ENSG00000086827|ENST00000200135
MASFVTEVLAHSGRLEKEDLGTRISRLTRRVEEIKGEVCNMISKKYSEFLPSMQSAQGLI
TQVDKLSEDIDLLKSRIESEVRRDLHVSTGEFTDLKQQLERDSVVLSLLKQLQEFSTAIE
EYNCALTEKKYVTGAQRLEEAQKCLKLLKSRKCFDLKILKSLSMELTIQKQNILYHLGEE
WQKLIVWKFPPSKDTSSLESYLQTELHLYTEQSHKEEKTPMPPISSVLLAFSVLGELHSK
LKSFGQMLLKYILRPLASCPSLHAVIESQPNIVIIRFESIMTNLEYPSPSEVFTKIRLVL
EVLQKQLLDLPLDTDLENEKTSTVPLAEMLGDMIWEDLSECLIKNCLVYSIPTNSSKLQQ
YEEIIQSTEEFENALKEMRFLKGDTTDLLKYARNINSHFANKKCQDVIVAARNLMTSEIH
NTVKIIPDSKINVPELPTPDEDNKLEVQKVSNTQYHEVMNLEPENTLDQHSFSLPTCRIS

ESVKKLMELAYQTLLEATTSSDQCAVQLFYSVRNIFHLFHDVVPTYHKENLQKLPQLAAI
HHNNCMYIAHHLLTLGHQFRLRLAPILCDGTATFVDLVPGFRRLGTECFLAQMRAQKGEL
LERLSSARNFSNMDDEENYSAASKAVRQVLHQLKRLGIVWQDVLPVNIYCKAMGTLLNTA
ISEVIGKITALEDISTEDGDRLYSLCKTVMDEGPQVFAPLSEESKNKKYQEEVPVYVPKW
MPFKELMMMLQASLQEIGDRWADGKGPLAAAFSSSEVKALIRALFQNTERRAAALAKIK*
>ENSG00000087510|ENST00000201031
MLWKITDNVKYEEDCEDRHDGSSNGNPRVPHLSSAGQHLYSPAPPLSHTGVAEYQPPPYF
PPPYQQLAYSQSADPYSHLGEAYAAAINPLHQPAPTGSQQQAWPGRQSQEGAGLPSHHGR
PAGLLPHLSGLEAGAVSARRDAYRRSDLLLPHAHALDAAGLAENLGLHDMPHQMDEVQNV
DDQHLLLHDQTVIRKGPISMTKNPLNLPCQKELVGAVMNPTEVFCSVPGRLSLLSSTSKY
KVTVAEVQRRLSPPECLNASLLGGVLRRAKSKNGGRSLREKLDKIGLNLPAGRRKAAHVT
LLTSLVEGEAVHLARDFAYVCEAEFPSKPVAEYLTRPHLGGRNEMAARKNMLLAAQQLCK
EFTELLSQDRTPHGTSRLAPVLETNIQNCLSHFSLITHGFGSQAICAAVSALQNYIKEAL
IVIDKSYMNPGDQSPADSNKTLEKMEKHRK*
>ENSG00000159885|ENST00000187879
MAKLYEAVTFKDVAVIFTEEELGLLDPAQRKLYRDVMLENFRNLLSVGGKIQTEMETVPE
AGTHEEFSCKQIWEQIASDLTRSQDTTISNSQLFEQDDNPSQIKARLSTVHTREKPFQGE
NCKQFFSDVSFFDLPQQLYSGEKSHTCDECGKSFCYISALHIHQRVHMGVKCYKCDVCGK
EFSQSSRLQTHQRVHTGEKPFKCEQCGKGFRCRSALKVHCKLHMREKPYNCEKCGKAFMH
NFQLQKHHRIHTGEKPFKCEICGKSFCLRSSLNRHCMVHTAEKLYKSEKYGRGFIDRLDL
HKHQMIHMGQKPYNCKECGKSFKWSSYLLVHQRVHTGEKPYKCEECGKGYISKSGLDFHH
RTHTGERSYNCDNCGKSFRHASSILNHKKLHCQRKPLKCEDCGKRLVCRSYCKDQQRDHS
GENPSKCEDCGKRYKRRLNLDIILSLFLNDI*
>ENSG00000083544|ENST00000196169
MLRLQMTDGHISCTAVEFSYMSKISLNTPPGTKVKLSGIVDIKNGFLLLNDSNTTVLGGE
VEHLIEKWELQRSLSKHNRSNIGTEGGPPPFVPFGQKCVSHVQVDSRELDRRKTLQVTMP
VKPTNDNDEFEKQRTAAIAEVAKSKETKTFGGGGGGARSNLNMNAAGNRNREVLQKEKST
KSEGKHEGVYRELVDEKALKHITEMGFSKEASRQALMDNGNNLEAALNVLLTSNKQKPVM
GPPLRGRGKGRGRIRSEDEEDLGNARPSAPSTLFDFLESKMGTLNVEEPKSQPQQLHQGQ
YRSSNTEQNGVKDNNHLRHPPRNDTRQPRNEKPPRFQRDSQNSKSVLEGSGLPRNRGSER
PSTSSVSEVWAEDRIKCDRPYSRYDRTKDTSYPLGSQHSDGAFKKRDNSMQSRSGKGPSF
AEAKENPLPQGSVDYNNQKRGKRESQTSIPDYFYDRKSQTINNEAFSGIKIEKHFNVNTD
YQNPVRSNSFIGVPNGEVEMPLKGRRIGPIKPAGPVTAVPCDDKIFYNSGPKRRSGPIKP
EKILESSIPMEYAKMWKPGDECFALYWEDNKFYRAEVEALHSSGMTAVVKFIDYGNYEEV
LLSNIKPIQTEAWEEEGTYDQTLEFRRGGDGQPRRSTRPTQQFYQPPRARN*
>ENSG00000077150|ENST00000189444
MESCYNPGLDGIIEYDDFKLNSSIVEPKEPAPETADGPYLVIVEQPKQRGFRFRYGCEGP
SHGGLPGASSEKGRKTYPTVKICNYEGPAKIEVDLVTHSDPPRAHAHSLVGKQCSELGIC
AVSVGPKDMTAQFNNLGVLHVTKKNMMGTMIQKLQRQRLRSRPQGLTEAEQRELEQEAKE
LKKVMDLSIVRLRFSAFLRASDGSFSLPLKPVISQPIHDSKSPGASNLKISRMDKTAGSV
RGGDEVYLLCDKVQKDDIEVRFYEDDENGWQAFGDFSPTDVHKQYAIVFRTPPYHKMKIE
RPVTVFLQLKRKRGGDVSDSKQFTYYPLVEDKEEVQRKRRKALPTFSQPFGGGSHMGGGS
GGAAGGYGGAGGGGSLGFFPSSLAYSPYQSGAGPMGCYPGGGGGAQMAATVPSRDSGEEA
AEPSAPSRTPQCEPQAPEMLQRAREYNARLFGLAQRSARALLDYGVTADARALLAGQRHL

LTAQDENGDTPLHLAIIHGQTSVIEQIVYVIHHAQDLGVVNLTNHLHQTPLHLAVITGQT
SVVSFLLRVGADPALLDRHGDSAMHLALRAGAGAPELLRALLQSGAPAVPQLLHMPDFEG
LYPVHLAVRARSPECLDLLVDSGAEVEATERQGGRTALHLATEMEELGLVTHLVTKLRAN
VNARTFAGNTPLHLAAGLGYPTLTRLLLKAGADIHAENEEPLCPLPSPPTSDSDSDSEGP
EKDTRSSFRGHTPLDLTCSTKVKTLLLNAAQNTMEPPLTPPSPAGPGLSLGDTALQNLEQ
LLDGPEAQGSWAELAERLGLRSLVDTYRQTTSPSGSLLRSYELAGGDLAGLLEALSDMGL
EEGVRLLRGPETRDKLPSTEVKEDSAYGSQSVEQEAEKLGPPPEPPGGLCHGHPQPQVH*
>ENSG00000085872|ENST00000198939
MEMPLPPDDQELRNVIDKLAQFVARNGPEFEKMTMEKQKDNPKFSFLFGGEFYSYYKCKL
ALEQQQLICKQQTPELEPAATMPPLPQPPLAPAAPIPPAQGAPSMDELIQQSQWNLQQQE
QHLLALRQEQVTAAVAHAVEQQMQKLLEETQLDMNEFDNLLQPIIDTCTKDAISAGKNWM
FSNAKSPPHCELMAGHLRNRITADGAHFELRLHLIYLINDVLHHWALTRGKSLPHSQRKQ
ARELLAALQKVVVPIYCTSFLAVEEDKQQKIARLLQLWEKNGYFDDSIIQQLQSPALGLG
QYQATLINEYSSVVQPVQLAFQQQIQTLKTQHEEFVTSLAQQQQQQQQQQQLQMPQMEA
EVKATPPPPAPPPAPAPAPAPAIPPTTQPDDSKPPIQMPGSSEYEAPGGVQDPAAAGPRGPG
PHDQIPPNKPPWFDQPHPVAPWGQQQPPEQPPYPHHQGGPPHCPPWNNSHEGMWGEQRGD
PGWNGQRDAPWNNQPDAAWNSQFEGPWNSQHEQPPWGGGQREPPFRMQRPPHFRGPFPPH
QQHPQFNQPPHPHNFNRFPPRFMQDDFPPRHPFERPPYPHRFDYPQGDFPAEMGPPHHHP
GHRMPHPGINEHPPWAGPQHPDFGPPPHGFNGQPPHMRRQGPPHINHDDPSLVPNVPYFD
LPAGLMAPLVKLEDHEYKPLDPKDIRLPPPMPPSERLLAAVEAFYSPPSHDRPRNSEGWE
QNGLYEFFRAKMRARRRKGQEKRNSGPSRSRSRSKSRGRSSSRSNSRSSKSSGSYSRSRS
RSCSRSYSRSRSRSRSRSRSSRSRSRSQSRSRSKSYSPGRRRRSRSRSPTPPSSAGLGSN
SAPPIPDSRLGEENKGHQMLVKMGWSGSGGLGAKEQGIQDPIKGGDVRDKWDQYKGVGVA
LDDPYENYRRNKSYSFIARMKARDECK*
>ENSG00000083812|ENST00000196482
MAFEDVAVYFSQEEWGLLDTAQRALYRRVMLDNFALVASLGLSTSRPRVVIQLERGEEPW
VPSGTDTTLSRTTYRRRNPGSWSLTEDRDVSGEWPRAFPDTPPGMTTSVFPVAGACHSVK
SLQRQRGASPSRERKPTGVSVIYWERLLLGSGSGQASVSLRLTSPLRPPEGVRLREKTLT
EHALLGRQPRTPERQKPCAQEVPGRTFGSAQDLEAAGGRGHHRMGAVWQEPHRLLGGQEP
STWDELGEALHAGEKSFECRACSKVFVKSSDLLKHLRTHTGERPYECAQCGKAFSQTSHL
TQHQRIHSGETPYACPVCGKAFRHSSSLVRHQRIHTAEKSFRCSECGKAFSHGSNLSQHR
KIHAGGRPYACAQCGRRFCRNSHLIQHERTHTGEKPFVCALCGAAFSQGSSLFKHQRVHT
GEKPFACPQCGRAFSHSSNLTQHQLLHTGERPFRCVDCGKAFAKGAVLLSHRRIHTGEKP
FVCTQCGRAFRERPALFHHQRIHTGEKTVRRSRASLHPQARSVAGASSEGAPAKETEPTP
ASGPAAVSQPAEV*
>ENSG00000086232|ENST00000199389
MQGGNSGVRKREEEGDGAGAVAAPPAIDFPAEGPDPEYDESDVPAEIQVLKEPLQQPTFP
FAVANQLLLVSLLEHLSHVHEPNPLRSRQVFKLLCQTFIKMGLLSSFTCSDEFSSLRLHH
NRAITHLMRSAKERVRQDPCEDISRIQKIRSREVALEAQTSRYLNEFEELAILGKGGYGR
VYKVRNKLDGQYYAIKKILIKGATKTVCMKVLREVKVLAGLQHPNIVGYHTAWIEHVHVI
QPRADRAAIELPSLEVLSDQEEDREQCGVKNDESSSSSIIFAEPTPEKEKRFGESDTENQ
NNKSVKYTTNLVIRESGELESTLELQENGLAGLSASSIVEQQLPLRRNSHLEESFTSTEE
SSEENVNFLGQTEAQYHLMLHIQMQLCELSLWDWIVERNKRGREYVDESACPYVMANVAT
KIFQELVEGVFYIHNMGIVHRDLKPRNIFLHGPDQQVKIGDFGLACTDILQKNTDWTNRN

GKRTPTHTSRVGTCLYASPEQLEGSEYDAKSDMYSLGVVLLELFQPFGTEMERAEVLTGL
RTGQLPESLRKRCPVQAKYIQHLTRRNSSQRPSAIQLLQSELFQNSGNVNLTLQMKIIEQ
EKEIAELKKQLNLLSQDKGVRDDGKDGGVG*
>ENSG00000083817|ENST00000196489
MAAAVLRDSTSVPVTAEAKLMGFTQGCVTFEDVAIYFSQEEWGLLDEAQRLLYRDVMLEN
FALITALVCWHGMEDEETPEQSVSVEGVPQVRTPEASPSTQKIQSCDMCVPFLTDILHLT
DLPGQELYLTGACAVFHQDQKHHSAEKPLESDMDKASFVQCCLFHESGMPFTSSEVGKDF
LAPLGILQPQAIANYEKPNKISKCEEAFHVGISHYKWSQCRRESSHKHTFFHPRVCTGKR
LYESSKCGKACCCECSLVQLQRVHPGERPYECSECGKSFSQTSHLNDHRRIHTGERPYVC
GQCGKSFSQRATLIKHHRVHTGERPYECGECGKSFSQSSNLIEHCRIHTGERPYECDECG
KAFGSKSTLVRHQRTHTGEKPYECGECGKLFRQSFSLVVHQRIHTTARPYECGQCGKSFS
LKCGLIQHQLIHSGARPFECDECGKSFSQRTTLNKHHKVHTAERPYVCGECGKAFMFKSK
LVRHQRTHTGERPFECSECGKFFRQSYTLVEHQKIHTGLRPYDCGQCGKSFIQKSSLIQH
QVVHTGERPYECGKCGKSFTQHSGLILHRKSHTVERPRDSSKCGKPYSPRSNIV*
>ENSG00000073861|ENST00000177694
MGIVEPGCGDMLTGTEPMPGSDEGRAPGADPQHRYFYPEPGAQDADERRGGGSLGSPYPG
GALVPAPPSRFLGAYAYPPRPQAAGFPGAGESFPPPADAEGYQPGEGYAAPDPRAGLYPG
PREDYALPAGLEVSGKLRVALNNHLLWSKFNQHQTEMIITKQGRRMFPFLSFTVAGLEPT
SHYRMFVDVVLVDQHHWRYQSGKWVQCGKAEGSMPGNRLYVHPDSPNTGAHWMRQEVSFG
KLKLTNNKGASNNVTQMIVLQSLHKYQPRLHIVEVNDGEPEAACNASNTHIFTFQETQFI
AVTAYQNAEITQLKIDNNPFAKGFRENFESMYTSVDTSIPSPPGPNCQFLGGDHYSPLLP
NQYPVPSRFYPDLPGQAKDVVPQAYWLGAPRDHSYEAEFRAVSMKPAFLPSAPGPTMSYY
RGQEVLAPGAGWPVAPQYPPKMGPASWFRPMRTLPMEPGPGGSEGRGPEDQGPPLVWTEI
APIRPESSDSGLGEGDSKRRRVSPYPSSGDSSSPAGAPSPFDKEAEGQFYNYFPN*
>ENSG00000064218|ENST00000190165
MNGYGSPYLYMGGPVSQPPRAPLQRTPKCARCRNHGVLSWLKGHKRYCRFKDCTCEKCIL
IIERQRVMAAQVALRRQQANESLESLIPDSLRALPGPPPPGDAVAAPQPPPASQPSQPQP
PRPAAELAAAAALRWTAEPQPGALQAQLAKPDLTEERLGDGKSADNTEVFSDKDTDQRSS
PDVAKSKGCFTPESPEIVSVEEGGYAVQKNGGNPESRPDSPKCHAEQNHLLIEGPSGTVS
LPFSLKANRPPLEVLKKIFPNQKPTVLELILKGCGGDLVSAVEVLLSSRSSVTGAERTSA
EPESLALPSNGHIFEHTLSSYPISSSKWSVGSAFRVPDTLRFSADSSNVVPSPLAGPLQP
PFPQPPRYPLMLRNTLARSQSSPFLPNDVTLWNTMTLQQQYQLRSQYVSPFPSNSTSVFR
SSPVLPARATEDPRISIPDDGCPFVSKQSIYTEDDYDERSDSSDSRTLNTSS*
>ENSG00000085721|ENST00000198767
MAAPLLHTRLPGDAAASSSAVKKLGASRTGISNMRALENDFFNSPPRKTVRFGGTVTEVL
LKYKKGETNDFELLKNQLLDPDIKDDQIINWLLEFRSSIMYLTKDFEQLISIILRLPWLN
RSQTVVEEYLAFLGNLVSAQTVFLRPCLSMIASHFVPPRVIIKEGDVDVSDSDDEDDNLP
ANFDTCHRALQIIARYVPSTPWFLMPILVEKFPFVRKSERTLECYVHNLLRISVYFPTLR
HEILELIIEKLLKLDVNASRQGIEDAEETATQTCGGTDSTEGLFNMDEDEETEHETKAGP
ERLDQMVHPVAERLDILMSLVLSYMKDVCYVDGKVDNGKTKDLYRDLINIFDKLLLPTHA
SCHVQFFMFYLCSFKLGFAEAFLEHLWKKLQDPSNPAIIRQAAGNYIGSFLARAKFIPLI
TVKSCLDLLVNWLHIYLNNQDSGTKAFCDVALHGPFYSACQAVFYTFVFRHKQLLSGNLK
EGLQYLQSLNFERIVMSQLNPLKICLPSVVNFFAAITNKYQLVFCYTIIERNNRQMLPVI
RSTAGGDSVQICTNPLDTFFPFDPCVLKRSKKFIDPIYQVWEDMSAEELQEFKKPMKKDI

VEDEDDDFLKGEVPQNDTVIGITPSSFDTHFRSPSSSVGSPPVLYMQPSPL*
>ENSG00000012504|ENST00000188403
MVMQFQGLENPIQISPHCSCTPSGFFMEMMSMKPAKGVLTEQVAGPLGQNLEVEPYSQYS
NVQFPQVQPQISSSSYYSNLGFYPQQPEEWYSPGIYELRRMPAETLYQGETEVAEMPVTK
KPRMGASAGRIKGDELCVVCGDRASGYHYNALTCEGCKGFFRRSITKNAVYKCKNGGNCV
MDMYMRRKCQECRLRKCKEMGMLAECLLTEIQCKSKRLRKNVKQHADQTVNEDSEGRDLR
QVTSTTKSCREKTELTPDQQTLLHFIMDSYNKQRMPQEITNKILKEEFSAEENFLILTEM
ATNHVQVLVEFTKKLPGFQTLDHEDQIALLKGSAVEAMFLRSAEIFNKKLPSGHSDLLEE
RIRNSGISDEYITPMFSFYKSIGELKMTQEEYALLTAIVILSPDRQYIKDREAVEKLQEP
LLDVLQKLCKIHQPENPQHFACLLGRLTELRTFNHHHAEMLMSWRVNDHKFTPLLCEIWD
VQ*
>ENSG00000079308|ENST00000171887
MSVSRTMEDSCELDLVYVTERIIAVSFPSTANEENFRSNLREVAQMLKSKHGGNYLLFNL
SERRPDITKLHAKVLEFGWPDLHTPALEKICSICKAMDTWLNADPHNVVVLHNKGNRGRI
GVVIAAYMHYSNISASADQALDRFAMKRFYEDKIVPIGQPSQRRYVHYFSGLLSGSIKMN
NKPLFLHHVIMHGIPNFESKGGCRPFLRIYQAMQPVYTSGIYNIPGDSQTSVCITIEPGL
LLKGDILLKCYHKKFRSPARDVIFRVQFHTCAIHDLGVVFGKEDLDDAFKDDRFPEYGKV
EFVFSYGPEKIQGMEHLENGPSVSVDYNTSDPLIRWDSYDNFSGHRDDGMEEVVGHTQGP
LDGSLYAKVKKKDSLHGSTGAVNATRPTLSATPNHVEHTLSVSSDSGNSTASTKTDKTDE
PVPGASSATAALSPQEKRELDRLLSGFGLEREKQGAMYHTQHLRSRPAGGSAVPSSGRHV
VPAQVHVNGGALASERETDILDDELPNQDGHSAGSMGTLSSLDGVTNTSEGGYPEALSPL
TNGLDKSYPMEPMVNGGGYPYESASRAGPAHAGHTAPMRPSYSAQEGLAGYQREGPHPAW
PQPVTTSHYAHDPSGMFRSQSFSEAEPQLPPAPVRGGSSREAVQRGLNSWQQQQQQQQP
RPPPRQQERAHLESLVASRPSPQPLAETPIPSLPEFPRAASQQEIEQSIETLNMLMLDLE
PASAAAPLHKSQSVPGAWPGASPLSSQPLSGSSRQSHPLTQSRSGYIPSGHSLGTPEPAP
RASLESVPPGRSYSPYDYQPCLAGPNQDFHSKSPASSSLPAFLPTTHSPPGPQQPPASLP
GLTAQPLLSPKEATSDPSRTPEEEPLNLEGLVAHRVAGVQAREKQPAEPPAPLRRRAASD
GQYENQSPEATSPRSPGVRSPVQCVSPELALTIALNPGGRPKEPHLHSYKEAFEEMEGTS
PSSPPPSGVRSPPGLAKTPLSALGLKPHNPADILLHPTGVTRRRIQPEEDEGKVVVRLSE
EPRSYVESVARTAVAGPRAQDSEPKSFSAPATQAYGHEIPLRNGTLGGSFVSPSPLSTSS
PILSADSTSVGSFPSGESSDQGPRTPTQPLLESGFRSGSLGQPSPSAQRNYQSSSPLPTV
GSSYSSPDYSLQHFSSSPESQARAQFSVAGVHTVPGSPQARHRTVGTNTPPSPGFGWRAI
NPSMAAPSSPSLSHHQMMGPPGTGFHGSTVSSPQSSAATTPGSPSLCRHPAGVYQVSGLH
NKVATTPGSPSLGRHPGAHQGNLASGLHSNAIASPGSPSLGRHLGGSGSVVPGSPCLDRH
VAYGGYSTPEDRRPTLSRQSSASGYQAPSTPSFPVSPAYYPGLSSPATSPSPDSAAFRQG
SPTPALPEKRRMSVGDRAGSLPNYATINGKVSSPVASGMSSPSGGSTVSFSHTLPDFSKY
SMPDNSPETRAKVKFVQDTSKYWYKPEISREQAIALLKDQEPGAFIIRDSHSFRGAYGLA
MKVSSPPPTIMQQNKKGDMTHELVRHFLIETGPRGVKLKGCPNEPNFGSLSALVYQHSII
PLALPCKLVIPNRDPTDESKDSSGPANSTADLLKQGAACNVLFVNSVDMESLTGPQAISK
ATSETLAADPTPAATIVHFKVSAQGITLTDNQRKLFFRRHYPLNTVTFCDLDPQERKWMK
TEGGAPAKLFGFVARKQGSTTDNACHLFAELDPNQPASAIVNFVSKVMLNAGQKR*
>ENSG00000068097|ENST00000184956
MAAVQVVGSWPSVQPREAPREAIPERGNGFRLLSARLCALRPDDSSSARTEIHLLFDQLI
SENYSEGSGVAPEDVSALLVQACRLVPLNQNHLVSKVSQLIHHLLNRLQVIVDEQHLDFL

LAYTISAIHQCSSWTHREILQALAALVYCNGSKCQKYLPELLGNTGLLMKLSDLAQSDPE
VRRAAVHCMANLCLSVPGQPYLEEPYQNVCFQAFLTILQSPKSSDMDDITFCMLLQNALK
GIQSLLNGGRMKLTQTDELGALLAVLKKFMFHGLPGLNIEMPTVLYPTPLPQYDGRTPIK
PQQSESSASRPTLNKKKKSKVKPKKIQQGEEEEKESSGEIEAAPVTGTGRVNLHEGNTWC
PSSLGVQSLPLDGSGAAEKDGVSSSFSSSSWKRVSSSESDFSDAEGGMQSKMRSYQAKVR
QGALVCFLSTIKSIEKKVLYGYWSAFIPDTPELGSPQSVSLMTLTLKDPSPKTRACALQV
LSAILEGSKQFLSVAEDTSDHRRAFTPFSVMIACSIRELHRCLLLALVAESSSQTVTQII
KCLANLVSNAPYDRLKLSLLTKVWNQIKPYIRHKDVNVRVSSLTLLGAIVSTHAPLPEVQ
LLLQQPCSSGLGNSNSATPHLSPPDWWKKAPAGPSLEETSVSSPKGSSEPCWLIRLCISI
VVLPKEDSCSGSDAGSAAGSTYEPSPMRLEALQVLTLLARGYFSMTQAYLMELGEVICKC
MGEADPSIQLHGAKLLEELGTGLIQQYKPDSTAAPDQRAPVFLVVMFWTMMLNGPLPRAL
QNSEHPTLQASACDALSSILPEAFSNLPNDRQMLCITVLLGLNDSKNRLVKAATSRALGV
YVLFPCLRQDVIFVADAANAILMSLEDKSLNVRAKAAWSLGNLTDTLIVNMETPDPSFQE
EFSGLLLLKMLRSAIEASKDKDKVKSNAVRALGNLLHFLQPSHIEKPTFAEIIEESIQAL
ISTVLTEAAMKVRWNACYAMGNVFKNPALPLGTAPWTSQAYNALTSVVTSCKNFKVRIRS
AAALSVPGKREQYGSVDQYARIWNALVTALQKSEDTIDFLEFKYCVSLRTQICQALIHLL
SLASASDLPCMKETLELSGNMVQSYILQFLKSGAEGDDTGAPHSPQERDQMVRMALKHMG
SIQAPTGDTARRAIMGFLEEILAVCFDSSGSQGALPGLTNQ*
>ENSG00000069399|ENST00000164227
MPRCPAGAMDEGPVDLRTRPKAAGLPGAALPLRKRPLRAPSPEPAAPRGAAGLVVPLDPL
RGGCDLPAVPGPPHGLARPEALYYPGALLPLYPTRAMGSPFPLVNLPTPLYPMMCPMEHP
LSADIAMATRADEDGDTPLHIAVVQGNLPAVHRLVNLFQQGGRELDIYNNLRQTPLHLAV
ITTLPSVVRLLVTAGASPMALDRHGQTAAHLACEHRSPTCLRALLDSAAPGTLDLEARNY
DGLTALHVAVNTECQETVQLLLERGADIDAVDIKSGRSPLIHAVENNSLSMVQLLLQHGA
NVNAQMYSGSSALHSASGRGLLPLVRTLVRSGADSSLKNCHNDTPLMVARSRRVIDILRG
KATRPASTSQPDPSPDRSANTSPESSSRLSSNGLLSASPSSSPSQSPPRDPPGFPMAPPN
FFLPSPSPPAFLPFAGVLRGPGRPVPPSPAPGGS*
>ENSG00000076650|ENST00000170564
MAARDSDSEEDLVSYGTGLEPLEEGERPKKPIPLQDQTVRDEKGRYKRFHGAFSGGFSAG
YFNTVGSKEGWTPSTFVSSRQNRADKSVLGPEDFMDEEDLSEFGIAPKAIVTTDDFASKT
KDRIREKARQLAAATAPIPGATLLDDLITPAKLSVGFELLRKMGWKEGQGVGPRVKRRPR
RQKPDPGVKIYGCALPPGSSEGSEGEDDDYLPDNVTFAPKDVTPVDFTPKDNVHGLAYKG
LDPHQALFGTSGEHFNLFSGGSERAGDLGEIGLNKGRKLGISGQAFGVGALEEEDDDIYA
TETLSKYDTVLKDEEPGDGLYGWTAPRQYKNQKESEKDLRYVGKILDGFSLASKPLSSKK
IYPPPELPRDYRPVHYFRPMVAATSENSHLLQVLSESAGKATPDPGTHSKHQLNASKRAE
LLGETPIQGSATSVLEFLSQKDKERIKEMKQATDLKAAQLKARSLAQNAQSSRAQLSPAA
AAGHCSWNMALGGGTATLKASNFKPFAKDPEKQKRYDEFLVHMKQGQKDALERCLDPSMT
EWERGRERDEFARAALLYASSHSTLSSRFTHAKEEDDSDQVEVPRDQENDVGDKQSAVKM
KMFGKLTRDTFEWHPDKLLCKRFNVPDPYPDSTLVGLPRVKRDKYSVFNFLTLPETASLP
TTQASSEKVSQHRGPDKSRKPSRWDTSKHEKKEDSISEFLSLARSKAEPPKQQSSPLVNK
EEEHAPELSANQTVNKDVDAQAEGEGSRPSMDLFRAIFASSSDEKSSSSEDEQGDSEDDQ
AGSGEANFQSSQDTDLGETSSVAHALVPAPQEPPPSFPIQKMQIDEREEFGPRLPPVFCP
NARQTLEVPQKEKHKKNKDKHKAKKEHRRKKEKKKKHRKHKHKGKQKNKKPEKSSSSESS
DSSDSQSDEETADVSPQELLRRLKSLPLRRQ*

>ENSG00000065970|ENST00000162391
MASDLESSLTSIDWLPQLTLRATIEKLGSASQAGPPGSSRKCSPGSPTDPNATLSKDEAA
VHQDGKPRYSYATLITYAINSSPAKKMTLSEIYRWICDNFPYYKNAGIGWKNSIRHNLSL
NKCFRKVPRPRDDPGKGSYWTIDTCPDISRKRRHPPDDDLSQDSPEQEASKSPRGGVAGS
GEASLPPEGNPQMSLQSPTSIASYSQGTGSVDGGAVAAGASGRESAEGPPPLYNTNHDFK
FSYSEINFQDLSWSFRNLYKSMLEKSSSSSQHGFSSLLGDIPPSNNYYMYQQQQPPPPQQ
QQQQQQPPQPPPQQSQPQQQQAPAQGPSAVGGAPPLHTPSTDGCTPPGGKQAGAEGYGPP
PVMAMHPPPLQHGGYHPHQHHPHSHPAQQPPPPQPQAQGQAPINNTGFAFPSDWCSNIDS
LKESFKMVNRLNWSSIEQSQFSELMESLRQAEQKNWTLDQHHIANLCDSLNHFLTQTGHV
PPQGGTHRPPAPARIADSCALTSGKQESAMSQVNSYGHPQAPHLYPGPSPMYPIPTQDSA
GYNRPAHHMVPRPSVPPPGANEEIPDDFDWDLIT*
>ENSG00000075975|ENST00000170447
MSTKQITCRYFMHGVCREGSQCLFSHDLANSKPSTICKYYQKGYCAYGTRCRYDHTRPSA
AAGGAVGTMAHSVPSPAFHSPHPPSEVTASIVKTNSHEPGKREKRTLVLRDRNLSGMAER
KTQPSMVSNPGSCSDPQPSPEMKPHSYLDAIRSGLDDVEASSSYSNEQQLCPYAAAGECR
FGDACVYLHGEVCEICRLQVLHPFDPEQRKAHEKICMLTFEHEMEKAFAFQASQDKVCSI
CMEVILEKASASERRFGILSNCNHTYCLSCIRQWRCAKQFENPIIKSCPECRVISEFVIP
SVYWVEDQNKKNELIEAFKQGMGKKACKYFEQGKGTCPFGSKCLYRHAYPDGRLAEPEKP
RKQLSSQGTVRFFNSVRLWDFIENRESRHVPNNEDVDMTELGDLFMHLSGVESSEP*
>ENSG00000079313|ENST00000170168
MLRSTGFFRAIDCPYWSGAPGGPCRRPYCHFRHRGARGSGAPGDGGEAPPAAGLGYDPYN
PELPKPPAQRENGTLGLGEEPRPDVLELELVNQAIEAVRSEVELEQRRYRELLETTREHR
SAEAPALAPRGPNASPTVGPDEDAFPLAFDYSPGSHGLLSPDAGYQPTPLAAPAEPGSKY
SLASLDRGQGRGGGGGGALEYVPKAVSQPRRHSRPVPSGKYVVDNSRPPTDLEYDPLSNY
SARHLSRASSRDERAAKRPRGSRGSEPYTPAPKKLCDPFGSCDARFSDSEDEAATVPGNE
PTTASTPKARADPEIKATGQPPSKEGLEAEGGGLRETKETAVQCDVGDLQPPPAKPASPA
QVQSSQDGGCPKEGKPKKKKTGAPPAPSCKDGAQGKDKTKDKGRGRPVEKPRADKKGPQA
SSPRRKAERPEGTKKKPSSATPVATSGKGRPDRPARRPSPTSGDSRPAAGRGPPRPLQLP
DRKSTKAPSGKLVERKARSLDEGASQDAPKLKKRALSHADLFGDESEDEAAGPGVPSVWP
SALPSLSSDSDSDSDSSLGFPEAQGPPKRLKASPPPSPAPSSSSSSSSSTSSAGADVDYS
ALEKEVDFDSDPMEECLRIFNESTSVKTEDRGRLARQPPKEEKSEEKGLSGLTTLFPGQK
RRISHLSKQGQEVEPPRRGPAVPPARPPTAQEVCYLRAQQAQRASASLLQAPARLAEKSP
SVHISAPGEKRRIAHIPNPRLAAAPTGAKRTLAASGSQSSNGPEPGGQQLKTRTLSGMAS
KTTTTIIPKRIAHSPSLQSLKKPIIPKEFGGKVPTVIRQRYLNLFIEECLKFCTSNQEAI
EKALNEEKVAYDRSPSKNIYLNVAVNTLKKLRGLAPSAVPGLSKTSGRRVVSHEVVLGGR
LAAKTSFSLSRPSSPRVEDLKGAALYSRLREYLLTQDQLKENGYPFPHPERPGGAIIFTA
EEKRPKDSSCRTCCRCGTEYLVSSSGRCIRDEECYYHWGRLRRNRVAGGWETQYMCCSAA
AGSVGCQVAKQHVQDGRKERLEGFVKTFEKELSGDTHPGIYALDCEMSYTTYGLELTRVT
VVDTDVHVVYDTFVKPDNEIVDYNTRFSGVTEADLADTSVTLRDVQAVLLSMFSADTILI
GHSLESDLLALKVIHSTVVDTSVLFPHRLGLPYKRSLRNLMADYLRQIIQDNVDGHSSSE
DAGACMHLVIWKVREDAKTKR*
>ENSG00000086589|ENST00000199814
MATSLGSNTYNRQNWEDADFPILCQTCLGENPYIRMTKEKYGKECKICARPFTVFRWCPG
VRMRFKKTEVCQTCSKLKNVCQTCLLDLEYGLPIQVRDAGLSFKDDMPKSDVNKEYYTQN

MEREISNSDGTRPVGMLGKATSTSDMLLKLARTTPYYKRNRPHICSFWVKGECKRGEECP
YRHEKPTDPDDPLADQNIKDRYYGINDPVADKLLKRASTMPRLDPPEDKTITTLYVGGLG
DTITETDLRNHFYQFGEIRTITVVQRQQCAFIQFATRQAAEVAAEKSFNKLIVNGRRLNV
KWGRSQAARGKEKEKDGTTDSGIKLEPVPGLPGALPPPPAAEEEASANYFNLPPSGPPAV
VNIALPPPPGIAPPPPPGFGPHMFHPMGPPPPFMRAPGPIHYPSQDPQRMGAHAGKHSSP
*
>ENSG00000087077|ENST00000200457
MSGPTWLPPKQPEPARAPQGRAIPRGTPGPPPAHGAALQPHPRVNFCPLPSEQCYQAPGG
PEDRGPAWVGSHGVLQHTQGLPADRGGLRPGSLDAEIDLLSSTLAELNGGRGHASRRPDR
QAYEPPPPPAYRTGSLKPNPASPLPASPYGGPTPASYTTASTPAGPAFPVQVKVAQPVRG
CGPPRRGASQASGPLPGPHFPLPGRGEVWGPGYRSQREPGPGAKEEAAGVSGPAGRGRGG
EHGPQVPLSQPPEDELDRLTKKLVHDMNHPPSGEYFGQCGGCGEDVVGDGAGVVALDRVF
HVGCFVCSTCRAQLRGQHFYAVERRAYCEGCYVATLEKCATCSQPILDRILRAMGKAYHP
GCFTCVVCHRGLDGIPFTVDATSQIHCIEDFHRKFAPRCSVCGGAIMPEPGQEETVRIVA
LDRSFHIGCYKCEECGLLLSSEGECQGCYPLDGHILCKACSAWRIQELSATVTTDC*
>ENSG00000072506|ENST00000168216
MAAACRSVKGLVAVITGGASGLGLATAERLVGQGASAVLLDLPNSGGEAQAKKLGNNCVF
APADVTSEKDVQTALALAKGKFGRVDVAVNCAGIAVASKTYNLKKGQTHTLEDFQRVLDV
NLMGTFNVIRLVAGEMGQNEPDQGGQRGVIINTASVAAFEGQVGQAAYSASKGGIVGMTL
PIARDLAPIGIRVMTIAPGLFGTPLLTSLPEKVCNFLASQVPFPSRLGDPAEYAHLVQAI
IENPFLNGEVIRLDGAIRMQP*
>ENSG00000172995|ENST00000187397
MSEQGDLNQAIAEEGGTEQETATPENGIVKSESLDEEEKLELQRRLEAQNQERRKSKSGA
GKGKLTRSLAVCEESSARPGGESLQDQESIHLQLSSFSSLQEEDKSRKDDSEREKEKDKN
KDKTSEKPKIRMLSKDCSQEYTDSTGIDLHEFLINTLKNNSRDRMILLKMEQEIIDFIAD
NNNHYKKFPQMSSYQRMLVHRVAAYFGLDHNVDQTGKSVIINKTSSTRIPEQRFCEHLKD
EKGEESQKRFILKRDNSSIDKEDNQQNRMHPFRDDRRSKSIEEREEEYQRVRERIFAHDS
VCSQESLFVENSRLLEDSNICNETYKKRQLFRGNRDGSGRTSGSRQSSSENELKWSDHQR
AWSSTDSDSSNRNLKPAMTKTASFGGITVLTRGDSTSSTRSTGKLSKAGSESSSSAGSSG
SLSRTHPPLQSTPLVSGVAAGSPGCVPYPENGIGGQVAPSSTSYILLPLEAATGIPPGSI
LLNPHTGQPFVNPDGTPAIYNPPTSQQPLRSAMVGQSQQQPPQQQPSPQPQQQVQPPQPQ
MAGPLVTQRDDVATQFGQMTLSRQSSGETPEPPSGPVYPSSLMPQPAQQPSYVIASTGQQ
LPTGGFSGSGPPISQQVLQPPPSPQGFVQQPPPAQMPVYYYPSGQYPTSTTQQYRPMAPV
QYNAQRSQQMPQAAQQAGYQPVLSGQQGFQGLIGVQQPPQSQNVINNQQGTPVQSVMVSY
PTMSSYQVPMTQGSQGLPQQSYQQPIMLPNQAGQGSLPATGMPVYCNVTPPTPQNNLRLI
GPHCPSSTVPVMSASCRTNCASMSNAGWQVKF*
>ENSG00000047188|ENST00000161863
MSRPSSVSPRQPAPGGGGGGGPSPCGPGGGGRAKGLKDIRIDEEVKIAVNIALERFRYGD
QREMEFPSSLTSTERAFIHRLSQSLGLVSKSKGKGANRYLTVKKKDGSETAHAMMTCNLT
HNTKHAVRSLIQRFPVTNKERTELLPKTERGNVFAVEAENREMSKTSGRLNNGIPQIPVK
RGESEFDSFRQSLPVFEKQEEIVKIIKENKVVLIVGETGSGKTTQIPQFLLDDCFKNGIP
CRIFCTQPRRLAAIAVAERVAAERRERIGQTIGYQIRLESRVSPKTLLTFCTNGVLLRTL
MAGDSTLSTVTHVIVDEVHERDRFSDFLLTKLRDLLQKHPTLKLILSSAALDVNLFIRYF
GSCPVIYIQGRPFEVKEMFLEDILRTTGYTNKEMLKYKKEKQQEEKQQTTLTEWYSAQEN

SFKPESQRQRTVLNVTDEYDLLDDGGDAVFSQLTEKDVNCLEPWLIKEMDACLSDIWLHK
DIDAFAQVFHLILTENVSVDYRHSETSATALMVAAGRGFASQVEQLISMGANVHSKASNG
WMALDWAKHFGQTEIVDLLESYSATLEFGNLDESSLVQTNGSDLSAEDRELLKAYHHSFD
DEKVDLDLIMHLLYNICHSCDAGAVLIFLPGYDEIVGLRDRILFDDKRFADSTHRYQVFM
LHSNMQTSDQKKVLKNPPAGVRKIILSTNIAETSITVNDVVFVIDSGKVKEKSFDALNFV
TMLKMVWISKASAIQRKGRAGRCRPGICFRLFSRLRFQNMLEFQTPELLRMPLQELCLHT
KLLAPVNCPIADFLMKAPEPPPALIVRNAVQMLKTIDAMDTWEDLTELGYHLADLPVEPH
LGKMVLCAVVLKCLDPILTIACTLAYRDPFVLPTQASQKRAAMLCRKRFTAGAFSDHMAL
LRAFQAWQKARSDGWERAFCEKNFLSQATMEIIIGMRTQLLGQLRASGFVRARGGGDIRD
VNTNSENWAVVKAALVAGMYPNLVHVDRENLVLTGPKEKKVRFHPASVLSQPQYKKIPPA
NGQAAAIKALPTDWLIYDEMTRAHRIANIRCCSAVTPVTILVFCGPARLASNALQEPSSF
RVDGIPNDSSDSEMEDKTTANLAALKLDEWLHFTLEPEAASLLLQLRQKWHSLFLRRMRA
PSKPWSQVDEATIRAIIAVLSTEEQSAGLQQPSGIGQRPRPMSSEELPLASSWRSNNSRK
SSADTEFSDECTTAERVLMKSPSPALHPPQKYKDRGILHPKRGTEDRSDQSSLKSTDSSS
YPSPCASPSPPSSGKGSKSPSPRPNMPVRYFIMKSSNLRNLEISQQKGIWSTTPSNERKL
NRAFWESSIVYLVFSVQGSGHFQGFSRMSSEIGREKSQDWGSAGLGGVFKVEWIRKESLP
FQFAHHLLNPWNDNKKVQISRDGQELEPLVGEQLLQLWERLPLGEKNTTD*
>ENSG00000083845|ENST00000196551
MTEWETAAPAVAETPDIKLFGKWSTDDVQINDISLQDYIAVKEKYAKYLPHSAGRYAAKR
FRKAQCPIVERLTNSMMMHGRNNGKKLMTVRIVKHAFEIIHLLTGENPLQVLVNAIINSG
PREDSTRIGRAGTVRRQAVDVSPLRRVNQAIWLLCTGAREAAFRNIKTIAECLADELINA
AKGSSNSYAIKKKDELERVAKSNR*
>ENSG00000086189|ENST00000199320
MPKVKSGAIGRRRGRQEQRRELKSAGGLMFNTGIGQHILKNPLIINSIIDKAALRPTDVV
LEVGPGTGNMTVKLLEKAKKVVACELDPRLVAELHKRVQGTPVASKLQVLVGDVLKTDLP
FFDTCVANLPYQISSPFVFKLLLHRPFFRCAILMFQREFALRLVAKPGDKLYCRLSINTQ
LLARVDHLMKVGKNNFRPPPKVESSVVRIEPKNPPPPINFQEWDGLVRITFVRKNKTLSA
AFKSSAVQQLLEKNYRIHCSVHNIIIPEDFSIADKIQQILTSTGFSDKRARSMDIDDFIR
LLHGFNAEGIHFS*
>ENSG00000070444|ENST00000174618
MSIETLLEAARFLEWQAQQQQRAREEQERLRLEQEREQEQKKANSLARLAHTLPVEEPRM
EAPPLPLSPPAPPPAPPPPLATPAPLTVIPIPVVTNSPQPLPPPPPLPAAAQPLPLAPRQ
PALVGAPGLSIKEPAPLPSRPQVPTPAPLLPDSKATIPPNGSPKPLQPLPTPVLTIAPHP
GVQPQLAPQQPPPPTLGTLKLAPAEEVKSSEQKKRPGGIGTREVHNKLEKNRRAHLKECF
ETLKRNIPNVDDKKTSNLSVLRTALRYIQSLKRKEKEYEHEMERLAREKIATQQRLAELK
HELSQWMDVLEIDRVLRQTGQPEDDQASTSTASEGEDNIDEDMEEDRAGLGPPKLSHRPQ
PELLKSTLPPPSTTPAPLPPHPHPHPHSVALPPAHLPVQQQQPQQKTPLPAPPPPPAAPA
QTLVPAPAHLVATAGGGSTVIAHTATTHASVIQTVNHVLQGPGGKHIAHIAPSAPSPAVQ
LAPATPPIGHITVHPATLNHVAHLGSQLPLYPQPVAVSHIAHTLSHQQVNGTAGLGPPAT
VMAKPAVGAQVVHHPQLVGQTVLNPVTMVTMPSFPVSTLKLA*
>ENSG00000072110|ENST00000193403
MDHYDSQQTNDYMQPEEDWDRDLLLDPAWEKQQRKTFTAWCNSHLRKAGTQIENIEEDFR
DGLKLMLLLEVISGERLAKPERGKMRVHKISNVNKALDFIASKGVKLVSIGAEEIVDGNV
KMTLGMIWTIILRFAIQDISVEETSAKEGLLLWCQRKTAPYKNVNIQNFHISWKDGLGFC

ALIHRHRPELIDYGKLRKDDPLTNLNTAFDVAEKYLDIPKMLDAEDIVGTARPDEKAIMT
YVSSFYHAFSGAQKAETAANRICKVLAVNQENEQLMEDYEKLASDLLEWIRRTIPWLENR
VPENTMHAMQQKLEDFRDYRRLHKPPKVQEKCQLEINFNTLQTKLRLSNRPAFMPSEGRM
VSDINNAWGCLEQVEKGYEEWLLNEIRRLERLDHLAEKFRQKASIHEAWTDGKEAMLRQK
DYETATLSEIKALLKKHEAFESDLAAHQDRVEQIAAIAQELNELDYYDSPSVNARCQKIC
DQWDNLGALTQKRREALERTEKLLETIDQLYLEYAKRAAPFNNWMEGAMEDLQDTFIVHT
IEEIQGLTTAHEQFKATLPDADKERLAILGIHNEVSKIVQTYHVNMAGTNPYTTITPQEI
NGKWDHVRQLVPRRDQALTEEHARQQHNERLRKQFGAQANVIGPWIQTKMEEIGRISIEM
HGTLEDQLSHLRQYEKSIVNYKPKIDQLEGDHQLIQEALIFDNKHTNYTMEHIRVGWEQL
LTTIARTINEVENQILTRDAKGISQEQMNEFRASFNHFDRDHSGTLGPEEFKACLISLGY
DIGNDPQGEAEFARIMSIVDPNRLGVVTFQAFIDFMSRETADTDTADQVMASFKILAGDK
NYITMDELRRELPPDQAEYCIARMAPYTGPDSVPGALDYMSFSTALYGESDL*
>ENSG00000086504|ENST00000199706
MPLHKYPVWLWKRLQLREGICSRLPGHYLRSLEEERTPTPVHYRPHGAKFKINPKNGQRE
RVEDVPIPIYFPPESQRGLWGGEGWILGQIYANNDKLSKRLKKVWKPQLFEREFYSEILD
KKFTVTVTMRTLDLIDEAYGLDFYILKTPKEDLCSKFGMDLKRGMLLRLARQDPQLHPED
PERRAAIYDKYKEFAIPEEEAEWVGLTLEEAIEKQRLLEEKDPVPLFKIYVAELIQQLQQ
QALSEPAVVQKRASGQ*
>ENSG00000088305|ENST00000201963
MEPSPEPPSLESMKGDTRHLNGEEDAGGREDSILVNGACSDQSSDSPPILEAIRTPEIRG
RRSSSRLSKREVSSLLSYTQDLTGDGDGEDGDGSDTPVMPKLFRETRTRSESPAVRTRNN
NSVSSRERHRPSPRSTRGRQGRNHVDESPVEFPATRSLRRRATASAGTPWPSPPSSYLTI
DLTDDTEDTHGTPQSSSTPYARLAQDSQQGGMESPQVEADSGDGDSSEYQDGKEFGIGDL
VWGKIKGFSWWPAMVVSWKATSKRQAMSGMRWVQWFGDGKFSEVSADKLVALGLFSQHFN
LATFNKLVSYRKAMYHALEKARVRAGKTFPSSPGDSLEDQLKPMLEWAHGGFKPTGIEGL
KPNNTQPENKTRRRTADDSATSDYCPAPKRLKTNCYNNGKDRGDEDQSREQMASDVANNK
SSLEDGCLSCGRKNPVSFHPLFEGGLCQTCRDRFLELFYMYDDDGYQSYCTVCCEGRELL
LCSNTSCCRCFCVECLEVLVGTGTAAEAKLQEPWSCYMCLPQRCHGVLRRRKDWNVRLQA
FFTSDTGLEYEAPKLYPAIPAARRRPIRVLSLFDGIATGYLVLKELGIKVGKYVASEVCE
ESIAVGTVKHEGNIKYVNDVRNITKKNIEEWGPFDLVIGGSPCNDLSNVNPARKGLYEGT
GRLFFEFYHLLNYSRPKEGDDRPFFWMFENVVAMKVGDKRDISRFLECNPVMIDAIKVSA
AHRARYFWGNLPGMNRPVIASKNDKLELQDCLEYNRIAKLKKVQTITTKSNSIKQGKNQL
FPVVMNGKEDVLWCTELERIFGFPVHYTDVSNMGRGARQKLLGRSWSVPVIRHLFAPLKD
YFACE*
>ENSG00000065883|ENST00000181839
MPSSSDTALGGGGGLSWAEKKLEERRKRRRFLSPQQPPLLLPLLQPQLLQPPPPPPPLLF
LAAPGTAAAAAAAAAASSSCFSPGPPLEVKRLARGKRRAGGRQKRRRGPRAGQEAEKRRV
FSLPQPQQDGGGGASSGGGVTPLVEYEDVSSQSEQGLLLGGASAATAATAAGGTGGSGGS
PASSSGTQRRGEGSERRPRRDRRSSSGRSKERHREHRRRDGQRGGSEASKSRSRHSHSGE
ERAEVAKSGSSSSSGGRRKSASATSSSSSSRKDRDSKAHRSRTKSSKEPPSAYKEPPKAY
REDKTEPKAYRRRRSLSPLGGRDDSPVSHRASQSLRSRKSPSPAGGGSSPYSRRLPRSPS
PYSRRRSPSYSRHSSYERGGDVSPSPYSSSSWRRSRSPYSPVLRRSGKSRSRSPYSSRHS
RSRSRHRLSRSRSRHSSISPSTLTLKSSLAAELNKNKKARAAEAARAAEAAKAAEATKAA
EAAAKAAKASNTSTPTKGNTETSASASQTNHVKDVKKIKIEHAPSPSSGGTLKNDKAKTK

PPLQVTKVENNLIVDKATKKAVIVGKESKSAATKEESVSLKEKTKPLTPSIGAKEKEQHV
ALVTSTLPPLPLPPMLPEDKEADSLRGNISVKAVKKEVEKKLRCLLADLPLPPELPGGDD
LSKSPEEKKTATQLHSKRRPKICGPRYGETKEKDIDWGKRCVDKFDIIGIIGEGTYGQVY
KARDKDTGEMVALKKVRLDNEKEGFPITAIREIKILRQLTHQSIINMKEIVTDKEDALDF
KKDKGAFYLVFEYMDHDLMGLLESGLVHFNENHIKSFMRQLMEGLDYCHKKNFLHRDIKC
SNILLNNRGQIKLADFGLARLYSSEESRPYTNKVITLWYRPPELLLGEERYTPAIDVWSC
GCILGELFTKKPIFQANQELAQLELISRICGSPCPAVWPDVIKLPYFNTMKPKKQYRRKL
REEFVFIPAAALDLFDYMLALDPSKRCTAEQALQCEFLRDVEPSKMPPPDLPLWQDCHEL
WSKKRRRQKQMGMTDDVSTIKAPRKDLSLGLDDSRTNTPQGVLPSSQLKSQGSSNVAPVK
TGPGQHLNHSELAILLNLLQSKTSVNMADFVQVLNIKVNSETQQQLNKINLPAGILATGE
KQTDPSTPQQESSKPLGGIQPSSQTIQPKVETDAAQAAVQSAFAVLLTQLIKAQQSKQKD
VLLEERENGSGHEASLQLRPPPEPSTPVSGQDDLIQHQDMRILELTPEPDRPRILPPDQR
PPEPPEPPPVTEEDLDYRTENQHVPTTSSSLTDPHAGVKAALLQLLAQHQPQDDPKREGG
IDYQAGDTYVSTSDYKDNFGSSSFSSAPYVSNDGLGSSSAPPLERRSFIGNSDIQSLDNY
STASSHSGGPPQPSAFSESFPSSVAGYGDIYLNAGPMLFSGDKDHRFEYSHGPIAVLANS
SDPSTGPESTHPLPAKMHNYNYGGNLQENPSGPSLMHGQTWTSPAQGPGYSQGYRGHIST
STGRGRGRGLPY*
>ENSG00000101751|ENST00000217800
VHDQVLPTPNASSRVIVHVDLDCFYAQVEMISNPELKDKPLGVQQKYLVVTCNYEARKLG
VKKLMNVRDAKEKCPQLVLVNGEDLTRYREMSYKVTEMVEKRLQQLQSDELSAVTVSGHV
YNNQSINLLDVLHIRLLVGSQIAAEMREAMYNQLGLTGCAGVASNKLLAKLVSGVFKPNQ
QTVLLPESCQHLIHSLNHIKEIPGIGYKTAKCLEALGINSVRDLQTFSPKILEKELGISV
AQRIQKLSFGEDNSPVILSGPPQSFSEEDSFKKCSSEVEAKNKIEELLASLLNRVCQDGR
KPHTVRLIIRRYSSEKHYGRESRQCPIPSHVIQKLGTGNYDVMTPMVDILMKLFRNMVNV
KMPFHLTLLSVCFCNLKALNTAKKGLIDYYLMPSLSTTSRSGKHSFKMKDTHMEDFPKDK
ETNRDFLPSGRIESTRTRESPLDTTNFSKEKDINEFPLCSLPEGVDQEVFKQLPVDIQEE
ILSGKSREKFQGKGSVSCPLHASRGVLSFFSKKQMQDIPINPRDHLSSSKQVSSVSPCEP
GTSGFNSSSSSYMSSQKDYSYYLDNRLKDERISQGPKEPQGFHFTNSNPAVSAFHSFPNL
QSEQLFSRNHTTDSHKQTVATDSHEGLTENREPDSVDEKITFPSDIDPQVFYELPEAVQK
ELLAEWKRAGSDFHIGHK*
>ENSG00000100138|ENST00000215956
MTEADVNPKAYPLADAHLTKKLLDLVQQSCNYKQLRKGANEATKTLNRGISEFIVMAADA
EPLEIILHLPLLCEDKNVPYVFVRSKQALGRACGVSRPVIACSVTIKEGSQLKQQIQSIQ
QSIERLLV*
>ENSG00000100823|ENST00000216714
MPKRGKKGAVAEDGDELRTEPEAKKSKTAAKKNDKEAAGEGPALYEDPPDQKTSPSGKPA
TLKICSWNVDGLRAWIKKKGLDWVKEEAPDILCLQETKCSENKLPAELQELPGLSHQYWS
APSDKEGYSGVGLLSRQCPLKVSYGIGDEEHDQEGRVIVAEFDSFVLVTAYVPNAGRGLV
RLEYRQRWDEAFRKFLKGLASRKPLVLCGDLNVAHEEIDLRNPKGNKKNAGFTPQERQGF
GELLQAVPLADSFRHLYPNTPYAYTFWTYMMNARSKNVGWRLDYFLLSHSLLPALCDSKI
RSKALGSDHCPITLYLAL*
>ENSG00000100316|ENST00000216146
MSHRKFSAPRHGSLGFLPRKRSSRHRGKVKSFPKDDPSKPVHLTAFLGYKAGMTHIVREV
DRPGSKVNKKEVVEAVTIVETPPMVVVGIVGYVETPRGLRTFKTVFAEHISDECKRRFYK

NWHKSKKKAFTKYCKKWQDEDGKKQLEKDFSSMKKYCQVIRVIAHTQMRLLPLRQKKAHL
MEIQVNGGTVAEKLDWARERLEQQVPVNQVFGQDEMIDVIGVTKGKGYKGVTSRWHTKKL
PRKTHRGLRKVACIGAWHPARVAFSVARAGQKGYHHRTEINKKIYKIGQGYLIKDGKLIK
NNASTDYDLSDKSINPLGGFVHYGEVTNDFVMLKGCVVGTKKRVLTLRKSLLVQTKRRAL
EKIDLKFIDTTSKFGHGRFQTMEEKKAFMGPLKKDRIAKEEGA*
>ENSG00000092067|ENST00000206513
MSHGTYYECEPRGGQQPLEFSGGRAGPGELGDMCEHEASIDLSAYIESGEEQLLSDLFAV
KPAPEARGLKGPGTPAFPHYLPPDPRPFAYPPHTFGPDRKALGPGIYSSPGSYDPRAVAV
KEEPRGPEGSRAASRGSYNPLQYQVAHCGQTAMHLPPTLAAPGQPLRVLKAPLATAAPPC
SPLLKAPSPAGPLHKGKKAVNKDSLEYRLRRERNNIAVRKSRDKAKRRILETQQKVLEYM
AENERLRSRVEQLTQELDTLRNLFRQIPEAANLIKGVGGCS*
>ENSG00000089048|ENST00000202816
MSSKQEIMSDQRFRRVAKDPRFWEMPEKDRKVKIDKRFRAMFHDKKFKLNYAVDKRGRPI
SHSTTEDLKRFYDLSDSDSNLSGEDSKALSQKKIKKKKTQTKKEIDSKNLVEKKKETKKA
NHKGSENKTDLDNSIGIKKMKTSCKFKIDSNISPKKDSKEFTQKNKKEKKNIVQHTTDSS
LEEKQRTLDSGTSEIVKSPRIECSKTRREMQSVVQLIMTRDSDGYENSTDGEMCDKDALE
EDSESVSEIGSDEESENEITSVGRASGDDDGSEDDEEEDEDEEEDEDEDSEDDDKSDSGP
DLARGKGNIETSSEDEDDTADLFPEESGFEHAWRELDKDAPRADEITRRLAVCNMDWDRL
KAKDLLALFNSFKPKGGVIFSVKIYPSEFGKERMKEEQVQGPVELLSIPEDAPEKDWTSR
EKLRDYQFKRLKYYYAVVDCDSPETASKIYEDCDGLEFESSCSFIDLRFIPDDITFDDEP
KDVASEVNLTAYKPKYFTSAAMGTSTVEITWDETDHERITMLNRKFKKEELLDMDFQAYL
ASSSEDEEEIEEELQGDDGVNVEEDGKTKKSQKDDEEQIAKYRQLLQVIQEKEKKGKEND
MEMEIKWVPGLKESAEEMVKNKLEGKDKLTPWEQFLEKKKEKKRLKRKQKALAEEASEEE
LPSDVDLNDPYFAEEVKQIGINKKSVKSAKDGTSPEEEIEIERQKAEMALLMMDEDEDSK
KHFNYNKIVEHQNLSKKKKKQLMKKKELIEDDFEVNVNDARFQAMYTSHLFNLDPSDPNF
KKTKAMEKILEEKARQRERKEQELTQAIKKKESEIEKESQRKSIDPALSMLIKSIKTKTE
QFQARKKQKVK*
>ENSG00000119314|ENST00000210227
MDASPSPFSLPKKLNELSARRGSDELLSSGIINGPFTMNSSTPSTANGNDSKKFKRDRPP
CSPSRVLHLRKIPCDVTEAEIISLGLPFGKVTNLLMLKGKSQAFLEMASEEAAVTMVNYY
TPITPHLRSQPVYIQYSNHRELKTDNLPNQARAQAALQAVSAVQSGSLALSGGPSNEGTV
LPG
>ENSG00000089195|ENST00000203001
MEGSGEQPGPQPQHPGDHRIRDGDFVVLKREDVFKAVQVQRRKKVTFEKQWFYLDNVIGH
SYGTAFEVTSGGSLQPKKKREEPTAETKEAGTDNRNIVDDGKSQKLTQDDIKALKDKGIK
GEEIVQQLIENSTTFRDKTEFAQDKYIKKKKKKYEAIITVVKPSTRILSIMYYAREPGKI
NHMRYDTLAQMLTLGNIRAGNKMIVMETCAGLVLGAMMERMGGFGSIIQLYPGGGPVRAA
TACFGFPKSFLSGLYEFPLNKVDSLLHGTFSAKMLSSEPKDSALVEESNGTLEEKQASEQ
ENEDSMAEAPESNHPEDQETMETISQDPEHKGPKERGSKKDYIQEKQRRQEEQRKRHLEA
AALLSERNADGLIVASRFHPTPLLLSLLDFVAPSRPFVVYCQYKEPLLECYTKLRERGGV
INLRLSETWLRNYQVLPDRSHPKLLMSGGGGYLLSGFTVAMDNLKADTSLKSNASTLESH
ETEEPAAKKRKCPESDS*
>ENSG00000096654|ENST00000211936
MEDLSSPDSTLLQGGHNLLSSASFQEAVTFKDVIVDFTQEEWKQLDPGQRDLFRDVTLEN

YTHLVSIGLQVSKPDVISQLEQGTEPWIMEPSIPVGTCADWETRLENSVSAPEPDISEEE
LSPEVIVEKHKRDDSWSSNLLESWEYEGSLERQQANQQTLPKEIKVTEKTIPSWEKGPVN
NEFGKSVNVSSNLVTQEPSPEETSTKRSIKQNSNPVKKEKSCKCNECGKAFSYCSALIRH
QRTHTGEKPYKCNECEKAFSRSENLINHQRIHTGDKPYKCDQCGKGFIEGPSLTQHQRIH
TGEKPYKCDECGKAFSQRTHLVQHQRIHTGEKPYTCNECGKAFSQRGHFMEHQKIHTGEK
PFKCDECDKTFTRSTHLTQHQKIHTGEKTYKCNECGKAFNGPSTFIRHHMIHTGEKPYEC
NECGKAFSQHSNLTQHQKTHTGEKPYDCAECGKSFSYWSSLAQHLKIHTGEKPYKCNECG
KAFSYCSSLTQHRRIHTREKPFECSECGKAFSYLSNLNQHQKTHTQEKAYECKECGKAFI
RSSSLAKHERIHTGEKPYQCHECGKTFSYGSSLIQHRKIHTGERPYKCNECGRAFNQNIH
LTQHKRIHTGAKPYECAECGKAFRHCSSLAQHQKTHTEEKPYQCNKCEKTFSQSSHLTQH
QRIHTGEKPYKCNECDKAFSRSTHLTEHQNTHTGEKPYNCNECRKTFSQSTYLIQHQRIH
SGEKPFGCNDCGKSFRYRSALNKHQRLHPGI*
>ENSG00000101150|ENST00000217121
MDSAGQDINLNSPNKGLLSDSMTDVPVDTGVAARTPAVEGLTEAEEEELRAELTKVEEEI
VTLRQVLAAKERHCGELKRRLGLSTLGELKQNLSRSWHDVQVSSAYVKTSEKLGEWNEKV
TQSDLYKKTQETLSQAGQKTSAALSTVGSAISRKLGDMRAHPFSHSFSSYSIRHSISMPA
MRNSATFKSFEDRVGTIKSKVVGDRENGSDNLPSSAGSGDKPLSDPAPF*
>ENSG00000100625|ENST00000216513
MSSSSPTGQIASAADIKQENGMESASEGQEAHREVAGGAAVGLSPPAPAPFPLEPGDAAT
AAARVSGEEGAVAAAAAGAAADQVQLHSELLGRHHHAAAAAAQTPLAFSPDHVACVCEAL
QQGGNLDRLARFLWSLPQSDLLRGNESLLKARALVAFHQGIYPELYSILESHSFESANHP
LLQQLWYKARYTEAERARGRPLGAVDKYRLRRKFPLPRTIWDGEETVYCFKEKSRNALKE
LYKQNRYPSPAEKRHLAKITGLSLTQVSNWFKNRRQRDRNPSETQSKSESDGNPSTEDES
SKGHEDLSPHPLSSSSDGITNLSLSSHMEPVYMQQIGNAKISLSSSGVLLNGSLVPASTS
PVFLNGNSFIQGPSGVILNGLNVGNTQAVALNPPKMSSNIVSNGISMTDILGSTSQDVKE
FKVLQSSANSATTTSYSPSVPVSFPGLIPSTEVKREGIQTVASQDGGSVVTFTTPVQINQ
YGIVQIPNSGANSQFLNGSIGFSPLQLPPVSVAASQGNISVSSSTSDGSTFTSESTTVQQ
GKVFLSSLAPSAVVYTVPNTGQTIGSVKQEGLERSLVFSQLMPVNQNAQVNANLSSENIS
GSGLHPLASSLVNVSPTHNFSLSPSTLLNPTELNRDIADSQPMSAPVASKSTVTSVSNTN
YATLQNCSLITGQDLLSVPMTQAALGEIVPTAEDQVGHPSPAVHQDFVQEHRLVLQSVAN
MKENFLSNSESKATSSLMMLDSKSKYVLDGMVDTVCEDLETDKKELAKLQTVQLDEDMQD
L*
>ENSG00000099326|ENST00000215057
MRPAVLGSPDRAPPEDEGPVMVKLEDSEEEGEAALWDPGPEAARLRFRCFRYEEATGPQE
ALAQLRELCRQWLRPEVRSKEQMLELLVLEQFLGALPPEIQARVQGQRPGSPEEAAALVD
GLRREPGGPRRWVTVQVQGQEVLSEKMEPSSFQPLPETEPPTPEPGPKTPPRTMQESPLG
LQVKEESEVTEDSDFLESGPLAATQESVPTLLPEEAQRCGTVLDQIFPHSKTGPEGPSWR
EHPRALWHEEAGGIFSPGFALQLGSISAGPGSVSPHLHVPWDLGMAGLSGQIQSPSREGG
FAHALLLPSDLRSEQDPTDEDPCRGVGPALITTRWRSPRGRSRGRPSTGGGVVRGGRCDV
CGKVFSQRSNLLRHQKIHTGERPFVCSECGRSFSRSSHLLRHQLTHTEERPFVCGDCGQG
FVRSARLEEHRRVHTGEQPFRCAECGQSFRQRSNLLQHQRIHGDPPGPGAKPPAPPGAPE
PPGPFPCSECRESFARRAVLLEHQAVHTGDKSFGCVECGERFGRRSVLLQHRRVHSGERP
FACAECGQSFRQRSNLTQHRRIHTGERPFACAECGKAFRQRPTLTQHLRVHTGEKPFACP
ECGQRFSQRLKLTRHQRTHTGEKPYHCGECGLGFTQVSRLTEHQRIHTGERPFACPECGQ

SFRQHANLTQHRRIHTGERPYACPECGKAFRQRPTLTQHLRTHRREKPFACQDCGRRFHQ
STKLIQHQRVHSAE*
>ENSG00000101883|ENST00000217999
MARSLVHDTVFYCLSVYQVKISPTPQLGAASSAEGHVGQGAPGLMGNMNPEGGVNHENGM
NRDGGMIPEGGGGNQEPRQQPQPPPEEPAQAAMEGPQPENMQPRTRRTKFTLLQVEELES
VFRHTQYPDVPTRRELAENLGVTEDKVRVWFKNKRARCRRHQRELMLANELRADPDDCVY
IVVD*
>ENSG00000101104|ENST00000217073
MNASGSGYPLASLYVGDLHPDVTEAMLYEKFSPAGPILSIRVCRDVATRRSLGYAYINFQ
QPADAERALDTMNFEMLKGQPIRIMWSQRDPGLRKSGVGNIFIKNLEDSIDNKALYDTFS
TFGNILSCKVACDEHGSRGFGFVHFETHEAAQQAINTMNGMLLNDRKVFVGHFKSRRERE
AELGARALEFTNIYVKNLPVDVDEQGLQDLFSQFGKMLSVKVMRDNSGHSRCFGFVNFEK
HEEAQKAVVHMNGKEVSGRLLYAGRAQKRVERQNELKRRFEQMKQDRLRRYQGVNLYVKN
LDDSIDDDKLRKEFSPYGVITSAKVMTEGGHSKGFGFVCFSSPEEATKAVTEMNGRIVGT
KPLYVALAQRKEERKAILTNQYMQRLSTMRTLSNPLLGSFQQPSSYFLPAMPQPPAQAAY
YGCGPVTPTQPAPRWTSQPPRPSCASMVRPPVVPRRPPAHISSVRQASTQVPRTVPHTQR
VANIGTQTTGPSGVGCCTPGRPLLPCKCSSAAHSTYRVQEPAVHIPGQEPLTASMLAAAP
LHEQKQMIGERLYPLIHDVHTQLAGKITGMLLEIDNSELLLMLESPESLHAKIDEAVAVL
QAHQAMEQPKAYMH*
>ENSG00000092201|ENST00000216297
MAVTLDKDAYYRRVKRLYSNWRKGEDEYANVDAIVVSVGVDEEIVYAKSTALQTWLFGYE
LTDTIMVFCDDKIIFMASKKKVEFLKQIANTKGNENANGAPAITLLIREKNESNKSSFDK
MIEAIKESKNGKKIGVFSKDKFPGEFMKSWNDCLNKEGFDKIDISAVVAYTIAVKEDGEL
NLMKKAASITSEVFNKFFKERVMEIVDADEKVRHSKLAESVEKAIEEKKYLAGADPSTVE
MCYPPIIQSGGNYNLKFSVVSDKNHMHFGAITCAMGIRFKSYCSNLVRTLMVDPSQEVQE
NYNFLLQLQEELLKELRHGVKICDVYNAVMDVVKKQKPELLNKITKNLGFGMGIEFREGS
LVINSKNQYKLKKGMVFSINLGFSDLTNKEGKKPEEKTYALFIGDTVLVDEDGPATVLTS
VKKKVKNVGIFLKNEDEEEEEEEKDEAEDLLGRGSRAALLTERTRNEMTAEEKRRAHQKE
LAAQLNEEAKRRLTEQKGEQQIQKARKSNVSYKNPSLMPKEPHIREMKIYIDKKYETVIM
PVFGIATPFHIATIKNISMSVEGDYTYLRINFYCPGSALGRNEGNIFPNPEATFVKEITY
RASNIKAPGEQTVPALNLQNAFRIIKEVQKRYKTREAEEKEKEGIVKQDSLVINLNRSNP
KLKDLYIRPNIAQKRMQGSLEAHVNGFRFTSVRGDKVDILYNNIKHALFQPCDGEMIIVL
HFHLKNAIMFGKKRHTDVQFYTEVGEITTDLGKHQHMHDRDDLYAEQMEREMRHKLKTAF
KNFIEKVEALTKEELEFEVPFRDLGFNGAPYRSTCLLQPTSSALVNATEWPPFVVTLDEV
ELIHFERVQFHLKNFDMVIVYKDYSKKVTMINAIPVASLDPIKEWLNSCDLKYTEGVQSL
NWTKIMKTIVDDPEGFFEQGGWSFLEPEGEGSDAEEGDSESEIEDETFNPSEDDYEEEEE
DSDEDYSSEAEESDYSKESLGSEEESGKDWDELEEEARKADRESRYEEEEEQSRSMSRKR
KASVHSSGRGSNRGSRHSSAPPKKKRK*
>ENSG00000100297|ENST00000216122
MSGFDDPGIFYSDSFGGDAQADEGQARKSQLQRRFKEFLRQYRVGTDRTGFTFKYRDELK
RHYNLGEYWIEVEMEDLASFDEDLADYLYKQPAEHLQLLEEAAKEVADEVTRPRPSGEEV
LQDIQVMLKSDASPSSIRSLKSDMMSHLVKIPGIIIAASAVRAKATRISIQCRSCRNTLT
NIAMRPGLEGYALPRKCNTDQAGRPKCPLDPYFIMPDKCKCVDFQTLKLQELPDAVPHGE
MPRHMQLYCDRYLCDKVVPGNRVTIMGIYSIKKFGLTTSRGRDRVGVGIRSSYIRVLGIQ

VDTDGSGRSFAGAVSPQEEEEFRRLAALPNVYEVISKSIAPSIFGGTDMKKAIACLLFGG
SRKRLPDGLTRRGDINLLMLGDPGTAKSQLLKFVEKCSPIGVYTSGKGSSAAGLTASVMR
DPSSRNFIMEGGAMVLADGGVVCIDEFDKMREDDRVAIHEAMEQQTISIAKAGITTTLNS
RCSVLAAANSVFGRWDETKGEDNIDFMPTILSRFDMIFIVKDEHNEERDVMLAKHVITLH
VSALTQTQAVEGEIDLAKLKKFIAYCRVKCGPRLSAEAAEKLKNRYIIMRSGARQHERDS
DRRSSIPITVRQLEAIVRIAEALSKMKLQPFATEADVEEALRLFQVSTLDAALSGTLSGV
EGFTSQEDQEMLSRIEKQLKRRFAIGSQVSEHSIIKDFTKQKYPEHAIHKVLQLMLRRGE
IQHRMQRKVLYRLK*
>ENSG00000100442|ENST00000216330
MAAAVPQRAWTVEQLRSEQLPKKDIIKFLQEHGSDSFLAEHKLLGNIKNVAKTANKDHLV
TAYNHLFETKRFKGTESISKVSEQVKNVKLNEDKPKETKSEETLDEGPPKYTKSVLKKGD
KTNFPKKGDVVHCWYTGTLQDGTVFDTNIQTSAKKKKNAKPLSFKVGVGKVIRGWDEALL
TMSKGEKARLEIEPEWAYGKKGQPDAKIPPNAKLTFEVELVDID*
>ENSG00000020256|ENST00000216923
MNASSEGESFAGSVQIPGGTTVLVELTPDIHICGICKQQFNNLDAFVAHKQSGCQLTGTS
AAAPSTVQFVSEETVPATQTQTTTRTITSETQTITVSAPEFVFEHGYQTYLPTESNENQT
ATVISLPAKSRTKKPTTPPAQKRLNCCYPGCQFKTAYGMKDMERHLKIHTGDKPHKCEVC
GKCFSRKDKLKTHMRCHTGVKPYKCKTCDYAAADSSSLNKHLRIHSDERPFKCQICPYAS
RNSSQLTVHLRSHTGDAPFQCWLCSAKFKISSDLKRHMRVHSGEKPFKCEFCNVRCTMKG
NLKSHIRIKHSGNNFKCPHCDFLGDSKATLRKHSRVHQSEHPEKCSECSYSCSSKAALRI
HERIHCTDRPFKCNYCSFDTKQPSNLSKHMKKFHGDMVKTEALERKDTGRQSSRQVAKLD
AKKSFHCDICDASFMREDSLRSHKRQHSEYSESKNSDVTVLQFQIDPSKQPATPLTVGHL
QVPLQPSQVPQFSEGRVKIIVGHQVPQANTIVQAAAAAVNIVPPALVAQNPEELPGNSRL
QILRQVSLIAPPQSSRCPSEAGAMTQPAVLLTTHEQTDGATLHQTLIPTASGGPQEGSGN
QTFITSSGITCTDFEGLNALIQEGTAEVTVVSDGGQNIAVATTAPPVFSSSSQQELPKQT
YSIIQGAAHPALLCPADSIPD*
>ENSG00000099817|ENST00000215587
MDDEEETYRLWKIRKTIMQLCHDRGYLVTQDELDQTLEEFKAQSGDKPSEGRPRRTDLTV
LVAHNDDPTDQMFVFFPEEPKVGIKTIKVYCQRMQEENITRALIVVQQGMTPSAKQSLVD
MAPKYILEQFLQQELLINITEHELVPEHVVMTKEEVTELLARYKLRENQLPRIQAGDPVA
RYFGIKRGQPLLSVSAGGEDHPAQ*
>ENSG00000100226|ENST00000216044
MATERSRSAMDSPVPASMFAPEPSSPGAARAAAAAARLHGGFDSDCSEDGEALNGEPELD
LTSKLVLVSPTSEQYDSLLRQMWERMDEGCGETIYVIGQGSDGTEYGLSEADMEASYATV
KSMAEQIEADVILLRERQEAGGRVRDYLVRKRVGDNDFLEVRVAVVGNVDAGKSTLLGVL
THGELDNGRGFARQKLFRHKHEIESGRTSSVGNDILGFDSEGNVVNKPDSHGGSLEWTKI
CEKSTKVITFIDLAGHEKYLKTTVFGMTGHLPDFCMLMVGSNAGIVGMTKEHLGLALALN
VPVFVVVTKIDMCPANILQETLKLLQRLLKSPGCRKIPVLVQSKDDVIVTASNFSSERMC
PIFQISNVTGENLDLLKMFLNLLSPRTSYREEEPAEFQIDDTYSVPGVGTVVSGTTLRGL
IKLNDTLLLGPDPLGNFLSIAVKSIHRKRMPVKEVRGGQTASFALKKIKRSSIRKGMVMV
SPRLNPQASWEFEAEILVLHHPTTISPRYQAMVHCGSIRQTATILSMDKDCLRTGDKATV
HFRFIKTPEYLHIDQRLVFREGRTKAVGTITKLLQTTNNSPMNSKPQQIKMQSTKKGPLT
KRDEGGPSGGPAVGAPPPGDEASSVGAGQPAASSNLQPQPKPSSGGRRRGGQRHKVKSQG
ACVTPASGC*

>ENSG00000101138|ENST00000217109
MYRTKVGLKDRQQLYKLIISQLLYDGYISIANGLINEIKPQSVCAPSEQLLHLIKLGMEN
DDTAVQYAIGRSDTVAPGTGIDLEFDADVQTMSPEASEYETCYVTSHKGPCRVATYSRDG
QLIATGSADASIKILDTERMLAKSAMPIEVMMNETAQQNMENHPVIRTLYDHVDEVTCLA
FHPTEQILASGSRDYTLKLFDYSKPSAKRAFKYIQEAEMLRSISFHPSGDFILVGTQHPT
LRLYDINTFQCFVSCNPQDQHTDAICSVNYNSSANMYVTGSKDGCIKLWDGVSNRCITTF
EKAHDGAEVCSAIFSKNSKYILSSGKDSVAKLWEISTGRTLVRYTGAGLSGRQVHRTQAV
FNHTEDYVLLPDERTISLCCWDSRTAERRNLLSLGHNNIVRCIVHSPTNPGFMTCSDDFR
ARFWYRRSTTD*
>ENSG00000090060|ENST00000216277
MPFPVTTQGSQQTQPPQKHYGITSPISLAAPKETDCVLTQKLIETLKPFGVFEEEEELQR
RILILGKLNNLVKEWIREISESKNLPQSVIENVGGKIFTFGSYRLGVHTKGADIDALCVA
PRHVDRSDFFTSFYDKLKLQEEVKDLRAVEEAFVPVIKLCFDGIEIDILFARLALQTIPE
DLDLRDDSLLKNLDIRCIRSLNGCRVTDEILHLVPNIDNFRLTLRAIKLWAKRHNIYSNI
LGFLGGVSWAMLVARTCQLYPNAIASTLVHKFFLVFSKWEWPNPVLLKQPEECNLNLPVW
DPRVNPSDRYHLMPIITPAYPQQNSTYNVSVSTRMVMVEEFKQGLAITDEILLSKAEWSK
LFEAPNFFQKYKHYIVLLASAPTEKQRLEWVGLVESKIRILVGSLEKNEFITLAHVNPQS
FPAPKENPDKEEFRTMWVIGLVFKKTENSENLSVDLTYDIQSFTDTVYRQAINSKMFEVD
MKIAAMHVKRKQLHQLLPNHVLQKKKKHSTEGVKLTALNDSSLDLSMDSDNSMSVPSPTS
ATKTSPLNSSGSSQGRNSPAPAVTAASVTNIQATEVSVPQVNSSESSGGTSSESIPQTAT
QPAISPPPKPTVSRVVSSTRLVNPPPRSSGNAATSGNAATKIPTPIVGVKRTSSPHKEES
PKKTKTEEDETSEDANCLALSGHDKTEAKEQLDTETSTTQSETIQTAASLLASQKTSSTD
LSDIPALPANPIPVIKNSIKLRLNR*
>ENSG00000100281|ENST00000216106
MAYDDSVKKEDCFDGDHTFEDIGLAAGRSQREKKRSYKDFLREEEEIAAQVRNSSKKKLK
DSELYFLGTDTHKKKRKHSSDDYYYGDISSLESSQKKKKKSSPQSTDTAMDLLKAITSPL
AAGSKPSKKTGEKSSGSSSHSESKKEHHRKKVSGSSGELPLEDGGSHKSKKMKPLYVNTE
TLTLREPDGLKMKLILSPKEKGSSSVDEESFQYPSQQATVKKSSKKSARDEQGALLLGHE
LQSFLKTARKKHKSSSDAHSSPGPEGCGSDASQFAESHSANLDLSGLEPILVESDSSSGG
ELEAGELVIDDSYREIKKKKKSKKSKKKKDKEKHKEKRHSKSKRSLGLSAVPVGEVTVTS
GPPPSIPYAGAAAPPLPLPGLHTDGHSEKKKKKEEKDKERERGEKPKKKNMSAYQVFCKE
YRVTIVADHPGIDFGELSKKLAEVWKQLPEKDKLIWKQKAQYLQHKQNKAEATTVKRKAS
SSEGSMKVKASSVGVLSPQKKSPPTTMLLPASPAKAPETEPIDVAAHLQLLGESLSLIGH
RLQETEGMVAVSGSLSVLLDSIICALGPLACLTTQLPELNGCPKQVLSNTLDNIAYIMPG
L*
>ENSG00000100220|ENST00000216038
MSRSYNDELQFLEKINKNCWRIKKGFVPNMQVEGVFYVNDALEKLMFEELRNACRGGGVG
GFLPAMKQIGNVAALPGIVHRSIGLPDVHSGYGFAIGNMAAFDMNDPEAVVSPGGVGFDI
NCGVRLLRTNLDESDVQPVKEQLAQAMFDHIPVGVGSKGVIPMNAKDLEEALEMGVDWSL
REGYAWAEDKEHCEEYGRMLQADPNKVSARAKKRGLPQLGTLGAGNHYAEIQVVDEIFNE
YAAKKMGIDHKGQVCVMIHSGSRGLGHQVATDALVAMEKAMKRDKIIVNDRQLACARIAS
PEGQDYLKGMAAAGNYAWVNRSSMTFLTRQAFAKVFNTTPDDLDLHVIYDVSHNIAKVEQ
HVVDGKERTLLVHRKGSTRAFPPHHPLIAVDYQLTGQPVLIGGTMGTCSYVLTGTEQGMT
ETFGTTCHGAGRALSRAKSRRNLDFQDVLDKLADMGIAIRVASPKLVMEEAPESYKNVTD

VVNTCHDAGISKKAIKLRPIAVIKG*
>ENSG00000100028|ENST00000215829
MSIGVPIKVLHEAEGHIVTCETNTGEVYRGKLIEAEDNMNCQMSNITVTYRDGRVAQLEQ
VYIRGSKIRFLILPDMLKNAPMLKSMKNKNQGSGAGRGKAAILKAQVAARGRGRGMGRGN
IFQKRR*
>ENSG00000273841|ENST00000217893
MESGKTASPKSMPKDAQMMAQILKDMGITEYEPRVINQMLEFAFRYVTTILDDAKIYSSH
AKKATVDADDVRLAIQCRADQSFTSPPPRDFLLDIARQRNQTPLPLIKPYSGPRLPPDRY
CLTAPNYRLKSLQKKASTSAGRITVPRLSVGSVTSRPSTPTLGTPTPQTMSVSTKVGTPM
SLTGQRFTVQMPTSQSPAVKASIPATSAVQNVLINPSLIGSKNILITTNMMSSQNTANES
SNALKRKREDDDDDDDDDDDYDNL*
>ENSG00000100664|ENST00000216554
MSVNVNRSVSDQFYRYKMPRLIAKVEGKGNGIKTVIVNMVDVAKALNRPPTYPTKYFGCE
LGAQTQFDVKNDRYIVNGSHEANKLQDMLDGFIKKFVLCPECENPETDLHVNPKKQTIGN
SCKACGYRGMLDTHHKLCTFILKNPPENSDSGTGKKEKEKKNRKGKDKENGSVSSSETPP
PPPPPNEINPPPHTMEEEEDDDWGEDTTEEAQRRRMDEISDHAKVLTLSDDLERTIEERV
NILFDFVKKKKEEGVIDSSDKEIVAEAERLDVKAMGPLVLTEVLFNEKIREQIKKYRRHF
LRFCHNNKKAQRYLLHGLECVVAMHQAQLISKIPHILKEMYDADLLEEEVIISWSEKASK
KYVSKELAKEIRVKAEPFIKWLKEAEEESSGGEEEDEDENIEVVYSKAASVPKVETVKSD
NKDDDIDIDAI*
>ENSG00000101104|ENST00000217074
MNASGSGYPLASLYVGDLHPDVTEAMLYEKFSPAGPILSIRVCRDVATRRSLGYAYINFQ
QPADAERALDTMNFEMLKGQPIRIMWSQRDPGLRKSGVGNIFIKNLEDSIDNKALYDTFS
TFGNILSCKVACDEHGSRGFGFVHFETHEAAQQAINTMNGMLLNDRKVFVGHFKSRRERE
AELGARALEFTNIYVKNLPVDVDEQGLQDLFSQFGKMLSVKVMRDNSGHSRCFGFVNFEK
HEEAQKAVVHMNGKEVSGRLLYAGRAQKRVERQNELKRRFEQMKQDRLRRYQGVNLYVKN
LDDSIDDDKLRKEFSPYGVITSAKKRRQRP*
>ENSG00000091640|ENST00000206020
MADLLGSILSSMEKPPSLGDQETRRKAREQAARLKKLQEQEKQQKVEFRKRMEKEVSDFI
QDSGQIKKKFQPMNKIERSILHDVVEVAGLTSFSFGEDDDCRYVMIFKKEFAPSDEELDS
YRRGEEWDPQKAEEKRKLKELAQRQEEEAAQQGPVVVSPASDYKDKYSHLIGKGAAKDAA
HMLQANKTYGCVPVANKRDTRSIEEAMNEIRAKKRLRQSGEELPPTS*
>ENSG00000100426|ENST00000216268
MENNLKTCPKEDGDFVSDKIKFKIEEEDDDGIPPDSLERMDFKSEQEDMKQTDSGGERAG
LGGTGCSCKPPGKYLSAESEDDYGALFSQYSSTLYDVAMEAVTQSLLSSRNMSSRKKSPA
WKHFFISPRDSTKAICMYCVKEFSRGKNEKDLSTSCLMRHVRRAHPTVLIQENGSVSAVS
SFPSPSLLLPPQPADAGDLSTILSPIKLVQKVASKIPSPDRITEESVSVVSSEEISSDMS
VSEKCGREEALVGSSPHLPALHYDEPAENLAEKSLPLPKSTSGSRRRSAVWKHFYLSPLD
NSKAVCIHCMNEFSRGKNGKDLGTSCLIRHMWRAHRAIVLQENGGTGIPPLYSTPPTLLP
SLLPPEGELSSVSSSPVKPVRESPSASSSPDRLTEDLQSHLNPGDGLMEDVAAFSSSDDI
GEASASSPEKQQADGLSPRLFESGAIFQQNKKVMKRLKSEVWHHFSLAPMDSLKAECRYC
GCAISRGKKGDVGTSCLMRHLYRRHPEVVGSQKGFLGASLANSPYATLASAESSSSKLTD
LPTVVTKNNQVMFPVNSKKTSKLWNHFSICSADSTKVVCLHCGRTISRGKKPTNLGTSCL
LRHLQRFHSNVLKTEVSETARPSSPDTRVPRGTELSGASSFDDTNEKFYDSHPVAKKITS

LIAEMIALDLQPYSFVDNVGFNRLLEYLKPQYSLPAPSYFSRTAIPGMYDNVKQIIMSHL
KEAESGVIHFTSGIWMSNQTREYLTLTAHWVSFESPARPRCDDHHCSALLDVSQVDCDYS
GNSIQKQLECWWEAWVTSTGLQVGITVTDNASIGKTLNEGEHSSVQCFSHTVNLIVSEAI
KSQRMVQNLLSLARKICERVHRSPKAKEKLAELQREYALPQHHLIQDVPSKWSTSFHMLE
RLIEQKRAINEMSVECNFRELISCDQWEVMQSVCRALKPFEAASREMSTQMSTLSQVIPM
VHILNRKVEMLFEETMGIDTMLRSLKEAMVSRLSATLHDPRYVFATLLDPRYKASLFTEE
EAEQYKQDLIRELELMNSTSEDVAASHRCDAGSPSKDSAAEENLWSLVAKVKKKDPREKL
PEAMVLAYLEEEVLEHSCDPLTYWNLKKASWPGLSALAVRFLGCPPSIVPSEKLFNTPTE
NGSLGQSRLMMEHFEKLIFLKVNLPLIYFQY*
>ENSG00000100410|ENST00000216252
MAKHHPDLIFCRKQAGVAIGRLCEKCDGKCVICDSYVRPCTLVRICDECNYGSYQGRCVI
CGGPGVSDAYYCKECTIQEKDRDGCPKIVNLGSSKTDLFYERKKYGFKKR*
>ENSG00000101210|ENST00000217182
MGKEKTHINIVVIGHVDSGKSTTTGHLIYKCGGIDKRTIEKFEKEAAEMGKGSFKYAWVL
DKLKAERERGITIDISLWKFETTKYYITIIDAPGHRDFIKNMITGTSQADCAVLIVAAGV
GEFEAGISKNGQTREHALLAYTLGVKQLIVGVNKMDSTEPAYSEKRYDEIVKEVSAYIKK
IGYNPATVPFVPISGWHGDNMLEPSPNMPWFKGWKVERKEGNASGVSLLEALDTILPPTR
PTDKPLRLPLQDVYKIGGIGTVPVGRVETGILRPGMVVTFAPVNITTEVKSVEMHHEALS
EALPGDNVGFNVKNVSVKDIRRGNVCGDSKSDPPQEAAQFTSQVIILNHPGQISAGYSPV
IDCHTAHIACKFAELKEKIDRRSGKKLEDNPKSLKSGDAAIVEMVPGKPMCVESFSQYPP
LGRFAVRDMRQTVAVGVIKNVEKKSGGAGKVTKSAQKAQKAGK*
>ENSG00000100836|ENST00000216727
MAAAAAAAAAAGAAGGRGSGPGRRRHLVPGAGGEAGEGAPGGAGDYGNGLESEELEPEEL
LLEPEPEPEPEEEPPRPRAPPGAPGPGPGSGAPGSQEEEEEPGLVEGDPGDGAIEDPELE
AIKARVREMEEEAEKLKELQNEVEKQMNMSPPPGNAGPVIMSIEEKMEADARSIYVGNVD
YGATAEELEAHFHGCGSVNRVTILCDKFSGHPKGFAYIEFSDKESVRTSLALDESLFRGR
QIKVIPKRTNRPGISTTDRGFPRARYRARTTNYNSSRSRFYSGFNSRPRGRVYRGRARAT
SWYSPY*
>ENSG00000184436|ENST00000215742
MPRHCSAAGCCTRDTRETRNRGISFHRLPKKDNPRRGLWLANCQRLDPSGQGLWDPASEY
IYFCSKHFEEDCFELVGISGYHRLKEGAVPTIFESFSKLRRTTKTKGHSYPPGPAEVSRL
RRCRKRCSEGRGPTTPFSPPPPADVTCFPVEEASAPATLPASPAGRLEPGLSSPFSDLLG
PLGAQADEAGCSAQPSPERQPSPLEPRPVSPSAYMLRLPPPAGAYIQNEHSYQVGSALLW
KRRAEAALDALDKAQRQLQACKRREQRLRLRLTKLQQERAREKRAQADARQTLKEHVQDF
AMQLSSSMA*
>ENSG00000123569|ENST00000217926
MLRTEVPRLPRSTTAIVWSCHLMATASAMAGPSSETTSEEQLITQEPKEANSTTSQKQSK
QRKRGRHGPRRCHSNCRGDSFATYFRRVLKQVHQGLSLSREAVSVMDSLVHDILDRIATE
AGHLARSTKRQTITAWETRMAVRLLLPGQMGKLAESEGTKAVLRTSLYAIQQQRK*
>ENSG00000100485|ENST00000216373
MQQAPQPYEFFSEENSPKWRGLLVSALRKVQEQVHPTLSANEESLYYIEELIFQLLNKLC
MAQPRTVQDVEERVQKTFPHPIDKWAIADAQSAIEKRKRRNPLLLPVDKIHPSLKEVLGY
KVDYHVSLYIVAVLEYISADILKLAGNYVFNIRHYEISQQDIKVSMCADKVLMDMFDQDD
IGLVSLCEDEPSSSGELNYYDLVRTEIAEERQYLRELNMIIKVFREAFLSDRKLFKPSDI

EKIFSNISDIHELTVKLLGLIEDTVEMTDESSPHPLAGSCFEDLAEEQAFDPYETLSQDI
LSPEFHEHFNKLMARPAVALHFQSIADGFKEAVRYVLPRLMLVPVYHCWHYFELLKQLKA
CSEEQEDRECLNQAITALMNLQGSMDRIYKQYSPRRRPGDPVCPFYSHQLRSKHLAIKKM
NEIQKNIDGWEGKDIGQCCNEFIMEGPLTRIGAKHERHIFLFDGLMISCKPNHGQTRLPG
YSSAEYRLKEKFVMRKIQICDKEDTCEHKHAFELVSKDENSIIFAAKSAEEKNNWMAALI
SLHYRSTLDRMLDSVLLKEENEQPLRLPSPEVYRFVVKDSEENIVFEDNLQSRSGIPIIK
GGTVVKLIERLTYHMYADPNFVRTFLTTYRSFCKPQELLSLLIERFEIPEPEPTDADKLA
IEKGEQPISADLKRFRKEYVQPVQLRILNVFRHWVEHHFYDFERDLELLERLESFISSVR
GKAMKKWVESIAKIIRRKKQAQANGVSHNITFESPPPPIEWHISKPGQFETFDLMTLHPI
EIARQLTLLESDLYRKVQPSELVGSVWTKEDKEINSPNLLKMIRHTTNLTLWFEKCIVEA
ENFEERVAVLSRIIEILQVFQDLNNFNGVLEIVSAVNSVSVYRLDHTFEALQERKRKILD
EAVELSQDHFKKYLVKLKSINPPCVPFFGIYLTNILKTEEGNNDFLKKKGKDLINFSKRR
KVAEITGEIQQYQNQPYCLRIEPDMRRFFENLNPMGSASEKEFTDYLFNKSLEIEPRNCK
QPPRFPRKSTFSLKSPGIRPNTGRHGSTSGTLRGHPTPLEREPCKISFSRIAETELESTV
SAPTSPNTPSTPPVSASSDLSVFLDVDLNSSCGSNSIFAPVLLPHSKSFFSSCGSLHKLS
EEPLIPPPLPPRKKFDHDASNSKGNMKSDDDPPAIPPRQPPPPKVKPRVPVPTGAFDGPL
HSPPPPPPRDPLPDTPPPVPLRPPEHFINCPFNLQPPPLGHLHRDSDWLRDISTCPNSPS
TPPSTPSPRVPRRCYVLSSSQNNLAHPPAPPVPPRQNSSPHLPKLPPKTYKRELSHPPLY
RLPLLENAETPQ*
>ENSG00000100941|ENST00000216832
MAVAVRTLQEQLEKAKESLKNVDENIRKLTGRDPNDVRPIQARLLALSGPGGGRGRGSLL
LRRGFSDSGGGPPAKQRDLEGAVSRLGGERRTRRESRQESDPEDDDVKKPALQSSVVATS
KERTRRDLIQDQNMDEKGKQRNRRIFGLLMGTLQKFKQESTVATERQKRRQEIEQKLEVQ
AEEERKQVENERRELFEERRAKQTELRLLEQKVELAQLQEEWNEHNAKIIKYIRTKTKPH
LFYIPGRMCPATQKLIEESQRKMNALFEGRRIEFAEQINKMEARPRRQSMKEKEHQVVRN
EEQKAEQEEGKVAQREEELEETGNQHNDVEIEEAGEEEEKEIAIVHSDAEKEQEEEEQKQ
EMEVKMEEETEVRESEKQQDSQPEEVMDVLEMVENVKHVIADQEVMETNRVESVEPSENE
ASKELEPEMEFEIEPDKECKTLSPGKENVSALDMEKESEEKEEKESEPQPEPVAQPQPQS
QPQLQLQSQSQPVLQSQPPSQPEDLSLAVLQPTPQVTQEQGHLLPERKDFPVESVKLTEV
PVEPVLTVHPESKSKTKTRSRSRGRARNKTSKSRSRSSSSSSSSSSSTSSSSGSSSSSGS
SSSRSSSSSSSSTSGSSSRDSSSSTSSSSESRSRSRGRGHNRDRKHRRSVDRKRRDTSGL
ERSHKSSKGGSSRDTKGSKDKNSRSDRKRSISESSRSGKRSSRSERDRKSDRKDKRR*
>ENSG00000101115|ENST00000217086
MSRRKQAKPQHINSEEDQGEQQPQQQTPEFADAAPAAPAAGELGAPVNHPGNDEVASEDE
ATVKRLRREETHVCEKCCAEFFSISEFLEHKKNCTKNPPVLIMNDSEGPVPSEDFSGAVL
SHQPTSPGSKDCHRENGGSSEDMKEKPDAESVVYLKTETALPPTPQDISYLAKGKVANTN
VTLQALRGTKVAVNQRSADALPAPVPGANSIPWVLEQILCLQQQQLQQIQLTEQIRIQVN
MWASHALHSSGAGADTLKTLGSHMSQQVSAAVALLSQKAGSQGLSLDALKQAKLPHANIP
SATSSLSPGLAPFTLKPDGTRVLPNVMSRLPSALLPQAPGSVLFQSPFSTVALDTSKKGK
GKPPNISAVDVKPKDEAALYKHKCKYCSKVFGTDSSLQIHLRSHTGERPFVCSVCGHRFT
TKGNLKVHFHRHPQVKANPQLFAEFQDKVAAGNGIPYALSVPDPIDEPSLSLDSKPVLVT
TSVGLPQNLSSGTNPKDLTGGSLPGDLQPGPSPESEGGPTLPGVGPNYNSPRAGGFQGSG
TPEPGSETLKLQQLVENIDKATTDPNECLICHRVLSCQSSLKMHYRTHTGERPFQCKICG
RAFSTKGNLKTHLGVHRTNTSIKTQHSCPICQKKFTNAVMLQQHIRMHMGGQIPNTPLPE

NPCDFTGSEPMTVGENGSTGAICHDDVIESIDVEEVSSQEAPSSSSKVPTPLPSIHSASP
TLGFAMMASLDAPGKVGPAPFNLQRQGSRENGSVESDGLTNDSSSLMGDQEYQSRSPDIL
ETTSFQALSPANSQAESIKSKSPDAGSKAESSENSRTEMEGRSSLPSTFIRAPPTYVKVE
VPGTFVGPSTLSPGMTPLLAAQPRRQAKQHGCTRCGKNFSSASALQIHERTHTGEKPFVC
NICGRAFTTKGNLKVHYMTHGANNNSARRGRKLAIENTMALLGTDGKRVSEIFPKEILAP
SVNVDPVVWNQYTSMLNGGLAVKTNEISVIQSGGVPTLPVSLGATSVVNNATVSKMDGSQ
SGISADVEKPSATDGVPKHQFPHFLEENKIAVS*
>ENSG00000101057|ENST00000217026
MSRRTRCEDLDELHYQDTDSDVPEQRDSKCKVKWTHEEDEQLRALVRQFGQQDWKFLASH
FPNRTDQQCQYRWLRVLNPDLVKGPWTKEEDQKVIELVKKYGTKQWTLIAKHLKGRLGKQ
CRERWHNHLNPEVKKSCWTEEEDRIICEAHKVLGNRWAEIAKMLPGRTDNAVKNHWNSTI
KRKVDTGGFLSESKDCKPPVYLLLELEDKDGLQSAQPTEGQGSLLTNWPSVPPTIKEEEN
SEEELAAATTSKEQEPIGTDLDAVRTPEPLEEFPKREDQEGSPPETSLPYKWVVEAANLL
IPAVGSSLSEALDLIESDPDAWCDLSKFDLPEEPSAEDSINNSLVQLQASHQQQVLPPRQ
PSALVPSVTEYRLDGHTISDLSRSSRGELIPISPSTEVGGSGIGTPPSVLKRQRKRRVAL
SPVTENSTSLSFLDSCNSLTPKSTPVKTLPFSPSQFLNFWNKQDTLELESPSLTSTPVCS
QKVVVTTPLHRDKTPLHQKHAAFVTPDQKYSMDNTPHTPTPFKNALEKYGPLKPLPQTPH
LEEDLKEVLRSEAGIELIIEDDIRPEKQKRKPGLRRSPIKKVRKSLALDIVDEDVKLMMS
TLPKSLSLPTTAPSNSSSLTLSGIKEDNSLLNQGFLQAKPEKAAVAQKPRSHFTTPAPMS
SAWKTVACGGTRDQLFMQEKARQLLGRLKPSHTSRTLILS*
>ENSG00000092607|ENST00000207157
MSSMEEIQVELQCADLWKRFHDIGTEMIITKAGRRMFPAMRVKITGLDPHQQYYIAMDIV
PVDNKRYRYVYHSSKWMVAGNADSPVPPRVYIHPDSLASGDTWMRQVVSFDKLKLTNNEL
DDQGHIILHSMHKYQPRVHVIRKDFSSDLSPTKPVPVGDGVKTFNFPETVFTTVTAYQNQ
QITRLKIDRNPFAKGFRDSGRNRTGLEAIMETYAFWRPPVRTLTFEDFTTMQKQQGGSTG
TSPTTSSTGTPSPSASSHLLSPSCSPPTFHLAPNTFNVGCRESQLCNLNLSDYPPCARSN
MAALQSYPGLSDSGYNRLQSGTTSATQPSETFMPQRTPSLISGIPTPPSLPGNSKMEAYG
GQLGSFPTSQFQYVMQAGNAASSSSSPHMFGGSHMQQSSYNAFSLHNPYNLYGYNFPTSP
RLAASPEKLSASQSTLLCSSPSNGAFGERQYLPSGMEHSMHMISPSPNNQQATNTCDGRQ
YGAVPGSSSQMSVHMV*
>ENSG00000100883|ENST00000216774
MVLADLGRKITSALRSLSNATIINEEVLNAMLKEVCTALLEADVNIKLVKQLRENVKSAI
DLEEMASGLNKRKMIQHAVFKELVKLVDPGVKAWTPTKGKQNVIMFVGLQGSGKTTTCSK
LAYYYQRKGWKTCLICADTFRAGAFDQLKQNATKARIPFYGSYTEMDPVIIASEGVEKFK
NENFEIIIVDTSGRHKQEDSLFEEMLQVANAIQPDNIVYVMDASIGQACEAQAKAFKDKV
DVASVIVTKLDGHAKGGGALSAVAATKSPIIFIGTGEHIDDFEPFKTQPFISKLLGMGDI
EGLIDKVNELKLDDNEALIEKLKHGQFTLRDMYEQFQNIMKMGPFSQILGMIPGFGTDFM
SKGNEQESMARLKKLMTIMDSMNDQELDSTDGAKVFSKQPGRIQRVARGSGVSTRDVQEL
LTQYTKFAQMVKKMGGIKGLFKGGDMSKNVSQSQMAKLNQQMAKMMDPRVLHHMGGMAGL
QSMMRQFQQGAAGNMKGMMGFNNM*
>ENSG00000090447|ENST00000204517
MEYFMVPTQKVPSLQHFRKTEKEVIGGLCSLANIPLTPETQRDQERRIRREIANSNERRR
MQSINAGFQSLKTLIPHTDGEKLSKAAILQQTAEYIFSLEQEKTRLLQQNTQLKRFIQEL
SGSSPKRRRAEDKDEGIGSPDIWEDEKAEDLRREMIELRQQLDKERSVRMMLEEQVRSLE

AHMYPEKLKVIAQQVQLQQQQEQVRLLHQEKLEREQQQLRTQLLPPPAPTHHPTVIVPAP
PPPPSHHINVVTMGPSSVINSVSTSRQNLDTIVQAIQHIEGTQEKQELEEEQRRAVIVKP
VRSCPEAPTSDTASDSEASDSDAMDQSREEPSGDGELP*
>ENSG00000023608|ENST00000216294
MGTPPGLQTDCEALLSRFQETDSVRFEDFTELWRNMKFGTIFCGRMRNLEKNMFTKEALA
LAWRYFLPPYTFQIRVGALYLLYGLYNTQLCQPKQKIRVALKDWDEVLKFQQDLVNAQHF
DAAYIFRKLRLDRAFHFTAMPKLLSYRMKKKIHRAEVTEEFKDPSDRVMKLITSDVLEEM
LNVHDHYQNMKHVISVDKSKPDKALSLIKDDFFDNIKNIVLEHQQWHKDRKNPSLKSKTN
DGEEKMEGNSQETERCERAESLAKIKSKAFSVVIQASKSRRHRQVKLDSSDSDSASGQGQ
VKATRKKEKKERLKPAGRKMSLRNKGNVQNIHKEDKPLSLSMPVITEEEENESLSGTEFT
ASKKRRKH*
>ENSG00000100353|ENST00000216190
MAKFMTPVIQDNPSGWGPCAVPEQFRDMPYQPFSKGDRLGKVADWTGATYQDKRYTNKYS
SQFGGGSQYAYFHEEDESSFQLVDTARTQKTAYQRNRMRFAQRNLRRDKDRRNMLQFNLQ
ILPKSAKQKERERIRLQKKFQKQFGVRQKWDQKSQKPRDSSVEVRSDWEVKEEMDFPQLM
KMRYLEVSEPQDIECCGALEYYDKAFDRITTRSEKPLRSIKRIFHTVTTTDDPVIRKLAK
TQGNVFATDAILATLMSCTRSVYSWDIVVQRVGSKLFFDKRDNSDFDLLTVSETANEPPQ
DEGNSFNSPRNLAMEATYINHNFSQQCLRMGKERYNFPNPNPFVEDDMDKNEIASVAYRY
RRWKLGDDIDLIVRCEHDGVMTGANGEVSFINIKTLNEWDSRHCNGVDWRQKLDSQRGAV
IATELKNNSYKLARWTCCALLAGSEYLKLGYVSRYHVKDSSRHVILGTQQFKPNEFASQI
NLSVENAWGILRCVIDICMKLEEGKYLILKDPNKQVIRVYSLPDGTFSSDEDEEEEEEEE
EEEEEEET*
>ENSG00000100030|ENST00000215832
MAAAAAAGAGPEMVRGQVFDVGPRYTNLSYIGEGAYGMVCSAYDNVNKVRVAIKKISPFE
HQTYCQRTLREIKILLRFRHENIIGINDIIRAPTIEQMKDVYIVQDLMETDLYKLLKTQH
LSNDHICYFLYQILRGLKYIHSANVLHRDLKPSNLLLNTTCDLKICDFGLARVADPDHDH
TGFLTEYVATRWYRAPEIMLNSKGYTKSIDIWSVGCILAEMLSNRPIFPGKHYLDQLNHI
LGILGSPSQEDLNCIINLKARNYLLSLPHKNKVPWNRLFPNADSKALDLLDKMLTFNPHK
RIEVEQALAHPYLEQYYDPSDEPIAEAPFKFDMELDDLPKEKLKELIFEETARFQPGYRS
*
>ENSG00000100097|ENST00000215909
MACGLVASNLNLKPGECLRVRGEVAPDAKSFVLNLGKDSNNLCLHFNPRFNAHGDANTIV
CNSKDGGAWGTEQREAVFPFQPGSVAEVCITFDQANLTVKLPDGYEFKFPNRLNLEAINY
MAADGDFKIKCVAFD*
>ENSG00000096150|ENST00000211372
MSLVIPEKFQHILRVLNTNIDGRRKIAFAITAIKGVGRRYAHVVLRKADIDLTKRAGELT
EDEVERVITIMQNPRQYKIPDWFLNRQKDVKDGKYSQVLANGLDNKLREDLERLKKIRAH
RGLRHFWGLRVRGQHTKTTGRRGRTVGVSKKK*
>ENSG00000101916|ENST00000218032
MENMFLQSSMLTCIFLLISGSCELCAEENFSRSYPCDEKKQNDSVIAECSNRRLQEVPQT
VGKYVTELDLSDNFITHITNESFQGLQNLTKINLNHNPNVQHQNGNPGIQSNGLNITDGA
FLNLKNLRELLLEDNQLPQIPSGLPESLTELSLIQNNIYNITKEGISRLINLKNLYLAWN
CYFNKVCEKTNIEDGVFETLTNLELLSLSFNSLSHVPPKLPSSLRKLFLSNTQIKYISEE
DFKGLINLTLLDLSGNCPRCFNAPFPCVPCDGGASINIDRFAFQNLTQLRYLNLSSTSLR

KINAAWFKNMPHLKVLDLEFNYLVGEIASGAFLTMLPRLEILDLSFNYIKGSYPQHINIS
RNFSKLLSLRALHLRGYVFQELREDDFQPLMQLPNLSTINLGINFIKQIDFKLFQNFSNL
EIIYLSENRISPLVKDTRQSYANSSSFQRHIRKRRSTDFEFDPHSNFYHFTRPLIKPQCA
AYGKALDLSLNSIFFIGPNQFENLPDIACLNLSANSNAQVLSGTEFSAIPHVKYLDLTNN
RLDFDNASALTELSDLEVLDLSYNSHYFRIAGVTHHLEFIQNFTNLKVLNLSHNNIYTLT
DKYNLESKSLVELVFSGNRLDILWNDDDNRYISIFKGLKNLTRLDLSLNRLKHIPNEAFL
NLPASLTELHINDNMLKFFNWTLLQQFPRLELLDLRGNKLLFLTDSLSDFTSSLRTLLLS
HNRISHLPSGFLSEVSSLKHLDLSSNLLKTINKSALETKTTTKLSMLELHGNPFECTCDI
GDFRRWMDEHLNVKIPRLVDVICASPGDQRGKSIVSLELTTCVSDVTAVILFFFTFFITT
MVMLAALAHHLFYWDVWFIYNVCLAKVKGYRSLSTSQTFYDAYISYDTKDASVTDWVINE
LRYHLEESRDKNVLLCLEERDWDPGLAIIDNLMQSINQSKKTVFVLTKKYAKSWNFKTAF
YLALQRLMDENMDVIIFILLEPVLQHSQYLRLRQRICKSSILQWPDNPKAEGLFWQTLRN
VVLTENDSRYNNMYVDSIKQY*
>ENSG00000100312|ENST00000216139
MVEMLPTAILLVLAVSVVAKDNATCDGPCGLRFRQNPQGGVRIVGGKAAQHGAWPWMVSL
QIFTYNSHRYHTCGGSLLNSRWVLTAAHCFVGKNNVHDWRLVFGAKEITYGNNKPVKAPL
QERYVEKIIIHEKYNSATEGNDIALVEITPPISCGRFIGPGCLPHFKAGLPRGSQSCWVA
GWGYIEEKAPRPSSILMEARVDLIDLDLCNSTQWYNGRVQPTNVCAGYPVGKIDTCQGDS
GGPLMCKDSKESAYVVVGITSWGVGCARAKRPGIYTATWPYLNWIASKIGSNALRMIQSA
TPPPPTTRPPPIRPPFSHPISAHLPWYFQPPPRPLPPRPPAAQPRPPPSPPPPPPPPASP
LPPPPPPPPPTPSSTTKLPQGLSFAKRLQQLIEVLKGKTYSDGKNHYDMETTELPELTST
S*
>ENSG00000096717|ENST00000212015
MADEAALALQPGGSPSAAGADREAASSPAGEPLRKRPRRDGPGLERSPGEPGGAAPEREV
PAAARGCPGAAAAALWREAEAEAAAAGGEQEAQATAAAGEGDNGPGLQGPSREPPLADNL
YDEDDDDEGEEEEEAAAAAIGYRDNLLFGDEIITNGFHSCESDEEDRASHASSSDWTPRP
RIGPYTFVQQHLMIGTDPRTILKDLLPETIPPPELDDMTLWQIVINILSEPPKRKKRKDI
NTIEDAVKLLQECKKIIVLTGAGVSVSCGIPDFRSRDGIYARLAVDFPDLPDPQAMFDIE
YFRKDPRPFFKFAKEIYPGQFQPSLCHKFIALSDKEGKLLRNYTQNIDTLEQVAGIQRII
QCHGSFATASCLICKYKVDCEAVRGDIFNQVVPRCPRCPADEPLAIMKPEIVFFGENLPE
QFHRAMKYDKDEVDLLIVIGSSLKVRPVALIPSSIPHEVPQILINREPLPHLHFDVELLG
DCDVIINELCHRLGGEYAKLCCNPVKLSEITEKPPRTQKELAYLSELPPTPLHVSEDSSS
PERTSPPDSSVIVTLLDQAAKSNDDLDVSESKGCMEEKPQEVQTSRNVESIAEQMENPDL
KNVGSSTGEKNERTSVAGTVRKCWPNRVAKEQISRRLDGNQYLFLPPNRYIFHGAEVYSD
SEDDVLSSSSCGSNSDSGTCQSPSLEEPMEDESEIEEFYNGLEDEPDVPERAGGAGFGTD
GDDQEAINEAISVKQEVTDMNYPSNKS*
>ENSG00000100601|ENST00000216489
MGKMAAAVGSVATLATEPGEDAFRKLFRFYRQSRPGTADLEGVIDFSAAHAARGKGPGAQ
KVIKSQLNVSSVSEQNAYRAGLQPVSKWQAYGLKGYPGFIFIPNPFLPGYQWHWVKQCLK
LYSQKPNVCNLDKHMSKEETQDLWEQSKEFLRYKEATKRRPRSLLEKLRWVTVGYHYNWD
SKKYSADHYTPFPSDLGFLSEQVAAACGFEDFRAEAGILNYYRLDSTLGIHVDRSELDHS
KPLLSFSFGQSAIFLLGGLQRDEAPTAMFMHSGDIMIMSGFSRLLNHAVPRVLPNPEGEG
LPHCLEAPLPAVLPRDSMVEPCSMEDWQVCASYLKTARVNMTVRQVLATDQNFPLEPIED
EKRDISTEGFCHLDDQNSEVKRARINPDS*

>ENSG00000100462|ENST00000216350
MRGPNSGTEKGRLVIPEKQGFDFLCMPVFHPRFKREFIQEPAKNRPGPQTRSDLLLSGRA
FLLPLNQEDNTNLARVLTNHIHTGHHSSMFWMRVPLVAPEDLRDDIIENAPTTHTEEYSG
EEKTWMWWHNFRTLCDYSKRIAVALEIGADLPSNHVIDRWLGEPIKAAILPTSIFLTNKK
GFPVLSKMHQRLIFRLLKLEVQFIITGTNHHSEKEFCSYLQYLEYLSQNRPPPNAYELFA
KGYEDYLQSPLQPLMDNLESQTYEVFEKDPIKYSQYQQAIYKCLLDRVPEEEKDTNVQVL
MVLGAGRGPLVNASLRAAKQADRRIKLYAVEKNPNAVVTLENWQFEEWGSQVTVVSSDMR
EWVAPEKADIIVSELLGSFADNELSPECLDGAQHFLKDDGVSIPGEYTSFLAPISSSKLY
NEVRACREKDRDPEAQFEMPYVVRLHNFHQLSAPQPCFTFSHPNRDPMIDNNRYCTLEFP
VEVNTVLHGFAGYFETVLYQDITLSIRPETHSPGMFSWFPILFPIKQPITVREGQTICVR
FWRCSNSKKVWYEWAVTAPVCSAIHNPTGRSYTIGL*
>ENSG00000080824|ENST00000216281
MPEETQTQDQPMEEEEVETFAFQAEIAQLMSLIINTFYSNKEIFLRELISNSSDALDKIR
YESLTDPSKLDSGKELHINLIPNKQDRTLTIVDTGIGMTKADLINNLGTIAKSGTKAFME
ALQAGADISMIGQFGVGFYSAYLVAEKVTVITKHNDDEQYAWESSAGGSFTVRTDTGEPM
GRGTKVILHLKEDQTEYLEERRIKEIVKKHSQFIGYPITLFVEKERDKEVSDDEAEEKED
KEEEKEKEEKESEDKPEIEDVGSDEEEEKKDGDKKKKKKIKEKYIDQEELNKTKPIWTRN
PDDITNEEYGEFYKSLTNDWEDHLAVKHFSVEGQLEFRALLFVPRRAPFDLFENRKKKNN
IKLYVRRVFIMDNCEELIPEYLNFIRGVVDSEDLPLNISREMLQQSKILKVIRKNLVKKC
LELFTELAEDKENYKKFYEQFSKNIKLGIHEDSQNRKKLSELLRYYTSASGDEMVSLKDY
CTRMKENQKHIYYITGETKDQVANSAFVERLRKHGLEVIYMIEPIDEYCVQQLKEFEGKT
LVSVTKEGLELPEDEEEKKKQEEKKTKFENLCKIMKDILEKKVEKVVVSNRLVTSPCCIV
TSTYGWTANMERIMKAQALRDNSTMGYMAAKKHLEINPDHSIIETLRQKAEADKNDKSVK
DLVILLYETALLSSGFSLEDPQTHANRIYRMIKLGLGIDEDDPTADDTSAAVTEEMPPLE
GDDDTSRMEEVD*
>ENSG00000091831|ENST00000206249
MTMTLHTKASGMALLHQIQGNELEPLNRPQLKIPLERPLGEVYLDSSKPAVYNYPEGAAY
EFNAAAAANAQVYGQTGLPYGPGSEAAAFGSNGLGGFPPLNSVSPSPLMLLHPPPQLSPF
LQPHGQQVPYYLENEPSGYTVREAGPPAFYRPNSDNRRQGGRERLASTNDKGSMAMESAK
ETRYCAVCNDYASGYHYGVWSCEGCKAFFKRSIQGHNDYMCPATNQCTIDKNRRKSCQAC
RLRKCYEVGMMKGGIRKDRRGGRMLKHKRQRDDGEGRGEVGSAGDMRAANLWPSPLMIKR
SKKNSLALSLTADQMVSALLDAEPPILYSEYDPTRPFSEASMMGLLTNLADRELVHMINW
AKRVPGFVDLTLHDQVHLLECAWLEILMIGLVWRSMEHPGKLLFAPNLLLDRNQGKCVEG
MVEIFDMLLATSSRFRMMNLQGEEFVCLKSIILLNSGVYTFLSSTLKSLEEKDHIHRVLD
KITDTLIHLMAKAGLTLQQQHQRLAQLLLILSHIRHMSNKGMEHLYSMKCKNVVPLYDLL
LEMLDAHRLHAPTSRGGASVEETDQSHLATAGSTSSHSLQKYYITGEAEGFPATV*
>ENSG00000099995|ENST00000215793
MPAGPVQAVPPPPPVPTEPKQPTEEEASSKEDSAPSKPVVGIIYPPPEVRNIVDKTASFV
ARNGPEFEARIRQNEINNPKFNFLNPNDPYHAYYRHKVSEFKEGKAQEPSAAIPKVMQQQ
QQTTQQQLPQKVQAQVIQETIVPKEPPPEFEFIADPPSISAFDLDVVKLTAQFVARNGRQ
FLTQLMQKEQRNYQFDFLRPQHSLFNYFTKLVEQYTKILIPPKGLFSKLKKEAENPREVL
DQVCYRVEWAKFQERERKKEEEEKEKERVAYAQIDWHDFVVVETVDFQPNEQGNFPPPTT
PEELGARILIQERYEKFGESEEVEMEVESDEEDDKQEKAEEPPSQLDQDTQVQDMDEGSD
DEEEGQKVPPPPETPMPPPLPPTPDQVIVRKDYDPKASKPLPPAPAPDEYLVSPITGEKI

PASKMQEHMRIGLLDPRWLEQRDRSIREKQSDDEVYAPGLDIESSLKQLAERRTDIFGVE
ETAIGKKIGEEEIQKPEEKVTWDGHSGSMARTQQAAQANITLQEQIEAIHKAKGLVPEDD
TKEKIGPSKPNEIPQQPPPPSSATNIPSSAPPITSVPRPPTMPPPVRTTVVSAVPVMPRP
PMASVVRLPPGSVIAPMPPIIHAPRINVVPMPPSAPPIMAPRPPPMIVPTAFVPAPPVAP
VPAPAPMPPVHPPPPMEDEPTSKKLKTEDSLMPEEEFLRRNKGPVSIKVQVPNMQDKTEW
KLNGQVLVFTLPLTDQVSVIKVKIHEATGMPAGKQKLQYEGIFIKDSNSLAYYNMANGAV
IHLALKERGGRKK*
>ENSG00000101849|ENST00000217964
MTELAGASSSCCHRPAGRGAMQSVLHHFQRLRGREGGSHFINTSSPRGEAKMSITSDEVN
FLVYRYLQESGFSHSAFTFGIESHISQSNINGTLVPPAALISILQKGLQYVEAEISINED
GTVFDGRPIESLSLIDAVMPDVVQTRQQAFREKLAQQQASAAAAAAAATAAATAATTTSA
GVSHQNPSKNREATVNGEENRAHSVNNHAKPMEIDGEVEIPSSKATVLRGHESEVFICAW
NPVSDLLASGSGDSTARIWNLNENSNGGSTQLVLRHCIREGGHDVPSNKDVTSLDWNTNG
TLLATGSYDGFARIWTEDGNLASTLGQHKGPIFALKWNRKGNYILSAGVDKTTIIWDAHT
GEAKQQFPFHSAPALDVDWQNNTTFASCSTDMCIHVCRLGCDRPVKTFQGHTNEVNAIKW
DPSGMLLASCSDDMTLKIWSMKQEVCIHDLQAHNKEIYTIKWSPTGPATSNPNSNIMLAS
ASFDSTVRLWDIERGVCTHTLTKHQEPVYSVAFSPDGKYLASGSFDKCVHIWNTQSGNLV
HSYRGTGGIFEVCWNARGDKVGASASDGSVCVLDLRK*
>ENSG00000089127|ENST00000202917
MMDLRNTPAKSLDKFIEDYLLPDTCFRMQINHAIDIICGFLKERCFRGSSYPVCVSKVVK
GGSSGKGTTLRGRSDADLVVFLSPLTTFQDQLNRRGEFIQEIRRQLEACQRERAFSVKFE
VQAPRWGNPRALSFVLSSLQLGEGVEFDVLPAFDALGQLTGGYKPNPQIYVKLIEECTDL
QKEGEFSTCFTELQRDFLKQRPTKLKSLIRLVKHWYQNCKKKLGKLPPQYALELLTVYAW
ERGSMKTHFNTAQGFRTVLELVINYQQLCIYWTKYYDFKNPIIEKYLRRQLTKPRPVILD
PADPTGNLGGGDPKGWRQLAQEAEAWLNYPCFKNWDGSPVSSWILLAESNSADDETDDPR
RYQKYGYIGTHEYPHFSHRPSTLQAASTPQAEEDWTCTIL*
>ENSG00000100219|ENST00000216037
MVVVAAAPNPADGTPKVLLLSGQPASAAGAPAGQALPLMVPAQRGASPEAASGGLPQARK
RQRLTHLSPEEKALRRKLKNRVAAQTARDRKKARMSELEQQVVDLEEENQKLLLENQLLR
EKTHGLVVENQELRQRLGMDALVAEEEAEAKGNEVRPVAGSAESAALRLRAPLQQVQAQL
SPLQNISPWILAVLTLQIQSLISCWAFWTTWTQSCSSNALPQSLPAWRSSQRSTQKDPVP
YQPPFLCQWGRHQPSWKPLMN*
>ENSG00000100345|ENST00000216181
MAQQAADKYLYVDKNFINNPLAQADWAAKKLVWVPSDKSGFEPASLKEEVGEEAIVELVE
NGKKVKVNKDDIQKMNPPKFSKVEDMAELTCLNEASVLHNLKERYYSGLIYTYSGLFCVV
INPYKNLPIYSEEIVEMYKGKKRHEMPPHIYAITDTAYRSMMQDREDQSILCTGESGAGK
TENTKKVIQYLAYVASSHKSKKDQGELERQLLQANPILEAFGNAKTVKNDNSSRFGKFIR
INFDVNGYIVGANIETYLLEKSRAIRQAKEERTFHIFYYLLSGAGEHLKTDLLLEPYNKY
RFLSNGHVTIPGQQDKDMFQETMEAMRIMGIPEEEQMGLLRVISGVLQLGNIVFKKERNT
DQASMPDNTAAQKVSHLLGINVTDFTRGILTPRIKVGRDYVQKAQTKEQADFAIEALAKA
TYERMFRWLVLRINKALDKTKRQGASFIGILDIAGFEIFDLNSFEQLCINYTNEKLQQLF
NHTMFILEQEEYQREGIEWNFIDFGLDLQPCIDLIEKPAGPPGILALLDEECWFPKATDK
SFVEKVMQEQGTHPKFQKPKQLKDKADFCIIHYAGKVDYKADEWLMKNMDPLNDNIATLL
HQSSDKFVSELWKDVDRIIGLDQVAGMSETALPGAFKTRKGMFRTVGQLYKEQLAKLMAT

LRNTNPNFVRCIIPNHEKKAGKLDPHLVLDQLRCNGVLEGIRICRQGFPNRVVFQEFRQR
YEILTPNSIPKGFMDGKQACVLMIKALELDSNLYRIGQSKVFFRAGVLAHLEEERDLKIT
DVIIGFQACCRGYLARKAFAKRQQQLTAMKVLQRNCAAYLKLRNWQWWRLFTKVKPLLQV
SRQEEEMMAKEEELVKVREKQLAAENRLTEMETLQSQLMAEKLQLQEQLQAETELCAEAE
ELRARLTAKKQELEEICHDLEARVEEEEERCQHLQAEKKKMQQNIQELEEQLEEEESARQ
KLQLEKVTTEAKLKKLEEEQIILEDQNCKLAKEKKLLEDRIAEFTTNLTEEEEKSKSLAK
LKNKHEAMITDLEERLRREEKQRQELEKTRRKLEGDSTDLSDQIAELQAQIAELKMQLAK
KEEELQAALARVEEEAAQKNMALKKIRELESQISELQEDLESERASRNKAEKQKRDLGEE
LEALKTELEDTLDSTAAQQELRSKREQEVNILKKTLEEEAKTHEAQIQEMRQKHSQAVEE
LAEQLEQTKRVKANLEKAKQTLENERGELANEVKVLLQGKGDSEHKRKKVEAQLQELQVK
FNEGERVRTELADKVTKLQVELDNVTGLLSQSDSKSSKLTKDFSALESQLQDTQELLQEE
NRQKLSLSTKLKQVEDEKNSFREQLEEEEEAKHNLEKQIATLHAQVADMKKKMEDSVGCL
ETAEEVKRKLQKDLEGLSQRHEEKVAAYDKLEKTKTRLQQELDDLLVDLDHQRQSACNLE
KKQKKFDQLLAEEKTISAKYAEERDRAEAEAREKETKALSLARALEEAMEQKAELERLNK
QFRTEMEDLMSSKDDVGKSVHELEKSKRALEQQVEEMKTQLEELEDELQATEDAKLRLEV
NLQAMKAQFERDLQGRDEQSEEKKKQLVRQVREMEAELEDERKQRSMAVAARKKLEMDLK
DLEAHIDSANKNRDEAIKQLRKLQAQMKDCMRELDDTRASREEILAQAKENEKKLKSMEA
EMIQLQEELAAAERAKRQAQQERDELADEIANSSGKGALALEEKRRLEARIAQLEEELEE
EQGNTELINDRLKKANLQIDQINTDLNLERSHAQKNENARQQLERQNKELKVKLQEMEGT
VKSKYKASITALEAKIAQLEEQLDNETKERQAACKQVRRTEKKLKDVLLQVDDERRNAEQ
YKDQADKASTRLKQLKRQLEEAEEEAQRANASRRKLQRELEDATETADAMNREVSSLKNK
LRRGDLPFVVPRRMARKGAGDGSDEEVDGKADGAEAKPAE*
>ENSG00000090971|ENST00000205194
MAPSHLSVREMREDEKPLVLEMLKAGVKDTENRVALHALTRPPALLLLAAASSGLRFVLA
SFALALLLPVFLAVAAVKLGLRARWGSLPPPGGLGGPWVAVRGSGDVCGVLALAPGTNAG
DGARVTRLSVSRWHRRRGVGRRLLAFAEARARAWAGGMGEPRARLVVPVAVAAWGVGGML
EGCGYQAEGGWGCLGYTLVREFSKDL*
>ENSG00000101104|ENST00000217075
MVRPPVVPRRPPAHISSVRQASTQVPRTVPHTQRVANIGTQTTGPSGVGCCTPGRPLLPC
KCSSAAHSTYRVQEPAVHIPGQEPLTASMLAAAPLHEQKQMIGERLYPLIHDVHTQLAGK
ITGMLLEIDNSELLLMLESPESLHAKKRKSSLPWLPKGQCFWLSALRPCKL*
>ENSG00000100105|ENST00000215919
MERVNDASCGPSGCYTYQVSRHSTEMLHNLNQQRKNGGRFCDVLLRVGDESFPAHRAVLA
ACSEYFESVFSAQLGDGGAADGGPADVGGATAAPGGGAGGSRELEMHTISSKVFGDILDF
AYTSRIVVRLESFPELMTAAKFLLMRSVIEICQEVIKQSNVQILVPPARADIMLFRPPGT
SDLGFPLDMTNGAALAANSNGIAGSMQPEEEAARAAGAAIAGQASLPVLPGVDRLPMVAG
PLSPQLLTSPFPSVASSAPPLTGKRGRGRPRKANLLDSMFGSPGGLREAGILPCGLCGKV
FTDANRLRQHEAQHGVTSLQLGYIDLPPPRLGENGLPISEDPDGPRKRSRTRKQVACEIC
GKIFRDVYHLNRHKLSHSGEKPYSCPVCGLRFKRKDRMSYHVRSHDGSVGKPYICQSCGK
GFSRPDHLNGHIKQVHTSERPHKCQVWVGSSSGLPPLEPLPSDLPSWDFAQPALWRSSHS
VPDTAFSLSLKKSFPALENLGPAHSSNTLFCPAPPGYLRQGWTTPEGSRAFTQWPVG*
>ENSG00000089009|ENST00000202773
MAGEKVEKPDTKEKKPEAKKVDAGGKVKKGNLKAKKPKKGKPHCSRNPVLVRGIGRYSRS
AMYSRKAMYKRKYSAAKSKVEKKKKEKVLATVTKPVGGDKNGGTRVVKLRKMPRYYPTED

VPRKLLSHGKKPFSQHVRKLRASITPGTILIILTGRHRGKRVVFLKQLASGLLLVTGPLV
LNRVPLRRTHQKFVIATSTKIDISNVKIPKHLTDAYFKKKKLRKPRHQEGEIFDTEKEKY
EITEQRKIDQKAVDSQILPKIKAIPQLQGYLRSVFALTNGIYPHKLVF*
>ENSG00000100206|ENST00000216024
MKEDQVVAEEPGFQDEEESLFQDIDLLQKHGINVADIKKLKSVGICTIKGIQMTTRRALC
NVKGLSEAKVDKIKEAANKLIEPGFLTAFEYSEKRKMVFHITTGSQEFDKLLGGGIESMA
ITEAFGEFRTGKTQLSHTLCVTAQLPGAGGYPGGKIIFIDTENTFRPDRLRDIADRFNVD
HDAVLDNVLYARAYTSEHQMELLDYVAAKFHEEAGIFKLLIIDSIMALFRVDFSGRGELA
ERQQKLAQMLSRLQKISEEYNVAVFVTNQMTADPGATMTFQADPKKPIGGHILAHASTTR
ISLRKGRGELRIAKIYDSPEMPENEATFAITAGGIGDAKE*
>ENSG00000236011|ENST00000211377
MSRPLLITFTPATDPSDLWKDGQQQPQPEKPESTLDGAAALAFYEALIGDESSAPDSQRS
QTEPARERKRKKRRIMKAPAAEAVAEGASGRHGQGRSLEAEDKMTHRILRAAQEGDLPEL
RRLLEPHEAGGAGGNINARDAFWWTPLMCAARAGQGAAVSYLLGRGAAWVGVCELSGRDA
AQLAEEAGFPEVARMVRESHGETRSPENRSPTPSLQYCENCDTHFQDSNHRTSTAHLLSL
SQGPQPPNLPLGVPISSPGFKLLLRGGWEPGMGLGPRGEGRANPIPTVLKRDQEGLGYRS
APQPRVTHFPAWDTRAVAGRERPPRVATLSWREERRREEKDRAWERDLRTYMNLEF*
>ENSG00000100479|ENST00000216367
MAPERLRSRALSAFKLRGLLLRGEAIKYLTEALQSISELELEDKLEKIINAVEKQPLSSN
MIERSVVEAAVQECSQSVDETIEHVFNIIGAFDIPRFVYNSERKKFLPLLMTNHPAPNLF
GTPRDKAEMFRERYTILHQRTHRHELFTPPVIGSHPDESGSKFQLKTIETLLGSTTKIGD
AIVLGMITQLKEGKFFLEDPTGTVQLDLSKAQFHSGLYTEACFVLAEGWFEDQVFHVNAF
GFPPTEPSSTTRAYYGNINFFGGPSNTSVKTSAKLKQLEEENKDAMFVFLSDVWLDQVEV
LEKLRIMFAGYSPAPPTCFILCGNFSSAPYGKNQVQALKDSLKTLADIICEYPDIHQSSR
FVFVPGPEDPGFGSILPRPPLAESITNEFRQRVPFSVFTTNPCRIQYCTQEITVFREDLV
NKMCRNCVRFPSSNLAIPNHFVKTILSQGHLTPLPLYVCPVYWAYDYALRVYPVPDLLVI
ADKYDPFTTTNTECLCINPGSFPRSGFSFKVFYPSNKTVEDSKLQGF*
>ENSG00000108924|ENST00000226067
MEKMSRPLPLNPTFIPPPYGVLRSLLENPLKLPLHHEDAFSKDKDKEKKLDDESNSPTVP
QSAFLGPTLWDKTLPYDGDTFQLEYMDLEEFLSENGIPPSPSQHDHSPHPPGLQPASSAA
PSVMDLSSRASAPLHPGIPSPNCMQSPIRPGQLLPANRNTPSPIDPDTIQVPVGYEPDPA
DLALSSIPGQEMFDPRKRKFSEEELKPQPMIKKARKVFIPDDLKDDKYWARRRKNNMAAK
RSRDARRLKENQIAIRASFLEKENSALRQEVADLRKELGKCKNILAKYEARHGPL*
>ENSG00000104447|ENST00000220888
MVRKKNPPLRNVASEGEGQILEPIGTESKVSGKNKEFSADQMSENTDQSDAAELNHKEEH
SLHVQDPSSSSKKDLKSAVLSEKAGFNYESPSKGGNFPSFPHDEVTDRNMLAFSSPAAGG
VCEPLKSPQRAEADDPQDMACTPSGDSLETKEDQKMSPKATEETGQAQSGQANCQGLSPV
SVASKNPQVPSDGGVRLNKSKTDLLVNDNPDPAPLSPELQDFKCNICGYGYYGNDPTDLI
KHFRKYHLGLHNRTRQDAELDSKILALHNMVQFSHSKDFQKVNRSVFSGVLQDINSSRPV
LLNGTYDVQVTSGGTFIGIGRKTPDCQGNTKYFRCKFCNFTYMGNSSTELEQHFLQTHPN
KIKASLPSSEVAKPSEKNSNKSIPALQSSDSGDLGKWQDKITVKAGDDTPVGYSVPIKPL
DSSRQNGTEATSYYWCKFCSFSCESSSSLKLLEHYGKQHGAVQSGGLNPELNDKLSRGSV
INQNDLAKSSEGETMTKTDKSSSGAKKKDFSSKGAEDNMVTSYNCQFCDFRYSKSHGPDV
IVVGPLLRHYQQLHNIHKCTIKHCPFCPRGLCSPEKHLGEITYPFACRKSNCSHCALLLL

HLSPGAAGSSRVKHQCHQCSFTTPDVDVLLFHYESVHESQASDVKQEANHLQGSDGQQSV
KESKEHSCTKCDFITQVEEEISRHYRRAHSCYKCRQCSFTAADTQSLLEHFNTVHCQEQD
ITTANGEEDGHAISTIKEEPKIDFRVYNLLTPDSKMGEPVSESVVKREKLEEKDGLKEKV
WTESSSDDLRNVTWRGADILRGSPSYTQASLGLLTPVSGTQEQTKTLRDSPNVEAAHLAR
PIYGLAVETKGFLQGAPAGGEKSGALPQQYPASGENKSKDESQSLLRRRRGSGVFCANCL
TTKTSLWRKNANGGYVCNACGLYQKLHSTPRPLNIIKQNNGEQIIRRRTRKRLNPEALQA
EQLNKQQRGSNEEQVNGSPLERRSEDHLTESHQREIPLPSLSKYEAQGSLTKSHSAQQPV
LVSQTLDIHKRMQPLHIQIKSPQESTGDPGNSSSVSEGKGSSERGSPIEKYMRPAKHPNY
SPPGSPIEKYQYPLFGLPFVHNDFQSEADWLRFWSKYKLSVPGNPHYLSHVPGLPNPCQN
YVPYPTFNLPPHFSAVGSDNDIPLDLAIKHSRPGPTANGASKEKTKAPPNVKNEGPLNVV
KTEKVDRSTQDELSTKCVHCGIVFLDEVMYALHMSCHGDSGPFQCSICQHLCTDKYDFTT
HIQRGLHRNNAQVEKNGKPKE*
>ENSG00000108511|ENST00000225648
MSSYFVNSTFPVTLASGQESFLGQLPLYSSGYADPLRHYPAPYGPGPGQDKGFATSSYYP
PAGGGYGRAAPCDYGPAPAFYREKESACALSGADEQPPFHPEPRKSDCAQDKSVFGETEE
QKCSTPVYPWMQRMNSCNSSSFGPSGRRGRQTYTRYQTLELEKEFHYNRYLTRRRRIEIA
HALCLTERQIKIWFQNRRMKWKKESKLLSASQLSAEEEEEKQAE*
>ENSG00000106624|ENST00000223357
MAAVRGAPLLSCLLALLALCPGGRPQTVLTDDEIEEFLEGFLSELEPEPREDDVEAPPPP
EPTPRVRKAQAGGKPGKRPGTAAEVPPEKTKDKGKKGKKDKGPKVPKESLEGSPRPPKKG
KEKPPKATKKPKEKPPKATKKPKEKPPKATKKPKEKPPKATKKPPSGKRPPILAPSETLE
WPLPPPPSPGPEELPQEGGAPLSNNWQNPGEETHVEAREHQPEPEEETEQPTLDYNDQIE
REDYEDFEYIRRQKQPRPPPSRRRRPERVWPEPPEEKAPAPAPEERIEPPVKPLLPPLPP
DYGDGYVIPNYDDMDYYFGPPPPQKPDAERQTDEEKEELKKPKKEDSSPKEETDKWAVEK
GKDHKEPRKGEELEEEWTPTEKVKCPPIGMESHRIEDNQIRASSMLRHGLGAQRGRLNMQ
TGATEDDYYDGAWCAEDDARTQWIEVDTRRTTRFTGVITQGRDSSIHDDFVTTFFVGFSN
DSQTWVMYTNGYEEMTFHGNVDKDTPVLSELPEPVVARFIRIYPLTWNGSLCMRLEVLGC
SVAPVYSYYAQNEVVATDDLDFRHHSYKDMRQLMKVVNEECPTITRTYSLGKSSRGLKIY
AMEISDNPGEHELGEPEFRYTAGIHGNEVLGRELLLLLMQYLCREYRDGNPRVRSLVQDT
RIHLVPSLNPDGYEVAAQMGSEFGNWALGLWTEEGFDIFEDFPDLNSVLWGAEERKWVPY
RVPNNNLPIPERYLSPDATVSTEVRAIIAWMEKNPFVLGANLNGGERLVSYPYDMARTPT
QEQLLAAAMAAARGEDEDEVSEAQETPDHAIFRWLAISFASAHLTLTEPYRGGCQAQDYT
GGMGIVNGAKWNPRTGTINDFSYLHTNCLELSFYLGCDKFPHESELPREWENNKEALLTF
MEQVHRGIKGVVTDEQGIPIANATISVSGINHGVKTASGGDYWRILNPGEYRVTAHAEGY
TPSAKTCNVDYDIGATQCNFILARSNWKRIREIMAMNGNRPIPHIDPSRPMTPQQRRLQQ
RRLQHRLRLRAQMRLRRLNATTTLGPHTVPPTLPPAPATTLSTTIEPWGLIPPTTAGWEE
SETETYTEVVTEFGTEVEPEFGTKVEPEFETQLEPEFETQLEPEFEEEEEEEKEEEIATG
QAFPFTTVETYTVNFGDF*
>ENSG00000108256|ENST00000225388
MEEKPGQPQPQHHHSHHHPHHHPQQQQQQPHHHHHYYFYNHSHNHHHHHHHQQPHQYLQH
GAEGSPKAQPKPLKHEQKHTLQQHQETPKKKTGYGELNGNAGEREISLKNLSSDEATNPI
SRVLNGNQQVVDTSLKQTVKANTFGKAGIKTKNFIQKNSMDKKNGKSYENKSGENQSVDK
SDTIPIPNGVVTNNSGYITNGYMGKGADNDGSGSESGYTTPKKRKARRNSAKGCENLNIV
QDKIMQQETSVPTLKQGLETFKPDYSEQKGNRVDGSKPIWKYETGPGGTSRGKPAVGDML

RKSSDSKPGVSSKKFDDRPKGKHASAVASKEDSWTLFKPPPVFPVDNSSAKIVPKISYAS
KVKENLNKTIQNSSVSPTSSSSSSSSTGETQTQSSSRLSQVPMSALKSVTSANFSNGPVL
AGTDGNVYPPGGQPLLTTAANTLTPISSGTDSVLQDMSLTSAAVEQIKTSLFIYPSNMQT
MLLSTAQVDLPSQTDQQNLGDIFQNQWGLSFINEPSAGPETVTGKSSEHKVMEVTFQGEY
PATLVSQGAEIIPSGTEHPVFPKAYELEKRTSPQVLGSILKSGTTSESGALSLEPSHIGD
LQKADTSSQGALVFLSKDYEIESQNPLASPTNTLLGSAKEQRYQRGLERNDSWGSFDLRA
AIVYHTKEMESIWNLQKQDPKRIITYNEAMDSPDQ*
>ENSG00000013573|ENST00000228264
MAELYRVLEAGKIGIFESPTGTGKSLSLICGALSWLRDFEQKKREEEARLLETGTGPLHD
EKDESLCLSSSCEGAAGTPRPAGEPAWVTQFVQKKEERDLVDRLKAEQARRKQREERLQQ
LQHRVQLKYAAKRLRQEEEERENLLRLSREMLETGPEAERLEQLESGEEELVLAEYESDE
EKKVASRVDEDEDDLEEEHITKIYYCSRTHSQLAQFVHEVKKSPFGKDVRLVSLGSRQNL
CVNEDVKSLGSVQLINDRCVDMQRSRHEKKKGAEEEKPKRRRQEKQAACPFYNHEQMGLL
RDEALAEVKDMEQLLALGKEARACPYYGSRLAIPAAQLVVLPYQMLLHAATRQAAGIRLQ
DQVVIIDEAHNLIDTITGMHSVEVSGSQLCQAHSQLLQYVERYGKRLKAKNLMYLKQILY
LLEKFVAVLGGNIKQNPNTQSLSQTGTELKTINDFLFQSQIDNINLFKVQRYCEKSMISR
KLFGFTERYGAVFSSREQPKLAGFQQFLQSLQPRTTEALAAPADESQASTLRPASPLMHI
QGFLAALTTANQDGRVILSRQGSLSQSTLKFLLLNPAVHFAQVVKECRAVVIAGGTMQPV
SDFRQQLLACAGVEAERVVEFSCGHVIPPDNILPLVICSGISNQPLEFTFQKRELPQMMD
EVGRILCNLCGVVPGGVVCFFPSYEYLRQVHAHWEKGGLLGRLAARKKIFQEPKSAHQVE
QVLLAYSRCIQACGQERGQVTGALLLSVVGGKMSEGINFSDNLGRCVVMVGMPFPNIRSA
ELQEKMAYLDQTLPRAPGQAPPGKALVENLCMKAVNQSIGRAIRHQKDFASVVLLDQRYA
RPPVLAKLPAWIRARVEVKATFGPAIAAVQKFHREKSASS*
>ENSG00000105392|ENST00000221996
MMAYMNPGPHYSVNALALSGPSVDLMHQAVPYPSAPRKQRRERTTFTRSQLEELEALFAK
TQYPDVYAREEVALKINLPESRVQVWFKNRRAKCRQQRQQQKQQQQPPGGQAKARPAKRK
AGTSPRPSTDVCPDPLGISDSYSPPLPGPSGSPTTAVATVSIWSPASESPLPEAQRAGLV
ASGPSLTSAPYAMTYAPASAFCSSPSAYGSPSSYFSGLDPYLSPMVPQLGGPALSPLSGP
SVGPSLAQSPTSLSGQSYGAYSPVDSLEFKDPTGTWKFTYNPMDPLDYKDQSAWKFQIL*
>ENSG00000111087|ENST00000228682
MFNSMTPPPISSYGEPCCLRPLPSQGAPSVGTEGLSGPPFCHQANLMSGPHSYGPARETN
SCTEGPLFSSPRSAVKLTKKRALSISPLSDASLDLQTVIRTSPSSLVAFINSRCTSPGGS
YGHLSIGTMSPSLGFPAQMNHQKGPSPSFGVQPCGPHDSARGGMIPHPQSRGPFPTCQLK
SELDMLVGKCREEPLEGDMSSPNSTGIQDPLLGMLDGREDLEREEKREPESVYETDCRWD
GCSQEFDSQEQLVHHINSEHIHGERKEFVCHWGGCSRELRPFKAQYMLVVHMRRHTGEKP
HKCTFEGCRKSYSRLENLKTHLRSHTGEKPYMCEHEGCSKAFSNASDRAKHQNRTHSNEK
PYVCKLPGCTKRYTDPSSLRKHVKTVHGPDAHVTKRHRGDGPLPRAPSISTVEPKREREG
GPIREESRLTVPEGAMKPQPSPGAQSSCSSDHSPAGSAANTDSGVEMTGNAGGSTEDLSS
LDEGPCIAGTGLSTLRRLENLRLDQLHQLRPIGTRGLKLPSLSHTGTTVSRRVGPPVSLE
RRSSSSSSISSAYTVSRRSSLASPFPPGSPPENGASSLPGLMPAQHYLLRARYASARGGG
TSPTAASSLDRIGGLPMPPWRSRAEYPGYNPNAGVTRRASDPAQAADRPAPARVQRFKSL
GCVHTPPTVAGGGQNFDPYLPTSVYSPQPPSITENAAMDARGLQEEPEVGTSMVGSGLNP
YMDFPPTDTLGYGGPEGAAAEPYGARGPGSLPLGPGPPTNYGPNPCPQQASYPDPTQETW
GEFPSHSGLYPGPKALGGTYSQCPRLEHYGGQVQVKPEQGCPVGSDSTGLAPCLNAHPSEG

PPHPQPLFSHYPQPSPPQYLQSGPYTQPPPDYLPSEPRPCLDFDSPTHSTGQLKAQLVCN
YVQSQQELLWEGGGREDAPAQEPSYQSPKFLGGSQVSPSRAKAPVNTYGPGFGPNLPNHK
SGSYPTPSPCHENFVVGANRASHRAAAPPRLLPPLPTCYGPLKVGGTNPSCGHPEVGRLG
GGPALYPPPEGQVCNPLDSLDLDNTQLDFVAILDEPQGLSPPPSHDQRGSSGHTPPPSGP
PNMAVGNMSVLLRSLPGETEFLNSSA*
>ENSG00000109132|ENST00000226382
MYKMEYSYLNSSAYESCMAGMDTSSLASAYADFSSCSQASGFQYNPIRTTFGATSGCPSL
TPGSCSLGTLRDHQSSPYAAVPYKLFTDHGGLNEKRKQRRIRTTFTSAQLKELERVFAET
HYPDIYTREELALKIDLTEARVQVWFQNRRAKFRKQERAAAAAAAAAKNGSSGKKSDSSR
DDESKEAKSTDPDSTGGPGPNPNPTPSCGANGGGGGGPSPAGAPGAAGPGGPGGEPGKGG
AAAAAAAAAAAAAAAAAAAAGGLAAAGGPGQGWAPGPGPITSIPDSLGGPFASVLSSLQR
PNGAKAALVKSSMF*
>ENSG00000104129|ENST00000220496
MAVTKELLQMDLYALLGIEEKAADKEVKKAYRQKALSCHPDKNPDNPRAAELFHQLSQAL
EVLTDAAARAAYDKVRKAKKQAAERTQKLDEKRKKVKLDLEARERQAQAQESEEEEESRS
TRTLEQEIERLREEGSRQLEEQQRLIREQIRQERDQRLRGKAENTEGQGTPKLKLKWKCK
KEDESKGGYSKDVLLRLLQKYGEVLNLVLSSKKPGTAVVEFATVKAAELAVQNEVGLVDN
PLKISWLEGQPQDAVGRSHSGLSKGSVLSERDYESLVMMRMRQAAERQQLIARMQQEDQE
GPPT*
>ENSG00000103460|ENST00000219746
MDVRFYPAAAGDPASLDFAQCLGYYGYSKFGNNNNYMNMAEANNAFFAASEQTFHTPSLG
DEEFEIPPITPPPESDPALGMPDVLLPFQALSDPLPSQGSEFTPQFPPQSLDLPSITISR
NLVEQDGVLHSSGLHMDQSHTQVSQYRQDPSLIMRSIVHMTDAARSGVMPPAQLTTINQS
QLSAQLGLNLGGASMPHTSPSPPASKSATPSPSSSINEEDADEANRAIGEKRAAPDSGKK
PKTPKKKKKDPNEPQKPVSAYALFFRDTQAAIKGQNPNATFGEVSKIVASMWDSLGEEQ
KQVYKRKTEAAKKEYLKALAAYRASLVSKAAAESAEAQTIRSVQQTLASTNLTSSLLLNT
PLSQHGTVSASPQTLQQSLPRSIAPKPLTMRLPMNQIVTSVTIAANMPSNIGAPLISSMG
TTMVGSAPSTQVSPSVQTQQHQMLQQQQQQQQQQQMQQMQQQQLQQHQMHQQIQQQMQQQ
HFQHHMQQHLQQQQQHLQQQINQQQLQQQLQQRLQLQQLQHMQHQSQPSPRQHSPVASQI
TSPIPAIGSPQPASQQHQSQIQSQTQTQVLSQVSIF*
>ENSG00000105258|ENST00000221859
MEPDGTYEPGFVGIRFCQECNNMLYPKEDKENRILLYACRNCDYQQEADNSCIYVNKITH
EVDELTQIIADVSQDPTLPRTEDHPCQKCGHKEAVFFQSHSARAEDAMRLYYVCTAPHCG
HRWTE*
>ENSG00000106479|ENST00000223210
MEPRESGKAPVTFDDITVYLLQEEWVLLSQQQKELCGSNKLVAPLGPTVANPELFRKFGR
GPEPWLGSVQGQRSLLEHHPGKKQMGYMGEMEVQGPTRESGQSLPPQKKAYLSHLSTGSG
HIEGDWAGRNRKLLKPRSIQKSWFVQFPWLIMNEEQTALFCSACREYPSIRDKRSRLIEG
YTGPFKVETLKYHAKSKAHMFCVNALAARDPIWAARFRSIRDPPGDVLASPEPLFTADCP
IFYPPGPLGGFDSMAELLPSSRAELEDPGGDGAIPAMYLDCISDLRQKEITDGIHSSSDI
NILYNDAVESCIQDPSAEGLSEEVPVVFEELPVVFEDVAVYFTREEWGMLDKRQKELYRD
VMRMNYELLASLGPAAAKPDLISKLERRAAPWIKDPNGPKWGKGRPPGNKKMVAVREADT
QASAADSALLPGSPVEARASCCSSSICEEGDGPRRIKRTYRPRSIQRSWFGQFPWLVIDP
KETKLFCSACIERPNLHDKSSRLVRGYTGPFKVETLKYHEVSKAHRLCVNTVEIKEDTPH

TALVPEISSDLMANMEHFFNAAYSIAYHSRPLNDFEKILQLLQSTGTVILGKYRNRTACT
QFIKYISETLKREILEDVRNSPCVSVLLDSSTDASEQACVGIYIRYFKQMEVKESYITLA
PLYSETADGYFETIVSALDELDIPFRKPGWVVGLGTDGSAMLSCRGGLVEKFQEVIPQLL
PVHCVAHRLHLAVVDACGSIDLVKKCDRHIRTVFKFYQSSNKRLNELQEGAAPLEQEIIR
LKDLNAVRWVASRRRTLHALLVSWPALARHLQRVAEAGGQIGHRAKGMLKLMRGFHFVKF
CHFLLDFLSIYRPLSEVCQKEIVLITEVNATLGRAYVALESLRHQAGPKEEEFNASFKDG
RLHGICLDKLEVAEQRFQADRERTVLTGIEYLQQRFDADRPPQLKNMEVFDTMAWPSGIE
LASFGNDDILNLARYFECSLPTGYSEEALLEEWLGLKTIAQHLPFSMLCKNALAQHCRFP
LLSKLMAVVVCVPISTSCCERGFKAMNRIRTDERTKLSNEVLNMLMMTAVNGVAVTEYDP
QPAIQHWYLTSSGRRFSHVYTCAQVPARSPASARLRKEEMGALYVEEPRTQKPPILPSRE
AAEVLKDCIMEPPERLLYPHTSQEAPGMS*
>ENSG00000256087|ENST00000221315
MINAQELLTLEDVTVEFTWEEWQLLGPFQKDLYRDVMLEIYSNLLSMGYQVSKPDALSKL
ERGEEPWTMEDERHSRICPENNEVDDHLQDHLENQRMLKSVEQYHEHNAFGNTASQTKSL
CLFRENHDTFELYIKTLKSNLSLVNQNKSCEINNSTKFSGDGKSFLHGNYEELYSAAKFS
VSTKANSTKSQVSKHQRTHEIEKNHVCSECGKAFVKKSQLTDHERVHTGEKPYGCTLCAK
VFSRKSRLNEHQRIHKREKSFICSECGKVFTMKSRLIEHQRTHTGEKPYICNECGKGFPG
KRNLIVHQRNHTGEKSYICSECGKGFTGKSMLIIHQRTHTGEKPYICSECGKGFTTKHYV
IIHQRNHTGEKPYICNECGKGFTMKSRMIEHQRTHTGEKPYICSECGKGFPRKSNLIVHQ
RNHTVEKSYLCSECGKGFTVKSMLIIHQRTHTGEKPYTCSECGKGFPLKSRLIVHQRTHT
GEKPYRCSECGKGFIVNSGLMLHQRTHTGEKPYICNECGKGFAFKSNLVVHQRTHTGEKP
FMCSECGKGFTMKRYLIVHQQIHTEEKSCICSECGRGFAKETELALHKQVHTGEKPYGCN
ECGKGFTMKSRLIVHQRTHTGEKPFVCSECRKAFSSKRNLIVHQRTHNGNKP*
>ENSG00000104856|ENST00000221452
MLRSGPASGPSVPTGRAMPSRRVARPPAAPELGALGSPDLSSLSLAVSRSTDELEIIDEY
IKENGFGLDGGQPGPGEGLPRLVSRGAASLSTVTLGPVAPPATPPPWGCPLGRLVSPAPG
PGPQPHLVITEQPKQRGMRFRYECEGRSAGSILGESSTEASKTLPAIELRDCGGLREVEV
TACLVWKDWPHRVHPHSLVGKDCTDGICRVRLRPHVSPRHSFNNLGIQCVRKKEIEAAIE
RKIQLGIDPYNAGSLKNHQEVDMNVVRICFQASYRDQQGQMRRMDPVLSEPVYDKKSTNT
SELRICRINKESGPCTGGEELYLLCDKVQKEDISVVFSRASWEGRADFSQADVHRQIAIV
FKTPPYEDLEIVEPVTVNVFLQRLTDGVCSEPLPFTYLPRDHDSYGVDKKRKRGMPDVLG
ELNSSDPHGIESKRRKKKPAILDHFLPNHGSGPFLPPSALLPDPDFFSGTVSLPGLEPPG
GPDLLDDGFAYDPTAPTLFTMLDLLPPAPPHASAVVCSGGAGAVVGETPGPEPLTLDSYQ
APGPGDGGTASLVGSNMFPNHYREAAFGGGLLSPGPEAT*
>ENSG00000103495|ENST00000219782
MFPVFPCTLLAPPFPVLGLDSRGVGGLMNSFPPPQGHAQNPLQVGAELQSRFFASQGCAQ
SPFQAAPAPPPTPQAPAAEPLQVDLLPVLAAAQESAAAAAAAAAAAAAVAAAPPAPAAAS
TVDTAALKQPPAPPPPPPPVSAPAAEAAPPASAATIAAAAATAVVAPTSTVAVAPVASAL
EKKTKSKGPYICALCAKEFKNGYNLRRHEAIHTGAKAGRVPSGAMKMPTMVPLSLLSVPQ
LSGAGGGGGEAGAGGGAAAVAAGGVVTTTASGKRIRKNHACEMCGKAFRDVYHLNRHKLS
HSDEKPYQCPVCQQRFKRKDRMSYHVRSHDGAVHKPYNCSHCGKSFSRPDHLNSHVRQVH
STERPFKCEKCEAAFATKDRLRAHTVRHEEKVPCHVCGKMLSSAYISDHMKVHSQGPHHV
CELCNKGFTTAAYLRIHAVKDHGLQAPRADRILCKLCSVHCKTPAQLAGHMQTHLGGAAP
PVPGDAPQPQPTC*

>ENSG00000111049|ENST00000228644
MDVMDGCQFSPSEYFYDGSCIPSPEGEFGDEFVPRVAAFGAHKAELQGSDEDEHVRAPTG
HHQAGHCLMWACKACKRKSTTMDRRKAATMRERRRLKKVNQAFETLKRCTTTNPNQRLPK
VEILRNAIRYIESLQELLREQVENYYSLPGQSCSEPTSPTSNCSDGMPECNSPVWSRKSS
TFDSIYCPDVSNVYATDKNSLSSLDCLSNIVDRITSSEQPGLPLQDLASLSPVASTDSQP
ATPGASSSRLIYHVL*
>ENSG00000109534|ENST00000226796
MSFRGGGRGGFNRGGGGGGFNRGGSSNHFRGGGGGGGGGNFRGGGRGGFGRGGGRGGFNK
GQDQGPPERVVLLGEFLHPCEDDIVCKCTTDENKVPYFNAPVYLENKEQIGKVDEIFGQL
RDFYFSVKLSENMKASSFKKLQKFYIDPYKLLPLQRFLPRPPGEKGPPRGGGRGGRGGGR
GGGGRGGGRGGGFRGGRGGGGGGFRGGRGGGFRGRGH*
>ENSG00000108829|ENST00000225972
MTKAGSKGGNLRDKLDGNELDLSLSDLNEVPVKELAALPKATILDLSCNKLTTLPSDFCG
LTHLVKLDLSKNKLQQLPADFGRLVNLQHLDLLNNKLVTLPVSFAQLKNLKWLDLKDNPL
DPVLAKVAGDCLDEKQCKQCANKVLQHMKAVQADQERERQRRLEVEREAEKKREAKQRAK
EAQERELRKREKAEEKERRRKEYDALKAAKREQEKKPKKEANQAPKSKSGSRPRKPPPRK
HTRSWAVLKLLLLLLLLFGVAGGLVACRVTELQQQPLCTSVNTIYDNAVQGLRRHEILQWV
LQTDSQQ*
>ENSG00000179943|ENST00000221665
MDDVPAPTPAPAPPAAAAPRVPFHCSECGKSFRYRSDLRRHFARHTALKPHACPRCGKGF
KHSFNLANHLRSHTGERPYRCSACPKGFRDSTGLLHHQVVHTGEKPYCCLVCELRFSSRS
SLGRHLKRQHRGVLPSPLQPGPGLPALSAPCSVCCNVGPCSVCGGSGAGGGEGPEGAGAG
LGSWGLAEAAAAAAASLPPFACGACARRFDHGRELAAHWAAHTDVKPFKCPRCERDFNAP
ALLERHKLTHDLQGPGAPPAQAWAAGPGAGPETAGEGTAAEAGDAPLASDRRLLLGPAGG
GVPKLGGLLPEGGGEAPAPAAAAEPSEDTLYQCDCGTFFASAAALASHLEAHSGPATYGC
GHCGALYAALAALEEHRRVSHGEGGGEEAATAAREREPASGEPPSGSGRGKKIFGCSECE
KLFRSPRDLERHVLVHTGEKPFPCLECGKFFRHECYLKRHRLLHGTERPFPCHICGKGFI
TLSNLSRHLKLHRGMD*
>ENSG00000109536|ENST00000226798
MAEYSYVKSTKLVLKGTKTKSKKKKSKDKKRKREEDEETQLDIVGIWWTVTNFGEISGTI
AIEMDKGTYIHALDNGLFTLGAPHKEVDEGPSPPEQFTAVKLSDSRIALKSGYGKYLGIN
SDGLVVGRSDAIGPREQWEPVFQNGKMALLASNSCFIRCNEAGDIEAKSKTAGEEEMIKI
RSCAERETKKKDDIPEEDKGNVKQCEINYVKKFQSFQDHKLKISKEDSKILKKARKDGFL
HETLLDRRAKLKADRYCK*
>ENSG00000105640|ENST00000222247
MKASGTLREYKVVGRCLPTPKCHTPPLYRMRIFAPNHVVAKSRFWYFVSQLKKMKKSSGE
IVYCGQVFEKSPLRVKNFGIWLRYDSRSGTHNMYREYRDLTTAGAVTQCYRDMGARHRAR
AHSIQIMKVEEIAASKCRRPAVKQFHDSKIKFPLPHRVLRRQHKPRFTTKRPNTFF*
>ENSG00000077348|ENST00000221233
MEEETHTDAKIRAENGTGSSPRGPGCSLRHFACEQNLLSRPDGSASFLQGDTSVLAGVYG
PAEVKVSKEIFNKATLEVILRPKIGLPGVAEKSRERLIRNTCEAVVLGTLHPRTSITVVL
QVVSDAGSLLACCLNAACMALVDAGVPMRALFCGVACALDSDGTLVLDPTSKQEKEARAV
LTFALDSVERKLLMSSTKGLYSDTELQQCLAAAQAASQHVFRFYRESLQRRYSKS*
>ENSG00000102879|ENST00000219150

MSRQVVRSSKFRHVFGQPAKADQCYEDVRVSQTTWDSGFCAVNPKFVALICEASGGGAFL
VLPLGKTGRVDKNAPTVCGHTAPVLDIAWCPHNDNVIASGSEDCTVMVWEIPDGGLMLPL
REPVVTLEGHTKRVGIVAWHTTAQNVLLSAGCDNVIMVWDVGTGAAMLTLGPEVHPDTIY
SVDWSRDGGLICTSCRDKRVRIIEPRKGTVVAEKDRPHEGTRPVRAVFVSEGKILTTGFS
RMSERQVALWDTKHLEEPLSLQELDTSSGVLLPFFDPDTNIVYLCGKGDSSIRYFEITSE
APFLHYLSMFSSKESQRGMGYMPKRGLEVNKCEIARFYKLHERRCEPIAMTVPRKSDLFQ
EDLYPPTAGPDPALTAEEWLGGRDAGPLLISLKDGYVPPKSRELRVNRGLDTGRRRAAPE
ASGTPSSDAVSRLEEEMRKLQATVQELQKRLDRLEETVQAK*
>ENSG00000123908|ENST00000220592
MYSGAGPALAPPAPPPPIQGYAFKPPPRPDFGTSGRTIKLQANFFEMDIPKIDIYHYELD
IKPEKCPRRVNREIVEHMVQHFKTQIFGDRKPVFDGRKNLYTAMPLPIGRDKVELEVTLP
GEGKDRIFKVSIKWVSCVSLQALHDALSGRLPSVPFETIQALDVVMRHLPSMRYTPVGRS
FFTASEGCSNPLGGGREVWFGFHQSVRPSLWKMMLNIDVSATAFYKAQPVIEFVCEVLDF
KSIEEQQKPLTDSQRVKFTKEIKGLKVEITHCGQMKRKYRVCNVTRRPASHQTFPLQQES
GQTVECTVAQYFKDRHKLVLRYPHLPCLQVGQEQKHTYLPLEVCNIVAGQRCIKKLTDNQ
TSTMIRATARSAPDRQEEISKLMRSASFNTDPYVREFGIMVKDEMTDVTGRVLQPPSILY
GGRNKAIATPVQGVWDMRNKQFHTGIEIKVWAIACFAPQRQCTEVHLKSFTEQLRKISRD
AGMPIQGQPCFCKYAQGADSVEPMFRHLKNTYAGLQLVVVILPGKTPVYAEVKRVGDTVL
GMATQCVQMKNVQRTTPQTLSNLCLKINVKLGGVNNILLPQGRPPVFQQPVIFLGADVTH
PPAGDGKKPSIAAVVGSMDAHPNRYCATVRVQQHRQEIIQDLAAMVRELLIQFYKSTRFK
PTRIIFYRDGVSEGQFQQVLHHELLAIREACIKLEKDYQPGITFIVVQKRHHTRLFCTDK
NERVGKSGNIPAGTTVDTKITHPTEFDFYLCSHAGIQGTSRPSHYHVLWDDNRFSSDELQ
ILTYQLCHTYVRCTRSVSIPAPAYYAHLVAFRARYHLVDKEHDSAEGSHTSGQSNGRDHQ
ALAKAVQVHQDTLRTMYFA*
>ENSG00000105996|ENST00000222718
MNYEFEREIGFINSQPSLAECLTSFPPVADTFQSSSIKTSTLSHSTLIPPPFEQTIPSLN
PGSHPRHGAGGRPKPSPAGSRGSPVPAGALQPPEYPWMKEKKAAKKTALLPAAAAAATAA
ATGPACLSHKESLEIADGSGGGSRRLRTAYTNTQLLELEKEFHFNKYLCRPRRVEIAALL
DLTERQVKVWFQNRRMKHKRQTQCKENQNSEGKCKSLEDSEKVEEDEEEKTLFEQALSVS
GALLEREGYTFQQNALSQQQAPNGHNGDSQSFPVSPLTSNEKNLKHFQHQSPTVPNCLST
MGQNCGAGLNNDSPEALEVPSLQDFSVFSTDSCLQLSDAVSPSLPGSLDSPVDISADSLD
FFTDTLTTIDLQHLNY*
>ENSG00000103194|ENST00000219473
MALHSPQYIFGDFSPDEFNQFFVTPRSSVELPPYSGTVLCGTQAVDKLPDGQEYQRIEFG
VDEVIEPSDTLPRTPSYSISSTLNPQAPEFILGCTASKITPDGITKEASYGSIDCQYPGS
ALALDGSSNVEAEVLENDGVSGGLGQRERKKKKKRPPGYYSYLKDGGDDSISTEALVNGH
ANSAVPNSVSAEDAEFMGDMPPSVTPRTCNSPQNSTDSVSDIVPDSPFPGALGSDTRTAG
QPEGGPGADFGQSCFPAEAGRDTLSRTAGAQPCVGTDTTENLGVANGQILESSGEGTATN
GVELHTTESIDLDPTKPESASPPADGTGSASGTLPVSQPKSWASLFHDSKPSSSSPVAYV
ETKYSPPAISPLVSEKQVEVKEGLVPVSEDPVAIKIAELLENVTLIHKPVSLQPRGLINK
GNWCYINATLQALVACPPMYHLMKFIPLYSKVQRPCTSTPMIDSFVRLMNEFTNMPVPPK
PRQALGDKIVRDIRPGAAFEPTYIYRLLTVNKSSLSEKGRQEDAEEYLGFILNGLHEEML
NLKKLLSPSNEKLTISNGPKNHSVNEEEQEEQGEGSEDEWEQVGPRNKTSVTRQADFVQT
PITGIFGGHIRSVVYQQSSKESATLQPFFTLQLDIQSDKIRTVQDALESLVARESVQGYT

TKTKQEVEISRRVTLEKLPPVLVLHLKRFVYEKTGGCQKLIKNIEYPVDLEISKELLSPG
VKNKNFKCHRTYRLFAVVYHHGNSATGGHYTTDVFQIGLNGWLRIDDQTVKVINQYQVVK
PTAERTAYLLYYRRVDLL*
>ENSG00000104472|ENST00000220913
MADVVVGKDKGGEQRLISLPLSRIRVIMKSSPEVSSINQEALVLTAKATELFVQCLATYS
YRHGSGKEKKVLTYSDLANTAQQSETFQFLADILPKKILASKYLKMLKEEKREEDEENDN
DNESDHDEADS*
>ENSG00000103199|ENST00000219478
MATVPGLQPLPTLEQDLEQEEILIVKVEEDFCLEEEPSVETEDPSPETFRQLFRLFCYQE
VAGPREALSRLWELCCRWLRPELRTKEQILELLVLEQFLTVLPGEIQARVREQQPESGEE
AVVLVEGLQRKPRKHRQRGSELLSDDEVPLGIGGQFLKHQAEAQPEDLSLEEEARFSSQQ
PPAQLSHRPQRGPLLWPERGPPAPRHQEMASASPFLSAWSQVPVNLEDVAVYLSGEEPRC
MDPAQRDAPLENEGPGIQLEDGGDGREDAPLRMEWYRVLSARCQGPGHPLPGQRPAPVRG
LVRPDQPRGGPPPGRRASHGADKPYTCPECGKGFSKTSHLTKHQRTHTGERPYKCLVCGK
GFSDRSNFSTHQRVHTGEKPYPCPECGKRFSQSSSLVIHRRTHSGERPYACTQCGKRFNN
SSHFSAHRRTHTGEKPYTCPACGRGFRRGTDLHKHQRTHMGAGSLPTLQPVAPGGPGAKA
*
>ENSG00000103966|ENST00000220325
MFSWMGRQAGGRERAGGADAVQTVTGGLRSLYLRKVLPLEEAYRFHEFHSPALEDADFEN
KPMILLVGQYSTGKTTFIRYLLEQDFPGMRIGPEPTTDSFIAVMYGETEGSTPGNALVVD
PKKPFRKLSRFGNAFLNRFMCSQLPNQVLKSISVIDSPGILSGEKQRISRGYDFCQVLQW
FAERVDRIILLFDAHKLDISDEFSEAIKAFRGQDDKIRVVLNKADQVDTQQLMRVYGALM
WSLGKVINTPEVLRVYIGSFWAQPLQNTDNRRLFEAEAQDLFRDIQSLPQKAAVRKLNDL
IKRARLAKVHAYIISYLKKEMPSVFGKENKKRELISRLPEIYIQLQREYQISAGDFPEVK
AMQEQLENYDFTKFHSLKPKLIEAVDNMLSNKISPLMNLISQEETSTPTQLVQGGAFDGT
TEGPFNQGYGEGAKEGADEEEWVVAKDKPVYDELFYTLSPINGKISGVNAKKEMVTSKLP
NSVLGKIWKLADCDCDGMLDEEEFALAKHLIKIKLDGYELPSSLPPHLVPPSHRKSLPKA
D*
>ENSG00000105880|ENST00000222598
MTGVFDRRVPSIRSGDFQAPFQTSAAMHHPSQESPTLPESSATDSDYYSPTGGAPHGYCS
PTSASYGKALNPYQYQYHGVNGSAGSYPAKAYADYSYASSYHQYGGAYNRVPSATNQPEK
EVTEPEVRMVNGKPKKVRKPRTIYSSFQLAALQRRFQKTQYLALPERAELAASLGLTQTQ
VKIWFQNKRSKIKKIMKNGEMPPEHSPSSSDPMACNSPQSPAVWEPQGSSRSLSHHPHAH
PPTSNQSPASSYLENSASWYTSAASSINSHLPPPGSLQHPLALASGTLY*
>ENSG00000108840|ENST00000225983
MNSPNESADGMSGREPSLEILPRTSLHSIPVTVEVKPVLPRAMPSSMGGGGGGSPSPVEL
RGALVGSVDPTLREQQLQQELLALKQQQQLQKQLLFAEFQKQHDHLTRQHEVQLQKHLKQ
QQEMLAAKQQQEMLAAKRQQELEQQRQREQQRQEELEKQRLEQQLLILRNKEKSKESAIA
STEVKLRLQEFLLSKSKEPTPGGLNHSLPQHPKCWGAHHASLDQSSPPQSGPPGTPPSYK
LPLPGPYDSRDDFPLRKTASEPNLKVRSRLKQKVAERRSSPLLRRKDGTVISTFKKRAVE
ITGAGPGASSVCNSAPGSGPSSPNSSHSTIAENGFTGSVPNIPTEMLPQHRALPLDSSPN
QFSLYTSPSLPNISLGLQATVTVTNSHLTASPKLSTQQEAERQALQSLRQGGTLTGKFMS
TSSIPGCLLGVALEGDGSPHGHASLLQHVLLLEQARQQSTLIAVPLHGQSPLVTGERVAT
SMRTVGKLPRHRPLSRTQSSPLPQSPQALQQLVMQQQHQQFLEKQKQQQLQLGKILTKTG

ELPRQPTTHPEETEEELTEQQEVLLGEGALTMPREGSTESESTQEDLEEEDEEDDGEEEE
DCIQVKDEEGESGAEEGPDLEEPGAGYKKLFSDAQPLQPLQVYQAPLSLATVPHQALGRT
QSSPAAPGGMKSPPDQPVKHLFTTGVVYDTFMLKHQCMCGNTHVHPEHAGRIQSIWSRLQ
ETGLLSKCERIRGRKATLDEIQTVHSEYHTLLYGTSPLNRQKLDSKKLLGPISQKMYAVL
PCGGIGVDSDTVWNEMHSSSAVRMAVGCLLELAFKVAAGELKNGFAIIRPPGHHAEESTA
MGFCFFNSVAITAKLLQQKLNVGKVLIVDWDIHHGNGTQQAFYNDPSVLYISLHRYDNGN
FFPGSGAPEEVGGGPGVGYNVNVAWTGGVDPPIGDVEYLTAFRTVVMPIAHEFSPDVVLV
SAGFDAVEGHLSPLGGYSVTARCFGHLTRQLMTLAGGRVVLALEGGHDLTAICDASEACV
SALLSVELQPLDEAVLQQKPNINAVATLEKVIEIQSKHWSCVQKFAAGLGRSLREAQAGE
TEEAETVSAMALLSVGAEQAQAAAAREHSPRPAEEPMEQEPAL*
>ENSG00000104907|ENST00000221504
MQGSSLWLSLTFRSARVLSRARFFEWQSPGLPNTAAMENGTGPYGEERPREVQETTVTEG
AAKIAFPSANEVFYNPVQEFNRDLTCAVITEFARIQLGAKGIQIKVPGEKDTQKVVVDLS
EQEEEKVELKESENLASGDQPRTAAVGEICEEGLHVLEGLAASGLRSIRFALEVPGLRSV
VANDASTRAVDLIRRNVQLNDVAHLVQPSQADARMLMYQHQRVSERFDVIDLDPYGSPAT
FLDAAVQAVSEGGLLCVTCTDMAVLAGNSGETCYSKYGAMALKSRACHEMALRIVLHSLD
LRANCYQRFVVPLLSISADFYVRVFVRVFTGQAKVKASARAKFSAACGPPVTPECEHCGQ
RHQLGGPMWAEPIHDLDFVGRVLEAVSANPGRFHTSERIRGVLSVITEELPDVPLYYTLD
QLSSTIHCNTPSLLQLRSALLHADFRVSLSHACKNAVKTDAPASALWDIMRCWEKECPVK
RERLSETSPAFRILSVEPRLQANFTIREDANPSSRQRGLKRFQANPEANWGPRPRARPGG
KAADEAMEERRRLLQNKRKEPPEDVAQRAARLKTFPCKRFKEGTCQRGDQCCYSHSPPTP
RVSADAAPDCPETSNQTPPGPGAAAGPGID*
>ENSG00000104517|ENST00000220959
MTSIHFVVHPLPGTEDQLNDRLREVSEKLNKYNLNSHPPLNVLEQATIKQCVVGPNHAAF
LLEDGRVCRIGFSVQPDRLELGKPDNNDGSKLNSNSGAGRTSRPGRTSDSPWFLSGSETL
GRLAGNTLGSRWSSGVGGSGGGSSGRSSAGARDSRRQTRVIRTGRDRGSGLLGSQPQPVI
PASVIPEELISQAQVVLQGKSRSVIIRELQRTNLDVNLAVNNLLSRDDEDGDDGDDTASE
SYLPGEDLMSLLDADIHSAHPSVIIDADAMFSEDISYFGYPSFRRSSLSRLGSSRVLLLP
LERDSELLRERESVLRLRERRWLDGASFDNERGSTSKEGEPNLDKKNTPVQSPVSLGEDL
QWWPDKDGTKFICIGALYSELLAVSSKGELYQWKWSESEPYRNAQNPSLHHPRATFLGLT
NEKIVLLSANSIRATVATENNKVATWVDETLSSVASKLEHTAQTYSELQGERIVSLHCCA
LYTCAQLENSLYWWGVVPFSQRKKMLEKARAKNKKPKSSAGISSMPNITVGTQVCLRNNP
LYHAGAVAFSISAGIPKVGVLMESVWNMNDSCRFQLRSPESLKNMEKASKTTEAKPESKQ
EPVKTEMGPPPSPASTCSDASSIASSASMPYKRRRSTPAPKEEEKVNEEQWSLREVVFVE
DVKNVPVGKVLKVDGAYVAVKFPGTSSNTNCQNSSGPDADPSSLLQDCRLLRIDELQVVK
TGGTPKVPDCFQRTPKKLCIPEKTEILAVNVDSKGVHAVLKTGNWVRYCIFDLATGKAEQ
ENNFPTSSIAFLGQNERNVAIFTAGQESPIILRDGNGTIYPMAKDCMGGIRDPDWLDLPP
ISSLGMGVHSLINLPANSTIKKKAAVIIMAVEKQTLMQHILRCDYEACRQYLMNLEQAVV
LEQNLQMLQTFISHRCDGNRNILHACVSVCFPTSNKETKEEEEAERSERNTFAERLSAVE
AIANAISVVSSNGPGNRAGSSSSRSLRLREMMRRSLRAAGLGRHEAGASSSDHQDPVSPP
IAPPSWVPDPPAMDPDGDIDFILAPAVGSLTTAATGTGQGPSTSTIPGPSTEPSVVESKD
RKANAHFILKLLCDSVVLQPYLRELLSAKDARGMTPFMSAVSGRAYPAAITILETAQKIA
KAEISSSEKEEDVFMGMVCPSGTNPDDSPLYVLCCNDTCSFTWTGAEHINQDIFECRTCG
LLESLCCCTECARVCHKGHDCKLKRTSPTAYCDCWEKCKCKTLIAGQKSARLDLLYRLLT

ATNLVTLPNSRGEHLLLFLVQTVARQTVEHCQYRPPRIREDRNRKTASPEDSDMPDHDLE
PPRFAQLALERVLQDWNALKSMIMFGSQENKDPLSASSRIGHLLPEEQVYLNQQSGTIRL
DCFTHCLIVKCTADILLLDTLLGTLVKELQNKYTPGRREEAIAVTMRFLRSVARVFVILS
VEMASSKKKNNFIPQPIGKCKRVFQALLPYAVEELCNVAESLIVPVRMGIARPTAPFTLA
STSIDAMQGSEELFSVEPLPPRPSSDQSSSSSQSQSSYIIRNPQQRRISQSQPVRGRDEE
QDDIVSADVEEVEVVEGVAGEEDHHDEQEEHGEENAEAEGQHDEHDEDGSDMELDLLAAA
ETESDSESNHSNQDNASGRRSVVTAATAGSEAGASSVPAFFSEDDSQSNDSSDSDSSSSQ
SDDIEQETFMLDEPLERTTNSSHANGAAQAPRSMQWAVRNTQHQRAASTAPSSTSTPAAS
SAGLIYIDPSNLRRSGTISTSAAAAAAALEASNASSYLTSASSLARAYSIVIRQISDLMG
LIPKYNHLVYSQIPAAVKLTYQDAVNLQNYVEEKLIPTWNWMVSIMDSTEAQLRYGSALA
SAGDPGHPNHPLHASQNSARRERMTAREEASLRTLEGRRRATLLSARQGMMSARGDFLNY
ALSLMRSHNDEHSDVLPVLDVCSLKHVAYVFQALIYWIKAMNQQTTLDTPQLERKRTREL
LELGIDNEDSEHENDDDTNQSATLNDKDDDSLPAETGQNHPFFRRSDSMTFLGCIPPNPF
EVPLAEAIPLADQPHLLQPNARKEDLFGRPSQGLYSSSASSGKCLMEVTVDRNCLEVLPT
KMSYAANLKNVMNMQNRQKKEGEEQPVLPEETESSKPGPSAHDLAAQLKSSLLAEIGLTE
SEGPPLTSFRPQCSFMGMVISHDMLLGRWRLSLELFGRVFMEDVGAEPGSILTELGGFEV
KESKFRREMEKLRNQQSRDLSLEVDRDRDLLIQQTMRQLNNHFGRRCATTPMAVHRVKVT
FKDEPGEGSGVARSFYTAIAQAFLSNEKLPNLECIQNANKGTHTSLMQRLRNRGERDRER
EREREMRRSSGLRAGSRRDRDRDFRRQLSIDTRPFRPASEGNPSDDPEPLPAHRQALGER
LYPRVQAMQPAFASKITGMLLELSPAQLLLLLASEDSLRARVDEAMELIIAHGRENGADS
ILDLGLVDSSEKVQENRKRHGSSRSVVDMDLDDTDDGDDNAPLFYQPGKRGFYTPRPGKN
TEARLNCFRNIGRILGLCLLQNELCPITLNRHVIKVLLGRKVNWHDFAFFDPVMYESLRQ
LILASQSSDADAVFSAMDLAFAIDLCKEEGGGQVELIPNGVNIPVTPQNVYEYVRKYAEH
RMLVVAEQPLHAMRKGLLDVLPKNSLEDLTAEDFRLLVNGCGEVNVQMLISFTSFNDESG
ENAEKLLQFKRWFWSIVEKMSMTERQDLVYFWTSSPSLPASEEGFQPMPSITIRPPDDQH
LPTANTCISRLYVPLYSSKQILKQKLLLAIKTKNFGFV*
>ENSG00000229809|ENST00000223459
MAPPPAPLLAPRPGETRPGCRKPGTVSFADVAVYFSPEEWGCLRPAQRALYRDVMQETYG
HLGALGFPGPKPALISWMEQESEAWSPAAQDPEKGERLGGARRGDVPNRKEEEPEEVPRA
KGPRKAPVKESPEVLVERNPDPAISVAPARAQPPKNAAWDPTTGAQPPAPIPSMDAQAGQ
RRHVCTDCGRRFTYPSLLVSHRRMHSGERPFPCPECGMRFKRKFAVEAHQWIHRSCSGGR
RGRRPGIRAVPRAPVRGDRDPPVLFRHYPDIFEECG*
>ENSG00000104835|ENST00000221431
MAASMARRLWPLLTRRGFRPRGGCISNDSPRRSFTTEKRNRNLLYEYAREGYSALPQLDI
ERFCACPEEAAHALELRKGELRSADLPAIISTWQELRQLQEQIRSLEEEKAAVTEAVRAL
LANQDSGEVQQDPKYQGLRARGREIRKELVHLYPREAQLEEQFYLQALKLPNQTHPDVPV
GDESQARVLHMVGDKPVFSFQPRGHLEIGEKLDIIRQKRLSHVSGHRSYYLRGAGALLQH
GLVNFTFNKLLRRGFTPMTVPDLLRGAVFEGCGMTPNANPSQIYNIDPARFKDLNLAGTA
EVGLAGYFMDHTVAFRDLPVRMVCSSTCYRAETNTGGQEPRGLYRVHHFTKVEMFGVTGPG
LEQSSQLLEEFLSLQMEILTELGLHFRVLDMPTQELGLPAYRKFDIEAWMPGRGRFGEVT
SASNCTDFQSRRLHIMFQTEAGELQFAHTVNATACAVPRLLIALLESNQQKDGSVLVPPA
LQSYLGTDRITAPTHVPLQYIGPNQPRKPGLPGQPAVS*
>ENSG00000213246|ENST00000225504
MALETVPKDLRHLRACLLCSLVKTIDQFEYDGCDNCDAYLQMKGNREMVYDCTSSSFDGI

IAMMSPEDSWVSKWQRVSNFKPGVYAVSVTGRLPQGIVRELKSRGVAYKSRDTAIKT*
>ENSG00000013503|ENST00000228347
MDVLAEEFGNLTPEQLAAPIPTVEEKWRLLPAFLKVKGLVKQHIDSFNYFINVEIKKIMK
ANEKVTSDADPMWYLKYLNIYVGLPDVEESFNVTRPVSPHECRLRDMTYSAPITVDIEYT
RGSQRIIRNALPIGRMPIMLRSSNCVLTGKTPAEFAKLNECPLDPGGYFIVKGVEKVILI
QEQLSKNRIIVEADRKGAVGASVTSSTHEKKSRTNMAVKQGRFYLRHNTLSEDIPIVIIF
KAMGVESDQEIVQMIGTEEHVMAAFGPSLEECQKAQIFTQMQALKYIGNKVRRQRMWGGG
PKKTKIEEARELLASTILTHVPVKEFNFRAKCIYTAVMVRRVILAQGDNKVDDRDYYGNK
RLELAGQLLSLLFEDLFKKFNSEMKKIADQVIPKQRAAQFDVVKHMRQDQITNGMVNAIS
TGNWSLKRFKMDRQGVTQVLSRLSYISALGMMTRISSQFEKTRKVSGPRSLQPSQWGMLC
PSDTPEGEACGLVKNLALMTHITTDMEDGPIVKLASNLGVEDVNLLCGEELSYPNVFLVF
LNGNILGVIRDHKKLVNTFRLMRRAGYINEFVSISTNLTDRCVYISSDGGRLCRPYIIVK
KQKPAVTNKHMEELAQGYRNFEDFLHESLVEYLDVNEENDCNIALYEHTINKDTTHLEIE
PFTLLGVCAGLIPYPHHNQSPRNTYQCAMGKQAMGTIGYNQRNRIDTLMYLLAYPQKPMV
KTKTIELIEFEKLPAGQNATVAVMSYSGYDIEDALVLNKASLDRGFGRCLVYKNAKCTLK
RYTNQTFDKVMGPMLDAATRKPIWRHEILDADGICSPGEKVENKQVLVNKSMPTVTQIPL
EGSNVPQQPQYKDVPITYKGATDSYIEKVMISSNAEDAFLIKMLLRQTRRPEIGDKFSSR
HGQKGVCGLIVPQEDMPFCDSGICPDIIMNPHGFPSRMTVGKLIELLAGKAGVLDGRFHY
GTAFGGSKVKDVCEDLVRHGYNYLGKDYVTSGITGEPLEAYIYFGPVYYQKLKHMVLDKM
HARARGPRAVLTRQPTEGRSRDGGLRLGEMERDCLIGYGASMLLLERLMISSDAFEVDVC
GQCGLLGYSGWCHYCKSSCHVSSLRIPYACKLLFQELQSMNIIPRLKLSKYNE*
>ENSG00000108298|ENST00000225430
MSMLRLQKRLASSVLRCGKKKVWLDPNETNEIANANSRQQIRKLIKDGLIIRKPVTVHSR
ARCRKNTLARRKGRHMGIGKRKGTANARMPEKVTWMRRMRILRRLLRRYRESKKIDRHMY
HSLYLKVKGNVFKNKRILMEHIHKLKADKARKKLLADQAEARRSKTKEARKRREERLQAK
KEEIIKTLSKEEETKK*
>ENSG00000102241|ENST00000218364
MSGTNLDGNDEFDEQLRMQELYGDGKDGDTQTDAGGEPDSLGQQPTDTPYEWDLDKKAWF
PKITEDFIATYQANYGFSNDGASSSTANVEDVHARTAEEPPQEKAPEPTDARKKGEKRKA
ESGWFHVEEDRNTNVYVSGLPPDITVDEFIQLMSKFGIIMRDPQTEEFKVKLYKDNQGNL
KGDGLCCYLKRESVELALKLLDEDEIRGYKLHVEVAKFQLKGEYDASKKKKKCKDYKKKL
SMQQKQLDWRPERRAGPSRMRHERVVIIKNMFHPMDFEDDPLVLNEIREDLRVECSKFGQ
IRKLLLFDRHPDGVASVSFRDPEEADYCIQTLDGRWFGGRQITAQAWDGTTDYQVEETSR
EREERLRGWEAFLNAPEANRGLRRSDSVSASERAGPSRARHFSEHPSTSKMNAQETATGM
AFEEPIDEKKFEKTEDGGEFEEGASENNAKESSPEKEAEEGCPEKESEEGCPKRGFEGSC
SQKESEEGNPVRGSEEDSPKKESKKKTLKNDCEENGLAKESEDDLNKESEEEVGPTKESE
EDDSEKESDEDCSEKQSEDGSEREFEENGLEKDLDEEGSEKELHENVLDKELEENDSENS
EFEDDGSEKVLDEEGSEREFDEDSDEKEEEEDTYEKVFDDESDEKEDEEYADEKGLEAAD
KKAEEGDADEKLFEESDDKEDEDADGKEVEDADEKLFEDDDSNEKLFDEEEDSSEKLFDD
SDERGTLGGFGSVEEGPLSTGSSFILSSDDDDDDI*
>ENSG00000105136|ENST00000221735
MAAAALRDPAQVPVAADLLTDHEEGYVTFEDVAVYFSQEEWRLLDDAQRLLYRNVMLENF
TLLASLGLASSKTHEITQLESWEEPFMPAWEVVTSAIPRGCWHGAEAEEAPEQIASVGLL
SSNIQQHQKQHCGEKPLKRQEGRVPVLRSCKVHLSEKSLQSREVGKALLISSGVLKHQVT

HTGEKSHRSSKSREAFHAGKRHYKCSECGKAFGQKYLLVQHQRLHAGKKTYECSECGKLF
RDMSNLFIHQIVHTGERPYGCSNCGKSFSRNAHLIEHQRVHTGEKPFTCSECGKAFRHNS
TLVQHHKIHTGVRPYECSECGKLFSFNSSLMKHQRIHTGERPYKCSECGKFYSHKSNLIK
HWRVHTGERPYKCSDCGKFFTQCSSLMQHQKVHTGEKPFKCNECGRFFRENSTLVRHQRV
HTGAKPYECRECGKFFSQSSTLMQHRKVHIGEKPFKCNECGRLFRENSSLVKHQRVHTGA
KPYECRECGKFFRHNSSLFKHRRIHTGEMQ*
>ENSG00000105968|ENST00000222690
MAGGKAGKDSGKAKAKAVSRSQRAGLQFPVGRIHRHLKTRTTSHGRVGATAAVYSAAILE
YLTAEVLELAGNASKDLKVKRITPRHLQLAIRGDEELDSLIKATIAGGEKRRCS*
>ENSG00000110104|ENST00000227520
MDTPLRRSRRLGGLRPESPESLTSVSRTRRALVEFESNPEETREPGSPPSVQRAGLGSPE
RPPKTSPGSPRLQQGAGLESPQGQPEPGAASPQRQQDLHLESPQRQPEYSPESPRCQPKP
SEEAPKCSQDQGVLASELAQNKEELTPGAPQHQLPPVPGSPEPYPGQQAPGPEPSQPLLE
LTPRAPGSPRGQHEPSKPPPAGETVTGGFGAKKRKGSSSQAPASKKLNKEELPVIPKGKP
KSGRVWKDRSKKRFSQMLQDKPLRTSWQRKMKERQERKLAKDFARHLEEEKERRRQEKKQ
RRAENLKRRLENERKAEVVQVIRNPAKLKRAKKKQLRSIEKRDTLALLQKQPPQQPAAKI
*
>ENSG00000105856|ENST00000222574
MVWEVKTNQMPNAVQKLLLVMDKRASGMNDSLELLQCNENLPSSPGYNSCDEHMELDDLP
ELQAVQSDPTQSGMYQLSSDVSHQEYPRSSWNQNTSDIPETTYRENEVDWLTELANIATS
PQSPLMQCSFYNRSSPVHIIATSKSLHSYARPPPVSSSSKSEPAFPHHHWKEETPVRHER
ANSESESGIFCMSSLSDDDDLGWCNSWPSTVWHCFLKGTRLCFHKGSNKEWQDVEDFARA
EGCDNEEDLQMGIHKGYGSDGLKLLSHEESVSFGESVLKLTFDPGTVEDGLLTVECKLDH
PFYVKNKGWSSFYPSLTVVQHGIPCCEVHIGDVCLPPGHPDAINFDDSGVFDTFKSYDFT
PMDSSAVYVLSSMARQRRASLSCGGPGGQDFARSGFSKNCGSPGSSQLSSNSLYAKAVKN
HSSGTVSATSPNKCKRPMNAFMLFAKKYRVEYTQMYPGKDNRAISVILGDRWKKMKNEER
RMYTLEAKALAEEQKRLNPDCWKRKRTNSGSQQH*
>ENSG00000105298|ENST00000221899
MGRDTRSRSRSAGRRGRRRQSQSGSRSRSRSHGRRNRRRREDEGRRRRRRRSRERRSDSE
EERWQRSGMRSRSPPRPKWHSRDGSSQSDSGEEQSRGQWARRRRRARSWSPSSSASSSAS
PGRSQSPRAAAAALSQQQSLQERLRLREERKQQEELMKAFETPEEKRARRLAKKEAKERK
KREKMGWGEEYMGYTNTDNPFGDNNLLGTFIWNKALEKKGISHLEEKELKERNKRIQEDN
RLELQKVKQLRLEREREKAMREQELEMLQREKEAEHFKTWEEQEDNFHLQQAKLRSKIRI
RDGRAKPIDLLAKYISAEDDDLAVEMHEPYTFLNGLTVADMEDLLEDIQVYMELEQGKNA
DFWRDMTTITEDEISKLRKLEASGKGPGERREGVNASVSSDVQSVFKGKTYNQLQVIFQG
IEGKIRAGGPNLDMGYWESLLQQLRAHMARARLRERHQDVLRQKLYKLKQEQGVESEPLF
PILKQEPQSPSRSLEPEDAAPTPPGPSSEGGPAEAEVDGATPTEGDGDGDGEGEGEGEAV
LMEEDLIQQSLDDYDAGRYSPRLLTAHELPLDAHVLEPDEDLQRLQLSRQQLQVTGDASE
SAEDIFFRRAKEGMGQDEAQFSVEMPLTGKAYLWADKYRPRKPRFFNRVHTGFEWNKYNQ
THYDFDNPPPKIVQGYKFNIFYPDLIDKRSTPEYFLEACADNKDFAILRFHAGPPYEDIA
FKIVNREWEYSHRHGFRCQFANGIFQLWFHFKRYRYRR*
>ENSG00000090612|ENST00000228289
MATRVRTASIWVPPLQERNSSWDRIRKLQGGQESILGQGTPGLQPLPGTPRQKQKSRRIEK
VLEWLFISQEQPKITKSWGPLSFMDVFVDFTWEEWQLLDPAQKCLYRSVMLENYSNLVSL

GYQHTKPDIIFKLEQGEELCMVQAQVPNQTCPNTVWKIDDLMDWHQENKDKLGSTAKSFE
CTTFGKLCLLSTKYLSRQKPHKCGTHGKSLKYIDFTSDYARNNPNGFQVHGKSFFHSKHE
QTVIGIKYCESIESGKTVNKKSQLMCQQMYMGEKPFGCSCCEKAFSSKSYLLVHQQTHAE
EKPYGCNECGKDFSSKSYLIVHQRIHTGEKLHECSECRKTFSFHSQLVIHQRIHTGENPY
ECCECGKVFSRKDQLVSHQKTHSGQKPYVCNECGKAFGLKSQLIIHERIHTGEKPYECNE
CQKAFNTKSNLMVHQRTHTGEKPYVCSDCGKAFTFKSQLIVHQGIHTGVKPYGCIQCGKG
FSLKSQLIVHQRSHTGMKPYVCNECGKAFRSKSYLIIHTRTHTGEKLHECNNCGKAFSFK
SQLIIHQRIHTGENPYECHECGKAFSRKYQLISHQRTHAGEKPYECTDCGKAFGLKSQLI
IHQRTHTGEKPFECSECQKAFNTKSNLIVHQRTHTGEKPYSCNECGKAFTFKSQLIVHKG
VHTGVKPYGCSQCAKTFSLKSQLIVHQRSHTGVKPYGCSECGKAFRSKSYLIIHMRTHTG
EKPHECRECGKSFSFNSQLIVHQRIHTGENPYECSECGKAFNRKDQLISHQRTHAGEKPY
GCSECGKAFSSKSYLIIHMRTHSGEKPYECNECGKAFIWKSLLIVHERTHAGVNPYKCSQ
CEKSFSGKLRLLVHQRMHTREKPYECSECGKAFIRNSQLIVHQRTHSGEKPYGCNECGKT
FSQKSILSAHQRTHTGEKPCKCTECGKAFCWKSQLIMHQRTHVDDKH*
>ENSG00000102978|ENST00000219252
MPYANQPTVRITELTDENVKFIIENTDLAVANSIRRVFIAEVPIIAIDWVQIDANSSVLH
DEFIAHRLGLIPLISDDIVDKLQYSRDCTCEEFCPECSVEFTLDVRCNEDQTRHVTSRDL
ISNSPRVIPVTSRNRDNDPNDYVEQDDILIVKLRKGQELRLRAYAKKGFGKEHAKWNPTA
GVAFEYDPDNALRHTVYPKPEEWPKSEYSELDEDESQAPYDPNGKPERFYYNVESCGSLR
PETIVLSALSGLKKKLSDLQTQLSHEIQSDVLTIN*
>ENSG00000128000|ENST00000221355
MPAYTHASPIHNTHKPYECKECGKYFSCGSNLIQHQSIHTGEKPYKCKECGKAFQLHIQL
TRHQKFHTGEKTFECKECGKAFNLPTQLNRHKNIHTVKKLFECKECGKSFNRSSNLTQHQ
SIHAGVKPYQCKECGKAFNRGSNLIQHQKIHSNEKPFVCRECEMAFRYHYQLIEHCRIHT
GEKPFECKECRKAFTLLTKLVRHQKIHMGEKPFECRECGKAFSLLNQLNRHKNIHTGEKP
FECKECGKSFNRSSNLIQHQSIHADVKPYECKECGKGFNRGANLIQHQKIHSNEKPFVCR
ECEMAFRYHYQLIQHCQIHTGGKPFECKECGKAFSLLTQLARHKNIHTGEKPFECKDCGK
AFNRGSNLVQHQSIHTGEKPYECKECGKAFRLHLQLSQHEKTHTGEKPFECKECGKFFRR
GSNLNQHRSIHTGKKPFECKECGKAFRLHMHLIRHQKFHTGEKPFECKECGKAFSLHTQL
NHHKNIHTGEKPFKCKECGKSFNRVSNLVQHQSIHAGVKPYECKECGKGFSRVSNLIQHQ
KTHSSAKPFVCKECRKTFRYHYQLTEHYRIHTGEKPFECKECGKAFGLLTQLAQHQIIHT
GEKPFKCKECGKAFNRGSNLVQPQSIHTGEKPYECKECGKAFRLHLQLSLHQKLVQVRNP
LNVRNVGQPSDISSNLLNIRKFILG*
>ENSG00000110851|ENST00000228437
MHHRMNEMNLSPVGMEQLTSSSVSNALPVSGSHLGLAASPTHSAIPAPGLPVAIPNLGPS
LSSLPSALSLMLPMGIGDRGVMCGLPERNYTLPPPPYPHLESSYFRTILPGILSYLADRP
PPQYIHPNSINVDGNTALSITNNPSALDPYQSNGNVGLEPGIVSIDSRSVNTHGAQSLHP
SDGHEVALDTAITMENVSRVTSPISTDGMAEELTMDGVAGEHSQIPNGSRSHEPLSVDSV
SNNLAADAVGHGGVIPMHGNGLELPVVMETDHIASRVNGMSDSALSDSIHTVAMSTNSVS
VALSTSHNLASLESVSLHEVGLSLEPVAVSSITQEVAMGTGHVDVSSDSLSFVSPSLQME
DSNSNKENMATLFTIWCTLCDRAYPSDCPEHGPVTFVPDTPIESRARLSLPKQLVLRQSI
VGAEVGVWTGETIPVRTCFGPLIGQQSHSMEVAEWTDKAVNHIWKIYHNGVLEFCIITTD
ENECNWMMFVRKARNREEQNLVAYPHDGKIFFCTSQDIPPENELLFYYSRDYAQQIGVPE
HPDVHLCNCGKECNSYTEFKAHLTSHIHNHLPTQGHSGSHGPSHSKERKWKCSMCPQAFI

SPSKLHVHFMGHMGMKPHKCDFCSKAFSDPSNLRTHLKIHTGQKNYRCTLCDKSFTQKAH
LESHMVIHTGEKNLKCDYCDKLFMRRQDLKQHVLIHTQERQIKCPKCDKLFLRTNHLKKH
LNSHEGKRDYVCEKCTKAYLTKYHLTRHLKTCKGPTSSSSAPEEEEEEDDSEEEDLADSVG
TEDCRINSAVYSADESLSAHK*
>ENSG00000104889|ENST00000221486
MDLSELERDNTGRCRLSSPVPAVCRKEPCVLGVDEAGRGPVLGPMVYAICYCPLPRLADL
EALKVADSKTLLESERERLFAKMEDTDFVGWALDVLSPNLISTSMLGRVKYNLNSLSHDT
ATGLIQYALDQGVNVTQVFVDTVGMPETYQARLQQSFPGIEVTVKAKADALYPVVSAASI
CAKVARDQAVKKWQFVEKLQDLDTDYGSGYPNDPKTKAWLKEHVEPVFGFPQFVRFSWRT
AQTILEKEAEDVIWEDSASENQEGLRKITSYFLNEGSQARPRSSHRYFLERGLESATSL*
>ENSG00000168066|ENST00000227503
MATGANATPLDFPSKKRKRSRWNQDTMEQKTVIPGMPTVIPPGLTREQERAYIVQLQIED
LTRKLRTGDLGIPPNPEDRSPSPEPIYNSEGKRLNTREFRTRKKLEEERHNLITEMVALN
PDFKPPADYKPPATRVSDKVMIPQDEYPEINFVGLLIGPRGNTLKNIEKECNAKIMIRGK
GSVKEGKVGRKDGQMLPGEDEPLHALVTANTMENVKKAVEQIRNILKQGIETPEDQNDLR
KMQLRELARLNGTLREDDNRILRPWQSSETRSITNTTVCTKCGGAGHIASDCKFQRPGDP
QSAQDKARMDKEYLSLMAELGEAPVPASVGSTSGPATTPLASAPRPAAPANNPPPPSLMS
TTQSRPPWMNSGPSESRPYHGMHGGGPGGPGGGPHSFPHPLPSLTGGHGGHPMQHNPNGP
PPPWMQPPPPPMNQGPHPPGHHGPPPMDQYLGSTPVGSGVYRLHQGKGMMPPPPMGMMPP
PPPPPSGQPPPPPSGPLPPWQQQQQQPPPPPPPSSSMASSTPLPWQQRSLPAAAMARAMR
VRTFRAHW*
>ENSG00000106031|ENST00000222753
MTASVLLHPRWIEPTVMFLYDNGGGLVADELNKNMEGAAAAAAAAAAAAAAGAGGGGFPH
PAAAAAGGNFSVAAAAAAAAAAAANQCRNLMAHPAPLAPGAASAYSSAPGEAPPSAAAAA
AAAAAAAAAAAAASSSGGPGPAGPAGAEAAKQCSPCSAAAQSSSGPAALPYGYFGSGYYP
CARMGPHPNAIKSCAQPASAAAAAAFADKYMDTAGPAAEEFSSRAKEFAFYHQGYAAGPY
HHHQPMPGYLDMPVVPGLGGPGESRHEPLGLPMESYQPWALPNGWNGQMYCPKEQAQPPH
LWKSTLPDVVSHPSDASSYRRGRKKRVPYTKVQLKELEREYATNKFITKDKRRRISATTN
LSERQVTIWFQNRRVKEKKVINKLKTTS*
>ENSG00000075856|ENST00000228284
MATAAETSASEPEAESKAGPKADGEEDEVKAARTRRKVLSRAVAAATYKTMGPAWDQQEE
GVSESDGDEYAMASSAESSPGEYEWEYDEEEEKNQLEIERLEEQLSINVYDYNCHVDLIR
LLRLEGELTKVRMARQKMSEIFPLTEELWLEWLHDEISMAQDGLDREHVYDLFEKAVKDY
ICPNIWLEYGQYSVGGIGQKGGLEKVRSVFERALSSVGLHMTKGLALWEAYREFESAIVE
AARLEKVHSLFRRQLAIPLYDMEATFAEYEEWSEDPIPESVIQNYNKALQQLEKYKPYEE
ALLQAEAPRLAEYQAYIDFEMKIGDPARIQLIFERALVENCLVPDLWIRYSQYLDRQLKV
KDLVLSVHNRAIRNCPWTVALWSRYLLAMERHGVDHQVISVTFEKALNAGFIQATDYVEI
WQAYLDYLRRRVDFKQDSSKELEELRAAFTRALEYLKQEVEERFNESGDPSCVIMQNWAR
IEARLCNNMQKARELWDSIMTRGNAKYANMWLEYYNLERAHGDTQHCRKALHRAVQCTSD
YPEHVCEVLLTMERTEGSLEDWDIAVQKTETRLARVNEQRMKAAEKEAALVQQEEEKAEQ
RKRARAEKKALKKKKKIRGPEKRGADEDDEKEWGDDEEEQPSKRRRVENSIPAAGETQNV
EVAAGPAGKCAAVDVEPPSKQKEKAASLKRDMPKVLHDSSKDSITVFVSNLPYSMQEPDT
KLRPLFEACGEVVQIRPIFSNRGDFRGYCYVEFKEEKSALQALEMDRKSVEGRPMFVSPC
VDKSKNPDFKVFRYSTSLEKHKLFISGLPFSCTKEELEEICKAHGTVKDLRLVTNRAGKP

KGLAYVEYENESQASQAVMKMDGMTIKENIIKVAISNPPQRKVPEKPETRKAPGGPMLLP
QTYGARGKGRTQLSLLPRALQRPSAAAPQAENGPAAAPAVAAPAATEAPKMSNADFAKLF
LRK*
>ENSG00000111046|ENST00000228641
MMMDLFETGSYFFYLDGENVTLQPLEVAEGSPLYPGSDGTLSPCQDQMPPEAGSDSSGEE
HVLAPPGLQPPHCPGQCLIWACKTCKRKSAPTDRRKAATLRERRRLKKINEAFEALKRRT
VANPNQRLPKVEILRSAISYIERLQDLLHRLDQQEKMQELGVDPFSYRPKQENLEGADFL
RTCSSQWPSVSDHSRGLVITAKEGGASIDSSASSSLRCLSSIVDSISSEERKLPCVEEVV
EK*
>ENSG00000105722|ENST00000222329
MKTPADTGFAFPDWAYKPESSPGSRQIQLWHFILELLRKEEYQGVIAWQGDYGEFVIKDP
DEVARLWGVRKCKPQMNYDKLSRALRYYYNKRILHKTKGKRFTYKFNFNKLVLVNYPFID
VGLAGGAVPQSAPPVPSGGSHFRFPPSTPSEVLSPTEDPRSPPACSSSSSSLFSAVVARR
LGRGSVSDCSDGTSELEEPLGEDPRARPPGPPDLGAFRGPPLARLPHDPGVFRVYPRPRG
GPEPLSPFPVSPLAGPGSLLPPQLSPALPMTPTHLAYTPSPTLSPMYPSGGGGPSGSGGG
SHFSFSPEDMKRYLQAHTQSVYNYHLSPRAFLHYPGLVVPQPQRPDKCPLPPMAPETPPV
PSSASSSSSSSSSPFKFKLQPPPLGRRQRAAGEKAVAGADKSGGSAGGLAEGAGALAPPP
PPPQIKVEPISEGESEEVEVTDISDEDEEDGEVFKTPRAPPAPPKPEPGEAPGASQCMPL
KLRFKRRWSEDCRLEGGGGPAGGFEDEGEDKKVRGEGPGEAGGPLTPRRVSSDLQHATAQ
LSLEHRDS*
>ENSG00000104408|ENST00000220849
MAEYDLTTRIAHFLDRHLVFPLLEFLSVKEIYNEKELLQGKLDLLSDTNMVDFAMDVYKN
LYSDDIPHALREKRTTVVAQLKQLQAETEPIVKMFEDPETTRQMQSTRDGRMLFDYLADK
HGFRQEYLDTLYRYAKFQYECGNYSGAAEYLYFFRVLVPATDRNALSSLWGKLASEILMQ
NWDAAMEDLTRLKETIDNNSVSSPLQSLQQRTWLIHWSLFVFFNHPKGRDNIIDLFLYQP
QYLNAIQTMCPHILRYLTTAVITNKDVRKRRQVLKDLVKVIQQESYTYKDPITEFVECLY
VNFDFDGAQKKLRECESVLVNDFFLVACLEDFIENARLFIFETFCRIHQCISINMLADKL
NMTPEEAERWIVNLIRNARLDAKIDSKLGHVVMGNNAVSPYQQVIEKTKSLSFRSQMLAM
NIEKKLNQNSRSEAPNWATQDSGFY*
>ENSG00000104852|ENST00000221448
MTQFLPPNLLALFAPRDPIPYLPPLEKLPHEKHHNQPYCGIAPYIREFEDPRDAPPPTRA
ETREERMERKRREKIERRQQEVETELKMWDPHNDPNAQGDAFKTLFVARVNYDTTESKLR
REFEVYGPIKRIHMVYSKRSGKPRGYAFIEYEHERDMHSAYKHADGKKIDGRRVLVDVER
GRTVKGWRPRRLGGGLGGTRRGGADVNIRHSGRDDTSRYDERDRDRDRERERRERSRERD
KERERRRSRSRDRRRRSRSRDKEERRRSRERSKDKDRDRKRRSSRSRERARRERERKEEL
RGGGGDMAEPSEAGDAPPDDGPPGELGPDGPDGPEEKGRDRDRERRRSHRSERERRRDRD
RDRDRDREHKRGERGSERGRDEARGGGGGQDNGLEGLGNDSRDMYMESEGGDGYLAPENG
YLMEAAPE*
>ENSG00000108518|ENST00000225655
MAGWNAYIDNLMADGTCQDAAIVGYKDSPSVWAAVPGKTFVNITPAEVGVLVGKDRSSFY
VNGLTLGGQKCSVIRDSLLQDGEFSMDLRTKSTGGAPTFNVTVTKTDKTLVLLMGKEGVH
GGLINKKCYEMASHLRRSQY*
>ENSG00000065057|ENST00000219066
MCSPQESGMTALSARMLTRSRSLGPGAGPRGCREEPGPLRRREAAAEARKSHSPVKRPRK

AQRLRVAYEGSDSEKGEGAEPLKVPVWEPQDWQQQLVNIRAMRNKKDAPVDHLGTEHCYD
SSAPPKVRRYQVLLSLMLSSQTKDQVTAGAMQRLRARGLTVDSILQTDDATLGKLIYPVG
FWRSKVKYIKQTSAILQQHYGGDIPASVAELVALPGVGPKMAHLAMAVAWGTVSGIAVDT
HVHRIANRLRWTKKATKSPEETRAALEEWLPRELWHEINGLLVGFGQQTCLPVHPRCHAC
LNQALCPAAQGL*
>ENSG00000104897|ENST00000221494
MDFQHRPGGKTGSGGVASSSESNRDRRERLRQLALETIDINKDPYFMKNHLGSYECKLCL
TLHNNEGSYLAHTQGKKHQTNLARRAAKEAKEAPAQPAPEKVKVEVKKFVKIGRPGYKVT
KQRDSEMGQQSLLFQIDYPEIAEGIMPRHRFMSAYEQRIEPPDRRWQYLLMAAEPYETIA
FKVPSREIDKAEGKFWTHWNRETKQFFLQFHFKMEKPPAPPSLPAGPPGVKRPPPPLMNG
LPPRPPLPESLPPPPPGGLPLPPMPPTGPAPSGPPGPPQLPPPAPGVHPPAPVVHPPASG
VHPPAPGVHPPAPGVHPPAPGVHPPTSGVHPPAPGVHPPAPGVHPPAPGVHPPAPGVHPP
APGVHPPPSAGVHPQAPGVHPAAPAVHPQAPGVHPPAPGMHPQAPGVHPQPPGVHPSAPG
VHPQPPGVHPSNPGVHPPTPMPPMLRPPLPSEGPGNIPPPPPTN*
>ENSG00000104064|ENST00000220429
MSLVDLGKKLLEAARAGQDDEVRILMANGAPFTTDWLGTSPLHLAAQYGHYSTTEVLLRA
GVSRDARTKVDRTPLHMAASEGHASIVEVLLKHGADVNAKDMLKMTALHWATEHNHQEVV
ELLIKYGADVHTQSKFCKTAFDISIDNGNEDLAEILQIAMQNQINTNPESPDTVTIHAAT
PQFIIGPGGVVNLTGLVSSENSSKATDETGVSAVQFGNSSTSVLATLAALAEASAPLSNS
SETPVVATEEVVTAESVDGAIQQVVSSGGQQVITIVTDGIQLGNLHSIPTSGIGQPIIVT
MPDGQQVLTVPATDIAEETVISEEPPAKRQCIEIIENRVESAEIEEREALQKQLDEANRE
AQKYRQQLLKKEQEAEAYRQKLEAMTRLQTNKEAV*
>ENSG00000107290|ENST00000224140
MSTCCWCTPGGASTIDFLKRYASNTPSGEFQTADEDLCYCLECVAEYHKARDELPFLHEV
LWELETLRLINHFEKSMKAEIGDDDELYIVDNNGEMPLFDITGQDFENKLRVPLLEILKY
PYLLLHERVNELCVEALCRMEQANCSFQVFDKHPGIYLFLVHPNEMVRRWAILTARNLGK
VDRDDYYDLQEVLLCLFKVIELGLLESPDIYTSSVLEKGKLILLPSHMYDTTNYKSYWLG
ICMLLTILEEQAMDSLLLGSDKQNDFMQSILHTMEREADDDSVDPFWPALHCFMVILDRL
GSKVWGQLMDPIVAFQTIINNASYNREIRHIRNSSVRTKLEPESYLDDMVTCSQIVYNYN
PEKTKKDSGWRTAICPDYCPNMYEEMETLASVLQSDIGQDMRVHNSTFLWFIPFVQSLMD
LKDLGVAYIAQVVNHLYSEVKEVLNQTDAVCDKVTEFFLLILVSVIELHRNKKCLHLLWV
SSQQWVEAVVKCAKLPTTAFTRSSEKSSGNCSKGTAMISSLSLHSMPSNSVQLAYVQLIR
SLLKEGYQLGQQSLCKRFWDKLNLFLRGNLSLGWQLTSQETHELQSCLKQIIRNIKFKAP
PCNTFVDLTSACKISPASYNKEESEQMGKTSRKDMHCLEASSPTFSKEPMKVQDSVLIKA
DNTIEGDNNEQNYIKDVKLEDHLLAGSCLKQSSKNIFTERAEDQIKISTRKQKSVKEISS
YTPKDCTSRNGPERGCDRGIIVSTRLLTDSSTDALEKVSTSNEDFSLKDDALAKTSKRKT
KVQKDEICAKLSHVIKKQHRKSTLVDNTINLDENLTVSNIESFYSRKDTGVQKGDGFIHN
LSLDPSGVLDDKNGEQKSQNNVLPKEKQLKNEELVIFSFHENNCKIQEFHVDGKELIPFT
EMTNASEKKSSPFKDLMTVPESRDEEMSNSTSVIYSNLTREQAPDISPKSDTLTDSQIDR
DLHKLSLLAQASVITFPSDSPQNSSQLQRKVKEDKRCFTANQNNVGDTSRGQVIIISDSD
DDDDERILSLEKLTKQDKICLEREHPEQHVSTVNSKEEKNPVKEEKTETLFQFEESDSQC
FEFESSSEVFSVWQDHPDDNNSVQDGEKKCLAPIANTTNGQGCTDYVSEVVKKGAEGIEE
HTRPRSISVEEFCEIEVKKPKRKRSEKPMAEDPVRPSSSVRNEGQSDTNKRDLVGNDFKS
IDRRTSTPNSRIQRATTVSQKKSSKLCTCTEPIRKVPVSKTPKKTHSDAKKGQNRSSNYL

SCRTTPAIVPPKKFRQCPEPTSTAEKLGLKKGPRKAYELSQRSLDYVAQLRDHGKTVGVV
DTRKKTKLISPQNLSVRNNKKLLTSQELQMQRQIRPKSQKNRRRLSDCESTDVKRAGSHT
AQNSDIFVPESDRSDYNCTGGTEVLANSNRKQLIKCMPSEPETIKAKHGSPATDDACPLN
QCDSVVLNGTVPTNEVIVSTSEDPLGGGDPTARHIEMAALKEGEPDSSSDAEEDNLFLTQ
NDPEDMDLCSQMENDNYKLIELIHGKDTVEVEEDSVSRPQLESLSGTKCKYKDCLETTKN
QGEYCPKHSEVKAADEDVFRKPGLPPPASKPLRPTTKIFSSKSTSRIAGLSKSLETSSAL
SPSLKNKSKGIQSILKVPQPVPLIAQKPVGEMKNSCNVLHPQSPNNSNRQGCKVPFGESK
YFPSSSPVNILLSSQSVSDTFVKEVLKWKYEMFLNFGQCGPPASLCQSISRPVPVRFHNY
GDYFNVFFPLMVLNTFETVAQEWLNSPNRENFYQLQVRKFPADYIKYWEFAVYLEECELA
KQLYPKENDLVFLAPERINEEKKDTERNDIQDLHEYHSGYVHKFRRTSVMRNGKTECYLS
IQTQENFPANLNELVNCIVISSLVTTQRKLKAMSLLGSRNQLARAVLNPNPMDFCTKDLL
TTTSERIIAYLRDFNEDQKKAIETAYAMVKHSPSVAKICLIHGPPGTGKSKTIVGLLYRL
LTENQRKGHSDENSNAKIKQNRVLVCAPSNAAVDELMKKIILEFKEKCKDKKNPLGNCGD
INLVRLGPEKSINSEVLKFSLDSQVNHRMKKELPSHVQAMHKRKEFLDYQLDELSRQRAL
CRGGREIQRQELDENISKVSKERQELASKIKEVQGRPQKTQSIIILESHIICCTLSTSGG
LLLESAFRGQGGVPFSCVIVDEAGQSCEIETLTPLIHRCNKLILVGDPKQLPPTVISMKA
QEYGYDQSMMARFCRLLEENVEHNMISRLPILQLTVQYRMHPDICLFPSNYVYNRNLKTN
RQTEAIRCSSDWPFQPYLVFDVGDGSERRDNDSYINVQEIKLVMEIIKLIKDKRKDVSFR
NIGIITHYKAQKTMIQKDLDKEFDRKGPAEVDTVDAFQGRQKDCVIVTCVRANSIQGSIG
FLASLQRLNVTITRAKYSLFILGHLRTLMENQHWNQLIQDAQKRGAIIKTCDKNYRHDAV
KILKLKPVLQRSLTHPPTIAPEGSRPQGGLPSSKLDSGFAKTSVAASLYHTPSDSKEITL
TVTSKDPERPPVHDQLQDPRLLKRMGIEVKGGIFLWDPQPSSPQHPGATPPTGEPGFPVV
HQDLSHIQQPAAVVAALSSHKPPVRGEPPAASPEASTCQSKCDDPEEELCHRREARAFSE
GEQEKCGSETHHTRRNSRWDKRTLEQEDSSSKKRKLL*
>ENSG00000174197|ENST00000219905
MEEKQQIILANQDGGTVAGAAPTFFVILKQPGNGKTDQGILVTNQDACALASSVSSPVKS
KGKICLPADCTVGGITVTLDNNSMWNEFYHRSTEMILTKQGRRMFPYCRYWITGLDSNLK
YILVMDISPVDNHRYKWNGRWWEPSGKAEPHVLGRVFIHPESPSTGHYWMHQPVSFYKLK
LTNNTLDQEGHIILHSMHRYLPRLHLVPAEKAVEVIQLNGPGVHTFTFPQTEFFAVTAYQ
NIQITQLKIDYNPFAKGFRDDGLNNKPQRDGKQKNSSDQEGNNISSSSGHRVRLTEGQGS
EIQPGDLDPLSRGHETSGKGLEKTSLNIKRDFLGFMDTDSALSEVPQLKQEISECLIASS
FEDDSRVASPLDQNGSFNVVIKEEPLDDYDYELGECPEGVTVKQEETDEETDVYSNSDDD
PILEKQLKRHNKVDNPEADHLSSKWLPSSPSGVAKAKMFKLDTGKMPVVYLEPCAVTRST
VKISELPDNMLSTSRKDKSSMLAELEYLPTYIENSNETAFCLGKESENGLRKHSPDLRVV
QKYPLLKEPQWKYPDISDSISTERILDDSKDSVGDSLSGKEDLGRKRTTMLKIATAAKVV
NANQNASPNVPGKRGRPRKLKLCKAGRPPKNTGKSLISTKNTPVSPGSTFPDVKPDLEDV
DGVLFVSFESKEALDIHAVDGTTEESSSLQASTTNDSGYRARISQLEKELIEDLKTLRHK
QVIHPGLQEVGLKLNSVDPTMSIDLKYLGVQLPLAPATSFPFWNLTGTNPASPDAGFPFV
SRTGKTNDFTKIKGWRGKFHSASASRNEGGNSESSLKNRSAFCSDKLDEYLENEGKLMET
SMGFSSNAPTSPVVYQLPTKSTSYVRTLDSVLKKQSTISPSTSYSLKPHSVPPVSRKAKS
QNRQATFSGRTKSSYKSILPYPVSPKQKYSHVILGDKVTKNSSGIISENQANNFVVPTLD
ENIFPKQISLRQAQQQQQQQQGSRPPGLSKSQVKLMDLEDCALWEGKPRTYITEERADVS
LTTLLTAQASLKTKPIHTIIRKRAPPCNNDFCRLGCVCSSLALEKRQPAHCRRPDCMFGC
TCLKRKVVLVKGGSKTKHFQRKAAHRDPVFYDTLGEEAREEEEGIREEEEQLKEKKKRKK

LEYTICETEPEQPVRHYPLWVKVEGEVDPEPVYIPTPSVIEPMKPLLLPQPEVLSPTVKG
KLLTGIKSPRSYTPKPNPVIREEDKDPVYLYFESMMTCARVRVYERKKEDQRQPSSSSSP
SPSFQQQTSCHSSPENHNNAKEPDSEQQPLKQLTCDLEDDSDKLQEKSWKSSCNEGESSS
TSYMHQRSPGGPTKLIEIISDCNWEEDRNKILSILSQHINSNMPQSLKVGSFIIELASQR
KSRGEKNPPVYSSRVKISMPSCQDQDDMAEKSGSETPDGPLSPGKMEDISPVQTDALDSV
RERLHGGKGLPFYAGLSPAGKLVAYKRKPSSSTSGLIQVASNAKVAASRKPRTLLPSTSN
SKMASSSGTATNRPGKNLKAFVPAKRPIAARPSPGGVFTQFVMSKVGALQQKIPGVSTPQ
TLAGTQKFSIRPSPVMVVTPVVSSEPVQVCSPVTAAVTTTTPQVFLENTTAVTPMTAISD
VETKETTYSSGATTTGVVEVSETNTSTSVTSTQSTATVNLTKTTGITTPVASVAFPKSLV
ASPSTITLPVASTASTSLVVVTAAASSSMVTTPTSSLGSVPIILSGINGSPPVSQRPENA
AQIPVATPQVSPNTVKRAGPRLLLIPVQQGSPTLRPVSNTQLQGHRMVLQPVRSPSGMNL
FRHPNGQIVQLLPLHQLRGSNTQPNLQPVMFRNPGSVMGIRLPAPSKPSETPPSSTSSSA
FSVMNPVIQAVGSSSAVNVITQAPSLLSSGASFVSQAGTLTLRISPPEPQSFASKTGSET
KITYSSGGQPVGTASLIPLQSGSFALLQLPGQKPVPSSILQHVASLQMKRESQNPDQKDE
TNSIKREQETKKVLQSEGEAVDPEANVIKQNSGAATSEETLNDSLEDRGDHLDEECLPEE
GCATVKPSEHSCITGSHTDQDYKDVNEEYGARNRKSSKEKVAVLEVRTISEKASNKTVQN
LSKVQHQKLGDVKVEQQKGFDNPEENSSEFPVTFKEESKFELSGSKVMEQQSNLQPEAKE
KECGDSLEKDRERWRKHLKGPLTRKCVGASQECKKEADEQLIKETKTCQENSDVFQQEQG
ISDLLGKSGITEDARVLKTECDSWSRISNPSAFSIVPRRAAKSSRGNGHFQGHLLLPGEQ
IQPKQEKKGGRSSADFTVLDLEEDDEDDNEKTDDSIDEIVDVVSDYQSEEVDDVEKNNCV
EYIEDDEEHVDIETVEELSEEINVAHLKTTAAHTQSFKQPSCTHISADEKAAERSRKAPP
IPLKLKPDYWSDKLQKEAEAFAYYRRTHTANERRRRGEMRDLFEKLKITLGLLHSSKVSK
SLILTRAFSEIQGLTDQADKLIGQKNLLTRKRNILIRKVSSLSGKTEEVVLKKLEYIYAK
QQALEAQKRKKKMGSDEFDISPRISKQQEGSSASSVDLGQMFINNRRGKPLILSRKKDQA
TENTSPLNTPHTSANLVMTPQGQLLTLKGPLFSGPVVAVSPDLLESDLKPQVAGSAVALP
ENDDLFMMPRIVNVTSLATEGGLVDMGGSKYPHEVPDSKPSDHLKDTVRNEDNSLEDKGR
ISSRGNRDGRVTLGPTQVFLANKDSGYPQIVDVSNMQKAQEFLPKKISGDMRGIQYKWKE
SESRGERVKSKDSSFHKLKMKDLKDSSIEMELRKVTSAIEEAALDSSELLTNMEDEDDTD
ETLTSLLNEIAFLNQQLNDDSVGLAELPSSMDTEFPGDARRAFISKVPPGSRATFQVEHL
GTGLKELPDVQGESDSISPLLLHLEDDDFSENEKQLAEPASEPDVLKIVIDSEIKDSLLS
NKKAIDGGKNTSGLPAEPESVSSPPTLHMKTGLENSNSTDTLWRPMPKLAPLGLKVANPS
SDADGQSLKVMPCLAPIAAKVGSVGHKMNLTGNDQEGRESKVMPTLAPVVAKLGNSGASP
SSAGK*
>ENSG00000105321|ENST00000221922
MAATLDLKSKEEKDAELDKRIEALRRKNEALIRRYQEIEEDRKKAELEGVAVTAPRKGRS
VEKENVAVESEKNLGPSRRSPGTPRPPGASKGGRTPPQQGGRAGMGRASRSWEGSPGEQP
RGGGAGGRGRRGRGRGSPHLSGAGDTSISDRKSKEWEERRRQNIEKMNEEMEKIAEYERN
QREGVLEPNPVRNFLDDPRRRSGPLEESERDRREESRRHGRNWGGPDFERVRCGLEHERQ
GRRAGLGSAGDMTLSMTGRERSEYLRWKQEREKIDQERLQRHRKPTGQWRREWDAEKTDG
MFKDGPVPAHEPSHRYDDQAWARPPKPPTFGEFLSQHKAEASSRRRRKSSRPQAKAAPRA
YSDHDDRWETKEGAASPAPETPQPTSPETSPKETPMQPPEIPAPAHRPPEDEGEENEGEE
DEEWEDISEDEEEEEIEVEEGDEEEPAQDHQAPEAAPTGIPCSEQAHGVPFSPEEPLLEP
QAPGTPSSPFSPPSGHQPVSDWGEEVELNSPRTTHLAGALSPGEAWPFESV*
>ENSG00000089157|ENST00000228306

MPREDRATWKSNYFLKIIQLLDDYPKCFIVGADNVGSKQMQQIRMSLRGKAVVLMGKNTM
MRKAIRGHLENNPALEKLLPHIRGNVGFVFTKEDLTEIRDMLLANKVPAAARAGAIAPCE
VTVPAQNTGLGPEKTSFFQALGITTKISRGTIEILSDVQLIKTGDKVGASEATLLNMLNI
SPFSFGLVIQQVFDNGSIYNPEVLDITEETLHSRFLEGVRNVASVCLQIGYPTVASVPHS
IINGYKRVLALSVETDYTFPLAEKVKAFLADPSAFVAAAPVAAATTAAPAAAAAPAKVEA
KEESEESDEDMGFGLFD*
>ENSG00000106004|ENST00000222726
MSSYFVNSFCGRYPNGPDYQLHNYGDHSSVSEQFRDSASMHSGRYGYGYNGMDLSVGRSG
SGHFGSGERARSYAASASAAPAEPRYSQPATSTHSPQPDPLPCSAVAPSPGSDSHHGGKN
SLSNSSGASADAGSTHISSREGVGTASGAEEDAPASSEQASAQSEPSPAPPAQPQIYPWM
RKLHISHDNIGGPEGKRARTAYTRYQTLELEKEFHFNRYLTRRRRIEIAHALCLSERQIK
IWFQNRRMKWKKDNKLKSMSMAAAGGAFRP*
>ENSG00000081386|ENST00000223428
MSPHPEAITDCVTLNTVGQLAEGGYPLRFSTLFQEQQKMNISQASVSFKDVTIEFTQEEW
QQMAPVQKNLYRDVMLENYSNLVSVGYCCFKPEVIFKLEQGEEPWFSEEEFSNQSHPKDY
RGDDLIKQNKKIKDKHLEQAICINNKTLTTEEEKVLGKPFTLHVAAVASTKMSCKCNSWE
VNLQSISEFIINNRNYSTKKIGCGNVCENSPFKINFEKTQTGEKFYEHNKNMKALNYNEN
LPKHPKFQTLEQAFECNKIGKAFNDKANCVKHNSSHTGETSSKDDEFRKNCDKKTLFDHR
RTGTGKKHLHLNQCGKSFEKSTVEEYNKLNMGIKHYELNPSGNNFNRKAHLTDPQTAVIE
ENPLVSNDRTQTWVKSSEYHENKKSYQTSVHRVRRRSHSMMKPYKCNECGKSFCQKGHLI
QHQRTHTGEKPFECSECGKTFSQKSHLSTHQRIHTAEKPYKCNECGKTFVQKSTLRGHQR
IHTGEKPYECSECGKTFVQKSTLRDHHRIHTGEKSFQCNQCGKTFGQKSNLRIHQRTHTG
EKTYQCNECEKSFWRKDHLIQHQKTHTGEKPFKCNECGKTFARTSTLRVHQRIHTGEKPF
KCNECGKKFVRKAILSDHQRIHTGEKPFQCNKCGKTFGQKSNLRIHQRTHSGEKSYECNE
YGKLCKKSTLSLYQKIQGEGNPY*
>ENSG00000109971|ENST00000227378
MSKGPAVGIDLGTTYSCVGVFQHGKVEIIANDQGNRTTPSYVAFTDTERLIGDAAKNQVA
MNPTNTVFDAKRLIGRRFDDAVVQSDMKHWPFMVVNDAGRPKVQVEYKGETKSFYPEEVS
SMVLTKMKEIAEAYLGKTVTNAVVTVPAYFNDSQRQATKDAGTIAGLNVLRIINEPTAAA
IAYGLDKKVGAERNVLIFDLGGGTFDVSILTIEDGIFEVKSTAGDTHLGGEDFDNRMVNH
FIAEFKRKHKKDISENKRAVRRLRTACERAKRTLSSSTQASIEIDSLYEGIDFYTSITRA
RFEELNADLFRGTLDPVEKALRDAKLDKSQIHDIVLVGGSTRIPKIQKLLQDFFNGKELN
KSINPDEAVAYGAAVQAAILSGDKSENVQDLLLLDVTPLSLGIETAGGVMTVLIKRNTTI
PTKQTQTFTTYSDNQPGVLIQVYEGERAMTKDNNLLGKFELTGIPPAPRGVPQIEVTFDI
DANGILNVSAVDKSTGKENKITITNDKGRLSKEDIERMVQEAEKYKAEDEKQRDKVSSKN
SLESYAFNMKATVEDEKLQGKINDEDKQKILDKCNEIINWLDKNQTAEKEEFEHQQKELE
KVCNPIITKLYQSAGGMPGGMPGGFPGGGAPPSGGASSGPTIEEVD*
>ENSG00000108826|ENST00000225969
MASVVLALRTRTAVTSLLSPTPATALAVRYASKKSGGSSKNLGGKSSGRRQGIKKMEGHY
VHAGNIIATQRHFRWHPGAHVGVGKNKCLYALEEGIVRYTKEVYVPHPRNTEAVDLITRL
PKGAVLYKTFVHVVPAKPEGTFKLVAML*
>ENSG00000105171|ENST00000221770
MIQAKLLKADLHGAIISVTKSKCPSYVGITGILLQETKHIFKIITKEDRLKVIPKLNCVF
TVETDGFISYIYGSKFQLRSSERSAKKFKAKGTIDL*

>ENSG00000107223|ENST00000224073
MAESDWDTVTVLRKKGPTAAQAKSKQAILAAQRRGEDVETSKKWAAGQNKQHSITKNTAK
LDRETEELHHDRVTLEVGKVIQQGRQSKGLTQKDLATKINEKPQVIADYESGRAIPNNQV
LGKIERAIGLKLRGKDIGKPIEKGPRAK*
>ENSG00000104112|ENST00000220478
MGFLGTGTWILVLVLPIQAFPKPGGSQDKSLHNRELSAERPLNEQIAEAEEDKIKKTYPP
ENKPGQSNYSFVDNLNLLKAITEKEKIEKERQSIRSSPLDNKLNVEDVDSTKNRKLIDDY
DSTKSGLDHKFQDDPDGLHQLDGTPLTAEDIVHKIAARIYEENDRAVFDKIVSKLLNLGL
ITESQAHTLEDEVAEVLQKLISKEANNYEEDPNKPTSWTENQAGKIPEKVTPMAAIQDGL
AKGENDETVSNTLTLTNGLERRTKTYSEDNFEELQYFPNFYALLKSIDSEKEAKEKETLI
TIMKTLIDFVKMMVKYGTISPEEGVSYLENLDEMIALQTKNKLEKNATDNISKLFPAPSE
KSHEETDSTKEEAAKMEKEYGSLKDSTKDDNSNPGGKTDEPKGKTEAYLEAIRKNIEWLK
KHDKKGNKEDYDLSKMRDFINKQADAYVEKGILDKEEAEAIKRIYSSL*
>ENSG00000105185|ENST00000221784
MADEELEALRRQRLAELQAKHGDPGDAAQQEAKHSRNEKQYLSPSSGSVGPGQVK*
>ENSG00000167384|ENST00000221327
MRACAGSTREAGSGAQDLSTLLCLEESMEEQDEKPPEPPKVCAQDSFLPQEIIIKVEGED
TGSLTIPSQEGVNFKIVTVDFTREEQGTCNPAQRTLDRDVILENHRDLVSWDLATAVGKK
DSTSKQRIFDEEPANGVKIERFTRDDPWLSSCEEVDDCKDQLEKQQEKQEILLQEVAFTQ
RKAVIHERVCKSDETGEKSGLNSSLFSSPVIPIRNHFHKHVSHAKKWHLNAAVNSHQKIN
ENETLYENNECGKPPQSIHLIQFTRTQTKDKSYGFSDRIQSFCHGTPLHIHEKIHGGGKT
FDFKECGQVLNPKISHNEQQRIPFEESQYKCSETSHSSSLTQNMRNNSEEKPFECNQCGK
SFSWSSHLVAHQRTHTGEKPYECSECGKSFSRSSHLVSHQRTHTGEKPYRCNQCGKSFSQ
SYVLVVHQRTHTGEKPYECNQCGKSFRQSYKLIAHQRTHTGEKPYECNQCGKSFIQSYKL
IAHQRIHTGEKPYECNQCGKSFSQSYKLVAHQRTHTGEKPFECNQCGKSFSWSSQLVAHQ
RTHTGEKPYECSECGKSFNRSSHLVMHQRIHTGEKPYECNQCGKSFSQSYVLVVHQRTHT
GEKPYECSQCGKSFRQSSCLTQHQRTHTGEKPFECNQCGKTFSLSARLIVHQRTHTGEKP
FTCIQCGKAFINSYKLIRHQATHTEEKLYECN*
>ENSG00000107105|ENST00000223951
METQLSNGPTCNNTANGPTTINNNCSSPVDSGNTEDSKTNLIVNYLPQNMTQEELKSLFG
SIGEIESCKLVRDKITGQSLGYGFVNYIDPKDAEKAINTLNGLRLQTKTIKVSYARPSSA
SIRDANLYVSGLPKTMTQKELEQLFSQYGRIITSRILVDQVTGISRGVGFIRFDKRIEAE
EAIKGLNGQKPPGATEPITVKFANNPSQKTNQAILSQLYQSPNRRYPGPLAQQAQRFRFS
PMTIDGMTSLAGINIPGHPGTGWCIFVYNLAPDADESILWQMFGPFGAVTNVKVIRDFNT
NKCKGFGFVTMTNYDEAAMAIASLNGYRLGDRVLQVSFKTNKTHKA*
>ENSG00000102977|ENST00000219251
MPGRCQSDAAMRVNGPASRAPAGWTSGSLHTGPRAGRPRAQARGVRGRGLLLRPRPAKEL
PLPRKGGAWAPAGNPGPLHPLGVAVGMAGSGRLVLRPWIRELILGSETPSSPRAGQLLED
AEAAVAGPSHAPDTSDVGATLLVSDGTHSVRCLVTREALDTSDWEEKEFGFRGTEGRLLL
LQDCGVHVQVAEGGAPAEFYLQVDRFSLLPTEQPRLRVPGCNQDLDVQKKLYDCLEEHLS
ESTSSNAGLSLSQLLDEMREDQEHQGALVCLAESCLTLEGPCTAPPVTHWAASRCKATGE
AVYTVPSSMLCISENDQLILSSLGPCQRTQGPELPPPDPALQDLSLTLIASPPSSPSSSG
TPALPGHMSSEESGTSISLLPALSLAAPDPGQRSSSQPSPAICSAPATLTPRSPHASRTP
SSPLQSCTPSLSPRSHVPSPHQALVTRPQKPSLEFKEFVGLPCKNRPPFPRTGATRGAQE

PCSVWEPPKRHRDGSAFQYEYEPPCTSLCARVQAVRLPPQLMAWALHFLMDAQPGSEPTP
M*
>ENSG00000105732|ENST00000222339
MPRATWANSKERSWAESERGPRDTGNGGSKAERHIQEIETGRGGDRAKAHRRQRMRLRGT
ERASLGPGRRLGDSRGTDMPGARAQGLAAAMTEESEETVLYIEHRYVCSECNQLYGSLEE
VLMHQNSHVPQQHFELVGVADPGVTVATDTASGTGLYQTLVQESQYQCLECGQLLMSPSQ
LLEHQELHLKMMAPQEAVPAEPSPKAPPLSSSTIHYECVDCKALFASQELWLNHRQTHLR
ATPTKAPAPVVLGSPVVLGPPVGQARVAVEHSYRKAEEGGEGATVPSAAATTTEVVTEVE
LLLYKCSECSQLFQLPADFLEHQATHFPAPVPESQEPALQQEVQASSPAEVPVSQPDPLP
ASDHSYELRNGEAIGRDRRGRRARRNNSGEAGGAATQELFCSACDQLFLSPHQLQQHLRS
HREGVFKCPLCSRVFPSPSSLDQHLGDHSSESHFLCVDCGLAFGTEALLLAHRRAHTPNP
LHSCPCGKTFVNLTKFLYHRRTHGVGGVPLPTTPVPPEEPVIGFPEPAPAETGEPEAPEP
PVSEETSAGPAAPGTYRCLLCSREFGKALQLTRHQRFVHRLERRHKCSICGKMFKKKSHV
RNHLRTHTGERPFPCPDCSKPFNSPANLARHRLTHTGERPYRCGDCGKAFTQSSTLRQHR
LVHAQHFPYRCQECGVRFHRPYRLLMHRYHHTGEYPYKCRECPRSFLLRRLLEVHQLVVH
AGRQPHRCPSCGAAFPSSLRLREHRCAAAAAQAPRRFECGTCGKKVGSAARLQAHEAAHA
AAGPGEVLAKEPPAPRAPRATRAPVASPAALGSTATASPAAPARRRGLECSECKKLFSTE
TSLQVHRRIHTGERPYPCPDCGKAFRQSTHLKDHRRLHTGERPFACEVCGKAFAISMRLA
EHRRIHTGERPYSCPDCGKSYRSFSNLWKHRKTHQQQHQAAVRQQLAEAEAAVGLAVMET
AVEALPLVEAIEIYPLAEAEGVQISG*
>ENSG00000106803|ENST00000223641
MPGPTPSGTNVGSSGRSPSKAVAARAAGSTVRQRKNASCGTRSAGRTTSAGTGGMWRFYT
EDSPGLKVGPVPVLVMSLLFIASVFMLHIWGKYTRS*
>ENSG00000108588|ENST00000225726
MKAFHTFCVVLLVFGSVSEAKFDDFEDEEDIVEYDDNDFAEFEDVMEDSVTESPQRVIIT
EDDEDETTVELEGQDENQEGDFEDADTQEGDTESEPYDDEEFEGYEDKPDTSSSKNKDPI
TIVDVPAHLQNSWESYYLEILMVTGLLAYIMNYIIGKNKNSRLAQAWFNTHRELLESNFT
LVGDDGTNKEATSTGKLNQENEHIYNLWCSGRVCCEGMLIQLRFLKRQDLLNVLARMMRP
VSDQVQIKVTMNDEDMDTYVFAVGTRKALVRLQKEMQDLSEFCSDKPKSGAKYGLPDSLA
ILSEMGEVTDGMMDTKMVHFLTHYADKIESVHFSDQFSGPKIMQEEGQPLKLPDTKRTLL
FTFNVPGSGNTYPKDMEALLPLMNMVIYSIDKAKKFRLNREGKQKADKNRARVEENFLKL
THVQRQEAAQSRREEKKRAEKERIMNEEDPEKQRRLEEAALRREQKKLEKKQMKMKQIKV
KAM*
>ENSG00000106459|ENST00000223190
MEEHGVTQTEHMATIEAHAVAQQVQQVHVATYTEHSMLSADEDSPSSPEDTSYDDSDILN
STAADEVTAHLAAAGPVGMAAAAAVATGKKRKRPHVFESNPSIRKRQQTRLLRKLRATLD
EYTTRVGQQAIVLCISPSKPNPVFKVFGAAPLENVVRKYKSMILEDLESALAEHAPAPQE
VNSELPPLTIDGIPVSVDKMTQAQLRAFIPEMLKYSTGRGKPGWGKESCKPIWWPEDIPW
ANVRSDVRTEEQKQRVSWTQALRTIVKNCYKQHGREDLLYAFEDQQTQTQATATHSIAHL
VPSQTVVQTFSNPDGTVSLIQVGTGATVATLADASELPTTVTVAQVNYSAVADGEVEQNW
ATLQGGEMTIQTTQASEATQAVASLAEAAVAASQEMQQGATVTMALNSEAAAHAVATLAE
ATLQGGGQIVLSGETAAAVGALTGVQDANGLVQIPVSMYQTVVTSLAQGNGPVQVAMAPV
TTRISDSAVTMDGQAVEVVTLEQ*
>ENSG00000011260|ENST00000225298

MPPERRRRMKLDRRTGAKPKRKPGMRPDWKAGAGPGGPPQKPAPSSQRKPPARPSAAAAA
IAVAAAEEERRLRQRNRLRLEEDKPAVERCLEELVFGDVENDEDALLRRLRGPRVQEHED
SGDSEVENEAKGNFPPQKKPVWVDEEDEDEEMVDMMNNRFRKDMMKNASESKLSKDNLKK
RLKEEFQHAMGGVPAWAETTKRKTSSDDESEEDEDDLLQRTGNFISTSTSLPRGILKMKN
CQHANAERPTVARISSVQFHPGAQIVMVAGLDNAVSLFQVDGKTNPKIQSIYLERFPIFK
ACFSANGEEVLATSTHSKVLYVYDMLAGKLIPVHQVRGLKEKIVRSFEVSPDGSFLLING
IAGYLHLLAMKTKELIGSMKINGRVAASTFSSDSKKVYASSGDGEVYVWDVNSRKCLNRF
VDEGSLYGLSIATSRNGQYVACGSNCGVVNIYNQDSCLQETNPKPIKAIMNLVTGVTSLT
FNPTTEILAIASEKMKEAVRLVHLPSCTVFSNFPVIKNKNISHVHTMDFSPRSGYFALGN
EKGKALMYRLHHYSDF*
>ENSG00000107960|ENST00000224950
MQPGSSRCEEETPSLLWGLDPVFLAFAKLYIRDILDMKESRQVPGVFLYNGHPIKQVDVL
GTVIGVRERDAFYSYGVDDSTGVINCICWKKLNTESVSAAPSAARELSLTSQLKKLQETI
EQKTKIEIGDTIRVRGSIRTYREEREIHATTYYKVDDPVWNIQIARMLELPTIYRKVYDQ
PFHSSALEKEEALSNPGALDLPSLTSLLSEKAKEFLMENRVQSFYQQELEMVESLLSLAN
QPVIHSASSDQVNFKKDTTSKAIHSIFKNAIQLLQEKGLVFQKDDGFDNLYYVTREDKDL
HRKIHRIIQQDCQKPNHMEKGCHFLHILACARLSIRPGLSEAVLQQVLELLEDQSDIVST
MEHYYTAF*
>ENSG00000006194|ENST00000219069
MASGPGSQEREGLLIVKLEEDCAWSQELPPPDPGPSPEASHLRFRRFRFQEAAGPREALS
RLQELCHGWLRPEMRTKEQILELLVLEQFLTILPQEIQSRVQELHPESGEEAVTLVEDMQ
RELGRLRQQVTNHGRGTEVLLEEPLPLETARESPSFKLEPMETERSPGPRLQELLGPSPQ
RDPQAVKERALSAPWLSLFPPEGNMEDKEMTGPQLPESLEDVAMYISQEEWGHQDPSKRA
LSRDTVQESYENVDSLESHIPSQEVPGTQVGQGGKLWDPSVQSCKEGLSPRGPAPGEEKF
ENLEGVPSVCSENIHPQVLLPDQARGEVPWSPELGRPHDRSQGDWAPPPEGGMEQALAGA
SSGRELGRPKELQPKKLHLCPLCGKNFSNNSNLIRHQRIHAAERLCMGVDCTEIFGGNPR
FLSLHRAHLGEEAHKCLECGKCFSQNTHLTRHQRTHTGEKPYQCNICGKCFSCNSNLHRH
QRTHTGEKPYKCPECGEIFAHSSNLLRHQRIHTGERPYKCPECGKSFSRSSHLVIHERTH
ERERLYPFSECGEAVSDSTPFLTNHGAHKAEKKLFECLTCGKSFRQGMHLTRHQRTHTGE
KPYKCTLCGENFSHRSNLIRHQRIHTGEKPYTCHECGDSFSHSSNRIRHLRTHTGERPYK
CSECGESFSRSSRLMSHQRTHTG*
>ENSG00000105866|ENST00000222584
MSDQKKEEEEEAAAAAAMATEGGKTSEPENNNKKPKTSGSQDSQPSPLALLAATCSKIGT
PGENQATGQQQIIIDPSQGLVQLQNQPQQLELVTTQLAGNAWQLVASTPPASKENNVSQP
ASSSSSSSSSNNGSASPTKTKSGNSSTPGQFQVIQVQNPSGSVQYQVIPQLQTVEGQQIQ
INPTSSSSLQDLQGQIQLISAGNNQAILTAANRTASGNILAQNLANQTVPVQIRPGVSIP
LQLQTLPGTQAQVVTTLPINIGGVTLALPVINNVAAGGGTGQVGQPAATADSGTSNGNQL
VSTPTNTTTSASTMPESPSSSTTCTTTASTSLTSSDTLVSSADTGQYASTSASSSERTIE
ESQTPAATESEAQSSSQLQPNGMQNAQDQSNSLQQVQIVGQPILQQIQIQQPQQQIIQAI
PPQSFQLQSGQTIQTIQQQPLQNVQLQAVNPTQVLIRAPTLTPSGQISWQTVQVQNIQSL
SNLQVQNAGLSQQLTITPVSSSGGTTLAQIAPVAVAGAPITLNTAQLASVPNLQTVSVAN
LGAAGVQVQGVPVTITSVAGQQQGQDGVKVQQATIAPVTVAVGGIANATIGAVSPDQLTQ
VHLQQGQQTSDQEVQPGKRLRRVACSCPNCREGEGRGSNEPGKKKQHICHIEGCGKVYGK
TSHLRAHLRWHTGERPFICNWMFCGKRFTRSDELQRHRRTHTGEKRFECPECSKRFMRSD

HLSKHVKTHQNKKGGGTALAIVTSGELDSSVTEVLGSPRIVTVAAISQDSNPATPNVSTN
MEEF*
>ENSG00000060138|ENST00000228251
MSEAGEATTTTTTTLPQAPTEAAAAAPQDPAPKSPVGSGAPQAAAPAPAAHVAGNPGGDA
APAATGTAAAASLATAAGSEDAEKKVLATKVLGTVKWFNVRNGYGFINRNDTKEDVFVHQ
TAIKKNNPRKYLRSVGDGETVEFDVVEGEKGAEAANVTGPDGVPVEGSRYAADRRRYRRG
YYGRRRGPPRNYAGEEEEEGSGSSEGFDPPATDRQFSGARNQLRRPQYRPQYRQRRFPPY
HVGQTFDRRSRVLPHPNRIQAGEIGEMKDGVPEGAQLQGPVHRNPTYRPRYRSRGPPRPR
PAPAVGEAEDKENQQATSGPNQPSVRRGYRRPYNYRRRPRPPNAPSQDGKEAKAGEAPTE
NPAPPTQQSSAE*
>ENSG00000106628|ENST00000223361
MFSEQAAQRAHTLLSPPSANNATFARVPVATYTNSSQPFRLGERSFSRQYAHIYATRLIQ
MRPFLENRAQQHWGSGVGVKKLCELQPEEKCCVVGTLFKAMPLQPSILREVSEEHNLLPQ
PPRSKYIHPDDELVLEDELQRIKLKGTIDVSKLVTGTVLAVFGSVRDDGKFLVEDYCFAD
LAPQKPAPPLDTDRFVLLVSGLGLGGGGGESLLGTQLLVDVVTGQLGDEGEQCSAAHVSR
VILAGNLLSHSTQSRDSINKAKYLTKKTQAASVEAVKMLDEILLQLSASVPVDVMPGEFD
PTNYTLPQQPLHPCMFPLATAYSTLQLVTNPYQATIDGVRFLGTSGQNVSDIFRYSSMED
HLEILEWTLRVRHISPTAPDTLECPHVYFCGNTPSFGSKIIRGPEDQTVLLVTVPDFSAT
QTACLVNLRSLACQPISFSGFGAEDDDLGGLGLGP*
>ENSG00000108654|ENST00000225792
MSGYSSDRDRGRDRGFGAPRFGGSRAGPLSGKKFGNPGEKLVKKKWNLDELPKFEKNFYQ
EHPDLARRTAQEVETYRRSKEITVRGHNCPKPVLNFYEANFPANVMDVIARQNFTEPTAI
QAQGWPVALSGLDMVGVAQTGSGKTLSYLLPAIVHINHQPFLERGDGPICLVLAPTRELA
QQVQQVAAEYCRACRLKSTCIYGGAPKGPQIRDLERGVEICIATPGRLIDFLECGKTNLR
RTTYLVLDEADRMLDMGFEPQIRKIVDQIRPDRQTLMWSATWPKEVRQLAEDFLKDYIHI
NIGALELSANHNILQIVDVCHDVEKDEKLIRLMEEIMSEKENKTIVFVETKRRCDELTRK
MRRDGWPAMGIHGDKSQQERDWVLNEFKHGKAPILIATDVASRGLDVEDVKFVINYDYPN
SSEDYIHRIGRTARSTKTGTAYTFFTPNNIKQVSDLISVLREANQAINPKLLQLVEDRGS
GRSRGRGGMKDDRRDRYSAGKRGGFNTFRDRENYDRGYSSLLKRDFGAKTQNGVYSAANY
TNGSFGSNFVSAGIQTSFRTGNPTGTYQNGYDSTQQYGSNVPNMHNGMNQQAYAYPATAA
APMIGYPMPTGYSQ*
>ENSG00000103152|ENST00000219431
MVTPALQMKKPKQFCRRMGQKKQRPARAGQPHSSSDAAQAPAEQPHSSSDAAQAPCPRER
CLGPPTTPGPYRSIYFSSPKGHLTRLGLEFFDQPAVPLARAFLGQVLVRRLPNGTELRGR
IVETEAYLGPEDEAAHSRGGRQTPRNRGMFMKPGTLYVYIIYGMYFCMNISSQGDGACVL
LRALEPLEGLETMRQLRSTLRKGTASRVLKDRELCSGPSKLCQALAINKSFDQRDLAQDE
AVWLERGPLEPSEPAVVAAARVGVGHAGEWARKPLRFYVRGSPWVSVVDRVAEQDTQA*
>ENSG00000110060|ENST00000227474
MAYNDTDRNQTEKLLKRVRELEQEVQRLKKEQAKNKEDSNIRENSAGAGKTKRAFDFSAH
GRRHVALRIAYMGWGYQGFASQENTNNTIEEKLFEALTKTRLVESRQTSNYHRCGRTDKG
VSAFGQVISLDLRSQFPRGRDSEDFNVKEEANAAAEEIRYTHILNRVLPPDIRILAWAPV
EPSFSARFSCLERTYRYFFPRADLDIVTMDYAAQKYVGTHDFRNLCKMDVANGVINFQRT
ILSAQVQLVGQSPGEGRWQEPFQLCQFEVTGQAFLYHQVRCMMAILFLIGQGMEKPEIID
ELLNIEKNPQKPQYSMAVEFPLVLYDCKFENVKWIYDQEAQEFNITHLQQLWANHAVKTH

MLYSMLQGLDTVPVPCGIGPKMDGMTEWGNVKPSVIKQTSAFVEGVKMRTYKPLMDRPKC
QGLESRIQHFVRRGRIEHPHLFHEEETKAKRDCNDTLEEENTNLETPTKRVCVDTEIKSI
I*
>ENSG00000109320|ENST00000226574
MAEDDPYLGRPEQMFHLDPSLTHTIFNPEVFQPQMALPTADGPYLQILEQPKQRGFRFRY
VCEGPSHGGLPGASSEKNKKSYPQVKICNYVGPAKVIVQLVTNGKNIHLHAHSLVGKHCE
DGICTVTAGPKDMVVGFANLGILHVTKKKVFETLEARMTEACIRGYNPGLLVHPDLAYLQ
AEGGGDRQLGDREKELIRQAALQQTKEMDLSVVRLMFTAFLPDSTGSFTRRLEPVVSDAI
YDSKAPNASNLKIVRMDRTAGCVTGGEEIYLLCDKVQKDDIQIRFYEEEENGGVWEGFGD
FSPTDVHRQFAIVFKTPKYKDINITKPASVFVQLRRKSDLETSEPKPFLYYPEIKDKEEV
QRKRQKLMPNFSDSFGGGSGAGAGGGGMFGSGGGGGGTGSTGPGYSFPHYGFPTYGGITF
HPGTTKSNAGMKHGTMDTESKKDPEGCDKSDDKNTVNLFGKVIETTEQDQEPSEATVGNG
EVTLTYATGTKEESAGVQDNLFLEKAMQLAKRHANALFDYAVTGDVKMLLAVQRHLTAVQ
DENGDSVLHLAIIHLHSQLVRDLLEVTSGLISDDIINMRNDLYQTPLHLAVITKQEDVVE
DLLRAGADLSLLDRLGNSVLHLAAKEGHDKVLSILLKHKKAALLLDHPNGDGLNAIHLAM
MSNSLPCLLLLVAAGADVNAQEQKSGRTALHLAVEHDNISLAGCLLLEGDAHVDSTTYDG
TTPLHIAAGRGSTRLAALLKAAGADPLVENFEPLYDLDDSWENAGEDEGVVPGTTPLDMA
TSWQVFDILNGKPYEPEFTSDDLLAQGDMKQLAEDVKLQLYKLLEIPDPDKNWATLAQKL
GLGILNNAFRLSPAPSKTLMDNYEVSGGTVRELVEALRQMGYTEAIEVIQAASSPVKTTS
QAHSLPLSPASTRQQIDELRDSDSVCDSGVETSFRKLSFTESLTSGASLLTLNKMPHDYG
QEGPLEGKI*
>ENSG00000104824|ENST00000221419
MSRRLLPRAEKRRRRLEQRQQPDEQRRRSGAMVKMAAAGGGGGGGGRYYGGGSEGGRAPKR
LKTDNAGDQHGGGGGGGGGAGAAGGGGGGGENYDDPHKTPASPVVHIRGLIDGVVEADLVE
ALQEFGPISYVVVMPKKRQALVEFEDVLGACNAVNYAADNQIYIAGHPAFVNYSTSQKIS
RPGDSDDSRSVNSVLLFTILNPIYSITTDVLYTICNPCGPVQRIVIFRKNGVQAMVEFDS
VQSAQRAKASLNGADIYSGCCTLKIEYAKPTRLNVFKNDQDTWDYTNPNLSGQGDPGSNP
NKRQRQPPLLGDHPAEYGGPHGGYHSHYHDEGYGPPPPHYEGRRMGPPVGGHRRGPSRYG
PQYGHPPPPPPPPPEYGPHADSPVLMVYGLDQSKMNCDRVFNVFCLYGNVEKVKFMKSKPG
AAMVEMADGYAVDRAITHLNNNFMFGQKLNVCVSKQPAIMPGQSYGLEDGSCSYKDFSES
RNNRFSTPEQAAKNRIQHPSNVLHFFNAPLEVTEENFFEICDELGVKRPSSVKVFSGKSE
RSSSGLLEWESKSDALETLGFLNHYQMKNPNGPYPYTLKLCFSTAQHAS*
>ENSG00000105516|ENST00000222122
MARPVSDRTPAPLLLGGPAGTPPGGGALLGLRSLLQGTSKPKEPASCLLKEKERKAALPA
ATTPGPGLETAGPADAPAGAVVGGGSPRGRPGPVPAPGLLAPLLWERTLPFGDVEYVDLD
AFLLEHGLPPSPPPPGGPSPEPSPARTPAPSPGPGSCGSASPRSSPGHAPARAALGTASG
HRAGLTSRDTPSPVDPDTVEVLMTFEPDPADLALSSIPGHETFDPRRHRFSEEELKPQPI
MKKARKIQVPEEQKDEKYWSRRYKNNEAAKRSRDARRLKENQISVRAAFLEKENALLRQE
VVAVRQELSHYRAVLSRYQAQHGAL*
>ENSG00000106603|ENST00000223336
MMWQKYAGSRRSMPLGARILFHGVFYAGGFAIVYYLIQKFHSRALYYKLAVEQLQSHPEA
QEALGPPLNIHYLKLIDRENFVDIVDAKLKIPVSGSKSEGLLYVHSSRGGPFQRWHLDEV
FLELKDGQQIPVFKLSGENGDEVKKE*
>ENSG00000108528|ENST00000225665

MAATASAGAGGIDGKPRTSPKSVKFLFGGLAGMGATVFVQPLDLVKNRMQLSGEGAKTRE
YKTSFHALTSILKAEGLRGIYTGLSAGLLRQATYTTTRLGIYTVLFERLTGADGTPPGFL
LKAVIGMTAGATGAFVGTPAEVALIRMTADGRLPADQRRGYKNVFNALIRITREEGVLTL
WRGCIPTMARAVVVNAAQLASYSQSKQFLLDSGYFSDNILCHFCASMISGLVTTAASMPV
DIAKTRIQNMRMIDGKPEYKNGLDVLFKVVRYEGFFSLWKGFTPYYARLGPHTVLTFIFL
EQMNKAYKRLFLSG*
>ENSG00000109101|ENST00000226247
MVSLPPPQSDVTLPGPTRLEGERQGDLMQAPGLPGSPAPQSKHAGFSCSSFVSDGPPERT
PSLPPHSPRIASPGPEQVQGHCPAGPGPGPFRLSPSDKYPGFGFEEAAASSPGRFLKGSH
APFHPYKRPFHEDVFPEAETTLALKGHSFKTPGPLEAFEEIPVDVAEAEAFLPGFSAEAW
CNGLPYPSQEHGPQVLGSEVKVKPPVLESGAGMFCYQPPLQHMYCSSQPPFHQYSPGGGS
YPIPYLGSSHYQYQRMAPQASTDGHQPLFPKPIYSYSILIFMALKNSKTGSLPVSEIYNF
MTEHFPYFKTAPDGWKNSVRHNLSLNKCFEKVENKSGSSSRKGCLWALNPAKIDKMQEEL
QKWKRKDPIAVRKSMAKPEELDSLIGDKREKLGSPLLGCPPPGLSGSGPIRPLAPPAGLS
PPLHSLHPAPGPIPGKNPLQDLLMGHTPSCYGGQTYLHLSPGLAPPGPPQPLFPQPDGHLE
LRAQPGTPQDSPLPAHTPPSHSAKLLAEPSPARTMHDTLLPDGDLGTDLDAINPSLTDFD
FQGNLWEQLKDDSLALDPLVLVTSSPTSSSMPPPQPPPHCFPPGPCLTETGSGAGDLAAP
GSGGSGALGDLHLTTLYSAFMELEPTPPTAPAGPSVYLSPSSKPVALA*
>ENSG00000105202|ENST00000221801
MKPGFSPRGGGFGGRGGFGDRGGRGGRGGFGGGRGRGGGFRGRGRGGGGGGGGGGGGGRG
GGGFHSGGNRGRGRGGKRGNQSGKNVMVEPHRHEGVFICRGKEDALVTKNLVPGESVYGE
KRVSISEGDDKIEYRAWNPFRSKLAAAILGGVDQIHIKPGAKVLYLGAASGTTVSHVSDI
VGPDGLVYAVEFSHRSGRDLINLAKKRTNIIPVIEDARHPHKYRMLIAMVDVIFADVAQP
DQTRIVALNAHTFLRNGGHFVISIKANCIDSTASAEAVFASEVKKMQQENMKPQEQLTLE
PYERDHAVVVGVYRPPPKVKN*
>ENSG00000106006|ENST00000222728
MSSYFVNPTFPGSLPSGQDSFLGQLPLYQAGYDALRPFPASYGASSLPDKTYTSPCFYQQ
SNSVLACNRASYEYGASCFYSDKDLSGASPSGSGKQRGPGDYLHFSPEQQYKPDSSSGQG
KALHDEGADRKYTSPVYPWMQRMNSCAGAVYGSHGRRGRQTYTRYQTLELEKEFHFNRYL
TRRRRIEIANALCLTERQIKIWFQNRRMKWKKENKLINSTQPSGEDSEAKAGE*
>ENSG00000105793|ENST00000222511
MVHCSCVLFRKYGNFIDKLRLFTRGGSGGMGYPRLGGEGGKGGDVWVVAQNRMTLKQLKD
RYPRKRFVAGVGANSKISALKGSKGKDCEIPVPVGISVTDENGKIIGELNKENDRILVAQ
GGLGGKLLTNFLPLKGQKRIIHLDLKLIADVGLVGFPNAGKSSLLSCVSHAKPAIADYAF
TTLKPELGKIMYSDFKQISVADLPGLIEGAHMNKGMGHKFLKHIERTRQLLFVVDISGFQ
LSSHTQYRTAFETIILLTKELELYKEELQTKPALLAVNKMDLPDAQDKFHELMSQLQNPK
DFLHLFEKNMIPERTVEFQHIIPISAVTGEGIEELKNCIRKSLDEQANQENDALHKKQLL
NLWISDTMSSTEPPSKHAVTTSKMDII*
>ENSG00000102081|ENST00000218200
MEELVVEVRGSNGAFYKAFVKDVHEDSITVAFENNWQPDRQIPFHDVRFPPPVGYNKDIN
ESDEVEVYSRANEKEPCCWWLAKVRMIKGEFYVIEYAACDATYNEIVTIERLRSVNPNKP
ATKDTFHKIKLDVPEDLRQMCAKEAAHKDFKKAVGAFSVTYDPENYQLVILSINEVTSKR
AHMLIDMHFRSLRTKLSLIMRNEEASKQLESSRQLASRFHEQFIVREDLMGLAIGTHGAN
IQQARKVPGVTAIDLDEDTCTFHIYGEDQDAVKKARSFLEFAEDVIQVPRNLVGKVIGKN

GKLIQEIVDKSGVVRVRIEAENEKNVPQEEEIMPPNSLPSNNSRVGPNAPEEKKHLDIKE
NSTHFSQPNSTKVQRGMVPFVFVGTKDSIANATVLLDYHLNYLKEVDQLRLERLQIDEQL
RQIGASSRPPPNRTDKEKSYVTDDGQGMGRGSRPYRNRGHGRRGPGYTSGTNSEASNASE
TESDHRDELSDWSLAPTEEERESFLRRGDGRRRGGGGRGQGGRGRGGGFKGNDDHSRTDN
RPRNPREAKGRTTDGSLQIRVDCNNERSVHTKTLQNTSSEGSRLRTGKDRNQKKEKPDSV
DGQQPLVNGVP*
>ENSG00000106399|ENST00000223129
MVDMMDLPRSRINAGMLAQFIDKPVCFVGRLEKIHPTGKMFILSDGEGKNGTIELMEPLD
EEISGIVEVVGRVTAKATILCTSYVQFKEDSHPFDLGLYNEAVKIIHDFPQFYPLGIVQH
D*
>ENSG00000106948|ENST00000223791
MPTLGWLPENRDISEDQSSAEQTQALASQASQFLAKVESFERLIQAGRLMPQDQVKGFQR
LKAAHAALEEEYLKACREQHPAQPLAGSKGTPGRFDPRRELEAEIYRLGSCLEELKEHID
QTQQEPEPPGSDSALDSTPALPCLHQPTHLPAPSGQAPMPAIKTSCPEPATTTAAASTGP
CPLHVNVEVSSGNSEVEDRPQDPLARLRHKELQMEQVYHGLMERYLSVKSLPEAMRMEEE
EEGEEEEEEEGGGDSLEVDGVAATPGKAEATRVLPRQCPVQAEKSHGAPLEEATEKMVSM
KPPGFQASLARDGHMSGLGKAEAAPPGPGVPPHPPGTKSAASHQSSMTSLEGSGISERLP
QKPLHRGGGPHLEETWMASPETDSGFVGSETSRVSPLTQTPEHRLSHISTAGTLAQPFAA
SVPRDGASYPKARGSLIPRRATEPSTPRSQAQRYLSSPSGPLRQRAPNFSLERTLAAEMA
VPGSEFEGHKRISEQPLPNKTISPPPAPAPAAAPLPCGPTETIPSFLLTRAGRDQAICEL
QEEVSRLRLRLEDSLHQPLQGSPTRPASAFDRPARTRGRPADSPATWGSHYGSKSTERLP
GEPRGEEQIVPPGRQRARSSSVPREVLRLSLSSESELPSLPLFSEKSKTTKDSPQAARDG
KRGVGSAGWPDRVTFRGQYTGHEYHVLSPKAVPKGNGTVSCPHCRPIRTQDAGGAVTGDP
LGPPPADTLQCPLCGQVGSPPEADGPGSATSGAEKATTRRKASSTPSPKQRSKQAGSSPR
PPPGLWYLATAPPAPAPPAFAYISSVPIMPYPPAAVYYAPAGPTSAQPAAKWPPTASPPP
ARRHRHSIQLDLGDLEELNKALSRAVQAAESVRSTTRQMRSSLSADLRQAHSLRGSCLF*
>ENSG00000106344|ENST00000223073
MAGLTLFVGRLPPSARSEQLEELFSQVGPVKQCFVVTEKGSKACRGFGYVTFSMLEDVQR
ALKEITTFEGCKINVTVAKKKLRNKTKEKGKNENSECPKKEPKAKKAKVADKKARLIIRN
LSFKCSEDDLKTVFAQFGAVLEVNIPRKPDGKMRGFGFVQFKNLLEAGKALKGMNMKEIK
GRTVAVDWAVAKDKYKDTQSVSAIGEEKSHESKHQESVKKKGREEEDMEEEENDDDDDDD
DEEDGVFDDEDEEEENIESKVTKPVQIQKRAVKRPAPAKSSDHSEEDSDLEESDSIDDGE
ELAQSDTSTEEQEDKAVQVSNKKKRKLPSDVNEGKTVFIRNLSFDSEEEELGELLQQFGE
LKYVRIVLHPDTEHSKGCAFAQFMTQEAAQKCLLAASPENEAGGLKLDGRQLKVDLAVTR
DEAAKLQTTKVKKPTGTRNLYLAREGLIRAGTKAAEGVSAADMAKRERFELLKHQKLKDQ
NIFVSRTRLCLHNLPKAVDDKQLRKLLLSATSGEKGVRIKECRVMRDLKGVHGNMKGQSL
GYAFAEFQEHEHALKALRLINNNPEIFGPLKRPIVEFSLEDRRKLKMKELRIQRSLQKMR
SKPATGEPQKGQPEPAKDQQQKAAQHHTEEQSKVPPEQKRKAGSTSWTGFQTKAEVEQVE
LPDGKKRRKVLALPSHRGPKIRLRDKGKVKPVHPKKPKPQINQWKQEKQQLSSEQVSRKK
AKGNKTETRFNQLVEQYKQKLLGPSKGAPLAKRSKWFDS*
>ENSG00000102103|ENST00000218224
MPLPVALQTRLAKRGILKHLEPEPEEEIIAEDYDDDPVDYEATRLEGLPPSWYKVFDPSC
GLPYYWNADTDLVSWLSPHDPNSVVTKSAKKLRSSNADAEEKLDRSHDKSDRGHDKSDRS
HEKLDRGHDKSDRGHDKSDRDRERGYDKVDRERERDRERDRDRGYDKADREEGKERRHHR

REELAPYPKSKKAVSRKDEELDPMDPSSYSDAPRGTWSTGLPKRNEAKTGADTTAAGPLF
QQRPYPSPGAVLRANAEASRTKQQD*
>ENSG00000091651|ENST00000219097
MGSELIGRLAPRLGLAEPDMLRKAEEYLRLSRVKCVGLSARTTETSSAVMCLDLAASWMK
CPLDRAYLIKLSGLNKETYQSCLKSFECLLGLNSNIGIRDLAVQFSCIEAVNMASKILKS
YESSLPQTQQVDLDLSRPLFTSAALLSACKILKLKVDKNKMVATSGVKKAIFDRLCKQLE
KIGQQVDREPGDVATPPRKRKKIVVEAPAKEMEKVEEMPHKPQKDEDLTQDYEEWKRKIL
ENAASAQKATAE*
>ENSG00000105698|ENST00000222305
MDMLDPGLDPAASATAAAAASHDKGPEAEEGVELQEGGDGPGAEEQTAVAITSVQQAAFG
DHNIQYQFRTETNGGQVTYRVVQVTDGQLDGQGDTAGAVSVVSTAAFAGGQQAVTQVGVD
GAAQRPGPAAASVPPGPAAPFPLAVIQNPFSNGGSPAAEAVSGEARFAYFPASSVGDTTA
VSVQTTDQSLQAGGQFYVMMTPQDVLQTGTQRTIAPRTHPYSPKIDGTRTPRDERRRAQH
NEVERRRRDKINNWIVQLSKIIPDCNADNSKTGASKGGILSKACDYIRELRQTNQRMQET
FKEAERLQMDNELLRQQIEELKNENALLRAQLQQHNLEMVGEGTRQ*
>ENSG00000066336|ENST00000227163
MLQACKMEGFPLVPPQPSEDLVPYDTDLYQRQTHEYYPYLSSDGESHSDHYWDFHPHHVH
SEFESFAENNFTELQSVQPPQLQQLYRHMELEQMHVLDTPMVPPHPSLGHQVSYLPRMCL
QYPSLSPAQPSSDEEEGERQSPPLEVSDGEADGLEPGPGLLPGETGSKKKIRLYQFLLDL
LRSGDMKDSIWWVDKDKGTFQFSSKHKEALAHRWGIQKGNRKKMTYQKMARALRNYGKTG
EVKKVKKKLTYQFSGEVLGRGGLAERRHPPH*
>ENSG00000108561|ENST00000225698
MLPLLRCVPRVLGSSVAGLRAAAPASPFRQLLQPAPRLCTRPFGLLSVRAGSERRPGLLR
PRGPCACGCGCGSLHTDGDKAFVDFLSDEIKEERKIQKHKTLPKMSGGWELELNGTEAKL
VRKVAGEKITVTFNINNSIPPTFDGEEEPSQGQKVEEQEPELTSTPNFVVEVIKNDDGKK
ALVLDCHYPEDEVGQEDEAESDIFSIREVSFQSTGESEWKDTNYTLNTDSLDWALYDHLM
DFLADRGVDNTFADELVELSTALEHQEYITFLEDLKSFVKSQ*
>ENSG00000122386|ENST00000219091
MSADGGGIQDTQDKETPPEVPDRGHPHQEMPSKLGEAVPSGDTQESLHIKMEPEEPHSEG
ASQEDGAQGAWGWAPLSHGSKEKALFLPGGALPSPRIPVLSREGRTRDRQMAAALLTAWS
QMPVTFEDVALYLSREEWGRLDHTQQNFYRDVLQKKNGLSLGFPFSRPFWAPQAHGKGEA
SGSSRQAGDEKEWRGACTGAVEVGQRVQTSSVAALGNVKPFRTRAGRVQWGVPQCAQEAA
CGRSSGPAKDSGQPAEPDRTPDAAPPDPSPTEPQEYRVPEKPNEEEKGAPESGEEGLAPD
SEVGRKSYRCEQCGKGFSWHSHLVTHRRTHTGEKPYACTDCGKRFGRSSHLIQHQIIHTG
EKPYTCPACRKSFSHHSTLIQHQRIHTGEKPYVCDRCAKRFTRRSDLVTHQGTHTGAKPH
KCPICAKCFTQSSALVTHQRTHTGVKPYPCPECGKCFSQRSNLIAHNRTHTGEKPYHCLD
CGKSFSHSSHLTAHQRTHRGVRPYACPLCGKSFSRRSNLHRHEKIHTTGPKALAMLMLGA
AAAGALATPPPAPT*
>ENSG00000026025|ENST00000224237
MSTRSVSSSSYRRMFGGPGTASRPSSSRSYVTTSTRTYSLGSALRPSTSRSLYASSPGGV
YATRSSAVRLRSSVPGVRLLQDSVDFSLADAINTEFKNTRTNEKVELQELNDRFANYIDK
VRFLEQQNKILLAELEQLKGQGKSRLGDLYEEEMRELRRQVDQLTNDKARVEVERDNLAE
DIMRLREKLQEEMLQREEAENTLQSFRQDVDNASLARLDLERKVESLQEEIAFLKKLHEE
EIQELQAQIQEQHVQIDVDVSKPDLTAALRDVRQQYESVAAKNLQEAEEWYKSKFADLSE

AANRNNDALRQAKQESTEYRRQVQSLTCEVDALKGTNESLERQMREMEENFAVEAANYQD
TIGRLQDEIQNMKEEMARHLREYQDLLNVKMALDIEIATYRKLLEGEESRISLPLPNFSS
LNLRETNLDSLPLVDTHSKRTLLIKTVETRDGQVINETSQHHDDLE*
>ENSG00000105612|ENST00000222219
MIPLLLAALLCVPAGALTCYGDSGQPVDWFVVYKLPALRGSGEAAQRGLQYKYLDESSGG
WRDGRALINSPEGAVGRSLQPLYRSNTSQLAFLLYNDQPPQPSKAQDSSMRGHTKGVLLL
DHDGGFWLVHSVPNFPPPASSAAYSWPHSACTYGQTLLCVSFPFAQFSKMGKQLTYTYPW
VYNYQLEGIFAQEFPDLENVVKGHHVSQEPWNSSITLTSQAGAVFQSFAKFSKFGDDLYS
GWLAAALGTNLQVQFWHKTVGILPSNCSDIWQVLNVNQIAFPGPAGPSFNSTEDHSKWCV
SPKGPWTCVGDMNRNQGEEQRGGGTLCAQLPALWKAFQPLVKNYQPCNGMARKPSRAYKI
*
>ENSG00000104221|ENST00000220659
MPGRGRCPDCGSTELVEDSHYSQSQLVCSDCGCVVTEGVLTTTFSDEGNLREVTYSRSTG
ENEQVSRSQQRGLRRVRDLCRVLQLPPTFEDTAVAYYQQAYRHSGIRAARLQKKEVLVGC
CVLITCRQHNWPLTMGAICTLLYADLDVFSSTYMQIVKLLGLDVPSLCLAELVKTYCSSF
KLFQASPSVPAKYVEDKEKMLSRTMQLVELANETWLVTGRHPLPVITAATFLAWQSLQPA
DRLSCSLARFCKLANVDLPYPASSRLQELLAVLLRMAEQLAWLRVLRLDKRSVVKHIGDL
LQHRQSLVRSAFRDGTAEVETREKEPPGWGQGQGEGEVGNNSLGLPQGKRPASPALLLPP
CMLKSPKRICPVPPVSTVTGDENISDSEIEQYLRTPQEVRDFQRAQAARQAATSVPNPP*
>ENSG00000110925|ENST00000228515
MDAFTGSGLKRKFDDVDVGSSVSNSDDEISSSDSADSCDSLNPPTTASFTPTSILKRQKQ
LRRKNVRFDQVTVYYFARRQGFTSVPSQGGSSLGMAQRHNSVRSYTLCEFAQEQEVNHRE
ILREHLKEEKLHAKKMKLTKNGTVESVEADGLTLDDVSDEDIDVENVEVDDYFFLQPLPT
KRRRALLRASGVHRIDAEEKQELRAIRLSREECGCDCRLYCDPEACACSQAGIKCQVDRM
SFPCGCSRDGCGNMAGRIEFNPIRVRTHYLHTIMKLELESKRQVSRPAAPDEEPSPTASC
SLTGAQGSETQDFQEFIAENETAVMHLQSAEELERLKAEEDSSGSSASLDSSIESLGVCI
LEEPLAVPEELCPGLTAPILIQAQLPPGSSVLCFTENSDHPTASTVNSPSYLNSGPLVYY
QVEQRPVLGVKGEPGTEEGSASFPKEKDLNVFSLPVTSLVACSSTDPAALCKSEVGKTPT
LEALLPEDCNPEEPENEDFHPSWSPSSLPFRTDNEEGCGMVKTSQQNEDRPPEDSSLELP
LAV*
>ENSG00000005100|ENST00000225296
MPEEAGFPPAKRFRPGSGPPSRAGSFPPGRQVVMLLTAGSGGRGGGGGRRQQPPLAQPSA
SPYPEAVELQRRSLPIFQARGQLLAQLRNLDNAVLIGETGSGKTTQIPQYLYEGGISRQG
IIAVTQPRRVAAISLATRVSDEKRTELGKLVGYTVRFDDVTSEDTRIKFLTDGMLLREAI
SDSLLRKYSCVILDEAHERTIHTDVLFGVVKAAQKRRKELGKLPLKVIVMSATMDVDLFS
QYFNGAPVLYLEGRQHPIQVFYTKQPQNDYLHAALVSVFQIHQEAPSSQDILVFLTGQEE
IEAMSKTCRDIAKHLPDGCPAMLVLPLYASLPYAQQLRVFQGAPKGYRKVIISTNIAETS
ITITGIKYVVDTGMVKAKKYNPDSGLEVLAVQRVSKTQAWQRTGRAGREDSGICYRLYTE
DEFEKFDKMTVPEIQRCNLASVMLQLLAMKVPNVLTFDFMSKPSPDHIQAAIAQLDLLGA
LEHKDDQLTLTPMGRKMAAFPLEPKFAKTILMSPKFHCTEEILTIVSLLSVDSVLHNPPS
RREEVQGVRKKFISSEGDHMTLLNIYRTFKNLGGNKDWCKENFVNSKNMTLVAEVRAQLR
DICLKMSMPIASSRGDVESVRRCLAHSLFMSTAELQPDGTYATTDTHQPVAIHPSSVLFH
CKPACVVYTELLYTNKCYMRDLCVIDAQWLYEAAPEYFRRKLRTARN*
>ENSG00000106591|ENST00000223324

MALAMLVLVVSPWSAARGVLRNYWERLLRKLPQSRPGFPSPPWGPALAVQGPAMFTEPAN
DTSGSKENSSLLDSIFWMAAPKNRRTIEVNRCRRRNPQKLIKVKNNIDVCPECGHLKQKH
VLCAYCYEKVCKETAEIRRQIGKQEGGPFKAPTIETVVLYTGETPSEQDQGKRIIERDRK
RPSWFTQN*
>ENSG00000103550|ENST00000219837
MITKTHKVDLGLPEKKKKKKVVKEPETRYSVLNNDDYFADVSPLRATSPSKSVAHGQAPE
MPLVKKKKKKKKGVSTLCEEHVEPETTLPARRTEKSPSLRKQVFGHLEFLSGEKKNKKSP
LAMSHASGVKTSPDPRQGEEETRVGKKLKKHKKEKKGAQDPTAFSVQDPWFCEAREARDV
GDTCSVGKKDEEQAALGQKRKRKSPREHNGKVKKKKKIHQEGDALPGHSKPSRSMESSPR
KGSKKKPVKVEAPEYIPISDDPKASAKKKMKSKKKVEQPVIEEPALKRKKKKKRKESGVA
GDPWKEETDTDLEVVLEKKGNMDEAHIDQVRRKALQEEIDRESGKTEASETRKWTGTQFG
QWDTAGFENEDQKLKFLRLMGGFKNLSPSFSRPASTIARPNMALGKKAADSLQQNLQRDY
DRAMSWKYSRGAGLGFSTAPNKIFYIDRNASKSVKLED*
>ENSG00000129152|ENST00000250003
MELLSPPLRDVDLTAPDGSLCSFATTDDFYDDPCFDSPDLRFFEDLDPRLMHVGALLKPE
EHSHFPAAVHPAPGAREDEHVRAPSGHHQAGRCLLWACKACKRKTTNADRRKAATMRERR
RLSKVNEAFETLKRCTSSNPNQRLPKVEILRNAIRYIEGLQALLRDQDAAPPGAAAAFYA
PGPLPPGRGGEHYSGDSDASSPRSNCSDGMMDYSGPPSGARRRNCYEGAYYNEAPSEPRP
GKSAAVSSLDCLSSIVERISTESPAAPALLLADVPSESPPRRQEAAAPSEGESSGDPTQS
PDAAPQCPAGANPNPIYQVL*
>ENSG00000124216|ENST00000244050
MPRSFLVRKPSDPNRKPNYSELQDSNPEFTFQQPYDQAHLLAAIPPPEILNPTASLPMLI
WDSVLAPQAQPIAWASLRLQESPRVAELTSLSDEDSGKGSQPPSPPSPAPSSFSSTSVSS
LEAEAYAAFPGLGQVPKQLAQLSEAKDLQARKAFNCKYCNKEYLSLGALKMHIRSHTLPC
VCGTCGKAFSRPWLLQGHVRTHTGEKPFSCPHCSRAFADRSNLRAHLQTHSDVKKYQCQA
CARTFSRMSLLHKHQESGCSGCPR*
>ENSG00000124613|ENST00000244576
MESLRGNTAQGPTNEEDYKNEGQLSRQTKCPAQKKSSFENTVVRKVSVTLKEIFTGEEGP
ESSEFSLSPNLDAQQKIPKGHGSPISRKNSKDNSDLIKHQRLFSQRKPCKCNECEKAFSY
QSDLLVHSRIHGGEKPFECNKCGKSFSRSTHLIEHQRTHTGEKPYECNECGKAFSRSTHL
SLHQRIHTGEKPYECSECGKAFSRSTNLSQHQRTHTQERPYKCNECGKAFGDRSTIIQHQ
RIHTGENPYECSKCGKAFSWISSLTEHQRTHTGENPYECSECGKVFSRSSSLTEHQRIHS
GEKPHECRVCGKGFSRSSSLIIHQRTHTGEKPYKCNDCGKAFCQSSTLIRHQHLHTKE*
>ENSG00000121417|ENST00000240731
MLGFPPGRPQLPVQLRPQTRMATALRDPASVPIATEVLFKLTQGSVTFEDVAVYFSWEEW
DLLDEAQKHLYFDVMLENFALTSSLGCWCGVEHEETPSEQRISGERVPQFRTSKEGSSSQ
NADSCEICCLVLRDILHLAEHQGTNCGQKLHTCGKQFYISANLQQHQRQHITEAPFRSYV
DTASFTQSCIVHVSEKPFTCREIRKDFLANMRFLHQDATQTGEKPNNSNKCAVAFYSGKS
HHNWGKCSKAFSHIDTLVQDQRILTREGLFECSKCGKACTRRCNLIQHQKVHSEERPYEC
NECGKFFTYYSSFIIHQRVHTGERPYACPECGKSFSQIYSLNSHRKVHTGERPYECGECG
KSFSQRSNLMQHRRVHTGERPYECSECGKSFSQNFSLIYHQRVHTGERPHECNECGKSFS
RSSSLIHHRRLHTGERPYECSKCGKSFKQSSSFSSHRKVHTGERPYVCGECGKSFSHSSN
LKNHQRVHTGERPVECSECSKSFSCKSNLIKHLRVHTGERPYECSECGKSFSQSSSLIQH
RRVHTGKRPYQCSQCGKSFGCKSVLIQHQRVHIGEKP*

>ENSG00000111786|ENST00000229390
MSGWADERGGEGDGRIYVGNLPTDVREKDLEDLFYKYGRIREIELKNRHGLVPFAFVRFE
DPRDAEDAIYGRNGYDYGQCRLRVEFPRTYGGRGGWPRGGRNGPPTRRSDFRVLVSGLPP
SGSWQDLKDHMREAGDVCYADVQKDGVGMVEYLRKEDMEYALRKLDDTKFRSHEGETSYI
RVYPERSTSYGYSRSRSGSRGRDSPYQSRGSPHYFSPFRPY*
>ENSG00000083307|ENST00000251808
MSQESDNNKRLVALVPMPSDPPFNTRRAYTSEDEAWKSYLENPLTAATKAMMSINGDEDS
AAALGLLYDYYKVPRDKRLLSVSKASDSQEDQEKRNCLGTSEAQSNLSGGENRVQVLKTV
PVNLSLNQDHLENSKREQYSISFPESSAIIPVSGITVVKAEDFTPVFMAPPVHYPRGDGE
EQRVVIFEQTQYDVPSLATHSAYLKDDQRSTPDSTYSESFKDAATEKFRSASVGAEEYMY
DQTSSGTFQYTLEATKSLRQKQGEGPMTYLNKGQFYAITLSETGDNKCFRHPISKVRSVV
MVVFSEDKNRDEQLKYWKYWHSRQHTAKQRVLDIADYKESFNTIGNIEEIAYNAVSFTWD
VNEEAKIFITVNCLSTDFSSQKGVKGLPLMIQIDTYSYNNRSNKPIHRAYCQIKVFCDKG
AERKIRDEERKQNRKKGKGQASQTQCNSSSDGKLAAIPLQKKSDITYFKTMPDLHSQPVL
FIPDVHFANLQRTGQVYYNTDDEREGGSVLVKRMFRPMEEEFGPVPSKQMKEEGTKRVLL
YVRKETDDVFDALMLKSPTVKGLMEAISEKYGLPVEKIAKLYKKSKKGILVNMDDNIIEH
YSNEDTFILNMESMVEGFKVTLMEI*
>ENSG00000124374|ENST00000244221
MNGSNMANTSPSVKSKEDQGLSGHDEKENPFAEYMWMENEEDFNRQVEEELQEQDFLDRC
FQEMLDEEDQDWFIPSRDLPQAMGQLQQQLNGLSVSEGHDSEDILSKSNLNPDAKEFIPG
EKY*
>ENSG00000129484|ENST00000250416
MAARRRRSTGGGRARALNESKRVNNGNTAPEDSSPAKKTRRCQRQESKKMPVAGGKANKD
RTEDKQDGMPGRSWASKRVSESVKALLLKGKAPVDPECTAKVGKAHVYCEGNDVYDVMLN
QTNLQFNNNKYYLIQLLEDDAQRNFSVWMRWGRVGKMGQHSLVACSGNLNKAKEIFQKKF
LDKTKNNWEDREKFEKVPGKYDMLQMDYATNTQDEEETKKEESLKSPLKPESQLDLRVQE
LIKLICNVQAMEEMMMEMKYNTKKAPLGKLTVAQIKAGYQSLKKIEDCIRAGQHGRALME
ACNEFYTRIPHDFGLRTPPLIRTQKELSEKIQLLEALGDIEIAIKLVKTELQSPEHPLDQ
HYRNLHCALRPLDHESYEFKVISQYLQSTHAPTHSDYTMTLLDLFEVEKDGEKEAFREDL
HNRMLLWHGSRMSNWVGILSHGLRIAPPEAPITGYMFGKGIYFADMSSKSANYCFASRLK
NTGLLLLSEVALGQCNELLEANPKAEGLLQGKHSTKGLGKMAPSSAHFVTLNGSTVPLGP
ASDTGILNPDGYTLNYNEYIVYNPNQVRMRYLLKVQFNFLQLW*
>ENSG00000123136|ENST00000242776
MAEQDVENDLLDYDEEEEPQAPQESTPAPPKKDIKGSYVSIHSSGFRDFLLKPELLRAIV
DCGFEHPSEVQHECIPQAILGMDVLCQAKSGMGKTAVFVLATLQQIEPVNGQVTVLVMCH
TRELAFQISKEYERFSKYMPSVKVSVFFGGLSIKKDEEVLKKNCPHVVVGTPGRILALVR
NRSFSLKNVKHFVLDECDKMLEQLDMRRDVQEIFRLTPHEKQCMMFSATLSKDIRPVCRK
FMQDPMEVFVDDETKLTLHGLQQYYVKLKDSEKNRKLFDLLDVLEFNQVIIFVKSVQRCM
ALAQLLVEQNFPAIAIHRGMAQEERLSRYQQFKDFQRRILVATNLFGRGMDIERVNIVFN
YDMPEDSDTYLHRVARAGRFGTKGLAITFVSDENDAKILNDVQDRFEVNVAELPEEIDIS
TYIEQSR*
>ENSG00000103449|ENST00000251020
MSRRKQAKPQHFQSDPEVASLPRRDGDTEKGQPSRPTKSKDAHVCGRCCAEFFELSDLLL
HKKNCTKNQLVLIVNENPASPPETFSPSPPPDNPDEQMNDTVNKTDQVDCSDLSEHNGLD

REESMEVEAPVANKSGSGTSSGSHSSTAPSSSSSSSSSSGGGGSSSTGTSAITTSLPQLG
DLTTLGNFSVINSNVIIENLQSTKVAVAQFSQEARCGGASGGKLAVPALMEQLLALQQQQ
IHQLQLIEQIRHQILLLASQNADLPTSSSPSQGTLRTSANPLSTLSSHLSQQLAAAAGLA
QSLASQSASISGVKQLPPIQLPQSSSGNTIIPSNSGSSPNMNILAAAVTTPSSEKVASSA
GASHVSNPAVSSSSSPAFAISSLLSPASNPLLPQQASANSVFPSPLPNIGTTAEDLNSLS
ALAQQRKSKPPNVTAFEAKSTSDEAFFKHKCRFCAKVFGSDSALQIHLRSHTGERPFKCN
ICGNRFSTKGNLKVHFQRHKEKYPHIQMNPYPVPEHLDNIPTSTGIPYGMSIPPEKPVTS
WLDTKPVLPTLTTSVGLPLPPTLPSLIPFIKTEEPAPIPISHSATSPPGSVKSDSGGPES
ATRNLGGLPEEAEGSTLPPSGGKSEESGMVTNSVPTASSSVLSSPAADCGPAGSATTFTN
PLLPLMSEQFKAKFPFGGLLDSAQASETSKLQQLVENIDKKATDPNECIICHRVLSCQSA
LKMHYRTHTGERPFKCKICGRAFTTKGNLKTHYSVHRAMPPLRVQHSCPICQKKFTNAVV
LQQHIRMHMGGQIPNTPVPDSYSESMESDTGSFDEKNFDDLDNFSDENMEDCPEGSIPDT
PKSADASQDSLSSSPLPLEMSSIAALENQMKMINAGLAEQLQASLKSVENGSIEGDVLTN
DSSSVGGDMESQSAGSPAISESTSSMQALSPSNSTQEFHKSPSIEEKPQRAVPSEFANGL
SPTPVNGGALDLTSSHAEKIIKEDSLGILFPFRDRGKFKNTACDICGKTFACQSALDIHY
RSHTKERPFICTVCNRGFSTKGNLKQHMLTHQMRDLPSQLFEPSSNLGPNQNSAVIPANS
LSSLIKTEVNGFVHVSPQDSKDTPTSHVPSGPLSSSATSPVLLPALPRRTPKQHYCNTCG
KTFSSSSALQIHERTHTGEKPFACTICGRAFTTKGNLKVHMGTHMWNSTPARRGRRLSVD
GPMTFLGGNPVKFPEMFQKDLAARSGSGDPSSFWNQYAAALSNGLAMKANEISVIQNGGI
PPIPGSLGSGNSSPVSGLTGNLERLQNSEPNAPLAGLEKMASSENGTNFRFTRFVEDSKE
IVTS*
>ENSG00000124380|ENST00000244227
MGRSRSRSPRRERRRSRSTSRERERRRRERSRSRERDRRRSRSRSPHRRRSRSPRRHRST
SPSPSRLKERRDEEKKETKETKSKERQITEEDLEGKTEEEIEMMKLMGFASFDSTKGKKV
DGSVNAYAINVSQKRKYRQYMNRKGGFNRPLDFIA*
>ENSG00000114784|ENST00000232905
MSTIQNLQSFDPFADATKGDDLLPAGTEDYIHIRIQQRNGRKTLTTVQGIADDYDKKKLV
KAFKKKFACNGTVIEHPEYGEVIQLQGDQRKNICQFLLEVGIVKEEQLKVHGF*
>ENSG00000124562|ENST00000244520
MPKFYCDYCDTYLTHDSPSVRKTHCSGRKHKENVKDYYQKWMEEQAQSLIDKTTAAFQQG
KIPPTPFSAPPPAGAMIPPPPSLPGPPRPGMMPAPHMGGPPMMPMMGPPPPGMMPVGPAP
GMRPPMGGHMPMMPGPPMMRPPARPMMVPTRPGMTRPDR*
>ENSG00000111358|ENST00000228955
MVLGNSHLFMNRSNKLAVIASHIQESRFLYPGKNGRLGDFFGDPGNPPEFNPSGSKDGKY
ELLTSANEVIVEEIKDLMTKSDIKGQHTETLLAGSLAKALCYIHRMNKEVKDNQEMKSRI
LVIKAAEDSALQYMNFMNVIFAAQKQNILIDACVLDSDSGLLQQACDITGGLYLKVPQMP
SLLQYLLWVFLPDQDQRSQLILPPPVHVDYRAACFCHRNLIEIGYVCSVCLSIFCNFSPI
CTTCETAFKISLPPVLKAKKKKLKVSA*
>ENSG00000115816|ENST00000234170
MAAVKEPLEFHAKRPWRPEEAVEDPDEEDEDNTSEAENGFSLEEVLRLGGTKQDYLMLAT
LDENEEVIDGGKKGAIDDLQQGELEAFIQNLNLAKYTKASLVEEDEPAEKENSSKKEVKI
PKINNKNTAESQRTSVNKVKNKNRPEPHSDENGSTTPKVKKDKQNIFEFFERQTLLLRPG
GKWYDLEYSNEYSLKPQPQDVVSKYKTLAQKLYQHEINLFKSKTNSQKGASSTWMKAIVS
SGTLGDRMAAMILLIQDDAVHTLQFVETLVNLVKKKGSKQQCLMALDTFKELLITDLLPD

NRKLRIFSQRPFDKLEQLSSGNKDSRDRRLILWYFEHQLKHLVAEFVQVLETLSHDTLVT
TKTRALTVAHELLCNKPEEEKALLVQVVNKLGDPQNRIATKASHLLETLLCKHPNMKGVV
SGEVERLLFRSNISSKAQYYAICFLNQMALSHEESELANKLITVYFCFFRTCVKKKDVES
KMLSALLTGVNRAYPYSQTGDDKVREQIDTLFKVLHIVNFNTSVQALMLLFQVMNSQQTI
SDRYYTALYRKMLDPGLMTCSKQAMFLNLVYKSLKADIVLRRVKAFVKRLLQVTCQQMPP
FICGALYLVSEILKAKPGLRSQLDDHPESDDEENFIDANDDEDMEKFTDADKETEIVKKL
ETEETVPETDVETKKPEVASWVHFDNLKGGKQLNKYDPFSRNPLFCGAENTSLWELKKLS
VHFHPSVALFAKTILQGNYIQYSGDPLQDFTLMRFLDRFVYRNPKPHKGKENTDSVVMQP
KRKHFIKDIRHLPVNSKEFLAKEESQIPVDEVFFHRYYKKVAVKEKQKRDADEESIEDVD
DEEFEELIDTFEDDNCFSSGKDDMDFAGNVKKRTKGAKDNTLDEDSEGSDDELGNLDDDE
VSLGSMDDEEFAEVDEDGGTFMDVLDDESESVPELEVHSKVSTKKSKRKGTDDFDFAGSF
QGPRKKKRNLNDSSLFVSAEEFGHLLDENMGSKFDNIGMNAMANKDNASLKQLRWEAERD
DWLHNRDAKSIIKKKKHFKKKRIKTTQKTKKQRK*
>ENSG00000124788|ENST00000244769
MKSNQERSNECLPPKKREIPATSRSSEEKAPTLPSDNHRVEGTAWLPGNPGGRGHGGGRH
GPAGTSVELGLQQGIGLHKALSTGLDYSPPSAPRSVPVATTLPAAYATPQPGTPVSPVQY
AHLPHTFQFIGSSQYSGTYASFIPSQLIPPTANPVTSAVASAAGATTPSQRSQLEAYSTL
LANMGSLSQTPGHKAEQQQQQQQQQQQQHQHQQQQQQQQQQQQQQHLSRAPGLITPGSPP
PAQQNQYVHISSSPQNTGRTASPPAIPVHLHPHQTMIPHTLTLGPPSQVVMQYADSGSHF
VPREATKKAESSRLQQAIQAKEVLNGEMEKSRRYGAPSSADLGLGKAGGKSVPHPYESRH
VVVHPSPSDYSSRDPSGVRASVMVLPNSNTPAADLEVQQATHREASPSTLNDKSGLHLGK
PGHRSYALSPHTVIQTTHSASEPLPVGLPATAFYAGTQPPVIGYLSGQQQAITYAGSLPQ
HLVIPGTQPLLIPVGSTDMEASGAAPAIVTSSPQFAAVPHTFVTTALPKSENFNPEALVT
QAAYPAMVQAQIHLPVVQSVASPAAAPPTLPPYFMKGSIIQLANGELKKVEDLKTEDFIQ
SAEISNDLKIDSSTVERIEDSHSPGVAVIQFAVGEHRAQVSVEVLVEYPFFVFGQGWSSC
CPERTSQLFDLPCSKLSVGDVCISLTLKNLKNGSVKKGQPVDPASVLLKHSKADGLAGSR
HRYAEQENGINQGSAQMLSENGELKFPEKMGLPAAPFLTKIEPSKPAATRKRRWSAPESR
KLEKSEDEPPLTLPKPSLIPQEVKICIEGRSNVGK*
>ENSG00000122859|ENST00000242462
MTPQPSGAPTVQVTRETERSFPRASEDEVTCPTSAPPSPTRTRGNCAEAEEGGCRGAPRK
LRARRGGRSRPKSELALSKQRRSRRKKANDRERNRMHNLNSALDALRGVLPTFPDDAKLT
KIETLRFAHNYIWALTQTLRIADHSLYALEPPAPHCGELGSPGGSPGDWGSLYSPVSQAG
SLSPAASLEERPGLLGATFSACLSPGSLAFSDFL*
>ENSG00000115421|ENST00000238714
MKEMSANTVLDSQRQQKHYGITSPISLASPKEIDHIYTQKLIDAMKPFGVFEDEEELNHR
LVVLGKLNNLVKEWISDVSESKNLPPSVVATVGGKIFTFGSYRLGVHTKGADIDALCVAP
RHVERSDFFQSFFEKLKHQDGIRNLRAVEDAFVPVIKFEFDGIEIDLVFARLAIQTISDN
LDLRDDSRLRSLDIRCIRSLNGCRVTDEILHLVPNKETFRLTLRAVKLWAKRRGIYSNML
GFLGGVSWAMLVARTCQLYPNAAASTLVHKFFLVFSKWEWPNPVLLKQPEESNLNLPVWD
PRVNPSDRYHLMPIITPAYPQQNSTYNVSTSTRTVMVEEFKQGLAVTDEILQGKSDWSKL
LEPPNFFQKYRHYIVLTASASTEENHLEWVGLVESKIRVLVGNLERNEFITLAHVNPQSF
PGNKEHHKDNNYVSMWFLGIIFRRVENAESVNIDLTYDIQSFTDTVYRQANNINMLKEGM
KIEATHVKKKQLHHYLPAEILQKKKKQSLSDVNRSSGGLQSKRLSLDSSCLDSSRDTDNG
TPFNSPASKSDSPSVGETERNSAEPAAVIVEKPLSVPPAQGLSIPVIGAKVDSTVKTVSP

PTVCTIPTVVGRNVIPRITTPHNPAQGQPHLNGMSNITKTVTPKRSHSPSIDGTPKRLKD
VEKFIRLESTFKDPRTAEERKRKSVDAIGGESMPIPTIDTSRKKRLPSKELPDSSSPVPA
NNIRVIKNSIRLTLNR*
>ENSG00000113916|ENST00000232014
MASPADSCIQFTRHASDVLLNLNRLRSRDILTDVVIVVSREQFRAHKTVLMACSGLFYSI
FTDQLKCNLSVINLDPEINPEGFCILLDFMYTSRLNLREGNIMAVMATAMYLQMEHVVDT
CRKFIKASEAEMVSAIKPPREEFLNSRMLMPQDIMAYRGREVVENNLPLRSAPGCESRAF
APSLYSGLSTPPASYSMYSHLPVSSLLFSDEEFRDVRMPVANPFPKERALPCDSARPVPG
EYSRPTLEVSPNVCHSNIYSPKETIPEEARSDMHYSVAEGLKPAAPSARNAPYFPCDKAS
KEEERPSSEDEIALHFEPPNAPLNRKGLVSPQSPQKSDCQPNSPTESCSSKNACILQASG
SPPAKSPTDPKACNWKKYKFIVLNSLNQNAKPEGPEQAELGRLSPRAYTAPPACQPPMEP
ENLDLQSPTKLSASGEDSTIPQASRLNNIVNRSMTGSPRSSSESHSPLYMHPPKCTSCGS
QSPQHAEMCLHTAGPTFPEEMGETQSEYSDSSCENGAFFCNECDCRFSEEASLKRHTLQT
HSDKPYKCDRCQASFRYKGNLASHKTVHTGEKPYRCNICGAQFNRPANLKTHTRIHSGEK
PYKCETCGARFVQVAHLRAHVLIHTGEKPYPCEICGTRFRHLQTLKSHLRIHTGEKPYHC
EKCNLHFRHKSQLRLHLRQKHGAITNTKVQYRVSATDLPPELPKAC*
>ENSG00000111615|ENST00000229214
MASPSLERPEKGAGKSEFRNQKPKPENQDESELLTVPDGWKEPAFSKEDNPRGLLEESSF
ATLFPKYREAYLKECWPLVQKALNEHHVNATLDLIEGSMTVCTTKKTFDPYIIIRARDLI
KLLARSVSFEQAVRILQDDVACDIIKIGSLVRNKERFVKRRQRLIGPKGSTLKALELLTN
CYIMVQGNTVSAIGPFSGLKEVRKVVLDTMKNIHPIYNIKSLMIKRELAKDSELRSQSWE
RFLPQFKHKNVNKRKEPKKKTVKKEYTPFPPPQPESQIDKELASGEYFLKANQKKRQKME
AIKAKQAEAISKRQEERNKAFIPPKEKPIVKPKEASTETKIDVASIKEKVKKAKNKKLGA
LTAEEIALKMEADEKKKKKKK*
>ENSG00000105373|ENST00000246802
MAAGGSGVGGKRSSKSDADSGFLGLRPTSVDPALRRRRRGPRNKKRGWRRLAQEPLGLEV
DQFLEDVRLQERTSGGLLSEAPNEKLFFVDTGSKEKGLTKKRTKVQKKSLLLKKPLRVDL
ILENTSKVPAPKDVLAHQVPNAKKLRRKEQLWEKLAKQGELPREVRRAQARLLNPSATRA
KPGPQDTVERPFYDLWASDNPLDRPLVGQDEFFLEQTKKKGVKRPARLHTKPSQAPAVEV
APAGASYNPSFEDHQTLLSAAHEVELQRQKEAEKLERQLALPATEQAATQESTFQELCEG
LLEESDGEGEPGQGEGPEAGDAEVCPTPARLATTEKKTEQQRRREKAVHRLRVQQAALRA
ARLRHQELFRLRGIKAQVALRLAELARRQRRRQARREAEADKPRRLGRLKYQAPDIDVQL
SSELTDSLRTLKPEGNILRDRFKSFQRRNMIEPRERAKFKRKYKVKLVEKRAFREIQL*
>ENSG00000122592|ENST00000242159
MSSSYYVNALFSKYTAGASLFQNAEPTSCSFAPNSQRSGYGAGAGAFASTVPGLYNVNSP
LYQSPFASGYGLGADAYGNLPCASYDQNIPGLCSDLAKGACDKTDEGALHGAAEANFRIY
PWMRSSGPDRKRGRQTYTRYQTLELEKEFHFNRYLTRRRRIEIAHALCLTERQIKIWFQN
RRMKWKKEHKDEGPTAAAAPEGAVPSAAATAAADKADEEDDDEEEEDEEE*
>ENSG00000113722|ENST00000231656
MYVGYVLDKDSPVYPGPARPASLGLGPQAYGPPAPPPAPPQYPDFSSYSHVEPAPAPPTA
WGAPFPAPKDDWAAAYGPGPAAPAASPASLAFGPPPDFSPVPAPPGPGPGLLAQPLGGPG
TPSSPGAQRPTPYEWMRRSVAAGGGGGSGKTRTKDKYRVVYTDHQRLELEKEFHYSRYIT
IRRKSELAANLGLTERQVKIWFQNRRAKERKVNKKKQQQQQPPQPPMAHDITATPAGPSL
GGLCPSNTSLLATSSPMPVKEEFLP*

>ENSG00000129194|ENST00000250055
MALPGSSQDQAWSLEPPAATAAASSSSGPQEREGAGSPAAPGTLPLEKVKRPMNAFMVWS
SAQRRQMAQQNPKMHNSEISKRLGAQWKLLDEDEKRPFVEEAKRLRARHLRDYPDYKYRP
RRKAKSSGAGPSRCGQGRGNLASGGPLWGPGYATTQPSRGFGYRPPSYSTAYLPGSYGSS
HCKLEAPSPCSLPQSDPRLQGELLPTYTHYLPPGSPTPYNPPLAGAPMPLTHL*
>ENSG00000128311|ENST00000249042
MVHQVLYRALVSTKWLAESIRTGKLGPGLRVLDASWYSPGTREARKEYLERHVPGASFFD
IEECRDTASPYEMMLPSEAGFAEYVGRLGISNHTHVVVYDGEHLGSFYAPRVWWMFRVFG
HRTVSVLNGGFRNWLKEGHPVTSEPSRPEPAVFKATLDRSLLKTYEQVLENLESKRFQLV
DSRSQGRFLGTEPEPDAVGLDSGHIRGAVNMPFMDFLTEDGFEKGPEELRALFQTKKVDL
SQPLIATCRKGVTACHVALAAYLCGKPDVAVYDGSWSEWFRRAPPESRVSQGKSEKA*
>ENSG00000117614|ENST00000236273
MAAIAASEVLVDSAEEGSLAAAAELAAQKREQRLRKFRELHLMRNEARKLNHQEVVEEDK
RLKLPANWEAKKARLEWELKEEEKKKECAARGEDYEKVKLLEISAEDAERWERKKKRKNP
DLGFSDYAAAQLRQYHRLTKQIKPDMETYERLREKHGEEFFPTSNSLLHGTHVPSTEEID
RMVIDLEKQIEKRDKYSRRRPYNDDADIDYINERNAKFNKKAERFYGKYTAEIKQNLERG
TAV*
>ENSG00000108406|ENST00000251241
MSRFPAVAGRAPRRQEEGERSRDLQEERLSAVCIADREEKGCTSQEGGTTPTFPIQKQRK
KIIQAVRDNSFLIVTGNTGSGKTTQLPKYLYEAGFSQHGMIGVTQPRKVAAISVAQRVAE
EMKCTLGSKVGYQVRFDDCSSKETAIKYMTDGCLLKHILGDPNLTKFSVIILDEAHERTL
TTDILFGLLKKLFQEKSPNRKEHLKVVVMSATMELAKLSAFFGNCPIFDIPGRLYPVREK
FCNLIGPRDRENTAYIQAIVKVTMDIHLNEMAGDILVFLTGQFEIEKSCELLFQMAESVD
YDYDVQDTTLDGLLILPCYGSMTTDQQRRIFLPPPPGIRKCVISTNISATSLTIDGIRYV
VDGGFVKQLNHNPRLGLDILEVVPISKSEALQRSGRAGRTSSGKCFRIYSKDFWNQCMPD
HVIPEIKRTSLTSVVLTLKCLAIHDVIRFPYLDPPNERLILEALKQLYQCDAIDRSGHVT
RLGLSMVEFPLPPHLTCAVIKAASLDCEDLLLPIAAMLSVENVFIRPVDPEYQKEAEQRH
RELAAKAGGFNDFATLAVIFEQCKSSGAPASWCQKHWIHWRCLFSAFRVEAQLRELIRKL
KQQSDFPKETFEGPKHEVLRRCLCAGYFKNVARRSVGRTFCTMDGRGSPVHIHPSSALHE
QETKLEWIIFHEVLVTTKVYARIVCPIRYEWVRDLLPKLHEFNAHDLSSVARREVREDAR
RRWTNKENVKQLKDGISKDVLKKMQRRNDDKSISDARARFLERKQQRTQDHSDTRKETG*
>ENSG00000123384|ENST00000243077
MLTPPLLLLLPLLSALVAAAIDAPKTCSPKQFACRDQITCISKGWRCDGERDCPDGSDEA
PEICPQSKAQRCQPNEHNCLGTELCVPMSRLCNGVQDCMDGSDEGPHCRELQGNCSRLGC
QHHCVPTLDGPTCYCNSSFQLQADGKTCKDFDECSVYGTCSQLCTNTDGSFICGCVEGYL
LQPDNRSCKAKNEPVDRPPVLLIANSQNILATYLSGAQVSTITPTSTRQTTAMDFSYANE
TVCWVHVGDSAAQTQLKCARMPGLKGFVDEHTINISLSLHHVEQMAIDWLTGNFYFVDDI
DDRIFVCNRNGDTCVTLLDLELYNPKGIALDPAMGKVFFTDYGQIPKVERCDMDGQNRTK
LVDSKIVFPHGITLDLVSRLVYWADAYLDYIEVVDYEGKGRQTIIQGILIEHLYGLTVFE
NYLYATNSDNANAQQKTSVIRVNRFNSTEYQVVTRVDKGGALHIYHQRRQPRVRSHACEN
DQYGKPGGCSDICLLANSHKARTCRCRSGFSLGSDGKSCKKPEHELFLVYGKGRPGIIRG
MDMGAKVPDEHMIPIENLMNPRALDFHAETGFIYFADTTSYLIGRQKIDGTERETILKDG
IHNVEGVAVDWMGDNLYWTDDGPKKTISVARLEKAAQTRKTLIEGKMTHPRAIVVDPLNG
WMYWTDWEEDPKDSRRGRLERAWMDGSHRDIFVTSKTVLWPNGLSLDIPAGRLYWVDAFY

DRIETILLNGTDRKIVYEGPELNHAFGLCHHGNYLFWTEYRSGSVYRLERGVGGAPPTVT
LLRSERPPIFEIRMYDAQQQQVGTNKCRVNNGGCSSLCLATPGSRQCACAEDQVLDADGV
TCLANPSYVPPPQCQPGEFACANSRCIQERWKCDGDNDCLDNSDEAPALCHQHTCPSDRF
KCENNRCIPNRWLCDGDNDCGNSEDESNATCSARTCPPNQFSCASGRCIPISWTCDLDDD
CGDRSDESASCAYPTCFPLTQFTCNNGRCININWRCDNDNDCGDNSDEAGCSHSCSSTQF
KCNSGRCIPEHWTCDGDNDCGDYSDETHANCTNQATRPPGGCHTDEFQCRLDGLCIPLRW
RCDGDTDCMDSSDEKSCEGVTHVCDPSVKFGCKDSARCISKAWVCDGDNDCEDNSDEENC
ESLACRPPSHPCANNTSVCLPPDKLCDGNDDCGDGSDEGELCDQCSLNNGGCSHNCSVAP
GEGIVCSCPLGMELGPDNHTCQIQSYCAKHLKCSQKCDQNKFSVKCSCYEGWVLEPDGES
CRSLDPFKPFIIFSNRHEIRRIDLHKGDYSVLVPGLRNTIALDFHLSQSALYWTDVVEDK
IYRGKLLDNGALTSFEVVIQYGLATPEGLAVDWIAGNIYWVESNLDQIEVAKLDGTLRTT
LLAGDIEHPRAIALDPRDGILFWTDWDASLPRIEAASMSGAGRRTVHRETGSGGWPNGLT
VDYLEKRILWIDARSDAIYSARYDGSGHMEVLRGHEFLSHPFAVTLYGGEVYWTDWRTNT
LAKANKWTGHNVTVVQRTNTQPFDLQVYHPSRQPMAPNPCEANGGQGPCSHLCLINYNRT
VSCACPHLMKLHKDNTTCYEFKKFLLYARQMEIRGVDLDAPYYNYIISFTVPDIDNVTVL
DYDAREQRVYWSDVRTQAIKRAFINGTGVETVVSADLPNAHGLAVDWVSRNLFWTSYDTN
KKQINVARLDGSFKNAVVQGLEQPHGLVVHPLRGKLYWTDGDNISMANMDGSNRTLLFSG
QKGPVGLAIDFPESKLYWISSGNHTINRCNLDGSGLEVIDAMRSQLGKATALAIMGDKLW
WADQVSEKMGTCSKADGSGSVVLRNSTTLVMHMKVYDESIQLDHKGTNPCSVNNGDCSQL
CLPTSETTRSCMCTAGYSLRSGQQACEGVGSFLLYSVHEGIRGIPLDPNDKSDALVPVSG
TSLAVGIDFHAENDTIYWVDMGLSTISRAKRDQTWREDVVTNGIGRVEGIAVDWIAGNIY
WTDQGFDVIEVARLNGSFRYVVISQGLDKPRAITVHPEKGYLFWTEWGQYPRIERSRLDG
TERVVLVNVSISWPNGISVDYQDGKLYWCDARTDKIERIDLETGENREVVLSSNNMDMFS
VSVFEDFIYWSDRTHANGSIKRGSKDNATDSVPLRTGIGVQLKDIKVFNRDRQKGTNVCA
VANGGCQQLCLYRGRGQRACACAHGMLAEDGASCREYAGYLLYSERTILKSIHLSDERNL
NAPVQPFEDPEHMKNVIALAFDYRAGTSPGTPNRIFFSDIHFGNIQQINDDGSRRITIVE
NVGSVEGLAYHRGWDTLYWTSYTTSTITRHTVDQTRPGAFERETVITMSGDDHPRAFVLD
ECQNLMFWTNWNEQHPSIMRAALSGANVLTLIEKDIRTPNGLAIDHRAEKLYFSDATLDK
IERCEYDGSHRYVILKSEPVHPFGLAVYGEHIFWTDWVRRAVQRANKHVGSNMKLLRVDI
PQQPMGIIAVANDTNSCELSPCRINNGGCQDLCLLTHQGHVNCSCRGGRILQDDLTCRAV
NSSCRAQDEFECANGECINFSLTCDGVPHCKDKSDEKPSYCNSRRCKKTFRQCSNGRCVS
NMLWCNGADDCGDGSDEIPCNKTACGVGEFRCRDGTCIGNSSRCNQFVDCEDASDEMNCS
ATDCSSYFRLGVKGVLFQPCERTSLCYAPSWVCDGANDCGDYSDERDCPGVKRPRCPLNY
FACPSGRCIPMSWTCDKEDDCEHGEDETHCNKFCSEAQFECQNHRCISKQWLCDGSDDCG
DGSDEAAHCEGKTCGPSSFSCPGTHVCVPERWLCDGDKDCADGADESIAAGCLYNSTCDD
REFMCQNRQCIPKHFVCDHDRDCADGSDESPECEYPTCGPSEFRCANGRCLSSRQWECDG
ENDCHDQSDEAPKNPHCTSQEHKCNASSQFLCSSGRCVAEALLCNGQDDCGDSSDERGCH
INECLSRKLSGCSQDCEDLKIGFKCRCRPGFRLKDDGRTCADVDECSTTFPCSQRCINTH
GSYKCLCVEGYAPRGGDPHSCKAVTDEEPFLIFANRYYLRKLNLDGSNYTLLKQGLNNAV
ALDFDYREQMIYWTDVTTQGSMIRRMHLNGSNVQVLHRTGLSNPDGLAVDWVGGNLYWCD
KGRDTIEVSKLNGAYRTVLVSSGLREPRALVVDVQNGYLYWTDWGDHSLIGRIGMDGSSR
SVIVDTKITWPNGLTLDYVTERIYWADAREDYIEFASLDGSNRHVVLSQDIPHIFALTLF
EDYVYWTDWETKSINRAHKTTGTNKTLLISTLHRPMDLHVFHALRQPDVPNHPCKVNNGG
CSNLCLLSPGGGHKCACPTNFYLGSDGRTCVSNCTASQFVCKNDKCIPFWWKCDTEDDCG

DHSDEPPDCPEFKCRPGQFQCSTGICTNPAFICDGDNDCQDNSDEANCDIHVCLPSQFKC
TNTNRCIPGIFRCNGQDNCGDGEDERDCPEVTCAPNQFQCSITKRCIPRVWVCDRDNDCV
DGSDEPANCTQMTCGVDEFRCKDSGRCIPARWKCDGEDDCGDGSDEPKEECDERTCEPYQ
FRCKNNRCVPGRWQCDYDNDCGDNSDEESCTPRPCSESEFSCANGRCIAGRWKCDGDHDC
ADGSDEKDCTPRCDMDQFQCKSGHCIPLRWRCDADADCMDGSDEEACGTGVRTCPLDEFQ
CNNTLCKPLAWKCDGEDDCGDNSDENPEECARFVCPPNRPFRCKNDRVCLWIGRQCDGTD
NCGDGTDEEDCEPPTAHTTHCKDKKEFLCRNQRCLSSSLRCNMFDDCGDGSDEEDCSIDP
KLTSCATNASICGDEARCVRTEKAAYCACRSGFHTVPGQPGCQDINECLRFGTCSQLCNN
TKGGHLCSCARNFMKTHNTCKAEGSEYQVLYIADDNEIRSLFPGHPHSAYEQAFQGDESV
RIDAMDVHVKAGRVYWTNWHTGTISYRSLPPAAPPTTSNRHRRQIDRGVTHLNISGLKMP
RGIAIDWVAGNVYWTDSGRDVIEVAQMKGENRKTLISGMIDEPHAIVVDPLRGTMYWSDW
GNHPKIETAAMDGTLRETLVQDNIQWPTGLAVDYHNERLYWADAKLSVIGSIRLNGTDPI
VAADSKRGLSHPFSIDVFEDYIYGVTYINNRVFKIHKFGHSPLVNLTGGLSHASDVVLYH
QHKQPEVTNPCDRKKCEWLCLLSPSGPVCTCPNGKRLDNGTCVPVPSPTPPPDAPRPGTC
NLQCFNGGSCFLNARRQPKCRCQPRYTGDKCELDQCWEHCRNGGTCAASPSGMPTCRCPT
GFTGPKCTQQVCAGYCANNSTCTVNQGNQPQCRCLPGFLGDRCQYRQCSGYCENFGTCQM
AADGSRQCRCTAYFEGSRCEVNKCSRCLEGACVVNKQSGDVTCNCTDGRVAPSCLTCVGH
CSNGGSCTMNSKMMPECQCPPHMTGPRCEEHVFSQQQPGHIASILIPLLLLLLLVLVAGV
VFWYKRRVQGAKGFQHQRMTNGAMNVEIGNPTYKMYEGGEPDDVGGLLDADFALDPDKPT
NFTNPVYATLYMGGHGSRHSLASTDEKRELLGRGPEDEIGDPLA*
>ENSG00000123358|ENST00000243050
MPCIQAQYGTPAPSPGPRDHLASDPLTPEFIKPTMDLASPEAAPAAPTALPSFSTFMDGY
TGEFDTFLYQLPGTVQPCSSASSSASSTSSSSATSPASASFKFEDFQVYGCYPGPLSGPV
DEALSSSGSDYYGSPCSAPSPSTPSFQPPQLSPWDGSFGHFSPSQTYEGLRAWTEQLPKA
SGPPQPPAFFSFSPPTGPSPSLAQSPLKLFPSQATHQLGEGESYSMPTAFPGLAPTSPHL
EGSGILDTPVTSTKARSGAPGGSEGRCAVCGDNASCQHYGVRTCEGCKGFFKRTVQKNAK
YICLANKDCPVDKRRRNRCQFCRFQKCLAVGMVKEVVRTDSLKGRRGRLPSKPKQPPDAS
PANLLTSLVRAHLDSGPSTAKLDYSKFQELVLPHFGKEDAGDVQQFYDLLSGSLEVIRKW
AEKIPGFAELSPADQDLLLESAFLELFILRLAYRSKPGEGKLIFCSGLVLHRLQCARGFG
DWIDSILAFSRSLHSLLVDVPAFACLSALVLITDRHGLQEPRRVEELQNRIASCLKEHVA
AVAGEPQPASCLSRLLGKLPELRTLCTQGLQRIFYLKLEDLVPPPPIIDKIFMDTLPF*
>ENSG00000076242|ENST00000231790
MSFVAGVIRRLDETVVNRIAAGEVIQRPANAIKEMIENCLDAKSTSIQVIVKEGGLKLIQ
IQDNGTGIRKEDLDIVCERFTTSKLQSFEDLASISTYGFRGEALASISHVAHVTITTKTA
DGKCAYRASYSDGKLKAPPKPCAGNQGTQITVEDLFYNIATRRKALKNPSEEYGKILEVV
GRYSVHNAGISFSVKKQGETVADVRTLPNASTVDNIRSIFGNAVSRELIEIGCEDKTLAF
KMNGYISNANYSVKKCIFLLFINHRLVESTSLRKAIETVYAAYLPKNTHPFLYLSLEISP
QNVDVNVHPTKHEVHFLHEESILERVQQHIESKLLGSNSSRMYFTQTLLPGLAGPSGEMV
KSTTSLTSSSTSGSSDKVYAHQMVRTDSREQKLDAFLQPLSKPLSSQPQAIVTEDKTDIS
SGRARQQDEEMLELPAPAEVAAKNQSLEGDTTKGTSEMSEKRGPTSSNPRKRHREDSDVE
MVEDDSRKEMTAACTPRRRIINLTSVLSLQEEINEQGHEVLREMLHNHSFVGCVNPQWAL
AQHQTKLYLLNTTKLSEELFYQILIYDFANFGVLRLSEPAPLFDLAMLALDSPESGWTEE
DGPKEGLAEYIVEFLKKKAEMLADYFSLEIDEEGNLIGLPLLIDNYVPPLEGLPIFILRL
ATEVNWDEEKECFESLSKECAMFYSIRKQYISEESTLSGQQSEVPGSIPNSWKWTVEHIV

YKALRSHILPPKHFTEDGNILQLANLPDLYKVFERC*
>ENSG00000090661|ENST00000251363
MLSSFNEWFWQDRFWLPPNVTWTELEDRDGRVYPHPQDLLAALPLALVLLAMRLAFERFI
GLPLSRWLGVRDQTRRQVKPNATLEKHFLTEGHRPKEPQLSLLAAQCGLTLQQTQRWFRR
RRNQDRPQLTKKFCEASWRFLFYLSSFVGGLSVLYHESWLWAPVMCWDRYPNQTLKPSLY
WWYLLELGFYLSLLIRLPFDVKRKDFKEQVIHHFVAVILMTFSYSANLLRIGSLVLLLHD
SSDYLLEACKMVNYMQYQQVCDALFLIFSFVFFYTRLVLFPTQILYTTYYESISNRGPFF
GYYFFNGLLMLLQLLHVFWSCLILRMLYSFMKKGQMEKDIRSDVEESDSSEEAAAAQEPL
QLKNGAAGGPRPAPTDGPRSRVAGRLTNRHTTAT*
>ENSG00000256223|ENST00000248211
MDAKSLTAWSRTLVTFKDVFVDFTREEWKLLDTAQQIVYRNVMLENYKNLVSLGYQLTKP
DVILRLEKGEEPWLVEREIHQETHPDSETAFEIKSSVSSRSIFKDKQSCDIKMEGMARND
LWYLSLEEVWKCRDQLDKYQENPERHLRQVAFTQKKVLTQERVSESGKYGGNCLLPAQLV
LREYFHKRDSHTKSLKHDLVLNGHQDSCASNSNECGQTFCQNIHLIQFARTHTGDKSYKC
PDNDNSLTHGSSLGISKGIHREKPYECKECGKFFSWRSNLTRHQLIHTGEKPYECKECGK
SFSRSSHLIGHQKTHTGEEPYECKECGKSFSWFSHLVTHQRTHTGDKLYTCNQCGKSFVH
SSRLIRHQRTHTGEKPYECPECGKSFRQSTHLILHQRTHVRVRPYECNECGKSYSQRSHL
VVHHRIHTGLKPFECKDCGKCFSRSSHLYSHQRTHTGEKPYECHDCGKSFSQSSALIVHQ
RIHTGEKPYECCQCGKAFIRKNDLIKHQRIHVGEETYKCNQCGIIFSQNSPFIVHQIAHT
GEQFLTCNQCGTALVNTSNLIGYQTNHIRENAY*
>ENSG00000133937|ENST00000238558
MPASMFSIDNILAARPRCKDSVLPVAHSAAAPVVFPALHGDSLYGASGGASSDYGAFYPR
PVAPGGAGLPAAVSGSRLGYNNYFYGQLHVQAAPVGPACCGAVPPLGAQQCSCVPTPPGY
EGPGSVLVSPVPHQMLPYMNVGTLSRTELQLLNQLHCRRKRRHRTIFTDEQLEALENLFQ
ETKYPDVGTREQLARKVHLREEKVEVWFKNRRAKWRRQKRSSSEESENAEKWNKTSSSKA
SPEKREEEGKSDLDSDS*
>ENSG00000126226|ENST00000246505
MAHITINQYLQQVYEAIDSRDGASCAELVSFKHPHVANPRLQMASPEEKCQQVLEPPYDE
MFAAHLRCTYAVGNHDFIEAYKCQTVIVQYPLSFMAAVPHRTHAVDYLGLETRKHWASCS
FLQLERNDSVLEQKLVSALSLGTSFLRAFQAHKEENWALPVMYAVALDLRVFANNADQQL
VKKGKSKVGDMLEKAAELLMSCFRVCASDTRAGIEDSKKWGMLFLVNQLFKIYFKINKLH
LCKPLIRAIDSSNLKDDYSTAQRVTYKYYVGRKAMFDSDFKQAEEYLSFAFEHCHRSSQK
NKRMILIYLLPVKMLLGHMPTVELLKKYHLMQFAEVTRAVSEGNLLLLHEALAKHEAFFI
RCGIFLILEKLKIITYRNLFKKVYLLLKTHQLSLDAFLVALKFMQVEDVDIDEVQCILAN
LIYMGHVKGYISHQHQKLVVSKQNPFPPLSTVC*
>ENSG00000126070|ENST00000246314
MCEVLDIHNIDEQPRPLTDSHRVKFTKEIKGLKVEVTHCGTMRRKYRVCNVTRRPASHQT
FPLQLENGQTVERTVAQYFREKYTLQLKYPHLPCLQVGQEQKHTYLPLEVCNIVAGQRCI
KKLTDNQTSTMIKATARSAPDRQEEISRLVRSANYETDPFVQEFQFKVRDEMAHVTGRVL
PAPMLQYGGRNRTVATPSHGVWDMRGKQFHTGVEIKMWAIACFATQRQCREEILKGFTDQ
LRKISKDAGMPIQGQPCFCKYAQGADSVEPMFRHLKNTYSGLQLIIVILPGKTPVYAEVK
RVGDTLLGMATQCVQVKNVIKTSPQTLSNLCLKINVKLGGINNILVPHQRPSVFQQPVIF
LGADVTHPPAGDGKKPSIAAVVGSMDAHPSRYCATVRVQRPRQEIIQDLASMVRELLIQF
YKSTRFKPTRIIFYRDGVSEGQFRQVLYYELLAIREACISLEKDYQPGITYIVVQKRHHT

RLFCADRTERVGRSGNIPAGTTVDTDITHPYEFDFYLCSHAGIQGTSRPSHYHVLWDDNC
FTADELQLLTYQLCHTYVRCTRSVSIPAPAYYAHLVAFRARYHLVDKEHDSAEGSHVSGQ
SNGRDPQALAKAVQIHQDTLRTMYFA*
>ENSG00000071626|ENST00000233078
MNNSGADEIGKLFVGGLDWSTTQETLRSYFSQYGEVVDCVIMKDKTTNQSRGFGFVKFKD
PNCVGTVLASRPHTLDGRNIDPKPCTPRGMQPERTRPKEGWQKGPRSDNSKSNKIFVGGI
PHNCGETELREYFKKFGVVTEVVMIYDAEKQRPRGFGFITFEDEQSVDQAVNMHFHDIMG
KKVEVKRAEPRDSKSQAPGQPGASQWGSRVVPNAANGWAGQPPPTWQQGYGPQGMWVPAG
QAIGGYGPPPAGRGAPPPPPPFTSYIVSTPPGGFPPPQGFPQGYGAPPQFSFGYGPPPPP
PDQFAPPGVPPPPATPGAAPLAFPPPPSQAAPDMSKPPTAQPDFPYGQYAGYGQDLSGFG
QGFSDPSQQPPSYGGPSVPGSGGPPAGGSGFGRGQNHNVQGFHPYRR*
>ENSG00000120948|ENST00000240185
MSEYIRVTEDENDEPIEIPSEDDGTVLLSTVTAQFPGACGLRYRNPVSQCMRGVRLVEGI
LHAPDAGWGNLVYVVNYPKDNKRKMDETDASSAVKVKRAVQKTSDLIVLGLPWKTTEQDL
KEYFSTFGEVLMVQVKKDLKTGHSKGFGFVRFTEYETQVKVMSQRHMIDGRWCDCKLPNS
KQSQDEPLRSRKVFVGRCTEDMTEDELREFFSQYGDVMDVFIPKPFRAFAFVTFADDQIA
QSLCGEDLIIKGISVHISNAEPKHNSNRQLERSGRFGGNPGGFGNQGGFGNSRGGGAGLG
NNQGSNMGGGMNFGAFSINPAMMAAAQAALQSSWGMMGMLASQQNQSGPSGNNQNQGNMQ
REPNQAFGSGNNSYSGSNSGAAIGWGSASNAGSGSGFNGGFGSSMDSKSSGWGM*
>ENSG00000124092|ENST00000243914
MAATEISVLSEQFTKIKELELMPEKGLKEEEKDGVCREKDHRSPSELEAERTSGAFQDSV
LEEEVELVLAPSEESEKYILTLQTVHFTSEAVELQDMSLLSIQQQEGVQVVVQQPGPGLL
WLEEGPRQSLQQCVAISIQQELYSPQEMEVLQFHALEENVMVASEDSKLAVSLAETTGLI
KLEEEQEKNQLLAERTKEQLFFVETMSGDERSDEIVLTVSNSNVEEQEDQPTAGQADAEK
AKSTKNQRKTKGAKGTFHCDVCMFTSSRMSSFNRHMKTHTSEKPHLCHLCLKTFRTVTLL
RNHVNTHTGTRPYKCNDCNMAFVTSGELVRHRRYKHTHEKPFKCSMCKYASVEASKLKRH
VRSHTGERPFQCCQCSYASRDTYKLKRHMRTHSGEKPYECHICHTRFTQSGTMKIHILQK
HGENVPKYQCPHCATIIARKSDLRVHMRNLHAYSAAELKCRYCSAVFHERYALIQHQKTH
KNEKRFKCKHCSYACKQERHMTAHIRTHTGEKPFTCLSCNKCFRQKQLLNAHFRKYHDAN
FIPTVYKCSKCGKGFSRWINLHRHSEKCGSGEAKSAASGKGRRTRKRKQTILKEATKGQK
EAAKGWKEAANGDEAAAEEASTTKGEQFPGEMFPVACRETTARVKEEVDEGVTCEMLLNT
MDK*
>ENSG00000137776|ENST00000249736
MAAATGAVAASAASGQAEGKKITDLRVIDLKSELKRRNLDITGVKTVLISRLKQAIEEEG
GDPDNIELTVSTDTPNKKPTKGKGKKHEADELSGDASVEDDAFIKDCELENQEAHEQDGN
DELKDSEEFGENEEENVHSKELLSAEENKRAHELIEAEGIEDIEKEDIESQEIEAQEGED
DTFLTAQDGEEEENEKEGSLAEADHTAHEEMEAHTTVKEAEDDNISVTIQAEDAITLDFD
GDDLLETGKNVKITDSEASKPKDGQDAIAQSPEKESKDYEMNANHKDGKKEDCVKGDPVE
KEARESSKKAESGDKEKDTLKKGPSSTGASGQAKSSSKESKDSKTSSKDDKGSTSSTSGS
SGSSTKNIWVSGLSSNTKAADLKNLFGKYGKVLSAKVVTNARSPGAKCYGIVTMSSSTEV
SRCIAHLHRTELHGQLISVEKVKGDPSKKEMKK
>ENSG00000112561|ENST00000230323
MASRIGLRMQLMREQAQQEEQRERMQQQAVMHYMQQQQQQQQQLGGPPTPAINTPVHFQ
SPPPVPGEVLKVQSYLENPTSYHLQQSQHQKVREYLSETYGNKFAAHISPAQGSPKPPPA

ASPGVRAGHVLSSSAGNSAPNSPMAMLHIGSNPERELDDVIDNIMRLDDVLGYINPEMQM
PNTLPLSSSHLNVYSSDPQVTASLVGVTSSSCPADLTQKRELTDAESRALAKERQKKDNH
NLIERRRRFNINDRIKELGMLIPKANDLDVRWNKGTILKASVDYIRRMQKDLQKSRELEN
HSRRLEMTNKQLWLRIQELEMQARVHGLPTTSPSGMNMAELAQQVVKQELPSEEGPGEAL
MLGAEVPDPEPLPALPPQAPLPLPTQPPSPFHHLDFSHSLSFGGREDEGPPGYPEPLAPG
HGSPFPSLSKKDLDLMLLDDSLLPLASDPLLSTMSPEASKASSRRSSFSMEEGDVL*
>ENSG00000112306|ENST00000230050
MAEEGIAAGGVMDVNTALQEVLKTALIHDGLARGIREAAKALDKRQAHLCVLASNCDEPM
YVKLVEALCAEHQINLIKVDDNKKLGEWVGLCKIDREGKPRKVVGCSCVVVKDYGKESQA
KDVIEEYFKCKK*
>ENSG00000274618|ENST00000244537
MSGRGKGGKGLGKGGAKRHRKVLRDNIQGITKPAIRRLARRGGVKRISGLIYEETRGVLK
VFLENVIRDAVTYTEHAKRKTVTAMDVVYALKRQGRTLYGFGG*
>ENSG00000122180|ENST00000241651
MELYETSPYFYQEPRFYDGENYLPVHLQGFEPPGYERTELTLSPEAPGPLEDKGLGTPEH
CPGQCLPWACKVCKRKSVSVDRRRAATLREKRRLKKVNEAFEALKRSTLLNPNQRLPKVE
ILRSAIQYIERLQALLSSLNQEERDLRYRGGGGPQPGVPSECSSHSASCSPEWGSALEFS
ANPGDHLLTADPTDAHNLHSLTSIVDSITVEDVSVAFPDETMPN*
>ENSG00000124610|ENST00000244573
MSETVPPAPAASAAPEKPLAGKKAKKPAKAAAASKKKPAGPSVSELIVQAASSSKERGGV
SLAALKKALAAAGYDVEKNNSRIKLGIKSLVSKGTLVQTKGTGASGSFKLNKKASSVETK
PGASKVATKTKATGASKKLKKATGASKKSVKTPKKAKKPAATRKSSKNPKKPKTVKPKKV
AKSPAKAKAVKPKAAKARVTKPKTAKPKKAAPKKK*
>ENSG00000215397|ENST00000246104
MPRSFLVKKIKGDGFQCSGVPAPTYHPLETAYVLPGARGPPGDNGYAPHRLPPSSYDADQ
KPGLELAPAEPAYPPAAPEEYSDPESPQSSLSARYFRGEAAVTDSYSMDAFFISDGRSRR
RRGGGGGDAGGSGDAGGAGGRAGRAGAQAGGGHRHACAECGKTYATSSNLSRHKQTHRSL
DSQLARKCPTCGKAYVSMPALAMHLLTHNLRHKCGVCGKAFSRPWLLQGHMRSHTGEKPF
GCAHCGKAFADRSNLRAHMQTHSAFKHYRCRQCDKSFALKSYLHKHCEAACAKAAEPPPP
TPAGPAS*
>ENSG00000006468|ENST00000242066
MDGFYDQQVPYMVTNSQRGRNCNEKPTNVRKRKFINRDLAHDSEELFQDLSQLQETWLAE
VAFHGLPLKIKKEPHSPCSEISSACSQEQPFKFSYGEKCLYNVSAYDQKPQVGMRPSNPP
TPSSTPVSPLHHASPNSTHTPKPDRAFPAHLPPSQSIPDSSYPMDHRFRRQLSEPCNSFP
PLPTMPREGRPMYQRQMSEPNIPFPPQGFKQEYHDPVYEHNTMVGSAASQSFPPPLMIKQ
EPRDFAYDSEVPSCHSIYMRQEGFLAHPSRTEGCMFEKGPRQFYDDTCVVPEKFDGDIKQ
EPGMYREGPTYQRRGSLQLWQFLVALLDDPSNSHFIAWTGRGMEFKLIEPEEVARRWGIQ
KNRPAMNYDKLSRSLRYYYEKGIMQKVAGERYVYKFVCDPEALFSMAFPDNQRPLLKTDM
ERHINEEDTVPLSHFDESMAYMPEGGCCNPHPYNEGYVY*
>ENSG00000119203|ENST00000238112
MSAIPAEESDQLLIRPLGAGQEVGRSCIILEFKGRKIMLDCGIHPGLEGMDALPYIDLID
PAEIDLLLISHFHLDHCGALPWFLQKTSFKGRTFMTHATKAIYRWLLSDYVKVSNISADD
MLYTETDLEESMDKIETINFHEVKEVAGIKFWCYHAGHVLGAAMFMIEIAGVKLLYTGDF
SRQEDRHLMAAEIPNIKPDILIIESTYGTHIHEKREEREARFCNTVHDIVNRGGRGLIPV

FALGRAQELLLILDEYWQNHPELHDIPIYYASSLAKKCMAVYQTYVNAMNDKIRKQININ
NPFVFKHISNLKSMDHFDDIGPSVVMASPGMMQSGLSRELFESWCTDKRNGVIIAGYCVE
GTLAKHIMSEPEEITTMSGQKLPLKMSVDYISFSAHTDYQQTSEFIRALKPPHVILVHGE
QNEMARLKAALIREYEDNDEVHIEVHNPRNTEAVTLNFRGEKLAKVMGFLADKKPEQGQR
VSGILVKRNFNYHILSPCDLSNYTDLAMSTVKQTQAIPYTGPFNLLCYQLQKLTGDVEEL
EIQEKPALKVFKNITVIQEPGMVVLEWLANPSNDMYADTVTTVILEVQSNPKIRKGAVQK
VSKKLEMHVYSKRLEIMLQDIFGEDCVSVKDDSILSVTVDGKTANLNLETRTVECEEGSE
DDESLREMVELAAQRLYEALTPVH*
>ENSG00000092345|ENST00000250863
MAAPSCGGDRKARLTPSLPHESTANPETPNSTISREASTQSSSAATSQGYILPEGKIMPN
TVFVGGIDVRMDETEIRSFFARYGSVKEVKIITDRTGVSKGYGFVSFFNDVDVQKIVESQ
INFHGKKLKLGPAIRKQNLCAYHVQPRPLVFNHPPPPQFQNVWTNPNTETYMQPTTTMNP
ITQYVQAYPTYPNSPVQVITGYQLPVYNYQMPPQWPVGEQRSYVVPPAYSAVNYHCNEVD
PGAEVVPNECSVHEATPPSGNGPQKKSVDRSIQTVVSCLFNPENRLRNSVVTQDDYFKDK
RVHHFRRSRAMLKSV*
>ENSG00000122008|ENST00000241436
MDSTKEKCDSYKDDLLLRMGLNDNKAGMEGLDKEKINKIIMEATKGSRFYGNELKKEKQV
NQRIENMMQQKAQITSQQLRKAQLQVDRFAMELEQSRNLSNTIVHIDMDAFYAAVEMRDN
PELKDKPIAVGSMSMLSTSNYHARRFGVRAAMPGFIAKRLCPQLIIVPPNFDKYRAVSKE
VKEILADYDPNFMAMSLDEAYLNITKHLEERQNWPEDKRRYFIKMGSSVENDNPGKEVNK
LSEHERSISPLLFEESPSDVQPPGDPFQVNFEEQNNPQILQNSVVFGTSAQEVVKEIRFR
IEQKTTLTASAGIAPNTMLAKVCSDKNKPNGQYQILPNRQAVMDFIKDLPIRKVSGIGKV
TEKMLKALGIITCTELYQQRALLSLLFSETSWHYFLHISLGLGSTHLTRDGERKSMSVER
TFSEINKAEEQYSLCQELCSELAQDLQKERLKGRTVTIKLKNVNFEVKTRASTVSSVVST
AEEIFAIAKELLKTEIDADFPHPLRLRLMGVRISSFPNEEDRKHQQRSIIGFLQAGNQAL
SATECTLEKTDKDKFVKPLEMSHKKSFFDKKRSERKWSHQDTFKCEAVNKQSFQTSQPFQ
VLKKKMNENLEISENSDDCQILTCPVCFRAQGCISLEALNKHVDECLDGPSISENFKMFS
CSHVSATKVNKKENVPASSLCEKQDYEAHPKIKEISSVDCIALVDTIDNSSKAESIDALS
NKHSKEECSSLPSKSFNIEHCHQNSSSTVSLENEDVGSFRQEYRQPYLCEVKTGQALVCP
VCNVEQKTSDLTLFNVHVDVCLNKSFIQELRKDKFNPVNQPKESSRSTGSSSGVQKAVTR
TKRPGLMTKYSTSKKIKPNNPKHTLDIFFK*
>ENSG00000118245|ENST00000236979
MSTSRKLKSHGMRRSKSRSPHKGVKRGGSKRKYRKGNLKSRKRGDDANRNYRSHL*
>ENSG00000104626|ENST00000250263
MEDPQSKEPAGEAVALALLESPRPEGGEEPPRPSPEETQQCKFDGQETKGSKFITSSASD
FSDPVYKEIAITNGCINRMSKEELRAKLSEFKLETRGVKDVLKKRLKNYYKKQKLMLKES
NFADSYYDYICIIDFEATCEEGNPPEFVHEIIEFPVVLLNTHTLEIEDTFQQYVRPEINT
QLSDFCISLTGITQDQVDRADTFPQVLKKVIDWMKLKELGTKYKYSLLTDGSWDMSKFLN
IQCQLSRLKYPPFAKKWINIRKSYGNFYKVPRSQTKLTIMLEKLGMDYDGRPHCGLDDSK
NIARIAVRMLQDGCELRINEKMHAGQLMSVSSSLPIEGTPPPQMPHFRK*
>ENSG00000123307|ENST00000242994
MSKTFVKSKEMGELVNTPSWMDKGLGSQNEVKEEESRPGTYGMLSSLTEEHDSIEEEEEE
EEDGEKPKRRGPKKKKMTKARLERFRARRVKANARERTRMHGLNDALDNLRRVMPCYSKT
QKLSKIETLRLARNYIWALSEVLETGQTPEGKGFVEMLCKGLSQPTSNLVAGCLQLGPQS

VLLEKHEDKSPICDSAISVHNFNYQSPGLPSPPYGHMETHLLHLKPQVFKSLGESSFGSH
LPDCSTPPYEGPLTPPLSISGNFSLKQDGSPDLEKSYSFMPHYPSSSLSSGHVHSTPFQA
GTPRYDVPIDMSYDSYPHHGIGTQLNTVFTE*
>ENSG00000115541|ENST00000233893
MAGQAFRKFLPLFDRVLVERSAAETVTKGGIMLPEKSQGKVLQATVVAVGSGSKGKGGEI
QPVSVKVGDKVLLPEYGGTKVVLDDKDYFLFRDGDILGKYVD*
>ENSG00000129514|ENST00000250448
MLGTVKMEGHETSDWNSYYADTQEAYSSVPVSNMNSGLGSMNSMNTYMTMNTMTTSGNMT
PASFNMSYANPGLGAGLSPGAVAGMPGGSAGAMNSMTAAGVTAMGTALSPSGMGAMGAQQ
AASMNGLGPYAAAMNPCMSPMAYAPSNLGRSRAGGGGDAKTFKRSYPHAKPPYSYISLIT
MAIQQAPSKMLTLSEIYQWIMDLFPYYRQNQQRWQNSIRHSLSFNDCFVKVARSPDKPGK
GSYWTLHPDSGNMFENGCYLRRQKRFKCEKQPGAGGGGGSGSGGSGAKGGPESRKDPSGA
SNPSADSPLHRGVHGKTGQLEGAPAPGPAASPQTLDHSGATATGGASELKTPASSTAPPI
SSGPGALASVPASHPAHGLAPHESQLHLKGDPHYSFNHPFSINNLMSSSEQQHKLDFKAY
EQALQYSPYGSTLPASLPLGSASVTTRSPIEPSALEPAYYQGVYSRPVLNTS*
>ENSG00000115844|ENST00000234198
MTGVFDSLVADMHSTQIAASSTYHQHQQPPSGGGAGPGGNSSSSSSLHKPQESPTLPVST
ATDSSYYTNQQHPAGGGGGGGSPYAHMGSYQYQASGLNNVPYSAKSSYDLGYTAAYTSYA
PYGTSSSPANNEPEKEDLEPEIRIVNGKPKKVRKPRTIYSSFQLAALQRRFQKTQYLALP
ERAELAASLGLTQTQVKIWFQNRRSKFKKMWKSGEIPSEQHPGASASPPCASPPVSAPAS
WDFGVPQRMAGGGGPGSGGSGAGSSGSSPSSAASAFLGNYPWYHQTSGSASHLQATAPLL
HPTQTPQPHHHHHHHGGGGAPVSAGTIF*
>ENSG00000260027|ENST00000239165
MSSLYYANTLFSKYPASSSVFATGAFPEQTSCAFASNPQRPGYGAGSGASFAASMQGLYP
GGGGMAGQSAAGVYAAGYGLEPSSFNMHCAPFEQNLSGVCPGDSAKAAGAKEQRDSDLAA
ESNFRIYPWMRSSGTDRKRGRQTYTRYQTLELEKEFHYNRYLTRRRRIEIAHTLCLTERQ
IKIWFQNRRMKWKKENKTAGPGTTGQDRAEAEEEEEE*
>ENSG00000120075|ENST00000239151
MSSYFVNSFSGRYPNGPDYQLLNYGSGSSLSGSYRDPAAMHTGSYGYNYNGMDLSVNRSS
ASSSHFGAVGESSRAFPAPAQEPRFRQAASSCSLSSPESLPCTNGDSHGAKPSASSPSDQ
ATSASSSANFTEIDEASASSEPEEAASQLSSPSLARAQPEPMATSTAAPEGQTPQIFPWM
RKLHISHDMTGPDGKRARTAYTRYQTLELEKEFHFNRYLTRRRRIEIAHALCLSERQIKI
WFQNRRMKWKKDNKLKSMSLATAGSAFQP*
>ENSG00000123144|ENST00000242784
MAARGRRAEPQGREAPGPAGGGGGGSRWAESGSGTSPESGDEEVSGAGSSPVSGGVNLFA
NDGSFLELFKRKMEEEQRQRQEEPPPGPQRPDQSAAAAGPGDPKRKGGPGSTLSFVGKRR
GGNKLALKTGIVAKKQKTEDEVLTSKGDAWAKYMAEVKKYKAHQCGDDDKTRPLVK*
>ENSG00000122085|ENST00000241527
MAAFGRQVLDWHRLIPLTWACMARQTPHLGEQRRTTASLLRKLTTASNGGVIEELSCVRS
NNYVQEPECRRNLVQCLLEKQGTPVVQGSLELERVMSSLLDMGFSNAHINELLSVRRGAS
LQQLLDIISEFILLGLNPEPVCVVLKKSPQLLKLPIMQMRKRSSYLQKLGLGEGKLKRVL
YCCPEIFTMRQQDINDTVRLLKEKCLFTVQQVTKILHSCPSVLREDLGQLEYKFQYAYFR
MGIKHPDIVKSEYLQYSLTKIKQRHIYLERLGRYQTPDKKGQTQIPNPLLKDILRVSEAE
FLARTACTSVEEFQVFKKLLAREEEESESSTSDDKRASLDEDEDDDDEEDNDEDDNDEDD

DDEDDDEAEDNDEDEDDDEEE*
>ENSG00000125457|ENST00000245551
MGEPSREEYKIQSFDAETQQLLKTALKVACFETEDGEYSVCQRSYSNCSRLMPSRCNTQY
RDPGAVDLEKVANVIVDHSLQDCVFSKEAGRMCYAIIQAESKQAGQSVFRRGLLNRLQQE
YQAREQLRARSLQGWVCYVTFICNIFDYLRVNNMPMMALVNPVYDCLFRLAQPDSLSKEE
EVDCLVLQLHRVGEQLEKMNGQRMDELFVLIRDGFLLPTGLSSLAQLLLLEIIEFRAAGW
KTTPAAHKYYYSEVSD*
>ENSG00000020577|ENST00000251091
MMFRDQVGVLAGWFKGWNECEQTVALLSLLKRVSQTQARFLQLCLEHSLADCAELHVLER
EANSPGIINQWQQESKDKVISLLLTHLPLLKPGNLDAKVEYMKLLPKILAHSIEHNQHIE
ESRQLLSYALIHPATSLEDRSALAMWLNHLEDRTSTSFGGQNRGRSDSVDYGQTHYYHQR
QNSDDKLNGWQNSRDSGICINASNWQDKSMGCENGHVPLYSSSSVPTTINTIGTSTSTNV
PAWLKSLRLHKYAALFSQMTYEEMMALTECQLEAQNVTKGARHKIVISIQKLKERQNLLK
SLERDIIEGGSLRIPLQELHQMILTPIKAYSSPSTTPEARRREPQAPRQPSLMGPESQSP
DCKDGAAATGATATPSAGASGGLQPHQLSSCDGELAVAPLPEGDLPGQFTRVMGKVCTQL
LVSRPDEENISSYLQLIDKCLIHEAFTETQKKRLLSWKQQVQKLFRSFPRKTLLDISGYR
QQRNRGFGQSNSLPTAGSVGGGMGRRNPRQYQIPSRNVPSARLGLLGTSGFVSSNQRNTT
ATPTIMKQGRQNLWFANPGGSNSMPSRTHSSVQRTRSLPVHTSPQNMLMFQQPEFQLPVT
EPDINNRLESLCLSMTEHALGDGVDRTSTI*
>ENSG00000016082|ENST00000230658
MGDMGDPPKKKRLISLCVGCGNQIHDQYILRVSPDLEWHAACLKCAECNQYLDESCTCFV
RDGKTYCKRDYIRLYGIKCAKCSIGFSKNDFVMRARSKVYHIECFRCVACSRQLIPGDEF
ALREDGLFCRADHDVVERASLGAGDPLSPLHPARPLQMAAEPISARQPALRPHVHKQPEK
TTRVRTVLNEKQLHTLRTCYAANPRPDALMKEQLVEMTGLSPRVIRVWFQNKRCKDKKRS
IMMKQLQQQPNDKTNIQGMTGTPMVAASPERHDGGLQANPVEVQSYQPPWKVLSDFALQ
SDIDQPAFQQLVNFSEGGPGSNSTGSEVASMSSQLPDTPNSMVASPIEA*
>ENSG00000111405|ENST00000229003
MRACISLVLAVLCGLAWAEDHKESEPLPQLEEETEEALASNLYSAPTSCQGRCYEAFDKH
HQCHCNARCQEFGNCCKDFESLCSDHEVSHSSDAITKEEIQSISEKIYRADTNKAQKEDI
VLNSQNCISPSETRNQVDRCPKPLFTYVNEKLFSKPTYAAFINLLNNYQRATGHGEHFSA
QELAEQDAFLREIMKTAVMKELYSFLHHQNRYGSEQEFVDDLKNMWFGLYSRGNEEGDSS
GFEHVFSGEVKKGKVTGFHNWIRFYLEEKEGLVDYYSHIYDGPWDSYPDVLAMQFNWDGY
YKEVGSAFIGSSPEFEFALYSLCFIARPGKVCQLSLGGYPLAVRTYTWDKSTYGNGKKYI
ATAYIVSST*
>ENSG00000124383|ENST00000244230
MAPQVWRRRTLERCLTEVGKATGRPECFLTIQEGLASKFTSLTKVLYDFNKILENGRIHG
SPLQKLVIENFDDEQIWQQLELQNEPILQYFQNAVSETINDEDISLLPESEEQEREEDGS
EIEADDKEDLEDLEEEEVSDMGNDDPEMGERAENSSKSDLRKSPVFSDEDSDLDFDISKL
EQQSKVQNKGQGKPREKSIVDDKFFKLSEMEAYLENIEKEEERKDDNDEEEEDIDFFEDI
DSDEDEGGLFGSKKLKSGKSSRNLKYKDFFDPVESDEDITNVHDDELDSNKEDDEIAEEE
AEELSISETDEDDDLQENEDNKQHKESLKRVTFALPDDAETEDTGVLNVKKNSDEVKSSF
EKRQEKMNEKIASLEKELLEKKPWQLQGEVTAQKRPENSLLEETLHFDHAVRMAPVITEE
TTLQLEDIIKQRIRDQAWDDVVRKEKPKEDAYEYKKRLTLDHEKSKLSLAEIYEQEYIKL
NQQKTAEEENPEHVEIQKMMDSLFLKLDALSNFHFIPKPPVPEIKVVSNLPAITMEEVAP

VSVSDAALLAPEEIKEKNKAGDIKTAAEKTATDKKRERRKKKYQKRMKIKEKEKRRKLLE
KSSVDQAGKYSKTVASEKLKQLTKTGKASFIKDEGKDKALKSSQAFFSKLQDQVKMQIND
AKKTEKKKKKRQDISVHKLKL*
>ENSG00000102103|ENST00000247140
MPLPVALQTRLAKRGILKHLEPEPEEEIIAEDYDDDPVDYEATRLEGLPPSWYKVFDPSC
GLPYYWNADTDLVSWLSPHDPNSVVTKSAKKLRSSNAAVSRKDEELDPMDPSSYSDAPRG
TWSTGLPKRNEAKTGADTTAAGPLFQQRPYPSPGAVLRANAEASRTKQQD*
>ENSG00000123091|ENST00000242719
MGNCLKSPTSDDISLLHESQSDRASFGEGTEPDQEPPPPYQEQVPVPVYHPTPSQTRLAT
QLTEEEQIRIAQRIGLIQHLPKGVYDPGRDGSEKKIRECVICMMDFVYGDPIRFLPCMHI
YHLDCIDDWLMRSFTCPSCMEPVDAALLSSYETN*
>ENSG00000122691|ENST00000242261
MMQDVSSSPVSPADDSLSNSEEEPDRQQPPSGKRGGRKRRSSRRSAGGGAGPGGAAGGGV
GGGDEPGSPAQGKRGKKSAGCGGGGGAGGGGGSSSGGGSPQSYEELQTQRVMANVRERQR
TQSLNEAFAALRKIIPTLPSDKLSKIQTLKLAARYIDFLYQVLQSDELDSKMASCSYVAH
ERLSYAFSVWRMEGAWSMSASH*
>ENSG00000123685|ENST00000243440
MSQGLPAAGSVLQRSVAAPGNQPQPQPQQQSPEDDDRKVRRREKNRVAAQRSRKKQTQKA
DKLHEEYESLEQENTMLRREIGKLTEELKHLTEALKEHEKMCPLLLCPMNFVPVPPRPDP
VAGCLPR*
>ENSG00000198342|ENST00000242804
MIVFGGEDRSDLFLPDSQTNEERKQYDSVAFEDVAVNFTQEEWALLGPSQKSLYRDVMWE
TIRNLDCIGMKWEDTNIEDQHRNPRRSLRCHIIERFSESRQPDSTVNEKPPGVDPCKSSV
CGEIMGCSFLNCYITFDAGHKPDECQEYGEKPHTHKQCGTAFNYHHSFQTQERPHTGKKR
YDCKECGKTFSSSGNLRRHIIVQRGGGPYICKLCGKAFFWPSLFRMHERTHTGEKPYECK
QCCKAFPIYSSYLRHERTHTGEKPYECKHCSKAFPDYSSYVRHERTHTGEKPYKCKRCGR
AFSVSSSLRIHERTHTGEKPYECKQCGKAFHHLGSFQRHMIRHTGDGPHKCKICGKGFDC
PSSLQSHERTHTGEKPYECKQCGKALSHHSSFRSHMIMHTGDGPHKCKVCGKAFIYPSVF
QGHERTHTGEKPYECKECGKAFRISSSLRRHETTHTGEKPYKCKCGKAFIDFYSFQNHET
THTGEKPYECKECGKAFSCFTYLSQHRRTHMAEKPYECKTCKKAFSHFGNLKVHERIHTG
EKPYECKECRKAFSWLTCLLRHERIHTGKKSYECQQCGKAFTRSRFLRGHEKTHTGEKMH
ECKECGKALSSLSSLHRHKRTHWRDTL*
>ENSG00000106546|ENST00000242057
MNSSSANITYASRKRRKPVQKTVKPIPAEGIKSNPSKRHRDRLNTELDRLASLLPFPQDV
INKLDKLSVLRLSVSYLRAKSFFDVALKSSPTERNGGQDNCRAANFREGLNLQEGEFLLQ
ALNGFVLVVTTDALVFYASSTIQDYLGFQQSDVIHQSVYELIHTEDRAEFQRQLHWALNP
SQCTESGQGIEEATGLPQTVVCYNPDQIPPENSPLMERCFICRLRCLLDNSSGFLAMNFQ
GKLKYLHGQKKKGKDGSILPPQLALFAIATPLQPPSILEIRTKNFIFRTKHKLDFTPIGC
DAKGRIVLGYTEAELCTRGSGYQFIHAADMLYCAESHIRMIKTGESGMIVFRLLTKNNRW
TWVQSNARLLYKNGRPDYIIVTQRPLTDEEGTEHLRKRNTKLPFMFTTGEAVLYEATNPF
PAIMDPLPLRTKNGTSGKDSATTSTLSKDSLNPSSLLAAMMQQDESIYLYPASSTSSTAP
FENNFFNESMNECRNWQDNTAPMGNDTILKHEQIDQPQDVNSFAGGHPGLFQDSKNSDLY
SIMKNLGIDFEDIRHMQNEKFFRNDFSGEVDFRDIDLTDEILTYVQDSLSKSPFIPSDYQ
QQQSLALNSSCMVQEHLHLEQQQQHHQKQVVVEPQQQLCQKMKHMQVNGMFENWNSNQFV

PFNCPQQDPQQYNVFTDLHGISQEFPYKSEMDSMPYTQNFISCNQPVLPQHSKCTELDYP
MGSFEPSPYPTTSSLEDFVTCLQLPENQKHGLNPQSAIITPQTCYAGAVSMYQCQPEPQH
THVGQMQYNPVLPGQQAFLNKFQNGVLNETYPAELNNINNTQTTTHLQPLHHPSEARPFP
DLTSSGFL*
>ENSG00000091010|ENST00000230732
MMAMNSKQPFGMHPVLQEPKFSSLHSGSEAMRRVCLPAPQLQGNIFGSFDESLLARAEAL
AAVDIVSHGKNHPFKPDATYHTMSSVPCTSTSSTVPISHPAALTSHPHHAVHQGLEGDLL
EHISPTLSVSGLGAPEHSVMPAQIHPHHLGAMGHLHQAMGMSHPHTVAPHSAMPACLSDV
ESDPRELEAFAERFKQRRIKLGVTQADVGAALANLKIPGVGSLSQSTICRFESLTLSHNN
MIALKPVLQAWLEEAEAAYREKNSKPELFNGSERKRKRTSIAAPEKRSLEAYFAIQPRPS
SEKIAAIAEKLDLKKNVVRVWFCNQRQKQKRMKYSAVH*
>ENSG00000126705|ENST00000247087
MRVKPQGLVVTSSAVCSSPDYLREPKYYPGGPPTPRPLLPTRPPASPPDKAFSTHAFSEN
PRPPPRRDPSTRRPPVLAKGDDPLPPRAARPVSQARCPTPVGDGSSSRRCWDNGRVNLRP
VVQLIDIMKDLTRLSQDLQHSGVHLDCGGLRLSRPPAPPPGDLQYSFFSSPSLANSIRSP
EERATPHAKSERPSHPLYEPEPEPRDSPQPGQGHSPGATAAATGLPPEPEPDSTDYSELA
DADILSELASLTCPEAQLLEAQALEPPSPEPEPQLLDPQPRFLDPQALEPLGEALELPPL
QPLADPLGLPGLALQALDTLPDSLESQLLDPQALDPLPKLLDVPGRRLEPQQPLGHCPLA
EPLRLDLCSPHGPPGPEGHPKYALRRTDRPKILCRRRKAGRGRKADAGPEGRLLPLPMPT
GLVAALAEPPPPPPPPPPALPGPGPVSVPELKPESSQTPVVSTRKGKCRGVRRMVVKMAK
IPVSLGRRNKTTYKVSSLSSSLSVEGKELGLRVSAEPTPLLKMKNNGRNVVVVFPPGEMP
IILKRKRGRPPKNLLLGPGKPKEPAVVAAEAATVAAATMAMPEVKKRRRRKQKLASPQPS
YAADANDSKAEYSDVLAKLAFLNRQSQCAGRCSPPRCWTPSEPESVHQAPDTQSISHFLH
RVQGFRRRGGKAGGFGGRGGGHAAKSARCSFSDFFEGIGKKKKVVAVAAAGVGGPGLTEL
GHPRKRGRGEVDAVTGKPKRKRRSRKNGTLFPEQVPSGPGFGEAGAEWAGDKGGGWAPHH
GHPGGQAGRNCGFQGTEARAFASTGLESGASGRGSYYSTGAPSGQTELSQERQNLFTGYF
RSLLDSDDSSDLLDFALSASRPESRKASGTYAGPPTSALPAQRGLATFPSRGAKASPVAV
GSSGAGADPSFQPVLSARQTFPPGRAASYGLTPAASDCRAAETFPKLVPPPSAMARSPTT
HPPANTYLPQYGGYGAGQSVFAPTKPFTGQDCANSKDCSFAYGSGNSLPASPSSAHSAGY
APPPTGGPCLPPSKASFFSSSEGAPFSGSAPTPLRCDSRASTVSPGGYMVPKGTTASATS
AASAASSSSSSFQPSPENCRQFAGASQWPFRQGYGGLDWASEAFSQLYNPSFDCHVSEPN
VILDISNYTPQKVKQQTAVSETFSESSSDSTQFNQPVGGGGFRRANSEASSSEGQSSLSS
LEKLMMDWNEASSAPGYNWNQSVLFQSSSKPGRGRRKKVDLFEASHLGFPTSASAAASGY
PSKRSTGPRQPRGGRGGGACSAKKERGGAAAKAKFIPKPQPVNPLFQDSPDLGLDYYSGD
SSMSPLPSQSRAFGVGERDPCDFIGPYSMNPSTPSDGTFGQGFHCDSPSLGAPELDGKHF
PPLAHPPTVFDAGLQKAYSPTCSPTLGFKEELRPPPTKLAACEPLKHGLQGASLGHAAAA
QAHLSCRDLPLGQPHYDSPSCKGTAYWYPPGSAARSPPYEGKVGTGLLADFLGRTEAACL
SAPHLASPPATPKADKEPLEMARPPGPPRGPAAAAAGYGCPLLSDLTLSPVPRDSLLPLQ
DTAYRYPGFMPQAHPGLGGGPKSGFLGPMAEPHPEDTFTVTSL*
>ENSG00000126653|ENST00000247026
MAIPGRQYGLILPKKTQQLHPVLQKPSVFGNDSDDDDETSVSESLQREAAKKQAMKQTKL
EIQKALAEDATVYEYDSIYDEMQKKKEENNPKLLLGKDRKPKYIHNLLKAVEIRKKEQEK
RMEKKIQREREMEKGEFDDKEAFVTSAYKKKLQERAEEEEREKRAAALEACLDVTKQKDL
SGFYRHLLNQAVGEEEVPKCSFREARSGIKEEKSRGFSNEVSSKNRIPQEKCILQTDVKV

EENPDADSDFDAKSSADDEIEETRVNCRREKVIETPENDFKHHRSQNHSRSPSEERGHST
RHHTKGSRTSRGHEKREDQHQQKQSRDQENHYTDRDYRKERDSHRHREASHRDSHWKRHE
QEDKPRARDQRERSDRVWKREKDREKYSQREQERDRQQNDQNRPSEKGEKEEKSKAKEEH
MKVRKERYENNDKYRDREKREVGVQSSERNQDRKESSPNSRAKDKFLDQERSNKMRNMAK
DKERNQEKPSNSESSLGAKHRLTEEGQEKGKEQERPPEAVSKFAKRNNEETVMSARDRYL
ARQMARVNAKTYIEKEDD*
>ENSG00000033800|ENST00000249636
MADSAELKQMVMSLRVSELQVLLGYAGRNKHGRKHELLTKALHLLKAGCSPAVQMKIKEL
YRRRFPQKIMTPADLSIPNVHSSPMPATLSPSTIPQLTYDGHPASSPLLPVSLLGPKHEL
ELPHLTSALHPVHPDIKLQKLPFYDLLDELIKPTSLASDNSQRFRETCFAFALTPQQVQQ
ISSSMDISGTKCDFTVQVQLRFCLSETSCPQEDHFPPNLCVKVNTKPCSLPGYLPPTKNG
VEPKRPSRPINITSLVRLSTTVPNTIVVSWTAEIGRNYSMAVYLVKQLSSTVLLQRLRAK
GIRNPDHSRALIKEKLTADPDSEIATTSLRVSLLCPLGKMRLTIPCRALTCSHLQCFDAT
LYIQMNEKKPTWVCPVCDKKAPYEHLIIDGLFMEILKYCTDCDEIQFKEDGTWAPMRSKK
EVQEVSASYNGVDGCLSSTLEHQVASHHQSSNKNKKVEVIDLTIDSSSDEEEEEPSAKRT
CPSLSPTSPLNNKGILSLPHQASPVSRTPSLPAVDTSYINTSLIQDYRHPFHMTPMPYDL
QGLDFFPFLSGDNQHYNTSLLAAAAAAVSDDQDLLHSSRFFPYTSSQMFLDQLSAGGSTS
LPTTNGSSSGSNSSLVSSNSLRESHSHTVTNRSSTDTASIFGIIPDIISLD*
>ENSG00000111364|ENST00000238146
MEHVTEGSWESLPVPLHPQVLGALRELGFPYMTPVQSATIPLFMRNKDVAAEAVTGSGKT
LAFVIPILEILLRREEKLKKSQVGAIIITPTRELAIQIDEVLSHFTKHFPEFSQILWIGG
RNPGEDVERFKQQGGNIIVATPGRLEDMFRRKAEGLDLASCVRSLDVLVLDEADRLLDMG
FEASINTILEFLPKQRRTGLFSATQTQEVENLVRAGLRNPVRVSVKEKGVAASSAQKTPS
RLENYYMVCKADEKFNQLVHFLRNHKQEKHLVFFSTCACVEYYGKALEVLVKGVKIMCIH
GKMKYKRNKIFMEFRKLQSGILVCTDVMARGIDIPEVNWVLQYDPPSNASAFVHRCGRTA
RIGHGGSALVFLLPMEESYINFLAINQKCPLQEMKPQRNTADLLPKLKSMALADRAVFEK
GMKAFVSYVQAYAKHECNLIFRLKDLDFASLARGFALLRMPKMPELRGKQFPDFVPVDVN
TDTIPFKDKIREKQRQKLLEQQRREKTENEGRRKFIKNKAWSKQKAKKEKKKKMNEKRKR
EEGSDIEDEDMEELLNDTRLLKKLKKGKITEEEFEKGLLTTGKRTIKTVDLGISDLEDDC
*
>ENSG00000128534|ENST00000249299
MTSALENYINRTVAVITSDGRMIVGTLKGFDQTINLILDESHERVFSSSQGVEQVVLGLY
IVRGDNVAVIGEIDEETDSALDLGNIRAEPLNSVAH*
>ENSG00000124831|ENST00000244815
MTSPAAAQSREIDCLSPEAQKLAEARLAAKRAARAEAREIRMKELERQQKEEDSERYSRR
SRRNTSASDEDERMSVGSRGSLRVEERPEKDFTEKGSRNMPGLSAATLASLGGTSSRRGS
GDTSISIDTEASIREIKDSLAEVEEKYKKAMVSNAQLDNEKTNFMYQVDTLKDMLLELEE
QLAESRRQYEEKNKEFEREKHAHSILQFQFAEVKEALKQREEMLEKHGIILNSEIATNGE
TSDTLNNVGYQGPTKMTKEELNALKSTGDGTLGRASEVEVKNEIVANVGKREILHNTEKE
QHTEDTVKDCVDIEVFPAGENTEDQKSSEDTAPFLGTLAGATYEEQVQSQILESSSLPEN
TVQVESNEVMGAPDDRTRTPLEPSNCWSDLDGGNHTENVGEAAVTQVEEQAGTVASCPLG
HSDDTVYHDDKCMVEVPQELETSTGHSLEKEFTNQEAAEPKEVPAHSTEVGRDHNEEEGE
ETGLRDEKPIKTEVPGSPAGTEGNCQEATGPSTVDTQNEPLDMKEPDEEKSDQQGEALDS
SQKKTKNKKKKNKKKKSPVPVETLKDVKKELTYQNTDLSEIKEEEQVKSTDRKSAVEAQN

EVTENPKQKIAAESSENVDCPENPKIKLDGKLDQEGDDVQTAAEEVLADGDTLDFEDDTV
QSSGPRAGGEELDEGVAKDNAKIDGATQSSPAEPKSEDADRCTLPEHESPSQDISDACEA
ESTERCEMSEHPSQTVRKALDSNSLENDDLSAPGREPGHFNPESREDTRGGNEKGKSKED
CTMS*
>ENSG00000115297|ENST00000233638
MEPGMLGPHNLPHHEPISFGIDQILSGPETPGGGLGLGRGGQGHGENGAFSGGYHGASGY
GPAGSLAPLPGSSGVGPGGVIRVPAHRPLPVPPPAGGAPAVPGPSGLGGAGGLAGLTFPW
MDSGRRFAKDRLTAALSPFSGTRRIGHPYQNRTPPKRKKPRTSFSRSQVLELERRFLRQK
YLASAERAALAKALRMTDAQVKTWFQNRRTKWRRQTAEEREAERHRAGRLLLHLQQDALP
RPLRPPLPPDPLCLHNSSLFALQNLQPWAEDNKVASVSGLASVV*
>ENSG00000167840|ENST00000250076
MEPPGPVRGPLQDSSWYEPSAELVQTRMAVSLTAAETLALQGTQGQEKMMMMGPKEEEQS
CEYETRLPGNHSTSQEIFRQRFRHLRYQETPGPREALSQLRVLCCEWLRPEKHTKEQILE
FLVLEQFLTILPEELQSWVRGHHPKSGEEAVTVLEDLEKGLEPEPQVPGPAHGPAQEEPW
EKKESLGAAQEALSIQLQPKETQPFPKSEQVYLHFLSVVTEDGPEPKDKGSLPQPPITEV
ESQVFSEKLATDTSTFEATSEGTLELQQRNPKAERLRWSPAQEESFRQMVVIHKEIPTGK
KDHECSECGKTFIYNSHLVVHQRVHSGEKPYKCSDCGKTFKQSSNLGQHQRIHTGEKPFE
CNECGKAFRWGAHLVQHQRIHSGEKPYECNECGKAFSQSSYLSQHRRIHSGEKPFICKEC
GKAYGWCSELIRHRRVHARKEPSH*
>ENSG00000120837|ENST00000240055
MTMDGDSSTTDASQLGISADYIGGSHYVIQPHDDTEDSMNDHEDTNGSKESFREQDIYLP
IANVARIMKNAIPQTGKIAKDAKECVQECVSEFISFITSEASERCHQEKRKTINGEDILF
AMSTLGFDSYVEPLKLYLQKFREAMKGEKGIGGAVTATDGLSEELTEEAFTNQLPAGLIT
TDGQQQNVMVYTTSYQQISGVQQIQFS*
>ENSG00000112855|ENST00000230771
MPLLGLLPRRAWASLLSQLLRPPCASCTGAVRCQSQVAEAVLTSQLKAHQEKPNFIIKTP
KGTRDLSPQHMVVREKILDLVISCFKRHGAKGMDTPAFELKETLTEKYGEDSGLMYDLKD
QGGELLSLRYDLTVPFARYLAMNKVKKMKRYHVGKVWRRESPTIVQGRYREFCQCDFDIA
GQFDPMIPDAECLKIMCEILSGLQLGDFLIKVNDRRIVDGMFAVCGVPESKFRAICSSID
KLDKMAWKDVRHEMVVKKGLAPEVADRIGDYVQCHGGVSLVEQMFQDPRLSQNKQALEGL
GDLKLLFEYLTLFGIADKISFDLSLARGLDYYTGVIYEAVLLQTPTQAGEEPLNVGSVAA
GGRYDGLVGMFDPKGHKVPCVGLSIGVERIFYIVEQRMKTKGEKVRTTETQVFVATPQKN
FLQERLKLIAELWDSGIKAEMLYKNNPKLLTQLHYCESTGIPLVVIIGEQELKEGVIKIR
SVASREEVAIKRENFVAEIQKRLSES*
>ENSG00000113272|ENST00000231198
MWGACKVKVHDSLATISITLRRYLRLGATMAKSKFEYVRDFEADDTCLAHCWVVVRLDGR
NFHRFAEKHNFAKPNDSRALQLMTKCAQTVMEELEDIVIAYGQSDEYSFVFKRKTNWFKR
RASKFMTHVASQFASSYVFYWRDYFEDQPLLYPPGFDGRVVVYPSNQTLKDYLSWRQADC
HINNLYNTVFWALIQQSGLTPVQAQGRLQGTLAADKNEILFSEFNINYNNELPMYRKGTV
LIWQKVDEVMTKEIKLPTEMEGKKMAVTRTRTKPVPLHCDIIGDAFWKEHPEILDEDS*
>ENSG00000111247|ENST00000228843
MVRPVRHKKPVNYSQFDHSDSDDDFVSATVPLNKKSRTAPKELKQDKPKPNLNNLRKEEI
PVQEKTPKKRLPEGTFSIPASAVPCTKMALDDKLYQRDLEVALALSVKELPTVTTNVQNS
QDKSIEKHGSSKIETMNKSPHISNCSVASDYLDLDKITVEDDVGGVQGKRKAASKAAAQQ

RKILLEGSDGDSANDTEPDFAPGEDSEDDSDFCESEDNDEDFSMRKSKVKEIKKKEVKVK
SPVEKKEKKSKSKCNALVTSVDSAPAAVKSESQSLPKKVSLSSDTTRKPLEIRSPSAESK
KPKWVPPAASGGSRSSSSPLVVVSVKSPNQSLRLGLSRLARVKPLHPNATST*
>ENSG00000074800|ENST00000234590
MSILKIHAREIFDSRGNPTVEVDLFTSKGLFRAAVPSGASTGIYEALELRDNDKTRYMGK
GVSKAVEHINKTIAPALVSKKLNVTEQEKIDKLMIEMDGTENKSKFGANAILGVSLAVCK
AGAVEKGVPLYRHIADLAGNSEVILPVPAFNVINGGSHAGNKLAMQEFMILPVGAANFRE
AMRIGAEVYHNLKNVIKEKYGKDATNVGDEGGFAPNILENKEGLELLKTAIGKAGYTDKV
VIGMDVAASEFFRSGKYDLDFKSPDDPSRYISPDQLADLYKSFIKDYPVVSIEDPFDQDD
WGAWQKFTASAGIQVVGDDLTVTNPKRIAKAVNEKSCNCLLLKVNQIGSVTESLQACKLA
QANGWGVMVSHRSGETEDTFIADLVVGLCTGQIKTGAPCRSERLAKYNQLLRIEEELGSK
AKFAGRNFRNPLAK*
>ENSG00000112578|ENST00000230340
MPKFKAARGVGGQEKHAPLADQILAGNAVRAGVREKRRGRGTGEAEEEYVGPRLSRRILQ
QARQQQEELEAEHGTGDKPAAPRERTTRLGPRMPQDGSDDEDEEWPTLEKAATMTAAGHH
AEVVVDPEDERAIEMFMNKNPPARRTLADIIMEKLTEKQTEVETVMSEVSGFPMPQLDPR
VLEVYRGVREVLSKYRSGKLPKAFKIIPALSNWEQILYVTEPEAWTAAAMYQATRIFASN
LKERMAQRFYNLVLLPRVRDDVAEYKRLNFHLYMALKKALFKPGAWFKGILIPLCESGTC
TLREAIIVGSIITKCSIPVLHSSAAMLKIAEMEYSGANSIFLRLLLDKKYALPYRVLDAL
VFHFLGFRTEKRELPVLWHQCLLTLVQRYKADLATDQKEALLELLRLQPHPQLSPEIRRE
LQSAVPRDVEDVPITVE*
>ENSG00000170954|ENST00000243643
MAFTQLTFRDVAIEFSQDEWKCLNSTQRTLYRDVMLENYRNLVSLDLSRNCVIKELAPQQ
EGNPGEVFHTVTLEQHEKHDIEEFCFREIKKKIHDFDCQWRDDERNCNKVTTAPKENLTC
RRDQRDRRGIGNKSIKHQLGLSFLPHPHELQQFQAEGKIYECNHVEKSVNHGSSVSPPQI
ISSTIKTHVSNKYGTDFICSSLLTQEQKSCIREKPYRYIECDKALNHGSHMTVRQVSHSG
EKGYKCDLCGKVFSQKSNLARHWRVHTGEKPYKCNECDRSFSRNSCLALHRRVHTGEKPY
KCYECDKVFSRNSCLALHQKTHIGEKPYTCKECGKAFSVRSTLTNHQVIHSGKKPYKCNE
CGKVFSQTSSLATHQRIHTGEKPYKCNECGKVFSQTSSLARHWRIHTGEKPYKCNECGKV
FSYNSHLASHRRVHTGEKPYKCNECGKAFSVHSNLTTHQVIHTGEKPYKCNQCGKGFSVH
SSLTTHQVIHTGEKPYKCNECGKSFSVRPNLTRHQIIHTGKKPYKCSDCGKSFSVRPNLF
RHQIIHTKEKPYKRN*
>ENSG00000123737|ENST00000243498
MKETPLSNCERRFLLRAIEEKKRLDGRQTYDYRNIRISFGTDYGCCIVELGKTRVLGQVS
CELVSPKLNRATEGILFFNLELSQMAAPAFEPGRQSDLLVKLNRLMERCLRNSKCIDTES
LCVVAGEKVWQIRVDLHLLNHDGNIIDAASIAAIVALCHFRRPDVSVQGDEVTLYTPEER
DPVPLSIHHMPICVSFAFFQQGTYLLVDPNEREERVMDGLLVIAMNKHREICTIQSSGGI
MLLKDQVLRCSKIAGVKVAEITELILKALENDQKVRKEGGKFGFAESIANQRITAFKMEK
APIDTSDVEEKAEEIIAEAEPPSEVVSTPVLWTPGTAQIGEGVENSWGDLEDSEKEDDEG
GGDQAIILDGIKMDTGVEVSDIGSQDAPIILSDSEEEEMIILEPDKNPKKIRTQTTSAKQ
EKAPSKKPVKRRKKKRAAN*
>ENSG00000108788|ENST00000246912
MTEPGASPEDPWVKASPVGAHAGEGRAGRARARRGAGRRGASLLSPKSPTLSVPRGCRED
SSHPACAKVEYAYSDNSLDPGLFVESTRKGSVVSRANSIGSTSASSVPNTDDEDSDYHQE

AYKESYKDRRRRAHTQAEQKRRDAIKRGYDDLQTIVPTCQQQDFSIGSQKLSKAIVLQKT
IDYIQFLHKEKKKQEEEVSTLRKDVTALKIMKVNYEQIVKAHQDNPHEGEDQVSDQVKFN
VFQGIMDSLFQSFNASISVASFQELSACVFSWIEEHCKPQTLREIVIGVLHQLKNQLY*
>ENSG00000111144|ENST00000228740
MPEIVDTCSLASPASVCRTKHLHLRCSVDFTRRTLTGTAALTVQSQEDNLRSLVLDTKDL
TIEKVVINGQEVKYALGERQSYKGSPMEISLPIALSKNQEIVIEISFETSPKSSALQWLT
PEQTSGKEHPYLFSQCQAIHCRAILPCQDTPSVKLTYTAEVSVPKELVALMSAIRDGETP
DPEDPSRKIYKFIQKVPIPCYLIALVVGALESRQIGPRTLVWSEKEQVEKSAYEFSETES
MLKIAEDLGGPYVWGQYDLLVLPPSFPYGGMENPCLTFVTPTLLAGDKSLSNVIAHEISH
SWTGNLVTNKTWDHFWLNEGHTVYLERHICGRLFGEKFRHFNALGGWGELQNSVKTFGET
HPFTKLVVDLTDIDPDVAYSSVPYEKGFALLFYLEQLLGGPEIFLGFLKAYVEKFSYKSI
TTDDWKDFLYSYFKDKVDVLNQVDWNAWLYSPGLPPIKPNYDMTLTNACIALSQRWITAK
EDDLNSFNATDLKDLSSHQLNEFLAQTLQRAPLPLGHIKRMQEVYNFNAINNSEIRFRWL
RLCIQSKWEDAIPLALKMATEQGRMKFTRPLFKDLAAFDKSHDQAVRTYQEHKASMHPVT
AMLVGKDLKVD*
>ENSG00000130182|ENST00000252463
MGPRASLSRLRELCGHWLRPALHTKKQILELLVLEQFLSVLPPHLLGRLQGQPLRDGEEV
VLLLEGIHREPSHAGPLDFSCNAGKSCPRADVTLEEKGCASQVPSHSPKKELPAEEPSVL
GPSDEPPRPQPRAAQPAEPGQWRLPPSSKQPLSPGPQKTFQALQESSPQGPSPWPEESSR
DQELAAVLECLTFEDVPENKAWPAHPLGFGSRTPDKEEFKQEEPKGAAWPTPILAESQAD
SPGVPGEPCAQSLGRGAAASGPGEDGSLLGSSEILEVKVAEGVPEPNPELQFICADCGVS
FPQLSRLKAHQLRSHPAGRSFLCLCCGKSFGRSSILKLHMRTHTDERPHACHLCGHRFRQ
SSHLSKHLLTHSSEPAFLCAECGRGFQRRASLVQHLLAHAQDQKPPCAPESKAEAPPLTD
VLCSHCGQSFQRRSSLKRHLRIHARDKDRRSSEGSGSRRRDSDRRPFVCSDCGKAFRRSE
HLVAHRRVHTGERPFSCQACGRSFTQSSQLVSHQRVHTGEKPYACPQCGKRFVRRASLAR
HLLTHGGPRPHHCTQCGKSFGQTQDLARHQRSHTGEKPCRCSECGEGFSQSAHLARHQRI
HTGEKPHACDTCGHRFRNSSNLARHRRSHTGERPYSCQTCGRSFRRNAHLRRHLATHAEP
GQEQAEPPQECVECGKSFSRSCNLLRHLLVHTGARPYSCTQCGRSFSRNSHLLRHLRTHA
RETLY*
>ENSG00000111331|ENST00000228928
MDLYSTPAAALDRFVARRLQPRKEFVEKARRALGALAAALRERGGRLGAAAPRVLKTVKG
GSSGRGTALKGGCDSELVIFLDCFKSYVDQRARRAEILSEMRASLESWWQNPVPGLRLTF
PEQSVPGALQFRLTSVDLEDWMDVSLVPAFNVLGQAGSGVKPKPQVYSTLLNSGCQGGEH
AACFTELRRNFVNIRPAKLKNLILLVKHWYHQVCLQGLWKETLPPVYALELLTIFAWEQG
CKKDAFSLAEGLRTVLGLIQQHQHLCVFWTVNYGFEDPAVGQFLQRQLKRPRPVILDPAD
PTWDLGNGAAWHWDLLAQEAASCYDHPCFLRGMGDPVQSWKGPGLPRAGCSGLGHPIQLD
PNQKTPENSKSLNAVYPRAGSKPPSCPAPGPTGAASIVPSVPGMALDLSQIPTKELDRFI
QDHLKPSPQFQEQVKKAIDIILRCLHENCVHKASRVSKGGSFGRGTDLRDGCDVELIIFL
NCFTDYKDQGPRRAEILDEMRAQLESWWQDQVPSLSLQFPEQNVPEALQFQLVSTALKSW
TDVSLLPAFDAVGQLSSGTKPNPQVYSRLLTSGCQEGEHKACFAELRRNFMNIRPVKLKN
LILLVKHWYRQVAAQNKGKGPAPASLPPAYALELLTIFAWEQGCRQDCFNMAQGFRTVLG
LVQQHQQLCVYWTVNYSTEDPAMRMHLLGQLRKPRPLVLDPADPTWNVGHGSWELLAQEA
AALGMQACFLSRDGTSVQPWDVMPALLYQTPAGDLDKFISEFLQPNRQFLAQVNKAVDTI
CSFLKENCFRNSPIKVIKVVKGGSSAKGTALRGRSDADLVVFLSCFSQFTEQGNKRAEII

SEIRAQLEACQQERQFEVKFEVSKWENPRVLSFSLTSQTMLDQSVDFDVLPAFDALGQLV
SGSRPSSQVYVDLIHSYSNAGEYSTCFTELQRDFIISRPTKLKSLIRLVKHWYQQCTKIS
KGRGSLPPQHGLELLTVYAWEQGGKDSQFNMAEGFRTVLELVTQYRQLCIYWTINYNAKD
KTVGDFLKQQLQKPRPIILDPADPTGNLGHNARWDLLAKEAAACTSALCCMGRNGIPIQP
WPVKAAV*
>ENSG00000122304|ENST00000241808
MVRYRVRSLSERSHEVYRQQLHGQEQGHHGQEEQGLSPEHVEVYERTHGQSHYRRRHCSR
RRLHRIHRRQHRSCRRRKRRSCRHRRRHRRGCRTRKRTCRRH*
>ENSG00000100441|ENST00000251343
MPTWGARPASPDRFAVSAEAENKVREQQPHVERIFSVGVSVLPKDCPDNPHIWLQLEGPK
ENASRAKEYLKGLCSPELQDEIHYPPKLHCIFLGAQGFFLDCLAWSTSAHLVPRAPGSLM
ISGLTEAFVMAQSRVEELAERLSWDFTPGPSSGASQCTGVLRDFSALLQSPGDAHREALL
QLPLAVQEELLSLVQEASSGQGPGALASWEGRSSALLGAQCQGVRAPPSDGRESLDTGSM
GPGDCRGARGDTYAVEKEGGKQGGPREMDWGWKELPGEEAWEREVALRPQSVGGGARESA
PLKGKALGKEEIALGGGGFCVHREPPGAHGSCHRAAQSRGASLLQRLHNGNASPPRVPSP
PPAPEPPWHCGDRGDCGDRGDVGDRGDKQQGMARGRGPQWKRGARGGNLVTGTQRFKEAL
QDPFTLCLANVPGQPDLRHIVIDGSNVAMVHGLQHYFSSRGIAIAVQYFWDRGHRDITVF
VPQWRFSKDAKVRESHFLQKLYSLSLLSLTPSRVMDGKRISSYDDRFMVKLAEETDGIIV
SNDQFRDLAEESEKWMAIIRERLLPFTFVGNLFMVPDDPLGRNGPTLDEFLKKPARTQGS
SKAQHPSRGFAEHGKQQQGREEEKGSGGIRKTRETERLRRQLLEVFWGQDHKVDFILQRE
PYCRDINQLSEALLSLNF*
>ENSG00000126524|ENST00000246868
MSIFTPTNQIRLTNVAVVRMKRAGKRFEIACYKNKVVGWRSGVEKDLDEVLQTHSVFVNV
SKGQVAKKEDLISAFGTDDQTEICKQILTKGEVQVSDKERHTQLEQMFRDIATIVADKCV
NPETKRPYTVILIERAMKDIHYSVKTNKSTKQQALEVIKQLKEKMKIERAHMRLRFILPV
NEGKKLKEKLKPLIKVIESEDYGQQLEIVCLIDPGCFREIDELIKKETKGKGSLEVLNLK
DVEEGDEKFE*
>ENSG00000121075|ENST00000240335
MLQDKGLSESEEAFRAPGPALGEASAANAPEPALAAPGLSGAALGSPPGPGADVVAAAAA
EQTIENIKVGLHEKELWKKFHEAGTEMIITKAGRRMFPSYKVKVTGMNPKTKYILLIDIV
PADDHRYKFCDNKWMVAGKAEPAMPGRLYVHPDSPATGAHWMRQLVSFQKLKLTNNHLDP
FGHIILNSMHKYQPRLHIVKADENNAFGSKNTAFCTHVFPETSFISVTSYQNHKITQLKI
ENNPFAKGFRGSDDSDLRVARLQSKEYPVISKSIMRQRLISPQLSATPDVGPLLGTHQAL
QHYQHENGAHSQLAEPQDLPLSTFPTQRDSSLFYHCLKRRDGTRHLDLPCKRSYLEAPSS
VGEDHYFRSPPPYDQQMLSPSYCSEVTPREACMYSGSGPEIAGVSGVDDLPPPPLSCNMW
TSVSPYTSYSVQTMETVPYQPFPTHFTATTMMPRLPTLSAQSSQPPGNAHFSVYNQLSQS
QVRERGPSASFPRERGLPQGCERKPPSPHLNAANEFLYSQTFSLSRESSLQYHSGMGTVE
NWTDG*
>ENSG00000126778|ENST00000247182
MSMLPSFGFTQEQVACVCEVLQQGGNLERLGRFLWSLPACDHLHKNESVLKAKAVVAFHR
GNFRELYKILESHQFSPHNHPKLQQLWLKAHYVEAEKLRGRPLGAVGKYRVRRKFPLPRT
IWDGEETSYCFKEKSRGVLREWYAHNPYPSPREKRELAEATGLTTTQVSNWFKNRRQRDR
AAEAKERENTENNNSSSNKQNQLSPLEGGKPLMSSSEEEFSPPQSPDQNSVLLLQGNMGH
ARSSNYSLPGLTASQPSHGLQTHQHQLQDSLLGPLTSSLVDLGS*

>ENSG00000125445|ENST00000245539
MAAPAVKVARGWSGLALGVRRAVLQLPGLTQVRWSRYSPEFKDPLIDKEYYRKPVEELTE
EEKYVRELKKTQLIKAAPAGKTSSVFEDPVISKFTNMMMIGGNKVLARSLMIQTLEAVKR
KQFEKYHAASAEEQATIERNPYTIFHQALKNCEPMIGLVPILKGGRFYQVPVPLPDRRRR
FLAMKWMITECRDKKHQRTLMPEKLSHKLLEAFHNQGPVIKRKHDLHKMAEANRALAHYR
WW*
>ENSG00000197757|ENST00000243108
MNSYFTNPSLSCHLAGGQDVLPNVALNSTAYDPVRHFSTYGAAVAQNRIYSTPFYSPQEN
VVFSSSRGPYDYGSNSFYQEKDMLSNCRQNTLGHNTQTSIAQDFSSEQGRTAPQDQKASI
QIYPWMQRMNSHSGVGYGADRRRGRQIYSRYQTLELEKEFHFNRYLTRRRRIEIANALCL
TERQIKIWFQNRRMKWKKESNLTSTLSGGGGGATADSLGGKEEKREETEEEKQKE*
>ENSG00000012223|ENST00000231751
MKLVFLVLLFLGALGLCLAGRRRSVQWCAVSQPEATKCFQWQRNMRKVRGPPVSCIKRDS
PIQCIQAIAENRADAVTLDGGFIYEAGLAPYKLRPVAAEVYGTERQPRTHYYAVAVVKKG
GSFQLNELQGLKSCHTGLRRTAGWNVPIGTLRPFLNWTGPPEPIEAAVARFFSASCVPGA
DKGQFPNLCRLCAGTGENKCAFSSQEPYFSYSGAFKCLRDGAGDVAFIRESTVFEDLSDE
AERDEYELLCPDNTRKPVDKFKDCHLARVPSHAVVARSVNGKEDAIWNLLRQAQEKFGKD
KSPKFQLFGSPSGQKDLLFKDSAIGFSRVPPRIDSGLYLGSGYFTAIQNLRKSEEEVAAR
RARVVWCAVGEQELRKCNQWSGLSEGSVTCSSASTTEDCIALVLKGEADAMSLDGGYVYT
AGKCGLVPVLAENYKSQQSSDPDPNCVDRPVEGYLAVAVVRRSDTSLTWNSVKGKKSCHT
AVDRTAGWNIPMGLLFNQTGSCKFDEYFSQSCAPGSDPRSNLCALCIGDEQGENKCVPNS
NERYYGYTGAFRCLAENAGDVAFVKDVTVLQNTDGNNNEAWAKDLKLADFALLCLDGKRK
PVTEARSCHLAMAPNHAVVSRMDKVERLKQVLLHQQAKFGRNGSDCPDKFCLFQSETKNL
LFNDNTECLARLHGKTTYEKYLGPQYVAGITNLKKCSTSPLLEACEFLRK*
>ENSG00000129911|ENST00000250916
MSAAVACVDYFAADVLMAISSGAVVHRGRPGPEGAGPAAGLDVRAARREAASPGTPGPPP
PPPAASGPGPGAAAAPHLLAASILADLRGGPGAAPGGASPASSSSAASSPSSGRAPGAAP
SAAAKSHRCPFPDCAKAYYKSSHLKSHLRTHTGERPFACDWQGCDKKFARSDELARHHRT
HTGEKRFSCPLCSKRFTRSDHLAKHARRHPGFHPDLLRRPGARSTSPSDSLPCSLAGSPA
PSPAPSPAPAGL*
>ENSG00000123095|ENST00000242728
MDEGIPHLQERQLLEHRDFIGLDYSSLYMCKPKRSMKRDDTKDTYKLPHRLIEKKRRDRI
NECIAQLKDLLPEHLKLTTLGHLEKAVVLELTLKHLKALTALTEQQHQKIIALQNGERSL
KSPIQSDLDAFHSGFQTCAKEVLQYLSRFESWTPREPRCVQLINHLHAVATQFLPTPQLL
TQQVPLSKGTGAPSAAGSAAAPCLERAGQKLEPLAYCVPVIQRTQPSAELAAENDTDTDS
GYGGEAEARPDREKGKGAGASRVTIKQEPPGEDSPAPKRMKLDSRGGGSGGGPGGGAAAA
AAALLGPDPAAAAALLRPDAALLSSLVAFGGGGGAPFPQPAAAAAPFCLPFCFLSPSAAA
AYVQPFLDKSGLEKYLYPAAAAAPFPLLYPGIPAPAAAAAAAAAAAAAAAFPCLSSVLS
PPPEKAGAAAATLLPHEVAPLGAPHPQHPHGRTHLPFAGPREPGNPESSAQEDPSQPGKE
AP*
>ENSG00000122678|ENST00000242248
MLPKRRRARVGSPSGDAASSTPPSTRFPGVAIYLVEPRMGRSRRAFLTGLARSKGFRVLD
ACSSEATHVVMEETSAEEAVSWQERRMAAAPPGCTPPALLDISWLTESLGAGQPVPVECR
HRLEVAGPRKGPLSPAWMPAYACQRPTPLTHHNTGLSEALEILAEAAGFEGSEGRLLTFC

RAASVLKALPSPVTTLSQLQGLPHFGEHSSRVVQELLEHGVCEEVERVRRSERYQTMKLF
TQIFGVGVKTADRWYREGLRTLDDLREQPQKLTQQQKAGLQHHQDLSTPVLRSDVDALQQ
VVEEAVGQALPGATVTLTGGFRRGKLQGHDVDFLITHPKEGQEAGLLPRVMCRLQDQGLI
LYHQHQHSCCESPTRLAQQSHMDAFERSFCIFRLPQPPGAAVGGSTRPCPSWKAVRVDLV
VAPVSQFPFALLGWTGSKLFQRELRRFSRKEKGLWLNSHGLFDPEQKTFFQAASEEDIFR
HLGLEYLPPEQRNA*
>ENSG00000123415|ENST00000243112
MPQAFLLGSIHEPAGALMEPQPCPGSLAESFLEEELRLNAELSQLQFSEPVGIIYNPVEY
AWEPHRNYVTRYCQGPKEVLFLGMNPGPFGMAQTGVPFGEVSMVRDWLGIVGPVLTPPQE
HPKRPVLGLECPQSEGPRQSMGHEIKSELLMGGCSWIRGKIQCDRVQVRRPGFSSQL*
>ENSG00000113595|ENST00000231524
MATLVVNKLGAGVDSGRQGSRGTAVVKVLECGVCEDVFSLQGDKVPRLLLCGHTVCHDCL
TRLPLHGRAIRCPFDRQVTDLGDSGVWGLKKNFALLELLERLQNGPIGQYGAAEESIGIS
GESIIRCDEDEAHLASVYCTVCATHLCSECSQVTHSTKTLAKHRRVPLADKPHEKTMCSQ
HQVHAIEFVCLEEGCQTSPLMCCVCKEYGKHQGHKHSVLEPEANQIRASILDMAHCIRTF
TEEISDYSRKLVGIVQHIEGGEQIVEDGIGMAHTEHVPGTAENARSCIRAYFYDLHETLC
RQEEMALSVVDAHVREKLIWLRQQQEDMTILLSEVSAACLHCEKTLQQDDCRVVLAKQEI
TRLLETLQKQQQQFTEVADHIQLDASIPVTFTKDNRVHIGPKMEIRVVTLGLDGAGKTTI
LFKLKQDEFMQPIPTIGFNVETVEYKNLKFTIWDVGGKHKLRPLWKHYYLNTQAVVFVVD
SSHRDRISEAHSELAKLLTEKELRDALLLIFANKQDVAGALSVEEITELLSLHKLCCGRS
WYIQGCDARSGMGLYEGLDWLSRQLVAAGVLDVA*
>ENSG00000106211|ENST00000248553
MTERRVPFSLLRGPSWDPFRDWYPHSRLFDQAFGLPRLPEEWSQWLGGSSWPGYVRPLPP
AAIESPAVAAPAYSRALSRQLSSGVSEIRHTADRWRVSLDVNHFAPDELTVKTKDGVVEI
TGKHEERQDEHGYISRCFTRKYTLPPGVDPTQVSSSLSPEGTLTVEAPMPKLATQSNEIT
IPVTFESRAQLGGPEAAKSDETAAK*
>ENSG00000114626|ENST00000232744
MDTSDLFASCRKGDVGRVRYLLEQRDVEVNVRDKWDSTPLYYACLCGHEELVLYLLANGA
RCEANTFDGERCLYGALSDPIRRALRDYKQVTASCRRRDYYDDFLQRLLEQGIHSDVVFV
VHGKPFRVHRCVLGARSAYFANMLDTKWKGKSVVVLRHPLINPVAFGALLQYLYTGRLDI
GVEHVSDCERLAKQCQLWDLLSDLEAKCEKVSEFVASKPGTCVKVLTIEPPPADPRLRED
MALLADCALPPELRGDLWELPFPCPDGFNSCPDICFRVAGCSFLCHKAFFCGRSDYFRAL
LDDHFRESEEPATSGGPPAVTLHGISPDVFTHVLYYMYSDHTELSPEAAYDVLSVADMYL
LPGLKRLCGRSLAQMLDEDTVVGVWRVAKLFRLARLEDQCTEYMAKVIEKLVEREDFVEA
VKEEAAAVAARQETDSIPLVDDIRFHVASTVQTYSAIEEAQQRLRALEDLLVSIGLDC*
>ENSG00000099899|ENST00000252136
MSENLDNEGPKPMESCGQESSSALSCPTVSVPPAAPAALEEVEKEGAGAATGPGPQPGLY
SYIRDDLFTSEIFKLELQNVPRHASFSDVRRFLGRFGLQPHKTKLFGQPPCAFVTFRSAA
ERDKALRVLHGALWKGRPLSVRLARPKADPMARRRRQEGESEPPVTRVADVVTPLWTVPY
AEQLERKQLECEQVLQKLAKEIGSTNRALLPWLLEQRHKHNKACCPLEGVRPSPQQTEYR
NKCEFLVGVGVDGEDNTVGCRLGKYKGGTCAVAAPFDTVHIPEATKQVVKAFQEFIRSTP
YSAYDPETYTGHWKQLTVRTSRRHQAMAIAYFHPQKLSPEELAELKTSLAQHFTAGPGRA
SGVTCLYFVEEGQRKTPSQEGLPLEHVAGDRCIHEDLLGLTFRISPHAFFQVNTPAAEVL
YTVIQDWAQLDAGSMVLDVCCGTGTIGLALARKVKRVIGVELCPEAVEDARVNAQDNELS

NVEFHCGRAEDLVPTLVSRLASQHLVAILDPPRAGLHSKVILAIRRAKNLRRLLYVSCNP
RAAMGNFVDLCRAPSNRVKGIPFRPVKAVAVDLFPQTPHCEMLILFERVEHPNGTGVLGP
HSPPAQPTPGPPDNTLQETGTFPSS*
>ENSG00000124608|ENST00000244571
MAASVAAAARRLRRAIRRSPAWRGLSHRPLSSEPPAAKASAVRAAFLNFFRDRHGHRLVP
SASVRPRGDPSLLFVNAGMNQFKPIFLGTVDPRSEMAGFRRVANSQKCVRAGGHHNDLED
VGRDLSHHTFFEMLGNWAFGGEYFKEEACNMAWELLTQVYGIPEERLWISYFDGDPKAGL
DPDLETRDIWLSLGVPASRVLSFGPQENFWEMGDTGPCGPCTEIHYDLAGGVGAPQLVEL
WNLVFMQHNREADGSLQPLPQRHVDTGMGLERLVAVLQGKHSTYDTDLFSPLLNAIQQGC
RAPPYLGRVGVADEGRTDTAYRVVADHIRTLSVCISDGIFPGMSGPPLVLRRILRRAVRF
SMEILKAPPGFLGSLVPVVVETLGDAYPELQRNSAQIANLVSEDEAAFLASLERGRRIID
RTLRTLGPSDMFPAEVAWSLSLCGDLGLPLDMVELMLEEKGVQLDSAGLERLAQEEAQHR
ARQAEPVQKQGLWLDVHALGELQRQGVPPTDDSPKYNYSLRPSGSYEFGTCEAQVLQLYT
EDGTAVASVGKGQRCGLLLDRTNFYAEQGGQASDRGYLVRAGQEDVLFPVARAQVCGGFI
LHEAVAPECLRLGDQVQLHVDEAWRLGCMAKHTATHLLNWALRQTLGPGTEQQGSHLNPE
QLRLDVTTQTPLTPEQLRAVENTVQEAVGQDEAVYMEEVPLALTAQVPGLRSLDEVYPDP
VRVVSVGVPVAHALDPASQAALQTSVELCCGTHLLRTGAVGDLVIIGDRQLSKGTTRLLA
VTGEQAQQARELGQSLAQEVKAATERLSLGSRDVAEALRLSKDIGRLIEAVETAVMPQWQ
RRELLATVKMLQRRANTAIRKLQMGQAAKKTQELLERHSKGPLIVDTVSAESLSVLVKVV
RQLCEQAPSTSVLLLSPQPMGKVLCACQVAQGAMPTFTAEAWALAVCSHMGGKAWGSRVV
AQGTGSTTDLEAALSIAQTYALSQL*
>ENSG00000076248|ENST00000242576
MIGQKTLYSFFSPSPARKRHAPSPEPAVQGTGVAGVPEESGDAAAIPAKKAPAGQEEPGT
PPSSPLSAEQLDRIQRNKAAALLRLAARNVPVGFGESWKKHLSGEFGKPYFIKLMGFVAE
ERKHYTVYPPPHQVFTWTQMCDIKDVKVVILGQDPYHGPNQAHGLCFSVQRPVPPPPSLE
NIYKELSTDIEDFVHPGHGDLSGWAKQGVLLLNAVLTVRAHQANSHKERGWEQFTDAVVS
WLNQNSNGLVFLLWGSYAQKKGSAIDRKRHHVLQTAHPSPLSVYRGFFGCRHFSKTNELL
QKSGKKPIDWKEL*
>ENSG00000101901|ENST00000251943
MNNHQLELAKQLHKEGHLFYCTCRVLTCPGGQAKSIASAPGKCQDSAALTSTAFSGLDFGL
LSGYLHKQALVTATHPTCTLLFPSCHAFFPLPLTPTLYKMHKGWKNYCSQKSLNEASMDE
YLGSLGLFRKLTAKDASCLFRAISEQLFCSQVHHLEIRKACVSYMRENQQTFESYVEGSF
EKYLERLGDPKESAGQLEIRALSLIYNRDFILYRFPGKPPTYVTDNGYEDKILLCYSSSG
HYDSVYSKQFQSSAAVCQAVLYEILYKDVFVVDEEELKTAIKLFRSGSKKNRNNAVTGSE
DAHTDYKSSNQNRMEEWGACYNAENIPEGYNKGTEETKSPENPSKMPFPYKVLKALDPEI
YRNVEFDVWLDSRKELQKSDYMEYAGRQYYLGDKCQVCLESEGRYYNAHIQEVGNENNSV
TVFIEELAEKHVVPLANLKPVTQVMSVPAWNAMPSRKGRGYQKMPGGYVPEIVISEMDIK
QQKKMFKKIRGKEVYMTMAYGKGDPLLPPRLQHSMHYGHDPPMHYSQTAGNVMSNEHFHP
QHPSPRQGRGYGMPRNSSRFINRHNMPGPKVDFYPGPGKRCCQSYDNFSYRSRSFRRSHR
QMSCVNKESQYGFTPGNGQMPRGLEETITFYEVEEGDETAYPTLPNHGGPSTMVPATSGY
CVGRRGHSSGKQTLNLEEGNGQSENGRYHEEYLYRAEPDYETSGVYSTTASTANLSLQDR
KSCSMSPQDTVTSYNYPQKMMGNIAAVAASCANNVPAPVLSNGAAANQAISTTSVSSQNA
IQPLFVSPPTHGRPDTKVLQYYFNLGLQCYYHSYWHSMVYVPQMQQQLHVENYPVYTEPP
LVDQTVPQCYSEVRREDGIQAEASANDTFPNADSSSVPHGAVYYPVMSDPYGQPPLPGFD

SCLPVVPDYSCVPPWHPVGTAYGGSSQIHGAINPGPIGCIAPSPPASHYVPQGM*
>ENSG00000116132|ENST00000239461
MTSSYGHVLERQPALGGRLDSPGNLDTLQAKKNFSVSHLLDLEEAGDMVAAQADENVGEA
GRSLLESPGLTSGSDTPQQDNDQLNSEEKKKRKQRRNRTTFNSSQLQALERVFERTHYPD
AFVREDLARRVNLTEARVQVWFQNRRAKFRRNERAMLANKNASLLKSYSGDVTAVEQPIV
PRPAPRPTDYLSWGTASPYSAMATYSATCANNSPAQGINMANSIANLRLKAKEYSLQRNQ
VPTVN*
>ENSG00000118526|ENST00000237316
MSTGSLSDVEDLQEVEMLECDGLKMDSNKEFVTSNESTEESSNCENGSPQKGRGGLGKRR
KAPTKKSPLSGVSQEGKQVQRNAANARERARMRVLSKAFSRLKTTLPWVPPDTKLSKLDT
LRLASSYIAHLRQILANDKYENGYIHPVNLTWPFMVAGKPESDLKEVVTASRLCGTTAS*
>ENSG00000130584|ENST00000245663
MNNRKEDMEITSHYRHLLRELNEQRQHGVLCDVCVVVEGKVFKAHKNVLLGSSRYFKTLY
CQVQKTSEQATVTHLDIVTAQGFKAIIDFMYSAHLALTSRNVIEVMSAASFLQMTDIVQA
CHDFIKAALDISIKSDASDELAEFEIGASSSSSTEALISAVMAGRSISPWLARRTSPANS
SGDSAIASCHDGGSSYGKEDQEPKADGPDDVSSQPLWPGDVGYGPLRIKEEQVSPSQYGG
SELPSAKDGAVQNSFSEQSAGDAWQPTGRRKNRKNKETVRHITQQVEDDSRASSPVPSFL
PTSGWPFSSRDSNADLSVTEASSSDSRGERAELYAQVEEGLLGGEASYLGPPLTPEKDDA
LHQATAVANLRAALMSKNSLLSLKADVLGDDGSLLFEYLPRGAHSLSLNEFTVIRKKFKC
PYCSFSAMHQCILKRHMRSHTGERPYPCEICGKKFTRREHMKRHTLVHSKDKKYVCKVCS
RVFMSAASVGIRHGSRRHGVCTDCAGRGMAGPLDHGGGGGEGSPEALFPGDGPYLEDPED
PRGEAEELGEDDEGLAPEDALLADDKDEEDSPRPRSPPGGPDKDFAWLS*
>ENSG00000111783|ENST00000229387
MNWAAFGGSEFFIPEGIQIDSRCPLSRNITEWYHYYGIAVKESSQYYDVMYSKKGAAWVS
ETGKKEVSKQTVAYSPRSKLGTLLPEFPNVKDLNLPASLPEEKVSTFIMMYRTHCQRILD
TVIRANFDEVQSFLLHFWQGMPPHMLPVLGSSTVVNIVGVCDSILYKAISGVLMPTVLQA
LPDSLTQVIRKFAKQLDEWLKVALHDLPENLRNIKFELSRRFSQILRRQTSLNHLCQASR
TVIHSADITFQMLEDWRNVDLNSITKQTLYTMEDSRDEHRKLITQLYQEFDHLLEEQSPI
ESYIEWLDTMVDRCVVKVAAKRQGSLKKVAQQFLLMWSCFGTRVIRDMTLHSAPSFGSFH
LIHLMFDDYVLYLLESLHCQERANELMRAMKGEGSTAEVREEIILTEAAAPTPSPVPSFS
PAKSATSVEVPPPSSPVSNPSPEYTGLSTTGAMQSYTWSLTYTVTTAAGSPAENSQQLPC
MRNTHVPSSSVTHRIPVYPHREEHGYTGSYNYGSYGNQHPHPMQSQYPALPHDTAISGPL
HYAPYHRSSAQYPFNSPTSRMEPCLMSSTPRLHPTPVTPRWPEVPSANTCYTSPSVHSAR
YGNSSDMYTPLTTRRNSEYEHMQHFPGFAYINGEASTGWAK*
>ENSG00000125888|ENST00000246090
MDNMSPRLRAFLSEPIGEKDVCWVDGISHELAINLVTKGINKAYILLGQFLLMHKNEAEF
QRWLICCFGATECEAQQTSHCLKEWCACFL*
>ENSG00000129351|ENST00000250241
MRPMRIFVNDDRHVMAKHSSVYPTQEELEAVQNMVSHTERALKAVSDWIDEQEKGSSEQA
ESDNMDVPPEDDSKEGAGEQKTEHMTRTLRGVMRVGLVAKGLLLKGDLDLELVLLCKEKP
TTALLDKVADNLAIQLAAVTEDKYEILQSVDDAAIVIKNTKEPPLSLTIHLTSPVVREEM
EKVLAGETLSVNDPPDVLDRQKCLAALASLRHAKWFQARANGLKSCVIVIRVLRDLCTRV
PTWGPLRGWPLELLCEKSIGTANRPMGAGEALRRVLECLASGIVMPDGSGIYDPCEKEAT
DAIGHLDRQQREDITQSAQHALRLAAFGQLHKVLGMDPLPSKMPKKPKNENPVDYTVQIP

PSTTYAITPMKRPMEEDGEEKSPSKKKKKIQKKEEKAEPPQAMNALMRLNQLKPGLQYKL
VSQTGPVHAPIFTMSVEVDGNSFEASGPSKKTAKLHVAVKVLQDMGLPTGAEGRDSSKGE
DSAEETEAKPAVVAPAPVVEAVSTPSAAFPSDATAEQGPILTKHGKNPVMELNEKRRGLK
YELISETGGSHDKRFVMEVEVDGQKFQGAGSNKKVAKAYAALAALEKLFPDTPLALDANK
KKRAPVPVRGGPKFAAKPHNPGFGMGGPMHNEVPPPPNLRGRGRGGSIRGRGRGRGFGGA
NHGGYMNAGAGYGSYGYGGNSATAGYTGFV*
>ENSG00000115268|ENST00000233609
MSYEQLMQLYSARQRRRLNRGLRRKQHSLLKRLRKAKKEAPPMEKPEVVKTHLRDMIILP
EMVGSMVGVYNGKTFNQVEIKPEMIGHYLGEFSITYKPVKHGRPGIGATHSSRFIPLK*
>ENSG00000028839|ENST00000237264
MDADSDVALDILITNVVCVFRTRCHLNLRKIALEGANVIYKRDVGKVLMKLRKPRITATI
WSSGKIICTGATSEEEAKFGARRLARSLQKLGFQVIFTDFKVVNVLAVCNMPFEIRLPEF
TKNNRPHASYEPELHPAVCYRIKSLRATLQIFSTGSITVTGPNVKAVATAVEQIYPFVFE
SRKEIL*
>ENSG00000112118|ENST00000229854
MAGTVVLDDVELREAQRDYLDFLDDEEDQGIYQSKVRELISDNQYRLIVNVNDLRRKNEK
RANRLLNNAFEELVAFQRALKDFVASIDATYAKQYEEFYVGLEGSFGSKHVSPRTLTSCF
LSCVVCVEGIVTKCSLVRPKVVRSVHYCPATKKTIERRYSDLTTLVAFPSSSVYPTKDEE
NNPLETEYGLSVYKDHQTITIQEMPEKAPAGQLPRSVDVILDDDLVDKAKPGDRVQVVGT
YRCLPGKKGGYTSGTFRTVLIACNVKQMSKDAQPSFSAEDIAKIKKFSKTRSKDIFDQLA
KSLAPSIHGHDYVKKAILCLLLGGVERDLENGSHIRGDINILLIGDPSVAKSQLLRYVLC
TAPRAIPTTGRGSSGVGLTAAVTTDQETGERRLEAGAMVLADRGVVCIDEFDKMSDMDRT
AIHEVMEQGRVTIAKAGIHARLNARCSVLAAANPVYGRYDQYKTPMENIGLQDSLLSRFD
LLFIMLDQMDPEQDREISDHVLRMHRYRAPGEQDGDAMPLGSAVDILATDDPNFSQEDQQ
DTQIYEKHDNLLHGTKKKKEKMVSAAFMKKYIHVAKIIKPVLTQESATYIAEEYSRLRSQ
DSMSSDTARTSPVTARTLETLIRLATAHAKARMSKTVDLQDAEEAVELVQYAYFKKVLEK
EKKRKKRSEDESETEDEEEKSQEDQEQKRKRRKTRQPDAKDGDSYDPYDFSDTEEEMPQV
HTPKTADSQETKESQKVELSESRLKAFKVALLDVFREAHAQSIGMNRLTESINRDSEEPF
SSVEIQAALSKMQDDNQVMVSEGIIFLI*
>ENSG00000101452|ENST00000252011
MAAPVGPVKFWRPGTEGPGVSISEERQSLAENSGTTVVYNPYAALSIEQQRQKLPVFKLR
NHILYLIENYQTVVIVGETGCGKSTQIPQYLAEAGWTAEGRVVGVTQPRRVAAVTVAGRV
AEERGAVLGHEVGYCIRFDDCTDQLATRIKFLTDGMLVREMMVDPLLTKYSVIMLDEAHE
RTLYTDIAIGLLKKIQKKRGDLRLIVASATLDADKFRDFFNQNETSDPARDTCVILTVEG
RTFPVDIFYLQSPVPDYIKSTVETVVKIHQTEGDGDVLAFLTGQEEVETVVSMLIEQARA
LARTGMKRHLRVLPMYAGLPSFEQMKVFERVSRSVRKVIVATNVAETSITISGIVYVIDC
GFVKLRAYNPRTAIECLVVVPVSQASANQRAGRGGRSRSGKCYRLYTEEAFDKLPQSTVP
EMQRSNLAPVILQLKALGIDNVLRFHFMSPPPAQSMVQALELLYALGGLDKDCRLTEPLG
MRIAEFPLNPMFAKMLLESGNFGCSQEILSIAAMMQIQNIFVVPPNQKSHAIRVHRKFAV
EEGDHLTMLNIYEAFIKHNKDSKWCQEHFLNYKGLVRAATVREQLKKLLVKFQVPRKSSE
GDPDLVLRCIVSGFFANAARFHSTGAYRTIRDDHELHIHPASVLYAEKPPRWVIYNEVIQ
TSKYYMRDVTAIESAWLLELAPHFYQQGTHLSLKAKRAKVQDP*
>ENSG00000128710|ENST00000249501
MSFPNSSPAANTFLVDSLISACRSDSFYSSSASMYMPPPSADMGTYGMQTCGLLPSLAKR

EVNHQNMGMNVHPYIPQVDSWTDPNRSCRIEQPVTQQVPTCSFTTNIKEESNCCMYSDKR
NKLISAEVPSYQRLVPESCPVENPEVPVPGYFRLSQTYATGKTQEYNNSPEGSSTVMLQL
NPRGAAKPQLSAAQLQMEKKMNEPVSGQEPTKVSQVESPEAKGGLPEERSCLAEVSVSSP
EVQEKESKEEIKSDTPTSNWLTAKSGRKKRCPYTKHQTLELEKEFLFNMYLTRERRLEIS
KSVNLTDRQVKIWFQNRRMKLKKMSRENRIRELTANLTFS*
>ENSG00000105705|ENST00000247001
MSLKMDNRDVAGKANRWFGVAPPKSGKMNMNILHQEELIAQKKREIEAKMEQKAKQNQVA
SPQPPHPGEITNAHNSSCISNKFANDGSFLQQFLKLQKAQTSTDAPTSAPSAPPSTPTPS
AGKRSLLISRRTGLGLASLPGPVKSYSHAKQLPVAHRPSVFQSPDEDEEEDYEQWLEIKV
SPPEGAETRKVIEKLARFVAEGGPELEKVAMEDYKDNPAFAFLHDKNSREFLYYRKKVAE
IRKEAQKSQAASQKVSPPEDEEVKNLAEKLARFIADGGPEVETIALQNNRENQAFSFLYE
PNSQGYKYYRQKLEEFRKAKASSTGSFTAPDPGLKRKSPPEALSGSLPPATTCPASSTPA
PTIIPAPAAPGKPASAATVKRKRKSRWGPEEDKVELPPAELVQRDVDASPSPLSVQDLKG
LGYEKGKPVGLVGVTELSDAQKKQLKEQQEMQQMYDMIMQHKRAMQDMQLLWEKAVQQHQ
HGYDSDEEVDSELGTWEHQLRRMEMDKTREWAEQLTKMGRGKHFIGDFLPPDELEKFMET
FKALKEGREPDYSEYKEFKLTVENIGYQMLMKMGWKEGEGLGSEGQGIKNPVNKGTTTVD
GAGFGIDRPAELSKEDDEYEAFRKRMMLAYRFRPNPLNNPRRPYY*
>ENSG00000127337|ENST00000247843
MFKRMAEFGPDSGGRVKGVTIVKPIVYGNVARYFGKKREEDGHTHQWTVYVKPYRNEDMS
AYVKKIQFKLHESYGNPLRVVTKPPYEITETGWGEFEIIIKIFFIDPNERPVTLYHLLKL
FQSDTNAMLGKKTVVSEFYDEMIFQDPTAMMQQLLTTSRQLTLGAYKHETEFAELEVKTR
EKLEAAKKKTSFEIAELKERLKASRETINCLKNEIRKLEEDDQAKDI*
>ENSG00000114315|ENST00000232424
MPADIMEKNSSSPVAATPASVNTTPDKPKTASEHRKSSKPIMEKRRRARINESLSQLKTL
ILDALKKDSSRHSKLEKADILEMTVKHLRNLQRAQMTAALSTDPSVLGKYRAGFSECMNE
VTRFLSTCEGVNTEVRTRLLGHLANCMTQINAMTYPGQPHPALQAPPPPPPGPGGPQHAP
FAPPPPLVPIPGGAAPPPGGAPCKLGSQAGEAAKVFGGFQVVPAPDGQFAFLIPNGAFAH
SGPVIPVYTSNSGTSVGPNAVSPSSGPSLTADSMWRPWRN*
>ENSG00000077312|ENST00000243563
MAVPETRPNHTIYINNLNEKIKKDELKKSLYAIFSQFGQILDILVSRSLKMRGQAFVIFK
EVSSATNALRSMQGFPFYDKPMRIQYAKTDSDIIAKMKGTFVERDRKREKRKPKSQETPA
TKKAVQGGGATPVVGAVQGPVPGMPPMTQAPRIMHHMPGQPPYMPPPGMIPPPGLAPGQI
PPGAMPPQQLMPGQMPPAQPLSENPPNHILFLTNLPEETNELMLSMLFNQFPGFKEVRLV
PGRHDIAFVEFDNEVQAGAARDALQGFKITQNNAMKISFAKK*
>ENSG00000118246|ENST00000236980
MLTTLKPFGSVSVESKMNNKAGSFFWNLRQFSTLVSTSRTMRLCCLGLCKPKIVHSNWNI
LNNFHNRMQSTDIIRYLFQDAFIFKSDVGFQTKGISTLTALRIERLLYAKRLFFDSKQSL
VPVDKSDDELKKVNLNHEVSNEDVLTKETKPNRISSRKLSEECNSLSDVLDAFSKAPTFP
SSNYFTAMWTIAKRLSDDQKRFEKRLMFSHPAFNQLCEHMMREAKIMQYKYLLFSLHAIV
KLGIPQNTILVQTLLRVTQERINECDEICLSVLSTVLEAMEPCKNVHVLRTGFRILVDQQ
VWKIEDVFTLQVVMKCIGKDAPIALKRKLEMKALRELDRFSVLNSQHMFEVLAAMNHRSL
ILLDECSKVVLDNIHGCPLRIMINILQSCKDLQYHNLDLFKGLADYVAATFDIWKFRKVL
FILILFENLGFRPVGLMDLFMKRIVEDPESLNMKNILSILHTYSSLNHVYKCQNKEQFVE
VMASALTGYLHTISSENLLDAVYSFCLMNYFPLAPFNQLLQKDIISELLTSDDMKNAYKL

HTLDTCLKLDDTVYLRDIALSLPQLPRELPSSHTNAKVAEVLSSLLGGEGHFSKDVHLPH
NYHIDFEIRMDTNRNQVLPLSDVDTTSATDIQRVAVLCVSRSAYCLGSSHPRGFLAMKMR
HLNAMGFHVILVNNWEMDKLEMEDAVTFLKTKIYSVEALPVAAVNVQSTQ*
>ENSG00000124701|ENST00000244669
MAQKEEAAVATEAASQNGEDLENLDDPEKLKELIELPPFEIVTGERLPANFFKFQFRNVE
YSSGRNKTFLCYVVEAQGKGGQVQASRGYLEDEHAAAHAEEAFFNTILPAFDPALRYNVT
WYVSSSPCAACADRIIKTLSKTKNLRLLILVGRLFMWEEPEIQAALKKLKEAGCKLRIMK
PQDFEYVWQNFVEQEEGESKAFQPWEDIQENFLYYEEKLADILK*
>ENSG00000114767|ENST00000232888
MSATAAARKRGKPASGAGAGAGAGKRRRKADSAGDRGKSKGGGKMNEEISSDSESESLAP
RKPEEEEEEELEETAQEKKLRLAKLYLEQLRQQEEEKAEARAFEEDQVAGRLKEDVLEQR
GRLQKLVAKEIQAPASADIRVLRGHQLSITCLVVTPDDSAIFSAAKDCSIIKWSVESGRK
LHVIPRAKKGAEGKPPGHSSHVLCMAISSDGKYLASGDRSKLILIWEAQSCQHLYTFTGH
RDAVSGLAFRRGTHQLYSTSHDRSVKVWNVAENSYVETLFGHQDAVAALDALSRECCVTA
GGRDGTVRVWKIPEESQLVFYGHQGSIDCIHLINEEHMVSGADDGSVALWGLSKKRPLAL
QREAHGLRGEPGLEQPFWISSVAALLNTDLVATGSHSSCVRLWQCGEGFRQLDLLCDIPL
VGFINSLKFSSSGDFLVAGVGQEHRLGRWWRIKEARNSVCIIPLRRVPVPPAAGS*
>ENSG00000031698|ENST00000234677
MVLDLDLFRVDKGGDPALIRETQEKRFKDPGLVDQLVKADSEWRRCRFRADNLNKLKNLC
SKTIGEKMKKKEPVGDDESVPENVLSFDDLTADALANLKVSQIKKVRLLIDEAILKCDAE
RIKLEAERFENLREIGNLLHPSVPISNDEDVDNKVERIWGDCTVRKKYSHVDLVVMVDGF
EGEKGAVVAGSRGYFLKGVLVFLEQALIQYALRTLGSRGYIPIYTPFFMRKEVMQEVAQL
SQFDEELYKVIGKGSEKSDDNSYDEKYLIATSEQPIAALHRDEWLRPEDLPIKYAGLSTC
FRQEVGSHGRDTRGIFRVHQFEKIEQFVYSSPHDNKSWEMFEEMITTAEEFYQSLGIPYH
IVNIVSGSLNHAASKKLDLEAWFPGSGAFRELVSCSNCTDYQARRLRIRYGQTKKMMDKV
EFVHMLNATMCATTRTICAILENYQTEKGITVPEKLKEFMPPGLQELIPFVKPAPIEQEP
SKKQKKQHEGSKKKAAARDVTLENRLQNMEVTDA*
>ENSG00000111424|ENST00000229022
MEAMAASTSLPDPGDFDRNVPRICGVCGDRATGFHFNAMTCEGCKGFFRRSMKRKALFTC
PFNGDCRITKDNRRHCQACRLKRCVDIGMMKEFILTDEEVQRKREMILKRKEEEALKDSL
RPKLSEEQQRIIAILLDAHHKTYDPTYSDFCQFRPPVRVNDGGGSHPSRPNSRHTPSFSG
DSSSSCSDHCITSSDMMDSSSFSNLDLSEEDSDDPSVTLELSQLSMLPHLADLVSYSIQK
VIGFAKMIPGFRDLTSEDQIVLLKSSAIEVIMLRSNESFTMDDMSWTCGNQDYKYRVSDV
TKAGHSLELIEPLIKFQVGLKKLNLHEEEHVLLMAICIVSPDRPGVQDAALIEAIQDRLS
NTLQTYIRCRHPPPGSHLLYAKMIQKLADLRSLNEEHSKQYRCLSFQPECSMKLTPLVLE
VFGNEIS*
>ENSG00000117139|ENST00000235790
MGFAPGKAVGSHIRGHYERILNPYNLFLSGDSLRCLQKPNLTTDTKDKEYKPHDIPQRQS
VQPSETCPPARRAKRMRAEAMNIKIEPEETTEARTHNLRRRMGCPTPKCENEKEMKSSIK
QEPIERKDYIVENEKEKPKSRSKKATNAVDLYVCLLCGSGNDEDRLLLCDGCDDSYHTFC
LIPPLHDVPKGDWRCPKCLAQECSKPQEAFGFEQAARDYTLRTFGEMADAFKSDYFNMPV
HMVPTELVEKEFWRLVSTIEEDVTVEYGADIASKEFGSGFPVRDGKIKLSPEEEEYLDSG
WNLNNMPVMEQSVLAHITADICGMKLPWLYVGMCFSSFCWHIEDHWSYSINYLHWGEPKT
WYGVPGYAAEQLENVMKKLAPELFVSQPDLLHQLVTIMNPNTLMTHEVPVYRTNQCAGEF

VITFPRAYHSGFNQGFNFAEAVNFCTVDWLPLGRQCVEHYRLLHRYCVFSHDEMICKMAS
KADVLDVVVASTVQKDMAIMIEDEKALRETVRKLGVIDSERMDFELLPDDERQCVKCKTT
CFMSAISCSCKPGLLVCLHHVKELCSCPPYKYKLRYRYTLDDLYPMMNALKLRAESYNEW
ALNVNEALEAKINKKKSLVSFKALIEESEMKKFPDNDLLRHLRLVTQDAEKCASVAQQLL
NGKRQTRYRSGGGKSQNQLTVNELRQFVTQLYALPCVLSQTPLLKDLLNRVEDFQQHSQK
LLSEETPSAAELQDLLDVSFEFDVELPQLAEMRIRLEQARWLEEVQQACLDPSSLTLDDM
RRLIDLGVGLAPYSAVEKAMARLQELLTVSEHWDDKAKSLLKARPRHSLNSLATAVKEIE
EIPAYLPNGAALKDSVQRARDWLQDVEGLQAGGRVPVLDTLIELVTRGRSIPVHLNSLPR
LETLVAEVQAWKECAVNTFLTENSPYSLLEVLCPRCDIGLLGLKRKQRKLKEPLPNGKKK
STKLESLSDLERALTESKETASAMATLGEARLREMEALQSLRLANEGKLLSPLQDVDIKI
CLCQKAPAAPMIQCELCRDAFHTSCVAVPSISQGLRIWLCPHCRRSEKPPLEKILPLLAS
LQRIRVRLPEGDALRYMIERTVNWQHRAQQLLSSGNLKFVQDRVGSGLLYSRWQASAGQV
SDTNKVSQPPGTTSFSLPDDWDNRTSYLHSPFSTGRSCIPLHGVSPEVNELLMEAQLLQV
SLPEIQELYQTLLAKPSPAQQTDRSSPVRPSSEKNDCCRGKRDGINSLERKLKRRLEREG
LSSERWERVKKMRTP
>ENSG00000105821|ENST00000249270
MLLLPSAADGRGTAITHALTSASTLCQVEPVGRWFEAFVKRRNRNASASFQELEDKKELS
EESEDEELQLEEFPMLKTLDPKDWKNQDHYAVLGLGHVRYKATQRQIKAAHKAMVLKHHP
DKRKAAGEPIKEGDNDYFTCITKAYEMLSDPVKRRAFNSVDPTFDNSVPSKSEAKDNFFE
VFTPVFERNSRWSNKKNVPKLGDMNSSFEDVDIFYSFWYNFDSWREFSYLDEEEKEKAEC
RDERRWIEKQNRATRAQRKKEEMNRIRTLVDNAYSCDPRIKKFKEEEKAKKEAEKKAKAE
AKRKEQEAKEKQRQAELEAARLAKEKEEEEVRQQALLAKKEKDIQKKAIKKERQKLRNSC
KIEEINEQIRKEKEEAEARMRQASKNTEKSTGGGGNGSKNWSEDDLQLLIKAVNLFPAGT
NSRWEVIANYMNIHSSSGVKRTAKDVIGKAKSLQKLDPHQKDDINKKAFDKFKKEHGVVP
QADNATPSERFEGPYTDFTPWTTEEQKLLEQALKTYPVNTPERWEKIAEAVPGRTKKDCM
KRYKELVEMVKAKKAAQEQVLNASRAKK*
>ENSG00000124575|ENST00000244534
MSETAPLAPTIPAPAEKTPVKKKAKKAGATAGKRKASGPPVSELITKAVAASKERSGVSL
AALKKALAAAGYDVEKNNSRIKLGLKSLVSKGTLVQTKGTGASGSFKLNKKAASGEGKPK
AKKAGAAKPRKPAGAAKKPKKVAGAATPKKSIKKTPKKVKKPATAAGTKKVAKSAKKVKT
PQPKKAAKSPAKAKAPKPKAAKPKSGKPKVTKAKKAAPKKK*
>ENSG00000124766|ENST00000244745
MVQQTNNAENTEALLAGESSDSGAGLELGIASSPTPGSTASTGGKADDPSWCKTPSGHIK
RPMNAFMVWSQIERRKIMEQSPDMHNAEISKRLGKRWKLLKDSDKIPFIREAERLRLKHM
ADYPDYKYRPRKKVKSGNANSSSSAAASSKPGEKGDKVGGSGGGGHGGGGGGGSSNAGGG
GGGASGGGANSKPAQKKSCGSKVAGGAGGGVSKPHAKLILAGGGGGGKAAAAAAASFAAE
QAGAAALLPLGAAADHHSLYKARTPSASASASSAASASAALAAPGKHLAEKKVKRVYLFG
GLGTSSSPVGGVGAGADPSDPLGLYEEEGAGCSPDAPSLSGRSSAASSPAAGRSPADHRG
YASLRAASPAPSSAPSHASSSASSHSSSSSSSGSSSSDDEFEDDLLDLNPSSNFESMSLG
SFSSSSALDRDLDFNFEPGSGSHFEFPDYCTPEVSEMISGDWLESSISNLVFTY*
>ENSG00000131127|ENST00000240499
MELLTFRDVAIEFSPEEWKCLDPDQQNLYRDVMLENYRNLVSLGVAISNPDLVTCLEQRK
EPYNVKIHKIVARPPAMCSHFTQDHWPVQGIEDSFHKLILRRYEKCGHDNLQLRKGCKSL
NECKLQKGGYNEFNECLSTTQSKILQCKASVKVVSKFSNSNKRKTRHTGEKHFKECGKSF

QKFSHLTQHKVIHAGEKPYTCEECGKAFKWSLIFNEHKRIHTGEKPFTCEECGSIFTTSS
HFAKHKIIHTGEKPYKCEECGKAFNRFTTLTKHKRIHAGEKPITCEECRKIFTSSSNFAK
HKRIHTGEKPYKCEECGKAFNRSTTLTKHKRIHTGEKPYTCEECGKAFRQSSKLNEHKKV
HTGERPYKCDECGKAFGRSRVLNEHKKIHTGEKPYKCEECGKAFRRSTDRSQHKKIHSAD
KPYKCKECDKAFKQFSLLSQHKKIHTVDKPYKCKDCDKAFKRFSHLNKHKKIHT*
>ENSG00000108813|ENST00000240306
MTSLPCPLPGRDASKAVFPDLAPVPSVAAAYPLGLSPTTAASPNLSYSRPYGHLLSYPYT
EPANPGDSYLSCQQPAALSQPLCGPAEHPQELEADSEKPRLSPEPSERRPQAPAKKLRKP
RTIYSSLQLQHLNQRFQHTQYLALPERAQLAAQLGLTQTQVKIWFQNKRSKYKKLLKQNS
GGQEGDFPGRTFSVSPCSPPLPSLWDLPKAGTLPTSGYGNSFGAWYQHHSSDVLASPQMM
*
>ENSG00000122068|ENST00000241502
MNRFGTRLVGATATSSPPPKARSNENLDKIDMSLDDIIKLNRKEGKKQNFPRLNRRLLQQ
SGAQQFRMRVRWGIQQNSGFGKTSLNRRGRVMPGKRRPNGVITGLAARKTTGIRKGISPM
NRPPLSDKNIEQYFPVLKRKANLLRQNEGQRKPVAVLKRPSQLSRKNNIPANFTRSGNKL
NHQKDTRQATFLFRRGLKVQAQLNTEQLLDDVVAKRTRQWRTSTTNGGILTVSIDNPGAV
QCPVTQKPRLTRTAVPSFLTKREQSDVKKVPKGVPLQFDINSVGKQTGMTLNERFGILKE
QRATLTYNKGGSRFVTVG*
>ENSG00000123364|ENST00000243056
MTTSLLLHPRWPESLMYVYEDSAAESGIGGGGGGGGGGTGGAGGGCSGASPGKAPSMDGL
GSSCPASHCRDLLPHPVLGRPPAPLGAPQGAVYTDIPAPEAARQCAPPPAPPTSSSATLG
YGYPFGGSYYGCRLSHNVNLQQKPCAYHPGDKYPEPSGALPGDDLSSRAKEFAFYPSFAS
SYQAMPGYLDVSVVPGISGHPEPRHDALIPVEGYQHWALSNGWDSQVYCSKEQSQSAHLW
KSPFPDVVPLQPEVSSYRRGRKKRVPYTKVQLKELEKEYAASKFITKEKRRRISATTNLS
ERQVTIWFQNRRVKEKKVVSKSKAPHLHST*
>ENSG00000124541|ENST00000244496
MPGANYRAGAGAGAGARRPRGARDREEDGGGLEPAAVARDLLRGTSNMSFEELLELQSQV
GTKTYKQLVAGNSPKKQASRPPIQNACVADKHRPLEMSAKIRVPFLRQVVPISKKVARDP
RFDDLSGEYNPEVFDKTYQFLNDIRAKEKELVKKQLKKHLSGEEHEKLQQLLQRMEQQEM
AQQERKQQQELHLALKQERRAQAQQGHRPYFLKKSEQRQLALAEKFKELKRSKKLENFLS
RKRRRNAGKDRRHLPLSKE*
>ENSG00000137185|ENST00000252207
MNTNSKEVLSLGVQVPEAWEELLTMKVEAKSHLQWQESRLKRSNPLAREIFRRHFRQLCY
QETPGPREALTRLQELCYQWLRPHVSTKEQILDLLVLEQFLSILPKELQGWVREHCPESG
EEAVILLEDLERELDEPQHEMVAHRHRQEVLCKEMVPLAEQTPLTLQSQPKEPQLTCDSA
QKCHSIGETDEVTKTEDRELVLRKDCPKIVEPHGKMFNEQTWEVSQQDPSHGEVGEHKDR
IERQWGNLLGEGQHKCDECGKSFTQSSGLIRHQRIHTGERPYECNECGKAFSRSSGLFNH
RGIHNIQKRYHCKECGKVFSQSAGLIQHQRIHKGEKPYQCSQCSKSYSRRSFLIEHQRSH
TGERPHQCIECGKSFNRHCNLIRHQKIHTVAELV*
>ENSG00000125398|ENST00000245479
MNLLDPFMKMTDEQEKGLSGAPSPTMSEDSAGSPCPSGSGSDTENTRPQENTFPKGEPDL
KKESEEDKFPVCIREAVSQVLKGYDWTLVPMPVRVNGSSKNKPHVKRPMNAFMVWAQAAR
RKLADQYPHLHNAELSKTLGKLWRLLNESEKRPFVEEAERLRVQHKKDHPDYKYQPRRRK
SVKNGQAEAEEATEQTHISPNAIFKALQADSPHSSSGMSEVHSPGEHSGQSQGPPTPPTT

PKTDVQPGKADLKREGRPLPEGGRQPPIDFRDVDIGELSSDVISNIETFDVNEFDQYLPP
NGHPGVPATHGQVTYTGSYGISSTAATPASAGHVWMSKQQAPPPPPQQPPQAPPAPQAPP
QPQAAPPQQPAAPPQQPQAHTLTTLSSEPGQSQRTHIKTEQLSPSHYSEQQQHSPQQIAY
SPFNLPHYSPSYPPITRSQYDYTDHQNSSSYYSHAAGQGTGLYSTFTYMNPAQRPMYTPI
ADTSGVPSIPQTHSPQHWEQPVYTQLTRP*
>ENSG00000105298|ENST00000248420
MGRDTRSRSRSAGRRGRRRQSQSGSRSRSRSHGRRNRRRREDEGRRRRRRRSRERRSDSE
EERWQRSGMRSRSPPRPKWHSRDGSSQSDSGEEQSRGQWARRRRRARSWSPSSSASSSAS
PGRSQSPRAAAAALSQQQSLQERLRLREERKQQEELMKAFETPEEKRARRLAKKEAKERK
KREKMGWGEEYMGYTNTDNPFGDNNLLGTFIWNKALEKKGISHLEEKELKERNKRIQEDN
RLELQKVKQLRLEREREKAMREQELEMLQREKEAEHFKTWEEQEDNFHLQQAKLRSKIRI
RDGRAKPIDLLAKYISAEDDDLAVEMHEPYTFLNGLTVADMEDLLEDIQVYMELEQGKNA
DFWRDMTTITEDEISKLRKLEASGKGPGERREGVNASVSSDVQSVFKGKTYNQLQVIFQG
IEGKIRAGGPNLDMGYWESLLQQLRAHMARARLRERHQDVLRQKLYKLKQEQGVESEPLF
PILKQEPQSPSRSLEPEDAAPTPPGPSSEGGPAEAEVDGATPTEGDGDGDGEGEGEGEAV
LMEEDLIQQSLDDYDAGRYSPRLLTAHELPLDAHVLEPDEDLQRLQLSRQQLQVTGDASE
SAEDIFFRRAKEGMGQDEAQFSVEMPLTGKAYLWADKYRPRKPRFFNRVHTGFEWNKYNQ
THYDFDNPPPKIVQGYKFNIFYPDLIDKRSTPEYFLEACADNKDFAILRFHAGPPYEDIA
FKIVNREWEYSHRHGFRCQFANGIFQLWFHFKRYRYRR*
>ENSG00000120738|ENST00000239938
MAAAKAEMQLMSPLQISDPFGSFPHSPTMDNYPKLEEMMLLSNGAPQFLGAAGAPEGSGS
NSSSSSSGGGGGGGGGSNSSSSSSTFNPQADTGEQPYEHLTAESFPDISLNNEKVLVETS
YPSQTTRLPPITYTGRFSLEPAPNSGNTLWPEPLFSLVSGLVSMTNPPASSSSAPSPAAS
SASASQSPPLSCAVPSNDSSPIYSAAPTFPTPNTDIFPEPQSQAFPGSAGTALQYPPPAY
PAAKGGFQVPMIPDYLFPQQQGDLGLGTPDQKPFQGLESRTQQPSLTPLSTIKAFATQSG
SQDLKALNTSYQSQLIKPSRMRKYPNRPSKTPPHERPYACPVESCDRRFSRSDELTRHIR
IHTGQKPFQCRICMRNFSRSDHLTTHIRTHTGEKPFACDICGRKFARSDERKRHTKIHLR
QKDKKADKSVVASSATSSLSSYPSPVATSYPSPVTTSYPSPATTSYPSPVPTSFSSPGSS
TYPSPVHSGFPSPSVATTYSSVPPAFPAQVSSFPSSAVTNSFSASTGLSDMTATFSPRTI
EIC*
>ENSG00000115128|ENST00000233468
MAMQAAKRANIRLPPEVNRILYIRNLPYKITAEEMYDIFGKYGPIRQIRVGNTPETRGTA
YVVYEDIFDAKNACDHLSGFNVCNRYLVVLYYNANRAFQKMDTKKKEEQLKLLKEKYGIN
TDPPK*
>ENSG00000182521|ENST00000247219
MASAPWPERVPRLLAPRLPSYPPPPPTVGLRSMEQEETYLELYLDQCAAQDGLAPPRSPL
FSPVVPYDMYILNASNPDTAFNSNPEVKETSGDFSSVDLSFLPDEVTQENKDQPVISKHE
TEENSESQSPQSRLPSPSEQDVGLGLNSSSLSNSHSQLHPGDTDSVQPSPEKPNSDSLSL
ASITPMTPMTPISECCGIVPQLQNIVSTVNLACKLDLKKIALHAKNAEYNPKRFAAVIMR
IREPRTTALIFSSGKMVCTGAKSEEQSRLAARKYARVVQKLGFPARFLDFKIQNMVGSCD
VRFPIRLEGLVLTHQQFSSYEPELFPGLIYRMVKPRIVLLIFVSGKVVLTGAKERSEIYE
AFENIYPILKGFKKA*
>ENSG00000125166|ENST00000245206
MALLHSGRVLPGIAAAFHPGLAAAASARASSWWTHVEMGPPDPILGVTEAFKRDTNSKKM

NLGVGAYRDDNGKPYVLPSVRKAEAQIAAKNLDKEYLPIGGLAEFCKASAELALGENSEV
LKSGRFVTVQTISGTGALRIGASFLQRFFKFSRDVFLPKPTWGNHTPIFRDAGMQLQGYR
YYDPKTCGFDFTGAVEDISKIPEQSVLLLHACAHNPTGVDPRPEQWKEIATVVKKRNLFA
FFDMAYQGFASGDGDKDAWAVRHFIEQGINVCLCQSYAKNMGLYGERVGAFTMVCKDADE
AKRVESQLKILIRPMYSNPPLNGARIAAAILNTPDLRKQWLQEVKVMADRIIGMRTQLVS
NLKKEGSTHNWQHITDQIGMFCFTGLKPEQVERLIKEFSIYMTKDGRISVAGVTSSNVGY
LAHAIHQVTK*
>ENSG00000007372|ENST00000241001
MQNSHSGVNQLGGVFVNGRPLPDSTRQKIVELAHSGARPCDISRILQVSNGCVSKILGRY
YETGSIRPRAIGGSKPRVATPEVVSKIAQYKRECPSIFAWEIRDRLLSEGVCTNDNIPSV
SSINRVLRNLASEKQQMGADGMYDKLRMLNGQTGSWGTRPGWYPGTSVPGQPTQDGCQQQ
EGGGENTNSISSNGEDSDEAQMRLQLKRKLQRNRTSFTQEQIEALEKEFERTHYPDVFAR
ERLAAKIDLPEARIQVWFSNRRAKWRREEKLRNQRRQASNTPSHIPISSSFSTSVYQPIP
QPTTPVSSFTSGSMLGRTDTALTNTYSALPPMPSFTMANNLPMQPPVPSQTSSYSCMLPT
SPSVNGRSYDTYTPPHMQTHMNSQPMGTSGTTSTGLISPGVSVPVQVPGSEPDMSQYWPR
LQ*
>ENSG00000120690|ENST00000239882
MAAVVQQNDLVFEFASNVMEDERQLGDPAIFPAVIVEHVPGADILNSYAGLACVEEPNDM
ITESSLDVAEEEIIDDDDDDITLTVEASCHDGDETIETIEAAEALLNMDSPGPMLDEKRI
NNNIFSSPEDDMVVAPVTHVSVTLDGIPEVMETQQVQEKYADSPGASSPEQPKRKKGRKT
KPPRPDSPATTPNISVKKKNKDGKGNTIYLWEFLLALLQDKATCPKYIKWTQREKGIFKL
VDSKAVSRLWGKHKNKPDMNYETMGRALRYYYQRGILAKVEGQRLVYQFKEMPKDLIYIN
DEDPSSSIESSDPSLSSSATSNRNQTSRSRVSSSPGVKGGATTVLKPGNSKAAKPKDPVE
VAQPSEVLRTVQPTQSPYPTQLFRTVHVVQPVQAVPEGEAARTSTMQDETLNSSVQSIRT
IQAPTQVPVVVSPRNQQLHTVTLQTVPLTTVIASTDPSAGTGSQKFILQAIPSSQPMTVL
KENVMLQSQKAGSPPSIVLGPAQVQQVLTSNVQTICNGTVSVASSPSFSATAPVVTFSPR
SSQLVAHPPGTVITSVIKTQETKTLTQEVEKKESEDHLKENTEKTEQQPQPYVMVVSSSN
GFTSQVAMKQNELLEPNSF*
>ENSG00000226941|ENST00000250831
MVEADHPGKLFIGGLNRETNEKMLKAVFGKHGPISEVLLIKDRTSKSRGFAFITFENPAD
AKNAAKDMNGTSLHGKAIKVEQAKKPSFQSGGRRRPPASSRNRSPSGSLRSARGSSGGTR
GWLPSHEGHLDDGGYTPDLKMSYSRGLIPVKRGPSSRSGGPPPKKSAPSAVARSNSWMGS
QGPMSQRRENYGVPPRRATISSWRNDRMSTRHDGYATNDGNHPSCQETRDYAPPSRGYAY
RDNGHSNRDEHSSRGYRNHRSSRETRDYAPPSRGHAYRDYGHSRRDESYSRGYRNHRSSR
ETREYAPPSRGHGYRDYGHSRRHESYSRGYRNHPSSRETRDYAPPHRDYAYRDYGHSSWD
EHSSRGYSYHDGYGEALGRDHSEHLSGSSYRDALQRYGTSHGAPPARGPRMSYGGSTCHA
YSNTRDRYGRSWESYSSCGDFHYCDREHVCRKDQRNPPSLGRVLPDPREAYGSSSYVASI
VDGGESRSEKGDSSRY*
>ENSG00000123200|ENST00000242848
MSKIRRKVTVENTKTISDSTSRRPSVFERLGPSTGSTAETQCRNWLKTGNCLYGNTCRFV
HGPSPRGKGYSSNYRRSPERPTGDLRERMKNKRQDVDTEPQKRNTEESSSPVRKESSRGR
HREKEDIKITKERTPESEEENVEWETNRDDSDNGDINYDYVHELSLEMKRQKIQRELMKL
EQENMEKREEIIIKKEVSPEVVRSKLSPSPSLRKSSKSPKRKSSPKSSSASKKDRKTSAV
SSPLLDQQRNSKTNQSKKKGPRTPSPPPPIPEDIALGKKYKEKYKVKDRIEEKTRDGKDR

GRDFERQREKRDKPRSTSPAGQHHSPISSRHHSSSSQSGSSIQRHSPSPRRKRTPSPSYQ
RTLTPPLRRSASPYPSHSLSSPQRKQSPPRHRSPMREKGRHDHERTSQSHDRRHERREDT
RGKRDREKDSREEREYEQDQSSSRDHRDDREPRDGRDRRDARDTRDRRELRDSRDMRDSR
EMRDYSRDTKESRDPRDSRSTRDAHDYRDREGRDTHRKEDTYPEESRSYGRNHLREESSR
TEIRNESRNESRSEIRNDRMGRSRGRVPELPEKGSRGSRGSQIDSHSSNSNYHDSWETRS
SYPERDRYPERDNRDQARDSSFERRHGERDRRDNRERDQRPSSPIRHQGRNDELERDERR
EERRVDRVDDRRDERARERDRERERDRERERERERERDREREKERELERERARERERERE
KERDRERDRDRDHDREREREREREREKERERERERERERERERERERERERERERERARER
DKERERQRDWEDKDKGRDDRREKREEIREDRNPRDGHDERKSKKRYRNEGSPSPRQSPKR
RREHSPDSDAYNSGDDKNEKHRLLSQVVRPQESRSLSPSHLTEDRQGRWKEEDRKPERKE
SSRRYEEQELKEKVSSVDKQREQTEILESSRMRAQDIIGHHQSEDRETSDRAHDENKKKA
KIQKKPIKKKKEDDVGIERGNIETTSEDGQVFSPKKGQKKKSIEKKRKKSKGDSDISDEE
AAQQSKKKRGPRTPPITTKEELVEMCNGKNGILEDSQKKEDTAFSDWSDEDVPDRTEVTE
AEHTATATTPGSTPSPLSSLLPPPPPVATATATTVPATLAATTAAAATSFSTSAITISTS
ATPTNTTNNTFANEDSHRKCHRTRVEKVETPHVTIEDAQHRKPMDQKRSSSLGSNRSNRS
HTSGRLRSPSNDSAHRSGDDQSGRKRVLHSGSRDREKTKSLEITGERKSRIDQLKRGEPS
RSTSSDRQDSRSHSSRRSSPESDRQVHSRSGSFDSRDRLQERDRYEHDRERERERRDTRQ
REWDRDADKDWPRNRDRDRLRERERERERDKRRDLDRERERLISDSVERDRDRDRDRTFE
SSQIESVKRCEAKLEGEHERDLESTSRDSLALDKERMDKDLGSVQGFEETNKSERTESLE
GDDESKLDDAHSLGSGAGEGYEPISDDELDEILAGDAEKREDQQDEEKMPDPLDVIDVDW
SGLMPKHPKEPREPGAALLKFTPGAVMLRVGISKKLAGSELFAKVKETCQRLLEKPKDAD
NLFEHELGALNMAALLRKEERASLLSNLGPCCKALCFRRDSAIRKQLVKNEKGTIKQAYT
SAPMVDNELLRLSLRLFKRKTTCHAPGHEKTEDNKLSQSSIQQELCVS*
>ENSG00000039123|ENST00000230640
MADAFGDELFSVFEGDSTTAAGTKKDKEKDKGKWKGPPGSADKAGKRFDGKLQSESTNNG
KNKRDVDFEGTDEPIFGKKPRIEESITEDLSLADLMPRVKVQSVETVEGCTHEVALPAEE
DYLPLKPRVGKAAKEYPFILDAFQREAIQCVDNNQSVLVSAHTSAGKTVCAEYAIALALR
EKQRVIFTSPIKALSNQKYREMYEEFQDVGLMTGDVTINPTASCLVMTTEILRSMLYRGS
EVMREVAWVIFDEIHYMRDSERGVVWEETIILLPDNVHYVFLSATIPNARQFAEWICHLH
KQPCHVIYTDYRPTPLQHYIFPAGGDGLHLVVDENGDFREDNFNTAMQVLRDAGDLAKGD
QKGRKGGTKGPSNVFKIVKMIMERNFQPVIIFSFSKKDCEAYALQMTKLDFNTDEEKKMV
EEVFSNAIDCLSDEDKKLPQVEHVLPLLKRGIGIHHGGLLPILKETIEILFSEGLIKALF
ATETFAMGINMPARTVLFTNARKFDGKDFRWISSGEYIQMSGRAGRRGMDDRGIVILMVD
EKMSPTIGKQLLKGSADPLNSAFHLTYNMVLNLLRVEEINPEYMLEKSFYQFQHYRAIPG
VVEKVKNSEEQYNKIVIPNEESVVIYYKIRQQLAKLGKEIEEYIHKPKYCLPFLQPGRLV
KVKNEGDDFGWGVVVNFSKKSNVKPNSGELDPLYVVEVLLRCSKESLKNSATEAAKPAKP
DEKGEMQVVPVLVHLLSAISSVRLYIPKDLRPVDNRQSVLKSIQEVQKRFPDGIPLLDPI
DDMGIQDQGLKKVIQKVEAFEHRMYSHPLHNDPNLETVYTLCEKKAQIAIDIKSAKRELK
KARTVLQMDELKCRKRVLRRLGFATSSDVIEMKGRVACEISSADELLLTEMMFNGLFNDL
SAEQATALLSCFVFQENSSEMPKLTEQLAGPLRQMQECAKRIAKVSAEAKLEIDEETYLS
SFKPHLMDVVYTWATGATFAHICKMTDVFEGSIIRCMRRLEELLRQMCQAAKAIGNTELE
NKFAEGITKIKRDIVFAASLYL*
>ENSG00000113580|ENST00000231509
MDSKESLTPGREENPSSVLAQERGDVMDFYKTLRGGATVKVSASSPSLAVASQSDSKQRR

LLVDFPKGSVSNAQQPDLSKAVSLSMGLYMGETETKVMGNDLGFPQQGQISLSSGETDLK
LLEESIANLNRSTSVPENPKSSASTAVSAAPTEKEFPKTHSDVSSEQQHLKGQTGTNGGN
VKLYTTDQSTFDILQDLEFSSGSPGKETNESPWRSDLLIDENCLLSPLAGEDDSFLLEGN
SNEDCKPLILPDTKPKIKDNGDLVLSSPSNVTLPQVKTEKEDFIELCTPGVIKQEKLGTV
YCQASFPGANIIGNKMSAISVHGVSTSGGQMYHYDMNTASLSQQQDQKPIFNVIPPIPVG
SENWNRCQGSGDDNLTSLGTLNFPGRTVFSNGYSSPSMRPDVSSPPSSSSTATTGPPPKL
CLVCSDEASGCHYGVLTCGSCKVFFKRAVEGRQHNYLCAGRNDCIIDKIRRKNCPACRYR
KCLQAGMNLEARKTKKKIKGIQQATTGVSQETSENPGNKTIVPATLPQLTPTLVSLLEVI
EPEVLYAGYDSSVPDSTWRIMTTLNMLGGRQVIAAVKWAKAIPGFRNLHLDDQMTLLQYS
WMFLMAFALGWRSYRQSSANLLCFAPDLIINEQRMTLPCMYDQCKHMLYVSSELHRLQVS
YEEYLCMKTLLLLSSVPKDGLKSQELFDEIRMTYIKELGKAIVKREGNSSQNWQRFYQLT
KLLDSMHEVVENLLNYCFQTFLDKTMSIEFPEMLAEIITNQIPKYSNGNIKKLLFHQK*
>ENSG00000055332|ENST00000233057
MAGDLSAGFFMEELNTYRQKQGVVLKYQELPNSGPPHDRRFTFQVIIDGREFPEGEGRSK
KEAKNAAAKLAVEILNKEKKAVSPLLLTTTNSSEGLSMGNYIGLINRIAQKKRLTVNYEQ
CASGVHGPEGFHYKCKMGQKEYSIGTGSTKQEAKQLAAKLAYLQILSEETSVKSDYLSSG
SFATTCESQSNSLVTSTLASESSSEGDFSADTSEINSNSDSLNSSSLLMNGLRNNQRKAK
RSLAPRFDLPDMKETKYTVDKRFGMDFKEIELIGSGGFGQVFKAKHRIDGKTYVIKRVKY
NNEKAEREVKALAKLDHVNIVHYNGCWDGFDYDPETSDDSLESSDYDPENSKNSSRSKTK
CLFIQMEFCDKGTLEQWIEKRRGEKLDKVLALELFEQITKGVDYIHSKKLIHRDLKPSNI
FLVDTKQVKIGDFGLVTSLKNDGKRTRSKGTLRYMSPEQISSQDYGKEVDLYALGLILAE
LLHVCDTAFETSKFFTDLRDGIISDIFDKKEKTLLQKLLSKKPEDRPNTSEILRTLTVWK
KSPEKNERHTC*
>ENSG00000125931|ENST00000246139
MPTTSRPALDVKGGTSPAKEDANQEMSSVAYSNLAVKDRKAVAILHYPGVASNGTKASGA
PTSSSGSPIGSPTTTPPTKPPSFNLHPAPHLLASMHLQKLNSQYQGMAAATPGQPGEAGP
LQNWDFGAQAGGAESLSPSAGAQSPAIIDSDPVDEEVLMSLVVELGLDRANELPELWLGQ
NEFDFTADFPSSC*
>ENSG00000129204|ENST00000250066
MDMVENADSLQAQERKDILMKYDKGHRAGLPEDKGPEPVGINSSIDRFGILHETELPPVT
AREAKKIRREMTRTSKWMEMLGEWETYKHSSKLIDRVYKGIPMNIRGPVWSVLLNIQEIK
LKNPGRYQIMKERGKRSSEHIHHIDLDVRTTLRNHVFFRDRYGAKQRELFYILLAYSEYN
PEVGYCRDLSHITALFLLYLPEEDAFWALVQLLASERHSLPGFHSPNGGTVQGLQDQQEH
VVPKSQPKTMWHQDKEGLCGQCASLGCLLRNLIDGISLGLTLRLWDVYLVEGEQVLMPIT
SIALKVQQKRLMKTSRCGLWARLRNQFFDTWAMNDDTVLKHLRASTKKLTRKQGDLPPPA
KREQGSLAPRPVPASRGGKTLCKGYRQAPPGPPAQFQRPICSASPPWASRFSTPCPGGAV
REDTYPVGTQGVPSLALAQGGPQGSWRFLEWKSMPRLPTDLDIGGPWFPHYDFEWSCWVR
AISQEDQLATCWQAEHCGEVHNKDMSWPEEMSFTANSSKIDRQKVPTEKGATGLSNLGNT
CFMNSSIQCVSNTQPLTQYFISGRHLYELNRTNPIGMKGHMAKCYGDLVQELWSGTQKSV
APLKLRRTIAKYAPKFDGFQQQDSQELLAFLLDGLHEDLNRVHEKPYVELKDSDGRPDWE
VAAEAWDNHLRRNRSIIVDLFHGQLRSQVKCKTCGHISVRFDPFNFLSLPLPMDSYMDLE
ITVIKLDGTTPVRYGLRLNMDEKYTGLKKQLRDLCGLNSEQILLAEVHDSNIKNFPQDNQ
KVQLSVSGFLCAFEIPVPSSPISASSPTQIDFSSSPSTNGMFTLTTNGDLPKPIFIPNGM
PNTVVPCGTEKNFTNGMVNGHMPSLPDSPFTGYIIAVHRKMMRTELYFLSPQENRPSLFG

MPLIVPCTVHTRKKDLYDAVWIQVSWLARPLPPQEASIHAQDRDNCMGYQYPFTLRVVQK
DGNSCAWCPQYRFCRGCKIDCGEDRAFIGNAYIAVDWHPTALHLRYQTSQERVVDKHESV
EQSRRAQAEPINLDSCLRAFTSEEELGESEMYYCSKCKTHCLATKKLDLWRLPPFLIIHL
KRFQFVNDQWIKSQKIVRFLRESFDPSAFLVPRDPALCQHKPLTPQGDELSKPRILAREV
KKVDAQSSAGKEDMLLSKSPSSLSANISSSPKGSPSSSRKSGTSCPSSKNSSPNSSPRTL
GRSKGRLRLPQIGSKNKPSSSKKNLDASKENGAGQICELADALSRGHMRGGSQPELVTPQ
DHEVALANGFLYEHEACGNGCGDGYSNGQLGNHSEEDSTDDQREDTHIKPIYNLYAISCH
SGILSGGHYITYAKNPNCKWYCYNDSSCEELHPDEIDTDSAYILFYEQQGIDYAQFLPKI
DGKKMADTSSTDEDSESDYEKYSMLQ*
>ENSG00000116251|ENST00000234875
MAPVKKLVVKGGKKKKQVLKFTLDCTHPVEDGIMDAANFEQFLQERIKVNGKAGNLGGGV
VTIERSKSKITVTSEVPFSKRYLKYLTKKYLKKNNLRDWLRVVANSKESYELRYFQINQD
EEEEEDED*
>ENSG00000115163|ENST00000233505
MGPRRRSRKPEAPRRRSPSPTPTPGPSRRGPSLGASSHQHSRRRQGWLKEIRKLQKSTHL
LIRKLPFSRLAAEAFLVHLFEDAYLLTLHAGRVTLFPKDVQLARRIRGLEEGLG*
>ENSG00000124193|ENST00000244020
MPRVYIGRLSYNVREKDIQRFFSGYGRLLEVDLKNGYGFVEFEDSRDADDAVYELNGKEL
CGERVIVEHARGPRRDRDGYSYGSRSGGGGYSSRRTSGRDKYGPPVRTEYRLIVENLSSR
CSWQDLKDFMRQAGEVTYADAHKERTNEGVIEFRSYSDMKRALDKLDGTEINGRNIRLIE
DKPRTSHRRSYSGSRSRSRSRRRSRSRSRRSSRSRSRSISKSRSRSRSRSKGRSRSRSKG
RKSRSKSKSKPKSDRGSHSHSRSRSKDEYEKSRSRSRSRSPKENGKGDIKSKSRSRSQSR
SNSPLPVPPSKARSVSPPPKRATSRSRSRSRSKSRSRSRSSSRD*
>ENSG00000126368|ENST00000246672
MTTLDSNNNTGGVITYIGSSGSSPSRTSPESLYSDNSNGSFQSLTQGCPTYFPPSPTGSL
TQDPARSFGSIPPSLSDDGSPSSSSSSSSSSSSFYNGSPPGSLQVAMEDSSRVSPSKSTS
NITKLNGMVLLCKVCGDVASGFHYGVHACEGCKGFFRRSIQQNIQYKRCLKNENCSIVRI
NRNRCQQCRFKKCLSVGMSRDAVRFGRIPKREKQRMLAEMQSAMNLANNQLSSQCPLETS
PTQHPTPGPMGPSPPPAPVPSPLVGFSQFPQQLTPPRSPSPEPTVEDVISQVARAHREIF
TYAHDKLGSSPGNFNANHASGSPPATTPHRWENQGCPPAPNDNNTLAAQRHNEALNGLRQ
APSSYPPTWPPGPAHHSCHQSNSNGHRLCPTHVYAAPEGKAPANSPRQGNSKNVLLACPM
NMYPHGRSGRTVQEIWEDFSMSFTPAVREVVEFAKHIPGFRDLSQHDQVTLLKAGTFEVL
MVRFASLFNVKDQTVMFLSRTTYSLQELGAMGMGDLLSAMFDFSEKLNSLALTEEELGLF
TAVVLVSADRSGMENSASVEQLQETLLRALRALVLKNRPLETSRFTKLLLKLPDLRTLNN
MHSEKLLSFRVDAQ*
>ENSG00000125878|ENST00000246080
MAFALLRPVGAHVLYPDVRLLSEDEENRSESDASDQSFGCCEGPEAARRGPGPGGGRRAG
GGGGAGPVVVVRQRQAANARERDRTQSVNTAFTALRTLIPTEPVDRKLSKIETVRLASSY
IAHLANVLLLGDSADDGQPCFRAAGSAKGAVPAAADGGRQPRSICTFCLSNQRKGGGRRD
LGGSCLKVRGVAPLRGPRR*
>ENSG00000117751|ENST00000236412
MVQTAVVPVKKKRVEGPGSLGLEESGSRRMQNFAFSGGLYGGLPPTHSEAGSQPHGIHGT
ALIGGLPMPYPNLAPDVDLTPVVPSAVNMNPAPNPAVYNPEAVNEPKKKKYAKEAWPGKK
PTPSLLI*

>ENSG00000072756|ENST00000251607
MLRCLYHWHRPVLNRRWSRLCLPKQYLFTMKLQSPEFQSLFTEGLKSLTELFVKENHELR
IAGGAVRDLLNGVKPQDIDFATTATPTQMKEMFQSAGIRMINNRGEKHGTITARLHEENF
EITTLRIDVTTDGRHAEVEFTTDWQKDAERRDLTINSMFLGFDGTLFDYFNGYEDLKNKK
VRFVGHAKQRIQEDYLRILRYFRFYGRIVDKPGDHDPETLEAIAENAKGLAGISGERIWV
ELKKILVGNHVNHLIHLIYDLDVAPYIGLPANASLEEFDKVSKNVDGFSPKPVTLLASLF
KVQDDVTKLDLRLKIAKEEKNLGLFIVKNRKDLIKATDSSDPLKPYQDFIIDSREPDATT
RVCELLKYQGEHCLLKEMQQWSIPPFPVSGHDIRKVGISSGKEIGALLQQLREQWKKSGY
QMEKDELLSYIKKT*
>ENSG00000129824|ENST00000250784
MARGPKKHLKRVAAPKHWMLDKLTGVFAPRPSTGPHKLRECLPLIVFLRNRLKYALTGDE
VKKICMQRFIKIDGKVRVDVTYPAGFMDVISIEKTGEHFRLVYDTKGRFAVHRITVEEAK
YKLCKVRKITVGVKGIPHLVTHDARTIRYPDPVIKVNDTVQIDLGTGKIINFIKFDTGNL
CMVIGGANLGRVGVITNRERHPGSFDVVHVKDANGNSFATRLSNIFVIGNGNKPWISLPR
GKGIRLTVAEERDKRLATKQSSG*
>ENSG00000120832|ENST00000240050
MLWKLLLRSQSCRLCSFRKMRSPPKYRPFLACFTYTTDKQSSKENTRTVEKLYKCSVDIR
KIRRLKGWVLLEDETYVEEIANILQELGADETAVASILERCPEAIVCSPTAVNTQRKLWQ
LVCKNEEELIKLIEQFPESFFTIKDQENQKLNVQFFQELGLKNVVISRLLTAAPNVFHNP
VEKNKQMVRILQESYLDVGGSEANMKVWLLKLLSQNPFILLNSPTAIKETLEFLQEQGFT
SFEILQLLSKLKGFLFQLCPRSIQNSISFSKNAFKCTDHDLKQLVLKCPALLYYSVPVLE
ERMQGLLREGISIAQIRETPMVLELTPQIVQYRIRKLNSSGYRIKDGHLANLNGSKKEFE
ANFGKIQAKKVRPLFNPVAPLNVEE*
>ENSG00000204604|ENST00000243639
MALPQGLLTFRDVAIEFSQEEWKCLDPAQRTLYRDVMLENYRNLVSLEFWSVIPAKVQWC
HLRSLQPPLPRFKQFSCLSLPSTWDYRYLFQMHVEDVVVNRARQYRSDPHRDIAQTSKSS
HWRILFP*
>ENSG00000114853|ENST00000232974
MGRLNEQRLFQPDLCDVDLVLVPQRSVFPAHKGVLAAYSQFFHSLFTQNKQLQRVELSLE
ALAPGGLQQILNFIYTSKLLVNAANVHEVLSAASLLQMADIAASCQELLDARSLGPPGPG
TVALAQPAASCTPAAPPYYCDIKQEADTPGLPKIYAREGPDPYSVRVEDGAGTAGGTVPA
TIGPAQPFFKEEKEGGVEEAGGPPASLCKLEGGEELEEELGGSGTYSRREQSQIIVEVNL
NNQTLHVSTGPEGKPGAGPSPATVVLGREDGLQRHSDEEEEDDEEEEEEEEEEEGGGSGR
EEEEEEEGGSQGEEEEEEEEDGHSEQEEEEEEEEEEGPSEQDQESSEEEEGEEGEAGGKQG
PRGSRSSRADPPPHSHMATRSRENARRRGTPEPEEAGRRGGKRPKPPPGVASASARGPPA
TDGLGAKVKLEEKQHHPCQKCPRVFNNRWYLEKHMNVTHSRMQICDQCGKRFLLESELLL
HRQTDCERNIQCVTCGKAFKKLWSLHEHNKIVHGYAEKKFSCEICEKKFYTMAHVRKHMV
AHTKDMPFTCETCGKSFKRSMSLKVHSLQHSGEKPFRCENCNERFQYKYQLRSHMSIHIG
HKQFMCQWCGKDFNMKQYFDEHMKTHTGEKPYICEICGKSFTSRPNMKRHRRTHTGEKPY
PCDVCGQRFRFSNMLKAHKEKCFRVSHTLAGDGVPAAPGLPPTQPQAHALPLLPGLPQTL
PPPPHLPPPPPLFPTTASPGGRMNANN*
>ENSG00000125347|ENST00000245414
MPITRMRMRPWLEMQINSNQIPGLIWINKEEMIFQIPWKHAAKHGWDINKDACLFRSWAI
HTGRYKAGEKEPDPKTWKANFRCAMNSLPDIEEVKDQSRNKGSSAVRVYRMLPPLTKNQR

KERKSKSSRDAKSKAKRKSCGDSSPDTFSDGLSSSTLPDDHSSYTVPGYMQDLEVEQALT
PALSPCAVSSTLPDWHIPVEVVPDSTSDLYNFQVSPMPSTSEATTDEDEEGKLPEDIMKL
LEQSEWQPTNVDGKGYLLNEPGVQPTSVYGDFSCKEEPEIDSPGGDIGLSLQRVFTDLKN
MDATWLDSLLTPVRLPSIQAIPCAP*
>ENSG00000006576|ENST00000248550
MASKVTDAIVWYQKKEFLSVATTAPGPQQVLPGYCQCSLKDQGLFIQCLIGAYDQQIWEK
SVEQREIKFIKLGLRNKPKKTAHVKPDLIDVDLVRGSAFAKAKPESPWTSLTRKGIVRVV
FFPFFFRWWLQVTSKVIFFWLLVLYLLQVAAIVLFCSTSSPHSIPLTEVIGPIWLMLLLG
TVHCQIVSTRTPKPPLSTGGKRRRKLRKAAHLEVHREGDGSSTTDNTQEGAVQNHGTSTS
HSVGTVFRDLWHAAFFLSGSKKAKNSIDKSTETDNGYVSLDGKKTVKSGEDGIQNHEPQC
ETIRPEETAWNTGTLRNGPSKDTQRTITNVSDEVSSEEGPETGYSLRRHVDRTSEGVLRN
RKSHHYKKHYPNEDAPKSGTSCSSRCSSSRQDSESARPESETEDVLWEDLLHCAECHSSC
TSETDVENHQINPCVKKEYRDDPFHQSHLPWLHSSHPGLEKISAIVWEGNDCKKADMSVL
EISGMIMNRVNSHIPGIGYQIFGNAVSLILGLTPFVFRLSQATDLEQLTAHSASELYVIA
FGSNEDVIVLSMVIISFVVRVSLVWIFFFLLCVAERTYKQRLLFAKLFGHLTSARRARKS
EVPHFRLKKVQNIKMWLSLRSYLKRRGPQRSVDVIVSSAFLLTISVVFICCAQLLHVHEI
FLDCHYNWELVIWCISLTLFLLRFVTLGSETSKKYSNTSILLTEQINLYLKMEKKPNKKE
ELTLVNNVLKLATKLLKELDSPFRLYGLTMNPLLYNITQVVILSAVSGVISDLLGFNLKL
WKIKS*
>ENSG00000125676|ENST00000245838
MAAAAVVVPAEWIKNWEKSGRGEFLHLCRILSENKSHDSSTYRDFQQALYELSYHVIKGN
LKHEQASNVLSDISEFREDMPSILADVFCILDIETNCLEEKSKRDYFTQLVLACLYLVSD
TVLKERLDPETLESLGLIKQSQQFNQKSVKIKTKLFYKQQKFNLLREENEGYAKLIAELG
QDLSGSITSDLILENIKSLIGCFNLDPNRVLDVILEVFECRPEHDDFFISLLESYMSMCE
PQTLCHILGFKFKFYQEPNGETPSSLYRVAAVLLQFNLIDLDDLYVHLLPADNCIMDEHK
REIAEAKQIVRKLTMVVLSSEKMDEREKEKEKEEEKVEKPPDNQKLGLLEALLKIGDWQH
AQNIMDQMPPYYAASHKLIALAICKLIHITIEPLYRRVGVPKGAKGSPVNALQNKRAPKQ
AESFEDLRRDVFNMFCYLGPHLSHDPILFAKVVRIGKSFMKEFQSDGSKQEDKEKTEVIL
SCLLSITDQVLLPSLSLMDCNACMSEELWGMFKTFPYQHRYRLYGQWKNETYNSHPLLVK
VKAQTIDRAKYIMKRLTKENVKPSGRQIGKLSHSNPTILFDYILSQIQKYDNLITPVVDS
LKYLTSLNYDVLAYCIIEALANPEKERMKHDDTTISSWLQSLASFCGAVFRKYPIDLAGL
LQYVANQLKAGKSFDLLILKEVVQKMAGIEITEEMTMEQLEAMTGGEQLKAEGGYFGQIR
NTKKSSQRLKDALLDHDLALPLCLLMAQQRNGVIFQEGGEKHLKLVGKLYDQCHDTLVQF
GGFLASNLSTEDYIKRVPSIDVLCNEFHTPHDAAFFLSRPMYAHHISSKYDELKKSEKGS
KQQHKVHKYITSCEMVMAPVHEAVVSLHVSKVWDDISPQFYATFWSLTMYDLAVPHTSYE
REVNKLKVQMKAIDDNQEMPPNKKKKEKERCTALQDKLLEEEKKQMEHVQRVLQRLKLEK
DNWLLAKSTKNETITKFLQLCIFPRCIFSAIDAVYCARFVELVHQQKTPNFSTLLCYDRV
FSDIIYTVASCTENEASRYGRFLCCMLETVTRWHSDRATYEKECGNYPGFLTILRATGFD
GGNKADQLDYENFRHVVHKWHYKLTKASVHCLETGEYTHIRNILIVLTKILPWYPKVLNL
GQALERRVHKICQEEKEKRPDLYALAMGYSGQLKSRKSYMIPENEFHHKDPPPRNAVASV
QNGPGGGPSSSSIGSASKSDESSTEETDKSRERSQCGVKAVNKASSTTPKGNSSNGNSGS
NSNKAVKENDKEKGKEKEKEKKEKTPATTPEARVLGKDGKEKPKEERPNKDEKARETKER
TPKSDKEKEKFKKEEKAKDEKFKTTVPNAESKSTQEREREKEPSRERDIAKEMKSKENVK
GGEKTPVSGSLKSPVPRSDIPEPEREQKRRKIDTHPSPSHSSTVKDSLIELKESSAKLYI

NHTPPPLSKSKEREMDKKDLDKSRERSREREKKDEKDRKERKRDHSNNDREVPPDLTKRR
KEENGTMGVSKHKSESPCESPYPNEKDKEKNKSKSSGKEKGSDSFKSEKMDKISSGGKKE
SRHDKEKIEKKEKRDSSGGKEEKKHHKSSDKHR*
>ENSG00000108848|ENST00000240304
MISAAQLLDELMGRDRNLAPDEKRSNVRWDHESVCKYYLCGFCPAELFTNTRSDLGPCEK
IHDENLRKQYEKSSRFMKVGYERDFLRYLQSLLAEVERRIRRGHARLALSQNQQSSGAAG
PTGKNEEKIQVLTDKIDVLLQQIEELGSEGKVEEAQGMMKLVEQLKEERELLRSTTSTIE
SFAAQEKQMEVCEVCGAFLIVGDAQSRVDDHLMGKQHMGYAKIKATVEELKEKLRKRTEE
PDRDERLKKEKQEREEREKERERERERERKRRREEEEREKERARDRERRKRSRSRSRHS
SRTSDRRCSRSRDHKRSRSRERRRSRSRDRRRSRSHDRSERKHRSRSRDRRRSKSRDRKS
YKHRSKSRDREQDRKSKEKEKRGSDDKKSSVKSGSREKQSEDTNTESKESDTKNEVNGTS
EDIKSEGDTQSN*
>ENSG00000120094|ENST00000239174
MDYNRMNSFLEYPLCNRGPSAYSAHSAPTSFPPSSAQAVDSYASEGRYGGGLSSPAFQQN
SGYPAQQPPSTLGVPFPSSAPSGYAPAACSPSYGPSQYYPLGQSEGDGGYFHPSSYGAQL
GGLSDGYGAGGAGPGPYPPQHPPYGNEQTASFAPAYADLLSEDKETPCPSEPNTPTARTF
DWMKVKRNPPKTAKVSEPGLGSPSGLRTNFTTRQLTELEKEFHFNKYLSRARRVEIAATL
ELNETQVKIWFQNRRMKQKKREREEGRVPPAPPGCPKEAAGDASDQSTCTSPEASPSSVT
S*
>ENSG00000122877|ENST00000242480
MMTAKAVDKIPVTLSGFVHQLSDNIYPVEDLAATSVTIFPNAELGGPFDQMNGVAGDGMI
NIDMTGEKRSLDLPYPSSFAPVSAPRNQTFTYMGKFSIDPQYPGASCYPEGIINIVSAGI
LQGVTSPASTTASSSVTSASPNPLATGPLGVCTMSQTQPDLDHLYSPPPPPPPYSGCAGD
LYQDPSAFLSAATTSTSSSLAYPPPPSYPSPKPATDPGLFPMIPDYPGFFPSQCQRDLHG
TAGPDRKPFPCPLDTLRVPPPLTPLSTIRNFTLGGPSAGVTGPGASGGSEGPRLPGSSSA
AAAAAAAAAYNPHHLPLRPILRPRKYPNRPSKTPVHERPYPCPAEGCDRRFSRSDELTRH
IRIHTGHKPFQCRICMRNFSRSDHLTTHIRTHTGEKPFACDYCGRKFARSDERKRHTKIH
LRQKERKSSAPSASVPAPSTASCSGGVQPGGTLCSSNSSSLGGGPLAPCSSRTRTP*
>ENSG00000125952|ENST00000246163
MSDNDDIEVESDEEQPRFQSAADKRAHHNALERKRRDHIKDSFHSLRDSVPSLQGEKLYF
LFWKLCTPVLHRQSLMQKCHTFISSYQVHKKKECKI*
>ENSG00000095002|ENST00000233146
MAVQPKETLQLESAAEVGFVRFFQGMPEKPTTTVRLFDRGDFYTAHGEDALLAAREVFKT
QGVIKYMGPAGAKNLQSVVLSKMNFESFVKDLLLVRQYRVEVYKNRAGNKASKENDWYLA
YKASPGNLSQFEDILFGNNDMSASIGVVGVKMSAVDGQRQVGVGYVDSIQRKLGLCEFPD
NDQFSNLEALLIQIGPKECVLPGGETAGDMGKLRQIIQRGGILITERKKADFSTKDIYQD
LNRLLKGKKGEQMNSAVLPEMENQVAVSSLSAVIKFLELLSDDSNFGQFELTTFDFSQYM
KLDIAAVRALNLFQGSVEDTTGSQSLAALLNKCKTPQGQRLVNQWIKQPLMDKNRIEERL
NLVEAFVEDAELRQTLQEDLLRRFPDLNRLAKKFQRQAANLQDCYRLYQGINQLPNVIQA
LEKHEGKHQKLLLAVFVTPLTDLRSDFSKFQEMIETTLDMDQVENHEFLVKPSFDPNLSE
LREIMNDLEKKMQSTLISAARDLGLDPGKQIKLDSSAQFGYYFRVTCKEEKVLRNNKNFS
TVDIQKNGVKFTNSKLTSLNEEYTKNKTEYEEAQDAIVKEIVNISSGYVEPMQTLNDVLA
QLDAVVSFAHVSNGAPVPYVRPAILEKGQGRIILKASRHACVEVQDEIAFIPNDVYFEKD
KQMFHIITGPNMGGKSTYIRQTGVIVLMAQIGCFVPCESAEVSIVDCILARVGAGDSQLK

GVSTFMAEMLETASILRSATKDSLIIIDELGRGTSTYDGFGLAWAISEYIATKIGAFCMF
ATHFHELTALANQIPTVNNLHVTALTTEETLTMLYQVKKGVCDQSFGIHVAELANFPKHV
IECAKQKALELEEFQYIGESQGYDIMEPAAKKCYLEREQGEKIIQEFLSKVKQMPFTEMS
EENITIKLKQLKAEVIAKNNSFVNEIISRIKVTT*
>ENSG00000111145|ENST00000228741
MESAITLWQFLLQLLLDQKHEHLICWTSNDGEFKLLKAEEVAKLWGLRKNKTNMNYDKLS
RALRYYYDKNIIKKVIGQKFVYKFVSFPEILKMDPHAVEISRESLLLQDSDCKASPEGRE
AHKHGLAALRSTSRNEYIHSGLYSSFTINSLQNPPDAFKAIKTEKLEEPPEDSPPVEEVR
TVIRFVTNKTDKHVTRPVVSLPSTSEAAAASAFLASSVSAKISSLMLPNAASISSASPFS
SRSPSLSPNSPLPSEHRSLFLEAACHDSDSLEPLNLSSGSKTKSPSLPPKAKKPKGLEIS
APPLVLSGTDIGSIALNSPALPSGSLTPAFFTAQTPNGLLLTPSPLLSSIHFWSSLSPVA
PLSPARLQGPSTLFQFPTLLNGHMPVPIPSLDRAASPVLLSSNSQKS*
>ENSG00000115942|ENST00000234296
MSKPELKEDKMLEVHFVGDDDVLNHILDREGGAKLKKERAQLLVNPKKIIKKPEYDLEED
DQEVLKDQNYVEIMGRDVQESLKNGSATGGGNKVYSFQNRKHSEKMAKLASELAKTPQKS
VSFSLKNDPEITINVPQSSKGHSASDKVQPKNNDKSEFLSTAPRSLRKRLIVPRSHSDSE
SEYSASNSEDDEGVAQEHEEDTNAVIFSQKIQAQNRVVSAPVGKETPSKRMKRDKTSDLV
EEYFEAHSSSKVLTSDRTLQKLKRAKLDQQTLRNLLSKVSPSFSAELKQLNQQYEKLFHK
WMLQLHLGFNIVLYGLGSKRDLLERFRTTMLQDSIHVVINGFFPGISVKSVLNSITEEVL
DHMGTFRSILDQLDWIVNKFKEDSSLELFLLIHNLDSQMLRGEKSQQIIGQLSSLHNIYL
IASIDHLNAPLMWDHAKQSLFNWLWYETTTYSPYTEETSYENSLLVKQSGSLPLSSLTHV
LRSLTPNARGIFRLLIKYQLDNQDNPSYIGLSFQDFYQQCREAFLVNSDLTLRAQLTEFR
DHKLIRTKKGTDGVEYLLIPVDNGTLTDFLEKEEEEA*
>ENSG00000126602|ENST00000246957
MARELRALLLWGRRLRPLLRAPALAAVPGGKPILCPRRTTAQLGPRRNPAWSLQAGRLFS
TQTAEDKEEPLHSIISSTESVQGSTSKHEFQAETKKLLDIVARSLYSEKEVFIRELISNA
SDALEKLRHKLVSDGQALPEMEIHLQTNAEKGTITIQDTGIGMTQEELVSNLGTIARSGS
KAFLDALQNQAEASSKIIGQFGVGFYSAFMVADRVEVYSRSAAPGSLGYQWLSDGSGVFE
IAEASGVRTGTKIIIHLKSDCKEFSSEARVRDVVTKYSNFVSFPLYLNGRRMNTLQAIWM
MDPKDVREWQHEEFYRYVAQAHDKPRYTLHYKTDAPLNIRSIFYVPDMKPSMFDVSRELG
SSVALYSRKVLIQTKATDILPKWLRFIRGVVDSEDIPLNLSRELLQESALIRKLRDVLQQ
RLIKFFIDQSKKDAEKYAKFFEDYGLFMREGIVTATEQEVKEDIAKLLRYESSALPSGQL
TSLSEYASRMRAGTRNIYYLCAPNRHLAEHSPYYEAMKKKDTEVLFCFEQFDELTLLHLR
EFDKKKLISVETDIVVDHYKEEKFEDRSPAAECLSEKETEELMAWMRNVLGSRVTNVKVT
LRLDTHPAMVTVLEMGAARHFLRMQQLAKTQEERAQLLQPTLEINPRHALIKKLNQLRAS
EPGLAQLLVDQIYENAMIAAGLVDDPRAMVGRLNELLVKALERH*
>ENSG00000116833|ENST00000236914
MSSNSDTGDLQESLKHGLTPIVSQFKMVNYSYDEDLEELCPVCGDKVSGYHYGLLTCESC
KGFFKRTVQNNKRYTCIENQNCQIDKTQRKRCPYCRFQKCLSVGMKLEAVRADRMRGGRN
KFGPMYKRDRALKQQKKALIRANGLKLEAMSQVIQAMPSDLTISSAIQNIHSASKGLPLN
HAALPPTDYDRSPFVTSPISMTMPPHGSLQGYQTYGHFPSRAIKSEYPDPYTSSPESIMG
YSYMDSYQTSSPASIPHLILELLKCEPDEPQVQAKIMAYLQQEQANRSKHEKLSTFGLMC
KMADQTLFSIVEWARSSIFFRELKVDDQMKLLQNCWSELLILDHIYRQVVHGKEGSIFLV
TGQQVDYSIIASQAGATLNNLMSHAQELVAKLRSLQFDQREFVCLKFLVLFSLDVKNLEN

FQLVEGVQEQVNAALLDYTMCNYPQQTEKFGQLLLRLPEIRAISMQAEEYLYYKHLNGDV
PYNNLLIEMLHAKRA*
>ENSG00000189298|ENST00000252211
MARELSESTALDAQSTEDQMELLVIKVEEEEAGFPSSPDLGSEGSRERFRGFRYPEAAGP
REALSRLRELCRQWLQPEMHSKEQILELLVLEQFLTILPGNLQSWVREQHPESGEEVVVL
LEYLERQLDEPAPQVSGVDQGQELLCCKMALLTPAPGSQSSQFQLMKALLKHESVGSQPL
QDRVLQVPVLAHGGCCREDKVVASRLTPESQGLLKVEDVALTLTPEWTQQDSSQGNLCRD
EKQENHGSLVSLGDEKQTKSRDLPPAEELPEKEHGKISCHLREDIAQIPTCAEAGEQEGR
LQRKQKNATGGRRHICHECGKSFAQSSGLSKHRRIHTGEKPYECEECGKAFIGSSALVIH
QRVHTGEKPYECEECGKAFSHSSDLIKHQRTHTGEKPYECDDCGKTFSQSCSLLEHHRIH
TGEKPYQCSMCGKAFRRSSHLLRHQRIHTGDKNVQEPEQGEAWKSRMESQLENVETPMSY
KCNECERSFTQNTGLIEHQKIHTGEKPYQCNACGKGFTRISYLVQHQRSHVGKNILSQ*
>ENSG00000256683|ENST00000243644
MIQAQESITLEDVAVDFTWEEWQLLGAAQKDLYRDVMLENYSNLVAVGYQASKPDALFKL
EQGEQLWTIEDGIHSGACSDIWKVDHVLERLQSESLVNRRKPCHEHDAFENIVHCSKSQF
LLGQNHDIFDLRGKSLKSNLTLVNQSKGYEIKNSVEFTGNGDSFLHANHERLHTAIKFPA
SQKLISTKSQFISPKHQKTRKLEKHHVCSECGKAFIKKSWLTDHQVMHTGEKPHRCSLCE
KAFSRKFMLTEHQRTHTGEKPYECPECGKAFLKKSRLNIHQKTHTGEKPYICSECGKGFI
QKGNLIVHQRIHTGEKPYICNECGKGFIQKTCLIAHQRFHTGKTPFVCSECGKSCSQKSG
LIKHQRIHTGEKPFECSECGKAFSTKQKLIVHQRTHTGERPYGCNECGKAFAYMSCLVKH
KRIHTREKQEAAKVENPPAERHSSLHTSDVMQEKNSANGATTQVPSVAPQTSLNISGLLA
NRNVVLVGQPVVRCAASGDNRGFAQDRNLVNAVNVVVPSVINYVLFYVTENP*
>ENSG00000074319|ENST00000251968
MAVSESQLKKMVSKYKYRDLTVRETVNVITLYKDLKPVLDSYVFNDGSSRELMNLTGTIP
VPYRGNTYNIPICLWLLDTYPYNPPICFVKPTSSMTIKTGKHVDANGKIYLPYLHEWKHP
QSDLLGLIQVMIVVFGDEPPVFSRPISASYPPYQATGPPNTSYMPGMPGGISPYPSGYPP
NPSGYPGCPYPPGGPYPATTSSQYPSQPPVTTVGPSRDGTISEDTIRASLISAVSDKLRW
RMKEEMDRAQAELNALKRTEEDLKKGHQKLEEMVTRLDQEVAEVDKNIELLKKKDEELSS
ALEKMENQSENNDIDEVIIPTAPLYKQILNLYAEENAIEDTIFYLGEALRRGVIDLDVFL
KHVRLLSRKQFQLRALMQKARKTAGLSDLY*
>ENSG00000102098|ENST00000251900
MGQTVNEDSMDVKKENQEKTPQSSTSSVQRDDFHWEEYLKETGSISAPSECFRQSQIPPV
NDFKVGMKLEARDPRNATSVCIATVIGITGARLRLRLDGSDNRNDFWRLVDSPDIQPVGT
CEKEGDLLQPPLGYQMNTSSWPMFLLKTLNGSEMASATLFKKEPPKPPLNNFKVGMKLEA
IDKKNPYLICPATIGDVKGDEVHITFDGWSGAFDYWCKYDSRDIFPAGWCRLTGDVLQPP
GTSVPIVKNIAKTESSPSEASQHSMQSPQKTTLILPTQQVRRSSRIKPPGPTAVPKRSSS
VKNITPRKKGPNSGKKEKPLPVICSTSAASLKSLTRDRGMLYKDVASGPCKIVMSTVCVY
VNKHGNFGPHLDPKRIQQLPDHFGPGPVNVVLRRIVQACVDCALETKTVFGYLKPDNRGG
EVITASFDGETHSIQLPPVNSASFALRFLENFCHSLQCDNLLSSQPFSSSRGHTHSSAEH
DKNQSAKEDVTERQSTKRSPQQTVPYVVPLSPKLPKTKEYASEGEPLFAGGSAIPKEENL
SEDSKSSSLNSGNYLNPACRNPMYIHTSVSQDFSRSVPGTTSSPLVGDISPKSSPHEVKF
QMQRKSEAPSYIAVPDPSVLKQGFSKDPSTWSVDEVIQFMKHTDPQISGPLADLFRQHEI
DGKALFLLKSDVMMKYMGLKLGPALKLCYYIEKLKEGKYS*
>ENSG00000079785|ENST00000233084

MAAFSEMGVMPEIAQAVEEMDWLLPTDIQAESIPLILGGGDVLMAAETGSGKTGAFSIPV
IQIVYETLKDQQEGKKGKTTIKTGASVLNKWQMNPYDRGSAFAIGSDGLCCQSREVKEWH
GCRATKGLMKGKHYYEVSCHDQGLCRVGWSTMQASLDLGTDKFGFGFGGTGKKSHNKQFD
NYGEEFTMHDTIGCYLDIDKGHVKFSKNGKDLGLAFEIPPHMKNQALFPACVLKNAELKF
NFGEEEFKFPPKDGFVALSKAPDGYIVKSQHSGNAQVTQTKFLPNAPKALIVEPSRELAE
QTLNNIKQFKKYIDNPKLRELLIIGGVAARDQLSVLENGVDIVVGTPGRLDDLVSTGKLN
LSQVRFLVLDEADGLLSQGYSDFINRMHNQIPQVTSDGKRLQVIVCSATLHSFDVKKLSE
KIMHFPTWVDLKGEDSVPDTVHHVVVPVNPKTDRLWERLGKSHIRTDDVHAKDNTRPGAN
SPEMWSEAIKILKGEYAVRAIKEHKMDQAIIFCRTKIDCDNLEQYFIQQGGGPDKKGHQF
SCVCLHGDRKPHERKQNLERFKKGDVRFLICTDVAARGIDIHGVPYVINVTLPDEKQNYV
HRIGRVGRAERMGLAISLVATEKEKVWYHVCSSRGKGCYNTRLKEDGGCTIWYNEMQLLS
EIEEHLNCTISQVEPDIKVPVDEFDGKVTYGQKRAAGGGSYKGHVDILAPTVQELAALEK
EAQTSFLHLGYLPNQLFRTF*
>ENSG00000121406|ENST00000240719
MAEAALVITPQGHVTFEDIAVYFSQEEWGLLDEAQRCLYHDVMLENFSLMASVGCLHGIE
AEEAPSEQTLSAQGVSQARTPKLGPSIPNAHSCEMCILVMKDILYLSEHQGTLPWQKPYT
SVASGKWFSFGSNLQQHQNQDSGEKHIRKEESSALLLNSCKIPLSDNLFPCKDVEKDFPT
ILGLLQHQTTHSRQEYAHRSRETFQQRRYKCEQVFNEKVHVTEHQRVHTGEKAYKRREYG
KSLNSKYLFVEHQRTHNAEKPYVCNICGKSFLHKQTLVGHQQRIHTRERSYVCIECGKSL
SSKYSLVEHQRTHNGEKPYVCNVCGKSFRHKQTFVGHQQRIHTGERPYVCMECGKSFIHS
YDRIRHQRVHTGEGAYQCSECGKSFIYKQSLLDHHRIHTGERPYECKECGKAFIHKKRLL
EHQRIHTGEKPYVCIICGKSFIRSSDYMRHQRIHTGERAYECSDCGKAFISKQTLLKHHK
IHTRERPYECSECGKGFYLEVKLLQHQRIHTREQLCECNECGKVFSHQKRLLEHQKVHTG
EKPCECSECGKCFRHRTSLIQHQKVHSGERPYNCTACEKAFIYKNKLVEHQRIHTGEKPY
ECGKCGKAFNKRYSLVRHQKVHITEEP*
>ENSG00000119812|ENST00000238823
MECDLMETDILESLEDLGYKGPLLEDGALSQAVSAGASSPEFTKLCAWLVSELRVLCKLE
ENVQATNSPSEAEEFQLEVSGLLGEMNCPYLSLTSGDVTKRLLIQKNCLLLLTYLISELE
AARMLCVNAPPKKAQEGGGSEVFQELKGICIALGMSKPPANITMFQFFSGIEKKLKETLA
KVPPNHVGKPLLKKPMGPAHWEKIEAINQAIANEYEVRRKLLIKRLDVTVQSFGWSDRAK
SQTEKLAKVYQPKRSVLSPKTTISVAHLLAARQDLSKILRTSSGSIREKTACAINKVLMG
RVPDRGGRPNEIEPPPPEMPPWQKRQDGPQQQTGGRGGGRGGYEHSSYGGRGGHEQGGGR
GGRGGYDHGGRGGGRGNKHQGGWTDGGSGGGGGYQDGGYRDSGFQPGGYHGGHSSGGYQG
GGYGGFQTSSSYTGSGYQGGGYQQDNRYQDGGHHGDRGGGRGGRGGRGGRGGRAGQGGGW
GGRGSQNYHQGGQFEQHFQHGGYQYNHSGFGQGRHYTS*
>ENSG00000105193|ENST00000251453
MPSKGPLQSVQVFGRKKTATAVAHCKRGNGLIKVNGRPLEMIEPRTLQYKLLEPVLLLGK
ERFAGVDIRVRVKGGGHVAQIYAIRQSISKALVAYYQKYVDEASKKEIKDILIQYDRTLL
VADPRRCESKKFGGPGARARYQKSYR*
>ENSG00000128709|ENST00000249499
MLGGSAGRLKMSSSGTLSNYYVDSLIGHEGDEVFAARFGPPGPGAQGRPAGVADGPAATA
AEFASCSFAPRSAVFSASWSAVPSQPPAAAAMSGLYHPYVPPPPLAASASEPGRYVRSWM
EPLPGFPGGAGGGGGGGGGGPGRGPSPGPSGPANGRHYGIKPETRAAPAPATAASTTSSS
STSLSSSSKRTECSVARESQGSSGPEFSCNSFLQEKAAAATGGTGPGAGIGAATGTGGSS

EPSACSDHPIPGCSLKEEEKQHSQPQQQQLDPNNPAANWIHARSTRKKRCPYTKYQTLEL
EKEFLFNMYLTRDRRYEVARILNLTERQVKIWFQNRRMKMKKMSKEKCPKGD*
>ENSG00000122547|ENST00000242108
MGSTLGCHRSIPRDPSDLSHSRKFSAACNFSNILVNQERLNINTATEEELMTLPGVTRAV
ARSIVEYREYIGGFKKVEDLALVSGVGATKLEQVKFEICVSSKGSSAQHSPSSLRRDLLA
EQQPHHLATAVPLTPRVNINTATPAQLMSVRGLSEKMALSIVDFRREHGPFRSVEDLVRM
DGINAAFLDRIRHQVFAERSRPPSTHTNGGLTFTAKPHPSPTSLSLQSEDLDLPPGGPTQ
IISTRPSVEAFGGTRDGRPVLRLATWNLQGCSVEKANNPGVREVVCMTLLENSIKLLAVQ
ELLDREALEKFCTELNQPTLPNIRKWKGPRGCWKAVVAEKPSSQLQKGAGYAGFLWDAAA
GMELRDAGSQESSPSNGHGKLAGPSPYLGRFKVGSHDLTLVNLHLAALTLLGSENPSKNH
SDGHRLASFAQTLQETLKGEKDVIILGDFGQGPDSNDYDILRKEKFHHLIPAHTFTNIST
KNPQGSKSLDNIWISKSLKKVFTGHWAVVREGLTNPWIPDNWSWGGVASEHCPVLAEFYT
EKDWSKKDAPRNGSGVALERSEANIKHER*
>ENSG00000196453|ENST00000247930
MENQRSSPLSFPSVPQEETLRQAPAGLPRETLFQSRVLPPKEIPSLSPTIPRQGSLPQTS
SAPKQETSGRMPHVLQKGPSLLCSAASEQETSLQGPLASQEGTQYPPPAAAEQEVSLLSH
SPHHQEAPVHSPEAPEKDPLTLSPTVPETDMDPLLQSPVSQKDTPFQISSAVQKEQPLPT
AEITRLAVWAAVQAVERKLEAQAMRLLTLEGRTGTNEKKIADCEKTAVEFANHLESKWVV
LGTLLQEYGLLQRRLENMENLLKNRNFWILRLPPGSNGEVPKVPVTFDDVAVHFSEQEWG
NLSEWQKELYKNVMRGNYESLVSMDYAISKPDLMSQMERGERPTMQEQEDSEEGETPTDP
SAAHDGIVIKIEVQTNDEGSESLETPEPLMGQVEEHGFQDSELGDPCGEQPDLDMQEPEN
TLEESTEGSSEFSELKQMLVQQRNCTEGIVIKTEEQDEEEEEEEEDELPQHLQSLGQLSG
RYEASMYQTPLPGEMSPEGEESPPPLQLGNPAVKRLAPSVHGERHLSENRGASSQQQRNR
RGERPFTCMECGKSFRLKINLIIHQRNHIKEGPYECAECEISFRHKQQLTLHQRIHRVRG
GCVSPERGPTFNPKHALKPRPKSPSSGSGGGGPKPYKCPECDSSFSHKSSLTKHQITHTG
ERPYTCPECKKSFRLHISLVIHQRVHAGKHEVSFICSLCGKSFSRPSHLLRHQRTHTGER
PFKCPECEKSFSEKSKLTNHCRVHSRERPHACPECGKSFIRKHHLLEHRRIHTGERPYHC
AECGKRFTQKHHLLEHQRAHTGERPYPCTHCAKCFRYKQSLKYHLRTHTGE*
>ENSG00000129173|ENST00000250024
MENEKENLFCEPHKRGLMKTPLKESTTANIVLAEIQPDFGPLTTPTKPKEGSQGEPWTPT
ANLKMLISAVSPEIRNRDQKRGLFDNRSGLPEAKDCIHEHLSGDEFEKSQPSRKEKSLGL
LCHKFLARYPNYPNPAVNNDICLDEVAEELNVERRRIYDIVNVLESLHMVSRLAKNRYTW
HGRHNLNKTLGTLKSIGEENKYAEQIMMIKKKEYEQEFDFIKSYSIEDHIIKSNTGPNGH
PDMCFVELPGVEFRAASVNSRKDKSLRVMSQKFVMLFLVSTPQIVSLEVAAKILIGEDHV
EDLDKSKFKTKIRRLYDIANVLSSLDLIKKVHVTEERGRKPAFKWTGPEISPNTSGSSPV
IHFTPSDLEVRRSSKENCAKNLFSTRGKPNFTRHPSLIKLVKSIESDRRKINSAPSSPIK
TNKAESSQNSAPFPSKMAQLAAICKMQLEEQSSESRQKVKVQLARSGPCKPVAPLDPPVN
AEMELTAPSLIQPLGMVPLIPSPLSSAVPLILPQAPSGPSYAIYLQPTQAHQSVTPPQGL
SPTVCTTHSSKATGSKDSTDATTEKAANDTSKASASTRPGSLLPAPERQGAKSRTREPAG
ERGSKRASMLEDSGSKKKFKEDLKGLENVSATLFPSGYLIPLTQCSSLGAESILSGKENS
SALSPNHRIYSSPIAGVIPVTSSELTAVNFPSFHVTPLKLMVSPTSVAAVPVGNSPALAS
SHPVPIQNPSSAIVNFTLQHLGLISPNVQLSASPGSGIVPVSPRIESVNVAPENAGTQQG
RATNYDSPVPGQSQPNGQSVAVTGAQQPVPVTPKGSQLVAESFFRTPGGPTKPTSSSCMD
FEGANKTSLGTLFVPQRKLEVSTEDVH*

>ENSG00000105671|ENST00000247003
MAGFAELGLSSWLVEQCRQLGLKQPTPVQLGCIPAILEGRDCLGCAKTGSGKTAAFVLPI
LQKLSEDPYGIFCLVLTPTRELAYQIAEQFRVLGKPLGLKDCIIVGGMDMVAQALELSRK
PHVVIATPGRLADHLRSSNTFSIKKIRFLVMDEADRLLEQGCTDFTVDLEAILAAVPARR
QTLLFSATLTDTLRELQGLATNQPFFWEAQAPVSTVEQLDQRYLLVPEKVKDAYLVHLIQ
RFQDEHEDWSIIIFTNTCKTCQILCMMLRKFSFPTVALHSMMKQKERFAALAKFKSSIYR
ILIATDVASRGLDIPTVQVVINHNTPGLPKIYIHRVGRTARAGRQGQAITLVTQYDIHLV
HAIEEQIKKKLEEFSVEEAEVLQILTQVNVVRRECEIKLEAAHFDEKKEINKRKQLILEG
KDPDLEAKRKAELAKIKQKNRRFKEKVEETLKRQKAGRAGHKGRPPRTPSGSHSGPVPSQ
GLV*
>ENSG00000124795|ENST00000244776
MSASAPAAEGEGTPTQPASEKEPEMPGPREESEEEEDEDDEEEEEEEKGKGQKLCEIERI
HFFLSKKKTDELRNLHKLLYNRPGTVSSLKKNVGQFSGFPFEKGSVQYKKKEEMLKKFRN
AMLKSICEVLDLERSGVNSELVKRILNFLMHPKPSGKPLPKSKKTCSKGSKKERNSSGMA
RKAKRTKCPEILSDESSSDEDEKKNKEESSDDEDKESEEEPPKKTAKREKPKQKATSKSK
KSVKSANVKKADSSTTKKNQNSSKKESESEDSSDDEPLIKKLKKPPTDEELKETIKKLLA
SANLEEVTMKQICKKVYENYPTYDLTERKDFIKTTVKELIS*
>ENSG00000125207|ENST00000245255
MTGRARARARGRARGQETAQLVGSTASQQPGYIQPRPQPPPAEGELFGRGRQRGTAGGTA
KSQGLQISAGFQELSLAERGGRRRDFHDLGVNTRQNLDHVKESKTGSSGIIVRLSTNHFR
LTSRPQWALYQYHIDYNPLMEARRLRSALLFQHEDLIGKCHAFDGTILFLPKRLQQKVTE
VFSKTRNGEDVRITITLTNELPPTSPTCLQFYNIIFRRLLKIMNLQQIGRNYYNPNDPID
IPSHRLVIWPGFTTSILQYENSIMLCTDVSHKVLRSETVLDFMFNFYHQTEEHKFQEQVS
KELIGLVVLTKYNNKTYRVDDIDWDQNPKSTFKKADGSEVSFLEYYRKQYNQEITDLKQP
VLVSQPKRRRGPGGTLPGPAMLIPELCYLTGLTDKMRNDFNVMKDLAVHTRLTPEQRQRE
VGRLIDYIHKNDNVQRELRDWGLSFDSNLLSFSGRILQTEKIHQGGKTFDYNPQFADWSK
ETRGAPLISVKPLDNWLLIYTRRNYEAANSLIQNLFKVTPAMGMQMRKAIMIEVDDRTEA
YLRVLQQKVTADTQIVVCLLSSNRKDKYDAIKKYLCTDCPTPSQCVVARTLGKQQTVMAI
ATKIALQMNCKMGGELWRVDIPLKLVMIVGIDCYHDMTAGRRSIAGFVASINEGMTRWFS
RCIFQDRGQELVDGLKVCLQAALRAWNSCNEYMPSRIIVYRDGVGDGQLKTLVNYEVPQF
LDCLKSIGRGYNPRLTVIVVKKRVNTRFFAQSGGRLQNPLPGTVIDVEVTRPEWYDFFIV
SQAVRSGSVSPTHYNVIYDNSGLKPDHIQRLTYKLCHIYYNWPGVIRVPAPCQYAHKLAF
LVGQSIHREPNLSLSNRLYYL*
>ENSG00000113430|ENST00000231357
MSYPQFGYPYSSAPQFLMATNSLSTCCESGGRTLADSGPAASAQAPVYCPVYESRLLATA
RHELNSAAALGVYGGPYGGSQGYGNYVTYGSEASAFYSLNSFDSKDGSGSAHGGLAPAAA
AYYPYEPALGQYPYDRYGTMDSGTRRKNATRETTSTLKAWLQEHRKNPYPTKGEKIMLAI
ITKMTLTQVSTWFANARRRLKKENKMTWPPRNKCADEKRPYAEGEEEEGGEEEAREEPLK
SSKNAEPVGKEEKELELSDLDDFDPLEAEPPACELKPPFHSLDGGLERVPAAPDGPVKEA
SGALRMSLAAGGGAALDEDLERARSCLRSAAAGPEPLPGAEGGPQVCEAKLGFVPAGASA
GLEAKPRIWSLAHTATAAAAAATSLSQTEFPSCMLKRQGPAAPAAVSSAPATSPSVALPH
SGALDRHQDSPVTSLRNWVDGVFHDPILRHSTLNQAWATAKGALLDPGPLGRSLGAGANV
LTAPLARAFPPAVPQDAPAAGAARELLALPKAGGKPFCA*
>ENSG00000092853|ENST00000251195

MTGEVGSEVHLEINDPNVISQEEADSPSDSGQGSYETIGPLSEGDSDEEIFVSKKLKNRK
VLQDSDSETEDTNASPEKTTYDSAEEENKENLYAGKNTKIKRIYKTVADSDESYMEKSLY
QENLEAQVKPCLELSLQSGNSTDFTTDRKSSKKHIHDKEGTAGKAKVKSKRRLEKEERKM
EKIRQLKKKETKNQEDDVEQPFNDSGCLLVDKDLFETGLEDENNSPLEDEESLESIRAAV
KNKVKKHKKKEPSLESGVHSFEEGSELSKGTTRKERKAARLSKEALKQLHSETQRLIRES
ALNLPYHMPENKTIHDFFKRKPRPTCHGNAMALLKSSKYQSSHHKEIIDTANTTEMNSDH
HSKGSEQTTGAENEVETNALPVVSKETQIITGSDESCRKDLVKNEELEIQEKQKQSDIRP
SPGDSSVLQQESNFLGNNHSEECQVGGLVAFEPHALEGEGPQNPEETDEKVEEPEQQNKS
SAVGPPEKVRRFTLDRLKQLGVDVSIKPRLGADEDSFVILEPETNRELEALKQRFWKHAN
PAAKPRAGQTVNVNVIVKDMGTDGKEELKADVVPVTLAPKKLDGASHTKPGEKLQVLKAK
LQEAMKLRRFEERQKRQALFKLDNEDGFEEEEEEEEEMTDESEEDGEEKVEKEEKEEELE
EEEEKEEEEEEEEGNQETAEFLLSSEEIETKDEKEMDKENNDGSSEIGKAVGFLSVPKSLS
SDSTLLLFKDSSSKMGYFPTEEKSETDENSGKQPSKLDEDDSCSLLTKESSHNSSFELIG
STIPSYQPCNRQTGRGTSFFPTAGGFRSPSPGLFRASLVSSASKSSGKLSEPSLPIEDSQ
DLYNASPEPKTLFLGAGDFQFCLEDDTQSQLLDADGFLNVRNHRNQYQALKPRLPLASMD
ENAMDANMDELLDLCTGKFTSQAEKHLPRKSDKKENMEELLNLCSGKFTSQDASTPASSE
LNKQEKESSMGDPMEEALALCSGSFPTDKEEEDEEEEFGDFRLVSNDNEFDSDEDEHSDS
GNDLALEDHEDDDEEELLKRSEKLKRQMRLRKYLEDEAEVSGSDVGSEDEYDGEEIDEYE
EDVIDEVLPSDEELQSQIKKIHMKTMLDDDKRQLRLYQERYLADGDLHSDGPGRMRKFRW
KNIDDASQMDLFHRDSDDDQTEEQLDESEARWRKERIEREQWLRDMAQQGKITAEEEEEI
GEDSQFMILAKKVTAKALQKNASRPMVIQESKSLLRNPFEAIRPGSAQQVKTGSLLNQPK
AVLQKLAALSDHNPSAPRNSRNFVFHTLSPVKAEAAKESSKSQKIPEKDSDWLTWSGAPI
PGFFRLSFDPHG*
>ENSG00000067248|ENST00000251636
MGGKNKKHKAPAAAVVRAAVSASRAKSAEAGIAGEAQSKKPVSRPATAAAAAAGSREPRV
KQGPKIYSFNSTNDSSGPANLDKSILKVVINNKLEQRIIGVINEHKKQNNDKGMISGRLT
AKKLQDLYMALQAFSFKTKDIEDAMTNTLLYGGDLHSALDWLCLNLSDDALPEGFSQEFE
EQQPKSRPKFQSPQIQATISPPLQPKTKTYEEDPKSKPKKEEKNMEVNMKEWILRYAEQQ
NEEEKNENSKSLEEEEKFDPNERYLHLAAKLLDAKEQAATFKLEKNKQGQKEAQEKIRKF
QREMETLEDHPVFNPAMKISHQQNERKKPPVATEGESALNFNLFEKSAAATEEEKDKKKE
PHDVRNFDYTARSWTGKSPKQFLIDWVRKNLPKSPNPSFEKVPVGRYWKCRVRVIKSEDD
VLVVCPTILTEDGMQAQHLGATLALYRLVKGQSVHQLLPPTYRDVWLEWSDAEKKREELN
KMETNKPRDLFIAKLLNKLKQQQQQQQQHSENKRENSEDPEESWENLVSDEDFSALSLES
ANVEDLEPVRNLFRKLQSTPKYQKLLKERQQLPVFKHRDSIVETLKRHRVVVVAGETGSG
KSTQVPHFLLEDLLLNEWEASKCNIVCTQPRRISAVSLANRVCDELGCENGPGGRNSLCG
YQIRMESRACESTRLLYCTTGVLLRKLQEDGLLSNVSHVIVDEVHERSVQSDFLLIILKE
ILQKRSDLHLILMSATVDSEKFSTYFTHCPILRISGRSYPVEVFHLEDIIEETGFVLEKD
SEYCQKFLEEEEEVTINVTSKAGGIKKYQEYIPVQTGAHADLNPFYQKYSSRTQHAILYM
NPHKINLDLILELLAYLDKSPQFRNIEGAVLIFLPGLAHIQQLYDLLSNDRRFYSERYKV
IALHSILSTQDQAAAFTLPPPGVRKIVLATNIAETGITIPDVVFVIDTGRTKENKYHESS
QMSSLVETFVSKASALQRQGRAGRVRDGFCFRMYTRERFEGFMDYSVPEILRVPLEELCL
HIMKCNLGSPEDFLSKALDPPQLQVISNAMNLLRKIGACELNEPKLTPLGQHLAALPVNV
KIGKMLIFGAIFGCLDPVATLAAVMTEKSPFTTPIGRKDEADLAKSALAMADSDHLTIYN
AYLGWKKARQEGGYRSEITYCRRNFLNRTSLLTLEDVKQELIKLVKAAGFSSSTTSTSWE

GNRASQTLSFQEIALLKAVLVAGLYDNVGKIIYTKSVDVTEKLACIVETAQGKAQVHPSS
VNRDLQTHGWLLYQEKIRYARVYLRETTLITPFPVLLFGGDIEVQHRERLLSIDGWIYFQ
APVKIAVIFKQLRVLIDSVLRKKLENPKMSLENDKILQIITELIKTENN*
>ENSG00000125970|ENST00000246194
MSLKLQASNVTNKNDPKSINSRVFIGNLNTALVKKSDVETIFSKYGRVAGCSVHKGYAFV
QYSNERHARAAVLGENGRVLAGQTLDINMAGEPKPDRPKGLKRAASAIYSGYIFDYDYYR
DDFYDRLFDYRGRLSPVPVPRAVPVKRPRVTVPLVRRVKTNVPVKLFARSTAVTTSSAKI
KLKSSELQAIKTELTQIKSNIDALLSRLEQIAAEQKANPDGKKKGDGGGAGGGGGGGGSG
GGGSGGGGGGGSSRPPAPQENTTSEAGLPQGEARTRDDGDEEGLLTHSEEELEHSQDTDA
DDGALQ*
>ENSG00000105939|ENST00000242351
MADPEVCCFITKILCAHGGRMALDALLQEIALSEPQLCEVLQVAGPDRFVVLETGGEAGI
TRSVVATTRARVCRRKYCQRPCDNLHLCKLNLLGRCNYSQSERNLCKYSHEVLSEENFKV
LKNHELSGLNKEELAVLLLQSDPFFMPEICKSYKGEGRQQICNQQPPCSRLHICDHFTRG
NCRFPNCLRSHNLMDRKVLAIMREHGLNPDVVQNIQDICNSKHMQKNPPGPRAPSSHRRN
MAYRARSKSRDRFFQGSQEFLASASASAERSCTPSPDQISHRASLEDAPVDDLTRKFTYL
GSQDRARPPSGSSKATDLGGTSQAGTSQRFLENGSQEDLLHGNPGSTYLASNSTSAPNWK
SLTSWTNDQGARRKTVFSPTLPAARSSLGSLQTPEAVTTRKGTGLLSSDYRIINGKSGTQ
DIQPGPLFNNNADGVATDITSTRSLNYKSTSSGHREISSPRIQDAGPASRDVQATGRIAD
DADPRVALVNDSLSDVTSTTSSRVDDHDSEEICLDHLCKGCPLNGSCSKVHFHLPYRWQM
LIGKTWTDFEHMETIEKGYCNPGIHLCSVGSYTINFRVMSCDSFPIRRLSTPSSVTKPAN
SVFTTKWIWYWKNESGTWIQYGEEKDKRKNSNVDSSYLESLYQSCPRGVVPFQAGSRNYE
LSFQGMIQTNIASKTQKDVIRRPTFVPQWYVQQMKRGPDHQPAKTSSVSLTATFRPQEDF
CFLSSKKYKLSEIHHLHPEYVRVSEHFKASMKNFKIEKIKKIENSELLDKFTWKKSQMKE
EGKLLFYATSRAYVESICSNNFDSFLHETHENKYGKGIYFAKDAIYSHKNCPYDAKNVVM
FVAQVLVGKFTEGNITYTSPPPQFDSCVDTRSNPSVFVIFQKDQVYPQYVIEYTEDKACV
IS*
>ENSG00000116731|ENST00000235372
MNQNTTEPVAATETLAEVPEHVLRGLPEEVRLFPSAVDKTRIGVWATKPILKGKKFGPFV
GDKKKRSQVKNNVYMWEVYYPNLGWMCIDATDPEKGNWLRYVNWACSGEEQNLFPLEINR
AIYYKTLKPIAPGEELLVWYNGEDNPEIAAAIEEERASARSKRSSPKSRKGKKKSQENKN
KGNKIQDIQLKTSEPDFTSANMRDSAEGPKEDEEKPSASALEQPATLQEVASQEVPPELA
TPAPAWEPQPEPDERLEAAACEVNDLGEEEEEEEEEDEEEEEDDDDDELEDEGEEEASMP
NENSVKEPEIRCDEKPEDLLEEPKTTSEETLEDCSEVTPAMQIPRTKEEANGDVFETFMF
PCQHCERKFTTKQGLERHMHIHISTVNHAFKCKYCGKAFGTQINRRRHERRHEAGLKRKP
SQTLQPSEDLADGKASGENVASKDDSSPPSLGPDCLIMNSEKASQDTINSSVVEENGEVK
ELHPCKYCKKVFGTHTNMRRHQRRVHERHLIPKGVRRKGGLEEPQPPAEQAQATQNVYVP
STEPEEEGEADDVYIMDISSNISENLNYYIDGKIQTNNNTSNCDVIEMESASADLYGINC
LLTPVTVEITQNIKTTQVPVTEDLPKEPLGSTNSEAKKRRTASPPALPKIKAETDSDPMV
PSCSLSLPLSISTTEAVSFHKEKSVYLSSKLKQLLQTQDKLTPAGISATEIAKLGPVCVS
APASMLPVTSSRFKRRTSSPPSSPQHSPALRDFGKPSDGKAAWTDAGLTSKKSKLESHSD
SPAWSLSGRDERETVSPPCFDEYKMSKEWTASSAFSSVCNQQPLDLSSGVKQKAEGTGKT
PVQWESVLDLSVHKKHCSDSEGKEFKESHSVQPTCSAVKKRKPTTCMLQKVLLNEYNGID
LPVENPADGTRSPSPCKSLEAQPDPDLGPGSGFPAPTVESTPDVCPSSPALQTPSLSSGQ

LPPLLIPTDPSSPPPCPPVLTVATPPPPLLPTVPLPAPSSSASPHPCPSPLSNATAQSPL
PILSPTVSPSPSPIPPVEPLMSAASPGPPTLSSSSSSSSSSSSFSSSSSSSSSPSPPPLSA
ISSVVSSGDNLEASLPMISFKQEELENEGLKPREEPQSAAEQDVVVQETFNKNFVCNVCE
SPFLSIKDLTKHLSIHAEEWPFKCEFCVQLFKDKTDLSEHRFLLHGVGNIFVCSVCKKEF
AFLCNLQQHQRDLHPDKVCTHHEFESGTLRPQNFTDPSKAHVEHMQSLPEDPLETSKEEE
ELNDSSEELYTTIKIMASGIKTKDPDVRLGLNQHYPSFKPPPFQYHHRNPMGIGVTATNF
TTHNIPQTFTTAIRCTKCGKGVDNMPELHKHILACASASDKKRYTPKKNPVPLKQTVQPK
NGVVVLDNSGKNAFRRMGQPKRLNFSVELSKMSSNKLKLNALKKKNQLVQKAILQKNKSA
KQKADLKNACESSSHICPYCNREFTYIGSLNKHAAFSCPKKPLSPPKKKVSHSSKKGGHS
SPASSDKNSNSNHRRRTADAEIKMQSMQTPLGKTRARSSGPTQVPLPSSSFRSKQNVKFA
ASVKSKKPSSSSLRNSSPIRMAKITHVEGKKPKAVAKNHSAQLSSKTSRSLHVRVQKSKA
VLQSKSTLASKKRTDRFNIKSRERSGGPVTRSLQLAAAADLSENKREDGSAKQELKDFSY
SLRLASRCSPPAAPYITRQYRKVKAPAAAQFQGPFFKE*
>ENSG00000126767|ENST00000247161
MDPSVTLWQFLLQLLREQGNGHIISWTSRDGGEFKLVDAEEVARLWGLRKNKTNMNYDKL
SRALRYYYDKNIIRKVSGQKFVYKFVSYPEVAGCSTEDCPPQPEVSVTSTMPNVAPAAIH
AAPGDTVSGKPGTPKGAGMAGPGGLARSSRNEYMRSGLYSTFTIQSLQPQPPPHPRPAVV
LPSAAPAGAAAPPSGSRSTSPSPLEACLEAEEAGLPLQVILTPPEAPNLKSEELNVEPGL
GRALPPEVKVEGPKEELEVAGERGFVPETTKAEPEVPPQEGVPARLPAVVMDTAGQAGGH
AASSPEISQPQKGRKPRDLELPLSPSLLGGPGPERTPGSGSGSGLQAPGPALTPSLLPTH
TLTPVLLTPSSLPPSIHFWSTLSPIAPRSPAKLSFQFPSSGSAQVHIPSISVDGLSTPVV
LSPGPQKP*
>ENSG00000107372|ENST00000237937
MAQETNQTPGPMLCSTGCGFYGNPRTNGMCSVCYKEHLQRQQNSGRMSPMGTASGSNSPT
SDSASVQRADTSLNNCEGAAGSTSEKSRNVPVAALPVTQQMTEMSISREDKITTPKTEVS
EPVVTQPSPSVSQPSTSQSEEKAPELPKPKKNRCFMCRKKVGLTGFDCRCGNLFCGLHRY
SDKHNCPYDYKAEAAAKIRKENPVVVAEKIQRI*
>ENSG00000126003|ENST00000246229
MTTFFTSVPPWIQDAKQEEEVGWKLVPRPRGREAESQVKCQCEISGTPFSNGEKLRPHSL
PQPEQRPYSCPQLHCGKAFASKYKLYRHMATHSAQKPHQCMYCDKMFHRKDHLRNHLQTH
DPNKEALHCSECGKNYNTKLGYRRHLAMHAASSGDLSCKVCLQTFESTQALLEHLKAHSR
RVAGGAKEKKHPCDHCDRRFYTRKDVRRHLVVHTGRKDFLCQYCAQRFGRKDHLTRHVKK
SHSQELLKIKTEPVDMLGLLSCSSTVSVKEELSPVLCMASRDVMGTKAFPGMLPMGMYGA
HIPTMPSTGVPHSLVHNTLPMGMSYPLESSPISSPAQLPPKYQLGSTSYLPDKLPKVEVD
SFLAELPGSLSLSSAEPQPASPQPAAAAALLDEALLAKSPANLSEALCAANVDFSHLLGF
LPLNLPPCNPPGATGGLVMGYSQAEAQPLLTTLQAQPQDSPGAGGPLNFGPLHSLPPVFT
SGLSSTTLPRFHQAFQ*
>ENSG00000121297|ENST00000240587
MPRRKQQAPRRAAAYVSEELKAAALVDEGLDPEEHTADGEPSAKYMCPEKELARACPSYQ
NSPAAEFSCHEMDSESHISETSDRMADFESGSIKNEEETKEVTVPLEDTTVSDSLEQMKA
VYNNFLSNSYWSNLNLNLHQPSSEKNNGSSSSSSSSSSSCGSGSFDWHQSAMAKTLQQVS
QSRMLPEPSLFSTVQLYRQSSKLYGSIFTGASKFRCKDCSAAYDTLVELTVHMNETGHYR
DDNHETDNNNPKRWSKPRKRSLLEMEGKEDAQKVLKCMYCGHSFESLQDLSVHMIKTKHY
QKVPLKEPVTPVAAKIIPATRKKASLELELPSSPDSTGGTPKATISDTNDALQKNSNPYI

TPNNRYGHQNGASYAWHFEARKSQILKCMECGSSHDTLQELTAHMMVTGHFIKVTNSAMK
KGKPIVETPVTPTITTLLDEKVQSVPLAATTFTSPSNTPASISPKLNVEVKKEVDKEKAV
TDEKPKQKDKPGEEEEKCDISSKYHYLTENDLEESPKGGLDILKSLENTVTSAINKAQNG
TPSWGGYPSIHAAYQLPNMMKLSLGSSGKSTPLKPMFGNSEIVSPTKNQTLVSPPSSQTS
PMPKTNFHAMEELVKKVTEKVAKVEEKMKEPDGKLSPPKRATPSPCSSEVGEPIKMEASS
DGGFRSQENSPSPPRDGCKDGSPLAEPVENGKELVKPLASSLSGSTAIITDHPPEQPFVN
PLSALQSVMNIHLGKAAKPSLPALDPMSMLFKMSNSLAEKAAVATPPPLQSKKADHLDRY
FYHVNNDQPIDLTKGKSDKGCSLGSVLLSPTSTAPATSSSTVTTAKTSAVVSFMSNSPLR
ENALSDISDMLKNLTESHTSKSSTPSSISEKSDIDGATLEEAEESTPAQKRKGRQSNWNP
QHLLILQAQFAASLRQTSEGKYIMSDLSPQERMHISRFTGLSMTTISHWLANVKYQLRRT
GGTKFLKNLDTGHPVFFCNDCASQIRTPSTYISHLESHLGFRLRDLSKLSTEQINSQIAQ
TKSPSEKMVTSSPEEDLGTSYQCKLCNRTFASKHAVKLHLSKTHGKSPEDHLLYVSELEK
Q*
>ENSG00000114735|ENST00000232854
MELWGRMLWALLSGPGRRGSTRGWAFSSWQPQPPLAGLSSAIELVSHWTGVFEKRGIPEA
RESSEYIVAHVLGAKTFQSLRPALWTQPLTSQQLQCIRELSSRRLQRMPVQYILGEWDFQ
GLSLRMVPPVFIPRPETEELVEWVLEEVAQRSHAVGSPGSPLILEVGCGSGAISLSLLSQ
LPQSRVIAVDKREAAISLTHENAQRLRLQDRIWIIHLDMTSERSWTHLPWGPMDLIVSNP
PYVFHQDMEQLAPEIRSYEDPAALDGGEEGMDIITHILALAPRLLKDSGSIFLEVDPRHP
ELVSSWLQSRPDLYLNLVAVRRDFCGRPRFLHIRRSGP*
>ENSG00000113196|ENST00000231121
MNLVGSYAHHHHHHHPHPAHPMLHEPFLFGPASRCHQERPYFQSWLLSPADAAPDFPAGG
PPPAAAAAATAYGPDARPGQSPGRLEALGGRLGRRKGSGPKKERRRTESINSAFAELREC
IPNVPADTKLSKIKTLRLATSYIAYLMDVLAKDAQSGDPEAFKAELKKADGGRESKRKRE
LQQHEGFPPALGPVEKRIKGRTGWPQQVWALELNQ*
>ENSG00000120068|ENST00000239144
MSSYFVNSLFSKYKTGESLRPNYYDCGFAQDLGGRPTVVYGPSSGGSFQHPSQIQEFYHG
PSSLSTAPYQQNPCAVACHGDPGNFYGYDPLQRQSLFGAQDPDLVQYADCKLAAASGLGE
EAEGSEQSPSPTQLFPWMRPQAAAGRRRGRQTYSRYQTLELEKEFLFNPYLTRKRRIEVS
HALGLTERQVKIWFQNRRMKWKKENNKDKFPSSKCEQEELEKQKLERAPEAADEGDAQKG
DKK*
>ENSG00000116455|ENST00000235090
MRKETPPPLVPPAAREWNLPPNAPACMERQLEAARYRSDGALLLGASSLSGRCWAGSLWL
FKDPCAAPNEGFCSAGVQTEAGVADLTWVGERGILVASDSGAVELWELDENETLIVSKFC
KYEHDDIVSTVSVLSSGTQAVSGSKDICIKVWDLAQQVVLSSYRAHAAQVTCVAASPHKD
SVFLSCSEDNRILLWDTRCPKPASQIGCSAPGYLPTSLAWHPQQSEVFVFGDENGTVSLV
DTKSTSCVLSSAVHSQCVTGLVFSPHSVPFLASLSEDCSLAVLDSSLSELFRSQAHRDFV
RDATWSPLNHSLLTTVGWDHQVVHHVVPTEPLPAPGPASVTE*
>ENSG00000128604|ENST00000249375
MNQSIPVAPTPPRRVRLKPWLVAQVNSCQYPGLQWVNGEKKLFCIPWRHATRHGPSQDGD
NTIFKAWAKETGKYTEGVDEADPAKWKANLRCALNKSRDFRLIYDGPRDMPPQPYKIYEV
CSNGPAPTDSQPPEDYSFGAGEEEEEEEELQRMLPSLSLTEDVKWPPTLQPPTLRPPTLQ
PPTLQPPVVLGPPAPDPSPLAPPPGNPAGFRELLSEVLEPGPLPASLPPAGEQLLPDLLI
SPHMLPLTDLEIKFQYRGRPPRALTISNPHGCRLFYSQLEATQEQVELFGPISLEQVRFP

SPEDIPSDKQRFYTNQLLDVLDRGLILQLQGQDLYAIRLCQCKVFWSGPCASAHDSCPNP
IQREVKTKLFSLEHFLNELILFQKGQTNTPPPFEIFFCFGEEWPDRKPREKKLITVQVVP
VAARLLLEMFSGELSWSADSIRLQISNPDLKDRMVEQFKELHHIWQSQQRLQPVAQAPPG
AGLGVGQGPWPMHPAGMQ*
>ENSG00000120149|ENST00000239243
MASPSKGNDLFSPDEEGPAVVAGPGPGPGGAEGAAEERRVKVSSLPFSVEALMSDKKPPK
EASPLPAESASAGATLRPLLLSGHGAREAHSPGPLVKPFETASVKSENSEDGAAWMQEPG
RYSPPPRHMSPTTCTLRKHKTNRKPRTPFTTSQLLALERKFRQKQYLSIAERAEFSSSLN
LTETQVKIWFQNRRAKAKRLQEAELEKLKMAAKPMLPSSFSLPFPISSPLQAASIYGASY
PFHRPVLPIPPVGLYATPVGYGMYHLS*
>ENSG00000119950|ENST00000239007
MERVKMINVQRLLEAAEFLERREREREСEHGYASSFPSMPSPRLQHSKPPRRLSRAQKHSSG
SSNTSTANRSTHNELEKNRRAHLRLCLERLKVLIPLGPDCTRHTTLGLLNKAKAHIKKLE
EAERKSQHQLENLEREQRFLKWRLEQLQGPQEMERIRMDSIGSTISSDRSDSEREEIEVD
VESTEFSHGEVDNISTTSISDIDDHSSLPSIGSDEGYSSASVKLSFTS*
>ENSG00000116062|ENST00000234420
MSRQSTLYSFFPKSPALSDANKASARASREGGRAAAAPGASPSPGGDAAWSEAGPGPRPL
ARSASPPKAKNLNGGLRRSVAPAAPTSCDFSPGDLVWAKMEGYPWWPCLVYNHPFDGTFI
REKGKSVRVHVQFFDDSPTRGWVSKRLLKPYTGSKSKEAQKGGHFYSAKPEILRAMQRAD
EALNKDKIKRLELAVCDEPSEPEEEEEMEVGTTYVTDKSEEDNEIESEEEVQPKTQGSRR
SSRQIKKRRVISDSESDIGGSDVEFKPDTKEEGSSDEISSGVGDSESEGLNSPVKVARKR
KRMVTGNGSLKRKSSRKETPSATKQATSISSETKNTLRAFSAPQNSESQAHVSGGGDDSS
RPTVWYHETLEWLKEEKRRDEHRRRPDHPDFDASTLYVPEDFLNSCTPGMRKWWQIKSQN
FDLVICYKVGKFYELYHMDALIGVSELGLVFMKGNWAHSGFPEIAFGRYSDSLVQKGYKV
ARVEQTETPEMMEARCRKMAHISKYDRVVRREICRIITKGTQTYSVLEGDPSENYSKYLL
SLKEKEEDSSGHTRAYGVCFVDTSLGKFFIGQFSDDRHCSRFRTLVAHYPPVQVLFEKGN
LSKETKTILKSSLSCSLQEGLIPGSQFWDASKTLRTLLEEEYFREKLSDGIGVMLPQVLK
GMTSESDSIGLTPGEKSELALSALGGCVFYLKKCLIDQELLSMANFEEYIPLDSDTVSTT
RSGAIFTKAYQRMVLDAVTLNNLEIFLNGTNGSTEGTLLERVDTCHTPFGKRLLKQWLCA
PLCNHYAINDRLDAIEDLMVVPDKISEVVELLKKLPDLERLLSKIHNVGSPLKSQNHPDS
RAIMYEETTYSKKKIIDFLSALEGFKVMCKIIGIMEEVADGFKSKILKQVISLQTKNPEG
RFPDLTVELNRWDTAFDHEKARKTGLITPKAGFDSDYDQALADIRENEQSLLEYLEKQRN
RIGCRTIVYWGIGRNRYQLEIPENFTTRNLPEEYELKSTKKGCKRYWTKTIEKKLANLIN
AEERRDVSLKDCMRRLFYNFDKNYKDWQSAVECIAVLDVLLCLANYSRGGDGPMCRPVIL
LPEDTPPFLELKGSRHPCITKTFFGDDFIPNDILIGCEEEEQENGKAYCVLVTGPNMGGK
STLMRQAGLLAVMAQMGCYVPAEVCRLTPIDRVFTRLGASDRIMSGESTFFVELSETASI
LMHATAHSLVLVDELGRGTATFDGTAIANAVVKELAETIKCRTLFSTHYHSLVEDYSQNV
AVRLGHMACMVENECEDPSQETITFLYKFIKGACPKSYGFNAARLANLPEEVIQKGHRKA
REFEKMNQSLRLFREVCLASERSTVDAEAVHKLLTLIKEL*
>ENSG00000113643|ENST00000231572
MDVLVSECSARLLQQEEEIKSLTAEIDRLKNCGCLGASPNLEQLQEENLKLKYRLNILRK
SLQAERNKPTKNMINIISRLQEVFGHAIKAAYPDLENPPLLVTPSQQAKFGDYQCNSAMG
ISQMLKTKEQKVNPREIAENITKHLPDNECIEKVEIAGPGFINVHLRKDFVSEQLTSLLV
NGVQLPALGENKKVIVDFSSPNIAKEMHVGHLRSTIIGESISRLFEFAGYDVLRLNHVGD

WGTQFGMLIAHLQDKFPDYLTVSPPIGDLQVFYKESKKRFDTEEEFKKRAYQCVVLLQGK
NPDITKAWKLICDVSRQELNKIYDALDVSLIERGESFYQDRMNDIVKEFEDRGFVQVDDG
RKIVFVPGCSIPLTIVKSDGGYTYDTSDLAAIKQRLFEEKADMIIYVVDNGQSVHFQTIF
AAAQMIGWYDPKVTRVFHAGFGVVLGEDKKKFKTRSGETVRLMDLLGEGLKRSMDKLKEK
ERDKVLTAEELNAAQTSVAYGCIKYADLSHNRLNDYIFSFDKMLDDRGNTAAYLLYAFTR
IRSIARLANIDEEMLQKAARETKILLDHEKEWKLGRCILRFPEILQKILDDLFLHTLCDY
IYELATAFTEFYDSCYCVEKDRQTGKILKVNMWRMLLCEAVAAVMAKGFDILGIKPVQRM
*
>ENSG00000129071|ENST00000249910
MGTTGLESLSLGDRGAAPTVTSSERLVPDPPNDLRKEDVAMELERVGEDEEQMMIKRSSE
CNPLLQEPIASAQFGATAGTECRKSVPCGWERVVKQRLFGKTAGRFDVYFISPQGLKFRS
KSSLANYLHKNGETSLKPEDFDFTVLSKRGIKSRYKDCSMAALTSHLQNQSNNSNWNLRT
RSKCKKDVFMPPSSSSELQESRGLSNFTSTHLLLKEDEGVDDVNFRKVRKPKGKVTILKG
IPIKKTKKGCRKSCSGFVQSDSKRESVCNKADAESEPVAQKSQLDRTVCISDAGACGETL
SVTSEENSLVKKKERSLSSGSNFCSEQKTSGIINKFCSAKDSEHNEKYEDTFLESEEIGT
KVEVVERKEHLHTDILKRGSEMDNNCSPTRKDFTGEKIFQEDTIPRTQIERRKTSLYFSS
KYNKEALSPPRRKAFKKWTPPRSPFNLVQETLFHDPWKLLIATIFLNRTSGKMAIPVLWK
FLEKYPSAEVARTADWRDVSELLKPLGLYDLRAKTIVKFSDEYLTKQWKYPIELHGIGKY
GNDSYRIFCVNEWKQVHPEDHKLNKYHDWLWENHEKLSLS*
>ENSG00000128713|ENST00000249504
MNDFDECGQSAASMYLPGCAYYVAPSDFASKPSFLSQPSSCQMTFPYSSNLAPHVQPVRE
VAFRDYGLERAKWPYRGGGGGGSAGGGSSGGGPGGGGGGAGGYAPYYAAAAAAAAAAAAA
EEAAMQRELLPPAGRRPDVLFKAPEPVCAAPGPPHGPAGAASNFYSAVGRNGILPQGFDQ
FYEAAPGPPFAGPQPPPPPAPPQPEGAADKGDPRTGAGGGGGSPCTKATPGSEPKGAAEG
SGGDGEGPPGEAGAEKSSSAVAPQRSRKKRCPYTKYQIRELEREFFFNVYINKEKRLQLS
RMLNLTDRQVKIWFQNRRMKEKKLNRDRLQYFTGNPLF*
>ENSG00000114942|ENST00000236957
MGFGDLKSPAGLQVLNDYLADKSYIEGYVPSQADVAVFEAVSSPPPADLCHALRWYNHIK
SYEKEKASLPGVKKALGKYGPADVEDTTGSGATDSKDDDDIDLFGSDDEEESEEAKRLRE
ERLAQYESKKAKKPALVAKSSILLDVKPWDDETDMAKLEECVRSIQADGLVWGSSKLVPV
GYGIKKLQIQCVVEDDKVGTDMLEEQITAFEDYVQSMDVAAFNKI*
>ENSG00000129028|ENST00000249861
MPARCVAAHCGNTTKSGKSLFRFPKDRAVRLLWDRFVRGCRADWYGGNDRSVICSDHFAP
ACFDVSSVIQKNLRFSQRLRLVAGAVPTLHRVPAPAPKRGEEGDQAGRLDTRGELQAARH
SEAAPGPVSCTRPRAGKQAAASQITCENELVQTQPHADNPSNTVTSVPTHCEEGPVHKST
QISLKRPRHRSVGIQAKVKAFGKRLCNATTQTEELWSRTSSLFDIYSSDSETDTDWDIKS
EQSDLSYMAVQVKEETC*
>ENSG00000111704|ENST00000229307
MSVDPACPQSLPCFEASDCKESSPMPVICGPEENYPSLQMSSAEMPHTETVSPLPSSMDL
LIQDSPDSSTSPKGKQPTSAEKSVAKKEDKVPVKKQKTRTVFSSTQLCVLNDRFQRQKYL
SLQQMQELSNILNLSYKQVKTWFQNQRMKSKRWQKNNWPKNSNGVTQKASAPTYPSLYSS
YHQGCLVNPTGNLPMWSNQTWNNSTWSNQTQNIQSWSNHSWNTQTWCTQSWNNQAWNSPF
YNCGEESLQSCMQFQPNSPASDLEAALEAAGEGLNVIQQTTRYFSTPQTMDLFLNYSMNM
QPEDV*

>ENSG00000130803|ENST00000247956
MAALSPTFATSTQDSTCLQDSEFPVSSKDHSCPQNLDLFVCSGLEPHTPSVGSQESVTFQ
DVAVDFTEKEWPLLDSSQRKLYKDVMLENYSNLTSLGYQVGKPSLISHLEQEEEPRTEER
GAHQGACADWETPSKTKWSLLMEDIFGKETPSGVTMERAGLGEKSTEYAHLFEVFGMDPH
LTQPMGRHAGKRPYHRRDYGVAFKGRPHLTQHMSMYDGRKMHECHQCQKAFTTSASLTRH
RRIHTGEKPYECSDCGKAFNDPSALRSHARTHLKEKPFDCSQCGNAFRTLSALKIHMRVH
TGERPYKCDQCGKAYGRSCHLIAHKRTHTGERPYECHDCGKAFQHPSHLKEHVRNHTGEK
PYACTQCGKAFRWKSNFNLHKKNHMVEKTYECKECGKSFGDLVSRRKHMRIHIVKKPVEC
RQCGKTFRNQSILKTHMNSHTGEKPYGCDLCGKAFSASSNLTAHRKIHTQERRYECAACG
KVFGDYLSRRRHMSVHLVKKRVECRQCGKAFRNQSTLKTHMRSHTGEKPYECDHCGKAFS
IGSNLNVHRRIHTGEKPYECLVCGKAFSDHSSLRSHVKTHRGEKLFVSSVWKRLQ*
>ENSG00000129245|ENST00000250113
MGGLASGGDVEPGLPVEVRGSNGAFYKGFVKDVHEDSVTIFFENNWQSERQIPFGDVRLP
PPADYNKEITEGDEVEVYSRANEQEPCGWWLARVRMMKGDFYVIEYAACDATYNEIVTLE
RLRPVNPNPLATKGSFFKVTMAVPEDLREACSNENVHKEFKKALGANCIFLNITNSELFI
LSTTEAPVKRASLLGDMHFRSLRTKLLLMSRNEEATKHLETSKQLAAAFQEEFTVREDLM
GLAIGTHGANIQQARKVPGVTAIELGEETCTFRIYGETPEACRQARSYLEFSEDSVQVPR
NLVGKVIGKNGKVIQEIVDKSGVVRVRVEGDNDKKNPREEGMVPFIFVGTRENISNAQAL
LEYHLSYLQEVEQLRLERLQIDEQLRQIGLGFRPPGSGRGSGGSDKAGYSTDESSSSSLH
ATRTYGGSYGGRGRGRRTGGPAYGPSSDVSTASETESEKREEPNRAGPGDRDPPTRGEES
RRRPTGGRGRGPPPAPRPTSRYNSSSISSVLKDPDSNPYSLLDTSEPEPPVDSEPGEPPP
ASARRRRSRRRRTDEDRTVMDGGLESDGPNMTENGLEDESRPQRRNRSRRRRNRGNRTDG
SISGDRQPVTVADYISRAESQSRQRPPLERTKPSEDSLSGQKGDSVSKLPKGPSENGELS
APLELGSMVNGVS*
>ENSG00000159905|ENST00000251269
MISPSLELLHSGLCKFPEVEGKMTTFKEAVTFKDVAVVFTEEELGLLDPAQRKLYRDVML
ENFRNLLSVGNQPFHQDTFHFLGKEKFWKMKTTSQREGNSGGKIQIEMETVPEAGPHEEW
SCQQIWEQIASDLTRSQNSIRNSSQFFKEGDVPCQIEARLSISHVQQKPYRCNECKQSFS
DVSVFDLHQQSHSGEKSHTCGECGKSFCYSPALHIHQRVHMGEKCYKCDVCGKEFNQSSH
LQTHQRVHTGEKPFKCGQCGKGFHSRSALNVHCKLHTGEKPYNCEECGKAFIHDSQLQEH
QRIHTGEKPFKCDICGKSFRVRSRLNRHSMVHTGEKPFRCDTCGKNFRQRSALNSHSMVH
IEEKPYKCEQCGKGFICRRDFCKHQMVHTGEKPYNCKECGKTFRWSSCLLNHQQVHSGQK
SFKCEECGKGFYTNSRRSSHQRSHNGEKPYNCEECGKDYKRRLDLEFHQRVHTGERPYNC
KECGKSFGWASCLLKHQRLHSGEKPFKCEECGKRFTQSSQLHSHQTCHTGEKLYKCEQCE
KGYNSKFNLDMHQRVHGGERPYNCKECGKSFGWASCLLKHQRLHSGEKPLKSGVWEEIYS
EFTASFTSVSLCGRKAI*
>ENSG00000121068|ENST00000240328
MREPALAASAMAYHPFHAPRPADFPMSAFLAAAQPSFFPALALPPGALAKPLPDPGLAGA
AAAAAAAAAAAEAGLHVSALGPHPPAAHLRSLKSLEPEDEVEDDPKVTLEAKELWDQFHK
LGTEMVITKSGRRMFPPFKVRVSGLDKKAKYILLMDIVAADDCRYKFHNSRWMVAGKADP
EMPKRMYIHPDSPATGEQWMAKPVAFHKLKLTNNISDKHGFTILNSMHKYQPRFHIVRAN
DILKLPYSTFRTYVFPETDFIAVTAYQNDKITQLKIDNNPFAKGFRDTGNGRREKRKQLT
LPSLRLYEEHCKPERDGAESDASSCDPPPAREPPTSPGAAPSPLRLHRARAEEKSCAADS
DPEPERLSEERAGAPLGRSPAPDSASPTRLTEPERARERRSPERGKEPAESGGDGPFGLR

SLEKERAEARRKDEGRKEAAEGKEQGLAPLVVQTDSASPLGAGHLPGLAFSSHLHGQQFF
GPLGAGQPLFLHPGQFTMGPGAFSAMGMGHLLASVAGGGNGGGGGPGTAAGLDAGGLGPA
ASAASTAAPFPFHLSQHMLASQGIPMPTFGGLFPYPYTYMAAAAAAASALPATSAAAAAA
AAAGSLSRSPFLGSARPRLRFSPYQIPVTIPPSTSLLTTGLASEGSKAAGGNSREPSPLP
ELALRKVGAPSRGALSPSGSAKEAANELQSIQRLVSGLESQRALSPGRESPK*
>ENSG00000108771|ENST00000251642
MELRSYQWEVIMPALEGKNIIIWLPTGAGKTRAAAYVAKRHLETVDGAKVVVLVNRVHLV
TQHGEEFRRMLDGRWTVTTLSGDMGPRAGFGHLARCHDLLICTAELLQMALTSPEEEEHV
ELTVFSLIVVDECHHTHKDTVYNVIMSQYLELKLQRAQPLPQVLGLTASPGTGGASKLDG
AINHVLQLCANLDTWCIMSPQNCCPQLQEHSQQPCKQYNLCHRRSQDPFGDLLKKLMDQI
HDHLEMPELSRKFGTQMYEQQVVKLSEAAALAGLQEQRVYALHLRRYNDALLIHDTVRAV
DALAALQDFYHREHVTKTQILCAERRLLALFDDRKNELAHLATHGPENPKLEMLEKILQR
QFSSSNSPRGIIFTRTRQSAHSLLLWLQQQQGLQTVDIRAQLLIGAGNSSQSTHMTQRDQ
QEVIQKFQDGTLNLLVATSVAEEGLDIPHCNVVVRYGLLTNEISMVQARGRARADQSVYA
FVATEGSRELKRELINEALETLMEQAVAAVQKMDQAEYQAKIRDLQQAALTKRAAQAAQR
ENQRQQFPVEHVQLLCINCMVAVGHGSDLRKVEGTHHVNVNPNFSNYYNVSRDPVVINKV
FKDWKPGGVISCRNCGEVWGLQMIYKSVKLPVLKVRSMLLETPQGRIQAKKWSRVPFSVP
DFDFLQHCAENLSDLSLD*
>ENSG00000112365|ENST00000230122
MAETSPEPSGQLVVHSDAHSDTVLASFEDQRKKGFLCDITLIVENVHFRAHKALLAASSE
YFSMMFAEEGEIGQSIYMLEGMVADTFGILLEFIYTGYLHASEKSTEQILATAQFLKVYD
LVKAYTDFQNNHSSPKPTTLNTAGAPVVVISNKKNDPPKRKRGRPKKVNTLQEEKSELAA
EEEIQLRVNNSVQNRQNFVVKGDSGVLNEQIAAKEKEESEPTCEPSREEEMPVEKDENYD
PKTEDGQASQSRYSKRRIWRSVKLKDYKLVGDQEDHGSAKRICGRRKRPGGPEARCKDCG
KVFKYNHFLAIHQRSHTGERPFKCNECGKGFAQKHSLQVHTRMHTGERPYTCTVCSKALT
TKHSLLEHMSLHSGQKSFTCDQCGKYFSQNRQLKSHYRVHTGHSLPECKDCHRKFMDVSQ
LKKHLRTHTGEKPFTCEICGKSFTAKSSLQTHIRIHRGEKPYSCGICGKSFSDSSAKRRH
CILHTGKKPFSCPECNLQFARLDNLKAHLKIHSKEKHASDASSISGSSNTEEVRNILQLQ
PYQLSTSGEQEIQLLVTDSVHNINFMPGPSQGISIVTAESSQNMTADQAANLTLLTQQPE
QLQNLILSAQQEQTEHIQSLNMIESQMGPSQTEPVHVITLSKETLEHLHAHQEQTEELHL
ATSTSDPAQHLQLTQEPGPPPPTHHVPQPTPLGQEQS*
>ENSG00000185404|ENST00000243810
MAGGGSDLSTRLFTEDQDVDEGLVYDTVFKHFKRHKLEISNAIKKTFPFLEGLRDRELIT
NKMFEDSEDSCRNLVPVQRVVYNVLSELEKTFNLSVLEALFSEVNMQEYPDLIHIYKSFK
NAIQDKLSFQESDRKEREERPDIKLSLKQGEVPESPEARKESDQACGKMDTVDIANNSTL
GKPKRKRRKKKGHGWSRMGTRTQKNNQQNDNSKADGQLVSSEKKANMNLKDLSKIRGRKR
GKPGTHFTQSDRAPQKRVRSRASRKHKDETVDFQAPLLPVTCGGVKGILHKEKLEQGTLA
KCIQTEDGKWFTPMEFEIKGGYARSKNWRLSVRCGGWPLRRLMEEGSLPNPPRIYYRNKK
RILKSQNNSSVDPCMRNLDECEVCRDGGELFCCDTCSRVFHEDCHIPPVESEKTPWNCIF
CRMKESPGSQQCCQESEVLERQMCPEEQLKCEFLLLKVYCCSESSFFAKIPYYYYIREAC
QGLKEPMWLDKIKKRLNEHGYPQVEGFVQDMRLIFQNHRASYKYKDFGQMGLRLEAEFEK
DFKEVFAIQETNGNS*
>ENSG00000127311|ENST00000247815
MARSSPYLRQLQGPLLPPRDLVEEDDDYLNDDVEEDEESVFIDAEELCSGGVKAGSLPGC

LRVSICDENTQETCKVFGRFPITGAWWRVKVQVKPVVGSRSYQYQVQGFPSYFLQSDMSP
PNQKHICALFLKECEVSSDDVNKFLTWVKEVSNYKNLNFENLRETLRTFHKETGRKDQKQ
PTQNGQEELFLDNEMSLPLENTIPFRNVMTALQFPKIMEFLPVLLPRHFKWIIGSGSKEM
LKEIEEILGTHPWKLGFSKITYREWKLLRCEASWIAFCQCESLLQLMTDLEKNALIMYSR
LKQICREDGHTYVEVNDLTLTLSNHMSFHAASESLKFLKDIGVVTYEKSCVFPYDLYHAE
RAIAFSICDLMKKPPWHLCVDVEKVLASIHTTKPENSSDDALNESKPDEVRLENPVDVVD
TQDNGDHIWTNGENEINAEISEVQLDQDQVEVPLDRDQVAALEMICSNPVTVISGKGGCG
KTTIVSRLFKHIEQLEEREVKKACEDFEQDQNASEEWITFTEQSQLEADKAIEVLLTAPT
GKAAGLLRQKTGLHAYTLCQVNYSFYSWTQTMMTTNKPWKFSSVRVLVVDEGSLVSVGIF
KSVLNLLCEHSKLSKLIILGDIRQLPSIEPGNLLKDLFETLKSRNCAIELKTNHRAESQL
IVDNATRISRRQFPKFDAELNISDNPTLPISIQDKTFIFVRLPEEDASSQSSKTNHHSCL
YSAVKTLLQENNLQNAKTSQFIAFRRQDCDLINDCCCKHYTGHLTKDHQSRLVFGIGDKI
CCTRNAYLSDLLPENISGSQQNNDLDASSEDFSGTLPDFAKNKRDFESNVRLCNGEIFFI
TNDVTDVTFGKRRSLTINNMAGLEVTVDFKKLMKYCRIKHAWARTIHTFQGSEEQTVVYV
VGKAGRQHWQHVYTAVTRGRCRVYVIAEESQLRNAIMKNSFPRKTRLKHFLQSKLSSSGA
PPADFPSPRKSSGDSGGPSTPSASPLPVVTDHAMTNDVTWSEASSPDERTLTFAERWQLS
SPDGVDTDDDLPKSRASKRTCGVNDDESPSKIFMVGESPQVSSRLQNLRLNNLIPRQLFK
PTDNQET*
>ENSG00000100968|ENST00000250373
MGAASCEDEELEFKLVFGEEKEAPPLGAGGLGEELDSEDAPPCCRLALGEPPPYGAAPIG
IPRPPPPRPGMHSPPPRPAPSPGTWESQPARSVRLGGPGGGAGGAGGGRVLECPSIRITS
ISPTPEPPAALEDNPDAWGDGSPRDYPPPEGFGGYREAGGQGGGAFFSPSPGSSSLSSWS
FFSDASDEAALYAACDEVESELNEAASRFGLGSPLPSPRASPRPWTPEDPWSLYGPSPGG
RGPEDSWLLLSAPGPTPASPRPASPCGKRRYSSSGTPSSASPALSRRGSLGEEGSEPPPP
PPLPLARDPGSPGPFDYVGAPPAESIPQKTRRTSSEQAVALPRSEEPASCNGKLPLGAEE
SVAPPGGSRKEVAGMDYLAVPSPLAWSKARIGGHSPIFRTSALPPLDWPLPSQYEQLELR
IEVQPRAHHRAHYETEGSRGAVKAAPGGHPVVKLLGYSEKPLTLQMFIGTADERNLRPHA
FYQVHRITGKMVATASYEAVVSGTKVLEMTLLPENNMAANIDCAGILKLRNSDIELRKGE
TDIGRKNTRVRLVFRVHVPQGGGKVVSVQAASVPIECSQRSAQELPQVEAYSPSACSVRG
GEELVLTGSNFLPDSKVVFIERGPDGKLQWEEEATVNRLQSNEVTLTLTVPEYSNKRVSR
PVQVYFYVSNGRRKRSPTQSFRFLPVICKEEPLPDSSLRGFPSASATPFGTDMDFSPPRP
PYPSYPHEDPACETPYLSEGFGYGMPPLYPQTGPPPSYRPGLRMFPETRGTTGCAQPPAV
SFLPRPFPSDPYGGRGSSFSLGLPFSPPAPFRPPPLPASPPLEGPFPSQSDVHPLPAEGY
NKVGPGYGPGEGAPEQEKSRGGYSSGFRDSVPIQGITLEEVSEIIGRDLSGFPAPPGEEP
PA*
>ENSG00000158055|ENST00000236255
MWMNSILPIFLFRSVRLLKNDPVNLQKFSYTSEDEAWKTYLENPLTAATKAMMRVNGDDD
SVAALSFLYDYYMGPKEKRILSSSTGGRNDQGKRYYHGMEYETDLTPLESPTHLMKFLTE
NVSGTPEYPDLLKKNNLMSLEGALPTPGKAAPLPAGPSKLEAGSVDSYLLPTTDMYDNGS
LNSLFESIHGVPPTQRWQPDSTFKDDPQESMLFPDILKTSPEPPCPEDYPSLKSDFEYTL
GSPKAIHIKSGESPMAYLNKGQFYPVTLRTPAGGKGLALSSNKVKSVVMVVFDNEKVPVE
QLRFWKHWHSRQPTAKQRVIDVADCKENFNTVEHIEEVAYNALSFVWNVNEEAKVFIGVN
CLSTDFSSQKGVKGVPLNLQIDTYDCGLGTERLVHRAVCQIKIFCDKGAERKMRDDERKQ
FRRKVKCPDSSNSGVKGCLLSGFRGNETTYLRPETDLETPPVLFIPNVHFSSLQRSGGAA

PSAGPSSSNRLPLKRTCSPFTEEFEPLPSKQAKEGDLQRVLLYVRRETEEVFDALMLKTP
DLKGLRNAISEKYGFPEENIYKVYKKCKRGILVNMDNNIIQHYSNHVAFLLDMGELDGKI
QIILKEL*
>ENSG00000125870|ENST00000246071
MDIRPNHTIYINNMNDKIKKEELKRSLYALFSQFGHVVDIVALKTMKMRGQAFVIFKELG
SSTNALRQLQGFPFYGKPMRIQYAKTDSDIISKMRGTFADKEKKKEKKKAKTVEQTATTT
NKKPGQGTPNSANTQGNSTPNPQVPDYPPNYILFLNNLPEETNEMMLSMLFNQFPGFKEV
RLVPGRHDIAFVEFENDGQAGAARDALQGFKITPSHAMKITYAKK*
>ENSG00000100227|ENST00000252115
MADISLDELIRKRGAAAKGRLNARPGVGGVRSRVGIQQGLLSQSTRTATFQQRFDARQKI
GLSDARLKLGVKDAREKLLQKDARFRIKGKVQDAREMLNSRKQQTTVPQKPRQVADAREK
ISLKRSSPAAFINPPIGTVTPALKLTKTIQVPQQKAMAPLHPHPAGMRINVVNNHQAKQN
LYDLDEDDDGIASVPTKQMKFAASGGFLHHMAGLSSSKLSMSKALPLTKVVQNDAYTAPA
LPSSIRTKALTNMSRTLVNKEEPPKELPAAEPVLSPLEGTKMTVNNLHPRVTEEDIVELF
CVCGALKRARLVHPGVAEVVFVKKDDAITAYKKYNNRCLDGQPMKCNLHMNGNVITSDQP
ILLRLSDSPSMKKESELPRRVNSASSSNPPAEVDPDTILKALFKSSGASVTTQPTEFKIK
L*
>ENSG00000128652|ENST00000249440
MLFEQGQQALELPECTMQKAAYYENPGLFGGYGYSKTTDTYGYSTPHQPYPPPAAASSLD
TDYPGSACSIQSSAPLRAPAHKGAELNGSCMRPGTGNSQGGGGGSQPPGLNSEQQPPQPP
PPPPTLPPSSPTNPGGGVPAKKPKGGPNASSSSATISKQIFPWMKESRQNSKQKNSCATA
GESCEDKSPPGPASKRVRTAYTSAQLVELEKEFHFNRYLCRPRRVEMANLLNLTERQIKI
WFQNRRMKYKKDQKAKGILHSPASQSPERSPPLGGAAGHVAYSGQLPPVPGLAYDAPSPP
AFAKSQPNMYGLAAYTAPLSSCLPQQKRYAAPEFEPHPMASNGGGFASANLQGSPVYVGG
NFVESMAPASGPVFNLGHLSHPSSASVDYSCAAQIPGNHHHGPCDPHPTYTDLSAHHSSQ
GRLPEAPKLTHL*
>ENSG00000127022|ENST00000247461
MEGKWLLCMLLVLGTAIVEAHDGHDDDVIDIEDDLDDVIEEVEDSKPDTTAPPSSPKVTY
KAPVPTGEVYFADSFDRGTLSGWILSKAKKDDTDDEIAKYDGKWEVEEMKESKLPGDKGL
VLMSRAKHHAISAKLNKPFLFDTKPLIVQYEVNFQNGIECGGAYVKLLSKTPELNLDQFH
DKTPYTIMFGPDKCGEDYKLHFIFRHKNPKTGIYEEKHAKRPDADLKTYFTDKKTHLYTL
ILNPDNSFEILVDQSVVNSGNLLNDMTPPVNPSREIEDPEDRKPEDWDERPKIPDPEAVK
PDDWDEDAPAKIPDEEATKPEGWLDDEPEYVPDPDAEKPEDWDEDMDGEWEAPQIANPRC
ESAPGCGVWQRPVIDNPNYKGKWKPPMIDNPSYQGIWKPRKIPNPDFFEDLEPFRMTPFS
AIGLELWSMTSDIFFDNFIICADRRIVDDWANDGWGLKKAADGAAEPGVVGQMIEAAEER
PWLWVVYILTVALPVFLVILFCCSGKKQTSGMEYKKTDAPQPDVKEEEEEKEEEKDKGDE
EEEGEEKLEEKQKSDAEEDGGTVSQEEEDRKPKAEEDEILNRSPRNRKPRRE*
>ENSG00000119705|ENST00000238688
MAASAARGAAALRRSINQPVAFVRRIPWTAASSQLKEHFAQFGHVRRCILPFDKETGFHR
GLGWVQFSSEEGLRNALQQENHIIDGVKVHTRRPKLPQTSDDEKKDF*
>ENSG00000112592|ENST00000230354
MDQNNSLPPYAQGLASPQGAMTPGIPIFSPMMPYGTGLTPQPIQNTNSLSILEEQQRQQQ
QQQQQQQQQQQQQQQQQQQQQQQQQQQQQQQQQQQQQQAVAAAAVQQSTSQQATQGTSGQAPQ
LFHSQTLTTAPLPGTTPLYPSPMTPMTPITPATPASESSGIVPQLQNIVSTVNLGCKLDL

KTIALRARNAEYNPKRFAAVIMRIREPRTTALIFSSGKMVCTGAKSEEQSRLAARKYARV
VQKLGFPAKFLDFKIQNMVGSCDVKFPIRLEGLVLTHQQFSSYEPELFPGLIYRMIKPRI
VLLIFVSGKVVLTGAKVRAEIYEAFENIYPILKGFRKTT*
>ENSG00000111269|ENST00000228865
MDDSKVVGGKVKKPGKRGRKPAKIDLKAKLERSRQSARECRARKKLRYQYLEELVSSRER
AICALREELEMYKQWCMAMDQGKIPSEIKALLTGEEQNKSQQNSSRHTKAGKTDANSNSW
*
>ENSG00000111701|ENST00000229304
MTSEKGPSTGDPTLRRRIEPWEFDVFYDPRELRKEACLLYEIKWGMSRKIWRSSGKNTTN
HVEVNFIKKFTSERDFHPSMSCSITWFLSWSPCWECSQAIREFLSRHPGVTLVIYVARLF
WHMDQQNRQGLRDLVNSGVTIQIMRASEYYHCWRNFVNYPPGDEAHWPQYPPLWMMLYAL
ELHCIILSLPPCLKISRRWQNHLTFFRLHLQNCHYQTIPPHILLATGLIHPSVAWR*
>ENSG00000100722|ENST00000251038
MEIGTEISRKIRSAIKGKLQELGAYVDEELPDYIMVMVANKKSQDQMTEDLSLFLGNNTI
RFTVWLHGVLDKLRSVTTEPSSLKSSDTNIFDSNVPSNKSNFSRGDERRHEAAVPPLAIP
SARPEKRDSRVSTSSQESKTTNVRQTYDDGAATRLMSTVKPLREPAPSEDVIDIKPEPDD
LIDEDLNFVQENPLSQKKPTVTLTYGSSRPSIEIYRPPASRNADSGVHLNRLQFQQQQNS
IHAAKQLDMQSSWVYETGRLCEPEVLNSLEETYSPFFRNNSEKMSMEDENFRKRKLPVVS
SVVKVKKFNHDGEEEEEDDDYGSRTGSISSSVSVPAKPERRPSLPPSKQANKNLILKAIS
EAQESVTKTTNYSTVPQKQTLPVAPRTRTSQEELLAEVVQGQSRTPRISPPIKEEETKGD
SVEKNQGTQQRQLLSRLQIDPVMAETLQMSQDYYDMESMVHADTRSFILKKPKLSEEVVV
APNQESGMKTADSLRVLSGHLMQTRDLVQPDKPASPKFIVTLDGVPSPPGYMSDQEEDMC
FEGMKPVNQTAASNKGLRGLLHPQQLHLLSRQLEDPNGSFSNAEMSELSVAQKPEKLLER
CKYWPACKNGDECAYHHPISPCKAFPNCKFAEKCLFVHPNCKYDAKCTKPDCPFTHVSRR
IPVLSPKPAVAPPAPPSSSQLCRYFPACKKMECPFYHPKHCRFNTQCTRPDCTFYHPTIN
VPPRHALKWIRPQTSE*
>ENSG00000121211|ENST00000240488
MSKKKGLSAEEKRTRMMEIFSETKDVFQLKDLEKIAPKEKGITAMSVKEVLQSLVDDGMV
DCERIGTSNYYWAFPSKALHARKHKLEVLESQLSEGSQKHASLQKSIEKAKIGRCETEER
TRLAKELSSLRDQREQLKAEVEKYKDCDPQVVEEIRQANKVAKEAANRWTDNIFAIKSWA
KRKFGFEENKIDRTFGIPEDFDYID*
>ENSG00000116035|ENST00000234392
MGDGGAERDRGPARRAESGGGGGRCGDRSGAGDLRADGGGHSPTEVAGTSASSPAGSRES
GADSDGQPGPGEADHCRRILVRDAKGTIREIVLPKGLDLDRPKRTRTSFTAEQLYRLEME
FQRCQYVVGRERTELARQLNLSETQVKVWFQNRRTKQKKDQSRDLEKRASSSASEAFATS
NILRLLEQGRLLSVPRAPSLLALTPSLPGLPASHRGTSLGDPRNSSPRLNPLSSASASPP
LPPPLPAVCFSSAPLLDLPAGYELGSSAFEPYSWLERKVGSASSCKKANT*
>ENSG00000127528|ENST00000248071
MALSEPILPSFSTFASPCRERGLQERWPRAEPESGGTDDDLNSVLDFILSMGLDGLGAEA
APEPPPPPPPPAFYYPEPGAPPPYSAPAGGLVSELLRPELDAPLGPALHGRFLLAPPGRL
VKAEPPEADGGGGYGCAPGLTRGPRGLKREGAPGPAASCMRGPGGRPPPPPDTPPLSPDG
PARLPAPGPRASFPPPFGGPGFGAPGPGLHYAPPAPPAFGLFDDAAAAAAALGLAPPAAR
GLLTPPASPLELLEAKPKRGRRSWPRKRTATHTCSYAGCGKTYTKSSHLKAHLRTHTGEK
PYHCNWDGCGWKFARSDELTRHYRKHTGHRPFQCHLCDRAFSRSDHLALHMKRHM*

>ENSG00000123407|ENST00000243103
MGEHNLLNPGFVGPLVNIHTGDTFYFPNFRASGAQLPGLPSLSYPRRDNVCSLSWPSAEP
CNGYPQPYLGSPVSLNPPFGRTCELARVEDGKGYYREPCAEGGGGGLKREERGRDPGAGP
GAALLPLEPSGPPALGFKYDYAAGGGGGDGGGGAGPPHDPPSCQSLESDSSSSLLNEGNK
GAGAGDPGSLVSPLNPGGGLSASGAPWYPINSRSRKKRKPYSKLQLAELEGEFLVNEFIT
RQRRRELSDRLNLSDQQVKIWFQNRRMKKKRLLLREQALSFF*
>ENSG00000105717|ENST00000251203
MAAPPRPAPSPPAPRRLDTSDVLQQIMAITDQSLDEAQARKHALNCHRMKPALFSVLCEI
KEKTVVSIRGIQDEDPPDAQLLRLDNMLLAEGVCRPEKRGRGGAVARAGTATPGGCPNDN
SIEHSDYRAKLSQIRQIYHSELEKYEQACREFTTHVTNLLQEQSRMRPVSPKEIERMVGA
IHGKFSAIQMQLKQSTCEAVMTLRSRLLDARRKRRNFSKQATEVLNEYFYSHLNNPYPSE
EAKEELARKGGLTISQVSNWFGNKRIRYKKNMGKFQEEATIYTGKTAVDTTEVGVPGNHA
SCLSTPSSGSSGPFPLPSAGDAFLTLRTLASLQPPPGGGCLQSQAQGSWQGATPQPATAS
PAGDPGSINSSTSN*
>ENSG00000117395|ENST00000236051
MDTPPLSDSESESDESLVTDRELQDAFSRGLLKPGLNVVLEGPKKAVNDVNGLKQCLAEF
KRDLEWVERLDVTLGPVPEIGGSEAPAPQNKDQKAVDPEDDFQREMSFYRQAQAAVLAVL
PRLHQLKVPTKRPTDYFAEMAKSDLQMQKIRQKLQTKQAAMERSEKAKQLRALRKYGKKV
QTEVLQKRQQEKAHMMNAIKKYQKGFSDKLDFLEGDQKPLAQRKKAGAKGQQMRKGPSAK
RRYKNQKFGFGGKKKGSKWNTRESYDDVSSFRAKTAHGRGLKRPGKKGSNKRPGKRTREK
MKNRTH*
>ENSG00000178982|ENST00000248342
MAMFEQMRANVGKLLKGIDRYNPENLATLERYVETQAKENAYDLEANLAVLKLYQFNPAF
FQTTVTAQILLKALTNLPHTDFTLCKCMIDQAHQEERPIRQILYLGDLLETCHFQAFWQA
LDENMDLLEGITGFEDSVRKFICHVVGITYQHIDRWLLAEMLGDLSDSQLKVWMSKYGWS
ADESGQIFICSQEESIKPKNIVEKIDFDSVSSIMASSQ*
>ENSG00000122728|ENST00000242310
MRPGCDLLLRAAATVTAAIMSDSDSEEDSSGGGPFTLAGILFGNISGAGQLEGESVLDDE
CKKHLAGLGALGLGSLITELTANEELTGTGGALVNDEGWIRSTEDAVDYSDINEVAEDES
QRHQQTMGSLQPLYHSDYDEDDYDADCEDIDCKLMPPPPPPPGPMKKDKDQDAITCVSES
GEDIILPSIIAPSFLASEKVDFSSYSDSESEMGPQEATQAESEDGKLTLPLAGIMQHDAT
KLLPSVTELFPEFRPGKVLRFLHLFGPGKNVPSVWRSARRKRKKHRELIQEEQIQEVECS
VESEVSQKSLWNYDYAPPPPPEQCLADDEITMMVPVESKFSQSTGDVDKVTDTKPRVAEW
RYGPARLWYDMLGVSEDGSGFDYGFKLRKTQHEPVIKSRMMEEFRKLEESNGTDLLADEN
FLMVTQLHWEDSIIWDGEDIKHKGTKPQGASLAGWLPSIKTRNVMAYNVQQGFAPTLDDD
KPWYSIFPIDNEDLVYGRWEDNIIWDAQAMPRLLEPPVLALDPNDENLILEIPDEKEEAT
SNSPSKESKKESSLKKSRILLGKTGVIREEPQQNMSQPEVKDPWNLSNDEYYFPKQQGLR
GTFGGNIIQHSIPAMELWQPFFPTHMGPIKIRQFHRPPLKKYSFGALSQPGPHSVQPLLK
HIKKKAKMREQERQASGGGELFFMRTPQDLTGKDGDLILAEYSEENGPLMMQVGMATKIK
NYYKRKPGKDPGAPDCKYGETVYCHTSPFLGSLHPGQLLQALENNLFRAPVYLHKMPETD
FLIIRTRQGYYIRELVDIFVVGQQCPLFEVPGPNSRRANMHIRDFLQVFIYRLFWKSKDR
PRRIRMEDIKKAFPSHSESSIRKRLKLCADFKRTGMDSNWWVLKSDFRLPTEEEIRAKVS
PEQCCAYYSMIAAKQRLKDAGYGEKSFFAPEEENEEDFQMKIDDEVHAAPWNTTRAFIAA
MKGKCLLEVTGVADPTGCGEGFSYVKIPNKPTQQKDDKEPQAVKKTVTGTDADLRRLSLK

NAKQLLRKFGVPEEEIKKLSRWEVIDVVRTMSTEQAHSGEGPMSKFARGSRFSVAEHQER
YKEECQRIFDLQNKVLSSTEVLSTDTDSISAEDSDFEEMGKNIENMLQNKKTSSQLSREW
EEQERKELRRMLLVAGSAASGNNHRDDVTASMTSLKSSATGHCLKIYRTFRDEEGKEYVR
CETVRKPAVIDAYVRIRTTKDEKFIQKFALFDEKHREEMRKERRRIQEQLRRLKRNQEKE
KLKGPPEKKPKKMKERPDLKLKCGACGAIGHMRTNKFCPLYYQTNVPPSKPVAMTEEQEE
ELEKTVIHNDNEELIKVEGTKIVFGKQLIENVHEVRRKSLVLKFPKQQLPPKKKRRVGTT
VHCDYLNIPHKSIHRRRTDPMVTLSSILESIINDMRDLPNTHPFHTPVNAKVVKDYYKII
TRPMDLQTLRENVRKCLYPSREEFREHLELIVKNSATYNGPKHSLTQISQSMLDLCDEKL
KEKEDKLARLEKAINPLLDDDDQVAFSFILDNIVTQKMMAVPDSWPFHHPVNKKFVPDYY
KMIVNPVDLETIRKNISKHKYQSRESFLDDVNLILANSVKYNGPESQYTKTAQEIVNICY
QTITEYDEHLTQLEKDICTAKEAALEEAELESLDPMTPGPYTSQPPDMYDTNTSLSTSRD
ASVFQDESNLSVLDISTATPEKQMCQGQGRLGEEDSDVDVEGYDDEEEDGKPKPPAPEGG
DGDLADEEEGTVQQPEASVLYEDLLISEGEDDEEDAGSDEEGDNPFSAIQLSESGSDSDV
GYGGIRPKQPFMLQHASGEHKDGHGK*
>ENSG00000121413|ENST00000240727
MLPLEKAFASPRSSPAPPDLPTPGSAAGVQQEEPETIPERTPADLEFSRLRFREFVYQEA
AGPHQTLARLHELCRQWLMPEARSKEQMLELLVLEQFLGILPDKVRPWVVAQYPESCKKA
ASLVEGLADVLEEPGMLLGSPAGSSSILSDGVYERHMDPLLLPGELASPSQALGAGEIPA
PSETPWLSPDPLFLEQRRVREAKTEEDGPANTEQKLKSFPEDPQHLGEWGHLDPAEENLK
SYRKLLLWGYQLSQPDAASRLDTEELRLVERDPQGSSLPEGGRRQESAGCACEEAAPAGV
LPELPTEAPPGDALADPPSGTTEEEEEQPGKAPDPQDPQDAESDSATGSQRQSVIQQPAP
DRGTAKLGTKRPHPEDGDGQSLEGVSSSGDSAGLEAGQGPGADEPGLSRGKPYACGECGE
AFAWLSHLMEHHSSHGGRKRYACQGCWKTFHFSLALAEHQKTHEKEKSYALGGARGPQPS
TREAQAGARAGGPPESVEGEAPPAPPEAQR*
>ENSG00000118503|ENST00000237289
MAEQVLPQALYLSNMRKAVKIRERTPEDIFKPTNGIIHHFKTMHRYTLEMFRTCQFCPQF
REIIHKALIDRNIQATLESQKKLNWCREVRKLVALKTNGDGNCLMHATSQYMWGVQDTDL
VLRKALFSTLKETDTRNFKFRWQLESLKSQEFVETGLCYDTRNWNDEWDNLIKMASTDTP
MARSGLQYNSLEEIHIFVLCNILRRPIIVISDKMLRSLESGSNFAPLKVGGIYLPLHWPA
QECYRYPIVLGYDSHHFVPLVTLKDSGPEIRAVPLVNRDRGRFEDLKVHFLTDPENEMKE
KLLKEYLMVIEIPVQGWDHGTTHLINAAKLDEANLPKEINLVDDYFELVQHEYKKWQENS
EQGRREGHAQNPMEPSVPQLSLMDVKCETPNCPFFMSVNTQPLCHECSERRQKNQNKLPK
LNSKPGPEGLPGMALGASRGEAYEPLAWNPEESTGGPHSAPPTAPSPFLFSETTAMKCRS
PGCPFTLNVQHNGFCERCHNARQLHASHAPDHTRHLDPGKCQACLQDVTRTFNGICSTCF
KRTTAEASSSLSTSLPPSCHQRSKSDPSRLVRSPSPHSCHRAGNDAPAGCLSQAARTPGD
RTGTSKCRKAGCVYFGTPENKGFCTLCFIEYRENKHFAAASGKVSPTASRFQNTIPCLGR
ECGTLGSTMFEGYCQKCFIEAQNQRFHEAKRTEEQLRSSQRRDVPRTTQSTSRPKCARAS
CKNILACRSEELCMECQHPNQRMGPGAHRGEPAPEDPPKQRCRAPACDHFGNAKCNGYCN
ECFQFKQMYG*
>ENSG00000132005|ENST00000254325
MATQAYTELQAAPPPSQPPQAPPQAQPQPPPPPPPAAPQPPQPPTAAATPQPQYVTELQS
PQPQAQPPGGQKQYVTELPAVPAPSQPTGAPTPSPAPQQYIVVTVSEGAMRASETVSEAS
PGSTASQTGVPTQVVQQVQGTQQRLLVQTSVQAKPGHVSPLQLTNIQVPQQALPTQRLVV
QSAAPGSKGGQVSLTVHGTQQVHSPPEQSPVQANSSSSKTAGAPTGTVPQQLQVHGVQQS

VPVTQERSVVQATPQAPKPGPVQPLTVQGLQPVHVAQEVQQLQQVPVPHVYSSQVQYVEG
GDASYTASAIRSSTYSYPETPLYTQTASTSYYEAAGTATQVSTPATSQAVASSGSMPMYV
SGSQVVASSTSTGAGASNSSGGGGSGGGGGGGGGGGGGGSGSTGGGGSGAGTYVIQGGYM
LGSASQSYSHTTRASPATVQWLLDNYETAEGVSLPRSTLYCHYLLHCQEQKLEPVNAASF
GKLIRSVFMGLRTRRLGTRGNSKYHYYGLRIKASSPLLRLMEDQQHMAMRGQPFSQKQRL
KPIQKMEGMTNGVAVGQQPSTGLSDISAQVQQYQQFLDASRSLPDFTELDLQGKVLPEGV
GPGDIKAFQVLYREHCEAIVDVMVNLQFTLVETLWKTFWRYNLSQPSEAPPLAVHDEAEK
RLPKAILVLLSKFEPVLQWTKHCDNVLYQGLVEILIPDVLRPIPSALTQAIRNFAKSLES
WLTHAMVNIPEEMLRVKVAAAGAFAQTLRRYTSLNHLAQAARAVLQNTAQINQMLSDLNR
VDFANVQEQASWVCRCEDRVVQRLEQDFKVTLQQQNSLEQWAAWLDGVVSQVLKPYQGSA
GFPKAAKLFLLKWSFYSSMVIRDLTLRSAASFGSFHLIRLLYDEYMYYLIEHRVAQAKGE
TPIAVMGEFANLATSLNPLDPDKDEEEEEEEEESEDELPQDISLAAGGESPALGPETLEPP
AKLARTDARGLFVQALPSS*
>ENSG00000134001|ENST00000256383
MPGLSCRFYQHKFPEVEDVVMVNVRSIAEMGAYVSLLEYNNIEGMILLSELSRRRIRSIN
KLIRIGRNECVVVIRVDKEKGYIDLSKRRVSPEEAIKCEDKFTKSKTVYSILRHVAEVLE
YTKDEQLESLFQRTAWVFDDKYKRPGYGAYDAFKHAVSDPSILDSLDLNEDEREVLINNI
NRRLTPQAVKIRADIEVACYGYEGIDAVKEALRAGLNCSTENMPIKINLIAPPRYVMTTT
TLERTEGLSVLSQAMAVIKEKIEEKRGVFNVQMEPKVVTDTDETELARQMERLERENAEV
DGDDDAEEMEAKAED*
>ENSG00000115112|ENST00000263707
MLFWHTQPEHYNQHNSGSYLRDVLALPIFKQEEPQLSPENEARLPPLQYVLCAATSPAVK
LHEETLTYLNQGQSYEIRLLENRKLGDFQDLNTKYVKSIIRVVFHDRRLQYTEHQQLEGW
RWSRPGDRILDIDIPLSVGILDPRASPTQLNAVEFLWDPAKRASAFIQVHCISTEFTPRK
HGGEKGVPFRVQIDTFKQNENGEYTEHLHSASCQIKVFKPKGADRKQKTDREKMEKRTAQ
EKEKYQPSYETTILTECSPWPDVAYQVNSAPSPSYNGSPNSFGLGEGNASPTHPVEALPV
GSDHLLPSASIQDAQQWLHRNRFSQFCRLFASFSGADLLKMSRDDLVQICGPADGIRLFN
AIKGRNVRPKMTIYVCQELEQNRVPLQQKRDGSGDSNLSVYHAIFLEELTTLELIEKIAN
LYSISPQHIHRVYRQGPTGIHVVVSNEMVQNFQDESCFVLSTIKAESNDGYHIILKCGL*
>ENSG00000037897|ENST00000257848
MAAETRNVAGAEAPPPQKRYYRQRAHSNPMADHTLRYPVKPEEMDWSELYPEFFAPLTQN
QSHDDPKDKKEKRAQAQVEFADIGCGYGGLLADKDVLPLPRPTFQADKAQVANHQSHPAS
RICLRAKSWGAGVYHNRCAGATRLDVHSFRRAPTV*
>ENSG00000093009|ENST00000263201
MFVSDFRKEFYEVVQSQRVLLFVASDVDALCACKILQALFQCDHVQYTLVPVSGWQELET
AFLEHKEQFHYFILINCGANVDLLDILQPDEDTIFFVCDTHRPVNVVNVYNDTQIKLLIK
QDDDLEVPAYEDIFRDEEEDEEHSGNDSDGSEPSEKRTRLEEEIVEQTMRRRQRREWEAR
RRDILFDYEQYEYHGTSSAMVMFELAWMLSKDLNDMLWWAIVGLTDQWVQDKITQMKYVT
DVGVLQRHVSRHNHRNEDEENTLSVDCTRISFEYDLRLVLYQHWSLHDSLCNTSYTAARF
KLWSVHGQKRLQEFLADMGLPLKQVKQKFQAMDISLKENLREMIEESANKFGMKDMRVQT
FSIHFGFKHKFLASDVVFATMSLMESPEKDGSGTDHFIQALDSLSRSNLDKLYHGLELAK
KQLRATQQTIASCLCTNLVISQGPFLYCSLMEGTPDVMLFSRPASLSLLSKHLLKSFVCS
TKNRRCKLLPLVMAAPLSMEHGTVTVVGIPPETDSSDRKNFFGRAFEKAAESTSSRMLHN
HFDLSVIELKAEDRSKFLDALISLLS*

>ENSG00000134438|ENST00000256852
MHLPGCAPAMADGSFSLAGHLLRSPGGSTSRLHSIEAILGFTKDDGILGTFPAERGARGA
KERDRRLGARPACPKAPEEGSEPSPPPAPAPAPEYEGVVPEPTG*
>ENSG00000088205|ENST00000263239
MSHLPMKLLRKKIEKRNLKLRQRNLKFQGASNLTLSETQNGDVSEETMGSRKVKKSKQKP
MNVGLSETQNGGMSQEAVGNIKVTKSPQKSTVLTNGEAAMQSSNSESKKKKKKKRKMVND
AEPDTKKAKTENKGKSEEESAETTKETENNVEKPDNDEDESEVPSLPLGLTGAFEDTSFA
SLCNLVNENTLKAIKEMGFTNMTEIQHKSIRPLLEGRDLLAAAKTGSGKTLAFLIPAVEL
IVKLRFMPRNGTGVLILSPTRELAMQTFGVLKELMTHHVHTYGLIMGGSNRSAEAQKLGN
GINIIVATPGRLLDHMQNTPGFMYKNLQCLVIDEADRILDVGFEEELKQIIKLLPTRRQT
MLFSATQTRKVEDLARISLKKEPLYVGVDDDKANATVDGLEQGYVVCPSEKRFLLLFTFL
KKNRKKKLMVFFSSCMSVKYHYELLNYIDLPVLAIHGKQKQNKRTTTFFQFCNADSGTLL
CTDVAARGLDIPEVDWIVQYDPPDDPKEYIHRVGRTARGLNGRGHALLILRPEELGFLRY
LKQSKVPLSEFDFSWSKISDIQSQLEKLIEKNYFLHKSAQEAYKSYIRAYDSHSLKQIFN
VNNLNLPQVALSFGFKVPPFVDLNVNSNEGKQKKRGGGGGFGYQKTKKVEKSKIFKHISK
KSSDSRQFSH*
>ENSG00000133773|ENST00000256151
MAPVRRSAKWRPGGIEARGEGVSTVGYRNKNVRQKTWRPNHPQAFVGSVREGQGFAFRRK
LKIQQSYKKLLRKEKKAQTSLESQFTDRYPDNLKHLYLAEEERHRKQARKVDHPLSEQVH
QPLLEEQCSIDEPLFEDQCSFDQPQPEEQCIKTVNSFTIPKKNKKKTSNQKAQEEYEQIQ
AKRAAKKQEFERRKQEREEAQRQYKKKKMEVFKILNKKTKKGQPNLNVQMEYLLQKIQEK
C*
>ENSG00000055044|ENST00000264279
MLVLFETSVGYAIFKVLNEKKLQEVDSLWKEFETPEKANKIVKLKHFEKFQDTAEALAAF
TALMEGKINKQLKKVLKKIVKEAHEPLAVADAKLGGVIKEKLNLSCIHSPVVNELMRGIR
SQMDGLIPGVEPREMAAMCLGLAHSLSRYRLKFSADKVDTMIVQAISLLDDLDKELNNYI
MRCREWYGWHFPELGKIISDNLTYCKCLQKVGDRKNYASAKLSELLPEEVEAEVKAAAEI
SMGTEVSEEDICNILHLCTQVIEISEYRTQLYEYLQNRMMAIAPNVTVMVGELVGARLIA
HAGSLLNLAKHAASTVQILGAEKALFRALKSRRDTPKYGLIYHASLVGQTSPKHKGKISR
MLAAKTVLAIRYDAFGEDSSSAMGVENRAKLEARLRTLEDRGIRKISGTGKALAKTEKYE
HKSEVKTYDPSGDSTLPTCSKKRKIEQVDKEDEITEKKAKKAKIKVKVEEEEEEKVAEEE
ETSVKKKKKRGKKKHIKEEPLSEEEPCTSTAIASPEKKKKKKKKRENED*
>ENSG00000079134|ENST00000261600
MSPTPPLFSLPEARTRFTKSTREALNNKNIKPLLSTFSQVPGSENEKKCTLDQAFRGILE
EEIINHSSCENVLAIISLAIGGVTEGICTASTPFVLLGDVLDCLPLDQCDTIFTFVEKNV
ATWKSNTFYSAGKNYLLRMCNDLLRRLSKSQNTVFCGRIQLFLARLFPLSEKSGLNLQSQ
FNLENVTVFNTNEQESTLGQKHTEDREEGMDVEEGEMGDEEAPTTCSIPIDYNLYRKFWS
LQDYFRNPVQCYEKISWKTFLKYSEEVLAVFKSYKLDDTQASRKKMEELKTGGEHVYFAK
FLTSEKLMDLQLSDSNFRRHILLQYLILFQYLKGQVKFKSSNYVLTDEQSLWIEDTTKSV
YQLLSENPPDGERFSKMVEHILNTEENWNSWKNEGCPSFVKERTSDTKPTRIIRKRTAPE
DFLGKGPTKKILMGNEELTRLWNLCPDNMEACKSETREHMPTLEEFFEEAIEQADPENMV
ENEYKAVNNSNYGWRALRLLARRSPHFFQPTNQQFKSLPEYLENMVIKLAKELPPPSEEI
KTGEDEDEEDNDALLKENESPDVRRDKPVTGEQIEVFANKLGEQWKILAPYLEMKDSEIR
QIECDSEDMKMRAKQLLVAWQDQEGVHATPENLINALNKSGLSDLAESLTNDNETNS*

>ENSG00000129933|ENST00000262815
MAAQAAAAAQAAAAQAAQAEAADSWYLALLGFAEHFRTSSPPKIRLCVHCLQAVFPFKPP
QRIEARTHLQLGSVLYHHTKNSEQARSHLEKAWLISQQIPQFEDVKFEAASLLSELYCQE
NSVDAAKPLLRKAIQISQQTPYWHCRLLFQLAQLHTLEKDLVSACDLLGVGAEYARVVGS
EYTRALFLLSKGMLLLMERKLQEVHPLLTLCGQIVENWQGNPIQKESLRVFFLVLQVTHY
LDAGQVKSVKPCLKQLQQCIQTISTLHDDEILPSNPADLFHWLPKEHMCVLVYLVTVMHS
MQAGYLEKAQKYTDKALMQLEKLKMLDCSPILSSFQVILLEHIIMCRLVTGHKATALQEI
SQVCQLCQQSPRLFSNHAAQLHTLLGLYCVSVNCMDNAEAQFTTALRLTNHQELWAFIVT
NLASVYIREGNRHQEVLYSLLERINPDHSFPVSSHCLRAAAFYVRGLFSFFQGRYNEAKR
FLRETLKMSNAEDLNRLTACSLVLLGHIFYVLGNHRESNNMVVPAMQLASKIPDMSVQLW
SSALLRDLNKACGNAMDAHEAAQMHQNFSQQLLQDHIEACSLPEHNLITWTDGPPPVQFQ
AQNGPNTSLASLL*
>ENSG00000076067|ENST00000262031
MLLSVTSRPGISTFGYNRNNKKPYVSLAQQMAPPSPSNSTPNSSSGSNGNDQLSKTNLYI
RGLQPGTTDQDLVKLCQPYGKIVSTKAILDKTTNKCKGYGFVDFDSPSAAQKAVTALKAS
GVQAQMAKQQEQDPTNLYISNLPLSMDEQELEGMLKPFGQVISTRILRDTSGTSRGVGFA
RMESTEKCEAIITHFNGKYIKTPPGVPAPSDPLLCKFADGGPKKRQNQGKFVQNGRAWPR
NADMGVMALTYDPTTALQNGFYPAPYNITPNRMLAQSALSPYLSSPVSSYQRVTQTSPLQ
VPNPSWMHHHSYLMQPSGSVLTPGMDHPISLQPASMMGPLTQQLGHLSLSSTGTYMPTAA
AMQGAYISQYTPVPSSSVSVEESSGQQNQVAVDAPSEHGVYSFQFNK*
>ENSG00000108349|ENST00000264645
MADRRRQRASQDTEDEESGASGSDSGGSPLRGGGSCSGSAGGGGSGSLPSQRGGRTGALH
LRRVESGGAKSAEESECESEDGIEGDAVLSDYESAEDSEGEEGEYSEEENSKVELKSEAN
DAVNSSTKEEKGEEKPDTKSTVTGERQSGDGQESTEPVENKVGKKGPKHLDDDEDRKNPA
YIPRKGLFFEHDLRGQTQEEEVRPKGRQRKLWKDEGRWEHDKFREDEQAPKSRQELIALY
GYDIRSAHNPDDIKPRRIRKPRYGSPPQRDPNWNGERLNKSHRHQGLGGTLPPRTFINRN
AAGTGRMSAPRNYSRSGGFKEGRAGFRPVEAGGQHGGRSGETVKHEISYRSRRLEQTSVR
DPSPEADAPVLGSPEKEEAASEPPAAAPDAAPPPPDRPIEKKSYSRARRTRTKVGDAVKL
AEEVPPPPEGLIPAPPVPETTPTPPTKTGTWEAPVDSSTSGLEQDVAQLNIAEQNWSPGQ
PSFLQPRELRGMPNHIHMGAGPPPQFNRMEEMGVQGGRAKRYSSQRQRPVPEPPAPPVHI
SIMEGHYYDPLQFQGPIYTHGDSPAPLPPQGMLVQPGMNLPHPGLHPHQTPAPLPNPGLY
PPPVSMSPGQPPPQQLLAPTYFSAPGVMNFGNPSYPYAPGALPPPPPPHLYPNTQAPSQV
YGGVTYYNPAQQQVQPKPSPPRRTPQPVTIKPPPPEVVSRGSS*
>ENSG00000102870|ENST00000262525
MEPETALWGPDLQGPEQSPNDAHRGAESENEEESPRQESSGEEIIMGDPAQSPESKDSTE
MSLERSSQDPSVPQNPPTPLGHSNPLDHQIPLDPPAPEVVPTPSDWTKACEASWQWGALT
TWNSPPVVPANEPSLRELVQGRPAGAEKPYICNECGKSFSQWSKLLRHQRIHTGERPNTC
SECGKSFTQSSHLVQHQRTHTGEKPYKCPDCGKCFSWSSNLVQHQRTHTGEKPYKCTECE
KAFTQSTNLIKHQRSHTGEKPYKCGECRRAFYRSSDLIQHQATHTGEKPYKCPECGKRFG
QNHNLLKHQKIHAGEKPYRCTECGKSFIQSSELTQHQRTHTGEKPYECLECGKSFGHSST
LIKHQRTHLREDPFKCPVCGKTFTLSATLLRHQRTHTGERPYKCPECGKSFSVSSNLINH
QRIHRGERPYICADCGKSFIMSSTLIRHQRIHTGEKPYKCSDCGKSFIRSSHLIQHRRTH
TGEKPYKCPECGKSFSQSSNLITHVRTHMDENLFVCSDCGKAFLEAHELEQHRVIHERGK
TPARRAQGDSLLGLGDPSLLTPPPGAKPHKCLVCGKGFNDEGIFMQHQRIHIGENPYKNA

DGLIAHAAPKPPQLRSPRLPFRGNSYPGAAEGRAEAPGQPLKPPEGQEGFSQRRGLLSSK
TYICSHCGESFLDRSVLLQHQLTHGNEKPFLFPDYRIGLGEGAGPSPFLSGKPFKCPECK
QSFGLSSELLLHQKVHAGGKSSQKSPELGKSSSVLLEHLRSPLGARPYRCSDCRASFLDR
VALTRHQETHTQEKPPNPEDPPPEAVTLSTDQEGEGETPTPTESSSHGEGQNPKTLVEEK
PYLCPECGAGFTEVAALLLHRSCHPGVSL*
>ENSG00000105278|ENST00000262961
MATSQYFDFAQGGGPQYSAQPPTLPLPTVGASYTAQPTPGMDPAVNPAFPPAAPAGYGGY
QPHSGQDFAYGSRPQEPVPTATTMATYQDSYSYGQSAAARSYEDRPYFQSAALQSGRMTA
ADSGQPGTQEACGQPSPHGSHSHAQPPQQAPIVESGQPASTLSSGYTYPTATGVQPESSA
SIVTSYPPPSYNPTCTAYTAPSYPNYDASVYSAASPFYPPAQPPPPPGPPQQLPPPPAPA
GSGSSPRADSKPPLPSKLPRPKAGPRQLQLHYCDICKISCAGPQTYREHLGGQKHRKKEA
AQKTGVQPNGSPRGVQAQLHCDLCAVSCTGADAYAAHIRGSKHQKVFKLHAKLGKPIPTL
EPALATESPPGAEAKPTSPTGPSVCASSRPALAKRPVASKALCEGPPEPQAAGCRPQWGK
PAQPKLEGPGAPTQGGSKEAPAGCSDAQPVGPEYVEEVFSDEGRVLRFHCKLCECSFNDL
NAKDLHVRGRRHRLQYRKKVNPDLPIATEPSSRARKVLEERMRKQRHLAEERLEQLRRWH
AERRRLEEEPPQDVPPHAPPDWAQPLLMGRPESPASAPLQPGRRPASSDDRHVMCKHATI
YPTEQELLAVQRAVSHAERALKLVSDTLAEEDRGRREEEGDKRSSVAPQTRVLKGVMRVG
ILAKGLLLRGDRNVRLALLCSEKPTHSLLRRIAQQLPRQLQMVTEDEYEVSSDPEANIVI
SSCEEPRMQVTISVTSPLMREDPSTDPGVEEPQADAGDVLSPKKCLESLAALRHARWFQA
RASGLQPCVIVIRVLRDLCRRVPTWGALPAWAMELLVEKAVSSAAGPLGPGDAVRRVLEC
VATGTLLTDGPGLQDPCERDQTDALEPMTLQEREDVTASAQHALRMLAFRQTHKVLGMDL
LPPRHRLGARFRKRQRGPGEGEEGAGEKKRGRRGGEGLV*
>ENSG00000115866|ENST00000264161
MPSASASRKSQEKPREIMDAAEDYAKERYGISSMIQSQEKPDRVLVRVRDLTIQKADEVV
WVRARVHTSRAKGKQCFLVLRQQQFNVQALVAVGDHASKQMVKFAANINKESIVDVEGVV
RKVNQKIGSCTQQDVELHVQKIYVISLAEPRLPLQLDDAVRPEAEGEEEGRATVNQDTRL
DNRVIDLRTSTSQAVFRLQSGICHLFRETLINKGFVEIQTPKIISAASEGGANVFTVSYF
KNNAYLAQSPQLYKQMCICADFEKVFSIGPVFRAEDSNTHRHLTEFVGLDIEMAFNYHYH
EVMEEIADTMVQIFKGLQERFQTEIQTVNKQFPCEPFKFLEPTLRLEYCEALAMLREAGV
EMGDEDDLSTPNEKLLGHLVKEKYDTDFYILDKYPLAVRPFYTMPDPRNPKQSNSYDMFM
RGEEILSGAQRIHDPQLLTERALHHGIDLEKIKAYIDSFRFGAPPHAGGGIGLERVTMLF
LGLHNVRQTSMFPRDPKRLTP*
>ENSG00000130522|ENST00000252818
METPFYGDEALSGLGGGASGSGGSFASPGRLFPGAPPTAAAGSMMKKDALTLSLSEQVAA
ALKPAAAPPPTPLRADGAPSAAPPDGLLASPDLGLLKLASPELERLIIQSNGLVTTTPTS
SQFLYPKVAASEEQEFAEGFVKALEDLHKQNQLGAGAAAAAAAAAAGGPSGTATGSAPPG
ELAPAAAAPEAPVYANLSSYAGGAGGAGGAATVAFAAEPVPFPPPPPPGALGPPRLAALK
DEPQTVPDVPSFGESPPLSPIDMDTQERIKAERKRLRNRIAASKCRKRKLERISRLEEKV
KTLKSQNTELASTASLLREQVAQLKQKVLSHVNSGCQLLPQHQVPAY*
>ENSG00000012822|ENST00000262059
MEESPLSRAPSRGGVNFLNVARTYIPNTKVECHYTLPPGTMPSASDWIGIFKVEAACVRD
YHTFVWSSVPESTTDGSPIHTSVQFQASYLPKPGAQLYQFRYVNRQGQVCGQSPPFQFRE
PRPMDELVTLEEADGGSDILLVVPKATVLQNQLDESQQERNDLMQLKLQLEGQVTELRSR
VQELERALATARQEHTELMEQYKGISRSHGEITEERDILSRQQGDHVARILELEDDIQTI

SEKVLTKEVELDRLRDTVKALTREQEKLLGQLKEVQADKEQSEAELQVAQQENHHLNLDL
KEAKSWQEEQSAQAQRLKDKVAQMKDTLGQAQQRVAELEPLKEQLRGAQELAASSQQKAT
LLGEELASAAAARDRTIAELHRSRLEVAEVNGRLAELGLHLKEEKCQWSKERAGLLQSVE
AEKDKILKLSAEILRLEKAVQEERTQNQVFKTELAREKDSSLVQLSESKRELTELRSALR
VLQKEKEQLQEEKQELLEYMRKLEARLEKVADEKWNEDATTEDEEAAVGLSCPAALTDSE
DESPEDMRLPPYGLCERGDPGSSPAGPREASPLVVISQPAPISPHLSGPAEDSSSDSAED
EKSVLMAAVQSGGEEANLLLPELGSAFYDMASGFTVGTLSETSTGGPATPTWKECPICKE
RFPAESDKDALEDHMDGHFFFSTQDPFTFE*
>ENSG00000130856|ENST00000253159
MGLCGLLERCWLHHDPDGVLTLNAENTNYAYQVPNFHKCEICLLSFPKESQFQRHMRDHE
RNDKPHRCDQCPQTFNVEFNLTLHKCTHSGEDPTCPVCNKKFSRVASLKAHIMLHEKEEN
LICSECGDEFTLQSQLAVHMEEHRQELAGTRQHACKACKKEFETSSELKEHMKTHYKIRV
SSTRSYNRNIDRSGFTYSCPHCGKTFQKPSQLTRHIRIHTGERPFKCSECGKAFNQKGAL
QTHMIKHTGEKPHACAFCPAAFSQKGNLQSHVQRVHSEVKNGPTYNCTECSCVFKSLGSL
NTHISKMHMGGPQNSTSSTETAHVLTATLFQTLPLQQTEAQATSASSQPSSQAVSDVIQQ
LLELSEPAPVESGQSPQPGQQLSITVGINQDILQQALENSGLSSIPAAAHPNDSCHAKTS
APHAQNPDVSSVSNEQTDPTDAEQEKEQESPEKLDKKEKKMIKKKSPFLPGSIREENGVR
WHVCPYCAKEFRKPSDLVRHIRIHTHEKPFKCPQCFRAFAVKSTLTAHIKTHTGIKAFKC
QYCMKSFSTSGSLKVHIRLHTGVRPFACPHCDKKFRTSGHRKTHIASHFKHTELRKMRHQ
RKPAKVRVGKTNIPVPDIPLQEPILITDLGLIQPIPKNQFFQSYFNNNFVNEADRPYKCF
YCHRAYKKSCHLKQHIRSHTGEKPFKCSQCGRGFVSAGVLKAHIRTHTGLKSFKCLICNG
AFTTGGSLRRHMGIHNDLRPYMCPYCQKTFKTSLNCKKHMKTHRYELAQQLQQHQQAASI
DDSTVDQQSMQASTQMQVEIESDELPQTAEVVAANPEAMLDLEPQHVVGTEEAGLGQQLA
DQPLEADEDGFVAPQDPLRGHVDQFEEQSPAQQSFEPAGLPQGFTVTDTYHQQPQFPPVQ
QLQDSSTLESQALSTSFHQQSLLQAPSSDGMNVTTRLIQESSQEELDLQAQGSQFLEDNE
DQSRRSYRCDYCNKGFKKSSHLKQHVRSHTGEKPYKCKLCGRGFVSSGVLKSHEKTHTGV
KAFSCSVCNASFTTNGSLTRHMATHMSMKPYKCPFCEEGFRTTVHCKKHMKRHQTVPSAV
SATGETEGGDICMEEEEEHSDRNASRKSRPEVITFTEEETAQLAKIRPQESATVSEKVLV
QSAAEKDRISELRDKQAELQDEPKHANCCTYCPKSFKKPSDLVRHVRIHTGEKPYKCDEC
GKSFTVKSTLDCHVKTHTGQKLFSCHVCSNAFSTKGSLKVHMRLHTGAKPFKCPHCELRF
RTSGRRKTHMQFHYKPDPKKARKPMTRSSSEGLQPVNLLNSSSTDPNVFIMNNSVLTGQF
DQNLLQPGLVGQAILPASVSAGGDLTVSLTDGSLATLEGIQLQLAANLVGPNVQISGIDA
ASINNITLQIDPSILQQTLQQGNLLAQQLTGEPGLAPQNSSLQTSDSTVPASVVIQPISG
LSLQPTVTSANLTIGPLSEQDSVLTTNSSGTQDLTQVMTSQGLVSPSGGPHEITLTINNS
SLSQVLAQAAGPTATSSSGSPQEITLTISELNTTSGSLPSTTPMSPSAISTQNLVMSSSG
VGGDASVTLTLADTQGMLSGGLDTVTLNITSQGQQFPALLTDPSLSGQGGAGSPQVILVS
HTPQSASAACEEIAYQVAGVSGNLAPGNQPEKEGRAHQCLECDRAFSSAAVLMHHSKEVH
GRERIHGCPVCRKAFKRATHLKEHMQTHQAGPSLSSQKPRVFKCDTCEKAFAKPSQLERH
SRIHTGERPFHCTLCEKAFNQKSALQVHMKKHTGERPYKCAYCVMGFTQKSNMKLHMKRA
HSYAGALQESAGHPEQDGEELSRTLHLEEVVQEAAGEWQALTHVF*
>ENSG00000089902|ENST00000262241
MPAMVEKGPEVSGKRRGRNNAAASASAAAASAAASAACASPAATAASGAAASSASAAAAS
AAAAPNNGQNKSLAAAAPNGNSSSNSWEEGSSGSSSDEEHGGGGMRVGPQYQAVVPDFDP
AKLARRSQERDNLGMLVWSPNQNLSEAKLDEYIAIAKEKHGYNMEQALGMLFWHKHNIEK

SLADLPNFTPFPDEWTVEDKVLFEQAFSFHGKTFHRIQQMLPDKSIASLVKFYYSWKKTR
TKTSVMDRHARKQKREREESEDELEEANGNNPIDIEVDQNKESKKEVPPTETVPQVKKEK
HSTQAKNRAKRKPPKGMFLSQEDVEAVSANATAATTVLRQLDMELVSVKRQIQNIKQTNS
ALKEKLDGGIEPYRLPEVIQKCNARWTTEEQLLAVQAIRKYGRDFQAISDVIGNKSVVQV
KNFFVNYRRRFNIDEVLQEWEAEHGKEETNGPSNQKPVKSPDNSIKMPEEEDEAPVLDVR
YASAS*
>ENSG00000086758|ENST00000262854
MKVDRTKLKKTPTEAPADCRALIDKLKVCNDEQLLLELQQIKTWNIGKCELYHWVDLLDR
FDGILADAGQTVENMSWMLVCDRPEREQLKMLLLAVLNFTALLIEYSFSRHLYSSIEHLT
TLLASSDMQVVLAVLNLLYVFSKRSNYITRLGSDKRTPLLTRLQHLAESWGGKENGFGLA
ECCRDLHMMKYPPSATTLHFEFYADPGAEVKIEKRTTSNTLHYIHIEQLDKISESPSEIM
ESLTKMYSIPKDKQMLLFTHIRLAHGFSNHRKRLQAVQARLHAISILVYSNALQESANSI
LYNGLIEELVDVLQITDKQLMEIKAASLRTLTSIVHLERTPKLSSIIDCTGTASYHGFLP
VLVRNCIQAMIDPSMDPYPHQFATALFSFLYHLASYDAGGEALVSCGMMEALLKVIKFLG
DEQDQITFVTRAVRVVDLITNLDMAAFQSHSGLSIFIYRLEHEVDLCRKECPFVIKPKIQ
RPNTTQEGEEMETDMDGVQCIPQRAALLKSMLNFLKKAIQDPAFSDGIRHVMDGSLPTSL
KHIISNAEYYGPSLFLLATEVVTVFVFQEPSLLSSLQDNGLTDVMLHALLIKDVPATREV
LGSLPNVFSALCLNARGLQSFVQCQPFERLFKVLLSPDYLPAMRRRRSSDPLGDTASNLG
SAVDELMRHQPTLKTDATTAIIKLLEEICNLGRDPKYICQKPSIQKADGTATAPPPRSNH
AAEEASSEDEEEEEVQAMQSFNSTQQNETEPNQQVVGTEERIPIPLMDYILNVMKFVESI
LSNNTTDDHCQEFVNQKGLLPLVTILGLPNLPIDFPTSAACQAVAGVCKSILTLSHEPKV
LQEGLLQLDSILSSLEPLHRPIESPGGSVLLRELACAGNVADATLSAQATPLLHALTAAH
AYIMMFVHTCRVGQSEIRSISVNQWGSQLGLSVLSKLSQLYCSLVWESTVLLSLCTPNSL
PSGCEFGQADMQKLVPKDEKAGTTQGGKRSDGEQDGAAGSMDASTQGLLEGIGLDGDTLA
PMETDEPTASDSKGKSKITPAMAARIKQIKPLLSASSRLGRALAELFGLLVKLCVGSPVR
QRRSHHAASTTTAPTPAARSTASALTKLLTKGLSWQPPPYTPTPRFRLTFFICSVGFTSP
MLFDERKYPYHLMLQKFLCSGGHNALFETFNWALSMGGKVPVSEGLEHSDLPDGTGEFLD
AWLMLVEKMVNPTTVLESPHSLPAKLPGGVQNFPQFSALRFLVVTQKAAFTCIKNLWNRK
PLKVYGGRMAESMLAILCHILRGEPVIRERLSKEKEGSRGEEDTGQEEGGSRREPQVNQQ
QLQQLMDMGFTREHAMEALLNTSTMEQATEYLLTHPPPIMGGVVRDLSMSEEDQMMRAIA
MSLGQDIPMDQRAESPEEVACRKEEEERKAREKQEEEEAKCLEKFQDADPLEQDELHTFT
DTMLPGCFHLLDELPDTVYRVCDLIMTAIKRNGADYRDMILKQVVNQVWEAADVLIKAAL
PLTTSDTKTVSEWISQMATLPQASNLATRILLLTLLFEELKLPCAWVVESSGILNVLIKL
LEVVQPCLQAAKEQKEVQTPKWITPVLLLIDFYEKTAISSKRRAQMTKYLQSNSNNWRWF
DDRSGRWCSYSASNNSTIDSAWKSGETSVRFTAGRRRYTVQFTTMVQVNEETGNRRPVML
TLLRVPRLNKNSKNSNGQELEKTLEESKEMDIKRKENKGNDTPLALESTNTEKETSLEET
KIGEILIQGLTEDMVTVLIRACVSMLGVPVDPDTLHATLRLCLRLTRDHKYAMMFAELKS
TRMILNLTQSSGFNGFTPLVTLLLRHIIEDPCTLRHTMEKVVRSAATSGAGSTTSGVVSG
SLGSREINYILRVLGPAACRNPDIFTEVANCCIRIALPAPRGSGTASDDEFENLRIKGPN
AVQLVKTTPLKPSPLPVIPDTIKEVIYDMLNALAAYHAPEEADKSDPKPGVMTQEVGQLL
QDMGDDVYQQYRSLTRQSSDFDTQSGFSINSQVFAADGASTETSASGTSQGEASTPEESR
DGKKDKEGDRASEEGKQKGKGSKPLMPTSTILRLLAELVRSYVGIATLIANYSYTVGQSE
LIKEDCSVLAFVLDHLLPHTQNAEDKDTPALARLFLASLAAAGSGTDAQVALVNEVKAAL
GRALAMAESTEKHARLQAVMCIISTIMESCPSTSSFYSSATAKTQHNGMNNIIRLFLKKG

LVNDLARVPHSLDLSSPNMANTVNAALKPLETLSRIVNQPSSLFGSKSASSKNKSEQDAQ
GASQDSSSNQQDPGEPGEAEVQEEDHDVTQTEVADGDIMDGEAETDSVVIAGQPEVLSSQ
EMQVENELEDLIDELLERDGGSGNSTIIVSRSGEDESQEDVLMDEAPSNLSQASTLQANR
EDSMNILDPEDEEEHTQEEDSSGSNEDEDDSQDEEEEEEEDEEDDQEDDEGEEGDEDDDD
DGSEMELDEDYPDMNASPLVRFERFDREDDLIIEFDNMFSSATDIPPSPGNIPTTHPLMV
RHADHSSLTLGSGSSTTRLTQGIGRSQRTLRQLTANTGHTIHVHYPGNRQPNPPLILQRL
LGPSAAADILQLSSSLPLQSRGRARLLVGNDDVHIIARSDDELLDDFFHDQSTATSQAGT
LSSIPTALTRWTEECKVLDAESMHDCVSVVKVSIVNHLEFLRDEELEERREKRRKQLAEE
ETKITDKGKEDKENRDQSAQCTASKSNDSTEQNLSDGTPMPDSYPTTPSSTDAATSESKE
TLGTLQSSQQQPTLPTPPALGEVPQELQSPAGEGGSSTQLLMPVEPEELGPTRPSGEAET
TQMELSPAPTITSLSPERAEDSDALTAVSSQLEGSPMDTSSLASCTLEEAVGDTSAAGSS
EQPRAGSSTPGDAPPAVAEVQGRSDGSGESAQPPEDSSPPASSESSSTRDSAVAISGADS
RGILEEPLPSTSSEEEDPLAGISLPEGVDPSFLAALPDDIRREVLQNQLGIRPPTRTAPS
TNSSAPAVVGNPGVTEVSPEFLAALPPAIQEEVLAQQRAEQQRRELAQNASSDTPMDPVT
FIQTLPSDLRRSVLEDMEDSVLAVMPPDIAAEAQALRREQEARQRQLMHERLFGHSSTSA
LSAILRSPAFTSRLSGNRGVQYTRLAVQRGGTFQMGGSSSHNRPSGSNVDTLLRLRGRLL
LDHEALSCLLVLLFVDEPKLNTSRLHRVLRNLCYHAQTRHWVIRSLLSILQRSSESELCI
ETPKLTTSEEKGKKSSKSCGSSSHENRPLDLLHKMESKSSNQLSWLSVSMDAALGCRTNI
FQIQRSGGRKHTEKHASGGSTVHIHPQAAPVVCRHVLDTLIQLAKVFPSHFTQQRTKETN
CESDRERGNKACSPCSSQSSSSGICTDFWDLLVKLDNMNVSRKGKNSVKSVPVSAGGEGE
TSPYSLEASPLGQLMNMLSHPVIRRSSLLTEKLLRLLSLISIALPENKVSEAQANSGSGA
SSTTTATSTTSTTTTTAASTTPTPPTAPTPVTSAPALVAATAISTIVVAASTTVTTPTTA
TTTVSISPTTKGSKSPAKVSDGGSSSTDFKMVSSGLTENQLQLSVEVLTSHSCSEEGLED
AANVLLQLSRGDSGTRDTVLKLLLNGARHLGYTLCKQIGTLLAELREYNLEQQRRAQCET
LSPDGLPEEQPQTTKLKGKMQSRFDMAENVVIVASQKRPLGGRELQLPSMSMLTSKTSTQ
KFFLRVLQVIIQLRDDTRRANKKAKQTGRLGSSGLGSASSIQAAVRQLEAEADAIIQMVR
EGQRARRQQQAATSESSQSEASVRREESPMDVDQPSPSAQDTQSIASDGTPQGEKEKEER
PPELPLLSEQLSLDELWDMLGECLKELEESHDQHAVLVLQPAVEAFFLVHATERESKPPV
RDTRESQLAHIKDEPPPLSPAPLTPATPSSLDPFFSREPSSMHISSSLPPDTQKFLRFAE
THRTVLNQILRQSTTHLADGPFAVLVDYIRVLDFDVKRKYFRQELERLDEGLRKEDMAVH
VRRDHVFEDSYRELHRKSPEEMKNRLYIVFEGEEGQDAGGLLREWYMIISREMFNPMYAL
FRTSPGDRVTYTINPSSHCNPNHLSYFKFVGRIVAKAVYDNRLLECYFTRSFYKHILGKS
VRYTDMESEDYHFYQGLVYLLENDVSTLGYDLTFSTEVQEFGVCEVRDLKPNGANILVTE
ENKKEYVHLVCQMRMTGAIRKQLAAFLEGFYEIIPKRLISIFTEQELELLISGLPTIDID
DLKSNTEYHKYQSNSIQIQWFWRALRSFDQADRAKFLQFVTGTSKVPLQGFAALEGMNGI
QKFQIHRDDRSTDRLPSAHTCFNQLDLPAYESFEKLRHMLLLAIQECSEGFGLA*
>ENSG00000131914|ENST00000254231
MGSVSNQQFAGGCAKAAEEAPEEAPEDAARAADEPQLLHGAGICKWFNVRMGFGFLSMTA
RAGVALDPPVDVFVHQSKLHMEGFRSLKEGEAVEFTFKKSAKGLESIRVTGPGGVFCIGS
ERRPKGKSMQKRRSKGDRCYNCGGLDHHAKECKLPPQPKKCHFCQSISHMVASCPLKAQQ
GPSAQGKPTYFREEEEEIHSPTLLPEAQN*
>ENSG00000130303|ENST00000252593
MASTSYDYCRVPMEDGDKRCKLLLGIGILVLLIIVILGVPLIIFTIKANSEACRDGLRAV
MECRNVTHLLQQELTEAQKGFQDVEAQAATCNHTVMALMASLDAEKAQGQKKVEELEGEI

TTLNHKLQDASAEVERLRRENQVLSVRIADKKYYPSSQDSSSAAAPQLLIVLLGLSALLQ
*
>ENSG00000113300|ENST00000261951
MPKEKYEPPDPRRMYTIMSSEEAANGKKSHWAELEISGKVRSLSASLWSLTHLTALHLSD
NSLSRIPSDIAKLHNLVYLDLSSNKIRSLPAELGNMVSLRELHLNNNLLRVLPFELGKLF
QLQTLGLKGNPLTQDILNLYQEPDGTRRLLNYLLDNLSGTAKRITTEQPPPRSWIMLQEP
DRTRPTALFSVMCYNVLCDKYATRQLYGYCPSWALNWDYRKKAIIQEILSCNADIVSLQE
VETEQYYSFFLVELKERGYNGFFSPKSRARTMSEQERKHVDGCAIFFKTEKFTLVQKHTV
EFNQLAMANSEGSEAMLNRVMTKDNIGVAVLLELRKESIEMPSGKPHLGTEKQLILVANA
HMHWDPEYSDVKLVQTMMFLSEVKNIIDKASRNLKSSVLGEFGTIPLVLCADLNSLPDSG
VVEYLSTGGVETNHKDFKELRYNESLTNFSCHGKNGTTNGRITHGFKLQSAYESGLMPYT
NYTFDFKGIIDYIFYSKPQLNTLGILGPLDHHWLVENNISGCPHPLIPSDHFSLFAQLEL
LLPFLPQVNGIHLPGRR*
>ENSG00000135945|ENST00000258428
MRRGGWRKRAENDGWETWGGYMAAKVQKLEEQFRSDAAMQKDGTSSTIFSGVAIYVNGYT
DPSAEELRKLMMLHGGQYHVYYSRSKTTHIIATNLPNAKIKELKGEKVIRPEWIVESIKA
GRLLSYIPYQLYTKQSSVQKGLSFNPVCRPEDPLPGPSNIAKQLNNRVNHIVKKIETENE
VKVNGMNSWNEEDENNDFSFVDLEQTSPGRKQNGIPHPRGSTAIFNGHTPSSNGALKTQD
CLVPMVNSVASRLSPAFSQEEDKAEKSSTDFRDCTLQQLQQSTRNTDALRNPHRTNSFSL
SPLHSNTKINGAHHSTVQGPSSTKSTSSVSTFSKAAPSVPSKPSDCNFISNFYSHSRLHH
ISMWKCELTEFVNTLQRQSNGIFPGREKLKKMKTGRSALVVTDTGDMSVLNSPRHQSCIM
HVDMDCFFVSVGIRNRPDLKGKPVAVTSNRGTGRAPLRPGANPQLEWQYYQNKILKGKAA
DIPDSSLWENPDSAQANGIDSVLSRAEIASCSYEARQLGIKNGMFFGHAKQLCPNLQAVP
YDFHAYKEVAQTLYETLASYTHNIEAVSCDEALVDITEILAETKLTPDEFANAVRMEIKD
QTKCAASVGIGSNILLARMATRKAKPDGQYHLKPEEVDDFIRGQLVTNLPGVGHSMESKL
ASLGIKTCGDLQYMTMAKLQKEFGPKTGQMLYRFCRGLDDRPVRTEKERKSVSAEINYGI
RFTQPKEAEAFLLSLSEEIQRRLEATGMKGKRLTLKIMVRKPGAPVETAKFGGHGICDNI
ARTVTLDQATDNAKIIGKAMLNMFHTMKLNISDMRGVGIHVNQLVPTNLNPSTCPSRPSV
QSSHFPSGSYSVRDVFQVQKAKKSTEEEHKEVFRAAVDLEISSASRTCTFLPPFPAHLPT
SPDTNKAESSGKWNGLHTPVSVQSRLNLSIEVPSPSQLDQSVLEALPPDLREQVEQVCAV
QQAESHGDKKKEPVNGCNTGILPQPVGTVLLQIPEPQESNSDAGINLIALPAFSQVDPEV
FAALPAELQRELKAAYDQRQRQGENSTHQQSASASVPKNPLLHLKAAVKEKKRNKKKKTI
GSPKRIQSPLNNKLLNSPAKTLPGACGSPQKLIDGFLKHEGPPAEKPLEELSASTSGVPG
LSSLQSDPAGCVRPPAPNLAGAVEFNDVKTLLREWITTISDPMEEDILQVVKYCTDLIEE
KDLEKLDLVIKYMKRLMQQSVESVWNMAFDFILDNVQVVLQQTYGSTLKVT*
>ENSG00000125618|ENST00000263334
MPHNSIRSGHGGLNQLGGAFVNGRPLPEVVRQRIVDLAHQGVRPCDISRQLRVSHGCVSK
ILGRYYETGSIRPGVIGGSKPKVATPKVVEKIGDYKRQNPTMFAWEIRDRLLAEGVCDND
TVPSVSSINRIIRTKVQQPFNLPMDSCVATKSLSPGHTLIPSSAVTPPESPQSDSLGSTY
SINGLLGIAQPGSDKRKMDDSDQDSCRLSIDSQSSSSGPRKHLRTDAFSQHHLEPLECPF
ERQHYPEAYASPSHTKGEQGLYPLPLLNSTLDDGKATLTPSNTPLGRNLSTHQTYPVVAD
PHSPFAIKQETPEVSSSSSTPSSLSSSAFLDLQQVGSGVPPFNAFPHAASVYGQFTGQAL
LSGREMVGPTLPGYPPHIPTSGQGSYASSAIAGMVAGSEYSGNAYGHTPYSSYSEAWRFP
NSSLLSSPYYYSSTSRPSAPPTTATAFDHL*

>ENSG00000136450|ENST00000258962
MSGGGVIRGPAGNNDCRIYVGNLPPDIRTKDIEDVFYKYGAIRDIDLKNRRGGPPFAFVE
FEDPRDAEDAVYGRDGYDYDGYRLRVEFPRSGRGTGRGGGGGGGGGAPRGRYGPPSRRSE
NRVVVSGLPPSGSWQDLKDHMREAGDVCYADVYRDGTGVVEFVRKEDMTYAVRKLDNTKF
RSHEGETAYIRVKVDGPRSPSYGRSRSRSRSRSRSRSRSNSRSRSYSPRRSRGSPRYSPR
HSRSRSRT*
>ENSG00000137760|ENST00000260318
MDSNHQSNYKLSKTEKKFLRKQIKAKHTLLRHEGIETVSYATQSLVVANGGLGNGVSRNQ
LLPVLEKCGLVDALLMPPNKPYSFARYRTTEESKRAYVTLNGKEVVDDLGQKITLYLNFV
EKVQWKELRPQALPPGLMVVEEIISSEEEKMLLESVDWTEDTDNQNSQKSLKHRRVKHFG
YEFHYENNNVDKDKPLSGGLPDICESFLEKWLRKGYIKHKPDQMTINQYEPGQDCHGF*
>ENSG00000135482|ENST00000257940
MPDRDSYANGTGSSGGGPGGGGSEEASGAGVGSGGASSDAICRDFLRNVCKRGKRCRYRH
PDMSEVSNLGVSKNEFIFCHDFQNKECSRPNCRFIHGSKEDEDGYKKTGELPPRLRQKVA
AGLGLSPADLPNGKEEVPICRDFLKGDCQRGAKCKFRHLQRDFEFDARGGGGTGGGSTGS
VLPGRRHDLYDIYDLPDRGFEDHEPGPKRRRGGCCPPDGPHFESYEYSLAPPRGVECRLL
EEENAMLRKRVEELKKQVSNLLATNEVLLEQNAQFRNQAKVITLSSTAPATEQTLAPTVG
TVATFNHGIAQTHTTLSSQALQPRPVSQQELVAPAGAPAAPPTNAAPPAAPPPPPPHLTP
EITPLSAALAQTIAQGMAPPPVSMAPVAVSVAPVAPVAVSMAQPLAGITMSHTTTPMVTY
PIASQSMRITAMPH*
>ENSG00000130332|ENST00000252622
MADKEKKKKESILDLSKYIDKTIRVKFQGGREASGILKGFDPLLNLVLDGTIEYMRDPDD
QYKLTEDTRQLGLVVCRGTSVVLICPQDGMEAIPNPFIQQQDA*
>ENSG00000131469|ENST00000253788
MGKFMKPGKVVLVLAGRYSGRKAVIVKNIDDGTSDRPYSHALVAGIDRYPRKVTAAMGKK
KIAKRSKIKSFVKVYNYNHLMPTRYSVDIPLDKTVVNKDVFRDPALKRKARREAKVKFEE
RYKTGKNKWFFQKLRF*
>ENSG00000132603|ENST00000254941
MRPLTEEETRVMFEKIAKYIGENLQLLVDRPDGTYCFRLHNDRVYYVSEKIMKLAANISG
DKLVSLGTCFGKFTKTHKFRLHVTALDYLAPYAKGFGVAAKSTQDCRKVDPMAIVVFHQA
DIGEYVRHEETLT*
>ENSG00000131408|ENST00000253727
MSSPTTSSLDTPLPGNGPPQPGAPSSSPTVKEEGPEPWPGGPDPDVPGTDEASSACSTDW
VIPDPEEEPERKRKKGPAPKMLGHELCRVCGDKASGFHYNVLSCEGCKGFFRRSVVRGGA
RRYACRGGGTCQMDAFMRRKCQQCRLRKCKEAGMREQCVLSEEQIRKKKIRKQQQESQSQ
SQSPVGPQGSSSSASGPGASPGGSEAGSQGSGEGEGVQLTAAQELMIQQLVAAQLQCNKR
SFSDQPKVTPWPLGADPQSRDARQQRFAHFTELAIISVQEIVDFAKQVPGFLQLGREDQI
ALLKASTIEIMLLETARRYNHETECITFLKDFTYSKDDFHRAGLQVEFINPIFEFSRAMR
RLGLDDAEYALLIAINIFSADRPNVQEPGRVEALQQPYVEALLSYTRIKRPQDQLRFPRM
LMKLVSLRTLSSVHSEQVFALRLQDKKLPPLLSEIWDVHE*
>ENSG00000137876|ENST00000260443
MRIEKCYFCSGPIYPGHGMMFVRNDCKVFRFCKSKCHKNFKKKRNPRKVRWTKAFRKAAG
KELTVDNSFEFEKRRNEPIKYQRELWNKTIDAMKRVEEIKQKRQAKFIMNRLKKNKELQK
VQDIKEVKQNIHLIRAPLAGKGKQLEEKMVQQLQEDVDMEDAP*

>ENSG00000130544|ENST00000252840
MAAVVLPPTAALSSLFPASQREGHTEGGELVNELLKSWLKGLVTFEDVAVEFTQEEWALL
DPAQRTLYRDVMLENCRNLASLGNQVDKPRLISQLEQEDKVMTEERGILSGTCPDVENPF
KAKGLTPKLHVFRKEQSRNMKMERNHLGATLNECNQCFKVFSTKSSLTRHRKIHTGERPY
GCSECGKSYSSRSYLAVHKRIHNGEKPYECNDCGKTFSSRSYLTVHKRIHNGEKPYECSD
CGKTFSNSSYLRPHLRIHTGEKPYKCNQCFREFRTQSIFTRHKRVHTGEGHYVCNQCGKA
FGTRSSLSSHYSIHTGEYPYECHDCGRTFRRRSNLTQHIRTHTGEKPYTCNECGKSFTNS
FSLTIHRRIHNGEKSYECSDCGKSFNVLSSVKKHMRTHTGKKPYECNYCGKSFTSNSYLS
VHTRMHNRQM*
>ENSG00000127804|ENST00000263092
MALSKSMHARNRYKDKPPDFAYLASKYPDFKQHVQINLNGRVSLNFKDPEAVRALTCTLL
REDFGLSIDIPLERLIPTVPLRLNYIHWVEDLIGHQDSDKSTLRRGIDIGTGASCIYPLL
GATLNGWYFLATEVDDMCFNYAKKNVEQNNLSDLIKVVKVPQKTLLMDALKEESEIIYDF
CMCNPPFFANQLEAKGVNSRNPRRPPPSSVNTGGITEIMAEGGELEFVKRIIHDSLQLKK
RLRWYSCMLGKKCSLAPLKEELRIQGVPKVTYTEFCQGRTMRWALAWSFYDDVTVPSPPS
KRRKLEKPRKPITFVVLASVMKELSLKASPLRSETAEGIVVVTTWIEKILTDLKVQHKRV
PCGKEEVSLFLTAIENSWIHLRRKKRERVRQLREVPRAPEDVIQALEEKKPTPKESGNSQ
ELARGPQERTPCGPALREGEAAAVEGPCPSQESLSQEENPEPTEDERSEEKGGVEVLESC
QGSSNGAQDQEASEQFGSPVAERGKRLPGVAGQYLFKCLINVKKEVDDALVEMHWVEGQN
RDLMNQLCTYIRNQIFRLVAVN*
>ENSG00000131849|ENST00000254166
MALPSPQVLMGLPALLMGPAQHTSWPCGSAVPTLKSMVTFEDVAVYFSQEEWELLDAAQR
HLYHSVMLENLELVTSLGSWHGVEGEGAHPKQNVSVEVLQVRIPNADPSTKKANSCDMCG
PFLKDILHLAEHQGTQSEEKPYTCGACGRDFWLNANLHQHQKEHSGGKPFRWYKDRDALM
KSSKVHLSENPFTCREGGKVILGSCDLLQLQAVDSGQKPYSNLGQLPEVCTTQKLFECSN
CGKAFLKSSTLPNHLRTHSEEIPFTCPTGGNFLEEKSILGNKKFHTGEIPHVCKECGKAF
SHSSKLRKHQKFHTEVKYYECIACGKTFNHKLTFVHHQRIHSGERPYECDECGKAFSNRS
HLIRHEKVHTGERPFECLKCGRAFSQSSNFLRHQKVHTQVRPYECSQCGKSFSRSSALIQ
HWRVHTGERPYECSECGRAFNNNSNLAQHQKVHTGERPFECSECGRDFSQSSHLLRHQKV
HTGERPFECCDCGKAFSNSSTLIQHQKVHTGQRPYECSECRKSFSRSSSLIQHWRIHTGE
KPYECSECGKAFAHSSTLIEHWRVHTKERPYECNECGKFFSQNSILIKHQKVHTGEKPYK
CSECGKFFSRKSSLICHWRVHTGERPYECSECGRAFSSNSHLVRHQRVHTQERPYECIQC
GKAFSERSTLVRHQKVHTRERTYECSQCGKLFSHLCNLAQHKKIHT*
>ENSG00000099956|ENST00000263121
MMMMALSKTFGQKPVKFQLEDDGEFYMIGSEVGNYLRMFRGSLYKRYPSLWRRLATVEER
KKIVASSHGKKTKPNTKDHGYTTLATSVTLLKASEVEEILDGNDEKYKAVSISTEPPTYL
SFDDHDPAVIHENASQPEVLVPIRLDMEIDGQKLRDAFTWNMNEKLMTPEMFSEILCDDL
DLNPLTFVPAIASAIRQQIESYPTDSILEDQSDQRVIIKLNIHVGNISLVDQFEWDMSEK
ENSPEKFALKLCSELGLGGEFVTTIAYSIRGQLSWHQKTYAFSENPLPTVEIAIRNTGDA
DQWCPLLETLTDAEMEKKIRDQDRNTRRMRRLANTAPAW*
>ENSG00000137492|ENST00000260045
MPNFCAAPNCTRKSTQSDLAFFRFPRDPARCQKWVENCRRADLEDKTPDQLNKHYRLCAK
HFETSMICRTSPYRTVLRDNAIPTIFDLTSHLNNPHSRHRKRIKELSEDEIRTLKQKKID
ETSEQEQKHKETNNSNAQNPSEEEGEGQDEDILPLTLEEKENKEYLKSLFEILILMGKQN

IPLDGHEADEIPEGLFTPDNFQALLECRINSGEEVLRKRFETTAVNTLFCSKTQQRQMLE
ICESCIREETLREVRDSHFFSIITDDVVDIAGEEHLPVLVRFVDESHNLREEFIGFLPYE
ADAEILAVKFHTMITEKWGLNMEYCRGQAYIVSSGFSSKMKVVASRLLEKYPQAIYTLCS
SCALNMWLAKSVPVMGVSVALGTIEEVCSFFHRSPQLLLELDNVISVLFQNSKERGKELK
EICHSQWTGRHDAFEILVELLQALVLCLDGINSDTNIRWNNYIAGRAFVLCSAVSDFDFI
VTIVVLKNVLSFTRAFGKNLQGQTSDVFFAAGSLTAVLHSLNEVMENIEVYHEFWFEEAT
NLATKLDIQMKLPGKFRRAHQGNLESQLTSESYYKETLSVPTVEHIIQELKDIFSEQHLK
ALKCLSLVPSVMGQLKFNTSEEHHADMYRSDLPNPDTLSAELHCWRIKWKHRGKDIELPS
TIYEALHLPDIKFFPNVYALLKVLCILPVMKVENERYENGRKRLKAYLRNTLTDQRSSNL
ALLNINFDIKHDLDLMVDTYIKLYTSKSELPTDNSETVENT*
>ENSG00000134107|ENST00000256495
MERIPSAQPPPACLPKAPGLEHGDLPGMYPAHMYQVYKSRRGIKRSEDSKETYKLPHRLI
EKKRRDRINECIAQLKDLLPEHLKLTTLGHLEKAVVLELTLKHVKALTNLIDQQQQKIIA
LQSGLQAGELSGRNVETGQEMFCSGFQTCAREVLQYLAKHENTRDLKSSQLVTHLHRVVS
ELLQGGTSRKPSDPAPKVMDFKEKPSSPAKGSEGPGKNCVPVIQRTFAHSSGEQSGSDTD
TDSGYGGESEKGDLRSEQPCFKSDHGRRFTMGERIGAIKQESEEPPTKKNRMQLSDDEGH
FTSSDLISSPFLGPHPHQPPFCLPFYLIPPSATAYLPMLEKCWYPTSVPVLYPGLNASAA
ALSSFMNPDKISAPLLMPQRLPSPLPAHPSVDSSVLLQALKPIPPLNLETKD*
>ENSG00000130844|ENST00000253144
MAQGLVTFADVAIDFSQEEWACLNSAQRDLYWDVMLENYSNLVSLDLESAYENKSLPTEK
NIHEIRASKRNSDRRSKSLGRNWICEGTLERPQRSRGRYVNQMIINYVKRPATREGTPPR
THQRHHKENSFECKDCGKAFSRGYQLSQHQKIHTGEKPYECKECKKAFRWGNQLTQHQKI
HTGEKPYECKDCGKAFRWGSSLVIHKRIHTGEKPYECKDCGKAFRRGDELTQHQRFHTGE
KDYECKDCGKTFSRVYKLIQHKRIHSGEKPYECKDCGKAFICGSSLIQHKRIHTGEKPYE
CQECGKAFTRVNYLTQHQKIHTGEKPHECKECGKAFRWGSSLVKHERIHTGEKPYKCTEC
GKAFNCGYHLTQHERIHTGETPYKCKECGKAFIYGSSLVKHERIHTGVKPYGCTECGKSF
SHGHQLTQHQKTHSGAKSYECKECGKACNHLNHLREHQRIHNS*
>ENSG00000136327|ENST00000258829
MATSGRLSFTVRSLLDLPEQDAQHLPRREPEPRAPQPDPCAAWLDSERGHYPSSDESSLE
TSPPDSSQRPSARPASPGSDAEKRKKRRVLFSKAQTLELERRFRQQRYLSAPEREQLASL
LRLTPTQVKIWFQNHRYKLKRARAPGAAESPDLAASAELHAAPGLLRRVVVPVLVRDGQP
CGGGGGGEVGTAAAQEKCGAPPAAACPLPGYPAFGPGSALGLFPAYQHLASPALVSWNW*
>ENSG00000111249|ENST00000261726
MAANVGSMFQYWKRFDLRRLQKELNSVASELSARQEESEHSHKHLIELRREFKKNVPEEI
REMVAPVLKSFQAEVVALSKRSQEAEAAFLSVYKQLIEAPDPVPVFEAARSLDDRLQPPS
FDPSGQPRRDLHTSWKRNPELLSPKEQREGTSPAGPTLTEGSRLPGIPGKALLTETLLQR
NEAEKQKGLQEVQITLAARLGEAEEKIKVLHSALKATQAELLELRRKYDEEAASKADEVG
LIMTNLEKANQRAEAAQREVESLREQLASVNSSIRLACCSPQGPSGDKVNFTLCSGPRLE
AALASKDREILRLLKDVQHLQSSLQELEEASANQIADLERQLTAKSEAIEKLEEKLQAQS
DYEEIKTELSILKAMKLASSTCSLPQGMAKPEDSLLIAKEAFFPTQKFLLEKPSLLASPE
EDPSEDDSIKDSLGTEQSYPSPQQLPPPPGPEDPLSPSPGQPLLGPSLGPDGTRTFSLSP
FPSLASGERLMMPPAAFKGEAGGLLVFPPAFYGAKPPTAPATPAPGPEPLGGPEPADGGG
GGAAGPGAEEEQLDTAEIAFQVKEQLLKHNIGQRVFGHYVLGLSQGSVSEILARPKPWRK
LTVKGKEPFIKMKQFLSDEQNVLALRTIQVRQRGSITPRIRTPETGSDDAIKSILEQAKK

EIESQKGGEPKTSVAPLSIANGTTPASTSEDAIKSILEQARREMQAQQQALLEMEVAPRG
RSVPPSPPERPSLATASQNGAPALVKQEEGSGGPAQAPLPVLSPAAFVQSIIRKVKSEIG
DAGYFDHHWASDRGLLSRPYASVSPSLSSSSSSGYSGQPNGRAWPRGDEAPVPPEDEAAA
GAEDEPPRTGELKAEGATAEAGARLPYYPAYVPRTLKPTVPPLTPEQYELYMYREVDTLE
LTRQVKEKLAKNGICQRIFGEKVLGLSQGSVSDMLSRPKPWSKLTQKGREPFIRMQLWLS
DQLGQAVGQQPGASQASPTEPRSSPSPPPSPTEPEKSSQEPLSLSLESSKENQQPEGRSS
SSLSGKMYSGSQAPGGIQEIVAMSPELDTYSITKRVKEVLTDNNLGQRLFGESILGLTQG
SVSDLLSRPKPWHKLSLKGREPFVRMQLWLNDPHNVEKLRDMKKLEKKAYLKRRYGLIST
GSDSESPATRSECPSPCLQPQDLSLLQIKKPRVVLAPEEKEALRKAYQLEPYPSQQTIEL
LSFQLNLKTNTVINWFHNYRSRMRREMLVEGTQDEPDLDPSGGPGILPPGHSHPDPTPQS
PDSETEDQKPTVKELELQEGPEENSTPLTTQDKAQVRIKQEQMEEDAEEEAGSQPQDSGE
LDKGQGPPKEEHPDPPGNDGLPKVAPGPLLPGGSTPDCPSLHPQQESEAGERLHPDPLSF
KSASESSRCSLEVSLNSPSAASSPGLMMSVSPVPSSSAPISPSPPGAPPAKVPSASPTAD
MAGALHPSAKVNPNLQRRHEKMANLNNIIYRVERAANREEALEWEF*
>ENSG00000132541|ENST00000254878
MSSLIRRVISTAKAPGAIGPYSQAVLVDRTIYISGQIGMDPSSGQLVSGGVAEEAKQALK
NMGEILKAAGCDFTNVVKTTVLLADINDFNTVNEIYKQYFKSNFPARAAYQVAALPKGSR
IEIEAVAIQGPLTTASL*
>ENSG00000060688|ENST00000263694
MIEQQKRKGPELPLVPVKRQRHELLLGAGSGPGAGQQQATPGALLQAGPPRCSSLQAPIM
LLSGHEGEVYCCKFHPNGSTLASAGFDRLILLWNVYGDCDNYATLKGHSGAVMELHYNTD
GSMLFSASTDKTVAVWDSETGERVKRLKGHTSFVNSCYPARRGPQLVCTGSDDGTVKLWD
IRKKAAIQTFQNTYQVLAVTFNDTSDQIISGGIDNDIKVWDLRQNKLTYTMRGHADSVTG
LSLSSEGSYLLSNAMDNTVRVWDVRPFAPKERCVKIFQGNVHNFEKNLLRCSWSPDGSKI
AAGSADRFVYVWDTTSRRILYKLPGHAGSINEVAFHPDEPIIISASSDKRLYMGEIQ*
>ENSG00000161016|ENST00000262584
MGRVIRGQRKGAGSVFRAHVKHRKGAARLRAVDFAERHGYIKGIVKDIIHDPGRGAPLAK
VVFRDPYRFKKRTELFIAAEGIHTGQFVYCGKKAQLNIGNVLPVGTMPEGTIVCCLEEKP
GDRGKLARASGNYATVISHNPETKKTRVKLPSGSKKVISSANRAVVGVVAGGGRIDKPIL
KAGRAYHKYKAKRNCWPRVRGVAMNPVEHPFGGGNHQHIGKPSTIRRDAPAGRKVGLIAA
RRTGRLRGTKTVQEKEN*
>ENSG00000136169|ENST00000258672
MGEKNGDAKTFWMELEDDGKVDFIFEQVQNVLQSLKQKIKDGSATNKEYIQAMILVNEAT
IINSSTSIKDPMPVTQKEQENKSNAFPSTSCENSFPEDCTFLTTENKEILSLEDKVVDFR
EKDSSSNLSYQSHDCSGACLMKMPLNLKGENPLQLPIKCHFQRRHAKTNSHSSALHVSYK
TPCGRSLRNVEEVFRYLLETECNFLFTDNFSFNTYVQLARNYPKQKEVVSDVDISNGVES
VPISFCNEIDSRKLPQFKYRKTVWPRAYNLTNFSSMFTDSCDCSEGCIDITKCACLQLTA
RNAKTSPLSSDKITTGYKYKRLQRQIPTGIYECSLLCKCNRQLCQNRVVQHGPQVRLQVF
KTEQKGWGVRCLDDIDRGTFVCIYSGRLLSRANTEKSYGIDENGRDENTMKNIFSKKRKL
EVACSDCEVEVLPLGLETHPRTAKTEKCPPKFSNNPKELTVETKYDNISRIQYHSVIRDP
ESKTAIFQHNGKKMEFVSSESVTPEDNDGFKPPREHLNSKTKGAQSRLCFFCECLPLLPV
SLSLAQYL*
>ENSG00000130700|ENST00000252997
MYQSLALAASPRQAAYADSGSFLHAPGAGSPMFVPPARVPSMLSYLSGCEPSPQPPELAA

RPGWAQTATADSSAFGPGSPHPPAAHPPGATAFPFAHSPSGPGSGGSAGGRDGSAYQGAL
LPREQFAAPLGRPVGTSYSATYPAYVSPDVAQSWTAGPFDGSVLHGLPGRRPTFVSDFLE
EFPGEGRECVNCGALSTPLWRRDGTGHYLCNACGLYHKMNGVNRPLVRPQKRLSSSRRAG
LCCTNCHTTNTTLWRRNSEGEPVCNACGLYMKLHGVPRPLAMKKESIQTRKRKPKTIAKA
RGSSGSTRNASASPSAVASTDSSAATSKAKPSLASPVCPGPSMAPQASGQEDDSLAPGHL
EFKFEPEDFAFPSTAPSPQAGLRGALRQEAWCALALA*
>ENSG00000132471|ENST00000254806
MALNKNHSEGGGVIVNNTESILMSYDHVELTFNDMKNVPEAFKGTKKGTVYLTPYRVIFL
SKGKDAMQSFMMPFYLMKDCEIKQPVFGANYIKGTVKAEAGGGWEGSASYKLTFTAGGAI
EFGQRMLQVASQASRGEVPSGAYGYSYMPSGAYVYPPPVANGMYPCPPGYPYPPPPPEFY
PGPPMMDGAMGYVQPPPPPYPGPMEPPVSGPDVPSTPAAEAKAAEAAASAYYNPGNPHNV
YMPTSQPPPPPYYPPEDKKTQ*
>ENSG00000131196|ENST00000253506
MPSTSFPVPSKFPLGPAAAVFGRGETLGPAPRAGGTMKSAEEEHYGYASSNVSPALPLPT
AHSTLPAPCHNLQTSTPGIIPPADHPSGYGAALDGGPAGYFLSSGHTRPDGAPALESPRI
EITSCLGLYHNNNQFFHDVEVEDVLPSSKRSPSTATLSLPSLEAYRDPSCLSPASSLSSR
SCNSEASSYESNYSYPYASPQTSPWQSPCVSPKTTDPEEGFPRGLGACTLLGSPRHSPST
SPRASVTEESWLGARSSRPASPCNKRKYSLNGRQPPYSPHHSPTPSPHGSPRVSVTDDSW
LGNTTQYTSSAIVAAINALTTDSSLDLGDGVPVKSRKTTLEQPPSVALKVEPVGEDLGSP
PPPADFAPEDYSSFQHIRKGGFCDQYLAVPQHPYQWAKPKPLSPTSYMSPTLPALDWQLP
SHSGPYELRIEVQPKSHHRAHYETEGSRGAVKASAGGHPIVQLHGYLENEPLMLQLFIGT
ADDRLLRPHAFYQVHRITGKTVSTTSHEAILSNTKVLEIPLLPENSMRAVIDCAGILKLR
NSDIELRKGETDIGRKNTRVRLVFRVHVPQPSGRTLSLQVASNPIECSQRSAQELPLVEK
QSTDSYPVVGGKKMVLSGHNFLQDSKVIFVEKAPDGHHVWEMEAKTDRDLCKPNSLVVEI
PPFRNQRITSPVHVSFYVCNGKRKRSQYQRFTYLPANVPIIKTEPTDDYEPAPTCGPVSQ
GLSPLPRPYYSQQLAMPPDPSSCLVAGFPPCPQRSTLMPAAPGVSPKLHDLSPAAYTKGV
ASPGHCHLGLPQPAGEAPAVQDVPRPVATHPGSPGQPPPALLPQQ*
>ENSG00000105447|ENST00000253237
MAARKGRRRTCETGEPMEAESGDTSSEGPAQVYLPGRGPPLREGEELVMDEEAYVLYHRA
QTGAPCLSFDIVRDHLGDNRTELPLTLYLCAGTQAESAQSNRLMMLRMHNLHGTKPPPSE
GSDEEEEEEDEEDEEERKPQLELAMVPHYGGINRVRVSWLGEEPVAGVWSEKGQVEVFAL
RRLLQVVEEPQALAAFLRDEQAQMKPIFSFAGHMGEGFALDWSPRVTGRLLTGDCQKNIH
LWTPTDGGSWHVDQRPFVGHTRSVEDLQWSPTENTVFASCSADASIRIWDIRAAPSKACM
LTTATAHDGDVNVISWSRREPFLLSGGDDGALKIWDLRQFKSGSPVATFKQHVAPVTSVE
WHPQDSGVFAASGADHQITQWDLAVERDPEAGDVEADPGLADLPQQLLFVHQGETELKEL
HWHPQCPGLLVSTALSGFTIFRTISV*
>ENSG00000131051|ENST00000253363
MADDIDIEAMLEAPYKKDENKLSSANGHEERSKKRKKSKSRSRSHERKRSKSKERKRSRD
RERKKSKSRERKRSRSKERRRSRSRSRDRRFRGRYRSPYSGPKFNSAIRGKIGLPHSIKL
SRRRSRSKSPFRKDKSPVREPIDNLTPEERDARTVFCMQLAARIRPRDLEEFFSTVGKVR
DVRMISDRNSRRSKGIAYVEFVDVSSVPLAIGLTGQRVLGVPIIVQASQAEKNRAAAMAN
NLQKGSAGPMRLYVGSLHFNITEDMLRGIFEPFGRIESIQLMMDSETGRSKGYGFITFSD
SECAKKALEQLNGFELAGRPMKVGHVTERTDASSASSFLDSDELERTGIDLGTTGRLQLM
ARLAEGTGLQIPPAAQQALQMSGSLAFGAVAEFSFVIDLQTRLSQQTEASALAAAASVQP

LATQCFQLSNMFNPQTEEEVGWDTEIKDDVIEECNKHGGVIHIYVDKNSAQGNVYVKCPS
IAAAIAAVNALHGRWFAGKMITAAYVPLPTYHNLFPDSMTATQLLVPSRR*
>ENSG00000130810|ENST00000253107
MGQSGRSRHQKRARAQAQLRNLEAYAANPHSFVFTRGCTGRNIRQLSLDVRRVMEPLTAS
RLQVRKKNSLKDCVAVAGPLGVTHFLILSKTETNVYFKLMRLPGGPTLTFQVKKYSLVRD
VVSSLRRHRMHEQQFAHPPLLVLNSFGPHGMHVKLMATMFQNLFPSINVHKVNLNTIKRC
LLIDYNPDSQELDFRHYSIKVVPVGASRGMKKLLQEKFPNMSRLQDISELLATGAGLSES
EAEPDGDHNITELPQAVAGRGNMRAQQSAVRLTEIGPRMTLQLIKVQEGVGEGKVMFHSF
VSKTEEELQAILEAKEKKLRLKAQRQAQQAQNVQRKQEQREAHRKKSLEGMKKARVGGSD
EEASGIPSRTASLELGEDDDEQEDDDIEYFCQAVGEAPSEDLFPEAKQKRLAKSPGRKRK
RWEMDRGRGRLCDQKFPKTKDKSQGAQARRGPRGASRDGGRGRGRGRPGKRVA*
>ENSG00000072858|ENST00000264852
MRGCLRLALLCALPWLLLAASPGHPAKSPRQPPAPRRDPFDAARGADFDHVYSGVVNLST
ENIYSFNYTSQPDQVTAVRVYVNSSSENLNYPVLVVVRQQKEVLSWQVPLLFQGLYQRSY
NYQEVSRTLCPSEATNETGPLQQLIFVDVASMAPLGAQYKLLVTKLKHFQLRTNVAFHFT
ASPSQPQYFLYKFPKDVDSVIIKVVSEMAYPCSVVSVQNIMCPVYDLDHNVEFNGVYQSM
TKKAAITLQKKDFPGEQFFVVFVIKPEDYACGGSFFIQEKENQTWNLQRKKNLEVTIVPS
IKESVYVKSSLFSVFIFLSFYLGCLLVGFVHYLRFQRKSIDGSFGSNDGSGNMVASHPIA
ASTPEGSNYGTIDESSSSPGRQMSSSDGGPPGQSDTDSSVEESDFDTMPDIESDKNIIRT
KMFLYLSDLSRKDRRIVSKKYKIYFWNIITIAVFYALPVIQLVITYQTVVNVTGNQDICY
YNFLCAHPLGVLSAFNNILSNLGHVLLGFLFLLIVLRRDILHRRALEAKDIFAVEYGIPK
HFGLFYAMGIALMMEGVLSACYHVCPNYSNFQFDTSFMYMIAGLCMLKLYQTRHPDINAS
AYSAYASFAVVIMVTVLGVVFGKNDVWFWVIFSAIHVLASLALSTQIYYMGRFKIDLGIF
RRAAMVFYTDCIQQCSRPLYMDRMVLLVVGNLVNWSFALFGLIYRPRDFASYMLGIFICN
LLLYLAFYIIMKLRSSEKVLPVPLFCIVATAVMWAAALYFFFQNLSSWEGTPAESREKNR
ECILLDFFDDHDIWHFLSATALFFSFLVLLTLDDDLDVVRRDQIPVF*
>ENSG00000105610|ENST00000264834
MATAETALPSISTLTALGPFPDTQDDFLKWWRSEEAQDMGPGPPDPTEPPLHVKSEDQPG
EEEDDERGADATWDLDLLLTNFSGPEPGGAPQTCALAPSEASGAQYPPPPETLGAYAGGP
GLVAGLLGSEDHSGWVRPALRARAPDAFVGPALAPAPAPEPKALALQPVYPGPGAGSSGG
YFPRTGLSVPAASGAPYGLLSGYPAMYPAPQYQGHFQLFRGLQGPAPGPATSPSFLSCLG
PGTVGTGLGGTAEDPGVIAETAPSKRGRRSWARKRQAAHTCAHPGCGKSYTKSSHLKAHL
RTHTGEKPYACTWEGCGWRFARSDELTRHYRKHTGQRPFRCQLCPRAFSRSDHLALHMKR
HL*
>ENSG00000136381|ENST00000258886
MDAPKAGYAFEYLIETLNDSSHKKFFDVSKLGTKYDVLPYSIRVLLEAAVRNCDGFLMKK
EDVMNILDWKTKQSNVEVPFFPARVLLQDFTGIPAMVDFAAMREAVKTLGGDPEKVHPAC
PTDLTVDHSLQIDFSKCAIQNAPNPGGGDLQKAGKLSPLKVQPKKLPCRGQTTCRGSCDS
GELGRNSGTFSSQIENTPILCPFHLQPVPEPETVLKNQEVEFGRNRERLQFFKWSSRVFK
NVAVIPPGTGMAHQINLEYLSRVVFEEKDLLFPDSVVGTDSHITMVNGLGILGWGVGGIE
TEAVMLGLPVSLTLPEVVGCELTGSSNPFVTSIDVVLGITKHLRQVGVAGKFVEFFGSGV
SQLSIVDRTTIANMCPEYGAILSFFPVDNVTLKHLEHTGFSKAKLESMETYLKAVKLFRN
DQNSSGEPEYSQVIQINLNSIVPSVSGPKRPQDRVAVTDMKSDFQACLNEKVGFKGFQIA
AEKQKDIVSIHYEGSEYKLSHGSVVIAAVISCTNNCNPSVMLAAGLLAKKAVEAGLRVKP

YIRTSLSPGSGMVTHYLSSSGVLPYLSKLGFEIVGYGCSICVGNTAPLSDAVLNAVKQGD
LVTCGILSGNKNFEGRLCDCVRANYLASPPLVVAYAIAGTVNIDFQTEPLGTDPTGKNIY
LHDIWPSREEVHRVEEEHVILSMFKALKDKIEMGNKRWNSLEAPDSVLFPWDLKSTYIRC
PSFFDKLTKEPIALQAIENAHVLLYLGDSVTTDHISPAGSIARNSAAAKYLTNRGLTPRE
FNSYGARRGNDAVMTRGTFANIKLFNKFIGKPAPKTIHFPSGQTLDVFEAAELYQKEGIP
LIILAGKKYGSGNSRDWAAKGPYLLGVKAVLAESYEKIHKDHLIGIGIAPLQFLPGENAD
SLGLSGRETFSLTFPEELSPGITLNIQTSTGKVFSVIASFEDDVEITLYKHGGLLNFVAR
KFS*
>ENSG00000197123|ENST00000255746
MAKRPGSPGSREMGLLTFRDVVIEFSLEEWQCLDHAQQNLYRDVMLENYRNLVSLGIAVS
KPDLITCLEQNKEPWNIKRNEMVTKHPVMCSHFTQDLPPELGIKDSLQKVIPRRYGKSGH
DNLQVKTCKSMGECEVQKGGCNEVNQCLSTTQNKIFQTHKCVKVFGKFSNSNRHKTRHTG
KKHFKCKKYGKSFCMVSQLHQHQIIHTRENSYQCEECGKPFNCSSTLSKHKRIHTGEKPY
RCEECGKAFTWSSTLTKHRRIHTGEKPYTCEECGQAFSRSSTLANHKRIHTGEKPYTCEE
CGKAFSLSSSLTYHKRIHTGEKPYTCEECGKAFNCSSTLKKHKIIHTGEKPYKCKECGKA
FAFSSTLNTHKRIHTGEEPYKCEECDKAFKWSSSLANHKSMHTGEKPYKCE*
>ENSG00000100603|ENST00000261531
MALTSFLPAPTQLSQDQLEAEEKARSQRSRQTSLVSSRREPPPYGYRKGWIPRLLEDFGD
GGAFPEIHVAQYPLDMGRKKKMSNALAIQVDSEGKIKYDAIARQGQSKDKVIYSKYTDLV
PKEVMNADDPDLQRPDEEAIKEITEKTRVALEKSVSQKVAAAMPVRAADKLAPAQYIRYT
PSQQGVAFNSGAKQRVIRMVEMQKDPMEPPRFKINKKIPRGPPSPPAPVMHSPSRKMTVK
EQQEWKIPPCISNWKNAKGYTIPLDKRLAADGRGLQTVHINENFAKLAEALYIADRKARE
AVEMRAQVERKMAQKEKEKHEEKLREMAQKARERRAGIKTHVEKEDGEARERDEIRHDRR
KERQHDRNLSRAAPDKRSKLQRNENRDISEVIALGVPNPRTSNEVQYDQRLFNQSKGMDS
GFAGGEDEIYNVYDQAWRGGKDMAQSIYRPSKNLDKDMYGDDLEARIKTNRFVPDKEFSG
SDRRQRGREGPVQFEEDPFGLDKFLEEAKQHGGSKRPSDSSRPKEHEHEGKKRRKE*
>ENSG00000104967|ENST00000263257
MEPEAPDSRKRPLETPPEVVCTKRSNTGEEGEYFLKVLIPSYAAGSIIGKGGQTIVQLQK
ETGATIKLSKSKDFYPGTTERVCLVQGTAEALNAVHSFIAEKVREIPQAMTKPEVVNILQ
PQTTMNPDRAKQAKLIVPNSTAGLIIGKGGATVKAVMEQSGAWVQLSQKPEGINLQERVV
TVSGEPEQVHKAVSAIVQKVQEDPQSSSCLNISYANVAGPVANSNPTGSPYASPADVLPA
AAAASAAAASGLLGPAGLAGVGAFPAALPAFSGTDLLAISTALNTLASYGYNTNSLGLGL
NSAAASGVLAAVAAGANPAAAAAANLLASYAGEAGAGPAGGAAPPPPPPPGALGSFALAA
AANGYLGAGAGGGAGGGGGPLVAAAAAAGAAGGFLTAEKLAAESAKELVEIAVPENLVGA
ILGKGGKTLVEYQELTGARIQISKKGEFLPGTRNRRVTITGSPAATQAAQYLISQRVTYE
QGVRASNPQKVG*
>ENSG00000073282|ENST00000264731
MNFETSRCATLQYCPDPYIQRFVETPAHFSWKESYYRSTMSQSTQTNEFLSPEVFQHIWD
FLEQPICSVQPIDLNFVDEPSEDGATNKIEISMDCIRMQDSDLSDPMWPQYTNLGLLNSM
DQQIQNGSSSTSPYNTDHAQNSVTAPSPYAQPSSTFDALSPSPAIPSNTDYPGPHSFDVS
FQQSSTAKSATWTYSTELKKLYCQIAKTCPIQIKVMTPPPQGAVIRAMPVYKKAEHVTEV
VKRCPNHELSREFNEGQIAPPSHLIRVEGNSHAQYVEDPITGRQSVLVPYEPPQVGTEFT
TVLYNFMCNSSCVGGMNRRPILIIVTLETRDGQVLGRRCFEARICACPGRDRKADEDSIR
KQQVSDSTKNGDGTKRPFRQNTHGIQMTSIKKRRSPDDELLYLPVRGRETYEMLLKIKES

LELMQYLPQHTIETYRQQQQQQHQHLLQKQTSIQSPSSYGNSSPPLNKMNSMNKLPSVSQ
LINPQQRNALTPTTIPDGMGANIPMMGTHMPMAGDMNGLSPTQALPPPLSMPSTSHCTPP
PPYPTDCSIVSFLARLGCSSCLDYFTTQGLTTIYQIEHYSMDDLASLKIPEQFRHAIWKG
ILDHRQLHEFSSPSHLLRTPSSASTVSVGSSETRGERVIDAVRFTLRQTISFPPRDEWND
FNFDMDARRNKQQRIKEEGE*
>ENSG00000256294|ENST00000262894
MTTLKEAVTFKDVAVVFTEEELRLLDLAQRKLYREVMLENFRNLLSVGHQSLHRDTFHFL
KEEKFWMMETATQREGNLGGKIQMEMETVSESGTHEGLFSHQTWEQISSDLTRFQDSMVN
SFQFSKQDDMPCQVDAGLSIIHVRQKPSEGRTCKKSFSDVSVLDLHQQLQSREKSHTCDE
CGKSFCYSSALRIHQRVHMGEKLYNCDVCGKEFNQSSHLQIHQRIHTGEKPFKCEQCGKG
FSRRSGLYVHRKLHTGVKPHICEKCGKAFIHDSQLQEHQRIHTGEKPFKCDICCKSFRSR
ANLNRHSMVHMREKPFRCDTCGKSFGLKSALNSHRMVHTGEKRYKCEECGKRFIYRQDLY
KHQIDHTGEKPYNCKECGKSFRWASGLSRHVRVHSGETTFKCEECGKGFYTNSQRYSHQR
AHSGEKPYRCEECGKGYKRRLDLDFHQRVHRGEKPYNCKECGKSFGWASCLLNHQRIHSG
EKPFKCEECGKRFTQNSQLYTHRRVHSGEKPFKCEECGKRFTQNSQLYSHRRVHTGVKPY
KCEECGKGFNSKFNLDMHQRVHTGERPYNCKECGKSFSRASSILNHKRLHGDEKPFKCEE
CGKRFTENSQLHSHQRVHTGEKPYKCEKCGKSFRWASTHLTHQRLHSREKLLQCEDCGKS
IVHSSCLKDQQRDQSGEKTSKCEDCGKRYKRRLNLDTLLSLFLNDT*
>ENSG00000132837|ENST00000255189
MLRPGAQLLRGLLLRSCPLQGSPGRPRSVCGREGEEKPPLSAETQWKDRAETVIIGGGCV
GVSLAYHLAKAGMKDVVLLEKSELTAGSTWHAAGLTTYFHPGINLKKIHYDSIKLYEKLE
EETGQVVGFHQPGSIRLATTPVRVDEFKYQMTRTGWHATEQYLIEPEKIQEMFPLLNMNK
VLAGLYNPGDGHIDPYSLTMALAAGARKCGALLKYPAPVTSLKARSDGTWDVETPQGSMR
ANRIVNAAGFWAREVGKMIGLEHPLIPVQHQYVVTSTISEVKALKRELPVLRDLEGSYYL
RQERDGLLFGPYESQEKMKVQDSWVTNGVPPGFGKELFESDLDRIMEHIKAAMEMVPVLK
KADIINVVNGPITYSPDILPMVGPHQGVRNYWVAIGFGYGIIHAGGVGKYLSDWILHGEP
PFDLIELDPNRYGKWTTTQYTEAKARESYGFNNIVGYPKEERFAGRPTQRVSGLYQRLES
KCSMGFHAGWEQPHWFYKPGQDTQYRPSFRRTNWFEPVGSEYKQVMQRVAVTDLSPFGKF
NIKGQDSIRLLDHLFANVIPKVGFTNISHMLTPKGRVYAELTVSHQSPGEFLLITGSGSE
LHDLRWIEEEAVKGGYDVEIKNITDELGVLGVAGPQARKVLQKLTSEDLSDDVFKFLQTK
SLKVSNIPVTAIRISYTGELGWELYHRREDSVALYDAIMNAGQEEGIDNFGTYAMNALRL
EKAFRAWGLEMNCDTNPLEAGLEYFVKLNKPADFIGKQALKQIKAKGLKRRLVCLTLATD
DVDPEGNESIWYNGKVVGNTTSGSYSYSIQKSLAFAYVPVQLSEVGQQVEVELLGKNYPA
VIIQEPLVLTEPTRNRLQKKGGKDKT*
>ENSG00000121454|ENST00000263726
MMQSATVPAEGAVKGLPEMLGVPMQQIPQCAGCNQHILDKFILKVLDRHWHSSCLKCADC
QMQLADRCFSRAGSVYCKEDFFKRFGTKCTACQQGIPPTQVVRKAQDFVYHLHCFACIIC
NRQLATGDEFYLMEDGRLVCKEDYETAKQNDDSEAGAKRPRTTITAKQLETLKNAYKNSP
KPARHVREQLSSETGLDMRVVQVWFQNRRAKEKRLKKDAGRHRWGQFYKSVKRSRGSSKQ
EKESSAEDCGVSDSELSFREDQILSELGHTNRIYGNVGDVTGGQLMNGSFSMDGTGQSYQ
DLRDGSPYGIPQSPSSISSLPSHAPLLNGLDYTVDSNLGIIAHAGQGVSQTLRAMAGGPT
SDISTGSSVGYPDFPTSPGSWLDEMDHPPF*
>ENSG00000135638|ENST00000258106
MCLAGCTPRKAAAPGRGALPRARLPRTAPAAATMFQPAAKRGFTIESLVAKDGGTGGGTG

GGGAGSHLLAAAASEEPLRPTALNYPHPSAAEAAFVSGFPAAAAAGAGRSLYGGPELVFP
EAMNHPALTVHPAHQLGASPLQPPHSFFGAQHRDPLHFYPWVLRNRFFGHRFQASDVPQD
GLLLHGPFARKPKRIRTAFSPSQLLRLERAFEKNHYVVGAERKQLAGSLSLSETQVKVWF
QNRRTKYKRQKLEEEGPESEQKKKGSHHINRWRIATKQANGEDIDVTSND*
>ENSG00000136243|ENST00000258742
MAICQFFLQGRCRFGDRCWNEHPGARGAGGGRQQPQQQPSGNNRRGWNTTSQRYSNVIQP
SSFSKSTPWGGSRDQEKPYFSSFDSGASTNRKEGFGLSENPFASLSPDEQKDEKKLLEGI
VKDMEVWESSGQWMFSVYSPVKKKPNISGFTDISPEELRLEYHNFLTSNNLQSYLNSVQR
LINQWRNRVNELKSLNISTKVALLSDVKDGVNQAAPAFGFGSSQAATFMSPGFPVNNSSS
DNAQNFSFKTNSGFAAASSGSPAGFGSSPAFGAAASTSSGISTSAPAFGFGKPEVTSAAS
FSFKSPAASSFGSPGFSGLPASLATGPVRAPVAPAFGGGSSVAGFGSPGSHSHTAFSKPS
SDTFGNSSISTSLSASSSIIATDNVLFTPRDKLTVEELEQFQSKKFTLGKIPLKPPPLEL
LNV*
>ENSG00000135930|ENST00000258416
MNNKFDALKDDDSGDHDQNEENSTQKDGEKEKTERDKNQSSSKRKAVVPGPAEHPLQYNY
TFWYSRRTPGRPTSSQSYEQNIKQIGTFASVEQFWRFYSHMVRPGDLTGHSDFHLFKEGI
KPMWEDDANKNGGKWIIRLRKGLASRCWENLILAMLGEQFMVGEEICGAVVSVRFQEDII
SIWNKTASDQATTARIRDTLRRVLNLPPNTIMEYKTHTDSIKMPGRLGPQRLLFQNLWKP
RLNVP*
>ENSG00000197037|ENST00000262941
MAEAPQQQLGIPVVKLEKELPWGRGREDPSPETFRLRFRQFRYQEAAGPQEALRELQELC
RRWLRPELHTKEQILELLVLEQFLTILPREFYAWIREHGPESGKALAAMVEDLTERALEA
KAVPCHRQGEQEETALCRGAWEPGIQLGPVEVKPEWGMPPGEGVQGPDPGTEEQLSQDPG
DETRAFQEQGGGSKEKEAKPPQEDLKGALVALTSERFGEASLQGPGLGRVCEQEPGGPAG
SAPGLPPPQHGAIPLPDEVKTHSSFWKPFQCPECGKGFSRSSNLVRHQRTHEEKSYGCVE
CGKGFTLREYLMKHQRTHLGKRPYVCSECWKTFSQRHHLEVHQRSHTGEKPYKCGDCWKS
FSRRQHLQVHRRTHTGEKPYTCECGKSFSRNANLAVHRRAHTGEKPYGCQVCGKRFSKGE
RLVRHQRIHTGEKPYHCPACGRSFNQRSILNRHQKTQHRQEPLVQ*
>ENSG00000037474|ENST00000264670
MGRRSRGRRLQQQQRPEDAEDGAEGGGKRGEAGWEGGYPEIVKENKLFEHYYQELKIVPE
GEWGQFMDALREPLPATLRITGYKSHAKEILHCLKNKYFKELEDLEVDGQKVEVPQPLSW
YPEELAWHTNLSRKILRKSPHLEKFHQFLVSETESGNISRQEAVSMIPPLLLNVRPHHKI
LDMCAAPGSKTTQLIEMLHADMNVPFPEGFVIANDVDNKRCYLLVHQAKRLSSPCIMVVN
HDASSIPRLQIDVDGRKEILFYDRILCDVPCSGDGTMRKNIDVWKKWTTLNSLQLHGLQL
RIATRGAEQLAEGGRMVYSTCSLNPIEDEAVIASLLEKSEGALELADVSNELPGLKWMPG
ITQWKVMTKDGQWFTDWDAVPHSRHTQIRPTMFPPKDPEKLQAMHLERCLRILPHHQNTG
GFFVAVLVKKSSMPWNKRQPKLQGKSAETRESTQLSPADLTEGKPTDPSKLESPSFTGTG
DTEIAHATEDLENNGSKKDGVCGPPPSKKMKLFGFKEDPFVFIPEDDPLFPPIEKFYALD
PSFPRMNLLTRTTEGKKRQLYMVSKELRNVLLNNSEKMKVINTGIKVWCRNNSGEEFDCA
FRLAQEGIYTLYPFINSRIITVSMEDVKILLTQENPFFRKLSSETYSQAKDLAKGSIVLK
YEPDSANPDALQCPIVLCGWRGKASIRTFVPKNERLHYLRMMGLEVLGEKKKEGVILTNE
SAASTGQPDNDVTEGQRAGEPNSPDAEEANSPDVTAGCDPAGVHPPR*
>ENSG00000086666|ENST00000261749
MAQETNHSQVPMLCSTGCGFYGNPRTNGMCSVCYKEHLQRQNSSNGRISPPATSVSSLSE

SLPVQCTDGSVPEAQSALDSTSSSMQPSPVSNQSLLSESVASSQLDSTSVDKAVPETEDV
QASVSDTAQQPSEEQSKSLEKPKQKKNRCFMCRKKVGLTGFECRCGNVYCGVHRYSDVHN
CSYNYKADAAEKIRKENPVVVGEKIQKI*
>ENSG00000109787|ENST00000261438
MLMFDPVPVKQEAMDPVSVSYPSNYMESMKPNKYGVIYSTPLPEKFFQTPEGLSHGIQME
PVDLTVNKRSSPPSAGNSPSSLKFPSSHRRASPGLSMPSSSPPIKKYSPPSPGVQPFGVP
LSMPPVMAAALSRHGIRSPGILPVIQPVVVQPVPFMYTSHLQQPLMVSLSEEMENSSSSM
QVPVIESYEKPISQKKIKIEPGIEPQRTDYYPEEMSPPLMNSVSPPQALLQENHPSVIVQ
PGKRPLPVESPDTQRKRRIHRCDYDGCNKVYTKSSHLKAHRRTHTGEKPYKCTWEGCTWK
FARSDELTRHFRKHTGIKPFQCPDCDRSFSRSDHLALHRKRHMLV*
>ENSG00000009307|ENST00000261443
MSFDPNLLHNNGHNGYPNGTSAALRETGVIEKLLTSYGFIQCSERQARLFFHCSQYNGNL
QDLKVGDDVEFEVSSDRRTGKPIAVKLVKIKQEILPEERMNGQEVFYLTYTPEDVEGNVQ
LETGDKINFVIDNNKHTGAVSARNIMLLKKKQARCQGVVCAMKEAFGFIERGDVVKEIFF
HYSEFKGDLETLQPGDDVEFTIKDRNGKEVATDVRLLPQGTVIFEDISIEHFEGTVTKVI
PKVPSKNQNDPLPGRIKVDFVIPKELPFGDKDTKSKVTLLEGDHVRFNISTDRRDKLERA
TNIEVLSNTFQFTNEAREMGVIAAMRDGFGFIKCVDRDVRMFFHFSEILDGNQLHIADEV
EFTVVPDMLSAQRNHAIRIKKLPKGTVSFHSHSDHRFLGTVEKEATFSNPKTTSPNKGKE
KEAEDGIIAYDDCGVKLTIAFQAKDVEGSTSPQIGDKVEFSISDKQRPGQQVATCVRLLG
RNSNSKRLLGYVATLKDNFGFIETANHDKEIFFHYSEFSGDVDSLELGDMVEYSLSKGKG
NKVSAEKVNKTHSVNGITEEADPTIYSGKVIRPLRSVDPTQTEYQGMIEIVEEGDMKGEV
YPFGIVGMANKGDCLQKGESVKFQLCVLGQNAQTMAYNITPLRRATVECVKDQFGFINYE
VGDSKKLFFHVKEVQDGIELQAGDEVEFSVILNQRTGKCSACNVWRVCEGPKAVAAPRPD
RLVNRLKNITLDDASAPRLMVLRQPRGPDNSMGFGAERKIRQAGVID*
>ENSG00000068784|ENST00000263736
MSSLPRRAKVQVQDVVLKDEFSSFSELSSASEEDDKEDSAWEPQKKVPRSRKQPPPKESK
PKRMPRVKKNAPQISDGSEVVVVKEELNSSVAIADTALEDRKNKLDTVQTLKTAKTKQKC
AAQPHTVRRTKKLKVEEETSKASNLEGESNSSETPSTSTVWGGTCKKEENDDDFTFGQSA
LKKIKTETYPQGQPVKFPANANSTKEEVEMNWDMVQVLSERTNIEPWVCANIIRLFNDDN
TIPFIIRYRKELINNLDADSLREVQQTLEELRAVAKKVHSTIQKIKKEGKMSECLLKAML
NCKTFEELEHVSAPYKTGSKGTKAQRARQLGLEGAARALLEKPGELSLLSYIRPDVKGLS
TLQDIEIGVQHILADMIAKDKDTLDFIRNLCQKRHVCIQSSLAKVSSKKVNEKDVDKFLL
YQHFSCNIRNIHHHQILAINRGENLKVLTVKVNISDGVKDEFCRWCIQNRWRPRSFARPE
LMKILYNSLNDSFKRLIYPLLCREFRAKLTSDAEKESVMMFGRNLRQLLLTSPVPGRTLM
GVDPGYKHGCKLAIISPTSQILHTDVVYLHCGQGFREAEKIKTLLLNFNCSTVVIGNGTA
CRETEAYFADLIMKNYFAPLDVVYCIVSEAGASIYSVSPEANKEMPGLDPNLRSAVSIAR
RVQDPLAELVKIEPKHIGVGMYQHDVSQTLLKATLDSVVEECVSFVGVDINICSEVLLRH
IAGLNANRAKNIIEWREKNGPFINREQLKKVKGLGPKSFQQCAGFIRINQDYIRTFCSQQ
TETSGQIQGVAVTSSADVEVTNEKQGKKKSKTAVNVLLKPNPLDQTCIHPESYDIAMRFL
SSIGGTLYEVGKPEMQQKINSFLEKEGMEKIAERLQTTVHTLQVIIDGLSQPESFDFRTD
FDKPDFKRSIVCLEDLQIGTVLTGKVENATLFGIFVDIGVGKSGLIPIRNVTEAKLSKTK
KRRSLGLGPGERVEVQVLNIDIPRSRITLDLIRVL*
>ENSG00000135698|ENST00000258169
MAAERKTRLSKNLLRMKFMQRGLDSETKKQLEEEEKKIISEEHWYLDLPELKEKESFIIE

EQSFLLCEDLLYGRMSFRGFNPEVEKLMLQMNAKHKAEEVEDETVELDVSDEEMARRYET
LVGTIGKKFARKRDHANYEEDENGDITPIKAKKMFLKPQD*
>ENSG00000059378|ENST00000263549
MAQAGVVGEVTQVLCAAGGALELPELRRRLRMGLSADALERLLRQRGRFVVAVRAGGAAA
APERVVLAASPLRLCRAHQGSKPGCVGLCAQLHLCRFMVYGACKFLRAGKNCRNSHSLTT
EHNLSVLRTHGVDHLSYNELCQLLFQNDPWLLPEICQHYNKGDGPHGSCAFQKQCIKLHI
CQYFLQGECKFGTSCKRSHDFSNSENLEKLEKLGMSSDLVSRLPTIYRNAHDIKNKSSAP
SRVPPLFVPQGTSERKDSSGSVSPNTLSQEEGDQICLYHIRKSCSFQDKCHRVHFHLPYR
WQFLDRGKWEDLDNMELIEEAYCNPKIERILCSESASTFHSHCLNFNAMTYGATQARRLS
TASSVTKPPHFILTTDWIWYWSDEFGSWQEYGRQGTVHPVTTVSSSDVEKAYLAYCTPGS
DGQAATLKFQAGKHNYELDFKAFVQKNLVYGTTKKVCRRPKYVSPQDVTTMQTCNTKFPG
PKSIPDYWDSSALPDPGFQKITLSSSSEEYQKVWNLFNRTLPFYFVQKIERVQNLALWEV
YQWQKGQMQKQNGGKAVDERQLFHGTSAIFVDAICQQNFDWRVCGVHGTSYGKGSYFARD
AAYSHHYSKSDTQTHTMFLARVLVGEFVRGNASFVRPPAKEGWSNAFYDSCVNSVSDPSI
FVIFEKHQVYPEYVIQYTTSSKPSVTPSILLALGSLFSSRQ*
>ENSG00000136271|ENST00000258772
MEDSEALGFEHMGLDPRLLQAVTDLGWSRPTLIQEKAIPLALEGKDLLARARTGSGKTAA
YAIPMLQLLLHRKATGPVVEQAVRGLVLVPTKELARQAQSMIQQLATYCARDVRVANVSA
AEDSVSQRAVLMEKPDVVVGTPSRILSHLQQDSLKLRDSLELLVVDEADLLFSFGFEEEL
KSLLCHLPRIYQAFLMSATFNEDVQALKELILHNPVTLKLQESQLPGPDQLQQFQVVCET
EEDKFLLLYALLKLSLIRGKSLLFVNTLERSYRLRLFLEQFSIPTCVLNGELPLRSRCHI
ISQFNQGFYDCVIATDAEVLGAPVKGKRRGRGPKGDKASDPEAGVARGIDFHHVSAVLNF
DLPPTPEAYIHRAGRTARANNPGIVLTFVLPTEQFHLGKIEELLSGENRGPILLPYQFRM
EEIEGFRYRCRDAMRSVTKQAIREARLKEIKEELLHSEKLKTYFEDNPRDLQLLRHDLPL
HPAVVKPHLGHVPDYLVPPALRGLVRPHKKRKKLSSSCRKAKRAKSQNPLRSFKHKGKKF
RPTAKPS*
>ENSG00000128829|ENST00000263791
MAGGRGAPGRGRDEPPESYPQRQDHELQALEAIYGADFQDLRPDACGPVKEPPEINLVLY
PQGLTGEEVYVKVDLRVKCPPTYPDVVPEIELKNAKGLSNESVNLLKSRLEELAKKHCGE
VMIFELAYHVQSFLSEHNKPPPKSFHEEMLERRAQEEQQRLLEAKRKEEQEQREILHEIQ
RRKEEIKEEKKRKEMAKQERLEIASLSNQDHTSKKDPGGHRTAAILHGGSPDFVGNGKHR
ANSSGRSRRERQYSVCNSEDSPGSCEILYFNMGSPDQLMVHKGKCIGSDEQLGKLVYNAL
ETATGGFVLLYEWVLQWQKKMGPFLTSQEKEKIDKCKKQIQGTETEFNSLVKLSHPNVVR
YLAMNLKEQDDSIVVDILVEHISGVSLAAHLSHSGPIPVHQLRRYTAQLLSGLDYLHSNS
VVHKVLSASNVLVDAEGTVKITDYSISKRLADICKEDVFEQTRVRFSDNALPYKTGKKGD
VWRLGLLLLSLSQGQECGEYPVTIPSDLPADFQDFLKKCVCLDDKERWSPQQLLKHSFIN
PQPKMPLVEQSPEDSEGQDYVETVIPSNRLPSAAFFSETQRQFSRYFIEFEELQLLGKGA
FGAVIKVQNKLDGCCYAVKRIPINPASRQFRRIKGEVTLLSRLHHENIVRYYNAWIERHE
RPAGPGTPPPDSGPLAKDDRAARGQPASDTDGLDSVEAAAPPPILSSSVEWSTSGERSAS
ARFPATGPGSSDDEDDDEDEHGGVFSQSFLPASDSESDIIFDNEDENSKSQNQDEDCNEK
NGCHESEPSVTTEAVHYLYIQMEYCEKSTLRDTIDQGLYRDTVRLWRLFREILDGLAYIH
EKGMIHRDLKPVNIFLDSDDHVKIGDFGLATDHLAFSADSKQDDQTGDLIKSDPSGHLTG
MVGTALYVSPEVQGSTKSAYNQKVDLFSLGIIFFEMSYHPMVTASERIFVLNQLRDPTSP
KFPEDFDDGEHAKQKSVISWLLNHDPAKRPTATELLKSELLPPPQMEESELHEVLHHTLT

NVDGKAYRTMMAQIFSQRISPAIDYTYDSDILKGNFSIRTAKMQQHVCETIIRIFKRHGA
VQLCTPLLLPRNRQIYEHNEAALFMDHSGMLVMLPFDLRIPFARYVARNNILNLKRYCIE
RVFRPRKLDRFHPKELLECAFDIVTSTTNSFLPTAEIIYTIYEIIQEFPALQERNYSIYL
NHTMLLKAILLHCGIPEDKLSQVYIILYDAVTEKLTRREVEAKFCNLSLSSNSLCRLYKF
IEQKGDLQDLMPTINSLIKQKTGIAQLVKYGLKDLEEVVGLLKKLGIKLQVLINLGLVYK
VQQHNGIIFQFVAFIKRRQRAVPEILAAGGRYDLLIPQFRGPQALGPVPTAIGVSIAIDK
ISAAVLNMEESVTISSCDLLVVSVGQMSMSRAINLTQKLWTAGITAEIMYDWSQSQEELQ
EYCRHHEITYVALVSDKEGSHVKVKSFEKERQTEKRVLETELVDHVLQKLRTKVTDERNG
REASDNLAVQNLKGSFSNASGLFEIHGATVVPIVSVLAPEKLSASTRRRYETQVQTRLQT
SLANLHQKSSEIEILAVDLPKETILQFLSLEWDADEQAFNTTVKQLLSRLPKQRYLKLVC
DEIYNIKVEKKVSVLFLYSYRDDYYRILF*
>ENSG00000137944|ENST00000260508
MFLAQRSLCSLSGRAKFLKTISSSKILGFSTSAKMSLKFTNAKRIEGLDSNVWIEFTKLA
ADPSVVNLGQGFPDISPPTYVKEELSKIAAIDSLNQYTRGFGHPSLVKALSYLYEKLYQK
QIDSNKEILVTVGAYGSLFNTIQALIDEGDEVILIVPFYDCYEPMVRMAGATPVFIPLRS
KPVYGKRWSSSDWTLDPQELESKFNSKTKAIILNTPHNPLGKVYNREELQVIADLCIKYD
TLCISDEVYEWLVYSGNKHLKIATFPGMWERTITIGSAGKTFSVTGWKLGWSIGPNHLIK
HLQTVQQNTIYTCATPLQEALAQAFWIDIKRMDDPECYFNSLPKELEVKRDRMVRLLESV
GLKPIVPDGGYFIIADVSLLDPDLSDMKNNEPYDYKFVKWMTKHKKLSAIPVSAFCNSET
KSQFEKFVRFCFIKKDSTLDAAEEIIKAWSVQKS*
>ENSG00000169951|ENST00000252797
MAPPLAPLPPRDPNGAGPEWREPGAVSFADVAVYFCREEWGCLRPAQRALYRDVMRETYG
HLSALGIGGNKPALISWVEEEAELWGPAAQDPEVAKCQTQTDPADSRNKKKERQREGTGA
LEKPDPVAAGSPGLKSPQAPSAGPPYGWEQLSKAPHRGRPSLCAHPPVPRADQRHGCYVC
GKSFAWRSTLVEHVYSHTGEKPFHCTDCGKGFGHASSLSKHRAIHRGERPHRCLECGRAF
TQRSALTSHLRVHTGEKPYGCADCGRRFSQSSALYQHRRVHSGETPFPCPDCGRAFAYPS
DLRRHVRTHTGEKPYPCPDCGRCFRQSSEMAAHRRTHSGEKPYPCPQCGRRFGQKSAVAK
HQWVHRPGAGGHRGRVAGRLSVTLTPGHGDLDPPVGFQLYPEIFQECG*
>ENSG00000115267|ENST00000263642
MSNGYSTDENFRYLISCFRARVKMYIQVEPVLDYLTFLPAEVKEQIQRTVATSGNMQAVE
LLLSTLEKGVWHLGWTREFVEALRRTGSPLAARYMNPELTDLPSPSFENAHDEYLQLLNL
LQPTLVDKLLVRDVLDKCMEEELLTIEDRNRIAAAENNGNESGVRELLKRIVQKENWFSA
FLNVLRQTGNNELVQELTGSDCSESNAEIENLSQVDGPQVEEQLLSTTVQPNLEKEVWGM
ENNSSESSFADSSVVSESDTSLAEGSVSCLDESLGHNSNMGSDSGTMGSDSDEENVAARA
SPEPELQLRPYQMEVAQPALEGKNIIICLPTGSGKTRVAVYIAKDHLDKKKKASEPGKVI
VLVNKVLLVEQLFRKEFQPFLKKWYRVIGLSGDTQLKISFPEVVKSCDIIISTAQILENS
LLNLENGEDAGVQLSDFSLIIIDECHHTNKEAVYNNIMRHYLMQKLKNNRLKKENKPVIP
LPQILGLTASPGVGGATKQAKAEEHILKLCANLDAFTIKTVKENLDQLKNQIQEPCKKFA
IADATREDPFKEKLLEIMTRIQTYCQMSPMSDFGTQPYEQWAIQMEKKAAKEGNRKERVC
AEHLRKYNEALQINDTIRMIDAYTHLETFYNEEKDKKFAVIEDDSDEGGDDEYCDGDEDE
DDLKKPLKLDETDRFLMTLFFENNKMLKRLAENPEYENEKLTKLRNTIMEQYTRTEESAR
GIIFTKTRQSAYALSQWITENEKFAEVGVKAHHLIGAGHSSEFKPMTQNEQKEVISKFRT
GKINLLIATTVAEEGLDIKECNIVIRYGLVTNEIAMVQARGRARADESTYVLVAHSGSGV
IEHETVNDFREKMMYKAIHCVQNMKPEEYAHKILELQMQSIMEKKMKTKRNIAKHYKNNP

SLITFLCKNCSVLACSGEDIHVIEKMHHVNMTPEFKELYIVRENKALQKKCADYQINGEI
ICKCGQAWGTMMVHKGLDLPCLKIRNFVVVFKNNSTKKQYKKWVELPITFPNLDYSECCL
FSDED*
>ENSG00000084092|ENST00000264230
MLPARLPFRLLSLFLRGSAPTAARHGLREPLLERRCAAASSFQHSSSLGRELPYDPVDTE
GFGEGGDMQERFLFPEYILDPEPQPTREKQLQELQQQQEEEERQRQQRREERRQQNLRAR
SREHPVVGHPDPALPPSGVNCSGCGAELHCQDAGVPGYLPREKFLRTAEADGGLARTVCQ
RCWLLSHHRRALRLQVSREQYLELVSAALRRPGPSLVLYMVDLLDLPDALLPDLPALVGP
KQLIVLGNKVDLLPQDAPGYRQRLRERLWEDCARAGLLLAPGHQGPQRPVKDEPQDGENP
NPPNWSRTVVRDVRLISAKTGYGVEELISALQRSWRYRGDVYLVGATNAGKSTLFNTLLE
SDYCTAKGSEAIDRATISPWPGTTLNLLKFPICNPTPYRMFKRHQRLKKDSTQAEEDLSE
QEQNQLNVLKKHGYVVGRVGRTFLYSEEQKDNIPFEFDADSLAFDMENDPVMGTHKSTKQ
VELTAQDVKDAHWFYDTPGITKENCILNLLTEKEVNIVLPTQSIVPRTFVLKPGMVLFLG
AIGRIDFLQGNQSAWFTVVASNILPVHITSLDRADALYQKHAGHTLLQIPMGGKERMAGF
PPLVAEDIMLKEGLGASEAVADIKFSSAGWVSVTPNFKDRLHLRGYTPEGTVLTVRPPLL
PYIVNIKGQRIKKSVAYKTKKPPSLMYNVRKKKGKINV*
>ENSG00000075426|ENST00000264716
MYQDYPGNFDTSSRGSSGSPAHAESYSSGGGGQQKFRVDMPGSGSAFIPTINAITTSQDL
QWMVQPTVITSMSNPYPRSHPYSPLPGLASVPGHMALPRPGVIKTIGTTVGRRRRDEQLS
PEEEEKRRIRRERNKLAAAKCRNRRRELTEKLQAETEELEEEKSGLQKEIAELQKEKEKL
EFMLVAHGPVCKISPEERRSPPAPGLQPMRSGGGSVGAVVVKQEPLEEDSPSSSSAGLDK
AQRSVIKPISIAGGFYGEEPLHTPIVVTSTPAVTPGTSNLVFTYPSVLEQESPASPSESC
SKAHRRSSSSGDQSSDSLNSPTLLAL*
>ENSG00000134686|ENST00000257118
MENELPVPHTSSSACATSSTSGASSSSGCNNSSSGGSGRPTGPQISVYSGIPDRQTVQVI
QQALHRQPSTAAQYLQQMYAAQQQHLMLQTAALQQQHLSSAQLQSLAAVQQASLVSNRQG
STSGSNVSAQAPAQSSSINLAASPAAAQLLNRAQSVNSAAASGIAQQAVLLGNTSSPALT
ASQAQMYLRAQMLIFTPTATVATVQPELGTGSPARPPTPAQVQNLTLRTQQTPAAAASGP
TPTQPVLPSLALKPTPGGSQPLPTPAQSRNTAQASPAGAKPGIADSVMEPHKKGDGNSSV
PGSMEGRAGLSRTVPAVAAHPLIAPAYAQLQPHQLLPQPSSKHLQPQFVIQQQPQPQQQQ
PPPQQSRPVLQAEPHPQLASVSPSVALQPSSEAHAMPLGPVTPALPLQCPTANLHKPGGS
QQCHPPTPDTGPQNGHPEGVPHTPQRRFQHTSAVILQLQPASPPQQCVPDDWKEVAPGEK
SVPETRSGPSPHQQAIVTAMPGGLPVPTSPNIQPSPAHETGQGIVHALTDLSSPGMTSGN
GNSASSIAGTAPQNGENKPPQAIVKPQILTHVIEGFVIQEGAEPFPVGRSSLLVGNLKKK
YAQGFLPEKLPQQDHTTTTDSEMEEPYLQESKEEGAPLKLKCELCGRVDFAYKFKRSKRF
CSMACAKRYNVGCTKRVGLFHSDRSKLQKAGAATHNRRRASKASLPPLTKDTKKQPTGTV
PLSVTAALQLTHSQEDSSRCSDNSSYEEPLSPISASSSTSRRRQGQRDLELPDMHMRDLV
GMGHHFLPSEPTKWNVEDVYEFIRSLPGCQEIAEEFRAQEIDGQALLLLKEDHLMSAMNI
KLGPALKIYARISMLKDS*
>ENSG00000084463|ENST00000261167
MGRRSTSSTKSGKFMNPTDQARKEARKRELKKNKKQRMMVRAAVLKMKDPKQIIRDMEKL
DEMEFNPVQQPQLNEKVLKDKRKKLRETFERILRLYEKENPDIYKELRKLEVEYEQKRAQ
LSQYFDAVKNAQHVEVESIPLPDMPHAPSNILIQDIPLPGAQPPSILKKTSAYGPPTRAV
SILPLLGHGVPRLPPGRKPPGPPPGPPPPQVVQMYGRKVGFALDLPPRRRDEDMLYSPEL

AQRGHDDDVSSTSEDDGYPEDMDQDKHDDSTDDSDTDKSDGESDGDEFVHRDNGERDNNE
EKKSGLSVRFADMPGKSRKKKKNMKELTPLQAMMLRMAGQEIPEEGREVEEFSEDDDEDD
SDDSEAEKQSQKQHKEESHSDGTSTASSQQQAPPQSVPPSQIQAPPMPGPPPLGPPPAPP
LRPPGPPTGLPPGPPPGAPPFLRPPGMPGLRGPLPRLLPPGPPPGRPPGPPPGPPPGLPP
GPPPRGPPPRLPPPAPPGIPPPRPGMMRPPLVPPLGPAPPGLFPPAPLPNPGVLSAPPNL
IQRPKADDTSAATIEKKATATISAKPQITNPKAEITRFVPTALRVRRENKGATAAPQRKS
EDDSAVPLAKAAPKSGPSVPVSVQTKDDVYEAFMKEMEGLL*
>ENSG00000134644|ENST00000257075
MSVACVLKRKAVLWQDSFSPHLKHHPQEPANPNMPVVLTSGTGSQAQPQPAANQALAAGT
HSSPVPGSIGVAGRSQDDAMVDYFFQRQHGEQLGGGGSGGGGYNNSKHRWPTGDNIHAEH
QVRSMDELNHDFQALALEGRAMGEQLLPGKKFWETDESSKDGPKGIFLGDQWRDSAWGTS
DHSVSQPIMVQRRPGQSFHVNSEVNSVLSPRSESGGLGVSMVEYVLSSSPGDSCLRKGGF
GPRDADSDENDKGEKKNKGTFDGDKLGDLKEEGDVMDKTNGLPVQNGIDADVKDFSRTPG
NCQNSANEVDLLGPNQNGSEGLAQLTSTNGAKPVEDFSNMESQSVPLDPMEHVGMEPLQF
DYSGTQVPVDSAAATVGLFDYNSQQQLFQRPNALAVQQLTAAQQQQYALAAAHQPHIGLA
PAAFVPNPYIISAAPPGTDPYTAGLAAAATLGPAVVPHQYYGVTPWGVYPASLFQQQAAA
AAAATNSANQQTTPQAQQGQQQVLRGGASQRPLTPNQNQQGQQTDPLVAAAAVNSALAFG
QGLAAGMPGYPVLAPAAYYDQTGALVVNAGARNGLGAPVRLVAPAPVIISSSAAQAAVAA
AAASANGAAGGLAGTTNGPFRPLGTQQPQPQPQQQPNNNLASSSFYGNNSLNSNSQSSSL
FSQGSAQPANTSLGFGSSSSLGATLGSALGGFGTAVANSNTGSGSRRDSLTGSSDLYKRT
SSSLTPIGHSFYNGLSFSSSPGPVGMPLPSQGPGHSQTPPPSLSSHGSSSSLNLGGLTNG
SGRYISAAPGAEAKYRSASSASSLFSPSSTLFSSSRLRYGMSDVMPSGRSRLLEDFRNNR
YPNLQLREIAGHIMEFSQDQHGSRFIQLKLERATPAERQLVFNEILQAAYQLMVDVFGNY
VIQKFFEFGSLEQKLALAERIRGHVLSLALQMYGCRVIQKALEFIPSDQQNEMVRELDGH
VLKCVKDQNGNHVVQKCIECVQPQSLQFIIDAFKGQVFALSTHPYGCRVIQRILEHCLPD
QTLPILEELHQHTEQLVQDQYGNYVIQHVLEHGRPEDKSKIVAEIRGNVLVLSQHKFASN
VVEKCVTHASRTERAVLIDEVCTMNDGPHSALYTMMKDQYANYVVQKMIDVAEPGQRKIV
MHKIRPHIATLRKYTYGKHILAKLEKYYMKNGVDLGPICGPPNGII*
>ENSG00000065150|ENST00000261574
MPEDQVGKLEATENTISAMAAAAAEQQQFYLLLGNLLSPDNVVRKQAEETYENIPGQSKI
TFLLQAIRNTTAAEEARQMAAVLLRRLLSSAFDEVYPALPSDVQTAIKSELLMIIQMETQ
SSMRKKVCDIAAELARNLIDEDGNNQWPEGLKFLFDSVSSQNVGLREAALHIFWNFPGIF
GNQQQHYLDVIKRMLVQCMQDQEHPSIRTLSARATAAFILANEHNVALFKHFADLLPGFL
QAVNDSCYQNDDSVLKSLVEIADTVPKYLRPHLEATLQLSLKLCGDTSLNNMQRQLALEV
IVTLSETAAAMLRKHTNIVAQTIPQMLAMMVDLEEDEDWANADELEDDDFDSNAVAGESA
LDRMACGLGGKLVLPMIKEHIMQMLQNPDWKYRHAGLMALSAIGEGCHQQMEGILNEIVN
FVLLFLQDPHPRVRYAACNAVGQMATDFAPGFQKKFHEKVIAALLQTMEDQGNQRVQAHA
AAALINFTEDCPKSLLIPYLDNLVKHLHSIMVLKLQELIQKGTKLVLEQVVTSIASVADT
AEEKFVPYYDLFMPSLKHIVENAVQKELRLLRGKTIECISLIGLAVGKEKFMQDASDVMQ
LLLKTQTDFNDMEDDDPQISYMISAWARMCKILGKEFQQYLPVVMGPLMKTASIKPEVAL
LDTQDMENMSDDDGWEFVNLGDQQSFGIKTAGLEEKSTACQMLVCYAKELKEGFVEYTEQ
VVKLMVPLLKFYFHDGVRVAAAESMPLLLECARVRGPEYLTQMWHFMCDALIKAIGTEPD
SDVLSEIMHSFAKCIEVMGDGCLNNEHFEELGGILKAKLEEHFKNQELRQVKRQDEDYDE
QVEESLQDEDDNDVYILTKVSDILHSIFSSYKEKVLPWFEQLLPLIVNLICPHRPWPDRQ

WGLCIFDDVIEHCSPASFKYAEYFLRPMLQYVCDNSPEVRQAAAYGLGVMAQYGGDNYRP
FCTEALPLLVRVIQSADSKTKENVNATENCISAVGKIMKFKPDCVNVEEVLPHWLSWLPL
HEDKEEAVQTFNYLCDLIESNHPIVLGPNNTNLPKIFSIIAEGEMHEAIKHEDPCAKRLA
NVVRQVQTSGGLWTECIAQLSPEQQAAIQELLNSA*
>ENSG00000135899|ENST00000258381
MFTMTRAMEEALFQHFMHQKLGIAYAIHKPFPFFEGLLDNSIITKRMYMESLEACRNLIP
VSRVVHNILTQLERTFNLSLLVTLFSQINLREYPNLVTIYRSFKRVGASYEWQSRDTPIL
LEAPTGLAEGSSLHTPLALPPPQPPQPSCSPCAPRVSEPGTSSQQSDEILSESPSPSDPV
LPLPALIQEGRSTSVTNDKLTSKMNAEEDSEEMPSLLTSTVQVASDNLIPQIRDKEDPQE
MPHSPLGSMPEIRDNSPEPNDPEEPQEVSSTPSDKKGKKRKRCIWSTPKRRHKKKSLPGG
TASSRHGIQKKLKRVDQVPQKKDDSTCNSTVETRAQKARTECARKSRSEEIIDGTSEMNE
GKRSQKTPSTPRRVTQGAASPGHGIQEKLQVVDKVTQRKDDSTWNSEVMMRVQKARTKCA
RKSRLKEKKKEKDICSSSKRRFQKNIHRRGKPKSDTVDFHCSKLPVTCGEAKGILYKKKM
KHGSSVKCIRNEDGTWLTPNEFEVEGKGRNAKNWKRNIRCEGMTLGELLKRKNSDECEVC
CQGGQLLCCGTCPRVFHEDCHIPPVEAKRMLWSCTFCRMKRSSGSQQCHHVSKTLERQMQ
PQDQLKCEFLLLKAYCHPQSSFFTGIPFNIRDYGEPFQEAMWLDLVKERLITEMYTVAWF
VRDMRLMFRNHKTFYKASDFGQVGLDLEAEFEKDLKDVLGFHEANDGGFWTLP*
>ENSG00000110063|ENST00000263579
MADAAPQLGKRKRELDVEEAHAASTEEKEAGVGNGTCAPVRLPFSGFRLQKVLRESARDK
IIFLHGKVNEASGDGDGEDAVVILEKTPFQVEQVAQLLTGSPELQLQFSNDIYSTYHLFP
PRQLNDVKTTVVYPATEKHLQKYLRQDLRLIRETGDDYRNITLPHLESQSLSIQWVYNIL
DKKAEADRIVFENPDPSDGFVLIPDLKWNQQQLDDLYLIAICHRRGIRSLRDLTPEHLPL
LRNILHQGQEAILQRYRMKGDHLRVYLHYLPSYYHLHVHFTALGFEAPGSGVERAHLLAE
VIENLECDPRHYQQRTLTFALRADDPLLKLLQEAQQS*
>ENSG00000116016|ENST00000263734
MTADKEKKRSSSERRKEKSRDAARCRRSKETEVFYELAHELPLPHSVSSHLDKASIMRLA
ISFLRTHKLLSSVCSENESEAEADQQMDNLYLKALEGFIAVVTQDGDMIFLSENISKFMG
LTQVELTGHSIFDFTHPCDHEEIRENLSLKNGSGFGKKSKDMSTERDFFMRMKCTVTNRG
RTVNLKSATWKVLHCTGQVKVYNNCPPHNSLCGYKEPLLSCLIIMCEPIQHPSHMDIPLD
SKTFLSRHSMDMKFTYCDDRITELIGYHPEELLGRSAYEFYHALDSENMTKSHQNLCTKG
QVVSGQYRMLAKHGGYVWLETQGTVIYNPRNLQPQCIMCVNYVLSEIEKNDVVFSMDQTE
SLFKPHLMAMNSIFDSSGKGAVSEKSNFLFTKLKEEPEELAQLAPTPGDAIISLDFGNQN
FEESSAYGKAILPPSQPWATELRSHSTQSEAGSLPAFTVPQAAAPGSTTPSATSSSSSCS
TPNSPEDYYTSLDNDLKIEVIEKLFAMDTEAKDQCSTQTDFNELDLETLAPYIPMDGEDF
QLSPICPEERLLAENPQSTPQHCFSAMTNIFQPLAPVAPHSPFLLDKFQQQLESKKTEPE
HRPMSSIFFDAGSKASLPPCCGQASTPLSSMGGRSNTQWPPDPPLHFGPTKWAVGDQRTE
FLGAAPLGPPVSPPHVSTFKTRSAKGFGARGPDVLSPAMVALSNKLKLKRQLEYEEQAFQ
DLSGGDPPGGSTSHLMWKRMKNLRGGSCPLMPDKPLSANVPNDKFTQNPMRGLGHPLRHL
PLPQPPSAISPGENSKSRFPPQCYATQYQDYSLSSAHKVSGMASRLLGPSFESYLLPELT
RYDCEVNVPVLGSSTLLQGGDLLRALDQAT*
>ENSG00000136451|ENST00000258963
XLNRHKLSHSDEKPFECPICNQRFKRKDRMTYHVRSHEGGITKPYTCSVCGKGFSRPDHL
SCHVKHVHSTERPFKCQFSSLMQTCTAAFATKDRLRTHMVRHEGKVSCNICGKLLSAAYI
TSHLKTHGQSQSINCNTCKQGISKTCMSEETSNQKQQQQQQQQQQQQQQQQQQHVTSWPG

KQVETLRLWEEAVKARKKEAANLCQTSTAATTPVTLTTPFSITSSVSSGTMSNPVTVAAA
MSMRSPVNVSSAVNITSPMNIGHPVTITSPLSMTSPLTLTTPVNLPTPVTAPVNIAHPVT
ITSPMNLPTPMTLAAPLNIAMRPVESMPFLPQALPTSPPW*
>ENSG00000085276|ENST00000264674
MILDEFYNVKFCIDASQPDVGSWLKYIRFAGCYDQHNLVACQINDQIFYRVVADIAPGEE
LLLFMKSEDYPHETMAPDIHEERQYRCEDCDQLFESKAELADHQKFPCSTPHSAFSMVEE
DFQQKLESENDLQEIHTIQECKECDQVFPDLQSLEKHMLSHTEEREYKCDQCPKAFNWKS
NLIRHQMSHDSGKHYECENCAKQVFTDPSNLQRHIRSQHVGARAHACPECGKTFATSSGL
KQHKHIHSSVKPFICEVCHKSYTQFSNLCRHKRMHADCRTQIKCKDCGQMFSTTSSLNKH
RRFCEGKNHFAAGGFFGQGISLPGTPAMDKTSMVNMSHANPGLADYFGANRHPAGLTFPT
APGFSFSFPGLFPSGLYHRPPLIPASSPVKGLSSTEQTNKSQSPLMTHPQILPATQDILK
ALSKHPSVGDNKPVELQPERSSEERPFEKISDQSESSDLDDVSTPSGSDLETTSGSDLES
DIESDKEKFKENGKMFKDKVSPLQNLASINNKKEYSNHSIFSPSLEEQTAVSGAVNDSIK
AIASIAEKYFGSTGLVGLQDKKVGALPYPSMFPLPFFPAFSQSMYPFPDRDLRSLPLKME
PQSPGEVKKLQKGSSESPFDLTTKRKDEKPLTPVPSKPPVTPATSQDQPLDLSMGSRSRA
SGTKLTEPRKNHVFGGKKGSNVESRPASDGSLQHARPTPFFMDPIYRVEKRKLTDPLEAL
KEKYLRPSPGFLFHPQFQLPDQRTWMSAIENMAEKLESFSALKPEASELLQSVPSMFNFR
APPNALPENLLRKGKERYTCRYCGKIFPRSANLTRHLRTHTGEQPYRCKYCDRSFSISSN
LQRHVRNIHNKEKPFKCHLCDRCFGQQTNLDRHLKKHENGNMSGTATSSPHSELESTGAI
LDDKEDAYFTEIRNFIGNSNHGSQSPRNVEERMNGSHFKDEKALVTSQNSDLLDDEEVED
EVLLDEEDEDNDITGKTGKEPVTSNLHEGNPEDDYEETSALEMSCKTSPVRYKEEEYKSG
LSALDHIRHFTDSLKMRKMEDNQYSEAELSSFSTSHVPEELKQPLHRKSKSQAYAMMLSL
SDKESLHSTSHSSSNVWHSMARAAAESSAIQSISHV*
>ENSG00000168827|ENST00000264263
MRLLGAAAVAALGRGRAPASLGWQRKQVNWKACRWSSSGVIPNEKIRNIGISAHIDSGKT
TLTERVLYYTGRIAKMHEVKGKDGVGAVMDSMELERQRGITIQSAATYTMWKDVNINIID
TPGHVDFTIEVERALRVLDGAVLVLCAVGGVQCQTMTVNRQMKRYNVPFLTFINKLDRMG
SNPARALQQMRSKLNHNAAFMQIPMGLEGNFKGIVDLIEERAIYFDGDFGHFLRDFLPLL
WNWDRRSGSQIVRYGEIPAELRAAATDHRQELIECVANSDEQLGEMFLEEKIPSISDLKL
AIRRATLKRSFTPVFLGSALKNKGVQPLLDAVLEYLPNPSEVQNYAILNKEDDSKEKTKI
LMNSSRDNSHPFVGLAFKLEVGRFGQLTYVRSYQGELKKGDTIYNTRTRKKVRLQRLARM
HADMMEDVEEVYAGDICALFGIDCASGDTFTDKANSGLSMESIHVPDPVISIAMKPSNKN
DLEKFSKGIGRFTREDPTFKVYFDTENKETVISGMGELHLEIYAQRLEREYGCPCITGKP
KVAFRETITAPVPFDFTHKKQSGGAGQYGKVIGVLEPLDPEDYTKLEFSDETFGSNIPKQ
FVPAVEKGFLDACEKGPLSGHKLSGLRFVLQDGAHHMVDSNEISFIRAGEGALKQALANA
TLCILEPIMAVEVVAPNEFQGQVIAGINRRHGVITGQDGVEDYFTLYADVPLNDMFGYST
ELRSCTEGKGEYTMEYSRYQPCLPSTQEDVINKYLEATGQLPVKKGKAKN*
>ENSG00000067369|ENST00000263801
MDPTGSQLDSDFSQQDTPCLIIEDSQPESQVLEDDSGSHFSMLSRHLPNLQTHKENPVLD
VVSNPEQTAGEERGDGNSGFNEHLKENKVADPVDSSNLDTCGSISQVIEQLPQPNRTSSV
LGMSVESAPAVEEEKGEELEQKEKEKEEDTSGNTTHSLGAEDTASSQLGFGVLELSQSQD
VEENTVPYEVDKEQLQSVTTNSGYTRLSDVDANTAIKHEEQSNEDIPIAEQSSKDIPVTA
QPSKDVHVVKEQNPPPARSEDMPFSPKASVAAMEAKEQLSAQELMESGLQIQKSPEPEVL
STQEDLFDQSNKTVSSDGCSTPSREEGGCSLASTPATTLHLLQLSGQRSLVQDSLSTNSS

DLVAPSPDAFRSTPFIVPSSPTEQEGRQDKPMDTSVLSEEGGEPFQKKLQSGEPVELENP
PLLPESTVSPQASTPISQSTPVFPPGSLPIPSQPQFSHDIFIPSPSLEEQSNDGKKDGDM
HSSSLTVECSKTSEIEPKNSPEDLGLSLTGDSCKLMLSTSEYSQSPKMESLSSHRIDEDG
ENTQIEDTEPMSPVLNSKFVPAENDSILMNPAQDGEVQLSQNDDKTKGDDTDTRDDISIL
ATGCKGREETVAEDVCIDLTCDSGSQAVPSPATRSEALSSVLDQEEAMEIKEHHPEEGSS
GSEVEEIPETPCESQGEELKEENMESVPLHLSLTETQSQGLCLQKEMPKKECSEAMEVET
SVISIDSPQKLAILDQELEHKEQEAWEEATSEDSSVVIVDVKEPSPRVDVSCEPLEGVEK
CSDSQSWEDIAPEIEPCAENRLDTKEEKSVEYEGDLKSGTAETEPVEQDSSQPSLPLVRA
DDPLRLDQELQQPQTQEKTSNSLTEDSKMANAKQLSSDAEAQKLGKPSAHASQSFCESSS
ETPFHFTLPKEGDIIPPLTGATPPLIGHLKLEPKRHSTPIGISNYPESTIATSDVMSESM
VETHDPILGSGKGDSGAAPDVDDKLCLRMKLVSPETEASEESLQFNLEKPATGERKNGST
AVAESVASPQKTMSVLSCICEARQENEARSEDPPTTPIRGNLLHFPSSQGEEEKEKLEGD
HTIRQSQQPMKPISPVKDPVSPASQKMVIQGPSSPQGEAMVTDVLEDQKEGRSTNKENPS
KALIERPSQNNIGIQTMECSLRVPETVSAATQTIKNVCEQGTSTVDQNFGKQDATVQTER
GSGEKPVSAPGDDTESLHSQGEEEFDMPQPPHGHVLHRHMRTIREVRTLVTRVITDVYYV
DGTEVERKVTEETEEPIVECQECETEVSPSQTGGSSGDLGDISSFSSKASSLHRTSSGTS
LSAMHSSGSSGKGAGPLRGKTSGTEPADFALPSSRGGPGKLSPRKGVSQTGTPVCEEDGD
AGLGIRQGGKAPVTPRGRGRRGRPPSRTTGTRETAVPGPLGIEDISPNLSPDDKSFSRVV
PRVPDSTRRTDVGAGALRRSDSPEIPFQAAAGPSDGLDASSPGNSFVGLRVVAKWSSNGY
FYSGKITRDVGAGKYKLLFDDGYECDVLGKDILLCDPIPLDTEVTALSEDEYFSAGVVKG
HRKESGELYYSIEKEGQRKWYKRMAVILSLEQGNRLREQYGLGPYEAVTPLTKAADISLD
NLVEGKRKRRSNVSSPATPTASSSSSTTPTRKITESPRASMGVLSGKRKLITSEEERSPA
KRGRKSATVKPGAVGAGEFVSPCESGDNTGEPSALEEQRGPLPLNKTLFLGYAFLLTMAT
TSDKLASRSKLPDGPTGSSEEEEEFLEIPPFNKQYTESQLRAGAGYILEDFNEAQCNTAY
QCLLIADQHCRTRKYFLCLASGIPCVSHVWVHDSCHANQLQNYRNYLLPAGYSLEEQRIL
DWQPRENPFQNLKVLLVSDQQQNFLELWSEILMTGGAASVKQHHSSAHNKDIALGVFDVV
VTDPSCPASVLKCAEALQLPVVSQEWVIQCLIVGERIGFKQHPKYKHDYVSH*
>ENSG00000117222|ENST00000264515
MNLELLESFGQNYPEEADGTLDCISMALTCTFNRWGTLLAVGCNDGRIVIWDFLTRGIAK
IISAHIHPVCSLCWSRDGHKLVSASTDNIVSQWDVLSGDCDQRFRFPSPILKVQYHPRDQ
NKVLVCPMKSAPVMLTLSDSKHVVLPVDDDSDLNVVASFDRRGEYIYTGNAKGKILVLKT
DSQDLVASFRVTTGTSNTTAIKSIEFARKGSCFLINTADRIIRVYDGREILTCGRDGEPE
PMQKLQDLVNRTPWKKCCFSGDGEYIVAGSARQHALYIWEKSIGNLVKILHGTRGELLLD
VAWHPVRPIIASISSGVVSIWAQNQVENWSAFAPDFKELDENVEYEERESEFDIEDEDKS
EPEQTGADAAEDEEVDVTSVDPIAAFCSSDEELEDSKALLYLPIAPEVEDPEENPYGPPP
DAVQTSLMDEGASSEKKRQSSADGSQPPKKKPKTTNIELQGVPNDEVHPLLGVKGDGKSK
KKQAGRPKGSKGKEKDSPFKPKLYKGDRGLPLEGSAKGKVQAELSQPLTAGGAISELL*
>ENSG00000169955|ENST00000252799
MTDPSLGLTVPMAPPLAPLPPRDPNGAGSEWRKPGAVSFADVAVYFSREEWGCLRPAQRA
LYRDVMRETYGHLGALGESPTCLPGPCASTGPAAPLGAACGVGGPGAGQAASSQRGVCVL
LPQESEAASRRSSPGWRRRPNCGIRLPRIRRWRSVRQKRTQQIPETRKRKDKGKGREPWR
SPTLWPPGLLG*
>ENSG00000135100|ENST00000257555
MVSKLSQLQTELLAALLESGLSKEALIQALGEPGPYLLAGEGPLDKGESCGGGRGELAEL

PNGLGETRGSEDETDDDGEDFTPPILKELENLSPEEAAHQKAVVETLLQEDPWRVAKMVK
SYLQQHNIPQREVVDTTGLNQSHLSQHLNKGTPMKTQKRAALYTWYVRKQREVAQQFTHA
GQGGLIEEPTGDELPTKKGRRNRFKWGPASQQILFQAYERQKNPSKEERETLVEECNRAE
CIQRGVSPSQAQGLGSNLVTEVRVYNWFANRRKEEAFRHKLAMDTYSGPPPGPGPGPALP
AHSSPGLPPPALSPSKVHGVRYGQPATSETAEVPSSSGGPLVTVSTPLHQVSPTGLEPSH
SLLSTEAKLVSAAGGPLPPVSTLTALHSLEQTSPGLNQQPQNLIMASLPGVMTIGPGEPA
SLGPTFTNTGASTLVIGLASTQAQSVPVINSMGSSLTTLQPVQFSQPLHPSYQQPLMPPV
QSHVTQSPFMATMAQLQSPHALYSHKPEVAQYTHTGLLPQTMLITDTTNLSALASLTPTK
QVFTSDTEASSESGLHTPASQATTLHVPSQDPASIQHLQPAHRLSASPTVSSSSLVLYQS
SDSSNGQSHLLPSNHSVIETFISTQMASSSQ*
>ENSG00000130935|ENST00000253247
MAALEEEFTLSSVVLSAGPEGLLGVEQSDKTDQFLVTDSGRTVILYKVSDQKPLGSWSVK
QGQIITCPAVCNFQTGEYVVVHDNKVLRIWNNEDVNLDKVFKATLSAEVYRILSVQGTEP
LVLFKEGAVRGLEALLADPQQKIETVISDEEVIKWTKFFVVFRHPVLIFITEKHGNYFAY
VQMFNSRILTKYTLLLGQDENSVIKSFTASVDRKFISLMSLSSDGCIYETLIPIRPADPE
KNQSLVKSLLLKAVVSGNARNGVALTALDQDHVAVLGSPLAASKECLSVWNIKFQTLQTS
KELPQGTSGQLWYYGEHLFMLHGKSLTVIPYKCEVSSLAGALGKLKHSQDPGTHVVSHFV
NWETPQGCGLGFQNSEQSRRILRRRKIEVSLQPEVPPSKQLLSTIMKDSEKHIEVEVRKF
LALKQTPDFHTVIGDTVTGLLERCKAEPSFYPRNCLMQLIQTHVLSYSLCPDLMEIALKK
KDVQLLQLCLQQFPDIPESVTCACLKIFLSIGDDSLQETDVNMESVFDYSINSVHDEKME
EQTEILQNGFNPEEDKCNNCDQELNKKPQDETKESTSCPVVQKRAALLNAILHSAYSETF
LLPHLKDIPAQHITLFLKYLYFLYLKCSENATMTLPGIHPPTLNQIMDWICLLLDANFTV
VVMMPEAKRLLINLYKLVKSQISVYSELNKIEVSFRELQKLNQEKNNRGLYSIEVLELF*
>ENSG00000011590|ENST00000262630
MSLPPIRLPSPYGSDRLVQLAARLRPALCDTLITVGSQEFPAHSLVLAGVSQQLGRRGQW
ALGEGISPSTFAQLLNFVYGESVELQPGELRPLQEAARALGVQSLEEACWRARGDRAKKP
DPGLKKHQEEPEKPSRNPERELGDPGEKQKPEQVSRTGGREQEMLHKHSPPRGRPEMAGA
TQEAQQEQTRSKEKRLQAPVGQRGADGKHGVLTWLRENPGGSEESLRKLPGPLPPAGSLQ
TSVTPRPSWAEAPWLVGGQPALWSILLMPPRYGIPFYHSTPTTGAWQEVWREQRIPLSLN
APKGLWSQNQLASSSPTPGSLPQGPAQLSPGEMEESDQGHTGALATCAGHEDKAGCPPRP
HPPPAPPARSRPYACSVCGKRFSLKHQMETHYRVHTGEKPFSCSLCPQRSRDFSAMTKHL
RTHGAAPYRCSLCGAGCPSLASMQAHMRGHSPSQLPPGWTIRSTFLYSSSRPSRPSTSPC
CPSSSTT*
>ENSG00000082258|ENST00000264157
MASGRGASSRWFFTREQLENTPSRRCGVEADKELSCRQQAANLIQEMGQRLNVSQLTINT
AIVYMHRFYMHHSFTKFNKNIISSTALFLAAKVEEQARKLEHVIKVAHACLHPLEPLLDT
KCDAYLQQTQELVILETIMLQTLGFEITIEHPHTDVVKCTQLVRASKDLAQTSYFMATNS
LHLTTFCLQYKPTVIACVCIHLACKWSNWEIPVSTDGKHWWEYVDPTVTLELLDELTHEF
LQILEKTPNRLKKIRNWRANQAARKPKVDGQVSETPLLGSSLVQNSILVDSVTGVPTNPS
FQKPSTSAFPAPVPLNSGNISVQDSHTSDNLSMLATGMPSTSYGLSSHQEWPQHQDSART
EQLYSQKQETSLSGSQYNINFQQGPSISLHSGLHHRPDKISDHSSVKQEYTHKAGSSKHH
GPISTTPGIIPQKMSLDKYREKRKLETLDLDVRDHYIAAQVEQQHKQGQSQAASSSSVTS
PIKMKIPIANTEKYMADKKEKSGSLKLRIPIPPTDKSASKEELKMKIKVSSSERHSSSDE
GSGKSKHSSPHISRDHKEKHKEHPSSRHHTSSHKHSHSHSGSSSGGSKHSADGIPPTVLR

SPVGLSSDGISSSSSSSSRKRLHVNDASHNHHSKMSKSSKSSGSSSSSSSSSVKQYISSHNS
VFNHPLPPPPPVTYQVGYGHLSTLVKLDKKPVETNGPDANHEYSTSSQHMDYKDTFDMLD
SLLSAQGMNM*
>ENSG00000213780|ENST00000259895
MESTPSRGLNRVHLQCRNLQEFLGGLSPGVLDRLYGHPATCLAVFRELPSLAKNWVMRML
FLEQPLPQAAVALWVKKEFSKAQEESTGLLSGLRIWHTQLLPGGLQGLILNPIFRQNLRI
ALLGGGKAWSDDTSQLGPDKHARDVPSLDKYAEERWEVVLHFMVGSPSAAVSQDLAQLLS
QAGLMKSTEPGEPPCITSAGFQFLLLDTPAQLWYFMLQYLQTAQSRGMDLVEILSFLFQL
SFSTLGKDYSVEGMSDSLLNFLQHLREFGLVFQRKRKSRRYYPTRLAINLSSGVSGAGGT
VHQPGFIVVETNYRLYAYTESELQIALIALFSEMLYRFPNMVVAQVTRESVQQAIASGIT
AQQIIHFLRTRAHPVMLKQTPVLPPTITDQIRLWELERDRLRFTEGVLYNQFLSQVDFEL
LLAHARELGVLVFENSAKRLMVVTPAGHSDVKRFWKRQKHSS*
>ENSG00000118900|ENST00000262376
MSEPHRVQFTSLPGSLNPAFLKKSRKEEAGAGEQHQDCEPAAAAVRITLTLFEPDHKRCP
EFFYPELVKNIRGKVKGLQPGDKKKDLSDPFNDEEKERHKVEALARKFEEKYGGKKRRKD
RIQDLIDMGYGYDESDSFIDNSEAYDELVPASLTTKYGGFYINSGTLQFRQASESEDDFI
KEKKKKSPKKRKLKEGGEKIKKKKKDDTYDKEKKSKKSKFSKAGFTALNASKEKKKKKYS
GALSVKEMLKKFQKEKEAQKKREEEHKPVAVPSAEAQGLRELEGASDPLLSLFGSTSDND
LLQAATAMDSLTDLDLEHLLSESPEGSPFRDMDDGSDSLGVGLDQEFRQPSSLPEGLPAP
LEKRVKELAQAARAAEGESRQKFFTQDINGILLDIEAQTRELSSQVRSGVYAYLASFLPC
SKDALLKRARKLHLYEQGGRLKEPLQKLKEAIGRAMPEQMAKYQDECQAHTQAKVAKMLE
EEKDKEQRDRICSDEEEDEEKGGRRIMGPRKKFQWNDEIRELLCQVVKIKLESQDLERNN
KAQAWEDCVKGFLDAEVKPLWPKGWMQARTLFKESRRGHGHLTSILAKKKVMAPSKIKVK
ESSTKPDKKVSVPSGQIGGPIALPSDHQTGGLSIGASSRELPSQASGGLANPPPVNLEDS
LDEDLIRNPASSVEAVSKELAALNSRAAGNSEFTLPAPSKAPAEKVGGVLCTEEKRNFAK
PSPSAPPPASSLQSPLNFLAEQALALGQSSQEKKPESSGYKELSCQAPLNKGLPEVHQSK
AKHHSLPRTSHGPQVAVPVPGPQVKVFHAGTQQQKNFTPPSPFANKLQGPKASPTQCHRS
LLQLVKTAAKGQGFHPSAPATSGGLSASSSSSHKTPASSSSALSHPAKPHSVSSAGSSYK
NNPFASSISKHGVSSGSSSSGGTPVQSSVSGSLVPGIQPPSVGQATSRPVPSSAGKKMPV
SQKLTLVAPPGGPNGDSSGGTQGVAKLLTSPSLKPSAVSSVTSSTSLSKGASGTVLLAGS
SLMASPYKSSSPKLSGAMSSNSLGIITPVPIPVHVLSFSADSSAKAGVSKDAIVTGPAPG
SFHHGLGHSLLAGLHSSPPHAAPLPHAAVPTHIPQSLPGASQLHGKGPAVPRKL*
>ENSG00000103994|ENST00000263805
MPVGRIECPSSPSFPRDISHECRVCGVTEVGLSAYAKHISGQLHKDNVDAQEREDDGKGE
EEEEDYFDKELIQLIKQRKEQSRQDEPSNSNQEINSDDRRPQWRREDRIPYQDRESYSQP
AWHHRGPPQRDWKWEKDGFNNTRKNSFPHSLRNGGGPRGRSGWHKGVAGGSSTWFHNHSN
SGGGWLSNSGAVDWNHNGTGRNSSWLSEGTGGFSSWHMNNSNGNWKSSVRSTNNWNYSGP
GDKFQPGRNRNSNCQMEDMTMLWNKKSNKSNKYSHDRYNWQRQENDKLGTVATYRGPSEG
FTSDKFPSEGLLDFNFEQLESQTTKQADTATSKVSGKNGSAAREKPRRWTPYPSQKTLDL
QSGLKDITGNKSEMIEKPLFDFSLITTGIQEPQTDETRNSPTQKTQKEIHTGSLNHKASS
DSAASFEVVRQCPTAEKPEQEHTPNKMPSLKSPLLPCPATKSLSQKQDPKNISKNTKTNF
FSPGEHSNPSNKPTVEDNHGPYISKLRSSCPHVLKGNKSTFGSQKQSGDNLNDTLRKAKE
VLQCHESLQNPLLSTSKSTRNYAKASRNVEESEKGSLKIEFQVHALEDESDGETSDTEKH
GTKIGTLGSATTELLSGSTRTADEKEEDDRILKTSRELSTSPCNPIVRQKESELQMTSAA

SPHPGLLLDLKTSLEDAQVDDSIKSHVSYETEGFESASLDAELQKSDISQPSGPLLPELS
KLGFPASLQRDLTRHISLKSKTGVHLPEPNLNSARRIRNISGHRKSETEKESGLKPTLRQ
ILNASRRNVNWEQVIQQVTKKKQELGKGLPRFGIEMVPLVQNEQEALDLDGEPDLSSLEG
FQWEGVSISSSPGLARKRSLSESSVIMDRAPSVYSFFSEEGTGKENEPQQMVSPSNSLRA
GQSQKATMHLKQEVTPRAASLRTGERAENVATQRRHSAQLSSDHIIPLMHLAKDLNSQER
SIPPSENQNSQESNGEGNCLSSSASSALAISSLADAATDSSCTSGAEQNDGQSIRKKRRA
TGDGSSPELPSLERKNKRRKIKGKKERSQVDQLLNISLREEELSKSLQCMDNNLLQARAA
LQTAYVEVQRLLMLKQQITMEMSALRTHRIQILQGLQETYEPSEHPDQVPCSLTRERRNS
RSQTSIDAALLPTPFFPLFLEPPSSHVSPSPTGASLQITTSPTFQTHGSVPAPDSSVQIK
QEPMSPEQDENVNAVPPSSACNVSKELLEANREISDSCPVYPVITARLSLPESTESFHEP
SQELKFSVEQRNTRNRENSPSSQSAGLSSINKEGEEPTKGNSGSEACTSSFLRLSFASET
PLEKEPHSPADQPEQQAESTLTSAETRGSKKKKKLRKKKSLRAAHVPENSDTEQDVLTVK
PVRKVKAGKLIKGGKVTTSTWEDSRTGREQESVRDEPDSDSSLEVLEIPNPQLEVVAIDS
SESGEEKPDSPSKKDIWNSTEQNPLETSRSGCDEVSSTSEIGTRYKDGIPVSVAETQTVI
SSIKGSKNSSEISSEPGDDDEPTEGSFEGHQAAVNAIQIFGNLLYTCSADKTVRVYNLVS
RKCIGVFEGHTSKVNCLLVTQTSGKNAALYTGSSDHTIRCYNVKSRECVEQLQLEDRVLC
LHSRWRILYAGLANGTVVTFNIKNNKRLEIFECHGPRAVSCLATAQEGARKLLVVGSYDC
TISVRDARNGLLLRTLEGHSKTILCMKVVNDLVFSGSSDQSVHAHNIHTGELVRIYKGHN
HAVTVVNILGKVMVTACLDKFVRVYELQSHDRLQVYGGHKDMIMCMTIHKSMIYTGCYDG
SIQAVRLNLMQNYRCWWHGCSLIFGVVDHLKQHLLTDHTNPNFQTLKCRWKNCDAFFTAR
KGSKQDAAGHIERHAEDDSKIDS*
>ENSG00000059728|ENST00000264444
MAAAVRMNIQMLLEAADYLERREREAEHGYASMLPYNNKDRDALKRRNKSKKNNSSSRST
HNEMEKNRRAHLRLCLEKLKGLVPLGPESSRHTTLSLLTKAKLHIKKLEDCDRKAVHQID
QLQREQRHLKRQLEKLGIERIRMDSIGSTVSSERSDSDREEIDVDVESTDYLTGDLDWSS
SSVSDSDERGSMQSLGSDEGYSSTSIKRIKLQDSHKACLGL*
>ENSG00000120800|ENST00000261637
MKTKPVSHKTENTYRFLTFAERLGNVNIDIIHRIDRTASYEEEVETYFFEGLLKWRELNL
TEHFGKFYKEVIDKCQSFNQLVYHQNEIVQSLKTHLQVKNSFAYQPLLDLVVQLARDLQM
DFYPHFPEFFLTITSILETQDTELLEWAFTSLSYLYKYLWRLMVKDMSSIYSMYSTLLAH
KKLHIRNFAAESFTFLMRKVSDKNALFNLMFLDLDKHPEKVEGVGQLLFEMCKGVRNMFH
SCTGQAVKLILRKLGPVTETETQLPWMLIGETLKNMVKSTVSYISKEHFGTFFECLQESL
LDLHTKVTKTNCCESSEQIKRLLETYLILVKHGSGTKIPTPADVCKVLSQTLQVASLSTS
CWETLLDVISALILGENVSLPETLIKETIEKIFESRFEKRLIFSFSEVMFAMKQFEQLFL
PSFLSYIVNCFLIDDAVVKDEALAILAKLILNKAAPPTAGSMAIEKYPLVFSPQMVGFYI
KQKKTRSKGRNEQFPVLDHLLSIIKLPPNKDTTYLSQSWAALVVLPHIRPLEKEKVIPLV
TGFIEALFMTVDKGSFGKGNLFVLCQAVNTLLSLEESSELLHLVPVERVKNLVLTFPLEP
SVLLLTDLYYQRLALCGCKGPLSQEALMELFPKLQANISTGVSKIRLLTIRILNHFDVQL
PESMEDDGLSERQSVFAILRQAELVPATVNDYREKLLHLRKLRHDVVQTAVPDGPLQEVP
LRYLLGMLYINFSALWDPVIELISSHAHEMENKQFWKVYYEHLEKAATHAEKELQNDMTD
EKSVGDESWEQTQEGDVGALYHEQLALKTDCQERLDHTNFRFLLWRALTKFPERVEPRSR
ELSPLFLRFINNEYYPADLQVAPTQDLRRKGKGMVAEEIEEEPAAGDDEELEEEAVPQDE
SSQKKKTRRAAAKQLIAHLQVFSKFSNPRALYLESKLYELYLQLLLHQDQMVQKITLDCI
MTYKHPHVLPYRENLQRLLEDRSFKEEIVHFSISEDNAVVKTAHRADLFPILMRILYGRM

KNKTGSKTQGKSASGTRMAIVLRFLAGTQPEEIQIFLDLLFEPVRHFKNGECHSAVIQAV
EDLDLSKVLPLGRQHGILNSLEIVLKNISHLISAYLPKILQILLCMTATVSHILDQREKI
QLRFINPLKNLRRLGIKMVTDIFLDWESYQFRTEEIDAVFHGAVWPQISRLGSESQYSPT
PLLKLISIWSRNARYFPLLAKQKPGHPECDILTNVFAILSAKNLSDATASIVMDIVDDLL
NLPDFEPTETVLNLLVTGCVYPGIAENIGESITIGGRLILPHVPAILQYLSKTTISAEKV
KKKKNRAQVSKELGILSKISKFMKDKEQSSVLITLLLPFLHRGNIAEDTEVDILVTVQNL
LKHCVDPTSFLKPIAKLFSVIKNKLSRKLLCTVFETLSDFESGLKYITDVVKLNAFDQRH
LDDINFDVRFETFQTITSYIKEMQIVDVNYLIPVMHNCFYNLELGDMSLSDNASMCLMSI
IKKLAALNVTEKDYREIIHRSLLEKLRKGLKSQTESIQQDYTTILSCLIQTFPNQLEFKD
LVQLTHYHDPEMDFFENMKHIQIHRRARALKKLAKQLMEGKVVLSSKSLQNYIMPYAMTP
IFDEKMLKHENITTAATEIIGAICKHLSWSAYMYYLKHFIHVLQTGQINQKLGVSLLVIV
LEAFHFDHKTLEEQMGKIENEENAIEAIELPEPEAMELERVDEEEKEYTCKSLSDNGQPG
TPDPADSGGTSAKESECITKPVSFLPQNKEEIERTIKNIQGTITGDILPRLHKCLASTTK
REEEHKLVKSKVVNDEEVVRVPLAFAMVKLMQSLPQEVMEANLPSILLKVCALLKNRAQE
IRDIARSTLAKIIEDLGVHFLLYVLKELQTTLVRGYQVHVLTFTVHMLLQGLTNKLQVGD
LDSCLDIMIEIFNHELFGAVAEEKEVKQILSKVMEARRSKSYDSYEILGKFVGKDQVTKL
ILPLKEILQNTTSLKLARKVHETLRRITVGLIVNQEMTAESILLLSYGLISENLPLLTEK
EKNPVAPAPDPRLPPQSCLLLPPTPVRGGQKAVVSRKTNMHIFIESGLRLLHLSLKTSKI
KSSGECVLEMLDPFVSLLIDCLGSMDVKVITGALQCLIWVLRFPLPSIETKAEQLTKHLF
LLLKDYAKLGAARGQNFHLVVNCFKCVTILVKKVKSYQITEKQLQVLLAYAEEDIYDTSR
QATAFGLLKAILSRKLLVPEIDEVMRKVSKLAVSAQSEPARVQCRQVFLKYILDYPLGDK
LRPNLEFMLAQLNYEHETGRESTLEMIAYLFDTFPQGLLHENCGMFFIPLCLMTINDDSA
TCKKMASMTIKSLLGKISLEKKDWLFDMVTTWFGAKKRLNRQLAALICGLFVESEGVDFE
KRLGTVLPVIEKEIDPENFKDIMEETEEKAADRLLFSFLTLITKLIKECNIIQFTKPAET
LSKIWSHVHSHLRHPHNWVWLTAAQIFGLLFASCQPEELIQKWNTKKTKKHLPEPVAIKF
LASDLDQKMKSISLASCHQLHSKFLDQSLGEQVVKNLLFAAKVLYLLELYCEDKQSKIKE
DLEEQEALEDGVACADEKAESDGEEKEEVKEELGRPATLLWLIQKLSRIAKLEAAYSPRN
PLKRTCIFKFLGAVAMDLGIDKVKPYLPMIIAPLFRELNSTYSEQDPLLKNLSQEIIELL
KKLVGLESFSLAFASVQKQANEKRALRKKRKALEFVTNPDIAAKKKMKKHKNKSEAKKRK
IEFLRPGYKAKRQKSHSLKDLAMVE*
>ENSG00000101104|ENST00000255136
MNASGSGYPLASLYVGDLHPDVTEAMLYEKFSPAGPILSIRVCRDVATRRSLGYAYINFQ
QPADAERALDTMNFEMLKGQPIRIMWSQRDPGLRKSGVGNIFIKNLEDSIDNKALYDTFS
TFGNILSCKVACDEHGSRGFGFVHFETHEAAQQAINTMNGMLLNDRKVFVGHFKSRRERE
AELGARALEFTNIYVKNLPVDVDEQGLQDLFSQFGKMLSVKVMRDNSGHSRCFGFVNFEK
HEEAQKAVVHMNGKEVSGRLLYAGRAQKRVERQNELKRRFEQMKQDRLRRYQGVNLYVKN
LDDSIDDDKLRKEFSPYGVITSAKVMTEGGHSKGFGFVCFSSPEEATKAVTEMNGRIVGT
KPLYVALAQRKEERKAILTNQYMQRLSTMRTLSNPLLGSFQQPSSYFLPAMPQPPAQAAY
YGCGPVTPTQPAPRWTSQPPRPSCASMVRPPVVPRRPPAHISSVRQASTQVPRTVPHTQR
VANIGTQTTGPSGVGCCTPGRPLLPCKCSSAAHSTYRVQEPAVHIPGQEPLTASMLAAAP
LHEQKQMIGERLYPLIHDVHTQLAGKITGMLLEIDNSELLLMLESPESLHAKIDEAVAVL
QAHQAMEQPKAYMH*
>ENSG00000115966|ENST00000264110
MKFKLHVNSARQYKDLWNMSDDKPFLCTAPGCGQRFTNEDHLAVHKHKHEMTLKFGPARN

DSVIVADQTPTPTRFLKNCEEVGLFNELASPFENEFKKASEDDIKKMPLDLSPLATPIIR
SKIEEPSVVETTHQDSPLPHPESTTSDEKEVPLAQTAQPTSAIVRPASLQVPNVLLTSSD
SSVIIQQAVPSPTSSTVITQAPSSNRPIVPVPGPFPLLLHLPNGQTMPVAIPASITSSNV
HVPAAVPLVRPVTMVPSVPGIPGPSSPQPVQSEAKMRLKAALTQQHPPVTNGDTVKGHGS
GLVRTQSEESRPQSLQQPATSTTETPASPAHTTPQTQSTSGRRRRAANEDPDEKRRKFLE
RNRAAASRCRQKRKVWVQSLEKKAEDLSSLNGQLQSEVTLLRNEVAQLKQLLLAHKDCPV
TAMQKKSGYHTADKDDSSEDISVPSSPHTEAIQHSSVSTSNGVSSTSKAEAVATSVLTQM
ADQSTEPALSQIVMAPSSQSQPSGS*
>ENSG00000163781|ENST00000260810
MSRNDKEPFFVKFLKSSDNSKCFFKALESIKEFQSEEYLQIITEEEALKIKENDRSLYIC
DPFSGVVFDHLKKLGCRIVGPQVVIFCMHHQRCVPRAEHPVYNMVMSDVTISCTSLEKEK
REEVHKYVQMMGGRVYRDLNVSVTHLIAGEVGSKKYLVAANLKKPILLPSWIKTLWEKSQ
EKKITRYTDINMEDFKCPIFLGCIICVTGLCGLDRKEVQQLTVKHGGQYMGQLKMNECTH
LIVQEPKGQKYECAKRWNVHCVTTQWFFDSIEKGFCQDESIYKTEPRPEAKTMPNSSTPT
SQINTIDSRTLSDVSNISNINASCVSESICNSLNSKLEPTLENLENLDVSAFQAPEDLLD
GCRIYLCGFSGRKLDKLRRLINSGGGVRFNQLNEDVTHVIVGDYDDELKQFWNKSAHRPH
VVGAKWLLECFSKGYMLSEEPYIHANYQPVEIPVSHKPESKAALLKKKNSSFSKKDFAPS
EKHEQADEDLLSQYENGSSTVVEAKTSEARPFNDSTHAEPLNDSTHISLQEENQSSVSHC
VPDVSTITEEGLFSQKSFLVLGFSNENESNIANIIKENAGKIMSLLSRTVADYAVVPLLG
CEVEATVGEVVTNTWLVTCIDYQTLFDPKSNPLFTPVPVMTGMTPLEDCVISFSQCAGAE
KESLTFLANLLGASVQEYFVRKSNAKKGMFASTHLILKERGGSKYEAAKKWNLPAVTIAW
LLETARTGKRADESHFLIENSTKEERSLETEITNGINLNSDTAEHPGTRLQTHRKTVVTP
LDMNRFQSKAFRAVVSQHARQVAASPAVGQPLQKEPSLHLDTPSKFLSKDKLFKPSFDVK
DALAALETPGRPSQQKRKPSTPLSEVIVKNLQLALANSSRNAVALSASPQLKEAQSEKEE
APKPLHKVVVCVSKKLSKKQSELNGIAASLGADYRWSFDETVTHFIYQGRPNDTNREYKS
VKERGVHIVSEHWLLDCAQECKHLPESLYPHTYNPKMSLDISAVQDGRLCNSRLLSAVSS
TKDDEPDPLILEENDVDNMATNNKESAPSNGSGKNDSKGVLTQTLEMRENFQKQLQEIMS
ATSIVKPQGQRTSLSRSGCNSASSTPDSTRSARSGRSRVLEALRQSRQTVPDVNTEPSQN
EQIIWDDPTAREERARLASNLQWPSCPTQYSELQVDIQNLEDSPFQKPLHDSEIAKQAVC
DPGNIRVTEAPKHPISEELETPIKDSHLIPTPQAPSIAFPLANPPVAPHPREKIITIEET
HEELKKQYIFQLSSLNPQERIDYCHLIEKLGGLVIEKQCFDPTCTHIVVGHPLRNEKYLA
SVAAGKWVLHRSYLEACRTAGHFVQEEDYEWGSSSILDVLTGINVQQRRLALAAMRWRKK
IQQRQESGIVEGAFSGWKVILHVDQSREAGFKRLLQSGGAKVLPGHSVPLFKEATHLFSD
LNKLKPDDSGVNIAEAAAQNVYCLRTEYIADYLMQESPPHVENYCLPEAISFIQNNKELG
TGLSQKRKAPTEKNKIKRPRVH*
>ENSG00000130921|ENST00000253233
MSTVGLFHFPTPLTRICPAPWGLRLWEKLTLLSPGIAVTPVQMAGKKDYPALLSLDENEL
EEQFVKGHGPGGQATNKTSNCVVLKHIPSGIVVKCHQTRSVDQNRKLARKILQEKVDVFY
NGENSPVHKEKREAAKKKQERKKRAKETLEKKKLLKELWESSKKVH*
>ENSG00000061936|ENST00000261674
MYGASGGRAKPERKSGAKEEAGPGGAGGGGSRVELLVFGYACKLFRDDERALAQEQGQHL
IPWMGDHKILIDRYDGRGHLHDLSEYDAEYSTWNRDYQLSEEEARIEALCDEERYLALHT
DLLEEEARQEEEYKRLSEALAEDGSYNAVGFTYGSDYYDPSEPTEEEEPSKQREKNEAEN
LEENEEPFVAPLGLSVPSDVELPPTAKMHAIIERTASFVCRQGAQFEIMLKAKQARNSQF

DFLRFDHYLNPYYKFIQKAMKEGRYTVLAENKSDEKKKSGVSSDNEDDDDEEDGNYLHPS
LFASKKCNRLEELMKPLKVVDPDHPLAALVRKAQADSSTPTPHNADGAPVQPSQVEYTAD
STVAAMYYSYYMLPDGTYCLAPPPPGIDVTTYYSTLPAGVTVSNSPGVTTTAPPPPGTTP
LPPPTTAETSSGATSTTTTTSALAPVAAIIPPPPDVQPVIDKLAEYVARNGLKFETSVRA
KNDQRFEFLQPWHQYNAYYEFKKQFFLQKEGGDSMQAVSAPEEAPTDSAPEKPSDAGEDG
APEDAAEVGARAGSGGKKEASSSKTVPDGKLVKASFAPISFAIKAKENDLLPLEKNRVKL
DDDSDDDEESKEGQESSSSAANTNPAVAPPCVVVEEKKPQLTQEELEAKQAKQKLEDRLA
AAAREKLAQASKESKEKQLQAERKRKAALFLQTLKNPLPEAEAGKIEESPFSVEESSTTP
CPLLTGGRPLPTLEVKPPDRPSSKSKDPPREEEKEKKKKKHKKRSRTRSRSPKYHSSSKS
RSRSHSKAKHSLPSAYRTVRRSRSRSRSPRRRAHSPERRREERSVPTAYRVSRSPGASRK
RTRSRSPHEKKKKRRSRSRTKSKARSQSVSPSKQAAPRPAAPAAHSAHSASVSPVESRGS
SQERSRGVSQEKEAQISSAIVSSVQSKITQDLMAKVRAMLAASKNLQTSAS*
>ENSG00000173253|ENST00000259622
MADPQAGSAAGDWEIDVESLELEEDVCGAPRSTPPGPSPPPADGDCEDDEDDDGVDEDAE
EEGDGEEAGASPGMPGQPEQRGGPQPRPPLAPQASPAGTGPRERCTPAGGGAEPRKLSRT
PKCARCRNHGVVSCLKGHKRFCRWRDCQCANCLLVVERQRVMAAQVALRRQQATEDKKGL
SGKQNNFERKAVYQRQVRAPSLLAKSILEVLLGLFYSYYVYIMNHL*
>ENSG00000114166|ENST00000263754
MSEAGGAGPGGCGAGAGAGAGPGALPPQPAALPPAPPQGSPCAAAAGGSGACGPATAVAA
AGTAEGPGGGGSARIAVKKAQLRSAPRAKKLEKLGVYSACKAEESCKCNGWKNPNPSPTP
PRADLQQIIVSLTESCRSCSHALAAHVSHLENVSEEEMNRLLGIVLDVEYLFTCVHKEED
ADTKQVYFYLFKLLRKSILQRGKPVVEGSLEKKPPFEKPSIEQGVNNFVQYKFSHLPAKE
RQTIVELAKMFLNRINYWHLEAPSQRRLRSPNDDISGYKENYTRWLCYCNVPQFCDSLPR
YETTQVFGRTLLRSVFTVMRRQLLEQARQEKDKLPLEKRTLILTHFPKFLSMLEEEVYSQ
NSPIWDQDFLSASSRTSQLGIQTVINPPPVAGTISYNSTSSSLEQPNAGSSSPACKASSG
LEANPGEKRKMTDSHVLEEAKKPRVMGDIPMELINEVMSTITDPAAMLGPETNFLSAHSA
RDEAARLEERRGVIEFHVVGNSLNQKPNKKILMWLVGLQNVFSHQLPRMPKEYITRLVFD
PKHKTLALIKDGRVIGGICFRMFPSQGFTEIVFCAVTSNEQVKGYGTHLMNHLKEYHIKH
DILNFLTYADEYAIGYFKKQGFSKEIKIPKTKYVGYIKDYEGATLMGCELNPRIPYTEFS
VIIKKQKEIIKKLIERKQAQIRKVYPGLSCFKDGVRQIPIESIPGIRETGWKPSGKEKSK
EPRDPDQLYSTLKSILQQVKSHQSAWPFMEPVKRTEAPGYYEVIRFPMDLKTMSERLKNR
YYVSKKLFMADLQRVFTNCKEYNPPESEYYKCANILEKFFFSKIKEAGLIDK*
>ENSG00000024422|ENST00000263277
MFSWLKRGGARGQQPEAIRTVTSALKELYRTKLLPLEEHYRFGAFHSPALEDADFDGKPM
VLVAGQYSTGKTSFIQYLLEQEVPGSRVGPEPTTDCFVAVMHGDTEGTVPGNALVVDPDK
PFRKLNPFGNTFLNRFMCAQLPNQVLESISIIDTPGILSGAKQRVSRGYDFPAVLRWFAE
RVDLIILLFDAHKLEISDEFSEAIGALRGHEDKIRVVLNKADMVETQQLMRVYGALMWAL
GKVVGTPEVLRVYIGSFWSQPLLVPDNRRLFELEEQDLFRDIQGLPRHAALRKLNDLVKR
ARLVRVHAYIISYLKKEMPSVFGKENKKKQLILKLPVIFAKIQLEHHISPGDFPDCQKMQ
ELLMAHDFTKFHSLKPKLLEALDEMLTHDIAKLMPLLRQEELESTEVGVQGGAFEGTHMG
PFVERGPDEAMEDGEEGSDDEAEWVVTKDKSKYDEIFYNLAPADGKLSGSKAKTWMVGTK
LPNSVLGRIWKLSDVDRDGMLDDEEFALASHLIEAKLEGHGLPANLPRRLVPPSKRRHKG
SAE*
>ENSG00000125630|ENST00000263331

MDPGSRWRNLPSGPSLKHLTDPSYGIPREQQKAALQELTRAHVESFNYAVHEGLGLAVQA
IPPFEFAFKDERISFTILDAVISPPTVPKGTICKEANVYPAECRGRRSTYRGKLTADINW
AVNGISKGIIKQFLGYVPIMVKSKLCNLRNLPPQALIEHHEEAEEMGGYFIINGIEKVIR
MLIMPRRNFPIAMIRPKWKTRGPGYTQYGVSMHCVREEHSAVNMNLHYLENGTVMLNFIY
RKELFFLPLGFALKALVSFSDYQIFQELIKGKEDDSFLRNSVSQMLRIVMEEGCSTQKQV
LNYLGECFRVKLNVPDWYPNEQAAEFLFNQCICIHLKSNTEKFYMLCLMTRKLFALAKGE
CMEDNPDSLVNQEVLTPGQLFLMFLKEKLEGWLVSIKIAFDKKAQKTSVSMNTDNLMRIF
TMGIDLTKPFEYLFATGNLRSKTGLGLLQDSGLCVVADKLNFIRYLSHFRCVHRGADFAK
MRTTTVRRLLPESWGFLCPVHTPDGEPCGLMNHLTAVCEVVTQFVYTASIPALLCNLGVT
PIDGAPHRSYSECYPVLLDGVMVGWVDKDLAPGIADSLRHFKVLREKRIPPWMEVVLIPM
TGKPSLYPGLFLFTTPCRLVRPVQNLALGKEELIGTMEQIFMNVAIFEDEVFAGVTTHQE
LFPHSLLSVIANFIPFSDHNQSPRNMYQCQMGKQTMGFPLLTYQDRSDNKLYRLQTPQSP
LVRPSMYDYYDMDNYPIGTNAIVAVISYTGYDMEDAMIVNKASWERGFAHGSVYKSEFID
LSEKIKQGDSSLVFGIKPGDPRVLQKLDDDGLPFIGAKLQYGDPYYSYLNLNTGESFVMY
YKSKENCVVDNIKVCSNDTGSGKFKCVCITMRVPRNPTIGDKFASRHGQKGILSRLWPAE
DMPFTESGMVPDILFNPHGFPSRMTIGMLIESMAGKSAALHGLCHDATPFIFSEENSALE
YFGEMLKAAGYNFYGTERLYSGISGLELEADIFIGVVYYQRLRHMVSDKFQVRTTGARDR
VTNQPIGGRNVQGGIRFGEMERDALLAHGTSFLLHDRLFNCSDRSVAHVCVKCGSLLSPL
LEKPPPSWSAMRNRKYNCTLCSRSDTIDTVSVPYVFRYFVAELAAMNIKVKLDVV*
>ENSG00000133111|ENST00000255476
MEAQGVAEGAGPGAASGVPHPAALAPAAAPTLAPASVAAAASQFTLLVMQPCAGQDEAAA
PGGSVGAGKPVRYLCEGAGDGEEEAGEDEADLLDTSDPPGGGESAASLEDLEDEETHSGG
EGSSGGARRRGSGGGSMSKTCTYEGCSETTSQVAKQRKPWMCKKHRNKMYKDKYKKKKSD
QALNCGGTASTGSAGNVKLEESADNILSIVKQRTGSFGDRPARPTLLEQVLNQKRLSLLR
SPEVVQFLQKQQQLLNQQVLEQRQQQFPGTSM*
>ENSG00000105497|ENST00000262259
MPADVNLSQKPQVLGPEKQDGSCEASVSFEDVTVDFSREEWQQLDPAQRCLYRDVMLELY
SHLFAVGYHIPNPEVIFRMLKEKEPRVEEAEVSHQRCQEREFGLEIPQKEISKKASFQKD
MVGEFTRDGSWCSILEELRLDADRTKKDEQNQIQPMSHSAFFNKKTLNTESNCEYKDPGK
MIRTRPHLASSQKQPQKCCLFTESLKLNLEVNGQNESNDTEQLDDVVGSGQLFSHSSSDA
CSKNIHTGETFCKGNQCRKVCGHKQSLKQHQIHTQKKPDGCSECGGSFTQKSHLFAQQRI
HSVGNLHECGKCGKAFMPQLKLSVYLTDHTGDIPCICKECGKVFIQRSELLTHQKTHTRK
KPYKCHDCGKAFFQMLSLFRHQRTHSREKLYECSECGKGFSQNSTLIIHQKIHTGERQYA
CSECGKAFTQKSTLSLHQRIHSGQKSYVCIECGQAFIQKAHLIVHQRSHTGEKPYQCHNC
GKSFISKSQLDIHHRIHTGEKPYECSDCGKTFTQKSHLNIHQKIHTGERHHVCSECGKAF
NQKSILSMHQRIHTGEKPYKCSECGKAFTSKSQFKEHQRIHTGEKPYVCTECGKAFNGRS
NFHKHQITHTRERPFVCYKCGKAFVQKSELITHQRTHMGEKPYECLDCGKSFSKKPQLKV
HQRIHTGERPYVCSECGKAFNNRSNFNKHQTTHTRDKSYKCSYSVKGFTKQ*
>ENSG00000087302|ENST00000261700
MFRRKLTALDYHNPAGFNCKDETEFRNFIVWLEDQKIRHYKIEDRGNLRNIHSSDWPKFF
EKYLRDVNCPFKIQDRQEAIDWLLGLAVRLEYGDNAEKYKDLVPDNSKTADNATKNAEPL
INLDVNNPDFKAGVMALANLLQIQRHDDYLVMLKAIRILVQERLTQDAVAKANQTKEGLP
VALDKHILGFDTGDAVLNEAAQILRLLHIEELRELQTKINEAIVAVQAIIADPKTDHRLG
KVGR*

>ENSG00000102935|ENST00000262383
MHKKRVEEGEASDFSLAWDSSVTAAGGLEGEPECDQKTSRALEDRNSVTSQEERNEDDED
MEDESIYTCDHCQQDFESLADLTDHRAHRCPGDGDDDPQLSWVASSPSSKDVASPTQMIG
DGCDLGLGEEEGGTGLPYPCQFCDKSFIRLSYLKRHEQIHSDKLPFKCTYCSRLFKHKRS
RDRHIKLHTGDKKYHCHECEAAFSRSDHLKIHLKTHSSSKPFKCTVCKRGFSSTSSLQSH
MQAHKKNKEHLAKSEKEAKKDDFMCDYCEDTFSQTEELEKHVLTRHPQLSEKADLQCIHC
PEVFVDENTLLAHIHQAHANQKHKCPMCPEQFSSVEGVYCHLDSHRQPDSSNHSVSPDPV
LGSVASMSSATPDSSASVERGSTPDSTLKPLRGQKKMRDDGQGWTKVVYSCPYCSKRDFN
SLAVLEIHLKTIHADKPQQSHTCQICLDSMPTLYNLNEHVRKLHKNHAYPVMQFGNISAF
HCNYCPEMFADINSLQEHIRVSHCGPNANPSDGNNAFFCNQCSMGFLTESSLTEHIQQAH
CSVGSAKLESPVVQPTQSFMEVYSCPYCTNSPIFGSILKLTKHIKENHKNIPLAHSKKSK
AEQSPVSSDVEVSSPKRQRLSASANSISNGEYPCNQCDLKFSNFESFQTHLKLHLELLLR
KQACPQCKEDFDSQESLLQHLTVHYMTTSTHYVCESCDKQFSSVDDLQKHLLDMHTFVLY
HCTLCQEVFDSKVSIQVHLAVKHSNEKKMYRCTACNWDFRKEADLQVHVKHSHLGNPAKA
HKCIFCGETFSTEVELQCHITTHSKKYNCKFCSKAFHAIILLEKHLREKHCVFDAATENG
TANGVPPMATKKAEPADLQGMLLKNPEAPNSHEASEDDVDASEPMYGCDICGAAYTMEVL
LQNHRLRDHNIRPGEDDGSRKKAEFIKGSHKCNVCSRTFFSENGLREHLQTHRGPAKHYM
CPICGERFPSLLTLTEHKVTHSKSLDTGTCRICKMPLQSEEEFIEHCQMHPDLRNSLTGF
RCVVCMQTVTSTLELKIHGTFHMQKLAGSSAASSPNGQGLQKLYKCALCLKEFRSKQDLV
KLDVNGLPYGLCAGCMARSANGQVGGLAPPEPADRPCAGLRCPECSVKFESAEDLESHMQ
VDHRDLTPETSGPRKGTQTSPVPRKKTYQCIKCQMTFENEREIQIHVANHMIEEGINHEC
KLCNQMFDSPAKLLCHLIEHSFEGMGGTFKCPVCFTVFVQANKLQQHIFAVHGQEDKIYD
CSQCPQKFFFQTELQNHTMSQHAQ*
>ENSG00000137273|ENST00000259806
MTTEGGPPPAPLRRACSPVPGALQAALMSPPPAAAAAAAAAPETTSSSSSSSSASCASSS
SSSNSASAPSAACKSAGGGGAGAGSGGAKKASSGLRRPEKPPYSYIALIVMAIQSSPSKR
LTLSEIYQFLQARFPFFRGAYQGWKNSVRHNLSLNECFIKLPKGLGRPGKGHYWTIDPAS
EFMFEEGSFRRRPRGFRRKCQALKPMYHRVVSGLGFGASLLPQGFDFQAPPSAPLGCHSQ
GGYGGLDMMPAGYDAGAGAPSHAHPHHHHHHHVPHMSPNPGSTYMASCPVPAGPGGVGAA
GGGGGGDYGPDSSSSPVPSSPAMASAIECHSPYTSPAAHWSSPGASPYLKQPPALTPSSN
PAASAGLHSSMSSYSLEQSYLHQNAREDLSVGLPRYQHHSTPVCDRKDFVLNFNGISSFH
PSASGSYYHHHHQSVCQDIKPCVM*
>ENSG00000138231|ENST00000260803
MRVAVAGCCHGELDKIYETLALAERRGPGPVDLLLCCGDFQAVRNEADLRCMAVPPKYRH
MQTFYRYYSGEKKAPVLTLFIGGNHEASNHLQELPYGGWVAPNIYYLGLAGVVKYRGVRI
GGISGIFKSHDYRKGHFECPPYNSSTIRSIYHVRNIEVYKLKQLKQPIDIFLSHDWPRSI
YHYGNKKQLLKTKSFFRQEVENNTLGSPAASELLEHLKPTYWFSAHLHVKFAALMQHQAK
DKGQTARATKFLALDKCLPHRDFLQILEIEHDPSAPDYLEYDIEWLTILRATDDLINVTG
RLWNMPENNGLHARWDYSATEEGMKEVLEKLNHDLKVPCNFSVTAACYDPSKPQTQMQLI
HRINPQTTEFCAQLGIIDINVRLQKSKEEHHVCGEYEEQDDVESNDSGEDQSEYNTDTSA
LSSINPDEIMLDEEEDEDSIVSAHSGMNTPSVEPSDQASEFSASFSDVRILPGSMIVSSD
DTVDSTIDREGKPGGTVESGNGEDLTKVPLKRLSDEHEPEQRKKIKRRNQAIYAAVDDDD
DDAA*
>ENSG00000126603|ENST00000262366

MHSLDEPLDLKLSITKLRAAREKRERTLGVVRPRALHRELGLVDDSPTPGSPGSPPSGFL
LNSKFPEKVEGRFSAAPLVDLSLSPPSGLDSPNGSSSLSPERQGNGDLPPVPSASDFQPL
RYLDGVPSSFQFFLPLGSGGALHLPASSFLTPPKDKCLSPDLPLPKQLVCRWAKCNQLFE
LLQDLVDHVNDYHVKPEKDAGYCCHWEGCARHGRGFNARYKMLIHIRTHTNEKPHRCPTC
SKSFSRLENLKIHNRSHTGEKPYVCPYEGCNKRYSNSSDRFKHTRTHYVDKPYYCKMPGC
HKRYTDPSSLRKHIKAHGHFVSHEQQELLQLRPPPKPPLPAPDGGPYVSGAQIIIPNPAA
LFGGPGLPGLPLPLAPGPLDLSALACGNGGGSGGGGGMGPGLPGPVLPLNLAKNPLLPSP
FGAGGLGLPVVSLLAGAAGGKAEGEKGRGSVPTRALGMEGHKTPLERTESSCSRPSPDGL
PLLPGTVLDLSTGVNSAASSPEALAPGWVVIPPGSVLLKPAVVN*
>ENSG00000123268|ENST00000262053
MEDSHKSTTSETAPQPGSAVQGAHISHIAQQVSSLSESEESQDSSDSIGSSQKAHGILAR
RPSYRKILKDLSSEDTRGRKGDGENSGVSAAVTSMSVPTPIYQTSSGQYIAIAPNGALQL
ASPGTDGVQGLQTLTMTNSGSTQQGTTILQYAQTSDGQQILVPSNQVVVQTASGDMQTYQ
IRTTPSATSLPQTVVMTSPVTLTSQTTKTDDPQLKREIRLMKNREAARECRRKKKEYVKC
LENRVAVLENQNKTLIEELKTLKDLYSNKSV*
>ENSG00000168610|ENST00000264657
MAQWNQLQQLDTRYLEQLHQLYSDSFPMELRQFLAPWIESQDWAYAASKESHATLVFHNL
LGEIDQQYSRFLQESNVLYQHNLRRIKQFLQSRYLEKPMEIARIVARCLWEESRLLQTAA
TAAQQGGQANHPTAAVVTEKQQMLEQHLQDVRKRVQDLEQKMKVVENLQDDFDFNYKTLK
SQGDMQDLNGNNQSVTRQKMQQLEQMLTALDQMRRSIVSELAGLLSAMEYVQKTLTDEEL
ADWKRRQQIACIGGPPNICLDRLENWITSLAESQLQTRQQIKKLEELQQKVSYKGDPIVQ
HRPMLEERIVELFRNLMKSAFVVERQPCMPMHPDRPLVIKTGVQFTTKVRLLVKFPELNY
QLKIKVCIDKDSGDVAALRGSRKFNILGTNTKVMNMEESNNGSLSAEFKHLTLREQRCGN
GGRANCDASLIVTEELHLITFETEVYHQGLKIDLETHSLPVVVISNICQMPNAWASILWY
NMLTNNPKNVNFFTKPPIGTWDQVAEVLSWQFSSTTKRGLSIEQLTTLAEKLLGPGVNYS
GCQITWAKFCKENMAGKGFSFWVWLDNIIDLVKKYILALWNEGYIMGFISKERERAILST
KPPGTFLLRFSESSKEGGVTFTWVEKDISGKTQIQSVEPYTKQQLNNMSFAEIIMGYKIM
DATNILVSPLVYLYPDIPKEEAFGKYCRPESQEHPEADPGSAAPYLKTKFICVTPTTCSN
TIDLPMSPRTLDSLMQFGNNGEGAEPSAGGQFESLTFDMELTSECATSPM*
>ENSG00000135972|ENST00000258455
MAAPCVSYGGAVSYRLLLWGRGSLARKQGLWKTAAPELQTNVRSQILRLRHTAFVIPKKN
VPTSKRETYTEDFIKKQIEEFNIGKRHLANMMGEDPETFTQEDIDRAIAYLFPSGLFEKR
ARPVMKHPEQIFPRQRAIQWGEDGRPFHYLFYTGKQSYYSLMHDVYGMLLNLEKHQSHLQ
AKSLLPEKTVTRDVIGSRWLIKEELEEMLVEKLSDLDYMQFIRLLEKLLTSQCGAAEEEF
VQRFRRSVTLESKKQLIEPVQYDEQGMAFSKSEGKRKTAKAEAIVYKHGSGRIKVNGIDY
QLYFPITQDREQLMFPFHFVDRLGKHDVTCTVSGGGRSAQAGAIRLAMAKALCSFVTEDE
VEWMRQAGLLTTDPRVRERKKPGQEGARRKFTWKKR*
>ENSG00000119707|ENST00000261973
MSFPPHLNRPPMGIPALPPGIPPPQFPGFPPPVPPGTPMIPVPMSIMAPAPTVLVPTVSM
VGKHLGARKDHPGLKAKENDENCGPTTTVFVGNISEKASDMLIRQLLAKCGLVLSWKRVQ
GASGKLQAFGFCEYKEPESTLRALRLLHDLQIGEKKLLVKVDAKTKAQLDEWKAKKKASN
GNARPETVTNDDEEALDEETKRRDQMIKGAIEVLIREYSSELNAPSQESDSHPRKKKKEK
KEDIFRRFPVAPLIPYPLITKEDINAIEMEEDKRDLISREISKFRDTHKKLEEEKGKKEK
ERQEIEKERRERERERERERERRERERERERERERERFKEKERERERERDRDRDRTKERDRD

RDRERDRDRDRERSSDRNKDRSRSREKSRDRERERERERERERERERERERERERERERERE
RERERERKDKKRDREEDEEDAYERRKLERKLREKEAAYQERLKNWEIRERKKTREYEKEAE
REEERRREMAKEAKRLKEFLEDYDDDRDDPKYYRGSALQKRLRDREKEMEADERDRKREK
EELEEIRQRLLAEGHPDPDAELQRMEQEAERRRQPQIKQEPESEEEEEEKQEKEEKREEP
MEEEEEPEQKPCLKPTLRPISSAPSVSSASGNATPNTPGDESPCGIIIPHENSPDQQQPE
EHRPKIGLSLKLGASNSPGQPNSVKRKKLPVDSVFNKFEDEDSDDVPRKRKLVPLDYGED
DKNATKGTVNTEEKRKHIKSLIEKIPTAKPELFAYPLDWSIVDSILMERRIRPWINKKII
EYIGEEEATLVDFVCSKVMAHSSPQSILDDVAMVLDEEAEVFIVKMWRLLIYETEAKKIG
LVK*
>ENSG00000005339|ENST00000262367
MAENLLDGPPNPKRAKLSSPGFSANDSTDFGSLFDLENDLPDELIPNGGELGLLNSGNLV
PDAASKHKQLSELLRGGSGSSINPGIGNVSASSPVQQGLGGQAQGQPNSANMASLSAMGK
SPLSQGDSSAPSLPKQAASTSGPTPAASQALNPQAQKQVGLATSSPATSQTGPGICMNAN
FNQTHPGLLNSNSGHSLINQASQGQAQVMNGSLGAAGRGRGAGMPYPTPAMQGASSSVLA
ETLTQVSPQMTGHAGLNTAQAGGMAKMGITGNTSPFGQPFSQAGGQPMGATGVNPQLASK
QSMVNSLPTFPTDIKNTSVTNVPNMSQMQTSVGIVPTQAIATGPTADPEKRKLIQQQLVL
LLHAHKCQRREQANGEVRACSLPHCRTMKNVLNHMTHCQAGKACQVAHCASSRQIISHWK
NCTRHDCPVCLPLKNASDKRNQQTILGSPASGIQNTIGSVGTGQQNATSLSNPNPIDPSS
MQRAYAALGLPYMNQPQTQLQPQVPGQQPAQPQTHQQMRTLNPLGNNPMNIPAGGITTDQ
QPPNLISESALPTSLGATNPLMNDGSNSGNIGTLSTIPTAAPPSSTGVRKGWHEHVTQDL
RSHLVHKLVQAIFPTPDPAALKDRRMENLVAYAKKVEGDMYESANSRDEYYHLLAEKIYK
IQKELEEKRRSRLHKQGILGNQPALPAPGAQPPVIPQAQPVRPPNGPLSLPVNRMQVSQG
MNSFNPMSLGNVQLPQAPMGPRAASPMNHSVQMNSMGSVPGMAISPSRMPQPPNMMGAHT
NNMMAQAPAQSQFLPQNQFPSSSGAMSVGMGQPPAQTGVSQGQVPGAALPNPLNMLGPQA
SQLPCPPVTQSPLHPTPPPASTAAGMPSLQHTTPPGMTPPQPAAPTQPSTPVSSSGQTPT
PTPGSVPSATQTQSTPTVQAAAQAQVTPQPQTPVQPPSVATPQSSQQQPTPVHAQPPGTP
LSQAAASIDNRVPTPSSVASAETNSQQPGPDVPVLEMKTETQAEDTEPDPGESKGEPRSE
MMEEDLQGASQVKEETDIAEQKSEPMEVDEKKPEVKVEVKEEEESSSNGTASQSTSPSQP
RKKIFKPEELRQALMPTLEALYRQDPESLPFRQPVDPQLLGIPDYFDIVKNPMDLSTIKR
KLDTGQYQEPWQYVDDVWLMFNNAWLYNRKTSRVYKFCSKLAEVFEQEIDPVMQSLGYCC
GRKYEFSPQTLCCYGKQLCTIPRDAAYYSYQNRYHFCEKCFTEIQGENVTLGDDPSQPQT
TISKDQFEKKKNDTLDPEPFVDCKECGRKMHQICVLHYDIIWPSGFVCDNCLKKTGRPRK
ENKFSAKRLQTTRLGNHLEDRVNKFLRRQNHPEAGEVFVRVVASSDKTVEVKPGMKSRFV
DSGEMSESFPYRTKALFAFEEIDGVDVCFFGMHVQEYGSDCPPPNTRRVYISYLDSIHFF
RPRCLRTAVYHEILIGYLEYVKKLGYVTGHIWACPPSEGDDYIFHCHPPDQKIPKPKRLQ
EWYKKMLDKAFAERIIHDYKDIFKQATEDRLTSAKELPYFEGDFWPNVLEESIKELEQEE
EERKKEESTAASETTEGSQGDSKNAKKKNNKKTNKNKSSISRANKKKPSMPNVSNDLSQK
LYATMEKHKEVFFVIHLHAGPVINTLPPIVDPDPLLSCDLMDGRDAFLTLARDKHWEFSS
LRRSKWSTLCMLVELHTQGQDRFVYTCNECKHHVETRWHCTVCEDYDLCINCYNTKSHAH
KMVKWGLGLDDEGSSQGEPQSKSPQESRRLSIQRCIQSLVHACQCRNANCSLPSCQKMKR
VVQHTKGCKRKTNGGCPVCKQLIALCCYHAKHCQENKCPVPFCLNIKHKLRQQQIQHRLQ
QAQLMRRRMATMNTRNVPQQSLPSPTSAPPGTPTQQPSTPQTPQPPAQPQPSPVSMSPAG
FPSVARTQPPTTVSTGKPTSQVPAPPPPAQPPPAAVEAARQIEREAQQQQHLYRVNINNS
MPPGRTGMGTPGSQMAPVSLNVPRPNQVSGPVMPSMPPGQWQQAPLPQQQPMPGLPRPVI

SMQAQAAVAGPRMPSVQPPRSISPSALQDLLRTLKSPSSPQQQQQVLNILKSNPQLMAAF
IKQRTAKYVANQPGMQPQPGLQSQPGMQPQPGMHQQPSLQNLNAMQAGVPRPGVPPQQQA
MGGLNPQGQALNIMNPGHNPNMASMNPQYREMLRRQLLQQQQQQQQQQQQQQQQQQGSAG
MAGGMAGHGQFQQPQGPGGYPPAMQQQQRMQQHLPLQGSSMGQMAAQMGQLGQMGQPGLG
ADSTPNIQQALQQRILQQQQMKQQIGSPGQPNPMSPQQHMLSGQPQASHLPGQQIATSLS
NQVRSPAPVQSPRPQSQPPHSSPSPRIQPQPSPHHVSPQTGSPHPGLAVTMASSIDQGHL
GNPEQSAMLPQLNTPSRSALSSELSLVGDTTGDTLEKFVEGL*
>ENSG00000130811|ENST00000253108
MPTGDFDSKPSWADQVEEEGEDDKCVTSELLKGIPLATGDTSPEPELLPGAPLPPPKEVI
NGNIKTVTEYKIDEDGKKFKIVRTFRIETRKASKAVARRKNWKKFGNSEFDPPGPNVATT
TVSDDVSMTFITSKEDLNCQEEEDPMNKLKGQKIVSCRICKGDHWTTRCPYKDTLGPMQK
ELAEQLGLSTGEKEKLPGELEPVQATQNKTGKYVPPSLRDGASRRGESMQPNRRADDNAT
IRVTNLSEDTRETDLQELFRPFGSISRIYLAKDKTTGQSKGFAFISFHRREDAARAIAGV
SGFGYDHLILNVEWAKPSTN*
>ENSG00000175387|ENST00000262160
MSSILPFTPPVVKRLLGWKKSAGGSGGAGGGEQNGQEEKWCEKAVKSLVKKLKKTGRLDE
LEKAITTQNCNTKCVTIPSTCSEIWGLSTPNTIDQWDTTGLYSFSEQTRSLDGRLQVSHR
KGLPHVIYCRLWRWPDLHSHHELKAIENCEYAFNLKKDEVCVNPYHYQRVETPVLPPVLV
PRHTEILTELPPLDDYTHSIPENTNFPAGIEPQSNYIPETPPPGYISEDGETSDQQLNQS
MDTGSPAELSPTTLSPVNHSLDLQPVTYSEPAFWCSIAYYELNQRVGETFHASQPSLTVD
GFTDPSNSERFCLGLLSNVNRNATVEMTRRHIGRGVRLYYIGGEVFAECLSDSAIFVQSP
NCNQRYGWHPATVCKIPPGCNLKIFNNQEFAALLAQSVNQGFEAVYQLTRMCTIRMSFVK
GWGAEYRRQTVTSTPCWIELHLNGPLQWLDKVLTQMGSPSVRCSSMS*
>ENSG00000133740|ENST00000256117
XLLQEAKDGVLDLKAAADTLAVRQKRRIYDITNVLEGIDLIEKKSKNSIQWKGVGAGCNT
KEVIDRLRYLKAEIEDLELKERELDQQKLWLQQSIKNVMDDSINNRFSYVTHEDICNCFN
GDTLLAIQAPSGTQLEVPIPEMGQNGQKKYQINLKSHSGPIHVLLINKESSSSKPVVFPV
PPPDDLTQPSSQSLTPVTPQKSSMATQNLPEQHVSERSQALQQTSATDISSAGSISGDII
DELMSSDGK*
>ENSG00000105556|ENST00000264819
MAEASSLGRQSPRVVSCLEHSLCPGEPGLQTTAVVSMGSGDHQFNLAEILSQNYSVRGEC
EEASRCPDKPKEELEKDFISQSNDMPFDELLALYGYEASDPISDRESEGGDVAPNLPDMT
LDKEQIAKDLLSGEEEEETQSSADDLTPSVTSHEASDLFPNRSGSRFLADEDREPGSSAS
SDTEEDSLPANKCKKEIMVGPQFQADLSNLHLNRHCEKIYENEDQLLWDPSVLPEREVEE
FLYRAVKRRWHEMAGPQLPEGEAVKDSEQALYELVKCNFNVEEALRRLRFNVKVIRDGLC
AWSEEECRNFEHGFRVHGKNFHLIQANKVRTRSVGECVEYYYLWKKSERYDYFAQQTRLG
RRKYVPSGTTDADQDLDGSDPDGPGRPRPEQDTLTGMRTDPLSVDGTAGGLDEPGVASDG
LPSSEPGPCSFQQLDESPAVPLSHRPPALADPASYQPAVTAPEPDASPRLAVDFALPKEL
PLISSHVDLSGDPEETVAPAQVALSVTEFGLIGIGDVNPFLAAHPTCPAPGLHSEPLSHC
NVMTC*
>ENSG00000120656|ENST00000263974
MNQFGPSALINLSNFSSIKPEPASTPPQGSMANSTAVVKIPGTPGAGGRLSPENNQVLTK
KKLQDLVREVDPNEQLDEDVEEMLLQIADDFIESVVTAACQLARHRKSSTLEVKDVQLHL
ERQWNMWIPGFGSEEIRPYKKACTTEAHKQRMALIRKTTKK*

>ENSG00000130741|ENST00000253039
MAGGEAGVTLGQPHLSRQDLTTLDVTKLTPLSHEVISRQATINIGTIGHVAHGKSTVVKA
ISGVHTVRFKNELERNITIKLGYANAKIYKLDDPSCPRPECYRSCGSSTPDEFPTDIPGT
KGNFKLVRHVSFVDCPGHDILMATMLNGAAVMDAALLLIAGNESCPQPQTSEHLAAIEIM
KLKHILILQNKIDLVKESQAKEQYEQILAFVQGTVAEGAPIIPISAQLKYNIEVVCEYIV
KKIPVPPRDFTSEPRLIVIRSFDVNKPGCEVDDLKGGVAGGSILKGVLKVGQEIEVRPGI
VSKDSEGKLMCKPIFSKIVSLFAEHNDLQYAAPGGLIGVGTKIDPTLCRADRMVGQVLGA
VGALPEIFTELEISYFLLRRLLGVRTEGDKKAAKVQKLSKNEVLMVNIGSLSTGGRVSAV
KADLGKIVLTNPVCTEVGEKIALSRRVEKHWRLIGWGQIRRGVTIKPTVDDD*
>ENSG00000131759|ENST00000254066
MASNSSSCPTPGGGHLNGYPVPPYAFFFPPMLGGLSPPGALTTLQHQLPVSGYSTPSPAT
IETQSSSSEEIVPSPPSPPPLPRIYKPCFVCQDKSSGYHYGVSACEGCKGFFRRSIQKNM
VYTCHRDKNCIINKVTRNRCQYCRLQKCFEVGMSKESVRNDRNKKKKEVPKPECSESYTL
TPEVGELIEKVRKAHQETFPALCQLGKYTTNNSSEQRVSLDIDLWDKFSELSTKCIIKTV
EFAKQLPGFTTLTIADQITLLKAACLDILILRICTRYTPEQDTMTFSDGLTLNRTQMHNA
GFGPLTDLVFAFANQLLPLEMDDAETGLLSAICLICGDRQDLEQPDRVDMLQEPLLEALK
VYVRKRRPSRPHMFPKMLMKITDLRSISAKGAERVITLKMEIPGSMPPLIQEMLENSEGL
DTLSGQPGGGGRDGGGLAPPPGSCSPSLSPSSNRSSPATHSP*
>ENSG00000117707|ENST00000261454
MPDHDSTALLSRQTKRRRVDIGVKRTVGTASAFFAKARATFFSAMNPQGSEQDVEYSVVQ
HADGEKSNVLRKLLKRANSYEDAMMPFPGATIISQLLKNNMNKNGGTEPSFQASGLSSTG
SEVHQEDICSNSSRDSPPECLSPFGRPTMSQFDMDRLCDEHLRAKRARVENIIRGMSHSP
SVALRGNENEREMAPQSVSPRESYRENKRKQKLPQQQQQSFQQLVSARKEQKREERRQLK
QQLEDMQKQLRQLQEKFYQIYDSTDSENDEDGNLSEDSMRSEILDARAQDSVGRSDNEMC
ELDPGQFIDRARALIREQEMAENKPKREGNNKERDHGPNSLQPEGKHLAETLKQELNTAM
SQVVDTVVKVFSAKPSRQVPQVFPPLQIPQARFAVNGENHNFHTANQRLQCFGDVIIPNP
LDTFGNVQMASSTDQTEALPLVVRKNSSDQSASGPAAGGHHQPLHQSPLSATTGFTTSTF
RHPFPLPLMAYPFQSPLGAPSGSFSGKDRASPESLDLTRDTTSLRTKMSSHHLSHHPCSP
AHPPSTAEGLSLSLIKSECGDLQDMSEISPYSGSAMQEGLSPNHLKKAKLMFFYTRYPSS
NMLKTYFSDVKFNRCITSQLIKWFSNFREFYYIQMEKYARQAINDGVTSTEELSITRDCE
LYRALNMHYNKANDFEVPERFLEVAQITLREFFNAIIAGKDVDPSWKKAIYKVICKLDSE
VPEIFKSPNCLQELLHE*
>ENSG00000126351|ENST00000264637
MEQKPSKVECGSDPEENSARSPDGKRKRKNGQCSLKTSMSGYIPSYLDKDEQCVVCGDKA
TGYHYRCITCEGCKGFFRRTIQKNLHPTYSCKYDSCCVIDKITRNQCQLCRFKKCIAVGM
AMDLVLDDSKRVAKRKLIEQNRERRRKEEMIRSLQQRPEPTPEEWDLIHIATEAHRSTNA
QGSHWKQRRKFLPDDIGQSPIVSMPDGDKVDLEAFSEFTKIITPAITRVVDFAKKLPMFS
ELPCEDQIILLKGCCMEIMSLRAAVRYDPESDTLTLSGEMAVKREQLKNGGLGVVSDAIF
ELGKSLSAFNLDDTEVALLQAVLLMSTDRSGLLCVDKIEKSQEAYLLAFEHYVNHRKHNI
PHFWPKLLMKEREVQSSILYKGAAAEGRPGGSLGVHPEGQQLLGMHVVQGPQVRQLEQQL
GEAGSLQGPVLQHQSPKSPQQRLLELLHRSGILHARAVCGEDDSSEADSPSSSEEEPEVC
EDLAGNAASP*
>ENSG00000264424|ENST00000255381
MSSDSEMAIFGEAAPFLRKSEKERIEAQNKPFDAKTSVFVVDPKESYVKAIVQSREGGKV

TAKTEAGATVTVKEDQVFSMNPPKYDKIEDMAMMTHLHEPAVLYNLKERYAAWMIYTYSG
LFCVTVNPYKWLPVYNPEVVTAYRGKKRQEAPPHIFSISDNAYQFMLTDRENQSILITGE
SGAGKTVNTKRVIQYFATIAVTGEKKKEEPASGKMQGTLEDQIISANPLLEAFGNAKTVR
NDNSSRFGKFIRIHFGATGKLASADIETYLLEKSRVTFQLKAERSYHIFYQILSNKKPEL
IEMLLITTNPYDFAFVSQGEITVPSIDDQEELMATDSAVDILGFTADEKVAIYKLTGAVM
HYGNMKFKQKQREEQAEPDGTEVADKAAYLTSLNSADLLKSLCYPRVKVGNEFVTKGQTV
QQVYNAVGALAKAIYEKMFLWMVTRINQQLDTKQPRQYFIGVLDIAGFEIFDFNSLEQLC
INFTNEKLQQFFNHHMFVLEQEEYKKEGIEWEFIDFGMDLAACIELIEKPMGIFSILEEE
CMFPKATDTSFKNKLYEQHLGKSNNFQKPKPAKGKPEAHFSLVHYAGTVDYNIAGWLDKN
KDPLNETVVGLYQKSAMKTLAFLFSGAQTAEAEGGGGKKGGKKKGSSFQTVSALFRENLN
KLMTNLRSTHPHFVRCIIPNETKTPGAMEHELVLHQLRCNGVLEGIRICRKGFPSRILYA
DFKQRYKVLNASAIPEGQFIDSKKASEKLLGSIEIDHTQYKFGHTKVFFKAGLLGTLEEM
RDEKLAQLITRTQAICRGFLMRVEFRKMMERRESIFCIQYNIRAFMNVKHWPWMKLYFKI
KPLLKSAETEKEMANMKEEFEKTKEELAKTEAKRKELEEKMVTLMQEKNDLQLQVQAEAD
ALADAEERCDQLIKTKIQLEAKIKEVTERAEDEEEINAELTAKKRKLEDECSELKKDIDD
LELTLAKVEKEKHATENKVKNLTEEMAGLDETIAKLTKEKKALQEAHQQTLDDLQMEEDK
VNTLTKAKTKLEQQVDDLEGSLEQEKKLCMDLERAKRKLEGDLKLAQESTMDTENDKQQL
NEKLKKKEFEMSNLQGKIEDEQALAIQLQKKIKELQARIEELEEEIEAERASRAKAEKQR
SDLSRELEEISERLEEAGGATSAQIEMNKKREAEFQKMRRDLEESTLQHEATAAALRKKH
ADSVAELGEQIDSLQRVKQKLEKEKSELKMEINDLASNMETVSKAKANFEKMCRTLEDQL
SEIKTKEEEQQRLINELSAQKARLHTESGEFSRQLDEKDAMVSQLSRGKQAFTQQIEELK
RQLEEETKAKSTLAHALQSARHDCDLLREQYEEEQEAKAELQRGMSKANSEVAQWRTKYE
TDAIQRTEELEEAKKKLAQRLQDAEEHVEAVNSKCASLEKTKQRLQNEVEDLMIDVERSN
AACIALDKKQRNFDKVLAEWKQKYEETQAELEASQKESRSLSTELFKVKNAYEESLDHLE
TLKRENKNLQQEISDLTEQIAEGGKHIHELEKVKKQLDHEKSELQTSLEEAEASLEHEEG
KILRIQLELNQVKSEIDRKIAEKDEELDQLKRNHLRVVESMQSTLDAEIRSRNDALRIKK
KMEGDLNEMEIQLNHANRQAAEALRNLRNTQGILKDTQLHLDDAIRGQDDLKEQLAMVER
RANLMQAEVEELRASLERTERGRKMAEQELLDASERVQLLHTQNTSLINTKKKLETDISQ
IQGEMEDIVQEARNAEEKAKKAITDAAMMAEELKKEQDTSAHLERMKKNMEQTVKDLQLR
LDEAEQLALKGGKKQIQKLEARVRELESEVESEQKHNVEAVKGLRKHERRVKELTYQTEE
DRKNILRLQDLVDKLQTKVKAYKRQAEEAEEQSNVNLAKFRKLQHELEEAKERADIAESQ
VNKLRVKSREVHTKVISEE*
>ENSG00000104738|ENST00000262105
MSSPASTPSRRGSRRGRATPAQTPRSEDARSSPSQRRRGEDSTSTGELQPMPTSPGVDLQ
SPAAQDVLFSSPPQMHSSAIPLDFDVSSPLTYGTPSSRVEGTPRSGVRGTPVRQRPDLGS
AQKGLQVDLQSDGAAAEDIVASEQSLGQKLVIWGTDVNVAACKENFQRFLQRFIDPLAKE
EENVGIDITEPLYMQRLGEINVIGEPFLNVNCEHIKSFDKNLYRQLISYPQEVIPTFDMA
VNEIFFDRYPDSILEHQIQVRPFNALKTKNMRNLNPEDIDQLITISGMVIRTSQLIPEMQ
EAFFQCQVCAHTTRVEMDRGRIAEPSVCGRCHTTHSMALIHNRSLFSDKQMIKLQESPED
MPAGQTPHTVILFAHNDLVDKVQPGDRVNVTGIYRAVPIRVNPRVSNVKSVYKTHIDVIH
YRKTDAKRLHGLDEEAEQKLFSEKRVELLKELSRKPDIYERLASALAPSIYEHEDIKKGI
LLQLFGGTRKDFSHTGRGKFRAEINILLCGDPGTSKSQLLQYVYNLVPRGQYTSGKGSSA
VGLTAYVMKDPETRQLVLQTGALVLSDNGICCIDEFDKMNESTRSVLHEVMEQQTLSIAK
AGIICQLNARTSVLAAANPIESQWNPKKTTIENIQLPHTLLSRFDLIFLLLDPQDEAYDR

RLAHHLVALYYQSEEQAEEELLDMAVLKDYIAYAHSTIMPRLSEEASQALIEAYVDMRKI
GSSRGMVSAYPRQLESLIRLAEAHAKVRLSNKVEAIDVEEAKRLHREALKQSATDPRTGI
VDISILTTGMSATSRKRKEELAEALKKLILSKGKTPALKYQQLFEDIRGQSDIAITKDMF
EEALRALADDDFLTVTGKTVRLL*
>ENSG00000100811|ENST00000262238
MASGDTLYIATDGSEMPAEIVELHEIEVETIPVETIETTVVGEEEEEDDDDEDGGGGDHG
GGGGHGHAGHHHHHHHHHHHPPMIALQPLVTDDPTQVHHHQEVILVQTREEVVGGDDSDG
LRAEDGFEDQILIPVPAPAGGDDDYIEQTLVTVAAAGKSGGGGSSSSGGGRVKKGGGKKS
GKKSYLSGGAGAAGGGGADPGNKKWEQKQVQIKTLEGEFSVTMWSSDEKKDIDHETVVEE
QIIGENSPPDYSEYMTGKKLPPGGIPGIDLSDPKQLAEFARMKPRKIKEDDAPRTIACPH
KGCTKMFRDNSAMRKHLHTHGPRVHVCAECGKAFVESSKLKRHQLVHTGEKPFQCTFEGC
GKRFSLDFNLRTHVRIHTGDRPYVCPFDGCNKKFAQSTNLKSHILTHAKAKNNQ*
>ENSG00000105364|ENST00000253099
MLQFVRAGARAWLRPTGSQGLSSLAEEAARATENPEQVASEGLPEPVLRKVELPVPTHRR
PVQAWVESLRGFEQERVGLADLHPDVFATAPRLDILHQVAMWQKNFKRISYAKTKTRAEV
RGGGRKPWPQKGTGRARHGSIRSPLWRGGGVAHGPRGPTSYYYMLPMKVRALGLKVALTV
KLAQDDLHIMDSLELPTGDPQYLTELAHYRRWGDSVLLVDLTHEEMPQSIVEATSRLKTF
NLIPAVGLNVHSMLKHQTLVLTLPTVAFLEDKLLWQDSRYRPLYPFSLPYSDFPRPLPHA
TQGPAATPYHC*
>ENSG00000124789|ENST00000262077
MASGAGGVGGGGGGKIRTRRCHQGPIKPYQQGRQQHQGILSRVTESVKNIVPGWLQRYFN
KNEDVCSCSTDTSEVPRWPENKEDHLVYADEESSNITDGRITPEPAVSNTEEPSTTSTAS
NYPDVLTRPSLHRSHLNFSMLESPALHCQPSTSSAFPIGSSGFSLVKEIKDSTSQHDDDN
ISTTSGFSSRASDKDITVSKNTSLPPLWSPEAERSHSLSQHTATSSKKPAFNLSAFGTLS
PSLGNSSILKTSQLGDSPFYPGKTTYGGAAAAVRQSKLRNTPYQAPVRRQMKAKQLSAQS
YGVTSSTARRILQSLEKMSSPLADAKRIPSIVSSPLNSPLDRSGIDITDFQAKREKVDSQ
YPPVQRLMTPKPVSIATNRSVYFKPSLTPSGEFRKTNQRIDNKCSTGYEKNMTPGQNREQ
RESGFSYPNFSLPAANGLSSGVGGGGGKMRRERTRFVASKPLEEEEMEVPVLPKISLPIT
SSSLPTFNFSSPEITTSSPSPINSSQALTNKVQMTSPSSTGSPMFKFSSPIVKSTEANVL
PPSSIGFTFSVPVAKTAELSGSSSTLEPIISSSAHHVTTVNSTNCKKTPPEDCEGPFRPA
EILKEGSVLDILKSPGFASPKIDSVAAQPTATSPVVYTRPAISSFSSSGIGFGESLKAGS
SWQCDTCLLQNKVTDNKCIACQAAKLSPRDTAKQTGIETPNKSGKTTLSASGTGFGDKFK
PVIGTWDCDTCLVQNKPEAIKCVACETPKPGTCVKRALTLTVVSESAETMTASSSSCTVT
TGTLGFGDKFKRPIGSWECSVCCVSNNAEDNKCVSCMSEKPGSSVPASSSSTVPVSLPSG
GSLGLEKFKKPEGSWDCELCLVQNKADSTKCLACESAKPGTKSGFKGFDTSSSSSNSAAS
SSFKFGVSSSSSGPSQTLTSTGNFKFGDQGGFKIGVSSDSGSINPMSEGFKFSKPIGDFK
FGVSSESKPEEVKKDSKNDNFKFGLSSGLSNPVSLTPFQFGVSNLGQEEKKEELPKSSSA
GFSFGTGVINSTPAPANTIVTSENKSSFNLGTIETKSASVAPFTCKTSEAKKEEMPATKG
GFSFGNVEPASLPSASVFVLGRTEEKQQEPVTSTSLVFGKKADNEEPKCQPVFSFGNSEQ
TKDENSSKSTFSFSMTKPSEKESEQPAKATFAFGAQTSTTADQGAAKPVFSFLNNSSSSS
STPATSAGGGIFGSSTSSSNPPVATFVFGQSSNPVSSSAFGNTAESSTSQSLLFSQDSKL
ATTSSTGTAVTPFVFGPGASSNNTTTSGFGFGATTTSSSAGSSFVFGTGPSAPSASPAFG
ANQTPTFGQSQGASQPNPPGFGSISSSTALFPTGSQPAPPTFGTVSSSSQPPVFGQQPSQ
SAFGSGTTPNSSSAFQFGSSTTNFNFTNNSPSGVFTFGANSSTPAASAQPSGSGGFPFNQ

SPAAFTVGSNGKNVFSSSGTSFSGRKIKTAVRRRK*
>ENSG00000119614|ENST00000261980
MTGKAGEALSKPKSETVAKSTSGGAPARCTGFGIQEILGLNKEPPSSHPRAALDGLAPGH
LLAARSVLSPAGVGGMGLLGPGGLPGFYTQPTFLEVLSDPQSVHLQPLGRASGPLDTSQT
ASSDSEDVSSSDRKMSKSALNQTKKRKKRRHRTIFTSYQLEELEKAFNEAHYPDVYAREM
LAMKTELPEDRIQVWFQNRRAKWRKREKCWGRSSVMAEYGLYGAMVRHSIPLPESILKSA
KDGIMDSCAPWLLGMHKKSLEAAAESGRKPEGERQALPKLDKMEQDERGPDAQAAISQEE
LRENSIAVLRAKAQEHSTKVLGTVSGPDSLARSTEKPEEEEAMDEDRPAERLSPPQLEDM
A*
>ENSG00000112182|ENST00000257749
MSVDEKPDSPMYVYESTVHCTNILLGLNDQRKKDILCDVTLIVERKEFRAHRAVLAACSE
YFWQALVGQTKNDLVVSLPEEVTARGFGPLLQFAYTAKLLLSRENIREVIRCAEFLRMHN
LEDSCFSFLQTQLLNSEDGLFVCRKDAACQRPHEDCENSAGEEEDEEEETMDSETAKMAC
PRDQMLPEPISFEAAAIPVAEKEEALLPEPDVPTDTKESSEKDALTQYPRYKKYQLACTK
NVYNASSHSTSGFASTFREDNSSNSLKPGLARGQIKSEPPSEENEEESITLCLSGDEPDA
KDRAGDVEMDRKQPSPAPTPTAPAGAACLERSRSVASPSCLRSLFSITKSVELSGLPSTS
QQHFARSPACPFDKGITQGDLKTDYTPFTGNYGQPHVGQKEVSNFTMGSPLRGPGLEALC
KQEGELDRRSVIFSSSACDQVSTSVHSYSGVSSLDKDLSEPVPKGLWVGAGQSLPSSQAY
SHGGLMADHLPGRMRPNTSCPVPIKVCPRSPPLETRTRTSSSCSSYSYAEDGSGGSPCSL
PLCEFSSSPCSQGARFLATEHQEPGLMGDGMYNQVRPQIKCEQSYGTNSSDESGSFSEAD
SESCPVQDRGQEVKLPFPVDQITDLPRNDFQMMIKMHKLTSEQLEFIHDVRRRSKNRIAA
QRCRKRKLDCIQNLECEIRKLVCEKEKLLSERNQLKACMGELLDNFSCLSQEVCRDIQSP
EQIQALHRYCPVLRPMDLPTASSINPAPLGAEQNIAASQCAVGENVPCCLEPGAAPPGPP
WAPSNTSENCTSGRRLEGTDPGTFSERGPPLEPRSQTVTVDFCQEMTDKCTTDEQPRKDY
T*
>ENSG00000138376|ENST00000260947
MPDNRQPRNRQPRIRSGNEPRSAPAMEPDGRGAWAHSRAALDRLEKLLRCSRCTNILREP
VCLGGCEHIFCSNCVSDCIGTGCPVCYTPAWIQDLKINRQLDSMIQLCSKLRNLLHDNEL
SDLKEDKPRKSLFNDAGNKKNSIKMWFSPRSKKVRYVVSKASVQTQPAIKKDASAQQDSY
EFVSPSPPADVSERAKKASARSGKKQKKKTLAEINQKWNLEAEKEDGEFDSKEESKQKLV
SFCSQPSVISSPQINGEIDLLASGSLTESECFGSLTEVSLPLAEQIESPDTKSRNEVVTP
EKVCKNYLTSKKSLPLENNGKRGHHNRLSSPISKRCRTSILSTSGDFVKQTVPSENIPLP
ECSSPPSCKRKVGGTSGRKNSNMSDEFISLSPGTPPSTLSSSSYRRVMSSPSAMKLLPNM
AVKRNHRGETLLHIASIKGDIPSVEYLLQNGSDPNVKDHAGWTPLHEACNHGHLKVVELL
LQHKALVNTTGYQNDSPLHDAAKNGHVDIVKLLLSYGASRNAVNIFGLRPVDYTDDESMK
SLLLLPEKNESSSASHCSVMNTGQRRDGPLVLIGSGLSSEQQKMLSELAVILKAKKYTEF
DSTVTHVVVPGDAVQSTLKCMLGILNGCWILKFEWVKACLRRKVCEQEEKYEIPEGPRRS
RLNREQLLPKLFDGCYFYLWGTFKHHPKDNLIKLVTAGGGQILSRKPKPDSDVTQTINTV
AYHARPDSDQRFCTQYIIYEDLCNYHPERVRQGKVWKAPSSWFIDCVMSFELLPLDS*
>ENSG00000131732|ENST00000254037
MTRWARVSTTYNKRPLPATSWEDMKKGSFEGTSQNLPKRKQLEANRLSLKNDAPQAKHKK
NKKKKEYLNEDVNGFMEYLRQNSQMVHNGQIIATDSEEVREEIAVALKKDSRREGRRLKR
QAAKKNAMVCFHCRKPGHGIADCPAALENQDMGTGICYRCGSTEHEITKCKAKVDPALGE
FPFAKCFVCGEMGHLSRSCPDNPKGLYADGGGCKLCGSVEHLKKDCPESQNSERMVTVGR

WAKGMSADYEEILDVPKPQKPKTKIPKVVNF*
>ENSG00000075151|ENST00000264211
MNSQPQTRSPFFQRPQIQPPRATIPNSSPSIRPGAQTPTAVYQANQHIMMVNHLPMPYPV
PQGPQYCIPQYRHSGPPYVGPPQQYPVQPPGPGPFYPGPGPGDFPNAYGTPFYPSQPVYQ
SAPIIVPTQQQPPPAKREKKTIRIRDPNQGGKDITEEIMSGGGSRNPTPPIGRPTSTPTP
PQQLPSQVPEHSPVVYGTVESAHLAASTPVTAASDQKQEEKPKPDPVLKSPSPVLRLVLS
GEKKEQEGQTSETTAIVSIAELPLPPSPTTVSSVARSTIAAPTSSALSSQPIFTTAIDDR
CELSSPREDTIPIPSLTSCTETSDPLPTNENDDDICKKPCSVAPNDIPLVSSTNLINEIN
GVSEKLSATESIVEIVKQEVLPLTLELEILENPPEEMKLECIPAPITPSTVPSFPPTPPT
PPASPPHTPVIVPAAATTVSSPSAAITVQRVLEEDESIRTCLSEDAKEIQNKIEVEADGQ
TEEILDSQNLNSRRSPVPAQIAITVPKTWKKPKDRTRTTEEMLEAELELKAEEELSIDKV
LESEQDKMSQGFHPERDPSDLKKVKAVEENGEEAEPVRNGAESVSEGEGIDANSGSTDSS
GDGVTFPFKPESWKPTDTEGKKQYDREFLLDFQFMPACIQKPEGLPPISDVVLDKINQPK
LPMRTLDPRILPRGPDFTPAFADFGRQTPGGRGVPLLNVGSRRSQPGQRREPRKIITVSV
KEDVHLKKAENAWKPSQKRDSQADDPENIKTQELFRKVRSILNKLTPQMFNQLMKQVSGL
TVDTEERLKGVIDLVFEKAIDEPSFSVAYANMCRCLVTLKVPMADKPGNTVNFRKLLLNR
CQKEFEKDKADDDVFEKKQKELEAASAPEERTRLHDELEEAKDKARRRSIGNIKFIGELF
KLKMLTEAIMHDCVVKLLKNHDEESLECLCRLLTTIGKDLDFEKAKPRMDQYFNQMEKIV
KERKTSSRIRFMLQDVIDLRLCNWVSRRADQGPKTIEQIHKEAKIEEQEEQRKVQQLMTK
EKRRPGVQRVDEGGWNTVQGAKNSRVLDPSKFLKITKPTIDEKIQLVPKAQLGSWGKGSS
GGAKASETDALRSSASSLNRFSALQPPAPSGSTPSTPVEFDSRRTLTSRGSMGREKNDKP
LPSATARPNTFMRGGSSKDLLDNQSQEEQRREMLETVKQLTGGVDVERNSTEAERNKTRE
SAKPEISAMSAHDKAALSEEELERKSKSIIDEFLHINDFKEAMQCVEELNAQGLLHVFVR
VGVESTLERSQITRDHMGQLLYQLVQSEKLSKQDFFKGFSETLELADDMAIDIPHIWLYL
AELVTPMLKEGGISMRELTIEFSKPLLPVGRAGVLLSEILHLLCKQMSHKKVGALWREAD
LSWKDFLPEGEDVHNFLLEQKLDFIESDSPCSSEALSKKELSAEELYKRLEKLIIEDKAN
DEQIFDWVEANLDEIQMSSPTFLRALMTAVCKAAIIADSSTFRVDTAVIKQRVPILLKYL
DSDTEKELQALYALQASIVKLDQPANLLRMFFDCLYDEEVISEDAFYKWESSKDPAEQNG
KGVALKSVTAFFTWLREAEEESEDN*
>ENSG00000132475|ENST00000254810
MARTKQTARKSTGGKAPRKQLATKAARKSAPSTGGVKKPHRYRPGTVALREIRRYQKSTE
LLIRKLPFQRLVREIAQDFKTDLRFQSAAIGALQEASEAYLVGLFEDTNLCAIHAKRVTI
MPKDIQLARRIRGERA*
>ENSG00000134046|ENST00000256429
MRAHPGGGRCCPEQEEGESAAGGSGAGGDSAIEQGGQGSALAPSPVSGVRREGARGGGRG
RGRWKQAGRGGGVCGRGRGRGRGRGRGRGRGRGRGRPPSGGSGLGGDGGGCGGGGSGGGG
APRREPVPFPSGSAGPGPRGPRATESGKRMDCPALPPGWKKEEVIRKSGLSAGKSDVYYF
SPSGKKFRSKPQLARYLGNTVDLSSFDFRTGKMMPSKLQKNKQRLRNDPLNQNKGKPDLN
TTLPIRQTASIFKQPVTKVTNHPSNKVKSDPQRMNEQPRQLFWEKRLQGLSASDVTEQII
KTMELPKGLQGVGPGSNDETLLSAVASALHTSSAPITGQVSAAVEKNPAVWLNTSQPLCK
AFIVTDEDIRKQEERVQQVRKKLEEALMADILSRAADTEEMDIEMDSGDEA*
>ENSG00000105486|ENST00000263274
MQRSIMSFFHPKKEGKAKKPEKEASNSSRETEPPPKAALKEWNGVVSESDSPVKRPGRKA
ARVLGSEGEEEDEALSPAKGQKPALDCSQVSPPRPATSPENNASLSDTSPMDSSPSGIPK

RRTARKQLPKRTIQEVLEEQSEDEDREAKRKKEEEEEETPKESLTEAEVATEKEGEDGDQ
PTTPPKPLKTSKAETPTESVSEPEVATKQELQEEEEQTKPPRRAPKTLSSFFTPRKPAVK
KEVKEEEPGAPGKEGAAEGPLDPSGYNPAKNNYHPVEDACWKPGQKVPYLAVARTFEKIE
EVSARLRMVETLSNLLRSVVALSPPDLLPVLYLSLNHLGPPQQGLELGVGDGVLLKAVAQ
ATGRQLESVRAEAAEKGDVGLVAENSRSTQRLMLPPPPLTASGVFSKFRDIARLTGSAST
AKKIDIIKGLFVACRHSEARFIARSLSGRLRLGLAEQSVLAALSQAVSLTPPGQEFPPAM
VDAGKGKTAEARKTWLEEQGMILKQTFCEVPDLDRIIPVLLEHGLERLPEHCKLSPGIPL
KPMLAHPTRGISEVLKRFEEAAFTCEYKYDGQRAQIHALEGGEVKIFSRNQEDNTGKYPD
IISRIPKIKLPSVTSFILDTEAVAWDREKKQIQPFQVLTTRKRKEVDASEIQVQVCLYAF
DLIYLNGESLVREPLSRRRQLLRENFVETEGEFVFATSLDTKDIEQIAEFLEQSVKDSCE
GLMVKTLDVDATYEIAKRSHNWLKLKKDYLDGVGDTLDLVVIGAYLGRGKRAGRYGGFLL
ASYDEDSEELQAICKLGTGFSDEELEEHHQSLKALVLPSPRPYVRIDGAVIPDHWLDPSA
VWEVKCADLSLSPIYPAARGLVDSDKGISLRFPRFIRVREDKQPEQATTSAQVACLYRKQ
SQIQNQQGEDSGSDPEDTY*
>ENSG00000101544|ENST00000262198
MFQIPVENLDNIRKVRKKVKGILVDIGLDSCKELLKDLKGFDPGEKYFHNTSWGDVSLWE
PSGKKVRYRTKPYCCGLCKYSTKVLTSFKNHLHRYHEDEIDQELVIPCPNCVFASQPKVV
GRHFRMFHAPVRKVQNYTVNILGETKSSRSDVISFTCLKCNFSNTLYYSMKKHVLVAHFH
YLINSYFGLRTEEMGEQPKTNDTVSIEKIPPPDKYYCKKCNANASSQDALMYHILTSDIH
RDLENKLRSVISEHIKRTGLLKQTHIAPKPAAHLAAPANGSAPSAPAQPPCFHLALPQNS
PSPAAGQPVTVAQGAPGSLTHSPPAAGQSHMTLVSSPLPVGQNSLTLQPPAPQPVFLSHG
VPLHQSVNPPVLPLSQPVGPVNKSVGTSVLPINQTVRPGVLPLTQPVGPINRPVGPGVLP
VSPSVTPGVLQAVSPGVLSVSRAVPSGVLPAGQMTPAGQMTPAGVIPGQTATSGVLPTGQ
MVQSGVLPVGQTAPSRVLPPGQTAPLRVISAGQVVPSGLLSPNQTVSSSAVVPVNQGVNS
GVLQLSQPVVSGVLPVGQPVRPGVLQLNQTVGTNILPVNQPVRPGASQNTTFLTSGSILR
QLIPTGKQVNGIPTYTLAPVSVTLPVPPGGLATVAPPQMPIQLLPSGAAAPMAGSMPGMP
SPPVLVNAAQSVFVQASSSAADTNQVLKQAKQWKTCPVCNELFPSNVYQVHMEVAHKHSE
SKSGEKLEPEKLAACAPFLKWMREKTVRCLSCKCLVSEEELIHHLLMHGLGCLFCPCTFH
DIKGLSEHSRNRHLGKKKLPMDYSNRGFQLDVDANGNLLFPHLDFITILPKEKLGEREVY
LAILAGIHSKSLVPVYVKVRPQAEGTPGSTGKRVSTCPFCFGPFVTTEAYELHLKERHHI
MPTVHTVLKSPAFKCIHCCGVYTGNMTLAAIAVHLVRCRSAPKDSSSDLQAQPGFIHNSE
LLLVSGEVMHDSSFSVKRKLPDGHLGAEDQRHGEEQPPILNADAAPGPEKVTSVVPFKRQ
RNESRTEGPIVKDEALQILALDPKKYEGRSYEEKKQFLKDYFHKKPYPSKKEIELLSSLF
WVWKIDVASFFGKRRYICMKAIKNHKPSVLLGFDMSELKNVKHRLNFEYEP*
>ENSG00000101746|ENST00000261592
MESERDMYRQFQDWCLRTYGDSGKTKTVTRKKYERIVQLLNGSESSSTDNAKFKFWVKSK
GFQLGQPDEVRGGGGGAKQVLYVPVKTTDGVGVDEKLSLRRVAVVEDFFDIIYSMHVETG
PNGEQIRKHAGQKRTYKAISESYAFLPREAVTRFLMSCSECQKRMHLNPDGTDHKDNGKP
PTLVTSMIDYNMPITMAYMKHMKLQLLNSQQDEDESSIESDEFDMSDSTRMSAVNSDLSS
NLEERMQSPQNLHGQQDDDSAAESFNGNETLGHSSIASGGTHSREMGDSNSDGKTGLEQD
EQPLNLSDSPLSAQLTSEYRIDDHNSNGKNKYKNLLISDLKMEREARENGSKSPAHSYSS
YDSGKNESVDRGAEDLSLNRGDEDEDDHEDHDDSEKVNETDGVEAERLKAFNMFVRLFVD
ENLDRMVPISKQPKEKIQAIIDSCRRQFPEYQERARKRIRTYLKSCRRMKRSGFEMSRPI
PSHLTSAVAESILASACESESRNAAKRMRLERQQDESAPADKQCKPEATQATYSTSAVPG

SQDVLYINGNGTYSYHSYRGLGGGLLNLNDASSSGPTDLSMKRQLATSSGSSSSSNSRPQ
LSPTEINAVRQLVAGYRESAAFLLRSADELENLILQQN*
>ENSG00000141002|ENST00000263346
MSRRALRRLRGEQRGQEPLGPGALHFDLRDDDDAEEEGPKRELGVRRPGGAGKEGVRVNN
RFELINIDDLEDDPVVNGERSGCALTDAVAPGNKGRGQRGNTESKTDGDDTETVPSEQSH
ASGKLRKKKKKQKNKKSSTGEASENGLEDIDRILERIEDSTGLNRPGPAPLSSRKHVLYV
EHRHLNPDTELKRYFGARAILGEQRPRQRQRVYPKCTWLTTPKSTWPRYSKPGLSMRLLE
SKKGLSFFAFEHSEEYQQAQHKFLVAVESMEPNNIVVLLQTSPYHVDSLLQLSDACRFQE
DQEMARDLVERALYSMECAFHPLFSLTSGACRLDYRRPENRSFYLALYKQMSFLEKRGCP
RTALEYCKLILSLEPDEDPLCMLLLIDHLALRARNYEYLIRLFQEWEAHRNLSQLPNFAF
SVPLAYFLLSQQTDLPECEQSSARQKASLLIQQALTMFPGVLLPLLESCSVRPDASVSSH
RFFGPNAEISQPPALSQLVNLYLGRSHFLWKEPATMSWLEENVHEVLQAVDAGDPAVEAC
ENRRKVLYQRAPRNIHRHVILSEIKEAVAALPPDVTTQSVMGFDPLPPSDTIYSYVRPER
LSPISHGNTIALFFRSLLPNYTMEGERPEEGVAGGLNRNQGLNRLMLAVRDMMANFHLND
LEAPHEDDAEGEGEWD*
>ENSG00000102974|ENST00000264010
MEGDAVEAIVEESETFIKGKERKTYQRRREGGQEEDACHLPQNQTDGGEVVQDVNSSVQM
VMMEQLDPTLLQMKTEVMEGTVAPEAEAAVDDTQIITLQVVNMEEQPINIGELQLVQVPV
PVTVPVATTSVEELQGAYENEVSKEGLAESEPMICHTLPLPEGFQVVKVGANGEVETLEQ
GELPPQEDPSWQKDPDYQPPAKKTKKTKKSKLRYTEEGKDVDVSVYDFEEEQQEGLLSEV
NAEKVVGNMKPPKPTKIKKKGVKKTFQCELCSYTCPRRSNLDRHMKSHTDERPHKCHLCG
RAFRTVTLLRNHLNTHTGTRPHKCPDCDMAFVTSGELVRHRRYKHTHEKPFKCSMCDYAS
VEVSKLKRHIRSHTGERPFQCSLCSYASRDTYKLKRHMRTHSGEKPYECYICHARFTQSG
TMKMHILQKHTENVAKFHCPHCDTVIARKSDLGVHLRKQHSYIEQGKKCRYCDAVFHERY
ALIQHQKSHKNEKRFKCDQCDYACRQERHMIMHKRTHTGEKPYACSHCDKTFRQKQLLDM
HFKRYHDPNFVPAAFVCSKCGKTFTRRNTMARHADNCAGPDGVEGENGGETKKSKRGRKR
KMRSKKEDSSDSENAEPDLDDNEDEEEPAVEIEPEPEPQPVTPAPPPAKKRRGRPPGRTN
QPKQNQPTAIIQVEDQNTGAIENIIVEVKKEPDAEPAEGEEEEAQPAATDAPNGDLTPEM
ILSMMDR*
>ENSG00000100902|ENST00000261479
MSRGSSAGFDRHITIFSPEGRLYQVEYAFKAINQGGLTSVAVRGKDCAVIVTQKKVPDKL
LDSSTVTHLFKITENIGCVMTGMTADSRSQVQRARYEAANWKYKYGYEIPVDMLCKRIAD
ISQVYTQNAEMRPLGCCMILIGIDEEQGPQVYKCDPAGYYCGFKATAAGVKQTESTSFLE
KKVKKKFDWTFEQTVETAITCLSTVLSIDFKPSEIEVGVVTVENPKFRILTEAEIDAHLV
ALAERD*
>ENSG00000131381|ENST00000253699
MASLDDPGEVREGFLCPLCLKDLQSFYQLHSHYEEEHSGEDRDVKGQIKSLVQKAKKAKD
RLLKREGDDRAESGTQGYESFSYGGVDPYMWEPQELGAVRSHLSDFKKHRAARIDHYVVE
VNKLIIRLEKLTAFDRTNTESAKIRAIEKSVVPWVNDQDVPFCPDCGNKFSIRNRRHHCR
LCGSIMCKKCMELISLPLANKLTSASKESLSTHTSPSQSPNSVHGSRRGSISSMSSVSSV
LDEKDDDRIRCCTHCKDTLLKREQQIDEKEHTPDIVKLYEKLRLCMEKVDQKAPEYIRMA
ASLNAGETTYSLEHASDLRVEVQKVYELIDALSKKILTLGLNQDPPPHPSNLRLQRMIRY
SATLFVQEKLLGLMSLPTKEQFEELKKKRKEEMERKRAVERQAALESQRRLEERQSGLAS
RAANGEVASLRRGPAPLRKAEGWLPLSGGQGQSEDSDPLLQQIHNITSFIRQAKAAGRMD

EVRTLQENLRQLQDEYDQQQTEKAIELSRRQAEEEDLQREQLQMLRERELEREREQFRVA
SLHTRTRSLDFREIGPFQLEPSREPRTHLAYALDLGSSPVPSSTAPKTPSLSSTQPTRVW
SGPPAVGQERLPQSSMPQQHEGPSLNPFDEEDLSSPMEEATTGPPAAGVSLDPSARILKE
YNPFEEEDEEEEAVAGNPFIQPDSPAPNPFSEEDEHPQQRLSSPLVPGNPFEEPTCINPF
EMDSDSGPEAEEPIEEELLLQQIDNIKAYIFDAKQCGRLDEVEVLTENLRELKHTLAKQK
GGTD*
>ENSG00000114446|ENST00000264538
MTAALAVVTTSGLEDGVPRSRGEGTGEVVLERGPGAAYHMFVVMEDLVEKLKLLRYEEEF
LRKSNLKAPSRHYFALPTNPGEQFYMFCTLAAWLINKAGRPFEQPQEYDDPNATISNILS
ELRSFGRTADFPPSKLKSGYGEHVCYVLDCFAEEALKYIGFTWKRPIYPVEELEEESVAE
DDAELTLNKVDEEFVEEETDNEENFIDLNVLKAQTYHLDMNETAKQEDILESTTDAAEWS
LEVERVLPQLKVTIRTDNKDWRIHVDQMHQHRSGIESALKETKGFLDKLHNEITRTLEKI
SSREKYINNQLENLVQEYRAAQAQLSEAKERYQQGNGGVTERTRLLSEVMEELEKVKQEM
EEKGSSMTDGAPLVKIKQSLTKLKQETVEMDIRIGIVEHTLLQSKLKEKSNMTRNMHATV
IPEPATGFY*
>ENSG00000135457|ENST00000257915
MAWALKLPLADEVIESGLVQDFDASLSGIGQELGAGAYSMSDVLALPIFKQEESSLPPDN
ENKILPFQYVLCAATSPAVKLHDETLTYLNQGQSYEIRMLDNRKLGELPEINGKLVKSIF
RVVFHDRRLQYTEHQQLEGWRWNRPGDRILDIDIPMSVGIIDPRANPTQLNTVEFLWDPA
KRTSVFIQVHCISTEFTMRKHGGEKGVPFRVQIDTFKENENGEYTEHLHSASCQIKVFKP
KGADRKQKTDREKMEKRTPHEKEKYQPSYETTILTECSPWPEITYVNNSPSPGFNSSHSS
FSLGEGNGSPNHQPEPPPPVTDNLLPTTTPQEAQQWLHRNRFSTFTRLFTNFSGADLLKL
TRDDVIQICGPADGIRLFNALKGRMVRPRLTIYVCQESLQLREQQQQQQQQQKHEDGDS
NGTFFVYHAIYLEELTAVELTEKIAQLFSISPCQISQIYKQGPTGIHVLISDEMIQNFQE
EACFILDTMKAETNDSYHIILK*
>ENSG00000115947|ENST00000264169
MSSRKSKSNSLIHTECLSQVQRILRERFCRQSPHSNLFGVQVQYKHLSELLKRTALHGES
NSVLIIGPRGSGKTMLINHALKELMEIEEVSENVLQVHLNGLLQINDKIALKEITRQLNL
ENVVGDKVFGSFAENLSFLLEALKKGDRTSSCPVIFILDEFDLFAHHKNQTLLYNLFDIS
QSAQTPIAVIGLTCRLDILELLEKRVKSRFSHRQIHLMNSFGFPQYVKIFKEQLSLPAEF
PDKVFAEKWNENVQYLSEDRSVQEVLQKHFNISKNLRSLHMLLMLALNRVTASHPFMTAV
DLMEASQLCSMDSKANIVHGLSVLEICLIIAMKHLNDIYEEEPFNFQMVYNEFQKFVQRK
AHSVYNFEKPVVMKAFEHLQQLELIKPMERTSGNSQREYQLMKLLLDNTQIMNALQKYPN
CPTDVRQWATSSLSWL*
>ENSG00000132326|ENST00000254657
MNGYAEFPPSPSNPTKEPVEPQPSQVPLQEDVDMSSGSSGHETNENCSTGRDSQGSDCDD
SGKELGMLVEPPDARQSPDTFSLMMAKSEHNPSTSGCSSDQSSKVDTHKELIKTLKELKV
HLPADKKAKGKASTLATLKYALRSVKQVKANEEYYQLLMSSEGHPCGADVPSYTVEEMES
VTSEHIVKNADMFAVAVSLVSGKILYISDQVASIFHCKRDAFSDAKFVEFLAPHDVGVFH
SFTSPYKLPLWSMCSGADSFTQECMEEKSFFCRVSVRKSHENEIRYHPFRMTPYLVKVRD
QQGAESQLCCLLLAERVHSGYEAPRIPPEKRIFTTTHTPNCLFQDVDERAVPLLGYLPQD
LIETPVLVQLHPSDRPLMLAIHKKILQSGGQPFDYSPIRFRARNGEYITLDTSWSSFINP
WSRKISFIIGRHKVRVGPLNEDVFAAHPCTEEKALHPSIQELTEQIHRLLLQPVPHSGSS
GYGSLGSNGSHEHLMSQTSSSDSNGHEDSRRRRAEICKNGNKTKNRSHYSHESGEQKKKS

VTEMQTNPPAEKKAVPAMEKDSLGVSFPEELACKNQPTCSYQQISCLDSVIRYLESCNEA
ATLKRKCEFPANVPALRSSDKRKATVSPGPHAGEAEPPSRVNSRTGVGTHLTSLALPGKA
ESVASLTSQCSYSSTIVHVGDKKPQPELEMVEDAASGPESLDCLAGPALACGLSQEKEPF
KKLGLTKEVLAAHTQKEEQSFLQKFKEIRKLSIFQSHCHYYLQERSKGQPSERTAPGLRN
TSGIDSPWKKTGKNRKLKSKRVKPRDSSESTGSGGPVSARPPLVGLNATAWSPSDTSQSS
CPAVPFPAPVPAAYSLPVFPAPGTVAAPPAPPHASFTVPAVPVDLQHQFAVQPPPFPAPL
APVMAFMLPSYSFPSGTPNLPQAFFPSQPQFPSHPTLTSEMASASQPEFPSRTSIPRQPC
ACPATRATPPSAMGRASPPLFQSRSSSPLQLNLLQLEEAPEGGTGAMGTTGATETAAVGA
DCKPGTSRDQQPKAPLTRDEPSDTQNSDALSTSSGLLNLLLNEDLCSASGSAASESLGSG
SLGCDASPSGAGSSDTSHTSKYFGSIDSSENNHKAKMNTGMEESEHFIKCVLQDPIWLLM
ADADSSVMMTYQLPSRNLEAVLKEDREKLKLLQKLQPRFTESQKQELREVHQWMQTGGLP
AAIDVAECVYCENKEKGNICIPYEEDIPSLGLSEVSDTKEDENGSPLNHRIEEQT*
>ENSG00000059691|ENST00000263985
MAAPMLRWGCRGRRWAFARVDGGSCHRRGAPTGSTSNQIRGESSVAQQPLHTAQKTRKGE
HKWAAVVGLEIHAQISSNSKLFSGSQVRFSAPPNSLVSFFDASLPGTLPVLNRRCVEAAV
MTGLALNCHINKKSLFDRKHYFYADLPAGYQITQQRLPIAVNGSLIYGVCAGKKQSQVIP
KTVRIKQIQLEQDSGKSLHDNLRSQTLIDLNRAGVGLLEVVLEPDMSCGEEAATAVRELQ
LILQALGTSQANMAEGQLRVDANISVHHPGEPLGVRTEVKNLNSIRFLAKAIDYEIQRQI
NELENGGEILNETRSFHHKLGCTMSMRDKEGKQDYRFMPEPNLPPLVLYDATSLPAGADP
QQVINIDQIRETLPELPSVTREKLVQQYGMLLEHSFTLLNEVGLLEFFQNVIKETRAEPK
KVTSWVLNTFLGYLKQQNLAVSESPVTPSALAELLDLLDSRTISSSAAKQVFEELWKREG
KTPGQIVSEKQLELMQDQGALEQLCHSVMEAHPQVVMDVKNRNPRAINKLIGLVRKATQS
RADPVMIKEILEKKLSL*
>ENSG00000130749|ENST00000253048
MEAAPGTPPPPPSESPPPPSPPPPSTPSPPPCSPDARPATPHLLHHRLPLPDDREDGELE
EGELEDDGAEETQDTSGGPERSRKEKGEKHHSDSDEEKSHRRLKRKRKKEREKEKRRSKK
RRKSKHKRHASSSDDFSDFSDDSDFSPSEKGHRKYREYSPPYAPSHQQYPPSHATPLPKK
AYSKMDSKSYGMYEDYENEQYGEYEGDEEEDMGKEDYDDFTKELNQYRRAKEGSSRGRGS
RGRGRGYRGRGSRGGSRGRGMGRGSRGRGRGSMGGDHPEDEEDFYEEEMDYGESEEPMGD
DDYDEYSKELNQYRRSKDSRGRGLSRGRGRGSRGRGKGMGRGRGRGGSRGGMNKGGMNDD
EDFYDEDMGDGGGGSYRSRDHDKPHQQSDKKGKVICKYFVEGRCTWGDHCNFSHDIELPK
KRELCKFYITGFCARAENCPYMHGDFPCKLYHTTGNCINGDDCMFSHDPLTEETRELLDK
MLADDAEAGAEDEKEVEELKKQGINPLPKPPPGVGLLPTPPRPPGPQAPTSPNGRPMQGG
PPPPPPPPPPPPGPPQMPMPVHEPLSPQQLQQQDMYNKKIPSLFEIVVRPTGQLAEKLGV
RFPGPGGPPGPMGPGPNMGPPGPMGGPMHPDMHPDMHPDMHPDMHADMHADMPMGPGMNP
GPPMGPGGPPMMPYGPGDSPHSGMMPPIPPAQNFYENFYQQQEGMEMEPGLLGDAEDYGH
YEELPGEPGEHLFPEHPLEPDSFSEGGPPGRPKPGAGVPDFLPSAQRALYLRIQQKQQEE
EERARRLAESSKQDRENEEGDTGNWYSSDEDEGGSSVTSILKTLRQQTSSRPPASVGELS
SSGLGDPRLQKGHPTGSRLADPRLSRDPRLTRHVEASGGSGPGDSGPSDPRLARALPTSK
PEGSLHSSPVGPSSSKGSGPPPTEEEEGERALREKAVNIPLDPLPGHPLRDPRSQLQQFS
HIKKDVTLSKPSFARTVLWNPEDLIPLPIPKQDAVPPVPAALQSMPTLDPRLHRAATAGP
PNARQRPGASTDSSTQGANLPDFELLSRILKTVNATGSSAAPGSSDKPSDPRVRKAPTDP
RLQKPTDSTASSRAAKPGPAEAPSPTASPSGDASPPATAPYDPRVLAAGGLGQGGGGGQS
SVLSGISLYDPRTPNAGGKATEPAADTGAQPKGAEGNGKSSASKAKEPPFVRKSALEQPE

TGKAGADGGTPTDRYNSYNRPRPKAAAAPAATTATPPPEGAPPQPGVHNLPVPTLFGTVK
QTPKTGSGSPFAGNSPAREGEQDAASLKDVFKGFDPTASPFCQ*
>ENSG00000136603|ENST00000259119
MENLQTNFSLVQGSTKKLNGMGDDGSPPAKKMITDIHANGKTINKVPTVKKEHLDDYGEA
PVETDGEHVKRTCTSVPETLHLNPSLKHTLAQFHLSSQSSLGGPAAFSARHSQESMSPTV
FLPLPSPQVLPGPLLIPSDSSTELTQTVLEGESISCFQVGGEKRLCLPQVLNSVLREFTL
QQINTVCDELYIYCSRCTSDQLHILKVLGILPFNAPSCGLITLTDAQRLCNALLRPRTFP
QNGSVLPAKSSLAQLKETGSAFEVEHECLGKCQGLFAPQFYVQPDAPCIQCLECCGMFAP
QTFVMHSHRSPDKRTCHWGFESAKWHCYLHVNQKYLGTPEEKKLKIILEEMKEKFSMRSG
KRNQSKTDAPSGMELQSWYPVIKQEGDHVSQTHSFLHPSYYLYMCDKVVAPNVSLTSAVS
QSKELTKTEASKSISRQSEKAHSSGKLQKTVSYPDVSLEEQEKMDLKTSRELCSRLDASI
SNNSTSKRKSESATCNLVRDINKVGIGLVAAASSPLLVKDVICEDDKGKIMEEVMRTYLK
QQEKLNLILQKKQQLQMEVKMLSSSKSMKELTEEQQNLQKELESLQNEHAQRMEEFYVEQ
KDLEKKLEQIMKQKCTCDSNLEKDKEAEYAGQLAELRQRLDHAEADRQELQDELRQEREA
RQKLEMMIKELKLQILKSSKTAKE*
>ENSG00000133606|ENST00000255977
MAEAATPGTTATTSGAGAAAATAAAASPTPIPTVTAPSLGAGGGGGGSDGSGGGWTKQVT
CRYFMHGVCKEGDNCRYSHDLSDSPYSVVCKYFQRGYCIYGDRCRYEHSKPLKQEEATAT
ELTTKSSLAASSSLSSIVGPLVEMNTGEAESRNSNFATVGAGSEDWVNAIEFVPGQPYCG
RTAPSCTEAPLQGSVTKEESEKEQTAVETKKQLCPYAAVGECRYGENCVYLHGDSCDMCG
LQVLHPMDAAQRSQHIKSCIEAHEKDMELSFAVQRSKDMVCGICMEVVYEKANPSERRFG
ILSNCNHTYCLKCIRKWRSAKQFESKIIKSCPECRITSNFVIPSEYWVEEKEEKQKLILK
YKEAMSNKACRYFDEGRGSCPFGGNCFYKHAYPDGRREEPQRQKVGTSSRYRAQRRNHFW
ELIEERENSNPFDNDEEEVVTFELGEMLLMLLAAGGDDELTDSEDEWDLFHDELEDFYDL
DL*
>ENSG00000014164|ENST00000262577
MEEKEILRRQIRLLQGLIDDYKTLHGNAPAPGTPAASGWQPPTYHSGRAFSARYPRPSRR
GYSSHHGPSWRKKYSLVNRPPGPSDPPADHAVRPLHGARGGQPPVPQQHVLERQVQLSQG
QNVVIKVKPPSKSGSASASGAQRGSLEEFEETPWSDQRPREGEGEPPRGQLQPSRPTRAR
GTCSVEDPLLVCQKEPGKPRMVKSVGSVGDSPREPRRTVSESVIAVKASFPSSALPPRTG
VALGRKLGSHSVASCAPQLLGDRRVDAGHTDQPVPSGSVGGPARPASGPRQAREASLVVT
CRTNKFRKNNYKWVAASSKSPRVARRALSPRVAAENVCKASAGMANKVEKPQLIADPEPK
PRKPATSSKPGSAPSKYKWKASSPSASSSSSFRWQSEASSKDHASQLSPVLSRSPSGDRP
AVGHSGLKPLSGETPLSAYKVKSRTKIIRRRSSTSLPGDKKSGTSPAATAKSHLSLRRRQ
ALRGKSSPVLKKTPNKGLVQVTTHRLCRLPPSRAHLPTKEASSLHAVRTAPTSKVIKTRY
RIVKKTPASPLSAPPFPLSLPSWRARRLSLSRSLVLNRLRPVASGGGKAQPGSPWWRSKG
YRCIGGVLYKVSANKLSKTSGQPSDAGSRPLLRTGRLDPAGSCSRSLASRAVQRSLAIIR
QARQRREKRKEYCMYYNRFGRCNRGERCPYIHDPEKVAVCTRFVRGTCKKTDGTCPFSHH
VSKEKMPVCSYFLKGICSNSNCPYSHVYVSRKAEVCSDFLKGYCPLGAKCKKKHTLLCPD
FARRGACPRGAQCQLLHRTQKRHSRRAATSPAPGPSDATARSRVSASHGPRKPSASQRPT
RQTPSSAALTAAAVAAPPHCPGGSASPSSSKASSSSSSSSSPPASLDHEAPSLQEAALAA
ACSNRLCKLPSFISLQSSPSPGAQPRVRAPRAPLTKDSGKPLHIKPRL*
>ENSG00000130684|ENST00000252979
MGPQGARRQAFLAFGDVTVDFTQKEWRLLSPAQRALYREVTLENYSHLVSLGILHSKPEL

IRRLEQGEVPWGEERRRRPGPCAGIYAEHVLRPKNLGLAHQRQQQLQFSDQSFQSDTAEG
QEKEKSTKPMAFSSPPLRHAVSSRRRNSVVEIESSQGQRENPTEIDKVLKGIENSRWGAF
KCAERGQDFSRKMMVIIHKKAHSRQKLFTCRECHQGFRDESALLLHQNTHTGEKSYVCSV
CGRGFSLKANLLRHQRTHSGEKPFLCKVCGRGYTSKSYLTVHERTHTGEKPYECQECGRR
FNDKSSYNKHLKAHSGEKPFVCKECGRGYTNKSYFVVHKRIHSGEKPYRCQECGRGFSNK
SHLITHQRTHSGEKPFACRQCKQSFSVKGSLLRHQRTHSGEKPFVCKDCERSFSQKSTLV
YHQRTHSGEKPFVCRECGQGFIQKSTLVKHQITHSEEKPFVCKDCGRGFIQKSTFTLHQR
THSEEKPYGCRECGRRFRDKSSYNKHLRAHLGEKRFFCRDCGRGFTLKPNLTIHQRTHSG
EKPFMCKQCEKSFSLKANLLRHQWTHSGERPFNCKDCGRGFILKSTLLFHQKTHSGEKPF
ICSECGQGFIWKSNLVKHQLAHSGKQPFVCKECGRGFNWKGNLLTHQRTHSGEKPFVCNV
CGQGFSWKRSLTRHHWRIHSKEKPFVCQECKRGYTSKSDLTVHERIHTGERPYECQECGR
KFSNKSYYSKHLKRHLREKRFCTGSVGEASS*
>ENSG00000070366|ENST00000263073
MAEGLERVRISASELRGILATLAPQAGSRENMKELKEARPRKDNRRPDLEIYKPGLSRLR
NKPKIKEPPGSEEFKDEIVNDRDCSAVENGTQPVKDVCKELNNQEQNGPIDPENNRGQES
FPRTAGQEDRSLKIIKRTKKPDLQIYQPGRRLQTVSKESASRVEEEEVLNQVEQLRVEED
ECRGNVAKEEVANKPDRAEIEKSPGGGRVGAAKGEKGKRMGKGEGVRETHDDPARGRPGS
AKRYSRSDKRRNRYRTRSTSSAGSNNSAEGAGLTDNGCRRRRQDRTKERPRLKKQVSVSS
TDSLDEDRIDEPDGLGPRRSSERKRHLERNWSGRGEGEQKNSAKEYRGTLRVTFDAEAMN
KESPMVRSARDDMDRGKPDKGLSSGGKGSEKQESKNPKQELRGRGRGILILPAHTTLSVN
SAGSPESAPLGPRLLFGSGSKGSRSWGRGGTTRRLWDPNNPDQKPALKTQTPQLHFLDTD
DEVSPTSWGDSRQAQASYYKFQNSDNPYYYPRTPGPASQYPYTGYNPLQYPVGPTNGVYP
GPYYPGYPTPSGQYVCSPLPTSTMSPEEVEQHMRNLQQQELHRLLRVADNQELQLSNLLS
RDRISPEGLEKMAQLRAELLQLYERCILLDIEFSDNQNVDQILWKNAFYQVIEKFRQLVK
DPNVENPEQIRNRLLELLDEGSDFFDSLLQKLQVTYKFKLEDYMDGLAIRSKPLRKTVKY
ALISAQRCMICQGDIARYREQASDTANYGKARSWYLKAQHIAPKNGRPYNQLALLAVYTR
RKLDAVYYYMRSLAASNPILTAKESLMSLFEETKRKAEQMEKKQHEEFDLSPDQWRKGKK
STFRHVGDDTTRLEIWIHPSHPRSSQGTESGKDSEQENGLGSLSPSDLNKRFILSFLHAH
GKLFTRIGMETFPAVAEKVLKEFQVLLQHSPSPIGSTRMLQLMTINMFAVHNSQLKDCFS
EECRSVIQEQAAALGLAMFSLLVRRCTCLLKESAKAQLSSPEDQDDQDDIKVSSFVPDLK
ELLPSVKVWSDWMLGYPDTWNPPPTSLDLPSHVAVDVWSTLADFCNILTAVNQSEVPLYK
DPDDDLTLLILEEDRLLSGFVPLLAAPQDPCYVEKTSDKVIAADCKRVTVLKYFLEALCG
QEEPLLAFKGGKYVSVAPVPDTMGKEMGSQEGTRLEDEEEDVVIEDFEEDSEAEGSGGED
DIRELRAKKLALARKIAEQQRRQEKIQAVLEDHSQMRQMELEIRPLFLVPDTNGFIDHLA
SLARLLESRKYILVVPLIVINELDGLAKGQETDHRAGGYARVVQEKARKSIEFLEQRFES
RDSCLRALTSRGNELESIAFRSEDITGQLGNNDDLILSCCLHYCKDKAKDFMPASKEEPI
RLLREVVLLTDDRNLRVKALTRNVPVRDIPAFLTWAQVG*
>ENSG00000122965|ENST00000261741
MSRLIVKNLPNGMKEERFRQLFAAFGTLTDCSLKFTKDGKFRKFGFIGFKSEEEAQKAQK
HFNKSFIDTSRITVEFCKSFGDPAKPRAWSKHAQKPSQPKQPPKDSTTPEIKKDEKKKKV
AGQLEKLKEDTEFQEFLSVHQRRAQAATWANDGLDAEPSKGKSKPASDYLNFDSDSGQES
EEEGAGEDLEEEASLEPKAAVQKELSDMDYLKSKMVKAGSSSSSEEEESEDEAVHCDEGS
EAEEEDSSATPVLQERDSKGAGQEQGMPAGKKRPPEARAETEKPANQKEPTTCHTVKLRG
APFNVTEKNVMEFLAPLKPVAIRIVRNAHGNKTGYIFVDFSNEEEVKQALKCNREYMGGR

YIEVFREKNVPTTKGAPKNTTKSWQGRILGENEEEEDLAESGRLFVRNLPYTSTEEDLEK
LFSKYGPLSELHYPIDSLTKKPKGFAFITFMFPEHAVKAYSEVDGQVFQGRMLHVLPSTI
KKEASEDASALGSSSYKKKKEAQDKANSASSHNWNTLFMGPNAVADAIAQKYNATKSQVF
DHETKGSVAVRVALGETQLVQEVRRFLIDNGVSLDSFSQAAAERSKTVILVKNLPAGTLA
AQLQETFGHFGSLGRVLLPEGGITAIVEFLEPLEARKAFRHLAYSKFHHVPLYLEWAPVG
VFSSTAPQKKKLQDTPSEPMEKDPAEPETVPDGETPEDENPTEEGADNSSAKMEEEEEEE
EEEEESLPGCTLFIKNLNFDTTEEKLKEVFSKVGTVKSCSISKKKNKAGVLLSMGFGFVE
YRKPEQAQKALKQLQGHVVDGHKLEVRISERATKPAVTLARKKQVPRKQTTSKILVRNIP
FQAHSREIRELFSTFGELKTVRLPKKMTGTGTHRGFGFVDFLTKQDAKRAFNALCHSTHL
YGRRLVLEWADSEVTLQALRRKTAAHFHEPPKKKRSVVLDEILEQLEGSDSDSEEQTLQL
*
>ENSG00000132382|ENST00000254718
MESRDPAQPMSPGEATQSGARPADRYGLLKHSREFLDFFWDIAKPEQETRLAATEKLLEY
LRGRPKGSEMKYALKRLITGLGVGRETARPCYSLALAQLLQSFEDLPLCSILQQIQEKYD
LHQVKKAMLRPALFANLFGVLALFQSGRLVKDQEALMKSVKLLQALAQYQNHLQEQPRKA
LVDILSEVSKATLQEILPEVLKADLNIILSSPEQLELFLLAQQKVPSKLKKLVGSVNLFS
DENVPRLVNVLKMAASSVKKDRKLPAIALDLLRLALKEDKFPRFWKEVVEQGLLKMQFWP
ASYLCFRLLGAALPLLTKEQLHLVMQGDVIRHYGEHVCTAKLPKQFKFAPEMDDYVGTFL
EGCQDDPERQLAVLVAFSSVTNQGLPVTPTFWRVVRFLSPPALQGYVAWLRAMFLQPDLD
SLVDFSTNNQKKAQDSSLHMPERAVFRLRKWIIFRLVSIVDSLHLEMEEALTEQVARFCL
FHSFFVTKKPTSQIPETKHPFSFPLENQAREAVSSAFFSLLQTLSTQFKQAPGQTQGGQP
WTYHLVQFADLLLNHSHNVTTVTPFTAQQRQAWDRMLQTLKELEAHSAEARAAAFQHLLL
LVGIHLLKSPAESCDLLGDIQTCIRKSLGEKPRRSRTKTIDPQEPPWVEVLVEILLALLA
QPSHLMRQVARSVFGHICSHLTPRALQLILDVLNPETSEDENDRVVVTDDSDERRLKGAE
DKSEEGEDNRSSESEEESEGEESEEEERDGDVDQGFREQLMTVLQAGKALGGEDSENEEE
LGDEAMMALDQSLASLFAEQKLRIQARRDEKNKLQKEKALRRDFQIRVLDLVEVLVTKQP
ENALVLELLEPLLSIIRRSLRSSSSKQEQDLLHKTARIFTHHLCRARRYCHDLGERAGAL
HAQVERLVQQAGRQPDSPTALYHFNASLYLLRVLKGNTAEGCVHETQEKQKAGTDPSHMP
TGPQAASCLDLNLVTRVYSTALSSFLTKRNSPLTVPMFLSLFSRHPVLCQSLLPILVQHI
TGPVRPRHQACLLLQKTLSMREVRSCFEDPEWKQLMGQVLAKVTENLRVLGEAQTKAQHQ
QALSSLELLNVLFRTCKHEKLTLDLTVLLGVLQGQQQSLQQGAHSTGSSRLHDLYWQAMK
TLGVQRPKLEKKDAKEIPSATQSPISKKRKKKGFLPETKKRKKRKSEDGTPAEDGTPAAT
GGSQPPSMGRKKRNRTKAKVPAQANGTPTTKSPAPGAPTRSPSTPAKSPKLQKKNQKPSQ
VNGAPGSPTEPAGQKQHQKALPKKGVLGKSPLSALARKKARLSLVIRSPSLLQSGAKKKA
QVRKAGKP*
>ENSG00000089280|ENST00000254108
MASNDYTQQATQSYGAYPTQPGQGYSQQSSQPYGQQSYSGYSQSTDTSGYGQSSYSSYGQ
SQNTGYGTQSTPQGYGSTGGYGSSQSSQSSYGQQSSYPGYGQQPAPSSTSGSYGSSSQSS
SYGQPQSGSYSQQPSYGGQQQSYGQQQSYNPPQGYGQQNQYNSSSGGGGGGGGGGNYGQD
QSSMSSGGGSGGGYGNQDQSGGGGSGGYGQQDRGGRGRGGSGGGGGGGGGGYNRSSGGYE
PRGRGGGRGGRGGMGGSDRGGFNKFGGPRDQGSRHDSEQDNSDNNTIFVQGLGENVTIES
VADYFKQIGIIKTNKKTGQPMINLYTDRETGKLKGEATVSFDDPPSAKAAIDWFDGKEFS
GNPIKVSFATRRADFNRGGGNGRGGRGRGGPMGRGGYGGGGSGGGGRGGFPSGGGGGGGQ
QRAGDWKCPNPTCENMNFSWRNECNQCKAPKPDGPGGGPGGSHMGGNYGDDRRGGRGGYD

RGGYRGRGGDRGGFRGGRGGGDRGGFGPGKMDSRGEHRQDRRERPY*
>ENSG00000135903|ENST00000258387
MTTLAGAVPRMMRPGPGQNYPRSGFPLEVSTPLGQGRVNQLGGVFINGRPLPNHIRHKIV
EMAHHGIRPCVISRQLRVSHGCVSKILCRYQETGSIRPGAIGGSKPKQVTTPDVEKKIEE
YKRENPGMFSWEIRDKLLKDAVCDRNTVPSVSSISRILRSKFGKGEEEEADLERKEAEES
EKKAKHSIDGILSERGKALVSGVSSH*
>ENSG00000135900|ENST00000258383
MASGLVRLLQQGHRCLLAPVAPKLVPPVRGVKKGFRAAFRFQKELERQRLLRCPPPPVRR
SEKPNWDYHAEIQAFGHRLQENFSLDLLKTAFVNSCYIKSEEAKRQQLGIEKEAVLLNLK
SNQELSEQGTSFSQTCLTQFLEDEYPDMPTEGIKNLVDFLTGEEVVCHVARNLAVEQLTL
SEEFPVPPAVLQQTFFAVIGALLQSSGPERTALFIRDFLITQMTGKELFEMWKIINPMGL
LVEELKKRNVSAPESRLTRQSGGTTALPLYFVGLYCDKKLIAEGPGETVLVAEEEAARVA
LRKLYGFTENRRPWNYSKPKETLRAEKSITAS*
>ENSG00000131652|ENST00000253952
MERAVPLAVPLGQTEVFQALQRLHMTIFSQSVSPCGKFLAAGNNYGQIAIFSLSSALSSE
AKEESKKPVVTFQAHDGPVYSMVSTDRHLLSAGDGEVKAWLWAEMLKKGCKELWRRQPPY
RTSLEVPEINALLLVPKENSLILAGGDCQLHTMDLETGTFTRVLRGHTDYIHCLALRERS
PEVLSGGEDGAVRLWDLRTAKEVQTIEVYKHEECSRPHNGRWIGCLATDSDWMVCGGGPA
LTLWHLRSSTPTTIFPIRAPQKHVTFYQDLVLTAAGNSCRVDVFTNLGYRAFSLSF*
>ENSG00000048991|ENST00000264160
MRMSDTVTVKDETATMKDLEAEVKDTTRVENLIKSENYGKILVEKNEHCIENNIDLQRPL
QSFGQTGKRSKSSSKLKLVRSLAVCEESPPPPAPEISQENQEKIQIQLTQSFEKEEKPSK
DEAEKEKASDKLPRKMLSRDSSQEYTDSTGIDLHEFLVNTLKNNPRDRMMLLKLEQEILD
FIGNNESPRKKFPPMTSYHRMLLHRVAAYFGLDHNVDQSGKSVIVNKTSNTRIPDQKFNE
HIKDDKGEDFQKRYILKRDNSSFDKDDNQMRIRLKDDRRSKSIEEREEEYQRARDRIFSQ
DSLCSQENYIIDKRLQDEDASSTQQRRQIFRVNKDASGRSTNSHQSSTENELKYSEPRPW
SSTDSDSSLRNLKPAVTKASSFSGISVLTRGDSSGSSKSIGRLSKTGSESSGSVGSSTGS
LSHIQQPLPGTALSQSSHGAPVVYPTVSTHSSLSFDGGLNGQVASPSTSFFLLPLEAAGI
PPGSILINPQTGQPFINPDGSPVVYNPPMTQQPVRSQVPGPPQPPLPAPPQQPAANHIFS
QDNLGSQFSHMSLARQPSADGSDPHAAMFQSTVVLQSPQQSGYIMTAAPPPHPPPPPPPP
PPPPPLPPGQPVPTAGYPASGHPVSQPVLQQQGYIQQPSPQMPACYCAPGHYHSSQPQYR
PVPSVHYNSHLNQPLPQPAQQTGYQVIPNQQQNYQGIVGVQQPQSQSLVSGQPNSIGNQI
QGVVIPYTSVPTYQVSLPQGSQGIPHQTYQQPVMFPNQSNQGSMPTTGMPVYYSVIPPGQ
QNNLSSSVGYLQHPGSEQVQFPRTTSPCSSQQLQGHQCTAGPPPPPGGGMVMMQLSVPNN
PQSCAHSPPQWKQNKYYCDHQRGQKCVEFSSVDNIVQHSPQLSSPIISPAQSPAPAQLST
LKTVRPSGPPLSIMPQFSRPFVPGQGDSRYPLLGQPLQYNPPAVLHGHIPNQQGQPGSRH
GNRGRRQAKKAASTDLGAGETVVGKVLEITELPDGITRMEAEKLFGELFKIGAKIRWLRD
PQSQPRRHPLCCGSGDNTANPERSKPSDLASTYTVLATFPSISAAQNALKKQINSVNKFK
LRTSKKHYDFHILERASSQ*
>ENSG00000132300|ENST00000254630
MAVVSAVRWLGLRSRLGQPLTGRRAGLCEQARSCRFYSGSATLSKVEGTDVTGIEEVVIP
KKKTWDKVAVLQALASTVNRDTTAVPYVFQDDPYLMPASSLESRSFLLAKKSGENVAKFI
INSYPKYFQKDIAEPHIPCLMPEYFEPQIKDISEAALKERIELRKVKASVDMFDQLLQAG
TTVSLETTNSLLDLLCYYGDQEPSTDYHFQQTGQSEALEEENDETSRRKAGHQFGVTWRA

KNNAERIFSLMPEKNEHSYCTMIRGMVKHRAYEQALNLYTELLNNRLHADVYTFNALIEA
TVCAINEKFEEKWSKILELLRHMVAQKVKPNLQTFNTILKCLRRFHVFARSPALQVLREM
KAIGIEPSLATYHHIIRLFDQPGDPLKRSSFIIYDIMNELMGKRFSPKDPDDDKFFQSAM
SICSSLRDLELAYQVHGLLKTGDNWKFIGPDQHRNFYYSKFFDLICLMEQIDVTLKWYED
LIPSAYFPHSQTMIHLLQALDVANRLEVIPKIWKDSKEYGHTFRSDLREEILMLMARDKH
PPELQVAFADCAADIKSAYESQPIRQTAQDWPATSLNCIAILFLRAGRTQEAWKMLGLFR
KHNKIPRSELLNELMDSAKVSNSPSQAIEVVELASAFSLPICEGLTQRVMSDFAINQEQK
EALSNLTALTSDSDTDSSSDSDSDTSEGK*
>ENSG00000099381|ENST00000262519
MDQEGGGDGQKAPSFQWRNYKLIVDPALDPALRRPSQKVYRYDGVHFSVNDSKYIPVEDL
QDPRCHVRSKNRDFSLPVPKFKLDEFYIGQIPLKEVTFARLNDNVRETFLKDMCRKYGEV
EEVEILLHPRTRKHLGLARVLFTSTRGAKETVKNLHLTSVMGNIIHAQLDIKGQQRMKYY
ELIVNGSYTPQTVPTGGKALSEKFQGSGAATETAESRRRSSSDTAAYPAGTTAVGTPGNG
TPCSQDTSFSSSRQDTPSSFGQFTPQSSQGTPYTSRGSTPYSQDSAYSSSTTSTSFKPRR
SENSYQDAFSRRHFSASSASTTASTAIAATTAATASSSASSSSLSSSSSSSSSSSSSQFR
SSDANYPAYYESWNRYQRHTSYPPRRATREEPPGAPFAENTAERFPPSYTSYLPPEPSRP
TDQDYRPPASEAPPPEPPEPGGGGGGGGPSPEREEVRTSPRPASPARSGSPAPETTNESV
PFAQHSSLDSRIEMLLKEQRSKFSFLASDTEEEEENSSMVLGARDTGSEVPSGSGHGPCT
PPPAPANFEDVAPTGSGEPGATRESPKANGQNQASPCSSGDDMEISDDDRGGSPPPAPTP
PQQPPPPPPPPPPPPPYLASLPLGYPPHQPAYLLPPRPDGPPPPEYPPPPPPPPHIYDFV
NSLELMDRLGAQWGGMPMSFQMQTQMLTRLHQLRQGKGLIAASAGPPGGAFGEAFLPFPP
PQEAAYGLPYALYAQGQEGRGAYSREAYHLPMPMAAEPLPSSSVSGEEARLPPREEAELA
EGKTLPTAGTVGRVLAMLVQEMKSIMQRDLNRKMVENVAFGAFDQWWESKEEKAKPFQNA
AKQQAKEEDKEKTKLKEPGLLSLVDWAKSGGTTGIEAFAFGSGLRGALRLPSFKVKRKEP
SEISEASEEKRPRPSTPAEEDEDDPEQEKEAGEPGRPGTKPPKRDEERGKTQGKHRKSFA
LDSEGEEASQESSSEKDEEDDEEDEEDEDREEAVDTTKKETEVSDGEDEESDSSSKCSLY
ADSDGENDSTSDSESSSSSSSSSSSSSSSSSSSSSSSSSSSESSSEDEEEEERPAALPSASPP
PREVPVPTPAPVEVPVPERVAGSPVTPLPEQEASPARPAGPTEESPPSAPLRPPEPPAGP
PAPAPRPDERPSSPIPLLPPPKKRRKTVSFSAIEVVPAPEPPPATPPQAKFPGPASRKAP
RGVERTIRNLPLDHASLVKSWPEEVSRGGRSRAGGRGRLTEEEEAEPGTEVDLAVLADLA
LTPARRGLPALPAVEDSEATETSDEAERPRPLLSHILLEHNYALAVKPTPPAPALRPPEP
VPAPAALFSSPADEVLEAPEVVVAEAEEPKPQQLQQQREEGEEEGEEEGEEEEEESSDSS
SSSDGEGALRRRSLRSHARRRRPPPPPPPPPPRAYEPRSEFEQMTILYDIWNSGLDSEDM
SYLRLTYERLLQQTSGADWLNDTHWVHHTITNLTTPKRKRRPQDGPREHQTGSARSEGYY
PISKKEKDKYLDVCPVSARQLEGVDTQGTNRVLSERRSEQRRLLSAIGTSAIMDSDLLKL
NQLKFRKKKLRFGRSRIHEWGLFAMEPIAADEMVIEYVGQNIRQMVADMREKRYVQEGIG
SSYLFRVDHDTIIDATKCGNLARFINHCCTPNCYAKVITIESQKKIVIYSKQPIGVDEEI
TYDYKFPLEDNKIPCLCGTESCRGSLN*
>ENSG00000095139|ENST00000264028
MVLLAAAVCTKAGKAIVSRQFVEMTRTRIEGLLAAFPKLMNTGKQHTFVETESVRYVYQP
MEKLYMVLITTKNSNILEDLETLRLFSRVIPEYCRALEENEISEHCFDLIFAFDEIVALG
YRENVNLAQIRTFTEMDSHEEKVFRAVRETQEREAKAEMRRKAKELQQARRDAERQGKKA
PGFGGFGSSAVSGGSTAAMITETIIETDKPKVAPAPARPSGPSKALKLGAKGKEVDNFVD
KLKSEGETIMSSSMGKRTSEATKMHAPPINMESVHMKIEEKITLTCGRDGGLQNMELHGM

IMLRISDDKYGRIRLHVENEDKKGVQLQTHPNVDKKLFTAESLIGLKNPEKSFPVNSDVG
VLKWRLQTTEESFIPLTINCWPSESGNGCDVNIEYELQEDNLELNDVVITIPLPSGVGAP
VIGEIDGEYRHDSRRNTLEWCLPVIDAKNKSGSLEFSIAGQPNDFFPVQVSFVSKKNYCN
IQVTKVTQVDGNSPVRFSTETTFLVDKYEIL*
>ENSG00000138593|ENST00000261847
MDRAPTEQNVKLSAEVEPFIPQKKSPDTFMIPMALPNDNGSVSGVEPTPIPSYLITCYPF
VQENQSNRQFPLYNNDIRWQQPNPNPTGPYFAYPIISAQPPVSTEYTYYQLMPAPCAQVM
GFYHPFPTPYSNTFQAANTVNAITTECTERPSQLGQVFPLSSHRSRNSNRGSVVPKQQLL
QQHIKSKRPLVKNVATQKETNAAGPDSRSKIVLLVDASQQTDFPSDIANKSLSETTATML
WKSKGRRRRASHPTAESSSEQGASEADIDSDSGYCSPKHSNNQPAAGALRNPDSGTMNHV
ESSMCAGGVNWSNVTCQATQKKPWMEKNQTFSRGGRQTEQRNNSQDEDGFQELNENGNAK
DENIQQKLSSKVLDDLPENSPINIVQTPIPITTSVPKRAKSQKKKALAAALATAQEYSEI
SMEQKKLQEALSKAAGKKNKTPVQLDLGDMLAALEKQQQAMKARQITNTRPLSYTVVTAA
SFHTKDSTNRKPLTKSQPCLTSFNSVDIASSKAKKGKEKEIAKLKRPTALKKVILKEREE
KKGRLTVDHNLLGSEEPTEMHLDFIDDLPQEIVSQEDTGLSMPSDTSLSPASQNSPYCMT
PVSQGSPASSGIGSPMASSTITKIHSKRFREYCNQVLCKEIDECVTLLLQELVSFQERIY
QKDPVRAKARRRLVMGLREVTKHMKLNKIKCVIISPNCEKIQSKGGLDEALYNVIAMARE
QEIPFVFALGRKALGRCVNKLVPVSVVGIFNYFGAESLFNKLVELTEEARKAYKDMVAAM
EQEQAEEALKNVKKVPHHMGHSRNPSAASAISFCSVISEPISEVNEKEYETNWRNMVETS
DGLEASENEKEVSCKHSTSEKPSKLPFDTPPIGKQPSLVATGSTTSATSAGKSTASDKEE
VKPDDLEWASQQSTETGSLDGSCRDLLNSSITSTTSTLVPGMLEEEEDEDEEEEEDYTHE
PISVEVQLNSRIESWVSETQRTMETLQLGKTLNGSEEDNVEQSGEEEAEAPEVLEPGMDS
EAWTADQQASPGQQKSSNCSSLNKEHSDSNYTTQTT*
>ENSG00000111142|ENST00000261220
MAGVEEVAASGSHLNGDLDPDDREEGAASTAEEAAKKKRRKKKKSKGPSAGEQEPDKESG
ASVDEVARQLERSALEDKERDEDDEAKVQTDPPSVPICDLYPNGVFPKGQECEYPPTQDG
RTAAWRTTSEEKKALDQASEEIWNDFREAAEAHRQVRKYVMSWIKPGMTMIEICEKLEDC
SRKLIKENGLNAGLAFPTGCSLNNCAAHYTPNAGDTTVLQYDDICKIDFGTHISGRIIDC
AFTVTFNPKYDTLLKAVKDATNTGIKCAGIDVRLCDVGEAIQEVMESYEVEIDGKTYQVK
PIRNLNGHSIGQYRIHAGKTVPIVKGGEATRMEEGEVYAIETFGSTGKGVVHDDMECSHY
MKNFDVGHVPIRLPRTKHLLNVINENFGTLAFCRRWLDRLGESKYLMALKNLCDLGIVDP
YPPLCDIKGSYTAQFEHTILLRPTCKEVVSRGDDY*
>ENSG00000187109|ENST00000261182
MADIDNKEQSELDQDLDDVEEVEEEETGEETKLKARQLTVQMMQNPQILAALQERLDGLV
ETPTGYIESLPRVVKRRVNALKNLQVKCAQIEAKFYEEVHDLERKYAVLYQPLFDKRFEI
INAIYEPTEEECEWKPDEEDEISEELKEKAKIEDEKKDEEKEDPKGIPEFWLTVFKNVDL
LSDMVQEHDEPILKHLKDIKVKFSDAGQPMSFVLEFHFEPNEYFTNEVLTKTYRMRSEPD
DSDPFSFDGPEIMGCTGCQIDWKKGKNVTLKTIKKKQKHKGRGTVRTVTKTVSNDSFFNF
FAPPEVPESGDLDDDAEAILAADFEIGHFLRERIIPRSVLYFTGEAIEDDDDDYDEEGEE
ADEEGEEEGDEENDPDYDPKKDQNPAECKQQ*
>ENSG00000129566|ENST00000262715
MEKLHGHVSAHPDILSLENRCLAMLPDLQPLEKLHQHVSTHSDILSLKNQCLATLPDLKT
MEKPHGYVSAHPDILSLENQCLATLSDLKTMEKPHGHVSAHPDILSLENRCLATLSSLKS
TVSASPLFQSLQISHMTQADLYRVNNSNCLLSEPPSWRAQHFSKGLDLSTCPIALKSISA

TETAQEATLGRWFDSEEKKGAETQMPSYSLSLGEEEEVEDLAVKLTSGDSESHPEPTDHV
LQEKKMALLSLLCSTLVSEVNMNNTSDPTLAAIFEICRELALLEPEFILKASLYARQQLN
VRNVANNILAIAAFLPACRPHLRRYFCAIVQLPSDWIQVAELYQSLAEGDKNKLVPLPAC
LRTAMTDKFAQFDEYQLAKYNPRKHRAKRHPRRPPRSPGMEPPFSHRCFPRYIGFLREEQ
RKFEKAGDTVSEKKNPPRFTLKKLVQRLHIHKPAQHVQALLGYRYPSNLQLFSRSRLPGP
WDSSRAGKRMKLSRPETWERELSLRGNKASVWEELIENGKLPFMAMLRNLCNLLRVGISS
RHHELILQRLQHAKSVIHSRQFPFRFLNAHDAIDALEAQLRNQALPFPSNITLMRRILTR
NEKNRPRRRFLCHLSRQQLRMAMRIPVLYEQLKREKLRVHKARQWKYDGEMLNRYRQALE
TAVNLSVKHSLPLLPGRTVLVYLTDANADRLCPKSNPQGPPLNYALLLIGMMITRAEQVD
VVLCGGDTLKTAVLKAEEGILKTAIKLQAQVQEFDENDGWSLNTFGKYLLSLAGQRVPVD
RVILLGQSMDDGMINVAKQLYWQRVNSKCLFVGILLRRVQYLSTDLNPNDVTLSGCTDAI
LKFIAEHGASHLLEHVGQMDKIFKIPPPPGKTGVQSLRPLEEDTPSPLAPVSQQGWRSIR
LFISSTFRDMHGERDLLLRSVLPALQARAAPHRISLHGIDLRWGVTEEETRRNRQLEVCL
GEVENAQLFVGILGSRYGYIPPSYNLPDHPHFHWAQQYPSGRSVTEMEVMQFLNRNQRLQ
PSAQALIYFRDSSFLSSVPDAWKSDFVSESEEAARRISELKSYLSRQKGITCRRYPCEWG
GVAAGRPYVGGLEEFGQLVLQDVWNMIQKLYLQPGALLEQPVSIPDDDLVQATFQQLQKP
PSPARPRLLQDTVQRLMLPHGRLSLVTGQSGQGKTAFLASLVSALQAPDGAKVASLVFFH
FSGARPDQGLALTLLRRLCTYLRGQLKEPGALPSTYRSLVWELQQRLLPKSAESLHPGQT
QVLIIDGADRLVDQNGQLISDWIPKKLPRCVHLVLSVSSDAGLGETLEQSQGAHVLALGP
LEASARARLVREELALYGKRLEESPFNNQMRLLLVKRESGRPLYLRLVTDHLRLFTLYEQ
VSERLRTLPATVPLLLQHILSTLEKEHGPDVLPQALTALEVTRSGLTVDQLHGVLSVWRT
LPKGTKSWEEAVAAGNSGDPYPMGPFACLVQSLRSLLGEGPLERPGARLCLPDGPLRTAA
KRCYGKRPGLEDTAHILIAAQLWKTCDADASGTFRSCPPEALGDLPYHLLQSGNRGLLSK
FLTNLHVVAAHLELGLVSRLLEAHALYASSVPKEEQKLPEADVAVFRTFLRQQASILSQY
PRLLPQQAANQPLDSPLCHQASLLSRRWHLQHTLRWLNKPRTMKNQQSSSLSLAVSSSPT
AVAFSTNGQRAAVGTANGTVYLLDLRTWQEEKSVVSGCDGISACLFLSDDTLFLTAFDGL
LELWDLQHGCRVLQTKAHQYQITGCCLSPDCRLLATVCLGGCLKLWDTVRGQLAFQHTYP
KSLNCVAFHPEGQVIATGSWAGSISFFQVDGLKVTKDLGAPGASIRTLAFNVPGGVVAVG
RLDSMVELWAWREGARLAAFPAHHGFVAAALFLHAGCQLLTAGEDGKVQVWSGSLGRPRG
HLGSLSLSPALSVALSPDGDRVAVGYRADGIRIYKISSGSQGAQGQALDVAVSALAWLSP
KVLVSGAEDGSLQGWALKECSLQSLWLLSRFQKPVLGLATSQELLASASEDFTVQLWPRQ
LLTRPHKAEDFPCGTELRGHEGPVSCCSFSTDGGSLATGGRDRSLLCWDVRTPKTPVLIH
SFPACHRDWVTGCAWTKDNLLISCSSDGSVGLWDPESGQRLGQFLGHQSAVSAVAAVEEH
VVSVSRDGTLKVWDHQGVELTSIPAHSGPISHCAAAMEPRAAGQPGSELLVVTVGLDGAT
RLWHPLLVCQTHTLLGHSGPVRAAAVSETSGLMLTASEDGSVRLWQVPKEADDTCIPRSS
AAVTAVAWAPDGSMAVSGNQAGELILWQEAKAVATAQAPGHIGALIWSSAHTFFVLSADE
KISEWQVKLRKGSAPGNLSLHLNRILQEDLGVLTSLDWAPDGHFLILAKADLKLLCMKPG
DAPSEIWSSYTENPMILSTHKEYGIFVLQPKDPGVLSFLRQKESGEFEERLNFDINLENP
SRTLISITQAKPESESSFLCASSDGILWNLAKCSPEGEWTTGNMWQKKANTPETQTPGTD
PSTCRESDASMDSDASMDSEPTPHLKTRQRRKIHSGSVTALHVLPELLVTASKDRDVKLW
ERPSMQLLGLFRCEGSVSCLEPWLGANSTLQLAVGDVQGNVYFLNWE*
>ENSG00000166986|ENST00000262027
MRLFVSDGVPGCLPVLAAAGRARGRAEVLISTVGPEDCVVPFLTRPKVPVLQLDSGNYLF
STSAICRYFFLLSGWEQDDLTNQWLEWEATELQPALSAALYYLVVQGKKGEDVLGSVRRA

LTHIDHSLSRQNCPFLAGETESLADIVLWGALYPLLQDPAYLPEELSALHSWFQTLSTQE
PCQRAAETVLKQQGVLALRPYLQKQPQPSPAEGRAVTNEPEEEELATLSEEEIAMAVTAW
EKGLESLPPLRPQQNPVLPVAGERNVLITSALPYVNNVPHLGNIIGCVLSADVFARYSRL
RQWNTLYLCGTDEYGTATETKALEEGLTPQEICDKYHIIHADIYRWFNISFDIFGRTTTP
QQTKITQDIFQQLLKRGFVLQDTVEQLRCEHCARFLADRFVEGVCPFCGYEEARGDQCDK
CGKLINAVELKKPQCKVCRSCPVVQSSQHLFLDLPKLEKRLEEWLGRTLPGSDWTPNAQF
ITRSWLRDGLKPRCITRDLKWGTPVPLEGFEDKVFYVWFDATIGYLSITANYTDQWERWW
KNPEQVDLYQFMAKDNVPFHSLVFPCSALGAEDNYTLVSHLIATEYLNYEDGKFSKSRGV
GVFGDMAQDTGIPADIWRFYLLYIRPEGQDSAFSWTDLLLKNNSELLNNLGNFINRAGMF
VSKFFGGYVPEMVLTPDDQRLLAHVTLELQHYHQLLEKVRIRDALRSILTISRHGNQYIQ
VNEPWKRIKGSEADRQRAGTVTGLAVNIAALLSVMLQPYMPTVSATIQAQLQLPPPACSI
LLTNFLCTLPAGHQIGTVSPLFQKLENDQIESLRQRFGGGQAKTSPKPAVVETVTTAKPQ
QIQALMDEVTKQGNIVRELKAQKADKNEVAAEVAKLLDLKKQLAVAEGKPPEAPKGKKKK
*
>ENSG00000173473|ENST00000254480
MAAAAGGGGPGTAVGATGSGIAAAAAGLAVYRRKDGGPATKFWESPETVSQLDSVRVWLG
KHYKKYVHADAPTNKTLAGLVVQLLQFQEDAFGKHVTNPAFTKLPAKCFMDFKAGGALCH
ILGAAYKYKNEQGWRRFDLQNPSRMDRNVEMFMNIEKTLVQNNCLTRPNIYLIPDIDLKL
ANKLKDIIKRHQGTFTDEKSKASHHIYPYSSSQDDEEWLRPVMRKEKQVLVHWGFYPDSY
DTWVHSNDVDAEIEDPPIPEKPWKVHVKWILDTDIFNEWMNEEDYEVDENRKPVSFRQRI
STKNEEPVRSPERRDRKASANARKRKHSPSPPPPTPTESRKKSGKKGQASLYGKRRSQKE
EDEQEDLTKDMEDPTPVPNIEEVVLPKNVNLKKDSENTPVKGGTVADLDEQDEETVTAGG
KEDEDPAKGDQSRSVDLGEDNVTEQTNHIIIPSYASWFDYNCIHVIERRALPEFFNGKNK
SKTPEIYLAYRNFMIDTYRLNPQEYLTSTACRRNLTGDVCAVMRVHAFLEQWGLVNYQVD
PESRPMAMGPPPTPHFNVLADTPSGLVPLHLRSPQVPAAQQMLNFPEKNKEKPVDLQNFG
LRTDIYSKKTLAKSKGASAGREWTEQETLLLLEALEMYKDDWNKVSEHVGSRTQDECILH
FLRLPIEDPYLENSDASLGPLAYQPVPFSQSGNPVMSTVAFLASVVDPRVASAAAKAALE
EFSRVREEVPLELVEAHVKKVQEAARASGKVDPTYGLESSCIAGTGPDEPEKLEGAEEEK
MEADPDGQQPEKAENKVENETDEGDKAQDGENEKNSEKEQDSEVSEDTKSEEKETEENKE
LTDTCKERESDTGKKKVEHEISEGNVATAAAAALASAATKAKHLAAVEERKIKSLVALLV
ETQMKKLEIKLRHFEELETIMDREKEALEQQRQQLLTERQNFHMEQLKYAELRARQQMEQ
QQHGQNPQQAHQHSGGPGLAPLGAAGHPGMMPHQQPPPYPLMHHQMPPPHPPQPGQIPGP
GSMMPGQHMPGRMIPTVAANIHPSGSGPTPPGMPPMPGNILGPRVPLTAPNGMYPPPPQQ
QPPPPPPADGVPPPPAPGPPASAAP*
>ENSG00000132740|ENST00000255078
MASAAVESFVTKQLDLLELERDAEVEERRSWQENISLKELQSRGVCLLKLQVSSQRTGLY
GRLLVTFEPRRYGSAAALPSNSFTSGDIVGLYDAANEGSQLATGILTRVTQKSVTVAFDE
SHDFQLSLDRENSYRLLKLANDVTYRRLKKALIALKKYHSGPASSLIEVLFGRSAPSPAS
EIHPLTFFNTCLDTSQKEAVLFALSQKELAIIHGPPGTGKTTTVVEIILQAVKQGLKVLC
CAPSNIAVDNLVERLALCKQRILRLGHPARLLESIQQHSLDAVLARSDSAQIVADIRKDI
DQVFVKNKKTQDKREKSNFRNEIKLLRKELKEREEAAMLESLTSANVVLATNTGASADGP
LKLLPESYFDVVVIDECAQALEASCWIPLLKARKCILAGDHKQLPPTTVSHKAALAGLSL
SLMERLAEEYGARVVRTLTVQYRMHQAIMRWASDTMYLGQLTAHSSVARHLLRDLPGVAA
TEETGVPLLLVDTAGCGLFELEEEDEQSKGNPGEVRLVSLHIQALVDAGVPARDIAVVSP

YNLQVDLLRQSLVHRHPELEIKSVDGFQGREKEAVILSFVRSNRKGEVGFLAEDRRINVA
VTRARRHVAVICDSRTVNNHAFLKTLVEYFTQHGEVRTAFEYLDDIVPENYSHENSQGSS
HAATKPQGPATSTRTGSQRQEGGQEAAAPARQGRKKPAGKSLASEAPSQPSLNGGSPEGV
ESQDGVDHFRAMIVEFMASKKMQLEFPPSLNSHDRLRVHQIAEEHGLRHDSSGEGKRRFI
TVSKRAPRPRAALGPPAGTGGPAPLQPVPPTPAQTEQPPREQRGPDQPDLRTLHLERLQR
VRSAQGQPASKEQQASGQQKLPEKKKKKAKGHPATDLPTEEDFEALVSAAVKADNTCGFA
KCTAGVTTLGQFCQLCSRRYCLSHHLPEIHGCGERARAHARQRISREGVLYAGSGTKNGS
LDPAKRAQLQRRLDKKLSELSNQRTSRRKERGT*
>ENSG00000137547|ENST00000260102
MAGPLQGGGARALDLLRGLPRVSLANLKPNPGSKKPERRPRGRRRGRKCGRGHKGERQRG
TRPRLGFEGGQTPFYIRIPKYGFNEGHSFRRQYKPLSLNRLQYLIDLGRVDPSQPIDLTQ
LVNGRGVTIQPLKRDYGVQLVEEGADTFTAKVNIEVQLASELAIAAIEKNGGVVTTAFYD
PRSLDIVCKPVPFFLRGQPIPKRMLPPEELVPYYTDAKNRGYLADPAKFPEARLELARKY
GYILPDITKDELFKMLCTRKDPRQIFFGLAPGWVVNMADKKILKPTDENLLKYYTS*
>ENSG00000138095|ENST00000260665
MAALLRSARWLLRAGAAPRLPLSLRLLPGGPGRLHAASYLPAARAGPVAGGLLSPARLYA
IAAKEKDIQEESTFSSRKISNQFDWALMRLDLSVRRTGRIPKKLLQKVFNDTCRSGGLGG
SHALLLLRSCGSLLPELKLEERTEFAHRIWDTLQKLGAVYDVSHYNALLKVYLQNEYKFS
PTDFLAKMEEANIQPNRVTYQRLIASYCNVGDIEGASKILGFMKTKDLPVTEAVFSALVT
GHARAGDMENAENILTVMRDAGIEPGPDTYLALLNAYAEKGDIDHVKQTLEKVEKSELHL
MDRDLLQIIFSFSKAGYPQYVSEILEKVTCERRYIPDAMNLILLLVTEKLEDVALQILLA
CPVSKEDGPSVFGSFFLQHCVTMNTPVEKLTDYCKKLKEVQMHSFPLQFTLHCALLANKT
DLAKALMKAVKEEGFPIRPHYFWPLLVGRRKEKNVQGIIEILKGMQELGVHPDQETYTDY
VIPCFDSVNSARAILQENGCLSDSDMFSQAGLRSEAANGNLDFVLSFLKSNTLPISLQSI
RSSLLLGFRRSMNINLWSEITELLYKDGRYCQEPRGPTEAVGYFLYNLIDSMSDSEVQAK
EEHLRQYFHQLEKMNVKIPENIYRGIRNLLESYHVPELIKDAHLLVESKNLDFQKTVQLT
SSELESTLETLKAENQPIRDVLKQLILVLCSEENMQKALELKAKYESDMVTGGYAALINL
CCRHDKVEDALNLKEEFDRLDSSAVLDTGKYVGLVRVLAKHGKLQDAINILKEMKEKDVL
IKDTTALSFFHMLNGAALRGEIETVKQLHEAIVTLGLAEPSTNISFPLVTVHLEKGDLST
ALEVAIDCYEKYKVLPRIHDVLCKLVEKGETDLIQKAMDFVSQEQGEMVMLYDLFFAFLQ
TGNYKEAKKIIETPGIRARSARLQWFCDRCVANNQVETLEKLVELTQKLFECDRDQMYYN
LLKLYKINGDWQRADAVWNKIQEENVIPREKTLRLLAEILREGNQEVPFDVPELWYEDEK
HSLNSSSASTTEPDFQKDILIACRLNQKKGAYDIFLNAKEQNIVFNAETYSNLIKLLMSE
DYFTQAMEVKAFAETHIKGFTLNDAANSRLIITQVRRDYLKEAVTTLKTVLDQQQTPSRL
AVTRVIQALAMKGDVENIEVVQKMLNGLEDSIGLSKMVFINNIALAQIKNNNIDAAIENI
ENMLTSENKVIEPQYFGLAYLFRKVIEEQLEPAVEKISIMAERLANQFAIYKPVTDFFLQ
LVDAGKVDDARALLQRCGAIAEQTPILLLFLLRNSRKQGKASTVKSVLELIPELNEKEEA
YNSLMKSYVSEKDVTSAKALYEHLTAKNTKLDDLFLKRYASLLKYAGEPVPFIEPPESFE
FYAQQLRKLRENSS*
>ENSG00000079841|ENST00000264839
MSSAVGPRGPRPPTVPPPMQELPDLSHLTEEERNIIMAVMDRQKEEEEKEEAMLKCVVRD
MAKPAACKTPRNAENQPHQPSPRLHQQFESYKEQVRKIGEEARRYQGEHKDDAPTCGICH
KTKFADGCGHLCSYCRTKFCARCGGRVSLRSNNEDKVVMWVCNLCRKQQEILTKSGAWFF
GSGPQQTSQDGTLSDTATGAGSEVPREKKARLQERSRSQTPLSTAAASSQDAAPPSAPPD

RSKGAEPSQQALGPEQKQASSRSRSEPPRERKKTPGLSEQNGKGALKSERKRVPKTSAQP
VEGAVEERERKERRESRRLEKGRSQDYPDTPEKRDEGKAADEEKQRKEEDYQTRYRSDPN
LARYPVKPPPEEQQMRMHARVSRARHERRHSDVALPRTEAGAALPEGKAGKRAPAAARAS
PPDSPRAYSAERTAETRAPGAKQLTNHSPPAPRHGPVPAEAPELKAQEPLRKQSRLDPSS
AVLMRKAKREKVETMLRNDSLSSDQSESVRPSPPKPHRSKRGGKKRQMSVSSSEEEGVST
PEYTSCEDVELESESVSEKGDLDYYWLDPATWHSRETSPISSHPVTWQPSKEGDRLIGRV
ILNKRTTMPKDSGALLGLKVVGGKMTDLGRLGAFITKVKKGSLADVVGHLRAGDEVLEWN
GKPLPGATNEEVYNIILESKSEPQVEIIVSRPIGDIPRIPESSHPPLESSSSSFESQKME
RPSISVISPTSPGALKDAPQVLPGQLSVKLWYDKVGHQLIVNVLQATDLPARVDGRPRNP
YVKMYFLPDRSDKSKRRTKTVKKILEPKWNQTFVYSHVHRRDFRERMLEITVWDQPRVQE
EESEFLGEILIELETALLDDEPHWYKLQTHDESSLPLPQPSPFMPRRHIHGESSSKKLQR
SQRISDSDISDYEVDDGIGVVPPVGYRSSARESKSTTLTVPEQQRTTHHRSRSVSPHRGN
DQGKPRSRLPNVPLQRSLDEIHPTRRSRSPTRHHDASRSPVDHRTRDVDSQYLSEQDSEL
LMLPRAKRGRSAECLHTTRHLVRHYKTLPPKMPLLQSSSHWNIYSELQPFLDRARSASTN
CLRPDTSLHSPERERMHRQRSPTQSPPADTSFSSRRGRQLPQVPVRSGSIEQASLVVEER
TRQMKMKVHRFKQTTGSGSSQELDREQYSKYNIHKDQYRSCDNVSAKSSDSDVSDVSAIS
RTSSASRLSSTSFMSEQSERPRGRISSFTPKMQGRRMGTSGRSIMKSTSVSGEMYTLEHN
DGSQSDTAVGTVGAGGKKRRSSLSAKVVAIVSRRSRSTSQLSQTESGHKKLKSTIQRSTE
TGMAAEMRKMVRQPSRESTDGSINSYSSEGNLIFPGVRLGADSQFSDFLDGLGPAQLVGR
QTLATPAMGDIQIGMEDKKGQLEVEVIRARSLTQKPGSKSTPAPYVKVYLLENGACIAKK
KTRIARKTLDPLYQQSLVFDESPQGKVLQVIVWGDYGRMDHKCFMGVAQILLEELDLSSM
VIGWYKLFPPSSLVDPTLTPLTRRASQSSLESSTGPPCIRS*
>ENSG00000136960|ENST00000259486
MARRSSFQSCQIISLFTFAVGVNICLGFTAHRIKRAEGWEEGPPTVLSDSPWTNISGSCK
GRCFELQEAGPPDCRCDNLCKSYTSCCHDFDELCLKTARGWECTKDRCGEVRNEENACHC
SEDCLARGDCCTNYQVVCKGESHWVDDDCEEIKAAECPAGFVRPPLIIFSVDGFRASYMK
KGSKVMPNIEKLRSCGTHSPYMRPVYPTKTFPNLYTLATGLYPESHGIVGNSMYDPVFDA
TFHLRGREKFNHRWWGGQPLWITATKQGVKAGTFFWSVVIPHERRILTILQWLTLPDHER
PSVYAFYSEQPDFSGHKYGPFGPEESSYGSPFTPAKRPKRKVAPKRRQERPVAPPKKRRR
KIHRMDHYAAETRQDKMTNPLREIDKIVGQLMDGLKQLKLHRCVNVIFVGDHGMEDVTCD
RTEFLSNYLTNVDDITLVPGTLGRIRSKFSNNAKYDPKAIIANLTCKKPDQHFKPYLKQH
LPKRLHYANNRRIEDIHLLVERRWHVARKPLDVYKKPSGKCFFQGDHGFDNKVNSMQTVF
VGYGSTFKYKTKVPPFENIELYNVMCDLLGLKPAPNNGTHGSLNHLLRTNTFRPTMPEEV
TRPNYPGIMYLQSDFDLGCTCDDKVEPKNKLDELNKRLHTKGSTEERHLLYGRPAVLYRT
RYDILYHTDFESGYSEIFLMPLWTSYTVSKQAEVSSVPDHLTSCVRPDVRVSPSFSQNCL
AYKNDKQMSYGFLFPPYLSSSPEAKYDAFLVTNMVPMYPAFKRVWNYFQRVLVKKYASER
NGVNVISGPIFDYDYDGLHDTEDKIKQYVEGSSIPVPTHYYSIITSCLDFTQPADKCDGP
LSVSSFILPHRPDNEESCNSSEDESKWVEELMKMHTARVRDIEHLTSLDFFRKTSRSYPE
ILTLKTYLHTYESEI*
>ENSG00000121988|ENST00000264159
MPRVHNIKKSLTPHISCVTNESDNLLDFLPDRLRAKLLPFQKDGIIFALKRNGRCMVADE
MGLGKTIQAIGITYFYKEEWPLLIVVPSSLRYPWTEEIEKWIPELSPEEINVIQNKTDVR
RMSTSKVTVLGYGLLTADAKTLIDALNNQNFKVVIVDESHYMKSRNATRSRILLPIVQKA
RRAILLTGTPALGRPEELFMQIEALFPQKFGRWTDYAKRYCNAHIRYFGKRPQWDCRGAS

NLNELHQLLSDIMIRRLKTEVLTQLPPKVRQRIPFDLPSAAAKELNTSFEEWEKIMRTPN
SGAMETVMGLITRMFKQTAIAKAGAVKDYIKMMLQNDSLKFLVFAHHLSMLQACTEAVIE
NKTRYIRIDGSVSSSERIHLVNQFQKDPDTRVAILSIQAAGQGLTFTAASHVVFAELYWD
PGHIKQAEDRAHRIGQCSSVNIHYLIANGTLDTLMWGMLNRKAQVTGSTLNGRKEKIQAE
EGDKEKWDFLQFAEAWTPNDSSEELRKEALFTHFEKEKQHDIRSFFVPQPKKRQLMTSCD
ESKRFREENTVVSSDPTKTAARDIIDYESDVEPETKRLKLAASEDHCSPSEETPSQSKQI
RTPLVESVQEAKAQLTTPAFPVEGWQCSLCTYINNSELPYCEMCETPQGSAVMQIDSLNH
IQDKNEKDDSQKDTSKKVQTISDCEKQALAQSEPGQLADSKEETPKIEKEDGLTSQPGNE
QWKSSDTLPVYDTLMFCASRNTDRIHIYTKDGKQMSCNFIPLDIKLDLWEDLPASFQLKQ
YRSLILRFVREWSSLTAMKQRIIRKSGQLFCSPILALEEITKQQTKQNCTKRYITKEDVA
VASMDKVKNVGGHVRLITKESRPRDPFTKKLLEDGACVPFLNPYTVQADLTVKPSTSKGY
LQAVDNEGNPLCLRCQQPTCQTKQACKANSWDSRFCSLKCQEEFWIRSNNSYLRAKVFET
EHGVCQLCNVNAQELFLRLRDAPKSQRKNLLYATWTSKLPLEQLNEMIRNPGEGHFWQVD
HIKPVYGGGGQCSLDNLQTLCTVCHKERTARQAKERSQVRRQSLASKHGSDITRFLVKK*
>ENSG00000124942|ENST00000257247
MEKEETTRELLLPNWQGSGSHGLTIAQRDDGVFVQEVTQNSPAARTGVVKEGDQIVGATI
YFDNLQSGEVTQLLNTMGHHTVGLKLHRKGDRSPEPGQTWTREVFSSCSSEVVLNTPQPS
ALECKDQNKQKEASSQAGAVSVSTPNAGL*
>ENSG00000067596|ENST00000262415
MAVAVAMAGALIGSEPGPAEELAKLEYLSLVSKVCTELDNHLGINDKDLAEFVISLAEKN
TTFDTFKASLVKNGAEFTDSLISNLLRLIQTMRPPAKPSTSKDPVVKPKTEKEKLKELFP
VLCQPDNPSVRTMLDEDDVKVAVDVLKELEALMPSAAGQEKQRDAEHRDRTKKKKRSRSR
DRNRDRDRDRERNRDRDHKRRHRSRSRSRSRTRERNKVKSRYRSRSRSQSPPKDRKDRDK
YGERNLDRWRDKHVDRPPPEEPTIGDIYNGKVTSIMQFGCFVQLEGLRKRWEGLVHISEL
RREGRVANVADVVSKGQRVKVKVLSFTGTKTSLSMKDVDQETGEDLNPNRRRNLVGETNE
ETSMRNPDRPTHLSLVSAPEVEDDSLERKRLTRISDPEKWEIKQMIAANVLSKEEFPDFD
EETGILPKVDDEEDEDLEIELVEEEPPFLRGHTKQSMDMSPIKIVKNPDGSLSQAAMMQS
ALAKERRELKQAQREAEMDSIPMGLNKHWVDPLPDAEGRQIAANMRGIGMMPNDIPEWKK
HAFGGNKASYGKKTQMSILEQRESLPIYKLKEQLVQAVHDNQILIVIGETGSGKTTQITQ
YLAEAGYTSRGKIGCTQPRRVAAMSVAKRVSEEFGCCLGQEVGYTIRFEDCTSPETVIKY
MTDGMLLRECLIDPDLTQYAIIMLDEAHERTIHTDVLFGLLKKTVQKRQDMKLIVTSATL
DAVKFSQYFYEAPIFTIPGRTYPVEILYTKEPETDYLDASLITVMQIHLTEPPGDILVFL
TGQEEIDTACEILYERMKSLGPDVPELIILPVYSALPSEMQTRIFDPAPPGSRKVVIATN
IAETSLTIDGIYYVVDPGFVKQKVYNSKTGIDQLVVTPISQAQAKQRAGRAGRTGPGKCY
RLYTERAYRDEMLTTNVPEIQRTNLASTVLSLKAMGINDLLSFDFMDAPPMETLITAMEQ
LYTLGALDDEGLLTRLGRRMAEFPLEPMLCKMLIMSVHLGCSEEMLTIVSMLSVQNVFYR
PKDKQALADQKKAKFHQTEGDHLTLLAVYNSWKNNKFSNPWCYENFIQARSLRRAQDIRK
QMLGIMDRHKLDVVSCGKSTVRVQKAICSGFFRNAAKKDPQEGYRTLIDQQVVYIHPSSA
LFNRQPEWVVYHELVLTTKEYMREVTTIDPRWLVEFAPAFFKVSDPTKLSKQKKQQRLEP
LYNRYEEPNAWRISRAFRRR*
>ENSG00000135336|ENST00000257789
MATSSMSKGCFVFKPNSKKRKISLPIEDYFNKGKNEPEDSKLRFETYQLIWQQMKSENER
LQEELNKNLFDNLIEFLQKSHSGFQKNSRDLGGQIKLREIPTAALVLGVNVTDHDLTFGS
LTEALQNNVTPYVVSLQAKDCPDMKHFLQKLISQLMDCCVDIKSKEEESVHVTQRKTHYS

MDSLSSWYMTVTQKTDPKMLSKKRTTSSQWQSPPVVVILKDMESFATKVLQDFIIISSQH
LHEFPLILIFGIATSPIIIHRLLPHAVSSLLCIELFQSLSCKEHLTTVLDKLLLTTQFPF
KINEKVLQVLTNIFLYHDFSVQNFIKGLQLSLLEHFYSQPLSVLCCNLPEAKRRINFLSN
NQCENIRRLPSFRRYVEKQASEKQVALLTNERYLKEETQLLLENLHVYHMNYFLVLRCLH
KFTSSLPKYPLGRQIRELYCTCLEKNIWDSEEYASVLQLLRMLAKDELMTILEKCFKVFK
SYCENHLGSTAKRIEEFLAQFQSLDAETKEEEDASGSQPKGLQKTDLYHLQKSLLEMKEL
RRSKKQTKFEVLRENVVNFIDCLVREYLLPPETQPLHEVVYFSAAHALREHLNAAPRIAL
HTALNNPYYYLKNEALKSEEGCIPNIAPDICIAYKLHLECSRLINLVDWSEAFATVVTAA
EKMDANSATSEEMNEIIHARFIRAVSELELLGFIKPTKQKTDHVARLTWGGC*
>ENSG00000130813|ENST00000253110
MSQEGVELEKSVRRLREKFHGKVSSKKAGALMRKFGSDHTGVGRSIVYGVKQKDGQELSN
DLDAQDPPEDMKQDRDIQAVATSLLPLTEANLRMFQRAQDDLIPAVDRQFACSSCDHVWW
RRVPQRKEVSRCRKCRKRYEPVPADKMWGLAEFHCPKCRHNFRGWAQMGSPSPCYGCGFP
VYPTRILPPRWDRDPDRRSTHTHSCSAADCYNRREPHVPGTSCAHPKSRKQNHLPKVLHP
SNPHISSGSTVATCLSQGGLLEDLDNLILEDLKEEEEEEEEVEDEEGGPRE*
>ENSG00000140853|ENST00000262510
MDPVGLQLGNKNLWSCLVRLLTKDPEWLNAKMKFFLPNTDLDSRNETLDPEQRVILQLNK
LHVQGSDTWQSFIHCVCMQLEVPLDLEVLLLSTFGYDDGFTSQLGAEGKSQPESQLHHGL
KRPHQSCGSSPRRKQCKKQQLELAKKYLQLLRTSAQQRYRSQIPGSGQPHAFHQVYVPPI
LRRATASLDTPEGAIMGDVKVEDGADVSISDLFNTRVNKGPRVTVLLGKAGMGKTTLAHR
LCQKWAEGHLNCFQALFLFEFRQLNLITRFLTPSELLFDLYLSPESDHDTVFQYLEKNAD
QVLLIFDGLDEALQPMGPDGPGPVLTLFSHLCNGTLLPGCRVMATSRPGKLPACLPAEAA
MVHMLGFDGPRVEEYVNHFFSAQPSREGALVELQTNGRLRSLCAVPALCQVACLCLHHLL
PDHAPGQSVALLPNMTQLYMQMVLALSPPGHLPTSSLLDLGEVALRGLETGKVIFYAKDI
APPLIAFGATHSLLTSFCVCTGPGHQQTGYAFTHLSLQEFLAALHLMASPKVNKDTLTQY
VTLHSRWVQRTKARLGLSDHLPTFLAGLASCTCRPFLSHLAQGNEDCVGAKQAAVVQVLK
KLATRKLTGPKVVELCHCVDETQEPELASLTAQSLPYQLPFHNFPLTCTDLATLTNILEH
REAPIHLDFDGCPLEPHCPEALVGCGQIENLSFKSRKCGDAFAEALSRSLPTMGRLQMLG
LAGSKITARGISHLVKALPLCPQLKEVSFRDNQLSDQVVLNIVEVLPHLPRLRKLDLSSN
SICVSTLLCLARVAVTCPTVRMLQAREADLIFLLSPPTETTAELQRAPDLQESDGQRKGA
QSRSLTLRLQKCQLQVHDAEALIALLQEGPHLEEVDLSGNQLEDEGCRLMAEAASQLHIA
RKLDLSNNGLSVAGVHCVLRAVSACWTLAELHISLQHKTVIFMFAQEPEEQKGPQERAAF
LDSLMLQMPSELPLSSRRMRLTHCGLQEKHLEQLCKALGGSCHLGHLHLDFSGNALGDEG
AARLAQLLPGLGALQSLNLSENGLSLDAVLGLVRCFSTLQWLFRLDISFESQHILLRGDK
TSRDMWATGSLPDFPAAAKFLGFRQRCIPRSLCLSECPLEPPSLTRLCATLKDCPGPLEL
QLSCEFLSDQSLETLLDCLPQLPQLSLLQLSQTGLSPKSPFLLANTLSLCPRVKKVDLRS
LHHATLHFRSNEEEEGVCCGRFTGCSLSQEHVESLCWLLSKCKDLSQVDLSANLLGDSGL
RCLLECLPQVPISGLLDLSHNSISQESALYLLETLPSCPRVREASVNLGSEQSFRIHFSR
EDQAGKTLRLSECSFRPEHVSRLATGLSKSLQLTELTLTQCCLGQKQLAILLSLVGRPAG
LFSLRVQEPWADRARVLSLLEVCAQASGSVTEISISETQQQLCVQLEFPRQEENPEAVAL
RLAHCDLGAHHSLLVGQLMETCARLQQLSLSQVNLCEDDDASSLLLQSLLLSLSELKTFR
LTSSCVSTEGLAHLASGLGHCHHLEELDLSNNQFDEEGTKALMRALEGKWMLKRLDLSHL
LLNSSTLALLTHRLSQMTCLQSLRLNRNSIGDVGCCHLSEALRAATSLEELDLSHNQIGD
AGVQHLATILPGLPELRKIDLSGNSISSAGGVQLAESLVLCRRLEELMLGCNALGDPTAL

GLAQELPQHLRVLHLPFSHLGPGGALSLAQALDGSPHLEEISLAENNLAGGVLRFCMELP
LLRQIDLVSCKIDNQTAKLLTSSFTSCPALEVILLSWNLLGDEAAAELAQVLPQMGRLKR
VDLEKNQITALGAWLLAEGLAQGSSIQVIRLWNNPIPCDMAQHLKSQEPRLDFAFFDNQP
QAPWGT*
>ENSG00000135473|ENST00000257931
MNFEGLDPGLAEYAPAMHSALDPVLDAHLNPSLLQNVELDPEGVALEALPVQESVHIMEG
VYSELHSVVAEVGVPVSVSHFDLHEEMLWVGSHGGHATSFFGPALERYSSFQVNGSDDIR
QIQSLENGILFLTKNNLKYMARGGLIIFDYLLDENEDMHSLLLTDSSTLLVGGLQNHIIE
IDLNTVQETQKYAVETPGVTIMRQTNRFFFCGHTSGKVSLRDLRTFKVEHEFDAFSGSLS
DFDVHGNLLAACGFSSRLTGLACDRFLKVYDLRMMRAITPLQVHVDPAFLRFIPTYTSRL
AIISQSGQCQFCEPTGLANPADIFHVNPVGPLLMTFDVSASKQALAFGDSEGCVHLWTDS
PEPSFNPYSRETEFALPCLVDSLPPLDWSQDLLPLSLIPVPLTTDTLLSDWPAANSAPAP
RRAPPVDAEILRTMKKVGFIGYAPNPRTRLRNQIPYRLKESDSEFDSFSQVTESPVGREE
EPHLHMVSKKYRKVTIKYSKLGLEDFDFKHYNKTLFAGLEPHIPNAYCNCMIQVLYFLEP
VRCLIQNHLCQKEFCLACELGFLFHMLDLSRGDPCQGNNFLRAFRTIPEASALGLILADS
DEASGKGNLARLIQRWNRFILTQLHQDMQELEIPQAYRGAGGSFCSSGDSVIGQLFSCEM
ENCSLCRCGSETVRASSTLLFTLSYPDGSKSDKTGKNYDFAQVLKRSICLDQNTQAWCDT
CEKYQPTIQTRNIRHLPDILVINCEVNSSKEADFWRMQAEVAFKMAVKKHGGEISKNKEF
ALADWKELGSPEGVLVCPSIEELKNVWLPFSIRMKMTKNKGLDVCNWTDGDEMQWGPARA
EEEHGVYVYDLMATVVHILDSRTGGSLVAHIKVGETYHQRKEGVTHQQWYLFNDFLIEPI
DKHEAVQFDMNWKVPAILYYVKRNLNSRYNLNIKNPIEASVLLAEASLARKQRKTHTTFI
PLMLNEMPQIGDLVGLDAEFVTLNEEEAELRSDGTKSTIKPSQMSVARITCVRGQGPNEG
IPFIDDYISTQEQVVDYLTQYSGIKPGDLDAKISSKHLTTLKSTYLKLRFLIDIGVKFVG
HGLQKDFRVINLMVPKDQVLDTVYLFHMPRKRMISLRFLAWYFLDLKIQGETHDSIEDAR
TALQLYRKYLELSKNGTEPESFHKVLKGLYEKGRKMDWKVPEPEGQTSPKNAAVFSSVLA
L*
>ENSG00000134440|ENST00000256854
MVLAELYVSDREGSDATGDGTKEKPFKTGLKALMTVGKEPFPTIYVDSQKENERWNVISK
SQLKNIKKMWHREQMKSESREKKEAEDSLRREKNLEEAKKITIKNDPSLPEPKCVKIGAL
EGYRGQRVKVFGWVHRLRRQGKNLMFLVLRDGTGYLQCVLADELCQCYNGVLLSTESSVA
VYGMLNLTPKGKQAPGGHELSCDFWELIGLAPAGGADNLINEESDVDVQLNNRHMMIRGE
NMSKILKARSMVTRCFRDHFFDRGYYEVTPPTLVQTQVEGGATLFKLDYFGEEAFLTQSS
QLYLETCLPALGDVFCIAQSYRAEQSRTRRHLAEYTHVEAECPFLTFDDLLNRLEDLVCD
VVDRILKSPAGSIVHELNPNFQPPKRPFKRMNYSDAIVWLKEHDVKKEDGTFYEFGEDIP
EAPERLMTDTINEPILLCRFPVEIKSFYMQRCPEDSRLTESVDVLMPNVGEIVGGSMRIF
DSEEILAGYKREGIDPTPYYWYTDQRKYGTCPHGGYGLGLERFLTWILNRYHIRDVCLYP
RFVQRCTP*
>ENSG00000085760|ENST00000263629
MNQKLLKLENLLRFHTIYRQLHSLCQRRALRQWRHGFSSAYPVWTAQLCAWPWPTDVLTG
AALSQYRLLVTKKEEGPWKSQLSSTKSKKVVEVWIGMTIEELARAMEKNTDYVYEALLNT
DIDIDSLEADSHLDEVWIKEVITKAGMKLKWSKLKQDKVRKNKDAVRRPQADPALLTPRS
PVVTIMGHVDHGKTTLLDKFRKTQVAAVETGGITQHIGAFLVSLPSGEKITFLDTPGHAA
FSAMRARGAQVTDIVVLVVAADDGVMKQTVESIQHAKDAQVPIILAVNKCDKAEADPEKV
KKELLAYDVVCEDYGGDVQAVPVSALTGDNLMALAEATVALAEMLELKADPNGPVEGTVI

ESFTDKGRGLVTTAIIQRGTLRKGSVLVAGKCWAKVRLMFDENGKTIDEAYPSMPVGITG
WRDLPSAGEEILEVESEPRAREVVDWRKYEQEQEKGQEDLKIIEEKRKEHKEAHQKAREK
YGHLLWKKRSILRFLERKEQIPLKPKEKRERDSNVLSVIIKGDVDGSVEAILNIIDTYDA
SHECELELVHFGVGDVSANDVNLAETFDGVIYGFNVNAGNVIQQSAAKKGVKIKLHKIIY
RLVEDLQEELSSRLPCAVEEHPVGEASILATFSVTEGKKKVPVAGCRVQKGQLEKQKKFK
LTRNGHVIWKGSLTSLKHHKDDISIVKTGMDCGLSLDEDNMEFQVGDRIVCYEEKQIQAK
TSWDPGF*
>ENSG00000131981|ENST00000254301
MADNFSLHDALSGSGNPNPQGWPGAWGNQPAGAGGYPGASYPGAYPGQAPPGAYPGQAPP
GAYPGAPGAYPGAPAPGVYPGPPSGPGAYPSSGQPSATGAYPATGPYGAPAGPLIVPYNL
PLPGGVVPRMLITILGTVKPNANRIALDFQRGNDVAFHFNPRFNENNRRVIVCNTKLDNN
WGREERQSVFPFESGKPFKIQVLVEPDHFKVAVNDAHLLQYNHRVKKLNEISKLGISGDI
DLTSASYTMI*
>ENSG00000120802|ENST00000261210
MPEFLEDPSVLTKDKLKSELVANNVTLPAGEQRKDVYVQLYLQHLTARNRPPLPAGTNSK
GPPDFSSDEEREPTPVLGSGAAAAGRSRAAVGRKATKKTDKPRQEDKDDLDVTELTNEDL
LDQLVKYGVNPGPIVGTTRKLYEKKLLKLREQGTESRSSTPLPTISSSAENTRQNGSNDS
DRYSDNEEDSKIELKLEKREPLKGRAKTPVTLKQRRVEHNQVSLVLLPPCTGINNLLTTL
IHVLAFNG*
>ENSG00000132846|ENST00000255198
MRSGEPACTMDQARGLDDAAARGGQCPGLGPAPTPTPPGRLGAPYSEAWGYFHLAPGRPG
HPSGHWATCRLCGEQVGRGPGFHAGTSALWRHLRSAHRRELESSGAGSSPPAAPCPPPPG
PAAAPEGDWARLLEQMGALAVRGSRRERELERRELAVEQGERALERRRRALQEEERAAAQ
ARRELQAEREALQARLRDVSRREGALGWAPAAPPPLKDDPEGDRDGCVITKVLL*
>ENSG00000126952|ENST00000263032
MRRNTQDENMRKWFKVTIPYGIKYDKAWLMNSIQSNCSVPFTPVDFHYIRNRACFFVQVA
SAASALKDVSYKIYDDENQKICIFVSHFTAPYSVKNKLKPGQMEMLKLTMNKRYNVSQQA
LDLQNLRFDPDLMGRDIDIILNRRNCMAATLKITERNFPELLSLNLCNNKLYQLDGLSDI
TEKAPKVKTLNLSKNKLESAWELGKVKGLKLEELWLEGNPLCSTFSDQSAYVSAIRDCFP
KLLRLDGRELSAPVIVDIDSSETMKPCKENFTGSETLKHLVLQFLQQSNLCKYFKDSRNI
KILKDPYLQRKLLKHTKCPRNVDSLSALPETQHDFTSILVDMWYQTVNTCFLPRAGPESQ
RWWCLLSLKWKDGLRVLILPSCGPSSLPLAAIPVCAS*
>ENSG00000137656|ENST00000260210
MAAAPPLSKAEYLKRYLSGADAGVDRGSESGRKRRKKRPKPGGAGGKGMRIVDDDVSWTA
ISTTKLEKEEEEDDGDLPVVAEFVDERPEEVKQMEAFRSSAKWKLLGGHNEDLPSNRHFR
HDTPDSSPRRVRHGTPDPSPRKDRHDTPDPSPRRARHDTPDPSPLRGARHDSDTSPPRRI
RHDSSDTSPPRRRARHDSPDPSPPRRPQHNSSGASPRRVRHDSPDPSPPRRARHGSSDISS
PRRVHNNSPDTSRRTLGSSDTQQLRRARHDSPDLAPNVTYSLPRTKSGKAPERASSKTSP
HWKESGASHLSFPKNSKYEYDPDISPPRKKQAKSHFGDKKQLDSKGDCQKATDSDLSSPR
HKQSPGHQDSDSDLSPPRNRPRHRSSDSDLSPPRRRQRTKSSDSDLSPPRRSQPPGKKAA
HMYSGAKTGLVLTDIQREQQELKEQDQETMAFEAEFQYAETVFRDKSGRKRNLKLERLEQ
RRKAEKDSERDELYAQWGKGLAQSRQQQQNVEDAMKEMQKPLARYIDDEDLDRMLREQER
EGDPMANFIKKNKAKENKNKKVRPRYSGPAPPPNRFNIWPGYRWDGVDRSNGFEQKRFAR
LASKKAVEELAYKWSVEDM*

>ENSG00000076604|ENST00000262395
MPGFDYKFLEKPKRRLLCPLCGKPMREPVQVSTCGHRFCDTCLQEFLSEGVFKCPEDQLP
LDYAKIYPDPELEVQVLGLPIRCIHSEEGCRWSGPLRHLQGHLNTCSFNVIPCPNRCPMK
LSRRDLPAHLQHDCPKRRLKCEFCGCDFSGEAYESHEGMCPQESVYCENKCGARMMRRLL
AQHATSECPKRTQPCTYCTKEFVFDTIQSHQYQCPRLPVACPNQCGVGTVAREDLPGHLK
DSCNTALVLCPFKDSGCKHRCPKLAMARHVEESVKPHLAMMCALVSRQRQELQELRRELE
ELSVGSDGVLIWKIGSYGRRLQEAKAKPNLECFSPAFYTHKYGYKLQVSAFLNGNGSGEG
THLSLYIRVLPGAFDNLLEWPFARRVTFSLLDQSDPGLAKPQHVTETFHPDPNWKNFQKP
GTWRGSLDESSLGFGYPKFISHQDIRKRNYVRDDAVFIRAAVELPRKILS*
>ENSG00000130254|ENST00000252542
MAETLPGSGDSGPGTASLGPGVAETGTRRLSELRVIDLRAELKKRNLDTGGNKSVLMERL
KKAVKEEGQDPDEIGIELEATSKKSAKRCVKGLKMEEEGTEDNGLEDDSRDGQEDMEASL
ENLQNMGMMDMSVLDETEVANSSAPDFGEDGTDGLLDSFCDSKEYVAAQLRQLPAQPPEH
AVDGEGFKNTLETSSLNFKVTPDIEESLLEPENEKILDILGETCKSEPVKEESSELEQPF
AQDTSSVGPDRKLAEEEDLFDSAHPEEGDLDLASESTAHAQSSKADSLLAVVKREPAEQP
GDGERTDCEPVGLEPAVEQSSAASELAEASSEELAEAPTEAPSPEARDSKEDGRKFDFDA
CNEVPPAPKESSTSEGADQKMSSFKEEKDIKPIIKDEKGRVGSGSGRNLWVSGLSSTTRA
TDLKNLFSKYGKVVGAKVVTNARSPGARCYGFVTMSTSDEATKCISHLHRTELHGRMISV
EKAKNEPAGKKLSDRKECEVKKEKLSSVDRHHSVEIKIEKTVIKKEEKIEKKEEKKPEDI
KKEEKDQDELKPGPTNRSRVTKSGSRGMERTVVMDKSKGEPVISVKTTSRSKERSSKSQD
RKSESKEKRDILSFDKIKEQRERERQRQREREIRETERRREREQREREQRLEAFHERKEK
ARLQRERLQLECQRQRLERERMERERLERERMRVERERRKEQERIHREREELRRQQEQLR
YEQERRPGRRPYDLDRRDDAYWPEGKRVAMEDRYRADFPRPDHRFHDFDHRDRGQYQDHA
IDRREGSRPMMGDHRDGQHYGDDRHGHGGPPERHGRDSRDGWGGYGSDKRLSEGRGLPPP
PRGGRDWGEHNQRLEEHQARAWQGAMDAGAASREHARWQGGERGLSGPSGPGHMASRGGV
AGRGGFAQGGHSQGHVVPGGGLEGGGVASQDRGSRVPHPHPHPPPYPHFTRRY*
>ENSG00000132485|ENST00000254821
MSTKNFRVSDGDWICPDKKCGNVNFARRTSCNRCGREKTTEAKMMKAGGTEIGKTLAEKS
RGLFSANDWQCKTCSNVNWARRSECNMCNTPKYAKLEERTGYGGGFNERENVEYIEREES
DGEYDEFGRKKKKYRGKAVGPASILKEVEDKESEGEEEDEDEDLSKYKLDEDEDEDDADL
SKYNLDASEEEDSNKKKSNRRSRSKSRSSHSRSSSRSSSPSSSRSRSRSRSRSSSSSQSR
SRSSSRERSRSRGSKSRSSSRSHRGSSSPRKRSYSSSSSSPERNRKRSRSRSSSSGDRKK
RRTRSRSPESQVIGENTKQP*
>ENSG00000011451|ENST00000263381
MEGSLAGSLAAPDRPQGPERLPGPAPRENIEGGAEAAEGEGGIFRSTRYLPVTKEGPRDI
LDGRGGISVANFDPGTFSLMRCDFCGAGFDTRAGLSSHARAHLRDFGITNWELTVSPINI
LQELLATSAAEQPPSPLGREPGGPPGSFLTSRRPRLPLTVPFPPTWAEDPGPAYGDASGP
EPARDIRCEFCGEFFENRKGLSSHARSHLRQMGVTEWYVNGSPIDTLREILKRRTQSRPG
GPPNPPGPSPKALAKMMGGAGPGSSLEARSPSDLHISPLAKKLPPPPGSPLGHSPTASPP
PTARKMFPGLAAPSLPKKLKPEQIRVEIKREMLPGALHGELHPSEGPWGAPREDMTPLNL
SSRAEPVRDIRCEFCGEFFENRKGLSSHARSHLRQMGVTEWSVNGSPIDTLREILKKKSK
PCLIKKEPPAGDLAPALAEDGPPTVAPGPVQSPLPLSPLAGRPGKPGAGPAQVPRELSLT
PITGAKPSATGYLGSVAAKRPLQEDRLLPAEVKAKTYIQTELPFKAKTLHEKTSHSSTEA
CCELCGLYFENRKALASHARAHLRQFGVTEWCVNGSPIETLSEWIKHRPQKVGAYRSYIQ

GGRPFTKKFRSAGHGRDSDKRPSLGLAPGGLAVVGRSAGGEPGPEAGRAADGGERPLAAS
PPGTVKAEEHQRQNINKFERRQARPPDASAARGGEDTNDLQQKLEEVRQPPPRVRPVPSL
VPRPPQTSLVKFVGNIYTLKCRFCEVEFQGPLSIQEEWVRHLQRHILEMNFSKADPPPEE
SQAPQAQTAAAEAP*
>ENSG00000083093|ENST00000261584
MDEPPGKPLSCEEKEKLKEKLAFLKREYSKTLARLQRAQRAEKIKHSIKKTVEEQDCLSQ
QDLSPQLKHSEPKNKICVYDKLHIKTHLDEETGEKTSITLDVGPESFNPGDGPGGLPIQR
TDDTQEHFPHRVSDPSGEQKQKLPSRRKKQQKRTFISQERDCVFGTDSLRLSGKRLKEQE
EISSKNPARSPVTEIRTHLLSLKSELPDSPEPVTEINEDSVLIPPTAQPEKGVDTFLRRP
NFTRATTVPLQTLSDSGSSQHLEHIPPKGSSELTTHDLKNIRFTSPVSLEAQGKKMTVST
DNLLVNKAISKSGQLPTSSNLEANISCSLNELTYNNLPANENQNLKEQNQTEKSLKSPSD
TLDGRNENLQESEILSQPKSLSLEATSPLSAEKHSCTVPEGLLFPAEYYVRTTRSMSNCQ
RKVAVEAVIQSHLDVKKKGFKNKNKDASKNLNLSNEETDQSEIRMSGTCTGQPSSRTSQK
LLSLTKVSSPAGPTEDNDLSRKAVAQAPGRRYTGKRKSACTPASDHCEPLLPTSSLSIVN
RSKEEVTSHKYQHEKLFIQVKGKKSRHQKEDSLSWSNSAYLSLDDDAFTAPFHRDGMLSL
KQLLSFLSITDFQLPDEDFGPLKLEKVKSCSEKPVEPFESKMFGERHLKEGSCIFPEELS
PKRMDTEMEDLEEDLIVLPGKSHPKRPNSQSQHTKTGLSSSILLYTPLNTVAPDDNDRPT
TDMCSPAFPILGTTPAFGPQGSYEKASTEVAGRTCCTPQLAHLKDSVCLASDTKQFDSSG
SPAKPHTTLQVSGRQGQPTCDCDSVPPGTPPPIESFTFKENQLCRNTCQELHKHSVEQTE
TAELPASDSINPGNLQLVSELKNPSGSCSVDVSAMFWERAGCKEPCIITACEDVVSLWKA
LDAWQWEKLYTWHFAEVPVLQIVPVPDVYNLVCVALGNLEIREIRALFCSSDDESEKQVL
LKSGNIKAVLGLTKRRLVSSSGTLSDQQVEVMTFAEDGGGKENQFLMPPEETILTFAEVQ
GMQEALLGTTIMNNIVIWNLKTGQLLKKMHIDDSYQASVCHKAYSEMGLLFIVLSHPCAK
ESESLRSPVFQLIVINPKTTLSVGVMLYCLPPGQAGRFLEGDVKDHCAAAILTSGTIAIW
DLLLGQCTALLPPVSDQHWSFVKWSGTDSHLLAGQKDGNIFVYHYS*
>ENSG00000136231|ENST00000258729
MNKLYIGNLSENAAPSDLESIFKDAKIPVSGPFLVKTGYAFVDCPDESWALKAIEALSGK
IELHGKPIEVEHSVPKRQRIRKLQIRNIPPHLQWEVLDSLLVQYGVVESCEQVNTDSETA
VVNVTYSSKDQARQALDKLNGFQLENFTLKVAYIPDEMAAQQNPLQQPRGRRGLGQRGSS
RQGSPGSVSKQKPCDLPLRLLVPTQFVGAIIGKEGATIRNITKQTQSKIDVHRKENAGAA
EKSITILSTPEGTSAACKSILEIMHKEAQDIKFTEEIPLKILAHNNFVGRLIGKEGRNLK
KIEQDTDTKITISPLQELTLYNPERTITVKGNVETCAKAEEEIMKKIRESYENDIASMNL
QAHLIPGLNLNALGLFPPTSGMPPPTSGPPSAMTPPYPQFEQSETETVHLFIPALSVGAI
IGKQGQHIKQLSRFAGASIKIAPAEAPDAKVRMVIITGPPEAQFKAQGRIYGKIKEENFV
SPKEEVKLEAHIRVPSFAAGRVIGKGGKTVNELQNLSSAEVVVPRDQTPDENDQVVVKIT
GHFYACQVAQRKIQEILTQVKQHQQQKALQSGPPQSRRK*
>ENSG00000119772|ENST00000264709
MPAMPSSGPGDTSSSAAEREEDRKDGEEQEEPRGKEERQEPSTTARKVGRPGRKRKHPPV
ESGDTPKDPAVISKSPSMAQDSGASELLPNGDLEKRSEPQPEEGSPAGGQKGGAPAEGEG
AAETLPEASRAVENGCCTPKEGRGAPAEAGKEQKETNIESMKMEGSRGRLRGGLGWESSL
RQRPMPRLTFQAGDPYYISKRKRDEWLARWKREAEKKAKVIAGMNAVEENQGPGESQKVE
EASPPAVQQPTDPASPTVATTPEPVGSDAGDKNATKAGDDEPEYEDGRGFGIGELVWGKL
RGFSWWPGRIVSWWMTGRSRAAEGTRWVMWFGDGKFSVVCVEKLMPLSSFCSAFHQATYN
KQPMYRKAIYEVLQVASSRAGKLFPVCHDSDESDTAKAVEVQNKPMIEWALGGFQPSGPK

GLEPPEEEKNPYKEVYTDMWVEPEAAAYAPPPPAKKPRKSTAEKPKVKEIIDERTRERLV
YEVRQKCRNIEDICISCGSLNVTLEHPLFVGGMCQNCKNCFLECAYQYDDDGYQSYCTIC
CGGREVLMCGNNNCCRCFCVECVDLLVGPGAAQAAIKEDPWNCYMCGHKGTYGLLRRRED
WPSRLQMFFANNHDQEFDPPKVYPPVPAEKRKPIRVLSLFDGIATGLLVLKDLGIQVDRY
IASEVCEDSITVGMVRHQGKIMYVGDVRSVTQKHIQEWGPFDLVIGGSPCNDLSIVNPAR
KGLYEGTGRLFFEFYRLLHDARPKEGDDRPFFWLFENVVAMGVSDKRDISRFLESNPVMI
DAKEVSAAHRARYFWGNLPGMNRPLASTVNDKLELQECLEHGRIAKFSKVRTITTRSNSI
KQGKDQHFPVFMNEKEDILWCTEMERVFGFPVHYTDVSNMSRLARQRLLGRSWSVPVIRH
LFAPLKEYFACV*
>ENSG00000196757|ENST00000254321
MPCCSHRSCREDPGTSESREMDPVAFEDVAVNFTQEEWTLLDISQKNLFREVMLETFRNL
TSIGKKWSDQNIEYEYQNPRRSFRSLIEEKVNEIKEDSHCGETFTQVPDDRLNFQEKKAS
PEVKSCDSFVCAEVGIGNSSFNMSIRGDTGHKAYEYQEYGPKPYKCQQPKNKKAFRYRPS
IRTQERDHTGEKPYACKVCGKTFIFHSSIRRHMVMHSGDGTYKCKFCGKAFHSFSLYLIH
ERTHTGEKPYECKQCGKSFTYSATLQIHERTHTGEKPYECSKCDKAFHSSSSYHRHERSH
MGEKPYQCKECGKAFAYTSSLRRHERTHSGKKPYECKQYGEGLSYLISFQTHIRMNSGER
PYKCKICGKGFYSAKSFQTHEKTHTGEKRYKCKQCGKAFNLSSSFRYHERIHTGEKPYEC
KQCGKAFRSASQLRVHGGTHTGEKPYECKECGKAFRSTSHLRVHGRTHTGEKPYECKECG
KAFRYVKHLQIHERTEKHIRMPSGERPYKCSICEKGFYSAKSFQTHEKTHTGEKPYECNQ
CGKAFRCCNSLRYHERTHTGEKPYECKQCGKAFRSASHLRMHERTHTGEKPYECKQCGKA
FSCASNLRKHGRTHTGEKPYECKQCGKAFRSASNLQMHERTHTGEKPYECKECEKAFCKF
SSFQIHERKHRGEKPYECKHCGNGFTSAKILQIHARTHIGEKHYECKECGKAFNYFSSLH
IHARTHMGEKPYECKDCGKAFS*
>ENSG00000104131|ENST00000261868
MAAAAAAAGDSDSWDADAFSVEDPVRKVGGGGTAGGDRWEGEDEDEDVKDNWDDDDDEKK
EEAEVKPEVKISEKKKIAEKIKEKERQQKKRQEEIKKRLEEPEEPKVLTPEEQLADKLRL
KKLQEESDLELAKETFGVNNAVYGIDAMNPSSRDDFTEFGKLLKDKITQYEKSLYYASFL
EVLVRDVCISLEIDDLKKITNSLTVLCSEKQKQEKQSKAKKKKKGVVPGGGLKATMKDDL
ADYGGYDGGYVQDYEDFM*
>ENSG00000132383|ENST00000254719
MVGQLSEGAIAAIMQKGDTNIKPILQVINIRPITTGNSPPRYRLLMSDGLNTLSSFMLAT
QLNPLVEEEQLSSNCVCQIHRFIVNTLKDGRRVVILMELEVLKSAEAVGVKIGNPVPYNE
GLGQPQVAPPAPAASPAASSRPQPQNGSSGMGSTVSKAYGASKTFGKAAGPSLSHTSGGT
QSKVVPIASLTPYQSKWTICARVTNKSQIRTWSNSRGEGKLFSLELVDESGEIRATAFNE
QVDKFFPLIEVNKVYYFSKGTLKIANKQFTAVKNDYEMTFNNETSVMPCEDDHHLPTVQF
DFTGIDDLENKSKDSLVDIIGICKSYEDATKITVRSNNREVAKRNIYLMDTSGKVVTATL
WGEDADKFDGSRQPVLAIKGARVSDFGGRSLSVLSSSTIIANPDIPEAYKLRGWFDAEGQ
ALDGVSISDLKSGGVGGSNTNWKTLYEVKSENLGQGDKPDYFSSVATVVYLRKENCMYQA
CPTQDCNKKVIDQQNGLYRCEKCDTEFPNFKYRMILSVNIADFQENQWVTCFQESAEAIL
GQNAAYLGELKDKNEQAFEEVFQNANFRSFIFRVRVKVETYNDESRIKATVMDVKPVDYR
EYGRRLVMSIRRSALM*
>ENSG00000138443|ENST00000261017
MAELQMLLEEEIPGGRRALFDSYTNLERVADYCENNYIQSADKQRALEETKAYTTQSLAS
VAYLINTLANNVLQMLDIQASQLRRMESSINHISQTVDIHKEKVARREIGILTTNKNTSR

THKIIAPANLERPVRYIRKPIDYTILDDIGHGVKVSTQNMKMGGLPRTTPPTQKPPSPPM
SGKGTLGRHSPYRTLEPVRPPVVPNDYVPSPTRNMAPSQQSPVRTASVNQRNRTYSSSGS
SGGSHPSSRSSSRENSGSGSVGVPIAVPTPSPPSVFPGHPVQFYSMNRPASRHTPPTIGG
SLPYRRPPSITSQTSLQNQMNGGPFYSQNPVSLAPPPPSILQVTPQLPLMGFVARVQENI
SDTPPPPPPVEEPVFDESPPPPPPPEDYEEEEAAVVEYSDPYAEEDPPWAPRSYLEKVVA
IYDYTKDKEDELSFQEGAIIYVIKKNDDGWYEGVMNGVTGLFPGNYVESIMHYSE*
>ENSG00000116017|ENST00000263620
MKLQAVMETLLQRQQRARQELEARQQLPPDPPAAPPGRARAAPDEDREPESARMQRAQMA
ALAAMRAAAAGLGHPASPGGSEDGPPGSEEEDAAREGTPGSPGRGREGPGEEHFEDMASD
EDMKPKWEEEEMEEDLGEDEEEEEEDYEDEEEEEDEEGLGPPGPASLGTTALFPRKAQPP
QAFRGDGVPRVLGGQERPGPGPAHPGGAAHVAPQLQPPDHGDWTYEEQFKQLYELDGDPK
RKEFLDDLFSFMQKRGTPVNRIPIMAKQVLDLFMLYVLVTEKGGLVEVINKKLWREITKG
LNLPTSITSAAFTLRTQYMKYLYPYECEKRGLSNPNELQAAIDSNRREGRRQSFGGSLFA
YSPGGAHGMLSSPKLPVSSLGLAASTNGSSITPAPKIKKEEDSAIPITVPGRLPVSLAGH
PVVAAQAAAVQAAAAQAAVAAQAAALEQLREKLESAEPPEKKMALVADEQQRLMQRALQQ
NFLAMAAQLPMSIRINSQASESRQDSAVNLTGTNGSNSISMSVEINGIMYTGVLFAQPPA
PTPTSAPNKGGGGGGGSSSNAGGRGGNTGTSGGQAGPAGLSTPSTSTSNNSLP*
>ENSG00000051341|ENST00000264233
MNLLRRSGKRRRSESGSDSFSGSGGDSSASPQFLSGSVLSPPPGLGRCLKAAAAGECKPT
VPDYERDKLLLANWGLPKAVLEKYHSFGVKKMFEWQAECLLLGQVLEGKNLVYSAPTSAG
KTLVAELLILKRVLEMRKKALFILPFVSVAKEKKYYLQSLFQEVGIKVDGYMGSTSPSRH
FSSLDIAVCTIERANGLINRLIEENKMDLLGMVVVDELHMLGDSHRGYLLELLLTKICYI
TRKSASCQADLASSLSNAVQIVGMSATLPNLELVASWLNAELYHTDFRPVPLLESVKVGN
SIYDSSMKLVREFEPMLQVKGDEDHVVSLCYETICDNHSVLLFCPSKKWCEKLADIIARE
FYNLHHQAEGLVKPSECPPVILEQKELLEVMDQLRRLPSGLDSVLQKTVPWGVAFHHAGL
TFEERDIIEGAFRQGLIRVLAATSTLSSGVNLPARRVIIRTPIFGGRPLDILTYKQMVGR
AGRKGVDTVGESILICKNSEKSKGIALLQGSLKPVRSCLQRREGEEVTGSMIRAILEIIV
GGVASTSQDMHTYAACTFLAASMKEGKQGIQRNQESVQLGAIEACVMWLLENEFIQSTEA
SDGTEGKVYHPTHLGSATLSSSLSPADTLDIFADLQRAMKGFVLENDLHILYLVTPMFED
WTTIDWYRFFCLWEKLPTSMKRVAELVGVEEGFLARCVKGKVVARTERQHRQMAIHKRFF
TSLVLLDLISEVPLREINQKYGCNRGQIQSLQQSAAVYAGMITVFSNRLGWHNMELLLSQ
FQKRLTFGIQRELCDLVRVSLLNAQRARVLYASGFHTVADLARANIVEVEVILKNAVPFK
SARKAVDEEEEAVEERRNMRTIWVTGRKGLTEREAAALIVEEARMILQQDLVEMGVQWNP
CALLHSSTCSLTHSESEVKEHTFISQTKSSYKKLTSKNKSNTIFSDSYIKHSPNIVQDLN
KSREHTSSFNCNFQNGNQEHQTCSIFRARKRASLDINKEKPGASQNEGKTSDKKVVQTFS
QKTKKAPLNFNSEKMSRSFRSWKRRKHLKRSRDSSPLKDSGACRIHLQGQTLSNPSLCED
PFTLDEKKTEFRNSGPFAKNVSLSGKEKDNKTSFPLQIKQNCSWNITLTNDNFVEHIVTG
SQSKNVTCQATSVVSEKGRGVAVEAEKINEVLIQNGSKNQNVYMKHHDIHPINQYLRKQS
HEQTSTITKQKNIIERQMPCEAVSSYINRDSNVTINCERIKLNTEENKPSHFQALGDDIS
RTVIPSEVLPSAGAFSKSEGQHENFLNISRLQEKTGTYTTNKTKNNHVSDLGLVLCDFED
SFYLDTQSEKIIQQMATENAKLGAKDTNLAAGIMQKSLVQQNSMNSFQKECHIPFPAEQH
PLGATKIDHLDLKTVGTMKQSSDSHGVDILTPESPIFHSPILLEENGLFLKKNEVSVTDS
QLNSFLQGYQTQETVKPVILLIPQKRTPTGVEGECLPVPETSLNMSDSLLFDSFSDDYLV
KEQLPDMQMKEPLPSEVTSNHFSDSLCLQEDLIKKSNVNENQDTHQQLTCSNDESIIFSE

MDSVQMVEALDNVDIFPVQEKNHTVVSPRALELSDPVLDEHHQGDQDGGDQDERAEKSKL
TGTRQNHSFIWSGASFDLSPGLQRILDKVSSPLENEKLKSMTINFSSLNRKNTELNEEQE
VISNLETKQVQGISFSSNNEVKSKIEMLENNANHDETSSLLPRKESNIVDDNGLIPPTPI
PTSASKLTFPGILETPVNPWKTNNVLQPGESYLFGSPSDIKNHDLSPGSRNGFKDNSPIS
DTSFSLQLSQDGLQLTPASSSSESLSIIDVASDQNLFQTFIKEWRCKKRFSISLACEKIR
SLTSSKTATIGSRFKQASSPQEIPIRDDGFPIKGCDDTLVVGLAVCWGGRDAYYFSLQKE
QKHSEISASLVPPSLDPSLTLKDRMWYLQSCLRKESDKECSVVIYDFIQSYKILLLSCGI
SLEQSYEDPKVACWLLDPDSQEPTLHSIVTSFLPHELPLLEGMETSQGIQSLGLNAGSEH
SGRYRASVESILIFNSMNQLNSLLQKENLQDVFRKVEMPSQYCLALLELNGIGFSTAECE
SQKHIMQAKLDAIETQAYQLAGHSFSFTSSDDIAEVLFLELKLPPNREMKNQGSKKTLGS
TRRGIDNGRKLRLGRQFSTSKDVLNKLKALHPLPGLILEWRRITNAITKVVFPLQREKCL
NPFLGMERIYPVSQSHTATGRITFTEPNIQNVPRDFEIKMPTLVGESPPSQAVGKGLLPM
GRGKYKKGFSVNPRCQAQMEERAADRGMPFSISMRHAFVPFPGGSILAADYSQLELRILA
HLSHDRRLIQVLNTGADVFRSIAAEWKMIEPESVGDDLRQQAKQICYGIIYGMGAKSLGE
QMGIKENDAACYIDSFKSRYTGINQFMTETVKNCKRDGFVQTILGRRRYLPGIKDNNPYR
KAHAERQAINTIVQGSAADIVKIATVNIQKQLETFHSTFKSHGHREGMLQSDQTGLSRKR
KLQGMFCPIRGGFFILQLHDELLYEVAEEDVVQVAQIVKNEMESAVKLSVKLKVKVKIGA
SWGELKDFDV*
>ENSG00000132603|ENST00000254940
MRPLTEEETRVMFEKIAKYIGENLQLLVDRPDGTYCFRLHNDRVYYVSEKIMKLAANISG
DKLVSLGTCFGKFTKTHKFRLHVTALDYLAPYAKYKVWIKPGAEQSFLYGNHVLKSGLGR
ITENTSQYQGVVVYSMADIPLGFGVAAKSTQDCRKVDPMAIVVFHQADIGEYVRHEETLT
*
>ENSG00000137996|ENST00000260563
MAGPRVEVDGSIMEGGGQILRVSTALSCLLGLPLRVQKIRAGRSTPGLSSGGWKSKIKVL
TRPQHLSGLEMIRDLCDGQLEGAEIGSTEITFTPEKIKGGIHTADTKTAGSVCLLMQVSM
PCVLFAASPSELHLKGGTNAEMAPQIDYTVMVFKPIVEKFGFIFNCDIKTRGYYPKGGGE
VIVRMSPVKQLNPINLTERGCVTKIYGRAFVAGVLPFKVAKDMAAAAVRCIRKEIRDLYV
NIQPVQEPKDQAFGNGNGIIIIAETSTGCLFAGSSLGKRGVNADKVGIEAAEMLLANLRH
GGTVDEYLQDQLIVFMALANGVSRIKTGPVTLHTQTAIHFAEQIAKAKFIVKKSEDEEDA
AKDTYIIECQGIGMTNPNL*
>ENSG00000116254|ENST00000262450
MRGPVGTEEELPRLFAEEMENEDEMSEEEDGGLEAFDDFFPVEPVSLPKKKKPKKLKENK
CKGKRKKKEGSNDELSENEEDLEEKSESEGSDYSPNKKKKKKLKDKKEKKAKRKKKDEDE
DDNDDGCLKEPKSSGQLMAEWGLDDVDYLFSEEDYHTLTNYKAFSQFLRPLIAKKNPKIP
MSKMMTVLGAKWREFSANNPFKGSSAAAAAAAVAAAVETVTISPPLAVSPPQVPQPVPIR
KAKTKEGKGPGVRKKIKGSKDGKKKGKGKKTAGLKFRFGGISNKRKKGSSSEEDEREESD
FDSASIHSASVRSECSAALGKKSKRRRKKKRIDDGDGYETDHQDYCEVCQQGGEIILCDT
CPRAYHLVCLDPELEKAPEGKWSCPHCEKEGIQWEPKDDDDEEEEGGCEEEEDDHMEFCR
VCKDGGELLCCDACPSSYHLHCLNPPLPEIPNGEWLCPRCTCPPLKGKVQRILHWRWTEP
PAPFMVGLPGPDVEPSLPPPKPLEGIPEREFFVKWAGLSYWHCSWVKELQLELYHTVMYR
NYQRKNDMDEPPPFDYGSGDEDGKSEKRKNKDPLYAKMEERFYRYGIKPEWMMIHRILNH
SFDKKGDVHYLIKWKDLPYDQCTWEIDDIDIPYYDNLKQAYWGHRELMLGEDTRLPKRLL
KKGKKLRDDKQEKPPDTPIVDPTVKFDKQPWYIDSTGGTLHPYQLEGLNWLRFSWAQGTD

TILADEMGLGKTVQTIVFLYSLYKEGHSKGPYLVSAPLSTIINWEREFEMWAPDFYVVTY
TGDKESRSVIRENEFSFEDNAIRSGKKVFRMKKEVQIKFHVLLTSYELITIDQAILGSIE
WACLVVDEAHRLKNNQSKFFRVLNSYKIDYKLLLTGTPLQNNLEELFHLLNFLTPERFNN
LEGFLEEFADISKEDQIKKLHDLLGPHMLRRLKADVFKNMPAKTELIVRVELSQMQKKYY
KFILTRNFEALNSKGGGNQVSLLNIMMDLKKCCNHPYLFPVAAVEAPVLPNGSYDGSSLV
KSSGKLMLLQKMLKKLRDEGHRVLIFSQMTKMLDLLEDFLEYEGYKYERIDGGITGGLRQ
EAIDRFNAPGAQQFCFLLSTRAGGLGINLATADTVIIYDSDWNPHNDIQAFSRAHRIGQN
KKVMIYRFVTRASVEERITQVAKRKMMLTHLVVRPGLGSKSGSMTKQELDDILKFGTEEL
FKDDVEGMMSQGQRPVTPIPDVQSSKGGNLAASAKKKHGSTPPGDNKDVEDSSVIHYDDA
AISKLLDRNQDATDDTELQNMNEYLSSFKVAQYVVREEDGVEEVEREIIKQEENVDPDYW
EKLLRHHYEQQQEDLARNLGKGKRIRKQVNYNDASQEDQEWQDELSDNQSEYSIGSEDED
EDFEERPEGQSGRRQSRRQLKSDRDKPLPPLLARVGGNIEVLGFNARQRKAFLNAIMRWG
MPPQDAFNSHWLVRDLRGKSEKEFRAYVSLFMRHLCEPGADGAETFADGVPREGLSRQHV
LTRIGVMSLVRKKVQEFEHVNGKYSTPDLIPEGPEGKKSGEVISSDPNTPVPASPAHLLP
APLGLPDKMEAQLGYMDEKDPGAQKPRQPLEVQALPAALDRVESEDKHESPASKERAREE
RPEETEKAPPSPEQLPREEVLPEKEKILDKLELSLIHSRGDSSELRPDDTKAEEKEPIET
QQNGDKEEDDEGKKEDKKGKFKFMFNIADGGFTELHTLWQNEERAAVSSGKIYDIWHRRH
DYWLLAGIVTHGYARWQDIQNDPRYMILNEPFKSEVHKGNYLEMKNKFLARRFKLLEQAL
VIEEQLRRAAYLNMTQDPNHPAMALNARLAEVECLAESHQHLSKESLAGNKPANAVLHKV
LNQLEELLSDMKADVTRLPSMLSRIPPVAARLQMSERSILSRLTNRAGDPTIQQGAFGSS
QMYSNNFGPNFRGPGPGGIVNYNQMPLGPYVTDI*
>ENSG00000134574|ENST00000256996
MAPKKRPETQKTSEIVLRPRNKRSRSPLELEPEAKKLCAKGSGPSRRCDSDCLWVGLAGP
QILPPCRSIVRTLHQHKLGRASWPSVQQGLQQSFLHTLDSYRILQKAAPFDRRATSLAWH
PTHPSTVAVGSKGGDIMLWNFGIKDKPTFIKGIGAGGSITGLKFNPLNTNQFYASSMEGT
TRLQDFKGNILRVFASSDTINIWFCSLDVSASSRMVVTGDNVGNVILLNMDGKELWNLRM
HKKKVTHVALNPCCDWFLATASVDQTVKIWDLRQVRGKASFLYSLPHRHPVNAACFSPDG
ARLLTTDQKSEIRVYSASQWDCPLGLIPHPHRHFQHLTPIKAAWHPRYNLIVVGRYPDPN
FKSCTPYELRTIDVFDGNSGKMMCQLYDPESSGISSLNEFNPMGDTLASAMGYHILIWSQ
EEARTRK*
>ENSG00000115946|ENST00000263657
MESEMETQSARAEEGFTQVTRKGGRRAKKRQAEQLSAAGEGGDAGRMDTEEARPAKRPVF
PPLCGDGLLSGKEETRKIPVPANRYTPLKENWMKIFTPIVEHLGLQIRFNLKSRNVEIRT
CKETKDVSALTKAADFVKAFILGFQVEDALALIRLDDLFLESFEITDVKPLKGDHLSRAI
GRIAGKGGKTKFTIENVTRTRIVLADVKVHILGSFQNIKMARTAICNLILGNPPSKVYGN
IRAVASRSADRF*
>ENSG00000186868|ENST00000262410
MAEPRQEFEVMEDHAGTYGLGDRKDQGGYTMHQDQEGDTDAGLKESPLQTPTEDGSEEPG
SETSDAKSTPTAEDVTAPLVDEGAPGKQAAAQPHTEIPEGTTAEEAGIGDTPSLEDEAAG
HVTQEPESGKVVQEGFLREPGPPGLSHQLMSGMPGAPLLPEGPREATRQPSGTGPEDTEG
GRHAPELLKHQLLGDLHQEGPPLKGAGGKERPGSKEEVDEDRDVDESSPQDSPPSKASPA
QDGRPPQTAAREATSIPGFPAEGAIPLPVDFLSKVSTEIPASEPDGPSVGRAKGQDAPLE
FTFHVEITPNVQKEQAHSEEHLGRAAFPGAPGEGPEARGPSLGEDTKEADLPEPSEKQPA
AAPRGKPVSRVPQLKARMVSKSKDGTGSDDKKAKTSTRSSAKTLKNRPCLSPKHPTPGSS

DPLIQPSSPAVCPEPPSSPKYVSSVTSRTGSSGAKEMKLKGADGKTKIATPRGAAPPGQK
GQANATRIPAKTPPAPKTPPSSGEPPKSGDRSGYSSPGSPGTPGSRSRTPSLPTPPTREP
KKVAVVRTPPKSPSSAKSRLQTAPVPMPDLKNVKSKIGSTENLKHQPGGGKVQIINKKLD
LSNVQSKCGSKDNIKHVPGGGSVQIVYKPVDLSKVTSKCGSLGNIHHKPGGGQVEVKSEK
LDFKDRVQSKIGSLDNITHVPGGGNKKIETHKLTFRENAKAKTDHGAEIVYKSPVVSGDT
SPRHLSNVSSTGSIDMVDSPQLATLADEVSASLAKQGL*
>ENSG00000130699|ENST00000252996
MAAGSDLLDEVFFNSEVDEKVVSDLVGSLESQLAASAAHHHHLAPRTPEVRAAAAGALGN
HVVSGSPAGAAGAGPAAPAEGAPGAAPEPPPAGRARPGGGGPQRPGPPSPRRPLVPAGPA
PPAAKLRPPPEGSAGSCAPVPAAAAVAAGPEPAPAGPAKPAGPAALAARAGPGPGPGPGP
GPGPGPGKPAGPGAAQTLNGSAALLNSHHAAAPAVSLVNNGPAALLPLPKPAAPGTVIQT
PPFVGAAAPPAPAAPSPPAAPAPAAPAAAPPPPPPAPATLARPPGHPAGPPTAAPAVPPP
AAAQNGGSAGAAPAPAPAAGGPAGVSGQPGPGAAAAAPAPGVKAESPKRVVQAAPPAAQT
LAASGPASTAASMVIGPTMQGALPSPAAVPPPAPGTPTGLPKGAAGAVTQSLSRTPTATT
SGIRATLTPTVLAPRLPQPPQNPTNIQNFQLPPGMVLVRSENGQLLMIPQQALAQMQAQA
HAQPQTTMAPRPATPTSAPPVQISTVQAPGTPIIARQVTPTTIIKQVSQAQTTVQPSATL
QRSPGVQPQLVLGGAAQTASLGTATAVQTGTPQRTVPGATTTSSAATETMENVKKCKNFL
STLIKLASSGKQSTETAANVKELVQNLLDGKIEAEDFTSRLYRELNSSPQPYLVPFLKRS
LPALRQLTPDSAAFIQQSQQQPPPPTSQATTALTAVVLSSSVQRTAGKTAATVTSALQPP
VLSLTQPTQVGVGKQGQPTPLVIQQPPKPGALIRPPQVTLTQTPMVALRQPHNRIMLTTP
QQIQLNPLQPVPVVKPAVLPGTKALSAVSAQAAAAQKNKLKEPGGGSFRDDDDINDVASM
AGVNLSEESARILATNSELVGTLTRSCKDETFLLQAPLQRRILEIGKKHGITELHPDVVS
YVSHATQQRLQNLVEKISETAQQKNFSYKDDDRYEQASDVRAQLKFFEQLDQIEKQRKDE
QEREILMRAAKSRSRQEDPEQLRLKQKAKEMQQQELAQMRQRDANLTALAAIGPRKKRKV
DCPGPGSGAEGSGPGSVVPGSSGVGTPRQFTRQRITRVNLRDLIFCLENERETSHSLLLY
KAFLK*
>ENSG00000005156|ENST00000262327
MSLAFKIFFPQTLRALSRKELCLFRKHHWRDVRQFSQWSETDLLHGHPLFLRRKPVLSFQ
GSHLRSRATYLVFLPGLHVGLCSGPCEMAEQRFCVDYAKRGTAGCKKCKEKIVKGVCRIG
KVVPNPFSESGGDMKEWYHIKCMFEKLERARATTKKIEDLTELEGWEELEDNEKEQITQH
IADLSSKAAGTPKKKAVVQAKLTTTGQVTSPVKGASFVTSTNPRKFSGFSAKPNNSGEAP
SSPTPKRSLSSSKCDPRHKDCLLREFRKLCAMVADNPSYNTKTQIIQDFLRKGSAGDGFH
GDVYLTVKLLLPGVIKTVYNLNDKQIVKLFSRIFNCNPDDMARDLEQGDVSETIRVFFEQ
SKSFPPAAKSLLTIQEVDEFLLRLSKLTKEDEQQQALQDIASRCTANDLKCIIRLIKHDL
KMNSGAKHVLDALDPNAYEAFKASRNLQDVVERVLHNAQEVEKEPGQRRALSVQASLMTP
VQPMLAEACKSVEYAMKKCPNGMFSEIKYDGERVQVHKNGDHFSYFSRSLKPVLPHKVAH
FKDYIPQAFPGGHSMILDSEVLLIDNKTGKPLPFGTLGVHKKAAFQDANVCLFVFDCIYF
NDVSLMDRPLCERRKFLHDNMVEIPNRIMFSEMKRVTKALDLADMITRVIQEGLEGLVLK
DVKGTYEPGKRHWLKVKKDYLNEGAMADTADLVVLGAFYGQGSKGGMMSIFLMGCYDPGS
QKWCTVTKCAGGHDDATLARLQNELDMVKISKDPSKIPSWLKVNKIYYPDFIVPDPKKAA
VWEITGAEFSKSEAHTADGISIRFPRCTRIRDDKDWKSATNLPQLKELYQLSKEKADFTV
VAGDEGSSTTGGSSEENKGPSGSAVSRKAPSKPSASTKKAEGKLSNSNSKDGNMQTAKPS
AMKVGEKLATKSSPVKVGEKRKAADETLCQTKRRPASEQRGRTVPAGRR*
>ENSG00000103319|ENST00000263026

MADEDLIFRLEGVDGGQSPRAGHDGDSDGDSDDEEGYFICPITDDPSSNQNVNSKVNKYY
SNLTKSERYSSSGSPANSFHFKEAWKHAIQKAKHMPDPWAEFHLEDIATERATRHRYNAV
TGEWLDDEVLIKMASQPFGRGAMRECFRTKKLSNFLHAQQWKGASNYVAKRYIEPVDRDV
YFEDVRLQMEAKLWGEEYNRHKPPKQVDIMQMCIIELKDRPGKPLFHLEHYIEGKYIKYN
SNSGFVRDDNIRLTPQAFSHFTFERSGHQLIVVDIQGVGDLYTDPQIHTETGTDFGDGNL
GVRGMALFFYSHACNRICESMGLAPFDLSPRERDAVNQNTKLLQSAKTILRGTEEKCGSP
QVRTLSGSRPPLLRPLSENSGDENMSDVTFDSLPSSPSSATPHSQKLDHLHWPVFSDLDN
MASRDHDHLDNHRESENSGDSGYPSEKRGELDDPEPREHGHSYSNRKYESDEDSLGSSGR
VCVEKWNLLNSSRLHLPRASAVALEVQRLNALDLEKKIGKSILGKVHLAMVRYHEGGRFC
EKGEEWDQESAVFHLEHAANLGELEAIVGLGLMYSQLPHHILADVSLKETEENKTKGFDY
LLKAAEAGDRQSMILVARAFDSGQNLSPDRCQDWLEALHWYNTALEMTDCDEGGEYDGMQ
DEPRYMMLAREAEMLFTGGYGLEKDPQRSGDLYTQAAEAAMEAMKGRLANQYYQKAEEAW
AQMEE*
>ENSG00000101665|ENST00000262158
MFRTKRSALVRRLWRSRAPGGEDEEEGAGGGGGGGELRGEGATDSRAHGAGGGGPGRAGC
CLGKAVRGAKGHHHPHPPAAGAGAAGGAEADLKALTHSVLKKLKERQLELLLQAVESRGG
TRTACLLLPGRLDCRLGPGAPAGAQPAQPPSSYSLPLLLCKVFRWPDLRHSSEVKRLCCC
ESYGKINPELVCCNPHHLSRLCELESPPPPYSRYPMDFLKPTADCPDAVPSSAETGGTNY
LAPGGLSDSQLLLEPGDRSHWCVVAYWEEKTRVGRLYCVQEPSLDIFYDLPQGNGFCLGQ
LNSDNKSQLVQKVRSKIGCGIQLTREVDGVWVYNRSSYPIFIKSATLDNPDSRTLLVHKV
FPGFSIKAFDYEKAYSLQRPNDHEFMQQPWTGFTVQISFVKGWGQCYTRQFISSCPCWLE
VIFNSR*
>ENSG00000130382|ENST00000252674
MDNQCTVQVRLELGHRAQLRKKPTTEGFTHDWMVFVRGPEQCDIQHFVEKVVFWLHDSFP
KPRRVCKEPPYKVEESGYAGFIMPIEVHFKNKEEPRKVCFTYDLFLNLEGNPPVNHLRCE
KLTFNNPTTEFRYKLLRAGGVMVMPEGADTVSRPSPDYPMLPTIPLSAFSDPKKTKPSHG
SKDANKESSKTSKPHKVTKEHRERPRKDSESKSSSKELEREQAKSSKDTSRKLGEGRLPK
EEKAPPPKAAFKEPKMALKETKLESTSPKGGPPPPPPPPPRASSKRPATADSPKPSAKKQ
KKSSSKGSRSAPGTSPRTSSSSSFSDKKPAKDKSSTRGEKVKAESEPREAKKALEVEESN
SEDEASFKSESAQSSPSNSSSSSDSSSDSDFEPSQNHSQGPLRSMVEDLQSEESDEDDSS
SGEEAAGKTNPGRDSRLSFSDSESDNSADSSLPSREPPPPQKPPPPNSKVSGRRSPESCS
KPEKILKKGTYDKAYTDELVELHRRLMALRERNVLQQIVNLIEETGHFNVTNTTFDFDLF
SLDETTVRKLQSCLEAVAT*
>ENSG00000131470|ENST00000253789
MSKGRAEAAAGAAGILLRYLQEQNRPYSSQDVFGNLQREHGLGKAVVVKTLEQLAQQGKI
KEKMYGKQKIYFADQDQFDMVSDADLQVLDGKIVALTAKVQSLQQSCRYMEAEMQKEIQE
LKKECAGYRERLKNIKAATNHVTPEEKEQVYRERQKYCKEWRKRKRMATELSDAILEGYP
KSKKQFFEEVGIETDEDYNVTLPDP*
>ENSG00000135363|ENST00000257818
MEGSAVTVLERGGASSPAERRSKRRRRSGGDGGGGGGARAPEGVRAPAAGQPRATKGAPP
PPGTPPPSPMSSAIERKSLDPSEEPVDEVLQIPPSLLTCGGCQQNIGDRYFLKAIDQYWH
EDCLSCDLCGCRLGEVGRRLYYKLGRKLCRRDYLRLFGQDGLCASCDKRIRAYEMTMRVK
DKVYHLECFKCAACQKHFCVGDRYLLINSDIVCEQDIYEWTKINGMI*
>ENSG00000135373|ENST00000257831

MILEGGGVMNLNPGNNLLHQPPAWTDSYSTCNVSSGFFGGQWHEIHPQYWTKYQVWEWLQ
HLLDTNQLDANCIPFQEFDINGEHLCSMSLQEFTRAAGTAGQLLYSNLQHLKWNGQCSSD
LFQSTHNVIVKTEQTEPSIMNTWKDENYLYDTNYGSTVDLLDSKTFCRAQISMTTTSHLP
VAESPDMKKEQDPPAKCHTKKHNPRGTHLWEFIRDILLNPDKNPGLIKWEDRSEGVFRFL
KSEAVAQLWGKKKNNSSMTYEKLSRAMRYYYKREILERVDGRRLVYKFGKNARGWRENEN
*
>ENSG00000005007|ENST00000262803
MSVEAYGPSSQTLTFLDTEEAELLGADTQGSEFEFTDFTLPSQTQTPPGGPGGPGGGGAG
GPGGAGAGAAAGQLDAQVGPEGILQNGAVDDSVAKTSQLLAELNFEEDEEDTYYTKDLPI
HACSYCGIHDPACVVYCNTSKKWFCNGRGNTSGSHIVNHLVRAKCKEVTLHKDGPLGETV
LECYNCGCRNVFLLGFIPAKADSVVVLLCRQPCASQSSLKDINWDSSQWQPLIQDRCFLS
WLVKIPSEQEQLRARQITAQQINKLEELWKENPSATLEDLEKPGVDEEPQHVLLRYEDAY
QYQNIFGPLVKLEADYDKKLKESQTQDNITVRWDLGLNKKRIAYFTLPKTDSDMRLMQGD
EICLRYKGDLAPLWKGIGHVIKVPDNYGDEIAIELRSSVGAPVEVTHNFQVDFVWKSTSF
DRMQSALKTFAVDETSVSGYIYHKLLGHEVEDVIIKCQLPKRFTAQGLPDLNHSQVYAVK
TVLQRPLSLIQGPPGTGKTVTSATIVYHLARQGNGPVLVCAPSNIAVDQLTEKIHQTGLK
VVRLCAKSREAIDSPVSFLALHNQIRNMDSMPELQKLQQLKDETGELSSADEKRYRALKR
TAERELLMNADVICCTCVGAGDPRLAKMQFRSILIDESTQATEPECMVPVVLGAKQLILV
GDHCQLGPVVMCKKAAKAGLSQSLFERLVVLGIRPIRLQVQYRMHPALSAFPSNIFYEGS
LQNGVTAADRVKKGFDFQWPQPDKPMFFYVTQGQEEIASSGTSYLNRTEAANVEKITTKL
LKAGAKPDQIGIITPYEGQRSYLVQYMQFSGSLHTKLYQEVEIASVDAFQGREKDFIILS
CVRANEHQGIGFLNDPRRLNVALTRARYGVIIVGNPKALSKQPLWNHLLNYYKEQKVLVE
GPLNNLRESLMQFSKPRKLVNTINPGARFMTTAMYDAREAIIPGSVYDRSSQGRPSSMYF
QTHDQIGMISAGPSHVAAMNIPIPFNLVMPPMPPPGYFGQANGPAAGRGTPKGKTGRGGR
QKNRFGLPGPSQTNLPNSQASQDVASQPFSQGALTQGYISMSQPSQMSQPGLSQPELSQD
SYLGDEFKSQIDVALSQDSTYQGERAYQHGGVTGLSQY*
>ENSG00000131013|ENST00000253329
MAVLLETTLGDVVIDLYTEERPRACLNFLKLCKIKYYNYCLIHNVQRDFIIQTGDPTGTG
RGGESIFGQLYGDQASFFEAEKVPRIKHKKKGTVSMVNNGSDQHGSQFLITTGENLDYLD
GVHTVFGEVTEGMDIIKKINETFVDKDFVPYQDIRINHTVILDDPFDDPPDLLIPDRSPE
PTREQLDSGRIGADEEIDDFKGRSAEEVEEIKAEKEAKTQAILLEMVGDLPDADIKPPEN
VLFVCKLNPVTTDEDLEIIFSRFGPIRSCEVIRDWKTGESLCYAFIEFEKEEDCEKAFFK
MDNVLIDDRRIHVDFSQSVAKVKWKGKGGKYTKSDFKEYEKEQDKPPNLVLKDKVKPKQD
TKYDLILDEQAEDSKSSHSHTSKKHKKKTHHCSEEKEDEDYMPIKNTNQDIYREMGFGHY
EEEESCWEKQKSEKRDRTQNRSRSRSRERDGHYSNSHKSKYQTDLYERERSKKRDRSRSP
KKSKDKEKSKYR*
>ENSG00000186812|ENST00000261333
MAVESGVISTLIPQDPPEQELILVKVEDNFSWDEKFKQNGSTQSCQELFRQQFRKFCYQE
TPGPREALSRLQELCYQWLMPELHTKEQILELLVLEQFLSILPEELQIWVQQHNPESGEE
AVTLLEDLEREFDDPGQQVPASPQGPAVPWKDLTCLRASQESTDIHLQPLKTQLKSWKPC
LSPKSDCENSETATKEGISEEKSQGLPQEPSFRGIKLSRPPKASSAIRWECVSPGSFPGD
IIAAEATHSTISCFAINTLPATILPSKNVNRKYFS*
>ENSG00000135374|ENST00000257832
MLDSVTHSTFLPNASFCDPLMSWTDLFSNEEYYPAFEHQTACDSYWTSVHPEYWTKRHVW

EWLQFCCDQYKLDTNCISFCNFNISGLQLCSMTQEEFVEAAGLCGEYLYFILQNIRTQGY
SFFNDAEESKATIKDYADSNCLKTSGIKSQDCHSHSRTSLQSSHLWEFVRDLLLSPEENC
GILEWEDREQGIFRVVKSEALAKMWGQRKKNDRMTYEKLSRALRYYYKTGILERVDRRLV
YKFGKNAHGWQEDKL*
>ENSG00000106511|ENST00000262041
MEHPLFGCLRSPHATAQGLHPFSQSSLALHGRSDHMSYPELSTSSSSCIIAGYPNEEGMF
ASQHHRGHHHHHHHHHHHHHHQQQQHQALQTNWHLPQMSSPPSAARHSLCLQPDSGGPPEL
GSSPPVLCSNSSSLGSSTPTGAACAPGDYGRQALSPAEAEKRSGGKRKSDSSDSQEGNYK
SEVNSKPRKERTAFTKEQIRELEAEFAHHNYLTRLRRYEIAVNLDLTERQVKVWFQNRRM
KWKRVKGGQQGAAAREKELVNVKKGTLLPSELSGIGAATLQQTGDSIANEDSHDSDHSSE
HAHL*
>ENSG00000136718|ENST00000259239
MLRREARLRREYLYRKAREEAQRSAQERKERLRRALEENRLIPTELRREALALQGSLEFD
DAGGEGVTSHVDDEYRWAGVEDPKVMITTSRDPSSRLKMFAKELKLVFPGAQRMNRGRHE
VGALVRACKANGVTDLLVVHEHRGTPVGLIVSHLPFGPTAYFTLCNVVMRHDIPDLGTMS
EAKPHLITHGFSSRLGKRVSDILRYLFPVPKDDSHRVITFANQDDYISFRHHVYKKTDHR
NVELTEVGPRFELKLYMIRLGTLEQEATADVEWRWHPYTNTARKRVFLSTE*
>ENSG00000115750|ENST00000263663
MDLEEAEEFKERCTQCAAVSWGLTDEGKYYCTSCHNVTERYQEVTNTDLIPNTQIKALNR
GLKKKNNTEKGWDWYVCEGFQYILYQQAEALKNLGVGPELKNDVLHNFWKRYLQKSKQAY
CKNPVYTTGRKPTVLEDNLSHSDWASEPELLSDVSCPPFLESGAESQSDIHTRKPFPVSK
ASQSETSVCSGSLDGVEYSQRKEKGIVKMTMPQTLAFCYLSLLWQREAITLSDLLRFVEE
DHIPYINAFQHFPEQMKLYGRDRGIFGIESWPDYEDIYKKTVEVGTFLDLPRFPDITEDC
YLHPNILCMKYLMEVNLPDEMHSLTCHVVKMTGMGEVDFLTFDPIAKMAKTVKYDVQAVA
IIVVVLKLLFLLDDSFEWSLSNLAEKHNEKNKKDKPWFDFRKWYQIMKKAFDEKKQKWEE
ARAKYLWKSEKPLYYSFVDKPVAYKKREMVVNLQKQFSTLVESTATAGKKSPSSFQFNWT
EEDTDRTCFHGHSLQGVLKEKGQSLLTKNSLYWLSTQKFCRCYCTHVTTYEESNYSLSYQ
FILNLFSFLLRIKTSLLHEEVSLVEKKLFEKKYSVKRKKSRSKKVRRH*
>ENSG00000138738|ENST00000264808
MLGMYVPDRFSLKSSRVQDGMGLYTARRVRKGEKFGPFAGEKRMPEDLDENMDYRLMWEV
RGSKGEVLYILDATNPRHSNWLRFVHEAPSQEQKNLAAIQEGENIFYLAVEDIETDTELL
IGYLDSDMEAEEEEQQIMTVIKEGEVENSRRQSTAGRKDRLGCKEDYACPQCESSFTSED
ILAEHLQTLHQKPTEEKEFKCKNCGKKFPVKQALQRHVLQCTAKSSLKESSRSFQCSVCN
SSFSSASSFEQHQETCRGDARFVCKADSCGKRLKSKDALKRHQENVHTGDPKKKLICSVC
NKKCSSASSLQEHRKIHEIFDCQECMKKFISANQLKRHMITHSEKRPYNCEICNKSFKRL
DQVGAHKVIHSEDKPYKCKLCGKGFAHRNVYKNHKKTHSEERPFQCEECKALFRTPFSLQ
RHLLIHNSERTFKCHHCDATFKRKDTLNVHVQVVHERHKKYRCELCNKAFVTPSVLRSHK
KTHTGEKEKICPYCGQKFASSGTLRVHIRSHTGERPYQCPYCEKGFSKNDGLKMHIRTHT
REKPYKCSECSKAFSQKRGLDEHKRTHTGEKPFQCDVCDLAFSLKKMLIRHKMTHNPNRP
LAECQFCHKKFTRNDYLKVHMDNIHGVADS*
>ENSG00000135679|ENST00000258149
MVRSRQMCNTNMSVPTDGAVTTSQIPASEQETLVRPKPLLLKLLKSVGAQKDTYTMKEVL
FYLGQYIMTKRLYDEKQQHIVYCSNDLLGDLFGVPSFSVKEHRKIYTMIYRNLVVVNQQE
SSDSGTSVSENRCHLEGGSDQKDLVQELQEEKPSSSHLVSRPSTSSRRRAISETEENSDE

LSGERQRKRHKSDSISLSFDESLALCVIREICCERSSSSESTGTPSNPDLDAGVSEHSGD
WLDQDSVSDQFSVEFEVESLDSEDYSLSEEGQELSDEDDEVYQVTVYQAGESDTDSFEED
PEISLADYWKCTSCNEMNPPLPSHCNRCWALRENWLPEDKGKDKGEISEKAKLENSTQAE
EGFDVPDCKKTIVNDSRESCVEENDDKITQASQSQESEDYSQPSTSSSIIYSSQEDVKEF
EREETQDKEESVESSLPLNAIEPCVICQGRPKNGCIVHGKTGHLMACFTCAKKLKKRNKP
CPVCRQPIQMIVLTYFP*
>ENSG00000126254|ENST00000262633
MAGAGPAPGLPGAGGPVVPGPGAGIPGKSGEERLKEMEAEMALFEQEVLGAPVPGIPTAV
PAVPTVPTVPTVEAMQVPAAPVIRPIIATNTYQQVQQTLEARAAAAATVVPPMVGGPPFV
GPVGFGPGDRSHLDSPEAREAMFLRRAAVAPQRAPILRPAFVPHVLQRADSALSSAAAGP
RPMALRPPHQALVGPPLPGPPGPPMMLPPMARAPGPPLGSMAALRPPLEEPAAPRELGLG
LGLGLKEKEEAVVAAAAGLEEASAAVAVGAGGAPAGPAVIGPSLPLALAMPLPEPEPLPL
PLEVVRGLLPPLRIPELLSLRPRPRPPRPEPPPGLMALEVPEPLGEDKKKGKPEKLKRCI
RTAAGSSWEDPSLLEWDADDFRIFCGDLGNEVNDDILARAFSRFPSFLKAKVIRDKRTGK
TKGYGFVSFKDPSDYVRAMREMNGKYVGSRPIKLRKSMWKDRNLDVVRKKQKEKKKLGLR
*
>ENSG00000073050|ENST00000262887
MPEIRLRHVVSCSSQDSTHCAENLLKADTYRKWRAAKAGEKTISVVLQLEKEEQIHSVDI
GNDGSAFVEVLVGSSAGGAGEQDYEVLLVTSSFMSPSESRSGSNPNRVRMFGPDKLVRAA
AEKRWDRVKIVCSQPYSKDSPFGLSFVRFHSPPDKDEAEAPSQKVTVTKLGQFRVKEEDE
SANSLRPGALFFSRINKTSPVTASDPAGPSYAAATLQASSAASSASPVSRAIGSTSKPQE
SPKGKRKLDLNQEEKKTPSKPPAQLSPSVPKRPKLPAPTRTPATAPVPARAQGAVTGKPR
GEGTEPRRPRAGPEELGKILQGVVVVLSGFQNPFRSELRDKALELGAKYRPDWTRDSTHL
ICAFANTPKYSQVLGLGGRIVRKEWVLDCHRMRRRLPSQRYLMAGPGSSSEEDEASHSGG
SGDEAPKLPQKQPQTKTKPTQAAGPSSPQKPPTPEETKAASPVLQEDIDIEGVQSEGQDN
GAEDSGDTEDELRRVAEQKEHRLPPGQEENGEDPYAGSTDENTDSEEHQEPPDLPVPELP
DFFQGKHFFLYGEFPGDERRKLIRYVTAFNGELEDYMSDRVQFVITAQEWDPSFEEALMD
NPSLAFVRPRWIYSCNEKQKLLPHQLYGVVPQA*
>ENSG00000137270|ENST00000259803
MEPDDFDSEDKEILSWDINDVKLPQNVKKTDWFQEWPDSYAKHIYSSEDKNAQRHLSSWA
MRNTNNHNSRILKKSCLGVVVCGRDCLAEEGRKIYLRPAICDKARQKQQRKRCPNCDGPL
KLIPCRGHGGFPVTNFWRHDGRFIFFQSKGEHDHPKPETKLEAEARRAMKKVNTAPSSVS
LSLKGSTETRSLPGETQSQGSLPLTWSFQEGVQLPGSYSGHLIANTPQQNSLNDCFSFSK
SYGLGGITDLTDQTSTVDPMKLYEKRKLSSSRTYSSGDLLPPSASGVYSDHGDLQAWSKN
AALGRNHLADNCYSNYPFPLTSWPCSFSPSQNSSEPFYQQLPLEPPAAKTGCPPLWPNPA
GNLYEEKVHVDFNSYVQSPAYHSPQEDPFLFTYASHPHQQYSLPSKSSKWDFEEEMTYLG
LDHCNNDMLLNLCPLR*
>ENSG00000089116|ENST00000261731
MMVHCAGCERPILDRFLLNVLDRAWHIKCVQCCECKTNLSEKCFSREGKLYCKNDFFRRF
GTKCAGCAQGISPSDLVRKARSKVFHLNCFTCMVCNKQLSTGEELYVIDENKFVCKDDYL
SSSSLKEGSLNSVSSCTDRSLSPDLQDALQDDPKETDNSTSSDKETANNENEEQNSGTKR
RGPRTTIKAKQLETLKAAFAATPKPTRHIREQLAQETGLNMRVIQVWFQNRRSKERRMKQ
LSALGARRHAFFRSPRRMRPLGGRLDESEMLGSTPYTYYGDYQGDYYAPGSNYDFFAHGP
PSQAQSPADSSFLAASGPGSTPLGALEPPLAGPHAADNPRFTDMISHPDTPSPEPGLPGT

LHPMPGEVFSGGPSPPFPMSGTSGYSGPLSHPNPELNEAAVW*
>ENSG00000100320|ENST00000262829
MEKKKMVTQGNQEPTTTPDAMVQPFTTIPFPPPPQNGIPTEYGVPHTQDYAGQTGEHNLT
LYGSTQAHGEQSSNSPSTQNGSLTTEGGAQTDGQQSQTQSSENSESKSTPKRLHVSNIPF
RFRDPDLRQMFGGFGFVTFENSADADRAREKLHGTVVEGRKIEVNNATARVMTNKKMVTP
YANGWKLSPVVGAVYGPELYAASSFQADVSLGNDAAVPLSGRGGINTYIPLIIPGFPYPT
AATTAAAFRGAHLRGRGRTVYGAVRAVPPTAIPAYPGVVYQDGFYGADLYGGYAAYRYAQ
PATATAATAAAAAAAAYSDGYGRVYTADPYHALAPAASYGVGAVASLYRGGYSRFAPY*
>ENSG00000130711|ENST00000253008
MMGSVLPAEALVLKTGLKAPGLALAEVITSDILHSFLYGRWRNVLGEQLFEDKSHHASPK
TAFTAEVLAQSFSGEVQKLSSLVLPAEVIIAQSSIPGEGLGIFSKTWIKAGTEMGPFTGR
VIAPEHVDICKNNNLMWEVFNEDGTVRYFIDASQEDHRSWMTYIKCARNEQEQNLEVVQI
GTSIFYKAIEMIPPDQELLVWYGNSHNTFLGIPGVPGLEEDQKKNKHEDFHPADSAAGPA
GRMRCVICHRGFNSRSNLRSHMRIHTLDKPFVCRFCNRRFSQSSTLRNHVRLHTGERPYK
CQVCQSAYSQLAGLRAHQKSARHRPPSTALQAHSPALPAPHAHAPALAAAAAAAAAAAAH
HLPAMVL*
>ENSG00000105229|ENST00000262971
MAAELVEAKNMVMSFRVSDLQMLLGFVGRSKSGLKHELVTRALQLVQFDCSPELFKKIKE
LYETRYAKKNSEPAPQPHRPLDPLTMHSTYDRAGAVPRTPLAGPNIDYPVLYGKYLNGLG
RLPAKTLKPEVRLVKLPFFNMLDELLKPTELVPQNNEKLQESPCIFALTPRQVELIRNSR
ELQPGVKAVQVVLRICYSDTSCPQEDQYPPNIAVKVNHSYCSVPGYYPSNKPGVEPKRPC
RPINLTHLMYLSSATNRITVTWGNYGKSYSVALYLVRQLTSSELLQRLKTIGVKHPELCK
ALVKEKLRLDPDSEIATTGVRVSLICPLVKMRLSVPCRAETCAHLQCFDAVFYLQMNEKK
PTWMCPVCDKPAPYDQLIIDGLLSKILSECEDADEIEYLVDGSWCPIRAEKERSCSPQGA
ILVLGPSDANGLLPAPSVNGSGALGSTGGGGPVGSMENGKPGADVVDLTLDSSSSSEDEE
EEEEEEEDEDEEGPRPKRRCPFQKGLVPAC*
>ENSG00000133250|ENST00000255616
MEEKPSGPIPDMLATAEPSSSETDKEVLSPAVPAAAPSSSMSEEPGPEQAATPPVWERGG
AGGMQQGSSPAPDSCQPGPGPSPGLTSIVSGTSEDLRPPRRRPPPGKQIPCSSPGCCLSF
PSVRDLAQHLRTHCPPTQSLEGKLFRCSALSCTETFPSMQELVAHSKLHYKPNRYFKCEN
CLLRFRTHRSLFKHLHVCAEHAQSPAPPPPPALDREPPAPERPPEVDPASAPGLPFPLLE
PFTTPAPAPTGPFLPYLNPAPFGLSPPRLRPFLAAAPGPPASSAAVWKKSQGAGSSPRRP
QGGSDAPSGACR*
>ENSG00000181381|ENST00000260184
MGSKDHAVFFREMTQLILNEMPKAGYSSILNDFVESNFFVIDGDSLLVTCLGVKSFKWGQ
NLHFFYLVECYLVDLLSNGGQFTIVFFKDAEYAYFDFPELLSLRTALILHLQHNTNIDVQ
TEFSGCLSQDWKLFLEQHYPYFLIVSEEGLSDLQTYLFNFLIIHSWGMKVNVVLSSGHES
DTLRFYAYTMESTDRNQTFSKENETVIQSAYKSLIQHLEEIRVLVLATHFEHLKWNDMME
EAYQTLFLLQHLWSEGSDIQRVLCVTSCSLSLRMYHRVLVHSNCLSLQEVEDFCRLRCLC
VAFQLHLPLSQRACSRVITCSWIRNSDSFLKMNKWCEYFILSNLNVFGCWNLNLNHVSDL
YDEQLLKNIAFYYEFESTQEPHLNLGDSIRRDYEDLWNVVSHLVKEFNVGKSFPLRTTRR
HFLRQEKSVIQEISLEKMPSVGFIPMTSAVIDEFVGDMMKDLPILKSDDPVVPSLFKQKT
SDELLHWHAQRLLSDDYDRIKCHVDEQSRDPHVLDFLKKIQDYQQFYGKSLESISTKVIV
TQTTRPKEDSSGASGEILQNTKPHQITKKSKKKSFLKEDQNKAQQNDDLLFSIEEEMKNN

LHSGIRKLEDYLTSCASNSVKFGVEMLGLIACFKAWKKHCRGEGKISKDLSIAVQMMKRI
HSLLERYPEILEAEHHQYIAKCLKYLGFNDLANSLDPTLIGDDKNKKKYSIDIGPARFQL
QYMGHYLIRDERKDRDPRVQDFIPNAWQQELLDVVDKNESAVIVAPTSSGKTYASYYCME
KVLRESDVGVVVYVAPAKSLVGQVAATVENRFTKTLPAGRTLCGAFTRDYCHNVLNCQVL
ITVPECFEILLLAPHRQKWVERIRYVIFDEVHYLGREVGAKFWELLLVIIRCPFLVLSAT
INNPNLLTKWLQSVKQYWKQADKIMEEKCISEKQADKCLNFLQDHSYKNQSYEVRLVLCG
ERYNDLEKHICSVKHDDVYFDHFHPCAALTTDIIEKYGFPPDLTLTPQESIQLYDTMAQV
WETWPRAQELCPEEFILFKNKIVIKKLDARKYEENLKAELTNWIKNGQVKKVKRVLKNLS
PDSLSSSKDMVKMFPLLVEKLRQMDKLPAIFFLFKNDDVGKRAGSVCTFLEKTETKSHPH
TECHSYVFAIDEVLEKVRKTQKRITKKNPKKAEKLERKKVYRAEYINFLENLKILEISED
CTYADVKALHTEITRNKDSTLERVLPRVRFTRHGKELKALAQRGIGYHHSSMYFKEKEFV
EILFVKGLIRVVTATETLALGIHMPCKSVVFAQDSVYLDALNYRQMSGRAGRRGQDLLGN
VYFFDIPLPKIKRLLASSVPELRGQFPLSITLVLRLMLLASKGDDPEDAKAKVLSVLKHS
LLSFKRRRAMETLKLYFLFSLQLLIKEDYLNKKGNPKKFAGLASYLHGHEPSNLVFVNFL
KRGLFHNLCKPAWKGSQQFSQDVMEKLVLVLANLFGRKYIPAKFQNANLSFSQSKVILAE
LPEDFKAALYEYNLAVMKDFASFLLIASKSVNMKKEHQLPLSRIKFTGKECEDSQLVSHL
MSCKKGRVAISPFVCLSGNTDNDLLRPETINQVILRTVGVSGTQAPLLWPWKLDNRGRRM
PLNAYVLNFYKHNCLTRLDQKNGMRMGQLLKCLKDFAFNIQAISDSLSELCENKRDNVVL
AFKQLSQTFYEKLQEMQIQMSQNHLE*
>ENSG00000134744|ENST00000257177
MEESKTLKSENHEPKKNVICEESKAVQVIGNQTLKARNDKSVKEIENSSPNRNSSKKNKQ
NDICIEKTEVKSCKVNAANLPGPKDLGLVLRDQSHCKAKKFPNSPVKAEKATISQAKSEK
ATSLQAKAEKSPKSPNSVKAEKASSYQMKSEKVPSSPAEAEKGPSLLLKDMRQKTELQQI
GKKIPSSFTSVDKVNIEAVGGEKCALQNSPRSQKQQTCTDNTGDSDDSASGIEDVSDDLS
KMKNDESNKENSSEMDYLENATVIDESALTPEQRLGLKQAEERLERDHIFRLEKRSPEYT
NCRYLCKLCLIHIENIQGAHKHIKEKRHKKNILEKQEESELRSLPPPSPAHLAALSVAVI
ELAKEHGITDDDLRVRQEIVEEMSKVITTFLPECSLRLYGSSLTRFALKSSDVNIDIKFP
PKMNHPDLLIKVLGILKKNVLYVDVESDFHAKVPVVVCRDRKSGLLCRVSAGNDMACLTT
DLLTALGKIEPVFIPLVLAFRYWAKLCYIDSQTDGGIPSYCFALMVMFFLQQRKPPLLPC
LLGSWIEGFDPKRMDDFQLKGIVEEKFVKWECNSSSATEKNSIAEENKAKADQPKDDTKK
TETDNQSNAMKEKHGKSPLALETPNRVSLGQLWLELLKFYTLDFALEEYVICVRIQDILT
RENKNWPKRRIAIEDPFSVKRNVARSLNSQLVYEYVVERFRAAYRYFACPQTKGGNKSTV
DFKKREKGKISNKKPVKSNNMATNGCILLGETTEKINAEREQPVQCDEMDCTSQRCIIDN
NNLLVNELDFADHGQDSSSLSTSKSSEIEPKLDKKQDDLAPSETCLKKELSQCNCIDLSK
SPDPDKSTGTDCRSNLETESSHQSVCTDTSATSCNCKATEDASDLNDDDNLPTQELYYVF
DKFILTSGKPPTIVCSICKKDGHSKNDCPEDFRKIDLKPLPPMTNRFREILDLVCKRCFD
ELSPPCSEQHNREQILIGLEKFIQKEYDEKARLCLFGSSKNGFGFRDSDLDICMTLEGHE
NAEKLNCKEIIENLAKILKRHPGLRNILPITTAKVPIVKFEHRRSGLEGDISLYNTLAQH
NTRMLATYAAIDPRVQYLGYTMKVFAKRCDIGDASRGSLSSYAYILMVLYFLQQRKPPVI
PVLQEIFDGKQIPQRMVDGWNAFFFDKTEELKKRLPSLGKNTESLGELWLGLLRFYTEEF
DFKEYVISIRQKKLLTTFEKQWTSKCIAIEDPFDLNHNLGAGVSRKMTNFIMKAFINGRK
LFGTPFYPLIGREAEYFFDSRVLTDGELAPNDRCCRVCGKIGHYMKDCPKRKSSLLFRLK
KKDSEEEKEGNEEEKDSRDVLDPRDLHDTRDFRDPRDLRCFICGDAGHVRRECPEVKLAR
QRNSSVAAAQLVRNLVNAQQVAGSAQQQGDQSIRTRQSSECSESPSYSPQPQPFPQNSSQ

SAAITQPSSQPGSQPKLGPPQQGAQPPHQVQMPLYNFPQSPPAQYSPMHNMGLLPMHPLQ
IPAPSWPIHGPVIHSAPGSAPSNIGLNDPSIIFAQPAARPVAIPNTSHDGHWPRTVAPNS
LVNSGAVGNSEPGFRGLTPPIPWEHAPRPHFPLVPASWPYGLHQNFMHQGNARFQPNKPF
YTQDRCATRRCRERCPHPPRGNVSE*
>ENSG00000136270|ENST00000258770
MAAHLVKRCTCLLREAARQAPAMAPVGRLRLAWVAHKTLTSSATSPISHLPGSLMEPVEK
ERASTPYIEKQVDHLIKKATRPEELLELLGGSHDLDSNQAAMVLIRLSHLLSEKPEDKGL
LIQDAHFHQLLCLLNSQIASVWHGTLSKLLGSLYALGIPKASKELQSVEQEVRWRMRKLK
YKHLAFLAESCATLSQEQHSQELLAELLTHLERRWTEIEDSHTLVTVMMKVGHLSEPLMN
RLEDKCLELVEHFGPNELRKVLVMLAAQSRRSVPLLRAISYHLVQKPFSLTKDVLLDVAY
AYGKLSFHQTQVSQRLATDLLSLMPSLTSGEVAHCAKSFALLKWLSLPLFEAFAQHVLNR
AQDITLPHLCSVLLAFARLNFHPDQEDQFFSLVHEKLGSELPGLEPALQVDLVWALCVLQ
QAREAELQAVLHPEFHIQFLGGKSQKDQNTFQKLLHINATALLEYPEYSGPLLPASAVAP
GPSALDRKVTPLQKELQETLKGLLGSADKGSLEVATQYGWVLDAEVLLDSDGEFLPVRDF
VAPHLAQPTGSQSPPPGSKRLAFLRWEFPNFNSRSKDLLGRFVLARRHIVAAGFLIVDVP
FYEWLELKSEWQKGAYLKDKMRKAVAEELAK*
>ENSG00000072310|ENST00000261646
MDEPPFSEAALEQALGEPCDLDAALLTDIEDMLQLINNQDSDFPGLFDPPYAGSGAGGTD
PASPDTSSPGSLSPPPATLSSSLEAFLSGPQAAPSPLSPPQPAPTPLKMYPSMPAFSPGP
GIKEESVPLSILQTPTPQPLPGALLPQSFPAPAPPQFSSTPVLGYPSPPGGFSTGSPPGN
TQQPLPGLPLASPPGVPPVSLHTQVQSVVPQQLLTVTAAPTAAPVTTTVTSQIQQVPVLL
QPHFIKADSLLLTAMKTDGATVKAAGLSPLVSGTTVQTGPLPTLVSGGTILATVPLVVDA
EKLPINRLAAGSKAPASAQSRGEKRTAHNAIEKRYRSSINDKIIELKDLVVGTEAKLNKS
AVLRKAIDYIRFLQHSNQKLKQENLSLRTAVHKSKSLKDLVSACGSGGNTDVLMEGVKTE
VEDTLTPPPSDAGSPFQSSPLSLGSRGSGSGGSGSDSEPDSPVFEDSKAKPEQRPSLHSR
GMLDRSRLALCTLVFLCLSCNPLASLLGARGLPSPSDTTSVYHSPGRNVLGTESRDGPGW
AQWLLPPVVWLLNGLLVLVSLVLLFVYGEPVTRPHSGPAVYFWRHRKQADLDLARGDFAQ
AAQQLWLALRALGRPLPTSHLDLACSLLWNLIRHLLQRLWVGRWLAGRAGGLQQDCALRV
DASASARDAALVYHKLHQLHTMGKHTGGHLTATNLALSALNLAECAGDAVSVATLAEIYV
AAALRVKTSLPRALHFLTRFFLSSARQACLAQSGSVPPAMQWLCHPVGHRFFVDGDWSVL
STPWESLYSLAGNPVDPLAQVTQLFREHLLERALNCVTQPNPSPGSADGDKEFSDALGYL
QLLNSCSDAAGAPAYSFSISSSMATTTGVDPVAKWWASLTAVVIHWLRRDEEAAERLCPL
VEHLPRVLQESERPLPRAALHSFKAARALLGCAKAESGPASLTICEKASGYLQDSLATTP
ASSSIDKAVQLFLCDLLLVVRTSLWRQQQPPAPAPAAQGTSSRPQASALELRGFQRDLSS
LRRLAQSFRPAMRRVFLHEATARLMAGASPTRTHQLLDRSLRRRAGPGGKGGAVAELEPR
PTRREHAEALLLASCYLPPGFLSAPGQRVGMLAEAARTLEKLGDRRLLHDCQQMLMRLGG
GTTVTSS*
>ENSG00000129315|ENST00000261900
MEGERKNNNKRWYFTREQLENSPSRRFGVDPDKELSYRQQAANLLQDMGQRLNVSQLTIN
TAIVYMHRFYMIQSFTQFPGNSVAPAALFLAAKVEEQPKKLEHVIKVAHTCLHPQESLPD
TRSEAYLQQVQDLVILESIILQTLGFELTIDHPHTHVVKCTQLVRASKDLAQTSYFMATN
SLHLTTFSLQYTPPVVACVCIHLACKWSNWEIPVSTDGKHWWEYVDATVTLELLDELTHE
FLQILEKTPNRLKRIWNWRACEAAKKTKADDRGTDEKTSEQTILNMISQSSSDTTIAGLM
SMSTSTTSAVPSLPVSEESSSNLTSVEMLPGKRWLSSQPSFKLEPTQGHRTSENLALTGV

DHSLPQDGSNAFISQKQNSKSVPSAKVSLKEYRAKHAEELAAQKRQLENMEANVKSQYAY
AAQNLLSHHDSHSSVILKMPIEGSENPERPFLEKADKTALKMRIPVAGGDKAASSKPEEI
KMRIKVHAAADKHNSVEDSVTKSREHKEKHKTHPSNHHHHHNHHSHKHSHSQLPVGTGNK
RPGDPKHSSQTSNLAHKTYSLSSSFSSSSSTRKRGPSEETGGAVFDHPAKIAKSTKSSSL
NFSFPSLPTMGQMPGHSSDTSGLSFSQPSCKTRVPHSKLDKGPTGANGHNTTQTIDYQDT
VNMLHSLLSAQGVQPTQPTAFEFVRPYSDYLNPRSGGISSRSGNTDKPRPPPLPSEPPPP
LPPLPK*

>ENSG00000067066|ENST00000264052

MAGGGGDLSTRRLNECISPVANEMNHLPAHSHDLQRMFTEDQGVDDRLLYDIVFKHFKRN
KVEISNAIKKTFPFLEGLRDRDLITNKMFEDSQDSCRNLVPVQRVVYNVLSELEKTFNLP
VLEALFSDVNMQEYPDLIHIYKGFENVIHDKLPLQESEEEEREERSGLQLSLEQGTGENS
FRSLTWPPSGSPSHAGTTPPENGLSEHPCETEQINAKRKDTTSDKDDSLGSQQTNEQCAQ
KAEPTESCEQIAVQVNNGDAGREMPCPLPCDEESPEAELHNHGIQINSCSVRLVDIKKEK
PFSNSKVECQAQARTHHNQASDIIVISSEDSEGSTDVDEPLEVFISAPRSEPVINNDNPL
ESNDEKEGQEATCSRPQIVPEPMDFRKLSTFRESFKKRVIGQDHDFSESSEEEAPAEASS
GALRSKHGEKAPMTSRSTSTWRIPSRKRRFSSSDFSDLSNGEELQETCSSSLRRGSGSQP
QEPENKKCSCVMCFPKGVPRSQEARTESSQASDMMDTMDVENNSTLEKHSGKRRKKRRHR
SKVNGLQRGRKKDRPRKHLTLNNKVQKKRWQQRGRKANTRPLKRRRKRGPRIPKDENINF
KQSELPVTCGEVKGTLYKERFKQGTSKKCIQSEDKKWFTPREFEIEGDRGASKNWKLSIR
CGGYTLKVLMENKFLPEPPSTRKKRILESHNNTLVDPCEEHKKKNPDASVKFSEFLKKCS
ETWKTIFAKEKGKFEDMAKADKAHYEREMKTYIPPKGEKKKKFKDPNAPKRPPLAFFLFC
SEYRPKIKGEHPGLSIDDVVKKLAGMWNNTAAADKQFYEKKAAKLKEKYKKDIAAYRAKG
KPNSAKKRVVKAEKSKKKKEEEEDEEDEQEEENEEDDDK*

>ENSG00000132394|ENST00000254730

MAGRRVNVNVGVLGHIDSGKTALARALSTTASTAAFDKQPQSRERGITLDLGFSCFSVPL
PARLRSSLPEFQAAPEAEPEPGEPLLQVTLVDCPGHASLIRTIIGGAQIIDLMMLVIDVT
KGMQTQSAECLVIGQIACQKLVVVLNKIDLLPEGKRQAAIDKMTKKMQKTLENTKFRGAP
IIPVAAKPGGPEAPETEAPQGIPELIELLTSQISIPTRDPSGPFLMSVDHCFSIKGQGTV
MTGTILSGSISLGDSVEIPALKVVKKVKSMQMFHMPITSAMQGDRLGICVTQFDPKLLER
GLVCAPESLHTVHAALISVEKIPYFRGPLQTKAKFHITVGHETVMGRLMFFSPAPDNFDQ
EPILDSFNFSQEYLFQEQYLSKDLTPAVTDNDEADKKAGQATEGHCPRQQWALVEFEKPV
TCPRLCLVIGSRLDADIHTNTCRLAFHGILLHGLEDRNYADSFLPRLKVYKLKHKHGLVE
RAMDDYSVIGRSLFKKETNIQLFVGLKVHLSTGELGIIDSAFGQSGKFKIHIPGGLSPES
KKILTPALKKRARAGRGEATRQEESAERSEPSQHVVLSLTFKRYVFDTHKRMVQSP*

>ENSG00000107951|ENST00000263063

MAVPGVGLLTRLNLCARRRTRVQRPIVRLLSCPGTVAKDLRRDEQPSGSVETGFEDKIPK
RRFSEMQNERREQAQRTVLIHCPEKISENKFLKYLSQFGPINNHFFYESFGLYAVVEFCQ
KESIGSLQNGTHTPSTAMETAIPFRSRFFNLKLKNQTSERSRVRSSNQLPRSNKQLFELL
CYAESIDDQLNTLLKEFQLTEENTKLRYLTCSLIEDMAAAYFPDCIVRPFGSSVNTFGKL
GCDLDMFLDLDETRNLSAHKISGNFLMEFQVKNVPSERIATQKILSVLGECLDHFGPGCV
GVQKILNARCPLVRFSHQASGFQCDLTTNNRIALTSSELLYIYGALDSRVRALVFSVRCW
ARAHSLTSSIPGAWITNFSLTMMVIFFLQRRSPPILPTLDSLKTLADAEDKCVIEGNNCT
FVRDLSRIKPSQNTETLELLLKEFFEYFGNFAFDKNSINIRQGREQNKPDSSPLYIQNPF
ETSLNISKNVSQSQLQKFVDLARESAWILQQEDTDRPSISSNRPWGLVSLLLPSAPNRKS

FTKKKSNKFAIETVKNLLESLKGNRTENFTKTSGKRTISTQT*
>ENSG00000135114|ENST00000257570
MALMQELYSTPASRLDSFVAQWLQPHREWKEEVLDAVRTVEEFLRQEHFQGKRGLDQDVR
VLKVVKVGSFGNGTVLRSTREVELVAFLSCFHSFQEAAKHHKDVLRLIWKTMWQSQDLLD
LGLEDLRMEQRVPDALVFTIQTRGTAEPITVTIVPAYRALGPSLPNSQPPPEVYVSLIKA
CGGPGNFCPSFSELQRNFVKHRPTKLKSLLRLVKHWYQQYVKARSPRANLPPLYALELLT
IYAWEMGTEEDENFMLDEGFTTVMDLLLEYEVICIYWTKYYTLHNAIIEDCVRKQLKKER
PIILDPADPTLNVAEGYRWDIVAQRASQCLKQDCCYDNRENPISSWNVKRARDIHLTVEQ
RGYPDFNLIVNPYEPIRKVKEKIRRTRGYSGLQRLSFQVPGSERQLLSSRCSLAKYGIFS
HTHIYLLETIPSEIQVFVKNPDGGSYAYAINPNSFILGLKQQIEDQQGLPKKQQQLEFQG
QVLQDWLGLGIYGIQDSDTLILSKKKGEALFPAS*
>ENSG00000123411|ENST00000262032
MHTPPALPRRFQGGGRVRTPGSHRQGKDNLERDPSGGCVPDFLPQAQDSNHFIMESLFCE
SSGDSSLEKEFLGAPVGPSVSTPNSQHSSPSRSLSANSIKVEMYSDEESSRLLGPDERLL
EKDDSVIVEDSLSEPLGYCDGSGPEPHSPGGIRLPNGKLKCDVCGMVCIGPNVLMVHKRS
HTGERPFHCNQCGASFTQKGNLLRHIKLHSGEKPFKCPFCNYACRRRDALTGHLRTHSVS
SPTVGKPYKCNYCGRSYKQQSTLEEHKERCHNYLQSLSTEAQALAGQPGDEIRDLEMVPD
SMLHSSSERPTFIDRLANSLTKRKRSTPQKFVGEKQMRFSLSDLPYDVNSGGYEKDVELV
AHHSLEPGFGSSLAFVGAEHLRPLRLPPTNCISELTPVISSVYTQMQPLPGRLELPGSRE
AGEGPEDLADGGPLLYRPRGPLTDPGASPSNGCQDSTDTESNHEDRVAGVVSLPQGPPPQ
PPPTIVVGRHSPAYAKEDPKPQEGLLRGTPGPSKEVLRVVGESGEPVKAFKCEHCRILFL
DHVMFTIHMGCHGFRDPFECNICGYHSQDRYEFSSHIVRGEHKVG*
>ENSG00000135111|ENST00000257566
MSLSMRDPVIPGTSMAYHPFLPHRAPDFAMSAVLGHQPPFFPALTLPPNGAAALSLPGAL
AKPIMDQLVGAAETGIPFSSLGPQAHLRPLKTMEPEEEVEDDPKVHLEAKELWDQFHKRG
TEMVITKSGRRMFPPFKVRCSGLDKKAKYILLMDIIAADDCRYKFHNSRWMVAGKADPEM
PKRMYIHPDSPATGEQWMSKVVTFHKLKLTNNISDKHGFTLAFPSDHATWQGNYSFGTQT
ILNSMHKYQPRFHIVRANDILKLPYSTFRTYLFPETEFIAVTAYQNDKITQLKIDNNPFA
KGFRDTGNGRREKRKQLTLQSMRVFDERHKKENGTSDESSSEQAAFNCFAQASSPAASTV
GTSNLKDLCPSEGESDAEAESKEEHGPEACDAAKISTTTSEEPCRDKGSPAVKAHLFAAE
RPRDSGRLDKASPDSRHSPATISSSTRGLGAEERRSPVREGTAPAKVEEARALPGKEAFA
PLTVQTDAAAAHLAQGPLPGLGFAPGLAGQQFFNGHPLFLHPSQFAMGGAFSSMAAAGMG
PLLATVSGASTGVSGLDSTAMASAAAAQGLSGASAATLPFHLQQHVLASQGLAMSPFGSL
FPYPYTYMAAAAAASSAAASSSVHRHPFLNLNTMRPRLRYSPYSIPVPVPDGSSLLTTAL
PSMAAAAGPLDGKVAALAASPASVAVDSGSELNSRSSTLSSSSMSLSPKLCAEKEAATSE
LQSIQRLVSGLEAKPDRSRSASP*
>ENSG00000101347|ENST00000262878
MQRADSEQPSKRPRCDDSPRTPSNTPSAEADWSPGLELHPDYKTWGPEQVCSFLRRGGFE
EPVLLKNIRENEITGALLPCLDESRFENLGVSSLGERKKLLSYIQRLVQIHVDTMKVIND
PIHGHIELHPLLVRIIDTPQFQRLRYIKQLGGGYYVFPGASHNRFEHSLGVGYLAGCLVH
ALGEKQPELQISERDVLCVQIAGLCHDLGHGPFSHMFDGRFIPLARPEVKWTHEQGSVMM
FEHLINSNGIKPVMEQYGLIPEEDICFIKEQIVGPLESPVEDSLWPYKGRPENKSFLYEI
VSNKRNGIDVDKWDYFARDCHHLGIQNNFDYKRFIKFARVCEVDNELRICARDKEVGNLY
DMFHTRNSLHRRAYQHKVGNIIDTMITDAFLKADDYIEITGAGGKKYRISTAIDDMEAYT

KLTDNIFLEILYSTDPKLKDAREILKQIEYRNLFKYVGETQPTGQIKIKREDYESLPKEV
ASAKPKVLLDVKLKAEDFIVDVSQLLPEKFAEQLIRVYCKKVDRKSLYAARQYFVQWCAD
RNFTKPQDGDVIAPLITPQKKEWNDSTSVQNPTRLREASKSRVQLFKDDPM*
>ENSG00000121417|ENST00000254182
MLENFALTSSLGLISSWSHVVAQLGLGEVPSVLHRMFMTPASARWDQRGPGLHEWHLGKG
MSSGCWCGVEHEETPSEQRISGERVPQFRTSKEGSSSQNADSCEICCLVLRDILHLAEHQ
GTNCGQKLHTCGKQFYISANLQQHQRQHITEAPFRSYVDTASFTQSCIVHVSEKPFTCRE
IRKDFLANMRFLHQDATQTGEKPNNSNKCAVAFYSGKSHHNWGKCSKAFSHIDTLVQDQR
ILTREGLFECSKCGKACTRRCNLIQHQKVHSEERPYECNECGKFFTYYSSFIIHQRVHTG
ERPYACPECGKSFSQIYSLNSHRKVHTGERPYECGECGKSFSQRSNLMQHRRVHTGERPY
ECSECGKSFSQNFSLIYHQRVHTGERPHECNECGKSFSRSSSLIHHRRLHTGERPYECSK
CGKSFKQSSSFSSHRKVHTGERPYVCGECGKSFSHSSNLKNHQRVHTGERPVECSECSKS
FSCKSNLIKHLRVHTGERPYECSECGKSFSQSSSLIQHRRVHTGKRPYQCSQCGKSFGCK
SVLIQHQRVHIGEKP*
>ENSG00000127586|ENST00000262315
MEDYEQELCGVEDDFHNQFAAELEVLAELEGASTPSPSGVPLFTAGRPPRTFEEALARGD
AASSPAPAASVGSSQGGARKRQVDADLQPAGSLPHAPRIKRPRLQVVKRLNFRSEEMEEP
PPPDSSPTDITPPPSPEDLAELWGHGVSEAAADVGLTRASPAARNPVLRRPPILEDYVHV
TSTEGVRAYLVLRADPMAPGVQGSLLHVPWRGGGQLDLLGVSLASLKKQVDGERRERLLQ
EAQKLSDTLHSLRSGEEEAAQPLGAPEEEPTDGQDASSHCLWVDEFAPRHYTELLSDDFT
NRCLLKWLKLWDLVVFGHERPSRKPRPSVEPARVSKEATAPGKWKSHEQVLEEMLEAGLD
PSQRPKQKVALLCGPPGLGKTTLAHVIARHAGYSVVEMNASDDRSPEVFRTRIEAATQME
SVLGAGGKPNCLVIDEIDGAPVAAINVLLSILNRKGPQEVGPQGPAVPSGGGRRRRAEGG
LLMRPIICICNDQFAPSLRQLKQQAFLLHFPPTLPSRLVQRLQEVSLRQGMRADPGVLAA
LCEKTDNDIRACINTLQFLYSRGQRELSVRDVQATRVGLKDQRRGLFSVWQEVFQLPRAQ
RRRVGQDPALPADTLLLGDGDAGSLTSASQRFYRVLHAAASAGEHEKVVQGLFDNFLRLR
LRDSSLGAVCVALDWLAFDDLLAGAAHHSQSFQLLRYPPFLPVAFHVLFASSHTPRITFP
SSQQEAQNRMSQMRNLIQTLVSGIAPATRSRATPQALLLDALCLLLDILAPKLRPVSTQL
YSTREKQQLASLVGTMLAYSLTYRQERTPDGQYIYRLEPNVEELCRFPELPARKPLTYQT
KQLIAREIEVEKMRRAEASARVENSPQVDGSPPGLEGLLGGIGEKGVHRPAPRNHEQRLE
HIMRRAAREEQPEKDFFGRVVVRSTAVPSAGDTAPEQDSVERRMGTAVGRSEVWFRFNEG
VSNAVRRSLYIRDLL*
>ENSG00000204531|ENST00000259915
MAGHLASDFAFSPPPGGGGDGPGGPEPGWVDPRTWLSFQGPPGGPGIGPGVGPGSEVWGI
PPCPPPYEFCGGMAYCGPQVGVGLVPQGGLETSQPEGEAGVGVESNSDGASPEPCTVTPG
AVKLEKEKLEQNPEESQDIKALQKELEQFAKLLKQKRITLGYTQADVGLTLGVLFGKVFS
QTTICRFEALQLSFKNMCKLRPLLQKWVEEADNNENLQEICKAETLVQARKRKRTSIENR
VRGNLENLFLQCPKPTLQQISHIAQQLGLEKDVVRVWFCNRRQKGKRSSSDYAQREDFEA
AGSPFSGGPVSFPLAPGPHFGTPGYGSPHFTALYSSVPFPEGEAFPPVSVTTLGSPMHSN
*
>ENSG00000029153|ENST00000261178
MAAEEEAAAGGKVLREENQCIAPVVSSRVSPGTRPTAMGSFSSHMTEFPRKRKGSDSDPS
QEAHSQTEKRRRDKMNNLIEELSAMIPQCNPMARKLDKLTVLRMAVQHLRSLKGLTNSYV
GSNYRPSFLQDNELRHLILKTAEGFLFVVGCERGKILFVSKSVSKILNYDQASLTGQSLF

DFLHPKDVAKVKEQLSSFDISPREKLIDAKTGLQVHSNLHAGRTRVYSGSRRSFFCRIKS
CKISVKEEHGCLPNSKKKEHRKFYTIHCTGYLRSWPPNIVGMEEERNSKKDNSNFTCLVA
IGRLQPYIVPQNSGEINVKPTEFITRFAVNGKFVYVDQRATAILGYLPQELLGTSCYEYF
HQDDHNNLTDKHKAVLQSKEKILTDSYKFRAKDGSFVTLKSQWFSFTNPWTKELEYIVSV
NTLVLGHSEPGEASFLPCSSQSSEESSRQSCMSVPGMSTGTVLGAGSIGTDIANEILDLQ
RLQSSSYLDDSSPTGLMKDTHTVNCRSMSNKELFPPSPSEMGELEATRQNQSTVAVHSHE
PLLSDGAQLDFDALCDNDDTAMAAFMNYLEAEGGLGDPGDFSDIQWTL*
>ENSG00000131668|ENST00000253968
MQRPGEPGAARFGPPEGCADHRPHRYRSFMIEEILTEPPGPKGAAPAAAAAAAGELLKFG
VQALLAARPFHSHLAVLKAEQAAVFKFPLAPLGCSGLSSALLAAGPGLPGAAGAPHLPLE
LQLRGKLEAAGPGEPGTKAKKGRRSRTVFTELQLMGLEKRFEKQKYLSTPDRIDLAESLG
LSQLQVKTWYQNRRMKWKKIVLQGGGLESPTKPKGRPKKNSIPTSEQLTEQERAKDAEKP
AEVPGEPSDRSRED*
>ENSG00000114251|ENST00000264634
MKKSIGILSPGVALGMAGSAMSSKFFLVALAIFFSFAQVVIEANSWWSLGMNNPVQMSEV
YIIGAQPLCSQLAGLSQGQKKLCHLYQDHMQYIGEGAKTGIKECQYQFRHRRWNCSTVDN
TSVFGRVMQIGSRETAFTYAVSAAGVVNAMSRACREGELSTCGCSRAARPKDLPRDWLWG
GCGDNIDYGYRFAKEFVDARERERIHAKGSYESARILMNLHNNEAGRRTVYNLADVACKC
HGVSGSCSLKTCWLQLADFRKVGDALKEKYDSAAAMRLNSRGKLVQVNSRFNSPTTQDLV
YIDPSPDYCVRNESTGSLGTQGRLCNKTSEGMDGCELMCCGRGYDQFKTVQTERCHCKFH
WCCYVKCKKCTEIVDQFVCK*
>ENSG00000076003|ENST00000264156
MDLAAAAEPGAGSQHLEVRDEVAEKCQKLFLDFLEEFQSSDGEIKYLQLAEELIRPERNT
LVVSFVDLEQFNQQLSTTIQEEFYRVYPYLCRALKTFVKDRKEIPLAKDFYVAFQDLPTR
HKIRELTSSRIGLLTRISGQVVRTHPVHPELVSGTFLCLDCQTVIRDVEQQFKYTQPNIC
RNPVCANRRRFLLDTNKSRFVDFQKVRIQETQAELPRGSIPRSLEVILRAEAVESAQAGD
KCDFTGTLIVVPDVSKLSTPGARAETNSRVSGVDGYETEGIRGLRALGVRDLSYRLVFLA
CCVAPTNPRFGGKELRDEEQTAESIKNQMTVKEWEKVFEMSQDKNLYHNLCTSLFPTIHG
NDEVKRGVLLMLFGGVPKTTGEGTSLRGDINVCIVGDPSTAKSQFLKHVEEFSPRAVYTS
GKASSAAGLTAAVVRDEESHEFVIEAGALMLADNGVCCIDEFDKMDVRDQVAIHEAMEQQ
TISITKAGVKATLNARTSILAAANPISGHYDRSKSLKQNINLSAPIMSRFDLFFILVDEC
NEVTDYAIARRIVDLHSRIEESIDRVYSLDDIRRYLLFARQFKPKISKESEDFIVEQYKH
LRQRDGSGVTKSSWRITVRQLESMIRLSEAMARMHCCDEVQPKHVKEAFRLLNKSIIRVE
TPDVNLDQEEEIQMEVDEGAGGINGHADSPAPVNGINGYNEDINQESAPKASLRLGFSEY
CRISNLIVLHLRKVEEEEDESALKRSELVNWYLKEIESEIDSEEELINKKRIIEKVIHRL
THYDHVLIELTQAGLKGSTEGSESYEEDPYLVVNPNYLLED*
>ENSG00000124279|ENST00000264669
MALITLRKNLYRLSDFQMHRALAALKNKPLNHVHKVVKERLCPWLCSRQPEPFGVKFHHA
HCKKFHSKNGNDLHPLGGPVFSQVSDCDRLEQNVKNEESQMFYRRLSNLTSSEEVLSFIS
TMETLPDTMAAGALQRICEVEKKDGDQGLPKEILENSIFQALCFQFEKEPSQLSNTSLVT
ALQALILLHVDPQSSLLLNLVAECQNRLRKGGMEVRNLCILGESLITLHSSGCVTLELII
NQLQGEKLETFTPEDIVALYRILQACTEKVDEHQTFLNKINNFSLSIVSNLSPKLISQML
TALVVLDQSQAFPLIIKLGKYVVRHVPHFTNEELRRVLEAFIYFGHHDTFFTKALEHRVA
AVCLTLDPEVVCRVMEYCSRELILSKPILNAVAETFVCQTEKFSPRQISALMEPFGKLNY

LPPNASALFRKLENVLFTHFNYFPPKSLLKLLHSCSLNECHPVNFLAKIFKPLFLQRLQG
KESHLDTLSRAQLTQLFLASVLECPFYKGPKLLPKYQVKSFLTPCCSLETPVDSQLYRYV
KIGLTNLLGARLYFAPKVLTPYCYTIDVEIKLDEEGFVLPSTANEDIHKRIALCIDGPKR
FCSNSKHLLGKEAIKQRHLQLLGYQVVQIPYHEIGMLKSRRELVEYLQRKLFSQNTVHWL
QE*
>ENSG00000138035|ENST00000260604
MAACRYCCSCLRLRPLSDGPFLLPRRDRALTQLQVRALWSSAGSRAVAVDLGNRKLEISS
GKLARFADGSAVVQSGDTAVMVTAVSKTKPSPSQFMPLVVDYRQKAAAAGRIPTNYLRRE
IGTSDKEILTSRIIDRSIRPLFPAGYFYDTQVLCNLLAVDGVNEPDVLAINGGKYKSRD*
>ENSG00000136527|ENST00000259043
XPIADVSIVYDQQSRRSRGFAFVYFENVDDAKEAKERANGMELDGRRIRVDFSITKRPHT
PTPGIYMGRPTYGSSRRRDYYDRGYDRGYDDRDYYSRSYRRRSPSPYYSRGGYRSRSRSR
SYSPRRY*
>ENSG00000132465|ENST00000254801
MKNHLLFWGVLAVFIKAVHVKAQEDERIVLVDNKCKCARITSRIIRSSEDPNEDIVERNI
RIIVPLNNRENISDPTSPLRTRFVYHLSDLCKKCDPTEVELDNQIVTATQSNICDEDSAT
ETCYTYDRNKCYTAVVPLVYGGETKMVETALTPDACYPD*
>ENSG00000132591|ENST00000254928
MAAPSWRGARLVQSVLRVWQVGPHVARERVIPFSSLLGFQRRCVSCVAGSAFSGPRLASA
SRSNGQGSALDHFLGFSQPDSSVTPCVPAVSMNRDEQDVLLVHHPDMPENSRVLRVVLLG
APNAGKSTLSNQLLGRKVFPVSRKVHTTRCQALGVITEKETQVILLDTPGIISPGKQKRH
HLELSLLEDPWKSMESADLVVVLVDVSDKWTRNQLSPQLLRCLTKYSQIPSVLVMNKVDC
LKQKSVLLELTAALTEGVVNGKKLKMRQAFHSHPGTHCPSPAVKDPNTQSVGNPQRIGWP
HFKEIFMLSALSQEDVKTLKQYLLTQAQPGPWEYHSAVLTSQTPEEICANIIREKLLEHL
PQEVPYNVQQKTAVWEEGPGGELVIQQKLLVPKESYVKLLIGPKGHVISQIAQEAGHDLM
DIFLCDVDIRLSVKLLK*
>ENSG00000135870|ENST00000258349
MPVQAPQWTDFLSCPICTQTFDETIRKPISLGCGHTVCKMCLNKLHRKACPFDQTTINTD
IELLPVNSALLQLVGAQVPEQQPITLCSGVEDTKHYEEAKKCVEELALYLKPLSSARGVG
LNSTTQSVLSRPMQRKLVTLVHCQLVEEEGRIRAMRAARSLGERTVTELILQHQNPQQLS
SNLWAAVRARGCQFLGPAMQEEALKLVLLALEDGSALSRKVLVLFVVQRLEPRFPQASKT
SIGHVVQLLYRASCFKVTKRDEDSSLMQLKEEFRTYEALRREHDSQIVQIAMEAGLRIAP
DQWSSLLYGDQSHKSHMQSIIDKLQTPASFAQSVQELTIALQRTGDPANLNRLRPHLELL
ANIDPSPDAPPPTWEQLENGLVAVRTVVHGLVDYIQNHSKKGADQQQPPQHSKYKTYMCR
DMKQRGGCPRGASCTFAHSQEELEKFRKMNKRLVPRRPLSASLGQLNEVGLPSAAILPDE
GAVDLPSRKPPALPNGIVSTGNTVTQLIPRGTDPSYDSSLKPGKIDHLSSSAPGSPPDLL
ESVPKSISALPVNPHSIPPRGPADLPPMPVTKPLQMVPRGSQLYPAQQTDVYYQDPRGAA
PPFEPAPYQQGMYYTPPPQCVSRFVRPPPSAPEPAPPYLDHYPPYLQERVVNSQYGTQPQ
QYPPIYPSHYDGRRVYPAPSYTREEIFRESPIPIEIPPAAVPSYVPESRERYQQIESYYP
VAPHPTQIRPSYLREPPYSRLPPPPQPHPSLDELHRRRKEIMAQLEERKVISPPPFAPSP
TLPPTFHPEEFLDEDLKVAGKYKGNDYSQYSPWSCDTIGSYIGTKDAKPKDVVAAGSVEM
MNVESKGMRDQRLDLQRRAAETSDDDLIPFGDRPTVSRFGAISRTSKTIYQGAGPMQAMA
PQGAPTKSINISDYSPYGTHGGWGASPYSPHQNIPSQGHFSERERISMSEVASHGKPLPS
AEREQLRLELQQLNHQISQQTQLRGLEAVSNRLVLQREANTLAGQSQPPPPPPPKWPGMI

SSEQLSLELHQVEREIGKRTRELSMENQCSLDMKSKLNTSKQAENGQPEPQNKVPAEDLT
LTFSDVPNGSALTQENISLLSNKTSSLNLSEDPEGGGDNNDSQRSGVTPSSAP*
>ENSG00000059122|ENST00000253928
MPLPEPSEQEGESVKAGQEPSPKPGTDVIPAAPRKPREFSKLVLLTASDQDEDGVGSKPQ
EVHCVLSLEMAGPATLASTLQILPVEEQGGVVQPALEMPEQKCSKLDAAAPQSLEFLRTP
FGGRLLVLESFLYKQEKAVGDKVYWKCRQHAELGCRGRAITRGLRATVMRGHCHAPDEQG
LEARRQREKLPSLALPEGLGEPQGPEGPGGRVEEPLEGVGPWQCPEEPEPTPGLVLSKPA
LEEEEAPRALSLLSLPPKKRSILGLGQARPLEFLRTCYGGSFLVHESFLYKREKAVGDKV
YWTCRDHALHGCRSRAITQGGQRVTVMRGHCHQPDMEGLEARRQQEKAVETLQAGQDGPGS
QVDTLLRGVDSLLYRRGPGPLTLTRPRPRKRAKVEDQELPTQPEAPDEHQDMDADPGGPE
FLKTPLGGSFLVYESFLYRREKAAGEKVYWTCRDQARMGCRSRAITQGRRVTVMRGHCHP
PDLGGLEALRQREKRPNTAQRGSPGGPEFLKTPLGGSFLVYESFLYRREKAAGEKVYWTC
RDQARMGCRSRAITQGRRVMVMRRHCHPPDLGGLEALRQREHFPNLAQWDSPDPLRPLEF
LRTSLGGRFLVHESFLYRKEKAAGEKVYWMCRDQARLGCRSRAITQGHRIMVMRSHCHQP
DLAGLEALRQRERLPTTAQQEDPEKIQVQLCFKTCSPESQQIYGDIKDVRLDGESQ*
>ENSG00000090861|ENST00000261772
MDSTLTASEIRQRFIDFFKRNEHTYVHSSATIPLDDPTLLFANAGMNQFKPIFLNTIDPS
HPMAKLSRAANTQKCIRAGGKHNDLDDVGKDVYHHTFFEMLGSWSFGDYFKELACKMALE
LLTQEFGIPIERLYVTYFGGDEAAGLEADLECKQIWQNLGLDDTKILPGNMKDNFWEMGD
TGPCGPCSEIHYDRIGGRDAAHLVNQDDPNVLEIWNLVFIQYNREADGILKPLPKKSIDT
GMGLERLVSVLQNKMSNYDTDLFVPYFEAIQKGTGARPYTGKVGAEDADGIDMAYRVLAD
HARTITVALADGGRPDNTGRGYVLRRILRRAVRYAHEKLNASRGFFATLVDVVVQSLGDA
FPELKKDPDMVKDIINEEEVQFLKTLSRGRRILDRKIQSLGDSKTIPGDTAWLLYDTYGF
PVDLTGLIAEEKGLVVDMDGFEEERKLAQLKSQGKGAGGEDLIMLDIYAIEELRARGLEV
TDDSPKYNYHLDSSGSYVFENTVATVMALRREKMFVEEVSTGQECGVVLDKTCFYAEQGG
QIYDEGYLVKVDDSSEDKTEFTVKNAQVRGGYVLHIGTIYGDLKVGDQVWLFIDEPRRRP
IMSNHTATHILNFALRSVLGEADQKGSLVAPDRLRFDFTAKGAMSTQQIKKAEEIANEMI
EAAKAVYTQDCPLAAAKAIQGLRAVFDETYPDPVRVVSIGVPVSELLDDPSGPAGSLTSV
EFCGGTHLRNSSHAGAFVIVTEEAIAKGIRRIVAVTGAEAQKALRKAESLKKCLSVMEAK
VKAQTAPNKDVQREIADLGEALATAVIPQWQKDELRETLKSLKKVMDDLDRASKADVQKR
VLEKTKQFIDSNPNQPLVILEMESGASAKALNEALKLFKMHSPQTSAMLFTVDNEAGKIT
CLCQVPQNAANRGLKASEWVQQVSGLMDGKGGGKDVSAQATGKNVGCLQEALQLATSFAQ
LRLGDVKN*
>ENSG00000108953|ENST00000264335
MDDREDLVYQAKLAEQAERYDEMVESMKKVAGMDVELTVEERNLLSVAYKNVIGARRASW
RIISSIEQKEENKGGEDKLKMIREYRQMVETELKLICCDILDVLDKHLIPAANTGESKVF
YYKMKGDYHRYLAEFATGNDRKEAAENSLVAYKAASDIAMTELPPTHPIRLGLALNFSVF
YYEILNSPDRACRLAKAAFDDAIAELDTLSEESYKDSTLIMQLLRDNLTLWTSDMQGDGE
EQNKEALQDVEDENQ*
>ENSG00000132275|ENST00000254605
MFEEPEWAEAAPVAAGLGPVISRPPPAASSQNKGSKRRQLLATLRALEAASLSQHPPSLC
ISDSEEEEEERKKKCPKKASFASASAEVGKKGKKKCQKQGPPCSDSEEEVERKKKCHKQA
LVGSDSAEDEKRKRKCQKHAPINSAQHLDNVDQTGPKAWKGSTTNDPPKQSPGSTSPKPP
HTLSRKQWRNRQKNKRRCKNKFQPPQVPDQAPAEAPTEKTEVSPVPRTDSHEARAGALRA

RMAQRLDGARFRYLNEQLYSGPSSAAQRLFQEDPEAFLLYHRGFQSQVKKWPLQPVDRIA
RDLRQRPASLVVADFGCGDCRLASSIRNPVHCFDLASLDPRVTVCDMAQVPLEDESVDVA
VFCLSLMGTNIRDFLEEANRVLKPGGLLKVAEVSSRFEDVRTFLRAVTKLGFKIVSKDLT
NSHFFLFDFQKTGPPLVGPKAQLSGLQLQPCLYKRR*
>ENSG00000197343|ENST00000252713
MEEIPAQEAAGSPRVQFQSLETQSECLSPEPQFVQDTDMEQGLTGDGETREENKLLIPKQ
KISEEVHSYKVRVGRLKHDITQVPETREVYKSEDRLERLQEILRKFLYLEREFRQITISK
ETFTSEKNNECHEPEKSFSLDSTIDADQRVLRIQNTDDNDKYDMSFNQNSASGKHEHLNL
TEDFQSSECKESLMDLSHLNKWESIPNTEKSYKCDVCGKIFHQSSALTRHQRIHTREKPY
KCKECEKSFSQSSSLSRHKRIHTREKPYKCEASDKSCEASDKSCSPSSGIIQHKKIHTRA
KSYKCSSCERVFSRSVHLTQHQKIHKEMPCKCKTVCGSDFCHTSYLLEHQRVHHEEKAYEY
DEYGLAYIKQQGIHFREKPYTCSECGKDFRLNSHLIQHQRIHTGEKAHECNECGKAFSQT
SCLIQHHKMHRKEKSYECNEYEGSFSHSSDLILQQEVLTRQKAFDCDVWEKNSSQRAHLV
QHQSIHTKENS*
>ENSG00000172466|ENST00000261332
MSAQSVEEDSILIIPTPDEEEKILRVKLEEDPDGEEGSSIPWNHLPDPEIFRQRFRQFGY
QDSPGPREAVSQLRELCRLWLRPETHTKEQILELVVLEQFVAILPKELQTWVRDHHPENG
EEAVTVLEDLESELDDPGQPVSLRRRKREVLVEDMVSQEEAQGLPSSELDAVENQLKWAS
WELHSLRHCDDDGRTENGALAPKQELPSALESHEVPGTLNMGVPQIFKYGETCFPKGRFE
RKRNPSRKKQHICDECGKHFSQGSALILHQRIHSGEKPYGCVECGKAFSRSSILVQHQRV
HTGEKPYKCLECGKAFSQNSGLINHQRIHTGEKPYECVQCGKSYSQSSNLFRHQRRHNAE
KLLNVVKV*
>ENSG00000104872|ENST00000262265
MANPKLLGMGLSEAEAIGADSARFEELLLQASKELQQAQTTRPESTQIQPQPGFCIKTNS
SEGKVFINICHSPSIPPPADVTEEELLQMLEEDQAGFRIPMSLGEPHAELDAKGQGCTAY
DVAVNSDFYRRMQNSDFLRELVITIAREGLEDKYNLQLNPEWRMMKNRPFMGSISQQNIR
SEQRPRIQELGDLYTPAPGRAESGPEKPHLNLWLEAPDLLLAEVDLPKLDGALGLSLEIG
ENRLVMGGPQQLYHLDAYIPLQINSHESKAAFHRKRKQLMVAMPLLPVPS*
>ENSG00000070761|ENST00000262498
MFKNTFQSGFLSILYSIGSKPLQIWDKKVRNGHIKRITDNDIQSLVLEIEGTNVSTTYIT
CPADPKKTLGIKLPFLVMIIKNLKKYFTFEVQVLDDKNVRRRFRASNYQSTTRVKPFICT
MPMRLDDGWNQIQFNLLDFTRRAYGTNYIETLRVQIHANCRIRRVYFSDRLYSEDELPAE
FKLYLPVQNKAKQ*
>ENSG00000084234|ENST00000263574
MAATGTAAAAATGRLLLLLLVGLTAPALALAGYIEALAANAGTGFAVAEPQIAMFCGKLN
MHVNIQTGKWEPDPTGTKSCFETKEEVLQYCQEMYPELQITNVMEANQRVSIDNWCRRDK
KQCKSRFVTPFKCLVGEFVSDVLLVPEKCQFFHKERMEVCENHQHWHTVVKEACLTQGMT
LYSYGMLLPCGVDQFHGTEYVCCPQTKIIGSVSKEEEEEDEEEEEEEDEEEDYDVYKSEF
PTEADLEDFTEAAVDEDDEDEEEGEEVVEDRDYYYDTFKGDDYNEENPTEPGSDGTMSDK
EITHDVKAVCSQEAMTGPCRAVMPRWYFDLSKGKCVRFIYGGCGGNRNNFESEDYCMAVC
KAMIPPTPLPTNDVDVYFETSADDNEHARFQKAKEQLEIRHRNRMDRVKKEWEEAELQAK
NLPKAERQTLIQHFQAMVKALEKEAASEKQQLVETHLARVEAMLNDRRRMALENYLAALQ
SDPPRPHRILQALRRYVRAENKDRLHTIRHYQHVLAVDPEKAAQMKSQVMTHLHVIEERR
NQSLSLLYKVPYVAQEIQEEIDELLQEQRADMDQFTASISETPVDVRVSSEESEEIPPFH

PFHPFPALPENEDTQPELYHPMKKGSGVGEQDGGLIGAEEKVINSKNKVDENMVIDETLD
VKEMIFNAERVGGLEEERESVGPLREDFSLSSSALIGLLVIAVAIATVIVISLVMLRKRQ
YGTISHGIVEVDPMLTPEERHLNKMQNHGYENPTYKYLEQMQI*
>ENSG00000103241|ENST00000262426
MSSAPEKQQPPHGGGGGGGGGGGAAMDPASSGPSKAKKTNAGIRRPEKPPYSYIALIVMA
IQSSPTKRLTLSEIYQFLQSRFPFFRGSYQGWKNSVRHNLSLNECFIKLPKGLGRPGKGH
YWTIDPASEFMFEEGSFRRRPRGFRRKCQALKPMYSMMNGLGFNHLPDTYGFQGSAGGLS
CPPNSLALEGGLGMMNGHLPGNVDGMALPSHSVPHLPSNGGHSYMGGCGGAAAGEYPHHD
SSVPASPLLPTGAGGVMEPHAVYSGSAAAWPPSASAALNSGASYIKQQPLSPCNPAANPL
SGSLSTHSLEQPYLHQNSHNAPAELQGIPRYHSQSPSMCDRKEFVFSFNAMASSSMHSAG
GGSYYHQQVTYQDIKPCVM*
>ENSG00000068654|ENST00000263857
MLISKNMPWRRLQGISFGMYSAEELKKLSVKSITNPRYLDSLGNPSANGLYDLALGPADS
KEVCSTCVQDFSNCSGHLGHIELPLTVYNPLLFDKLYLLLRGSCLNCHMLTCPRAVIHLL
LCQLRVLEVGALQAVYELERILNRFLEENPDPSASEIREELEQYTTEIVQNNLLGSQGAH
VKNVCESKSKLIALFWKAHMNAKRCPHCKTGRSVVRKEHNSKLTITFPAMVHRTAGQKDS
EPLGIEEAQIGKRGYLTPTSAREHLSALWKNEGFFLNYLFSGMDDDGMESRFNPSVFFLD
FLVVPPSRYRPVSRLGDQMFTNGQTVNLQAVMKDVVLIRKLLALMAQEQKLPEEVATPTT
DEEKDSLIAIDRSFLSTLPGQSLIDKLYNIWIRLQSHVNIVFDSEMDKLMMDKYPGIRQI
LEKKEGLFRKHMMGKRVDYAARSVICPDMYINTNEIGIPMVFATKLTYPQPVTPWNVQEL
RQAVINGPNVHPGASMVINEDGSRTALSAVDMTQREAVAKQLLTPATGAPKPQGTKIVCR
HVKNGDILLLNRQPTLHRPSIQAHRARILPEEKVLRLHYANCKAYNADFDGDEMNAHFPQ
SELGRAEAYVLACTDQQYLVPKDGQPLAGLIQDHMVSGASMTTRGCFFTREHYMELVYRG
LTDKVGRVKLLSPSILKPFPLWTGKQVVSTLLINIIPEDHIPLNLSGKAKITGKAWVKET
PRSVPGFNPDSMCESQVIIREGELLCGVLDKAHYGSSAYGLVHCCYEIYGGETSGKVLTC
LARLFTAYLQLYRGFTLGVEDILVKPKADVKRQRIIEESTHCGPQAVRAALNLPEAASYD
EVRGKWQDAHLGKDQRDFNMIDLKFKEEVNHYSNEINKACMPFGLHRQFPENSLQMMVQS
GAKGSTVNTMQISCLLGQIELEGRRPPLMASGKSLPCFEPYEFTPRAGGFVTGRFLTGIK
PPEFFFHCMAGREGLVDTAVKTSRSGYLQRCIIKHLEGLVVQYDLTVRDSDGSVVQFLYG
EDGLDIPKTQFLQPKQFPFLASNYEVIMKSQHLHEVLSRADPKKALHHFRAIKKWQSKHP
NTLLRRGAFLSYSQKIQEAVKALKLESENRNGRSPGTQEMLRMWYELDEESRRKYQKKAA
ACPDPSLSVWRPDIYFASVSETFETKVDDYSQEWAAQTEKSYEKSELSLDRLRTLLQLKW
QRSLCEPGEAVGLLAAQSIGEPSTQMTLNTFHFAGRGEMNVTLGIPRLREILMVASANIK
TPMMSVPVLNTKKALKRVKSLKKQLTRVCLGEVLQKIDVQESFCMEEKQNKFQVYQLRFQ
FLPHAYYQQEKCLRPEDILRFMETRFFKLLMESIKKKNNKASAFRNVNTRRATQRDLDNA
GELGRSRGEQEGDEEEEGHIVDAEAEEGDADASDAKRKEKQEEEVDYESEEEEEREGEEN
DDEDMQEERNPHREGARKTQEQDEEVGLGTEEDPSLPALLTQPRKPTHSQEPQGPEAMER
RVQAVREIHPFIDDYQYDTEESLWCQVTVKLPLMKINFDMSSLVVSLAHGAVIYATKGIT
RCLLNETTNNKNEKELVLNTEGINLPELFKYAEVLDLRRLYSNDIHAIANTYGIEAALRV
IEKEIKDVFAVYGIAVDPRHLSLVADYMCFEGVYKPLNRFGIRSNSSPLQQMTFETSFQF
LKQATMLGSHDELRSPSACLVVGKVVRGGTGLFELKQPLR*
>ENSG00000105058|ENST00000263384
MEAYEQVQKGPLKLKGVAELGVTKRKKKKKDKDKAKLLEAMGTSKKNEEEKRRGLDKRTP
AQAAFEKMQEKRQMERILKKASKTHKQRVEDFNRHLDTLTEHYDIPKVSWTK*

>ENSG00000119041|ENST00000263956
MSGFSPELIDYLEGKISFEEFERRREERKTREKKSLQEKGKLSAEENPDDSEVPSSSGIN
STKSQDKDVNEGETSDGVRKSVHKVFASMLGENEDDEEEEEEEEEEEEEEEETPEQPTAGD
VFVLEMVLNRETKKMMKEKRPRSKLPRALRGLMGEANIRFARGEREEAILMCMEIIRQAP
LAYEPFSTLAMIYEDQGDMEKSLQFELIAAHLNPSDTEEWVRLAEMSLEQDNIKQAIFCY
TKALKYEPTNVRYLWERSSLYEQMGDHKMAMDGYRRILNLLSPSDGERFMQLARDMAKSY
YEANDVTSAINIIDEAFSKHQGLVSMEDVNIAAELYISNKQYDKALEIITDFSGIVLEKK
TSEEGTSEENKAPENVTCTIPDGVPIDITVKLMVCLVHLNILEPLNPLLTTLVEQNPEDM
GDLYLDVAEAFLDVGEYNSALPLLSALVCSERYNLAVVWLRHAECLKALGYMERAAESYG
KVVDLAPLHLDARISLSTLQQQLGQPEKALEALEPMYDPDTLAQDANAAQQELKLLLHRS
TLLFSQGKMYGYVDTLLTMLAMLLKVAMNRAQVCLISSSKSGERHLYLIKVSRDKISDSN
DQESANCDAKAIFAVLTSVLTKDDWWNLLLKAIYSLCDLSRFQEAELLVDSSLEYYSFYD
DRQKRKELEYFGLSAAILDKNFRKAYNYIRIMVMENVNKPQLWNIFNQVTMHSQDVRHHR
FCLRLMLKNPENHALCVLNGHNAFVSGSFKHALGQYVQAFRTHPDEPLYSFCIGLTFIHM
ASQKYVLRRHALIVQGFSFLNRYLSLRGPCQESFYNLGRGLHQLGLIHLAIHYYQKALEL
PPLVVEGIELDQLDLRRDIAYNLSLIYQSSGNTGMAQTLLYTYCSI*
>ENSG00000134758|ENST00000261593
MAEDLSAATSYTEDDFYCPVCQEVLKTPVRTTACQHVFCRKCFLTAMRESGAHCPLCRGN
VTRRERACPERALDLENIMRKFSGSCRCCAKQIKFYRMRHHYKSCKKYQDEYGVSSIIPN
FQISQDSVGNSNRSETSTSDNTETYQENTSSSGHPTFKCPLCQESNFTRQRLLDHCNSNH
LFQIVPVTCPICVSLPWGDPSQITRNFVSHLNQRHQFDYGEFVNLQLDEETQYQTAVEES
FQVNI*
>ENSG00000130818|ENST00000253115
MAAADLSHGHYLSGDPVCLHEEKTPAGRIVADCLTDCYQDSVTFDDVAVDFTQEEWTLLD
STQRSLYSDVMLENYKNLATVGGQIIKPSLISWLEQEESRTVQGGVLQGWEMRLETQWSI
LQQDFLRGQTSIGIQLEGKHNGRELCDCEQCGEVFSEHSCLKTHVRTQSTGNTHDCNQYG
KDFLTLCEKTSTGEKLSEFNQSEKIFSLTPNIVYQRTSTQEKSFECSHCGKSFINESYLQ
AHMRTHNGEKLYEWRNYGPGFIDSTSLSVLIETLNAKKPYKCKECGKGYRYPAYLSIHMR
THTGEKPYECKECGKAFNYSNSFQIHGRTHTGEKPYVCKECGKAFTQYSGLSMHVRSHSG
DKPYECKECGKSFLTSSRLIQHIRTHTGEKPFVCVECGKAFAVSSNLSGHLRTHTEEKAC
ECKICGKVFGYPSCLNNHMRTHSAQKPYTCKECGKAFNYSTHLKIHMRIHTGEKPYECKQ
CGKAFSHSSSFQIHERTHTGEKPYECKECGKAFTCSSSFRIHEKTHTEEKPYKCQQCGKA
YSHPRSLRRHEQIH*
>ENSG00000136319|ENST00000258821
MMADEEEEVKPILQKLQELVDQLYSFRDCYFETHSVEDAGRKQQDVQKEMEKTLQQMEEV
VGSVQGKAQVLMLTGKALNVTPDYSPKAEELLSKAVKLEPELVEAWNQLGEVYWKKGDVA
AAHTCFSGALTHCRNKVSLQNLSMVLRQLRTDTEDEHSHHVMDSVRQAKLAVQMDVHDGR
SWYILGNSYLSLYFSTGQNPKISQQALSAYAQAEKVDRKASSNPDLHLNRATLHKYEESY
GEALEGFSRAAALDPAWPEPRQREQQLLEFLDRLTSLLESKGKVKTKKLQSMLGSLRPAH
LGPCSDGHYQSASGQKVTLELKPLSTLQPGVNSGAVILGKVVFSLTTEEKVPFTFGLVDS
DGPCYAVMVYNIVQSWGVLIGDSVAIPEPNLRLHRIQHKGKDYSFSSVRVETPLLLVVNG
KPQGSSSQAVATVASRPQCE*
>ENSG00000130726|ENST00000253024
MAASAAAASAAAASAASGSPGPGEGSAGGEKRSTAPSAAASASASAAASSPAGGGAEALE

LLEHCGVCRERLRPEREPRLLPCLHSACSACLGPAAPAAANSSGDGGAAGDGTVVDCPVC
KQQCFSKDIVENYFMRDSGSKAATDAQDANQCCTSCEDNAPATSYCVECSEPLCETCVEA
HQRVKYTKDHTVRSTGPAKSRDGERTVYCNVHKHEPLVLFCESCDTLTCRDCQLNAHKDH
QYQFLEDAVRNQRKLLASLVKRLGDKHATLQKSTKEVRSSIRQVSDVQKRVQVDVKMAIL
QIMKELNKRGRVLVNDAQKVTEGQQERLERQHWTMTKIQKHQEHILRFASWALESDNNTA
LLLSKKLIYFQLHRALKMIVDPVEPHGEMKFQWDLNAWTKSAEAFGKIVAERPGTNSTGP
APMAPPRAPGPLSKQGSGSSQPMEVQEGYGFGSGDDPYSSAEPHVSGVKRSRSGEGEVSG
LMRKVPRVSLERLDLDLTADSQPPVFKVFPGSTTEDYNLIVIERGAAAAATGQPGTAPAG
TPGAPPLAGMAIVKEEETEAAIGAPPTATEGPETKPVLMALAEGPGAEGPRLASPSGSTS
SGLEVVAPEGTSAPGGGPGTLDDSATICRVCQKPGDLVMCNQCEFCFHLDCHLPALQDVP
GEEWSCSLCHVLPDLKEEDGSLSLDGADSTGVVAKLSPANQRKCERVLLALFCHEPCRPL
HQLATDSTFSLDQPGGTLDLTLIRARLQEKLSPPYSSPQEFAQDVGRMFKQFNKLTEDKA
DVQSIIGLQRFFETRMNEAFGDTKFSAVLVEPPPMSLPGAGLSSQELSGGPGDGP*
>ENSG00000138083|ENST00000260653
MVFRSPLDLYSSHFLLPNFADSHHRSILLASSGGGNGAGGGGGAGGGSGGGNGAGGGGAG
GAGGGGGGGSRAPPEELSMFQLPTLNFSPEQVASVCETLEETGDIERLGRFLWSLPVAPG
ACEAINKHESILRARAVVAFHTGNFRDLYHILENHKFTKESHGKLQAMWLEAHYQEAEKL
RGRPLGPVDKYRVRKKFPLPRTIWDGEQKTHCFKERTRSLLREWYLQDPYPNPSKKRELA
QATGLTPTQVGNWFKNRRQRDRAAAAKNRLQHQAIGPSGMRSLAEPGCPTHGSAESPSTA
ASPTTSVSSLTERADTGTSILSVTSSDSECDV*
>ENSG00000082175|ENST00000263463
MTELKAKGPRAPHVAGGPPSPEVGSPLLCRPAAGPFPGSQTSDTLPEVSAIPISLDGLLF
PRPCQGQDPSDEKTQDQQSLSDVEGAYSRAEATRGAGGSSSSPPEKDSGLLDSVLDTLLA
PSGPGQSQPSPPACEVTSSWCLFGPELPEDPPAAPATQRVLSPLMSRSGCKVGDSSGTAA
AHKVLPRGLSPARQLLLPASESPHWSGAPVKPSPQAAAVEVEEEDGSESEESAGPLLKGK
PRALGGAAAGGGAAAVPPGAAAGGVALVPKEDSRFSAPRVALVEQDAPMAPGRSPLATTV
MDFIHVPILPLNHALLAARTRQLLEDESYDGGAGAASAFAPPRSSPCASSTPVAVGDFPD
CAYPPDAEPKDDAYPLYSDFQPPALKIKEEEEGAEASARSPRSYLVAGANPAAFPDFPLG
PPPPLPPRATPSRPGEAAVTAAPASASVSSASSSGSTLECILYKAEGAPPQQGPFAPPPC
KAPGASGCLLPRDGLPSTSASAAAAGAAPALYPALGLNGLPQLGYQAAVLKEGLPQVYPP
YLNYLRPDSEASQSPQYSFESLPQKICLICGDEASGCHYGVLTCGSCKVFFKRAMEGQHN
YLCAGRNDCIVDKIRRKNCPACRLRKCCQAGMVLGGFRNLHIDDQITLIQYSWMSLMVFG
LGWRSYKHVSGQMLYFAPDLILNEQRMKESSFYSLCLTMWQIPQEFVKLQVSQEEFLCMK
VLLLLNTIPLEGLRSQTQFEEMRSSYIRELIKAIGLRQKGVVSSSQRFYQLTKLLDNLHD
LVKQLHLYCLNTFIQSRALSVEFPEMMSEVIAAQLPKILAGMVKPLLFHKK*
>ENSG00000089220|ENST00000261313
MPVDLSKWSGPLSLQEVDEQPQHPLHVTYAGAAVDELGKVLTPTQVKNRPTSISWDGLDS
GKLYTLVLTDPDAPSRKDPKYREWHHFLVVNMKGNDISSGTVLSDYVGSGPPKGTGLHRY
VWLVYEQDRPLKCDEPILSNRSGDHRGKFKVASFRKKYELRAPVAGTCYQAEWDDYVPKL
YEQLSGK*
>ENSG00000135899|ENST00000258382
MFTMTRAMEEALFQHFMHQKLGIAYAIHKPFPFFEGLLDNSIITKRMYMESLEACRNLIP
VSRVVHNILTQLERTFNLSLLVTLFSQINLREYPNLVTIYRSFKRVGASYEWQSRDTPIL
LEAPTGLAEGSSLHTPLALPPPQPPQPSCSPCAPRVSEPGTSSQQSDEILSESPSPSDPV

LPLPALIQEGRSTSVTNDKLTSKMNAEEDSEEMPSLLTSTVQVASDNLIPQIRDKEDPQE
MPHSPLGSMPEIRDNSPEPNDPEEPQEVSSTPSDKKGKKRKRCIWSTPKRRHKKKSLPGG
TASSRHGIQKKLKRVDQVPQKKDDSTCNSTVETRAQKARTECARKSRSEEIIDGTSEMNE
GKRSQKTPSTPRRVTQGAASPGHGIQEKLQVVDKVTQRKDDSTWNSEVMMRVQKARTKCA
RKSRLKEKKKEKDICSSSKRRFQKNIHRRGKPKSDTVDFHCSKLPVTCGEAKGILYKKKM
KHGSSVKCIRNEDGTWLTPNEFEVEGKGRNAKNWKRNIRCEGMTLGELLKSGLLLCPPRI
NLKRELNSK*
>ENSG00000119042|ENST00000260926
MERRSESPCLRDSPDRRSGSPDVKGPPPVKVARLEQNGSPMGARGRPNGAVAKAVGGLMI
PVFCVVEQLDGSLEYDNREEHAEFVLVRKDVLFSQLVETALLALGYSHSSAAQAQGIIKL
GRWNPLPLSYVTDAPDATVADMLQDVYHVVTLKIQLQSCSKLEDLPAEQWNHATVRNALK
ELLKEMNQSTLAKECPLSQSMISSIVNSTYYANVSATKCQEFGRWYKKYKKIKVERVERE
NLSDYCVLGQRPMHLPNMNQLASLGKTNEQSPHSQIHHSTPIRNQVPALQPIMSPGLLSP
QLSPQLVRQQIAMAHLINQQIAVSRLLAHQHPQAINQQFLNHPPIPRAVKPEPTNSSVEV
SPDIYQQVRDELKRASVSQAVFARVAFNRTQGLLSEILRKEEDPRTASQSLLVNLRAMQN
FLNLPEVERDRIYQDERERSMNPNVSMVSSASSSPSSSRTPQAKTSTPTTDLPIKVDGAN
INITAAIYDEIQQEMKRAKVSQALFAKVAANKSQGWLCELLRWKENPSPENRTLWENLCT
IRRFLNLPQHERDVIYEEESRHHHSERMQHVVQLPPEPVQVLHRQQSQPAKESSPPREEA
PPPPPPTEDSCAKKPRSRTKISLEALGILQSFIHDVGLYPDQEAIHTLSAQLDLPKHTII
KFFQNQRYHVKHHGKLKEHLGSAVDVAEYKDEELLTESEENDSEEGSEEMYKVEAEEENA
DKSKAAPAEIDQR*
>ENSG00000133980|ENST00000256362
MTSRNQLVQKVLQELQEAVECEGLEGLIGASLEAKQVLSSFTLPTCREGGPGLQVLEVDS
VALSLYPEDAPRNMLPLVCKGEGSLLFEAASMLLWGDAGLSLELRARTVVEMLLHRHYYL
QGMIDSKVMLQAVRYSLCSEESPEMTSLPPATLEAIFDADVKASCFPSSFSNVWHLYALA
SVLQRNIYSIYPMRNLKIRPYFNRVIRPRRCDHVPSTLHIMWAGQPLTSHFFRHQYFAPV
VGLEEVEAEGAPGVAPALPALAPLSSPAKTLELLNREPGLSYSHLCERYSVTKSTFYRWR
RQSQEHRQKVAARFSAKHFLQDSFHRGGVVPLQQFLQRFPEISRSTYYAWKHELLGSGTC
PALPPREVLGMEELEKLPEEQVAEEELECSALAVSSPGMVLMQRAKLYLEHCISLNTLVP
YRCFKRRFPGISRSTYYNWRRKALRRNPSFKPAPALSAAGTPQLASVGEGAVIPWKSEAE
EGAGNATGEDPPAPGELLPLRMPLSRWQRRLRRAARRQVLSGHLPFCRFRLRYPSLSPSA
FWVWKSLARGWPRGLSKLQVPVPTLGKGGQEAEEKQEKEAGRDVTAVMAPPVGASSEDVE
GGPSREGALQEGATAQGQPHSGPLLSQPVVAAAGGRDGRMLVMDMIATTKFKAQAKLFLQ
KRFQSKSFPSYKEFSALFPLTARSTYYMWKRALYDGLTLVDG*
>ENSG00000069667|ENST00000261523
MNEGAPGDSDLETEARVPWSIMGHCLRTGQARMSATPTPAGEGARRDELFGILQILHQCI
LSSGDAFVLTGVCCSWRQNGKPPYSQKEDKEVQTGYMNAQIEIIPCKICGDKSSGIHYGV
ITCEGCKGFFRRSQQSNATYSCPRQKNCLIDRTSRNRCQHCRLQKCLAVGMSRDAVKFGR
MSKKQRDSLYAEVQKHRMQQQQRDHQQQPGEAEPLTPTYNISANGLTELHDDLSNYIDGH
TPEGSKADSAVSSFYLDIQPSPDQSGLDINGIKPEPICDYTPASGFFPYCSFTNGETSPT
VSMAELEHLAQNISKSHLETCQYLREELQQITWQTFLQEEIENYQNKQREVMWQLCAIKI
TEAIQYVVEFAKRIDGFMELCQNDQIVLLKAGSLEVVFIRMCRAFDSQNNTVYFDGKYAS
PDVFKSLGCEDFISFVFEFGKSLCSMHLTEDEIALFSAFVLMSADRSWLQEKVKIEKLQQ
KIQLALQHVLQKNHREDGILTKLICKVSTLRALCGRHTEKLMAFKAIYPDIVRLHFPPLY

KELFTSEFEPAMQIDG*
>ENSG00000075131|ENST00000261881
MLEPQENGVIDLPDYEHVEDETFPPFPPPASPERQDGEGTEPDEESGNGAPVRVPPKRTV
KRNIPKLDAQRLISERGLPALRHVFDKAKFKGKGHEAEDLKMLIRHMEHWAHRLFPKLQF
EDFIDRVEYLGSKKEVQTCLKRIRLDLPILHEDFVSNNDEVAENNEHDVTSTELDPFLTN
LSESEMFASELSRSLTEEQQQRIERNKQLALERRQAKLLSNSQTLGNDMLMNTPRAHTVE
EVNTDEDQKEESNGLNEDILDNPCNDAIANTLNEEETLLDQSFKNVQQQLDATSRNITEA
R*
>ENSG00000109832|ENST00000263576
MASLLWGGDAGAAESERLNSHFSNLSQPRKNLWGIKSTAVRNIDGSINNINEDDEEDVVD
LAANSLLNKLIHQSLVESSHRVEVLQKDPSSPLYSVKTFEELRLKEELLKGIYAMGFNRP
SKIQEMALPMMLAHPPQNLIAQSQSGTGKTAAFVLAMLSRVNALELFPQCLCLAPTYELA
LQTGRVVEQMGKFCVDVQVMYAIRGNRIPRGTDITKQIIIGTPGTVLDWCFKLKLIDLTK
IRVFVLDEADVMIDTQGFSDHSIRIQRALPSECQMLLFSATFEDSVWHFAERIIPDPNVI
KLRKEELTLNNIRQYYVLCEHRKDKYQALCNIYGSITIGQAIIFCQTRRNAKWLTVEMIQ
DGHQVSLLSGELTVEQRASIIQRFRDGKEKVLITTNVCARGIDVKQVTIVVNFDLPVKQG
EEPDYETYLHRIGRTGRFGKKGLAFNMIEVDELPSLMKIQDHFNSSIKQLNAEDMDEIEK
IDY*
>ENSG00000134748|ENST00000257181
MANRTVKDAHSIHGTNPQYLVEKIIRTRIYESKYWKEECFGLTAELVVDKAMELRFVGGV
YGGNIKPTPFLCLTLKMLQIQPEKDIIVEFIKNEDFKYVRMLGALYMRLTGTAIDCYKYL
EPLYNDYRKIKSQNRNGEFELMHVDEFIDELLHSERVCDIILPRLQKRYVLEEAEQLEPR
VSALEEDMDDVESSEEEEEEDEKLERVPSPDHRRRSYRDLDKPRRSPTLRYRRSRSRSPR
RRSRSPKRRSPSPRRERHRSKSPRRHRSRSRDRRHRSRSKSPGHHRSHRHRSHSKSPERS
KKSHKKSRRGNE*
>ENSG00000136504|ENST00000259021
MPRRKRNAGSSSDGTEDSDFSTDLEHTDSSESDGTSRRSARVTRSSARLSQSSQDSSPVR
NLQSFGTEEPAYSTRRVTRSQQQPTPVTPKKYPLRQTRSSGSETEQVVDFSDRETKNTAD
HDESPPRTPTGNAPSSESDIDISSPNVSHDESIAKDMSLKDSGSDLSHRPKRRRFHESYN
FNMKCPTPGCNSLGHLTGKHERHFSISGCPLYHNLSADECKVRAQSRDKQIEERMLSHRQ
DDNNRHATRHQAPTERQLRYKEKVAELRKKRNSGLSKEQKEKYMEHRQTYGNTREPLLEN
LTSEYDLDLFRRAQARASEDLEKLRLQGQITEGSNMIKTIAFGRYELDTWYHSPYPEEYA
RLGRLYMCEFCLKYMKSQTILRRHMAKCVWKHPPGDEIYRKGSISVFEVDGKKNKIYCQN
LCLLAKLFLDHKTLYYDVEPFLFYVMTEADNTGCHLIGYFSKEKNSFLNYNVSCILTMPQ
YMRQGYGKMLIDFSYLLSKVEEKVGSPERPLSDLGLISYRSYWKEVLLRYLHNFQGKEIS
IKEISQETAVNPVDIVSTLQALQMLKYWKGKHLVLKRQDLIDEWIAKEAKRSNSNKTMDP
SCLKWTPPKGT*
>ENSG00000131910|ENST00000254227
MSTSQPGACPCQGAASRPAILYALLSSSLKAVPRPRSRCLCRQHRPVQLCAPHRTCREAL
DVLAKTVAFLRNLPSFWQLPPQDQRRLLQGCWGPLFLLGLAQDAVTFEVAEAPVPSILKK
ILLEEPSSSGGSGQLPDRPQPSLAAVQWLQCCLESFWSLELSPKEYACLKGTILFNPDVP
GLQAASHIGHLQQEAHWVLCEVLEPWCPAAQGRLTRVLLTASTLKSIPTSLLGDLFFRPI
IGDVDIAGLLGDMLLLR*
>ENSG00000138646|ENST00000264350

MERRSRRKSRRNGRSTAGKAAATQPAKSPGAQLWLFPSAAGLHRALLRRVEVTRQLCCSP
GRLAVLERGGAGVQVHQLLAGSGGARTPKCIKLGKNMKIHSVDQGAEHMLILSSDGKPFE
YDNYSMKHLRFESILQEKKIIQITCGDYHSLALSKGGELFAWGQNLHGQLGVGRKFPSTT
TPQIVEHLAGVPLAQISAGEAHSMALSMSGNIYSWGKNECGQLGLGHTESKDDPSLIEGL
DNQKVEFVACGGSHSALLTQDGLLFTFGAGKHGQLGHNSTQNELRPCLVAELVGYRVTQI
ACGRWHTLAYVSDLGKVFSFGSGKDGQLGNGGTRDQLMPLPVKVSSSEELKLESHTSEKE
LIMIAGGNQSILLWIKKENSYVNLKRTIPTLNEGTVKRWIADVETKRWQSTKREIQEIFS
SPACLTGSFLRKKRRTTEMMPVYLDLNKARNIFKELTQKDWITNMITTCLKDNLLKRLPFH
SPPQEALEIFFLLPECPMMHISNNWESLVVPFAKVVCKMSDQSSLVLEEYWATLQESTFS
KLVQMFKTAVICQLDYWDESAEENGNVQALLEMLKKLHRVNQVKCQLPESIFQVDELLHR
LNFFVEVCRRYLWKMTVDASENVQCCVIFSHFPFIFNNLSKIKLLHTDTLLKIESKKHKA
YLRSAAIEEERESEFALRPTFDLTVRRNHLIEDVLNQLSQFENEDLRKELWVSFSGEIGY
DLGGVKKEFFYCLFAEMIQPEYGMFMYPEGASCMWFPVKPKFEKKRYFFFGVLCGLSLFN
CNVANLPFPLALFKKLLDQMPSLEDLKELSPDLGKNLQTLLDDEGDNFEEVFYIHFNVHW
DRNDTNLIPNGSSITVNQTNKRDYVSKYINYIFNDSVKAVYEEFRRGFYKMCDEDIIKLF
HPEELKDVIVGNTDYDWKTFEKNARYEPGYNSSHPTIVMFWKAFHKLTLEEKKKFLVFLT
GTDRLQMKDLNNMKITFCCPESWNERDPIRALTCFSVLFLPKYSTMETVEEALQEAINNN
RGFG*
>ENSG00000118156|ENST00000263351
XHSFQWLRNLPGCPKSKGNNVFVVHKPSAVPSREGSESGPGPSSGSPSEESPPGPGGGLE
DALPFPAALLRVPAEAPSDPRSASGEDDPCAPKKVKVDCDSFLCQNPGEPGLQEAQKAGG
LPADASPLFRQLFLKSQEPLVSHEQMQVFQMITKSQRIFSHAQVAAVSSQLPAPEGKPAA
LRPLQGPWPQQPPPLAPAVDSLHAGPGNPEAEGSPARRRKTTPGVPREASPGSTRRDAKG
GLKVAAVPTPLAAPSLDPSRNPDISSLAKQLRSSKGTLDLEDIFPSTGQRQTQLGGEEPP
GASLPGKQAPAENGAASRITKGEKGPACSRGGGYRLLGNPRAPRFSGFRKEKAKMDMCCA
ASPSQVAMASFSSAGPPADPSKSKLTIFSRIQGGNIYRLPHPVKEENVAGRGNQQNGSPT
DWTKPRSTFVCKNCSQMFYTEKGLSSHMCFHSDQWPSPRGKQEPQVFGTEFCKPLRQVLR
PEGDRHSPPGTKKPLDPTAAAPLVVPQSIPVVPVTRHIGSMAMIQTKTVAQCVEYYYIWK
KMIKFDCGRAPGLEKRVKREPEEVERTEEKVPCSPRERPSHHPTPKLKTKSYRRESILSS
SPNAGSKRTPELLGSAESQGIFPCRECERVFDKIKSRNAHMKRHRLQDHVEPIIRVKWPV
KPFQLKEEELGADIGPLQW*
>ENSG00000113048|ENST00000261413
MAASIVRRGMLLARQVVLPQLSPAGKRYLLSSAYVDSHKWEAREKEHYCLADLASLMDKT
FERKLPVSSLTISRLIDNISSREEIDHAEYYLYKFRHSPNCWYLRNWTIHTWIRQCLKYD
AQDKALYTLVNKVQYGIFPDNFTFNLLMDSFIKKENYKDALSVVFEVMMQEAFEVPSTQL
LSLYVLFHCLAKKTDFSWEEERNFGASLLLPGLKQKNSVGFSSQLYGYALLGKVELQQGL
RAVYHNMPLIWKPGYLDRALQVMEKVAASPEDIKLCREALDVLGAVLKALTSADGASEEQ
SQNDEDNQGSEKLVEQLDIEETEQSKLPQYLERFKALHSKLQALGKIESEGLLSLTTQLV
KEKLSTCEAEDIATYEQNLQQWHLDLVQLIQREQQQREQAKQEYQAQKAAKASA*
>ENSG00000135314|ENST00000257765
MDMGTSALSKKPWWTLPQNFHAPMVFHMEEDQEELIFGHGDTYLRCIEVHSHTLIQLESW
FTATGQTRVTVVGPHRARQWLLHMFCCVGSQDSYHHARGLEMLERVRSQPLTNDDLVTSI
SVPPYTGDLSLAPRISGTVCLSVPQPSPYQVIGCSGFHLSSLYP*
>ENSG00000071564|ENST00000262965

MNQPQRMAPVGTDKELSDLLDFSMMFPLPVTNGKGRPASLAGAQFGGSGLEDRPSSGSWG
SGDQSSSSFDPSRTFSEGTHFTESHSSLSSSTFLGPGLGGKSGERGAYASFGRDAGVGGL
TQAGFLSGELALNSPGPLSPSGMKGTSQYYPSYSGSSRRRAADGSLDTQPKKVRKVPPGL
PSSVYPPSSGEDYGRDATAYPSAKTPSSTYPAPFYVADGSLHPSAELWSPPGQAGFGPML
GGGSSPLPLPPGSGPVGSSGSSSTFGGLHQHERMGYQLHGAEVNGGLPSASSFSSAPGAT
YGGVSSHTPPVSGADSLLGSRGTTAGSSGDALGKALASIYSPDHSSNNFSSSPSTPVGSP
QGLAGTSQWPRAGAPGALSPSYDGGLHGLQSKIEDHLDEAIHVLRSHAVGTAGDMHTLLP
GHGALASGFTGPMSLGGRHAGLVGGSHPEDGLAGSTSLMHNHAALPSQPGTLPDLSRPPD
SYSGLGRAGATAAASEIKREEKEDEENTSAADHSEEEKKELKAPRARTSPDEDEDDLLPP
EQKAEREKERRVANNARERLRVRDINEAFKELGRMCQLHLNSEKPQTKLLILHQAVSVIL
NLEQQVRERNLNPKAACLKRREEEKVSGVVGDPQMVLSAPHPGLSEAHNPAGHM*
>ENSG00000104903|ENST00000264824
MCPPQAQAEVGPTMTEKAEMVCAPSPAPAPPPKPASPGPPQVEEVGHRGGSSPPRLPPGV
PVISLGHSRPPGVAMPTTELGTLRPPLLQLSTLGTAPPTLALHYHPHPFLNSVYIGPAGP
FSIFPSSRLKRRPSHCELDLAEGHQPQKVARRVFTNSRERWRQQNVNGAFAELRKLLPTH
PPDRKLSKNEVLRLAMKYIGFLVRLLRDQAAALAAGPTPPGPRKRPVHRVPDDGARRGSG
RRAEAAARSQPAPPADPDGSPGGAARPIKMEQTALSPEVR*
>ENSG00000137449|ENST00000259997
MPPPSPDSENGFYPGLPSSMNPAFFPSFSPVSPHGCTGLSVPTSGGGGGGFGGPFSATAV
PPPPPPAMNIPQQQPPPPAAPQQPQSRRSPVSPQLQQQHQAAAAAFLQQRNSYNHHQPLL
KQSPWSNHQSSGWGTGSMSWGAMHGRDHRRTGNMGIPGTMNQISPLKKPFSGNVIAPPKF
TRSTPSLTPKSWIEDNVFRTDNNSNTLLPLQVRSSLQLPAWGSDSLQDSWCTAAGTSRID
QDRSRMYDSLNMHSLENSLIDIMRAEHDPLKGRLSYPHPGTDNLLMLNARSYGRRRGRSS
LFPIDDGLLDDGHSDQVGVLNSPTCYSAHQNGERIERFSRKVFVGGLPPDIDEDEITASF
RRFGPLVVDWPHKAESKSYFPPKGYAFLLFQEESSVQALIDACIEEDGKLYLCVSSPTIK
DKPVQIRPWNLSDSDFVMDGSQPLDPRKTIFVGGVPRPLRAVELAMIMDRLYGGVCYAGI
DTDPELKYPKGAGRVAFSNQQSYIAAISARFVQLQHGDIDKRVEVKPYVLDDQMCDECQG
ARCGGKFAPFFCANVTCLQYYCEFCWANIHSRAGREFHKPLVKEGADRPRQIHFRWN*
>ENSG00000125492|ENST00000263610
MEGSNGFGIDSILSHRAGSPALPKGDPLLGDCRSPLELSPRSESSSDCSSPASPGRDCLE
TGTPRPGGASGPGLDSHLQPGQLSAPAQSRTVTSSFLIRDILADCKPLAACAPYSSSGQP
AAPEPGGRLAAKAAEDFRDKLDKSGSNASSDSEYKVKEEGDREISSSRDSPPVRLKKPRK
ARTAFTDHQLAQLERSFERQKYLSVQDRMELAASLNLTDTQVKTWYQNRRTKWKRQTAVG
LELLAEAGNYSALQRMFPSPYFYPQSLVSNLDPGAALYLYRGPSAPPPALQRPLVPRILI
HGLQGASEPPPPLPPLAGVLPRAAQPR*
>ENSG00000108651|ENST00000261708
MAEIIQERIEDRLPELEQLERIGLFSHAEIKAIIKKASDLEYKIQRRTLFKEDFINYVQY
EINLLELIQRRRTRIGYSFKKDEIENSIVHRVQGVFQRASAKWKDDVQLWLSYVAFCKKW
ATKTRLSKVFSAMLAIHSNKPALWIMAAKWEMEDRLSSESARQLFLRALRFHPECPKLYK
EYFRMELMHAEKLRKEKEEFEKASMDVENPDYSEEILKGELAWIIYKNSVSIIKGAEFHV
SLLSIAQLFDFAKDLQKEIYDDLQALHTDDPLTWDYVARRELEIESQTEEQPTTKQAKAV
EVGRKEERCCAVYEEAVKTLPTEAMWKCYITFCLERFTKKSNSGFLRGKRLERTMTVFRK
AHELKLLSECQYKQLSVSLLCYNFLREALEVAVAGTELFRDSGTMWQLKLQVLIESKSPD
IAMLFEEAFVHLKPQVCLPLWISWAEWSEGAKSQEDTEAVFKKALLAVIGADSVTLKNKY

LDWAYRSGGYKKARAVFKSLQESRPFSVDFFRKMIQFEKEQESCNMANIREYYERALREF
GSADSDLWMDYMKEELNHPLGRPENCGQIYWRAMKMLQGESAEAFVAKHAMHQTGHL*
>ENSG00000131876|ENST00000254193
MVKLTAELIEQAAQYTNAVRDRELDLRGYKIPVIENLGATLDQFDAIDFSDNEIRKLDGF
PLLRRLKTLLVNNNRICRIGEGLDQALPCLTELILTNNSLVELGDLDPLASLKSLTYLSI
LRNPVTNKKHYRLYVIYKVPQVRVLDFQKVKLKERQEAEKMFKGKRGAQLAKDIARRSKT
FNPGAGLPTDKKKGGPSPGDVEAIKNAIANASTLAEVERLKGLLQSGQIPGRERRSGPTD
DGEEEMEEDTVTNGS*
>ENSG00000101040|ENST00000262975
MDISTRSKDPGSAERTAQKRKFPSPPHSSNGHSPQDTSTSPIKKKKKPGLLNSNNKEQSE
LRHGPFYYMKQPLTTDPVDVVPQDGRNDFYCWVCHREGQVLCCELCPRVYHAKCLRLTSE
PEGDWFCPECEKITVAECIETQSKAMTMLTIEQLSYLLKFAIQKMKQPGTDAFQKPVPLE
QHPDYAEYIFHPMDLCTLEKNAKKKMYGCTEAFLADAKWILHNCIIYNGGNHKLTQIAKV
VIKICEHEMNEIEVCPECYLAACQKRDNWFCEPCSNPHPLVWAKLKGFPFWPAKALRDKD
GQVDARFFGQHDRAWVPINNCYLMSKEIPFSVKKTKSIFNSAMQEMEVYVENIRRKFGVF
NYSPFRTPYTPNSQYQMLLDPTNPSAGTAKIDKQEKVKLNFDMTASPKILMSKPVLSGGT
GRRISLSDMPRSPMSTNSSVHTGSDVEQDAEKKATSSHFSASEESMDFLDKSTASPASTK
TGQAGSLSGSPKPFSPQLSAPITTKTDKTSTTGSILNLNLDRSKAEMDLKELSESVQQQS
TPVPLISPKRQIRSRFQLNLDKTIESCKAQLGINEISEDVYTAVEHSDSEDSEKSDSSDS
EYISDDEQKSKNEPEDTEDKEGCQMDKEPSAVKKKPKPTNPVEIKEELKSTSPASEKADP
GAVKDKASPEPEKDFSEKAKPSPHPIKDKLKGKDETDSPTVHLGLDSDSESELVIDLGED
HSGREGRKNKKEPKEPSPKQDVVGKTPPSTTVGSHSPPETPVLTRSSAQTSAAGATATTS
TSSTVTVTAPAPAATGSPVKKQRPLLPKETAPAVQRVVWNSSTVQQKEITQSPSTSTITL
VTSTQSSPLVTSSGSMSTLVSSVNADLPIATASADVAADIAKYTSKMMDAIKGTMTEIYN
DLSKNTTGSTIAEIRRLRIEIEKLQWLHQQELSEMKHNLELTMAEMRQSLEQERDRLIAE
VKKQLELEKQQAVDETKKKQWCANCKKEAIFYCCWNTSYCDYPCQQAHWPEHMKSCTQSA
TAPQQEADAEVNTETLNKSSQGSSSSTQSAPSETASASKEKETSAEKSKESGSTLDLSGS
RETPSSILLGSNQGSVSKRCDKQPAYAPTTTDHQPHPNYPAQKYHSRSNKSSWSSSDEKR
GSTRSDHNTSTSTKSLLPKESRLDTFWD*
>ENSG00000110844|ENST00000261897
MPPPGIPPPFPPMGLPPMSQRPPAIPPMPPGILPPMLPPMGAPPPLTQIPGMVPPMMPGM
LMPAVPVTAATAPGADTASSAVAGTGPPRALWSEHVAPDGRIYYYNADDKQSVWEKPSVL
KSKAELLLSQCPWKEYKSDTGKPYYYNNQSKESRWTRPKDLDDLEVLVKQEAAGKQQQQL
PQTLQPQPPQPQPDPPPVPPGPTPVPTGLLEPEPGGSEDCDVLEATQPLEQGFLQQLEEG
PSSSGQHQPQQEEEESKPEPERSGLSWSNREKAKQAFKELLRDKAVPSNASWEQAMKMVV
TDPRYSALPKLSEKKQAFNAYKAQREKEEKEEARLRAKEAKQTLQHFLEQHERMTSTTRY
RRAEQTFGELEVWAVVPERDRKEVYDDVLFFLAKKEKEQAKQLRRRNIQALKSILDGMSS
VNFQTTWSQAQQYLMDNPSFAQDHQLQNMDKEDALICFEEHIRALEREEEEEERERARLRE
RRQQRKNREAFQTFLDELHETGQLHSMSTWMELYPAVSTDVRFANMLGQPGSTPLDLFKF
YVEELKARFHDEKKIIKDILKVNTAFEDFAHVISFDKRAAALDAGNIKLTFNSLLEKAEA
REREREKEEARRMRRREAAFRSMLRQAVPALELGTAWEEVRERFVCDSAFEQITLESERI
RLFREFLQVLEQTECQHLHTKGRKHGRKGKKHHHKRSHSPSGSESEEEELPPPSLRPPKR
RRRNPSESGSEPSSSLDSVESGGAALGGRGSPSSHLLGADHGLRKAKKPKKKTKKRRHKS
NSPESETDPEEKAGKESDEKEQEQDKDRELQQAELPNRSPGFGIKKEKTGWDTSESELSE

GELERRRRTLLQQLDDHQ*
>ENSG00000062194|ENST00000264779
MAQHDFAPAWLNFPTPPSSTKVLTSSLKSSLNFEKHSENFAWTENRYDVNRRRHNSSDGF
DSAIGRPNGGNFGRKEKNGWRTHGRNGTENINHRGGYHGGSSRSRSSIFHAGKSQGLHEN
NIPDNETGRKEDKRERKQFEAEDFPSLNPEYEREPNHNKSLAAGVWEYPPNPKSRAPRML
VIKKGNTKDLQLSGFPVVGNLPSQPVKNGTGPSVYKGLVPKPAAPPTKPTQWKSQTKENK
VGTSFPHESTFGVGNFNAFKSTAKNFSPSTNSVKECNRSNSSSPVDKLNQQPRLTKLTRM
RTDKKSEFLKALKRDRVEEEHEDESRAGSEKDDDSFNLHNSNSTHQERDINRNFDENEIP
QENGNASVISQQIIRSSTFPQTDVLSSSLEAEHRLLKEMGWQEDSENDETCAPLTEDEMR
EFQVISEQLQKNGLRKNGILKNGLICDFKFGPWKNSTFKPTTENDDTETSSSDTSDDDDV
*
>ENSG00000132510|ENST00000254846
MHRAVDPPGARAAREAFALGGLSCAGAWSSCPPHPPPRSAWLPGGRCSASIGQPPLPAPL
PPSHGSSSGHPSKPYYAPGAPTPRPLHGKLESLHGCVQALLREPAQPGLWEQLGQLYESE
HDSEEATRCYHSALRYGGSFAELGPRIGRLQQAQLWNFHTGSCQHRAKVLPPLEQVWNLL
HLEHKRNYGAKRGGPPVKRAAEPPVVQPVPPAALSGPSGEEGLSPGGKRRRGCNSEQTGL
PPGLPLPPPPLPPPPPPPPPPPPLPGLATSPPFQLTKPGLWSTLHGDAWGPERKGSAPP
ERQEQRHSLPHPYPYPAPAYTAHPPGHRLVPAAPPGPGPRPPGAESHGCLPATRPPGSDL
RESRVQRSRMDSSVSPAATTACVPYAPSRPPGLPGTTTSSSSSSSSNTGLRGVEPNPGIP
GADHYQTPALEVSHHGRLGPSAHSSRKPFLGAPAATPHLSLPPGPSSPPPPPCPRLLRPP
PPPAWLKGPACRAAREDGEILEELFFGTEGPPRPAPPPLPHREGFLGPPASRFSVGTQDS
HTPPTPPTPTTSSSNSNSGSHSSSPAGPVSFPPPPYLARSIDPLPRPPSPAQNPQDPPLV
PLTLALPPAPPSSCHQNTSGSFRRPESPRPRVSFPKTPEVGPGPPPGPLSKAPQPVPPGV
GELPARGPRLFDFPPTPLEDQFEEPAEFKILPDGLANIMKMLDESIRKEEEQQQHEAGVA
PQPPLKEPFASLQSPFPTDTAPTTTAPAVAVTTTTTTTTTTATQEEEKKPPPALPPPPP
LAKFPPPSQPQPPPPPPPSPASLLKSLASVLEGQKYCYRGTGAAVSTRPGPLPTTQYSPG
PPSGATALPPTSAAPSAQGSPQPSASSSSQFSTSGGPWARERRAGEEPVPGPMTPTQPPP
PLSLPPARSESEVLEEISRACETLVERVGRSATDPADPVDTAEPADSGTERLLPPAQAKE
EAGGVAAVSGSCKRRQKEHQKEHRRHRRACKDSVGRRPREGRAKAKAKVPKEKSRRVLGN
LDLQSEEIQGREKSRPDLGGASKAKPPTAPAPPSAPAPSAQPTPPSASVPGKKAREEAPG
PPGVSRADMLKLRSLSEGPPKELKIRLIKVESGDKETFIASEVEERRLRMADLTISHCAA
DVVRASRNAKVKGKFRESYLSPAQSVKPKINTEEKLPREKLNPPTPSIYLESKRDAFSPV
LLQFCTDPRNPITVIRGLAGSLRLNLGLFSTKTLVEASGEHTVEVRTQVQQPSDENWDLT
GTRQIWPCESSRSHTTIAKYAQYQASSFQESLQEEKESEDEESEEPDSTTGTPPSSAPDP
KNHHIIKFGTNIDLSDAKRWKPQLQELLKLPAFMRVTSTGNMLSHVGHTILGMNTVQLYM
KVPGSRTPGHQENNNFCSVNINIGPGDCEWFAVHEHYWETISAFCDRHGVDYLTGSWWPI
LDDLYASNIPVYRFVQRPGDLVWINAGTVHWVQATGWCNNIAWNVGPLTAYQYQLALERY
EWNEVKNVKSIVPMIHVSWNVARTVKISDPDLFKMIKFCLLQSMKHCQVQRESLVRAGKK
IAYQGRVKDEPAYYCNECDVEVFNILFVTSENGSRNTYLVHCEGCARRRSAGLQGVVVLE
QYRTEELAQAYDAFTLVRARRARGQRRRALGQAAGTGFGSPAAPFPEPPPAFSPQAPAST
SR*
>ENSG00000100764|ENST00000261303
MGQSQSGGHGPGGGKKDDKDKKKKYEPPVPTRVGKKKKKTKGPDAASKLPLVTPHTQCRL
KLLKLERIKDYLLMEEEFIRNQEQMKPLEEKQEEERSKVDDLRGTPMSVGTLEEIIDDNH

AIVSTSVGSEHYVSILSFVDKDLLEPGCSVLLNHKVHAVIGVLMDDTDPLVTVMKVEKAP
QETYADIGGLDNQIQEIKESVELPLTHPEYYEEMGIKPPKGVILYGPPGTGKTLLAKAVA
NQTSATFLRVVGSELIQKYLGDGPKLVRELFRVAEEHAPSIVFIDEIDAIGTKRYDSNSG
GEREIQRTMLELLNQLDGFDSRGDVKVIMATNRIETLDPALIRPGRIDRKIEFPLPDEKT
KKRIFQIHTSRMTLADDVTLDDLIMAKDDLSGADIKAICTEAGLMALRERRMKVTNEDFK
KSKENVLYKKQEGTPEGLYL*
>ENSG00000055609|ENST00000262189
MSSEEDKSVEQPQPPPPPPEEPGAPAPSPAAADKRPRGRPRKDGASPFQRARKKPRSRGK
TAVEDEDSMDGLETTETETIVETEIKEQSAEEDAEAEVDNSKQLIPTLQRSVSEESANSL
VSVGVEAKISEQLCAFCYCGEKSSLGQGDLKQFRITPGFILPWRNQPSNKKDIDDNSNGT
YEKMQNSAPRKQRGQRKERSPQQNIVSCVSVSTQTASDDQAGKLWDELSLVGLPDAIDIQ
ALFDSTGTCWAHHRCVEWSLGVCQMEEPLLVNVDKAVVSGSTERCAFCKHLGATIKCCEE
KCTQMYHYPCAAGAGTFQDFSHIFLLCPEHIDQAPERSKEDANCAVCDSPGDLLDQFFCT
TCGQHYHGMCLDIAVTPLKRAGWQCPECKVCQNCKQSGEDSKMLVCDTCDKGYHTFCLQP
VMKSVPTNGWKCKNCRICIECGTRSSSQWHHNCLICDNCYQQQDNLCPFCGKCYHPELQK
DMLHCNMCKRWVHLECDKPTDHELDTQLKEEYICMYCKHLGAEMDRLQPGEEVEIAELTT
DYNNEMEVEGPEDQMVFSEQAANKDVNGQESTPGIVPDAVQVHTEEQQKSHPSESLDTDS
LLIAVSSQHTVNTELEKQISNEVDSEDLKMSSEVKHICGEDQIEDKMEVTENIEVVTHQI
TVQQEQLQLLEEPETVVSREESRPPKLVMESVTLPLETLVSPHEESISLCPEEQLVIERL
QGEKEQKENSELSTGLMDSEMTPTIEGCVKDVSYQGGKSIKLSSETESSFSSSADISKAD
VSSSPTPSSDLPSHDMLHNYPSALSSSAGNIMPTTYISVTPKIGMGKPAITKRKFSPGRP
RSKQGAWSTHNTVSPPSWSPDISEGREIFKPRQLPGSAIWSIKVGRGSGFPGKRRPRGAG
LSGRGGRGRSKLKSGIGAVVLPGVSTADISSNKDDEENSMHNTVVLFSSSDKFTLNQDMC
VVCGSFGQGAEGRLLACSQCGQCYHPYCVSIKITKVVLSKGWRCLECTVCEACGKATDPG
RLLLCDDCDISYHTYCLDPPLQTVPKGGWKCKWCVWCRHCGATSAGLRCEWQNNYTQCAP
CASLSSCPVCYRNYREEDLILQCRQCDRWMHAVCQNLNTEEEVENVADIGFDCSMCRPYM
PASNVPSSDCCESSLVAQIVTKVKELDPPKTYTQDGVCLTESGMTQLQSLTVTVPRRKRS
KPKLKLKIINQNSVAVLQTPPDIQSEHSRDGEMDDSREGELMDCDGKSESSPEREAVDDE
TKGVEGTDGVKKRKRKPYRPGIGGFMVRQRSRTGQGKTKRSVIRKDSSGSISEQLPCRDD
GWSEQLPDTLVDESVSVTESTEKIKKRYRKRKNKLEETFPAYLQEAFFGKDLLDTSRQSK
ISLDNLSEDGAQLLYKTNMNTGFLDPSLDPLLSSSSAPTKSGTHGPADDPLADISEVLNT
DDDILGIISDDLAKSVDHSDIGPVTDDPSSLPQPNVNQSSRPLSEEQLDGILSPELDKMV
TDGAILGKLYKIPELGGKDVEDLFTAVLSPANTQPTPLPQPPPPTQLLPIHNQDAFSRMP
LMNGLIGSSPHLPHNSLPPGSGLGTFSAIAQSSYPDARDKNSAFNPMASDPNNSWTSSAP
TVEGENDTMSNAQRSTLKWEKEEALGEMATVAPVLYTNINFPNLKEEFPDWTTRVKQIAK
LWRKASSQERAPYVQKARDNRAALRINKVQMSNDSMKRQQQQDSIDPSSRIDSELFKDPL
KQRESEHEQEWKFRQQMRQKSKQQAKIEATQKLEQVKNEQQQQQQQQFGSQHLLVQSGSD
TPSSGIQSPLTPQPGNGNMSPAQSFHKELFTKQPPSTPTSTSSDDVFVKPQAPPPPPAPS
RIPIQDSLSQAQTSQPPSPQVFSPGSSNSRPPSPMDPYAKMVGTPRPPPVGHSFSRRNSA
APVENCTPLSSVSRPLQMNETTANRPSPVRDLCSSSTTNNDPYAKPPDTPRPVMTDQFPK
SLGLSRSPVVSEQTAKGPIAAGTSDHFTKPSPRADVFQRQRIPDSYARPLLTPAPLDSGP
GPFKTPMQPPPSSQDPYGSVSQASRRLSVDPYERPALTPRPIDNFSHNQSNDPYSQPPLT
PHPAVNESFAHPSRAFSQPGTISRPTSQDPYSQPPGTPRPVVDSYSQSSGTARSNTDPYS
QPPGTPRPTTVDPYSQQPQTPRPSTQTDLFVTPVTNQRHSDPYAHPPGTPRPGISVPYSQ

PPATPRPRISEGFTRSSMTRPVLMPNQDPFLQAAQNRGPALPGPLVRPPDTCSQTPRPPG
PGLSDTFSRVSPSAARDPYDQSPMTPRSQSDSFGTSQTAHDVADQPRPGSEGSFCASSNS
PMHSQGQQFSGVSQLPGPVPTSGVTDTQNTVNMAQADTEKLRQRQKLREIILQQQQQKKI
AGRQEKGSQDSPAVPHPGPLQHWQPENVNQAFTRPPPPYPGNIRSPVAPPLGPRYAVFPK
DQRGPYPPDVASMGMRPHGFRFGFPGGSHGTMPSQERFLVPPQQIQGSGVSPQLRRSVSV
DMPRPLNNSQMNNPVGLPQHFSPQSLPVQQHNILGQAYIELRHRAPDGRQRLPFSAPPGS
VVEASSNLRHGNFIPRPDFPGPRHTDPMRRPPQGLPNQLPVHPDLEQVPPSQQEQGHSVH
SSSMVMRTLNHPLGGEFSEAPLSTSVPSETTSDNLQITTQPSDGLEEKLDSDDPSVKELD
VKDLEGVEVKDLDDEDLENLNLDTEDGKVVELDTLDNLETNDPNLDDLLRSGEFDIIAYT
DPELDMGDKKSMFNEELDLPIDDKLDNQCVSVEPKKKEQENKTLVLSDKHSPQKKSTVTN
EVKTEVLSPNSKVESKCETEKNDENKDNVDTPCSQASAHSDLNDGEKTSLHPCDPDLFEK
RTNRETAGPSANVIQASTQLPAQDVINSCGITGSTPVLSSLLANEKSDNSDIRPSGSPPP
PTLPASPSNHVSSLPPFIAPPGRVLDNAMNSNVTVVSRVNHVFSQGVQVNPGLIPGQSTV
NHSLGTGKPATQTGPQTSQSGTSSMSGPQQLMIPQTLAQQNRERPLLLEEQPLLLQDLLD
QERQEQQQQRQMQAMIRQRSEPFFPNIDFDAITDPIMKAKMVALKGINKVMAQNNLGMPP
MVMSRFPFMGQVVTGTQNSEGQNLGPQAIPQDGSITHQISRPNPPNFGPGFVNDSQRKQY
EEWLQETQQLLQMQQKYLEEQIGAHRKSKKALSAKQRTAKKAGREFPEEDAEQLKHVTEQ
QSMVQKQLEQIRKQQKEHAELIEDYRIKQQQQCAMAPPTMMPSVQPQPPLIPGATPPTMS
QPTFPMVPQQLQHQQHTTVISGHTSPVRMPSLPGWQPNSAPAHLPLNPPRIQPPIAQLPI
KTCTPAPGTVSNANPQSGPPPRVEFDDNNPFSESFQERERKERLREQQERQRIQLMQEVD
RQRALQQRMEMEQHGMVGSEISSSRTSVSQIPFYSSDLPCDFMQPLGPLQQSPQHQQQMG
QVLQQQNIQQGSINSPSTQTFMQTNERRQVGPPSFVPDSPSIPVGSPNFSSVKQGHGNLS
GTSFQQSPVRPSFTPALPAAPPVANSSLPCGQDSTITHGHSYPGSTQSLIQLYSDIIPEE
KGKKKRTRKKKRDDDAESTKAPSTPHSDITAPPTPGISETTSTPAVSTPSELPQQADQES
VEPVGPSTPNMAAGQLCTELENKLPNSDFSQATPNQQTYANSEVDKLSMETPAKTEEIKL
EKAETESCPGQEEPKLEEQNGSKVEGNAVACPVSSAQSPPHSAGAPAAKGDSGNELLKHL
LKNKKSSSLLNQKPEGSICSEDDCTKDNKLVEKQNPAEGLQTLGAQMQGGFGCGNQLPKT
DGGSETKKQRSKRTQRTGEKAAPRSKKRKKDEEEKQAMYSSTDTFTHLKQQNNLSNPPTP
PASLPPTPPPMACQKMANGFATTEELAGKAGVLVSHEVTKTLGPKPFQLPFRPQDDLLAR
ALAQQGPKTVDVPASLPTPPHNNQEELRIQDHCGDRDTPDSFVPSSSPESVVGVEVSRYPD
LSLVKEEPPEPVPSPIIPILPSTAGKSSESRRNDIKTEPGTLYFASPFGPSPNGPRSGLI
SVAITLHPTAAENISSVVAAFSDLLHVRIPNSYEVSSAPDVPSMGLVSSHRINPGLEYRQ
HLLLRGPPPGSANPPRLVSSYRLKQPNVPFPPTSNGLSGYKDSSHGIAESAALRPQWCCH
CKVVILGSGVRKSFKDLTLLNKDSRESTKRVEKDIVFCSNNCFILYSSTAQAKNSENKES
IPSLPQSPMRETPSKAFHQYSNNISTLDVHCLPQLPEKASPPASPPIAFPPAFEAAQVEA
KPDELKVTVKLKPRLRAVHGGFEDCRPLNKKWRGMKWKKWSIHIVIPKGTFKPPCEDEID
EFLKKLGTSLKPDPVPKDYRKCCFCHEEGDGLTDGPARLLNLDLDLWVHLNCALWSTEVY
ETQAGALINVELALRRGLQMKCVFCHKTGATSGCHRFRCTNIYHFTCAIKAQCMFFKDKT
MLCPMHKPKGIHEQELSYFAVFRRVYVQRDEVRQIASIVQRGERDHTFRVGSLIFHTIGQ
LLPQQMQAFHSPKALFPVGYEASRLYWSTRYANRRCRYLCSIEEKDGRPVFVIRIVEQGH
EDLVLSDISPKGVWDKILEPVACVRKKSEMLQLFPAYLKGEDLFGLTVSAVARIAESLPG
VEACENYTFRYGRNPLMELPLAVNPTGCARSEPKMSAHVKRFVLRPHTLNSTSTSKSFQS
TVTGELNAPYSKQFVHSKSSQYRKMKTEWKSNVYLARSRIQGLGLYAARDIEKHTMVIEY
IGTIIRNEVANRKEKLYESQNRGVYMFRMDNDHVIDATLTGGPARYINHSCAPNCVAEVV

TFERGHKIIISSSRRIQKGEELCYDYKFDFEDDQHKIPCHCGAVNCRKWMN*
>ENSG00000131931|ENST00000254250
MVQSCSAYGCKNRYDKDKPVSFHKFPLTRPSLCKEWEAAVRRKNFKPTKYSSICSEHFTP
DCFKRECNNKLLKENAVPTIFLCTEPHDKKEDLLEPQEQLPPPPLPPPVSQVDAAIGLLM
PPLQTPVNLSVFCDHNYTVEDTMHQRKRIHQLEQQVEKLRKKLKTAQQRCRRQERQLEKL
KEVVHFQKEKDDVSERGYVILPNDYFEIVEVPA*
>ENSG00000136492|ENST00000259008
MSSMWSEYTIGGVKIYFPYKAYPSQLAMMNSILRGLNSKQHCLLESPTGSGKSLALLCSA
LAWQQSLSGKPADEGVSEKAEVQLSCCCACHSKDFTNNDMNQGTSRHFNYPSTPPSERNG
TSSTCQDSPEKTTLAAKLSAKKQASIYRDENDDFQVEKKRIRPLETTQQIRKRHCFGTEV
HNLDAKVDSGKTVKLNSPLEKINSFSPQKPPGHCSRCCCSTKQGNSQESSNTIKKDHTGK
SKIPKIYFGTRTHKQIAQITRELRRTAYSGVPMTILSSRDHTCVHPEVVGNFNRNEKCME
LLDGKNGKSCYFYHGVHKISDQHTLQTFQGMCKAWDIEELVSLGKKLKACPYYTARELIQ
DADIIFCPYNYLLDAQIRESMDLNLKEQVVILDEAHNIEDCARESASYSVTEVQLRFARD
ELDSMVNNNIRKKDHEPLRAVCCSLINWLEANAEYLVERDYESACKIWSGNEMLLTLHKM
GITTATFPILQGHFSAVLQKEEKISPIYGKEEAREVPVISASTQIMLKGLFMVLDYLFRQ
NSRFADDYKIAIQQTYSWTNQIDISDKNGLLVLPKNKKRSRQKTAVHVLNFWCLNPAVAF
SDINGKVQTIVLTSGTLSPMKSFSSELGVTFTIQLEANHIIKNSQVWVGTIGSGPKGRNL
CATFQNTETFEFQDEVGALLLSVCQTVSQGILCFLPSYKLLEKLKERWLSTGLWHNLELV
KTVIVEPQGGEKTNFDELLQVYYDAIKYKGEKDGALLVAVCRGKVSEGLDFSDDNARAVI
TIGIPFPNVKDLQVELKRQYNDHHSKLRGLLPGRQWYEIQAYRALNQALGRCIRHRNDWG
ALILVDDRFRNNPSRYISGLSKWVRQQIQHHSTFESALESLAEFSKKHQKVLNVSIKDRT
NIQDNESTLEVTSLKYSTSPYLLEAASHLSPENFVEDEAKICVQELQCPKIITKNSPLPS
SIISRKEKNDPVFLEEAGKAEKIVISRSTSPTFNKQTKRVSWSSFNSLGQYFTGKIPKAT
PELGSSENSASSPPRFKTEKMESKTVLPFTDKCESSNLTVNTSFGSCPQSETIISSLKID
ATLTRKNHSEHPLCSEEALDPDIELSLVSEEDKQSTSNRDFETEAEDESIYFTPELYDPE
DTDEEKNDLAETDRGNRLANNSDCILAKDLFEIRTIKEVDSAREVKAEDCIDTKLNGILH
IEESKIDDIDGNVKTTWINELELGKTHEIEIKNFKPSPSKNKGMFPGFK*
>ENSG00000130675|ENST00000252971
MEKSKNFRIDALLAVDPPRAASAQSAPLALVTSLAAAASGTGGGGGGGGASGGTSGSCSP
ASSEPPAAPADRLRAESPSPPRLLAAHCALLPKPGFLGAGGGGGGTGGGHGGPHHHAHPG
AAAAAAAAAAAAAAGGLALGLHPGGAQGGAGLPAQAALYGHPVYGYSAAAAAAALAGQHP
ALSYSYPQVQGAHPAHPADPIKLGAGTFQLDQWLRASTAGMILPKMPDFNSQAQSNLLGK
CRRPRTAFTSQQLLELEHQFKLNKYLSRPKRFEVATSLMLTETQVKIWFQNRRMKWKRSK
KAKEQAAQEAEKQKGGGGGAGKGGAEEPGAEELLGPPAPGDKGSGRRLRDLRDSDPEEDE
DEDDEDHFPYSNGASVHAASSDCSSEDDSPPPRPSHQPAPQ*
>ENSG00000109674|ENST00000264596
MVEGPGCTLNGEKIRARVLPGQAVTGVRGSALRSLQGRALRLAASTVVVSPQAAALNNDS
SQNVLSLFNGYVYSGVETLGKELFMYFGPKALRIHFGMKGFIMINPLEYKYKNGASPVLE
VQLTKDLICFFDSSVELRNSMESQQRIRMMKELDVCSPEFSFLRAESEVKKQKGRMLGDV
LMDQNVLPGVGNIIKNEALFDSGLHPAVKVCQLTDEQIHHLMKMIRDFSILFYRCRKAGL
ALSKHYKVYKRPNCGQCHCRITVCRFGDNNRMTYFCPHCQKENPQHVDICKLPTRNTIIS
WTSSRVDHVMDSVARKSEEHWTCVVCTLINKPSSKACDACLTSRPIDSVLKSEENSTVFS
HLMKYPCNTFGKPHTEVKINRKTAFGTTTLVLTDFSNKSSTLERKTKQNQILDEEFQNSP

PASVCLNDIQHPSKKTTNDITQPSSKVNISPTISSESKLFSPAHKKPKTAQYSSPELKSC
NPGYSNSELQINMTDGPRTLNPDSPRCSKHNRLCILRVVGKDGENKGRQFYACPLPREAQ
CGFFEWADLSFPFCNHGKRSTMKTVLKIGPNNGKNFFVCPLGKEKQCNFFQWAENGPGIK
IIPGC*
>ENSG00000136870|ENST00000259395
MMENYGNLVSLDVLNRDKDEEPTVKQEIEEIEEEVEPQGVIVTRIKSEIDQDPMGRETFE
LVGRLDKQRGIFLWEIPRESLTQEQRMFRENTNIIRKRPNSEEKCHKCEECGKGFVRKAH
FIQHQRVHTGEKPFQCNECGKSFSRSSFVIEHQRIHTGERPYECNYCGKTFSVSSTLIRH
QRIHTGERPYQCNQCKQSFSQRRSLVKHQRIHTGEKPHKCSDCGKAFSWKSHLIEHQRTH
TGEKPYHCTKCKKSFSRNSLLVEHQRIHTGERPHKCGECGKAFRLSTYLIQHQKIHTGEK
PFLCIECGKSFSRSSFLIEHQRIHTGERPYQCKECGKSFSQLCNLTRHQRIHTGDKPHKC
EECGKAFSRSSGLIQHQRIHTREKTYPYNETKESFDPNCSLVIQQEVYPKEKSYKCDECG
KTFSVSAHLVQHQRIHTGEKPYLCTVCGKSFSRSSFLIEHQRIHTGERPYLCRQCGKSFS
QLCNLIRHQGVHTGNKPHKCDECGKAFSRNSGLIQHQRIHTGEKPYKCEKCDKSFSQQRS
LVNHQKIHAEVKTQETHECDACGEAFNCRISLIQHQKLHTAWMQ*
>ENSG00000137709|ENST00000260264
MESPRTAKGGRDIKMSGDVADSTDARSTLSQVEPGNDRNGLDFNRQIKTEDLSDSLQQTL
SHRPCHLSQGPAMMSGNQMSGLNASPCQDMASLHPLQQLVLVPGHLQSVSQFLLSQTQPG
QQGLQPNLLPFPQQQSGLLLPQTGPGLASQAFGHPGLPGSSLEPHLEASQHLPVPKHLPS
SGGADEPSDLEELEKFAKTFKQRRIKLGFTQGDVGLAMGKLYGNDFSQTTISRFEALNLS
FKNMCKLKPLLEKWLNDAESSPSDPSVSTPSSYPSLSEVFGRKRKKRTSIETNIRLTLEK
RFQDNPKPSSEEISMIAEQLSMEKEVVRVWFCNRRQKEKRINCPVATPIKPPVYNSRLVS
PSGSLGPLSVPPVHSTMPGTVTSSCSPGNNSRPSSPGSGLHASSPTASQNNSKAAVNSAS
SFNSSGSWYRWNHSTYLH*
>ENSG00000073146|ENST00000262794
MLSLAAKLVAFFWRTADTPREEAGQLEPELAEGDTKLKTVRGVVTRYCSDYGMIDDMIYF
SSDAVTSRVLLNVGQEVIAVVEENKVSNGLKAIRVEAVSDKWEDDSRNHGSPSDCGPRVL
IGCVTSLVEGAGCISQTTYFSLESVCEGFEPCKGDWVEAEYRIRPGTWSSEATSVKPLRY
KRVDKVCISSLCGRNGVLEESIFFTLDSLKLPDGYTPRRGDVVNAVVVESSQSCYVWRAL
CMTLVKRRDAAPVHEATHFYGTILLKNKGDIEVTQVTHFGTLKEGRSKTMVIWIENKGDI
PQNLVSCKLAGWDKSKQFRFQMLDKDQMCPVVSFVSVPEKENSSDENINSLNSHTKNKTS
QMSESSLVNNRGISPGDCTCKGENGEKDNILSRKQMTEPEPGGLVPPGGKTFIVVICDGK
NPGRCKELLLLCFSDFLIGRYLEVNVISGEESLIAAREPFSWKKLKSSQALTSAKTTVVV
TAQKRNSRRQLPSFLPQYPIPDRLRKCVEQKIDILTFQPLLAELLNMSNYKEKFSTLLWL
EEIYAEMELKEYNMSGIILRRNGDLLVLEVPGLAEGRPSLYAGDKLILKTQEYNGHAIEY
ISYVTEIHEEDVTLKINPEFEQAYNFEPMDVEFTYNRTTSRRCHFALEHVIHLGVKVLFP
EEIILQSPQVTGNWNHAQDTKSSGQSTSKKNRKTMTDQAEHGTEERRVGDKDLPVLAPFT
AEMSDWVDEIQTPKARKMEFFNPVLNENQKLAVKRILSGDCRPLPYILFGPPGTGKTVTI
IEAVLQVHFALPDSRILVCAPSNSAADLVCLRLHESKVLQPATMVRVNATCRFEEIVIDA
VKPYCRDGEDIWKASRFRIIITTCSSSGLFYQIGVRVGHFTHVFVDEAGQASEPECLIPL
GLMSDISGQIVLAGDPMQLGPVIKSRLAMAYGLNVSFLERLMSRPAYQRDENAFGACGAH
NPLLVTKLVKNYRSHEALLMLPSRLFYHRELEVCADPTVVTSLLGWEKLPKKGFPLIFHG
VRGSEAREGKSPSWFNPAEAVQVLRYCCLLAHSISSQVSASDIGVITPYRKQVEKIRILL
RNVDLMDIKVGSVEEFQGQEYLVIIISTVRSNEDRFEDDRYFLGFLSNSKRFNVAITRPK

ALLIVLGNPHVLVRDPCFGALLEYSITNGVYMGCDLPPALQSLQNCGEGVADPSYPVVPE
STGPEKHQEPS*
>ENSG00000087274|ENST00000264758
MNGDSRAAVVTSPPPTTAPHKERYFDRVDENNPEYLRERNMAPDLRQDFNMMEQKKRVSM
ILQSPAFCEELESMIQEQFKKGKNPTGLLALQQIADFMTTNVPNVYPAAPQGGMAALNMS
LGMVTPVNDLRGSDSIAYDKGEKLLRCKLAAFYRLADLFGWSQLIYNHITTRVNSEQEHF
LIVPFGLLYSEVTASSLVKINLQGDIVDRGSTNLGVNQAGFTLHSAIYAARPDVKCVVHI
HTPAGAAVSAMKCGLLPISPEALSLGEVAYHDYHGILVDEEEKVLIQKNLGPKSKVLILR
NHGLVSVGESVEEAFYYIHNLVVACEIQVRTLASAGGPDNLVLLNPEKYKAKSRSPGSPV
GEGTGSPPKWQIGEQEFEALMRMLDNLGYRTGYPYRYPALREKSKKYSDVEVPASVTGYS
FASDGDSGTCSPLRHSFQKQQREKTRWLNSGRGDEASEEGQNGSSPKSKTKVWTNITHDH
VKPLLQSLSSGVCVPSCITNCLWTKEDGHRTSTSAVPNLFVPLNTNPKEVQEMRNKIREQ
NLQDIKTAGPQSQVLCGVVMDRSLVQGELVTASKAIIEKEYQPHVIVSTTGPNPFTTLTD
RELEEYRREVERKQKGSEENLDEAREQKEKSPPDQPAVPHPPPSTPIKLEEDLVPEPTTG
DDSDAATFKPTLPDLSPDEPSEALGFPMLEKEEEAHRPPSPTEAPTEASPEPAPDPAPVA
EEAAPSAVEEGAAADPGSDGSPGKSPSKKKKKFRTPSFLKKSKKKSDS*
>ENSG00000137804|ENST00000260359
MIIPSLEELDSLKYSDLQNLAKSLGLRANLRATKLLKALKGYIKHEARKGNENQDESQTS
ASSCDETEIQISNQEEAERQPLGHVTKTRRRCKTVRVDPDSQNHEKQESQDLRATAKVPS
PPDEHQEAENAVSSGNRDSKVPSEGKKSLYTDESSKPGKNKRTAITTPNFKKLHEAHFKE
MESIDQYIERKKKHFEEHNSMNELKQQPINKGGVRTPVPPRGRLSVASTPISQRRSQGRS
CGPASQSTLGLKGSLKRSAISAAKTGVRFSAATKDNEHKRSLTKTPARKSAHVTVSGGTP
KGEAVLGTHKLKTITGNSAAVITPFKLTTEATQTPVSNKKPVFDLKASLSRPLNYEPHKG
KLKPWGQSKENNYLNQHVNRINFYKKTYKQPHLQTKEEQRKKREQERKEKKAKVLGMRRG
LILAED*
>ENSG00000063046|ENST00000262056
MAASAKKKNKKGKTISLTDFLAEDGGTGGGSTYVSKPVSWADETDDLEGDVSTTWHSNDD
DVYRAPPIDRSILPTAPRAAREPNIDRSRLPKSPPYTAFLGNLPYDVTEESIKEFFRGLN
ISAVRLPREPSNPERLKGFGYAEFEDLDSLLSALSLNEESLGNRRIRVDVADQAQDKDRD
DRSFGRDRNRDSDKTDTDWRARPATDSFDDYPPRRGDDSFGDKYRDRYDSDRYRDGYRDG
YRDGPRRDMDRYGGRDRYDDRGSRDYDRGYDSRIGSGRRAFGSGYRRDDDYRGGGDRYED
RYDRRDDRSWSSRDDYSRDDYRRDDRGPPQRPKLNLKPRSTPKEDDSSASTSQSTRAASI
FGGAKPVDTAAREREVEERLQKEQEKLQRQLDEPKLERRPRERHPSWRSEETQERERSRT
GSESSQTGTSTTSSRNARRRESEKSLENETLNKEEDCHSPTSKPPKPDQPLKVMPAPPPK
ENAWVKRSSNPPARSQSSDTEQQSPTSGGGKVAPAQPSEEGPGRKDENKVDGMNAPKGQT
GNSSRGPGDGGNRDHWKESDRKDGKKDQDSRSAPEPKKPEENPASKFSSASKYAALSVDG
EDENEGEDYAE*
>ENSG00000075292|ENST00000264447
MSRPRFNPRGDFPLQRPRAPNPSGMRPPGPFMRPGSMGLPRFYPAGRARGIPHRFAGHES
YQNMGPQRMNVQVTQHRTDPRLTKEKLDFHEAQQKKGKPHGSRWDDEPHISASVAVKQSS
VTQVTEQSPKVQSRYTKESASSILASFGLSNEDLEELSRYPDEQLTPENMPLILRDIRMR
KMGRRLPNLPSQSRNKETLGSEAVSSNVIDYGHASKYGYTEDPLEVRIYDPEIPTDEVEN
EFQSQQNISASVPNPNVICNSMFPVEDVFRQMDFPGESSNNRSFFSVESGTKMSGLHISG
GQSVLEPIKSVNQSINQTVSQTMSQSLIPPSMNQQPFSSELISSVSQQERIPHEPVINSS

NVHVGSRGSKKNYQSQADIPIRSPFGIVKASWLPKFSHADAQKMKRLPTPSMMNDYYAAS
PRIFPHLCSLCNVECSHLKDWIQHQNTSTHIESCRQLRQQYPDWNPEILPSRRNEGNRKE
NETPRRRSHSPSPRRSRRSSSSHRFRRSRSPMHYMYRPRSRSPRICHRFISRYRSRSRSR
SPYRIRNPFRGSPKCFRSVSPERMSRRSVRSSDRKKALEDVVQRSGHGTEFNKQKHLEAA
DKGHSPAQKPKTSSGTKPSVKPTSATKSDSNLGGHSIRCKSKNLEDDTLSECKQVSDKAV
SLQRKLRKEQSLHYGSVLLITELPEDGCTEEDVRKLFQPFGKVNDVLIVPYRKEAYLEME
FKEAITAIMKYIETTPLTIKGKSVKICVPGKKKAQNKEVKKKTLESKKVSASTLKRDADA
SKAVEIVTSTSAAKTGQAKASVAKVNKSTGKSASSVKSVVTVAVKGNKASIKTAKSGGKK
SLEAKKTGNVKNKDSNKPVTIPENSEIKTSIEVKATENCAKEAISDAALEATENEPLNKE
TEEMCVMLVSNLPNKGYSVEEVYDLAKPFGGLKDILILSSHKKAYIEINRKAAESMVKFY
TCFPVLMDGNQLSISMAPENMNIKDEEAIFITLVKENDPEANIDTIYDRFVHLDNLPEDG
LQCVLCVGLQFGKVDHHVFISNRNKAILQLDSPESAQSMYSFLKQNPQNIGDHMLTCSLS
PKIDLPEVQIEHDPELEKESPGLKNSPIDESEVQTATDSPSVKPNELEEESTPSIQTETL
VQQEEPCEEEAEKATCDSDFAVETLELETQGEEVKEEIPLVASASVSIEQFTENAEECAL
NQQMFNSDLEKKGAEIINPKTALLPSDSVFAEERNLKGILEESPSEAEDFISGITQTMVE
AVAEVEKNETVSEILPSTCIVTLVPGIPTGDEKTVDKKNISEKKGNMDEKEEKEFNTKET
RMDLQIGTEKAEKNEGRMDAEKVEKMAAMKEKPAENTLFKAYPNKGVGQANKPDETSKTS
ILAVSDVSSSKPSIKAVIVSSPKAKATVSKTENQKSFPKSVPRDQINAEKKLSAKEFGLL
KPTSARSGLAESSSKFKPTQSSLTRGGSGRISALQGKLSKLDYRDITKQSQETEARPSIM
KRDDSNNKTLAEQNTKNPKSTTGRSSKSKEEPLFPFNLDEFVTVDEVIEEVNPSQAKQNP
LKGKRKETLKNVPFSELNLKKKKGKTSTPRGVEGELSFVTLDEIGEEEDAAAHLAQALVT
VDEVIDEEELNMEEMVKNSNSLFTLDELIDQDDCISHSEPKDVTVLSVAEEQDLLKQERL
VTVDEIGEVEELPLNESADITFATLNTKGNEGDTVRDSIGFISSQVPEDPSTLVTVDEIQ
DDSSDLHLVTLDEVTEEDEDSLADFNNLKEELNFVTVDEVGEEEDGDNDLKVELAQSKND
HPTDKKGNRKKRAVDTKKTKLESLSQVGPVNENVMEEDLKTMIERHLTAKTPTKRVRIGK
TLPSEKAVVTEPAKGEEAFQMSEVDEESGLKDSEPERKRKKTEDSSSGKSVASDVPEELD
FLVPKAGFFCPICSLFYSGEKAMTNHCKSTRHKQNTEKFMAKQRKEKEQNEAEERSSR*
>ENSG00000138385|ENST00000260956
MAENGDNEKMAALEAKICHQIEYYFGDFNLPRDKFLKEQIKLDEGWVPLEIMIKFNRLNR
LTTDFNVIVEALSKSKAELMEISEDKTKIRRSPSKPLPEVTDEYKNDVKNRSVYIKGFPT
DATLDDIKEWLEDKGQVLNIQMRRTLHKAFKGSIFVVFDSIESAKKFVETPGQKYKETDL
LILFKDDYFAKKNEERKQNKVEAKLRAKQEQEAKQKLEEDAEMKSLEEKIGCLLKFSGDL
DDQTCREDLHILFSNHGEIKWIDFVRGAKEGIILFKEKAKEALGKAKDANNGNLQLRNKE
VTWEVLEGEVEKEALKKIIEDQQESLNKWKSKGRRFKGKGKGNKAAQPGSGKGKVQFQGK
KTKFASDDEHDEHDENGATGPVKRAREETDKEEPASKQQKTENGAGDQ*
>ENSG00000077097|ENST00000264331
MAKSGGCGAGAGVGGGNGALTWVTLFDQNNAAKKEESETANKNDSSKKLSVERVYQKKTQ
LEHILLRPDTYIGSVEPLTQFMWVYDEDVGMNCREVTFVPGLYKIFDEILVNAADNKQRD
KNMTCIKVSIDPESNIISIWNNGKGIPVVEHKVEKVYVPALIFGQLLTSSNYDDDEKKVT
GGRNGYGAKLCNIFSTKFTVETACKEYKHSFKQTWMNNMMKTSEAKIKHFDGEDYTCITF
QPDLSKFKMEKLDKDIVALMTRRAYDLAGSCRGVKVMFNGKKLPVNGFRSYVDLYVKDKL
DETGVALKVIHELANERWDVCLTLSEKGFQQISFVNSIATTKGGRHVDYVVDQVVGKLIE
VVKKKNKAGVSVKPFQVKNHIWVFINCLIENPTFDSQTKENMTLQPKSFGSKCQLSEKFF
KAASNCGIVESILNWVKFKAQTQLNKKCSSVKYSKIKGIPKLDDANDAGGKHSLECTLIL

TEGDSAKSLAVSGLGVIGRDRYGVFPLRGKILNVREASHKQIMENAEINNIIKIVGLQYK
KSYDDAESLKTLRYGKIMIMTDQDQDGSHIKGLLINFIHHNWPSLLKHGFLEEFITPIVK
ASKNKQELSFYSIPEFDEWKKHIENQKAWKIKYYKGLGTSTAKEAKEYFADMERHRILFR
YAGPEDDAAITLAFSKKKIDDRKEWLTNFMEDRRQRRLHGLPEQFLYGTATKHLTYNDFI
NKELILFSNSDNERSIPSLVDGFKPGQRKVLFTCFKRNDKREVKVAQLAGSVAEMSAYHH
GEQALMMTIVNLAQNFVGSNNINLLQPIGQFGTRLHGGKDAASPRYIFTMLSTLARLLFP
AVDDNLLKFLYDDNQRVEPEWYIPIIPMVLINGAEGIGTGWACKLPNYDAREIVNNVRRM
LDGLDPHPMLPNYKNFKGTIQELGQNQYAVSGEIFVVDRNTVEITELPVRTWTQVYKEQV
LEPMLNGTDKTPALISDYKEYHTDTTVKFVVKMTEEKLAQAEAAGLHKVFKLQTTLTCNS
MVLFDHMGCLKKYETVQDILKEFFDLRLSYYGLRKEWLVGMLGAESTKLNNQARFILEKI
QGKITIENRSKKDLIQMLVQRGYESDPVKAWKEAQEKAAEEDETQNQHDDSSSDSGTPSG
PDFNYILNMSLWSLTKEKVEELIKQRDAKGREVNDLKRKSPSDLWKEDLAAFVEELDKVE
SQEREDVLAGMSGKAIKGKVGKPKVKKLQLEETMPSPYGRRIIPEITAMKADASKKLLKK
KKGDLDTAAVKVEFDEEFSGAPVEGAGEEALTPSVPINKGPKPKREKKEPGTRVRKTPTS
SGKPSAKKVKKRNPWSDDESKSESDLEETEPVVIPRDSLLRRAAAERPKYTFDFSEEEDD
DADDDDDDNNDLEELKVKASPITNDGEDEFVPSDGLDKDEYTFSPGKSKATPEKSLHDKK
SQDFGNLFSFPSYSQKSEDDSAKFDSNEEDSASVFSPSFGLKQTDKVPSKTVAAKKGKPS
SDTVPKPKRAPKQKKVVEAVNSDSDSEFGIPKKTTTPKGKGRGAKKRKASGSENEGDYNP
GRKTSKTTSKKPKKTSFDQDSDVDIFPSDFPTEPPSLPRTGRARKEVKYFAESDEEEDDV
DFAMFN*
>ENSG00000103479|ENST00000262133
MPSGGDQSPPPPPPPPAAAASDEEEEDDGEAEDAAPPAESPTPQIQQRFDELCSRLNMDE
AARAEAWDSYRSMSESYTLEGNDLHWLACALYVACRKSVPTVSKGTVEGNYVSLTRILKC
SEQSLIEFFNKMKKWEDMANLPPHFRERTERLERNFTVSAVIFKKYEPIFQDIFKYPQEE
QPRQQRGRKQRRQPCTVSEIFHFCWVLFIYAKGNFPMISDDLVNSYHLLLCALDLVYGNA
LQCSNRKELVNPNFKGLSEDFHAKDSKPSSDPPCIIEKLCSLHDGLVLEAKGIKEHFWKP
YIRKLYEKKLLKGKEENLTGFLEPGNFGESFKAINKAYEEYVLSVGNLDERIFLGEDAEE
EIGTLSRCLNAGSGTETAERVQMKNILQQHFDKSKALRISTPLTGVRYIKENSPCVTPVS
TATHSLSRLHTMLTGLRNAPSEKLEQILRTCSRDPTQAIANRLKEMFEIYSQHFQPDEDF
SNCAKEIASKHFRFAEMLYYKVLESVIEQEQKRLGDMDLSGILEQDAFHRSLLACCLEVV
TFSYKPPGNFPFITEIFDVPLYHFYKVIEVFIRAEDGLCREVVKHLNQIEEQILDHLAWK
PESPLWEKIRDNENRVPTCEEVMPPQNLERADEICIAGSPLTPRRVTEVRADTGGLGRSI
TSPTTLYDRYSSPPASTTRRRLFVENDSPSDGGTPGRMPPQPLVNAVPVQNVSGETVSVT
PVPGQTLVTMATATVTANNGQTVTIPVQGIANENGGITFFPVQVNVGGQAQAVTGSIQPL
SAQALAGSLSSQQVTGTTLQVPGQVAIQQISPGGQQQKQGQSVTSSSNRPRKTSSLSLFF
RKVYHLAAVRLRDLCAKLDISDELRKKIWTCFEFSIIQCPELMMDRHLDQLLMCAIYVMA
KVTKEDKSFQNIMRCYRTQPQARSQVYRSVLIKGKRKRRNSGSSDSRSHQNSPTELNKDR
TSRDSSPVMRSSSTLPVPQPSSAPPTPTRLTGANSDMEEEERGDLIQFYNNIYIKQIKTF
AMKYSQANMDAPPLSPYPFVRTGSPRRIQLSQNHPVYISPHKNETMLSPREKIFYYFSNS
PSKRLREINSMIRTGETPTKKRGILLEDGSESPAKRICPENHSALLRRLQDVANDRGSH*
>ENSG00000125618|ENST00000263335
MPHNSIRSGHGGLNQLGGAFVNGRPLPEVVRQRIVDLAHQGVRPCDISRQLRVSHGCVSK
ILGRYYETGSIRPGVIGGSKPKVATPKVVEKIGDYKRQNPTMFAWEIRDRLLAEGVCDND
TVPSVSSINRIIRTKVQQPFNLPMDSCVATKSLSPGHTLIPSSAVTPPESPQSDSLGSTY

SINGLLGIAQPGSDKRKMDDSDQDSCRLSIDSQSSSSGPRKHLRTDAFSQHHLEPLECPF
ERQHYPEAYASPSHTKGEQGERWWGPRCPDTHPTSPPADRAAMPPLPSQAWWQEVNTLAM
PMATPPTPPTARPGASPTPAC*
>ENSG00000115207|ENST00000264720
MDTCGVGYVALGEAGPVGNMTVVDSPGQEVLNQLDVKTSSEMTSAEASVEMSLPTPLPGF
EDSPDQRRLPPEQESLSRLEQPDLSSEMSKVSKPRASKPGRKRGGRTRKGPKRPQQPNPP
SAPLVPGLLDQSNPLSTPMPKKRGRKSKAELLLLKLSKDLDRPESQSPKRPPEDFETPSG
ERPRRRAAQVALLYLQELAEELSTALPAPVSCPEGPKVSSPTKPKKIRQPAACPGGEEVD
GAPRDEDFFLQVEAEDVEESEGPSESSSEPEPVVPRSTPRGSTSGKQKPHCRGMAPNGLP
NHIMAPVWKCLHLTKDFREQKHSYWEFAEWIPLAWKWHLLSELEAAPYLPQEEKSPLFSV
QREGLPEDGTLYRINRFSSITAHPERWDVSFFTGGPLWALDWCPVPEGAGASQYVALFSS
PDMNETHPLSQLHSGPGLLQLWGLGTLQQESCPGNRAHFVYGIACDNGCIWDLKFCPSGA
WELPGTPRKAPLLPRLGLLALACSDGKVLLFSLPHPEALLAQQPPDAVKPAIYKVQCVAT
LQVGSMQATDPSECGQCLSLAWMPTRPHQHLAAGYYNGMVVFWNLPTNSPLQRIRLSDGS
LKLYPFQCFLAHDQAVRTLQWCKANSHFLVSAGSDRKIKFWDLRRPYEPINSIKRFLSTE
LAWLLPYNGVTVAQDNCYASYGLCGIHYIDAGYLGFKAYFTAPRKGTVWSLSGSDWLGTI
AAGDISGELIAAILPDMALNPINVKRPVERRFPIYKADLIPYQDSPEGPDHSSASSGVPN
PPKARTYTETVNHHYLLFQDTDLGSFHDLLRREPMLRMQEGEGHSQLCLDRLQLEAIHKV
RFSPNLDSYGWLVSGGQSGLVRIHFVRGLASPLGHRMQLESRAHFNAMFQPSSPTRRPGF
SPTSHRLLPTP*
>ENSG00000054267|ENST00000264183
MKALDEPPYLTVGTDVSAKYRGAFCEAKIKTAKRLVKVKVTFRHDSSTVEVQDDHIKGPL
KVGAIVEVKNLDGAYQEAVINKLTDASWYTVVFDDGDEKTLRRSSLCLKGERHFAESETL
DQLPLTNPEHFGTPVIGKKTNRGRRSNHIPEEESSSSSSDEDEDDRKQIDELLGKVVCVD
YISLDKKKALWFPALVVCPDCSDEIAVKKDNILVRSFKDGKFTSVPRKDVHEITSDTAPK
PDAVLKQAFEQALEFHKSRTIPANWKTELKEDSSSSEAEEEEEEEDDEKEKEDNSSEEEE
EIEPFPEERENFLQQLYKFMEDRGTPINKRPVLGYRNLNLFKLFRLVHKLGGFDNIESGA
VWKQVYQDLGIPVLNSAAGYNVKCAYKKYLYGFEEYCRSANIEFQMALPEKVVNKQCKEC
ENVKEIKVKEENETEIKEIKMEEERNIIPREEKPIEDEIERKENIKPSLGSKKNLLESIP
THSDQEKEVNIKKPEDNENLDDKDDDTTRVDESLNIKVEAEEEKAKSGDETNKEEDEDDE
EAEEEEEEEEEEEDEDDDDNNEEEEFECYPPGMKVQVRYGRGKNQKMYEASIKDSDVEGG
EVLYLVHYCGWNVRYDEWIKADKIVRPADKNVPKIKHRKKIKNKLDKEKDKDEKYSPKNC
KLRRLSKPPFQTNPSPEMVSKLDLTDAKNSDTAHIKSIEITSILNGLQASESSAEDSEQE
DERGAQDMDNNGKEESKIDHLTNNRNDLISKEEQNSSSLLEENKVHADLVISKPVSKSPE
RLRKDIEVLSEDTDYEEDEVTKKRKDVKKDTTDKSSKPQIKRGKRRYCNTEECLKTGSPG
KKEEKAKNKESLCMENSSNSSSDEDEEETKAKMTPTKKYNGLEEKRKSLRTTGFYSGFSE
VAEKRIKLLNNSDERLQNSRAKDRKDVWSSIQGQWPKKTLKELFSDSDTEAAASPPHPAP
EEGVAEESLQTVAEEESCSPSVELEKPPPVNVDSKPIEEKTVEVNDRKAEFPSSGSNSVL
NTPPTTPESPSSVTVTEGSRQQSSVTVSEPLAPNQEEVRSIKSETDSTIEVDSVAGELQD
LQSEGNSSPAGFDASVSSSSSNQPEPEHPEKACTGQKRVKDAQGGGSSSKKQKRSHKATV
VNNKKKGKGTNSSDSEELSAGESITKSQPVKSVSTGMKSHSTKSPARTQSPGKCGKNGDK
DPDLKEPSNRLPKVYKWSFQMSDLENMTSAERITILQEKLQEIRKHYLSLKSEVASIDRR
RKRLKKKERESAATSSSSSSPSSSSITAAVMLTLAEPSMSSASQNGMSVECR*
>ENSG00000074966|ENST00000264316

MILSSYNTIQSVFCCCCCCSVQKRQMRTQISLSTDEELPEKYTQRRRPWLSQLSNKKQSN
TGRVQPSKRKPLPPLPPSEVAEEKIQVKALYDFLPREPCNLALRRAEEYLILEKYNPHWW
KARDRLGNEGLIPSNYVTENKITNLEIYEWYHRNITRNQAEHLLRQESKEGAFIVRDSRH
LGSYTISVFMGARRSTEAAIKHYQIKKNDSGQWYVAERHAFQSIPELIWYHQHNAAGLMT
RLRYPVGLMGSCLPATAGFSYEKWEIDPSELAFIKEIGSGQFGVVHLGEWRSHIQVAIKA
INEGSMSEEDFIEEAKVMMKLSHSKLVQLYGVCIQRKPLYIVTEFMENGCLLNYLRENKG
KLRKEMLLSVCQDICEGMEYLERNGYIHRDLAARNCLVSSTCIVKISDFGMTRYVLDDEY
VSSFGAKFPIKWSPPEVFLFNKYSSKSDVWSFGVLMWEVFTEGKMPFENKSNLQVVEAIS
EGFRLYRPHLAPMSIYEVMYSCWHEKPEGRPTFAELLRAVTEIAETW*
>ENSG00000101654|ENST00000262173
MANSAKAEEYEKMSLEQAKASVNSETESSFNINENTTASGTGLSEKTSVCRQVDIARKRK
EFEDDLVKESSSCGKDTPSKKRKLDPEIVPEEKDCGDAEGNSKKRKRETEDVPKDKSSTG
DGTQNKRKIALEDVPEKQKNLEEGHSSTVAAHYNELQEVGLEKRSQSRIFYLRNFNNWMK
SVLIGEFLEKVRQKKKRDITVLDLGCGKGGDLLKWKKGRINKLVCTDIADVSVKQCQQRY
EDMKNRRDSEYIFSAEFITADSSKELLIDKFRDPQMCFDICSCQFVCHYSFESYEQADMM
LRNACERLSPGGYFIGTTPNSFELIRRLEASETESFGNEIYTVKFQKKGDYPLFGCKYDF
NLEGVVDVPEFLVYFPLLNEMAKKYNMKLVYKKTFLEFYEEKIKNNENKMLLKRMQALEP
YPANESSKLVSEKVDDYEHAAKYMKNSQVRLPLGTLSKSEWEATSIYLVFAFEKQQ*
>ENSG00000105793|ENST00000257659
MVHCSCVLFRKYGNFIDKLRLFTRGGSGGMGYPRLGGEGGKGGDVWVVAQNRMTLKQLKD
RYPRKRFVAGVGANSKFPNAGKSSLLSCVSHAKPAIADYAFTTLKPELGKIMYSDFKQIS
VADLPGLIEGAHMNKGMGHKFLKHIERTRQLLFVVDISGFQLSSHTQYRTAFETIILLTK
ELELYKEELQTKPALLAVNKMDLPDAQDKFHELMSQLQNPKDFLHLFEKNMIPERTVEFQ
HIIPISAVTGEGIEELKNCIRKSLDEQANQENDALHKKQLLNLWISDTMSSTEPPSKHAV
TTSKMDII*
>ENSG00000112246|ENST00000262901
MKEKSKNAARTRREKENSEFYELAKLLPLPSAITSQLDKASIIRLTTSYLKMRVVFPEGL
GEAWGHSSRTSPLDNVGRELGSHLLQTLDGFIFVVAPDGKIMYISETASVHLGLSQVELT
GNSIYEYIHPADHDEMTAVLTAHQPYHSHFVQEYEIERSFFLRMKCVLAKRNAGLTCGGY
KVIHCSGYLKIRQYSLDMSPFDGCYQNVGLVAVGHSLPPSAVTEIKLHSNMFMFRASLDM
KLIFLDSRVAELTGYEPQDLIEKTLYHHVHGCDTFHLRCAHHLLLVKGQVTTKYYRFLAK
HGGWVWVQSYATIVHNSRSSRPHCIVSVNYVLTDTEYKGLQLSLDQISASKPAFSYTSSS
TPTMTDNRKGAKSRLSSSKSKSRTSPYPQYSGFHTERSESDHDSQWGGSPLTDTASPQLL
DPADRPGSQHDASCAYRQFSDRSSLCYGFALDHSRLVEERHFHTQACEGGRCEAGRYFLG
TPQAGREPWWGSRAALPLTKASPESREAYENSMPHIASVHRIHGRGHWDEDSVVSSPDPG
SASESGDRYRTEQYQSSPHEPSKIETLIRATQQMIKEEENRLQLRKAPSDQLASINGAGK
KHSLCFANYQQPPPTGEVCHGSALANTSPCDHIQQREGKMLSPHENDYDNSPTALSRISS
PNSDRISKSSLILAKDYLHSDISPHQTAGDHPTVSPNCFGSHRQYFDKHAYTLTGYALEH
LYDSETIRNYSLGCNGSHFDVTSHLRMQPDPAQGHKGTSVIITNGS*
>ENSG00000005189|ENST00000261377
MEPEREGTERHPRKVRESRQAPNKLVGAAEAMKAGWDLEESQPEAKKARLSTILFTDNCE
VTHDQLCELLKYAVLGKSNVPKPSWCQLFHQNHLNNVVVFVLQGMSQLHFYRFYLEFGCL
RKAFRHKFRLPPPSSDFLADVVGLQTEQRAGDLPKTMEGPLPSNAKAAINLQDDPIIQKY
GSKKVGLTRCLLTKEEMRTFHFPLQGFPDCENFLLTKCNGSIADNSPLFGLDCEMCLTSK

GRELTRISLVAEGGCCVMDELVKPENKILDYLTSFSGITKKILNPVTTKLKDVQRQLKAL
LPPDAVLVGHSLDLDLRALKMIHPYVIDTSLLYVREQGRRFKLKFLAKVILGKDIQCPDR
LGHDATEDARTILELARYFLKHGPKKIAELNLEALANHQEIQAAGQEPKNTAEVLQHPNT
SVLECLDSVGQKLLFLTRETDAGELPSSRNCQTIKCLSNKEVLEQARVEIPLFPFSIVQF
SFKAFSPVLTEEMNKRMRIKWTEISTVYAGPFSKNCNLRALKRLFKSFGPVQSMTFVLET
RQPHLCIQYEVLEAAQLAIESLDGILVDGICIKVQRPVTELTLDCDTLVNELEGDSENQG
SIYLSGVSETFKEQLLQEPRLFLGLEAVILPKDLKSGKQKKYCFLKFKSFGSAQQALNIL
TGKDWKLKGRHALTPRHLHAWLRGLPPESTRLPGLRVVPPPFEQEALQTLKLDHPKIAAW
RWSRKIGKLYNSLCPGTLCLILLPGTKSTHGSLSGLGLMGIKEEEESAGPGLCS*
>ENSG00000080603|ENST00000262518
MQSSPSPAHPQLPVLQTQMVSDGMTGSNPVSPASSSSPASSGAGGISPQHIAQDSSLDGP
PGPPDGATVPLEGFSLSQAADLANKGPKWEKSHAEIAEQAKHEAEIETRIAELRKEGFWS
LKRLPKVPEPPRPKGHWDYLCEEMQWLSADFAQERRWKRGVARKVVRMVIRHHEEQRQKE
ERARREEQAKLRRIASTMAKDVRQFWSNVEKVVQFKQQSRLEEKRKKALDLHLDFIVGQT
EKYSDLLSQSLNQPLTSSKAGSSPCLGSSSAASSPPPPASRLDDEDGDFQPQEDEEEDDE
ETIEVEEQQEGNDAEAQRREIELLRREGELPLEELLRSLPPQLLEGPSSPSQTPSSHDSD
TRDGPEEGAEEEPPQVLEIKPPPSAVTQRNKQPWHPDEDDEEFTANEEEAEDEEDTIAAE
EQLEGEVDHAMELSELAREGELSMEELLQQYAGAYAPGSGSSEDEDEDEVDANSSDCEPE
GPVEAEEPPQEDSSSQSDSVEDRSEDEEDEHSEEEETSGSSASEESESEESEDAQSQSQA
DEEEEDDDFGVEYLLARDEEQSEADAGSGPPTPGPTTLGPKKEITDIAAAAESLQPKGYT
LATTQVKTPIPLLLRGQLREYQHIGLDWLVTMYEKKLNGILADEMGLGKTIQTISLLAHL
ACEKGNWGPHLIIVPTSVMLNWEMELKRWCPSFKILTYYGAQKERKLKRQGWTKPNAFHV
CITSYKLVLQDHQAFRRKNWRYLILDEAQNIKNFKSQRWQSLLNFNSQRRLLLTGTPLQN
SLMELWSLMHFLMPHVFQSHREFKEWFSNPLTGMIEGSQEYNEGLVKRLHKVLRPFLLRR
VKVDVEKQMPKKYEHVIRCRLSKRQRCLYDDFMAQTTTKETLATGHFMSVINILMQLRKV
CNHPNLFDPRPVTSPFITPGICFSTASLVLRATDVHPLQRIDMGRFDLIGLEGRVSRYEA
DTFLPRHRLSRRVLLEVATAPDPPPRPKPVKMKVNRMLQPVPKQEGRTVVVVNNPRAPLG
PVPVRPPPGPELSAQPTPGPVPQVLPASLMVSASPAGPPLIPASRPPGPVLLPPLQPNSG
SLPQVLPSPLGVLSGTSRPPTPTLSLKPTPPAPVRLSPAPPPGSSSLLKPLTVPPGYTFP
PAAATTTSTTTATATTTAVPAPTPAPQRLILSPDMQARLPSGEVVSIGQLASLAQRPVAN
AGGSKPLTFQIQGNKLTLTGAQVRQLAVGQPRPLQRNVVHLVSAGGQHHLISQPAHVALI
QAVAPTPGPTPVSVLPSSTPSTTPAPTGLSLPLAANQVPPTMVNNTGVVKIVVRQAPRDG
LTPVPPLAPAPRPPSSGLPAVLNPRPTLTPGRLPTPTLGTARAPMPTPTLVRPLLKLVHS
PSPEVSASAPGAAPLTISSPLHVPSSLPGPASSPMPIPNSSPLASPVSSTVSVPLSSSLP
ISVPTTLPAPASAPLTIPISAPLTVSASGPALLTSVTPPLAPVVPAAPGPPSLAPSGASP
SASALTLGLATAPSLSSSQTPGHPLLLAPTSSHVPGLNSTVAPACSPVLVPASALASPFP
SAPNPAPAQASLLAPASSASQALATPLAPMAAPQTAILAPSPAPPLAPLPVLAPSPGAAP
VLASSQTPVPVMAPSSTPGTSLASASPVPAPTPVLAPSSTQTMLPAPVPSPLPSPASTQT
LALAPALAPTLGGSSPSQTLSLGTGNPQGPFPTQTLSLTPASSLVPTPAQTLSLAPGPPL
GPTQTLSLAPAPPLAPASPVGPAPAHTLTLAPASSSASLLAPASVQTLTLSPAPVPTLGP
AAAQTLALAPASTQSPASQASSLVVSASGAAPLPVTMVSRLPVSKDEPDTLTLRSGPPSP
PSTATSFGGPRPRRQPPPPPRSPFYLDSLEEKRKRQRSERLERIFQLSEAHGALAPVYGT
EVLDFCTLPQPVASPIGPRSPGPSHPTFWTYTEAAHRAVLFPQQRLDQLSEIIERFIFVM
PPVEAPPPSLHACHPPPWLAPRQAAFQEQLASELWPRARPLHRIVCNMRTQFPDLRLIQY

DCGKLQTLAVLLRQLKAEGHRVLIFTQMTRMLDVLEQFLTYHGHLYLRLDGSTRVEQRQA
LMERFNADKRIFCFILSTRSGGVGVNLTGADTVVFYDSDWNPTMDAQAQDRCHRIGQTRD
VHIYRLISERTVEENILKKANQKRMLGDMAIEGGNFTTAYFKQQTIRELFDMPLEEPSSS
SVPSAPEEEEETVASKQTHILEQALCRAEDEEDIRAATQAKAEQVAELAEFNENDGFPAG
EGEEAGRPGAEDEEMSRAEQEIAALVEQLTPIERYAMKFLEASLEEVSREELKQAEEQVE
AARKDLDQAKEEVFRLPQEEEEGPGAGDESSCGTGGGTHRRSKKAKAPERPGTRVSERLR
GARAETQGANHTPVISAHQTRSTTTPPRCSPARERVPRPAPRPRPTPASAPAAIPALVPV
PVSAPVPISAPNPITILPVHILPSPPPPSQIPPCSSPACTPPPACTPPPAHTPPPAQTCL
VTPSSPLLLGPPSVPISASVTNLPLGLRPEAELCAQALASPESLELASVASSETSSLSLV
PPKDLLPVAVEILPVSEKNLSLTPSAPSLTLEAGSIPNGQEQEAPDSAEGTTLTVLPEGE
ELPLCVSESNGLELPPSAASDEPLQEPLEADRTSEELTEAKTPTSSPEKPQELVTAEVAA
PSTSSSATSSPEGPSPARPPRRRTSADVEIRGQGTGRPGQPPGPKVLRKLPGRLVTVVEE
KELVRRRRQQRGAASTLVPGVSETSASPGSPSVRSMSGPESSPPIGGPCEAAPSSSLPTP
PQQPFIARRHIELGVTGGGSPENGDGALLAITPPAVKRRRGRPPKKNRSPADAGRGVDEA
PSSTLKGKTNGADPVPGPETLIVADPVLEPQLIPGPQPLGPQPVHRPNPLLSPVEKRRRG
RPPKARDLPIPGTISSAGDGNSESRTQPPPHPSPLTPLPPLLVCPTATVANTVTTVTIST
SPPKRKRGRPPKNPPSPRPSQLPVLDRDSTSVLESCGLGRRRQPQGQGESEGSSSDEDGS
RPLTRLARLRLEAEGMRGRKSGGSMVVAVIQDDLDLADSGPGGLELTPPVVSLTPKLRST
RLRPGSLVPPLETEKLPRKRAGAPVGGSPGLAKRGRLQPPSPLGPEGSVEESEAEASGEE
EEGDGTPRRRPGPRRLVGTTNQGDQRILRSSAPPSLAGPAVSHRGRKAKT*
>ENSG00000118620|ENST00000261560
MENLKSGVYPLKEASGCPGADRNLLVYSFYEKGPLTFRDVAIEFSLEEWQCLDTAQQDLY
RKVMLENYRNLVFLAGIAVSKPDLITCLEQGKEPWNMKRHAMVDQPPVTYSHFAQDLWPE
QGIKDSFQEVILRRYGKCGHEDLQLRTGCKSVDECNLHKECYDELNQCLTTTQSEIFQYD
KYVNVFYKFSNPNIQKIRHTGKKPFKCKKCDKSFCMLLHLTQHKRIHIRENSYQCEECGK
VFNWFSTLTRHRRIHTGEKPYKCEQCGKAFKQSSTLTTHKIIHTGEKPYRCEECGKTFNR
SSHLTTHKRIHTGEKPYRCEECGRAFNRSSHLTTHKIIHTGEKPYKCEECGKAFNQSSTL
TTHKIIHAGEKPYKCEECGKAFYRFSYLTKHKIIHTGEKFYKCEECGKGFNWSSTLTKHK
RIHTGEKPYKCEQCGKAFNESSNLTAHKIIHTGEKPYKCEECGKAFNRSPKLTAHKVIHS
GEKPYKCEECGKAFNQFSNLTKHKITHIGDTSYKYLECDKAFSQSSTLTKHKVIHTGEKP
YNCEEYGKAFNQSSNLIEQSNSYWRETLQM*
>ENSG00000120658|ENST00000261488
MVDAGGVENITQLPQELPQMMAAAADGLGSIAIDTTQLNMSVTDPTAWATAMNNLGMVPV
GLPGQQLVSDSICVPGFDPSLNMMTGITPINPMIPGLGLVPPPPPTEVAVVKEIIHCKSC
TLFPQNPNLPPPSTRERPPGCKTVFVGGLPENATEEIIQEVFEQCGDITAIRKSKKNFCH
IRFAEEFMVDKAIYLSGYRMRLGSSTDKKDSGRLHVDFAQARDDFYEWECKQRMRAREER
HRRKLEEDRLRPPSPPAIMHYSEHEAALLAEKLKDDSKFSEAITVLLSWIERGEVNRRSA
NQFYSMVQSANSHVRRLMNEKATHEQEMEEAKENFKNALTGILTQFEQIVAVFNASTRQK
AWDHFSKAQRKNIDIWRKHSEELRNAQSEQLMGIRREEEMEMSDDENCDSPTKKMRVDES
ALAAQAYALKEENDSLRWQLDAYRNEVELLKQEKEQLFRTEENLTKDQQLQFLQQTMQGM
QQQLLTIQEELNNKKSELEQAKEEQSHTQALLKVLQEQLKGTKELVETNGHSHEDSNEIN
VLTVALVNQDRENNIEKRSQGLKSEKEALLIGIISTFLHVHPFGANIEYLWSYMQQLDSK
ISANEIEMLLMRLPRMFKQEFTGVGATLEKRWKLCAFEGIKTT*
>ENSG00000138382|ENST00000260953

MKKVRLKELESRLQQVDGFEKPKLLLEQYPTRPHIAACMLYTIHNTYDDIENKVVADLGC
GCGVLSIGTAMLGAGLCVGFDIDEDALEIFNRNAEEFELTNIDMVQCDVCLLSNRMSKSF
DTVIMNPPFGTKNNKGTDMAFLKTALEMARTAVYSLHKSSTREHVQKKAAEWKIKIDIIA
ELRYDLPASYKFHKKKSVDIEVDLIRFSF*
>ENSG00000057468|ENST00000263187
MLRPEISSTSPSAPAVSPSSGETRSPQGPRYNFGLQETPQSRPSVQVVSASTCPGTSGAA
GDRSSSSSSLPCPAPNSRPAQGSYFGNKRAYAENTVASNFTFGASSSSARDTNYPQTLKT
PLSTGNPQRSGYKSWTPQVGYSASSSSAISAHSPSVIVAVVEGRGLARGEIGMASIDLKN
PQIILSQFADNTTYAKVITKLKILSPLEIIMSNTACAVGNSTKLFTLITENFKNVNFTTI
QRKYFNETKGLEYIEQLCIAEFSTVLMEVQSKYYCLAAVAALLKYVEFIQNSVYAPKSLK
ICFQGSEQTAMIDSSSAQNLELLINNQDYRNNHTLFGVLNYTKTPGGSRRLRSNILEPLV
DIETINMRLDCVQELLQDEELFFGLQSVISRFLDTEQLLSVLVQIPKQDTVNAAESKITN
LIYLKHTLELVDPLKIAMKNCNTPLLRAYYGSLEDKRFGIILEKIKTVINDDARYMKGCL
NMRTQKCYAVRSNINEFLDIARRTYTEIVDDIAGMISQLGEKYSLPLRTSFSSARGFFIQ
MTTDCIALPSDQLPSEFIKISKVKNSYSFTSADLIKMNERCQESLREIYHMTYMIVCKLL
SEIYEHIHCLYKLSDTVSMLDMLLSFAHACTLSDYVRPEFTDTLAIKQGWHPILEKISAE
KPIANNTYVTEGSNFLIITGPNMSGKSTYLKQIALCQIMAQIGSYVPAEYSSFRIAKQIF
TRISTDDDIETNSSTFMKEMKEIAYILHNANDKSLILIDELGRGTNTEEGIGICYAVCEY
LLSLKAFTLFATHFLELCHIDALYPNVENMHFEVQHVKNTSRNKEAILYTYKLSKGLTEE
KNYGLKAAEVSSLPPSIVLDAKEITTQITRQILQNQRSTPEMERQRAVYHLATRLVQTAR
NSQLDPDSLRIYLSNLKKKYKEDFPRTEQVPEKTEE*
>ENSG00000092470|ENST00000263795
MSRSGAAAEKADSRQRPQMKVNEYKENQNIAYVSLRPAQTTVLIKTAKVYLAPFSLSNYQ
LDQLMCPKSLSEKNSNNEVACKKTKIKKTCRRIIPPKMKNTSSKAESTLQNSSSAVHTES
NKLQPKRTADAMNLSVDVESSQDGDSDEDTTPSLDFSGLSPYERKRLKNISENADFFASL
QLSESAARLREMIEKRQPPKSKRKKPKRENGIGCRRSMRLLKVDPSGVSLPAAPTPPTLV
ADETPLLPPGPLEMTSENQEDNNERFKGFLHTWAGMSKPSSKNTEKGLSSIKSYKANLNG
MVISEDTVYKVTTGPIFSMALHPSETRTLVAVGAKFGQVGLCDLTQQPKEDGVYVFHPHS
QPVSCLYFSPANPAHILSLSYDGTLRCGDFSRAIFEEVYRNERSSFSSFDFLAEDASTLI
VGHWDGNMSLVDRRTPGTSYEKLTSSSMGKIRTVHVHPVHRQYFITAGLRDTHIYDARRL
NSRRSQPLISLTEHTKSIASAYFSPLTGNRVVTTCADCNLRIFDSSCISSKIPLLTTIRH
NTFTGRWLTRFQAMWDPKQEDCVIVGSMAHPRRVEIFHETGKRVHSFGGEYLVSVCSINA
MHPTRYILAGGNSSGKIHVFMNEKSC*
>ENSG00000071082|ENST00000264258
MAPAKKGGEKKKGRSAINEVVTREYTINIHKRIHGVGFKKRAPRALKEIRKFAMKEMGTP
DVRIDTRLNKAVWAKGIRNVPYRIRVRLSRKRNEDEDSPNKLYTLVTYVPVTTFKNLQTV
NVDEN*
>ENSG00000117450|ENST00000262746
MSSGNAKIGHPAPNFKATAVMPDGQFKDISLSDYKGKYVVFFFYPLDFTFVCPTEIIAFS
DRAEEFKKLNCQVIGASVDSHFCHLAWVNTPKKQGGLGPMNIPLVSDPKRTIAQDYGVLK
ADEGISFRGLFIIDDKGILRQITVNDLPVGRSVDETLRLVQAFQFTDKHGEVCPAGWKPG
SDTIKPDVQKSKEYFSKQK*
>ENSG00000100813|ENST00000262710
MWRRKHPRTSGGTRGVLSGNRGVEYGSGRGHLGTFEGRWRKLPKMPEAVGTDPSTSRKMA

ELEEVTLDGKPLQALRVTDLKAALEQRGLAKSGQKSALVKRLKGALMLENLQKHSTPHAA
FQPNSQIGEEMSQNSFIKQYLEKQQELLRQRLEREAREAAELEEASAESEDEMIHPEGVA
SLLPPDFQSSLERPELELSRHSPRKSSSISEEKGDSDDEKPRKGERRSSRVRQARAAKLS
EGSQPAEEEEDQETPSRNLRVRADRNLKTEEEEEEEEEEEEEDDEEEEGDDEGQKSREAPI
LKEFKEEGEEIPRVKPEEMMDERPKTRSQEQEVLERGGRFTRSQEEARKSHLARQQQEKE
MKTTSPLEEEEREIKSSQGLKEKSKSPSPPRLTEDRKKASLVALPEQTASEEETPPPLLT
KEASSPPPHPQLHSEEEIEPMEGPAPAVLIQLSPPNTDADTRELLVSQHTVQLVGGLSPL
SSPSDTKAESPAEKVPEESVLPLVQKSTLADYSAQKDLEPESDRSAQPLPLKIEELALAK
GITEECLKQPSLEQKEGRRASHTLLPSHRLKQSADSSSSRSSSSSSSSSRSRSRSPDSSG
SRSHSPLRSKQRDVAQARTHANPRGRPKMGSRSTSESRSRSRSRSRSASSNSRKSLSPGV
SRDSSTSYTETKDPSSGQEVATPPVPQLQVCEPKERTSTSSSSVQARRLSQPESAEKHVT
QRLQPERGSPKKCEAEEAEPPAATQPQTSETQTSHLPESERIHHTVEEKEEVTMDTSENR
PENDVPEPPMPIADQVSNDDRPEGSVEDEEKKESSLPKSFKRKISVVSATKGVPAGNSDT
EGGQPGRKRRWGASTATTQKKPSISITTESLKSLIPDIKPLAGQEAVVDLHADDSRISED
ETERNGDDGTHDKGLKICRTVTQVVPAEGQENGQREEEEEEKEPEAEPPVPPQVSVEVAL
PPPAEHEVKKVTLGDTLTRRSISQQKSGVSITIDDPVRTAQVPSPPRGKISNIVHISNLV
RPFTLGQLKELLGRTGTLVEEAFWIDKIKSHCFVTYSTVEEAVATRTALHGVKWPQSNPK
FLCADYAEQDELDYHRGLLVDRPSETKTEEQGIPRPLHPPPPPPVQPPQHPRAEQREQER
AVREQWAEREREMERRERTRSEREWDRDKVREGPRSRSRSRDRRRKERAKSKEKKSEKKE
KAQEEPPAKLLDDLFRKTKAAPCIYWLPLTDSQIVQKEAERAERAKEREKRRKEQEEEEQ
KEREKEAERERNRQLEREKRREHSRERDRERERERERDRGDRDRDRERDRERGRERDRRD
TKRHSRSRSRSTPVRDRGGRR*
>ENSG00000135097|ENST00000257552
METDAPQPGLASPDSPHDPCKMFIGGLSWQTTQEGLREYFGQFGEVKECLVMRDPLTKRS
RGFGFVTFMDQAGVDKVLAQSRHELDSKTIDPKVAFPRRAQPKMVTRTKKIFVGGLSVNT
TVEDVKQYFEQFGKVDDAMLMFDKTTNRHRGFGFVTFESEDIVEKVCEIHFHEINNKMVE
CKKAQPKEVMSPTGSARGRSRVMPYGMDAFMLGIGMLGYPGFQATTYASRSYTGLAPGYT
YQFPEFRVERTPLPSAPVLPELTAIPLTAYGPMAAAAAAAAVVRGTGSHPWTMAPPPGST
PSRTGGFLGTTSPGPMAELYGAANQDSGVSSYISAASPAPSTGFGHSLGGPLIATAFTNG
YH*
>ENSG00000053254|ENST00000261302
MGPVMPPSKKPESSGISVSSGLSQCYGGSGFSKALQEDDDLDFSLPDIRLEEGAMEDEEL
TNLNWLHESKNLLKSFGESVLRSVSPVQDLDDDTPPSPAHSDMPYDARQNPNCKPPYSFS
CLIFMAIEDSPTKRLPVKDIYNWILEHFPYFANAPTGWKNSVRHNLSLNKCFKKVDKERS
QSIGKGSLWCIDPEYRQNLIQALKKTPYHPHPHVFNTPPTCPQAYQSTSGPPIWPGSTFF
KRNGALLQDPDIDAASAMMLLNTPPEIQAGFPPGVIQNGARVLSRGLFPGVRPLPITPIG
VTAAMRNGITSCRMRTESEPSCGSPVVSGDPKEDHNYSSAKSSNARSTSPTSDSISSSSS
SADDHYEFATKGSQEGSEGSEGSFRSHESPSDTEEDDRKHSQKEPKDSLGDSGYASQHKK
RQHFAKARKVPSDTLPLKKRRTEKPPESDDEEMKEAAGSLLHLAGIRSCLNNITNRTAKG
QKEQKETTKN*
>ENSG00000186951|ENST00000262735
MVDTESPLCPLSPLEAGDLESPLSEEFLQEMGNIQEISQSIGEDSSGSFGFTEYQYLGSC
PGSDGSVITDTLSPASSPSSVTYPVVPGSVDESPSGALNIECRICGDKASGYHYGVHACE
GCKGFFRRTIRLKLVYDKCDRSCKIQKKNRNKCQYCRFHKCLSVGMSHNAIRFGRMPRSE

KAKLKAEILTCEHDIEDSETADLKSLAKRIYEAYLKNFNMNKVKARVILSGKASNNPPFV
IHDMETLCMAEKTLVAKLVANGIQNKEAEVRIFHCCQCTSVETVTELTEFAKAIPGFANL
DLNDQVTLLKYGVYEAIFAMLSSVMNKDGMLVAYGNGFITREFLKSLRKPFCDIMEPKFD
FAMKFNALELDDSDISLFVAAIICCGDRPGLLNVGHIEKMQEGIVHVLRLHLQSNHPDDI
FLFPKLLQKMADLRQLVTEHAQLVQIIKKTESDAALHPLLQEIYRDMY*
>ENSG00000083844|ENST00000263095
MAAAVLTDRAQVSVTFDDVAVTFTKEEWGQLDLAQRTLYQEVMLENCGLLVSLGCPVPKA
ELICHLEHGQEPWTRKEDLSQDTCPGDKGKPKTTEPTTCEPALSEGISLQGQVTQGNSVD
SQLGQAEDQDGLSEMQEGHFRPGIDPQEKSPGKMSPECDGLGTADGVCSRIGQEQVSPGD
RVRSHNSCESGKDPMIQEEENNFKCSECGKVFNKKHLLAGHEKIHSGVKPYECTECGKTF
IKSTHLLQHHMIHTGERPYECMECGKAFNRKSYLTQHQRIHSGEKPYKCNECGKAFTHRS
NFVLHNRRHTGEKSFVCTECGQVFRHRPGFLRHYVVHSGENPYECLECGKVFKHRSYLMW
HQQTHTGEKPYECSECGKVFLESAALIHHYVIHTGEKPFECLECGKAFNHRSYLKRHQRI
HTGEKPFVCSECGKAFTHCSTFILHKRAHTGEKPFECKECGKAFSNRKDLIRHFSIHTGE
KPYECVECGKAFTRMSGLTRHKRIHSGEKPYECVECGKSFCWSTNLIRHAIIHTGEKPYK
CSECGKAFSRSSSLTQHQRMHTGKNPISVTDVGRPFTSGQTSVTLRELLLGKDFLNVTTE
ANILPEETSSSASDQPYQRETPQVSSL*
>ENSG00000132463|ENST00000254799
MAGTRWVLGALLRGCGCNCSSCRRTGAACLPFYSAAGSIPSGVSGRRRLLLLLGAAAAAA
SQTRGLQTGPVPPGRLAGPPAVATSAAAAAAASYSALRASLLPQSLAAAAAVPTRSYSQE
SKTTYLEDLPPPPEYELAPSKLEEEVDDVFLIRAQGLPWSCTMEDVLNFFSDCRIRNGEN
GIHFLLNRDGKRRGDALIEMESEQDVQKALEKHRMYMGQRYVEVYEINNEDVDALMKSLQ
VKSSPVVNDGVVRLRGLPYSCNEKDIVDFFAGLNIVDITFVMDYRGRRKTGEAYVQFEEP
EMANQALLKHREEIGNRYIEIFPSRRNEVRTHVGSYKGKKIASFPTAKYITEPEMVFEEH
EVNEDIQPMTAFESEKEIELPKEVPEKLPEAADFGTTSSLHFVHMRGLPFQANAQDIINF
FAPLKPVRITMEYSSSGKATGEADVHFETHEDAVAAMLKDRSHVHHRYIELFLNSCPKGK
*
>ENSG00000130402|ENST00000252699
MVDYHAANQSYQYGPSSAGNGAGGGGSMGDYMAQEDDWDRDLLLDPAWEKQQRKTFTAWC
NSHLRKAGTQIENIDEDFRDGLKLMLLLEVISGERLPKPERGKMRVHKINNVNKALDFIA
SKGVKLVSIGAEEIVDGNAKMTLGMIWTIILRFAIQDISVEETSAKEGLLLWCQRKTAPY
KNVNVQNFHISWKDGLAFNALIHRHRPELIEYDKLRKDDPVTNLNNAFEVAEKYLDIPKM
LDAEDIVNTARPDEKAIMTYVSSFYHAFSGAQKAETAANRICKVLAVNQENEHLMEDYEK
LASDLLEWIRRTIPWLEDRVPQKTIQEMQQKLEDFRDYRRVHKPPKVQEKCQLEINFNTL
QTKLRLSNRPAFMPSEGKMVSDINNGWQHLEQAEKGYEEWLLNEIRRLERLDHLAEKFRQ
KASIHEAWTDGKEAMLKHRDYETATLSDIKALIRKHEAFESDLAAHQDRVEQIAAIAQEL
NELDYYDSHNVNTRCQKICDQWDALGSLTHSRREALEKTEKQLEAIDQLHLEYAKRAAPF
NNWMESAMEDLQDMFIVHTIEEIEGLISAHDQFKSTLPDADREREAILAIHKEAQRIAES
NHIKLSGSNPYTTVTPQIINSKWEKVQQLVPKRDHALLEEQSKQQSNEHLRRQFASQANV
VGPWIQTKMEEIGRISIEMNGTLEDQLSHLKQYERSIVDYKPNLDLLEQQHQLIQEALIF
DNKHTNYTMEHIRVGWEQLLTTIARTINEVENQILTRDAKGISQEQMQEFRASFNHFDKD
HGGALGPEEFKACLISLGYDVENDRQGEAEFNRIMSLVDPNHSGLVTFQAFIDFMSRETT
DTDTADQVIASFKVLAGDKNFITAEELRRELPPDQAEYCIARMAPYQGPDAVPGALDYKS
FSTALYGESDL*

>ENSG00000134030|ENST00000256413
MENSSAASASSEAGSSRSQEIEELERFIDSYVLEYQVQGLLADKTEGDGESERTQSHISQ
WTADCSEPLDSSCSFSRGRAPPQQNGSKDNSLDMLGTDIWAANTFDSFSGATWDLQPEKL
DFTQFHRKVRHTPKQPLPHIDREGCGKGKLEDGDGINLNDIEKVLPAWQGYHPMPHEVEI
AHTKKLFRRRRNDRRRQQRPPGGNKPQQHGDHQPGSAKHNRDHQKSYQGGSAPHPSGRPT
HHGYSQNRRWHHGNMKHPPGDKGEAGAHRNAKETMTIENPKLEDTAGDTGHSSLEAPRSP
DTLAPVASERLPPQQSGGPEVETKRKDSILPERIGERPKITLLQSSKDRLRRRLKEKDEV
AVETTTPQQNKMDKLIEILNSMRNNSSDVDTKLTTFMEEAQNSTNSEEMLGEIVRTIYQK
AVSDRSFAFTAAKLCDKMALFMVEGTKFRSLLLNMLQKDFTVREELQQQDVERWLGFITF
LCEVFGTMRSSTGEPFRVLVCPIYTCLRELLQSQDVKEDAVLCCSMELQSTGRLLEEQLP
EMMTELLASARDKMLCPSESMLTRSLLLEVIELHANSWNPLTPPITQYYNRTIQKLTA*
>ENSG00000130305|ENST00000252594
MGLYAAAAGVLAGVESRQGSIKGLVYSSNFQNVKQLYALVCETQRYSAVLDAVIASAGLL
RAEKKLRPHLAKVLVYELLLGKGFRGGGGRWKALLGRHQARLKAELARLKVHRGVSRNED
LLEVGSRPGPASQLPRFVRVNTLKTCSDDVVDYFKRQGFSYQGRASSLDDLRALKGKHFL
LDPLMPELLVFPAQTDLHEHPLYRAGHLILQDRASCLPAMLLDPPPGSHVIDACAAPGNK
TSHLAALLKNQGKIFAFDLDAKRLASMATLLARAGVSCCELAEEDFLAVSPSDPRYHEVH
YILLDPSCSGSGMPSRQLEEPGAGTPSPVRLHALAGFQQRALCHALTFPSLQRLVYSTCS
LCQEENEDVVRDALQQNPGAFRLAPALPAWPHRGLSTFPGAEHCLRASPETTLSSGFFVA
VIERVEVPR*
>ENSG00000132604|ENST00000254942
MAAGAGTAGPASGPGVVRDPAASQPRKRPGREGGEGARRSDTMAGGGGSSDGSGRAAGRR
ASRSSGRARRGRHEPGLGGPAERGAGEARLEEAVNRWVLKFYFHEALRAFRGSRYGDFRQ
IRDIMQALLVRPLGKEHTVSRLLRVMQCLSRIEEGENLDCSFDMEAELTPLESAINVLEM
IKTEFTLTEAVVESSRKLVKEAAVIICIKNKEFEKASKILKKHMSKDPTTQKLRNDLLNI
IREKNLAHPVIQNFSYETFQQKMLRFLESHLDDAEPYLLTMAKKALKSESAASSTGKEDK
QPAPGPVEKPPREPARQLRNPPTTIGMMTLKAAFKTLSGAQDSEAAFAKLDQKDLVLPTQ
ALPASPALKNKRPRKDENESSAPADGEGGSELQPKNKRMTISRLVLEEDSQSTEPSAGLN
SSQEAASAPPSKPTVLNQPLPGEKNPKVPKGKWNSSNGVEEKETWVEEDELFQVQAAPDE
DSTTNITKKQKWTVEESEWVKAGVQKYGEGNWAAISKNYPFVNRTAVMIKDRWRTMKRLG
MN*
>ENSG00000138073|ENST00000260643
MGRRRAPELYRAPFPLYALQVDPSTGLLIAAGGGGAAKTGIKNGVHFLQLELINGRLSAS
LLHSHDTETRATMNLALAGDILAAGQDAHCQLLRFQAHQQQGNKAEKAGSKEQGPRQRKG
AAPAEKKCGAETQHEGLELRVENLQAVQTDFSSDPLQKVVCFNHDNTLLATGGTDGYVRV
WKVPSLEKVLEFKAHEGEIEDLALGPDGKLVTVGRDLKASVWQKDQLVTQLHWQENGPTF
SSTPYRYQACRFGQVPDQPAGLRLFTVQIPHKRLRQPPPCYLTAWDGSNFLPLRTKSCGH
EVVSCLDVSESGTFLGLGTVTGSVAIYIAFSLQCLYYVREAHGIVVTDVAFLPEKGRGPE
LLGSHETALFSVAVDSRCQLHLLPSRRSVPVWLLLLLCVGLIIVTILLLQSAFPGFL*
>ENSG00000146963|ENST00000263545
MVIHSQLKKIQGASERMGDTTRQRIKFSDDRVCKSHLLNCCPHDVLSGTRMDLGECLKVH
DLALRADYEIASKEQDFFFELDAMDHLQSFIADCDRRTEVAKKRLAETQEEISAEVAAKA
ERVHELNEEIGKLLAKVEQLGAEGNVEESQKVMDEVEKARAKKREAEEVYRNSMPASSFQ
QQKLRVCEVCSAYLGLHDNDRRLADHFGGKLHLGFIEIREKLEELKRVVAEKQEKRNQER

LKRREERERERREEREKLRRSRSHSKNPKRSRSREHRRHRSRSMSRERKRRTRSKSREKRHRH
RSRSSSRSRSRSHQRSRHSSRDRSRERSKRRSSKERFRDQDLASCDRDRSSRDRSPRDRD
RKDKKRSYESANGRSEDRRSSEEREAGEI*
>ENSG00000132109|ENST00000254436
MASAARLTMMWEEVTCPICLDPFVEPVSIECGHSFCQECISQVGKGGGSVCPVCRQRFLL
KNLRPNRQLANMVNNLKEISQEAREGTQGERCAVHGERLHLFCEKDGKALCWVCAQSRKH
RDHAMVPLEEAAQEYQEKLQVALGELRRKQELAEKLEVEIAIKRADWKKTVETQKSRIHA
EFVQQKNFLVEEEQRQLQELEKDEREQLRILGEKEAKLAQQSQALQELISELDRRCHSSA
LELLQEVIIVLERSESWNLKDLDITSPELRSVCHVPGLKKMLRTCAVHITLDPDTANPWL
ILSEDRRQVRLGDTQQSIPGNEERFDSYPMVLGAQHFHSGKHYWEVDVTGKEAWDLGVCR
DSVRRKGHFLLSSKSGFWTIWLWNKQKYEAGTYPQTPLHLQVPPCQVGIFLDYEAGMVSF
YNITDHGSLIYSFSECAFTGPLRPFFSPGFNDGGKNTAPLTLCPLNIGSQGSTDY*
>ENSG00000132467|ENST00000254803
MVGRSRRRGAAKWAAVRAKAGPTLTDENGDDLGLPPSPGDTSYYQDQVDDFHEARSRAAL
AKGWNEVQSGDEEDGEEEEEEVLALDMDDEDDEDGGNAGEEEEEENADDDGGSSVQSEAE
ASVDPSLSWGQRKKLYYDTDYGSKSRGRQSQQEAEEEEREEEEEAQIIQRRLAQALQEDD
FGVAWVEAFAKPVPQVDEAETRVVKDLAKVSVKEKLKMLRKESPELLELIEDLKVKLTEV
KDELEPLLELVEQGIIPPGKGSQYLRTKYNLYLNYCSNISFYLILKARRVPAHGHPVIER
LVTYRNLINKLSVVDQKLSSEIRHLLTLKDDAVKKELIPKAKSTKPKPKSVSKTSAAACA
VTDLSDDSDFDEKAKLKYYKEIEDRQKLKRKKEENSTEEQALEDQNAKRAITYQIAKNRG
LTPRRKKIDRNPRVKHREKFRRAKIRRRGQVREVRKEEQRYSGELSGIRAGVKKSIKLK*
>ENSG00000135372|ENST00000257829
MHRKKVDNRIRILIENGVAERQRSLFVVVGDRGKDQVVILHHMLSKATVKARPSVLWCYK
KELGFSSHRKKRMRQLQKKIKNGTLNIKQDDPFELFIAATNIRYCYYNETHKILGNTFGM
CVLQDFEALTPNLLARTVETVEGGGLVVILLRTMNSLKQLYTVTMDVHSRYRTEAHQDVV
GRFNERFILSLASCKKCLVIDDQLNILPISSHVATMEALPPQTPDESLGPSDLELRELKE
SLQDTQPVGVLVDCCKTLDQAKAVLKFIEGISEKTLRSTVALTAARGRGKSAALGLAIAG
AVAFGYSNIFVTSPSPDNLHTLFEFVFKGFDALQYQEHLDYEIIQSLNPEFNKAVIRVNV
FREHRQTIQYIHPADAVKLGQAELVVIDEAAAIPLPLVKSLLGPYLVFMASTINGYEGTG
RSLSLKLIQQLRQQSAQSQVSTTAENKTTTTARLASARTLYEVSLQESIRYAPGDAVEKW
LNDLLCLDCLNITRIVSGCPLPEACELYYVNRDTLFCYHKASEVFLQRLMALYVASHYKN
SPNDLQMLSDAPAHHLFCLLPPVPPTQNALPEVLAVIQVCLEGEISRQSILNSLSRGKKA
SGDLIPWTVSEQFQDPDFGGLSGGRVVRIAVHPDYQGMGYGSRALQLLQMYYEGRFPCLE
EKVLETPQEIHTVSSEAVSLLEEVITPRKDLPPLLLKLNERPAERLDYLGVSYGLTPRLL
KFWKRAGFVPVYLRQTPNDLTGEHSCIMLKTLTDEDEADQGGWLAAFWKDFRRRFLALLS
YQFSTFSPSLALNIIQNRNMGKPAQPALSREELEALFLPYDLKRLEMYSRNMVDYHLIMD
MIPAISRIYFLNQLGDLALSAAQSALLLGIGLQHKSVDQLEKEIELPSGQLMGLFNRIIR
KVVKLFNEVQEKAIEEQMVAAKDVVMEPTMKTLSDDLDEAAKEFQEKHKKEVGKLKSMDL
SEYIIRGDDEEWNEVLNKAGPNASIISLKSDKKRKLEAKQEPKQSKKLKNRETKNKKDMK
LKRKK*
>ENSG00000134758|ENST00000257190
MAEDLSAATSYTEDDFYCPVCQEVLKTPVRTTACQHVNRSETSTSDNTETYQENTSSSGH
PTFKCPLCQESNFTRQRLLDHCNSNHLFQIVPVTCPICVSLPWGDPSQITRNFVSHLNQR
HQFDYGEFVNLQLDEETQYQTAVEESFQVNI*

>ENSG00000100393|ENST00000263253
MAENVVEPGPPSAKRPKLSSPALSASASDGTDFGSLFDLEHDLPDELINSTELGLTNGGD
INQLQTSLGMVQDAASKHKQLSELLRSGSSPNLNMGVGGPGQVMASQAQQSSPGLGLINS
MVKSPMTQAGLTSPNMGMGTSGPNQGPTQSTGMMNSPVNQPAMGMNTGMNAGMNPGMLAA
GNGQGIMPNQVMNGSIGAGRGRQNMQYPNPGMGSAGNLLTEPLQQGSPQMGGQTGLRGPQ
PLKMGMMNNPNPYGSPYTQNPGQQIGASGLGLQIQTKTVLSNNLSPFAMDKKAVPGGGMP
NMGQQPAPQVQQPGLVTPVAQGMGSGAHTADPEKRKLIQQQLVLLLHAHKCQRREQANGE
VRQCNLPHCRTMKNVLNHMTHCQSGKSCQVAHCASSRQIISHWKNCTRHDCPVCLPLKNA
GDKRNQQPILTGAPVGLGNPSSLGVGQQSAPNLSTVSQIDPSSIERAYAALGLPYQVNQM
PTQPQVQAKNQQNQQPGQSPQGMRPMSNMSASPMGVNGGVGVQTPSLLSDSMLHSAINSQ
NPMMSENASVPSLGPMPTAAQPSTTGIRKQWHEDITQDLRNHLVHKLVQAIFPTPDPAAL
KDRRMENLVAYARKVEGDMYESANNRAEYYHLLAEKIYKIQKELEEKRRTRLQKQNMLPN
AAGMVPVSMNPGPNMGQPQPGMTSNGPLPDPSMIRGSVPNQMMPRITPQSGLNQFGQMSM
AQPPIVPRQTPPLQHHGQLAQPGALNPPMGYGPRMQQPSNQGQFLPQTQFPSQGMNVTNI
PLAPSSGQAPVSQAQMSSSSCPVNSPIMPPGSQGSHIHCPQLPQPALHQNSPSPVPSRTP
TPHHTPPSIGAQQPPATTIPAPVPTPPAMPPGPQSQALHPPPRQTPTPPTTQLPQQVQPS
LPAAPSADQPQQQPRSQQSTAASVPTPTAPLLPPQPATPLSQPAVSIEGQVSNPPSTSST
EVNSQAIAEKQPSQEVKMEAKMEVDQPEPADTQPEDISESKVEDCKMESTETEERSTELK
TEIKEEEDQPSTSATQSSPAPGQSKKKIFKPEELRQALMPTLEALYRQDPESLPFRQPVD
PQLLGIPDYFDIVKSPMDLSTIKRKLDTGQYQEPWQYVDDIWLMFNNAWLYNRKTSRVYK
YCSKLSEVFEQEIDPVMQSLGYCCGRKLEFSPQTLCCYGKQLCTIPRDATYYSYQNRYHF
CEKCFNEIQGESVSLGDDPSQPQTTINKEQFSKRKNDTLDPELFVECTECGRKMHQICVL
HHEIIWPAGFVCDGCLKKSARTRKENKFSAKRLPSTRLGTFLENRVNDFLRRQNHPESGE
VTVRVVHASDKTVEVKPGMKARFVDSGEMAESFPYRTKALFAFEEIDGVDLCFFGMHVQE
YGSDCPPPNQRRVYISYLDSVHFFRPKCLRTAVYHEILIGYLEYVKKLGYTTGHIWACPP
SEGDDYIFHCHPPDQKIPKPKRLQEWYKKMLDKAVSERIVHDYKDIFKQATEDRLTSAKE
LPYFEGDFWPNVLEESIKELEQEEEERKREENTSNESTDVTKGDSKNAKKKNNKKTSKNK
SSLSRGNKKKPGMPNVSNDLSQKLYATMEKHKEVFFVIRLIAGPAANSLPPIVDPDPLIP
CDLMDGRDAFLTLARDKHLEFSSLRRAQWSTMCMLVELHTQSQDRFVYTCNECKHHVETR
WHCTVCEDYDLCITCYNTKNHDHKMEKLGLGLDDESNNQQAAATQSPGDSRRLSIQRCIQ
SLVHACQCRNANCSLPSCQKMKRVVQHTKGCKRKTNGGCPICKQLIALCCYHAKHCQENK
CPVPFCLNIKQKLRQQQLQHRLQQAQMLRRRMASMQRTGVVGQQQGLPSPTPATPTTPTG
QQPTTTPQTPQPTSQPQPTPPNSMPPYLPRTQAAGPVSQGKAAGQVTPPTPPQTAQPPLPG
PPPAAVEMAMQIQRAAETQRQMAHVQIFQRPIQHQMPPMTPMAPMGMNPPPMTRGPSGHL
EPGMGPTGMQQQPPWSQGGLPQPQQLQSGMPRPAMMSVAQHGQPLNMAPQPGLGQVGISP
LKPGTVSQQALQNLLRTLRSPSSPLQQQQVLSILHANPQLLAAFIKQRAAKYANSNPQPI
PGQPGMPQGQPGLQPPTMPGQQGVHSNPAMQNMNPMQAGVQRAGLPQQQPQQQLQPPMGG
MSPQAQQMNMNHNTMPSQFRDILRRQQMMQQQQQQGAGPGIGPGMANHNQFQQPQGVGYP
PQQQQRMQHHMQQMQQGNMGQIGQLPQALGAEAGASLQAYQQRLLQQQMGSPVQPNPMSP
QQHMLPNQAQSPHLQGQQIPNSLSNQVRSPQPVPSPRPQSQPPHSSPSPRMQPQPSPHHV
SPQTSSPHPGLVAAQANPMEQGHFASPDQNSMLSQLASNPGMANLHGASATDLGLSTDNS
DLNSNLSQSTLDIH*
>ENSG00000138398|ENST00000260970
MGIKVQRPRCFFDIAINNQPAGRVVFELFSDVCPKTCENFRCLCTGEKGTGKSTQKPLHY

KSCLFHRVVKDFMVQGGDFSEGNGRGGESIYGGFFEDESFAVKHNKEFLLSMANRGKDTN
GSQFFITTKPTPHLDGHHVVFGQVISGQEVVREIENQKTDAASKPFAEVRILSCGELIPK
SKVKKEEKKRHKSSSSSSSSSSDSDSSSDSQSSSDSSDSESATEEKSKKRKKKHRKNSRK
HKKEKKKRKKSKKSASSESEAENLEAQPQSTVRPEEIPPIPENRFLMRKSPPKADEKERK
NRERERERECNPPNSQPASYQRRLLVTRSGRKIKGRGPRRYRTPSRSRSRDRFRRSETPP
HWRQEMQRAQRMRVSSGERWIKGDKSELNEIKENQRSPVRVKERKITDHRNVSESPNRKN
EKEKKVKDHKSNSKERDIRRNSEKDDKYKNKVKKRAKSKSRSKSKEKSKSKERDSKHNRN
EEKRMRSRSKGRDHENVKEKEKQSDSKGKDQERSRSKEKSKQLESKSNEHDHSKSKEKDR
RAQSRSRECDITKGKHSYNSRTRERSRSRDRSRRVRSRTHDRDRSRSKEYHRYREQEYRR
RGRSRSRERRTPPGRSRSKDRRRRRRDSRSSEREESQSRNKDKYRNQESKSSHRKENSES
EKRMYSKSRDHNSSNNSREKKADRDQSPFSKIKQSSQDNELKSSMLKNKEDEKIRSSVEK
ENQKSKGQENDHVHEKNKKFDHESSPGTDEDKSG*
>ENSG00000153922|ENST00000284049
MNGHSDEESVRNSSGESSQSDDDSGSASGSGSGSSSGSSSDGSSSQSGSSDSDSGSESGS
QSESESDTSRENKVQAKPPKVDGAEFWKSSPSILAVQRSAILKKQQQQQQQQQHQASSNS
GSEEDSSSSEDSDDSSSEVKRKKHKDEDWQMSGSGSPSQSGSDSESEEEREKSSCDETES
DYEPKNKVKSRKPQNRSKSKNGKKILGQKKRQIDSSEEDDDEEDYDNDKRSSRRQATVNV
SYKEDEEMKTDSDDLLEVCGEDVPQPEEEEFETIERFMDCRIGRKGATGATTTIYAVEAD
GDPNAGFEKNKEPGEIQYLIKWKGWSHIHNTWETEETLKQQNVRGMKKLDNYKKKDQETK
RWLKNASPEDVEYYNCQQELTDDLHKQYQIVERIIAHSNQKSAAGYPDYYCKWQGLPYSE
CSWEDGALISKKFQACIDEYFSRNQSKTTPFKDCKVLKQRPRFVALKKQPSYIGGHEGLE
LRDYQLNGLNWLAHSWCKGNSCILADEMGLGKTIQTISFLNYLFHEHQLYGPFLLVVPLS
TLTSWQREIQTWASQMNAVVYLGDINSRNMIRTHEWTHHQTKRLKFNILLTTYEILLKDK
AFLGGLNWAFIGVDEAHRLKNDDSLLYKTLIDFKSNHRLLITGTPLQNSLKELWSLLHFI
MPEKFSSWEDFEEEHGKGREYGYASLHKELEPFLLRRVKKDVEKSLPAKVEQILRMEMSA
LQKQYYKWILTRNYKALSKGSKGSTSGFLNIMMELKKCCNHCYLIKPPDNNEFYNKQEAL
QHLIRSSGKLILLDKLLIRLRERGNRVLIFSQMVRMLDILAEYLKYRQFPFQRLDGSIKG
ELRKQALDHFNAEGSEDFCFLLSTRAGGLGINLASADTVVIFDSDWNPQNDLQAQARAHR
IGQKKQVNIYRLVTKGSVEEDILERAKKKMVLDHLVIQRMDTTGKTVLHTGSAPSSSTPF
NKEELSAILKFGAEELFKEPEGEEQEPQEMDIDEILKRAETHENEPGPLTVGDELLSQFK
VANFSNMDEDDIELEPERNSKNWEEIIPEDQRRRLEEEERQKELEEIYMLPRMRNCAKQI
SFNGSEGRRSRSRRYSGSDSDSISEGKRPKKRGRPRTIPRENIKGFSDAEIRRFIKSYKK
FGGPLERLDAIARDAELVDKSETDLRRLGELVHNGCIKALKDSSSGTERTGGRLGKVKGP
TFRISGVQVNAKLVISHEEELIPLHKSIPSDPEERKQYTIPCHTKAAHFDIDWGKEDDSN
LLIGIYEYGYGSWEMIKMDPDLSLTHKILPDDPDKKPQAKQLQTRADYLIKLLSRDLAKK
EALSGAGSSKRRKARAKKNKAMKSIKVKEEIKSDSSPLPSEKSDEDDDKLSESKSDGRER
SKKSSVSDAPVHITASGEPVPISEESEELDQKTFSICKERMRPVKAALKQLDRPEKGLSE
REQLEHTRQCLIKIGDHITECLKEYTNPEQIKQWRKNLWIFVSKFTEFDARKLHKLYKHA
IKKRQESQQNSDQNSNLNPHVIRNPDVERLKENTNHDDSSRDSYSSDRHLTQYHDHHKDR
HQGDSYKKSDSRKRPYSSFSNGKDHRDWDHYKQDSRYYSDREKHRKLDDHRSRDHRSNLE
GSLKDRSHSDHRSHSDHRLHSDHRSSSEYTHHKSSRDYRYHSDWQMDHRASSSGPRSPLD
QRSPYGSRSPFEHSVEHKSTPEHTWSSRKT*
>ENSG00000174460|ENST00000310164
MASIIARVGNSRRLNAPLPPWAHSMLRSLGRSLGPIMASMADRNMKLFSGRVVPAQGEET

FENWLTQVNGVLPDWNMSEEEKLKRLMKTLRGPAREVMRVLQATNPNLSVADFLRAMKLV
FGESESSVTAHGKFFNTLQAQGEKASLYVIRLEVQLQNAIQAGIIAEKDANRTRLQQLLL
GGELSRDLRLRLKDFLRMYANEQERLPNFLELIRMVREEEDWDDAFIKRKRPKRSESMVE
RAVSPVAFQGSPPIVIGSADCNVIEIDDTLDDSDEDVILVESQDPPLPSWGAPPLRDRAR
PQDEVLVIDSPHNSRAQFPSTSGGSGYKNNGPGEMRRARKRKHTIRCSYCGEEGHSKETC
DNESDKAQVFENLIITLQELTHTEMERSRVAPGEYNDFSEPL*
>ENSG00000162419|ENST00000294409
MANAEVSVPVGDVVVVPTEGNEGENPEDTKTQVILQLQPVQQGLFIDGHFYNRIYEAGSE
NNTAVVAVETHTIHKIEEGIDTGTIEANEDMEIAYPITCGESKAILLWKKFVCPGINVKC
VKFNDQLISPKHFVHLAGKSTLKDWKRAIRLGGIMLRKMMDSGQIDFYQHDKVCSNTCRS
TKFDLLISSARAPVPGQQTSVVQTPTSADGSITQIAISEESMEEAGLEWNSALTAAVTMA
TEEGVKKDSEEISEDTLMFWKGIADVGLMEEVVCNIQKEIEELLRGVQQRLIQAPFQVTD
AAVLNNVAHTFGLMDTVKKVLDNRRNQVEQGEEQFLYTLTDLERQLEEQKKQGQDHRLKS
QTVQNVVLMPVSTPKPPKRPRLQRPASTTVLSPSPPVQQPQFTVISPITITPVGQSFSMG
NIPVATLSQGSSPVTVHTLPSGPQLFRYATVVSSAKSSSPDTVTIHPSSSLALLSSTAMQ
DGSTLGNMTTMVSPVELVAMESGLTSAIQAVESTSEDGQTIIEIDPAPDPEAEDTEGKAV
ILETELRTEEKVVAEMEEHQHQVHNVEIVVLED*
>ENSG00000196705|ENST00000311048
MDDLKYGVYPLKEASGCPGAERNLLVYSYFEKETLTFRDVAIEFSLEEWECLNPAQQNLY
MNVMLENYKNLVFLGVAVSKQDPVTCLEQEKEPWNMKRHEMVDEPPAMCSYFTKDLWPEQ
DIKDSFQQVILRRYGKCEHENLQLRKGSASVDEYKVHKEGYNELNQCLTTTQSKIFPCDK
YVKVFHKFLNANRHKTRHTGKKPFKCKKCGKSFCMLLHLSQHKRIHIRENSYQCEECGKA
FKWFSTLTRHKRIHTGEKPFKCEECGKAFKQSSTLTTHKIIHTGEKPYRCEECGKAFNRS
SHLTTHKIIHTGEKPYKCEECGKAFNQSSTLSTHKFIHAGEKPYKCEECDKAFNRFSYLT
KHKIIHTGEKSYKCEECGKGFNWSSTLTKHKRIHTGEKPYKCEVCGKAFNESSNLTTHKM
IHTGEKPYKCEECGKAFNRSPQLTAHKIIHTGEKPYKCEECGKAFSQSSILTTHKRIHTG
EKPYKCEECGKAFNRSSNLTKHKIIHTGEKSYKCEECGKAFNQSSTLTKHRKIHTRQKPY
NCEECDNTFNQSSNLIKQNNSYWRETLQMSRMWESL*
>ENSG00000166173|ENST00000299213
MAQSGGEARPGPKTAVQIRVAIQEAEDVDELEDEEEGAETRGAGDPARYLSPGWGSASEE
EPSRGHSGTTASGGENEREDLEQEWKPPDEELIKKLVDQIEFYFSDENLEKDAFLLKHVR
RNKLGYVSVKLLTSFKKVKHLTRDWRTTAHALKYSVVLELNEDHRKVRRTTPVPLFPNEN
LPSKMLLVYDLYLSPKLWALATPQKNGRVQEKVMEHLLKLFGTFGVISSVRILKPGRELP
PDIRRISSRYSQVGTQECAIVEFEEVEAAIKAHEFMITESQGKENMKAVLIGMKPPKKKP
AKDKNHDEEPTASIHLNKSLNKRVEELQYMGDESSANSSSDPESNPTSPMAGRRHAATNK
LSPSGHQNLFLSPNASPCTSPWSSPLAQRKGVSRKSPLAEEGRLNCSTSPEIFRKCMDYS
SDSSVTPSGSPWVRRRRQAEMGTQEKSPGTSPLLSRKMQTADGLPVGVLRLPRGPDNTRG
FHGHERSRACV*
>ENSG00000169371|ENST00000308588
MEELSQALASSFSVSQDLNSTAAPHPRLSQYKSKYSSLEQSERRRRLLELQKSKRLDYVN
HARRLAEDDWTGMESEEENKKDDEEMDIDTVKKLPKHYANQLMLSEWLIDVPSDLGQEWI
VVVCPVGKRALIVASRGSTSAYTKSGYCVNRFSSLLPGGNRRNSTAKDYTILDCIYNEVN
QTYYVLDVMCWRGHPFYDCQTDFRFYWMHSKLPEEEGLGEKTKLNPFKFVGLKNFPCTPE
SLCDVLSMDFPFEVDGLLFYHKQTHYSPGSTPLVGWLRPYMVSDVLGVAVPAGPLTTKPD

YAGHQLQQIMEHKKSQKEGMKEKLTHKASENGHYELEHLSTPKLKGSSHSPDHPGCLMEN
*
>ENSG00000169612|ENST00000304191
MTDTAEAVPKFEEMFASRFTENDKEYQEYLKRPPESPPIVEEWNSRAGGNQRNRGNRLQD
NRQFRGRDNRWGWPSDNRSNQWHGRSWGNNYPQHRQEPYYPQQYGHYGYNQRPPYGYY*
>ENSG00000167658|ENST00000309311
MVNFTVDQIRAIMDKKANIRNMSVIAHVDHGKSTLTDSLVCKAGIIASARAGETRFTDTR
KDEQERCITIKSTAISLFYELSENDLNFIKQSKDGAGFLINLIDSPGHVDFSSEVTAALR
VTDGALVVVDCVSGVCVQTETVLRQAIAERIKPVLMMNKMDRALLELQLEPEELYQTFQR
IVENVNVIISTYGEGESGPMGNIMIDPVLGTVGFGSGLHGWAFTLKQFAEMYVAKFAAKG
EGQLGPAERAKKVEDMMKKLWGDRYFDPANGKFSKSATSPEGKKLPRTFCQLILDPIFKV
FDAIMNFKKEETAKLIEKLDIKLDSEDKDKEGKPLLKAVMRRWLPAGDALLQMITIHLPS
PVTAQKYRCELLYEGPPDDEAAMGIKSCDPKGPLMMYISKMVPTSDKGRFYAFGRVFSGL
VSTGLKVRIMGPNYTPGKKEDLYLKPIQRTILMMGRYVEPIEDVPCGNIVGLVGVDQFLV
KTGTITTFEHAHNMRVMKFSVSPVVRVAVEAKNPADLPKLVEGLKRLAKSDPMVQCIIEE
SGEHIIAGAGELHLEICLKDLEEDHACIPIKKSDPVVSYRETVSEESNVLCLSKSPNKHN
RLYMKARPFPDGLAEDIDKGEVSARQELKQRARYLAEKYEWDVAEARKIWCFGPDGTGPN
ILTDITKGVQYLNEIKDSVVAGFQWATKEGALCEENMRGVRFDVHDVTLHADAIHRGGGQ
IIPTARRCLYASVLTAQPRLMEPIYLVEIQCPEQVVGGIYGVLNRKRGHVFEESQVAGTP
MFVVKAYLPVNESFGFTADLRSNTGGQAFPQCVFDHWQILPGDPFDNSSRPSQVVAETRK
RKGLKEGIPALDNFLDKL*
>ENSG00000155858|ENST00000286307
MEERERGARSAGAGSPARPPSPRLDVSSDSFDPLLALYAPRLPPIPYPNAPCFNNVAEYE
SFLRTGVRGGGRGRGRARGAAAGSGVPAAPGPSGRTRRRPDAPAPDPERIQRLRRLMVAK
EEGDGAAGAGRRGPGRSRKAPRNVLTRMPLHEGSPLGELHRCIREGVKVNVHIRTFKGLR
GVCTGFLVAFDKFWNMALTDVDETYRKPVLGKAYERDSSLTLTRLFDRLKLQDSSKKEAD
SKSAVEDSTLSRYSQTSTWKLASVWGRADTGRGSHKRSRSVPSSLQASAREESRSELSGR
TTRTDGSSVGGTFSRATTLSRGQSRKKKRKPKVDYQQVFTRHINQIFIRGENVLLVHLAQ
*
>ENSG00000130305|ENST00000310326
MGLYAAAAGVLAGVESRQGSIKGLVYSSNFQNVKQLYALVCETQRYSAVLDAVIASAGLL
RAEKKLRPHLAKVLVYELLLGKGFRGGGGRWKALLGRHQARLKAELARLKVHRGVSRNED
LLEVGSRPGPASQLPRFVRVNTLKTCSDDVVDYFKRQGFSYQGRASSLDDLRALKGKHFL
LDPLMPELLVFPAQTDLHEHPLYRAGHLILQDRASCLPAMLLDPPPGSHVIDACAAPGNK
TSHLAALLKNQGKIFAFDLDAKRLASMATLLARAGVSCCELAEEDFLAVSPSDPRYHEVH
YILLDPSCSGSGMPSRQLEEPGAGTPSPVRLHALAGFQQRALCHALTFPSLQRLVYSTCS
LCQEENEDVVRDALQQNPGAFRLAPALPAWPHRGLSTFPGAEHCLRASPETTLSSGFFVA
VIERVEVPSLTGQSISTRTHTQPSPKEKEETAKSRSRCLHTALHIAEAPG*
>ENSG00000164463|ENST00000296953
MPQPSVSGMDPPFGDAFRSHTFSEQTLMSTDLLANSSDPDFMYELDREMNYQQNPRDNFL
SLEDCKDIENLESFTDVLDNEGALTSNWEQWDTYCEDLTKYTKLTSCDIWGTKEVDYLGL
DDFSSPYQDEEVISKTPTLAQLNSEDSQSVSDSLYYPDSLFSVKQNPLPSSFPGKKITSR
AAAPVCSSKTLQAEVPLSDCVQKASKPTSSTQIMVKTNMYHNEKVNFHVECKDYVKKAKV
KINPVQQSRPLLSQIHTDAAKENTCYCGAVAKRQEKKGMEPLQGHATPALPFKETQELLL

SPLPQEGPGSLAAGESSSLSASTSVSDSSQKKEEHNYSLFVSDNLGEQPTKCSPEEDEED
EEDVDDEDHDEGFGSEHELSENEEEEEEEEDYEDDKDDDISDTFSEPGYENDSVEDLKEV
TSISSRKRGKRRYFWEYSEQLTPSQQERMLRPSEWNRDTLPSNMYQKNGLHHGKYAVKKS
RRTDVEDLTPNPKKLLQIGNELRKLNKVISDLTPVSELPLTARPRSRKEKNKLASRACRL
KKKAQYEANKVKLWGLNTEYDNLLFVINSIKQEIVNRVQNPRDERGPNMGQKLEILIKDT
LGLPVAGQTSEFVNQVLEKTAEGNPTGGLVGLRIPTSKV*
>ENSG00000109685|ENST00000312087
MEFSIKQSPLSVQSVVKCIKMKQAPEILGSANGKTPSCEVNRECSVFLSKAQLSSSLQEG
VMQKFNGHDALPFIPADKLKDLTSRVFNGEPGAHDAKLRFESQEMKGIGTPPNTTPIKNG
SPEIKLKITKTYMNGKPLFESSICGDSAADVSQSEENGQKPENKARRNRKRSIKYDSLLE
QGLVEAALVSKISSPSDKKIPAKKESCPNTGRDKDHLLKYNVGDLVWSKVSGYPWWPCMV
SADPLLHSYTKLKGQKKSARQYHVQFFGDAPERAWIFEKSLVAFEGEGQFEKLCQESAKQ
APTKAEKIKLLKPISGKLRAQWEMGIVQAEEAASMSVEERKAKFTFLYVGDQLHLNPQVA
KEAGIAAESLGEMAESSGVSEEAAENPKSVREECIPMKRRRRAKLCSSAETLESHPDIGK
STPQKTAEADPRRGVGSPPGRKKTTVSMPRSRKGDAASQFLVFCQKHRDEVVAEHPDASG
EEIEELLRSQWSLLSEKQRARYNTKFALVAPVQAEEDSGNVNGKKRNHTKRIQDPTEDAE
AEDTPRKRLRTDKHSLRKRDTITDKTARTSSYKAMEAASSLKSQAATKNLSDACKPLKKR
NRASTAASSALGFSKSSSPSASLTENELLWEPTPVKLDLNPAALYCT*
>ENSG00000153560|ENST00000283628
MAWVLKMDEVIESGLVHDFDASLSGIGQELGAGAYSMSDVLALPIFKQEDSSLPLDGETE
HPPFQYVMCAATSPAVKLHDETLTYLNQGQSYEIRMLDNRKMGDMPEINGKLVKSIIRVV
FHDRRLQYTEHQQLEGWKWNRPGDRLLDLDIPMSVGIIDTRTNPSQLNAVEFLWDPAKRT
SAFIQVHCISTEFTPRKHGGEKGVPFRIQVDTFKQNENGEYTDHLHSASCQIKVFKPKGA
DRKQKTDREKMEKRTAHEKEKYQPSYDTTILTEMRLEPIIEDAVEHEQKKSSKRTLPADY
GDSLAKRGSCSPWPDAPTAYVNNSPSPAPTFTSPQQSTCSVPDSNSSSPNHQGDGASQTS
GEQIQPSATIQETQQWLLKNRFSSYTRLFSNFSGADLLKLTKEDLVQICGAADGIRLYNS
LKSRSVRPRLTIYVCREQPSSTVLQGQQQAASSASENGSGAPYVYHAIYLEEMIASEVAR
KLALVFNIPLHQINQVYRQGPTGIHILVSDQMVQNFQDESCFLFSTVKAESSDGIHIILK
*
>ENSG00000180818|ENST00000303460
MTCPRNVTPNSYAEPLAAPGGGERYSRSAGMYMQSGSDFNCGVMRGCGLAPSLSKRDEGS
SPSLALNTYPSYLSQLDSWGDPKAAYRLEQPVGRPLSSCSYPPSVKEENVCCMYSAEKRA
KSGPEAALYSHPLPESCLGEHEVPVPSYYRASPSYSALDKTPHCSGANDFEAPFEQRASL
NPRAEHLESPQLGGKVSFPETPKSDSQTPSPNEIKTEQSLAGPKGSPSESEKERAKAADS
SPDTSDNEAKEEIKAENTTGNWLTAKSGRKKRCPYTKHQTLELEKEFLFNMYLTRERRLE
ISKTINLTDRQVKIWFQNRRMKLKKMNRENRIRELTSNFNFT*
>ENSG00000113387|ENST00000265073
MPKSKELVSSSSSGSDSDSEVDKKLKRKKQVAPEKPVKKQKTGETSRALSSSKQSSSSRD
DNMFQIGKMRYVSVRDFKGKVLIDIREYWMDPEGEMKPGRKGISLNPEQWSQLKEQISDI
DDAVRKL*
>ENSG00000167380|ENST00000300823
MNMFKEAVTFKDVAVAFTEEELGLLGPAQRKLYRDVMVENFRNLLSVGHPPFKQDVSPIE
RNEQLWIMTTATRRQGNLDTLLVKALLLYDLAQT*
>ENSG00000167526|ENST00000311528

MAPSRNGMVLKPHFHKDWQRRVATWFNQPARKIRRRKARQAKARRIAPRPASGPIRPIVR
CPTVRYHTKVRAGRGFSLEELRVAGIHKKVARTIGISVDPRRRNKSTESLQANVQRLKEY
RSKLILFPRKPSAPKKGDSSAEELKLATQLTGPVMPVRNVYKKEKARVITEEEKNFKAFA
SLRMARANARLFGIRAKRAKEAAEQDVEKKK*
>ENSG00000147596|ENST00000276594
MALPRPSEAVPQDKVCYPPESSPQNLAAYYTPFPSYGHYRNSLATVEEDFQPFRQLEAAA
SAAPAMPPFPFRMAPPLLSPGLGLQREPLYDLPWYSKLPPWYPIPHVPREVPPFLSSSHE
YAGASSEDLGHQIIGGDNESGPCCGPDTLIPPPPADASLLPEGLRTSQLLPCSPSKQSED
GPKPSNQEGKSPARFQFTEEDLHFVLYGVTPSLEHPASLHHAISGLLVPPDSSGSDSLPQ
TLDKDSLQLPEGLCLMQTVFGEVPHFGVFCSSFIAKGVRFGPFQGKVVNASEVKTYGDNS
VMWEIFEDGHLSHFIDGKGGTGNWMSYVNCARFPKEQNLVAVQCQGHIFYESCKEIHQNQ
ELLVWYGDCYEKFLDIPVSLQVTEPGKQPSGPSEESAEGYRCERCGKVFTYKYYRDKHLK
YTPCVDKGDRKFPCSLCKRSFEKRDRLRIHILHVHEKHRPHKCSTCGKCFSQSSSLNKHM
RVHSGDRPYQCVYCTKRFTASSILRTHIRQHSGEKPFKCKYCGKSFASHAAHDSHVRRSH
KEDDGCSCSICGKIFSDQETFYSHMKFHEDY*
>ENSG00000140451|ENST00000268043
MLSGIEAAAGEYEDSELRCRVAVEELSPGGQPRRRQALRTAELSLGRNERRELMLRLQAP
GPAGRPRCFPLRAARLFTRFAEAGRSTLRLPAHDTPGAGAVQLLLSDCPPDRLRRFLRTL
RLKLAAAPGPGPASARAQLLGPRPRDFVTISPVQPEERRLRAATRVPDTTLVKRPVEPQA
GAEPSTEAPRWPLPVKRLSLPSTKPQLSEEQAAVLRAVLKGQSIFFTGSAGTGKSYLLKR
ILGSLPPTGTVATASTGVAACHIGGTTLHAFAGIGSGQAPLAQCVALAQRPGVRQGWLNC
QRLVIDEISMVEADLFDKLEAVARAVRQQNKPFGGIQLIICGDFLQLPPVTKGSQPPRFC
FQSKSWKRCVPVTLELTKVWRQADQTFISLLQAVRLGRCSDEVTRQLQATASHKVGRDGI
VATRLCTHQDDVALTNERRLQELPGKVHRFEAMDSNPELASTLDAQCPVSQLLQLKLGAQ
VMLVKNLSVSRGLVNGARGVVVGFEAEGRGLPQVRFLCGVTEVIHADRWTVQATGGQLLS
RQQLPLQLAWAMSIHKSQGMTLDCVEISLGRVFASGQAYVALSRARSLQGLRVLDFDPMA
VRCDPRVLHFYATLRRGRSLSLESPDDDEAASDQENMDPIL*
>ENSG00000114686|ENST00000264995
MPGWRLLTQVGAQVLGRLGDGLGAALGPGNRTHIWLFVRGLHGKSGTWWDEHLSEENVPF
IKQLVSDEDKAQLASKLCPLKDEPWPIHPWEPGSFRVGLIALKLGMMPLWTKDGQKHVVT
LLQVQDCHVLKYTSKENCNGKMATLSVGGKTVSRFRKATSILEFYRELGLPPKQTVKIFN
ITDNAAIKPGTPLYAAHFRPGQYVDVTAKTIGKGFQGVMKRWGFKGQPATHGQTKTHRRP
GAVATGDIGRVWPGTKMPGKMGNIYRTEYGLKVWRINTKHNIIYVNGSVPGHKNCLVKVK
DSKLPAYKDLGKNLPFPTYFPDGDEEELPEDLYDENVCQPGAPSITFA*
>ENSG00000167967|ENST00000301727
MEGAMAVRVTAAHTAEAQAEAGREAGEGAVAAVAAALAPSGFLGLPAPFSEEDEDDVHRC
GRCQAEFTALEDFVQHKIQKACQRAPPEALPATPATTALLGQEVVPAAPGPEEPITVAHI
VVEAASLAADISHASDLVGGGHIKEVIVAAEAELGDGEMAEAPGSPRQQGLGLAGEGEQA
QVKLLVNKDGRYVCALCHKTFKTGSILKAHMVTHSSRKDHECKLCGASFRTKGSLIRHHR
RHTDERPYKCSKCGKSFRESGALTRHLKSLTPCTEKIRFSVSKDVVVSKEDARAGSGAGA
AGLGTATSSVTGEPIETSPVIHLVTDAKGTVIHEVHVQMQELSLGMKALAPEPPVSQELP
CSSEGSRENLLHQAMQNSGIVLERAAGEEGALEPAPAAGSSPQPLAVAAPQLPVLEVQPL
ETQVASEASAVPRTHPCPQCSETFPTAATLEAHKRGHTGPRPFACAQCGKAFPKAYLLKK
HQEVHVRERRFRCGDCGKLYKTIAHVRGHRRVHSDERPYPCPKCGKRYKTKNAQQVHFRT

HLEEKPHVCQFCSRGFREKGSLVRHVRHHTGEKPFKCYKCGRGFAEHGTLNRHLRTKGGC
LLEVEELLVSEDSPAAATTVLTEDPHTVLVEFSSVVADTQEYIIEATADDAETSEATEII
EGTQTEVDSHIMKVVQQIVHQASAGHQIIVQNVTMDEETALGPEAAAADTITIATPESLT
EQVAMTLASAISEGTVLAARAGTSGTEQATVTMVSSEDIEILEHAGELVIASPEGQLEVQ
TVIV*
>ENSG00000165583|ENST00000311798
MNGDDAFVRRPRVGSQIPEKMQKHPWRQVCDRGIHLVNLSPFWKVGREPASSIKALLCGR
GEARAFDDIAKYFSEKEWEKMKASEKIIYVYMKRKYEAMTKLGFKATLPPFMRNKRVADF
QGNDFDNDPNRGNQVEHPQMTFGRLQGIFPKITPEKPAEEGNDSKGVPEASGPQNNGKQL
RPSGKLNTSEKVNKTSGPKRGKHAWTHRVRERKQLVIYEEISDPQEDDE*
>ENSG00000174282|ENST00000311403
MPPPAEVTDPSHAPAVLRQLNEQRLRGLFCDVTLIAGDTKFPAHRSVLAASSPFFREALL
TSAPLPLPPATGGAAPNPATTTAASSSSSSSSSSSSSSSSASSSSSSSSSSSPPPASPPAS
SPPRVLELPGVPAAAFSDVLNFIYSARLALPGGGGDGAAVAEIGALGRRLGISRLQGLGE
GGDAWVPPTPAPMATSQPEEDSFGPGPRPAGEWEGDRAEAQAPDLQCSLPRRPLPCPQCG
KSFIHPKRLQTHEAQCRRGASTRGSTGLGAGGAGPGGPAGVDASALPPPVGFRGGPEHVV
KVVGGHVLYVCAACERSYVTLSSLKRHSNVHSWRRKYPCRYCEKVFALAEYRTKHEVWHT
GERRYQCIFCWETFVTYYNLKTHQRAFHGISPGLLASEKTPNGGYKPKLNTLKLYRLLPM
RAAKRPYKTYSQGAPEAPLSPTLNTPAPVAMPASPPPGPPPAPEPGPPPSVITFAHPAPS
VIVHGGSSSGGGGSGTASTGGSQAASVITYTAPPRPPKKREYPPPPPEPAATPTSPATAV
SPATAAGPAMATTTEEAKGRNPRAGRTLTYTAKPVGGIGGGGGPPTGAGRGPSQLQAPPP
LCQITVRIGEEAIVKRRISETDLRPGELSGEEMEESEEDEEEEDEEEEEEDEEESKAGGE
DQLWRPYYSYKPKRKAGAAGGASVGGSGLPRGRRPPRWRQKLERRSWEETPAAESPAGRA
RTERRHRCGDCAQTFTTLRKLRKHQEAHGGGSHSSRAGRRPSTRFTCPHCAKVCKTAAAL
SRHGQRHAAERPGGTPTPVIAYSKGSAGTRPGDVKEEAPQEMQVSSSSGEAGGGSTAAEE
ASETASLQDPIISGGEEPPVVASGGSYVYPPVQEFPLALIGGGREPGGGRGKSGSEGPVG
AGEGDRMEGIGAAKVTFYPEPYPLVYGPQLLAAYPYNFSNLAALPVALNMVLPDEKGAGA
LPFLPGVFGYAVNPQAAPPAPPTPPPPTLPPPIPPKGEGERAGVERTQKGDVG*
>ENSG00000146834|ENST00000310512
MIEMAAEKEPFLVPAPPPPLKDESGGGGGPTVPPHQEAASGELRGGTERGPGRCAPSAGS
PAAAVGRESPGAAATSSSGPQAQQHRGGGPQAQSHGEARLSDPPGRAAPPDVGEERRGGG
GTELGPPAPPRPRNGYQPHRPPGGGGGKRRNSCNVGGGGGGFKHPAFKRRRRVNSDCDSV
LPSNFLLGGNIFDPLNLNSLLDEEVSRTLNAETPKSSPLPAKGRDPVEILIPKDITDPLS
LNTCTDEGHVVLASPLKTGRKRHRHRGQHHQQQQAAGGSESHPVPPTAPLTPLLHGEGAS
QQPRHRGQNRDAPQPYELNTAINCRDEVVSPLPSALQGPSGSLSAPPAASVISAPPSSSS
RHRKRRRTSSKSEAGARGGGQGSKEKGRGSWGGRHHHHHPLPAAGFKKQQRKFQYGNYCK
YYGYRNPSCEDGRLRVLKPEWFRGRDVLDLGCNVGHLTLSIACKWGPSRMVGLDIDSRLI
HSARQNIRHYLSEELRLPPQTLEGDPGAEGEEGTTTVRKRSCFPASLTASRGPIAAPQVP
LDGADTSVFPNNVVFVTGNYVLDRDDLVEAQTPEYDVVLCLSLTKWVHLNWGDEGLKRMF
RRIYRHLRPGGILVLEPQPWSSYGKRKTLTETIYKNYYRIQLKPEQFSSYLTSPDVGFSS
YELVATPHNTSKGFQRPVYLFHKARSPSH*
>ENSG00000175329|ENST00000308700
MCAEVGPALCRGMERNSLGCCEAPKKLSLSFSIEAILKRPARRSDMDRPEGPGEEGPGEA
AASGSGLEKPPKDQPQEGRKSKRRVRTTFTTEQLHELEKIFHFTHYPDVHIRSQLAARIN

LPEARVQIWFQNQRAKWRKQEKIGNLGAPQQLSEASVALPTNLDVAGPTWTSTALRRLAP
PTSCCPSAQDQLASAWFPAWITLLPAHPWETQPVPGLPIHQTCIPVLCILPPPHPKWGSI
CATST*
>ENSG00000168286|ENST00000303596
MPGFTCCVPGCYNNSHRDKALHFYTFPKDAELRRLWLKNVSRAGVSGCFSTFQPTTGHRL
CSVHFQGGRKTYTVRVPTIFPLRGVNERKVARRPAGAAAARRRQQQQQQQQQQQQQQQQQ
QQQQQQQQQQQQSSPSASTAQTAQLQPNLVSASAAVLLTLQATVDSSQAPGSVQPAPITP
TGEDVKPIDLTVQVEFAAAEGAAAAAAASELQAATAGLEAAECPMGPQLVVVGEEGFPDT
GSDHSYSLSSGTTEEELLRKLNEQRDILALMEVKMKEMKGSIRHLRLTEAKLREELREKD
RLLAMAVIRKKHGM*
>ENSG00000171425|ENST00000270451
MLVLPSPCPQPLAFSSVETMEGPPRRTCRSPEPGPSSSIGSPQASSPPRPNHYLLIDTQG
VPYTVLVDEESQREPGASGAPGQKKCYSCPVCSRVFEYMSYLQRHSITHSEVKPFECDIC
GKAFKRASHLARHHSIHLAGGGRPHGCPLCPRRFRDAGELAQHSRVHSGERPFQCPHCPR
RFMEQNTLQKHTRWKHP*
>ENSG00000173559|ENST00000307849
MNRVNDPLIFIRDIKPGLKNLNVVFIVLEIGRVTKTKDGHEVRSCKVADKTGSITISVWD
EIGGLIQPGDIIRLTRGYASMWKGCLTLYTGRGGELQKIGDLGAVQAAAMRDSIHYYPGN
DLHPDLEEPSSLGV*
>ENSG00000172845|ENST00000310015
MTAPEKPVKQEEMAALDVDSGGGGGGGGGHGEYLQQQQQHGNGAVAAAAAAQDTQPSPLA
LLAATCSKIGPPSPGDDEEEAAAAAGAPAAAGATGDLASAQLGGAPNRWEVLSATPTTIK
DEAGNLVQIPSAATSSGQYVLPLQNLQNQQIFSVAPGSDSSNGTVSSVQYQVIPQIQSAD
GQQVQIGFTGSSDNGGINQESSQIQIIPGSNQTLLASGTPSANIQNLIPQTGQVQVQGVA
IGGSSFPGQTQVVANVPLGLPGNITFVPINSVDLDSLGLSGSSQTMTAGINADGHLINTG
QAMDSSDNSERTGERVSPDINETNTDTDLFVPTSSSSQLPVTIDSTGILQQNTNSLTTSS
GQVHSSDLQGNYIQSPVSEETQAQNIQVSTAQPVVQHLQLQESQQPTSQAQIVQGITPQT
IHGVQASGQNISQQALQNLQLQLNPGTFLIQAQTVTPSGQVTWQTFQVQGVQNLQNLQIQ
NTAAQQITLTPVQTLTLGQVAAGGAFTSTPVSLSTGQLPNLQTVTVNSIDSAGIQLHPGE
NADSPADIRIKEEEPDPEEWQLSGDSTLNTNDLTHLRVQVVDEEGDQQHQEGKRLRRVAC
TCPNCKEGGGRGTNLGKKKQHICHIPGCGKVYGKTSHLRAHLRWHSGERPFVCNWMYCGK
RFTRSDELQRHRRTHTGEKKFVCPECSKRFMRSDHLAKHIKTHQNKKGIHSSSTVLASVE
AARDDTLITAGGTTLILANIQQGSVSGIGTVNTSATSNQDILTNTEIPLQLVTVSGNETM
E*
>ENSG00000159200|ENST00000313806
MEDGVAGPQLGAAAEAAEAAEARARPGVTLRPFAPLSGAAEADEGGGDWSFIDCEMEEVD
LQDLPSATIACHLDPRVFVDGLCRAKFESLFRTYDKDITFQYFKSFKRVRINFSNPFSAA
DARLQLHKTEFLGKEMKLYFAQTLHIGSSHLAPPNPDKQFLISPPASPPVGWKQVEDATP
VINYDLLYAISKLGPGEKYELHAATDTTPSVVVHVCESDQEKEEEEEMERMRRPKPKIIQ
TRRPEYTPIHLS*
>ENSG00000120802|ENST00000266732
MPEFLEDPSVLTKDKLKSELVANNVTLPAGEQRKDVYVQLYLQHLTARNRPPLPAGTNSK
GPPDFSSDEEREPTPVLGSGAAAAGRSRAAVGRKATKKTDKPRQEDKDDLDVTELTNEDL
LDQLVKYGVNPGPIVGTTRKLYEKKLLKLREQGTESRSSTPLPTISSSAENTRQNGSNDS

DRYSDNEEGKKKEHKKVKSTRDIVPFSELGTTPSGGGFFQGISFPEISTRPPLGSTELQA
AKKVHTSKGDLPREPLVATNLPGRGQLQKLASERNLFISCKSSHDRCLEKSSSSSSQPEH
SAMLVSTAASPSLIKETTTGYYKDIVENICGREKSGIQPLCPERSHISDQSPLSSKRKAL
EESESSQLISPPLAQAIRDYVNSLLVQGGVGSLPGTSNSMPPLDVENIQKRIDQSKFQET
EFLSPPRKVPRLSEKSVEERDSGSFVAFQNIPGSELMSSFAKTVVSHSLTTLGLEVAKQS
QHDKIDASELSFPFHESILKVIEEEWQQVDRQLPSLACKYPVSSREATQILSVPKVDDEI
LGFISEATPLGGIQAASTESCNQQLDLALCRAYEAAASALQIATHTAFVAKAMQADISQA
AQILSSDPSRTHQALGILSKTYDAASYICEAAFDEVKMAAHTMGNATVGRRYLWLKDCKI
NLASKNKLASTPFKGGTLFGGEVCKVIKKRGNKH*
>ENSG00000154743|ENST00000284995
MAEAVFHAPKRKRRVYETYESPLPIPFGQDHGPLKEFKIFRAEMINNNVIVRNAEDIEQL
YGKGYFGKGILSRSRPSFTISDPKLVAKWKDMKTNMPIITSKRYQHSVEWAAELMRRQGQ
DESTVRRILKDYTKPLEHPPVKRNEEAQVHDKLNSGMVSNMEGTAGGERPSVVNGDSGKS
GGVGDPREPLGCLQEGSGCHPTTESFEKSVREDASPLPHVCCCKQDALILQRGLHHEDGS
QHIGLLHPGDRGPDHEYVLVEEAECAMSEREAAPNEELVQRNRLICRRNPYRIFEYLQLS
LEEAFFLVYALGCLSIYYEKEPLTIVKLWKAFTVVQPTFRTTYMAYHYFRSKGWVPKVGL
KYGTDLLLYRKGPPFYHASYSVIIELVDDHFEGSLRRPLSWKSLAALSRVSVNVSKELML
CYLIKPSTMTDKEMESPECMKRIKVQEVILSRWVSSRERSDQDDL*
>ENSG00000123064|ENST00000306014
MAADKGPAAGPRSRAAMAQWRKKKGLRKRRGAASQARGSDSEDGEFEIQAEDDARARKLG
PGRPLPTFPTSECTSDVEPDTREMVRAQNKKKKKSGGFQSMGLSYPVFKGIMKKGYKVPT
PIQRKTIPVILDGKDVVAMARTGSGKTACFLLPMFERLKTHSAQTGARALILSPTRELAL
QTLKFTKELGKFTGLKTALILGGDRMEDQFAALHENPDIIIATPGRLVHVAVEMSLKLQS
VEYVVFDEADRLFEMGFAEQLQEIIARLPGGHQTVLFSATLPKLLVEFARAGLTEPVLIR
LDVDTKLNEQLKTSFFLVREDTKAAVLLHLLHNVVRPQDQTVVFVATKHHAEYLTELLTT
QRVSCAHIYSALDPTARKINLAKFTLGKCSTLIVTDLAARGLDIPLLDNVINYSFPAKGK
LFLHRVGRVARAGRSGTAYSLVAPDEIPYLLDLHLFLGRSLTLARPLKEPSGVAGVDGML
GRVPQSVVDEEDSGLQSTLEASLELRGLARVADNAQQQYVRSRPAPSPESIKRAKEMDLV
GLGLHPLFSSRFEEEELQRLRLVDSIKNYRSRATIFEINASSRDLCSQVMRAKRQKDRKA
IARFQQGQQGRQEQQEGPVGPAPSRPALQEKQPEKEEEEEAGESVEDIFSEVVGRKRQRS
GPNRGAKRRREEARQRDQEFYIPYRPKDFDSERGLSISGEGGAFEQQAAGAVLDLMGDEA
QNLTRGRQQLKWDRKKKRFVGQSGGQEDKKKIKTESGRYISSSYKRDLYQKWKQKQKIDDR
DSDEEGASDRRGPERRGGKRDRGQGASRPHAPGTPAGRVRPELKTKQQILKQRRRAQKLH
FLQRGGLKQLSARNRRRVQELQQGAFGRGARSKKGKMRKRM*
>ENSG00000178952|ENST00000313511
MTTMAAATLLRATPHFSGLAAGRTFLLQGLLRLLKAPALPLLCRGLAVEAKKTYVRDKPH
VNVGTIGHVDHGKTTLTAAITKILAEGGGAKFKKYEEIDNAPEERARGITINAAHVEYST
AARHYAHTDCPGHADYVKNMITGTAPLDGCILVVAANDGPMPQTREHLLLARQIGVEHVV
VYVNKADAVQDSEMVELVELEIRELLTEFGYKGEETPVIVGSALCALEGRDPELGLKSVQ
KLLDAVDTYIPVPARDLEKPFLLPVEAVYSVPGRGTVVTGTLERGILKKGDECELLGHSK
NIRTVVTGIEMFHKSLERAEAGDNLGALVRGLKREDLRRGLVMVKPGSIKPHQKVEAQVY
ILSKEEGGRHKPFVSHFMPVMFSLTWDMACRIILPPEKELAMPGEDLKFNLILRQPMILE
KGQRFTLRDGNRTIGTGLVTNTLAMTEEEKNIKWG*
>ENSG00000089876|ENST00000284690

MEEEGLECPNSSSEKRYFPESLDSSDGDEEEVLACEDLELNPFDGLPYSSRYYKLLKERE
DLPIWKEKYSFMENLLQNQIVIVSGDAKCGKSAQVPQWCAEYCLSIHYQHGGVICTQVHK
QTVVQLALRVADEMDVNIGHEVGYVIPFENCCTNETILRYCTDDMLQREMMSNPFLGSYG
VIILDDIHERSIATDVLLGLLKDVLLARPELKLIINSSPHLISKLNSYYGNVPVIEVKNK
HPVEVVYLSEAQKDSFESILRLIFEIHHSGEKGDIVVFLACEQDIEKVCETVYQGSNLNP
DLGELVVVPLYPKEKCSLFKPLDETEKRCQVYQRRVVLTTSSGEFLIWSNSVRFVIDVGV
ERRKVYNPRIRANSLVMQPISQSQAEIRKQILGSSSSGKFFCLYTEEFASKDMTPLKPAE
MQEANLTSMVLFMKRIDIAGLGHCDFMNRPAPESLMQALEDLDYLAALDNDGNLSEFGII
MSEFPLDPQLSKSILASCEFDCVDEVLTIAAMVTAPNCFSHVPHGAEEAALTCWKTFLHP
EGDHFTLISIYKAYQDTTLNSSSEYCVEKWCRDYFLNCSALRMADVIRAELLEIIKRIEL
PYAEPAFGSKENTLNIKKALLSGYFMQIARDVDGSGNYLMLTHKQVAQLHPLSGYSITKK
MPEWVLFHKFSISENNYIRITSEISPELFMQLVPQYYFSNLPPSESKDILQQVVDHLSPV
STMNKEQQMCETCPETEQRCTLQ*
>ENSG00000167004|ENST00000300289
MRLRRLALFPGVALLLAAARLAAASDVLELTDDNFESRISDTGSAGLMLVEFFAPWCGHC
KRLAPEYEAAATRLKGIVPLAKVDCTANTNTCNKYGVSGYPTLKIFRDGEEAGAYDGPRT
ADGIVSHLKKQAGPASVPLRTEEEFKKFISDKDASIVGFFDDSFSEAHSEFLKAASNLRD
NYRFAHTNVESLVNEYDDNGEGIILFRPSHLTNKFEDKTVAYTEQKMTSGKIKKFIQENI
FGICPHMTEDNKDLIQGKDLLIAYYDVDYEKNAKGSNYWRNRVMMVAKKFLDAGHKLNFA
VASRKTFSHELSDFGLESTAGEIPVVAIRTAKGEKFVMQEEFSRDGKALERFLQDYFDGN
LKRYLKSEPIPESNDGPVKVVVAENFDEIVNNENKDVLIEFYAPWCGHCKNLEPKYKELG
EKLSKDPNIVIAKMDATANDVPSPYEVRGFPTIYFSPANKKLNPKKYEGGRELSDFISYL
QREATNPPVIQEEKPKKKKKAQEDL*
>ENSG00000171861|ENST00000304478
MAALVRPARFVVRPLLQVVQAWDLDARRWVRALRRSPVKVVFPSGEVVEQKRAPGKQPRK
APSEASAQEQREKQPLEESASRAPSTWEESGLRYDKAYPGDRRLSSVMTIVKSRPFREKQ
GKILLEGRRLISDALKAGAVPKMFFFSRLEYLKELPVDKLKGVSLIKVKFEDIKDWSDLV
TPQGIMGIFAKPDHVKMTYPKTQLQHSLPLLLICDNLRDPGNLGTILRSAAGAGCSKVLL
TKGCVDAWEPKVLRAGMGAHFRMPIINNLEWETVPNYLPPDTRVYVADNCGLYAQAEMSN
KASDHGWVCDQRVMKFHKYEEEEDVETGASQDWLPHVEVQSYDSDWTEAPAAVVIGGETY
GVSLESLQLAESTGGKRLLIPVVPGVDSLNSAMAASILLFEGKRQLRGRAEDLSRDRSYH
*
>ENSG00000130584|ENST00000302995
MNNRKEDMEITSHYRHLLRELNEQRQHGVLCDVCVVVEGKVFKAHKNVLLGSSRYFKTLY
CQVQKTSEQATVTHLDIVTAQGFKAIIDFMYSAHLALTSRNVIEVMSAASFLQMTDIVQA
CHDFIKAALDISIKSDASDELAEFEIGASSSSSTEALISAVMAGRSISPWLARRTSPANS
SGDSAIASCHDGGSSYGKEDQEPKADGPDDVSSQPLWPGDVGYGPLRIKEEQVSPSQYGG
SELPSAKDGAVQNSFSEQSAGDAWQPTGRRKNRKNKETVRHITQQVEDDSRASSPVPSFL
PTSGWPFSSRDSNADLSVTEASSSDSRGERAELYAQVEEGLLGGEASYLGPPLTPEKDDA
LHQATAVANLRAALMSKNSLLSLKADVLGDDGSLLFEYLPRGAHSLSLNEFTVIRKKFKC
PYCSFSAMHQCILKRHMRSHTGERPYPCEICGKKFTRREHMKRHTLVHSKDKKYVCKVCS
RVFMSAASVGIRHGSRRHGVCTDCAGRGMAGPLDHGGGGGEGSPEALFPGDGPYLEDPED
PRGEAEELGEDDEGLAPEDALLADDKDEEDSPRPRSPPGGPDKDFAWLS*
>ENSG00000169989|ENST00000304337

MAEASVDASTLPVTVKKKKSLSIEEKIDIINAVESGKKKAEIAAEYGIKKNSLSSIMKNK
DKVLEAFESLRFDPKRKRLRTAFYTDLEEALMRWYRIAQCLNVPVNGPMLRLKANDFAQK
LGHNDFKCSNGWLDRFKSRYGLVFRAQPVEATGVPVDPSTVWYQNVLPYYLNDYHPKNVF
NIKETGLLYRMLPTNTFAFKGETCSVGKLCKDRITLVVGTNMDGSEKLPLLVIGKKRTPH
CFKGLKSLPVCYEANRMAWMTSDVFEQWMRKLDEEFQAQQRRVVIFVESFPAHPEVKNLK
SIELAFFPSCLSSKCIAMKQGVIKSLKIKYRHCLIKKFLSSVEGSKEFTFSLLDAVDTLH
LCWRAVTPETIVKSYEEAGFKSQKGESDITNAEKDTGLDLVADALGAGVEFPEGLSIEEY
AALDDDLETCEAAPNGDSICTKESKSDETGFYTSDEEDDDGSPGTELPLPSKSEAITALD
TLKKFLRSQDMNDGLQNSLADLENFINSLSPK*
>ENSG00000051180|ENST00000267868
MAMQMQLEANADTSVEEESFGPQPISRLEQCGINANDVKKLEEAGFHTVEAVAYAPKKEL
INIKGISEAKADKILAEAAKLVPMGFTTATEFHQRRSEIIQITTGSKELDKLLQGGIETG
SITEMFGEFRTGKTQICHTLAVTCQLPIDRGGGEGKAMYIDTEGTFRPERLLAVAERYGL
SGSDVLDNVAYARAFNTDHQTQLLYQASAMMVESRYALLIVDSATALYRTDYSGRGELSA
RQMHLARFLRMLLRLADEFGVAVVITNQVVAQVDGAAMFAADPKKPIGGNIIAHASTTRL
YLRKGRGETRICKIYDSPCLPEAEAMFAINADGVGDAKD*
>ENSG00000140525|ENST00000300027
MDQKILSLAAEKTADKLQEFLQTLREGDLTNLLQNQAVKGKVAGALLRAIFKGSPCSEEA
GTLRRRKIYTCCIQLVESGDLQKEIASEIIGLLMLEAHHFPGPLLVELANEFISAVREGS
LVNGKSLELLPIILTALATKKENLAYGKGVLSGEECKKQLINTLCSGRWDQQYVIQLTSM
FKDVPLTAEEVEFVVEKALSMFSKMNLQEIPPLVYQLLVLSSKGSRKSVLEGIIAFFSAL
DKQHNEEQSGDELLDVVTVPSGELRHVEGTIILHIVFAIKLDYELGRELVKHLKVGQQGD
SNNNLSPFSIALLLSVTRIQRFQDQVLDLLKTSVVKSFKDLQLLQGSKFLQNLVPHRSYV
STMILEVVKNSVHSWDHVTQGLVELGFILMDSYGPKKVLDGKTIETSPSLSRMPNQHACK
LGANILLETFKIHEMIRQEILEQVLNRVVTRASSPISHFLDLLSNIVMYAPLVLQSCSSK
VTEAFDYLSFLPLQTVQRLLKAVQPLLKVSMSMRDCLILVLRKAMFANQLDARKSAVAGF
LLLLKNFKVLGSLSSSQCSQSLSVSQVHVDVHSHYNSVANETFCLEIMDSLRRCLSQQAD
VRLMLYEGFYDVLRRNSQLANSVMQTLLSQLKQFYEPKPDLLPPLKLEACILTQGDKISL
QEPLDYLLCCIQHCLAWYKNTVIPLQQGEEEEEEEEAFYEDLDDILESITNRMIKSELED
FELDKSADFSQSTSIGIKNNICAFLVMGVCEVLIEYNFSISSFSKNRFEDILSLFMCYKK
LSDILNEKAGKAKTKMANKTSDSLLSMKFVSSLLTALFRVLLWRYTSIPTSVEESGKKEK
GKSISLLCLEGLQKIFSAVQQFYQPKIQQFLRALDVTDKEGEEREDADVSVTQRTAFQIR
QFQRSLLNLLSSQEEDFNSKEALLLVTVLTSLSKLLEPSSPQFVQMLSWTSKICKENSRE
DALFCKSLMNLLFSLHVSYKSPVILLRDLSQDIHGHLGDIDQDVEVEKTNHFAIVNLRTA
APTVCLLVLSQAEKVLEEVDWLITKLKGQVSQETLSEEASSQATLPNQPVEKAIIMQLGT
LLTFFHELVQTALPSGSCVDTLLKDLCKMYTTLTALVRYYLQVCQSSGGIPKNMEKLVKL
SGSHLTPLCYSFISYVQNKSKSLNYTGEKKEKPAAVATAMARVLRETKPIPNLIFAIEQY
EKFLIHLSKKSKVNLMQHMKLSTSRDFKIKGNILDMVLREDGEDENEEGTASEHGGQNKE
PAKKKRKK*
>ENSG00000171604|ENST00000302517
MSSLGGGSQDAGGSSSSSTNGSGGSGSSGPKAGAADKSAVVAAAAPASVADDTPPPERRN
KSGIISEPLNKSLRRSRPLSHYSSFGSSGGSGGGSMMGGESADKATAAAAAASLLANGHD
LAAAMAVDKSNPTSKHKSGAVASLLSKAERATELAAEGQLTLQQFAQSTEMLKRVVQEHL
PLMSEAGAGLPDMEAVAGAEALNGQSDFPYLGAFPINPGLFIMTPAGVFLAESALHMAGL

AEYPMQGELASAISSGKKKRKRCGMCAPCRRRINCEQCSSCRNRKTGHQICKFRKCEELK
KKPSAALEKVMLPTGAAFRWFQ*
>ENSG00000143947|ENST00000272317
MQIFVKTLTGKTITLEVEPSDTIENVKAKIQDKEGIPPDQQRLIFAGKQLEDGRTLSDYN
IQKESTLHLVLRLRGGAKKRKKKSYTTPKKNKHKRKKVKLAVLKYYKVDENGKISRLRRE
CPSDECGAGVFMASHFDRHYCGKCCLTYCFNKPEDK*
>ENSG00000169016|ENST00000307236
MNPSPSKIRINLEDNVQYVSMRKALKVKRPRFDVSLVYLTRKFMDLVRSAPGGILDLNKV
ATKLGVRKRRVYDITNVLDGIDLVEKKSKNHIRWIGSDLSNFGAVPQQKKLQEELSDLSA
MEDALDELIKDCAQQLFELTDDKENERLAYVTYQDIHSIQAFHEQIVIAVKAPAETRLDV
PAPREDSITVHIRSTNGPIDVYLCEVEQGQTSNKRSEGVGTSSSESTHPEGPEEEENPQQ
SEELLEVSN*
>ENSG00000152217|ENST00000282030
MESRETLSSSRQRGGESDFLPVSSAKPPAAPGCAGEPLLSTPGPGKGIPVGGERMEPEEE
DELGSGRDVDSNSNADSEKWVAGDGLEEQEFSIKEANFTEGSLKLKIQTTKRAKKPPKNL
ENYICPPEIKITIKQSGDQKVSRAGKNSKATKEEERSHSKKKLLTASDLAASDLKGFQPQ
AYERPQKHSTLHYDTGLPQDFTGDTLKPKHQQKSSSQNHMDWSTNSDSGPVTQNCFISPE
SGRETASTSKIPALEPVASFAKAQGKKGSAGNTWSQLSNNNKDLLLGGVAPSPSSHSSPA
PPSSSAECNGLQPLVDQDGGGTKEPPEPPTVGSKKKSSKKDVISQTIPNPDLDWVKNAQK
AFDNTEGKREGYSADSAQEASPARQNVSSASNPENDSSHVRITIPIKAPSLDPTNHKRKK
RQSIKAVVEKIMPEKALASGITMSSEVVNRILSNSEGNKKDPRVPKLSKMIENESPSVGL
ETGGNAEKVIPGGVSKPRKPPMVMTPPTCTDHSPSRKLPEIQHPKFAAKRRWTCSKPKPS
TMLREAVMATSDKLMLEPPSAYPITPSSPLYTNTDSLTVITPVKKKRGRPKKQPLLTVET
IHEGTSTSPVSPISREFPGTKKRKRRRNLAKLAQLVPGEDKPMSEMKFHKKVGKLGVLDK
KTIKTINKMKTLKRKNILNQILSCSSSVALKAKAPPETSPGAAAIESKLGKQINVSKRGT
IYIGKKRGRKPRAELPPPSEEPKTAIKHPRPVSSQPDVPAVPSNFQSLVASSPAAMHPLS
TQLGGSNGNLSPASTETNFSELKTMPNLQPISALPTKTQKGIHSGTWKLSPPRLMANSPS
HLCEIGSLKEITLSPVSESHSEETIPSDSGIGTDNNSTSDQAEKSSESRRRYSFDFCSLD
NPEAIPSDTSTKNRHGHRQKHLIVDNFLAHESLKKPKHKRKRKSLQNRDDLQFLADLEEL
ITKFQVFRISHRSYTFYHENPYPSIFRINFDHYYPVPYIQYDPLLYLRRTSDLKSKKKRG
RPAKTNDTMTKVPFLQGFSYPIPSGSYYAPYGMPYTSMPMMNLGYYGQYPAPLYLSHTLG
AASPFMRPTVPPPQFHTNSHVKMSGAAKHKAKHGVHLQGPVSMGLGDMQPSLNPPKVGSA
SLSSGRLHKRKHKHKHKHKEDRILGTHDNLSGLFAGKATGFSSHILSERLSSADKELPLV
SEKNKHKEKQKHQHSEAGHKASKNNFEVDTLSTLSLSDAQHWTQAKEKGDLSSEPVDSCT
KRYSGSGGDGGSTRSENLDVFSEMNPSNDKWDSDVSGSKRRSYEGFGTYREKDIQAFKMN
RKERSSYDSSMSPGMPSPHLKVDQTAVHSKNEGSVPTMMTRKKPAAVDSVTIPPAPVLSL
LAASAATSDAVGSSLKKRFKRREIEAIQCEVRKMCNYTKILSTKKNLDHVNKILKAKRLQ
RQSKTGNNFVKKRRGRPRKQPTQFDEDSRDQMPVLEKCIDLPSKRGQKPSLSPLVLEPAA
SQDTIMATIEAVIHMAREAPPLPPPPPPPLPPPPPPPLPPPPPLPKTPRGGKRKHKPQAP
AQPPQQSPPQQPLPQEEEVKAKRQRKSRGSESEVLP*
>ENSG00000108312|ENST00000302904
MNGEADCPTDLEMAAPKGQDRWSQEDMLTLLECMKNNLPSNDSSKFKTTESHMDWEKVAF
KDFSGDMCKLKWVEISNEVRKFRTLTELILDAQEHVKNPYKGKKLKKHPDFPKKPLTPYF
RFFMEKRAKYAKLHPEMSNLDLTKILSKKYKELPEKKKMKYIQDFQREKQEFERNLARFR

EDHPDLIQNAKKSDIPEKPKTPQQLWYTHEKKVYLKVRPDATTKEVKDSLGKQWSQLSDK
KRLKWIHKALEQRKEYEEIMRDYIQKHPELNISEEGITKSTLTKAERQLKDKFDGRPTKP
PPNSYSLYCAELMANMKDVPSTERMVLCSQQWKLLSQKEKDAYHKKCDQKKKDYEVELLR
FLESLPEEEQQRVLGEEKMLNINKKQATSPASKKPAQEGGKGGSEKPKRPVSAMFIFSEE
KRRQLQEERPELSESELTRLLARMWNDLSEKKKAKYKAREAALKAQSERKPGGEREERGK
LPESPKRAEEIWQQSVIGDYLARFKNDRVKALKAMEMTWNNMEKKEKLMWIKKAAEDQKR
YERELSEMRAPPAATNSSKKMKFQGEPKKPPMNGYQKFSQELLSNGELNHLPLKERMVEI
GSRWQRISQSQKEHYKKLAEEQQKQYKVHLDLWVKSLSPQDRAAYKEYISNKRKSMTKLR
GPNPKSSRTTLQSKSESEEDDEEDEDDEDEDEEEEDDENGDSSEDGGDSSESSSEDESED
GDENEEDDEDEDDDEDDDEDEDNESEGSSSSSSSSGDSSDSDSN*
>ENSG00000156030|ENST00000286523
MNLQAQPKAQNKRKRCLFGGQEPAPKEQPPPLQPPQQSIRVKEEQYLGHEGPGGAVSTSQ
PVELPPPSSLALLNSVVYGPERTSAAMLSQQVASVKWPNSVMAPGRGPERGGGGGVSDSS
WQQQPGQPPPHSTWNCHSLSLYSATKGSPHPGVGVPTYYNHPEALKREKAGGPQLDRYVR
PMMPQKVQLEVGRPQAPLNSFHAAKKPPNQSLPLQPFQLAFGHQVNRQVFRQGPPPPNPV
AAFPPQKQQQQQQPQQQQQQQAALPQMPLFENFYSMPQQPSQQPQDFGLQPAGPLGQSH
LAHHSMAPYPFPPNPDMNPELRKALLQDSAPQPALPQVQIPFPRRSRRLSKEGILPPSAL
DGAGTQPGQEATGNLFLHHWPLQQPPPGSLGQPHPEALGFPLELRESQLLPDGERLAPNG
REREAPAMGSEEGMRAVSTGDCGQVLRGGVIQSTRRRRRASQEANLLTLAQKAVELASLQ
NAKDGSGSEEKRKSVLASTTKCGVEFSEPSLATKRAREDSGMVPLIIPVSVPVRTVDPTE
AAQAGGLDEDGKGPEQNPAEHKPSVIVTRRRSTRIPGTDAQAQAEDMNVKLEGEPSVRKP
KQRPRPEPLIIPTKAGTFIAPPVYSNITPYQSHLRSPVRLADHPSERSFELPPYTPPPIL
SPVREGSGLYFNAIISTSTIPAPPPITPKSAHRTLLRTNSAEVTPPVLSVMGEATPVSIE
PRINVGSRFQAEIPLMRDRALAAADPHKADLVWQPWEDLESSREKQRQVEDLLTAACSSI
FPGAGTNQELALHCLHESRGDILETLNKLLLKKPLRPHNHPLATYHYTGSDQWKMAERKL
FNKGIAIYKKDFFLVQKLIQTKTVAQCVEFYYTYKKQVKIGRNGTLTFGDVDTSDEKSAQ
EEVEVDIKTSQKFPRVPLPRRESPSEERLEPKREVKEPRKEGEEEVPEIQEKEEQEEGRE
RSRRAAAVKATQTLQANESASDILILRSHESNAPGSAGGQASEKPREGTGKSRRALPFSE
KKKKTETFSKTQNQENTFPCKKCGR*
>ENSG00000145088|ENST00000273668
MNSAAGFSHLDRRERVLKLGESFEKQPRCAFHTVRYDFKPASIDTSSEGYLEVGEGEQVT
ITLPNIEGSTPPVTVFKGSKKPYLKECILIINHDTGECRLEKLSSNITVKKTRVEGSSKI
QYRKEQQQQQMWNSARTPNLVKHSPSEDKMSPASPIDDIERELKAEASLMDQMSSCDSSS
DSKSSSSSSSEDSSSDSEDEDCKSSTSDTGNCVSGHPTMTQYRIPDIDASHNRFRDNSGL
LMNTLRNDLQLSESGSDSDD*
>ENSG00000141664|ENST00000269499
MLRMKLPLKPTHPAEPPPEAEEPEADARPGAKAPSRRRRDCRPPPPPPPPAGPSRGPLPP
PPPPRGLGPPVAGGAAAGAGMPGGGGGPSAALREQERVYEWFGLVLGSAQRLEFMCGLLD
LCNPLELRFLGSCLEDLARKDYHYLRDSEAKANGLSDPGPLADFREPAVRSRLIVYLALL
GSENREAAGRLHRLLPQVDSVLKSLRAARGEGSRGGAEDERGEDGDGEQDAEKDGSGPEG
GIVEPRVGGGLGSRAQEELLLLFTMASLHPAFSFHQRVTLREHLERLRAALRGGPEDAEV
EVEPCKFAGPRAQNNSAHGDYMQNNESSLIEQAPIPQDGLTVAPHRAQREAVHIEKIMLK
GVQRKRADKYWEYTFKVNWSDLSVTTVTKTHQELQEFLLKLPKELSSETFDKTILRALNQ
GSLKREERRHPDLEPILRQLFSSSSQAFLQSQKVHSFFQSISSDSLHSINNLQSSLKTSK

ILEHLKEDSSEASSQEEDVLQHAIIHKKHTGKSPIVNNIGTSCSPLDGLTMQYSEQNGIV
DWRKQSCTTIQHPEHCVTSADQHSAEKRSLSSINKKKGKPQTEKEKIKKTDNRLNSRING
IRLSTPQHAHGGTVKDVNLDIGSGHDTCGETSSESYSSPSSPRHDGRESFESEEEKDRDT
DSNSEDSGNPSTTRFTGYGSVNQTVTVKPPVQIASLGNENGNLLEDPLNSPKYQHISFMP
TLHCVMHNGAQKSEVVVPAPKPADGKTIGMLVPSPVAISAIRESANSTPVGILGPTACTG
ESEKHLELLASPLPIPSTFLPHSSTPALHLTVQRLKLPPPQGSSESCTVNIPQQPPGSLS
IASPNTAFIPIHNPGSFPGSPVATTDPITKSASQVVGLNQMVPQIEGNTGTVPQPTNVKV
VLPAAGLSAAQPPASYPLPGSPLAAGVLPSQNSSVLSTAATSPQPASAGISQAQATVPPA
VPTHTPGPAPSPSPALTHSTAQSDSTSYISAVGNTNANGTVVPPQQMGSGPCGSCGRRCS
CGTNGNLQLNSYYYPNPMPGPMYRVPSFFTLPSICNGSYLNQAHQSNGNQLPFFLPQTPY
ANGLVHDPVMGSQANYGMQQMAGFGRFYPVYPAPNVVANTSGSGPKKNGNVSCYNCGVSG
HYAQDCKQSSMEANQQGTYRLRYAPPLPPSNDTLDSAD*
>ENSG00000170345|ENST00000303562
MMFSGFNADYEASSSRCSSASPAGDSLSYYHSPADSFSSMGSPVNAQDFCTDLAVSSANF
IPTVTAISTSPDLQWLVQPALVSSVAPSQTRAPHPFGVPAPSAGAYSRAGVVKTMTGGRA
QSIGRRGKVEQLSPEEEEKRRIRRERNKMAAAKCRNRRRELTDTLQAETDQLEDEKSALQ
TEIANLLKEKEKLEFILAAHRPACKIPDDLGFPEEMSVASLDLTGGLPEVATPESEEAFT
LPLLNDPEPKPSVEPVKSISSMELKTEPFDDFLFPASSRPSGSETARSVPDMDLSGSFYA
ADWEPLHSGSLGMGPMATELEPLCTPVVTCTPSCTAYTSSFVFTYPEADSFPSCAAAHRK
GSSSNEPSSDSLSSPTLLAL*
>ENSG00000073111|ENST00000265056
MAESSESFTMASSPAQRRRGNDPLTSSPGRSSRRTDALTSSPGRDLPPFEDESEGLLGTE
GPLEEEEDGEELIGDGMERDYRAIPELDAYEAEGLALDDEDVEELTASQREAAERAMRQR
DREAGRGLGRMRRGLLYDSDEEDEERPARKRRQVERATEDGEEDEEMIESIENLEDLKGH
SVREWVSMAGPRLEIHHRFKNFLRTHVDSHGHNVFKERISDMCKENRESLVVNYEDLAAR
EHVLAYFLPEAPAELLQIFDEAALEVVLAMYPKYDRITNHIHVRISHLPLVEELRSLRQL
HLNQLIRTSGVVTSCTGVLPQLSMVKYNCNKCNFVLGPFCQSQNQEVKPGSCPECQSAGP
FEVNMEETIYQNYQRIRIQESPGKVAAGRLPRSKDAILLADLVDSCKPGDEIELTGIYHN
NYDGSLNTANGFPVFATVILANHVAKKDNKVAVGELTDEDVKMITSLSKDQQIGEKIFAS
IAPSIYGHEDIKRGLALALFGGEPKNPGGKHKVRGDINVLLCGDPGTAKSQFLKYIEKVS
SRAIFTTGQGASAVGLTAYVQRHPVSREWTLEAGALVLADRGVCLIDEFDKMNDQDRTSI
HEAMEQQSISISKAGIVTSLQARCTVIAAANPIGGRYDPSLTFSENVDLTEPIISRFDIL
CVVRDTVDPVQDEMLARFVVGSHVRHHPSNKEEEGLANGSAAEPAMPNTYGVEPLPQEVL
KKYIIYAKERVHPKLNQMDQDKVAKMYSDLRKESMATGSIPITVRHIESMIRMAEAHARI
HLRDYVIEDDVNMAIRVMLESFIDTQKFSVMRSMRKTFARYLSFRRDNNELLLFILKQLV
AEQVTYQRNRFGAQQDTIEVPEKDLVDKARQINIHNLSAFYDSELFRMNKFSHDLKRKMI
LQQF*
>ENSG00000166888|ENST00000300134
MSLWGLVSKMPPEKVQRLYVDFPQHLRHLLGDWLESQPWEFLVGSDAFCCNLASALLSDT
VQHLQASVGEQGEGSTILQHISTLESIYQRDPLKLVATFRQILQGEKKAVMEQFRHLPMP
FHWKQEELKFKTGLRRLQHRVGEIHLLREALQKGAEAGQVSLHSLIETPANGTGPSEALA
MLLQETTGELEAAKALVLKRIQIWKRQQQLAGNGAPFEESLAPLQERCESLVDIYSQLQQ
EVGAAGGELEPKTRASLTGRLDEVLRTLVTSCFLVEKQPPQVLKTQTKFQAGVRFLLGLR
FLGAPAKPPLVRADMVTEKQARELSVPQGPGAGAESTGEIINNTVPLENSIPGNCCSALF

KNLLLKKIKRCERKGTESVTEEKCAVLFSASFTLGPGKLPIQLQALSLPLVVIVHGNQDN
NAKATILWDNAFSEMDRVPFVVAERVPWEKMCETLNLKFMAEVGTNRGLLPEHFLFLAQK
IFNDNSLSMEAFQHRSVSWSQFNKEILLGRGFTFWQWFDGVLDLTKRCLRSYWSDRLIIG
FISKQYVTSLLLNEPDGTFLLRFSDSEIGGITIAHVIRGQDGSPQIENIQPFSAKDLSIR
SLGDRIRDLAQLKNLYPKKPKDEAFRSHYKPEQMGKDGRGYVPATIKMTVERDQPLPTPE
LQMPTMVPSYDLGMAPDSSMSMQLGPDMVPQVYPPHSHSIPPYQGLSPEESVNVLSAFQE
PHLQMPPSLGQMSLPFDQPHPQGLLPCQPQEHAVSSPDPLLCSDVTMVEDSCLSQPVTAF
PQGTWIGEDIFPPLLPPTEQDLTKLLLEGQGESGGGSLGAQPLLQPSHYGQSGISMSHMD
LRANPSW*
>ENSG00000180855|ENST00000301547
MASVALEDVAVNFTREEWALLGPCQKNLYKDVMQETIRNLDCVVMKWKDQNIEDQYRYPR
KNLRCRMLERFVESKDGTQCGETSSQIQDSIVTKNTLPGVGPCESSMRGEKVMGHSSLNC
YIRVGAGHKPHEYHECGEKPDTHKQRGKAFSYHNSFQTHERLHTGKKPYDCKECGKSFSS
LGNLQRHMAVQRGDGPYKCKLCGKAFFWPSLLHMHERTHTGEKPYECKQCSKAFSFYSSY
LRHERTHTGEKPYECKQCSKAFPFYSSYLRHERTHTGEKPYKCKQCSKAFPDSSSCLIHE
RTHTGEKPYTCKQCGKAFSVSGSLQRHETTHSAEKPYACQQCGKAFHHLGSFQRHMIRHT
GNGPHKCKICGKGFDCPSSLQSHERTHTGEKPYECKQCGKALSHRSSFRSHMIMHTGDGP
HKCKVCGKAFVYPSVFQRHERTHTAEKPYKCKQCGKAYRISSSLRRHETTHTGEKPYKCK
LGKACIDFCSFQNHKTTHTGEKPYECKECGKAFSRFRYLSRHKRTHTGEKPYECKTCRKA
FGHYDNLKVHERIHSGEKPYECKECGKAFSWLTCFLRHERIHMREKSYECPQCGKAFTHS
RFLQGHEKTHTGENPYECKECGKAFASLSSLHRHKKTHWKKTHTGENPYECKECGKAFAS
LSSLHRHKKTH*
>ENSG00000151379|ENST00000281047
MDNLRETFLSLEDGLGSSDSPGLLSSWDWKDRAGPFELNQASPSQSLSPAPSLESYSSSP
CPAVAGLPCEHGGASSGGSEGCSVGGASGLVEVDYNMLAFQPTHLQGGGGPKAQKGTKVR
MSVQRRRKASEREKLRMRTLADALHTLRNYLPPVYSQRGQPLTKIQTLKYTIKYIGELTD
LLNRGREPRAQSA*
>ENSG00000163412|ENST00000295612
MRGERRPLWEEESNAKGGVWKMKVPKDSTSTVWKELLLATIGEQFTDCAAADDEVIGVSV
SVRDREDVVQVWNVNASLVGEATVLEKIYELLPHITFKAVFYKPHEEHHAFEGGRGKH*
>ENSG00000141367|ENST00000269122
MAQILPIRFQEHLQLQNLGINPANIGFSTLTMESDKFICIREKVGEQAQVVIIDMNDPSN
PIRRPISADSAIMNPASKVIALKAGKTLQIFNIEMKSKMKAHTMTDDVTFWKWISLNTVA
LVTDNAVYHWSMEGESQPVKMFDRHSSLAGCQIINYRTDAKQKWLLLTGISAQQNRVVGA
MQLYSVDRKVSQPIEGHAASFAQFKMEGNAEESTLFCFAVRGQAGGKLHIIEVGTPPTGN
QPFPKKAVDVFFPPEAQNDFPVAMQISEKHDVVFLITKYGYIHLYDLETGTCIYMNRISG
ETIFVTAPHEATAGIIGVNRKGQVLSVCVEEENIIPYITNVLQNPDLALRMAVRNNLAGA
EELFARKFNALFAQGNYSEAAKVAANAPKGILRTPDTIRRFQSVPAQPGQTSPLLQYFGI
LLDQGQLNKYESLELCRPVLQQGRKQLLEKWLKEDKLECSEELGDLVKSVDPTLALSVYL
RANVPNKVIQCFAETGQVQKIVLYAKKVGYTPDWIFLLRNVMRISPDQGQQFAQMLVQDE
EPLADITQIVDVFMEYNLIQQCTAFLLDALKNNRPSEGPLQTRLLEMNLMHAPQVADAIL
GNQMFTHYDRAHIAQLCEKAGLLQRALEHFTDLYDIKRAVVHTHLLNPEWLVNYFGSLSV
EDSLECLRAMLSANIRQNLQICVQVASKYHEQLSTQSLIELFESFKSFEGLFYFLGSIVN
FSQDPDVHFKYIQAACKTGQIKEVERICRESNCYDPERVKNFLKEAKLTDQLPLIIVCDR

FDFVHDLVLYLYRNNLQKYIEIYVQKVNPSRLPVVIGGLLDVDCSEDVIKNLILVVRGQF
STDELVAEVEKRNRLKLLLPWLEARIHEGCEEPATHNALAKIYIDSNNNPERFLRENPYY
DSRVVGKYCEKRDPHLACVAYERGQCDLELINVCNENSLFKSLSRYLVRRKDPELWGSVL
LESNPYRRPLIDQVVQTALSETQDPEEVSVTVKAFMTADLPNELIELLEKIVLDNSVFSE
HRNLQNLLILTAIKADRTRVMEYINRLDNYDAPDIANIAISNELFEEAFAIFRKFDVNTS
AVQVLIEHIGNLDRAYEFAERCNEPAVWSQLAKAQLQKGMVKEAIDSYIKADDPSSYMEV
VQAANTSGNWEELVKYLQMARKKARESYVETELIFALAKTNRLAELEEFINGPNNAHIQQ
VGDRCYDEKMYDAAKLLYNNVSNFGRLASTLVHLGEYQAAVDGARKANSTRTWKEVCFAC
VDGKEFRLAQMCGLHIVVHADELEELINYYQDRGYFEELITMLEAALGLERAHMGMFTEL
AILYSKFKPQKMREHLELFWSRVNIPKVLRAAEQAHLWAELVFLYDKYEEYDNAIITMMN
HPTDAWKEGQFKDIITKVANVELYYRAIQFYLEFKPLLLNDLLMVLSPRLDHTRAVNYFS
KVKQLPLVKPYLRSVQNHNNKSVNESLNNLFITEEDYQALRTSIDAYDNFDNISLAQRLE
KHELIEFRRIAAYLFKGNNRWKQSVELCKKDSLYKDAMQYASESKDTELAEELLQWFLQE
EKRECFGACLFTCYDLLRPDVVLETAWRHNIMDFAMPYFIQVMKEYLTKVDKLDASESLR
KEEEQATETQPIVYGQPQLMLTAGPSVAVPPQAPFGYGYTAPPYGQPQPGFGYSM*
>ENSG00000173404|ENST00000310227
MPRGFLVKRSKKSTPVSYRVRGGEDGDRALLLSPSCGGARAEPPAPSPVPGPLPPPPPAE
RAHAALAAALACAPGPQPPPQGPRAAHFGNPEAAHPAPLYSPTRPVSREHEKHKYFERSF
NLGSPVSAESFPTPAALLGGGGGGGASGAGGGGTCGGDPLLFAPAELKMGTAFSAGAEAA
RGPGPGPPLPPAAALRPPGKRPPPPTAAEPPAKAVKAPGAKKPKAIRKLHFEDEVTTSPV
LGLKIKEGPVEAPRGRAGGAARPLGEFICQLCKEEYADPFALAQHKCSRIVRVEYRCPEC
AKVFSCPANLASHRRWHKPRPAPAAARAPEPEAAARAEAREAPGGGSDRDTPSPGGVSES
GSEDGLYECHHCAKKFRRQAYLRKHLLAHHQALQAKGAPLAPPAEDLLALYPGPDEKAPQ
EAAGDGEGAGVLGLSASAECHLCPVCGESFASKGAQERHLRLLHAAQVFPCKYCPATFYS
SPGLTRHINKCHPSENRQVILLQVPVRPAC*
>ENSG00000175324|ENST00000311351
MNYMPGTASLIEDIDKKHLVLLRDGRTLIGFLRSIDQFANLVLHQTVERIHVGKKYGDIP
RGIFVVRGENVVLLGEIDLEKESDTPLQQVSIEEILEEQRVEQQTKLEAEKLKVQALKDR
GLSIPRADTLDEY*
>ENSG00000189091|ENST00000302516
MFLYNLTLQRATGISFAIHGNFSGTKQQEIVVSRGKILELLRPDPNTGKVHTLLTVEVFG
VIRSLMAFRLTGGTKDYIVVGSDSGRIVILEYQPSKNMFEKIHQETFGKSGCRRIVPGQF
LAVDPKGRAVMISAIEKQKLVYILNRDAAARLTISSPLEAHKANTLVYHVVGVDVGFENP
MFACLEMDYEEADNDPTGEAAANTQQTLTFYELDLGLNHVVRKYSEPLEEHGNFLITVPG
GSDGPSGVLICSENYITYKNFGDQPDIRCPIPRRRNDLDDPERGMIFVCSATHKTKSMFF
FLAQTEQGDIFKITLETDEDMVTEIRLKYFDTVPVAAAMCVLKTGFLFVASEFGNHYLYQ
IAHLGDDDEEPEFSSAMPLEEGDTFFFQPRPLKNLVLVDELDSLSPILFCQIADLANEDT
PQLYVACGRGPRSSLRVLRHGLEVSEMAVSELPGNPNAVWTVRRHIEDEFDAYIIVSFVN
ATLVLSIGETVEEVTDSGFLGTTPTLSCSLLGDDALVQVYPDGIRHIRADKRVNEWKTPG
KKTIVKCAVNQRQVVIALTGGELVYFEMDPSGQLNEYTERKEMSADVVCMSLANVPPGEQ
RSRFLAVGLVDNTVRIISLDPSDCLQPLSMQALPAQPESLCIVEMGGTEKQDELGERGSI
GFLYLNIGLQNGVLLRTVLDPVTGDLSDTRTRYLGSRPVKLFRVRMQGQEAVLAMSSRSW
LSYSYQSRFHLTPLSYETLEFASGFASEQCPEGIVAISTNTLRILALEKLGAVFNQVAFP
LQYTPRKFVIHPESNNLIIIETDHNAYTEATKAQRKQQMAEEMVEAAGEDERELAAEMAA

AFLNENLPESIFGAPKAGNGQWASVIRVMNPIQGNTLDLVQLEQNEAAFSVAVCRFSNTG
EDWYVLVGVAKDLILNPRSVAGGFVYTYKLVNNGEKLEFLHKTPVEEVPAAIAPFQGRVL
IGVGKLLRVYDLGKKKLLRKCENKHIANYISGIQTIGHRVIVSDVQESFIWVRYKRNENQ
LIIFADDTYPRWVTTASLLDYDTVAGADKFGNICVVRLPPNTNDEVDEDPTGNKALWDRG
LLNGASQKAEVIMNYHVGETVLSLQKTTLIPGGSESLVYTTLSGGIGILVPFTSHEDHDF
FQHVEMHLRSEHPPLCGRDHLSFRSYYFPVKNVIDGDLCEQFNSMEPNKQKNVSEELDRT
PPEVSKKLEDIRTRYAF*
>ENSG00000156469|ENST00000287025
MALSAQQIPRWFNSVKLRSLINAAQLTKRFTRPARTLLHGFSAQPQISSDNCFLQWGFKT
YRTSSLWNSSQSTSSSSQENNSAQSSLLPSMNEQSQKTQNISSFDSELFLEELDELPPLS
PMQPISEEEAIQIIADPPLPPASFTLRDYVDHSETLQKLVLLGVDLSKIEKHPEAANLLL
RLDFEKDIKQMLLFLKDVGIEDNQLGAFLTKNHAIFSEDLENLKTRVAYLHSKNFSKADV
AQMVRKAPFLLNFSVERLDNRLGFFQKELELSVKKTRDLVVRLPRLLTGSLEPVKENMKV
YRLELGFKHNEIQHMITRIPKMLTANKMKLTETFDFVHNVMSIPHHIIVKFPQVFNTRLF
KVKERHLFLTYLGRAQYDPAKPNYISLDKLVSIPDEIFCEEIAKASVQDFEKFLKTL*
>ENSG00000158373|ENST00000289316
MPEPTKSAPAPKKGSKKAVTKAQKKDGKKRKRSRKESYSVYVYKVLKQVHPDTGISSKAM
GIMNSFVNDIFERIAGEASRLAHYNKRSTITSREIQTAVRLLLPGELAKHAVSEGTKAVT
KYTSSK*
>ENSG00000138768|ENST00000264904
MNFLRGVMGGQSAGPQHTEAETIQKLCDRVASSTLLDDRRNAVRALKSLSKKYRLEVGIQ
AMEHLIHVLQTDRSDSEIIGYALDTLYNIISNEEEEEVDDVEEENSTRQSEDLGSQFTEI
FIKQQENVTLLLSLLEEFDFHVRWPGVKLLTSLLKQLGPQVQQIILVSPMGVSRLMDLLA
DSREVIRNDGVLLLQALTRSNGAIQKIVAFENAFERLLDIISEEGNSDGGIVVEDCLILL
QNLLKNNNSNQNFFKEGSYIQRMKPWFEVGDENSGWSAQKVTNLHLMLQLVRVLVSPTNP
PGATSSCQKAMFQCGLLQQLCTILMATGVPADILTETINTVSEVIRGCQVNQDYFASVNA
PSNPPRPAIVVLLMSMVNERQPFVLRCAVLYCFQCFLYKNQKGQGEIVSTLLPSTIDATG
NSVSAGQLLCGGLFSTDSLSNWCAAVALAHALQENATQKEQLLRVQLATSIGNPPVSLLQ
QCTNILSQGDKIDRRGSKIQTRVGLLMLLCTWLSNCPIAVTHFLHNSANVPFLTGQIAEN
LGEEEQLVQGLCALLLGISIYFNDNSLESYMKEKLKQLIEKRIGKENFIEKLGFISKHEL
YSRASQKPQPNFPSPEYMIFDHEFTKLVKELEGVITKAIYKSSEEDKKEEEVKKTLEQHD
NIVTHYKNMIREQDLQLEELRQQVSTLKCQNEQLQTAVTQQVSQIQQHKDQYNLLKIQLG
KDNQHQGSYSEGAQMNGIQPEEIGRLREEIEELKRNQELLQSQLTEKDSMIENMKSSQTS
GTNEQSSAIVSARDSEQVAELKQELATLKSQLNSQSVEITKLQTEKQELLQKTEAFAKSV
EVQGETETIIATKTTDVEGRLSALLQETKELKNEIKALSEERTAIKEQLDSSNSTIAILQ
TEKDKLELEITDSKKEQDDLLVLLADQDQKILSLKNKLKDLGHPVEEEDELESGDQEDED
DESEDPGKDLDHI*
>ENSG00000143390|ENST00000290524
MAEDEPDAKSPKTGGRAPPGGAEAGEPTTLLQRLRGTISKAVQNKVEGILQDVQKFSDND
KLYLYLQLPSGPTTGDKSSEPSTLSNEEYMYAYRWIRNHLEEHTDTCLPKQSVYDAYRKY
CESLACCRPLSTANFGKIIREIFPDIKARRLGGRGQSKYCYSGIRRKTLVSMPPLPGLDL
KGSESPEMGPEVTPAPRDELVEAACALTCDWAERILKRSFSSIVEVARFLLQQHLISARS
AHAHVLKAMGLAEEDEHAPRERSSKPKNGLENPEGGAHKKPERLAQPPKDLEARTGAGPL
ARGERKKSVVESSAPGANNLQVNALVARLPLLLPRAPRSLIPPIPVSPPILAPRLSSGAL

KVATLPLSSRAGAPPAAVPIINMILPTVPALPGPGPGPGRAPPGGLTQPRGTENREVGIG
GDQGPHDKGVKRTAEVPVSEASGQAPPAKAAKQDIEDTASDAKRKRGRPRKKSGGSGERN
STPLKSAAAMESAQSSRLPWETWGSGGEGNSAGGAERPGPMGEAEKGAVLAQGQGDGTVS
KGGRGPGSQHTKEAEDKIPLVPSKVSVIKGSRSQKEAFPLAKGEVDTAPQGNKDLKEHVL
QSSLSQEHKDPKATPP*
>ENSG00000156650|ENST00000287239
MVKLANPLYTEWILEAIQKIKKQKQRPSEERICHAVSTSHGLDKKTVSEQLELSVQDGSV
LKVTNKGLASYKDPDNPGRFSSVKPGTFPKSAKGSRGSCNDLRNVDWNKLLRRAIEGLEE
PNGSSLKNIEKYLRSQSDLTSTTNNPAFQQRLRLGAKRAVNNGRLLKDGPQYRVNYGSLD
GKGAPQYPSAFPSSLPPVSLLPHEKDQPRADPIPICSFCLGTKESNREKKPEELLSCADC
GSSGHPSCLKFCPELTTNVKALRWQCIECKTCSACRVQGRNADNMLFCDSCDRGFHMECC
DPPLSRMPKGMWICQVCRPKKKGRKLLHEKAAQIKRRYAKPIGRPKNKLKQRLLSVTSDE
GSMNAFTGRGSPGRGQKTKVCTTPSSGHAASGKDSSSRLAVTDPTRPGATTKITTTSTYI
SASTLKVNKKTKGLIDGLTKFFTPSPDGRRSRGEIIDFSKHYRPRKKVSQKQSCTSHVLA
TGTTQKLKPPPSSLPPPTPISGQSPSSQKSSTATSSPSPQSSSSQCSVPSLSSLTTNSQL
KALFDGLSHIYTTQGQSRKKGHPSYAPPKRMRRKTELSSTAKSKAHFFGKRDIRSRFISH
SSSSSWGMARGSIFKAIAHFKRTTFLKKHRMLGRLKYKVTPQMGTPSPGKGSLTDGRIKP
DQDDDTEIKINIKQESADVNVIGNKDVVTEEDLDVFKQAQELSWEKIECESGVEDCGRYP
SVIEFGKYEIQTWYSSPYPQEYARLPKLYLCEFCLKYMKSKNILLRHSKKCGWFHPPANE
IYRRKDLSVFEVDGNMSKIYCQNLCLLAKLFLDHKTLYYDVEPFLFYVLTKNDEKGCHLV
GYFSKEKLCQQKYNVSCIMIMPQHQRQGFGRFLIDFSYLLSRREGQAGSPEKPLSDLGRL
SYLAYWKSVILEYLYHHHERHISIKAISRATGMCPHDIATTLQHLHMIDKRDGRFVIIRR
EKLILSHMEKLKTCSRANELDPDSLRWTPILISNAAVSEEEREAEKEAERLMEQASCWEK
EEQEILSTRANSRQSPAKVQSKNKYLHSPESRPVTGERGQLLELSKESSEEEEEEEDEEE
EEEEEEEEEDEEEEEEEEEEEEEENIQSSPPRLTKPQSVAIKRKRPFVLKKKRGRKRRRI
NSSVTTETISETTEVLNEPFDNSDEERPMPQLEPTCEIEVEEDGRKPVLRKAFQHQPGKK
RQTEEEEGKDNHCFKNADPCRNNMNDDSSNLKEGSKDNPEPLKCKQVWPKGTKRGLSKWR
QNKERKTGFKLNLYTPPETPMEPDEQVTVEEQKETSEGKTSPSPIRIEEEVKETGEALLP
QEENRREETCAPVSPNTSPGEKPEDDLIKPEEEEEEEEEEEEEEEEEEGEEEEGGGNVEK
DPDGAKSQEKEEPEISTEKEDSARLDDHEEEEEEDEEPSHNEDHDADDEDDSHMESAEVE
KEELPRESFKEVLENQETFLDLNVQPGHSNPEVLMDCGVDLTASCNSEPKELAGDPEAVP
ESDEEPPPGEQAQKQDQKNSKEVDTEFKEGNPATMEIDSETVQAVQSLTQESSEQDDTFQ
DCAETQEACRSLQNYTRADQSPQIATTLDDCQQSDHSSPVSSVHSHPGQSVRSVNSPSVP
ALENSYAQISPDQSAISVPSLQNMETSPMMDVPSVSDHSQQVVDSGFSDLGSIESTTENY
ENPSSYDSTMGGSICGNGSSQNSCSYSNLTSSSLTQSSCAVTQQMSNISGSCSMLQQTSI
SSPPTCSVKSPQGCVVERPPSSSQQLAQCSMAANFTPPMQLAEIPETSNANIGLYERMGQ
SDFGAGHYPQPSATFSLAKLQQLTNTLIDHSLPYSHSAAVTSYANSASLSTPLSNTGLVQ
LSQSPHSVPGGPQAQATMTPPPNLTPPPMNLPPPLLQRNMAASNIGISHSQRLQTQIASK
GHISMRTKSASLSPAAATHQSQIYGRSQTVAMQGPARTLTMQRGMNMSVNLMPAPAYNVN
SVNMNMNTLNAMNGYSMSQPMMNSGYHSNHGYMNQTPQYPMQMQMGMMGTQPYAQQPMQT
PPHGNMMYTAPGHHGYMNTGMSKQSLNGSYMRR*
>ENSG00000166823|ENST00000300057
MAQPLCPPLSESWMLSAAWGPTRRPPPSDKDCGRSLVSSPDSWGSTPADSPVASPARPGT
LRDPRAPSVGRRGARSSRLGSGGQRQSASEREKLRMRTLARALHELRRFLPPSVAPAGQSL

TKIETLRLAIRYIGHLSAVLGLSEESLQRRCRQRGDAGSPRGCPLCPDDCPAQMQTRTQA
EGQGQGRGLGLVSAVRAGASWGSPPACPGARAAPEPRDPPALFAEAACPEGQAMEPSPPS
PLLPGDVLALLETWMPLSPLEWLPEEPK*
>ENSG00000051596|ENST00000265097
MAVPAAAMGPSALGQSGPGSMAPWCSVSSGPSRYVLGMQELFRGHSKTREFLAHSAKVHS
VAWSCDGRRLASGSFDKTASVFLLEKDRLVKENNYRGHGDSVDQLCWHPSNPDLFVTASG
DKTIRIWDVRTTKCIATVNTKGENINICWSPDGQTIAVGNKDDVVTFIDAKTHRSKAEEQ
FKFEVNEISWNNDNNMFFLTNGNGCINILSYPELKPVQSINAHPSNCICIKFDPMGKYFA
TGSADALVSLWDVDELVCVRCFSRLDWPVRTLSFSHDGKMLASASEDHFIDIAEVETGDK
LWEVQCESPTFTVAWHPKRPLLAFACDDKDGKYDSSREAGTVKLFGLPNDS*
>ENSG00000174547|ENST00000310999
MSKLGRAARGLRKPEVGGVIRAIVRAGLAMPGPPLGPVLGQRGVSINQFCKEFNERTKDI
KEGIPLPTKILVKPDRTFEIKIGQPTVSYFLKAAAGIEKGARQTGKEVAGLVTLKHVYEI
ARIKAQDEAFALQDVPLSSVVRSIIGSARSLGIRVVKDLSSEELAAFQKERAIFLAAQKE
ADLAAQEEAAKK*
>ENSG00000171824|ENST00000304457
MAPPSTREPRVLSATSATKSDGEMVLPGFPDADSFVKFALGSVVAVTKASGGLPQFGDEY
DFYRSFPGFQAFCETQGDRLLQCMSRVMQYHGCRSNIKDRSKVTELEDKFDLLVDANDVI
LERVGILLDEASGVNKNQQPVLPAGLQVPKTVVSSWNRKAAEYGKKAKSETFRLLHAKNI
IRPQLKFREKIDNSNTPFLPKIFIKPNAQKPLPQALSKERRERPQDRPEDLDVPPALADF
IHQQRTQQVEQDMFAHPYQYELNHFTPADAVLQKPQPQLYRPIEETPCHFISSLDELVEL
NEKLLNCQEFAVDLEHHSYRSFLGLTCLMQISTRTEDFIIDTLELRSDMYILNESLTDPA
IVKVFHGADSDIEWLQKDFGLYVVNMFDTHQAARLLNLGRHSLDHLLKLYCNVDSNKQYQ
LADWRIRPLPEEMLSYARDDTHYLLYIYDKMRLEMWERGNGQPVQLQVVWQRSRDICLKK
FIKPIFTDESYLELYRKQKKHLNTQQLTAFQLLFAWRDKTARREDESYGYVLPNHMMLKI
AEELPKEPQGIIACCNPVPPLVRQQINEMHLLIQQAREMPLLKSEVAAGVKKSGPLPSAE
RLENVLFGPHDCSHAPPDGYPIIPTSGSVPVQKQASLFPDEKEDNLLGTTCLIATAVITL
FNEPSAEDSKKGPLTVAQKKAQNIMESFENPFRMISNRWKLAQVQVQKDSKEAVKKKAAE
QTAAREQAKEACKAAAEQAISVRQQVVLENAAKKRERATSDPRTTEQKQEKKRLKISKKP
KDPEPPEKEFTPYDYSQSDFKAFAGNSKSKVSSQFDPNKQTPSGKKCIAAKKIKQSVGNK
SMSFPTGKSDRGFRYNWPQR*
>ENSG00000075702|ENST00000270301
MAAVGSGGYARNDAGEKLPSVMAGVPARRGQSSPPPAPPICLRRRTRLSTASEETVQNRV
SLEKVLGITAQNSSGLTCDPGTGHVAYLAGCVVVILDPKENKQQHIFNTARKSLSALAFS
PDGKYIVTGENGHRPAVRIWDVEEKNQVAEMLGHKYGVACVAFSPNMKHIVSMGYQHDMV
LNVWDWKKDIVVASNKVSCRVIALSFSEDSSYFVTVGNRHVRFWFLEVSTETKVTSTVPL
VGRSGILGELHNNIFCGVACGRGRMAGSTFCVSYSGLLCQFNEKRVLEKWINLKVSLSSC
LCVSQELIFCGCTDGIVRIFQAHSLHYLANLPKPHYLGVDVAQGLEPSFLFHRKAEAVYP
DTVALTFDPIHQWLSCVYKDHSIYIWDVKDINRVGKVWSELFHSSYVWNVEVYPEFEDQR
ACLPSGSFLTCSSDNTIRFWNLDSSPDSHWQKNIFSNTLLKVVYVENDIQHLQDMSHFPD
RGSENGTPMDVKAGVRVMQVSPDGQHLASGDRSGNLRIHELHFMDELVKVEAHDAEVLCL
EYSKPETGLTLLASASRDRLIHVLNVEKNYNLEQTLDDHSSSITAIKFAGNRDIQMISCG
ADKSIYFRSAQQGSDGLHFVRTHHVAEKTTLYDMDIDITQKYVAVACQDRNVRVYNTVNG
KQKKCYKGSQGDEGSLLKVHVDPSGTFLATSCSDKSISVIDFYSGECIAKMFGHSEIITS

MKFTYDCHHLITVSGDSCVFIWHLGPEITNCMKQHLLEIDHRQQQQHTNDKKRSGHPRQD
TYVSTPSEIHSLSPGEQTEDDLEEECEPEEMLKTPSKDSLDPDPRCLLTNGKLPLWAKRL
LGDDDVADGLAFHAKRSYQPHGRWAERAGQEPLKTILDAQDLDCYFTPMKPESLENSILD
SLEPQSLASLLSESESPQEAGRGHPSFLPQQKESSEASELILYSLEAEVTVTGTDSQYCR
KEVEAGPGDQQGDSYLRVSSDSPKDQSPPEDSGESEADLECSFAAIHSPAPPPDPAPRFA
TSLPHFPGCAGPTEDELSLPEGPSVPSSSLPQTPEQEKFLRHHFETLTESPCRALGDVEA
SEAEDHFFNPRLSISTQFLSSLQKASRFTHTFPPRATQCLVKSPEVKLMDRGGSQPRAGT
GYASPDRTHVLAAGKAEETLEAWRPPPPCLTSLASCVPASSVLPTDRNLPTPTSAPTPGL
AQGVHAPSTCSYMEATASSRARISRSISLGDSEGPIVATLAQPLRRPSSVGELASLGQEL
QAITTATTPSLDSEGQEPALRSWGNHEARANLRLTLSSACDGLLQPPVDTQPGVTVPAVS
FPAPSPVEESALRLHGSAFRPSLPAPESPGLPAHPSNPQLPEARPGIPGGTASLLEPTSG
ALGLLQGSPARWSEPWVPVEALPPSPLELSRVGNILHRLQTTFQEALDLYRVLVSSGQVD
TGQQQARTELVSTFLWIHSQLEAECLVGTSVAPAQALPSPGPPSPPTLYPLASPDLQALL
EHYSELLVQAVRRKARGH*
>ENSG00000146648|ENST00000275493
MRPSGTAGAALLALLAALCPASRALEEKKVCQGTSNKLTQLGTFEDHFLSLQRMFNNCEV
VLGNLEITYVQRNYDLSFLKTIQEVAGYVLIALNTVERIPLENLQIIRGNMYYENSYALA
VLSNYDANKTGLKELPMRNLQEILHGAVRFSNNPALCNVESIQWRDIVSSDFLSNMSMDF
QNHLGSCQKCDPSCPNGSCWGAGEENCQKLTKIICAQQCSGRCRGKSPSDCCHNQCAAGC
TGPRESDCLVCRKFRDEATCKDTCPPLMLYNPTTYQMDVNPEGKYSFGATCVKKCPRNYV
VTDHGSCVRACGADSYEMEEDGVRKCKKCEGPCRKVCNGIGIGEFKDSLSINATNIKHFK
NCTSISGDLHILPVAFRGDSFTHTPPLDPQELDILKTVKEITGFLLIQAWPENRTDLHAF
ENLEIIRGRTKQHGQFSLAVVSLNITSLGLRSLKEISDGDVIISGNKNLCYANTINWKKL
FGTSGQKTKIISNRGENSCKATGQVCHALCSPEGCWGPEPRDCVSCRNVSRGRECVDKCN
LLEGEPREFVENSECIQCHPECLPQAMNITCTGRGPDNCIQCAHYIDGPHCVKTCPAGVM
GENNTLVWKYADAGHVCHLCHPNCTYGCTGPGLEGCPTNGPKIPSIATGMVGALLLLLVV
ALGIGLFMRRRHIVRKRTLRRLLQERELVEPLTPSGEAPNQALLRILKETEFKKIKVLGS
GAFGTVYKGLWIPEGEKVKIPVAIKELREATSPKANKEILDEAYVMASVDNPHVCRLLGI
CLTSTVQLITQLMPFGCLLDYVREHKDNIGSQYLLNWCVQIAKGMNYLEDRRLVHRDLAA
RNVLVKTPQHVKITDFGLAKLLGAEEKEYHAEGGKVPIKWMALESILHRIYTHQSDVWSY
GVTVWELMTFGSKPYDGIPASEISSILEKGERLPQPPICTIDVYMIMVKCWMIDADSRPK
FRELIIEFSKMARDPQRYLVIQGDERMHLPSPTDSNFYRALMDEEDMDDVVDADEYLIPQ
QGFFSSPSTSRTPLLSSLSATSNNSTVACIDRNGLQSCPIKEDSFLQRYSSDPTGALTED
SIDDTFLPVPEYINQSVPKRPAGSVQNPVYHNQPLNPAPSRDPHYQDPHSTAVGNPEYLN
TVQPTCVNSTFDSPAHWAQKGSHQISLDNPDYQQDFFPKEAKPNGIFKGSTAENAEYLRV
APQSSEFIGA*
>ENSG00000167771|ENST00000301459
MPSVMEKPSAGSGILSRSRAKTVPNGGQPHSEDDSSEEEHSHDSMIRVGTNYQAVIPECK
PESPARYSNKELKGMLVWSPNHCVSDAKLDKYIAMAKEKHGYNIEQALGMLLWHKHDVEK
SLADLANFTPFPDEWTVEDKVLFEQAFGFHGKCFQRIQQMLPDKLIPSLVKYYYSWKKTR
SRTSVMDRQARRLGGRKDKEDSDELEEGRGGVSEGEPDPADPKREPLPSRPLNARPGPGK
KEVQVSQYRHHPLRTRRRPPKGMYLSPEGLTAVSGSPDLANLTLRGLDSQLISLKRQVQS
MKQTNSSLRQALEGGIDPLRPPEANTKFNSRWTTDEQLLAVQAIRRYGKDFGAIAEVIGN
KTLTQVKTFFVSYRRRFNLEEVLQEWEAEQDGAPGAPVPMEEARRGAPLPAPALEEDDEV

QITSVSTSVPRSVPPAPPPPPPPTSLSQPPPLLRPPLPTAPTLLRQPPPLQQGRFLQPRL
APNQPPPPLIRPALAAPRHSARPGPQPPPTLIGTPLEPPAPSL*
>ENSG00000112033|ENST00000311565
MEQPQEEAPEVREEEEKEEVAEAEGAPELNGGPQHALPSSSYTDLSRSSSPPSLLDQLQM
GCDGASCGSLNMECRVCGDKASGFHYGVHACEGCKGFFRRTIRMKLEYEKCERSCKIQKK
NRNKCQYCRFQKCLALGMSHNAIRFGRMPEAEKRKLVAGLTANEGSQYNPQVADLKAFSK
HIYNAYLKNFNMTKKKARSILTGKASHTAPFVIHDIETLWQAEKGLVWKQLVNGLPPYKE
ISVHVFYRCQCTTVETVRELTEFAKSIPSFSSLFLNDQVTLLKYGVHEAIFAMLASIVNK
DGLLVANGSGFVTREFLRSLRKPFSDIIEPKFEFAVKFNALELDDSDLALFIAAIILCGD
RPGLMNVPRVEAIQDTILRALEFHLQANHPDAQYLFPKLLQKMADLRQLVTEHAQMMQRI
KKTETETSLHPLLQEIYKDMY*
>ENSG00000076043|ENST00000265881
MLGGSLGSRLLRGVGGSHGRFGARGVREGGAAMAAGESMAQRMVWVDLEMTGLDIEKDQI
IEMACLITDSDLNILAEGPNLIIKQPDELLDSMSDWCKEHHGKSGLTKAVKESTITLQQA
EYEFLSFVRQQTPPGLCPLAGNSVHEDKKFLDKYMPQFMKHLHYRIIDVSTVKELCRRWY
PEEYEFAPKKAASHRALDDISESIKELQFYRNNIFKKKIDEKKRKIIENGENEKTVS*
>ENSG00000147183|ENST00000276127
MSYPTKEGSDTAGNAHKNSENEPPNDCSTDIESPSADPNMIYQVETNPINREPGTATSQE
DVVPQAAENSELETEIQKDQREEDLKEELLLLQTPIPRKLVSHKPLNDRSRSHSGKVEMK
ANNFPINHKTRFRLSTSWRVPFINSHEIRSMILHLLCDRYFSQAAGCQNTMWVKRKYIAC
LYHPNSFTHHERAITFRRPSRVHYYRPLTERMTSGKFCKSTDTKGKCRFRAIVRSVLFVS
QIQIESIFNIKGFVDILTYIHTMNVMITNTNNGWKYFCPICGRLFNTYSELRQHSCSSSG
N*
>ENSG00000143867|ENST00000272223
MGSKTLPAPVPIHPSLQLTNYSFLQAVNGLPTVPSDHLPNLYGFSALHAVHLHQWTLGYP
AMHLPRSSFSKVPGTVSSLVDARFQLPAFPWFPHVIQPKPEITAGGSVPALKTKPRFDFA
NLALAATQEDPAKLGRGEGPGSPAGGLGALLDVTKLSPEKKPTRGRLPSKTKKEFVCKFC
GRHFTKSYNLLIHERTHTDERPYTCDICHKAFRRQDHLRDHRYIHSKEKPFKCQECGKGF
CQSRTLAVHKTLHSQVKELKTSKIKC*
>ENSG00000164609|ENST00000297151
MSATVVDAVNAAPLSGSKEMSLEEPKKMTREDWRKKKELEEQRKLGNAPAEVDEEGKDIN
PHIPQYISSVPWYIDPSKRPTLKHQRPQPEKQKQFSSSGEWYKRGVKENSIITKYRKGAC
ENCGAMTHKKKDCFERPRRVGAKFTGTNIAPDEHVQPQLMFDYDGKRDRWNGYNPEEHMK
IVEEYAKVDLAKRTLKAQKLQEELASGKLVEQANSPKHQWGEEEPNSQMEKDHNSEDEDE
DKYADDIDMPGQNFDSKRRITVRNLRIREDIAKYLRNLDPNSAYYDPKTRAMRENPYANA
GKNPDEVSYAGDNFVRYTGDTISMAQTQLFAWEAYDKGSEVHLQADPTKLELLYKSFKVK
KEDFKEQQKESILEKYGGQEHLDAPPAELLLAQTEDYVEYSRHGTVIKGQERAVACSKYE
EDVKIHNHTHIWGSYWKEGRWGYKCCHSFFKYSYCTGEAGKEIVNSEECIINEITGEESV
KKPQTLMELHQEKLKEEKKKKKKKKKHRKSSSDSDDEEKKHEKLKKALNAEEARLLHVK
ETMQIDERKRPYNSMYETREPTEEEMEAYRMKRQRPDDPMASFLGQ*
>ENSG00000148584|ENST00000282641
MESNHKSGDGLSGTQKEAALRALVQRTGYSLVQENGQRKYGGPPPGWDAAPPERGCEIFI
GKLPRDLFEDELIPLCEKIGKIYEMRMMMDFNGNNRGYAFVTFSNKVEAKNAIKQLNNYE
IRNGRLLGVCASVDNCRLFVGGIPKTKKREEILSEMKKVTEGVVDVIVYPSAADKTKNRG

FAFVEYESHRAAAMARRKLLPGRIQLWGHGIAVDWAEPEVEVDEDTMSSVKILYVRNLML
STSEEMIEKEFNNIKPGAVERVKKIRDYAFVHFSNREDAVEAMKALNGKVLDGSPIEVTL
AKPVDKDSYVRYTRGTGGRGTMLQGEYTYSLGQVYDPTTTYLGAPVFYAPQTYAAIPSLH
FPATKGHLSNRAIIRAPSVRGAAGVRGLGGRGYLAYTGLGRGYQVKGDKREDKLYDILPG
MELTPMNPVTLKPQGIKLAPQILEEICQKNNWGQPVYQLHSAIGQDQRQLFLYKITIPAL
ASQNPAIHPFTPPKLSAFVDEAKTYAAEYTLQTLGIPTDGGDGTMATAAAAATAFPGYAV
PNATAPVSAAQLKQAVTLGQDLAAYTTYEVYPTFAVTARGDGYGTF*
>ENSG00000153113|ENST00000309190
MNPTETKAVKTEPEKKSQSTKLSVVHEKKSQEGKPKEHTEPKSLPKQASDTGSNDAHNKK
AVSRSAEQQPSEKSTEPKTKPQDMISAGGESVAGITAISGKPGDKKKEKKSLTPAVPVES
KPDKPSGKSGMDAALDDLIDTLGGPEETEEENTTYTGPEVSDPMSSTYIEELGKREVTIP
PKYRELLAKKEGITGPPADSSKPIGPDDAIDALSSDFTCGSPTAAGKKTEKEESTEVLKA
QSAGTVRSAAPPQEKKRKVEKDTMSDQALEALSASLGTRQAEPELDLRSIKEVDEAKAKE
EKLEKCGEDDETIPSEYRLKPATDKDGKPLLPEPEEKPKPRSESELIDELSEDFDRSECK
EKPSKPTEKTEESKAAAPAPVSEAVCRTSMCSIQSAPPEPATLKGTVPDDAVEALADSLG
KKEADPEDGKPVMDKVKEKAKEEDREKLGEKEETIPPDYRLEEVKDKDGKPLLPKESKEQ
LPPMSEDFLLDALSEDFSGPQNASSLKFEDAKLAAAISEVVSQTPASTTQAGAPPRDTSQ
SDKDLDDALDKLSDSLGQRQPDPDENKPMEDKVKEKAKAEHRDKLGERDDTIPPEYRHLL
DDNGQDKPVKPPTKKSEDSKKPADDQDPIDALSGDLDSCPSTTETSQNTAKDKCKKAASS
SKAPKNGGKAKDSAKTTEETSKPKDD*
>ENSG00000163584|ENST00000295830
MAPQKDRKPKRSTWRFNLDLTHPVEDGIFDSGNFEQFLREKVKVNGKTGNLGNVVHIERF
KNKITVVSEKQFSKRYLKYLTKKYLKKNNLRDWLRVVASDKETYELRYFQISQDEDESES
ED*
>ENSG00000143418|ENST00000271688
MLQTLYDYFWWERLWLPVNLTWADLEDRDGRVYAKASDLYITLPLALLFLIVRYFFELYV
ATPLAALLNIKEKTRLRAPPNATLEHFYLTSGKQPKQVEVELLSRQSGLSGRQVERWFRR
RRNQDRPSLLKKFREASWRFTFYLIAFIAGMAVIVDKPWFYDMKKVWEGYPIQSTIPSQY
WYYMIELSFYWSLLFSIASDVKRKDFKEQIIHHVATIILISFSWFANYIRAGTLIMALHD
SSDYLLESAKMFNYAGWKNTCNNIFIVFAIVFIITRLVILPFWILHCTLVYPLELYPAFF
GYYFFNSMMGVLQLLHIFWAYLILRMAHKFITGKLVEDERSDREETESSEGEEAAAGGGA
KSRPLANGHPILNNNHRKND*
>ENSG00000078328|ENST00000311745
MLASQGVLLHPYGVPMIVPAAPYLPGLIQGNQEAAAAPDTMAQPYASAQFAPPQNGIPAE
YTAPHPHPAPEYTGQTTVPEHTLNLYPPAQTHSEQSPADTSAQTVSGTATQTDDAAPTDG
QPQTQPSENTENKSQPKRLHVSNIPFRFRDPDLRQMFGQFGKILDVEIIFNERGSKGFGF
VTFENSADADRAREKLHGTVVEGRKIEVNNATARVMTNKKTVNPYTNGWKLNPVVGAVYS
PEFYAGTVLLCQANQEGSSMYSAPSSLVYTSAMPGFPYPAATAAAAYRGAHLRGRGRTVY
NTFRAAAPPPPIPAYGGVVYQEPVYGNKLLQGGYAAYRYAQPTPATAAAYSDSYGRVYAA
DPYHHALAPAPTYGVGAMNAFAPLTDAKTRSHADDVGLVLSSLQASIYRGGYNRFAPY*
>ENSG00000147140|ENST00000276079
MQSNKTFNLEKQNHTPRKHHQHHHQQQHQQQQQPPPPPIPANGQQASSQNEGLTIDLK
NFRKPGEKTFTQRSRLFVGNLPPDITEEEMRKLFEKYGKAGEVFIHKDKGFGFIRLETRT
LAEIAKVELDNMPLRGKQLRVRFACHSASLTVRNLPQYVSNELLEEAFSVFGQVERAVVI

VDDRGRPSGKGIVEFSGKPAARKALDRCSEGSFLLTTFPRPVTVEPMDQLDDEEGLPEKL
VIKNQQFHKEREQPPRFAQPGSFEYEYAMRWKALIEMEKQQQDQVDRNIKEAREKLEMEM
EAARHEHQVMLMRQDLMRRQEELRRMEELHNQEVQKRKQLELRQEEERRRREEEMRRQQE
EMMRRQQEGFKGTFPDAREQEIRMGQMAMGGAMGINNRGAMPPAPVPAGTPAPPGPATMM
PDGTLGLTPPTTERFGQAATMEGIGAIGGTPPAFNRAAPGAEFAPNKRRRY*
>ENSG00000239789|ENST00000285298
MSVVRSSVHARWIVGKVIGTKMQKTAKVRVTRLVLDPYLLKYFNKRKTYFAHDALQQCTV
GDIVLLRALPVPRAKHVKHELAEIVFKVGKVIDPVTGKPCAGTTYLESPLSSETTQLSKN
LEELNISSAQ*
>ENSG00000168269|ENST00000306268
MSSFDLPAPSPPRCSPQFPSIGQEPPEMNLYYENFFHPQGVPSPQRPSFEGGGEYGATPN
PYLWFNGPTMTPPPYLPGPNASPFLPQAYGVQRPLLPSVSGLGGSDLGWLPIPSQEELMK
LVRPPYSYSALIAMAIHGAPDKRLTLSQIYQYVADNFPFYNKSKAGWQNSIRHNLSLNDC
FKKVPRDEDDPGKGNYWTLDPNCEKMFDNGNFRRKRKRKSDVSSSTASLALEKTESSLPV
DSPKTTEPQDILDGASPGGTTSSPEKRPSPPPSGAPCLNSFLSSMTAYVSGGSPTSHPLV
TPGLSPEPSDKTGQNSLTFNSFSPLTNLSNHSGGGDWANPMPTNMLSYGGSVLSQFSPHF
YNSVNTSGVLYPREGTEV*
>ENSG00000158711|ENST00000289703
MDSAITLWQFLLQLLQKPQNKHMICWTSNDGQFKLLQAEEVARLWGIRKNKPNMNYDKLS
RALRYYYVKNIIKKVNGQKFVYKFVSYPEILNMDPMTVGRIEGDCESLNFSEVSSSSKDV
ENGGKDKPPQPGAKTSSRNDYIHSGLYSSFTLNSLNSSNVKLFKLIKTENPAEKLAEKKS
PQEPTPSVIKFVTTPSKKPPVEPVAATISIGPSISPSSEETIQALETLVSPKLPSLEAPT
SASNVMTAFATTPPISSIPPLQEPPRTPSPPLSSHPDIDTDIDSVASQPMELPENLSLEP
KDQDSVLLEKDKVNNSSRSKKPKGLELAPTLVITSSDPSPLGILSPSLPTASLTPAFFSQ
VACSLFMVSPLLSFICPFKQIQNLYTQVCFLLLRFVLERLCVTVM*
>ENSG00000106459|ENST00000311967
MEEHGVTQTEHMATIEAHAVAQQVQQVHVATYTEHSMLSADEDSPSSPEDTSYDDSDILN
STAADEVTAHLAAAGPVGMAAAAAVATGKKRKRPHVFESNPSIRKRQQTRLLRKLRATLD
EYTTRVGQQAIVLCISPSKPNPVFKVFGAAPLENVVRKYKSMILEDLESALAEHAPAPQE
VNSELPPLTIDGIPVSVDKMTQAQLRAFIPEMLKYSTGRGKPGWGKESCKPIWWPEDIPW
ANVRSDVRTEEQKQRVSWTQALRTIVKNCYKQHGREDLLYAFEDQQTQTQATATHSIAHL
VPSQTVVQTFSNPDGTVSLIQVGTGATVATLADASELPTTVTVAQVNYSAVADGEVEQNW
ATLQGGEMTIQTTQASEATQAVASLAEAAVAASQEMQQGATVTMALNSEAAAHAVATLAE
ATLQGGGQIVLSGETAAAVGALTGVQDANGLFMADRAGRKWILTDKATGLVQIPVSMYQT
VVTSLAQGNGPVQVAMAPVTTRISDSAVTMDGQAVEVVTLEQ*
>ENSG00000166889|ENST00000300146
MFRYESLEDCPLDEDEDAFQGLGEEDEEIDQFNDDTFGSGAVDDDWQEAHERLAELEEKL
PVAVNEQTGNGERDEMDLLGDHEENLAERLSKMVIENELEDPAIMRAVQTRPVLQPQPGS
LNSSIWDGSEVLRRIRGPLLAQEMPTVSVLEYALPQRPPQGPEDDRDLSERALPRRSTSP
IIGSPPVRAVPIGTPPKQMAVPSFTQQILCPKPVHVRPPMPPRYPAPYGERMSPNQLCSV
PNSSLLGHPFPPSVPPVLSPLQRAQLLGGAQLQPGRMSPSQFARVPGFVGSPLAAMNPKL
LQGRVGQMLPPAPGFRAFFSAPPSATPPPQQHPPGPGPHLQNLRSQAPMFRPDTTHLHPQ
HRRLLHQRQQQNRSQHRNLNGAGDRGSHRSSHQDHLRKDPYANLMLQREKDWVSKIQMMQ
LQSTDPYLDDFYYQNYFEKLEKLSAAEEIQGDGPKKERTKLITPQVAKLEHAYKPVQFEG

SLGKLTVSSVNNPRKMIDAVVTSRSEDDETKEKQVRDKRRKTLVIIEKTYSLLLDVEDYE
RRYLLSLEEERPALMDDRKHKICSMYDNLRGKLPGQERPSDDHFVQIMCIRKGKRMVARI
LPFLSTEQAADILMTTARNLPFLIKKDAQDEVLPCLLSPFSLLLYHLPSVSITSLLRQLM
NLPQSAATPALSNPHLTAVLQNKFGLSLLLILLSRGEDLQSSDPATESTQNNQWTEVMFM
ATRELLRIPQAALAKPISIPTNLVSLFSRYVDRQKLNLLETKLQLVQGIR*
>ENSG00000172216|ENST00000303004
MQRLVAWDPACLPLPPPPPAFKSMEVANFYYEADCLAAAYGGKAAPAAPPAARPGPRPPA
GELGSIGDHERAIDFSPYLEPLGAPQAPAPATATDTFEAAPPAPAPAPASSGQHHDFLSD
LFSDDYGGKNCKKPAEYGYVSLGRLGAAKGALHPGCFAPLHPPPPPPPPPAELKAEPGFE
PADCKRKEEAGAPGGGAGMAAGFPYALRAYLGYQAVPSGSSGSLSTSSSSSPPGTPSPAD
AKAPPTACYAGAAPAPSQVKSKAKKTVDKHSDEYKIRRERNNIAVRKSRDKAKMRNLETQ
HKVLELTAENERLQKKVEQLSRELSTLRNLFKQLPEPLLASSGHC*
>ENSG00000147180|ENST00000276123
MDSGGGSLGLHTPDSRMAHTMIMQDFVAGMAGTAHIDGDHIVVSVPEAVLVSDVVTDDGI
TLDHGLAAEVVHGPDIITETDVVTEGVIVPEAVLEADVAIEEDLEEDDGDHILTSELITE
TVRVPEQVFVADLVTGPNGHLEHVVQDCVSGVDSPTMVSEEVLVTNSDTETVIQAAGGVP
GSTVTIKTEDDDDDDVKSTSEDYLMISLDDVGEKLEHMGNTPLKIGSDGSQEDAKEDGFG
SEVIKVYIFKAEAEDDVEIGGTEIVTESEYTSGHSVAGVLDQSRMQREKMVYMAVKDSSQ
EEDDIRDERRVSRRYEDCQASGNTLDSALESRSSTAAQYLQICDGINTNKVLKQKAKKRR
RGETRQWQTAVIIGPDGQPLTVYPCHICTKKFKSRGFLKRHMKNHPDHLMRKKYQCTDCD
FTTNKKVSFHNHLESHKLINKVDKTHEFTEYTRRYREASPLSSNKLILRDKEPKMHKCKY
CDYETAEQGLLNRHLLAVHSKNFPHVCVECGKGFRHPSELKKHMRTHTGEKPYQCQYCIF
RCADQSNLKTHIKSKHGNNLPYKCEHCPQAFGDERELQRHLDLFQGHKTHQCPHCDHKST
NSSDLKRHIISVHTKDFPHKCEVCDKGFHRPSELKKHSDIHKGRKIHQCRHCDFKTSDPF
ILSGHILSVHTKDQPLKCKRCKRGFRQQNELKKHMKTHTGRKIYQCEYCEYSTTDASGFK
RHVISIHTKDYPHRCEFCKKGFRRPSEKNQHIMRHHKEALM*
>ENSG00000181444|ENST00000302017
MRETLEALSSLGFSVGQPEMAPQSEPREGSHNAQEQMSSSREERALGVCSGHEAPTPEEG
AHTEQAEAPCRGQACSAQKAQPVGTCPGEEWMIRKVKVEDEDQEAEEEVEWPQHLSLLPS
PFPAPDLGHLAAAYKLEPGAPGALSGLALSGWGPMPEKPYGCGECERRFRDQLTLRLHQR
LHRGEGPCACPDCGRSFTQRAHMLLHQRSHRGERPFPCSECDKRFSKKAHLTRHLRTHTG
ERPYPCAECGKRFSQKIHLGSHQKTHTGERPFPCTECEKRFRKKTHLIRHQRIHTGERPY
QCAQCARSFTHKQHLVRHQRVHQTAGPARPSPDSSASPHSTAPSPTPSFPGPKPFACSDC
GLSFGWKKNLATHQCLHRSEGRPFGCDECALGATVDAPAAKPLASAPGGPGCGPGSDPVV
PQRAPSGERSFFCPDCGRGFSHGQHLARHPRVHTGERPFACTQCDRRFGSRPNLVAHSRA
HSGARPFACAQCGRRFSRKSHLGRHQAVHTGSRPHACAVCARSFSSKTNLVRHQAIHTGS
RPFSCPQCGKSFSRKTHLVRHQLIHGEAAHAAPDAALAAPAWSAPPEVAPPPLFF*
>ENSG00000140470|ENST00000268070
MCDGALLPPLVLPVLLLLVWGLDPGTAVGDAAADVEVVLPWRVRPDDVHLPPLPAAPGPR
RRRRPRTPPAAPRARPGERALLLHLPAFGRDLYLQLRRDLRFLSRGFEVEEAGAARRRGR
PAELCFYSGRVLGHPGSLVSLSACGAAGGLVGLIQLGQEQVLIQPLNNSQGPFSGREHLI
RRKWSLTPSPSAEAQRPEQLCKVLTEKKKPTWGRPSRDWRERRNAIRLTSEHTVETLVVA
DADMVQYHGAEAAQRFILTVMNMVYNMFQHQSLGIKINIQVTKLVLLRQRPAKLSIGHHG
ERSLESFCHWQNEEYGGARYLGNNQVPGGKDDPPLVDAAVFVTRTDFCVHKDEPCDTVGI

AYLGGVCSAKRKCVLAEDNGLNLAFTIAHELGHNLGMNHDDDHSSCAGRSHIMSGEWVKG
RNPSDLSWSSCSRDDLENFLKSKVSTCLLVTDPRSQHTVRLPHKLPGMHYSANEQCQILF
GMNATFCRNMEHLMCAGLWCLVEGDTSCKTKLDPPLDGTECGADKWCRAGECVSKTPIPE
HVDGDWSPWGAWSMCSRTCGTGARFRQRKCDNPPPGPGGTHCPGASVEHAVCENLPCPKG
LPSFRDQQCQAHDRLSPKKKGLLTAVVVDDKPCELYCSPLGKESPLLVADRVLDGTPCGP
YETDLCVHGKCQKIGCDGIIGSAAKEDRCGVCSGDGKTCHLVKGDFSHARGTALKDSGKG
SINSDWKIELPGEFQIAGTTVRYVRRGLWEKISAKGPTKLPLHLMVLLFHDQDYGIHYEY
TVPVNRTAENQSEPEKPQDSLFIWTHSGWEGCSVQCGGGERRTIVSCTRIVNKTTTLVND
SDCPQASRPEPQVRRCNLHPCQSRWVAGPWSPCSATCEKGFQHREVTCVYQLQNGTHVAT
RPLYCPGPRPAAVQSCEGQDCLSIWEASEWSQCSASCGKGVWKRTVACTNSQGKCDASTR
PRAEEACEDYSGCYEWKTGDWSTCSSTCGKGLQSRVVQCMHKVTGRHGSECPALSKPAPY
RQCYQEVCNDRINANTITSPRLAALTYKCTRDQWTVYCRVIREKNLCQDMRWYQRCCQTC
RDFYANKMRQPPPNS*
>ENSG00000171163|ENST00000306601
MASSPAVDVSCRRREKRRQLDARRSKCRIRLGGHMEQWCLLKERLGFSLHSQLAKFLLDR
YTSSGCVLCAGPEPLPPKGLQYLVLLSHAHSRECSLVPGLRGPGGQDGGLVWECSAGHTF
SWGPSLSPTPSEAPKPASLPHTTRRSWCSEATSGQELADLESEHDERTQEARLPRRVGPP
PETFPPPGEEEGEEEEDNDEDEEEMLSDASLWTYSSSPDDSEPDAPRLLPSPVTCTPKEG
ETPPAPAALSSPLAVPALSASSLSSRAPPPAEVRVQPQLSRTPQAAQQTEALASTGSQAQ
SAPTPAWDEDTAQIGPKRIRKAAKRELMPCDFPGCGRIFSNRQYLNHHKKYQHIHQKSFS
CPEPACGKSFNFKKHLKEHMKLHSDTRDYICEFCARSFRTSSNLVIHRRIHTGEKPLQCE
ICGFTCRQKASLNWHQRKHAETVAALRFPCEFCGKRFEKPDSVAAHRSKSHPALLLAPQE
SPSGPLEPCPSISAPGPLGSSEGSRPSASPQAPTLLPQQ*
>ENSG00000146007|ENST00000274712
MASGSGTKNLDFRRKWDKDEYEKLAEKRLTEEREKKDGKPVQPVKRELLRHRDYKVDLES
KLGKTIVITKTTPQSEMGGYYCNVCDCVVKDSINFLDHINGKKHQRNLGMSMRVERSTLD
QVKKRFEVNKKKMEEKQKDYDFEERMKELREEEEKAKAYKKEKQKEKKRRAEEDLTFEED
DEMAAVMGFSGFGSTKKSY*
>ENSG00000042286|ENST00000307864
MGSQVSVESGALHVVIVGGGFGGIAAASQLQALNVPFMLVDMKDSFHHNVAALRASVETG
FAKKTFISYSVTFKDNFRQGLVVGIDLKNQMVLLQGGEALPFSHLILATGSTGPFPGKFN
EVSSQQAAIQAYEDMVRQVQRSRFIVVVGGGSAGVEMAAEIKTEYPEKEVTLIHSQVALA
DKELLPSVRQEVKEILLRKGVQLLLSERVSNLEELPLNEYREYIKVQTDKGTEVATNLVI
LCTGIKINSSAYRKAFESRLASSGALRVNEHLQVEGHSNVYAIGDCADVRTPKMAYLAGL
HANIAVANIVNSVKQRPLQAYKPGALTFLLSMGRNDGVGQISGFYVGRLMVRLTKSRDLF
VSTSWKTMRQSPP*
>ENSG00000139351|ENST00000266743
MVSSGKKYSRKSGKPSVEDQFTRAYDFETEDKKDLSGSEEDVIEGKTAVIEKRRKKRSSA
GVVEDMGGEVQNMLEGVGVDINKALLAKRKRLEMYTKASLKTSNQKIEHVWKTQQDQRQK
LNQEYSQQFLTLFQQWDLDMQKAEEQEEKILNMFRQQQKILQQSRIVQSQRLKTIKQLYE
QFIKSMEELEKNHDNLLTGAQNEFKKEMAMLQKKIMMETQQQEIASVRKSLQSMLF*
>ENSG00000108424|ENST00000290158
MELITILEKTVSPDRLELEAAQKFLERAAVENLPTFLVELSRVLANPGNSQVARVAAGLQ
IKNSLTSKDPDIKAQYQQRWLAIDANARREVKNYVLQTLGTETYRPSSASQCVAGIACAE

IPVNQWPELIPQLVANVTNPNSTEHMKESTLEAIGYICQDIDPEQLQDKSNEILTAIIQG
MRKEEPSNNVKLAATNALLNSLEFTKANFDKESERHFIMQVVCEATQCPDTRVRVAALQN
LVKIMSLYYQYMETYMGPALFAITIEAMKSDIDEVALQGIEFWSNVCDEEMDLAIEASEA
AEQGRPPEHTSKFYAKGALQYLVPILTQTLTKQDENDDDDDWNPCKAAGVCLMLLATCCE
DDIVPHVLPFIKEHIKNPDWRYRDAAVMAFGCILEGPEPSQLKPLVIQAMPTLIELMKDP
SVVVRDTAAWTVGRICELLPEAAINDVYLAPLLQCLIEGLSAEPRVASNVCWAFSSLAEA
AYEAADVADDQEEPATYCLSSSFELIVQKLLETTDRPDGHQNNLRSSAYESLMEIVKNSA
KDCYPAVQKTTLVIMERLQQVLQMESHIQSTSDRIQFNDLQSLLCATLQNVLRKVQHQDA
LQISDVVMASLLRMFQSTAGSGGVQEDALMAVSTLVEVLGGEFLKYMEAFKPFLGIGLKN
YAEYQVCLAAVGLVGDLCRALQSNIIPFCDEVMQLLLENLGNENVHRSVKPQILSVFGDI
ALAIGGEFKKYLEVVLNTLQQASQAQVDKSDYDMVDYLNELRESCLEAYTGIVQGLKGDQ
ENVHPDVMLVQPRVEFILSFIDHIAGDEDHTDGVVACAAGLIGDLCTAFGKDVLKLVEAR
PMIHELLTEGRRSKTNKAKTLATWATKELRKLKNQA*
>ENSG00000141738|ENST00000309156
MELDLSPPHLSSSPEDLCPAPGTPPGTPRPPDTPLPEEVKRSQPLLIPTTGRKLREEERR
ATSLPSIPNPFPELCSPPSQSPILGGPSSARGLLPRDASRPHVVKVYSEDGACRSVEVAA
GATARHVCEMLVQRAHALSDETWGLVECHPHLALERGLEDHESVVEVQAAWPVGGDSRFV
FRKNFAKYELFKSSPHSLFPEKMVSSCLDAHTGISHEDLIQNFLNAGSFPEIQGFLQLRG
SGRKLWKRFFCFLRRSGLYYSTKGTSKDPRHLQYVADVNESNVYVVTQGRKLYGMPTDFG
FCVKPNKLRNGHKGLRIFCSEDEQSRTCWLAAFRLFKYGVQLYKNYQQAQSRHLHPSCLG
SPPLRSASDNTLVAMDFSGHAGRVIENPREALSVALEEAQAWRKKTNHRLSLPMPASGTS
LSAAIHRTQLWFHGRISREESQRLIGQQGLVDGLFLVRESQRNPQGFVLSLCHLQKVKHY
LILPSEEEGRLYFSMDDGQTRFTDLLQLVEFHQLNRGILPCLLRHCCTRVAL*
>ENSG00000159216|ENST00000300305
MASDSIFESFPSYPQCFMRECILGMNPSRDVHDASTSRRFTPPSTALSPGKMSEALPLGA
PDAGAALAGKLRSGDRSMVEVLADHPGELVRTDSPNFLCSVLPTHWRCNKTLPIAFKVVA
LGDVPDGTLVTVMAGNDENYSAELRNATAAMKNQVARFNDLRFVGRSGRGKSFTLTITVF
TNPPQVATYHRAIKITVDGPREPRRHRQKLDDQTKPGSLSFSERLSELEQLRRTAMRVSP
HHPAPTPNPRASLNHSTAFNPQPQSQMQDTRQIQPSPPWSYDQSYQYLGSIASPSVHPAT
PISPGRASGMTTLSAELSSRLSTAPDLTAFSDPRQFPALPSISDPRMHYPGAFTYSPTPV
TSGIGIGMSAMGSATRYHTYLPPPYPGSSQAQGGPFQASSPSYHLYYGASAGSYQFSMVG
GERSPPRILPPCTNASTGSALLNPSLPNQSDVVEAEGSHSNSPTNMAPSARLEEAVWRPY
*
>ENSG00000173480|ENST00000312026
MAAAAPRRPTQQGTVTFEDVAVNFSQEEWCLLSEAQRCLYRDVMLENLALISSLGCWCGS
KDEEAPCKQRISVQRESQSRTPRAGVSPKKAHPCEMCGLILEDVFHFADHQETHHKQKLN
RSGACGKNLDDTAYLHQHQKQHIGEKFYRKSVREASFVKKRKLRVSQEPFVFREFGKDVL
PSSGLCQEAAAVEKTDSETMHGPPFQEGKTNYSCGKRTKAFSTKHSVIPHQKLFTRDGCY
VCSDCGKSFSRYVSFSNHQRDHTAKGPYDCGECGKSYSRKSSLIQHQRVHTGKTAYPCEE
CGKSFSQKGSLISHQRVHTGERPYECREYGKSFGQKGNLIQHQQGHTGERAYHCGECGKS
FRQKFCFINHQRVHTGERPYKCGECGKSFGQKGNLVQHQRGHTGERPYECKECGKSFRYR
SHLTEHQRLHTGERPYNCRECGKLFNRKYHLLVHERVHTGERPYACEVCGKLFGNKNCVT
IHQRIHTGERPYECNECGKSFLSSSALHVHKRVHSGQKPYKCSECGKSFAECSSLIKHRR
IHTGERPYECTKCGKTFQRSSTLLHHQSSHRRKAL*

>ENSG00000124831|ENST00000308482
MDMGTQGSGRKRLPNRERLTAEDDALNQIAREAEARLAAKRAARAEAREIRMKELERQQK
EIYQVQKKYYGLDTKWGDIEQWMEDSERYSRRSRRNTSASDEDERMSVGSRGSLRSQPDL
EYGGPYAWTNGYDGELYGSQSLNRRSGRPSCLYSAARPSGSYRASVLDEGSFGGTRRGST
SGSRAPSEYSGHLNSSSRASSRASSARASPVVEERPEKDFTEKGSRNMPGLSAATLASLG
GTSSRRGSGDTSISIDTEASIREIKELNELKDQIQDVEGKYMQGLKEMKDSLAEVEEKYK
KAMVSNAQLDNEKTNFMYQVDTLKDMLLELEEQLAESRRQYEEKNKEFEREKHAHSILQF
QFAEVKEALKQREEMLEEIRQLQQKQASSIREISDLQETIEWKDKKIGALERQKEFFDSV
RSERDDLREEVVMLKEELKKHGIILNSEIATNGETSDTLNNVGYQGPTKMTKEELNALKS
TGDGTLDIRLKKLVDERECLLEQIKKLKGQLEERQKIGKLDNLRSEDDVLENGTDMHVMD
LQRDANRQISDLKFKLAKSEQEITALEQNVIRLESQVSRYKSAAENAEKIEDELKAEKRK
LQRELRSALDKTEELEVSNGHLVKRLEKMKANRSALLSQQ*
>ENSG00000167005|ENST00000300291
MSVVPPNRSQTGWPRGVTQFGNKYIQQTKPLTLERTINLYPLTNYTFGTKEPLYEKDSSV
AARFQRMREEFDKIGMRRTVEGVLIVHEHRLPHVLLLQLGTTFFKLPGGELNPGEDEVEG
LKRLMTEILGRQDGVLQDWVIDDCIGNWWRPNFEPPQYPYIPAHITKPKEHKKLFLVQLQ
EKALFAVPKNYKLVAAPLFELYDNAPGYGPIISSLPQLLSRFNFIYN*
>ENSG00000131845|ENST00000282286
MAAAVLMDRVQSCVTFEDVFVYFSREEWELLEEAQRFLYRDVMLENFALVATLGFWCEAE
HEAPSEQSVSVEGVSQVRTAESGLFQKAHPCEMCDPLLKDILHLAEHQGSHLTQKLCTRG
LCRRRFSFSANFYQHQKQHNGENCFRGDDGGASFVKSCTVHMLGRSFTCREEGMDLPDSS
GLFQHQTTYNRVSPCRRTECMESFPHSSSLRQHQGDYDGQMLFSCGDEGKAFLDTFTLLD
SQMTHAEVRPFRCLPCGNVFKEKSALINHRKIHSGEISHVCKECGKAFIHLHHLKMHQKF
HTGKRHYTCSECGKAFSRKDTLVQHQRVHTGERSYDCSECGKAYSRSSHLVQHQRIHTGE
RPYKCNKCGKAFSRKDTLVQHQRFHTGERPYECSECGKFFSQSSHLIEHWRIHTGARPYE
CIECGKFFSHNSSLIKHRRVHTGARSYVCSKCGKAFGCKDTLVQHQIIHTGARPYECSEC
GKAFSRKDTLVQHQKIHTGERPYECGECGKFFSHSSNLIVHQRIHTGAKPYECNECGKCF
SHNSSLILHQRVHTGARPYVCSECGKAYISSSHLVQHKKVHTGARPYECSECGKFFSRNS
GLILHQRVHTGEKPYVCSECGKAYSRSSHLVRHQKAHTGERAHECNSFGGPLAASLKLV*
>ENSG00000144642|ENST00000273139
MGKRLDQPQMYPQYTYYYPHYLQTKQSYAPAPHPMAPPSPSTNSSSNNSSNNSSGEQLSK
TNLYIRGLPPGTTDQDLIKLCQPYGKIVSTKAILDKNTNQCKGYGFVDFDSPAAAQKAVA
SLKANGVQAQMAKQQEQDPTNLYISNLPISMDEQELENMLKPFGHVISTRILRDANGVSR
GVGFARMESTEKCEVVIQHFNGKYLKTPPGIPAPSEPLLCKFADGGQKKRQNQSKYTQNG
RPWPREGEAGMALTYDPTAAIQNGFYSSPYSIATNRMIPQTSITPFIAASPVSTYQVQST
SWMPHPPYVMQPTGAVITPTMDHPMSMQPANMMGPLTQQMNHLSLGTTGTYMTAAAPMQG
TYIPQYTPVPPTAVSIEGVVADTSPQTVAPSSQDTSGQQQQIAVDTSNEHAPAYSYQQSK
*
>ENSG00000161813|ENST00000293618
MLLFVEQVASKGTGLNPNAKVWQEIAPGNTDATPVTHGTESSWHEIAATSGAHPEGNAEL
SEDICKEYEVMYSSSCETTRNTTGIEESTDGMILGPEDLSYQIYDVSGESNSAVSTEDLK
ECLKKQLEFCFSRENLSKDLYLISQMDSDQFIPIWTVANMEEIKKLTTDPDLILEVLRSS
PMVQVDEKGEKVRPSHKRCIVILREIPETTPIEEVKGLFKSENCPKVISCEFAHNSNWYI
TFQSDTDAQQAFKYLREEVKTFQGKPIMARIKAINTFFAKNGYRLMDSSIYSHPIQTQAQ

YASPVFMQPVYNPHQQYSVYSIVPQSWSPNPTPYFETPLAPFPNGSFVNGFNSPGSYKTN
AAAMNMGRPFQKNRRTLFRGRRRREDDRISRPHPSTAESKAPTPKFDLLASNFPPLPGSS
SRMPGELVLENRMSDVVKGVYKEKDNEELTISCPVPADEQTECTSAQQLNMSTSSPCAAE
LTALSTTQQEKDLIEDSSVQKDGLNQTTIPVSPPSTTKPSRASTASPCNNNINAATAVAL
QEPRKLSYAEVCQKPPKEPSSVLVQPLRELRSNVVSPTKNEDNGAPENSVEKPHEKPEAR
ASKDYSGFRGNIIPRGAAGKIREQRRQFSHRAIPQGVTRRNGKEQYVPPRSPK*
>ENSG00000198028|ENST00000301480
MAYCLTNCYQYSVTFEDTAVDFTQEEWILLDPVQRNLYRDVMLENYENVAKVGFQLFKPS
VISWLEEEELRTLQQGVLQDWAIKHQTSVSALQQEFWKIQTSNGIQMDLVTFDSVAVEFT
QEEWTLLDPAQRNLYSDVMLENYKNLSSVGYQLFKPSLISWLEEEEELSTLPRVLQEWKM
CLKTKGPALWQDNFCLKTLNGIQLARNQNGEELYDCKQCEDVFCKHPCLKTNMSTQNRGN
TSECIQYAKDLLSLYNKTSTIRKVSVFSKHGKSFRLILNVQVQRKCTQDKSFEGTDYGKA
FIYQSYLEAHRKTQSGEKLNEWKQCGEAFTHSTSHAVNVETHIIKNPYECKECGKDFRYP
THLNNHMQTHIGIKPYKCKHCGKTFTVPSGFLEHVRTHTGEKPYGCKECGKAFGTSAGLI
EHIRCHAREKTFKCDHCGKAFISYPSLFGHLRVHNGEKPYEHKEYGKAFGTSSGVIEDRR
SNTGQKRFDCDQCGKVFVSFSSLFAHLRTHTGEKPFKCYKCGKPFTSSACLRIHMRTHTE
ERLYQCKKCGKAFTKCSYLTKHLRTHAGEKPYECMKCGKAFTERSYLTKHLRRHSGEKPY
ECKKCGKAFTERSDLTKHLRRHTGDKPYEYKDCGKAFVVSSSLVDHLRTHTGYKPYKCNA
CEKAYSRSCVLTQHLKTHAAEKTSECNACGNSFRNSMCFHDRLKTLTKIKPYKCKDCGKA
FTCHSDLTNHVRIHTGEKPYKCKECGKAFRTSSGRIQHLRTHMGEKPFECDQCGKAFASF
SARIAHLKTH*
>ENSG00000152284|ENST00000282111
MPQLGGGGGGGGGGSGGGGGSSAGAAGGGDDLGANDELIPFQDEGGEEQEPSSDSASAQR
DLDEVKSSLVNESENQSSSSDSEAERRPQPVRDTFQKPRDYFAEVRRPQDSAFFKGPPYP
GYPFLMIPDLSSPYLSNGPLSPGGARTYLQMKWPLLDVPSSATVKDTRSPSPAHLSNKVP
VVQHPHHMHPLTPLITYSNDHFSPGSPPTHLSPEIDPKTGIPRPPHPSELSPYYPLSPGA
VGQIPHPLGWLVPQQGQPMYSLPPGGFRHPYPALAMNASMSSLVSSRFSPHMVAPAHPGL
PTSGIPHPAIVSPIVKQEPAPPSLSPAVSVKSPVTVKKEEEKKPHVKKPLNAFMLYMKEM
RAKVVAECTLKESAAINQILGRKWHNLSREEQAKYYELARKERQLHSQLYPTWSARDNYG
KKKKRKREKQLSQTQSQQQVQEAEGALASKSKKPCVQYLPPEKPCDSPASSHGSMLDSPA
TPSAALASPAAPAATHSEQAQPLSLTTKPETRAQLALHSAAFLSAKAAASSSGQMGSQPP
LLSRPLPLGSMPTALLASPPSFPATLHAHQALPVLQAQPLSLVTKSAH*
>ENSG00000114416|ENST00000305586
MMKGEFYVIEYAACDATYNEIVTFERLRPVNQNKTVKKNTFFKCTVDVPEDLREACANEN
AHKDFKKAVGACRIFYHPETTQLMILSASEATVKRVNILSDMHLRSIRTKLMLMSRNEEA
TKHLECTKQLAAAFHEEFVVREDLMGLAIGTHGSNIQQARKVPGVTAIELDEDTGTFRIY
GESADAVKKARGFLEFVEDFIQVPRNLVGKVIGKNGKVIQEIVDKSGVVRVRIEGDNENK
LPREDGMVPFVFVGTKESIGNVQVLLEYHIAYLKEVEQLRMERLQIDEQLRQIGSRSYSG
RGRGRRGPNYTSGYGTNSELSNPSETESERKDELSDWSLAGEDDRDSRHQRDSRRRPGGR
GRSVSGGRGRGGPRGGKSSISSVLKDPDSNPYSLLDNTESDQTADTDASESHHSTNRRRR
SRRRRTDEDAVLMDGMTESDTASVNENGLVTVADYISRAESQSRQRNLPRETLAKNKKEM
AKDVIEEHGPSEKAINGPTSASGDDISKLQRTPGEEKINTLKEENTQEAAVLNGVS*
>ENSG00000184492|ENST00000306507
MNLPRAERPRSTPQRSLRDSDGEDGKIDVLGEEEDEDEVEDEEEEASQKFLEQSLQPGLQ

VARWGGVALPREHIEGGGPSDPSEFGTEFRAPPRSAAASEDARQPAKPPYSYIALITMAI
LQSPHKRLTLSGICAFISGRFPYYRRKFPAWQNSIRHNLSLNDCFVKIPREPGHPGKGTY
WSLDPASQDMFDNGSFLRRRKRFKRHQLTPGAHLPHPFPLPAAHAALHNPRPGPLLGAPA
LPQPVPGAYPNTAPGRRPYALLHPHPPRYLLLSAPAYAGAPKKAEGADLATPGTLPVLQP
SLGPQPWEEGKGLASPPGGGCISFSIESIMQGVRGAGTGAAQSLSPTAWSYCPLLQRPSS
LSDNFAATAAASGGGLRQRLRSHQGRGAGRAPVGRVGAAAVSGGGRGL*
>ENSG00000164896|ENST00000297532
MRRPRGEPGPRAPRPTEGATCAGPGESWSPSPNSMLRVLLSAQTSPARLSGLLLIPPVQP
CCLGPSKWGDRPVGGGPSAGPVQGLQRLLEQAKSPGELLRWLGQNPSKVRAHHYSVALRR
LGQLLGSRPRPPPVEQVTLQDLSQLIIRNCPSFDIHTIHVCLHLAVLLGFPSDGPLVCAL
EQERRLRLPPKPPPPLQPLLRGGQGLEAALSCPRFLRYPRQHLISSLAEARPEELTPHVM
VLLAQHLARHRLREPQLLEAIAHFLVVQETQLSSKVVQKLVLPFGRLNYLPLEQQFMPCL
ERILAREAGVAPLATVNILMSLCQLRCLPFRALHFVFSPGFINYISGTPHALIVRRYLSL
LDTAVELELPGYRGPRLPRRQQVPIFPQPLITDRARCKYSHKDIVAEGLRQLLGEEKYRQ
DLTVPPGYCTDFLLCASSSGAVLPVRTQDPFLPYPPRSCPQGQAASSATTRDPAQRVVLV
LRERWHFCRDGRVLLGSRALRERHLGLMGYQLLPLPFEELESQRGLPQLKSYLRQKLQAL
GLRWGPEGG*
>ENSG00000162714|ENST00000294753
MPTALCPRVLAPKESEEPRKMRSPPGENPSPQGELPSPESSRRLFRRFRYQEAAGPREAL
QRLWDLCGGWLRPERHTKEQILELLVLEQFLAILPREIQSWVRAQEPESGEQAVAAVEAL
EREPGRPWQWLKHCEDPVVIDDGDSPLDQEQEQLPVEPHSDLAKNQDAQPITLAQCLGLP
SRPPSQLSGDPVLQDAFLLQEENVRDTQQVTTLQLPPSRVSPFKDMILCFSEEDWSLLDP
AQTGFYGEFIIGEDYGVSMPPNDLAAQPDLSQGEENEPRVPELQDLQGKEVPQVSYLDSP
SLQPFQVEERRKREELQVPEFQACPQTVVPQNTYPAGGNPRSLENSLDEEVTIEIVLSSS
GDEDSQHGPYCTEELGSPTEKQRSLPASHRSSTEAGGEVQTSKKSYVCPNCGKIFRWRVN
FIRHLRSRREQEKPHECSVCGELFSDSEDLDGHLESHEAQKPYRCGACGKSFRLNSHLLS
HRRIHLQPDRLQPVEKREQAASEDADKGPKEPLENGKAKLSFQCCECGKAFQRHDHLARH
RSHFHLKDKARPFQCRYCVKSFTQNYDLLRHERLHMKRRSKQALNSY*
>ENSG00000186184|ENST00000302979
MEEDQELERKISGLKTSMAEGERKTALEMVQAAGTDRHCVTFVLHEEDHTLGNSLRYMIM
KNPEVEFCGYTTTHPSESKINLRIQTRGTLPAVEPFQRGLNELMNVCQHVLDKFEASIKD
YKDQKASRNESTF*
>ENSG00000174243|ENST00000308025
MAGELADKKDRDASPSKEERKRSRTPDRERDRDRDRKSSPSKDRKRHRSRDRRRGGSRSR
SRSRSKSAERERRHKERERDKERDRNKKDRDRDKDGHRRDKDRKRSSLSPGRGKDFKSRK
DRDSKKDEEDEHGDKKPKAQPLSLEELLAKKKAEEEAEAKPKFLSKAEREAEALKRRQQE
VEERQRMLEEERKKRKQFQDLGRKMLEDPQERERRERRERMERETNGNEDEEGRQKIREE
KDKSKELHAIKERYLGGIKKRRRTRHLNDRKFVFEWDASEDTSIDYNPLYKERHQVQLLG
RGFIAGIDLKQQKREQSRFYGDLMEKRRTLEEKEQEEARLRKLRKKEAKQRWDDRHWSQK
KLDEMTDRDWRIFREDYSITTKGGKIPNPIRSWKDSSLPPHILEVIDKCGYKEPTPIQRQ
AIPIGLQNRDIIGVAETGSGKTAAFLIPLLVWITTLPKIDRIEESDQGPYAIILAPTREL
AQQIEEETIKFGKPLGIRTVAVIGGISREDQGFRLRMGCEIVIATPGRLIDVLENRYLVL
SRCTYVVLDEADRMIDMGFEPDVQKILEHMPVSNQKPDTDEAEDPEKMLANFESGKHKYR
QTVMFTATMPPAVERLARSYLRRPAVVYIGSAGKPHERVEQKVFLMSESEKRKKLLAILE

QGFDPPIIIFVNQKKGCDVLAKSLEKMGYNACTLHGGKGQEQREFALSNLKAGAKDILVA
TDVAGRGIDIQDVSMVVNYDMAKNIEDYIHRIGRTGRAGKSGVAITFLTKEDSAVFYELK
QAILESPVSSCPPELANHPDAQHKPGTILTKKRREETIFA*
>ENSG00000145414|ENST00000274054
MEVVEAAAAQLETLKFNGTDFGVGEGPAAPSPGSAPVPGTQPPLQSFEGSPDAGQTVEVK
PAGEQPLQPVLNAVAAGTPAPQPQPPAESPACGDCVTSPGAAEPARAPDSLETSDSDSDS
DSETDSDSSSSSSSSSSSSSSSSSSCISLPPVLSDGDDDLQIEKENKNFPLKTKDELLLN
ELPSVEELTIILPEDIELKPLGMVSSIIEQLVIIESMTNLPPVNEETVIFKSDRQAAGKI
FEIFGPVAHPFYVLRFNSSDHIESKGIKIKETMYFAPSMKDFTQYIFTEKLKQDKGSDAS
WKNDQEPPPEALDFSDDEKEKEAKQRKKSQIQGRKKLKSEFNEPGEDFTEVHQNWNAHSS
ASEHAKGYRNREFTRGFSRARYPRSCHGRPPPQHFYNSEHMVSQETSGFPSQRQNNPIMP
QYPFPLPVFDMHNFPLRPPPPPPPPPVNMGWATPNMAAHPLLNLPYSLPPPPPPPPLPPP
PSSGDSNSHFGPYY*
>ENSG00000147601|ENST00000276602
MAEDVSSAAPSPRGCADGRDADPTEEQMAETERNDEEQFECQELLECQVQVGAPEEEEEE
EEDAGLVAEAEAVAAGWMLDFLCLSLCRAFRDGRSEDFRRTRNSAEAIIHGLSSLTACQL
RTIYICQFLTRIAAGKTLDAQFENDERITPLESALMIWGSIEKEHDKLHEEIQNLIKIQA
IAVCMENGNFKEAEEVFERIFGDPNSHMPFKSKLLMIISQKDTFHSFFQHFSYNHMMEKI
KSYVNYVLSEKSSTFLMKAAAKVVESKRTRTITSQDKPSGNDVEMETEANLDTRKRSHKN
LFLSKLQHGTQQQDLNKKERRVGTPQSTKKKKESRRATESRIPVSKSQPVTPEKHRARKR
QAWLWEEDKNLRSGVRKYGEGNWSKILLHYKFNNRTSVMLKDRWRTMKKLKLISSDSED*
>ENSG00000107949|ENST00000299130
MASRSKRRAVESGVPQPPDPPVQRDEEEEKEVENEDEDDDSDKEKDEEDEVIDEEVNIE
FEAYSLSDNDYDGIKKLLQQLFLKAPVNTAELTDLLIQQNHIGSVIKQTDVSEDSNDDMD
EDEVFGFISLLNLTERKGTQCVEQIQELVLRFCEKNCEKSMVEQLDKFLNDTTKPVGLLL
SERFINVPPQIALPMYQQLQKELAGAHRTNKPCGKCYFYLLISKTFVEAGKNNSKKKPSN
KKKAALMFANAEEEFFYEEQGKPEVLGGPDTRRGLEPVPIQHNGWSVPPVLE*
>ENSG00000143748|ENST00000281701
MKPRPAGFVDNKLKQRVIQYLTSNKCGKYVDIGVLASDLQRVYSIDYGRRKRNAFRIQVE
KVFSIISSEKELKNLTELEDEHLAKRARQGEEDNEYTESYSDDDSSMEDYPDPQSANHMN
SSLLSLYRKGNPDSVSNTPEMEQRETTSSTPRISSKTGSIPLKTPAKDSEGGWFIDKTPS
VKKDSFFLDLSCEKSNPKKPITEIQDSKDSSLLESDMKRKGKLKNKGSKRKKEDLQEVDG
EIEAVLQKKAKARGLEFQISNVKFEDVGGNDMTLKEVCKMLIHMRHPEVYHHLGVVPPRG
VLLHGPPGCGKTLLAHAIAGELDLPILKVAAPEIVSGVSGESEQKLRELFEQAVSNAPCI
IFIDEIDAITPKREVASKDMERRIVAQLLTCMDDLNNVAATARVLVIGATNRPDSLDPAL
RRAGRFDREICLGIPDEASRERILQTLCRKLRLPQAFDFCHLAHLTPGFVGADLMALCRE
AAMCAVNRVLMKLQEQQKKNPEMEDLPSKGVQEERLGTEPTSETQDELQRLLGLLRDQDP
LSEEQMQGLCIELNDFIVALSSVQPSAKREGFVTVPNVTWADIGALEDIREELTMAILAP
VRNPDQFKALGLVTPAGVLLAGPPGCGKTLLAKAVANESGLNFISVKGPELLNMYVGESE
RAVRQVFQRAKNSAPCVIFFDEVDALCPRRSDRETGASVRVVNQLLTEMDGLEARQQVFI
MAATNRPDIIDPAILRPGRLDKTLFVGLPPPADRLAILKTITKNGTKPPLDADVNLEAIA
GDLRCDCYTGADLSALVREASICALRQEMARQKSGNEKGELKVSHKHFEEAFKKVRSSIS
KKDQIMYERLQESLSR*
>ENSG00000198440|ENST00000291598

MSKDLVTFGDVAVNFSQEEWEWLNPAQRNLYRKVMLENYRSLVSLGVSVSKPDVISLLEQ
GKEPWMVKKEGTRGPCPDWEYVFKNSEFSSKQETYEESSKVVTVGARHLSYSLDYPSLRE
DCQSEDWYKNQLGSQEVHLSQLIITHKEILPEVQNKEYNKSWQTFHQDTIFDIQQSFPTK
EKAHKHEPQKKSYRKKSVEMKHRKVYVEKKLLKCNDCEKVFNQSSSLTLHQRIHTGEKPY
ACVECGKTFSQSANLAQHKRIHTGEKPYECKECRKAFSQNAHLAQHQRVHTGEKPYQCKE
CKKAFSQIAHLTQHQRVHTGERPFECIECGKAFSNGSFLAQHQRIHTGEKPYVCNVCGKA
FSHRGYLIVHQRIHTGERPYECKECRKAFSQYAHLAQHQRVHTGEKPYECKVCRKAFSQI
AYLDQHQRVHTGEKPYECIECGKAFSNSSSLAQHQRSHTGEKPYMCKECRKTFSQNAGLA
QHQRIHTGEKPYECNVCGKAFSYSGSLTLHQRIHTGERPYECKDCRKSFRQRAHLAHHER
IHTMESFLTLSSPSPSTSNQLPRPVGFIS*
>ENSG00000160633|ENST00000292123
MAETLSGLGDSGAAGAAALSSASSETGTRRLSDLRVIDLRAELRKRNVDSSGNKSVLMER
LKKAIEDEGGNPDEIEITSEGNKKTSKRSSKGRKPEEEGVEDNGLEENSGDGQEDVETSL
ENLQDIDIMDISVLDEAEIDNGSVADCVEDDDADNLQESLSDSRELVEGEMKELPEQLQE
HAIEDKETINNLDTSSSDFTILQEIEEPSLEPENEKILDILGETCKSEPVKEESSELEQP
FAQDTSSVGPDRKLAEEEDLFDSAHPEEGDLDLASESTAHAQSSKADSLLAVVKREPAEQ
PGDGERTDCEPVGLEPAVEQSSAASELAEASSEELAEAPTEAPSPEARDSKEDGRKFDFD
ACNEVPPAPKESSTSEGADQKMSSPEDDSDTKRLSKEEKGRSSCGRNFWVSGLSSTTRAT
DLKNLFSKYGKVVGAKVVTNARSPGARCYGFVTMSTAEEATKCINHLHKTELHGKMISVE
KAKNEPVGKKTSDKRDSDGKKEKSSNSDRSTNLKRDDKCDRKDDAKKGDDGSGEKSKDQD
DQKPGPSERSRATKSGSRGTERTVVMDKSKGVPVISVKTSGSKERASKSQDRKSASREKR
SVVSFDKVKEPRKSRDSESHSRVRERSEREQRMQAQWEREERERLEIARERLAFQRQRLE
RERMERERLERERMHVEHERRREQERIHREREELRRQQELRYEQERRPAVRRPYDLDRRD
DAYWPEAKRAALDERYHSDFNRQDRFHDFDHRDRGRYPDHSVDRREGSRSMMGEREGQHY
PERHGGPERHGRDSRDGWGGYGSDKRMSEGRGLPPPPRRDWGDHGRREDDRSWQGTADGG
MMDRDHKRWQGGERSMSGHSGPGHMMNRGGMSGRGSFAPGGASRGHPIPHGGMQGGFGGQ
SRGSRPSDARFTRRY*
>ENSG00000172172|ENST00000306185
MSSFSRAPQQWATFARIWYLLDGKMQPPGKLAAMASIRLQGLHKPVYHALSDCGDHVVIM
NTRHIAFSGNKWEQKVYSSHTGYPGGFRQVTAAQLHLRDPVAIVKLAIYGMLPKNLHRRT
MMERLHLFPDEYIPEDILKNLVEELPQPRKIPKRLDEYTQEEIDAFPRLWTPPEDYRL*
>ENSG00000188612|ENST00000314523
MADEKPKEGVKTENNDHINLKVAGQDGSVVQFKIKRHTPLSKLMKAYCERQLEMEDEDTI
DVFQQQTGGVY*
>ENSG00000164032|ENST00000296417
MAGGKAGKDSGKAKTKAVSRSQRAGLQFPVGRIHRHLKSRTTSHGRVGATAAVYSAAILE
YLTAEVLELAGNASKDLKVKRITPRHLQLAIRGDEELDSLIKATIAGGGVIPHIHKSLIG
KKGQQKTV*
>ENSG00000140948|ENST00000268616
MASNHPAFSFHQKQVLRQELTQIQSSLNGGGGHGGKGAPGPGGALPTCPACHKITPRTEA
PVSSSVSNSLENALHTSAHSTEESLPKRPLGKHSKVSVEKIDLKGLSHTKNDRNVECSFEV
LWSDSSITSVTKSSSEVTEFISKLCQLYPEENLEKLIPCLAGPDAFYVERNHVDLDSGLR
YLASLPSHVLKNDHVRRFLSTSSPPQQLQSPSPGNPSLSKVGTVMGVSGRPVCGVAGIPS
SQSGAQHHGQHPAGSAAPLPHCSHAGSAGSALAYRTQMDTSPAILMPSSLQTPQTQEQNG

ILDWLRKLRLHKYYPVFKQLSMEKFLSLTEEDLNKFESLTMGAKKKLKTQLELEKEKSER
RCLNPSAPPLVTSSGVARVPPTSHVGPVQSGRGSHAAELRVEVEQPHHQLPREGSSSEYS
SSSSSPMGVQAREESSDSAEENDRRVEIHLESSDKEKPVMLLNHFTSSSARPTAQVLPVQ
NEASSNPSGHHPLPPQMLSAASHITPIRMLNSVHKPERGSADMKLLSSSVHSLLSLEERN
KGSGPRSSMKVDKSFGSAMMDVLPASAPHQPVQVLSGLSESSSMSPTVSFGPRTKVVHAS
TLDRVLKTAQQPALVVETSTAATGTPSTVLHAARPPIKLLLSSSVPADSAISGQTSCPNN
VQISVPPAIINPRTALYTANTKVAFSAMSSMPVGPLQGGFCANSNTASPSSHPSTSFANM
ATLPSCPAPSSSPALSSVPESSFYSSSGGGGSTGNIPASNPNHHHHHHHQQPPAPPQPAP
PPPGCIVCTSCGCSGSCGSSGLTVSYANYFQHPFSGPSVFTFPFLPFSPMCSSGYVSAQQ
YGGGSTFPVVHAPYSSSGTPDPVLSGQSTFAVPPMQNFMAGTAGVYQTQGLVGSSNGSSH
KKSGNLSCYNCGATGHRAQDCKQPSMDFNRPGTFRLKYAPPAESLDSTD*
>ENSG00000173914|ENST00000310046
MVKLFIGNLPREATEQEIRSLFEQYGKVLECDIIKNYGFVHIEDKTAAEDAIRNLHHYKL
HGVNINVEASKNKSKASTKLHVGNISPTCTNQELRAKFEEYGPVIECDIVKDYAFVHMER
AEDAVEAIRGLDNTEFQGKRMHVQLSTSRLRTAPGMGDQSGCYRCGKEGHWSKECPVDRT
GRVADFTEQYNEQYGAVRTPYTMGYGESMYYNDAYGALDYYKRYRVRSYEAVAAAAAASA
YNYAEQTMSHLPQVQSTTVTSHLNSTSVDPYDRHLLPNSGAAATSAAMAAAAATTSSYYG
RDRSPLRRAAAMLPTVGEGYGYGPESELSQASAATRNSLYDMARYEREQYVDRARYSAF*
>ENSG00000160201|ENST00000291552
MAEYLASIFGTEKDKVNCSFYFKIGACRHGDRCSRLHNKPTFSQTIALLNIYRNPQNSSQ
SADGLRCAVSDVEMQEHYDEFFEEVFTEMEEKYGEVEEMNVCDNLGDHLVGNVYVKFRRE
EDAEKAVIDLNNRWFNGQPIHAELSPVTDFREACCRQYEMGECTRGGFCNFMHLKPISRE
LRRELYGRRRKKHRSRSRSRERRSRSRDRGRGGGGGGGGGGGGRERDRRRSRDRERSGRF
*
>ENSG00000146618|ENST00000275461
MAAYPESCVDTTVLDFVADLSLASPRRPLLCDFAPGVSLGDPALALREGRPRRMARFEEG
DPEEEECEVDQGDGEEEEEEERGRGVSLLGRPKRKRVITYAQRQAANIRERKRMFNLNEA
FDQLRRKVPTFAYEKRLSRIETLRLAIVYISFMTELLESCEKKESG*
>ENSG00000170892|ENST00000302937
MLVVEVANGRSLVWGAEAVQALRERLGVGGRTVGALPRGPRQNSRLGLPLLLMPEEARLL
AEIGAVTLVSAPRPDSRHHSLALTSFKRQQEESFQEQSALAAEARETRRQELLEKITEGQ
AAKKQKLEQASGASSSQEAGSSQAAKEDETSDGQASGEQEEAGPSSSQAGPSNGVAPLPR
SALLVQLATARPRPVKARPLDWRVQSKDWPHAGRPAHELRYSIYRDLWERGFFLSAAGKF
GGDFLVYPGDPLRFHAHYIAQCWAPEDTIPLQDLVAAGRLGTSVRKTLLLCSPQPDGKVV
YTSLQWASLQ*
>ENSG00000115041|ENST00000295225
MQPAKEVTKASDGSLLGDLGHTPLSKKEGIKWQRPRLSRQALMRCCLVKWILSSTAPQGS
DSSDSELELSTVRHQPEGLDQLQAQTKFTKKELQSLYRGFKNECPTGLVDEDTFKLIYAQ
FFPQGDATTYAHFLFNAFDADGNGAIHFEDFVVGLSILLRGTVHEKLKWAFNLYDINKDG
YITKEEMLAIMKSIYDMMGRHTYPILREDAPAEHVERFFEKMDRNQDGVVTIEEFLEACQ
KDENIMSSMQLFENVI*
>ENSG00000165494|ENST00000298281
MSEQTPAEAGAAGAREDACRDYQSSLEDLTFNSKPHINMLTILAEENLPFAKEIVSLIEA
QTAKAPSSEKLPVMYLMDSIVKNVGREYLTAFTKNLVATFICVFEKVDENTRKSLFKLRS

TWDEIFPLKKLYALDVRVNSLDPAWPIKPLPPNVNTSSIHVNPKFLNKSPEEPSTPGTVV
SSPSISTPPIVPDIQKNLTQEQLIRQQLLAKQKQLLELQQKKLELELEQAKAQLAVSLSV
QQETSNLGPGSAPSKLHVSQIPPMAVKAPHQVPVQSEKSRPGPSLQIQDLKGTNRDPRLN
RISQHSHGKDQSHRKEFLMNTLNQSDTKTSKTIPSEKLNSSKQEKSKSGEKITKKELDQL
DSKSKSKSKSPSPLKNKLSHTKDLKNQESESMRLSDMNKRDPRLKKHLQDKTDGKDDDVK
EKRKTAEKKDKDEHMKSSEHRLAGSRNKIINGIVQKQDTITEESEKQGTKPGRSSTRKRS
RSRSPKSRSPIIHSPKRRDRRSPKRRQRSMSPTSTPKAGKIRQSGAKQSHMEEFTPPSRE
DRNAKRSTKQDIRDPRRMKKTEEERPQETTNQHSTKSGTEPKENVENWQSSKSAKRWKSG
WEENKSLQQVDEHSKPPHLRHRESWSSTKGILSPRAPKQQQHRLSVDANLQIPKELTLAS
KRELLQKTSERLASGEITQDDFLVVVHQIRQLFQYQEGVREEQRSPFNDRFPLKRPRYED
SDKPFVDSPASRFAGLDTNQRLTALAEDRPLFDGPSRPSVARDGPTKMIFEGPNKLSPRI
DGPPTPASLRFDGSPGQMGGGGPLRFEGPQGQLGGGCPLRFEGPPGPVGTPLRFEGPIGQ
AGGGGFRFEGSPGLRFEGSPGGLRFEGPGGQPVGGLRFEGHRGQPVGGLRFEGPHGQPVG
GLRFDNPRGQPVGGLRFEGGHGPSGAAIRFDGPHGQPGGGIRFEGPLLQQGVGMRFEGPH
GQSVAGLRFEGQHNQLGGNLRFEGPHGQPGVGIRFEGPLVQQGGGMRFEGPSVPGGGLRI
EGPLGQGGPRFEGCHALRFDGQPGQPSLLPRFDGLHGQPGPRFERTPGQPGPQRFDGPPG
QQVQPRFDGVPQRFDGPQHQQASRFDIPLGLQGTRFDNHPSQRLESVSFNQTGPYNDPPG
NAFNAPSQGLQFQRHEQIFDSPQGPNFNGPHGPGNQSFSNPLNRASGHYFDEKNLQSSQF
GNFGNIPAPMTVGNIQASQQVLSGVAQPVAFGQGQQFLPVHPQNPGFVQNPSGALPKAYP
DNHLSQVDVNELFSKLLKTGILKLSQTDSATTQVSEVTAQPPPEEEEDQNEDQDVPDLTN
FTVEELKQRYDSVINRLYTGIQCYSCGMRFTTSQTDVYADHLDWHYRQNRTEKDVSRKVT
HRRWYYSLTDWIEFEEIADLEERAKSQFFEKVHEEVVLKTQEAAKEKEFQSVPAGPAGAV
ESCEICQEQFEQYWDEEEEEWHLKNAIRVDGKIYHPSCYEDYQNTSSFDCTPSPSKTPVE
NPLNIMLNIVKNELQEPCDSPKVKEERIDTPPACTEESIATPSEIKTENDTVESV*
>ENSG00000139613|ENST00000267064
MAVRKKDGGPNVKYYEAADTVTQFDNVRLWLGKNYKKYIQAEPPTNKSLSSLVVQLLQFQ
EEVFGKHVSNAPLTKLPIKCFLDFKAGGSLCHILAAAYKFKSDQGWRRYDFQNPSRMDRN
VEMFMTIEKSLVQNNCLSRPNIFLCPEIEPKLLGKLKDIIKRHQGTVTEDKNNASHVVYP
VPGNLEEEEWVRPVMKRDKQVLLHWGYYPDSYDTWIPASEIEASVEDAPTPEKPRKVHAK
WILDTDTFNEWMNEEDYEVNDDKNPVSRRKKISAKTLTDEVNSPDSDRRDKKGGNYKKRK
RSPSPSPTPEAKKKNAKKGPSTPYTKSKRGHREEEQEDLTKDMDEPSPVPNVEEVTLPKT
VNTKKDSESAPVKGGTMTDLDEQEDESMETTGKDEDENSTGNKGEQTKNPDLHEDNVTEQ
THHIIIPSYAAWFDYNSVHAIERRALPEFFNGKNKSKTPEIYLAYRNFMIDTYRLNPQEY
LTSTACRRNLAGDVCAIMRVHAFLEQWGLINYQVDAESRPTPMGPPPTSHFHVLADTPSG
LVPLQPKTPQQTSASQQMLNFPDKGKEKPTDMQNFGLRTDMYTKKNVPSKSKAAASATRE
WTEQETLLLLEALEMYKDDWNKVSEHVGSRTQDECILHFLRLPIEDPYLEDSEASLGPLA
YQPIPFSQSGNPVMSTVAFLASVVDPRVASAAAKSALEEFSKMKEEVPTALVEAHVRKVE
EAAKVTGKADPAFGLESSGIAGTTSDEPERIEESGNDEARVEGQATDEKKEPKEPREGGG
AIEEEAKEKTSEAPKKDEEKGKEGDSEKESEKSDGDPIVDPEKEKEPKEGQEEVLKEVVE
SEGERKTKVERDIGEGNLSTAAAAALAAAAVKAKHLAAVEERKIKSLVALLVETQMKKLE
IKLRHFEELETIMDREREALEYQRQQLLADRQAFHMEQLKYAEMRARQQHFQQMHQQQQQ
PPPALPPGSQPIPPTGAAGPPAVHGLAVAPASVVPAPAGSGAPPGSLGPSEQIGQAGSTA
GPQQQQPAGAPQPGAVPPGVPPPGPHGPSPFPNQQTPPSMMPGAVPGSGHPGVAGNAPLG
LPFGMPPPPPPPAPSIIPFGSLADSISINLPAPPNLHGHHHHLPFAPGTLPPPNLPVSMA

NPLHPNLPATTTMPSSLPLGPGLGSAAAQSPAIVAAVQGNLLPSASPLPDPGTPLPPDPT
APSPGTVTPVPPPQ*
>ENSG00000141579|ENST00000269394
MSLLKERKPKKPHYIPRPPGKPFKYKCFQCPFTCNEKSHLFNHMKYGLCKNSITLVSEQD
RVPKCPKSNSLDPKQTNQPDATAKPASSKSVANGLSAFDSKLQHSSAREDIKENLELQAR
GTHRCLGQKPALHRASPCKSPAPEAALGAQPALEGAARPSAFVPVGEHRLKGPDNAEAPE
TLALHNPTAKAVSFHTKSAFHTPGYPWKAGSPFLPPEFPHKISSTKGLGAISPYMHPTIP
EYPPHFYTEHGLATIYSPYLLAGSSPECDAPLLSVYGTQDPRHFLPHPGPIPKHLAPSPA
TYDHYRFFQQYPSNLPIPYGFYRPESAFSSYGLRLPPVTGLTRDQSSHLLEEATLVYPAS
SPSRLNPSDPNRKHVEFESPIPEAKDSSKAGQRDTEGSKMSPRAGSAATGSPGRPSPTDF
MQTSQTCEGLYDLSNKAASSALGRLYPPEQSLTAFRPVKKSTECLPAQAAETTAESPVSL
NVVNGDPPAPTGSASLVSEAAPSSPDDSSGMGPLNLSKKSEINLAATHEPTYQGSPQAET
ASFSELQDLPLNLSVKDPCNTQAPRPAFPGRPRAAEPAAAVPQKTGTEGSEDGPSHPETK
PGSLDGDGAPPTGPGEEAPDACAVDSSEEQKQTAAVALCQLAAYSPRNIRVGDGDAAAPE
PACRQDTPTLSSMESQEAQCDLRPKGQKRTSLRDAGKSQQGAKKAKLQDTARVFTLRRRA
RVS*
>ENSG00000156853|ENST00000287461
MAPPSAPLPAQGPGKARPSRKRGRRPRALKFVDVAVYFSPEEWGCLRPAQRALYRDVMRE
TYGHLGALGCAGPKPALISWLERNTDDWEPAALDPQEYPRGLTVQRKSRTRKKNGEKEVF
PPKEAPRKGKRGRRPSKPRLIPRQTSGGPICPDCGCTFPDHQALESHKCAQNLKKPYPCP
DCGRRFSYPSLLVSHRRAHSGECPYVCDQCGKRFSQRKNLSQHQVIHTGEKPYHCPDCGR
CFRRSRSLANHRTTHTGEKPHQCPSCGRRFAYPSLLAIHQRTHTGEKPYTCLECNRRFRQ
RTALVIHQRIHTGEKPYPCPDCERRFSSSSRLVSHRRVHSGERPYACEHCEARFSQRSTL
LQHQLLHTGEKPYPCPDCGRAFRRSGSLAIHRSTHTEEKLHACDDCGRRFAYPSLLASHR
RVHSGERPYACDLCSKRFAQWSHLAQHQLLHTGEKPFPCLECGRCFRQRWSLAVHKCSPK
APNCSPRSAIGGSSQRGNAH*
>ENSG00000119718|ENST00000266126
MPGSAAKGSELSERIESFVETLKRGGGPRSSEEMARETLGLLRQIITDHRWSNAGELMEL
IRREGRRMTAAQPSETTVGNMVRRVLKIIREEYGRLHGRSDESDQQESLHKLLTSGGLNE
DFSFHYAQLQSNIIEAINELLVELEGTMENIAAQALEHIHSNEVIMTIGFSRTVEAFLKE
AARKRKFHVIVAECAPFCQGHEMAVNLSKAGIETTVMTDAAIFAVMSRVNKVIIGTKTIL
ANGALRAVTGTHTLALAAKHHSTPLIVCAPMFKLSPQFPNEEDSFHKFVAPEEVLPFTEG
DILEKVSVHCPVFDYVPPELITLFISNIGGNAPSYIYRLMSELYHPDDHVL*
>ENSG00000165462|ENST00000298231
MDYSYLNSYDSCVAAMEASAYGDFGACSQPGGFQYSPLRPAFPAAGPPCPALGSSNCALG
ALRDHQPAPYSAVPYKFFPEPSGLHEKRKQRRIRTTFTSAQLKELERVFAETHYPDIYTR
EELALKIDLTEARVQVWFQNRRAKFRKQERAASAKGAAGAAGAKKGEARCSSEDDDSKES
TCSPTPDSTASLPPPPAPGLASPRLSPSPLPVALGSGPGPGPGPQPLKGALWAGVAGGGG
GGPGAGAAELLKAWQPAESGPGPFSGVLSSFHRKPGPALKTNLF*
>ENSG00000196867|ENST00000301318
MRGAASASVREPTPLPGRGAPRTKPRAGRGPTVGTPATLALPARGRPRSRNGLASKGQRG
AAPTGPGHRALPSRDTALPQERNKKLEAVGTGIEPKAMSQGLVTFGDVAVDFSQEEWEWL
NPIQRNLYRKVMLENYRNLASLGLCVSKPDVISSLEQGKEPWTVKRKMTRAWCPDLKAVW
KIKELPLKKDFCEGKLSQAVITERLTSYNLEYSLLGEHWDYDALFETQPGLVTIKNLAVD

FRQQLHPAQKNFCKNGIWENNSDLGSAGHCVAKPDLVSLLEQEKEPWMVKRELTGSLFSG
QRSVHETQELFPKQDSYAEGVTDRTSNTKLDCSSFRENWDSDYVFGRKLAVGQETQFRQE
PITHNKTLSKERERTYNKSGRWFYLDDSEEKVHNRDSIKNFQKSSVVIKQTGIYAGKKLF
KCNECKKTFTQSSSLTVHQRIHTGEKPYKCNECGKAFSDGSSFARHQRCHTGKKPYECIE
CGKAFIQNTSLIRHWRYYHTGEKPFDCIDCGKAFSDHIGLNQHRRIHTGEKPYKCDVCHK
SFRYGSSLTVHQRIHTGEKPYECDVCRKAFSHHASLTQHQRVHSGEKPFKCKECGKAFRQ
NIHLASHLRIHTGEKPFECAECGKSFSISSQLATHQRIHTGEKPYECKVCSKAFTQKAHL
AQHQKTHTGEKPYECKECGKAFSQTTHLIQHQRVHTGEKPYKCMECGKAFGDNSSCTQHQ
RLHTGQRPYECIECGKAFKTKSSLICHRRSHTGEKPYECSVCGKAFSHRQSLSVHQRIHS
GKKPYECKECRKTFIQIGHLNQHKRVHTGERSYNYKKSRKVFRQTAHLAHHQRIHTGESS
TCPSLPSTSNPVDLFPKFLWNPSSLPSP*
>ENSG00000171466|ENST00000293648
MSAFDMSHDFFFCLTSEWEIQPRTKRSSLQQGFLKNQIFTGIQMQTRSYSGWKLCENCGE
VFSEQFCLKTHMRAQNGGNTFEGNCYGKDSISVHKEASIGQELSKFNPCGKVFTLTPGLA
VHLEILNGRQPYKCKECGKGFKYFASLDNHMGIHIGEKLCEFQECERAITTSSHLKQCVA
VHTGKKSEKTKNCGKSFTNFSQLSAHAKTHKGEKSFECKECGRSFRNSSSFNVHIQIHTG
IKPHKCTECGKAFTRSTHLTQHVRTHTGIKPYECKECGQAFTQYTGLAIHIRNHTGEKPY
QCKECGKAFNRSSTLTQHRRIHTGEKPYECVECGKTFITSSHRSKHLKTHSGER*
>ENSG00000186496|ENST00000306346
MSAKLGKSSSLLTQTSEECNGILTEKMEEEEQTCDPDSSLHWSSSYSPETFRQQFRQFGY
QDSPGPHEALSRLWELCHLWLRPEVHTKEQILELLVLEQFLAILPKELQAWVQKHHPENG
EETVTMLEDVERELDGPKQIFFGRRKDMIAEKLAPSEITEELPSSQLMPVKKQLQGASWE
LQSLRPHDEDIKTTNVKSASRQKTSLGIELHCNVSNILHMNGSQSSTYRGTYEQDGRFEK
RQGNPSWKKQQKCDECGKIFSQSSALILHQRIHSGKKPYACDECAKAFSRSAILIQHRRT
HTDSKYEHAIAEAQKNMYFKTRLKCPRSLSGGR*
>ENSG00000144048|ENST00000272444
MRNSETLERGVGGCRVFSCLGSYPGIEGAGLALLADLALGGRLLGTHMSQWHHPRSGWGR
RRDFSGRSSAKKKGGNHIPERWKDYLPVGQRMPGTRFIAFKVPLQKSFEKKLAPEECFSP
LDLFNKIREQNEELGLIIDLTYTQRYYKPEDLPETVPYLKIFTVGHQVPDDETIFKFKHA
VNGFLKENKDNDKLIGVHCTHGLNRTGYLICRYLIDVEGVRPDDAIELFNRCRGHCLERQ
NYIEDLQNGPIRKNWNSSVPRSSDFEDSAHLMQPVHNKPVKQGPRYNLHQIQGHSAPRHF
HTQTQSLQQSVRKFSENPHVYQRHHLPPPGPPGEDYSHRRYSWNVKPNASRAAQDRRRWY
PYNYSRLSYPACWEWTQ*
>ENSG00000125945|ENST00000314011
MAATLLMAGSQAPVTFEDMAMYLTREEWRPLDAAQRDLYRDVMQENYGNVVSLDFEIRSE
NEVNPKQEISEDVQFGTTSERPAENAEENPESEEGFESGDRSERQWGDLTAEEWVSYPLQ
PVTDLLVHKEVHTGIRYHICSHCGKAFSQISDLNRHQKTHTGDRPYKCYECGKGFSRSSH
LIQHQRTHTGERPYDCNECGKSFGRSSHLIQHQTIHTGEKPHKCNECGKSFCRLSHLIQH
QRTHSGEKPYECEECGKSFSRSSHLAQHQRTHTGEKPYECNECGRGFSERSDLIKHYRVH
TGERPYKCDECGKNFSQNSDLVRHRRAHTGEKPYHCNECGENFSRISHLVQHQRTHTGEK
PYECNACGKSFSRSSHLITHQKIHTGEKPYECNECWRSFGERSDLIKHQRTHTGEKPYEC
VQCGKGFTQSSNLITHQRVHTGEKPYECTECEKSFSRSSALIKHKRVHTD*
>ENSG00000187098|ENST00000314557
MLEMLEYNHYQVQTHLENPTKYHIQQAQRQQVKQYLSTTLANKHANQVLSLPCPNQPGDH

VMPPVPGSSAPNSPMAMLTLNSNCEKEGFYKFEEQNRAESECPGMNTHSRASCMQMDDVI
DDIISLESSYNEEILGLMDPALQMANTLPVSGNLIDLYGNQGLPPPGLTISNSCPANLPN
IKRELTESEARALAKERQKKDNHNLIERRRRFNINDRIKELGTLIPKSNDPDMRWNKGTI
LKASVDYIRKLQREQQRAKELENRQKKLEHANRHLLLRIQELEMQARAHGLSLIPSTGLC
SPDLVNRIIKQEPVLENCSQDLLQHHADLTCTTTLDLTDGTITFNNNLGTGTEANQAYSV
PTKMGSKLEDILMDDTLSPVGVTDPLLSSVSPGASKTSSRRSSMSMEETEHTC*
>ENSG00000109819|ENST00000264867
MAWDMCNQDSESVWSDIECAALVGEDQPLCPDLPELDLSELDVNDLDTDSFLGGLKWCSD
QSEIISNQYNNEPSNIFEKIDEENEANLLAVLTETLDSLPVDEDGLPSFDALTDGDVTTD
NEASPSSMPDGTPPPQEAEEPSLLKKLLLAPANTQLSYNECSGLSTQNHANHNHRIRTNP
AIVKTENSWSNKAKSICQQQKPQRRPCSELLKYLTTNDDPPHTKPTENRNSSRDKCTSKK
KSHTQSQSQHLQAKPTTLSLPLTPESPNDPKGSPFENKTIERTLSVELSGTAGLTPPTTP
PHKANQDNPFRASPKLKSSCKTVVPPPSKKPRYSESSGTQGNNSTKKGPEQSELYAQLSK
SSVLTGGHEERKTKRPSLRLFGDHDYCQSINSKTEILINISQELQDSRQLENKDVSSDWQ
GQICSSTDSDQCYLRETLEASKQVSPCSTRKQLQDQEIRAELNKHFGHPSQAVFDDEADK
TGELRDSDFSNEQFSKLPMFINSGLAMDGLFDDSEDESDKLSYPWDGTQSYSLFNVSPSC
SSFNSPCRDSVSPPKSLFSQRPQRMRSRSRSFSRHRSCSRSPYSRSRSRSPGSRSSSRSC
YYYESSHYRHRTHRNSPLYVRSRSRSPYSRRPRYDSYEEYQHERLKREEYRREYEKRESE
RAKQRERQRQKAIEERRVIYVGKIRPDTTRTELRDRFEVFGEIEECTVNLRDDGDSYGFI
TYRYTCDAFAALENGYTLRRSNETDFELYFCGRKQFFKSNYADLDSNSDDFDPASTKSKY
DSLDFDSLLKEAQRSLRR*
>ENSG00000169689|ENST00000306704
MEGAGAGSGFRKELVSRLLHLHFKDDKTKEAAVRGVRQAQAEDALRVDVDQLEKVLPQLL
LDF*
>ENSG00000124459|ENST00000269973
MTKSKEAVTFKDVAVVFSEEELQLLDLAQRKLYRDVMLENFRNVVSVGHQSTPDGLPQLE
REEKLWMMKMATQRDNSSGAKNLKEMETLQEVGLRYLPHEELFCSQIWQQITRELIKYQD
SVVNIQRTGCQLEKRDDLHYKDEGFSNQSSHLQVHRVHTGEKPYKGEHCVKSFSWSSHLQ
INQRAHAGEKPYKCEKCDNAFRRFSSLQAHQRVHSRAKSYTNDASYRSFSQRSHLPHHQR
VPTGENPYKYEECGRNVGKSSHCQAPLIVHTGEKPYKCEECGVGFSQRSYLQVHLKVHTG
KKPYKCEECGKSFSWRSRLQAHERIHTGEKPYKCNACGKSFSYSSHLNIHCRIHTGEKPY
KCEECGKGFSVGSHLQAHQISHTGEKPYKCEECGKGFCRASNLLDHQRGHTGEKPYQCDA
CGKGFSRSSDFNIHFRVHTGEKPYKCEECGKGFSQASNLLAHQRGHTGEKPYKCGTCGKG
FSRSSDLNVHCRIHTGEKPYKCERCGKAFSQFSSLQVHQRVHTGEKPYQCAECGKGFSVG
SQLQAHQRCHTGEKPYQCEECGKGFCRASNFLAHRGVHTGEKPYRCDVCGKRFRQRSYLQ
AHQRVHTGERPYKCEECGKVFSWSSYLQAHQRVHTGEKPYKCEECGKGFSWSSSLIIHQR
VHADDEGDKDFPSSEDSHRKTR*
>ENSG00000168028|ENST00000301821
MSGALDVLQMKEEDVLKFLAAGTHLGGTNLDFQMEQYIYKRKSDGIYIINLKRTWEKLLL
AARAIVAIENPADVSVISSRNTGQRAVLKFAAATGATPIAGRFTPGTFTNQIQAAFREPR
LLVVTDPRADHQPLTEASYVNLPTIALCNTDSPLRYVDIAIPCNNKGAHSVGLMWWMLAR
EVLRMRGTISREHPWEVMPDLYFYRDPEEIEKEEQAAAEKAVTKEEFQGEWTAPAPEFTA
TQPEVADWSEGVQVPSVPIQQFPTEDWSAQPATEDWSAAPTAQATEWVGATTDWS*
>ENSG00000163508|ENST00000295743

MQLGEQLLVSSVNLPGAHFYPLESARGGSGGSAGHLPSAAPSPQKLDLDKASKKFSGSLS
CEAVSGEPAAASAGAPAAMLSDTDAGDAFASAAAVAKPGPPDGRKGSPCGEEELPSAAAA
AAAAAAAAAATARYSMDSLSSERYYLQSPGPQGSELAAPCSLFPYQAAAGAPHGPVYPAP
NGARYPYGSMLPPGGFPAAVCPPGRAQFGPGAGAGSGAGGSSGGGGGPGTYQYSQGAPLY
GPYPGAAAAGSCGGLGGLGVPGSGFRAHVYLCNRPLWLKFHRHQTEMIITKQGRRMFPFL
SFNINGLNPTAHYNVFVEVVLADPNHWRFQGGKWVTCGKADNNMQGNKMYVHPESPNTGS
HWMRQEISFGKLKLTNNKGANNNNTQMIVLQSLHKYQPRLHIVEVTEDGVEDLNEPSKTQ
TFTFSETQFIAVTAYQNTDITQLKIDHNPFAKGFRDNYDSSHQIVPGGRYGVQSFFPEPF
VNTLPQARYYNGERTVPQTNGLLSPQQSEEVANPPQRWLVTPVQQPGTNKLDISSYESEY
TSSTLLPYGIKSLPLQTSHALGYYPDPTFPAMAGWGGRGSYQRKMAAGLPWTSRTSPTVF
SEDQLSKEKVKEEIGSSWIETPPSIKSLDSNDSGVYTSACKRRRLSPSNSSNENSPSIKC
EDINAEEYSKDTSKGMGGYYAFYTTP*
>ENSG00000164920|ENST00000297565
MGSKALPAPIPLHPSLQLTNYSFLQAVNTFPATVDHLQGLYGLSAVQTMHMNHWTLGYPN
VHEITRSTITEMAAAQGLVDARFPFPALPFTTHLFHPKQGAIAHVLPALHKDRPRFDFAN
LAVAATQEDPPKMGDLSKLSPGLGSPISGLSKLTPDRKPSRGRLPSKTKKEFICKFCGRH
FTKSYNLLIHERTHTDERPYTCDICHKAFRRQDHLRDHRYIHSKEKPFKCQECGKGFCQS
RTLAVHKTLHMQESPHKCPTCGRTFNQRSNLKTHLLTHTDIKPYSCEQCGKVFRRNCDLR
RHSLTHTPRQDF*
>ENSG00000151615|ENST00000281321
MMMMSLNSKQAFSMPHGGSLHVEPKYSALHSTSPGSSAPIAPSASSPSSSSNAGGGGGGG
GGGGGGGGRSSSSSSSGSSGGGGSEAMRRACLPTPPSNIFGGLDESLLARAEALAAVDIV
SQSKSHHHHPPHHSPFKPDATYHTMNTIPCTSAASSSSVPISHPSALAGTHHHHHHHHHH
HHQPHQALEGELLEHLSPGLALGAMAGPDGAVVSTPAHAPHMATMNPMHQAALSMAHAHG
LPSHMGCMSDVDADPRDLEAFAERFKQRRIKLGVTQADVGSALANLKIPGVGSLSQSTIC
RFESLTLSHNNMIALKPILQAWLEEAEKSHREKLTKPELFNGAEKKRKRTSIAAPEKRSL
EAYFAIQPRPSSEKIAAIAEKLDLKKNVVRVWFCNQRQKQKRMKYSAGI*
>ENSG00000143977|ENST00000272348
MSKAHPPELKKFMDKKLSLKLNGGRHVQGILRGFDPFMNLVIDECVEMATSGQQNNIGMV
VIRGNSIIMLEALERV*
>ENSG00000162924|ENST00000295025
MASGAYNPYIEIIEQPRQRGMRFRYKCEGRSAGSIPGEHSTDNNRTYPSIQIMNYYGKGK
VRITLVTKNDPYKPHPHDLVGKDCRDGYYEAEFGQERRPLFFQNLGIRCVKKKEVKEAII
TRIKAGINPFNVPEKQLNDIEDCDLNVVRLCFQVFLPDEHGNLTTALPPVVSNPIYDNRA
PNTAELRICRVNKNCGSVRGGDEIFLLCDKVQKDDIEVRFVLNDWEAKGIFSQADVHRQV
AIVFKTPPYCKAITEPVTVKMQLRRPSDQEVSESMDFRYLPDEKDTYGNKAKKQKTTLLF
QKLCQDHVETGFRHVDQDGLELLTSGDPPTLASQSAGITVNFPERPRPGLLGSIGEGRYF
KKEPNLFSHDAVVREMPTGVSSQAESYYPSPGPISSGLSHHASMAPLPSSSWSSVAHPTP
RSGNTNPLSSFSTRTLPSNSQGIPPFLRIPVGNDLNASNACIYNNADDIVGMEASSMPSA
DLYGISDPNMLSNCSVNMMTTSSDSMGETDNPRLLSMNLENPSCNSVLDPRDLRQLHQMS
SSSMSAGANSNTTVFVSQSDAFEGSDFSCADNSMINESGPSNSTNPNSHGFVQDSQYSGI
GSMQNEQLSDSFPYEFFQV*
>ENSG00000169385|ENST00000304625
MVPKLFTSQICLLLLLGLLAVEGSLHVKPPQFTWAQWFETQHINMTSQQCTNAMQVINNY

QRRCKNQNTFLLTTFANVVNVCGNPNMTCPSNKTRKNCHHSGSQVPLIHCNLTTPSPQNI
SNCRYAQTPANMFYIVACDNRDQRRDPPQYPVVPVHLDRII*
>ENSG00000241945|ENST00000291576
MKFAYRFSNLLGTVYRRGNLNFTCDGNSVISPVGNRVTVFDLKNNKSDTLPLATRYNVKC
VGLSPDGRLAIIVDEGGDALLVSLVCRSVLHHFHFKGSVHSVSFSPDGRKFVVTKGNIAQ
MYHAPGKKREFNAFVLDKTYFGPYDETTCIDWTDDSRCFVVGSKDMSTWVFGAERWDNLI
YYALGGHKDAIVACFFESNSLDLYSLSQDGVLCMWQCDTPPEGLRLKPPAGWKADLLQRE
EEEEEEEDQEGDRETTIRGKATPAEEEKTGKVKYSRLAKYFFNKEGDFNNLTAAAFHKKS
HLLVTGFASGIFHLHELPEFNLIHSLSISDQSIASVAINSSGDWIAFGCSGLGQLLVWEW
QSESYVLKQQGHFNSMVALAYSPDGQYIVTGGDDGKVKVWNTLSGFCFVTFTEHSSGVTG
VTFTATGYVVVTSSMDGTVRAFDLHRYRNFRTFTSPRPTQFSCVAVDASGEIVSAGAQDS
FEIFVWSMQTGRLLDVLSGHEGPISGLCFNPMKSVLASASWDKTVRLWDMFDSWRTKETL
ALTSDALAVTFRPDGAELAVATLNSQITFWDPENAVQTGSIEGRHDLKTGRKELDKITAK
HAAKGKAFTALCYSADGHSILAGGMSKFVCIYHVREQILMKRFEISCNLSLDAMEEFLNR
RKMTEFGNLALIDQDAGQEDGVAIPLPGVRKGDMSSRHFKPEIRVTSLRFSPTGRCWAAT
TTEGLLIYSLDTRVLFDPFELDTSVTPGRVREALRQQDFTRAILMALRLNESKLVQEALE
AVPRGEIEVVTSSLPELYVEKVLEFLASSFEVSRHLEFYLLWTHKLLMLHGQKLKSRAGT
LLPVIQFLQKSIQRHLDDLSKLCSWNHYNMQYALAVSKQRGTKRSLDPLGSEEEAEASED
DSLHLLGGGGRDSEEEMLA*
>ENSG00000123064|ENST00000314045
MAADKGPAAGPRSRAAMAQWRKKKGLRKRRGAASQARGSDSEDGEFEIQAEDDARARKLG
PGRPLPTFPTSECTSDVEPDTREMVRAQNKKKKKSGGFQSMGLSYPVFKGIMKKGYKVPT
PIQRKTIPVILDGKDVVAMARTGSGKTACFLLPMFERLKTHSAQTGARALILSPTRELAL
QTLKFTKELGKFTGLKTALILGGDRMEDQFAALHENPDIIIATPGRLVHVAVEMSLKLQS
VEYVVFDEADRLFEMGFAEQLQEIIARLPGGHQTVLFSATLPKLLVEFARAGLTEPVLIR
LDVDTKLNEQLKTSFFLVREDTKAAVLLHLLHNVVRPQDQTVVFVATKHHAEYLTELLTT
QRVSCAHIYSALDPTARKINLAKFTLGKCSTLIVTDLAARGLDIPLLDNVINYSFPAKGK
LFLHRVGRVARAGRSGTAYSLVAPDEIPYLLDLHLFLGRSLTLARPLKEPSGVAGVDGML
GRVPQSVVDEEDSGLQSTLEASLELRGLARVADNAQQQYVRSRPAPSPESIKRAKEMDLV
GLGLHPLFSSRFEEEELQRLRLVDSIKNYRSRATIFEINASSRDLCSQVMRAKRQKDRKA
IARFQQGQQGRQEQQEGPVGPAPSRPALQEKQPEKEEEEEAGESVEDIFSEVVGRKRQRS
GPNRGAKRRREEARQRDQEFYIPYRPKDFDSERGLSISGEGGAFEQQAAGAVLDLMGDEA
QNLTRGRQQLKWDRKKKRFVGQSGQEDKKKIKTESGRYISSSYKRDLYQKWKQKQKIDDR
DSDEEGASDRRGPERRGGKRDRGQAGASRPHAPGTPAGRVRPELKTKQQILKQRRRAQKL
HFLQRGGLKQLSARNRRRVQELQQGAFGRGARSKKGKMRKRM*
>ENSG00000196967|ENST00000292841
MPANWTSPQKSSALAPEDHGSSYEGSVSFRDVAIDFSREEWRHLDPSQRNLYRDVMLETY
SHLLSVGYQVPEAEVVMLEQGKEPWALQGERPRQSCPGEKLWDHNQCRKILSYKQVSSQP
QKMYPGEKAYECAKFEKIFTQKSQLKVHLKVLAGEKLYVCIECGKAFVQKPEFIIHQKTH
MREKPFKCNECGKSFFQVSSLFRHQRIHTGEKLYECSQCGKGFSYNSDLSIHEKIHTGER
HHECTDCGKAFTQKSTLKMHQKIHTGERSYICIECGQAFIQKTHLIAHRRIHTGEKPYEC
SNCGKSFISKSQLQVHQRVHTRVKPYICTEYGKVFSNNSNLVTHKKVQSREKSSICTECG
KAFTYRSELIIHQRIHTGEKPYECSDCGKAFTQKSALTVHQRIHTGEKSYICMKCGLAFI
QKAHLIAHQIIHTGEKPHKCGHCGKLFTSKSQLHVHKRIHTGEKPYMCNKCGKAFTNRSN

LITHQKTHTGEKSYICSKCGKAFTQRSDLITHQRIHTGEKPYECNTCGKAFTQKSHLNIH
QKIHTGERQYECHECGKAFNQKSILIVHQKIHTGEKPYVCTECGRAFIRKSNFITHQRIH
TGEKPYECSDCGKSFTSKSQLLVHQPVHTGEKPYVCAECGKAFSGRSNLSKHQKTHTGEK
PYICSECGKTFRQKSELITHHRIHTGEKPYECSDCGKSFTKKSQLQVHQRIHTGEKPYVC
AECGKAFTDRSNLNKHQTTHTGDKPYKCGICGKGFVQKSVFSVHQSSHA*
>ENSG00000095564|ENST00000265990
MAVSRLDRLFILLDTGTTPVTRKAAAQQLGEVVKLHPHELNNLLSKVLIYLRSANWDTRI
AAGQAVEAIVKNVPEWNPVPRTRQEPTSESSMEDSPTTERLNFDRFDICRLLQHGASLLG
SAGAEFEVQDEKSGEVDPKERIARQRKLLQKKLGLNMGEAIGMSTEELFNDEDLDYTPTS
ASFVNKQPTLQAAELIDSEFRAGMSNRQKNKAKRMAKLFAKQRSRDAVETNEKSNDSTDG
EPEEKRRKIANVVINQSANDSKVLIDNIPDSSSLIEETNEWPLESFCEELCNDLFNPSWE
VRHGAGTGLREILKAHGKSGGKMGDSTLEEMIQQHQEWLEDLVIRLLCVFALDRFGDFVS
DEVVAPVRETCAQTLGVVLKHMNETGVHKTVDVLLKLLTQEQWEVRHGGLLGIKYALAVR
QDVINTLLPKVLTRIIEGLQDLDDDVRAVAAASLVPVVESLVYLQTQKVPFIINTLWDAL
LELDDLTASTNSIMTLLSSLLTYPQVQQCSIQQSLTVLVPRVWPFLHHTISSVRRAALET
LFTLLSTQDQNSSSWLIPILPDMLRHIFQFCVLESSQEILDLIHKVWMELLSKASVQYVV
AAACPWMGAWLCLMMQPSHLPIDLNMLLEVKARAKEKTGGKVRQGQSQNKEVLQEYIAGA
DTIMEDPATRDFVVMRARMMAAKLLGALCCCICDPGVNVVTQEIKPAESLGQLLLFHLNS
KSALQRISVALVICEWAALQKECKAVTLAVQPRLLDILSEHLYYDEIAVPFTRMQNECKQ
LISSLADVHIEVGNRVNNNVLTIDQASDLVTTVFNEATSSFDLNPQVLQQLDSKRQQVQM
TVTETNQEWQVLQLRVHTFAACAVVSLQQLPEKLNPIIKPLMETIKKEENTLVQNYAAQC
IAKLLQQCTTRTPCPNSKIIKNLCSSLCVDPYLTPCVTCPVPTQSGQENSKGSTSEKDGM
HHTVTKHRGIITLYRHQKAAFAITSRRGPTPKAVKAQIADLPAGSSGNILVELDEAQKPY
LVQRRGAEFALTTIVKHFGGEMAVKLPHLWDAMVGPLRNTIDINNFDGKSLLDKGDSPAQ
ELVNSLQVFETAAASMDSELHPLLVQHLPHLYMCLQYPSTAVRHMAARCVGVMSKIATME
TMNIFLEKVLPWLGAIDDSVKQEGAIEALACVMEQLDVGIVPYIVLLVVPVLGRMSDQTD
SVRFMATQCFATLIRLMPLEAGIPDPPNMSAELIQLKAKERHFLEQLLDGKKLENYKIPV
PINAELRKYQQDGVNWLAFLNKYKLHGILCDDMGLGKTLQSICILAGDHCHRAQEYARSK
LAECMPLPSLVVCPPTLTGHWVDEVGKFCSREYLNPLHYTGPPTERIRLQHQVKRHNLIV
ASYDVVRNDIDFFRNIKFNYCILDEGHVIKNGKTKLSKAVKQLTANYRIILSGTPIQNNV
LELWSLFDFLMPGFLGTERQFAARYGKPILASRDARSSSREQEAGVLAMDALHRQVLPFL
LRRMKEDVLQDLPPKIIQDYYCTLSPLQVQLYEDFAKSRAKCDVDETVSSATLSEETEKP
KLKATGHVFQALQYLRKLCNHPALVLTPQHPEFKTTAEKLAVQNSSLHDIQHAPKLSALK
QLLLDCGLGNGSTSESGTESVVAQHRILIFCQLKSMLDIVEHDLLKPHLPSVTYLRLDGS
IPPGQRHSIVSRFNNDPSIDVLLLTTHVGGLGLNLTGADTVVFVEHDWNPMRDLQAMDRA
HRIGQKRVVNVYRLITRGTLEEKIMGLQKFKMNIANTVISQENSSLQSMGTDQLLDLFTL
DKDGKAEKADTSTSGKASMKSILENLSDLWDQEQYDSEYSLENFMHSLK*
>ENSG00000125850|ENST00000278780
MPKVFLVKRRSLGVSVRSWDELPDEKRADTYIPVGLGRLLHDPPEDCRSDGGSSSGSGSS
SAGEPGGAESSSSPHAPESETPEPGDAEGPDGHLATKQRPVARSKIKFTTGTCSDSVVHS
CDLCGKGFRLQRMLNRHLKCHNQVKRHLCTFCGKGFNDTFDLKRHVRTHTGIRPYKCNVC
NKAFTQRCSLESHLKKIHGVQQQYAYKQRRDKLYVCEDCGYTGPTQEDLYLHVNSAHPGS
SFLKKTSKKLAALLQGKLTSAHQENTSLSEEEERK*
>ENSG00000129347|ENST00000312962

MGHRTAMPEPRGSSQLRVNAAFAARYNRYREREELQRLKDRYGDRDSSSDSSSESDSSDE
RVEFDPQQERDFYKTLSLLKKKDPRIYQKDATFYNRTASSSDSEEDPEALEKQKKVRPMY
LKDYERKVILEKAGKYVDEENSDGETSNHRLQETSSQSYVEEQKQLKESFRAFVEDSEDE
DGAGEGGSSLLQKRAKTRQEKAQEEADYIEWLKGQKEIRNPDSLKELTHLKEYWNDPELD
EGERFLRDYILNKRYEEEEEEEEDEEEMEEEEGVHGPPVQLAVDDSSDEGELFLKKQEDF
EQKYNFRFEEPDSASVKTYPRSIASSVRRKDERRKEKREETRERKKREKAKKQEELKQLK
NLKRKEILAKLEKLRKVTGNEMLGLEEGDLEDDFDPAQHDQLMQKCFGDEYYGAVEEEKP
QFEEEEGLEDDWNWDTWDGPEQEGDWSQQELHCEDPNFNMDADYDPSQPRKKKREAPLTG
KKKRKSPFAAAVGQEKPVFEPGDKTFEEYLDEYYRLDYEDIIDDLPCRFKYRTVVPCDFG
LSTEEILAADDKELNRWCSLKKTCMYRSEQEELRDKRAYSQKAQNSWKKRQVFKSLCREE
AETPAEATGKPQRDEAGPQRQLPALDGSLMGPESPPAQEEEAPVSPHKKPAPQKRRRAKK
ARLLGPTVMLGGCEFSRQRLQAFGLNPKRLHFRQLGRQRRKQQGPKNSS*
>ENSG00000116731|ENST00000311066
MNQNTTEPVAATETLAEVPEHVLRGLPEEVRLFPSAVDKTRIGVWATKPILKGKKFGPFV
GDKKKRSQVKNNVYMWEVYYPNLGWMCIDATDPEKGNWLRYVNWACSGEEQNLFPLEINR
AIYYKTLKPIAPGEELLVWYNGEDNPEIAAAIEEERASARSKRSSPKSRKGKKKSQENKN
KGNKIQDIQLKTSEPDFTSANMRDSAEGPKEDEEKPSASALEQPATLQEVASQEVPPELA
TPAPAWEPQPEPDERLEAAACEVNDLGEEEEEEEEEDEEEEEDDDDDELEDEGEEEASMP
NENSVKEPEIRCDEKPEDLLEEPKTTSEETLEDCSEVTPAMQIPRTKEEANGDVFETFMF
PCQHCERKFTTKQGLERHMHIHISTVNHAFKCKYCGKAFGTQINRRRHERRHEAGLKRKP
SQTLQPSEDLADGKASGENVASKDDSSPPSLGPDCLIMNSEKASQDTINSSVVEENGEVK
ELHPCKYCKKVFGTHTNMRRHQRRVHERHLIPKGVRRKGGLEEPQPPAEQAQATQNVYVP
STEPEEEGEADDVYIMDISSNISENLNYYIDGKIQTNNNTSNCDVIEMESASADLYGINC
LLTPVTVEITQNIKTTQVPVTEDLPKEPLGSTNSEAKKRRTASPPALPKIKAETDSDPMV
PSCSLSLPLSISTTEAVSFHKEKSVYLSSKLKQLLQTQDKLTPAGISATEIAKLGPVCVS
APASMLPVTSSRFKRRTSSPPSSPQHSPALRDFGKPSDGKAAWTDAGLTSKKSKLESHSD
SPAWSLSGRDERETVSPPCFDEYKMSKEWTASSAFSSVCNQQPLDLSSGVKQKAEGTGKT
PVQWESVLDLSVHKKHCSDSEGKEFKESHSVQPTCSAVKKRKPTTCMLQKVLLNEYNGID
LPVENPADGTRSPSPCKSLEAQPDPDLGPGSGFPAPTVESTPDVCPSSPALQTPSLSSGQ
LPPLLIPTDPSSPPPCPPVLTVATPPPPLLPTVPLPAPSSSASPHPCPSPLSNATAQSPL
PILSPTVSPSPSPIPPVEPLMSAASPGPPTLSSSSSSSSSSSFSSSSSSSSPSPPPLSA
ISSVVSSGDNLEASLPMISFKQEELENEGLKPREEPQSAAEQDVVVQETFNKNFVCNVCE
SPFLSIKDLTKHLSIHAEEWPFKCEFCVQLFKDKTDLSEHRFLLHGVGNIFVCSVCKKEF
AFLCNLQQHQRDLHPDKVCTHHEFESGTLRPQNFTDPSKAHVEHMQSLPEDPLETSKEEE
ELNDSSEELYTTIKIMASGIKTKDPDVRLGLNQHYPSFKPPPFQYHHRNPMGIGVTATNF
TTHNIPQTFTTAIRCTKCGKGVDNMPELHKHILACASASDKKRYTPKKNPVPLKQTVQPK
NGVVVLDNSGKNAFRRMGQPKRLNFSVELSKMSSNKLKLNALKKKNQLVQKAILQKNKSA
KQKADLKNACESSSHICPYCNREFTYIGSLNKHAAFSCPKKPLSPPKKKVSHSSKKGGHS
SPASSDKNSNSNHRRRTADAEIKMQSMQTPLGKTRARSSGPTQVPLPSSSFRSKQNVKFA
ASVKSKKPSSSSLRNSSPIRMAKITHVEGKKPKAVAKNHSAQLSSKTSRSLHVRVQKSKA
VLQSKSTLASKKRTDRFNIKSRERSGGPVTRSLQLAAAADLSENKREDGSAKQELKDFRN
FL*
>ENSG00000147601|ENST00000276603
MAEDVSSAAPSPRGCADGRDADPTEEQMAETERNDEEQFECQELLECQVQVGAPEEEEEE

EEDAGLVAEAEAVAAGWMLDFLCLSLCRAFRDGRSEDFRRTRNSAEAIIHGLSSLTACQL
RTIYICQFLTRIAAGKTLDAQFENDERITPLESALMIWGSIEKEHDKLHEEIQNLIKIQA
IAVCMENGNFKEAEEVFERIFGDPNSHMPFKSKLLMIISQKDTFHSFFQHFSYNHMMEKI
KSYVNYVLSEKSSTFLMKAAAKVVESKRTRTITSQDKPSGNDVEMETEANLDTRKSVSDK
QSAVTESSEGTVSLLRSHKNLFLSKLQHGTQQQDLNKKERRVGTPQSTKKKKESRRATES
RIPVSKSQPVTPEKHRARKRQAWLWEEDKNLRSGVRKYGEGNWSKILLHYKFNNRTSVML
KDRWRTMKKLKLISSDSED*
>ENSG00000146731|ENST00000275603
MAAVKTLNPKAEVARAQAALAVNISAARGLQDVLRTNLGPKGTMKMLVSGAGDIKLTKDG
NVLLHEMQIQHPTASLIAKVATAQDDITGDGTTSNVLIIGELLKQADLYISEGLHPRIIT
EGFEAAKEKALQFLEEVKVSREMDRETLIDVARTSLRTKVHAELADVLTEAVVDSILAIK
KQDEPIDLFMIEIMEMKHKSETDTSLIRGLVLDHGARHPDMKKRVEDAYILTCNVSLEYE
KTEVNSGFFYKSAEEREKLVKAERKFIEDRVKKIIELKRKVCGDSDKGFVVINQKGIDPF
SLDALSKEGIVALRRAKRRNMERLTLACGGVALNSFDDLSPDCLGHAGLVYEYTLGEEKF
TFIEKCNNPRSVTLLIKGPNKHTLTQIKDAVRDGLRAVKNAIDDGCVVPGAGAVEVAMAE
ALIKHKPSVKGRAQLGVQAFADALLIIPKVLAQNSGFDLQETLVKIQAEHSESGQLVGVD
LNTGEPMVAAEVGVWDNYCVKKQLLHSCTVIATNILLVDEIMRAGMSSLKG*
>ENSG00000164610|ENST00000297157
MSSRPGREDVGAAGARRPREPPEQELQRRREQKRRRHDAQQLQQLKHLESFYEKPPPGLI
KEDETKPEDCIPDVPGNEHAREFLAHAPTKGLWMPLGKEVKVMQCWRCKRYGHRTGDKEC
PFFIKGNQKLEQFRVAHEDPMYDIIRDNKRHEKDVRIQQLKQLLEDSTSDEDRSSSSSSE
GKEKHKKKKKKEKHKKRKKEKKKKKKRKHKSSKSNEGSDSE*
>ENSG00000149136|ENST00000278412
MAETLEFNDVYQEVKGSMNDGRLRLSRQGIIFKNSKTGKVDNIQAGELTEGIWRRVALGH
GLKLLTKNGHVYKYDGFRESEFEKLSDFFKTHYRLELMEKDLCVKGWNWGTVKFGGQLLS
FDIGDQPVFEIPLSNVSQCTTGKNEVTLEFHQNDDAEVSLMEVRFYVPPTQEDGVDPVEA
FAQNVLSKADVIQATGDAICIFRELQCLTPRGRYDIRIYPTFLHLHGKTFDYKIPYTTVL
RLFLLPHKDQRQMFFVISLDPPIKQGQTRYHFLILLFSKDEDISLTLNMNEEEVEKRFEG
RLTKNMSGSLYEMVSRVMKALVNRKITVPGNFQGHSGAQCITCSYKASSGLLYPLERGFI
YVHKPPVHIRFDEISFVNFARGTTTTRSFDFEIETKQGTQYTFSSIEREEYGKLFDFVNA
KKLNIKNRGLKEGMNPSYDEYADSDEDQHDAYLERMKEEGKIREENANDSSDDSGEETDE
SFNPGEEEEDVAEEFDSNASASSSSNEGDSDRDEKKRKQLKKAKMAKDRKSRKKPVEVKK
GKDPNAPKRPMSAYMLWLNASREKIKSDHPGISITDLSKKAGEIWKGMSKEKKEEWDRKA
EDARRDYEKAMKEYEGGRGESSKRDKSKKKKKVKVKMEKKSTPSRGSSSKSSSRQLSESF
KSKEFVSSDESSSGENKSKKKRRRSEDSEEEELASTPPSSEDSASGSDE*
>ENSG00000128626|ENST00000308018
MSWSGLLHGLNTSLTCGPALVPRLWATCSMATLNQMHRLGPPKRPPRKLGPTEGRPQLKG
VVLCTFTRKPKKPNSANRKCCRVRLSTGREAVCFIPGEGHTLQEHQIVLVEGGRTQDLPG
VKLTVVRGKYDCGHVQKK*
>ENSG00000196597|ENST00000289032
XFINVDQSARTRGRCVCGKTPSCGDRKSCINSSFITCYCFTKPELIFTLEQGEDPWLLEK
EKGFLSRNSPEDSQPDEISEKSPENQGKHLLQVLFTNKLLTTEQEISGKPHNRDINIFRA
RMMPCKCDIAGSACQGLSLMAPHCQYSKEKAHERNVCDKWLISIKDGRTNTQEKSFAYSK
IVKTLHHKEEVIQHQTIQTLGQDFEYNESRKAFLEKAALVTSNSTHPKGKSYNFNKFGEN

KYDKSTFIIPQNMNPEKSHYEFNDTGNCFCRITHKTLTGGKSFSQKSHIREHHRVHIGVK
PFEYGKSFNRNSTLPVHQRTHATDKYSDYHPCTETFSYQSTFSVHQKVHIRAKPYEYNEC
GKSCSMNSHLIWPQKSHTGEKPYECPECGKAFSEKSRLRKHQRTHTGEKPYKCDGCDKAF
SAKSGLRIHQRTHTGEKPFECHECGKSFNYKSILIVHQRTHTGEKPFECNECGKSFSHMS
GLRNHRRTHTGERPYKCDECGKAFKLKSGLRKHHRTHTGEKPYKCNQCGKAFGQKSQLRG
HHRIHTGEKPYKCNHCGEAFSQKSNLRVHHRTHTGEKPYQCEECGKTFRQKSNLRGHQRT
HTGEKPYECNECGKAFSEKSVLRKHQRTHTGEKPYNCNQCGEAFSQKSNLRVHQRTHTGE
KPYKCDKCGRTFSQKSSLREHQKAHPGD*
>ENSG00000164944|ENST00000297591
MAVDSAMELLFLDTFKHPSAEQSSHIDVVRFPCVVYINEVRVIPPGVRAHSSLPDNRAYG
ETSPHTFQLDLFFNNVSKPSAPVFDRLGSLEYDENTSIIFRPNSKVNTDGLVLRGWYNCL
TLAIYGSVDRVISHDRDSPPPPPPPPPPPQPQPSLKRNPKHADGEKEDQFNGSPPRPQPR
GPRTPPGPPPPDDDEDDPVPLPVSGDKEEDAPHREDYFEPISPDRNSVPQEGQYSDEGEV
EEEQQEEGEEDEDDVDVEEEEDEDEDDRRTVDSIPEEEEEDEEEEGEEDEEGEGDDGYEQ
ISSDEDGIADLERETFKYPNFDVEYTAEDLASVPPMTYDPYDRELVPLLYFSCPYKTTFE
IEISRMKDQGPDKENSGAIEASVKLTELLDLYREDRGAKWVTALEEIPSLIIKGLSYLQL
KNTKQDSLGQLVDWTMQALNLQVALRQPIALNVRQLKAGTKLVSSLAECGAQGVTGLLQA
GVISGLFELLFADHVSSSLKLNAFKALDSVISMTEGMEAFLRGRQNEKSGYQKLLELILL
DQTVRVVTAGSAILQKCHFYEVLSEIKRLGDHLAEKTSSLPNHSEPDHDTDAGLERTNPE
YENEVEASMDMDLLESSNISEGEIERLINLLEEVFHLMETAPHTMIQQPVKSFPTMARIT
GPPERDDPYPVLFRYLHSHHFLELVTLLLSIPVTSAHPGVLQATKDVLKFLAQSQKGLLF
FMSEYEATNLLIRALCHFYDQDEEEGLQSDGVIDDAFALWLQDSTQTLQCITELFSHFQR
CTASEETDHSDLLGTLHNLYLITFNPVGRSAVGHVFSLEKNLQSLITLMEYYSKEALGDS
KSKKSVAYNYACILILVVVQSSSDVQMLEQHAASLLKLCKADENNAKLQELGKWLEPLKN
LRFEINCIPNLIEYVKQNIDNLMTPEGVGLTTALRVLCNVACPPPPVEGQQKDLKWNLAV
IQLFSAEGMDTFIRVLQKLNSILTQPWRLHVNMGTTLHRVTTISMARCTLTLLKTMLTEL
LRGGSFEFKDMRVPSALVTLHMLLCSIPLSGRLDSDEQKIQNDIIDILLTFTQGVNEKLT
ISEETLANNTWSLMLKEVLSSILKVPEGFFSGLILLSELLPLPLPMQTTQVIEPHDISVA
LNTRKLWSMHLHVQAKLLQEIVRSFSGTTCQPIQHMLRRICVQLCDLASPTALLIMRTVL
DLIVEDLQSTSEDKEKQYTSQTTRLLALLDALASHKACKLAILHLINGTIKGDERYAEIF
QDLLALVRSPGDSVIRQQCVEYVTSILQSLCDQDIALILPSSSEGSISELEQLSNSLPNK
ELMTSICDCLLATLANSESSYNCLLTCVRTMMFLAEHDYGLFHLKSSLRKNSSALHSLLK
RVVSTFSKDTGELASSFLEFMRQILNSDTIGCCGDDNGLMEVEGAHTSRTMSINAAELKQ
LLQSKEESPENLFLELEKLVLEHSKDDDNLDSLLDSVVGLKQMLESSGDPLPLSDQDVEP
VLSAPESLQNLFNNRTAYVLADVMDDQLKSMWFTPFQAEEIDTDLDLVKVDLIELSEKCC
SDFDLHSELERSFLSEPSSPGRTKTTKGFKLGKHKHETFITSSGKSEYIEPAKRAHVVPP
PRGRGRGGFGQGIRPHDIFRQRKQNTSRPPSMHVDDFVAAESKEVVPQDGIPPPKRPLKV
SQKISSRGGFSGNRGGRGAFHSQNRFFTPPASKGNYSRREGTRGSSWSAQNTPRGNYNES
RGGQSNFNRGPLPPLRPLSSTGYRPSPRDRASRGRGGLGPSWASANSGSGGSRGKFVSGG
SGRGRHVRSFTR*
>ENSG00000125885|ENST00000265187
MNGEYRGRGFGRGRFQSWKRGRGGGNFSGKWREREHRPDLSKTTGKRTSEQTPQFLLSTK
TPQSMQSTLDRFIPYKGWKLYFSEVYSDSSPLIEKIQAFEKFFTRHIDLYDKDEIERKGS
ILVDFKELTEGGEVTNLIPDIATELRDAPEKTLACMGLAIHQVLTKDLERHAAELQAQEG

LSNDGETMVNVPHIHARVYNYEPLTQLKNVRANYYGKYIALRGTVVRVSNIKPLCTKMAF
LCAACGEIQSFPLPDGKYSLPTKCPVPVCRGRSFTALRSSPLTVTMDWQSIKIQELMSDD
QREAGRIPRTIECELVHDLVDSCVPGDTVTITGIVKVSNAEEANSISNSKGQKTKSSEDG
CKHGMLMEFSLKDLYAIQEIQAEENLFKLIVNSLCPVIFGHELVKAGLALALFGGSQKYA
DDKNRIPIRGDPHILVVGDPGLGKSQMLQAACNVAPRGVYVCGNTTTTSGLTVTLSKDSS
SGDFALEAGALVLGDQGICGIDEFDKMGNQHQALLEAMEQQSISLAKAGVVCSLPARTSI
IAAANPVGGHYNKAKTVSENLKMGSALLSRFDLVFILLDTPNEHHDHLLSEHVIAIRAGK
QRTISSATVARMNSQDSNTSVLEVVSEKPLSERLKVVPGETIDPIPHQLLRKYIGYARQY
VYPRLSTEAARVLQDFYLELRKQSQRLNSSPITTRQLESLIRLTEARARLELREEATKED
AEDIVEIMKYSMLGTYSDEFGNLDFERSQHGSGMSNRSTAKRFISALNNVAERTYNNIFQ
FHQLRQIAKELNIQVADFENFIGSLNDQGYLLKKGPKVYQLQTM*
>ENSG00000174306|ENST00000309060
MASKRKSTTPCMIPVKTVVLQDASMEAQPAETLPEGPQQDLPPEASAASSEAAQNPSSTD
GSTLANGHRSTLDGYLYSCKYCDFRSHDMTQFVGHMNSEHTDFNKDPTFVCSGCSFLAKT
PEGLSLHNATCHSGEASFVWNVAKPDNHVVVEQSIPESTSTPDLAGEPSAEGADGQAEII
ITKTPIMKIMKGKAEAKKIHTLKENVPSQPVGEALPKLSTGEMEVREGDHSFINGAVPVS
QASASSAKNPHAANGPLIGTVPVLPAGIAQFLSLQQQPPVHAQHHVHQPLPTAKALPKVM
IPLSSIPTYNAAMDSNSFLKNSFHKFPYPTKAELCYLTVVTKYPEEQLKIWFTAQRLKQG
ISWSPEEIEDARKKMFNTVIQSVPQPTITVLNTPLVASAGNVQHLIQAALPGHVVGQPEG
TGGGLLVTQPLMANGLQATSSPLPLTVTSVPKQPGVAPINTVCSNTTSAVKVVNAAQSLL
TACPSITSQAFLDASIYKNKKSHEQLSALKGSFCRNQFPGQSEVEHLTKVTGLSTREVRK
WFSDRRYHCRNLKGSRAMIPGDHSSIIIDSVPEVSFSPSSKVPEVTCIPTTATLATHPSA
KRQSWHQTPDFTPTKYKERAPEQLRALESSFAQNPLPLDEELDRLRSETKMTRREIDSWF
SERRKKVNAEETKKAEENASQEEEEAAEDEGGEEDLASELRVSGENGSLEMPSSHILAER
KVSPIKINLKNLRVTEANGRNEIPGLGACDPEDDESNKLAEQLPGKVSCKKTAQQRHLLR
QLFVQTQWPSNQDYDSIMAQTGLPRPEVVRWFGDSRYALKNGQLKWYEDYKRGNFPPGLL
VIAPGNRELLQDYYMTHKMLYEEDLQNLCDKTQMSSQQVKQWFAEKMGEETRAVADTGSE
DQGPGTGELTAVHKGMGDTYSEVSENSESWEPRVPEASSEPFDTSSPQAGRQLETD*
>ENSG00000171786|ENST00000302101
MMLNSDTMELDLPPTHSETESGFSDCGGGAGPDGAGPGGPGGGQARGPEPGEPGRKDLQH
LSREERRRRRRATAKYRTAHATRERIRVEAFNLAFAELRKLLPTLPPDKKLSKIEILRLA
ICYISYLNHVLDV*
>ENSG00000153048|ENST00000311052
MSSEPPPPPQPPTHQASVGLLDTPRSRERSPSPLRGNVVPSPLPTRRTRTFSATVRASQG
PVYKGVCKCFCRSKGHGFITPADGGPDIFLHISDVEGEYVPVEGDEVTYKMCSIPPKNEK
LQAVEVVITHLAPGTKHETWSGHVISS*
>ENSG00000104177|ENST00000267836
MADANKAEVPGATGGDSPHLQPAEPPGEPRREPHPAEAEKQQPQHSSSSNGVKMENDESA
KEEKSDLKEKSTGSKKANRFHPYSKDKNSGAGEKKGPNRNRVFISNIPYDMKWQAIKDLM
REKVGEVTYVELFKDAEGKSRGCGVVEFKDEEFVKKALETMNKYDLSGRPLNIKEDPDGE
NARRALQRTGGSFPGGHVPDMGSGLMNLPPSILNNPNIPPEVISNLQAGRLGSTIFVANL
DFKVGWKKLKEVFSIAGTVKRADIKEDKDGKSRGMGTVTFEQAIEAVQAISMFNGQFLFD
RPMHVKMDDKSVPHEEYRSHDGKTPQLPRGLGGIGMGLGPGGQPISASQLNIGGVMGNLG
PGGMGMDGPGFGGMNRIGGGIGFGGLEAMNSMGGFGGVGRMGELYRGAMTSSMERDFGRG

DIGINRGFGDSFGRLGGGMGSMNSVTGGMGMGLDRMSSSFDRMGPGIGAILERSIDMDRG
FLSGPMGSGMRERIGSKGNQIFVRNLPFDLTWQKLKEKFSQCGHVMFAEIKMENGKSKGC
GTVRFDSPESAEKACRIMNGIKISGREIDVRLDRNA*
>ENSG00000141644|ENST00000269471
MAEDWLDCPALGPGWKRREVFRKSGATCGRSDTYYQSPTGDRIRSKVELTRYLGPACDLT
LFDFKQGILCYPAPKAHPVAVASKKRKKPSRPAKTRKRQVGPQSGEVRKEAPRDETKADT
DTAPASFPAPGCCENCGISFSGDGTQRQRLKTLCKDCRAQRIAFNREQRMFKRVGCGECA
ACQVTEDCGACSTCLLQLPHDVASGLFCKCERRRCLRIVERSRGCGVCRGCQTQEDCGHC
PICLRPPRPGLRRQWKCVQRRCLRGKHARRKGGCDSKMAARRRPGAQPLPPPPPSQSPEP
TEPQPYTNRRQNRKCGACAACLRRMDCGRCDFCCDKPKFGGSNQKRQKCRWRQCLQFAMK
RLLPSVWSESEDGAGSPPPYRRRKRPSSARRHHLGPTLKPTLATRTAQPDHTQAPTKQEA
GGGFVLPPPGTDLVFLREGASSPVQVPGPVAASTEALLQAVDPGLPSVKQEPPDPEEDKE
ENKDDSASKLAPEEEAGGAGTPVITEIFSLGGTRFRDTAVWLPSLQGRHSGREDGCKVWE
TEDTVEPTSTSWNPRGWPGTHVSLSPPPASMMWVSCRRSWCPSSQS*
>ENSG00000029153|ENST00000311001
MAAEEEAAAGGKVLREENQCIAPVVSSRVSPGTRPTAMGSFSSHMTEFPRKRKGSDSDPS
QSGIMTEKVVEKLSQNPLTYLLSTRIEISASSGSREAHSQTEKRRRDKMNNLIEELSAMI
PQCNPMARKLDKLTVLRMAVQHLRSLKGLTNSYVGSNYRPSFLQDNELRHLILKTAEGFL
FVVGCERGKILFVSKSVSKILNYDQASLTGQSLFDFLHPKDVAKVKEQLSSFDISPREKL
IDAKTGLQVHSNLHAGRTRVYSGSRRSFFCRIKSCKISVKEEHGCLPNSKKKEHRKFYTI
HCTGYLRSWPPNIVGMEEERNSKKDNSNFTCLVAIGRLQPYIVPQNSGEINVKPTEFITR
FAVNGKFVYVDQRATAILGYLPQELLGTSCYEYFHQDDHNNLTDKHKAVLQSKEKILTDS
YKFRAKDGSFVTLKSQWFSFTNPWTKELEYIVSVNTLVLGHSEPGEASFLPCSSQSSEES
SRQSCMSVPGMSTGTVLGAGSIGTDIANEILDLQRLQSSSYLDDSSPTGLMKDTHTVNCR
SMSNKELFPPSPSEMGELEATRQNQSTVAVHSHEPLLSDGAQLDFDALCDNDDTAMAAFM
NYLEAEGGLGDPGDFSDIQWTL*
>ENSG00000164379|ENST00000296839
MKLEVFVPRAAHGDKQGSDLEGAGGSDAPSPLSAAGDDSLGSDGDCAANSPAAGGGARDT
QGDGEQSAGGGPGAEEAIPAAAAAAVVAEGAEAGAAGPGAGGAGSGEGARSKPYTRRPKP
PYSYIALIAMAIRDSAGGRLTLAEINEYLMGKFPFFRGSYTGWRNSVRHNLSLNDCFVKV
LRDPSRPWGKDNYWMLNPNSEYTFADGVFRRRRKRLSHRAPVPAPGLRPEEAPGLPAAPP
PAPAAPASPRMRSPARQEERASPAGKFSSSFAIDSILRKPFRSRRLRDTAPGTTLQWGAA
PCPPLPAFPALLPAAPCRALLPLCAYGAGEPARLGAREAEVPPTAPPLLLAPLPAAAPAK
PLRGPAAGGAHLYCPLRLPAALQAASVRRPGPHLPYPVETLLA*
>ENSG00000173276|ENST00000310826
MEGLLHYINPAHAISLLSALNEERLKGQLCDVLLIVGDQKFRAHKNVLAASSEYFQSLFT
NKENESQTVFQLDFCEPDAFDNVLNYIYSSSLFVEKSSLAAVQELGYSLGISFLTNIVSK
TPQAPFPTCPNRKKVFVEDDENSSQKRSVIVCQSRNEAQGKTVSQNQPDVSHTSRPSPSI
AVKANTNKPHVPKPIEPLHNLSLTEKSWPKDSSVVYAKSLEHSGSLDDPNRISLVKRNAV
LPSKPLQDREAMDDKPGVSGQLPKGKALELALKRPRPPVLSVCSSSETPYLLKETNKGNG
QGEDRNLLYYSKLGLVIPSSGSGSGNQSIDRSGPLVKSLLRRSLSMDSQVPVYSPSIDLK
SSQGSSSVSSDAPGNVLCALSQKSSLKDCSEKTALDDRPQVLQPHRLRSFSASQSTDREG
ASPVTEVRIKTEPSSPLSDPSDIIRVTVGDAATTAAASSSSVTRDLSLKTEDDQKDMSRL
PAKRRFQADRRLPFKKLKVNEHGSPVSEDNFEEGSSPTLLDADFPDSDLNKDEFGELEGT

RPNKKFKCKHCLKIFRSTAGLHRHVNMYHNPEKPYACDICHKRFHTNFKVWTHCQTQHGI
VKNPSPASSSHAVLDEKFQRKLIDIVREREIKKALIIKLRRGKPGFQGQSSSQAQQVIKR
NLRSRAKGAYICTYCGKAYRFLSQFKQHIKMHPGEKPLGVNKVAKPKEHAPLASPVENKE
VYQCRLCNAKLSSLLEQGSHERLCRNAAVCPYCSLRFFSPELKQEHESKCEYKKLTCLEC
MRTFKSSFSIWRHQVEVHNQNNMAPTENFSLPVLDHNGDVTGSSRPQSQPEPNKVNHIVT
TKDDNVFSDSSEQVNFDSEDSSCLPEDLSLSKQLKIQVKEEPVEEAEEEAPEASTAPKEA
GPSKEASLWPCEKCGKMFTVHKQLERHQELLCSVKPFICHVCNKAFRTNFRLWSHFQSHM
SQASEESAHKESEVCPVPTNSPSPPPLPPPPPLPKIQPLEPDSPTGLSENPTPATEKLFV
PQESDTLFYHAPPLSAITFKRQFMCKLCHRTFKTAFSLWSHEQTHN*
>ENSG00000147121|ENST00000298190
MAMSQESLTFKDVFVDFTLEEWQQLDSAQKNLYRDVMLENYSHLVSVGYLVAKPDVIFRL
GPGEESWMADGGTPVRTCAEVWQVDEQIDHYKESQDKLPWQAAFIGKETLKDESGQESRT
CRKSIYLSTEFDSVRQRLPKYYSWEKAFKTSFKLSWSKWKLCKKER*
>ENSG00000174231|ENST00000304992
MAGVFPYRGPGNPVPGPLAPLPDYMSEEKLQEKARKWQQLQAKRYAEKRKFGFVDAQKED
MPPEHVRKIIRDHGDMTNRKFRHDKRVYLGALKYMPHAVLKLLENMPMPWEQIRDVPVLY
HITGAISFVNEIPWVIEPVYISQWGSMWIMMRREKRDRRHFKRMRFPPFDDEEPPLDYAD
NILDVEPLEAIQLELDPEEDAPVLDWFYDHQPLRDSRKYVNGSTYQRWQFTLPMMSTLYR
LANQLLTDLVDDNYFYLFDLKAFFTSKALNMAIPGGPKFEPLVRDINLQDEDWNEFNDIN
KIIIRQPIRTEYKIAFPYLYNNLPHHVHLTWYHTPNVVFIKTEDPDLPAFYFDPLINPIS
HRHSVKSQEPLPDDDEEFELPEFVEPFLKDTPLYTDNTANGIALLWAPRPFNLRSGRTRR
ALDIPLVKNWYREHCPAGQPVKVRVSYQKLLKYYVLNALKHRPPKAQKKRYLFRSFKATK
FFQSTKLDWVEVGLQVCRQGYNMLNLLIHRKNLNYLHLDYNFNLKPVKTLTTKERKKSRF
GNAFHLCREVLRLTKLVVDSHVQYRLGNVDAFQLADGLQYIFAHVGQLTGMYRYKYKLMR
QIRMCKDLKHLIYYRFNTGPVGKGPGCGFWAAGWRVWLFFMRGITPLLERWLGNLLARQF
EGRHSKGVAKTVTKQRVESHFDLELRAAVMHDILDMMPEGIKQNKARTILQHLSEAWRCW
KANIPWKVPGLPTPIENMILRYVKAKADWWTNTAHYNRERIRRGATVDKTVCKKNLGRLT
RLYLKAEQERQHNYLKDGPYITAEEAVAVYTTTVHWLESRRFSPIPFPPLSYKHDTKLLI
LALERLKEAYSVKSRLNQSQREELGLIEQAYDNPHEALSRIKRHLLTQRAFKEVGIEFMD
LYSHLVPVYDVEPLEKITDAYLDQYLWYEADKRRLFPPWIKPADTEPPPLLVYKWCQGIN
NLQDVWETSEGECNVMLESRFEKMYEKIDLTLLNRLLRLIVDHNIADYMTAKNNVVINYK
DMNHTNSYGIIRGLQFASFIVQYYGLVMDLLVLGLHRASEMAGPPQMPNDFLSFQDIATE
AAHPIRLFCRYIDRIHIFFRFTADEARDLIQRYLTEHPDPNNENIVGYNNKKCWPRDARM
RLMKHDVNLGRAVFWDIKNRLPRSVTTVQWENSFVSVYSKDNPNLLFNMCGFECRILPKC
RTSYEEFTHKDGVWNLQNEVTKERTAQCFLRVDDESMQRFHNRVRQILMASGSTTFTKIV
NKWNTALIGLMTYFREAVVNTQELLDLLVKCENKIQTRIKIGLNSKMPSRFPPVVFYTPK
ELGGLGMLSMGHVLIPQSDLRWSKQTDVGITHFRSGMSHEEDQLIPNLYRYIQPWESEFI
DSQRVWAEYALKRQEAIAQNRRLTLEDLEDSWDRGIPRINTLFQKDRHTLAYDKGWRVRT
DFKQYQVLKQNPFWWTHQRHDGKLWNLNNYRTDMIQALGGVEGILEHTLFKGTYFPTWEG
LFWEKASGFEESMKWKKLTNAQRSGLNQIPNRRFTLWWSPTINRANVYVGFQVQLDLTGI
FMHGKIPTLKISLIQIFRAHLWQKIHESIVMDLCQVFDQELDALEIETVQKETIHPRKSY
KMNSSCADILLFASYKWNVSRPSLLADSKDVMDSTTTQKYWIDIQLRWGDYDSHDIERYA
RAKFLDYTTDNMSIYPSPTGVLIAIDLAYNLHSAYGNWFPGSKPLIQQAMAKIMKANPAL
YVLRERIRKGLQLYSSEPTEPYLSSQNYGELFSNQIIWFVDDTNVYRVTIHKTFEGNLTT

KPINGAIFIFNPRTGQLFLKIIHTSVWAGQKRLGQLAKWKTAEEVAALIRSLPVEEQPKQ
IIVTRKGMLDPLEVHLLDFPNIVIKGSELQLPFQACLKVEKFGDLILKATEPQMVLFNLY
DDWLKTISSYTAFSRLILILRALHVNNDRAKVILKPDKTTITEPHHIWPTLTDEEWIKVE
VQLKDLILADYGKKNNVNVASLTQSEIRDIILGMEISAPSQQRQQIAEIEKQTKEQSQLT
ATQTRTVNKHGDEIITSTTSNYETQTFSSKTEWRVRAISAANLHLRTNHIYVSSDDIKET
GYTYILPKNVLKKFICISDLRAQIAGYLYGVSPPDNPQVKEIRCIVMVPQWGTHQTVHLP
GQLPQHEYLKEMEPLGWIHTQPNESPQLSPQDVTTHAKIMADNPSWDGEKTIIITCSFTP
GSCTLTAYKLTPSGYEWGRQNTDKGNNPKGYLPSHYERVQMLLSDRFLGFFMVPAQSSWN
YNFMGVRHDPNMKYELQLANPKEFYHEVHRPSHFLNFALLQEGEVYSADREDLYA*
>ENSG00000160199|ENST00000291547
MMATQTLSIDSYQDGQQMQVVTELKTEQDPNCSEPDAEGVSPPPVESQTPMDVDKQAIYR
HPLFPLLALLFEKCEQSTQGSEGTTSASFDVDIENFVRKQEKEGKPFFCEDPETDNLMVK
AIQVLRIHLLELEKVNELCKDFCSRYIACLKTKMNSETLLSGEPGSPYSPVQSQQIQSAI
TGTISPQGIVVPASALQQGNVAMATVAGGTVYQPVTVVTPQGQVVTQTLSPGTIRIQNSQ
LQLQLNQDLSILHQDDGSSKNKRGVLPKHATNVMRSWLFQHIGHPYPTEDEKKQIAAQTN
LTLLQVNNWFINARRRILQPMLDSSCSETPKTKKKTAQNRPVQRFWPDSIASGVAQPPPS
ELTMSEGAVVTITTPVNMNVDSLQSLSSDGATLAVQQVMMAGQSEDESVDSTEEDAGALA
PAHISGLVLENSDSLQ*
>ENSG00000180596|ENST00000314332
MPEPAKSAPAPKKGSKKAVTKAQKKDGKKRKRSRKESYSVYVYKVLKQVHPDTGISSKAM
GIMNSFVNDIFERIAGEASRLAHYNKRSTITSREIQTAVRLLLPGELAKHAVSEGTKAVT
KYTSSK*
>ENSG00000196678|ENST00000300005
MATKRLARQLGLIRRKSIAPANGNLGRSKSKQLFDYLIVIDFESTCWNDGKHHHSQEIIE
FPAVLLNTSTGQIDSEFQAYVQPQEHPILSEFCMELTGIKQAQVDEGVPLKICLSQFCKW
IHKIQQQKNIIFATGISEPSASEVKLCAFVTWSDWDLGVCLEYECKRKQLLKPVFLNSWI
DLRATYKLFYRRKPKGLSGALQEVGIEFSGREHSGLDDSRNTALLAWKMIRDGCVMKITR
SLNKGPFLLPSWTWNSDLASGDQHAFLQQEFGCGTYRTLLQKPNMSKQEKGNILWLTMVW
LSLACLQRKNYNDCMLNTASQTVTTEKF*
>ENSG00000170549|ENST00000302006
MSFPQLGYPQYLSAAGPGAYGGERPGVLAAAAAAAAAASSGRPGAAELGGGAGAAAVTSV
LGMYAAAGPYAGAPNYSAFLPYAADLSLFSQMGSQYELKDNPGVHPATFAAHTAPAYYPY
GQFQYGDPGRPKNATRESTSTLKAWLNEHRKNPYPTKGEKIMLAIITKMTLTQVSTWFAN
ARRRLKKENKVTWGARSKDQEDGALFGSDTEGDPEKAEDDEEIDLESIDIDKIDEHDGDQ
SNEDDEDKAEAPHAPAAPSALARDQGSPLAAADVLKPQDSPLGLAKEAPEPGSTRLLSPG
AAAGGLQGAPHGKPKIWSLAETATSPDGAPKASPPPPAGHPGAHGPSAGAPLQHPAFLPS
HGLYTCHIGKFSNWTNSAFLAQGSLLNMRSFLGVGAPHAAPHGPHLPAPPPPQPPVAIAP
GALNGDKASVRSSPTLPERDLVPRPDSPAQQLKSPFQPVRDNSLAPQEGTPRILAALPSA
*
>ENSG00000177683|ENST00000313516
MKRDSWVPSKYQFLCSDHFTPDSLDIRWGIRYLKQTAVPTIFSLPEDNQGKDPSKKKSQK
KNLEDEKEVCPKAKSEESFVLNETKKNIVNTDVPHQHPELLHSSSLVKPPAPKTGSIQNN
MLTLNLVKQHTGKPESTLETSVNQDTGRGGFHTCFENLNSTTITLTTSNSESIHQSLETQ
EVLEVTTSHLANPNFTSNSMEIKSAQENPFLFSTINQTVEELNTNKESVIAIFVPAENSK

PSVNSFISAQKETTEMEDTDIEDSLYKDVDYGTEVLQIEHSYCRQDINKEHLWQKVSKLH
SKITLLELKEQQTLGRLKSLEALIRQLKQENWLSEENVKIIENHFTTYEVTMI*
>ENSG00000155090|ENST00000285407
MLNFGASLQQTAEERMEMISERPKESMYSWNKTAEKSDFEAVEALMSMSCSWKSDFKKYV
ENRPVTPVSDLSEEENLLPGTPDFHTIPAFCLTPPYSPSDFEPSQVSNLMAPAPSTVHFK
SLSDTAKPHIAAPFKEEEKSPVSAPKLPKAQATSVIRHTADAQLCNHQTCPMKAASILNY
QNNSFRRRTHLNVEAARKNIPCAAVSPNRSKCERNTVADVDEKASAALYDFSVPSSETVI
CRSQPAPVSPQQKSVLVSPPAVSAGGVPPMPVICQMVPLPANNPVVTTVVPSTPPSQPPA
VCPPVVFMGTQVPKGAVMFVVPQPVVQSSKPPVVSPNGTRLSPIAPAPGFSPSAAKVTPQ
IDSSRIRSHICSHPGCGKTYFKSSHLKAHTRTHTGEKPFSCSWKGCERRFARSDELSRHR
RTHTGEKKFACPMCDRRFMRSDHLTKHARRHLSAKKLPNWQMEVSKLNDIALPPTPAPTQ
*
>ENSG00000158545|ENST00000301011
MDVAESPERDPHSPEDEEQPQGLSDDDILRDSGSDQDLDGAGVRASDLEDEESAARGPSQ
EEEDNHSDEEDRASEPKSQDQDSEVNELSRGPTSSPCEEEGDEGEEDRTSDLRDEASSVT
RELDEHELDYDEEVPEEPAPAVQEDEAEKAGAEDDEEKGEGTPREEGKAGVQSVGEKESL
EAAKEKKKEDDDGEIDDGEIDDDDLEEGEVKDPSDRKVRPRPTCRFFMKGNCTWGMNCRF
IHPGVNDKGNYSLITKADPFPPNGAPPLGPHPLMPANPWGGPVVDEILPPPPPEPPTESA
WERGLRHAKEVLKKATIRKEQEPDFEEKRFTVTIGEDEREFDKENEVFRDWNSRIPRDVR
DTVLEPYADPYYDYEIERFWRGGQYENFRVQYTETEPYHNYRERERERERENRQRERERE
RERDRERERRQRERERERERERDKERQRRKEEWERERAKRDEKDRQHRDRDREKEREKEK
GKPKPRSPQPPSRQAEPPKKEAATTGPQVKRADEWKDPWRRSKSPKKKLGVSVSPSRARR
RRKTSASSASASNSSRSSSRSSSYSGSGSSRSRSRSSSYSSYSSRSSRHSSFSGSRSRSR
SFSSSPSPSPTPSPHRPSIRTKGEPAPPPGKAGEKSVKKPAPPPAPPQATKTTAPVPEPT
KPGDPREARRKERPARTPPRRRTLSGSGSGSGSSYSGSSSRSRSLSVSSVSSVSSATSSS
SSAHSVDSEDMYADLASPVSSASSRSPAPAQTRKEKGKSKKEDGVKEEKRKRDSSTQPPK
SAKPPAGGKSSQQPSTPQQAPPGQPQQGTFVAHKEIKLTLLNKAADKGSRKRYEPSDKDR
QSPPPAKRPNTSPDRGSRDRKSGGRLGSPKPERQRGQNSKAPAAPADRKRQLSPQSKSSS
KVTSVPGKASDPGAASTKSGKASTLSRREELLKQLKAVEDAIARKRAKIPGKA*
>ENSG00000144635|ENST00000273130
MAAVGRVGSFGSSPPGLSSTYTGGPLGNEIASGNGGAAAGDDEDGQNLWSCILSEVSTRS
RSKLPAGKNVLLLGEDGAGKTSLIRKIQGIEEYKKGRGLEYLYLNVHDEDRDDQTRCNVW
ILDGDLYHKGLLKFSLDAVSLKDTLVMLVVDMSKPWTALDSLQKWASVVREHVDKLKIPP
EEMKQMEQKLIRDFQEYVEPGEDFPASPQRRNTASQEDKDDSVVLPLGADTLTHNLGIPV
LVVCTKCDAISVLEKEHDYRDEHFDFIQSHIRKFCLQYGAALIYTSVKENKNIDLVYKYI
VQKLYGFPYKIPAVVVEKDAVFIPAGWDNDKKIGILHENFQTLKAEDNFEDIITKPPVRK
FVHEKEIMAEDDQVFLMKLQSLLAKQPPTAAGRPVDASPRVPGGSPRTPNRSVSSNVASV
SPIPAGSKKIDPNMKAGATSEGVLANFFNSLLSKKTGSPGGPGVSGGSPAGGAGGGSSGL
PPSTKKSGQKPVLDVHAELDRITRKPVTVSPTTPTSPTEGEAS*
>ENSG00000115677|ENST00000310931
MSSVAVLTQESFAEHRSGLVPQQIKVATLNSEEESDPPTYKDAFPPLPEKAACLESAQEP
AGAWGNKIRPIKASVITQVFHVPLEERKYKDMNQFGEGEQAKICLEIMQRTGAHLELSLA
KDQGLSIMVSGKLDAVMKARKDIVARLQTQASATVAIPKEHHRFVIGKNGEKLQDLELKT
ATKIQIPRPDDPSNQIKITGTKEGIEKARHEVLLISAEQDKRAVERLEVEKAFHPFIAGP

YNRLVGEIMQETGTRINIPPPSVNRTEIVFTGEKEQLAQAVARIKKIYEEKKKKTTTIAV
EVKKSQHKYVIGPKGNSLQEILERTGVSVEIPPSDSISETVILRGEPEKLGQALTEVYAK
ANSFTVSSVAAPSWLHRFIIGKKGQNLAKITQQMPKVHIEFTEGEDKITLEGPTEDVNVA
QEQIEGMVKDLINRMDYVEINIDHKFHRHLIGKSGANINRIKDQYKVSVRIPPDSEKSNL
IRIEGDPQGVQQAKRELLELASRMENERTKDLIIEQRFHRTIIGQKGERIREIRDKFPEV
IINFPDPAQKSDIVQLRGPKNEVEKCTKYMQKMVADLVENSYSISVPIFKQFHKNIIGKG
GANIKKIREESNTKIDLPAENSNSETIIITGKRANCEAARSRILSIQKDLANIAEVEVSI
PAKLHNSLIGTKGRLIRSIMEECGGVHIHFPVEGSGSDTVVIRGPSSDVEKAKKQLLHLA
EEKQTKSFTVDIRAKPEYHKFLIGKGGGKIRKVRDSTGARVIFPAAEDKDQDLITIIGKE
DAVREAQKELEALIQNLDNVVEDSMLVDPKHHRHFVIRRGQVLREIAEEYGGVMVSFPRS
GTQSDKVTLKGAKDCVEAAKKRIQEIIEDLEAQVTLECAIPQKFHRSVMGPKGSRIQQIT
RDFSVQIKFPDREENAVHSTEPVVQENGDEAGEGREAKDCDPGSPRRCDIIIISGRKEKC
EAAKEALEALVPVTIEVEVPFDLHRYVIGQKGSGIRKMMDEFEVNIHVPAPELQSDIIAI
TGLAANLDRAKAGLLERVKELQAEQEDRALRSFKLSVTVDPKYHPKIIGRKGAVITQIRL
EHDVNIQFPDKDDGNQPQDQITITGYEKNTEAARDAILRIVGELEQMVSEDVPLDHRVHA
RIIGARGKAIRKIMDEFKVDIRFPQSGAPDPNCVTVTGLPENVEEAIDHILNLEEEYLAD
VVDSEALQVYMKPPAHEEAKAPSRGFVVRDAPWTASSSEKAPDMSSSEEFPSFGAQVAPK
TLPWGPKR*
>ENSG00000148090|ENST00000303617
MAAAVAAAPGALGSLHAGGARLVAACSAWLCPGLRLPGSLAGRRAGPAIWAQGWVPAAGG
PAPKRGYSSEMKTEDELRVRHLEEENRGIVVLGINRAYGKNSLSKNLIKMLSKAVDALKS
DKKVRTIIIRSEVPGIFCAANLPVPTIAAIDGLALGGGLELALACDIRVAASSAKMGLVE
TKLAIIPGGGGTQRLPRAIGMSLAKELIFSARVLDGKEAKAVGLISHVLEQNQEGDAAYR
KALDLAREFLPQGPVAMRVAKLAINQGMEVDLVTGLAIEEACYAQTIPTKDRLEGLLAFK
EKRPPRYKGE*
>ENSG00000138382|ENST00000308099
MKKVRLKELESRLQQVDGFEKPKLLLEQYPTRPHIAACMLYTIHNTYDDIENKVVADLGC
GCGVLSIGTAMLGAGLCVGFDIDEDALEIFNRNAEEFELTNIDMVQCDVCLLSNRMSKSF
DTVIMNPPFGTKNNKELRYDLPASYKFHKKKSVDIEVDLIRFSF*
>ENSG00000150753|ENST00000280326
MASMGTLAFDEYGRPFLIIKDQDRKSRLMGLEALKSHIMAAKAVANTMRTSLGPNGLDKM
MVDKDGDVTVTNDGATILSMMDVDHQIAKLMVELSKSQDDEIGDGTTGVVVLAGALLEEA
EQLLDRGIHPIRIADGYEQAARVAIEHLDKISDSVLVDIKDTEPLIQTAKTTLGSKVVNS
CHRQMAEIAVNAVLTVADMERRDVDFELIKVEGKVGGRLEDTKLIKGVIVDKDFSHPQMP
KKVEDAKIAILTCPFEPPKPKTKHKLDVTSVEDYKALQKYEKEKFEEMIQQIKETGANLA
ICQWGFDDEANHLLLQNNLPAVRWVGGPEIELIAIATGGRIVPRFSELTAEKLGFAGLVQ
EISFGTTKDKMLVIEQCKNSRAVTIFIRGGNKMIIEEAKRSLHDALCVIRNLIRDNRVVY
GGGAAEISCALAVSQEADKCPTLEQYAMRAFADALEVIPMALSENSGMNPIQTMTEVRAR
QVKEMNPALGIDCLHKGTNDMKQQHVIETLIGKKQQISLATQMVRMILKIDDIRKPGESE
E*
>ENSG00000167528|ENST00000301042
MQAEDRSQFGSAAEMLSEQTAALGTGWESMNVQLDGAEPQVERGSQEERPWRTVPGPLEH
LCCDLEEEPQSLQEKAQSAPWVPAIPQEGNTGDWEMAAALLAAGSQGLVTIKDVSLCFSQ
EEWRSLDPSQTDFYGEYVMQENCGIVVSLRFPIPKLDMLSQLEGGEEQWVPDPQDLEERD

ILRVTYTGDGSEHEGDTPELEAEPPRMLSSVSEDTVLWNPEHDESWDSMPSSSRGMLLGP
PFLQEDSFSNLLCSTEMDSLLRPHTCPQCGKQFVWGSHLARHQQTHTGERPYSCLKCEKT
FGRRHHLIRHQKTHLHDKTSRCSECGKNFRCNSHLASHQRVHAEGKSCKGQEVGESPGTR
KRQRAPPVPKCHVCTECGKSFGRRHHLVRHWLTHTGEKPFQCPRCEKSFGRKHHLDRHLL
THQGQSPRNSWDRGTSVF*
>ENSG00000167978|ENST00000301740
MYNGIGLPTPRGSGTNGYVQRNLSLVRGRRGERPDYKGEEELRRLEAALVKRPNPDILDH
ERKRRVELRCLELEEMMEEQGYEEQQIQEKVATFRLMLLEKDVNPGGKEETPGQRPAVTE
THQLAELNEKKNERLRAAFGISDSYVDGSSFDPQRRAREAKQPAPEPPKPYSLVRESSSS
RSPTPKQKKKKKKKDRGRRSESSSPRRERKKSSKKKKHRSESESKKRKHRSPTPKSKRKS
KDKKRKRSRSTTPAPKSRRAHRSTSADSASSSDTSRSRSRSAAAKTHTTALAGRSPSPAS
GRRGEGDAPFSEPGTTSTQRPSSPETATKQPSSPYEDKDKDKKEKSATRPSPSPERSSTG
PEPPAPTPLLAERHGGSPQPLATTPLSQEPVNPPSEASPTRDRSPPKSPEKLPQSSSSES
SPPSPQPTKVSRHASSSPESPKPAPAPGSHREISSSPTSKNRSHGRAKRDKSHSHTPSRR
MGRSRSPATAKRGRSRSRTPTKRGHSRSRSPQWRRSRSAQRWGRSRSPQRRGRSRSPQRP
GWSRSRNTQRRGRSRSARRGRSHSRSPATRGRSRSRTPARRGRSRSRTPARRRSRSRTPT
RRRSRSRTPARRGRSRSRTPARRRSRTRSPVRRRSRSRSPARRSGRSRSRTPARRGRSRS
RTPARRGRSRSRTPARRSGRSRSRTPARRGRSRSRTPRRGRSRSRSLVRRGRSHSRTPQR
RGRSGSSSERKNKSRTSQRRSRSNSSPEMKKSRISSRRSRSLSSPRSKAKSRLSLRRSLS
GSSPCPKQKSQTPPRRSRSGSSQPKAKSRTPPRRSRSSSSPPPKQKSKTPSRQSHSSSSP
HPKVKSGTPPRQGSITSPQANEQSVTPQRRSCFESSPDPELKSRTPSRHSCSGSSPPRVK
SSTPPRQSPSRSSSPQPKVKAIISPRQRSHSGSSSPSPSRVTSRTTPRRSRSVSPCSNVE
SRLLPRYSHSGSSSPDTKVKPETPPRQSHSGSISPYPKVKAQTPPGPSLSGSKSPCPQEK
SKDSLVQSCPGSLSLCAGVKSSTPPGESYFGVSSLQLKGQSQTSPDHRSDTSSPEVRQSH
SESPSLQSKSQTSPKGGRSRSSSPVTELASRSPIRQDRGEFSASPMLKSGMSPEQSRFQS
DSSSYPTVDSNSLLGQSRLETAESKEKMALPPQEDATASPPRQKDKFSPFPVQDRPESSL
VFKDTLRTPPRERSGAGSSPETKEQNSALPTSSQDEELMEVVEKSEEPAGQILSHLSSEL
KEMSTSNFESSPEVEERPAVSLTLDQSQSQASLEAVEVPSMASSWGGPHFSPEHKELSNS
PLRENSFGSPLEFRNSGPLGTEMNTGFSSEVKEDLNGPFLNQLETDPSLDMKEQSTRSSG
HSSSELSPDAVEKAGMSSNQSISSPVLDAVPRTPSRERSSSASSPEMKDGLPRTPSRRSR
SGSSPGLRDGSGTPSRHSLSGSSPGMKDIPRTPSRGRSECDSSPEPKALPQTPRPRSRSP
SSPELNNKCLTPQRERSGSESSVDQKTVARTPLGQRSRSGSSQELDVKPSASPQERSESD
SSPDSKAKTRTPLRQRSRSGSSPEVDSKSRLSPRRSRSGSSPEVKDKPRAAPRAQSGSDS
SPEPKAPAPRALPRRSRSGSSSKGRGPSPEGSSSTESSPEHPPKSRTARRGSRSSPEPKT
KSRTPPRRRSSRSSPELTRKARLSRRSRSASSSPETRSRTPPRHRRSPSVSSPEPAEKSR
SSRRRRSASSPRTKTTSRRGRSPSPKPRGLQRSRSRSRREKTRTTRRRDRSGSSQSTSRR
RQRSRSRSRVTRRRRGGSGYHSRSPARQESSRTSSRRRRGRSRTPPTSRKRSRSRTSPAP
WKRSRSRASPATHRRSRSRTPLISRRRSRSRTSPVSRRRSRSRTSVTRRRSRSRASPVSR
RRSRSRTPPVTRRRSRSRTPTTRRRSRSRTPPVTRRRSRSRTPPVTRRRSRSRTSPITRR
RSRSRTSPVTRRRSRSRTSPVTRRRSRSRTSPVTRRRSRSRTPPAIRRRSRSRTPLLPRK
RSRSRSPLAIRRRSRSRTPRTARGKRSLTRSPPAIRRRSASGSSSDRSRSATPPATRNHS
GSRTPPVALNSSRMSCFSRPSMSPTPLDRCRSPGMLEPLGSSRTPMSVLQQAGGSMMDGP
GPRIPDHQRTSVPENHAQSRIALALTAISLGTARPPPSMSAAGLAARMSQVPAPVPLMSL
RTAPAANLASRIPAASAAAMNLASARTPAIPTAVNLADSRTPAAAAAMNLASPRTAVAPS

AVNLADPRTPTAPAVNLAGARTPAALAALSLTGSGTPPTAANYPSSSRTPQAPASANLVG
PRSAHATAPVNIAGSRTAAALAPASLTSARMAPALSGANLTSPRVPLSAYERVSGRTSPP
LLDRARSRTPPSAPSQSRMTSERAPSPSSRMGQAPSQSLLPPAQDQPRSPVPSAFSDQSR
CLIAQTTPVAGSQSLSSGAVATTTSSAGDHNGMLSVPAPGVPHSDVGEPPASTGAQQPSA
LAALQPAKERRSSSSSSSSSSSSSSSSSSSSSSSSSGSSSSDSEGSSLPVQPEVALKRVP
SPTPAPKEAVREGRPPEPTPAKRKRRSSSSSSSSSSSSSSSSSSSSSSSSSSSSSSSSSS
SSSSSSSSPSPAKPGPQALPKPASPKKPPPGERRSRSPRKPIDSLRDSRSLSYSPVERRR
PSPQPSPRDQQSSSSERGSRRGQRGDSRSPSHKRRRETPSPRPMRHRSSRSP*
>ENSG00000105750|ENST00000300540
MGPLTFRDVAIEFSLKEWQCLDTAQRNLYRNVMLENYRNLVFLGITVSKPDLITCLEQGK
EAWSMKRHEIMVAKPTESYSVTQSGMQ*
>ENSG00000120093|ENST00000311626
MQKATYYDNAAAALFGGYSSYPGSNGFGFDVPPQPPFQAATHLEGDYQRSACSLQSLGNA
APHAKSKELNGSCMRPGLAPEPLSAPPGSPPPSAAPTSATSNSSNGGGPSKSGPPKCGPG
TNSTLTKQIFPWMKESRQTSKLKNNSPGTAEGCGGGGGGGGGGGSGGSGGGGGGGGGGDK
SPPGSAASKRARTAYTSAQLVELEKEFHFNRYLCRPRRVEMANLLNLSERQIKIWFQNRR
MKYKKDQKAKGLASSSGGPSPAGSPPQPMQSTAGFMNALHSMTPSYESPSPPAFGKAHQN
AYALPSNYQPPLKGCGAPQKYPPTPAPEYEPHVLQANGGAYGTPTMQGSPVYVGGGGYAD
PLPPPAGPSLYGLNHLSHHPSGNLDYNGAPPMAPSQHHGPCEPHPTYTDLSSHHAPPPQG
RIQEAPKLTHL*
>ENSG00000172171|ENST00000306049
MSGSVLFTAGERWRCFLTPSRSSLYWALHNFCCRKKSTTPKKITPNVTFCDENAKEPENA
LDKLFSSEQQASILHVLNTASTKELEAFRLLRGRRSINIVEHRENFGPFQNLESLMNVPL
FKYKSTVQVCNSILCPKTGREKRKSPENRFLRKLLKPDIERERLKNCLGSP*
>ENSG00000125686|ENST00000300651
MKAQGETEESEKLSKMSSLLERLHAKFNQNRPWSETIKLVRQVMEKRVVMSSGGHQHLVS
CLETLQKALKVTSLPAMTDRLESIARQNGLGSHLSASGTECYITSDMFYVEVQLDPAGQL
CDVKVAHHGENPVSCPELVQQLREKNFDEFSKHLKGLVNLYNLPGDNKLKTKMYLALQSL
EQDLSKMAIMYWKATNAGPLDKILHGSVGYLTPRSGGHLMNLKYYVSPSDLLDDKTASPI
ILHENNVSRSLGMNASVTIEGTSAVYKLPIAPLIMGSHPVDNKWTPSFSSITSANSVDLP
ACFFLKFPQPIPVSRAFVQKLQNCTGIPLFETQPTYAPLYELITQFELSKDPDPIPLNHN
MRFYAALPGQQHCYFLNKDAPLPDGRSLQGTLVSKITFQHPGRVPLILNLIRHQVAYNTL
IGSCVKRTILKEDSPGLLQFEVCPLSESRFSVSFQHPVNDSLVCVVMDVQDSTHVSCKLY
KGLSDALICTDDFIAKVVQRCMSIPVTMRAIRRKAETIQADTPALSLIAETVEDMVKKNL
PPASSPGYGMTTGNNPMSGTTTPTNTFPGGPITTLFNMSMSIKDRHESVGHGEDFSKVSQ
NPILTSLLQITGNGGSTIGSSPTPPHHTPPPVSSMAGNTKNHPMLMNLLKDNPAQDFSTL
YGSSPLERQNSSSGSPRMEICSGSNKTKKKKSSRLPPEKPKHQTEDDFQRELFSMDVDSQ
NPIFDVNMTADTLDTPHITPAPSQCSTPPTTYPQPVPHPQPSIQRMVRLSSSDSIGPDVT
DILSDIAEEASKLPSTSDDCPAIGTPLRDSSSSGHSQSTLFDSDVFQTNNNENPYTDPAD
LIADAAGSPSSDSPTNHFFHDGVDFNPDLLNSQSQSGFGEEYFDESSQSGDNDDFKGFAS
QALNTLGVPMLGGDNGETKFKGNNQADTVDFSIISVAGKALAPADLMEHHSGSQGPLLTT
GDLGKEKTQKRVKEGNGTSNSTLSGPGLDSKPGKRSRTPSNDGKSKDKPPKRKKADTEGK
SPSHSSSNRPFTPPTSTGGSKSPGSAGRSQTPPGVATPPIPKITIQIPKGTVMVGKPSSH
SQYTSSGSVSSSGSKSHHSHSSSSSSSASTSGKMKSSKSEGSSSSKLSSSMYSSQGSSGS

SQSKNSSQSGGKPGSSPITKHGLSSGSSSTKMKPQGKPSSLMNPSLSKPNISPSHSRPPG
GSDKLASPMKPVPGTPPSSKAKSPISSGSGGSHMSGTSSSSGMKSSSGLGSSGSLSQKTP
PSSNSCTASSSSFSSSGSSMSSSQNQHGSSKGKSPSRNKKPSLTAVIDKLKHGVVTSGPG
GEDPLDGQMGVSTNSSSHPMSSKHNMSGGEFQGKREKSDKDKSKVSTSGSSVDSSKKTSE
SKNVGSTGVAKIIISKHDGGSPSIKAKVTLQKPGESSGEGLRPQMASSKNYGSPLISGST
PKHERGSPSHSKSPAYTPQNLDSESESGSSIAEKSYQNSPSSDDGIRPLPEYSTEKHKKH
KKEKKKVKDKDRDRDRDKDRDKKKSHSIKPESWSKSPISSDQSLSMTSNTILSADRPSRL
SPDFMIGEEDDDLMDVALIGN*
>ENSG00000164062|ENST00000296456
MERQVLLSEPEEAAALYRGLSRQPALSAACLGPEVTTQYGGQYRTVHTEWTQRDLERMEN
IRFCRQYLVFHDGDSVVFAGPAGNSVETRGELLSRESPSGTMKAVLRKAGGTGPGEEKQF
LEVWEKNRKLKSFNLSALEKHGPVYEDDCFGCLSWSHSETHLLYVAEKKRPKAESFFQTK
ALDVSASDDEIARLKKPDQAIKGDQFVFYEDWGENMVSKSIPVLCVLDVESGNISVLEGV
PENVSPGQAFWAPGDAGVVFVGWWHEPFRLGIRFCTNRRSALYYVDLIGGKCELLSDDSL
AVSSPRLSPDQCRIVYLQYPSLIPHHQCSQLCLYDWYTKVTSVVVDVVPRQLGENFSGIY
CSLLPLGCWSADSQRVVFDSAQRSRQDLFAVDTQVGTVTSLTAGGSGGSWKLLTIDQDLM
VAQFSTPSLPPTLKVGFLPSAGKEQSVLWVSLEEAEPIPDIHWGIRVLQPPPEQENVQYA
GLDFEAILLQPGSPPDKTQVPMVVMPHGGPHSSFVTAWMLFPAMLCKMGFAVLLVNYRGS
TGFGQDSILSLPGNVGHQDVKDVQFAVEQVLQEEHFDASHVALMGGSHGGFISCHLIGQY
PETYRACVARNPVINIASMLGSTDIPDWCVVEAGFPFSSDCLPDLSVWAEMLDKSPIRYI
PQVKTPLLLMLGQEDRRVPFKQGMEYYRALKTRNVPVRLLLYPKSTHALSEVEVESDSFM
NAVLWLRTHLGS*
>ENSG00000166169|ENST00000299206
MDPRGILKAFPKRQKIHADASSKVLAKIPRREEGEEAEEWLSSLRAHVVRTGIGRARAEL
FEKQIVQHGGQLCPAQGPGVTHIVVDEGMDYERALRLLRLPQLPPGAQLVKSAWLSLCLQ
ERRLVDVAGFSIFIPSRYLDHPQPSKAEQDASIPPGTHEALLQTALSPPPPPTRPVSPPQ
KAKEAPNTQAQPISDDEASDGEETQVSAADLEALISGHYPTSLEGDCEPSPAPAVLDKWV
CAQPSSQKATNHNLHITEKLEVLAKAYSVQGDKWRALGYAKAINALKSFHKPVTSYQEAC
SIPGIGKRMAEKIIEILESGHLRKLDHISESVPVLELFSNIWGAGTKTAQMWYQQGFRSL
EDIRSQASLTTQQAIGLKHYSDFLERMPREEATEIEQTVQKAAQAFNSGLLCVACGSYRR
GKATCGDVDVLITHPDGRSHRGIFSRLLDSLRQEGFLTDDLVSQEENGQQQKYLGVCRLP
GPGRRHRRLDIIVVPYSEFACALLYFTGSAHFNRSMRALAKTKGMSLSEHALSTAVVRNT
HGCKVGPGRVLPTPTEKDVFRLLGLPYREPAERDW*
>ENSG00000161036|ENST00000292616
MGPLSARLLMQRGRPKSDRLGKIRSLDLSGLELLSEHLDPKLLCRLTQLQELDLSNNHLE
TLPDNLGLSHLRVLRCANNQLGDVTALCQFPKLEELSLEGNPFLTVNDNLKVSFLLPTLR
KVNGKDASSTYSQVENLNRELTSRVTAHWEKFMATLGPEEEAEKAQADFVKSAVRDVRYG
PESLSEFTQWRVRMISEELVAASRTQVQKANSPEKPPEAGAAHKPRARLAALKRPDDVPL
SLSPSKRACASPSAQVEGSPVAGSDGSQPAVKLEPLHFLQCHSKNNSPQDLETQLWACAF
EPAWEEGATSQTVATCGGEAVCVIDCQTGIVLHKYKAPGEEFFSVAWTALMVVTQAGHKK
RWSVLAAAGLRGLVRLLHVRAGFCCGVIRAHKKAIATLCFSPAHETHLFTASYDKRIILW
DIGVPNQDYEFQASQLLTLDTTSIPLRLCPVASCPDARLLAGCEGGCCCWDVRLDQPQKR
RVCEVEFVFSEGSEASGRRVDGLAFVNEDIVASKGSGLGTICLWSWRQTWGGRGSQSTVA
VVVLARLQWSSTELAYFSLSACPDKGIVLCGDEEGNVWLYDVSNILKQPPLLPAALQAPT

QILKWPQPWALGQVVTKTMVNTVVANASFTYLTALTDSNIVAIWGRM*
>ENSG00000136068|ENST00000295956
MPVTEKDLAEDAPWKKIQQNTFTRWCNEHLKCVNKRIGNLQTDLSDGLRLIALLEVLSQK
RMYRKYHQRPTFRQMQLENVSVALEFLDRESIKLVSIDSKAIVDGNLKLILGLVWTLILH
YSISMPVWEDEGDDDAKKQTPKQRLLGWIQNKIPYLPITNFNQNWQDGKALGALVDSCAP
GLCPDWESWDPQKPVDNAREAMQQADDWLGVPQVITPEEIIHPDVDEHSVMTYLSQFPKA
KLKPGAPLKPKLNPKKARAYGRGIEPTGNMVKQPAKFTVDTISAGQGDVMVFVEDPEGNK
EEAQVTPDSDKNKTYSVEYLPKVTGLHKVTVLFAGQHISKSPFEVSVDKAQGDASKVTAK
GPGLEAVGNIANKPTYFDIYTAGAGVGDIGVEVEDPQGKNTVELLVEDKGNQVYRCVYKP
MQPGPHVVKIFFAGDTIPKSPFVVQVGEACNPNACRASGRGLQPKGVRIRETTDFKVDTK
AAGSGELGVTMKGPKGLEELVKQKDFLDGVYAFEYYPSTPGRYSIAITWGGHHIPKSPFE
VQVGPEAGMQKVRAWGPGLHGGIVGRSADFVVESIGSEVGSLGFAIEGPSQAKIEYNDQN
DGSCDVKYWPKEPGEYAVHIMCDDEDIKDSPYMAFIHPATGGYNPDLVRAYGPGLEKSGC
IVNNLAEFTVDPKDAGKAPLKIFAQDGEGQRIDIQMKNRMDGTYACSYTPVKAIKHTIAV
VWGGVNIPHSPYRVNIGQGSHPQKVKVFGPGVERSGLKANEPTHFTVDCTEAGEGDVSVG
IKCDARVLSEDEEDVDFDIIHNANDTFTVKYVPPAAGRYTIKVLFASQEIPASPFRVKVD
PSHDASKVKAEGPGLSKAGVENGKPTHFTVYTKGAGKAPLNVQFNSPLPGDAVKDLDIID
NYDYSHTVKYTPTQQGNMQVLVTYGGDPIPKSPFTVGVAAPLDLSKIKLNGLENRVEVGK
DQEFTVDTRGAGGQGKLDVTILSPSRKVVPCLVTPVTGRENSTAKFIPREEGLYAVDVTY
DGHPVPGSPYTVEASLPPDPSKVKAHGPGLEGGLVGKPAEFTIDTKGAGTGGLGLTVEGP
CEAKIECSDNGDGTCSVSYLPTKPGEYFVNILFEEVHIPGSPFKADIEMPFDPSKVVASG
PGLEHGKVGEAGLLSVDCSEAGPGALGLEAVSDSGTKAEVSIQNNKDGTYAVTYVPLTAG
MYTLTMKYGGELVPHFPARVKVEPAVDTSRIKVFGPGIEGKDVFREATTDFTVDSRPLTQ
VGGDHIKAHIANPSGASTECFVTDNADGTYQVEYTPFEKGLHVVEVTYDDVPIPNSPFKV
AVTEGCQPSRVQAQGPGLKEAFTNKPNVFTVVTRGAGIGGLGITVEGPSESKINCRDNKD
GSCSAEYIPFAPGDYDVNITYGGAHIPGSPFRVPVKDVVDPSKVKIAGPGLGSGVRARVL
QSFTVDSSKAGLAPLEVRVLGPRGLVEPVNVVDNGDGTHTVTYTPSQEGPYMVSVKYADE
EIPRSPFKVKVLPTYDASKVTASGPGLSSYGVPASLPVDFAIDARDAGEGLLAVQITDQE
GKPKRAIVHDNKDGTYAVTYIPDKTGRYMIGVTYGGDDIPLSPYRIRATQTGDASKCLAT
GPGIASTVKTGEEVGFVVDAKTAGKGKVTCTVLTPDGTEAEADVIENEDGTYDIFYTAAK
PGTYVIYVRFGGVDIPNSPFTVMATDGEVTAVEEAPVNACPPGFRPWVTEEAYVPVSDMN
GLGFKPFDLVIPFAVRKGEITGEVHMPSGKTATPEIVDNKDGTVTVRYAPTEVGLHEMHI
KYMGSHIPESPLQFYVNYPNSGSVSAYGPGLVYGVANKTATFTIVTEDAGEGGLDLAIEG
PSKAEISCIDNKDGTCTVTYLPTLPGDYSILVKYNDKHIPGSPFTAKITDDSRRCSQVKL
GSAADFLLDISETDLSSLTASIKAPSGRDEPCLLKRLPNNHIGISFIPREVGEHLVSIKK
NGNHVANSPVSIMVVQSEIGDARRAKVYGRGLSEGRTFEMSDFIVDTRDAGYGGISLAVE
GPSKVDIQTEDLEDGTCKVSYFPTVPGVYIVSTKFADEHVPGSPFTVKISGEGRVKESIT
RTSRAPSVATVGSICDLNLKIPEINSSDMSAHVTSPSGRVTEAEIVPMGKNSHCVRFVPQ
EMGVHTVSVKYRGQHVTGSPFQFTVGPLGEGGAHKVRAGGPGLERGEAGVPAEFSIWTRE
AGAGGLSIAVEGPSKAEITFDDHKNGSCGVSYIAQEPGNYEVSIKFNDEHIPESPYLVPV
IAPSDDARRLTVMSLQESGLKVNQPASFAIRLNGAKGKIDAKVHSPSGAVEECHVSELEP
DKYAVRFIPHENGVHTIDVKFNGSHVVGSPFKVRVGEPGQAGNPALVSAYGTGLEGGTTG
IQSEFFINTTRAGPGTLSVTIEGPSKVKMDCQETPEGYKVMYTPMAPGNYLISVKYGGPN
HIVGSPFKAKVTGQRLVSPGSANETSSILVESVTRSSTETCYSAIPKASSDASKVTSKGA

GLSKAFVGQKSSFLVDCSKAGSNMLLIGVHGPTTPCEEVSMKHVGNQQYNVTYVVKERGD
YVLAVKWGEEHIPGSPFHVTVP*
>ENSG00000159086|ENST00000290178
MFRKARRVNVRKRNDSEEEERERDEEQEPPPLLPPPGTGEEAGPGGGDRAPGGESLLGPG
PSPPSALTPGLGAEAGGGFPGGAEPGNGLKPRKRPRENKEVPRASLLSFQDEEEENEEVF
KVKKSSYSKKIVKLLKKEYKEDLEKSKIKTELNSSAESEQPLDKTGHVKDTNQEDGVIIS
EHGEDEMDMESEKEEEKPKTGGAFSNALSSLNVLRPGEIPDAAFIHAARKKRQMARELGD
FTPHDNEPGKGRLVREDENDASDDEDDDEKRRIVFSVKEKSQRQKIAEEIGIEGSDDDAL
VTGEQDEELSRWEQEQIRKGINIPQVQASQPAEVNMYYQNTYQTMPYGSSYGIPYSYTAY
GSSDAKSQKTDNTVPFKTPSNEMTPVTIDLVKKQLKDRLDSMKELHKTNRQQHEKHLQSR
VDSTRAIERLEGSSGGIGERYKFLQEMRGYVQDLLECFSEKVPLINELESAIHQLYKQRA
SRLVQRRQDDIKDESSEFSSHSNKALMAPNLDSFGRDRALYQEHAKRRIAEREARRTRRR
QAREQTGKMADHLEGLSSDDEETSTDITNFNLEKDRISKESGKVFEDVLESFYSIDCIKS
QFEAWRSKYYTSYKDAYIGLCLPKLFNPLIRLQLLTWTPLEAKCRDFENMLWFESLLFYG
CEEREQEKDDVDVALLPTIVEKVILPKLTVIAENMWDPFSTTQTSRMVGITLKLINGYPS
VVNAENKNTQVYLKALLLRMRRTLDDDVFMPLYPKNVLENKNSGPYLFFQRQFWSSVKVI
KPPFQRGSCPIPRRKECCSERPRRIWTDRPCVVFS*
>ENSG00000166831|ENST00000300069
MSNLKPDGEHGGSTGTGSGAGSGGALEEEVRTLFVSGLPVDIKPRELYLLFRPFKGYEGS
LIKLTARQPVGFVIFDSRAGAEAAKNALNGIRFDPENPQTLRLEFAKANTKMAKSKLMAT
PNPSNVHPALGAHFIARDPYDLMGAALIPASPEAWAPYPLYTTELTPAISHAAFTYPTAT
AAAAALHAQVRWYPSSDTTQQGWKYRQFC*
>ENSG00000125944|ENST00000302271
MANQVNGNAVQLKEEEEPMDTSSVTHTEHYKTLIEAGLPQKVAERLDEIFQTGLVAYVDL
DERAIDALREFNEEGALSVLQQFKESDLSHVQNKSAFLCGVMKTYRQREKQGSKVQESTK
GPDEAKIKALLERTGYTLDVTTGQRKYGGPPPDSVYSGVQPGIGTEVFVGKIPRDLYEDE
LVPLFEKAGPIWDLRLMMDPLSGQNRGYAFITFCGKEAAQEAVKLCDSYEIRPGKHLGVC
ISVANNRLFVGSIPKNKTKENILEEFSKVTEGLVDVILYHQPDDKKKNRGFCFLEYEDHK
SAAQARRRLMSGKVKVWGNVVTVEWADPVEEPDPEVMAKVKVLFVRNLATTVTEEILEKS
FSEFGKLERVKKLKDYAFVHFEDRGAAVKAMDEMNGKEIEGEEIEIVLAKPPDKKRKERQ
AARQASRSTAYEDYYYHPPPRMPPPIRGRGRGGGRGGYGYPPDYYGYEDYYDDYYGYDYH
DYRGGYEDPYYGYDDGYAVRGRGGGRGGRGAPPPPRGRGAPPPRGRAGYSQRGAPLGPPR
GSRGGRGGPAQQQRGRGSRGSRGNRGGNVGGKRKADGYNQPDSKRRQTNNQQNWGSQPIA
QQPLQQGGDYSGNYGYNNDNQEFYQDTYGQQWK*
>ENSG00000134627|ENST00000299001
MSGRARVKARGIARSPSATEVGRIQASPLPRSVDLSNNEASSSNGFLGTSRISTNDKYGI
SSGDAGSTFMERGVKNKQDFMDLSICTREKLAHVRNCKTGSSGIPVKLVTNLFNLDFPQD
WQLYQYHVTYIPDLASRRLRIALLYSHSELSNKAKAFDGAILFLSQKLEEKVTELSSETQ
RGETIKMTITLKRELPSSSPVCIQVFNIIFRKILKKLSMYQIGRNFYNPSEPMEIPQHKL
SLWPGFAISVSYFERKLLFSADVSYKVLRNETVLEFMTALCQRTGLSCFTQTCEKQLIGL
IVLTRYNNRTYSIDDIDWSVKPTHTFQKRDGTEITYVDYYKQQYDITVSDLNQPMLVSLL
KKKRNDNSEAQLAHLIPELCFLTGLTDQATSDFQLMKAVAEKTRLSPSGRQQRLARLVDN
IQRNTNARFELETWGLHFGSQISLTGRIVPSEKILMQDHICQPVSAADWSKDIRTCKILN
AQSLNTWLILCSDRTEYVAESFLNCLRRVAGSMGFNVDYPKIIKVQENPAAFVRAIQQYV

DPDVQLVMCILPSNQKTYYDSIKKYLSSDCPVPSQCVLARTLNKQGMMMSIATKIAMQMT
CKLGGELWAVEIPLKSLMVVGIDVCKDALSKDVMVVGCVASVNPRITRWFSRCILQRTMT
DVADCLKVFMTGALNKWYKYNHDLPARIIVYRAGVGDGQLKTLIEYEVPQLLSSVAESSS
NTSSRLSVIVVRKKCMPRFFTEMNRTVQNPPLGTVVDSEATRNEWYDFYLISQVACRGTV
SPTYYNVIYDDNGLKPDHMQRLTFKLCHLYYNWPGIVSVPAPCQYAHKLTFLVAQSIHKE
PSLELANHLFYL*
>ENSG00000078687|ENST00000301624
MATGSAQGNFTGHTKKTNGNNGTNGALVQSPSNQSALGAGGANSNGSAARVWGVATGSSS
GLAHCSVSGGDGKMDTMIGDGRSQNCWGASNSNAGINLNLNPNANPAAWPVLGHEGTVAT
GNPSSICSPVSAIGQNMGNQNGNPTGTLGAWGNLLPQESTEPQTSTSQNVSFSAQPQNLN
TDGPNNTNPMNSSPNPINAMQTNGLPNWGMAVGMGAIIPPHLQGLPGANGSSVSQVSGGS
AEGISNSVWGLSPGNPATGNSNSGFSQGNGDTVNSALSAKQNGSSSAVQKEGSGGNAWDS
GPPAGPGILAWGRGSGNNGVGNIHSGAWGHPSRSTSNGVNGEWGKPPNQHSNSDINGKGS
TGWESPSVTSQNPTVQPGGEHMNSWAKAASSGTTASEGSSDGSGNHNEGSTGREGTGEGR
RRDKGIIDQGHIQLPRNDLDPRVLSNTGWGQTPVKQNTAWEFEESPRSERKNDNGTEAWG
CAATQASNSGGKNDGSIMNSTNTSSVSGWVNAPPAAVPANTGWGDSNNKAPSGPGVWGDS
ISSTAVSTAAAAKSGHAWSGAANQEDKSPTWGEPPKPKSQHWGDGQRSNPAWSAGGGDWA
DSSSVLGHLGDGKKNGSGWDADSNRSGSGWNDTTRSGNSGWGNSTNTKANPGTNWGETLK
PGPQQNWASKPQDNNVSNWGGAASVKQTGTGWIGGPVPVKQKDSSEATGWEEPSPPSIRR
KMEIDDGTSAWGDPSNYNNKTVNMWDRNNPVIQSSTTTNTTTTTTTTSNTTHRVETPPP
HQAGTQLNRSPLLGPGRKVSSGWGEMPNVHSKTENSWGEPSSPSTLVDNGTAAWGKPPSS
GSGWGDHPAEPPVAFGRAGAPVAASALCKPASKSMQEGWGSGGDEMNLSTSQWEDEEGDV
WNNAASQESTSSCSSWGNAPKKGLQKGMKTSGKQDEAWIMSRLIKQLTDMGFPREPAEEA
LKSNNMNLDQAMSALLEKKVDVDKRGLGVTDHNGMAAKPLGCRPPISKESSVDRPTFLDK
DGGLVEEPTPSPFLPSPSLKLPLSHSALPSQALGGIASGLGMQNLNSSRQIPSGNLGMFG
NSGAAQARTMQQPPQPPVQPLNSSQPSLRAQVPQFLSPQVQAQLLQFAAKNIGLNPALLT
SPINPQHMTMLNQLYQLQLAYQRLQIQQQMLQAQRNVSGSMRQQEQQVARTITNLQQQIQ
QHQRQLAQALLVKQPPPPPPPPHLSLHPSAGKSAMDSFPSHPQTPGLPDLQTKEQQSSPN
TFAPYPLAGLNPNMNVNSMDMTGGLSVKDPSQSQSRLPQWTHPNSMDNLPSAASPLEQNP
SKHGAIPGGLSIGPPGKSSIDDSYGRYDLIQNSESPASPPVAVPHSWSRAKSDSDKISNG
SSINWPPEFHPGVPWKGLQNIDPENDPDVTPGSVPTGPTINTTIQDVNRYLLKSGGKLSD
IKSTWSSGPTSHTQASLSHELWKVPRNSTAPTRPPPGLTNPKPSSTWGASPLGWTSSYSS
GSAWSTDTSGRTSSWLVLRNLTPQIDGSTLRTLCLQHGPLITFHLNLTQGNAVVRYSSKE
EAAKAQKSLHMCVLGNTTILAEFAGEEEVNRFLAQGQALPPTSSWQSSSASSQPRLSAAG
SSHGLVRSDAGHWNAPCLGGKGSSELLWGGVPQYSSSLWGPPSADDSRVIGSPTPLTTLL
PGDLLSGESL*
>ENSG00000142556|ENST00000270649
MIKTQESLTLEDVAVEFSWEEWQLLDTAQKNLYRDVMVENYNHLVSLGYQTSKPDVLSKL
AHGQEPWTTDAKIQNKNCPGIGKVDSHLQEHSPNQRLLKSVQQCNGQNTLRNIVHLSKTH
FPIVQNHDTFDLYRKNLKSSLSLINQKRRHGINNPVEFIGGEKTLLHGKHERTHTKTRFS
ENAKCIHTKFQVFKHQRTQKIEKPHACIECEQTFLRKSQLIYHENICIQENPGSGQCEKL
SRSVLFTKHLKTNTTDKICIPNEYRKGSTVKSSLITHQQTHTEEKSYMCSECGKGFTMKR
YLIAHQRTHSGEKPYVCKECGKGFTVKSNLIVHQRTHTGEKPYICSECGKGFTMKRYLVV
HQRTHTGEKPYMCSECGKGFTVKSNLIVHQRSHTGEKSYICSECGKGFTVKRTLVIHQRT

HTGEKSYICNECGKGFTTKRTLIIHQRTHTGEKPYECNECGKAFSQKICLIQHERCHTGK
TPFVCTECGKSYSHKYGLITHQRIHTGEKPYECNECGKAFTTKSVLNVHQRTHTGERPYG
CSDCEKAFSHLSNLVKHKKMHTREMGRISQVENSCNGESQLLPYK*
>ENSG00000174576|ENST00000311034
MYRSTKGASKARRDQINAEIRNLKELLPLAEADKVRLSYLHIMSLACIYTRKGVFFAGGT
PLAGPTGLLSAQELEDIVAALPGFLLVFTAEGKLLYLSESVSEHLGHSMVDLVAQGDSIY
DIIDPADHLTVRQQLTLPSALDTDRLFRCRFNTSKSLRRQSAGNKLVLIRGRFHAHPPGA
YWAGNPVFTAFCAPLEPRPRPGPGPGPGPASLFLAMFQSRHAKDLALLDISESVLIYLGF
ERSELLCKSWYGLLHPEDLAHASAQHYRLLAESGDIQAEMVVRLQAKTGGWAWIYCLLYS
EGPEGPITANNYPISDMEAWSLRQQLNSEDTQAAYVLGTPTMLPSFPENILSQEECSSTN
PLFTAALGAPRSTSFPSAPELSVVSASEELPRPSKELDFSYLTFPSGPEPSLQAELSKDL
VCTPPYTPHQPGGCAFLFSLHEPFQTHLPTPSSTLQEQLTPSTATFSDQLTPSSATFPDP
LTSPLQGQLTETSVRSYEDQLTPCTSTFPDQLLPSTATFPEPLGSPAHEQLTPPSTAFQA
HLDSPSQTFPEQLSPNPTKTYFAQEGCSFLYEKLPPSPSSPGNGDCTLLALAQLRGPLSV
DVPLVPEGLLTPEASPVKQSFFHYSEKEQNEIDRLIQQISQLAQGMDRPFSAEAGTGGLE
PLGGLEPLDSNLSLSGAGPPVLSLDLKPWKCQELDFLADPDNMFLEETPVEDIFMDLSTP
DPSEEWGSGDPEAEGPGGAPSPCNNLSPEDHSFLEDLATYETAFETGVSAFPYDGFTDEL
HQLQSQVQDSFHEDGSGGEPTF*
>ENSG00000154710|ENST00000284957
MSLKSERRGIHVDQSDLLCKKGCGYYGNPAWQGFCSKCWREEYHKARQKQIQEDWELAER
LQREEEEAFASSQSSQGAQSLTFSKFEEKKTNEKTRKVTTVKKFFSASSRVGSKKEIQEA
KAPSPSINRQTSIETDRVSKEFIEFLKTFHKTGQEIYKQTKLFLEGMHYKRDLSIEEQSE
CAQDFYHNVAERMQTRGKVPPERVEKIMDQIEKYIMTRLYKYVFCPETTDDEKKDLAIQK
RIRALRWVTPQMLCVPVNEDIPEVSDMVVKAITDIIEMDSKRVPRDKLACITKCSKHIFN
AIKITKNEPASADDFLPTLIYIVLKGNPPRLQSNIQYITRFCNPSRLMTGEDGYYFTNLC
CAVAFIEKLDAQSLNLSQEDFDRYMSGQTSPRKQEAESWSPDACLGVKQMYKNLDLLSQL
NERQERIMNEAKKLEKDLIDWTDGIAREVQDIVEKYPLEIKPPNQPLAAIDSENVENDKL
PPPLQPQVYAG*
>ENSG00000141644|ENST00000269468
MAEDWLDCPALGPGWKRREVFRKSGATCGRSDTYYQSPTGDRIRSKVELTRYLGPACDLT
LFDFKQGILCYPAPKAHPVAVASKKRKKPSRPAKTRKRQVGPQSGEVRKEAPRDETKADT
DTAPASFPAPGCCENCGISFSGDGTQRQRLKTLCKDCRAQRIAFNREQRMFKRVGCGECA
ACQVTEDCGACSTCLLQLPHDVASGLFCKCERRRCLRIVERSRGCGVCRGCQTQEDCGHC
PICLRPPRPGLRRQWKCVQRRCLRGKHARRKGGCDSKMAARRRPGAQPLPPPPPSQSPEP
TEPHPRALAPSPPAEFIYYCVDEDELQPYTNRRQNRKCGACAACLRRMDCGRCDFCCDKP
KFGGSNQKRQKCRWRQCLQFAMKRLLPSVWSESEDGAGSPPPYRRRKRPSSARRHHLGPT
LKPTLATRTAQPDHTQAPTKQEAGGGFVLPPPGTDLVFLREGASSPVQVPGPVAASTEAL
LQEAQCSGLSWVVALPQVKQEKADTQDEWTPGTAVLTSPVLVPGCPSKAVDPGLPSVKQE
PPDPEEDKEENKDDSASKLAPEEEAGGAGTPVITEIFSLGGTRFRDTAVWLPRSKDLKKP
GARKQ*
>ENSG00000165512|ENST00000298299
MRLAKPKAGISRSSSQGKAYENKRKTGRQRQKWGMTIRFDSSFSRLRRSLDDKPYKCTEC
EKSFSQSSTLFQHQKIHTGKKSHKCADCGKSFFQSSNLIQHRRIHTGEKPYKCDECGESF
KQSSNLIQHQRIHTGEKPYQCDECGRCFSQSSHLIQHQRTHTGEKPYQCSECGKCFSQSS

HLRQHMKVHKEEKPRKTRGKNIRVKTHLPSWKAGTGRKSVAGLR*
>ENSG00000124831|ENST00000289175
MTSPAAAQSREIDCLSPEAQKLAEARLAAKRAARAEAREIRMKELERQQKEVEERPEKDF
TEKGSRNMPGLSAATLASLGGTSSRRGSGDTSISIDTEASIREIKDSLAEVEEKYKKAMV
SNAQLDNEKTNFMYQVDTLKDMLLELEEQLAESRRQYEEKNKEFEREKHAHSILQFQFAE
VKEALKQREEMLEKHGIILNSEIATNGETSDTLNNVGYQGPTKMTKEELNALKSTGDGTL
GRASEVEVKNEIVANVGKREILHNTEKEQHTEDTVKDCVDIEVFPAGENTEDQKSSEDTA
PFLGTLAGATYEEQVQSQILESSSLPENTVQVESNEVMGAPDDRTRTPLEPSNCWSDLDG
GNHTENVGEAAVTQVEEQAGTVASCPLGHSDDTVYHDDKCMVEVPQELETSTGHSLEKEF
TNQEAAEPKEVPAHSTEVGRDHNEEEGEETGLRDEKPIKTEVPGSPAGTEGNCQEATGPS
TVDTQNEPLDMKEPDEEKSDQQGEALDSSQKKTKNKKKKNKKKKSPVPVETLKDVKKELT
YQNTDLSEIKEEEQVKSTDRKSAVEAQNEVTENPKQKIAAESSENVDCPENPKIKLDGKL
DQEGDDVQTAAEEVLADGDTLDFEDDTVQSSGPRAGGEELDEGVAKDNAKIDGATQSSPA
EPKSEDADRCTLPEHESPSQDISDACEAESTERCEMSEHPSQTVRKALDSNSLENDDLSA
PGREPGHFNPESREDTRGGNEKGKSKEDCTMS*
>ENSG00000166508|ENST00000303887
MALKDYALEKEKVKKFLQEFYQDDELGKKQFKYGNQLVRLAHREQVALYVDLDDVAEDDP
ELVDSICENARRYAKLFADAVQELLPQYKEREVVNKDVLDVYIEHRLMMEQRSRDPGMVR
SPQNQYPAELMRRFELYFQGPSSNKPRVIREVRADSVGKLVTVRGIVTRVSEVKPKMVVA
TYTCDQCGAETYQPIQSPTFMPLIMCPSQECQTNRSGGRLYLQTRGSRFIKFQEMKMQEH
SDQVPVGNIPRSITVLVEGENTRIAQPGDHVSVTGIFLPILRTGFRQVVQGLLSETYLEA
HRIVKMNKSEDDESGAGELTREELRQIAEEDFYEKLAASIAPEIYGHEDVKKALLLLLVG
GVDQSPRGMKIRGNINICLMGDPGVAKSQLLSYIDRLAPRSQYTTGRGSSGVGLTAAVLR
DSVSGELTLEGGALVLADQGVCCIDEFDKMAEADRTAIHEVMEQQTISIAKAGILTTLNA
RCSILAAANPAYGRYNPRRSLEQNIQLPAALLSRFDLLWLIQDRPDRDNDLRLAQHITYV
HQHSRQPPSQFEPLDMKLMRRYIAMCREKQPMVPESLADYITAAYVEMRREAWASKDATY
TSARTLLAILRLSTALARLRMVDVVEKEDVNEAIRLMEMSKDSLLGDKGQTARTQRPADV
IFATVRELVSGGRSVRFSEAEQRCVSRGFTPAQFQAALDEYEELNVWQVNASRTRITFV*
>ENSG00000152430|ENST00000282278
MTELEYPKGSSIMPAAGTMYLTTSTGYPYTYHNGVAYFHTPEVTSVPPPWPSRSVCSSPV
MVAQPIYQQPAYHYQATTQYLPGQWQWSVPQPSASSAPFLYLQPSEVIYQPVEIAQDGGC
VPPPLSLMETSVPEPYSDHGVQATYHQVYAPSAITMPAPVMQPEPIKTVWSIHY*
>ENSG00000162613|ENST00000294623
MADYSTVPPPSSGSAGGGGGGGGGGGGVNDAFKDALQRARQIAAKIGGDAGTSLNSNDYGY
GGQKRPLEDGDQPDAKKVAPQNDSFGTQLPPMHQQQRSVMTEEYKVPDGMVGFIIGRGGE
QISRIQQESGCKIQIAPDSGGLPERSCMLTGTPESVQSAKRLLDQIVEKGRPAPGFHHGD
GPGNAVQEIMIPASKAGLVIGKGGETIKQLQERAGVKMVMIQDGPQNTGADKPLRITGDP
YKVQQAKEMVLELIRDQGGFREVRNEYGSRIGGNEGIDVPIPRFAVGIVIGRNGEMIKKI
QNDAGVRIQFKPDDGTTPERIAQITGPPDRCQHAAEIITDLLRSVQAGNPGGPGPGGRGR
GRGQGNWNMGPPGGLQEFNFIVPTGKTGLIIGKGGETIKSISQQSGARIELQRNPPPNAD
PNMKLFTIRGTPQQIDYARQLIEEKIGGPVNPLGPPVPHGPHGVPGPHGPPGPPGPGTPM
GPYNPAPYNPGPPGPAPHGPPAPYAPQGWGNAYPHWQQQAPPDPAKAGTDPNSAAWAAYY
AHYYQQQAQPPPAAPAPGAPTTTQTNGQGDQQNPAPAGQVDYTKAWEEYYKKMGQAVPAPT
GAPPGGQPDYSAAWAEYYRQQAAYYAQTSPQGMPQHPPAPQCRFDPASIELAL*

>ENSG00000160685|ENST00000292176
MGSPEDDLIGIPFPDHSSELLSCLNEQRQLGHLCDLTIRTQGLEYRTHRAVLAACSHYFK
KLFTEGGGGAVMGAGGSGTATGGAGAGVCELDFVGPEALGALLEFAYTATLTTSSANMPA
VLQAARLLEIPCVIAACMEILQGSGLEAPSPDEDDCERARQYLEAFATATASGVPNGEDS
PPQVPLPPPPPPPPRPVARRSRKPRKAFLQTKGARANHLVPEVPTVPAHPLTYEEEEVAG
RVGSSGGSGPGDSYSPPTGTASPPEGPQSYEPYEGEEEEEELVYPPAYGLAQGGGPPLSP
EELGSDEDAIDPDLMAYLSSLHQDNLAPGLDSQDKLVRKRRSQMPQECPVCHKIIHGAGK
LPRHMRTHTGEKPFACEVCGVRFTRNDKLKIHMRKHTGERPYSCPHCPARFLHSYDLKNH
MHLHTGDRPYECHLCHKAFAKEDHLQRHLKGQNCLEVRTRRRRKDDAPPHYPPPSTAAAS
PAGLDLSNGHLDTFRLSLARFWEQSAPTGPPVSTPGPPDDDEEEGAPTTPQAEGAMESS*
>ENSG00000172239|ENST00000306846
MSDGFDRAPGAGRGRSRGLGRGGGGPEGGGFPNGAGPAERARHQPPQPKAPGFLQPPPLR
QPRTTPPPGAQCEVPASPQRPSRPGALPEQTRPLRAPPSSQDKIPQQNSESAMAKPQVVV
APVLMSKLSVNAPEFYPSGYSSSYTESYEDGCEDYPTLSEYVQDFLNHLTEQPGSFETEI
EQFAETLNGCVTTDDALQELVELIYQQATSIPNFSYMGARLCNYLSHHLTISPQSGNFRQ
LLLQRCRTEYEVKDQAAKGDEVTRKRFHAFVLFLGELYLNLEIKGTNGQVTRADILQVGL
RELLNALFSNPMDDNLICAVKLLKLTGSVLEDAWKEKGKMDMEEIIQRIENVVLDANCSR
DVKQMLLKLVELRSSNWGRVHATSTYREATPENDPNYFMNEPTFYTSDGVPFTAADPDYQ
EKYQELLEREDFFPDYEENGTDLSGAGDPYLDDIDDEMDPEIEEAYEKFCLESERKRKQ*
>ENSG00000161277|ENST00000292894
MPKYCRAPNCSNTAGRLGADNRPVSFYKFPLKDGPRLQAWLQHMGCEHWVPSCHQHLCSE
HFTPSCFQWRWGVRYLRPDAVPSIFSRGPPAKSQRRTRSTQKPVSPPPPLQKNTPLPQSP
AIPVSGPVRLVVLGPTSGSPKTVATMLLTPLAPAPTPERSQPEVPAQQAQTGLGPVLGAL
QRRVRRLQRCQERHQAQLQALERLAQQLHGESLLARARRGLQRLTTAQTLGPEESQTFTI
ICGGPDIAMVLAQDPAPATVDAKPELLDTRIPSA*
>ENSG00000168813|ENST00000311921
MEESSSVAMLVPDIGEQEAILTAESIISPSLEIDEQRKTKPDPLIHVIQKLSKIVENEKS
QKCLLIGKKRPRSSAATHSLETQELCEIPAKVIQSPAADTRRAEMSQTNFTPDTLAQNEG
KAMSYQCSLCKFLSSSFSVLKDHIKQHGQQNEVILMCSECHITSRSQEELEAHVVNDHDN
DANIHTQSKAQQCVSPSSSLCRKTTERNETIPDIPVSVDNLQTHTVQTASVAEMGRRKWY
AYEQYGMYRCLFCSYTCGQQRMLKTHAWKHAGEVDCSYPIFENENEPLGLLDSSAAAAPG
GVDAVVIAIGESELSIHNGPSVQVQICSSEQLSSSSPLEQSAERGVHLSQSVTLDPNEEE
MLEVISDAEENLIPDSLLTSAQKIISSSPNKKGHVNVIVERLPSAEETLSQKRFLMNTEM
EEGKDLSLTEAQIGREGMDDVYRADKCTVDIGGLIIGWSSSEKKDELMNKGLATDENAPP
GRRRTNSESLRLHSLAAEALVTMPIRAAELTRANLGHYGDINLLDPDTSQRQVDSTLAAY
SKMMSPLKNSSDGLTSLNQSNSTLVALPEGRQELSDGQVKTGISMSLLTVIEKLRERTDQ
NASDDDILKELQDNAQCQPNSDTSLSGNNVVEYIPNAERPYRCRLCHYTSGNKGYIKQHL
RVHRQRQPYQCPICEHIADNSKDLESHMIHHCKTRIYQCKQCEESFHYKSQLRNHEREQH
SLPDTLSIATSNEPRISSDTADGKCVQEGNKSSVQKQYRCDVCDYTSTTYVGVRNHRRIH
NSDKPYRCSLCGYVCSHPPSLKSHMWKHASDQNYNYEQVNKAINDAISQSGRVLGKSPGK
TQLKSSEESADPVTGSSENAVSSSELMSQTPSEVLGTNENEKLSPTSNTSYSLEKISSLA
PPSMEYCVLLFCCCICGFESTSKENLLDHMKEHEGEIVNIILNKDHNTALNTN*
>ENSG00000113742|ENST00000265085
MGDYGFGVLVQSNTGNKSAFPVRFHPHLQPPHHHQNATPSPAAFINNNTAANGSSAGSAW

LFPAPATHNIQDEILGSEKAKSQQQEQQDPLEKQQLSPSPGQEAGILPETEKAKSEENQG
DNSSENGNGKEKIRIESPVLTGFDYQEATGLGTSTQPLTSSASSLTGFSNWSAAIAPSSS
TIINEDASFFHQGGVPAASANNGALLFQNFPHHVSPGFGGSFSPQIGPLSQHHPHHPHFQ
HHHSQHQQQRRSPASPHPPPFTHRNAAFNQLPHLANNLNKPPSPWSSYQSPSPTPSSSWS
PGGGGYGGWGGSQGRDHRRGLNGGITPLNSISPLKKNFASNHIQLQKYARPSSAFAPKSW
MEDSLNRADNIFPFPDRPRTFDMHSLESSLIDIMRAENDTIKGRLNYSYPGSDSSLLINA
RTYGRRRGQSSLFPMEDGFLDDGRGDQPLHSGLGSPHCFSHQNGERVERYSRKVFVGGLP
PDIDEDEITASFRRFGPLIVDWPHKAESKSYFPPKGYAFLLFQDESSVQALIDACIEEDG
KLYLCVSSPTIKDKPVQIRPWNLSDSDFVMDGSQPLDPRKTIFVGGVPRPLRAVELAMIM
DRLYGGVCYAGIDTDPELKYPKGAGRVAFSNQQSYIAAISARFVQLQHGEIDKRVEVKPY
VLDDQLCDECQGARCGGKFAPFFCANVTCLQYYCEYCWAAIHSRAGREFHKPLVKEGGDR
PRHISFRWN*
>ENSG00000196943|ENST00000267425
MGQGPRSPHKVGRRFPAGGKRGRGAKGSGRPLPGRKRQPWPPPDGRSEPAPDSHPHLSPE
ALGYFRRALSALKEAPETGEERDLMVHNIMKEVETQALALSTNRTGSEMLQELLGFSPLK
PLCRVWAALRSNLRTVACHRCGVHVLQSALLQLPRLLGSAAEEEEEEEEDGKDGPTETLE
ELVLGLAAEVCDDFLVYCGDTHGSFVVRTLLQVLGGTILESERARPRGSQSSEAQKTPAQ
ECKPADFEVPETFLNRLQDLSSSFLKDIAVFITDKISSFCLQVALQVLHRKLPQFCAHLC
NAVIGYLSTRGSSVDGSPLLLFLRDQTSSRLLEQVLLVLEPPRLQSLFEEHLQGQLQTLA
AHPIANFPLQRLLDAVTTPELLSPVFEELSPVLEAVLAQGHPGVVIALVGACRRVGAYQA
KVLQLLLEAFHCAEPSSRQVACVPLFATLMAYEVYYGLTEEEGAVPAEHQVAMAAARALG
DVTVLGSLLLQHLLHFSTPGLVLRSLGALTGPQLLSLAQSPAGSHVLDAILTSPSVTRKL
RRRVLQNLKGQYVALACSRHGSRVLDAIWSGAALRARKEIAAELGEQNQELIRDPFGHHV
ARNVALTTFLKRREAWEQQQGAVAKRRRALNSILED*
>ENSG00000147133|ENST00000276072
MGPGCDLLLRTAATITAAAIMSDTDSDEDSAGGGPFSLAGFLFGNINGAGQLEGESVLDD
ECKKHLAGLGALGLGSLITELTANEELTGTDGALVNDEGWVRSTEDAVDYSDINEVAEDE
SRRYQQTMGSLQPLCHSDYDEDDYDADCEDIDCKLMPPPPPPPGPMKKDKDQDSITGVSE
NGEGIILPSIIAPSSLASEKVDFSSSSDSESEMGPQEATQAESEDGKLTLPLAGIMQHDA
TKLLPSVTELFPEFRPGKVLRFLRLFGPGKNVPSVWRSARRKRKKKHRELIQEEQIQEVE
CSVESEVSQKSLWNYDYAPPPPPEQCLSDDEITMMAPVESKFSQSTGDIDKVTDTKPRVA
EWRYGPARLWYDMLGVPEDGSGFDYGFKLRKTEHEPVIKSRMIEEFRKLEENNGTDLLAD
ENFLMVTQLHWEDDIIWDGEDVKHKGTKPQRASLAGWLPSSMTRNAMAYNVQQGFAATLD
DDKPWYSIFPIDNEDLVYGRWEDNIIWDAQAMPRLLEPPVLTLDPNDENLILEIPDEKEE
ATSNSPSKESKKESSLKKSRILLGKTGVIKEEPQQNMSQPEVKDPWNLSNDEYYYPKQQG
LRGTFGGNIIQHSIPAVELRQPFFPTHMGPIKLRQFHRPPLKKYSFGALSQPGPHSVQPL
LKHIKKKAKMREQERQASGGGEMFFMRTPQDLTGKDGDLILAEYSEENGPLMMQVGMATK
IKNYYKRKPGKDPGAPDCKYGETVYCHTSPFLGSLHPGQLLQAFENNLFRAPIYLHKMPE
TDFLIIRTRQGYYIRELVDIFVVGQQCPLFEVPGPNSKRANTHIRDFLQVFIYRLFWKSK
DRPRRIRMEDIKKAFPSHSESSIRKRLKLCADFKRTGMDSNWWVLKSDFRLPTEEEIRAM
VSPEQCCAYYSMIAAEQRLKDAGYGEKSFFAPEEENEEDFQMKIDDEVRTAPWNTTRAFI
AAMKGKCLLEVTGVADPTGCGEGFSYVKIPNKPTQQKDDKEPQPVKKTVTGTDADLRRLS
LKNAKQLLRKFGVPEEEIKKLSRWEVIDVVRTMSTEQARSGEGPMSKFARGSRFSVAEHQ
ERYKEECQRIFDLQNKVLSSTEVLSTDTDSSSAEDSDFEEMGKNIENMLQNKKTSSQLSR

EREEQERKELQRMLLAAGSAASGNNHRDDDTASVTSLNSSATGRCLKIYRTFRDEEGKEY
VRCETVRKPAVIDAYVRIRTTKDEEFIRKFALFDEQHREEMRKERRRIQEQLRRLKRNQE
KEKLKGPPEKKPKKMKERPDLKLKCGACGAIGHMRTNKFCPLYYQTNAPPSNPVAMTEEQ
EEELEKTVIHNDNEELIKVEGTKIVLGKQLIESADEVRRKSLVLKFPKQQLPPKKKRRVG
TTVHCDYLNRPHKSIHRRRTDPMVTLSSILESIINDMRDLPNTYPFHTPVNAKVVKDYYK
IITRPMDLQTLRENVRKRLYPSREEFREHLELIVKNSATYNGPKHSLTQISQSMLDLCDE
KLKEKEDKLARLEKAINPLLDDDDQVAFSFILDNIVTQKMMAVPDSWPFHHPVNKKFVPD
YYKVIVNPMDLETIRKNISKHKYQSRESFLDDVNLILANSVKYNGPESQYTKTAQEIVNV
CYQTLTEYDEHLTQLEKDICTAKEAALEEAELESLDPMTPGPYTPQPPDLYDTNTSLSMS
RDASVFQDESNMSVLDIPSATPEKQVTQEGEDGDGDLADEEEGTVQQPQASVLYEDLLMS
EGEDDEEDAGSDEEGDNPFSAIQLSESGSDSDVGSGGIRPKQPRMLQENTRMDMENEESM
MSYEGDGGEASHGLEDSNISYGSYEEPDPKSNTQDTSFSSIGGYEVSEEEEDEEEEEQRS
GPSVLSQVHLSEDEEDSEDFHSIAGDSDLDSDE*
>ENSG00000164256|ENST00000296682
MSPEKSQEESPEEDTERTERKPMVKDAFKDISIYFTKEEWAEMGDWEKTRYRNVKRNYNA
LITIGLRATRPAFMCHRRQAIKLQVDDTEDSDEEWTPRQQVKPPWMALRVEQRKHQKGMP
KASFSNESSLKELSRTANLLNASGSEQAQKPVSPSGEASTSGQHSRLKLELRKKETERKM
YSLRERKGHAYKEVSEPQDDDYLYCEMCQNFFIDSCAAHGPPTFVKDSAVDKGHPNRSAL
SLPPGLRIGPSGIPQAGLGVWNEASDLPLGLHFGPYEGRITEDEEAANNGYSWLITKGRN
CYEYVDGKDKSWANWMRYVNCARDDEEQNLVAFQYHRQIFYRTCRVIRPGCELLVWYGDE
YGQELGIKWGSKWKKELMAGREPKPEIHPCPSCCLAFSSQKFLSQHVERNHSSQNFPGPS
ARKLLQPENPCPGDQNQEQQYPDPHSRNDKTKGQEIKERSKLLNKRTWQREISRAFSSPP
KGQMGSCRVGKRIMEEESRTGQKVNPGNTGKLFVGVGISRIAKVKYGECGQGFSVKSDVI
THQRTHTGEKLYVCRECGRGFSWKSHLLIHQRIHTGEKPYVCRECGRGFSWQSVLLTHQR
THTGEKPYVCRECGRGFSRQSVLLTHQRRHTGEKPYVCRECGRGFSRQSVLLTHQRRHTG
EKPYVCRECGRGFSWQSVLLTHQRTHTGEKPYVCRECGRGFSWQSVLLTHQRTHTGEKPY
VCRECGRGFSNKSHLLRHQRTHTGEKPYVCRECGRGFRDKSHLLRHQRTHTGEKPYVCRE
CGRGFRDKSNLLSHQRTHTGEKPYVCRECGRGFSNKSHLLRHQRTHTGEKPYVCRECGRG
FRNKSHLLRHQRTHTGEKPYVCRECGRGFSDRSSLCYHQRTHTGEKPYVCREDE*
>ENSG00000157349|ENST00000288071
MATDSWALAVDEQEAAAESLSNLHLKEEKIKPDTNGAVVKTNANAEKTDEEEKEDRAAQS
LLNKLIRSNLVDNTNQVEVLQRDPNSPLYSVKSFEELRLKPQLLQGVYAMGFNRPSKIQE
NALPLMLAEPPQNLIAQSQSGTGKTAAFVLAMLSQVEPANKYPQCLCLSPTYELALQTGK
VIEQMGKFYPELKLAYAVRGNKLERGQKISEQIVIGTPGTVLDWCSKLKFIDPKKIKVFV
LDEADVMIATQGHQDQSIRIQRMLPRNCQMLLFSATFEDSVWKFAQKVVPDPNVIKLKRE
EETLDTIKQYYVLCSSRDEKFQALCNLYGAITIAQAMIFCHTRKTASWLAAELSKEGHQV
ALLSGEMMVEQRAAVIERFREGKEKVLVTTNVCARGIDVEQVSVVINFDLPVDKDGNPDN
ETYLHRIGRTGRFGKRGLAVNMVDSKHSMNILNRIQEHFNKKIERLDTDDLDEIEKIAN*
>ENSG00000064490|ENST00000303088
MELTQPAEDLIQTQQTPASELGDPEDPGEEAADGSDTVVLSLFPCTPEPVNPEPDASVSS
PQAGSSLKHSTTLTNRQRGNEVSALPATLDSLSIHQLAAQGELDQLKEHLRKGDNLVNKP
DERGFTPLIWASAFGEIETVRFLLEWGADPHILAKERESALSLASTGGYTDIVGLLLERD
VDINIYDWNGGTPLLYAVRGNHVKCVEALLARGADLTTEADSGYTPMDLAVALGYRKVQQ
VIENHILKLFQSNLVPADPE*

>ENSG00000139154|ENST00000266508
MAAAITDMADLEELSRLSPLPPGSPGSAARGRAEPPEEEEEEEEEEEEEAEAEAVAALLLN
GGSGGGGGGGGGGVGGGEAETMSEPSPESASQAGEDEDEEEDDEEEEDESSSSGGGEEES
SAESLVGSSGGSSSDETRSLSPGAASSSSGDGDGKEGLEEPKGPRGSQGGGGGGSSSSSV
VSSGGDEGYGTGGGGSSATSGGRRGSLEMSSDGEPLSRMDSEDSISSTIMDVDSTISSGR
STPAMMNGQGSTTSSSKNIAYNCCWDQCQACFNSSPDLADHIRSIHVDGQRGGVFVCLWK
GCKVYNTPSTSQSWLQRHMLTHSGDKPFKCVVGGCNASFASQGGLARHVPTHFSQQNSSK
VSSQPKAKEESPSKAGMNKRRKLKNKRRRSLPRPHDFFDAQTLDAIRHRAICFNLSAHIE
SLGKGHSVVFHSTVIAKRKEDSGKIKLLLHWMPEDILPDVWVNESERHQLKTKVVHLSKL
PKDTALLLDPNIYRTMPQKRLKR*
>ENSG00000167088|ENST00000300413
MKLVRFLMKLSHETVTIELKNGTQVHGTITGVDVSMNTHLKAVKMTLKNREPVQLETLSI
RGNNIRYFILPDSLPLDTLLVDVEPKVKSKKREAVAGRGRGRGRGRGRGRGRGRGGPRR*
>ENSG00000153779|ENST00000283891
MEAAADGPAETQSPVEKDSPAKTQSPAQDTSIMSRNNADTGRVLALPEHKKKRKGNLPAE
SVKILRDWMYKHRFKAYPSEEEKQMLSEKTNLSLLQISNWFINARRRILPDMLQQRRNDP
IIGHKTGKDAHATHLQSTEASVPAKSGPSGPDNVQSLPLWPLPKGQMSREKQPDPESAPS
QKLTGIAQPKKKVKVSVTSPSSPELVSPEEHADFSSFLLLVDAAVQRAAELELEKKQEPN
P*
>ENSG00000257923|ENST00000292535
MLCVAGARLKRELDATATVLANRQDESEQSRKRLIEQSREFKKNTPEDLRKQVAPLLKSF
QGEIDALSKRSKEAEAAFLNVYKRLIDVPDPVPALDLGQQLQLKVQRLHDIETENQKLRE
TLEEYNKEFAEVKNQEVTIKALKEKIREYEQTLKNQAETIALEKEQKLQNDFAEKERKLQ
ETQMSTTSKLEEAEHKVQSLQTALEKTRTELFDLKTKYDEETTAKADEIEMIMTDLERAN
QRAEVAQREAETLREQLSSANHSLQLASQIQKAPDVEQAIEVLTRSSLEVELAAKEREIA
QLVEDVQRLQASLTKLRENSASQISQLEQQLSAKNSTLKQLEEKLKGQADYEEVKKELNI
LKSMEFAPSEGAGTQDAAKPLEVLLLEKNRSLQSENAALRISNSDLSGSARRKGKDQPES
RRPGSLPAPPPSQLPRNPGEQASNTNGTHQFSPAGLSQDFFSSSLASPSLPLASTGKFAL
NSLLQRQLMQSFYSKAMQEAGSTSMIFSTGPYSTNSISSQSPLQQSPDVNGMAPSPSQSE
SAGSVSEGEEMDTAEIARQVKEQLIKHNIGQRIFGHYVLGLSQGSVSEILARPKPWNKLT
VRGKEPFHKMKQFLSDEQNILALRSIQGRQRENPGQSLNRLFQEVPKRRNGSEGNITTRI
RASETGSDEAIKSILEQAKRELQVQKTAEPAQPSSASGSGNSDDAIRSILQQARREMEAQ
QAALDPALKQAPLSQSDITILTPKLLSTSPMPTVSSYPPLAISLKKPSAAPEAGASALPN
PPALKKEAQDAPGLDPQGAADCAQGVLRQVKNEVGRSGAWKDHWWSAVQPERRNAASSEE
AKAEETGGGKEKGSGGSGGGSQPRAERSQLQGPSSSEYWKEWPSAESPYSQSSELSLTGA
SRSETPQNSPLPSSPIVPMSKPTKPSVPPLTPEQYEVYMYQEVDTIELTRQVKEKLAKNG
ICQRIFGEKVLGLSQGSVSDMLSRPKPWSKLTQKGREPFIRMQLWLNGELGQGVLPVQGQ
QQGPVLHSVTSLQDPLQQGCVSSESTPKTSASCSPAPESPMSSSESVKSLTELVQQPCPP
IEASKDSKPPEPSDPPASDSQPTTPLPLSGHSALSIQELVAMSPELDTYGITKRVKEVLT
DNNLGQRLFGETILGLTQGSVSDLLARPKPWHKLSLKGREPFVRMQLWLNDPNNVEKLMD
MKRMEKKAYMKRRHSSVSDSQPCEPPSVGTEYSQGASPQPQHQLKKPRVVLAPEEKEALK
RAYQQKPYPSPKTIEDLATQLNLKTSTVINWFHNYRSRIRRELFIEEIQAGSQGQAGASD
SPSARSGRAAPSSEGDSCDGVEATEGPGSADTEEPKSQGEAEREEVPRPAEQTEPPPSGT
PGPDDARDDDHEGGPVEGPGPLPSPASATATAAPAAPEDAATSAAAAPGEGPAAPSSAPP

PSNSSSSSAPRRPSSLQSLFGLPEAAGARDSRDNPLRKKKAANLNSIIHRLEKAASREEP
IEWEF*
>ENSG00000151065|ENST00000280665
MAAVAAGGLVGKGRDISLAALQRHDPYINRIVDVASQVALYTFGHRANEWEKTDVEGTLF
VYTRSASPKHGFTIMNRLSMENRTEPITKDLDFQLQDPFLLYRNARLSIYGIWFYDKEEC
QRIAELMKNLTQYEQLKAHQGTGAGISPVILNSGEGKEVDILRMLIKAKDEYTKCKTCSE
PKKITSSSAIYDNPNLIKPIPVKPSENQQQRIPQPNQTLDPEPQHLSLTALFGKQDKATC
QETVEPPQTLHQQQQQQQQQQEKLPIRQGVVRSLSYEEPRRHSPPIEKQLCPAIQKLMVR
SADLHPLSELPENRPCENGSTHSAGEFFTGPVQPGSPHNIGTSRGVQNASRTQNLFEKLQ
STPGAANKCDPSTPAPASSAALNRSRAPTSVTPVAPGKGLAQPPQAYFNGSLPPQTVGHQ
AHGREQSTLPRQTLPISGSQTGSSGVISPQELLKKLQIVQQEQQLHASNRPALAAKFPVL
AQSSGTGKPLESWINKTPNTEQQTPLFQVISPQRIPATAAPSLLMSPMVFAQPTSVPPKE
RESGLLPVGGQEPPAAATSLLLPIQSPEPSVITSSPLTKLQLQEALLYLIQNDDNFLNII
YEAYLFSMTQAAMKKTM*
>ENSG00000070501|ENST00000265421
MSKRKAPQETLNGGITDMLTELANFEKNVSQAIHKYNAYRKAASVIAKYPHKIKSGAEAK
KLPGVGTKIAEKIDEFLATGKLRKLEKIRQDDTSSSINFLTRVSGIGPSAARKFVDEGIK
TLEDLRKNEDKLNHHQRIGLKYFGDFEKRIPREEMLQMQDIVLNEVKKVDSEYIATVCGS
FRRGAESSGDMDVLLTHPSFTSESTKQPKLLHQVVEQLQKVHFITDTLSKGETKFMGVCQ
LPSKNDEKEYPHRRIDIRLIPKDQYYCGVLYFTGSDIFNKNMRAHALEKGFTINEYTIRP
LGVTGVAGEPLPVDSEKDIFDYIQWKYREPKDRSE*
>ENSG00000112658|ENST00000265354
MLPTQAGAAAALGRGSALGGSLNRTPTGRPGGGGGTRGANGGRVPGNGAGLGPGRLEREA
AAAAATTPAPTAGALYSGSEGDSESGEEEELGAERRGLKRSLSEMEIGMVVGGPEASAAA
TGGYGPVSGAVSGAKPGKKTRGRVKIKMEFIDNKLRRYTTFSKRKTGIMKKAYELSTLTG
TQVLLLVASETGHVYTFATRKLQPMITSETGKALIQTCLNSPDSPPRSDPTTDQRMSATG
FEETDLTYQVSESDSSGETKDTLKPAFTVTNLPGTTSTIQTAPSTSTTMQVSSGPSFPIT
NYLAPVSASVSPSAVSSANGTVLKSTGSGPVSSGGLMQLPTSFTLMPGGAVAQQVPVQAI
QVHQAPQQASPSRDSSTDLTQTSSSGTVTLPATIMTSSVPTTVGGHMMYPSPHAVMYAPT
SGLGDGSLTVLNAFSQAPSTMQVSHSQVQEPGGVPQVFLTASSGTVQIPVSAVQLHQMAV
IGQQAGSSSNLTELQVVNLDTAHSTKSE*
>ENSG00000175279|ENST00000309048
MEEEAETEEQQRFSYQQRLKAAVHYTVGCLCEEVALDKEMQFSKQTIAAISELTFRQCEN
FAKDLEMFARHAKRTTINTEDVKLLARRSNSLLKYITDKSEEIAQINLERKAQKKKKSED
GSKNSRQPAEAGVVESEN*
>ENSG00000122026|ENST00000272274
MTNTKGKRRGTRYMFSRPFRKHGVVPLATYMRIYKKGDIVDIKGMGTVQKGMPHKCYHGK
TGRVYNVTQHAVGIVVNKQVKGKILAKRINVRIEHIKHSKSRDSFLKRVKENDQKKKEAK
EKGTWVQLKRQPAPPREAHFVRTNGKEPELLEPIPYEFMA*
>ENSG00000089154|ENST00000300648
MAADTQVSETLKRFAGKVTTASVKERREILSELGKCVAGKDLPEGAVKGLCKLFCLTLHR
YRDAASRRALQAAIQQLAEAQPEATAKNLLHSLQSSGIGSKAGVPSKSSGSAALLALTWT
CLLVRIVFPSRAKRQGDIWNKLVEVQCLLLLEVLGGSHKHAVDGAVKKLTKLWKENPGLV
EQYLSAILSLEPNQNYAGMLGLLVQFCTSHKEMDVVSQHKSALLDFYMKNILMSKVKPPK

YLLDSCAPLLRYLSHSEFKDLILPTIQKSLLRSPENVIETISSLLASVTLDLSQYAMDIV
KGLAGHLKSNSPRLMDEAVLALRNLARQCSDSSAMESLTKHLFAILGGSEGKLTVVAQKM
SVLSGIGSVSHHVVSGPSSQVLNGIVAELFIPFLQQEVHEGTLVHAVSVLALWCNRFTME
VPKKLTEWFKKAFSLKTSTSAVRHAYLQCMLASYRGDTLLQALDLLPLLIQTVEKAASQS
TQVPTITEGVAAALLLLKLSVADSQAEAKLSSFWQLIVDEKKQVFTSEKFLVMASEDALC
TVLHLTERLFLDHPHRLTGNKVQQYHRALVAVLLSRTWHVRRQAQQTVRKLLSSLGGFKL
AHGLLEELKTVLSSHKVLPLEALVTDAGEVTEAGKAYVPPRVLQEALCVISGVPGLKGDV
TDTEQLAQEMLIISHHPSLVAVQSGLWPALLARMKIDPEAFITRHLDQIIPRMTTQSPLN
QSSMNAMGSLSVLSPDRVLPQLISTITASVQNPALRLVTREEFAIMQTPAGELYDKSIIQ
SAQQDSIKKANMKRENKAYSFKEQIIELELKEEIKKKKGIKEEVQLTSKQKEMLQAQLDR
EAQVRRRLQELDGELEAALGLLDIILAKNPSGLTQYIPVLVDSFLPLLKSPLAAPRIKNP
FLSLAACVMPSRLKALGTLVSHVTLRLLKPECVLDKSWCQEELSVAVKRAVMLLHTHTIT
SRVGKGEPGAAPLSAPAFSLVFPFLKMVLTEMPHHSEEEEEWMAQILQILTVQAQLRASP
NTPPGRVDENGPELLPRVAMLRLLTWVIGTGSPRLQVLASDTLTTLCASSSGDDGCAFAE
QEEVDVLLCALQSPCASVRETVLRGLMELHMVLPAPDTDEKNGLNLLRRLWVVKFDKEEE
IRKLAERLWSMMGLDLQPDLCSLLIDDVIYHEAAVRQAGAEALSQAVARYQRQAAEVMGR
LMEIYQEKLYRPPPVLDALGRVISESPPDQWEARCGLALALNKLSQYLDSSQVKPLFQFF
VPDALNDRHPDVRKCMLDAALATLNTHGKENVNSLLPVFEEFLKNAPNDASYDAVRQSVV
VLMGSLAKHLDKSDPKVKPIVAKLIAALSTPSQQVQESVASCLPPLVPAIKEDAGGMIQR
LMQQLLESDKYAERKGAAYGLAGLVKGLGILSLKQQEMMAALTDAIQDKKNFRRREGALF
AFEMLCTMLGKLFEPYVVHVLPHLLLCFGDGNQYVREAADDCAKAVMSNLSAHGVKLVLP
SLLAALEEESWRTKAGSVELLGAMAYCAPKQLSSCLPNIVPKLTEVLTDSHVKVQKAGQQ
ALRQIGSVIRNPEILAIAPVLLDALTDPSRKTQKCLQTLLDTKFVHFIDAPSLALIMPIV
QRAFQDRSTDTRKMAAQIIGNMYSLTDQKDLAPYLPSVTPGLKASLLDPVPEVRTVSAKA
LGAMVKGMGESCFEDLLPWLMETLTYEQSSVDRSGAAQGLAEVMAGLGVEKLEKLMPEIV
ATASKVDIAPHVRDGYIMMFNYLPITFGDKFTPYVGPIIPCILKALADENEFVRDTALRA
GQRVISMYAETAIALLLPQLEQGLFDDLWRIRFSSVQLLGDLLFHISGVTGKMTTETASE
DDNFGTAQSNKAIITALGVERRNRVLAGLYMGRSDTQLVVRQASLHVWKIVVSNTPRTLR
EILPTLFGLLLGFLASTCADKRTIAARTLGDLVRKLGEKILPEIIPILEEGLRSQKSDER
QGVCIGLSEIMKSTSRDAVLYFSESLVPTARKALCDPLEEVREAAAKTFEQLHSTIGHQA
LEDILPFLLKQLDDEEVSEFALDGLKQVMAIKSRVVLPYLVPKLTTPPVNTRVLAFLSSV
AGDALTRHLGVILPAVMLALKEKLGTPDEQLEMANCQAVILSVEDDTGHRIIIEDLLEAT
RSPEVGMRQAAAIILNIYCSRSKADYTSHLRSLVSGLIRLFNDSSPVVLEESWDALNAIT
KKLDAGNQLALIEELHKEIRLIGNESKGEHVPGFCLPKKGVTSILPVLREGVLTGSPEQK
EEAAKALGLVIRLTSADALRPSVVSITGPLIRILGDRFSWNVKAALLETLSLLLAKVGIA
LKPFLPQLQTTFTKALQDSNRGVRLKAADALGKLISIHIKVDPLFTELLNGIRAMEDPGV
RDTMLQALRFVIQGAGAKVDAVIRKNIVSLLLSMLGHDEDNTRISSAGCLGELCAFLTEE
ELSAVLQQCLLADVSGIDWMVRHGRSLALSVAVNVAPGRLCAGRYSSDVQEMILSSATAD
RIPIAVSGVRGMGFLMRHHIETGGGQLPAKLSSLFVKCLQNPSSDIRLVAEKMIWWANKD
PLPPLDPQAIKPILKALLDNTKDKNTVVRAYSDQAIVNLLKMRQGEEVFQSLSKILDVAS
LEVLNEVNRRSLKKLASQADSTEQVDDTILT*
>ENSG00000181378|ENST00000295729
MMLTQAPSSVVRSRNSRNHTVNSGGSCLSASTVAIPAINDSSAAMSACSTISAQPASSMD
TQMHSPKKQERVNKRVIWGIEVAEELHWKGWELGKETTRNLVLKNRSLKLQKMKYRYQYK

GSRTQCHSLEPRKQALFKTKQNKQKKPLTCHIKASECLKYVQYE*
>ENSG00000173894|ENST00000269399
MEELSSVGEQVFAAECILSKRLRKGKLEYLVKWRGWSSKHNSWEPEENILDPRLLLAFQK
KEHEKEVQNRKRGKRPRGRPRKLTAMSSCSRRSKLKVGGCAGYADPTSQHPLGVGGRQRE
GLGPSGRGWHFCQQSVPLLGKQEPPFFLSLSFCCQGPQPAESSSPPLPGASCFSLSCTPL
CWVAGSNCCRQALFPPRGSLGDGKEQEACVQ*
>ENSG00000063978|ENST00000314289
MSTRKRRGGAINSRQAQKRTREATSTPEISLEAEPIELVETAGDEIVDLTCESLEPVVVD
LTHNDSVVIVDERRRPRRNARRLPQDHADSCVVSSDDEELSRDRDVYVTTHTPRNARDEG
ATGLRPSGTVSCPICMDGYSEIVQNGRLIVSTECGHVFCSQCLRDSLKNANTCPTCRKKI
NHKRYHPIYI*
>ENSG00000145241|ENST00000273853
MAASGLDHLKNGYRRRFCRPSRARDINTEQGQNVLEILQDCFEEKSLANDFSTNSTKSVP
NSTRKIKDTCIQSPSKECQKSHPKSVPVSSKKKEASLQFVVEPSEATNRSVQAHEVHQKI
LATDVSSKNTPDSKKISSRNINDHHSEADEEFYLSVGSPSVLLDAKTSVSQNVIPSSAQK
RETYTFENSVNMLPSSTEVSVKTKKRLNFDDKVMLKKIEIDNKVSDEEDKTSEGQERKPS
GSSQNRIRDSEYEIQRQAKKSFSTLFLETVKRKSESSPIVRHAATAPPHSCPPDDTKLIE
DEFIIDESDQSFASRSWITIPRKAGSLKQRTISPAESTALLQGRKSREKHHNILPKTLAN
DKHSHKPHPVETSQPSDKTVLDTSYALIGETVNNYRSTKYEMYSKNAEKPSRSKRTIKQK
QRRKFMAKPAEEQLDVGQSKDENIHTSHITQDEFQRNSDRNMEEHEEMGNDCVSKKQMPP
VGSKKSSTRKDKEESKKKRFSSESKNKLVPEEVTSTVTKSRRISRRPSDWWVVKSEESPV
YSNSSVRNELPMHHNSSRKSTKKTNQSSKNIRKKTIPLKRQKTATKGNQRVQKFLNAEGS
GGIVGHDEISRCSLSEPLESDEADLAKKKNLDCSRSTRSSKNEDNIMTAQNVPLKPQTSG
YTCNIPTESNLDSGEHKTSVLEESGPSRLNNNYLMSGKNDVDDEEVHGSSDDSKQSKVIP
KNRIHHKLVLPSNTPNVRRTKRTRLKPLEYWRGERIDYQGRPSGGFVISGVLSPDTISSK
RKAKENIGKVNKKSNKKRICLDNDERKTNLMVNLGIPLGDPLQPTRVKDPETREIILMDL
VRPQDTYQFFVKHGELKVYKTLDTPFFSTGKLILGPQEEKGKQHVGQDILVFYVNFGDLL
CTLHETPYILSTGDSFYVPSGNYYNIKNLRNEESVLLFTQIKR*
>ENSG00000137166|ENST00000307972
MMVESASETIRSAPSGQNGVGSLSGQADGSSGGATGTTASGTGREVTTGADSNGEMSPAE
LLHFQQQQALQVARQFLLQQASGLSSPGNNDSKQSASAVQVPVSVAMMSPQMLTPQQMQQ
ILSPPQLQALLQQQQALMLQQLQEYYKKQQEQLHLQLLTQQQAGKPQPKEALGNKQLAFQ
QQLLQMQQLQQQHLLNLQRQGLVSLQPNQASGPLQTLPQAAVCPTDLPQLWKGEGAPGQP
AEDSVKQEGLDLTGTAATATSFAAPPKVSPPLSHHTLPNGQPTVLTSRRDSSSHEETPGS
HPLYGHGECKWPGCETLCEDLGQFIKHLNTEHALDDRSTAQCRVQMQVVQQLEIQLAKES
ERLQAMMAHLHMRPSEPKPFSQPLNPVPGSSSFSKVTVSAADSFPDGLVHPPTSAAAPVT
PLRPPGLGSASLHGGGPARRRSSDKFCSPISSELAQNHEFYKNADVRPPFTYASLIRQAI
LETPDRQLTLNEIYNWFTRMFAYFRRNTATWKNAVRHNLSLHKCFVRVENVKGAVWTVDE
REYQKRRPPKMTGSPTLVKNMISGLSYGALNASYQAALAESSFPLLNSPGMLNPGSASSL
LPLSHDDVGAPVEPLPSNGSSSPPRLSPPQYSHQVQVKEEPAEAEEDRQPGPPLGAPNPS
ASGPPEDRDLEEELPGEELS*
>ENSG00000159592|ENST00000290795
MAQHDFVPAWLNFSTPQSAKSPTATFEKHGEHLPRGEGRFGVSRRRHNSSDGFFNNGPLR
TAGDSWHQPSLFRHDSVDSGVSKGAYAGITGNPSGWHSSSRGHDGMSQRSGGGTGNHRHW

NGSFHSRKGCAFQEKPPMEIREEKKEDKVEKLQFEEEDFPSLNPEAGKQHQPCRPIGTPS
GVWENPPSAKQPSKMLVIKKVSKEDPAAAFSAAFTSPGSHHANGNKLSSVVPSVYKNLVP
KPVPPPSKPNAWKANRMEHKSGSLSSSRESAFTSPISVTKPVVLASGAALSSPKESPSST
TPPIEISSSRLTKLTRRTTDRKSEFLKTLKDDRNGDFSENRDCDKLEDLEDNSTPEPKEN
GEEGCHQNGLALPVVEEGEVLSHSLEAEHRLLKAMGWQEYPENDENCLPLTEDELKEFHM
KTEQLRRNGFGKNGFLQSRSSSLFSPWRSTCKAEFEDSDTETSSSETSDDDAWK*
>ENSG00000169174|ENST00000302118
MGTVSSRRSWWPLPLLLLLLLLLGPAGARAQEDEDGDYEELVLALRSEEDGLAEAPEHGT
TATFHRCAKDPWRLPGTYVVVLKEETHLSQSERTARRLQAQAARRGYLTKILHVFHGLLP
GFLVKMSGDLLELALKLPHVDYIEEDSSVFAQSIPWNLERITPPRYRADEYQPPDGGSLV
EVYLLDTSIQSDHREIEGRVMVTDFENVPEEDGTRFHRQASKCDSHGTHLAGVVSGRDAG
VAKGASMRSLRVLNCQGKGTVSGTLIGLEFIRKSQLVQPVGPLVVLLPLAGGYSRVLNAA
CQRLARAGVVLVTAAGNFRDDACLYSPASAPEVITVGATNAQDQPVTLGTLGTNFGRCVD
LFAPGEDIIGASSDCSTCFVSQSGTSQAAAHVAGIAAMMLSAEPELTLAELRQRLIHFSA
KDVINEAWFPEDQRVLTPNLVAALPPSTHGAGWQLFCRTVWSAHSGPTRMATAVARCAPD
EELLSCSSFSRSGKRRGERMEAQGGKLVCRAHNAFGGEGVYAIARCCLLPQANCSVHTAP
PAEASMGTRVHCHQQGHVLTGCSSHWEVEDLGTHKPPVLRPRGQPNQCVGHREASIHASC
CHAPGLECKVKEHGIPAPQEQVTVACEEGWTLTGCSALPGTSHVLGAYAVDNTCVVRSRD
VSTTGSTSEGAVTAVAICCRSRHLAQASQELQ*
>ENSG00000172716|ENST00000308377
MEANQCPLVVEPSYPDLVINVGEVTLGEENRKKLQKIQRDQEKERVMRAACALLNSGGGV
IRMAKKVEHPVEMGLDLEQSLRELIQSSDLQAFFETKQQGRCFYIFVKSWSSGPFPEDRS
VKPRLCSLSSSLYRRSETSVRSMDSREAFCFLKTKRKPKILEEGPFHKIHKGVYQELPNS
DPADPNSDPADLIFQKDYLEYGEILPFPESQLVEFKQFSTKHFQEYVKRTIPEYVPAFAN
TGGGYLFIGVDDKSREVLGCAKENVDPDSLRRKIEQAIYKLPCVHFCQPQRPITFTLKIV
NVLKRGELYGYACMIRVNPFCCAVFSEAPNSWIVEDKYVCSLTTEKWVGMMTDTDPDLLQ
LSEDFECQLSLSSGPPLSRPVYSKKGLEHKKELQQLLFSVPPGYLRYTPESLWRDLISEH
RGLEELINKQMQPFFRGILIFSRSWAVDLNLQEKPGVICDALLIAQNSTPILYTILREQD
AEGQDYCTRTAFTLKQKLVNMGGYTGKVCVRAKVLCLSPESSAEALEAAVSPMDYPASYS
LAGTQHMEALLQSLVIVLLGFRSLLSDQLGCEVLNLLTAQQYEIFSRSLRKNRELFVHGL
PGSGKTIMAMKIMEKIRNVFHCEAHRILYVCENQPLRNFISDRNICRAETRKTFLRENFE
HIQHIVIDEAQNFRTEDGDWYGKAKSITRRAKGGPGILWIFLDYFQTSHLDCSGLPPLSD
QYPREELTRIVRNADPIAKYLQKEMQVIRSNPSFNIPTGCLEVFPEAEWSQGVQGTLRIK
KYLTVEQIMTCVADTCRRFFDRGYSPKDVAVLVSTAKEVEHYKYELLKAMRKKRVVQLSD
ACDMLGDHIVLDSVRRFSGLERSIVFGIHPRTADPAILPNVLICLASRAKQHLYIFPWGG
H*
>ENSG00000140488|ENST00000287202
MAAAPGGSAQPAGPGPRLGFSTADSGVGMSGLNPGPAVPMKDHDAIKLFVGQIPRGLDEQ
DLKPLFEEFGRIYELTVLKDRLTGLHKGCAFLTYCARDSALKAQSALHEQKTLPGMNRPI
QVKPAASEGRGEDRKLFVGMLGKQQGEEDVRRLFQPFGHIEECTVLRSPDGTSKGCAFVK
FGSQGEAQAAIRGLHGSRTMAGASSSLVVKLADTDRERALRRMQQMAGHLGAFHPAPLPL
GACGAYTTAILQHQAALLAAAQGPGLGPVAAVAAQMQHVAAFSLVAAPLLPAAAANSPPG
SGPGTLPGLPAPIGVNGFGPLTPQTNGQPGSDTLYNNGLSPYPAQSPGVADPLQQAYAGM
HHYAAAYPSAYAPVSTAFPQQPSALPQQQREGPEGCNLFIYHLPQEFGDAELIQTFLPFG

AVVSAKVFVDRATNQSKCFGFVSFDNPTSAQTAIQAMNGFQIGMKRLKVQLKRPKDANRP
Y*
>ENSG00000205581|ENST00000288344
MPKRKVSSAEGAAKEEPKRRSARLSAKPPAKVEAKPKKAAAKILQTPK*
>ENSG00000101161|ENST00000266079
MNKKKKPFLGMPAPLGYVPGLGRGATGFTTRSDIGPARDANDPVDDRHAPPGKRTVGDQM
KKNQAADDDDEDLNDTNYDEFNGYAGSLFSSGPYEKDDEEADAIYAALDKRMDERRKERR
EQREKEEIEKYRMERPKIQQQFSDLKRKLAEVTEEEWLSIPEVGDARNKRQRNPRYEKLT
PVPDSFFAKHLQTGENHTSVDPRQTQFGGLNTPYPGGLNTPYPGGMTPGLMTPGTGELDM
RKIGQARNTLMDMRLSQVSDSVSGQTVVDPKGYLTDLNSMIPTHGGDINDIKKARLLLKS
VRETNPHHPPAWIASARLEEVTGKLQVARNLIMKGTEMCPKSEDVWLEAARLQPGDTAKA
VVAQAVRHLPQSVRIYIRAAELETDIRAKKRVLRKALEHVPNSVRLWKAAVELEEPEDAR
IMLSRAVECCPTSVELWLALARLETYENARKVLNKARENIPTDRHIWITAAKLEEANGNT
QMVEKIIDRAITSLRANGVEINREQWIQDAEECDRAGSVATCQAVMRAVIGIGIEEEDRK
HTWMEDADSCVAHNALECARAIYAYALQVFPSKKSVWLRAAYFEKNHGTRESLEALLQRA
VAHCPKAEVLWLMGAKSKWLAGDVPAARSILALAFQANPNSEEIWLAAVKLESENDEYER
ARRLLAKARSSAPTARVFMKSVKLEWVQDNIRAAQDLCEEALRHYEDFPKLWMMKGQIEE
QKEMMEKAREAYNQGLKKCPHSTPLWLLLSRLEEKIGQLTRARAILEKSRLKNPKNPGLW
LESVRLEYRAGLKNIANTLMAKALQECPNSGILWSEAIFLEARPQRRTKSVDALKKCEHD
PHVLLAVAKLFWSQRKITKAREWFHRTVKIDSDLGDAWAFFYKFELQHGTEEQQEEVRKR
CESAEPRHGELWCAVSKDIANWQKKIGDILRLVAGRIKNTF*
>ENSG00000109118|ENST00000268756
MWEKMETKTIVYDLDTSGGLMEQIQALLAPPKTDEAEKRSRKPEKEPRRSGRATNHDSCD
SCKEGGDLLCCDHCPAAFHLQCCNPPLSEEMLPPGEWMCHRCTVRRKKREQKKELGHVNG
LVDKSGKRTTSPSSDTDLLDRSASKTELKAIAHARILERRASRPGTPTSSASTETPTSEQ
NDVDEDIIDVDEEPVAAEPDYVQPQLRRPFELLIAAAMERNPTQFQLPNELTCTTALPGS
SKRRRKEETTGKNVKKTQHELDHNGLVPLPVKVCFTCNRSCRVAPLIQCDYCPLLFHMDC
LEPPLTAMPLGRWMCPNHIEHVVLNQKNMTLSNRCQVFDRFQDTVSQHVVKVDFLNRIHK
KHPPNRRVLQSVKRRSLKVPDAIKSQYQFPPPLIAPAAIRDGELICNGIPEESQMHLLNS
EHLATQAEQQEWLCSVVALQCSILKHLSAKQMPSHWDSEQTEKADIKPVIVTDSSVTTSL
QTADKTPTPSHYPLSCPSGISTQNSLSCSPPHQSPALEDIGCSSCAEKSKKTPCGTANGP
VNTEVKANGPHLYSSPTDSTDPRRLPGANTPLPGLSHRQGWPRPLTPPAAGGLQNHTVGI
IVKTENATGPSSCPQRSLVPVPSLPPSIPSSCASIENTSTLQRKTVQSQIGPPLTDSRPL
GSPPNATRVLTPPQAAGDGILATTANQRFSSPAPSSGECCSALG*
>ENSG00000174738|ENST00000312521
MEVNAGGVIAYISSSSSASSPASCHSEGSENSFQSSSSSVPSSPNSSNSDTNGNPKNGDL
ANIEGILKNDRIDCSMKTSKSSAPGMTKSHSGVTKFSGMVLLCKVCGDVASGFHYGVHAC
EGCKGFFRRSIQQNIQYKKCLKNENCSIMRMNRNRCQQCRFKKCLSVGMSRDAVRFGRIP
KREKQRMLIEMQSAMKTMMNSQFSGHLQNDTLVEHHEQTALPAQEQLRPKPQLEQENIKS
SSPPSSDFAKEEVIGMVTRAHKDTFMYNQEQQENSAESMQPQRGERIPKNMEQYNLNHDH
CGNGLSSHFPCSESQQHLNGQFKGRNIMHYPNGHAICIANGHCMNFSNAYTQRVCDRVPI
DGFSQNENKNSYLCNTGGRMHLVCPMSKSPYVDPHKSGHEIWEEFSMSFTPAVKEVVEFA
KRIPGFRDLSQHDQVNLLKAGTFEVLMVRFASLFDAKERTVTFLSGKKYSVDDLHSMGAG
DLLNSMFEFSEKLNALQLSDEEMSLFTAVVLVSADRSGIENVNSVEALQETLIRALRTLI

MKNHPNEASIFTKLLLKLPDLRSLNNMHSEELLAFKVHP*
>ENSG00000163877|ENST00000296215
MKAVKSERERGSRRRHRDGDVVLPAGVVVKQERLSPEVAPPAHRRPDHSGGSPSPPTSEP
ARSGHRGNRARGVSRSPPKKKNKASGRRSKSPRSKRNRSPHHSTVKVKQEREDHPRRGRE
DRQHREPSEQEHRRARNSDRDRHRGHSHQRRTSNERPGSGQGQGRDRDTQNLQAQEEERE
FYNARRREHRQRNDVGGGGSESQELVPRPGGNNKEKEVPAKEKPSFELSGALLEDTNTFR
GVVIKYSEPPEARIPKKRWRLYPFKNDEVLPVMYIHRQSAYLLGRHRRIADIPIDHPSCS
KQHAVFQYRLVEYTRADGTVGRRVKPYIIDLGSGNGTFLNNKRIEPQRYYELKEKDVLKF
GFSSREYVLLHESSDTSEIDRKDDEDEEEEEEVSDS*
>ENSG00000141431|ENST00000269197
MKDKRKKKDRTWAEAARLALEKHPNSPMTAKQILEVIQKEGLKETSGTSPLACLNAMLHT
NTRIGDGTFFKIPGKSGLYALKKEESSCPADGTLDLVCESELDGTDMAEANAHGEENGVC
SKQVTDEASSTRDSSLTNTAVQSKLVSSFQQHTKKALKQALRQQQKRRNGVSMMVNKTVP
RVVLTPLKVSDEQSDSPSGSESKNGEADSSDKEMKHGQKSPTGKQTSQHLKRLKKSGLGH
LKWTKAEDIDIETPGSILVNTNLRALINKHTFASLPQHFQQYLLLLLPEVDRQMGSDGIL
RLSTSALNNEFFAYAAQGWKQRLAEGEFTPEMQLRIRQEIEKEKKTEPWKEKFFERFYGE
KLGMSREESVKLTTGPNNAGAQSSSSCGTSGLPVSAQTALAEQQPKSMKSPASPEPGFCA
TLCPMVEIPPKDIMAELESEDILIPEESVIQEEIAEEVETSICECQDENHKTIPEFSEEA
ESLTNSHEEPQIAPPEDNLESCVMMNDVLETLPHIEVKIEGKSESPQEEMTVVIDQLEVC
DSLIPSTSSMTHVSDTEHKESETAVETSTPKIKTGSSSLEGQFPNEGIAIDMELQSDPEE
QLSENACISETSFSSESPEGACTSLPSPGGETQSTSEESCTPASLETTFCSEVSSTENTD
KYNQRNSTDENFHASLMSEISPISTSPEISEASLMSNLPLTSEASPVSNLPLTSETSPMS
DLPLTSETSSVSSMLLTSETTFVSSLPLPSETSPISNSSINERMAHQQRKSPSVSEEPLS
PQKDESSATAKPLGENLTSQQKNLSNTPEPIIMSSSSIAPEAFPSEDLHNKTLSQQTCKS
HVDTEKPYPASIPELASTEMIKVKNHSVLQRTEKKVLPSPLELSVFSEGTDNKGNELPSA
KLQDKQYISSVDKAPFSEGSRNKTHKQGSTQSRLETSHTSKSSEPSKSPDGIRNESRDSE
ISKRKTAEQHSFGICKEKRARIEDDQSTRNISSSSPPEKEQPPREEPRVPPLKIQLSKIG
PPFIIKSQPVSKPESRASTSTSVSGGRNTGARTLADIKARAQQARAQREAAAAAAVAAAA
SIVSGAMGSPGEGGKTRTLAHIKEQTKAKLFAKHQARAHLFQTSKETRLPPPLSSKEGPP
NLEVSSTPETKMEGSTGVIIVNPNCRSPSNKSAHLRETTTVLQQSLNPSKLPETATDLSV
HSSDENIPVSHLSEKIVSSTSSENSSVPMLFNKNSVPVSVCSTAISGAIKEHPFVSSVDK
SSVLMSVDSANTTISACNISMLKTIQGTDTPCIAIIPKCIESTPISATTEGSSISSSMDD
KQLLISSSSASNLVSTQYTSVPTPSIGNNLPNLSTSSVLIPPMGINNRFPSEKIAIPGSE
EQATVSMGTTVRAALSCSDSVAVTDSLVAHPTVAMFTGNMLTINSYDSPPKLSAESLDKN
SGPRNRADNSGKPQQPPGGFAPAAINRSIPCKVIVDHSTTLTSSLSLTVSVESSEASLDL
QGRPVRTEASVQPVACPQVSVISRPEPVANEGIDHSSTFIAASAAKQDSKTLPATCTSLR
ELPLVPDKLNEPTAPSHNFAEQARGPAPFKSEADTTCSNQYNPSNRICWNDDGMRSTGQP
LVTHSGSSKQKEYLEQSCPKAIKTEHANYLNVSELHPRNLVTNVALPVKSELHEADKGFR
MDTEDFPGPELPPPAAEGASSVQQTQNMKASTSSPMEEAISLATDALKRVPGAGSSGCRL
SSVEANNPLVTQLLQGNLPLEKVLPQPRLGAKLEINRLPLPLQTTSVGKTAPERNVEIPP
SSPNPDGKGYLAGTLAPLQMRKRENHPKKRVARTVGEHTQVKCEPGKLLVEPDVKGVPCV
ISSGISQLGHSQPFKQEWLNKHSMQNRIVHSPEVKQQKRLLPSCSFQQNLFHVDKNGGFH
TDAGTSHRQQFYQMPVAARGPIPTAALLQASSKTPVGCNAFAFNRHLEQKGLGEVSLSSA
PHQLRLANMLSPNMPMKEGDEVGGTAHTMPNKALVHPPPPPPPPPPPPLALPPPPPPPPP

LPPPLPNAEVPSDQKQPPVTMETTKRLSWPQSTGICSNIKSEPLSFEEGLSSSCELGMKQ
VSYDQNEMKEQLKAFALKSADFSSYLLSEPQKPFTQLAAQKMQVQQQQQLCGNYPTIHFG
STSFKRAASAIEKSIGILGSGSNPATGLSGQNAQMPVQNFADSSNADELELKCSCRLKAM
IVCKGCGAFCHDDCIGPSKLCVACLVVR*
>ENSG00000163682|ENST00000295955
MKTILSNQTVDIPENVDITLKGRTVIVKGPRGTLRRDFNHINVELSLLGKKKKRLRVDKW
WGNRKELATVRTICSHVQNMIKGVTLGFRYKMRSVYAHFPINVVIQENGSLVEIRNFLGE
KYIRRVRMRPGVACSVSQAQKDELILEGNDIELVSNSAALIQQATTVKNKDIRKFLDGIY
VSEKGTVQQADE*
>ENSG00000174748|ENST00000307839
MGAYKYIQELWRKKQSDVMRFLLRVRCWQYRQLSALHRAPRPTRPDKARRLGYKAKQGYV
IYRIRVRRGGRKRPVPKGATYGKPVHHGVNQLKFARSLQSVAEERAGRHCGALRVLNSYW
VGEDSTYKFFEVILIDPFHKAIRRNPDTQWITKPVHKHREMRGLTSAGRKSRGLGKGHKF
HHTIGGSRRAAWRRRNTLQLHRYR*
>ENSG00000124571|ENST00000265351
MAMDQVNALCEQLVKAVTVMMDPNSTQRYRLEALKFCEEFKEKCPICVPCGLRLAEKTQV
AIVRHFGLQILEHVVKFRWNGMSRLEKVYLKNSVMELIANGTLNILEEENHIKDALSRIV
VEMIKREWPQHWPDMLIELDTLSKQGETQTELVMFILLRLAEDVVTFQTLPPQRRRDIQQ
TLTQNMERIFSFLLNTLQENVNKYQQVKTDTSQESKAQANCRVGVAALNTLAGYIDWVSM
SHITAENCKLLEILCLLLNEQELQLGAAECLLIAVSRKGKLEDRKPLMVLFGDVAMHYIL
SAAQTADGGGLVEKHYVFLKRLCQVLCALGNQLCALLGADSDVETPSNFGKYLESFLAFT
THPSQFLRSSTQMTWGALFRHEILSRDPLLLAIIPKYLRASMTNLVKMGFPSKTDSPSCE
YSRFDFDSDEDFNAFFNSSRAQQGEVMRLACRLDPKTSFQMAGEWLKYQLSTFLDAGSVN
SCSAVGTGEGSLCSVFSPSFVQWEAMTLFLESVITQMFRTLNREEIPVNDGIELLQMVLN
FDTKDPLILSCVLTNVSALFPFVTYRPEFLPQVFSKLFSSVTFETVEESKAPRTRAVRNV
RRHACSSIIKMCRDYPQLVLPNFDMLYNHVKQLLSNELLLTQMEKCALMEALVLISNQFK
NYERQKVFLEELMAPVASIWLSQDMHRVLSDVDAFIAYVGTDQKSCDPGLEDPCGLNRAR
MSFCVYSILGVVKRTCWPTDLEEAKAGGFVVGYTSSGNPIFRNPCTEQILKLLDNLLALI
RTHNTLYAPEMLAKMAEPFTKALDMLDAEKSAILGLPQPLLELNDSPVFKTVLERMQRFF
STLYENCFHILGKAGPSMQQDFYTVEDLATQLLSSAFVNLNNIPDYRLRPMLRVFVKPLV
LFCPPEHYEALVSPILGPLFTYLHMRLSQKWQVINQRSLLCGEDEAADENPESQEMLEEQ
LVRMLTREVMDLITVCCVSKKGADHSSAPPADGDDEEMMATEVTPSAMAELTDLGKCLMK
HEDVCTALLITAFNSLAWKDTLSCQRTTSQLCWPLLKQVLSGTLLADAVTWLFTSVLKGL
QMHGQHDGCMASLVHLAFQIYEALRPRYLEIRAVMEQIPEIQKDSLDQFDCKLLNPSLQK
VADKRRKDQFKRLIAGCIGKPLGEQFRKEVHIKNLPSLFKKTKPMLETEVLDNDGGGLAT
IFEP*
>ENSG00000138668|ENST00000313899
MSEEQFGGDGAAAAATAAVGGSAGEQEGAMVAATQGAAAAAGSGAGTGGGTASGGTEGGS
AESEGAKIDASKNEEDEGHSNSSPRHSEAATAQREEWKMFIGGLSWDTTKKDLKDYFSKF
GEVVDCTLKLDPITGRSRGFGFVLFKESESVDKVMDQKEHKLNGKVIDPKRAKAMKTKEP
VKKIFVGGLSPDTPEEKIREYFGGFGEVESIELPMDNKTNKRRGFCFITFKEEEPVKKIM
EKKYHNVGLSKCEIKVAMSKEQYQQQQQWGSRGGFAGRARGRGGGPSQNWNQGYSNYWNQ
GYGNYGYNSQGYGGYGGYDYTGYNNYYGYGDYSNQQSGYGKVSRRGGHQNSYKPY*
>ENSG00000131115|ENST00000313040

MPSQNYDLPQKKQEKMTKFQEAVTFKDVAVVFSREELRLLDLTQRKLYRDVMVENFKNLV
AVGHLPFQPDMVSQLEAEEKLWMMETETQRSSKHQNKMETLQKFALKYLSNQELSCWQIW
KQVASELTRCLQGKSSQLLQGDSIQVSENENNIMNPKGDSSIYIENQEFPFWRTQHSCGN
TYLSESQIQSRGKQIDVKNNLQIHEDFMKKSPFHEHIKTDTEPKPCKGNEYGKIISDGSN
QKLPLGEKPHPCGECGRGFSYSPRLPLHPNVHTGEKCFSQSSHLRTHQRIHPGEKLNRCH
ESGDCFNKSSFHSYQSNHTGEKSYRCDSCGKGFSSSTGLIIHYRTHTGEKPYKCEECGKC
FSQSSNFQCHQRVHTEEKPYKCEECGKGFGWSVNLRVHQRVHRGEKPYKCEECGKGFTQA
AHFHIHQRVHTGEKPYKCDVCGKGFSHNSPLICHRRVHTGEKPYKCEACGKGFTRNTDLH
IHFRVHTGEKPYKCKECGKGFSQASNLQVHQNVHTGEKRFKCETCGKGFSQSSKLQTHQR
VHTGEKPYRCDVCGKDFSYSSNLKLHQVIHTGEKPYKCEECGKGFSWRSNLHAHQRVHSG
EKPYKCEQCDKSFSQAIDFRVHQRVHTGEKPYKCGVCGKGFSQSSGLQSHQRVHTGEKPY
KCDVCGKGFRYSSQFIYHQRGHTGEKPYKCEECGKGFGRSLNLRHHQRVHTGEKPHICEE
CGKAFSLPSNLRVHLGVHTREKLFKCEECGKGFSQSARLEAHQRVHTGEKPYKCDICDKD
FRHRSRLTYHQKVHTGKKL*
>ENSG00000214753|ENST00000301785
MEVKRLKVTELRSELQRRGLDSRGLKVDLAQRLQEALDAEMLEDEAGGGGAGPGGACKAE
PRPVAASGGGPGGDEEEDEEEEEEDEEALLEDEDEEPPPAQALGQAAQPPPEPPEAAAME
AAAEPDASEKPAEATAGSGGVNGGEEQGLGKREEDEPEERSGDETPGSEVPGDKAAEEQG
DDQDSEKSKPAGSDGERRGVKRQRDEKDEHGRAYYEFREEAYHSRSKSPLPPEEEAKDEE
EDQTLVNLDTYTSDLHFQVSKDRYGGQPLFSEKFPTLWSGARSTYGVTKGKVCFEAKVTQ
NLPMKEGCTEVSLLRVGWSVDFSRPQLGEDEFSYGFDGRGLKAENGQFEEFGQTFGENDV
IGCFANFETEEVELSFSKNGEDLGVAFWISKDSLADRALLPHVLCKNCVVELNFGQKEEP
FFPPPEEFVFIHAVPVEERVRTAVPPKTIEECEVILMVGLPGSGKTQWALKYAKENPEKR
YNVLGAETVLNQMRMKGLEEPEMDPKSRDLLVQQASQCLSKLVQIASRTKRNFILDQCNV
YNSGQRRKLLLFKTFSRKVVVVVPNEEDWKKRLELRKEVEGDDVPESIMLEMKANFSLPE
KCDYMDEVTYGELEKEEAQPIVTKYKEEARKLLPPSEKRTNRRNNRNKRNRQNRSRGQGY
VGGQRRGYDNRAYGQQYWGQPGNRGGYRNFYDRYRGDYDRFYGRDYEYNRYRDYYRQYNR
DWQSYYYHHPQDRDRYYRNYYGYQGYR*
>ENSG00000168502|ENST00000306329
METLNGPAGGGAPDAKLQPPGQHHRHHHLHPVAERRRLHRAPSPARPFLKDLHARPAAPG
PAVPSSGRAPAPAAPRSPNLAGKAPPSPGSLAAPGRLSRRSGGVPGAKDKPPPGAGARAA
GGAKAALGSRRAARVAPAEPLSRAGKPPGAEPPSAAAKGRKAKRGSRAPPARTVGPPTPA
ARIPAVTLAVTSVAGSPARCSRISHTDSSSDLSDCPSEPLSDEQRLLPAASSDAESGTGS
SDREPPRGAPTPSPAARGAPPGSPEPPALLAAPLAAGACPGGRSIPSGVSGGFAGPGVAE
DVRGRSPPERPVPGTPKEPSLGEQSRLVPAAEEEELLREMEELRSENDYLKDELDELRAE
MEEMRDSYLEEDVYQLQELRRELDRANKNCRILQYRLRKAEQKSLKVAETGQVDGELIRS
LEQDLKVAKDVSVRLHHELKTVEEKRAKAEDENETLRQQMIEVEISKQALQNELERLKES
SLKRRSTREMYKEKKTFNQDDSADLRCQLQFAKEEAFLMRKKMAKLGREKDELEQELQKY
KSLYGDVDSPLPTGEAGGPPSTREAELKLRLKLVEEEANILGRKIVELEVENRGLKAEME
DMRGQQEREGPGRDHAPSIPTSPFGDSLESSTELRRHLQFVEEEAELLRRSISEIEDHNR
QLTHELSKFKFEPPREPGWLGEGASPGAGGGAPLQEELKSARLQISELSGKVLKLQHENH
ALLSNIQRCDLAAHLGLRAPSPRDSDAESDAGKKESDGEESRLPQPKREGPVGGESDSEE
MFEKTSGFGSGKPSEASEPCPTELLKAREDSEYLVTLKHEAQRLERTVERLITDTDSFLH
DAGLRGGAPLPGPGLQGEEEQGEGDQQEPQLLGTINAKMKAFKKELQAFLEQVNRIGDGL

SPLPHLTESSSFLSTVTSVSRDSPIGNLGKELGPDLQSRLKEQLEWQLGPARGDERESLR
LRAARELHRRADGDTGSHGLGGQTCFSLEMEEEHLYALRWKELEMHSLALQNTLHERTWS
DEKNLMQQELRSLKQNIFLFYVKLRWLLKHWRQGKQMEEEGEEFTEGEHPETLSRLGELG
VQGGHQADGPDHDSDRGCGFPVGEHSPHSRVQIGDHSLRLQTADRGQPHKQVVENQQLFS
AFKALLEDFRAELREDERARLRLQQQYASDKAAWDVEWAVLKCRLEQLEEKTENKLGELG
SSAESKGALKKEREVHQKLLADSHSLVMDLRWQIHHSEKNWNREKVELLDRLDRDRQEWE
RQKKEFLWRIEQLQKENSPRRGGSFLCDQKDGNVRPFPHQGSLRMPRPVAMWPCADADSI
PFEDRPLSKLKESDRCSASENLYLDALSLDDEPEEPPAHRPEREFRNRLPEEEENHKGNL
QRAVSVSSMSEFQRLMDISPFLPEKGLPSTSSKEDVTPPLSPDDLKYIEEFNKSWDYTPN
RGHNGGGPDLWADRTEVGRAGHEDSTEPFPDSSWYLTTSVTMTTDTMTSPEHCQKQPLRS
HVLTEQSGLRVLHSPPAVRRVDSITAAGGEGPFPTSRARGSPGDTKGGPPEPMLSRWPCT
SPRHSRDYVEGARRPLDSPLCTSLGFASPLHSLEMSKNLSDDMKEVAFSVRNAICSGPGE
LQVKDMACQTNGSRTMGTQTVQTISVGLQTEALRGSGVTSSPHKCLTPKAGGGATPVSSP
SRSLRSRQVAPAIEKVQAKFERTCCSPKYGSPKLQRKPLPKADQPNNRTSPGMAQKGYSE
SAWARSTTTRESPVHTTINDGLSSLFNIIDHSPVVQDPFQKGLRAGSRSRSAEPRPELGP
GQETGTNSRGRSPSPIGVGSEMCREEGGEGTPVKQDLSAPPGYTLTENVARILNKKLLEH
ALKEERRQAAHGPPGLHSDSHSLGDTAEPGPMENQTVLLTAPWGL*
>ENSG00000132170|ENST00000287820
MGETLGDSPIDPESDSFTDTLSANISQEMTMVDTEMPFWPTNFGISSVDLSVMEDHSHSF
DIKPFTTVDFSSISTPHYEDIPFTRTDPVVADYKYDLKLQEYQSAIKVEPASPPYYSEKT
QLYNKPHEEPSNSLMAIECRVCGDKASGFHYGVHACEGCKGFFRRTIRLKLIYDRCDLNC
RIHKKSRNKCQYCRFQKCLAVGMSHNAIRFGRMPQAEKEKLLAEISSDIDQLNPESADLR
ALAKHLYDSYIKSFPLTKAKARAILTGKTTDKSPFVIYDMNSLMMGEDKIKFKHITPLQE
QSKEVAIRIFQGCQFRSVEAVQEITEYAKSIPGFVNLDLNDQVTLLKYGVHEIIYTMLAS
LMNKDGVLISEGQGFMTREFLKSLRKPFGDFMEPKFEFAVKFNALELDDSDLAIFIAVII
LSGDRPGLLNVKPIEDIQDNLLQALELQLKLNHPESSQLFAKLLQKMTDLRQIVTEHVQL
LQVIKKTETDMSLHPLLQEIYKDLY*
>ENSG00000175097|ENST00000311485
MSLQMVTVSNNIALIQPGFSLMNFDGQVFFFGQKGWPKRSCPTGVFHLDVKHNHVKLKPT
IFSKDSCYLPPLRYPATCTFKGSLESEKHQYIIHGGKTPNNEVSDKIYVMSIVCKNNKKV
TFRCTEKDLVGDVPEARYGHSINVVYSRGKSMGVLFGGRSYMPSTHRTTEKWNSVADCLP
CVFLVDFEFGCATSYILPELQDGLSFHVSIAKNDTIYILGGHSLANNIRPANLYRIRVDL
PLGSPAVNCTVLPGGISVSSAILTQTNNDEFVIVGGYQLENQKRMICNIISLEDNKIEIR
EMETPDWTPDIKHSKIWFGSNMGNGTVFLGIPGDNKQVVSEGFYFYMLKCAEDDTNEEQT
TFTNSQTSTEDPGDSTPFEDSEEFCFSAEANSFDGDDEFDTYNEDDEEDESETGYWITCC
PTCDVDINTWVPFYSTELNKPAMIYCSHGDGHWVHAQCMDLAERTLIHLSAGSNKYYCNE
HVEIARALHTPQRVLPLKKPPMKSLRKKGSGKILTPAKKSFLRRLFD*
>ENSG00000198169|ENST00000292562
MAATFQLPGHQEMPLTFQDVAVYFSQAEGRQLGPQQRALYRDVMLENYGNVASLGFPVPK
PELISQLEQGKELWVLNLLGAEEPDILKSCQKDSEVGTKKELSILNQKFSEEVKTPEFVS
RRLLRDNAQAAEFREAWGREGKLKERVGNSAGQSLNKPNIHKRVLTEATVGRERSLGERT
QECSAFDRNLNLDQNVVRLQRNKTGERVFKCDICSKTFKYNSDLSRHQRSHTGEKPYECG
RCGRAFTHSSNLVLHHHIHTGNKPFKCDECGKTFGLNSHLRLHRRIHTGEKPFGCGECGK
AFSRSSTLIQHRIIHTGEKPYKCNECGRGFSQSPQLTQHQRIHTGEKPHECSHCGKAFSR

SSSLIQHERIHTGEKPHKCNQCGKAFSQSSSLFLHHRVHTGEKPYVCNECGRAFGFNSHL
TEHVRIHTGEKPYVCNECGKAFRRSSTLVQHRRVHTGEKPYQCVECGKAFSQSSQLTLHQ
RVHTGEKPYDCGDCGKAFSRRSTLIQHQKVHSGETRKCRKHGPAFVHGSSLTADGQIPTG
EKHGRAFNHGANLILRWTVHTGEKSFGCNEYGKAFSPTSRPTEDQIMHAGEKPYKCQECG
NAFSGKSTLIQHQVTHTGQKPCHCSVYGKAFSQSSQLTPPQQTRVGEKPALNDGSKRYFI
HIKKIFQERHF*
>ENSG00000113595|ENST00000274327
MATLVVNKLGAGVDSGRQGSRGTAVVKVLECGVCEDVFSLQGDKVPRLLLCGHTVCHDCL
TRLPLHGRAIRCPFDRQVTDLGDSGVWGLKKNFALLELLERLQNGPIGQYGAAEESIGIS
GESIIRCDEDEAHLASVYCTVCATHLCSECSQVTHSTKTLAKHRRVPLADKPHEKTMCSQ
HQVHAIEFVCLEEGCQTSPLMCCVCKEYGKHQGHKHSVLEPEANQIRASILDMAHCIRTF
TEEISDYSRKLVGIVQHIEGGEQIVEDGIGMAHTEHVPGTAENARSCIRAYFYDLHETLC
RQEEMALSVVDAHVREKLIWLRQQQEDMTILLSEVSAACLHCEKTLQQDDCRVVLAKQEI
TRLLETLQKQQQQFTEVADHIQLDASIPVTFTKDNRVHIGPKMEIRVVTLGLDGAGKTTI
LFKLKQDEFMQPIPTIGFNVETVEYKNLKFTIWDVGGKHKLRPLWKHYYLNTQAVVFVVD
SSHRDRISEAHSELAKLLTEKELRDALLLIFANKQDVAGALSVEEITELLSLHKLCCGRS
CFSDNM*
>ENSG00000174444|ENST00000307961
MACARPLISVYSEKGESSGKNVTLPAVFKAPIRPDIVNFVHTNLRKNNRQPYAVSELAGH
QTSAESWGTGRAVARIPRVRGGGTHRSGQGAFGNMCRGGRMFAPTKTWRRWHRRVNTTQK
RYAICSALAASALPALVMSKGHRIEEVPELPLVVEDKVEGYKKTKEAVLLLKKLKAWNDI
KKVYASQRMRAGKGKMRNRRRIQRRGPCIIYNEDNGIIKAFRNIPGITLLNVSKLNILKL
APGGHVGRFCIWTESAFRKLDELYGTWRKAASLKSNYNLPMHKMINTDLSRILKSPEIQR
ALRAPRKKIHRRVLKKNPLKNLRIMLKLNPYAKTMRRNTILRQARNHKLRVDKAAAAAAA
LQAKSDEKAAVAGKKPVVGKKGKKAAVGVKKQKKPLVGKKAAATKKPAPEKKPAEKKPTT
EEKKPAA*
>ENSG00000091009|ENST00000265271
MLIEDVDALKSWLAKLLEPICDADPSALANYVVALVKKDKPEKELKAFCADQLDVFLQKE
TSGFVDKLFESLYTKNYLPLLEPVKPEPKPLVQEKEEIKEEVFQEPAEEERDGRKKKYPS
PQKTRSESSERRTREKKREDGKWRDYDRYYERNELYREKYDWRRGRSKSRSKSRGLSRSR
SRSRGRSKDRDPNRNVEHRERSKFKSERNDLESSYVPVSAPPPNSSEQYSSGAQSIPSTV
TVIAPAHHSENTTESWSNYYNNHSSSNSFGRNLPPKRRCRDYDERGFCVLGDLCQFDHGN
DPLVVDEVALPSMIPFPPPPPGLPPPPPPGMLMPPMPGPGPGPGPGPGPGPGPGPGPGHS
MRLPVPQGHGQPPPSVVLPIPRPPITQSSLINSRDQPGTSAVPNLASVGTRLPPPLPQNL
LYTVSERQPMYSREHGAAASERLQLGTPPPLLAARLVPPRNLMGSSIGYHTSVSSPTPLV
PDTYEPDGYNPEAPSITSSGRSQYRQFFSRTQTQRPNLIGLTSGDMDVNPRAANIVIQTE
PPVPVSINSNITRVVLEPDSRKRAMSGLEGPLTKKPWLGKQGNNNQNKPGFLRKNQYTNT
KLEVKKIPQELNNITKLNEHFSKFGTIVNIQVAFKGDPEAALIQYLTNEEARKAISSTEA
VLNNRFIRVLWHRENNEQPTLQSSAQLLLQQQQTLSHLSQQHHHLPQHLHQQQVLVAQSA
PSTVHGGIQKMMSKPQTSGAYVLNKVPVKHRLGHAGGNQSDASHLLNQSGGAGEDCQIFS
TPGHPKMIYSSSNLKTPSKLCSGSKSHDVQEVLKKKQEAMKLQQDMRKKRQEVLEKQIEC
QKMLISKLEKNKNMKPEERANIMKTLKELGEKISQLKDELKTSSAVSTPSKVKTKTEAQK
ELLDTELDLHKRLSSGEDTTELRKKLSQLQVEAARLGILPVGRGKTMSSQGRGRGRGRGG
RGRGSLNHMVVDHRPKALTVGGFIEEEKEDLLQHFSTANQGPKFKDRRLQISWHKPKVPS

ISTETEEEEVKEEETETSDLFLPDDDDEDEDEYESRSWRR*
>ENSG00000082515|ENST00000265229
MWYLAKLIRGMSIDQALAQLEFNDKKGAKIIKEVLLEAQDMAVRDHNVEFRSNLYIAEST
SGRGQCLKRIRYHGRGRFGIMEKVYCHYFVKLVEGPPPPPEPPKTAVAHAKEYIQQLRSR
TIVHTL*
>ENSG00000161265|ENST00000292879
MAEYLASIFGTEKDKVNCSFYFKIGVCRHGDRCSRLHNKPTFSQEVFTELQEKYGEIEEM
NVCDNLGDHLVGNVYVKFRREEDGERAVAELSNRWFNGQAVHGNVPEVASATSCICGPFP
RTSRGSSMGGDPGAGHPRGSILATIPERGTIGVPLITGMAASEALAPLPFTPNRDRCSWQ
DLSSKPPSLSCPILPRLPGSIM*
>ENSG00000169548|ENST00000302097
MGDIFLCKKVESPKKNLRESKQREEDDEDPDLIYVGVEHVHRDAEVLFVGMISNSKPVVS
NILNRVTPGSNSRRKKGHFRQYPAHVSQPANHVTSMAKAIMPVSLSEGRSTDSPVTMKSS
SEPGYKMSSPQVVSPSSSDSLPPGTQCLVGAMVSGGGRNESSPDSKRLSTSDINSRDSKR
VKLRDGIPGVPSLAVVPSDMSSTISTNTPSQGICNSSNHVQNGVTFPWPDANGKAHFNLT
DPERASESALAMTDISSLASQNKTFDPKKENPIVLLSDFYYGQHKGDGQPEQKTHTTFKC
LSCVKVLKNIKFMNHMKHHLEFEKQRNDSWEDHTTCQHCHRQFPTPFQLQCHIDSVHIAM
GPSAVCKICELSFETDQVLLQHMKDHHKPGEMPYVCQVCHYRSSVFADVETHFRTCHENT
KNLLCLFCLKLFKTAIPYMNHCWRHSRRRVLQCSKCRLQFLTLKEEIEHKTKDHQTFKKP
EQLQGLPSETKVIIQTSVQPGSSGMASVIVSNTDPQSSPVKTKKKTAMNTRDSRLPCSKD
SS*
>ENSG00000115507|ENST00000282549
MMSYLKQPPYGMNGLGLAGPAMDLLHPSVGYPATPRKQRRERTTFTRSQLDVLEALFAKT
RYPDIFMREEVALKINLPESRVQVWFKNRRAKCRQQQQSGSGTKSRPAKKKSSPVRESSG
SESSGQFTPPAVSSSASSSSSASSSSANPAAAAAAGLGGNPVAAASSLSTPAASSIWSPA
SISPGSAPASVSVPEPLAAPSNTSCMQRSVAAGAATAAASYPMSYGQGGSYGQGYPTPSS
SYFGGVDCSSYLAPMHSHHHPHQLSPMAPSSMAGHHHHHPHAHHPLSQSSGHHHHHHHHH
HQGYGGSGLAFNSADCLDYKEPGAAAASSAWKLNFNSPDCLDYKDQASWRFQVL*
>ENSG00000118263|ENST00000309446
MDVLASYSIFQELQLVHDTGYFSALPSLEETWQQTCLELERYLQTEPRRISETFGEDLDC
FLHASPPPCIEESFRRLDPLLLPVEAAICEKSSAVDILLSRDKLLSETCLSLQPASSSLD
SYTAVNQAQLNAVTSLTPPSSPELSRHLVKTSQTLSAVDGTVTLKLVAKKAALSSVKVGG
VATAAAAVTAAGAVKSGQSDSDQGGLGAEACPENKKRVHRCQFNGCRKVYTKSSHLKAHQ
RTHTGEKPYKCSWEGCEWRFARSDELTRHYRKHTGAKPFKCNHCDRCFSRSDHLALHMKR
HI*
>ENSG00000168070|ENST00000301896
MSLKPFTYPFPETRFLHAGPNVYKFKIRYGKSIRGEEIENKEVITQELEDSVRVVLGNLD
NLQPFATEHFIVFPYKSKWERVSHLKFKHGEIILIPYPFVFTLYVEMKWFHENLSPGKPI
SDSPLGLVPVEKKAVGAVMRKRKHMDEPSSPSRPGLDRIGKEKPNKDCRRLWPLISLMSR
NKILSGDTACQGELSHPCSTTHLHLRSEQPPASLGF*
>ENSG00000107949|ENST00000278100
MASRSKRRAVESGVPQPPDPPVQRDEEEEKEVENEDEDDDDSDKEKDEEDEVIDEEVNIE
FEAYSLSDNDYDGIKKLLQQLFLKAPVNTAELTDLLIQQNHIGSVIKQTDVSEDSNDDMD
EDEVFGFISLLNLTERKGTQCVEQIQELVLRFCEKNCEKSMVEQLDKFLNDTTKPVGLLL

SERFINVPPQIALPMYQQLQKELAGAHRTNKPCGKCYFYLLISKTFVEAGKNNSKKKPSN
KKKAALMFANAEEEFFYEKAILKFNYSVQEESDTCLGGKWSFDDVPMTPLRTVMLIPGDK
MNEIMDKLKEYLSV*
>ENSG00000010072|ENST00000295050
MDDDLMLALRLQEEWNLQEAERDHAQESLSLVDASWELVDPTPDLQALFVQFNDQFFWGQ
LEAVEVKWSVRMTLCAGICSYEGKGGMCSIRLSEPLLKLRPRKDLVETLLHEMIHAYLFV
TNNDKDREGHGPEFCKHMHRINSLTGANITVYHTFHDEVDEYRRHWWRCNGPCQHRPPYY
GYVKRATNREPSAHDYWWAEHQKTCGGTYIKIKEPENYSKKGKGKAKLGKEPVLAAENKD
KPNRGEAQLVIPFSGKGYVLGETSNLPSPGKLITSHAINKTQDLLNQNHSANAVRPNSKI
KVKFEQNGSSKNSHLVSPAVSNSHQNVLSNYFPRVSFANQKAFRGVNGSPRISVTVGNIP
KNSVSSSSQRRVSSSKISLRNSSKVTESASVMPSQDVSGSEDTFPNKRPRLEDKTVFDNF
FIKKEQIKSSGNDPKYSTTTAQNSSSSSSQSKMVNCPVCQNEVLESQINEHLDWCLEGDS
IKVKSEESL*
>ENSG00000140968|ENST00000268638
MCDRNGGRRLRQWLIEQIDSSMYPGLIWENEEKSMFRIPWKHAGKQDYNQEVDASIFKAW
AVFKGKFKEGDKAEPATWKTRLRCALNKSPDFEEVTDRSQLDISEPYKVYRIVPEEEQKC
KLGVATAGCVNEVTEMECGRSEIDELIKEPSVDDYMGMIKRSPSPPEACRSQLLPDWWAQ
QPSTGVPLVTGYTTYDAHHSAFSQMVISFYYGGKLVGQATTTCPEGCRLSLSQPGLPGTK
LYGPEGLELVRFPPADAIPSERQRQVTRKLFGHLERGVLLHSSRQGVFVKRLCQGRVFCS
GNAVVCKGRPNKLERDEVVQVFDTSQFFRELQQFYNSQGRLPDGRVVLCFGEEFPDMAPL
RSKLILVQIEQLYVRQLAEEAGKSCGAGSVMQAPEEPPPDQVFRMFPDICASHQRSFFRE
NQQITV*
>ENSG00000150593|ENST00000280154
MDVENEQILNVNPADPDNLSDSLFSGDEENAGTEEIKNEINGNWISASSINEARINAKAK
RRLRKNSSRDSGRGDSVSDSGSDALRSGLTVPTSPKGRLLDRRSRSGKGRGLPKKGGAGG
KGVWGTPGQVYDVEEVDVKDPNYDDDQENCVYETVVLPLDERAFEKTLTPIIQEYFEHGD
TNEVAEMLRDLNLGEMKSGVPVLAVSLALEGKASHREMTSKLLSDLCGTVMSTTDVEKSF
DKLLKDLPELALDTPRAPQLVGQFIARAVGDGILCNTYIDSYKGTVDCVQARAALDKATV
LLSMSKGGKRKDSVWGSGGGQQSVNHLVKEIDMLLKEYLLSGDISEAEHCLKELEVPHFH
HELVYEAIIMVLESTGESTFKMILDLLKSLWKSSTITVDQMKRGYERIYNEIPDINLDVP
HSYSVLERFVEECFQAGIISKQLRDLCPSRGRKRFVSEGDGGRLKPESY*
>ENSG00000173011|ENST00000310074
MAELGKKYCVYCLAEVSPLRFRCTECQDIELCPECFSAGAEIGHHRRYHGYQLVDGGRFT
LWGPEAEGGWTSREEQLLLDAIEQFGFGNWEDMAAHVGASRTPQEVMEHYVSMYIHGNLG
KACIPDTIPNRVTDHTCPSGGPLSPSLTTPLPPLDISVAEQQQLGYMPLRDDYEIEYDQD
AETLISGLSVNYDDDDVEIELKRAHVDMYVRKLKERQRRKNIARDYNLVPAFLGKDKKEK
EKALKRKITKEEKELRLKLRPLYQFMSCKEFDDLFENMHKEKMLRAKIRELQRYRRNGIT
KMEESAEYEAARHKREKRKENKNLAGSKRGKEDGKDSEFAAIENLPGFELLSDREKVLCS
SLNLSPARYVTVKTIIIKDHLQKRQGIPSKSRLPSYLDKVLKKRILNFLTESGWISRDAS
*
>ENSG00000155660|ENST00000286091
MRPRKAFLLLLLLGLVQLLAVAGAEGPDEDSSNRENAIEDEEEEEEEEDDDEEEDDLEVKE
ENGVLVLNDANFDNFVADKDTVLLEFYAPWCGHCKQFAPEYEKIANILKDKDPPIPVAKI
DATSASVLASRFDVSGYPTIKILKKGQAVDYEGSRTQEEIVAKVREVSQPDWTPPPEVTL

VLTKENFDEVVNDADIILVEFYAPWCGHCKKLAPEYEKAAKELSKRSPPIPLAKVDATAE
TDLAKRFDVSGYPTLKIFRKGRPYDYNGPREKYGIVDYMIEQSGPPSKEILTLKQVQEFL
KDGDDVIIIGVFKGESDPAYQQYQDAANNLREDYKFHHTFSTEIAKFLKVSQGQLVVMQP
EKFQSKYEPRSHMMDVQGSTQDSAIKDFVLKYALPLVGHRKVSNDAKRYTRRPLVVVYYS
VDFSFDYRAATQFWRSKVLEVAKDFPEYTFAIADEEDYAGEVKDLGLSESGEDVNAAILD
ESGKKFAMEPEEFDSDTLREFVTAFKKGKLKPVIKSQPVPKNNKGPVKVVVGKTFDSIVM
DPKKDVLIEFYAPWCGHCKQLEPVYNSLAKKYKGQKGLVIAKMDATANDVPSDRYKVEGF
PTIYFAPSGDKKNPVKFEGGDRDLEHLSKFIEEHATKLSRTKEEL*
>ENSG00000150347|ENST00000309334
MAPNLKGRPRKKKPCPQRRDSFSGVKDSNNNSDGKAVAKVKCEARSALTKPKNNHNCKKV
SNEEKPKVAIGEECRADEQAFLVALYKYMKERKTPIERIPYLGFKQINLWTMFQAAQKLG
GYETITARRQWKHIYDELGGNPGSTSAATCTRRHYERLILPYERFIKGEEDKPLPPIKPR
KQENSSQENENKTKVSGTKRIKHEIPKSKKEKENAPKPQDAAEVSSEQEKEQETLISQKS
IPEPLPAADMKKKIEGYQEFSAKPLASRVDPEKDNETDQGSNSEKVAEEAGEKGPTPPLP
SAPLAPEKDSALVPGASKQPLTSPSALVDSKQESKLCCFTESPESEPQEASFPSFPTTQP
PLANQNETEDDKLPAMADYIANCTVKVDQLGSDDIHNALKQTPKVLVVQSFDMFKDKDLT
GPMNENHGLNYTPLLYSRGNPGIMSPLAKKKLLSQVSGASLSSSYPYGSPPPLISKKKLI
ARDDLCSSLSQTHHGQSTDHMAVSRPSVIQHVQSFRSKPSEERKTINDIFKHEKLSRSDP
HRCSFSKHHLNPLADSYVLKQEIQEGKDKLLEKRALPHSHMPSFLADFYSSPHLHSLYRH
TEHHLHNEQTSKYPSRDMYRESENSSFPSHRHQEKLHVNYLTSLHLQDKKSAAAEAPTDD
QPTDLSLPKNPHKPTGKVLGLAHSTTGPQESKGISQFQVLGSQSRDCHPKACRVSPMTMS
GPKKYPESLSRSGKPHHVRLENFRKMEGMVHPILHRKMSPQNIGAARPIKRSLEDLDLVI
AGKKARAVSPLDPSKEVSGKEKASEQESEGSKAAHGGHSGGGSEGHKLPLSSPIFPGLYS
GSLCNSGLNSRLPAGYSHSLQYLKNQTVLSPLMQPLAFHSLVMQRGIFTSPTNSQQLYRH
LAAATPVGSSYGDLLHNSIYPLAAINPQAAFPSSQLSSVHPSTKL*
>ENSG00000160917|ENST00000292476
MQEIIASVDHIKFDLEIAVEQQLGAQPLPFPGMDKSGAAVCEFFLKAACGKGGMCPFRHI
SGEKTVVCKHWLRGLCKKGDQCEFLHEYDMTKMPECYFYSKFGECSNKECPFLHIDPESK
IKDCPWYDRGFCKHGPLCRHRHTRRVICVNYLVGFCPEGPSCKFMHPRFELPMGTTEQPP
LPQQTQPPAKQSNNPPLQRSSSLIQLTSQNSSPNQQRTPQVIGVMQSQNSSAGNRGPRPL
EQVTCYKCGEKGHYANRCTKGHLAFLSGQ*
>ENSG00000164548|ENST00000297071
MSDVEENNFEGRESRSQSKSPTGTPARVKSESRSGSRSPSRVSKHSESHSRSRSKSRSRS
RRHSHRRYTRSRSHSHSHRRRSRSRSYTPEYRRRRSRSHSPMSNRRRHTGSRANPDPNTC
LGVFGLSLYTTERDLREVFSRYGPLSGVNVVYDQRTGRSRGFAFVYFERIDDSKEAMERA
NGMELDGRRIRVDYSITKRAHTPTPGIYMGRPTHSGGGGGGGGGGGGGGGGRRRDSYYDR
GYDRGYDRYEDYDYRYRRRSPSPYYSRYRSRSRSRSYSPRRY*
>ENSG00000168101|ENST00000304301
MSTAAVPELKQISRVEAMRLGPGWSHSCHAMLYAANPGQLFGRIPMRFSVLMQMRFDGLL
GFPGGFVDRRFWSLEDGLNRVLGLGLGCLRLTEADYLSSHLTEGPHRVVAHLYARQLTLE
QLHAVEISAVHSRDHGLEVLGLVRVPLYTQKDRVGGFPNFLSNAFVSTAKCQLLFALKVL
NMMPEEKLVEALAAATEKQKKALEKLLPASS*
>ENSG00000147854|ENST00000276893
MWIQVRTIDGSKTCTIEDVSRKATIEELRERVWALFDVRPECQRLFYRGKQLENGYTLFD

YDVGLNDIIQLLVRPDPDHLPGTSTQIEAKPCSNSPPKVKKAPRVGPSNQPSTSARARLI
DPGFGIYKVNELVDARDVGLGAWFEAHIHSVTRASDGQSRGKTPLKNGSSCKRTNGNIKH
KSKENTNKLDSVPSTSNSDCVAADEDVIYHIQYDEYPESGTLEMNVKDLRPRARTILKWN
ELNVGDVVMVNYNVESPGQRGFWFDAEITTLKTISRTKKELRVKIFLGGSEGTLNDCKII
SVDEIFKIERPGAHPLSFADGKFLRRNDPECDLCGGDPEKKCHSCSCRVCGGKHEPNMQL
LCDECNVAYHIYCLNPPLDKVPEEEYWYCPSCKTDSSEVVKAGERLKMSKKKAKMPSAST
ESRRDWGRGMACVGRTRECTIVPSNHYGPIPGIPVGSTWRFRVQVSEAGVHRPHVGGIHG
RSNDGAYSLVLAGGFADEVDRGDEFTYTGSGGKNLAGNKRIGAPSADQTLTNMNRALALN
CDAPLDDKIGAESRNWRAGKPVRVIRSFKGRKISKYAPEEGNRYDGIYKVVKYWPEISSS
HGFLVWRYLLRRDDVEPAPWTSEGIERSRRLCLRLQYPAGYPSDKEGKKPKGQSKKQPSG
TTKRPISDDDCPSASKVYKASDSAEAIEAFQLTPQQQHLIREDCQNQKLWDEVLSHLVEG
PNFLKKLEQSFMCVCCQELVYQPVTTECFHNVCKDCLQRSFKAQVFSCPACRHDLGQNYI
MIPNEILQTLLDLFFPGYSKGR*
>ENSG00000144535|ENST00000273009
MSHPDYRMNLRPLGTPRGVSAVAGPHDIGASPGDKKSKNRSTRGKKKSIFETYMSKEDVS
EGLKRGTLIQGVLRINPKKFHEAFIPSPDGDRDIFIDGVVARNRALNGDLVVVKLLPEEH
WKVVKPESNDKETEAAYESDIPEELCGHHLPQQSLKSYNDSPDVIVEAQFDGSDSEDGHG
ITQNVLVDGVKKLSVCVSEKGREDGDAPVTKDETTCISQDTRALSEKSLQRSAKVVYILE
KKHSRAATGFLKLLADKNSELFRKYALFSPSDHRVPRIYVPLKDCPQDFVARPKDYANTL
FICRIVDWKEDCNFALGQLAKSLGQAGEIEPETEGILTEYGVDFSDFSSEVLECLPQGLP
WTIPPEEFSKRRDLRKDCIFTIDPSTARDLDDALSCKPLADGNFKVGVHIADVSYFVPEG
SDLDKVAAERATSVYLVQKVVPMLPRLLCEELCSLNPMSDKLTFSVIWTLTPEGKILDEW
FGRTIIRSCTKLSYEHAQSMIESPTEKIPAKELPPISPEHSSEEVHQQNADKDGAAHLQA
SHSPSAEDAEAQPSTEERLPETRGICDRDPDTRLFFLQQQSRVLEAKPQNTIRVEEQTTQ
LQI*
>ENSG00000143889|ENST00000272249
XGSHHKVSVSPVVHVRGLCESVVEADLVEALEKFGTICYVMMMPFKRQALVEFENIDSAK
ECVTFAADEPVYIAGQQAFFNYSTSKRITRPGNTDDPSGGNKVLLLSIQNPLYPITVDVL
YTVCNPVGKVQRIVIFKRNGIQAMVEFESVLCAQKAKAALNGADIYAGCCTLKIEYARPT
RLNVIRNDNDSWDYTKPYLGRRGCCWLMLKLLERSPL*
>ENSG00000196263|ENST00000308031
MNVEVVKVMPQDLVTFKDVAIDFSQEEWQWMNPAQKRLYRSMMLENYQSLVSLGLCISKP
YVISLLEQGREPWEMTSEMTRSPFSDWESIYVTQELPLKQFMYDDACMEGITSYGLECST
FEENWKWEDLFEKQMGSHEMFSKKEIITHKETITKETEFKYTKFGKCIHLENIEESIYNH
TSDKKSFSKNSMVIKHKKVYVGKKLFKCNECDKTFTHSSSLTVHFRIHTGEKPYACEECG
KAFKQRQHLAQHHRTHTGEKLFECKECRKAFKQSEHLIQHQRIHTGEKPYKCKECRKAFR
QPAHLAQHQRIHTGEKPYECKECGKAFSDGSSFARHQRCHTGKRPYECIECGKAFRYNTS
FIRHWRSYHTGEKPFNCIDCGKAFSVHIGLILHRRIHTGEKPYKCGVCGKTFSSGSSRTV
HQRIHTGEKPYECDICGKDFSHHASLTQHQRVHSGEKPYECKECGKAFRQNVHLVSHLRI
HTGEKPYECKECGKAFRISSQLATHQRIHTGEKPYECIECGNAFKQRSHLAQHQKTHTGE
KPYECNECGKAFSQTSNLTQHQRIHTGEKPYKCTECGKAFSDSSSCAQHQRLHTGQRPYQ
CFECGKAFRRKLSLICHQRSHTGEEP*
>ENSG00000153944|ENST00000284073
MEANGSQGTSGSANDSQHDPGKMFIGGLSWQTSPDSLRDYFSKFGEIRECMVMRDPTTKR

SRGFGFVTFADPASVDKVLGQPHHELDSKTIDPKVAFPRRAQPKMVTRTKKIFVGGLSAN
TVVEDVKQYFEQFGKVEDAMLMFDKTTNRHRGFGFVTFENEDVVEKVCEIHFHEINNKMV
ECKKAQPKEVMFPPGTRGRARGLPYTMDAFMLGMGMLGYPNFVATYGRGYPGFAPSYGYQ
FPGFPAAAYGPVAAAAVAAARGSGSNPARPGGFPGANSPGPVADLYGPASQDSGVGNYIS
AASPQPGSGFGHGIAGPLIATAFTNGYH*
>ENSG00000147488|ENST00000276480
MDAEAEDKTLRTRSKGTEVPMDSLIQELSVAYDCSMAKKRTAEDQALGVPVNKRKSLLMK
PRHYSPKADCQEDRSDRTEDDGPLETHGHSTAEEIMIKPMDESLLSTAQENSSRKEDRYS
CYQELMVKSLMHLGKFEKNVSVQTVSENLNDSGIQSLKAESDEADECFLIHSDDGRDKID
DSQPPFCSSDDNESNSESAENGWDSGSNFSEETKPPRVPKYVLTDHKKDLLEVPEIKTEG
DKFIPCENRCDSETERKDPQNALAEPLDGNAQPSFPDVEEEDSESLAVMTEEGSDLEKAK
GNLSLLEQAIALQAERGCVFHNTYKELDRFLLEHLAGERRQTKVIDMGGRQIFNNKHSPR
PEKRETKCPIPGCDGTGHVTGLYPHHRSLSGCPHKVRVPLEILAMHENVLKCPTPGCTGR
GHVNSNRNTHRSLSGCPIAAAEKLAMSQDKNQLDSPQTGQCPDQAHRTSLVKQIEFNFPS
QAITSPRATVSKEQEKFGKVPFDYASFDAQVFGKRPLIQTVQGRKTPPFPESKHFPNPVK
FPNRLPSAGAHTQSPGRASSYSYGQCSEDTHIAAAAAILNLSTRCREATDILSNKPQSLH
AKGAEIEVDENGTLDLSMKKNRILDKSAPLTSSNTSIPTPSSSPFKTSSILVNAAFYQAL
CDQEGWDTPINYSKTHGKTEEEKEKDPVSSLENLEEKKFPGEASIPSPKPKLHARDLKKE
LITCPTPGCDGSGHVTGNYASHRSVSGCPLADKTLKSLMAANSQELKCPTPGCDGSGHVT
GNYASHRSLSGCPRARKGGVKMTPTKEEKEDPELKCPVIGCDGQGHISGKYTSHRTASGC
PLAAKRQKENPLNGASLSWKLNKQELPHCPLPGCNGLGHVNNVFVTHRSLSGCPLNAQVI
KKGKVSEELMTIKLKATGGIESDEEIRHLDEEIKELNESNLKIEADMMKLQTQITSMESN
LKTIEEENKLIEQNNESLLKELAGLSQALISSLADIQLPQMGPISEQNFEAYVNTLTDMY
SNLERDYSPECKALLESIKQAVKGIHV*
>ENSG00000140876|ENST00000268533
MSRLGLPEEPVRNSLLDDAKARLRKYDIGGKYSHLPYNKYSVLLPLVAKEGKLHLLFTVR
SEKLRRAPGEVCFPGGKRDPTDMDDAATALREAQEEVGLRPHQVEVVCCLVPCLIDTDTL
ITPFVGLIDHNFQAQPNPAEVKDVFLVPLAYFLHPQVHDQHYVTRLGHRFINHIFEYTNP
EDGVTYQIKGMTANLAVLVAFIILEKKPTFEVQFNLNDVLASSEELFLKVHKKATSRL*
>ENSG00000167625|ENST00000301215
MAEVVAEVAEMPTQMSPGAVEMSTPMSAEMMEMSTEVTEMTPGEALASSLFFQHHQFMCS
ECGSLYNTLEEVLSHQEQHMLAVSEEEALTTQNVGLEPELVPGAEGPFQCGECSQLILSP
GELLAHQDAHLRESANQIQYQCWDCQELFPSPELWVAHRKAQHLSATVAEPPVPPPLPPP
TPLPPPSPPSEVKMEPYECPECSTLCATPEEFLEHQGTHFDSLEKEERNGLEEEEEDDEE
DEEDDEEMEDEEAMAEVGDDAVGGDESTAGWAQGCGDCPQHQPSAGARRQHRRTAHSPAS
ATHPFHCSQCQRSFSSANRLQAHGRAHVGGTHECTTCSKVFKKAASLEQHLRLHRGEARY
LCVDCGRGFGTELTLVAHRRAHTANPLHRCRCGKTFSNMTKFLYHRRTHAGKSGAPPTGA
TAPPAPAEPTPPPPPPAPPAQLPCPQCSKSFASASRLSRHRRAVHGPPERRHRCGVCGKG
FKKLIHVRNHLRTHTGERPFQCHSCGKTFASLANLSRHQLTHTGARPYQCLDCGKRFTQS
SNLQQHRRLHLRPVAFARAPRLPITGLYNKSPYYCGTCGRWFRAMAGLRLHQRVHARART
LTLQPPRSPSPAPPPPPEPQQTIMCTELGETIAIIETSQPLALEDTLQLCQAALGASEAG
GLLQLDTAFV*
>ENSG00000179134|ENST00000314471
MMFRDQVGILAGWFKGWNECEQTVALLSLLKRVTRTQARFLQLCLEHSLADCNDIHLLES

EANSAAIVSQWQQESKEKVVSLLLSHLPLLQPGNTEAKSEYMRLLQKVLAYSIESNAFIE
ESRQLLSYALIHPATTLEDRNALALWLSHLEERLASGFRSRPEPSYHSRQGSDEWGGPAE
LGPGEAGPGWQDKPPRENGHVPFHPSSSVPPAINSIGSNANTGLPCQIHPSPLKRSMSLI
PTSPQVPGEWPSPEELGARAAFTTPDHAPLSPQSSVASSGSEQTEEQGSSRNTFQEDGSG
MKDVPSWLKSLRLHKYAALFSQMSYEEMMTLTEQHLESQNVTKGARHKIALSIQKLRERQ
SVLKSLEKDVLEGGNLRNALQELQQIIITPIKAYSVLQATVAAATTTPTAKDGAPGEPPL
PGAEPPLAHPGTDKGTEAKDPPAVENYPPPPAPAPTDGSEPAPAPVADGDIPSQFTRVMG
KVCTQLLVSRPDEENITSYLQLIEKCLTHEAFTETQKKRLLSWKQQVLKLLRTFPRKAAL
EMQNYRQQKGWAFGSNSLPIAGSVGMGVARRTQRQFPMPPRALPPGRMGLLSPSGIGGVS
PRHALTSPSLGGQGRQNLWFANPGGSNSMPSQSRSSVQRTHSLPVHSSPQAILMFPPDCP
VPGPDLEINPTLESLCLSMTEHALGDGTDKTSTI*
>ENSG00000168439|ENST00000305218
MEQVNELKEKGNKALSVGNIDDALQCYSEAIKLDPHNHVLYSNRSAAYAKKGDYQKAYED
GCKTVDLKPDWGKGYSRKAAALEFLNRFEEAKRTYEEGLKHEANNPQLKEGLQNMEARLA
ERKFMNPFNMPNLYQKLESDPRTRTLLSDPTYRELIEQLRNKPSDLGTKLQDPRIMTTLS
VLLGVDLGSMDEEEEIATPPPPPPPKKETKPEPMEEDLPENKKQALKEKELGNDAYKKKD
FDTALKHYDKAKELDPTNMTYITNQAAVYFEKGDYNKCRELCEKAIEVGRENREDYRQIA
KAYARIGNSYFKEEKYKDAIHFYNKSLAEHRTPDVLKKCQQAEKILKEQERLAYINPDLA
LEEKNKGNECFQKGDYPQAMKHYTEAIKRNPKDAKLYSNRAACYTKLLEFQLALKDCEEC
IQLEPTFIKGYTRKAAALEAMKDYTKAMDVYQKALDLDSSCKEAADGYQRCMMAQYNRHD
SPEDVKRRAMADPEVQQIMSDPAMRLILEQMQKDPQALSEHLKNPVIAQKIQKLMDVGLI
AIR*
>ENSG00000122359|ENST00000265447
MPPIGLDNVATYAGQFNQDYLSGMAANMSGTFGGANMPNLYPGAPGAGYPPVPPGGFGQP
PSAQQPVPPYGMYPPPGGNPPSRMPSYPPYPGAPVPGQPMPPPGQQPPGAYPGQPPVTYP
GQPPVPLPGQQQPVPSYPGYPGSGTVTPAVPPTQFGSRGTITDAPGFDPLRDAEVLRKAM
KGFGTDEQAIIDCLGSRSNKQRQQILLSFKTAYGKDLIKDLKSELSGNFEKTILALMKTP
VLFDIYEIKEAIKGVGTDEACLIEILASRSNEHIRELNRAYKAEFKKTLEEAIRSDTSGH
FQRLLISLSQGNRDESTNVDMSLAQRDAQELYAAGENRLGTDESKFNAVLCSRSRAHLVA
VFNEYQRMTGRDIEKSICREMSGDLEEGMLAVVKCLKNTPAFFAERLNKAMRGAGTKDRT
LIRIMVSRSETDLLDIRSEYKRMYGKSLYHDISGDTSGDYRKILLKICGGND*
>ENSG00000165643|ENST00000298466
MASRCSEPYPEVSRIPTVRGCNGSLSGALSCCEDSARGSGPPKAPTVAEGPSSCLRRNVI
SERERRKRMSLSCERLRALLPQFDGRREDMASVLEMSVQFLRLASALGPSQEQHAILASS
KEMWHSLQEDVLQLTLSSQIQAGVPDPGTGASSGTRTPDVKAFLESPWSLDPASASPEPV
PHILASSRQWDPASCTSLGTDKCEALLGLCQVRGGLPPFSEPSSLVPWPPGRSLPKAVRP
PLSWPPFSQQQTLPVMSGEALGWLGQAGPLAMGAAPLGEPAKEDPMLAQEAGSALGSDVD
DGTSFLLTAGPSSWPGEWGPGFRAGPPA*
>ENSG00000158805|ENST00000289816
MGHCRLCHGKFSSRSLRSISERAPGASMERPSAEERVLVRDFQRLLGVAVRQDPTLSPFV
CKSCHAQFYQCHSLLKSFLQRVNASPAGRRKPCAKVGAQPPTGAEEGACLVDLITSSPQC
LHGLVGWVHGHAASCGALPHLQRTLSSEYCGVIQVVWGCDQGHDYTMDTSSSCKAFLLDS
ALAVKWPWDKETAPRLPQHRGWNPGDAPQTSQGRGTGTPVGAETKTLPSTDVAQPPSDSD
AVGPRSGFPPQPSLPLCRAPGQLGEKQLPSSTSDDRVKDEFSDLSEGDVLSEDENDKKQN

AQSSDESFEPYPERKVSGKKSESKEAKKSEEPRIRKKPGPKPGWKKKLRCEREELPTIYK
CPYQGCTAVYRGADGMKKHIKEHHEEVRERPCPHPGCNKVFMIDRYLQRHVKLIHTEVRN
YICDECGQTFKQRKHLLVHQMRHSGAKPLQCEVCGFQCRQRASLKYHMTKHKAETELDFA
CDQCGRRFEKAHNLNVHMSMVHPLTQTQDKALPLEAEPPPGPPSPSVTTEGQAVKPEPT*
>ENSG00000009950|ENST00000313375
MAGALAGLAAGLQVPRVAPSPDSDSDTDSEDPSLRRSAGGLLRSQVIHSGHFMVSSPHSD
SLPRRRDQEGSVGPSDFGPRSIDPTLTRLFECLSLAYSGKLVSPKWKNFKGLKLLCRDKI
RLNNAIWRAWYIQYVKRRKSPVCGFVTPLQGPEADAHRKPEAVVLEGNYWKRRIEVVMRE
YHKWRIYYKKRLRKPSREDDLLAPKQAEGRWPPPEQWCKQLFSSVVPVLLGDPEEEPGGR
QLLDLNCFLSDISDTLFTMTQSGPSPLQLPPEDAYVGNADMIQPDLTPLQPSLDDFMDIS
DFFTNSRLPQPPMPSNFPEPPSFSPVVDSLFSSGTLGPEVPPASSAMTHLSGHSRLQARN
SCPGPLDSSAFLSSDFLLPEDPKPRLPPPPVPPPLLHYPPPAKVPGLEPCPPPPFPPMAP
PTALLQEEPLFSPRFPFPTVPPAPGVSPLPAPAAFPPTPQSVPSPAPTPFPIELLPLGYS
EPAFGPCFSMPRGKPPAPSPRGQKASPPTLAPATASPPTTAGSNNPCLTQLLTAAKPEQA
LEPPLVSSTLLRSPGSPQETVPEFPCTFLPPTPAPTPPRPPPGPATLAPSRPLLVPKAER
LSPPAPSGSERRLSGDLSSMPGPGTLSVRVSPPQPILSRGRPDSNKTENRRITHISAEQK
RRFNIKLGFDTLHGLVSTLSAQPSLKVSKATTLQKTAEYILMLQQERAGLQEEAQQLRDE
IEELNAAINLCQQQLPATGVPITHQRFDQMRDMFDDYVRTRTLHNWKFWVFSILIRPLFE
SFNGMVSTASVHTLRQTSLAWLDQYCSLPALRPTVLNSLRQLGTSTSILTDPGRIPEQAT
RAVTEGTLGKPL*
>ENSG00000163312|ENST00000295488
MDECGSRIRRRVSLPKRNRPSLGCIFGAPTAAELVPGDEGKEEEEMVAENRRRKTAGVLP
VEVQPLLLSDSPECLVLGGGDTNPDLLRHMPTDRGVGDQPNDSEVDMFGDYDSFTENSFI
AQVDDLEQKYMQLPEHKKHATDFATENLCSESIKNKLSITTIGNLTELQTDKHTENQSGY
EGVTIEPGADLLYDVPSSQAIYFENLQNSSNDLGDHSMKERDWKSSSHNTVNEELPHNCI
EQPQQNDESSSKVRTSSDMNRRKSIKDHLKNAMTGNAKAQTPIFSRSKQLKDTLLSEEIN
VAKKTVESSSNDLGPFYSLPSKVRDLYAQFKGIEKLYEWQHTCLTLNSVQERKNLIYSLP
TSGGKTLVAEILMLQELLCCRKDVLMILPYVAIVQEKISGLSSFGIELGFFVEEYAGSKG
RFPPTKRREKKSLYIATIEKGHSLVNSLIETGRIDSLGLVVVDELHMIGEGSRGATLEMT
LAKILYTSKTTQIIGMSATLNNVEDLQKFLQAEYYTSQFRPVELKEYLKINDTIYEVDSK
AENGMTFSRLLNYKYSDTLKKMDPDHLVALVTEVIPNYSCLVFCPSKKNCENVAEMICKF
LSKEYLKHKEKEKCEVIKNLKNIGNGNLCPVLKRTIPFGVAYHHSGLTSDERKLLEEAYS
TGVLCLFTCTSTLAAGVNLPARRVILRAPYVAKEFLKRNQYKQMIGRAGRAGIDTIGESI
LILQEKDKQQVLELITKPLENCYSHLVQEFTKGIQTLFLSLIGLKIATNLDDIYHFMNGT
FFGVQQKVLLKEKSLWEITVESLRYLTEKGLLQKDTIYKSEEEVQYNFHITKLGRASFKG
TIDLAYCDILYRDLKKGLEGLVLESLLHLIYLTTPYDLVSQCNPDWMIYFRQFSQLSPAE
QNVAAILGVSESFIGKKASGQAIGKKVDKNVVNRLYLSFVLYTLLKETNIWTVSEKFNMP
RGYIQNLLTGTASFSSCVLHFCEELEEFWVYRALLVELTKKLTYCVKAELIPLMEVTGVL
EGRAKQLYSAGYKSLMHLANANPEVLVRTIDHLSRRQAKQIVSSAKMLLHEKAEALQEEV
EELLRLPSDFPGAVASSTDKA*
>ENSG00000170889|ENST00000302907
MPVARSWVCRKTYVTPRRPFEKSRLDQELKLIGEYGLRNKREVWRVKFTLAKIRKAAREL
LTLDEKDPRRLFEGNALLRRLVRIGVLDEGKMKLDYILGLKIEDFLERRLQTQVFKLGLA
KSIHHARVLIRQRHIRVRKQVVNIPSFIVRLDSQKHIDFSLRSPYGGGRPGRVKRKNAKK

GQGGAGAGDDEEED*
>ENSG00000063244|ENST00000308924
MSDFDEFERQLNENKQERDKENRHRKRSHSRSRSRDRKRRSRSRDRRNRDQRSASRDRRR
RSKPLTRGAKEEHGGLIRSPRHEKKKKVRKYWDVPPPGFEHITPMQYKAMQAAGQIPATA
LLPTMTPDGLAVTPTPVPVVGSQMTRQARRLYVGNIPFGITEEAMMDFFNAQMRLGGLTQ
APGNPVLAVQINQDKNFAFLEFRSVDETTQAMAFDGIIFQGQSLKIRRPHDYQPLPGMSE
NPSVYVPGVVSTVVPDSAHKLFIGGLPNYLNDDQVKELLTSFGPLKAFNLVKDSATGLSK
GYAFCEYVDINVTDQAIAGLNGMQLGDKKLLVQRASVGAKNATLVSPPSTINQTPVTLQV
PGLMSSQVQMGGHPTEVLCLMNMVLPEELLDDEEYEEIVEDVRDECSKYGLVKSIEIPRP
VDGVEVPGCGKIFVEFTSVFDCQKAMQGLTGRKFANRVVVTKYCDPDSYHRRDFW*
>ENSG00000173933|ENST00000310092
MVKLFIGNLPREATEQEIRSLFEQYGKVLECDIIKNYGFVHIEDKTAAEDAIRNLHHYKL
HGVNINVEASKNKSKTSTKLHVGNISPTCTNKELRAKFEEYGPVIECDIVKDYAFVHMER
AEDAVEAIRGLDNTEFQGKRMHVQLSTSRLRTAPGMGDQSGCYRCGKEGHWSKECPIDRS
GRVADLTEQYNEQYGAVRTPYTMSYGDSLYYNNAYGALDAYYKRCRAARSYEAVAAAAAS
VYNYAEQTLSQLPQVQNTAMASHLTSTSLDPYDRHLLPTSGAAATAAAAAAAAAAVTAAS
TSYYGRDRSPLRRATAPVPTVGEGYGYGHESELSQASAAARNSLYDMARYEREQYADRAR
YSAF*
>ENSG00000172346|ENST00000306149
MTSESTSPPVVPPLHSPKSPVWPTFPFHREGSRVWERGGVPPRDLPSPLPTKRTRTYSAT
ARASAGPVFKGVCKQFSRSQGHGFITPENGSEDIFVHVSDIEGEYVPVEGDEVTYKMCPI
PPKNQKFQAVEVVLTQLAPHTPHETWSGQVVGS*
>ENSG00000146909|ENST00000275820
MAASRSAGEAGPGGSQGRVVRMKRRGGRGPRRGPAGGGEKALKRLKLAVEEFVHATSEGE
APGGCEGRGAPVSFRPGGRKSRKELRKEKRHLRKARRLQRTAGPEQGPGLGGRSGAEEAS
GHRQDTEERARPAPSRDPSPPRKPRPSRVKAKATAATAKTRPSAAATAAARKRALLAANE
EEDREIRKLERCLGLNKRKKKDGSSSVPLSFARDGLDYILGALESGKNSGLYDSSGEEEE
DAGQTLPESDLESDSQDESEEEEEGDVEKEKKAQEAEAQSEDDDEDTEEEQGEEKEKGAQ
EKRRGKRVRFAEDEEKSENSSEDGDITDKSLCGSGEKYIPPHVRQAEETVDFKKKEELER
LKKHVKGLLNRLSEPNMASISGQLEELYMAHSRKDMNDTLTSALMGACVTASAMPSRLMM
EHVLLVSILHHTVGIEVGAHFLEAVVRKFDAIYKYGSEGKECDNLFTVIAHLYNFHVVQS
LLIFDILKKLIGTFTEKDIELILLMLKNVGFSLRKDDALSLKELITEAQTKASGAGSEFQ
DQTRIRFMLETMLALKNNDMRKIPGYDPEPVEKLRKLQRALVRNAGSGSETQLRVSWDSV
LSAEQTGRWWIVGSAWSGAPMIDNSHHTHLQKQLVGTVSSKILELARKQRMNTDIRRNIF
CTIMTSEDFLDAFEKLLKLGLKDQQEREIIHVLMDCCLQEKTYNPFYAFLASKFCEYERR
FQMTFQFSIWDKFRDLENLPATNFSNLVHLVAHLLKTKSLSLSILKVVEFSELDKPRVRF
LRKVLSILLMETEVEDLSLIFTRVSDNPKLGVLREGLKLFISHFLLKNAQAHRSADEANV
LREKADLATKCLQGKASLRM*
>ENSG00000277075|ENST00000303910
MSGRGKQGGKARAKAKTRSSRAGLQFPVGRVHRLLRKGNYSERVGAGAPVYLAAVLEYLT
AEILELAGNAARDNKKTRIIPRHLQLAIRNDEELNKLLGRVTIAQGGVLPNIQAVLLPKK
TESHHKAKGK*
>ENSG00000163161|ENST00000285398
MGKRDRADRDKKKSRKRHYEDEEDDEEDAPGNDPQEAVPSAAGKQVDESGTKVDEYGAKD

YRLQMPLKDDHTSRPLWVAPDGHIFLEAFSPVYKYAQDFLVAIAEPVCRPTHVHEYKLTA
YSLYAAVSVGLQTSDITEYLRKLSKTGVPDGIMQFIKLCTVSYGKVKLVLKHNRYFVESC
HPDVIQHLLQDPVIRECRLRNSEGEATELITETFTSKSAISKTAESSGGPSTSRVTDPQG
KSDIPMDLFDFYEQMDKDEEEEEETQTVSFEVKQEMIEELQKRCIHLEYPLLAEYDFRND
SVNPDINIDLKPTAVLRPYQEKSLRKMFGNGRARSGVIVLPCGAGKSLVGVTAACTVRKR
CLVLGNSAVSVEQWKAQFKMWSTIDDSQICRFTSDAKDKPIGCSVAISTYSMLGHTTKRS
WEAERVMEWLKTQEWGLMILDEVHTIPAKMFRRVLTIVQAHCKLGLTATLVREDDKIVDL
NFLIGPKLYEANWMELQNNGYIAKVQCAEVWCPMSPEFYREYVAIKTKKRILLYTMNPNK
FRACQFLIKFHERRNDKIIVFADNVFALKEYAIRLNKPYIYGPTSQGERMQILQNFKHNP
KINTIFISKVGDTSFDLPEANVLIQISSHGGSRRQEAQRLGRVLRAKKGMVAEEYNAFFY
SLVSQDTQEMAYSTKRQRFLVDQGYSFKVITKLAGMEEEDLAFSTKEEQQQLLQKVLAAT
DLDAEEEVVAGEFGSRSSQASRRFGTMSSMSGADDTVYMEYHSSRSKAPSKHVHPLFKRF
RK*
>ENSG00000041988|ENST00000307896
MPKSCAARQCCNRYSSRRKQLTFHRFPFSRPELLKEWVLNIGRGNFKPKQHTVICSEHFR
PECFSAFGNRKNLKHNAVPTVFAFQDPTQVRENTDPASERGNASSSQKEKVLPEAGAGED
SPGRNMDTALEELQLPPNAEGHVKQVSPRRPQATEAVGRPTGPAGLRRTPNKQPSDHSYA
LLDLDSLKKKLFLTLKENEKLRKRLQAQRLVMRRMSSRLRACKGHQGLQARLGPEQQS*
>ENSG00000111605|ENST00000266679
MADGVDHIDIYADVGEEFNQEAEYGGHDQIDLYDDVISPSANNGDAPEDRDYMDTLPPTV
GDDVGKGAAPNVVYTYTGKRIALYIGNLTWWTTDEDLTEAVHSLGVNDILEIKFFENRAN
GQSKGFALVGVGSEASSKKLMDLLPKRELHGQNPVVTPCNKQFLSQFEMQSRKTTQSGQM
SGEGKAGPPGGSSRAAFPQGGRGRGRFPGAVPGGDRFPGPAGPGGPPPPFPGNLIKHLVK
GTRPLFLETRIPWHMGHSIEEIPIFGLKAGQTPPRPPLGPPGPPGPPGPPPPGQVLPPPL
AGPPNRGDRPPPPVLFPGQPFGQPPLGPLPPGPPPPVPGYGPPPGPPPPQQGPPPPPGPF
PPRPPGPLGPPLTLAPPPHLPGPPPGAPPPAPHVNPAFFPPPTNSGMPTSDSRGPPPTDP
YGRPPPYDRGDYGPPGREMDTARTPLSEAEFEEIMNRNRAISSSAISRAVSDASAGDYGS
AIETLVTAISLIKQSKVSADDRCKVLISSLQDCLHGIESKSYGSGSRRERSRERDHSRSR
EKSRRHKSRSRDRHDDYYRERSRERERHRDRDRDRDRERDREREYRHR*
>ENSG00000168556|ENST00000302327
MLGQQQQQLYSSAALLTGERSRLLTCYVQDYLECVESLPHDMQRNVSVLRELDNKYQETL
KEIDDVYEKYKKEDDLNQKKRLQQLLQRALINSQELGDEKIQIVTQMLELVENRARQMEL
HSQCFQDPAESERASDKAKMDSSQPERSSRRPRRQRTSESRDLCHMANGIEDCDDQPPKE
KKSKSAKKKKRSKAKQEREASPVEFAIDPNEPTYCLCNQVSYGEMIGCDNEQCPIEWFHF
SCVSLTYKPKGKWYCPKCRGDNEKTMDKSTEKTKKDRRSR*
>ENSG00000101216|ENST00000266068
MATPDVSVHMEEVVVVTTPDTAVDGSGVEGVKTVLVTTNLAPHGGDLTEDNMETENAAAA
AAAAFTASSQLKEAVLVKMAEEGENLEAEIVYPITCGDSRANLIWRKFVCPGINVKCVQY
DEHVISPKEFVHLAGKSTLKDWKRAIRMNGIMLRKIMDSGELDFYQHDKVCSNTCRSTKI
DLSGARVSLSSPTSAEYIPLTPAAADVNGSPATITIETCEDPGDWTAAIGDDTFTFWRGL
KDAGLLDEVIQEFHQELVETMRGLQQRVQDPPLQLRDAVLLNNIVQNFGMLDLVKKVLAS
HKCQMDRSREQYARDLAALEQQCDEHRRRAKELKHKSQHLSNVLMTLTPVSLPPPVKRPR
LARATSGPAAMASQVLTQSAQLALGPGVPVPQLTSVPLGKVVSTLPSTVLGKGSLQAPPA
SSPASPLLGGYTVLASSGSTYPSTVEIHPDASSLTVLSTAAVQDGSTVFKVVSPLQLLTL

PGLGPTLQNVAQASPGSSTIVTVPAGAAPGPEEHTATIEVAAMAEDHERK*
>ENSG00000100105|ENST00000266269
MERVNDASCGPSGCYTYQVSRHSTEMLHNLNQQRKNGGRFCDVLLRVGDESFPAHRAVLA
ACSEYFESVFSAQLGDGGAADGGPADVGGATAAPGGGAGGSRELEMHTISSKVFGDILDF
AYTSRIVVRLESFPELMTAAKFLLMRSVIEICQEVIKQSNVQILVPPARADIMLFRPPGT
SDLGFPLDMTNGAALAANSNGIAGSMQPEEEAARAAGAAIAGQASLPVLPGVDRLPMVAG
PLSPQLLTSPFPSVASSAPPLTGKRGRGRPRKANLLDSMFGSPGGLREAGILPCGLCGKV
FTDANRLRQHEAQHGVTSLQLGYIDLPPPRLGENGLPISEDPDGPRKRSRTRKQVACEIC
GKIFRDVYHLNRHKLSHSGEKPYSCPVCGLRFKRKDRMSYHVRSHDGSVGKPYICQSCGK
GFSRPDHLNGHIKQVHTSERPHKCQTCNASFATRDRLRSHLACHEDKVPCQVCGKYLRAA
YMADHLKKHSEGPSNFCSICNRGFSSASYLKVHVKTHHGVPLPQVSRHQEPILNGGAAFH
CARTYGNKEGQKCSHQDPIESSDSYGDLSDASDLKTPEKQSANGSFSCDMAVPKNKMESD
GEKKYPCPECGSFFRSKSYLNKHIQKVHVRALGGPLGDLGPALGSPFSPQQNMSLLESFG
FQIVQSAFASSLVDPEVDQQPMGPEGK*
>ENSG00000154328|ENST00000284503
MPEGPLVRKFHHLVSPFVGQQVVKTGGSSKKLQPASLQSLWLQDTQVHGKKLFLRFDLDE
EMGPPGSSPTPEPPQKEVQKEGAADPKQVGEPSGQKTLDGSSRSAELVPQGEDDSEYLER
DAPAGDAGRWLRVSFGLFGSVWVNDFSRAKKANKRGDWRDPSPRLVLHFGGGGFLAFYNC
QLSWSSSPVVTPTCDILSEKFHRGQALEALGQAQPVCYTLLDQRYFSGLGNIIKNEALYR
AGIHPLSLGSVLSASRREVLVDHVVEFSTAWLQGKFQGRPQHTQVYQKEQCPAGHQVMKE
AFGPEDGLQRLTWWCPQCQPQLSEEPEQCQFS*
>ENSG00000106682|ENST00000265753
MADFDTYDDRAYSSFGGGRGSRGSAGGHGSRSQKELPTEPPYTAYVGNLPFNTVQGDIDA
IFKDLSIRSVRLVRDKDTDKFKGFCYVEFDEVDSLKEALTYDGALLGDRSLRVDIAEGRK
QDKGGFGFRKGGPDDRGMGSSRESRGGWDSRDDFNSGFRDDFLGGRGGSRPGDRRTGPPM
GSRFRDGPPLRGSNMDFREPTEEERAQRPRLQLKPRTVATPLNQVANPNSAIFGGARPRE
EVVQKEQE*
>ENSG00000147592|ENST00000276590
MAAVLQRVERLSNRVVRVLGCNPGPMTLQGTNTYLVGTGPRRILIDTGEPAIPEYISCLK
QALTEFNTAIQEIVVTHWHRDHSGGIGDICKSINNDTTYCIKKLPRNPQREEIIGNGEQQ
YVYLKDGDVIKTEGATLRVLYTPGHTDDHMALLLEEENAIFSGDCILGEGTTVFEDLYDY
MNSLKELLKIKADIIYPGHGPVIHNAEAKIQQYISHRNIREQQILTLFRENFEKSFTVME
LVKIIYKNTPENLHEMAKHNLLLHLKKLEKEGKIFSNTDPDKKWKAHL*
>ENSG00000151014|ENST00000280614
MFHSPRRLCSALLQRDAPGLRRLPAPGLRRPLSPPAAVPRPASPRLLAAASAASGAARSC
SRTVCSMGTGTSRLYSALAKTLNSSAASQHPEYLVSPDPEHLEPIDPKELLEECRAVLHT
RPPRFQRDFVDLRTDCPSTHPPIRVMQWNILAQALGEGKDNFVQCPVEALKWEERKCLIL
EEILAYQPDILCLQEVDHYFDTFQPLLSRLGYQGTFFPKPWSPCLDVEHNNGPDGCALFF
LQNRFKLVNSANIRLTAMTLKTNQVAIAQTLECKESGRQFCIAVTHLKARTGWERFRSAQ
GCDLLQNLQNITQGAKIPLIVCGDFNAEPTEEVYKHFASSSLNLNSAYKLLSADGQSEPP
YTTWKIRTSGECRHTLDYIWYSKHALNVRSALDLLTEEQIGPNRLPSFNYPSDHLSLVCD
FSFTEESDGLS*
>ENSG00000198633|ENST00000301085
MALTQGQLSFSDVAIEFSQEEWKCLDPGQKALYRDVMLENYRNLVSLGICLPDLSVTSML

EQKRDPWTLQSEVKIINNPDGRECIKGVNTDFSLLAWDARVFRVEAWRASGGAEEPTGPN
FVGNDSPLPSPLALLPFPSVSASLGDAKRARLTAEGRGHGTLRAGPAPRPRSAAPHAPDA
VQRWPPEQRLPRS*
>ENSG00000160224|ENST00000291582
MATDAALRRLLRLHRTEIAVAVDSAFPLLHALADHDVVPEDKFQETLHLKEKEGCPQAFH
ALLSWLLTQDSTAILDFWRVLFKDYNLERYGRLQPILDSFPKDVDLSQPRKGRKPPAVPK
ALVPPPRLPTKRKASEEARAAAPAALTPRGTASPGSQLKAKPPKKPESSAEQQRLPLGNG
IQTMSASVQRAVAMSSGDVPGARGAVEGILIQQVFESGGSKKCIQVGGEFYTPSKFEDSG
SGKNKARSSSGPKPLVRAKGAQGAAPGGGEARLGQQGSVPAPLALPSDPQLHQKNEDECA
VCRDGGELICCDGCPRAFHLACLSPPLREIPSGTWRCSSCLQATVQEVQPRAEEPRPQEP
PVETPLPPGLRSAGEEVRGPPGEPLAGMDTTLVYKHLPAPPSAAPLPGLDSSALHPLLCV
GPEGQQNLAPGARCGVCGDGTDVLRCTHCAAAFHWRCHFPAGTSRPGTGLRCRSCSGDVT
PAPVEGVLAPSPARLAPGPAKDDTASHEPALHRDDLESLLSEHTFDGILQWAIQSMARPA
APFPS*
>ENSG00000152942|ENST00000305138
MNQVTDWVDPSFDDFLECSGVSTITATSLGVNNSSHRRKNGPSTLESSRFPARKRGNLSS
LEQIYGLENSKEYLSENEPWVDKYKPETQHELAVHKKKIEEVETWLKAQVLERQPKQGGS
ILLITGPPGCGKTTTLKILSKEHGIQVQEWINPVLPDFQKDDFKGMFNTESSFHMFPYQS
QIAVFKEFLLRATKYNKLQMLGDDLRTDKKIILVEDLPNQFYRDSHTLHEVLRKYVRIGR
CPLIFIISDSLSGDNNQRLLFPKEIQEECSISNISFNPVAPTIMMKFLNRIVTIEANKNG
GKITVPDKTSLELLCQGCSGDIRSAINSLQFSSSKGENNLRPRKKGMSLKSDAVLSKSKR
RKKPDRVFENQEVQAIGGKDVSLFLFRALGKILYCKRASLTELDSPRLPSHLSEYERDTL
LVEPEEVVEMSHMPGDLFNLYLHQNYIDFFMEIDDIVRASEFLSFADILSGDWNTRSLLR
EYSTSIATRGVMHSNKARGYAHCQGGGSSFRPLHKPQWFLINKKYRENCLAAKALFPDFC
LPALCLQTQLLPYLALLTIPMRNQAQISFIQDIGRLPLKRHFGRLKMEALTDREHGMIDP
DSGDEAQLNGGHSAEESLGEPTQATVPETWSLPLSQNSASELPASQPQPFSAQGDMEENI
IIEDYESDGT*
>ENSG00000162624|ENST00000294638
MQILSRCQGLMSEECGRTTALAAGRTRKGAGEEGLVSPEGAGDEDSCSSSAPLSPSSSPR
SMASGSGCPPGKCVCNSCGLEIVDKYLLKVNDLCWHVRCLSCSVCRTSLGRHTSCYIKDK
DIFCKLDYFRRYGTRCSRCGRHIHSTDWVRRAKGNVYHLACFACFSCKRQLSTGEEFALV
EEKVLCRVHYDCMLDNLKREVENGNGISVEGALLTEQDVNHPKPAKRARTSFTADQLQVM
QAQFAQDNNPDAQTLQKLAERTGLSRRVIQVWFQNCRARHKKHVSPNHSSSTPVTAVPPS
RLSPPMLEEMAYSAYVPQDGTMLTALHSYMDAHSPTTLGLQPLLPHSMTQLPISHT*
>ENSG00000149311|ENST00000278616
MSLVLNDLLICCRQLEHDRATERKKEVEKFKRLIRDPETIKHLDRHSDSKQGKYLNWDAV
FRFLQKYIQKETECLRIAKPNVSASTQASRQKKMQEISSLVKYFIKCANRRAPRLKCQEL
LNYIMDTVKDSSNGAIYGADCSNILLKDILSVRKYWCEISQQQWLELFSVYFRLYLKPSQ
DVHRVLVARIIHAVTKGCCSQTDGLNSKFLDFFSKAIQCARQEKSSSGLNHILAALTIFL
KTLAVNFRIRVCELGDEILPTLLYIWTQHRLNDSLKEVIIELFQLQIYIHHPKGAKTQEK
GAYESTKWRSILYNLYDLLVNEISHIGSRGKYSSGFRNIAVKENLIELMADICHQVFNED
TRSLEISQSYTTTQRESSDYSVPCKRKKIELGWEVIKDHLQKSQNDFDLVPWLQIATQLI
SKYPASLPNCELSPLLMILSQLLPQQRHGERTPYVLRCLTEVALCQDKRSNLESSQKSDL
LKLWNKIWCITFRGISSEQIQAENFGLLGAIIQGSLVEVDREFWKLFTGSACRPSCPAVC

CLTLALTTSIVPGTVKMGIEQNMCEVNRSFSLKESIMKWLLFYQLEGDLENSTEVPPILH
SNFPHLVLEKILVSLTMKNCKAAMNFFQSVPECEHHQKDKEELSFSEVEELFLQTTFDKM
DFLTIVRECGIEKHQSSIGFSVHQNLKESLDRCLLGLSEQLLNNYSSEITNSETLVRCSR
LLVGVLGCYCYMGVIAEEEAYKSELFQKAKSLMQCAGESITLFKNKTNEEFRIGSLRNMM
QLCTRCLSNCTKKSPNKIASGFFLRLLTSKLMNDIADICKSLASFIKKPFDRGEVESMED
DTNGNLMEVEDQSSMNLFNDYPDSSVSDANEPGESQSTIGAINPLAEEYLSKQDLLFLDM
LKFLCLCVTTAQTNTVSFRAADIRRKLLMLIDSSTLEPTKSLHLHMYLMLLKELPGEEYP
LPMEDVLELLKPLSNVCSLYRRDQDVCKTILNHVLHVVKNLGQSNMDSENTRDAQGQFLT
VIGAFWHLTKERKYIFSVRMALVNCLKTLLEADPYSKWAILNVMGKDFPVNEVFTQFLAD
NHHQVRMLAAESINRLFQDTKGDSSRLLKALPLKLQQTAFENAYLKAQEGMREMSHSAEN
PETLDEIYNRKSVLLTLIAVVLSCSPICEKQALFALCKSVKENGLEPHLVKKVLEKVSET
FGYRRLEDFMASHLDYLVLEWLNLQDTEYNLSSFPFILLNYTNIEDFYRSCYKVLIPHLV
IRSHFDEVKSIANQIQEDWKSLLTDCFPKILVNILPYFAYEGTRDSGMAQQRETATKVYD
MLKSENLLGKQIDHLFISNLPEIVVELLMTLHEPANSSASQSTDLCDFSGDLDPAPNPPH
FPSHVIKATFAYISNCHKTKLKSILEILSKSPDSYQKILLAICEQAAETNNVYKKHRILK
IYHLFVSLLLKDIKSGLGGAWAFVLRDVIYTLIHYINQRPSCIMDVSLRSFSLCCDLLSQ
VCQTAVTYCKDALENHLHVIVGTLIPLVYEQVEVQKQVLDLLKYLVIDNKDNENLYITIK
LLDPFPDHVVFKDLRITQQKIKYSRGPFSLLEEINHFLSVSVYDALPLTRLEGLKDLRRQ
LELHKDQMVDIMRASQDNPQDGIMVKLVVNLLQLSKMAINHTGEKEVLEAVGSCLGEVGP
IDFSTIAIQHSKDASYTKALKLFEDKELQWTFIMLTYLNNTLVEDCVKVRSAAVTCLKNI
LATKTGHSFWEIYKMTTDPMLAYLQPFRTSRKKFLEVPRFDKENPFEGLDDINLWIPLSE
NHDIWIKTLTCAFLDSGGTKCEILQLLKPMCEVKTDFCQTVLPYLIHDILLQDTNESWRN
LLSTHVQGFFTSCLRHFSQTSRSTTPANLDSESEHFFRCCLDKKSQRTMLAVVDYMRRQK
RPSSGTIFNDAFWLDLNYLEVAKVAQSCAAHFTALLYAEIYADKKSMDDQEKRSLAFEEG
SQSTTISSLSEKSKEETGISLQDLLLEIYRSIGEPDSLYGCGGGKMLQPITRLRTYEHEA
MWGKALVTYDLETAIPSSTRQAGIIQALQNLGLCHILSVYLKGLDYENKDWCPELEELHY
QAAWRNMQWDHCTSVSKEVEGTSYHESLYNALQSLRDREFSTFYESLKYARVKEVEEMCK
RSLESVYSLYPTLSRLQAIGELESIGELFSRSVTHRQLSEVYIKWQKHSQLLKDSDFSFQ
EPIMALRTVILEILMEKEMDNSQRECIKDILTKHLVELSILARTFKNTQLPERAIFQIKQ
YNSVSCGVSEWQLEEAQVFWAKKEQSLALSILKQMIKKLDASCAANNPSLKLTYTECLRV
CGNWLAETCLENPAVIMQTYLEKAVEVAGNYDGESSDELRNGKMKAFLSLARFSDTQYQR
IENYMKSSEFENKQALLKRAKEEVGLLREHKIQTNRYTVKVQRELELDELALRALKEDRK
RFLCKAVENYINCLLSGEEHDMWVFRLCSLWLENSGVSEVNGMMKRDGMKIPTYKFLPLM
YQLAARMGTKMMGGLGFHEVLNNLISRISMDHPHHTLFIILALANANRDEFLTKPEVARR
SRITKNVPKQSSQLDEDRTEAANRIICTIRSRRPQMVRSVEALCDAYIILANLDATQWKT
QRKGINIPADQPITKLKNLEDVVVPTMEIKVDHTGEYGNLVTIQSFKAEFRLAGGVNLPK
IIDCVGSDGKERRQLVKGRDDLRQDAVMQQVFQMCNTLLQRNTETRKRKLTICTYKVVPL
SQRSGVLEWCTGTVPIGEFLVNNEDGAHKRYRPNDFSAFQCQKKMMEVQKKSFEEKYEVF
MDVCQNFQPVFRYFCMEKFLDPAIWFEKRLAYTRSVATSSIVGYILGLGDRHVQNILINE
QSAELVHIDLGVAFEQGKILPTPETVPFRLTRDIVDGMGITGVEGVFRRCCEKTMEVMRN
SQETLLTIVEVLLYDPLFDWTMNPLKALYLQQRPEDETELHPTLNADDQECKRNLSDIDQ
SFNKVAERVLMRLQEKLKGVEEGTVLSVGGQVNLLIQQAIDPKNLSRLFPGWKAWV*
>ENSG00000173041|ENST00000309683
MPGPPGSLEMGPLTFRDVAIEFSLEEWQCLDTAQRNLYRKVMFENYRNLVFLGIAVSKPH

LITCLEQGKEPWNRKRQEMVAKPPVIYSHFTEDLWPEHSIKDSFQKVILRGYGKCGHENL
QLRISCKSVDESKVFKEGYNELNQCLRTTQSKIFQCDKYVKVFHKFSNSNSHKKRNTGKK
VFKCKECGKSFCMLSHLTQHIRIHTRENSYKCEECGKVLNWFSELIKHKGIHMGEKPYKC
EECGKAFNQSSTLIKHKKIHIEEKPFKCEECGKAFSLFSILSKHKIIHTGDKPYKCDECH
KAFNWFATLTNHKRIHTGEKPFKCEECGKDFNQFSNLTKHKKIHTGEKPYKCEECGKAFN
QFANLTRHKKIHTGEKSYKCEECGKAFIQSSNLTEHMRIHTGEKPYKCEECGKAFNGCSS
LTRHKRIHTRENTYKCEECGKGFTLFSTLTNHKVIHTGEKSYKCDECGNVFNWPATLANH
KRIHAREKPYKCEECGKAFNRSSHLTRHKKIHTGEKLYKPEKCDNNFDNT*
>ENSG00000163497|ENST00000295727
MRQSGASQPLLINMYLPDPVGDGLFKDGKNPSWGPLSPAVQKGSGQIQLWQFLLELLADR
ANAGCIAWEGGHGEFKLTDPDEVARRWGERKSKPNMNYDKLSRALRYYYDKNIMSKVHGK
RYAYRFDFQGLAQACQPPPAHAHAAAAAAAAAAAAQDGALYKLPAGLAPLPFPGLSKLNL
MAASAGVAPAGFSYWPGPGPAATAAAATAALYPSPSLQPPPGPFGAVAAASHLGGHYH*
>ENSG00000178913|ENST00000313368
MSKSKDDAPHELESQFILRLPPEYASTVRRAVQSGHVNLKDRLTIELHPDGRHGIVRVDR
VPLASKLVDLPCVMESLKTIDKKTFYKTADICQMLVSTVDGDLYPPVEEPVASTDPKASK
KKDKDKEKKFIWNHGITLPLKNVRKRRFRKTAKKKYIESPDVEKEVKRLLSTDAEAVSTR
WEIIAEDETKEAENQGLDISSPGMSGHRQGHDSLEHDELREIFNDLSSSSEDEDETQHQD
EEDINIIDTEEDLERQLQDKLNESDEQHQENEGTNQLVMGIQKQIDNMKGKLQETQDRAK
RQEDLIMKVENLALKNRFQAVLDELKQKEDREKEQLSSLQEELESLLEK*
>ENSG00000172336|ENST00000303151
MAENREPRGAVEAELDPVEYTLRKRLPSRLPRRPNDIYVNMKTDFKAQLARCQKLLDGGA
RGQNACSEIYIHGLGLAINRAINIALQLQAGSFGSLQVAANTSTVELVDELEPETDTREP
LTRIRNNSAIHIRVFRVTPK*
>ENSG00000168916|ENST00000306315
MSVNISTAGKGVDPNTVDTYDSGDDWEIGVGNLIIDLDADLEKDRQKFEMNNSTTTTSSS
NSKDCGGPASSGAGATAALADGLKFASVQASAPQGNSHKETSKSKVKRSKTSKDANKSLP
SAALYGIPEISSTGKRQEVQGRPGEATGMNSALGQSVSSGGSGNPNSNSTSTSTSAATAG
AGSCGKSKEEKPGKSQSSRGAKRDKDAGKSRKDKHDLLQGHQNGSGSQAPSGGHLYGFGA
KSNGGGASPFHCGGTGSGSVAAAGEVSKSAPDSGLMGNSMLVKKEEEEEESHRRIKKLKT
EKVDPLFTVPAPPPPISSSLTPQILPSYFSPSSSNIAAPVEQLLVRTRSVGVNTCEVGVV
TEPECLGPCEPGTSVNLEGIVWHETEEGVLVVNVTWRNKTYVGTLLDCTKHDWAPPRFCE
SPTSDLEMRGGRGRGKRARSAAAAPGSEASFTESRGLQNKNRGGANGKGRRGSLNASGRR
TPPNCAAEDIKASPSSTNKRKNKPPMELDLNSSSEDNKPGKRVRTNSRSTPTTPQGKPET
TFLDQGCSSPVLIDCPHPNCNKKYKHINGLRYHQAHAHLDPENKLEFEPDSEDKISDCEE
GLSNVALECSEPSTSVSAYDQLKAPASPGAGNPPGTPKGKRELMSNGPGSIIGAKAGKNS
GKKKGLNNELNNLPVISNMTAALDSCSAADGSLAAEMPKLEAEGLIDKKNLGDKEKGKKA
TNCKTDKNLSKLKSARPIAPAPAPTPPQLIAIPTATFTTTTTGTIPGLPSLTTTVVQATP
KSPPLKPIQPKPTIMGEPITVNPALVSLKDKKKKEKRKLKDKEGKETGSPKMDAKLGKLE
DSKGASKDLPGHFLKDHLNKNEGLANGLSESQESRMASIKAEADKVYTFTDNAPSPSIGS
ASRLECSTLVNGQAPMAPLHVLTQNGAESSAAKTSSPAYSDISDAADDGGSDSRSEGMRS
KASSPSDIISSKDSVVKGHSSTTAQSSQLKESHSPYYHSYDPYYSPSYMHPGQVGAPAAG
NSGSTQGMKIKKESEEDAEKKDKAEQLDSKKVDHNSASLQPQHQSVITQRHPALAQSLYY
GQYAYGLYMDQKSLMATSPAYRQQYEKYYEDQRLAEQKMAQTGRGDCERKSELPLKELGK

EETKQKNMPSATISKAPSTPEPNKNHSKLGPSVPNKTEETGKSQLLSNHQQQLQADSFKA
KQMENHQLIKEAVEMKSVMDSMKQTGVDPTSRFKQDPDSRTWHHYVYQPKYLDQQKSEEL
DREKKLKEDSPRKTPNKESGVPSLPVSLTSIKEEPKEAKHPDSQSMEESKLKNDDRKTPV
NWKDSRGTRVAVSSPMSQHQSYIQYLHAYPYPQMYDPSHPAYRAVSPVLMHSYPGAYLSP
GFHYPVYGKMSGREETEKVNTSPSVNTKTTTESKALDLLQQHANQYRSKSPAPVEKATAE
REREAERERDRHSPFGQRHLHTHHHTHVGMGYPLIPGQYDPFQGLTSAALVASQQVAAQA
SASGMFPGQRRE*
>ENSG00000170561|ENST00000302057
MSYPQGYLYQAPGSLALYSCPAYGASALAAPRSEELARSASGSAFSPYPGSAAFTAQAAT
GFGSPLQYSADAAAAAAGFPSYMGAPYDAHTTGMTGAISYHPYGSAAYPYQLNDPAYRKN
ATRDATATLKAWLNEHRKNPYPTKGEKIMLAIITKMTLTQVSTWFANARRRLKKENKMTW
APRNKSEDEDEDEGDATRSKDESPDKAQEGTETSAEDEGISLHVDSLTDHSCSAESDGEK
LPCRAGDPLCESGSECKDKYDDLEDDEDDDEEGERGLAPPKPVTSSPLTGLEAPLLSPPP
EAAPRGGRKTPQGSRTSPGAPPPASKPKLWSLAEIATSDLKQPSLGPGCGPPGLPAAAAP
ASTGAPPGGSPYPASPLLGRPLYYTSPFYGNYTNYGNLNAALQGQGLLRYNSAAAAPGEA
LHTAPKAASDAGKAGAHPLESHYRSPGGGYEPKKDASEGCTVVGGGVQPYL*
>ENSG00000165526|ENST00000298317
MAAPRWSASGPWIRGNGQGCGSLFTLVSKPFCAAAAASTAINAQRLAEKLRAQKREQDTK
KEPVSTNAVQRRVQEIVRFTRQLQRVHPNVLAKALTRGILHQDKNLVVINKPYGLPVHGG
PGVQLCITDVLPILAKMLHGHKAEPLHLCHRLDKETTGVMVLAWDKDMAHQVQELFRTRQ
VVKKYWAITVHVPMPSAGVVDIPIVEKEAQGQQQHHKMTLSPSYRMDDGKMVKVRRSRNA
QVAVTQYQVLSSTLSSALVELQPITGIKHQLRVHLSFGLDCPILGDHKYSDWNRLAPQKL
SVGTLKKLGLEQSKARYIPLHLHARQLILPALGSGKEELNLVCKLPRFFVHSLHRLRLEM
PNEDQNENNEAKCLGAQ*
>ENSG00000145912|ENST00000314397
MTKIKADPDGPEAQAEACSGERTYQELLVNQNPIAQPLASRRLTRKLYKCIKKAVKQKQI
RRGVKEVQKFVNKGEKGTWVQPQAPSAPPV*
>ENSG00000172789|ENST00000312492
MSSYVANSFYKQSPNIPAYNMQTCGNYGSASEVQASRYCYGGLDLSITFPPPAPSNSLHG
VDMAANPRAHPDRPACSAAAAPGHAPGRDEAAPLNPGMYSQKAARPALEERAKSSGEIKE
EQAQTGQPAGLSQPPAPPQIYPWMTKLHMSHETDGKRSRTSYTRYQTLELEKEFHFNRYL
TRRRRIEIANNLCLNERQIKIWFQNRRMKWKKDSKMKSKEAL*
>ENSG00000096746|ENST00000265866
MDWVMKHNGPNDASDGTVRLRGLPFGCSKEEIVQFFQGLEIVPNGITLTMDYQGRSTGEA
FVQFASKEIAENALGKHKERIGHRYIEIFRSSRSEIKGFYDPPRRLLGQRPGPYDRPIGG
RGGYYGAGRGSMYDRMRRGGDGYDGGYGGFDDYGGYNNYGYGNDGFDDRMRDGRGMGGHG
YGGAGDASSGFHGGHFVHMRGLPFRATENDIANFFSPLNPIRVHIDIGADGRATGEADVE
FVTHEDAVAAMSKDKNNMQHRYIELFLNSTPGGGSGMGGSGMGGYGRDGMDNQGGYGSVG
RMGMGNNYSGGYGTPDGLGGYGRGGGGSGGYYGQGGMSGGGWRGMY*
>ENSG00000101210|ENST00000298049
MGKEKTHINIVVIGHVDSGKSTTTGHLIYKCGGIDKRTIEKFEKEAAEMGKGSFKYAWVL
DKLKAERERGITIDISLWKFETTKYYITIIDAPGHRDFIKNMITGTSQADCAVLIVAAGV
GEFEAGISKNGQTREHALLAYTLGVKQLIVGVNKMDSTEPAYSEKRYDEIVKEVSAYIKK
IGYNPATVPFVPISGWHGDNMLEPSPNMPWFKGWKVERKEGNASGVSLLEALDTILPPTR

PTDKPLRLPLQDVYKIGGIGTVPVGRVETGILRPGMVVTFAPVNITTEVKSVEMHHEALS
EALPGDNVGFNVKNVSVKDIRRGNVCGDSKSDPPQEAAQFTSQVIILNHPGQISAGYSPV
IDCHTAHIACKFAELKEKIDRRSGKKLEDNPKSLKSGDAAIVEMVPGKPMCVESFSQYPP
LGRFAVRDMRQTVAVGVIKNVEKKSGGAGKALGQRLDARHQGPVEVLQEERRPRPRLPRP
ALATLSARFTNKLSDP
>ENSG00000163918|ENST00000296273
MQAFLKGTSISTKPPLTKDRGVAASAGSSGENKKAKPVPWVEKYRPKCVDEVAFQEEVVA
VLKKSLEGADLPNLLFYGPPGTGKTSTILAAARELFGPELFRLRVLELNASDERGIQVVR
EKVKNFAQLTVSGSRSDGKPCPPFKIVILDEADSMTSAAQAALRRTMEKESKTTRFCLIC
NYVSRIIEPLTSRCSKFRFKPLSDKIQQQRLLDIAKKENVKISDEGIAYLVKVSEGDLRK
AITFLQSATRLTGGKEITEKVITDIAGVIPAEKIDGVFAACQSGSFDKLEAVVKDLIDEG
HAATQLVNQLHDVVVENNLSDKQKSIITEKLAEVDKCLADGADEHLQLISLCATVMQQLS
QNC*
>ENSG00000140525|ENST00000310775
MDQKILSLAAEKTADKLQEFLQTLREGDLTNLLQNQAVKGKVAGALLRAIFKGSPCSEEA
GTLRRRKIYTCCIQLVESGDLQKEIASEIIGLLMLEAHHFPGPLLVELANEFISAVREGS
LVNGKSLELLPIILTALATKKENLAYGKGVLSGEECKKQLINTLCSGRWDQQYVIQLTSM
FKDVPLTAEEVEFVVEKALSMFSKMNLQEIPPLVYQLLVLSSKGSRKSVLEGIIAFFSAL
DKQHNEEQSGDELLDVVTVPSGELRHVEGTIILHIVFAIKLDYELGRELVKHLKVGQQGD
SNNNLSPFSIALLLSVTRIQRFQDQVLDLLKTSVVKSFKDLQLLQGSKFLQNLVPHRSYV
STMILEVVKNSVHSWDHVTQGLVELGFILMDSYGPKKVLDGKTIETSPSLSRMPNQHACK
LGANILLETFKIHEMIRQEILEQVLNRVVTRASSPISHFLDLLSNIVMYAPLVLQSCSSK
VTEAFDYLSFLPLQTVQRLLKAVQPLLKVSMSMRDCLILVLRKAMFANQLDARKSAVAGF
LLLLKNFKVLGSLSSSQCSQSLSVSQVHVDVHSHYNSVANETFCLEIMDSLRRCLSQQAD
VRLMLYEGFYDVLRRNSQLANSVMQTLLSQLKQFYEPKPDLLPPLKLEACILTQGDKISL
QEPLDYLLCCIQHCLAWYKNTVIPLQQGEEEEEEEEAFYEDLDDILESITNRMIKSELED
FELDKSADFSQSTSIGIKNNICAFLVMGVCEVLIEYNFSISSFSKNRFEDILSLFMCYKK
LSDILNEKAGKAKTKMANKTSDSLLSMKFVSSLLTALFRDSIQSHQESLSVLRSSNEFMR
YAVNVALQKVQQLKETGHVSGPDGQNPEKIFQNLCDITRVLLWRYTSIPTSVEESGKKEK
GKSISLLCLEGLQKIFSAVQQFYQPKIQQFLRALDVTDKEGEEREDADVSVTQRTAFQIR
QFQRSLLNLLSSQEEDFNSKEALLLVTVLTSLSKLLEPSSPQFVQMLSWTSKICKENSRE
DALFCKSLMNLLFSLHVSYKSPVILLRDLSQDIHGHLGDIDQDVEVEKTNHFAIVNLRTA
APTVCLLVLSQAEKVLEEVDWLITKLKGQVSQETLSEEASSQATLPNQPVEKAIIMQLGT
LLTFFHELVQTALPSGSCVDTLLKDLCKMYTTLTALVRYYLQVCQSSGGIPKNMEKLVKL
SGSHLTPLCYSFISYVQNKSKSLNYTGEKKEKPAAVATAMARVLRETKPIPNLIFAIEQY
EKFLIHLSKKSKVNLMQHMKLSTSRDFKIKGNILDMVLREDGEDENEEGTASEHGGQNKE
PAKKKRKK*
>ENSG00000159217|ENST00000290341
MNKLYIGNLNESVTPADLEKVFAEHKISYSGQFLVKSGYAFVDCPDEHWAMKAIETFSGK
VELQGKRLEIEHSVPKKQRSRKIQIRNIPPQLRWEVLDSLLAQYGTVENCEQVNTESETA
VVNVTYSNREQTRQAIMKLNGHQLENHALKVSYIPDEQIAQGPENGRRGGFGSRGQPRQG
SPVAAGAPAKQQQVDIPLRLLVPTQYVGAIIGKEGATIRNITKQTQSKIDVHRKENAGAA
EKAISVHSTPEGCSSACKMILEIMHKEAKDTKTADEVPLKILAHNNFVGRLIGKEGRNLK
KVEQDTETKITISSLQDLTLYNPERTITVKGAIENCCRAEQEIMKKVREAYENDVAAMSL

QSHLIPGLNLAAVGLFPASSSAVPPPPSSVTGAAPYSSFMQAPEQEMVQVFIPAQAVGAI
IGKKGQHIKQLSRFASASIKIAPPETPDSKVRMVIITGPPEAQFKAQGRIYGKLKEENFF
GPKEEVKLETHIRVPASAAGRVIGKGGKTVNELQNLTAAEVVVPRDQTPDENDQVIVKII
GHFYASQMAQRKIRDILAQVKQQHQKGQSNQAQARRK*
>ENSG00000005075|ENST00000292614
MNAPPAFESFLLFEGEKKITINKDTKVPNACLFTINKEDHTLGNIIKSQLLKDPQVLFAG
YKVPHPLEHKIIIRVQTTPDYSPQEAFTNAITDLISELSLLEERFRVAIKDKQEGIE*
>ENSG00000168769|ENST00000305737
MEQDRTNHVEGNRLSPFLIPSPPICQTEPLATKLQNGSPLPERAHPEVNGDTKWHSFKSY
YGIPCMKGSQNSRVSPDFTQESRGYSKCLQNGGIKRTVSEPSLSGLLQIKKLKQDQKANG
ERRNFGVSQERNPGESSQPNVSDLSDKKESVSSVAQENAVKDFTSFSTHNCSGPENPELQ
ILNEQEGKSANYHDKNIVLLKNKAVLMPNGATVSASSVEHTHGELLEKTLSQYYPDCVSI
AVQKTTSHINAINSQATNELSCEITHPSHTSGQINSAQTSNSELPPKPAAVVSEACDADD
ADNASKLAAMLNTCSFQKPEQLQQQKSVFEICPSPAENNIQGTTKLASGEEFCSGSSSNL
QAPGGSSERYLKQNEMNGAYFKQSSVFTKDSFSATTTPPPPSQLLLSPPPPLPQVPQLPS
EGKSTLNGGVLEEHHHYPNQSNTTLLREVKIEGKPEAPPSQSPNPSTHVCSPSPMLSERP
QNNCVNRNDIQTAGTMTVPLCSEKTRPMSEHLKHNPPIFGSSGELQDNCQQLMRNKEQEI
LKGRDKEQTRDLVPPTQHYLKPGWIELKAPRFHQAESHLKRNEASLPSILQYQPNLSNQM
TSKQYTGNSNMPGGLPRQAYTQKTTQLEHKSQMYQVEMNQGQSQGTVDQHLQFQKPSHQV
HFSKTDHLPKAHVQSLCGTRFHFQQRADSQTEKLMSPVLKQHLNQQASETEPFSNSHLLQ
HKPHKQAAQTQPSQSSHLPQNQQQQQKLQIKNKEEILQTFPHPQSNNDQQREGSFFGQTK
VEECFHGENQYSKSSEFETHNVQMGLEEVQNINRRNSPYSQTMKSSACKIQVSCSNNTHL
VSENKEQTTHPELFAGNKTQNLHHMQYFPNNVIPKQDLLHRCFQEQEQKSQQASVLQGYK
NRNQDMSGQQAAQLAQQRYLIHNHANVFPVPDQGGSHTQTPPQKDTQKHAALRWHLLQKQ
EQQQTQQPQTESCHSQMHRPIKVEPGCKPHACMHTAPPENKTWKKVTKQENPPASCDNVQ
QKSIIETMEQHLKQFHAKSLFDHKALTLKSQKQVKVEMSGPVTVLTRQTTAAELDSHTPA
LEQQTTSSEKTPTKRTAASVLNNFIESPSKLLDTPIKNLLDTPVKTQYDFPSCRCVGKCQ
KCTETHGVYPELANLSSDMGFSFFF*
>ENSG00000168468|ENST00000293709
MAELMLLSEIADPTRFFTDNLLSPEDWGLQNSTLYSGLDEVAEEQTQLFRCPEQDVPFDG
SSLDVGMDVSPSEPPWELLPIFPDLQVKSEPSSPCSSSSLSSESSRLSTEPSSEALGVGE
VLHVKTESLAPPLCLLGDDPTSSFETVQINVIPTSDDSSDVQTKIEPVSPCSSVNSEASL
LSADSSSQAFIGEEVLEVKTESLSPSGCLLWDVPAPSLGAVQISMGPSLDGSSGKALPTR
KPPLQPKPVVLTTVPMPSRAVPPSTTVLLQSLVQPPPVSPVVLIQGAIRVQPEGPAPSLP
RPERKSIVPAPMPGNSCPPEVDAKLLKRQQRMIKNRESACQSRRKKKEYLQGLEARLQAV
LADNQQLRRENAALRRRLEALLAENSELKLGSGNRKVVCIMVFLLFIAFNFGPVSISEPP
SAPISPRMNKGEPQPRRHLLGFSEQEPVQGVEPLQGSSQGPKEPQPSPTDQPSFSNLTAF
PGGAKELLLRDLDQLFLSSDCRHFNRTESLRLADELSGWVQRHQRGRRKIPQRAQERQKS
QPRKKSPPVKAVPIQPPGPPERDSVGQLQLYRHPDRSQPAFLDAIDRREDTFYVVSFRRD
HLLLPAISHNKTSRPKMSLVMPAMAPNETLSGRGAPGDYEEMMQIECEVMDTRVIHIKTS
TVPPSLRKQPSPTPGNATGGPLPVSAASQAHQASHQPLYLNHP*
>ENSG00000160213|ENST00000291568
MMCGAPSATQPATAETQHIADQVRSQLEEKENKKFPVFKAVSFKSQVVAGTNYFIKVHVG
DEDFVHLRVFQSLPHENKPLTLSNYQTNKAKHDELTYF*

>ENSG00000164105|ENST00000296504
MNGFTPDEMSRGGDAAAAVAAVVAAAAAAASAGNGTGAGTGAEVPGAGAVSAAGPPGAAG
PGPGQLCCLREDGERCGRAAGNASFSKRIQKSISQKKVKIELDKSARHLYICDYHKNLIQ
SVRNRRKRKGSDDDGGDSPVQDIDTPEVDLYQLQVNTLRRYKRHFKLPTRPGLNKAQLVE
IVGCHFRSIPVNEKDTLTYFIYSVKNDKNKSDLKVDSGVH*
>ENSG00000175325|ENST00000308304
MEAERRRQAEKPKKGRVGSNLLPERHPATGTPTTTVDSSAPPCRRLPGAGGGRSRFSPQG
GQRGRPHSRRRHRTTFSPVQLEQLESAFGRNQYPDIWARESLARDTGLSEARIQVWFQNR
RAKQRKQERSLLQPLAHLSPAAFSSFLPESTACPYSYAAPPPPVTCFPHPYSHALPSQPS
TGGAFALSHQSEDWYPTLHPAPAGHLPCPPPPPMLPLSLEPSKSWN*
>ENSG00000142528|ENST00000270617
MAEEFVTLKDVGMDFTLGDWEQLGLEQGDTFWDTALDNCQDLFLLDPPRPNLTSHPDGSE
DLEPLAGGSPEATSPDVTETKNSPLMEDFFEEGFSQEIIEMLSKDGFWNSNFGEACIEDT
WLDSLLGDPESLLRSDIATNGESPTECKSHELKRGLSPVSTVSTGEDSMVHNVSEKTLTP
AKSKEYRGEFFSYSDHSQQDSVQEGEKPYQCSECGKSFSGSYRLTQHWITHTREKPTVHQ
ECEQGFDRNASLSVYPKTHTGYKFYVCNEYGTTFSQSTYLWHQKTHTGEKPCKSQDSDHP
PSHDTQPGEHQKTHTDSKSYNCNECGKAFTRIFHLTRHQKIHTRKRYECSKCQATFNLRK
HLIQHQKTHAAKTTSECQECGKIFRHSSLLIEHQALHAGEEPYKCNERGKSFRHNSTLKI
HQRVHSGEKPYKCSECGKAFHRHTHLNEHRRIHTGYRPHKCQECVRSFSRPSHLMRHQAI
HTAEKPYSCAECKETFSDNNRLVQHQKMHTVKTPYECQECGERFICGSTLKCHESVHARE
KQGFFVSGKILDQNPEQKEKCFKCNKCEKTFSCSKYLTQHERIHTRGVKPFECDQCGKAF
GQSTRLIHHQRIHSRVRLYKWGEQGKAISSASLIKLQSFHTKEHPFKCNECGKTFSHSAH
LSKHQLIHAGENPFKCSKCDRVFTQRNYLVQHERTHARKKPLVCNECGKTFRQSSCLSKH
QRIHSGEKPYVCDYCGKAFGLSAELVRHQRIHTGEKPYVCQECGKAFTQSSCLSIHRRVH
TGEKPYRCGECGKAFAQKANLTQHQRIHTGEKPYSCNVCGKAFVLSAHLNQHLRVHTQET
LYQCQRCQKAFRCHSSLSRHQRVHNKQQYCL*
>ENSG00000162076|ENST00000293981
MPLPEPSEQEGESVKASQEPSPKPGTEVIPAAPRKPRKFSKLVLLTASKDSTKVAGAKRK
GVHCVMSLGVPGPATLAKALLQTHPEAQRAIEAAPQEPEQKRSRQDPGTDRTEDSGLAAG
PPEAAGENFAPCSVAPGKSL*
>ENSG00000198353|ENST00000303406
MIMSSYLMDSNYIDPKFPPCEEYSQNSYIPEHSPEYYGRTRESGFQHHHQELYPPPPPRP
SYPERQYSCTSLQGPGNSRGHGPAQAGHHHPEKSQSLCEPAPLSGASASPSPAPPACSQP
APDHPSSAASKQPIVYPWMKKIHVSTVNPNYNGGEPKRSRTAYTRQQVLELEKEFHYNRY
LTRRRRIEIAHSLCLSERQIKIWFQNRRMKWKKDHRLPNTKVRSAPPAGAAPSTLSAATP
GTSEDHSQSATPPEQQRAEDITRL*
>ENSG00000159263|ENST00000290399
MKEKSKNAAKTRREKENGEFYELAKLLPLPSAITSQLDKASIIRLTTSYLKMRAVFPEGL
GDAWGQPSRAGPLDGVAKELGSHLLQTLDGFVFVVASDGKIMYISETASVHLGLSQVELT
GNSIYEYIHPSDHDEMTAVLTAHQPLHHHLLQEYEIERSFFLRMKCVLAKRNAGLTCSGY
KVIHCSGYLKIRQYMLDMSLYDSCYQIVGLVAVGQSLPPSAITEIKLYSNMFMFRASLDL
KLIFLDSRVTEVTGYEPQDLIEKTLYHHVHGCDVFHLRYAHHLLLVKGQVTTKYYRLLSK
RGGWVWVQSYATVVHNSRSSRPHCIVSVNYVLTEIEYKELQLSLEQVSTAKSQDSWRTAL
STSQETRKLVKPKNTKMKTKLRTNPYPPQQYSSFQMDKLECGQLGNWRASPPASAAAPPE

LQPHSESSDLLYTPSYSLPFSYHYGHFPLDSHVFSSKKPMLPAKFGQPQGSPCEVARFFL
STLPASGECQWHYANPLVPSSSSPAKNPPEPPANTARHSLVPSYEAPAAAVRRFGEDTAP
PSFPSCGHYREEPALGPAKAARQAARDGARLALARAAPECCAPPTPEAPGAPAQLPFVLL
NYHRVLARRGPLGGAAPAASGLACAPGGPEAATGALRLRHPSPAATSPPGAPLPHYLGAS
VIITNGR*
>ENSG00000005302|ENST00000312196
MSASEGMKFKFHSGEKVLCFEPDPTKARVLYDAKIVDVIVGKDEKGRKIPEYLIHFNGWN
RSWDRWAAEDHVLRDTDENRRLQRKLARKAVARLRSTGRKKKRCRLPGVDSVLKGLPTEE
KDENDENSLSSSSDCSENKDEEISEESDIEEKTEVKEEPELQTRREMEERTITIEIPEVL
KKQLEDDCYYINRRKRLVKLPCQTNIITILESYVKHFAINAAFSANERPRHHHVMPHANM
NVHYIPAEKNVDLCKEMVDGLRITFDYTLPLVLLYPYEQAQYKKVTSSKFFLPIKESATS
TNRSQEELSPSPPLLNPSTPQSTESQPTTGEPATPKRRKAEPEALQSLRRSTRHSANCDR
LSESSASPQPKRRQQDTSASMPKLFLHLEKKTPVHSRSSSPIPLTPSKEGSAVFAGFEGR
RTNEINEVLSWKLVPDNYPPGDQPPPPSYIYGAQHLLRLFVKLPEILGKMSFSEKNLKAL
LKHFDLFLRFLAEYHDDFFPESAYVAACEAHYSTKNPRAIY*
>ENSG00000153879|ENST00000284000
MSKISQQNSTPGVNGISVIHTQAHASGLQQVPQLVPAGPGGGGKAVAPSKQSKKSSPMDR
NSDEYRQRRERNNMAVKKSRLKSKQKAQDTLQRVNQLKEENERLEAKIKLLTKELSVLKD
LFLEHAHNLADNVQSISTENTTADGDNAGQ*
>ENSG00000075914|ENST00000265564
MASVTLSEAEKVYIVHGVQEDLRVDGRGCEDYRCVEVETDVVSNTSGSARVKLGHTDILV
GVKAEMGTPKLEKPNEGYLEFFVDCSASATPEFEGRGGDDLGTEIANTLYRIFNNKSSVD
LKTLCISPREHCWVLYVDVLLLECGGNLFDAISIAVKAALFNTRIPRVRVLEDEEGSKDI
ELSDDPYDCIRLSVENVPCIVTLCKIGYRHVVDATLQEEACSLASLLVSVTSKGVVTCMR
KVGKGSLDPESIFEMMETGKRVGKVLHASLQSVVHKEESLGPKRQKVGFLG*
>ENSG00000166860|ENST00000300101
MGMRIKLQSTNHPNNLLKELNKCRLSETMCDVTIVVGSRSFPAHKAVLACAAGYFQNLFL
NTGLDAARTYVVDFITPANFEKVLSFVYTSELFTDLINVGVIYEVAERLGMEDLLQACHS
TFPDLESTARAKPLTSTSESHSGTLSCPSAEPAHPLGELRGGGDYLGADRNYVLPSDAGG
SYKEEEKNVASDANHSLHLPQPPPPPPKTEDHDTPAPFTSIPSMMTQPLLGTVSTGIQTS
TSSCQPYKVQSNGDFSKNSFLTPDNAVDITTGTNSCLSNSEHSKDPGFGQMDELQLEDLG
DDDLQFEDPAEDIGTTEEVIELSDDSEDELAFGENDNRENKAMPCQVCKKVLEPNIQLIR
QHARDHVDLLTGNCKVCETHFQDRNSRVTHVLSHIGIFLFSCDMCETKFFTQWQLTLHRR
DGIFENNIIVHPNDPLPGKLGLFSGAASPELKCAACGKVLAKDFHVVRGHILDHLNLKGQ
ACSVCDQRHLNLCSLMWHTLSHLGISVFSCSVCANSFVDWHLLEKHMAVHQSLEDALFHC
RLCSQSFKSEAAYRYHVSQHKCNSGLDARPGFGLQHPALQKRKLPAEEFLGEELALQGQP
GNSKYSCKVCGKRFAHTSEFNYHRRIHTGEKPYQCKVCHKFFRGRSTIKCHLKTHSGALM
YRCTVCGHYSSTLNLMSKHVGVHKGSLPPDFTIEQTFMYIIHSKEADKNPDS*
>ENSG00000153266|ENST00000283268
MASSASLETMVPPACPRAGASPATSKTLAFSIERIMAKTSEPRAPFEPRPGALEADGSQG
KKLLNLCSPLPCMIPLQPLGYEVPSKTLLSYSELWKSSLRAGGGGGGGGGGGGGGGAPV
CGASGLCKTNCGVCCKAELGLAPSALPAGRVIKPQVINQAVGLPASGSLYYFNYLDSTAY
PPSELLSGHLFPSGLLNAQAPAALAAHPKLFLLENAKLAGLAADKFPHPAPYPHKERLPA
PLEQVLKENSALTAERGGVKGHSKLPGGSADGKPKNFTCEVCGKVFNAHYNLTRHMPVHT

GARPFVCKVCGKGFRQASTLCRHKIIHTQEKPHKCNQCGKAFNRSSTLNTHIRIHAGYKP
FVCEFCGKGFHQKGNYKNHKLTHSGEKQYKCTICNKAFHQVYNLTFHMHTHNDKKPFTCA
TCGKGFCRNFDLKKHVRKLHDSVGPAAPSAKDLTRTVQS*
>ENSG00000125952|ENST00000284165
MSDNDDIEVESDEEQPRFQSAADKRAHHNALERKRRDHIKDSFHSLRDSVPSLQGEKASR
AQILDKATEYIQYMRRKNHTHQQDIDDLKRQNALLEQQGEHPSSWGSWPCCAPARSGFGT
WACRVRASHGVCAQ*
>ENSG00000169710|ENST00000306749
MEEVVIAGMSGKLPESENLQEFWDNLIGGVDMVTDDDRRWKAGLYGLPRRSGKLKDLSRF
DASFFGVHPKQAHTMDPQLRLLLEVTYEAIVDGGINPDSLRGTHTGVWVGVSGSETSEAL
SRDPETLVGYSMVGCQRAMMANRLSFFFDFRGPSIALDTACSSSLMALQNAYQAIHSGQC
PAAIVGGINVLLKPNTSVQFLRLGMLSPEGTCKAFDTAGNGYCRSEGVVAVLLTKKSLAR
RVYATILNAGTNTDGFKEQGVTFPSGDIQEQLIRSLYQSAGVAPESFEYIEAHGTGTKVG
DPQELNGITRALCATRQEPLLIGSTKSNMGHPEPASGLAALAKVLLSLEHGLWAPNLHFH
SPNPEIPALLDGRLQVVDQPLPVRGGNVGINSFGFGGSNVHIILRPNTQPPPAPAPHATL
PRLLRASGRTPEAVQKLLEQGLRHSQDLAFLSMLNDIAAVPATAMPFRGYAVLGGERGGP
EVQQVPAGERPLWFICSGMGTQWRGMGLSLMRLDRFRDSILRSDEAVKPFGLKVSQLLLS
TDESTFDDIVHSFVSLTAIQIGLIDLLSCMGLRPDGIVGHSLGEVACGYADGCLSQEEAV
LAAYWRGQCIKEAHLPPGAMAAVGLSWEECKQRCPPGVVPACHNSKDTVTISGPQAPVFE
FVEQLRKEGVFAKEVRTGGMAFHSYFMEAIAPPLLQELKKVIREPKPRSARWLSTSIPEA
QWHSSLARTSSAEYNVNNLVSPVLFQEALWHVPEHAVVLEIAPHALLQAVLKRGLKPSCT
IIPLMKKDHRDNLEFFLAGIGRLHLSGIDANPNALFPPVEFPAPRGTPLISPLIKWDHSL
AWDVPAAEDFPNGSGSPSAAIYNIDTSSESPDHYLVDHTLDGRVLFPATGYLSIVWKTLA
RALGLGVEQLPVVFEDVVLHQATILPKTGTVSLEVRLLEASRAFEVSENGNLVVSGKVYQ
WDDPDPRLFDHPESPTPNPTEPLFLAQAEVYKELRLRGYDYGPHFQGILEASLEGDSGRL
LWKDNWVSFMDTMLQMSILGSAKHGLYLPTRVTAIHIDPATHRQKLYTLQDKAQVADVVV
SRWLRVTVAGGVHISGLHTESAPRRQQEQQVPILEKFCFTPHTEEGCLSERAALQEELQL
CKGLVQALQTKVTQQGLKMVVPGLDGAQIPRDPSQQELPRLLSAACRLQLNGNLQLELAQ
VLAQERPKLPEDPLLSGLLDSPALKACLDTAVENMPSLKMKVVEVLAGHGHLYSRIPGLL
SPHPLLQLSYTATDRHPQALEAAQAELQQHDVAQGQWDPADPAPSALGSADLLVCNCAVA
ALGDPASALSNMVAALREGGFLLLHTLLRGHPLGDIVAFLTSTEPQYGQGILSQDAWESL
FSRVSLRLVGLKKSFYGSTLFLCRRPTPQDSPIFLPVDDTSFRWVESLKGILADEDSSRP
VWLKAINCATSGVVGLVNCLRREPGGNRLRCVLLSNLSSTSHVPEVDPGSAELQKVLQGD
LVMNVYRDGAWGAFRHFLLEEDKPEEPTAHAFVSTLTRGDLSSIRWVCSSLRHAQPTCPG
AQLCTVYYASLNFRDIMLATGKLSPDAIPGKWTSQDSLLGMEFSGRDASGKRVMGLVPAK
GLATSVLLSPDFLWDVPSNWTLEEAASVPVVYSTAYYALVVRGRVRPGETLLIHSGSGGV
GQAAIAIALSLGCRVFTTVGSAEKRAYLQARFPQLDSTSFANSRDTSFEQHVLWHTGGKG
VDLVLNSLAEEKLQASVRCLATHGRFLEIGKFDLSQNHPLGMAIFLKNVTFHGVLLDAFF
NESSADWREVWALVQAGIRDGVVRPLKCTVFHGAQVEDAFRYMAQGKHIGKVVVQVLAEE
PEAVLKGAKPKLMSAISKTFCPAHKSYIIAGGLGGFGLELAQWLIQRGVQKLVLTSRSGI
RTGYQAKQVRRWRRQGVQVQVSTSNISSLEGARGLIAEAAQLGPVGGVFNLAVVLRDGLL
ENQTPEFFQDVCKPKYSGTLNLDRVTREACPELDYFVVFSSVSCGRGNAGQSNYGFANSA
MERICEKRRHEGLPGLAVQWGAIGDVGILVETMSTNDTIVSGTLPQRMASCLEVLDLFLN
QPHMVLSSFVLAEKAAAYRDRDSQRDLVEAVAHILGIRDLAAVNLDSSLADLGLDSLMSV

EVRQTLERELNLVLSVREVRQLTLRKLQELSSKADEASELACPTPKEDGLAQQQTQLNLR
SLLVNPEGPTLMRLNSVQSSERPLFLVHPIEGSTTVFHSLASRLSIPTYGLQCTRAAPLD
SIHSLAAYYIDCIRQVQPEGPYRVAGYSYGACVAFEMCSQLQAQQSPAPTHNSLFLFDGS
PTYVLAYTQSYRAKLTPGCEAEAETEAICFFVQQFTDMEHNRVLEALLPLKGLEERVAAA
VDLIIKSHQGLDRQELSFAARSFYYKLRAAEQYTPKAKYHGNVMLLRAKTGGAYGEDLGA
DYNLSQVCDGKVSVHVIEGDHRTLLEGSGLESIISIIHSSLAEPRVSVREG*
>ENSG00000156110|ENST00000286621
MAAAEEEPKPKKLKVEAPQALRENILFGMGNPLLDISAVVDKDFLDKYSLKPNDQILAED
KHKELFDELVKKFKVEYHAGGSTQNSIKVAQWMIQQPHKAATFFGCIGIDKFGEILKRKA
AEAHVDAHYYEQNEQPTGTCAACITGDNRSLIANLAAANCYKKEKHLDLEKNWMLVEKAR
VCYIAGFFLTVSPESVLKVAHHASENNRIFTLNLSAPFISQFYKESLMKVMPYVDILFGN
ETEAATFAREQGFETKDIKEIAKKTQALPKMNSKRQRIVIFTQGRDDTIMATESEVTAFA
VLDQDQKEIIDTNGAGDAFVGGFLSQLVSDKPLTECIRAGHYAASIIIRRTGCTFPEKPD
FH*
>ENSG00000171223|ENST00000302754
MCTKMEQPFYHDDSYTATGYGRAPGGLSLHDYKLLKPSLAVNLADPYRSLKAPGARGPGP
EGGGGGSYFSGQGSDTGASLKLASSELERLIVPNSNGVITTTPTPPGQYFYPRGGGSGGG
AGGAGGGVTEEQEGFADGFVKALDDLHKMNHVTPPNVSLGATGGPPAGPGGVYAGPEPPP
VYTNLSSYSPASASSGGAGAAVGTGSSYPTTTISYLPHAPPFAGGHPAQLGLGRGASTFK
EEPQTVPEARSRDATPPVSPINMEDQERIKVERKRLRNRLAATKCRKRKLERIARLEDKV
KTLKAENAGLSSTAGLLREQVAQLKQKVMTHVSNGCQLLLGVKGHAF*
>ENSG00000106028|ENST00000265304
MFRRPVLQVLRQFVRHESETTTSLVLERSLNRVHLLGRVGQDPVLRQVEGKNPVTIFSLA
TNEMWRSGDSEVYQLGDVSQKTTWHRISVFRPGLRDVAYQYVKKGSRIYLEGKIDYGEYM
DKNNVRRQATTIIADNIIFLSDQTKEKE*
>ENSG00000162437|ENST00000294428
MAAAAGDGGGEGGAGLGSAAGLGPGPGLRGQGPSAEAHEGAPDPMPAALHPEEVAARLQR
MQRELSNRRKILVKNLPQDSNCQEVHDLLKDYDLKYCYVDRNKRTAFVTLLNGEQAQNAI
QMFHQYSFRGKDLIVQLQPTDALLCITNVPISFTSEEFEELVRAYGNIERCFLVYSEVTG
HSKGYGFVEYMKKDFAAKARLELLGRQLGASALFAQWMDVNLLASELIHSKCLCIDKLPS
DYRDSEELLQIFSSVHKPVFCQLAQDEGSYVGGFAVVEYSTAEQAEEVQQAADGMTIKGS
KVQVSFCAPGAPGRSTLAALIAAQRVMHSNQKGLLPEPNPVQIMKSLNNPAMLQVLLQPQ
LCGRAVKPAVLGTPHSLPHLMNPSISPAFLHLNKAHQSSVMGNTSNLFLQNLSHIPLAQQ
QLMKFENIHTNNKPGLLGEPPAVVLQTALGIGSVLPLKKELGHHHGEAHKTSSLIPTQTT
ITAGMGMLPFFPNQHIAGQAGPGHSNTQEKQPATVGMAEGNFSGSQPYLQSFPNLAAGSL
LVGHHKQQQSQPKGTEISSGAASKNQTSLLGEPPKEIRLSKNPYLNLASVLPSVCLSSPA
SKTTLHKTGIASSILDAISQGSESQHALEKCIAYSPPFGDYAQVSSLRNEKRGSSYLISA
PEGGSVECVDQHSQGTGAYYMETYLKKKRVY*
>ENSG00000079308|ENST00000310858
MVPFQQRKDCVLEGSTRVTPSVQPHLQPIRNMSVSRTMEDSCELDLVYVTERIIAVSFPS
TANEENFRSNLREVAQMLKSKHGGNYLLFNLSERRPDITKLHAKVLEFGWPDLHTPALEK
ICSICKAMDTWLNADPHNVVVLHNKGNRGRIGVVIAAYMHYSNISASADQALDRFAMKRF
YEDKIVPIGQPSQRRYVHYFSGLLSGSIKMNNKPLFLHHVIMHGIPNFESKGGCRPFLRI
YQAMQPVYTSGIYNIPGDSQTSVCITIEPGLLLKGDILLKCYHKKFRSPARDVIFRVQFH

TCAIHDLGVVFGKEDLDDAFKDDRFPEYGKVEFVFSYGPEKIQGMEHLENGPSVSVDYNT
SDPLIRWDSYDNFSGHRDDGMEDGNKQNTNSQSIGSISGGLEDQYTWPDTHWPSQS*
>ENSG00000197976|ENST00000313871
MAAATIVHDTSEAVELCPAYGLYLKPITKMTISVALPQLKQPGKSISNWEVMERLKGMVQ
NHQFSTLRISKSTMDFIRFEGEVENKSLVKSFLACLDGKTIKLSGFSDILKVRAAEFKID
FPTRHDWDSFFRDAKDMNETLPGERPDTIHLEGLPCKWFALKESGSEKPSEDVLVKVFEK
FGEIRNVDIPMLDPYREEMTGRNFHTFSFGGHLNFEAYVQYREYMGFIQAMSALRGMKLM
YKGEDGKAVACNIKVSFDSTKHLSDASIKKRQLERQKLQELEQQREEQKRREKEAEERQR
AEERKQKELEELERERKREEKLRKREQKQRDRELRRNQKKLEKLQAEEQKQLQEKIKLEE
RKLLLAQRNLQSIRLIAELLSRAKAVKLREQEQKEEKLRLQQQEERRRLQEAELRRVEEE
KERALGLQRKERELRERLLSILLSKKPDDSHTHDELGVAHADLLQPVLDILQTVSSGCVS
ATTLHPLGGQPPAGAPKESPAHPEADGAPKSVNGSVAEEAPCKEVQSSCRVVPEDGSPEK
RCPGGVLSCIPDNNQQPKGIPACEQNVSRKDTRSEQDKCNREPSKGRGRATGDGLADRHK
RERSRARRASSREDGRPRKERRPHKKHAYKDDSPRRRSTSPDHTRSRRSHSKDRHRRERS
RERRGSASRKHSRHRRRSERSRSRSPSRHRSTWNR*
>ENSG00000176165|ENST00000313071
MLDMGDRKEVKMIPKSSFSINSLVPEAVQNDNHHASHGHHNSHHPQHHHHHHHHHHHPPP
PAPQPPPPPQQQQPPPPPPPAPQPPQTRGAPAADDDKGPQQLLLPPPPPPPPAAALDGAK
ADGLGGKGEPGGGPGELAPVGPDEKEKGAGAGGEEKKGAGEGGKDGEGGKEGEKKNGKYE
KPPFSYNALIMMAIRQSPEKRLTLNGIYEFIMKNFPYYRENKQGWQNSIRHNLSLNKCFV
KVPRHYDDPGKGNYWMLDPSSDDVFIGGTTGKLRRRSTTSRAKLAFKRGARLTSTGLTFM
DRAGSLYWPMSPFLSLHHPRASSTLSYNGTTSAYPSHPMPYSSVLTQNSLGNNHSFSTAN
GLSVDRLVNGEIPYATHHLTAAALAASVPCGLSVPCSGTYSLNPCSVNLLAGQTSYFFPH
VPHPSMTSQSSTSMSARAASSSTSPQAPSTLPCESLRPSLPSFTTGLSGGLSDYFTHQNQ
GSSSNPLIH*
>ENSG00000151247|ENST00000280892
MLDLTSRGQVGTSRRMAEAACSAHFLETTPTPNPPTTEEEKTESNQEVANPEHYIKHPLQ
NRWALWFFKNDKSKTWQANLRLISKFDTVEDFWALYNHIQLSSNLMPGCDYSLFKDGIEP
MWEDEKNKRGGRWLITLNKQQRRSDLDRFWLETLLCLIGESFDDYSDDVCGAVVNVRAKG
DKIAIWTTECENREAVTHIGRVYKERLGLPPKIVIGYQSHADTATKSGSTTKNRFVV*
>ENSG00000173258|ENST00000309235
MQAVVPLNKMTAISPEPQTLASTEQNEVPRVVTSGEQEAILRGNAADAESFRQRFRWFCY
SEVAGPRKALSQLWELCNQWLRPDIHTKEQILELLVFEQFLTILPGEIRIWVKSQHPESS
EEVVTLIEDLTQMLEEKDPVSQDSTVSQEENSKEDKMVTVCPNTESCESITLKDVAVNFS
RGEWKKLEPFQKELYKEVLLENLRNLEFLDFPVSKLELISQLKWVELPWLLEEVSKSSRL
DESALDKIIERCLRDDDHGLMEESQQYCGSSEEDHGNQGNSKGRVAQNKTLGSGSRGKKF
DPDKSPFGHNFKETSDLIKHLRVYLRKKSRRYNESKKPFSFHSDLVLNRKEKTAGEKSRK
SNDGGKVLSHSSALTEHQKRQKIHLGDRSQKCSKCGIIFIRRSTLSRRKTPMCEKCRKDS
CQEAALNKDEGNESGEKTHKCSKCGKAFGYSASLTKHRRIHTGEKPYMCNECGKAFSDSS
SLTPHHRTHSGEKPFKCDDCGKGFTLSAHLIKHQRIHTGEKPYKCKDCGRPFSDSSSLIQ
HQRIHTGEKPYTCSNCGKSFSHSSSLSKHQRIHTGEKPYKCGECGKAFRQNSCLTRHQRI
HTGEKPYLCNDCGMTFSHFTSVIYHQRLHSGEKPYKCNQCEKAFPTHSLLSRHQRIHTGV
KPYKCKECGKSFSQSSSLNEHHRIHTGEKPYECNYCGATFSRSSILVEHLKIHTGRREYE
CNECEKTFKSNSGLIRHRGFHSAE*

>ENSG00000164587|ENST00000312037
MAPRKGKEKKEEQVISLGPQVAEGENVFGVCHIFASFNDTFVHVTDLSGKETICRVTGGM
KVKADRDESSPYAAMLAAQDVAQRCKELGITALHIKLRATGGNRTKTPGPGAQSALRALA
RSGMKIGRIEDVTPIPSDSTRRKGGRRGRRL*
>ENSG00000175390|ENST00000309828
MATPAVPVSAPPATPTPVPAAAPASVPAPTPAPAAAPVPAAAPASSSDPAAAAAATAAPG
QTPASAQAPAQTPAPALPGPALPGPFPGGRVVRLHPVILASIVDSYERRNEGAARVIGTL
LGTVDKHSVEVTNCFSVPHNESEDEVAVDMEFAKNMYELHKKVSPNELILGWYATGHDIT
EHSVLIHEYYSREAPNPIHLTVDTSLQNGRMSIKAYVSTLMGVPGRTMGVMFTPLTVKYA
YYDTERIGVDLIMKTCFSPNRVIGLSSDLQQVGGASARIQDALSTVLQYAEDVLSGKVSA
DNTVGRFLMSLVNQVPKIVPDDFETMLNSNINDLLMVTYLANLTQSQIALNEKLVNL*
>ENSG00000157554|ENST00000288319
MASTIKEALSVVSEDQSLFECAYGTPHLAKTEMTASSSSDYGQTSKMSPRVPQQDWLSQP
PARVTIKMECNPSQVNGSRNSPDECSVAKGGKMVGSPDTVGMNYGSYMEEKHMPPPNMTT
NERRVIVPADPTLWSTDHVRQWLEWAVKEYGLPDVNILLFQNIDGKELCKMTKDDFQRLT
PSYNADILLSHLHYLRETPLPHLTSDDVDKALQNSPRLMHARNTGGAAFIFPNTSVYPEA
TQRITTRPDLPYEPPRRSAWTGHGHPTPQSKAAQPSPSTVPKTEDQRPQLDPYQILGPTS
SRLANPGSGQIQLWQFLLELLSDSSNSSCITWEGTNGEFKMTDPDEVARRWGERKSKPNM
NYDKLSRALRYYYDKNIMTKVHGKRYAYKFDFHGIAQALQPHPPESSLYKYPSDLPYMGS
YHAHPQKMNFVAPHPPALPVTSSSFFAAPNPYWNSPTGGIYPNTRLPTSHMPSHLGTYY*
>ENSG00000147586|ENST00000276585
MAALCRTRAVAAESHFLRVFLFFRPFRGVGTESGSESGSSNAKEPKTRAGGFASALERHS
ELLQKVEPLQKGSPKNVESFASMLRHSPLTQMGPAKDKLVIGRIFHIVENDLYIDFGGKF
HCVCRRPEVDGEKYQKGTRVRLRLLDLELTSRFLGATTDTTVLEANAVLLGIQESKDSRS
KEEHHEK*
>ENSG00000139767|ENST00000267260
MASVQQGEKQLFEKFWRGTFKAVATPRPESIIVASITARKPLPRTEPQNNPVVPAQDGPS
EKLGQHLATEPLGTNSWERDKTCRELGATRGHSASHDKDLTPPPSSRGKKKKKKSTRKKR
RRSSSYSPSPVKKKKKKSSKKHKRRRSFSKKRRHSSSSPKSKRRDEKRHKKQSRSRPRKS
HRHRHHRCPSRSQSSESRPSSCESRHRGRSPEEGQKSRRRHSRRCSKTLCKDSPEAQSSR
PPSQPLQMLGYLSARGVITGSGSAADLFTKTASPLTTSRGRSQEYDSGNDTSSPPSTQTS
SARSRGQEKGSPSGGLSKSRELNSGNTSDSGNSFTTSSPQNKGAMLENLSPTSRGRESRG
FQSPCLECAEVKKSSLVPSTARSSPMKGCSRSSSYASTRSSSHSSRSPNPRASPRYTQSR
STSSEKRSYSRSPSYSSKSGKRSPPSRSSRSRRSPSYSRYSPSRERDPKYSEKDSQQRER
ERARRRRRSYSPMRKRRRDSPSHLEARRITSARKRPIPYYRPSPSSSGSLSSTSSWYSSS
SSRSASRSYSRSRSRSRSRRRSRTRTSSSSSSRSPSPGSRSRSRSRSRSRSRSRSQSRSY
SSADSYSSTRR*
>ENSG00000106948|ENST00000312033
MASSETEIRWAEPGLGKGPQRRRWAWAEDKRDVDRSSSQSWEEERLFPNATSPELLEDFR
LAQQHLPPLEWDPHPQPDGHQDSESGETSGEEAEAEDVDSPASSHEPLAWLPQQGRQLDM
TEEEPDGTLGSLEVEEAGESSSRLGYEAGLSLEGHGNTSPMALGHGQARGWVASGEQASG
DKLSEHSEVNPSVELSPARSWSSGTVSLDHPSDSLDSTWEGETDGPQPTALAETLPEGPS
HHLLSPDGRTGGSVARATPMEFQDSSAPPAQSPQHATDRWRRETTRFFCPQPKEHIWKQT
KTSPKPLPSRFIGSISPLNPQPRPTRQGRPLPRQGATLAGRSSSNAPKYGRGQLNYPLPD

FSKVGPRVRFPKDESYRPPKSRSHNRKPQAPARPLIFKSPAEIVQEVLLSSGEAALAKDT
PPAHPITRVPQEFQTPEQATELVHQLQEDYHRLLTKYAEAENTIDQLRLGAKVNLFSDPP
QPNHSIHTGMVPQGTKVLSFTIPQPRSAEWWPGPAEDPQASAASGWPSARGDLSPSSLTS
MPTLGWLPENRDISEDQSSAEQTQALASQASQFLAKVESFERLIQAGRLMPQDQVKGFQR
LKAAHAALEEEYLKACREQHPAQPLAGSKGTPGRFDPRRELEAEIYRLGSCLEELKEHID
QTQQEPEPPGSDSALDSTPALPCLHQPTHLPAPSGQAPMPAIKTSCPEPATTTAAASTGP
CPLHVNVEVSSGNSEVEDRPQDPLARLRHKELQMEQVYHGLMERYLSVKSLPEAMRMEEE
EEGEEEEEEEGGGDSLEVDGVAATPGKAEATRVLPRQCPVQAEKSHGAPLE*
>ENSG00000044115|ENST00000302763
MTAVHAGNINFKWDPKSLEIRTLAVERLLEPLVTQVTTLVNTNSKGPSNKKRGRSKKAHV
LAASVEQATENFLEKGDKIAKESQFLKEELVAAVEDVRKQGDLMKAAAGEFADDPCSSVK
RGNMVRAARALLSAVTRLLILADMADVYKLLVQLKVVEDGILKLRNAGNEQDLGIQYKAL
KPEVDKLNIMAAKRQQELKDVGHRDQMAAARGILQKNVPILYTASQACLQHPDVAAYKAN
RDLIYKQLQQAVTGISNAAQATASDDASQHQGGGGGELAYALNNFDKQIIVDPLSFSEER
FRPSLEERLESIISGAALMADSSCTRDDRRERIVAECNAVRQALQDLLSEYMGNAGRKER
SDALNSAIDKMTKKTRDLRRQLRKAVMDHVSDSFLETNVPLLVLIEAAKNGNEKEVKEYA
QVFREHANKLIEVANLACSISNNEEGVKLVRMSASQLEALCPQVINAALALAAKPQSKLA
QENMDLFKEQWEKQVRVLTDAVDDITSIDDFLAVSENHILEDVNKCVIALQEKDVDGLDR
TAGAIRGRAARVIHVVTSEMDNYEPGVYTEKVLEATKLLSNTVMPRFTEQVEAAVEALSS
DPAQPMDENEFIDASRLVYDGIRDIRKAVLMIRTPEELDDSDFETEDFDVRSRTSVQTED
DQLIAGQSARAIMAQLPQEQKAKIAEQVASFQEEKSKLDAEVSKWDDSGNDIIVLAKQMC
MIMMEMTDFTRGKGPLKNTSDVISAAKKIAEAGSRMDKLGRTIADHCPDSACKQDLLAYL
QRIALYCHQLNICSKVKAEVQNLGGELVVSGVDSAMSLIQAAKNLMNAVVQTVKASYVAS
TKYQKSQGMASLNLPAVSWKMKAPEKKPLVKREKQDETQTKIKRASQKKHVNPVQALSEF
KAMDSI*
>ENSG00000145216|ENST00000306932
MSAGEVERLVSELSGGTGGDEEEEWLYGDENEVERPEEENASANPPSGIEDETAENGVPK
PKVTETEDDSDSDSDDDEDDVHVTIGDIKTGAPQYGSYGTAPVNLNIKTGGRVYGTTGTK
VKGVDLDAPGSINGVPLLEVDLDSFEDKPWRKPGADLSDYFNYGFNEDTWKAYCEKQKRI
RMGLEVIPVTSTTNKITVQQGRTGNSEKETALPSTKAEFTSPPSLFKTGLPPSRRLPGAI
DVIGQTITISRVEGRRRANENSNIQVLSERSATEVDNNFSKPPPFFPPGAPPTHLPPPPF
LPPPPTVSTAPPLIPPPGFPPPPGAPPPSLIPTIESGHSSGYDSRSARAFPYGNVAFPHL
PGSAPSWPSLVDTSKQWDYYARREKDRDRERDRDRERDRDRDRERERTRERERERDHSPT
PSVFNSDEERYRYREYAERGYERHRASREKEERHRERRHREKEETRHKSSRSNSRRRHES
EEGDSHRRHKHKKSKRSKEGKEAGSEPAPEQESTEATPAE*
>ENSG00000134852|ENST00000309964
MLFTVSCSKMSSIVDRDDSSIFDGLVEEDDKDKAKRVSRNKSEKKRRDQFNVLIKELGSM
LPGNARKMDKSTVLQKSIDFLRKHKEITAQSDASEIRQDWKPTFLSNEEFTQLMLEALDG
FFLAIMTDGSIIYVSESVTSLLEHLPSDLVDQSIFNFIPEGEHSEVYKILSTHLLESDSL
TPEYLKSKNQLEFCCHMLRGTIDPKEPSTYEYVKFIGNFKSLNSVSSSAHNGFEGTIQRT
HRPSYEDRVCFVATVRLATPQFIKEMCTVEEPNEEFTSRHSLEWKFLFLDHRAPPIIGYL
PFEVLGTSGYDYYHVDDLENLAKCHEHLMQYGKGKSCYYRFLTKGQQWIWLQTHYYITYH
QWNSRPEFIVCTHTVVSYAEVRAERRRELGIEESLPETAADKSQDSGSDNRINTVSLKEA
LERFDHSPTPSASSRSSRKSSHTAVSDPSSTPTKIPTDTSTPPRQHLPAHEKMVQRRSSF

SSQSINSQSVGSSLTQPVMSQATNLPIPQGMSQFQFSAQLGAMQHLKDQLEQRTRMIEAN
IHRQQEELRKIQEQLQMVHGQGLQMFLQQSNPGLNFGSVQLSSGNSSNIQQLAPINMQGQ
VVPTNQIQSGMNTGHIGTTQHMIQQQTLQSTSTQSQQNVLSGHSQQTSLPSQTQSTLTAP
LYNTMVISQPAAGSMVQIPSSMPQNSTQSAAVTTFTQDRQIRFSQGQQLVTKLVTAPVAC
GAVMVPSTMLMGQVVTAYPTFATQQQQSQTLSVTQQQQQQSSQEQQLTSVQQPSQAQLTQ
PPQQFLQTSRLLHGNPSTQLILSAAFPLQQSTFPQSHHQQHQSQQQQQLSRHRTDSLPDP
SKVQPQ*
>ENSG00000105197|ENST00000314349
MASALSLGNKCDPFLRCVLCRGGGALQGPRGRGPDDFESQLSPPGSARRLVRSKRACGNP
PDAFGLSRASVHPPLPRVSIGCSSGPGRAKRERVGGAAWRQRKMAASAAVFSRLRSGLRL
GSRGLCTRLATPPRRAPDQAAEIGSRGSTKAQGPQQQPGSEGPSYAKKVALWLAGLLGAG
GTVSVVYIFGNNPVDENGAKIPDEFDNDPILVQQLRRTYKYFKDYRQMIIEPTSPCLLPD
PLQEPYYQPPYTLVLELTGVLLHPEWSLATGWRFKKRPGIETLFQQLAPLYEIVIFTSET
GMTAFPLIDSVDPHGFISYRLFRDATRYMDGHHVKDISCLNRDPARVVVVDCKKEAFRLQ
PYNGVALRPWDGNSDDRVLLDLSAFLKTIALNGVEDVRTVLEHYALEDDPLAAFKQRQSR
LEQEEQQRLAELSKSNKQNLFLGSLTSRLWPRSKQP*
>ENSG00000150316|ENST00000279839
MTTAARPTFEPARGGRGKGEGDLSQLSKQYSSRDLPSHTKIKYRQTTQDAPEEVRNRDFR
RELEERERAAAREKNRDRPTREHTTSSSVSKKPRLDQIPAANLDADDPLTDEEDEDFEEE
SDDDDTAALLAELEKIKKERAEEQARKEQEQKAEEERIRMENILSGNPLLNLTGPSQPQA
NFKVKRRWDDDVVFKNCAKGVDDQKKDKRFVNDTLRSEFHKKFMEKYIK*
>ENSG00000106948|ENST00000307564
MASSETEIRWAEPGLGKGPQRRRWAWAEDKRDVDRSSSQSWEEERLFPNATSPELLEDFR
LAQQHLPPLEWDPHPQPDGHQDSESGETSGEEAEAEDVDSPASSHEPLAWLPQQGRQLDM
TEEEPDGTLGSLEVEEAGESSSRLGYEAGLSLEGHGNTSPMALGHGQARGWVASGEQASG
DKLSEHSEVNPSVELSPARSWSSGTVSLDHPSDSLDSTWEGETDGPQPTALAETLPEGPS
HHLLSPDGRTGGSVARATPMEFQDSSAPPAQSPQHATDRWRRETTRFFCPQPKEHIWKQT
KTSPKPLPSRFIGSISPLNPQPRPTRQGRPLPRQGATLAGRSSSNAPKYGRGQLNYPLPD
FSKVGPRVRFPKDESYRPPKSRSHNRKPQAPARPLIFKSPAEIVQEVLLSSGEAALAKDT
PPAHPITRVPQEFQTPEQATELVHQLQEDYHRLLTKYAEAENTIDQLRLGAKVNLFSDPP
QPNHSIHTGMVPQGTKVLSFTIPQPRSAEWWPGPAEDPQASAASGWPSARGDLSPSSLTS
MPTLGWLPENRDISEDQSSAEQTQALASQASQFLAKVESFERLIQAGRLMPQDQVKGFQR
LKAAHAALEEEYLKACREQHPAQPLAGSKGTPGRFDPRRELEAEIYRLGSCLEELKEHID
QTQQEPEPPGSDSALDSTPALPCLHQPTHLPAPSGQAPMPAIKTSCPEPATTTAAASTGP
CPLHVNVEVSSGNSEVEDRPQDPLARLRHKELQMEQVYHGLMERYLSVKSLPEAMRMEEE
EEGEEEEEEEGGGDSLEVDGVAATPGKAEATRVLPRQCPVQAEKSHGAPLEEATEKMVSM
KPPGFQASLARDGHMSGLGKAEAAPPGPGVPPHPPGTKSAASHQSSMTSLEGSGISERLP
QKPLHRGGGPHLEETWMASPETDSGFVGSETSRVSPLTQTPEHRLSHISTAGTLAQPFAA
SVPRDGASYPKARGSLIPRRATEPSTPRSQAQRYLSSPSGPLRQRAPNFSLERTLAAEMA
VPGSEFEGHKRISEQPLPNKTISPPPAPAPAAAPLPCGPTETIPSFLLTRAGRDQAICEL
QEEVSRLRLRLEDSLHQPLQGSPTRPASAFDRPARTRGRPADSPATWGSHYGSKSTERLP
GEPRGEEQIVPPGRQRARSSSVPREVLRLSLSSESELPSLPLFSEKSKTTKDSPQAARDG
KRGVGSAGWPDRVTFRGQYTGHEYHVLSPKAVPKGNGTVSCPHCRPIRTQDAGGAVTGDP
LGPPPADTLQCPLCGQVGSPPEADGPGSATSGAEKATTRRKASSTPSPKQRSKQAGSSPR

PPPGLWYLATAPPAPAPPAFAYISSVPIMPYPPAAVYYAPAGPTSAQPAAKWPPTASPPP
ARRHRHSIQLDLGDLEELNKALSRAVQAAESVRSTTRQMRSSLSADLRQAHSLRGSCLF*
>ENSG00000164342|ENST00000296795
MRQTLPCIYFWGGLLPFGMLCASSTTKCTVSHEVADCSHLKLTQVPDDLPTNITVLNLTH
NQLRRLPAANFTRYSQLTSLDVGFNTISKLEPELCQKLPMLKVLNLQHNELSQLSDKTFA
FCTNLTELHLMSNSIQKIKNNPFVKQKNLITLDLSHNGLSSTKLGTQVQLENLQELLLSN
NKIQALKSEELDIFANSSLKKLELSSNQIKEFSPGCFHAIGRLFGLFLNNVQLGPSLTEK
LCLELANTSIRNLSLSNSQLSTTSNTTFLGLKWTNLTMLDLSYNNLNVVGNDSFAWLPQL
EYFFLEYNNIQHLFSHSLHGLFNVRYLNLKRSFTKQSISLASLPKIDDFSFQWLKCLEHL
NMEDNDIPGIKSNMFTGLINLKYLSLSNSFTSLRTLTNETFVSLAHSPLHILNLTKNKIS
KIESDAFSWLGHLEVLDLGLNEIGQELTGQEWRGLENIFEIYLSYNKYLQLTRNSFALVP
SLQRLMLRRVALKNVDSSPSPFQPLRNLTILDLSNNNIANINDDMLEGLEKLEILDLQHN
NLARLWKHANPGGPIYFLKGLSHLHILNLESNGFDEIPVEVFKDLFELKIIDLGLNNLNT
LPASVFNNQVSLKSLNLQKNLITSVEKKVFGPAFRNLTELDMRFNPFDCTCESIAWFVNW
INETHTNIPELSSHYLCNTPPHYHGFPVRLFDTSSCKDSAPFELFFMINTSILLIFIFIV
LLIHFEGWRISFYWNVSVHRVLGFKEIDRQTEQFEYAAYIIHAYKDKDWVWEHFSSMEKE
DQSLKFCLEERDFEAGVFELEAIVNSIKRSRKIIFVITHHLLKDPLCKRFKVHHAVQQAI
EQNLDSIILVFLEEIPDYKLNHALCLRRGMFKSHCILNWPVQKERIGAFRHKLQVALGSK
NSVH*
>ENSG00000143742|ENST00000304786
MPQYQTWEEFSRAAEKLYLADPMKARVVLKYRHSDGNLCVKVTDDLVCLVYKTDQAQDVK
KIEKFHSQLMRLMVAKEARNVTMETE*
>ENSG00000168874|ENST00000306279
MKHIPVLEDGPWKTVCVKELNGLKKLKRKGKEPARRANGYKTFRLDLEAPEPRAVATNGL
RDRTHRLQPVPVPVPVPVPVAPAVPPRGGTDTAGERGGSRAPEVSDARKRCFALGAVGPG
LPTPPPPPPPAPQSQAPGGPEAQPFREPGLRPRILLCAPPARPAPSAPPAPPAPPESTVR
PAPPTRPGESSYSSISHVIYNNHQDSSASPRKRPGEATAASSEIKALQQTRRLLANARER
TRVHTISAAFEALRKQVPCYSYGQKLSKLAILRIACNYILSLARLADLDYSADHSNLSFS
ECVQRCTRTLQAEGRAKKRKE*
>ENSG00000197008|ENST00000307355
MGPLTFMDVAIEFSLEEWQCLDTAQRNVYRHVMLENYRNLVFLDLITCLEQGKEPWNMKR
HEMVVAKHSALCSRFAQDLWLEQNIKDSFQKVTLSRYGKYGHKNLQLRKGCKSVDECKGH
QGGFNGLNQCLKITTSKIFQCNKYVKVMHKFSNSNRHKIRHTENKHFRCKECDKSLCMLS
RLTQHKKIHTRENFYKCEECGKTFNWSTNLSKPKKIHTGEKPYKCEVCGKAFHQSSILTK
HKIIRTGEKPYKCAHCGKAFKQSSHLTRHKIIHTEEKPYKCEQCGKVFKQSPTLTKHQII
YTGEEPYKCEECGKAFNLS*
>ENSG00000182150|ENST00000288985
MQPGSAPPPGRMDPSAPQPRAETSGKDIWHPGERCLAPSPDNGKLCEASIKSITVDENGK
SFAVVLYADFQERKIPLKQLQEVKFVKDCPRNLIFDDEDLEKPYFPNRKFPSSSVAFKLS
DNGDSIPYTINRYLRDYQREGTRFLYGHYIHGGGCILGDDMGLGKTVQVISFLAAVLHKK
GTREDIENNMPEFLLRSMKKEPLSSTAKKMFLIVAPLSVLYNWKDELDTWGYFRVTVLHG
NRKDNELIRVKQRKCEIALTTYETLRLCLDELNSLEWSAVIVDEAHRIKNPKARVTEVMK
ALKCNVRIGLTGTILQNNMKELWCVMDWAVPGLLGSGTYFKKQFSDPVEHGQRHTATKRE
LATGRKAMQRLAKKMSGWFLRRTKTLIKDQLPKKEDRMVYCSLTDFQKAVYQTVLETEDV

TLILQSSEPCTCRSGQKRRNCCYKTNSHGETVKTLYLSYLTVLQKVANHVALLQAASTSK
QQETLIKRICDQVFSRFPDFVQKSKDAAFETLSDPKYSGKMKVLQQLLNHCRKNRDKVLL
FSFSTKLLDVLQQYCMASGLDYRRLDGSTKSEERLKIVKEFNSTQDVNICLVSTMAGGLG
LNFVGANVVVLFDPTWNPANDLQAIDRAYRIGQCRDVKVLRLISLGTVEEIMYLRQIYKQ
QLHCVVVGSENAKRYFEAVQGSKEHQGELFGIHNLFKFRSQGSCLTKDILEV*
>ENSG00000145592|ENST00000274242
MTKGTSSFGKRRNKTHTLCRRCGSKAYHLQKSTCGKCGYPAKRKRKYNWSAKAKRRNTTG
TGRMRHLKIVYRRFRHGFREGTTPKPKRAAVAASSSS*
>ENSG00000142025|ENST00000269945
MEPSDMPAGYHCPLDSAPWDETRDPQSTELIPRRAISRSPTCARCRNHGVTAHLKGHKRL
CLFQACECHKCVLILERRRVMAAQVALRRQQEAQLKKHLMRRGEASPKAPNHFRKGTTQP
QVPSGKENIAPQPQTPHGAVLLAPTPPGKNSCGPLLLSHPPEASPLSWTPVPPGPWVPGH
WLPPGFSMPPPVVCRLLYQEPAVSLPPFPGFDPGTSLQLPTHGPFTTCPGSHPVLTAPLS
GEPQGPPSQPRTHSTLILQPCGTPDPLQLQPQASGASCLARTSGPSEWQLQQEAAEALVG
LKDSSQAPRVTPSVPPNPAWISLLHPCGPPAPAGGRGFQPVGPCLRPSPAPSVALHIGRL
GSISLLS*
>ENSG00000048405|ENST00000265827
MPLRDKYCQTDHHHHGCCEPVYILEPGDPPLLQQPLQTSKSGIQQIIECFRSGTKQLKHI
LLKDVDTIFECKLCRSLFRGLPNLITHKKFYCPPSLQMDDNLPDVNDKQSQAINDLLEAI
YPSVDKREYIIKLEPIETNQNAVFQYISRTDNPIEVTESSSTPEQTEVQIQETSTEQSKT
VPVTDTEVETVEPPPVEIVTDEVAPTSDEQPQESQADLETSDNSDFGHQLICCLCRKEFN
SRRGVRRHIRKVHKKKMEELKKYIETRKNPNQSSKGRSKNVLVPLSRSCPVCCKSFATKA
NVRRHFDEVHRGLRRDSITPDIATKPGQPLFLDSISPKKSFKTRKQKSSSKAEYNLTACK
CLLCKRKYSSQIMLKRHMQIVHKITLSGTNSKREKGPNNTANSSEIKVKVEPADSVESSP
PSITHSPQNELKGTNHSNEKKNTPAAQKNKVKQDSESPKSTSPSAAGGQQKTRKPKLSAG
FDFKQLYCKLCKRQFTSKQNLTKHIELHTDGNNIYVKFYKCPLCTYETRRKRDVIRHITV
VHKKSSRYLGKITASLEIRAIKKPIDFVLNKVAKRGPSRDEAKHSDSKHDGTSNSPSKKY
EVADVGIEVKVTKNFSLHRCNKCGKAFAKKTYLEHHKKTHKANASNSPEGNKTKGRSTRS
KALV*
>ENSG00000161960|ENST00000293831
MSASQDSRSRDNGPDGMEPEGVIESNWNEIVDSFDDMNLSESLLRGIYAYGFEKPSAIQQ
RAILPCIKGYDVIAQAQSGTGKTATFAISILQQIELDLKATQALVLAPTRELAQQIQKVV
MALGDYMGASCHACIGGTNVRAEVQKLQMEAPHIIVGTPGRVFDMLNRRYLSPKYIKMFV
LDEADEMLSRGFKDQIYDIFQKLNSNTQVVLLSATMPSDVLEVTKKFMRDPIRILVKKEE
LTLEGIRQFYINVEREEWKLDTLCDLYETLTITQAVIFINTRRKVDWLTEKMHARDFTVS
AMHGDMDQKERDVIMREFRSGSSRVLITTDLLARGIDVQQVSLVINYDLPTNRENYIHRI
GRGGRFGRKGVAINMVTEEDKRTLRDIETFYNTSIEEMPLNVADLI*
>ENSG00000163002|ENST00000295119
MAAFAVEPQGPALGSEPMMLGSPTSPKPGVNAQFLPGFLMGDLPAPVTPQPRSISGPSVG
VMEMRSPLLAGGSPPQPVVPAHKDKSGAPPVRSIYDDISSPGLGSTPLTSRRQPNISVMQ
SPLVGVTSTPGTGQSMFSPASIGQPRKTTLSPAQLDPFYTQGDSLTSEDHLDDSWVTVFG
FPQASASYILLQFAQYGNILKHVMSNTGNWMHIRYQSKLQARKALSKDGRIFGESIMIGV
KPCIDKSVMESSDRCALSSPSLAFTPPIKTLGTPTQPGSTPRISTMRPLATAYKASTSDY
QVISDRQTPKKDESLVSKAMEYMFGW*

>ENSG00000134323|ENST00000281043
MPSCSTSTMPGMICKNPDLEFDSLQPCFYPDEDDFYFGGPDSTPPGEDIWKKFELLPTPP
LSPSRGFAEHSSEPPSWVTEMLLENELWGSPAEEDAFGLGGLGGLTPNPVILQDCMWSGF
SAREKLERAVSEKLQHGRGPPTAGSTAQSPGAGAASPAGRGHGGAAGAGRAGAALPAELA
HPAAECVDPAVVFPFPVNKREPAPVPAAPASAPAAGPAVASGAGIAAPAGAPGVAPPRPG
GRQTSGGDHKALSTSGEDTLSDSDDEDDEEEDEEEEIDVVTVEKRRSSSNTKAVTTFTIT
VRPKNAALGPGRAQSSELILKRCLPIHQQHNYAAPSPYVESEDAPPQKKIKSEASPRPLK
SVIPPKAKSLSPRNSDSEDSERRRNHNILERQRRNDLRSSFLTLRDHVPELVKNEKAAKV
VILKKATEYVHSLQAEEHQLLLEKEKLQARQQQLLKKIEHARTC*
>ENSG00000172059|ENST00000305883
MHTPDFAGPDDARAVDIMDICESILERKRHDSERSTCSILEQTDMEAVEALVCMSSWGQR
SQKGDLLRIRPLTPVSDSGDVTTTVHMDAATPELPKDFHSLSTLCITPPQSPDLVEPSTR
TPVSPQVTDSKACTATDVLQSSAVVARALSGGAERGLLGLEPVPSSPCRAKGTSVIRHTG
ESPAACFPTIQTPDCRLSDSREGEEQLLGHFETLQDTHLTDSLLSTNLVSCQPCLHKSGG
LLLTDKGQQAGWPGAVQTCSPKNYENDLPRKTTPLISVSVPAPPVLCQMIPVTGQSSMLP
AFLKPPPQLSVGTVRPILAQAAPAPQPVFVGPAVPQGAVMLVLPQGALPPPAPCAANVMA
AGNTKLLPLAPAPVFITSSQNCVPQVDFSRRRNYVCSFPGCRKTYFKSSHLKAHLRTHTG
EKPFNCSWDGCDKKFARSDELSRHRRTHTGEKKFVCPVCDRRFMRSDHLTKHARRHMTTK
KIPGWQAEVGKLNRIASAESPGSPLVSMPASA*
>ENSG00000164600|ENST00000297142
MLTLPFDESVVMPESQMCRKFSRECEDQKQIKKPESFSKQIVLRGKSIKRAPGEETEKEE
EEEDREEEDENGLPRRRGLRKKKTTKLRLERVKFRRQEANARERNRMHGLNDALDNLRKV
VPCYSKTQKLSKIETLRLAKNYIWALSEILRIGKRPDLLTFVQNLCKGLSQPTTNLVAGC
LQLNARSFLMGQGGEAAHHTRSPYSTFYPPYHSPELTTPPGHGTLDNSKSMKPYNYCSAY
ESFYESTSPECASPQFEGPLSPPPINYNGIFSLKQEETLDYGKNYNYGMHYCAVPPRGPL
GQGAMFRLPTDSHFPYDLHLRSQSLTMQDELNAVFHN*
>ENSG00000140319|ENST00000267884
MVLLESEQFLTELTRLFQKCRTSGSVYITLKKYDGRTKPIPKKGTVEGFEPADNKCLLRA
TDGKKKISTVVSSKEVNKFQMAYSNLLRANMDGLKKRDKKNKTKKTKAAAAAAAAAPAAA
ATAPTTAATTAATAAQ*
>ENSG00000165799|ENST00000298690
MAPARAGFCPLLLLLLLGLWVAEIPVSAKPKGMTSSQWFKIQHMQPSPQACNSAMKNINK
HTKRCKDLNTFLHEPFSSVAATCQTPKIACKNGDKNCHQSHGAVSLTMCKLTSGKHPNCR
YKEKRQNKSYVVACKPPQKKDSQQFHLVPVHLDRVL*
>ENSG00000112531|ENST00000275262
MVGEMETKEKPKPTPDYLMQLMNDKKLMSSLPNFCGIFNHLERLLDEEISRVRKDMYNDT
LNGSTEKRSAELPDAVGPIVQLQEKLYVPVKEYPDFNFVGRILGPRGLTAKQLEAETGCK
IMVRGKGSMRDKKKEEQNRGKPNWEHLNEDLHVLITVEDAQNRAEIKLKRAVEEVKKLLV
PAAEGEDSLKKMQLMELAILNGTYRDANIKSPALAFSLAATAQAAPRIITGPAPVLPPAA
LRTPTPAGPTIMPLIRQIQTAVMPNGTPHPTAAIVPPGPEAGLIYTPYEYPYTLAPATSI
LEYPIEPSGVLEWIEMPVMPDISAH*
>ENSG00000140009|ENST00000267525
MDIKNSPSSLNSPSSYNCSQSILPLEHGSIYIPSSYVDSHHEYPAMTFYSPAVMNYSIPS
NVTNLEGGPGRQTTSPNVLWPTPGHLSPLVVHRQLSHLYAEPQKSPWCEARSLEHTLPVN

RETLKRKVSGNRCASPVTGPGSKRDAHFCAVCSDYASGYHYGVWSCEGCKAFFKRSIQGH
NDYICPATNQCTIDKNRRKSCQACRLRKCYEVGMVKCGSRRERCGYRLVRRQRSADEQLH
CAGKAKRSGGHAPRVRELLLDALSPEQLVLTLLEAEPPHVLISRPSAPFTEASMMMSLTK
LADKELVHMISWAKKIPGMYPLVTATQDADSSRKLAHLLNAVTDALVWVIAKSGISSQQQ
SMRLANLLMLLSHVRHASNKGMEHLLNMKCKNVVPVYDLLLEMLNAHVLRGCKSSITGSE
CSPAEDSKSKEGSQNPQSQ*
>ENSG00000060138|ENST00000279550
MSEAGEATTTTTTTLPQAPTEAAAAAPQDPAPKSPVGSGAPQAAAPAPAAHVAGNPGGDA
APAATGTAAAASLATAAGSEDAEKKVLATKVLGTVKWFNVRNGYGFINRNDTKEDVFVHQ
TAIKKNNPRKYLRSVGDGETVEFDVVEGEKGAEAANVTGPDGVPVEGSRYAADRRRYRRG
YYGRRRGPPRNAGEIGEMKDGVPEGAQLQGPVHRNPTYRPRYRSRGPPRPRPAPAVGEAE
DKENQQATSGPNQPSVRRGYRRPYNYRRRPRPPNAPSQDGKEAKAGEAPTENPAPPTQQS
SAE*
>ENSG00000149187|ENST00000310513
MNGTLDHPDQPDLDAIKMFVGQVPRTWSEKDLRELFEQYGAVYEINVLRDRSQNPPQSKG
CCFVTFYTRKAALEAQNALHNMKVLPGMHHPIQMKPADSEKNNAVEDRKLFIGMISKKCT
ENDIRVMFSSFGQIEECRILRGPDGLSRGCAFVTFTTRAMAQTAIKAMHQAQTMEGCSSP
MVVKFADTQKDKEQKRMAQQLQQQMQQISAASVWGNLAGLNTLGPQYLALLQQTASSGNL
NTLSSLHPMGGLNAMQLQNLAALAAAASAAQNTPSGTNALTTSSSPLSVLTSSGSSPSSS
SSNSVNPIASLGALQTLAGATAGLNVGSLAGMAALNGGLGSSGLSNGTGSTMEALTQAYS
GIQQYAAAALPTLYNQNLLTQQSIGAAGSQKEGPEGANLFIYHLPQEFGDQDLLQMFMPF
GNVVSAKVFIDKQTNLSKCFGFVSYDNPVSAQAAIQSMNGFQIGMKRLKVQLKRSKNDSK
PY*
>ENSG00000107864|ENST00000265997
MQDDLLMDKSKTQPQPQQQQRQQQQPQPESSVSEAPSTPLSSETPKPEENSAVPALSPAA
APPAPNGPDKMQMESPLLPGLSFHQPPQQPPPPQEPAAPGASLSPSFGSTWSTGTTNAVE
DSFFQGITPVNGTMLFQNFPHHVNPVFGGTFSPQIGLAQTQHHQQPPPPAPAPQPAQPAQ
PPQAQPPQQRRSPASPSQAPYAQRSAAAAYGHQPIMTSKPSSSSAVAAAAAAAAASSASS
SWNTHQSVNAAWSAPSNPWGGLQAGRDPRRAVGVGVGVGVGVPSPLNPISPLKKPFSSNV
IAPPKFPRAAPLTSKSWMEDNAFRTDNGNNLLPFQDRSRPYDTFNLHSLENSLMDMIRTD
HEPLKGKHYPPSGPPMSFADIMWRNHFAGRMGINFHHPGTDNIMALNNAFLDDSHGDQAL
SSGLSSPTRCQNGERVERYSRKVFVGGLPPDIDEDEITASFRRFGPLVVDWPHKAESKSY
FPPKGYAFLLFQEESSVQALIDACLEEDGKLYLCVSSPTIKDKPVQIRPWNLSDSDFVMD
GSQPLDPRKTIFVGGVPRPLRAVELAMIMDRLYGGVCYAGIDTDPELKYPKGAGRVAFSN
QQSYIAAISARFVQLQHNDIDKRVEVKPYVLDDQMCDECQGTRCGGKFAPFFCANVTCLQ
YYCEYCWASIHSRAGREFHKPLVKEGGDRPRHVPFRWS*
>ENSG00000154832|ENST00000285106
MEGDGSDPEPPDAGEDSKSENGENAPIYCICRKPDINCFMIGCDNCNEWFHGDCIRITEK
MAKAIREWYCRECREKDPKLEIRYRHKKSRERDGNERDSSEPRDEGGGRKRPVPDPDLQR
RAGSGTGVGAMLARGSASPHKSSPQPLVATPSQHHQQQQQIKRSARMCGECEACRRTED
CGHCDFCRDMKKFGGPNKIRQKCRLRQCQLRARESYKYFPSSLSPVTPSESLPRPRRPLP
TQQQPQPSQKLGRIREDEGAVASSTVKEPPEATATPEPLSDEDLPLDPDLYQDFCAGAFD
DHGLPWMSDTEESPFLDPALRKRAVKVKHVKRREKKSEKKKEERYKRHRQKQKHKDKWKH
PERADAKDPASLPQCLGPGCVRPAQPSSKYCSDDCGMKLAANRIYEILPQRIQQWQQSPC

IAEEHGKKLLERIRREQQSARTRLQEMERRFHELEAIILRAKQQAVREDEESNEGDSDDT
DLQIFCVSCGHPINPRVALRHMERCYAKYESQTSFGSMYPTRIEGATRLFCDVYNPQSKT
YCKRLQVLCPEHSRDPKVPADEVCGCPLVRDVFELTGDFCRLPKRQCNRHYCWEKLRRAE
VDLERVRVWYKLDELFEQERNVRTAMTNRAGLLALMLHQTIQHDPLTTDLRSSADR*
>ENSG00000140521|ENST00000268124
MSRLLWRKVAGATVGPGPVPAPGRWVSSSVPASDPSDGQRRRQQQQQQQQQQQQQPQQPQ
VLSSEGGQLRHNPLDIQMLSRGLHEQIFGQGGEMPGEAAVRRSVEHLQKHGLWGQPAVPL
PDVELRLPPLYGDNLDQHFRLLAQKQSLPYLEAANLLLQAQLPPKPPAWAWAEGWTRYGP
EGEAVPVAIPEERALVFDVEVCLAEGTCPTLAVAISPSAWYSWCSQRLVEERYSWTSQLS
PADLIPLEVPTGASSPTQRDWQEQLVVGHNVSFDRAHIREQYLIQGSRMRFLDTMSMHMA
ISGLSSFQRSLWIAAKQGKHKVQPPTKQGQKSQRKARRGPAISSWDWLDISSVNSLAEVH
RLYVGGPPLEKEPRELFVKGTMKDIRENFQDLMQYCAQDVWATHEVFQQQLPLFLERCPH
PVTLAGMLEMGVSYLPVNQNWERYLAEAQGTYEELQREMKKSLMDLANDACQLLSGERYK
EDPWLWDLEWDLQEFKQKKAKKVKKEPATASKLPIEGAGAPGDPMDQEDLGPCSEEEEFQ
QDVMARACLQKLKGTTELLPKRPQHLPGHPGWYRKLCPRLDDPAWTPGPSLLSLQMRVTP
KLMALTWDGFPLHYSERHGWGYLVPGRRDNLAKLPTGTTLESAGVVCPYRAIESLYRKHC
LEQGKQQLMPQEAGLAEEFLLTDNSAIWQTVEELDYLEVEAEAKMENLRAAVPGQPLALT
ARGGPKDTQPSYHHGNGPYNDVDIPGCWFFKLPHKDGNSCNVGSPFAKDFLPKMEDGTLQ
AGPGGASGPRALEINKMISFWRNAHKRISSQMVVWLPRSALPRAVIRHPDYDEEGLYGAI
LPQVVTAGTITRRAVEPTWLTASNARPDRVGSELKAMVQAPPGYTLVGADVDSQELWIAA
VLGDAHFAGMHGCTAFGWMTLQGRKSRGTDLHSKTATTVGISREHAKIFNYGRIYGAGQP
FAERLLMQFNHRLTQQEAAEKAQQMYAATKGLRWYRLSDEGEWLVRELNLPVDRTEGGWI
SLQDLRKVQRETARKSQWKKWEVVAERAWKGGTESEMFNKLESIATSDIPRTPVLGCCIS
RALEPSAVQEEFMTSRVNWVVQSSAVDYLHLMLVAMKWLFEEFAIDGRFCISIHDEVRYL
VREEDRYRAALALQITNLLTRCMFAYKLGLNDLPQSVAFFSAVDIDRCLRKEVTMDCKTP
SNPTGMERRYGIPQGEALDIYQIIELTKGSLEKRSQPGP*
>ENSG00000145191|ENST00000273783
MAAPVVAPPGVVVSRANKRSGAGPGGSGGGGARGAEEEPPPPLQAVLVADSFDRRFFPIS
KDQPRVLLPLANVALIDYTLEFLTATGVQETFVFCCWKAAQIKEHLLKSKWCRPTSLNVV
RIITSELYRSLGDVLRDVDAKALVRSDFLLVYGDVISNINITRALEEHRLRRKLEKNVSV
MTMIFKESSPSHPTRCHEDNVVVAVDSTTNRVLHFQKTQGLRRFAFPLSLFQGSSDGVEV
RYDLLDCHISICSPQVAQLFTDNFDYQTRDDFVRGLLVNEEILGNQIHMHVTAKEYGARV
SNLHMYSAVCADVIRRWVYPLTPEANFTDSTTQSCTHSRHNIYRGPEVSLGHGSILEENV
LLGSGTVIGSNCFITNSVIGPGCHIGDNVVLDQTYLWQGVRVAAGAQIHQSLLCDNAEVK
ERVTLKPRSVLTSQVVVGPNITLPEGSVISLHPPDAEEDEDDGEFSDDSGADQEKDKVKM
KGYNPAEVGAAGKGYLWKAAGMNMEEEEELQQNLWGLKINMEEESESESEQSMDSEEPDS
RGGSPQMDDIKVFQNEVLGTLQRGKEENISCDNLVLEINSLKYAYNISLKEVMQVLSHVV
LEFPLQQMDSPLDSSRYCALLLPLLKAWSPVFRNYIKRAADHLEALAAIEDFFLEHEALG
ISMAKVLMAFYQLEILAEETILSWFSQRDTTDKGQQLRKNQQLQRFIQWLKEAEEESSED
D*
>ENSG00000163884|ENST00000296233
MVDHLLPVDENFSSPKCPVGYLGDRLVGRRAYHMLPSPVSEDDSDASSPCSCSSPDSQAL
CSCYGGGLGTESQDSILDFLLSQATLGSGGGSGSSIGASSGPVAWGPWRRAAAPVKGEHF
CLPEFPLGDPDDVPRPFQPTLEEIEEFLEENMEPGVKEVPEGNSKDLDACSQLSAGPHKS

HLHPGSSGRERCSPPPGGASAGGAQGPGGGPTPDGPIPVLLQIQPVPVKQESGTGPASPG
QAPENVKVAQLLVNIQGQTFALVPQVVPSSNLNLPSKFVRIAPVPIAAKPVGSGPLGPGP
AGLLMGQKFPKNPAAELIKMHKCTFPGCSKMYTKSSHLKAHLRRHTGEKPFACTWPGCGW
RFSRSDELSRHRRSHSGVKPYQCPVCEKKFARSDHLSKHIKVHRFPRSSRSVRSVN*
>ENSG00000132170|ENST00000309576
MTMVDTEMPFWPTNFGISSVDLSVMEDHSHSFDIKPFTTVDFSSISTPHYEDIPFTRTDP
VVADYKYDLKLQEYQSAIKVEPASPPYYSEKTQLYNKPHEEPSNSLMAIECRVCGDKASG
FHYGVHACEGCKGFFRRTIRLKLIYDRCDLNCRIHKKSRNKCQYCRFQKCLAVGMSHNAI
RFGRMPQAEKEKLLAEISSDIDQLNPESADLRALAKHLYDSYIKSFPLTKAKARAILTGK
TTDKSPFVIYDMNSLMMGEDKIKFKHITPLQEQSKEVAIRIFQGCQFRSVEAVQEITEYA
KSIPGFVNLDLNDQVTLLKYGVHEIIYTMLASLMNKDGVLISEGQGFMTREFLKSLRKPF
GDFMEPKFEFAVKFNALELDDSDLAIFIAVIILSGDRPGLLNVKPIEDIQDNLLQALELQ
LKLNHPESSQLFAKLLQKMTDLRQIVTEHVQLLQVIKKTETDMSLHPLLQEIYKDLY*
>ENSG00000069667|ENST00000309157
MNEGAPGDSDLETEARVPWSIMGHCLRTGQARMSATPTPAGEGARSSSTCSSLSRLFWSQ
LEHINWDGATAKNFINLREFFSFLLPALRKAQIEIIPCKICGDKSSGIHYGVITCEGCKG
FFRRSQQSNATYSCPRQKNCLIDRTSRNRCQHCRLQKCLAVGMSRDAVKFGRMSKKQRDS
LYAEVQKHRMQQQQRDHQQQPGEAEPLTPTYNISANGLTELHDDLSNYIDGHTPEGSKAD
SAVSSFYLDIQPSPDQSGLDINGIKPEPICDYTPASGFFPYCSFTNGETSPTVSMAELEH
LAQNISKSHLETCQYLREELQQITWQTFLQEEIENYQNKQREVMWQLCAIKITEAIQYVV
EFAKRIDGFMELCQNDQIVLLKAGSLEVVFIRMCRAFDSQNNTVYFDGKYASPDVFKSLG
CEDFISFVFEFGKSLCSMHLTEDEIALFSAFVLMSADRSWLQEKVKIEKLQQKIQLALQH
VLQKNHREDGILTKLICKVSTLRALCGRHTEKLMAFKAIYPDIVRLHFPPLYKELFTSEF
EPAMQIDG*
>ENSG00000163684|ENST00000295959
MPAPAATYERVVYKNPSEYHYMKVCLEFQDCGVGLNAAQFKQLLISAVKDLFGEVDAALP
LDILTYEEKTLSAILRICSSGLVKLWSSLTLLGSYKGKKCAFRVIQVSPFLLALSGNSRE
LVLD*
>ENSG00000122026|ENST00000311549
MTNTKGKRRGTRYMFSRPFRKHGVVPLATYMRIYKKGDIVDIKGMGTVQKGMPHKCYHGK
TGRVYNVTQHAVGIVVNKQVKGKILAKRINVRIEHIKHSKSRDSFLKRVKENDQKKKEAK
EKGTWVQLKRQPAPPREAHFVRTNGKEPELLEPIPYEFMA*
>ENSG00000172888|ENST00000310898
MLQTTWPQESVTFEDVAVYFTQNQWASLDPAQRALYGEVMLENYANVASLVAFPFPKPAL
ISHLERGEAPWGPDPWDTEILRGISQGGESWIKNEGLVIKQEASEETELHRMPVGGLLRN
VSQHFDFKRKALKQTFNLNPNLILRGGSVEVRSLRPA*
>ENSG00000005889|ENST00000304543
MDEDGLELQQEPNSFFDATGADGTHMDGDQIVVEVQETVFVSDVVDSDITVHNFVPDDPD
SVVIQDVIEDVVIEDVQCPDIMEEADVSETVIIPEQVLDSDVTEEVSLAHCTVPDDVLAS
DITSASMSMPEHVLTGDSIHVSDVGHVGHVGHVEHVVHDSVVEAEIVTDPLTTDVVSEEV
LVADCASEAVIDANGIPVDQQDDDKGNCEDYLMISLDDAGKIEHDGSSGMTMDTESEIDP
CKVDGTCPEVIKVYIFKADPGEDDLGGTVDIVESEPENDHGVELLDQNSSIRVPREKMVY
MTVNDSQPEDEDLNVAEIADEVYMEVIVGEEDAAAAAAAAAVHEQQMDDNEIKTFMPIAW
AAAYGNNSDGIENRNGTASALLHIDESAGLGRLAKQKPKKRRRPDSRQYQTAIIIGPDGH

PLTVYPCMICGKKFKSRGFLKRHMKNHPEHLAKKKYRCTDCDYTTNKKISLHNHLESHKL
TSKAEKAIECDECGKHFSHAGALFTHKMVHKEKGANKMHKCKFCEYETAEQGLLNRHLLA
VHSKNFPHICVECGKGFRHPSELKKHMRIHTGEKPYQCQYCEYRSADSSNLKTHVKTKHS
KEMPFKCDICLLTFSDTKEVQQHALIHQESKTHQCLHCDHKSSNSSDLKRHIISVHTKDY
PHKCDMCDKGFHRPSELKKHVAAHKGKKMHQCRHCDFKIADPFVLSRHILSVHTKDLPFR
CKRCRKGFRQQSELKKHMKTHSGRKVYQCEYCEYSTTDASGFKRHVISIHTKDYPHRCEY
CKKGFRRPSEKNQHIMRHHKEVGLP*
>ENSG00000170430|ENST00000306010
MLGQPAPLERFASRRPQVLAVRTVCDLVLGKMDKDCEMKRTTLDSPLGKLELSGCEQGLH
EIKLLGKGTSAADAVEVPAPAAVLGGPEPLMQCTAWLNAYFHQPEAIEEFPVPALHHPVF
QQESFTRQVLWKLLKVVKFGEVISYQQLAALAGNPKAARAVGGAMRGNPVPILIPCHRVV
CSSGAVGNYSGGLAVKEWLLAHEGHRLGKPGLGGSSGLAGAWLKGAGATSGSPPAGRN*
>ENSG00000004534|ENST00000266022
MWGDSRPANRTGPFRGSQEERFAPGWNRDYPPPPLKSHAQERHSGNFPGRDSLPFDFQGH
SGPPFANVEEHSFSYGARDGPHGDYRGGEGPGHDFRGGDFSSSDFQSRDSSQLDFRGRDI
HSGDFRDREGPPMDYRGGDGTSMDYRGREAPHMNYRDRDAHAVDFRGRDAPPSDFRGRGT
YDLDFRGRDGSHADFRGRDLSDLDFRAREQSRSDFRNRDVSDLDFRDKDGTQVDFRGRGS
GTTDLDFRDRDTPHSDFRGRHRSRTDQDFRGREMGSCMEFKDREMPPVDPNILDYIQPST
QDREHSGMNVNRREESTHDHTIERPAFGIQKGEFEHSETREGETQGVAFEHESPADFQNS
QSPVQDQDKSQLSGREEQSSDAGLFKEEGGLDFLGRQDTDYRSMEYRDVDHRLPGSQMFG
YGQSKSFPEGKTARDAQRDLQDQDYRTGPSEEKPSRLIRLSGVPEDATKEEILNAFRTPD
GMPVKNLQLKEYNTGYDYGYVCVEFSLLEDAIGCMEANQGTLMIQDKEVTLEYVSSLDFW
YCKRCKANIGGHRSSCSFCKNPREVTEAKQELITYPQPQKTSIPAPLEKQPNQPLRPADK
EPEPRKREEGQESRLGHQKREAERYLPPSRREGPTFRRDRERESWSGETRQDGESKTIML
KRIYRSTPPEVIVEVLEPYVRLTTANVRIIKNRTGPMGHTYGFIDLDSHAEALRVVKILQ
NLDPPFSIDGKMVAVNLATGKRRNDSGDHSDHMHYYQGKKYFRDRRGGGRNSDWSSDTNR
QGQQSSSDCYIYDSATGYYYDPLAGTYYDPNTQQEVYVPQDPGLPEEEEIKEKKPTSQGK
SSSKKEMSKRDGKEKKDRGVTRFQENASEGKAPAEDVFKKPLPPTVKKEESPPPPKVVNP
LIGLLGEYGGDSDYEEEEEEEEQTPPPQPRTAQPQKREEQTKKENEEDKLTDWNKLACLLC
RRQFPNKEVLIKHQQLSDLHKQNLEIHRKIKQSEQELAYLERREREGKFKGRGNDRREKL
QSFDSPERKRIKYSRETDSDRKLVDKEDIDTSSKGGCVQQATGWRKGTGLGYGHPGLASS
EEAEGRMRGPSVGASGRTSKRQSNETYRDAVRRVMFARYKELD*
>ENSG00000140044|ENST00000267569
MVAGWPATPPAMMPGQIPDPSVTTGSLPGLGPLTGLPSSALTVEELKYADIRNLGAMIAP
LHFLEVKLGKRPQPVKSELDEEEERRKRRREKNKVAAARCRNKKKERTEFLQRESERLEL
MNAELKTQIEELKQERQQLILMLNRHRPTCIVRTDSVKTPESEGNPLLEQLEKK*
>ENSG00000148704|ENST00000277905
MFGKPDKMDVRCHSDAEAARVSKNAHKESRESKGAEGNLPAAFLKEPQGAFSASGAAEDC
NKSKSNSAADPDYCRRILVRDAKGSIREIILPKGLDLDRPKRTRTSFTAEQLYRLEMEFQ
RCQYVVGRERTELARQLNLSETQANSEENNERFKRGIKKQKKKRKKEPANDESRRGDSGG
RGWQPL*
>ENSG00000166349|ENST00000299440
MAASFPPTLGLSSAPDEIQHPHIKFSEWKFKLFRVRSFEKTPEEAQKEKKDSFEGKPSLE
QSPAVLDKADGQKPVPTQPLLKAHPKFSKKFHDNEKARGKAIHQANLRHLCRICGNSFRA

DEHNRRYPVHGPVDGKTLGLLRKKEKRATSWPDLIAKVFRIDVKADVDSIHPTEFCHNCW
SIMHRKFSSAPCEVYFPRNVTMEWHPHTPSCDICNTARRGLKRKSLQPNLQLSKKLKTVL
DQARQARQHKRRAQARISSKDVMKKIANCSKIHLSTKLLAVDFPEHFVKSISCQICEHIL
ADPVETNCKHVFCRVCILRCLKVMGSYCPSCRYPCFPTDLESPVKSFLSVLNSLMVKCPA
KECNEEVSLEKYNHHISSHKESKEIFVHINKGGRPRQHLLSLTRRAQKHRLRELKLQVKA
FADKEEGGDVKSVCMTLFLLALRARNEHRQADELEAIMQGKGSGLQPAVCLAIRVNTFLS
CSQYHKMYRTVKAITGRQIFQPLHALRNAEKVLLPGYHHFEWQPPLKNVSSSTDVGIIDG
LSGLSSSVDDYPVDTIAKRFRYDSALVSALMDMEEDILEGMRSQDLDDYLNGPFTVVVKE
SCDGMGDVSEKHGSGPVVPEKAVRFSFTIMKITIAHSSQNVKVFEEAKPNSELCCKPLCL
MLADESDHETLTAILSPLIAEREAMKSSELMLELGGILRTFKFIFRGTGYDEKLVREVEG
LEASGSVYICTLCDATRLEASQNLVFHSITRSHAENLERYEVWRSNPYHESVEELRDRVK
GVSAKPFIETVPSIDALHCDIGNAAEFYKIFQLEIGEVYKNPNASKEERKRWQATLDKHL
RKKMNLKPIMRMNGNFARKLMTKETVDAVCELIPSEERHEALRELMDLYLKMKPVWRSSC
PAKECPESLCQYSFNSQRFAELLSTKFKYRYEGKITNYFHKTLAHVPEIIERDGSIGAWA
SEGNESGNKLFRRFRKMNARQSKCYEMEDVLKHHWLYTSKYLQKFMNAHNALKTSGFTMN
PQASLGDPLGIEDSLESQDSMEF*
>ENSG00000039600|ENST00000311371
MERARPEPPPQPRPLRPAPPPLPVEGTSFWAAAMEPPPSSPTLSAAASATLASSCGEAVA
SGLQPAVRRLLQVKPEQVLLLPQPQAQNEEAAASSAQARLLQFRPDLRLLQPPTASDGAT
SRPELHPVQPLALHVKAKKQKLGPSLDQSVGPRGAVETGPRASRVVKLEGPGPALGYFRG
DEKGKLEAEEVMRDSMQGGAGKSPAAIREGVIKTEEPERLLEDCRLGAEPASNGLVHGSA
EVILAPTSGAFGPHQQDLRIPLTLHTVPPGARIQFQGAPPSELIRLTKVPLTPVPTKMQS
LLEPSVKIETKDVPLTVLPSDAGIPDTPFSKDRNGHVKRPMNAFMVWARIHRPALAKANP
AANNAEISVQLGLEWNKLSEEQKKPYYDEAQKIKEKHREEFPGWVYQPRPGKRKRFPLSV
SNVFSGTTQNIISTNPTTVYPYRSPTYSVVIPSLQNPITHPVVHALTVGLPLAMEIFRVQ
CQNALVIMKTGTQNMRVSFQL*
>ENSG00000143995|ENST00000272369
MAQRYDDLPHYGGMDGVGIPSTMYGDPHAARSMQPVHHLNHGPPLHSHQYPHTAHTNAMA
PSMGSSVNDALKRDKDAIYGHPLFPLLALIFEKCELATCTPREPGVAGGDVCSSESFNED
IAVFAKQIRAEKPLFSSNPELDNLMIQAIQVLRFHLLELEKVHELCDNFCHRYISCLKGK
MPIDLVIDDREGGSKSDSEDITRSANLTDQPSWNRDHDDTASTRSGGTPGPSSGGHTSHS
GDNSSEQGDGLDNSVASPSTGDDDDPDKDKKRHKKRGIFPKVATNIMRAWLFQHLTHPYP
SEEQKKQLAQDTGLTILQVNNWFINARRRIVQPMIDQSNRAVSQGTPYNPDGQPMGGFVM
DGQQHMGIRAPGPMSGMGMNMGMEGQWHYM*
>ENSG00000161914|ENST00000293771
MAERALEPEAEAEAEAGAGGEAAAEEGAAGRKARGRPRLTESDRARRRLESRKKYDVRRV
YLGEAHGPWVDLRRRSGWSDAKLAAYLISLERGQRSGRHGKPWEQVPKKPKRKKRRRRNV
NCLKNVVIWYEDHKHRCPYEPHLAELDPTFGLYTTAVWQCEAGHRYFQDLHSPLKPLSDS
DPDSDKVGNGLVAGSSDSSSSGSASDSEESPEGQPVKAAAAAAAATPTSPVGSSGLITQE
GVHIPFDVHHVESLAEQGTPLCSNPAGNGPEALETVVCVPVPVQVGAGPSALFENVPQEA
LGEVVASCPMPGMVPGSQVIIIAGPGYDALTAEGIHLNMAAGSGVPGSGLGEEVPCAMME
GVAAYTQTEPEGSQPSTMDATAVAGIETKKEKEDLCLLKKEEKEEPVAPELATTVPESAE
PEAEADGEELDGSDMSAIIYEIPKEPEKRRRSKRSRVMDADGLLEMFHCPYEGCSQVYVA
LSSFQNHVNLVHRKGKTKVCPHPGCGKKFYLSNHLRRHMIIHSGVREFTCETCGKSFKRK

NHLEVHRRTHTGETPLQCEICGYQCRQRASLNWHMKKHTAEVQYNFTCDRCGKRFEKLDS
VKFHTLKSHPDHKPT*
>ENSG00000113013|ENST00000297185
MISASRAAAARLVGAAASRGPTAARHQDSWNGLSHEAFRLVSRRDYASEAIKGAVVGIDL
GTTNSCVAVMEGKQAKVLENAEGARTTPSVVAFTADGERLVGMPAKRQAVTNPNNTFYAT
KRLIGRRYDDPEVQKDIKNVPFKIVRASNGDAWVEAHGKLYSPSQIGAFVLMKMKETAEN
YLGHTAKNAVITVPAYFNDSQRQATKDAGQISGLNVLRVINEPTAAALAYGLDKSEDKVI
AVYDLGGGTFDISILEIQKGVFEVKSTNGDTFLGGEDFDQALLRHIVKEFKRETGVDLTK
DNMALQRVREAAEKAKCELSSSVQTDINLPYLTMDSSGPKHLNMKLTRAQFEGIVTDLIR
RTIAPCQKAMQDAEVSKSDIGEVILVGGMTRMPKVQQTVQDLFGRAPSKAVNPDEAVAIG
AAIQGGVLAGDVTDVLLLDVTPLSLGIETLGGVFTKLINRNTTIPTKKSQVFSTAADGQT
QVEIKVCQGEREMAGDNKLLGQFTLIGIPPAPRGVPQIEVTFDIDANGIVHVSAKDKGTG
REQQIVIQSSGGLSKDDIENMVKNAEKYAEEDRRKKERVEAVNMAEGIIHDTETKMEEFK
DQLPADECNKLKEEISKMRELLARKDSETGENIRQAASSLQQASLKLFEMAYKKMASERE
GSGSSGTGEQKEDQKEEKQ*
>ENSG00000152795|ENST00000295470
MEVPPRLSHVPPPLFPSAPATLASRSLSHWRPRPPRQLAPLLPSLAPSSARQGARRAQRH
VTAQQPSRLAGGAAIKGGRRRRPDLFRRHFKSSSIQRSAAAAAATRTARQHPPADSSVTM
EDMNEYSNIEEFAEGSKINASKNQQDDGKMFIGGLSWDTSKKDLTEYLSRFGEVVDCTIK
TDPVTGRSRGFGFVLFKDAASVDKVLELKEHKLDGKLIDPKRAKALKGKEPPKKVFVGGL
SPDTSEEQIKEYFGAFGEIENIELPMDTKTNERRGFCFITYTDEEPVKKLLESRYHQIGS
GKCEIKVAQPKEVYRQQQQQQKGGRGAAAGGRGGTRGRGRGQGQNWNQGFNNYYDQGYGN
YNSAYGGDQNYSGYGGYDYTGYNYGNYGYGQGYADYSGQQSTYGKASRGGGNHQNNYQPY
*
>ENSG00000126456|ENST00000309877
MGTPKPRILPWLVSQLDLGQLEGVAWVNKSRTRFRIPWKHGLRQDAQQEDFGIFQAWAEA
TGAYVPGRDKPDLPTWKRNFRSALNRKEGLRLAEDRSKDPHDPHKIYEFVNSGVGDFSQP
DTSPDTNGGGSTSDTQEDILDELLGNMVLAPLPDPGPPSLAVAPEPCPQPLRSPSLDNPT
PFPNLGPSENPLKRLLVPGEEWEFEVTAFYRGRQVFQQTISCPEGLRLVGSEVGDRTLPG
WPVTLPDPGMSLTDRGVMSYVRHVLSCLGGGLALWRAGQWLWAQRLGHCHTYWAVSEELL
PNSGHGPDGEVPKDKEGGVFDLGPFIVDLITFTEGSGRSPRYALWFCVGESWPQDQPWTK
RLVMVKVVPTCLRALVEMARVGGASSLENTVDLHISNSHPLSLTSDQYKAYLQDLVEGMD
FQGPGES*
>ENSG00000156239|ENST00000303775
MAGENFATPFHGHVGRGAFSDVYEPAEDTFLLLDALEAAAAELAGVEICLEVGSGSGVVS
AFLASMIGPQALYMCTDINPEAAACTLETARCNKVHIQPVITDLVKGLLPRLTEKVDLLV
FNPPYVVTPPQEVGSHGIEAAWAGGRNGREVMDRFFPLVPDLLSPRGLFYLVTIKENNPE
EILKIMKTKGLQGTTALSRQAGQETLSVLKFTKS*
>ENSG00000175376|ENST00000312234
MSQATKRKHVVKEVLGEHIVPSDQQQIVRVLRTPGNNLHEVETAQGQRFLVSMPSKYRKN
IWIKRGDFLIVDPIEEGEKVKAEISFVLCKDHVRSLQKEGFWPEAFSEVAEKHNNRNRQT
QPELPAEPQLSGEESSSEDDSDLFVNTNRRQYHESEEESEEEEAA*
>ENSG00000157657|ENST00000288466
MNQPGGAAAPQADGASAAGRKSTASRERLKRSQKSTKVEGPEPVPAEASLSAEQGTMTEV

KVKTELPDDYIQEVIWQGEAKEEKKAVSKDGTSDVPAEICVVIGGVRNQQTLGSYECGIC
GKKYKYYNCFQTHVRAHRDTEATSGEGASQSNNFRYTCDICGKKYKYYSCFQEHRDLHAV
DVFSVEGAPENRADPFDQGVVATDEVKEEPPEPFQKIGPMNNITSDIFKKKEVRQCQKRE
TGNYTCEFCGKQYKYYTPYQEHVALHAPIKSAFSRRVEGKAQNHFEETNSSSQNSSEPYT
CGACGIQFQFYNNLLEHMQSHAADNENNIASNQSRSPPAVVEEKWKPQAQRNSANNTTTS
GLTPNSMIPEKERQNIAERLLRVMCADLGALSVVSGKEFLKLAQTLVDSGARYGAFSVTE
ILGNFNTLALKHLPRMYNQVKVKVTCALGSNACLGIGVTCHSQSVGPDSCYILTAYQAEG
NHIKSYVLGVKGADIRDSGDLVHHWVQNVLSEFVMSEIRTVYVTDCRVSTSAFSKAGMCL
RCSACALNSVVQSVLSKRTLQARSMHEVIELLNVCEDLAGSTGLAKETFGSLEETSPPPC
WNSVTDSLLLVHERYEQICEFYSRAKKMNLIQSLNKHLLSNLAAILTPVKQAVIELSNES
QPTLQLVLPTYVRLEKLFTAKANDAGTVSKLCHLFLEALKENFKVHPAHKVAMILDPQQK
LRPVPPYQHEEIIGKVCELINEVKESWAEEADFEPAAKKPRSAAVENPAAQEDDRLGKNE
VYDYLQEPLFQATPDLFQYWSCVTQKHTKLAKLAFWLLAVPAVGARSGCVNMCEQALLIK
RRRLLSPEDMNKLMFLKSNML*
>ENSG00000133026|ENST00000269243
MAQRTGLEDPERYLFVDRAVIYNPATQADWTAKKLVWIPSERHGFEAASIKEERGDEVMV
ELAENGKKAMVNKDDIQKMNPPKFSKVEDMAELTCLNEASVLHNLKDRYYSGLIYTYSGL
FCVVINPYKNLPIYSENIIEMYRGKKRHEMPPHIYAISESAYRCMLQDREDQSILCTGES
GAGKTENTKKVIQYLAHVASSHKGRKDHNIPGELERQLLQANPILESFGNAKTVKNDNSS
RFGKFIRINFDVTGYIVGANIETYLLEKSRAVRQAKDERTFHIFYQLLSGAGEHLKSDLL
LEGFNNYRFLSNGYIPIPGQQDKDNFQETMEAMHIMGFSHEEILSMLKVVSSVLQFGNIS
FKKERNTDQASMPENTVAQKLCHLLGMNVMEFTRAILTPRIKVGRDYVQKAQTKEQADFA
VEALAKATYERLFRWLVHRINKALDRTKRQGASFIGILDIAGFEIFELNSFEQLCINYTN
EKLQQLFNHTMFILEQEEYQREGIEWNFIDFGLDLQPCIDLIERPANPPGVLALLDEECW
FPKATDKTFVEKLVQEQGSHSKFQKPRQLKDKADFCIIHYAGKVDYKADEWLMKNMDPLN
DNVATLLHQSSDRFVAELWKDVDRIVGLDQVTGMTETAFGSAYKTKKGMFRTVGQLYKES
LTKLMATLRNTNPNFVRCIIPNHEKRAGKLDPHLVLDQLRCNGVLEGIRICRQGFPNRIV
FQEFRQRYEILTPNAIPKGFMDGKQACERMIRALELDPNLYRIGQSKIFFRAGVLAHLEE
ERDLKITDIIIFFQAVCRGYLARKAFAKKQQQLSALKVLQRNCAAYLKLRHWQWWRVFTK
VKPLLQVTRQEEELQAKDEELLKVKEKQTKVEGELEEMERKHQQLLEEKNILAEQLQAET
ELFAEAEEMRARLAAKKQELEEILHDLESRVEEEEERNQILQNEKKKMQAHIQDLEEQLD
EEEGARQKLQLEKVTAEAKIKKMEEEILLLEDQNSKFIKEKKLMEDRIAECSSQLAEEEE
KAKNLAKIRNKQEVMISDLEERLKKEEKTRQELEKAKRKLDGETTDLQDQIAELQAQIDE
LKLQLAKKEEELQGALARGDDETLHKNNALKVVRELQAQIAELQEDFESEKASRNKAEKQ
KRDLSEELEALKTELEDTLDTTAAQQELRTKREQEVAELKKALEEETKNHEAQIQDMRQR
HATALEELSEQLEQAKRFKANLEKNKQGLETDNKELACEVKVLQQVKAESEHKRKKLDAQ
VQELHAKVSEGDRLRVELAEKASKLQNELDNVSTLLEEAEKKGIKFAKDAASLESQLQDT
QELLQEETRQKLNLSSRIRQLEEEKNSLQEQQEEEEEARKNLEKQVLALQSQLADTKKKV
DDDLGTIESLEEAKKKLLKDAEALSQRLEEKALAYDKLEKTKNRLQQELDDLTVDLDHQR
QVASNLEKKQKKFDQLLAEEKSISARYAEERDRAEAEAREKETKALSLARALEEALEAKE
EFERQNKQLRADMEDLMSSKDDVGKNVHELEKSKRALEQQVEEMRTQLEELEDELQATED
AKLRLEVNMQAMKAQFERDLQTRDEQNEEKKRLLIKQVRELEAELEDERKQRALAVASKK
KMEIDLKDLEAQIEAANKARDEVIKQLRKLQAQMKDYQRELEEARASRDEIFAQSKESEK
KLKSLEAEILQLQEELASSERARRHAEQERDELADEITNSASGKSALLDEKRRLEARIAQ

LEEELEEEQSNMELLNDRFRKTTLQVDTLNAELAAERSAAQKSDNARQQLERQNKELKAK
LQELEGAVKSKFKATISALEAKIGQLEEQLEQEAKERAAANKLVRRTEKKLKEIFMQVED
ERRHADQYKEQMEKANARMKQLKRQLEEAEEEATRANASRRKLQRELDDATEANEGLSRE
VSTLKNRLRRGGPISFSSSRSGRRQLHLEGASLELSDDDTESKTSDVNETQPPQSE*
>ENSG00000165392|ENST00000298139
MSEKKLETTAQQRKCPEWMNVQNKRCAVEERKACVRKSVFEDDLPFLEFTGSIVYSYDAS
DCSFLSEDISMSLSDGDVVGFDMEWPPLYNRGKLGKVALIQLCVSESKCYLFHVSSMSVF
PQGLKMLLENKAVKKAGVGIEGDQWKLLRDFDIKLKNFVELTDVANKKLKCTETWSLNSL
VKHLLGKQLLKDKSIRCSNWSKFPLTEDQKLYAATDAYAGFIIYRNLEILDDTVQRFAIN
KEEEILLSDMNKQLTSISEEVMDLAKHLPHAFSKLENPRRVSILLKDISENLYSLRRMII
GSTNIETELRPSNNLNLLSFEDSTTGGVQQKQIREHEVLIHVEDETWDPTLDHLAKHDGE
DVLGNKVERKEDGFEDGVEDNKLKENMERACLMSLDITEHELQILEQQSQEEYLSDIAYK
STEHLSPNDNENDTSYVIESDEDLEMEMLKHLSPNDNENDTSYVIESDEDLEMEMLKSLE
NLNSGTVEPTHSKCLKMERNLGLPTKEEEEDDENEANEGEEDDDKDFLWPAPNEEQVTCL
KMYFGHSSFKPVQWKVIHSVLEERRDNVAVMATGYGKSLCFQYPPVYVGKIGLVISPLIS
LMEDQVLQLKMSNIPACFLGSAQSENVLTDIKLGKYRIVYVTPEYCSGNMGLLQQLEADI
GITLIAVDEAHCISEWGHDFRDSFRKLGSLKTALPMVPIVALTATASSSIREDIVRCLNL
RNPQITCTGFDRPNLYLEVRRKTGNILQDLQPFLVKTSSHWEFEGPTIIYCPSRKMTQQV
TGELRKLNLSCGTYHAGMSFSTRKDIHHRFVRDEIQCVIATIAFGMGINKADIRQVIHYG
APKDMESYYQEIGRAGRDGLQSSCHVLWAPADINLNRHLLTEIRNEKFRLYKLKMMAKME
KYLHSSRCRRQIILSHFEDKQVQKASLGIMGTEKCCDNCRSRLDHCYSMDDSEDTSWDFG
PQAFKLLSAVDILGEKFGIGLPILFLRGSNSQRLADQYRRHSLFGTGKDQTESWWKAFSR
QLITEGFLVEVSRYNKFMKICALTKKGRNWLHKANTESQSLILQANEELCPKKLLLPSSK
TVSSGTKEHCYNQVPVELSTEKKSNLEKLYSYKPCDKISSGSNISKKSIMVQSPEKAYSS
SQPVISAQEQETQIVLYGKLVEARQKHANKMDVPPAILATNKILVDMAKMRPTTVENVKR
IDGVSEGKAAMLAPLLEVIKHFCQTNSVQTDLFSSTKPQEEQKTSLVAKNKICTLSQSMA
ITYSLFQEKKMPLKSIAESRILPLMTIGMHLSQAVKAGCPLDLERAGLTPEVQKIIADVI
RNPPVNSDMSKISLIRMLVPENIDTYLIHMAIEILKHGPDSGLQPSCDVNKRRCFPGSEE
ICSSSKRSKEEVGINTETSSAERKRRLPVWFAKGSDTSKKLMDKTKRGGLFS*
>ENSG00000149273|ENST00000278572
MAVQISKKRKFVADGIFKAELNEFLTRELAEDGYSGVEVRVTPTRTEIIILATRTQNVLG
EKGRRIRELTAVVQKRFGFPEGSVELKIMVMVTGYPLLPLKLYAEKVATRGLCAIAQAES
LRYKLLGGLAVRRACYGVLRFIMESGAKGCEVVVSGKLRGQRAKSMKFVDGLMIHSGDPV
NYYVDTAVRHVLLRQGVLGIKVKIMLPWDPTGKIGPKKPLPDHVSIVEPKDEILPTTPIS
EQKGGKPEPPAMPQPVPTA*
>ENSG00000161970|ENST00000293842
MKFNPFVTSDRSKNRKRHFNAPSHIRRKIMSSPLSKELRQKYNVRSMPIRKDDEVQVVRG
HYKGQQIGKVVQVYRKKYVIYIERVQREKANGTTVHVGIHPSKVVITRLKLDKDRKKILE
RKAKSRQVGKEKGKYKEETIEKMQE*
>ENSG00000145425|ENST00000274065
MAVGKNKRLTKGGKKGAKKKVVDPFSKKDWYDVKAPAMFNIRNIGKTLVTRTQGTKIASD
GLKGRVFEVSLADLQNDEVAFRKFKLITEDVQGKNCLTNFHGMDLTRDKMCSMVKKWQTM
IEAHVDVKTTDGYLLRLFCVGFTKKRNNQIRKTSYAQHQQVRQIRKKMMEIMTREVQTND
LKEVVNKLIPDSIGKDIEKACQSIYPLHDVFVRKVKMLKKPKFELGKLMELHGEGSSSGK

ATGDETGAKVERADGYEPPVQESV*
>ENSG00000140464|ENST00000268058
MEPAPARSPRPQQDPARPQEPTMPPPETPSEGRQPSPSPSPTERAPASEEEFQFLRCQQC
QAEAKCPKLLPCLHTLCSGCLEASGMQCPICQAPWPLGADTPALDNVFFESLQRRLSVYR
QIVDAQAVCTRCKESADFWCFECEQLLCAKCFEAHQWFLKHEARPLAELRNQSVREFLDG
TRKTNNIFCSNPNHRTPTLTSIYCRGCSKPLCCSCALLDSSHSELKCDISAEIQQRQEEL
DAMTQALQEQDSAFGAVHAQMHAAVGQLGRARAETEELIRERVRQVVAHVRAQERELLEA
VDARYQRDYEEMASRLGRLDAVLQRIRTGSALVQRMKCYASDQEVLDMHGFLRQALCRLR
QEEPQSLQAAVRTDGFDEFKVRLQDLSSCITQGKDAAVSKKASPEAASTPRDPIDVDLPE
EAERVKAQVQALGLAEAQPMAVVQSVPGAHPVPVYAFSIKGPSYGEDVSNTTTAQKRKCS
QTQCPRKVIKMESEEGKEARLARSSPEQPRPSTSKAVSPPHLDGPPSPRSPVIGSEVFLP
NSNHVASGAGEAEERVVVISSSEDSDAENSSSRELDDSSSESSDLQLEGPSTLRVLDENL
ADPQAEDRPLVFFDLKIDNETQKISQLAAVNRESKFRVVIQPEAFFSIYSKAVSLEVGLQ
HFLSFLSSMRRPILACYKLWGPGLPNFFRALEDINRLWEFQEAISGFLAALPLIRERVPG
ASSFKLKNLAQTYLARNMSERSAMAAVLAMRDLCRLLEVSPGPQLAQHVYPFSSLQCFAS
LQPLVQAAVLPRAEARLLALHNVSFMELLSAHRRDRQGGLKKYSRYLSLQTTTLPPAQPA
FNLQALGTYFEGLLEGPALARAEGVSTPLAGRGLAERASQQS*
>ENSG00000161940|ENST00000293805
MGSPAAPEGALGYVREFTRHSSDVLGNLNELRLRGILTDVTLLVGGQPLRAHKAVLIACS
GFFYSIFRGRAGVGVDVLSLPGGPEARGFAPLLDFMYTSRLRLSPATAPAVLAAATYLQM
EHVVQACHRFIQASYEPLGISLRPLEAEPPTPPTAPPPGSPRRSEGHPDPPTESRSCSQG
PPSPASPDPKACNWKKYKYIVLNSQASQAGSLVGERSSGQPCPQARLPSGDEASSSSSSS
SSSSEEGPIPGPQSRLSPTAATVQFKCGAPASTPYLLTSQAQDTSGSPSERARPLPGSEF
FSCQNCEAVAGCSSGLDSLVPGDEDKPYKCQLCRSSFRYKGNLASHRTVHTGEKPYHCSI
CGARFNRPANLKTHSRIHSGEKPYKCETCGSRFVQVAHLRAHVLIHTGEKPYPCPTCGTR
FRHLQTLKSHVRIHTGEKPYHCDPCGLHFRHKSQLRLHLRQKHGAATNTKVHYHILGGP*
>ENSG00000172348|ENST00000306764
MRGESYFIGMRSPGQQGHVPEDGGLFLLCCIDRDWAVTRCFAEEAFQAITDFNDLPNSLF
ACNVHQSVFEGEESKEKFEGLFRTYDDCVTFQLFKSFRRVRINFSNPKSAARARIELHET
QFRGKKLKLYFAQVQTPETDGDKLHLAPPQPAKQFLISPPSSPPVGWQPINDATPVLNYD
LLYAVAKLGPGEKYELHAGTESTPSVVVHVCDSDIEEEEDPKTSPKPKIIQTRRPGLPPS
VSN*
>ENSG00000160888|ENST00000292433
MEVQKEAQRIMTLSVWKMYHSRMQRGGLRLHRSLQLSLVMRSARELYLSAKVEALEPEVS
LPAALPSDPRLHPPREAESTAETATPDGEHPFPEPMDTQEAPTAEETSACCAPRPAKVSR
KRRSSSLSDGGDAGLVPSKKARLEEKEEEEGASSEVADRLQPPPAQAEGAFPNLARVLQR
RFSGLLNCSPAAPPTAPPACEAKPACRPADSMLNVLVRAVVAF*
>ENSG00000141543|ENST00000269349
MATTATMATSGSARKRLLKEEDMTKVEFETSEEVDVTPTFDTMGLREDLLRGIYAYGFEK
PSAIQQRAIKQIIKGRDVIAQSQSGTGKTATFSISVLQCLDIQVRETQALILAPTRELAV
QIQKGLLALGDYMNVQCHACIGGTNVGEDIRKLDYGQHVVAGTPGRVFDMIRRRSLRTRA
IKMLVLDEADEMLNKGFKEQIYDVYRYLPPATQVVLISATLPHEILEMTNKFMTDPIRIL
VKRDELTLEGIKQFFVAVEREEWKFDTLCDLYDTLTITQAVIFCNTKRKVDWLTEKMREA
NFTVSSMHGDMPQKERESIMKEFRSGASRVLISTDVWARGLDVPQVSLIINYDLPNNREL

YIHRIGRSGRYGRKGVAINFVKNDDIRILRDIEQYYSTQIDEMPMNVADLI*
>ENSG00000173545|ENST00000308683
MATYTCITCRVAFRDADMQRAHYKTDWHRYNLRRKVASMAPVTAEGFQERVRAQRAVAEE
ESKGSATYCTVCSKKFASFNAYENHLKSRRHVELEKKAVQAVNRKVEMMNEKNLEKGLGV
DSVDKDAMNAAIQQAIKAQPSMSPKKAPPAPAKEARNVVAVGTGGRGTHDRDPSEKPPRL
QWFEQQAKKLAKQQEEDSEEEEEDLDGDDWEDIDSDEELECEDTEAMDDVVEQDAEEEEA
EEGPPLGAIPITDCLFCSHHSSSLMKNVAHMTKDHSFFIPDIEYLSDIKGLIKYLGEKVG
VGKICLWCNEKGKSFYSTEAVQAHMNDKSHCKLFTDGDAALEFADFYDFRSSYPDHKEGE
DPNKAEELPSEKNLEYDDETMELILPSGARVGHRSLMRYYKQRFGLSRAVAVAKNRKAVG
RVLQQYRALGWTGSTGAALMRERDMQYVQRMKSKWMLKTGMKNNATKQMHFRVQVRF*
>ENSG00000161298|ENST00000292928
MPLQGSVSFKDVTVDFTQEEWQQLDPAQKALYRDVMLENYCHFVSVGFHMAKPDMIRKLE
QGEELWTQRIFPSYSYLEEDGKTEDVLVKFKEYQDRHSRPLIFINHKKLIKERSNIYGKT
FTLGKNRISKTILCEYKPDGKVLKNISELVIRNISPIKEKFGDSTGWEKSLLNTKHEKIH
PAVNLHKQTERVLSGKQELIQHQKVQAPEQPFDHNECEKSFLMKGMLFTHTRAHRGERTF
EYNKDGIAFIEKSSLSVHPSNLMEKKPSAYNKYGKFLCRKPVFIMPQRPQTEEKPFHCPY
CGNNFRRKSYLIEHQRIHTGEKPYVCNQCGKAFRQKTALTLHEKTHIEGKPFICIDCGKS
FRQKATLTRHHKTHTGEKAYECPQCGSAFRKKSYLIDHQRTHTGEKPYQCNECGKAFIQK
TTLTVHQRTHTGEKPYICNECGKSFCQKTTLTLHQRIHTGEKPYICNECGKSFRQKAILT
VHHRIHTGEKSNGCPQCGKAFSRKSNLIRHQKTHTGEKPYECKQCGKFFSCKSNLIVHQK
THKVETTGIQ*
>ENSG00000168496|ENST00000305885
MGIQGLAKLIADVAPSAIRENDIKSYFGRKVAIDASMSIYQFLIAVRQGGDVLQNEEGET
TSHLMGMFYRTIRMMENGIKPVYVFDGKPPQLKSGELAKRSERRAEAEKQLQQAQAAGAE
QEVEKFTKRLVKVTKQHNDECKHLLSLMGIPYLDAPSEAEASCAALVKAGKVYAAATEDM
DCLTFGSPVLMRHLTASEAKKLPIQEFHLSRILQELGLNQEQFVDLCILLGSDYCESIRG
IGPKRAVDLIQKHKSIEEIVRRLDPNKYPVPENWLHKEAHQLFLEPEVLDPESVELKWSE
PNEEELIKFMCGEKQFSEERIRSGVKRLSKSRQGSTQGRLDDFFKVTGSLSSAKRKEPEP
KGSTKKKAKTGAAGKFKRGK*
>ENSG00000164778|ENST00000297375
MEENDPKPGEAAAAVEGQRQPESSPGGGSGGGGGSSPGEADTGRRRALMLPAVLQAPGNH
QHPHRITNFFIDNILRPEFGRRKDAGTCCAGAGGGRGGGAGGEGGASGAEGGGGAGGSEQ
LLGSGSREPRQNPPCAPGAGGPLPAAGSDSPGDGEGGSKTLSLHGGAKKGGDPGGPLDGS
LKARGLGGGDLSVSSDSDSSQAGANLGAQPMLWPAWVYCTRYSDRPSSGPRSRKPKKKNP
NKEDKRPRTAFTAEQLQRLKAEFQTNRYLTEQRRQSLAQELSLNESQIKIWFQNKRAKIK
KATGNKNTLAVHLMAQGLYNHSTTAKEGKSDSE*
>ENSG00000159917|ENST00000291182
MTKFQEAVTFKDVAVAFTEEELGLLDSAQRKLYRDVMLENFRNLVSVGHQSFKPDMISQL
EREEKLWMKELQTQRGKHSGDRNQNEMATLHKAGLRCFSLGELSCWQIKRHIASKLARSQ
DSMINIEGKSSQFPKHHDSPCQVGAGESIQASVDDNCLVNHIGDHSSIIENQEFPTGKVP
NSWSKIYLNETQNYQRSCKQTQMKNKLCIFAPYVDIFSCISHHHDDNIVHKRDKVHSNSD
CGKDTLKVSPLTQRSIHTGQKTYQGNECEEAFNDSSSLELHKQVHLGKKSPACSTHEKDT
SYSSGIPVQQSVRTGKKRYWCHECGKGFSQSSNLQTHQRVHTGEKPYTCHECGKSFNQSS
HLYAHLPIHTGEKPYRCDSCGKGFSRSTDLNIHCRVHTGEKPYKCEVCGKGFTQRSHLQA

HERIHTGEKPYKCGDCGKRFSCSSNLHTHQRVHTEEKPYKCDECGKCFSLSFNLHSHQRV
HTGEKPYKCEECGKGFSSASSFQSHQRVHTGEKPFRCNVCGKGFSQSSYFQAHQRVHTGE
KPYKCEVCGKRFNWSLNLHNHQRVHTGEKPYKCEECGKGFSQASNLQAHQSVHTGEKPFK
CDACQKRFSQASHLQAHQRVHTGEKPYKCDTCGKAFSQRSNLQVHQIIHTGEKPFKCEEC
GKEFSWSAGLSAHQRVHTGEKPYTCQQCGKGFSQASHFHTHQRVHTGERPYICDVCCKGF
SQRSHLIYHQRVHTGGNL*
>ENSG00000164508|ENST00000297012
MSGRGKQGGKARAKSKSRSSRAGLQFPVGRIHRLLRKGNYAERIGAGAPVYLAAVLEYLT
AEILELAGNASRDNKKTRIIPRHLQLAIRNDEELNKLLGGVTIAQGGVLPNIQAVLLPKK
TESHHHKAQSK*
>ENSG00000113648|ENST00000304332
MSSRGGKKKSTKTSRSAKAGVIFPVGRMLRYIKKGHPKYRIGVGAPVYMAAVLEYLTAEI
LELAGNAARDNKKGRVTPRHILLAVANDEELNQLLKGVTIASGGVLPNIHPELLAKKRGS
KGKLEAIITPPPAKKAKSPSQKKPVSKKAGGKKGARKSKKQGEVSKAASADSTTEGTPAD
GFTVLSTKSLFLGQKLNLIHSEISNLAGFEVEAIINPTNADIDLKDDLGNTLEKKGGKEF
VEAVLELRKKNGPLEVAGAAVSAGHGLPAKFVIHCNSPVWGADKCEELLEKTVKNCLALA
DDKKLKSIAFPSIGSGRNGFPKQTAAQLILKAISSYFVSTMSSSIKTVYFVLFDSESIGI
YVQEMAKLDAN*
>ENSG00000198046|ENST00000292069
MPSARGKSKSKAPITFGDLAIYFSQEEWEWLSPIQKDLYEDVMLENYRNLVSLGLSFRRP
NVITLLEKGKAPWMVEPVRRRRAPDSGSKCETKKLPPNQCNKSGQSICQKLVSAQQKAPT
RKSGCNKNSVLVKPKKGHSGKKPLKCNDCGKTFSRSFSLKLHQNIHTGEKPFECSNCRKA
FRQISSILLHQRIHSGKKSHECNKCGESFNQRTTLILHMRIHDGKEILDCGKALSQCQSF
NIHQKIHVVGNVCQCRKCGKAFNQMSSLLLHKKIHNGKKTHKYNKCGRGFKKKSVFVVHK
RIHAGEKIPENAKALSQSLQQRSHHLENPFKCRKCGKLFNRISPLMLHQRIHTSEKPYKC
DKCDKFFRRLSTLILHLRIHNGEKLYRCNKCEKVCNRHSSLIQHQKVHTKKKKLFECKEC
GKMFSGTANLKIHQNIHSEEKPFKCNKCSKVFGRQSFLIEHQRIHTGEKPYQCEECGKAF
SHRISLTRHKRIHTEDRPYECDQCGKAFSQSAHLAQHERIHTGEKPYTCKTCGKAFSQRT
SLILHERSHTGEKPYECNECGKAFSSGSDLIRHQRSHSSEKPYECSKCGKAYSRSSSLIR
HQNTHSEEKA*
>ENSG00000161082|ENST00000292672
MARLTESEARRQQQQLLQPRPSPVGSSGPEPPGGQPDGMKDLDAIKLFVGQIPRHLDEKD
LKPLFEQFGRIYELTVLKDPYTGMHKGCAFLTYCARDSAIKAQTALHEQKTLPGMARPIQ
VKPADSESRGGRDRKLFVGMLNKQQSEEDVLRLFQPFGVIDECTVLRGPDGSSKGCAFVK
FSSHTEAQAAIHALHGSQTMPGASSSLVVKFADTDKERTLRRMQQMVGQLGILTPSLTLP
FSPYSAYAQALMQQQTTVLSTSGSYLSPGVAFSPCHIQQIGAVSLNGLPATPIAPASGLH
SPPLLGTTAVPGLVAPITNGFAGVVPFPGGHPALETVYANGLVPYPAQSPTVAETLHPAF
SGVQQYTAMYPTAAITPIAHSVPQPPPLLQQQQREGPEGCNLFIYHLPQEFGDTELTQMF
LPFGNIISSKVFMDRATNQSKCFGFVSFDNPASAQAAIQAMNGFQIGMKRLKVQLKRPKD
PGHPY*
>ENSG00000147130|ENST00000314425
MDPSDFPSPFDPLTLPEKPLAGDLPVDMEFGEDLLESQTAPTRGWAPPGPSPSSGALDLL
DTPAGLEKDPGVLDGATELLGLGGLLYKAPSPPEVDHGPEGTLAWDAGDQTLEPGPGGQT
PEVVPPDPGAGANSCSPEGLLEPLAPDSPITLQSPHIEEEETTSIATARRGSPGQEEELP

QGQPQSPNAPPSPSVGETLGDGINSSQTKPGGSSPPAHPSLPGDGLTAKASEKPPERKRS
ERVRRAEPPKPEVVDSTESIPVSDEDSDAMVDDPNDEDFVPFRPRRSPRMSLRSSVSQRA
GRSAVGTKMTCAHCRTPLQKGQTAYQRKGLPQLFCSSSCLTTFSKKPSGKKTCTFCKKEI
WNTKDSVVAQTGSGGSFHEFCTSVCLSLYEAQQQRPIPQSGDPADATRCSICQKTGEVLH
EVSNGSVVHRLCSDSCFSKFRANKGLKTNCCDQCGAYIYTKTGSPGPELLFHEGQQKRFC
NTTCLGAYKKKNTRVYPCVWCKTLCKNFEMLSHVDRNGKTSLFCSLCCTTSYKVKQAGLT
GPPRPCSFCRRSLSDPCYYNKVDRTVYQFCSPSCWTKFQRTSPEGGIHLSCHYCHSLFSG
KPEVLDWQDQVFQFCCRDCCEDFKRLRGVVSQCEHCRQEKLLHEKLRFSGVEKSFCSEGC
VLLYKQDFTKKLGLCCITCTYCSQTCQRGVTEQLDGSTWDFCSEDCKSKYLLWYCKAARC
HACKRQGKLLETIHWRGQIRHFCNQQCLLRFYSQQNQPNLDTQSGPESLLNSQSPESKPQ
TPSQTKVENSNTVRTPEENGNLGKIPVKTRSAPTAPTPPPPPPPATPRKNKAAMCKPLMQ
NRGVSCKVEMKSKGSQTEEWKPQVIVLPIPVPIFVPVPMHLYCQKVPVPFSMPIPVPVPM
FLPTTLESTDKIVETIEELKVKIPSNPLEADILAMAEMIAEAEELDKASSDLCDLVSNQS
AEGLLEDCDLFGPARDDVLAMAVKMANVLDEPGQDLEADFPKNPLDINPSVDFLFDCGLV
GPEDVSTEQDLPRTMRKGQKRLVLSESCSRDSMSSQPSCTGLNYSYGVNAWKCWVQSKYA
NGETSKGDELRFGPKPMRIKEDILACSAAELNYGLAQFVREITRPNGERYEPDSIYYLCL
GIQQYLLENNRMVNIFTDLYYLTFVQELNKSLSTWQPTLLPNNTVFSRVEEEHLWECKQL
GVYSPFVLLNTLMFFNTKFFGLQTAEEHMQLSFTNVVRQSRKCTTPRGTTKVVSIRYYAP
VRQRKGRDTGPGKRKREDEAPILEQRENRMNPLRCPVKFYEFYLSKCPESLRTRNDVFYL
QPERSCIAESPLWYSVIPMDRSMLESMLNRILAVREIYEELGRPGEEDLD*
>ENSG00000148411|ENST00000277554
MSQMLHIEIPNFGNTVLGCLNEQRLLGLYCDVSIVVKGQAFKAHRAVLAASSLYFRDLFS
GNSKSAFELPGSVPPACFQQILSFCYTGRLTMTASEQLVVMYTAGFLQIQHIVERGTDLM
FKVSSPHCDSQTAVIEDAGSEPQSPCNQLQPAAAAAAPYVVSPSVPIPLLTRVKHEAMEL
PPAGPGLAPKRPLETGPRDGVAVAAGAAVAAGTAPLKLPRVSYYGVPSLATLIPGIQQMP
YPQGERTSPGASSLPTTDSPTSYHNEEDEEDDEAYDTMVEEQYGQMYIKASGSYAVQEKP
EPVPLESRSCVLIRRDLVALPASLISQIGYRCHPKLYSEGDPGEKLELVAGSGVYITRGQ
LMNCHLCAGVKHKVLLRRLLATFFDRNTLANSCGTGIRSSTSDPSRKPLDSRVLNAVKLY
CQNFAPSFKESEMNVIAADMCTNARRVRKRWLPKIKSMLPEGVEMYRTVMGSAAASVPLD
PEFPPAAAQVFEQRIYAERRGDAATIVALRTDAVNVDLSAAANPAFDAGEEVDGAGSVIQ
EVAAPEPLPADGQSPPQPFEQGGGGPSRPQTPAAAARRPEGTYAGTL*
>ENSG00000106603|ENST00000310564
MMWQKYAGSRRSMPLGARILFHGVFYAGGFAIVYYLIQKFHSRALYYKLAVEQLQSHPEA
QEALGPPLNIHYLKLIDRENFVDIVDAKLKIPVSGSKSEGLLYVHSSRGGPFQRWHLDEV
FLELKDGQQIPVFKLSGENGDEVKKE*
>ENSG00000204519|ENST00000282296
MPAPVGRRSPPSPRSSMAAVALRDSAQGMTFEDVAIYFSQEEWELLDESQRFLYCDVMLE
NFAHVTSLGYCHGMENEAIASEQSVSIQVRTSKGNTPTQKTHLSEIKMCVPVLKDILPAA
EHQTTSPVQKSYLGSTSMRGFCFSADLHQHQKHYNEEEPWKRKVDEATFVTGCRFHVLNY
FTCGEAFPAPTDLLQHEATPSGEEPHSSSSKHIQAFFNAKSYYKWGEYRKASSHKHTLVQ
HQSVCSEGGLYECSKCEKAFTCKNTLVQHQQIHTGQKMFECSECEESFSKKCHLILHKII
HTGERPYECSDREKAFIHKSEFIHHQRRHTGGVRHECGECRKTFSYKSNLIEHQRVHTGE
RPYECGECGKSFRQSSSLFRHQRVHSGERPYQCCECGKSFRQIFNLIRHRRVHTGEMPYQ
CSDCGKSFSCKSELIQHQRIHSGERPYECRECGKSFRQFSNLIRHRSIHTGDRPYECSEC

EKSFSRKFILIQHQRVHTGERPYECSECGKSFTRKSDLIQHRRIHTGTRPYECSECGKSF
RQRSGLIQHRRLHTGERPYECSECGKSFSQSASLIQHQRVHTGERPYECSECGKSFSQSS
SLIQHQRGHTGERPYECSQCGKPFTHKSDLIQHQRVHTGERPYECSECGKSFSRKSNLIR
HRRVHTEERP*
>ENSG00000163666|ENST00000295934
MSPSLQEGAQLGENKPSTCSFSIERILGLDQKKDCVPLMKPHRPWADTCSSSGKDGNLCL
HVPNPPSGISFPSVVDHPMPEERASKYENYFSASERLSLKRELSWYRGRRPRTAFTQNQI
EVLENVFRVNCYPGIDIREDLAQKLNLEEDRIQIWFQNRRAKLKRSHRESQFLMAKKNFN
TNLLE*
>ENSG00000143751|ENST00000272091
MAEAAALVWIRGPGFGCKAVRCASGRCTVRDFIHRHCQDQNVPVENFFVKCNGALINTSD
TVQHGAVYSLEPRLCGGKGGFGSMLRALGAQIEKTTNREACRDLSGRRLRDVNHEKAMAE
WVKQQAEREAEKEQKRLERLQRKLVEPKHCFTSPDYQQQCHEMAERLEDSVLKGMQAASS
KMVSAEISENRKRQWPTKSQTDRGASAGKRRCFWLGMEGLETAEGSNSESSDDDSEEAPS
TSGMGFHAPKIGSNGVEMAAKFPSGSQRARVVNTDHGSPEQLQIPVTDSGRHILEDSCAE
LGESKEHMESRMVTETEETQEKKAESKEPIEEEPTGAGLNKDKETEERTDGERVAEVAPE
ERENVAVAKLQESQPGNAVIDKETIDLLAFTSVAELELLGLEKLKCELMALGLKCGGTLQ
ERAARLFSVRGLAKEQIDPALFAKPLKGKKK*
>ENSG00000137513|ENST00000281038
MLGVRCLLRSVRFCSSAPFPKHKPSAKLSVRDALGAQNASGERIKIQGWIRSVRSQKEVL
FLHVNDGSSLESLQVVADSGLDSRELNFGSSVEVQGQLIKSPSKRQNVELKAEKIKVIGN
CDAKDFPIKYKERHPLEYLRQYPHFRCRTNVLGSILRIRSEATAAIHSFFKDSGFVHIHT
PIITSNDSEGAGELFQLEPSGKLKVPEENFFNVPAFLTVSGQLHLEVMSGAFTQVFTFGP
TFRAENSQSRRHLAEFYMIEAEISFVDSLQDLMQVIEELFKATTMMVLSKCPEDVELCHK
FIAPGQKDRLEHMLKNNFLIISYTEAVEILKQASQNFTFTPEWGADLRTEHEKYLVKHCG
NIPVFVINYPLTLKPFYMRDNEDGPQHTVAAVDLLVPGVGELFGGGLREERYHFLEERLA
RSGLTEVYQWYLDLRRFGSVPHGGFGMGFERYLQCILGVDNIKDVIPFPRFPHSCLL*
>ENSG00000149480|ENST00000278823
MAANMYRVGDYVYFENSSSNPYLVRRIEELNKTANGNVEAKVVCLFRRRDISSSLNSLAD
SNAREFEEESKQPGVSEQQRHQLKHRELFLSRQFESLPATHIRGKCSVTLLNETDILSQY
LEKEDCFFYSLVFDPVQKTLLADQGEIRVGCKYQAEIPDRLVEGESDNRNQQKMEMKVWD
PDNPLTDRQIDQFLVVARAVGTFARALDCSSSIRQPSLHMSAAAASRDITLFHAMDTLQR
NGYDLAKAMSTLVPQGGPVLCRDEMEEWSASEAMLFEEALEKYGKDFNDIRQDFLPWKSL
ASIVQFYYMWKTTDRYIQQKRLKAAEADSKLKQVYIPTYTKPNPNQIISVGSKPGMNGAG
FQKGLTCESCHTTQSAQWYAWGPPNMQCRLCASCWIYWKKYGGLKTPTQLEGATRGTTEP
HSRGHLSRPEAQSLSPYTTSANRAKLLAKNRQTFLLQTTKLTRLARRMCRDLLQPRRAAR
RPYAPINANAIKAECSIRLPKAAKTPLKIHPLVRLPLATIVKDLVAQAPLKPKTPRGTKT
PINRNQLSQNRGLGGIMVKRAYETMAGAGVPFSANGRPLASGIRSSSQPAAKRQKLNPAD
APNPVVFVATKDTRALRKALTHLEMRRAARRPNLPLKVKPTLIAVRPPVPLPAPSHPAST
NEPIVLED*
>ENSG00000125351|ENST00000276201
MKEEKEHRPKEKRVTLLTPAGATGSGGGTSGDSSKGEDKQDRNKEKKEALSKVVIRRLPP
TLTKEQLQEHLQPMPEHDYFEFFSNDTSLYPHMYARAYINFKNQEDIILFRDRFDGYVFL
DNKGQEYPAIVEFAPFQKAAKKKTKKRDTKVGTIDDDPEYRKFLESYATDNEKMTSTPET

LLEEIEAKNRELIAKKTTPLLSFLKNKQRMREEKREERRRREIERKRQREEERRKWKEEE
KRKRKDIEKLKKIDRIPERDKLKDEPKIKVHRFLLQAVNQKNLLKKPEKGDEKELDKREK
AKKLDKENLSDERASGQSCTLPKRSDSELKDEKPKRPEDESGRDYREREREYERDQERIL
RERERLKRQEEERRRQKERYEKEKTFKRKEEEMKKEKDTLRDKGKKAESTESIGSSEKTE
KKEEVVKRDRIRNKDRPAMQLYQPGARSRNRLCPPDDSTKSGDSAAERKQESGISHRKEG
GEE*
>ENSG00000124920|ENST00000278836
MEVVDETEALQRFFEGHDINGALEPSNIDTSILEEYISKEDASDLCFPDISAPASSASYS
HGQPAMPGSSGVHHLSPPGGGPSPGRHGPLPPPGYGTPLNCNNNNGMGAAPKPFPGGTGP
PIKAEPKAPYAPGTLPDSPPDSGSEAYSPQQVNEPHLLRTITPETLCHVGVPSRLEHPPP
PPAHLPGPPPPPPPPPHYPVLQRDLYMKAEPPIPHYAAMGQGLVPTDLHHTQQSQMLHQL
LQQHGAELPTHPSKKRKHSESPPSTLNAQMLNGMIKQEPGTVTALPLHPTRAPSPPWPPQ
GPLSPGPGSLPLSIARVQTPPWHPPGAPSPGLLQDSDSLSGSYLDPNYQSIKWQPHQQNK
WATLYDANYKELPMLTYRVDADKGFNFSVGDDAFVCQKKNHFQVTVYIGMLGEPKYVKTP
EGLKPLDCFYLKLHGVKLEALNQSINIEQSQSDRSKRPFNPVTVNLPPEQVTKVTVGRLH
FSETTANNMRKKGKPNPDQRYFMLVVALQAHAQNQNYTLAAQISERIIVRASNPGQFESD
SDVLWQRAQVPDTVFHHGRVGINTDRPDEALVVHGNVKVMGSLMHPSDLRAKEHVQEVDT
TEQLKRISRMRLVHYRYKPEFAASAGIEATAPETGVIAQEVKEILPEAVKDTGDMVFANG
KTIENFLVVNKERIFMENVGAVKELCKLTDNLETRIDELERWSHKLAKLRRLDSLKSTGS
SGAFSHAGSQFSRAGSVPHKKRPPKVASKSSSVVPDQACISQRFLQGTIIALVVVMAFSV
VSMSTLYVLSLRTEEDLVDTDGSFAVSTSCLLALLRPQPPGGSEALCPWSSQSFGTTQLR
QSPLTTGLPGIQPSLLLVTTSLTSSAPGSAVRTLDMCSSHPCPVICCSSPTTNPTTGPSL
GPSFNPGHVLSPSPSPSTNRSGPSQMALLPVTNIRAKSWGLSVNGIGHSKHHKSLEPLAS
PAVPFPGGQGKAKNSPSLGFHGRARRGALQSSVGPAEPTWAQGQSASLLAEPVPSLTSIQ
VLENSMSITSQYCAPGDACRPGNFTYHIPVSSGTPLHLSLTLQMNSSSPVSVVLCSLRSK
EEPCEEGSLPQSLHTHQDTQGTSHRWPITILSFREFTYHFRVALLGQANCSSEALAQPAT
DYHFHFYRLCD*
>ENSG00000137309|ENST00000311487
MSESSSKSSQPLASKQEKDGTEKRGRGRPRKQPPVSPGTALVGSQKEPSEVPTPKRPRGR
PKGSKNKGAAKTRKTTTTPGRKPRGRPKKLEKEEEEGISQESSEEEQ*
>ENSG00000171634|ENST00000306378
MRGRRGRPPKQPAAPAAERCAPAPPPPPPPPTSGPIGGLRSRHRGSSRGRWAAAQAEVAP
KTRLSSPRGGSSSRRKPPPPPPAPPSTSAPGRGGRGGGGGRTGGGGGGGHLARTTAARRA
VNKVVYDDHESEEEEEEEDMVSEEEEEEDGDAEETQDSEDDEEDEMEEDDDDSDYPEEME
DDDDDASYCTESSFRSHSTYSSTPGRRKPRVHRPRSPILEEKDIPPLEFPKSSEDLMVPN
EHIMNVIAIYEVLRNFGTVLRLSPFRFEDFCAALVSQEQCTLMAEMHVVLLKAVLREEDT
SNTTFGPADLKDSVNSTLYFIDGMTWPEVLRVYCESDKEYHHVLPYQEAEDYPYGPVENK
IKVLQFLVDQFLTTNIAREELMSEGVIQYDDHCRVCHKLGDLLCCETCSAVYHLECVKPP
LEEVPEDEWQCEVCVAHKVPGVTDCVAEIQKNKPYIRHEPIGYDRSRRKYWFLNRRLIIE
EDTENENEKKIWYYSTKVQLAELIDCLDKDYWEAELCKILEEMREEIHRHMDITEDLTNK
ARGSNKSFLAAANEEILESIRAKKGDIDNVKSPEETEKDKNETENDSKDAEKNREEFEDQ
SLEKDSDDKTPDDDPEQGKSEVGDFKSEKSNGELSESPGAGKGASGSTRIITRLRNPDSK
LSQLKSQQVAAAAHEANKLFKEGKEVLVVNSQGEISRLSTKKEVIMKGNINNYFKLGQEG
KYRVYHNQYSTNSFALNKHQHREDHDKRRHLAHKFCLTPAGEFKWNGSVHGSKVLTISTL

RLTITQLENNIPSSFLHPNWASHRANWIKAVQMCSKPREFALALAILECAVKPVVMLPIW
RESLGHTRLHRMTSIEREEKEKVKKKEKKQEEEETMQQATWVKYTFPVKHQVWKQKGEEY
RVTGYGGWSWISKTHVYRFVPKLPGNTNVNYRKSLEGTKNNMDENMDESDKRKCSRSPKK
IKIEPDSEKDEVKGSDAAKGADQNEMDISKITEKKDQDVKELLDSDSDKPCKEEPMEVDD
DMKTESHVNCQESSQVDVVNVSEGFHLRTSYKKKTKSSKLDGLLERRIKQFTLEEKQRLE
KIKLEGGIKGIGKTSTNSSKNLSESPVITKAKEGCQSDSMRQEQSPNANNDQPEDLIQGC
SESDSSVLRMSDPSHTTNKLYPKDRVLDDVSIRSPETKCPKQNSIENDIEEKVSDLASRG
QEPSKSKTKGNDFFIDDSKLASADDIGTLICKNKKPLIQEESDTIVSSSKSALHSSVPKS
TNDRDATPLSRAMDFEGKLGCDSESNSTLENSSDTVSIQDSSEEDMIVQNSNESISEQFR
TREQDVEVLEPLKCELVSGESTGNCEDRLPVKGTEANGKKPSQQKKLEERPVNKCSDQIK
LKNTTDKKNNENRESEKKGQRTSTFQINGKDNKPKIYLKGECLKEISESRVVSGNVEPKV
NNINKIIPENDIKSLTVKESAIRPFINGDVIMEDFNERNSSETKSHLLSSSDAEGNYRDS
LETLPSTKESDSTQTTTPSASCPESNSVNQVEDMEIETSEVKKVTSSPITSEEESNLSND
FIDENGLPINKNENVNGESKRKTVITEVTTMTSTVATESKTVIKVEKGDKQTVVSSTENC
AKSTVTTTTTTVTKLSTPSTGGSVDIISVKEQSKTVVTTTVTDSLTTTGGTLVTSMTVSK
EYSTRDKVKLMKFSRPKKTRSGTALPSYRKFVTKSSKKSIFVLPNDDLKKLARKGGIREV
PYFNYNAKPALDIWPYPSPRPTFGITWRYRLQTVKSLAGVSLMLRLLWASLRWDDMAAKA
PPGGGTTRTETSETEITTTEIIKRRDVGPYGIRSEYCIRKIICPIGVPETPKETPTPQRK
GLRSSALRPKRPETPKQTGPVIIETWVAEEELELWEIRAFAERVEKEKAQAVEQQAKKRL
EQQKPTVIATSTTSPTSSTTSTISPAQKVMVAPISGSVTTGTKMVLTTKVGSPATVTFQQ
NKNFHQTFATWVKQGQSNSGVVQVQQKVLGIIPSSTGTSQQTFTSFQPRTATVTIRPNTS
GSGGTTSNSQVITGPQIRPGMTVIRTPLQQSTLGKAIIRTPVMVQPGAPQQVMTQIIRGQ
PVSTAVSAPNTVSSTPGQKSLTSATSTSNIQSSASQPPRPQQGQVKLTMAQLTQLTQGHG
GNQGLTVVIQGQGQTTGQLQLIPQGVTVLPGPGQQLMQAAMPNGTVQRFLFTPLATTATT
ASTTTTTVSTTAAGTGEQRQSKLSPQMQVHQDKTLPPAQSSSVGPAEAQPQTAQPSAQPQ
PQTQPQSPAQPEVQTQPEVQTQTTVSSHVPSEAQPTHAQSSKPQVAAQSQPQSNVQGQSP
VRVQSPSQTRIRPSTPSQLSPGQQSQVQTTTSQPIPIQPHTSLQIPSQGQPQSQPQVQSS
TQTLSSGQTLNQVTVSSPSRPQLQIQQPQPQVIAVPQLQQQVQVLSQIQSQVVAQIQAQQ
SGVPQQIKLQLPIQIQQSSAVQTHQIQNVVTVQAASVQEQLQRVQQLRDQQQKKKQQQIE
IKREHTLQASNQSEIIQKQVVMKHNAVIEHLKQKKSMTPAEREENQRMIVCNQVMKYILD
KIDKEEKQAAKKRKREESVEQKRSKQNATKLSALLFKHKEQLRAEILKKRALLDKDLQIE
VQEELKRDLKIKKEKDLMQLAQATAVAAPCPPVTPAPPAPPAPPPSPPPPPAVQHTGLLS
TPTLPAASQKRKREEEKDSSSKSKKKKMISTTSKETKKDTKLYCICKTPYDESKFYIGCD
RCQNWYHGRCVGILQSEAELIDEYVCPQCQSTEDAMTVLTPLTEKDYEGLKRVLRSLQAH
KMAWPFLEPVDPNDAPDYYGVIKEPMDLATMEERVQRRYYEKLTEFVADMTKIFDNCRYY
NPSDSPFYQCAEVLESFFVQKLKGFKASRSHNNKLQSTAS*
>ENSG00000167513|ENST00000301019
MEQRRVTDFFARRRPGPPRIAPPKLACRTPSPARPALRAPASATSGSRKRARPPAAPGRD
QARPPARRRLRLSVDEVSSPSTPEAPDIPACPSPGQKIKKSTPAAGQPPHLTSAQDQDTI
SELASCLQRARELGARVRALKASAQDAGESCTPEAEGRPEEPCGEKAPAYQRFHALAQPG
LPGLVLPYKYQVLAEMFRSMDTIVGMLHNRSETPTFAKVQRGVQDMMRRRFEECNVGQIK
TVYPASYRFRQERSVPTFKDGTRRSDYQLTIEPLLEQEADGAAPQLTASRLLQRRQIFSQ
KLVEHVKEHHKAFLASLSPAMVVPEDQLTRWHPRFNVDEVPDIEPAALPQPPATEKLTTA
QEVLARARNLISPRMEKALSQLALRSAAPSSPGSPRPALPATPPATPPAASPSALKGVSQ

DLLERIRAKEAQKQLAQMTRCPEQEQRLQRLERLPELARVLRSVFVSERKPALSMEVACA
RMVGSCCTIMSPGEMEKHLLLLSELLPDWLSLHRIRTDTYVKLDKAADLAHITARLAHQT
RAEEGL*
>ENSG00000144161|ENST00000272570
MDFENLFSKPPNPALGKTATDSDERIDDEIDTEVEETQEEKIKLECEQIPKKFRHSAISP
KSSLHRKSRSKDYDVYSDNDICSQESEDNFAKELQQYIQAREMANAAQPEESTKKEGVKD
TPQAAKQKNKNLKAGHKNGKQKKMKRKWPGPGNKGSNALLRNSGSQEEDGKPKEKQQHLS
QAFINQHTVERKGKQICKYFLERKCIKGDQCKFDHDAEIEKKKEMCKFYVQGYCTRGENC
LYLHNEYPCKFYHTGTKCYQGEYCKFSHAPLTPETQELLAKVLDTEKKSCK*
>ENSG00000163565|ENST00000295809
MGKKYKNIVLLKGLEVINDYHFRMVKSLLSNDLKLNLKMREEYDKIQIADLMEEKFRGDA
GLGKLIKIFEDIPTLEDLAETLKKEKLKVKGPALSRKRKKEVDATSPAPSTSSTVKTEGA
EATPGAQKRKKSTKEKAGPKGSKVSEEQTQPPSPAGAGMSTAMGRSPSPKTSLSAPPNSS
STENPKTVAKCQVTPRRNVLQKRPVIVKVLSTTKPFEYETPEMEKKIMFHATVATQTQFF
HVKVLNTSLKEKFNGKKIIIISDYLEYDSLLEVNEESTVSEAGPNQTFEVPNKIINRAKE
TLKIDILHKQASGNIVYGVFMLHKKTVNQKTTIYEIQDDRGKMDVVGTGQCHNIPCEEGD
KLQLFCFRLRKKNQMSKLISEMHSFIQIKKKTNPRNNDPKSMKLPQEQRQLPYPSEASTT
FPESHLRTPQMPPTTPSSSFFTKKSEDTISKMNDFMRMQILKEGSHFPGPFMTSIGPAES
HPHTPQMPPSTPSSSFLTTKSEDTISKMNDFMRMQILKEGSHFPGPFMTSIGPAESHPHT
PQMPPSTPSSSFLTTLKPRLKTEPEEVSIEDSAQSDLKEVMVLNATESFVYEPKEQKKMF
HATVATENEVFRVKVFNIDLKEKFTPKKIIAIANYVCRNGFLEVYPFTLVADVNADRNME
IPKGLIRSASVTPKINQLCSQTKGSFVNGVFEVHKKNVRGEFTYYEIQDNTGKMEVVVHG
RLTTINCEEGDKLKLTCFELAPKSGNTGELRSVIHSHIKVIKTRKNKKDILNPDSSMETS
PDFFF*
>ENSG00000145331|ENST00000273962
MSSEMLPAFIETSNVDKKQGINEDQEESQKPRLGEGCEPISKRQMKKLIKQKQWEEQREL
RKQKRKEKRKRKKKLERQCQMEPNSDGHDRKRVRRDVVHSTLRLIIDCSFDHLMVLKDIKK
LHKQIQRCYAENRRALHPVQFYLTSHGGQLKKNMDENDKGWVNWKDIHIKPEHYSELIKK
EDLIYLTSDSPNILKELDESKAYVIGGLVDHNHHKGLTYKQASDYGINHAQLPLGNFVKM
NSRKVLAVNHVFEIILEYLETRDWQEAFFTILPQRKGAVPTDKACESASHDNQSVRMEEG
GSDSDSSEEEYSRNELDSPHEEKQDKENHTESTVNSLPH*
>ENSG00000154582|ENST00000284811
MDGEEKTYGGCEGPDAMYVKLISSDGHEFIVKREHALTSGTIKAMLSGPGQFAENETNEV
NFREIPSHVLSKVCMYFTYKVRYTNSSTEIPEFPIAPEIALELLMAANFLDC*
>ENSG00000170027|ENST00000307630
MVDREQLVQKARLAEQAERYDDMAAAMKNVTELNEPLSNEERNLLSVAYKNVVGARRSSW
RVISSIEQKTSADGNEKKIEMVRAYREKIEKELEAVCQDVLSLLDNYLIKNCSETQYESK
VFYLKMKGDYYRYLAEVATGEKRATVVESSEKAYSEAHEISKEHMQPTHPIRLGLALNYS
VFYYEIQNAPEQACHLAKTAFDDAIAELDTLNEDSYKDSTLIMQLLRDNLTLWTSDQQDD
DGGEGNN*
>ENSG00000180573|ENST00000314088
MSGRGKQGGKARAKAKSRSSRAGLQFPVGRVHRLLRKGNYAERVGAGAPVYLAAVLEYLT
AEILELAGNAARDNKKTRIIPRHLQLAIRNDEELNKLLGRVTIAQGGVLPNIQAVLLPKK
TESHHKAKGK*

>ENSG00000172183|ENST00000306072
MAGSREVVAMDCEMVGLGPHRESGLARCSLVNVHGAVLYDKFIRPEGEITDYRTRVSGVT
PQHMVGATPFAVARLEILQLLKGKLVVGHDLKHDFQALKEDMSGYTIYDTSTDRLLWREA
KLDHCRRVSLRVLSERLLHKSIQNSLLGHSSVEDARATMELYQISQRIRARRGLPRLAVS
D*
>ENSG00000088876|ENST00000278772
MMLPYPSALGDQYWEEILLPKNGENVETMKKLTQNHKAKGLPSNDTDCPQKKEGKAQIVV
PVTFRDVTVIFTEAEWKRLSPEQRNLYKEVMLENYRNLLSLAEPKPEIYTCSSCLLAFSC
QQFLSQHVLQIFLGLCAENHFHPGNSSPGHWKQQGQQYSHVSCWFENAEGQERGGGSKPW
SARTEERETSRAFPSPLQRQSASPRKGNMVVETEPSSAQRPNPVQLDKGLKELETLRFGA
INCREYEPDHNLESNFITNPRTLLGKKPYICSDCGRSFKDRSTLIRHHRIHSMEKPYVCS
ECGRGFSQKSNLSRHQRTHSEEKPYLCRECGQSFRSKSILNRHQWTHSEEKPYVCSECGR
GFSEKSSFIRHQRTHSGEKPYVCLECGRSFCDKSTLRKHQRIHSGEKPYVCRECGRGFSQ
NSDLIKHQRTHLDEKPYVCRECGRGFCDKSTLIIHERTHSGEKPYVCGECGRGFSRKSLL
LVHQRTHSGEKHYVCRECRRGFSQKSNLIRHQRTHSNEKPYICRECGRGFCDKSTLIVHE
RTHSGEKPYVCSECGRGFSRKSLLLVHQRTHSGEKHYVCRECGRGFSHKSNLIRHQRTH*
>ENSG00000171161|ENST00000306562
MFATSGAVAAGKPYSCSECGKSFCYSSVLLRHERAHGGDGRFRCLECGERCARAADLRAH
RRTHAGQTLYICSECGQSFRHSGRLDLHLGAHRQRCRTCPCRTCGRRFPHLPALLLHRRR
QHLPERPRRCPLCARTFRQSALLFHQARAHPLGTTSDPAAPPHRCAQCPRAFRSGAGLRS
HARIHVSRSPTRPRVSDAHQCGVCGKCFGKSSTLTRHLQTHSGEKPFKCPECGKGFLESA
TLVRHQRTHTGEKPYACGDCGRCFSESSTLLRHRRSHQGERPHACATCGKGFGQRSDLVV
HQRIHTGEKPFACPECGRRFSDRSDLTKHRRTHTGEKPYRCELCGKRFTCVSNLNVHRRN
HAGHKPHKCPECSKAFSVASKLALHRKTHLGERPAECAECGKCFSHSRSLSQHQRAHTRA
RTAAAVAIQSAVGTALVFEGPAEQEKPGFSVS*
>ENSG00000006634|ENST00000265728
MNSGAMRIHSKGHFQGGIQVKNEKNRPSLKSLKTDNRPEKSKCKPLWGKVFYLDLPSVTI
SEKLQKDIKDLGGRVEEFLSKDISYLISNKKEAKFAQTLGRISPVPSPESAYTAETTSPH
PSHDGSSFKSPDTVCLSRGKLLVEKAIKDHDFIPSNSILSNALSWGVKILHIDDIRYYIE
QKKKELYLLKKSSTSVRDGGKRVGSGAQKTRTGRLKKPFVKVEDMSQLYRPFYLQLTNMP
FINYSIQKPCSPFDVDKPSSMQKQTQVKLRIQTDGDKYGGTSIQLQLKEKKKKGYCECCL
QKYEDLETHLLSEQHRNFAQSNQYQVVDDIVSKLVFDFVEYEKDTPKKKRIKYSVGSLSP
VSASVLKKTEQKEKVELQHISQKDCQEDDTTVKEQNFLYKETQETEKKLLFISEPIPHPS
NELRGLNEKMSNKCSMLSTAEDDIRQNFTQLPLHKNKQECILDISEHTLSENDLEELRVD
HYKCNIQASVHVSDFSTDNSGSQPKQKSDTVLFPAKDLKEKDLHSIFTHDSGLITINSSQ
EHLTVQAKAPFHTPPEEPNECDFKNMDSLPSGKIHRKVKIILGRNRKENLEPNAEFDKRT
EFITQEENRICSSPVQSLLDLFQTSEEKSEFLGFTSYTEKSGICNVLDIWEEENSDNLLT
AFFSSPSTSTFTGF*
>ENSG00000144895|ENST00000273435
MAPSTPLLTVRGSEGLYMVNGPPHFTESTVFPRESGKNCKVCIFSKDGTLFAWGNGENVT
NKGLLHSFDLLKAVCLEFSPKNTVLATWQPYTTSKDGTAGIPNLQLYDVKTGTCLKSFIQ
KKMQNWCPSWSEDETLCARNVNNEVHFFENNNFNTIANKLHLQKINDFVLSPGPQPYKVA
VYVPGSKGAPSFVRLYQYPNFAGPHAALANKSFFKADKVTMLWNKKATAVLVIASTDVDK
TGASYYGEQTLHYIATNGESAVVQLPKNGPIYDVVWNSSSTEFCAVYGFMPAKATIFNLK

CDPVFDFGTGPRNAAYYSPHGHILVLAGFGNLRGQMEVWDVKNYKLISKPVASDSTYFAW
CPDGEHILTATCAPRLRVNNGYKIWHYTGSILHKYDVPSNAELWQVSWQPFLDGIFPAKT
ITYQAVPSEVPNEEPKVATAYRPPALRNKPITNSKLHEEEPPQNMKPQSGNDKPLSKTAL
KNQRKHEAKKAAKQEARSDKSPDLAPTPAPQSTPRNTVSQSISGDPEIDKKIKNLKKKLK
AIEQLKEQAATGKQLEKNQLEKIQKETALLQELEDLELGI*
>ENSG00000164458|ENST00000296946
MSSPGTESAGKSLQYRVDHLLSAVENELQAGSEKGDPTERELRVGLEESELWLRFKELTN
EMIVTKNGRRMFPVLKVNVSGLDPNAMYSFLLDFVAADNHRWKYVNGEWVPGGKPEPQAP
SCVYIHPDSPNFGAHWMKAPVSFSKVKLTNKLNGGGQIMLNSLHKYEPRIHIVRVGGPQR
MITSHCFPETQFIAVTAYQNEEITALKIKYNPFAKAFLDAKERSDHKEMMEEPGDSQQPG
YSQWGWLLPGTSTLCPPANPHPQFGGALSLPSTHSCDRYPTLRSHRSSPYPSPYAHRNNS
PTYSDNSPACLSMLQSHDNWSSLGMPAHPSMLPVSHNASPPTSSSQYPSLWSVSNGAVTP
GSQAAAVSNGLGAQFFRGSPAHYTPLTHPVSAPSSSGSPLYEGAAAATDIVDSQYDAAAQ
GRLIASWTPVSPPSM*
>ENSG00000018869|ENST00000301310
MSLGSELFRDVAIVFSQEEWQWLAPAQRDLYRDVMLETYSNLVSLGLAVSKPDVISFLEQ
GKEPWMVERVVSGGLCPVLESRYDTKELFPKQHVYEVESPQWEIMESLTSYGLECSSFQD
DWECRNQFDRQQGNPDRHFHQMIIRHEEMPTFDQHASLTFYQKIHTREKPFGYNKCRKDF
WQKELLINHQGIYTNEKPYKCKECGKAFKYGSRLIQHENIHSGKKPYECKECGKAFNSGS
NFIQHQRVHTGEKPYECKDCEKAFSRSSQLIEHQRTHTGEKPYQCKECGKAFNRISHLKV
HYRIHTGEKPYACKECGKTFSHRSQLIQHQTVHTGRKLYECKECGKAFNQGSTLIRHQRI
HTGEKPYECKVCGKAFRVSSQLKQHQRIHTGEKPYQCKVCGRAFKRVSHLTVHYRIHTGE
KPYECKECGKAFSHCSQLIHHQVIHTEKKPYEYKECEKTLSHDSTTVQPQRMHNRETHVN
IINVEKPSISSYPLLIIREFMLASNHMNGSNGESPLA*
>ENSG00000124160|ENST00000290231
MNTAPSRPSPTRRDPYGFGDSRDSRRDRSPIRGSPRREPRDGRNGRDARDSRDIRDPRDL
RDHRHSRDLRDHRDSRSVRDVRDVRDLRDFRDLRDSRDFRDQRDPMYDRYRDMRDSRDPM
YRREGSYDRYLRMDDYCRRKDDSYFDRYRDSFDGRGPPGPESQSRAKERLKREERRREEL
YRQYFEEIQRRFDAERPVDCSVIVVNKQTKDYAESVGRKVRDLGMVVDLIFLNTEVSLSQ
ALEDVSRGGSPFAIVITQQHQIHRSCTVNIMFGTPQEHRNMPQADAMVLVARNYERYKNE
CREKEREEIARQAAKMADEAILQERERGGPEEGVRGGHPPAIQSLINLLADNRYLTAEET
DKIINYLRERKERLMRSSTDSLPGPISRQPLGATSGASLKTQPSSQPLQSGQVLPSATPT
PSAPPTSQQELQAKILSLFNSGTVTANSSSASPSVAAGNTPNQNFSTAANSQPQQRSQAS
GNQPPSILGQGGSAQNMGPRPGAPSQGLFGQPSSRLAPASNMTSQRPVSSTGINFDNPSV
QKALDTLIQSGPALSHLVSQTTAQMGQPQAPMGSYQRHY*
>ENSG00000102034|ENST00000308167
MAITLQPSDLIFEFASNGMDDDIHQLEDPSVFPAVIVEQVPYPDLLHLYSGLELDDVHNG
IITDGTLCMTQDQILEGSFLLTDDNEATSHTMSTAEVLLNMESPSDILDEKQIFSTSEML
PDSDPAPAVTLPNYLFPASEPDALNRAGDTSDQEGHSLEEKASREESAKKTGKSKKRIRK
TKGNRSTSPVTDPSIPIRKKSKDGKGSTIYLWEFLLALLQDRNTCPKYIKWTQREKGIFK
LVDSKAVSKLWGKQKNKPDMNYETMGRALRYYYQRGILAKVEGQRLVYQFKEMPKDLVVI
EDEDESSEATAAPPQASTASVASASTTRRTSSRVSSRSAPQGKGSSSWEKPKIQHVGLQP
SASLELGPSLDEEIPTTSTMLVSPAEGQVKLTKAVSASSVPSNIHLGVAPVGSGSALTLQ
TIPLTTVLTNGPPASTTAPTQLVLQSVPAASTFKDTFTLQASFPLNASFQDSQVAAPGAP

LILSGLPQLLAGANRPTNPAPPTVTGAGPAGPSSQPPGTVIAAFIRTSGTTAAPRVKEGP
LRSSSYVQGMVTGAPMEGLLVPEETLRELLRDQAHLQPLPTQVVSRGSHNPSLLGNQTLS
PPSRPTVGLTPVAELELSSGSGSLLMAEPSVTTSGSLLTRSPTPAPFSPFNPTSLIKMEP
HDI*
>ENSG00000140986|ENST00000268661
MSHRKFSAPRHGHLGFLPHKRSHRHRGKVKTWPRDDPSQPVHLTAFLGYKAGMTHTLREV
HRPGLKISKREEVEAVTIVETPPLVVVGVVGYVATPRGLRSFKTIFAEHLSDECRRRFYK
DWHKSKKKAFTKACKRWRDTDGKKQLQKDFAAMKKYCKVIRVIVHTQMKLLPFRQKKAHI
MEIQLNGGTVAEKVAWAQARLEKQVPVHSVFSQSEVIDVIAVTKGRGVKGVTSRWHTKKL
PRKTHKGLRKVACIGAWHPARVGCSIARAGQKGYHHRTELNKKIFRIGRGPHMEDGKLVK
NNASTSYDVTAKSITPLGGFPHYGEVNNDFVMLKGCIAGTKKRVITLRKSLLVHHSRQAV
ENIELKFIDTTSKFGHGRFQTAQEKRAFMGPQKKHLEKETPETSGDL*
>ENSG00000167232|ENST00000300619
MPGTPGSLEMGLLTFRDVAIEFSPEEWQCLDTAQQNLYRNVMLENYRNLAFLGIALSKPD
LITYLEQGKEPWNMKQHEMVDEPTGICPHFPQDFWPEQSMEDSFQKVLLRKYEKCGHENL
QLRKGCKSVDECKVHKEGYNKLNQCLTTAQSKVFQCGKYLKVFYKFLNSNRHTIRHTGKK
CFKCKKCVKSFCIRLHKTQHKCVYITEKSCKCKECEKTFHWSSTLTNHKEIHTEDKPYKC
EECGKAFKQLSTLTTHKIICAKEKIYKCEECGKAFLWSSTLTRHKRIHTGEKPYKCEECG
KAFSHSSTLAKHKRIHTGEKPYKCEECGKAFSRSSTLAKHKRIHTGEKPYKCKECGKAFS
NSSTLANHKITHTEEKPYKCKECDKAFKRLSTLTKHKIIHAGEKLYKCEECGKAFNRSSN
LTIHKFIHTGEKPYKCEECGKAFNWSSSLTKHKRFHTREKPFKCKECGKAFIWSSTLTRH
KRIHTGEKPYKCEECGKAFRQSSTLTKHKIIHTGEKPYKFEECGKAFRQSLTLNKHKIIH
SREKPYKCKECGKAFKQFSTLTTHKIIHAGKKLYKCEECGKAFNHSSSLSTHKIIHTGEK
SYKCEECGKAFLWSSTLRRHKRIHTGEKPYKCEECGKAFSHSSALAKHKRIHTGEKPYKC
KECGKAFSNSSTLANHKITHTEEKPYKCKECDKTFKRLSTLTKHKIIHAGEKLYKCEECG
KAFNRSSNLTIHKFIHTGEKPYKCEECGKAFNWSSSLTKHKRIHTREKPFKCKECGKAFI
WSSTLTRHKRIHTGEKPYKCEECGKAFSRSSTLTKHKTIHTGEKPYKCKECGKAFKHSSA
LAKHKIIHAGEKLYKCEECGKAFNQSSNLTTHKIIHTKEKPSKSEECDKAFIWSSTLTEH
KRIHTREKTYKCEECGKAFSQPSHLTTHKRMHTGEKPYKCEECGKAFSQSSTLTTHKIIH
TGEKPYKCEECGKAFRKSSTLTEHKIIHTGEKPYKCEECGKAFSQSSTLTRHTRMHTGEK
PYKCEECGKAFNRSSKLTTHKIIHTGEKPYKCEECGKAFISSSTLNGHKRIHTREKPYKC
EECGKAFSQSSTLTRHKRLHTGEKPYKCGECGKAFKESSALTKHKIIHTGEKPYKCEKCG
KAFNQSSILTNHKKIHTITPVIPLLWEAEAGGSRGQEMETILANTVKPLLY*
>ENSG00000139083|ENST00000266427
XMTYEKMSRALRHYYKLNIIRKEPGQRLLFRFLFFLPRGSGLFLLLLGLG*
>ENSG00000169413|ENST00000304677
MVLCFPLLLLLLVLWGPVCPLHAWPKRLTKAHWFEIQHIQPSPLQCNRAMSGINNYTQHC
KHQNTFLHDSFQNVAAVCDLLSIVCKNRRHNCHQSSKPVNMTDCRLTSGKYPQCRYSAAA
QYKFFIVACDPPQKSDPPYKLVPVHLDSIL*
>ENSG00000084093|ENST00000309042
MATQVMGQSSGGGGLFTSSGNIGMALPNDMYDLHDLSKAELAAPQLIMLANVALTGEVNG
SCCDYLVGEERQMAELMPVGDNNFSDSEEGEGLEESADIKGEPHGLENMELRSLELSVVE
PQPVFEASGAPDIYSSNKDLPPETPGAEDKGKSSKTKPFRCKPCQYEAESEEQFVHHIRV
HSAKKFFVEESAEKQAKARESGSSTAEEGDFSKGPIRCDRCGYNTNRYDHYTAHLKHHTR

AGDNERVYKCIICTYTTVSEYHWRKHLRNHFPRKVYTCGKCNYFSDRKNNYVQHVRTHTG
ERPYKCELCPYSSSQKTHLTRHMRTHSGEKPFKCDQCSYVASNQHEVTRHARQVHNGPKP
LNCPHCDYKTADRSNFKKHVELHVNPRQFNCPVCDYAASKKCNLQYHFKSKHPTCPNKTM
DVSKVKLKKTKKREADLPDNITNEKTEIEQTKIKGDVAGKKNEKSVKAEKRDVSKEKKPS
NNVSVIQVTTRTRKSVTEVKEMDVHTGSNSEKFSKTKKSKRKLEVDSHSLHGPVNDEESS
TKKKKKVESKSKNNSQEVPKGDSKVEENKKQNTCMKKSTKKKTLKNKSSKKSSKPPQKEP
VEKGSAQMDPPQMGPAPTEAVQKGPVQVEPPPPMEHAQMEGAQIRPAPDEPVQMEVVQEG
PAQKELLPPVEPAQMVGAQIVLAHMELPPPMETAQTEVAQMGPAPMEPAQMEVAQVESAP
MQVVQKEPVQMELSPPMEVVQKEPVQIELSPPMEVVQKEPVKIELSPPIEVVQKEPVQME
LSPPMGVVQKEPAQREPPPPREPPLHMEPISKKPPLRKDKKEKSNMQSERARKEQVLIEV
GLVPVKDSWLLKESVSTEDLSPPSPPLPKENLREEASGDQKLLNTGEGNKEAPLQKVGAE
EADESLPGLAANINESTHISSSGQNLNTPEGETLNGKHQTDSIVCEMKMDTDQNTRENLT
GINSTVEEPVSPMLPPSAVEEREAVSKTALASPPATMAANESQEIDEDEGIHSHEGSDLS
DNMSEGSDDSGLHGARPVPQESSRKNAKEALAVKAAKGDFVCIFCDRSFRKGKDYSKHLN
RHLVNVYYLEEAAQGQE*
>ENSG00000083223|ENST00000277141
MGNEHISVHPENSDCIQADVNSDDYKGDKVYHPETGRKNEKEKVGRKGKHLLTVDQKRGE
HVVCGSTRNNESESTLDLEGFQNPTAKECEGLATLDNKADLDGESTEGTEELEDSLNHFT
HSVQGQTSEMIPSDEEEEDDEEEEEEEEPRLTINQREDEDGMANEDELDNTYTGSGDEDA
LSEEDDELGEAAKYEDVKECGKHVERALLVELNKISLKEENVCEEKNSPVDQSDFFYEFS
KLIFTKGKSPTVVCSLCKREGHLKKDCPEDFKRIQLEPLPPLTPKFLNILDQVCIQCYKD
FSPTIIEDQAREHIRQNLESFIRQDFPGTKLSLFGSSKNGFGFKQSDLDVCMTINGLETA
EGLDCVRTIEELARVLRKHSGLRNILPITTAKVPIVKFFHLRSGLEVDISLYNTLALHNT
RLLSAYSAIDPRVKYLCYTMKVFTKMCDIGDASRGSLSSYAYTLMVLYFLQQRNPPVIPV
LQEIYKGEKKPEIFVDGWNIYFFDQIDELPTYWSECGKNTESVGQLWLGLLRFYTEEFDF
KEHVISIRRKSLLTTFKKQWTSKYIVIEDPFDLNHNLGAGLSRKMTNFIMKAFINGRRVF
GIPVKGFPKDYPSKMEYFFDPDVLTEGELAPNDRCCRICGKIGHFMKDCPMRRKVRRRRD
QEDALNQRYPENKEKRSKEDKEIHNKYTEREVSTKEDKPIQCTPQKAKPMRAAADLGREK
ILRPPVEKWKRQDDKDLREKRCFICGREGHIKKECPQFKGSSGSLSSKYMTQGKASAKRT
QQES*
>ENSG00000166598|ENST00000299767
MRALWVLGLCCVLLTFGSVRADDEVDVDGTVEEDLGKSREGSRTDDEVVQREEEAIQLDG
LNASQIRELREKSEKFAFQAEVNRMMKLIINSLYKNKEIFLRELISNASDALDKIRLISL
TDENALSGNEELTVKIKCDKEKNLLHVTDTGVGMTREELVKNLGTIAKSGTSEFLNKMTE
AQEDGQSTSELIGQFGVGFYSAFLVADKVIVTSKHNNDTQHIWESDSNEFSVIADPRGNT
LGRGTTITLVLKEEASDYLELDTIKNLVKKYSQFINFPIYVWSSKTETVEEPMEEEEAAK
EEKEESDDEAAVEEEEEEKKPKTKKVEKTVWDWELMNDIKPIWQRPSKEVEEDEYKAFYK
SFSKESDDPMAYIHFTAEGEVTFKSILFVPTSAPRGLFDEYGSKKSDYIKLYVRRVFITD
DFHDMMPKYLNFVKGVVDSDDLPLNVSRETLQQHKLLKVIRKKLVRKTLDMIKKIADDKY
NDTFWKEFGTNIKLGVIEDHSNRTRLAKLLRFQSSHHPTDITSLDQYVERMKEKQDKIYF
MAGSSRKEAESSPFVERLLKKGYEVIYLTEPVDEYCIQALPEFDGKRFQNVAKEGVKFDE
SEKTKESREAVEKEFEPLLNWMKDKALKDKIEKAVVSQRLTESPCALVASQYGWSGNMER
IMKAQAYQTGKDISTNYYASQKKTFEINPRHPLIRDMLRRIKEDEDDKTVLDLAVVLFET
ATLRSGYLLPDTKAYGDRIERMLRLSLNIDPDAKVEEEPEEEPEETAEDTTEDTEQDEDE

EMDVGTDEEEETAKESTAEKDEL*
>ENSG00000159111|ENST00000290208
MISAHCNLHLPGSSDSPASASQVAGITGRLPTLQTVRYGSKAVTRHRRVMHFQRQKLMAV
TEYIPPKPAIHPSCLPSPPSPPQEEIGLIRLLRREIAAVFQDNRMIAVCQNVALSAEDKL
LMRHQLRKHKILMKVFPNQVLKPFLEDSKYQNLLPLFVGHNMLLVSEEPKVKEMVRILRT
VPFLPLLGGCIDDTILSRQGFINYSKLPSLPLVQGELVGGLTCLTAQTHSLLQHQPLQLT
TLLDQYIREQREKDSVMSANGKPDPDTVPDS*
>ENSG00000141994|ENST00000309061
MAEGTAEAPLENGGGDSGAGALERGVAPIKRQYLTTKEQFHQFLEAKGQEKTCRETEVG
DPAGNELAEPEAKRIRLEDGQTADGQTEEAAEPGEQLQTQKRARGQNKGRPHVKPTNYDK
NRLCPSLIQESAAKCFFGDRCRFLHDVGRYLETKPADLGPRCVLFETFGRCPYGVTCRFA
GAHLRPEGQNLVQEELAARGTQPPSIRNGLDKALQQQLRKREVRFERAEQALRRFSQGPT
PAAAVPEGTAAEGAPRQENCGAQQVPAGPGTSTPPSSPVRTCGPLTDEDVVRLRPCEKKR
LDIRGKLYLAPLTTCGNLPFRRICKRFGADVTCGEMAVCTNLLQGQMSEWALLKRHQCED
IFGVQLEGAFPDTMTKCAELLSRTVEVDFVDINVGCPIDLVYKKGGGCALMNRSTKFQQI
VRGMNQVLDVPLTVKIRTGVQERVNLAHRLLPELRDWGVALVTLHGRSREQRYTKLADWQ
YIEECVQAASPMPLFGNGDILSFEDANRAMQTGVTGIMIARGALLKPWLFTEIKEQRHWD
ISSSERLDILRDFTNYGLEHWGSDTQGVEKTRRFLLEWLSFLCRYVPVGLLERLPQRINE
RPPYYLGRDYLETLMASQKAADWIRISEMLLGPVPPSFAFLPKHKANAYK*
>ENSG00000174740|ENST00000312600
MGSGEPNPAGKKKKYLKAALYVGDLDPDVTEDMLYKKFRPAGPLRFTRICRDPVTRSPLG
YGYVNFRFPADAEWALNTMNFDLINGKPFRLMWSQPDDRLRKSGVGNIFIKNLDKSIDNR
ALFYLFSAFGNILSCKVVCDDNGSKGYAYVHFDSLAAANRAIWHMNGVRLNNRQVYVGRF
KFPEERAAEVRTRDRATFTNVFVKNIGDDIDDEKLKELFCEYGPTESVKVIRDASGKSKG
FGFVRYETHEAAQKAVLDLHGKSIDGKVLYVGRAQKKIERLAELRRRFERLRLKEKSRPP
GVPIYIKNLDETINDEKLKEEFSSFGSISRAKVMMEVGQGKGFGVVCFSSFEEATKAVDE
MNGRIVGSKPLHVTLGQARRRC*
>ENSG00000037241|ENST00000265100
MKFNPFVTSDRSKNRKRHFNAPSHVRRKIMSSPLSKELRQKYNVRSMPIRKDDEVQVVRG
HYKGQQIGKVVQVYRKKYVIYIERVQREKANGTTVHVGIHPSKVVITRLKLDKDRKKILE
RKAKSRQVGKEKGKYKEELIEKMQE*
>ENSG00000156273|ENST00000286800
MSLSENSVFAYESSVHSTNVLLSLNDQRKKDVLCDVTIFVEGQRFRAHRSVLAACSSYFH
SRIVGQADGELNITLPEEVTVKGFEPLIQFAYTAKLILSKENVDEVCKCVEFLSVHNIEE
SCFQFLKFKFLDSTADQQECPRKKCFSSHCQKTDLKLSLLDQRDLETDEVEEFLENKNVQ
TPQCKLRRYQGNAKASPPLQDSASQTYESMCLEKDAALALPSLCPKYRKFQKAFGTDRVR
TGESSVKDIHASVQPNERSENECLGGVPECRDLQVMLKCDESKLAMEPEETKKDPASQCP
TEKSEVTPFPHNSSIDPHGLYSLSLLHTYDQYGDLNFAGMQNTTVLTEKPLSGTDVQEKT
FGESQDLPLKSDLGTREDSSVASSDRSSVEREVAEHLAKGFWSDICSTDTPCQMQLSPAV
AKDGSEQISQKRSECPWLGIRISESPEPGQRTFTTLSSVNCPFISTLSTEGCSSNLEIGN
DDYVSEPQQEPCPYACVISLGDDSETDTEGDSESCSAREQECEVKLPFNAQRIISLSRND
FQSLLKMHKLTPEQLDCIHDIRRRSKNRIAAQRCRKRKLDCIQNLESEIEKLQSEKESLL
KERDHILSTLGETKQNLTGLCQKVCKEAALSQEQIQILAKYSAADCPLSFLISEKDKSTP
DGELALPSIFSLSDRPPAVLPPCARGNSEPGYARGQESQQMSTATSEQAGPAEQCRQSGG

ISDFCQQMTDKCTTDE*
>ENSG00000175879|ENST00000313173
MSSYFVNPLYSKYKAAAAAAAAAGEAINPTYYDCHFAPEVGGRHAAAAAALQLYGNSAAG
FPHAPPQAHAHPHPSPPPSGTGCGGREGRGQEYFHPGGGSPAAAYQAAPPPPPHPPPPPP
PPPCGGIACHGEPAKFYGYDNLQRQPIFTTQQEAELVQYPDCKSSSGNIGEDPDHLNQSS
SPSQMFPWMRPQAAPGRRRGRQTYSRFQTLELEKEFLFNPYLTRKRRIEVSHALALTERQ
VKIWFQNRRMKWKKENNKDKFPVSRQEVKDGETKKEAQELEEDRAEGLTN*
>ENSG00000165496|ENST00000298283
MGRRPARCYRYCKNKPYPKSRFCRGVPDAKIRIFDLGRKKAKVDEFPLGGHMVSDEYEQL
SSEALEAARICANKYMVKSCGRDGFHMRVRLHPFHVIRINKMLSCAGADRLQTGMRGAFG
KPQGTVARVHIGQVIMSIRTKLQNEEHVIEALRRAKFKFPGRQKIHISKKWGFTKFNADE
FEDMVAKKCLIPDGCGVKYVPSHGPLDKWRVLHS*
>ENSG00000145354|ENST00000273986
MVLESVARIVKVQLPAYLKRLPVPESITGFARLTVSEWLRLLPFLGVLALLGYLAVRPFL
PKKKQQKDSLINLKIQKENPKVVNEINIEDLCLTKAAYCRCWRSKTFPACDGSHNKHNEL
TGDNVGPLILKKKEV*
>ENSG00000122729|ENST00000309951
MSNPFAHLAEPLDPVQPGKKFFNLNKLEDSRYGRLPFSIRVLLEAAIRNCDEFLVKKQDI
ENILHWNVTQHKNIEVPFKPARVILQDFTGVPAVVDFAAMRDAVKKLGGDPEKINPVCPA
DLVIDHSIQVDFNRRADSLQKNQDLEFERNRERFEFLKWGSQAFHNMRIIPPGSGIIHQV
NLEYLARVVFDQDGYYYPDSLVGTDSHTTMIDGLGILGWGVGGIEAEAVMLGQPISMVLP
QVIGYRLMGKPHPLVTSTDIVLTITKHLRQVGVVGKFVEFFGPGVAQLSIADRATIANMC
PEYGATAAFFPVDEVSITYLVQTGRDEEKLKYIKKYLQAVGMFRDFNDPSQDPDFTQVVE
LDLKTVVPCCSGPKRPQDKVAVSDMKKDFESCLGAKQGFKGFQVAPEHHNDHKTFIYDNT
EFTLAHGSVVIAAITSCTNTSNPSVMLGAGLLAKKAVDAGLNVMPYIKTSLSPGSGVVTY
YLQESGVMPYLSQLGFDVVGYGCMTCIGNSGPLPEPVVEAITQGDLVAVGVLSGNRNFEG
RVHPNTRANYLASPPLVIAYAIAGTIRIDFEKEPLGVNAKGQQVFLKDIWPTRDEIQAVE
RQYVIPGMFKEVYQKIETVNESWNALATPSDKLFFWNSKSTYIKSPPFFENLTLDLQPPK
SIVDAYVLLNLGDSVTTDHISPAGNIARNSPAARYLTNRGLTPREFNSYGSRRGNDAVMA
RGTFANIRLLNRFLNKQAPQTIHLPSGEILDVFDAAERYQQAGLPLIVLAGKEYGAGSSR
DWAAKGPFLLGIKAVLAESYERIHRSNLVGMGVIPLEYLPGENADALGLTGQERYTIIIP
ENLKPQMKVQVKLDTGKTFQAVMRFDTDVELTYFLNGGILNYMIRKMAK*
>ENSG00000173431|ENST00000308227
MAPARAGCCPLLLLLLGLWVAEVLVRAKPKDMTSSQWFKTQHVQPSPQACNSAMSIINKY
TERCKDLNTFLHEPFSSVAITCQTPNIACKNSCKNCHQSHGPMSLTMGELTSGKYPNCRY
KEKHLNTPYIVACDPPQQGDPGYPLVPVHLDKVV*
>ENSG00000164113|ENST00000296513
MASNNHWFQSSQVPSFAQMLKKNLPVQPATKTITTPTGWSSESYGLSKMASKVTQVTGNF
PEPLLSKNLSSISNPVLPPKKIPKEFIMKYKRGEINPVSALHQFAQMQRVQLDLKETVTT
GNVMGPYFAFCAVVDGIQYKTGLGQNKKESRSNAAKLALDELLQLDEPEPRILETSGPPP
FPAEPVVLSELAYVSKVHYEGRHIQYAKISQIVKERFNQLISNRSEYLKYSSSLAAFIIE
RAGQHEVVAIGTGEYNYSQDIKPDGRVLHDTHAVVTARRSLLRYFYRQLLLFYSKNPAMM
EKSIFCTEPTSNLLTLKQNINICLYMNQLPKGSAQIKSQLRLNPHSISAFEANEELCLHV
AVEGKIYLTVYCPKDGVNRISSMSSSDKLTRWEVLGVQGALLSHFIQPVYISSILIGDGN

CSDTRGLEIAIKQRVDDALTSKLPMFYLVNRPHISLVPSAYPLQMNLEYKFLSLNWAQGD
VSLEIVDGLSGKITESSPFKSGMSMASRLCKAAMLSRFNLLAKEAKKELLEAGTYHAAKC
MSASYQEAKCKLKSYLQQHGYGSWIVKSPCIEQFNM*
>ENSG00000164252|ENST00000312916
MASEAPSPPRSPPPPTSPEPELAQLRRKVEKLERELRSCKRQVREIEKLLHHTERLYQNA
ESNNQELRTQVEELSKILQRGRNEDNKKSDVEVQTENHAPWSISDYFYQTYYNDVSLPNK
VTELSDQQDQAIETSILNSKDHLQVENDAYPGTDRTENVKYRQVDHFASNSQEPASALAT
EDTSLEGSSLAESLRAAAEAAVSQTGFSYDENTGLYFDHSTGFYYDSENQLYYDPSTGIY
YYCDVESGRYQFHSRVDLQPYPTSSTKQSKDKKLKKKRKDPDSSATNEEKDLNSEDQKAF
SVEHTSCNEEENFANMKKKAKIGIHHKNSPPKVTVPTSGNTIESPLHENISNSTSFKDEK
IMETDSEPEEGEITDSQTEDSYDEAITSEGNVTAEDSEDEDEDKIWPPCIRVIVIRSPVL
QIGSLFIITAVNPATIGREKDMEHTLRIPEVGVSKFHAEIYFDHDLQSYVLVDQGSQNGT
IVNGKQILQPKTKCDPYVLEHGDEVKIGETVLSFHIHPGSDTCDGCEPGQVRAHLRLDKK
DESFVGPTLSKEEKELERRKELKKIRVKYGLQNTEYEDEKTLKNPKYKDRAGKRREQVGS
EGTFQRDDAPASVHSEITDSNKGRKMLEKMGWKKGEGLGKDGGGMKTPIQLQLRRTHAGL
GTGKPSSFEDVHLLQNKNKKNWDKARERFTENFPETKPQKDDPGTMPWVKGTLE*
>ENSG00000133895|ENST00000312049
MGLKAAQKTLFPLRSIDDVVRLFAAELGREEPDLVLLSLVLGFVEHFLAVNRVIPTNVPE
LTFQPSPAPDPPGGLTYFPVADLSIIAALYARFTAQIRGAVDLSLYPREGGVSSRELVKK
VSDVIWNSLSRSYFKDRAHIQSLFSFITGTKLDSSGVAFAVVGACQALGLRDVHLALSED
HAWVVFGPNGEQTAEVTWHGKGNEDRRGQTVNAGVAERSWLYLKGSYMRCDRKMEVAFMV
CAINPSIDLHTDSLELLQLQQKLLWLLYDLGHLERYPMALGNLADLEELEPTPGRPDPLT
LYHKGIASAKTYYRDEHIYPYMYLAGYHCRNRNVREALQAWADTATVIQDYNYCREDEEI
YKEFFEVANDVIPNLLKEAASLLEAGEERPGEQSQGTQSQGSALQDPECFAHLLRFYDGI
CKWEEGSPTPVLHVGWATFLVQSLGRFEGQVRQKVRIVSREAEAAEAEEPWGEEAREGRR
RGPRRESKPEEPPPPKKPALDKGLGTGQGAVSGPPRKPPGTVAGTARGPEGGSTAQVPAP
TASPPPEGPVLTFQSEKMKGMKELLVATKINSSAIKLQLTAQSQVQMKKQKVSTPSDYTL
SFLKRQRKGL*
>ENSG00000156482|ENST00000287038
MVAAKKTKKSLESINSRLQLVMKSGKYVLGYKQTLKMIRQGKAKLVILANNCPALRKSEI
EYYAMLAKTGVHHYSGNNIELGTACGKYYRVCTLAIIDPGDSDIIRSMPEQTGEK*
>ENSG00000152518|ENST00000282388
MSTTLLSAFYDVDFLCKTEKSLANLNLNNMLDKKAVGTPVAAAPSSGFAPGFLRRHSASN
LHALAHPAPSPGSCSPKFPGAANGSSCGSAAAGGPTSYGTLKEPSGGGGTALLNKENKFR
DRSFSENGDRSQHLLHLQQQQKGGGGSQINSTRYKTELCRPFEESGTCKYGEKCQFAHGF
HELRSLTRHPKYKTELCRTFHTIGFCPYGPRCHFIHNADERRPAPSGGASGDLRAFGTRD
ALHLGFPREPRPKLHHSLSFSGFPSGHHQPPGGLESPLLLDSPTSRTPPPPSCSSASSCS
SSASSCSSASAASTPSGAPTCCASAAAAAAAALLYGTGGAEDLLAPGAPCAACSSASCAN
NAFAFGPELSSLITPLAIQTHNFAAVAAAAYYRSQQQQQQQGLAPPAQPPAPPSATLPAG
AAAPPSPPFSFQLPRRLSDSPVFDAPPSPPDSLSDRDSYLSGSLSSGSLSGSESPSLDPG
RRLPIFSRLSISDD*
>ENSG00000196357|ENST00000304116
MAQGLVTFRDVAIEFSLEEWKCLEPAQRDLYREVTLENFGHLASLGLSISKPDVVSLLEQ
GKEPWMIANDVTGPWCPDLESRCEKFLQKDIFEIGAFNWEIMESLKCSDLEGSDFRADWE

CEGQFERQVNEECYFKQVNVTYGHMPVFQHHTSHTVRQSRETGEKLMECHECGKAFSRGS
HLIQHQKIHTGEKPFGCKECGKAFSRASHLVQHQRIHTGEKPYDCKDCGKAFGRTSELIL
HQRLHTGVKPYECKECGKTFRQHSQLILHQRTHTGEKPYVCKDCGKAFIRGSQLTVHRRI
HTGARPYECKECGKAFRQHSQLTVHQRIHTGEKPYECKECGKGFIHSSEVTRHQRIHSGE
KPYECKECGKAFRQHAQLTRHQRVHTGDRPYECKDCGKAFSRSSYLIQHQRIHTGDKPYE
CKECGKAFIRVSQLTHHQRIHTCEKPYECRECGMAFIRSSQLTEHQRIHPGIKPYECREC
GQAFILGSQLIEHYRIHTG*
>ENSG00000188868|ENST00000293725
MDAVAFEDVAVNFTQEEWALLGPSQKNLYRYVMQETIRNLDCIRMIWEEQNTEDQYKNPR
RNLRCHMVERFSESKDSSQCGETFSLIRDSIVNNSICPGEDPCQSAECEEVIMGHLSLNS
HIRVDSGHKPHEYQEYGEKPHTHKQRGKAFSYHHSFQSRGRPHTGKKRYECKECGKTFSS
RRNLRRHMVVQGGNRPYKCKLCGKAFFWPSLLRMHERTHTGEKPYECKQCSKAFPFYSSY
RRHERMHTGEKPYECKQCSKALPDSSSYIRHERTHTGEKPYTCKQCGKAFSVSSSLRRHE
TTHSAEKPYECKQCGKTFHHLGSFQIHMKRHTGDRPHKCKICGKGFDRPSLVRYHERIHT
GEKPYECKQCGKTLSHSSSFRRHMIMHTGGGPHKCKICGKAFVYPSVCQRHEKSHSGEKP
YECKQCGKALSHSSSFRRHMVMHTGDGPNKCKVCGKAFVYPSVCQRHEKTHWRETI*
>ENSG00000118156|ENST00000314121
MDQYSLGDEGALPSEMHLPSFSESQGLNCSDTLNRDLGPNTRGFLYAGLSGLDPDPSLPT
PDMSSEVLEDNLDTLSLYSGKDSDSVKLLEEYADSESQASLQDLGLGVLKAKEADEGGRA
TSGSARKGKRQHSSPQNPLLDCSLCGKVFSSASSLSKHYLTHSQERKHVCKICSKAFKRQ
DHLTGHMLTHQKTKPFVCIEQGCSKSYCDYRSLRRHYEVHHGLCILKEAPPEEEACGDSP
HAHESAGQPPPSSLRSLVPPEARSPGSLLPHRDLLRRIVSSIVHQKTPSPGPAPAGASDS
EGRNTACPCPASSGSSSCTPAGPHAAPAALDTELPEEPCLPQKEPATDVFTAPNSRAAEN
GAPDPPEPEPDTALLQARSTAECWPEGGSVPACLPLFRGQTVPASSQPSSHSFQWLRNLP
GCPKSKGNNVFVVHKPSAVPSREGSESGPGPSSGSPSEESPPGPGGGLEDALPFPAALLR
VPAEAPSDPRSASGEDDPCAPKKVKVDCDSFLCQNPGEPGLQEAQKAGGLPADASPLFRQ
LFLKSQEPLVSHEQMQVFQMITKSQRIFSHAQVAAVSSQLPAPEGKPAALRPLQGPWPQQ
PPPLAPAVDSLHAGPGNPEAEGSPARRRKTTPGVPREASPGSTRRDAKGGLKVAAVPTPL
AAPSLDPSRNPDISSLAKQLRSSKGTLDLEDIFPSTGQRQTQLGGEEPPGASLPGKQAPA
ENGAASRITKGEKGPACSRGGGYRLLGNPRAPRFSGFRKEKAKMDMCCAASPSQVAMASF
SSAGPPADPSKSKLTIFSRIQGGNIYRLPHPVKEENVAGRGNQQNGSPTDWTKPRSTFVC
KNCSQMFYTEKGLSSHMCFHSDQWPSPRGKQEPQVFGTEFCKPLRQVLRPEGDRHSPPGT
KKPLDPTAAAPLVVPQSIPVVPVTRHIGSMAMMEWCSRWLQATTCGSKLLQGQEKDGEER
DSKESSQQRKRKKRPPPSTAGEPGPAGCHQSRLRSPMFLVDCLLKGLFQCSPYTPPPMLS
PIREGSGVYFNTLCSTSTQASPDQLISSMLDQVDGSFGICVVKDDTKISIEPHINIGSRF
QAEIPELQERSLAGTDEHVASLVWKPWGDMMISSETQDRVTELCNVACSSVMPGGGTNLE
LALHCLHEAQGNVQVALETLLLRGPHKPRTHLLADYRYTGSDVWTPIEKRLFKKAFYAHK
KDFYLIHKMIQTKTVAQCVEYYYIWKKMIKFDCGRAPGLEKRVKREPEEVERTEEKVPCS
PRERPSHHPTPKLKTKSYRRESILSSSPNAGSKRTPELLGSAESQGIFPCRECERVFDKI
KSRNAHMKRHRLQDHVEPIIRVKWPVKPFQLKEEELGADIGPLQW*
>ENSG00000187754|ENST00000298181
MNGDDAFARRPRAGAQIPEKIQKSFDDIAKYFSKKEWEKMKSLEKISYVYMKRKYEAMTK
LGFKATLPPFMHNTGATDLQGNDFDNDRNQGNQVERPQMTFCRLQRIFPKIMPKKPAEEG
NDSKGVPEASGSQNDGKHLCPPGKPSTSEKINKTSGPKRGKHAWTHRLRERKQLVIYEEI

SDPEEDDE*
>ENSG00000197050|ENST00000304239
MARKLVMFRDVAIDFSQEEWECLDSAQRDLYRDVMLENYSNLVSLDLPSRCASKDLSPEK
NTYETELSQWEMSDRLENCDLEESNSRDYLEAKGKMEKQQENQKEYFRQGMIIYDKMSIF
NQHTYLSQHSRCHSTEKPYKCKECGKAFRRASHLTQHQSIHTGEKPYECKQCGKAFSRDS
QLSLHQRLHTGEKPYACKECGKAFTQSSQLILHHRIHTGEKPYKCEECGKAFIRSSQLTR
HQKVHTGEKPYECKECGKAFTQNSQLTLHQRLHTGEKLYECKECRKVFTQLSQLILHKRI
HTGEKPYECKECGKAFICGSQLSQHQKIHNGEKPYECKECGRAFIRGSLLMQHQRIHTGE
KPYKCEECGKAFIRGSQLTQHQRIHTNEKPYECKECGKMFSHGSQLTQHQRIHTGEKPYQ
CKECGKAFNRGSLLTRHQRIHTGEKPYECKECGKTFSRGSELTQHERIHTAFLAGQQDRS
DIYMSRRFTKSIFPVFFSVPTMWTSKLELRYSHGDFENGIWVLWIMEQKYRRSLQP*
>ENSG00000165832|ENST00000298746
MAASEAAVVSSPSLKTDTSPVLETAGTVAAMAATPSARAAAAVVAAAARTGSEARVSKAA
LATKLLSLSGVFAVHKPKGPTSAELLNRLKEKLLAEAGMPSPEWTKRKKQTLKIGHGGTL
DSAARGVLVVGIGSGTKMLTSMLSGSKRYTAIGELGKATDTLDSTGRVTEEKPYDKITQE
DIEGILQKFTGNIMQVPPLYSALKKDGQRLSTLMKRGEVVEAKPARPVTVYSISLQKFQP
PFFTLDVECGGGFYIRSLVSDIGKELSSCANVLELTRTKQGPFTLEEHALPEDKWTIDDI
AQSLEHCSSLFPAELALKKSKPESNEQVLSCEYITLNEPKREDDVIKTC*
>ENSG00000140987|ENST00000304926
MRDNRAVSLCQQEWMCPGPAQRALYRGATQRKDSHVSLATGVPWGYEETKTLLAILSSSQ
FYGKLQTCQQNSQIYRAMAEGLWEQGFLRTPEQCRTKFKSLQLSYRKVRRGRVPEPCIFY
EEMNALSGSWASAPPMASDAVPGQEGSDIEAGELNHQNGEPTEVEDGTVDGADRDEKDFR
NPGQEVRKLDLPVLFPNRLGFEFKNEIKKENLKWDDSEEVEINKALQRKSRGVYWHSELQ
KGLESEPTSRRQCRNSPGESEEKTPSQEKMSHQSFCARDKACTHILCGKNCSQSVHSPHK
PALKLEKVSQCPECGKTFSRSSYLVRHQRIHTGEKPHKCSECGKGFSERSNLTAHLRTHT
GERPYQCGQCGKSFNQSSSLIVHQRTHTGEKPYQCIVCGKRFNNSSQFSAHRRIHTGESP
YKCAVCGKIFNNSSHFSAHRKTHTGEKPYRCSHCERGFTKNSALTRHQTVHMKAVLSSQE
GRDAL*
>ENSG00000160294|ENST00000291688
MNPTNPFSGQQPSAFSASSSNVGTLPSKPPFRFGQPSLFGQNSTLSGKSSGFSQVSSFPA
SSGVSHSSSVQTLGFTQTSSVGPFSGLEHTSTFVATSGPSSSSVLGNTGFSFKSPTSVGA
FPSTSAFGQEAGEIVNSGFGKTEFSFKPLENAVFKPILGAESEPEKTQSQIASGFFTFSH
PISSAPGGLAPFSFPQVTSSSATTSNFTFSKPVSSNNSLSAFTPALSNQNVEEEKRGPKS
IFGSSNNSFSSFPVSSAVLGEPFQASKAGVRQGCEEAVSQVEPLPSLMKGLKRKEDQDRS
PRRHGHEPAEDSDPLSRGDHPPDKRPVRLNRPRGGTLFGRTIQDVFKSNKEVGRLGNKEA
KKETGFVESAESDHMAIPGGNQSVLAPSRIPGVNKEEETESREKKEDSLRGTPARQSNRS
ESTDSLGGLSPSEVTAIQCKNIPDYLNDRTILENHFGKIAKVQRIFTRRSKKLAVVHFFD
HASAALARKKGKSLHKDMAIFWHRKKISPNKKPFSLKEKKPGDGEVSPSTEDAPFQHSPL
GKAAGRTGASSLLNKSSPVKKPSLLKAHQFEGDSFDSASEGSEGLGPCVLSLSTLIGTVA
ETSKEKYRLLDQRDRIMRQARVKRTDLDKARTFVGTCLDMCPEKERYMRETRSQLSVFEV
VPGTDQVDHAAAVKEYSRSSADQEEPLPHELRPLPVLSRTMDYLVTQIMDQKEGSLRDWY
DFVWNRTRGIRKDITQQHLCDPLTVSLIEKCTRFHIHCAHFMCEEPMSSFDAKINNENMT
KCLQSLKEMYQDLRNKGVFCASEAEFQGYNVLLSLNKGDILREVQQFHPAVRNSSEVKFA
VQAFAALNSNNFVRFFKLVQSASYLNACLLHCYFSQIRKDALRALNFAYTVSTQRSTIFP

LDGVVRMLLFRDCEEATDFLTCHGLTVSDGCVELNRSAFLEPEGLSKTRKSVFITRKLTV
SVGEIVNGGPLPPVPRHTPVCSFNSQNKYIGESLAAELPVSTQRPGSDTVGGGRGEECGV
EPDAPLSSLPQSLPAPAPSPVPLPPVLALTPSVAPSLFQLSVQPEPPPPEPVPMYSDEDL
AQVVDELIQEALQRDCEEVGSAGAAYAAAALGVSNAAMEDLLTAATTGILRHIAAEEVSK
ERERREQERQRAEEERLKQERELVLSELSQGLAVELMERVMMEFVRETCSQELKNAVETD
QRVRVARCCEDVCAHLVDLFLVEEIFQTAKETLQELQCFCKYLQRWREAVTARKKLRRQM
RAFPAAPCCVDVSDRLRALAPSAECPIAEENLARGLLDLGHAGRLGISCTRLRRLRNKTA
HQMKVQHFYQQLLSDVAWASLDLPSLVAEHLPGRQEHVFWKLVLVLPDVEEQSPESCGRI
LANWLKVKFMGDEGSVDDTSSDAGGIQTLSLFNSLSSKGDQMISVNVCIKVAHGALSDGA
IDAVETQKDLLGASGLMLLLPPKMKSEDMAEEDVYWLSALLQLKQLLQAKPFQPALPLVV
LVPSPGGDAVEKEVEDGLMLQDLVSAKLISDYTVTEIPDTINDLQGSTKVLQAVQWLVSH
CPHSLDLCCQTLIQYVEDGIGHEFSGRFFHDRRERRLGGLASQEPGAIIELFNSVLQFLA
SVVSSEQLCDLSWPVTEFAEAGGSRLLPHLHWNAPEHLAWLKQAVLGFQLPQMDLPPLGA
PWLPVCSMVVQYASQIPSSRQTQPVLQSQVENLLHRTYCRWKSKSPSPVHGAGPSVMEIP
WDDLIALCINHKLRDWTPPRLPVTSEALSEDGQICVYFFKNDLKKYDVPLSWEQARLQTQ
KELQLREGRLAIKPFHPSANNFPIPLLHMHRNWKRSTECAQEGRIPSTEDLMRGASAEEL
LAQCLSSSLLLEKEENKRFEDQLQQWLSEDSGAFTDLTSLPLYLPQTLVSLSHTIEPVMK
TSVTTSPQSDMMREQLQLSEATGTCLGERLKHLERLIRSSREEEVASELHLSALLDMVDI
*
>ENSG00000146143|ENST00000274891
MEFSGRKWRKLRLAGDQRNASYPHCLQFYLQPPSENISLIEFENLAIDRVKLLKSVENLG
VSYVKGTEQYQSKLESELRKLKFSYRENLEDEYEPRRRDHISHFILRLAYCQSEELRRWF
IQQEMDLLRFRFSILPKDKIQDFLKDSQLQFEAVSIFL*
>ENSG00000123405|ENST00000312156
MSPCPPQQSRNRVIQLSTSELGEMELTWQEIMSITELQGLNAPSEPSFEPQAPAPYLGPP
PPTTYCPCSIHPDSGFPLPPPPYELPASTSHVPDPPYSYGNMAIPVSKPLSLSGLLSEPL
QDPLALLDIGLPAGPPKPQEDPESDSGLSLNYSDAESLELEGTEAGRRRSEYVEMYPVEY
PYSLMPNSLAHSNYTLPAAETPLALEPSSGPVRAKPTARGEAGSRDERRALAMKIPFPTD
KIVNLPVDDFNELLARYPLTESQLALVRDIRRRGKNKVAAQNCRKRKLETIVQLERELER
LTNERERLLRARGEADRTLEVMRQQLTELYRDIFQHLRDESGNSYSPEEYALQQAADGTI
FLVPRGTKMEATD*
>ENSG00000122512|ENST00000265849
MERAESSSTEPAKAIKPIDRKSVHQICSGQVVLSLSTAVKELVENSLDAGATNIDLKLKD
YGVDLIEVSDNGCGVEEENFEGLTLKHHTSKIQEFADLTQVETFGFRGEALSSLCALSDV
TISTCHASAKVGTRLMFDHNGKIIQKTPYPRPRGTTVSVQQLFSTLPVRHKEFQRNIKKE
YAKMVQVLHAYCIISAGIRVSCTNQLGQGKRQPVVCTGGSPSIKENIGSVFGQKQLQSLI
PFVQLPPSDSVCEEYGLSCSDALHNLFYISGFISQCTHGVGRSSTDRQFFFINRRPCDPA
KVCRLVNEVYHMYNRHQYPFVVLNISVDSECVDINVTPDKRQILLQEEKLLLAVLKTSLI
GMFDSDVNKLNVSQQPLLDVEGNLIKMHAADLEKPMVEKQDQSPSLRTGEEKKDVSISRL
REAFSLRHTTENKPHSPKTPEPRRSPLGQKRGMLSSSTSGAISDKGVLRPQKEAVSSSHG
PSDPTDRAEVEKDSGHGSTSVDSEGFSIPDTGSHCSSEYAASSPGDRGSQEHVDSQEKAP
KTDDSFSDVDCHSNQEDTGCKFRVLPQPTNLATPNTKRFKKEEILSSSDICQKLVNTQDM
SASQVDVAVKINKKVVPLDFSMSSLAKRIKQLHHEAQQSEGEQNYRKFRAKICPGENQAA
EDELRKEISKTMFAEMEIIGQFNLGFIITKLNEDIFIVDQHATDEKYNFEMLQQHTVLQG

QRLIAPQTLNLTAVNEAVLIENLEIFRKNGFDFVIDENAPVTERAKLISLPTSKNWTFGP
QDVDELIFMLSDSPGVMCRPSRVKQMFASRACRKSVMIGTALNTSEMKKLITHMGEMDHP
WNCPHGRPTMRHIANLGVISQN*
>ENSG00000151090|ENST00000280696
MNYCMQEIYEVHPAAGSNCYMQSTDYYAYFEDSPGYSGCDAQAVPSNNIYMEQAWAVNQP
YTCSYPGNMFKSKDSDLDMALNQYSQPEYFTEEKPTFSQVQSPSYSQKKGYIPSYLDKDE
LCVVCGDKATGYHYRCITCEGCKGFFRRTIQKNLHPSYSCKYEGKCVIDKVTRNQCQECR
FKKCIYVGMATDLVLDDSKRLAKRKLIEENREKRRREELQKSIGHKPEPTDEEWELIKTV
TEAHVATNAQGSHWKQKRKFLPEDIGQAPIVNAPEGGKVDLEAFSHFTKIITPAITRVVD
FAKKLPMFCELPCEDQIILLKGCCMEIMSLRAAVRYDPESETLTLNGEMAVTRGQLKNGG
LGVVSDAIFDLGMSLSSFNLDDTEVALLQAVLLMSSDRPGLACVERIEKYQDSFLLAFEH
YINYRKHHVTHFWPKLLMKVTDLRMIGACHASRFLHMKVECPTELFPPLFLEVFED*
>ENSG00000055483|ENST00000312010
MPIVDKLKEALKPGRKDSADDGELGKLLASSAKKVLLQKIEFEPASKSFSYQLEALKSKY
VLLNPKTEGASRHKSGDDPPARRQGSEHTYESCGDGVPAPQKVLFPTERLSLRWERVFRV
GAGLHNLGNTCFLNATIQCLTYTPPLANYLLSKEHARSCHQGSFCMLCVMQNHIVQAFAN
SGNAIKPVSFIRDLKKIARHFRFGNQEDAHEFLRYTIDAMQKACLNGCAKLDRQTQATTL
VHQIFGGYLRSRVKCSVCKSVSDTYDPYLDVALEIRQAANIVRALELFVKADVLSGENAY
MCAKCKKKVPASKRFTIHRTSNVLTLSLKRFANFSGGKITKDVGYPEFLNIRPYMSQNNG
DPVMYGLYAVLVHSGYSCHAGHYYCYVKASNGQWYQMNDSLVHSSNVKVVLNQQAYVLFY
LRIPGSKKSPEGLISRTGSSSLPGRPSVIPDHSKKNIGNGIISSPLTGKRQDSGTMKKPH
TTEEIGVPISRNGSTLGLKSQNGCIPPKLPSGSPSPKLSQTPTHMPTILDDPGKKVKKPA
PPQHFSPRTAQGLPGTSNSNSSRSGSQRQGSWDSRDVVLSTSPKLLATATANGHGLKGND
ESAGLDRRGSSSSSPEHSASSDSTKAPQTPRSGAAHLCDSQETNCSTAGHSKTPPSGADS
KTVKLKSPVLSNTTTEPASTMSPPPAKKLALSAKKASTLWRATGNDLRPPPPSPSSDLTH
PMKTSHPVVASTWPVHRARAVSPAPQSSSRLQPPFSPHPTLLSSTPKPPGTSEPRSCSSI
STALPQVNEDLVSLPHQLPEASEPPQSPSEKRKKTFVGEPQRLGSETRLPQHIREATAAP
HGKRKRKKKKRPEDTAASALQEGQTQRQPGSPMYRREGQAQLPAVRRQEDGTQPQVNGQQ
VGCVTDGHHASSRKRRRKGAEGLGEEGGLHQDPLRHSCSPMGDGDPEAMEESPRKKKKKK
RKQETQRAVEEDGHLKCPRSAKPQDAVVPESSSCAPSANGWCPGDRMGLSQAPPVSWNGE
RESDVVQELLKYSSDKAYGRKVLTWDGKMSAVSQDAIEDSRQARTETVVDDWDEEFDRGK
EKKIKKFKREKRRNFNAFQKLQTRRNFWSVTHPAKAASLSYRR*
>ENSG00000162300|ENST00000294258
MGLCKCPKRKVTNLFCFEHRVNVCEHCLVANHAKCIVQSYLQWLQDSDYNPNCRLCNIPL
ASRETTRLVCYDLFHWACLNERAAQLPRNTAPAGYQCPSCNGPIFPPTNLAGPVASALRE
KLATVNWARAGLGLPLIDEVVSPEPEPLNTSDFSDWSSFNASSTPGPEEVDSASAAPAFY
SQAPRPPASPGRPEQHTVIHMGNPEPLTHAPRKVYDTRDDDRTPGLHGDCDDDKYRRRPA
LGWLARLLRSRAGSRKRPLTLLQRAGLLLLLGLLGFLALLALMSRLGRAAADSDPNLDPL
MNPHIRVGPS*
>ENSG00000166526|ENST00000303915
METQADLVSQEPQALLDSALPSKVPAFSDKDSLGDEMLAAALLKAKSQELVTFEDVAVYF
IRKEWKRLEPAQRDLYRDVMLENYGNVFSLDRETRTENDQEISEDTRSHGVLLGRFQKDI
SQGLKFKEAYEREVSLKRPLGNSPGERLNRKMPDFGQVTVEEKLTPRGERSEKYNDFGNS
FTVNSNLISHQRLPVGDRPHKCDECSKSFNRTSDLIQHQRIHTGEKPYECNECGKAFSQS

SHLIQHQRIHTGEKPYECSDCGKTFSCSSALILHRRIHTGEKPYECNECGKTFSWSSTLT
HHQRIHTGEKPYACNECGKAFSRSSTLIHHQRIHTGEKPYECNECGKAFSQSSHLYQHQR
IHTGEKPYECMECGGKFTYSSGLIQHQRIHTGENPYECSECGKAFRYSSALVRHQRIHTG
EKPLNGIGMSKSSLRVTTELNIREST*
>ENSG00000140836|ENST00000268489
MEGCDSPVVSGKDNGCGIPQHQQWTELNSTHLPDKPSSMEQSTGESHGPLDSLRAPFNER
LAESTASAGPPSEPASKEVTCNECSASFASLQTYMEHHCPSARPPPPLREESASDTGEEG
DEESDVENLAGEIVYQPDGSAYIVESLSQLTQGGGACGSGSGSGPLPSLFLNSLPGAGGK
QGDPSCAAPVYPQIINTFHIASSFGKWFEGPDQAFPNTSALAGLSPVLHSFRVFDVRHKS
NKDYLNSDGSAKSSCVSKDVPNNVDLSKFDGFVLYGKRKPILMCFLCKLSFGYVRSFVTH
AVHDHRMTLSEDERKILSNKNISAIIQGIGKDKEPLVSFLEPKNKNFQHPLVSTANLIGP
GHSFYGKFSGIRMEGEEALPAGSAAGPEQPQAGLLTPSTLLNLGGLTSSVLKTPITSVPL
GPLASSPTKSSEGKDSGAAEGEKQEVGDGDCFSEKVEPAEEEAEEEEEEEEAEEEEEEEE
EEEEEEEDEGCKGLFPSELDEELEDRPHEEPGAAAGSSSKKDLALSNQSISNSPLMPNVL
QTLSRGTASTSSNSASSFVVFDGANRRNRLSFNSEGVRANVAEGGRRLDFADESANKDNA
TAPEPNESTEGDDGGFVPHHQHAGSLCELGVGECPSGSGVECPKCDTVLGSSRSLGGHMT
MMHSRNSCKTLKCPKCNWHYKYQQTLEAHMKEKHPEPGGSCVYCKSGQPHPRLARGESYT
CGYKPFRCEVCNYSTTTKGNLSIHMQSDKHLNNMQNLQNGGGEQVFSHTAGAAAAAVAAA
AAAANISSSCGAPSPTKPKTKPTWRCEVCDYETNVARNLRIHMTSEKHMHNMMLLQQNMT
QIQHNRHLGLGSLPSPAEAELYQYYLAQNMNLPNLKMDSAASDAQFMMSGFQLDPAGPMA
AMTPALVGGEIPLDMRLGGGQLVSEELMNLGESFIQTNDPSLKLFQCAVCNKFTTDNLDM
LGLHMNVERSLSEDEWKAVMGDSYQCKLCRYNTQLKANFQLHCKTDKHVQKYQLVAHIKE
GGKANEWRLKCVAIGNPVHLKCNACDYYTNSLEKLRLHTVNSRHEASLKLYKHLQQHESG
VEGESCYYHCVLCNYSTKAKLNLIQHVRSMKHQRSESLRKLQRLQKGLPEEDEDLGQIFT
IRRCPSTDPEEAIEDVEGPSETAADPEELAKDQEGGASSSQAEKELTDSPATSKRISFPG
SSESPLSSKRPKTAEEIKPEQMYQCPYCKYSNADVNRLRVHAMTQHSVQPMLRCPLCQDM
LNNKIHLQLHLTHLHSVAPDCVEKLIMTVTTPEMVMPSSMFLPAAVPDRDGNSNLEEAGK
QPETSEDLGKNILPSASTEQSGDLKPSPADPGSVREDSGFICWKKGCNQVFKTSAALQTH
FNEVHAKRPQLPVSDRHVYKYRCNQCSLAFKTIEKLQLHSQYHVIRAATMCCLCQRSFRT
FQALKKHLETSHLELSEADIQQLYGGLLANGDLLAMGDPTLAEDHTIIVEEDKEEESDLE
DKQSPTGSDSGSVQEDSGSEPKRALPFRKGPNFTMEKFLDPSRPYKCTVCKESFTQKNIL
LVHYNSVSHLHKLKRALQESATGQPEPTSSPDNKPFKCNTCNVAYSQSSTLEIHMRSVLH
QTKARAAKLEAASGSSNGTGNSSSISLSSSTPSPVSTSGSNTFTTSNPSSAGIAPSSNLL
SQVPTESVGMPPLGNPIGANIASPSEPKEANRKKLADMIASRQQQQQQQQQQQQQQQQQQ
QAQTLAQAQAQVQAHLQQELQQQAALIQSQLFNPTLLPHFPMTTETLLQLQQQQHLLFPF
YIPSAEFQLNPEVSLPVTSGALTLTGTGPGLLEDLKAQVQVPQQSHQQILPQQQQNQLSI
AQSHSALLQPSQHPEKKNKLVIKEKEKESQRERDSAEGGEGNTGPKETLPDALKAKEKKE
LAPGGGSEPSMLPPRIASDARGNATKALLENFGFELVIQYNENKQKVQKKNGKTDQGENL
EKLECDSCGKLFSNILILKSHQEHVHQNYFPFKQLERFAKQYRDHYDKLYPLRPQTPEPP
PPPPPPPPPPLPAAPPQPASTPAIPASAPPITSPTIAPAQPSVPLTQLSMPMELPIFSPL
MMQTMPLQTLPAQLPPQLGPVEPLPADLAQLYQHQLNPTLLQQQNKRPRTRITDDQLRVL
RQYFDINNSPSEEQIKEMADKSGLPQKVIKHWFRNTLFKERQRNKDSPYNFSNPPITSLE
ELKIDSRPPSPEPPKQEYWGSKRSSRTRFTDYQLRVLQDFFDANAYPKDDEFEQLSNLLN
LPTRVIVVWFQNARQKARKNYENQGEGKDGERRELTNDRYIRTSNLNYQCKKCSLVFQRI

FDLIKHQKKLCYKDEDEEGQDDSQNEDSMDAMEILTPTSSSCSTPMPSQAYSAPAPSANN
TASSAFLQLTAEAEELATFNSKTEAGDEKPKLAEAPSAQPNQTQEKQGQPKPELQQQEQP
EQKTNTPQQKLPQLVSLPSLPQPPPQAPPPQCPLPQSSPSPSQLSHLPLKPLHTSTPQQL
ANLPPQLIPYQCDQCKLAFPSFEHWQEHQQLHFLSAQNQFIHPQFLDRSLDMPFMLFDPS
NPLLASQLLSGAIPQIPASSATSPSTPTSTMNTLKRKLEEKASASPGENDSGTGGEEPQR
DKRLRTTITPEQLEILYQKYLLDSNPTRKMLDHIAHEVGLKKRVVQVWFQNTRARERKGQ
FRAVGPAQAHRRCPFCRALFKAKTALEAHIRSRHWHEAKRAGYNLTLSAMLLDCDGGLQM
KGDIFDGTSFSHLPPSSSDGQGVPLSPVSKTMELSPRTLLSPSSIKVEGIEDFESPSMSS
VNLNFDQTKLDNDDCSSVNTAITDTTTGDEGNADNDSATGIATETKSSSAPNEGLTKAAM
MAMSEYEDRLSSGLVSPAPSFYSKEYDNEGTVDYSETSSLADPCSPSPGASGSAGKSGDS
GDRPGQKRFRTQMTNLQLKVLKSCFNDYRTPTMLECEVLGNDIGLPKRVVQVWFQNARAK
EKKSKLSMAKHFGINQTSYEGPKTECTLCGIKYSARLSVRDHIFSQQHISKVKDTIGSQL
DKEKEYFDPATVRQLMAQQELDRIKKANEVLGLAAQQQGMFDNTPLQALNLPTAYPALQG
IPPVLLPGLNSPSLPGFTPSNTALTSPKPNLMGLPSTTVPSPGLPTSGLPNKPSSASLSS
PTPAQATMAMGPQQPPQQQQQQQQPQVQQPPPPPAAQPPPTPQLPLQQQQQRKDKDSEKV
KEKEKAHKGKGEPLPVPKKEKGEAPTATAATISAPLPTMEYAVDPAQLQALQAALTSDPT
ALLTSQFLPYFVPGFSPYYAPQIPGALQSGYLQPMYGMEGLFPYSPALSQALMGLSPGSL
LQQYQQYQQSLQEAIQQQQQRQLQQQQQQKVQQQQPKASQTPVPPGAPSPDKDPAKESPK
PEEQKNTPREVSPLLPKLPEEPEAESKSADSLYDPFIVPKVQYKLVCRKCQAGFSDEEAA
RSHLKSLCFFGQSVVNLQEMVLHVPTGGGGGGSGGGGGGGGGGGGGGSYHCLACESALCG
EEALSQHLESALHKHRTITRAARNAKEHPSLLPHSACFPDPSTASTSQSAAHSNDSPPPP
SAAAPSSASPHASRKSWPQVVSRASAAKPPSFPPLSSSSTVTSSSCSTSGVQPSMPTDDY
SEESDTDLSQKSDGPASPVEGPKDPSCPKDSGLTSVGTDTFRL*
>ENSG00000165684|ENST00000298532
MDVDAEREKITQEIKELERILDPGSSGSHVEISESSLESDSEADSLPSEDLDPADPPISE
EERWGEASNDEDDPKDKTLPEDPETCLQLNMVYQEVIQEKLAEANLLLAQNREQQEELMR
DLAGSKGTKVKDGKSLPPSTYMGHFMKPYFKDKVTGVGPPANEDTREKAAQGIKAFEELL
VTKWKNWEKALLRKSVVSDRLQRLLQPKLLKLEYLHQKQSKVSSELERQALEKQGREAEK
EIQDINQLPEEALLGNRLDSHDWEKISNINFEGSRSAEEIRKFWQNSEHPSINKQEWSRE
EEERLQAIAAAHGHLEWQKIAEELGTSRSAFQCLQKFQQHNKALKRKEWTEEEDRMLTQL
VQEMRVGSHIPYRRIVYYMEGRDSMQLIYRWTKSLDPGLKKGYWAPEEDAKLLQAVAKYG
EQDWFKIREEVPGRSDAQCRDRYLRRLHFSLKKGRWNLKEEEQLIELIEKYGVGHWAKIA
SELPHRSGSQCLSKWKIMMGKKQGLRRRRRRARHSVRWSSTSSSGSSSGSSGGSSSSSSS
SSEEDEPEQAQAGEGDRALLSPQYMVPDMDLWVPARQSTSQPWRGGAGAWLGGPAASLSP
PKGSSASQGGSKEASTTAAAPGEETSPVQVPARAHGPVPRSAQASHSADTRPAGAEKQAL
EGGRRLLTVPVETVLRVLRANTAARSCTQKEQLRQPPLPTSSPGVSSGDSVARSHVQWLR
HRATQSGQRRWRHALHRRLLNRRLLLAVTPWVGDVVVPCTQASQRPAVVQTQADGLREQL
QQARLASTPVFTLFTQLFHIDTAGCLEVVRERKALPPRLPQAGARDPPVHLLQASSSAQS
TPGHLFPNVPAQEASKSASHKGSRRLASSRVERTLPQASLLASTGPRPKPKTVSELLQEK
RLQEARAREATRGPVVLPSQLLVSSSVILQPPLPHTPHGRPAPGPTVLNVPLSGPGAPAA
AKPGTSGSWQEAGTSAKDKRLSTMQALPLAPVFSEAEGTAPAASQAPALGPGQISVSCPE
SGLGQSQAPAASRKQGLPEAPPFLPAAPSPTPLPVQPLSLTHIGGPHVATSVPLPVTWVL
TAQGLLPVPVPAVVSLPRPAGTPGPAGLLATLLPPLTETRAAQGPRAPALSSSWQPPANM
NREPEPSCRTDTPAPPTHALSQSPAEADGSVAFVPGEAQVAREIPEPRTSSHADPPEAEP

PWSGRLPAFGGVIPATEPRGTPGSPSGTQEPRGPLGLEKLPLRQPGPEKGALDLEKPPLP
QPGPEKGALDLGLLSQEGEAATQQWLGGQRGVRVPLLGSRLPYQPPALCSLRALSGLLLH
KKALEHKATSLVVGGEAERPAGALQASLGLVRGQLQDNPAYLLLRARFLAAFTLPALLAT
LAPQGVRTTLSVPSRVGSESEDEDLLSELELADRDGQPGCTTATCPIQGAPDSGKCSASS
CLDTSNDPDDLDVLRTRHARHTRKRRRLV*
>ENSG00000146223|ENST00000304734
MISSCTTRKMAEQEQRKIPLVPENLLKKRKAYQALKATQAKQALLAKKEQKKGKGLRFKR
LESFLHDSWRQKRDKVRLRRLEVKPHALELPDKHSLAFVVRIERIDGVSLLVQRTIARLR
LKKIFSGVFVKVTPQNLKMLRIVEPYVTWGFPNLKSVRELILKRGQAKVKNKTIPLTDNT
VIEEHLGKFGVICLEDLIHEIAFPGKHFQEISWFLCPFHLSVARHATKNRVGFLKEMGTP
GYRGERINQLIRQLN*
>ENSG00000170365|ENST00000302085
MNVTSLFSFTSPAVKRLLGWKQGDEEEKWAEKAVDALVKKLKKKKGAMEELEKALSCPGQ
PSNCVTIPRSLDGRLQVSHRKGLPHVIYCRVWRWPDLQSHHELKPLECCEFPFGSKQKEV
CINPYHYKRVESPVLPPVLVPRHSEYNPQHSLLAQFRNLGQNEPHMPLNATFPDSFQQPN
SHPFPHSPNSSYPNSPGSSSSTYPHSPTSSDPGSPFQMPADTPPPAYLPPEDPMTQDGSQ
PMDTNMMAPPLPSEINRGDVQAVAYEEPKHWCSIVYYELNNRVGEAFHASSTSVLVDGFT
DPSNNKNRFCLGLLSNVNRNSTIENTRRHIGKGVHLYYVGGEVYAECLSDSSIFVQSRNC
NYHHGFHPTTVCKIPSGCSLKIFNNQEFAQLLAQSVNHGFETVYELTKMCTIRMSFVKGW
GAEYHRQDVTSTPCWIEIHLHGPLQWLDKVLTQMGSPHNPISSVS*
>ENSG00000163577|ENST00000295822
MADEIDFTTGDAGASSTYPMQCSALRKNGFVVLKGRPCKIVEMSTSKTGKHGHAKVHLVG
IDIFTGKKYEDICPSTHNMDVPNIKRNDYQLICIQDGYLSLLTETGEVREDLKLPEGELG
KEIEGKYNAGEDVQVSVMCAMSEEYAVAIKPCK*
>ENSG00000174279|ENST00000308618
MMERIRKEMILMERGLHSPTAGKRFSNLSNSAGNAVLEALENSQHPARLSPRLPSAPLHS
ALGELPAKGKFEIDTLFNLQHTGSESTVSSEISSAAESRKKPGHYSEAAAEADMSSDVEV
GCSALRSPGGLGAAQLKENNGKGYAESGSAAGTTTSASGSGLGSLHGGSGGSGGSAALGG
SGSGADQVRRYRTAFTREQIARLEKEFYRENYVSRPRRCELAAALNLPETTIKVWFQNRR
MKDKRQRLAMSWPHPADPSFYTYMMTHAAATGSLPYPFHSHVPLHYYPHVGVTAAAAAAA
ASGAAAAAASSPFATSIRPLDTFRALSHPYSRPELLCSFRHPGLYQAPAAAAGLNSAASAA
AAAAAAAAAASSAAAAGAPPSGGSAPCSCLSCHSSQSAAAAAAAAAAALGSRGGGGGGGG
GGGGGGGGAGAGGGSDFGCSAAAPRSESGFLPYSAAVLSKTAVSPPDQRDEAPLTR*
>ENSG00000139546|ENST00000266987
MSEEEQGSGTTTGCGLPSIEQMLAANPGKTPISLLQEYGTRIGKTPVYDLLKAEGQAHQP
NFTFRVTVGDTSCTGQGPSKKAAKHKAAEVALKHLKGGSMLEPALEDSSSFSPLDSSLPE
DIPVFTAAAAATPVPSVVLTRSPPMELQPPVSPQQSECNPVGALQELVVQKGWRLPEYTV
TQESGPAHRKEFTMTCRVERFIEIGSGTSKKLAKRNAAAKMLLRVHTVPLDARDGNEVEP
DDDHFSIGVGSRLDGLRNRGPGCTWDSLRNSVGEKILSLRSCSLGSLGALGPACCRVLSE
LSEEQAFHVSYLDIEELSLSGLCQCLVELSTQPATVCHGSATTREAARGEAARRALQYLK
IMAGSK*
>ENSG00000169249|ENST00000307771
MAAPEKMTFPEKPSHKKYRAALKKEKRKKRRQELARLRDSGLSQKEEEEDTFIEEQQLEE
EKLLERERQRLHEEWLLREQKAQEEFRIKKEKEEAAKKRQEEQERKLKEQWEEQQRKERE

EEEQKRQEKKEKEEALQKMLDQAENELENGTTWQNPEPPVDFRVMEKDRANCPFYSKTGA
CRFGDRCSRKHNFPTSSPTLLIKSMFTTFGMEQCRRDDYDPDASLEYSEEETYQQFLDFY
EDVLPEFKNVGKVIQFKVSCNLEPHLRGNVYVQYQSEEECQAALSLFNGRWYAGRQLQCE
FCPVTRWKMAICGLFEIQQCPRGKHCNFLHVFRNPNNEFWEANRDIYLSPDRTGSSFGKN
SERRERMGHHDDYYSRLRGRRNPSPDHSYKRNGESERKSSRHRGKKSHKRTSKSRERHNS
RSRGRNRDRSRDRSRGRGSRSRSRSRSRRSRRSRSQSSSRSRSRGRRRSGNRDRTVQSPK
SK*
>ENSG00000162231|ENST00000294172
MADEGKSYSEHDDERVNFPQRKKKGRGPFRWKYGEGNRRSGRGGSGIRSSRLEEDDGDVA
MSDAQDGPRVRYNPYTTRPNRRGDTWHDRDRIHVTVRRDRAPPERGGAGTSQDGTSKNWF
KITIPYGRKYDKAWLLSMIQSKCSVPFTPIEFHYENTRAQFFVEDASTASALKAVNYKIL
DRENRRISIIINSSAPPHTILNELKPEQVEQLKLIMSKRYDGSQQALDLKGLRSDPDLVA
QNIDVVLNRRSCMAATLRIIEENIPELLSLNLSNNRLYRLDDMSSIVQKAPNLKILNLSG
NELKSERELDKIKGLKLEELWLDGNSLCDTFRDQSTYISAIRERFPKLLRLDGHELPPPI
AFDVEAPTTLPPCKGSYFGTENLKSLVLHFLQQYYAIYDSGDRQGLLDAYHDGACCSLSI
PFIPQNPARSSLAEYFKDSRNVKKLKDPTLRFRLLKHTRLNVVAFLNELPKTQHDVNSFV
VDISAQTSTLLCFSVNGVFKEVDGKSRDSLRAFTRTFIAVPASNSGLCIVNDELFVRNAS
SEEIQRAFAMPAPTPSSSPVPTLSPEQQEMLQAFSTQSGMNLEWSQKCLQDNNWDYTRSA
QAFTHLKAKGEIPEVAFMK*
>ENSG00000116120|ENST00000281828
MPTVSVKRDLLFQALGRTYTDEEFDELCFEFGLELDEITSEKEIISKEQGNVKAAGASDV
VLYKIDVPANRYDLLCLEGLVRGLQVFKERIKAPVYKRVMPDGKIQKLIITEETAKIRPF
AVAAVLRNIKFTKDRYDSFIELQEKLHQNICRKRALVAIGTHDLDTLSGPFTYTAKRPSD
IKFKPLNKTKEYTACELMNIYKTDNHLKHYLHIIENKPLYPVIYDSNGVVLSMPPIINGD
HSRITVNTRNIFIECTGTDFTKAKIVLDIIVTMFSEYCENQFTVEAAEVVFPNGKSHTFP
ELAYRKEMVRADLINKKVGIRETPENLAKLLTRMYLKSEVIGDGNQIEIEIPPTRADIIH
ACDIVEDAAIAYGYNNIQMTLPKTYTIANQFPLNKLTELLRHDMAAAGFTEALTFALCSQ
EDIADKLGVDISATKAVHISNPKTAEFQVARTTLLPGLLKTIAANRKMPLPLKLFEISDI
VIKDSNTDVGAKNYRHLCAVYYNKNPGFEIIHGLLDRIMQLLDVPPGEDKGGYVIKASEG
PAFFPGRCAEIFARGQSVGKLGVLHPDVITKFELTMPCSSLEINVGPFL*
>ENSG00000113649|ENST00000296702
MAERGGDGGESERFNPGELRMAQQQALRFRGPAPPPNAVMRGPPPLMRPPPPFGMMRGPP
PPPRPPFGRPPFDPNMPPMPPPGGIPPPMGPPHLQRPPFMPPPMSSMPPPPGMMFPPGMP
PVTAPGTPALPPTEEIWVENKTPDGKVYYYNARTRESAWTKPDGVKVIQQSELTPMLAAQ
AQVQAQAQAQAQAQAQAQAQAQAQAQAQAQAQAQAQAQAQAQAQAQAQAQAQAQAQAQAQ
AQVQAQVQAQVQAQAVGASTPTTSSPAPAVSTSTSSSTPSSTTSTTTTATSVAQTVSTPT
TQDQTPSSAVSVATPTVSVSTPAPTATPVQTVPQPHPQTLPPAVPHSVPQPTTAIPAFPP
VMVPPFRVPLPGMPIPLPGVAMMQIVSCPYVKTVATTKTGVLPGMAPPIVPMIHPQVAIA
ASPATLAGATAVSEWTEYKTADGKTYYYNNRTLESTWEKPQELKEKEKLEEKIKEPIKEP
SEEPLPMETEEEDPKEEPIKEIKEEPKEEEMTEEEKAAQKAKPVATAPIPGTPWCVVWTG
DERVFFYNPTTRLSMWDRPDDLIGRADVDKIIQEPPHKKGMEELKKLRHPTPTMLSIQKW
QFSMSAIKEEQELMEEINEDEPVKAKKRKRDDNKDIDSEKEAAMEAEIKAARERAIVPLE
ARMKQFKDMLLERGVSAFSTWEKELHKIVFDPRYLLLNPKERKQVFDQYVKTRAEEERRE
KKNKIMQAKEDFKKMMEEAKFNPRATFSEFAAKHAKDSRFKAIEKMKDREALFNEFVAAA

RKKEKEDSKTRGEKIKSDFFELLSNHHLDSQSRWSKVKDKVESDPRYKAVDSSSMREDLF
KQYIEKIAKNLDSEKEKELERQARIEASLREREREVQKARSEQTKEIDREREQHKREEAI
QNFKALLSDMVRSSDVSWSDTRRTLRKDHRWESGSLLEREEKEKLFNEHIEALTKKKREH
FRQLLDETSAITLTSTWKEVKKIIKEDPRCIKFSSSDRKKQREFEEYIRDKYITAKADFR
TLLKETKFITYRSKKLIQESDQHLKDVEKILQNDKRYLVLDCVPEERRKLIVAYVDDLDR
RGPPPPPTASEPTRRSTK*
>ENSG00000101191|ENST00000266070
MDDKGDPSNEEAPKAIKPTSKEFRKTWGFRRTTIAKREGAGDAEADPLEPPPPQQQLGLS
LRRSGRQPKRTERVEQFLTIARRRGRRSMPVSLEDSGEPTSCPATDAETASEGSVESASE
TRSGPQSASTAVKERPASSEKVKGGDDHDDTSDSDSDGLTLKELQNRLRRKREQEPTERP
LKGIQSRLRKKRREEGPAETVGSEASDTVEGVLPSKQEPENDQGVVSQAGKDDRESKLEG
KAAQDIKDEEPGDLGRPKPECEGYDPNALYCICRQPHNNRFMICCDRCEEWFHGDCVGIS
EARGRLLERNGEDYICPNCTILQVQDETHSETADQQEAKWRPGDADGTDCTSIGTIEQKS
SEDQGIKGRIEKAANPSGKKKLKIFQPVIEAPGASKCIGPGCCHVAQPDSVYCSNDCILK
HAAATMKFLSSGKEQKPKPKEKMKMKPEKPSLPKCGAQAGIKISSVHKRPAPEKKETTVK
KAVVVPARSEALGKEAACESSTPSWASDHNYNAVKPEKTAAPSPSLLYKSTKEDRRSEEK
AAAMAASKKTAPPGSAVGKQPAPRNLVPKKSSFANVAAATPAIKKPPSGFKGTIPKRPWL
SATPSSGASAARQAGPAPAAATAASKKFPGSAALVGAVRKPVVPSVPMASPAPGRLGAMS
AAPSQPNSQIRQNIRRSLKEILWKRVNDSDDLIMTENEVGKIALHIEKEMFNLFQVTDNR
YKSKYRSIMFNLKDPKNQGLFHRVLREEISLAKLVRLKPEELVSKELSTWKERPARSVME
SRTKLHNESKKTAPRQEAIPDLEDSPPVSDSEEQQESARAVPEKSTAPLLDVFSSMLKDT
TSQHRAHLFDLNCKICTGQVPSAEDEPAPKKQKLSASVKKEDLKSKHDSSAPDPAPDSAD
EVMPEAVPEVASEPGLESASHPNVDRTYFPGPPGDGHPEPSPLEDLSPCPASCGSGVVTT
VTVSGRDPRTAPSSSCTAVASAASRPDSTHMVEARQDVPKPVLTSVMVPKSILAKPSSSP
DPRYLSVPPSPNISTSESRSPPEGDTTLFLSRLSTIWKGFINMQSVAKFVTKAYPVSGCF
DYLSEDLPDTIHIGGRIAPKTVWDYVGKLKSSVSKELCLIRFHPATEEEEVAYISLYSYF
SSRGRFGVVANNNRHVKDLYLIPLSAQDPVPSKLLPFEGPGLESPRPNIILGLVICQKIK
RPANSGELDKMDEKRTRLQPEEADVPAYPKVATVPQSEKKPSKYPLCSADAAVSTTPPGS
PPPPPPLPEPPVLKVLSSLKPAAPSPATAATTAAAASTAASSTASSASKTASPLEHILQT
LFGKKKSFDPSAREPPGSTAGLPQEPKTTAEDGVPAPPLLDPIVQQFGQFSKDKALEEEE
DDRPYDPEEEYDPERAFDTQLVERGRRHEVERAPEAAAAEREEVAYDPEDETILEEAKVT
VDDLPNRMCADVRRNSVERPAEPVAGAATPSLVEQQKMLEELNKQIEEQKRQLEEQEEAL
RQQRAAVGVSMAHFSVSDALMSPPPKSSLPKAELFQQEQQSADKPASLPPASQASNHRDP
RQARRLATETGEGEGEPLSRLSARGAQGALPERDASRGGLVGQAPMPVPEEKEPASSPWA
SGEKPPAGSEQDGWKAEPGEGTRPATVGDSSARPARRVLLPTPPCGALQPGFPLQHDGER
DPFTCPGFASQDKALGSAQYEDPRNLHSAGRSSSPAGETEGDREPQARPGEGTAPLPPPG
QKVGGSQPPFQGQREPGPHALGMSGLHGPNFPGPRGPAPPFPEENIASNDGPRGPPPARF
GAQKGPIPSLFSGQHGPPPYGDSRGPSPSYLGGPRGVAPSQFEERKDPHGEKREFQDAPY
NEVTGAPAQFEGTEQAPFLGSRGGAPFQFGGQRRPLLSQLKGPRGGPPPSQFGGQRGPPP
GHFVGPRGPHPSQFETARGPHPNQFEGPRGQAPNFMPGPRGIQPQQFEDQRVHSPPRFTN
QRAPAPLQFGGLRGSAPFSEKNEQTPSRFHFQGQAPQVMKPGPRPLLELPSHPPQHRKDR
WEEAGPPSALSSSAPGQGPEADGQWASADFREGKGHEYRNQTFEGRQRERFDVGPKEKPL
EEPDAQGRASEDRRRERERGRNWSRERDWDRPREWDRHRDKDSSRDWDRNRERSANRDRE
READRGKEWDRSRERSRNRERERDRRRDRDRSRSRERDRDKARDRERGRDRKDRSKSKES

ARDPKPEASRASDAGTASQA*
>ENSG00000139579|ENST00000267023
MTTETFVKDIKPGLKNLNLIFIVLETGRVTKTKDGHEVRTCKVADKTGSINISVWDDVGN
LIQPGDIIRLTKGYASVFKGCLTLYTGRGGDLQKIGEFCMVYSEVPNFSEPNPEYSTQQA
PNKAVQNDSNPSASQPTTGPSAASPASENQNGNGLSAPPGPGGGPHPPHTPSHPPSTRIT
RSQPNHTPAGPPGPSSNPVSNGKETRRSSKR*
>ENSG00000080298|ENST00000302303
MQTSETGSDTGSTVTLQTSVASQAAVPTQVVQQVPVQQQVQQVQTVQQVQHVYPAQVQYV
EGSDTVYTNGAIRTTTYPYTETQMYSQNTGGNYFDTQGSSAQVTTVVSSHSMVGTGGIQM
GVTGGQLISSSGGTYLIGNSMENSGHSVTHTTRASPATIEMAIETLQKSDGLSTHRSSLL
NSHLQWLLDNYETAEGVSLPRSTLYNHYLRHCQEHKLDPVNAASFGKLIRSIFMGLRTRR
LGTRGNSKYHYYGIRVKPDSPLNRLQEDMQYMAMRQQPMQQKQRYKPMQKVDGVADGFTG
SGQQTGTSVEQTVIAQSQHHQQFLDASRALPEFGEVEISSLPDGTTFEDIKSLQSLYREH
CEAILDVVVNLQFSLIEKLWQTFWRYSPSTPTDGTTITESRSESTSFPIHFHG*
>ENSG00000082516|ENST00000285873
MGQEPRTLPPSPNWYCARCSDAVPGGLFGFAARTSVFLVRVGPGAGESPGTPPFRVIGEL
VGHTERVSGFTFSHHPGQYNLCATSSDDGTVKIWDVETKTVVTEHALHQHTISTLHWSPR
VKDLIVSGDEKGVVFCYWFNRNDSQHLFIEPRTIFCLTCSPHHEDLVAIGYKDGIVVIID
ISKKGEVIHRLRGHDDEIHSIAWCPLPGEDCLSINQEETSEEAEITNGNAVAQAPVTKGC
YLATGSKDQTIRIWSCSRGRGVMILKLPFLKRRGGGIDPTVKERLWLTLHWPSNQPTQLV
SSCFGGELLQWDLTQSWRRKYTLFSASSEGQNHSRIVFNLCPLQTEDDKQLLLSTSMDRD
VKCWDIATLECSWTLPSLGGFAYSLAFSSVDIGSLAIGVGDGMIRVWNTLSIKNNYDVKN
FWQGVKSKVTALCWHPTKEGCLAFGTDDGKVGLYDTYSNKPPQISSTYHKKTVYTLAWGP
PVPPMSLGGEGDRPSLALYSCGGEGIVLQHNPWKLSGEAFDINKLIRDTNSIKYKLPVHT
EISWKADGKIMALGNEDGSIEIFQIPNLKLICTIQQHHKLVNTISWHHEHGSQPELSYLM
ASGSNNAVIYVHNLKTVIESSPESPVTITEPYRTLSGHTAKITSVAWSPHHDGRLVSASY
DGTAQVWDALREEPLCNFRGHRGRLLCVAWSPLDPDCIYSGADDFCVHKWLTSMQDHSRP
PQGKKSIELEKKRLSQPKAKPKKKKKPTLRTPVKLESIDGNEEESMKENSGPVENGVSDQ
EGEEQAREPELPCGLAPAVSREPVICTPVSSGFEKSKVTINNKVILLKKEPPKEKPETLI
KKRKARSLLPLSTSLDHRSKEELHQDCLVLATAKHSRELNEDVSADVEERFHLGLFTDRA
TLYRMIDIEGKGHLENGHPELFHQLMLWKGDLKGVLQTAAERGELTDNLVAMAPAAGYHV
WLWAVEAFAKQLCFQDQYVKAASHLLSIHKVYEAVELLKSNHFYREAIAIAKARLRPEDP
VLKDLYLSWGTVLERDGHYAVAAKCYLGATCAYDAAKVLAKKGDAASLRTAAELAAIVGE
DELSASLALRCAQELLLANNWVGAQEALQLHESLQGQRLVFCLLELLSRHLEEKQLSEGK
SSSSYHTWNTGTEGPFVERVTAVWKSIFSLDTPEQYQEAFQKLQNIKYPSATNNTPAKQL
LLHICHDLTLAVLSQQMASWDEAVQALLRAVVRSYDSGSFTIMQEVYSAFLPDGCDHLRD
KLGDHQSPATPAFKSLEAFFLYGRLYEFWWSLSRPCPNSSVWVRAGHRTLSVEPSQQLDT
ASTEETDPETSQPEPNRPSELDLRLTEEGERMLSTFKELFSEKHASLQNSQRTVAEVQET
LAEMIRQHQKSQLCKSTANGPDKNEPEVEAEQPLCSSQSQCKEEKNEPLSLPELTKRLTE
ANQRMAKFPESIKAWPFPDVLECCLVLLLIRSHFPGCLAQEMQQQAQELLQKYGNTKTYR
RHCQTFCM*
>ENSG00000180535|ENST00000314018
MKTKNRPPRRRAPVQDTEATPGEGTPDGSLPNPGPEPAKGLRSRPARAAARAPGEGRRRR
PGPSGPGGRRDSSIQRRLESNERERQRMHKLNNAFQALREVIPHVRADKKLSKIETLTLA

KNYIKSLTATILTMSSSRLPGLEGPGPKLYQHYQQQQQVAGGALGATEAQPQGHLQRYST
QIHSFREGT*
>ENSG00000159208|ENST00000290363
MDSPSSVSSYSSYSLSSSFPTSPVNSDFGFPSDSEREDKGAHGPRPDTVGQRGGSRPSPG
PIRCRHRSKVSGNQHTPSHPKQRGSASPMAGSGAKRSRDGELETSLNTQGCTTEGDLLFA
QKCKELQGFIPPLTDLLNGLKMGRFERGLSSFQQSVAMDRIQRIVGVLQKPQMGERYLGT
LLQVEGMLKTWFPQIAAQKSSLGGGKHQLTKHFPSHHSDSAASSPASPMEKMDQTQLGHL
ALKPKQPWHLTQWPAMNLTWIHTTPICNPPLSSPGTISFSHGPLGTGTGIGVILFLQHGV
QPFTHSAPTTPVPPTTASPVIPGEPMKLSGEGPRCYSLPVTLPSDWSYTLSPPSLPTLAR
KMTIGHREQQRSHPPVAADAHLLNL*
>ENSG00000156639|ENST00000287218
MGDAGSERSKAPSLPPRCPCGFWGSSKTMNLCSKCFADFQKKQPDDDSAPSTSNSQSDLF
SEETTSDNNNTSITTPTLSPSQQPLPTELNVTSPSKEECGPCTDTAHVSLITPTKRSCGT
DSQSENEASPVKRPRLLENTERSEETSRSKQKSRRRCFQCQTKLELVQQELGSCRCGYVF
CMLHRLPEQHDCTFDHMGRGREEAIMKMVKLDRKVGRSCQRIGEGCS*
>ENSG00000056487|ENST00000313237
MELQSRPEALAVELARHQNGDLKKQLHERQPRIAALSDKQALGTITAVPVTGPQVSSLQR
LAGQGAAVLPQVRPKTLIPDSLPVAPGRDRPPKQPPTFQKATVVSVKNPSPALPTANNTV
SHVPAPGSQPQALAEPAALASPLSSAGVAYAIISTSPSNAAAMAPSTAVSVVSDSIKVQP
LLISADNKPPPRLLSSPHPATHHCPLHPSSLPLTPPSPSLSPSPLHGIFQVIIIQPQVQT
QPESTAESRPPTEEPSQGAQATKKKKEDRPPTQENPEKIAFMVALGLVTTEHLEEIQSKR
QERKRRSTANPAYSGLLETERKRLASNYLNNPLFLTARANEDPCWKNEITHDEHCAACKR
GANLQPCGTCPGAYHLSCLEPPLKTAPKGVWVCPRCQQKALKKDEGVPWTGMLAIVHSYV
THKTVKEEEKQKLLQRGSELQNEHQQLEERDRRLASAVQKCLELKTSLLARQRGTQSSLD
RLRALLRLIQGEQLLQVTMTTTSPAPLLAGPWTKPSVAATHPTVQHPQGHN*
>ENSG00000168002|ENST00000301788
MFYHISLEHEILLHPRYFGPNLLNTVKQKLFTEVEGTCTGKYGFVIAVTTIDNIGAGVIQ
PGRGFVLYPVKYKAIVFRPFKGEVVDAVVTQVNKVGLFTEIGPMSCFISRHSIPSEMEFD
PNSNPPCYKTMDEDIVIQQDDEIRLKIVGTRVDKNDIFAIGSLMDDYLGLVS*
>ENSG00000151789|ENST00000281523
MRNIMYFGGTCQSPALPALVRPPAPPLQPSLDIKPFLPFPLDTAAAVNLFPNFNAMDPIQ
KAVINHTFGVPLPHRRKQIISCNICQLRFNSDSQAAAHYKGTKHAKKLKALEAMKNKQKS
VTAKDSAKTTFTSITTNTINTSSDKTDGTAGTPAISTTTTVEIRKSSVMTTEITSKVEKS
PTTATGNSSCPSTETEEEKAKRLLYCSLCKVAVNSASQLEAHNSGTKHKTMLEARNGSGT
IKAFPRAGVKGKGPVNKGNTGLQNKTFHCEICDVHVNSETQLKQHISSRRHKDRAAGKPP
KPKYSPYNKLQKTAHPLGVKLVFSKEPSKPLAPRILPNPLAAAAAAAAVAVSSPFSLRTA
PAATLFQTSALPPALLRPAPGPIRTAHTPVLFAPY*
>ENSG00000167074|ENST00000266304
MSDAGGGKKPPVDPQAGPGPGPGRAAGERGLSGSFPLVLKKLMENPPREARLDKEKGKEK
LEEDEAAAASTMAVSASLMPPIWDKTIPYDGESFHLEYMDLDEFLLENGIPASPTHLAHN
LLLPVAELEGKESASSSTASPPSSSTAIFQPSETVSSTESSLEKERETPSPIDPNCVEVD
VNFNPDPADLVLSSVPGGELFNPRKHKFAEEDLKPQPMIKKAKKVFVPDEQKDEKYWTRR
KKNNVAAKRSRDARRLKENQITIRAAFLEKENTALRTEVAELRKEVGKCKTIVSKYETKY
GPL*

>ENSG00000084234|ENST00000278756
MLRAPGELPRQAARCSLCRLGPGRGRAFFKWRCLPASVDRGNPLWALAANAGTGFAVAEP
QIAMFCGKLNMHVNIQTGKWEPDPTGTKSCFETKEEVLQYCQEMYPELQITNVMEANQRV
SIDNWCRRDKKQCKSRFVTPFKCLVGEFVSDVLLVPEKCQFFHKERMEVCENHQHWHTVV
KEACLTQGMTLYSYGMLLPCGVDQFHGTEYVCCPQTKIIGSVSKEEEEEDEEEEEEEDEE
EDYDVYKSEFPTEADLEDFTEAAVDEDDEDEEEGEEVVEDRDYYYDTFKGDDYNEENPTE
PGSDGTMSDKEITHDVKAVCSQEAMTGPCRAVMPRWYFDLSKGKCVRFIYGGCGGNRNNF
ESEDYCMAVCKAMIPPTPLPTNDVDVYFETSADDNEHARFQKAKEQLEIRHRNRMDRVKK
EWEEAELQAKNLPKAERQTLIQHFQAMVKALEKEAASEKQQLVETHLARVEAMLNDRRRM
ALENYLAALQSDPPRPHRILQALRRYVRAENKDRLHTIRHYQHVLAVDPEKAAQMKSQVM
THLHVIEERRNQSLSLLYKVPYVAQEIQEEIDELLQEQRADMDQFTASISETPVDVRVSS
EESEEIPPFHPFHPFPALPENEGSGVGEQDGGLIGAEEKVINSKNKVDENMVIDETLDVK
EMIFNAERVGGLEEERESVGPLREDFSLSSSALIGLLVIAVAIATVIVISLVMLRKRQYG
TISHGIVEVDPMLTPEERHLNKMQNHGYENPTYKYLEQMQI*
>ENSG00000055950|ENST00000299179
MTARGTPSRFLASVLHNGLGRYVQQLQRLSFSVSRDGASSRGAREFVEREVIDFARRNPG
VVIYVNSRPCCVPRVVAEYLNGAVREESIHCKSVEEISTLVQKLADQSGLDVIRIRKPFH
TDNPSIQGQWHPFTNKPTTFRGLRPREVQDPAPAQDTGLRLSAVAPQILLPGWPDPISVQ
SSDLPWGNTHYRPEPLSSTTWL*
>ENSG00000065457|ENST00000307921
MWTADEIAQLCYEHYGIRLPKKGKPEPNHEWTLLAAVVKIQSPADKACDTPDKPVQVTKE
VVSMGTGTKCIGQSKMRKNGDILNDSHAEVIARRSFQRYLLHQLQLAATLKEDSIFVPGT
QKGVWKLRRDLIFVFFSSHTPCGDASIIPMLEFEDQPCCPVFRNWAHNSSVEASSNLEAP
GNERKCEDPDSPVTKKMRLEPGTAAREVTNGAAHHQSFGKQKSGPISPGIHSCDLTVEGL
ATVTRIAPGSAKVIDVYRTGAKCVPGEAGDSGKPGAAFHQVGLLRVKPGRGDRTRSMSCS
DKMARWNVLGCQGALLMHLLEEPIYLSAVVIGKCPYSQEAMQRALIGRCQNVSALPKGFG
VQELKILQSDLLFEQSRSAVQAKRADSPGRLVPCGAAISWSAVPEQPLDVTANGFPQGTT
KKTIGSLQARSQISKVELFRSFQKLLSRIARDKWPHSLRVQKLDTYQEYKEAASSYQEAW
STLRKQVFGSWIRNPPDYHQFK*
>ENSG00000156709|ENST00000287295
MFRCGGLAAGALKQKLVPLVRTVCVRSPRQRNRLPGNLFQRWHVPLELQMTRQMASSGAS
GGKIDNSVLVLIVGLSTVGAGAYAYKTMKEDEKRYNERISGLGLTPEQKQKKAALSASEG
EEVPQDKAPSHVPFLLIGGGTAAFAAARSIRARDPGARVLIVSEDPELPYMRPPLSKELW
FSDDPNVTKTLRFKQWNGKERSIYFQPPSFYVSAQDLPHIENGGVAVLTGKKVVQLDVRD
NMVKLNDGSQITYEKCLIATGGTPRSLSAIDRAGAEVKSRTTLFRKIGDFRSLEKISREV
KSITIIGGGFLGSELACALGRKARALGTEVIQLFPEKGNMGKILPEYLSNWTMEKVRREG
VKVMPNAIVQSVGVSSGKLLIKLKDGRKVETDHIVAAVGLEPNVELAKTGGLEIDSDFGG
FRVNAELQARSNIWVAGDAACFYDIKLGRRRVEHHDHAVVSGRLAGENMTGAAKPYWHQS
MFWSDLGPDVGYEAIGLVDSSLPTVGVFAKATAQDNPKSATEQSGTGIRSESETESEASE
ITIPPSTPAVPQAPVQGEDYGKGVIFYLRDKVVVGIVLWNIFNRMPIARKIIKDGEQHED
LNEVAKLFNIHED*
>ENSG00000175643|ENST00000312499
MAAAADSFSGGPAGVRLPRSPPLKVLAEQLRRDAEGGPGAWRLSRAAAGRGPLDLAAVWM
QGRVVMADRGEARLRDPSGDFSVRGLERVPRGRPCLVPGKYVMVMGVVQACSPEPCLQAV

KMTDLSDNPIHESMWELEVEDLHRNIP*
>ENSG00000049883|ENST00000308077
MVRDSMAAAFRPSNRVLLQALQILVYPGVGGSGSVSCRCPLGAKRYLLTDNVVKLKEFQQ
KKVAVACNLSGTKETYFRNLKKKLTQNKLILKGELITLLHLCESRDHVELAKNVIYRYHA
ENKNFTLGEYKFGPLFVRLCYELDLEESAVELMKDQHLRGFFSDSTSFNILMDMLFIKGK
YKSALQVLIEMKNQDVKFTKDTYVLAFAICYKLNSPESFKICTTLREEALLKGEILSRRA
SCFAVALALNQNEMAKAVSIFSQIMNPESIACINLNIIIHIQSNMLENLIKTLKNAAEGN
LSKFVKRHVFSEEVLAKVREKVKDVPALVAKFDEIYGTLHITGQVTTDSLDAVLCHTPRD
RKSHTLLLNKRMVSRRTFQPLSQSLLAE*
>ENSG00000187790|ENST00000267430
MSGRQRTLFQTWGSSISRSSGTPGCSSGTERPQSPGSSKAPLPAAAEAQLESDDDVLLVA
AYEAERQLCLENGGFCTSAGALWIYPTNCPVRDYQLHISRAALFCNTLVCLPTGLGKTFI
AAVVMYNFYRWFPSGKVVFMAPTKPLVTQQIEACYQVMGIPQSHMAEMTGSTQASTRKEI
WCSKRVLFLTPQVMVNDLSRGACPAAEIKCLVIDEAHKALGNYAYCQVVRELVKYTNHFR
ILALSATPGSDIKAVQQVITNLLIGQIELRSEDSPDILTYSHERKVEKLIVPLGEELAAI
QKTYIQILESFARSLIQRNVLMRRDIPNLTKYQIILARDQFRKNPSPNIVGIQQGIIEGE
FAICISLYHGYELLQQMGMRSLYFFLCGIMDGTKGMTRSKNELGRNEDFMKLYNHLECMF
ARTRSTSANGISAIQQGDKNKKFVYSHPKLKKLEEVVIEHFKSWNAENTTEKKRDETRVM
IFSSFRDSVQEIAEMLSQHQPIIRVMTFVGHASGKSTKGFTQKEQLEVVKQFRDGGYNTL
VSTCVGEEGLDIGEVDLIICFDSQKSPIRLVQRMGRTGRKRQGRIVIILSEGREERIYNQ
SQSNKRSIYKAISSNRQVLHFYQRSPRMVPDGINPKLHKMFITHGVYEPEKPSRNLQRKS
SIFSYRDGMRQSSLKKDWFLSEEEFKLWNRLYRLRDSDEIKEITLPQVQFSSLQNEENKP
AQESTTGIHQLSLSEWRLWQDHPLPTHQVDHSDRCRHFIGLMQMIEGMRHEEGECSYELE
VESYLQMEDVTSTFIAPRNESNNLASDTFITHKKSSFIKNINQGSSSSVIESDEECAEIV
KQTHIKPTKIVSLKKKVSKEIKKDQLKKENNHGIIDSVDNDRNSTVENIFQEDLPNDKRT
SDTDEIAATCTINENVIKEPCVLLTECQFTNKSTSSLAGNVLDSGYNSFNDEKSVSSNLF
LPFEEELYIVRTDDQFYNCHSLTKEVLANVERFLSYSPPPLSGLSDLEYEIAKGTALENL
LFLPCAEHLRSDKCTCLLSHSAVNSQQNLELNSLKCINYPSEKSCLYDIPNDNISDEPSL
CDCDVHKHNQNENLVPNNRVQIHRSPAQNLVGENNHDVDNSDLPVLSTDQDESLLLFEDV
NTEFDDVSLSPLNSKSESLPVSDKTAISETPLVSQFLISDELLLDNNSELQDQITRDANS
FKSRDQRGVQEEKVKNHEDIFDCSRDLFSVTFDLGFCSPDSDDEILEHTSDSNRPLDDLY
GRYLEIKEISDANYVSNQALIPRDHSKNFTSGTVIIPSNEDMQNPNYVHLPLSAAKNEEL
LSPGYSQFSLPVQKKVMSTPLSKSNTLNSFSKIRKEILKTPDSSKEKVNLQRFKEALNST
FDYSEFSLEKSKSSGPMYLHKSCHSVEDGQLLTSNESEDDEIFRRKVKRAKGNVLNSPED
QKNSEVDSPLHAVKKRRFPINRSELSSSDESENFPKPCSQLEDFKVCNGNARRGIKVPKR
QSHLKHVARKFLDDEAELSEEDAEYVSSDENDESENEQDSSLLDFLNDETQLSQAINDSE
MRAIYMKSLRSPMMNNKYKMIHKTHKNINIFSQIPEQDETYLEDSFCVDEEESCKGQSSE
EEVCVDFNLITDDCFANSKKYKTRRAVMLKEMMEQNCAHSKKKLSRIILPDDSSEEENNV
NDKRESNIAVNPSTVKKNKQQDHCLNSVPSGSSAQSKVRSTPRVNPLAKQSKQTSLNLKD
TISEVSDFKPQNHNEVQSTTPPFTTVDSQKDCRKFPVPQKDGSALEDSSTSGASCSKSRP
HLAGTHTSLRLPQEGKGTCILVGGHEITSGLEVISSLRAIHGLQVEVCPLNGCDYIVSNR
MVVERRSQSEMLNSVNKNKFIEQIQHLQSMFERICVIVEKDREKTGDTSRMFRRTKSYDS
LLTTLIGAGIRILFSSCQEETADLLKELSLVEQRKNVGIHVPTVVNSNKSEALQFYLSIP
NISYITALNMCHQFSSVKRMANSSLQEISMYAQVTHQKAEEIYRYIHYVFDIQMLPNDLN

QDRLKSDI*
>ENSG00000043039|ENST00000281437
MHCHAELRLSSPGQLKAARRRYKTFMIDEILSKETCDYFEKLSLYSVCPSLVVRPKPLHS
CTGSPSLRAYPLLSVITRQPTVISHLVPATPGIAQALSCHQVTEAVSAEAPGGEALASSE
SETEQPTPRQKKPRRSRTIFTELQLMGLEKKFQKQKYLSTPDRLDLAQSLGLTQLQVKTW
YQNRRMKWKKMVLKGGQEAPTKPKGRPKKNSIPTSEEIEAEEKMNSQAQGQEQLEPSQGQ
EELCEAQEPKARDVPLEMAEPPDPPQELPIPSSEPPPLS*
>ENSG00000173039|ENST00000308639
MDELFPLIFPAEPAQASGPYVEIIEQPKQRGMRFRYKCEGRSAGSIPGERSTDTTKTHPT
IKINGYTGPGTVRISLVTKDPPHRPHPHELVGKDCRDGFYEAELCPDRCIHSFQNLGIQC
VKKRDLEQAISQRIQTNNNPFQEEQRGDYDLNAVRLCFQVTVRDPSGRPLRLPPVLSHPI
FDNRAPNTAELKICRVNRNSGSCLGGDEIFLLCDKVQKEDIEVYFTGPGWEARGSFSQAD
VHRQVAIVFRTPPYADPSLQAPVRVSMQLRRPSDRELSEPMEFQYLPDTDDRHRIEEKRK
RTYETFKSIMKKSPFSGPTDPRPPPRRIAVPSRSSASVPKPAPQPYPFTSSLSTINYDEF
PTMVFPSGQISQASALAPAPPQVLPQAPAPAPAPAMVSALAQAPAPVPVLAPGPPQAVAP
PAPKPTQAGEGTLSEALLQLQFDDEDLGALLGNSTDPAVFTDLASVDNSEFQQLLNQGIP
VAPHTTEPMLMEYPEAITRLVTGAQRPPDPAPAPLGAPGLPNGLLSGDEDFSSIADMDFS
ALLSQISS*
>ENSG00000078403|ENST00000307729
MVSSDRPVSLEDEVSHSMKEMIGGCCVCSDERGWAENPLVYCDGHGCSVAVHQACYGIVQ
VPTGPWFCRKCESQERAARVRCELCPHKDGALKRTDNGGWAHVVCALYIPEVQFANVSTM
EPIVLQSVPHDRYNKTCYICDEQGRESKAATGACMTCNKHGCRQAFHVTCAQFAGLLCEE
EGNGADNVQYCGYCKYHFSKLKKSKRGSNRSYDQSLSDSSSHSQDKHHEKEKKKYKEKDK
HKQKHKKQPEPSPALVPSLTVTTEKTYTSTSNNSISGSLKRLEDTTARFTNANFQEVSAH
TSSGKDVSETRGSEGKGKKSSAHSSGQRGRKPGGGRNPGTTVSAASPFPQGSFSGTPGSV
KSSSGSSVQSPQDFLSFTDSDLRNDSYSHSQQSSATKDVHKGESGSQEGGVNSFSTLIGL
PSTSAVTSQPKSFENSPGDLGNSSLPTAGYKRAQTSGIEEETVKEKKRKGNKQSKHGPGR
PKGNKNQENVSHLSVSSASPTSSVASAAGSITSSSLQKSPTLLRNGSLQSLSVGSSPVGS
EISMQYRHDGACPTTTFSELLNAIHNGIYNSNDVAVSFPNVVSGSGSSTPVSSSHLPQQS
SGHLQQVGALSPSAVSSAAPAVATTQANTLSGSSLSQAPSHMYGNRSNSSMAALIAQSEN
NQTDQDLGDNSRNLVGRGSSPRGSLSPRSPVSSLQIRYDQPGNSSLENLPPVAASIEQLL
ERQWSEGQQFLLEQGTPSDILGMLKSLHQLQVENRRLEEQIKNLTAKKERLQLLNAQLSV
PFPTITANPSPSHQIHTFSAQTAPTTDSLNSSKSPHIGNSFLPDNSLPVLNQDLTSSGQS
TSSSSALSTPPPAGQSPAQQGSGVSGVQQVNGVTVGALASGMQPVTSTIPAVSAVGGIIG
ALPGNQLAINGIVGALNGVMQTPVTMSQNPTPLTHTTVPPNATHPMPATLTNSASGLGLL
SDQQRQILIHQQQFQQLLNSQQLTPEQHQAFLYQLMQHHHQQHHQPELQQLQIPGPTQIP
INNLLAGTQAPPLHTATTNPFLTIHGDNASQKVARLSDKTGPVAQEKS*
>ENSG00000165584|ENST00000298396
MNGDDTFARRPTVGAQIPEKIQKAFDDIAKYFSKEEWEKMKVSEKIVYVYMKRKYEAMTK
LGFKAILPSFMRNKRVTDFQGNDFDNDPNRGNQVQRPQMTFGRLQGIFPKIMPKKPAEEG
NVSKEVPEASGPQNDGKQLCPPGKPTTSEKINMISGPKRGEHAWTHRLRERKQLVIYEEI
SDPEEDDE*
>ENSG00000135679|ENST00000299252
MCNTNMSVPTDGAVTTSQIPASEQETLVRPKPLLLKLLKSVGAQKDTYTMKEDLDAGVSE

HSGDWLDQDSVSDQFSVEFEVESLDSEDYSLSEEGQELSDEDDEVYQVTVYQAGESDTDS
FEEDPEISLADYWKCTSCNEMNPPLPSHCNRCWALRENWLPEDKGKDKGEISEKAKLENS
TQAEEGFDVPDCKKTIVNDSRESCVEENDDKITQASQSQESEDYSQPSTSSSIIYSSQED
VKEFEREETQDKEESVESSLPLNAIEPCVICQGRPKNGCIVHGKTGHLMACFTCAKKLKK
RNKPCPVCRQPIQMIVLTYFP*
>ENSG00000151353|ENST00000281017
MPSAFSVSSFPVSIPAVLTQTDWTEPWLMGLATFHALCVLLTCLSSRSYRLQIGHFLCLV
ILVYCAEYINEAAAMNWRLFSKYQYFDSRGMFISIVFSAPLLVNAMIIVVMWVWKTLNVM
TDLKNAQERRKEKKRRRKED*
>ENSG00000174173|ENST00000309922
MAAFLKMSVSVNFFRPFTRFLVPFTLHRKRNNLTILQRYMSSKIPAVTYPKNESTPPSEE
LELDKWKTTMKSSVQEECVSTISSSKDEDPLAATREFIEMWRLLGREVPEHITEEELKTL
MECVSNTAKKKYLKYLYTKEKVKKARQIKKEMKAAAREEAKNIKLLETTEEDKQKNFLFL
RLWDRNMDIAMGWKGAQAMQFGQPLVFDMAYENYMKRKELQNTVSQLLESEGWNRRNVDP
FHIYFCNLKIDGALHRELVKRYQEKWDKLLLTSTEKSHVDLFPKDSIIYLTADSPNVMTT
FRHDKVYVIGSFVDKSMQPGTSLAKAKRLNLATECLPLDKYLQWEIGNKNLTLDQMIRIL
LCLKNNGNWQEALQFVPKRKHTGFLEISQHSQEFINRLKKAKT*
>ENSG00000139746|ENST00000267229
MVSKMIIENFEALKSWLSKTLEPICDADPSALAKYVLALVKKDKSEKELKALCIDQLDVF
LQKETQIFVEKLFDAVNTKSYLPPPEQPSSGSLKVEFFPHQEKDIKKEEITKEEEREKKF
SRRLNHSPPQSSSRYRENRSRDERKKDDRSRKRDYDRNPPRRDSYRDRYNRRRGRSRSYS
RSRSRSWSKERLRERDRDRSRTRSRSRTRSRERDLVKPKYDLDRTDPLENNYTPVSSVPS
ISSGHYPVPTLSSTITVIAPTHHGNNTTESWSEFHEDQVDHNSYVRPPMPKKRCRDYDEK
GFCMRGDMCPFDHGSDPVVVEDVNLPGMLPFPAQPPVVEGPPPPGLPPPPPILTPPPVNL
RPPVPPPGPLPPSLPPVTGPPPPLPPLQPSGMDAPPNSATSSVPTVVTTGIHHQPPPAPP
SLFTADTYDTDGYNPEAPSITNTSRPMYRHRVHAQRPNLIGLTSGDMDLPPREKPPNKSS
MRIVVDSESRKRTIGSGEPGVPTKKTWFDKPNFNRTNSPGFQKKVQFGNENTKLELRKVP
PELNNISKLNEHFSRFGTLVNLQVAYNGDPEGALIQFATYEEAKKAISSTEAVLNNRFIK
VYWHREGSTQQLQTTSPKPLVQQPILPVVKQSVKERLGPVPSSTIEPAEAQSASSDLPQV
LSTSTGLTKTVYNPAALKAAQKTLLVSTSAVDNNEAQKKKQEALKLQQDVRKRKQEILEK
HIETQKMLISKLEKNKTMKSEDKAEIMKTLEVLTKNITKLKDEVKAASPGRCLPKSIKTK
TQMQKELLDTELDLYKKMQAGEEVTELRRKYTELQLEAAKRGILSSGRGRGIHSRGRGAV
HGRGRGRGRGRGVPGHAVVDHRPRALEISAFTESDREDLLPHFAQYGEIEDCQIDDSSLH
AVITFKTRAEAEAAAVHGARFKGQDLKLAWNKPVTNISAVETEEVEPDEEEFQEESLVDD
SLLQDDDEEEEDNESRSWRR*
>ENSG00000150347|ENST00000279873
MEPNSLQWVGSPCGLHGPYIFYKAFQFHLEGKPRILSLGDFFFVRCTPKDPICIAELQLL
WEERTSRQLLSSSKLYFLPEDTPQGRNSDHGEDEVIAVSEKVIVKLEDLVKWVHSDFSKW
RCGFHAGPVKTEALGRNGQKEALLKYRQSTLNSGLNFKDVLKEKADLGEDEEETNVIVLS
YPQYCRYRSMLKRIQDKPSSILTDQFALALGGIAVVSRNPQILYCRDTFDHPTLIENESI
CDEFAPNLKGRPRKKKPCPQRRDSFSGVKDSNNNSDGKAVAKVKCEARSALTKPKNNHNC
KKVSNEEKPKVAIGEECRADEQAFLVALYKYMKERKTPIERIPYLGFKQINLWTMFQAAQ
KLGGYETITARRQWKHIYDELGGNPGSTSAATCTRRHYERLILPYERFIKGEEDKPLPPI
KPRKQENSSQENENKTKVSGTKRIKHEIPKSKKEKENAPKPQDAAEVSSEQEKEQETLIS

QKSIPEPLPAADMKKKIEGYQEFSAKPLASRVDPEKDNETDQGSNSEKVAEEAGEKGPTP
PLPSAPLAPEKDSALVPGASKQPLTSPSALVDSKQESKLCCFTESPESEPQEASFPSFPT
TQPPLANQNETEDDKLPAMADYIANCTVKVDQLGSDDIHNALKQTPKVLVVQSFDMFKDK
DLTGPMNENHGLNYTPLLYSRGNPGIMSPLAKKKLLSQVSGASLSSSYPYGSPPPLISKK
KLIARDDLCSSLSQTHHGQSTDHMAVSRPSVIQHVQSFRSKPSEERKTINDIFKHEKLSR
SDPHRCSFSKHHLNPLADSYVLKQEIQEGKDKLLEKRALPHSHMPSFLADFYSSPHLHSL
YRHTEHHLHNEQTSKYPSRDMYRESENSSFPSHRHQEKLHVNYLTSLHLQDKKSAAAEAP
TDDQPTDLSLPKNPHKPTGKVLGLAHSTTGPQESKGISQFQVLGSQSRDCHPKACRVSPM
TMSGPKKYPESLSRSGKPHHVRLENFRKMEGMVHPILHRKMSPQNIGAARPIKRSLEDLD
LVIAGKKARAVSPLDPSKEVSGKEKASEQESEGSKAAHGGHSGGGSEGHKLPLSSPIFPG
LYSGSLCNSGLNSRLPAGYSHSLQYLKNQTVLSPLMQPLAFHSLVMQRGIFTSPTNSQQL
YRHLAAATPVGSSYGDLLHNSIYPLAAINPQAAFPSSQLSSVHPSTKL*
>ENSG00000100416|ENST00000290846
MQALRHVVCALSGGVDSAVAALLLRRRGYQVTGVFMKNWDSLDEHGVCTADKDCEDAYRV
CQILDIPFHQVSYVKEYWNDVFSDFLNEYEKGRTPNPDIVCNKHIKFSCFFHYAVDNLGA
DAIATGHYARTSLEDEEVFEQKHVKKPEGLFRNRFEVRNAVKLLQAADSFKDQTFFLSQV
SQDALRRTIFPLGGLTKEFVKKIAAENRLHHVLQKKESMGMCFIGKRNFEHFLLQYLQPR
PGHFISIEDNKVLGTHKGWFLYTLGQRANIGGLREPWYVVEKDSVKGDVFVAPRTDHPAL
YRDLLRTSRVHWIAEEPPAALVRDKMMECHFRFRHQMALVPCVLTLNQDGTVWVTAVQAV
RALATGQFAVFYKGDECLGSGKILRLGPSAYTLQKGQRRAGMATESPSDSPEDGPGLSPL
L*
>ENSG00000101040|ENST00000311275
MDISTRSKDPGSAERTAQKRKFPSPPHSSNGHSPQDTSTSPIKKKKKPGLLNSNNKEQSE
LRHGPFYYMKQPLTTDPVDVVPQDGRNDFYCWVCHREGQVLCCELCPRVYHAKCLRLTSE
PEGDWFCPECEKITVAECIETQSKAMTMLTIEQLSYLLKFAIQKMKQPGTDAFQKPVPLE
QHPDYAEYIFHPMDLCTLEKNAKKKMYGCTEAFLADAKWILHNCIIYNGGNHKLTQIAKV
VIKICEHEMNEIEVCPECYLAACQKRDNWFCEPCSNPHPLVWAKLKGFPFWPAKALRDKD
GQVDARFFGQHDRAWVPINNCYLMSKEIPFSVKKTKSIFNSAMQEMEVYVENIRRKFGVF
NYSPFRTPYTPNSQYQMLLDPTNPSAGTAKIDKQEKVKLNFDMTASPKILMSKPVLSGGT
GRRISLSDMPRSPMSTNSSVHTGSDVEQDAEKKATSSHFSASEESMDFLDKSTASPASTK
TGQAGSLSGSPKPFSPQLSAPITTKTDKTSTTGSILNLNLDRSKAEMDLKELSESVQQQS
TPVPLISPKRQIRSRFQLNLDKTIESCKAQLGINEISEDVYTAVEHSDSEDSEKSDSSDS
EYISDDEQKSKNEPEDTEDKEGCQMDKEPSAVKKKPKPTNPVEIKEELKSTSPASEKADP
GAVKDKASPEPEKDFSEKAKPSPHPIKDKLKGKDETDSPTVHLGLDSDSESELVIDLGED
HSGREGRKNKKEPKEPSPKQDVVGKTPPSTTVGSHSPPETPVLTRSSAQTSAAGATATTS
TSSTVTVTAPAPAATGSPVKKQRPLLPKETAPAVQRVVWNSSSKFQTSSQKWHMQKMQRQ
QQQQQQQNQQQQPQSSQGTRYQTRQAVKAVQQKEITQSPSTSTITLVTSTQSSPLVTSSG
SMSTLVSSVNADLPIATASADVAADIAKYTSKMMDAIKGTMTEIYNDLSKNTTGSTIAEI
RRLRIEIEKLQWLHQQELSEMKHNLELTMAEMRQSLEQERDRLIAEVKKQLELEKQQAVD
ETKKKQWCANCKKEAIFYCCWNTSYCDYPCQQAHWPEHMKSCTQSATAPQQEADAEVNTE
TLNKSSQGSSSSTQSAPSETASASKEKETSAEKSKESGSTLDLSGSRETPSSILLGSNQG
SDHSRSNKSSWSSSDEKRGSTRSDHNTSTSTKSLLPKESRLDTFWD*
>ENSG00000167766|ENST00000301096
MHGRKDDAQKQPVKNQLGLNPQSHLPELQLFQAEGKIYKYDHMEKSVNSSSLVSPPQRIS

STVKTHISHTYECNFVDSLFTQKEKANIGTEHYKCSERGKAFHQGLHFTIHQIIHTKETQ
FKCDICGKIFNKKSNLASHQRIHTGEKPYKCNECGKVFHNMSHLAQHRRIHTGEKPYKCN
ECGKVFNQISHLAQHQRIHTGEKPYKCNECGKVFHQISHLAQHRTIHTGEKPYECNKCGK
VFSRNSYLVQHLIIHTGEKPYRCNVCGKVFHHISHLAQHQRIHTGEKPYKCNECGKVFSH
KSSLVNHWRIHTGEKPYKCNECGKVFSHKSSLVNHWRIHTGEKPYKCNECGKVFSRNSYL
AQHLIIHAGEKPYKCDECDKAFSQNSHLVQHHRIHTGEKPYKCDECGKVFSQNSYLAYHW
RIHTGEKAYKCNECGKVFGLNSSLAHHRKIHTGEKPFKCNECGKAFSMRSSLTNHHAIHT
GEKHFKCNECGKLFRDNSYLVRHQRFHAGKKSNTCN*
>ENSG00000169953|ENST00000304790
MAHVSSETQDVSPKDELTASEASTRSPLCEHTFPGDSDLRSMIEEHAFQVLSQGSLLESP
SYTVCVSEPDKDDDFLSLNFPRKLWKIVESDQFKSISWDENGTCIVINEELFKKEILETK
APYRIFQTDAIKSFVRQLNLYGFSKIQQNFQRSAFLATFLSEEKESSVLSKLKFYYNPNF
KRGYPQLLVRVKRRIGVKNASPISTLFNEDFNKKHFRAGANMENHNSALAAEASEESLFS
ASKNLNMPLTRESSVRQIIANSSVPIRSGFPPPSPSTSVGPSEQIATDQHAILNQLTTIH
MHSHSTYMQARGHIVNFITTTTSQYHIISPLQNGYFGLTVEPSAVPTRYPLVSVNEAPYR
NMLPAGNPWLQMPTIADRSAAPHSRLALQPSPLDKYHPNYN*
>ENSG00000117751|ENST00000311772
MAAAANSGSSLPLFDCPTWAGKPPPGLHLDVVKGDKLIEKLIIDEKKYYLFGRNPDLCDF
TIDHQSCSRVHAALVYHKHLKRVFLIDLNSTHGTFLGHIRLEPHKPQQIPIDSTVSFGAS
TRAYTLREKPQTLPSAVKGDEKMGGEDDELKGLLGLPEEETELDNLTEFNTAHNKRISTL
TIEEGNLDIQRPKRKRKNSRVTFSEDDEIINPEDVDPSVGRFRNMVQTAVVPVKKKRVEG
PGSLGLEESGSRRMQNFAFSGGLYGGLPPTHSEAGSQPHGIHGTALIGGLPMPYPNLAPD
VDLTPVVPSAVNMNPAPNPAVYNPEAVNEPKKKKYAKEAWPGKKPTPSLLI*
>ENSG00000253729|ENST00000314191
MAGSGAGVRCSLLRLQETLSAADRCGAALAGHQLIRGLGQECVLSSSPAVLALQTSLVFS
RDFGLLVFVRKSLNSIEFRECREEILKFLCIFLEKMGQKIAPYSVEIKNTCTSVYTKDRA
AKCKIPALDLLIKLLQTFRSSRLMDEFKIGELFSKFYGELALKKKIPDTVLEKVYELLGL
LGEVHPSEMINNAENLFRAFLGELKTQMTSAVREPKLPVLAGCLKGLSSLLCNFTKSMEE
DPQTSREIFNFVLKAIRPQIDLKRYAVPSAGLRLFALHASQFSTCLLDNYVSLFEVLLKW
CAHTNVELKKAALSALESFLKQVSNMVAKNAEMHKNKLQYFMEQFYGIIRNVDSNNKELS
IAIRGYGLFAGPCKVINAKDVDFMYVELIQRCKQMFLTQTDTGDDRVYQMPSFLQSVASV
LLYLDTVPEVYTPVLEHLVVMQIDSFPQYSPKMQLVCCRAIVKVFLALAAKGPVLRNCIS
TVVHQGLIRICSKPVVLPKGPESESEDHRASGEVRTGKWKVPTYKDYVDLFRHLLSSDQM
MDSILADEAFFSVNSSSESLNHLLYDEFVKSVLKIVEKLDLTLEIQTVGEQENGDEAPGV
WMIPTSDPAANLHPAKPKDFSAFINLVEFCREILPEKQAEFFEPWVYSFSYELILQSTRL
PLISGFYKLLSITVRNAKKIKYFEGVSPKSLKHSPEDPEKYSCFALFVKFGKEVAVKMKQ
YKDELLASCLTFLLSLPHNIIELDVRAYVPALQMAFKLGLSYTPLAEVGLNALEEWSIYI
DRHVMQPYYKDILPCLDGYLKTSALSDETKNNWEVSALSRAAQKGFNKVVLKHLKKTKNL
SSNEAISLEEIRIRVVQMLGSLGGQINKNLLTVTSSDEMMKSYVAWDREKRLSFAVPFRE
MKPVIFLDVFLPRVTELALTASDRQTKVAACELLHSMVMFMLGKATQMPEGGQGAPPMYQ
LYKRTFPVLLRLACDVDQVTRQLYEPLVMQLIHWFTNNKKFESQDTVALLEAILDGIVDP
VDSTLRDFCGRCIREFLKWSIKQITPQQQEKSPVNTKSLFKRLYSLALHPNAFKRLGASL
AFNNIYREFREEESLVEQFVFEALVIYMESLALAHADEKSLGTIQQCCDAIDHLCRIIEK
KHVSLNKAKKRRLPRGFPPSASLCLLDLVKWLLAHCGRPQTECRHKSIELFYKFVPLLPG

NRSPNLWLKDVLKEEGVSFLINTFEGGGCGQPSGILAQPTLLYLRGPFSLQATLCWLDLL
LAALECYNTFIGERTVGALQVLGTEAQSSLLKAVAFFLESIAMHDIIAAEKCFGTGAAGN
RTSPQEGERYNYSKCTVVVRIMEFTTTLLNTSPEGWKLLKKDLCNTHLMRVLVQTLCEPA
SIGFNIGDVQVMAHLPDVCVNLMKALKMSPYKDILETHLREKITAQSIEELCAVNLYGPD
AQVDRSRLAAVVSACKQLHRAGLLHNILPSQSTDLHHSVGTELLSLVYKGIAPGDERQCL
PSLDLSCKQLASGLLELAFAFGGLCERLVSLLLNPAVLSTASLGSSQGSVIHFSHGEYFY
SLFSETINTELLKNLDLAVLELMQSSVDNTKMVSAVLNGMLDQSFRERANQKHQGLKLAT
TILQHWKKCDSWWAKDSPLETKMAVLALLAKILQIDSSVSFNTSHGSFPEVFTTYISLLA
DTKLDLHLKGQAVTLLPFFTSLTGGSLEELRRVLEQLIVAHFPMQSREFPPGTPRFNNYV
DCMKKFLDALELSQSPMLLELMTEVLCREQQHVMEELFQSSFRRIARRGSCVTQVGLLES
VYEMFRKDDPRLSFTRQSFVDRSLLTLLWHCSLDALREFFSTIVVDAIDVLKSRFTKLNE
STFDTQITKKMGYYKILDVMYSRLPKDDVHAKESKINQVFHGSCITEGNELTKTLIKLCY
DAFTENMAGENQLLERRRLYHCAAYNCAISVICCVFNELKFYQGFLFSEKPEKNLLIFEN
LIDLKRRYNFPVEVEVPMERKKKYIEIRKEAREAANGDSDGPSYMSSLSYLADSTLSEEM
SQFDFSTGVQSYSYSSQDPRPATGRFRRREQRDPTVHDDVLELEMDELNRHECMAPLTAL
VKHMHRSLGPPQGEEDSVPRDLPSWMKFLHGKLGNPIVPLNIRLFLAKLVINTEEVFRPY
AKHWLSPLLQLAASENNGGEGIHYMVVEIVATILSWTGLATPTGVPKDEVLANRLLNFLM
KHVFHPKRAVFRHNLEIIKTLVECWKDCLSIPYRLIFEKFSGKDPNSKDNSVGIQLLGIV
MANDLPPYDPQCGIQSSEYFQALVNNMSFVRYKEVYAAAAEVLGLILRYVMERKNILEES
LCELVAKQLKQHQNTMEDKFIVCLNKVTKSFPPLADRFMNAVFFLLPKFHGVLKTLCLEV
VLCRVEGMTELYFQLKSKDFVQVMRHRDDERQKVCLDIIYKMMPKLKPVELRELLNPVVE
FVSHPSTTCREQMYNILMWIHDNYRDPESETDNDSQEIFKLAKDVLIQGLIDENPGLQLI
IRNFWSHETRLPSNTLDRLLALNSLYSPKIEVHFLSLATNFLLEMTSMSPDYPNPMFEHP
LSECEFQEYTIDSDWRFRSTVLTPMFVETQASQGTLQTRTQEGSLSARWPVAGQIRATQQ
QHDFTLTQTADGRSSFDWLTGSSTDPLVDHTSPSSDSLLFAHKRSERLQRAPLKSVGPDF
GKKRLGLPGDEVDNKVKGAAGRTDLLRLRRRFMRDQEKLSLMYARKGVAEQKREKEIKSE
LKMKQDAQVVLYRSYRHGDLPDIQIKHSSLITPLQAVAQRDPIIAKQLFSSLFSGILKEM
DKFKTLSEKNNITQKLLQDFNRFLNTTFSFFPPFVSCIQDISCQHAALLSLDPAAVSAGC
LASLQQPVGIRLLEEALLRLLPAELPAKRVRGKARLPPDVLRWVELAKLYRSIGEYDVLR
GIFTSEIGTKQITQSALLAEARSDYSEAAKQYDEALNKQDWVDGEPTEAEKDFWELASLD
CYNHLAEWKSLEYCSTASIDSENPPDLNKIWSEPFYQETYLPYMIRSKLKLLLQGEADQS
LLTFIDKAMHGELQKAILELHYSQELSLLYLLQDDVDRAKYYIQNGIQSFMQNYSSIDVL
LHQSRLTKLQSVQALTEIQEFISFISKQGNLSSQVPLKRLLNTWTNRYPDAKMDPMNIWD
DIITNRCFFLSKIEEKLTPLPEDNSMNVDQDGDPSDRMEVQEQEEDISSLIRSCKFSMKM
KMIDSARKQNNFSLAMKLLKELHKESKTRDDWLVSWVQSYCRLSHCRSRSQGCSEQVLTV
LKTVSLLDENNVSSYLSKNILAFRDQNILLGTTYRIIANALSSEPACLAEIEEDKARRIL
ELSGSSSEDSEKVIAGLYQRAFQHLSEAVQAAEEEAQPPSWSCGPAAGVIDAYMTLADFC
DQQLRKEEENASVIDSAELQAYPALVVEKMLKALKLNSNEARLKFPRLLQIIERYPEETL
SLMTKEISSVPCWQFISWISHMVALLDKDQAVAVQHSVEEITDNYPQAIVYPFIISSESY
SFKDTSTGHKNKEFVARIKSKLDQGGVIQDFINALDQLSNPELLFKDWSNDVRAELAKTP
VNKKNIEKMYERMYAALGDPKAPGLGAFRRKFIQTFGKEFDKHFGKGGSKLLRMKLSDFN
DITNMLLLKMNKDSKPPGNLKECSPWMSDFKVEFLRNELEIPGQYDGRGKPLPEYHVRIA
GFDERVTVMASLRRPKRIIIRGHDEREHPFLVKGGEDLRQDQRVEQLFQVMNGILAQDSA
CSQRALQLRTYSVVPMTSRLGLIEWLENTVTLKDLLLNTMSQEEKAAYLSDPRAPPCEYK

DWLTKMSGKHDVGAYMLMYKGANRTETVTSFRKRESKVPADLLKRAFVRMSTSPEAFLAL
RSHFASSHALICISHWILGIGDRHLNNFMVAMETGGVIGIDFGHAFGSATQFLPVPELMP
FRLTRQFINLMLPMKETGLMYSIMVHALRAFRSDPGLLTNTMDVFVKEPSFDWKNFEQKM
LKKGGSWIQEINVAEKNWYPRQKICYAKRKLAGANPAVITCDELLLGHEKAPAFRDYVAV
ARGSKDHNIRAQEPESGLSEETQVKCLMDQATDPNILGRTWEGWEPWM*
>ENSG00000166529|ENST00000292450
MMTKVLGMAPVLGPRPPQEQVGPLMVKVEEKEEKGKYLPSLEMFRQRFRQFGYHDTPGPR
EALSQLRVLCCEWLRPEIHTKEQILELLVLEQFLTILPQELQAWVQEHCPESAEEAVTLL
EDLERELDEPGHQVSTPPNEQKPVWEKISSSGTAKESPSSMQPQPLETSHKYESWGPLYI
QESGEEQEFAQDPRKVRDCRLSTQHEESADEQKGSEAEGLKGDIISVIIANKPEASLERQ
CVNLENEKGTKPPLQEAGSKKGRESVPTKPTPGERRYICAECGKAFSNSSNLTKHRRTHT
GEKPYVCTKCGKAFSHSSNLTLHYRTHLVDRPYDCKCGKAFGQSSDLLKHQRMHTEEAPY
QCKDCGKAFSGKGSLIRHYRIHTGEKPYQCNECGKSFSQHAGLSSHQRLHTGEKPYKCKE
CGKAFNHSSNFNKHHRIHTGEKPYWCHHCGKTFCSKSNLSKHQRVHTGEGEAP*
>ENSG00000140939|ENST00000268605
MGNAQERPSETIDRERKRLVETLQADSGLLLDALLARGVLTGPEYEALDALPDAERRVRR
LLLLVQGKGEAACQELLRCAQRTAGAPDPAWDWQHVGPGYRDRSYDPPCPGHWTPEAPGS
GTTCPGLPRASDPDEAGGPEGSEAVQSGTPEEPEPELEAEASKEAEPEPEPEPELEPEAE
AEPEPELEPEPDPEPEPDFEERDESEDS*
>ENSG00000240682|ENST00000273541
MARNAEKAMTALARFRQAQLEEGKVKERRPFLASECTELPKAEKWRRQIIGEISKKVAQI
QNAGLGEFRIRDLNDEINKLLREKGHWEVRIKELGGPDYGKVGPKMLDHEGKEVPGNRGY
KYFGAAKDLPGVRELFEKERQVRWLMPVIPALWEAEAGGSQALPPPRKTRAELMKAIDFE
YYGYLDEDDGVIVPLEQEYEKKLRAELVEKWKAEREARLARGEKEEEEEEEEEINIYAVT
EEESDEEGSQEKGGDDSQQKFIAHVPVPSQQEIEEALVRRKKMELLQKYASETLQAQSEE
ARRLLGY*
>ENSG00000131116|ENST00000300811
MTETREPAETGGYASLEEDDEDLSPGPEHSSDSEYTLSEPDSEEEEDEEEEEEETTDDPE
YDPGYKVKQRLGGGRGGPSRRAPRAAQPPAQPCQLCGRSPLGEAPPGTPPCRLCCPATAP
QEAPAPEGRALGEEEEEPPRAGEGRPAGREEEEEEEEEGTYHCTECEDSFDNLGELHGHF
MLHARGEV*
>ENSG00000163468|ENST00000295688
MMGHRPVLVLSQNTKRESGRKVQSGNINAAKTIADIIRTCLGPKSMMKMLLDPMGGIVMT
NDGNAILREIQVQHPAAKSMIEISRTQDEEVGDGTTSVIILAGEMLSVAEHFLEQQMHPT
VVISAYRKALDDMISTLKKISIPVDISDSDMMLNIINSSITTKAISRWSSLACNIALDAV
KMVQFEENGRKEIDIKKYARVEKIPGGIIEDSCVLRGVMINKDVTHPRMRRYIKNPRIVL
LDSSLEYKKGESQTDIEITREEDFTRILQMEEEYIQQLCEDIIQLKPDVVITEKGISDLA
QHYLMRANITAIRRVRKTDNNRIARACGARIVSRPEELREDDVGTGAGLLEIKKIGDEYF
TFITDCKDPKACTILLRGASKEILSEVERNLQDAMQVCRNVLLDPQLVPGGGASEMAVAH
ALTEKSKAMTGVEQWPYRAVAQALEVIPRTLIQNCGASTIRLLTSLRAKHTQENCETWGV
NGETGTLVDMKELGIWEPLAVKLQTYKTAVETAVLLLRIDDIVSGHKKKGDDQSRQGGAP
DAGQE*
>ENSG00000148840|ENST00000278070
MAARRGRRDGVAPPPSGGPGPDPGGGARGSGWGSRSQAPYGTLGAVSGGEQVLLHEEAGD

SGFVSLSRLGPSLRDKDLEMEELMLQDETLLGTMQSYMDASLISLIEDFGSLGESRLSLE
DQNEVSLLTALTEILDNADSENLSPFDSIPDSELLVSPREGSSLHKLLTLSRTPPERDLI
TPVDPLGPSTGSSRGSGVEMSLPDPSWDFSPPSFLETSSPKLPSWRPPRSRPRWGQSPPP
QQRSDGEEEEEVASFSGQILAGELDNCVSSIPDFPMHLACPEEEDKATAAEMAVPAAGDE
SISSLSELVRAMHPYCLPNLTHLASLEDELQEQPDDLTLPEGCVVLEIVGQAATAGDDLE
IPVVVRQVSPGPRPVLLDDSLETSSALQLLMPTLESETEAAVPKVTLCSEKEGLSLNSEE
KLDSACLLKPREVVEPVVPKEPQNPPANAAPGSQRARKGRKKKSKEQPAACVEGYARRLR
SSSRGQSTVGTEVTSQVDNLQKQPQEELQKESGPLQGKGKPRAWARAWAAALENSSPKNL
ERSAGQSSPAKEGPLDLYPKLADTIQTNPIPTHLSLVDSAQASPMPVDSVEADPTAVGPV
LAGPVPVDPGLVDLASTSSELVEPLPAEPVLINPVLADSAAVDPAVVPISDNLPPVDAVP
SGPAPVDLALVDPVPNDLTPVDPVLVKSRPTDPRRGAVSSALGGSAPQLLVESESLDPPK
TIIPEVKEVVDSLKIESGTSATTHEARPRPLSLSEYRRRRQQRQAETEERSPQPPTGKWP
SLPETPTGLADIPCLVIPPAPAKKTALQRSPETPLEICLVPVGPSPASPSPEPPVSKPVA
SSPTEQVPSQEMPLLARPSPPVQSVSPAVPTPPSMSAALPFPAGGLGMPPSLPPPPLQPP
SLPLSMGPVLPDPFTHYAPLPSWPCYPHVSPSGYPCLPPPPTVPLVSGTPGAYAVPPTCS
VPWAPPPAPVSPYSSTCTYGPLGWGPGPQHAPFWSTVPPPPLPPASIGRAVPQPKMESRG
TPAGPPENVLPLSMAPPLSLGLPGHGAPQTEPTKVEVKPVPASPHPKHKVSALVQSPQMK
ALACVSAEGVTVEEPASERLKPETQETRPREKPPLPATKAVPTPRQSTVPKLPAVHPARL
RKLSFLPTPRTQGSEDVVQAFISEIGIEASDLSSLLEQFEKSEAKKECPPPAPADSLAVG
NSGGVDIPQEKRPLDRLQAPELANVAGLTPPATPPHQLWKPLAAVSLLAKAKSPKSTAQE
GTLKPEGVTEAKHPAAVRLQEGVHGPSRVHVGSGDHDYCVRSRTPPKKMPALVIPEVGSR
WNVKRHQDITIKPVLSLGPAAPPPPCIAASREPLDHRTSSEQADPSAPCLAPSSLLSPEA
SPCRNDMNTRTPPEPSAKQRSMRCYRKACRSASPSSQGWQGRRGRNSRSVSSGSNRTSEA
SSSSSSSSSSSRSRSRSLSPPHKRWRRSSCSSSGRSRRCSSSSSSSSSSSSSSSSSSSSR
SRSRSPSPRRRSDRRRRYSSYRSHDHYQRQRVLQKERAIEERRVVFIGKIPGRMTRSELK
QRFSVFGEIEECTIHFRVQGDNYGFVTYRYAEEAFAAIESGHKLRQADEQPFDLCFGGRR
QFCKRSYSDLDSNREDFDPAPVKSKFDSLDFDTLLKQAQKNLRR*
>ENSG00000170608|ENST00000302177
MLGSVKMEAHDLAEWSYYPEAGEVYSPVTPVPTMAPLNSYMTLNPLSSPYPPGGLPASPL
PSGPLAPPAPAAPLGPTFPGLGVSGGSSSSGYGAPGPGLVHGKEMPKGYRRPLAHAKPPY
SYISLITMAIQQAPGKMLTLSEIYQWIMDLFPYYRENQQRWQNSIRHSLSFNDCFVKVAR
SPDKPGKGSYWALHPSSGNMFENGCYLRRQKRFKLEEKVKKGGSGAATTTRNGTGSAAST
TTPAATVTSPPQPPPPAPEPEAQGGEDVGALDCGSPASSTPYFTGLELPGELKLDAPYNF
NHPFSINNLMSEQTPAPPKLDVGFGGYGAEGGEPGVYYQGLYSRSLLNAS*
>ENSG00000144029|ENST00000272418
MATAVRAVGCLPVLCSGTAGHLLGRQCSLNTLPAASILAWKSVLGNGHLSSLGTRDTHPY
ASLSRALQTQCCISSPSHLMSQQYRPYSFFTKLTADELWKGALAETGAGAKKGRGKRTKK
KKRKDLNRGQIIGEGRYGFLWPGLNVPLMKNGAVQTIAQRSKEEQEKVEADMIQQREEWD
RKKKMKVKRERGWSGNSWGGISLGPPDPGPCGETYEDFDTRILEVRNVFTMTAKEGRKKS
IRVLVAVGNGKGAAGFSIGKATDRMDAFRKAKNRAVHHLHYIERYEDHTIFHDISLRFKR
THIKMKKQPKGYGLRCHRAIITICRLIGIKDMYAKVSGSINMLSLTQGLFRGLSRQETHQ
QLADKKGLHVVEIREECGPLPIVVASPRGPLRKDPEPEDEVPDVKLDWEDVKTAQGMKRS
VWSNLKRAAT*
>ENSG00000170684|ENST00000303809

MSRRKAGSAPRRVEPAPAANPDDEMEMQDLVIELKPEPDAQPQQAPRLGPFSPKEVSSAG
RFGGEPHHSPGPMPAGAALLALGPRNPWTLWTPLTPNYPDRQPWTDKHPDLLTCGRCLQT
FPLEAITAFMDHKKLGCQLFRGPSRGQGSEREELKALSCLRCGKQFTVAWKLLRHAQWDH
GLSIYQTESEAPEAPLLGLAEVAAAVSAVVGPAAEAKSPRASGSGLTRRSPTCPVCKKTL
SSFSNLKVHMRSHTGERPYACDQCPYACAQSSKLNRHKKTHRQVPPQSPLMADTSQEQAS
AAPPEPAVHAAAPTSTLPCSGGEGAGAAATAGVQEPGAPGSGAQAGPGGDTWGAITTEQR
TDPANSQKASPKKMPKSGGKSRGPGGSCEFCGKHFTNSSNLTVHRRSHTGERPYTCEFCN
YACAQSSKLNRHRRMHGMTPGSTRFECPHCHVPFGLRATLDKHLRQKHPEAAGEA*
>ENSG00000172687|ENST00000311015
MDDLRYGVYPVKGASGYPGAERNLLEYSYFEKGPLTFRDVVIEFSQEEWQCLDTAQQDLY
RKVMLENFRNLVFLGIDVSKPDLITCLEQGKDPWNMKRHSMVATPPESGVLKFPTIIILL
SRCFFQSVNICFIYLEP*
>ENSG00000215421|ENST00000299687
MMDSENKPENDEDEKINKEAQDLTKLSSHNEDGGPVSDVIASFPENSMGKRGFSESSNSD
SVVIGEDRNKHASKRRKLDEAEPLKSGKQGICRLETSESSVTEGGIALDETGKETFLSDC
TVGGTCLPNALSPSCNFSTIDVVSLKTDTEKTSAQEMVSLDLERESPFPPKEISVSCTIG
NVDTVLKCSICGHLFSSCSDLEKHAESHMQQPKEHTCCHCSHKAESSSALHMHIKQAHGP
QKVFSCDLCGFQCSEENLLNAHYLGKTHLRRQNLAARGGFVQILTKQPFPKKSRTMATKN
VHSKPRTSKSIAKNSDSKGLRNVGSTFKDFRGSISKQSGSSSELLVEMMPSRNTLSQEVE
IVEEHVTSLGLAQNPENQSRKLDTLVTSEGLLEKLESTKNTLQAAHGNSVTSRPRPERNI
LVLGNSFRRRSSTFTLKGQAKKRFNLLGIKRGTSETQRMYMKHLRTQMKTHDAESVLKHL
EACSSVQRVCVTTSETQEAEQGQGSARPPDSGLHSLTVKPASGSQTLCACTDCGQVATNR
TDLEIHVKRCHAREMKFYCRTCDFSSMSRRDLDEHLHSNQHQQTASVLSCQCCSFISLDE
INLRDHMKEKHNMHFLCTPCNLFFLSEKDVEEHKATEKHINSLVQPKTLQSSNSDLVLQT
LPLSTLESENAKESMDDSGKASQEEPLKSRVSHGNEVRHSSKPQFQCKKCFYKTRSSTVL
TRHIKLRHGQDYHFLCKACNLYSLSKEGMEKHIKRSKHLENAKKNNIGLSFEECIERVCI
GANDKKEEFDVSGNGRIEGHIGVQLQEHSYLEKGMLASEELSQSGGSTKDDELASTTTPK
RGRPKGNISRTCSHCGLLASSITNLTVHIRRKHSHQYSYLCKVCKYYTVTKGDMERHCAT
KKHKGRVEIEASGKHSSDIIVGPEGGSLEAGKKNAGSAVTMSDEHANKPAESPTSVLEKP
DRGNSIEAEVENVFHSLDGEVNSHLLDKKEQISSEPEDFAQPGDVYSQRDVTGTGENKCL
HCEFSAHSSASLELHVKRKHTKEFEFYCMACDYYAVTRREMTRHAATEKHKMKRQSYLNS
ANVEAGSADMSKNIIMPEEEHQQNSEEFQIISGQPSDTLKSRNAADCSILNENTNLDMSK
VLCAADSVEVETEEESNFNEDHSFCETFQQAPVKDKVRKPEEMMSLTMSSNYGSPSRFQN
ENSGSSALNCETAKKNHEISNDAGELRVHCEGEGGNAGDGGGVVPHRHLCPVTLDGERSA
ESPVLVVTRITREQGNLESGGQNRVARGHGLEDLKGVQEDPVLGNKEILMNSQHETEFIL
EEDGPASDSTVESSDVYETIISIDDKGGQAMYSFGRFDSSIIRIKNPEDGELIDQSEEGLI
ATGVRISELPLKDCAQGVKKKKSEGSSIGESTRIRCDDCGFLADGLSGLNVHIAMKHPTK
EKHFHCLLCGKSFYTESNLHQHLASAGHMRNEQASVEELPEGGATFKCVKCTEPFDSEQN
LFLHIKGQHEELLREVNKYIVEDTEQINREREENQGNVCKYCGKMCRSSNSMAFLAHIRT
HTGSKPFKCKICHFATAQLGDARNHVKRHLGMREYKCHVCGVAFVMKKHLNTHLLGKHGV
GTPKERKFTCHLCDRSFTEKWALNNHMKLHTGEKPFKCTWPTCHYSFLTASAMKDHYRTH
TGEKSFLCDLCGFAGGTRHALTKHRRQHTGEKPFKCDECNFASTTQSHLTRHKRVHTGEK
PYRCPWCDYRSNCAENIRKHILHTGKHEGVKMYNCPKCDYGTNVPVEFRNHLKEQHPDIE
NPDLAYLHAGIVSKSYECRLKGQGATFVETDSPFTAAALAEEPLVKEKPLRSSRRPAPP

EQVQQVIIFQGYDGEFALDPSVEETAAATLQTLAMAGQVARVVHITEDGQVIATSQSGAH
VGSVVPGPILPEQLADGATQVVVVGGSMEGHGMDESLSPGGAVIQQVTKQEILNLSEAGV
APPEASSALDALLCAVTELGEVEGRAGLEEQGRPGAKDVLIQLPGQEVSHVAADPEAPEI
QMFPQAQESPAAVEVLTQVVHPSAAMASQERAQVAFKKMVQGVLQFAVCDTAAAGQLVKD
GVTQVVVSEEGAVHMVAGEGAQIIMQEAQGEHMDLVESDGEISQIIVTEELVQAMVQESS
GGFSEGTTHYILTELPPGVQDEPGLYSHTVLETADSQELLQAGATLGTEAGAPSRAEQLA
SVVIYTQEGSSAAAAIQSQRESSELQEA*
>ENSG00000162367|ENST00000294339
MTERPPSEAARSDPQLEGRDAAEASMAPPHLVLLNGVAKETSRAAAAEPPVIELGARGGP
GGGPAGGGGAARDLKGRDAATAEARHRVPTTELCRPPGPAPAPAPASVTAELPGDGRMVQ
LSPPALAAPAAPGRALLYSLSQPLASLGSGFFGEPDAFPMFTTNNRVKRRPSPYEMEITD
GPHTKVVRRIFTNSRERWRQQNVNGAFAELRKLIPTHPPDKKLSKNEILRLAMKYINFLA
KLLNDQEEEGTQRAKTGKDPVVGAGGGGGGGGGGAPPDDLLQDVLSPNSSCGSSLDGAAS
PDSYTEEPAPKHTARSLHPAMLPAADGAGPR*
>ENSG00000163631|ENST00000295897
MKWVTFISLLFLFSSAYSRGVFRRDAHKSEVAHRFKDLGEENFKALVLIAFAQYLQQCPF
EDHVKLVNEVTEFAKTCVADESAENCDKSLHTLFGDKLCTVATLRETYGEMADCCAKQEP
ERNECFLQHKDDNPNLPRLVRPEVDVMCTAFHDNEETFLKKYLYEIARRHPYFYAPELLF
FAKRYKAAFTECCQAADKAACLLPKLDELRDEGKASSAKQRLKCASLQKFGERAFKAWAV
ARLSQRFPKAEFAEVSKLVTDLTKVHTECCHGDLLECADDRADLAKYICENQDSISSKLK
ECCEKPLLEKSHCIAEVENDEMPADLPSLAADFVESKDVCKNYAEAKDVFLGMFLYEYAR
RHPDYSVVLLLRLAKTYETTLEKCCAAADPHECYAKVFDEFKPLVEEPQNLIKQNCELFE
QLGEYKFQNALLVRYTKKVPQVSTPTLVEVSRNLGKVGSKCCKHPEAKRMPCAEDYLSVV
LNQLCVLHEKTPVSDRVTKCCTESLVNRRPCFSALEVDETYVPKEFNAETFTFHADICTL
SEKERQIKKQTALVELVKHKPKATKEQLKAVMDDFAAFVEKCCKADDKETCFAEEGKKLV
AASQAALGL*
>ENSG00000168517|ENST00000307275
MMATPNQTACNAESPVALEEAKTSGAPGSPQTPPERHDSGGSLPLTPRMESHSEDEDLAG
AVGGLGWNSRSPRTQSPGGCSAEAVLARKKHRRRPSKRKRHWRPYLELSWAEKQQRDERQ
SQRASRVREEMFAKGQPVAPYNTTQFLMNDRDPEEPNLDVPHGISHPGSSGESEAGDSDG
RGRAHGEFQRKDFSETYERFHTESLQGRSKQELVRDYLELEKRLSQAEEETRRLQQLQAC
TGQQSCRQVEELAAEVQRLRTENQRLRQENQMWNREGCRCDEEPGT*
>ENSG00000144231|ENST00000272645
MAAGGSDPRAGDVEEDASQLIFPKEFETAETLLNSEVHMLLEHRKQQNESAEDEQELSEV
FMKTLNYTARFSRFKNRETIASVRSLLLQKKLHKFELACLANLCPETAEESKALIPSLEG
RFEDEELQQILDDIQTKRSFQY*
>ENSG00000069011|ENST00000265340
MDAFKGGMSLERLPEGLRPPPPPPHDMGPAFHLARPADPREPLENSASESSDTELPEKER
GGEPKGPEDSGAGGTGCGGADDPAKKKKQRRQRTHFTSQQLQELEATFQRNRYPDMSMRE
EIAVWTNLTEPRVRVWFKNRRAKWRKRERNQQLDLCKGGYVPQFSGLVQPYEDVYAAGYS
YNNWAAKSLAPAPLSTKSFTFFNSMSPLSSQSMFSAPSSISSMTMPSSMGPGAVPGMPNS
GLNNINNLTGSSLNSAMSPGACPYGTPASPYSVYRDTCNSSLASLRLKSKQHSSFGYGGL
QGPASGLNACQYNS*
>ENSG00000163728|ENST00000296015

MDRDLLRQSLNCHGSSLLSLLRSEQQDNPHFRSLLGSAAEPARGPPPQHPLQGRKEKRVD
NIEIQKFISKKADLLFALSWKSDAPATSEINEDSEDHYAIMPPLEQFMEIPSMDRRELFF
RDIERGDIVIGRISSIREFGFFMVLICLGSGIMRDIAHLEITALCPLRDVPSHSNHGDPL
SYYQTGDIIRAGIKDIDRYHEKLAVSLYSSSLPPHLSGIKLGVISSEELPLYYRRSVELN
SNSLESYENVMQSSLGFVNPGVVEFLLEKLGIDESNPPSLMRGLQSKNFSEDDFASALRK
KQSASWALKCVKIGVDYFKVGRHVDAMNEYNKALEIDKQNVEALVARGALYATKGSLNKA
IEDFELALENCPTHRNARKYLCQTLVERGGQLEEEEKFLNAESYYKKALALDETFKDAED
ALQKLHKYMQKSLELREKQAEKEEKQKTKKIETSAEKLRKLLKEEKRLKKKRRKSTSSSS
VSSADESVSSSSSSSSSGHKRHKKHKRNRSESSRSSRRHSSRASSNQIDQNRKDECYPVP
ANTSASFLNHKQEVEKLLGKQDRLQYEKTQIKEKDRCPLSSSSLEIPDDFGGRSEDPRDF
YNSYKTQAGSSKTEKPYKSERHFSSRRNSSDSFCRNSEDKIYGYRRFEKDIEGRKEHYRR
WEPGSVRHSTSPASSEYSWKSVEKYKKYAHSGSRDFSRHEQRYRLNTNQGEYEREDNYGE
DIKTEVPEEDALSSKEHSESSVKKNLPQNLLNIFNQIAEFEKEKGNKSKN*
>ENSG00000006704|ENST00000265755
MALLGKRCDVPTNGCGPDRWNSAFTRKDEIITSLVSALDSMCSALSKLNAEVACVAVHDE
SAFVVGTEKGRMFLNARKELQSDFLRFCRGPPWKDPEAEHPKKVQRGEGGGRSLPRSSLE
HGSDVYLLRKMVEEVFDVLYSEALGRASVVPLPYERLLREPGLLAVQGLPEGLAFRRPAE
YDPKALMAILEHSHRIRFKLKRPLEDGGRDSKALVELNGVSLIPKGSRDCGLHGQAPKVP
PQDLPPTATSSSMASFLYSTALPNHAIRELKQEAPSCPLAPSDLGLSRPMPEPKATGAQD
FSDCCGQKPTGPGGPLIQNVHASKRILFSIVHDKSEKWDAFIKETEDINTLRECVQILFN
SRYAEALGLDHMVPVPYRKIACDPEAVEIVGIPDKIPFKRPCTYGVPKLKRILEERHSIH
FIIKRMFDERIFTGNKFTKDTTKLEPASPPEDTSAEVSRATVLDLAGNARSDKGSMSEDC
GPGTSGELGGLRPIKIEPEDLDIIQVTVPDPSPTSEEMTDSMPGHLPSEDSGYGMEMLTD
KGLSEDARPEERPVEDSHGDVIRPLRKQVELLFNTRYAKAIGISEPVKVPYSKFLMHPEE
LFVVGLPEGISLRRPNCFGIAKLRKILEASNSIQFVIKRPELLTEGVKEPIMDSQGTASS
LGFSPPALPPERDSGDPLVDESLKRQGFQENYDARLSRIDIANTLREQVQDLFNKKYGEA
LGIKYPVQVPYKRIKSNPGSVIIEGLPPGIPFRKPCTFGSQNLERILAVADKIKFTVTRP
FQGLIPKPDEDDANRLGEKVILREQVKELFNEKYGEALGLNRPVLVPYKLIRDSPDAVEV
TGLPDDIPFRNPNTYDIHRLEKILKAREHVRMVIINQLQPFAEICNDAKVPAKDSSIPKR
KRKRVSEGNSVSSSSSSSSSSSSNPDSVASANQISLVQWPMYMVDYAGLNVQLPGPLNY*
>ENSG00000174177|ENST00000312060
MCQVGEDYGEPAPEEPPPAPRPSREQKCVKCKEAQPVVVIRAGDAFCRDCFKAFYVHKFR
AMLGKNRLIFPGEKVLLAWSGGPSSSSMVWQVLEGLSQDSAKRLRFVAGVIFVDEGAACG
QSLEERSKTLAEVKPILQATGFPWHVVALEEVFSLPPSVLWCSAQELVGSEGAYKAAVDS
FLQQQHVLGAGGGPGPTQGEEQPPQPPLDPQNLARPPAPAQTEALSQLFCSVRTLTAKEE
LLQTLRTHLILHMARAHGYSKVMTGDSCTRLAIKLMTNLALGRGAFLAWDTGFSDERHGD
VVVVRPMRDHTLKEVAFYNRLFSVPSVFTPAVDTKAPEKASIHRLMEAFILRLQTQFPST
VSTVYRTSEKLVKGPRDGPAAGDSGPRCLLCMCALDVDAADSATAFGAQTSSRLSQMQSP
IPLTETRTPPGPCCSPGVGWAQRCGQGACRREDPQACIEEQLCYSCRVNMKDLGLGLAGD
PGLSD*
>ENSG00000164093|ENST00000306732
MNCMKGPLHLEHRAAGTKLSAVSSSSCHHPQPLAMASVLAPGQPRSLDSSKHRLEVHTIS
DTSSPEAAEKDKSQQGKNEDVGAEDPSKKKRQRRQRTHFTSQQLQELEATFQRNRYPDMS
TREEIAVWTNLTEARVRVWFKNRRAKWRKRERNQQAELCKNGFGPQFNGLMQPYDDMYPG

YSYNNWAAKGLTSASLSTKSFPFFNSMNVNPLSSQSMFSPPNSISSMSMSSSMVPSAVTG
VPGSSLNSLNNLNNLSSPSLNSAVPTPACPYAPPTPPYVYRDTCNSSLASLRLKAKQHSS
FGYASVQNPASNLSACQYAVDRPV*
>ENSG00000140598|ENST00000268206
MVLNSLDKMIQLQKNTANIRNICVLAHVDHGKTTLADCLISSNGIISSRLAGKLRYMDSR
EDEQIRGITMKSSAISLHYATGNEEYLINLIDSPGHVDFSSEVSTAVRICDGCIIVVDAV
EGVCPQTQAVLRQAWLENIRPVLVINKIDRLIVELKFTPQEAYSHLKNILEQINALTGTL
FTSKVLEERAERETESQVNPNSEQGEQVYDWSTGLEDTDDSHLYFSPEQGNVVFTSAIDG
WGFGIEHFARIYSQKIGIKKEVLMKTLWGDYYINMKAKKIMKGDQAKGKKPLFVQLILEN
IWSLYDAVLKKDKDKIDKIVTSLGLKIGAREARHSDPKVQINAICSQWLPISHAVLAMVC
QKLPSPLDITAERVERLMCTGSQTFDSFPPETQALKAAFMKCGSEDTAPVIIFVSKMFAV
DAKALPQNKPRPLTQEEIAQRRERARQRHAEKLAAAQGQAPLEPTQDGSAIETCPKGEEP
RGDEQQVESMTPKPVLQEENNQESFIAFARVFSGVARRGKKIFVLGPKYSPLEFLRRVPL
GFSAPPDGLPQVPHMAYCALENLYLLMGRELEYLEEVPPGNVLGIGGLQDFVLKSATLCS
LPSCPPFIPLNFEATPIVRVAVEPKHPSEMPQLVKGMKLLNQADPCVQILIQETGEHVLV
TAGEVHLQRCLDDLKERFAKIHISVSEPIIPFRETITKPPKVDMVNEEIGKQQKVAVIHQ
MKEDQSKIPEGIQVDSDGLITITTPNKLATLSVRAMPLPEEVTQILEENSDLIRSMEQLT
SSLNEGENTHMIHQKTQEKIWEFKGKLEQHLTGRRWRNIVDQIWSFGPRKCGPNILVNKS
EDFQNSVWTGPADKASKEASRYRDLGNSIVSGFQLATLSGPMCEEPLMGVCFVLEKWDLS
KFEEQGASDLAKEGQEENETCSGGNENQELQDGCSEAFEKRTSQKGESPLTDCYGPFSGQ
LIATMKEACRYALQVKPQRLMAAMYTCDIMATGDVLGRVYAVLSKREGRVLQEEMKEGTD
MFIIKAVLPVAESFGFADEIRKRTSGLASPQLVFSHWEIIPSDPFWVPTTEEEYLHFGEK
ADSENQARKYMNAVRKRKGLYVEEKIVEHAEKQRTLSKNK*
>ENSG00000148337|ENST00000277465
MFSQQQQQQLQQQQQQLQQLQQQQLQQQQLQQQQLLQLQQLLQQSPPQAPLPMAVSRGLP
PQQPQQPLLNLQGTNSASLLNGSMLQRALLLQQLQGLDQFAMPPATYDTAGLTMPTATLG
NLRGYGMASPGLAAPSLTPPQLATPNLQQFFPQATRQSLLGPPPVGVPMNPSQFNLSGRN
PQKQARTSSSTTPNRKDSSSQTMPVEDKSDPPEGSEEAAEPRMDTPEDQDLPPCPEDIAK
EKRTPAPEPEPCEASELPAKRLRSSEEPTEKEPPGQLQVKAQPQARMTVPKQTQTPDLLP
EALEAQVLPRFQPRVLQVQAQVQSQTQPRIPSTDTQVQPKLQKQAQTQTSPEHLVLQQKQ
VQPQLQQEAEPQKQVQPQVQPQAHSQGPRQVQLQQEAEPLKQVQPQVHTQAQPSVQPQEH
PPAQVSVQPPEQTHEQPHTQPQVSLLAPEQTPVVVHVCGLEMPPDAVEAGGGMEKTLPEP
VGTQVSMEEIQNESACGLDVGECENRAREMPGVWGAGGSLKVTILQSSDSRAFSTVPLTP
VPRPSDSVSSTPAATSTPSKQALQFFCYICKASCSSQQEFQDHMSEPQHQQRLGEIQHMS
QACLLSLLPVPRDVLETEDEEPPPRRWCNTCQLYYMGDLIQHRRTQDHKIAKQSLRPFCT
VCNRYFKTPRKFVEHVKSQGHKDKAKELKSLEKEIAGQDEDHFITVDAVGCFEGDEEEEE
DDEDEEEIEVEEELCKQVRSRDISREEWKGSETYSPNTAYGVDFLVPVMGYICRICHKFY
HSNSGAQLSHCKSLGHFENLQKYKAAKNPSPTTRPVSRRCAINARNALTALFTSSGRPPS
QPNTQDKTPSKVTARPSQPPLPRRSTRLKT*
>ENSG00000128394|ENST00000308521
MKPHFRNTVERMYRDTFSYNFYNRPILSRRNTVWLCYEVKTKGPSRPRLDAKIFRGQVYS
QPEHHAEMCFLSWFCGNQLPAYKCFQITWFVSWTPCPDCVAKLAEFLAEHPNVTLTISAA
RLYYYWERDYRRALCRLSQAGARVKIMDDEEFAYCWENFVYSEGQPFMPWYKFDDNYAFL
HRTLKEILRNPMEAMYPHIFYFHFKNLRKAYGRNESWLCFTMEVVKHHSPVSWKRGVFRN

QVDPETHCHAERCFLSWFCDDILSPNTNYEVTWYTSWSPCPECAGEVAEFLARHSNVNLT
IFTARLYYFWDTDYQEGLRSLSQEGASVEIMGYKDFKYCWENFVYNDDEPFKPWKGLKYN
FLFLDSKLQEILE*
>ENSG00000163694|ENST00000295971
MTAEDSTAAMSSDSAAGSSAKVPEGVAGAPNEAALLALMERTGYSMVQENGQRKYGGPPP
GWEGPHPQRGCEVFVGKIPRDVYEDELVPVFEAVGRIYELRLMMDFDGKNRGYAFVMYCH
KHEAKRAVRELNNYEIRPGRLLGVCCSVDNCRLFIGGIPKMKKREEILEEIAKVTEGVLD
VIVYASAADKMKNRGFAFVEYESHRAAAMARRKLMPGRIQLWGHQIAVDWAEPEIDVDED
VMETVKILYVRNLMIETTEDTIKKSFGQFNPGCVERVKKIRDYAFVHFTSREDAVHAMNN
LNGTELEGSCLEVTLAKPVDKEQYSRYQKAARGGGAAEAAQQPSYVYSCDPYTLAYYGYP
YNALIGPNRDYFVKAGSIRGRGRGAAGNRAPGPRGSYLGGYSAGRGIYSRYHEGKGKQQE
KGYELVPNLEIPTVNPVAIKPGTVAIPAIGAQYSMFPAAPAPKMIEDGKIHTVEHMISPI
AVQPDPASAAAAAAAAAAAAAAVIPTVSTPPPFQGRPITPVYTVAPNVQRIPTAGIYGAS
YVPFAAPATATIATLQKNAAAAAAMYGGYAGYIPQAFPAAAIQVPIPDVYQTY*
>ENSG00000125084|ENST00000293549
MGLWALLPGWVSATLLLALAALPAALAANSSGRWWGIVNVASSTNLLTDSKSLQLVLEPS
LQLLSRKQRRLIRQNPGILHSVSGGLQSAVRECKWQFRNRRWNCPTAPGPHLFGKIVNRG
CRETAFIFAITSAGVTHSVARSCSEGSIESCTCDYRRRGPGGPDWHWGGCSDNIDFGRLF
GREFVDSGEKGRDLRFLMNLHNNEAGRTTVFSEMRQECKCHGMSGSCTVRTCWMRLPTLR
AVGDVLRDRFDGASRVLYGNRGSNRASRAELLRLEPEDPAHKPPSPHDLVYFEKSPNFCT
YSGRLGTAGTAGRACNSSSPALDGCELLCCGRGHRTRTQRVTERCNCTFHWCCHVSCRNC
THTRVLHECL*
>ENSG00000087269|ENST00000314262
MAKAKKVGARRKASGAPAGARGGPAKANSNPFEVKVNRQKFQILGRKTRHDVGLPGVSRA
RALRKRTQTLLKEYKERDKSNVFRDKRFGEYNSNMSPEEKMMKRFALEQQRHHEKKSIYN
LNEDEELTHYGQSLADIEKHNDIVDSDSDAEDRGTLSAELTAAHFGGGGGLLHKKTQQEG
EEREKPKSRKELIEELIAKSKQEKRERQAQREDALELTEKLDQDWKEIQTLLSHKTPKSE
NRDKKEKPKPDAYDMMVRELGFEMKAQPSNRMKTEAELAKEEQEHLRKLEAERLRRMLGK
DEDENVKKPKHMSADDLNDGFVLDKDDRRLLSYKDGKMNVEEDVQEEQSKEASDPESNEE
EGDSSGGEDTEESDSPDSHLDLESNVESEEENEKPAKEQRQTPGKGLISGKERAGKATRD
ELPYTFAAPESYEELRSLLLGRSMEEQLLVVERIQKCNHPSLAEGNKAKLEKLFGFLLEY
VGDLATDDPPDLTVIDKLVVHLYHLCQMFPESASDAIKFVLRDAMHEMEEMIETKGRAAL
PGLDVLIYLKITGLLFPTSDFWHPVVTPALVCLSQLLTKCPILSLQDVVKGLFVCCLFLE
YVALSQRFIPELINFLLGILYIATPNKASQGSTLVHPFRALGKNSELLVVSAREDVATWQ
QSSLSLRWASRLRAPTSTEANHIRLSCLAVGLALLKRCVLMYGSLPSFHAIMGPLQALLT
DHLADCSHPQELQELCQSTLTEMESQKQLCRPLTCEKSKPVPLKLFTPRLVKVLEFGRKQ
GSSKEEQERKRLIHKHKREFKGAVREIRKDNQFLARMQLSEIMERDAERKRKVKQLFNSL
ATQEGEWKALKRKKFKK*
>ENSG00000141510|ENST00000269305
MEEPQSDPSVEPPLSQETFSDLWKLLPENNVLSPLPSQAMDDLMLSPDDIEQWFTEDPGP
DEAPRMPEAAPPVAPAPAAPTPAAPAPAPSWPLSSSVPSQKTYQGSYGFRLGFLHSGTAK
SVTCTYSPALNKMFCQLAKTCPVQLWVDSTPPPGTRVRAMAIYKQSQHMTEVVRRCPHHE
RCSDSDGLAPPQHLIRVEGNLRVEYLDDRNTFRHSVVVPYEPPEVGSDCTTIHYNYMCNS
SCMGGMNRRPILTIITLEDSSGNLLGRNSFEVRVCACPGRDRRTEEENLRKKGEPHHELP

PGSTKRALPNNTSSSPQPKKKPLDGEYFTLQIRGRERFEMFRELNEALELKDAQAGKEPG
GSRAHSSHLKSKKGQSTSRHKKLMFKTEGPDSD*
>ENSG00000152454|ENST00000282308
MAAAELTAPAQGIVTFEDVAVYFSWKEWGLLDEAQKCLYHDVMLENLTLTTSLGGSGAGD
EEAPYQQSTSPQRVSQVRIPKALPSPQKTNPCEICGPVLRQILHLVEHQGTHHGQKLYTD
GACRKQLQFTAYLHQHQKQHVGQKHFRSNGGRDMFLSSCTFEVSGKPFTCKEVGKDFLVR
SRFLQQQAAHTRKKSNRTKSAVAFHSVKNHYNWGECVKAFSYKHVRVQHQGDLIRERSYM
CSECGKSFSTSCSLSDHLRVHTSEKPYTCGECGKSYRQSSSLITHRRIHTGVRPHQCDEC
GKLFNRKYDLLIHQRVHTGERPYKCSECGKSFSHSSSLITHQRIHTGMRPYECSECGKSF
IHSSSLITHQRVHTGTRPYMCSECGKSFSQSCHLIKHRRLHIGEGPYECSECGKLFTYRS
RFFQHQRVHTGVRSHECHECGKLFSRKFDLIVHERVHTGERPYECSECGKSFTCKSYLIS
HWKVHTGARPYECGECGKSFTHSSTLLQHQRVHTGERPYECNECGKFFSQSSSLIRHRRS
HTGERPYECSECWKSFSNHSSLVKHRRVHTGERPYECSECGKSFSQSSNLTNHQRIHSGE
RPYECSDCGKFFTFNSNLLKHQNVHKG*
>ENSG00000104679|ENST00000265806
LALLCLDGVFLSSAENDFVHRIQEELDRFLLQKQLSKVLLFPPLSSRLRYLIHRTAENFD
LLSSFSVGEGWKRRTVICHQDIRVPSSDGLSGPCRAPASCPSRYHGPRPISNQGAAAVPR
GARAGRWYRGRKPDQPLYVPRVLRRQEEWGLTSTSVLKREAPAGRDPEEPGDVGAGDPNS
DQGLPVLMTQGTEDLKGPGQRCENEPLLDPVGPEPLGPESQSGKGDMVEMATRFGSTLQL
DLEKGKESLLEKRLVAEEEEDEEEVEEDGPSSCSEDDYSELLQEITDNLTKKEIQIEKIH
LDTSSFVEELPGEKDLAHVVEIYDFEPALKTEDLLATFSEFQEKGFRIQWVDDTHALGIF
PCLASAAEALTREFSVLKIRPLTQGTKQSKLKALQRPKLLRLVKERPQTNATVARRLVAR
ALGLQHKKKERPAVRGPLPP*
>ENSG00000156304|ENST00000286835
MDAVNAFNQELFSLMDMKPPISRAKMILITKAAIKAIKLYKHVVQIVEKFIKKCKPEYKV
PGLYVIDSIVRQSRHQFGTDKDVFGPRFSKNITATFQYLYLCPSEDKSKIVRVLNLWQKN
GVFKIEIIQPLLDMAAGTSNAAPVAENVTNNEGSPPPPVKVSSEPPTQATPNSVPAVPQL
PSSDAFAAVAQLFQTTQGQQLQQILQTFQQPPKPQSPALDNAVMAQVQAITAQLKTTPTQ
PSEQKAAFPPPEQKTAFDKKLLDRFDYDDEPEAVEESKKEDTTAVTTTAPAAAVPPAPTA
TVPAAAAPAAASPPPPQAPFGFPGDGMQQPAYTQHQNMDQFQPRMMGIQQDPMHHQVPLP
PNGQMPGFGLLPTPPFPPMAQPVIPPTPPVQQPFQASFQAQNEPLTQKPHQQEMEVEQPC
IQEVKRHMSDNRKSRSRSASRSPKRRRSRSGSRSRRSRHRRSRSRSRDRRRHSPRSRSQE
RRDREKERERRQKGLPQVKPETASVCSTTLWVGQLDKRTTQQDVASLLEEFGPIESINMI
PPRGCAYIVMVHRQDAYRALQKLSRGNYKVNQKSIKIAWALNKGIKADYKQYWDVELGVT
YIPWDKVKPEELESFCEGGMLDSDTLNPDWKGIPKKPENEVAQNGGAETSHTEPVSPIPK
PLPVPVPPIPVPAPITVPPPQVPPHQPGPPVVGALQPPAFTPPLGIPPPGFGPGVPPPPP
PPPFLRPGFNPMHLPPGFLPPGPPPPITPPVSIPPPHTPPISIPNSTIAGINEDTTKDLS
IGNPIPTVVSGARGNAESGDSVKMYGSAVPPAAPTNLPTPPVTQPVSLLGTQGVAPGPVI
GLQAPSTGLLGARPGLIPLQRPPGMPPPHLQRFPLMPPRPMPPHMMHRGPPPGPGGFAMP
PPHGMKGPFPPHGPFVRPGGMPGLGGPGPGPGGPEDRDGRQQPPQQPQQQPQPQAPQQPQ
QQQQQQPPPSQQPPPTQQQPQQFRNDNRQQFNSGRDQERFGRRSFGNRVENDRERYGNRN
DDRDNSNRDRREWGRRSPDRDRHRDLEERNRRSSGHRDRERDSRDRESRREKEEARGKEK
PEVTDRAGGNKTVEPPISQVGNVDTASELEKGVSEAAVLKPSEELPAEATSSVEPEKDSG
SAAEAPR*

>ENSG00000170577|ENST00000303077
MSMLPTFGFTQEQVACVCEVLQQGGNIERLGRFLWSLPACEHLHKNESVLKAKAVVAFHR
GNFRELYKILESHQFSPHNHAKLQQLWLKAHYIEAEKLRGRPLGAVGKYRVRRKFPLPRS
IWDGEETSYCFKEKSRSVLREWYAHNPYPSPREKRELAEATGLTTTQVSNWFKNRRQRDR
AAEAKERENNENSNSNSHNPLNGSGKSVLGSSEDEKTPSGTPDHSSSSPALLLSPPPPGL
PSLHSLGHPPGPSAVPVPVPGGGGADPLQHHHGLQDSILNPMSANLVDLGS*
>ENSG00000197020|ENST00000305570
MLENYRNLVFLGIALTKPDLITCLEQGKEPWNIKRHEMVAKPPVICSHFPQDLWAEQDIK
DSFQEAILKKYGKYGHDNLQLQKGCKSVDECKVHKEHDNKLNQCLITTQSNIFQCDPSAK
VFHTFSNSNRHKIRHTRKKPFKCKKCEKSFCMLLHLTQHKRFHITENSYQCKDCGKAFNW
FSTLTTHRRIHTGEKPYKCEECGKAFNRSSHLTTHKIIHTGEKPYRCEECGKAFNRSSHL
TTHKRIHTGVKPYKCTECGKAFNRSSHLTTHRIIHTGEKPYKCEECGKAFNQSSTLTTHK
ITHAGEKPYKCEECGKAFYRFSYLTKHKTSHTGEKFYKCEECGKGFNWSSALTKHKRIHT
GEKPYKCEECGKAFNESSNLTTHKMIHTGEKPYKCDECGKAFNRSSQLTAHKMIHTGEKP
YKCEECGKAFNRSSTLTKHKITHTGEKSYKWEECGKDFNQSLSLIKQNNSYWRETLQM*
>ENSG00000140259|ENST00000267812
MSVPSALMKQPPIQSTAGAVPVRNEKGEISMEKVKVKRYVSGKRPDYAPMESSDEEDEEF
QFIKKAKEQEAPEEQEEDSSSDPRLRRLQNRISEDVEERLARHRKIVEPEVVGESDSEV
EGDAWRMEREDSSEEEEEEIDDEEIERRRGMMRQRAQERKNEEMEVMEVEDEGRSGEESE
SESEYEEYTDSEDEMEPRLKPVFIRKKDRVTVQEREAEALKQKELEQEAKRMAEERRKYT
LKIVEEETKKELEENKRSLAALDALNTDDENDEEEYEAWKVRELKRIKRDREDREALEKE
KAEIERMRNLTEEERRAELRANGKVITNKAVKGKYKFLQKYYHRGAFFMDEDEEVYKRDF
SAPTLEDHFNKTILPKVMQVKNFGRSGRTKYTHLVDQDTTSFDSAWGQESAQNTKFFKQK
AAGVRDVFERPSAKKRKTT*
>ENSG00000181163|ENST00000296930
MEDSMDMDMSPLRPQNYLFGCELKADKDYHFKVDNDENEHQLSLRTVSLGAGAKDELHIV
EAEAMNYEGSPIKVTLATLKMSVQPTVSLGGFEITPPVVLRLKCGSGPVHISGQHLVAVE
EDAESEDEEEEDVKLLSISGKRSAPGGGSKVPQKKVKLAADEDDDDDDEEDDDEDDDDDD
FDDEEAEEKAPVKKSIRDTPAKNAQKSNQNGKDSKPSSTPRSKGQESFKKQEKTPKTPKG
PSSVEDIKAKMQASIEKGGSLPKVEAKFINYVKNCFRMTDQEAIQDLWQWRKSL*
>ENSG00000175592|ENST00000312562
MFRDFGEPGPSSGNGGGYGGPAQPPAAAQAAQQKFHLVPSINTMSGSQELQWMVQPHFLG
PSSYPRPLTYPQYSPPQPRPGVIRALGPPPGVRRRPCEQISPEEEERRRVRRERNKLAAA
KCRNRRKELTDFLQAETDKLEDEKSGLQREIEELQKQKERLELVLEAHRPICKIPEGAKE
GDTGSTSGTSSPPAPCRPVPCISLSPGPVLEPEALHTPTLMTTPSLTPFTPSLVFTYPST
PEPCASAHRKSSSSSGDPSSDPLGSPTLLAL*
>ENSG00000139445|ENST00000299162
MIESDTSSIMSGIIRNSGQNHHPSPQEYRLLATTSDDDLPGDLQSLSWLTAVDVPRLQQM
ASGRVDLGGPCVPHPHPGALAGVADLHVGATPSPLLHGPAGMAPRGMPGLGPITGHRDSM
SQFPVGGQPSSGLQDPPHLYSPATQPQFPLPPGAQQCPPVGLYGPPFGVRPPYPQPHVAV
HSSQELHPKHYPKPIYSYSCLIAMALKNSKTGSLPVSEIYSFMKEHFPYFKTAPDGWKNS
VRHNLSLNKCFEKVENKMSGSSRKGCLWALNLARIDKMEEEMHKWKRKDLAAIHRSMANP
EELDKLISDRPESCRRPGKPGEPEAPVLTHATTVAVAHGCLAVSQLPPQPLMTLSLQSVP
LHHQVQPQAHLAPDSPAPAQTPPLHALPDLSPSPLPHPAMGRAPVDFINISTDMNTEVDA

LDPSIMDFALQGNLWEEMKDEGFSLDTLGAFADSPLGCDLGASGLTPASGGSDQSFPDLQ
VTGLYTAYSTPDSVAASGTSSSSQYLGAQGNKPIALL*
>ENSG00000105127|ENST00000269701
MDQGYGGYGAWSAGPANTQGAYGTGVASWQGYENYNYYGAQNTSVTTGATYSYGPASWEA
AKANDGGLAAGAPAMHMASYGPEPCTDNSDSLIAKINQRLDMMSKEGGRGGSGGGGEGIQ
DRESSFRFQPFESYDSRPCLPEHNPYRPSYSYDYEFDLGSDRNGSFGGQYSECRDPARER
GSLDGFMRGRGQGRFQDRSNPGTFMRSDPFVPPAASSEPLSTPWNELNYVGGRGLGGPSP
SRPPPSLFSQSMAPDYGVMGMQGAGGYDSTMPYGCGRSQPRMRDRDRPKRRGFDRFGPDG
TGRKRKQFQLYEEPDTKLARVDSEGDFSENDDAAGDFRSGDEEFKGEDELCDSGRQRGEK
EDEDEDVKKRREKQRRRDRTRDRAADRIQFACSVCKFRSFDDEEIQKHLQSKFHKETLRF
ISTKLPDKTVEFLQEYIVNRNKKIEKRRQELMEKETAKPKPDPFKGIGQEHFFKKIEAAH
CLACDMLIPAQPQLLQRHLHSVDHNHNRRLAAEQFKKTSLHVAKSVLNNRHIVKMLEKYL
KGEDPFTSETVDPEMEGDDNLGGEDKKETPEEVAADVLAEVITAAVRAVDGEGAPAPESS
GEPAEDEGPTDTAEAGSDPQAEQLLEEQVPCGTAHEKGVPKARSEAAEAGNGAETMAAEA
ESAQTRVAPAPAAADAEVEQTDAESKDAVPTE*
>ENSG00000272886|ENST00000294241
MEALSRAGQEMSLAALKQHDPYITSIADLTGQVALYTFCPKANQWEKTDIEGTLFVYRRS
ASPYHGFTIVNRLNMHNLVEPVNKDLEFQLHEPFLLYRNASLSIYSIWFYDKNDCHRIAK
LMADVVEEETRRSQQAARDKQSPSQANGCSDHRPIDILEMLSRAKDEYERSAPSGHKHLT
VEELFGTSLPKEQPAVVGLDSEEMERLPGDASQKEPNSFLPFPFEQLGGAPQSETLGVPS
AAHHSVQPEITTPVLITPASITQSNEKHAPTYTIPLSPVLSPTLPAEAPTAQVPPSLPRN
STMMQAVKTTPRQRSPLLNQPVPELSHASLIANQSPFRAPLNVTNTAGTSLPSVDLLQKL
RLTPQHDQIQTQPLGKGAMVASFSPAAGQLATPESFIEPPSKTAAARVAASASLSNMVLA
PLQSMQQNQDPEVFVQPKVLSSAIPVAGAPLVTATTTAVSSVLLAPSVFQQTVTRSSDLE
RKASSPSPLTIGTPESQRKPSIILSKSQLQDTLIHLIKNDSSFLSTLHEVYLQVLTKNKD
NHNL*
>ENSG00000180257|ENST00000270457
MLREEATKKSKEKEPGMALPQGRLTFRDVAIEFSLEEWKCLNPAQRALYRAVMLENYRNL
EFVGEENVPPDMRNLHLSILALSGFIFSFVILPHTCF*
>ENSG00000151702|ENST00000281428
MEGGLAGERARESPVDCSVSKCSKLVGGGESNPMNYNSYMDEKNGPPPPNMTTNERRVIV
PADPTLWTQEHVRQWLEWAIKEYSLMEIDTSFFQNMDGKELCKMNKEDFLRATTLYNTEV
LLSHLSYLRESSLLAYNTTSHTDQSSRLSVKEDPSYDSVRRGAWGNNMNSGLNKSPPLGG
AQTISKNTEQRPQPDPYQILGPTSSRLANPGSGQIQLWQFLLELLSDSANASCITWEGTN
GEFKMTDPDEVARRWGERKSKPNMNYDKLSRALRYYYDKNIMTKVHGKRYAYKFDFHGIA
QALQPHPTESSMYKYPSDISYMPSYHAHQQKVNFVPPHPSSMPVTSSSFFGAASQYWTSP
TGGIYPNPNVPRHPNTHVPSHLGSYY*
>ENSG00000155636|ENST00000286070
MDEAGSSASGGGFRPGVDSLDEPPNSRIFLVISKYTPESVLRERFSPFGDIQDIWVVRDK
HTKESKGIAFVKFARSSQACRAMEEMHGQCLGPNDTKPIKVFIAQSRSSGSHRDVEDEEL
TRIFVMIPKSYTEEDLREKFKVYGDIEYCSIIKNKVTGESKGLGYVRYLKPSQAAQAIEN
CDRSFRAILAEPKNKASESSEQDYYSNMRQEALGHEPRVNMFPFEQQSEFSSFDKNDSRG
QEAISKRLSVVSRVPFTEEQLFSIFDIVPGLEYCEVQRDPYSNYGHGVVQYFNVASAIYA
KYKLHGFQYPPGNRIGVSFIDDGSNATDLLRKMATQMVAAQLASMVWNNPSQQQFMQFGG

SSGSQLPQIQTDVVLPSCKKKAPAETPVKERLFIVFNPHPLPLDVLEDIFCRFGNLIEVY
LVSGKNVGYAKYADRISANDAIATLHGKILNGVRLKVMLADSPREESNKRQRTY*
>ENSG00000148400|ENST00000277541
MPPLLAPLLCLALLPALAARGPRCSQPGETCLNGGKCEAANGTEACVCGGAFVGPRCQDP
NPCLSTPCKNAGTCHVVDRRGVADYACSCALGFSGPLCLTPLDNACLTNPCRNGGTCDLL
TLTEYKCRCPPGWSGKSCQQADPCASNPCANGGQCLPFEASYICHCPPSFHGPTCRQDVN
ECGQKPGLCRHGGTCHNEVGSYRCVCRATHTGPNCERPYVPCSPSPCQNGGTCRPTGDVT
HECACLPGFTGQNCEENIDDCPGNNCKNGGACVDGVNTYNCRCPPEWTGQYCTEDVDECQ
LMPNACQNGGTCHNTHGGYNCVCVNGWTGEDCSENIDDCASAACFHGATCHDRVASFYCE
CPHGRTGLLCHLNDACISNPCNEGSNCDTNPVNGKAICTCPSGYTGPACSQDVDECSLGA
NPCEHAGKCINTLGSFECQCLQGYTGPRCEIDVNECVSNPCQNDATCLDQIGEFQCICMP
GYEGVHCEVNTDECASSPCLHNGRCLDKINEFQCECPTGFTGHLCQYDVDECASTPCKNG
AKCLDGPNTYTCVCTEGYTGTHCEVDIDECDPDPCHYGSCKDGVATFTCLCRPGYTGHHC
ETNINECSSQPCRHGGTCQDRDNAYLCFCLKGTTGPNCEINLDDCASSPCDSGTCLDKID
GYECACEPGYTGSMCNINIDECAGNPCHNGGTCEDGINGFTCRCPEGYHDPTCLSEVNEC
NSNPCVHGACRDSLNGYKCDCDPGWSGTNCDINNNECESNPCVNGGTCKDMTSGYVCTCR
EGFSGPNCQTNINECASNPCLNQGTCIDDVAGYKCNCLLPYTGATCEVVLAPCAPSPCRN
GGECRQSEDYESFSCVCPTGWQGQTCEVDINECVLSPCRHGASCQNTHGGYRCHCQAGYS
GRNCETDIDDCRPNPCHNGGSCTDGINTAFCDCLPGFRGTFCEEDINECASDPCRNGANC
TDCVDSYTCTCPAGFSGIHCENNTPDCTESSCFNGGTCVDGINSFTCLCPPGFTGSYCQH
DVNECDSQPCLHGGTCQDGCGSYRCTCPQGYTGPNCQNLVHWCDSSPCKNGGKCWQTHTQ
YRCECPSGWTGLYCDVPSVSCEVAAQRQGVDVARLCQHGGLCVDAGNTHHCRCQAGYTGS
YCEDLVDECSPSPCQNGATCTDYLGGYSCKCVAGYHGVNCSEEIDECLSHPCQNGGTCLD
LPNTYKCSCPRGTQGVHCEINVDDCNPPVDPVSRSPKCFNNGTCVDQVGGYSCTCPPGFV
GERCEGDVNECLSNPCDARGTQNCVQRVNDFHCECRAGHTGRRCESVINGCKGKPCKNGG
TCAVASNTARGFICKCPAGFEGATCENDARTCGSLRCLNGGTCISGPRSPTCLCLGPFTG
PECQFPASSPCLGGNPCYNQGTCEPTSESPFYRCLCPAKFNGLLCHILDYSFGGGAGRDI
PPPLIEEACELPECQEDAGNKVCSLQCNNHACGWDGGDCSLNFNDPWKNCTQSLQCWKYF
SDGHCDSQCNSAGCLFDGFDCQRAEGQCNPLYDQYCKDHFSDGHCDQGCNSAECEWDGLD
CAEHVPERLAAGTLVVVVLMPPEQLRNSSFHFLRELSRVLHTNVVFKRDAHGQQMIFPYY
GREEELRKHPIKRAAEGWAAPDALLGQVKASLLPGGSEGGRRRRELDPMDVRGSIVYLEI
DNRQCVQASSQCFQSATDVAAFLGALASLGSLNIPYKIEAVQSETVEPPPPAQLHFMYVA
AAAFVLLFFVGCGVLLSRKRRRQHGQLWFPEGFKVSEASKKKRREPLGEDSVGLKPLKNA
SDGALMDDNQNEWGDEDLETKKFRFEEPVVLPDLDDQTDHRQWTQQHLDAADLRMSAMAP
TPPQGEVDADCMDVNVRGPDGFTPLMIASCSGGGLETGNSEEEEDAPAVISDFIYQGASL
HNQTDRTGETALHLAARYSRSDAAKRLLEASADANIQDNMGRTPLHAAVSADAQGVFQIL
IRNRATDLDARMHDGTTPLILAARLAVEGMLEDLINSHADVNAVDDLGKSALHWAAAVNN
VDAAVVLLKNGANKDMQNNREETPLFLAAREGSYETAKVLLDHFANRDITDHMDRLPRDI
AQERMHHDIVRLLDEYNLVRSPQLHGAPLGGTPTLSPPLCSPNGYLGSLKPGVQGKKVRK
PSSKGLACGSKEAKDLKARRKKSQDGKGCLLDSSGMLSPVDSLESPHGYLSDVASPPLLP
SPFQQSPSVPLNHLPGMPDTHLGIGHLNVAAKPEMAALGGGGRLAFETGPPRLSHLPVAS
GTSTVLGSSSGGALNFTVGGSTSLNGQCEWLSRLQSGMVPNQYNPLRGSVAPGPLSTQAP
SLQHGMVGPLHSSLAASALSQMMSYQGLPSTRLATQPHLVQTQQVQPQNLQMQQQNLQPA
NIQQQQSLQPPPPPPQPHLGVSSAASGHLGRSFLSGEPSQADVQPLGPSSLAVHTILPQE

SPALPTSLPSSLVPPVTAAQFLTPPSQHSYSSPVDNTPSHQLQVPEHPFLTPSPESPDQW
SSSSPHSNVSDWSEGVSSPPTSMQSQIARIPEAFK*
>ENSG00000168453|ENST00000312841
MESTPSFLKGTPTWEKTAPENGIVRQEPGSPPRDGLHHGPLCLGEPAPFWRGVLSTPDSW
LPPGFPQGPKDMLPLVEGEGPQNGERKVNWLGSKEGLRWKEAMLTHPLAFCGPACPPRCG
PLMPEHSGGHLKSDPVAFRPWHCPFLLETKILERAPFWVPTCLPPYLVSGLPPEHPCDWP
LTPHPWVYSGGQPKVPSAFSLGSKGFYYKDPSIPRLAKEPLAAAEPGLFGLNSGGHLQRA
GEAERPSLHQRDGEMGAGRQQNPCPLFLGQPDTVPWTSWPACPPGLVHTLGNVWAGPGDG
NLGYQLGPPATPRCPSPEPPVTQRGCCSSYPPTKGGGLGPCGKCQEGLEGGASGASEPSE
EVNKASGPRACPPSHHTKLKKTWLTRHSEQFECPRGCPEVEERPVARLRALKRAGSPEVQ
GAMGSPAPKRPPDPFPGTAEQGAGGWQEVRDTSIGNKDVDSGQHDEQKGPQDGQASLQDP
GLQDIPCLALPAKLAQCQSCAQAAGEGGGHACHSQQVRRSPLGGELQQEEDTATNSSSEE
GPGSGPDSRLSTGLAKHLLSGLGDRLCRLLRREREALAWAQREGQGPAVTEDSPGIPRCC
SRCHHGLFNTHWRCPRCSHRLCVACGRVAGTGRAREKAGFQEQSAEECTQEAGHAACSLM
LTQFVSSQALAELSTAMHQVWVKFDIRGHCPCQADARVWAPGDAGQQKESTQKTPPTPQP
SCNGDTHRTKSIKEETPDSAETPAEDRAGRGPLPCPSLCELLASTAVKLCLGHERIHMAF
APVTPALPSDDRITNILDSIIAQVVERKIQEKALGPGLRAGPGLRKGLGLPLSPVRPRLP
PPGALLWLQEPQPCPRRGFHLFQEHWRQGQPVLVSGIQRTLQGNLWGTEALGALGGQVQA
LSPLGPPQPSSLGSTTFWEGFSWPELRPKSDEGSVLLLHRALGDEDTSRVENLAASLPLP
EYCALHGKLNLASYLPPGLALRPLEPQLWAAYGVSPHRGHLGTKNLCVEVADLVSILVHA
DTPLPAWHRAQKDFLSGLDGEGLWSPGSQVSTVWHVFRAQDAQRIRRFLQMVQGLVSTVS
VTQHFLSPETSALSAQLCHQGPSLPPDCHLLYAQMDWAVFQAVKVAVGTLQEAK*
>ENSG00000113318|ENST00000265081
MSRRKPASGGLAASSSAPARQAVLSRFFQSTGSLKSTSSSTGAADQVDPGAAAAAAAAAA
AAPPAPPAPAFPPQLPPHIATEIDRRKKRPLENDGPVKKKVKKVQQKEGGSDLGMSGNSE
PKKCLRTRNVSKSLEKLKEFCCDSALPQSRVQTESLQERFAVLPKCTDFDDISLLHAKNA
VSSEDSKRQINQKDTTLFDLSQFGSSNTSHENLQKTASKSANKRSKSIYTPLELQYIEMK
QQHKDAVLCVECGYKYRFFGEDAEIAARELNIYCHLDHNFMTASIPTHRLFVHVRRLVAK
GYKVGVVKQTETAALKAIGDNRSSLFSRKLTALYTKSTLIGEDVNPLIKLDDAVNVDEIM
TDTSTSYLLCISENKENVRDKKKGNIFIGIVGVQPATGEVVFDSFQDSASRSELETRMSS
LQPVELLLPSALSEQTEALIHRATSVSVQDDRIRVERMDNIYFEYSHAFQAVTEFYAKDT
VDIKGSQIISGIVNLEKPVICSLAAIIKYLKEFNLEKMLSKPENFKQLSSKMEFMTINGT
TLRNLEILQNQTDMKTKGSLLWVLDHTKTSFGRRKLKKWVTQPLLKLREINARLDAVSEV
LHSESSVFGQIENHLRKLPDIERGLCSIYHKKCSTQEFFLIVKTLYHLKSEFQAIIPAVN
SHIQSDLLRTVILEIPELLSPVEHYLKILNEQAAKVGDKTELFKDLSDFPLIKKRKDEIQ
GVIDEIRMHLQEIRKILKNPSAQYVTVSGQEFMIEIKNSAVSCIPTDWVKVGSTKAVSRF
HSPFIVENYRHLNQLREQLVLDCSAEWLDFLEKFSEHYHSLCKAVHHLATVDCIFSLAKV
AKQGDYCRPTVQEERKIVIKNGRHPVIDVLLGEQDQYVPNNTDLSEDSERVMIITGPNMG
GKSSYIKQVALITIMAQIGSYVPAEEATIGIVDGIFTRMGAADNIYKGQSTFMEELTDTA
EIIRKATSQSLVILDELGRGTSTHDGIAIAYATLEYFIRDVKSLTLFVTHYPPVCELEKN
YSHQVGNYHMGFLVSEDESKLDPGAAEQVPDFVTFLYQITRGIAARSYGLNVAKLADVPG
EILKKAAHKSKELEGLINTKRKRLKYFAKLWTMHNAQDLQKWTEEFNMEETQTSLLH*
>ENSG00000104320|ENST00000265433
MWKLLPAAGPAGGEPYRLLTGVEYVVGRKNCAILIENDQSISRNHAVLTANFSVTNLSQT

DEIPVLTLKDNSKYGTFVNEEKMQNGFSRTLKSGDGITFGVFGSKFRIEYEPLVACSSCL
DVSGKTALNQAILQLGGFTVNNWTEECTHLVMVSVKVTIKTICALICGRPIVKPEYFTEF
LKAVESKKQPPQIESFYPPLDEPSIGSKNVDLSGRQERKQIFKGKTFIFLNAKQHKKLSS
AVVFGGGEARLITEENEEEHNFFLAPGTCVVDTGITNSQTLIPDCQKKWIQSIMDMLQRQ
GLRPIPEAEIGLAVIFMTTKNYCDPQGHPSTGLKTTTPGPSLSQGVSVDEKLMPSAPVNT
TTYVADTESEQADTWDLSERPKEIKVSKMEQKFRMLSQDAPTVKESCKTSSNNNSMVSNT
LAKMRIPNYQLSPTKLPSINKSKDRASQQQQTNSIRNYFQPSTKKRERDEENQEMSSCKS
ARIETSCSLLEQTQPATPSLWKNKEQHLSENEPVDTNSDNNLFTDTDLKSIVKNSASKSH
AAEKLRSNKKREMDDVAIEDEVLEQLFKDTKPELEIDVKVQKQEEDVNVRKRPRMDIETN
DTFSDEAVPESSKISQENEIGKKRELKEDSLWSAKEISNNDKLQDDSEMLPKKLLLTEFR
SLVIKNSTSRNPSGINDDYGQLKNFKKFKKVTYPGAGKLPHIIGGSDLIAHHARKNTELE
EWLRQEMEVQNQHAKEESLADDLFRYNPYLKRRR*
>ENSG00000239306|ENST00000310137
MKIFVGNVDGADTTPEELAALFAPYGTVMSCAVMKQFAFVHMRENAGALRAIEALHGHEL
RPGRALVVEMSRPRPLNTWKIFVGNVSAACTSQELRSLFERRGRVIECDVVKDYAFVHME
KEADAKAAIAQLNGKEVKGKRINVELSTKGQKKGPGLAVQSGDKTKKPGAGDTAFPGTGG
FSATFDYQQAFGNSTGGFDGQARQPTPPFFGRDRSPLRRSPPRASYVAPLTAQPATYRAQ
PSVSLGAAYRAQPSASLGVGYRTQPMTAQAASYRAQPSVSLGAPYRGQLASPSSQSAAAS
SLGPYGGAQPSASALSSYGGQAAAASSLNSYGAQGSSLASYGNQPSSYGAQAASSYGVRA
AASSYNTQGAASSLGSYGAQAASYGAQSAASSLAYGAQAASYNAQPSASYNAQSAPYAAQ
QAASYSSQPAAYVAQPATAAAYASQPAAYAAQATTPMAGSYGAQPVVQTQLNSYGAQASM
GLSGSYGAQSAAAATGSYGAAAAYGAQPSATLAAPYRTQSSASLAASYAAQQHPQAAASY
RGQPGNAYDGAGQPSAAYLSMSQGAVANANSTPPPYERTRLSPPRASYDDPYKKAVAMSK
RYGSDRRLAELSDYRRLSESQLSFRRSPTKSSLDYRRLPDAHSDYARYSGSYNDYLRAAQ
MHSGYQRRM*
>ENSG00000152133|ENST00000281932
MSRKSGIGHEASLKRKAEEKLESYRKKIHMKNQAEEKAAEQFRMRLKNKQDEMKLEGDLR
RSQRACQQLDVQKNIQVPREAWYWLRLEEETEEDEEEKEQDEDEYKSEDLSVLEKLQILT
SYLREEHLYCIWCGTAYEDKEDLSSNCPGPTSADHD*
>ENSG00000158417|ENST00000289371
MGKKQKNKSEDSTKDDIDLDALAAEIEGAGAAKEQEPQKSKGKKKKEKKKQDFDEDDILK
ELEELSLEAQGIKADRETVAVKPTENNEEEFTSKDKKKKGQKGKKQSFDDNDSEELEDKD
SKSKKTAKPKVEMYSGSDDDDDFNKLPKKAKGKAQKSNKKWDGSEEDEDNSKKIKERSRI
NSSGESGDESDEFLQSRKGQKKNQKNKPGPNIESGNEDDDASFKIKTVAQKKAEKKERER
KKRDEEKAKLRKLKEKEELETGKKDQSKQKESQRKFEEETVKSKVTVDTGVIPASEEKAE
TPTAAEDDNEGDKKKKDKKKKKGEKEEKEKEKKKGPSKATVKAMQEALAKLKEEEERQKR
EEEERIKRLEELEAKRKEEERLEQEKRERKKQKEKERKERLKKEGKLLTKSQREARARAE
ATLKLLQAQGVEVPSKDSLPKKRPIYEDKKRKKIPQQLESKEVSESMELCAAVEVMEQGV
PEKEETPPPVEPEEEEDTEDAGLDDWEAMASDEETEKVEGNKVHIEVKENPEEEEEEEEE
EEEDEESEEEEEEEGESEGSEGDEEDEKVSDEKDSGKTLDKKPSKEMSSDSEYDSDDDRT
KEERAYDKAKRRIEKRRLEHSKNVNTEKLRAPIICVLGHVDTGKTKILDKLRHTHVQDGE
AGGITQQIGATNVPLEAINEQTKMIKNFDRENVRIPGMLIIDTPGHESFSNLRNRGSSLC
DIAILVVDIMHGLEPQTIESINLLKSKKCPFIVALNKIDRLYDWKKSPDSDVAATLKKQK
KNTKDEFEERAKAIIVEFAQQGLNAALFYENKDPRTFVSLVPTSAHTGDGMGSLIYLLVE

LTQTMLSKRLAHCEELRAQVMEVKALPGMGTTIDVILINGRLKEGDTIIVPGVEGPIVTQ
IRGLLLPPPMKELRVKNQYEKHKEVEAAQGVKILGKDLEKTLAGLPLLVAYKEDEIPVLK
DELIHELKQTLNAIKLEEKGVYVQASTLGSLEALLEFLKTSEVPYAGINIGPVHKKDVMK
ASVMLEHDPQYAVILAFDVRIERDAQEMADSLGVRIFSAEIIYHLFDAFTKYRQDYKKQK
QEEFKHIAVFPCKIKILPQYIFNSRDPIVMGVTVEAGQVKQGTPMCVPSKNFVDIGIVTS
IEINHKQVDVAKKGQEVCVKIEPIPGESPKMFGRHFEATDILVSKISRQSIDALKDWFRD
EMQKSDWQLIVELKKVFEII*
>ENSG00000166478|ENST00000299606
MLLAQINRDSQGMTEFPGGGMEAQHVTLCLTEAVTVADGDNLENMEGVSLQAVTLADGST
AYIQHNSKDAKLIDGQVIQLEDGSAAYVQHVPIPKSNSYDQSALQAVQLEDGTTAYIHHA
VQVPQSDTILAIQADGTVAGLHTGDATIDPDTISALEQYAAKVSIDGSESVAGTGMIGEN
EQEKKMQIVLQGHATRVTAKSQQSGEKAFRCEYDGCGKLYTTAHHLKVHERSHTGDRPYQ
CEHAGCGKAFATGYGLKSHVRTHTGEKPYRCSEDNCTKSFKTSGDLQKHIRTHTGERPFK
CPFEGCGRSFTTSNIRKVHVRTHTGERPYYCTEPGCGRAFASATNYKNHVRIHTGEKPYV
CTVPGCDKRFTEYSSLYKHHVVHTHSKPYNCNHCGKTYKQISTLAMHKRTAHNDTEPIEE
EQEAFFEPPPGQGEDVLKGSQITYVTGVEGDDVVSTQVATVTQSGLSQQVTLISQDGTQH
VNISQADMQAIGNTITMVTQDGTPITVPAHDAVISSAGTHSVAMVTAEGTEGEQVAIVAQ
DLAAFHTASSEMGHQQHSHHLVTTETRPLTLVATSNGTQIAVQLGEQPSLEEAIRIASRI
QQGETPGLDD*
>ENSG00000164338|ENST00000296792
MAGYKPVAIQTYPILGEKITQDTLYWNNYKTPVQIKEFGAVSKVDFSPQPPYNYAVTASS
RIHIYGRYSQEPIKTFSRFKDTAYCATFRQDGRLLVAGSEDGGVQLFDISGRAPLRQFEG
HTKAVHTVDFTADKYHVVSGADDYTVKLWDIPNSKEILTFKEHSDYVRCGCASKLNPDLF
ITGSYDHTVKMFDARTSESVLSVEHGQPVESVLLFPSGGLLVSAGGRYVKVWDMLKGGQL
LVSLKNHHKTVTCLCLSSSGQRLLSGSLDRKVKVYSTTSYKVVHSFDYAASILSLALAHE
DETIVVGMTNGILSVKHRKSEAKKESLPRRRRPAYRTFIKGKNYMKQRDDILINRPAKKH
LELYDRDLKHFRISKALDRVLDPTCTIKTPEITVSIIKELNRRGVLANALAGRDEKEISH
VLNFLIRNLSQPRFAPVLINAAEIIIDIYLPVIGQSPVVDKKFLLLQGLVEKEIDYQREL
LETLGMMDMLFATMRRKEGTSVLEHTSDGFPENKKIES*
>ENSG00000140955|ENST00000268624
MASASQGADDDGSRRKPRLAASLQISPQPRPWRPLPAQAQSAWGPAPAPATYRAEGGWPQ
VSVLRDSGPGAGAGVGELGAARAWENLGEQMGKAPRVPVPPAGLSLPLKDPPASQAVSLL
TEYAASLGIFLLFREDQPPGKVFKYRAPGGEELKAMCLWQVEVLRAFLLRTGWRGLWRGD
LDLGPDSSWANRLPFLLICEMESRIPAVEELGPCFPFSVSAELDGVVCPAGTANSKTEAK
QQAALSALCYIRSQLENPESPQTSSRPPLAPLSVENILTHEQRCAALVSAGFDLLLDERS
PYWACKGTVAGVILERGWAVSAPSCTEIPRARGHVKEIYKLVALGTGSSCCAGWLEFSGQ
QLHDCHGLVIARRALLRFLFRQLLLATQGGPKGKEQSVLAPQPGPGPPFTLKPRVFLHLY
ISNTPKGAARDIYLPPTSEGGLPHSPPMRLQAHVLGQLKPVCYVAPSLCDTHVGCLSASD
KLARWAVLGLGGALLAHLVSPLYSTSLILADSCHDPPTLSRAIHTRPCLDSVLGPCLPPP
YVRTALHLFAGPPVAPSEPTPDTCRGLSLNWSLGDPGIEVVDVATGRVKANAALGPPSRL
CKASFLRAFHQAARAVGKPYLLALKTYEAAKAGPYQEARRQLSLLLDQQGLGAWPSKPLV
GKFRN*
>ENSG00000120733|ENST00000314358
MADAAASPVGKRLLLLFADTAASASASAPAAAAASGDPGPALRTRAWRAGTVRAMSGAVP

QDLAIFVEFDGCNWKQHSWVKVHAEEVIVLLLEGSLVWAPREDPVLLQGIRVSIAQWPAL
TFTPLVDKLGLGSVVPVEYLLDRELRFLSDANGLHLFQMGTDSQNQILLEHAALRETVNA
LISDQKLQEIFSRGPYSVQGHRVKIYQPEGEEGWLYGVVSHQDSITRLMEVSVTESGEIK
SVDPRLIHVMLMDNSAPQSEGGTLKAVKSSKGKKKRESIEGKDGRRRKSASDSGCDPASK
KLKGDRGEVDSNGSDGGEASRGPWKGGNASGEPGLDQRAKQPPSTFVPQINRNIRFATYT
KENGRTLVVQDEPVGGDTPASFTPYSTATGQTPLAPEVGGAENKEAGKTLEQVGQGIVAS
AAVVTTASSTPNTVRISDTGLAAGTVPEKQKGSRSQASGENSRNSILASSGFGAPLPSSS
QPLTFGSGRSQSNGVLATENKPLGFSFGCSSAQEAQKDTDLSKNLFFQCMSQTLPTSNYF
TTVSESLADDSSSRDSFKQSLESLSSGLCKGRSVLGTDTKPGSKAGSSVDRKVPAESMPT
LTPAFPRSLLNARTPENHENLFLQPPKLSREEPSNPFLAFVEKVEHSPFSSFASQASGSS
SSATTVTSKVAPSWPESHSSADSASLAKKKPLFITTDSSKLVSGVLGSALTSGGPSLSAM
GNGRSSSPTSSLTQPIEMPTLSSSPTEERPTVGPGQQDNPLLKTFSNVFGRHSGGFLSSP
ADFSQENKAPFEAVKRFSLDERSLACRQDSDSSTNSDLSDLSDSEEQLQAKTGLKGIPEH
LMGKLGPNGERSAELLLGKSKGKQAPKGRPRTAPLKVGQSVLKDVSKVKKLKQSGEPFLQ
DGSCINVAPHLHKCRECRLERYRKFKEQEQDDSTVACRFFHFRRLIFTRKGVLRVEGFLS
PQQSDPDAMNLWIPSSSLAEGIDLETSKYILANVGDQFCQLVMSEKEAMMMVEPHQKVAW
KRAVRGVREMCDVCETTLFNIHWVCRKCGFGVCLDCYRLRKSRPRSETEEMGDEEVFSWL
KCAKGQSHEPENLMPTQIIPGTALYNIGDMVHAARGKWGIKANCPCISRQNKSVLRPAVT
NGMSQLPSINPSASSGNETTFSGGGGPAPVTTPEPDHVPKADSTDIRSEEPLKTDSSASN
SNSELKAIRPPCPDTAPPSSALHWLADLATQKAKEETKEAGSLRSVLNKESHSPFGLDSF
NSTAKVSPLTPKLFNSLLLGPTASNNKTEGSSLRDLLHSGPGKLPQTPLDTGIPFPPVFS
TSSAGVKSKASLPNFLDHIIASVVENKKTSDASKRACNLTDTQKEVKEMVMGLNVLDPHT
SHSWLCDGRLLCLHDPSNKNNWKIFRECWKQGQPVLVSGVHKKLKSELWKPEAFSQEFGD
QDVDLVNCRNCAIISDVKVRDFWDGFEIICKRLRSEDGQPMVLKLKDWPPGEDFRDMMPT
RFEDLMENLPLPEYTKRDGRLNLASRLPSYFVRPDLGPKMYNAYGLITAEDRRVGTTNLH
LDVSDAVNVMVYVGIPIGEGAHDEEVLKTIDEGDADEVTKQRIHDGKEKPGALWHIYAAK
DAEKIRELLRKVGEEQGQENPPDHDPIHDQSWYLDQTLRKRLYEEYGVQGWAIVQFLGDA
VFIPAGAPHQVHNLYSCIKVAEDFVSPEHVKHCFRLTQEFRHLSNTHTNHEDKLQVKNII
YHAVKDAVGTLKAHESKLARS*
>ENSG00000066136|ENST00000308733
MSTEGGFGGTSSSDAQQSLQSFWPRVMEEIRNLTVKDFRVQELPLARIKKIMKLDEDVKM
ISAEAPVLFAKAAQIFITELTLRAWIHTEDNKRRTLQRNDIAMAITKFDQFDFLIDIVPR
DELKPPKRQEEVRQSVTPAEPVQYYFTLAQQPTAVQVQGQQQGQQTTSSTTTIQPGQIII
AQPQQGQTTPVTMQVGEGQQVQIVQAQPQGQAQQAQSGTGQTMQVMQQIITNTGEIQQIP
VQLNAGQLQYIRLAQPVSGTQVVQGQIQTLATNAQQGQRNASQGKPRRCLKETLQITQTE
VQQGQQQFSQFTDGQRNSVQQARVSELTGEAEPREVKATGNSTPCTSSLPTTHPPSHRAG
ASCVCCSQPQQSSTSPPPSDALQWVVVEVSGTPNQLETHRELHAPLPGMTSLSPLHPSQQ
LYQIQQVTMPAGQDLAQPMFIQSANQPSDGQAPQVTGD*
>ENSG00000115875|ENST00000313117
MSRYGRYGGETKVYVGNLGTGAGKGELERAFSYYGPLRTVWIARNPPGFAFVEFEDPRDA
EDAVRGLDGKVICGSRVRVELSTGMPRRSRFDRPPARRPFDPNDRCYECGEKGHYAYDCH
RYSRRRRSRSRSRSHSRSRGRRYSRSRSRSRGRRSRSASPRRSRSISLRRSRSASLRRSR
SGSIKGSRYFQSPSRSRSRSRSISRPRSSRSKSRSPSPKRSRSPSGSPRRSASPERMD*
>ENSG00000162244|ENST00000294189

MAKSKNHTTHNQSRKWHRNGIKKPRSQRYESLKGVDPKFLRNMRFAKKHNKKGLKKMQAN
NAKAMSARAEAIKALVKPKEVKPKIPKGVSRKLDRLAYIAHPKLGKRARARIAKGLRLCR
PKAKAKAKAKDQTKAQAAAPASVPAQAPKRTQAPTKASE*
>ENSG00000159409|ENST00000290583
MKEPDAIKLFVGQIPRHLEEKDLKPIFEQFGRIFELTVIKDKYTGLHKGCAFLTYCARDS
ALKAQSALHEQKTLPGMNRPIQVKPADSESRGEDRKLFVGMLGKQQTDEDVRKMFEPFGT
IDECTVLRGPDGTSKGCAFVKFQTHAEAQAAINTLHSSRTLPGASSSLVVKFADTEKERG
LRRMQQVATQLGMFSPIALQFGAYSAYTQALMQQQAALVAAHSAYLSPMATMAAVQMQHM
AAINANGLIATPITPSSGTSTPPAIAATPVSAIPAALGVNGYSPVPTQPTGQPAPDALYP
NGVHPYPAQSPAAPVDPLQQAYAGMQHYTAAYPAAYSLVAPAFPQPPALVAQQPPPPPQQ
QQQQQQQQQQQQQREGPDGCNIFIYHLPQEFTDSEILQMFVPFGHVISAKVFVDRATNQS
KCFGFVSFDNPASAQAAIQAMNGFQIGMKRLKVQLKRPKDANRPY*
>ENSG00000168152|ENST00000302236
MTRSCSAVGCSTRDTVLSRERGLSFHQFPTDTIQRSKWIRAVNRVDPRSKKIWIPGPGAI
LCSKHFQESDFESYGIRRKLKKGAVPSVSLYKIPQGVHLKGKARQKILKQPLPDNSQEVA
TEDHNYSLKTPLTIGAEKLAEVQQMLQVSKKRLISVKNYRMIKKRKGLRLIDALVEEKLL
SEETECLLRAQFSDFKWELYNWRETDEYSAEMKQFACTLYLCSSKVYDYVRKILKLPHSS
ILRTWLSKCQPSPGFNSNIFSFLQRRVENGDQLYQYCSLLIKSMPLKQQLQWDPSSHSLQ
GFMDFGLGKLDADETPLASETVLLMAVGIFGHWRTPLGYFFVNRASGYLQAQLLRLTIGK
LSDIGITVLAVTSDATAHSVQMAKALGIHIDGDDMKCTFQHPSSSSQQIAYFFDSCHLLR
LIRNAFQNFQSIQFINGIAHWQHLVELVALEEQELSNMERIPSTLANLKNHVLKVNSATQ
LFSESVASALEYLLSLDLPPFQNCIGTIHFLRLINNLFDIFNSRNCYGKGLKGPLLPETY
SKINHVLIEAKTIFVTLSDTSNNQIIKGKQKLGFLGFLLNAESLKWLYQNYVFPKVMPFP
YLLTYKFSHDHLELFLKMLRQVLVTSSSPTCMAFQKAYYNLETRYKFQDEVFLSKVSIFD
ISIARRKDLALWTVQRQYGVSVTKTVFHEEGICQDWSHCSLSEALLDLSDHRRNLICYAG
YVANKLSALLTCEDCITALYASDLKASKIGSLLFVKKKNGLHFPSESLCRVINICERVVR
THSRMAIFELVSKQRELYLQQKILCELSGHINLFVDVNKHLFDGEVCAINHFVKLLKDII
ICFLNIRAKNVAQNPLKHHSERTDMKTLSRKHWSSVQDYKCSSFANTSSKFRHLLSNDGY
PFK*
>ENSG00000155438|ENST00000285814
MATFSGPAGPILSLNPQEDVEFQKEVAQVRKRITQRKKQEQLTPGVVYVRHLPNLLDETQ
IFSYFSQFGTVTRFRLSRSKRTGNSKGYAFVEFESEDVAKIVAETMNNYLFGERLLECHF
MPPEKVHKELFKDWNIPFKQPSYPSVKRYNRNRTLTQKLRMEERFKKKERLLRKKLAKKG
IDYDFPSLILQKTESISKTNRQTSTKGQVLRKKKKKVSGTLDTPEKTVDSQGPTPVCTPT
FLERRKSQVAELNDDDKDDEIVFKQPISCVKEEIQETQTPTHSRKKRRRSSNQ*
>ENSG00000112232|ENST00000281156
MEEEKYLPELMAEKDSLDPSFVHASRLLAEEIEKFQGSDGKKEDEEKKYLDVISNKNIKL
SERVLIPVKQYPKFNFVGKLLGPRGNSLKRLQEETGAKMSILGKGSMRDKAKEEELRKSG
EAKYAHLSDELHVLIEVFAPPGEAYSRMSHALEEIKKFLVPDYNDEIRQEQLRELSYLNG
SEDSGRGRGIRGRGIRIAPTAPSRGRGGAIPPPPPPGRGVLTPRGSTVTRGALPVPPVAR
GVPTPRARGAPTVPGYRAPPPPAHEAYEEYGYDDGYGGEYDDQTYETYDNSYATQTQSVP
EYYDYGHGVSEDAYDSYAPEEWATTRSSLKAPPQRSARGGYREHPYGRY*
>ENSG00000156508|ENST00000309268
MGKEKTHINIVVIGHVDSGKSTTTGHLIYKCGGIDKRTIEKFEKEAAEMGKGSFKYAWVL

DKLKAERERGITIDISLWKFETSKYYVTIIDAPGHRDFIKNMITGTSQADCAVLIVAAGV
GEFEAGISKNGQTREHALLAYTLGVKQLIVGVNKMDSTEPPYSQKRYEEIVKEVSTYIKK
IGYNPDTVAFVPISGWNGDNMLEPSANMPWFKGWKVTRKDGNASGTTLLEALDCILPPTR
PTDKPLRLPLQDVYKIGGIGTVPVGRVETGVLKPGMVVTFAPVNVTTEVKSVEMHHEALS
EALPGDNVGFNVKNVSVKDVRRGNVAGDSKNDPPMEAAGFTAQVIILNHPGQISAGYAPV
LDCHTAHIACKFAELKEKIDRRSGKKLEDGPKFLKSGDAAIVDMVPGKPMCVESFSDYPP
LGRFAVRDMRQTVAVGVIKAVDKKAAGAGKVTKSAQKAQKAK*
>ENSG00000139718|ENST00000267197
MENSHPPHHHHQQPPPQPGPSGERRNHHWRSYKLMIDPALKKGHHKLYRYDGQHFSLAMS
SNRPVEIVEDPRVVGIWTKNKELELSVPKFKIDEFYVGPVPPKQVTFAKLNDNIRENFLR
DMCKKYGEVEEVEILYNPKTKKHLGIAKVVFATVRGAKDAVQHLHSTSVMGNIIHVELDT
KGETRMRFYELLVTGRYTPQTLPVGELDAVSPIVNETLQLSDALKRLKDGGLSAGCGSGS
SSVTPNSGGTPFSQDTAYSSCRLDTPNSYGQGTPLTPRLGTPFSQDSSYSSRQPTPSYLF
SQDPAVTFKARRHESKFTDAYNRRHEHHYVHNSPAVTAVAGATAAFRGSSDLPFGAVGGT
GGSSGPPFKAQPQDSATFAHTPPPAQATPAPGFKSAFSPYQTPVAHFPPPPEEPTATAAF
GARDSGEFRRAPAPPPLPPAEPLAKEKPGTPPGPPPPDTNSMELGGRPTFGWSPEPCDSP
GTPTLESSPAGPEKPHDSLDSRIEMLLKEQRTKLLFLREPDSDTELQMEGSPISSSSSQL
SPLAPFGTNSQPGFRGPTPPSSRPSSTGLEDISPTPLPDSDEDEELDLGLGPRPPPEPGP
PDPAGLLSQTAEVALDLVGDRTPTSEKMDEGQQSSGEDMEISDDEMPSAPITSADCPKPM
VVTPGAAAVAAPSVLAPTLPLPPPPGFPPLPPPPPPPPPQPGFPMPPPLPPPPPPPPPAH
PAVTVPPPPLPAPPGVPPPPILPPLPPFPPGLFPVMQVDMSHVLGGQWGGMPMSFQMQTQ
VLSRLMTGQGACPYPPFMAAAAAAASAGLQFVNLPPYRGPFSLSNSGPGRGQHWPPLPKF
DPSVPPPGYMPRQEDPHKATVDGVLLVVLKELKAIMKRDLNRKMVEVVAFRAFDEWWDKK
ERMAKASLTPVKSGEHKDEDRPKPKDRIASCLLESWGKGEGLGYEGLGLGIGLRGAIRLP
SFKVKRKEPPDTTSSGDQKRLRPSTSVDEEDEESERERDRDMADTPCELAKRDPKGVGVR
RRPARPLELDSGGEEDEKESLEEEQESTEEEEEAEEEEEEEDDDDDSDDRDESENDDED
TALSEASEKDEGDSDEEETVSIVTSKAEATSSSESSESSEFESSSESSPSSSEDEEEVVA
REEEEEEEEEEMVAEESMASAGPEDFEQDGEEAALAPGAPAVDSLGMEEEVDIETEAVAP
EERPSMLDEPPLPVGVEEPADSREPPEEPGLSQEGAMLLSPEPPAKEVEARPPLSPERAP
EHDLEVEPEPPMMLPLPLQPPLPPPRPPRPPSPPPEPETTDASHPSVPPEPLAEDHPPHT
PGLCGSLAKSQSTETVPATPGGEPPLSGGSSGLSLSSPQVPGSPFSYPAPSPSLSSGGLP
RTPGRDFSFTPTFSEPSGPLLLPVCPLPTGRRDERSGPLASPVLLETGLPLPLPLPLPLP
LALPAVLRAQARAPTPLPPLLPAPLASCPPPMKRKPGRPRRSPPSMLSLDGPLVRPPAGA
ALGRELLLLPGQPQTPVFPSTHDPRTVTLDFRNAGIPAPPPPLPPQPPPPPPPPPVEPTK
LPFKELDNQWPSEAIPPGPRGRDEVTEEYMELAKSRGPWRRPPKKRHEDLVPPAGSPELS
PPQPLFRPRSEFEEMTILYDIWNGGIDEEDIRFLCVTYERLLQQDNGMDWLNDTLWVYHP
STSLSSAKKKKRDDGIREHVTGCARSEGFYTIDKKDKLRYLNSSRASTDEPPADTQGMSI
PAQPHASTRAGSERRSEQRRLLSSFTGSCDSDLLKFNQLKFRKKKLKFCKSHIHDWGLFA
MEPIAADEMVIEYVGQNIRQVIADMREKRYEDEGIGSSYMFRVDHDTIIDATKCGNFARF
INHSCNPNCYAKVITVESQKKIVIYSKQHINVNEEITYDYKFPIEDVKIPCLCGSENCRG
TLN*
>ENSG00000144655|ENST00000273153
MTGLLKRKFDQLDEDNSSVSSSSSSSGCQSRSCSPSSSVSRAWDSEEEGPWDQMPLPDRD
FCGPRSFTPLSILKRARRERPGRVAFDGITVFYFPRCQGFTSVPSRGGCTLGMALRHSAC

RRFSLAEFAQEQARARHEKLRQRLKEEKLEMLQWKLSAAGVPQAEAGLPPVVDAIDDASV
EEDLAVAVAGGRLEEVSFLQPYPARRRRALLRASGVRRIDREEKRELQALRQSREDCGCH
CDRICDPETCSCSLAGIKCQMDHTAFPCGCCREGCENPMGRVEFNQARVQTHFIHTLTRL
QLEQEAESFRELEAPAQGSPPSPGEEALVPTFPLAKPPMNNELGDNSCSSDMTDSSTASS
SASGTSEAPDCPTHPGLPGPGFQPGVDDDSLARILSFSDSDFGGEEEEEEEGSVGNLDNL
SCFHPADIFGTSDPGGLASWTHSYSGCSFTSGVLDENANLDASCFLNGGLEGSREGSLPG
TSVPPSMDAGRSSSVDLSLSSCDSFELLQALPDYSLGPHYTSQKVSDSLDNIEAPHFPLP
GLSPPGDASSCFLESLMGFSEPAAEALDPFIDSQFEDTVPASLMEPVPV*
>ENSG00000163902|ENST00000296255
MEAPAAGLFLLLLLGTWAPAPGSASSEAPPLINEDVKRTVDLSSHLAKVTAEVVLAHLGG
GSTSRATSFLLALEPELEARLAHLGVQVKGEDEEENNLEVRETKIKGKSGRFFTVKLPVA
LDPGAKISVIVETVYTHVLHPYPTQITQSEKQFVVFEGNHYFYSPYPTKTQTMRVKLASR
NVESYTKLGNPTRSEDLLDYGPFRDVPAYSQDTFKVHYENNSPFLTITSMTRVIEVSHWG
NIAVEENVDLKHTGAVLKGPFSRYDYQRQPDSGISSIRSFKTILPAAAQDVYYRDEIGNV
STSHLLILDDSVEMEIRPRFPLFGGWKTHYIVGYNLPSYEYLYNLGDQYALKMRFVDHVF
DEQVIDSLTVKIILPEGAKNIEIDSPYEISRAPDELHYTYLDTFGRPVIVAYKKNLVEQH
IQDIVVHYTFNKVLMLQEPLLVVAAFYILFFTVIIYVRLDFSITKDPAAEARMKVACITE
QVLTLVNKRIGLYRHFDETVNRYKQSRDISTLNSGKKSLETEHKALTSEIALLQSRLKTE
GSDLCDRVSEMQKLDAQVKELVLKSAVEAERLVAGKLKKDTYIENEKLISGKRQELVTKI
DHILDAL*
>ENSG00000170325|ENST00000304538
MSAYSVPSTFAQASLPVHNQVLPSIESVDGSDPLATLQTPLGRLEAKEEEDEDEDEDTEE
DEEEDGEDTDLDDWEPDPPRPFDPHDLWCEECNNAHASVCPKHGPLHPIPNRPVLTRARA
SLPLVLYIDRFLGGVFSKRRIPKRTQFGPVEGPLVRGSELKDCYIHLKVSLDKGDRKERD
LHEDLWFELSDETLCNWMMFVRPAQNHLEQNLVAYQYGHHVYYTTIKNVEPKQELKVWYA
ASYAEFVNQKIHDISEEERKVLREQEKNWPCYECNRRFISSEQLQQHLNSHDEKLDVFSR
TRGRGRGRGKRRFGPGRRPGRPPKFIRLEITSENGEKSDDGTQDLLHFPTKEQFDEAEPA
TLNGLDQPEQTTIPIPQLPQETQSSLEHEPETHTLHLQPQHEESVVPTQSTLTADDMRRA
KRIRNAALQHLFIRKSFRPFKCLQCGKAFREKDKLDQHLRFHGREGNCPLTCDLCNKGFI
SSTSLESHMKLHSDQKTYSCIFCPESFDRLDLLKDHVAIHINDGYFTCPTCKKRFPDFIQ
VKKHVRSFHSEKIYQCTECDKAFCRPDKLRLHMLRHSDRKDFLCSTCGKQFKRKDKLREH
MQRMHNPEREAKKADRISRSKTFKPRITSTDYDSFTFKCRLCMMGFRRRGMLVNHLSKRH
PDMKIEEVPELTLPIIKPNRDYFCQYCDKVYKSASKRKAHILKNHPGAELPPSIRKLRPA
GPGEPDPMLSTHTQLTGTIATPPVCCPHCSKQYSSKTKMVQHIRKKHPEFAQLSNTIHTP
LTTAVISATPAVLTTDSATGETVVTTDLLTQAMTELSQTLTTDYRTPQGDYQRIQYIPVS
QSASGLQQPQHIQLQVVQVASVSGQPLSPSAQQAQQGLSPSHIQGSSSTQGQALQQQQQQ
QQNSSVQHTYLPSAWNSFRGYSSEIQMMTLPPGQFVITDSGVATPVTTGQVKAVTSGHYV
LSESQSELEEKQTSALSGGVQVEPPAHSDSLDPQTNSQQQTTQYIITTTTNGNGSSEVHI
TKP*
>ENSG00000175691|ENST00000314531
MDCVIFEEVAVNFTPEEWALLDHAQRSLYRDVMLETCRNLASLDCYIYVRTSGSSSQRDV
FGNGISNDEEIVKFTGSDSWSIFGENWRFDNTGDQHQIPQRHLRSQLGRLCESNEGHQCG
ETLSQTANLLVHKSYPTEAKPSECTKCGKAFENRQRSHTGQRPCKECGQACSCLSCQSPP
MKTQTVEKPCNCQDSRTASVTYVKSLSSKKSYECQKCGKAFICPSSFRGHVNSHHGQKTH

ACKVCGKTFMYYSYLTRHVRTHTGEKPYECKECGKAFSCPSYFREHVRTHTGEKPYECKH
CGKSFSCYSSFRDHVRTHTGEKPCQCKHCGKAFTCYSSLREHGRTHSGEKPYECKECGKA
FRYPSSLRAHMRMHTGEKPYVCKQCGKAFGCPTYFRRHVKTHSGVKPYQCKECGKAYSFS
SSLRIHVRTHTGEKPFECKHCGKAFSCHSSLREHVRTHSGEKPYECNQCGKAFSHAQYFQ
KHVRSHSGVKPYECTECGKAYSCSSSLRVHVRTHTGERPYECKQCGKTFRYLASLQAHVR
THAGA*
>ENSG00000143815|ENST00000272163
MPSRKFADGEVVRGRWPGSSLYYEVEILSHDSTSQLYTVKYKDGTELELKENDIKPLTSF
RQRKGGSTSSSPSRRRGSRSRSRSRSPGRPPKSARRSASASHQADIKEARREVEVKLTPL
ILKPFGNSISRYNGEPEHIERNDAPHKNTQEKFSLSQESSYIATQYSLRPRREEVKLKEI
DSKEEKYVAKELAVRTFEVTPIRAKDLEFGGVPGVFLIMFGLPVFLFLLLLMCKQKDPSL
LNFPPPLPALYELWETRVFGVYLLWFLIQVLFYLLPIGKVVEGTPLIDGRRLKYRLNGFY
AFILTSAVIGTSLFQGVEFHYVYSHFLQFALAATVFCVVLSVYLYMRSLKAPRNDLSPAS
SGNAVYDFFIGRELNPRIGTFDLKYFCELRPGLIGWVVINLVMLLAEMKIQDRAVPSLAM
ILVNSFQLLYVVDALWNEEALLTTMDIIHDGFGFMLAFGDLVWVPFIYSFQAFYLVSHPN
EVSWPMASLIIVLKLCGYVIFRGANSQKNAFRKNPSDPKLAHLKTIHTSTGKNLLVSGWW
GFVRHPNYLGDLIMALAWSLPCGFNHILPYFYIIYFTMLLVHREARDEYHCKKKYGVAWE
KYCQRVPYRIFPYIY*
>ENSG00000142065|ENST00000270001
MAHGSVTFRDVAIDFSQEEWEFLDPAQRDLYRDVMWENYSNFISLGPSISKPDVITLLDE
ERKEPGMVVREGTRRYCPDLESRYRTNTLSPEKDIYEIYSFQWDIMERIKSYSLQGSIFR
NDWECKSKIEGEKEQQEGYFGQVKITSEKMTTYKRHNFLTEYQIVHNGEKVYECKECRKT
FIRRSTLSQHLRIHTGEKPYKCKECGQAFRQRAHLIRHHKLHTGEKPYECKECGKAFTVL
QELTQHQRLHTGEKPYECKECGKAFRVHQQLARHQRIHTGEKPYECKDCGKTFRQCTHLT
RHQRLHTAEKLYECKECGKAFVCGPDLRVHQKIHFGEKPYECKECGKAFRICQQLTVHQS
IHTGEKPYECKECGKTFRLRQQLVRHQRIHTREKPYECMECWKTFSSYSQLISHQSIHIG
ERPYECEECGKAFRLLSQLTQHQSIHTGEKPYECKECRKPFRLLSQLTQHQSIHTGEKPY
ECKECGKAFRLYSFLTQHQRIHTGEKPYKCKECKKAFRQHSHLTQHQKIHNGI*
>ENSG00000169554|ENST00000303660
MKQPIMADGPRCKRRKQANPRRKNVVNYDNVVDTGSETDEEDKLHIAEDDGIANPLDQET
SPASVPNHESSPHVSQALLPREEEEDEIREGGVEHPWHNNEILQASVDGPEMKEDYDTMG
PEATIQTAINNGTVKNANCTSDFEEYFAKRKLEERDGHAVSIEEYLQRSDTAIIYPEAPE
ELSRLGTPEANGQEENDLPPGTPDAFAQLLTCPYCDRGYKRLTSLKEHIKYRHEKNEENF
SCPLCSYTFAYRTQLERHMVTHKPGTDQHQMLTQGAGNRKFKCTECGKAFKYKHHLKEHL
RIHSGEKPYECPNCKKRFSHSGSYSSHISSKKCIGLISVNGRMRNNIKTGSSPNSVSSSP
TNSAITQLRNKLENGKPLSMSEQTGLLKIKTEPLDFNDYKVLMATHGFSGTSPFMNGGLG
ATSPLGVHPSAQSPMQHLGVGMEAPLLGFPTMNSNLSEVQKVLQIVDNTVSRQKMDCKAE
EISKLKGYHMKDPCSQPEEQGVTSPNIPPVGLPVVSHNGATKSIIDYTLEKVNEAKACLQ
SLTTDSRRQISNIKKEKLRTLIDLVTDDKMIENHNISTPFSCQFCKESFPGPIPLHQHER
YLCKMNEEIKAVLQPHENIVPNKAGVFVDNKALLLSSVLSEKGMTSPINPYKDHMSVLKA
YYAMNMEPNSDELLKISIAVGLPQEFVKEWFEQRKVYQYSNSRSPSLERSSKPLAPNSNP
PTKDSLLPRSPVKPMDSITSPSIAELHNSVTNCDPPLRLTKPSHFTNIKPVEKLDHSRSN
TPSPLNLSSTSSKNSHSSSYTPNSFSSEELQAEPLDLSLPKQMKEPKSIIATKNKTKASS
ISLDHNSVSSSSENSDEPLNLTFIKKEFSNSNNLDNKSTNPVFSMNPFSAKPLYTALPPQ

SAFPPATFMPPVQTSIPGLRPYPGLDQMSFLPHMAYTYPTGAATFADMQQRRKYQRKQGF
QGELLDGAQDYMSGLDDMTDSDSCLSRKKIKKTESGMYACDLCDKTFQKSSSLLRHKYEH
TGKRPHQCQICKKAFKHKHHLIEHSRLHSGEKPYQCDKCGKRFSHSGSYSQHMNHRYSYC
KREAEEREAAEREAREKGHLEPTELLMNRAYLQSITPQGYSDSEERESMPRDGESEKEHE
KEGEDGYGKLGRQDGDEEFEEEEEESENKSMDTDPETIRDEEETGDHSMDDSSEDGKMET
KSDHEEDNMEDGM*
>ENSG00000139343|ENST00000266735
MSLPLNPKPFLNGLTGKPVMVKLKWGMEYKGYLVSVDGYMNMQLANTEEYIDGALSGHLG
EVLIRCNNVLYIRGVEEEEEDGEMRE*
>ENSG00000103343|ENST00000268655
MAAKMEITLSSNTEASSKQERHIIAKLEEKRGPPLQKNCPDPELCRQSFRRFCYQEVSGP
QEALSQLRQLCRQWLQPELHTKEQILELLVMEQFLTILPPEIQARVRHRCPMSSKEIVTL
VEDFHRASKKPKQWVAVCMQGQKVLLEKTGSQLGEQELPDFQPQTPRRDLRESSPAEPSQ
AGAYDRLSPHHWEKSPLLQEPTPKLAGTEAPRMRSDNKENPQQEGAKGAKPCAVSAGRSK
GNGLQNPEPRGANMSEPRLSRRQVSSPNAQKPFAHYQRHCRVEYISSPLKSHPLRELKKS
KGGKRSLSNRLQHLGHQPTRSAKKPYKCDDCGKSFTWNSELKRHKRVHTGERPYTCGECG
NCFGRQSTLKLHQRIHTGEKPYQCGQCGKSFRQSSNLHQHHRLHHGD*
>ENSG00000164900|ENST00000297537
MQRAGGGSAPGGNGGGGGGGPGTAFSIDSLIGPPPPRSGHLLYTGYPMFMPYRPLVLPQA
LAPAPLPAGLPPLAPLASFAGRLTNTFCAGLGQAVPSMVALTTALPSFAEPPDAFYGPQE
LAAAAAAAAATAARNNPEPGGRRPEGGLEADELLPAREKVAEPPPPPPPHFSETFPSLPA
EGKVYSSDEEKLEASAGDPAGSEQEEEGSGGDSEDDGFLDSSAGGPGALLGPKPKLKGSL
GTGAEEGAPVTAGVTAPGGKSRRRRTAFTSEQLLELEKEFHCKKYLSLTERSQIAHALKL
SEVQVKIWFQNRRAKWKRIKAGNVSSRSGEPVRNPKIVVPIPVHVNRFAVRSQHQQMEQG
ARP*
>ENSG00000060339|ENST00000265872
MAQFGGQKNPPWATQFTATAVSQPAALGVQQPSLLGASPTIYTQQTALAAAGLTTQTPAN
YQLTQTAALQQQAAAAAAALQQQYSQPQQALYSVQQQLQQPQQTLLTQPAVALPTSLSLS
TPQPTAQITVSYPTPRSSQQQTQPQKQRVFTGVVTKLHDTFGFVDEDVFFQLSAVKGKTP
QVGDRVLVEATYNPNMPFKWNAQRIQTLPNQNQSQTQPLLKTPPAVLQPIAPQTTFGVQT
QPQPQSLLQAQISAASITPLLQTQPQPLLQQPQQKAGLLQPPVRIVSQPQPARRLDPPSR
FSGRNDRGDQVPNRKDDRSRERERERRRSRERSPQRKRSRERSPRRERERSPRRVRRVVP
RYTVQFSKFSLDCPSCDMMELRRRYQNLYIPSDFFDAQFTWVDAFPLSRPFQLGNYCNFY
VMHREVESLEKNMAILDPPDADHLYSAKVMLMASPSMEDLYHKSCALAEDPQELRDGFQH
PARLVKFLVGMKGKDEAMAIGGHWSPSLDGPDPEKDPSVLIKTAIRCCKALTGIDLSVCT
QWYRFAEIRYHRPEETHKGRTVPAHVETVVLFFPDVWHCLPTRSEWETLSRGYKQQLVEK
LQGERKEADGEQDEEEKDDGEAKEISTPTHWSKLDPKTMKVNDLRKELESRALSSKGLKS
QLIARLTKQLKVEEQKEEQKELEKSEKEEDEDDDRKSEDDKEEEERKRQEEIERQRRERR
YILPDEPAIIVHPNWAAKSGKFDCSIMSLSVLLDYRLEDNKEHSFEVSLFAELFNEMLQR
DFGVRIYKSLLSLPEKEDKKEKDKKSKKDERKDKKEERDDETDEPKPKRRKSGDDKDKKE
DRDERKKEDKRKDDSKDDDETEEDNNQDEYDPMEAEEAEDEEDDRDEEEMTKRDDKRDIN
RYCKERPSKDKEKEKTQMITINRDLLMAFVYFDQSHCGYLLEKDLEEILYTLGLHLSRAQ
VKKLLNKVVLRESCFYRKLTDTSKDEENHEESESLQEDMLGNRLLLPTPTVKQESKDVEE
NVGLIVYNGAMVDVGSLLQKLEKSEKVRAEVEQKLQLLEEKTDEDEKTILNLENSNKSLS

GELREVKKDLSQLQENLKISENMNLQFENQMNKTIRNLSTVMDEIHTVLKKDNVKNEDKD
QKSKENGASV*
>ENSG00000178662|ENST00000314499
MSGILKRKFEEVDGSSPCSSVRESDDEVSSSESADSGDSVNPSTSSHFTPSSILKREKRL
RTKNVHFSCVTVYYFTRRQGFTSVPSQGGSTLGMSSRHNSVRQYTLGEFAREQERLHREM
LREHLREEKLNSLKLKMTKNGTVESEEASTLTLDDISDDDIDLDNTEVDEYFFLQPLPTK
KRRALLRASGVKKIDVEEKHELRAIRLSREDCGCDCRVFCDPDTCTCSLAGIKCQVDRMS
FPCGCTKEGCSNTAGRIEFNPIRVRTHFLHTIMKLELEKNREQQIPTLNGCHSEISAHSS
SMGPVAHSVEYSIADSFEIETEPQAAVLHLQSAEELDCQGEEEEEEEDGSSFCSGVTDSS
TQSLAPSESDEEEEEEEEEEEEEEDDDDDKGDGFVEGLGTHAEVVPLPSVLCYSDGTAVHE
SHAKNASFYANSSTLYYQIDSHIPGTPNQISENYSERDTVKNGTLSLVPYTMTPEQFVDY
ARQAEEAYGASHYPAANPSVIVCCSSSENDSGVPCNSLYPEHRSNHPQVEFHSYLKGPSQ
EGFVSALNGDSHISEHPAENSLSLAEKSILHEECIKSPVVETVPV*
>ENSG00000065427|ENST00000302445
MAAVQAAEVKVDGSEPKLSKNELKRRLKAEKKVAEKEAKQKELSEKQLSQATAAATNHTT
DNGVGPEEESVDPNQYYKIRSQAIHQLKVNGEDPYPHKFHVDISLTDFIQKYSHLQPGDH
LTDITLKVAGRIHAKRASGGKLIFYDLRGEGVKLQVMANSRNYKSEEEFIHINNKLRRGD
IIGVQGNPGKTKKGELSIIPYEITLLSPCLHMLPHLHFGLKDKETRYRQRYLDLILNDFV
RQKFIIRSKIITYIRSFLDELGFLEIETPMMNIIPGGAVAKPFITYHNELDMNLYMRIAP
ELYHKMLVVGGIDRVYEIGRQFRNEGIDLTHNPEFTTCEFYMAYADYHDLMEITEKMVSG
MVKHITGSYKVTYHPDGPEGQAYDVDFTPPFRRINMVEELEKALGMKLPETNLFETEETR
KILDDICVAKAVECPPPRTTARLLDKLVGEFLEVTCINPTFICDHPQIMSPLAKWHRSKE
GLTERFELFVMKKEICNAYTELNDPMRQRQLFEEQAKAKAAGDDEAMFIDENFCTALEYG
LPPTAGWGMGIDRVAMFLTDSNNIKEVLLFPAMKPEDKKENVATTDTLESTTVGTSV*
>ENSG00000163320|ENST00000309534
MERFVVTAPPARNRSKTALYVTPLDRVTEFGGELHEDGGKLFCTSCNVVLNHVRKSAISD
HLKSKTHTKRKAEFEEQNVRKKQRPLTASLQCNSTAQTEKVSVIQDFVKMCLEANIPLEK
ADHPAVRAFLSRHVKNGGSIPKSDQLRRAYLPDGYENENQLLNSQDC*
>ENSG00000171469|ENST00000302851
MAAIYLSRGFFSREPICPFEEKTKVERMVEDYLASGYQDSVTFDDVAVDFTPEEWALLDT
TEKYLYRDVMLENYMNLASVEWEIQPRTKRSSLQQGFLKNQIFSGIQMTRGYSGWKLCDC
KNCGEVFREQFCLKTHMRVQNGGNTSEGNCYGKDTLSVHKEASTGQELSKFNPCGKVFTL
TPGLAVHLEVLNARQPYKCKECGKGFKYFASLDNHMGIHTDEKLCEFQEYGRAVTASSHL
KQCVAVHTGKKSKKTKKCGKSFTNFSQLYAPVKTHKGEKSFECKECGRSFRNSSCLNDHI
QIHTGIKPHKCTYCGKAFTRSTQLTEHVRTHTGIKPYECKECGQAFAQYSGLSIHIRSHS
GKKPYQCKECGKAFTTSTSLIQHTRIHTGEKPYECVECGKTFITSSRRSKHLKTHSGEKP
FVCKICGKAFLYSSRLNVHLRTHTGEKPFVCKECGKAFAVSSRLSRHERIHTGEKPYECK
DMSVTI*
>ENSG00000074219|ENST00000311227
MGEPRAGAALDDGSGWTGSEEGSEEGTGGSEGAGGDGGPDAEGVWSPDIEQSFQEALAIY
PPCGRRKIILSDEGKMYGRNELIARYIKLRTGKTRTRKQVSSHIQVLARRKSREIQSKLK
DQVSKDKAFQTMATMSSAQLISAPSLQAKLGPTGPQASELFQFWSGGSGPPWNVPDVKPF
SQTPFTLSLTPPSTDLPGYEPPQALSPLPPPTPSPPAWQARGLGTARLQLVEFSAFVEPP
DAVDSYQRHLFVHISQHCPSPGAPPLESVDVRQIYDKFPEKKGGLRELYDRGPPHAFFLV

KFWADLNWGPSGEEAGAGGSISSGGFYGVSSQYESLEHMTLTCSSKVCSFGKQVVEKVET
ERAQLEDGRFVYRLLRSPMCEYLVNFLHKLRQLPERYMMNSVLENFTILQVVTNRDTQEL
LLCTAYVFEVSTSERGAQHHIYRLVRD*
>ENSG00000020633|ENST00000308873
MRIPVDPSTSRRFTPPSPAFPCGGGGGKMGENSGALSAQAAVGPGGRARPEVRSMVDVLA
DHAGELVRTDSPNFLCSVLPSHWRCNKTLPVAFKVVALGDVPDGTVVTVMAGNDENYSAE
LRNASAVMKNQVARFNDLRFVGRSGRGKSFTLTITVFTNPTQVATYHRAIKVTVDGPREP
RRHRQKLEDQTKPFPDRFGDLERLRMRVTPSTPSPRGSLSTTSHFSSQPQTPIQGTSELN
PFSDPRQFDRSFPTLPTLTESRFPDPRMHYPGAMSAAFPYSATPSGTSISSLSVAGMPAT
SRFHHTYLPPPYPGAPQNQSGPFQANPSPYHLYYGTSSGSYQFSMVAGSSSGGDRSPTRM
LASCTSSAASVAAGNLMNPSLGGQSDGVEADGSHSNSPTALSTPGRMDEAVWRPY*
>ENSG00000152926|ENST00000282869
MKRHEMVAKHLVMFYYFAQHLWPEQNIRDSFQKVTLRRYRKCGYENLQLRKGCKSVVECK
QHKGDYSGLNQCLKTTLSKIFQCNKYVEVFHKISNSNRHKMRHTENKHFKCKECRKTFCM
LSHLTQHKRIQTRVNFYKCEAYGRAFNWSSTLNKHKRIHTGEKPYKCKECGKAFNQTSHL
IRHKRIHTEEKPYKCEECGKAFNQSSTLTTHNIIHTGEIPYKCEKCVRAFNQASKLTEHK
LIHTGEKRYECEECGKAFNRSSKLTEHKYIHTGEKLYKCEECGKAFNQSSTLTTHKRIHS
GEKPYKCEECGKAFKQFSNLTDHKKIHTGEKPYKCEECGKAFNQLSNLTRHKVIHTGEKP
YKCGECGKAFNQSSALNTHKIIHTGENPHKCRESGKVFHLSSKLSTCKKIHTGEKLYKCE
ECGKAFNRSSTLIGHKRIHTGEKPYKCEECGKAFNQSSTLTTHKIIHTEEKQYKCDECGK
AST*
>ENSG00000152804|ENST00000282728
MQYPHPGPAAGAVGVPLYAPTPLLQPAHPTPFYIEDILGRGPAAPTPAPTLPSPNSSFTS
LVSPYRTPVYEPTPIHPAFSHHSAAALAAAYGPGGFGGPLYPFPRTVNDYTHALLRHDPL
GKPLLWSPFLQRPLHKRKGGQVRFSNDQTIELEKKFETQKYLSPPERKRLAKMLQLSERQ
VKTWFQNRRAKWRRLKQENPQSNKKEELESLDSSCDQRQDLPSEQNKGASLDSSQCSPSP
ASQEDLESEISEDSDQEVDIEGDKSYFNAG*
>ENSG00000165097|ENST00000297792
MATPRGRTKKKASFDHSPDSLPLRSSGRQAKKKATETTDEDEDGGSEKKYRKCEKAGCTA
TCPVCFASASERCAKNGYTSRWYHLSCGEHFCNECFDHYYRSHKDGYDKYTTWKKIWTSN
GKTEPSPKAFMADQQLPYWVQCTKPECRKWRQLTKEIQLTPQIAKTYRCGMKPNTAIKPE
TSDHCSLPEDLEALTPQKCIPHIIVRGLVRIRCVQEVERILYFMTRKGLINTGVLSVGAD
QYLLPKDYHNKSVIIIGAGPAGLAAARQLHNFGIKVTVLEAKDRIGGRVWDDKSFKGVTV
GRGAQIVNGCINNPVALMCEQVSARSWDHNEFFAQFAGDHTLLTPGYSVIIEKLAEGLDI
QLKSPVQCIDYSGDEVQVTTTDGTGYSAQKVLVTVPLALLQKGAIQFNPPLSEKKMKAIN
SLGAGIIEKIALQFPYRFWDSKVQGADFFGHVPPSASKRGLFAVFYDMDPQKKHSVLMSV
IAGEAVASVRTLDDKQVLQQCMATLRELFKEQEVPDPTKYFVTRWSTDPWIQMAYSFVKT
GGSGEAYDIIAEDIQGTVFFAGEATNRHFPQTVTGAYLSGVREASKIAAF*
>ENSG00000129534|ENST00000310806
MIATPLKHSRIYLPPEASSQRRNLPMDAIFFDSIPSGTLTPVKDLVKYQNSSLKLNDHKK
NQFLKMTTFNNKNIFQSTMLTEATTSNSSLDISAIKPNKDGLKNKANYESPGKIFLRMKE
KVLRDKQEQPSRNSSLLEPQKSGNNETFTPNRVEKKKLQHTYLCEEKENNKSFQSDDSSL
RASVQGVPLESSNNDIFLPVKQKIQCQQEKKAPLHNLTYELPTLNQEQENFLAVEARNKT
LTRAQLAKQIFHSKESIVATTKSKKDTFVLESVDSADEQFQNTNAETLSTNCIPIKNGSL

LMVSDSERTTEGTSQQKVKEGNGKTVPGETGLPGSMKDTCKIVLATPRLHITIPRRSKRN
ISKLSPPRIFQTVTNGLKKNQVVQLQEWMIKSINNNTAICVEGKLIDVTNIYWHSNVIIE
RIEHNKLRTISGNVYILKGMIDQISMKEAGYPNYLIRKFMFGFPENWKEHIDNFLEQLRA
GEKNREKTKQKQKTGRSVRDIRKSMKNDARENQTDTAQRATTTYDFDCDNLELKSNKHSE
SPGATELNMCHSNCQNKPTLRFPDDQVNNTIQNGGGDDLSNQELIGKKEYKMSSKKLKIG
ERTNERIIKSQKQETTEELDVSIDILTSREQFFSDEERKYMAINQKKAYILVTPLKSRKV
IEQRCMRYNLSAGTIKAVTDFVIPECQKKSPISKSMGTLENTFEGHKSKNKEDCDERDLL
TVNRKIKISNLEKEQMLTSDFKKNTRLLPKLKKIENQVAMSFYKHQSSPDLSSEESETEK
EIKRKAEVKKTKAGNTKEAVVHLRKSTRNTSNIPVILEPETEESENEFYIKQKKARPSVK
ETLQKSGVRKEFPITEAVGSDKTNRHPLECLPGLIQDKEWNEKELQKLHCAFASLPKHKP
GFWSEVAAAVGSRSPEECQRKYMENPRGKGSQKHVTKKKPANSKGQNGKRGDADQKQTIK
ITAKVGTLKRKQQMREFLEQLPKDDHDDFFSTTPLQHQRILLPSFQDSEDDDDILPNMDK
NPTTPSSVIFPLVKTPQCQHVSPGMLGSINRNDCDKYVFRMQKYHKSNGGIVWGNIKKKL
VETDFSTPTPRRKTPFNTDLGENSGIGKLFTNAVESLDEEEKDYYFSNSDSA*
>ENSG00000172238|ENST00000306011
MSRLLHAEEWAEVKELGDHHRQPQPHHLPQPPPPPQPPATLQAREHPVYPPELSLLDSTD
PRAWLAPTLQGICTARAAQYLLHSPELGASEAAAPRDEVDGRGELVRRSSGGASSSKSPG
PVKVREQLCKLKGGVVVDELGCSRQRAPSSKQVNGVQKQRRLAANARERRRMHGLNHAFD
QLRNVIPSFNNDKKLSKYETLQMAQIYINALSELLQTPSGGEQPPPPPASCKSDHHHLRT
AASYEGGAGNATAAGAQQASGGSQRPTPPGSCRTRFSAPASAGGYSVQLDALHFSTFEDS
ALTAMMAQKNLSPSLPGSILQPVQEENSKTSPRSHRSDGEFSPHSHYSDSDEAS*
>ENSG00000172262|ENST00000306938
MEAEETMECLQEFPEHHKMILDRLNEQREQDRFTDITLIVDGHHFKAHKAVLAACSKFFY
KFFQEFTQEPLVEIEGVSKMAFRHLIEFTYTAKLMIQGEEEANDVWKAAEFLQMLEAIKA
LEVRNKENSAPLEENTTGKNEAKKRKIAETSNVITESLPSAESEPVEIEVEIAEGTIEVE
DEGIETLEEVASAKQSVKYIQSTGSSDDSALALLADITSKYRQGDRKGQIKEDGCPSDPT
SKQEHMKSHSTESFKCEICNKRYLRESAWKQHLNCYHLEEGGVSKKQRTGKKIHVCQYCE
KQFDHFGHFKEHLRKHTGEKPFECPNCHERFARNSTLKCHLTACQTGVGAKKGRKKLYEC
QVCNSVFNSWDQFKDHLVIHTGDKPNHCTLCDLWFMQGNELRRHLSDAHNISERLVTEEV
LSVETRVQTEPVTSMTIIEQVGKVHVLPLLQVQVDSAQVTVEQVHPDLLQDSQVHDSHMS
ELPEQVQVSYLEVGRIQTEEGTEVHVEELHVERVNQMPVEVQTELLEADLDHVTPEIMNQ
EERESSQADAAEAAREDHEDAEDLETKPTVDSEAEKAENEDRTALPVLE*
>ENSG00000172809|ENST00000311111
MPRKIEEIKDFLLTARRKDAKSVKIKKNKDNVKFKVRCSRYLYTLVITDKEKAEKLKQSL
PPGLAVKELK*
>ENSG00000174332|ENST00000312233
MAEARTSLSAHCRGPLATGLHPDLDLPGRSLATPAPSCYLLGSEPSSGLGLQPETHLPEG
SLKRCCVLGLPPTSPASSSPCASSDVTSIIRSSQTSLVTCVNGLRSPPLTGDLGGPSKRA
RPGPASTDSHEGSLQLEACRKASFLKQEPADEFSELFGPHQQGLPPPYPLSQLPPGPSLG
GLGLGLAGRVVAGRQACRWVDCCAAYEQQEELVRHIEKSHIDQRKGEDFTCFWAGCVRRY
KPFNARYKLLIHMRVHSGEKPNKCMFEGCSKAFSRLENLKIHLRSHTGEKPYLCQHPGCQ
KAFSNSSDRAKHQRTHLDTKPYACQIPGCSKRYTDPSSLRKHVKAHSAKEQQVRKKLHAG
PDTEADVLTECLVLQQLHTSTQLAASDGKGGCGLGQELLPGVYPGSITPHNGLASGLLPP
AHDVPSRHHPLDATTSSHHHLSPLPMAESTRDGLGPGLLSPIVSPLKGLGPPPLPPSSQS

HSPGGQPFPTLPSKPSYPPFQSPPPPPLPSPQGYQGSFHSIQSCFPYGDCYRMAEPAAGG
DGLVGETHGFNPLRPNGYHSLSTPLPATGYEALAEASCPTALPQQPSEDVVSSGPEDCGF
FPNGAFDHCLGHIPSIYTDT*
>ENSG00000171456|ENST00000306058
MGVVNFVFRASEDLQVLENYSDAPMTPKQILQVIEAEGLKEMSGTSPLACLNAMLHSNSR
GGEGLFYKLPGRISLFTLKKDALQWSRHPATVEGEEPEDTADVESCGSNEASTVSGENDV
SLDETSSNASCSTESQSRPLSNPRDSYRASSQANKQKKKTGVMLPRVVLTPLKVNGAHVE
SASGFSGCHADGESGSPSSSSSGSLALGSAAIRGQAEVTQDPAPLLRGFRKPATGQMKRN
RGEEIDFETPGSILVNTNLRALINSRTFHALPSHFQQQLLFLLPEVDRQVGTDGLLRLSS
SALNNEFFTHAAQSWRERLADGEFTHEMQVRIRQEMEKEKKVEQWKEKFFEDYYGQKLGL
TKEESLQQNVGQEEAEIKSGLCVPGESVRIQRGPATRQRDGHFKKRSRPDLRTRARRNLY
KKQESEQAGVAKDAKSVASDVPLYKDGEAKTDPAGLSSPHLPGTSSAAPDLEGPEFPVES
VASRIQAEPDNLARASASPDRIPSLPQETVDQEPKDQKRKSFEQAASASFPEKKPRLEDR
QSFRNTIESVHTEKPQPTKEEPKVPPIRIQLSRIKPPWVVKGQPTYQICPRIIPTTESSC
RGWTGARTLADIKARALQVRGARGHHCHREAATTAIGGGGGPGGGGGATDEGGGRGSSS
GDGGEACGHPEPRGGPSTPGKCTSDLQRTQLLPPYPLNGEHTQAGTAMSRARREDLPSLR
KEESCLLQRATVGLTDGLGDASQLPVAPTGDQPCQALPLLSSQTSVAERLVEQPQLHPDV
RTECESGTTSWESDDEEQGPTVPADNGPIPSLVGDDTLEKGTGQALDSHPTMKDPVNVTP
SSTPESSPTDCLQNRAFDDELGLGGSCPPMRESDTRQENLKTKALVSNSSLHWIPIPSND
EVVKQPKPESREHIPSVEPQVGEEWEKAAPTPPALPGDLTAEEGLDPLDSLTSLWTVPSR
GGSDSNGSYCQQVDIEKLKINGDSEALSPHGESTDTASDFEGHLTEDSSEADTREAAVTK
GSSVDKDEKPNWNQSAPLSKVNGDMRLVTRTDGMVAPQSWVSRVCAVRQKIPDSLLLAST
EYQPRAVCLSMPGSSVEATNPLVMQLLQGSLPLEKVLPPAHDDSMSESPQVPLTKDQSHG
SLRMGSLHGLGKNSGMVDGSSPSSLRALKEPLLPDSCETGTGLARIEATQAPGAPQKNCK
AVPSFDSLHPVTNPITSSRKLEEMDSKEQFSSFSCEDQKEVRAMSQDSNSNAAPGKSPGD
LTTSRTPRFSSPNVISFGPEQTGRALGDQSNVTGQGKKLFGSGNVAATLQRPRPADPMPL
PAEIPPVFPSGKLGPSTNSMSGGVQTPREDWAPKPHAFVGSVKNEKTFVGGPLKANAENR
KATGHSPLELVGHLEGMPFVMDLPFWKLPREPGKGLSEPLEPSSLPSQLSIKQAFYGKLS
KLQLSSTSFNYSSSSPTFPKGLAGSVVQLSHKANFGASHSASLSLQMFTDSSTVESISLQ
CACSLKAMIMCQGCGAFCHDDCIGPSKLCVLCLVVR*
>ENSG00000141570|ENST00000269385
MELSAVGERVFAAEALLKRRIRKGRMEYLVKWKGWSQKYSTWEPEENILDARLLAAFEER
EREMELYGPKKRGPKPKTFLLKAQAKAKAKTYEFRSDSARGIRIPYPGRSPQDLASTSRA
REGLRNMGLSPPASSTSTSSTCRAEAPRDRDRDRDRDRERDRERERERERERERERERER
GTSRVDDKPSSPGDSSKKRGPKPRKELPDPSQRPLGEPSAGLGEYLKGRKLDDTPSGAGK
FPAGHSVIQLARRQDSDLVQCGVTSPSSAEATGKLAVDTFPARVIKHRAAFLEAKGQGAL
DPNGTRVRHGSGPPSSGGGLYRDMGAQGGRPSLIARIPVARILGDPEEESWSPSLTNLEK
VVVTDVTSNFLTVTIKESNTDQGFFKEKR*
>ENSG00000127603|ENST00000289893
MQKGLLDQDTGLVLLESQVIMSGLIAPETGENLSLEEGIARNLINPQMYQQLRELQDALA
LISRLTESRGPLSVVEAIEKRIISETVGLKILEAHLATGGFSLSPSENCINLEEAFHQGL
ISAWLHSVLESYLRTSKNLIDPNTAEKIGLLDLMQRCIVHQESGFKLLPVKQLAGGMVSL
KSGRKVSIFRAVQEGLIDRQVTVRLLEAQLFAGGIVDPRTGHRLTVEEAVRHNLIDQDMA
CAILIRQLQTGGIIDTVTGQRLTIDEAVSNDLVAAKIALVILESLWSFMGLLWPESGEIL

PITDALEQGIVSTELAHKILSNRQHIKALFLPATTEILSWKKAIESGILDRDLANNLKSI
CIPDVMPHMQLADSAEQNINPGAAVLPCSKSHPKATASQSENLLFQLMTHSYINVQNGQR
LLLLDKELMETLTSRDEYQTSPPKVVEIGHQRQKTPEGLQESANVKISGTFSSGWTVRLP
EFQFSSQNKEYPDREDCTTEKGKKTTVETEDSSVENPEQDLFVEQKERNPNIDALKVINK
VKLEVQRQLIGTQREDQTAVSVRENASRGHLLTIPPAEAEGVPLVVDKDVFSVETPKKEH
QPLRNTSFTCQNEQAHTLETEYIHDETGGSHIKPQSKKLQVQVKKTLGIKLELKSETDGN
VHPLDKKEMLKKTFLAKDDHKESQEAQNIAGGSMMMSEKTDEEDSGREIFLSCSHPLELL
EEATLNVLSAQLLDGGIFHEQTGQKLLLNEAISRGIVPSHTAVKLMEKLNMFQGFFDSQT
CESLTTEEVINEGLMDEKLLHNVLMADKAISGVLDPRTQTLCSVKDAVTVGLLDKETATR
ILERQVVTGGIIDLKRGKKVSVTLASTLGLVDVADQPELINLEKASKGRDAEKTVRERLI
SLQMETTGLIDPDSKAPLTVVQSIDRGLLEREEAVRLLTKQVVDGGIIHHISGMRLSVDN
AFRHGLIGEDLAEKLKRVENLNIHQIFNPETKENISLPKAIKLDLITSDLKREIQEVQAF
TGNFVDLISGQRLTLAEAKKEGLLTNEAVLSPGMMHGIVDPENCRIVPYSELVKKCKIDI
ESGQRYLEVIPFSDIKDGVSDKVLTLSQAIQLGKVDFASTLKVLEAQANTGGIIDTATGK
RLTLASALEEKLVDENMVRIIASHQVLNGGIVDIFSDQRVTLVEAIEKRLISPELANMIQ
IDSSEFSDHRAQIEKQEGIEVCALQNEFLGKDMLIACNQTAEMSCNKVEESERLFQVENQ
SAQEKVKVRVSDGEQAKKSREISLKEFGCKDQRKPRMSSDAKEFISIINPHNLKGKSLGQ
VSLTHPYSECDFKLKEVARNNMGNDTNEEQEKAVTKIEIISHMKQSTSCLDSEEIRENQG
EVILEVQETYCETSGKLPSEQVLQQPMNARVKSKREKREVIVEESIRTCKPAFLSEEKLY
QETAIRDEHDSHIKSQPREMTSSEKGKEADTEMGFSITFKIEESSSQVVPQGISVKHLDA
LTLFSSKQANEGKVNNLSLCLTLKPEENLSREIACGAQSEPFPCMTPRPEGLHYQESDGK
AQVTGPSQISKTDKSFQGTTRQETNYQDSWVTSKTKETKHQISSSNECKEKSYQEVSFDP
ARGLKLEEITVSRPDSKEVRYLEFSDRKDLHHQGSKSDDKLCGTLKSEIATQELTGEKFL
EMANPNVAGLEAGSIEDIVTQRGSRVLGSFLPEKLFKGVSQKENTGQQNAIISPTVLETS
EEKTVSLTVCSAVKTEKTPQEKLRESPGSEQTPFMTAPEGKGNGGVNPEPFRATQNVFTR
QLCLEHDEKLVSYLSLLRNIEMRTKQIQPLELNLAELQDLLCQAKVLERELKDLTTLVSQ
ELECVNQIIISQPQEVPAQLLKALEKDAKNLQKSLSSVSDTWNSRLLHFQNAVEIEKTKV
LNQHTQLEGRLQDLRAWVGNKNLILNSKGSNSEIDVDSLNLCLQQYEDLKQPMAERKAQL
DALAFDIQFFISEHAQDLSPQQNRQMLRLLNELQRSFQDILEQTAAQVDALQGHLQQMEQ
EALVKTLQKQQNTCHQQLEDLCSWVGQAERALAGHQGRTTQQDLSALQKNQSDLKDLQDD
IQNRATSFATVVKDIEGFMEENQTKLSPRELTALREKLHQAKEQYEALQEETRVAQKELE
EAVTSALQQETEKSKAAKELAENKKKIDALLDWVTSVGSSGGQLLTNLPGMEQLSGASLE
KGALDTTDGYMGVNQAPEKLDKQCEMMKARHQELLSQQQNFILATQSAQAFLDQHGHNLT
PEEQQMLQQKLGELKEQYSTSLAQSEAELKQVQTLQDELQKFLQDHKEFESWLERSEKEL
ENMHKGGSSPETLPSLLKRQGSFSEDVISHKGDLRFVTISGQKVLDMENSFKEGKEPSEI
GNLVKDKLKDATERYTALHSKCTRLGSHLNMLLGQYHQFQNSADSLQAWMQACEANVEKL
LSDTVASDPGVLQEQLATTKQLQEELAEHQVPVEKLQKVARDIMEIEGEPAPDHRHVQET
TDSILSHFQSLSYSLAERSSLLQKAIAQSQSVQESLESLLQSIGEVEQNLEGKQVSSLSS
GVIQEALATNMKLKQDIARQKSSLEATREMVTRFMETADSTTAAVLQGKLAEVSQRFEQL
CLQQQEKESSLKKLLPQAEMFEHLSGKLQQFMENKSRMLASGNQPDQDITHFFQQIQELN
LEMEDQQENLDTLEHLVTELSSCGFALDLCQHQDRVQNLRKDFTELQKTVKEREKDASSC
QEQLDEFRKLVRTFQKWLKETEGSIPPTETSMSAKELEKQIEHLKSLLDDWASKGTLVEE
INCKGTSLENLIMEITAPDSQGKTGSILPSVGSSVGSVNGYHTCKDLTEIQCDMSDVNLK
YEKLGGVLHERQESLQAILNRMEEVHKEANSVLQWLESKEEVLKSMDAMSSPTKTETVKA

QAESNKAFLAELEQNSPKIQKVKEALAGLLVTYPNSQEAENWKKIQEELNSRWERATEVT
VARQRQLEESASHLACFQAAESQLRPWLMEKELMMGVLGPLSIDPNMLNAQKQQVQFMLK
EFEARRQQHEQLNEAAQGILTGPGDVSLSTSQVQKELQSINQKWVELTDKLNSRSSQIDQ
AIVKSTQYQELLQDLSEKVRAVGQRLSVQSAISTQPEAVKQQLEETSEIRSDLEQLDHEV
KEAQTLCDELSVLIGEQYLKDELKKRLETVALPLQGLEDLAADRINRLQAALASTQQFQQ
MFDELRTWLDDKQSQQAKNCPISAKLERLQSQLQENEEFQKSLNQHSGSYEVIVAEGESL
LLSVPPGEEKRTLQNQLVELKNHWEELSKKTADRQSRLKDCMQKAQKYQWHVEDLVPWIE
DCKAKMSELRVTLDPVQLESSLLRSKAMLNEVEKRRSLLEILNSAADILINSSEADEDGI
RDEKAGINQNMDAVTEELQAKTGSLEEMTQRLREFQESFKNIEKKVEGAKHQLEIFDALG
SQACSNKNLEKLRAQQEVLQALEPQVDYLRNFTQGLVEDAPDGSDASQLLHQAEVAQQEF
LEVKQRVNSGCVMMENKLEGIGQFHCRVREMFSQLADLDDELDGMGAIGRDTDSLQSQIE
DVRLFLNKIHVLKLDIEASEAECRHMLEEEGTLDLLGLKRELEALNKQCGKLTERGKARQ
EQLELTLGRVEDFYRKLKGLNDATTAAEEAEALQWVVGTEVEIINQQLADFKMFQKEQVD
PLQMKLQQVNGLGQGLIQSAGKDCDVQGLEHDMEEINARWNTLNKKVAQRIAQLQEALLH
CGKFQDALEPLLSWLADTEELIANQKPPSAEYKVVKAQIQEQKLLQRLLDDRKATVDMLQ
AEGGRIAQSAELADREKITGQLESLESRWTELLSKAAARQKQLEDILVLAKQFHETAEPI
SDFLSVTEKKLANSEPVGTQTAKIQQQIIRHKALEEDIENHATDVHQAVKIGQSLSSLTS
PAEQGVLSEKIDSLQARYSEIQDRCCRKAALLDQALSNARLFGEDEVEVLNWLAEVEDKL
SSVFVKDFKQDVLHRQHADHLALNEEIVNRKKNVDQAIKNGQALLKQTTGEEVLLIQEKL
DGIKTRYADITVTSSKALRTLEQARQLATKFQSTYEELTGWLREVEEELATSGGQSPTGE
QIPQFQQRQKELKKEVMEHRLVLDTVNEVSRALLELVPWRAREGLDKLVSDANEQYKLVS
DTIGQRVDEIDAAIQRSQQYEQAADAELAWVAETKRKLMALGPIRLEQDQTTAQLQVQKA
FSIDIIRHKDSMDELFSHRSEIFGTCGEEQKTVLQEKTESLIQQYEAISLLNSERYARLE
RAQVLVNQFWETYEELSPWIEETRALIAQLPSPAIDHEQLRQQQEEMRQLRESIAEHKPH
IDKLLKIGPQLKELNPEEGEMVEEKYQKAENMYAQIKEEVRQRALALDEAVSQSTQITEF
HDKIEPMLETLENLSSRLRMPPLIPAEVDKIRECISDNKSATVELEKLQPSFEALKRRGE
ELIGRSQGADKDLAAKEIQDKLDQMVFFWEDIKARAEEREIKFLDVLELAEKFWYDMAAL
LTTIKDTQDIVHDLESPGIDPSIIKQQVEAAETIKEETDGLHEELEFIRILGADLIFACG
ETEKPEVRKSIDEMNNAWENLNKTWKERLEKLEDAMQAAVQYQDTLQAMFDWLDNTVIKL
CTMPPVGTDLNTVKDQLNEMKEFKVEVYQQQIEMEKLNHQGELMLKKATDETDRDIIREP
LTELKHLWENLGEKIAHRQHKLEGALLALGQFQHALEELMSWLTHTEELLDAQRPISGDP
KVIEVELAKHHVLKNDVLAHQATVETVNKAGNELLESSAGDDASSLRSRLEAMNQCWESV
LQKTEEREQQLQSTLQQAQGFHSEIEDFLLELTRMESQLSASKPTGGLPETAREQLDTHM
ELYSQLKAKEETYNQLLDKGRLMLLSRDDSGSGSKTEQSVALLEQKWHVVSSKMEERKSK
LEEALNLATEFQNSLQEFINWLTLAEQSLNIASPPSLILNTVLSQIEEHKVFANEVNAHR
DQIIELDQTGNQLKFLSQKQDVVLIKNLLVSVQSRWEKVVQRSIERGRSLDDARKRAKQF
HEAWKKLIDWLEDAESHLDSELEISNDPDKIKLQLSKHKEFQKTLGGKQPVYDTTIRTGR
ALKEKTLLPEDSQKLDNFLGEVRDKWDTVCGKSVERQHKLEEALLFSGQFMDALQALVDW
LYKVEPQLAEDQPVHGDLDLVMNLMDAHKVFQKELGKRTGTVQVLKRSGRELIENSRDDT
TWVKGQLQELSTRWDTVCKLSVSKQSRLEQALKQAEVFRDTVHMLLEWLSEAEQTLRFRG
ALPDDTEALQSLIDTHKEFMKKVEEKRVDVNSAVAMGEVILAVCHPDCITTIKHWITIIR
ARFEEVLTWAKQHQQRLETALSELVANAELLEELLAWIQWAETTLIQRDQEPIPQNIDRV
KALIAEHQTFMEEMTRKQPDVDRVTKTYKRKNIEPTHAPFIEKSRSGGRKSLSQPTPPPM
PILSQSEAKNPRINQLSARWQQVWLLALERQRKLNDALDRLEELKEFANFDFDVWRKKYM

RWMNHKKSRVMDFFRRIDKDQDGKITRQEFIDGILASKFPTTKLEMTAVADIFDRDGDGY
IDYYEFVAALHPNKDAYRPTTDADKIEDEVTRQVAQCKCAKRFQVEQIGENKYRFFLGNQ
FGDSQQLRLVRILRSTVMVRVGGGWMALDEFLVKNDPCRARGRTNIELREKFILPEGASQ
GMTPFRSRGRRSKPSSRAASPTRSSSSASQSNHSCTSMPSSPATPASGTKVIPSSGSKLK
RPTPTFHSSRTSLAGDTSNSSSPASTGAKTNRADPKKSASRPGSRAGSRAGSRASSRRGS
DASDFDLLETQSACSDTSESSAAGGQGNSRRGLNKPSKIPTMSKKTTTASPRTPGPKR*
>ENSG00000137834|ENST00000288840
MFRSKRSGLVRRLWRSRVVPDREEGGSGGGGGGDEDGSLGSRAEPAPRAREGGGCGRSEV
RPVAPRRPRDAVGQRGAQGAGRRRRAGGPPRPMSEPGAGAGSSLLDVAEPGGPGWLPESD
CETVTCCLFSERDAAGAPRDASDPLAGAALEPAGGGRSREARSRLLLLEQELKTVTYSLL
KRLKERSLDTLLEAVESRGGVPGGCVLVPRADLRLGGQPAPPQLLLGRLFRWPDLQHAVE
LKPLCGCHSFAAAADGPTVCCNPYHFSRLCGPESPPPPYSRLSPRDEYKPLDLSDSTLSY
TETEATNSLITAPGEFSDASMSPDATKPSHWCSVAYWEHRTRVGRLYAVYDQAVSIFYDL
PQGSGFCLGQLNLEQRSESVRRTRSKIGFGILLSKEPDGVWAYNRGEHPIFVNSPTLDAP
GGRALVVRKVPPGYSIKVFDFERSGLQHAPEPDAADGPYDPNSVRISFAKGWGPCYSRQF
ITSCPCWLEILLNNPR*
>ENSG00000168495|ENST00000306433
MSEGNAAGEPSTPGGPRPLLTGARGLIGRRPAPPLTPGRLPSIRSRDLTLGGVKKKTFTP
NIISRKIKEEPKEEVTVKKEKRERDRDRQREGHGRGRGRPEVIQSHSIFEQGPAEMMKKK
GNWDKTVDVSDMGPSHIINIKKEKRETDEETKQILRMLEKDDFLDDPGLRNDTRNMPVQL
PLAHSGWLFKEENDEPDVKPWLAGPKEEDMEVDIPAVKVKEEPRDEEEEAKMKAPPKAAR
KTPGLPKDVSVAELLRELSLTKEEELLFLQLPDTLPGQPPTQDIKPIKTEVQGEDGQVVL
IKQEKDREAKLAENACTLADLTEGQVGKLLIRKSGRVQLLLGKVTLDVTMGTACSFLQEL
VSVGLGDSRTGEMTVLGHVKHKLVCSPDFESLLDHKHR*
>ENSG00000175550|ENST00000312515
MPSKKKKYNARFPPARIKKIMQTDEEIGKVAAAVPVIISRALELFLESLLKKACQVTQSR
NAKTMTTSHLKQCIELEQQFDFLKDLVASVPDMQGDGEDNHMDGDKGARRGRKPGSGGRK
NGGMGTKSKDKKLSGTDSEQEDESEDTDTDGEEETSQPPPQASHPSAHFQSPPTPFLPFA
STLPLPPAPPGPSAPDEEDEEDYDS*
>ENSG00000165271|ENST00000297990
MGPAPAGEQLRGATGEPEVMEPALEGTGKEGKKASSRKRTLAEPPAKGLLQPVKLSRAEL
YKEPTNEELNRLRETEILFHSSLLRLQVEELLKEVRLSEKKKDRIDAFLREVNQRVVRVP
SVPETELTDQAWLPAGVRVPLHQVPYAVKGCFRFLPPAQVTVVGSYLLGTCIRPDINVDV
ALTMPREILQDKDGLNQRYFRKRALYLAHLAHHLAQDPLFGSVCFSYTNGCHLKPSLLLR
PRGKDERLVTVRLHPCPPPDFFRPCRLLPTKNNVRSAWYRGQSPAGDGSPEPPTPRYNTW
VLQDTVLESHLQLLSTILSSAQGLKDGVALLKVWLRQRELDKGQGGFTGFLVSMLVVFLV
STRKIHTTMSGYQVLRSVLQFLATTDLTVNGISLCLSSDPSLPALADFHQAFSVVFLDSS
GHLNLCADVTASTYHQVQHEARLSMMLLDSRADDGFHLLLMTPKPMIRAFDHVLHLRPLS
RLQAACHRLKLWPELQDNGGDYVSAALGPLTTLLEQGLGARLNLLAHSRPPVPEWDISQD
PPKHKDSGTLTLGLLLRPEGLTSVLELGPEADQPEAAKFRQFWGSRSELRRFQDGAIREA
VVWEAASMSQKRLIPHQVVTHLLALHADIPETCVHYVGGPLDALIQGLKETSSTGEEALV
AAVRCYDDLSRLLWGLEGLPLTVSAVQGAHPVLRYTEVFPPTPVRPAFSFYETLRERSSL
LPRLDKPCPAYVEPMTVVCHLEGSGGQWPQDAEAVQRVRAAFQLRLAELLTQQHGLQCRAT
ATHTDVLKDGFVFRIRVAYQREPQILKEVQSPEGMISLRDTAASLRLERDTRQLPLLTSA

LHGLQQQHPAFSGVARLAKRWVRAQLLGEGFADESLDLVAAALFLHPEPFTPPSSPQVGF
LRFLFLVSTFDWKNNPLFVNLNNELTVEEQVEIRSGFLAARAQLPVMVIVTPQDRKNSVW
TQDGPSAQILQQLVVLAAEALPMLEKQLMDPRGPGDIRTVFRPPLDIYDVLIRLSPRHIP
RHRQAVDSPAASFCRGLLSQPGPSSLMPVLGYDPPQLYLTQLREAFGDLALFFYDQHGGE
VIGVLWKPTSFQPQPFKASSTKGRMVMSRGGELVMVPNVEAILEDFAVLGEGLVQTVEAR
SERWTV*
>ENSG00000007545|ENST00000293925
MTVKLGDGGSGEDGLKKLGKRAADEESLEGEGAGGADAAEESSGTKRDEKTPRAGADGPP
APPGAPQAPSPPQGSPQDQHHFLRSSVRPQSKRPRKDPPSAVGSGNAGGSGPRGKGAEGG
GSSSGNVSGVAPAAPAGGSRSSSRNLGSSGGEKEEGKKVRRQWESWSTEDKNTFFEGLYE
HGKDFEAIQNNIALKYKKKGKPASMVKNKEQVRHFYYRTWHKITKYIDFDHVFSRGLKKS
SQELYGLICYGELRKKIGGCMDDKNATKLNELIQVGATTVRYKGRNLRIKAPMCRALKKL
CDPDGLSDEEDQKPVRLPLKVPIELQPRNNHAWARVQSLAQNPRLRMIVELHRKVSSLIE
FLKQKWALHEVRVRKTLEERQLQDSCSAPMQEKVTLHLFPGENCTLTPLPGVARVVHSKA
FCTVHWQEGGRCKQSAKDAHVLPPAQILGIQSGQGTARGQVKCPRSGAEGKGVGRPPPAA
DALQSSGESSPESAPGEGAALSLSSPDAPDRPPPRHQDTGPCLEKTPAEGRDSPTREPGA
LPCACGQLPDLEDELSLLDPLPRYLKSCQDLIVPEQCRCADTRPGSEQPPLGGAASPEVL
APVSKEAADLAPTGPSPRPGPGLLLDVCTKDLADAPAEELQEKGSPAGPPPSQGQPAARP
PKEVPASRLAQQLREEGWNLQTSESLTLAEVYLMMGKPSKLQLEYDWLGPGRQDPRPGSL
PTALHKQRLLSCLLKLISTEVNPKLALEANTISTASVRPAQEEQSMTPPGKVVTVSSRSP
RCPRNQASLRSSKTFPPSSAPCSSGLRNPPRPLLVPGPSSTGSNDSDGGLFAVPTTLPPN
SRHGKLFSPSKEAELTFRQHLNSISMQSDFFLPKPRKLRNRHLRKPLVVQRTLLPRPSEN
QSHNVCSFSILSNSSVTGRGSFRPIQSSLTKAALSRPIVPKVLPPQATSHLASAIDLAAT
SAGILSGNPLPALDTEGLSGISPLSSDEVTGAISGQDSTGTHQDGDTLPTVGGSDPFVSI
PSRPEQEPVADSFQGSSVLSLSELPKAPLQNGLSIPLSSSESSSTRLSPPDVSALLDISL
PGPPEDALSQGEPATHISDSIIEIAISSGQYGEGVPLSPAKLNGSDSSKSLPSPSSSPQP
HWIASPTHDPQWYPSDSTDSSLSSLFASFISPEKSRKMLPTPIGTNSGTSLLGPSLLDGN
SRDSFVSRSLADVAEVVDSQLVCMMNENSIDYISRFNDLAQELSIAEPGRREALFDGGGG
GPAVSDLSQ*
>ENSG00000186416|ENST00000304449
MEKILQMAEGIDIGEMPSYDLVLSKPSKGQKRHLSTCDGQNPPKKQAGSKFHARPRFEPV
HFVASSSKDERQEDPYGPQTKEVNEQTHFASMPRDIYQDYTQDSFSIQDGNSQYCDSSGF
ILTKDQPVTANMYFDSGNPAPSTTSQQANSQSTPEPSPSQTFPESVVAEKQYFIEKLTAT
IWKNLSNPEMTSGSDKINYTYMLTRCIQACKTNPEYIYAPLKEIPPADIPKNKKLLTDGY
ACEVRCQNIYLTTGYAGSKNGSRDRATELAVKLLQKRIEVRVVRRKFKHTFGEDLVVCQI
GMSSYEFPPALKPPEDLVVLGKDASGQPIFNASAKHWTNFVITENANDAIGILNNSASFN
KMSIEYKYEMMPNRTWRCRVFLQDHCLAEGYGTKKTSKHAAADEALKILQKTQPTYPSVK
SSQCHTGSSPRGSGKKKDIKDLVVYENSSNPVCTLNDTAQFNRMTVEYVYERMTGLRWKC
KVILESEVIAEAVGVKKTVKYEAAGEAVKTLKKTQPTVINNLKKGAVEDVISRNEIQGRS
AEEAYKQQIKEDNIGNQLLRKMGWTGGGLGKSGEGIREPISVKEQHKREGLGLDVERVNK
IAKRDIEQIIRNYARSESHTDLTFSRELTNDERKQIHQIAQKYGLKSKSHGVGHDRYLVV
GRKRRKEDLLDQLKQEGQVGHYELVMPQAN*
>ENSG00000143486|ENST00000271764
MFAKAFRVKSNTAIKGSDRRKLRADVTTAFPTLGTDQVSELVPGKEELNIVKLYAHKGDA

VTVYVSGGNPILFELEKNLYPTVYTLWSYPDLLPTFTTWPLVLEKLVGGADLMLPGLVMP
PAGLPQVQKGDLCAISLVGNRAPVAIGVAAMSTAEMLTSGLKGRGFSVLHTYQDHLWRSG
NKSSPPSIAPLALDSADLSEEKGSVQMDSTLQGDMRHMTLEGEEENGEVHQAREDKSLSE
APEDTSTRGLNQDSTDSKTLQEQMDELLQQCFLHALKCRVKKADLPLLTSTFLGSHMFSC
CPEGRQLDIKKSSYKKLSKFLQQMQQEQIIQVKELSKGVESIVAVDWKHPRITSFVIPEP
SPTSQTIQEGSREQPYHPPDIKPLYCVPASMTLLFQESGHKKGSFLEGSEVRTIVINYAK
KNDLVDADNKNLVRLDPILCDCILEKNEQHTVMKLPWDSLLTRCLEKLQPAYQVTLPGQE
PIVKKGRICPIDITLAQRASNKKVTVVRNLEAYGLDPYSVAAILQQRCQASTTVNPAPGA
KDSLQVQIQGNQVHHLGWLLLEEYQLPRKHIQGLEKALKPGKKK*
>ENSG00000100354|ENST00000301923
MQTNEGEVSEESSSKVEQEDFVMEGHGKTPPPGEESKQEKEQEREEQLMEDKKRKKEDKK
KKEATQKVTEQKTKVPEVTKPSLSQPTAASPIGSSPSPPVNGGNNAKRVAVPNGQPPSAA
RYMPREVPPRFRCQQDHKVLLKRGQPPPPSCMLLGGGAGPPPCTAPGANPNNAQVTGALL
QSESGTAPVWSKSTPPAPDNGTSAWGEPNESSPGWGEMDDTGASTTGWGNTPANAPNAMK
PNSKSMQDGWGESDGPVTGARHPSWEEEEDGGVWNTTGSQGSASSHNSASWGQGGKKQMK
CSLKGGNNDSWMNPLAKQFSNMGLLSQTEDNPSSKMDLSVGSLSDKKFDVDKRAMNLGDF
NDIMRKDRSGFRPPNSKDMGTTDSGPYFEKGGSHGLFGNSTAQSRGLHTPVQPLNSSPSL
RAQVPPQFISPQVSASMLKQFPNSGLSPGLFNVGPQLSPQQIAMLSQLPQIPQFQLACQL
LLQQQQQQLLQNQRKISQAVRQQQEQQLARMVSALQQQQQQQRQPGMKHSPSHPVGPK
PHLDNMVPNALNVGLPDLQTKGPIPGYGSGFSSGGMDYGMVGGKEAGTESRFKQWTSMME
GLPSVATQEANMHKNGAIVAPGKTRGGSPYNQFDIIPGDTLGGHTGPAGDSWLPAKSPPT
NKIGSKSSNASWPPEFQPGVPWKGIQNIDPESDPYVTPGSVLGGTATSPIVDTDHQLLRD
NTTGSNSSLNTSLPSPGAWPYSASDNSFTNVHSTSAKFPDYKSTWSPDPIGHNPTHLSNK
MWKNHISSRNTTPLPRPPPGLTNPKPSSPWSSTAPRSVRGWGTQDSRLASASTWSDGGSV
RPSYWLVLHNLTPQIDGSTLRTICMQHGPLLTFHLNLTQGTALIRYSTKQEAAKAQTALH
MCVLGNTTILAEFATDDEVSRFLAQAQPPTPAATPSAPAAGWQSLETGQNQSDPVGPALN
LFGGSTGLGQWSSSAGGSSGADLAGASLWGPPNYSSSLWGVPTVEDPHRMGSPAPLLPGD
LLGGGSDSI*
>ENSG00000161904|ENST00000293760
MAGLSDLELRRELQALGFQPGPITDTTRDVYRNKLRRLRGEARLRDEERLREEARPRGEE
RLREEARLREDAPLRARPAAASPRAEPWLSQPASGSAYATPGAYGDIRPSAASWVGSRGL
AYPARPAQLRRRASVRGSSEEDEDARTPDRATQGPGLAARRWWAASPAPARLPSSLLGPD
PRPGLRATRAGPAGAARARPEVGRRLERWLSRLLLWASLGLLLVFLGILWVKMGKPSAPQ
EAEDNMKLLPVDCERKTDEFCQAKQKAALLELLHELYNFLAIQAGNFECGNPENLKSKCI
PVMEAQEYIANVTSSSSAKFEAALTWILSSNKDVGIWLKGEDQSELVTTVDKVVCLESAH
PRMGVGCRLSRALLTAVTNVLIFFWCLAFLWGLLILLKYRWRKLEEEEQAMYEMVKKIID
VVQDHYVDWEQDMERYPYVGILHVRDSLIPPQSRRRMKRVWDRAVEFLASNESRIQTESH
RVAGEDMLVWRWTKPSSFSDSER*
>ENSG00000147421|ENST00000287701
MLSSFPVVLLETMSHYTDEPRFTIEQIDLLQRLRRTGMTKHEILHALETLDRLDQEHSDK
FGRRSSYGGSSYGNSTNNVPASSSTATASTQTQHSGMSPSPSNSYDTSPQPCTTNQNGRE
NNERLSTSNGKMSPTRYHANSMGQRSYSFEASEEDLDVDDKVEELMRRDSSVIKEEIKAF
LANRRISQAVVAQVTGISQSRISHWLLQQGSDLSEQKKRAFYRWYQLEKTNPGATLSMRP
APIPIEDPEWRQTPPPVSATSGTFRLRRGSRFTWRKECLAVMESYFNENQYPDEAKREEI

ANACNAVIQKPGKKLSDLERVTSLKVYNWFANRRKEIKRRANIEAAILESHGIDVQSPGG
HSNSDDVDGNDYSEQDDSTSHSDHQDPISLAVEMAAVNHTILALARQGANEIKTEALDDD
*
>ENSG00000156735|ENST00000287322
MSALRRSGYGPSDGPSYGRYYGPGGGDVPVHPPPPLYPLRPEPPQPPISWRVRGGGPAET
TWLGEGGGGDGYYPSGGAWPEPGRAGGSHQEQPPYPSYNSNYWNSTARSRAPYPSTYPVR
PELQGQSLNSYTNGAYGPTYPPGPGANTASYSGAYYAPGYTQTSYSTEVPSTYRSSGNSP
TPVSRWIYPQQDCQTEAPPLRGQVPGYPPSQNPGMTLPHYPYGDGNRSVPQSGPTVRPQE
DAWASPGAYGMGGRYPWPSSAPSAPPGNLYMTESTSPWPSSGSPQSPPSPPVQQPKDSSY
PYSQSDQSMNRHNFPCSVHQYESSGTVNNDDSDLLDSQVQYSAEPQLYGNATSDHPNNQD
QSSSLPEECVPSDESTPPSIKKIIHVLEKVQYLEQEVEEFVGKKTDKAYWLLEEMLTKEL
LELDSVETGGQDSVRQARKEAVCKIQAILEKLEKKGL*
>ENSG00000171970|ENST00000306908
MDSVVFEDVAVDFTLEEWALLDSAQRDLYRDVMLETFRNLASVDDGTQFKANGSVSLQDM
YGQEKSKEQTIPNFTGNNSCAYTLEKNCEGYGTEDHHKNLRNHMVDRFCTHNEGNQYGEA
IHQMPDLTLHKKVSAGEKPYECTKCRTVFTHLSSLKRHVKSHCGRKAPPGEECKQACICP
SHLHSHGRTDTEEKPYKCQACGQTFQHPRYLSHHVKTHTAEKTYKCEQCRMAFNGFASFT
RHVRTHTKDRPYKCQECGRAFIYPSTFQRHMTTHTGEKPYKCQHCGKAFTYPQAFQRHEK
THTGEKPYECKQCGKTFSWSETLRVHMRIHTGDKLYKCEHCGKAFTSSRSFQGHLRTHTG
EKPYECKQCGKAFTWSSTFREHVRIHTQEQLYKCEQCGKAFTSSRSFRGHLRTHTGEKPY
ECKQCGKTFTWSSTFREHVRIHTQEQLHKCEHCGKAFTSSRAFQGHLRMHTGEKPYECKQ
CGKTFTWSSTLHNHVRMHTGEKPHKCKQCGMSFKWHSSFRNHLRMHTGQKSHECQSYSKA
FSCQVILSKTSESTH*
>ENSG00000164134|ENST00000296543
MPAVSLPPKENALFKRILRCYEHKQYRNGLKFCKQILSNPKFAEHGETLAMKGLTLNCLG
KKEEAYELVRRGLRNDLKSHVCWHVYGLLQRSDKKYDEAIKCYRNALKWDKDNLQILRDL
SLLQIQMRDLEGYRETRYQLLQLRPAQRASWIGYAIAYHLLEDYEMAAKILEEFRKTQQT
SPDKVDYEYSELLLYQNQVLREAGLYREALEHLCTYEKQICDKLAVEETKGELLLQLCRL
EDAADVYRGLQERNPENWAYYKGLEKALKPANMLERLKIYEEAWTKYPRGLVPRRLPLNF
LSGEKFKECLDKFLRMNFSKGCPPVFNTLRSLYKDKEKVAIIEELVVGYETSLKSCRLFN
PNDDGKEEPPTTLLWVQYYLAQHYDKIGQPSIALEYINTAIESTPTLIELFLVKAKIYKH
AGNIKEAARWMDEAQALDTADRFINSKCAKYMLKANLIKEAEEMCSKFTREGTSAVENLN
EMQCMWFQTECAQAYKAMNKFGEALKKCHEIERHFIEITDDQFDFHTYCMRKITLRSYVD
LLKLEDVLRQHPFYFKAARIAIEIYLKLHDNPLTDENKEHEADTANMSDKELKKLRNKQR
RAQKKAQIEEEKKNAEKEKQQRNQKKKKDDDDEEIGGPKEELIPEKLAKVETPLEEAIKF
LTPLKNLVKNKIETHLFAFEIYFRKEKFLLMLQSVKRAFAIDSSHPWLHECMIRLFNTAV
CESKDLSDTVRTVLKQEMNRLFGATNPKNFNETFLKRNSDSLPHRLSAAKMVYYLDPSSQ
KRAIELATTLDESLTNRNLQTCMEVLEALYDGSLGDCKEAAEIYRANCHKLFPYALAFMP
PGYEEDMKITVNGDSSAEAEELANEI*
>ENSG00000167085|ENST00000300408
MAAKVFESIGKFGLALAVAGGVVNSALYNVDAGHRAVIFDRFRGVQDIVVGEGTHFLIPW
VQKPIIFDCRSRPRNVPVITGSKDLQNVNITLRILFRPVASQLPRIFTSIGEDYDERVLP
SITTEILKSVVARFDAGELITQRELVSRQVSDDLTERAATFGLILDDVSLTHLTFGKEFT
EAVEAKQVAQQEAERARFVVEKAEQQKKAAIISAEGDSKAAELIANSLATAGDGLIELRK

LEAAEDIAYQLSRSRNITYLPAGQSVLLQLPQ*
>ENSG00000149922|ENST00000279386
MYHPRELYPSLGAGYRLGPAQPGADSSFPPALAEGYRYPELDTPKLDCFLSGMEAAPRTL
AAHPPLPLLPPAMGTEPAPSAPEALHSLPGVSLSLENRELWKEFSSVGTEMIITKAGRRM
FPACRVSVTGLDPEARYLFLLDVIPVDGARYRWQGRRWEPSGKAEPRLPDRVYIHPDSPA
TGAHWMRQPVSFHRVKLTNSTLDPHGHLILHSMHKYQPRIHLVRAAQLCSQHWGGMASFR
FPETTFISVTAYQNPQITQLKIAANPFAKGFRENGRNCKRERDARVKRKLRGPEPAATEA
YGSGDTPGGPCDSTLGGDIRESDPEQAPAPGEATAAPAPLCGGPSAEAYLLHPAAFHGAP
SHLPTRSPSFPEAPDSGRSAPYSAAFLELPHGSGGSGYPAAPPAVPFAPHFLQGGPFPLP
YTAPGGYLDVGSKPMY*
>ENSG00000168505|ENST00000306318
MSAAFPPSLMMMQRPLGSSTAFSIDSLIGSPPQPSPGHFVYTGYPMFMPYRPVVLPPPPP
PPPALPQAALQPALPPAHPHHQIPSLPTGFCSSLAQGMALTSTLMATLPGGFSASPQHQE
AAAARKFAPQPLPGGGNFDKAEALQADAEDGKGFLAKEGSLLAFSAAETVQASLVGAVRG
QGKDESKVEDDPKGKEESFSLESDVDYSSDDNLTGQAAHKEEDPGHALEETPPSSGAAGS
TTSTGKNRRRRTAFTSEQLLELEKEFHCKKYLSLTERSQIAHALKLSEVQVKIWFQNRRA
KWKRVKAGNANSKTGEPSRNPKIVVPIPVHVSRFAIRSQHQQLEQARP*
>ENSG00000110315|ENST00000265981
MGQQISDQTQLVINKLPEKVAKHVTLVRESGSLTYEEFLGRVAELNDVTAKVASGQEKHL
LFEVQPGSDSSAFWKVVVRVVCTKINKSSGIVEASRIMNLYQFIQLYKDITSQAAGVLAQ
SSTSEEPDENSSSVTSCQASLWMGRVKQLTDEEECCICMDGRADLILPCAHSFCQKCIDK
WSDRHRNCPICRLQMTGANESWVVSDAPTEDDMANYILNMADEAGQPHRP*
>ENSG00000152977|ENST00000282928
MLLDAGPQYPAIGVTTFGASRHHSAGDVAERDVGLGINPFADGMGAFKLNPSSHELASAG
QTAFTSQAPGYAAAAALGHHHHPGHVGSYSSAAFNSTRDFLFRNRGFGDAAAAASAQHSL
FAASAGGFGGPHGHTDAAGHLLFPGLHEQAAGHASPNVVNGQMRLGFSGDMYPRPEQYGQ
VTSPRSEHYAAPQLHGYGPMNVNMAAHHGAGAFFRYMRQPIKQELICKWIEPEQLANPKK
SCNKTFSTMHELVTHVTVEHVGGPEQSNHICFWEECPREGKPFKAKYKLVNHIRVHTGEK
PFPCPFPGCGKVFARSENLKIHKRTHTGEKPFKCEFEGCDRRFANSSDRKKHMHVHTSDK
PYLCKMCDKSYTHPSSLRKHMKVHESSSQGSQPSPAASSGYESSTPPTIVSPSTDNPTTS
SLSPSSSAVHHTAGHSALSSNFNEWYV*
>ENSG00000164296|ENST00000296736
MANKGNKKRRQFSLEEKMKVVGAVDSGKRKGDVAKEFGITPSTLSTFLKDRTKFEEKVRE
ASVGPQRKRMRSALYDDIDKAVFAWFQEIHAKNILVTGSVIRKKALNLANMLGYDNFQAS
VGWLNRFRDRHGIALKAVCREDSDRLMNGLGIDKINEWHAGEIIKLIADYSPDDIFNADE
TGVFFQLLPQHTLAAKGDHCRGGKKAKQRLTALFCCNASGTEKMRPLIVGRSASPHCLKN
IHSLPCDYRANQWAWMTRDLFNEWLMQVDARMKRAERRILLLIDNCSAHNMLPHLERIQV
GYLPSNCTAVLQPLNLGIIHTMKVLYQSHLLKQILLKLNSSEDQEEVDIKQAIDMIAAAW
WSVKPSTVVKCWQKAGIVPMEFAECDTESAASEPDIAIEKLWHTVAIATCVPNEVNFQDF
VTADDDLIISQDTDIIQDMVAGENTSEAGSEDEGEVSLPEQPKVTITEAISSVQKLRQFL
STCVDIPDAIFGQLNGIDEYLMKRVTQTLIDSKITDFLQTK*
>ENSG00000168872|ENST00000302243
MATDSWALAVDEQEAAVKSMTNLQIKEEKVKADTNGIIKTSTTAEKTDEEEKEDRAAQSL
LNKLIRSNLVDNTNQVEVLQRDPNSPLYSVKSFEELRLKPQLLQGVYAMGFNRPSKIQEN

ALPMMLAEPPQNLIAQSQSGTGKTAAFVLAMLSRVEPSDRYPQCLCLSPTYELALQTGKV
IEQMGKFYPELKLAYAVRGNKLERGQKISEQIVIGTPGTVLDWCSKLKFIDPKKIKVFVL
DEADVMIATQGHQDQSIRIQRMLPRNCQMLLFSATFEDSVWKFAQKVVPDPNVIKLKREE
ETLDTIKQYYVLCSSRDEKFQALCNLYGAITIAQAMIFCHTRKTASWLAAELSKEGHQVA
LLSGEMMVEQRAAVIERFREGKEKVLVTTNVCARGIDVEQVSVVINFDLPVDKDGNPDNE
TYLHRIGRTGRFGKRGLAVNMVDSKHSMNILNRIQEHFNKKIERLDTDDLDEIEKIAN*
>ENSG00000169856|ENST00000305901
MNAQLTMEAIGELHGVSHEPVPAPADLLGGSPHARSSVAHRGSHLPPAHPRSMGMASLLD
GGSGGGDYHHHHRAPEHSLAGPLHPTMTMACETPPGMSMPTTYTTLTPLQPLPPISTVSD
KFPHHHHHHHHHHHPHHHQRLAGNVSGSFTLMRDERGLASMNNLYTPYHKDVAGMGQSLS
PLSSSGLGSIHNSQQGLPHYAHPGAAMPTDKMLTPNGFEAHHPAMLGRHGEQHLTPTSAG
MVPINGLPPHHPHAHLNAQGHGQLLGTAREPNPSVTGAQVSNGSNSGQMEEINTKEVAQR
ITTELKRYSIPQAIFAQRVLCRSQGTLSDLLRNPKPWSKLKSGRETFRRMWKWLQEPEFQ
RMSALRLAACKRKEQEHGKDRGNTPKKPRLVFTDVQRRTLHAIFKENKRPSKELQITISQ
QLGLELSTVSNFFMNARRRSLDKWQDEGSSNSGNSSSSSSTCTKA*
>ENSG00000147677|ENST00000276682
MYSRHAQRIPPGKKMASRKEGTGSTATSSSSTAGAAGKGKGKGGSGDSAVKQVQIDGLVV
LKIIKHYQEEGQGTEVVQGVLLGLVVEDRLEITNCFPFPQHTEDDADFDEVQYQMEMMRS
LRHVNIDHLHVGWYQSTYYGSFVTRALLDSQFSYQHAIEESVVLIYDPIKTAQGSLSLKA
YRLTPKLMEVCKEKDFSPEALKKANITFEYMFEEVPIVIKNSHLINVLMWELEKKSAVAD
KHELLSLASSNHLGKNLQLLMDRVDEMSQDIVKYNTYMRNTSKQQQQKHQYQQRRQQENM
QRQSRGEPPLPEEDLSKLFKPPQPPARMDSLLIAGQINTYCQNIKEFTAQNLGKLFMAQA
LQEYNN*
>ENSG00000167721|ENST00000301364
MAAHRPGPLKQQNKAHKGGRHRGRGSAQRDGKGRLALKTLSKKVRKELSRVDQRHRASQL
RKQKKEAVLAEKRQLGGKDGPPHQVLVVPLHSRISLPEAMQLLQDRDTGTVHLNELGNTQ
NFMLLCPRLKHRWFFTSARPGDLHVVLDMAKVADTILFLLDPLEGWDSTGDYCLSCLFAQ
GLPTYTLAVQGISGLPLKKQIDTRKKLSKAVEKRFPHDKLLLLDTQQEAGMLLRQLANQK
QQHLAFRDRRAYLFAHAVDFVPSEENNLVGTLKISGYVRGQTLNVNRLLHIVGYGDFQMK
QIDAPGDPFPLNPRGIKPQKDPDMAMEICATDAVDDMEEGLKVLMKADPGRQESLQAEVI
PDPMEGEQTWPTEEELSEAKDFLKESSKVVKKVPKGTSSYQAEWILDGGSQSGGEGDEYE
YDDMEHEDFMEEESQDESSEEEEEYETMTIGESVHDDLYDKKVDEEAEAKMLEKYKQERL
EEMFPDEVDTPRDVAARIRFQKYRGLKSFRTSPWDPKENLPQDYARIFQFQNFTNTRKSI
FKEVEEKEVEGAEVGWYVTLHVSEVPVSVVECFRQGTPLIAFSLLPHEQKMSVLNMVVRR
DPGNTEPVKAKEELIFHCGFRRFRASPLFSQHTAADKHKLQRFLTADMALVATVYAPITF
PPASVLLFKQKSNGMHSLIATGHLMSVDPDRMVIKRVVLSGHPFKIFTKMAVVRYMFFNR
EDVLWFKPVELRTKWGRRGHIKEPLGTHGHMKCSFDGKLKSQDTVLMNLYKRVFPKWTYD
PYVPEPVPWLKSEISSTVPQGGME*
>ENSG00000214944|ENST00000296794
MELSCSEAPLYGQMMIYAKFDKNVYLPEDAEFYFTYDGSHQRHVMIAERIEDNVLQSSVP
GHGLQETVTVSVCLCSEGYSPVTMGSGSVTYYVDNMACRLARLLVTQANRLTACSHQTLLT
PFALTAGALPALDEELVLALTHLELPLEWTVLGSSSLEVSSHRESLLHLAMRWGLAKLSQ
FFLCLPGGVQALALPNEEGATPLDLALREGHSKLVEDVTNFQGRWSPSFSRVQLSEEASL
HYIHSSETLTLTLNHTAEHLLEADIKLFRKYFWDRAFLVKAFEPEARPEERTAMPSSGAE

TEEEIKNSVSSRSAAEKEDIKRVKSLVVQHNEHEDQHSLDLDRSFDILKKSKPPSTLLAA
GRLSDMLNGGDEVYANCMVIDQVGDLDISYINIEGITATTSPESRGCTLWPQSSKHTLPT
ETSPSVYPLSENVEGTAHTEAQQSFMSPSSSCASNLNLSFGWHGFEKEQSHLKKRSSSLD
ALDADSEGEGHSEPSHICYTPGSQSSSRTGIPSGDELDSFETNTEPDFNISRAESLPLSS
NLQSKESLLSGVRSRSYSCSSPKISLGKTRLVRELTVCSSSEEQRAYSLSEPPRENRIQE
EEWDKYIIPAKSESEKYKVSRTFSFLMNRMTSPRNKSKTKSKDAKDKEKLNRHQFAPGTF
SGVLQCLVCDKTLLGKESLQCSNCNANVHKGCKDAAPACTKKFQEKYNKNKPQTILGNSS
FRDIPQPGLSLHPSSSVPVGLPTGRRETVGQVHPLSRSVPGTTLESFRRSATSLESESDH
NSCRSRSHSDELLQSMGSSPSTESFIMEDVVDSSLWSDLSSDAQEFEAESWSLVVDPSFC
NRQEKDVIKRQDVIFELMQTEMHHIQTLFIMSEIFRKGMKEELQLDHSTVDKIFPCLDEL
LEIHRHFFYSMKERRQESCAGSDRNFVIDRIGDILVQQFSEENASKMKKIYGEFCCHHKE
AVNLFKELQQNKKFQNFIKLRNSNLLARRRGIPECILLVTQRITKYPVLVERILQYTKER
TEEHKDLRKALCLIKDMIATVDLKVNEYEKNQKWLEILNKIENKTYTKLKNGHVFRKQAL
MSEERTLLYDGLVYWKTATGRFKDILALLLTDVLLFLQEKDQKYIFAAVDQKPSVISLQK
LIAREVANEERGMFLISASSAGPEMYEIHTNSKEERNNWMRRIQQAVESCPEEKGGRTSE
SDEDKRKAEARVAKIQQCQEILTNQDQQICAYLEEKLHIYAELGELSGFEDVHLEPHLLI
KPDPGEPPQAASLLAAALKEAESLQVAVKASQMGAVSQSCEDSCGDSVLADTLSSHDVPG
SPTASLVTGGREGRGCSDVDPGIQGVVTDLAVSDAGEKVECRNFPGSSQSEIIQAIQNLT
RLLYSLQAALTIQDSHIEIHRLVLQQQEGLSLGHSILRGGPLQDQKSRDADRQHEELANV
HQLQHQLQQEQRRWLRRCEQQQRAQATRESWLQERERECQSQEELLLRSRGELDLQLQEY
QHSLERLREGQRLVEREQARMRAQQSLLGHWKHGRQRSLPAVLLPGGPEVMELNRSESLC
HENSFFINEALVQMSFNTFNKLNPSVIHQDATYPTTQSHSDLVRTSEHQVDLKVDPSQPS
NVSHKLWTAAGSGHQILPFHESSKDSCKNGN*
>ENSG00000172468|ENST00000307393
MAHVSSETQDVSPKDELTASEASTRSPLCEHTFPGDSDLRSMIEEHAFQVLSQGSLLESP
SYTVCVSEPDKDDDFLSLNFPRKLWKIVESDQFKSISWDENGTCIVINEELFKKEILETK
APYRIFQTDAIKSFVRQLNLYGFSKIQQNFQRSAFLATFLSEEKESSVLSKLKFYYNPNF
KRGYPQLLVRVKRRIGVKNASPISTLFNEDFNKKHFRAGANMENHNSALAAEASEESLFS
ASKNLNMPLTRESSVRQIIANSSVPIRSGFPPPSPSTSVGPSEQIATDQHAILNQLTTIH
MHSHSTYMQARGHIVNFITTTTSQYHIISPLQNGYFGLTVEPSAVPTRYPLVSVNEAPYR
NMLPAGNPWLQMPTIADRSAAPHSRLALQPSPLDKYHPNYN*
>ENSG00000180806|ENST00000303450
MSATGPISNYYVDSLISHDNEDLLASRFPATGAHPAAARPSGLVPDCSDFPSCSFAPKPA
VFSTSWAPVPSQSSVVYHPYGPQPHLGADTRYMRTWLEPLSGAVSFPSFPAGGRHYALKP
DAYPGRRADCGPGEGRSYPDYMYGSPGELRDRAPQTLPSPEADALAGSKHKEEKADLDPS
NPVANWIHARSTRKKRCPYTKYQTLELEKEFLFNMYLTRDRRYEVARVLNLTERQVKIWF
QNRRMKMKKMNKEKTDKEQS*
>ENSG00000153814|ENST00000283928
MTGIAAASFFSNTCRFGGCGLHFPTLADLIEHIEDNHIDTDPRVLEKQELQQPTYVALSY
INRFMTDAARREQESLKKKIQPKLSLTLSSSVSRGNVSTPPRHSSGSLTPPVTPPITPSS
SFRSSTPTGSEYDEEEVDYEESDSDESWTTESAISSEAILSSMCMNGGEEKPFACPVPGC
KKRYKNVNGIKYHAKNGHRTQIRVRKPFKCRCGKSYKTAQGLRHHTINFHPPVSAEIIRK
MQQ*
>ENSG00000170581|ENST00000314128

MAQWEMLQNLDSPFQDQLHQLYSHSLLPVDIRQYLAVWIEDQNWQEAALGSDDSKATMLF
FHFLDQLNYECGRCSQDPESLLLQHNLRKFCRDIQPFSQDPTQLAEMIFNLLLEEKRILI
QAQRAQLEQGEPVLETPVESQQHEIESRILDLRAMMEKLVKSISQLKDQQDVFCFRYKIQ
AKGKTPSLDPHQTKEQKILQETLNELDKRRKEVLDASKALLGRLTTLIELLLPKLEEWKA
QQQKACIRAPIDHGLEQLETWFTAGAKLLFHLRQLLKELKGLSCLVSYQDDPLTKGVDLR
NAQVTELLQRLLHRAFVVETQPCMPQTPHRPLILKTGSKFTVRTRLLVRLQEGNESLTVE
VSIDRNPPQLQGFRKFNILTSNQKTLTPEKGQSQGLIWDFGYLTLVEQRSGGSGKGSNKG
PLGVTEELHIISFTVKYTYQGLKQELKTDTLPVVIISNMNQLSIAWASVLWFNLLSPNLQ
NQQFFSNPPKAPWSLLGPALSWQFSSYVGRGLNSDQLSMLRNKLFGQNCRTEDPLLSWAD
FTKRESPPGKLPFWTWLDKILELVHDHLKDLWNDGRIMGFVSRSQERRLLKKTMSGTFLL
RFSESSEGGITCSWVEHQDDDKVLIYSVQPYTKEVLQSLPLTEIIRHYQLLTEENIPENP
LRFLYPRIPRDEAFGCYYQEKVNLQERRKYLKHRLIVVSNRQVDELQQPLELKPEPELES
LELELGLVPEPELSLDLEPLLKAGLDLGPELESVLESTLEPVIEPTLCMVSQTVPEPDQG
PVSQPVPEPDLPCDLRHLNTEPMEIFRNCVKIEEIMPNGDPLLAGQNTVDEVYVSRPSHF
YTDGPLMPSDF*
>ENSG00000070950|ENST00000264926
MDSLAESRWPPGLAVMKTIDDLLRCGICFEYFNIAMIIPQCSHNYCSLCIRKFLSYKTQC
PTCCVTVTEPDLKNNRILDELVKSLNFARNHLLQFALESPAKSPASSSSKNLAVKVYTPV
ASRQSLKQGSRLMDNFLIREMSGTSELLIKENKSKFSPQKEASPAAKTKETRSVEEIAP
DPSEAKRPEPPSTSTLKQVTKVDCPVCGVNIPESHINKHLDSCLSREEKKESLRSSVHKR
KPLPKTVYNLLSDRDLKKKLKEHGLSIQGNKQQLIKRHQEFVHMYNAQCDALHPKSAAEI
VREIENIEKTRMRLEASKLNESVMVFTKDQTEKEIDEIHSKYRKKHKSEFQLLVDQARKG
YKKIAGMSQKTVTITKEDESTEKLSSVCMGQEDNMTSVTNHFSQSKLDSPEELEPDREED
SSSCIDIQEVLSSSESDSCNSSSSDIIRDLLEEEEAWEASHKNDLQDTEISPRQNRRTRA
AESAEIEPRNKRNRN*
>ENSG00000164438|ENST00000296921
MEAPASAQTPHPHEPISFGIDQILNSPDQDSAPAPRGPDGASYLGGPPGGRPGATYPSLP
ASFAGLGAPFEDAGSYSVNLSLAPAGVIRVPAHRPLPGAVPPPLPSALPAMPSVPTVSSL
GGLNFPWMESSRRFVKDRFTAAAALTPFTVTRRIGHPYQNRTPPKRKKPRTSFSRVQICE
LEKRFHRQKYLASAERAALAKSLKMTDAQVKTWFQNRRTKWRRQTAEEREAERQQASRLM
LQLQHDAFQKSLNDSIQPDPLCLHNSSLFALQNLQPWEEDSSKVPAVTSLV*
>ENSG00000247596|ENST00000305533
MAHQTGIHATEELKEFFAKARAGSVRLIKVVIEDEQLVLGASQEPVGRWDQDYDRAVLPL
LDAQQPCYLLYRLDSQNAQGFEWLFLAWSPDNSPVRLKMLYAATRATVKKEFGGGHIKDE
LFGTVKDDLSFAGYQKHLSSCAAPAPLTSAERELQQIRINEVKTEISVESKHQTLQGLAF
PLQPEAQRALQQLKQKMVNYIQMKLDLERETIELVHTEPTDVAQLPSRVPRDAARYHFFL
YKHTHEGDPLESVVFIYSMPGYKCSIKERMLYSSCKSRLLDSVEQDFHLEIAKKIEIGDG
AELTAEFLYDEVHPKQHAFKQAFAKPKGPGGKRGHKRLIRGPGENGDDS*
>ENSG00000165495|ENST00000298282
MMQHASPAPALTMMATQNVPPPPYQDSPQMTATAQPPSKAQAVHISAPSAAASTPVPSAP
IDPQAQLEADKRAVYRHPLFPLLTLLFEKCEQATQGSECITSASFDVDIENFVHQQEQEH
KPFFSDDPELDNLMVKAIQVLRIHLLELEKVNELCKDFCNRYITCLKTKMHSDNLLRNDL
GGPYSPNQPSINLHSQDLLQNSPNSMSGVSNNPQGIVVPASALQQGNIAMTTVNSQVVSG
GALYQPVTMVTSQGQVVTQAIPQGAIQIQNTQVNLDLTSLLDNEDKKSKNKRGVLPKHAT

NIMRSWLFQHLMHPYPTEDEKRQIAAQTNLTLLQVNNWFINARRRILQPMLDASNPDPAP
KAKKIKSQHRPTQRFWPNSIAAGVLQQQGGAPGTNPDGSINLDNLQSLSSDSATMAMQQA
MMAAHDDSLDGTEEEDEDEMEEEEEEELEEEVDELQTTNVSDLGLEHSDSLE*
>ENSG00000166188|ENST00000299237
MSESWQQPPQTQPQQPQPPQPQHHAEPPPALAEHTLPPGTAENPLGCAVYGILLQPDPGL
QPPQHAPLQAAGEPGPKCGVCGHDLAHLSSPHEHQCLAGHDRSFQCTQCLKIFHQATDLL
EHQCVQAEQKPFVCGVCKMGFSLLTSLAQHHSSHSGLVKCSICEKTYKPAEAAEPATTAA
PSLPAAPAPSTVTPAEQADKPYSCPICQKPFKHLSELSRHERIHTGEKPYKCTLCDKSFS
QSSHLVHHKRTHSSERPYKCAVCEKTFKHRSHLVRHMYAHSGEHHLFRCNVCELHFKESS
ELLQHPCTPSGERPFRCGECQKAFKRPSDLRQHERTHSAERPFKCDLCPMGFKQQYALMR
HRRTHKTEEPFKCGLCEKGFGQPSHLLYHQHVHTLETLFKCPVCQKGFDQSAELLRHKCL
PGAAERPFKCPVCNKAYKRASALQKHQLAHCAAAEKPLRCTLCERRFFSSSEFVQHRCDP
AREKPLKCPDCEKRFKYASDLQRHRRVHTGEKPYKCPNCDKAFKQREHLNKHQGVHAREQ
QFKCVWCGERFLDVALLQEHSAQHSAAAAAAEGAYQVAACLP*
>ENSG00000166716|ENST00000299927
MGDMKTPDFDDLLAAFDIPDPTSLDAKEAIQTPSEENESPLKPPGICMDESVSLSHSGSA
PDVPAVSVIVKNTSRQESFEAEKDHITPSLLHNGFRGSDLPPDPHNCGKFDSTFMNGDSA
RSFPGKLEPPKSEPLPTFNQFSPISSPEPEDPIKDNGFGIKPKHSDSYFPPPLGCGAVGG
PVLEALAKFPVPELHMFDHFCKKEPKPEPLPLGSQQEHEQSGQNTVEPHKDPDATRFFGE
ALEFNSHPSNSIGESKGLARELGTCSSVPPRQRLKPAHSKLSSCVAALVALQAKRVASVT
KEDQPGHTKDLSGPTKESSKGSPKMPKSPKSPRSPLEATRKSIKPSDSPRSICSDSSSKG
SPSVAASSPPAIPKVRIKTIKTSSGEIKRTVTRILPDPDDPSKSPVGSPLGSAIAEAPSE
MPGDEVPVEEHFPEAGTNSGSPQGARKGDESMTKASDSSSPSCSSGPRVPKGAAPGSQTG
KKQQSTALQASTLAPANLLPKAVHLANLNLVPHSVAASVTAKSSVQRRSQPQLTQMSVPL
VHQVKKAAPLIVEVFNKVLHSSNPVPLYAPNLSPPADSRIHVPASGYCCLECGDAFALEK
SLSQHYGRRSVHIEVLCTLCSKTLLFFNKCSLLRHARDHKSKGLVMQCSQLLVKPISADQ
MFVSAPVNSTAPAAPAPSSSPKHGLTSGSASPPPPALPLYPDPVRLIRYSIKCLECHKQM
RDYMVLAAHFQRTTEETEGLTCQVCQMLLPNQCSFCAHQRIHAHKSPYCCPECGVLCRSA
YFQTHVKENCLHYARKVGYRCIHCGVVHLTLALLKSHIQERHCQVFHKCAFCPMAFKTAS
STADHSATQHPTQPHRPSQLIYKCSCEMVFNKKRHIQQHFYQNVSKTQVGVFKCPECPLL
FVQKPELMQHVKSTHGVPRNVDELSSLQSSADTSSSRPGSRVPTEPPATSVAARSSSLPS
GRWGRPEAHRRVEARPRLRNTGWTCQECQEWVPDRESYVSHMKKSHGRTLKRYPCRQCEQ
SFHTPNSLRKHIRNNHDTVKKFYTCGYCTEDSPSFPRPSLLESHISLMHGIRNPDLSQTS
KVKPPGGHSPQVNHLKRPVSGVGDAPGTSNGATVSSTKRHKSLFQCAKCSFATDSGLEFQ
SHIPQHQVDSSTAQCLLCGLCYTSASSLSRHLFIVHKVRDQEEEEEEEAAAAEMAVEVAE
PEEGSGEEVPMETRENGLEECAGEPLSADPEARRLLGPAPEDDGGHNDHSQPQASQDQDS
HTLSPQV*
>ENSG00000168875|ENST00000306087
MSKPSDHIKRPMNAFMVWSRGQRRKMAQENPKMHNSEISKRLGAEWKLLSEAEKRPYIDE
AKRLRAQHMKEHPDYKYRPRRKPKNLLKKDRYVFPLPYLGDTDPLKAAGLPVGASDGLLS
APEKARAFLPPASAPYSLLDPAQFSSSAIQKMGEVPHTLATGALPYASTLGYQNGAFGSL
SCPSQHTHTHPSPTNPGYVVPCNCTAWSASTLQPPVAYILFPGMTKTGIDPYSSAHATAM
*
>ENSG00000170689|ENST00000311177

MSISGTLSSYYVDSIISHESEDAPPAKFPSGQYASSRQPGHAEHLEFPSCSFQPKAPVFG
ASWAPLSPHASGSLPSVYHPYIQPQGVPPAESRYLRTWLEPAPRGEAAPGQGQAAVKAEP
LLGAPGELLKQGTPEYSLETSAGREAVLSNQRPGYGDNKICEGSEDKERPDQTNPSANWL
HARSSRKKRCPYTKYQTLELEKEFLFNMYLTRDRRHEVARLLNLSERQVKIWFQNRRMKM
KKMNKEQGKE*
>ENSG00000145908|ENST00000274599
MMKSQGLVSFKDVAVDFTQEEWQQLDPSQRTLYRDVMLENYSHLVSMGYPVSKPDVISKL
EQGEEPWIIKGDISNWIYPDEYQADGRQDRKSNLHNSQSCILGTVSFHHKILKGVTRDGS
LCSILKVCQGDGQLQRFLENQDKLFRQVTFVNSKTVTEASGHKYNPLGKIFQECIETDIS
IQRFHKYDAFKKNLKPNIDLPSCYKSNSRKKPDQSFGGGKSSSQSEPNSNLEKIHNGVIP
FDDNQCGNVFRNTQSLIQYQNVETKEKSCVCVTCGKAFAKKSQLIVHQRIHTGKKPYDCG
ACGKAFSEKFHLVVHQRTHTGEKPYDCSECGKAFSQKSSLIIHQRVHTGEKPYECSECGK
AFSQKSPLIIHQRIHTGEKPYECRECGKAFSQKSQLIIHHRAHTGEKPYECTECGKAFCE
KSHLIIHKRIHTGEKPYKCAQCEEAFSRKTELITHQLVHTGEKPYECTECGKTFSRKSQL
IIHQRTHTGEKPYKCSECGKAFCQKSHLIGHQRIHTGEKPYICTECGKAFSQKSHLPGHQ
RIHTGEKPYICAECGKAFSQKSDLVLHQRIHTGERPYQCAICGKAFIQKSQLTVHQRIHT
VVKS*
>ENSG00000175467|ENST00000312397
MGSSKKHRGEKEAAGTTAAAGTGGATEQPPRHREHKKHKHRSGGSGGSGGERRKRSRERG
GERGSGRRGAEAEARSSTHGRERSQAEPSERRVKREKRDDGYEAAASSKTSSGDASSLSI
EETNKLRAKLGLKPLEVNAIKKEAGTKEEPVTADVINPMALRQREELREKLAAAKEKRLL
NQKLGKIKTLGEDDPWLDDTAAWIERSRQLQKEKDLAEKRAKLLEEMDQEFGVSTLVEEE
FGQRRQDLYSARDLQGLTVEHAIDSFREGETMILTLKDKGVLQEEEDVLVNVNLVDKERA
EKNVELRKKKPDYLPYAEDESVDDLAQQKPRSILSKYDEELEGERPHSFRLEQGGTADGL
RERELEEIRAKLRLQAQSLSTVGPRLASEYLTPEEMVTFKKTKRRVKKIRKKEKEVVVRA
DDLLPLGDQTQDGDFGSRLRGRGRRRVSEVEEEKEPVPQPLPSDDTRVENMDISDEEEGG
APPPGSPQVLEEDEAELELQKQLEKGRRLRQLQQLQQLRDSGEKVVEIVKKLESRQRGWE
EDEDPERKGAIVFNATSEFCRTLGEIPTYGLAGNREEQEELMDFERDEERSANGGSESDG
EENIGWSTVNLDEEKQQQDFSASSTTILDEEPIVNRGLAAALLLCQNKGLLETTVQKVAR
VKAPNKSLPSAVYCIEDKMAIDDKYSRREEYRGFTQDFKEKDGYKPDVKIEYVDETGRKL
TPKEAFRQLSHRFHGKGSGKMKTERRMKKLDEEALLKKMSSSDTPLGTVALLQEKQKAQK
TPYIVLSGSGKSMNANTITK*
>ENSG00000188290|ENST00000304952
MAADTPGKPSASPMAGAPASASRTPDKPRSAAEHRKSSKPVMEKRRRARINESLAQLKTL
ILDALRKESSRHSKLEKADILEMTVRHLRSLRRVQVTAALSADPAVLGKYRAGFHECLAE
VNRFLAGCEGVPADVRSRLLGHLAACLRQLGPSRRPASLSPAAPAEAPAPEVYAGRPLLP
SLGGPFPLLAPPLLPGLTRALPAAPRAGPQGPGGPWRPWLR*
>ENSG00000157036|ENST00000287675
MAIKSIASRLRGSRRFLSGFVAGAVVGAAGAGLAALQFFRSQGAEGALTGKQPDGSAEKA
VLEQFGFPLTGTEARCYTNHALSYDQAKRVPRWVLEHISKSKIMGDADRKHCKFKPDPNI
PPTFSAFNEDYVGSGWSRGHMAPAGNNKFSSKAMAETFYLSNIVPQDFDNNSGYWNRIEM
YCRELTERFEDVWVVSGPLTLPQTRGDGKKIVSYQVIGEDNVAVPSHLYKVILARRSSVS
TEPLALGAFVVPNEAIGFQPQLTEFQVSLQDLEKLSGLVFFPHLDRTSDIRNICSVDTCK
LLDFQEFTLYLSTRKIEGARSVLRLEKIMENLKNAEIEPDDYFMSRYEKKLEELKAKEQS

GTQIRKPS*
>ENSG00000151846|ENST00000281589
MNPSTPSYPTASLYVGDLHPDVTEAMLYEKFSPAGPILSIRICRDLITSGSSNYAYVNFQ
HTKDAEHALDTMNFDVIKGKPVRIMWSQRDPSLRKSGVGNIFVKNLDKSINNKALYDTVS
AFGNILSCNVVCDENGSKGYGFVHFETHEAAERAIKKMNGMLLNGRKVFVGQFKSRKERE
AELGARAKEFPNVYIKNFGEDMDDERLKDLFGKFGPALSVKVMTDESGKSKGFGFVSFER
HEDAQKAVDEMNGKELNGKQIYVGRAQKKVERQTELKRTFEQMKQDRITRYQVVNLYVKN
LDDGIDDERLRKAFSPFGTITSAKVMMEGGRSKGFGFVCFSSPEEATKAVTEMNGRIVAT
KPLYVALAQRKEERQAYLTNEYMQRMASVRAVPNQRAPPSGYFMTAVPQTQNHAAYYPPS
QIARLRPSPRWTAQGARPHPFQNKPSAIRPGAPRVPFSTMRPASSQVPRVMSTQRVANTS
TQTVGPRPAAAAAAAATPAVRTVPRYKYAAGVRNPQQHRNAQPQVTMQQLAVHVQGQETL
TASRLASAPPQKQKQMLGERLFPLIQAMHPTLAGKITGMLLEIDNSELLYMLESPESLRS
KVDEAVAVLQAHQAKEATQKAVNSATGVPTV*
>ENSG00000081177|ENST00000312994
MSRQNLVALTVTTLLGVAVGGFVLWKGIQRRRRSKTSPVTQQPQQKVLGSRELPPPEDDQ
LHSSAPRSSWKERILKAKVVTVSQEAEWDQIEPLLRSELEDFPVLGIDCEWVNLEGKASP
LSLLQMASPSGLCVLVRLPKLICGGKTLPRTLLDILADGTILKVGVGCSEDASKLLQDYG
LVVRGCLDLRYLAMRQRNNLLCNGLSLKSLAETVLNFPLDKSLLLRCSNWDAETLTEDQV
IYAARDAQISVALFLHLLGYPFSRNSPGEKNDDHSSWRKVLEKCQGVVDIPFRSKGMSRL
GEEVNGEATESQQKPRNKKSKMDGMVPGNHQGRDPRKHKRKPLGVGYSARKSPLYDNCFL
HAPDGQPLCTCDRRKAQWYLDKGIGELVSEEPFVVKLRFEPAGRPESPGDYYLMVKENLC
VVCGKRDSYIRKNVIPHEYRKHFPIEMKDHNSHDVLLLCTSCHAISNYYDNHLKQQLAKE
FQAPIGSEEGLRLLEDPERRQVRSGARALLNAESLPTQRKEELLQALREFYNTDVVTEEM
LQEAASLETRISNENYVPHGLKVVQCHSQGGLRSLMQLESRWRQHFLDSMQPKHLPQQWS
VDHNHQKLLRKFGEDLPIQLS*
>ENSG00000155966|ENST00000286437
MKFKRRHQAFPSFFKMKVSLPSDPSCVEEILRESQHLTPGFTLQKWNDPTTRASTKSVSF
KSMLEDDLKLSSDEDDLEPVKTLTTQCTATELYQAVEKAKPRNNPVNPPLATPQPPPAVQ
ASGGSGSSSESESSSESDSDTESSTTDSESNEAPRVATPEPEPPSTNKWQLDKWLNKVTS
QNKSFICGQNETPMETISLPPPIIQPMEVQMKVKTNASQVPAEPKERPLLSLIREKARPR
PTQKIPETKALKHKLSTTSETVSQRTIGKKQPKKVEKNTSTDEFTWPKPNITSSTPKEKE
SVELHDPPRGRNKATAHKPAPRKEPRPNIPLAPEKKKYRGPGKIVPKSREFIETDSSTSD
SNTDQEETLQIKVLPPCIISGGNTAKSKEICGASLTLSTLMSSSGSNNNLSISNEEPTFS
PIPVMQTEILSPLRDHENLKNLWVKIDLDLLSRVPGHSSLHAAPAKPDHKETATKPKRQT
AVTAVEKPAPKGKRKHKPIEVAEKIPEKKQRLEEATTICLLPPCISPAPPHKPPNTRENN
SSRRANRRKEEKLFPPPLSPLPEDPPRRRNVSGNNGPFGQDKNIAMTGQITSTKPKRTEG
KFCATFKGISVNEGDTPKKASSATITVTNTAIATATVTATAIVTTTVTATATATATTTTT
TTTISTITSTITTGLMDSSHLEMTSWAALPLLSSSSTNVRRPKLTFDDSVHNADYYMQEA
KKLKHKADALFEKFGKAVNYADAALSFTECGNAMERDPLEAKSPYTMYSETVELLRYAMR
LKNFASPLASDGDKKLAVLCYRCLSLLYLRMFKLKKDHAMKYSRSLMEYFKQNASKVAQI
PSPWVSNGKNTPSPVSLNNVSPINAMGNCNNGPVTIPQRIHHMAASHVNITSNVLRGYEH
WDMADKLTRENKEFFGDLDTLMGPLTQHSSMTNLVRYVRQGLCWLRIDAHLL*
>ENSG00000165819|ENST00000298717
MSDTWSSIQAHKKQLDSLRERLQRRRKQDSGHLDLRNPEAALSPTFRSDSPVPTAPTSGG

PKPSTASAVPELATDPELEKKLLHHLSDLALTLPTDAVSICLAISTPDAPATQDGVESLL
QKFAAQELIEVKRGLLQDDAHPTLVTYADHSKLSAMMGAVAEKKGPGEVAGTVTGQKRRA
EQDSTTVAAFASSLVSGLNSSASEPAKEPAKKSRKHAASDVDLEIESLLNQQSTKEQQSK
KVSQEILELLNTTTAKEQSIVEKFRSRGRAQVQEFCDYGTKEECMKASDADRPCRKLHFR
RIINKHTDESLGDCSFLNTCFHMDTCKYVHYEIDACMDSEAPGSKDHTPSQELALTQSVG
GDSSADRLFPPQWICCDIRYLDVSILGKFAVVMADPPWDIHMELPYGTLTDDEMRRLNIP
VLQDDGFLFLWVTGRAMELGRECLNLWGYERVDEIIWVKTNQLQRIIRTGRTGHWLNHGK
EHCLVGVKGNPQGFNQGLDCDVIVAEVRSTSHKPDEIYGMIERLSPGTRKIELFGRPHNV
QPNWITLGNQLDGIHLLDPDVVARFKQRYPDGIISKPKNL*
>ENSG00000175395|ENST00000302609
MNKFQGPVTLKDVIVEFTKEEWKLLTPAQRTLYKDVMLENYSHLVSVGYHVNKPNAVFKL
KQGKEPWILEVEFPHRGFPEDLWSIHDLEARYQESQAGNSRNGELTKHQKTHTTEKACEC
KECGKFFCQKSALIVHQHTHSKGKSYDCDKCGKSFSKNEDLIRHQKIHTRDKTYECKECK
KIFYHLSSLSRHLRTHAGEKPYECNQCEKSFYQKPHLTEHQKTHTGEKPFECTECGKFFY
VKAYLMVHQKTHTGEKPYECKECGKAFSQKSHLTVHQRMHTGEKPYKCKECGKFFSRNSH
LKTHQRSHTGEKPYECKECRKCFYQKSALTVHQRTHTGEKPFECNKCGKTFYYKSDLTKH
QRKHTGEKPYECTECGKSFAVNSVLRLHQRTHTGEKPYACKECGKSFSQKSHFIIHQRKH
TGEKPYECQECGETFIQKSQLTAHQKTHTKKRNAEK*
>ENSG00000169871|ENST00000306085
MVSHGSSPSLLEALSSDFLACKICLEQLRAPKTLPCLHTYCQDCLAQLADGGRVRCPECR
ETVPVPPEGVASFKTNFFVNGLLDLVKARACGDLRAGKPACALCPLVGGTSTGGPATARC
LDCADDLCQACADGHRCTRQTHTHRVVDLVGYRAGWYDEEARERQAAQCPQHPGEALRFL
CQPCSQLLCRECRLDPHLDHPCLPLAEAVRARRPGLEGLLAGVDNNLVELEAARRVEKEA
LARLREQAARVGTQVEEAAEGVLRALLAQKQEVLGQLRAHVEAAEEAARERLAELEGREQ
VARAAAAFARRVLSLGREAEILSLEGAIAQRLRQLQGCPWAPGPAPCLLPQLELHPGLLD
KNCHLLRLSFEEQQPQKDGGKDGAGTQGGEESQSRREDEPKTERQGGVQPQAGDGAQTPK
EEKAQTTREEGAQTLEEDRAQTPHEDGGPQPHRGGRPNKKKKFKGRLKSISREPSPALGP
NLDGSGLLPRPIFYCSFPTRMPGDKRSPRITGLCPFGPREILVADEQNRALKRFSLNGDY
KGTVPVPEGCSPCSVAALQSAVAFSASARLYLINPNGEVQWRRALSLSQASHAVAALPSG
DRVAVSVAGHVEVYNMEGSLATRFIPGGKASRGLRALVFLTTSPQGHFVGSDWQQNSVVI
CDGLGQVVGEYKGPGLHGCQPGSVSVDKKGYIFLTLREVNKVVILDPKGSLLGDFLTAYH
GLEKPRVTTMVDGRYLVVSLSNGTIHIFRVRSPDS*
>ENSG00000164902|ENST00000297540
MALEVGDMEDGQLSDSDSDMTVAPSDRPLQLPKVLGGDSAMRAFQNTATACAPVSHYRAV
ESVDSSEESFSDSDDDSCLWKRKRQKCFNPPPKPEPFQFGQSSQKPPVAGGKKINNIWGA
VLQEQNQDAVATELGILGMEGTIDRSRQSETYNYLLAKKLRKESQEHTKDLDKELDEYMH
GGKKMGSKEEENGQGHLKRKRPVKDRLGNRPEMNYKGRYEITAEDSQEKVADEISFRLQE
PKKDLIARVVRIIGNKKAIELLMETAEVEQNGGLFIMNGSRRRTPGGVFLNLLKNTPSIS
EEQIKDIFYIENQKEYENKKAARKRRTQVLGKKMKQAIKSLNFQEDDDTSRETFASDTNE
ALASLDESQEGHAEAKLEAEEAIEVDHSHDLDIF*
>ENSG00000159409|ENST00000290585
MKEPDAIKLFVGQIPRHLEEKDLKPIFEQFGRIFELTVIKDKYTGLHKGCAFLTYCARDS
ALKAQSALHEQKTLPGMNRPIQVKPADSESRGEDRKLFVGMLGKQQTDEDVRKMFEPFGT
IDECTVLRGPDGTSKGCAFVKFQTHAEAQAAINTLHSSRTLPGASSSLVVKFADTEKERG

LRRMQQVATQLGMFSPIALQFGAYSAYTQALMQQQAALVAAHSAYLSPMATMAAVQMQHM
AAINANGLIATPITPSSAQSPAAPVDPLQQAYAGMQHYTAAYPAAYSLVAPAFPQPPALV
AQQPPPPPQQQQQQQQQQQQQQQREGPDGCNIFIYHLPQEFTDSEILQMFVPFGHVISAK
VFVDRATNQSKCFGFVSFDNPASAQAAIQAMNGFQIGMKRLKVQLKRPKDANRPY*
>ENSG00000196345|ENST00000273320
MTTAGRGNLGLIPRSTAFQKQEGRLTVKQEPANQTWGQGSSLQKNYPPVCEIFRLHFRQL
CYHEMSGPQEALSRLRELCRWWLMPEVHTKEQILELLVLEQFLSILPGELRTWVQLHHPE
SGEEAVAVVEDFQRHLSGSEEVSAPAQKQEMHFEETTALGTTKESPPTSPLSGGSAPGAH
LEPPYDPGTHHLPSGDFAQCTSPVPTLPQVGNSGDQAGATVLRMVRPQDTVAYEDLSVDY
TQKKWKSLTLSQRALQWNMMPENHHSMASLAGENMMKGSELTPKQEFFKGSESSNRTSGG
LFGVVPGAAETGDVCEDTFKELEGQTSDEEGSRLENDFLEITDEDKKKSTKDRYDKYKEV
GEHPPLSSSPVEHEGVLKGQKSYRCDECGKAFNRSSHLIGHQRIHTGEKPYECNECGKTF
RQTSQLIVHLRTHTGEKPYECSECGKAYRHSSHLIQHQRLHNGEKPYKCNECAKAFTQSS
RLTDHQRTHTGEKPYECNECGEAFIRSKSLARHQVLHTGKKPYKCNECGRAFCSNRNLID
HQRIHTGEKPYECSECGKAFSRSKCLIRHQSLHTGEKPYKCSECGKAFNQNSQLIEHERI
HTGEKPFECSECGKAFGLSKCLIRHQRLHTGEKPYKCNECGKSFNQNSHLIIHQRIHTGE
KPYECNECGKVFSYSSSLMVHQRTHTGEKPYKCNDCGKAFSDSSQLIVHQRVHTGEKPYE
CSECGKAFSQRSTFNHHQRTHTGEKSSGLAWSVS*
>ENSG00000155508|ENST00000285896
MPAALVENSQVICEVWASNLEEEMRKIREIVLSYSYIAMDTEFPGVVVRPIGEFRSSIDY
QYQLLRCNVDLLKIIQLGLTFTNEKGEYPSGINTWQFNFKFNLTEDMYSQDSIDLLANSG
LQFQKHEEEGIDTLHFAELLMTSGVVLCDNVKWLSFHSGYDFGYMVKLLTDSRLPEEEHE
FFHILNLFFPSIYDVKYLMKSCKNLKGGLQEVADQLDLQRIGRQHQAGSDSLLTGMAFFR
MKELFFEDSIDDAKYCGRLYGLGTGVAQKQNEDVDSAQEKMSILAIINNMQQ*
>ENSG00000186468|ENST00000296674
MGKCRGLRTARKLRSHRRDQKWHDKQYKKAHLGTALKANPFGGASHAKGIVLEKVGVEAK
QPNSAIRKCVRVQLIKNGKKITAFVPNDGCLNFIEENDEVLVAGFGRKGHAVGDIPGVRF
KVVKVANVSLLALYKGKKERPRS*
>ENSG00000127993|ENST00000265732
MASSGGELGSLFDHHVQRAVCDTRAKYREGRRPRAVKVYTINLESQYLLIQGVPAVGVMK
ELVERFALYGAIEQYNALDEYPAEDFTEVYLIKFMNLQSARTAKRKMDEQSFFGGLLHVC
YAPEFETVEETRKKLQMRKAYVVKTTENKDHYVTKKKLVTEHKDTEDFRQDFHSEMSGFC
KAALNTSAGNSNPYLPYSCELPLCYFSSKCMCSSGGPVDRAPDSSKDGRNHHKTMGHYNH
NDSLRKTQINSLKNSVACPGAQKAITSSEAVDRFMPRTTQLQERKRRREDDRKLGTFLQT
NPTGNEIMIGPLLPDISKVDMHDDSLNTTANLIRHKLKEVISSVPKPPEDKPEDVHTSHP
LKQRRRI*
>ENSG00000169926|ENST00000307145
MAAAAYVDHFAAECLVSMSSRAVVHGPREGPESRPEGAAVAATPTLPRVEERRDGKDSAS
LFVVARILADLNQQAPAPAPAERREGAAARKARTPCRLPPPAPEPTSPGAEGAAAAPPSP
AWSEPEPEAGLEPEREPGPAGSGEPGLRQRVRRGRSRADLESPQRKHKCHYAGCEKVYGK
SSHLKAHLRTHTGERPFACSWQDCNKKFARSDELARHYRTHTGEKKFSCPICEKRFMRSD
HLTKHARRHANFHPGMLQRRGGGSRTGSLSDYSRSDASSPTISPASSP*
>ENSG00000175213|ENST00000311764
MEEAEELLLEGKKALQLAREPRLGLDLGWNPSGEGCTQGLKDVPPEPTRDILALKSLPRG

LALGPSLAKEQRLGVWCVGDPLQPGLLWGPLEEESASKEKGEGVKPRQEENLSLGPWGDV
CACEQSSGWTSLVQRGRLESEGNVAPVRISERLHLQVYQLVLPGSELLLWPQPSSEGPSL
TQPGLDKEAAVAVVTEVESAVQQEVASPGEDAAEPCIDPGSQSPSGIQAENMVSPGLKFP
TQDRISKDSQPLGPLLQDGDVDEECPAQAQMPPELQSNSATQQDPDGSGASFSSSARGTQ
PHGYLAKKLHSPSDQCPPRAKTPEPGAQQSGFPTLSRSPPGPAGSSPKQGRRYRCGECGK
AFLQLCHLKKHAFVHTGHKPFLCTECGKSYSSEESFKAHMLGHRGVRPFPCPQCDKAYGT
QRDLKEHQVVHSGARPFACDQCGKAFARRPSLRLHRKTHQVPAAPAPCPCPVCGRPLANQ
GSLRNHMRLHTGEKPFLCPHCGRAFRQRGNLRGHLRLHTGERPYRCPHCADAFPQLPELR
RHLISHTGEAHLCPVCGKALRDPHTLRAHERLHSGERPFPCPQCGRAYTLATKLRRHLKS
HLEDKPYRCPTCGMGYTLPQSLRRHQLSHRPEAPCSPPSVPSAASEPTVVLLQAEPQLLD
THREEEVSPARDVVEVTISESQEKCFVVPEEPDAAPSLVLIHKDMGLGAWAEVVEVEMGT
*
>ENSG00000039600|ENST00000265007
MERARPEPPPQPRPLRPAPPPLPVEGTSFWAAAMEPPPSSPTLSAAASATLASSCGEAVA
SGLQPAVRRLLQVKPEQVLLLPQPQAQNEEAAASSAQARLLQFRPDLRLLQPPTASDGAT
SRPELHPVQPLALHVKAKKQKLGPSLDQSVGPRGAVETGPRASRVVKLEGPGPALGYFRG
DEKGKLEAEEVMRDSMQGGAGKSPAAIREGVIKTEEPERLLEDCRLGAEPASNGLVHGSA
EVILAPTSGAFGPHQQDLRIPLTLHTVPPGARIQFQGAPPSELIRLTKVPLTPVPTKMQS
LLEPSVKIETKDVPLTVLPSDAGIPDTPFSKDRNGHVKRPMNAFMVWARIHRPALAKANP
AANNAEISVQLGLEWNKLSEEQKKPYYDEAQKIKEKHREEFPGWVYQPRPGKRKRFPLSV
SNVFSGTTQNIISTNPTTVYPYRSPTYSVVIPSLQNPITHPVGETSPAIQLPTPAVQSPS
PVTLFQPSVSSAAQVAVQDPSLPVYPALPPQRFTGPSQTDTHQLHSEATHTVKQPTPVSL
ESANRISSSASTAHARFATSTIQPPREYSSVSPCPRSAPIPQASPIPHPHVYQPPPLGHP
ATLFGTPPRFSFHHPYFLPGPHYFPSSTCPYSRPPFGYGNFPSSMPECLSYYEDRYPKHE
GIFSTLNRDYSFRDYSSECTHSENSRSCENMNGTSYYNSHSHSGEENLNPVPQLDIGTLE
NVFTAPTSTPSSIQQVNVTDSDEEEEEKVLRDL*
>ENSG00000141946|ENST00000269834
MNNSQGRVTFEDVTVNFTQGEWQRLNPEQRNLYRDVMLENYSNLVSVGQGETTKPDVILR
LEQGKEPWLEEEEVLGSGRAEKNGDIGGQIWKPKDVKESLAREVPSINKETLTTQKGVEC
DGSKKILPLGIDDVSSLQHYVQNNSHDDNGYRKLVGNNPSKFVGQQLKCNACRKLFSSKS
RLQSHLRRHACQKPFECHSCGRAFGEKWKLDKHQKTHAEERPYKCENCGNAYKQKSNLFQ
HQKMHTKEKPYQCKTCGKAFSWKSSCINHEKIHNAKKSYQCNECEKSFRQNSTLIQHKKV
HTGQKPFQCTDCGKAFIYKSDLVKHQRIHTGEKPYKCSICEKAFSQKSNVIDHEKIHTGK
RAYECDLCGNTFIQKKNLIQHKKIHTGEKPYECNRCGKAFFQKSNLHSHQKTHSGERTYR
CSECGKTFIRKLNLSLHKKTHTGQKPYGCSECGKAFADRSYLVRHQKRIHSR*
>ENSG00000152455|ENST00000313519
MEYYLVKWKGWPDSTNTWEPLQNLKCPLLLQQFSNDKHNYLSQVKKGKAITPKDNNKTLK
PAIAEYIVKKAKQRIALQRWQDELNRRKNHKGMIFVENTVDLEGPPSDFYYINEYKPAPG
ISLVNEATFGCSCTDCFFQKCCPAEAGVLLAYNKNQQIKIPPGTPIYECNSRCQCGPDCP
NRIVQKGTQYSLCIFRTSNGRGWGVKTLVKIKRMSFVMEYVGEVITSEEAERRGQFYDNK
GITYLFDLDYESDEFTVDAARYGNVSHFVNHSCDPNLQVFNVFIDNLDTRLPRIALFSTR
TINAGEELTFDYQMKGSGDISSDSIDHSPAKKRVRTVCKCGAVTCRGYLN*
>ENSG00000164347|ENST00000296805
MLTNLRIFAMSHQTIPSVYINNICCYKIRASLKRLKPHVPLGRNCSSLPGLIGNDIKSLH

SIINPPIAKIRNIGIMAHIDAGKTTTTERILYYSGYTRSLGDVDDGDTVTDFMAQERERG
ITIQSAAVTFDWKGYRVNLIDTPGHVDFTLEVERCLRVLDGAVAVFDASAGVEAQTLTVW
RQADKHNIPRICFLNKMDKTGASFKYAVESIREKLKAKPLLLQLPIGEAKTFKGVVDVVM
KEKLLWNCNSNDGKDFERKPLLEMNDPELLKETTEARNALIEQVADLDDEFADLVLEEFS
ENFDLLPAEKLQTAIHRVTLAQTAVPVLCGSALKNKGIQPLLDAVTMYLPSPEERNYEFL
QWYKDDLCALAFKVLHDKQRGPLVFMRIYSGTIKPQLAIHNINGNCTERISRLLLPFADQ
HVEIPSLTAGNIALTVGLKHTATGDTIVSSKSSALAAARRAEREGEKKHRQNNEAERLLL
AGVEIPEPVFFCTIEPPSLSKQPDLEHALKCLQREDPSLKVRLDPDSGQTVLCGMGELHI
EIIHDRIKREYGLETYLGPLQVAYRETILNSVRATDTLDRTLGDKRHLVTVEVEARPIET
SSVMPVIEFEYAESINEGLLKVSQEAIENGIHSACLQGPLLGSPIQDVAITLHSLTIHPG
TSTTMISACVSRCVQKALKKADKQVLEPLMNLEVTVARDYLSPVLADLAQRRGNIQEIQT
RQDNKVVIGFVPLAEIMGYSTVLRTLTSGSATFALELSTYQAMNPQDQNTLLNRRSGLT*
>ENSG00000253293|ENST00000283921
MSARKGYLLPSPNYPTTMSCSESPAANSFLVDSLISSGRGEAGGGGGGAGGGGGGGYYAH
GGVYLPPAADLPYGLQSCGLFPTLGGKRNEAASPGSGGGGGGLGPGAHGYGPSPIDLWLD
APRSCRMEPPDGPPPPPQQQPPPPPQPPQPAPQATSCSFAQNIKEESSYCLYDSADKCPK
VSATAAELAPFPRGPPPDGCALGTSSGVPVPGYFRLSQAYGTAKGYGSGGGGAQQLGAGP
FPAQPPGRGFDLPPALASGSADAARKERALDSPPPPTLACGSGGGSQGDEEAHASSSAAE
ELSPAPSESSKASPEKDSLGNSKGENAANWLTAKSGRKKRCPYTKHQTLELEKEFLFNMY
LTRERRLEISRSVHLTDRQVKIWFQNRRMKLKKMNRENRIRELTANFNFS*
>ENSG00000170166|ENST00000306324
MVMSSYMVNSKYVDPKFPPCEEYLQGGYLGEQGADYYGGGAQGADFQPPGLYPRPDFGEQ
PFGGSGPGPGSALPARGHGQEPGGPGGHYAAPGEPCPAPPAPPPAPLPGARAYSQSDPKQ
PPSGTALKQPAVVYPWMKKVHVNSVNPNYTGGEPKRSRTAYTRQQVLELEKEFHFNRYLT
RRRRIEIAHTLCLSERQIKIWFQNRRMKWKKDHKLPNTKGRSSSSSSSSSCSSSVAPSQH
LQPMAKDHHTDLTTL*
>ENSG00000005513|ENST00000293894
MLDMSEARSQPPCSPSGTASSMSHVEDSDSDAPPSPAGSEGLGRAGVAVGGARGDPAEAA
DERFPACIRDAVSQVLKGYDWSLVPMPVRGGGGGALKAKPHVKRPMNAFMVWAQAARRKL
ADQYPHLHNAELSKTLGKLWRLLSESEKRPFVEEAERLRVQHKKDHPDYKYQPRRRKSAK
AGHSDSDSGAELGPHPGGGAVYKAEAGLGDGHHHGDHTGQTHGPPTPPTTPKTELQQAGA
KPELKLEGRRPVDSGRQNIDFSNVDISELSSEVMGTMDAFDVHEFDQYLPLGGPAPPEPG
QAYGGAYFHAGASPVWAHKSAPSASASPTETGPPRPHIKTEQPSPGHYGDQPRGSPDYGS
CSGQSSATPAAPAGPFAGSQGDYGDLQASSYYGAYPGYAPGLYQYPCFHSPRRPYASPLL
NGLALPPAHSPTSHWDQPVYTTLTRP*
>ENSG00000249961|ENST00000313294
MESEDTKKTQEMKTDLNLLLECLKYQMDNAFSQKEALVTIHSICQQNSNASVYFREIGGL
MFVKNLAKSSEHSMVKEAALYTLGAIAEKNVYCQQTLCTSELFEDLTWFLSNDSNINLKR
MSVYVILVLVSNNRTGQTLVRETGCITVLSRLFRTVISKHELDLSDKNVFQSYQLWSSVC
STLCVCVNNPQNDENQMFCCSLFPHANEWLKNCTTPEIIRPICSFIGLTLANNTYVQKYF
VSVGGLDVLSQVLMQLESDSHETLSSAKLAVVVTKTVDACIADNPTFGIVLSKYHIVSKL
LALLLHESLDSGEKFSIMLTLGHCTEDCAEKLSLSLGEYPFDENETQQLKDISVKENNLE
EHWRKAKEILHRIEQLEREGNEEEIQRENYQDNISSMNISIQNTWKHLHADRIGRGSKAE
DEDKSHSRQLQSYKSHGVMSKACTNDDQMKTPLKSANPVHACYRESEQNKTLYKAKSSCN

QNLHEETTFEKNFVSQSRSIYIMFRYNK*
>ENSG00000161634|ENST00000293371
MRFMTLLFLTALAGALVCAYDPEAASAPGSGNPCHEASAAQKENAGEDPGLARQAPKPRK
QRSSLLEKGLDGAKKAVGGLGKLGKDAVEDLESVGKGAVHDVKDVLDSVL*
>ENSG00000171453|ENST00000304004
MAASQAVEEMRSRVVLGEFGVRNVHTTDFPGNYSGYDDAWDQDRFEKNFRVDVVHMDENS
LEFDMVGIDAAIANAFRRILLAEVPTMAVEKVLVYNNTSIVQDEILAHRLGLIPIHADPR
LFEYRNQGDEEGTEIDTLQFRLQVRCTRNPHAAKDSSDPNELYVNHKVYTRHMTWIPLGN
QADLFPEGTIRPVHDDILIAQLRPGQEIDLLMHCVKGIGKDHAKFSPVATASYRLLPDIT
LLEPVEGEAAEELSRCFSPGVIEVQEVQGKKVARVANPRLDTFSREIFRNEKLKKVVRLA
RVRDHYICKKDLLAAVAHTCNPSTLGGQGEWITGSRERDHPG*
>ENSG00000269404|ENST00000270632
MLALEAAQLDGPHFSCLYPDGVFYDLDSCKHSSYPDSEGAPDSLWDWTVAPPVPATPYEA
FDPAAAAFSHPQAAQLCYEPPTYSPAGNLELAPSLEAPGPGLPAYPTENFASQTLVPPAY
APYPSPVLSEEEDLPLDSPALEVSDSESDEALVAGPEGKGSEGLARSCACTSSCWGY*
>ENSG00000169840|ENST00000302945
MPRSFLVDSLVLREAGEKKAPEGSPPPLFPYAVPPPHALHGLSPGACHARKAGLLCVCPL
CVTASQLHGPPGPPALPLLKASFPPFGSQYCHAPLGRQHSAVSPGVAHGPAAAAAAAALY
QTSYPLPDPRQFHCISVDSSSNQLPSSKRMRTAFTSTQLLELEREFASNMYLSRLRRIEI
ATYLNLSEKQVKIWFQNRRVKHKKEGKGSNHRGGGGGGAGGGGSAPQGCKCASLSSAKCS
EDDDELPMSPSSSGKDDRDLTVTP*
>ENSG00000196150|ENST00000292579
MAAARLLPVPAGPQPLSFQAKLTFEDVAVLLSQDEWDRLCPAQRGLYRNVMMETYGNVVS
LGLPGSKPDIISQLERGEDPWVLDRKGAKKSQGLWSDYSDNLKYDHTTACTQQDSLSCPW
ECETKGESQNTDLSPKPLISEQTVILGKTPLGRIDQENNETKQSFCLSPNSVDHREVQVL
SQSMPLTPHQAVPSGERPYMCVECGKCFGRSSHLLQHQRIHTGEKPYVCSVCGKAFSQSS
VLSKHRRIHTGEKPYECNECGKAFRVSSDLAQHHKIHTGEKPHECLECRKAFTQLSHLIQ
HQRIHTGERPYVCPLCGKAFNHSTVLRSHQRVHTGEKPHRCNECGKTFSVKRTLLQHQRI
HTGEKPYTCSECGKAFSDRSVLIQHHNVHTGEKPYECSECGKTFSHRSTLMNHERIHTEE
KPYACYECGKAFVQHSHLIQHQRVHTGEKPYVCGECGHAFSARRSLIQHERIHTGEKPFQ
CTECGKAFSLKATLIVHLRTHTGEKPYECNSCGKAFSQYSVLIQHQRIHTGEKPYECGEC
GRAFNQHGHLIQHQKVHRKL*
>ENSG00000072756|ENST00000280591
MLRCLYHWHRPVLNRRWSRLCLPKQYLFTMKLQSPEFQSLFTEGLKSLTELFVKENHELR
IAGGAVRDLLNGVKPQDIDFATTATPTQMKEMFQSAGIRMINNRGEKHGTITARLHEENF
EITTLRIDVTTDGRHAEVEFTTDWQKDAERRDLTINSMFLGFDGTLFDYFNGYEDLKNKK
VRFVGHAKQRIQEDYLRILRYFRFYGRIVDKPGDHDPETLEAIAENAKGLAGISGERIWV
ELKKILVGLPANASLEEFDKVSKNVDGFSPKPVTLLASLFKVQDDVTKLDLRLKIAKEEK
NLGLFIVKNRKDLIKATDSSDPLKPYQDFIIDSREPDATTRVCELLKYQGEHCLLKEMQQ
WSIPPFPVSGHDIRKVGISSGKEIGALLQQLREQWKKSGYQMEKDELLSYIKKT*
>ENSG00000155846|ENST00000309241
MAGNDCGALLDEELSSFFLNYLADTQGGGSGEEQLYADFPELDLSQLDASDFDSATCFGE
LQWCPENSETEPNQYSPDDSELFQIDSENEALLAELTKTLDDIPEDDVGLAAFPALDGGD
ALSCTSASPAPSSAPPSPAPEKPSAPAPEVDELSLLQKLLLATSYPTSSSDTQKEGTAWR

QAGLRSKSQRPCVKADSTQDKKAPMMQSQSRSCTELHKHLTSAQCCLQDRGLQPPCLQSP
RLPAKEDKEPGEDCPSPQPAPASPRDSLALGRADPGAPVSQEDMQAMVQLIRYMHTYCLP
QRKLPPQTPEPLPKACSNPSQQVRSRPWSRHHSKASWAEFSILRELLAQDVLCDVSKPYR
LATPVYASLTPRSRPRPPKDSQASPGRPSSVEEVRIAASPKSTGPRPSLRPLRLEVKREV
RRPARLQQQEEEDEEEEEEEEEEEKEEEEEWGRKRPGRGLPWTKLGRKLESSVCPVRRSR
RLNPELGPWLTFADEPLVPSEPQGALPSLCLAPKAYDVERELGSPTDEDSGQDQQLLRGP
QIPALESPCESGCGDMDEDPSCPQLPPRDSPRCLMLALSQSDPTFGKKSFEQTLTVELCG
TAGLTPPTTPPYKPTEEDPFKPDIKHSLGKEIALSLPSPEGLSLKATPGAAHKLPKKHPE
RSELLSHLRHATAQPASQAGQKRPFSCSFGDHDYCQVLRPEGVLQRKVLRSWEPSGVHLE
DWPQQGAPWAEAQAPGREEDRSCDAGAPPKDSTLLRDHEIRASLTKHFGLLETALEEEDL
ASCKSPEYDTVFEDSSSSSGESSFLPEEEEEEGEEEEEDDEEEDSGVSPTCSDHCPYQSP
PSKANRQLCSRSRSSSGSSPCHSWSPATRRNFRCESRGPCSDRTPSIRHARKRREKAIGE
GRVVYIQNLSSDMSSRELKRRFEVFGEIEECEVLTRNRRGEKYGFITYRCSEHAALSLTK
GAALRKRNEPSFQLSYGGLRHFCWPRYTDYDSNSEEALPASGKSKYEAMDFDSLLKEAQQ
SLH*
>ENSG00000165156|ENST00000297857
MASRRKSTTPCMVLASEQDPDLELISDLDEGPPVLTPVENTRAESISSDEEVHESVDSDN
QQNKKVEGGYECKYCTFQTPDLNMFTFHVDSEHPNVVLNSSYVCVECNFLTKRYDALSEH
NLKYHPGEENFKLTMVKRNNQTIFEQTINDLTFDGSFVKEENAEQAESTEVSSSGISISK
TPIMKMMKNKVENKRIAVHHNSVEDVPEEKENEIKPDREEIVENPSSSASESNTSTSIVN
RIHPSTASTVVTPAAVLPGLAQVITAVSAQQNSNLIPKVLIPVNSIPTYNAALDNNPLLL
NTYNKFPYPTMSEITVLSAQAKYTEEQIKIWFSAQRLKHGVSWTPEEVEEARRKQFNGTV
HTVPQTITVIPTHISTGSNGLPSILQTCQIVGQPGLVLTQVAGTNTLPVTAPIALTVAGV
PSQNNIQKSQVPAAQPTAETKPATAAVPTSQSVKHETALVNPDSFGIRAKKTKEQLAELK
VSYLKNQFPHDSEIIRLMKITGLTKGEIKKWFSDTRYNQRNSKSNQCLHLNNDSSTTIII
DSSDETTESPTVGTAQPKQSWNPFPDFTPQKFKEKTAEQLRVLQASFLNSSVLTDEELNR
LRAQTKLTRREIDAWFTEKKKSKALKEEKMEIDESNAGSSKEEAGETSPADESGAPKSGS
TGKICKKTPEQLHMLKSAFVRTQWPSPEEYDKLAKESGLARTDIVSWFGDTRYAWKNGNL
KWYYYYQSANSSSMNGLSSLRKRGRGRPKGRGRGRPRGRPRGSKRINNWDRGPSLIKFKT
GTAILKDYYLKHKFLNEQDLDELVNKSHMGYEQVREWFAERQRRSELGIELFEENEEEDE
VIDDQEEDEEETDDSDTWEPPRHVKRKLSKSDD*
>ENSG00000150990|ENST00000308736
MGKLRRRYNIKGRQQAGPGPSKGPPEPPPVQLELEDKDTLKGVDASNALVLPGKKKKKTK
APPLSKKEKKPLTKKEKKVLQKILEQKEKKSQRAEMLQKLSEVQASEAEMRLFYTTSKLG
TGNRMYHTKEKADEVVAPGQEKISSLSGAHRKRRRWPSAEEEEEEEEESESELEEESELD
EDPAAEPAEAGVGTTVAPLPPAPAPSSQPVPAGMTVPPPPAAAPPLPRALAKPAVFIPVN
RSPEMQEERLKLPILSEEQVIMEAVAEHPIVIVCGETGSGKTTQVPQFLYEAGFSSEDSI
IGVTEPRRVAAVAMSQRVAKEMNLSQRVVSYQIRYEGNVTEETRIKFMTDGVLLKEIQKD
FLLLRYKVVIIDEAHERSVYTDILIGLLSRIVTLRAKRNLPLKLLIMSATLRVEDFTQNP
RLFAKPPPVIKVESRQFPVTVHFNKRTPLEDYSGECFRKVCKIHRMLPAGGILVFLTGQA
EVHALCRRLRKAFPPSRARPQEKDDDQKDSVEEMRKFKKSRARAKKARAEVLPQINLDHY
SVLPAGEGDEDREAEVDEEEGALDSDLDLDLGDGGQDGGEQPDASLPLHVLPLYSLLAPE
KQAQVFKPPPEGTRLCVVATNVAETSLTIPGIKYVVDCGKVKKRYYDRVTGVSSFRVTWV
SQASADQRAGRAGRTEPGHCYRLYSSAVFGDFEQFPPPEITRRPVEDLILQMKALNVEKV

INFPFPTPPSVEALLAAEELLIALGALQPPQKAERVKQLQENRLSCPITALGRTMATFPV
APRYAKMLALSRQHGCLPYAITIVASMTVRELFEELDRPAASDEELTRLKSKRARVAQMK
RTWAGQGASLKLGDLMVLLGAVGACEYASCTPQFCEANGLRYKAMMEIRRLRGQLTTAVN
AVCPEAELFVDPKMQPPTESQVTYLRQIVTAGLGDHLARRVQSEEMLEDKWRNAYKTPLL
DDPVFIHPSSVLFKELPEFVVYQEIVETTKMYMKGVSSVEVQWIPALLPSYCQFDKPLEE
PAPTYCPERGRVLCHRASVFYRVGWPLPAIEVDFPEGIDRYKHFARFLLEGQVFRKLASY
RSCLLSSPGTMLKTWARLQPRTESLLRALVAEKADCHEALLAAWKKNPKYLLAEYCEWLP
QAMHPDIEKAWPPTTVH*
>ENSG00000152467|ENST00000282326
MLPRPKAPASPRRPQTPTPSEQDADPGPASPRDTEAQRLRFRQFQYHVASGPHLALGQLW
TLCRQWLRPEARSKEQMLELLVLEQFLGALPSKMRTWVQSQGPRSCREAASLVEDLTQMC
QQEVLVSLDSVEPQDWSFGEEEDGKSPRSQKEPSQASELILDAVAAAPALPEESEWLETT
QLQQSLHTRAEAEAPRAPGLLGSRARLPLKPSIWDEPEDLLAGPSSDLRAEGTVISSPKG
PSAQRISPRRRNRNTDQSGRHQPSLKHTKGGTQEAVAGISVVPRGPRGGRPFQCADCGMV
FTWVTHFIEHQKTHREEGPFPCPECGKVFLHNSVLTEHGKIHLLEPPRKKAPRSKGPRES
VPPRDGAQGPVAPRSPKRPFQCSVCGKAFPWMVHLIDHQKLHTAHGHM*
>ENSG00000163623|ENST00000295886
MLAVGAMEGTRQSAFLLSSPPLAALHSMAEMKTPLYPAAYPPLPAGPPSSSSSSSSSSSP
SPPLGTHNPGGLKPPATGGLSSLGSPPQQLSAATPHGINDILSRPSMPVASGAALPSASP
SGSSSSSSSSASASSASAAAAAAAAAAAAASSPAGLLAGLPRFSSLSPPPPPPGLYFSPS
AAAVAAVGRYPKPLAELPGRTPIFWPGVMQSPPWRDARLACTPHQGSILLDKDGKRKHTR
PTFSGQQIFALEKTFEQTKYLAGPERARLAYSLGMTESQVKVWFQNRRTKWRKKHAAEMA
TAKKKQDSETERLKGASENEEEDDDYNKPLDPNSDDEKITQLLKKHKSSSGGGGGLLLHA
SEPESSS*
>ENSG00000145982|ENST00000274680
MVGSALRRGAHAYVYLVSKASHISRGHQHQAWGSRPPAAECATQRAPGSVVELLGKSYPQ
DDHSNLTRKVLTRVGRNLHNQQHHPLWLIKERVKEHFYKQYVGRFGTPLFSVYDNLSPVV
TTWQNFDSLLIPADHPSRKKGDNYYLNRTHMLRAHTSAHQWDLLHAGLDAFLVVGDVYRR
DQIDSQHYPIFHQLEAVRLFSKHELFAGIKDGESLQLFEQSSRSAHKQETHTMEAVKLVE
FDLKQTLTRLMAHLFGDELEIRWVDCYFPFTHPSFEMEINFHGEWLEVLGCGVMEQQLVN
SAGAQDRIGWAFGLGLERLAMILYDIPDIRLFWCEDERFLKQFCVSNINQKVKFQPLSKY
PAVINDISFWLPSENYAENDFYDLVRTIGGDLVEKVDLIDKFVHPKTHKTSHCYRITYRH
MERTLSQREVRHIHQALQEAAVQLLGVEGRF*
>ENSG00000168062|ENST00000301887
MHLCGGNGLLTQTDPKEQQRQLKKQKNRAAAQRSRQKHTDKADALHQQHESLEKDNLALR
KEIQSLQAELAWWSRTLHVHERLCPMDCASCSAPGLLGCWDQAEGLLGPGPQGQHGCREQ
LELFQTPGSCYPAQPLSPGPQPHDSPSLLQCPLPSLSLGPAVVAEPPVQLSPSPLLFASH
TGSSLQGSSSKLSALQPSLTAQTAPPQPLELEHPTRGKLGSSPDNPSSALGLARLQSREH
KPALSAATWQGLVVDPSPHPLLAFPLLSSAQVHF*
>ENSG00000172748|ENST00000308811
MPSPDSMTFEDIIVDFTQEEWALLDTSQRKLFQDVMLENISHLVSIGKQLCKSVVLSQLE
QVEKLSTQRISLLQGREVGIKHQEIPFIQHIYQKGTSTISTMRSHTQEDPFLCNDLGEDF
TQHIALTQNVITYMRTKHFVSKKFGKIFSDWLSFNQHKEIHTKCKSYGSHLFDYAFIQNS
ALRPHSVTHTREITLECRVCGKTFSKNSNLRRHEMIHTGEKPHGCHLCGKAFTHCSDLRK

HERTHTGEKPYGCHLCGKAFSKSSNLRRHEMIHTREKAQICHLCGKAFTHCSDLRKHERT
HLGDKPYGCLLCGKAFSKCSYLRQHERTHNGEKPYECHLCGKAFSHCSHLRQHERSHNGE
KPHGCHLCGKAFTESSVLKRHERIHTGEKPYECHVCGKAFTESSDLRRHERTHTGEKPYE
CHLCGKAFNHSSVLRRHERTHTGEKPYECNICGKAFNRSYNFRLHRRVHTGEKPYVCPLC
GKAFSKFFNLRQHERTHTKKAMNM*
>ENSG00000170948|ENST00000305625
MAKSSQRKQRDCVNQCKSKPGLSTSIPLRMSSYTFKRPVTRITPHPGNEVRYHQWEESLE
KPQQVCWQRRLQGLQAYSSAGELSSTLDLANTLQKLVPSYTGGSLLEDLASGLEHSCPMP
HLACSSDAVEIIPAEGVGISQLLCKQFLVTEEDIRKQEGKVKTVRERLAIALIADGLANE
AEKVRDQEGRPEKR*
>ENSG00000149016|ENST00000308436
MSLPIGSAEVASERVELWRSGFRWWQRCLCFCRYRRVAMAAVDSDVESLPRGGFRCCLCH
VTTANRPSLDAHLGGRKHRHLVELRAARKAQGLRSVFVSGFPRDVDSAQLSEYFLAFGPV
ASVVMDKDKGVFAIVEMGDVGAREAVLSQSQHSLGGHRLRVRPREQKEFQSPASKSPKGA
APDSHQLAKALAEAADVGAQMIKLVGLRELSEAERQLRSLVVALMQEVFTEFFPGCVVHP
FGSSINSFDVHGCDLDLFLDLGDLEEPQPVPKAPESPSLDSALASPLDPQALACTPASPP
DSQPPASPQDSEALDFETPSSSLAPQTPDSALASETLASPQSLPPASPLLEDREEGDLGK
ASELAETPKEEKAEGAAMLELVGSILRGCVPGVYRVQTVPSARRPVVKFCHRPSGLHGDV
SLSNRLALHNSRFLSLCSELDGRVRPLVYTLRCWAQGRGLSGSGPLLSNYALTLLVIYFL
QTRDPPVLPTVSQLTQKAGEGEQVEVDGWDCSFPRDASRLEPSINVEPLSSLLAQFFSCV
SCWDLRGSLLSLREGQALPVAGGLPSNLWEGLRLGPLNLQDPFDLSHNVAANVTSRVAGR
LQNCCRAAANYCRSLQYQRRSSRGRDWGLLPLLQPSSPSSLLSATPIPLPLAPFTQLTAA
LVQVFREALGCHIEQATKRTRSEGGGTGESSQGGTSKRLKVDGQKNCCEEGKEEQQGCAG
DGGEDRVEEMVIEVGEMVQDWAMQSPGQPGDLPLTTGKHGAPGEEGQPSHAALAERGPKG
HEAAQEWSQGEAGKGASLPSSASWRCALWHRVWQGRRRARRRLQQQTKEGAGGGAGTRAG
WLATEAQVTQELKGLSGGEERPETEPLLSFVASVSPADRMLTVTPLQDPQGLFPDLHHFL
QVFLPQAIRHLK*
>ENSG00000102984|ENST00000313565
MSRRKQTNPNKVHCDSEGDEEETTQDEVSSHTSEEDGGVVKVEKELENTEQPVGGNEVVE
HEVTGNLNSDPLLELCQCPLCQLDCGSREQLIAHVYQHTAAVVSAKSYMCPVCGRALSSP
GSLGRHLLIHSEDQRSNCAVCGARFTSHATFNSEKLPEVLNMESLPTVHNEGPSSAEGKD
IAFSPPVYPAGILLVCNNCAAYRKLLEAQTPSVRKWALRRQNEPLEVRLQRLERERTAKK
SRRDNETPEEREVRRMRDREAKRLQRMQETDEQRARRLQRDREAMRLKRANETPEKRQAR
LIREREAKRLKRRLEKMDMMLRAQFGQDPSAMAALAAEMNFFQLPVSGVELDSQLLGKMA
FEEQNSSSLH*
>ENSG00000171291|ENST00000304030
MLSLSPILLYTCEMFQDPVAFKDVAVNFTQEEWALLDISQKNLYREVMLETFWNLTSIGK
KWKDQNIEYEYQNPRRNFRSVTEEKVNEIKEDSHCGETFTPVPDDRLNFQKKKASPEVKS
CDSFVCEVGLGNSSSNMNIRGDTGHKACECQEYGPKPWKSQQPKKAFRYHPSLRTQERDH
TGKKPYACKECGKNIIYHSSIQRHMVVHSGDGPYKCKFCGKAFHCLSLYLIHERTHTGEK
PYECKQCGKSFSYSATHRIHERTHIGEKPYECQECGKAFHSPRSCHRHERSHMGEKAYQC
KECGKAFMCPRYVRRHERTHSRKKLYECKQCGKALSSLTSFQTHIRMHSGERPYECKTCG
KGFYSAKSFQRHEKTHSGEKPYKCKQCGKAFTRSGSFRYHERTHTGEKPYECKQCGKAFR
SAPNLQLHGRTHTGEKPYQCKECGKAFRSASQLRIHRRIHTGEKPYECKKCGKAFRYVQN

FRFHERTQTHKNALWRKTL*
>ENSG00000145912|ENST00000274606
MTKIKADPDGPEAQAEACSGERTYQELLVNQNPIAQPLASRRLTRKLYKCIKKAVKQKQI
RRGVKEVQKFVNKGEKGIMVLAGDTLPIEVYCHLPVMCEDRNLPYVYIPSKTDLGAAAGS
KRPTCVIMVKPHEEYQEAYDECLEEVQSLPLPL*
>ENSG00000123200|ENST00000282007
MSKIRRKVTVENTKTISDSTSRRPSVFERLGPSTGSTAETQCRNWLKTGNCLYGNTCRFV
HGPSPRGKGYSSNYRRSPERPTGDLRERMKNKRQDVDTEPQKRNTEESSSPVRKESSRGR
HREKEDIKITKERTPESEEENVEWETNRDDSDNGDINYDYVHELSLEMKRQKIQRELMKL
EQENMEKREEIIIKKEVSPEVVRSKLSPSPSLRKSSKSPKRKSSPKSSSASKKDRKTSAV
SSPLLDQQRNSKTNQSKKKGPRTPSPPPPIPEDIALGKKYKEKYKVKDRIEEKTRDGKDR
GRDFERQREKRDKPRSTSPAGQHHSPISSRHHSSSSQSGSSIQRHSPSPRRKRTPSPSYQ
RTLTPPLRRSASPYPSHSLSSPQRKQSPPRHRSPMREKGRHDHERTSQSHDRRHERREDT
RGKRDREKDSREEREYEQDQSSSRDHRDDREPRDGRDRRDARDTRDRRELRDSRDMRDSR
EMRDYSRDTKESRDPRDSRSTRDAHDYRDREGRDTHRKEDTYPEESRSYGRNHLREESSR
TEIRNESRNESRSEIRNDRMGRSRGRVPELPEKGSRGSRGSQIDSHSSNSNYHDSWETRS
SYPERDRYPERDNRDQARDSSFERRHGERDRRDNRERDQRPSSPIRHQGRNDELERDERR
EERRVDRVDDRRDERARERDRERERDREREREREREREREREKERELERERARERERERE
KERDRERDRDRDHDRERERERERDREKEREREREEREREREREREREREREREREREREARER
DKERERQRDWEDKDKGRDDRREKREEIREDRNPRDGHDERKSKKRYRNEGSPSPRQSPKR
RREHSPDSDAYNSGDDKNEKHRLLSQVVRPQESRSLSPSHLTEDRQGRWKEEDRKPERKE
SSRRYEEQELKEKVSSVDKQREQTEILESSRMRAQDIIGHHQSEDRETSDRAHDENKKKA
KIQKKPIKKKKEDDVGIERGNIETTSEDGQVFSPKKGQKKKSIEKKRKKSKGDSDISDEE
AAQQSKKKRGPRTPPITTKEELVEMCNGKNGILEDSQKKEDTAFSDWSDEDVPDRTEVTE
AEHTATATTPGSTPSPLSSLLPPPPPVATATATTVPATLAATTAAAATSFSTSAITISTS
ATPTNTTNNTFANEDSHRKCHRTRVEKVETPHVTIEDAQHRKPMDQKRSSSLGSNRSNRS
HTSGRLRSPSNDSAHRSGDDQSGRKRVLHSGSRDREKTKSLEITGERKSRIDQLKRGEPS
RSTSSDRQDSRSHSSRRSSPESDRQVHSRSGSFDSRDRLQERDRYEHDRERERERRDTRQ
REWDRDADKDWPRNRDRDRLRERERERERDKRRDLDRERERLISDSVERDRDRDRDRTFE
SSQIESVKRCEAKLEGEHERDLESTSRDSLALDKERMDKDLGSVQGFEETNKSERTESLE
AGDDESKLDDAHSLGSGAGEGYEPISDDELDEILAGDAEKREDQQDEEKMPDPLDVIDVD
WSGLMPKHPKEPREPGAALLKFTPGAVMLRVGISKKLAGSELFAKVKETCQRLLEKPKGS
FILL*
>ENSG00000171940|ENST00000302342
MQSKVTGNMPTQSLLMYMDGPEVIGSSLGSPMEMEDALSMKGTAVVPFRATQEKNVIQIE
GYMPLDCMFCSQTFTHSEDLNKHVLMQHRPTLCEPAVLRVEAEYLSPLDKSQVRTEPPKE
KNCKENEFSCEVCGQTFRVAFDVEIHMRTHKDSFTYGCNMCGRRFKEPWFLKNHMRTHNG
KSGARSKLQQGLESSPATINEVVQVHAAESISSPYKICMVCGFLFPNKESLIEHRKVHTK
KTAFGTSSAQTDSPQGGMPSSREDFLQLFNLRPKSHPETGKKPVRCIPQLDPFTTFQAWQ
LATKGKVAICQEVKESGQEGSTDNDDSSSEKELGETNKGSCAGLSQEKEKCKHSHGEAPS
VDADPKLPSSKEKPTHCSECGKAFRTYHQLVLHSRVHKKDRRAGAESPTMSVDGRQPGTC
SPDLAAPLDENGAVDRGEGGSEDGSEDGLPEGIHLDKNDDGGKIKHLTSSRECSYCGKFF
RSNYYLNIHLRTHTGEKPYKCEFCEYAAAQKTSLRYHLERHHKEKQTDVAAEVKNDGKNQ
DTEDALLTADSAQTKNLKRFFDGAKDVTGSPPAKQLKEMPSVFQNVLGSAVLSPAHKDTQ

DFHKNAADDSADKVNKNPTPAYLDLLKKRSAVETQANNLICRTKADVTPPPDGSTTHNLE
VSPKEKQTETAADCRYRPSVDCHEKPLNLSVGALHNCPAISLSKSLIPSITCPFCTFKTF
YPEVLMMHQRLEHKYNPDVHKNCRNKSLLRSRRTGCPPALLGKDVPPLSSFCKPKPKSAF
PAQSKSLPSAKGKQSPPGPGKAPLTSGIDSSTLAPSNLKSHRPQQNVGVQGAATRQQQSE
MFPKTSVSPAPDKTKRPETKLKPLPVAPSQPTLGSSNINGSIDYPAKNDSPWAPPGRDYF
CNRSASNTAAEFGEPLPKRLKSSVVALDVDQPGANYRRGYDLPKYHMVRGITSLLPQDCV
YPSQALPPKPRFLSSSEVDSPNVLTVQKPYGGSGPLYTCVPAGSPASSSTLEGKRPVSYQ
HLSNSMAQKRNYENFIGNAHYRPNDKKT*
>ENSG00000006576|ENST00000307305
MASKVTDAIVWYQKKIGAYDQQIWEKSVEQREIKGLRNKPKKTAHVKPDLIDVDLVRGSA
FAKAKPESPWTSLTRKGIVRVVFFPFFFRWWLQVTSKVIFFWLLVLYLLQVAAIVLFCST
SSPHSIPLTEVIGPIWLMLLLGTVHCQIVSTRTPKPPLSTGGKRRRKLRKAAHLEVHREG
DGSSTTDNTQEGAVQNHGTSTSHSVGTVFRDLWHAAFFLSGSKKAKNSIDKSTETDNGYV
SLDGKKTVKSGEDGIQNHEPQCETIRPEETAWNTGTLRNGPSKDTQRTITNVSDEVSSEE
GPETGYSLRRHVDRTSEGVLRNRKSHHYKKHYPNEDAPKSGTSCSSRCSSSRQDSESARP
ESETEDVLWEDLLHCAECHSSCTSETDVENHQINPCVKKEYRDDPFHQSHLPWLHSSHPG
LEKISAIVWEGNDCKKADMSVLEISGMIMNRVNSHIPGIGYQIFGNAVSLILGLTPFVFR
LSQATDLEQLTAHSASELYVIAFGSNEDVIVLSMVIISFVVRVSLVWIFFFLLCVAERTY
KQRLLFAKLFGHLTSARRARKSEVPHFRLKKVQNIKMWLSLRSYLKRRGPQRSVDVIVSS
AFLLTISVVFICCAQLLHVHEIFLDCHYNWELVIWCISLTLFLLRFVTLGSETSKKYSNT
SILLTEQINLYLKMEKKPNKKEELTLVNNVLKLATKLLKELDSPFRLYGLTMNPLLYNIT
QVVILSAVSGVISDLLGFNLKLWKIKS*
>ENSG00000163634|ENST00000295899
MGAVTDDEVIRKRLLIDGDGAGDDRRINLLVKSFIKWCNSGSQEEGYSQYQRMLSTLSQC
EFSMGKTLLVYDMNLREMENYEKIYKEIECSIAGAHEKIAECKKQILQAKRIRKNRQEYD
ALAKVIQHHPDRHETLKELEALGKELEHLSHIKESVEDKLELRRKQFHVLLSTIHELQQT
LENDEKLSEVEEAQEASMETDPKP*
>ENSG00000162702|ENST00000294740
MKIGSGFLSGGGGTGSSGGSGSGGGGSGGGGGGGSSGRRAEMEPTFPQGMVMFNHRLPPV
TSFTRPAGSAAPPPQCVLSSSTSAAPAAEPPPPPAPDMTFKKEPAASAAAFPSQRTSWGF
LQSLVSIKQEKPADPEEQQSHHHHHHHHYGGLFAGAEERSPGLGGGEGGSHGVIQDLSIL
HQHVQQQPAQHHRDVLLSSSSRTDDHHGTEEPKQDTNVKKAKRPKPESQGIKAKRKPSAS
SKPSLVGDGEGAILSPSQKPHICDHCSAAFRSSYHLRRHVLIHTGERPFQCSQCSMGFIQ
KYLLQRHEKIHSREKPFGCDQCSMKFIQKYHMERHKRTHSGEKPYKCDTCQQYFSRTDRL
LKHRRTCGEVIVKGATSAEPGSSNHTNMGNLAVLSQGNTSSSRRKTKSKSIAIENKEQKT
GKTNESQISNNINMQSYSVEMPTVSSSGGIIGTGIDELQKRVPKLIFKKGSRKNTDKNYL
NFVSPLPDIVGQKSLSGKPSGSLGIVSNNSVETIGLLQSTSGKQGQISSNYDDAMQFSKK
RRYLPTASSNSAFSINVGHMVSQQSVIQSAGVSVLDNEAPLSLIDSSALNAEIKSCHDKS
GIPDEVLQSILDQYSNKSESQKEDPFNIAEPRVDLHTSGEHSELVQEENLSPGTQTPSND
KASMLQEYSKYLQQAFEKSTNASFTLGHGFQFVSLSSPLHNHTLFPEKQIYTTSPLECGF
GQSVTSVLPSSLPKPPFGMLFGSQPGLYLSALDATHQQLTPSQELDDLIDSQKNLETSSA
FQSSSQKLTSQKEQKNLESSTGFQIPSQELASQIDPQKDIEPRTTYQIENFAQAFGSQFK
SGSRVPMTFITNSNGEVDHRVRTSVSDFSGYTNMMSDVSEPCSTRVKTPTSQSYR*
>ENSG00000153147|ENST00000283131

MSSAAEPPPPPPPESAPSKPAASIASGGSNSSNKGGPEGVAAQAVASAASAGPADAEMEE
IFDDASPGKQKEIQEPDPTYEEKMQTDRANRFEYLLKQTELFAHFIQPAAQKTPTSPLKM
KPGRPRIKKDEKQNLLSVGDYRHRRTEQEEDEELLTESSKATNVCTRFEDSPSYVKWGKL
RDYQVRGLNWLISLYENGINGILADEMGLGKTLQTISLLGYMKHYRNIPGPHMVLVPKST
LHNWMSEFKRWVPTLRSVCLIGDKEQRAAFVRDVLLPGEWDVCVTSYEMLIKEKSVFKKF
NWRYLVIDEAHRIKNEKSKLSEIVREFKTTNRLLLTGTPLQNNLHELWSLLNFLLPDVFN
SADDFDSWFDTNNCLGDQKLVERLHMVLRPFLLRRIKADVEKSLPPKKEVKIYVGLSKMQ
REWYTRILMKDIDILNSAGKMDKMRLLNILMQLRKCCNHPYLFDGAEPGPPYTTDMHLVT
NSGKMVVLDKLLPKLKEQGSRVLIFSQMTRVLDILEDYCMWRNYEYCRLDGQTPHDERQD
SINAYNEPNSTKFVFMLSTRAGGLGINLATADVVILYDSDWNPQVDLQAMDRAHRIGQTK
TVRVFRFITDNTVEERIVERAEMKLRLDSIVIQQGRLVDQNLNKIGKDEMLQMIRHGATH
VFASKESEITDEDIDGILERGAKKTAEMNEKLSKMGESSLRNFTMDTESSVYNFEGEDYR
EKQKIAFTEWIEPPKRERKANYAVDAYFREALRVSEPKAPKAPRPPKQPNVQDFQFFPPR
LFELLEKEILFYRKTIGYKVPRNPELPNAAQAQKEEQLKIDEAESLNDEELEEKEKLLTQ
GFTNWNKRDFNQFIKANEKWGRDDIENIAREVEGKTPEEVIEYSAVFWERCNELQDIEKI
MAQIERGEARIQRRISIKKALDTKIGRYKAPFHQLRISYGTNKGKNYTEEEDRFLICMLH
KLGFDKENVYDELRQCIRNSPQFRFDWFLKSRTAMELQRRCNTLITLIERENMELEEKEK
AEKKKRGPKPSTQKRKMDGAPDGRGRKKKLKL*
>ENSG00000151962|ENST00000281722
MNEENIDGTNGCSKVRTGIQNEAALLALMEKTGYNMVQENGQRKFGGPPPGWEGPPPPRG
CEVFVGKIPRDMYEDELVPVFERAGKIYEFRLMMEFSGENRGYAFVMYTTKEEAQLAIRI
LNNYEIRPGKFIGVCVSLDNCRLFIGAIPKEKKKEEILDEMKKVTEGVVDVIVYPSATDK
TKNRGFAFVEYESHRAAAMARRKLIPGTFQLWGHTIQVDWADPEKEVDEETMQRVKVLYV
RNLMISTTEETIKAEFNKFKPGAVERVKKLRDYAFVHFFNREDAVAAMSVMNGKCIDGAS
IEVTLAKPVNKENTWRQHLNGQISPNSENLIVFANKEESHPKTLGKLPTLPARLNGQHSP
SPPEVERCTYPFYPGTKLTPISMYSLKSNHFNSAVMHLDYYCNKNNWAPPEYYLYSTTSQ
DGKVLLVYKIVIPAIANGSQSYFMPDKLCTTLEDAKELAAQFTLLHLDYNFHRSSINSLS
PVSATLSSGTPSVLPYTSRPYSYPGYPLSPTISLANGSHVGQRLCISNQASFF*
>ENSG00000183434|ENST00000310125
MAKYVSLTEANEELKVLMDENQTSRPVAVHTSTVNPLGKQLLPKTFGQSSVNIDQQVVIG
MPQRPAASNIPVVGSPNPPSTHFASQNQHSYSSPPWAGQHNRKGEKNGMGLCRLSMKVWE
TVQRKGTTSCQEVVGELVAKFRAASNHASPNESAYDVKNIKRRTYDALNVLMAMNIISRE
KKKIKWIGLTTNSAQNCQNLRVERQKRLERIKQKQSELQQLILQQIAFKNLVLRNQYVEE
QVSQRPLPNSVIHVPFIIISSSKKTVINCSISDDKSEYLFKFNSSFEIHDDTEVLMWMGM
TFGLESGSCSAEDLKMARNLVPKALEPYVTEMAQGTFGGVFTTAGSRSNGTWLSASDLTN
IAIGMLATSSGGSQYSGSRVETPAVEEEEEEDNNDDDLSENDEDD*
>ENSG00000167395|ENST00000300850
MEDTPPSLSCSDCQRHFPSLPELSRHRELLHPSPNQDSEEADSIPRPYRCQQCGRGYRHP
GSLVNHRRTHETGLFPCTTCGKDFSNPMALKSHMRTHAPEGRRRHRPPRPKEATPHLQGE
TVSTDSWGQRLGSSEGWENQTKHTEETPDCESVPDPRAASGTWEDLPTRQREGLASHPGP
EDGADGWGPSTNSARAPPLPIPASSLLSNLEQYLAESVVNFTGGQEPTQSPPAEEERRYK
CSQCGKTYKHAGSLTNHRQSHTLGIYPCAICFKEFSNLMALKNHSRLHAQYRPYHCPHCP
RVFRLPRELLEHQQSHEGERQEPRWEEKGMPTTNGHTDESSQDQLPSAQMLNGSAELSTS
GELEDSGLEEYRPFRCGDCGRTYRHAGSLINHRKSHQTGVYPCSLCSKQLFNAAALKNHV

RAHHRPRQGVGENGQPSVPPAPLLLAETTHKEEEDPTTTLDHRPYKCSECGRAYRHRGSL
VNHRHSHRTGEYQCSLCPRKYPNLMALRNHVRVHCKAARRSADIGAEGAPSHLKVELPPD
PVEAEAAPHTDQDHVCKHEEEATDITPAADKTAAHICSICGLLFEDAESLERHGLTHGAG
EKENSRTETTMSPPRAFACRDCGKSYRHSGSLINHRQTHQTGDFSCGACAKHFHTMAAMK
NHLRRHSRRRSRRHRKRAGGASGGREAKLLAAESWTRELEDNEGLESPQDPSGESPHGAE
GNLESDGDCLQAESEGDKCGLERDETHFQGDKESGGTGEGLERKDASLLDNLDIPGEEGG
GTHFCDSLTGVDEDQKPATGQPNSSSHSANAVTGWQAGAAHTCSDCGHSFPHATGLLSHR
PCHPPGIYQCSLCPKEFDSLPALRSHFQNHRPGEATSAQPFLCCLCGMIFPGRAGYRLHR
RQAHSSSGMTEGSEEEGEEEGVAEAAPARSPPLQLSEAELLNQLQREVEALDSAGYGHIC
GCCGQTYDDLGSLERHHQSQSSGTTADKAPSPLGVAGDAMEMVVDSVLEDIVNSVSGEGG
DAKSQEGAGTPLGDSLCIQGGESLLEAQPRPFRCNQCGKTYRHGGSLVNHRKIHQTGDFL
CPVCSRCYPNLAAYRNHLRNHPRCKGSEPQVGPIPEAAGSSELQVGPIPEGGSNKPQHMA
EEGPGQAEVEKLQEELKVEPLEEVARVKEEVWEETTVKGEEIEPRLETAEKGCQTEASSE
RPFSCEVCGRSYKHAGSLINHRQSHQTGHFGCQACSKGFSNLMSLKNHRRIHADPRRFRC
SECGKAFRLRKQLASHQRVHMERRGGGGTRKATREDRPFRCGQCGRTYRHAGSLLNHRRS
HETGQYSCPTCPKTYSNRMALKDHQRLHSENRRRRAGRSRRTAVRCALCGRSFPGRGSLE
RHLREHEETEREPANGQGGLDGTAASEANLTGSQGLETQLGGAEPVPHLEDGVPRPGERS
QSPIRAASSEAPEPLSWGAGKAGGWPVGGGLGNHSGGWVPQFLTRSEEPEDSVHRSPCHA
GDCQLNGPTLSHMDSWDNRDNSSQLQPGSHSSCSQCGKTYCQSGSLLNHNTNKTDRHYCL
LCSKEFLNPVATKSHSHNHIDAQTFACPDCGKAFESHQELASHLQAHARGHSQVPAQMEE
ARDPKAGTGEDQVVLPGQGKAQEAPSETPRGPGESVERARGGQAVTSMAAEDKERPFRCT
QCGRSYRHAGSLLNHQKAHTTGLYPCSLCPKLLPNLLSLKNHSRTHTDPKRHCCSICGKA
FRTAARLEGHGRVHAPREGPFTCPHCPRHFRRRISFVQHQQQHQEEWTVAGSGAPVAPVT
GRGDLPLPPPPTPTTPLLDPSPQWPADLSFSL*
>ENSG00000163659|ENST00000295924
MEMETTEPEPDCVVQPPSPPDDFSCQMRLSEKITPLKTCFKKKDQKRLGTGTLRSLRPIL
NTLLESGSLDGVFRSRNQSTDENSLHEPMMKKAMEINSSCPPAENNMSVLIPDRTNVGDQ
IPEAHPSTEAPERVVPIQDHSFPSETLSGTVADSTPAHFQTDLLHPVSSDVPTSPDCLDK
VIDYVPGIFQENSFTIQYILDTSDKLSTELFQDKSEEASLDLVFELVNQLQYHTHQENGI
EICMDFLQGTCIYGRDCLKHHTVLPYHWQIKRTTTQKWQSVFNDSQEHLERFYCNPENDR
MRMKYGGQEFWADLNAMNVYETTEFDQLRRLSTPPSSNVNSIYHTVWKFFCRDHFGWREY
PESVIRLIEEANSRGLKEVRFMMWNNHYILHNSFFRREIKRRPLFRSCFILLPYLQTLGG
VPTQAPPPLEATSSSQIICPDGVTSANFYPETWVYMHPSQDFIQVPVSAEDKSYRIIYNL
FHKTVPEFKYRILQILRVQNQFLWEKYKRKKEYMNRKMFGRDRIINERHLFHGTSQDVVD
GICKHNFDPRVCGKHATMFGQGSYFAKKASYSHNFSKKSSKGVHFMFLAKVLTGRYTMGS
HGMRRPPPVNPGSVTSDLYDSCVDNFFEPQIFVIFNDDQSYPYFVIQYEEVSNTVSI*
>ENSG00000175646|ENST00000312511
MARYRCCRSQSRSRYYRQRQRSRRRRRRSCQTRRRAMRCCRPRYRPRCRRH*
>ENSG00000172667|ENST00000311417
MILLQHAVLPPPKQPSPSPPMSVATRSTGTLQLPPQKPFGQEASLPLAGEEELSKGGEQD
CALEELCKPLYCKLCNVTLNSAQQAQAHYQGKNHGKKLRNYYAANSCPPPARMSNVVEPA
ATPVVPVPPQMGSFKPGGRVILATENDYCKLCDASFSSPAVAQAHYQGKNHAKRLRLAEA
QSNSFSESSELGQRRARKEGNEFKMMPNRRNMYTVQNNSAGPYFNPRSRQRIPRDLAMCV
TPSGQFYCSMCNVGAGEEMEFRQHLESKQHKSKVSEQRYRNEMENLGYV*

>ENSG00000165259|ENST00000297977
MNLRSVFTVEQQRILQRYYENGMTNQSKNCFQLILQCAQETKLDFSVVRTWVGNKRRKMS
SKNSESGTATTGTSLSAPDITVRNVVNIARPSSQQSSWTSANNDVIVTGIYSPASSSSRQ
GTNKHTDTQITEAHKIPIQKTATKNDTEFQLHIPVQRQVAHCKNASLLLGEKTIILSRQT
SVLNAGNSVFNHAKKNYGNSSVQASEMTVPQKPSVCHRPCKIEPVGIQRSYKPEHTGPAL
HNLCGQKPTIRDPYCRTQNLEIREVFSLAVSDYPQRILGGNAPQKPSSAEGNCLSIAMET
GDAEDEYAREEELASMRAQIPSYSRFYESGSSLRAENQSTTLPGPGRNMPNSQMVNIRDM
SDNVLYQNRNYHLTPRTSLHTASSTMYSNTNPLRSNFSPHFASSNQLRLSQNQNNYQISG
NLTVPWITGCSRKRALQDRTQFSDRDLATLKKYWDNGMTSLGSVCREKIEAVATELNVDC
EIVRTWIGNRRRKYRLMGIEVPPPRGGPADFSEQPESGSLSALTPGEEAGPEVGEDNDRN
DEVSICLSEGSSQEEPNEVVPNDARAHKEEDHHAVTTDNVKIEIIDDEESDMISNSEVEQ
VNSFLDYKNEEVKFIENELEIQKQKYFKLQTFVRSLILAMKADDKEQQQALLSDLPPELE
EMDFNHASLEPDDTSFSVSSLSEKNVSESL*
>ENSG00000157110|ENST00000287771
MNNGGKAEKENTPSEANLQEEEVRTLFVSGLPLDIKPRELYLLFRPFKGYEGSLIKLTSK
QPVGFVSFDSRSEAEAAKNALNGIRFDPEIPQTLRLEFAKANTKMAKNKLVGTPNPSTPL
PNTVPQFIAREPYELTVPALYPSSPEVWAPYPLYPAELAPALPPPAFTYPASLHAQLCEG
QTVRRSHPLSAPSPDSASLAWFPV*
>ENSG00000137074|ENST00000309615
MMRVCWLVRQDSRHQRIRLPHLEAVVIGRGPETKITDKKCSRQQEFEEEAKNPGLETHRK
RKRSGNSDSIERDAAQEAEAGTGLEPGSNSGQCSVPLKKGKDAPIKKESLGHWSQGLKIS
MQDPKMQVYKDEQVVVIKDKYPKARYHWLVLPWTSISSLKAVAREHLELLKHMHTVGEKV
IVDFAGSSKLRFRLGYHAIPSMSHVHLHVISQDFDSPCLKNKKHWNSFNTEYFLESQAVI
EMVQEAGRVTVRDGMPELLKLPLRCHECQQLLPSIPQLKEHLRKHWTQ*
>ENSG00000116580|ENST00000271883
MLPCKKRRTTVTESLQHKGNQEENNVDLESAVKPESDQVKDLSSVSLSWDPSHGRVAGFE
VQSLQDAGNQLGMEDTSLSSGMLTQNTNVPILEGVDVAISQGITLPSLESFHPLNIHIGK
GKLHATGSKRGKKMTLRPGPVTQEDRCDHLTLKEPFSGEPSEEVKEEGGKPQMNSEGEIP
SLPSGSQSAKPVSQPRKSTQPDVCASPQEKPLRTLFHQPEEEIEDGGLFIPMEEQDNEES
EKRRKKKKGTKRKRDGRGQEGTLAYDLKLDDMLDRTLEDGAKQHNLTAVNVRNILHEVIT
NEHVVAMMKAAISETEDMPMFEPKMTRSKLKEVVEKGVVIPTWNISPIKKANEIKPPQFV
DIHLEEDDSSDEEYQPDDEEEDETAEESLLESDVESTASSPRGAKKSRLRQSSEMTETDE
ESGILSEAEKVTTPAIRHISAEVVPMGPPPPPKPKQTRDSTFMEKLHAVDEELASSPVCM
DSFQPMDDSLIAFRTRSKMPLKDVPLGQLEAELQAPDITPDMYDPNTADDEDWKMWLGGL
MNDDVGNEDEADDDDDPEYNFLEDLDEPDTEDFRTDRAVRITKKEVNELMEELFETFQDE
MGFSNMEDDGPEEEECVAEPRPNFNTPQALRFEEPLANLLNEQHRTVKELFEQLKMKKSS
AKQLQEVEKVKPQSEKVHQTLILDPAQRKRLQQQMQQHVQLLTQIHLLATCNPNLNPEAT
TTRIFLKELGTFAQSSIALHHQYNPKFQTLFQPCNLMGAMQLIEDFSTHVSIDCSPHKTV
KKTANEFPCLPKQVAWILATSKVFMYPELLPVCSLKAKNPQDKIVFTKAEDNLLALGLKH
FEGTEFPNPLISKYLLTCKTAHQLTVRIKNLNMNRAPDNIIKFYKKTKQLPVLGKCCEEI
QPHQWKPPIEREEHRLPFWLKASLPSIQEELRHMADGAREVGNMTGTTEINSDRSLEKDN
LELGSESRYPLLLPKGVVLKLKPVATRFPRKAWRQKRSSVLKPLLIQPSPSLQPSFNPGK
TPARSTHSEAPPSKMVLRIPHPIQPATVLQTVPGVPPLGVSGGESFESPAALPAVPPEAR
TSFPLSESQTLLSSAPVPKVMLPSLAPSKFRKPYVRRRPSKRRGVKASPCMKPAPVIHHP

ASVIFTVPATTVKIVSLGGGCNMIQPVNAAVAQSPQTIPITTLLVNPTSFPCPLNQSLVA
SSVSPLIVSGNSVNLPIPSTPEDKAHVNVDIACAVADGENAFQGLEPKLEPQELSPLSAT
VFPKVEHSPGPPLADAECQEGLSENSACRWTVVKTEEGRQALEPLPQGIQESLNNPTPGD
LEEIVKMEPEEAREEISGSPERDICDDIKVEHAVELDTGAPSEELSSAGEVTKQTVLQKE
EERSQPTKTPSSSQEPPDEGTSGTDVNKGSSKNALSSMDPEVRLSSPPGKPEDSSSVDGQ
SVGTPVGPETGGEKNGPEEEEEEDFDDLTQDEEDEMSSASEESVLSVPELQETMEKLTWL
ASERRMSQEGESEEENSQEENSEPEEEEEEEEAEGMESLQKEDEMTDEAVGDSAEKPPTFA
SPETAPEVETSRTPPGESIKAAGKGRNNHRARNKRGSRARASKDTSKLLLLYDEDILERD
PLREQKDLAFAQAYLTRVREALQHIPGKYEDFLQVIYEFESSTQRRTAVDLYKSLQILLQ
DWPQLLKDFAAFLLPEQALACGLFEEQQAFEKSRKFLRQLEICFAENPSHHQKIIKVLQG
CADCLPQEITELKTQMWQLLKGHDHLQDEFSIFFDHLRPAASRMGDFEEINWTEEKEYEF
DGFEEVALPDVEEEEEPPKIPTASKNKRKKEIGVQNHDKETEWPDGAKDCACSCHEGGPD
SKLKKSKRRSCSHCSSKVCDSKSYKSKEPHELVGSSPHREASPMPGAKEAGQGKDMMEEE
APEERESTEATQSRTVRTTRKGEMPVSGLAVGSTLPSPREVTVTERLLLDGPPPHSPETP
QFPPTTGAVLYTVKRNQVGPEVRSCPKASPRLQKEREGQKAVSESEALMLVWDASETEKL
PGTVEPPASFLSPVSSKTRDAGRRHVSGKPDTQERWLPSSRARVKTRDRTCPVHESPSGI
DTSETSPKAPRGGLAKDSGTQAKGPEGEQQPKAAEATVCANNSKVSSTGEKVVLWTREAD
RVILTMCQEQGAQPQTFNIISQQLGNKTPAEVSHRFRELMQLFHTACEASSEDEDDATST
SNADQLSDHGDLLSEEELDE*
>ENSG00000164002|ENST00000296380
MAETREEETVSAEASGFSDLSDSEFLEFLDLEDAQESKALVNMPGPSSESLGKDDKPISL
QNWKRGLDILSPMERFHLKYLYVTDLATQNWCELQTAYGKELPGFLAPEKAAVLDTGASI
HLARELELHDLVTVPVTTKEDAWAIKFLNILLLIPTLQSEGHIREFPVFGEGEGVLLVGV
IDELHYTAKGELELAELKTRRRPMLPLEAQKKKDCFQVSLYKYIFDAMVQGKVTPASLIH
HTKLCLEKPLGPSVLRHAQQGGFSVKSLGDLMELVFLSLTLSDLPVIDILKIEYIHQETA
TVLGTEIVAFKEKEVRAKVQHYMAYWMGHREPQGVDVEEAWKCRTCTYADICEWRKGSGV
LSSTLAPQVKKAK*
>ENSG00000159140|ENST00000300278
MATNIEQIFRSFVVSKFREIQQELSSGRNEGQLNGETNTPIEGNQAGDAAASARSLPNEE
IVQKIEEVLSGVLDTELRYKPDLKEGSRKSRCVSVQTDPTDEIPTKKSKKHKKHKNKKKK
KKKEKEKKYKRQPEESESKTKSHDDGNIDLESDSFLKFDSEPSAVALELPTRAFGPSETN
ESPAVVLEPPVVSMEVSEPHILETLKPATKTAELSVVSTSVISEQSEQSVAVMPEPSMTK
ILDSFAAAPVPTTTLVLKSSEPVVTMSVEYQMKSVLKSVESTSPEPSKIMLVEPPVAKVL
EPSETLVVSSETPTEVYPEPSTSTTMDFPESSAIEALRLPEQPVDVPSEIADSSMTRPQE
LPELPKTTALELQESSVASAMELPGPPATSMPELQGPPVTPVLELPGPSATPVPELPGPL
STPVPELPGPPATAVPELPGPSVTPVPQLSQELPGLPAPSMGLEPPQEVPEPPVMAQELP
GLPLVTAAVELPEQPAVTVAMELTEQPVTTTELEQPVGMTTVEHPGHPEVTTATGLLGQP
EATMVLELPGQPVATTALELPGQPSVTGVPELPGLPSATRALELSGQPVATGALELPGPL
MAAGALEFSGQSGAAGALELLGQPLATGVLELPGQPGAPELPGQPVATVALEISVQSVVT
TSELSTMTVSQSLEVPSTTALESYNTVAQELPTTLVGETSVTVGVDPLMAPESHILASNT
METHILASNTMDSQMLASNTMDSQMLASNTMDSQMLASSTMDSQMLATSSMDSQMLATSS
MDSQMLATSTMDSQMLATSSMDSQMLATSSMDSQMLATSSMDSQMLATSSMDSQMLATST
MDSQMLATSTMDSQMLATSSMDSQMLASGTMDSQMLASGTMDAQMLASGTMDAQMLASST
QDSAMLGSKSPDPYRLAQDPYRLAQDPYRLGHDPYRLGHDAYRLGQDPYRLGHDPYRLTP

DPYRMSPRPYRIAPRSYRIAPRPYRLAPRPLMLASRRSMMMSYAAERSMMSSYERSMMSY
ERSMMSPMAERSMMSAYERSMMSAYERSMMSPMAERSMMSAYERSMMSAYERSMMSPMAD
RSMMSMGADRSMMSSYSAADRSMMSSYSAADRSMMSSYTADRSMMSMAADSYTDSYTDTY
TEAYMVPPLPPEEPPTMPPLPPEEPPMTPPLPPEEPPEGPALPTEQSALTAENTWPTEVP
SSPSEESVSQPEPPVSQSEISEPSAVPTDYSVSASDPSVLVSEAAVTVPEPPPEPESSIT
LTPVESAVVAEEHEVVPERPVTCMVSETPAMSAEPTVLASEPPVMSETAETFDSMRASGH
VASEVSTSLLVPAVTTPVLAESILEPPAMAAPESSAMAVLESSAVTVLESSTVTVLESST
VTVLEPSVVTVPEPPVVAEPDYVTIPVPVVSALEPSVPVLEPAVSVLQPSMIVSEPSVSV
QESTVTVSEPAVTVSEQTQVIPTEVAIESTPMILESSIMSSHVMKGINLSSGDQNLAPEI
GMQEIALHSGEEPHAEEHLKGDFYESEHGINIDLNINNHLIAKEMEHNTVCAAGTSPVGE
IGEEKILPTSETKQRTVLDTYPGVSEADAGETLSSTGPFALEPDATGTSKGIEFTTASTL
SLVNKYDVDLSLTTQDTEHDMVISTSPSGGSEADIEGPLPAKDIHLDLPSNNNLVSKDTE
EPLPVKESDQTLAALLSPKESSGGEKEVPPPPKETLPDSGFSANIEDINEADLVRPLLPK
DMERLTSLRAGIEGPLLASDVGRDRSAASPVVSSMPERASESSSEEKDDYEIFVKVKDTH
EKSKKNKNRDKGEKEKKRDSSLRSRSKRSKSSEHKSRKRTSESRSRARKRSSKSKSHRSQ
TRSRSRSRRRRRSSRSRSKSRGRRSVSKEKRKRSPKHRSKSRERKRKRSSSRDNRKTVRA
RSRTPSRRSRSHTPSRRRRSRSVGRRRSFSISPSRRSRTPSRRSRTPSRRSRTPSRRSRT
PSRRSRTPSRRSRTPSRRRRSRSVVRRRSFSISPVRLRRSRTPLRRRFSRSPIRRKRSRS
SERGRSPKRLTDLDKAQLLEIAKANAAAMCAKAGVPLPPNLKPAPPPTIEEKVAKKSGGA
TIEELTEKCKQIAQSKEDDDVIVNKPHVSDEEEEEPPFYHHPFKLSEPKPIFFNLNIAAA
KPTPPKSQVTLTKEFPVSSGSQHRKKEADSVYGEWVPVEKNGEENKDDDNVFSSNLPSEG
RVKRQGRVRRQMKQPAASHLTVTRCNSLCGTKPQSEKHRIAENSVITSLPNIGPSLHLWE
GSPRYNYLASRFASRLYSSRFWW*
>ENSG00000146109|ENST00000274849
MEAEESEKAATEQEPLEGTEQTLDAEEEQEESEEAACGSKKRVVPGIVYLGHIPPRFRPL
HVRNLLSAYGEVGRVFFQAEDRFVRRKKKAAAAAGGKKRSYTKDYTEGWVEFRDKRIAKR
VAASLHNTPMGARRRSPFRYDLWNLKYLHRFTWSHLSEHLAFERQVRRQRLRAEVAQAKR
ETDFYLQSVERGQRFLAADGDPARPDGSWTFAQRPTEQELRARKAARPGGRERARLATAQ
DKARSNKGLLARIFGAPPPSESMEGPSLVRDS*
>ENSG00000167548|ENST00000301067
MDSQKLAGEDKDSEPAADGPAASEDPSATESDLPNPHVGEVSVLSSGSPRLQETPQDCSG
GPVRRCALCNCGEPSLHGQRELRRFELPFDWPRCPVVSPGGSPGPNEAVLPSEDLSQIGF
PEGLTPAHLGEPGGSCWAHHWCAAWSAGVWGQEGPELCGVDKAIFSGISQRCSHCTRLGA
SIPCRSPGCPRLYHFPCATASGSFLSMKTLQLLCPEHSEGAAYLEEARCAVCEGPGELCD
LFFCTSCGHHYHGACLDTALTARKRAGWQCPECKVCQACRKPGNDSKMLVCETCDKGYHT
FCLKPPMEELPAHSWKCKACRVCRACGAGSAELNPNSEWFENYSLCHRCHKAQGGQTIRS
VAEQHTPVCSRFSPPEPGDTPTDEPDALYVACQGQPKGGHVTSMQPKEPGPLQCEAKPLG
KAGVQLEPQLEAPLNEEMPLLPPPEESPLSPPPEESPTSPPPEASRLSPPPEELPASPLP
EALHLSRPLEESPLSPPPEESPLSPPPESSPFSPLEESPLSPPEESPPSPALETPLSPPP
EASPLSPPFEESPLSPPPEELPTSPPPEASRLSPPPEESPMSPPPEESPMSPPPEASRLF
PPFEESPLSPPPEESPLSPPPEASRLSPPPEDSPMSPPPEESPMSPPPEVSRLSPLPVVS
RLSPPPEESPLSPPPEESPTSPPPEASRLSPPPEDSPTSPPPEDSPASPPPEDSLMSLPL
EESPLLPLPEEPQLCPRSEGPHLSPRPEEPHLSPRPEEPHLSPQAEEPHLSPQPEEPCLC
AVPEEPHLSPQAEGPHLSPQPEELHLSPQTEEPHLSPVPEEPCLSPQPEESHLSPQSEEP

CLSPRPEESHLSPELEKPPLSPRPEKPPEEPGQCPAPEELPLFPPPGEPSLSPLLGEPAL
SEPGEPPLSPLPEELPLSPSGEPSLSPQLMPPDPLPPPLSPIITAAAPPALSPLGELEYP
FGAKGDSDPESPLAAPILETPISPPPEANCTDPEPVPPMILPPSPGSPVGPASPILMEPL
PPQCSPLLQHSLVPQNSPPSQCSPPALPLSVPSPLSPIGKVVGVSDEAELHEMETEKVSE
PECPALEPSATSPLPSPMGDLSCPAPSPAPALDDFSGLGEDTAPLDGIDAPGSQPEPGQT
PGSLASELKGSPVLLDPEELAPVTPMEVYPECKQTAGQGSPCEEQEEPRAPVAPTPPTLI
KSDIVNEISNLSQGDASASFPGSEPLLGSPDPEGGGSLSMELGVSTDVSPARDEGSLRLC
TDSLPETDDSLLCDAGTAISGGKAEGEKGRRRSSPARSRIKQGRSSSFPGRRRPRGGAHG
GRGRGRARLKSTASSIETLVVADIDSSPSKEEEEEDDDTMQNTVVLFSNTDKFVLMQDMC
VVCGSFGRGAEGHLLACSQCSQCYHPYCVNSKITKVMLLKGWRCVECIVCEVCGQASDPS
RLLLCDDCDISYHTYCLDPPLLTVPKGGWKCKWCVSCMQCGAASPGFHCEWQNSYTHCGP
CASLVTCPICHAPYVEEDLLIQCRHCERWMHAGCESLFTEDDVEQAADEGFDCVSCQPYV
VKPVAPVAPPELVPMKVKEPEPQYFRFEGVWLTETGMALLRNLTMSPLHKRRQRRGRLGL
PGEAGLEGSEPSDALGPDDKKDGDLDTDELLKGEGGVEHMECEIKLEGPVSPDVEPGKEE
TEESKKRKRKPYRPGIGGFMVRQRKSHTRTKKGPAAQAEVLSGDGQPDEVIPADLPAEGA
VEQSLAEGDEKKKQQRRGRKKSKLEDMFPAYLQEAFFGKELLDLSRKALFAVGVGRPSFG
LGTPKAKGDGGSERKELPTSQKGDDGPDIADEESRGLEGKADTPGPEDGGVKASPVPSDP
EKPGTPGEGMLSSDLDRISTEELPKMESKDLQQLFKDVLGSEREQHLGCGTPGLEGSRTP
LQRPFLQGGLPLGNLPSSSPMDSYPGLCQSPFLDSRERGGFFSPEPGEPDSPWTGSGGTT
PSTPTTPTTEGEGDGLSYNQRSLQRWEKDEELGQLSTISPVLYANINFPNLKQDYPDWSS
RCKQIMKLWRKVPAADKAPYLQKAKDNRAAHRINKVQKQAESQINKQTKVGDIARKTDRP
ALHLRIPPQPGALGSPPPAAAPTIFIGSPTTPAGLSTSADGFLKPPAGSVPGPDSPGELF
LKLPPQVPAQVPSQDPFGLAPAYPLEPRFPTAPPTYPPYPSPTGAPAQPPMLGASSRPGA
GQPGEFHTTPPGTPRHQPSTPDPFLKPRCPSLDNLAVPESPGVGGGKASEPLLSPPPFGE
SRKALEVKKEELGASSPSYGPPNLGFVDSPSSGTHLGGLELKTPDVFKAPLTPRASQVEP
QSPGLGLRPQEPPPAQALAPSPPSHPDIFRPGSYTDPYAQPPLTPRPQPPPPESCCALPP
RSLPSDPFSRVPASPQSQSSSQSPLTPRPLSAEAFCPSPVTPRFQSPDPYSRPPSRPQSR
DPFAPLHKPPRPQPPEVAFKAGSLAHTSLGAGGFPAALPAGPAGELHAKVPSGQPPNFVR
SPGTGAFVGTPSPMRFTFPQAVGEPSLKPPVPQPGLPPPHGINSHFGPGPTLGKPQSTNY
TVATGNFHPSGSPLGPSSGSTGESYGLSPLRPPSVLPPPAPDGSLPYLSHGASQRSGITS
PVEKREDPGTGMGSSLATAELPGTQDPGMSGLSQTELEKQRQRQRLRELLIRQQIQRNTL
RQEKETAAAAAGAVGPPGSWGAEPSSPAFEQLSRGQTPFAGTQDKSSLVGLPPSKLSGPI
LGPGSFPSDDRLSRPPPPATPSSMDVNSRQLVGGSQAFYQRAPYPGSLPLQQQQQQLWQQ
QQATAATSMRFAMSARFPSTPGPELGRQALGSPLAGISTRLPGPGEPVPGPAGPAQFIEL
RHNVQKGLGPGGTPFPGQGPPQRPRFYPVSEDPHRLAPEGLRGLAVSGLPPQKPSAPPAP
ELNNSLHPTPHTKGPTLPTGLELVNRPPSSTELGRPNPLALEAGKLPCEDPELDDDFDAH
KALEDDEELAHLGLGVDVAKGDDELGTLENLETNDPHLDDLLNGDEFDLLAYTDPELDTG
DKKDIFNEHLRLVESANEKAEREALLRGVEPGPLGPEERPPPAADASEPRLASVLPEVKP
KVEEGGRHPSPCQFTIATPKVEPAPAANSLGLGLKPGQSMMGSRDTRMGTGPFSSSGHTA
EKASFGATGGPPAHLLTPSPLSGPGGSSLLEKFELESGALTLPGGPAASGDELDKMESSL
VASELPLLIEDLLEHEKKELQKKQQLSAQLQPAQQQQQQQQHSLLSAPGPAQAMSLPHE
GSSPSLAGSQQQLSLGLAGARQPGLPQPLMPTQPPAHALQQRLAPSMAMVSNQGHMLSGQ
HGGQAGLVPQQSSQPVLSQKPMGTMPPSMCMKPQQLAMQQQLANSFFPDTDLDKFAAEDI
IDPIAKAKMVALKGIKKVMAQGSIGVAPGMNRQQVSLLAQRLSGGPSSDLQNHVAAGSGQ

ERSAGDPSQPRPNPPTFAQGVINEADQRQYEEWLFHTQQLLQMQLKVLEEQIGVHRKSRK
ALCAKQRTAKKAGREFPEADAEKLKLVTEQQSKIQKQLDQVRKQQKEHTNLMAEYRNKQQ
QQQQQQQQQQQQHSAVLALSPSQSPRLLTKLPGQLLPGHGLQPPQGPPGGQAGGLRLTPG
GMALPGQPGGPFLNTALAQQQQQQHSGGAGSLAGPSGGFFPGNLALRSLGPDSRLLQERQ
LQLQQQRMQLAQKLQQQQQQQQQQQHLLGQVAIQQQQQQGPGVQTNQALGPKPQGLMPPS
SHQGLLVQQLSPQPPQGPQGMLGPAQVAVLQQQHPGALGPQGPHRQVLMTQSRVLSSPQL
AQQGQGLMGHRLVTAQQQQQQQQHQQQGSMAGLSHLQQSLMSHSGQPKLSAQPMGSLQQL
QQQQQLQQQQQLQQQQQQQLQQQQQLQQQQLQQQQQQQQLQQQQQQQLQQQQQQLQQQQQ
QQQQQFQQQQQQQQMGLLNQSRTLLSPQQQQQQQVALGPGMPAKPLQHFSSPGALGPTLL
LTGKEQNTVDPAVSSEATEGPSTHQGGPLAIGTTPESMATEPGEVKPSLSGDSQLLLVQP
QPQPQPSSLQLQPPLRLPGQQQQQVSLLHTAGGGSHGQLGSGSSSEASSVPHLLAQPSVS
LGDQPGSMTQNLLGPQQPMLERPMQNNTGPQPPKPGPVLQSGQGLPGVGIMPTVGQLRAQ
LQGVLAKNPQLRHLSPQQQQQLQALLMQRQLQQSQAVRQTPPYQEPGTQTSPLQGLLGCQ
PQLGGFPGPQTGPLQELGAGPRPQGPPRLPAPPGALSTGPVLGPVHPTPPPSSPQEPKRP
SQLPSPSSQLPTEAQLPPTHPGTPKPQGPTLEPPPGRVSPAAAQLADTLFSKGLGPWDPP
DNLAETQKPEQSSLVPGHLDQVNGQVVPEASQLSIKQEPREEPCALGAQSVKREANGEPI
GAPGTSNHLLLAGPRSEAGHLLLQKLLRAKNVQLSTGRGSEGLRAEINGHIDSKLAGLEQ
KLQGTPSNKEDAAARKPLTPKPKRVQKASDRLVSSRKKLRKEDGVRASEALLKQLKQELS
LLPLTEPAITANFSLFAPFGSGCPVNGQSQLRGAFGSGALPTGPDYYSQLLTKNNLSNPP
TPPSSLPPTPPPSVQQKMVNGVTPSEELGEHPKDAASARDSERALRDTSEVKSLDLLAAL
PTPPHNQTEDVRMESDEDSDSPDSIVPASSPESILGEEAPRFPHLGSGRWEQEDRALSPV
IPLIPRASIPVFPDTKPYGALGLEVPGKLPVTTWEKGKGSEVSVMLTVSAAAAKNLNGVM
VAVAELLSMKIPNSYEVLFPESPARAGTEPKKGEAEGPGGKEKGLEGKSPDTGPDWLKQF
DAVLPGYTLKSQLDILSLLKQESPAPEPPTQHSYTYNVSNLDVRQLSAPPPEEPSPPPSP
LAPSPASPPTEPLVELPTEPLAEPPVPSPLPLASSPESARPKPRARPPEEGEDSRPPRLK
KWKGVRWKRLRLLLTIQKGSGRQEDEREVAEFMEQLGTALRPDKVPRDMRRCCFCHEEGD
GATDGPARLLNLDLDLWVHLNCALWSTEVYETQGGALMNVEVALHRGLLTKCSLCQRTGA
TSSCNRMRCPNVYHFACAIRAKCMFFKDKTMLCPMHKIKGPCEQELSSFAVFRRVYIERD
EVKQIASIIQRGERLHMFRVGGLVFHAIGQLLPHQMADFHSATALYPVGYEATRIYWSLR
TNNRRCCYRCSIGENNGRPEFVIKVIEQGLEDLVFTDASPQAVWNRIIEPVAAMRKEADM
LRLFPEYLKGEELFGLTVHAVLRIAESLPGVESCQNYLFRYGRHPLMELPLMINPTGCAR
SEPKILTHYKRPHTLNSTSMSKAYQSTFTGETNTPYSKQFVHSKSSQYRRLRTEWKNNVY
LARSRIQGLGLYAAKDLEKHTMVIEYIGTIIRNEVANRREKIYEEQNRGIYMFRINNEHV
IDATLTGGPARYINHSCAPNCVAEVVTFDKEDKIIIISSRRIPKGEELTYDYQFDFEDDQ
HKIPCHCGAWNCRKWMN*
>ENSG00000170100|ENST00000306502
MLENYENLASVGHHLFQPSVIYWLEQEEELRAGRRAVLQEWRLKTKGPALRQDRSWFRAS
NETQTARSHNGGQLCDRTQCGEAFSEHSGLSTHVRTQNTGDSCVSNHYERDFFIPCQKTL
FKIGEQFSVLGQCGKAFSSTPNVVSQQACTRDRSLDYSSCGEVFLNQSYLQARAGSHNGE
ETWKWKPCGKALTHSMGCATPVEMHAVRNPHVCRECGKAFRYTAYLTGRVQVHPGEKPCE
LEECGKASPVSSSLTQHVRIHAAEKPCECKECGKAFTGLSGLSKHVQTDPGQKPYECKDC
GKACGGFYLLNEHGKTHTREKPFACVVCGKYFRNSSCLNNHVRIHTGIKPYTCSYCGKAF
TVRCGLTRHVRTHTGEKPYTCKDCGKAFCTSSGLTEHVRTHTGEKPYECKDCGKSFTVSS
SLTEHARIHTGEKPYECKQCGKAFTGRSGLTKHMRTHTGEKPYECKDCGKAYNRVYLLNE

HVKTHTEEKPFICTVCRKSFRNSSCLNKHIQIHTGIKPYECKDCGKTFTVSSSLTEHIRT
HTGEKPYECKVCGKAFTTSSHLIVHIRTHTGEKPYICKECGKAFASSSHLIEHRRTHTGE
KPYICNECGKAFRASSHLHKHGRIHTGQKPYKCKECGKAYNRFYLLKEHLKTYTEEQVFV
CKDCGKSFKNSSCLNHHTQIHTDEKPF*
>ENSG00000167394|ENST00000300849
MEVEAAEARSPAPGYKRSGRRYKCLSCTKTFPNAPRAARHAATHGPADCSEEVAEVKPKP
ETEAKAEEASGEKVSGSAAKPRPYACPLCPKAYKTAPELRSHGRSHTGEKPFPCPECGRR
FMQPVCLRVHLASHAGELPFRCAHCPKAYGALSKLKIHQRGHTGERPYACADCGKSFADP
SVFRKHRRTHAGLRPYSCERCGKAYAELKDLRNHERSHTGERPFLCSECGKSFSRSSSLT
CHQRIHAAQKPYRCPACGKGFTQLSSYQSHERTHSGEKPFLCPRCGRMFSDPSSFRRHQR
AHEGVKPYHCEKCGKDFRQPADLAMHRRVHTGDRPFKCLQCDKTFVASWDLKRHALVHSG
QRPFRCEECGRAFAERASLTKHSRVHSGERPFHCNACGKSFVVSSSLRKHERTHRSSEAA
GVPPAQELVVGLALPVGVAGESSAAPAAGAGLGDPPAGLLGLPPESGGVMATQWQVVGMT
VEHVECQDAGVREAPGPLEGAGEAGGEEADEKPPQFVCRECKETFSTMTLLRRHERSHPE
LRPFPCTQCGKSFSDRAGLRKHSRTHSSVRPYTCPHCPKAFLSASDLRKHERTHPVPMGT
PTPLEPLVALLGMPEEGPA*
>ENSG00000167981|ENST00000301744
MASMPPTPEAQGPILFEDLAVYFSQEECVTLHPAQRSLSKDGTKESLEDAALMGEEGKPE
INQQLSLESMELDELALEKYPIAAPLVPYPEKSSEDGVGNPEAKILSGTPTYKRRVISLL
VTIENHTPLVELSEYLGTNTLSEILDSPWEGAKNVYKCPECDQNFSDHSYLVLHQKIHSG
EKKHKCGDCGKIFNHRANLRTHRRIHTGEKPYKCAKCSASFRQHSHLSRHMNSHVKEKPY
TCSICGRGFMWLPGLAQHQKSHSAENTYESTNCDKHFNEKPNLALPEETFVSGPQYQHTK
CMKSFRQSLYPALSEKSHDEDSERCSDDGDNFFSFSKFKPLQCPDCDMTFPCFSELISHQ
NIHTEERPHKCKTCEESFALDSELACHQKSHMLAEPFKCTVCGKTFKSNLHLITHKRTHI
KNTT*
>ENSG00000071462|ENST00000265758
MASRGRRPEHGGPPELFYDETEARKYVRNSRMIDIQTRMAGRALELLYLPENKPCYLLDI
GCGTGLSGSYLSDEGHYWVGLDISPAMLDEAVDREIEGDLLLGDMGQGIPFKPGTFDGCI
SISAVQWLCNANKKSENPAKRLYCFFASLFSVLVRGSRAVLQLYPENSEQLELITTQATK
AGFSGGMVVDYPNSAKAKKFYLCLFSGPSTFIPEGLSENQDEVEPRESVFTNERFPLRMS
RRGMVRKSRAWVLEKKERHRRQGREVRPDTQYTGRKRKPRF*
>ENSG00000029153|ENST00000266503
MAAEEEAAAGGKVLREENQCIAPVVSSRVSPGTRPTAMGSFSSHMTEFPRKRKGSDSDPS
QSGIMTEKVVEKLSQNPLTYLLSTRIEISASSGSRVEDGEHQVKMKAFREAHSQTEKRRR
DKMNNLIEELSAMIPQCNPMARKLDKLTVLRMAVQHLRSLKGLTNSYVGSNYRPSFLQDN
ELRHLILKTAEGFLFVVGCERGKILFVSKSVSKILNYDQASLTGQSLFDFLHPKDVAKVK
EQLSSFDISPREKLIDAKTGLQVHSNLHAGRTRVYSGSRRSFFCRIKSCKISVKEEHGCL
PNSKKKEHRKFYTIHCTGYLRSWPPNIVGMEEERNSKKDNSNFTCLVAIGRLQPYIVPQN
SGEINVKPTEFITRFAVNGKFVYVDQRATAILGYLPQELLGTSCYEYFHQDDHNNLTDKH
KAVLQSKEKILTDSYKFRAKDGSFVTLKSQWFSFTNPWTKELEYIVSVNTLVLGHSEPGE
ASFLPCSSQSSEESSRQSCMSVPGMSTGTVLGAGSIGTDIANEILDLQRLQSSSYLDDSS
PTGLMKDTHTVNCRSMSNKELFPPSPSEMGELEATRQNQSTVAVHSHEPLLSDGAQLDFD
ALCDNDDTAMAAFMNYLEAEGGLGDPGDFSDIQWTL*
>ENSG00000161551|ENST00000301399

MKNATIVMSVRREQGSSSGEGSLSFEDVAVGFTREEWQFLDQSQKVLYKEVMLENYINLV
SIGYRGTKPDSLFKLEQGEPPGIAEGAAHSQICPGFVIQSRRYAGKDSDAFGGYGRSCLH
IKRDKTLTGVKYHRCVKPSSPKSQLNDLQKICAGGKPHECSVCGRAFSRKAQLIQHQRTE
RGEKPHGCGECGKTFMRKIQLTEHQRTHTGEKPHECSECGKAFSRKSQLMVHQRTHTGEK
PYRCSKCGKAFSRKCRLNRHQRSHTGEKLYGCSVCGKAFSQKAYLTAHQRLHTGDKPYKC
SDCGRTFYFKSDLTRHQRIHTGEKPYECSECEKAFRSKSKLIQHQRTHTGERPYSCRECG
KAFAHMSVLIKHEKTHIRETAINSLTVEKPSSRSHTSLYMSELIQEQKTVNTVPIEMPSS
GTPPLLNKSERLVGRNVVIVEQPFPRNQAFVVNQEFEQRISLTNEVNVAPSVINYILYLT
DIVSE*
>ENSG00000197275|ENST00000297592
MRRSAAPSQLQGNSFKKPKFIPPGRSNPGLNEEITKLNPDIKLFEGVAINNTFLPSQNDL
RICSLNLPSEESTREINNRDNCSGKYCFEAPTLATLDPPHTVQTWMRRHRLVPVHYR*
>ENSG00000141448|ENST00000269216
MALTDGGWCLPKRFGAAGADASDSRAFPAREPSTPPSPISSSSSSCSRGGERGPGGASNC
GTPQLDTEAAAGPPARSLLLSSYASHPFGAPHGPSAPGVAGPGGNLSSWEDLLLFTDLDQ
AATASKLLWSSRGAKLSPFAPEQPEEMYQTLAALSSQGPAAYDGAPGGFVHSAAAAAAAA
AAASSPVYVPTTRVGSMLPGLPYHLQGSGSGPANHAGGAGAHPGWPQASADSPPYGSGGG
AAGGGAAGPGGAGSAAAHVSARFPYSPSPPMANGAAREPGGYAAAGSGGAGGVSGGGSSL
AAMGGREPQYSSLSAARPLNGTYHHHHHHHHHHPSPYSPYVGAPLTPAWPAGPFETPVLH
SLQSRAGAPLPVPRGPSADLLEDLSESRECVNCGSIQTPLWRRDGTGHYLCNACGLYSKM
NGLSRPLIKPQKRVPSSRRLGLSCANCHTTTTTLWRRNAEGEPVCNACGLYMKLHGVPRP
LAMKKEGIQTRKRKPKNINKSKTCSGNSNNSIPMTPTSTSSNSDDCSKNTSPTTQPTASG
AGAPVMTGAGESTNPENSELKYSGQDGLYIGVSLASPAEVTSSVRPDSWCALALA*
>ENSG00000189180|ENST00000307441
MCPRGYPEQSNWMSSPLYSTEVEQKSQESVSFKDVTVGFTQEEWQHLDPSQRALYRDVML
ENYSNLVSVGYCVHKPEVIFRLQQGEEPWKQEEEFPSQSFPEVWTADHLKERSQENQSKH
LWEVVFINNEMLTKEQGDVIGIPFNVDVSSFPSRKMFCQCDSCGMSFNTVSELVISKINY
LGKKSDEFNACGKLLLNIKHDETHTQEKNEVLKNRNTLSHHEETLQHEKIQTLEHNFEYS
ICQETLLEKAVFNTQKRENAEENNCDYNEFGRTLCDSSSLLFHQISPSRDNHYEFSDCEK
FLCVKSTLSKPHGVSMKHYDCGESGNNFRRKLCLSHLQKGDKGEKHFECNECGKAFWEKS
HLTRHQRVHTGQKPFQCNECEKAFWDKSNLTKHQRSHTGEKPFECNECGKAFSHKSALTL
HQRTHTGEKPYQCNACGKTFCQKSDLTKHQRTHTGLKPYECYECGKSFRVTSHLKVHQRT
HTGEKPFECLECGKSFSEKSNLTQHQRIHIGDKSYECNACGKTFYHKSLLTRHQIIHTGW
KPYECYECGKTFCLKSDLTVHQRTHTGQKPFACPECGKFFSHKSTLSQHYRTHTGEKPYE
CHECGKIFYNKSYLTKHNRTHTGEKPYECNECGKAFYQKSQLTQHQRIHIGEKPYKCNEC
GKAFCHKSALIVHQRTHTQEKPYKCNECGKSFCVKSGLIFHERKHTGEKPYECNECGKFF
RHKSSLTVHHRAHTGEKSCQCNECGKIFYRKSELAQHQRSHTGEKPYECNTCRKTFSQKS
NLIVHQRRHIGENLMNEMDIRNFQPQVSLHNASEYSHCGESPDDILNVQ*
>ENSG00000149054|ENST00000278319
MQPLSKLMAISKPRNLSLREQREVLRADMSWQQETNPVVETHDSEASRQKFRHFQYLKVS
GPHEALSQLWELCLQWLRPEIHTKKQIIELLVLEQFLAILPEEVRTWVNLQHPNNSKDMV
TLIEDVIEMLEDEDMPCKDSALQMGSIKEKMKAGSRTGKPQEPVTFKDVVVEFSKEEWGQ
LDSAVKNLYRNVMLENFRNLNSLRKAHLLSKPFESLKLESKKKRWIMEKEIPRKTIFDMK
SISGEESSHGVIMTRLTESGHPSSDAWKGENWLYRNQKKWDINLPQEAFIPETIYTEEED

FECSENKKSFDINSVSSICAIQVGIPSRKGSPKCDKFKTYFKFNLDSVGKQHSEYEYGND
LSLSTDIRHQKSHTTMNSYECYQCGKAFCRSSSLIRHQIIHTGEKPYKCSECGRFFNRRT
NLTKHQKLHAEAKACTSNKCGKAFSKSEDSNNPTLHFGNNFYQCVNCGKSFNRSSSLIRH
QMIHTGEKPFKCKECSKAFNRSSNLVKHQKLHTRDKS*
>ENSG00000167315|ENST00000285093
MALLRGVFVVAAKRTPFGAYGGLLKDFTATDLSEFAAKAALSAGKVSPETVDSVIMGNVL
QSSSDAIYLARHVGLRVGIPKETPALTINRLCGSGFQSIVNGCQEICVKEAEVVLCGGTE
SMSQAPYCVRNVRFGTKLGSDIKLEDSLWVSLTDQHVQLPMAMTAENLAVKHKISREECD
KYALQSQQRWKAANDAGYFNDEMAPIEVKTKKGKQTMQVDEHARPQTTLEQLQKLPPVFK
KDGTVTAGNASGVADGAGAVIIASEDAVKKHNFTPLARIVGYFVSGCDPSIMGIGPVPAI
SGALKKAGLSLKDMDLVEVNEAFAPQYLAVERSLDLDISKTNVNGGAIALGHPLGGSGSR
ITAHLVHELRRRGGKYAVGSACIGGGQGIAVIIQSTA*
>ENSG00000147889|ENST00000304494
MEPAAGSSMEPSADWLATAAARGRVEEVRALLEAGALPNAPNSYGRRPIQVMMMGSARVA
ELLLLHGAEPNCADPATLTRPVHDAAREGFLDTLVVLHRAGARLDVRDAWGRLPVDLAEE
LGHRDVARYLRAAAGGTRGSNHARIDAAEGPSDIPD*
>ENSG00000188033|ENST00000311437
MRRNSSLSFQMERPLEEQVQSKWSSSQGRTGTGGSDVLQMQNSEHHGQSIKTQTDSISLE
DVAVNFTLEEWALLDPGQRNIYRDVMRATFKNLACIGEKWKDQDIEDEHKNQGRNLRSPM
VEALCENKEDCPCGKSTSQIPDLNTNLETPTGLKPCDCSVCGEVFMHQVSLNRHMRSHTE
QKPNECHEYGEKPHKCKECGKTFTRSSSIRTHERIHTGEKPYECKECGKAFAFLFSFRNH
IRIHTGETPYECKECGKAFRYLTALRRHEKNHTGEKPYKCKQCGKAFIYYQPFLTHERTH
TGEKPYECKQCGKAFSCPTYLRSHEKTHTGEKPFVCRECGRAFFSHSSLRKHVKTHTGVQ
PYTCKKCGEAFKSSSSCEVHERTHFGEKPYECKQCGKAFNSSSYLQLHERVHTGEKTYEC
KECGKAFLYSTHFRIHERTHTREKPYECKQCGRVFIYFSHLRRHERSHTGVKPCECKQCG
KAFTCLNSLKVHKRIHTGERPFQCRQCGKAFSYSKSLHVHERTHSRQKP*
>ENSG00000173928|ENST00000312423
MPAAGPPLLLLGTPGSGKTALLFAAALEAAGEGQGPVLFLTRRPLQSMPRGTGTTLDPMR
LQKIRFQYPPSTRELFRLLCSAHEAPGPAPSLLLLDGLEEYLAEDPEPQEAAYLIALLLD
TAAHFSHRLGPGRDCGLMVALQTQEEAGSGDVLHLALLQRYFPAQCWLQPDAPGPGEHGL
RACLEPGGLGPRTEWWVTFRSDGEMMIAPWPTQAGDPSSGKGSSSGGQP*
>ENSG00000173757|ENST00000293328
MAVWIQAQQLQGEALHQMQALYGQHFPIEVRHYLSQWIESQAWDSVDLDNPQENIKATQL
LEGLVQELQKKAEHQVGEDGFLLKIKLGHYATQLQNTYDRCPMELVRCIRHILYNEQRLV
REANNGSSPAGSLADAMSQKHLQINQTFEELRLVTQDTENELKKLQQTQEYFIIQYQESL
RIQAQFGPLAQLSPQERLSRETALQQKQVSLEAWLQREAQTLQQYRVELAEKHQKTLQLL
RKQQTIILDDELIQWKRRQQLAGNGGPPEGSLDVLQSWCEKLAEIIWQNRQQIRRAEHLC
QQLPIPGPVEEMLAEVNATITDIISALVTSTFIIEKQPPQVLKTQTKFAATVRLLVGGKL
NVHMNPPQVKATIISEQQAKSLLKNENTRNDYSGEILNNCCVMEYHQATGTLSAHFRNMS
LKRIKRSDRRGAESVTEEKFTILFESQFSVGGNELVFQVKTLSLPVVVIVHGSQDNNATA
TVLWDNAFAEPGRVPFAVPDKVLWPQLCEALNMKFKAEVQSNRGLTKENLVFLAQKLFNN
SSSHLEDYSGLSVSWSQFNRENLPGRNYTFWQWFDGVMEVLKKHLKPHWNDGAILGFVNK
QQAHDLLINKPDGTFLLRFSDSEIGGITIAWKFDSQERMFWNLMPFTTRDFSIRSLADRL
GDLNYLIYVFPDRPKDEVYSKYYTPVPCESATAKAVDGYVKPQIKQVVPEFVNASADAGG

GSATYMDQAPSPAVCPQAHYNMYPQNPDSVLDTDGDFDLEDTMDVARRVEELLGRPMDSQ
WIPHAQS*
>ENSG00000079102|ENST00000265814
MISVKRNTWRALSLVIGDCRKKGNFEYCQDRTEKHSTMPDSPVDVKTQSRLTPPTMPPPP
TTQGAPRTSSFTPTTLTNGTSHSPTALNGAPSPPNGFSNGPSSSSSSSLANQQLPPACGA
RQLSKLKRFLTTLQQFGNDISPEIGERVRTLVLGLVNSTLTIEEFHSKLQEATNFPLRPF
VIPFLKANLPLLQRELLHCARLAKQNPAQYLAQHEQLLLDASTTSPVDSSELLLDVNENG
KRRTPDRTKENGFDREPLHSEHPSKRPCTISPGQRYSPNNGLSYQPNGLPHPTPPPPQHY
RLDDMAIAHHYRDSYRHPSHRDLRDRNRPMGLHGTRQEEMIDHRLTDREWAEEWKHLDHL
LNCIMDMVEKTRRSLTVLRRCQEADREELNYWIRRYSDAEDLKKGGGSSSSHSRQQSPVN
PDPVALDAHREFLHRPASGYVPEEIWKKAEEAVNEVKRQAMTELQKAVSEAERKAHDMIT
TERAKMERTVAEAKRQAAEDALAVINQQEDSSESCWNCGRKASETCSGCNTARYCGSFCQ
HKDWEKHHHICGQTLQAQQQGDTPAVSSSVTPNSGAGSPMDTPPAATPRSTTPGTPSTIE
TTPR*
>ENSG00000162227|ENST00000294168
MSEREERRFVEIPRESVRLMAESTGLELSDEVAALLAEDVCYRLREATQNSSQFMKHTKR
RKLTVEDFNRALRWSSVEAVCGYGSQEALPMRPAREGELYFPEDREVNLVELALATNIPK
GCAETAVRVHVSYLDGKGNLAPQGSVPSAVSSLTDDLLKYYHQVTRAVLGDDPQLMKVAL
QDLQTNSKIGALLPYFVYVVSGVKSVSHDLEQLHRLLQVARSLFRNPHLCLGPYVRCLVG
SVLYCVLEPLAASINPLNDHWTLRDGAALLLSHIFWTHGDLVSGLYQHILLSLQKILADP
VRPLCCHYGAVVGLHALGWKAVERVLYPHLSTYWTNLQAVLDDYSVSNAQVKADGHKVYG
AILVAVERLLKMKAQAAEPNRGGPGGRGCRRLDDLPWDSLLFQESSSGGGAEPSFGSGLP
LPPGGAGPEDPSLSVTLADIYRELYAFFGDSLATRFGTGQPAPTAPRPPGDKKEPAAAPD
SVRKMPQLTASAIVSPHGDESPRGSGGGGPASASGPAASESRPLPRVHRARGAPRQQGPG
TGTRDVFQKSRFAPRGAPHFRFIIAGRQAGRRCRGRLFQTAFPAPYGPSPASRYVQKLPM
IGRTSRPARRWALSDYSLYLPL*
>ENSG00000130511|ENST00000270061
MYAKGGKGSAVPSDSQAREKLALYVYEYLLHIGAQKSAQTFLSEIRWEKNITLGEPPGFL
HSWWCVFWDLYCAAPDRREACEHSGEAKAFQDYSAAAAPSPVMGSMAPGDTMAAGSMAAG
FFQGPPGSQPSPHNPNAPMMGPHGQPFMSPRFPGGPRPTLRMPSQPPAGLPGSQPLLPGA
MEPSPRAQGHPSMGGPMQRVTPPRGMASVGPQSYGGGMRPPPNSLAGPGLPAMNMGPGVR
GPWASPSGNSIPYSSSSPGSYTGPPGGGGPPGTPIMPSPGDSTNSSENMYTIMNPIGQGA
GRANFPLGPGPEGPMAAMSAMEPHHVNGSLGSGDMDGLPKSSPGAVAGLSNAPGTPRDDG
EMAAAGTFLHPFPSESYSPGMTMSV*
>ENSG00000106246|ENST00000292478
MDFVRLARLFARARPMGLFILQHLDPCRARWAGGREGLMRPMWAPFSSSSSQLPLGQERQ
ENTGSLGSDPSHSNSTATQEEDEEEEESFGTLSDKYSSRRLFRKSAAQFHNLRFGERRDE
QMEPEPKLWRGRRNTPYWYFLQCKHLIKEGKLVEALDLFERQMLKEERLQPMESNYTVLI
GGCGRVGYLKKAFNLYNQMKKRDLEPSDATYTALFNVCAESPWKDSALQSALKLRQQLQA
KNFELNLKTYHALLKMAAKCADLRMCLDVFKEIIHKGHVVTEETFSFLLMGCIQDKKTGF
RYALQVWRLMLSLGLQPSRDSYNLLLVAARDCGLGDPQVASELLLKPREEATVLQPPVSR
QRPRRTAQAKAGNLMSAMLHVEALERQLFLEPSQALGPPEPPEARVPGKAQPEVDTKAEP
SHTAALTAVALKPPPVELEVNLLTPGAVPPTVVSFGTVTTPADRLALIGGLEGFLSKMAE
HRQQPDIRTLTLLAEVVESGSPAESLLLALLDEHQVEADLTFFNTLVRKKSKLGDLEGAK

ALLPVLAKRGLVPNLQTFCNLAIGCHRPKDGLQLLTDMKKSQVTPNTHIYSALINAAIRK
LNYTYLISILKDMKQNRVPVNEVVIRQLEFAAQYPPTFDRYQGKNTYLEKIDGFRAYYKQ
WLTVMPAEETPHPWQKFRTKPQGDQDTGKEADDGCALGGR*
>ENSG00000244405|ENST00000306376
MDGFYDQQVPFMVPGKSRSEECRGRPVIDRKRKFLDTDLAHDSEELFQDLSQLQEAWLAE
AQVPDDEQFVPDFQSDNLVLHAPPPTKIKRELHSPSSELSSCSHEQALGANYGEKCLYNY
CAYDRKPPSGFKPLTPPTTPLSPTHQNPLFPPPQATLPTSGHAPAAGPVQGVGPAPAPHS
LPEPGPQQQTFAVPRPPHQPLQMPKMMPENQYPSEQRFQRQLSEPCHPFPPQPGVPGDNR
PSYHRQMSEPIVPAAPPPPQGFKQEYHDPLYEHGVPGMPGPPAHGFQSPMGIKQEPRDYC
VDSEVPNCQSSYMRGGYFSSSHEGFSYEKDPRLYFDDTCVVPERLEGKVKQEPTMYREGP
PYQRRGSLQLWQFLVTLLDDPANAHFIAWTGRGMEFKLIEPEEVARRWGIQKNRPAMNYD
KLSRSLRYYYEKGIMQKVAGERYVYKFVCDPDALFSMAFPDNQRPFLKAESECHLSEEDT
LPLTHFEDSPAYLLDMDRCSSLPYAEGFAY*
>ENSG00000142684|ENST00000270812
MGRSRRTGAHRAHSLARQMKAKRRRPDLDEIHRELRPQGSARPQPDPNAEFDPDLPGGGL
HRCLACARYFIDSTNLKTHFRSKDHKKRYEGVRRGLMDGCSADRALTWSAPNSCFSFSQA
EAAERRALQSGRGGEGSGYGILCAPQAAGSAHGSVH*
>ENSG00000143793|ENST00000272139
MFGSSRGGVRGGQDQFNWEDVKTDKQRENYLGNSLMAPVGRWQKGRDLTWYAKGRAPCAG
PSREEELAAVREAEREALLAALGYKNVKKQPTGLSKEDFAEVCKREGGDPEEKGVDRLLG
LGSASGSVGRVAMSREDKEAAKLGLSVFTHHRVESGGPGTSAASARRKPRAEDQTESSCE
SHRKSKKEKKKKKKRKHKKEKKKKDKEHRRPAEATSSPTSPERPRHHHHDSDSNSPCCKR
RKRGHSGDRRSPSRRWHDRGSEA*
>ENSG00000066422|ENST00000312938
MSSEESYRAILRYLTNEREPYAPGTEGNVKRKIRKAAACYVVRGGTLYYQRRQRHRKTFA
ELEVVLQPERRRDLIEAAHLGPGGTHHTRHQTWHYLSKTYWWRGILKQVKDYIKQCSKCQ
EKLDRSRPISDVSEMLEELGLDLESGEESNESEDDLSNFTSSPTTASKPAKKKPVSKHEL
VFVDTKGVVKRSSPKHCQAVLKQLNEQRLSNQFCDVTLLIEGEEYKAHKSVLSANSEYFR
DLFIEKGAVSSHEAVVDLSGFCKASFLPLLEFAYTSVLSFDFCSMADVAILARHLFMSEV
LEICESVHKLMEEKQLTVYKKGEVQTVASTQDLRVQNGGTAPPVASSEGTTTSLPTELGD
CEIVLLVNGELPEAEQNGEVGRQPEPQVSSEAESALSSVGCIADSHPEMESVDLITKNNQ
TELETSNNRENNTVSNIHPKLSKENVISSSPEDSGMGNDISAEDICAEDIPKHRQKVDQP
LKDQENLVASTAKTDFGPDDDTYRSRLRQRSVNEGAYIRLHKGMEKKLQKRKAVPKSAVQ
QVAQKLVQRGKKMKQPKRDAKENTEEASHKCGECGMVFQRRYALIMHKLKHERARDYKCP
LCKKQFQYSASLRAHLIRHTRKDAPSSSSSNSTSNEASGTSSEKGRTKREFICSICGRTL
PKLYSLRIHMLKHTGVKPHACQVCGKTFIYKHGLKLHQSLHQSQKQFQCELCVKSFVTKR
SLQEHMSIHTGESKYLCSVCGKSFHRGSGLSKHFKKHQPKPEVRGYHCTQCEKSFFEARD
LRQHMNKHLGVKPFQCQFCDKCYSWKKDWYSHVKSHSVTEPYRCNICGKEFYEKALFRRH
VKKATHGKKGRAKQNLERVCEKCGRKFTQLREYRRHMNNHEGVKPFECLTCGVAWADARS
LKRHVRTHTGERPYVCPVCSEAYIDARTLRKHMTKFHRDYVPCKIMLEKDTLQFHNQGTQ
VAHAVSILTAGMQEQESSGPQELETVVVTGETMEALEAVAATEEYPSVSTLSDQSIMQVV
NYVLAQQQGQKLSEVAEAIQTVKVEVAHISGGE*
>ENSG00000087903|ENST00000303657
MQNSEGGADSPASVALRPSAAAPPVPASPQRVLVQAASSNPKGAQMQPISLPRVQQVPQQ

VQPVQHVYPAQVQYVEGGDAVYTNGAIRTAYTYNPEPQMYAPSSTASYFEAPGGAQVTVA
ASSPPAVPSHSMVGITMDVGGSPIVSSAGAYLIHGGMDSTRHSLAHTSRSSPATLEMAIE
NLQKSEGITSHKSGLLNSHLQWLLDNYETAEGVSLPRSSLYNHYLRHCQEHKLDPVNAAS
FGKLIRSVFMGLRTRRLGTRGNSKYHYYGIRLKPDSPLNRLQEDTQYMAMRQQPMHQKPR
YRPAQKTDSLGDSGSHSGLHSTPEQTMAVQSQHHQQYIDVSHVFPEFPAPDLGSFLLQDG
VTLHDVKALQLVYRRHCEATVDVVMNLQFHYIEKLWLSFWNSKASSSDGPTSLPASDEDP
EGAVLPKDKLISLCQCDPILRWMRSCDHILYQALVEILIPDVLRPVPSTLTQAIRNFAKS
LEGWLTNAMSDFPQQVIQTKVGVVSAFAQTLRRYTSLNHLAQAARAVLQNTSQINQMLSD
LNRVDFANVQEQASWVCQCEESVVQRLEQDFKLTLQQQSSLDQWASWLDSVVTQVLKQHA
GSPSFPKAARQFLLKWSFYSSMVIRDLTLRSAASFGSFHLIRLLYDEYMFYLVEHRVAEA
TGETPIAVMGEFNDLASLSLTLLDKDDMGDEQRGSEAGPDARSLGEPLVKRERSDPNHSL
QGI*
>ENSG00000166794|ENST00000300026
MLRLSERNMKVLLAAALIAGSVFFLLLPGPSAADEKKKGPKVTVKVYFDLRIGDEDVGRV
IFGLFGKTVPKTVDNFVALATGEKGFGYKNSKFHRVIKDFMIQGGDFTRGDGTGGKSIYG
ERFPDENFKLKHYGPGWVSMANAGKDTNGSQFFITTVKTAWLDGKHVVFGKVLEGMEVVR
KVESTKTDSRDKPLKDVIIADCGKIEVEKPFAIAKE*
>ENSG00000100722|ENST00000302216
MEIGTEISRKIRSAIKGKLQELGAYVDEELPDYIMVMVANKKSQDQMTEDLSLFLGNNTI
RFTVWLHGVLDKLRSVTTEPSSLKSSDTNIFDSNVPSNKSNFSRGDERRHEAAVPPLAIP
SARPEKRDSRVSTSSQESKTTNVRQTYDDGAATRLMSTVKPLREPAPSEDVIDIKPEPDD
LIDEDLNFVQENPLSQKKPTVTLTYGSSRPSIEIYRPPASRNADSGVHLNRLQFQQQQNS
IHAAKQLDMQSSWVYETGRLCEPEVLNSLEETYSPFFRNNSEKMSMEDENFRKRKLPVVS
SVVKVKKFNHDGEEEEEDDDYGSRTGSISSSVSVPAKPERRPSLPPSKQANKNLILKAIS
EAQESVTKTTNYSTVPQKQTLPVAPRTRTSQEELLAEVVQGQSRTPRISPPIKEEETKGD
SVEKNQAEMSELSVAQKPEKLLERCKYWPACKNGDECAYHHPISPCKAFPNCKFAEKCLF
VHPNCKYDAKCTKPDCPFTHVSRRIPVLSPKPVAPPAPPSSSQLCRYFPACKKMECPFYH
PKHCRFNTQCTRPDCTFYHPTINVPPRHALKWIRPQTSE*
>ENSG00000163923|ENST00000296277
MSSHKTFTIKRFLAKKQKQNRPIPQWIQMKPGSKIRYNSKRRHWRRTKLGL*
>ENSG00000172379|ENST00000303329
MATPAAVNPPEMASDIPGSVTLPVAPMAATGQVRMAGAMPARGGKRRSGMDFDDEDGEGP
SKFSRENHSEIERRRRNKMTQYITELSDMVPTCSALARKPDKLTILRMAVSHMKSMRGTG
NKSTDGAYKPSFLTEQELKHLILEAADGFLFVVAAETGRVIYVSDSVTPVLNQPQSEWFG
STLYEQVHPDDVEKLREQLCTSENSMTGRILDLKTGTVKKEGQQSSMRMCMGSRRSFICR
MRCGNAPLDHLPLNRITTMRKRFRNGLGPVKEGEAQYAVVHCTGYIKAWPPAGMTIPEED
ADVGQGSKYCLVAIGRLQVTSSPVCMDMNGMSVPTEFLSRHNSDGIITFVDPRCISVIGY
QPQDLLGKDILEFCHPEDQSHLRESFQQVVKLKGQVLSVMYRFRTKNREWMLIRTSSFTF
QNPYSDEIEYIICTNTNVKQLQQQQAELEVHQRDGLSSYDLSQVPVPNLPAGVHEAGKSV
EKADAIFSQERDPRFAEMFAGISASEKKMMSSASAAGTQQIYSQGSPFPSGHSGKAFSSS
VVHVPGVNDIQSSSSTGQNMSQISRQLNQSQVAWTGSRPPFPGQQIPSQSSKTQSSPFGI
GTSHTYPADPSSYSPLSSPATSSPSGNAYSSLANRTPGFAESGQSSGQFQGRPSEVWSQW
QSQHHGQQSGEQHSHQQPGQTEVFQDMLPMPGDPTQGTGNYNIEDFADLGMFPPFSE*
>ENSG00000164104|ENST00000296503

MGKGDPNKPRGKMSSYAFFVQTCREEHKKKHPDSSVNFAEFSKKCSERWKTMSAKEKSKF
EDMAKSDKARYDREMKNYVPPKGDKKGKKKDPNAPKRPPSAFFLFCSEHRPKIKSEHPGL
SIGDTAKKLGEMWSEQSAKDKQPYEQKAAKLKEKYEKDIAAYRAKGKSEAGKKGPGRPTG
SKKKNEPEDEEEEEEEEEDEDEEEEEDEDEE*
>ENSG00000171574|ENST00000306910
MAGEAEAQLDPSLQGLVMFEDVTVYFSREEWGLLNVTQKGLYRDVMLENFALVSSLGLAP
SRSPVFTQLEDDEQSWVPSWVDVTPVSRAEARRGFGLDGLCRVEDERAHPEHLKSYRVIQ
HQDTHSEGKPRRHTEHGAAFPPGSSCGQQQEVHVAEKLFKCSDCGKVFLKAFALLDHLIT
HSEERPFRCPTGRSAFKKSAHINPRKIHTGETAHVCNECGKAFSYPSKLRKHQKVHTGIK
PFKCSDCGKTFNRKDALVLHQRIHTGERPYECSKCGKTFSVLSTLIRHRKVHIGERPYEC
TECGKFFKYNNSFILHQRVHTGERPFECKQCGKGYVTRSGLYQHWKVHTGERPYECSLCG
KTFTTRSYRNRHQQFHTEERSYECTECGKAFKHSSTLLQHKKVHTPERRQEDRAHGKVVS
C*
>ENSG00000164967|ENST00000297613
MEHYRKAGSVELPAPSPMPQLPPDTLEMRVRDGSKIRNLLGLALGRLEGGSARHVVFSGS
GRAAGKAVSCAEIVKRRVPGLHQLTKLRFLQTEDSWVPASPDTGLDPLTVRRHVPAVWVL
LSRDPLDPNECGYQPPGAPPGLGSMPSSSCGPRSRRRARDTRS*
>ENSG00000121057|ENST00000314126
MAIQFRSLFPLALPGMLALLGWWWFFSRKKGHVSSHDEQQVEAGAVQLRADPAIKEPLPV
EDVCPKVVSTPPSVTEPPEKELSTVSKLPAEPPALLQTHPPCRRSESSGILPNTTDMRLR
PGTRRDDSTKLELALTGGEAKSIPLECPLSSPKGVLFSSKSAEVCKQDSPFSRVPRKVQP
GYPVVPAEKRSSGERARETGGAEGTGDAVLGEKVLEEALLSREHVLELENSKGPSLASLE
GEEDKGKSSSSQVVGPVQEEEYVAEKLPSRFIESAHTELAKDDAAPAPPVADAKAQDRGV
EGELGNEESLDRNEEGLDRNEEGLDRNEESLDRNEEGLDRNEEIKRAAFQIISQVISEAT
EQVLATTVGKVAGRVCQASQLQGQKEESCVPVHQKTVLGPDTAEPATAEAAVAPPDAGLP
LPGLPAEGSPPPKTYVSCLKSLLSSPTKDSKPNISAHHISLASCLALTTPSEELPDRAGI
LVEDATCVTCMSDSSQSVPLVASPGHCSDSFSTSGLEDSCTETSSSPRDKAITPPLPEST
VPFSNGVLKGELSDLGAEDGWTMDAEADHSGVAAPPPGKRGTLITRCPGFFEC*
>ENSG00000168438|ENST00000307731
MSAAIAALAASYGSGSGSESDSDSESSRCPLPAADSLMHLTKSPSSKPSLAVAVDSAPEV
AVKEDLETGVHLDPAVKEVQYNPTYETMFAPEFGPENPFRTQQMAAPRNMLSGYAEPAHI
NDFMFEQQRRTFATYGYALDPSLDNHQVSAKYIGSVEEAEKNQGLTVFETGQKKTEKRKK
FKENDASNIDGFLGPWAKYVDEKDVAKPSEEEQKELDEITAKRQKKGKQEEEKPGEEKTI
LHVKEMYDYQGRSYLHIPQDVGVNLRSTMPPEKCYLPKKQIHVWSGHTKGVSAVRLFPLS
GHLLLSCSMDCKIKLWEVYGERRCLRTFIGHSKAVRDICFNTAGTQFLSAAYDRYLKLWD
TETGQCISRFTNRKVPYCVKFNPDEDKQNLFVAGMSDKKIVQWDIRSGEIVQEYDRHLGA
VNTIVFVDENRRFVSTSDDKSLRVWEWDIPVDFKYIAEPSMHSMPAVTLSPNGKWLACQS
MDNQILIFGAQNRFRLNKKKIFKGHMVAGYACQVDFSPDMSYVISGDGNGKLNIWDWKTT
KLYSRFKAHDKVCIGAVWHPHETSKVITCGWDGLIKLWD*
>ENSG00000172000|ENST00000307635
MDTVVFEDVVVDFTLEEWALLNPAQRKLYRDVMLETFKHLASVDNEAQLKASGSISQQDT
SGEKLSLKQKIEKFTRKNIWASLLGKNWEEHSVKDKHNTKERHLSRNPRVERPCKSSKGN
KRGRTFRKTRNCNRHLRKNCCTSVRRYECSQCGKLFTHSSSLIRHKRAHSGQKLYKCKEC
GKAFSRPSYLQTHEKTHSGEKPYACQSCGKTFLRSHSLTEHVRTHTGEKPYECGQCGKGF

SCPKSFRAHVMMHAGGRPYECKHCGKAFRCQKSFRVHMIMHAGGRPYECKQCGKAYCWAT
SFQRHVRIHNGEKPYKCGKCGKAFGWPSSLHKHARTHAKKKPVSGGSVGKSSARPRPSTD
VKSQTREKVYKCETCGKTYGWSSSLHKHERKHTGEKPVNAASVGKPSGGLCSSKNVRTQI
GQKPSKCEKCGKAFSCPKAFQGHVRSHTGKKSCTSK*
>ENSG00000106638|ENST00000305632
MELSQMSELMGLSVLLGLLALMATAAVARGWLRAGEERSGRPACQKANGFPPDKSSGSKK
QKQYQRIRKEKPQQHNFTHRLLAAALKSHSGNISCMDFSSNGKYLATCADDRTIRIWSTK
DFLQREHRSMRANVELDHATLVRFSPDCRAFIVWLANGDTLRVFKMTKREDGGYTFTATP
EDFPKKHKAPVIDIGIANTGKFIMTASSDTTVLIWSLKGQVLSTINTNQMNNTHAAVSPC
GRFVASCGFTPDVKVWEVCFGKKGEFQEVVRAFELKGHSAAVHSFAFSNDSRRMASVSKD
GTWKLWDTDVEYKKKQDPYLLKTGRFEEAAGAAPCRLALSPNAQVLALASGSSIHLYNTR
RGEKEECFERVHGECIANLSFDITGRFLASCGDRAVRLFHNTPGHRAMVEEMQGHLKRAS
NESTRQRLQQQLTQAQETLKSLGALKK*
>ENSG00000092330|ENST00000267415
MATPLVAGPAALRFAAAASWQVVRGRCVEHFPRVLEFLRSLRAVAPGLVRYRHHERLCMG
LKAKVVVELILQGRPWAQVLKALNHHFPESGPIVRDPKATKQDLRKILEAQETFYQQVKQ
LSEAPVDLASKLQELEQEYGEPFLAAMEKLLFEYLCQLEKALPTPQAQQLQDVLSWMQPG
VSITSSLAWRQYGVDMGWLLPECSVTDSVNLAEPMEQNPPQQQRLALHNPLPKAKPGTHL
PQGPSSRTHPEPLAGRHFNLAPLGRRRVQSQWASTRGGHKERPTVMLFPFRNLGSPTQVI
SKPESKEEHAIYTADLAMGTRAASTGKSKSPCQTLGGRALKENPVDLPATEQKENCLDCY
MDPLRLSLLPPRARKPVCPPSLCSSVITIGDLVLDSDEEENGQGEGKESLENYQKTKFDT
LIPTLCEYLPPSGHGAIPVSSCDCRDSSRPL*
>ENSG00000082258|ENST00000295238
MASGRGASSRWFFTREQLENTPSRRCGVEADKELSCRQQAANLIQEMGQRLNVSQLTINT
AIVYMHRFYMHHSFTKFNKNIISSTALFLAAKVEEQARKLEHVIKVAHACLHPLEPLLDT
KCDAYLQQTQELVILETIMLQTLGFEITIEHPHTDVVKCTQLVRASKDLAQTSYFMATNS
LHLTTFCLQYKPTVIACVCIHLACKWSNWEIPVSTDGKHWWEYVDPTVTLELLDELTHEF
LQILEKTPNRLKKIRNWRANQAARKPKVDGQVSETPLLGSSLVQNSILVDSVTGVPTNPS
FQKPSTSAFPAPVPLNSGNISVQDSHTSDNLSMLATGMPSTSYGLSSHQEWPQHQDSART
EQLYSQKQETSLSGSQYNINFQQGPSISLHSGLHHRPDKISDHSSVKQEYTHKAGSSKHH
GPISTTPGIIPQKMSLDKYREKRKLETLDLDVRDHYIAAQVEQQHKQGQSQAASSSSVTS
PIKMKIPIANTEKYMADKKEKSGSLKLRIPIPPTDKSASKEELKMKIKVSSSERHSSSDE
GSGKSKHSSPHISRDHKEKHKEHPSSRHHTSSHKHSHSHSGSSSGGSKHSADGIPPTVLR
SPVGLSSDGISSSSSSSRKRLHVNDASHNHHSKMSKSSKSSGGLRTSQHPRETGQEASGD
QRS*
>ENSG00000168795|ENST00000307750
MDFPGHFEQIFQQLNYQRLHGQLCDCVIVVGNRHFKAHRSVLAACSTHFRALFSVAEGDQ
TMNMIQLDSEVVTAEAFAALIDMMYTSTLMLGESNVMDVLLAASHLHLNSVVKACKHYLT
TRTLPMSPPSERVQEQSARMQRSFMLQQLGLSIVSSALNSSQNGEEQPAPMSSSMRSNLD
QRTPFPMRRLHKRKQSAEERARQRLRPSIDESAISDVTPENGPSGVHSREEFFSPDSLKI
VDNPKADGMTDNQEDSAIMFDQSFGTQEDAQVPSQSDNSAGNMAQLSMASRATQVETSFD
QEAAPEKSSFQCENPEVGLGEKEHMRVVVKSEPLSSPEPQDEVSDVTSQAEGSESVEVEG
VVVSAEKIDLSPESSDRSFSDPQSSTDRVGDIHILEVTNNLEHKSTFSISNFLNKSRGNN
FTANQNNDDNIPNTTSDCRLESEAPYLLSPEAGPAGGPSSAPGSHVENPFSEPADSHFVR

PMQEVMGLPCVQTSGYQGGEQFGMDFSRSGLGLHSSFSRVMIGSPRGGASNFPYYRRIAP
KMPVVTSVRSSQIPENSTSSQLMMNGATSSFENGHPSQPGPPQLTRASADVLSKCKKALS
EHNVLVVEGARKYACKICCKTFLTLTDCKKHIRVHTGEKPYACLKCGKRFSQSSHLYKHS
KTTCLRWQSSNLPSTLL*
>ENSG00000125520|ENST00000266077
MERPPPRAAGRDPSALRAEAPWLRAEGPGPRAAPVTVPTPPQGSSVGGGFAGLEFARPQE
SEPRASDLGAPRTWTGAAAGPRTPSAHIPVPAQRATPGKARLDEVMAAAALTSLSTSPLL
LGAPVAAFSPEPGLEPWKEALVRPPGSYSSSSNSGDWGWDLASDQSSPSTPSPPLPPEAA
HFLFGEPTLRKRKSPAQVMFQCLWKSCGKVLSTASAMQRHIRLVHLGRQAEPEQSDGEED
FYYTELDVGVDTLTDGLSSLTPVSPTASMPPAFPRLELPELLEPPALPSPLRPPAPPLPP
PPVLSTVANPQSCHSDRVYQGCLTPARLEPQPTEVGACPPALSSRIGVTLRKPRGDAKKC
RKVYGMERRDLWCTACRWKKACQRFLD*
>ENSG00000131503|ENST00000297183
MDVNSSKYPSLLLHSQEEKTSTATSKTQTRLEGEVTPNSLSTSYKTVSLPLSSPNIKLNL
TSPKRGQKREEGWKEVVRRSKKLSVPASVVSRIMGRGGCNITAIQDVTGAHIDVDKQKDK
NGERMITIRGGTESTRYAVQLINALIQDPAKELEDLIPKNHIRTPASTKSIHANFSSGVG
TTAASSKNAFPLGAPTLVTSQATTLSTFQPANKLNKNVPTNVRSSFPVSLPLAYPHPHFA
LLAAQTMQQIRHPRLPMAQFGGTFSPSPNTWGPFPVRPVNPGNTNSSPKHNNTSRLPNQN
GTVLPSESAGLATASCPITVSSVVAASQQLCVTNTRTPSSVRKQLFACVPKTSPPATVIS
SVTSTCSSLPSVSSAPITSGQAPTTFLPASTSQAQLSSQKMESFSAVPPTKEKVSTQDQP
MANLCTPSSTANSCSSSASNTPGAPETHPSSSPTPTSSNTQEEAQPSSVSDLSPMSMPFA
SNSEPAPLTLTSPRMVAADNQDTSNLPQLAVPAPRVSHRMQPRGSFYSMVPNATIHQDPQ
SIFVTNPVTLTPPQGPPAAVQLSSAVNIMNGSQMHINPANKSLPPTFGPATLFNHFSSLF
DSSQVPANQGWGDGPLSSRVATDASFTVQSAFLGNSVLGHLENMHPDNSKAPGFRPPSQR
VSTSPVATSAPPTLGQPKGVSASQDRKIPPPIGTERLARIRQGGSVAQAPAGTSFVAPVG
HSGIWSFGVNAVSEGLSGWSQSVMGNHPMHQQLSDPSTFSQHQPMERDDSGMVAPSNIFH
QPMASGFVDFSKGLPISMYGGTIIPSHPQLADVPGGPLFNGLHNPDPAWNPMIKVIQNST
ECTDAQQIWPGTWAPHIGNMHLKYVN*
>ENSG00000174796|ENST00000311638
MVKCCSAIGCASRCLPNSKLKGLTFHVFPTDENIKRKWVLAMKRLDVNAAGIWEPKKGDV
LCSRHFKKTDFDRSAPNIKLKPGVIPSIFDSPYHLQGKREKLHCRKNFTLKTVPATNYNH
HLVGASSCIEEFQSQFIFEHSYSVMDSPKKLKHKLDHVIGELEDTKESLRNVLDREKRFQ
KSLRKTIRELKDECLISQETANRLDTFCWDCCQESIEQDYIS*
>ENSG00000164330|ENST00000313708
MFGIQESIQRSGSSMKEEPLGSGMNAVRTWMQGAGVLDANTAAQSGVGLARAHFEKQPPS
NLRKSNFFHFVLALYDRQGQPVEIERTAFVGFVEKEKEANSEKTNNGIHYRLQLLYSNGI
RTEQDFYVRLIDSMTKQAIVYEGQDKNPEMCRVLLTHEIMCSRCCDKKSCGNRNETPSDP
VIIDRFFLKFFLKCNQNCLKNAGNPRDMRRFQVVVSTTVNVDGHVLAVSDNMFVHNNSKH
GRRARRLDPSEGTPSYLEHATPCIKAISPSEGWTTGGATVIIIGDNFFDGLQVIFGTMLV
WSELITPHAIRVQTPPRHIPGVVEVTLSYKSKQFCKGTPGRFIYTALNEPTIDYGFQRLQ
KVIPRHPGDPERLPKEVILKRAADLVEALYGMPHNNQEIILKRAADIAEALYSVPRNHNQ
LPALANTSVHAGMMGVNSFSGQLAVNVSEASQATNQGFTRNSSSVSPHGYVPSTTPQQTN
YNSVTTSMNGYGSAAMSNLGGSPTFLNGSAANSPYAIVPSSPTMASSTSLPSNCSSSSGI
FSFSPANMVSAVKQKSAFAPVVRPQTSPPPTCTSTNGNSLQAISGMIVPPM*

>ENSG00000149016|ENST00000278279
MAAVDSDVESLPRGGFRCCLCHVTTANRPSLDAHLGGRKHRHLVELRAARKAQGLRSVFV
SGFPRDVDSAQLSEYFLAFGPVASVVMDKDKPVPKAPESPSLDSALASPLDPQALACTPA
SPPDSQPPASPQDSEALDFETPSSSLAPQTPDSALASETLASPQS
>ENSG00000113648|ENST00000312469
MSSRGGKKKSTKTSRSAKAGVIFPVGRMLRYIKKGHPKYRIGVGAPVYMAAVLEYLTAEI
LELAGNAARDNKKGRVTPRHILLAVANDEELNQLLKGVTIASGGVLPNIHPELLAKKRGS
KGKLEAIITPPPAKKAKSPSQKKPVSKKAGGKKGARKSKKKQGEVSKAASADSTTEGTPA
DGFTVLSTKSLFLGQKLQVVQADIASIDSDAVVHPTNTDFYIGGEVGNTLEKKGGKEFVE
AVLELRKKNGPLEVAGAAVSAGHGLPAKFVIHCNSPVWGADKCEELLEKTVKNCLALADD
KKLKSIAFPSIGSGRNGFPKQTAAQLILKAISSYFVSTMSSSIKTVYFVLFDSESIGIYV
QEMAKLDAN*
>ENSG00000171530|ENST00000306388
MADPRVRQIKIKTGVVKRLVKEKVMYEKEAKQQEEKIEKMRAEDGENYDIKKQAEILQES
RMMIPDCQRRLEAAYLDLQRILVSKDFCVMFKFFFSIQYMESQKFKYLTLRTSWQYFLFT
CPLYRYILK*
>ENSG00000148737|ENST00000277945
LTVNASMSRFPPHMVPPHHTLHTTGIPHPAIVTPTVKQESSQSDVGSLHSSKHQDSKKEE
EKKKPHIKKPLNAFMLYMKEMRAKVVAECTLKESAAINQILGRRWHALSREEQAKYYELA
RKERQLHMQLYPGWSARDNYGKKKKRKRDKQPGETNDLSAPKKCRARFGLDQQNNWCGPC
RRKKKCVRYIQGEGSCLSPPSSDGSLL
>ENSG00000142534|ENST00000270625
MADIQTERAYQKQPTIFQNKKRVLLGETGKEKLPRYYKNIGLGFKTPKEAIEGTYIDKKC
PFTGNVSIRGRILSGVVTKMKMQRTIVIRRDYLHYIRKYNRFEKRHKNMSVHLSPCFRDV
QIGDIVTVGECRPLSKTVRFNVLKVTKAAGTKKQFQKF*
>ENSG00000147679|ENST00000309822
MKITRQKHAKKHLGFFRNNFGVREPYQILLDGTFCQAALRGRIQLREQLPRYLMGETQLC
TTRCVLKELETLGKDLYGAKLIAQKCQVRNCPHFKNAVSGSECLLSMVEEGNPHHYFVAT
QDQNLSVKVKKKPGVPLMFIIQNTMVLDKPSPKTIAFVKAVESGQLVSVHEKESIKHLKE
EQGLVKNTEQSRRKKRKKISGPNPLSCLKKKKKAPDTQSSASEKKRKRKRIRNRSNPKVL
SEKQNAEGE*
>ENSG00000170860|ENST00000306024
MADDVDQQQTTNTVEEPLDLIRLSLDERIYVKMRNDRELRGRLHAYDQHLNMILGDVEET
VTTIEIDEETYEEIYKSTKRNIPMLFVRGDGVVLVAPPLRVG*
>ENSG00000176472|ENST00000314228
MLERGAESAAGATDPSPTGKEPVTKEAPHQGPPQKPSQSAPGPTASAGSPPRPRRRPPPQ
RPHRCPDCDKAFSYPSKLATHRLAHGGARPHPCPDCPKAFSYPSKLAAHRLTHSGARPHP
CPHCPKSFGHRSKLAAHLWTHAPTRPYPCPDCPKSFCYPSKLAAHRHTHHATDARPYPCP
HCPKAFSFPSKLAAHRLCHDPPTAPGSQATAWHRCSSCGQAFGQRRLLLLHQRSHHQVEH
KGERD*
>ENSG00000170631|ENST00000276816
MPSLRTRREEAEMELSVPGPSPWTPAAQARVRDAPAVTHPGSAACGTPCCSDTELEAICP
HYQQPDCDTRTEDKEFLHKEDIHEDLESQAEISENYAGDVSQVPELGDLCDDVSERDWGV
PEGRRLPQSLSQEGDFTPAAMGLLRGPLGEKDLDCNGFDSRFSLSPNLMACQEIPTEERP

HPYDMGGQSFQHSVDLTGHEGVPTAESPLICNECGKTFQGNPDLIQRQIVHTGEASFMCD
DCGKTFSQNSVLKNRHRSHMSEKAYQCSECGKAFRGHSDFSRHQSHHSSERPYMCNECGK
AFSQNSSLKKHQKSHMSEKPYECNECGKAFRRSSNLIQHQRIHSGEKPYVCSECGKAFRR
SSNLIKHHRTHTGEKPFECGECGKAFSQSAHLRKHQRVHTGEKPYECNDCGKPFSRVSNL
IKHHRVHTGEKPYKCSDCGKAFSQSSSLIQHRRIHTGEKPHVCNVCGKAFSYSSVLRKHQ
IIHTGEKPYRCSVCGKAFSHSSALIQHQGVHTGDKPYACHECGKTFGRSSNLILHQRVHT
GEKPYECTECGKTFSQSSTLIQHQRIHNGLKPHECNQCGKAFNRSSNLIHHQKVHTGEKP
YTCVECGKGFSQSSHLIQHQIIHTGERPYKCSECGKAFSQRSVLIQHQRIHTGVKPYDCA
ACGKAFSQRSKLIKHQLIHTRE*
>ENSG00000198131|ENST00000269829
MEARSMLVPPQASVCFEDVAMAFTQEEWEQLDLAQRTLYREVTLETWEHIVSLGLFLSKS
DVISQLEQEEDLCRAEQEAPRDWKATLEENRLNSEKDRAREELSHHVEVYRSGPEEPPSL
VLGKVQDQSNQLREHQENSLRFMVLTSERLFAQREHCELELGGGYSLPSTLSLLPTTLPT
STGFPKPNSQVKELKQNSAFINHEKNGADGKHCESHQCARAFCQSIYLSKLGNVETGKKN
PYEYIVSGDSLNYGSSLCFHGRTFSVKKSDDCKDYGNLFSHSVSLNEQKPVHFGKSQYEC
DECRETCSESLCLVQTERSGPGETPFRCEERCAAFPMASSFSDCNIIQTTEKPSVCNQCG
KSFSCCKLIHQRTHTGEKPFECTQCGKSFSQSYDLVIHQRTHTGEKPYECDLCGKSFTQR
SKLITHQRIHTGEKPYQCIECRKSFRWNSNLIVHQRIHTGEKPYECTHCGKSFSQSYELV
THKRTHTGEKPFKCTQCGKSFSQKYDLVVHQRTHTGEKPYECNLCGKSFSQSSKLITHQR
IHTGEKPYQCIECGKSFRWNSNLVIHQRIHTGEKPYDCTHCGKSFSQSYQLVAHKRTHTG
EKPYECNECGKAFNRSTQLIRHLQIHTGEKPYKCNQCNKAFARSSYLVMHQRTHTGEKPF
ECSQCGKAFSGSSNLLSHHRIHSGEKPYECSDCGKSFRQQSQLVVHRRTHTGEKP*
>ENSG00000169918|ENST00000307050
MVSSVLPNPTSAECWAALLHDPMTLDMDAVLSDFVRSTGAEPGLARDLLEGKNWDLTAAL
SDYEQLRQVHTANLPHVFNEGRGPKQPEREPQPGHKVERPCLQRQDDIAQEKRLSRGISH
ASSAIVSLARSHVASECNNEQFPLEMPIYTFQLPDLSVYSEDFRSFIERDLIEQATMVAL
EQAGRLNWWSTVCTSCKRLLPLATTGDGNCLLHAASLGMWGFHDRDLVLRKALYTMMRTG
AEREALKRRWRWQQTQQNKEEEWEREWTELLKLASSEPRTHFSKNGGTGGGVDNSEDPVY
ESLEEFHVFVLAHILRRPIVVVADTMLRDSGGEAFAPIPFGGIYLPLEVPPNRCHCSPLV
LAYDQAHFSALVSMEQRDQQREQAVIPLTDSEHKLLPLHFAVDPGKDWEWGKDDNDNARL
AHLILSLEAKLNLLHSYMNVTWIRIPSETRAPLAQPESPTASAGEDVQSLADSLDSDRDS
VCSNSNSNNGKNGKDKEKEKQRKEKDKTRADSVANKLGSFSKTLGIKLKKNMGGLGGLVH
GKMGRANSANGKNGDSAERGKEKKAKSRKGSKEESGASASTSPSEKTTPSPTDKAAGASP
AEKGGGPRGDAWKYSTDVKLSLNILRAAMQGERKFIFAGLLLTSHRHQFHEEMIGYYLTS
AQERFSAEQEQRRRDAATAAAAAAAAAAATAKRPPRRPETEGVPVPERASPGPPTQLVLK
LKERPSPGPAAGRAARAAAGGTASPGGGARRASASGPVPGRSPPAPARQSVIHVQASGAR
DEACAPAVGALRPCATYPQQNRSLSSQSYSPARAAALRTVNTVESLARAVPGALPGAAGT
AGAAEHKSQTYTNGFGALRDGLEFADADAPTARSNGECGRGGPGPVQRRCQRENCAFYGR
AETEHYCSYCYREELRRRREARGARP*
>ENSG00000171540|ENST00000306422
MLSHADLLDARLGMKDAAELLGHREAVKCRLGVGGSDPGGHPGDLAPNSDPVEGATLLPG
EDITTVGSTPASLAVSAKDPDKQPGPQGGPNPSQAGQQQGQQKQKRHRTRFTPAQLNELE
RSFAKTHYPDIFMREELALRIGLTESRVQVWFQNRRAKWKKRKKTTNVFRAPGTLLPTPG
LPQFPSAAAAAAAAMGDSLCSFHANDTRWAAAAMPGVSQLPLPPALGRQQAMAQSLSQCS

LAAGPPPNSMGLSNSLAGSNGAGLQSHLYQPAFPGMVPASLPGPSNVSGSPQLCSSPDSS
DVWRGTSIASLRRKALEHTVSMSFT*
>ENSG00000163939|ENST00000296302
MGSKRRRATSPSSSVSGDFDDGHHSVSTPGPSRKRRRLSNLPTVDPIAVCHELYNTIRDY
KDEQGRLLCELFIRAPKRRNQPDYYEVVSQPIDLMKIQQKLKMEEYDDVNLLTADFQLLF
NNAKSYYKPDSPEYKAACKLWDLYLRTRNEFVQKGEADDEDDDEDGQDNQGTVTEGSSPA
YLKEILEQLLEAIVVATNPSGRLISELFQKLPSKVQYPDYYAIIKEPIDLKTIAQRIQNG
SYKSIHAMAKDIDLLAKNAKTYNEPGSQVFKDANSIKKIFYMKKAEIEHHEMAKSSLRMR
TPSNLAAARLTGPSHSKGSLGEERNPTSKYYRNKRAVQGGRLSAITMALQYGSESEEDAA
LAAARYEEGESEAESITSFMDVSNPFYQLYDTVRSCRNNQGQLIAEPFYHLPSKKKYPDY
YQQIKMPISLQQIRTKLKNQEYETLDHLECDLNLMFENAKRYNVPNSAIYKRVLKLQQVM
QAKKKELARRDDIEDGDSMISSATSDTGSAKRKSKKNIRKQRMKILFNVVLEAREPGSGR
RLCDLFMVKPSKKDYPDYYKIILEPMDLKIIEHNIRNDKYAGEEGMIEDMKLMFRNARHY
NEEGSQVYNDAHILEKLLKEKRKELGPLPDDDDMASPKLKLSRKSGISPKKSKYMTPMQQ
KLNEVYEAVKNYTDKRGRRLSAIFLRLPSRSELPDYYLTIKKPMDMEKIRSHMMANKYQD
IDSMVEDFVMMFNNACTYNEPESLIYKDALVLHKVLLETRRDLEGDEDSHVPNVTLLIQE
LIHNLFVSVMSHQDDEGRCYSDSLAEIPAVDPNFPNKPPLTFDIIRKNVENNRYRRLDLF
QEHMFEVLERARRMNRTDSEIYEDAVELQQFFIKIRDELCKNGEILLSPALSYTTKHLHN
DVEKERKEKLPKEIEEDKLKREEEKREAEKSEDSSGAAGLSGLHRTYSQDCSFKNSMYHV
GDYVYVEPAEANLQPHIVCIERLWEDSAGEKWLYGCWFYRPNETFHLATRKFLEKEVFKS
DYYNKVPVSKILGKCVVMFVKEYFKLCPENFRDEDVFVCESRYSAKTKSFKKIKLWTMPI
SSVRFVPRDVPLPVVRVASVFANADKGDDEKNTDNSEDSRAEDNFNLEKEKEDVPVEMSN
GEPGCHYFEQLHYNDMWLKVGDCVFIKSHGLVRPRVGRIEKVWVRDGAAYFYGPIFIHPE
ETEHEPTKMFYKKEVFLSNLEETCPMTCILGKCAVLSFKDFLSCRPTEIPENDILLCESR
YNESDKQMKKFKGLKRFSLSAKVVDDEIYYFRKPIVPQKEPSPLLEKKIQLLEAKFAELE
GGDDDIEEMGEEDSEVIEPPSLPQLQTPLASELDLMPYTPPQSTPKSAKGSAKKEGSKRK
INMSGYILFSSEMRAVIKAQHPDYSFGELSRLVGTEWRNLETAKKAEYEERAAKVAEQQE
RERAAQQQQPSASPRAGTPVGALMGVVPPPTPMGMLNQQLTPVAGMMGGYPPGLPPLQGP
VDGLVSMGSMQPLHPGGPPPHHLPPGVPGLPGIPPPGVMNQGVAPMVGTPAPGGSPYGQQ
VGVLGPPGQQAPPPYPGPHPAGPPVIQQPTTPMFVAPPPKTQRLLHSEAYLKYIEGLSAE
SNSISKWDQTLAARRRDVHLSKEQESRLPSHWLKSKGAHTTMADALWRLRDLMLRDTLNI
RQAYNLENV*
>ENSG00000187955|ENST00000309791
MKIFQRKMRYWLLPPFLAIVYFCTIVQGQVAPPTRLRYNVISHDSIQISWKAPRGKFGGY
KLLVTPTSGGKTNQLNLQNTATKAIIQGLMPDQNYTVQIIAYNKDKESKPAQGQFRIKDL
EKRKDPKPRVKVVDRGNGSRPSSPEEVKFVCQTPAIADIVILVDGSWSIGRFNFRLVRHF
LENLVTAFDVGSEKTRIGLAQYSGDPRIEWHLNAFSTKDEVIEAVRNLPYKGGNTLTGLA
LNYIFENSFKPEAGSRTGVSKIGILITDGKSQDDIIPPSRNLRESGVELFAIGVKNADVN
ELQEIASEPDSTHVYNVAEFDLMHTVVESLTRTLCSRVEEQDREIKASAHAITGPPTELI
TSEVTARSFMVNWTHAPGNVEKYRVVYYPTRGGKPDEVVVDGTVSSTVLKNLMSLTEYQI
AVFAIYAHTASEGLRGTETTLALPMASDLLLYDVTENSMRVKWDAVPGASGYLILYAPLT
EGLAGDEKEMKIGETHTDIELSGLLPNTEYTVTVYAMFGEEASDPVTGQETTLALSPPRN
LRISNVGSNSARLTWDPTSRQINGYRIVYNNADGTEINEVEVDPITTFPLKGLTPLTEYT
IAIFSIYDEGQSEPLTGVFTTEEVPAQQYLEIDEVTTDSFRVTWHPLSADEGLHKLMWIP

VYGGKTEEVVLKEEQDSHVIEGLEPGTEYEVSLLAVLDDGSESEVVTAVGTTLDSFWTEP
ATTIVPTTSVTSVFQTGIRNLVVGDETTSSLRVKWDISDSDVQQFRVTYMTAQGDPEEEV
IGTVMVPGSQNNLLLKPLLPDTEYKVTVTPIYTDGEGVSVSAPGKTLPSSGPQNLRVSEE
WYNRLRITWDPPSSPVKGYRIVYKPVSVPGPTLETFVGADINTILITNLLSGMDYNVKIF
ASQASGFSDALTGMVKTLFLGVTNLQAKHVEMTSLCAHWQVHRHATAYRVVIESLQDRQK
QESTVGGGTTRHCFYGLQPDSEYKISVYTKLQEIEGPSVSIMEKTQSLPTRPPTFPPTIP
PAKEVCKAAKADLVFMVDGSWSIGDENFNKIISFLYSTVGALNKIGTDGTQVAMVQFTDD
PRTEFKLNAYKTKETLLDAIKHISYKGGNTKTGKAIKYVRDTLFTAESGTRRGIPKVIVV
ITDGRSQDDVNKISREMQLDGYSIFAIGVADADYSELVSIGSKPSARHVFFVDDFDAFKK
IEDELITFVCETASATCPVVHKDGIDLAGFKMMEMFGLVEKDFSSVEGVSMEPGTFNVFP
CYQLHKDALVSQPTRYLHPEGLPSDYTISFLFRILPDTPQEPFALWEILNKNSDPLVGVI
LDNGGKTLTYFNYDQSGDFQTVTFEGPEIRKIFYGSFHKLHIVVSETLVKVVIDCKQVGE
KAMNASANITSDGVEVLGKMVRSRGPGGNSAPFQLQMFDIVCSTSWANTDKCCELPGLRD
DESCPDLPHSCSCSETNEVALGPAGPPGGPGLRGPKGQQGEPGPKGPDGPRGEIGLPGPQ
GPPGPQGPSGLSIQGMPGMPGEKGEKGDTGLPGPQGIPGGVGSPGRDGSPGQRGLPGKDG
SSGPPGPPGPIGIPGTPGVPGITGSMGPQGALGPPGVPGAKGERGERGDLQSQAMVRSVA
RQVCEQLIQSHMARYTAILNQIPSHSSSIRTVQGPPGEPGRPGSPGAPGEQGPPGTPGFP
GNAGVPGTPGERGLTGIKGEKGNPGVGTQGPRGPPGPAGPSGESRPGSPGPPGSPGPRGP
PGHLGVPGPQGPSGQPGYCDPSSCSAYGVRDLIPYNDYQH*
>ENSG00000146414|ENST00000275233
MSSRRKRAPPVRVDEEKRQQLHWNMHEDRRNEPIIISDDDEQPCPGSDTSSAHYIILSDS
LKEEVAHRDKKRCSKVVSFSKPIEKEETVGIFSPLSVKLNIVISPYHFDNSWKAFLGELT
LQLLPAQSLIENFSERSITLMSSESSNQFLIYVHSKGEDVEKQKKEPMSICDKGILVESS
FSGEMLEDLGWLQKKRRIKLYQKPEGNHIIKVGIYLLEAGLAKLDFLSDANSRMKKFNQL
MKKVMEKLHNSIIPDVLEEDEDDPESEPEGQDIDELYHFVKQTHQQETQSIQVDVQHPAL
IPVLRPYQREAVNWMLQQECFRSSPATESALHFLWREIVTSEGLKLYYNPYTGCIIREYP
NSGPQLLGGILADEMGLGKTVEVLALILTHTRQDVKQDALTLPEGKVVNYFIPSHYFGGK
LKKTEIQNIEFEPKEKVQCPPTRVMILTAVKEMNGKKGVSILSIYKYVSSIYRYDVQRNR
SLLKRMLKCLIFEGLVKQIKGHGFSGTFTLGKNYKEEDICDKTKKQAVGSPRKIQKETRK
SGNKDTDSEYLPSDTSDDDDDPYYYYYKSRRNRSKLRKKLVPSTKKGKSQPFINPDSQGH
CPATSDSGITDVAMSKSTCISEFNQEHETEDCAESLNHADSDVPPSNTMSPFNTSDYRFE
CICGELDQIDRKPRVQCLKCHLWQHAKCVNYDEKNLKIKPFYCPHCLVAMEPVSTRATLI
ISPSSICHQWVDEINRHVRSSSLRVLVYQGVKKDGFLQPHFLAEQDIVIITYDVLRSELN
YVDIPHSNSEDGRRLRNQKRYMAIPSPLVAVEWWRICLDEAQMVECPTVKAAEMAQRLSG
INRWCISGTPVQRGLEDLFGLVVFLGIEPYCVKHWWVRLLYRPYCKKNPQHLYSFIAKIL
WRSAKKDVIDQIQIPPQTEEIHWLHFSPVERHFYHRQHEVCCQDVVVKLRKISDWALKLS
SLDRRTVTSILYPLLRLRQACCHPQAVRGEFLPLQKSTMTMEELLTSLQKKCGTECEEAH
RQLVCALNGLAGIHIIKGEYALAAELYREVLRSSEEHKGKLKTDSLQRLHATHNLMELLI
ARHPGIPPTLRDGRLEEEAKQLREHYMSKCNTEVAEAQQALYPVQQTIHELQRKIHSNSP
WWLNVIHRAIEFTIDEELVQRVRNEITSNYKQQTGKLSMSEKFRDCRGLQFLLTTQMEEL
NKCQKLVREAVKNLEGPPSRNVIESATVCHLRPARLPLNCCVFCKADELFTEYESKLFSN
TVKGQTAIFEEMIEDEEGLVDDRAPTTTRGLWAISETERSMKAILSFAKSHRFDVEFVDE
GSTSMDLFEAWKKEYKLLHEYWMALRNRVSAVDELAMATERLRVRDPREPKPNPPVLHII
EPHEVEQNRIKLLNDKAVATSQLQKKLGQLLYLTNLEKSQDKTSGGVNPEPCPICARQLG

KQWAVLTCGHCFCNECISIIIEQYSVGSHRSSIKCAICRQTTSHKEISYVFTSEKANQEE
DIPVKGSHSTKVEAVVRTLMKIQLRDPGAKALVFSTWQDVLDIISKALTDNNMEFAQISR
VKTFQENLSAFKRDPQINILLLPLHTGSNGLTIIEATHVLLVEPILNPAHELQAIGRVHR
IGQTKPTIVHRFLIKATIEERMQAMLKTAERSHTNSSAKHSEASVLTVADLADLFTKETE
ELE*
>ENSG00000135374|ENST00000312319
MPSLPHSHRVMLDSVTHSTFLPNASFCDPLMSWTDLFSNEEYYPAFEHQTACDSYWTSVH
PEYWTKRHVWEWLQFCCDQYKLDTNCISFCNFNISGLQLCSMTQEEFVEAAGLCGEYLYF
ILQNIRTQGYSFFNDAEESKATIKDYADSNCLKTSGIKSQDCHSHSRTSLQSSHLWEFVR
DLLLSPEENCGILEWEDREQGIFRVVKSEALAKMWGQRKKNDRMTYEKLSRALRYYYKTG
ILERVDRRLVYKFGKNAHGWQEDKL*
>ENSG00000006576|ENST00000275575
MASKVTDAIVWYQKKIGAYDQQIWEKSVEQREIKGLRNKPKKTAHVKPDLIDVDLVRGSA
FAKAKPESPWTSLTRKGIVRVVFFPFFFRWWLQVTSKVIFFWLLVLYLLQVAAIVLFCST
SSPHSIPLTEVIGPIWLMLLLGTVHCQIVSTRTPKPPLSTGGKRRRKLRKAAHLEVHREG
DGSSTTDNTQEGAVQNHGTSTSHSVGTVFRDLWHAAFFLSGSKKAKNSIDKSTETDNGYV
SLDGKKTVKSGEDGIQNHEPQCETIRPEETAWNTGTLRNGPSKDTQRTITNVSDEVSSEE
GPETGYSLRRHVDRTSEGVLRNRKSHHYKKHYPNEDAPKSGTSCSSRCSSSRQDSESARP
ESETEDVLWEDLLHCAECHSSCTSETDVENHQINPCVKKEYRDDPFHQSHLPWLHSSHPG
LEKISAIVWEGNDCKKADMSVLEISGMIMNRVNSHIPGIGYQIFGNAVSLILGLTPFVFR
LSQATDLEQLTAHSASELYVIAFGSNEDVIVLSMVIISFVVRVSLVWIFFFLLCVAERTY
KQRLLFAKLFGHLTSARRARKSEVPHFRLKKVQNIKMWLSLRSYLKRRGPQRSVDVIVSS
AFLLTISVVFICCAQINLYLKMEKKPNKKEELTLVNNVLKLATKLLKELDSPFRLYGLTM
NPLLYNITQVVILSAVSGVISDLLGFNLKLWKIKS*
>ENSG00000114026|ENST00000302008
MPARALLPRRMGHRTLASTPALWASIPCPRSELRLDLVLPSGQSFRWREQSPAHWSGVLA
DQVWTLTQTEEQLHCTVYRGDKSQASRPTPDELEAVRKYFQLDVTLAQLYHHWGSVDSHF
QEVAQKFQGVRLLRQDPIECLFSFICSSNNNIARITGMVERLCQAFGPRLIQLDDVTYHG
FPSLQALAGPEVEAHLRKLGLGYRARYVSASARAILEEQGGLAWLQQLRESSYEEAHKAL
CILPGVGTKVADCICLMALDKPQAVPVDVHMWHIAQRDYSWHPTTSQAKGPSPQTNKELG
NFFRSLWGPYAGWAQALCQVITTFMTFLGPHRLDQMPPEELQTSSSRLGGPPWQCI*
>ENSG00000152433|ENST00000282282
MAEMNPAQGHVVFEDVAIYFSQEEWGHLDEAQRLLYRDVMLENLALLSSLGCCHGAEDEE
APLEPGVSVGVSQVMAPKPCLSTQNTQPCETCSSLLKDILRLAEHDGTHPEQGLYTCPAH
LHQHQKEQIREKLSRGDGGRPTFVKNHRVHMAGKTFLCSECGKAFSHKHKLSDHQKIHTG
ERTYKCSKCGILFMERSTLNRHQRTHTGERPYECNECGKAFLCKSHLVRHQTIHSGERPY
ECSECGKLFMWSSTLITHQRVHTGKRPYGCSECGKFFKCNSNLFRHYRIHTGKRSYGCSE
CGKFFMERSTLSRHQRVHTGERPYECNECGKFFSLKSVLIQHQRVHTGERPYECSECGKA
FLTKSHLICHQTVHTAAKQCSECGKFFRYNSTLLRHQKVHTG*
>ENSG00000160961|ENST00000292530
MESVTFEDVAVEFIQEWALLDSARRSLCKYRMLDQCRTLASRGTPPCKPSCVSQLGQRAE
PKATERGILRATGVAWESQLKPEELPSMQDLLEEASSRDMQMGPGLFLRMQLVPSIEERE
TPLTREDRPALQEPPWSLGCTGLKAAMQIQRVVIPVPTLGHRNPWVARDSAVPARDPAWL
QEDKVEEEAMAPGLPTACSQEPVTFADVAVVFTPEEWVFLDSTQRSLYRDVMLENYRNLA

SVADQLCKPNALSYLEERGEQWTTDRGVLSDTCAEPQCQPQEAIPSQDTFTEILSIDVKG
EQPQPGEKLYKYNELEKPFNSIEPLFQYQRIHAGEASCECQEIRNSFFQSAHLIVPEKIR
SGDKSYACNKCEKSFRYSSDLIRHEKTHTAEKCFDCQECGQAFKYSSNLRRHMRTHTGEK
PFECSQCGKTFTRNFNLILHQRNHTGEKPYECKDCGKAFNQPSSLRSHVRTHTGEKPFEC
SQCGKAFREHSSLKTHLRTHTREKPYECNQCGKPFRTSTHLNVHKRIHTGEKLYECATCG
QVLSRLSTLKSHMRTHTGEKPYVCQECGRAFSEPSSLRKHARTHSGKKPYACQECGRAFG
QSSHLIVHVRTHSAGRPYQCNQCEKAFRHSSSLTVHKRTHVGRETIRNGSLPLSMSHPYC
GPLAN*
>ENSG00000204920|ENST00000270014
MTTFKEAVTFKDVAVVFTEEELGLLDPAQRKLYRDVMLENFRNLLSVGHQPFHQDTCHFL
REEKFWMMGTATQREGNSGGKIQTELESVPEAGAHEEWSCQQIWEQIAKDLTRSQDSIIN
NSQFFENGDVPSQVEAGLPTIHTGQKPSQGGKCKQSISDVPIFDLPQQLYSEEKSYTCDE
CGKSICYISALHVHQRVHVGEKLFMCDVCGKEFSQSSHLQTHQRVHTGEKPFKCEQCGKG
FSRRSALNVHRKLHTGEKPYICEACGKAFIHDSQLKEHKRIHTGEKPFKCDICGKTFYFR
SRLKSHSMVHTGEKPFRCDTCDKSFHQRSALNRHCMVHTGEKPYRCEQCGKGFIGRLDFY
KHQVVHTGEKPYNCKECGKSFRWSSCLLNHQRVHSGEKSFKCEECGKGFYTNSQLSSHQR
SHSGEKPYKCEECGKGYVTKFNLDLHQRVHTGERPYNCKECGKNFSRASSILNHKRLHCQ
KKPFKCEDCGKRLVHRTYRKDQPRDYSGENPSKCEDCGRRYKRRLNLDILLSLFLNDT*
>ENSG00000177842|ENST00000314529
MFQTAWRQEPVTFEDVAVYFTQNEWASLDSVQRALYREVMLENYANVASLAFPFTTPVLV
SQLEQGELPWGLDPWEPMGREALRGICPGDEARTEKEGLTPKDHVSKETESFRLMVGGLP
GNVSQHLDFGSSLEQPQGHWIIKTKSKRRHFTDTSARHHEAYEVKNGEKFEKLGKNISVS
TQLTTNQTNPSGQISYECGQCGRYFIQMADFHRHEKCHTGEKSFECKECGKYFRYNSLLI
RHQIIHTGKKPFKCKECGKGLSSDTALIQHQRIHTGEKPYECKECGKAFSSSSSVFLQHQR
FHTGEKLYECNECWKTFSCSSSFTVHQRMHTGEKPYECKECGKRLSSNTALTQHQRIHTG
EKPFECKECGKAFNQKITLIQHQRVHTGEKPYECKVCGKTFSWCGRFILHQKLHTQKTPV
QA*
>ENSG00000153767|ENST00000283875
MADPDVLTEVPAALKRLAKYVIRGFYGIEHALALDILIRNSCVKEEDMLELLKFDRKQLR
SVLNNLKGDKFIKCRMRVETAADGKTTRHNYYFINYRTLVNVVKYKLDHMRRRIETDERD
STNRASFKCPVCSSTFTDLEANQLFDPMTGTFRCTFCHTEVEEDESAMPKKDARTLLARF
NEQIEPIYALLRETEDVNLAYEILEPEPTEIPALKQSKDHAATTAGAASLAGGHHREAWA
TKGPSYEDLYTQNVVINMDDQEDLHRASLEGKSAKERPIWLRESTVQGAYGSEDMKEGGI
DMDAFQEREEGHAGPDDNEEVMRALLIHEKKTSSAMAGSVGAAAPVTAANGSDSESETSE
SDDDSPPRPAAVAVHKREEDEEEDDEFEEVADDPIVMVAGRPFSYSEVSQRPELVAQMTP
EEKEAYIAMGQRMFEDLFE*
>ENSG00000145736|ENST00000274400
MDEEPERTKRWEGGYERTWEILKEDESGSLKATIEDILFKAKRKRVFEHHGQVRLGMMRH
LYVVVDGSRTMEDQDLKPNRLTCTLKLLEYFVEEYFDQNPISQIGIIVTKSKRAEKLTEL
SGNPRKHITSLKKAVDMTCHGEPSLYNSLSIAMQTLKHMPGHTSREVLIIFSSLTTCDPS
NIYDLIKTLKAAKIRVSVIGLSAEVRVCTVLARETGGTYHVILDESHYKELLTHHVSPPP
ASSSSECSLIRMGFPQHTIASLSDQDAKPSFSMAHLDGNTEPGLTLGGYFCPQCRAKYCE
LPVECKICGLTLVSAPHLARSYHHLFPLDAFQEIPLEEYNGERFCYGCQGELKDQHVYVC
AVCQNVFCVDCDVFVHDSLHCCPGCIHKIPAPSGV*

>ENSG00000149050|ENST00000278314
MAVTFEDVTIIFTWEEWKFLDSSQKRLYREVMWENYTNVMSVENWNESYKSQEEKFRYLE
YENFSYWQGWWNAGAQMYENQNYGETVQGTDSKDLTQQDRSQCQEWLILSTQVPGYGNYE
LTFESKSLRNLKYKNFMPWQSLETKTTQDYGREIYMSGSHGFQGGRYRLGISRKNLSMEK
EQKLIVQHSYIPVEEALPQYVGVICQEDLLRDSMEEKYCGCNKCKGIYYWNSRCVFHKRN
QPGENLCQCSICKACFSQRSDLYRHPRNHIGKKLYGCDEVDGNFHQSSGVHFHQRVHIGE
VPYSCNACGKSFSQISSLHNHQRVHTEEKFYKIECDKDLSRNSLLHIHQRLHIGEKPFKC
NQCGKSFNRSSVLHVHQRVHTGEKPYKCDECGKGFSQSSNLRIHQLVHTGEKSYKCEDCG
KGFTQRSNLQIHQRVHTGEKPYKCDDCGKDFSHSSDLRIHQRVHTGEKPYTCPECGKGFS
KSSKLHTHQRVHTGEKPYKCEECGKGFSQRSHLLIHQRVHTGEKPYKCHDCGKGFSHSSN
LHIHQRVHTGEKPYQCAKCGKGFSHSSALRIHQRVHAGEKPYKCREYYKGFDHNSHLHNN
HRRGNL*
>ENSG00000105364|ENST00000307422
MLQFVRAGARAWLRPTGSQGLSSLAEEAARATENPEQVASEGLPEPVLRKVELPVPTHRR
PVQAWVESLRGFEQERVGLADLHPDVFATAPRLDILHQVAMWQKNFKRISYAKTKTRAEV
RGGGRKPWPQKGTGRARHGSIRSPLWRGGGVAHGPRGPTSYYYMLPMKVRALGLKVALTV
KLAQDDLHIMDSLELPTGDPQYLTELAHYRRWGDSVLLVDLTHEEMPQSIVEATSRLKTF
NLIPAVGLNVHSMLKHQTLVLTLPTVAFLEDKLLWQDSRYRPLYPFSLPYSDFPRPLPHA
TQGPAATPYHC*
>ENSG00000163064|ENST00000295206
MEEQQPEPKSQRDSALGAAAAATPGGLSLSLSPGASGSSGSGSDGDSVPVSPQPAPPSPP
AAPCLPPLAHHPHLPPHPPPPPPQHLAAPAHQPQPAAQLHRTTNFFIDNILRPDFGCKKE
QPPPQLLVAAAARGGAGGGGRVERDRGQTAAGRDPVHPLGTRAPGAASLLCAPDANCGPP
DGSQPAAAGAGASKAGNPAAAAAAAAAAVAAAAAAAAAKPSDTGGGGSGGGAGSPGAQGT
KYPEHGNPAILLMGSANGGPVVKTDSQQPLVWPAWVYCTRYSDRPSSGPRTRKLKKKKNE
KEDKRPRTAFTAEQLQRLKAEFQANRYITEQRRQTLAQELSLNESQIKIWFQNKRAKIKK
ATGIKNGLALHLMAQGLYNHSTTTVQDKDESE*
>ENSG00000159184|ENST00000290295
MEPGNYATLDGAKDIEGLLGAGGGRNLVAHSPLTSHPAAPTLMPAVNYAPLDLPGSAEPP
KQCHPCPGVPQGTSPAPVPYGYFGGGYYSCRVSRSSLKPCAQAATLAAYPAETPTAGEEY
PSRPTEFAFYPGYPGTYQPMASYLDVSVVQTLGAPGEPRHDSLLPVDSYQSWALAGGWNS
QMCCQGEQNPPGPFWKAAFADSSGQHPPDACAFRRGRKKRIPYSKGQLRELEREYAANKF
ITKDKRRKISAATSLSERQITIWFQNRRVKEKKVLAKVKNSATP*
>ENSG00000173894|ENST00000310942
MEELSSVGEQVFAAECILSKRLRKGKLEYLVKWRGWSSKHNSWEPEENILDPRLLLAFQK
KEHEKEVQNRKRGKRPRGRPRKLTAMSSCSRRSKLKEPDAPSKSKSSSSSSSSTSSSSSS
DEEDDSDLDAKRGPRGRETHPVPQKKAQILVAKPELKDPIRKKRGRKPLPPEQKATRRPV
SLAKVLKTARKDLGAPASKLPPPLSAPVAGLAALKAHAKEACGGPSAMATPENLASLMKG
MASSPGRGGISWQSSIVHYMNRMTQSQAQAASRLALKAQATNKCGLGLDLKVRTQKGELG
MSPPGSKIPKAPSGGAVEQKVGNTGGPPHTHGASRVPAGCPGPQPAPTQELSLQVLDLQS
VKNGMPGVGLLARHATATKGVPATNPAPGKGTGSGLIGASGATMPTDTSKSEKLASRAVA
PPTPASKRDCVKGSATPSGQESRTAPGEARKAATLPEMSAGEESSSSDSDPDSASPPSTG
QNPSVSVQTSQDWKPTRSLIEHVFVTDVTANLITVTVKESPTSVGFFNLRHY*
>ENSG00000164362|ENST00000310581

MPRAPRCRAVRSLLRSHYREVLPLATFVRRLGPQGWRLVQRGDPAAFRALVAQCLVCVPW
DARPPPAAPSFRQVSCLKELVARVLQRLCERGAKNVLAFGFALLDGARGGPPEAFTTSVR
SYLPNTVTDALRGSGAWGLLLRRVGDDVLVHLLARCALFVLVAPSCAYQVCGPPLYQLGA
ATQARPPPHASGPRRRLGCERAWNHSVREAGVPLGLPAPGARRRGGSASRSLPLPKRPRR
GAAPEPERTPVGQGSWAHPGRTRGPSDRGFCVVSPARPAEEATSLEGALSGTRHSHPSVG
RQHHAGPPSTSRPPRPWDTPCPPVYAETKHFLYSSGDKEQLRPSFLLSSLRPSLTGARRL
VETIFLGSRPWMPGTPRRLPRLPQRYWQMRPLFLELLGNHAQCPYGVLLKTHCPLRAAVT
PAAGVCAREKPQGSVAAPEEEDTDPRRLVQLLRQHSSPWQVYGFVRACLRRLVPPGLWGS
RHNERRFLRNTKKFISLGKHAKLSLQELTWKMSVRDCAWLRRSPGVGCVPAAEHRLREEI
LAKFLHWLMSVYVVELLRSFFYVTETTFQKNRLFFYRKSVWSKLQSIGIRQHLKRVQLRE
LSEAEVRQHREARPALLTSRLRFIPKPDGLRPIVNMDYVVGARTFRREKRAERLTSRVKA
LFSVLNYERARRPGLLGASVLGLDDIHRAWRTFVLRVRAQDPPPELYFVKVDVTGAYDTI
PQDRLTEVIASIIKPQNTYCVRRYAVVQKAAHGHVRKAFKSHVSTLTDLQPYMRQFVAHL
QETSPLRDAVVIEQSSSLNEASSGLFDVFLRFMCHHAVRIRGKSYVQCQGIPQGSILSTL
LCSLCYGDMENKLFAGIRRDGLLLRLVDDFLLVTPHLTHAKTFLRTLVRGVPEYGCVVNL
RKTVVNFPVEDEALGGTAFVQMPAHGLFPWCGLLLDTRTLEVQSDYSSYARTSIRASLTF
NRGFKAGRNMRRKLFGVLRLKCHSLFLDLQVNSLQTVCTNIYKILLLQAYRFHACVLQLP
FHQQVWKNPTFFLRVISDTASLCYSILKAKNAGMSLGAKGAAGPLPSEAVQWLCHQAFLL
KLTRHRVTYVPLLGSLRTAQTQLSRKLPGTTLTALEAAANPALPSDFKTILD*
>ENSG00000139168|ENST00000266529
MSGGLAPSKSTVYVSNLPFSLTNNDLYRIFSKYGKVVKVTIMKDKDTRKSKGVAFILFLD
KDSAQNCTRAINNKQLFGRVIKASIAIDNGRAAEFIRRRNYFDKSKCYECGESGHLSYAC
PKNMLGEREPPKKKEKKKKKKAPEPEEEIEEVEESEDEGEDPALDSLSQAIAFQQAKIEE
EQKKWKPSSGVPSTSDDSRRPRIKKSTYFSDEEELSD*
>ENSG00000166526|ENST00000299667
METQADLVSQEPQALLDSALPSKVPAFSDKDSLGDEMLAAALLKAKSQELVTFEDVAVYF
IRKEWKRLEPAQRDLYRDVMLENYGNVFSLDRETRTENDQEISEDTRSHGVLLGRFQKDI
SQGLKFKEAYEREVSLKRPLGNSPGERLNRKMPDFGQVTVEEKLTPRGERSEKYNDFGNS
FTVNSNLISHQRLPVGDRPHKCDECSKSFNRTSDLIQHQRIHTGEKPYECNECGKAFSQS
SHLIQHQRIHTGEKPYECSDCGKTFSCSSALILHRRIHTGEKPYECNECGKTFSWSSTLT
HHQRIHTGEKPYACNECGKAFSRSSTLIHHQRIHTGEKPYECNECGKAFSQSSHLYQHQR
IHTGEKPYECMECGGKFTYSSGLIQHQRIHTGENPYECSECGKAFRYSSALVRHQRIHTG
EKPLNGIGMSKSSLRVTTELNIREST*
>ENSG00000125485|ENST00000310532
MAPDLASQRHSESFPSVNSRPNVILPGREGRREGLPPGGGTRGSLVPTRPVPPSPAPLGT
SPYSWSRSGPGRGGGAGSSRVPRGVPGPAVCAPGSLLHHASPTQTMAAADGSLFDNPRTF
SRRPPAQASRQAKATKRKYQASSEAPPAKRRNETSFLPAKKTSVKETQRTFKGNAQKMFS
PKKHSVSTSDRNQEERQCIKTSSLFKNNPDIPELHRPVVKQVQEKVFTSAAFHELGLHPH
LISTINTVLKMSSMTSVQKQSIPVLLEGRDALVRSQTGSGKTLAYCIPVVQSLQAMESKI
QRSDGPYALVLVPTRELALQSFDTVQKLLKPFTWIVPGVLMGGEKRKSEKARLRKGINIL
ISTPGRLVDHIKSTKNIHFSRLRWLVFDEADRILDLGFEKDITVILNAVNAECQKRQNVL
LSATLTEGVTRLADISLHDPVSISVLDKSHDQLNPKDKAVQEVCPPPAGDKLDSFAIPES
LKQHVTVVPSKLRLVCLAAFILQKCKFEEDQKMVVFFSSCELVEFHYSLFLQTLLSSSGA
PASGQLPSASMRLKFLRLHGGMEQEERTAVFQEFSHSRRGVLLCT*

>ENSG00000181610|ENST00000313608
MAGSRLETVGSIFSRTRDLVRAGVLKEKPLWFDVYDAFPPLREPVFQRPRVRYGKAKAPI
QDIWYHEDRIRAKFYSVYGSGQRAFDLFNPNFKSTCQRFVEKYTELQKLGETDEEKLFVE
TGKALLAEGVILRRVGEARTQHGGSHVSRKSEHLSVRPQTALEENETQKEVPQDQHLEAP
ADQSKGLLPP*
>ENSG00000142611|ENST00000270722
MRSKARARKLAKSDGDVVNNMYEPNRDLLASHSAEDEAEDSAMSPIPVGPPSPFPTSEDF
TPKEGSPYEAPVYIPEDIPIPADFELRESSIPGAGLGVWAKRKMEAGERLGPCVVVPRAA
AKETDFGWEQILTDVEVSPQEGCITKISEDLGSEKFCVDANQAGAGSWLKYIRVACSCDD
QNLTMCQISEQIYYKVIKDIEPGEELLVHVKEGVYPLGTVPPGLDEEPTFRCDECDELFQ
SKLDLRRHKKYTCGSVGAALYEGLAEELKPEGLGGGSGQAHECKDCERMFPNKYSLEQHM
VIHTEEREYKCDQCPKAFNWKSNLIRHQMSHDSGKRFECENCVKVFTDPSNLQRHIRSQH
VGARAHACPDCGKTFATSSGLKQHKHIHSTVKPFICEVCHKSYTQFSNLCRHKRMHADCR
TQIKCKDCGQMFSTTSSLNKHRRFCEGKNHYTPGGIFAPGLPLTPSPMMDKAKPSPSLNH
ASLGFNEYFPSRPHPGSLPFSTAPPTFPALTPGFPGIFPPSLYPRPPLLPPTSLLKSPLN
HTQDAKLPSPLGNPALPLVSAVSNSSQGTTAAAGPEEKFESRLEDSCVEKLKTRSSDMSD
GSDFEDVNTTTGTDLDTTTGTGSDLDSDVDSDPDKDKGKGKSAEGQPKFGGGLAPPGAPN
SVAEVPVFYSQHSFFPPPDEQLLTATGAAGDSIKAIASIAEKYFGPGFMGMQEKKLGSLP
YHSAFPFQFLPNFPHSLYPFTDRALAHNLLVKAEPKSPRDALKVGGPSAECPFDLTTKPK
DVKPILPMPKGPSAPASGEEQPLDLSIGSRARASQNGGGREPRKNHVYGERKLGAGEGLP
QVCPARMPQQPPLHYAKPSPFFMDPIYSRVEKRKVTDPVGALKEKYLRPSPLLFHPQMSA
IETMTEKLESFAAMKADSGSSLQPLPHHPFNFRSPPPTLSDPILRKGKERYTCRYCGKIF
PRSANLTRHLRTHTGEQPYRCKYCDRSFSISSNLQRHVRNIHNKEKPFKCHLCNRCFGQQ
TNLDRHLKKHEHENAPVSQHPGVLTNHLGTSASSPTSESDNHALLDEKEDSYFSEIRNFI
ANSEMNQASTRTEKRADMQIVDGSAQCPGLASEKQEDVEEEDDDDLEEDDEDSLAGKSQD
DTVSPAPEPQAAYEDEEDEEPAASLAVGFDHTRRCAEDHEGGLLALEPMPTFGKGLDLRR
AAEEAFEVKDVLNSTLDSEALKHTLCRQAKNQAYAMMLSLSEDTPLHTPSQGSLDAWLKV
TGATSESGAFHPINHL*
>ENSG00000140262|ENST00000267811
MNPQQQRMAAIGTDKELSDLLDFSAMFSPPVNSGKTRPTTLGSSQFSGSGIDERGGTTSW
GTSGQPSPSYDSSRGFTDSPHYSDHLNDSRLGAHEGLSPTPFMNSNLMGKTSERGSFSLY
SRDTGLPGCQSSLLRQDLGLGSPAQLSSSGKPGTAYYSFSATSSRRRPLHDSAALDPLQA
KKVRKVPPGLPSSVYAPSPNSDDFNRESPSYPSPKPPTSMFASTFFMQDGTHNSSDLWSS
SNGMSQPGFGGILGTSTSHMSQSSSYGNLHSHDRLSYPPHSVSPTDINTSLPPMSSFHRG
STSSSPYVAASHTPPINGSDSILGTRGNAAGSSQTGDALGKALASIYSPDHTSSSFPSNP
STPVGSPSPLTGTSQWPRPGGQAPSSPSYENSLHSLQSRMEDRLDRLDDAIHVLRNHAVG
PSTSLPAGHSDIHSLLGPSHNAPIGSLNSNYGGSSLVASSRSASMVGTHREDSVSLNGNH
SVLSSTVTTSSTDLNHKTQENYRGGLQSQSGTVVTTEIKTENKEKDENLHEPPSSDDMKS
DDESSQKDIKVSSRGRTSSTNEDEDLNPEQKIEREKERRMANNARERLRVRDINEAFKEL
GRMCQLHLKSEKPQTKLLILHQAVAVILSLEQQVRERNLNPKAACLKRREEEKVSAVSAE
PPTTLPGTHPGLSETTNPMGHM*
>ENSG00000165030|ENST00000297689
MQLRKMQTVKKEQASLDASSNVDKMMVLNSALTEVSEDSTTGEELLLSEGSVGKNKSSAC
RRKREFIPDEKKDAMYWEKRRKNNEAAKRSREKRRLNDLVLENKLIALGEENATLKAELL

SLKLKFGLISSTAYAQEIQKLSNSTAVYFQDYQTSKSNVSSFVDEHEPSMVSSSCISVIK
HSPQSSLSDVSEVSSVEHTQESSVQGSCRSPENKFQIIKQEPMELESYTREPRDDRGSYT
ASIYQNYMGNSFSGYSHSPPLLQVNRSSSNSPRTSETDDGVVGKSSDGEDEQQVPKGPIH
SPVELKHVHATVVKVPEVNSSALPHKLRIKAKAMQIKVEAFDNEFEATQKLSSPIDMTSK
RHFELEKHSAPSMVHSSLTPFSVQVTNIQDWSLKSEHWHQKELSGKTQNSFKTGVVEMKD
SGYKVSDPENLYLKQGIANLSAEVVSLKRLIATQPISASDSG*
>ENSG00000152439|ENST00000282292
MAAATLRDPAQQGYVTFEDVAVYFSQEEWRLLDDAQRLLYRNVMLENFTLLASLGLASSK
THEITQLESWEEPFMPAWEVVTSAILRGSWQGAKAEAAAEQSASVEVPSSNVQQHQKQHC
GEKPLKRQEGRVPVLRSCRVHLSEKSLQSREVGKDLLTSSGVLKHQVTHTGEKSHRSSKS
REAFHAGKRHYKCSECGKAFGQKYLLVQHQRLHTGEKPYECSECGKLFSHKSNLFIHQIV
HTGERPYGCSDCGKSFSRNADLIQHQRVHTGEKPFTCSECGKAFRHNSTLVQHHRIHTGV
RPYECSECGKLFSFNSSLMKHQRVHTGERPYKCSECGKFYSHKSSLINHWRVHTGERPYE
CSECGKFFSQSSSLMQHRKVHTGEKPFKCNECGRFFSENSSLVKHQRVHTGAKPYECREC
GKFFRHSSSLVKHRRIHTGEIQ*
>ENSG00000155307|ENST00000285670
MEIRLDTLSASLGRSSTLNNCNLEDKLAWYEGEAYMWHHWKPFPENPLWTCLDFQIAQVG
PWDHCSSCIRHTRLKSSCSDMDLLHSWRSSSFGNFDRFRNNSLSKPDDSTEAHEGDPTNG
SGEQSKTSNNGGGLGKKMRAISWTMKKKVGKKYIKALSEEKDEEDGENAHPYRNSDPVIG
THTEKVSLKASDSMDSLYSGQSSSSGITSCSDGTSNRDSFRLDDDGPYSGPFCGRARVHT
DFTPSPYDTDSLKIKKGDIIDIICKTPMGMWTGMLNNKVGNFKFIYVDVISEEEAAPKKI
KANRRSNSKKSKTLQEFLERIHLQEYTSTLLLNGYETLEDLKDIKESHLIELNIENPDDR
RRLLSAAENFLEEEIIQEQENEPEPLSLSSDISLNKSQLDDCPRDSGCYISSGNSDNGKE
DLESENLSDMVHKIIITEPSD*
>ENSG00000179950|ENST00000313352
MENGQSTAAKLGLPPLTPEQQEALQKAKKYAMEQSIKSVLVKQTIAHQQQQLTNLQMAAQ
RQRALAIMCRVYVGSIYYELGEDTIRQAFAPFGPIKSIDMSWDSVTMKHKGFAFVEYEVP
EAAQLALEQMNSVMLGGRNIKVGRPSNIGQAQPIIDQLAEEARAFNRIYVASVHQDLSDD
DIKSVFEAFGKIKSCTLARDPTTGKHKGYGFIEYEKAQSSQDAVSSMNLFDLGGQYLRVG
KAVTPPMPLLTPATPGGLPPAAAVAAAAATAKITAQEAVAGAAVLGTLGTPGLVSPALTL
AQPLGTLPQAVMAAQAPGVITGVTPARPPIPVTIPSVGVVNPILASPPTLGLLEPKKEKE
EEELFPESERPEMLSEQEHMSISGSSARHMVMQKLLRKQESTVMVLRNMVDPKDIDDDLE
GEVTEECGKFGAVNRVIIYQEKQGEEEDAEIIVKIFVEFSIASETHKAIQALNGRWFAGR
KVVAEVYDQERFDNSDLSA*
>ENSG00000166337|ENST00000299424
MSCSGSGADPEAAPASAASAPGPAPPVSAPAALPSSTAAENKASPAGTAGGPGAGAAAGG
TGPLAARAGEPAERRGAAPVSAGGAAPPEGAISNGVYVLPSAANGDVKPVVSSTPLVDFL
MQLEDYTPTIPDAVTGYYLNRAGFEASDPRIIRLISLAAQKFISDIANDALQHCKMKGTA
SGSSRSKSKDRKYTLTMEDLTPALSEYGINVKKPHYFT*
>ENSG00000114026|ENST00000302003
MPARALLPRRMGHRTLASTPALWASIPCPRSELRLDLVLPSGQSFRWREQSPAHWSGVLA
DQVWTLTQTEEQLHCTVYRGDKSQASRPTPDELEAVRKYFQLDVTLAQLYHHWGSVDSHF
QEVAQKFQGVRLLRQDPIECLFSFICSSNNNIARITGMVERLCQAFGPRLIQLDDVTYHG
FPSLQALAGPEVEAHLRKLGLGYRARYVSASARAILEEQGGLAWLQQLRESSYEEAHKAL

CILPGVGTKVADCICLMALDKPQAVPVDVHMWHIAQRDYSWHPTTSQAKGPSPQTNKELG
NFFRSLWGPYAGWAQATPPSYRCCSVPTCANPAMLRSHQQSAERVPKGRKARWGTLDKEI
PQAPSPPFPTSLSPSPPSLMLGRGLPVTTSKARHPQIKQSVCTTRWGGGY*
>ENSG00000139800|ENST00000267294
MFLKAGRGNKVPPVRVYGPDCVVLMEPPLSKRNPPALRLADLATAQVQPLQNMTGFPALA
GPPAHSQLRAAVAHLRLRDLGADPGVATTPLGPEHMAQASTLGLSPPSQAFPAHPEAPAA
AARAAALVAHPGAGSYPCGGGSSGAQPSAPPPPAPPLPPTPSPPPPPPPPPPPALSGYTT
TNSGGGGSSGKGHSRDFVLRRDLSATAPAAAMHGAPLGGEQRSGTGSPQHPAPPPHSAGM
FISASGTYAGPDGSGGPALFPALHDTPGAPGGHPHPLNGQMRLGLAAAAAAAAAELYGRA
EPPFAPRSGDAHYGAVAAAAAAALHGYGAVNLNLNLAAAAAAAAAGPGPHLQHHAPPPAP
PPPPAPAQHPHQHHPHLPGAAGAFLRYMRQPIKQELICKWIDPDELAGLPPPPPPPPPPP
PPPPAGGAKPCSKTFGTMHELVNHVTVEHVGGPEQSSHVCFWEDCPREGKPFKAKYKLIN
HIRVHTGEKPFPCPFPGCGKVFARSENLKIHKRTHTGEKPFKCEFDGCDRKFANSSDRKK
HSHVHTSDKPYYCKIRGCDKSYTHPSSLRKHMKIHCKSPPPSPGPLGYSSVGTPVGAPLS
PVLDPARSHSSTLSPQVTNLNEWYVCQASGAPSHLHTPSSNGTTSETEDEEIYGNPEVVR
TIH*
>ENSG00000014138|ENST00000265465
MSASAQQLAEELQIFGLDCEEALIEKLVELCVQYGQNEEGMVGELIAFCTSTHKVGLTSE
ILNSFEHEFLSKRLSKARHSTCKDSGHAGARDIVSIQELIEVEEEEEILLNSYTTPSKGS
QKRAISTPETPLTKRSVSTRSPHQLLSPSSFSPSATPSQKYNSRSNRGEVVTSFGLAQGV
SWSGRGGAGNISLKVLGCPEALTGSYKSMFQKLPDIREVLTCKIEELGSELKEHYKIEAF
TPLLAPAQEPVTLLGQIGCDSNGKLNNKSVILEGDREHSSGAQIPVDLSELKEYSLFPGQ
VVIMEGINTTGRKLVATKLYEGVPLPFYQPTEEDADFEQSMVLVACGPYTTSDSITYDPL
LDLIAVINHDRPDVCILFGPFLDAKHEQVENCLLTSPFEDIFKQCLRTIIEGTRSSGSHL
VFVPSLRDVHHEPVYPQPPFSYSDLSREDKKQVQFVSEPCSLSINGVIFGLTSTDLLFHL
GAEEISSSSGTSDRFSRILKHILTQRSYYPLYPPQEDMAIDYESFYVYAQLPVTPDVLII
PSELRYFVKDVLGCVCVNPGRLTKGQVGGTFARLYLRRPAADGAERQSPCIAVQVVRI*
>ENSG00000178279|ENST00000312693
MDTQTHSLPITHTQLHSNSQPQSRTCTRHCQTFSQSCRQSHRGSRSQSSSQSPASHRNPT
GAHSSSGHQSQSPNTSPPPKRHKKTMNSHHSPMRPTILHCRCPKNRKNLEGKLKKKKMAK
RIQQVYKTKTRSSGWKSN*
>ENSG00000141076|ENST00000314423
MGEFKVHRVRFFNYVPSGIRCVAYNNQSNRLAVSRTDGTVEIYNLSANYFQEKFFPGHES
RATEALCWAEGQRLFSAGLNGEIMEYDLQALNIKYAMDAFGGPIWSMAASPSGSQLLVGC
EDGSVKLFQITPDKIQFERNFDRQKSRILSLSWHPSGTHIAAGSIDYISVFDVKSGSAVH
KMIVDRQYMGVSKRKCIVWGVAFLSDGTIISVDSAGKVQFWDSATGTLVKSHLIANADVQ
SIAVADQEDSFVVGTAEGTVFHFQLVPVTSNSSEKQWVRTKPFQHHTHDVRTVAHSPTAL
ISGGTDTHLVFRPLMEKVEVKNYDAALRKITFPHRCLISCSKKRQLLLFQFAHHLELWRL
GSTVATGKNGDTLPLSKNADHLLHLKTKGPENIICSCISPCGSWIAYSTVSRFFLYRLNY
EHDNISLKRVSKMPAFLRSALQILFSEDSTKLFVASNQGALHIVQLSGGSFKHLHAFQPQ
SGTVEAMCLLAVSPDGNWLAASGTSAGVHVYNVKQLKLHCTVPAYNFPVTAMAIAPNTNN
LVIAHSDQQVFEYSIPDKQYTDWSRTVQKQGFHHLWLQRDTPITHISFHPKRPMHILLHD
AYMFCIIDKSLPLPNDKTLLYNPFPPTNESDVIRRRTAHAFKISKIYKPLLFMDLLDERT
LVAVERPLDDIIAQLPPPIKKKKFGT*

>ENSG00000163840|ENST00000296161
MASHLRPPSPLLVRVYKSGPRVRRKLESYFQSSKSSGGGECTVSTQEHEAPGTFRVEFSE
RAAKERVLKKGEHQILVDEKPVPIFLVPTENSIKKNTRPQISSLTQSQAETPSGDMHQHE
GHIPNAVDSCLQKIFLTVTADLNCNLFSKEQRAYITTLCPSIRKMEGHDGIEKVCGDFQD
IERIHQFLSEQFLESEQKQQFSPSMTERKPLSQQERDSCISPSEPETKAEQKSNYFEVPL
PYFEYFKYICPDKINSIEKRFGVNIEIQESSPNMVCLDFTSSRSGDLEAARESFASEFQK
NTEPLKQECVSLADSKQANKFKQELNHQFTKLLIKEKGGELTLLGTQDDISAAKQKISEA
FVKIPVKLFAANYMMNVIEVDSAHYKLLETELLQEISEIEKRYDICSKVSEKGQKTCILF
ESKDRQVDLSVHAYASFIDAFQHASCQLMREVLLLKSLGKERKHLHQTKFADDFRKRHPN
VHFVLNQESMTLTGLPNHLAKAKQYVLKGGGMSSLAGKKLKEGHETPMDIDSDDSKAASP
PLKGSVSSEASELDKKEKGICVICMDTISNKKVLPKCKHEFCAPCINKAMSYKPICPTCQ
TSYGIQKGNQPEGSMVFTVSRDSLPGYESFGTIVITYSMKAGIQTEEHPNPGKRYPGIQR
TAYLPDNKEGRKVLKLLYRAFDQKLIFTVGYSRVLGVSDVITWNDIHHKTSRFGGPEMYG
YPDPSYLKRVKEELKAKGIE*
>ENSG00000013563|ENST00000309585
MHYPTALLFLILANGAQAFRICAFNAQRLTLAKVAREQVMDTLVRILARCDIMVLQEVVD
SSGSAIPLLLRELNRFDGSGPYSTLSSPQLGRSTYMETYVYFYRSHKTQVLSSYVYNDED
DVFAREPFVAQFSLPSNVLPSLVLVPLHTTPKAVEKELNALYDVFLEVSQHWQSKDVILL
GDFNADCASLTKKRLDKLELRTEPGFHWVIADGEDTTVRASTHCTYDRVVLHGERCRSLL
HTAAAFDFPTSFQLTEEEALNISDHYPVEVELKLSQAHSVQPLSLTVLLLLSLLSPQLCP
AA*
>ENSG00000007392|ENST00000293872
MSAQAQMRALLDQLMGTARDGDETRQRVKFTDDRVCKSHLLDCCPHDILAGTRMDLGECT
KIHDLALRADYEIASKERDLFFELDAMDHLESFIAECDRRTELAKKRLAETQEEISAEVS
AKAEKVHELNEEIGKLLAKAEQLGAEGNVDESQKILMEVEKVRAKKKEAEEEYRNSMPAS
SFQQQKLRVCEVCSAYLGLHDNDRRLADHFGGKLHLGFIQIREKLDQLRKTVAEKQEKRN
QDRLRRREEREREERLSRRSGSRTRDRRRSRSRDRRRRRSRSTSRERRKLSRSRSRDRHR
RHRSRSRSHSRGHRRASRDRSAKYKFSRERASREESWESGRSERGPPDWRLESSNGKMAS
RRSEEKEAGEI*
>ENSG00000172292|ENST00000305747
MAGILAWFWNERFWLPHNVTWADLKNTEEATFPQAEDLYLAFPLAFCIFMVRLIFERFVA
KPCAIALNIQANGPQIAPPNAILEKVFTAITKHPDEKRLEGLSKQLDWDVRSIQRWFRQR
RNQEKPSTLTRFCESMWRFSFYLYVFTYGVRFLKKTPWLWNTRHCWYNYPYQPLTTDLHY
YYILELSFYWSLMFSQFTDIKRKDFGIMFLHHLVSIFLITFSYVNNMARVGTLVLCLHDS
ADALLEAAKMANYAKFQKMCDLLFVMFAVVFITTRLGIFPLWVLNTTLFESWEIVGPYPS
WWVFNLLLLLVQGLNCFWSYLIVKIACKAVSRGKVSKDDRSDIESSSDEEDSEPPGKNPH
TATTTNGTSGTNGYLLTGSCSMDD*
>ENSG00000012061|ENST00000300853
MDPGKDKEGVPQPSGPPARKKFVIPLDEDEVPPGVAKPLFRSTQSLPTVDTSAQAAPQTY
AEYAISQPLEGAGATCPTGSEPLAGETPNQALKPGAKSNSIIVSPRQRGNPVLKFVRNVP
WEFGDVIPDYVLGQSTCALFLSLRYHNLHPDYIHGRLQSLGKNFALRVLLVQVDVKDPQQ
ALKELAKMCILADCTLILAWSPEEAGRYLETYKAYEQKPADLLMEKLEQDFVSRVTECLT
TVKSVNKTDSQTLLTTFGSLEQLIAASREDLALCPGLGPQKARRLFDVLHEPFLKVP*
>ENSG00000166454|ENST00000299575

MAASEAAAAAGSAALAAGARAVPAATTGAAAAASGPWVPPGPRLRGSRPRPAGATQQPAV
PAPPAGELIQPSVSELSRAVRTNILCTVRGCGKILPNSPALNMHLVKSHRLQDGIVNPTI
RKDLKTGPKFYCCPIEGCPRGPERPFSQFSLVKQHFMKMHAEKKHKCSKCSNSYGTEWDL
KRHAEDCGKTFRCTCGCPYASRTALQSHIYRTGHEIPAEHRDPPSKKRKMENCAQNQKLS
NKTIESLNNQPIPRPDTQELEASEIKLEPSFEDSCGSNTDKQTLTTPPRYPQKLLLPKPK
VALVKLPVMQFSVMPVFVPTADSSAQPVVLGVDQGSATGAVHLMPLSVGTLILGLDSEAC
SLKESLPLFKIANPIAGEPISTGVQVNFGKSPSNPLQELGNTCQKNSISSINVQTDLSYA
SQNFIPSAQWATADSSVSSCSQTDLSFDSQVSLPISVHTQTFLPSSKVTSSIAAQTDAFM
DTCFQSGGVSRETQTSGIESPTDDHVQMDQAGMCGDIFESVHSSYNVATGNIISNSLVAE
TVTHSLLPQNEPKTLNQDIEKSAPIINFSAQNSMLPSQNMTDNQTQTIDLLSDLENILSS
NLPAQTLDHRSLLSDTNPGPDTQLPSGPAQNPGIDFDIEEFFSASNIQTQTEESELSTMT
TEPVLESLDIETQTDFLLADTSAQSYGCRGNSNFLGLEMFDTQTQTDLNFFLDSSPHLPL
GSILKHSSFSVSTDSSDTETQTEGVSTAKNIPALESKVQLNSTETQTMSSGFETLGSLFF
TSNETQTAMDDFLLADLAWNTMESQFSSVETQTSAEPHTVSNF*
>ENSG00000178403|ENST00000313341
MFVKSETLELKEEEDVLVLLGSASPALAALTPLSSSADEEEEEEPGASGGARRQRGAEAG
QGARGGVAAGAEGCRPARLLGLVHDCKRRPSRARAVSRGAKTAETVQRIKKTRRLKANNR
ERNRMHNLNAALDALREVLPTFPEDAKLTKIETLRFAHNYIWALTETLRLADHCGGGGGG
LPGALFSEAVLLSPGGASAALSSSGDSPSPASTWSCTNSPAPSSSVSSNSTSPYSCTLSP
ASPAGSDMDYWQPPPPDKHRYAPHLPIARDCI*
>ENSG00000089157|ENST00000313104
MPREDRATWKSNYFLKIIQLLDDYPKCFIVGADNVGSKQMQQIRMSLRGKAVVLMGKNTM
MRKAIRGHLENNPALEKLLPHIRGNVGFVFTKEDLTEIRDMLLANKVPAAARAGAIAPCE
VTVPAQNTGLGPEKTSFFQALGITTKISRGTIEILGVRNVASVCLQIGYPTVASVPHSII
NGYKRVLALSVETDYTFPLAEKVKAFLADPSAFVAAAPVAAATTAAPAAAAAPAKVEAKE
ESEESDEDMGFGLFD*
>ENSG00000168228|ENST00000302874
MAASRNGFEAVEAEGSAGCRGSSGMEVVLPLDPAVPAPLCPHGPTLLFVKVTQGKEETRR
FYACSACRDRKDCNFFQWEDEKLSGARLAAREAHNRRCQPPLSRTQCVERYLKFIELPLT
QRKFCQTCQQLLLPDDWGQHSEHQVLGNVSITQLRRPSQLLYPLENKKTNAQYLFADRSC
QFLVDLLSALGFRRVLCVGTPRLHELIKLTASGDKKSNIKSLLLDIDFRYSQFYMEDSFC
HYNMFNHHFFDGKTALEVCRAFLQEDKGEGIIMVTDPPFGGLVEPLAITFKKLIAMWKEG
QSQDDSHKELPIFWIFPYFFESRICQFFPSFQMLDYQVDYDNHALYKHGKTGRKQSPVRI
FTNIPPNKIILPTEEGYRFCSPCQRYVSLENQHCELCNSCTSKDGRKWNHCFLCKKCVKP
SWIHCSICNHCAVPDHSCEGPKHGCFICGELDHKRSTCPNIATSKRANKAVRKQKQRKSN
KMKMETTKGQSMNHTSATRRKKRRERAHQYLGS*
>ENSG00000160113|ENST00000291442
MAMVTGGWGGPGGDTNGVDKAGGYPRAAEDDSASPPGAASDAEPGDEERPGLQVDCVVCG
DKSSGKHYGVFTCEGCKSFFKRSIRRNLSYTCRSNRDCQIDQHHRNQCQYCRLKKCFRVG
MRKEAVQRGRIPHSLPGAVAASSGSPPGSALAAVASGGDLFPGQPVSELIAQLLRAEPYP
AAAGRFGAGGGAAGAVLGIDNVCELAARLLFSTVEWARHAPFFPELPVADQVALLRLSWS
ELFVLNAAQAALPLHTAPLLAAAGLHAAPMAAERAVAFMDQVRAFQEQVDKLGRLQVDSA
EYGCLKAIALFTPDACGLSDPAHVESLQEKAQVALTEYVRAQYPSQPQRFGRLLLRLPAL
RAVPASLISQLFFMRLVGKTPIETLIRDMLLSGSTFNWPYGSGQ*

>ENSG00000172732|ENST00000308110
MAAPVRLGRKRPLPACPNPLFVRWLTEWRDEATRSRRRTRFVFQKALRSLRRYPLPLRSG
KEAKILQHFGDGLCRMLDERLQRHRTSGGDHAPDSPSGENSPAPQGRLAEVQDSSMPVPA
QPKAGGSGSYWPARHSGARVILLVLYREHLNPNGHHFLTKEELLQRCAQKSPRVAPGSAR
PWPALRSLLHRNLVLRTHQPARYSLTPEGLELAQKLAESEGLSLLNVGIGPKEPPGEETA
VPGAASAELASEAGVQQQPLELRPGEYRVLLCVDIGETRGGGHRPELLRELQRLHVTHTV
RKLHVGDFVWVAQETNPRDPANPGELVLDHIVERKRLDDLCSSIIDGRFREQKFRLKRCG
LERRVYLVEEHGSVHNLSLPESTLLQAVTNTQVIDGFFVKRTADIKESAAYLALLTRGLQ
RLYQGHTLRSRPWGTPGNPESGAMTSPNPLCSLLTFSDFNAGAIKNKAQSVREVFARQLM
QVRGVSGEKAAALVDRYSTPASLLAAYDACATPKEQETLLSTIKCGRLQRNLGPALSRTL
SQLYCSYGPLT*
>ENSG00000168298|ENST00000304218
MSETAPAAPAAPAPAEKTPVKKKARKSAGAAKRKASGPPVSELITKAVAASKERSGVSLA
ALKKALAAAGYDVEKNNSRIKLGLKSLVSKGTLVQTKGTGASGSFKLNKKAASGEAKPKA
KKAGAAKAKKPAGAAKKPKKATGAATPKKSAKKTPKKAKKPAAAAGAKKAKSPKKAKAAK
PKKAPKSPAKAKAVKPKAAKPKTAKPKAAKPKKAAAKKK*
>ENSG00000139352|ENST00000266744
MESSAKMESGGAGQQPQPQPQQPFLPPAACFFATAAAAAAAAAAAAQSAQQQQQQQQQ
QQAPQLRPAADGQPSGGGHKSAPKQVKRQRSSSPELMRCKRRLNFSGFGYSLPQQQPAAV
ARRNERERNRVKLVNLGFATLREHVPNGAANKKMSKVETLRSAVEYIRALQQLLDEHDAV
SAAFQAGVLSPTISPNYSNDLNSMAGSPVSSYSSDEGSYDPLSPEEQELLDFTNWF*
>ENSG00000148143|ENST00000277225
MEVLQCDGCDFRAPSYEDLKAHIQDVHTAFLQPTDVAEDNVNELRCGSVNASNQTEVEFS
SIKDEFAIAEDLSGQNATSLGTGGYYGHSPGYYGQHIAANPKPTNKFFQCKFCVRYFRSK
NLLIEHTRKVHGAQAEGSSSGPPVPGSLNYNIMMHEGFGKVFSCQFCTYKSPRRARIIKH
QKMYHKNNLKETTAPPPAPAPMPDPVVPPVSLQDPCKELPAEVVERSILESMVKPLTKSR
GNFCCEWCSYQTPRRERWCDHMMKKHRSMVKILSSLRQQQEGTNLPDVPNKSAPSPTSNS
TYLTMNAASREIPNTTVSNFRGSMGNSIMRPNSSASKFSPMSYPQMKPKSPHNSGLVNLT
ERSRYGMTDMTNSSADLETNSMLNDSSSDEELNEIDSENGLSAMDHQTSGLSAEQLMGSD
GNKLLETKGIPFRRFMNRFQCPFCPFLTMHRRSISRHIENIHLSGKTAVYKCDECPFTCK
SSLKLGAHKQCHTGTTSDWDAVNSQSESISSSLNEGVVSYESSSINGRKSGVMLDPLQQQ
QPPQPPPPPPPPPPSQPQPLQQPQPPQLQPPHQVPPQPQTQPPPTQQPQPPTQAAPLHPY
KCTMCNYSTTTLKGLRVHQQHKHSFCDNLPKFEGQPSSLPLENETDSHPSSSNTVKKSQT
SILGLSSKNNFVAKASRKLANDFPLDLSPVKKRTRIDEIASNLQSKINQTKQQEDAVINV
EDDEEEEEDNEVEIEVELDREEEPTEPIIEVPTSFSAQQIWVRDTSEPQKEPNFRNITHD
YNATNGAEIELTLSEDEEDYYGSSTNLKDHQVSNTALLNTQTPIYGTEHNSENTDFGDSG
RLYYCKHCDFNNKSARSVSTHYQRMHPYIKFSFRYILDPNDHSAVYRCLECYIDYTNFED
LQQHYGEHHPEAMNVLNFDHSDLIYRCRFCSYTSPNVRSLMPHYQRMHPTVKINNAMIFS
SYVVEQQEGLNTESQTLREILNSAPKNMATSTPVARGGGLPATFNKNTPKTFTPECENQK
DPLVNTVVVYDCDVCSFASPNMHSVLVHYQKKHPEEKASYFRIQKTMRMVSVDRGSALSQ
LSFEVGAPMSPKMSNMGSPPPPQPPPPDLSTELYYCKHCSYSNRSVVGVLVHYQKRHPEI
KVTAKYIRQAPPTAAMMRGVEGPQGSPRPPAPIQQLNRSSSERDGPPVENEMFFCQHCDY
GNRTVKGVLIHYQKKHRDFKANADVIRQHTATIRSLCDRNQKKPASCVLVSPSNLERDKT
KLRALKCRQCSYTSPYFYALRKHIKKDHPALKATVTSIMRWAFLDGLIEAGYHCEWCIYS

HTEPNGLLLHYQRRHPEHYVDYTYMATKLWAGPDPSPPSLTMPAEAKTYRCRDCVFEAVS
IWDITNHYQAFHPWAMNGDESVLLDIIKEKDAVEKPILSSEELAGPVNCENSIPTPFPEQ
EAECPEDARLSPEKSLQLASANPAISSTPYQCTVCQSEYNNLHGLLTHYGKKHPGMKVKA
ADFAQDIDINPGAVYKCRHCPYINTRIHGVLTHYQKRHPSIKVTAEDFVHDVEQSADISQ
NDVEETSRIFKQGYGAYRCKLCPYTHGTLEKLKIHYEKYHNQPEFDVFSQSPPKLPVPLE
PEMTTEVSPSQVSITEEEVGEEPVSTSHFSTSHLVSHTVFRCQLCKYFCSTRKGIARHYR
IKHNNVRAQPEGKNNLFKCALCAYTNPIRKGLAAHYQKRHDIDAYYTHCLAASRTISDKP
NKVIIPSPPKDDSPQLSEELRRAVEKKKCSLCSFQSFSKKGIVSHYMKRHPGVFPKKQHA
SKLGGYFTAVYADEHEKPTLMEEEERGNFEKAEVEGEAQEIEWLPFRCIKCFKLSFSTAE
LLCMHYTDHHSRDLKRDFIILGNGPRLQNSTYQCKHCDSKLQSTAELTSHLNIHNEEFQK
RAKRQERRKQLLSKQKYADGAFADFKQERPFGHLEEVPKIKERKVVGYKCKFCVEVHPTL
RAICNHLRKHVQYGNVPAVSAAVKGLRSHERSHLALAMFTREDKYSCQYCSFVSAFRHNL
DRHMQTHHGHHKPFRCKLCSFKSSYNSRLKTHILKAHAGEHAYKCSWCSFSTMTISQLKE
HSLKVHGKALTLPRPRIVSLLSSHSHHSSQKATPAEEVEDSNDSSYSEPPDVQQQLNHYQ
SAALARNNSRVSPVPLSGAAAGTEQKTEAVLHCEFCEFSSGYIQSIRRHYRDKHGGKKLF
KCKDCSFYTGFKSAFTMHVEAGHSAVPEEGPKDLRCPLCLYHTKYKRNMIDHIVLHREER
VVPIEVCRSKLSKYLQGVVFRCDKCTFTCSSDESLQQHIEKHNELKPYKCQLCYYETKHT
EELDSHLRDEHKVSRNFELVGRVNLDQLEQMKEKMESSSSDDEDKEEEMNSKAEDRELMR
FSDHGAALNTEKRFPCEFCGRAFSQGSEWERHVLRHGMALNDTKQVSREEIHPKEIMENS
VKMPSIEEKEDDEAIGIDFSLKNETVAICVVTADKSLLENAEAKKE*
>ENSG00000165934|ENST00000298875
MTSIIKLTTLSGVQEESALCYLLQVDEFRFLLDCGWDEHFSMDIIDSLRKHVHQIDAVLL
SHPDPLHLGALPYAVGKLGLNCAIYATIPVYKMGQMFMYDLYQSRHNTEDFTLFTLDDVD
AAFDKIQQLKFSQIVNLKGKGHGLSITPLPAGHMIGGTIWKIVKDGEEEIVYAVDFNHKR
EIHLNGCSLEMLSRPSLLITDSFNATYVQPRRKQRDEQLLTNVLETLRGDGNVLIAVDTA
GRVLELAQLLDQIWRTKDAGLGVYSLALLNNVSYNVVEFSKSQVEWMSDKLMRCFEDKRN
NPFQFRHLSLCHGLSDLARVPSPKVVLASQPDLECGFSRDLFIQWCQDPKNSIILTYRTT
PGTLARFLIDNPSEKITEIELRKRVKLEGKELEEYLEKEKLKKEAAKKLEQSKEADIDSS
DESDIEEDIDQPSAHKTKHDLMMKGEGSRKGSFFKQAKKSYPMFPAPEERIKWDEYGEII
KPEDFLVPELQATEEEKSKLESGLTNGDEPMDQDLSDVPTKCISTTESIEIKARVTYIDY
EGRSDGDSIKKIINQMKPRQLIIVHGPPEASQDLAECCRAFGGKDIKVYMPKLHETVDAT
SETHIYQVRLKDSLVSSLQFCKAKDAELAWIDGVLDMRVSKVDTGVILEEGELKDDGEDS
EMQVEAPSDSSVIAQQKAMKSLFGDDEKETGEESEIIPTLEPLPPHEVPGHQSVFMNEPR
LSDFKQVLLREGIQAEFVGGVLVCNNQVAVRRTETGRIGLEGCLCQDFYRIRDLLYEQYA
IV*
>ENSG00000159556|ENST00000290759
MVDIIFHYPFLGAMGDHSKKKPGTAMCVGCGSQIHDQFILRVSPDLEWHAACLKCAECSQ
YLDETCTCFVRDGKTYCKRDYVRLFGIKCAKCQVGFSSSDLVMRARDSVYHIECFRCSVC
SRQLLPGDEFSLREHELLCRADHGLLLERAAAGSPRSPGPLPGARGLHLPDAGSGRQPAL
RPHVHKQTEKTTRVRTVLNEKQLHTLRTCYAANPRPDALMKEQLVEMTGLSPRVIRVWFQ
NKRCKDKKKSILMKQLQQQHSDKTSLQGLTGTPLVAGSPIRHENAVQGSAVEVQTYQPP
WKALSEFALQSDLDQPAFQQLVSFSESGSLGNSSGSDVTSLSSQLPDTPNSMVPSPVET*
>ENSG00000121417|ENST00000299871
MLGFPPGRPQLPVQLRPQTRMATALRDPASVPIATEVLFKLTQGSVTFEDVAVYFSWEEW

DLLDEAQKHLYFDVMLENFALTSSLGLISSWSHVVAQLGLGEVPSVLHRMFMTPASARWD
QRGPGLHEWHLGKGMSSGCWCGVEHEETPSEQRISGERVPQFRTSKEGSSSQNADSCEIC
CLVLRDILHLAEHQGTNCGQKLHTCGKQFYISANLQQHQRQHITEAPFRSYVDTASFTQS
CIVHVSEKPFTCREIRKDFLANMRFLHQDATQTGEKPNNSNKCAVAFYSGKSHHNWGKCS
KAFSHIDTLVQDQRILTREGLFECSKCGKACTRRCNLIQHQKVHSEERPYECNECGKFFT
YYSSFIIHQRVHTGERPYACPECGKSFSQIYSLNSHRKVHTGERPYECGECGKSFSQRSN
LMQHRRVHTGERPYECSECGKSFSQNFSLIYHQRVHTGERPHECNECGKSFSRSSSLIHH
RRLHTGERPYECSKCGKSFKQSSSFSSHRKVHTGERPYVCGECGKSFSHSSNLKNHQRVH
TGERPVECSECSKSFSCKSNLIKHLRVHTGERPYECSECGKSFSQSSSLIQHRRVHTGKR
PYQCSQCGKSFGCKSVLIQHQRVHIGEKP*
>ENSG00000196110|ENST00000308650
MEEERKTAELQKNRIQDSVVFEDVAVDFTQEEWALLDLAQRNLYRDVMLENFQNLASLGY
PLHTPHLISQWEQEEDLQTVKRELIQGIFMGEHREGFETQLKTNESVASQDICGEKISNE
QKIVRFKRNDSWFSSLHENQESCGIDYQNKSHERHLRNHMVENIYECYEENQDGQTFSQV
PNLDSLKRNTEVKSCECHECGKAFVDHSSLKSHIRSHTGSKPYQCKECGKAFHFLACFKK
HMKTPTEEKPYECKECTKAFSCSSFFRAHMKIHIGKTNYECKECGKGFSCSSSLTEHKRI
HSGDKPYECKECGKAFSCSSSLSKHKRIHSGDKPYECKECGKAFSSSSHLIIHIRIHTGE
KPYECKECGKAFSESSKLTVHGRTHTGEKPYKCKECGKAYNCPSSLSIHMRKHTGEKPYE
CLECGKAFYLPTSLNTHVKNQSREKPYECKECGKAFSCPSSFRAHVRDHTGKIQYECKEC
GKTFSRSSSLTEHLRTHSGEKPYECKECGKAFISSSHLTVHIRTHTGEKPYECKKCGKAF
IYPSALRIHMRTHTGEKPYECKECGKAFRHSSYLTVHARMHTGEKPFECLECGKAFSCPS
SFRRHVRSHTGEKPYECKECGKAFVCPAYFRRHVKTHTRENI*
>ENSG00000116001|ENST00000282574
MEDEMPKTLYVGNLSRDVTEALILQLFSQIGPCKNCKMIMDTAGNDPYCFVEFHEHRHAA
AALAAMNGRKIMGKEVKVNWATTPSSQKKDTSSSTVVSTQRSQDHFHVFVGDLSPEITTE
DIKAAFAPFGRISDARVVKDMATGKSKGYGFVSFFNKWDAENAIQQMGGQWLGGRQIRTN
WATRKPPAPKSTYESNTKQLSYDEVVNQSSPSNCTVYCGGVTSGLTEQLMRQTFSPFGQI
MEIRVFPDKGYSFVRFNSHESAAHAIVSVNGTTIEGHVVKCYWGKETLDMINPVQQNQIG
YPQPYGQWGQWYGNAQQIGQYMPNGWQVPAYGMYGQAWNQQGFNQTQSSAPWMGPNYGVQ
PPQGQNGSMLPNQPSGYRVAGYETQ*
>ENSG00000141582|ENST00000269397
MELPAVGEHVFAVESIEKKRIRKGRVEYLVKWRGWSPKYNTWEPEENILDPRLLIAFQNR
ERQEQLMGYRKRGPKPKPLVVQVPTFARRSNVLTGLQDSSTDNRAKLDLGAQGKGQGHQY
ELNSKKHHQYQPHSKERAGKPPPPGKSGKYYYQLNSKKHHPYQPDPKMYDLQYQGGHKEA
PSPTCPDLGAKSHPPDKWAQGAGAKGYLGAVKPLAGAAGAPGKGSEKGPPNGMMPAPKEA
VTGNGIGGKMKIVKNKNKNGRIVIVMSKYMENGMQAVKIKSGEVAEGEARSPSHKKRAAD
ERHPPADRTFKKAAGAEEKKVEAPPKRREEEVSGVSDPQPQDAGSRKLSPTKEAFGEQPL
QLTTKPDLLAWDPARNTHPPSHHPHPHPHHHHHHHHHHHHAVGLNLSHVRKRCLSETHGE
REPCKKRLTARSISTPTCLGGSPAAERPADLPPAAALPQPEVILLDSDLDEPIDLRCVKT
RSEAGEPPSSLQVKPETPASAAVAVAAAAAPTTTAEKPPAEAQDEPAESLSEFKPFFGNI
IITDVTANCLTVTFKEYVTV*
>ENSG00000100802|ENST00000299088
MSFSATILFSPPSGSEARCCCCACKSETNGGNTGSQGGNPPPSTPITVTGHGLAVQSSEQ
LLHVIYQRVDKAVGLAEAALGLARANNELLKRLQEEVGDLRQGKVSIPDEDGESRAHSSP

PEEPGPLKESPGEAFKALSAVEEECDSVGSGVQVVIEELRQLGAASVGPGPLGFPATQRD
MRLPGCTLAASEAAPLLNPLVDDYVASEGAVQRVLVPAYAKQLSPATQLAIQRATPETGP
ENGTKLPPPRPEDMLNAAAALDSALEESGPGSTGELRHSLGLTVSPCRTRGSGQKNSRRK
RDLVLSKLVHNVHNHITNDKRFNGSESIKSSWNISVVKFLLEKLKQELVTSPHNYTDKEL
KGACVAYFLTKRREYRNSLNPFKGLKEKEEKKLRSRRYRLFANRSSIMRHFGPEDQRLWN
DVTEELMSDEEDSLNEPGVWVARPPRFRAQRLTELCYHLDANSKHGTKANRVYGPPSDRL
PSAEAQLLPPELYNPNFQEEEDEGGDENAPGSPSFDQPHKTCCPDLNSFIEIKVEKDE*
>ENSG00000110888|ENST00000298892
MEVQVSQASLGFELTSVEKSLREWSRLSREVIAWLCPSSPNFILNFPPPPSASSVSMVQL
FSSPFGYQSPSGHSEEEREGNMKSAKPQVNHSQHGESQRALSPLQSTLSSAASPSQAYET
YIENGLICLKHKIRNIEKKKLKLEDYKDRLKSGEHLNPDQLEAVEKYEEVLHNLEFAKEL
QKTFSGLSLDLLKAQKKAQRREHMLKLEAEKKKLRTILQVQYVLQNLTQEHVQKDFKGGL
NGAVYLPSKELDYLIKFSKLTCPERNESLSVEDQMEQSSLYFWDLLEGSEKAVVGTTYKH
LKDLLSKLLNSGYFESIPVPKNAKEKEVPLEEEMLIQSEKKTQLSKTESVKESESLMEFA
QPEIQPQEFLNRRYMTEVDYSNKQGEEQPWEADYARKPNLPKRWDMLTEPDGQEKKQESF
KSWEASGKHQEVSKPAVSLEQRKQDTSKLRSTLPEEQKKQEISKSKPSPSQWKQDTPKSK
AGYVQEEQKKQETPKLWPVQLQKEQDPKKQTPKSWTPSMQSEQNTTKSWTTPMCEEQDSK
QPETPKSWENNVESQKHSLTSQSQISPKSWGVATASLIPNDQLLPRKLNTEPKDVPKPVH
QPVGSSSTLPKDPVLRKEKLQDLMTQIQGTCNFMQESVLDFDKPSSAIPTSQPPSATPGS
PVASKEQNLSSQSDFLQEPLQATSSPVTCSSNACLVTTDQASSGSETEFMTSETPEVFNV
NAPLPPRKEQEIKESPYSPGYNQSFTTASTQTPPQCQLPSIHVEQTVHSQETANYHPDGT
IQVSNGSLAFYPAQTNVFPRPTQPFVNSRGSVRGCTRGGRLITNSYRSPGGYKGFDTYRG
LPSISNGNYSQLQFQAREYSGAPYSQRDNFQQCYKRGGTSGGPRANSRAGWSDSSQVSSP
ERDNETFNSGDSGQGDSRSMTPVDVPVTNPAATILPVHVYPLPQQMRVAFSAARTSNLAP
GTLDQPIVFDLLLNNLGETFDLQLGRFNCPVNGTYVFIFHMLKLAVNVPLYVNLMKNEEV
LVSAYANDGAPDHETASNHAILQLFQGDQIWLRLHRGAIYGSSWKYSTFSGYLLYQD*
>ENSG00000185947|ENST00000300870
MGLLTFRDVAVEFSLEEWEHLEPAQKNLYQDVMLENYRNLVSLGLVVSKPDLITFLEQRK
EPWNVKSEETVAIQPDVFSHYNKDLLTEHCTEASFQKVISRRHGSCDLENLHLRKRWKRE
ECEGHNGCYDEKTFKYDQFDESSVESLFHQQILSSCAKSYNFDQYRKVFTHSSLLNQQEE
IDIWGKHHIYDKTSVLFRQVSTLNSYRNVFIGEKNYHCNNSEKTLNQSSSPKNHQENYFL
EKQYKCKEFEEVFLQSMHGQEKQEQSYKCNKCVEVCTQSLKHIQHQTIHIRENSYSYNKY
DKDLSQSSNLRKQIIHNEEKPYKCEKCGDSLNHSLHLTQHQIIPTEEKPCKWKECGKVFN
LNCSLYLTKQQQIDTGENLYKCKACSKSFTRSSNLIVHQRIHTGEKPYKCKECGKAFRCS
SYLTKHKRIHTGEKPYKCKECGKAFNRSSCLTQHQTTHTGEKLYKCKVCSKSYARSSNLI
MHQRVHTGEKPYKCKECGKVFSRSSCLTQHRKIHTGENLYKCKVCAKPFTCFSNLIVHER
IHTGEKPYKCKECGKAFPYSSHLIRHHRIHTGEKPYKCKACSKSFSDSSGLTVHRRTHTG
EKPYTCKECGKAFSYSSDVIQHRRIHTGQRPYKCEECGKAFNYRSYLTTHQRSHTGERPY
KCEECGKAFNSRSYLTTHRRRHTGERPYKCDECGKAFSYRSYLTTHRRSHSGERPYKCEE
CGKAFNSRSYLIAHQRSHTREKL*
>ENSG00000048828|ENST00000277165
MGVQGFQDYIEKHCPSAVVPVELQKLARGSLVGGGRQRPPQTPLRLLVDADNCLHRLYGG
FYTDWVSGGQWNHMLGYLAALAKACFGGNIELFVFFNGALEKARLHEWVKRQGNERQTAQ
QIVSHVQNKGTPPPKVWFLPPVCMAHCIRLALIRFHVKVAQSIEDHHQEVIGFCRENGFH

GLVAYDSDYALCNIPYYFSAHALKLSRNGKSLTTSQYLMHEVAKQLDLNPNRFPIFAALL
GNHILPDEDLASFHWSLLGPEHPLASLKVRAHQLVLPPCDVVIKAVADYVRNIQDTSDLD
AIAKDVFQHSQSRTDDKVIRFKRAIGYYSATSKPMSFHPPHYLAARPGPFGMPGMVPPHV
PPQMLNIPQTSLQAKPVAPQVPSPGGAPGQGPYPYSLSEPAPLTLDTSGKNLTEQNSYSN
IPHEGKHTPLYERSSPINPAQSGSPNHVDSAYFPGSSTSSSSDNDEGSGGATNHISGNKI
GWEKTGSHSEPQARGDPGDQTKAEGSSTASSGSQLAEGKGSQMGTVQPIPCLLSMPTRNH
MDITTPPLPPVAPEVLRVAEHRHKKGLMYPYIFHVLTKGEIKIAVSIEDEANKDLPPAAL
LYRPVRQYVYGVLFSLAESRKKTERLAFRKNRLPPEFSPVIIKEWAAYKGKSPQTPELVE
ALAFREWTCPNLKRLWLGKAVEDKNRRMRAFLACMRSDTPAMLNPANVPTHLMVLCCVLR
YMVQWPGARILRRQELDAFLAQALSPKLYEPDQLQELKIENLDPRGIQLSALFMSGVDMA
LFANDACGQPIPWEHCCPWMYFDGKLFQSKLLKASREKTPLIDLCDGQADQAAKVEKMRQ
SVLEGLSFSRQSHTLPFPPPPALPFYPASAYPRHFGPVPPSQGRGRGFAGVCGFGGPYGE
TVATGPYRAFRVAAASGHCGAFSGSDSSRTSKSQGGVQPIPSQGGKLEIAGTVVGHWAGS
RRGRGGRGPFPLQVVSVGGPARGRPRGVISTPVIRTFGRGGRYYGRGYKNQAAIQGRPPY
AASAEEVAKELKSKSGESKSSAMSSDGSLAENGVMAEEKPAPQMNGSTGDARAPSHSESA
LNNDSKTCNTNPHLNALSTDSACRREAALEAAVLNKEE*
>ENSG00000141027|ENST00000268712
MSSSGYPPNQGAFSTEQSRYPPHSVQYTFPNTRHQQEFAVPDYRSSHLEVSQASQLLQQQ
QQQQLRRRPSLLSEFHPGSDRPQERRTSYEPFHPGPSPVDHDSLESKRPRLEQVSDSHFQ
RVSAAVLPLVHPLPEGLRASADAKKDPAFGGKHEAPSSPISGQPCGDDQNASPSKLSKEE
LIQSMDRVDREIAKVEQQILKLKKKQQQLEEEAAKPPEPEKPVSPPPVEQKHRSIVQIIY
DENRKKAEEAHKIFEGLGPKVELPLYNQPSDTKVYHENIKTNQVMRKKLILFFKRRNHAR
KQREQKICQRYDQLMEAWEKKVDRIENNPRRKAKESKTREYYEKQFPEIRKQREQQERFQ
RVGQRGAGLSATIARSEHEISEIIDGLSEQENNEKQMRQLSVIPPMMFDAEQRRVKFINM
NGLMEDPMKVYKDRQFMNVWTDHEKEIFKDKFIQHPKNFGLIASYLERKSVPDCVLYYYL
TKKNENYKALVRRNYGKRRGRNQQIARPSQEEKVEEKEEDKAEKTEKKEEEKKDEEEKDE
KEDSKENTKEKDKIDGTAEETEEREQATPRGRKTANSQGRRKGRITRSMTNEAAAASAAA
AAATEEPPPPLPPPPEPISTEPVETSRWTEEEMEVAKKGLVEHGRNWAAIAKMVGTKSEA
QCKNFYFNYKRRHNLDNLLQQHKQKTSRKPREERDVSQCESVASTVSAQEDEDIEASNEE
ENPEDSEVEAVKPSEDSPENATSRGNTEPAVELEPTTETAPSTSPSLAVPSTKPAEDESV
ETQVNDSISAETAEQMDVDQQEHSAEEGSVCDPPPATKADSVDVEVRVPENHASKVEGDN
TKERDLDRASEKVEPRDEDLVVAQQINAQRPEPQSDNDSSATCSADEDVDGEPERQRMFP
MDSKPSLLNPTGSILVSSPLKPNPLDLPQLQHRAAVIPPMVSCTPCNIPIGTPVSGYALY
QRHIKAMHESALLEEQRQRQEQIDLECRSSTSPCGTSKSPNREWEVLQPAPHQVITNLPE
GVRLPTTRPTRPPPPLIPSSKTTVASEKPSFIMGGSISQGTPGTYLTSHNQASYTQETPK
PSVGSISLGLPRQQESAKSATLPYIKQEEFSPRSQNSQPEGLLVRAQHEGVVRGTAGAIQ
EGSITRGTPTSKISVESIPSLRGSITQGTPALPQTGIPTEALVKGSISRMPIEDSSPEKG
REEAASKGHVIYEGKSGHILSYDNIKNAREGTRSPRTAHEISLKRSYESVEGNIKQGMSM
RESPVSAPLEGLICRALPRGSPHSDLKERTVLSGSIMQGTPRATTESFEDGLKYPKQIKR
ESPPIRAFEGAITKGKPYDGITTIKEMGRSIHEIPRQDILTQESRKTPEVVQSTRPIIEG
SISQGTPIKFDNNSGQSAIKHNVKSLITGPSKLSRGMPPLEIVPENIKVVERGKYEDVKA
GETVRSRHTSVVSSGPSVLRSTLHEAPKAQLSPGIYDDTSARRTPVSYQNTMSRGSPMMN
RTSDVTISSNKSTNHERKSTLTPTQRESIPAKSPVPGVDPVVSHSPFDPHHRGSTAGEVY
RSHLPTHLDPAMPFHRALDPAAAAYLFQRQLSPTPGYPSQYQLYAMENTRQTILNDYITS

QQMQVNLRPDVARGLSPREQPLGLPYPATRGIIDLTNMPPTILVPHPGGTSTPPMDRITY
IPGTQITFPPRPYNSASMSPGHPTHLAAAASAERERERERERERERERERIAAASSDLYLRP
GSEQPGRPGSHGYVRSPSPSVRTQETMLQQRPSVFQGTNGTSVITPLDPTAQLRIMPLPA
GGPSISQGLPASRYNTAADALAALVDAAASAPQMDVSKTKESKHEAARLEENLRSRSAAV
SEQQQLEQKTLEVEKRSVQCLYTSSAFPSGKPQPHSSVVYSEAGKDKGPPPKSRYEEELR
TRGKTTITAANFIDVIITRQIASDKDARERGSQSSDSSSSLSSHRYETPSDAIEVISPAS
SPAPPQEKLQTYQPEVVKANQAENDPTRQYEGPLHHYRPQQESPSPQQQLPPSSQAEGMG
QVPRTHRLITLADHICQIITQDFARNQVSSQTPQQPPTSTFQNSPSALVSTPVRTKTSNR
YSPESQAQSVHHQRPGSRVSPENLVDKSRGSRPGKSPERSHVSSEPYEPISPPQVPVVHE
KQDSLLLLSQRGAEPAEQRNDARSPGSISYLPSFFTKLENTSPMVKSKKQEIFRKLNSSG
GGDSDMAAAQPGTEIFNLPAVTTSGSVSSRGHSFADPASNLGLEDIIRKALMGSFDDKVE
DHGVVMSQPMGVVPGTANTSVVTSGETRREEGDPSPHSGGVCKPKLISKSNSRKSKSPIP
GQGYLGTERPSSVSSVHSEGDYHRQTPGWAWEDRPSSTGSTQFPYNPLTMRMLSSTPPTP
IACAPSAVNQAAPHQQNRIWEREPAPLLSAQYETLSDSDD*
>ENSG00000126432|ENST00000265462
MGLAGVCALRRSAGYILVGGAGGQSAAAAARRYSEGEWASGGVRSFSRAAAAMAPIKVGD
AIPAVEVFEGEPGNKVNLAELFKGKKGVLFGVPGAFTPGCSKTHLPGFVEQAEALKAKGV
QVVACLSVNDAFVTGEWGRAHKAEGKVRLLADPTGAFGKETDLLLDDSLVSIFGNRRLKR
FSMVVQDGIVKALNVEPDGTGLTCSLAPNIISQL*
>ENSG00000178764|ENST00000314393
MASKRKSTTPCMVRTSQVVEQDVPEEVDRAKEKGIGTPQPDVAKDSWAAELENSSKENEV
IEVKSMGESQSKKLQGGYECKYCPYSTQNLNEFTEHVDMQHPNVILNPLYVCAECNFTTK
KYDSLSDHNSKFHPGEANFKLKLIKRNNQTVLEQSIETTNHVVSITTSGPGTGDSDSGIS
VSKTPIMKPGKPKADAKKVPKKPEEITPENHVEGTARLVTDTAEILSRLGGVELLQDTLG
HVMPSVQLPPNINLVPKVPVPLNTTKYNSALDTNATMINSFNKFPYPTQAELSWLTAASK
HPEEHIRIWFATQRLKHGISWSPEEVEEARKKMFNGTIQSVPPTITVLPAQLAPTKVTQP
ILQTALPCQILGQTSLVLTQVTSGSTTVSCSPITLAVAGVTNHGQKRPLVTPQAAPEPKR
PHIAQVPEPPPKVANPPLTPASDRKKTKEQIAHLKASFLQSQFPDDAEVYRLIEVTGLAR
SEIKKWFSDHRYRCQRGIVHITSESLAKDQLAIAASRHGRTYHAYPDFAPQKFKEKTQGQ
VKILEDSFLKSSFPTQAELDRLRVETKLSRREIDSWFSERRKLRDSMEQAVLDSMGSGKK
GQDVGAPNGALSRLDQLSGAQLTSSLPSPSPAIAKSQEQVHLLRSTFARTQWPTPQEYDQ
LAAKTGLVRTEIVRWFKENRCLLKTGTVKWMEQYQHQPMADDHGYDAVARKATKPMAESP
KNGGDVVPQYYKDPKKLCEEDLEKLVTRVKVGSEPAKDCLPAKPSEATSDRSEGSSRDGQ
GSDENEESSVVDYVEVTVGEEDAISDRSDSWSQAAAEGVSELAESDSDCVPAEAGQA*
>ENSG00000143578|ENST00000271889
MDLGIPDLLDAWLEPPEDIFSTGSVLELGLHCPPPEVPVTRLQEQGLQGWKSGGDRGCGL
QESEPEDFLKLFIDPNEVYCSEASPGSDSGISEDPCHPDSPPAPRATSSPMLYEVVYEAG
ALERMQGETGPNVGLISIQLDQWSPAFMVPDSCMVSELPFDAHAHILPRAGTVAPVPCTT
LLPCQTLFLTDEEKRLLGQEGVSLPSHLPLTKAEERVLKKVRRKIRNKQSAQDSRRRKKE
YIDGLESRVAACSAQNQELQKKVQELERHNISLVAQLRQLQTLIAQTSNKAAQTSTCVLI
LLFSLALIILPSFSPFQSRPEAGSEDYQPHGVTSRNILTHKDVTENLETQVVESRLREPP
GAKDANGSTRTLLEKMGGKPRPSGRIRSVLHADEM*
>ENSG00000127483|ENST00000312239
MATDTSQGELVHPKALPLIVGAQLIHADKLGEKVEDSTMPIRRTVNSTRETPPKSKLAEG

EEEKPEPDISSEESVSTVEEQENETPPATSSEAEQPKGEPENEEKEENKSSEETKKDEKD
QSKEKEKKVKKTIPSWATLSASQLARAQKQTPMASSPRPKMDAILTEAIKACFQKSGASV
VAIRKYIIHKYPSLELERRGYLLKQALKRELNRGVIKQVKGKGASGSFVVVQKSRKTPQK
SRNRKNRSSAVDPEPQVKLEDVLPLAFTRLCEPKEASYSLIRKYVSQYYPKLRVDIRPQL
LKNALQRAVERGQLEQITGKGASGTFQLKKSGEKPLLGGSLMEYAILSAIAAMNEPKTCS
TTALKKYVLENHPGTNSNYQMHLLKKTLQKCEKNGWMEQISGKGFSGTFQLCFPYYPSPG
VLFPKKEPDDSRDEDEDEDESSEEDSEDEEPPPKRRLQKKTPAKSPGKAASVKQRGSKPA
PKVSAAQRGKARPLPKKAPPKAKTPAKKTRPSSTVIKKPSGGSSKKPATSARKEVKLPGK
GKSTMKKSFRVKK*
>ENSG00000173120|ENST00000308783
MRRRYEDDGISDDEIEGKRTFDLEEKLHTNKYNANFVTFMEGKDFNVEYIQRGGLRDPLI
FKNSDGLGIKMPDPDFTVNDVKMCVGSRRMVDVMDVNTQKGIEMTMAQWTRYYETPEEER
EKLYNVISLEFSHTRLENMVQRPSTVDFIDWVDNMWPRHLKESQTESTNAILEMQYPKVQ
KYCLMSVRGCYTDFHVDFGGTSVWYHIHQGGKVFWLIPPTAHNLELYENWLLSGKQGDIF
LGDRVSDCQRIELKQGYTFVIPSGWIHAVYTPTDTLVFGGNFLHSFNIPMQLKIYNIEDR
TRVPNKFRYPFYYEMCWYVLERYVYCITNRSHLTKEFQKESLSMDLELNGLESGNGDEEA
VDREPRRLSSRRSVLTSPVANGVNLDYDGLGKTCRSLPSLKKTLAGDSSSDCSRGSHNGQ
VWDPQCAPRKDRQVHLTHFELEGLRCLVDKLESLPLHKKCVPTGIEDEDALIADVKILLE
ELANSDPKLALTGVPIVQWPKRDKLKFPTRPKVRVPTIPITKPHTMKPAPRLTPVRPAAA
SPIVSGARRRRVRCRKCKACVQGECGVCHYCRDMKKFGGPGRMKQSCVLRQCLAPRLPHS
VTCSLCGEVDQNEETQDFEKKLMECCICNEIVHPGCLQMDGEGLLNEELPNCWECPKCYQ
EDSSEKAQKRKMEESDEEAVQAKVLRPLRSCDEPLTPPPHSPTSMLQLIHDPVSPRGMVT
RSSPGAGPSDHHSASRDERFKRRQLLRLQATERTMVREKENNPSGKKELSEVEKAKIRGS
YLTVTLQRPTKELHGTSIVPKLQAITASSANLRHSPRVLVQHCPARTPQRGDEEGLGGEE
EEEEEEEEDDSAEEGGAARLNGRGSWAQDGDESWMQREVWMSVFRYLSRRELCECMRVC
KTWYKWCCDKRLWTKIDLSRCKAIVPQALSGIIKRQPVSLDLSWTNISKKQLTWLVNRLP
GLKDLLLAGCSWSAVSALSTSSCPLLRTLDLRWAVGIKDPQIRDLLTPPADKPGQDNRSK
LRNMTDFRLAGLDITDATLRLIIRHMPLLSRLDLSHCSHLTDQSSNLLTAVGSSTRYSLT
ELNMAGCNKLTDQTLIYLRRIANVTLIDLRGCKQITRKACEHFISDLSINSLYCLSDEKL
IQKIS*
>ENSG00000134597|ENST00000305536
MNPLTKVKLINELNEREVQLGVADKVSWHSEYKDSAWIFLGGLPYELTEGDIICVFSQYG
EIVNINLVRDKKTGKSKGFCFLCYEDQRSTILAVDNFNGIKIKGRTIRVDHVSNYRAPKD
SEEIDDVTRQLQEKGCGARTPSPSLSESSEDEKPTKKHKKDKKEKKKKKKEKEKADREVQ
AEQPSSSSPRRKTVKEKDDTGPKKHSSKNSERAQKSEPREGQKLPKSRTAYSGGAEDLER
ELKKEKPKHEHKSSSRREAREEKTRIRDRGRSSDAHSSWYNGRSEGRSYRSRSRSRDKSH
RHKRARRSRERESSNPSDRWRH*
>ENSG00000273045|ENST00000302513
MSSTLGKLSNQVEETLPLLKKVPANYFHICSAILMGFSLSKSATQVSAIHMDSKVDDHLI
RGTEKSRLEPATQLFQNTKKIRLEDTNQENFTRIEGTGTGSLSGKALGSVVYVKESDGLE
MTDVE*
>ENSG00000115548|ENST00000312912
MVLTLGESWPVLVGRRFLSLSAADGSDGSHDSWDVERVAEWPWLSGTIRAVSHTDVTKKD
LKVCVEFDGESWRKRRWIEVYSLLRRAFLVEHNLVLAERKSPEISERIVQWPAITYKPLL

DKAGLGSITSVRFLGDQQRVFLSKDLLKPIQDVNSLRLSLTDNQIVSKEFQALIVKHLDE
SHLLKGDKNLVGSEVKIYSLDPSTQWFSATVINGNPASKTLQVNCEEIPALKIVDPSLIH
VEVVHDNLVTCGNSARIGAVKRKSSENNGTLVSKQAKSCSEASPSMCPVQSVPTTVFKEI
LLGCTAATPPSKDPRQQSTPQAANSPPNLGAKIPQGCHKQSLPEEISSCLNTKSEALRTK
PDVCKAGLLSKSSQIGTGDLKILTEPKGSCTQPKTNTDQENRLESVPQALTGLPKECLPT
KASSKAELEIANPPELQKHLEHAPSPSDVSNAPEVKAGVNSDSPNNCSGKKVEPSALACR
SQNLKESSVKVDNESCCSRSNNKIQNAPSRKSVLTDPAKLKKLQQSGEAFVQDDSCVNIV
AQLPKCRECRLDSLRKDKEQQKDSPVFCRFFHFRRLQFNKHGVLRVEGFLTPNKYDNEAI
GLWLPLTKNVVGIDLDTAKYILANIGDHFCQMVISEKEAMSTIEPHRQVAWKRAVKGVRE
MCDVCDTTIFNLHWVCPRCGFGVCVDCYRMKRKNCQQGAAYKTFSWLKCVKSQIHEPENL
MPTQIIPGKALYDVGDIVHSVRAKWGIKANCPCSNRQFKLFSKPASKEDLKQTSLAGEKP
TLGAVLQQNPSVLEPAAVGGEAASKPAGSMKPACPASTSPLNWLADLTSGNVNKENKEKQ
PTMPILKNEIKCLPPLPPLSKSSTVLHTFNSTILTPVSNNNSGFLRNLLNSSTGKTENGL
KNTPKILDDIFASLVQNKTTSDLSKRPQGLTIKPSILGFDTPHYWLCDNRLLCLQDPNNK
SNWNVFRECWKQGQPVMVSGVHHKLNSELWKPESFRKEFGEQEVDLVNCRTNEIITGATV
GDFWDGFEDVPNRLKNEKEPMVLKLKDWPPGEDFRDMMPSRFDDLMANIPLPEYTRRDGK
LNLASRLPNYFVRPDLGPKMYNAYGLITPEDRKYGTTNLHLDVSDAANVMVYVGIPKGQC
EQEEEVLKTIQDGDSDELTIKRFIEGKEKPGALWHIYAAKDTEKIREFLKKVSEEQGQEN
PADHDPIHDQSWYLDRSLRKRLHQEYGVQGWAIVQFLGDVVFIPAGAPHQVHNLYSCIKV
AEDFVSPEHVKHCFWLTQEFRYLSQTHTNHEDKLQVKNVIYHAVKDAVAMLKASESSFGK
P*
>ENSG00000105967|ENST00000265440
MTLDHQIINPTLKWSQPAVPSGGPLVQHAHTTLDSDAGLTENPLTKLLAIGKEDDNAQWH
MEDVIEDIIGMESSFKEEGADSPLLMQRTLSGSILDVYSGEQGISPINMGLTSASCPSSL
PMKREITETDTRALAKERQKKDNHNLIERRRRYNINYRIKELGTLIPKSNDPDMRWNKGT
ILKASVEYIKWLQKEQQRARELEHRQKKLEQANRRLLLRIQELEIQARTHGLPTLASLGT
VDLGAHVTKQQSHPEQNSVDYCQQLTVSQGPSPELCDQAIAFSDPLSYFTDLSFSAALKE
EQRLDGMLLDDTISPFGTDPLLSATSPAVSKESSRRSSFSSDDGDEL*
>ENSG00000160396|ENST00000291823
MSTIQSETDCYDIIEVLGKGTFGEVAKGWRRSTGEMVAIKILKNDAYRNRIIKNELKLLH
CMRGLDPEEAHVIRFLEFFHDALKFYLVFELLEQNLFEFQKENNFAPLPARHIRTVTLQV
LTALARLKELAIIHADLKPENIMLVDQTRCPFRVKVIDFGSASIFSEVRYVKEPYIQSRF
YRAPEILLGLPFCEKVDVWSLGCVMAELHLGWPLYPGNNEYDQVRYICETQGLPKPHLLH
AACKAHHFFKRNPHPDAANPWQLKSSADYLAETKVRPLERRKYMLKSLDQIETVNGGSVA
SRLTFPDREALAEHADLKSMVELIKRMLTWESHERISPSAALRHPFVSMQQLRSAHETTH
YYQLSLRSYRLSLQVEGKPPTPVVAAEDGTPYYCLAEEKEAAGMGSVAGSSPFFREEKAP
GMQRAIDQLDDLSLQEAGHGLWGETCTNAVSDMMVPLKAAITGHHVPDSGPEPILAFYSS
RLAGRHKARKPPAGSKSDSNFSNLIRLSQVSPEDDRPCRGSSWEEGEHLGASAEPLAILQ
RDEDGPNIDNMTMEAERPDPELFDPSSCPGEWLSEPDCTLESVRGPRAQGLPPRRSHQHG
PPRGATSFLQHVTGHH*
>ENSG00000142186|ENST00000270176
MWFFARDPVRDFPFELIPEPPEGGLPGPWALHRGRKKATGSPVSIFVYDVKPGAEEQTQV
AKAAFKRFKTLRHPNILAYIDGLETEKCLHVVTEAVTPLGIYLKARVEAGGLKELEISWG
LHQIVKALSFLVNDCSLIHNNVCMAAVFVDRAGEWKLGGLDYMYSAQGNGGGPPRKGIPE

LEQYDPPELADSSGRVVREKWSADMWRLGCLIWEVFNGPLPRAAALRNPGKIPKTLVPHY
CELVGANPKVRPNPARFLQNCRAPGGFMSNRFVETNLFLEEIQIKEPAEKQKFFQELSKS
LDAFPEDFCRHKVLPQLLTAFEFGNAGAVVLTPLFKVGKFLSAEEYQQKIIPVVVKMFSS
TDRAMRIRLLQQMEQFIQYLDEPTVNTQIFPHVVHGFLDTNPAIREQTVKSMLLLAPKLN
EANLNVELMKHFARLQAKDEQGPIRCNTTVCLGKIGSYLSASTRHRVLTSAFSRATRDPF
APSRVAGVLGFAATHNLYSMNDCAQKILPVLCGLTVDPEKSVRDQAFKAIRSFLSKLESV
SEDPTQLEEVEKDVHAASSPGMGGAAASWAGWAVTGVSSLTSKLIRSHPTTAPTETNIPQ
RPTPEGVPAPAPTPVPATPTTSGHWETQEEDKDTAEDSSTADRWDDEDWGSLEQEAESVL
AQQDDWSTGGQVSRASQVSNSDHKSSKSPESDWSSWEAEGSWEQGWQEPSSQEPPPDGTR
LASEYNWGGPESSDKGDPFATLSARPSTQPRPDSWGEDNWEGLETDSRQVKAELARKKRE
ERRREMEAKRAERKVAKGPMKLGARKLD*
>ENSG00000152234|ENST00000282050
MLSVRVAAAVVRALPRRAGLVSRNALGSSFIAARNFHASNTHLQKTGTAEMSSILEERIL
GADTSVDLEETGRVLSIGDGIARVHGLRNVQAEEMVEFSSGLKGMSLNLEPDNVGVVVFG
NDKLIKEGDIVKRTGAIVDVPVGEELLGRVVDALGNAIDGKGPIGSKTRRRVGLKAPGII
PRISVREPMQTGIKAVDSLVPIGRGQRELIIGDRQTGKTSIAIDTIINQKRFNDGSDEKK
KLYCIYVAIGQKRSTVAQLVKRLTDADAMKYTIVVSATASDAAPLQYLAPYSGCSMGEYF
RDNGKHALIIYDDLSKQAVAYRQMSLLLRRPPGREAYPGDVFYLHSRLLERAAKMNDAFG
GGSLTALPVIETQAGDVSAYIPTNVISITDGQIFLETELFYKGIRPAINVGLSVSRVGSA
AQTRAMKQVAGTMKLELAQYREVAAFAQFGSDLDAATQQLLSRGVRLTELLKQGQYSPMA
IEEQVAVIYAGVRGYLDKLEPSKITKFENAFLSHVVSQHQALLGTIRADGKISEQSDAKL
KEIVTNFLAGFEA*
>ENSG00000173451|ENST00000308086
MPTNCAAAGCATTYNKHINISFHRFPLDPKRRKEWVRLVRRKNFVPGKHTFLCSKHFEAS
CFDLTGQTRRLKMDAVPTIFDFCTHIKSMKLKSRNLLKKNNSCSPAGPSNLKSNISSQQV
LLEHSYAFRNPMEAKKRIIKLEKEIASLRRKMKTCLQKERRATRRWIKATCLVKNLEANS
VLPKGTSEHMLPTALSSLPLEDFKILEQDQQDKTLLSLNLKQTKSTFI*
>ENSG00000166441|ENST00000314138
MPSRLRKTRKLRGHVSHGHGRIGKHRKHPGGRGNAGGLHHHRINFDKYHPGYFGKVGMKH
YHLKRNQSFCPTVNLDKLWTLVSEQTRVNAAKNKTGAAPIIDVVRSGYYKVLGKGKLPKQ
PVIVKAKFFSRRAEEKIKSVGGACVLVA*
>ENSG00000164299|ENST00000296739
MASSAKSAEMPTISKTVNPTPDPHQEYLDPRITIALFEIGSHSPSSWGSLPFLKNSSHQV
TEQQTAQKFNNLLKEIKDILKNMAGFEEKITEAKELFEETNITEDVSAHKENIRGLDKIN
EMLSTNLPVSLAPEKEDNEKKQEMILETNITEDVSAHKENIRGLDKINEMLSTNLPVSLA
PEKEDNEKKQQMIMENQNSENTAQVFARDLVNRLEEKKVLNETQQSQEKAKNRLNVQEET
MKIRNNMEQLLQEAEHWSKQHTELSKLIKSYQKSQKDISETLGNNGVGFQTQPNNEVSAK
HELEEQVKKLSHDTYSLQLMAALLENECQILQQRVEILKELHHQKQGTLQEKPIQINYKQ
DKKNQKPSEAKKVEMYKQNKQAMKGTFWKKDRSCRSLDVCLNKKACNTQFNIHVARKALR
GKMRSASSLR*
>ENSG00000221994|ENST00000276054
MDRYQGNQDRVLRQVTVISRETLTDEMGSKYSAFGKMFNRCTDLAPLSQKFHKFDSCENS
LKSNSDLLNYNRSYARKNPTKRFRCGRPPKYNASCSVPEKEGFIHTGMEPYGDSQCEKVL
SHKQAHVQYKKFQAREKPNVCSMCGKAFIKKSQLIIHQRIHTGEKPYVCGDCRKAFSEKS

HLIVHQRIHTGEKPYECTKYGRAFSRKSPFTVHQRVHTGEKPYECFECPKAFSQKSHLII
HQRVHTREKPFECSECRKAFCEMSHLFIHQITHTGKKPYECTECGKTFPRKTQLIIHQRT
HTGEKPYKCGECGKTFCQQSHLIGHQRIHTGEKPYVCTDCGKAFSQKSHLTGHQRLHTGE
KPYMCTECGKSFSQKSPLIIHQRIHTGEKPYQCGECGKTFSQKSLLIIHLRVHTGEKPYE
CTECGRAFSLKSHLILHQRGHTGEKPYECSECGKAFCGKSPLIIHQKTHPREKTPECAES
GMTFFWKSQMITYQRRHTGEKPSRCSDCGKAFCQHVYFTGHQNPYRKDTLYIC*
>ENSG00000173744|ENST00000310078
MAASAKRKQEEKHLKMLRDMTGLPHNRKCFDCDQRGPTYVNMTVGSFVCTSCSGSLRGLN
PPHRVKSISMTTFTQQEIEFLQKHGNEVCKQIWLGLFDDRSSAIPDFRDPQKVKEFLQEK
YEKKRWYVPPEQAKVVASVHASISGSSASSTSSTPEVKPLKSLLGDSAPTLHLNKGTPSQ
SPVVGRSQGQQQEKKQFDLLSDLGSDIFAAPAPQSTATANFANFAHFNSHAAQNSANADF
ANFDAFGQSSGSSNFGGFPTASHSPFQPQTTGGSAASVNANFAHFDNFPKSSSADFGTFN
TSQSHQTASAVSKVSTNKAGLQTADKYAALANLDNIFSAGQGGDQGSGFGTTGKAPVGSV
VSVPSQSSASSDKYAALAELDSVFSSAATSSNAYTSTSNASSNVFGTVPVVASAQTQPAS
SSVPAPFGATPSTNPFVAAAGPSVASSTNPFQTNARGATAATFGTASMSMPTGFGTPAPY
SLPTSFSGSFQQPAFPAQAAFPQQTAFSQQPNGAGFAAFGQTKPVVTPFGQVAAAGVSSN
PFMTGAPTGQFPTGSSSTNPFL*
>ENSG00000165061|ENST00000297737
MKSSDIDQDLFTDSYCKVCSAQLISESQRVAHYESRKHASKVRLYYMLHPRDGGCPAKRL
RSENGSDADMVDKNKCCTLCNMSFTSAVVADSHYQGKIHAKRLKLLLGEKTPLKTTATPL
SPLKPPRMDTAPVVASPYQRRDSDRYCGLCAAWFNNPLMAQQHYDGKKHKKNAARVALLE
QLGTTLDMGELRGLRRNYRCTICSVSLNSIEQYHAHLKGSKHQTNLKNK*
>ENSG00000113407|ENST00000265112
MFEEKASSPSGKMGGEEKPIGAGEEKQKEGGKKKNKEGSGDGGRAELNPWPEYIYTRLEM
YNILKAEHDSILAEKAEKDSKPIKVTLPDGKQVDAESWKTTPYQIACGISQGLADNTVIA
KVNNVVWDLDRPLEEDCTLELLKFEDEEAQAVYWHSSAHIMGEAMERVYGGCLCYGPPIE
NGFYYDMYLEEGGVSSNDFSSLEALCKKIIKEKQAFERLEVKKETLLAMFKYNKFKCRIL
NEKVNTPTTTVYRCGPLIDLCRGPHVRHTGKIKALKIHKNSSTYWEGKADMETLQRIYGI
SFPDPKMLKEWEKFQEEAKNRDHRKIGRDQELYFFHELSPGSCFFLPKGAYIYNALIEFI
RSEYRKRGFQEVVTPNIFNSRLWMTSGHWQHYSENMFSFEVEKELFALKPMNCPGHCLMF
DHRPRSWRELPLRLADFGVLHRNELSGALTGLTRVRRFQQDDAHIFCAMEQIEDEIKGCL
DFLRTVYSVFGFSFKLNLSTRPEKFLGDIEVWDQAEKQLENSLNEFGEKWELNSGDGAFY
GPKIDIQIKDAIGRYHQCATIQLDFQLPIRFNLTYVSHDGDDKKRPVIVHRAILGSVERM
IAILTENYGGKWPFWLSPRQVMVVPVGPTCDEYAQKVRQQFHDAKFMADIDLDPGCTLNK
KIRNAQLAQYNFILVVGEKEKISGTVNIRTRDNKVHGERTISETIERLQQLKEFRSKQAE
EEF*
>ENSG00000161405|ENST00000293068
MEDIQTNAELKSTQEQSVPAESAAVLNDYSLTKSHEMENVDSGEGPANEDEDIGDDSMKV
KDEYSERDENVLKSEPMGNAEEPEIPYSYSREYNEYENIKLERHVVSFDSSRPTSGKMNC
DVCGLSCISFNVLMVHKRSHTGERPFQCNQCGASFTQKGNLLRHIKLHTGEKPFKCHLCN
YACQRRDALTGHLRTHSVEKPYKCEFCGRSYKQRSSLEEHKERCRTFLQSTDPGDTGTGW
GWVELSHLGIRLQDLNVPWCRLH*
>ENSG00000139726|ENST00000280557
MAADISESSGADCKGDPRNSAKLDADYPLRVLYCGVCSLPTEYCEYMPDVAKCRQWLEKN

FPNEFAKLTVENSPKQEAGISEGQGTAGEEEEKKKQKRGGRGQIKQKKKTVPQKVTIAKI
PRAKKKYVTRVCGLATFEIDLKEAQRFFAQKFSCGASVTGEDEIIIQGDFTDDIIDVIQE
KWPEVDDDSIEDLGEVKK*
>ENSG00000171863|ENST00000304921
MFSSSAKIVKPNGEKPDEFESGISQALLELEMNSDLKAQLRELNITAAKEIEVGGGRKAI
IIFVPVPQLKSFQKIQVRLVRELEKKFSGKHVVFIAQRRILPKPTRKSRTKNKQKRPRSR
TLTAVHDAILEDLVFPSEIVGKRIRVKLDGSRLIKVHLDKAQQNNVEHKVETFSGVYKKL
TGKDVNFEFPEFQL*
>ENSG00000166848|ENST00000300086
MAEAMDLGKDPNGPTHSSTLFVRDDGSSMSFYVRPSPAKRRLSTLILHGGGTVCRVQEPG
AVLLAQPGEALAEASGDFISTQYILDCVERNERLELEAYRLGPASAADTGSEAKPGALAE
GAAEPEPQRHAGRIAFTDADDVAILTYVKENARSPSSVTGNALWKAMEKSSLTQHSWQSL
KDRYLKHLRGQEHKYLLGDAPVSPSSQKLKRKAEEDPEAADSGEPQNKRTPDLPEEEYVK
EEIQENEEAVKKMLVEATREFEEVVVDESPPDFEIHITMCDDDPPTPEEDSETQPDEEEE
EEEEKVSQPEVGAAIKIIRQLMEKFNLDLSTVTQAFLKNSGELEATSAFLASGQRADGYP
IWSRQDDIDLQKDDEDTREALVKKFGAQNVARRIEFRKK*
>ENSG00000117748|ENST00000313433
MWNSNDGGAGWRRKRIAGGFSKRASLGSERRVVAGEEGRERSWGVWGSPAGRRRGRLGRL
GQCLKGRSLREPAGFSEAWDVAQALILLFKTGGFESYGSSSYGGAGGYTQSPGGFGSPAP
SQAEKKSRARAQHIVPCTISQLLSATLVDEVFRIGNVEISQVTIVGIIRHAEKAPTNIVY
KIDDMTAAPMDVRQWVDTDDTSSENTVVPPETYVKVAGHLRSFQNKKSLVAFKIMPLEDM
NEFTTHILEVINAHMVLSKANSQPSAGRAPISNPGMSEAGNFGGNSFMPANGLTVAQNQV
LNLIKACPRPEGLNFQDLKNQLKHMSVSSIKQAVDFLSNEGHIYSTVDDDHFKSTDAE*
>ENSG00000164736|ENST00000297316
MSSPDAGYASDDQSQTQSALPAVMAGLGPCPWAESLSPIGDMKVKGEAPANSGAPAGAAG
RAKGESRIRRPMNAFMVWAKDERKRLAQQNPDLHNAELSKMLGKSWKALTLAEKRPFVEE
AERLRVQHMQDHPNYKYRPRRRKQVKRLKRVEGGFLHGLAEPQAAALGPEGGRVAMDGLG
LQFPEQGFPAGPPLLPPHMGGHYRDCQSLGAPPLDGYPLPTPDTSPLDGVDPDPAFFAAP
MPGDCPAAGTYSYAQVSDYAGPPEPPAGPMHPRLGPEPAGPSIPGLLAPPSALHVYYGAM
GSPGAGGGRGFQMQPQHQHQHQHQHHPPGPGQPSPPPEALPCRDGTDPSQPAELLGEVDR
TEFEQYLHFVCKPEMGLPYQGHDSGVNLPDSHGAISSVVSDASSAVYYCNYPDV*
>ENSG00000170396|ENST00000302277
MECYYIVISSTHLSNGHFRNIKGVFRGPLSKNGNKTLDYAEKENTIAKALEDLKANFYCE
LCDKQYYKHQEFDNHINSYDHAHKQRLKELKQREFARNVASKSRKDERKQEKALQRLHKL
AELRKETVCAPGSGPMFKSTTVTVRENCNEISQRVVVDSVNNQQDFKYTLIHSEENTKDA
TTVAEDPESANNYTAKNNQVGDQAQGIHRHKIGFSFAFPKKASVKLESSAAAFSEYSDDA
SVGKGFSRKSRFVPSACHLQQSSPTDVLLSSEEKTNSFHPPEAMCRDKETVQTQEIKEVS
SEKDALLLPSFCKFQLQLSSDADNCQNSVPLADQIPLESVVINEDIPVSGNSFELLGNKS
TVLDMSNDCISVQATTEENVKHNEASTTEVENKNGPETLAPSNTEEVNITIHKKTNFCKR
QCEPFVPVLNKHRSTVLQWPSEMLVYTTTKPSISYSCNPLCFDFKSTKVNNNLDKNKPDL
KDLCSQQKQEDICMGPLSDYKDVSTEGLTDYEIGSSKNKCSQVTPLLADDILSSSCDSGK
NENTGQRYKNISCKIRETEKYNFTKSQIKQDTLDEKYNKIRLKETHEYWFHKSRRKKKRK
KLCQHHHMEKTKESETRCKMEAENSYTENAGKYLLEPISEKQYLAAEQLLDSHQLLDKRP
KSESISLSDNEEMCKTWNTEYNTYDTISSKNHCKKNTILLNGQSNATMIHSGKHNLTYSR

TYCCWKTKMSSCSQDHRSLVLQNDMKHMSQNQAVKRGYNSVMNESERFYRKRRQHSHSYS
SDESLNRQNHLPEEFLRPPSTSVAPCKPKKKRRRKRGRFHPGFETLELKENTDYPVKDNS
SLNPLDRLISEDKKEKMKPQEVAKIERNSEQTNQLRNKLSFHPNNLLPSETNGETEHLEM
ETTSGELSDVSNDPTTSVCVASAPTKEAIDNTLLEHKERSENINLNEKQIPFQVPNIERN
FRQSQPKSYLCHYELAEALPQGKMNETPTEWLRYNSGILNTQPPLPFKEAHVSGHTFVTA
EQILAPLALPEQALLIPLENHDKFKNVPCEVYQHILQPNMLANKVKFTFPPAALPPPSTP
LQPLPLQQSLCSTSVTTIHHTVLQQHAAAAAAAAAAAAAGTFKVLQPHQQFLSQIPALTR
TSLPQLSVGPVGPRLCPGNQPTFVAPPQMPIIPASVLHPSHLAFPSLPHALFPSLLSPHP
TVIPLQPLF*
>ENSG00000156925|ENST00000287538
MTMLLDGGPQFPGLGVGSFGAPRHHEMPNREPAGMGLNPFGDSTHAAAAAAAAAAFKLSP
AAAHDLSSGQSSAFTPQGSGYANALGHHHHHHHHHHHTSQVPSYGGAASAAFNSTREFLF
RQRSSGLSEAASGGGQHGLFAGSASSLHAPAGIPEPPSYLLFPGLHEQGAGHPSPTGHVD
NNQVHLGLRGELFGRADPYRPVASPRTDPYAAGAQFPNYSPMNMNMGVNVAAHHGPGAFF
RYMRQPIKQELSCKWIDEAQLSRPKKSCDRTFSTMHELVTHVTMEHVGGPEQNNHVCYWE
ECPREGKSFKAKYKLVNHIRVHTGEKPFPCPFPGCGKIFARSENLKIHKRTHTGEKPFKC
EFEGCDRRFANSSDRKKHMHVHTSDKPYICKVCDKSYTHPSSLRKHMKVHESQGSDSSPA
ASSGYESSTPPAIASANSKDTTKTPSAVQTSTSHNPGLPPNFNEWYV*
>ENSG00000136982|ENST00000313655
MKRTRDEVDATLQIAKLNAAELLPAVHCLGFGPGASGAAAGDFCLLELEPTLCQQLEDGH
SLVIRGDKDEQAVLCSKDKTYDLKIADTSNMLLFIPGCKTPDQLKKEDSHCNIIHTEIFG
FSNNYWELRRRRPKLKKLKKLLMENPYEGPDSQKEKDSNSSKYTTEDLLDQIQASEEEIM
TQLQVLNACKIGGYWRILEFDYEMKLLNHVTQLVDSESWSFGKVPLNTCLQELGPLEPEE
MIEHCLKCYGKKYVDEGEVYFELDADKICRAAARMLLQNAVKFNLAEFQEVWQQSVPEGM
VTSLDQLKGLALVDRHSRPEIIFLLKVDDLPEDNQERFNSLFSLREKWTEEDIAPYIQDL
CGEKQTIGALLTKYSHSSMQNGVKVYNSRRPIS*
>ENSG00000156127|ENST00000286639
MPHSSDSSDSSFSRSPPPGKQDSSDDVRRVQRREKNRIAAQKSRQRQTQKADTLHLESED
LEKQNAALRKEIKQLTEELKYFTSVLNSHEPLCSVLAASTPSPPEVVYSAHAFHQPHVSS
PRFQP*
>ENSG00000166902|ENST00000300151
MWRLLARASAPLLRVPLSDSWALLPASAGVKTLLPVPSFEDVSIPEKPKLRFIERAPLVP
KVRREPKNLSDIRGPSTEATEFTEGNFAILALGGGYLHWGHFEMMRLTINRSMDPKNMFA
IWRVPAPFKPITRKSVGHRMGGGKGAIDHYVTPVKAGRLVVEMGGRCEFEEVQGFLDQVA
HKLPFAAKAVSRGTLEKMRKDQEERERNNQNPWTFERIATANMLGIRKVLSPYDLTHKGK
YWGKFYMPKRV*
>ENSG00000205937|ENST00000301730
MDLSGVKKKSLLGVKENNKKSSTRAPSPTKRKDRSDEKSKDRSKDKGATKESSEKDRGRD
KTRKRRSASSGSSSTRSRSSSTSSSGSSTSTGSSSGSSSSSASSRSGSSSTSRSSSSSSS
SGSPSPSRRRHDNRRRSRSKSKPPKRDEKERKRRSPSPKPTKVHIGRLTRNVTKDHIMEI
FSTYGKIKMIDMPVERMHPHLSKGYAYVEFENPDEAEKALKHMDGGQIDGQEITATAVLA
PWPRPPPRRFSPPRRMLPPPPMWRRSPPRMRRRSRSPRRRSPVRRRSRSPGRRRHRSRSS
SNSSR*
>ENSG00000171735|ENST00000303635

MWRAEGKWLPKTSRKSVSQSVFCGTSTYCVLNTVPPIEDDHGNSNSSHVKIFLPKKLLEC
LPKCSSLPKERHRWNTNEEIAAYLITFEKHEEWLTTSPKTRPQNGSMILYNRKKVKYRKD
GYCWKKRKDGKTTREDHMKLKVQGVECLYGCYVHSSIIPTFHRRCYWLLQNPDIVLVHYL
NVPAIEDCGKPCGPILCSINTDKKEWAKWTKEELIGQLKPMFHGIKWTCSNGNSSSGFSV
EQLVQQILDSHQTKPQPRTHNCLCTGSLGAGGSVHHKCNSAKHRIISPKVEPRTGGYGSH
SEVQHNDVSEGKHEHSHSKGSSREKRNGKVAKPVLLHQSSTEVSSTNQVEVPDTTQSSPV
SISSGLNSDPDMVDSPVVTGVSGMAVASVMGSLSQSATVFMSEVTNEAVYTMSPTAGPNH
HLLSPDASQGLVLAVSSDGHKFAFPTTGSSESLSMLPTNVSEELVLSTTLDGGRKIPETT
MNFDPDCFLNNPKQGQTYGGGGLKAEMVSSNIRHSPPGERSFSFTTVLTKEIKTEDTSFE
QQMAKEAYSSSAAAVAASSLTLTAGSSLLPSGGGLSPSTTLEQMDFSAIDSNKDYTSSFS
QTGHSPHIHQTPSPSFFLQDASKPLPVEQNTHSSLSDSGGTFVMPTVKTEASSQTSSCSG
HVETRIESTSSLHLMQFQANFQAMTAEGEVTMETSQAAEGSEVLLKSGELQACSSEHYLQ
PETNGVIRSAGGVPILPGNVVQGLYPVAQPSLGNASNMELSLDHFDISFSNQFSDLINDF
ISVEGGSSTIYGHQLVSGDSTALSQSEDGARAPFTQAEMCLPCCSPQQGSLQLSSSEGGA
STMAYMHVAEVVSAASAQGTLGMLQQSGRVFMVTDYSPEWSYPEGGVKVLITGPWQEASN
NYSCLFDQISVPASLIQPGVLRCYCPAHDTGLVTLQVAFNNQIISNSVVFEYKARALPTL
PSSQHDWLSLDDNQFRMSILERLEQMERRMAEMTGSQQHKQASGGGSSGGGSGSGNGGSQ
AQCASGTGALGSCFESRVVVVCEKMMSRACWAKSKHLIHSKTFRGMTLLHLAAAQGYATL
IQTLIKWRTKHADSIDLELEVDPLNVDHFSCTPLMWACALGHLEAAVVLYKWDRRAISIP
DSLGRLPLGIARSRGHVKLAECLEHLQRDEQAQLGQNPRIHCPASEEPSTESWMAQWHSE
AISSPEIPKGVTVIASTNPELRRPRSEPSNYYSSESHKDYPAPKKHKLNPEYFQTRQEKL
LPTALSLEEPNIRKQSPSSKQSVPETLSPSEGVRDFSRELSPPTPETAAFQASGSQPVGK
WNSKDLYIGVSTVQVTGNPKGTSVGKEAAPSQVRPREPMSVLMMANREVVNTELGSYRDS
AENEECGQPMDDIQVNMMTLAEHIIEATPDRIKQENFVPMESSGLERTDPATISSTMSWL
ASYLADADCLPSAAQIRSAYNEPLTPSSNTSLSPVGSPVSEIAFEKPNLPSAADWSEFLS
ASTSEKVENEFAQLTLSDHEQRELYEAARLVQTAFRKYKGRPLREQQEVAAAVIQRCYRK
YKQYALYKKMTQAAILIQSKFRSYYEQKKFQQSRRAAVLIQKYYRSYKKCGKRRQARRTA
VIVQQKLRSSLLTKKQDQAARKIMRFLRRCRHSPLVDHRLYKRSERIEKGQGT*
>ENSG00000152422|ENST00000282268
MERKISRIHLVSEPSITHFLQVSWEKTLESGFVITLTDGHSAWTGTVSESEISQEADDMA
MEKGKYVGELRKALLSGAGPADVYTFNFSKESCYFFFEKNLKDVSFRLGSFNLEKVENPA
EVIRELICYCLDTIAENQAKNEHLQKENERLLRDWNDVQGRFEKCVSAKEALETDLYKRF
ILVLNEKKTKIRSLHNKLLNAAQEREKDIKQEGETAICSEMTADRDPVYDESTDEESENQ
TDLSGLASAAVSKDDSIISSLDVTDIAPSRKRRQRMQRNLGTEPKMAPQENQLQEKEKPD
SSLPETSKKEHISAENMSLETLRNSSPEDLFDEI*
>ENSG00000168661|ENST00000303586
MAHKYVGLQYHGSVTFEDVAIAFSQQEWESLDSSQRGLYRDVMLENYRNLVSMAGHSRSK
PHVIALLEQWKEPEVTVRKDGRRWCTDLQLEDDTIGCKEMPTSENCPSFALHQKISRQKP
RECQEYGKTLCQDSKPVQHERIHSSEKPNRCKECGKNFSNGHQLTIHQRLHVGEKPYKYE
KCGKAFISGSAFVKHGRIHTGEKPLKCKQCGKTISGSYQLTVHKSIHTGKKPYECGECGK
AFLVYGKLTRHQSTHTGEKPFGCEECGKAFSTFSYLVQHQRIHTSEKPYECKECGKAFST
SSPLAKHQRIHTGEKPYECKECGKSFTVYGQLTRHQSIHTGEKPFECKECGKAFRLSSFL
HAHQRIHAEIKPYGCKECGRTFSRASYLVQHGRLHTGEKPYECKECGKAFSTGSYLVQHQ
RIHTGEKPYECKECGKAFISRHQLTVHQRVHTGEKPYECKECGKAFRVHVHLTQHRKIHT

DVKPYECKECGKTFSRASYLVQHSRIHTGKKPYECKECGKAFSSGSYLVQHQRIHTGEKP
YECNKCGKAFTVYGQLIGHQSVHTGEKPFECKECGKAFRLNSFLTEHQRVHTGEKPFKCK
KCGKTFRYSSALKVHLRKHMSVIP*
>ENSG00000170748|ENST00000306904
MVEADRPGKLFIGGLNLETDEKALEAEFGKYGRIVEVLLMKDRETNKSRGFAFVTFESPA
DAKAAARDMNGKSLDGKAIKVAQATKPAFESSRRGPPPPRSRGRPRFLRGTRGGGGGPRR
SPSRGGPDDDGGYTADFDLRPSRAPMPMKRGPPPRRVGPPPKRAAPSGPARSSGGGMRGR
ALAVRGRDGYSGPPRREPLPPRRDPYLGPRDEGYSSRDGYSSRDYREPRGFAPSPGEYTH
RDYGHSSVRDDCPLRGYSDRDGYGGRDRDYGDHLSRGSHREPFESYGELRGAAPGRGTPP
SYGGGGRYEEYRGYSPDAYSGGRDSYSSSYGRSDRYSRGRHRVGRPDRGLSLSMERGCPP
QRDSYSRSGCRVPRGGGRLGGRLERGGGRSRY*
>ENSG00000171056|ENST00000304501
MASLLGAYPWPEGLECPALDAELSDGQSPPAVPRPPGDKGSESRIRRPMNAFMVWAKDER
KRLAVQNPDLHNAELSKMLGKSWKALTLSQKRPYVDEAERLRLQHMQDYPNYKYRPRRKK
QAKRLCKRVDPGFLLSSLSRDQNALPEKRSGSRGALGEKEDRGEYSPGTALPSLRGCYHE
GPAGGGGGGTPSSVDTYPYGLPTPPEMSPLDVLEPEQTFFSSPCQEEHGHPRRIPHLPGH
PYSPEYAPSPLHCSHPLGSLALGQSPGVSMMSPVPGCPPSPAYYSPATYHPLHSNLQAHL
GQLSPPPEHPGFDALDQLSQVELLGDMDRNEFDQYLNTPGHPDSATGAMALSGHVPVSQV
TPTGPTETSLISVLADATATYYNSYSVS*
>ENSG00000164815|ENST00000297431
MPHLENVVLCRESQVSILQSLFGERHHFSFPSIFIYGHTASGKTYVTQTLLKTLELPHVF
VNCVECFTLRLLLEQILNKLNHLSSSEDGCSTEITCETFNDFVRLFKQVTTAENLKDQTV
YIVLDKAEYLRDMEANLLPGFLRLQELADRNVTVLFLSEIVWEKFRPNTGCFEPFVLYFP
DYSIGNLQKILSHDHPPEYSADFYAAYINILLGVFYTVCRDLKELRHLAVLNFPKYCEPV
VKGEASERDTRKLWRNIEPHLKKAMQTVYLREISSSQWEKLQKDDTDPGQLKGLSAHTHV
ELPYYSKFILIAAYLASYNPARTDKRFFLKHHGKIKKTNFLKKHEKTSNHLLGPKPFPLD
RLLAILYSIVDSRVAPTANIFSQITSLVTLQLLTLVGHDDQLDGPKYKCTVSLDFIRAIA
RTVNFDIIKYLYDFL*
>ENSG00000214944|ENST00000296799
MDSDSDSPFNYSWPSFPKMKIRRRTSKQDRSFDILKKSKPPSTLLAAGRLSDMLNGGDEV
YANCMVIDQVGDLDISYINIEGITATTSPESRGCTLWPQSSKHTLPTETSPSVYPLSENV
EGTAHTEAQQSFMSPSSSCASNLNLSFGWHGFEKEQSHLKKRSSSLDALDADSEGEGHSE
PSHICYTPGSQSSSRTGIPSGDELDSFETNTEPDFNISRAESLPLSSNLQSKESLLSGVR
SRSYSCSSPKISLGKTRLVRELTVCSSSEEQRAYSLSEPPRENRIQEEEWDKYIIPAKSE
SEKYKVSRTFSFLMNRMTSPRNKSKTKSKDAKDKEKLNRHQFAPGTFSGVLQCLVCDKTL
LGKESLQCSNCNANVHKGCKDAAPACTKKFQEKYNKNKPQTILGNSSFRDIPQPGLSLHP
SSSVPVGLPTGRRETVGQVHPLSRSVPGTTLESFRRSATSLESESDHNSCRSRSHSDELL
QSMGSSPSTESFIMEDVVDSSLWSDLSSDAQEFEAESWSLVVDPSFCNRQEKDVIKRQDV
IFELMQTEMHHIQTLFIMSEIFRKGMKEELQLDHSTVDKIFPCLDELLEIHRHFFYSMKE
RRQESCAGSDRNFVIDRIGDILVQQFSEENASKMKKIYGEFCCHHKEAVNLFKELQQNKK
FQNFIKLRNSNLLARRRGIPECILLVTQRITKYPVLVERILQYTKERTEEHKDLRKALCL
IKDMIATVDLKVNEYEKNQKWLEILNKIENKTYTKLKNGHVFRKQALMSEERTLLYDGLV
YWKTATGRFKDILALLLTDVLLFLQEKDQKYIFAAVDQKPSVISLQKLIAREVANEERGM
FLISASSAGPEMYEIHTNSKEERNNWMRRIQQAVESCPEEKGGRTSESDEDKRKAEARVA

KIQQCQEILTNQDQQICAYLEEKLHIYAELGELSGFEDVHLEPHLLIKPDPGEPPQAASL
LAAALKEAESLQVAVKASQMGAVSQSCEDSCGDSVLADTLSSHDVPGSPTASLVTGGREG
RGCSDVDPGIQGVVTDLAVSDAGEKVECRNFPGSSQSEIIQAIQNLTRLLYSLQAALTIQ
DSHIEIHRLVLQQQEGLSLGHSILRGGPLQDQKSRDADRQHEELANVHQLQHQLQQEQRR
WLRRCEQQQRAQATRESWLQERERECQSQEELLLRSRGELDLQLQEYQHSLERLREGQRL
VEREQARMRAQQSLLGHWKHGRQRSLPAVLLPGGPEVMELNRSESLCHENSFFINEALVQ
MSFNTFNKLNPSVIHQDATYPTTQSHSDLVRTSEHQVDLKVDPSQPSNVSHKLWTAAGSG
HQILPFHESSKDSCKNDLDTSHTESPTPHDSNSHRPQLQAFITEAKLNLPTRTMTRQDGE
TGDGAKENIVYL*
>ENSG00000188171|ENST00000291750
MGPLQFRDVAIEFSLEEWHCLDTAQRNLYRNVMLENYSNLVFLGITVSKPDLITCLEQGR
KPLTMKRNEMIAKPSVSFLQVHSESQSPLHDI*
>ENSG00000146047|ENST00000274764
MPEVSSKGATISKKGFKKAVVKTQKKEGKKRKRTRKESYSIYIYKVLKQVHPDTGISSKA
MSIMNSFVTDIFERIASEASRLAHYSKRSTISSREIQTAVRLLLPGELAKHAVSEGTKAV
TKYTSSK*
>ENSG00000117877|ENST00000309424
MEEPQAGDAARFSCPPNFTAKPPASESPRFSLEALTGPDTELWLIQAPADFAPECFNGRH
VPLSGSQIVKGKLAGKRHRYRVLSSCPQAGEATLLAPSTEAGGGLTCASAPQGTLRILEG
PQQSLSGSPLQPIPASPPPQIPPGLRPRFCAFGGNPPVTGPRSALAPNLLTSGKKKKEMQ
VTEAPVTQEAVNGHGALEVDMALGSPEMDVRKKKKKKNQQLKEPEAAGPVGTEPTVETLE
PLGVLFPSTTKKRKKPKGKETFEPEDKTVKQEQINTEPLEDTVLSPTKKRKRQKGTEGME
PEEGVTVESQPQVKVEPLEEAIPLPPTKKRKKEKGQMAMMEPGTEAMEPVEPEMKPLESP
GGTMAPQQPEGAKPQAQAALAAPKKKTKKEKQQDATVEPETEVVGPELPDDLEPQAAPTS
TKKKKKKKERGHTVTEPIQPLEPELPGEGQPEARATPGSTKKRKKQSQESRMPETVPQEE
MPGPPLNSESGEEAPTGRDKKRKQQQQQPV*
>ENSG00000157259|ENST00000287957
MPLGLKPTCSVCKTTSSSMWKKGAQGEILCHHCTGRGGAGSGGAGSGAAGGTGGSGGGGF
GAATFASTSATPPQSNGGGGGKQSKQEIHRRSARLRNTKYKSAPAAEKKVSTKGKGRRHI
FKLKNPIKAPESVSTIITAESIFYKGVYYQIGDVVSVIDEQDGKPYYAQIRGFIQDQYCE
KSAALTWLIPTLSSPRDQFDPASYIIGPEEDLPRKMEYLEFVCHAPSEYFKSRSSPFPTV
PTRPEKGYIWTHVGPTPAITIKESVANHL*
>ENSG00000153560|ENST00000283629
MAWVLKMDEVIESGLVHDFDASLSGIGQELGAGAYSMSDVLALPIFKQEDSSLPLDGETE
HPPFQYVMCAATSPAVKLHDETLTYLNQGQSYEIRMLDNRKMGDMPEINGKLVKSIIRVV
FHDRRLQYTEHQQLEGWKWNRPGDRLLDLDIPMSVGIIDTRTNPSQLNAVEFLWDPAKRT
SAFIQVHCISTEFTPRKHGGEKGVPFRIQVDTFKQNENGEYTDHLHSASCQIKVFKPKGA
DRKQKTDREKMEKRTAHEKEKYQPSYDTTILTEMRLEPIIEDAVEHEQKKSSKRTLPADY
GDSLAKRGSCSPWPDAPTAYVNNSPSPAPTFTSPQQSTCSVPDSNSSSPNHQGDGASQTS
GEQIQPSATIQETQQWLLKNRFSSYTRLFSNFSGADLLKLTKEDLVQICGAADGIRLYNS
LKSRSVRPRLTIYVCREQPSSTVLQGQQQAASSASENGSGAPYVVYHAIYLEEMIASEVAR
KLALVFNIPLHQINQVYRQGPTGIHILVSDQMVQNFQDESCFLFSTVKAESSDGIHIILK
*
>ENSG00000105968|ENST00000308153

MAGGKAGKDSGKAKAKAVSRSQRAGLQFPVGRIHRHLKTRTTSHGRVGATAAVYSAAILE
YLTAEVLELAGNASKDLKVKRITPRHLQLAIRGDEELDSLIKATIAGGGVIPHIHKSLIG
KKGQQKTA*
>ENSG00000140829|ENST00000268482
MGDTSEDASIHRLEGTDLDCQVGGLICKSKSAASEQHVFKAPAPRPSLLGLDLLASLKRR
EREEKDDGEDKKKSKVSSYKDWEESKDDQKDAEEEGGDQAGQNIRKDRHYRSARVETPSH
PGGVSEEFWERSRQRERERREHGVYASSKEEKDWKKEKSRDRDYDRKRDRDERDRSRHSS
RSERDGGSERSSRRNEPESPRHRPKDAATPSRSTWEEEDSGYGSSRRSQWESPSPTPSYR
DSERSHRLSTRDRDRSVRGKYSDDTPLPTPSYKYNEWADDRRHLGSTPRLSRGRGRREEG
EEGISFDTEEERQQWEDDQRQADRDWYMMDEGYDEFHNPLAYSSEDYVRRREQHLHKQKQ
KRISAQRRQINEDNERWETNRMLTSGVVHRLEVDEDFEEDNAAKVHLMVHNLVPPFLDGR
IVFTKQPEPVIPVKDATSDLAIIARKGSQTVRKHREQKERKKAQHKHWELAGTKLGDIMG
VKKEEEPDKAVTEDGKVDYRTEQKFADHMKRKSEASSEFAKKKSILEQRQYLPIFAVQQE
LLTIIRDNSIVIVVGETGSGKTTQLTQYLHEDGYTDYGMIGCTQPRRVAAMSVAKRVSEE
MGGNLGEEVGYAIRFEDCTSENTLIKYMTDGILLRESLREADLDHYSAIIMDEAHERSLN
TDVLFGLLREVVARRSDLKLIVTSATMDAEKFAAFFGNVPIFHIPGRTFPVDILFSKTPQ
EDYVEAAVKQSLQVHLSGAPGDILIFMPGQEDIEVTSDQIVEHLEELENAPALAVLPIYS
QLPSDLQAKIFQKAPDGVRKCIVATNIAETSLTVDGIMFVIDSGYCKLKVFNPRIGMDAL
QIYPISQANANQRSGRAGRTGPGQCFRLYTQSAYKNELLTTTVPEIQRTNLANVVLLLKS
LGVQDLLQFHFMDPPPEDNMLNSMYQLWILGALDNTGGLTSTGRLMVEFPLDPALSKMLI
VSCDMGCSSEILLIVSMLSVPAIFYRPKGREEESDQIREKFAVPESDHLTYLNVYLQWKN
NNYSTIWCNDHFIHAKAMRKVREVRAQLKDIMVQQRMSLASCGTDWDIVRKCICAAYFHQ
AAKLKGIGEYVNIRTGMPCHLHPTSSLFGMGYTPDYIVYHELVMTTKEYMQCVTAVDGEW
LAELGPMFYSVKQAGKSRQENRRRAKEEASAMEEEMALAEEQLRARRQEQEKRSPLGSVR
STKIYTPGRKEQGEPMTPRRTPARFGL*
>ENSG00000169397|ENST00000304639
MVPKLFTSQICLLLLLGLMGVEGSLHARPPQFTRAQWFAIQHISLNPPRCTIAMRAINNY
RWRCKNQNTFLRTTFANVVNVCGNQSIRCPHNRTLNNCHRSRFRVPLLHCDLINPGAQNI
SNCTYADRPGRRFYVVACDNRDPRDSPRYPVVPVHLDTTI*
>ENSG00000174255|ENST00000308095
MSPKRDGLGTGDGLHSQVLQEQVSTGDNLHECDSQGPSKDTLVREGKTYKCKECGSVFNK
NSLLVRHQQIHTGVKPYECQECGKAFPEKVDFVRPMRIHTGEKPCKCVECGKVFNRRSHL
LCYRQIHTGEKPYECSECGKTFSYHSVFIQHRVTHTGEKLFGCKECGKTFYYNSSLTRHM
KIHTGEKPCKCSECGKTFTYRSVFFRHSMTHTAGKPYECKECGKGFYYSYSLTRHTRSHT
GEKPYECLEHRKDFGYHSAFAQQSKIHSGGKNL*
>ENSG00000172468|ENST00000309834
MAHVSSETQDVSPKDELTASEASTRSPLCEHTFPGDSDLRSMIEEHAFQVLSQGSLLESP
SYTVCVSEPDKDDDFLSLNFPRKLWKIVESDQFKSISWDENGTCIVINEELFKKEILETK
APYRIFQTDAIKSFVRQLNLYGFSKIQQNFQRSAFLATFLSEEKESSVLSKIRFTKMKLS
RSSTYENRYLCCNLHLKDESNYS*
>ENSG00000173825|ENST00000309880
MELSSKKKLHALSLAEKIQVLELLDESKMSQSEVARRFQVSQPQISRICKNKEKLLADWC
SGTANRERKRKRESKYSGIDEALLCWYHIARAKAWDVTGPMLLHKAKELADIMGQDFVPS
IGWLVRWKRRNNVGFGARHVLAPSFPPEPPPPGLTSQAQLPLSLKDFSPEDVFGCAELPL

LYRAVPGSFGACDQVQVLLCANSRGTEKRRVLLGGLQAAPRCFFGIRSEALPASYHPDLG
IPWLEWLAQFDRDMGQQGRQVALLLAARVVEELAGLPGLYHVKLLPLAASSTTPPLPSSV
VRAFKAHYRHRLLGKLAAIQSERDGTSLAEAGAGITVLDALHVASAAWAKVPPQLIFSSF
IQEGLAPGKTPPSSHKTSEMPPVPGGLSLEEFSRFVDLEGEEPRSGVCKEEIGTEDEKGD
REGAFEPLPTKADALRALGTLRRWFECNSTSPELFEKFYDCEEEVERLCCL*
>ENSG00000154227|ENST00000284382
MFWTFKEWFWLERFWLPPTIKWSDLEDHDGLVFVKPSHLYVTIPYAFLLLIIRRVFEKFV
ASPLAKSFGIKETVRKVTPNTVLENFFKHSTRQPLQTDIYGLAKKCNLTERQVERWFRSR
RNQERPSRLKKFQEACWRFAFYLMITVAGIAFLYDKPWLYDLWEVWNGYPKQPLLPSQYW
YYILEMSFYWSLLFRLGFDVKRKDFLAHIIHHLAAISLMSFSWCANYIRSGTLVMIVHDV
ADIWLESAKMFSYAGWTQTCNTLFFIFSTIFFISRLIVFPFWILYCTLILPMYHLEPFFS
YIFLNLQLMILQVLHLYWGYYILKMLNRCIFMKSIQDVRSDDEDYEEEEEEEEEEATKGK
EMDCLKNGLRAERHLIPNGQHGH*
>ENSG00000168769|ENST00000265149
MEQDRTNHVEGNRLSPFLIPSPPICQTEPLATKLQNGSPLPERAHPEVNGDTKWHSFKSY
YGIPCMKGSQNSRVSPDFTQESRGYSKCLQNGGIKRTVSEPSLSGLLQIKKLKQDQKANG
ERRNFGVSQERNPGESSQPNVSDLSDKKESVSSVAQENAVKDFTSFSTHNCSGPENPELQ
ILNEQEGKSANYHDKNIVLLKNKAVLMPNGATVSASSVEHTHGELLEKTLSQYYPDCVSI
AVQKTTSHINAINSQATNELSCEITHPSHTSGQINSAQTSNSELPPKPAAVVSEACDADD
ADNASKLAAMLNTCSFQKPEQLQQQKSVFEICPSPAENNIQGTTKLASGEEFCSGSSSNL
QAPGGSSERYLKQNEMNGAYFKQSSVFTKDSFSATTTPPPPSQLLLSPPPPLPQVPQLPS
EGKSTLNGGVLEEHHHYPNQSNTTLLREVKIEGKPEAPPSQSPNPSTHVCSPSPMLSERP
QNNCVNRNDIQTAGTMTVPLCSEKTRPMSEHLKHNPPIFGSSGELQDNCQQLMRNKEQEI
LKGRDKEQTRDLVPPTQHYLKPGWIELKAPRFHQAESHLKRNEASLPSILQYQPNLSNQM
TSKQYTGNSNMPGGLPRQAYTQKTTQLEHKSQMYQVEMNQGQSQGTVDQHLQFQKPSHQV
HFSKTDHLPKAHVQSLCGTRFHFQQRADSQTEKLMSPVLKQHLNQQASETEPFSNSHLLQ
HKPHKQAAQTQPSQSSHLPQNQQQQQKLQIKNKEEILQTFPHPQSNNDQQREGSFFGQTK
VEECFHGENQYSKSSEFETHNVQMGLEEVQNINRRNSPYSQTMKSSACKIQVSCSNNTHL
VSENKEQTTHPELFAGNKTQNLHHMQYFPNNVIPKQDLLHRCFQEQEQKSQQASVLQGYK
NRNQDMSGQQAAQLAQQRYLIHNHANVFPVPDQGGSHTQTPPQKDTQKHAALRWHLLQKQ
EQQQTQQPQTESCHSQMHRPIKVEPGCKPHACMHTAPPENKTWKKVTKQENPPASCDNVQ
QKSIIETMEQHLKQFHAKSLFDHKALTLKSQKQVKVEMSGPVTVLTRQTTAAELDSHTPA
LEQQTTSSEKTPTKRTAASVLNNFIESPSKLLDTPIKNLLDTPVKTQYDFPSCRCVGLDR
RVKLLGLKESSILVKKAKVLRDVLLLSGWFAEAAVKRSYCVWCGSELATPVRLQ*
>ENSG00000167986|ENST00000301764
MSYNYVVTAQKPTAVNGCVTGHFTSAEDLNLLIAKNTRLEIYVVTAEGLRPVKEVGMYGK
IAVMELFRPKGESKDLLFILTAKYNACILEYKQSGESIDIITRAHGNVQDRIGRPSETGI
IGIIDPECRMIGLRLYDGLFKVIPLDRDNKELKAFNIRLEELHVIDVKFLYGCQAPTICF
VYQDPQGRHVKTYEVSLREKEFNKGPWKQENVEAEASMVIAVPEPFGGAIIIGQESITYH
NGDKYLAIAPPIIKQSTIVCHNRVDPNGSRYLLGDMEGRLFMLLLEKEEQMDGTVTLKDL
RVELLGETSIAECLTYLDNGVVFVGSRLGDSQLVKLNVDSNEQGSYVVAMETFTNLGPIV
DMCVVDLERQGQGQLVTCSGAFKEGSLRIIRNGIGIHEHASIDLPGIKGLWPLRSDPNRE
TDDTLVLSFVGQTRVLMLNGEEVEETELMGFVDDQQTFFCGNVAHQQLIQITSASVRLVS
QEPKALVSEWKEPQAKNISVASCNSSQVVVAVGRALYYLQIHPQELRQISHTEMEHEVAC

LDITPLGDSNGLSPLCAIGLWTDISARILKLPSFELLHKEMLGGEIIPRSILMTTFESSH
YLLCALGDGALFYFGLNIETGLLSDRKKVTLGTQPTVLRTFRSLSTTNVFACSDRPTVIY
SSNHKLVFSNVNLKEVNYMCPLNSDGYPDSLALANNSTLTIGTIDEIQKLHIRTVPLYES
PRKICYQEVSQCFGVLSSRIEVQDTSGGTTALRPSASTQALSSSVSSSKLFSSSTAPHET
SFGEEVEVHNLLIIDQHTFEVLHAHQFLQNEYALSLVSCKLGKDPNTYFIVGTAMVYPEE
AEPKQGRIVVFQYSDGKLQTVAEKEVKGAVYSMVEFNGKLLASINSTVRLYEWTTEKELR
TECNHYNNIMALYLKTKGDFILVGDLMRSVLLLAYKPMEGNFEEIARDFNPNWMSAVEIL
DDDNFLGAENAFNLFVCQKDSAATTDEERQHLQEVGLFHLGEFVNVFCHGSLVMQNLGET
STPTQGSVLFGTVNGMIGLVTSLSESWYNLLLDMQNRLNKVIKSVGKIEHSFWRSFHTER
KTEPATGFIDGDLIESFLDISRPKMQEVVANLQYDDGSGMKREATADDLIKVVEELTRIH
*
>ENSG00000167881|ENST00000307877
MAAEKQVPGGGGGGGSGGGGGSGGGGSGGGRGAGGEENKENERPSAGSKANKEFGDSLSL
EILQIIKESQQQHGLRHGDFQRYRGYCSRRQRRLRKTLNFKMGNRHKFTGKKVTEELLTD
NRYLLLVLMDAERAWSYAMQLKQEANTEPRKRFHLLSRLRKAVKHAEELERLCESNRVDA
KTKLEAQAYTAYLSGMLRFEHQEWKAAIEAFNKCKTIYEKLASAFTEEQAVLYNQRVEEI
SPNIRYCAYNIGDQSAINELMQMRLRSGGTEGLLAEKLEALITQTRAKQAATMSEVEWRG
RTVPVKIDKVRIFLLGLADNEAAIVQAESEETKERLFESMLSECRDAIQVVREELKPDQK
QRDYILEGEPGKVSNLQYLHSYLTYIKLSTAIKRNENMAKGLQRALLQQQPEDDSKRSPR
PQDLIRLYDIILQNLVELLQLPGLEEDKAFQKEIGLKTLVFKAYRCFFIAQSYVLVKKWS
EALVLYDRVLKYANEVNSDAGAFKNSLKDLPDVQELITQVRSEKCSLQAAAILDANDAHQ
TETSSSQVKDNKPLVERFETFCLDPSLVTKQANLVHFPPGFQPIPCKPLFFDLALNHVAF
PPLEDKLEQKTKSGLTGYIKGIFGFRS*
>ENSG00000154556|ENST00000284776
MSYYQRPFSPSAYSLPASLNSSIVMQHGTSLDSTDTYPQHAQSLDGTTSSSIPLYRSSEE
EKRVTVIKAPHYPGIGPVDESGIPTAIRTTVDRPKDWYKTMFKQIHMVHKPDDDTDMYNT
PYTYNAGLYNPPYSAQSHPAAKTQTYRPLSKSHSDNSPNAFKDASSPVPPPHVPPPVPPL
RPRDRSSTEKHDWDPPDRKVDTRKFRSEPRSIFEYEPGKSSILQHERPASLYQSSIDRSL
ERPMSSASMASDFRKRRKSEPAVGPPRGLGDQSASRTSPGRVDLPGSSTTLTKSFTSSSP
SSPSRAKGGDDSKICPSLCSYSGLNGNPSSELDYCSTYRQHLDVPRDSPRAISFKNGWQM
ARQNAEIWSSTEETVSPKIKSRSCDDLLNDDCDSFPDPKVKSESMGSLLCEEDSKESCPM
AWGSPYVPEVRSNGRSRIRHRSARNAPGFLKMYKKMHRINRKDLMNSEVICSVKSRILQY
ESEQQHKDLLRAWSQCSTEEVPRDMVPTRISEFEKLIQKSKSMPNLGDDMLSPVTLEPPQ
NGLCPKRRFSIEYLLEEENQSGPPARGRRGCQSNALVPIHIEVTSDEQPRAHVEFSDSDQ
DGVVSDHSDYIHLEGSSFCSESDFDHFSFTSSESFYGSSHHHHHHHHHHHRHLISSCKGR
CPASYTRFTTMLKHERARHENTEEPRRQEMDPGLSKLAFLVSPVPFRRKKNSAPKKQTEK
AKCKASVFEALDSALKDICDQIKAEKKRGSLPDNSILHRLISELLPDVPERNSSLRALRR
SPLHQPLHPLPPDGAIHCPPYQNDCGRMPRSASFQDVDTANSSCHHQDRGGALQDRESPR
SYSSTLTDMGRSAPRERRGTPEKEKLPAKAVYDFKAQTSKELSFKKGDTVYILRKIDQNW
YEGEHHGRVGIFPISYVEKLTPPEKAQPARPPPPAQPGEIGEAIAKYNFNADTNVELSLR
KGDRVILLKRVDQNWYEGKIPGTNRQGIFPVSYVEVVKKNTKGAEDYPDPPIPHSYSSDR
IHSLSSNKPQRPVFTHENIQGGGEPFQALYNYTPRNEDELELRESDVIDVMEKCDDGWFV
GTSRRTKFFGTFPGNYVKRL*
>ENSG00000159387|ENST00000290552

MSFPHFGHPYRGASQFLASASSSTTCCESTQRSVSDVASGSTPAPALCCAPYDSRLLGSA
RPELGAALGIYGAPYAAAAAAQSYPGYLPYSPEPPSLYGALNPQYEFKEAAGSFTSSLAQ
PGAYYPYERTLGQYQYERYGAVELSGAGRRKNATRETTSTLKAWLNEHRKNPYPTKGEKI
MLAIITKMTLTQVSTWFANARRRLKKENKMTWAPKNKGGEERKAEGGEEDSLGCLTADTK
EVTASQEARGLRLSDLEDLEEEEEEEEEAEDEEVVATAGDRLTEFRKGAQSLPGPCAAAR
EGRLERRECGLAAPRFSFNDPSGSEEADFLSAETGSPRLTMHYPCLEKPRIWSLAHTATA
SAVEGAPPARPRPRSPECRMIPGQPPASARRLSVPRDSACDESSCIPKAFGNPKFALQGL
PLNCAPCPRRSEPVVQCQYPSGAEAG*
>ENSG00000172530|ENST00000286122
MMSEHDLADVVQIAVEDLSPDHPVVLENHVVTDEDEPALKRQRLEINCQDPSIKTICLRL
DSIEAKLQALEATCKSLEEKLDLVTNKQHSPIQVPMVAGSPLGATQTCNKVRCVVPQTTV
ILNNDRQNAIVAKMEDPLSNRAPDSLENVISNAVPGRRQNTIVVKVPGQEDSHHEDGESG
SEASDSVSSCGQAGSQSIGSNVTLITLNSEEDYPNGTWLGDENNPEMRVRCAIIPSDMLH
ISTNCRTAEKMALTLLDYLFHREVQAVSNLSGQGKHGKKQLDPLTIYGIRCHLFYKFGIT
ESDWYRIKQSIDSKCRTAWRRKQRGQSLAVKSFSRRTPNSSSYCPSEPMMSTPPPASELP
QPQPQPQALHYALANAQQVQIHQIGEDGQVQVGHLHIAQVPQGEQVQITQDSEGNLQIHH
VGQDGQLLEATRIPCLLAPSVFKASSGQVLQGAQLIAVASSDPAAAGVDGSPLQGSDIQV
QYVQLAPVSDHTAGAQTAEALQPTLQPEMQLEHGAIQIQ*
>ENSG00000140464|ENST00000268059
MEPAPARSPRPQQDPARPQEPTMPPPETPSEGRQPSPSPSPTERAPASEEEFQFLRCQQC
QAEAKCPKLLPCLHTLCSGCLEASGMQCPICQAPWPLGADTPALDNVFFESLQRRLSVYR
QIVDAQAVCTRCKESADFWCFECEQLLCAKCFEAHQWFLKHEARPLAELRNQSVREFLDG
TRKTNNIFCSNPNHRTPTLTSIYCRGCSKPLCCSCALLDSSHSELKCDISAEIQQRQEEL
DAMTQALQEQDSAFGAVHAQMHAAVGQLGRARAETEELIRERVRQVVAHVRAQERELLEA
VDARYQRDYEEMASRLGRLDAVLQRIRTGSALVQRMKCYASDQEVLDMHGFLRQALCRLR
QEEPQSLQAAVRTDGFDEFKVRLQDLSSCITQGKDAAVSKKASPEAASTPRDPIDVDLPE
EAERVKAQVQALGLAEAQPMAVVQSVPGAHPVPVYAFSIKGPSYGEDVSNTTTAQKRKCS
QTQCPRKVIKMESEEGKEARLARSSPEQPRPSTSKAVSPPHLDGPPSPRSPVIGSEVFLP
NSNHVASGAGEAEERVVVISSSEDSDAENSCMEPMETAEPQSSPAHSSPAHSSPAHSSPV
QSLLRAQGASSLPCGTYHPPAWPPHQPAEQAATPDAEPHSEPPDHQERPAVHRGIRYLLY
RAQRAIRLRHALRLHPQLHRAPIRTWSPHVVQASTPAITGPLNHPANAQEHPAQLQRGIS
PPHRIRGAVRSRSRSLRGSSHLSQWLNNFFALPFSSMASQLDMSSVVGAGESRAQTLGAG
VPPGDSVRGSMEASQVQVPLEASPITFPPPCAPERPPISPVPGARQAGL*
>ENSG00000169564|ENST00000303577
MDAGVTESGLNVTLTIRLLMHGKEVGSIIGKKGESVKRIREESGARINISEGNCPERIIT
LTGPTNAIFKAFAMIIDKLEEDINSSMTNSTAASRPPVTLRLVVPATQCGSLIGKGGCKI
KEIRESTGAQVQVAGDMLPNSTERAITIAGVPQSVTECVKQICLVMLETLSQSPQGRVMT
IPYQPMPASSPVICAGGQDRCSDAAGYPHATHDLEGPPLDAYSIQGQHTISPLDLAKLNQ
VARQQSHFAMMHGGTGFAGIDSSSPEVKGYWASLDASTQTTHELTIPNNLIGCIIGRQGA
NINEIRQMSGAQIKIANPVEGSSGRQVTITGSAASISLAQYLINARLSSEKGMGCS*
>ENSG00000143319|ENST00000313146
MSTLLLNLDFGEPPPKKALEGNAKHRNFVKKRRLLERRGFLSKKNQPPSKAPKLHSEPSK
KGETPTVDGTWKTPSFPKKKTAASSNGSGQPLDKKAAVSWLTPAPSKKADSVAAKVDLLG
EFQSALPKINSHPTRSQKKSSQKKSSKKNHPQKNAPQNSTQAHSENKCSGASQKLPRKMV

AIDCEMVGTGPKGHVSSLARCSIVNYNGDVLYDEYILPPCHIVDYRTRWSGIRKQHMVNA
TPFKIARGQILKILTGKIVVGHAIHNDFKALQYFHPKSLTRDTSHIPPLNRKADCPENAT
MSLKHLTKKLLNRDIQVGKSGHSSVEDAQATMELYKLVEVEWEEHLARNPPTD*
>ENSG00000175334|ENST00000312175
MTTSQKHRDFVAEPMGEKPVGSLAGIGEVLGKKLEERGFDKAYVVLGQFLVLKKDEDLFR
EWLKDTCGANAKQSRDCFGCLREWCDAFL*
>ENSG00000234414|ENST00000303902
MVEADHPGKLFIGGLNRETNEKMLKAVFGKHGPISEVLLIKDRTSKSRGFAFITFENPAD
AKNAAKDMNGKSLHGKAIKVEQAKKPSFQSGGRRRPPASSRNRSPSGSLRSARGSRGGTR
GWLPSHEGHLDDGGYTPDLKMSYSRGLIPVKRGPSSRSGGPPPKKSAPSAVARSNSWMGS
QGPMSQRRENYGVPPRRATISSWRNDRMSTRHDGYATNDGNHPSCQETRDYAPPSRGYAY
RDNGHSNRDEHSSRGYRNHRSSRETRDYAPPSRGHAYRDYGHSRRDESYSRGYRNRRSSR
ETREYAPPSRGHGYRDYGHSRRHESYSRGYSYHDGYGEALGRDHSEHLSGSSYRDALQRY
GTSHGAPPARGPRMSYGGSTCHAYSNTRDRYGRSWESYSSCGDFHYCDREHVCRKDQRNP
PSLGRVLPDPREACGSSSYVASIVDGGESRSEKGDSSRY*
>ENSG00000197566|ENST00000311331
MSLQDSTLSREGKPEGEIMAAVFFSVGRLSPEVTQPDEDLHLQAEETQLVKESVTFKDVA
IDFTLEEWRLMDPTQRNLHKDVMLENYRNLVSLGLAVSKPDMISHLENGKGPWVTVREIS
RIPYPDMEPKPATKKATRTKAISEDLSQEAILEKLTENGLWDSRMEGLWKWNDRILRLQN
NQENHLSQRIIPLKKTPTSQRGFRFESILIPEPGIATEELHSRCQTQEENFTENLNLITD
THLGKIICKEMKGSKAIRQTSELTLGKKSNNKEKPYKCSTCEKAFHYRSLLIQHQRTHTK
EKPYECNECGKTFSQPSYLSQHKKIHTGEKPYKCNECGKAFIASSSLMVHQRIHTKEKPY
QCNVCGKSFSQCARLNQHQRIQTGEKPYKCSECGKAFSDKSKLARHQETHNGEKPYKCDD
CGKAFRNKSYLSVHQKTHTEEKPYQCNECGKSFKNTTIFNVHQRIHTGEKPFRCNECGKA
YRSNSSLIVHIRTHTGEKPYECNECGKAFNRIANFTEHQRIHTGEKPYKCNECGKAFINY
SCLTVHHRMHTGEKPYKCTECGKAFMRSSSLIIHQRIHTEEKPYLCNECGESFRIKSHLT
VHQRIHTGEKPYKCTDCERAFTKMVNLKEHQKIHTGVKPYKCYDCGKSFRTKSYLIVHQR
THTGEKPYKCNECEKAFTNTSQLTVHQRRHTGEKPYKCNECGKVFTSNSGFNTHQRTHTG
EKPFKCNDCGKAFSQMVHVTEHQKIHSGEKPYKCDVCGKAFRRGSYLTVHWRTHTGEKPY
TCKECGKGCITLSQLTLHQRIHTGERPYKCEECGKAFRTNSDFTVHLRMHTGEKPYKCNE
CGKAFRSSSSLTVHQRIHQRETQLI*
>ENSG00000171532|ENST00000302584
MLTRLFSEPGLLSDVPKFASWGDGEDDEPRSDKGDAPPPPPPAPGPGAPGPARAAKPVPL
RGEEGTEATLAEVKEEGELGGEEEEEEEEEEEGLDEAEGERPKKRGPKKRKMTKARLERSK
LRRQKANARERNRMHDLNAALDNLRKVVPCYSKTQKLSKIETLRLAKNYIWALSEILRSG
KRPDLVSYVQTLCKGLSQPTTNLVAGCLQLNSRNFLTEQGADGAGRFHGSGGPFAMHPYP
YPCSRLAGAQCQAAGGLGGGAAHALRTHGYCAAYETLYAAAGGGGASPDYNSSEYEGPLS
PPLCLNGNFSLKQDSSPDHEKSYHYSMHYSALPGSRPTGHGLVFGSSAVRGGVHSENLLS
YDMHLHHDRGPMYEELNAFFHN*
>ENSG00000152969|ENST00000282924
MSKKGRSKGEKPEMETDAVQMANEELRAKLTSIQIEFQQEKSKVGKLRERLQEAKLEREQ
EQRRHTAYISELKAKLHEEKTKELQALREGLIRQHEQEAARTAKIKEGELQRLQATLNVL
RDGAADKVKTALLTEAREEARRAFDGERLRLQQEILELKAARKQAEEALSNCMQADKTKA
ADLRAAYQAHQDEVHRIKRECERDIRRLMDEIKGKDRVILALEKELGVQAGQTQKLLLQK

EALDEQLVQVKEAERHHSSPKRELPPGIGDMVELMGVQDQHMDERDVRRFQLKIAELNSV
IRKLEDRNTLLADERNELLKRSRETEVQLKPLVEKNKRMNKKNEDLLQSIQRMEEKIKNL
TRENVEMKEKLSAQASLKRHTSLNDLSLTRDEQEIEFLRLQVLEQQHVIDDLSLERERLL
RSKRHRGKSLKPPKKHVVETFFGFDEESVDSETLSETSYNTDRTDRTPATPEEDLDDATA
REEADLRFCQLTREYQALQRAYALLQEQVGGTLDAEREARTREQLQADLLRCQAKIEDLE
KLLVEKGQDSKWVEEKQLLIRTNQDLLEKIYRLEMEENQLKNEMQDAKDQNELLEFRVLE
LEVRDSICCKLSNGADILFEPKLKFM*
>ENSG00000118418|ENST00000275036
MPKRKSPENTEGKDGSKVTKQEPTRRSARLSAKPAPPKPEPKPRKTSAKKEPGAKISRGA
KGKKEEKQEAGKEGTEN*
>ENSG00000137871|ENST00000267807
MGDNPFQPKSNSKMAELFMECEEEELEPWQKKVKEVEDDDDDEPIFVGEISSSKPAISNI
LNRVNPSSYSRGLKNGALSRGITAAFKPTSQHYTNPTSNPVPASPINFHPESRSSDSSVI
VQPFSKPGYITNSSRVVSNKSSELLFDLTQDTGLSHYQGGPTLSMAGMSESSFLSKRPST
SEVNNVNPKKPKPSESVSGANSSAVLPSVKSPSVTSSQAMLAKGTNTSSNQSKNGTPFPR
ACPKCNIHFNLLDPLKNHMKYCCPDMINNFLGLAKTEFSSTVNKNTTIDSEKGKLIMLVN
DFYYGKHEGDVQEEQKTHTTFKCFSCLKILKNNIRFMNHMKHHLELEKQSSESWENHTTC
QHCYRQFPTPFQLQCHIESTHTPHEFSTICKICELSFETEHVLLQHMKDNHKPGEMPYVC
QVCNYRSSSFSDVETHFRTSHENTKNLLCPFCLKVIKIATPYMHHYMKHQKKGIHRCTKC
RLQFLTCKEKMDHKTQHHRTFIKPKQLEGLPPGTKVTIRASVGPLQSGASPTPSISASAS
TLQLSPPRTKNITAKNPAKSNTSKPNTVKSNASKPNTSKPNGSKSKYKPKISNMQKKQST
LASSNKKSKVNTALRNLRYRRGIHKCIECCSEIKDFANHFPTYVHCSFCRYNTSCSKAYV
NHMMSFHSNRPSKRFCIFKKHSENLRGITLVCLNCDFLSDVSGLDNMATHLSQHKTHTCQ
VVMQKVSVCIPTSEHLSELKKEAPAKEQEPVSKEIARPNMAERETETSNSESKQDKAASS
KEKNGCNANSFEGSSTTKSEESITVSDKENETCLADQETGSKNIVSCDSNIGADKVEKKK
QIQHVCQEMELKMCQSSENIILSDQIKDHNSSEARFSSKNIKDLRLASDNVSIDQFLRKR
HEPESVSSDVSEQGSIHLEPLTPSEVLEYEATEILQKGSGDPSAKTDEVVSDQTDDIPGG
NNPSTTEATVDLEDEKERS*
>ENSG00000176024|ENST00000293471
MIKSQESLTLEDVAVEFTWEEWQLLGPAQKDLYRDVMLENYSNLVSVGYQASKPDALFKL
EQGEPWTVENEIHSQICPEIKKVDNHLQMHSQKQRCLKRVEQCHKHNAFGNIIHQRKSDF
PLRQNHDTFDLHGKILKSNLSLVNQNKRYEIKNSVGVNGDGKSFLHAKHEQFHNEMNFPE
GGNSVNTNSQFIKHQRTQNIDKPHVCTECGKAFLKKSRLIYHQRVHTGEKPHGCSICGKA
FSRKSGLTEHQRNHTGEKPYECTECDKAFRWKSQLNAHQKIHTGEKSYICSDCGKGFIKK
SRLINHQRVHTGEKPHGCSLCGKAFSKRSRLTEHQRTHTGEKPYECTECDKAFRWKSQLN
AHQKAHTGEKSYICRDCGKGFIQKGNLIVHQRIHTGEKPYICNECGKGFIQKGNLLIHRR
THTGEKPYVCNECGKGFSQKTCLISHQRFHTGKTPFVCTECGKSCSHKSGLINHQRIHTG
EKPYTCSDCGKAFRDKSCLNRHRRTHTGERPYGCSDCGKAFSHLSCLVYHKGMLHAREKC
VGSVKLENPCSESHSLSHTRDLIQDKDSVNMVTLQMPSVAAQTSLTNSAFQAESKVAIVS
QPVARSSVSADSRICTE*
>ENSG00000184863|ENST00000287912
MAAALGASGGAGAGDDDFDQFDKPGAERSWRRRAADEDWDSELEDDLLGEDLLSGKKNQS
DLSDEELNDDLLQSDNEDEENFSSQGVTISLNATSGMVTSFELSDNTNDQSGEQESEYEQ
EQGEDELVYHKSDGSELYTQEYPEEGQYEGHEAELTEDQIEYVEEPEEEQLYTDEVLDIE

INEPLDEFTGGMETLELQKDIKEESDEEEEDDEESGRLRFKTERKEGTIIRLSDVTRERR
NIPETLGNFFACLPSSFTLISTSSIVLL*
>ENSG00000157429|ENST00000288177
MAAMPLKAQYQEMVTFEDVAVHFTKTEWTGLSPAQRALYRSVMLENFGNLTALGYPVPKP
ALISLLERGDMAWGLEAQDDPPAERTKNVCKDVETNIDSESTLIQGISEERDGMMSHGQL
KSVPQRTDFPETRNVEKHQDIPTVKNIQGKVPRIPCARKPFICEECGKSFSYFSYYARHQ
RIHTGEKPFECSECGKAFNGNSSLIRHQRIHTGERPYQCEECGRAFNDNANLIRHQRIHS
GDRPYYCTECGNSFTSSSEFVIHQRIHTGEKPYECNECGKAFVGNSPLLRHQKIHTGEKP
YECNECGKSFGRTSHLSQHQRIHTGEKPYSCKVCGQAFNFHTKLTRHQRIHSEEKPFDCV
DCGKAFSAQEQLKRHLRIHTQESSYVCDECGKALTSKRNLHQHQRIHTGEKPYECSKYEK
AFGTSSQLGHLEHVYSGEKPVLDICRFGLPEFFTPFYW*
>ENSG00000152601|ENST00000282486
MAVSVTPIRDTKWLTLEVCREFQRGTCSRPDTECKFAHPSKSCQVENGRVIACFDSLKGR
CSRENCKYLHPPPHLKTQLEINGRNNLIQQKNMAMLAQQMQLANAMMPGAPLQPVPMFSV
APSLATNASAAAFNPYLGPVSPSLVPAEILPTAPMLVTGNPGVPVPAAAAAAAQKLMRTD
RLEVCREYQRGNCNRGENDCRFAHPADSTMIDTNDNTVTVCMDYIKGRCSREKCKYFHPP
AHLQAKIKAAQYQVNQAAAAQAAATAAAMTQSAVKSLKRPLEATFDLGIPQAVLPPLPKR
PALEKTNGATAVFNTGIFQYQQALANMQLQQHTAFLPPVPMVHGATPATVSAATTSATSV
PFAATATANQIPIISAEHLTSHKYVTQM*
>ENSG00000164576|ENST00000297109
MNGFSTEEDSREGPPAAPAAAAPGYGGQSCCLIEDGERCVRPAGNASFSKRVQKSISQKKL
KLDIDKSVRHLYICDFHKNFIQSVRNKRKRKTSDDGGDSPEHDTDIPEVDLFQLQVNTLR
RYKRHYKLQTRPGFNKAQLAETVSRHFRNIPVNEKETLAYFIYMVKSNKSRLDQKSEGGK
QLE*
>ENSG00000186272|ENST00000307658
MLMDAGQDYMVFEDVAIHFSQEEWGILNDVQRHLHSDVMLENFALLSSVGCWHGAKDEEA
PSKQCVSVGVSQVTTLKPALSTQKAQPCETCSSLLKDILHLAEHDGTHPKRTAKLYLHQK
EHLREKLTRSDEGRPSFVNDSVHLAKRNLTCMQGGKDFTGDSDLQQQALHSGWKPHRDTH
GVEAFQSGQNNYSCTQCGKDFCHQHTLFEHQKIHTEERPYECSECGKLFRYNSDLIKHQR
NHTGERPYKCSECGKAFSLKYNVVQHQKIHTGERPYECSECGKAFLRKSHLLQHQRIHTR
PRPYVCSECGKAFLTQAHLVGHQKIHTGERPYGCNECGKYFMYSSALIRHQKVHTGERPF
YCCECGKFFMDSCTLIIHQRVHTGEKPYECNECGKFFRYRSTLIRHQKVHTGEKPYECSE
CGKFFMDTSTLIIHQRVHTGEKPYECNKCGKFFRYCFTLNRHQRVHSGERPYECSECGKF
FVDSCTLKSHQRVHTGERPFECSICGKSFRCRSTLDTHQRIHTGERPYECSECGKFFRHN
SNHIRHRRNHFGERSFECTECGRVFSQNSHLIRHQKVHTRERTYKCSKCGKFFMDSSTLI
SHERVHTGEKPYECSECGKVFRYNSSLIKHRRIHTGERPYQCSECGRVFNQNSHLIQHQK
VHTR*
>ENSG00000176293|ENST00000313434
MTPGVRVSTDPEQVTFEDVVVGFSQEEWGQLKPAQRTLYRDVMLDTFRLLVSVGHWLPKP
NVISLLEQEAELWAVESRLPQGVYPDLETRPKVKLSVLKQGISEEISNSVILVERFLWDG
LWYCRGEDTEGHWEWSCESLESLAVPVAFTPVKTPVLEQWQRNGFGENISLNPDLPHQPM
TPERQSPHTWGTRGKREKPDLNVLQKTCVKEKPYKCQECGKAFSHSSALIEHHRTHTGER
PYECHECLKGFRNSSALTKHQRIHTGEKPYKCTQCGRTFNQIAPLIQHQRTHTGEKPYEC
SECGKSFSFRSSFSQHERTHTGEKPYECSECGKAFRQSIHLTQHLRIHTGEKPYQCGECG

KAFSHSSSLTKHQRIHTGEKPYECHECGKAFTQITPLIQHQRTHTGEKPYECGECGKAFS
QSTLLTEHRRIHTGEKPYGCNECGKTFSHSSSLSQHERTHTGEKPYECSQCGKAFRQSTH
LTQHQRIHTGEKPYECNDCGKAFSHSSSLTKHQRIHTGEKPYECNQCGRAFSQLAPLIQH
QRIHTGEKPYECNQCGRAFSQSSLLIEHQRIHTKEKPYGCNECGKSFSHSSSLSQHERTH
TGEKPYECHDCGKSFRQSTHLTQHRRIHTGEKPYACRDCGKAFTHSSSLTKHQRTHTG*
>ENSG00000138795|ENST00000265165
MPQLSGGGGGGGGDPELCATDEMIPFKDEGDPQKEKIFAEISHPEEEGDLADIKSSLVNE
SEIIPASNGHEVARQAQTSQEPYHDKAREHPDDGKHPDGGLYNKGPSYSSYSGYIMMPNM
NNDPYMSNGSLSPPIPRTSNKVPVVQPSHAVHPLTPLITYSDEHFSPGSHPSHIPSDVNS
KQGMSRHPPAPDIPTFYPLSPGGVGQITPPLGWQGQPVYPITGGFRQPYPSSLSVDTSMS
RFSHHMIPGPPGPHTTGIPHPAIVTPQVKQEHPHTDSDLMHVKPQHEQRKEQEPKRPHIK
KPLNAFMLYMKEMRANVVAECTLKESAAINQILGRRWHALSREEQAKYYELARKERQLHM
QLYPGWSARDNYGKKKKRKREKLQESASGTGPRMTAAYI*
>ENSG00000107815|ENST00000311916
MWVLLRSGYPLRILLPLRGEWMGRRGLPRNLAPGPPRRRYRKETLQALDMPVLPVTATEI
RQYLRGHGIPFQDGHSCLRALSPFAESSQLKGQTGVTTSFSLFIDKTTGHFLCMTSLAEG
SWEDFQASVEGRGDGAREGFLLSKAPEFEDSEEVRRIWNRAIPLWELPDQEEVQLADTMF
GLTKVTDDTLKRFSVRYLRPARSLVFPWFSPGGSGLRGLKLLEAKCQGDGVSYEETTIPR
PSAYHNLFGLPLISRRDAEVVLTSRELDSLALNQSTGLPTLTLPRGTTCLPPALLPYLEQ
FRRIVFWLGDDLRSWEAAKLFARKLNPKRCFLVRPGDQQPRPLEALNGGFNLSRILRTAL
PAWHKSIVSFRQLREEVLGELSNVEQAAGLRWSRFPDLNRILKGHRKGELTVFTGPTGSG
KTTFISEYALDLCSQGVNTLWGSFEISNVRLARVMLTQFAEGRLEDQLDKYDHWADRFED
LPLYFMTFHGQQSIRTVIDTMQHAVYVYDICHVIIDNLQFMMGHEQLSTDRIAAQDYIIG
VFRKFATDNNCHVTLVIHPRKEDDDKELQTASIFGSAKASQEADNVLILQDRKLVTGPGK
RYLQVSKNRFDGDVGVFPLEFNKNSLTFSIPPKNKARLKKIKDDTGPVAKKPSSGKKGAT
TQNSEICSGQAPTPDQPDTSKRSK*
>ENSG00000174106|ENST00000308330
MAAAAASAPQQLSDEELFSQLRRYGLSPGPVTESTRPVYLKKLKKLREEEQQQHRSGGRG
NKTRNSNNNNTAAATVAAAGPAAAAAAGMGVRPVSGDLSYLRTPGGLCRISASGPESLLG
GPGGASAAPAAGSKVLLGFSSDESDVEASPRDQAGGGGRKDRASLQYRGLKAPPAPLAAS
EVTNSNSAERRKPHSWWGARRPAGPELQTPPGKDGAVEDEEGEGEDGEERDPETEEPLWA
SRTVNGSRLVPYSCRENYSDSEEEDDDDVASSRQVLKDDSLSRHRPRRTHSKPLPPLTAK
SAGGRLETSVQGGGGLAMNDRAAAAGSLDRSRNLEEAAAAEQGGGCDQVDSSPVPRYRVN
AKKLTPLLPPPLTDMDSTLDSSTGSLLKTNNHIGGGAFSVDSPRIYSNSLPPSAAVAASS
SLRINHANHTGSNHTYLKNTYNKPKLSEPEEELLQQFKREEVSPTGSFSAHYLSMFLLTA
ACLFFLILGLTYLGMRGTGVSEDGELSIENPFGETFGKIQESEKTLMMNTLYKLHDRLAQ
LAGDHECGSSSQRTLSVQEAAAYLKDLGPEYEGIFNTSLQWILENGKDVGIRCVGFGPEE
ELTNITDVQFLQSTRPLMSFWCRFRRAFVTVTHRLLLLCLGVVMVCVVLRYMKYRWTKEE
EETRQMYDMVVKIIDVLRSHNEACQENKDLQPYMPIPHVRDSLIQPHDRKKMKKVWDRAV
DFLAANESRVRTETRRIGGADFLVWRWIQPSASCDKILVIPSKVWQGQAFHLDRRNSPPN
SLTPCLKIRNMFDPVMEIGDQWHLAIQEAILEKCSDNDGIVHIAVDKNSREGCVYVKCLS
PEYAGKAFKALHGSWFDGKLVTVKYLRLDRYHHRFPQALTSNTPLKPSNKHMNSMSHLRL
RTGLTNSQGSS*
>ENSG00000144485|ENST00000272937

MAPPAAPGRDRVGREDEDGWETRGDRKARKPLVEKKRRARINESLQELRLLLAGAEVQAK
LENAEVLELTVRRVQGVLRGRAREREQLQAEASERFAAGYIQCMHEVHTFVSTCQAIDAT
VAAELLNHLLESMPLREGSSFQDLLGDALAGPPRAPGRSGWPAGGAPGSPIPSPPGPGDD
LCSDLEEAPEAELSQAPAEGPDLVPAALGSLTTAQIARSVWRPW*
>ENSG00000175105|ENST00000309495
MLCNKEFLGGHIVRHAQAHQKKGSFACVICGRKFRNRGLMQKHLKNHVKKIQRQQIAAAQ
QDDQEVTALEEINCSSSSISFENGNSDSKDLEVETLTASSEGNKEVIPEHVAEFIEIPIS
VPEDVIENVIENGSPNNSLNNVFKPLTECGDDYEEEEDEEGDYEEDDYDLNQETSVIHKI
NGTVCHPKDIYATDQEGNFKCPALGCVRIFKRIGFLNKHAMTVHPTDLNVRQTVMKWSKG
KCKFCQRQFEDSQHFIDHLNRHSYPNVYFCLHFNCNESFKLPFQLAQHTKSHRIFQAQCS
FPECHELFEDLPLLYEHEAQHYLSKTPESSAQPSETILWDVQTDSNPNQEKDSSSNEKQT
ISLPVSTSKSRKESTEPKTCIESMEKKTDSLVQNGNERSDDTVSNISLIDQKMPDIEPNS
ENNCSSSDIVNGHSEIEQTPLVSSDPALKIDTNRIRTENGSILPSVVPQEHNTLPVSQAP
SKPNLTSEHTSYGLILTKPYVRPLPPSYLDERYLSMPKRRKFLTDRVDACSDQDNVYKKS
VKRLRCGKCLTTYCNAEALEAHLAQKKCQTLFGFDSDDESA*
>ENSG00000196653|ENST00000296091
MLNMQGAEERDIRRETCPGWVNKNKPALEQDVCKIDSSGIVVKRFQEDEYQDSTFEEKYA
CEGMKENSPREIAESCLFQEGGFGRITFIHKEAPPEIISQGYNFEKSLLLTSSLVTRLRV
STEESLHQWETSNIQTNDISDQSKCPTLCTQKKSWKCNECGKTFTQSSSLTQHQRTHTGE
RPYTCEECGKAFSRSSFLVQHQRIHTGVKPYGCEQCGKTFRCRSFLTQHQRIHTGEKPYK
CNECGNSFRNHSHLTEHQRIHTGEKPYKCNRCGKAFNQNTHLIHHQRIHTGEKPYICSEC
GSSFRKHSNLTQHQRIHTGEKPHKCDECGKTFQTKANLSQHQRIHSGEKPYKCKECGKAF
CQSPSLIKHQRIHTGEKPYKCKECGKAFTQSTPLTKHQRIHTGERPYKCSECGKAFIQSI
CLIRHQRSHTGEKPYKCNECGKGFNQNTCLTQHMRIHTGEKPYKCKECGKAFAHSSSLTE
HHRTHTGEKLYKCSECEKTFRKYAHLSEHYRIHTGEKPYECIECGKFFRHSSVLFRHQKL
HSGD*
>ENSG00000164167|ENST00000296581
MSLRKQTPSDFLKQIIGRPVVVKLNSGVDYRGVLACLDGYMNIALEQTEEYVNGQLKNKY
GDAFIRGNNVLYISTQKRRM*
>ENSG00000170374|ENST00000303846
MASSLLEEEVHYGSSPLAMLTAACSKFGGSSPLRDSTTLGKAGTKKPYSVGSDLSASKTM
GDAYPAPFTSTNGLLSPAGSPPAPTSGYANDYPPFSHSFPGPTGTQDPGLLVPKGHSSSD
CLPSVYTSLDMTHPYGSWYKAGIHAGISPGPGNTPTPWWDMHPGGNWLGGGQGQGDGLQG
TLPTGPAQPPLNPQLPTYPSDFAPLNPAPYPAPHLLQPGPQHVLPQDVYKPKAVGNSGQL
EGSGGAKPPRGASTGGSGGYGGSGAGRSSCDCPNCQELERLGAAAAGLRKKPIHSCHIPG
CGKVYGKASHLKAHLRWHTGERPFVCNWLFCGKRFTRSDELERHVRTHTREKKFTCLLCS
KRFTRSDHLSKHQRTHGEPGPGPPPSGPKELGEGRSTGEEEASQTPRPSASPATPEKAPG
GSPEQSNLLEI*
>ENSG00000162676|ENST00000294702
MPRSFLVKSKKAHSYHQPRSPGPDYSLRLENVPAPSRADSTSNAGGAKAEPRDRLSPESQ
LTEAPDRASASPDSCEGSVCERSSEFEDFWRPPSPSASPASEKSMCPSLDEAQPFPLPFK
PYSWSGLAGSDLRHLVQSYRPCGALERGAGLGLFCEPAPEPGHPAALYGPKRAAGGAGAG
APGSCSAGAGATAGPGLGLYGDFGSAAAGLYERPTAAAGLLYPERGHGLHADKGAGVKVE
SELLCTRLLLGGGSYKCIKCSKVFSTPHGLEVHVRRSHSGTRPFACEMCGKTFGHAVSLE

QHKAVHSQERSFDCKICGKSFKRSSTLSTHLLIHSDTRPYPCQYCGKRFHQKSDMKKHTF
IHTGEKPHKCQVCGKAFSQSSNLITHSRKHTGFKPFGCDLCGKGFQRKVDLRRHRETQHG
LK*
>ENSG00000147124|ENST00000313116
MAANGDSPPWSPALAAEGRGSSCEASVSFEDVTVDFSKEEWQHLDPAQRRLYWDVTLENY
SHLLSVGYQIPKSEAAFKLEQGEGPWMLEGEAPHQSCSGEAIGKMQQQGIPGGIFFHCER
FDQPIGEDSLCSILEELWQDNDQLEQRQENQNNLLSHVKVLIKERGYEHKNIEKIIHVTT
KLVPSIKRLHNCDTILKHTLNSHNHNRNSATKNLGKIFGNGNNFPHSPSSTKNENAKTGA
NSCEHDHYEKHLSHKQAPTHHQKIHPEEKLYVCTECVMGFTQKSHLFEHQRIHAGEKSRE
CDKSNKVFPQKPQVDVHPSVYTGEKPYLCTQCGKVFTLKSNLITHQKIHTGQKPYKCSEC
GKAFFQRSDLFRHLRIHTGEKPYECSECGKGFSQNSDLSIHQKTHTGEKHYECNECGKAF
TRKSALRMHQRIHTGEKPYVCADCGKAFIQKSHFNTHQRIHTGEKPYECSDCGKSFTKKS
QLHVHQRIHTGEKPYICTECGKVFTHRTNLTTHQKTHTGEKPYMCAECGKAFTDQSNLIK
HQKTHTGEKPYKCNGCGKAFIWKSRLKIHQKSHIGERHYECKDCGKAFIQKSTLSVHQRI
HTGEKPYVCPECGKAFIQKSHFIAHHRIHTGEKPYECSDCGKCFTKKSQLRVHQKIHTGE
KPNICAECGKAFTDRSNLITHQKIHTREKPYECGDCGKTFTWKSRLNIHQKSHTGERHYE
CSKCGKAFIQKATLSMHQIIHTGKKPYACTECQKAFTDRSNLIKHQKMHSGEKRYKASD*
>ENSG00000110619|ENST00000278224
MADSSGQQGKGRRVQPQWSPPAGTQPCRLHLYNSLTRNKEVFIPQDGKKVTWYCCGPTVY
DASHMGHARSYISFDILRRVLKDYFKFDVFYCMNITDIDDKIIKRARQNHLFEQYREKRP
EAAQLLEDVQAALKPFSVKLNETTDPDKKQMLERIQHAVQLATEPLEKAVQSRLTGEEVN
SCVEVLLEEAKDLLSDWLDSTLGCDVTDNSIFSKLPKFWEGDFHRDMEALNVLPPDVLTR
VSEYVPEIVNFVQKIVDNGYGYVSNGSVYFDTAKFASSEKHSYGKLVPEAVGDQKALQEG
EGDLSISADRLSEKRSPNDFALWKASKPGEPSWPCPWGKGRPGWHIECSAMAGTLLGASM
DIHGGGFDLRFPHHDNELAQSEAYFENDCWVRYFLHTGHLTIAGCKMSKSLKNFITIKDA
LKKHSARQLRLAFLMHSWKDTLDYSSNTMESALQYEKFLNEFFLNVKDILRAPVDITGQF
EKWGEEEAELNKNFYDKKTAIHKALCDNVDTRTVMEEMRALVSQCNLYMAARKAVRKRPN
QALLENIALYLTHMLKIFGAVEEDSSLGFPVGGPGTSLSLEATVMPYLQVLSEFREGVRK
IAREQKVPEILQLSDALRDNILPELGVRFEDHEGLPTVVKLVDRNTLLKEREEKRRVEEE
KRKKKEEAARRKQEQEAAKLAKMKIPPSEMFLSETDKYSKFDENVSMVCPHMTWRAKSSA
KGKPRS*
>ENSG00000154719|ENST00000307301
MEALAMGSRALRLWLVAPGGGIKWRFIATSSASQLSPTELTEMRNDLFNKEKARQLSLTP
RTEKIEVKHVGKTDPGTVFVMNKNISTPYSCAMHLSEWYCRKSILALVDGQPWDMYKPLT
KSCEIKFLTFKDCDPGEVNKAYWRSCAMMMGCVIERAFKDEYMVNLVRAPEVPVISGAFC
YDVVLDSKLDEWMPTKENLRSFTKDAHALIYKDLPFETLEVEAKVALEIFQHSKYKVDFI
EEKASQNPERIVKLHRIGDFIDVSEGPLIPRTSICFQYEVSAVHNLQPTQPSLIRRFQGV
SLPVHLRAHFTIWDKLLERSRKMTPFPILLLFTTQSFFTTSPESYLLHGTVSE*
>ENSG00000164185|ENST00000296600
MERGKKKRISNKLQQTFHHSKEPTFLINQAGLLSSDSYSSLSPETESVNPGENIKTDTQK
KRPGTVILSKLSSRRIISESQLSPPVIPARRPGFRVCYICGREFGSQSIAIHEPQCLQKW
HIENSKLPKHLRRPEPSKPQSLSSSGSYSLQATNEAAFQSAQAQLLPCESCGRTFLPDHL
LVHHRSCKPKGEGPRAPHSNSSDHLTGLKKACSGTPARPRTVICYICGKEFGTLSLPIHE
PKCLEKWKMENDRLPVELHQPLPQKPQPLPNAQSSQAGPNQAQLVFCPHCSRIFTSDRLL

VHQRSCKTHPYGPKYQNLNLGSKGGLKEYTNSKQQRNRAAPSVTDKVIHATQDALGEPGG
ALCL*
>ENSG00000134138|ENST00000314177
MAQRYDELPHYGGMDGVGVPASMYGDPHAPRPIPPVHHLNHGPPLHATQHYGAHAPHPNV
MPASMGSAVNDALKRDKDAIYGHPLFPLLALVFEKCELATCTPREPGVAGGDVCSSDSFN
EDIAVFAKQVRAEKPLFSSNPELDNLMIQAIQVLRFHLLELEKVHELCDNFCHRYISCLK
GKMPIDLVIDERDGSSKSDHEELSGSSTNLADHNPSSWRDHDDATSTHSAGTPGPSSGGH
ASQSGDNSSEQGDGLDNSVASPGTGDDDDPDKDKKRQKKRGIFPKVATNIMRAWLFQHLT
VY*
>ENSG00000141564|ENST00000306801
MESEMLQSPLLGLGEEDEADLTDWNLPLAFMKKRHCEKIEGSKSLAQSWRMKDRMKTVSV
ALVLCLNVGVDPPDVVKTTPCARLECWIDPLSMGPQKALETIGANLQKQYENWQPRARYK
QSLDPTVDEVKKLCTSLRRNAKEERVLFHYNGHGVPRPTVNGEVWVFNKNYTQYIPLSIY
DLQTWMGSPSIFVYDCSNAGLIVKSFKQFALQREQELEVAAINPNHPLAQMPLPPSMKNC
IQLAACEATELLPMIPDLPADLFTSCLTTPIKIALRWFCMQKCVSLVPGVTLDLIEKIPG
RLNDRRTPLGELNWIFTAITDTIAWNVLPRDLFQKLFRQDLLVASLFRNFLLAERIMRSY
NCTPVSSPRLPPTYMHAMWQAWDLAVDICLSQLPTIIEEGTAFRHSPFFAEQLTAFQVWL
TMGVENRNPPEQLPIVLQVLLSQVHRLRALDLLGRFLDLGPWAVSLALSVGIFPYVLKLL
QSSARELRPLLVFIWAKILAVDSSCQADLVKDNGHKYFLSVLADPYMPAEHRTMTAFILA
VIVNSYHTGQEACLQGNLIAICLEQLNDPHPLLRQWVAICLGRIWQNFDSARWCGVRDSA
HEKLYSLLSDPIPEVRCAAVFALGTFVGNSAERTDHSTTIDHNVAMMLAQLVSDGSPMVR
KELVVALSHLVVQYESNFCTVALQFIEEEKNYALPSPATTEGGSLTPVRDSPCTPRLRSV
SSYGNIRAVATARSLNKSLQNLSLTEESGGAVAFSPGNLSTSSSASSTLGSPENEEHILS
FETIDKMRRASSYSSLNSLIGVSFNSVYTQIWRVLLHLAADPYPEVSDVAMKVLNSIAYK
ATVNARPQRVLDTSSLTQSAPASPTNKGVHIHQAGGSPPASSTSSSSLTNDVAKQPVSRD
LPSGRPGTTGPAGAQYTPHSHQFPRTRKMFDKGPEQTADDADDAAGHKSFISATVQTGFC
DWSARYFAQPVMKIPEEHDLESQIRKEREWRFLRNSRVRRQAQQVIQKGITRLDDQIFLN
RNPGVPSVVKFHPFTPCIAVADKDSICFWDWEKGEKLDYFHNGNPRYTRVTAMEYLNGQD
CSLLLTATDDGAIRVWKNFADLEKNPEMVTAWQGLSDMLPTTRGAGMVVDWEQETGLLMS
SGDVRIVRIWDTDREMKVQDIPTGADSCVTSLSCDSHRSLIVAGLGDGSIRVYDRRMALS
ECRVMTYREHTAWVVKASLQKRPDGHIVSVSVNGDVRIFDPRMPESVNVLQIVKGLTALD
IHPQADLIACGSVNQFTAIYNSSGELINNIKYYDGFMGQRVGAISCLAFHPHWPHLAVGS
NDYYISVYSVEKRVR*
>ENSG00000162761|ENST00000294816
MLDGLKMEENFQSAIDTSASFSSLLGRAVSPKSVCEGCQRVILDRFLLRLNDSFWHEQCV
QCASCKEPLETTCFYRDKKLYCKYDYEKLFAVKCGGCFEAIAPNEFVMRAQKSVYHLSCF
CCCVCERQLQKGDEFVLKEGQLLCKGDYEKERELLSLVSPAASDSGKSDDEESLCKSAHG
AGKGTAEEGKDHKRPKRPRTILTTQQRRAFKASFEVSSKPCRKVRETLAAETGLSVRVVQ
VWFQNQRAKMKKLARRQQQQQQDQQNTQRLSSAQTNGGGSAGMEGIMNPYTALPTPQQLL
AIEQSVYSSDPFRQGLTPPQMPGDHMHPYGAEPLFHDLDSDDTSLSNLGDCFLATSEAGP
LQSRVGNPIDHLYSMQNSYFTS*
>ENSG00000071794|ENST00000310053
MSWMFKRDPVWKYLQTVQYGVHGNFPRLSYPTFFPRFEFQDVIPPDDFLTSDEEVDSVLF
GSLRGHVVGLRYYTGVVNNNEMVALQRDPNNPYDKNAIKVNNVNGNQVGHLKKELAGALA

YIMDNKLAQIEGVVPFGANNAFTMPLHMTFWGKEENRKAVSDQLKKHGFKLGPAPKTLGF
NLESGWGSGRAGPSYSMPVHAAVQMTTEQLKTEFDKLFEDLKEDDKTHEMEPAEAIETPL
LPHQKQALAWMVSRENSKELPPFWEQRNDLYYNTITNFSEKDRPENVHGGILADDMGLGK
TLTAIAVILTNFHDGRPLPIERVKKNLLKKEYNVNDDSMKLGGNNTSEKADGLSKDASRC
SEQPSISDIKEKSKFRMSELSSSRPKRRKTAVQYIESSDSEEIETSELPQKMKGKLKNVQ
SETKGRAKAGSSKVIEDVAFACALTSSVPTTKKKMLKKGACAVEGSKKTDVEERPRTTLI
ICPLSVLSNWIDQFGQHIKSDVHLNFYVYYGPDRIREPALLSKQDIVLTTYNILTHDYGT
KGDSPLHSIRWLRVILDEGHAIRNPNAQQTKAVLDLESERRWVLTGTPIQNSLKDLWSLL
SFLKLKPFIDREWWHRTIQRPVTMGDEGGLRRLQSLIKNITLRRTKTSKIKGKPVLELPE
RKVFIQHITLSDEERKIYQSVKNEGRATIGRYFNEGTVLAHYADVLGLLLRLRQICCHTY
LLTNAVSSNGPSGNDTPEELRKKLIRKMKLILSSGSDEECAICLDSLTVPVITHCAHVFC
KPCICQVIQNEQPHAKCPLCRNDIHEDNLLECPPEELARDSEKKSDMEWTSSSKINALMH
ALTDLRKKNPNIKSLVVSQFTTFLSLIEIPLKASGFVFTRLDGSMAQKKRVESIQCFQNT
EAGSPTIMLLSLKAGGVGLNLSAASRVFLMDPAWNPAAEDQCFDRCHRLGQKQEVIITKF
IVKDSVEENMLKIQNKKRELAAGAFGTKKPNADEMKQAKINEIRTLIDL*
>ENSG00000165730|ENST00000298596
MARPVQLAPGSLALVLCRLEAQKAAGAAEEPGGRAVFRAFRRANARCFWNARLARAASRL
AFQGWLRRGVLLVRAPPACLQVLRDAWRRRALRPPRGFRIRAVGDVFPVQMNPITQSQFV
PLGEVLCCAISDMNTAQIVVTQESLLERLMKHYPGIAIPSEDILYTTLGTLIKERKIYHT
GEGYFIVTPQTYFITNTTTQENKRMLPSDESRLMPASMTYLVSMESCAESAQENAAPISH
CQSCQCFRDMHTQDVQEAPVAAEVTRKSHRGLGESVSWVQNGAVSVSAEHHICESTKPLP
YTRDKEKGKKFGFSLLWRSLSRKEKPKTEHSSFSAQFPPEEWPVRDEDDLDNIPRDVEHE
IIKRINPILTVDNLIKHTVLMQKYEEQKKYNSQGTSTDMLTIGHKYPSKEGVKKRQGLSA
KPQGQGHSRRDRHKARNQGSEFQPGSIRLEKHPKLPATQPIPRIKSPNEMVGQKPLGEIT
TVLGSHLIYKKRISNPFQGLSHRGSTISKGHKIQKTSDLKPSQTGPKEKPFQKPRSLDSS
RIFDGKAKEPYAEQPNDKMEAESIYINDPTVKPINDDFRGHLFSHPQQSMLQNDGKCCPF
MESMLRYEVYGGENEVIPEVLRKSHSHFDKLGETKQTPHSLPSRGASFSDRTPSACRLVD
NTIHQFQNLGLLDYPVGVNPLRQAARQDKDSEELLRKGFVQDAETTSLENEQLSNDDQAL
YQNEVEDDDGACSSLYLEEDDISENDDLRQMLPGHSQYSFTGGSQGNHLGKQKVIERSLT
EYNSTMERVESQVLKRNECYKPTGLHATPGESQEPNLSAESCGLNSGAQFGFNYEEEPSV
AKCVQASAPADERIFDYYSARKASFEAEVIQDTIGDTGKKPASWSQSPQNQEMRKHFPQK
FQLFNTSHMPVLAQDVQYEHSHLEGTENHSMAGDSGIDSPRTQSLGSNNSVILDGLKRRQ
NFLQNVEGTKSSQPLTSNSLLPLTPVINV*
>ENSG00000152942|ENST00000282891
MNQHELAVHKKKIEEVETWLKAQVLERQPKQGGSILLITGPPGCGKTTTLKILSKEHGIQ
VQEWINPVLPDFQKDDFKGMFNTESSFHMFPYQSQIAVFKEFLLRATKYNKLQMLGDDLR
TDKKIILVEDLPNQFYRDSHTLHEVLRKYVRIGRCPLIFIISDSLSGDNNQRLLFPKEIQ
EECSISNISFNPVAPTIMMKFLNRIVTIEANKNGGKITVPDKTSLELLCQGCSGDIRSAI
NSLQFSSSKGENNLRPRKKGMSLKSDAVLSKSKRRKKPDRVFENQEVQAIGGKDVSLFLF
RALGKILYCKRASLTELDSPRLPSHLSEYERDTLLVEPEEVVEMSHMPGDLFNLYLHQNY
IDFFMEIDDIVRASEFLSFADILSGDWNTRSLLREYSTSIATRGVMHSNKARGYAHCQGG
GSSFRPLHKPQWFLINKKYRENCLAAKALFPDFCLPALCLQTQLLPYLALLTIPMRNQAQ
ISFIQDIGRLPLKRHFGRLKMEALTDREHGMIDPDSGDEAQLNGGHSAEESLGEPTQATV
PETWSLPLSQNSASELPASQPQPFSAQGDMEENIIIEDYESDGT*

>ENSG00000161980|ENST00000293860
MLLFCPGCGNGLIVEEGQRCHRFACNTCPYVHNITRKVTNRKYPKLKEVDDVLGGAAAWE
NVDSTAESCPKCEHPRAYFMQLQTRSADEPMTTFYKCCNAQCGHRWRD*
>ENSG00000143368|ENST00000271628
MAAGPISERNQDATVYVGGLDEKVSEPLLWELFLQAGPVVNTHMPKDRVTGQHQGYGFVE
FLSEEDADYAIKIMNMIKLYGKPIRVNKASAHNKNLDVGANIFIGNLDPEIDEKLLYDTF
SAFGVILQTPKIMRDPDTGNSKGYAFINFASFDASDAAIEAMNGQYLCNRPITVSYAFKK
DSKGERHGSAAERLLAAQNPLSQADRPHQLFADAPPPPSAPNPVVSSLGSGLPPPGMPPP
GSFPPPVPPPGALPPGIPPAMPPPPMPPGAAGHGPPSAGTPGAGHPGHGHSHPHPFPPGG
MPHPGMSQMQLAHHGPHGLGHPHAGPPGSGGQPPPRPPPGMPHPGPPPMGMPPRGPPFGS
PMGHPGPMPPHGMRGPPPLMPPHGYTGPPRPPPYGYQRGPLPPPRPTPRPPVPPRGPLRG
PLPQ*
>ENSG00000153037|ENST00000282999
MACAAARSPADQDRFICIYPAYLNNKKTIAEGRRIPISKAVENPTATEIQDVCSAVGLNV
FLEKNKMYSREWNRDVQYRGRVRVQLKQEDGSLCLVQFPSHYTLSLTSGS*
>ENSG00000140548|ENST00000268154
MEGFMDSGTQTDAVVVLSLAQAAVLGLVSENELFGATISAEAFYPDLGPELSGAAMGEPE
PPGPDVYQLACNGRALEEPAEEEVLEVEAACEKHTRRKTRPPVRLVPKVKFEKVEEEEQE
VYEVSVPGDDKDAGPAEAPAEAASGGCDALVQSSAVKMIDLSAFSRKPRTLRHLPRTPRP
ELNVAPYDPHFPAPARDGFPEPSMALPGPEALPTECGFEPPHLAPLSDPEAPSMESPEPV
KPEQGFVWQEASEFEADTAGSTVERHKKAQLDRLDINVQIDDSYLVEAGDRQKRWQCRMC
EKSYTSKYNLVTHILGHNGIKPHSCPHCSKLFKQPSHLQTHLLTHQGTRPHKCQVCHKAF
TQTSHLKRHMLLHSEVKPYSCHFCGRGFAYPSELKAHEVKHESGRCHVCVECGLDFSTLT
QLKRHLASHQGPTLYQCLECDKSFHYRSQLQNHMLKHQNVRPFVCTECGMEFSQIHHLKQ
HSLTHKGVKEFKCEVCGREFTLQANMKRHMLIHTSVRPYQCHICFKTFVQKQTLKTHMIV
HSPVKPFKCKVCGKSFNRMYNLLGHMHLHAGSKPFKCPYCSSKFNLKGNLSRHMKVKHGV
MDIGLDSQDPMMELTGTDPSELDGQQEMEDFEENAYSYASVDSSAEASVLTEQAMKEMAY
YNVL*
>ENSG00000161847|ENST00000293677
MPLRPGSLVPEGAGFPKMAADVSVTHRPPLSPKSGAEVEAGDAAERRAPEEELPPLDPEE
IRKRLEHTERQFRNRRKILIRGLPGDVTNQEVHDLLSDYELKYCFVDKYKGTAFVTLLNG
EQAEAAINAFHQSRLRERELSVQLQPTDALLCVANLPPSLTQQQFEELVRPFGSLERCFL
VYSERTGQSKGYGFAEYMKKDSAARAKSDLLGKPLGPRTLYVHWTDAGQLTPALLHSRCL
CVDRLPPGFNDVDALCRALSAVHSPTFCQLACGQDGQLKGFAVLEYETAEMAEEAQQQAD
GLSLGGSHLRVSFCAPGPPGRSMLAALIAAQATALNRGKGLLPEPNILQLLNNLGPSASL
QLLLNPLLHGSAGGKQGLLGAPPAMPLLNGPALSTALLQLALQTQGQKKPGILGDSPLGA
LQPGAQPANPLLGELPAGGGLPPELPPRRGKPPPLLPSVLGPAGGDREALGLGPPAAQLT
PPPAPVGLRGSGLRGLQKDSGPLPTPPGVSLLGEPPKDYRIPLNPYLNLHSLLPASNLAG
KEARGWGGAGRSRRPAEGPPTNPPAPGGGSSSSKAFQLKSRLLSPLSSARLPPEPGLSDS
YSFDYPSDMGPRRLFSHPREPALGPHGPSRHKMSPPPSGFGERSSGGSGGGPLSHFYSGS
PTSYFTSGLQAGLKQSHLSKAIGSSPLGSGEGLLGLSPGPNGHSHLLKTPLGGQKRSFAH
LLPSPEPSPEGSYVGQHSQGLGGHYADSYLKRKRIF*
>ENSG00000187987|ENST00000289788
MAITLTLQTAEMQEGLLAVKVKEEEEEHSCGPESGLSRNNPHTREIFRRRFRQFCYQESP

GPREALQRLQELCHQWLRPEMHTKEQILELLVLEQFLTILPEELQAWVRQHRPVSGEEAV
TVLEDLERELDDPGEQVLSHAHEQEEFVKEKATPGAAQESSNDQFQTLEEQLGYNLREVC
PVQEIDGKAGTWNVELAPKREISQEVKSLIQVLGKQNGNITQIPEYGDTCDREGRLEKQR
VSSSVERPYICSECGKSFTQNSILIEHQRTHTGEKPYECDECGRAFSQRSGLFQHQRLHT
GEKRYQCSVCGKAFSQNAGLFHHLRIHTGEKPYQCNQCNKSFSRRSVLIKHQRIHTGERP
YECEECGKNFIYHCNLIQHRKVHPVAESS*
>ENSG00000061337|ENST00000265801
MGSVSSLISGHSFHSKHCRASQYKLRKSSHLKKLNRYSDGLLRFGFSQDSGHGKSSSKMG
KSEDFFYIKVSQKARGSHHPDYTALSSGDLGGQAGVDFDPSTPPKLMPFSNQLEMGSEKG
AVRPTAFKPVLPRSGAILHSSPESASHQLHPAPPDKPKEQELKPGLCSGALSDSGRNSMS
SLPTHSTSSSYQLDPLVTPVGPTSRFGGSAHNITQGIVLQDSNMMSLKALSFSDGGSKLG
HSNKADKGPSCVRSPISTDECSIQELEQKLLEREGALQKLQRSFEEKELASSLAYEERPR
RCRDELEGPEPKGGNKLKQASQKSQRAQQVLHLQVLQLQQEKRQLRQELESLMKEQDLLE
TKLRSYEREKTSFGPALEETQWEVCQKSGEISLLKQQLKESQTEVNAKASEILGLKAQLK
DTRGKLEGLELRTQDLEGALRTKGLELEVCENELQRKKNEAELLREKVNLLEQELQELRA
QAALARDMGPPTFPEDVPALQRELERLRAELREERQGHDQMSSGFQHERLVWKEEKEKVI
QYQKQLQQSYVAMYQRNQRLEKALQQLARGDSAGEPLEVDLEGADIPYEDIIATEI*
>ENSG00000056097|ENST00000265069
MIPICPVVSFTYVPSRLGEDAKMATGNYFGFTHSGAAAAAAAAQYSQQPASGVAYSHPTT
VASYTVHQAPVAAHTVTAAYAPAAATVAVARPAPVAVAAAATAAAYGGYPTAHTATDYGY
TQRQQEAPPPPPPATTQNYQDSYSYVRSTAPAVAYDSKQYYQQPTATAAAVAAAAQPQPS
VAETYYQTAPKAGYSQGATQYTQAQQTRQVTAIKPATPSPATTTFSIYPVSSTVQPVAAA
ATVVPSYTQSATYSTTAVTYSGTSYSGYEAAVYSAASSYYQQQQQQQKQAAAAAAAAAAT
AAWTGTTFTKKAPFQNKQLKPKQPPKPPQIHYCDVCKISCAGPQTYKEHLEGQKHKKKEA
ALKASQNTSSSNSSTRGTQNQLRCELCDVSCTGADAYAAHIRGAKHQKVVKLHTKLGKPI
PSTEPNVVSQATSSTAVSASKPTASPSSIAANNCTVNTSSVATSSMKGLTTTGNSSLNST
SNTKVSAVPTNMAAKKTSTPKINFVGGNKLQSTGNKAEDIKGTECVKSTPVTSAVQIPEV
KQDTVSEPVTPASLAALQSDVQPVGHDYVEEVRNDEGKVIRFHCKLCECSFNDPNAKEMH
LKGRRHRLQYKKKVNPDLQVEVKPSIRARKIQEEKMRKQMQKEEYWRRREEEERWRMEMR
RYEEDMYWRRMEEEQHHWDDRRRMPDGGYPHGPPGPLGLLGVRPGMPPQPQGPAPLRRPD
SSDDRYVMTKHATIYPTEEELQAVQKIVSITERALKLVSDSLSEHEKNKNKEGDDKKEGG
KDRALKGVLRVGVLAKGLLLRGDRNVNLVLLCSEKPSKTLLSRIAENLPKQLAVISPEKY
DIKCAVSEAAIILNSCVEPKMQVTITLTSPIIREENMREGDVTSGMVKDPPDVLDRQKCL
DALAALRHAKWFQARANGLQSCVIIIRILRDLCQRVPTWSDFPSWAMELLVEKAISSASS
PQSPGDALRRVFECISSGIILKGSPGLLDPCEKDPFDTLATMTDQQREDITSSAQFALRL
LAFRQIHKVLGMDPLPQMSQRFNIHNNRKRRRDSDGVDGFEAEGKKDKKDYDNF*
>ENSG00000196793|ENST00000306006
MASTITGSQDCIVNHRGEVDGEPELDISPCQQWGEASSPISRNRDSVMTLQSGCFENIES
ETYLPLKVSSQIDTQDSSVKFCKNEPQDHQESRRLFVMEESTERKVIKGESCSENLQVKL
VSDGQELASPLLNGEATCQNGQLKESLDPIDCNCKDIHGWKSQVVSCSQQRAHTEEKPCD
HNNCGKILNTSPDGHPYEKIHTAEKQYECSQCGKNFSQSSELLLHQRDHTEEKPYKCEQC
GKGFTRSSSLLIHQAVHTDEKPYKCDKCGKGFTRSSSLLIHHAVHTGEKPYKCDKCGKGF
SQSSKLHIHQRVHTGEKPYECEECGMSFSQRSNLHIHQRVHTGERPYKCGECGKGFSQSS
NLHIHRCIHTGEKPYQCYECGKGFSQSSDLRIHLRVHTGEKPYHCGKCGKGFSQSSKLLI

HQRVHTGEKPYECSKCGKGFSQSSNLHIHQRVHKKDPR*
>ENSG00000167862|ENST00000301585
MAATRCLRWGLSRAGVWLLPPPARCPRRALHKQKDGTEFKSIYSLDKLYPESQGSDTAWR
VPNGAKQADSDIPLDRLTISYCRSSGPGGQNVNKVNSKAEVRFHLATAEWIAEPVRQKIA
ITHKNKINRLGELILTSESSRYQFRNLADCLQKIRDMITEASQTPKEPTKEDVKLHRIRI
ENMNRERLRQKRIHSAVKTSRRVDMD*
>ENSG00000143761|ENST00000272102
MGNIFANLFKGLFGKKEMRILMVGLDAAGKTTILYKLKLGEIVTTIPTIGFNVETVEYKN
ISFTVWDVGGQDKIRPLWRHYFQNTQGLIFVVDSNDRERVNEAREELMRMLAEDELRDAV
LLVFANKQDLPNAMNAAEITDKLGLHSLRHRNWYIQATCATSGDGLYEGLDWLSNQLRNQ
K*
>ENSG00000167785|ENST00000301475
MAAVILPSTAAPSSLFPASQQKGHTQGGELVNELLTSWLRGLVTFEDVAVEFTQEEWALL
DPAQRTLYRDVMLENCRNLASLGCRVNKPSLISQLEQDKKVVTEERGILPSTCPDLETLL
KAKWLTPKKNVFRKEQSKGVKTERSHRGVKLNECNQCFKVFSTKSNLTQHKRIHTGEKPY
DCSQCGKSFSSRSYLTIHKRIHNGEKPYECNHCGKAFSDPSSLRLHLRIHTGEKPYECNQ
CFHVFRTSCNLKSHKRIHTGENHHECNQCGKAFSTRSSLTGHNSIHTGEKPYECHDCGKT
FRKSSYLTQHVRTHTGEKPYECNECGKSFSSSFSLTVHKRIHTGEKPYECSDCGKAFNNL
SAVKKHLRTHTGEKPYECNHCGKSFTSNSYLSVHKRIHNRWI*
>ENSG00000101916|ENST00000311912
MKESSLQNSSCSLGKETKKENMFLQSSMLTCIFLLISGSCELCAEENFSRSYPCDEKKQN
DSVIAECSNRRLQEVPQTVGKYVTELDLSDNFITHITNESFQGLQNLTKINLNHNPNVQH
QNGNPGIQSNGLNITDGAFLNLKNLRELLLEDNQLPQIPSGLPESLTELSLIQNNIYNIT
KEGISRLINLKNLYLAWNCYFNKVCEKTNIEDGVFETLTNLELLSLSFNSLSHVPPKLPS
SLRKLFLSNTQIKYISEEDFKGLINLTLLDLSGNCPRCFNAPFPCVPCDGGASINIDRFA
FQNLTQLRYLNLSSTSLRKINAAWFKNMPHLKVLDLEFNYLVGEIASGAFLTMLPRLEIL
DLSFNYIKGSYPQHINISRNFSKLLSLRALHLRGYVFQELREDDFQPLMQLPNLSTINLG
INFIKQIDFKLFQNFSNLEIIYLSENRISPLVKDTRQSYANSSSFQRHIRKRRSTDFEFD
PHSNFYHFTRPLIKPQCAAYGKALDLSLNSIFFIGPNQFENLPDIACLNLSANSNAQVLS
GTEFSAIPHVKYLDLTNNRLDFDNASALTELSDLEVLDLSYNSHYFRIAGVTHHLEFIQN
FTNLKVLNLSHNNIYTLTDKYNLESKSLVELVFSGNRLDILWNDDDNRYISIFKGLKNLT
RLDLSLNRLKHIPNEAFLNLPASLTELHINDNMLKFFNWTLLQQFPRLELLDLRGNKLLF
LTDSLSDFTSSLRTLLLSHNRISHLPSGFLSEVSSLKHLDLSSNLLKTINKSALETKTTT
KLSMLELHGNPFECTCDIGDFRRWMDEHLNVKIPRLVDVICASPGDQRGKSIVSLELTTC
VSDVTAVILFFFTFFITTMVMLAALAHHLFYWDVWFIYNVCLAKVKGYRSLSTSQTFYDA
YISYDTKDASVTDWVINELRYHLEESRDKNVLLCLEERDWDPGLAIIDNLMQSINQSKKT
VFVLTKKYAKSWNFKTAFYLALQRLMDENMDVIIFILLEPVLQHSQYLRLRQRICKSSIL
QWPDNPKAEGLFWQTLRNVVLTENDSRYNNMYVDSIKQY*
>ENSG00000139233|ENST00000266604
MAKSLRSKWKRKMRAEKRKKNAPKEASRLKSILKLDGDVLMKDVQEIATVVVPKPKHCQE
KMQCEVKDEKDDMKMETDIKRNKKTLLDQHGQYPIWMNQRQRKRLKAKREKRKGKSKAKA
VKVAKGLAW*
>ENSG00000187955|ENST00000297848
MKIFQRKMRYWLLPPFLAIVYFCTIVQGQVAPPTRLRYNVISHDSIQISWKAPRGKFGGY

KLLVTPTSGGKTNQLNLQNTATKAIIQGLMPDQNYTVQIIAYNKDKESKPAQGQFRIKDL
EKRKDPKPRVKVVDRGNGSRPSSPEEVKFVCQTPAIADIVILVDGSWSIGRFNFRLVRHF
LENLVTAFDVGSEKTRIGLAQYSGDPRIEWHLNAFSTKDEVIEAVRNLPYKGGNTLTGLA
LNYIFENSFKPEAGSRTGVSKIGILITDGKSQDDIIPPSRNLRESGVELFAIGVKNADVN
ELQEIASEPDSTHVYNVAEFDLMHTVVESLTRTLCSRVEEQDREIKASAHAITGPPTELI
TSEVTARSFMVNWTHAPGNVEKYRVVYYPTRGGKPDEVVVDGTVSSTVLKNLMSLTEYQI
AVFAIYAHTASEGLRGTETTLALPMASDLLLYDVTENSMRVKWDAVPGASGYLILYAPLT
EGLAGDEKEMKIGETHTDIELSGLLPNTEYTVTVYAMFGEEASDPVTGQETTLALSPPRN
LRISNVGSNSARLTWDPTSRQINGYRIVYNNADGTEINEVEVDPITTFPLKGLTPLTEYT
IAIFSIYDEGQSEPLTGVFTTEEVPAQQYLEIDEVTTDSFRVTWHPLSADEGLHKLMWIP
VYGGKTEEVVLKEEQDSHVIEGLEPGTEYEVSLLAVLDDGSESEVVTAVGTTLDSFWTEP
ATTIVPTTSVTSVFQTGIRNLVVGDETTSSLRVKWDISDSDVQQFRVTYMTAQGDPEEEV
IGTVMVPGSQNNLLLKPLLPDTEYKVTVTPIYTDGEGVSVSAPGKTLPSSGPQNLRVSEE
WYNRLRITWDPPSSPVKGYRIVYKPVSVPGPTLETFVGADINTILITNLLSGMDYNVKIF
ASQASGFSDALTGMVKTLFLGVTNLQAKHVEMTSLCAHWQVHRHATAYRVVIESLQDRQK
QESTVGGGTTRHCFYGLQPDSEYKISVYTKLQEIEGPSVSIMEKTQSLPTRPPTFPPTIP
PAKEVCKAAKADLVFMVDGSWSIGDENFNKIISFLYSTVGALNKIGTDGTQVAMVQFTDD
PRTEFKLNAYKTKETLLDAIKHISYKGGNTKTGKAIKYVRDTLFTAESGTRRGIPKVIVV
ITDGRSQDDVNKISREMQLDGYSIFAIGVADADYSELVSIGSKPSARHVFFVDDFDAFKK
IEDELITFVCETASATCPVVHKDGIDLAGFKMMEMFGLVEKDFSSVEGVSMEPGTFNVFP
CYQLHKDALVSQPTRYLHPEGLPSDYTISFLFRILPDTPQEPFALWEILNKNSDPLVGVI
LDNGGKTLTYFNYDQSGDFQTVTFEGPEIRKIFYGSFHKLHIVVSETLVKVVIDCKQVGE
KAMNASANITSDGVEVLGKMVRSRGPGGNSAPFQLQMFDIVCSTSWANTDKCCELPGLRD
DESCPDLPHSCSCSETNEVALGPAGPPGGPGLRGPKGQQGEPGPKGPDGPRGEIGLPGPQ
GPPGPQGPSGLSIQGMPGMPGEKGEKGDTGLPGPQGIPGGVGSPGRDGSPGQRGLPGKDG
SSGPPGPPGPIGIPGTPGVPGITGSMGPQGALGPPGVPGAKGERGERGDLQSQAMVRSVA
RQVCEQLIQSHMARYTAILNQIPSHSSSIRTVQGPPGEPGRPGSPGAPGEQGPPGTPGFP
GNAGVPGTPGERGLTGIKGEKGNPGVGTQGPRGPPGPAGPSGESRPGSPGPPGSPGPRGP
PGHLGVPGPQGPSGQPGYCDPSSCSAYGVRAPHPDQPEFTPVQDELEAMELWGPGV*
>ENSG00000171295|ENST00000304060
MDPVAFKDVAVNFTQEEWALLDISQRKLYREVMLETFRNLTSLGKRWKDQNIEYEHQNPR
RNFRSLIEEKVNEIKDDSHCGETFTPVPDDRLNFQEKKASPEVKSCESFVCGEVGLGNSS
FNMNIRGDIGHKAYEYQEYGPKPCKCQQPKKAFRYRPSFRTQERDHTGEKPNACKVCGKT
FISHSSVRRHMVMHSGDGPYKCKFCGKAFHCLRLYLIHERIHTGEKPCECKQCGKSFSYS
ATHRIHKRTHTGEKPYEYQECGKAFHSPRSYRRHERIHMGEKAYQCKECGKAFTCPRYVR
IHERTHSRKNLYECKQCGKALSSLTSFQTHVRLHSGERPYECKICGKDFCSVNSFQRHEK
IHSGEKPYKCKQCGKAFPHSSSLRYHERTHTGEKPYECKQCGKAFRSASHLRVHGRTHTG
EKPYECKECGKAFRYVNNLQSHERTQTHIRIHSGERRYKCKICGKGFYCPKSFQRHEKTH
TGEKLYECKQRSVVPSVVPVPFDIMKGLTLERSPINASNVGKPSELCQSFECMVGLTLKR
NPMSVSNDGKPSDLPHTFEYVVGHTMERSPMHVRNVGNPSDLPRTFEFMKGHKHT*
>ENSG00000153201|ENST00000283195
MRRSKADVERYIASVQGSTPSPRQKSMKGFYFAKLYYEAKEYDLAKKYICTYINVQERDP
KAHRFLGLLYELEENTDKAVECYRRSVELNPTQKDLVLKIAELLCKNDVTDGRAKYWLER
AAKLFPGSPAIYKLKEQLLDCEGEDGWNKLFDLIQSELYVRPDDVHVNIRLVEVYRSTKR

LKDAVAHCHEAERNIALRSSLEWNSCVVQTLKEYLESLQCLESDKSDWRATNTDLLLAYA
NLMLLTLSTRDVQESRELLQSFDSALQSVKSLGGNDELSATFLEMKGHFYMHAGSLLLKM
GQHSSNVQWRALSELAALCYLIAFQVPRPKIKLIKGEAGQNLLEMMACDRLSQSGHMLLN
LSRGKQDFLKEIVETFANKSGQSALYDALFSSQSPKDTSFLGSDDIGNIDVREPELEDLT
RYDVGAIRAHNGSLQHLTWLGLQWNSLPALPGIRKWLKQLFHHLPHETSRLETNAPESIC
ILDLEVFLLGVVYTSHLQLKEKCNSHHSSYQPLCLPLPVCKQLCTERQKSWWDAVCTLIH
RKAVPGNVAKLRLLVQHEINTLRAQEKHGLQPALLVHWAECLQKTGSGLNSFYDQREYIG
RSVHYWKKVLPLLKIIKKKNSIPEPIDPLFKHFHSVDIQASEIVEYEEDAHITFAILDAV
NGNIEDAVTAFESIKSVVSYWNLALIFHRKAEDIENDALSPEEQEECKNYLRKTRDYLIK
IIDDSDSNLSVVKKLPVPLESVKEMLNSVMQELEDYSEGGPLYKNGSLRNADSEIKHSTP
SPTRYSLSPSKSYKYSPKTPPRWAEDQNSLLKMICQQVEAIKKEMQELKLNSSNSASPHR
WPTENYGPDSVPDGYQGSQTFHGAPLTVATTGPSVYYSQSPAYNSQYLLRPAANVTPTKG
PVYGMNRLPPQQHIYAYPQQMHTPPVQSSSACMFSQEMYGPPALRFESPATGILSPRGDD
YFNYNVQQTSTNPPLPEPGYFTKPPIAAHASRSAESKTIEFGKTNFVQPMPGEGLRPSLP
TQAHTTQPTPFKFNSNFKSNDGDFTFSSPQVVTQPPPAAYSNSESLLGLLTSDKPLQGDG
YSGAKPIPGGQTIGPRNTFNFGSKNVSGISFTENMGSSQQKNSGFRRSDDMFTFHGPGKS
VFGTPTLETANKNHETDGGSAHGDDDDDGPHFEPVVPLPDKIEVKTGEEDEEEFFCNRAK
LFRFDVESKEWKERGIGNVKILRHKTSGKIRLLMRREQVLKICANHYISPDMKLTPNAGS
DRSFVWHALDYADELPKPEQLAIRFKTPEEAALFKCKFEEAQSILKAPGTNVAMASNQAV
RIVKEPTSHDNKDICKSDAGNLNFEFQVAKKEGSWWHCNSCSLKNASTAKKCVSCQNLNP
SNKELVGPPLAETVFTPKTSPENVQDRFALVTPKKEGHWDCSICLVRNEPTVSRCIACQN
TKSANKSGSSFVHQASFKFGQGDLPKPINSDFRSVFSTKEGQWDCSACLVQNEGSSTKCA
ACQNPRKQSLPATSIPTPASFKFGTSETSKTLKSGFEDMFAKKEGQWDCSSCLVRNEANA
TRCVACQNPDKPSPSTSVPAPASFKFGTSETSKAPKSGFEGMFTKKEGQWDCSVCLVRNE
ASATKCIACQNPGKQNQTTSAVSTPASSETSKAPKSGFEGMFTKKEGQWDCSVCLVRNEA
SATKCIACQNPGKQNQTTSAVSTPASSETSKAPKSGFEGMFTKKEGQWDCSVCLVRNEAS
ATKCIACQCPSKQNQTTAISTPASSEISKAPKSGFEGMFIRKGQWDCSVCCVQNESSSLK
CVACDASKPTHKPIAEAPSAFTLGSEMKLHDSSGSQVGTGFKSNFSEKASKFGNTEQGFK
FGHVDQENSPSFMFQGSSNTEFKSTKEGFSIPVSADGFKFGISEPGNQEKKSEKPLENGT
GFQAQDISGQKNGRGVIFGQTSSTFTFADLAKSTSGEGFQFGKKDPNFKGFSGAGEKLFS
SQYGKMANKANTSGDFEKDDDAYKTEDSDDIHFEPVVQMPEKVELVTGEEDEKVLYSQRV
KLFRFDAEVSQWKERGLGNLKILKNEVNGKLRMLMRREQVLKVCANHWITTTMNLKPLSG
SDRAWMWLASDFSDGDAKLEQLAAKFKTPELAEEFKQKFEECQRLLLDIPLQTPHKLVDT
GRAAKLIQRAEEMKSGLKDFKTFLTNDQTKVTEEENKGSGTGAAGASDTTIKPNPENTGP
TLEWDNYDLREDALDDSVSSSSVHASPLASSPVRKNLFRFGESTTGFNFSFKSALSPSKS
PAKLNQSGTSVGTDEESDVTQEEERDGQYFEPVVPLPDLVEVSSGEENEQVVFSHRAKLY
RYDKDVGQWKERGIGDIKILQNYDNKQVRIVMRRDQVLKLCANHRITPDMTLQNMKGTER
VWLWTACDFADGERKVEHLAVRFKLQDVADSFKKIFDEAKTAQEKDSLITPHVSRSSTPR
ESPCGKIAVAVLEETTRERTDVIQGDDVADATSEVEVSSTSETTPKAVVSPPKFVFGSES
VKSIFSSEKSKPFAFGNSSATGSLFGFSFNAPLKSNNSETSSVAQSGSESKVEPKKCELS
KNSDIEQSSDSKVKNLFASFPTEESSINYTFKTPEKAKEKKKPEDSPSDDDVLIVYELTP
TAEQKALATKLKLPPTFFCYKNRPDYVSEEEEDDEDFETAVKKLNGKLYLDGSEKCRPLE
ENTADNEKECIIVWEKKPTVEEKAKADTLKLPPTFFCGVCSDTDEDNGNGEDFQSELQKV
QEAQKSQTEEITSTTDSVYTGGTEVMVPSFCKSEEPDSITKSISSPSVSSETMDKPVDLS

TRKEIDTDSTSQGESKIVSFGFGSSTGLSFADLASSNSGDFAFGSKDKNFQWANTGAAVF
GTQSVGTQSAGKVGEDEDGSDEEVVHNEDIHFEPIVSLPEVEVKSGEEDEEILFKERAKL
YRWDRDVSQWKERGVGDIKILWHTMKNYYRILMRRDQVFKVCANHVITKTMELKPLNVSN
NALVWTASDYADGEAKVEQLAVRFKTKEVADCFKKTFEECQQNLMKLQKGHVSLAAELSK
ETNPVVFFDVCADGEPLGRITMELFSNIVPRTAENFRALCTGEKGFGFKNSIFHRVIPDF
VCQGGDITKHDGTGGQSIYGDKFEDENFDVKHTGPGLLSMANQGQNTNNSQFVITLKKAE
HLDFKHVVFGFVKDGMDTVKKIESFGSPKGSVCRRITITECGQI*
>ENSG00000143379|ENST00000271640
MSSLPGCIGLDAATATVESEEIAELQQAVVEELGISMEELRHFIDEELEKMDCVQQRKKQ
LAELETWVIQKESEVAHVDQLFDDASRAVTNCESLVKDFYSKLGLQYRDSSSEDESSRPT
EIIEIPDEDDDVLSIDSGDAGSRTPKDQKLREAMAALRKSAQDVQKFMDAVNKKSSSQDL
HKGTLSQMSGELSKDGDLIVSMRILGKKRTKTWHKGTLIAIQTVGPGKKYKVKFDNKGKS
LLSGNHIAYDYHPPADKLYVGSRVVAKYKDGNQVWLYAGIVAETPNVKNKLRFLIFFDDG
YASYVTQSELYPICRPLKKTWEDIEDISCRDFIEEYVTAYPNRPMVLLKSGQLIKTEWEG
TWWKSRVEEVDGSLVRILFLDDKRCEWIYRGSTRLEPMFSMKTSSASALEKKQGQLRTRP
NMGAVRSKGPVVQYTQDLTGTGTQFKPVEPPQPTAPPAPPFPPAPPLSPQAGDSDLESQL
AQSRKQVAKKSTSFRPGSVGSGHSSPTSPALSENVSGGKPGINQTYRSPLGSTASAPAPS
ALPAPPAPPVFHGMLERAPAEPSYRAPMEKLFYLPHVCSYTCLSRVRPMRNEQYRGKNPL
LVPLLYDFRRMTARRRVNRKMGFHVIYKTPCGLCLRTMQEIERYLFETGCDFLFLEMFCL
DPYVLVDRKFQPYKPFYYILDITYGKEDVPLSCVNEIDTTPPPQVAYSKERIPGKGVFIN
TGPEFLVGCDCKDGCRDKSKCACHQLTIQATACTPGGQINPNSGYQYKRLEECLPTGVYE
CNKRCKCDPNMCTNRLVQHGLQVRLQLFKTQNKGWGIRCLDDIAKGSFVCIYAGKILTDD
FADKEGLEMGDEYFANLDHIESVENFKEGYESDAPCSSDSSGVDLKDQEDGNSGTEDPEE
SNDDSSDDNFCKDEDFSTSSVWRSYATRRQTRGQKENGLSETTSKDSHPPDLGPPHIPVP
PSIPVGGCNPPSSEETPKNKVASWLSCNSVSEGGFADSDSHSSFKTNEGGEGRAGGSRME
AEKASTSGLGIKDEGDIKQAKKEDTDDRNKMSVVTESSRNYGYNPSPVKPEGLRRPPSKT
SMHQSRRLMASAQSNPDDVLTLSSSTESEGESGTSRKPTAGQTSATAVDSDDIQTISSGS
EGDDFEDKKNMTGPMKRQVAVKSTRGFALKSTHGIAIKSTNMASVDKGESAPVRKNTRQF
YDGEESCYIIDAKLEGNLGRYLNHSCSPNLFVQNVFVDTHDLRFPWVAFFASKRIRAGTE
LTWDYNYEVGSVEGKELLCCCGAIECRGRLL*
>ENSG00000124920|ENST00000265460
MHWLPAGHDINGALEPSNIDTSILEEYISKEDASDLCFPDISAPASSASYSHGQPAMPGS
SGVHHLSPPGGGPSPGRHGPLPPPGYGTPLNCNNNNGMGAAPKPFPGGTGPPIKAEPKAP
YAPGTLPDSPPDSGSEAYSPQQVNEPHLLRTITPETLCHVGVPSRLEHPPPPPAHLPGPP
PPPPPPPHYPVLQRDLYMKAEPPIPHYAAMGQGLVPTDLHHTQQSQMLHQLLQQHGAELP
THPSKKRKHSESPPSTLNAQMLNGMIKQEPGTVTALPLHPTRAPSPPWPPQGPLSPGPGS
LPLSIARVQTPPWHPPGAPSPGLLQDSDSLSGSYLDPNYQSIKWQPHQQNKWATLYDANY
KELPMLTYRVDADKGFNFSVGDDAFVCQKKNHFQVTVYIGMLGEPKYVKTPEGLKPLDCF
YLKLHGVKLEALNQSINIEQSQSDRSKRPFNPVTVNLPPEQVTKVTVGRLHFSETTANNM
RKKGKPNPDQRYFMLVVALQAHAQNQNYTLAAQISERIIVRASNPGQFESDSDVLWQRAQ
VPDTVFHHGRVGINTDRPDEALVVHGNVKVMGSLMHPSDLRAKEHVQEVDTTEQLKRISR
MRLVHYRYKPEFAASAGIEATAPETGVIAQEVKEILPEAVKDTGDMVFANGKTIENFLVV
NKERIFMENVGAVKELCKLTDNLETRIDELERWSHKLAKLRRLDSLKSTGSSGAFSHAGS
QFSRAGSVPHKKRPPKVASKSSSVVPDQACISQRFLQGTIIALVVVMAFSVVSMSTLYVL

SLRTEEDLVDTDGRSSQSFGTTQLRQSPLTTGLPGIQPSLLLVTTSLTSSAPGSAVRTLD
MCSSHPCPVICCSSPTTNPTTGPSLGPSFNPGHVLSPSPSPSTNRSGPSQMALLPVTNIR
AKSWGLSVNGIGHSKHHKSLEPLASPAVPFPGGQGKAKNSPSLGFHGRARRGALQSSVGP
AEPTWAQGQSEPVPSLTSIQVLENSMSITSQYCAPGDACRPGNFTYHIPVSSGTPLHLSL
TLQMNSSSPVSVVLCSLRSKEEPCEEGSLPQSLHTHQDTQGTSHRWPITILSFREFTYHF
RVALLGQANCSSEALAQPATDYHFHFYRLCD*
>ENSG00000215421|ENST00000309902
MMDSENKPENDEDEKINKEAQDLTKLSSHNEDGGPVSDVIASFPENSMGKRGFSESSNSD
SVVIGEDRNKHASKRRKLDEAEPLKSGKQGICRLETSESSVTEGGIALDETGKETFLSDC
TVGGTCLPNALSPSCNFSTIDVVSLKTDTEKTSAQEMVSLDLERESPFPPKEISVSCTIG
NVDTVLKCSICGHLFSSCSDLEKHAESHMQQPKEHTCCHCSHKAESSSALHMHIKQAHGP
QKVFSCDLCGFQCSEENLLNAHYLGKTHLRRQNLAARGGFVQILTKQPFPKKSRTMATKN
VHSKPRTSKSIAKNSDSKGLRNVGSTFKDFRGSISKQSGSSSELLVEMMPSRNTLSQEVE
IVEEHVTSLGLAQNPENQSRKLDTLVTSEGLLEKLESTKNTLQAAHGNSVTSRPRPERNI
LVLGNSFRRRSSTFTLKGGQAKKRFNLLGIKRGTSETQRMYMKHLRTQMKTHDAESVLKHL
EACSSVQRVCVTTSETQEAEQGQGSARPPDSGLHSLTVKPASGSQTLCACTDCGQVATNR
TDLEIHVKRCHAREMKFYCRTCDFSSMSRRDLDEHLHSNQHQQTASVLSCQCCSFISLDE
INLRDHMKEKHNMHFLCTPCNLFFLSEKDVEEHKATEKHINSLVQPKTLQSSNSDLVLQT
LPLSTLESENAKESMDDSGKASQEEPLKSRVSHGNEVRHSSKPQFQCKKCFYKTRSSTVL
TRHIKLRHGQDYHFLCKACNLYSLSKEGMEKHIKRSKHLENAKKNNIGLSFEECIERVCI
GANDKKEEFDVSGNGRIEGHIGVQLQEHSYLEKGMLASEELSQSGGSTKDDELASTTTPK
RGRPKGNISRTCSHCGLLASSITNLTVHIRRKHSHQYSYLCKVCKYYTVTKGDMERHCAT
KKHKGRVEIEASGKHSSDIIVGPEGGSLEAGKKNAGSAVTMSDEHANKPAESPTSVLEKP
DRGNSIEAEVENVFHSLDGEVNSHLLDKKEQISSEPEDFAQPGDVYSQRDVTGTGENKCL
HCEFSAHSSASLELHVKRKHTKEFEFYCMACDYYAVTRREMTRHAATEKHKMKRQSYLNS
ANVEAGSADMSKNIIMPEEEHQQNSEEFQIISGQPSDTLKSRNAADCSILNENTNLDMSK
VLCAADSVEVETEEESNFNEDHSFCETFQQAPVKDKVRKPEEMMSLTMSSNYGSPSRFQN
ENSGSSALNCETAKKNHEISNDAGELRVHCEGEGGNAGDGGGVVPHRHLCPVTLDGERSA
ESPVLVVTRITREQGNLESGGQNRVARGHGLEDLKGVQEDPVLGNKEILMNSQHETEFIL
EEDGPASDSTVESSDVYETIISIDDKGQAMYSFGRFDSSIIRIKNPEDGELIDQSEEGLI
ATGVRISELPLKDCAQGVKKKKSEGSSIGESTRIRCDDCGFLADGLSGLNVHIAMKHPTK
EKHFHCLLCGKSFYTESNLHQHLASAGHMRNEQASVEELPEGGATFKCVKCTEPFDSEQN
LFLHIKGQHEELLREVNKYIVEDTEQINREREENQGNVCKYCGKMCRSSNSMAFLAHIRT
HTGSKPFKCKICHFATAQLGDARNHVKRHLGMREYKCHVCGVAFVMKKHLNTHLLGKHGV
GTPKERVKVAYRKIGTLPGIQNNRNASSASEAQSLCEHFS*
>ENSG00000169800|ENST00000303766
MVEADHPGKLFIGGLNRETNEKMLKAVFGKHGPISEVLLIKDRTSKSRGFAFITFENPAD
AKNAAKDMNGTSLHGKAIKVEQAKKPSFQSGGRRRPPASSRNRSPSGSLRSARGSSGGTR
GWLPSHEGHLDDGGYTPDLKMSYSRGLIPVKRGPSSRSGGPPPKKSAPSAVARSNSWMGS
QGPMSQRRENYGVPPRRATISSWRNDRMSTRHDGYATNDGNHPSCQETRDYAPPSRGYAY
RDNGHSNRDEHSSRGYRNHRSSRETRDYAPPSRGHAYRDYGHSRRDESYSRGYRNHRSSR
ETREYAPPSRGHGYRDYGHSRRHESYSRGYRNHPSSRETRDYAPPHRDYAYRDYGHSSWD
EHSSRGYSYHDGYGEALGRDHSEHLSGSSYRDALQRYGTSHGAPPARGPRMSYGGSTCHA
YSNTRDRYGRSWESYSSCGDFHYCDREHVCRKDQRNPPSLGRVLPDPREAYGSSSYVASI

VDGGESRSEKGDSSRY*
>ENSG00000174953|ENST00000308361
MSYDYHQNWGRDGGPRSSGGGYGGGPAGGHGGNRGSGGGGGGGGGGRGGRGRHPGHLKGR
EIGMWYAKKQGQKNKEAERQERAVVHMDERREEQIVQLLNSVQAKNDKESEAQISWFAPE
DHGYGTEVSTKNTPCSENKLDIQEKKLINQEKKMFRIRNRSYIDRDSEYLLQENEPDGTL
DQKLLEDLQKKKNDLRYIEMQHFREKLPSYGMQKELVNLIDNHQVTVISGETGCGKTTQV
TQFILDNYIERGKGSACRIVCTQPRRISAISVAERVAAERAESCGSGNSTGYQIRLQSRL
PRKQGSILYCTTGIILQWLQSDPYLSSVSHIVLDEIHERNLQSDVLMTVVKDLLNFRSDL
KVILMSATLNAEKFSEYFGNCPMIHIPGFTFPVVEYLLEDVIEKIRYVPEQKEHRSQFKR
GFMQGHVNRQEKEEKEAIYKERWPDYVRELRRRYSASTVDVIEMMEDDKVDLNLIVALIR
YIVLEEEDGAILVFLPGWDNISTLHDLLMSQVMFKSDKFLIIPLHSLMPTVNQTQVFKRT
PPGVRKIVIATNIAETSITIDDVVYVIDGGKIKETHFDTQNNISTMSAEWVSKANAKQRK
GRAGRVQPGHCYHLYNGLRASLLDDYQLPEILRTPLEELCLQIKILRLGGIAYFLSRLMD
PPSNEAVLLSIRHLMELNALDKQEELTPLGVHLARLPVEPHIGKMILFGALFCCLDPVLT
IAASLSFKDPFVIPLGWEEARRRGFRYEKDYCWEYFLSSNTLQMLHNMKGQFAEHLLGAG
FVSSRNPKDPESNINSDNEKIIKAVICAGLYPKVAKIRLNLGKKRKMVKVYTKTDGLVAV
HPKSVNVEQTDFHYNWLIYHLKMRTSSIYLYDCTEVSPYCLLFFGGDISIQKDNDQETIA
VDEWIVFQSPARIAHLVKELRKELDILLQEKIESPHPVDWNDTKSRDCAVLSAIIDLIKT
QEKATPRNFPPRFQDGYYS*
>ENSG00000168348|ENST00000307169
MPRGFLVKRTKRTGGLYRVRLAERVFPLLGPQGAPPFLEEAPSASLPGAERATPPTREEP
GKGLTAEAAREQSGSPCRAAGVSPGTGGREGAEWRAGGREGPGPSPSPSPSPAKPAGAEL
RRAFLERCLSSPVSAESFPGGAAAVAAFSCSVAPAAAPTPGEQFLLPLRAPFPEPALQPD
PAPLSAALQSLKRAAGGERRGKAPTDCASGPAAAGIKKPKAMRKLSFADEVTTSPVLGLK
IKEEEPGAPSRGLGGSRTPLGEFICQLCKEQYADPFALAQHRCSRIVRVEYRCPECDKVF
SCPANLASHRRWHKPRPAAANAATVSSADGKPPSSSSSSSRDSGAIASFLAEGKENSRIE
RTADQHPQARDSSGADQHPDSAPRQGLQVLTHPEPPLPQGPYTEGVLGRRVPVPGSTSGG
RGSEIFVCPYCHKKFRRQAYLRKHLSTHEAGSARALAPGFGSERGAPLAFACPLCGAHFP
TADIREKHRLWHAVREELLLPALAGAPPETSGPSGPSDGSAQQIFSCKHCPSTFFSSPGL
TRHINKCHPSESRQVLLLQMPLRPGC*
>ENSG00000114026|ENST00000302036
MPARALLPRRMGHRTLASTPALWASIPCPRSELRLDLVLPSGQSFRWREQSPAHWSGVLA
DQVWTLTQTEEQLHCTVYRGDKSQASRPTPDELEAVRKYFQLDVTLAQLYHHWGSVDSHF
QEVAQKFQGVRLLRQDPIECLFSFICSSNNNIARITGMVERLCQAFGPRLIQLDDVTYHG
FPSLQALAGPEVEAHLRKLGLGYRARYVSASARAILEEQGGLAWLQQLRESSYEEAHKAL
CILPGVGTKVADCICLMALDKPQAVPVDVHMWHIAQRDYSWHPTTSQAKGPSPQTNKELG
NFFRSLWGPYAGWAQAGLLGNAFDGHQLLRPLIFCQDHLREGPPIGRGDSQGEELEPQLP
SSLSSIPYGFCDHCWTKDVDDPPLVTHPSPGSRDGHMTQAWPVKVVSPLATVIGHVMQAS
LLAL*
>ENSG00000143442|ENST00000271715
MADTDLFMECEEEELEPWQKISDVIEDSVVEDYNSVDKTTTVSVSQQPVSAPVPIAAHAS
VAGHLSTSTTVSSSGAQNSDSTKKTLVTLIANNNAGNPLVQQGGQPLILTQNPAPGLGTM
VTQPVLRPVQVMQNANHVTSSPVASQPIFITTQGFPVRNVRPVQNAMNQVGIVLNVQQGQ
TVRPITLVPAPGTQFVKPTVGVPQVFSQMTPVRPGSTMPVRPTTNTFTTVIPATLTIRST

VPQSQSQQTKSTPSTSTTPTATQPTSLGQLAVQSPGQSNQTTNPKLAPSFPSPPAVSIAS
FVTVKRPGVTGENSNEVAKLVNTLNTIPSLGQSPGPVVVSNNSSAHGSQRTSGPESSMKV
TSSIPVFDLQDGGRKICPRCNAQFRVTEALRGHMCYCCPEMVEYQKKGKSLDSEPSVPSA
AKPPSPEKTAPVASTPSSTPIPALSPPTKVPEPNENVGDAVQTKLIMLVDDFYYGRDGGK
VAQLTNFPKVATSFRCPHCTKRLKNNIRFMNHMKHHVELDQQNGEVDGHTICQHCYRQFS
TPFQLQCHLENVHSPYESTTKCKICEWAFESEPLFLQHMKDTHKPGEMPYVCQVCQYRSS
LYSEVDVHFRMIHEDTRHLLCPYCLKVFKNGNAFQQHYMRHQKRNVYHCNKCRLQFLFAK
DKIEHKLQHHKTFRKPKQLEGLKPGTKVTIRASRGQPRTVPVSSNDTPPSALQEAAPLTS
SMDPLPVFLYPPVQRSIQKRAVRKMSVMGRQTCLECSFEIPDFPNHFPTYVHCSLCRYST
CCSRAYANHMINNHVPRKSPKYLALFKNSVSGIKLACTSCTFVTSVGDAMAKHLVFNPSH
RSSSILPRGLTWIAHSRHGQTRDRVHDRNVKNMYPPPSFPTNKAATVKSAGATPAEPEEL
LTPLAPALPSPASTATPPPTPTHPQALALPPLATEGAECLNVDDQDEGSPVTQEPELASG
GGGSGGVGKKEQLSVKKLRVVLFALCCNTEQAAEHFRNPQRRIRRWLRRFQASQGENLEG
KYLSFEAEEKLAEWVLTQREQQLPVNEETLFQKATKIGRSLEGGFKISYEWAVRFMLRHH
LTPHARRAVAHTLPKDVAENAGLFIDFVQRQIHNQDLPLSMIVAIDEISLFLDTEVLSSD
DRKENALQTVGTGEPWCDVVLAILADGTVLPTLVFYRGQMDQPANMPDSILLEAKESGYS
DDEIMELWSTRVWQKHTACQRSKGMLVMDCHRTHLSEEVLAMLSASSTLPAVVPAGCSSK
IQPLDVCIKRTVKNFLHKKWKEQAREMADTACDSDVLLQLVLVWLGEVLGVIGDCPELVQ
RSFLVASVLPGPDGNINSPTRNADMQEELIASLEEQLKLSGEHSESSTPRPRSSPEETIE
PESLHQLFEGESETESFYGFEEADLDLMEI*
>ENSG00000141956|ENST00000269844
MPRRRPPASGAAQFPERIATRSPDPIPLCTFQRQPRAAPVQPPCRLFFVTFAGCGHRWRS
ESKPGWISRSRSGIALRAARPPGSSPPRPAAPRPPPPGGVVAEAPGDVVIPRPRVQPMRV
ARGGPWTPNPAFREAESWSQIGNQRVSEQLLETSLGNEVSDTEPLSPASAGLRRNPALPP
GPFAQNFSWGNQENLPPALGKIANGGGTGAGKAECGYETESHLLEPHEIPLNVNTHKFSD
CEFPYEFCTVCFSPFKLLGMSGVEGVWNQHSRSASMHTFLNHSATGIREAGCRKDMPVSE
MAEDGSEEIMFIWCEDCSQYHDSECPELGPVVMVKDSFVLSRARSWPASGHVHTQAGQGM
RGYEDRDRADPQQLPEAVPAGLVRRLSGQQLPCRSTLTWGRLCHLVAQGRSSLPPNLEIR
RLEDGAEGVFAITQLVKRTQFGPFESRRVAKWEKESAFPLKVFQKDGHPVCFDTSNEDDC
NWMMLVRPAAEAEHQNLTAYQHGSDVYFTTSRDIPPGTELRVWYAAFYAKKMDKPMLKQA
GSGVHAAGTPENSAPVESEPSQWACKVCSATFLELQLLNEHLLGHLEQAKSLPPGSQSEA
AAPEKEQDTPRGEPPAVPESENVATKEQKKKPRRGRKPKVSKAEQPLVIVEDKEPTEQVA
EIITEVPPDEPVSATPDERIMELVLGKLATTTTDTSSVPKFTHHQNNTITLKRSLILSSR
HGIRRKLIKQLGEHKRVYQCNICSKIFQNSSNLSRHVRSHGDKLFKCEECAKLFSRKESL
KQHVSYKHSRNEVDGEYRYRCGTCEKTFRIESALEFHNCRTDDKTFQCEMCFRFFSTNSN
LSKHKKKHGDKKFACEVCSKMFYRKDVMLDHQRRHLEGVRRVKREDLEAGGENLVRYKKE
PSGCPVCGKVFSCRSNMNKHLLTHGDKKYTCEICGRKFFRVDVLRDHIHVHFKDIALMDD
HQREEFIGKIGISSEENDDNSDESADSEPHKYSCKRCQLTFGRGKEYLKHIMEVHKEKGY
GCSICNRRFALKATYHAHMVIHRENLPDPNVQKYIHPCEICGRIFNSIGNLERHKLIHTG
VKSHACEQCGKSFARKDMLKEHMRVHDNVREYLCAECGKGMKTKHALRHHMKLHKGIKEY
ECKECHRRFAQKVNMLKHCKRHTGIKDFMCELCGKTFSERNTMETHKLIHTVGKQWTCSV
CDKKYVTEYMLQKHVQLTHDKVEAQSCQLCGTKVSTRASMSRHMRRKHPEVLAVRIDDLD
HLPETTTIDASSIGIVQPELTLEQEDLAEGKHGKAAKRSHKRKQKPEEEAGAPVPEDATF
SEYSEKETEFTGSVGDETNSAVQSIQQVVVTLGDPNVTTPSSSVGLTNITVTPITTAAAT

QFTNLQPVAVGHLTTPERQLQLDNSILTVTFDTVSGSAMLHNRQNDVQIHPQPEASNPQS
VAHFINLTTLVNSITPLGSQLSDQHPLTWRAVPQTDVLPPSQPQAPPQQAAQPQVQAEQQ
QQQMYSY*
>ENSG00000103932|ENST00000304330
MLSRPKPGESEVDLLHFQSQFLAAGAAPAVQLVKKGNRGGGDANSDRPPLQDHRDVVMLD
NLPDLPPALVPSPPKRARPSPGHCLPEDEDPEERLRRHDQHITAVLTKIIERDTSSVAVN
LPVPSGVAFPAVFLRSRDTQGKSATSGKRSIFAQEIAARRIAEAKGPSVGEVVPNVGPPE
GAVTCETPTPRNQGCQLPGSSHSFQGPNLVTGKGLRDQEAEQEAQTIHEENIARLQAMAP
EEILQEQQRLLAQLDPSLVAFLRSHSHTQEQTGETASEEQRPGGPSANVTKEEPLMSAFA
SEPRKRDKLEPEAPALALPVTPQKEWLHMDTVELEKLHWTQDLPPVRRQQTQERMQARFS
LQGELLAPDVDLPTHLGLHHHGEEAERAGYSLQELFHLTRSQVSQQRALALHVLAQVISR
AQAGEFGDRLAGSVLSLLLDAGFLFLLRFSLDDRVDGVIATAIRALRALLVAPGDEELLD
STFSWYHGALTFPLMPSQEDKEDEDEDEECPAGKAKRKSPEEESRPPPDLARHDVIKGLL
ATSLLPRLRYVLEVTYPGPAVVLDILAVLIRLARHSLESATRVLECPRLIETIVREFLPT
SWSPVGAGPTPSLYKVPCATAMKLLRVLASAGRNIAARLLSSFDLRSRLCRIIAEAPQEL
ALPPEEAEMLSTEALRLWAVAASYGQGGYLYRELYPVLMRALQVVPRELSTHPPQPLSMQ
RIASLLTLLTQLTLAAGSTPAETISDSAEASLSATPSLVTWTQVSGLQPLVEPCLRQTLK
LLSRPEMWRAVGPVPVACLLFLGAYYQAWSQQPSSCPEDWLQDMQRLSEELLLPLLSQPT
LGSLWDSLRHCSLLCNPLSCVPALEAPPSLVSLGCSGGCPRLSLAGSASPFPFLTALLSL
LNTLAQIHKGLCGQLAAILAAPGLQNYFLQCVAPGAAPHLTPFSAWALRHEYHLQYLALA
LAQKAAALQPLPATHAALYHGMALALLSRLLPGSEYLTHELLLSCVFRLEFLPERTSGGP
EAADFSDQLSLGSSRVPRCGQGTLLAQACQDLPSIRNCYLTHCSPARASLLASQALHRGE
LQRVPTLLLPMPTEPLLPTDWPFLPLIRLYHRASDTPSGLSPTDTMGTAMRVLQWVLVLE
SWRPQALWAVPPAARLARLMCVFLVDSELFRESPVQHLVAALLAQLCQPQVLPNLNLDCR
LPGLTSFPDLYANFLDHFEAVSFGDHLFGALVLLPLQRRFSVTLRLALFGEHVGALRALS
LPLTQLPVSLECYTVPPEDNLALLQLYFRTLVTGALRPRWCPVLYAVAVAHVNSFIFSQD
PQSSDEVKAARRSMLQKTWLLADEGLRQHLLHYKLPNSTLPEGFELYSQLPPLRQHYLQR
LTSTVLQNGVSET*
>ENSG00000139687|ENST00000267163
MPPKTPRKTAATAAAAAAEPPAPPPPPPPEEDPEQDSGPEDLPLVRLEFEETEEPDFTAL
CQKLKIPDHVRERAWLTWEKVSSVDGVLGGYIQKKKELWGICIFIAAVDLDEMSFTFTEL
QKNIEISVHKFFNLLKEIDTSTKVDNAMSRLLKKYDVLFALFSKLERTCELIYLTQPSSS
ISTEINSALVLKVSWITFLLAKGEVLQMEDDLVISFQLMLCVLDYFIKLSPPMLLKEPYK
TAVIPINGSPRTPRRGQNRSARIAKQLENDTRIIEVLCKEHECNIDEVKNVYFKNFIPFM
NSLGLVTSNGLPEVENLSKRYEEIYLKNKDLDARLFLDHDKTLQTDSIDSFETQRTPRKS
NLDEEVNVIPPHTPVRTVMNTIQQLMMILNSASDQPSENLISYFNNCTVNPKESILKRVK
DIGYIFKEKFAKAVGQGCVEIGSQRYKLGVRLYYRVMESMLKSEEERLSIQNFSKLLNDN
IFHMSLLACALEVVMATYSRSTSQNLDSGTDLSFPWILNVLNLKAFDFYKVIESFIKAEG
NLTREMIKHLERCEHRIMESLAWLSDSPLFDLIKQSKDREGPTDHLESACPLNLPLQNNH
TAADMYLSPVRSPKKKGSTTRVNSTANAETQATSAFQTQKPLKSTSLSLFYKKVYRLAYL
RLNTLCERLLSEHPELEHIIWTLFQHTLQNEYELMRDRHLDQIMMCSMYGICKVKNIDLK
FKIIVTAYKDLPHAVQETFKRVLIKEEEYDSIIVFYNSVFMQRLKTNILQYASTRPPTLS
PIPHIPRSPYKFPSSPLRIPGGNIYISPLKSPYKISEGLPTPTKMTPRSRILVSIGESFG
TSEKFQKINQMVCNSDRVLKRSAEGSNPPKPLKKLRFDIEGSDEADGSKHLPGESKFQQK

LAEMTSTRTRMQKQKMNDSMDTSNKEEK*
>ENSG00000174586|ENST00000311044
MESPRGWTLQVAPEEGQVLCNVKTATRGLSEGAVSGGWGAWENSTEVPREAGDGQRQQAT
LGAADEQGGPGRELGPADGGRDGAGPRSEPADRALRPSPLPEEPGCRCGECGKAFSQGSY
LLQHRRVHTGEKPYTCPECGKAFAWSSNLSQHQRIHSGEKPYACRECGKAFRAHSQLIHH
QETHSGLKPFRCPDCGKSFGRSTTLVQHRRTHTGEKPYECPECGKAFSWNSNFLEHRRVH
TGARPHACRDCGKAFSQSSNLAEHLKIHAGARPHACPDCGKAFVRVAGLRQHRRTHSSEK
PFPCAECGKAFRESSQLLQHQRTHTGERPFECAECGQAFVMGSYLAEHRRVHTGEKPHAC
AQCGKAFSQRSNLLSHRRTHSGAKPFACADCGKAFRGSSGLAHHRLSHTGERPFACAECG
KAFRGSSELRQHQRLHSGERPFVCAHCSKAFVRKSELLSHRRTHTGERPYACGECGKPFS
HRCNLNEHQKRHGGRAAP*
>ENSG00000154767|ENST00000285021
MARKRAAGGEPRGRELRSQKSKAKSKARREEEEEDAFEDEKPPKKSLLSKVSQGKRKRGC
SHPGGSADGPAKKKVAKVTVKSENLKVIKDEALSDGDDLRDFPSDLKKAHHLKRGATMNE
DSNEEEEESENDWEEVEELSEPVLGDVRESTAFSRSLLPVKPVEIEIETPEQAKTRERSE
KIKLEFETYLRRAMKRFNKGVHEDTHKVHLLCLLANGFYRNNICSQPDLHAIGLSIIPAR
FTRVLPRDVDTYYLSNLVKWFIGTFTVNAELSASEQDNLQTTLERRFAIYSARDDEELVH
IFLLILRALQLLTRLVLSLQPIPLKSATAKGKKPSKERLTADPGGSSETSSQVLENHTKP
KTSKGTKQEETFAKGTCRPSAKGKRNKGGRKKRSKPSSSEEDEGPGDKQEKATQRRPHGR
ERRVASRVSYKEESGSDEAGSGSDFELSSGEASDPSDEDSEPGPPKQRKAPAPQRTKAGS
KSASRTHRGSHRKDPSLPAASSSSSSSKRGKKMCSDGEKAEKRSIAGIDQWLEVFCEQEE
KWVCVDCVHGVVGQPLTCYKYATKPMTYVVGIDSDGWVRDVTQRYDPVWMTVTRKCRVDA
EWWAETLRPYQSPFMDREKKEDLEFQAKHMDQPLPTAIGLYKNHPLYALKRHLLKYEAIY
PETAAILGYCRGEAVYSRDCVHTLHSRDTWLKKARVVRLGEVPYKMVKGFSNRARKARLA
EPQLREENDLGLFGYWQTEEYQPPVAVDGKVPRNEFGNVYLFLPSMMPIGCVQLNLPNLH
RVARKLDIDCVQAITGFDFHGGYSHPVTDGYIVCEEFKDVLLTAWENEQAVIERKEKEKK
EKRALGNWKLLAKGLLIRERLKRRYGPKSEAAAPHTDAGGGLSSDEEEGTSSQAEAARIL
AASWPQNREDEEKQKLKGGPKKTKREKKAAASHLFPFEQL*
>ENSG00000083168|ENST00000265713
MVKLANPLYTEWILEAIKKVKKQKQRPSEERICNAVSSSHGLDRKTVLEQLELSVKDGTI
LKVSNKGLNSYKDPDNPGRIALPKPRNHGKLDNKQNVDWNKLIKRAVEGLAESGGSTLKS
IERFLKGQKDVSALFGGSAASGFHQQLRLAIKRAIGHGRLLKDGPLYRLNTKATNVDGKE
SCESLSCLPPVSLLPHEKDKPVAEPIPICSFCLGTKEQNREKKPEELISCADCGNSGHPS
CLKFSPELTVRVKALRWQCIECKTCSSCRDQGKNADNMLFCDSCDRGFHMECCDPPLTRM
PKGMWICQICRPRKKGRKLLQKKAAQIKRRYTNPIGRPKNRLKKQNTVSKGPFSKVRTGP
GRGRKRKITLSSQSASSSSEEGYLERIDGLDFCRDSNVSLKFNKKTKGLIDGLTKFFTPS
PDGRKARGEVVDYSEQYRIRKRGNRKSSTSDWPTDNQDGWDGKQENEERLFGSQEIMTEK
DMELFRDIQEQALQKVGVTGPPDPQVRCPSVIEFGKYEIHTWYSSPYPQEYSRLPKLYLC
EFCLKYMKSRTILQQHMKKCGWFHPPANEIYRKNNISVFEVDGNVSTIYCQNLCLLAKLF
LDHKTLYYDVEPFLFYVLTQNDVKGCHLVGYFSKEKHCQQKYNVSCIMILPQYQRKGYGR
FLIDFSYLLSKREGQAGSPEKPLSDLGRLSYMAYWKSVILECLYHQNDKQISIKKLSKLT
GICPQDITSTLHHLRMLDFRSDQFVIIRREKLIQDHMAKLQLNLRPVDVDPECLRWTPVI
VSNSVVSEEEEEEAEEGENEEPQCQERELEISVGKSVSHENKEQDSYSVESEKKPEVMAP
VSSTRLSKQVLPHDSLPANSQPSRRGRWGRKNRKTQERFGDKDSKLLLEETSSAPQEQYG

ECGEKSEATQEQYTESEEQLVASEEQPSQDGKPDLPKRRLSEGVEPWRGQLKKSPEALKC
RLTEGSERLPRRYSEGDRAVLRGFSESSEEEEEPESPRSSSPPILTKPTLKRKKPFLHRR
RRVRKRKHHNSSVVTETISETTEVLDEPFEDSDSERPMPRLEPTFEIDEEEEEEDENELF
PREYFRRLSSQDVLRCQSSSKRKSKDEEEDEESDDADDTPILKPVSLLRKRDVKNSPLEP
DTSTPLKKKKGWPKGKSRKPIHWKKRPGRKPGFKLSREIMPVSTQACVIEPIVSIPKAGR
KPKIQESEETVEPKEDMPLPEERKEEEEMQAEAEEAEEGEEEDAASSEVPAASPADSSNS
PETETKEPEVEEEEEKPRVSEEQRQSEEEQQELEEPEPEEEEDAAAETAQNDDHDADDED
DGHLESTKKKELEEQPTREDVKEEPGVQESFLDANMQKSREKIKDKEETELDSEEEQPSH
DTSVVSEQMAGSEDDHEEDSHTKEELIELKEEEEIPHSELDLETVQAVQSLTQEESSEHE
GAYQDCEETLAACQTLQSYTQADEDPQMSMVEDCHASEHNSPISSVQSHPSQSVRSVSSP
NVPALESGYTQISPEQGSLSAPSMQNMETSPMMDVPSVSDHSQQVVDSGFSDLGSIESTT
ENYENPSSYDSTMGGSICGNSSSQSSCSYGGLSSSSSLTQSSCVVTQQMASMGSSCSMMQ
QSSVQPAANCSIKSPQSCVVERPPSNQQQQPPPPPPQQPQPPPPQPQPAPQPPPPQQQPQ
QQPQPQPQQPPPPPPPQQQPPLSQCSMNNSFTPAPMIMEIPESGSTGNISIYERIPGDFG
AGSYSQPSATFSLAKLQQLTNTIMDPHAMPYSHSPAVTSYATSVSLSNTGLAQLAPSHPL
AGTPQAQATMTPPPNLASTTMNLTSPLLQCNMSATNIGIPHTQRLQGQMPVKGHISIRSK
SAPLPSAAAHQQQLYGRSPSAVAMQAGPRALAVQRGMNMGVNLMPTPAYNVNSMNMNTLN
AMNSYRMTQPMMNSSYHSNPAYMNQTAQYPMQMQMGMMGSQAYTQQPMQPNPHGNMMYTG
PSHHSYMNAAGVPKQSLNGPYMRR*
>ENSG00000170515|ENST00000303305
MSGEDEQQEQTIAEDLVVTKYKMGGDIANRVLRSLVEASSSGVSVLSLCEKGDAMIMEET
GKIFKKEKEMKKGIAFPTSISVNNCVCHFSPLKSDQDYILKEGDLVKIDLGVHVDGFIAN
VAHTFVVDVAQGTQVTGRKADVIKAAHLCAEAALRLVKPGNQNTQVTEAWNKVAHSFNCT
PIEGMLSHQLKQHVIDGEKTIIQNPTDQQKKDHEKAEFEVHEVYAVDVLVSSGEGKAKDA
GQRTTIYKRDPSKQYGLKMKTSRAFFSEVERRFDAMPFTLRAFEDEKKARMGVVECAKHE
LLQPFNVLYEKEGEFVAQFKFTVLLMPNGPMRITSGPFEPDLYKSEMEVQDAELKALLQS
SASRKTQKKKKKKASKTAENATSGETLEENEAGD*
>ENSG00000089225|ENST00000310346
MADADEGFGLAHTPLEPDAKDLPCDSKPESALGAPSKSPSSPQAAFTQQGMEGIKVFLHE
RELWLKFHEVGTEMIITKAGRRMFPSYKVKVTGLNPKTKYILLMDIVPADDHRYKFADNK
WSVTGKAEPAMPGRLYVHPDSPATGAHWMRQLVSFQKLKLTNNHLDPFGHIILNSMHKYQ
PRLHIVKADENNGFGSKNTAFCTHVFPETAFIAVTSYQNHKITQLKIENNPFAKGFRGSD
DMELHRMSRMQSKEYPVVPRSTVRQKVASNHSPFSSESRALSTSSNLGSQYQCENGVSGP
SQDLLPPPNPYPLPQEHSQIYHCTKRKEEECSTTDHPYKKPYMETSPSEEDSFYRSSYPQ
QQGLGASYRTESAQRQACMYASSAPPSEPVPSLEDISCNTWPSMPSYSSCTVTTVQPMDR
LPYQHFSAHFTSGPLVPRLAGMANHGSPQLGEGMFQHQTSVAHQPVVRQCGPQTGLQSPG
TLQPPEFLYSHGVPRTLSPHQYHSVHGVGMVPEWSDNS*
>ENSG00000149218|ENST00000278505
MGTARWLALGSLFALAGLLEGRLVGEEEAGFGECDKFFYAGTPPAGLAADSHVKICQRAE
GAERFATLYSTRDRIPVYSAFRAPRPAPGGAEQRWLVEPQIDDPNSNLEEAINEAEAITS
VNSLGSKQALNTDYLDSDYQRGQLYPFSLSSDVQVATFTLTNSAPMTQSFQERWYVNLHS
LMDRALTPQCGSGEDLYILTGTVPSDYRVKDKVAVPEFVWLAACCAVPGGGWAMGFVKHT
RDSDIIEDVMVKDLQKLLPFNPQLFQNNCGETEQDTEKMKKILEVVNQIQDEERMVQSQK
SSSPLSSTRSKRSTLLPPEASEGSSSFLGKLMGFIATPFIKLFQLIYYLVVAILKNIVYF

LWCVTKQVINGIESCLYRLGSATISYFMAIGEELVSIPWKVLKVVAKVIRALLRILCCLL
KAICRVLSIPVRVLVDVATFPVYTMGAIPIVCKDIALGLGGTVSLLFDTAFGTLGGLFQV
VFSVCKRIGYKVTFDNSGEL*
>ENSG00000162992|ENST00000295108
MTKSYSESGLMGEPQPQGPPSWTDECLSSQDEEHEADKKEDDLETMNAEEDSLRNGGEEE
DEDEDLEEEEEEEEEEDDDQKPKRRGPKKKKMTKARLERFKLRRMKANARERNRMHGLNAA
LDNLRKVVPCYSKTQKLSKIETLRLAKNYIWALSEILRSGKSPDLVSFVQTLCKGLSQPT
TNLVAGCLQLNPRTFLPEQNQDMPPHLPTASASFPVHPYSYQSPGLPSPPYGTMDSSHVF
HVKPPPHAYSAALEPFFESPLTDCTSPSFDGPLSPPLSINGNFSFKHEPSAEFEKNYAFT
MHYPAATLAGAQSHGSIFSGTAAPRCEIPIDNIMSFDSHSHHERVMSAQLNAIFHD*
>ENSG00000158941|ENST00000308511
MSQFKRQRINPLPGGRNFSGTASTSLLGPPPGLLTPPVATELSQNARHLQGGEKQRVFTG
IVTSLHDYFGVVDEEVFFQLSVVKGRLPQLGEKVLVKAAYNPGQAVPWNAVKVQTLSNQP
LLKSPAPPLLHVAALGQKQGILGAQPQLIFQPHRIPPLFPQKPLSLFQTSHTLHLSHLNR
FPARGPHGRLDQGRSDDYDSKKRKQRAGGEPWGAKKPRHDLPPYRVHLTPYTVDSPICDF
LELQRRYRSLLVPSDFLSVHLSWLSAFPLSQPFSLHHPSRIQVSSEKEAAPDAGAEPITA
DSDPAYSSKVLLLSSPGLEELYRCCMLFVDDMAEPRETPEHPLKQIKFLLGRKEEEAVLV
GGEWSPSLDGLDPQADPQVLVRTAIRCAQAQTGIDLSGCTKWWRFAEFQYLQPGPPRRLQ
TVVVYLPDVWTIMPTLEEWEALCQQKAAEAAPPTQEAQGETEPTEQAPDALEQAADTSRR
NAETPEATTQQETDTDLPEAPPPPLEPAVIARPGCVNLSLHGIVEDRRPKERISFEVMVL
AELFLEMLQRDFGYRVYKMLLSLPEKVVSPPEPEKEEAAKEEATKEEEAIKEEVVKEPKD
EAQNEGPATESEAPLKEDGLLPKPLSSGGEEEEKPRGEASEDLCEMALDPELLLLRDDGE
EEFAGAKLEDSEVRSVASNQSEMEFSSLQDMPKELDPSAVLPLDCLLAFVFFDANWCGYL
HRRDLERILLTLGIRLSAEQAKQLVSRVVTQNICQYRSLQYSRQEGLDGGLPEEVLFGNL
DLLPPPGKSTKPGAAPTEHKALVSHNGSLINVGSLLQRAEQQDSGRLYLENKIHTLELKL
EESHNRFSATEVTNKTLAAEMQELRVRLAEAEETARTAERQKSQLQRLLQELRRRLTPLQ
LEIQRVVEKADSWVEKEEPAPSN*
>ENSG00000114127|ENST00000264951
MGVPKFYRWISERYPCLSEVVKEHQIPEFDNLYLDMNGIIHQCSHPNDDDVHFRISDDKI
FTDIFHYLEVLFRIIKPRKVFFMAVDGVAPRAKMNQQRGRRFRSAKEAEDKIKKAIEKGE
TLPTEARFDSNCITPGTEFMARLHEHLKYFVNMKISTDKSWQGVTIYFSGHETPGEGEHK
IMEFIRSEKAKPDHDPNTRHCLYGLDADLIMLGLTSHEAHFSLLREEVRFGGKKTQRVCA
PEETTFHLLHLSLMREYIDYEFSVLKEKITFKYDIERIIDDWILMGFLVGNDFIPHLPHL
HINHDALPLLYGTYVTILPELGGYINESGHLNLPRFEKYLVKLSDFDREHFSEVFVDLKW
FESKVGNKYLNEAAGVAAEEARNYKEKKKLKGQENSLCWTALDKNEGEMITSKDNLEDET
EDDDLFETEFRQYKRTYYMTKMGVDVVSDDFLADQAACYVQAIQWILHYYYHGVQSWSWY
YPYHYAPFLSDIHNISTLKIHFELGKPFKPFEQLLAVLPAASKNLLPACYQHLMTNEDSP
IIEYYPPDFKTDLNGKQQEWEAVVLIPFIDEKRLLEAMETCNHSLKKEERKRNQHSECLM
CWYDRDTEFIYPSPWPEKFPAIERCCTRYKIISLDAWRVDINKNKITRIDQKALYFCGFP
TLKHIRHKFFLKKSGVQVFQQSSRGENMMLEILVDAESDELTVENVASSVLGKSVFVNWP
HLEEARVVAVSDGETKFYLEEPPGTQKLYSGRTAPPSKVVHLGDKEQSNWAKEVQGISEH
YLRRKGIIINETSAVVYAQLLTGRKYQINQNGEVRLEKQWSKQVVPFVYQTIVKDIRAFD
SRFSNIKTLDDLFPLRSMVFMLGTPYYGCTGEVQDSGDVITEGRIRVIFSIPCEPNLDAL
IQNQHKYSIKYNPGYVLASRLGVSGYLVSRFTGSIFIGRGSRRNPHGDHKANVGLNLKFN

KKNEEVPGYTKKVGSEWMYSSAAEQLLAEYLERAPELFSYIAKNSQEDVFYEDDIWPGEN
ENGAEKVQEIITWLKGHPVSTLSRSSCDLQILDAAIVEKIEEEVEKCKQRKNNKKVRVTV
KPHLLYRPLEQQHGVIPDRDAEFCLFDRVVNVRENFSVPVGLRGTIIGIKGANREADVLF
EVLFDEEFPGGLTIRCSPGRGYRLPTSALVNLSHGSRSETGNQKLTAIVKPQPAVHQHSS
SSSVSSGHLGALNHSPQSLFVPTQVPTKDDDEFCNIWQSLQGSGKMQYFQPTIQEKGAVL
PQEISQVNQHHKSGFNDNSVKYQQRKHDPHRKFKEECKSPKAECWSQKMSNKQPNSGIEN
FLASLNISKENEVQSSHHGEPPSEEHLSPQSFAMGTRMLKEILKIDGSNTVDHKNEIKQI
ANEIPVSSNRRDEYGLPSQPKQNKKLASYMNKPHSANEYHNVQSMDNMCWPAPSQIPPVS
TPVTELSRICSLVGMPQPDFSFLRMPQTMTVCQVKLSNGLLVHGPQCHSENEAKEKAALF
ALQQLGSLGMNFPLPSQVFANYPSAVPPGTIPPAFPPPTGWDHYGSNYALGAANIMPSSS
HLFGSMPWGPSVPVPGKPFHHTLYSGTMPMAGGIPGGVHNQFIPLQVTKKRVANKKNFEN
KEAQSSQATPVQTSQPDSSNIVKVSPRESSSASLKSSPIAQPASSFQVETASQGHSISHH
KSTPISSSRRKSRKLAVNFGVSKPSE*
>ENSG00000137693|ENST00000282441
MDPGQQPPPQPAPQGQGQPPSQPPQGQGPPSGPGQPAPAATQAAPQAPPAGHQIVHVRGD
SETDLEALFNAVMNPKTANVPQTVPMRLRKLPDSFFKPPEPKSHSRQASTDAGTAGALTP
QHVRAHSSPASLQLGAVSPGTLTPTGVVSGPAATPTAQHLRQSSFEIPDDVPLPAGWEMA
KTSSGQRYFLNHIDQTTTWQDPRKAMLSQMNVTAPTSPPVQQNMMNSASGPLPDGWEQAM
TQDGEIYYINHKNKTTSWLDPRLDPRFAMNQRISQSAPVKQPPPLAPQSPQGGVMGGSNS
NQQQQMRLQQLQMEKERLRLKQQELLRQAMRNINPSTANSPKCQELALRSQLPTLEQDGG
TQNPVSSPGMSQELRTMTTNSSDPFLNSGTYHSRDESTDSGLSMSSYSVPRTPDDFLNSV
DEMDTGDTINQSTLPSQQNRFPDYLEAIPGTNVDLGTLEGDGMNIEGEELMPSLQEALSS
DILNDMESVLAATKLDKESFLTWL*
>ENSG00000144026|ENST00000295208
MTFEDVAVEFSQWEWGQLNPAQKDLYREVMLENFRNLAILGLLVSKPYVICQLEEGGEPF
MVEREISTGAHSDWKRRSKSKESMPSWGISKEELFQVVSVEKHIQDVLQFSKLKAACGCD
GQLEMQQIKQERHLKQMSTIHKSATTLSRDYKWNGFGRSLGLRSVLVNQHSILMGEGSYK
CDTEFRQTLGGNNSQRTHPEKKSCKCNECGKSFHFQSELRRHQRCHTGEKPYECSDCGRA
FGHISSLIKHQRTHTGEKPYECSECGRAFSQSSSLVLHYRFHTGEKPYKCNECGRAFGHT
SSLIKHQRTHTGEKPYECRECGRTFSQSSSLIVHYRFHTGEKPYKCNKCGRAFSQSSSLT
QHYRFHTGEKPYKCNECGRAFAHTASLIKHQRSHAGKKTL*
>ENSG00000100294|ENST00000290429
MSVRVARVAWVRGLGASYRRGASSFPVPPPGAQGVAELLRDATGAEEEAPWAATERRMPG
QCSVLLFPGQGSQVVGMGRGLLNYPRVRELYAAARRVLGYDLLELSLHGPQETLDRTVHC
QPAIFVASLAAVEKLHHLQPSVIENCVAAAGFSVGEFAALVFAGAMEFAEGLYAVKIRAE
AMQEASEAVPSGMLSVLGQPQSKFNFACLEAREHCKSLGIENPVCEVSNYLFPDCRVISG
HQEALRFLQKNSSKFHFRRTRMLPVSGAFHTRLMEPAVEPLTQALKAVDIKKPLVSVYSN
VHAHRYRHPGHIHKLLAQQLVSPVKWEQTMHAIYERKKGRGFPQTFEVGPGRQLGAILKS
CNMQAWKSYSAVDVLQTLEHVDLDPQEPPR*
>ENSG00000169057|ENST00000303391
MVAGMLGLREEKSEDQDLQGLKDKPLKFKKVKKDKKEEKEGKHEPVQPSAHHSAEPAEAG
KAETSEGSGSAPAVPEASASPKQRRSIIRDRGPMYDDPTLPEGWTRKLKQRKSGRSAGKY
DVYLINPQGKAFRSKVELIAYFEKVGDTSLDPNDFDFTVTGRGSPSRREQKPPKKPKSPK
APGTGRGRGRPKGSGTTRPKAATSEGVQVKRVLEKSPGKLLVKMPFQTSPGGKAEGGGAT

TSTQVMVIKRPGRKRKAEADPQAIPKKRGRKPGSVVAAAAAEAKKKAVKESSIRSVQETV
LPIKKRKTRETVSIEVKEVVKPLLVSTLGEKSGKGLKTCKSPGRKSKESSPKGRSSSASS
PPKKEHHHHHHHSESPKAPVPLLPPLPPPPPEPESSEDPTSPPEPQDLSSSVCKEEKMPR
GGSLESDGCPKEPAKTQPAVATAATAAEKYKHRGEGERKDIVSSSMPRPNREEPVDSRTP
VTERVS*
>ENSG00000175595|ENST00000311895
MESGQPARRIAMAPLLEYERQLVLELLDTDGLVVCARGLGADRLLYHFLQLHCHPACLVL
VLNTQPAEEEYFINQLKIEGVEHLPRRVTNEITSNSRYEVYTQGGVIFATSRILVVDFLT
DRIPSDLITGILVYRAHRIIESCQEAFILRLFRQKNKRGFIKAFTDNAVAFDTGFCHVER
VMRNLFVRKLYLWPRFHVAVNSFLEQHKPEVVEIHVSMTPTMLAIQTAILDILNACLKEL
KCHNPSLEVEDLSLENAIGKPFDKTIRHYLDPLWHQLGAKTKSLVQDLKILRTLLQYLSQ
YDCVTFLNLLESLRATEKAFGQNSGWLFLDSSTSMFINARARVYHLPDAKMSKKEKISEK
MEIKEGEETKKELVLESNPKWEALTEVLKEIEAENKESEALGGPGQVLICASDDRTCSQL
RDYITLGAEAFLLRLYRKTFEKDSKAEEVWMKFRKEDSSKRIRKSHKRPKDPQNKERAST
KERTLKKKKRKLTLTQMVGKPEELEEEGDVEEGYRREISSSPESCPEEIKHEEFDVNLSS
DAAFGILKEPLTIIHPLLGCSDPYALTRVLHEVEPRYVVLYDAELTFVRQLEIYRASRPG
KPLRVYFLIYGGSTEEQRYLTALRKEKEAFEKLIREKASMVVPEEREGRDETNLDLVRGT
ASADVSTDTRKAGGQEQNGTQQSIVVDMREFRSELPSLIHRRGIDIEPVTLEVGDYILTP
EMCVERKSISDLIGSLNNGRLYSQCISMSRYYKRPVLLIEFDPSKPFSLTSRGALFQEIS
SNDISSKLTLLTLHFPRLRILWCPSPHATAELFEELKQSKPQPDAATALAITADSETLPE
SEKYNPGPQDFLLKMPGVNAKNCRSLMHHVKNIAELAALSQDELTSILGNAANAKQLYDF
IHTSFAEVVSKGKGKK*
>ENSG00000171443|ENST00000301073
MDTPSPDPLPSPLPGEEEKPLALSPPVPRGRRGRRPGGATSSNRTLKASLPRKRGRPPKS
GQEPPLVQVQGVTAPVGSSGGSDLLLIDDQGVPYTVSEGSAAGPEGSGPRKAPHFCPVCL
RAFPYLSDLERHSISHSELKPHQCKVCGKTFKRSSHLRRHCNIHAGLRPFRCPLCPRRFR
EAGELAHHHRVHSGERPYQCPICRLRFTEANTLRRHAKRKHPEAMGVPLCAPDPGSEPPW
DEEGIPATAGAEEEEETEGKGEPA*
>ENSG00000178460|ENST00000313616
MSNLKMKEAALIYLDRSGGLQKFIDDCKYYNDSKQSYAVYRFKILINPSDVVELDAELGN
HILHQPLKAAEVFQSVCFIAVKTLSLIGQLQTETQINIVLKLTHLPPLPSYGLDLCEFPL
DYTSQRFYMMQGIVIAMTTITKYTQGARFLCSDEACPLSKGFQYIRVHVPGATESATIRN
DFLCNLCASSLQEDRKFRVLGDKQIVEIIATKALRAFQGYSNNQPFRFQSLTIFLRDESV
NKMNIGNEYKIIGIPTCVKTSQTAVCIEANSITFCNSKVPSGISDNFRCLLSLTSSSCWK
FTAILANIFASQITPPGTYNLLKLCLLMSLVQTTDRNKELEDCLDILIITSDTLLIDRLL
NFSINLVPRGIRHLVSTEIFPTLSRNKYGTGAVSIQAGSALLAKGGICFIGDLASHKKDK
LEQLQTVLESRSITVYIPGKKFGEDIDQQMTFPVQCSFWSFVDVDSSSRRNAQKINTLIG
QMDCSLIPANLVEAFGLLINCNESSPCHPFLPTVQHTLNKAINPEGLFYAASRQFTTEDF
EKLLAFAKNLNVEFSLEAERMTHGYYLASRRIRTGSVCGSKLSASALKYLVFLSEAHARL
NLRNKVLKEDVLIAALLFETSLTLKYGNELNY*
>ENSG00000149657|ENST00000279068
MSGSSGTPYLGSKISLISKAQIRYEGILYTIDTDNSTVALAKVRSFGTEDRPTDRPAPPR
EEIYEYIIFRGSDIKDITVCEPPKAQHTLPQDPAIVQSSLGSASASPFQPHVPYSPFRGM
APYGPLAASSLLSQQYAASLGLGAGFPSIPVGKSPMVEQAVQTGSADNLNAKKLLPGKGT

TGTQLNGRQAQPSSKTASDVVQPAAVQAQGQVNDENRRPQRRRSGNRRTRNRSRGQNRPT
NVKENTIKFEGDFDFESANAQFNREELDKEFKKKLNFKDDKAEKGEEKDLAVVTQSAEAP
AEEDLLGPNCYYDKSKSFFDNISSELKTSSRRTTWAEERKLNTETFGVSGRFLRGRSSRG
GFRGGRGNGTTRRNPTSHRAGTGRV*
>ENSG00000167562|ENST00000301093
MGFLHVGQDGLELPTSGDPPASASQSAGITGVSHRTQPPCFEGLTSKDLVREEKTRKRKR
KAKESGMALLQGLLTFRDVAIEFSQEEWKCLDPAQRTLYRDVMLENYRNLVSLDTSSKCM
MKMFSSTGQGNTEVVHTGTLQIHASHHIGDTCFQEIEKDIHDFVFQWQENETNGHEALMT
KTKKLMSSTERHDQRHAGNKPIKNELGSSFHSHLPEVHIFHPEGKIGNQVEKAINDAFSV
SASQRISCRPKTRISNKYRNNFLQSSLLTQKREVHTREKSFQRNESGKAFNGSSLLKKHQ
IIHLGDKQYKCDVCGKDFHQKRYLACHRCHTGENPYTCNECGKTFSHNSALLVHKAIHTG
EKPYKCNECGKVFNQQSNLARHHRVHTGEKPYKCEECDKVFSRKSHLERHRRIHTGEKPY
KCKVCDKAFRRDSHLAQHTVIHTGEKPYKCNECGKTFVQNSSLVMHKVIHTGEKRYKCNE
CGKVFNHKSNLACHRRLHTGEKPYKCNECGKVFNRKSNLERHHRLHTGKKS*
>ENSG00000141384|ENST00000269142
MPAGLTEPAGAAPPAAVSASGTVTMAPAGALPVRVESTPVALGAVTKAPVSVCVEPTASQ
PLRSPVGTLVTKVAPVSAPPKVSSGPRLPAPQIVAVKAPNTTTIQFPANLQLPPGTVLIK
SNSGPLMLVSPQQTVTRAETTSNITSRPAVPANPQTVKICTVPNSSSQLIKKVAVTPVKK
LAQIGTTVVTTVPKPSSVQSVAVPTSVVTVTPGKPLNTVTTLKPSSLGASSTPSNEPNLK
AENSAAVQINLSPTMLENVKKCKNFLAMLIKLACSGSQSPEMGQNVKKLVEQLLDAKIEA
EEFTRKLYVELKSSPQPHLVPFLKKSVVALRQLLPNSQSFIQQCVQQTSSDMVIATCTTT
VTTSPVVTTTVSSSQSEKSIIVSGATAPRTVSVQTLNPLAGPVGAKAGVVTLHSVGPTAA
TGGTTAGTGLLQTSKPLVTSVANTVTTVSLQPEKPVVSGTAVTLSLPAVTFGETSGAAIC
LPSVKPVVSSAGTTSDKPVIGTPVQIKLAQPGPVLSQPAGIPQAVQVKQLVVQQPSGGNE
KQVTTISHSSTLTIQKCGQKTMPVNTIIPTSQFPPASILKQITLPGNKILSLQASPTQKN
RIKENVTSCFRDEDDINDVTSMAGVNLNEENACILATNSELVGTLIQSCKDEPFLFIGAL
QKRILDIGKKHDITELNSDAVNLISQATQERLRGLLEKLTAIAQHRMTTYKASENYILCS
DTRSQLKFLEKLDQLEKQRKDLEEREMLLKAAKSRSNKEDPEQLRLKQKAKELQQLELAQ
IQHRDANLTALAAIGPRKKRPLESGIEGLKDNLLASGTSSLTATKQLHRPRITRICLRDL
IFCMEQEREMKYSRALYLALLK*
>ENSG00000097007|ENST00000318560
MLEICLKLVGCKSKKGLSSSSSCYLEEALQRPVASDFEPQGLSEAARWNSKENLLAGPSE
NDPNLFVALYDFVASGDNTLSITKGEKLRVLGYNHNGEWCEAQTKNGQGWVPSNYITPVN
SLEKHSWYHGPVSRNAAEYLLSSGINGSFLVRESESSPGQRSISLRYEGRVYHYRINTAS
DGKLYVSSESRFNTLAELVHHHSTVADGLITTLHYPAPKRNKPTVYGVSPNYDKWEMERT
DITMKHKLGGGQYGEVYEGVWKKYSLTVAVKTLKEDTMEVEEFLKEAAVMKEIKHPNLVQ
LLGVCTREPPFYIITEFMTYGNLLDYLRECNRQEVNAVVLLYMATQISSAMEYLEKKNFI
HRDLAARNCLVGENHLVKVADFGLSRLMTGDTYTAHAGAKFPIKWTAPESLAYNKFSIKS
DVWAFGVLLWEIATYGMSPYPGIDLSQVYELLEKDYRMERPEGCPEKVYELMRACWQWNP
SDRPSFAEIHQAFETMFQESSISDEVEKELGKQGVRGAVSTLLQAPELPTKTRTSRRAAE
HRDTTDVPEMPHSKGQGESDPLDHEPAVSPLLPRKERGPPEGGLNEDERLLPKDKKTNLF
SALIKKKKKTAPTPPKRSSSFREMDGQPERRGAGEEEGRDISNGALAFTPLDTADPAKSP
KPSNGAGVPNGALRESGGSGFRSPHLWKKSSTLTSSRLATGEEEGGGSSSKRFLRSCSAS
CVPHGAKDTEWRSVTLPRDLQSTGRQFDSSTFGGHKSEKPALPRKRAGENRSDQVTRGTV

TPPPRLVKKNEEAADEVFKDIMESSPGSSPPNLTPKPLRRQVTVAPASGLPHKEEAGKGS
ALGTPAAAEPVTPTSKAGSGAPGGTSKGPAEESRVRRHKHSSESPGRDKGKLSRLKPAPP
PPPAASAGKAGGKPSQSPSQEAAGEAVLGAKTKATSLVDAVNSDAAKPSQPGEGLKKPVL
PATPKPQSAKPSGTPISPAPVPSTLPSASSALAGDQPSSTAFIPLISTRVSLRKTRQPPE
RIASGAITKGVVLDSTEALCLAISRNSEQMASHSAVLEAGKNLYTFCVSYVDSIQQMRNK
FAFREAINKLENNLRELQICPATAGSGPAATQDFSKLLSSVKEISDIVQR*
>ENSG00000145216|ENST00000358575
MSAGEVERLVSELSGGTGGDEEEEWLYGDENEVERPEEENASANPPSGIEDETAENGVPK
PKVTETEDDSDSDSDDDEDDVHVTIGDIKTGAPQYGSYGTAPVNLNIKTGGRVYGTTGTK
VKGVDLDAPGSINGVPLLEVDLDSFEDKPWRKPGADLSDYFNYGFNEDTWKAYCEKQKRI
RMGLEVIPVTSTTNKITAEDCTMEVTPGAEIQDGRFNLFKVQQGRTGNSEKETALPSTKA
EFTSPPSLFKTGLPPSRNSTSSQSQTSTASRKANSSVGKWQDRYGRAESPDLRRLPGAID
VIGQTITISRVEGRRRANENSNIQVLSERSATEVDNNFSKPPPFFPPGAPPTHLPPPPFL
PPPPTVSTAPPLIPPPGIPITVPPPGFPPPPGAPPPSLIPTIESGHSSGYDSRSARAFPY
GNVAFPHLPGSAPSWPSLVDTSKQWDYYARREKDRDRERDRDRERDRDRDRERERTRERE
RERDHSPTPSVFNSDEERYRYREYAERGYERHRASREKEERHRERRHREKEETRHKSSRS
NSRRRHESEEGDSHRRHKHKKSKRSKEGKEAGSEPAPEQESTEATPAE*
>ENSG00000177551|ENST00000320238
MMLSPDQAADSDHPSSAHSDPESLGGTDTKVLGSVSDLEPVEEAEGDGKGGSRAALYPHP
QQLSREEKRRRRRATAKYRSAHATRERIRVEAFNLAFAELRKLLPTLPPDKKLSKIEILR
LAICYISYLNHVLDV*
>ENSG00000112110|ENST00000367034
MALRSRFWGLFSVCRNPGCRFAALSTSSEPAAKPEVDPVENEAVAPEFTNRNPRNLELLS
VARKERGWRTVFPSREFWHRLRVIRTQHHVEALVEHQNGKVVVSASTREWAIKKHLYSTR
NVVACESIGRVLAQRCLEAGINFMVYQPTPWEAASDSMKRLQSAMTEGGVVLREPQRIYE
*
>ENSG00000197050|ENST00000337995
MARKLVMFRDVAIDFSQEEWECLDSAQRDLYRDVMLENYSNLVSLDLPSRCASKDLSPEK
NTYETELSQWEMSDRLENCDLEESNSRDYLEAKGKMEKQQENQKEYFRQGMIIYDKMSIF
NQHTYLSQHSRCHSTEKPYKCKECGKAFRRASHLTQHQSIHTGEKPYECKQCGKAFSRDS
QLSLHQRLHTGEKPYACKECGKAFTQSSQLILHHRIHTGEKPYKCEECGKAFIRSSQLTR
HQKVHTGEKPYECKECGKAFTQNSQLTLHQRLHTGEKLYECKECRKVFTQLSQLILHKRI
HTGEKPYECKECGKAFICGSQLSQHQKIHNGEKPYECKECGRAFIRGSLLMQHQRIHTGE
KPYKCEECGKAFIRGSQLTQHQRIHTNEKPYECKECGKMFSHGSQLTQHQRIHTGEKPYQ
CKECGKAFNRGSLLTRHQRIHTGEKPYECKECGKTFSRGSELTQHERIHTGEKPYECKEC
GKSFIRGSQLTQHQRIHTGEKPYECKECRMAFTQSSHLSQHQRLHTGEKPYVCNECGKAF
ARGLLLIQHQRIHTGEKPYQCKECGKAFIRGSQLTQHQRIHTGEKPYECKECGKAFSHGS
QLTLHQRIHTGEKPYECRECRKAFTQSSHLSRHQRIHTGEKPYQCKECGKAFTRGSQLTQ
HQRIHISEKSFEYKECGIDFSHGSQVYM*
>ENSG00000198807|ENST00000361487
MEPAFGEVNQLGGVFVNGRPLPNAIRLRIVELAQLGIRPCDISRQLRVSHGCVSKILARY
NETGSILPGAIGGSKPRVTTPTVVKHIRTYKQRDPGIFAWEIRDRLLADGVCDKYNVPSV
SSISRILRNKIGNLAQQGHYDSYKQHQPTPQPALPYNHIYSYPSPITAAAAKVPTPPGVP
AIPGSVAMPRTWPSSHSVTDILGIRSITDQVSDSSPYHSPKVEEWSSLGRNNFPAAAPHA

VNGLEKGALEQEAKYGGQAPNGLPAVGSFVSASSMAPYPTPAQVSPYMTYSAAPSGYVAGH
GWQHAGGTSLSPHNCDIPASLAFKGMQAAREGSHSVTASAL*
>ENSG00000113580|ENST00000343796
MDSKESLTPGREENPSSVLAQERGDVMDFYKTLRGGATVKVSASSPSLAVASQSDSKQRR
LLVDFPKGSVSNAQQPDLSKAVSLSMGLYMGETETKVMGNDLGFPQQGQISLSSGETDLK
LLEESIANLNRSTSVPENPKSSASTAVSAAPTEKEFPKTHSDVSSEQQHLKGQTGTNGGN
VKLYTTDQSTFDILQDLEFSSGSPGKETNESPWRSDLLIDENCLLSPLAGEDDSFLLEGN
SNEDCKPLILPDTKPKIKDNGDLVLSSPSNVTLPQVKTEKEDFIELCTPGVIKQEKLGTV
YCQASFPGANIIGNKMSAISVHGVSTSGGQMYHYDMNTASLSQQQDQKPIFNVIPPIPVG
SENWNRCQGSGDDNLTSLGTLNFPGRTVFSNGYSSPSMRPDVSSPPSSSSTATTGPPPKL
CLVCSDEASGCHYGVLTCGSCKVFFKRAVEGQHNYLCAGRNDCIIDKIRRKNCPACRYRK
CLQAGMNLEARKTKKKIKGIQQATTGVSQETSENPGNKTIVPATLPQLTPTLVSLLEVIE
PEVLYAGYDSSVPDSTWRIMTTLNMLGGRQVIAAVKWAKAIPGFRNLHLDDQMTLLQYSW
MFLMAFALGWRSYRQSSANLLCFAPDLIINEQRMTLPCMYDQCKHMLYVSSELHRLQVSY
EEYLCMKTLLLLSSVPKDGLKSQELFDEIRMTYIKELGKAIVKREGNSSQNWQRFYQLTK
LLDSMHEVVENLLNYCFQTFLDKTMSIEFPEMLAEIITNQIPKYSNGNIKKLLFHQK*
>ENSG00000108604|ENST00000323347
MGRRVGVEVTPRWAPQKCQGARPQRPGMSPGNRMPMAGLQVGPPAGSPFGAAAPLRPGMP
PTMMDPFRKRLLVPQAQPPMPAQRRGLKRRKMADKVLPQRIRELVPESQAYMDLLAFERK
LDQTIARKRMEIQEAIKKPLTQKRKLRIYISNTFSPSKAEGDSAGTAGTPGGTPAGDKVA
SWELRVEGKLLDDPSKQKRKFSSFFKSLVIELDKELYGPDNHLVEWHRMPTTQETDGFQV
KRPGDLNVKCTLLLMLDHQPPQYKLDPRLARLLGVHTQTRAAIMQALWLYIKHNQLQDGH
EREYINCNRYFRQIFSCGRLRFSEIPMKLAGLLQHPDPIVINHVISVDPNDQKKTACYDI
DVEVDDPLKAQMSNFLASTTNQQEIASLDVKIHETIESINQLKTQRDFMLSFSTDPQDFI
QEWLRSQRRDLKIITDVIGNPEEERRAAFYHQPWAQEAVGRHIFAKVQQRRQELEQVLGI
RLT*
>ENSG00000161405|ENST00000351680
MEDIQTNAELKSTQEQSVPAESAAVLNDYSLTKSHEMENVDSGEGPANEDEDIGDDSMKV
KDEYSERDENVLKSEPMGNAEEPEIPYSYSREYNEYENIKLERHVVSFDSSRPTSGKMNC
DVCGLSCISFNVLMVHKRSHTGERPFQCNQCGASFTQKGNLLRHIKLHTGEKPFKCHLCN
YACQRRDALTGHLRTHSASAEARHIKAEMGSERALVLDRLASNVAKRKSSMPQKFIGEKR
HCFDVNYNSSYMYEKESELIQTRMMDQAINNAISYLGAEALRPLVQTPPAPTSEMVPVIS
SMYPIALTRAEMSNGAPQELEKKSIHLPEKSVPSERGLSPNNSGHDSTDTDSNHEERQNH
IYQQNHMVLSRARNGMPLLKEVPRSYELLKPPPICPRDSVKVINKEGEVMDVYRCDHCRV
LFLDYVMFTIHMGCHGFRDPFECNMCGYRSHDRYEFSSHIARGEHRALLK*
>ENSG00000145736|ENST00000330280
MDEEPERTKRWEGGYERTWEILKEDESGSLKATIEDILFKAKRKRVFEHHGQVRLGMMRH
LYVVVDGSRTMEDQDLKPNRLTCTLKLLEYFVEEYFDQNPISQIGIIVTKSKRAEKLTEL
SGNPRKHITSLKKAVDMTCHGEPSLYNSLSIAMQTLKHMPGHTSREVLIIFSSLTTCDPS
NIYDLIKTLKAAKIRVSVIGLSAEVRVCTVLARETGGTYHVILDESHYKELLTHHVSPPP
ASSSSECSLIRMGFPQHTIASLSDQDAKPSFSMAHLDGNTEPGLTLGGYFCPQCRAKYCE
LPVECKICGLTLVSAPHLARSYHHLFPLDAFQEIPLEEYNGERFCYGCQGELKDQHVYVC
AVCQNVFCVDCDVFVHDSLHCCPGCIHKIPAPSGV*
>ENSG00000198026|ENST00000322927

MEENEVESSSDAAPGPGRPEEPSESGLGVGTSEAVSADSSDAAAAPGQAEADDSGVGQSS
DRGSRSQEEVSESSSSADPLPNSYLPDSSSVSHGPVAGVTGGPPALVHSSALPDPNMLVS
DCTASSSDLGSAIDKIIESTIGPDLIQNCITVTSAEDGGAETTRYLILQGPDDGAPMTSP
MSSSTLAHSLAAIEALADGPTSTSTCLEAQGGPSSPVQLPPASGAEEPDLQSLEAMMEVV
VVQQFKCKMCQYRSSTKATLLRHMRERHFRPVAAAAAAAGKKGRLRKWSTSTKSQEEEGP
EEEDDDDIVDAGAIDDLEEDSDYNPAEDEPRGRQLRLQRPTPSTPRPRRRPGRPRKLPRL
EISDLPDGVEGEPLVSSQSGQSPPEPQDPEAPSSSGPGHLVAMGKVSRTPVEAGVSQSDA
ENAAPSCPDEHDTLPRRRGRPSRRFLGKKYRKYYYKSPKPLLRPFLCRICGSRFLSHEDL
RFHVNSHEAGDPQLFKCLQCSYRSRRWSSLKEHMFNHVGSKPYKCDECSYTSVYRKDVIR
HAAVHSRDRKKRPDPTPKLSSFPCPVCGRVYPMQKRLTQHMKTHSTEKPHMCDKCGKSFK
KRYTFKMHLLTHIQAVANRRFKCEFCEFVCEDKKALLNHQLSHVSDKPFKCSFCPYRTFR
EDFLLSHVAVKHTGAKPFACEYCHFSTRHKKNLRLHVRCRHASSFEEWGRRHPEEPPSRR
RPFFSLQQIEELKQQHSAAPGPPPSSPGPPEIPPEATTFQSSEAPSLLCSDTLGGATIIY
QQGAEESTAMATQTALDLLLNMSAQRELGGTALQVAVVKSEDVEAGLASPGGQPSPEGAT
PQVVTLHVAEPGGGAAAESQLGPPDLPQITLAPGPFGGTGYSVITAPPMEEGTSAPGTPY
SEEPAGEAAQAVVVSDTLKEAGTHYIMATDGTQLHHIELTADGSISFPSPDALASGAKWP
LLQCGGLPRDGPEPPSPAKTHCVGDSQSSASSPPATSKALGLAVPPSPPSAATAASKKFS
CKICAEAFPGRAEMESHKRAHAGPGAFKCPDCPFSARQWPEVRAHMAQHSSLRPHQCSQC
SFASKNKKDLRRHMLTHTKEKPFACHLCGQRFNRNGHLKFHIQRLHSPDGRKSGTPTARA
PTQTPTQTIILNSDDETLATLHTALQSSHGVLGPERLQQALSQEHIIVAQEQTVTNQEEA
AYIQEITTADGQTVQHLVTSDNQVQYIISQDGVQHLLPQEYVVVPEGHHIQVQEGQITHI
QYEQGAPFLQESQIQYVPVSPGQQLVTQAQLEAAAHSAVTAVADAAMAQAQGLFGTDETV
PEHIQQLQHQGIEYDVITLADD*
>ENSG00000065427|ENST00000319410
MLTQAAVRLVRGSLRKTSWAEWGHRELRLGQLAPFTAPHKDKSFSDQRSELKRRLKAEKK
VAEKEAKQKELSEKQLSQATAAATNHTTDNGVGPEEESVDPNQYYKIRSQAIHQLKVNGE
DPYPHKFHVDISLTDFIQKYSHLQPGDHLTDITLKVAGRIHAKRASGGKLIFYDLRGEGV
KLQVMANSRNYKSEEEFIHINNKLRRGDIIGVQGNPGKTKKGELSIIPYEITLLSPCLHM
LPHLHFGLKDKETRYRQRYLDLILNDFVRQKFIIRSKIITYIRSFLDELGFLEIETPMMN
IIPGGAVAKPFITYHNELDMNLYMRIAPELYHKMLVVGGIDRVYEIGRQFRNEGIDLTHN
PEFTTCEFYMAYADYHDLMEITEKMVSGMVKHITGSYKVTYHPDGPEGQAYDVDFTPPFR
RINMVEELEKALGMKLPETNLFETEETRKILDDICVAKAVECPPPRTTARLLDKLVGEFL
EVTCINPTFICDHPQIMSPLAKWHRSKEGLTERFELFVMKKEICNAYTELNDPMRQRQLF
EEQAKAKAAGDDEAMFIDENFCTALEYGLPPTAGWGMGIDRVAMFLTDSNNIKEVLLFPA
MKPEDKKENVATTDTLESTTVGTSV*
>ENSG00000183735|ENST00000331710
MQSTSNHLWLLSDILGQGATANVFRGRHKKTGDLFAIKVFNNISFLRPVDVQMREFEVLK
KLNHKNIVKLFAIEEETTTRHKVLIMEFCPCGSLYTVLEEPSNAYGLPESEFLIVLRDVV
GGMNHLRENGIVHRDIKPGNIMRVIGEDGQSVYKLTDFGAARELEDDEQFVSLYGTEEYL
HPDMYERAVLRKDHQKKYGATVDLWSIGVTFYHAATGSLPFRPFEGPRRNKEVMYKIITG
KPSGAISGVQKAENGPIDWSGDMPVSCSLSRGLQVLLTPVLANILEADQEKCWGFDQFFA
ETSDILHRMVIHVFSLQQMTAHKIYIHSYNTATIFHELVYKQTKIISSNQELIYEGRRLV
LEPGRLAQHFPKTTEENPIFVVSREPLNTIGLIYEKISLPKVHPRYDLDGDASMAKAITG
VVCYACRIASTLLLYQELMRKGIRWLIELIKDDYNETVHKKTEVVITLDFCIRNIEKTVK

VYEKLMKINLEAAELGEISDIHTKLLRLSSSQGTIETSLQDIDSRLSPGGSLADAWAHQE
GTHPKDRNVEKLQVLLNCMTEIYYQFKKDKAERRLAYNEEQIHKFDKQKLYYHATKAMTH
FTDECVKKYEAFLNKSEEWIRKMLHLRKQLLSLTNQCFDIEEEVSKYQEYTNELQETLPQ
KMFTASSGIKHTMTPIYPSSNTLVEMTLGMKKLKEEMEGVVKELAENNHILERFGSLTMD
GGLRNVDCL*
>ENSG00000176222|ENST00000324394
FQVPLTFSDVAIDFSQEEWEYLNSDQRDLYRDVMLENYTNLVSLDFNFTTESNKLSSEKR
NYEVNAYHQETWKRNKTFNLMRFIFRTDPQYTIEFGRQQRPKVGCFSQMIFKKHKSLPLH
KRNNTREKSYECKEYKKGFRKYLHLTEHLRDHTGVIPYECNECGKAFVVFQHFIRHRKIH
TDLKPYECNGCEKAFRFYSQLIQHQIIHTGMKPYECKQCGKAFRRHSHLTEHQKIHVGLK
PFECKECGETFRLYRHMCLHQKIHHGVKPYKCKECGKAFGHRSSLYQHKKIHSGEKPYKC
EQCEKAFVRSYLLVEHQRSHTGEKPHECMECGKAFGKGSSLLKHKRIHSSEKLYDCKDCG
KAFCRGSQLTQHQRIHTGEKPHECKECGKTFKLHSYLIQHQIIHTDLKPYECKQCGKAFS
RVGDLKTHQSIHAGEKPYECKECGKTFRLNSQLIYHQTIHTGLKPYVCKECKKAFRSISG
LSQHKRIHTGEKPYECKECDKAFNRSDRLTQHETIHTGVKPQKCKECGKAFSHCYQLSQH
QRFHHGERLLM*
>ENSG00000080503|ENST00000357248
MSTPTDPGAMPHPGPSPGPGPSPGPILGPSPGPGPSPGSVHSMMGPSPGPPSVSHPMPTM
GSTDFPQEGMHQMHKPIDGIHDKGIVEDIHCGSMKGTGMRPPHPGMGPPQSPMDQHSQGY
MSPHPSPLGAPEHVSSPMSGGGPTPPQMPPSQPGALIPGDPQAMSQPNRGPSPFSPVQLH
QLRAQILAYKMLARGQPLPETLQLAVQGKRTLPGLQQQQQQQQQQQQQQQQQQQQQQQPQ
QQPPQPQTQQQQQPALVNYNRPSGPGPELSGPSTPQKLPVPAPGGRPSPAPPAAAQPPAA
AVPGPSVPQPAPGQPSPVLQLQQKQSRISPIQKPQGLDPVEILQEREYRLQARIAHRIQE
LENLPGSLPPDLRTKATVELKALRLLNFQRQLRQEVVACMRRDTTLETALNSKAYKRSKR
QTLREARMTEKLEKQQKIEQERKRRQKHQEYLNSILQHAKDFKEYHRSVAGKIQKLSKAV
ATWHANTEREQKKETERIEKERMRRLMAEDEEGYRKLIDQKKDRRLAYLLQQTDEYVANL
TNLVWEHKQAQAAKEKKKRRRRKKKAEENAEGGESALGPDGEPIDESSQMSDLPVKVTHT
ETGKVLFGPEAPKASQLDAWLEMNPGYEVAPRSDSEESDSDYEEEDEEEESSRQETEEKI
LLDPNSEEVSEKDAKQIIETAKQDVDDEYSMQYSARGSQSYYTVAHAISERVEKQSALLI
NGTLKHYQLQGLEWMVSLYNNNLNGILADEMGLGKTIQTIALITYLMEHKRLNGPYLIIV
PLSTLSNWTYEFDKWAPSVVKISYKGTPAMRRSLVPQLRSGKFNVLLTTYEYIIKDKHIL
AKIRWKYMIVDEGHRMKNHHCKLTQVLNTHYVAPRRILLTGTPLQNKLPELWALLNFLLP
TIFKSCSTFEQWFNAPFAMTGERVDLNEEETILIIRRLHKVLRPFLLRRLKKEVESQLPE
KVEYVIKCDMSALQKILYRHMQAKGILLTDGSEKDKKGKGGAKTLMNTIMQLRKICNHPY
MFQHIEESFAEHLGYSNGVINGAELYRASGKFELLDRILPKLRATNHRVLLFCQMTSLMT
IMEDYFAFRNFLYLRLDGTTKSEDRAALLKKFNEPGSQYFIFLLSTRAGGLGLNLQAADT
VVIFDSDWNPHQDLQAQDRAHRIGQQNEVRVLRLCTVNSVEEKILAAAKYKLNVDQKVIQ
AGMFDQKSSSHERRAFLQAILEHEEENEEEDEVPDDETLNQMIARREEEFDLFMRMDMDR
RREDARNPKRKPRLMEEDELPSWIIKDDAEVERLTCEEEEEKIFGRGSRQRRDVDYSDAL
TEKQWLRAIEDGNLEEMEEEVRLKKRKRRRNVDKDPAKEDVEKAKKRRGRPPAEKLSPNP
PKLTKQMNAIIDTVINYKDSSGRQLSEVFIQLPSRKELPEYYELIRKPVDFKKIKERIRN
HKYRSLGDLEKDVMLLCHNAQTFNLEGSQIYEDSIVLQSVFKSARQKIAKEEESEDESNE
EEEEEDEEESESEAKSVKVKIKLNKKDDKGRDKGKGKKRPNRGKAKPVVSDFDSDEEQDE
REQSEGSGTDDE*

>ENSG00000154556|ENST00000355634
MNTDSGGCARKRAAMSVTLTSVKRVQSSPNLLAAGRDSQSPDSAWRSYNDGNQETLNGDA
TYSSLAAKGFRSVRPNLQDKRSPTQSQITVNGNSGGAVSPMSYYQRPFSPSAYSLPASLN
SSIVMQHGTSLDSTDTYPQHAQSLDGTTSSSIPLYRSSEEEKRVTVIKAPHYPGIGPVDE
SGIPTAIRTTVDRPKDWYKTMFKQIHMVHKPDDDTDMYNTPYTYNAGLYNPPYSAQSHPA
AKTQTYRPLSKSHSDNSPNAFKDASSPVPPPHVPPPVPPLRPRDRSSTEKHDWDPPDRKV
DTRKFRSEPRSIFEYEPGKSSILQHERPASLYQSSIDRSLERPMSSASMASDFRKRRKSE
PAVGPPRGLGDQSASRTSPGRVDLPGSSTTLTKSFTSSSPSSPSRAKGGDDSKICPSLCS
YSGLNGNPSSELDYCSTYRQHLDVPRDSPRAISFKNGWQMARQNAEIWSSTEETVSPKIK
SRSCDDLLNDDCDSFPDPKVKSESMGSLLCEEDSKESCPMAWGSPYVPEVRSNGRSRIRH
RSARNAPGFLKMYKKMHRINRKDLMNSEVICSVKSRILQYESEQQHKDLLRAWSQCSTEE
VPRDMVPTRISEFEKLIQKSKSMPNLGDDMLSPVTLEPPQNGLCPKRRFSIEYLLEEENQ
SGPPARGRRGCQSNALVPIHIEVTSDEQPRAHVEFSDSDQDGVVSDHSDYIHLEGSSFCS
ESDFDHFSFTSSESFYGSSHHHHHHHHHHHRHLISSCKGRCPASYTRFTTMLKHERARHE
NTEEPRRQEMDPGLSKLAFLVSPVPFRRKKNSAPKKQTEKAKCKASVFEALDSALKDICD
QIKAEKKRGSLPDNSILHRLISELLPDVPERNSSLRALRRSPLHQPLHPLPPDGAIHCPP
YQNDCGRMPRSASFQDVDTANSSCHHQDRGGALQDRESPRSYSSTLTDMGRSAPRERRGT
PEKEKLPAKAVYDFKAQTSKELSFKKGDTVYILRKIDQNWYEGEHHGRVGIFPISYVEKL
TPPEKAQPARPPPPAQPGEIGEAIAKYNFNADTNVELSLRKGDRVILLKRVDQNWYEGKI
PGTNRQGIFPVSYVEVVKKNTKGAEDYPDPPIPHSYSSDRIHSLSSNKPQRPVFTHENIQ
GGGEPFQALYNYTPRNEDELELRESDVIDVMEKCDDGWFVGTSRRTKFFGTFPGNYVKRL
*
>ENSG00000100029|ENST00000335214
MGGLEKKKYERGSATNYITRNKARKKLQLSLADFRRLCILKGIYPHEPKHKKKVNKGSTA
ARTFYLIKDIRFLLHEPIVNKFREYKVFVRKLRKAYGKSEWNTVERLKDNKPNYKLDHII
KERYPTFIDALRDLDDALSMCFLFSTFPRTGKCHVQTIQLCRRLTVEFMHYIIAARALRK
VFLSIKGIYYQAEVLGQPIVWITPYAFSHDHPTDVDYRVMATFTEFYTTLLGFVNFRLYQ
LLNLHYPPKLEGQAQAEAKAGEGTYALDSESCMEKLAALSASLARVVVPATEEEAEVDEF
PTDGEEDRRKELEAQEKHKKLFEGLKFFLNREVPREALAFIIRSFGGEVSWDKSLCIGAT
YDVTDSRITHQIVDRPGQQTSVIGRCYVQPQWVFDSVNARLLLPVAEYFSGVQLPPHLSP
FVTEKEGDYVPPEKLKLLALQRGEDPGNLNESEEEEEEDDNNEGDGDEEGENEEEEEDAE
AGSEKEEEARLAALEEQRMEGKKPRVMAGTLKLEDKQRLAQEEESEAKRLAIMMMKKREK
YLYQKIMFGKRRKIREANKLAEKRKAHDEAVRSEKKAKKARPE*
>ENSG00000171163|ENST00000366471
MASSPAVDVSCRRREKRRQLDARRSKCRIRLGGHMEQWCLLKERLGFSLHSQLAKFLLDR
YTSSGCVLCAGPEPLPPKGLQYLVLLSHAHSRECSLVPGLRGPGGQDGGLVWECSAGHTF
SWGPSLSPTPSEAPKPASLPHTTRRSWCSEATSGQELADLESEHDERTQEARLPSSEPDA
PRLLPSPVTCTPKEGETPPAPAALSSPLAVPALSASSLSSRAPPPAEVRVQPQLSRTPQA
AQQTEALASTGSQAQSAPTPAWDEDTAQIGPKRIRKAAKRELMPCDFPGCGRIFSNRQYL
NHHKKYQHIHQKSFSCPEPACGKSFNFKKHLKEHMKLHSDTRDYICEFCARSFRTSSNLV
IHRRIHTGEKPLQCEICGFTCRQKASLNWHQRKHAETVAALRFPCEFCGKRFEKPDSVAA
HRSKSHPALLLAPQESPSGPLEPCPSISAPGPLGSSEGSRPSASPQAPTLLPQQ*
>ENSG00000197124|ENST00000358523
MLENYRNLVSLGLTVSKPELISRLEQRQEPWNVKRHETIAKPPAMSSHYTEDLLPEQCMQ

DSFQKVILRRYGSCGLEDLHLRKDGENVGECKDQKEIYNGLNQCLSTLPSKIFPYNKCVK
VFSKSSNLNRENIRHTTEKLFKCMQCGKVFKSHSGLSYHKIIHTEEKLCICEECGKTFKW
FSYLTKHKRIHTGEKPYKCEECGKAFNWCSSLTKHKRIHTGEKPYKCEECGKAFHWCSPF
VRHKKIHTGEKPYTCEDCGRAFNRHSHLTKHKTIHTGKKPYKCKECGKAFNHCSLLTIHE
RTHTGEKPYKCEECGKAFNSSSILTEHKVIHSGEKPYKCEKCDKVFKRFSYLTKHKRIHT
GEKPYKCEECGKAFNWSSILTEHKRIHTGEKPYNCEECGKAFNRCSHLTRHKKIHTAVKR
YKCEECGKAFKRCSHLNEHKRVQRGEKSCKYKKCGEAFNHCSNLTT*
>ENSG00000100413|ENST00000337566
MFVLVEMVDTVRIPPWQFERKLNDSIAEELNKKLANKVVYNVGLCICLFDITKLEDAYVF
PGDGASHTKVSLGFFDDILIPPESLQQPAKFDEAEQVWVWEYETEEGAHDLYMDTGEEIR
FRVVDESFVDTSPTGPSSADATTSSEELPKKEAPYTLVGSISEPGLGLLSWWTSN*
>ENSG00000197451|ENST00000358344
MSEAGEEQPMETTGATENGHEAVPEGESPAGAGTGAAAGAGGATAAPPSGNQNGAEGDQI
NASKNEEDAGKMFVGGLSWDTSKKDLKDYFTKFGEVVDCTIKMDPNTGRSRGFGFILFKD
AASVEKVLDQKEHRLDGRVIDPKKAMAMKKDPVKKIFVGGLNPEATEEKIREYFGEFGEI
EAIELPMDPKLNKRRGFVFITFKEEEPVKKVLEKKFHTVSGSKCEIKVAQPKEVYQQQQY
GSGGRGNRNRGNRGSGGGGGGGGQSQSWNQGYGNYWNQGYGYQQGYGPGYGGYDYSPYGY
YGYGPGYDYSQGSTNYGKSQRRGGHQNNYKPY*
>ENSG00000171703|ENST00000343484
MMGKEEEIARIARRLDKMVTKKSAEGAMDLLRELKAMPITLHLLQSTRVGMSVNALRKQS
SDEEVIALAKSLIKSWKKLLDASDAKARERGRGMPLPTSSRDASEAPDPSRKRPELPRAP
STPRITTFPPVPVTCDAVRNKCREMLTAALQTDHDHVAIGADCERLSAQIEECIFRDVGN
TDMKYKNRVRSRISNLKDAKNPDLRRNVLCGAITPQQIAVMTSEEMASDELKEIRKAMTK
EAIREHQMARTGGTQTDLFTCGKCRKKNCTYTQVQTRSSDEPMTTFVVCNECGNRWKFC*
>ENSG00000198987|ENST00000362117
Sequence unavailable
>ENSG00000120798|ENST00000330677
MATIEEIAHQIIEQQMGEIVTEQQTGQKIQIVTALDHNTQGKQFILTNHDGSTPSKVILA
RQDSTPGKVFLTTPDAAGVNQLFFTTPDLSAQHLQLLTDNSPDQGPNKVFDLCVVCGDKA
SGRHYGAVTCEGCKGFFKRSIRKNLVYSCRGSKDCIINKHHRNRCQYCRLQRCIAFGMKQ
DSVQCERKPIEVSREKSSNCAASTEKIYIRKDLRSPLTATPTFVTDSESTRSTGLLDSGM
FMNIHPSGVKTESAVLMTSDKAESCQGDLSTLANVVTSLANLGKTKDLSQNSNEMSMIES
LSNDDTSLCEFQEMQTNGDVSRAFDTLAKALNPGESTACQSSVAGMEGSVHLITGDSSIN
YTEKEGPLLSDSHVAFRLTMPSPMPEYLNVHYIGESASRLLFLSMHWALSIPSFQALGQE
NSISLVKAYWNELFTLGLAQCWQVMNVATILATFVNCLHNSLQQDAKVIAALIHFTRRAI
TDL*
>ENSG00000120802|ENST00000343315
MPEFLEDPSVLTKDKLKSELVANNVTLPAGEQRKDVYVQLYLQHLTARNRPPLPAGTNSK
GPPDFSSDEEREPTPVLGSGAAAAGRSRAAVGRKATKKTDKPRQEDKDDLDVTELTNEDL
LDQLVKYGVNPGPIVGTTRKLYEKKLLKLREQGTESRSSTPLPTISSSAENTRQNGSNDS
DRYSDNEEDSKIELKLEKREPLKGRAKTPVTLKQRRVEHNQVETSEHFRIDGPVISESTP
IAETIMASSNESLVVNRVTGNFKHASPILPITEFSDIPRRAPKKPLTRAEVGEKTEERRV
ERDILKEMFPYEASTPTGISASCRRPIKGAAGRPLELSDFRMEESFSSKYVPKYVPLADV
KSEKTKKGRSIPVWIKILLFVVVAVFLFLVYQAMETNQVNPFSNFLHVDPRKSN*

>ENSG00000176974|ENST00000352886
MGLDLPDGGHLTHGFMTDKKKISATSIFFESMPYKVNPDTGYINYDQLEENARLFHPKLI
IAGTSCYSRNLEYARLRKIADENGAYLMADMAHISGLVAAGVVPSPFEHCHVVTTTTHKT
LRGCRAGMIFYRKGVKSVDPKTGKEILYNLESLINSAVFPGLQGGPHNHAIAGVAVALKQ
AMTLEFKVYQHQVVANCRALSEALTELGYKIVTGGSDNHLILVDLRSKGTDGGRAEKVLE
ACSIACNKNTCPGDRSALRPSGLRLGTPALTSRGLLEKDFQKVAHFIHRGIELTLQIQSD
TGVRATLKEFKERLAGDKYQAAVQALREEVESFASLFPLPGLPDF*
>ENSG00000151322|ENST00000356141
MAPTKPSFQQDPSRRERITAQHPLPNQSECRKIYRYDGIYCESTYQNLQALRKEKSRDAA
RSRRGKENFEFYELAKLLPLPAAITSQLDKASIIRLTISYLKMRDFANQGDPPWNLRMEG
PPPNTSVKVIGAQRRRSPSALAIEVFEAHLGSHILQSLDGFVFALNQEGKFLYISETVSI
YLGLSQVELTGSSVFDYVHPGDHVEMAEQLGMKLPPGRGLLSQGTAEDGASSASSSSQSE
TPEPVESTSPSLLTTDNTLERSFFIRMKSTLTKRGVHIKSSGYKVIHITGRLRLRVSLSH
GRTVPSQIMGLVVVAHALPPPTINEVRIDCHMFVTRVNMDLNIIYCENRISDYMDLTPVD
IVGKRCYHFIHAEDVEGIRHSHLDLLNKGQCVTKYYRWMQKNGGYIWIQSSATIAINAKN
ANEKNIIWVNYLLSNPEYKDTPMDIAQLPHLPEKTSESSETSDSESDSKDTSGITEDNEN
SKSDEKGNQSENSEDPEPDRKKSGNACDNDMNCNDDGHSSSNPDSRDSDDSFEHSDFENP
KAGEDGFGALGAMQIKVERYVESESDLRLQNCESLTSDSAKDSDSAGEAGAQASSKHQKR
KKRRKRQKGGSASRRRLSSASSPGGLDAGLVEPPRLLSSPNSASVLKIKTEISEPINFDN
DSSIWNYPPNREISRNESPYSMTKPPSSEHFPSPQGGGGGGGGGGGLHVAIPDSVLTPPG
ADGAAARKTQFGASATAALAPVASDPLSPPLSASPRDKHPGNGGGGGGGGGAGGGGPSA
SNSLLYTGDLEALQRLQAGNVVLPLVHRVTGTLAATSTAAQRVYTTGTIRYAPAEVTLAM
QSNLLPNAHAVNFVDVNSPGFGLDPKTPMEMLYHHVHRLNMSGPFGGAVSAASLTQMPAG
NVFTTAEGLFSTLPFPVYSNGIHAAQTLERKED*
>ENSG00000130816|ENST00000340748
MPARTAPARVPTLAVPAISLPDDVRRRLKDLERDSLTEKECVKEKLNLLHEFLQTEIKNQ
LCDLETKLRKEELSEEGYLAKVKSLLNKDLSLENGAHAYNREVNGRLENGNQARSEARRV
GMADANSPPKPLSKPRTPRRSKSDGEAKPEPSPSPRITRKSTRQTTITSHFAKGPAKRKP
QEESERAKSDESIKEEDKDQDEKRRRVTSRERVARPLPAEEPERAKSGTRTEKEEERDEK
EEKRLRSQTKEPTPKQKLKEEPDREARAGVQADEDEDGDEKDEKKHRSQPKDLAAKRRPE
EKEPEKVNPQISDEKDEDEKEEKRRKTTPKEPTEKKMARAKTVMNSKTHPPKCIQCGQYL
DDPDLKYGQHPPDAVDEPQMLTNEKLSIFDANESGFESYEALPQHKLTCFSVYCKHGHLC
PIDTGLIEKNIELFFSGSAKPIYDDDPSLEGGVNGKNLGPINEWWITGFDGGEKALIGFS
TSFAEYILMDPSPEYAPIFGLMQEKIYISKIVVEFLQSNSDSTYEDLINKIETTVPPSGL
NLNRFTEDSLLRHAQFVVEQVESYDEAGDSDEQPIFLTPCMRDLIKLAGVTLGQRRAQAR
RQTIRHSTREKDRGPTKATTTKLVYQIFDTFFAEQIEKDDREDKENAFKRRRCGVCEVCQ
QPECGKCKACKDMVKFGGSGRSKQACQERRCPNMAMKEADDDEEVDDNIPEMPSPKKMHQ
GKKKKQNKNRISWVGEAVKTDGKKSYYKKVCIDAETLEVGDCVSVIPDDSSKPLYLARVT
ALWEDSSNGQMFHAHWFCAGTDTVLGATSDPLELFLVDECEDMQLSYIHSKVKVIYKAPS
ENWAMEGGMDPESLLEGDDGKTYFYQLWYDQDYARFESPPKTQPTEDNKFKFCVSCARLA
EMRQKEIPRVLEQLEDLDSRVLYYSATKNGILYRVGDGVYLPPEAFTFNIKLSSPVKRPR
KEPVDEDLYPEHYRKYSDYIKGSNLDAPEPYRIGRIKEIFCPKKSNGRPNETDIKIRVNK
FYRPENTHKSTPASYHADINLLYWSDEEAVVDFKAVQGRCTVEYGEDLPECVQVYSMGGP
NRFYFLEAYNAKSKSFEDPPNHARSPGNKGKGKGKGKGKPKSQACEPSEPEIEIKLPKLR

TLDVFSGCGGLSEGFHQAGISDTLWAIEMWDPAAQAFRLNNPGSTVFTEDCNILLKLVMA
GETTNSRGQRLPQKGDVEMLCGGPPCQGFSGMNRFNSRTYSKFKNSLVVSFLSYCDYYRP
RFFLLENVRNFVSFKRSMVLKLTLRCLVRMGYQCTFGVLQAGQYGVAQTRRRAIILAAAP
GEKLPLFPEPLHVFAPRACQLSVVVDDKKFVSNITRLSSGPFRTITVRDTMSDLPEVRNG
ASALEISYNGEPQSWFQRQLRGAQYQPILRDHICKDMSALVAARMRHIPLAPGSDWRDLP
NIEVRLSDGTMARKLRYTHHDRKNGRSSSGALRGVCSCVEAGKACDPAARQFNTLIPWCL
PHTGNRHNHWAGLYGRLEWDGFFSTTVTNPEPMGKQGRVLHPEQHRVVSVRECARSQGFP
DTYRLFGNILDKHRQVGNAVPPPLAKAIGLEIKLCMLAKARESASAKIKEEEAAKD*
>ENSG00000151322|ENST00000357798
MAPTKPSFQQDPSRRERLQALRKEKSRDAARSRRGKENFEFYELAKLLPLPAAITSQLDK
ASIIRLTISYLKMRDFANQGDPPWNLRMEGPPPNTSVKGIQMWKSELCMRKTPCEVIGAQ
RRRSPSALAIEVFEAHLGSHILQSLDGFVFALNQEGKFLYISETVSIYLGLSQVELTGSS
VFDYVHPGDHVEMAEQLGMKLPPGRGLLSQGTAEDGASSASSSSQSETPEPVESTSPSLL
TTDNTLERSFFIRMKSTLTKRGVHIKSSGYKVIHITGRLRLRVSLSHGRTVPSQIMGLVV
VAHALPPPTINEVRIDCHMFVTRVNMDLNIIYCENRISDYMDLTPVDIVGKRCYHFIHAE
DVEGIRHSHLDLLNKGQCVTKYYRWMQKNGGYIWIQSSATIAINAKNANEKNIIWVNYLL
SNPEYKDTPMDIAQLPHLPEKTSESSETSDSESDSKDTSGITEDNENSKSDEKGNQSENS
EDPEPDRKKSGNACDNDMNCNDDGHSSSNPDSRDSDDSFEHSDFENPKAGEDGFGALGAM
QIKVERYVESESDLRLQNCESLTSDSAKDSDSAGEAGAQASSKHQKRKKRRKRQKGGSAS
RRRLSSASSPGGLDAGLVEPPRLLSSPNSASVLKIKTEISEPINFDNDSSIWNYPPNREI
SRNESPYSMTKPPSSEHFPSPQGGGGGGGGGGGLHVAIPDSVLTPPGADGAAARKTQFGA
SATAALAPVASDPLSPPLSASPRDKHPGNGGGGGGGGGGAGGGGPSASNSLLYTGDLEAL
QRLQAGNVVLPLVHRVTGTLAATSTAAQRVYTTGTIRYAPAEVTLAMQSNLLPNAHAVNF
VDVNSPGFGLDPKTPMEMLYHHVHRLNMSGPFGGAVSAASLTQMPAGNVFTTAEGLFSTL
PFPVYSNGIHAAQTLERKED*
>ENSG00000115761|ENST00000345985
MQVSSLNEVKIYSLSCGKSLPEWLSDRKKRALQKKDVDVRRRIELIQDFEMPTVCTTIKV
SKDGQYILATGTYKPRVRCYDTYQLSLKFERCLDSEVVTFEILSDDYSKIVFLHNDRYIE
FHSQSGFYYKTRIPKFGRDFSYHYPSCDLYFVGASSEVYRLNLEQGRYLNPLQTDAAENN
VCDINSVHGLFATGTIEGRVECWDPRTRNRVGLLDCALNSVTADSEINSLPTISALKFNG
ALTMAVGTTTGQGKIFTSLEPEHDLNDVCLYPNSGMLLTANETPKMGIYYIPVLGPAPRW
CSFLDNLTEELEENPESTVYDDYKFVTKKDLENLGLTHLIGSPFLRAYMHGFFMDIRLYH
KVKLMVNPFAYEEYRKDKIRQKIEETRAQRVQLKKLPKVNKELALKLIEEEEEKQKSTWK
KKVKSLPNILTDDRFKVMFENPDFQVDEESEEFRLLNPLVSKISEKRKKKLRLLEQQELR
EKEEEEEPEGKPSDAESSESSDDEKAWVEEVRKQRRLLQQEEKVKRQERLKEDQQTVLKP
QFYEIKAGEEFRSFKDSATKQKLMNKTLEDRLKIEAKNGTLSVSDTTVGSKQLTFTLKRS
EQQKKQQEAEKLHRQERKRLRRSAGHLKSRHKRGRSFH*
>ENSG00000124813|ENST00000359524
MRIPVDPSTSRRFSPPSSSLQPGKMSDVSPVVAAQQQQQQQQQQQQQQQQQQQQQQQQEAA
AAAAAAAAAAAAAAVPRLRPPHDNRTMVEIIADHPAELVRTDSPNFLCSVLPSHWRCNK
TLPVAFKVVALGEVPDGTVVTVMAGNDENYSAELRNASAVMKNQVARFNDLRFVGRSGRG
KSFTLTITVFTNPPQVATYHRAIKVTVDGPREPRRHRQKLDDSKPSLFSDRLSDLGRIPH
PSMRVGVPPQNPRPSLNSAPSPFNPQGQSQITDPRQAQSSPPWSYDQSYPSYLSQMTSPS
IHSTTPLSSTRGTGLPAITDVPRRISDDDTATSDFCLWPSTLSKKSQAGASELGPFSDPR

QFPSISSLTESRFSNPRMHYPATFTYTPPVTSGMSLGMSATTHYHTYLPPPYPGSSQSQS
GPFQTSSTPYLYYGTSSGSYQFPMVPGGDRSPSRMLPPCTTTSNGSTLLNPNLPNQNDGV
DADGSHSSSPTVLNSSGRMDESVWRPY*
>ENSG00000196628|ENST00000356073
MHHQQRMAALGTDKELSDLLDFSAMFSPPVSSGKNGPTSLASGHFTGSNVEDRSSSGSWG
NGGHPSPSRNYGDGTPYDHMTSRDLGSHDNLSPPFVNSRIQSKTERGSYSSYGRESNLQG
CHQQSLLGGDMDMGNPGTLSPTKPGSQYYQYSSNNPRRRPLHSSAMEVQTKKVRKVPPGL
PSSVYAPSASTADYNRDSPGYPSSKPATSTFPSSFFMQDGHHSSDPWSSSSGMNQPGYAG
MLGNSSHIPQSSSYCSLHPHERLSYPSHSSADINSSLPPMSTFHRSGTNHYSTSSCTPPA
NGTDSIMANRGSGAAGSSQTGDALGKALASIYSPDHTNNSFSSNPSTPVGSPPSLSAGTA
VWSRNGGQASSSPNYEGPLHSLQSRIEDRLERLDDAIHVLRNHAVGPSTAMPGGHGDMHG
IIGPSHNGAMGGLGSGYGTGLLSANRHSLMVGTHREDGVALRGSHSLLPNQVPVPQLPVQ
SATSPDLNPPQDPYRGMPPGLQGQSVSSGSSEIKSDDEGDENLQDTKSSEDKKLDDDKKD
IKSITSNNDDEDLTPEQKAEREKERRMANNARERLRVRDINEAFKELGRMVQLHLKSDKP
QTKLLILHQAVAVILSLEQQVRERNLNPKAACLKRREEEKVSSEPPPLSLAGPHPGMGDA
SNHMGQM*
>ENSG00000005436|ENST00000321027
MAHRPKRTFRQRAADSSDSDGAEESPAEPGAPRELPVPGSAEEEPPSGGGRAQVAGLPHR
VRGPRGRGRVWASSRRATKAAPRADEGSESRTLDVSTDEEDKIHHSSESKDDQGLSSDSS
SSLGEKELSSTVKIPDAAFIQAARRKRELARAQDDYISLDVQHTSSISGMKRESEDDPES
EPDDHEKRIPFTLRPQTLRQRMAEESISRNEETSEESQEDEKQDTWEQQQMRKAVKIIEE
RDIDLSCGNGSSKVKKFDTSISFPPVNLEIIKKQLNTRLTLLQETHRSHLREYEKYVQDV
KSSKSTIQNLESSSNQALNCKFYKSMKIYVENLIDCLNEKIINIQEIESSMHALLLKQAM
TFMKRRQDELKHESTYLQQLSRKDETSTSGNFSVDEKTQWILEEIESRRTKRRQARVLSG
NCNHQEGTSSDDELPSAEMIDFQKSQGDILQKQKKVFEEVQDDFCNIQNILLKFQQWREK
FPDSYYEAFISLCIPKLLNPLIRVQLIDWNPLKLESTGLKEMPWFKSVEEFMDSSVEDSK
KESSSDKKVLSAIINKTIIPRLTDFVEFLWDPLSTSQTTSLITHCRVILEEHSTCENEVS
KSRQDLLKSIVSRMKKAVEDDVFIPLYPKSAVENKTSPHSKFQERQFWSGLKLFRNILLW
NGLLTDDTLQELGLGKLLNRYLIIALLNATPGPDVVKKCNQVAACLPEKWFENSAMRTSI
PQLENFIQFLLQSAHKLSRSEFRDEVEEIILILVKIKALNQAESFIGEHHLDHLKSLIKE
D*
>ENSG00000171817|ENST00000343599
MAHALVTFRDVAIDFSQKEWECLDTTQRKLYRDVMLENYNNLVSLGYSGSKPDVITLLEQ
GKEPCVVARDVTGRQCPGLLSRHKTKKLSSEKDIHEISLSKESIIEKSKTLRLKGSIFRN
EWQNKSEFEGQQGLKERSISQKKIVSKKMSTDRKRPSFTLNQRIHNSEKSCDSHLVQHGK
IDSDVKHDCKECGSTFNNVYQLTLHQKIHTGEKSCKCEKCGKVFSHSYQLTLHQRFHTGE
KPYECQECGKTFTLYPQLNRHQKIHTGKKPYMCKKCDKGFFSRLELTQHKRIHTGKKSYE
CKECGKVFQLIFYFKEHERIHTGKKPYECKECGKAFSVCGQLTRHQKIHTGVKPYECKEC
GKTFRLSFYLTEHRRTHAGKKPYECKECGKSFNVRGQLNRHKTIHTGIKPFACKVCEKAF
SYSGDLRVHSRIHTGEKPYECKECGKAFMLRSVLTEHQRLHTGVKPYECKECGKTFRVRS
QISLHKKIHTDVKPYKCVRCGKTFRFGFYLTEHQRIHTGEKPYKCKECGKAFIRRGNLKE
HLKIHSGLKPYDCKECGKSFSRRGQFTEHQKIHTGVKPYKCKECGKAFSRSVDLRIHQRI
HTGEKPYECKQCGKAFRLNSHLTEHQRIHTGEKPYECKVCRKAFRQYSHLYQHQKTHNVI
*

>ENSG00000139793|ENST00000343600
MALNVAPVRDTKWLTLEVCRQFQRGTCSRSDEECKFAHPPKSCQVENGRVIACFDSLKGR
CSRENCKYLHPPTHLKTQLEINGRNNLIQQKTAAAMLAQQMQFMFPGTPLHPVPTFPVGP
AIGTNTAISFAPYLAPVTPGVGLVPTEILPTTPVIVPGSPPVTVPGSTATQKLLRTDKLE
VCREFQRGNCARGETDCRFAHPADSTMIDTSDNTVTVCMDYIKGRCMREKCKYFHPPAHL
QAKIKAAQHQANQAAVAAQAAAAAATVMAFPPGALHPLPKRQALEKSNGTSAVFNPSVLH
YQQALTSAQLQQHAAFIPTDNSEIISRNGMECQESALRITKHCYCTYYPVSSSIELPQTA
C*
>ENSG00000213096|ENST00000357002
MPGPPRSLEMGLLTFRDVAIEFSLEEWQHLDIAQQNLYRNVMLENYRNLAFLGIAVSKPD
LITCLEQGKEPWNMKRHEMVDEPPGMCPHFAQDLWPEQGMEDSFQKAILRRYGKYGHENL
QLRKGCKSVDEYKVNKEGYNGLNQCFTTAQSKVFQCDKYLKVFYKFLNSNRPKIRHTEKK
SFKCKKRVKLFCMLSHKTQHKSIYHREKSYKCKECGKTFNWSSTLTNHRKIYTEEKPYKC
EEYNKSPKQLSTLTTHEIIHAGEKLYKCEECGEAFNRSSNLTTHKIIHTGEKPYKCEECG
KAFIWSSTLTEHKKIHTRKKPYKCEECGKAFIWSSTLTRHKRMHTGEKPYKCEECGKAFS
QSSTLTTHKIIHTGEKRYKCLECGKAFKQLSTLTTHKIIHVGEKLYKCEECGKGFNRSSN
LTTHKIIHTGEKPYKCEECGKAFIWSSTLTKHKRIHTREKPYKCEECGKAFIWSSTLTRH
KRMHTGEKPYKCEECGKSFSQSSTLTTHKIIHTGEKPYKCEECGKAFNWSSTLTKHKIIH
TEEKPYKCEKCGKAFKQSSILTNHKRIHTGEKPYKCEECGKSFNRSSTFTKHKVIHTGVK
PYKCEECGKAFFWSSTLTKHKRIHTGEQPYKWEKFGKAFNRSSHLTTDKITHWREILQV*
>ENSG00000199032|ENST00000362162
Sequence unavailable
>ENSG00000183733|ENST00000332372
MDPAPGVLDPRAAPPALLGTPQAEVLEDVLREQFGPLPQLAAVCRLKRLPSGGYSSTENL
QLVLERRRVANAKERERIKNLNRGFARLKALVPFLPQSRKPSKVDILKGATEYIQVLSDL
LEGAKDSKKQDPDEQSYSNNSSESHTSSARQLSRNITQHISCAFGLKNEEEGPWADGGSG
EPAHACRHSVMSTTEIISPTRSLDRFPEVELLSHRLPQV*
>ENSG00000072736|ENST00000349223
MTTANCGAHDELDFKLVFGEDGAPAPPPPGSRPADLEPDDCASIYIFNVDPPPSTLTTPL
CLPHHGLPSHSSVLSPSFQLQSHKNYEGTCEIPESKYSPLGGPKPFECPSIQITSISPNC
HQELDAHEDDLQINDPEREFLERPSRDHLYLPLEPSYRESSLSPSPASSISSRSWFSDAS
SCESLSHIYDDVDSELNEAAARFTLGSPLTSPGGSPGGCPGEETWHQQYGLGHSLSPRQS
PCHSPRSSVTDENWLSPRPASGPSSRPTSPCGKRRHSSAEVCYAGSLSPHHSPVPSPGHS
PRGSVTEDTWLNASVHGGSGLGPAVFPFQYCVETDIPLKTRKTSEDQAAILPGKLELCSD
DQGSLSPARETSIDDGLGSQYPLKKDSCGDQFLSVPSPFTWSKPKPGHTPIFRTSSLPPL
DWPLPAHFGQCELKIEVQPKTHHRAHYETEGSRGAVKASTGGHPVVKLLGYNEKPINLQM
FIGTADDRYLRPHAFYQVHRITGKTVATASQEIIIASTKVLEIPLLPENNMSASIDCAGI
LKLRNSDIELRKGETDIGRKNTRVRLVFRVHIPQPSGKVLSLQIASIPVECSQRSAQELP
HIEKYSINSCSVNGGHEMVVTGSNFLPESKIIFLEKGQDGRPQWEVEGKIIREKCQGAHI
VLEVPPYHNPAVTAAVQVHFYLCNGKRKKSQSQRFTYTPVLMKQEHREEIDLSSVPSLPV
PHPAQTQRPSSDSGCSHDSVLSGQRSLICSIPQTYASMVTSSHLPQLQCRDESVSKEQHM
IPSPIVHQPFQVTPTPPVGSSYQPMQTNVVYNGPTCLPINAASSQEFDSVLFQQDATLSG
LVNLGCQPLSSIPFHSSNSGSTGHLLAHTPHSVHTLPHLQSMGYHCSNTGQRSLSSPVAD
QITGQPSSQLQPITYGPSHSGSATTASPAASHPLASSPLSGPPSPQLQPMPYQSPSSGTA

SSPSPATRMHSGQHSTQAQSTGQGGLSAPSSLICHSLCDPASFPPDGATVSIKPEPEDRE
PNFATIGLQDITLDDDLFTSNNFDLLQLRPTFWPVPAGRYLRNLE*
>ENSG00000169953|ENST00000344884
MAHVSSETQDVSPKDELTASEASTRSPLCEHTFPGDSDLRSMIEEHAFQVLSQGSLLESP
SYTVCVSEPDKDDDFLSLNFPRKLWKIVESDQFKSISWDENGTCIVINEELFKKEILETK
APYRIFQTDAIKSFVRQLNLYGFSKIQQNFQRSAFLATFLSEEKESSVLSKIRFTKMKLS
RSSTYENRYLCCNLHLKDESNYS*
>ENSG00000164107|ENST00000359562
MSLVGGFPHHPVVHHEGYPFAAAAAAAAAAAASRCSHEENPYFHGWLIGHPEMSPPDYSM
ALSYSPEYASGAAGLDHSHYGGVPPGAGPPGLGGPRPVKRRGTANRKERRRTQSINSAFA
ELRECIPNVPADTKLSKIKTLRLATSYIAYLMDLLAKDDQNGEAEAFKAEIKKTDVKEEK
RKKELNEILKSTVSSNDKKTKGRTGWPQHVWALELKQ*
>ENSG00000196458|ENST00000360187
MIQSQISFEDVAVDFTLEEWQLLNPTQKNLYRDVMLENYSNLVFLEVWLDNPKMWLRDNQ
DNLKSMERGHKYDVFGKIFNSSINIVHVGLRSHKCGTGEKSLKCPFDLLIPKNNCERKKI
DELNKKLLFCIKPGRTHGGIKYCDCSTCRKSSNEEPWLTANHITHTGVYLCMECGRFFNK
KSQLVIHQRTHTGEKPYQCSECGKAFSQKSLLTVHQRTHSGEKPHGCSECQKAFSRKSLL
ILHQRIHTGEKPYGCSECGKAFSRKSQLKRHQITHTIEKPYSCSECGKAFSQKLKLITHQ
RAHTGEKPYPCSHCGKAFFWKSQLITHQRTHTGKKPYGCGECQKAFSRNSLLIRHQRIHT
GEKPYECNECGEAFIRKPQLIKHQITHTGEKNYRCSDCEEAFFKKSELIRHQKIHLGEKP
YGCIQCGKTFFGKSQLLTHHRTHTGEKPYECSECGKAFTQKSSLISHQRTHTGEKPYECS
ECRKTFSEKSSLIHHQRTHTGEKPFECSECRKAFAWKPQLLRHQRIHTGEKPYECSECGK
AFVQKVQLIKHQRNHTGEKTYGCSDCAKAFFEKAQLIIHQRIHTGERPYKCGECGKSFTR
KSHLMRHQRIHTGDKYYGCNECGTTFNRKSQLMIHQRNHII*
>ENSG00000171476|ENST00000337881
MSAETASGPTEDQVEILEYNFNKVDKHPDSTTLCLIAAEAGLSEEETQKWFKQRLAKWRR
SEGLPSECRSVTD*
>ENSG00000111142|ENST00000323666
MAGVEEVAASGSHLNGDLDPDDREEGAASTAEEAAKKKRRKKKKSKGPSAAGEQEPDKES
GASVDEVARQLERSALEDKERDEDDEDGDGDGDGATGKKKKKKKKKRGPKVQTDPPSVPI
CDLYPNGVFPKGQECEYPPTQDGRTAAWRTTSEEKKALDQASEEIWNDFREAAEAHRQVR
KYVMSWIKPGMTMIEICEKLEDCSRKLIKENGLNAGLAFPTGCSLNNCAAHYTPNAGDTT
VLQYDDICKIDFGTHISGRIIDCAFTVTFNPKYDTLLKAVKDATNTGIKCAGIDVRLCDV
GEAIQEVMESYEVEIDGKTYQVKPIRNLNGHSIGQYRIHAGKTVPIVKGGEATRMEEGEV
YAIETFGSTGKGVVHDDMECSHYMKNFDVGHVPIRLPRTKHLLNVINENFGTLAFCRRWL
DRLGESKYLMALKNLCDLGIVDPYPPLCDIKGSYTAQFEHTILLRPTCKEVVSRGDDY*
>ENSG00000012048|ENST00000352993
MDLSALRVEEVQNVINAMQKILECPICLELIKEPVSTKCDHIFCKFCMLKLLNQKKGPSQ
CPLCKNDITKRSLQESTRFSQLVEELLKIICAFQLDTGLEYANSYNFAKKENNSPEHLKD
EVSIIQSMGYRNRAKRLLQSEPENPSLQETSLSVQLSNLGTVRTLRTKQRIQPQKTSVYI
ELGSDSSEDTVNKATYCSVGDQELLQITPQGTRDEISLDSAKKGEAASGCESETSVSEDC
SGLSSQSDILTTQQRDTMQHNLIKLQQEMAELEAVLEQHGSQPSNSYPSIISDSSALEDL
RNPEQSTSEKAVLTSQKSSEYPISQNPEGLSADKFEVSADSSTSKNKEPGVERSSPSKCP
SLDDRWYMHSCSGSLQNRNYPSQEELIKVVDVEEQQLEESGPHDLTETSYLPRQDLEGTP

YLESGISLFSDDPESDPSEDRAPESARVGNIPSSTSALKVPQLKVAESAQSPAAAHTTDT
AGYNAMEESVSREKPELTASTERVNKRMSMVVSGLTPEEFMLVYKFARKHHITLTNLITE
ETTHVVMKTDAEFVCERTLKYFLGIAGGKWVVSYFWVTQSIKERKMLNEHDFEVRGDVVN
GRNHQGPKRARESQDRKIFRGLEICCYGPFTNMPTDQLEWMVQLCGASVVKELSSFTLGT
GVHPIVVVQPDAWTEDNGFHAIGQMCEAPVVTREWVLDSVALYQCQELDTYLIPQIPHSH
Y*
>ENSG00000168214|ENST00000348160
MAPVVTGKFGERPPPKRLTREAMRNYLKERGDQTVLILHAKVAQKSYGNEKRFFCPPPCV
YLMGSGWKKKKEQMERDGCSEQESQPCAFIGIGNSDQEMQQLNLEGKNYCTAKTLYISDS
DKRKHFMLSVKMFYGNSDDIGVFLSKRIKVISKPSKKKQSLKNADLCIASGTKVALFNRL
RSQTVSTRYLHVEGGNFHASSQQWGAFFIHLLDDDESEGEEFTVRDGYIHYGQTVKLVCS
VTGMALPRLIIRKVDKQTALLDADDPVSQLHKCAFYLKDTERMYLCLSQERIIQFQATPC
PKEPNKEMINDGASWTIISTDKAEYTFYEGMGPVLAPVTPVPVVESLQLNGGGDVAMLEL
TGQNFTPNLRVWFGDVEAETMYRCGESMLCVVPDISAFREGWRWVRQPVQVPVTLVRNDG
IIYSTSLTFTYTPEPGPRPHCSAAGAILRANSSQVPPNESNTNSEGSYTNASTNSTSVTS
STATVVS*
>ENSG00000197114|ENST00000355969
MDEESLESALQTYRAQLQQVELALGAGLDSSEQADLRQLQGDLKELIELTEASLVSVRKS
SLLAALDEERPGRQEDAEYQAFREAITEAVEAPAAARGSGSETVPKAEAGPESAAGGQEE
EEGEDEEELSGTKVSAPYYSSWGTLEYHNAMVVGTEEAEDGSAGVRVLYLYPTHKSLKPC
PFFLEGKCRFKENCRFSHGQVVSLDELRPFQDPDLSSLQAGSACLAKHQDGLWHAARITD
VDNGYYTVKFDSLLLREAVVEGDGILPPLRTEATESDSDSDGTGDSSYARVVGSDAVDSG
TCSSAFAGWEVHTRGIGSRLLTKMGYEFGKGLGRHAEGRVEPIHAVVLPRGKSLDQCVET
LQKQTRVGKAGTNKPPRCRGRGARPGGRPAPRNVFDFLNEKLQGQAPGALEAGAAPAGRR
SKDMYHASKSAKRALSLRLFQTEEKIERTQRDIRSIQEALARNAGRHSVASAQLQEKLAG
AQRQLGQLRAQEAGLQQEQRKADTHKKMTEF*
>ENSG00000101150|ENST00000351424
MDSAGQDINLNSPNKGLLSDSMTDVPVDTGVAARTPAVEGLTEAEEEELRAELTKVEEEI
VTLRQVLAAKERHCGELKRRLGLSTLGELKQNLSRSWHDVQVSSAYKKTQETLSQAGQKT
SAALSTVGSAISRKLGDMRAHPFSHSFSSYSIRHSISMPAMRNSATFKSFEDRVGTIKSK
VVGDRENGSDNLPSSAGSGDKPLSDPAPF*
>ENSG00000133026|ENST00000360416
MAQRTGLEDPERYLFVDRAVIYNPATQADWTAKKLVWIPSERHGFEAASIKEERGDEVMV
ELAENGKKAMVNKDDIQKMNPPKFSKVEDMAELTCLNEASVLHNLKDRYYSGLIYTYSGL
FCVVINPYKNLPIYSENIIEMYRGKKRHEMPPHIYAISESAYRCMLQDREDQSILCTGES
GAGKTENTKKVIQYLAHVASSHKGRKDHNIPQESPKPVKHQGELERQLLQANPILESFGN
AKTVKNDNSSRFGKFIRINFDVTGYIVGANIETYLLEKSRAVRQAKDERTFHIFYQLLSG
AGEHLKSDLLLEGFNNYRFLSNGYIPIPGQQDKDNFQETMEAMHIMGFSHEEILSMLKVV
SSVLQFGNISFKKERNTDQASMPENTVAQKLCHLLGMNVMEFTRAILTPRIKVGRDYVQK
AQTKEQADFAVEALAKATYERLFRWLVHRINKALDRTKRQGASFIGILDIAGFEIFELNS
FEQLCINYTNEKLQQLFNHTMFILEQEEYQREGIEWNFIDFGLDLQPCIDLIERPANPPG
VLALLDEECWFPKATDKTFVEKLVQEQGSHSKFQKPRQLKDKADFCIIHYAGKVDYKADE
WLMKNMDPLNDNVATLLHQSSDRFVAELWKDEIQNIQRASFYDSVSGLHEPPVDRIVGLD
QVTGMTETAFGSAYKTKKGMFRTVGQLYKESLTKLMATLRNTNPNFVRCIIPNHEKRAGK

LDPHLVLDQLRCNGVLEGIRICRQGFPNRIVFQEFRQRYEILTPNAIPKGFMDGKQACER
MIRALELDPNLYRIGQSKIFFRAGVLAHLEEERDLKITDIIIFFQAVCRGYLARKAFAKK
QQQLSALKVLQRNCAAYLKLRHWQWWRVFTKVKPLLQVTRQEEELQAKDEELLKVKEKQT
KVEGELEEMERKHQQLLEEKNILAEQLQAETELFAEAEEMRARLAAKKQELEEILHDLES
RVEEEEERNQILQNEKKKMQAHIQDLEEQLDEEEGARQKLQLEKVTAEAKIKKMEEEILL
LEDQNSKFIKEKKLMEDRIAECSSQLAEEEEKAKNLAKIRNKQEVMISDLEERLKKEEKT
RQELEKAKRKLDGETTDLQDQIAELQAQIDELKLQLAKKEEELQGALARGDDETLHKNNA
LKVVRELQAQIAELQEDFESEKASRNKAEKQKRDLSEELEALKTELEDTLDTTAAQQELR
TKREQEVAELKKALEEETKNHEAQIQDMRQRHATALEELSEQLEQAKRFKANLEKNKQGL
ETDNKELACEVKVLQQVKAESEHKRKKLDAQVQELHAKVSEGDRLRVELAEKASKLQNEL
DNVSTLLEEAEKKGIKFAKDAASLESQLQDTQELLQEETRQKLNLSSRIRQLEEEKNSLQ
EQQEEEEEARKNLEKQVLALQSQLADTKKKVDDDLGTIESLEEAKKKLLKDAEALSQRLE
EKALAYDKLEKTKNRLQQELDDLTVDLDHQRQVASNLEKKQKKFDQLLAEEKSISARYAE
ERDRAEAEAREKETKALSLARALEEALEAKEEFERQNKQLRADMEDLMSSKDDVGKNVHE
LEKSKRALEQQVEEMRTQLEELEDELQATEDAKLRLEVNMQAMKAQFERDLQTRDEQNEE
KKRLLIKQVRELEAELEDERKQRALAVASKKKMEIDLKDLEAQIEAANKARDEVIKQLRK
LQAQMKDYQRELEEARASRDEIFAQSKESEKKLKSLEAEILQLQEELASSERARRHAEQE
RDELADEITNSASGKSALLDEKRRLEARIAQLEEELEEEQSNMELLNDRFRKTTLQVDTL
NAELAAERSAAQKSDNARQQLERQNKELKAKLQELEGAVKSKFKATISALEAKIGQLEEQ
LEQEAKERAAANKLVRRTEKKLKEIFMQVEDERRHADQYKEQMEKANARMKQLKRQLEEA
EEEATRANASRRKLQRELDDATEANEGLSREVSTLKNRLRRGGPISFSSSRSGRRQLHLE
GASLELSDDDTESKTSDVNETQPPQSE*
>ENSG00000179115|ENST00000314606
MADGQVAELLLRRLEASDGGLDSAELAAELGMEHQAVVGAVKSLQALGEVIEAELRSTKH
WELTAEGEEIAREGSHEARVFRSIPPEGLAQSELMRLPSGKVGFSKAMSNKWIRVDKSAA
DGPRVFRVVDSMEDEVQRRLQLVRGGQAEKLGEKERSELRKRKLLAEVTLKTYWVSKGSA
FSTSISKQETELSPEMISSGSWRDRPFKPYNFLAHGVLPDSGHLHPLLKVRSQFRQIFLE
MGFTEMPTDNFIESSFWNFDALFQPQQHPARDQHDTFFLRDPAEALQLPMDYVQRVKRTH
SQGGYGSQGYKYNWKLDEARKNLLRTHTTSASARALYRLAQKKPFTPVKYFSIDRVFRNE
TLDATHLAEFHQIEGVVADHGLTLGHLMGVLREFFTKLGITQLRFKPAYNPYTEPSMEVF
SYHQGLKKWVEVGNSGVFRPEMLLPMGLPENVSVIAWGLSLERPTMIKYGINNIRELVGH
KVNLQMVYDSPLCRLDAEPRPPPTQEAA*
>ENSG00000180257|ENST00000357666
MLREEATKKSKEKEPGMALPQGRLTFRDVAIEFSLEEWKCLNPAQRALYRAVMLENYRNL
EFVDSSLKSMMEFSSTRHSITGEVIHTGTLQRHKSHHIGDFCFPEMKKDIHHFEFQWQEV
ERNGHEAPMTKIKKLTGSTDRSDHRHAGNKPIKDQLGLSFHSHLPELHMFQTKGKISNQL
DKSIGASSASESQRISCRLKTHISNKYGKNFLHSSFTQIQEICMREKPCQSNECGKAFNY
SSLLRRHHITHSREREYKCDVCGKIFNQKQYIVYHHRCHTGEKTYKCNECGKTFTQMSSL
VCHRRLHTGEKPYKCNECGKTFSEKSSLRCHRRLHTGEKPYKCNECGKTFGRNSALVIHK
AIHTGEKPYKCNECGKTFSQKSSLQCHHILHTGEKPYKCEECDNVYIRRSHLERHRKIHT
GEGSYKCKVCDKVFRSDSYLAEHQRVHTGEKPYKCNKCGRSFSRKSSLQYHHTLHTGEKP
YTCNECGKVFSRRENLARHHRLHAGEKPYKCEECDKVFSRRSHLERHRRIHTGEKPYKCK
VCDKAFRSDSCLANHTRVHTGEKPYKCNKCAKVFNQKGILAQHQRVHTGEKPYKCNECGK
VFNQKASLAKHQRVHTAEKPYKCNECGKAFTGQSTLIHHQAIHGCRETLQM*

>ENSG00000197114|ENST00000328969
MDEESLESALQTYRAQLQQVELALGAGLDSSEQADLRQLQGDLKELIELTEASLVSVRKS
SLLAALDEERPGRQEDAEYQAFREAITEAVEAPAAARGSGSETVPKAEAGPESAAGGQEE
EEGEDEEELSGTKVSAPYYSSWGTLEYHNAMVVGTEEAEDGSAGVRVLYLYPTHKSLKPC
PFFLEGKCRFKENCRFSHGQVVSLDELRPFQDPDLSSLQAGSACLAKHQDGLWHAARITD
VDNGYYTVKFDSLLLREAVVEGDGILPPLRTEATESDSDSDGTGDSSYARVVGSDAVDSA
QSSALCPSLAVVGSDAVDSGTCSSAFAGWEVHTRGIGSRLLTKMGYEFGKGLGRHAEGRV
EPIHAVVLPRGKSLDQCVETLQKQTRVGKAGTNKPPRCRGRGARPGGRPAPRNVFDFLNE
KLQGQAPGALEAGAAPAGRRSKDMYHASKSAKRALSLRLFQTEEKIERTQRDIRSIQEAL
ARNAGRHSVASAQLQEKLAGAQRQLGQLRAQEAGLQQEQRKADTHKKMTEF*
>ENSG00000138029|ENST00000317799
MTILTYPFKNLPTASKWALRFSIRPLSCSSQLRAAPAVQTKTKKTLAKPNIRNVVVVDGV
RTPFLLSGTSYKDLMPHDLARAALTGLLHRTSVPKEVVDYIIFGTVIQEVKTSNVAREAA
LGAGFSDKTPAHTVTMACISANQAMTTGVGLIASGQCDVIVAGGVELMSDVPIRHSRKMR
KLMLDLNKAKSMGQRLSLISKFRFNFLAPELPAVSEFSTSETMGHSADRLAAAFAVSRLE
QDEYALRSHSLAKKAQDEGLLSDVVPFKVPGKDTVTKDNGIRPSSLEQMAKLKPAFIKPY
GTVTAANSSFLTDGASAMLIMAEEKALAMGYKPKAYLRDFMYVSQDPKDQLLLGPTYATP
KVLEKAGLTMNDIDAFEFHEAFSGQILANFKAMDSDWFAENYMGRKTKVGLPPLEKFNNW
GGSLSLGHPFGATGCRLVMAAANRLRKEGGQYGLVAACAAGGQGHAMIVEAYPK*
>ENSG00000199017|ENST00000362147
Sequence unavailable
>ENSG00000154920|ENST00000338165
MALKKSSPSLDSGDSDSEELPTFAFLKKEPSSTKRRQPEREEKIVVVDISDCEASCPPAP
ELFSPPVPEIAETVTQTQPVRLLSSESEDEEEFIPLAQRLTCKFLTHKQLSPEDSSSPVK
SVLDHQNNEGASCDWKKPFPKIPEVPLHDTPERSAADNKDLILDPCCQLPAYLSTCPGQS
SSLAVTKTNSDILPPQKKTKPSQKVQGRGSHGCRQQRQARQKESTLRRQERKNAALVTRM
KAQRPEECLKHIIVVLDPVLLQMEGGGQLLGALQTMECRCVIEAQAVPCSVTWRRRAGPS
EDREDWVEEPTVLVLLRAEAFVSMIDNGKQGSLDSTMKGKETLQGFVTDITAKTAGKALS
LVIVDQEKCFSAQNPPRRGKQGANKQTKKQQQRQPEASIGSMVSRVDAEEALVDLQLHTE
AQAQIVQSWKELADFTCAFTKAVAEAPFKKLRDETTFSFCLESDWAGGVKVDLAGRGLAL
VWRRQIQQLNRVSLEMASAVVNAYPSPQLLVQAYQQCFSDKERQNLLADIQVRRGEGVTS
TSRRIGPELSRRIYLQMTTLQPHLSLDSAD*
>ENSG00000102081|ENST00000334557
MEELVVEVRGSNGAFYKAFVKDVHEDSITVAFENNWQPDRQIPFHDVRFPPPVGYNKDIN
ESDEVEVYSRANEKEPCCWWLAKVRMIKGEFYVIEYAACDATYNEIVTIERLRSVNPNKP
ATKDTFHKIKLDVPEDLRQMCAKEAAHKDFKKAVGAFSVTYDPENYQLVILSINEVTSKR
AHMLIDMHFRSLRTKLSLIMRNEEASKQLESSRQLASRFHEQFIVREDLMGLAIGTHGAN
IQQARKVPGVTAIDLDEDTCTFHIYGEDQDAVKKARSFLEFAEDVIQVPRNLVGLKI*
>ENSG00000117713|ENST00000324856
MAAQVAPAAASSLGNPPPPPPSELKKAEQQQREEAGGEAAAAAAAERGEMKAAAGQESEG
PAVGPPQPLGKELQDGAESNGGGGGGGAGSGGGPGAEPDLKNSNGNAGPRPALNNNLTEP
PGGGGGGSSDGVGAPPHSAAAALPPPAYGFGQPYGRSPSAVAAAAAAVFHQQHGGQQSPG
LAALQSGGGGGLEPYAGPQQNSHDHGFPNHQYNSYYPNRSAYPPPAPAYALSSPRGGTPG
SGAAAAAGSKPPPSSSASASSSSSSFAQQRFGAMGGGGPSAAGGGTPQPTATPTLNQLLT

SPSSARGYQGYPGGDYSGGPQDGGAGKGPADMASQCWGAAAAAAAAAAASGGAQQRSHHA
PMSPGSSGGGGQPLARTPQPSSPMDQMGKMRPQPYGGTNPYSQQQGPPSGPQQGHGYPGQ
PYGSQTPQRYPMTMQGRAQSAMGGLSYTQQIPPYGQQGPSGYGQQGQTPYYNQQSPHPQQ
QQPPYSQQPPSQTPHAQPSYQQQPQSQPPQLQSSQPPYSQQPSQPPHQQSPAPYPSQQST
TQQHPQSQPPYSQPQAQSPYQQQQPQQPAPSTLSQQAAYPQPQSQQSQQTAYSQQRFPPP
QELSQDSFGSQASSAPSMTSSKGGQEDMNLSLQSRPSSLPDLSGSIDDLPMGTEGALSPG
VSTSGISSSQGEQSNPAQSPFSPHTSPHLPGIRGPSPSPVGSPASVAQSRSGPLSPAAVP
GNQMPPRPPSGQSDSIMHPSMNQSSIAQDRGYMQRNPQMPQYSSPQPGSALSPRQPSGGQ
IHTGMGSYQQNSMGSYGPQGGQYGPQGGYPRQPNYNALPNANYPSAGMAGGINPMGAGGQ
MHGQPGIPPYGTLPPGRMSHASMGNRPYGPNMANMPPQVGSGMCPPPGGMNRKTQETAVA
MHVAANSIQNRPPGYPNMNQGGMMGTGPPYGQGINSMAGMINPQGPPYSMGGTMANNSAG
MAASPEMMGLGDVKLTPATKMNNKADGTPKTESKSKKSSSSTTTNEKITKLYELGGEPER
KMWVDRYLAFTEEKAMGMTNLPAVGRKPLDLYRLYVSVKEIGGLTQVNKNKKWRELATNL
NVGTSSSAASSLKKQYIQCLYAFECKIERGEDPPPDIFAAADSKKSQPKIQPPSPAGSGS
MQGPQTPQSTSSSMAEGGDLKPPTPASTPHSQIPPLPGMSRSNSVGIQDAFNDGSDSTFQ
KRNSMTPNPGYQPSMNTSDMMGRMSYEPNKDPYGSMRKAPGSDPFMSSGQGPNGGMGDPY
SRAAGPGLGNVAMGPRQHYPYGGPYDRVRTEPGIGPEGNMSTGAPQPNLMPSNPDSGMYS
PSRYPPQQQQQQQQRHDSYGNQFSTQGTPSGSPFPSQQTTMYQQQQQNYKRPMDGTYGPP
AKRHEGEMYSVPYSTGQGQPQQQQLPPAQPQPASQQQAAQPSPQQDVYNQYGNAYPATAT
AATERRPAGGPQNQFPFQFGRDRVSAPPGTNAQQNMPPQMMGGPIQASAEVAQQGTMWQG
RNDMTYNYANRQSTGSAPQGPAYHGVNRTDEMLHTDQRANHEGSWPSHGTRQPPYGPSAP
VPPMTRPPPSNYQPPPSMQNHIPQVSSPAPLPRPMENRTSPSKSPFLHSGMKMQKAGPPV
PASHIAPAPVQPPMIRRDITFPPGSVEATQPVLKQRRRLTMKDIGTPEAWRVMMSLKSGL
LAESTWALDTINILLYDDNSIMTFNLSQLPGLLELLVEYFRRCLIEIFGILKEYEVGDPG
QRTLLDPGRFSKVSSPAPMEGGEEEEELLGPKLEEEEEEEVVENDEEIAFSGKDKPASEN
SEEKLISKFDKLPVKIVQKNDPFVVDCSDKLGRVQEFDSGLLHWRIGGGDTTEHIQTHFE
SKTELLPSRPHAPCPPAPRKHVTTAEGTPGTTDQEGPPPDGPPEKRITATMDDMLSTRSS
TLTEDGAKSSEAIKESSKFPFGISPAQSHRNIKILEDEPHSKDETPLCTLLDWQDSLAKR
CVCVSNTIRSLSFVPGNDFEMSKHPGLLLILGKLILLHHKHPERKQAPLTYEKEEEQDQG
VSCNKVEWWWDCLEMLRENTLVTLANISGQLDLSPYPESICLPVLDGLLHWAVCPSAEAQ
DPFSTLGPNAVLSPQRLVLETLSKLSIQDNNVDLILATPPFSRLEKLYSTMVRFLSDRKN
PVCREMAVVLLANLAQGDSLAARAIAVQKGSIGNLLGFLEDSLAATQFQQSQASLLHMQN
PPFEPTSVDMMRRAARALLALAKVDENHSEFTLYESRLLDISVSPLMNSLVSQVICDVLF
LIGQS*
>ENSG00000035928|ENST00000349703
MDIRKFFGVIPSGKKLVSETVKKNEKTKSDEETLKAKKGIKEIKVNSSRKEDDFKQKQPS
KKKRIIYDSDSESEETLQVKNAKKPPEKLPVSSKPGKISRQDPVTYISETDEEDDFMCKK
AASKSKENGRSTNSHLGTSNMKKNEENTKTKNKPLSPIKLTPTSVLDYFGTGSVQRSNKK
MVASKRKELSQNTDESGLNDEAIAKQLQLDEDAELERQLHEDEEFARTLAMLDEEPKTKK
ARKDTEAGETFSSVQANLSKAEKHKYPHKVKTAQVSDERKSYSPRKQSKYESSKESQQHS
KSSADKIGEVSSPKASSKLAIMKRKEESSYKEIEPVASKRKENAIKLKGETKTPKKTKSS
PAKKESVSPEDSEKKRTNYQAYRSYLNREGPKALGSKEIPKGAENCLEGLIFVITGVLES
IERDEAKSLIERYGGKVTGNVSKKTNYLVMGRDSGQSKSDKAAALGTKIIDEDGLLNLIR
TMPGKKSKYEIAVETEMKKESKLERTPQKNVQGKRKISPSKKESESKKSRPTSKRDSLAK

TIKKETDVFWKSLDFKEQVAEETSGDSKARNLADDSSENKVENLLWVDKYKPTSLKTIIG
QQGDQSCANKLLRWLRNWQKSSSEDKKHAKFGKFSGKDDGSSFKAALLSGPPGVGKTTTA
SLVCQELGYSYVELNASDTRSKSSLKAIVAESLNNTSIKGFYSNGAASSVSTKHALIMDE
VDGMAGNEDRGGIQELIGLIKHTKIPIICMCNDRNHPKIRSLVHYCFDLRFQRPRVEQIK
GAMMSIAFKEGLKIPPPAMNEIILGANQDIRQVLHNLSMWCARSKALTYDQAKADSHRAK
KDIKMGPFDVARKVFAAGEETAHMSLVDKSDLFFHDYSIAPLFVQENYIHVKPVAAGGDM
KKHLMLLSRAADSICDGDLVDSQIRSKQNWSLLPAQAIYASVLPGELMRGYMTQFPTFPS
WLGKHSSTGKHDRIVQDLALHMSLRTYSSKRTVNMDYLSLLRDALVQPLTSQGVDGVQDV
VALMDTYYLMKEDFENIMEISSWGGKPSPFSKLDPKVKAAFTRAYNKEAHLTPYSLQAIK
ASRHSTSPSLDSEYNEELNEDDSQSDEKDQDAIETDAMIKKKTKSSKPSKPEKDKEPRKG
KGKSSKK*
>ENSG00000115310|ENST00000357732
MEDLDQSPLVSSSDSPPRPQPAFKYQFVREPEDEEEEEEEEEEDEDEDLEELEVLERKPA
AGLSAAPVPTAPAAGAPLMDFGNDFVPPAPRGPLPAAPPVAPERQPSWDPSPVSSTVPAP
SPLSAAAVSPSKLPEDDEPPARPPPPPPASVSPQAEPVWTPPAPAPAAPPSTPAAPKRRG
SSGSVDETLFALPAASEPVIRSSAVVDLLYWRDIKKTGVVFGASLFLLLSLTVFSIVSVT
AYIALALLSVTISFRIYKGVIQAIQKSDEGHPFRAYLESEVAISEELVQKYSNSALGHVN
CTIKELRRLFLVDDLVDSLKFAVLMWVFTYVGALFNGLTLLILALISLFSVPVIYERHQA
QIDHYLGLANKNVKDAMAKIQAKIPGLKRKAE*
>ENSG00000153944|ENST00000322684
MADLTSVLTSVMFSPSSKMFIGGLSWQTSPDSLRDYFSKFGEIRECMVMRDPTTKRSRGF
GFVTFADPASVDKVLGQPHHELDSKTIDPKVAFPRRAQPKMVTRTKKIFVGGLSANTVVE
DVKQYFEQFGKVEDAMLMFDKTTNRHRGFGFVTFENEDVVEKVCEIHFHEINNKMVECKK
AQPKEVMFPPGTRGRARGLPYTMDAFMLGMGMLGYPNFVATYGRGYPGFAPSYGYQFPDY
LPVSQDIIFIN*
>ENSG00000128573|ENST00000360232
MMQESATETISNSSMNQNGMSTLSSQLDAGSRDGRSSGDTSSEVSTVELLHLQQQQALQA
ARQLLLQQQTSGLKSPKSSDKQRPLQVPVSVAMMTPQVITPQQMQQILQQQVLSPQQLQA
LLQQQQAVMLQQQQLQEFYKKQQEQLHLQLLQQQQQQQQQQQQQQQQQQQQQQQQQQQQQ
QQQQQQQQQQHPGKQAKEQQQQQQQQQQLAAQQLVFQQQLLQMQQLQQQQHLLSLQRQG
LISIPPGQAALPVQSLPQAGLSPAEIQQLWKEVTGVHSMEDNGIKHGGLDLTTNNSSSTT
SSNTSKASPPITHHSIVNGQSSVLSARRDSSSHEETGASHTLYGHGVCKWPGCESICEDF
GQFLKHLNNEHALDDRSTAQCRVQMQVVQQLEIQLSKERERLQAMMTHLHMRPSEPKPSP
KPVSAYCFINSK*
>ENSG00000185177|ENST00000331162
MAKRPGPPGSREMGLLTFRDIAIEFSLEEWQCLDCAQRNLYRDVMLENYRNLVSLGIAVS
KPDLITCLEQNKESQNIKRNEMVAKHPVTRSHFTQDLQPEQGIKDSLQKVIPRTYGKCGH
EKLQFKKCCKSVGEYEVHKGGYSEVNQCLSTTQNKIFQTHKYVKVFGKFSNSNRDKTRYT
GNKHFKCNKYGKSFCMLSHLNQHQVIHTREKSYKCKECGKSFNCSSNHTTHKIIHTGEKP
YRCEECGKAFSWSANLTRHKRTHTGEKPYTCEECGQAFRRSSALTNHKRIHTGERPYKCE
ECGKAFSVSSTLTDHKRIHTGEKPCRCEECGKAFSWSSNLTRHKRIHTREKPYACEECGQ
AFSLSSNLMRHRRIHTGEKPYTCEECGQDFRRSSALTIHKRIHTGERPYKCEECGKVFSL
SSTLTDHKRIHTGERPYKCEECGKAFSLSSTLTDHKRIHTGERPYTCEECGKAFNCSSTL
MQHKRIHTGEKPYKCEECEQAFKWHSSLAKHKIIHTGEKPYKCE*

>ENSG00000176293|ENST00000359978
MELGSRRRSVGCRCRGLCLAVRREQVTFEDVVVGFSQEEWGQLKPAQRTLYRDVMLDTFR
LLVSVGHWLPKPNVISLLEQEAELWAVESRLPQGVYPDLETRPKVKLSVLKQGISEEISN
SVILVERFLWDGLWYCRGEDTEGHWEWSCESLESLAVPVAFTPVKTPVLEQWQRNGFGEN
ISLNPDLPHQPMTPERQSPHTWGTRGKREKPDLNVLQKTCVKEKPYKCQECGKAFSHSSA
LIEHHRTHTGERPYECHECLKGFRNSSALTKHQRIHTGEKPYKCTQCGRTFNQIAPLIQH
QRTHTGEKPYECSECGKSFSFRSSFSQHERTHTGEKPYECSECGKAFRQSIHLTQHLRIH
TGEKPYQCGECGKAFTHSSSLTKHQRTHTG*
>ENSG00000095794|ENST00000337656
MSKCARKKYIKTNPRQMTMETVESQHDGSITASLTESKSAHVQTQTGQNSIPALAQVAAI
AETDESAESEGVIDSHKRREILSRRPSYRKILNELSSDVPGVPKIEEERSEEEGTPPSIA
TMAVPTSIYQTSTGQYIAIAQGGTIQISNPGSDGVQGLQALTMTNSGAPPPGATIVQYAA
QSADGTQQFFVPGSQVVVQAATGDMPTYQIRAPTAALPQGVVMAASPGSLHSPQQLAEEA
TRKRELRLMKNREAARECRRKKKEYVKCLENRVAVLENQNKTLIEELKALKDLYCHKVE*
>ENSG00000181218|ENST00000366695
MSGRGKQGGKARAKAKSRSSRAGLQFPVGRVHRLLRKGNYSERVGAGAPVYLAAVLEYLT
AEILELAGNAARDNKKTRIIPRHLQLAIRNDEELNKLLGRVTIAQGGVLPNIQAVLLPKK
TESHHKAKGK*
>ENSG00000197587|ENST00000360032
MQHYGVNGYSLHAMNSLSAMYNLHQQAAQQAQHAPDYRPSVHALTLAERLADIILEARYG
SQHRKQRRSRTAFTAQQLEALEKTFQKTHYPDVVMRERLAMCTNLPEARVQVWFKNRRAK
FRKKQRSLQKEQLQKQKEAEGSHGEGKAEAPTPDTQLDTEQPPRLPGSDPPAELHLSLSE
QSASESAPEDQPDREEDPRAGAEDPKAEKSPGADSKGLGCKRGSPKADSPGSLTITPVAP
GGGLLGPSHSYSSSPLSLFRLQEQFRQHMAATNNLVHYSSFEVGGPAPAAAAAAAAVPYL
GVNMAPLGSLHCQSYYQSLSAAAAAHQGVWGSPLLPAPPAGLAPASATLNSKTTSIENLR
LRAKQHAASLGLDTLPN*
>ENSG00000179111|ENST00000317814
MVTRDRAENRDGPKMLKPLVEKRRRDRINRSLEELRLLLLERTRDQNLRNPKLEKAEILE
FAVGYLRERSRVEPPGVPRSPVQDAEALASCYLSGFRECLLRLAAFAHDASPAARAQLFS
ALHGYLRPKPPRPKPVDPRPPAPRPSLDPAAPALGPALHQRPPVHQGHPSPRCAWSPSLC
SPRAGDSGAPAPLTGLLPPPPPPHRQDGAPKAPLPPPPAFWRPWP*
>ENSG00000145687|ENST00000320672
MYGKGKSNSSAVPSDSQAREKLALYVVYEYLLHVGAQKSAQTFLSEIRWEKNITLGEPPGF
LHSWWCVFWDLYCAAPERRETCEHSSEAKAFHDYSAAAAPSPVLGNIPPGDGMPVGPVPP
GFFQPFMSPRYPGGPRPPLRIPNQALGGVPGSQPLLPSGMDPTRQQGHPNMGGPMQRMTP
PRGMVPLGPQNYGGAMRPPLNALGGPGMPGMNMGPGGGRPWPNPTNANSIPYSSASPGNY
VGPPGGGGPPGTPIMPSPADSTNSGDNMYTLMNAVPPGPNRPNFPMGPGSDGPMGGLGGM
ESHHMNGSLGSGDMDSISKNSPNNMSLSNQPGTPRDDGEMGGNFLNPFQSESYSPSMTMS
V*
>ENSG00000110693|ENST00000316399
MSSKQATSPFACAADGEDAMTQDLTSREKEEGSDQHVASHLPLHPIMHNKPHSEELPTLV
STIQQDADWDSVLSSQQRMESENNKLCSLYSFRNTSTSPHKPDEGSRDREIMTSVTFGTP
ERRKGSLADVVDTLKQKKLEEMTRTEQEDSSCMEKLLSKDWKEKMERLNTSELLGEIKGT
PESLAEKERQLSTMITQLISLREQLLAAHDEQKKLAASQIEKQRQQMDLARQQQEQIARQ

QQQLLQQQHKINLLQQQIQVQGHMPPLMIPIFPHDQRTLAAAAAAQQGFLFPPGITYKPG
DNYPVQFIPSTMAAAAASGLSPLQLQKGHVSHPQINQRLKGLSDRFGRNLDTFEHGGGHS
YNHKQIEQLYAAQLASMQVSPGAKMPSTPQPPNTAGTVSPTGIKNEKRGTSPVTQVKDEA
AAQPLNLSSRPKTAEPVKSPTSPTQNLFPASKTSPVNLPNKSSIPSPIGGSLGRGSSLDI
LSSLNSPALFGDQDTVMKAIQEARKMREQIQREQQQQQPHGVDGKLSSINNMGLNSCRNE
KERTRFENLGPQLTGKSNEDGKLGPGVIDLTRPEDAEGGATVAEARVYRDARGRASSEPH
IKRPMNAFMVWAKDERRKILQAFPDMHNSNISKILGSRWKSMSNQEKQPYYEEQARLSKI
HLEKYPNYKYKPRPKRTCIVDGKKLRIGEYKQLMRSRRQEMRQFFTVGQQPQIPITTGTG
VVYPGAITMATTTPSPQMTSDCSSTSASPEPSLPVIQSTYGMKTDGGSLAGNEMINGEDE
MEMYDDYEDDPKSDYSSENEAPEAVSAN*
>ENSG00000136574|ENST00000335135
MYQSLAMAANHGPPPGAYEAGGPGAFMHGAGAASSPVYVPTPRVPSSVLGLSYLQGGGAG
SASGGASGGSSGGAASGAGPGTQQGSPGWSQAGADGAAYTPPPVSPRFSFPGTTGSLAAA
AAAAAAREAAAYSSGGGAAGAGLAGREQYGRAGFAGSYSSPYPAYMADVGASWAAAAAAS
AGPFDSPVLHSLPGRANPAARHPNLDMFDDFSEGRECVNCGAMSTPLWRRDGTGHYLCNA
CGLYHKMNGINRPLIKPQRRLSASRRVGLSCANCQTTTTTLWRRNAEGEPVCNACGLYMK
LHGVPRPLAMRKEGIQTRKRKPKNLNKSKTPAAPSGSESLPPASGASSNSSNATTSSSEE
MRPIKTEPGLSSHYGHSSSVSQTFSVSAMSGHGPSIHPVLSALKLSPQGYASPVSQSPQT
SSKQDSWNSLVLADSHGDIITA*
>ENSG00000179300|ENST00000321110
MVEDLAASYIVLKLENEIRQAQVQWLMEENAALQAQIPELQKSQAAKEYDLLRKSSEAKE
PQKLPEHMNPPAAWEAQKTPEFKEPQKPPEPQDLLPWEPPAAWELQEAPAAPESLAPPAT
RESQKPPMAHEIPTVLEGQGPANTQDATIAQEPKNSEPQDPPNIEKPQEAPEYQETAAQL
EFLELPPPQEPLEPSNAQEFLELSAAQESLEGLIVVETSAASEFPQAPIGLEATDFPLQY
TLTFSGDSQKLPEFLVQLYSYMRVRGHLYPTEAALVSFVGNCFSGRAGWWFQLLLDIQSP
LLEQCESFIPVLQDTFDNPENMKDANQCIHQLCQGEGHVATHFHLIAQELNWDESTLWIQ
FQEGLASSIQDELSHTSPATNLSDLITQCISLEEKPDPNPLGKSSSAEGDGPESPPAENQ
PMQAAINCPHISEAEWVRWHKGRLCLYCGYPGHFARDCPVKPHQALQAGNIQACQ*
>ENSG00000144792|ENST00000322734
MRRKTRNFKHKTVKDNKVLTEGSDQESEKDNSQCCDPATNERVQAEKRQYVCTECGKAFS
QSANLTVHERIHTGEKPYKCKECGKAFSHSSNLVVHRRIHTGLKPYTCSECGKSFSGKSH
LIRHQGIHSGEKTYECKECGKAFSRSSGLISHHRVHTGEKPYSCIECGKAFSRSSNLTQH
QRMHRGKKVYKCKECGKTCGSNTKIMDHQRIHTGEKPYECDECGKTFILRKTLNEHQRLH
RREKPYKCNECGKAFTSNRNLVDHQRVHTGEKPYKCNECGKTFRQTSQVILHLRTHTKEK
PYKCSECGKAYRYSSQLIQHQRKHNEEKETS*
>ENSG00000103343|ENST00000344823
MAAKMEITLSSNTEASSKQERHIIAKLEEKRGPPLQKNCPDPELCRQSFRRFCYQEVSGP
QEALSQLRQLCRQWLQPELHTKEQILELLVMEQFLTILPPEIQARVRHRCPMSSKEIVTL
VEDFHRASKKPKQWVAVCMQGQKVLLEKTGSQLGEQELPDFQPQTPRRDLRESSPAEPSQ
AGAYDRLSPHHWEKSPLLQEPTPKLAGTELLIEKTDPNMATDELPCKLWLSFIA*
>ENSG00000188620|ENST00000357878
MPEPGPDAAGTASAQPQPPPPPPPAPKESPFSIKNLLNGDHHRPPPKPQPPPRTLFAPAS
AAAAAAAAAAAAAKGALEGAAGFALSQVGDLAFPRFEIPAQRFALPAHYLERSPAWWYPY
TLTPAGGHLPRPEASEKALLRDSSPASGTDRDSPEPLLKADPDHKELDSKSPDEIILEES

DSEESKKEGEAAPGAAGASVGAAAATPGAEDWKKGAESPEKKPACRKKKTRTVFSRSQVF
QLESTFDMKRYLSSSERAGLAASLHLTETQVKIWFQNRRNKWKRQLAAELEAANLSHAAA
QRIVRVPILYHENSAAEGAAAAAAGAPVPVSQPLLTFPHPVYYSHPVVSSVPLLRPV*
>ENSG00000185608|ENST00000333130
MTASVLRSISLALRPTSGLLGTWQTQLRETHQRASLLSFWELIPMRSEPLRKKKKVDPKK
DQEAKERLKRKIRKLEKATQELIPIEDFITPLKFLDKARERPQVELTFEETERRALLLKK
WSLYKQQERKMERDTIRAMLEAQQEALEELQLESPKLHAEAIKRDPNLFPFEKEGPHYTP
PIPNYQPPEGRYNDITKVYTQVEFKR*
>ENSG00000174405|ENST00000356922
MAASQTSQTVASHVPFADLCSTLERIQKSKGRAEKIRHFREFLDSWRKFHDALHKNHKDV
TDSFYPAMRLILPQLERERMAYGIKETMLAKLYIELLNLPRDGKDALKLLNYRTPTGTHG
DAGDFAMIAYFVLKPRCLQKGSLTIQQVNDLLDSIASNNSAKRKDLIKKSLLQLITQSSA
LEQKWLIRMIIKDLKLGVSQQTIFSVFHNDAAELHNVTTDLEKVCRQLHDPSVGLSDISI
TLFSAFKPMLAAIADIEHIEKDMKHQSFYIETKLDGERMQMHKDGDVYKYFSRNGYNYTD
QFGASPTEGSLTPFIHNAFKADIQICILDGEMMAYNPNTQTFMQKGTKFDIKRMVEDSDL
QTCYCVFDVLMVNNKKLGHETLRKRYEILSSIFTPIPGRIEIVQKTQAHTKNEVIDALNE
AIDKREEGIMVKQPLSIYKPDKRGEGWLKIKPEYVSGLMDELDILIVGGYWGKGSRGGMM
SHFLCAVAEKPPPGEKPSVFHTLSRVGSGCTMKELYDLGLKLAKYWKPFHRKAPPSSILC
GTEKPEVYIEPCNSVIVQIKAAEIVPSDMYKTGCTLRFPRIEKIRDDKEWHECMTLDDLE
QLRGKASGKLASKHLYIGGDDEPQEKKRKAAPKMKKVIGIIEHLKAPNLTNVNKISNIFE
DVEFCVMSGTDSQPKPDLENRIAEFGGYIVQNPGPDTYCVIAGSENIRVKNIILSNKHDV
VKPAWLLECFKTKSFVPWQPRFMIHMCPSTKEHFAREYDCYGDSYFIDTDLNQLKEVFSG
IKNSNEQTPEEMASLIADLEYRYSWDCSPLSMFRRHTVYLDSYAVINDLSTKNEGTRLAI
KALELRFHGAKVVSCLAEGVSHVIIGEDHSRVADFKAFRRTFKRKFKILKESWVTDSIDK
CELQEENQYLI*
>ENSG00000198453|ENST00000333987
MTSQSSVISNSCVTMERLSHMMERKAWCSQESALSEEEEDTTRPLETVTFKDVAVDLTQE
EWEQMKPAQRNLYRDVMLENYSNLVTVGCQVTKPDVIFKLEQEEEPWVMEEEMFGRHCPE
VWEVDEQIKKQQETLVRKVTSISKKILIKEKVIECKKVAKIFPLSSDIVTSRQSFYDCDS
LDKGLEHNLDLLRYEKGCVREKQSNEFGKPFYHCASYVVTPFKCNQCGQDFSHKFDLIRH
ERIHAGEKPYECKECGKAFSRKENLITHQKIHTGEKPYKCNECGKAFIQMSNLIRHHRIH
TGEKPYACKDCWKAFSQKSNLIEHERIHTGEKPYECKECGKSFSQKQNLIEHEKIHTGEK
PYACNECGRAFSRMSSVTLHMRSHTGEKPYKCNKCGKAFSQCSVFIIHMRSHTGEKPYVC
SECGKAFSQSSSLTVHMRNHTAEKPYECKECGKAFSRKENLITHQKIHTGEKPYECSECG
KAFIQMSNLIRHQRIHTGEKPYACTVCGKAFSQKSNLTEHEKIHTGEKPYHCNQCGKAFS
QRQNLLEHEKIHTGEKPFKCNECGKAFSRISSLTLHVRSHTGEKPYECNKCGKAFSQCSL
LIIHMRSHTGEKPFECNECGKAFSQRASLSIHKRGHTGERHQVY*
>ENSG00000094631|ENST00000334136
MTSTGQDSTTTRQRRSRQNPQSPPQDSSVTSKRNIKKGAVPRSIPNLAEVKKKGKMKKLG
QAMEEDLIVGLQGMDLNLEAEALAGTGLVLDEQLNEFHCLWDDSFPEGPERLHAIKEQLI
QEGLLDRCVSFQARFAEKEELMLVHSLEYIDLMETTQYMNEGELRVLADTYDSVYLHPNS
YSCACLASGSVLRLVDAVLGAEIRNGMAIIRPPGHHAQHSLMDGYCMFNHVAVAARYAQQ
KHRIRRVLIVDWDVHHGQGTQFTFDQDPSVLYFSIHRYEQGRFWPHLKASNWSTTGFGQG
QGYTINVPWNQVGMRDADYIAAFLHVLLPVALEFQPQLVLVAAGFDALQGDPKGEMAATP

AGFAQLTHLLMGLAGGKLILSLEGGYNLRALAEGVSASLHTLLGDPCPMLESPGAPCRSA
QASVSCALEALEPFWEVLVRSTETVERDNMEEDNVEESEEEGPWEPPVLPILTWPVLQSR
TGLVYDQNMMNHCNLWDSHHPEVPQRILRIMCRLEELGLAGRCLTLTPRPATEAELLTCH
SAEYVGHLRATEKMKTRELHRESSNFDSIYICPSTFACAQLATGAACRLVEAVLSGEVLN
GAAVVRPPGHHAEQDAACGFCFFNSVAVAARHAQTISGHALRILIVDWDVHHGNGTQHMF
EDDPSVLYVSLHRYDHGTFFPMGDEGASSQIGRAAGTGFTVNVAWNGPRMGDADYLAAWH
RLVLPIAYEFNPELVLVSAGFDAARGDPLGGCQVSPEGYAHLTHLLMGLASGRIILILEG
GYNLTSISESMAACTRSLLGDPPPLLTLPRPPLSGALASITETIQVHRRYWRSLRVMKVE
DREGPSSSKLVTKKAPQPAKPRLAERMTTREKKVLEAGMGKVTSASFGEESTPGQTNSET
AVVALTQDQPSEAATGGATLAQTISEAAIGGAMLGQTTSEEAVGGATPDQTTSEETVGGA
ILDQTTSEDAVGGATLGQTTSEEAVGGATLAQTTSEAAMEGATLDQTTSEEAPGGTELIQ
TPLASSTDHQTPPTSPVQGTTPQISPSTLIGSLRTLELGSESQGASESQAPGEENLLGEA
AGGQDMADSMLMQGSRGLTDQAIFYAVTPLPWCPHLVAVCPIPAAGLDVTQPCGDCGTIQ
ENWVCLSCYQVYCGRYINGHMLQHHGNSGHPLVLSYIDLSAWCYYCQAYVHHQALLDVKN
IAHQNKFGEDMPHPH*
>ENSG00000156313|ENST00000318842
MREPEELMPDSGAVFTFGKSKFAENNPGKFWFKNDVPVHLSCGDEHSAVVTGNNKLYMFG
SNNWGQLGLGSKSAISKPTCVKALKPEKVKLAACGRNHTLVSTEGGNVYATGGNNEGQLG
LGDTEERNTFHVISFFTSEHKIKQLSAGSNTSAALTEDGRLFMWGDNSEGQIGLKNVSNV
CVPQQVTIGKPVSWISCGYYHSAFVTTDGELYVFGEPENGKLGLPNQLLGNHRTPQLVSE
IPEKVIQVACGGEHTVVLTENAVYTFGLGQFGQLGLGTFLFETSEPKVIENIRDQTISYI
SCGENHTALITDIGLMYTFGDGRHGKLGLGLENFTNHFIPTLCSNFLRFIVKLVACGGCH
MVVFAAPHRGVAKEIEFDEINDTCLSVATFLPYSSLTSGNVLQRTLSARMRRRERERSPD
SFSMRRTLPPIEGTLGLSACFLPNSVFPRCSERNLQESVLSEQDLMQPEEPDYLLDEMTK
EAEIDNSSTVESLGETTDILNMTHIMSLNSNEKSLKLSPVQKQKKQQTIGELTQDTALTE
NDDSDEYEEMSEMKEGKACKQHVSQGIFMTQPATTIEAFSDEEVEIPEEKEGAEDSKGNG
IEEQEVEANEENVKVHGGRKEKTEILSDDLTDKAEDHEFSKTEELKLEDVDEEINAENVE
SKKKTVGDDESVPTGYHSKTEGAERTNDDSSAETIEKKEKANLEERAICEYNENPKGYML
DDADSSSLEILENSETTPSKDMKKTKKIFLFKRVPSINQKIVKNNNEPLPEIKSIGDQII
LKSDNKDADQNHMSQNHQNIPPTNTERRSKSCTIL*
>ENSG00000125952|ENST00000341653
MSDNDDIEVESDEEQPRFQSAADKRAHHNALERKRRDHIKDSFHSLRDSVPSLQGEKGTK
MKLTLPPVFPYEHLPFPTVFCHG*
>ENSG00000119574|ENST00000354590
MAAAEAVHHIHLQNFSRSLLETLNGQRLGGHFCDVTVRIREASLRAHRCVLAAGSPFFQD
KLLLGHSEIRVPPVVPAQTVRQLVEFLYSGSLVVAQGEALQVLTAASVLRIQTVIDECTQ
IIARARAPGTSAPTPLPTPVPPPLAPAQLRHRLRHLLAARPPGHPGAAHSRKQRQPARLQ
LPAPPTPAKAEGPDADPSLSAAPDDRGDEDDEESDDETDGEDGEGGGPGEGQAPPSFPDC
AAGFLTAAADSACEEPPAPTGLADYSGAGRDFLRGAGSAEDVFPDSYVSTWHDEDGAVPE
GCPTETPVQPDCILSGSRPPGVKTPGPPVALFPFHLGAPGPPAPPPSAPSGPAPAPPPAF
YPTLQPEAAPSTQLGEVPAPSAAPTTAPSGTPARTPGAEPPTYECSHCRKTFSSRKNYTK
HMFIHSGEKPHQCAVCWRSFSLRDYLLKHMVTHTGVRAFQCAVCAKRFTQKSSLNVHMRT
HRPERAPCPACGKVFSHRALLERHLAAHPAP*
>ENSG00000172086|ENST00000347055

MKHSKKTYDSFQDELEDYIKVQKARGLEPKTCFRKMKGDYLETCGYKGEVNSRPTYRMFD
QRLPSETIQTYPRSCNIPQTVENRLPQWLPAHDSRLRLDSLSYCQFTRDCFSEKPVPLNF
NQQEYICGSHGVEHRVYKHFSSDNSTSTHQASHKQIHQKRKRHPEEGREKSEEERSKHKR
KKSCEEIDLDKHKSIQRKKTEVEIETVHVSTEKLKNRKEKKSRDVVSKKEERKRTKKKKE
QGQERTEEEMLWDQSILGF*
>ENSG00000177508|ENST00000329734
MSFPQLGYQYIRPLYPSERPGAAGGSGGSAGARGGLGAGASELNASGSLSNVLSSVYGAP
YAAAAAAAAAQGYGAFLPYAAELPIFPQLGAQYELKDSPGVQHPAAAAAFPHPHPAFYPY
GQYQFGDPSRPKNATRESTSTLKAWLNEHRKNPYPTKGEKIMLAIITKMTLTQVSTWFAN
ARRRLKKENKMTWAPRSRTDEEGNAYGSEREEEDEEEDEEDGKRELELEEEELGGEEEDT
GGEGLADDDEDEEIDLENLDGAATEPELSLAGAARRDGDLGLGPISDSKNSDSEDSSEGL
EDRPLPVLSLAPAPPPVAVASPSLPSPPVSLDPCAPAPAPASALQKPKIWSLAETATSPD
NPRRSPPGAGGSPPGAAVAPSALQLSPAAAAAAAHRLVSAPLGKFPAWTNRPFPGPPPGP
RLHPLSLLGSAPPHLLGLPGAAGHPAAAAAFARPAEPEGGTDRCSALEVEKKLLKTAFQP
VPRRPQNHLDAALVLSALSSS*
>ENSG00000132819|ENST00000342690
MLLQPAPCAPSAGFPRPLAAPGAMHGSQKDTTFTKIFVGGLPYHTTDASLRKYFEGFGDI
EEAVVITDRQTGDYFCP*
>ENSG00000199035|ENST00000362165
Sequence unavailable
>ENSG00000105708|ENST00000344099
MDSVSFEDVAVNFTLEEWALLDSSQKKLYEDVMQETFKNLVCLGKKWEDQDIEDDHRNQG
KNRRCHMVERLCESRRGSKCGETTSQMPNVNINKETFTGAKPHECSFCGRDFIHHSSLNR
HMRSHTGQKPNEYQEYEKQPCKCKAVGKTFSYHHCFRKHERTHTGVKPYECKQCGKAFIY
YQPFQRHERTHAGQKPYECKQCGKTFIYYQSFQKHAHTGKKPYECKQCGKAFICYQSFQR
HKRTHTGEKPYECKQCGKAFSCPTYFRTHERTHTGEKPYKCKECGKAFSFLSSFRRHKRT
HSGEKPYECKECGKAFFYSASFRAHVIIHTGARPYKCKECGKAFNSSNSCRVHERTHIGE
KPYECKRCGKSFSWSISLRLHERTHTGEKPYECKQCHKTFSFSSSLREHETTHTGEKPYE
CKQCGKTFSFSSSLQRHERTHNAEKPYECKQCGKAFRCSSYFRIHERSHTGEKPYECKQC
GKVFIRSSSFRLHERTHTGEKPYECKLCGKTFSFSSSLREHEKIHTGNKPFECKQCGKAF
LRSSQIRLHERTHTGEKPYQCKQCGKAFISSSKFRMHERTHTGEKPYRCKQCGKAFRFSS
SVRIHERSHTGEKPYECKQCGKAFISSSHFRLHERTHMGEKV*
>ENSG00000128573|ENST00000350908
MMQESATETISNSSMNQNGMSTLSSQLDAGSRDGRSSGDTSSEVSTVELLHLQQQQALQA
ARQLLLQQQTSGLKSPKSSDKQRPLQVPVSVAMMTPQVITPQQMQQILQQQVLSPQQLQA
LLQQQQAVMLQQQQLQEFYKKQQEQLHLQLLQQQQQQQQQQQQQQQQQQQQQQQQQQQQQ
QQQQQQQQQQHPGKQAKEQQQQQQQQQLAAQQLVFQQQLLQMQQLQQQHLLSLQRQG
LISIPPGQAALPVQSLPQAGLSPAEIQQLWKEVTGVHSMEDNGIKHGGLDLTTNNSSSTT
SSNTSKASPPITHHSIVNGQSSVLSARRDSSSHEETGASHTLYGHGVCKWPGCESICEDF
GQFLKHLNNEHALDDRSTAQCRVQMQVVQQLEIQLSKERERLQAMMTHLHMRPSEPKPSP
KPLNLVSSVTMSKNMLETSPQSLPQTPTTPTAPVTPITQGPSVITPASVPNVGAIRRRHS
DKYNIPMSSEIAPNYEFYKNADVRPPFTYATLIRQAIMESSDRQLTLNEIYSWFTRTFAY
FRRNAATWKNAVRHNLSLHKCFVRVENVKGAVWTVDEVEYQKRRSQKITGSPTLVKNIPT
SLGYGAALNASLQAALAESSLPLLSNPGLINNASSGLLQAVHEDLNGSLDHIDSNGNSSP

GCSPQPHIHSIHVKEEPVIAEDEDCPMSLVTTANHSPELEDDREIEEEPLSEDLE*
>ENSG00000196092|ENST00000358127
MDLEKNYPTPRTSRTGHGGVNQLGGVFVNGRPLPDVVRQRIVELAHQGVRPCDISRQLRV
SHGCVSKILGRYYETGSIKPGVIGGSKPKVATPKVVEKIAEYKRQNPTMFAWEIRDRLLA
ERVCDNDTVPSVSSINRIIRTKVQQPPNQPVPASSHSIVSTGSVTQVSSVSTDSAGSSYS
ISGILGITSPSADTNKRKRDEGIQESPVPNGHSLPGRDFLRKQMRGDLFTQQQLEVLDRV
FERQHYSDIFTTTEPIKPEQTTEYSAMASLAGGLDDMKANLASPTPADIGSSVPGPQSYP
IVTGRDLASTTLPGYPPHVPPAGQGSYSAPTLTGMVPGSEFSGSPYSHPQYSSYNDSWRF
PNPGLLGSPYYYSAAARGAAPPAAATAYDRH*
>ENSG00000197102|ENST00000360184
MSEPGGGGGEDGSAGLEVSAVQNVADVSVLQKHLRKLVPLLLEDGGEAPAALEAALEEKS
ALEQMRKFLSDPQVHTVLVERSTLKEDVGDEGEEEKEFISYNINIDIHYGVKSNSLAFIK
RTPVIDADKPVSSQLRVLTLSEDSPYETLHSFISNAVAPFFKSYIRESGKADRDGDKMAP
SVEKKIAELEMGLLHLQQNIEIPEISLPIHPMITNVAKQCYERGEKPKVTDFGDKVEDPT
FLNQLQSGVNRWIREIQKVTKLDRDPASGTALQEISFWLNLERALYRIQEKRESPEVLLT
LDILKHGKRFHATVSFDTDTGLKQALETVNDYNPLMKDFPLNDLLSATELDKIRQALVAI
FTHLRKIRNTKYPIQRALRLVEAISRDLSSQLLKVLGTRKLMHVAYEEFEKVMVACFEVF
QTWDDEYEKLQVLLRDIVKRKREENLKMVWRINPAHRKLQARLDQMRKFRRQHEQLRAVI
VRVLRPQVTAVAQQNQGEVPEPQDMKVAEVLFDAADANAIEEVNLAYENVKEVDGLDVSK
EGTEAWEAAMKRYDERIDRVETRITARLRDQLGTAKNANEMFRIFSRFNALFVRPHIRGA
IREYQTQLIQRVKDDIESLHDKFKVQYPQSQACKMSHVRDLPPVSGSIIWAKQIDRQLTA
YMKRVEDVLGKGWENHVEGQKLKQDGDSFRMKLNTQEIFDDWARKVQQRNLGVSGRIFTI
ESTRVRGRTGNVLKLKVNFLPEIITLSKEVRNLKWLGFRVPLAIVNKAHQANQLYPFAIS
LIESVRTYERTCEKVEERNTISLLVAGLKKEVQALIAEGIALVWESYKLDPYVQRLAETV
FNFQEKVDDLLIIEEKIDLEVRSLETCMYDHKTFSEILNRVQKAVDDLNLHSYSNLPIWV
NKLDMEIERILGVRLQAGLRAWTQVLLGQAEDKAEVDMDTDAPQVSHKPGGEPKIKNVVH
ELRITNQVIYLNPPIEECRYKLYQEMFAWKMVVLSLPRIQSQRYQVGVHYELTEEEKFYR
NALTRMPDGPVALEESYSAVMGIVSEVEQYVKVWLQYQCLWDMQAENIYNRLGEDLNKWQ
ALLVQIRKARGTFDNAETKKEFGPVVIDYGKVQSKVNLKYDSWHKEVLSKFGQMLGSNMT
EFHSQISKSRQELEQHSVDTASTSDAVTFITYVQSLKRKIKQFEKQVELYRNGQRLLEKQ
RFQFPPSWLYIDNIEGEWGAFNDIMRRKDSAIQQQVANLQMKIVQEDRAVESRTTDLLTD
WEKTKPVTGNLRPEEALQALTIYEGKFGRLKDDREKCAKAKEALELTDTGLLSGSEERVQ
VALEELQDLKGVWSELSKVWEQIDQMKEQPWVSVQPRKLRQNLDALLNQLKSFPARLRQY
ASYEFVQRLLKGYMKINMLVIELKSEALKDRHWKQLMKRLHVNWVVSELTLGQIWDVDLQ
KNEAIVKDVLLVAQGEMALEEFLKQIREVWNTYELDLVNYQNKCRLIRGWDDLFNKVKEH
INSVSAMKLSPYYKVFEEDALSWEDKLNRIMALFDVWIDVQRRWVYLEGIFTGSADIKHL
LPVETQRFQSISTEFLALMKKVSKSPLVMDVLNIQGVQRSLERLADLLGKIQKALGEYLE
RERSSFPRFYFVGDEDLLEIIGNSKNVAKLQKHFKKMFAGVSSIILNEDNSVVLGISSRE
GEEVMFKTPVSITEHPKINEWLTLVEKEMRVTLAKLLAESVTEVEIFGKATSIDPNTYIT
WIDKYQAQLVVLSAQIAWSENVETALSSMGGGGDAAPLHSVLSNVEVTLNVLADSVLMEQ
PPLRRRKLEHLITELVHQRDVTRSLIKSKIDNAKSFEWLSQMRFYFDPKQTDVLQQLSIQ
MANAKFNYGFEYLGVQDKLVQTPLTDRCYLTMTQALEARLGGSPFGPAGTGKTESVKALG
HQLGRFVLVFNCDETFDFQAMGRIFVGLCQVGAWGCFDEFNRLEERMLSAVSQQVQCIQE
ALREHSNPNYDKTSAPITCELLNKQVKVSPDMAIFITMNPGYAGRSNLPDNLKKLFRSLA

MTKPDRQLIAQVMLYSQGFRTAEVLANKIVPFFKLCDEQLSSQSHYDFGLRALKSVLVSA
GNVKRERIQKIKREKEERGEAVDEGEIAENLPEQEILIQSVCETMVPKLVAEDIPLLFSL
LSDVFPGVQYHRGEMTALREELKKVCQEMYLTYGDGEEVGGMWVEKVLQLYQITQINHGL
MMVGPSGSGKSMAWRVLLKALERLEGVEGVAHIIDPKAISKDHLYGTLDPNTREWTDGLF
THVLRKIIDSVRGELQKRQWIVFDGDVDPEWVENLNSVLDDNKLLTLPNGERLSLPPNVR
IMFEVQDLKYATLATVSRCGMVWFSEDVLSTDMIFNNFLARLRSIPLDEGEDEAQRRRKG
KEDEGEEAASPMLQIQRDAATIMQPYFTSNGLVTKALEHAFQLEHIMDLTRLRCLGSLFS
MLHQACRNVAQYNANHPDFPMQIEQLERYIQRYLVYAILWSLSGDSRLKMRAELGEYIRR
ITTVPLPTAPNIPIIDYEVSISGEWSPWQAKVPQIEVETHKVAAPDVVVPTLDTVRHEAL
LYTWLAEHKPLVLCGPPGSGKTMTLFSALRALPDMEVVGLNFSSATTPELLLKTFDHYCE
YRRTPNGVVLAPVQLGKWLVLFCDEINLPDMDKYGTQRVISFIRQMVEHGGFYRTSDQTW
VKLERIQFVGACNPPTDPGRKPLSHRFLRHVPVVYVDYPGPASLTQIYGTFNRAMLRLIP
SLRTYAEPLTAAMVEFYTMSQERFTQDTQPHYIYSPREMTRWVRGIFEALRPLETLPVEG
LIRIWAHEALRLFQDRLVEDEERRWTDENIDTVALKHFPNIDREKAMSRPILYSNWLSKD
YIPVDQEELRDYVKARLKVFYEEELDVPLVLFNEVLDHVLRIDRIFRQPQGHLLLIGVSG
AGKTTLSRFVAWMNGLSVYQIKVHRKYTGEDFDEDLRTVLRRSGCKNEKIAFIMDESNVL
DSGFLERMNTLLANGEVPGLFEGDEYATLMTQCKEGAQKEGLMLDSHEELYKWFTSQVIR
NLHVVFTMNPSSEGLKDRAATSPALFNRCVLNWFGDWSTEALYQVGKEFTSKMDLEKPNY
IVPDYMPVVYDKLPQPPSHREAIVNSCVFVHQTLHQANARLAKRGGRTMAITPRHYLDFI
NHYANLFHEKRSELEEQQMHLNVGLRKIKETVDQVEELRRDLRIKSQELEVKNAAANDKL
KKMVKDQQEAEKKKVMSQEIQEQLHKQQEVIADKQMSVKEDLDKVEPAVIEAQNAVKSIK
KQHLVEVRSMANPPAAVKLALESICLLLGESTTDWKQIRSIIMRENFIPTIVNFSAEEIS
DAIREKMKKNYMSNPSYNYEIVNRASLACGPMVKWAIAQLNYADMLKRVEPLRNELQKLE
DDAKDNQQKANEVEQMIRDLEASIARYKEEYAVLISEAQAIKADLAAVEAKVNRSTALLK
SLSAERERWEKTSETFKNQMSTIAGDCLLSAAFIAYAGYFDQQMRQNLFTTWSHHLQQAN
IQFRTDIARTEYLSNADERLRWQASSLPADDLCTENAIMLKRFNRYPLIIDPSGQATEFI
MNEYKDRKITRTSFLDDAFRKNLESALRFGNPLLVQDVESYDPVLNPVLNREVRRTGGRV
LITLGDQDIDLSPSFVIFLSTRDPTVEFPPDLCSRVTFVNFTVTRSSLQSQCLNEVLKAE
RPDVDEKRSDLLKLQGEFQLRLRQLEKSLLQALNEVKGRILDDDTIITTLENLKREAAEV
TRKVEETDIVMQEVETVSQQYLPLSTACSSIYFTMESLKQIHFLYQYSLQFFLDIYHNVL
YENPNLKGVTDHTQRLSIITKDLFQVAFNRVARGMLHQDHITFAMLLARIKLKGTVGEPT
YDAEFQHFLRGNEIVLSAGSTPRIQGLTVEQAEAVVRLSCLPAFKDLIAKVQADEQFGIW
LDSSSPEQTVPYLWSEETPATPIGQAIHRLLLIQAFRPDRLLAMAHMFVSTNLGESFMSI
MEQPLDLTHIVGTEVKPNTPVLMCSVPGYDASGHVEDLAAEQNTQITSIAIGSAEGFNQA
DKAINTAVKSGRWVMLKNVHLAPGWLMQLEKKLHSLQPHACFRLFLTMEINPKVPVNLLR
AGRIFVFEPPPGVKANMLRTFSSIPVSRICKSPNERARLYFLLAWFHAIIQERLRYAPLG
WSKKYEFGESDLRSACDTVDTWLDDTAKGRQNISPDKIPWSALKTLMAQSIYGGRVDNEF
DQRLLNTFLERLFTTRSFDSEFKLACKVDGHKDIQMPDGIRREEFVQWVELLPDTQTPSW
LGLPNNAERVLLTTQGVDMISKMLKMQMLEDEDDLAYAETEKKTRTDSTSDGRPAWMRTL
HTTASNWLHLIPQTLSHLKRTVENIKDPLFRFFEREVKMGAKLLQDVRQDLADVVQVCEG
KKKQTNYLRTLINELVKGILPRSWSHYTVPAGMTVIQWVSDFSERIKQLQNISLAAASGG
AKELKNIHVCLGGLFVPEAYITATRQYVAQANSWSLEELCLEVNVTTSQGATLDACSFGV
TGLKLQGATCNNNKLSLSNAISTALPLTQLRWVKQTNTEKKASVVTLPVYLNFTRADLIF
TVDFEIATKEDPRSFYERGVAVLCTE*

>ENSG00000080802|ENST00000315544
MSRSPDAKEDPVECPLCMEPLEIDDINFFPCTCGYQICRFCWHRIRTDENGLCPACRKPY
PEDPAVYKPLSQEELQRIKNEKKQKQNERKQKISENRKHLASVRVVQKNLVFVVGLSQRL
ADPEVLKRPEYFGKFGKIHKVVINNSTSYAGSQGPSASAYVTYIRSEDALRAIQCVNNVV
VDGRTLKASLGTTKYCSYFLKNMQCPKPDCMYLHELGDEAASFTKEEMQAGKHQEYEQKL
LQELYKLNPNFLQLSTGSVDKNKNKVTPLQRYDTPIDKPSDSLSIGNGDNSQQISNSDTP
SPPPGLSKSNPVIPISSSNHSARSPFEGAVTESQSLFSDNFRHPNPIPSGLPPFPSSPQT
SSDWPTAPEPQSLFTSETIPVSSSTDWQAAFGFGSSKQPEDDLGFDPFDVTRKALADLIE
KELSVQDQPSLSPTSLQNSSSHTTTAKGPGSGFLHPAAATNANSLNSTFSVLPQRFPQFQ
QHRAVYNSFSFPGQAARYPWMAFPRNSIMHLNHTANPTSNSNFLDLNLPPQHNTGLGGIP
VAGEEEVKVSTMPLSTSSHSLQQGQQPTSLHTTVA*
>ENSG00000178209|ENST00000356346
MAGPLPDEQDFIQAYEEVREKYKDERDRVQKKTFTKWVNKHLIKAQRHISDLYEDLRDGH
NLISLLEVLSGDSLPREKGRMRFHKLQNVQIALDYLRHRQVKLVNIRNDDIADGNPKLTL
GLIWTIILHFQISDIQVSGQSEDMTAKEKLLLWSQRMVEGYQGLRCDNFTSSWRDGRLFN
AIIHRHKPLLIDMNKVYRQTNLENLDQAFSVAERDLGVTRLLDPEDVDVPQPDEKSIITY
VSSLYDAMPRVPDVQDGVRANELQLRWQEYRELVLLLLQWMRHHTAAFEERRFPSSFEEI
EILWSQFLKFKEMELPAKEADKNRSKGIYQSLEGAVQAGQLKVPPGYHPLDVEKEWGKLH
VAILEREKQLRSEFERLECLQRIVTKLQMEAGLCEEQLNQADALLQSDVRLLAAGKVPQR
AGEVERDLDKADSMIRLLFNDVQTLKDGRHPQGEQMYRRVYRLHERLVAIRTEYNLRLKA
GVAAPATQVAQVTLQSVQRRPELEDSTLRYLQDLLAWVEENQHRVDGAEWGVDLPSVEAQ
LGSHRGLHQSIEEFRAKIERARSDEGQLSPATRGAYRDCLGRLDLQYAKLLNSSKARLRS
LESLHSFVAAATKELMWLNEKEEEEVGFDWSDRNTNMTAKKESYSALMRELELKEKKIKE
LQNAGDRLLREDHPARPTVESFQAALQTQWSWMLQLCCCIEAHLKENAAYFQFFSDVREA
EGQLQKLQEALRRKYSCDRSATVTRLEDLLQDAQDEKEQLNEYKGHLSGLAKRAKAVVQL
KPRHPAHPMRGRLPLLAVCDYKQVEVTVHKGDECQLVGPAQPSHWKVLSSSGSEAAVPSV
CFLVPPPNQEAQEAVTRLEAQHQALVTLWHQLHVDMKSLLAWQSLRRDVQLIRSWSLATF
RTLKPEEQRQALHSLELHYQAFLRDSQDAGGFGPEDRLMAEREYGSCSHHYQQLLQSLEQ
GAQEESRCQRCISELKDIRLQLEACETRTVHRLRLPLDKEPARECAQRIAEQQKAQAEVE
GLGKGVARLSAEAEKVLALPEPSPAAPTLRSELELTLGKLEQVRSLSAIYLEKLKTISLV
IRGTQGAEEVLRAHEEQLKEAQAVPATLPELEATKASLKKLRAQAEAQQPTFDALRDELR
GAQEVGERLQQRHGERDVEVERWRERVAQLLERWQAVLAQTDVRQRELEQLGRQLRYYRE
SADPLGAWLQDARRRQEQIQAMPLADSQAVREQLRQEQALLEEIERHGEKVEECQRFAKQ
YINAIKDYELQLVTYKAQLEPVASPAKKPKVQSGSESVIQEYVDLRTHYSELTTLTSQYI
KFISETLRRMEEEERLAEQQRAEERERLAEVEAALEKQRQLAEAHAQAKAQAEREAKELQ
QRMQEEVVRREEAAVDAQQQKRSIQEELQQLRQSSEAEIQAKARQAEAAERSRLRIEEEI
RVVRLQLEATERQRGGAEGELQALRARAEEAEAQKRQAQEEAERLRRQVQDESQRKRQAE
VELASRVKAEAEAAREKQRALQALEELRLQAEEAERRLRQAEVERARQVQVALETAQRSA
EAELQSKRASFAEKTAQLERSLQEEHVAVAQLREEAERRAQQQAEAERAREEAERELERW
QLKANEALRLRLQAEEVAQQKSLAQAEAEKQKEEAEREARRRGKAEEQAVRQRELAEQEL
EKQRQLAEGTAQQRLAAEQELIRLRAETEQGEQQRQLLEEELARLQREAAAATQKRQELE
AELAKVRAEMEVLLASKARAEEESRSTSEKSKQRLEAEAGRFRELAEEAARLRALAEEAK
RQRQLAEEDAARQRAEAERVLAEKLAAIGEATRLKTEAEIALKEKEAENERLRRLAEDEA
FQRRRLEEQAAQHKADIEERLAQLRKASDSELERQKGLVEDTLRQRRQVEEEILALKASF

EKAAAGKAELELELGRIRSNAEDTLRSKEQAELEAARQRQLAAEEERRRREAEERVQKSL
AAEEEAARQRKAALEEVERLKAKVEEARRLRERAEQESARQLQLAQEAAQKRLQAEEKAH
AFAVQQKEQELQQTLQQEQSVLDQLRGEAEAARRAAEEAEEARVQAEREAAQSRRQVEEA
ERLKQSAEEQAQARAQAQAAAEKLRKEAEQEAARRAQAEQAALRQKQAADAEMEKHKKFA
EQTLRQKAQVEQELTTLRLQLEETDHQKNLLDEELQRLKAEATEAARQRSQVEEELFSVR
VQMEELSKLKARIEAENRALILRDKDNTQRFLQEEAEKMKQVAEEAARLSVAAQEAARLR
QLAEEDLAQQRALAEKMLKEKMQAVQEATRLKAEAELLQQQKELAQEQARRLQEDKEQMA
QQLAEETQGFQRTLEAERQRQLEMSAEAERLKLRVAEMSRAQARAEEDAQRFRKQAEEIG
EKLHRTELATQEKVTLVQTLEIQRQQSDHDAERLREAIAELEREKEKLQQEAKLLQLKSE
EMQTVQQEQLLQETQALQQSFLSEKDSLLQRERFIEQEKAKLEQLFQDEVAKAQQLREEQ
QRQQQQMEQERQRLVASMEEARRRQHEAEEGVRRKQEELQQLEQQRRQQEELLAEENQRL
REQLQLLEEQHRAALAHSEEVTASQVAATKTLPNGRDALDGPAAEAEPEHSFDGLRRKVS
AQRLQEAGILSAEELQRLAQGHTTVDELARREDVRHYLQGRSSIAGLLLKATNEKLSVYA
ALQRQLLSPGTALILLEAQAASGFLLDPVRNRRLTVNEAVKEGVVGPELHHKLLSAERAV
TGYKDPYTGQQISLFQAMQKGLIVREHGIRLLEAQIATGGVIDPVHSHRVPVDVAYRRGY
FDEEMNRVLADPSDDTKGFFDPNTHENLTYLQLLERCVEDPETGLCLLPLTDKAAKGGEL
VYTDSEARDVFEKATVSAPFGKFQGKTVTIWEIINSEYFTAEQRRDLLRQFRTGRITVEK
IIKIIITVVEEQEQKGRLCFEGLRSLVPAAELLESRVIDRELYQQLQRGERSVRDVAEVD
TVRRALRGANVIAGVWLEEAGQKLSIYNALKKDLLPSDMAVALLEAQAGTGHIIDPATSA
RLTVDEAVRAGLVGPEFHEKLLSAEKAVTGYRDPYTGQSVSLFQALKKGLIPREQGLRLL
DAQLSTGGIVDPSKSHRVPLDVACARGCLDEETSRALSAPRADAKAYSDPSTGEPATYGE
LQQRCRPDQLTGLSLLPLSEKAARARQEELYSELQARETFEKTPVEVPVGGFKGRTVTVW
ELISSEYFTAEQRQELLRQFRTGKVTVEKVIKILITIVEEVETLRQERLSFSGLRAPVPA
SELLASGVLSRAQFEQLKDGKTTVKDLSELGSVRTLLQGSGCLAGIYLEDTKEKVSIYEA
MRRGLLRATTAALLLEAQAATGFLVDPVRNQRLYVHEAVKAGVVGPELHEQLLSAEKAVT
GYRDPYSGSTISLFQAMQKGLVLRQHGIRLLEAQIATGGIIDPVHSHRVPVDVAYQRGYF
SEEMNRVLADPSDDTKGFFDPNTHENLTYRQLLERCVEDPETGLRLLPLKGAEKAEVVET
TQVYTEEETRRAFEETQIDIPGGGSHGGSTMSLWEVMQSDLIPEEQRAQLMADFQAGRVT
KERMIIIIIEIIEKTEIIRQQGLASYDYVRRRLTAEDLFEARIISLETYNLLREGTRSLR
EALEAESAWCYLYGTGSVAGVYLPGSRQTLSIYQALKKGLLSAEVARLLLEAQAATGFLL
DPVKGERLTVDEAVRKGLVGPELHDRLLSAERAVTGYRDPYTEQTISLFQAMKKELIPTE
EALRLLDAQLATGGIVDPRLGFHLPLEVAYQRGYLNKDTHDQLSEPSEVRSYVDPSTDER
LSYTQLLRRCRRDDGTGQLLLPLSDARKLTFRGLRKQITMEELVRSQVMDEATALQLREG
LTSIEEVTKNLQKFLEGTSCIAGVFVDATKERLSVYQAMKKGIIRPGTAFELLEAQAATG
YVIDPIKGLKLTVEEAVRMGIVGPEFKDKLLSAERAVTGYKDPYSGKLISLFQAMKKGLI
LKDHGIRLLEAQIATGGIIDPEESHRLPVEVAYKRGLFDEEMNEILTDPSDDTKGFFDPN
TEENLTYLQLMERCITDPQTGLCLLPLKEKKRERKTSSKSSVRKRRVVIVDPETGKEMSV
YEAYRKGLIDHQTYLELSEQECEWEEITISSSDGVVKSMIIDRRSGRQYDIDDAIAKNLI
DRSALDQYRAGTLSITEFADMLSGNAGGFRSRSSSVGSSSSYPISPAVSRTQLASWSDPT
EETGPVAGILDTETLEKVSITEAMHRNLVDNITGQRLLEAQACTGGIIDPSTGERFPVTD
AVNKGLVDKIMVDRINLAQKAFCGFEDPRTKTKMSAAQALKKGWLYYEAGQRFLEVQYLT
GGLIEPDTPGRVPLDEALQRGTVDARTAQKLRDVGAYSKYLTCPKTKLKISYKDALDRSM
VEEGTGLRLLEAAAQSTKGYYSPYSVSGSGSTAGSRTGSRTGSRAGSRRGSFDATGSGFS
MTFSSSSYSSSGYGRRYASGSSASLGGPESAVA*

>ENSG00000187821|ENST00000338875
MSDKLKERKVSRLSPNGTCALVVEASDSPTRHLGGPMAGKCPHGTLSVEESRVRTPVSHK
VIEKRRRDRINRCLNELGKTVPMALAKQGEPQEALAQIRSRVRSLVLSSATVPDQQALGR
CEGPFLLLQSSGKLEKAEILEMTVQYLRALHSADFPRGREKAELLAEFANYFHYGYHECM
KNLVHYLTTVERMETKDTKYARILAFLQSKARLGAEPAFPPLGSLPEPDFSYQLHPAGPE
FAGHSPGEAAVFPQGSGAGPFPWPPGAARSPALPYLPSAPVPLASPAQQHSPFLTPVQGL
DRHYLNLIGHAHPNALNLHTPQHPPVL*
>ENSG00000072518|ENST00000361128
MSSARTPLPTLNERDTEQPTLGHLDSKPSSKSNMIRGRNSATSADEQPHIGNYRLLKTIG
KGNFAKVKLARHILTGKEVAVKIIDKTQLNSSSLQKLFREVRIMKVLNHPNIVKLFEVIE
TEKTLYLVMEYASGGEVFDYLVAHGRMKEKEARAKFRQIVSAVQYCHQKFIVHRDLKAEN
LLLDADMNIKIADFGFSNEFTFGNKLDTFCGSPPYAAPELFQGKKYDGPEVDVWSLGVIL
YTLVSGSLPFDGQNLKELRERVLRGKYRIPFYMSTDCENLLKKFLILNPSKRGTLEQIMK
DRWMNVGHEDDELKPYVEPLPDYKDPRRTELMVSMGYTREEIQDSLVGQRYNEVMATYLL
LGYKSSELEGDTITLKPRPSADLTNSSAPSPSHKVQRSVSANPKQRRFSDQAAGPAIPTS
NSYSKKTQSNNAENKRPEEDRESGRKASSTAKVPASPLPGLERKKTTPTPSTNSVLSTST
NRSRNSPLLERASLGQASIQNGKDSTAPQRVPVASPSAHNISSSGGAPDRTNFPRGVSSR
STFHAGQLRQVRDQQNLPYGVTPASPSGHSQGRRGASGSIFSKFTSKFVRRNLSFRFARR
PHVVGSGGNDKEKEEFREAKPRSLRFTWSMKTTSSMEPNEMMREIRKVLDANSCQSELHE
KYMLLCMHGTPGHEDFVQWEMEVCKLPRLSLNGVRFKRISGTSMAFKNIASKIANELKL*
>ENSG00000072310|ENST00000355815
MDEPPFSEAALEQALGEPCDLDAALLTDIEGEVGAGRGRANGLDAPRAGADRGAMDCTFE
DMLQLINNQDSDFPGLFDPPYAGSGAGGTDPASPDTSSPGSLSPPPATLSSSLEAFLSGP
QAAPSPLSPPQPAPTPLKMYPSMPAFSPGPGIKEESVPLSILQTPTPQPLPGALLPQSFP
APAPPQFSSTPVLGYPSPPGGFSTGSPPGNTQQPLPGLPLASPPGVPPVSLHTQVQSVVP
QQLLTVTAAPTAAPVTTTVTSQIQQVPVLLQPHFIKADSLLLTAMKTDGATVKAAGLSPL
VSGTTVQTGPLPTLVSGGTILATVPLVVDAEKLPINRLAAGSKAPASAQSRGEKRTAHNA
IEKRYRSSINDKIIELKDLVVGTEAKLNKSAVLRKAIDYIRFLQHSNQKLKQENLSLRTA
VHKSKSLKDLVSACGSGGNTDVLMEGVKTEVEDTLTPPPSDAGSPFQSSPLSLGSRGSGS
GGSGSDSEPDSPVFEDSKAKPEQRPSLHSRGMLDRSRLALCTLVFLCLSCNPLASLLGAR
GLPSPSDTTSVYHSPGRNVLGTESRDGPGWAQWLLPPVVWLLNGLLVLVSLVLLFVYGEP
VTRPHSGPAVYFWRHRKQADLDLARGDFAQAAQQLWLALRALGRPLPTSHLDLACSLLWN
LIRHLLQRLWVGRWLAGRAGGLQQDCALRVDASASARDAALVYHKLHQLHTMGKHTGGHL
TATNLALSALNLAECAGDAVSVATLAEIYVAAALRVKTSLPRALHFLTRFFLSSARQACL
AQSGSVPPAMQWLCHPVGHRFFVDGDWSVLSTPWESLYSLAGNPVDPLAQVTQLFREHLL
ERALNCVTQPNPSPGSADGDKEFSDALGYLQLLNSCSDAAGAPAYSFSISSSMATTTGVD
PVAKWWASLTAVVIHWLRRDEEAAERLCPLVEHLPRVLQESERPLPRAALHSFKAARALL
GCAKAESGPASLTICEKASGYLQDSLATTPASSSIDKAVQLFLCDLLLVVRTSLWRQQQP
PAPAPAAQGTSSRPQASALELRGFQRDLSSLRRLAQSFRPAMRRVFLHEATARLMAGASP
TRTHQLLDRSLRRRAGPGGKGGAVAELEPRPTRREHAEALLLASCYLPPGFLSAPGQRVG
MLAEAARTLEKLGDRRLLHDCQQMLMRLGGGTTVTSS*
>ENSG00000182944|ENST00000332050
MASTDYSTYSQAAAQQGYSAYTAQPTQGYAQTTQAYGQQSYGTYGQPTDVSYTQAQTTAT
YGQTAYATSYGQPPTGYTTPTAPQAYSQPVQGYGTGAYDTTTATVTTTQASYAAQSAYGT

QPAYPAYGQQPAATAPTRPQDGNKPTETSQPQSSTGGYNQPSLGYGQSNYSYPQVPGSYP
MQPVTAPPSYPPTSYSSTQPTSYDQSSYSQQNTYGQPSSYGQQSSYGQQSSYGQQPPTSY
PPQTGSYSQAPSQYSQQSSSYGQQIYFVGSGRTKNGFETRAFSSLHGFQSAGERGGFNKP
GGPMDEGPDLDLGPPVDPDEDSDNSAIYVQGLNDSVTLDDLADFFKQCGVVKMNKRTGQP
MIHIYLDKETGKPKGDATVSYEDPPTAKAAVEWFDGKDFQGSKLKVSLARKKPPMNSMRG
GLPPREGRGMPPPLRGGPGGPGGPGGPMGRMGGRGGDRGGFPPRGPRGSRGNPSGGGNVQ
HRAGDWQCPNPGCGNQNFAWRTECNQCKAPKPEGFLPPPFPPPGGDRGRGGPGGMRGGRG
GLMDRGGPGGMFRGGRGGDRGGFRGGRGMDRGGFGGGRRGGPGGPPGPLMEQMGGRRGGR
GGPGKMDKGEHRQERRDRPY*
>ENSG00000189164|ENST00000356178
MAVGLCKAMSQGLVTFRDVALDFSQEEWEWLKPSQKDLYRDVMLENYRNLVWLGDGCSCC
STLRTTVTAEASSSSSSIQASFHSLALPLMSILA*
>ENSG00000185345|ENST00000366898
MIVFVRFNSSHGFPVEVDSDTSIFQLKEVVAKRQGVPADQLRVIFAGKELRNDWTVQNCD
LDQQSIVHIVQRPWRKGQEMNATGGDDPRNAAGGCEREPQSLTRVDLSSSVLPGDSVGLA
VILHTDSRKDSPPAGSPAGRSIYNSFYVYCKGPCQRVQPGKLRVQCSTCRQATLTLTQGP
SCWDDVLIPNRMSGECQSPHCPGTSAEFFFKCGAHPTSDKETSVALHLIATNSRNITCIT
CTDVRSPVLVFQCNSRHVICLDCFHLYCVTRLNDRQFVHDPQLGYSLPCVAGCPNSLIKE
LHHFRILGEEQYNRYQQYGAEECVLQMGGVLCPRPGCGAGLLPEPDQRKVTCEGGNGLGC
GFAFCRECKEAYHEGECSAVFEASGTTTQAYRVDERAAEQARWEAASKETIKKTTKPCPR
CHVPVEKNGGCMHMKCPQPQCRLEWCWNCGCEWNRVCMGDHWFDV*
>ENSG00000159216|ENST00000344691
MRIPVDASTSRRFTPPSTALSPGKMSEALPLGAPDAGAALAGKLRSGDRSMVEVLADHPG
ELVRTDSPNFLCSVLPTHWRCNKTLPIAFKVVALGDVPDGTLVTVMAGNDENYSAELRNA
TAAMKNQVARFNDLRFVGRSGRGKSFTLTITVFTNPPQVATYHRAIKITVDGPREPRRHR
QKLDDQTKPGSLSFSERLSELEQLRRTAMRVSPHHPAPTPNPRASLNHSTAFNPQPQSQM
QDTRQIQPSPPWSYDQSYQYLGSIASPSVHPATPISPGRASGMTTLSAELSSRLSTAPDL
TAFSDPRQFPALPSISDPRMHYPGAFTYSPTPVTSGIGIGMSAMGSATRYHTYLPPPYPG
SSQAQGGPFQASSPSYHLYYGASAGSYQFSMVGGERSPPRILPPCTNASTGSALLNPSLP
NQSDVVEAEGSHSNSPTNMAPSARLEEAVWRPY*
>ENSG00000085741|ENST00000322563
MRARPQVCEALLFALALQTGVCYGIKWLALSKTPSALALNQTQHCKQLEGLVSAQVQLCR
SNLELMHTVVHAAREVMKACRRAFADMRWNCSSIELAPNYLLDLERGTRESAFVYALSAA
AISHAIARACTSGDLPGCSCGPVPGEPPGPGNRWGGCADNLSYGLLMGAKFSDAPMKVKK
TGSQANKLMRLHNSEVGRQALRASLEMKCKCHGVSGSCSIRTCWKGLQELQDVAADLKTR
YLSATKVVHRPMGTRKHLVPKDLDIRPVKDSELVYLQSSPDFCMKNEKVGSHGTQDRQCN
KTSNGSDSCDLMCCGRGYNPYTDRVVERCHCKYHWCCYVTCRRCERTVERYVCK*
>ENSG00000153207|ENST00000326225
MAAERRCGSMRDLRAQVTSGLLPFPEVTLQALGEDEITLESVLRGKFAAGKNGLACLACG
PQLEVVNSITGERLSAYRFSGVNEQPPVVLAVKEFSWQKRTGLLIGLEETEGSVLCLYDL
GISKVVKAVVLPGRVTAIEPIINHGGASASTQHLHPSLRWLFGVAAVVTDVGQILLVDLC
LDDLSCNQNEVEASDLEVLTGIPAEVPHIRESVMRQGRHLCFQLVSPTGTAVSTLSYISR
TNQLAVGFSDGYLALWNMKSMKREYYIQLESGQVPVYAVTFQEPENDPRNCCYLWAVQST
QDSEGDVLSLHLLQLAFGNRKCLASGQILYEGLEYCEERYTLDLTGGMFPLRGQTSNTKL

LGCQSIEKFRSHGDREEGVNEALSPDTSVSVFTWQVNIYGQGKPSVYLGLFDINRWYHAQ
MPDSLRSGEYLHNCSYFALWSLESVVSRTSPHGILDILVHERSLNRGVPPSYPPPEQFFN
PSTYNFDATCLLNSGVVHLTCTGFQKETLTFLKKSGPSLNELIPDGYNRCLVAGLLSPRF
VDVQPSSLSQEEQLEAILSAAIQTSSLGLLTGYIRRWITEEQPNSATNLRFVLEWTWNKV
VLTKEEFDRLCVPLFDGSCHFMDPQTIQSIQQCYLLLSNLNIVLSCFASEAREITERGLI
DLSNKFVVSHLICQYAQVVLWFSHSGLLPEGIDDSVQLSRLCYNYPVIQNYYTSRRQKFE
RLSRGKWNPDCLMIDGLVSQLGERIEKLWKRDEGGTGKYPPASLHAVLDMYLLDGVTEAA
KHSITIYLLLDIMYSFPNKTDTPIESFPTVFAISWGQVKLIQGFWLIDHNDYESGLDLLF
HPATAKPLSWQHSKIIQAFMSQGEHRQALRYIQTMKPTVSSGNDVILHLTVLLFNRCMVE
AWNFLRQHCNRLNIEELLKHMYEVCQEMGLMEDLLKLPFTDTEQECLVKFLQSSASVQNH
EFLLVHHLQRANYVPALKLNQTLKINVMNDRDPRLRERSLARNSILDQYGKILPRVHRKL
AIERAKPYHLSTSSVFRLVSRPKPLSAVPKQVVTGTVLTRSVFINNVLSKIGEVWASKEP
INSTTPFNSSKIEEPSPIVYSLPAPELPEAFFGTPISKASQKISRLLDLVVQPVPRPSQC
SEFIQQSSMKSPLYLVSRSLPSSSQLKGSPQAISRASELHLLETPLVVKKAKSLAMSVTT
SGFSEFTPQSILRSTLRSTPLASPSPSPGRSPQRLKETRISFVEEDVHPKWIPGAADDSK
LEVFTTPKKCAVPVETEWLKSKDRTTSFFLNSPEKEHQEMDEGSQSLEKLDVSKGNSSVS
ITSDETTLEYQDAPSPEDLEETVFTASKPKSSSTALTTNVTEQTEKDGDKDVFASEVTPS
DLQKQMGNLEDAETKDLLVAAEAFSELNHLSPVQGTEASLCAPSVYEGKIFTQKSKVPVL
DEGLTSVETYTPAIRANDNKSMADVLGDGGNSSLTISEGPIVSERRLNQEVALNLKEDHE
VEVGVLKESVDLPEEKLPISDSPPDTQEIHVIEQEKLEAQDSGEEARNLSFNELYPSGTL
KLQYNFDTIDQQFCDLADNKDTAECDIAEVDGELFVAQSNFTLILEGEEGEVEPGDFASS
DVLPKAANTATEEKLVCSGENDNHGQIANLPSAVTSDQKSQKVDTLPYVPEPIKVAIAEN
LLDVIKDTRSKEITSDTMEQSIHETIPLVSQNIMCPTKLVKSAFKTAQETSTMTMNVSQV
DDVVSSKTRTRGQRIQNVNVKSAQQEASADVATPKMPGQSVRKKTRKAKEISEASENIYS
DVRGLSQNQQIPQNSVTPRRGRRKKEVNQDILENTSSVEQELQITTGRESKRLKSSQLLE
PAVEETTKKEVKVSSVTKRTPRRIKRSVENQESVEIINDLKVSTVTSPSRMIRKLRSTNL
DASENTGNKQDDKSSDKQLRIKHVRRVRGREVSPSDVREDSNLESSQLTVQAEFDMSAIP
RKRGRPRKINPSEDVGSKAVKEERSPKKKEAPSIRRRSTRNTPAKSENVDVGKPALGKSI
LVPNEELSMVMSSKKKLTKKTESQSQKRSLHSVSEERTDEMTHKETNEQEERLLATASFT
KSSRSSRTRSSKAILLPDLSEPNNEPLFSPASEVPRKAKAKKIEVPAQLKELVSDLSSQF
VISPPALRSRQKNTSNKNKLEDELKDDAQSVETLGKPKAKRIRTSKTKQASKNTEKESAW
SPPPIEIRLISPLASPADGVKSKPRKTTEVTGTGLGRNRKKLSSYPKQILRRKML*
>ENSG00000128739|ENST00000346403
MTVGKSSKMLQHIDYRMRCILQDGRIFIGTFKAFDKHMNLILCDCDEFRKIKPKNAKQPE
REEKRVLGLVLLRGENLVSMTVEGPPPKDTGIARVPLAGAAGGPGVGRAAGRGVPAGVPI
PQAPAGLAGPVRGVGGPSQQVMTPQGRGTVAAAAVAATASIAGAPTQYPPGRGTPPPPVG
RATPPPGIMAPPPGMRPPMGPPIGLPPARGTPIGMPPPGMRPPPPGIRGPPPPGMRPPRP
*
>ENSG00000186812|ENST00000330501
MAVESGVISTLIPQDPPEQELILVKVEDNFSWDEKFKQNGSTQSCQELFRQQFRKFCYQE
TPGPREALSRLQELCYQWLMPELHTKEQILELLVLEQFLSILPEELQIWVQQHNPESGEE
AVTLLEDLEREFDDPGQQVPASPQGPAVPWKDLTCLRASQESTDIHLQPLKTQLKSWKPC
LSPKSDCENSETATKEGISEEKSQGLPQEPSFRGISEHESNLVWKQGSATGEKLRSPSQG
GSFSQVIFTNKSLGKRDLYDEAERCLILTTDSIMCQKVPPEERPYRCDVCGHSFKQHSSL

TQHQRIHTGEKPYKCNQCGKAFSLRSYLIIHQRIHSGEKAYECSECGKAFNQSSALIRHR
KIHTGEKACKCNECGKAFSQSSYLIIHQRIHTGEKPYECNECGKTFSQSSKLIRHQRIHT
GERPYECNECGKAFRQSSELITHQRIHSGEKPYECSECGKAFSLSSNLIRHQRIHSGEEP
YQCNECGKTFKRSSALVQHQRIHSGDEAYICNECGKAFRHRSVLMRHQRVHTIK*
>ENSG00000173253|ENST00000358146
MADPQAGSAAGDWEIDVESLELEEDVCGAPRSTPPGPSPPPADGDCEDDEDDDGVDEDAE
EEGDGEEAGASPGMPGQPEQRGGPQPRPPLAPQASPAGTGPRERCTPAGGGAEPRKLSRT
PKCARCRNHGVVSCLKGHKRFCRWRDCQCANCLLVVERQRVMAAQVALRRQQATEDKKGL
SGKQNNFERKAVYQRQVRAPSLLAKSILEGYRPIPAETYVGGTFPLPPPVSDRMRKRRAF
ADKELENIMLEREYKEREMLETSQAAALFLPNRMVPGPDYNSYKSAYSPSPVEPPSKDFC
NFLPTCLDLTMQYSGSGNMELISSNVSVATTYRQYPLSSRFLVWPKCGPISDTLLYQQCL
LNATTSVQALKPGASWDLKGARVQDGLSAEQDMMPSKLEGSLVLPHTPEIQTTRSDLQGH
QAVPERSAFSPPRRNFSPIVDTDSLAAQGHVLTKISKENTRHPLPLRHNPFHSLFQQTLT
DKSGPELKTPFVKEAFEETPKKHRECLVKDNQKYTFTIDRCAKDLFVAKQVGTKLSVNEP
LSFSVESILKRPSSAITRVSQ*
>ENSG00000165732|ENST00000354185
MPGKLRSDAGLESDTAMKKGETLRKQTEEKEKKEKPKSDKTEEIAEEEETVFPKAKQVKK
KAEPSEVDMNSPKSKKAKKKEEPSQNDISPKTKSLRKKKEPIEKKVVSSKTKKVTKNEEP
SEEEIDAPKPKKMKKEKEMNGETREKSPKLKNGFPHPEPDCNPSEAASEESNSEIEQEIP
VEQKEGAFSNFPISEETIKLLKGRGVTFLFPIQAKTFHHVYSGKDLIAQARTGTGKTFSF
AIPLIEKLHGELQDRKRGRAPQVLVLAPTRELANQVSKDFSDITKKLSVACFYGGTPYGG
QFERMRNGIDILVGTPGRIKDHIQNGKLDLTKLKHVVLDEVDQMLDMGFADQVEEILSVA
YKKDSEDNPQTLLFSATCPHWVFNVAKKYMKSTYEQVDLIGKKTQKTAITVEHLAIKCHW
TQRAAVIGDVIRVYSGHQGRTIIFCETKKEAQELSQNSAIKQDAQSLHGDIPQKQREITL
KGFRNGSFGVLVATNVAARGLDIPEVDLVIQSSPPKDVESYIHRSGRTGRAGRTGVCICF
YQHKEEYQLVQVEQKAGIKFKRIGVPSATEIIKASSKDAIRLLDSVPPTAISHFKQSAEK
LIEEKGAVEALAAALAHISGATSVDQRSLINSNVGFVTMILQCSIEMPNISYAWKELKEQ
LGEEIDSKVKGMVFLKGKLGVCFDVPTASVTEIQEKWHDSRRWQLSVATEQPELEGPREG
YGGFRGQREGSRGFRGQRDGNRRFRGQREGSRGPRGQRSGGGNKSNRSQNKGQKRSFSKA
FGQ*
>ENSG00000124214|ENST00000360426
MKLGKKPMYKPVDPYSRMQSTYNYNMRGGAYPPRYFYPFPVPPLLYQVELSVGGQQFNGK
GKTRQAAKHDAAAKALRILQNEPLPERLEVNGRESEEENLNKSEISQVFEIALKRNLPVN
FEVARESGPPHMKNFVTKVSVGEFVGEGEGKSKKISKKNAAIAVLEELKKLPPLPAVERV
KPRIKKKTKPIVKPQTSPEYGQGINPISRLAQIQQAKKEKEPEYTLLTERGLPRRREFVM
QVKVGNHTAEGTGTNKKVAKRNAAENMLEILGFKVPQAQPTKPALKSEEKTPIKKPGDGR
KVTFFEPGSGDENGTSNKEDEFRMPYLSHQQLPAGILPMVPEVAQAVGVSQGHHTKDFTR
AAPNPAKATVTAMIARELLYGGTSPTAETILKNNISSGHVPHGPLTRPSEQLDYLSRVQG
FQVEYKDFPKNNKNEFVSLINCSSQPPLISHGIGKDVESCHDMAALNILKLLSELDQQST
EMPRTGNGPMSVCGRC*
>ENSG00000128645|ENST00000331462
MSSYLEYVSCSSSGGVGGDVLSLAPKFCRSDARPVALQPAFPLGNGDGAFVSCLPLAAAR
PSPSPPAAPARPSVPPPAAPQYAQCTLEGAYEPGAAPAAAAGGADYGFLGSGPAYDFPGV
LGRAADDGGSHVHYATSAVFSGGGSFLLSGQVDYAAFGEPGPFPACLKASADGHPGAFQT

ASPAPGTYPKSVSPASGLPAAFSTFEWMKVKRNASKKGKLAEYGAASPSSAIRTNFSTKQ
LTELEKEFHFNKYLTRARRIEIANCLHLNDTQVKIWFQNRRMKQKKREREGLLATAIPVA
PLQLPLSGTTPTKFIKNPGSPSQSQEPS*
>ENSG00000186871|ENST00000334463
MEASRRFPEAEALSPEQAAHYLRYVKEAKEATKNGDLEEAFKLFNLAKDIFPNEKVLSRI
QKIQEALEELAEQGDDEFTDVCNSGLLLYRELHNQLFEHQKEGIAFLYSLYRDGRKGGIL
ADDMGLGKTVQIIAFLSGMFDASLVNHVLLIMPTNLINTWVKEFIKWTPGMRVKTFHGPS
KDERTRNLNRIQQRNGVIITTYQMLINNWQQLSSFRGQEFVWDYVILDEAHKIKTSSTKS
AICARAIPASNRLLLTGTPIQNNLQELWSLFDFACQGSLLGTLKTFKMEYENPITRAREK
DATPGEKALGFKISENLMAIIKPYFLRRTKEDVQKKKSSNPEARLNEKNPDVDAICEMPS
LSRKNDLIIWIRLVPLQEEIYRKFVSLDHIKELLMETRSPLAELGVLKKLCDHPRLLSAR
ACCLLNLGTFSAQDGNEGEDSPDVDHIDQVTDDTLMEESGKMIFLMDLLKRLRDEGHQTL
VFSQSRQILNIIERLLKNRHFKTLRIDGTVTHLLEREKRINLFQQNKDYSVFLLTTQVGG
VGLTLTAATRVVIFDPSWNPATDAQAVDRVYRIGQKENVVVYRLITCGTVEEKIYRRQVF
KDSLIRQTTGEKKNPFRYFSKQELRELFTIEDLQNSVTQLQLQSLHAAQRKSDIKLDEHI
AYLQSLGIAGISDHDLMYTCDLSVKEELDVVEESHYIQQRVQKAQFLVEFESQNKEFLME
QQRTRNEGAWLREPVFPSSTKKKCPKLNKPQPQPSPLLSTHHTQEEDISSKMASVVIDDL
PKEGEKQDLSSIKVNVTTLQDGKGTGSADSIATLPKGFGSVEELCTNSSLGMEKSFATKN
EAVQKETLQEGPKQEALQEDPLESFNYVLSKSTKADIGPNLDQLKDDEILRHCNPWPIIS
ITNESQNAESNVSIIEIADDLSASHSALQDAQASEAKLEEEPSASSPQYACDFNLFLEDS
ADNRQNFSSQSLEHVEKENSLCGSAPNSRAGFVHSKTCLSWEFSEKDDEPEEVVVKAKIR
SKARRIVSDGEDEDDSFKDTSSINPFNTSLFQFSSVKQFDASTPKNDISPPGRFFSSQIP
SSVNKSMNSRRSLASRRSLINMVLDHVEDMEERLDDSSEAKGPEDYPEEGVEESSGEASK
YTEEDPSGETLSSENKSSWLMTSKPSALAQETSLGAPEPLSGEQLVGSPQDKAAEATNDY
ETLVKRGKELKECGKIQEALNCLVKALDIKSADPEVMLLTLSLYKQLNNN*
>ENSG00000198315|ENST00000330236
MAEESRKPSAPSPPDQTPEEDLVIVKVEEDHGWDQESSLHESNPLGQEVFRLRFRQLRYQ
ETLGPREALIQLRALCHQWLRPDLNTKEQILELLVLEQFLTILPEELQTLVKEHQLENGE
EVVTLLEDLERQIDILGRPVSARVHGHRVLWEEVVHSASAPEPPNTQLQSEATQHKSPVP
QESQERAMSTSQSPTRSQKGSSGDQEMTATLLTAGFQTLEKIEDMAVSLIREEWLLDPSQ
KDLCRDNRPENFRNMFSLGGETRSENRELASKQVISTGIQPHGETAAKCNGDVIRGLEHE
EARDLLGRLERQRGNPTQERRHKCDECGKSFAQSSGLVRHWRIHTGEKPYQCNVCGKAFS
YRSALLSHQDIHNKVKRYHCKECGKAFSQNTGLILHQRIHTGEKPYQCNQCGKAFSQSAG
LILHQRIHSGERPYECNECGKAFSHSSHLIGHQRIHTGEKPYECDECGKTFRRSSHLIGH
QRSHTGEKPYKCNECGRAFSQKSGLIEHQRIHTGERPYKCKECGKAFNGNTGLIQHLRIH
TGEKPYQCNECGKAFIQRSSLIRHQRIHSGEKSESISV*
>ENSG00000165280|ENST00000358901
MASGADSKGDDLSTAILKQKNRPNRLIVDEAINEDNSVVSLSQPKMDELQLFRGDTVLLK
GKKRREAVCIVLSDDTCSDEKIRMNRVVRNNLRVRLGDVISIQPCPDVKYGKRIHVLPID
DTVEGITGNLFEVYLKPYFLEAYRPIRKGDIFLVRGGMRAVEFKVVETDPSPYCIVAPDT
VIHCEGEPIKREDEEESLNEVGYDDIGGCRKQLAQIKEMVELPLRHPALFKAIGVKPPRG
ILLYGPPGTGKTLIARAVANETGAFFFLINGPEIMSKLAGESESNLRKAFEEAEKNAPAI
IFIDELDAIAPKREKTHGEVERRIVSQLLTLMDGLKQRAHVIVMAATNRPNSIDPALRRF
GRFDREVDIGIPDATGRLEILQIHTKNMKLADDVDLEQVANETHGHVGADLAALCSEAAL

QAIRKKMDLIDLEDETIDAEVMNSLAVTMDDFRWALSQSNPSALRETVVEVPQVTWEDIG
GLEDVKRELQELVQYPVEHPDKFLKFGMTPSKGVLFYGPPGCGKTLLAKAIANECQANFI
SIKGPELLTMWFGESEANVREIFDKARQAAPCVLFFDELDSIAKARGGNIGDGGGAADRV
INQILTEMDGMSTKKNVFIIGATNRPDIIDPAILRPGRLDQLIYIPLPDEKSRVAILKAN
LRKSPVAKDVDLEFLAKMTNGFSGADLTEICQRACKLAIRESIESEIRRERERQTNPSAM
EVEEDDPVPEIRRDHFEEAMRFARRSVSDNDIRKYEMFAQTLQQSRGFGSFRFPSGNQGG
AGPSQGSGGGTGGSVYTEDNDDDLYG*
>ENSG00000064607|ENST00000337018
MAARRITQETFDAVLQEKAKRYHMDASGEAVSETLQFKAQDLLRAVPRSRAEMYDDVHSD
GRYSLSGSVAHSRDAGREGLRSDVFPGPSFRSSNPSISDDSYFRKECGRDLEFSHSDSRD
QVIGHRKLGHFRSQDWKFALRGSWEQDFGHPVSQESSWSQEYSFGPSAVLGDFGSSRLIE
KECLEKESRDYDVDHPGEADSVLRGGSQVQARGRALNIVDQEGSLLGKGETQGLLTAKGG
VGKLVTLRNVSTKKIPTVNRITPKTQGTNQIQKNTPSPDVTLGTNPGTEDIQFPIQKIPL
GLDLKNLRLPRRKMSFDIIDKSDVFSRFGIEIIKWAGFHTIKDDIKFSQLFQTLFELETE
TCAKMLASFKCSLKPEHRDFCFFTIKFLKHSALKTPRVDNEFLNMLLDKGAVKTKNCFFE
IIKPFDKYIMRLQDRLLKSVTPLLMACNAYELSVKMKTLSNPLDLALALETTNSLCRKSL
ALLGQTFSLASSFRQEKILEAVGLQDIAPSPAAFPNFEDSTLFGREYIDHLKAWLVSSGC
PLQVKKAEPEPMREEEKMIPPTKPEIQAKAPSSLSDAVPQRADHRVVGTIDQLVKRVIEG
SLSPKERTLLKEDPAYWFLSDENSLEYKYYKLKLAEMQRMSENLRGADQKPTSADCAVRA
MLYSRAVRNLKKKLLPWQRRGLLRAQGLRGWKARRATTGTQTLLSSGTRLKHHGRQAPGL
SQAKPSLPDRNDAAKDCPPDPVGPSPQDPSLEASGPSPKPAGVDISEAPQTSSPCPSADI
DMKTMETAEKLARFVAQVGPEIEQFSIENSTDNPDLWFLHDQNSSAFKFYRKKVFELCPS
ICFTSSPHNLHTGGGDTTGSQESPVDLMEGEAEFEDEPPPREAELESPEVMPEEEDEDDE
DGGEEAPAPGGAGKSEGSTPADGLPGEAAEDDLAGAPALSQASSGTCFPRKRISSKSLKV
GMIPAPKRVCLIQEPKVHEPVRIAYDRPRGRPMSKKKKPKDLDFAQQKLTDKNLGFQMLQ
KMGWKEGHGLGSLGKGIREPVSVGTPSEGEGLGADGQEHKEDTFDVFRQRMMQMYRHKRA
NK*
>ENSG00000114861|ENST00000318779
MMQESGTETKSNGSAIQNGSGGSNHLLECGGLREGRSNGETPAVDIGAADLAHAQQQQQQ
WHLINHQPSRSPSSWLKRLISSPWELEVLQVPLWGAVAETKMSGPVCQPNPSPF*
>ENSG00000042088|ENST00000335725
MSQEGDYGRWTISSSDESEEEKPKPDKPSTSSLLCARQGAANEPRYTCSEAQKAAHKRKI
SPVKFSNTDSVLPPKRQKSGSQEDLGWCLSSSDDELQPEMPQKQAEKVVIKKEKDISAPN
DGTAQRTENHGAPACHRLKEEEDEYETSGEGQDIWDMLDKGNPFQFYLTRVSGVKPKYNS
GALHIKDILSPLFGTLVSSAQFNYCFDVDWLVKQYPPEFRKKPILLVHGDKREAKAHLHA
QAKPYENISLCQAKLDIAFGTHHTKMMLLLYEEGLRVVIHTSNLIHADWHQKTQGIWLSP
LYPRIADGTHKSGESPTHFKADLISYLMAYNAPSLKEWIDVIHKHDLSETNVYLIGSTPG
RFQGSQKDNWGHFRLKKLLKDHASSMPNAESWPVVGQFSSVGSLGADESKWLCSEFKESM
LTLGKESKTPGKSSVPLYLIYPSVENVRTSLEGYPAGGSLPYSIQTAEKQNWLHSYFHKW
SAETSGRSNAMPHIKTYMRPSPDFSKIAWFLVTSANLSKAAWGALEKNGTQLMIRSYELG
VLFLPSAFGLDSFKVKQKFFAGSQEPMATFPVPYDLPPELYGSKDRPWIWNIPYVKAPDT
HGNMWVPS*
>ENSG00000180035|ENST00000320159
MERAVEPWGPDLHRPEEREPQRGARTGLGSENVISQPNEFEHTPQEDDLGFKEEDLAPDH

EVGNASLKPEGIQNWDDLWVQREGLGKPQPRDRGPRLLGEPRWGQASSDRAAVCGECGKS
FRQMSDLVKHQRTHTGEKPYKCGVCGKGFGDSSARIKHQRTHSGEKPYRARPPAQGPPKI
PRSRIPAGERPTICGECGKSFRQSSDLVKHQRTHTGEKPYKCGICGKGFGDSSARIKHQR
THRGEQPPRPVVPRRQPSRAATAATQGPKAQDKPYICTDCGKRFVLSCSLLSHQRSHLGP
KPFGCDVCGKEFARGSDLVKHLRVHTGEKPYLCPECGKGFADSSARVKHLRTHSGERPHA
CPECDRTFSLSSTLLRHRLTHMEPQDFSFPGYPLPALIPSPPPPPLGTSPPLTPRSPSHS
GEPFGLPGLEPEPGGPQAGEPPPPLAGDKPHKCPECGKGFRRSSDLVKHHRVHTGEKPYL
CPECGKGFADSSARVKHLRTHRGERARPPPPSTLLRPHNPPGPVPMAPRPRVRAQPSGPS
QPHVCGFCGKEFPRSSDLVKHRRTHTGEKPYKCAECGKGFGDSSARIKHQRGHLVLTPFG
IGDGRARPLKQEAATGLE*
>ENSG00000117475|ENST00000329281
MTTKNLETKVTVTSSPIRGAGDGMETEEPPKSVEVTSGVQSRKHHSLQSPWKKAVPSESP
GVLQLGKMLTEKAMEVKAVRILVPKAAITHDIPNKNTKVKSLGHHKGEFLGQSEGVIEPN
KELSEVKNVLEKLKNSERRLLQDKEGLSNQLRVQTEVNRELKKLLVASVGDDLQYHFERL
AREKNQLILENEALGRNTAQLSEQLERMSIQCDVWRSKFLASRVMADELTNSRAALQRQN
RDAHGAIQDLLSEREQFRQEMIATQKLLEELLVSLQWGREQTYSPSVQPHSTAELALTNH
KLAKAVNSHLLGNVGINNQKKIPSTVEFCSTPAEKMAETVLRILDPVTCKESSPDNPFFE
SSPTTLLATKKNIGRFHPYTRYENITFNCCNHCRGELIAL*
>ENSG00000198795|ENST00000361524
MSRRKQAKPRSLKDPNCKLEDKTEDGEALDCKKRPEDGEELEDEAVHSCDSCLQVFESLS
DITEHKINQCQLTDGVDVEDDPTCSWPASSPSSKDQTSPSHGEGCDFGEEEGGPGLPYPC
QFCDKSFSRLSYLKHHEQSHSDKLPFKCTYCSRLFKHKRSRDRHIKLHTGDKKYHCSECD
AAFSRSDHLKIHLKTHTSNKPYKCAICRRGFLSSSSLHGHMQVHERNKDGSQSGSRMEDW
KMKDTQKCSQCEEGFDFPEDLQKHIAECHPECSPNEDRAALQCVYCHELFVEETSLMNHM
EQVHSGEKKNSCSICSESFHTVEELYSHMDSHQQPESCNHSNSPSLVTVGYTSVSSTTPD
SNLSVDSSTMVEAAPPIPKSRGRKRAAQQTPDMTGPSSKQAKVTYSCIYCNKQLFSSLAV
LQIHLKTMHLDKPEQAHICQYCLEVLPSLYNLNEHLKQVHEAQDPGLIVSAMPAIVYQCN
FCSEVVNDLNTLQEHIRCSHGFANPAAKDSNAFFCPHCYMGFLTDSSLEEHIRQVHCDLS
GSRFGSPVLGTPKEPVVEVYSCSYCTNSPIFNSVLKLNKHIKENHKNIPLALNYIHNGKK
SRALSPLSPVAIEQTSLKMMQAVGGAPARPTGEYICNQCGAKYTSLDSFQTHLKTHLDTV
LPKLTCPQCNKEFPNQESLLKHVTIHFMITSTYYICESCDKQFTSVDDLQKHLLDMHTFV
FFRCTLCQEVFDSKVSIQLHLAVKHSNEKKVYRCTSCNWDFRNETDLQLHVKHNHLENQG
KVHKCIFCGESFGTEVELQCHITTHSKKYNCKFCSKAFHAIILLEKHLREKHCVFETKTP
NCGTNGASEQVQKEEVELQTLLTNSQESHNSHDGSEEDVDTSEPMYGCDICGAAYTMETL
LQNHQLRDHNIRPGESAIVKKKAELIKGNYKCNVCSRTFFSENGLREHMQTHLGPVKHYM
CPICGERFPSLLTLTEHKVTHSKSLDTGNCRICKMPLQSEEEFLEHCQMHPDLRNSLTGF
RCVVCMQTVTSTLELKIHGTFHMQKTGNGSAVQTTGRGQHVQKLYKCASCLKEFRSKQDL
VKLDINGLPYGLCAGCVNLSKSASPGINVPPGTNRPGLGQNENLSAIEGKGKVGGLKTRC
SSCNVKFESESELQNHIQTIHRELVPDSNSTQLKTPQVSPMPRISPSQSDEKKTYQCIKC
QMVFYNEWDIQVHVANHMIDEGLNHECKLCSQTFDSPAKLQCHLIEHSFEGMGGTFKCPV
CFTVFVQANKLQQHIFSAHGQEDKIYDCTQCPQKFFFQTELQNHTMTQHSS*
>ENSG00000176102|ENST00000323959
MSGDGATEQAAEYVPEKVKKAEKKLEENPYDLDAWSILIREAQNQPIDKARKTYERLVAQ
FPSSGRFWKLYIEAEIKAKNYDKVEKLFQRCLMKVLHIDLWKCYLSYVRETKGKLPSYKE

KMAQAYDFALDKIGMEIMSYQIWVDYINFLKGVEAVGSYAENQRITAVRRVYQRGCVNPM
INIEQLWRDYNKYEEGINIHLAKKMIEDRSRDYMNARRVAKEYETVMKGLDRNAPSVPPQ
NTPQEAQQVDMWKKYIQWEKSNPLRTEDQTLITKRVMFAYEQCLLVLGHHPDIWYEAAQY
LEQSSKLLAEKGDMNNAKLFSDEAANIYERAISTLLKKNMLLYFAYADYEESRMKYEKVH
SIYNRLLAIEDIDPTLVYIQYMKFARRAEGIKSGRMIFKKAREDTRTRHHVYVTAALMEY
YCSKDKSVAFKIFELGLKKYGDIPEYVLAYIDYLSHLNEDNNTRVLFERVLTSGSLPPEK
SGEIWARFLAFESNIGDLASILKVEKRRFTAFKEEYEGKETALLVDRYKFMDLYPCSASE
LKALGYKDVSRAKLAAIIPDPVVAPSIVPVLKDEVDRKPEYPKPDTQQMIPFQPRHLAPP
GLHPVPGGVFPVPPAAVVLMKLLPPPICFQGPFVQVDELMEIFRRCKIPNTVEEAVRIIT
GGAPELAVEGNGPVESNAVLTKAVKRPNEDSDEDEEKGAVVPPVHDIYRARQQKRIR*
>ENSG00000163104|ENST00000354268
MNLFNLDRFRFEKRNKIEEAPEATPQPSQPGPSSPISLSAEEENAEGEVSRANTPDSDIT
EKTEDSSVPETPDNERKASISYFKNQRGIQYIDLSSDSEDVVSPNCSNTVQEKTFNKDTV
IIVSEPSEDEESQGLPTMARRNDDISELEDLSELEDLKDAKLQTLKELFPQRSDNDLLKL
IESTSTMDGAIAAALLMFGDAGGGPRKRKLSSSSEPYEEDEFNDDQSIKKTRLDHGEESN
ESAESSSNWEKQESIVLKLQKEFPNFDKQELREVLKEHEWMYTEALESLKVFAEDQDMQY
VSQSEVPNGKEVSSRSQNYPKNATKTKLKQKFSMKAQNGFNKKRKKNVFNPKRVVEDSEY
DSGSDVGSSLDEDYSSGEEVMEDGYKGKILHFLQDASIGELTLIPQCSQKKAQKITELRP
FNSWEALFTKMSKTNGLSEDLIWHCKTLIQERDVVIRLMNKCEDISNKLTKQVTMLTGNG
GGWNIEQPSILNQSLSLKPYQKVGLNWLALVHKHGLNGILADEMGLGKTIQAIAFLAYLY
QEGNNGPHLIVVPASTIDNWLREVNLWCPTLKVLCYYGSQEERKQIRFNIHSRYEDYNVI
VTTYNCAISSSDDRSLFRRLKLNYAIFDEGHMLKNMGSIRYQHLMTINANNRLLLTGTPV
QNNLLELMSLLNFVMPHMFSSSTSEIRRMFSSKTKSADEQSIYEKERIAHAKQIIKPFIL
RRVKEEVLKQLPPKKDRIELCAMSEKQEQLYLGLFNRLKKSINNLEKNTEMCNVMMQLRK
MANHPLLHRQYYTAEKLKEMSQLMLKEPTHCEANPDLIFEDMEVMTDFELHVLCKQYRHI
NNFQLDMDLILDSGKFRVLGCILSELKQKGDRVVLFSQFTMMLDILEVLLKHHQHRYLRL
DGKTQISERIHLIDEFNTDMDIFVFLLSTKAGGLGINLTSANVVILHDIDCNPYNDKQAE
DRCHRVGQTKEVLVIKLISQGTIEESMLKINQQKLKLEQDMTTVDEGDEGSMPADIATLL
KTSMGL*
>ENSG00000133226|ENST00000323848
MDAGFFRGTSAEQDNRFSNKQKKLLKQLKFAECLEKKVDMSKVNLEVIKPWITKRVTEIL
GFEDDVVIEFIFNQLEVKNPDSKMMQINLTGFLNGKNAREFMGELWPLLLSAQENIAGIP
SAFLELKKEEIKQRQIEQEKLASMKKQDEDKDKRDKEEKESSREKRERSRSPRRRKSRSP
SPRRRSSPVRRERKRSHSRSPRHRTKSRSPSPAPEKKEKTPELPEPSVKVKEPSVQEATS
TSDILKVPKPEPIPEPKEPSPEKNSKKEKEKEKTRPRSRSRSKSRSRTRSRSPSHTRPRR
RHRSRSRSYSPRRRPSPRRRPSPRRRTPPRRMPPPPRHRRSRSPVRRRRRSSASLSGSSS
SSSSSRSRSPPKKPPKRTSSPPRKTRRLSPSASPPRRRHRPSPPATPPPKTRHSPTPQQS
NRTRKSRVSVSPGRTSGKVTKHKGTEKRESPSPAPKPRKVELSESEEDKGGKMAAADSVQ
QRRQYRRQNQQSSSDSGSSSSSEDERPKRSHVKNGEVGRRRRHSPSRSASPSPRKRQKET
SPRGRRRRSPSPPPTRRRRSPSPAPPPRRRRTPTPPPRRRTPSPPPRRRSPSPRRYSPPI
QRRYSPSPPPKRRTASPPPPPKRRASPSPPPKRRVSHSPPPKQRSSPVTKRRSPSLSSKH
RKGSSPSRSTREARSPQPNKRHSPSPRPRAPQTSSSPPPVRRGASSSPQRRQSPSPSTRP
IRRVSRTPEPKKIKKAASPSPQSVRRVSSSRSVSGSPEPAAKKPPAPPSPVQSQSPSTNW
SPAVPVKKAKSPTPSPSPPRNSDQEGGGKKKKKKKDKKHKKDKKHKKHKKHKKEKAVAAA

AAAAVTPAAIAAATTTLAQEEPVAAPEPKKETESEAEDNLDDLEKHLREKALRSMRKAQV
SPQS*
>ENSG00000168066|ENST00000334944
MATGANATPLDFPSKKRKRSRWNQDTMEQKTVIPGMPTVIPPGLTREQERAYIVQLQIED
LTRKLRTGDLGIPPNPEDRSPSPEPIYNSEGKRLNTREFRTRKKLEEERHNLITEMVALN
PDFKPPADYKPPATRVSDKVMIPQDEYPEINFVGLLIGPRGNTLKNIEKECNAKIMIRGK
GSVKEGKVGRKDGQMLPGEDEPLHALVTANTMENVKKAVEQIRNILKQGIETPEDQNDLR
KMQLRELARLNGTLREDDNRILRPWQSSETRSITNTTVCTKCGGAGHIASDCKFQRPGDP
QSAQDKARMDKEYLSLMAELGEAPVPASVGSTSGPATTPLASAPRPAAPANNPPPPSLMS
TTQSRPPWMNSGPSESRPYHGMHGGGPGGPGGGPHSFPHPLPSLTGGHGGHPMQHNPNGP
PPPWMQPPPPPMNQGPHPPGHHGPPPMDQYLGSTPVGSGVYRLHQGKGMMPPPPMGMMPP
PPPPPSGQPPPPPSGPLPPWQQQQQQPPPPPPPSSSMASSTPLPWQQNTTTTTTSAGTGS
IPPWQQQQAAAAASPGAPQMQGNPTMVPLPPGVQPPLPPGAPPPPPPPPPGSAGMMIPPR
GGDGPSHESEDFPRPLVTLPGRQPQQRPWWTGWFGKAA*
>ENSG00000049618|ENST00000319584
MPPQYGQQGVSGYCQQGQQPYYSQQPQPPHLPPQAQYLPSQSQQRYQPQQDMSQEGYGTR
SQPPLAPGKPNHEDLNLIQQERPSSLPDLSGSIDDLPTGTEATLSSAVSASGSTSSQGDQ
SNPAQSPFSPHASPHLSSIPGGPSPSPVGSPVGSNQSRSGPISPASIPGSQMPPQPPGSQ
SESSSHPALSQSPMPQERGFMAGTQRNPQMAQYGPQQTGPSMSPHPSPGGQMHAGISSFQ
QSNSSGTYGPQMSQYGPQGNYSRPPAYSGVPSASYSGPGPGMGISANNQMHGQGPSQPCG
AVPLGRMPSAGMQNRPFPGNMSSMTPSSPGMSQQGGPGMGPPMPTVNRKAQEAAAAVMQA
AANSAQSRQGSFPGMNQSGLMASSSPYSQPMNNSSSLMNTQAPPYSMAPAMVNSSAASVG
LADMMSPGESKLPLPLKADGKEEGTPQPESKSKDSYSSQGISQPPTPGNLPVPSPMSPSS
ASISSFHGDESDSISSPGWPKTPSSPKSSSSTTTGEKITKVYELGNEPERKLWVDRYLTF
MEERGSPVSSLPAVGKKPLDLFRLYVCVKEIGGLAQVNKNKKWRELATNLNVGTSSSAAS
SLKKQYIQYLFAFECKIERGEEPPPEVFSTGDTKKQPKLQPPSPVVQMEPRACDTGGSGR
KASWSRPAPRARAGGSRHTQASGGRRQRHPGLQKPLDSLPPTQQVASPIPVPSLGKVSGN
IFVVKGK*
>ENSG00000153113|ENST00000341926
MNPTETKAIPVSQQMEGPHLPNKKKHKKQAVKTEPEKKSQSTKLSVVHEKKSQEGKPKEH
TEPKSLPKQASDTGSNDAHNKKAVSRSAEQQPSEKSTEPKTKPQDMISAGGESVAGITAI
SGKPGDKKKEKKSLTPAVPVESKPDKPSGKSGMDAALDDLIDTLGGPEETEEENTTYTGP
EVSDPMSSTYIEELGKREVTIPPKYRELLAKKEGITGPPADSSKPIGPDDAIDALSSDFT
CGSPTAAGKKTEKEESTEVLKAQSAGTVRSAAPPQEKKRKVEKDTMSDQALEALSASLGT
RQAEPELDLRSIKEVDEAKAKEEKLEKCGEDDETIPSEYRLKPATDKDGKPLLPEPEEKP
KPRSESELIDELSEDFDRSECKEKPSKPTEKTEESKAAAPAPVSEAVCRTSMCSIQSAPP
EPATLKGTVPDDAVEALADSLGKKEADPEDGKPVMDKVKEKAKEEDREKLGEKEETIPPD
YRLEEVKDKDGKPLLPKESKEQLPPMSEDFLLDALSEDFSGPQNASSLKFEDAKLAAAIS
EVVSQTPASTTQAGAPPRDTSQSDKDLDDALDKLSDSLGQRQPDPDENKPMEDKVKEKAK
AEHRDKLGERDDTIPPEYRHLLDDNGQDKPVKPPTKKSEDSKKPADDQDPIDALSGDLDS
CPSTTETSQNTAKDKCKKAASSSKAPKNGGKAKDSAKTTEETSKPKDD*
>ENSG00000162772|ENST00000366987
MMLQHPGQVSASEVSASAIVPCLSPPGSLVFEDFANLTPFVKEELRFAIQNKHLCHRMSS
ALESVTVSDRPLGVSITKAEVAPEEDERKKRRRERNKIAAAKCRNKKKEKTECLQKESEK

LESVNAELKAQIEELKNEKQHLIYMLNLHRPTCIVRAQNGRTPEDERNLFIQQIKEGTLQ
S*
>ENSG00000198830|ENST00000361427
MPKRKAEGDAKGDKAKVKDEPQRRSARLSAKPAPPKPEPKPKKAPAKKGEKVPKGKKGKA
DAGKEGNNPAENGDAKTDQAQKAEGAGDAK*
>ENSG00000047315|ENST00000314595
MYDADEDMQYDEDDDEITPDLWQEACWIVISSYFDEKGLVRQQLDSFDEFIQMSVQRIVE
DAPPIDLQAEAQHASGEVEEPPRYLLKFEQIYLSKPTHWERDGAPSPMMPNEARLRNLTY
SAPLYVDITKTVIKEGEEQLQTQHQKTFIGKIPIMLRSTYCLLNGLTDRDLCELNECPLD
PGGYFIINGSEKVLIAQEKMATNTVYVFAKKDSKYAYTGECRSCLENSSRPTSTIWVSML
ARGGQGAKKSAIGQRIVATLPYIKQEVPIIIVFRALGFVSDRDILEHIIYDFEDPEMMEM
VKPSLDEAFVIQEQNVALNFIGSRGAKPGVTKEKRIKYAKEVLQKEMLPHVGVSDFCETK
KAYFLGYMVHRLLLAALGRRELDDRDHYGNKRLDLAGPLLAFLFRGMFKNLLKEVRIYAQ
KFIDRGKDFNLELAIKTRIISDGLKYSLATGNWGDQKKAHQARAGVSQVLNRLTFASTLS
HLRRLNSPIGRDGKLAKPRQLHNTLWGMVCPAETPEGHAVGLVKNLALMAYISVGSQPSP
ILEFLEEWSMENLEEISPAAIADATKIFVNGCWVGIHKDPEQLMNTLRKLRRQMDIIVSE
VSMIRDIREREIRIYTDAGRICRPLLIVEKQKLLLKKRHIDQLKEREYNNYSWQDLVASG
VVEYIDTLEEETVMLAMTPDDLQEKEVAYCSTYTHCEIHPSMILGVCASIIPFPDHNQSP
RNTYQSAMGKQAMGVYITNFHVRMDTLAHVLYYPQKPLVTTRSMEYLRFRELPAGINSIV
AIASYTGYNQEDSVIMNRSAVDRGFFRSVFYRSYKEQESKKGFDQEEVFEKPTRETCQGM
RHAIYDKLDDDGLIAPGVRVSGDDVIIGKTVTLPENEDELESTNRRYTKRDCSTFLRTSE
TGIVDQVMVTLNQEGYKFCKIRVRSVRIPQIGDKFASRHGQKGTCGIQYRQEDMPFTCEG
ITPDIIINPHAIPSRMTIGHLIECLQGKVSANKGEIGDATPFNDAVNVQKISNLLSDYGY
HLRGNEVLYNGFTGRKITSQIFIGPTYYQRLKHMVDDKIHSRARGPIQILNRQPMEGRSR
DGGLRFGEMERDCQIAHGAAQFLRERLFEASDPYQVHVCNLCGIMAIANTRTHTYECRGC
RNKTQISLVRMPYACKLLFQELMSMSIAPRMMSV*
>ENSG00000134899|ENST00000355739
MGVQGLWKLLECSGRQVSPEALEGKILAVDISIWLNQALKGVRDRHGNSIENPHLLTLFH
RLCKLLFFRIRPIFVFDGDAPLLKKQTLVKRRQRKDLASSDSRKTTEKLLKTFLKRQAIK
TAFRSKRDEALPSLTQVRRENDLYVLPPLQEEEKHSSEEEDEKEWQERMNQKQALQEEFF
HNPQAIDIESEDFSSLPPEVKHEILTDMKEFTKRRRTLFEAMPEESDDFSQYQLKGLLKK
NYLNQHIEHVQKEMNQQHSGHIRRQYEDEGGFLKEVESRRVVSEDTSHYILIKGIQAKTV
AEVDSESLPSSSKMHGMSFDVKSSPCEKLKTEKEPDATPPSPRTLLAMQAALLGSSSEEE
LESENRRQARGRNAPAAVDEGSISPRTLSAIKRALDDDEDVKVCAGDDVQTGGPGAEEMR
INSSTENSDEGLKVRDGKGIPFTATLASSSVNSAEEHVASTNEGREPTDSVPKEQMSLVH
VGTEAFPISDESMIKDRKDRLPLESAVVRHSDAPGLPNGRELTPASPTCTNSVSKNETHA
EVLEQQNELCPYESKFDSSLLSSDDETKCKPNSASEVIGPVSLQETSSIVSVPSEAVDNV
ENVVSFNAKEHENFLETIQEQQTTESAGQDLISIPKAVEPMEIDSEESESDGSFIEVQSV
ISDEELQAEFPETSKPPSEQGEEELVGTREGEAPAESESLLRDNSERDDVDGEPQEAEKD
AEDSLHEWQDINLEELETLESNLLAQQNSLKAQKQQQERIAATVTGQMFLESQELLRLFG
IPYIQAPMEAEAQCAILDLTDQTSGTITDDSDIWLFGARHVYRNFFNKNKFVEYYQYVDF
HNQLGLDRNKLINLAYLLGSDYTEGIPTVGCVTAMEILNEFPGHGLEPLLKFSEWWHEAQ
KNPKIRPNPHDTKVKKKLRTLQLTPGFPNPAVAEAYLKPVVDDSKGSFLWGKPDLDKIRE
FCQRYFGWNRTKTDESLFPVLKQLDAQQTQLRIDSFFRLAQQEKEDAKRIKSQRLNRAVT

CMLRKEKEAAASEIEAVSVAMEKEFELLDKAKGKTQKRGITNTLEESSSLKRKRLSDSKG
KNTCGGFLGETCLSESSDGSSSEDAESSSLMNVQRRTAAKEPKTSASDSQNSVKEAPVKN
GGATTSSSSDSDDDGGKEKMVLVTARSVFGKKRRKLRRARGRKRKT*
>ENSG00000116560|ENST00000357214
MSRDRFRSRGGGGGGFHRRGGGGGRGGLHDFRSPPPGMGLNQNRGPMGPGPGQSGPKPPI
PPPPPHQQQQQPPPQQPPPQQPPPHQPPPHPQPHQQQQPPPPPQDSSKPVVAQGPGPAPG
VGSAPPASSSAPPATPPTSGAPPGSGPGPTPTPPPAVTSAPPGAPPPTPPSSGVPTTPPQ
AGGPPPPPAAVPGPGPGPKQGPGPGGPKGGKMPGGPKPGGGPGLSTPGGHPKPPHRGGGE
PRGGRQHHPPYHQQHHQGPPPGGPGGRSEEKISDSEGFKANLSLLRRPGEKTYTQRCRLF
VGNLPADITEDEFKRLFAKYGEPGEVFINKGKGFGFIKLESRALAEIAKAELDDTPMRGR
QLRVRFATHAAALSVRNLSPYVSNELLEEAFSQFGPIERAVVIVDDRGRSTGKGIVEFAS
KPAARKAFERCSEGVFLLTTTPRPVIVEPLEQLDDEDGLPEKLAQKNPMYQKERETPPRF
AQHGTFEYEYSQRWKSLDEMEKQQREQVEKNMKDAKDKLESEMEDAYHEHQANLLRQDLM
RRQEELRRMEELHNQEMQKRKEMQLRQEEERRRREEEMMIRQREMEEQMRRQREESYSRM
GYMDPRERDMRMGGGGAMNMGDPYGSGGQKFPPLGGGGGIGYEANPGVPPATMSGSMMGS
DMRTERFGQGGAGPVGGQGPRGMGPGTPAGYGRGREEYEGPNKKPRF*
>ENSG00000152942|ENST00000354868
MNQVTDWVDPSFDDFLECSGVSTITATSLGVNNSSHRRKNGPSTLESSRFPARKRGNLSS
LEQIYGLENSKEYLSENEPWVDKYKPETQHELAVHKKKIEEVETWLKAQVLERQPKQGGS
ILLITGPPGCGKTTTLKILSKEHGIQVQEWINPVLPDFQKDDFKGMFNTESSFHMFPYQS
QIAVFKEFLLRATKYNKLQMLGDDLRTDKKIILVEDLPNQFYRDSHTLHEVLRKYVRIGR
CPLIFIISDSLSGDNNQRLLFPKEIQEECSISNISFNPVAPTIMMKFLNRIVTIEANKNG
GKITVPDKTSLELLCQGCSGDIRSAINSLQFSSSKGENNLRPRKKGMSLKSDAVLSKSKR
RKKPDRVFENQEVQAIGGKDVSLFLFRALGKILYCKRASLTELDSPRLPSHLSEYERDTL
LVEPEEVVEMSHMPGDLFNLYLHQNYIDFFMEIDDIVRASEFLSFADILSGDWNTRSLLR
EYSTSIATRGVMHSNKARGYAHCQGGGSSFRPLHKPQWFLINKKYRENCLAAKALFPDFC
LPALCLQTQLLPYLALLTIPMRNQAQISFIQDIGRLPLKRHFGRLKMEALTDREHGMIDP
DSGDEAQLNGGHSAEESLGEPTQATVPETWSLPLSQNSASELPASQPQPFSAQGDMEENI
IIEDYESDGT*
>ENSG00000130921|ENST00000366329
MSTVGLFHFPTPLTRICPAPWGLRLWEKLTLLSPGIAVTPVQMAGKKDYPALLSLDENEL
EEQFVKGHGPGGQATNKTSNCVVLKHIPSGIVVKCHQTRSVDQNRKLARKILQEKVDVFY
NGENSPVHKEKREAAKKKQERKKRAKETLEKKKLLKELWESSKKVH*
>ENSG00000177971|ENST00000314852
MVRKLKFHEQKLLKQVDFLNWEVTDHNLHELRVLRRYRLQRREDYTRYNQLSRAVRELAR
RLRDLPERDQFRVRASAALLDKLYALGLVPTRGSLELCDFVTASSFCRRRLPTVLLKLRM
AQHLQAAVAFVEQGHVRVGPDVVTDPAFLVTRSMEDFVTWVDSSKIKRHVLEYNEERDDF
DLEA*
>ENSG00000099942|ENST00000354336
MSSARFDSSDRSAWYMGPVSRQEAQTRLQGQRHGMFLVRDSSTCPGDYVLSVSENSRVSH
YIINSLPNRRFKIGDQEFDHLPALLEFYKIHYLDTTTLIEPAPRYPSPPMGSVSAPNLPT
AEDNLEYVRTLYDFPGNDAEDLPFKKGEILVIIEKPEEQWWSARNKDGRVGMIPVPYVEK
LVRSSPHGKHGNRNSNSYGIPEPAHAYAQPQTTTPLPAVSGSPGAAITPLPSTQNGPVFA
KAIQKRVPCAYDKTALALEVGDIVKVTRMNINGQWEGEVNGRKGLFPFTHVKIFDPQNPD

ENE*
>ENSG00000100401|ENST00000356244
MASEDIAKLAETLAKTQVAGGQLSFKGKSLKLNTAEDAKDVIKEIEDFDSLEALRLEGNT
VGVEAARVIAKALEKKSELKRCHWSDMFTGRLRTEIPPALISLGEGLITAGAQLVELDLS
DNAFGPDGVQGFEALLKSSACFTLQELKLNNCGMGIGGGKILAAALTECHRKSSAQGKPL
ALKVFVAGRNRLENDGATALAEAFRVIGTLEEVHMPQNGINHPGITALAQAFAVNPLLRV
INLNDNTFTEKGAVAMAETLKTLRQVEVINFGDCLVRSKGAVAIADAIRGGLPKLKELNL
SFCEIKRDAALAVAEAMADKAELEKLDLNGNTLGEEGCEQLQEVLEGFNMAKVLASLSDD
EDEEEEEEGEEEEEEAEEEEEEDEEEEEEEEEEEEEEPQQRGQGEKSATPSRKILDPNTG
EPAPVLSSPPPADVSTFLAFPSPEKLLRLGPKSSVLIAQQTDTSDPEKVVSAFLKVSSVF
KDEATVRMAVQDAVDALMQKAFNSSSFNSNTFLTRLLVHMGLLKSEDKVKAIANLYGPLM
ALNHMVQQDYFPKALAPLLLAFVTKPNSALESCSFARHSLLQTLYKV*
>ENSG00000164651|ENST00000361443
MLAATCNKIGSPSPSPSSLSDSSSSFGKGFHPWKRSSSSSSASCNVVGSSLSSFGVSGAS
RNGGSSSAAAAAAAAAAAAAALVSDSFSCGGSPGSSAFSLTSSSAAAAAAAAAAAASSSP
FANDYSVFQAPGVSGGSGGGGGGGGGGGSSAHSQDGSHQPVFISKVHTSVDGLQGIYPRVG
MAHPYESWFKPSHPGLGAAGEVGSAGASSWWDVGAGWIDVQNPNSAAALPGSLHPAAGGL
QTSLHSPLGGYNSDYSGLSHSAFSSGASSHLLSPAGQHLMDGFKPVLPGSYPDSAPSPLA
GAGGSMLSAGPSAPLGGSPRSSARRYSGRATCDCPNCQEAERLGPAGASLRRKGLHSCHI
PGCGKVYGKTSHLKAHLRWHTGERPFVCNWLFCGKRFTRSDELQRHLRTHTGEKRFACPV
CNKRFMRSDHLSKHVKTHSGGGGGGGSAGSGSGGKKGSDTDSEHSAAGSPPCHSPELLQP
PEPGHRNGLE*
>ENSG00000090097|ENST00000355852
MSGSDGGLEEEPELSITLTLRMLMHGKEVGSIIGKKGETVKRIREQSSARITISEGSCPE
RITTITGSTAAVFHAVSMIAFKLDEDLCAAPANGGNVSRPPVTLRLVIPASQCGSLIGKA
GTKIKEIRETTGAQVQVAGDLLPNSTERAVTVSGVPDAIILCVRQICAVILESPPKGATI
PYHPSLSLGTVLLSANQGFSVQGQYGAVTPAEVTKLQQLSSHAVPFATPSVVPGLDPGTQ
TSSQEFLVPNDLIGCVIGRQGSKISEIRQMSGAHIKIGNQAEGAGERHVTITGSPVSIAL
AQYLITACLETAKSTSGGTPSSAPADLPAPFSPPLTALPTAPPGLLGTPYAISLSNFIGL
KPMPFLALPPASPGPPPGLAAYTAKMAAANGSKKAERQKFSPY*
>ENSG00000184735|ENST00000327968
MSHWAPEWKRAEANPRDLGASWDVRGSRGSGWSGPFGHQGPRAAGSREPPLCFKIKNNMV
GVVIGYSGSKIKDLQHSTNTKIQIINGESEAKVRIFGNREMKAKAKAAIETLIRKQESYN
SESSVDNAASQTPIGRNLGRNDIVGEAEPLSNWDRIRAAVVECEKRKWADLPPVKKNFYI
ESKATSCMSEMQVINWRKENFNITCDDLKSGEKRLIPKPTCRFKDAFQQYPDLLKSIIRV
GIVKPTPIQSQAWPIILQGIDLIVVAQTGTGKTLSYLMPGFIHLDSQPISREQRNGPGML
VLTPTRELALHVEAECSKYSYKGLKSICIYGGRNRNGQIEDISKGVDIIIATPGRLNDLQ
MNNSVNLRSITYLVIDEADKMLDMEFEPQIRKILLDVRPDRQTVMTSATWPDTVRQLALS
YLKDPMIVYVGNLNLVAVNTVKQNIIVTTEKEKRALTQEFVENMSPNDKVIMFVSQKHIA
DDLSSDFNIQGISAESLHGNSEQSDQERAVEDFKSGNIKILITTDIVSRGLDLNDVTHVY
NYDFPRNIDVYVHRVGYIGRTGKTGTSVTLITQRDSKMAGELIKILDRANQSVPEDLVVM
AEQYKLNQQKRHRETRSRKPGQRRKEFYFLS*
>ENSG00000163808|ENST00000326047
MAPGCKTELRSVTNGQSNQPSNEGDAIKVFVRIRPPAERSGSADGEQNLCLSVLSSTSLR

LHSNPEPKTFTFDHVADVDTTQESVFATVAKSIVESCMSGYNGTIFAYGQTGSGKTFTMM
GPSESDNFSHNLRGVIPRSFEYLFSLIDREKEKAGAGKSFLCKCSFIEIYNEQIYDLLDS
ASAGLYLREHIKKGVFVVGAVEQVVTSAAEAYQVLSGGWRNRRVASTSMNRESSRSHAVF
TITIESMEKSNEIVNIRTSLLNLVDLAGSERQKDTHAEGMRLKEAGNINRSLSCLGQVIT
ALVDVGNGKQRHVCYRDSKLTFLLRDSLGGNAKTAIIANVHPGSRCFGETLSTLNFAQRA
KLIKNKAVVNEDTQGNVSQLQAEVKRLKEQLAELASGQTPPESFLTRDKKKTNYMEYFQE
AMLFFKKSEQEKKSLIEKVTQLEDLTLKKEKFIQSNKMIVKFREDQIIRLEKLHKESRGG
FLPEEQDRLLSELRNEIQTLREQIEHHPRVAKYAMENHSLREENRRLRLLEPVKRAQEMD
AQTIAKLEKAFSEISGMEKSDKNQQGFSPKAQKEPCLFANTEKLKAQLLQIQTELNNSKQ
EYEEFKELTRKRQLELESELQSLQKANLNLENLLEATKACKRQEVSQLNKIHAETLKIIT
TPTKAYQLHSRPVPKLSPEMGSFGSLYTQNSSILDNDILNEPVPPEMNEQAFEAISEELR
TVQEQMSALQAKLDEEEHKNLKLQQHVDKLEHHSTQMQELFSSERIDWTKQQEELLSQLN
VLEKQLQETQTKNDFLKSEVHDLRVVLHSADKELSSVKLEYSSFKTNQEKEFNKLSERHM
HVQLQLDNLRLENEKLLESKACLQDSYDNLQEIMKFEIDQLSRNLQNFKKENETLKSDLN
NLMELLEAEKERNNKLSLQFEEDKENSSKEILKVLEAVRQEKQKETAKCEQQMAKVQKLE
ESLLATEKVISSLEKSRDSDKKVVADLMNQIQELRTSVCEKTETIDTLKQELKDINCKYN
SALVDREESRVLIKKQEVDILDLKETLRLRILSEDIERDMLCEDLAHATEQLNMLTEASK
KHSGLLQSAQEELTKKEALIQELQHKLNQKKEEVEQKKNEYNFKMRQLEHVMDSAAEDPQ
SPKTPPHFQTHLAKLLETQEQEIEDGRASKTSLEHLVTKLNEDREVKNAEILRMKEQLRE
MENLRLESQQLIEKNWLLQGQLDDIKRQKENSDQNHPDNQQLKNEQEESIKERLAKSKIV
EEMLKMKADLEEVQSALYNKEMECLRMTDEVERTQTLESKAFQEKEQLRSKLEEMYEERE
RTSQEMEMLRKQVECLAEENGKLVGHQNLHQKIQYVVRLKKENVRLAEETEKLRAENVFL
KEKKRSES*
>ENSG00000082175|ENST00000325455
MTELKAKGPRAPHVAGGPPSPEVGSPLLCRPAAGPFPGSQTSDTLPEVSAIPISLDGLLF
PRPCQGQDPSDEKTQDQQSLSDVEGAYSRAEATRGAGGSSSSPPEKDSGLLDSVLDTLLA
PSGPGQSQPSPPACEVTSSWCLFGPELPEDPPAAPATQRVLSPLMSRSGCKVGDSSGTAA
AHKVLPRGLSPARQLLLPASESPHWSGAPVKPSPQAAAVEVEEEDGSESEESAGPLLKGK
PRALGGAAAGGGAAAVPPGAAAGGVALVPKEDSRFSAPRVALVEQDAPMAPGRSPLATTV
MDFIHVPILPLNHALLAARTRQLLEDESYDGGAGAASAFAPPRSSPCASSTPVAVGDFPD
CAYPPDAEPKDDAYPLYSDFQPPALKIKEEEEGAEASARSPRSYLVAGANPAAFPDFPLG
PPPPLPPRATPSRPGEAAVTAAPASASVSSASSSGSTLECILYKAEGAPPQQGPFAPPPC
KAPGASGCLLPRDGLPSTSASAAAAGAAPALYPALGLNGLPQLGYQAAVLKEGLPQVYPP
YLNYLRPDSEASQSPQYSFESLPQKICLICGDEASGCHYGVLTCGSCKVFFKRAMEGQHN
YLCAGRNDCIVDKIRRKNCPACRLRKCCQAGMVLGGRKFKKFNKVRVVRALDAVALPQPV
GVPNESQALSQRFTFSPGQDIQLIPPLINLLMSIEPDVIYAGHDNTKPDTSSSLLTSLNQ
LGERQLLSVVKWSKSLPGFRNLHIDDQITLIQYSWMSLMVFGLGWRSYKHVSGQMLYFAP
DLILNEQRMKESSFYSLCLTMWQIPQEFVKLQVSQEEFLCMKVLLLLNTIPLEGLRSQTQ
FEEMRSSYIRELIKAIGLRQKGVVSSSQRFYQLTKLLDNLHDLVKQLHLYCLNTFIQSRA
LSVEFPEMMSEVIAAQLPKILAGMVKPLLFHKK*
>ENSG00000166012|ENST00000323981
MDKSGIDSLDHVTSDAVELANRSDNSSDSSLFKTQCIPYSPKGEKRNPIRKFVRTPESVH
ASDSSSDSSFEPIPLTIKAIFERFKNRKKRYKKKKKRRYQPTGRPRGRPEGRRNPIYSLI
DKKKQFRSRGSGFPFLESENEKNAPWRKILTFEQAVARGFFNYIEKLKYEHHLKESLKQM

NVGEDLENEDFDSRRYKFLDDDGSISPIEESTAEDEDATHLEDNECDIKLAGDSFIVSSE
FPVRLSVYLEEEDITEEAALSKKRATKAKNTGQRGLKM*
>ENSG00000189190|ENST00000338230
MMKEVLSTGQGNTEVIHTGTLQRYQSYHIGDFCFQEIEKEIHDIEFQCQEDERNGHEAPM
TKIKKLTGSTDQHDHRHAGNKPIKDQLGSSFYSHLPELHIIQIKGKIGNQFEKSTSDAPS
VSTSQRISPRPQIHISNNYGNNSPNSSLLPQKQEVYMREKSFQCNESGKAFNCSSLLRKH
QIPHLGDKQYKCDVCGKLFNHKQYLTCHCRCHTGEKPYKCNECGKSFSQVSSLTCHRRLH
TAVKSHKCNECGKIFGQNSALVIHKAIHTGEKPYKCNECDKAFNQQSNLARHRRIHTGEK
PYKCEECDKVFSRKSTLESHKRIHTGEKPYKCKVCDTAFTWNSQLARHKRIHTGEKTYKC
NECGKTFSHKSSLVCHHRLHGGEKSYKCKVCDKAFAWNSHLVRHTRIHSGGKPYKCNECG
KTFGQNSDLLIHKSIHTGEQPYKYEECEKVFSCGSTLETHKIIHTGEKPYKCKVCDKAFA
CHSYLAKHTRIHSGEKPYKCNECSKTFRLRSYLASHRRVHSGEKPYKCNECSKTFSQRSY
LHCHRRLHSGEKPYKCNECGKTFSHKPSLVHHRRLHTGEKSYKCTVCDKAFVRNSYLARH
TRIHTAEKPYKCNECGKAFNQQSQLSLHHRIHAGEKLYKCETCDKVFSRKSHLKRHRRIH
PGKKPYKCKVCDKTFGSDSHLKQHTGLHTGEKPYKCNECGKAFSKQSTLIHHQAVHGVGK
LD*
>ENSG00000062582|ENST00000317534
MAASVCSGLLGPRVLSWSRELPCAWRALHTSPVCAKNRAARVRVSKGDKPVTYEEAHAPH
YIAHRKGWLSLHTGNLDGEDHAAERTVEDVFLRKFMWGTFPGCLADQLVLKRRGNQLEIC
AVVLRQLSPHKYYFLVGYSETLLSYFYKCPVRLHLQTVPSKVVYKYL*
>ENSG00000177885|ENST00000316804
MEAIAKYDFKATADDELSFKRGDILKVLNEECDQNWYKAELNGKDGFIPKNYIEMKPHPW
FFGKIPRAKAEEMLSKQRHDGAFLIRESESAPGDFSLSVKFGNDVQHFKVLRDGAGKYFL
WVVKFNSLNELVDYHRSTSVSRNQQIFLRDIEQVPQQPTYVQALFDFDPQEDGELGFRRG
DFIHVMDNSDPNWWKGACHGQTGMFPRNYVTPVNRNV*
>ENSG00000178053|ENST00000359117
MRQMIRSFSEPFGRDLLSISDGRGRAHNRRGHNDGEDSLTHTDVSSFQTMDQMVSNMRNY
MQKLERNFGQLSVDPNGHSFCSSSVMTYSKIGDEPPKVFQASTQTRRAPGGIKETRKAMR
DSDSGLEKMAIGHHIHDRAHVIKKSKNKKTGDEEVNQEFINMNESDAHAFDEEWQSEVLK
YKPGRHNLGNTRMRSVGHENPGSRELKRREKPQQSPAIEHGRRSNVLGDKLHIKGSSVKS
NKK*
>ENSG00000139445|ENST00000355216
MPGLPCPALPCPAPPPAPSCLIAMALKNSKTGSLPVSEIYSFMKEHFPYFKTAPDGWKNS
VRHNLSLNKCFEKVENKMSGSSRKGCLWALNLARIDKMEEEMHKWKRKDLAAIHRSMANP
EELDKLISDRPESCRRPGKPGEPEAPVLTHATTVAVAHGCLAVSQLPPQPLMTLSLQSVP
LHHQVQPQAHLAPDSPAPAQTPPLHALPDLSPSPLPHPAMGRAPVDFINISTDMNTEVDA
LDPSIMDFALQGNLWEEMKDEGFSLDTLGAFADSPLGCDLGASGLTPASGGSDQSFPDLQ
VTGLYTAYSTPDSVAASGTSSSSQYLGAQGNKPIALL*
>ENSG00000115446|ENST00000357765
MLPSTSVNSLVQGNGVLNSRDAARHTAGAKRYKYLRRLFRFRQMDFEFAAWQMLYLFTSP
QRVYRNFHYRKQTKDQWARDDPAFLVLLSIWLCVSTIGFGFVLDMGFFETIKLLLWVVLI
DCVGVGLLIATLMWFISNKYLVKRQSRDYDVEWGYAFDVHLNAFYPLLVILHFIQLFFIN
HVILTDTFIGYLVGNTLWLVAVGYYIYVTFLGYSALPFLKNTVILLYPFAPLILLYGLSL
ALGWNFTHTLCSFYKYRVK*

>ENSG00000078900|ENST00000357733
MAQSTATSPDGGTTFEHLWSSLEPDSTYFDLPQSSRGNNEVVGGTDSSMDVFHLEGMTTS
VMAQFNLLSSTMDQMSSRAASASPYTPEHAASVPTHSPYAQPSSTFDTMSPAPVIPSNTD
YPGPHHFEVTFQQSSTAKSATWTYSPLLKKLYCQIAKTCPIQIKVSTPPPPGTAIRAMPV
YKKAEHVTDVVKRCPNHELGRDFNEGQSAPASHLIRVEGNNLSQYVDDPVTGRQSVVVPY
EPPQVGTEFTTILYNFMCNSSCVGGMNRRPILIIITLEMRDGQVLGRRSFEGRICACPGR
DRKADEDHYREQQALNESSAKNGAASKRAFKQSPPAVPALGAGVKKRRHGDEDTYYLQVR
GRENFEILMKLKESLELMELVPQPLVDSYRQQQQLLQRPPRDAQQPWPRSASQRRDEQQP
QRPVHGLGVPLHSATPLPRRPQPRQDLGALKIPEQYRMTIWRGLQDLKQGHDYSTAQQLL
RSSNAATISIGGSGELQRQRVMEAVHFRVRHTITIPNRGGPGGGPDEWADFGFDLPDCKA
RKQPIKEEFTEAEIH*
>ENSG00000102908|ENST00000354436
MPSDFISLLSADLDLESPKSLYSRESVYDLLPKELQLPPSRETSVASMSQTSGGEAGSPP
PAVVAADASSAPSSSSMGGACSSFTTSSSPTIYSTSVTDSKAMQVESCSSAVGVSNRGVS
EKQLTSNTVQQHPSTPKRHTVLYISPPPEDLLDNSRMSCQDEGCGLESEQSCSMWMEDSP
SNFSNMSTSSYNDNTEVPRKSRKRNPKQRPGVKRRDCEESNMDIFDADSAKAPHYVLSQL
TTDNKGNSKAGNGTLENQKGTGVKKSPMLCGQYPVKSEGKELKIVVQPETQHRARYLTEG
SRGSVKDRTQQGFPTVKLEGHNEPVVLQVFVGNDSGRVKPHGFYQACRVTGRNTTPCKEV
DIEGTTVIEVGLDPSNNMTLAVDCVGILKLRNADVEARIGIAGSKKKSTRARLVFRVNIM
RKDGSTLTLQTPSSPILCTQPAGVPEILKKSLHSCSVKGEEEVFLIGKNFLKGTKVIFQE
NVSDENSWKSEAEIDMELFHQNHLIVKVPPYHDQHITLPVSVGIYVVTNAGRSHDVQPFT
YTPDPAAAGALNVNVKKEISSPARPCSFEEAMKAMKTTGCNLDKVNIIPNALMTPLIPSS
MIKSEDVTPMEVTAEKRSSTIFKTTKSVGSTQQTLENISNIAGNGSFSSPSSSHLPSENE
KQQQIQPKAYNPETLTTIQTQDISQPGTFPAVSASSQLPNSDALLQQATQFQTRETQSRE
ILQSDGTVVNLSQLTEASQQQQQSPLQEQAQTLQQQISSNIFPSPNSVSQLQNTIQQLQA
GSFTGSTASGSSGSVDLVQQVLEAQQQLSSVLFSAPDGNENVQEQLSADIFQQVSQIQSG
VSPGMFSSTEPTVHTRPDNLLPGRAESVHPQSENTLSNQQQQQQQQQQVMESSAAMVMEM
QQSICQAAAQIQSELFPSTASANGNLQQSPVYQQTSHMMSALSTNEDMQMQCELFSSPPA
VSGNETSTTTTQQVATPGTTMFQTSSSGDGEETGTQAKQIQNSVFQTMVQMQHSGDNQPQ
VNLFSSTKSMMSVQNSGTQQQGNGLFQQGNEMMSLQSGNFLQQSSHSQAQLFHPQNPIAD
AQNLSQETQGSLFHSPNPIVHSQTSTTSSEQMQPPMFHSQSTIAVLQGSSVPQDQQSTNI
FLSQSPMNNLQTNTVAQEAFFAAPNSISPLQSTSNSEQQAAFQQQAPISHIQTPMLSQEQ
AQPPQQGLFQPQVALGSLPPNPMPQSQQGTMFQSQHSIVAMQSNSPSQEQQQQQQQQQQQ
QQQQQQSILFSNQNTMATMASPKQPPPNMIFNPNQNPMANQEQQNQSIFHQQSNMAPMNQ
EQQPMQFQSQSTVSSLQNPGPTQSESSQTPLFHSSPQIQLVQGSPSSQEQQVTLFLSPAS
MSALQTSINQQDMQQSPLYSPQNNMPGIQGATSSPQPQATLFHNTAGGTMNQLQNSPGSS
QQTSGMFLFGIQNNCSQLLTSGPATLPDQLMAISQPGQPQNEGQPPVTTLLSQQMPENSP
LASSINTNQNIEKIDLLVSLQNQGNNLTGSF*
>ENSG00000135486|ENST00000330752
MSKSESPKEPEQLRKLFIGGLSFETTDESLRSHFEQWGTLTDCVVMRDPNTKRSRGFGFV
TYATVEEVDAAMNARPHKVDGRVVEPKRAVSREDSQRPGAHLTVKKIFVGGIKEDTEEHH
LRDYFEQYGKIEVIEIMTDRGSGKKRGFAFVTFDDHDSVDKIVIQKYHTVNGHNCEVRKA
LSKQEMASASSSQRGRSGSGNFGGGRGGGFGGNDNFGRGGNFSGRGIGDGYNGFGNDGSN
FGGGGSYNDFGNYNNQSSNFGPMKGGNFGGRSSGPYGGGGQYFAKPRNQGGYGGSSSSSS

YGSGRRF*
>ENSG00000162642|ENST00000344356
MAAEEKDPLSYFAAYGSSSSGSSDEEDNIEPEETSRRTPDPAKSAGGCRNKAEKRLPGPD
ELFRSVTRPAFLYNPLNKQIDWERHVVKAPEEPPKEFKIWKSNYVPPPETYTTEKKPPPP
ELDMAIKWSNIYEDNGDDAPQNAKKARLLPEGEETLESALEDLEIQN*
>ENSG00000099622|ENST00000320936
MASDEGKLFVGGLSFDTNEQSLEQVFSKYGQISEVVVVKDRETQRSRGFGFVTFENIDDA
KDAMMAMNGKSVDGRQIRVDQAGKSSDNRSRGYRGGSAGGRGFFRGGRGRGRGFSRGGGD
RGYGGNRFESRSGGYGGSRDYYSSRSQSGGYSDRSSGGSYRDSYDSYATHNE*
>ENSG00000112183|ENST00000318204
MYVCLCVSVAKVTMADRAAAERACKDPNPIIDGRKANVNLAYLGAKPRIMQPGFAFGVQQ
LHPALIQRPFGIPAHYVYPQAFVQPGVVIPHVQPTAAAASTTPYIDYTGAAYAQYSAAAA
AAAAAAAYDQYPYAASPAAAGYVTAGGYGYAVQQPITAAAPGTAAAAAAAAAAAAAFGQY
QPQQLQTDRMQ*
>ENSG00000107371|ENST00000327304
MAEPASVAAESLAGSRARAARTVLGQVVLPGEELLLPEQEDAEGPGGAVERPLSLNARAC
SRVRVVCGPGLRRCGDRLLVTKCGRLRHKEPGSGSGGGVYWVDSQQKRYVPVKGDHVIGI
VTAKSGDIFKVDVGGSEPASLSYLSFEGATKRNRPNVQVGDLIYGQFVVANKDMEPEMVC
IDSCGRANGMGVIGQDGLLFKVTLGLIRKLLAPDCEIIQEVGKLYPLEIVFGMNGRIWVK
AKTIQQTLILANILEACEHMTSDQRKQIFSRLAES*
>ENSG00000189195|ENST00000342818
MARCGEGSAAPMVLLGSAGVCSKGLQRKGPCERRRLKATVSEQLSQDLLRLLREEFHTDV
TFSVGCTLFKAHKAVLLARVPDFYFHTIGQTSNSLTNQEPIAVENVEALEFRTFLQIIYS
SNRNIKNYEEEILRKKIMEIGISQKQLDISFPKCENSSDCSLQKHEIPEDISDRDDDFIS
NDNYDLEPASELGEDLLKLYVKPCCPDIDIFVDGKRFKAHRAILSARSSYFAAMLSGCWA
ESSQEYVTLQGISHVELNVMMHFIYGGTLDIPDKTNVGQILNMADMYGLEGLKEVAIYIL
RRDYCNFFQKPVPRTLTSILECLIIAHSVGVESLFADCMKWIVKHFARFWSERSFANIPP
EIQKSCLNMLIQSLVSIT*
>ENSG00000075407|ENST00000351773
MITSQGSVSFRDVTVGFTQEEWQHLDPAQRTLYRDVMLENYSHLVSVGYCIPKPEVILKL
EKGEEPWILEEKFPSQSHLELINTSRNYSIMKFNEFNKGGKCFCDEKHEIIHSEEEPSEY
NKNGNSFWLNEDLIWHQKIKNWEQSFEYNECGKAFPENSLFLVHKRGYTGQKTCKYTEHG
KTCDMSFFITHQQTHPRENHYGNECGENIFEESILLEHQSVYPFSQKLNLTPIQRTHSIN
NIIEYNECGTFFSEKLVLHLQQRTHTGEKPYECHECGKTFTQKSAHTRHQRTHTGGKPYE
CHECGKTFYKNSDLIKHQRIHTGERPYGCHECGKSFSEKSTLTQHQRTHTGEKPYECHEC
GKTFSFKSVLTVHQKTHTGEKPYECYACGKAFLRKSDLIKHQRIHTGEKPYECNECGKSF
SEKSTLTKHLRTHTGEKPYECIQCGKFFCYYSGFTEHLRRHTGEKPFGCNECGKTFRQKS
ALIVHQRTHIRQKPYGCNQCGKSFCVKSKLIAHHRTHTGEKPYECNVCGKSFYVKSKLTV
HQRIHLGRNPINVVNEGNYSG*
>ENSG00000065883|ENST00000340829
MPSSSDTALGGGGGLSWAEKKLEERRKRRRFLSPQQPPLLLPLLQPQLLQPPPPPPPLLF
LAAPGTAAAAAAAAAASSSCFSPGPPLEVKRLARGKRRAGGRQKRRRGPRAGQEAEKRRV
FSLPQPQQDGGGGASSGGGVTPLVEYEDVSSQSEQGLLLGGASAATAATAAGGTGGSGGS
PASSSGTQRRGEGSERRPRRDRRSSSGRSKERHREHRRRDGQRGGSEASKSRSRHSHSGE

ERAEVAKSGSSSSSGGRRKSASATSSSSSSRKDRDSKAHRSRTKSSKEPPSAYKEPPKAY
REDKTEPKAYRRRRSLSPLGGRDDSPVSHRASQSLRSRKSPSPAGGGSSPYSRRLPRSPS
PYSRRRSPSYSRHSSYERGGDVSPSPYSSSSWRRSRSPYSPVLRRSGKSRSRSPYSSRHS
RSRSRHRLSRSRSRHSSISPSTLTLKSSLAAELNKNKKARAAEAARAAEAAKAAEATKAA
EAAAKAAKASNTSTPTKGNTETSASASQTNHVKDVKKIKIEHAPSPSSGGTLKNDKAKTK
PPLQVTKVENNLIVDKATKKAVIVGKESKSAATKEESVSLKEKTKPLTPSIGAKEKEQHV
ALVTSTLPPLPLPPMLPEDKEADSLRGNISVKAVKKEVEKKLRCLLADLPLPPELPGGDD
LSKSPEEKKTATQLHSKRRPKICGPRYGETKEKDIDWGKRCVDKFDIIGIIGEGTYGQVY
KARDKDTGEMVALKKVRLDNEKEGFPITAIREIKILRQLTHQSIINMKEIVTDKEDALDF
KKDKGAFYLVFEYMDHDLMGLLESGLVHFNENHIKSFMRQLMEGLDYCHKKNFLHRDIKC
SNILLNNRGQIKLADFGLARLYSSEESRPYTNKVITLWYRPPELLLGEERYTPAIDVWSC
GCILGELFTKKPIFQANQELAQLELISRICGSPCPAVWPDVIKLPYFNTMKPKKQYRRKL
REEFVFIPAAALDLFDYMLALDPSKRCTAEQALQCEFLRDVEPSKMPPPDLPLWQDCHEL
WSKKRRRQKQMGMTDDVSTIKAPRKDLSLGLDDSRTNTPQGVLPSSQLKSQGSSNVAPGE
KQTDPSTPQQESSKPLGGIQPSSQTIQPKVETDAAQAAVQSAFAVLLTQLIKAQQSKQKD
VLLEERENGSGHEASLQLRPPPEPSTPVSGQDDLIQHQDMRILELTPEPDRPRILPPDQR
PPEPPEPPPVTEEDLDYRTENQHVPTTSSSLTDPHAGVKAALLQLLAQHQPQDDPKREGG
IDYQAGDTYVSTSDYKDNFGSSSFSSAPYVSNDGLGSSSAPPLERRSFIGNSDIQSLDNY
STASSHSGGPPQPSAFSESFPSSVAGYGDIYLNAGPMLFSGDKDHRFEYSHGPIAVLANS
SDPSTGPESTHPLPAKMHNYNYGGNLQENPSGPSLMHGQTWTSPAQGPGYSQGYRGHIST
STGRGRGRGLPY*
>ENSG00000215271|ENST00000357460
MVRGWEPPPGLDCAISEGHKSEGTMPPNKEASGLSSSPAGLICLPPISEELQLVWTQAAQ
TSELDSNEHLLKTFSYFPYPSLADIALLCLRYGLQMEKVKTWFMAQRLRCGISWSSEEIE
ETRARVVYRRDQLHFKSLLSFTHHAGRPPEEVPPPPVPAPEQVGIGIGPPTLSKPTQTKG
LKVEPEEPSQMPPLPQSHQKLKESLMTPGSGAFPYQSDFWQHLQSSGLSKEQAGRGPNQS
HGIGTASWNHSTTVPQPQARDKPPPIALIASSCKEESASSVTPSSSSTSSSFQVLANGAT
AASKPLQPLGCVPQSVSPSEQALPPHLEPAWPQGLRHNSVPGRVGPTEYLSPDMQRQRKT
KRKTKEQLAILKSFFLQCQWARREDYQKLEQITGLPRPEIIQWFGDTRYALKHGQLKWFR
DNAVPGAPSFQDPAIPTPPPSTRSLNERAETPPLPIPPPPPDIQPLERYWAAHQQLRETD
IPQLSQASRLSTQQVLDWFDSRLPQPAEVVVCLDEEEEEEEEELPEDDEEEEEEEEEDDD
DDDDDVIIQD*
>ENSG00000090097|ENST00000322099
MSGSDGGLEEEPELSITLTLRMLMHGKEVGSIIGKKGETVKRIREQSSARITISEGSCPE
RITTITGSTAAVFHAVSMIAFKLDEDLCAAPANGGNVSRPPVTLRLVIPASQCGSLIGKA
GTKIKEIRETTGAQVQVAGDLLPNSTERAVTVSGVPDAIILCVRQICAVILESPPKGATI
PYHPSLSLGTVLLSANQGFSVQGQYGAVTPAEVTKLQQLSSHAVPFATPSVVPGLDPGTQ
TSSQEFLVPNDLIGCVIGRQGSKISEIRQMSGAHIKIGNQAEGAGERHVTITGSPVSIAL
AQYLITACLETAKSTSGGTPSSAPADLPAPFSPPLTALPTAPPGLLGTPYAISLSNFIGL
KPMPFLALPPASPGPPPGLAAYTAKMAAANGSKKAERQKFSPY*
>ENSG00000189308|ENST00000340417
MEVVPAEVNSLLPEEIMDTGITLVDDDSIEAVIVSSPIPMETELEEIVNINSTGDSTATP
ISTEPITVYSNHTNQVAVNTTITKADSNTTVKPAFPSGLQKLGAQTPVTISANQIILNKV
SQTSDLKLGNQTLKPDGQKLILTTLGKSGSPIVLALPHSQLPQAQKVTTQAQSGDAKLPP

QQIKVVTIGGRPEVKPVIGVSALTPGSQLINTTTQPSVLQTQQLKTVQIAKKPRTPTSGP
VITKLIFAKPINSKAVTGQTTQVSPPVIAGRVLSQSTPGTPSKTITISESGVIGSTLNST
TQTPNKIAISPLKSPNKAVKSTVQTITVGGVSTSQFKTIIPLATAPNVQQIQVPGSKFHY
VRLVTATSASSSTQPVSQNPSTNTQPLQQAKPVVVNTTPVRMSVPIVSAQAVKQVVPKPI
NPTSQIVTTSQPQQRLIMPATPLPQIQPNLTNLPPGTVLAPAPGTGNVGYAVLPAQYVTQ
LQQSSYVSIASNSTFTGTSGIQTQARLPFNGIIPSESASRPRKPCNCTKSLCLKLYCDCF
ANGEFCNNCNCTNCYNNLEHENERQKAIKACLDRNPEAFKPKIGKGKEGESDRRHSKGCN
CKRSGCLKNYCECYEAKIMCSSICKCIGCKNFEESPERKTLMHLADAAEVRVQQQTAAKT
KLSSQISDLLTRPTPALNSGGGKLPFTFVTKEVAEATCNCLLAQAEQADKKGKSKAAAER
MILEEFGRCLMSVINSAGKAKSDPCAMNC*
>ENSG00000153207|ENST00000366508
MDSLAGLSPREAAGVLSLSCVGVCSTCAWAWPHGSMRDLRAQVTSGLLPFPEVTLQALGE
DEITLESVLRGKFAAGKNGLACLACGPQLEVVNSITGERLSAYRFSGVNEQPPVVLAVKE
FSWQKRTGLLIGLEETEGSVLCLYDLGISKVVKAVVLPGRVTAIEPIINHGGASASTQHL
HPSLRWLFGVAAVVTDVGQILLVDLCLDDLSCNQNEVEASDLEVLTGIPAEVPHIRESVM
RQGRHLCFQLVSPTGTAVSTLSYISRTNQLAVGFSDGYLALWNMKSMKREYYIQLESGQV
PVYAVTFQEPENDPRNCCYLWAVQSTQDSEGDVLSLHLLQLAFGNRKCLASGQILYEGLE
YCEERYTLDLTGGMFPLRGQTSNTKLLGCQSIEKFRSHGDREEGVNEALSPDTSVSVFTW
QVNIYGQGKPSVYLGLFDINRWYHAQMPDSLRSGEYLHNCSYFALWSLESVVSRTSPHGI
LDILVHERSLNRGVPPSYPPPEQFFNPSTYNFDATCLLNSGVVHLTCTGFQKETLTFLKK
SGPSLNELIPDGYNRCLVAGLLSPRFVDVQPSSLSQEEQLEAILSAAIQTSSLGLLTGYI
RRWITEEQPNSATNLRFVLEWTWNKVVLTKEEFDRLCVPLFDGSCHFMDPQTIQSIQQCY
LLLSNLNIVLSCFASEAREITERGLIDLSNKFVVSHLICQYAQVVLWFSHSGLLPEGIDD
SVQLSRLCYNYPVIQNYYTSRRQKFERLSRGKWNPDCLMIDGLVSQLGERIEKLWKRDEG
GTGKYPPASLHAVLDMYLLDGVTEAAKHSITIYLLLDIMYSFPNKTDTPIESFPTVFAIS
WGQVKLIQGFWLIDHNDYESGLDLLFHPATAKPLSWQHSKIIQAFMSQGEHRQALRYIQT
MKPTVSSGNDVILHLTVLLFNRCMVEAWNFLRQHCNRLNIEELLKHMYEVCQEMGLMEDL
LKLPFTDTEQECLVKFLQSSASVQNHEFLLVHHLQRANYVPALKLNQTLKINVMNDRDPR
LRERSLARNSILDQYGKILPRVHRKLAIERAKPYHLSTSSVFRLVSRPKPLSAVPKQVVT
GTVLTRSVFINNVLSKIGEVWASKEPINSTTPFNSSKIEEPSPIVYSLPAPELPEAFFGT
PISKASQKISRLLDLVVQPVPRPSQCSEFIQQSSMKSPLYLVSRSLPSSSQLKGSPQAIS
RASELHLLETPLVVKKAKSLAMSVTTSGFSEFTPQSILRSTLRSTPLASPSPSPGRSPQR
LKETRISFVEEDVHPKWIPGAADDSKLEVFTTPKKCAVPVETEWLKSKDRTTSFFLNSPE
KEHQEMDEGSQSLEKLDVSKGNSSVSITSDETTLEYQDAPSPEDLEETVFTASKPKSSST
ALTTNVTEQTEKDGDKDVFASEVTPSDLQKQMGNLEDAETKDLLVAAEAFSELNHLSPVQ
GTEASLCAPSVYEGKIFTQKSKVPVLDEGLTSVETYTPAIRANDNKSMADVLGDGGNSSL
TISEGPIVSERRLNQEVALNLKEDHEVEVGVLKESVDLPEEKLPISDSPPDTQEIHVIEQ
EKLEAQDSGEEARNLSFNELYPSGTLKLQYNFDTIDQQFCDLADNKDTAECDIAEVDGEL
FVAQSNFTLILEGEEGEVEPGDFASSDVLPKAANTATEEKLVCSGENDNHGQIANLPSAV
TSDQKSQKVDTLPYVPEPIKVAIAENLLDVIKDTRSKEITSDTMEQSIHETIPLVSQNIM
CPTKLVKSAFKTAQETSTMTMNVSQVDDVVSSKTRTRGQRIQNVNVKSAQQEASADVATP
KMPGQSVRKKTRKAKEISEASENIYSDVRGLSQNQQIPQNSVTPRRGRRKKEVNQDILEN
TSSVEQELQITTGRESKRLKSSQLLEPAVEETTKKEVKVSSVTKRTPRRIKRSVENQESV
EIINDLKVSTVTSPSRMIRKLRSTNLDASENTGNKQDDKSSDKQLRIKHVRRVRGREVSP

SDVREDSNLESSQLTVQAEFDMSAIPRKRGRPRKINPSEDVGSKAVKEERSPKKKEAPSI
RRRSTRNTPAKSENVDVGKPALGKSILVPNEELSMVMSSKKKLTKKTESQSQKRSLHSVS
EERTDEMTHKETNEQEERLLATASFTKSSRSSRTRSSKAILLPDLSEPNNEPLFSPASEV
PRKAKAKKIEVPAQLKELVSDLSSQFVISPPALRSRQKNTSNKNKLEDELKDDAQSVETL
GKPKAKRIRTSKTKQASKNTEKESAWSPPPIEIRLISPLASPADGVKSKPRKTTEVTGTG
LGRNRKKLSSYPKQILRRKML*
>ENSG00000188573|ENST00000338333
MKSAASSRGGGGGGRGGGGWGSWGGGRGGGGGAGKGGGGDGGGQGGKGGFGARARGFGGG
GRGRGRGGGDGKDRGGGGQRRGGVAKSKSRRRKGAMVVSVEPHRHEGVFIYRGAEDALVT
LNMVPGQSVYGERRVTVTEGGVKQEYRTWNPFRSKLAAAILGGVDQIHIKPKSKVLYLGA
ASGTTVSHVSDIIGPDGLVYAVEFSHRAGRDLVNVAKKRTNIIPVLEDARHPLKYRMLIG
MVDVIFADVAQPDQSRIVALNAHTFLRNGGHFLISIKANCIDSTASAEAVFASEVRKLQQ
ENLKPQEQLTLEPYERDHAVVVGVYRPLPKSSSK*
>ENSG00000159592|ENST00000355105
MAQHDFVPAWLNFSTPQSAKSPTATFEKHGEHLPRGEGRFGVSRRRHNSSDGFFNNGPLR
TAGDSWHQPSLFRHDSVDSGVSKGAYAGITGNPSGWHSSSRGHDGMSQRSGGGTGNHRHW
NGSFHSRKGCAFQEKPPMEIREEKKEDKVEKLQFEEEDFPSLNPEAGKQHQPCRPIGTPS
GVWENPPSAKQPSKMLVIKKVSKEDPAAAFSAAFTSPGSHHANGNKLSSVVPSVYKNLVP
KPVPPPSKPNAWKANRMEHKSGSLSSSRESAFTSPISVTKPVVLASGAALSSPKESPSST
TPPIEISSSRLTKLTRRTTDRKSEFLKTLKDDRNGDFSENRDCDKLEDLEDNSTPEPKEN
GEEGCHQNGLALPVVEEGEVLSHSLEAEHRLLKAMGWQEYPENDENCLPLTEDELKEFHM
KTEQLRRNGFGKNGFLQSRSSSLFSPWRSTCKAEFEDSDTETSSSETSDDDAWK*
>ENSG00000197728|ENST00000356464
MTKKRRNNGRAKKGRGHVQPIRCTNCARCVPKDKAIKKFVIRNIVEAAAVRDISEASVFD
AYVLPKLYVKLHYCVSCAIHSKVVRNRSREARKDRTPPPRFRPAGAAPRPPPKPM*
>ENSG00000188295|ENST00000343381
MVSGLRLASRSGEEGWLKPAVARLGPPRHRLRNLRTESPWRSRGSVLFCSGPGRAGRAAE
PLHPVCTCGRHFRRPEPCREPLASPIQDSVAFEDVAVNFTQEEWALLDSSQKNLYREVMQ
ETCRNLASVGSQWKDQNIEDHFEKPGKDIRNHIVQRLCESKEDGQYGEVVSQIPNLDLNE
NISTGLKPCECSICGKVFVRHSLLNRHILAHSGYKPYGEKQYKCEQCGKFFVSVPGVRRH
MIMHSGNPAYKCTICGKAFYFLNSVERHQRTHTGEKPYKCKQCGKAFTVSGSCLIHERTH
TGEKPYECKECGKTFRFSCSFKTHERTHTGERPYKCTKCDKAFSCSTSLRYHGSIHTGER
PYECKQCGKAFSRLSSLCNHRSTHTGEKPYECKQCDQAFSRLSSLHLHERIHTGEKPYEC
KKCGKAYTRSSHLTRHERSHDIEAGCSDSAYNPSTLGGQGVWIA*
>ENSG00000164683|ENST00000354724
MKRAHPEYSSSDSELDETIEVEKESADENGNLSSALGSMSPTTSSQILARKRRRGIIEKR
RRDRINNSLSELRRLVPSAFEKQGSAKLEKAEILQMTVDHLKMLHTAGGKGYFDAHALAM
DYRSLGFRECLAEVARYLSIIEGLDASDPLRVRLVSHLNNYASQREAASGAHAGLGHIPW
GTVFGHHPHIAHPLLLPQNGHGNAGTTASPTEPHHQGRLGSAHPEAPALRAPPSGSLGPV
LPVVTSASKLSPPLLSSVASLSAFPFSFGSFHLLSPNALSPSAPTQAANLGKPYRPWGTE
IGAF*
>ENSG00000182872|ENST00000329236
MSGSPSLTARAEKVSVDAGRGGGESLQEASPRLADHGSSSGGGWEVKRSQRLRRGPSSPR
RPYQDMEYERRGGRGDRTGRYGATDRSQDDGGENRSRDHDYRDMDYRSYPREYGSQEGKH

DYDDSSEEQSAEDSYEASPGSETQRRRRRRHRHSPTGPPGFPRDGDYRDQDYRTEQGEEE
EEEEDEEEEEKASNIVMLRMLPQAATEDDIRGQLQSHGVQAREVRLMRNKSSGQSRGFAF
VEFSHLQDATRWMEANQHSLNILGQKVSMHYSDPKPKINEDWLCNKCGVQNFKRREKCFK
CGVPKSEAEQKLPLGTRLDQQTLPLGGRELSQGLLPLPQPYQAQGVLASQALSQGSEPSS
ENANDTIILRNLNPHSTMDSILGALAPYAVLSSSNVRVIKDKQTQLNRGFAFIQLSTIVE
AAQLLQILQALHPPLTIDGKTINVEFAKGSKRDMASNEGSRISAASVASTAIAAAQWAIS
QASQGGEGTWATSEEPPVDYSYYQQDEGYGNSQGTESSLYAHGYLKGTKGPGITGTKGDP
TGAGPEASLEPGADSVSMQAFSRAQPGAAPGIYQQSAEASSSQGTAANSQSYTIMSPAVL
KSELQSPTHPSSALPPATSPTAQESYSQYPVPDVSTYQYDETSGYYYDPQTGLYYDPNSQ
YYYNAQSQQYLYWDGERRTYVPALEQSADGHKETGAPSKEGKEKKEKHKTKTAQQIAKDM
ERWARSLNKQKENFKNSFQPISSLRDDERRESATADAGYAILEKKGALAERQHTSMDLPK
LASDDRPSPPRGLVAAYSGESDSEEEQERGGPEREEKLTDWQKLACLLCRRQFPSKEALI
RHQQLSGLHKQNLEIHRRAHLSENELEALEKNDMEQMKYRDRAAERREKYGIPEPPEPKR
RKYGGISTASVDFEQPTRDGLGSDNIGSRMLQAMGWKEGSGLGRKKQGIVTPIEAQTRVR
GSGLGARGSSYGVTSTESYKETLHKTMVTRFNEAQ*
>ENSG00000020633|ENST00000338888
MASNSIFDSFPTYSPTFIRDPSTSRRFTPPSPAFPCGGGGGKMGENSGALSAQAAVGPGG
RARPEVRSMVDVLADHAGELVRTDSPNFLCSVLPSHWRCNKTLPVAFKVVALGDVPDGTV
VTVMAGNDENYSAELRNASAVMKNQVARFNDLRFVGRSGRGKSFTLTITVFTNPTQVATY
HRAIKVTVDGPREPRRHRQKLEDQTKPFPDRFGDLERLRMRVTPSTPSPRGSLSTTSHFS
SQPQTPIQGTSELNPFSDPRQFDRSFPTLPTLTESRFPDPRMHYPGAMSAAFPYSATPSG
TSISSLSVAGMPATSRFHHTYLPPPYPGAPQNQSGPFQANPSPYHLYYGTSSGSYQFSMV
AGSSSGGDRSPTRMLASCTSSAASVAAGNLMNPSLGGQSDGVEADGSHSNSPTALSTPGR
MDEAVWRPY*
>ENSG00000187098|ENST00000328528
MGHLENTSVVFPRAIFSLCEKETRKLTLCLFSRSSSAEHPGASKPPISSSSMTSRILLRQ
QLMREQMQEQERREQQQKLQAAQFMQQRVPVSQTPAINVSVPTTLPSATQVPMEVLKVQT
HLENPTKYHIQQAQRQQVKQYLSTTLANKHANQVLSLPCPNQPGDHVMPPVPGSSAPNSP
MAMLTLNSNCEKEGFYKFEEQNRAESECPGMNTHSRASCMQMDDVIDDIISLESSYNEEI
LGLMDPALQMANTLPVSGNLIDLYGNQGLPPPGLTISNSCPANLPNIKRELTESEARALA
KERQKKDNHNLIERRRRFNINDRIKELGTLIPKSNDPDMRWNKGTILKASVDYIRKLQRE
QQRAKELENRQKKLEHANRHLLLRIQELEMQARAHGLSLIPSTGLCSPDLVNRIIKQEPV
LENCSQDLLQHHADLTCTTTLDLTDGTITFNNNLGTGTEANQAYSVPTKMGSKLEDILMD
DTLSPVGVTDPLLSSVSPGASKTSSRRSSMSMEETEHTC*
>ENSG00000285043|ENST00000338110
MDASSSPWNPTPAPVSSPPLLLPIPAIVFIAVGIYLLLLGLVLLTRNCLLAQGCCADGSS
PCRKQGSSGPPDCCWTCAEACNFPLPSPAHFLDACCPQPTRADWAPRCPRCCPLCDCACT
CQLPDCQSLNCLCFEIKLR*
>ENSG00000105618|ENST00000321030
MSLADELLADLEEAAEEEEGGSYGEEEEEPAIEDVQEETQLDLSGDSVKTIAKLWDSKMF
AEIMMKIEEYISKQAKASEVMGPVEAAPEYRVIVDANNLTVEIENELNIIHKFIRDKYSK
RFPELESLVPNALDYIRTVKELGNSLDKCKNNENLQQILTNATIMVVSVTASTTQGQQLS
EEELERLEEACDMALELNASKHRIYEYVESRMSFIAPNLSIIIGASTAAKIMGVAGGLTN
LSKMPACNIMLLGAQRKTLSGFSSTSVLPHTGYIYHSDIVQSLPPDLRRKAARLVAAKCT

LAARVDSFHESTEGKVGYELKDEIERKFDKWQEPPPVKQVKPLPAPLDGQRKKRGGRRYR
KMKERLGLTEIRKQANRMSFGEIEEDAYQEDLGFSLGHLGKSGSGRVRQTQVNEATKARI
SKTLQRTLQKQSVVYGGKSTIRDRSSGTASSVAFTPLQGLEIVNPQAAEKKVAEANQKYF
SSMAEFLKVKGEKSGLMST*
>ENSG00000132153|ENST00000348968
MCVNPTPGGTISRASRDLLKEFPQPKNLLNSVIGRALGISHAKDKLVYVHTNGPKKKKVT
LHIKWPKSVEVEGYGSKKIDAERQAAAAACQLFKGWGLLGPRNELFDAAKYRVLADRFGS
PADSWWRPEPTMPPTSWRQLNPESIRPGGPGGLSRSLGREEEEDEEEELEEGTIDVTDFL
SMTQQDSHAPLRDSRGSSFEMTDDDSAIRALTQFPLPKNLLAKVIQIATSSSTAKNLMQF
HTVGTKTKLSTLTLLWPCPMTFVAKGRRKAEAENKAAALACKKLKSLGLVDRNNEPLTHA
MYNLASLRELGETQRRPCTIQVPEPILRKIETFLNHYPVESSWIAPELRLQSDDILPLGK
DSGPLSDPITGKPYVPLLEAEEVRLSQSLLELWRRRGPVWQEAPQLPVDPHRDTILNAIE
QHPVVVISGDTGCGKTTRIPQLLLERYVTEGRGARCNVIITQPRRISAVSVAQRVSHELG
PSLRRNVGFQVRLESKPPSRGGALLFCTVGILLRKLQSNPSLEGVSHVIVDEVHERDVNT
DFLLILLKGLQRLNPALRLVLMSATGDNERFSRYFGGCPVIKVPGFMYPVKEHYLEDILA
KLGKHQYLHRHRHHESEDECALDLDLVTDLVLHIDARGEPGGILCFLPGWQEIKGVQQRL
QEALGMHESKYLILPVHSNIPMMDQKAIFQQPPVGVRKIVLATNIAETSITINDIVHVVD
SGLHKEERYDLKTKVSCLETVWVSRANVIQRRGRAGRCQSGFAYHLFPRSRLEKMVPFQV
PEILRTPLENLVLQAKIHMPEKTAVEFLSKAVDSPNIKAVDEAVILLQEIGVLDQREYLT
TLGQRLAHISTDPRLAKAIVLAAIFRCLHPLLVVVSCLTRDPFSSSLQNRAEVDKVKALL
SHDSGSDHLAFVRAVAGWEEVLRWQDRSSRENYLEENLLYAPSLRFIHGLIKQFSENIYE
AFLVGKPSDCTLASAQCNEYSEEEELVKGVLMAGLYPNLIQVRQGKVTRQGKFKPNSVTY
RTKSGNILLHKSTINREATRLRSRWLTYFMAVKSNGSVFVRDSSQVHPLAVLLLTDGDVH
IRDDGRRATISLSDSDLLRLEGDSRTVRLLKELRRALGRMVERSLRSELAALPPSVQEEH
GQLLALLAELLRGPCGSFDVRKTADD*
>ENSG00000280987|ENST00000361059
MSKSFQQSSLSRDSQGHGRDLSAAGIGLLAAATQSLSMPASLGRMNQGTARLASLMNLGM
SSSLNQQGAHSALSSASTSSHNLQSIFNIGSRGPLPLSSQHRGDADQASNILASFGLSAR
DLDELSRYPEDKITPENLPQILLQLKRRRTEEGPTLSYGRDGRSATREPPYRVPRDDWEE
KRHFRRDSFDDRGPSLNPVLDYDHGSRSQESGYYDRMDYEDDRLRDGERCRDDSFFGETS
HNYHKFDSEYERMGRGPGPLQERSLFEKKRGAPPSSNIEDFHGLLPKGYPHLCSICDLPV
HSNKEWSQHINGASHSRRCQLLLEIYPEWNPDNDTGHTMGDPFMLQQSTNPAPGILGPPP
PSFHLGGPAVGPRGNLGAGNGNLQGPRHMQKGRVETSRVVHIMDFQRGKNLRYQLLQLVE
PFGVISNHLILNKINEAFIEMATTEDAQAAVDYYTTTPALVFGKPVRVHLSQKYKRIKKP
EGKPDQKFDQKQELGRVIHLSNLPHSGYSDSAVLKLAEPYGKIKNYILMRMKSQAFIEME
TREDAMAMVDHCLKKALWFQGRCVKVDLSEKYKKLVLRIPNRGIDLLKKDKSRKRSYSPD
GKESPSDKKSKTDGSQKTESSTEGKEQEEKSGEDGEKDTKDDQTEQEPNMLLESEDELLV
DEEEAAALLESGSSVGDETDLANLGDVASDGKKEPSDKAVKKDGSASAAAKKKLKKVDKI
EELDQENEAALENGIKNEENTEPGAESSENADDPNKDTSENADGQSDENKDDYTIPDEYR
IGPYQPNVPVGIDYVIPKTGFYCKLCSLFYTNEEVAKNTHCSSLPHYQKLKKFLNKLAEE
RRQKKET*
>ENSG00000156709|ENST00000346424
MFRCGGLAAGALKQKLVPLVRTVCVRSPRQRNRLPARALGTEVIQLFPEKGNMGKILPEY
LSNWTMEKVRREGVKVMPNAIVQSVGVSSGKLLIKLKDGRKVETDHIVAAVGLEPNVELA

KTGGLEIDSDFGGFRVNAELQARSNIWVAGDAACFYDIKLGRRRVEHHDHAVVSGRLAGE
NMTGAAKPYWHQSMFWSDLGPDVGYEAIGLVDSSLPTVGVFAKATAQDNPKSATEQSGTG
IRSESETESEASEITIPPSTPAVPQAPVQGEDYGKGVIFYLRDKVVVGIVLWNIFNRMPI
ARKIIKDGEQHEDLNEVAKLFNIHED*
>ENSG00000067048|ENST00000360160
MSHVVVKNDPELDQQLANLDLNSEKQSGGASTASKGRYIPPHLRNREASKGFHDKDSSGW
SCSKDKDAYSSFGSRDSRGKPGYFSERGSGSRGRFDDRGRSDYDGIGNRERPGFGRFERS
GHSRWCDKSVEDDWSKPLPPSERLEQELFSGGNTGINFEKYDDIPVEATGSNCPPHIENF
SDIDMGEIIMGNIELTRYTRPTPVQKHAIPIIKGKRDLMACAQTGSGKTAAFLLPILSQI
YTDGPGEALKAVKENGRYGRRKQYPISLVLAPTRELAVQIYEEARKFSYRSRVRPCVVYG
GADIGQQIRDLERGCHLLVATPGRLVDMMERGKIGLDFCKYLVLDEADRMLDMGFEPQIR
RIVEQDTMPPKGVRHTMMFSATFPKEIQMLARDFLDEYIFLAVGRVGSTSENITQKVVWV
EDLDKRSFLLDILGATGSDSLTLVFVETKKGADSLEDFLYHEGYACTSIHGDRSQRDREE
ALHQFRSGKSPILVATAVAARGLDISNVRHVINFDLPSDIEEYVHRIGRTGRVGNLGLAT
SFFNEKNMNITKDLLDLLVEAKQEVPSWLENMAYEHHYKGGSRGRSKSNRFSGGFGARDY
RQSSGSSSSGFGASRGSSSRSGGGGYGNSRGFGGGGYGGFYNSDGYGGNYNSQGVDWWGN
*
>ENSG00000179195|ENST00000337815
MIYKCPMCREFFSERADLFMHQKIHTAEKPHKCDKCDKGFFHISELHIHWRDHTGEKVYK
CDDCGKDFSTTTKLNRHKKIHTVEKPYKCYECGKAFNWSSHLQIHMRVHTGEKPYVCSEC
GRGFSNSSNLCMHQRVHTGEKPFKCEECGKAFRHTSSLCMHQRVHTGEKPYKCYECGKAF
SQSSSLCIHQRVHTGEKPYRCCGCGKAFSQSSSLCIHQRVHTGEKPFKCDECGKAFSQST
SLCIHQRVHTKERNHLKISVI*
>ENSG00000180667|ENST00000315927
MFGPAKGRHFGVHPAPGFPGGVSQQAAGTKAGPAGAWPVGSRTDTMWRLRCKAKDGTHVL
QGLSSRTRVRELQGQIAAITGIAPGGQRILVGYPPECLDLSNGDTILEDLPIQSGDMLII
EEDQTRPRSSPAFTKRGASSYVRETLPVLTRTVVPADNSCLFTSVYYVVEGGVLNPACAP
EMRRLIAQIVASDPDFYSEAILGKTNQEYCDWIKRDDTWGGAIEISILSKFYQCEICVVD
TQTVRIDRFGEDAGYTKRVLLIYDGIHYDPLQRNFPDPDTPPLTIFSSNDDIVLVQALEL
ADEARRRRQFTDVNRFTLRCMVCQKGLTGQAEAREHAKETGHTNFGEV*
>ENSG00000113460|ENST00000336767
MAATKRKRRGGFAVQAKKPKRNEIDAEPPAKRHATAEEVEEEERDRIPGPVCKGKWKNKE
RILIFSSRGINFRTRHLMQDLRMLMPHSKADTKMDRKDKLFVINEVCEMKNCNKCIYFEA
KKKQDLYMWLSNSPHGPSAKFLVQNIHTLAELKMTGNCLKGSRPLLSFDPAFDELPHYAL
LKELLIQIFSTPRYHPKSQPFVDHVFTFTILDNRIWFRNFQIIEEDAALVEIGPRFVLNL
IKIFQGSFGGPTLYENPHYQSPNMHRRVIRSITAAKYREKQQVKDVQKLRKKEPKTLLPH
DPTADVFVTPAEEKPIEIQWVKPEPKVDLKARKKRIYKRQRKMKQRMDSGKTK*
>ENSG00000080802|ENST00000361528
MSRSPDAKEDPVECPLCMEPLEIDDINFFPCTCGYQICRFCWHRIRTDENGLCPACRKPY
PEDPAVYKPLSQEELQRIKNEKKQKQNERKQKISENRKHLASVRVVQKNLVFVVGLSQRL
ADPEVLKRPEYFGKFGKIHKVVINNSTSYAGSQGPSASAYVTYIRSEDALRAIQCVNNVV
VDGRTLKASLGTTKYCSYFLKNMQCPKPDCMYLHELGDEAASFTKEEMQAGKHQEYEQKL
LQELYKLNPNFLQLSTGSVDKNKNKVTPLQSPIDKPSDSLSIGNGDNSQQISNSDTPSPP
PGLSKSNPVIPISSSNHSARSPFEGAVTESQSLFSDNFRHPNPIPSGLPPFPSSPQTSSD

WPTAPEPQSLFTSETIPVSSSTDWQAAFGFGSSKQPEDDLGFDPFDVTRKALADLIEKEL
SVQDQPSLSPTSLQNSSSHTTTAKGPGSGFLHPAAATNANSLNSTFSVLPQRFPQFQQHR
AVYNSFSFPGQAARYPWMAFPRNSIMHLNHTANPTSNSNFLDLNLPPQHNTGLGGIPVAG
IPASSGNSLDSLQDDNPPHWLKSLQALTEMDGPSAAPSQTHHSAPFSTQIPLHRASWNPY
PPPSNPSSFHSPPPGFQTAFRPPSKTPTDLLQSSTLDRH*
>ENSG00000188342|ENST00000340473
MAERGELDLTGAKQNTGVWLVKVPKYLSQQWAKASGRGEVGKLRIAKTQGRTEVSFTLNE
DLANIHDIGGKPASVSAPREHPFVLQSVGGQTLTVFTESSSDKLSLEGIVVQRAECRPAA
SENYMRLKRLQIEESSKPVRLSQQLDKVVTTNYKPVANHQYNIEYERKKKEDGKRARADK
QHVLDMLFSAFEKHQYYNLKDLVDITKQPVVYLKEILKEIGVQNVKGIHKNTWELKPEYR
HYQGEEKSD*
>ENSG00000198521|ENST00000357491
MPGHPGSWEMGPLTFMDVAIEFCLEEWQCLDIAQQNLYRNVMLENYRNLVFLGIAVSKPD
LITCLEQEKEPWEPMRRHEMVAKPPVMCSHFTQDFWPEQHIKDPFQKATLRRYKNCEHKN
VHLKKDHKSVDECKVHRGGYNGFNQCLPATQSKIFLFDKCVKAFHKFSNSNRHKISHTEK
KLFKCKECGKSFCMLPHLAQHKIIHTRVNFCKCEKCGKAFNCPSIITKHKRINTGEKPYT
CEECGKVFNWSSRLTTHKKNYTRYKLYKCEECGKAFNKSSILTTHKIIRTGEKFYKCKEC
AKAFNQSSNLTEHKKIHPGEKPYKCEECGKAFNWPSTLTKHKRIHTGEKPYTCEECGKAF
NQFSNLTTHKRIHTAEKFYKCTECGEAFSRSSNLTKHKKIHTEKKPYKCEECGKAFKWSS
KLTEHKLTHTGEKPYKCEECGKAFNWPSTLTKHNRIHTGEKPYKCEVCGKAFNQFSNLTT
HKRIHTAEKPYKCEECGKAFSRSSNLTKHKKIHIEKKPYKCEECGKAFKWSSKLTEHKIT
HTGEKPYKCEECGKAFNHFSILTKHKRIHTGEKPYKCEECGKAFTQSSNLTTHKKIHTGE
KFYKCEECGKAFTQSSNLTTHKKIHTGGKPYKCEECGKAFNQFSTLTKHKIIHTEEKPYK
CEECGKAFKWSSTLTKHKIIHTGEKPYKCEECGKAFKLSSTLSTHKIIHTGEKPYKCEKC
GKAFNRSSNLIEHKKIHTGEQPYKCEECGKAFNYSSHLNTHKRIHTKEQPYKCKECGKAF
NQYSNLTTHNKIHTGEKLYKPEDVTVILTTPQTFSNIK*
>ENSG00000185811|ENST00000331340
MDADEGQDMSQVSGKESPPVSDTPDEGDEPMPIPEDLSTTSGGQQSSKSDRVVASNVKVE
TQSDEENGRACEMNGEECAEDLRMLDASGEKMNGSHRDQGSSALSGVGGIRLPNGKLKCD
ICGIICIGPNVLMVHKRSHTGERPFQCNQCGASFTQKGNLLRHIKLHSGEKPFKCHLCNY
ACRRRDALTGHLRTHSVGKPHKCGYCGRSYKQRSSLEEHKERCHNYLESMGLPGTLYPVI
KEETNHSEMAEDLCKIGSERSLVLDRLASNVAKRKSSMPQKFLGDKGLSDTPYDSSASYE
KENEMMKSHVMDQAINNAINYLGAESLRPLVQTPPGGSEVVPVISPMYQLHKPLAEGTPR
SNHSAQDSAVENLLLLSKAKLVPSEREASPSNSCQDSTDTESNNEEQRSGLIYLTNHIAP
HARNGLSLKEEHRAYDLLRAASENSQDALRVVSTSGEQMKVYKCEHCRVLFLDHVMYTIH
MGCHGFRDPFECNMCGYHSQDRYEFSSHITRGEHRFHMS*
>ENSG00000135387|ENST00000341394
MPSATSHSGSGSKSSGPPPPSGSSGSEAAAGAGAAAPASQHPATGTGAVQTEAMKQILGV
IDKKLRNLEKKKGKLDDYQERMNKGERLNQDQLDAVSKYQEVTNNLEFAKELQRSFMALS
QDIQKTIKKTARREQLMREEAEQKRLKTVLELQYVLDKLGDDEVRTDLKQGLNGVPILSE
EELSLLDEFYKLVDPERDMSLRLNEQYEHASIHLWDLLEGKEKPVCGTTYKVLKEIVERV
FQSNYFDSTHNHQNGLCEEEEAASAPAVEDQVPEAEPEPAEEYTEQSEVESTEYVNRQFM
AETQFTSGEKEQVDEWTVETVEVVNSLQQQPQAASPSVPEPHSLTPVAQADPLVRRQRVQ
DLMAQMQGPYNFIQDSMLDFENQTLDPAIVSAQPMNPTQNMDMPQLVCPPVHSESRLAQP

NQVPVQPEATQVPLVSSTSEGYTASQPLYQPSHATEQRPQKEPIDQIQATISLNTDQTTA
SSSLPAASQPQVFQAGTSKPLHSSGINVNAAPFQSMQTVFNMNAPVPPVNEPETLKQQNQ
YQASYNQSFSSQPHQVEQTELQQEQLQTVVGTYHGSPDQSHQVTGNHQQPPQQNTGFPRS
NQPYYNSRGVSRGGSRGARGLMNGYRGPANGFRGGYDGYRPSFSNTPNSGYTQSQFSAPR
DYSGYQRDGYQQNFKRGSGQSGPRGAPRGRGGPPRPNRGMPQMNTQQVN*
>ENSG00000008441|ENST00000360105
MGATEPGGDTSSDEFHPFIEALLPHVRAFSYTWFNLQARKRKYFKKHEKRMSKDEERAVK
DELLGEKPEIKQKWASRLLAKLRKDIRPEFREDFVLTITGKKPPCCVLSNPDQKGKIRRI
DCLRQADKVWRLDLVMVILFKGIPLESTDGERLYKSPQCSNPGLCVQPHHIGVTIKELDL
YLAYFVHTPESGQSDSSNQQGDADIKPLPNGHLSFQDCFVTSGVWNVTELVRVSQTPVAT
ASGPNFSLADLESPSYYNINQVTLGRRSITSPPSTSTTKRPKSIDDSEMESPVDDVFYPG
TGRSPAAGSSQSSGWPNDVDAGSPRATASALHFPSTSIIQQSSPYFTHPTIRYHHHHGQD
SLKEFVQFVCSDGSGQATGQHSQRQAPPLPTGLSASDPGTATF*
>ENSG00000117036|ENST00000326786
MKAGCSIVEKPEGGGGYQFPDWAYKTESSPGSRQIQLWHFILELLQKEEFRHVIAWQQGE
YGEFVIKDPDEVARLWGRRKCKPQMNYDKLSRALRYYYNKRILHKTKGKRFTYKFNFNKL
VMPNYPFINIRSSGKIQTLLVGN*
>ENSG00000078328|ENST00000355637
MLASQGVLLHPYGVPMIVPAAPYLPGLIQGNQEAAAAPDTMAQPYASAQFAPPQNGIPAE
YTAPHPHPAPEYTGQTTVPEHTLNLYPPAQTHSEQSPADTSAQTVSGTATQTDDAAPTDG
QPQTQPSENTENKSQPKRLHVSNIPFRFRDPDLRQMFGQFGKILDVEIIFNERGSKGFGF
VTFENSADADRAREKLHGTVVEGRKIEVNNATARVMTNKKTVNPYTNGWKLNPVVGAVYS
PEFYAGTVLLCQANQEGSSMYSAPSSLVYTSAMPGFPYPAATAAAAYRGAHLRGRGRTVY
NTFRAAAPPPPIPAYGGVVYQEPVYGNKLLQGGYAAYRYAQPTPATAAAYSDRNQFVFVA
ADEISCNTSAVTDEFMLPTPTTTHLLQPPPTALVP*
>ENSG00000055917|ENST00000319801
MSQPIMVQRRSGQGFHGNSEVNAILSPRSESGGLGVSMVEYVLSSSPADKLDSRFRKGNF
GTRDAETDGPEKGDQKGKASPFEEDQNRDLKQGDDDDSKINGRGLPNGMDADCKDFNRTP
GSRQASPTEVVERLGPNTNPSEGLGPLPNPTANKPLVEEFSNPETQNLDAMEQVGLESLQ
FDYPGNQVPMDSSGATVGLFDYNSQQQLFQRTNALTVQQLTAAQQQQYALAAAQQPHIAG
VFSAGLAPAAFVPNPYIISAAPPGTDPYTAAGLAAAATLAGPAVVPPQYYGVPWGVYPAN
LFQQQAAAAANNTASQQAASQAQPGQQQVLRAGAGQRPLTPNQGQQGQQAESLAAAAAAN
PTLAFGQGLATGMPGYQVLAPTAYYDQTGALVVGPGARTGLGAPVRLMAPTPVLISSAAA
QAAAAAAAGGTASSLTGSTNGLFRPIGTQPPQQQQQQPSTNLQSNSFYGSSSLTNSSQSS
SLFSHGPGQPGSTSLGFGSGNSLGAAIGSALSGFGSSGGLTNGSGRYISAAPGAEAKYRS
ASSTSSLFSSSSQLFPPSRLRYNRSDIMPSGRSRLLEDFRNNRFPNLQLRDLIGHIVEFS
QDQHGSRFIQQKLERATPAERQMVFNEILQAAYQLMTDVFGNYVIQKFFEFGSLDQKLAL
ATRIRGHVLPLALQMYGCRVIQKALESISSDQQSEMVKELDGHVLKCVKDQNGNHVVQKC
IECVQPQSLQFIIDAFKGQVFVLSTHPYGCRVIQRILEHCTAEQTLPILEELHQHTEQLV
QDQYGNYVIQHVLEHGRPEDKSKIVSEIRGKVLALSQHKFASNVVEKCVTHASRAERALL
IDEVCCQNDGPHSALYTMMKDQYANYVVQKMIDMAEPAQRKIIMHKIRPHITTLRKYTYG
KHILAKLEKYYLKNSPDLGPIGGPPNGML*
>ENSG00000214274|ENST00000336811
MVMGLGVLLLVFVLGLGLTPPTLAQDNSRYTHFLTQHYDAKPQGRDDRYCESIMRRRGLT

SPCKDINTFIHGNKRSIKAICENKNGNPHRENLRISKSSFQVTTCKLHGGSPWPPCQYRA
TAGFRNVVVACENGLPVHLDQSIFRRP*
>ENSG00000163435|ENST00000359651
MAATCEISNIFSNYFSAMYSSEDSTLASVPPAATFGADDLVLTLSNPQMSLEGTEKASWL
GEQPQFWSKTQVLDWISYQVEKNKYDASAIDFSRCDMDGATLCNCALEELRLVFGPLGDQ
LHAQLRDLTSSSSDELSWIIELLEKDGMAFQEALDPGPFDQGSPFAQELLDDGQQASPYH
PGSCGAGAPSPGSSDVSTAGTGASRSSHSSDSGGSDVDLDPTDGKLFPSDGFRDCKKGDP
KHGKRKRGRPRKLSKEYWDCLEGKKSKHAPRGTHLWEFIRDILIHPELNEGLMKWENRHE
GVFKFLRSEAVAQLWGQKKKNSNMTYEKLSRAMRYYYKREILERVDGRRLVYKFGKNSSG
WKEEEVLQSRN*
>ENSG00000181873|ENST00000366711
MATAALLRGATPGRGGPVWRWRLRAAPRCRLAHSSCSPGGDPTAGAAWACFRLDGRTLLR
VRGPDAAPFLLGLLTNELPLPSPAAAGAPPAARAGYAHFLNVQGRTLYDVILYGLQEHSE
VSGFLLECDSSVQGALQKHLALYRIRRKVTVEPHPELRVWAVLPSSPEACGAASLQERAG
AAAILIRDPRTARMGWRLLTQDEGPALVPGGRLGDLWDYHQHRYLQGVPEGVRDLPPGVA
LPLESNLAFMNGVSFTKGCYIGQELTARTHHMGVIRKRLFPVRFLDPLPTSGITPGATVL
TASGQTVGKFRAGQGNVGLALLWSEKIKGPLHIRASEGAQVALAASVPDWWPTVSK*
>ENSG00000197238|ENST00000355057
MSGRGKGGKGLGKGGAKRHRKVLRDNIQGITKPAIRRLARRGGVKRISGLIYEETRGVLK
VFLENVIRDAVTYTEHAKRKTVTAMDVVYALKRQGRTLYGFGG*
>ENSG00000198793|ENST00000361445
MLGTGPAAATTAATTSSNVSVLQQFASGLKSRNEETRAKAAKELQHYVTMELREMSQEES
TRFYDQLNHHIFELVSSSDANERKGGILAIASLIGVEGGNATRIGRFANYLRNLLPSNDP
VVMEMASKAIGRLAMAGDTFTAEYVEFEVKRALEWLGADRNEGRRHAAVLVLRELAISVP
TFFFQQVQPFFDNIFVAVWDPKQAIREGAVAALRACLILTTQREPKEMQKPQWYRHTFEE
AEKGFDETLAKEKGMNRDDRIHGALLILNELVRISSMEGERLREEMEEITQQQLVHDKYC
KDLMGFGTKPRHITPFTSFQAVQPQQSNALVGLLGYSSHQGLMGFGTSPSPAKSTLVESR
CCRDLMEEKFDQVCQWVLKCRNSKNSLIQMTILNLLPRLAAFRPSAFTDTQYLQDTMNHV
LSCVKKEKERTAAFQALGLLSVAVRSEFKVYLPRVLDIIRAALPPKDFAHKRQKAMQVDA
TVFTCISMLARAMGPGIQQDIKELLEPMLAVGLSPALTAVLYDLSRQIPQLKKDIQDGLL
KMLSLVLMHKPLRHPGMPKGLAHQLASPGLTTLPEASDVGSITLALRTLGSFEFEGHSLT
QFVRHCADHFLNSEHKEIRMEAARTCSRLLTPSIHLISGHAHVVSQTAVQVVADVLSKLL
VVGITDPDPDIRYCVLASLDERFDAHLAQAENLQALFVALNDQVFEIRELAICTVGRLSS
MNPAFVMPFLRKMLIQILTELEHSGIGRIKEQSARMLGHLVSNAPRLIRPYMEPILKALI
LKLKDPDPDPNPGVINNVLATIGELAQVSGLEMRKWVDELFIIIMDMLQDSSLLAKRQVA
LWTLGQLVASTGYVVEPYRKYPTLLEVLLNFLKTEQNQGTRREAIRVLGLLGALDPYKHK
VNIGMIDQSRDASAVSLSESKSSQDSSDYSTSEMLVNMGNLPLDEFYPAVSMVALMRIFR
DQSLSHHHTMVVQAITFIFKSLGLKCVQFLPQVMPTFLNVIRVCDGAIREFLFQQLGMLV
SFVKSHIRPYMDEIVTLMREFWVMNTSIQSTIILLIEQIVVALGGEFKLYLPQLIPHMLR
VFMHDNSPGRIVSIKLLAAIQLFGANLDDYLHLLLPPIVKLFDAPEAPLPSRKAALETVD
RLTESLDFTDYASRIIHPIVRTLDQSPELRSTAMDTLSSLVFQLGKKYQIFIPMVNKVLV
RHRINHQRYDVLICRIVKGYTLADEEEDPLIYQHRMLRSGQGDALASGPVETGPMKKLHV
STINLQKAWGAARRVSKDDWLEWLRRLSLELLKDSSSPSLRSCWALAQAYNPMARDLFNA
AFVSCWSELNEDQQDELIRSIELALTSQDIAEVTQTLLNLAEFMEHSDKGPLPLRDDNGI

VLLGERAAKCRAYAKALHYKELEFQKGPTPAILESLISINNKLQQPEAAAGVLEYAMKHF
GELEIQATWYEKLHEWEDALVAYDKKMDTNKDDPELMLGRMRCLEALGEWGQLHQQCCEK
WTLVNDETQAKMARMAAAAAWGLGQWDSMEEYTCMIPRDTHDGAFYRAVLALHQDLFSLA
QQCIDKARDLLDAELTAMAGESYSRAYGAMVSCHMLSELEEVIQYKLVPERREIIRQIWW
ERLQGCQRIVEDWQKILMVRSLVVSPHEDMRTWLKYASLCGKSGRLALAHKTLVLLLGVD
PSRQLDHPLPTVHPQVTYAYMKNMWKSARKIDAFQHMQHFVQTMQQQAQHAIATEDQQHK
QELHKLMARCFLKLGEWQLNLQGINESTIPKVLQYYSAATEHDRSWYKAWHAWAVMNFEA
VLHYKHQNQARDEKKKLRHASGANITNATTAATTAATATTTASTEGSNSESEAESTENSP
TPSPLQKKVTEDLSKTLLMYTVPAVQGFFRSISLSRGNNLQDTLRVLTLWFDYGHWPDVN
EALVEGVKAIQIDTWLQVIPQLIARIDTPRPLVGRLIHQLLTDIGRYHPQALIYPLTVAS
KSTTTARHNAANKILKNMCEHSNTLVQQAMMVSEELIRVAILWHEMWHEGLEEASRLYFG
ERNVKGMFEVLEPLHAMMERGPQTLKETSFNQAYGRDLMEAQEWCRKYMKSGNVKDLTQA
WDLYYHVFRRISKQLPQLTSLELQYVSPKLLMCRDLELAVPGTYDPNQPIIRIQSIAPSL
QVITSKQRPRKLTLMGSNGHEFVFLLKGHEDLRQDERVMQLFGLVNTLLANDPTSLRKNL
SIQRYAVIPLSTNSGLIGWVPHCDTLHALIRDYREKKKILLNIEHRIMLRMAPDYDHLTL
MQKVEVFEHAVNNTAGDDLAKLLWLKSPSSEVWFDRRTNYTRSLAVMSMVGYILGLGDRH
PSNLMLDRLSGKILHIDFGDCFEVAMTREKFPEKIPFRLTRMLTNAMEVTGLDGNYRITC
HTVMEVLREHKDSVMAVLEAFVYDPLLNWRLMDTNTKGNKRSRTRTDSYSAGQSVEILDG
VELGEPAHKKTGTTVPESIHSFIGDGLVKPEALNKKAIQIINRVRDKLTGRDFSHDDTLD
VPTQVELLIKQATSHENLCQCYIGWCPFW*
>ENSG00000088305|ENST00000353855
MKGDTRHLNGEEDAGGREDSILVNGACSDQSSDSPPILEAIRTPEIRGRRSSSRLSKREV
SSLLSYTQDLTGDGDGEDGDGSDTPVMPKLFRETRTRSESPAVRTRNNNSVSSRERHRPS
PRSTRGRQGRNHVDESPVEFPATRSLRRRATASAGTPWPSPPSSYLTIDLTDDTEDTHGT
PQSSSTPYARLAQDSQQGGMESPQVEADSGDGDSSEYQDGKEFGIGDLVWGKIKGFSWWP
AMVVSWKATSKRQAMSGMRWVQWFGDGKFSEVSADKLVALGLFSQHFNLATFNKLVSYRK
AMYHALEKARVRAGKTFPSSPGDSLEDQLKPMLEWAHGGFKPTGIEGLKPNNTQPENKTR
RRTADDSATSDYCPAPKRLKTNCYNNGKDRGDEDQSREQMASDVANNKSSLEDGCLSCGR
KNPVSFHPLFEGGLCQTCRDRFLELFYMYDDDGYQSYCTVCCEGRELLLCSNTSCCRCFC
VECLEVLVGTGTAAEAKLQEPWSCYMCLPQRCHGVLRRRKDWNVRLQAFFTSDTGLEYEA
PKLYPAIPAARRRPIRVLSLFDGIATGYLVLKELGIKVGKYVASEVCEESIAVGTVKHEG
NIKYVNDVRNITKKNIEEWGPFDLVIGGSPCNDLSNVNPARKGLYEGTGRLFFEFYHLLN
YSRPKEGDDRPFFWMFENVVAMKVGDKRDISRFLECNPVMIDAIKVSAAHRARYFWGNLP
GMNRPVIASKNDKLELQDCLEYNRIAKLKKVQTITTKSNSIKQGKNQLFPVVMNGKEDVL
WCTELERIFGFPVHYTDVSNMGRGARQKLLGRSWSVPVIRHLFAPLKDYFACE*
>ENSG00000178209|ENST00000354958
MDPSRAIQNEISSLKDERDRVQKKTFTKWVNKHLIKAQRHISDLYEDLRDGHNLISLLEV
LSGDSLPREKGRMRFHKLQNVQIALDYLRHRQVKLVNIRNDDIADGNPKLTLGLIWTIIL
HFQISDIQVSGQSEDMTAKEKLLLWSQRMVEGYQGLRCDNFTSSWRDGRLFNAIIHRHKP
LLIDMNKVYRQTNLENLDQAFSVAERDLGVTRLLDPEDVDVPQPDEKSIITYVSSLYDAM
PRVPDVQDGVRANELQLRWQEYRELVLLLLQWMRHHTAAFEERRFPSSFEEIEILWSQFL
KFKEMELPAKEADKNRSKGIYQSLEGAVQAGQLKVPPGYHPLDVEKEWGKLHVAILEREK
QLRSEFERLECLQRIVTKLQMEAGLCEEQLNQADALLQSDVRLLAAGKVPQRAGEVERDL
DKADSMIRLLFNDVQTLKDGRHPQGEQMYRRVYRLHERLVAIRTEYNLRLKAGVAAPATQ

VAQVTLQSVQRRPELEDSTLRYLQDLLAWVEENQHRVDGAEWGVDLPSVEAQLGSHRGLH
QSIEEFRAKIERARSDEGQLSPATRGAYRDCLGRLDLQYAKLLNSSKARLRSLESLHSFV
AAATKELMWLNEKEEEEVGFDWSDRNTNMTAKKESYSALMRELELKEKKIKELQNAGDRL
LREDHPARPTVESFQAALQTQWSWMLQLCCCIEAHLKENAAYFQFFSDVREAEGQLQKLQ
EALRRKYSCDRSATVTRLEDLLQDAQDEKEQLNEYKGHLSGLAKRAKAVVQLKPRHPAHP
MRGRLPLLAVCDYKQVEVTVHKGDECQLVGPAQPSHWKVLSSSGSEAAVPSVCFLVPPPN
QEAQEAVTRLEAQHQALVTLWHQLHVDMKSLLAWQSLRRDVQLIRSWSLATFRTLKPEEQ
RQALHSLELHYQAFLRDSQDAGGFGPEDRLMAEREYGSCSHHYQQLLQSLEQGAQEESRC
QRCISELKDIRLQLEACETRTVHRLRLPLDKEPARECAQRIAEQQKAQAEVEGLGKGVAR
LSAEAEKVLALPEPSPAAPTLRSELELTLGKLEQVRSLSAIYLEKLKTISLVIRGTQGAE
EVLRAHEEQLKEAQAVPATLPELEATKASLKKLRAQAEAQQPTFDALRDELRGAQEVGER
LQQRHGERDVEVERWRERVAQLLERWQAVLAQTDVRQRELEQLGRQLRYYRESADPLGAW
LQDARRRQEQIQAMPLADSQAVREQLRQEQALLEEIERHGEKVEECQRFAKQYINAIKDY
ELQLVTYKAQLEPVASPAKKPKVQSGSESVIQEYVDLRTHYSELTTLTSQYIKFISETLR
RMEEEERLAEQQRAEERERLAEVEAALEKQRQLAEAHAQAKAQAEREAKELQQRMQEEVV
RREEAAVDAQQQKRSIQEELQQLRQSSEAEIQAKARQAEAAERSRLRIEEEIRVVRLQLE
ATERQRGGAEGELQALRARAEEAEAQKRQAQEEAERLRRQVQDESQRKRQAEVELASRVK
AEAEAAREKQRALQALEELRLQAEEAERRLRQAEVERARQVQVALETAQRSAEAELQSKR
ASFAEKTAQLERSLQEEHVAVAQLREEAERRAQQQAEAERAREEAERELERWQLKANEAL
RLRLQAEEVAQQKSLAQAEAEKQKEEAEREARRRGKAEEQAVRQRELAEQELEKQRQLAE
GTAQQRLAAEQELIRLRAETEQGEQQRQLLEEELARLQREAAAATQKRQELEAELAKVRA
EMEVLLASKARAEEESRSTSEKSKQRLEAEAGRFRELAEEAARLRALAEEAKRQRQLAEE
DAARQRAEAERVLAEKLAAIGEATRLKTEAEIALKEKEAENERLRRLAEDEAFQRRRLEE
QAAQHKADIEERLAQLRKASDSELERQKGLVEDTLRQRRQVEEEILALKASFEKAAAGKA
ELELELGRIRSNAEDTLRSKEQAELEAARQRQLAAEEERRRREAEERVQKSLAAEEEAAR
QRKAALEEVERLKAKVEEARRLRERAEQESARQLQLAQEAAQKRLQAEEKAHAFAVQQKE
QELQQTLQQEQSVLDQLRGEAEAARRAAEEAEEARVQAEREAAQSRRQVEEAERLKQSAE
EQAQARAQAQAAAEKLRKEAEQEAARRAQAEQAALRQKQAADAEMEKHKKFAEQTLRQKA
QVEQELTTLRLQLEETDHQKNLLDEELQRLKAEATEAARQRSQVEEELFSVRVQMEELSK
LKARIEAENRALILRDKDNTQRFLQEEAEKMKQVAEEAARLSVAAQEAARLRQLAEEDLA
QQRALAEKMLKEKMQAVQEATRLKAEAELLQQQKELAQEQARRLQEDKEQMAQQLAEETQ
GFQRTLEAERQRQLEMSAEAERLKLRVAEMSRAQARAEEDAQRFRKQAEEIGEKLHRTEL
ATQEKVTLVQTLEIQRQQSDHDAERLREAIAELEREKEKLQQEAKLLQLKSEEMQTVQQE
QLLQETQALQQSFLSEKDSLLQRERFIEQEKAKLEQLFQDEVAKAQQLREEQQRQQQME
QERQRLVASMEEARRRQHEAEEGVRRKQEELQQLEQQRRQQEELLAEENQRLREQLQLLE
EQHRAALAHSEEVTASQVAATKTLPNGRDALDGPAAEAEPEHSFDGLRRKVSAQRLQEAG
ILSAEELQRLAQGHTTVDELARREDVRHYLQGRSSIAGLLLKATNEKLSVYAALQRQLLS
PGTALILLEAQAASGFLLDPVRNRRLTVNEAVKEGVVGPELHHKLLSAERAVTGYKDPYT
GQQISLFQAMQKGLIVREHGIRLLEAQIATGGVIDPVHSHRVPVDVAYRRGYFDEEMNRV
LADPSDDTKGFFDPNTHENLTYLQLLERCVEDPETGLCLLPLTDKAAKGGELVYTDSEAR
DVFEKATVSAPFGKFQGKTVTIWEIINSEYFTAEQRRDLLRQFRTGRITVEKIIKIIITV
VEEQEQKGRLCFEGLRSLVPAAELLESRVIDRELYQQLQRGERSVRDVAEVDTVRRALRG
ANVIAGVWLEEAGQKLSIYNALKKDLLPSDMAVALLEAQAGTGHIIDPATSARLTVDEAV
RAGLVGPEFHEKLLSAEKAVTGYRDPYTGQSVSLFQALKKGLIPREQGLRLLDAQLSTGG

IVDPSKSHRVPLDVACARGCLDEETSRALSAPRADAKAYSDPSTGEPATYGELQQRCRPD
QLTGLSLLPLSEKAARARQEELYSELQARETFEKTPVEVPVGGFKGRTVTVWELISSEYF
TAEQRQELLRQFRTGKVTVEKVIKILITIVEEVETLRQERLSFSGLRAPVPASELLASGV
LSRAQFEQLKDGKTTVKDLSELGSVRTLLQGSGCLAGIYLEDTKEKVSIYEAMRRGLLRA
TTAALLLEAQAATGFLVDPVRNQRLYVHEAVKAGVVGPELHEQLLSAEKAVTGYRDPYSG
STISLFQAMQKGLVLRQHGIRLLEAQIATGGIIDPVHSHRVPVDVAYQRGYFSEEMNRVL
ADPSDDTKGFFDPNTHENLTYRQLLERCVEDPETGLRLLPLKGAEKAEVVETTQVYTEEE
TRRAFEETQIDIPGGGSHGGSTMSLWEVMQSDLIPEEQRAQLMADFQAGRVTKERMIIII
IEIIEKTEIIRQQGLASYDYVRRRLTAEDLFEARIISLETYNLLREGTRSLREALEAESA
WCYLYGTGSVAGVYLPGSRQTLSIYQALKKGLLSAEVARLLLEAQAATGFLLDPVKGERL
TVDEAVRKGLVGPELHDRLLSAERAVTGYRDPYTEQTISLFQAMKKELIPTEEALRLLDA
QLATGGIVDPRLGFHLPLEVAYQRGYLNKDTHDQLSEPSEVRSYVDPSTDERLSYTQLLR
RCRRDDGTGQLLLPLSDARKLTFRGLRKQITMEELVRSQVMDEATALQLREGLTSIEEVT
KNLQKFLEGTSCIAGVFVDATKERLSVYQAMKKGIIRPGTAFELLEAQAATGYVIDPIKG
LKLTVEEAVRMGIVGPEFKDKLLSAERAVTGYKDPYSGKLISLFQAMKKGLILKDHGIRL
LEAQIATGGIIDPEESHRLPVEVAYKRGLFDEEMNEILTDPSDDTKGFFDPNTEENLTYL
QLMERCITDPQTGLCLLPLKEKKRERKTSSKSSVRKRRVVIVDPETGKEMSVYEAYRKGL
IDHQTYLELSEQECEWEEITISSSDGVVKSMIIDRRSGRQYDIDDAIAKNLIDRSALDQY
RAGTLSITEFADMLSGNAGGFRSRSSSVGSSSSYPISPAVSRTQLASWSDPTEETGPVAG
ILDTETLEKVSITEAMHRNLVDNITGQRLLEAQACTGGIIDPSTGERFPVTDAVNKGLVD
KIMVDRINLAQKAFCGFEDPRTKTKMSAAQALKKGWLYYEAGQRFLEVQYLTGGLIEPDT
PGRVPLDEALQRGTVDARTAQKLRDVGAYSKYLTCPKTKLKISYKDALDRSMVEEGTGLR
LLEAAAQSTKGYYSPYSVSGSGSTAGSRTGSRTGSRAGSRRGSFDATGSGFSMTFSSSSY
SSSGYGRRYASGSSASLGGPESAVA*
>ENSG00000145741|ENST00000335895
MKETIMNQEKLAKLQAQVRIGGKGTARRKKKVVHRTATADDKKLQFSLKKLGVNNISGIE
EVNMFTNQGTVIHFNNPKVQASLAANTFTITGHAETKQLTEMLPSILNQLGADSLTSLRR
LAEALPKQSVDGKAPLATGEDDDDEVPDLVENFDEASKNEAN*
>ENSG00000189067|ENST00000339430
MSVPGPYQAATGPSSAPSAPPSYEETVAVNSYYPTPPAPMPGPTTGLVTGPDGKGMNPPS
YYTQPAPIPNNNPITVQTVYVQHPITFLDRPIQMCCPSCNKMIVSQLSYNAGALTWLSCG
SLCLLGCIAGCCFIPFCVDALQDVDHYCPNCRALLGTYKRL*
>ENSG00000002016|ENST00000358495
MSGTEEAILGGRDSHPAAGGGSVLCFGQCQYTAEEYQAIQKALRQRLGPEYISSRMAGGG
QKVCYIEGHRVINLANEMFGYNGWAHSITQQNVDFVDLNNGKFYVGVCAFVRVQLKDGSY
HEDVGYGVSEGLKSKALSLEKARKEAVTDGLKRALRSFGNALGNCILDKDYLRSLNKLPR
QLPLEVDLTKAKRQDLEPSVEEARYNSCRPNMALGHPQLQQVTSPSRPSHAVIPADQDCS
SRSLSSSAVESEATHQRKLRQKQLQQQFRERMEKQQVRVSTPSAEKSEAAPPAPPVTHST
PVTVSEPLLEKDFLAGVTQELIKTLEDNSEKWAVTPDAGDGVVKPSSRADPAQTSDTLAL
NNQMVTQNRTPHSVCHQKPQAKSGSWDLQTYSADQRTTGNWESHRKSQDMKKRKYDPS*
>ENSG00000073146|ENST00000354853
MSDISGQVTKLVKNYRSHEALLMLPSRLFYHRELEVCADPTVVTSLLGWEKLPKKGFPLI
FHGVRGSEAREGKSPSWFNPAEAVQVLRYCCLLAHSISSQVSASDIGVITPYRKQVRPAQ
ARLVL*

>ENSG00000168214|ENST00000345843
MGGCRKFGERPPPKRLTREAMRNYLKERGDQTVLILHAKVAQKSYGNEKRFFCPPPCVYL
MGSGWKKKKEQMERDGCSEQESQPCAFIGIGNSDQEMQQLNLEGKNYCTAKTLYISDSDK
RKHFMLSVKMFYGNSDDIGVFLSKRIKVISKPSKKKQSLKNADLCIASGTKVALFNRLRS
QTVSTRYLHVEGGNFHASSQQWGAFFIHLLDDDESEGEEFTVRDGYIHYGQTVKLVCSVT
GMALPRLIIRKVDKQTALLDADDPVSQLHKCAFYLKDTERMYLCLSQERIIQFQATPCPK
EPNKEMINDGASWTIISTDKAEYTFYEGMGPVLAPVTPVPVVESLQLNGGGDVAMLELTG
QNFTPNLRVWFGDVEAETMYRCGESMLCVVPDISAFREGWRWVRQPVQVPVTLVRNDGII
YSTSLTFTYTPEPGPRPHCSAAGAILRANSSQVPPNESNTNSEGSYTNASTNSTSVTSST
ATVVS*
>ENSG00000167554|ENST00000321287
MLCDEEAQKRKAKESGMALPQGRLTFMDVAIEFSQEEWKSLDPGQRALYRDVMLENYRNL
VFLGICLPDLSIISMLKQRREPLILQSQVKIVKNTDGRECVRSVNTGRSCVLGSNAENKP
IKNQLGLTLEAHLSELQLFQAGRKIYRSNQVEKFTNHRSSVSPLQKISSSFTTHIFNKYR
NDLIDFPLLPQEEKAYIRGKSYEYECSEDGEVFRVRASLTNHQVIHTAEKPYKCTECGKV
FSRNSHLVEHWRIHTGQKPYKCSECDKVFNRNSNLARHQRIHTGEKPHKCNECGKAFREC
SGLTTHLVIHTGEKPYKCNECGKNFRHKFSLTNHQRSHTAEKPYKCNECGKVFSLLSYLA
RHQIIHSTEKPYKCNECGRAFHKRPGLMAHLLIHTGEKPYKCNECDKVFGRKLYLTNHQR
IHTGERPYKCNACGKVFNQNPHLSRHRKIHAGENSLRTLQME*
>ENSG00000101361|ENST00000329276
MVLLHVLFEHAVGYALLALKEVEEISLLQPQVEESVLNLGKFHSIVRLVAFCPFASSQVA
LENANAVSEGVVHEDLRLLLETHLPSKKKKVLLGVGDPKIGAAIQEELGYNCQTGGVIAE
ILRGVRLHFHNLVKGLTDLSACKAQLGLGHSYSRAKVKFNVNRVDNMIIQSISLLDQLDK
DINTFSMRVREWYGYHFPELVKIINDNATYCRLAQFIGNRRELNEDKLEKLEELTMDGAK
AKAILDASRSSMGMDISAIDLINIESFSSRVVSLSEYRQSLHTYLRSKMSQVAPSLSALI
GEAVGARLIAHAGSLTNLAKYPASTVQILGAEKALFRALKTRGNTPKYGLIFHSTFIGRA
AAKNKGRISRYLANKCSIASRIDCFSEVPTSVFGEKLREQVEERLSFYETGEIPRKNLDV
MKEAMVQAEEAAAEITRKLEKQEKKRLKKEKKRLAALALASSENSSSTPEECEEMSEKPK
KKKKQKPQEVPQENGMEDPSISFSKPKKKKSFSKEELMSSDLEETAGSTSIPKRKKSTPK
EETVNDPEEAGHRSGSKKKRKFSKEEPVSSGPEEAVGKSSSKKKKKFHKASQED*
>ENSG00000124232|ENST00000343694
MDPAGAADPSVPPNPLTHLSLQDRSEMQLQSEADRRSLPGTWTRSSPEHTTILRGGVRRC
LQQQCEQTVRILHAKVAQKSYGNEKRFFCPPPCVYLSGPGWRVKPGQDQAHQAGETGPTV
CGYMGLDSASGSATETQKLNFEQQPDSREFGCAKTLYISDADKRKHFRLVLRLVLRGGRE
LGTFHSRLIKVISKPSQKKQSLKNTDLCISSGSKVSLFNRLRSQTVSTRYLSVEDGAFVA
SARQWAAFTLHLADGHSAQGDFPPREGYVRYGSLVQLVCTVTGITLPPMIIRKVAKQCAL
LDVDEPISQLHKCAFQFPGSPPGGGGTYLCLATEKVVQFQASPCPKEANRALLNDSSCWT
IIGTESVEFSFSTSLACTLEPVTPVPLISTLELSGGGDVATLELHGENFHAGLKVWFGDV
EAETMYRSPRSLVCVVPDVAAFCSDWRWLRAPITIPMSLVRADGLFYPSAFSFTYTPEYS
VRPGHPGVPEPATDADALLESIHQEFTRTNFHLFIQT*
>ENSG00000184260|ENST00000331380
MSGRGKQGGKARAKAKSRSSRAGLQFPVGRVHRLLRKGNYAERVGAGAPVYMAAVLEYLT
AEILELAGNAARDNKKTRIIPRHLQLAIRNDEELNKLLGKVTIAQGGVLPNIQAVLLPKK
TESHKAKSK*

>ENSG00000149187|ENST00000361904
MNGTLDHPDQPDLDAIKMFVGQVPRTWSEKDLRELFEQYGAVYEINVLRDRSQNPPQSKG
CCFVTFYTRKAALEAQNALHNMKVLPGMHHPIQMKPADSEKNNAVEDRKLFIGMISKKCT
ENDIRVMFSSFGQIEECRILRGPDGLSRGCAFVTFTTRAMAQTAIKAMHQAQTMEGCSSP
MVVKFADTQKDKEQKRMAQQLQQQMQQISAASVWGNLAGLNTLGPQYLALLQQTASSGNL
NTLSSLHPMGGLNAMQLQNLAALAAAASAAQNTPSGTNALTTSSSPLSVLTSSAGSSPSS
SSSNSVNPIASLGALQTLAGATAGLNVGSLAGMAALNGGLGSSGLSNGTGSTMEALTQAY
SGIQQYAAAALPTLYNQNLLTQQSIGAAGSQKEGPEGANLFIYHLPQEFGDQDLLQMFMP
FGNVVSAKVFIDKQTNLSKCFGFVSYDNPVSAQAAIQSMNGFQIGMKRLKVQLKRSKNDS
KPY*
>ENSG00000123358|ENST00000360284
MWLAKACWSIQSEMPCIQAQYGTPAPSPGPRDHLASDPLTPEFIKPTMDLASPEAAPAAP
TALPSFSTFMDGYTGEFDTFLYQLPGTVQPCSSASSSASSTSSSSATSPASASFKFEDFQ
VYGCYPGPLSGPVDEALSSSGSDYYGSPCSAPSPSTPSFQPPQLSPWDGSFGHFSPSQTY
EGLRAWTEQLPKASGPPQPPAFFSFSPPTGPSPSLAQSPLKLFPSQATHQLGEGESYSMP
TAFPGLAPTSPHLEGSGILDTPVTSTKARSGAPGGSEGRCAVCGDNASCQHYGVRTCEGC
KGFFKRTVQKNAKYICLANKDCPVDKRRRNRCQFCRFQKCLAVGMVKEVVRTDSLKGRRG
RLPSKPKQPPDASPANLLTSLVRAHLDSGPSTAKLDYSKFQELVLPHFGKEDAGDVQQFY
DLLSGSLEVIRKWAEKIPGFAELSPADQDLLLESAFLELFILRLAYRSKPGEGKLIFCSG
LVLHRLQCARGFGDWIDSILAFSRSLHSLLVDVPAFACLSALVLITDRHGLQEPRRVEEL
QNRIASCLKEHVAAVAGEPQPASCLSRLLGKLPELRTLCTQGLQRIFYLKLEDLVPPPPI
IDKIFMDTLPF*
>ENSG00000134317|ENST00000324907
MTQEYDNKRPVLVLQNEALYPQRRSYTSEDEAWKSFLENPLTAATKAMMSINGDEDSAAA
LGLLYDYYKVPRERRSSTAKPEVEHPEPDHSKRNSIPIVTEQPLISAGENRVQVLKNVPF
NIVLPHGNQLGIDKRGHLTAPDTTVTVSIATMPTHSIKTETQPHGFAVGIPPAVYHPEPT
ERVVVFDRNLNTDQFSSGAQAPNAQRRTPDSTFSETFKEGVQEVFFPSDLSLRMPGMNSE
DYVFDSVSGNNFEYTLEASKSLRQKPGDSTMTYLNKGQFYPITLKEVSSSEGIHHPISKV
RSVIMVVFAEDKSREDQLRHWKYWHSRQHTAKQRCIDIADYKESFNTISNIEEIAYNAIS
FTWDINDEAKVFISVNCLSTDFSSQKGVKGLPLNIQVDTYSYNNRSNKPVHRAYCQIKVF
CDKGAERKIRDEERKQSKRKVSDVKVPLLPSHKRMDITVFKPFIDLDTQPVLFIPDVHFA
NLQRGTHVLPIASEELEGEGSVLKRGPYGTEDDFAVPPSTKLARIEEPKRVLLYVRKESE
EVFDALMLKTPSLKGLMEAISDKYDVPHDKIGKIFKKCKKGILVNMDDNIVKHYSNEDTF
QLQIEEAGGSYKLTLTEI*
>ENSG00000110713|ENST00000355260
MFNKSFGTPFGGGTGGFGTTSTFGQNTGFGTTSGGAFGTSAFGSSNNTGGLFGNSQTKPG
GLFGTSSFSQPATSTSTGFGFGTSTGTANTLFGTASTGTSLFSSQNNAFAQNKPTGFGNF
GTSTSSGGLFGTTNTTSNPFGSTSGSLFGPSSFTAAPTGTTIKFNPPTGTDTMVKAGVST
NISTKHQCITAMKEYESKSLEELRLEDYQANRKGPQNQVGAGTTTGLFGSSPATSSATGL
FSSSTTNSGFAYGQNKTAFGTSTTGFGTNPGGLFGQQNQQTTSLFSKPFGQATTTQNTGF
SFGNTSTIGQPSTNTMGLFGVTQASQPGGLFGTATNTSTGTAFGTGTGLFGQTNTGFGAV
GSTLFGNNKLTTFGSSTTSAPSFGTTSGGLFGFGTNTSGNSIFGSKPAPGTLGTGLGAGF
GTALGAGQASLFGNNQPKIGGPLGTGAFGAPGFNTTTATLGFGAPQAPVALTDPNASAAQ
QAVLQQHINSLTYSPFGDSPLFRNPMSDPKKKEERLKPTNPAAQKALTTPTHYKLTPRPA

TRVRPKALQTTGTAKSHLFDGLDDDEPSLANGAFMPKKSIKKLVLKNLNNSNLFSPVNRD
SENLASPSEYPENGERFSFLSKPVDENHQQDGDEDSLVSHFYTNPIAKPIPQTPESAGNK
HSNSNSVDDTIVALNMRAALRNGLEGSSEETSFHDESLQDDREEIENNSYHMHPAGIILT
KVGYYTIPSMDDLAKITNEKGECIVSDFTIGRKGYGSIYFEGDVNLTNLNLDDIVHIRRK
EVVVYLDDNQKPPVGEGLNRKAEVTLDGVWPTDKTSRCLIKSPDRLADINYEGRLEAVSR
KQGAQFKEYRPETGSWVFKVSHFSKYGLQDSDEEEEEHPSKTSTKKLKTAPLPPASQTTP
LQMALNGKPAPPPQSQSPEVEQLGRVVELDSDMVDITQEPVLDTMLEESMPEDQEPVSAS
THIASSLGINPHVLQIMKASLLTDEEDVDMALDQRFSRLPSKADTSQEICSPRLPISASH
SSKTRSLVGGLLQSKFTSGAFLSPSVSVQECRTPRAASLMNIPSTSSWSVPPPLTSVFTM
PSPAPEVPLKTVGTRRQLGLVPREKSVTYGKGKLLMDMALFMGRSFRVGWGPNWTLANSG
EQLNGSHELENHQIADSMEFGFLPNPVAVKPLTESPFKVHLEKLSLRQRKPDEDMKLYQT
PLELKLKHSTVHVDELCPLIVPNLGVAVIHDYADWVKEASGDLPEAQIVKHWSLTWTLCE
ALWGHLKELDSQLNEPREYIQILERRRAFSRWLSCTATPQIEEEVSLTQKNSPVEAVFSY
LTGKRISEACSLAQQSGDHRLALLLSQFVGSQSVRELLTMQLVDWHQLQADSFIQDERLR
IFALLAGKPVWQLSEKKQINVCSQLDWKRSLAIHLWYLLPPTASISRALSMYEEAFQNTS
DSDRYACSPLPSYLEGSGCVIAEEQNSQTPLRDVCFHLLKLYSDSIREKAVRELLTRHCQ
LLETPESWAKETFLTQKLRVPAKWIHEAKAVRAHMESDKHLEALCLFKAEHWNRCHKLII
RHLASDAIINENYDYLKGFLEDLAPPERSSLIQDWETSGLVYLDYIRVIEMLRHIQQVDC
SGNDLEQLHIKVTSLCSRIEQIQCYSAKDRLAQSDMAKRVANLLRVVLSLHHPPDRTSDS
TPDPQRVPLRLLAPHIGRLPMPEDYAMDELRSLTQSYLRELAVGSL*
>ENSG00000065978|ENST00000321358
MSSEAETQQPPAAPPAAPALSAADTKPGTTGSGAGSGGPGGLTSAAPAGGDKKVIATKVL
GTVKWFNVRNGYGFINRNDTKEDVFVHQTAIKKNNPRKYLRSVGDGETVEFDVVEGEKGA
EAANVTGPGGVPVQGSKYAADRNHYRRYPRRRGPPRNYQQNYQNSESGEKNEGSESAPEG
QAQQRRPYRRRRFPPYYMRRPYGRRPQYSNPPVQGEVMEGADNQGAGEQGRPVRQNMYRG
YRPRFRRGPPRQRQPREDGNEEDKENQGDETQGQQPPQRRYRRNFNYRRRRPENPKPQDG
KETKAADPPAENSSAPEAEQGGAE*
>ENSG00000197343|ENST00000357864
MEEIPAQEAAGSPRVQFQSLETQSECLSPEPQFVQDTDMEQGLTGGILLRLPTTRIHSVN
SCPALSHTQASAFSGETLAVLTAGISKRWPKYRLPIDIARPCSETPFPRL*
>ENSG00000096384|ENST00000353801
MPEEVHHGEEEVETFAFQAEIAQLMSLIINTFYSNKEIFLRELISNASDALDKIRYESLT
DPSKLDSGKELKIDIIPNPQERTLTLVDTGIGMTKADLINNLGTIAKSGTKAFMEALQAG
ADISMIGQFGVGFYSAYLVAEKVVVITKHNDDEQYAWESSAGGSFTVRADHGEPIGRGTK
VILHLKEDQTEYLEERRVKEVVKKHSQFIGYPITLYLEKEREKEISDDEAEEEKGEKEEE
DKDDEEKPKIEDVGSDEEDDSGKDKKKKTKKIKEKYIDQEELNKTKPIWTRNPDDITQEE
YGEFYKSLTNDWEDHLAVKHFSVEGQLEFRALLFIPRRAPFDLFENKKKKNNIKLYVRRV
FIMDSCDELIPEYLNFIRGVVDSEDLPLNISREMLQQSKILKVIRKNIVKKCLELFSELA
EDKENYKKFYEAFSKNLKLGIHEDSTNRRRLSELLRYHTSQSGDEMTSLSEYVSRMKETQ
KSIYYITGESKEQVANSAFVERVRKRGFEVVYMTEPIDEYCVQQLKEFDGKSLVSVTKEG
LELPEDEEEKKKMEESKAKFENLCKLMKEILDKKVEKVTISNRLVSSPCCIVTSTYGWTA
NMERIMKAQALRDNSTMGYMMAKKHLEINPDHPIVETLRQKAEADKNDKAVKDLVVLLFE
TALLSSGFSLEDPQTHSNRIYRMIKLGLGIDEDEVAAEEPNAAVPDEIPPLEGDEDASRM
EEVD*

>ENSG00000249471|ENST00000336614
MTFEDVAVYFSQEEWGLLDTAQRALYRHVMLENFTLVTSLGLSTSRPRVVIQLERGEEPW
VPSGKDMTLARNTYGRLNSGSWSLTEDRDVSGEWPRAFPDTPPGMTTSVFPVADACHSVK
SLQRQPGASPSQERKPTGVSVIYWERLLLGSRSDQASISLRLTSPLRPPKSSRPREKTFT
EYRVPGRQPRTPERQKPCAQEVPGRAFGNASDLKAASGGRDRRMGAAWQEPHRLLGGQEP
STWDELGEALHAGEKSFECRACSKVFVKSSDLLKHLRTHTGERPYECTQCGKAFSQTSHL
TQHQRIHSGETPYACPVCGKAFRHSSSLVRHQRIHTAEKSFRCSECGKAFSHGSNLSQHR
KIHAGGRPYACAQCGRRFCRNSHLIQHERTHTGEKPFVCALCGAAFSQGSSLFLHQRVHT
GEKPFACAQCGRSFSRSSNLTQHQLLHTGERPFRCVDCGKGFAKGAVLLSHRRIHTGEKP
FVCTQCGRAFRERPALLHHQRIHTTEKTNAAAPDCTPGPGFLQGHHRKVRRGGKPSPVLK
PAKV*
>ENSG00000171703|ENST00000361317
MDLLRELKAMPITLHLLQSTRVGMSVNALRKQSSDEEVIALAKSLIKSWKKLLDASDAKA
RERGRGMPLPTSSRDASEAPDPSRKRPELPRAPSTPRITTFPPVPVTCDAVRNKCREMLT
AALQTDHDHVAIGADCERLSAQIEECIFRDVGNTDMKYKNRVRSRISNLKDAKNPDLRRN
VLCGAITPQQIAVMTSEEMASDELKEIRKAMTKEAIREHQMARTGGTQTDLFTCGKCRKK
NCTYTQVQTRSSDEPMTTFVVCNECGNRWKFC*
>ENSG00000155363|ENST00000357443
MPSKFSCRQLREAGQCFESFLVVRGLDMETDRERLRTIYNRDFKISFGTPAPGFSSMLYG
MKIANLAYVTKTRVRFFRLDRWADVRFPEKRRMKLGSDISKHHKSLLAKIFYDRAEYLHG
KHGVDVEVQGPHEARDGQLLIRLDLNRKEVLTLRLRNGGTQSVTLTHLFPLCRTPQFAFY
NEDQELPCPLGPGECYELHVHCKTSFVGYFPATVLWELLGPGESGSEGAGTFYIARFLAA
VAHSPLAAQLKPMTPFKRTRITGNPVVTNRIEEGERPDRAKGYDLELSMALGTYYPPPRL
RQLLPMLLQGTSIFTAPKEIAEIKAQLETALKWRNYEVKLRLLLHLEELQMEHDIRHYDL
ESVPMTWDPVDQNPRLLTLEVPGVTESRPSVLRGDHLFALLSSETHQEDPITYKGFVHKV
ELDRVKLSFSMSLLSRFVDGLTFKVNFTFNRQPLRVQHRALELTGRWLLWPMLFPVAPRD
VPLLPSDVKLKLYDRSLESNPEQLQAMRHIVTGTTRPAPYIIFGPPGTGKTVTLVEAIKQ
VVKHLPKAHILACAPSNSGADLLCQRLRVHLPSSIYRLLAPSRDIRMVPEDIKPCCNWDA
KKGEYVFPAKKKLQEYRVLITTLITAGRLVSAQFPIDHFTHIFIDEAGHCMEPESLVAIA
GLMEVKETGDPGGQLVLAGDPRQLGPVLRSPLTQKHGLGYSLLERLLTYNSLYKKGPDGY
DPQFITKLLRNYRSHPTILDIPNQLYYEGELQACADVVDRERFCRWAGLPRQGFPIIFHG
VMGKDEREGNSPSFFNPEEAATVTSYLKLLLAPSSKKGKARLSPRSVGVISPYRKQVEKI
RYCITKLDRELRGLDDIKDLKVGSVEEFQGQERSVILISTVRSSQSFVQLDLDFNLGFLK
NPKRFNVAVTRAKALLIIVGNPLLLGHDPDWKVFLEFCKENGGYTGCPFPAKLDLQQGQN
LLQGLSKLSPSTSGPHSHDYLPQEREGEGGLSLQVEPEWRNEL*
>ENSG00000197213|ENST00000358992
MAANWTLSWGQGGPCNSPGSDTPRSVASPETQLGNHDRNPETWHMNFRMFSCPEESDPIQ
ALRKLTELCHLWLRPDLHTKEQILDMLVMEQFMISMPQELQVLVKVNGVQSCKDLEDLLR
NNRRPKKWSIVNLLGKEYLMLNSDVEMAEAPASVRDDPRDVSSQWASSVNQMHPGTGQAR
REQQILPRVAALSRRQGEDFLLHKSIDVTGDPNSPRPKQTLEKDLKENREENPGLSSPEP
QLPKSPNLVRAKEGKEPQKRASVENVDADTPSACVVEREALTHSGNRGDALNLSSPKRSK
PDASSISQEEPQGEATPVGNRESPGQAEINPVHSPGPAGPVSHPDGQEAKALPPFACDVC
NKSFKYFSQLSIHRRSHTGDRPFQCDLCRKRFLQPSDLRVHQRVHTGERPYMCDVCQKRF
AHESTLQGHKRIHTGERPFKCKYCSKVFSHKGNLNVHQRTHSGEKPYKCPTCQKAFRQLG

TFKRHLKTHRETTSQ*
>ENSG00000169813|ENST00000356053
MMLGPEGGEGFVVKLRGLPWSCSVEDVQNFLSDCTIHDGAAGVHFIYTREGRQSGEAFVE
LGSEDDVKMALKKDRESMGHRYIEVFKSHRTEMDWVLKHSGPNSADSANDGFVRLRGLPF
GCTKEEIVQFFSGLEIVPNGITLPVDPEGKITGEAFVQFASQELAEKALGKHKERIGHRY
IEVFKSSQEEVRSYSDPPLKFMSVQRPGPYDRPGTARRYIGIVKQAGLERMRPGAYSTGY
GGYEEYSGLSDGYGFTTDLFGRDLSYCLSGMYDHRYGDSEFTVQSTTGHCVHMRGLPYKA
TENDIYNFFSPLNPVRVHIEIGPDGRVTGEADVEFATHEEAVAAMSKDRANMQHRYIELF
LNSTTGASNGAYSSQVMQGMGVSAAQATYSGLESQSVSGCYGAGYSGQNSMGGYD*
>ENSG00000116918|ENST00000366639
MSNKEGSGGFRKRKHDNFPHNQRREGKDVNSSSPVMLAFKSFQQELDARHDKYERLVKLS
RDITVESKRTIFLLHRITSAPDMEDILTESEIKLDGVRQKIFQVAQELSGEDMHQFHRAI
TTGLQEYVEAVSFQHFIKTRSLISMDEINKQLIFTTEDNGKENKTPSSDAQDKQFGTWRL
RVTPVDYLLGVADLTGELMRMCINSVGNGDIDTPFEVSQFLRQVYDGFSFIGNTGPYEVS
KKLYTLKQSLAKVENACYALKVRGSEIPKHMLADVFSVKTEMIDQEEGIS*
>ENSG00000113456|ENST00000325577
MPLLTQQIQDEDDQYSLVASLDNVRNLSTILKAIHFREHATCFATKNGIKVTVENAKCVQ
ANAFIQGL*
>ENSG00000166173|ENST00000344870
MAQSGGEARPGPKTAVQIRVAIQEAEDVDELEDEEEGAETRGAGDPARYLSPGWGSASEE
EPSRGHRNRSSVNSRTMLASFIVSSAPSTAPST*
>ENSG00000198466|ENST00000339656
MAAAVPRRPTQQGTVTFEDVAVNFSQEEWCLLSEAQRCLYRDVMLENLALISSLGCWCGS
KDEEAPCKQRISVQRESQSRTPRAGVSPKKAHPCEMCGLILEDVFHFADHQETHHKQKLN
RSGACGKNLDDTAYLHQHQKQHIGEKFYRKSVREASFVKKRKLRVSQEPFVFREFGKDVL
PSSGLCQEEAAVEKTDSETMHGPPFQEGKTNYSCGKRTKAFSTKHSVIPHQKLFTRDGCY
VCSDCGKSFSRYVSFSNHQRDHTAKGPYDCGECGKSYSRKSSLIQHQRVHTGQTAYPCEE
CGKSFSQKGSLISHQLVHTGEGPYECRECGKSFGQKGNLIQHQQGHTGERAYHCGECGKS
FRQKFCFINHQRVHTGERPYKCGECGKSFGQKGNLVHHQRGHTGERPYECKECGKSFRYR
SHLTEHQRLHTGERPYNCRECGKLFNRKYHLLVHERVHTGERPYACEVCGKLFGNKHSVT
IHQRIHTGERPYECSECGKSFLSSSALHVHKRVHSGQKPYKCSECGKSFSECSSLIKHRR
IHTGERPYECTKCGKTFQRSSTLLHHQSSHRRKAL*
>ENSG00000136628|ENST00000366923
MATLSLTVNSGDPPLGALLAVEHVKDDVSISVEEGKENILHVSENVIFTDVNSILRYLAR
VATTAGLYGSNLMEHTEIDHWLEFSATKLSSCDSFTSTINELNHCLSLRTYLVGNSLSLA
DLCVWATLKGNAAWQEQLKQKKAPVHVKRWFGFLEAQQAFQSVGTKWDVSTTKARVAPEK
KQDVGKFVELPGAEMGKVTVRFPPEASGYLHIGHAKAALLNQHYQVNFKGKLIMRFDDTN
PEKEKEDFEKVILEDVAMLHIKPDQFTYTSDHFETIMKYAEKLIQEGKAYVDDTPAEQMK
AEREQRIDSKHRKNPIEKNLQMWEEMKKGSQFGQSCCLRAKIDMSSNNGCMRDPTLYRCK
IQPHPRTGNKYNVYPTYDFACPIVDSIEGVTHALRTTEYHDRDEQFYWIIEALGIRKPYI
WEYSRLNLNNTVLSKRKLTWFVNEGLVDGWDDPRFPTVRGVLRRGMTVEGLKQFIAAQGS
SRSVVNMEWDKIWAFNKKVIDPVAPRYVALLKKEVIPVNVPEAQEEMKEVAKHPKNPEVG
LKPVWYSPKVFIEGADAETFSEGEMVTFINWGNLNITKIHKNADGKIISLDAKLNLENKD
YKKTTKVTWLAETTHALPIPVICVTYEHLITKPVLGKDEDFKQYVNKNSKHEELMLGDPC

LKDLKKGDIIQLQRRGFFICDQPYEPVSPYSCKEAPCVLIYIPDGHTKEMPTSGSKEKTK
VEATKNETSAPFKERPTPSLNNNCTTSEDSLVLYNRVAVQGDVVRELKAKKAPKEDVDAA
VKQLLSLKAEYKEKTGQEYKPGNPPAEIGQNISSNSSASILESKSLYDEVAAQGEVVRKL
KAEKSPKAKINEAVECLLSLKAQYKEKTGKEYIPGQPPLSQSSDSSPTRNSEPAGLETPE
AKVLFDKVASQGEVVRKLKTEKAPKDQVDIAVQELLQLKAQYKSLIGVEYKPVSATGAED
KDKKKKEKENKSEKQNKPQKQNDGQRKDPSKNQGGGLSSSGAGEGQGPKKQTRLGLEAKK
EENLADWYSQVITKSEMIEYHDISGCYILRPWAYAIWEAIKDFFDAEIKKLGVENCYFPM
FVSQSALEKEKTHVADFAPEVAWVTRSGKTELAEPIAIRPTSETVMYPAYAKWVQSHRDL
PIKLNQWCNVVRWEFKHPQPFLRTREFLWQEGHSAFATMEEAAEEVLQILDLYAQVYEEL
LAIPVVKGRKTEKEKFAGGDYTTTIEAFISASGRAIQGGTSHHLGQNFSKMFEIVFEDPK
IPGEKQFAYQNSWGLTTRTIGVMTMVHGDNMGLVLPPRVACVQVVIIPCGITNALSEEDK
EALIAKCNDYRRRLLSVNIRVRADLRDNYSPGWKFNHWELKGVPIRLEVGPRDMKSCQFV
AVRRDTGEKLTVAENEAETKLQAILEDIQVTLFTRASEDLKTHMVVANTMEDFQKILDSG
KIVQIPFCGEIDCEDWIKKTTARDQDLEPGAPSMGAKSLCIPFKPLCELQPGAKCVCGKN
PAKYYTLFGRSY*
>ENSG00000091831|ENST00000338799
MTMTLHTKASGMALLHQIQGNELEPLNRPQLKIPLERPLGEVYLDSSKPAVYNYPEGAAY
EFNAAAAANAQVYGQTGLPYGPGSEAAAFGSNGLGGFPPLNSVSPSPLMLLHPPPQLSPF
LQPHGQQVPYYLENEPSGYTVREAGPPAFYRPNSDNRRQGGRERLASTNDKGSMAMESAK
ETRYCAVCNDYASGYHYGVWSCEGCKAFFKRSIQGHNDYMCPATNQCTIDKNRRKSCQAC
RLRKCYEVGMMKGGIRKDRRGGRMLKHKRQRDDGEGRGEVGSAGDMRAANLWPSPLMIKR
SKKNSLALSLTADQMVSALLDAEPPILYSEYDPTRPFSEASMMGLLTNLADRELVHMINW
AKRVPGFVDLTLHDQVHLLECAWLEILMIGLVWRSMEHPGKLLFAPNLLLDRNQGKCVEG
MVEIFDMLLATSSRFRMMNLQGEEFVCLKSIILLNSGVYTFLSSTLKSLEEKDHIHRVLD
KITDTLIHLMAKAGLTLQQQHQRLAQLLLILSHIRHMSNKGMEHLYSMKCKNVVPLYDLL
LEMLDAHRLHAPTSRGGASVEETDQSHLATAGSTSSHSLQKYYITGEAEGFPATV*
>ENSG00000189120|ENST00000342234
MLTAVCGSLGSQHTEAPHASPPRLDLQPLQTYQGHTSPEAGDYPSPLQPGELQSLPLGPE
VDFSQGYELPGASSRVTCEDLESDSPLAPGPFSKLLQPDMSHHYESWFRPTHPGAEDGSW
WDLHPGTSWMDLPHTQGALTSPGHPGALQAGLGGYVGDHQLCAPPPHPHAHHLLPAAGGQ
HLLGPPDGAKALEVAAPESQGLDSSLDGAARPKGSRRSVPRSSGQTVCRCPNCLEAERLG
APCGPDGGKKKHLHNCHIPGCGKAYAKTSHLKAHLRWHSGDRPFVCNWLFCGKRFTRSDE
LQRHLQTHTGTKKFPCAVCSRVFMRSDHLAKHMKTHEGAKEEAAGAASGEGKAGGAVEPP
GGKGKREAEGSVAPSN*
>ENSG00000179172|ENST00000317869
MASNVTNKMDPHSMNSRVFIGNLNTLVVKKSDVEAIFSKYGKIAGCSVHKGFAFVQYDKE
KNARAAVAGEDGRMIASQVVDINLAAEPKVNRGNAGVKRSAAEMYGSSFDLDYGFQRDYY
DGMYSFPARVPPPPPIALAVVPSKRQRLSGNTSRRGKSGFNSKSGKRGSSKSGKLKGDDL
QAIKQELTQIKQKVDSLLENLEKIEKEQSKQEVEVKNAKSEEEQSSSSMKKDETHVKMES
EGGAEDSAEEGDPLDDDVNEDQGDDQLELIKDDEKEAEEGEDDRDSTNGQDDS*
>ENSG00000119950|ENST00000361248
MPSPRLQHSKPPRRLSRAQKHSSGSSNTSTANRRAHLRLCLERLKVLIPLGPDCTRHTTL
GLLNKAKAHIKKLEEAERKSQHQLENLEREQRFLKWRLEQLQGPQEMERIRMDSIGSTIS
SDRSDSEREEIEVDVESTEFSHGEVDNISTTSISDIDDHSSLPSIGSDEGYSSASVKLSF

TS*
>ENSG00000099783|ENST00000325495
MAAGVEAAAEVAATEIKMEEESGAPGVPSGNGAPGPKGEGERPAQNEKRKEKNIKRGGNR
FEPYANPTKRYRAFITNIPFDVKWQSLKDLVKEKVGEVTYVELLMDAEGKSRGCAVVEFK
MEESMKKAAEVLNKHSLSGRPLKVKEDPDGEHARRAMQKVMATTGGMGMGPGGPGMITIP
PSILNNPNIPNEIIHALQAGRLGSTVFVANLDYKVGWKKLKEVFSMAGVVVRADILEDKD
GKSRGIGTVTFEQSIEAVQAISMFNGQLLFDRPMHVKMDERALPKGDFFPPERPQQLPHG
LGGIGMGLGPGGQPIDANHLNKGIGMGNIGPAGMGMEGIGFGINKMGGMEGPFGGGMENM
GRFGSGMNMGRINEILSNALKRGEIIAKQGGGGGGGSVPGIERMGPGIDRLGGAGMERMG
AGLGHGMDRVGSEIERMGLVMDRMGSVERMGSGIERMGPLGLDHMASSIERMGQTMERIG
SGVERMGAGMGFGLERMAAPIDRVGQTIERMGSGVERMGPAIERMGLSMERMVPAGMGAG
LERMGPVMDRMATGLERMGANNLERMGLERMGANSLERMGLERMGANSLERMGPAMGPAL
GAGIERMGLAMGGGGGASFDRAIEMERGNFGGSFAGSFGGAGGHAPGVARKACQIFVRNL
PFDFTWKMLKDKFNECGHVLYADIKMENGKSKGCGVVKFESPEVAERACRMMNGMKLSGR
EIDVRIDRNA*
>ENSG00000196419|ENST00000359308
MSGWESYYKTEGDEEAEEEQEENLEASGDYKYSGRDSLIFLVDASKAMFESQSEDELTPF
DMSIQCIQSVYISKIISSDRDLLAVVFYGTEKDKNSVNFKNIYVLQELDNPGAKRILELD
QFKGQQGQKRFQDMMGHGSDYSLSEVLWVCANLFSDVQFKMSHKRIMLFTNEDNPHGNDS
AKASRARTKAGDLRDTGIFLDLMHLKKPGGFDISLFYRDIISIAEDEDLRVHFEESSKLE
DLLRKVRAKETRKRALSRLKLKLNKDIVISVGIYNLVQKALKPPPIKLYRETNEPVKTKT
RTFNTSTGGLLLPSDTKRSQIYGSRQIILEKEETEELKRFDDPGLMLMGFKPLVLLKKHH
YLRPSLFVYPEESLVIGSSTLFSALLIKCLEKEVAALCRYTPRRNIPPYFVALVPQEEEL
DDQKIQVTPPGFQLVFLPFADDKRKMPFTEKIMATPEQVGKMKAIVEKLRFTYRSDSFEN
PVLQQHFRNLEALALDLMEPEQAVDLTLPKVEAMNKRLGSLVDEFKELVYPPDYNPEGKV
TKRKHDNEGSGSKRPKVEYSEEELKTHISKGTLGKFTVPMLKEACRAYGLKSGLKKQELL
EALTKHFQD*
>ENSG00000165209|ENST00000348403
MRSIRSFANDDRHVMVKHSTIYPSPEELEAVQNMVSTVECALKHVSDWLDETNKGTKTEG
ETEVKKDEAGENYSKDQGGRTLCGVMRIGLVAKGLLIKDDMDLELVLMCKDKPTETLLNT
VKDNLPIQIQKLTEEKYQVEQCVNEASIIIRNTKEPTLTLKVILTSPLIRDELEKKDGEN
VSMKDPPDLLDRQKCLNALASLRHAKWFQARANGLKSCVIVLRILRDLCNRVPTWAPLKG
WPLELICEKSIGTCNRPLGAGEALRRVMECLASGILLPGGPGLHDPCERDPTDALSYMTI
QQKEDITHSAQHALRLSAFGQIYKVLEMDPLPSSKPFQKYSWSVTDKEGAGSSALKRPFE
DGLGDDKDPNKKMKRNLRKILDSKAIDLMNALMRLNQIRPGLQYKLLSQSGPVHAPVFTM
SVDVDGTTYEASGPSKKTAKLHVAVKVLQAMGYPTGFDADIECMSSDEKSDNESKNETVS
SNSSNNTGNSTTETSSTLEVRTQGPILTASGKNPVMELNEKRRGLKYELISETGGSHDKR
FVMEVEVDGQKFRGAGPNKKVAKASAALAALEKLFSGPNAANNKKKKIIPQAKGVVNTAV
SAAVQAVRGRGRGTLTRGAFVGATAAPGYIAPGYGTPYGYSTAAPAYGLPKRMVLLPVMK
FPTYPVPHYSFF*
>ENSG00000145216|ENST00000337488
MSAGEVERLVSELSGGTGGDEEEEWLYGGPWDVHVHSDLAKDLDENEVERPEEENASANP
PSGIEDETAENGVPKPKVTETEDDSDSDSDDDEDDVHVTIGDIKTGAPQYGSYGTAPVNL
NIKTGGRVYGTTGTKVKGVDLDAPGSINGVPLLEVDLDSFEDKPWRKPGADLSDYFNYGF

NEDTWKAYCEKQKRIRMGLEVIPVTSTTNKITAEDCTMEVTPGAEIQDGRFNLFKVQQGR
TGNSEKETALPSTKAEFTSPPSLFKTGLPPSRNSTSSQSQTSTASRKANSSVGKWQDRYG
RAESPDLRRLPGAIDVIGQTITISRVEGRRRANENSNIQVLSERSATEVDNNFSKPPPFF
PPGAPPTHLPPPPFLPPPPTVSTAPPLIPPPGFPPPPGAPPPSLIPTIESGHSSGYDSRS
ARAFPYGNVAFPHLPGSAPSWPSLVDTSKQWDYYARREKDRDRERDRDRERDRDRDRERE
RTREREREREDHSPTPSVFNSDEERYRYREYAERGYERHRASREKEERHRERRHREKEETR
HKSSRSNSRRRHESEEGDSHRRHKHKKSKRSKEGKEAGSEPAPEQESTEATPAE*
>ENSG00000132010|ENST00000334213
MMFQDSVAFEDVAVSFTQEEWALLDPSQKNLYRDVMQETFKNLTSVGKTWKVQNIEDEYK
NPRRNLSLMREKLCESKESHHCGESFNQIADDMLNRKTLPGITPCESSVCGEVGTGHSSL
NTHIRADTGHKSSEYQEYGENPYRNKECKKAFSYLDSFQSHDKACTKEKPYDGKECTETF
ISHSCIQRHRVMHSGDGPYKCKFCGKAFYFLNLCLIHERIHTGVKPYKCKQCGKAFTRST
TLPVHERTHTGVNADECKECGNAFSFPSEIRRHKRSHTGEKPYECKQCGKVFISFSSIQY
HKMTHTGEKPYECKQCGKAFRCGSHLQKHGRTHTGEKPYECRQCGKAFRCTSDLQRHEKT
HTEDKPYGCKQCGKGFRCASQLQIHERTHSGEKPHECKECGKVFKYFSSLRIHERTHTGE
KPHECKQCGKAFRYFSSLHIHERTHTGDKPYECKVCGKAFTCSSSIRYHERTHTGEKPYE
CKHCGKAFISNYIRYHERTHTGEKPYQCKQCGKAFIRASSCREHERTHTINR*
>ENSG00000178175|ENST00000318442
MQKEMKMIKDEDVHFDLAVKKTPSFPHCLQPVASRGKAPQRHPFPEALRGPFSQFRYEPP
PGDLDGFPGVFEGAGSRKRKSMPTKMPYNHPAEEVTLALHSEENKNHGLPNLPLLFPQPP
RPKYDSQMIDLCNVGFQFYRSLEHFGGKPVKQEPIKPSAVWPQPTPTPFLPTPYPYYPKV
HPGLMFPFFVPSSSPFPFSRHTFLPKQPPEPLLPRKAEPQESEETKQKVERVDVNVQIDD
SYYVDVGGSQKRWQCPTCEKSYTSKYNLVTHILGHSGIKPHACTHCGKLFKQLSHLHTHM
LTHQGTRPHKCQVCHKAFTQTSHLKRHMMQHSEVKPHNCRVCGRGFAYPSELKAHEAKHA
SGRENICVECGLDFPTLAQLKRHLTTHRGPIQYNCSECDKTFQYPSQLQNHMMKHKDIRP
YICSECGMEFVQPHHLKQHSLTHKGVKEHKCGICGREFTLLANMKRHVLIHTNIRAYQCH
LCYKSFVQKQTLKAHMIVHSDVKPFKCKLCGKEFNRMHNLMGHMHLHSDSKPFKCLYCPS
KFTLKGNLTRHMKVKHGVMERGLHSQGLGRGRIALAQTAGVLRSLEQEEPFDLSQKRRAK
VPVFQSDGESAQGSHCHEEEEEDNCYEVEPYSPGLAPQSQQLCTPEDLSTKSEHAPEVLE
EACKEEKEDASKGEWEKRSKGDLGAEGGQERDCAGRDECLSLRAFQSTRRGPSFSDYLYF
KHRDESLKELLERKMEKQAVLLGI*
>ENSG00000197223|ENST00000355848
MAGEEINEDYPVEIHEYLSAFENSIGAVDEMLKTMMSVSRNELLQKLDPLEQAKVDLVSA
YTLNSMFWVYLATQGVNPKEHPVKQELERIRVYMNRVKEITDKKKAGKLDRGAASRFVKN
ALWEPKSKNASKVANKGKSKS*
>ENSG00000197961|ENST00000320451
MAEIHNGGELCDFMENGEIFSEHSCLNAHMGTENTGDTYDCDEYGENFPMLHNSAPAGET
LSVLNQCRKAFSLPPNVHQRTWIGDKSFEYSDCEEAFVDQSHLQANRITHNGETLYEQKQ
CGRAFTYSTSHAVSVKMHTVEKPYECKECGKFFRYSSYLNSHMRTHTGEKPYECKECGKC
FTVSSHLVEHVRIHTGEKPYQCKECGRAFAGRSGLTKHVRIHTGEKPYECNECGKAYNRF
YLLTEHFKTHTEEKPFECKVCGKSFRSSSCLKNHFRIHTGIKPYKCKECGKAFTVSSSLH
NHVKIHTGEKPYECKDCGKAFATSSQLIEHIRTHTGEKPYICKECGKTFRASSHLQKHVR
IHTGEKPYICNECGKAYNRFYLLTKHLKTH*
>ENSG00000243660|ENST00000315429

XRPRPRTPSRGPSDLRFRGEAGLRRVFLKKAGGSVSFSDVAVGFTQEEWQHLDSAQRTPY
RDMMLENYSLLLSVGYCITKPEVVCKLEHGQVLWILEEESPSQSHLDCCIDDDLMEKRQE
NQDQHLQKVDFVNNKTLTMDRNGVLGKTFSLDTNPILSRKIRGNCDSSGMNLNNISELII
SNRSSFVRNP
>ENSG00000103495|ENST00000322945
MFPVFPCTLLAPPFPVLGLDSRGVGGLMNSFPPPQGHAQNPLQVGAELQSRFFASQGCAQ
SPFQAAPAPPPTPQAPAAEPLQVDLLPVLAAAQESAAAAAAAAAAAAAVAAAPPAPAAAS
TVDTAALKQPPAPPPPPPPVSAPAAEAAPPASAATIAAAAATAVVAPTSTVAVAPVASAL
EKKTKSKGPYICALCAKEFKNGYNLRRHEAIHTGAKAGRVPSGAMKMPTMVPLSLLSVPQ
LSGAGGGGGEAGAGGGAAAVAAGGVVTTTASGKRIRKNHACEMCGKAFRDVYHLNRHKLS
HSDEKPYQCPVCQQRFKRKDRMSYHVRSHDGAVHKPYNCSHCGKSFSRPDHLNSHVRQVH
STERPFKCEKCEAAFATKDRLRAHTVRHEEKVPCHVCGKMLSSAYISDHMKVHSQGPHHV
CELCNKGTGEVCPMAAAAAAAAAAAAAAVAAPPTAVGSLSGAEGVPVSSQPLPSQPW*
>ENSG00000105750|ENST00000345030
MGPLTFRDVAIEFSLKEWQCLDTAQRNLYRNVMLENYRNLVFLVMCSHFAQDLWPEQNIK
DSFQKVTLKRYGKCRHENLPLRKGCESMDECKMHKGGCNGLNQCLTATQSKIFQCDKYVK
VAHKFSNSNRHEIRHTKKKPFKCTKCGKSFGMISCLTEHSRIHTRVNFYKCEECGKAFNW
SSTLTKHKRIHTGEKPYKCEECGKAFNQSSNLIKHKKIHTGEKPYKCEECGKTFNRFSTL
TTHKIIHTGEKPYKCKECGKAFNRSSTLTTHRKIHTGEKPYKCEECGKAFKQSSNLTTHK
IIHTGEKPYKCKKCGKAFNQSAHLTTHEVIHTGEKPYKCEKCGKAFNHFSHLTTHKIIHT
GEKPYKCKECGKAFKHSSTLTKHKIIHTGEKPYKCKECEKAFNQSSKLTEHKKIHTGEKP
YECEKCGKAFNQSSNLTRHKKSHTEEKPYKCEECGKGFKWPSTLTIHKIIHTGEKPYKCE
ECGKAFNQSSKLTKHKKIHTGEKPYTCEECGKAFNQSSNLTKHKRIHTGEKPYKCEECDK
AFKWSSVLTKHKIIHTGEKLQI*
>ENSG00000174606|ENST00000360506
MSYNILSQDLLEDNSHLYRHCRRPVLHWSFRFPNILKEIKHFDADVLCLQEVQEDHYGAE
IRPSLESLGYHCEYKMRTGRKPDGCAICFKHSKFSLLSVNPVEFFRPDISLLDRDNVGLV
LLLQPKIPYAACPAICVANTHLLYNPRRGDIKLTQLAMLLAEISSVAHQKDGSFCPIVMC
GDFNSVPGSPLYSFIKEGKLNYEGLPIGKVSGQEQSSRGQRILSIPIWPPNLGISQNCVY
EVQQVPKVEKTDSDLTQTQLKQTEVLVTAEKLSSNLQHHFSLSSVYSHYFPDTGIPEVTT
CHSRSAITVDYIFYSAEKEDVAGHPGAEVALVGGLKLLARLSLLTEQDLWTVNGLPNENN
SSDHLPLLAKFRLEL*
>ENSG00000163867|ENST00000317538
MKEPLDGECGKAVVPQQELLDKIKEEPDNAQEYGCVQQPKTQESKLKIGGVSSVNERPIA
QQLNPGFQLSFASSGPSVLLPSVPAVAIKVFCSGCKKMLYKGQTAYHKTGSTQLFCSTRC
ITRHSSPACLPPPPKKTCTNCSKYKILNIPFYFTFFLVCILFSSNILLIL*
>ENSG00000140598|ENST00000359445
MVLNSLDKMIQLQKNTANIRNICVLAHVDHGNEEYLINLIDSPGHVDFSSEVSTAVRICD
GCIIVVDAVEGVCPQTQAVLRQAWLENIRPVLVINKIDRLIVELKFTPQEAYSHLKNILE
QINALTGTLFTSKVLEERAERETESQVNPNSEQGEQVYDWSTGLEDTDDSHLYFSPEQGN
VVFTSAIDGWGFGIEHFARIYSQKIGIKKEVLMKTLWGDYYINMKAKKIMKGDQAKGKKP
LFVQLILENIWSLYDAVLKKDKDKIDKIVTSLGLKIGAREARHSDPKVQINAICSQWLPI
SHAVLAMVCQKLPSPLDITAERVERLMCTGSQTFDSFPPETQALKAAFMKCGSEDTAPVI
IFVSKMFAVDAKALPQNKPRPLTQEEIAQRRERARQRHAEKLAAAQGQAPLEPTQDGSAI

ETCPKGEEPRGDEQQVESMTPKPVLQEENNQESFIAFARVFSGVARRGKKIFVLGPKYSP
LEFLRRVPLGFSAPPDGLPQVPHMAYCALENLYLLMGRELEYLEEVPPGNVLGIGGLQDF
VLKSATLCSLPSCPPFIPLNFEATPIVRVAVEPKHPSEMPQLVKGMKLLNQADPCVQILI
QETGEHVLVTAGEVHLQRCLDDLKERFAKIHISVSEPIIPFRETITKPPKVDMVNEEIGK
QQKVAVIHQMKEDQSKIPEGIQVDSDGLITITTPNKLATLSVRAMPLPEEVTQILEENSD
LIRSMEQLTSSLNEGENTHMIHQKTQEKIWEFKGKLEQHLTGRRWRNIVDQIWSFGPRKC
GPNILVNKSEDFQNSVWTGPADKASKEASRYRDLGNSIVSGFQLATLSGPMCEEPLMGVC
FVLEKWDLSKFEEQGASDLAKEGQEENETCSGGNENQELQDGCSEAFEKRTSQKGESPLT
DCYGPFSGQLIATMKEACRYALQVKPQRLMAAMYTCDIMATGDVLGRVYAVLSKREGRVL
QEEMKEGTDMFIIKAVLPVAESFGFADEIRKRTSGLASPQLVFSHWEIIPSDPFWVPTTE
EEYLHFGEKADSENQARKYMNAVRKRKGLYVEEKIVEHAEKQRTLSKNK*
>ENSG00000164683|ENST00000337919
MKRAHPEYSSSDSELDETIEVEKESADENGNLSSALGSMSPTTSSQILARKRRRGIIEKR
RRDRINNSLSELRRLVPSAFEKQVMEQGSAKLEKAEILQMTVDHLKMLHTAGGKGYFDAH
ALAMDYRSLGFRECLAEVARYLSIIEGLDASDPLRVRLVSHLNNYASQREAASGAHAGLG
HIPWGTVFGHHPHIAHPLLLPQNGHGNAGTTASPTEPHHQGRLGSAHPEAPALRAPPSGS
LGPVLPVVTSASKLSPPLLSSVASLSAFPFSFGSFHLLSPNALSPSAPTQAANLGKPYRP
WGTEIGAF*
>ENSG00000199024|ENST00000362154
Sequence unavailable
>ENSG00000177946|ENST00000314994
MPGERPTDATVIPSAKRERKAITLDLKLEVLRRFEAGEKLSQIAKALDLAISTVATIRDS
KEKIKASSQIATPLRASRLTRHRSAVMESMEQLLSLWLEDQSQPNATLSAAIVQEKAEFD
DLQREHGEGSQTERFHASQGWLVRFKECHCLPHFKMNSAAPSNKDMYTEMLKSIIEEGEY
TPQVSLT*
>ENSG00000197647|ENST00000344980
MMFQDSVAFEDVAVTFTQEEWALLDPSQKNLCRDVMQETFRNLASIGKKWKPQNIYVEYE
NLRRNLRIVGERLFESKEGHQHGEILTQVPDDMLKKTTTGVKSCESSVYGEVGSAHSSLN
RHIRDDTGHKAYEYQEYGQKPYKCKYCKKPFNCLSSVQTHERAHSGRKLYVCEECGKTFI
SHSNLQRHRIMHRGDGPYKCKFCGKALMFLSLYLIHKRTHTGEKPYQCKQCGKAFSHSSS
LRIHERTHTGEKPYKCNECGKAFHSSTCLHAHKRTHTGEKPYECKQCGKAFSSSHSFQIH
ERTHTGEKPYECKECGKAFKCPSSVRRHERTHSRKKPYECKHCGKVLSYLTSFQNHLGMH
TGEISHKCKICGKAFYSPSSLQTHEKTHTGEKPYKCNQCGKAFNSSSSFRYHERTHTGEK
PYECKQCGKAFRSASLLQTHGRTHTGEKPYACKECGKPFSNFSFFQIHERMHREEKPYEC
KGYGKTFSLPSLFHRHERTHTGGKTYECKQCGRSFNCSSSFRYHGRTHTGEKPYECKQCG
KAFRSASQLQIHGRTHTGEKPYECKQCGKAFGSASHLQMHGRTHTGEKPYECKQCGKSFG
CASRLQMHGRTHTGEKPYKCKQCGKAFGCPSNLRRHGRTHTGEKPYKCNQCGKVFRCSSQ
LQVHGRAHCIDTP*
>ENSG00000170802|ENST00000340553
MGPVIGMTPDKRAETPGAEKIAGLSQIYKMGSLPEAVDAARPKATLVDSESADDELTNLN
WLHESTNLLTNFSLGSEGLPIVSPLYDIEGDDVPSFGPACYQNPEKKSATSKPPYSFSLL
IYMAIEHSPNKCLPVKEIYSWILDHFPYFATAPTGWKNSVRHNLSLNKCFQKVERSHGKV
NGKGSLWCVDPEYKPNLIQALKKQPFSSASSQNGSLSPHYLSSVIKQNQVRNLKESDIDA
AAAMMLLNTSIEQGILECEKPLPLKTALQKKRSYGNAFHHPSAVRLQESDSLATSIDPKE

DHNYSASSMAAQRCASRSSVSSLSSVDEVYEFIPKNSHVGSDGSEGFHSEEDTDVDYEDD
PLGDSGYASQPCAKISEKGQSGKKMRKQTCQEIDEELKEAAGSLLHLAGIRTCLGSLIST
AKTQNQKQRKK*
>ENSG00000184967|ENST00000330579
MEREPGAAGVRRALGRRLEAVLASRSEANAVFDILAVLQSEDQEEIQEAVRTCSRLFGAL
LERGELFVGQLPSEEMVMTGSQGATRKYKVWMRHRYHSCCNRLGELLGHPSFQVKELALS
ALLKFVQLEGAHPLEKSKWEGNYLFPRELFKLVVGGLLSPEEDQSLLLSQFREYLDYDDT
RYHTMQAAVDAVARVTGQHPEVPPAFWNNAFTLLSAVSLPRREPTVSSFYVKRAELWDTW
KVAHLKEHRRVFQAMWLSFLKHKLPLSLYKKVLLIVHDAILPQLAQPTLMIDFLTRACDL
GGALSLLALNGLFILIHKHNLEYPDFYRKLYGLLDPSVFHVKYRARFFHLADLFLSSSHL
PAYLVAAFAKRLARLALTAPPEALLMVLPFICNLLRRHPACRVLVHRPHGPELDADPYDP
GEEDPAQSRALESSLWELQALQRHYHPEVSKAASVINQALSMPEVSIAPLLELTAYEIFE
RDLKKKGPEPVPLEFIPAQGLLGRPGELCAQHFTLS*
>ENSG00000126215|ENST00000352127
MDLDLLDLNPRIIAAIKKAKLKSVKEVLHFSGPDLKRLTNLSSPEVWHLLRTASLHLRGS
SILTALQLHQQKERFPTQHQRLSLGCPVLDALLRGGLPLDGITELAGRSSAGKTQLALQL
CLAVQFPRQHGGLEAGAVYICTEDAFPHKRLQQLMAQQPRLRTDVPGELLQKLRFGSQIF
IEHVADVDTLLECVNKKVPVLLSRGMARLVVIDSVAAPFRCEFDSQASAPRARHLQSLGA
TLRELSSAFQSPVLCINQVTEAMEEQGAAHGPLGFWDERVSPALGITWANQLLVRLLADR
LREEEAALGCPARTLRVLSAPHLPPSSCSYTISAEGVRGTPGTQSH*
>ENSG00000102034|ENST00000335997
MAITLQPSDLIFEFASNGMDDDIHQLEDPSVFPAVIVEQVPYPDLLHLYSGLELDDVHNG
IITDGTLCMTQDQILEGSFLLTDDNEATSHTMSTAEVLLNMESPSDILDEKQIFSTSEML
PDSDPAPAVTLPNYLFPASEPDALNRAGDTSDQEGHSLEEKASREESAKKTGKSKKRIRK
TKGNRSTSPVTDPSIPIRKKSKDGKGSTIYLWEFLLALLQDRNTCPKYIKWTQREKGIFK
LVDSKAVSKLWGKQKNKPDMNYETMGRALRYYYQRGILAKVEGQRLVYQFKEMPKDLVVI
EDEDESSEATAAPPQASTASVASASTTRRTSSRVSSRSAPQGKGSSSWEKPKIQHVGLQP
SASLELGPSLDEEIPTTSTMLVSPAEGQVKLTKAVSASSVPSNIHLGVAPVGSGSALTLQ
TIPLTTVLTNGPPASTTAPTQLVLQSVPAASTFKDTFTLQASFPLNASFQDSQVAAPGAP
LILSGLPQLLAGANRPTNPAPPTVTGAGPAGPSSQPPGTVIAAFIRTSGTTAAPRVKEGP
LRSSSYVQGMVTGAPMEGLLVPEETLRELLRDQAHLQPLPTQVVSRGSHNPSLLGNQTLS
PPSRPTVGLTPVAELELSSGSGSLLMAEPSVTTSGSLLTRSPTPAPFSPFNPTSLIKMEP
HDI*
>ENSG00000121057|ENST00000337714
MAIQFRSLFPLALPGMLALLGWWWFFSRKKGHVSSHDEQQVEAGAVQLRADPAIKEPLPV
EDVCPKVVSTPPSVTEPPEKELSTVSKLPAEPPALLQTHPPCRRSESSGILPNTTDMRLR
PGTRRDDSTKLELALTGGEAKSIPLECPLSSPKGVLFSSKSAEVCKQDSPFSRVPRKVQP
GYPVVPAEKRSSGERARETGGAEGTGDAVLGEKVLEEALLSREHVLELENSKGPSLASLE
GEEDKGKSSSSQVVGPVQEEEYVAEKLPSRFIESAHTELAKDDAAPAPPVADAKAQDRGV
EGELGNEESLDRNEEGLDRNEEGLDRNEESLDRNEEGLDRNEEIKRAAFQIISQVISEAT
EQVLATTVGKVAGRVCQASQLQGQKEESCVPVHQKTVLGPDTAEPATAEAAVAPPDAGLP
LPGLPAEGSPPPKTYVSCLKSLLSSPTKDSKPNISAHHISLASCLALTTPSEELPDRAGI
LVEDATCVTCMSDSSQSVPLVASPGHCSDSFSTSGLEDSCTETSSSPRDKAITPPLPEST
VPFSNGVLKGELSDLGAEDGWTMDAEADHSGGSDRNSMDSVDSCCSLKKTESFQNAQAGS

NPKKVDLIIWEIEVPKHLVGRLIGKQGRYVSFLKQTSGAKIYISTLPYTQSVQICHIEGS
QHHVDKALNLIGKKFKELNLTNIYAPPLPSLALPSLPMTSWLMLPDGITVEVIVVNQVNA
GHLFVQQHTHPTFHALRSLDQQMYLCYSQPGIPTLPTPVEITVICAAPGADGAWWRAQVV
ASYEETNEVEIRYVDYGGYKRVKVDVLRQIRSDFVTLPFQGAEVLLDSVMPLSDDDQFSP
EADAAMSEMTGNTALLAQVTSYSPTGLPLIQLWSVVGDEVVLINRSLVERGLAQWVDSYY
TSL*
>ENSG00000198157|ENST00000358130
MPKRKAAGQGDMRQEPKRRSARLSAMLVPVTPEVKPKRTSSSRKMKTKSDMMEENIDTSA
QAVAETKQEAVVEEDYNENAKNGEAKITEAPASEKEIVEVKEENIEDATEKGGEKKEAVA
AEVKNEEEDQKEDEEDQNEEKGEAGKEDKDEKGEEDGKEDKNGNEKGEDAKEKEDGKKGE
DGKGNGEDGKEKGEDEKEEEDRKETGDGKENEDGKEKGDKKEGKDVKVKEDEKEREDGKE
DEGGNEEEAGKEKEDLKEEEEGKEEDEIKEDDGKKEEPQSIV*
>ENSG00000111206|ENST00000361953
MKTSPRRPLILKRRRLPLPVQNAPSETSEEEPKRSPAQQESNQAEASKEVAESNSCKFPA
GIKIINHPTMPNTQVVAIPNNANIHSIITALTAKGKESGSSGPNKFILISCGGAPTQPPG
LRPQTQTSYDAKRTEVTLETLGPKPAARDVNLPRPPGALCEQKRETCADGEAAGCTINNS
LSNIQWLRKMSSDGLGSRSIKQEMEEKENCHLEQRQVKVEEPSRPSASWQNSVSERPPYS
YMAMIQFAINSTERKRMTLKDIYTWIEDHFPYFKHIAKPGWKNSIRHNLSLHDMFVRETS
ANGKVSFWTIHPSANRYLTLDQVFKQQKRPNPELRRNMTIKTELPLGARRKMKPLLPRVS
SYLVPIQFPVNQSLVLQPSVKVPLPLAASLMSSELARHSKRVRIAPKVLLAEEGIAPLSS
AGPGKEEKLLFGEGFSPLLPVQTIKEEEIQPGEEMPHLARPIKVESPPLEEWPSPAPSFK
EESSHSWEDSSQSPTPRPKKSYSGLRSPTRCVSEMLVIQHRERRERSRSRRKQHLLPPCV
DEPELLFSEGPSTSRWAAELPFPADSSDPASQLSYSQEVGGPFKTPIKETLPISSTPSKS
VLPRTPESWRLTPPAKVGGLDFSPVQTSQGASDPLPDPLGLMDLSTTPLQSAPPLESPQR
LLSSEPLDLISVPFGNSSPSDIDVPKPGSPEPQVSGLAANRSLTEGLVLDTMNDSLSKIL
LDISFPGLDEDPLGPDNINWSQFIPELQ*
>ENSG00000134954|ENST00000319397
MKAAVDLKPTLTIIKTEKVDLELFPSPDMECADVPLLTPSSKEMMSQALKATFSGFTKEQ
QRLGIPKDPRQWTETHVRDWVMWAVNEFSLKGVDFQKFCMNGAALCALGKDCFLELAPDF
VGDILWEHLEILQKEDVKPYQVNGVNPAYPESRYTSDYFISYGIEHAQCVPPSEFSEPSF
ITESYQTLHPISSEELLSLKYENDYPSVILRDPLQTDTLQNDYFAIKQEVVTPDNMCMGR
TSRGKLGGQDSFESIESYDSCDRLTQSWSSQSSFNSLQRVPSYDSFDSEDYPAALPNHKP
KGTFKDYVRDRADLNKDKPVIPAAALAGYTGSGPIQLWQFLLELLTDKSCQSFISWTGDG
WEFKLSDPDEVARRWGKRKNKPKMNYEKLSRGLRYYYDKNIIHKTAGKRYVYRFVCDLQS
LLGYTPEELHAMLDVKPDADE*
>ENSG00000149577|ENST00000324225
MFALGLPFLVLLVASVESHLGVLGPKNVSQKDAEFERTYVDEVNSELVNIYTFNHTVTRN
RTEGVRVSVNVLNKQKGAPLLFVVRQKEAVVSFQVPLILRGMFQRKYLYQKVERTLCQPP
TKNESEIQFFYVDVSTLSPVNTTYQLRVSRMDDFVLRTGEQFSFNTTAAQPQYFKYEFPE
GVDSVIVKVTSNKAFPCSVISIQDVLCPVYDLDNNVAFIGMYQTMTKKAAITVQRKDFPS
NSFYVVVVVKTEDQACGGSLPFYPFAEDEPVDQGHRQKTLSVLVSQAVTSEAYVSGMLFC
LGIFLSFYLLTVLLACWENWRQKKKTLLVAIDRACPESGHPRVLADSFPGSSPYEGYNYG
SFENVSGSTDGLVDSAGTGDLSYGYQGRSFEPVGTRPRVDSMSSVEEDDYDTLTDIDSDK
NVIRTKQYLYVADLARKDKRVLRKKYQIYFWNIATIAVFYALPVVQLVITYQTVVNVTGN

QDICYYNFLCAHPLGNLSAFNNILSNLGYILLGLLFLLIILQREINHNRALLRNDLCALE
CGIPKHFGLFYAMGTALMMEGLLSACYHVCPNYTNFQFDTSFMYMIAGLCMLKLYQKRHP
DINASAYSAYACLAIVIFFSVLGVVFGKGNTAFWIVFSIIHIIATLLLSTQLYYMGRWKL
DSGIFRRILHVLYTDCIRQCSGPLYVDRMVLLVMGNVINWSLAAYGLIMRPNDFASYLLA
IGICNLLLYFAFYIIMKLRSGERIKLIPLLCIVCTSVVWGFALFFFFQGLSTWQKTPAES
REHNRDCILLDFFDDHDIWHFLSSIAMFGSFLVLLTLDDDLDTVQRDKIYVF*
>ENSG00000284508|ENST00000347538
Sequence unavailable
>ENSG00000140955|ENST00000315906
MASASQGADDDGSRRKPRLAASLQISPQPRPWRPLPAQAQSAWGPAPAPATYRAEGGWPQ
VSVLRDSGPGAGAGVGELGAARAWENLGEQMGKAPRVPVPPAGLSLPLKDPPASQAVSLL
TEYAASLGIFLLFREDQPPGPCFPFSVSAELDGVVCPAGTANSKTEAKQQAALSALCYIR
SQLENPESPQTSSRPPLAPLSVENILTHEQRCAALVSAGFDLLLDERSPYWACKGTVAGV
ILEREIPRARGHVKEIYKLVALGTGSSCCAGWLEFSGQQLHDCHGLVIARRALLRFLFRQ
LLLATQGGPKGKEQSVLAPQPGPGPPFTLKPRVFLHLYISNTPKGAARDIYLPPTSEGGL
PHSPPMRLQAHVLGQLKPVCYVAPSLCDTHVGCLSASDKLARWAVLGLGGALLAHLVSPL
YSTSLILADSCHDPPTLSRAIHTRPCLDSVLGPCLPPPYVRTALHLFAGPPVAPSEPTPD
TCRGLSLNWSLGDPGIEVVDVATGRVKANAALGPPSRLCKASFLRAFHQAARAVGKPYLL
ALKTYEAAKAGPYQEARRQLSLLLDQQGLGAWPSKPLVGKFRN*
>ENSG00000124782|ENST00000334984
MTSSSPAGLEGSDLSSINTMMSAVMSVGKVTENGGSPQGIKSPSKPPGPNRIGRRNQETK
EEKSSYNCPLCEKICTTQHQLTMHIRQHNTDTGGADHSCSICGKSLSSASSLDRHMLVHS
GERPYKCTVCGQSFTTNGNMHRHMKIHEKDPNSATATAPPSPLKRRRLSSKRKLSHDAES
EREDPAPAKKMVEDGQSGDLEKKADEVFHCPVCFKEFVCKYGLETHMETHSDNPLRCDIC
CVTFRTHRGLLRHNALVHKQLPRDAMGRPFIQNNPSIPAGFHDLGFTDFSCRKFPRISQA
WCETNLRRCISEQHRFVCDTCDKAFPMLCSLALHKQTHVAADQGQEKPQATPLPGDALDQ
KGFLALLGLQHTKDVRPAPAEEPLPDDNQAIQLQTLKCQLPQDPGCTNLLSLSPFEAASL
GGSLTVLPATKDSIKHLSLQPFQKGFIIQPDSSIVVKPISGESAIELADIQQILKMAASA
PPQISLPPFSKAPAAPLQAIFKHMPPLKPKPLVTPRTVVATSTPPPLINAQQASPGCISP
SLPPPPLKLLKGSVEAASNAHLLQSKSGTQPHAATRLSLQQPRAELPGQPEMKTQLEQDS
IIEALLPLSMEAKIKQEITEGELKAFMTAPGGKKTPAMRKVLYPCRFCNQVFAFSGVLRA
HVRSHLGISPYQCNICDYIAADKAALIRHLRTHSGERPYICKICHYPFTVKANCERHLRK
KHLKATRKDIEKNIEYVSSSAAELVDAFCAPDTVCRLCGEDLKHYRALRIHMRTHCGRGL
GGGHKGRKPFECKECSAAFAAKRNCIHHILKQHLHVPEQDIESYVLAADGLGPAEAPAAE
ASGRGEDSGCAALGDCKPLTAFLEPQNGFLHRGPTQPPPPHVSIKLEPASSFAVDFNEPL
DFSQKGLALVQVKQENISFLSPSSLVPYDCSMEPIDLSIPKNFRKGDKDLATPSEAKKPE
EEAGSSEQPSPCPAPGPSLPVTLGPSGILESPMAPAPAATPEPPAQPLQGPVQLAVPIYS
SALVSSPPLVGSSALLSGTALLRPLRPKPPLLLPKPPVTEELPPLASIAQIISSVSSAPT
LLKTKVADPGPASTGSNTTASDSLGGSVPKAATTATPAATTSPKESSEPPAPASSPEAAS
PTEQGPAGTSKKRGRKRGMRSRPRANSGGVDLDSSGEFASIEKMLATTDTNKFSPFLQTA
EDNTQDEVAGAPADHHGPSDEEQGSPPEDKLLRAKRNSYTNCLQKITCPHCPRVFPWASS
LQRHMLTHTGQKPFPCQKCDAFFSTKSNCERHQLRKHGVTTCSLRRNGLIPQSKESDVGS
HDSTGERPYKCQTCERTFTLKHSLVRHQRIHQKARHAKHHGKDSDKEERGEEDSENESTH
SGNNAVSENEAELAPNASNHMAVTRSRKEGLASATKDCSHREEKVTAGWPSEPGQGDLNP

ESPAALGQDLLEPRSKRPAHPILATADGASQLVGME*
>ENSG00000197857|ENST00000355684
MDSVAFEDVAVNFTHEEWALLGPSQKNLYRDVMRETIRNLNCIGMKWENQNIDDQHQNLR
RNPRCDVVERFGKSKDGSQCGETLSQIRNSIVNKNTPARVDACGSSVNGEVIMGHSSLNC
YIRVDTGHKHRECHEYAEKSYTHKQCGKGLSYRHSFQTCERPHTGKKPYDCKECGKTFSS
PGNLRRHMVVKGGDGPYKCELCGKAFFWPSLLRMHERTHTGEKPYECKQCSKAFPVYSSY
LRHEKIHTGEKPYECKQCSKAFPDYSSYLRHERTHTGEKPYKCKQCGKAFSVSGSLRVHE
RIHTGEKPYTCKQCGKAFCHLGSFQRHMIMHSGDGPHKCKICGKGFDFPGSARIHEGTHT
LEKPYECKQCGKLLSHRSSFRRHMMAHTGDGPHKCTVCGKAFDSPSVFQRHERTHTGEKP
YECKQCGKAFRTSSSLRKHETTHTGEQPYKCKCGKAFSDLFSFQSHETTHSEEEPYECKE
CGKAFSSFKYFCRHERTHSEEKSYECQICGKAFSRFSYLKTHERTHTAEKPYECKQCRKA
FFWPSFLLRHERTHTGERPYECKHCGKAFSRSSFCREHERTHTGEKPYECKECGKAFSSL
SSFNRHKRTHWKDIL*
>ENSG00000003756|ENST00000347869
MGSDKRVSRTERSGRYGSIIDRDDRDERESRSRRRDSDYKRSSDDRRGDRYDDYRDYDSP
ERERERRNSDRSEDGYHSDGDYGEHDYRHDISDERESKTIMLRGLPITITESDIREMMES
FEGPQPADVRLMKRKTGVSRGFAFVEFYHLQDATSWMEANQKKLVIQGKHIAMHYSNPRP
KFEDWLCNKCCLNNFRKRLKCFRCGADKFDSEQEVPPGTTESVQSVDYYCDTIILRNIAP
HTVVDSIMTALSPYASLAVNNIRLIKDKQTQQNRGFAFVQLSSAMDASQLLQILQSLHPP
LKIDGKTIGVDFAKSARKDLVLSDGNRVSAFSVASTAIAAAQWSSTQSQSGEGGSVDYSY
LQPGQDGYAQYAQYSQDYQQFYQQQAGGLESDASSASGTAVTTTSAAVVSQSPQLYNQTS
NPPGSPTEEAQPSTSTSTQAPAASPTGVVPGTKYAVPDTSTYQYDESSGYYYDPTTGLYY
DPNSQYYYNSLTQQYLYWDGEKETYVPAAESSSHQQSGLPPAKEGKEKKEKPKSKTAQQI
AKDMERWAKSLNKQKENFKNSFQPVNSLREEERRESAAADAGFALFEKKGALAERQQLIP
ELVRNGDEENPLKRGLVAAYSGDSDNEEELVERLESEEEKLADWKKMACLLCRRQFPNKD
ALVRHQQLSDLHKQNMDIYRRSRLSEQELEALELREREMKYRDRAAERREKYGIPEPPEP
KRKKQFDAGTVNYEQPTKDGIDHSNIGNKMLQAMGWREGSGLGRKCQGITAPIEAQVRLK
GAGLGAKGSAYGLSGADSYKDAVRKAMFARFTEME*
>ENSG00000184357|ENST00000331442
MSETAPAETATPAPVEKSPAKKKATKKAAGAGAAKRKATGPPVSELITKAVAASKERNGL
SLAALKKALAAGGYDVEKNNSRIKLGLKSLVSKGTLVQTKGTGASGSFKLNKKAASGEAK
PKAKKAGAAKAKKPAGATPKKAKKAAGAKKAVKKTPKKAKKPAAAGVKKVAKSPKKAKAA
AKPKKATKSPAKPKAVKPKAAKPKAAKPKAAKPKAAKAKKAAAKKK*
>ENSG00000196378|ENST00000343459
MLLLLSDQLLLTALRKPNPQAMAALFLSAPPQAEVTFEDVAVYLSREEWGRLGPAQRGLY
RDVMLETYGNLVSLGVGPAGPKPGVISQLERGDEPWVLDVQGTSGKEHLRVNSPALGTRT
EYKELTSQETFGEEDPQGSEPVEACDHISKSEGSLEKLVEQRGPRAVTLTNGESSRESGG
NLRLLSRPVPDQRPHKCDICEQSFEQRSYLNNHKRVHRSKKTNTVRNSGEIFSANLVVKE
DQKIPTGKKLHYCSYCGKTFRYSANLVKHQRLHTEEKPYKCDECGKAFSQSCEFINHRRM
HSGEIPYRCDECGKTFTRRPNLMKHQRIHTGEKPYKCGECGKHFSAYSSLIYHQRIHTGE
KPYKCNDCGKAFSDGSILIRHRRTHTGEKPFECKECGKGFTQSSNLIQHQRIHTGEKPYK
CNECEKAFIQKTKLVEHQRSHTGEKPYECNDCGKVFSQSTHLIQHQRIHTGEKPYKCSEC
GKAFHNSSRLIHHQRLHHGEKPYRCSDCKKAFSQSTYLIQHRRIHTGEKPYKCSECGKAF
RHSSNMCQHQRIHLREDFSM*

>ENSG00000187772|ENST00000345080
MAEGGASKGGGEEPGKLPEPAEEESQVLRGTGHCKWFNVRMGFGFISMINREGSPLDIPV
DVFVHQSKLFMEGFRSLKEGEPVEFTFKKSSKGLESIRVTGPGGSPCLGSERRPKGKTLQ
KRKPKGDRCYNCGGLDHHAKECSLPPQPKKCHYCQSIMHMVANCPHKNVAQPPASSQGRQ
EAESQPCTSTLPREVGGGHGCTSPPFPQEARAEISERSGRSPQEASSTKSSIAPEEQSKK
GPSVQKRKKT*
>ENSG00000068305|ENST00000338042
MGRKKIQITRIMDERNRQVTFTKRKFGLMKKAYELSVLCDCEIALIIFNSSNKLFQYAST
DMDKVLLKYTEYNEPHESRTNSDIVETLRKKGLNGCESPDADDYFEHSPLSEDRFSKLNE
DSDFIFKRGPPGLPPQNFSMSVTVPVTSPNALSYTNPGSSLVSPSLAASSTLTDSSMLSP
PQTTLHRNVSPGAPQRPPSTGNAGGMLSTTDLTVPNGAGSSPVGNGFVNSRASPNLIGAT
GANSLGKVMPTKSPPPPGGGNLGMNSRKPDLRVVIPPSSKGMMPPLNTQRISSSQATQPL
ATPVVSVTTPSLPPQGLVYSAMPTAYNTDYSLTSADLSALQGFNSPGMLSLGQVSAWQQH
HLGQAALSSLVAGGQLSQGSNLSINTNQNISIKSEPISPPRDRMTPSGFQQQQQQQQQQQ
PPPPPQPQPQPPQPQPRQEMGRSPVDSLSSSSSSYDGSDREDPRGDFHSPIVLGRPPNTE
DRESPSVKRMRMDAWVT*
>ENSG00000136942|ENST00000348462
MAKIKARDLRGKKKEELLKQLDDLKVELSQLRVAKVTGGAASKLSKIRVVRKSIARVLTV
INQTQKENLRKFYKGKKYKPLDLRPKKTRAMRRRLNKHEENLKTKKQQRKERLYPLRKYA
VKA*
>ENSG00000119725|ENST00000324593
MLSDELESKPELLVQFVQNTSIPLGQGLVESEAKDITCLSLLPVTEASECSRLMLPDDTT
NHSNSSKEVPSSAVLRSLRVNVGPDGEETRAQTVQKSPEFLSTSESSSLLQDLQPSDSTS
FILLNLTRAGLGSSAEHLVFVQDEAEDSGNDFLSSESTDSSIPWFLRVQELAHDSLIAAT
RAQLAKNAKTSSNGENVHLGSGDGQSKDSGPLPQVEKKLKCTVEGCDRTFVWPAHFKYHL
KTHRNDRSFICPAEGCGKSFYVLQRLKVHMRTHNGEKPFMCHESGCGKQFTTAGNLKNHR
RIHTGEKPFLCEAQGCGRSFAEYSSLRKHLVVHSGEKPHQCQVCGKTFSQSGSRNVHMRK
HHLQLGAAGSQEQEQTEVLAEGSPRSLSSVPDVTHHLVTMQSGRQSYEVSVLTAVNPQEL
LNQGDLTERRT*
>ENSG00000182979|ENST00000331320
MAANMYRVGDYVYFENSSSNPYLIRRIEELNKTANGNVEAKVVCFYRRRDISSTLIALAD
KHATLSVCYKAGPGADNGEEGEIEEEMENPEMVDLPEKLKHQLRHRELFLSRQLESLPAT
HIRGKCSVTLLNETESLKSYLEREDFFFYSLVYDPQQKTLLADKGEIRVGNRYQADITDL
LKEGEEDGRDQSRLETQVWEAHNPLTDKQIDQFLVVARSVGTFARALDCSSSVRQPSLHM
SAAAASRDITLFHAMDTLHKNIYDISKAISALVPQGGPVLCRDEMEEWSASEANLFEEAL
EKYGKDFTDIQQDFLPWKSLTSIIEYYYMWKTTDRYVQQKRLKAAEAESKLKQVYIPNYN
KPNPNQISVNNVKAGVVNGTGAPGQSPGAGRACESCYTTQSYQWYSWGPPNMQCRLCASC
WTYWKKYGGLKMPTRLDGERPGPNRSNMSPHGLPARSSGSPKFAMKTRQAFYLHTTKLTR
IARRLCREILRPWHAARHPYLPINSAAIKAECTARLPEASQSPLVLKQAVRKPLEAVLRY
LETHPRPPKPDPVKSVSSVLSSLTPAKVAPVINNGSPTILGKRSYEQHNGVDGNMKKRLL
MPSRGLANHGQARHMGPSRNLLLNGKSYPTKVRLIRGGSLPPVKRRRMNWIDAPDDVFYM
ATEETRKIRKLLSSSETKRAARRPYKPIALRQSQALPPRPPPPAPVNDEPIVIED*
>ENSG00000197780|ENST00000338366
MADEEEDPTFEEENEEIGGGAEGGQGKRKRLFSKELRCMMYGFGDDQNPYTESVDILEDL

VIEFITEMTHKAMSIGRQGRVQVEDIVFLIRKDPRKFARVKDLLTMNEELKRARKAFDEA
NYGS*
>ENSG00000163939|ENST00000356770
MGSKRRRATSPSSSVSGDFDDGHHSVSTPGPSRKRRRLSNLPTVDPIAVCHELYNTIRDY
KDEQGRLLCELFIRAPKRRNQPDYYEVVSQPIDLMKIQQKLKMEEYDDVNLLTADFQLLF
NNAKSYYKPDSPEYKAACKLWDLYLRTRNEFVQKGEADDEDDDEDGQDNQGTVTEGSSPA
YLKEILEQLLEAIVVATNPSGRLISELFQKLPSKVQYPDYYAIIKEPIDLKTIAQRIQNG
SYKSIHAMAKDIDLLAKNAKTYNEPGSQVFKDANSIKKIFYMKKAEIEHHEMAKSSLRMS
NKRAVQGGRLSAITMALQYGSESEEDAALAAARYEEGESEAESITSFMDVSNPFYQLYDT
VRSCRNNQGQLIAEPFYHLPSKKKYPDYYQQIKMPISLQQIRTKLKNQEYETLDHLECDL
NLMFENAKRYNVPNSAIYKRVLKLQQVMQAKKKELARRDDIEDGDSMISSATSDTGSAKR
KSKKNIRKQRMKILFNVVLEAREPGSGRRLCDLFMVKPSKKDYPDYYKIILEPMDLKIIE
HNIRNDKYAGEEGMIEDMKLMFRNARHYNEEGSQVYNDAHILEKLLKEKRKELGPLPDDD
DMASPKLKLSRKSGISPKKSKYMTPMQQKLNEVYEAVKNYTDKRGRRLSAIFLRLPSRSE
LPDYYLTIKKPMDMEKIRSHMMANKYQDIDSMVEDFVMMFNNACTYNEPESLIYKDALVL
HKVLLETRRDLEGDEDSHVPNVTLLIQELIHNLFVSVMSHQDDEGRCYSDSLAEIPAVDP
NFPNKPPLTFDIIRKNVENNRYRRLDLFQEHMFEVLERARRMNRTDSEIYEDAVELQQFF
IKIRDELCKNGEILLSPALSYTTKHLHNDVEKERKEKLPKEIEEDKLKREEEKREAEKSE
DSSGAAGLSGLHRTYSQDCSFKNSMYHVGDYVYVEPAEANLQPHIVCIERLWEDSAGEKW
LYGCWFYRPNETFHLATRKFLEKEVFKSDYYNKVPVSKILGKCVVMFVKEYFKLCPENFR
DEDVFVCESRYSAKTKSFKKIKLWTMPISSVRFVPRDVPLPVVRVASVFANADKGDDEKN
TDNSEDSRAEDNFNLEKEKEDVPVEMSNGEPGCHYFEQLHYNDMWLKVGDCVFIKSHGLV
RPRVGRIEKVWVRDGAAYFYGPIFIHPEETEHEPTKMFYKKEVFLSNLEETCPMTCILGK
CAVLSFKDFLSCRPTEIPENDILLCESRYNESDKQMKKFKGLKRFSLSAKVVDDEIYYFR
KPIVPQKEPSPLLEKKIQLLEAKFAELEGGDDDIEEMGEEDSEVIEPPSLPQLQTPLASE
LDLMPYTPPQSTPKSAKGSAKKEGSKRKINMSGYILFSSEMRAVIKAQHPDYSFGELSRL
VGTEWRNLETAKKAEYEGMMGGYPPGLPPLQGPVDGLVSMGSMQPLHPGGPPPHHLPPGV
PGLPGIPPPGVMNQGVAPMVGTPAPGGSPYGQQVGVLGPPGQQAPPPYPGPHPAGPPVIQ
QPTTPMFVAPPPKTQRLLHSEAYLKYIEGLSAESNSISKWDQTLAARRRDVHLSKEQESR
LPSHWLKSKGAHTTMADALWRLRDLMLRDTLNIRQAYNLENV*
>ENSG00000114867|ENST00000342981
MNKAPQSTGPPPAPSPGLPQPAFPPGQTAPVVFSTPQATQMNTPSQPRQHFYPSRAQPPS
SAASRVQSAAPARPGPAAHVYPAGSQVMMIPSQISYPASQGAYYIPGQGRSTYVVPTQQY
PVQPGAPGFYPGASPTEFGTYAGAYYPAQGVQQFPTGVAPTPVLMNQPPQIAPKRERKTI
RIRDPNQGGKDITEEIMSGARTASTPTPPQTGGGLEPQANGETPQVAVIVRPDDRSQGAI
IADRPGLPGPEHSPSESQPSSPSPTPSPSPVLEPGSEPNLAVLSIPGDTMTTIQMSVEES
TPISRETGEPYRLSPEPTPLAEPILEVEVTLSKPVPESEFSSSPLQAPTPLASHTVEIHE
PNGMVPSEDLEPEVESSPELAPPPACPSESPVPIAPTAQPEELLNGAPSPPAVDLSPVSE
PEEQAKEVTASMAPPTIPSATPATAPSATSPAQEEEMEEEEEEEEGEAGEAGEAESEKGG
EELLPPESTPIPANLSQNLEAAAATQVAVSVPKRRRKIKELNKKEAVGDLLDAFKEANPA
VPEVENQPPAGSNPGPESEGSGVPPRPEEADETWDSKEDKIHNAENIQPGEQKYEYKSDQ
WKPLNLEEKKRYDREFLLGFQFIFASMQKPEGLPHISDVVLDKANKTPLRPLDPTRLQGI
NCGPDFTPSFANLGRTTLSTRGPPRGGPGGELPRGPQAGLGPRRSQQGPRKEPRKIIATV
LMTEDIKLNKAEKAWKPSSKRTAADKDRGEEDADGSKTQDLFRRVRSILNKLTPQMFQQL

MKQVTQLAIDTEERLKGVIDLIFEKAISEPNFSVAYANMCRCLMALKVPTTEKPTVTVNF
RKLLLNRCQKEFEKDKDDDEVFEKKQKEMDEAATAEERGRLKEELEEARDIARRRSLGNI
KFIGELFKLKMLTEAIMHDCVVKLLKNHDEESLECLCRLLTTIGKDLDFEKAKPRMDQYF
NQMEKIIKEKKTSSRIRFMLQDVLDLRGSNWVPRRGDQGPKTIDQIHKEAEMEEHREHIK
VQQLMAKGSDKRRGGPPGPPISRGLPLVDDGGWNTVPISKGSRPIDTSRLTKITKPGSID
SNNQLFAPGGRLSWGKGSSGGSGAKPSDAASEAARPATSTLNRFSALQQAVPTESTDNRR
VVQRSSLSRERGEKAGDRGDRLERSERGGDRGDRLDRARTPATKRSFSKEVEERSRERPS
QPEGLRKAASLTEDRDRGRDAVKREAALPPVSPLKAALSEEELEKKSKAIIEEYLHLNDM
KEAVQCVQELASPSLLFIFVRHGVESTLERSAIAREHMGQLLHQLLCAGHLSTAQYYQGL
YEILELAEDMEIDIPHVWLYLAELVTPILQEGGVPMGELFREITKPLRPLGKAASLLLEI
LGLLCKSMGPKKVGTLWREAGLSWKEFLPEGQDIGAFVAEQKVEYTLGEESEAPGQRALP
SEELNRQLEKLLKEGSSNQRVFDWIEANLSEQQIVSNTLVRALMTAVCYSAIIFETPLRV
DVAVLKARAKLLQKYLCDEQKELQALYALQALVVTLEQPPNLLRMFFDALYDEDVVKEDA
FYSWESSKDPAEQQGKGVALKSVTAFFKWLREAEEESDHN*
>ENSG00000278677|ENST00000359611
MSGRGKQGGKARAKAKTRSSRAGLQFPVGRVHRLLRKGNYAERVGAGAPVYLAAVLEYLT
AEILELAGNAARDNKKTRIIPRHLQLAIRNDEELNKLLGKVTIAQGGVLPNIQAVLLPKK
TESHHKAKGK*
>ENSG00000196437|ENST00000316950
MTESQGTVTFKDVAIDFTQEEWKRLDPAQRKLYRNVMLENYNNLITVGYPFTKPDVIFKL
EQEEEPWVMEEEVLRRHWQGEIWGVDEHQKNQDRLLRQVEVKFQKTLTEEKGNECQKKFA
NVFPLNSDFFPSRHNLYEYDLFGKCLEHNFDCHNNVKCLMRKEHCEYNEPVKSYGNSSSH
FVITPFKCNHCGKGFNQTLDLIRHLRIHTGEKPYECSNCRKAFSHKEKLIKHYKIHSREQ
SYKCNECGKAFIKMSNLIRHQRIHTGEKPYACKECEKSFSQKSNLIDHEKIHTGEKPYEC
NECGKAFSQKQSLIAHQKVHTGEKPYACNECGKAFPRIASLALHMRSHTGEKPYKCDKCG
KAFSQFSMLIIHVRIHTGEKPYECNECGKAFSQSSALTVHMRSHTGEKPYECKECRKAFS
HKKNFITHQKIHTREKPYECNECGKAFIQMSNLVRHQRIHTGEKPYICKECGKAFSQKSN
LIAHEKIHSGEKPYECNECGKAFSQKQNFITHQKVHTGEKPYDCNECGKAFSQIASLTLH
LRSHTGEKPYECDKCGKAFSQCSLLNLHMRSHTGEKPYVCNECGKAFSQRTSLIVHMRGH
TGEKPYECNKCGKAFSQSSSLTIHIRGHTGEKPFDCSKCGKAFSQISSLTLHMRKHTGEK
PYHCIECGKAFSQKSHLVRHQRIHTH*
>ENSG00000095794|ENST00000344351
MPTYQIRAPTAALPQGVVMAASPGSLHSPQQLAEEATRKRELRLMKNREAARECRRKKKE
YVKCLENRVAVLENQNKTLIEELKALKDLYCHKVE*
>ENSG00000114026|ENST00000352937
XVRKYFQLDVTLAQLYHHWGSVDSHFQEVAQKFQGVRLLRQDPIECLFSFICSSNNNIAR
ITGMVERLCQAFGPRLIQLDDVTYHGFPSLQALAGPEVEAHLRKLGLGYRARYVSASARA
ILEEQGGLAWLQQLRESSYEEAHKALCILPGVGTKGLLGNAFDGHQLLRPLIFCQDHLRE
GPPIGRGDSQGEELEPQLPSSLSSIPYGFCDHCWTKDVDDPPLVTHPSPGSRDGHMTQAW
PVKVVSPLATVIGHVMQASLLAL*
>ENSG00000143748|ENST00000340871
MKRKGKLKNKGSKRKKEDLQEVDGEIEAVLQKKAKARGLEFQISNVKFEDVGGNDMTLKE
VCKMLIHMRHPEVYHHLGVVPPRGVLLHGPPGCGKTLLAHAIAGAECSGMITAHCSFDFS
GSNDPPASASQELDLPILKVAAPEIVSGVSGESEQKLRELFEQAVSNAPCIIFIDEIDAI

TPKREVASKDMERRIVAQLLTCMDDLNNVAATARVLVIGATNRPDSLDPALRRAGRFDRE
ICLGIPDEASRERILQTLCRKLRLPQAFDFCHLAHLTPGFVGADLMALCREAAMCAVNRV
LMKLQEQQKKNPEMEDLPSKGVQEERLGTEPTSETQDELQRLLGLLRDQDPLSEEQMQGL
CIELNDFIVALSSVQPSAKREGFVTVPNVTWADIGALEDIREELTMAILAPVRNPDQFKA
LGLVTPAGVLLAGPPGCGKTLLAKAVANESGLNFISVKGPELLNMYVGESERAVRQVFQR
AKNSAPCVIFFDEVDALCPRRSDRETGASVRVVNQLLTEMDGLEARQQVFIMAATNRPDI
IDPAILRPGRLDKTLFVGLPPPADRLAILKTITKNGTKPPLDADVNLEAIAGDLRCDCYT
GADLSALVREASICALRQEMARQKSGNEKGELKVSHKHFEEAFKKVRSSISKKDQIMYER
LQESLSR*
>ENSG00000166938|ENST00000319212
MLQKREKVLLLRTFQGRTLRIVREHYLRPCVPCHSPLCPQPAACSHDGKLLSSDVTHYVI
PDWKVVQDYLEILEFPELKGIIFMQTACQAVQHQRGRRQYNKLRNLLKDARHDCILFANE
FQQCCYLPRERGESMEKWQTRSIYNAAVWYYHHCQDRMPIVMVTEDEEAIQQYGSETEGV
FVITFKNYLDNFWPDLKAAHELCDSILQSRRERENESQESHGKEYPEHLPLEVLEAGIKS
GRYIQGILNVNKHRAQIEAFVRLQGASSKDSDLVSDILIHGMKARNRSIHGDVVVVELLP
KNEWKGRTVALCENDCDDKASGESPSEPMPTGRVVGILQKNWRDYVVTFPSKEEVQSQGK
NAQKILVTPWDYRIPKIRISTQQAETLQDFRVVVRIDSWESTSVYPNGHFVRVLGRIGDL
EGEIATILVENSISVIPFSEAQMCEMPVNTPESPWKVSPEEEQKRKDLRKSHLVFSIDPK
GCEDVDDTLSVRTLNNGNLELGVHIADVTHFVAPNSYIDIEARTRATTYYLADRRYDMLP
SVLSADLCSLLGGVDRYAVSIMWELDKASYEIKKVWYGRTIIRSAYKLFYEAAQELLDGN
LSVVDDIPEFKDLDEKSRQAKLEELVWAIGKLTDIARHVRAKRDGCGALELEGVEVCVQL
DDKKNIHDLIPKQPLEVHETVAECMILANHWVAKKIWESFPHQALLRQHPPPHQEFFSEL
RECAKAKGFFIDTRSNKTLADSLDNANDPHDPIVNRLLRSMATQAMSNALYFSTGSCAEE
EFHHYGLALDKYTHFTSPIRRYSDIVVHRLLMAAISKDKKMEIKGNLFSNKDLEELCRHI
NNRNQAAQHSQKQSTELFQCMYFKDKDPATEERCISDGVIYSIRTNGVLLFIPRFGIKGA
AYLKNKDGLVISCGPDSCSEWKPGSLQRFQNKITSTTTDGESVTFHLFDHVTVRISIQAS
RCHSDTIRLEIISNKPYKIPNTELIHQSSPLLKSELVKEVTKSVEEAQLAQEVKVNIIQE
EYQEYRQTKGRSLYTLLEEIRDLALLDVSNNYGI*
>ENSG00000122779|ENST00000343526
MEVAVEKAVAAAAAASAAASGGPSAAPSGENEAESRQGPDSERGGEAARLNLLDTCAVCH
QNIQSRAPKLLPCLHSFCQRCLPAPQRYLMLPAPMLGSAETPPPVPAPGSPVSGSSPFAT
QVGVIRCPVCSQECAERHIIDNFFVKDTTEVPSSTVEKSNQVCTSCEDNAEANGFCVECV
EWLCKTCIRAHQRVKFTKDHTVRQKEEVSPEAVGVTSQRPVFCPFHKKEQLKLYCETCDK
LTCRDCQLLEHKEHRYQFIEEAFQNQKVIIDTLITKLMEKTKYIKFTGNQIQNRIIEVNQ
NQKQVEQDIKVAIFTLMVEINKKGKALLHQLESLAKDHRMKLMQQQQEVAGLSKQLEHVM
HFSKWAVSSGSSTALLYSKRLITYRLRHLLRARCDASPVTNNTIQFHCDPSFWAQNIINL
GSLVIEDKESQPQMPKQNPVVEQNSQPPSGLSSNQLSKFPTQISLAQLRLQHMQQQVMAQ
RQQVQRRPAPVGLPNPRMQGPIQQPSISHQQPPPRLINFQNHSPKPNGPVLPPHPQQLRY
PPNQNIPRQAIKPNPLQMAFLAQQAIKQWQISSGQGTPSTTNSTSSTPSSPTITSAAGYD
GKAFGSPMIDLSSPVGGSYNLPSLPDIDCSSTIMLDNIVRKDTNIDHGQPRPPSNRTVQS
PNSSVPSPGLAGPVTMTSVHPPIRSPSASSVGSRGSSGSSSKPAGADSTHKVPVVMLEPI
RIKQENSGPPENYDFPVVIVKQESDEESRPQNANYPRSILTSLLLNSSQSSTSEETVLRS
DAPDSTGDQPGLHQDNSSNGKSEWLDPSQKSPLHVGETRKEDDPNEDWCAVCQNGGELLC
CEKCPKVFHLSCHVPTLTNFPSGEWICTFCRDLSKPEVEYDCDAPSHNSEKKKTEGLVKL

TPIDKRKCERLLLFLYCHEMSLAFQDPVPLTVPDYYKIIKNPMDLSTIKKRLQEDYSMYS
KPEDFVADFRLIFQNCAEFNEPDSEVANAGIKLENYFEELLKNLYPEKRFPKPEFRNESE
DNKFSDDSDDDFVQPRKKRLKSIEERQLLK*
>ENSG00000164896|ENST00000353841
MRRPRGEPGPRAPRPTEGATCAGPGESCFPSDGPLVCALEQERRLRLPPKPPPPLQPLLR
GGQGLEAALSCPRFLRYPRQHLISSLAEARPEELTPHVMVLLAQHLARHRLREPQLLEAI
AHFLVVQETQLSSKVVQKLVLPFGRLNYLPLEQQFMPCLERILAREAGVAPLATVNILMS
LCQLRCLPFRALHFVFSPGFINYISGTPHALIVRRYLSLLDTAVELELPGYRGPRLPRRQ
QVPIFPQPLITDRARCKYSHKDIVAEGLRQLLGEEKYRQDLTVPPGYCTDFLLCASSSGA
VLPVRTQDPFLPYPPRSCPQGQAASSATTRDPAQRVVLVLRERWHFCRDGRVLLGSRALR
ERHLGLMGYQLLPLPFEELESQRGLPQLKSYLRQKLQALGLRWGPEGG*
>ENSG00000105732|ENST00000359044
MTEESEETVLYIEHRYVCSECNQLYGSLEEVLMHQNSHVPQQHFELVGVADPGVTVATDT
ASGTGLYQTLVQESQYQCLECGQLLMSPSQLLEHQELHLKMMAPQEAVPAEPSPKAPPLS
SSTIHYECVDCKALFASQELWLNHRQTHLRATPTKAPAPVVLGSPVVLGPPVGQARVAVE
HSYRKAEEGGEGATVPSAAATTTEVVTEVELLLYKCSECSQLFQLPADFLEHQATHFPAP
VPESQEPALQQEVQASSPAEVPVSQPDPLPASDHSYELRNGEAIGRDRRGRRARRNNSGE
AGGAATQELFCSACDQLFLSPHQLQQHLRSHREGVFKCPLCSRVFPSPSSLDQHLGDHSS
ESHFLCVDCGLAFGTEALLLAHRRAHTPNPLHSCPCGKTFVNLTKFLYHRRTHGVGGVPL
PTTPVPPEEPVIGFPEPAPAETGEPEAPEPPVSEETSAGPAAPGTYRCLLCSREFGKALQ
LTRHQRFVHRLERRHKCSICGKMFKKKSHVRNHLRTHTGERPFPCPDCSKPFNSPANLAR
HRLTHTGERPYRCGDCGKAFTQSSTLRQHRLVHAQHFPYRCQECGVRFHRPYRLLMHRYH
HTGEYPYKCRECPRSFLLRRLLEVHQLVVHAGRQPHRCPSCGAAFPSSLRLREHRCAAAA
AQAPRRFECGTCGKKVGSAARLQAHEAAHAAAGPGEVLAKEPPAPRAPRATRAPVASPAA
LGSTATASPAAPARRRGLECSECKKLFSTETSLQVHRRIHTGERPYPCPDCGKAFRQSTH
LKDHRRLHTGERPFACEVCGKAFAISMRLAEHRRIHTGERPYSCPDCGKSYRSFSNLWKH
RKTHQQQHQAAVRQQLAEAEAAVGLAVMETAVEALPLVEAIEIYPLAEAEGVQISG*
>ENSG00000181965|ENST00000314744
MPARLETCISDLDCASSSGSDLSGFLTDEEDCARLQQAASASGPPAPARRGAPNISRASE
VPGAQDDEQERRRRRGRTRVRSEALLHSLRRSRRVKANDRERNRMHNLNAALDALRSVLP
SFPDDTKLTKIETLRFAYNYIWALAETLRLADQGLPGGGARERLLPPQCVPCLPGPPSPA
SDAESWGSGAAAASPLSDPSSPAASEDFTYRPGDPVFSFPSLPKDLLHTTPCFIPYH*
>ENSG00000119866|ENST00000335712
MSRRKQGKPQHLSKREFSPEPLEAILTDDEPDHGPLGAPEGDHDLLTCGQCQMNFPLGDI
LIFIEHKRKQCNGSLCLEKAVDKPPSPSPIEMKKASNPVEVGIQVTPEDDDCLSTSSRGI
CPKQEHIAGKDEPSSYTCTTCKQPFTSAWFLLQHAQNTHGLRIYLESEHGSPLTPRVGIP
SGLGAECPSQPPLHGIHIADNNPFNLLRIPGSVSREASGLAEGRFPPTPPLFSPPPRHHL
DPHRIERLGAEEMALATHHPSAFDRVLRLNPMAMEPPAMDFSRRLRELAGNTSSPPLSPG
RPSPMQRLLQPFQPGSKPPFLATPPLPPLQSAPPPSQPPVKSKSCEFCGKTFKFQSNLVV
HRRSHTGEKPYKCNLCDHACTQASKLKRHMKTHMHKSSPMTVKSDDGLSTASSPEPGTSD
LVGSASSALKSVVAKFKSENDPNLIPENGDEEEEEDDEEEEEEEEEEEEEELTESERVDYG
FGLSLEAARHHENSSRGAVVGVGDESRALPDVMQGMVLSSMQHFSEAFHQVLGEKHKRGH
LAEAEGHRDTCDEDSVAGESDRIDDGTVNGRGCSPGESASGGLSKKLLLGSPSSLSPFSK
RIKLEKEFDLPPAAMPNTENVYSQWLAGYAASRQLKDPFLSFGDSRQSPFASSSEHSSEN

GSLRFSTPPGELDGGISGRSGTGSGGSTPHISGPGPGRPSSKEGRRSDTCEYCGKVFKNC
SNLTVHRRSHTGERPYKCELCNYACAQSSKLTRHMKTHGQVGKDVYKCEICKMPFSVYST
LEKHMKKWHSDRVLNNDIKTE*
>ENSG00000100219|ENST00000344347
MVVVAAAPNPADGTPKVLLLSGQPASAAGAPAGQALPLMVPAQRGASPEAASGGLPQARK
RQRLTHLSPEEKALRRKLKNRVAAQTARDRKKARMSELEQQVVDLEEENQKLLLENQLLR
EKTHGLVVENQELRQRLGMDALVAEEEAEAKGNEVRPVAGSAESAAGAGPVVTPPEHLPM
DSGGIDSSDSESDILLGILDNLDPVMFFKCPSPEPASLEELPEVYPEGPSSLPASLSLSV
GTSSAKLEAINELIRFDHIYTKPLVLEIPSETESQANVVVKIEEAPLSPSENDHPEFIVS
VKEEPVEDDLVPELGISNLLSSSHCPKPSSCLLDAYSDCGYGGSLSPFSDMSSLLGVNHS
WEDTFANELFPQLISV*
>ENSG00000126070|ENST00000324350
MEIGSAGPAGAQPLLMVPRRPGYGTMGKPIKLLANCFQVEIPKIDVYLYEVDIKPDKCPR
RVNREVVDSMVQHFKVTIFGDRRPVYDGKRSLYTANPLPVATTGVDLDVTLPGEGGKDRP
FKVSIKFVSRVSWHLLHEVLTGRTLPEPLELDKPISTNPVHAVDVVLRHLPSMKYTPVGR
SFFSAPEGYDHPLGGGREVWFGFHQSVRPAMWKMMLNIDERDLWQQCGE*
>ENSG00000196482|ENST00000366940
MSNKDRHIDSSCSSFIKTEPSSPASLTDSVNHHSPGGSSDASGSYSSTMNGHQNGLDSPP
LYPSAPILGGSGPVRKLYDDCSSTIVEDPQTKCEYMLNSMPKRLCLVCGDIASGYHYGVA
SCEACKAFFKRTIQGNIEYSCPATNECEITKRRRKSCQACRFMKCLKVGMLKEGVRLDRV
RGGRQKYKRRIDAENSPYLNPQLVQPAKKPYNKIVSHLLVAEPEKIYAMPDPTVPDSDIK
ALTTLCDLADRELVVIIGWAKHIPGFSTLSLADQMSLLQSAWMEILILGVVYRSLSFEDE
LVYADDYIMDEDQSKLAGLLDLNNAILQLVKKYKSMKLEKEEFVTLKAIALANSDSMHIE
DVEAVQKLQDVLHEALQDYEAGQHMEDPRRAGKMLMTLPLLRQTSTKAVQHFYNIKLEGK
VPMHKLFLEMLEAKV*
>ENSG00000198633|ENST00000332323
MALTQGQLSFSDVAIEFSQEEWKCLDPGQKALYRDVMLENYRNLVSLGEDNVRPEACICS
GICLPDLSVTSMLEQKRDPWTLQSEVKIINNPDGRECIKGVNTEKSSKLGSSAGNKSLKN
QHGLTLQLHLTEWQPFQAVRNIYGCKHVEKSISDNSSVSPVQISFFSVKTHIFNNYRNDF
LFSTLLPQEQKVHIREKPYGCNEHGKVFRVSSSLTNRQVIHIADKTYKCSDCGEIFSSNS
NFAQHQRIHTGEKPYKYNECGKVFNQNSHLAQHQKIHTGQKPYNNKECGKVFSHHAYLAQ
HRKIHTGEKPYKCSECGKAFSVCSSLTAHLVIHTGEKPYDCKECGKVFRHKSSLTTHQTV
HTGERPYKCNECGKGFSRIAFLARHRKVHTGEKPYKCNECGKVFIGNSRLARHRKIHTGG
RRYKCNECGKAFRTCSDLTAHLLIHTGEKPYECIDCGKVFRHKSSLTYHCRIHTGEKPYK
CNECGKVFSQNSNLQRHRKIHTGEKLYKCNECGKVFRQNSHLAQHRDIHTGEKPYSCNEC
GKVFRRNSHLVRHRNVHTGEKPYSCNECGKVFSRNSHLARHRNIHTGEKPHSCNECGKVF
SRNSHLARHRKIHTGEKLYKCNECSKVFSRNSRLAQHRNIHTGVKPYSCNECGKVFSKNS
ILVQHCSIHTREKP*
>ENSG00000161642|ENST00000352268
MILGSLSRAGPLPLLRQPPIMQPPLDLKQILPFPLEPAPTLGLFSNYSTMDPVQKAVLSH
TFGGPLLKTKRPVISCNICQIRFNSQSQAEAHYKGNRHARRVKGIEAAKTRGREPGVREP
GDPAPPGSTPTNGDGVAPRPGTKHKTILEARSGLGPIKAYPRLGPPTPGEPEAPAQDRTF
HCEICNVKVNSEVQLKQHISSRRHRDGVAGKPNPLLSRHKKSRGAGELAGTLTFSKELPK
SLAGGLLPSPLAVAAVMAAAAGSPLSLRPAPAAPLLQGPPITHPLLHPAPGPIRTAHGPI

LFSPY*
>ENSG00000165209|ENST00000360998
MVKHSTIYPSPEELEAVQNMVSTVECALKHVSDWLDETNKGTKTEGETEVKKDEAGENYS
KDQGGRTLCGVMRIGLVAKGLLIKDDMDLELVLMCKDKPTETLLNTVKDNLPIQIQKLTE
EKYQVEQCVNEASIIIRNTKEPTLTLKVILTSPLIRDELEKKDGENVSMKDPPDLLDRQK
CLNALASLRHAKWFQARANGLKSCVIVLRILRDLCNRVPTWAPLKGWPLELICEKSIGTC
NRPLGAGEALRRVMECLASGILLPGGPGLHDPCERDPTDALSYMTIQQKEDITHSAQHAL
RLSAFGQIYKVLEMDPLPSSKPFQKYSWSVTDKEGAGSSALKRPFEDGLGDDKDPNKKMK
RNLRKILDSKAIDLMNALMRLNQIRPGLQYKLLSQSGPVHAPVFTMSVDVDGTTYEASGP
SKKTAKLHVAVKVLQAMGYPTGFDADIECMSSDEKSDNESKNETVSSNSSNNTGNSTTET
SSTLEVRTQGPILTASGKNPVMELNEKRRGLKYELISETGGSHDKRFVMEVEVDGQKFRG
AGPNKKVAKASAALAALEKLFSGPNAANNKKKKIIPQAKGVVNTAVSAAVQAVRGRGRGT
LTRGAFVGATAAPGYIAPGYGTPYGYSTAAPAYGLPKRMVLLPVMKFPTYPVPHYSFF*
>ENSG00000181019|ENST00000320623
MVGRRALIVLAHSERTSFNYAMKEAAAAALKKKGWEVVESDLYAMNFNPIISRKDITGKL
KDPANFQYPAESVLAYKEGHLSPDIVAEQKKLEAADLVIFQFPLQWFGVPAILKGWFERV
FIGEFAYTYAAMYDKGPFRSKKAVLSITTGGSGSMYSLQGIHGDMNVILWPIQSGILHFC
GFQVLEPQLTYSIGHTPADARIQILEGWKKRLENIWDETPLYFAPSSLFDLNFQAGFLMK
KEVQDEEKNKKFGLSVGHHLGKSIPTDNQIKARK*
>ENSG00000197302|ENST00000316491
MGLLTFRDVAIEFSREEWEHLDSDQKLLYGDVMLENYGNLVSLGLAVSKPDLITFLEQRK
EPWNVKSAETVAIQPDIFSHDTQGLLRKKLIEASFQKVILDGYGSCGPQNLNLRKEWESE
GKIILW*
>ENSG00000164334|ENST00000335286
MKRRQKRKHLENEESQETAEKGGGMSKSQEDALQPGSTRVAKGWSQGVGEVTSTSEYCSC
VSSSRKLIHSGIQRIHRDSPQPQSPLAQVQERGETPPRSQHVSLSSYSSYKTCVSSLCVN
KEERGMKIYYMQVQMNKGVAVSWETEETLESLEKQPRMEEVTLSEVVRVGTPPSDVSTRN
LLSDSEPSGEEKEHEERTESDSLPGSPTVEDTPRAKTPDWLVTMENGFRCMACCRVFTTM
EALQEHVQFGIREGFSCHVFHLTMAQLTGNMESESTQDEQEEENGNEKEEEEKPEAKEEE
GQPTEEDLGLRRSWSQCPGCVFHSPKDRN*
>ENSG00000213020|ENST00000319783
MLREEAAQKRKGKEPGMALPQGRLTFRDVAIEFSLAEWKCLNPSQRALYREVMLENYRNL
EAVDISSKCMMKEVLSTGQGNTEVIHTGTLQRHESHHIGDFCFQEIEKEIHDIEFQCQED
ERNGLEAPMTKIKKLTGSTDQHDHRHAGNKPIKDQLGSSFYSHLPELHIFQIKGEIGNQL
EKSTNDAPSVSTFQRISCRPQTQISNNYGNNPLNSSLLPQKQEVHMREKSFQCNKSGKAF
NCSSLLRKHQIPHLGDKQYKCDVCGKLFNHEQYLACHDRCHTVEKPYKCKECGKTFSQES
SLTCHRRLHTGVKRYNCNECGKIFGQNSALLIDKAIDTGENPYKCNECDKAFNQQSQLSH
HRIHTGEKPYKCEECDKVFSRKSTIETHKRIHTGEKPYRCKVCDTAFTWHSQLARHRRIH
TAKKTYKCNECGKTFSHKSSLVCHHRLHGGEKSYKCKVCDKAFVWSSQLAKHTRIDCGEK
PYKCNECGKTFGQNSDLLIHKSIHTGEQPYKCDECEKVFSRKSSLETHKIGHTGEKPYKC
KVCDKAFACHSYLAKHTRIHSGEKPYKCNECSKTFSHRSYLVCHHRVHSGEKPYKCNECS
KTFSRRSSLHCHRRLHSGEKPYKCNECGNTFRHCSSLIYHRRLHTGEKSYKCTICDKAFV
RNSLLSRHTRIHTAEKPYKCNECGKAFNQQSHLSRHHRIHTGEKP*
>ENSG00000182944|ENST00000333395

MASTDYSTYSQAAAQQGYSAYTAQPTQGYAQTTQAYGQQSYGTYGQPTDVSYTQAQTTAT
YGQTAYATSYGQPPTGYTTPTAPQAYSQPVQGYGTGAYDTTTATVTTTQASYAAQSAYGT
QPAYPAYGQQPAATAPTRPQDGNKPTETSQPQSSTGGYNQPSLGYGQSNYSYPQVPGSYP
MQPVTAPPSYPPTSYSSTQPTSYDQSSYSQQNTYGQPSSYGQQSSYGQQSSYGQQPPTSY
PPQTGSYSQAPSQYSQQSSSYGQQSSFRQDHPSSMGVYGQESGGFSGPGENRSMSGPDNR
GRGRGGFDRGGMSRGGRGGGRGGMGLQSESLVYTSILKKYPYSVLSRQHNEKWD*
>ENSG00000135111|ENST00000349155
MSLSMRDPVIPGTSMAYHPFLPHRAPDFAMSAVLGHQPPFFPALTLPPNGAAALSLPGAL
AKPIMDQLVGAAETGIPFSSLGPQAHLRPLKTMEPEEEVEDDPKVHLEAKELWDQFHKRG
TEMVITKSGRRMFPPFKVRCSGLDKKAKYILLMDIIAADDCRYKFHNSRWMVAGKADPEM
PKRMYIHPDSPATGEQWMSKVVTFHKLKLTNNISDKHGFTILNSMHKYQPRFHIVRANDI
LKLPYSTFRTYLFPETEFIAVTAYQNDKITQLKIDNNPFAKGFRDTGNGRREKRKQLTLQ
SMRVFDERHKKENGTSDESSSEQAAFNCFAQASSPAASTVGTSNLKDLCPSEGESDAEAE
SKEEHGPEACDAAKISTTTSEEPCRDKGSPAVKAHLFAAERPRDSGRLDKASPDSRHSPA
TISSSTRGLGAEERRSPVREGTAPAKVEEARALPGKEAFAPLTVQTDAAAAHLAQGPLPG
LGFAPGLAGQQFFNGHPLFLHPSQFAMGGAFSSMAAAGMGPLLATVSGASTGVSGLDSTA
MASAAAAQGLSGASAATLPFHLQQHVLASQGLAMSPFGSLFPYPYTYMAAAAAASSAAAS
SSVHRHPFLNLNTMRPRLRYSPYSIPVPVPDGSSLLTTALPSMAAAAGPLDGKVAALAAS
PASVAVDSGSELNSRSSTLSSSSMSLSPKLCAEKEAATSELQSIQRLVSGLEAKPDRSRS
ASP*
>ENSG00000185238|ENST00000331079
MCSLASGATGGRGAVENEEDLPELSDSGDEAAWEDEDDADLPHGKQQTPCLFCNRLFTSA
EETFSHCKSEHQFNIDSMVHKHGLEFYGYIKLINFIRLKNPTVEYMNSIYNPVPWEKEEY
LKPVLEDDLLLQFDVEDLYEPVSVPFSYPNGLSENTSVVEKLKHMEARALSAEAALARAR
EDLQKMKQFAQDFVMHTDVRTCSSSTSVIADLQEDEDGVYFSSYGHYGIHEEMLKDKIRT
ESYRDFIYQNPHIFKDKVVLDVGCGTGILSMFAAKAGAKKVLGVDQSEILYQAMDIIRLN
KLEDTITLIKGKIEEVHLPVEKVDVIISEWMGYFLLFESMLDSVLYAKNKYLAKGGSVYP
DICTISLVAVSDVNKHADRIAFWDDVYGFKMSCMKKAVIPEAVVEVLDPKTLISEPCGIK
HIDCHTTSISDLEFSSDFTLKITRTSMCTAIAGYFDIYFEKNCHNRVVFSTGPQSTKTHW
KQTVFLLEKPFSVKAGEALKGKVTVHKNKKDPRSLTVTLTLNNSTQTYGLQ*
>ENSG00000196967|ENST00000356958
MLETYSHLLSVGYQVPEAEVVMLEQGKEPWALQGERPRQSCPGEKLWDHNQCRKILSYKQ
VSSQPQKMYPGEKAYECAKFEKIFTQKSQLKVHLKVLAGEKLYVCIECGKAFVQKPEFII
HQKTHMREKPFKCNECGKSFFQVSSLFRHQRIHTGEKLYECSQCGKGFSYNSDLSIHEKI
HTGERHHECTDCGKAFTQKSTLKMHQKIHTGERSYICIECGQAFIQKTHLIAHRRIHTGE
KPYECSNCGKSFISKSQLQVHQRVHTRVKPYICTEYGKVFSNNSNLVTHKKVQSREKSSI
CTECGKAFTYRSELIIHQRIHTGEKPYECSDCGKAFTQKSALTVHQRIHTGEKSYICMKC
GLAFIQKAHLIAHQIIHTGEKPHKCGHCGKLFTSKSQLHVHKRIHTGEKPYMCNKCGKAF
TNRSNLITHQKTHTGEKSYICSKCGKAFTQRSDLITHQRIHTGEKPYECNTCGKAFTQKS
HLNIHQKIHTGERQYECHECGKAFNQKSILIVHQKIHTGEKPYVCTECGRAFIRKSNFIT
HQRIHTGEKPYECSDCGKSFTSKSQLLVHQPVHTGEKPYVCAECGKAFSGRSNLSKHQKT
HTGEKPYICSECGKTFRQKSELITHHRIHTGEKPYECSDCGKSFTKKSQLQVHQRIHTGE
KPYVCAECGKAFTDRSNLNKHQTTHTGDKPYKCGICGKGFVQKSVFSVHQSSHA*
>ENSG00000175832|ENST00000319349

MERRMKAGYLDQQVPYTFSSKSPGNGSLREALIGPLGKLMDPGSLPPLDSEDLFQDLSHF
QETWLAEAQVPDSDEQFVPDFHSENLAFHSPTTRIKKEPQSPRTDPALSCSRKPPLPYHH
GEQCLYSSAYDPPRQIAIKSPAPGALGQSPLQPFPRAEQRNFLRSSGTSQPHPGHGYLGE
HSSVFQQPLDICHSFTSQGGGREPLPAPYQHQLSEPCPPYPQQSFKQEYHDPLYEQAGQP
AVDQGGVNGHRYPGAGVVIKQEQTDFAYDSDVTGCASMYLHTEGFSGPSPGDGAMGYGYE
KPLRPFPDDVCVVPEKFEGDIKQEGVGAFREGPPYQRRGALQLWQFLVALLDDPTNAHFI
AWTGRGMEFKLIEPEEVARLWGIQKNRPAMNYDKLSRSLRYYYEKGIMQKVAGERYVYKF
VCEPEALFSLAFPDNQRPALKAEFDRPVSEEDTVPLSHLDESPAYLPELAGPAQPFGPKG
GYSY*
>ENSG00000146463|ENST00000314607
MAEREVESGPRKRFEQKSGAVFDEIVENCGGIMDTEMSEDIDHNLTPTLDSMSYGMPNQT
GSENSLLDEDDYFLNSGDLAGIPVVGSDNEDEQDFSSKDNLVSSIHTDDSLEVERRVTQH
ESDNENEIQIQNKLKKDFPKQFDQVSVFKSIRKDFSLVRENSKETFSGKEKNRDLTYERE
KRLDKPHKDLDSRLKSSFFDKAANQVEETLHTHLPQTPETNFRDSSYPFANKESIGSELG
NSFASNIRIKEEPLDDEYDKAMAPQQGLLDKIKDEPDNAQEYSHGQQQKTQEGELKISAV
FSVSGSPLAPQLTTGFQPSLASSGMNKMLPSVPATAVRVSCSGCKKILQKGQTAYQRKGS
TQLFCSTLCLTGYTVPPARPPPPLTKKTCSSCSKDILNPKDVISAQFENTTTSKDFCSQS
CLSTYELKKKPIVTINTNSISTKCSMCQKNAVIRHEVNYQNVVHKLCSDACFSKFRSANN
LTMNCCENCGGYCYSGSGQCHMLQIEGQSKKFCSSSCITAYKQKSAKITPCALCKSLRSS
AEMIENTNSLGKTELFCSVNCLSAYRVKMVTSAGVQVQCNSCKTSAIPQYHLAMSDGSIR
NFCSYSCVVAFQNLFNKPTGMNSSVVPLSQGQVIVSIPTGSTVSAGGGSTSAVSPTSISS
SAAAGLQRLAAQSQHVGFARSVVKLKCQHCNRLFATKPELLDYKGKMFQFCGKNCSDEYK
KINNVMAMCEYCKIEKIVKETVRFSGADKSFCSEGCKLLYKHDLAKRWGNHCKMCSYCLQ
TSPKLVQNNLGGKVEEFCCEECMSKYTVLFYQMAKCDACKRQGKLSESLKWRGEMKHFCN
LLCILMFCNQQSVCDPPSQNNAANISMVQAASAGPPSLRKDSTPVIANVVSLASAPAAQP
TVNSNSVLQGAVPTVTAKIIGDASTQTDALKLPPSQPPRLLKNKALLCKPITQTKATSCK
PHTQNKECQTEDTPSQPQIIVVPVPVPVFVPIPLHLYTQYAPVPFGIPVPMPVPMLIPSS
MDSEDKVTESIEDIKEKLPTHPFEADLLEMAEMIAEDEEKKTLSQGESQTSEHELFLDTK
IFEKDQGSTYSGDLESEAVSTPHSWEEELNHYALKSNAVQEADSELKQFSKGETEQDLEA
DFPSDSFDPLNKGQGIQARSRTRRRHRDGFPQPRRRGRKKSIVAVEPRSLIQGAFQGCSV
SGMTLKYMYGVNAWKNWVQWKNAKEEQGDLKCGGVEQASSSPRSDPLGSTQDHALSQESS
EPGCRVRSIKLKEDILSCTFAELSLGLCQFIQEVRRPNGEKYDPDSILYLCLGIQQYLFE
NGRIDNIFTEPYSRFMIELTKLLKIWEPTILPNGYMFSRIEEEHLWECKQLGAYSPIVLL
NTLLFFNTKYFQLKNVTEHLKLSFAHVMRRTRTLKYSTKMTYLRFFPPLQKQESEPDKLT
VGKRKRNEDDEVPVGVEMAENTDNPLRCPVRLYEFYLSKCSESVKQRNDVFYLQPERSCV
PNSPMWYSTFPIDPGTLDTMLTRILMVREVHEELAKAKSEDSDVELSD*
>ENSG00000185591|ENST00000327443
MSDQDHSMDEMTAVVKIEKGVGGNNGGNGNGGGAFSQARSSSTGSSSSTGGGGQESQPSP
LALLAATCSRIESPNENSNNSQGPSQSGGTGELDLTATQLSQGANGWQIISSSSGATPTS
KEQSGSSTNGSNGSESSKNRTVSGGQYVVAAAPNLQNQQVLTGLPGVMPNIQYQVIPQFQ
TVDGQQLQFAATGAQVQQDGSGQIQIIPGANQQIITNRGSGGNIIAAMPNLLQQAVPLQG
LANNVLSGQTQYVTNVPVALNGNITLLPVNSVSAATLTPSSQAVTISSSGSQESGSQPVT
SGTTISSASLVSSQASSSSFFTNANSYSTTTTTSNMGIMNFTTSGSSGTNSQGQTPQRVS
GLQGSDALNIQQNQTSGGSLQAGQQKEGEQNQQTQQQQILIQPQLVQGGQALQALQAAPL

SGQTFTTQAISQETLQNLQLQAVPNSGPIIIRTPTVGPNGQVSWQTLQLQNLQVQNPQAQ
TITLAPMQGVSLGQTSSSNTTLTPIASAASIPAGTVTVNAAQLSSMPGLQTINLSALGTS
GIQVHPIQGLPLAIANAPGDHGAQLGLHGAGGDGIHDDTAGGEEGENSPDAQPQAGRRTR
REACTCPYCKDSEGRGSGDPGKKKQHICHIQGCGKVYGKTSHLRAHLRWHTGERPFMCTW
SYCGKRFTRSDELQRHKRTHTGEKKFACPECPKRFMRSDHLSKHIKTHQNKKGGPGVALS
VGTLPLDSGAGSEGSGTATPSALITTNMVAMEAICPEGIARLANSGINVMQVADLQSINI
SGNGF*
>ENSG00000145220|ENST00000343470
MVFFTCNACGESVKKIQVEKHVSVCRNCECLSCIDCGKDFWGDDYKNHVKCISEDQKYGG
KGYEGKTHKGDIKQQAWIQKISELIKRPNVSPKVRELLEQISAFDNVPRKKAKFQNWMKN
SLKVHNESILDQVWNIFSEASNSEPVNKEQDQRPLHPVANPHAEISTKVPASKVKDAVEQ
QGEVKKNKRERKEERQKKRKREKKELKLENHQENSRNQKPKKRKKGQEADLEAGGEEVPE
ANGSAGKRSKKKKQRKDSASEEEARVGAGKRKRRHSEVETDSKKKKMKLPEHPEGGEPED
DEAPAKGKFNWKGTIKAILKQAPDNEITIKKLRKKVLAQYYTVTDEHHRSEEELLVIFNK
KISKNPTFKLLKDKVKLVK*
>ENSG00000130119|ENST00000336470
MMKLRHKNKKPGEGSKGHKKISWPYPQPAKQNGKKATSKVPSAPHFVHPNDHANREAELK
KKWVEEMREKQQAAREQERQKRRTIESYCQDVLRRQEEFEHKEEVLQELNMFPQLDDEAT
RKAYYKEFRKVVEYSDVILEVLDARDPLGCRCFQMEEAVLRAQGNKKLVLVLNKIDLVPK
EVVEKWLDYLRNELPTVAFKASTQHQVKNLNRCSVPVDQASESLLKSKACFGAENLMRVL
GNYCRLGEVRTHIRVGVVGLPNVGKSSLINSLKRSRACSVGAVPGITKFMQEVYLDKFIR
LLDAPGIVPGPNSEVGTILRNCVHVQKLADPVTPVETILQRCNLEEISNYYGVSGFQTTE
HFLTAVAHRLGKKKKGGLYSQEQAAKAVLADWVSGKISFYIPPPATHTLPTHLSAEIVKE
MTEVFDIEDTEQANEDTMECLATGESDELLGDTDPLEMEIKLLHSPMTKIADAIENKTTV
YKIGDLTGYCTNPNRHQMGWAKRNVDHRPKSNSMVDVCSVDRRSVLQRIMETDPLQQGQA
LASALKNKKKMQKRADKIASKLSDSMMSALDLSGNADDGVGD*
>ENSG00000001167|ENST00000353205
MEQYTANSNSSTEQIVVQAGQIQQQVQGQPLMVQVSGGQLITSTGQPIMVQAVPGGQGQT
IMQVPVSGTQGLQQIQLVPPGQIQIQGGQAVQVQGQQGQTQQIIIQQPQTAVTAGQTQTQ
QQIAVQGQQVAQTAEGQTIVYQPVNADGTILQQVTVPVSGMITIPAASLAGAQIVQTGAN
TNTTSSGQGTVTVTLPVAGNVVNSGGMVMMVPGAGSVPAIQRIPLPGAEMLEEEPLYVNA
KQYHRILKRRQARAKLEAEGKIPKERRKYLHESRHRHAMARKRGEGGRFFSPKEKDSPHM
QDPNQADEEAMTQIIRVS*
>ENSG00000143633|ENST00000366651
MRVDSSADPTMSQEQGPGSSTPPSSPTLLDALLQNLYDFGGTEGETEQKKIIKKRENKKR
DVMASAALAAEPSPLPGSLIRGQRKSASSFFKELREERHCAPSGTPTGPEILAAAVPPSS
LKNNREQVEVVEFHSNKKRKLTPDHNKNTKANPSVLERDVDTQEFNLEKARLEVHRFGIT
GYGKGKERILEQERAIMLGAKPPKKSYVNYKVLQEQIKEKKAAKEEEKRLAQETDIFKKK
KRKGQEDRKSKKKSAPSILSNGRIGQVGKFKNGTLILSPVDIKKINSSRVAK*
>ENSG00000179348|ENST00000341105
MEVAPEQPRWMAHPAVLNAQHPDSHHPGLAHNYMEPAQLLPPDEVDVFFNHLDSQGNPYY
ANPAHARARVSYSPAHARLTGGQMCRPHLLHSPGLPWLDGGKAALSAAAAHHHNPWTVSP
FSKTPLHPSAAGGPGGPLSVYPGAGGGSGGGSGSSVASLTPTAAHSGSHLFGFPPTPPKE
VSPDPSTTGAASPASSSAGGSAARGEDKDGVKYQVSLTESMKMESGSPLRPGLATMGTQP

ATHHPIPTYPSYVPAAAHDYSSGLFHPGGFLGGPASSFTPKQRSKARSCSEGRECVNCGA
TATPLWRRDGTGHYLCNACGLYHKMNGQNRPLIKPKRRLSAARRAGTCCANCQTTTTTLW
RRNANGDPVCNACGLYYKLHNVNRPLTMKKEGIQTRNRKMSNKSKKSKKGAECFEELSKC
MQEKSSPFSAAALAGHMAPVGHLPPFSHSGHILPTPTPIHPSSSLSFGHPHPSSMVTAMG
*
>ENSG00000070814|ENST00000323668
MAEARKRRELLPLIYHHLLRAGYVRAAREVKEQSGQKCFLAQPVTLLDIYTHWQQTSELG
RKRKAEEDAALQAKKTRVSDPISTSESSEEEEEAEAETAKATPRLASTNSSVLGADLPSS
MKEKAKAETEKAGKTGNSMPHPATGKTVANLLSGKSPRKSAEPSANTTLVSETEEEGSVP
AFGAAAKPGMVSAGQADSSSEDTSSSSDETDVEVKASEKILQVRAASAPAKGTPGKGATP
APPGKAGAVASQTKAGKPEEDSESSSEESSDSEEETPAAKALLQAKASGKTSQVGAASAP
AKESPRKGAAPAPPGKTGPAVAKAQAGKREEDSQSSSEESDSEEEAPAQAKPSGKAPQVR
AASAPAKESPRKGAAPAPPRKTGPAAAQVQVGKQEEDSRSSSEESDSDREALAAMNAAQV
KPLGKSPQVKPASTMGMGPLGKGAGPVPPGKVGPATPSAQVGKWEEDSESSSEESSDSSD
GEVPTAVAPAQEKSLGNILQAKPTSSPAKGPPQKAGPVAVQVKAEKPMDNSESSEESSDS
ADSEEAPAAMTAAQAKPALKIPQTKACPKKTNTTASAKVAPVRVGTQAPRKAGTATSPAG
SSPAVAGGTQRPAEDSSSSEESDSEEEKTGLAVTVGQAKSVGKGLQVKAASVPVKGSLGQ
GTAPVLPGKTGPTVTQVKAEKQEDSESSEEESDSEEAAASPAQVKTSVKKTQAKANPAAA
RAPSAKGTISAPGKVVTAAAQAKQRSPSKVKPPVRNPQNSTVLARGPASVPSVGKAVATA
AQAQTGPEEDSGSSEEESDSEEEAETLAQVKPSGKTHQIRAALAPAKESPRKGAAPTPPG
KTGPSAAQAGKQDDSGSSSEESDSDGEAPAAVTSAQVIKPPLIFVDPNRSPAGPAATPAQ
AQAASTPRKARASESTARSSSSESEDEDVIPATQCLTPGIRTNVVTMPTAHPRIAPKASM
AGASSSKESSRISDGKKQEGPATQVSKKNPASLPLTQAALKVLAQKASEAQPPVARTQPS
SGVDSAVGTLPATSPQSTSVQAKGTNKLRKPKLPEVQQATKAPESSDDSEDSSDSSSGSE
EDGEGPQGAKSAHTLGPTPSRTETLVEETAAESSEDDVVAPSQSLLSGYMTPGLTPANSQ
ASKATPKLDSSPSVSSTLAAKDDPDGKQEAKPQQAAGMLSPKTGGKEAASGTTPQKSRKP
KKGAGNPQASTLALQSNITQCLLGQPWPLNEAQVQASVVKVLTELLEQERKKVVDTTKES
SRKGWESRKRKLSGDQPAARTPRSKKKKKLGAGEGGEASVSPEKTSTTSKGKAKRDKASG
DVKEKKGKGSLGSQGAKDEPEEELQKGMGTVEGGDQSNPKSKKEKKKSDKRKKDKEKKEK
KKKAKKASTKDSESPSQKKKKKKKKTAEQTV*
>ENSG00000080298|ENST00000358730
MQTSETGSDTGSTVTLQTSVASQAAVPTQVVQQVPVQQQVQQVQTVQQVQHVYPAQVQYV
EGSDTVYTNGAIRTTTYPYTETQMYSQNTGGNYFDTQGSSAQVTTVVSSHSMVGTGGIQM
GVTGGQLISSSGGTYLIGNSMENSGHSVTHTTRASPATIEMAIETLQKSDGLSTHRSSLL
NSHLQWLLDNYETAEGVSLPRSTLYNHYLRHCQEHKLDPVNAASFGKLIRSIFMGLRTRR
LGTRGNSKYHYYGIRVKPDSPLNRLQEDMQYMAMRQQPMQQKQRYKPMQKVDGVADGFTG
SGQQTGTSVEQTVIAQSQHHQQFLDASRALPEFGEVEISSLPDGTTFEDIKSLQSLYREH
CEAILDVVVNLQFSLIEKLWQTFWRYSPSTPTDGTTITESSNLSEIESRLPKAKLITLCK
HESILKWMCNCDHGMYQALVEILIPDVLRPIPSALTQAIRNFAKSLEGWLSNAMNNIPQR
MIQTKVAAVSAFAQTLRRYTSLNHLAQAARAVLQNTSQINQMLSDLNRVDFANVQEQASW
VCQCDDNMVQRLETDFKMTLQQQSTLEQWAAWLDNVMMQALKPYEGRPSFPKAARQFLLK
WSFYSSMVIRDLTLRSAASFGSFHLIRLLYDEYMFYLVEHRVAQATGETPIAVMGEVREA
ERAVTHWVIKNKPELHFSLNTLLIKTMVPNQVSLRARRDCGVIARVP*
>ENSG00000161642|ENST00000338010

MILGSLSRAGPLPLLRQPPIMQPPLDLKQILPFPLEPAPTLGLFSNYSTMDPVQKAVLSH
TFGGPLLKTKRPVISCNICQIRFNSQSQAEAHYKGNRHARRVKGIEAAKTRGREPGVREP
GDPAPPGSTPTNGDGVAPRPVSMENGLGPAPGSPEKQPGSPSPPSIPETGQGVTKGEGGT
PAPASLPGGSKEEEEKAKRLLYCALCKVAVNSLSQLEAHNKGTKHKTILEARSGLGPIKA
YPRLGPPTPGEPEAPAQDRTFHCEICNVKVNSEVQLKQHISSRRHRDGVAGKPNPLLSRH
KKSRGAGELAGTLTFSKELPKSLAGGLLPSPLAVAAVMAAAAGSPLSLRPAPAAPLLQGP
PITHPLLHPAPGPIRTAHGPILFSPY*
>ENSG00000099783|ENST00000348943
MAAGVEAAAEVAATEIKMEEESGAPGVPSGNGAPGPKGEGERPAQNEKRKEKNIKRGGNR
FEPYANPTKRYRAFITNIPFDVKWQSLKDLVKEKVGEVTYVELLMDAEGKSRGCAVVEFK
MEESMKKAAEVLNKHSLSGRPLKVKEDPDGEHARRAMQKAGRLGSTVFVANLDYKVGWKK
LKEVFSMAGVVVRADILEDKDGKSRGIGTVTFEQSIEAVQAISMFNGQLLFDRPMHVKMD
ERALPKGDFFPPERPQQLPHGLGGIGMGLGPGGQPIDANHLNKGIGMGNIGPAGMGMEGI
GFGINKMGGMEGPFGGGMENMGRFGSGMNMGRINEILSNALKRGEIIAKQGGGGGGGSVP
GIERMGPGIDRLGGAGMERMGAGLGHGMDRVGSEIERMGLVMDRMGSVERMGSGIERMGP
LGLDHMASSIERMGQTMERIGSGVERMGAGMGFGLERMAAPIDRVGQTIERMGSGVERMG
PAIERMGLSMERMVPAGMGAGLERMGPVMDRMATGLERMGANNLERMGLERMGANSLERM
GLERMGANSLERMGPAMGPALGAGIERMGLAMGGGGGASFDRAIEMERGNFGGSFAGSFG
GAGGHAPGVARKACQIFVRNLPFDFTWKMLKDKFNECGHVLYADIKMENGKSKGCGVVKF
ESPEVAERACRMMNGMKLSGREIDVRIDRNA*
>ENSG00000122026|ENST00000319826
MTNTKGKRRGTRYMFSRPFRKHGVVPLATYMRIYKKGDIVDIKGMGTVQKGMPHKCYHGK
TGRVYNVTQHAVGIVVNKQVKGKILAKRINVRIEHIKHSKSRDSFLKRVKENDQKKKEAK
EKGTWVQLKRQPAPPREAHFVRTNGKEPELLEPIPYEFMA*
>ENSG00000125952|ENST00000358664
MSDNDDIEVESDEEQPRFQSAADKRAHHNALERKRRDHIKDSFHSLRDSVPSLQGEKASR
AQILDKATEYIQYMRRKNHTHQQDIDDLKRQNALLEQQVRALEKARSSAQLQTNYPSSDN
SLYTNAKGSTISAFDGGSDSSSESEPEEPQSRKKLRMEAS*
>ENSG00000182944|ENST00000332035
MASTDYSTYSQAAAQQGYSAYTAQPTQGYAQTTQAYGQQSYGTYGQPTDVSYTQAQTTAT
YGQTAYATSYGQPPTGYTTPTAPQAYSQPVQGYGTGAYDTTTATVTTTQASYAAQSAYGT
QPAYPAYGQQPAATAPTSYSSTQPTSYDQSSYSQQNTYGQPSSYGQQSSYGQQSSYGQQP
PTSYPPQTGSYSQAPSQYSQQSSSYGQQSSFRQDHPSSMGVYGQESGGFSGPGENRSMSG
PDNRGRGRGGFDRGGMSRGGRGGGRGGMGSAGERGGFNKPGGPMDEGPDLDLGPPVDPDE
DSDNSAIYVQGLNDSVTLDDLADFFKQCGVVKMNKRTGQPMIHIYLDKETGKPKGDATVS
YEDPPTAKAAVEWFDGKDFQGSKLKVSLARKKPPMNSMRGGLPPREGRGMPPPLRGGPGG
PGGPGGPMGRMGGRGGDRGGFPPRGPRGSRGNPSGGGNVQHRAGDWQCPNPGCGNQNFAW
RTECNQCKAPKPEGFLPPPFPPPGGDRGRGGPGGMRGGRGGLMDRGGPGGMFRGGRGGDR
GGFRGGRGMDRGGFGGGRRGGPGGPPGPLMEQMGGRRGGRGGPGKMDKGEHRQERRDRPY
*
>ENSG00000171791|ENST00000333681
MAHAGRTGYDNREIVMKYIHYKLSQRGYEWDAGDVGAAPPGAAPAPGIFSSQPGHTPHPA
ASRDPVARTSPLQTPAAPGAAAGPALSPVPPVVHLTLRQAGDDFSRRYRRDFAEMSSQLH
LTPFTARGRFATVVEELFRDGVNWGRIVAFFEFGGVMCVESVNREMSPLVDNIALWMTEY

LNRHLHTWIQDNGGWDAFVELYGPSMRPLFDFSWLSLKTLLSLALVGACITLGAYLGHK*
>ENSG00000180346|ENST00000317005
MLGKRKRVVLTIKDKLDIIKKLEEGISFKKLSVVYGIGESTVRDIKKNKERIINYANSSD
PTSGVSKRKSMKSSTYEELDRVMIEWFNQQKTDGIPVSGTICAKQAKFFFDALGMEGDFN
ASSGWLTRFKQRHGIPKAAGKGTKLKGDETAAREFCGSFQEFVEKENLQPEQIYGADQTG
LFWKCLPSRTLTLETDQSTSGCRSSRERIIIMCCANATGLHKLNLCVVGKAKKPRAFKGT
DLSNLPVTYYSQKGAWIEQSVFRQWFEKYFVPQVQKHLKSKGLLEKAVLLLDFPPARPNE
EMLSSDDGRIIVKYLPPNVTSLIQPMSQGVLATVKRYYRAGLLQKYMDEGNDPKIFWKNL
TVLDAIYEVSRAWNMVKSSTITKAWKKLFPGNEENSGMNIDEGAILAANLATVLQNTEEC
EHVDIENIDQWFDSRSSDSSCQVLTDSESAEDQTKAAEQKPSSKSRKTELNPEKHISHKA
ALEWTENLLDYLEQQDDMLLSDKLVLRRLRTIIRKKQKIQNNKNH*
>ENSG00000101773|ENST00000327155
MNISGSSCGSPNSADTSSDFKDLWTKLKECHDREVQGLQVKVTKLKQERILDAQRLEEFF
TKNQQLREQQKVLHETIKVLEDRLRAGLCDRCAVTEEHMRKKQQEFENIRQQNLKLITEL
MNERNTLQEENKKLSEQLQQKIENDQQHQAAELECEEDVIPDSPITAFSFSGVNRLRRKE
NPHVRYIEQTHTKLEHSVCANEMRKVSKSSTHPQHNPNENEILVADTYDQSQSPMAKAHG
TSSYTPDKSSFNLATVVAETLGLGVQEESETQGPMSPLGDELYHCLEGNHKKQPFEESTR
NTEDSLRFSDSTSKTPPQEELPTRVSSPVFGATSSIKSGLDLNTSLSPSLLQPGKKKHLK
TLPFSNTCISRLEKTRSKSEDSALFTHHSLGSEVNKIIIQSSNKQILINKNISESLGEQN
RTEYGKDSNTDKHLEPLKSLGGRTSKRKKTEEESEHEVSCPQASFDKENAFPFPMDNQFS
MNGDCVMDKPLDLSDRFSAIQRQEKSQGSETSKNKFRQVTLYEALKTIPKGFSSSRKASD
GNCTLPKDSPGEPCSQECIILQPLNKCSPDNKPSLQIKEENAVFKIPLRPRESLETENVL
DDIKSAGSHEPIKIQTRSDHGGCELASVLQLNPCRTGKIKSLQNNQDVSFENIQWSIDPG
ADLSQYKMDVTVIDTKDGSQSKLGGETVDMDCTLVSETVLLKMKKQEQKGEKSSNEERKM
NDSLEDMFDRTTHEEYESCLADSFSQAADEEEELSTATKKLHTHGDKQDKVKQKAFVEPY
FKGDERETSLQNFPHIEVVRKKEERRKLLGHTCKECEIYYADMPAEEREKKLASCSRHRF
RYIPPNTPENFWEVGFPSTQTCMERGYIKEDLDPCPRPKRRQPYNAIFSPKGKEQKT*
>ENSG00000100813|ENST00000357481
MLSESKEGEEKEEVTMDTSENRPENDVPEPPMPIADQVSNDDRPEGSVEDEEKKESSLPK
SFKRKISVVSATKGVPAGNSDTEGGQPGRKRRWGASTATTQKKPSISITTESLKSLIPDI
KPLAGQEAVVDLHADDSRISEDETERNGDDGTHDKGLKICRTVTQVVPAEGQENGQREEE
EEEKEPEAEPPVPPQVSVEVALPPPAEHEVKKVTLGDTLTRRSISQQKSGVSITIDDPVR
TAQVPSPPRGKISNIVHISNLVRPFTLGQLKELLGRTGTLVEEAFWIDKIKSHCFVTYST
VEEAVATRTALHGVKWPQSNPKFLCADYAEQDELDYHRGLLVDRPSETKTEEQGIPRPLH
PPPPPPVQPPQHPRAEQREQERAVREQWAEREREMERRERTRSEREWDRDKVREGPRSRS
RSRDRRRKERAKSKEKKSEKKEKAQEEPPAKLLDDLFRKTKAAPCIYWLPLTDSQIVQKE
AERAERAKEREKRRKEQEEEEQKEREKEAERERNRQLEREKRREHSRERDRERERERERD
RGDRDRDRERDRERGRERDRRDTKRHSRSRSRSTPVRDRGGRR*
>ENSG00000100227|ENST00000339677
MADISLDELIRKRGAAAKGRLNARPGVGGVRSRVGIQQGLLSQSTRTATFQQRFDARQKI
GLSDARLKLGVKDAREKLLQKDARFRIKGKVQDAREMLNSRKQQTTVPQKPRQVADAREK
ISLKRSSPAAFINPPIGTVTPALKLTKTIQVGLRAVPLLCLVSGGAWVGSRSQASCPLPS
GSRPH*
>ENSG00000029993|ENST00000325307

MAKGDPKKPKGKMSAYAFFVQTCREEHKKKNPEVPVNFAEFSKKCSERWKTMSGKEKSKF
DEMAKADKVRYDREMKDYGPAKGGKKKKDPNAPKRPPSGFFLFCSEFRPKIKSTNPGISI
GDVAKKLGEMWNNLNDSEKQPYITKAAKLKEKYEKDVADYKSKGKFDGAKGPAKVARKKV
EEEDEEEEEEEEEEEEEEEDE*
>ENSG00000124614|ENST00000326199
MLMPKKNRIAIYELLFKEGVMVAKKDVHMPKHPELADKNVPNLHVMKAMQSLKSRGYVKE
QFAWRHFYWYLTNEGIQYLRDYLHLPPEIVPATLRRSRPETGRPRPKGLEGERPARLTRG
EADRDTYRRSAVPPGADKKAEAGAGSATEFQFRGGFGRGRGQPPQ*
>ENSG00000146701|ENST00000315758
MLSALARPASAALRRSFSTSAQNNAKVAVLGASGGIGQPLSLLLKNSPLVSRLTLYDIAH
TPGVAADLSHIETKAAVKGYLGPEQLPDCLKGCDVVVIPAGVPRKPGMTRDDLFNTNATI
VATLTAACAQHCPEAMICVIANPVNSTIPITAEVFKKHGVYNPNKIFGVTTLDIVRANTF
VAELKGLDPARVNVPVIGGHAGKTIIPLISQCTPKVDFPQDQLTALTGRIQEAGTEVVKA
KAGAGSATLSMAYAGARFVFSLVDAMNGKEGVVECSFVKSQETECTYFSTPLLLGKKGIE
KNLGIGKVSSFEEKMISDAIPELKASIKKGEDFVKTLK*
>ENSG00000088002|ENST00000323090
MASPPPFHSQKLPGEYFRYKGVPFPVGLYSLESISLAENTQDVRDDDIFIITYPKSGTTW
MIEIICLILKEGDPSWIRSVPIWERAPWCETIVGAFSLPDQYSPRLMSSHLPIQIFTKAF
FSSKAKVIYMGRNPRDVVVSLYHYSKIAGQLKDPGTPDQFLRDFLKGEVQFGSWFDHIKG
WLRMKGKDNFLFITYEELQQDLQGSVERICGFLGRPLGKEALGSVVAHSTFSAMKANTMS
NYTLLPPSLLDHRRGAFLRKGVCGDWKNHFTVAQSEAFDRAYRKQMRGMPTFPWDEDPEE
DGSPDPEPSPEPEPKPSLEPNTSLEREPRPNSSPSPSPGQASETPHPRPS*
>ENSG00000062370|ENST00000354340
MVTFKDVAVVFTEEELGLLDSVQRKLYRDVMLENFRNLLLVAHQPFKPDLISQLEREEKL
LMVETETPRDGCSGRKNQQKMESIQEVTVSYFSPKELSSRQTWQQSAGGLIRCQDFLKVF
QGKNSQLQEQGNSLGQVWAGIPVQISEDKNYIFTHIGNGSNYIKSQGYPSWRAHHSWRKM
YLKESHNYQCRCQQISMKNHFCKCDSVSWLSHHNDKLEVHRKENYSCHDCGEDIMKVSLL
NQESIQTEEKPYPCTGYRKAFSNDSSSEVHQQFHLEGKPYTYSSCGKGCNYSSLLHIHQN
IEREDDIENSHLKSYQRVHTEEKPCKCGEYGENFNHCSPLNTYELIHTGEMSYRHNIYEK
AFSHSLDLNSIFRVHTRDEPHEYEENENVFNQSSCLQVHQKIHTEEKLYTDIEYGKSFIC
SSNLDIQHRVHMEENSYNSEECGNGFSLASHFQDLQIVHTKEQPYKRYVCSNSFSHNLYL
QGHPKIHIGEKPRKEHGNGFNWSSKLKDHQRVHTGQKPYKCNICGKGFNHRSVLNVHQRV
HTGEKPYKCEECDKGFSRSSYLQAHQRVHTGEKPYKCEECGKGFSRNSYLQGHQRVHTGE
KPYKCEECGKGFSRSSHLQGHQRVHTGEKPFKCEECGKGFSWSFNLQIHQRVHTGEKPYK
CEECGKGFSKASTLLAHQRVHTGEKPYQCDECGKSFSQRSYLQSHQSVHSGERPYICEVC
GKGFSQRAYLQGHQRVHTRVKPYKCEMCGKGFSQSSRLEAHRRVHTGGKPYKCEVCTKGF
SESSRLQAHQRVHVEGRPYKCEQCGKGFSGYSSLQAHHRVHTGEKPYKCEVCGKGFSQRS
NLQAHQRVHTGEKPYKCDACGKGFRWSSGLLIHQRVHSSDKFYKSEDYGKDYPSSENLHR
NEDSVLF*
>ENSG00000171574|ENST00000322834
MAGEAEGLVMFEDVTVYFSREEWGLLNVTQKGLYRDVMLENFALVSSLGLAPSRSPVFTQ
LEDDEQSWVPSWVDVTPVSRAEARRGFGLGPACSCLSGLRLLIALLCICAELSTVGDSVR
VLKCAHILKQMVCVEWRMREPILSI*
>ENSG00000153234|ENST00000339562

MPCVQAQYGSSPQGASPASQSYSYHSSGEYSSDFLTPEFVKFSMDLTNTEITATTSLPSF
STFMDNYSTGYDVKPPCLYQMPLSGQQSSIKVEDIQMHNYQQHSHLPPQSEEMMPHSGSV
YYKPSSPPTPTTPGFQVQHSPMWDDPGSLHNFHQNYVATTHMIEQRKTPVSRLSLFSFKQ
SPPGTPVSSCQMRFDGPLHVPMNPEPAGSHHVVDGQTFAVPNPIRKPASMGFPGLQIGHA
SQLLDTQVPSPPSRGSPSNEGLCAVCGDNAACQHYGVRTCEGCKGFFKRTVQKNAKYVCL
ANKNCPVDKRRRNRCQYCRFQKCLAVGMVKEVVRTDSLKGRRGRLPSKPKSPQEPSPPSP
PVSLISALVRAHVDSNPAMTSLDYSRFQANPDYQMSGDDTQHIQQFYDLLTGSMEIIRGW
AEKIPGFADLPKADQDLLFESAFLELFVLRLAYRSNPVEGKLIFCNGVVLHRLQCVRGFG
EWIDSIVEFSSNLQNMNIDISAFSCIAALAMVTERHGLKEPKRVEELQNKIVNCLKDHVT
FNNGGLNRPNYLSKLLGKLPELRTLCTQGLQRIFYLKLEDLVPPPAIIDKLFLDTLPF*
>ENSG00000187325|ENST00000341864
MESGKMAPPKNAPRDALVMAQILKDMGITEYEPRVINQMLEFAFRYVTTILDDAKIYSSH
AKKPNVDADDVRLAIQCRADQSFTSPPPRDFLLDIARQKNQTPLPLIKPYAGPRLPPDRY
CLTAPNYRLKSLIKKGPNQGRLVPRLSVGAVSSKPTTPTIATPQTVSVPNKVATPMSVTS
QRFTVQIPPSQSTPVKPVPATTAVQNVLINPSMIGPKNILITTNMVSSQNTANEANPLKR
KHEDDDDNDIM*
>ENSG00000154727|ENST00000354828
MTKREAEELIEIEIDGTEKAECTEESIVEQTYAPAECVSQAIDINEPIGNLKKLLEPRLQ
CSLDAHEICLQDIQLDPERSLFDQGVKTDGTVQLSVQVISYQGIEPKLNILEIVKPADTV
EVVIDPDAHHAESEAHLVEEAQVITLDGTKHITTISDETSEQVTRWAAALEGYRKEQERL
GIPYDPIQWSTDQVLHWVVWVMKEFSMTDIDLTTLNISGRELCSLNQEDFFQRVPRGEIL
WSHLELLRKYVLASQEQQMNEIVTIDQPVQIIPASVQSATPTTIKVINSSAKAAKVQRAP
RISGEDRSSPGNRTGNNGQIQLWQFLLELLTDKDARDCISWVGDEGEFKLNQPELVAQKW
GQRKNKPTMNYEKLSRALRYYYDGDMICKVQGKRFVYKFVCDLKTLIGYSAAELNRLVTE
CEQKKLAKMQLHGIAQPVTAVALATASLQTEKDN*
>ENSG00000199161|ENST00000362291
Sequence unavailable
>ENSG00000111206|ENST00000342628
MKTSPRRPLILKRRRLPLPVQNAPSETSEEEPKRSPAQQESNQAEASKEVAESNSCKFPA
GIKIINHPTMPNTQVVAIPNNANIHSIITALTAKGKESGSSGPNKFILISCGGAPTQPPG
LRPQTQTSYDAKRTEVTLETLGPKPAARDVNLPRPPGALCEQKRETCADGEAAGCTINNS
LSNIQWLRKMSSDGLGSRSIKQEMEEKENCHLEQRQVKVEEPSRPSASWQNSVSERPPYS
YMAMIQFAINSTERKRMTLKDIYTWIEDHFPYFKHIAKPGWKNSIRHNLSLHDMFVRETS
ANGKVSFWTIHPSANRYLTLDQVFKPLDPGSPQLPEHLESQQKRPNPELRRNMTIKTELP
LGARRKMKPLLPRVSSYLVPIQFPVNQSLVLQPSVKVPLPLAASLMSSELARHSKRVRIA
PKVFGEQVVFGYMSKFFSGDLRDFGTPITSLFNFIFLCLSVLLAEEGIAPLSSAGPGKEE
KLLFGEGFSPLLPVQTIKEEEIQPGEEMPHLARPIKVESPPLEEWPSPAPSFKEESSHSW
EDSSQSPTPRPKKSYSGLRSPTRCVSEMLVIQHRERRERSRSRRKQHLLPPCVDEPELLF
SEGPSTSRWAAELPFPADSSDPASQLSYSQEVGGPFKTPIKETLPISSTPSKSVLPRTPE
SWRLTPPAKVGGLDFSPVQTSQGASDPLPDPLGLMDLSTTPLQSAPPLESPQRLLSSEPL
DLISVPFGNSSPSDIDVPKPGSPEPQVSGLAANRSLTEGLVLDTMNDSLSKILLDISFPG
LDEDPLGPDNINWSQFIPELQ*
>ENSG00000126804|ENST00000358738
MAKPSHSSYVLQQLNNQREWGFLCDCCIAIDDIYFQAHKAVLAACSSYFRMFFMNHQHST

AQLNLSNMKISAECFDLILQFMYLGKIMTAPSSFEQFKVAMNYLQLYNVPDCLEDIQDAD
CSSSKCSSSASSKQNSKMIFGVRMYEDTVARNGNEANRWCAEPSSTVNTPHNREADEESL
QLGNFPEPLFDVCKKSSVSKLSTPKERVSRRFGRSFTCDSCGFGFSCEKLLDEHVLTCTN
RHLYQNTRSYHRIVDIRDGKDSNIKAEFGEKDSSKTFSAQTDKYRGDTSQAADDSASTTG
SRKSSTVESEIASEEKSRAAERKRIIIKMEPEDIPTDELKDFNIIKVTDKDCNESTDNDE
LEDEPEEPFYRYYVEEDVSIKKSGRKTLKPRMSVSADERGGLENMRPPNNSSPVQEDAEN
ASCELCGLTITEEDLSSHYLAKHIENICACGKCGQILVKGRQLQEHAQRCGEPQDLTMNG
LGNTEEKMDLEENPDEQSEIRDMFVEMLDDFRDNHYQINSIQKKQLFKHSACPFRCPNCG
QRFETENLVVEHMSSCLDQDMFKSAIMEENERDHRRKHFCNLCGKGFYQRCHLREHYTVH
TKEKQFVCQTCGKQFLRERQLRLHNDMHKGMASGEIGPSKPVEK*
>ENSG00000056586|ENST00000357244
MPVQAAQWTEFLSCPICYNEFDENVHKPISLGCSHTVCKTCLNKLHRKACPFDQTAINTD
IDVLPVNFALLQLVGAQVPDHQSIKLSNLGENKHYEVAKKCVEDLALYLKPLSGGKGVAS
LNQSALSRPMQRKLVTLVNCQLVEEEGRVRAMRAARSLGERTVTELILQHQNPQQLSANL
WAAVRARGCQFLGPAMQEEALKLVLLALEDGSALSRKVLVLFVVQRLEPRFPQASKTSIG
HVVQLLYRASCFKVTKRDEDSSLMQLKEEFRSYEALRREHDAQIVHIAMEAGLRISPEQW
SSLLYGDLAHKSHMQSIIDKLQSPESFAKSVQELTIVLQRTGDPANLNRLRPHLELLANI
DPNPDAVSPTWEQLENAMVAVKTVVHGLVDFIQNYSRKGHETPQPQPNSKYKTSMCRDLR
QQGGCPRGTNCTFAHSQEELEKYRLRNKKINATVRTFPLLNKVGVNNTVTTTAGNVISVI
GSTETTGKIVPSTNGISNAENSVSQLISRSTDSTLRALETVKKVGKVGANGQNAAGPSAD
SVTENKIGSPPKTPVSNVAATSAGPSNVGTELNSVPQKSSPFLTRVPVYPPHSENIQYFQ
DPRTQIPFEVPQYPQTGYYPPPPTVPAGVAPCVPRFVRSNNVPESSLPPASMPYADHYST
FSPRDRMNSSPYQPPPPQPYGPVPPVPSGMYAPVYDSRRIWRPPMYQRDDIIRSNSLPPM
DVMHSSVYQTSLRERYNSLDGYYSVACQPPSEPRTTVPLPREPCGHLKTSCEEQIRRKPD
QWAQYHTQKAPLVSSTLPVATQSPTPPSPLFSVDFRADFSESVSGTKFEEDHLSHYSPWS
CGTIGSCINAIDSEPKDVIANSNAVLMDLDSGDVKRRVHLFETQRRTKEEDPIIPFSDGP
IISKWGAISRSSRTGYHTTDPVQATASQGSATKPISVSDYVPYVNAVDSRWSSYGNEATS
SAHYVERDRFIVTDLSGHRKHSSTGDLLSLELQQAKSNSLLLQREANALAMQQKWNSLDE
GRHLTLNLLSKEIELRNGELQSDYTEDATDTKPDRDIELELSALDTDEPDGQSEPIEEIL
DIQLGISSQNDQLLNGMAVENGHPVQQHQKEPPKQKKQSLGEDHVILEEQKTILPVTSCF
SQPLPVSISNASCLPITTSVSAGNLILKTHVMSEDKNDFLKPVANGKMVNS*
>ENSG00000116791|ENST00000340866
MATGQKLMRAVRVFEFGGPEVLKLRSDIAVPIPKDHQVLIKVHACGVNPVETYIRSGTYS
RKPLLPYTPGSDVAGVIEAVGDNASAFKKGDRVFTSSTISGGYAEYALAADHTVYKLPEK
LDFKQGAAIGIPYFTAYRALIHSACVKAGESVLVHGASGGVGLAACQIARAYGLKILGTA
GTEEGQKIVLQNGAHEVFNHREVNYIDKIKKYVGEKGIDIIIEMLANVNLSKDLSLLSHG
GRVIVVGSRGTIEINPRDTMAKESSIIGVTLFSSTKEEFQQYAAALQAGMEIGWLKPVIG
SQYPLEKVAEAHENIIHGSGATGKMILLL*
>ENSG00000198300|ENST00000326441
MLPPKHLSATKPKKSWAPNLYELDSDLTKEPDVIIGEGPTDSEFFHQRFRNLIYVEFVGP
RKTLIKLRNLCLDWLQPETRTKEEIIELLVLEQYLTIIPEKLKPWVRAKKPENCEKLVTL
LENYKEMYQPEDDNNSDVTSDDDMTRNRRESSPPHSVHSFSDRDWDRRGRSRDMEPRDRW
SHTRNPRSRMPPRDLSLPVVAKTSFEMDREDDRDSRAYESRSQDAESYQNVVDLAEDRKP
HNTIQDNMENYRKLLSLVQLAEDDGHSHMTQGHSSRSKRSAYPSTSRGLKTMPEAKKSTH

RRGICEDESSHGVIMEKFIKDVSRSSKSGRARESSDRSQRFPRMSDDNWKDISLNKRESV
IQQRVYEGNAFRGGFRFNSTLVSRKRVLERKRRYHFDTDGKGSIHDQKGCPRKKPFECGS
EMRKAMSVSSLSSLSSPSFTESQPIDFGAMPYVCDECGRSFSVISEFVEHQIMHTRENLY
EYGESFIHSVAVSEVQKSQVGGKRFECKDCGETFNKSAALAEHRKIHARGYLVECKNQEC
EEAFMPSPTFSELQKIYGKDKFYECRVCKETFLHSSALIEHQKIHFGDDKDNEREHERER
ERERGETFRPSPALNEFQKMYGKEKMYECKVCGETFLHSSSLKEHQKIHTRGNPFENKGK
VCEETFIPGQSLKRRQKTYNKEKLCDFTDGRDAFMQSSELSEHQKIHSRKNLFEGRGYEK
SVIHSGPFTESQKSHTITRPLESDEDEKAFTISSNPYENQKIPTKENVYEAKSYERSVIH
SLASVEAQKSHSVAGPSKPKVMAESTIQSFDAINHQRVRAGGNTSEGREYSRSVIHSLVA
SKPPRSHNGNELVESNEKGESSIYISDLNDKRQKIPARENPCEGGSKNRNYEDSVIQSVF
RAKPQKSVPGEGSGEFKKDGEFSVPSSNVREYQKARAKKKYIEHRSNETSVIHSLPFGEQ
TFRPRGMLYECQECGECFAHSSDLTEHQKIHDREKPSGSRNYEWSVIRSLAPTDPQTSYA
QEQYAKEQARNKCKDFRQFFATSEDLNTNQKIYDQEKSHGEESQGENTDGEETHSEETHG
QETIEDPVIQGSDMEDPQKDDPDDKIYECEDCGLGFVDLTDLTDHQKVHSRKCLVDSREY
THSVIHTHSISEYQRDYTGEQLYECPKCGESFIHSSFLFEHQRIHEQDQLYSMKGCDDGF
IALLPMKPRRNRAAERNPALAGSAIRCLLCGQGFIHSSALNEHMRLHREDDLLEQSQMAE
EAIIPGLALTEFQRSQTEERLFECAVCGESFVNPAELADHVTVHKNEPYEYGSSYTHTSF
LTEPLKGAIPFYECKDCGKSFIHSTVLTKHKELHLEEEEEDEAAAAAAAAAQEVEANVHV
PQVVLRIQGLNVEAAEPEVEAAEPEVEAAEPEVEAAEPNGEAEGPDGEAAEPIGEAGQPN
GEAEQPNGDADEPDGAGIEDPEERAEEPEGKAEEPEGDADEPDGVGIEDPEEGEDQEIQV
EEPYYDCHECTETFTSSTAFSEHLKTHASMIIFEPANAFGECSGYIERASTSTGGANQAD
EKYFKCDVCGQLFNDRLSLARHQNTHTG*
>ENSG00000162711|ENST00000366496
MKMASTRCKLARYLEDLEDVDLKKFKMHLEDYPPQKGCIPLPRGQTEKADHVDLATLMID
FNGEEKAWAMAVWIFAAINRRDLYEKAKRDEPKWGSDNARVSNPTVICQEDSIEEEWMGL
LEYLSRISICKMKKDYRKKYRKYVRSRFQCIEDRNARLGESVSLNKRYTRLRLIKEHRSQ
QEREQELLAIGKTKTCESPVSPIKMELLFDPDDEHSEPVHTVVFQGAAGIGKTILARKMM
LDWASGTLYQDRFDYLFYIHCREVSLVTQRSLGDLIMSCCPDPNPPIHKIVRKPSRILFL
MDGFDELQGAFDEHIGPLCTDWQKAERGDILLSSLIRKKLLPEASLLITTRPVALEKLQH
LLDHPRHVEILGFSEAKRKEYFFKYFSDEAQARAAFSLIQENEVLFTMCFIPLVCWIVCT
GLKQQMESGKSLAQTSKTTTAVYVFFLSSLLQPRGGSQEHGLCAHLWGLCSLAADGIWNQ
KILFEESDLRNHGLQKADVSAFLRMNLFQKEVDCEKFYSFIHMTFQEFFAAMYYLLEEEK
EGRTNVPGSRLKLPSRDVTVLLENYGKFEKGYLIFVVRFLFGLVNQERTSYLEKKLSCKI
SQQIRLELLKWIEVKAKAKKLQIQPSQLELFYCLYEMQEEDFVQRAMDYFPKIEINLSTR
MDHMVSSFCIENCHRVESLSLGFLHNMPKEEEEEEKEGRHLDMVQCVLPSSSHAACSHGL
VNSHLTSSFCRGLFSVLSTSQSLTELDLSDNSLGDPGMRVLCETLQHPGCNIRRLWLGRC
GLSHECCFDISLVLSSNQKLVELDLSDNALGDFGIRLLCVGLKHLLCNLKKLWLVNSGLT
SVCCSALSSVLSTNQNLTHLYLRGNTLGDKGIKLLCEGLLHPDCKLQVLELDNCNLTSHC
CWDLSTLLTSSQSLRKLSLGNNDLGDLGVMMFCEVLKQQSCLLQNLGLSEMYFNYETKSA
LETLQEEKPELTVVFEPSW*
>ENSG00000122482|ENST00000347275
MLIRQNLALDCKQKKSRSRSGSKKKMLTLPHGADEVYILRCRFCGLVFRGPLSVQEDWIK
HLQRHIVNANLPRTGAGMVEVTSLLKKPASITETSFSLLMAEAAS*
>ENSG00000122566|ENST00000360787

MEKTLETVPLERKKREKEQFRKLFIGGLSFETTEESLRNYYEQWGKLTDCVVMRDPASKR
SRGFGFVTFSSMAEVDAAMAARPHSIDGRVVEPKRAVAREESGKPGAHVTVKKLFVGGIK
EDTEEHHLRDYFEEYGKIDTIEIITDRQSGKKRGFGFVTFDDHDPVDKIVLQKYHTINGH
NAEVRKALSRQEMQEVQSSRSGRGGNFGFGDSRGGGGNFGPGPGSNFRGGSDGYGSGRGF
GDGYNGYGGGPGGGNFGGSPGYGGGRGGYGGGGPGYGNQGGGYGGGYDNYGGGNYGSGNY
NDFGNYNQQPSNYGPMKSGNFGGSRNMGGPYGGGNYGPGGSGGSGGYGGRSRY*
>ENSG00000083312|ENST00000337273
MVWDRQTKMEYEWKPDEQGLQQILQLLKESQSPDTTIQRTVQQKLEQLNQYPDFNNYLIF
VLTKLKSEDEPTRSLSGLILKNNVKAHFQNFPNGVTDFIKSECLNNIGDSSPLIRATVGI
LITTIASKGELQNWPDLLPKLCSLLDSEDYNTCEGAFGALQKICEDSAEILDSDVLDRPL
NIMIPKFLQFFKHSSPKIRSHAVACVNQFIISRTQALMLHIDSFIENLFALAGDEEPEVR
KNVCRALVMLLEVRMDRLLPHMHNIVEYMLQRTQDQDENVALEACEFWLTLAEQPICKDV
LVRHLPKLIPVLVNGMKYSDIDIILLKGDVEEDETIPDSEQDIRPRFHRSRTVAQQHDED
GIEEEDDDDDEIDDDDTISDWNLRKCSAAALDVLANVYRDELLPHILPLLKELLFHHEWV
VKESGILVLGAIAEGCMQGMIPYLPELIPHLIQCLSDKKALVRSITCWTLSRYAHWVVSQ
PPDTYLKPLMTELLKRILDSNKRVQEAACSAFATLEEEACTELVPYLAYILDTLVFAFSK
YQHKNLLILYDAIGTLADSVGHHLNKPEYIQMLMPPLIQKWNMLKDEDKDLFPLLECLSS
VATALQSGFLPYCEPVYQRCVNLVQKTLAQAMLNNAQPDQYEAPDKDFMIVALDLLSGLA
EGLGGNIEQLVARSNILTLMYQCMQDKMPEVRQSSFALLGDLTKACFQHVKPCIADFMPI
LGTNLNPEFISVCNNATWAIGEISIQMGIEMQPYIPMVLHQLVEIINRPNTPKTLLENTA
ITIGRLGYVCPQEVAPMLQQFIRPWCTSLRNIRDNEEKDSAFRGICTMISVNPSGVIQDF
IFFCDAVASWINPKDDLRDMFCKILHGFKNQVGDENWRRFSDQFPLPLKERLAAFYGV*
>ENSG00000085274|ENST00000356716
MQYSHHCEHLLERLNKQREAGFLCDCTIVIGEFQFKAHRNVLASFSEYFGAIYRSTSENN
VFLDQSQVKADGFQKLLEFIYTGTLNLDSWNVKEIHQAADYLKVEEVVTKCKIKMEDFAF
IANPSSTEISSITGNIELNQQTCLLTLRDYNNREKSEVSTDLIQANPKQGALAKKSSQTK
KKKKAFNSPKTGQNKTVQYPSDILENASVELFLDANKLPTPVVEQVAQINDNSELELTSV
VENTFPAQDIVHTVTVKRKRGKSQPNCALKEHSMSNIASVKSPYEAENSGEELDQRYSKA
KPMCNTCGKVFSEASSLRRHMRIHKGVKPYVCHLCGKAFTQCNQLKTHVRTHTGEKPYKC
ELCDKGFAQKCQLVFHSRMHHGEEKPYKCDVCNLQFATSSNLKIHARKHSGEKPYVCDRC
GQRFAQASTLTYHVRRHTGEKPYVCDTCGKAFAVSSSLITHSRKHTGEKPYICGICGKSF
ISSGELNKHFRSHTGERPFICELCGNSYTDIKNLKKHKTKVHSGADKTLDSSAEDHTLSE
QDSIQKSPLSETMDVKPSDMTLPLALPLGTEDHHMLLPVTDTQSPTSDTLLRSTVNGYSE
PQLIFLQQLY*
>ENSG00000149187|ENST00000358597
MNGTLDHPDQPDLDAIKMFVGQVPRTWSEKDLRELFEQYGAVYEINVLRDRSQNPPQSKG
CCFVTFYTRKAALEAQNALHNMKVLPGMHHPIQMKPADSEKNNAVEDRKLFIGMISKKCT
ENDIRVMFSSFGQIEECRILRGPDGLSRGCAFVTFTTRAMAQTAIKAMHQAQTMEGCSSP
MVVKFADTQKDKEQKRMAQQLQQQMQQISAASVWGNLAGLNTLGPQYLALYLQLLQQTAS
SGNLNTLSSLHPMGGLNAMQLQNLAALAAAASAAQNTPSGTNALTTSSSPLSVLTSSGSS
PSSSSSNSVNPIASLGALQTLAGATAGLNVGSLAGMAALNGGLGSSGLSNGTGSTMEALT
QAYSGIQQYAAAALPTLYNQNLLTQQSIGAAGSQKEGPEGANLFIYHLPQEFGDQDLLQM
FMPFGNVVSAKVFIDKQTNLSKCFGFVSYDNPVSAQAAIQSMNGFQIGMKRLKVQLKRSK
NDSKPY*

>ENSG00000159082|ENST00000357345
MAFSKGFRIYHKLDPPPFSLIVETRHKEECLMFESGAVAVLSSAEKEAIKGTYSKVLDAY
GLLGVLRLNLGDTMLHYLVLVTGCMSVGKIQESEVFRVTSTEFISLRIDSSDEDRISEVR
KVLNSGNFYFAWSASGISLDLSLNAHRSMQEQTTDNRFFWNQSLHLHLKHYGVNCDDWLL
RLMCGGVEIRTIYAAHKQAKACLISRLSCERAGTRFNVRGTNDDGHVANFVETEQVVYLD
DSVSSFIQIRGSVPLFWEQPGLQVGSHRVRMSRGFEANAPAFDRHFRTLKNLYGKQIIVN
LLGSKEGEHMLSKAFQSHLKASEHAADIQMVNFDYHQMVKGGKAEKLHSVLKPQVQKFLD
YGFFYFNGSEVQRCQSGTVRTNCLDCLDRTNSVQAFLGLEMLAKQLEALGLAEKPQLVTR
FQEVFRSMWSVNGDSISKIYAGTGALEGKAKLKDGARSVTRTIQNNFFDSSKQEAIDVLL
LGNTLNSDLADKARALLTTGSLRVSEQTLQSASSKVLKSMCENFYKYSKPKKIRVCVGTW
NVNGGKQFRSIAFKNQTLTDWLLDAPKLAGIQEFQDKRSKPTDIFAIGFEEMVELNAGNI
VSASTTNQKLWAVELQKTISRDNKYVLLASEQLVGVCLFVFIRPQHAPFIRDVAVDTVKT
GMGGATGNKGAVAIRMLFHTTSLCFVCSHFAAGQSQVKERNEDFIEIARKLSFPMGRMLF
SHDYVFWCGDFNYRIDLPNEEVKELIRQQNWDSLIAGDQLINQKNAGQVFRGFLEGKVTF
APTYKYDLFSDDYDTSEKCRTPAWTDRVLWRRRKWPFDRSAEDLDLLNASFQDESKILYT
WTPGTLLHYGRAELKTSDHRPVVALIDIDIFEVEAEERQNIYKEVIAVQGPPDGTVLVSI
KSSLPENNFFDDALIDELLQQFASFGEVILIRFVEDKMWVTFLEGSSALNVLSLNGKELL
NRTITIALKSPDWIKNLEEEMSLEKISIALPSSTSSTLLGEDAEVAADFDMEGDVDDYSA
EVEELLPQHLQPSSSSGLGTSPSSSPRTSPCQSPTISEGPVPSLPIRPSRAPSRTPGPPS
AQSSPIDAQPATPLPQKDPAQPLEPKRPPPPRPVAPPTRPAPPQRPPPPSGARSPAPTRK
EFGAPKSPGTTRKDNIGRSQPSPQAGLAGPGPAGYSTARPTIPPRAGVISAPQSHARASA
GRLTPESQSKTSETSKGSTFLPEPLKPQAAFPPQSSLPPPAQRLQEPLVPVAAPMPQSGP
QPNLETPPQPPPRSRSSHSLPSEASSQPQQEQPSG*
>ENSG00000054118|ENST00000354618
MSKTNKSKSGSRSSRSRSASRSRSRSFSKSRSRSRSLSRSRKRRLSSRSRSRSYSPAHNR
ERNHPRVYQNRDFRGHNRGYRRPYYFRGRNRGFYPWGQYNRGGYGNYRSNWQNYRQAYSP
RRGRSRSRSPKRRSPSPRSRSHSRNSDKSSSDRSRRSSSSRSSSNHSRVESSKRKSAKEK
KSSSKDSRPSQAAGDNQGDEAKEQTFSGGTSQDTKASESSKPWPDATYGTGSASRASAVS
ELSPRERSPALKSPLQSVVVRRRSPRPSPVPKPSPPLSSTSQMGSTLPSGAGYQSGTHQG
QFDHGSGSLSPSKKSPVGKSPPSTGSTYGSSQKEESAASGGAAYTKRYLEEQKTENGKDK
EQKQTNTDKEKIKEKGSFSDTGLGDGKMKSDSFAPKTDSEKPFRGSQSPKRYKLRDDFEK
KMADFHKEEMDDQDKDKAKGRKESEFDDEPKFMSKVIGANKNQEEEKSGKWEGLVYAPPG
KEKQRKTEELEEESFPERSKKEDRGKRSEGGHRGFVPEKNFRVTAYKAVQEKSSSPPPRK
TSESRDKLGAKGDFPTGKSSFSITREAQVNVRMDSFDEDLARPSGLLAQERKLCRDLVHS
NKKEQEFRSIFQHIQSAQSQRSPSELFAQHIVTIVHHVKEHHFGSSGMTLHERFTKYLKR
GTEQEAAKNKKSPEIHRRIDISPSTFRKHGLAHDEMKSPREPGYKAEGKYKDDPVDLRLD
IERRKKHKERDLKRGKSRESVDSRDSSHSRERSAEKTEKTHKGSKKQKKHRRARDRSRSS
SSSSQSSHSYKAEEYTEETEEREESTTGFDKSRLGTKDFVGPSERGGGRARGTFQFRARG
RGWGRGNYSGNNNNNSNNDFQKRNREEEWDPEYTPKSKKYYLHDDREGEGSDKWVSRGRG
RGAFPRGRGRFMFRKSSTSPKWAHDKFSGEEGEIEDDESGTENREEKDNIQPTTE*
>ENSG00000170004|ENST00000330494
MKAADTVILWARSKNDQLRISFPPGLCWGDRMPDKDDIRLLPSALGVKKRKRGPKKQKEN
KPGKPRKRKKRDSEEEFGSERDEYREKSESGGSEYGTGPGRKRRRKHREKKEKKTKRRKK
GEGDGGQKQVEQKSSATLLLTWGLEDVEHVFSEEDYHTLTNYKAFSQFMRPLIAKKNPKI

PMSKMMTILGAKWREFSANNPFKGSAAAVAAAAAAAAAAVAEQVSAAVSSATPIAPSGPP
ALPPPPAADIQPPPIRRAKTKEGKGPGHKRRSKSPRVPDGRKKLRGKKMAPLKIKLGLLG
GKRKKGGSYVFQSDEGPEPEAEESDLDSGSVHSASGRPDGPVRTKKLKRGRPGRKKKKVL
GCPAVAGEEEVDGYETDHQDYCEVCQQGGEIILCDTCPRAYHLVCLDPELDRAPEGKWSC
PHCEKEGVQWEAKEEEEEYEEEGEEEGEKEEEDDHMEYCRVCKDGGELLCCDACISSYHI
HCLNPPLPDIPNGEWLCPRCTCPVLKGRVQKILHWRWGEPPVAVPAPQQADGNPDVPPPR
PLQGRSEREFFVKWVGLSYWHCSWAKELQLEIFHLVMYRNYQRKNDMDEPPPLDYGSGED
DGKSDKRKVKDPHYAEMEEKYYRFGIKPEWMTVHRIINHSVDKKGNYHYLVKWRDLPYDQ
STWEEDEMNIPEYEEHKQSYWRHRELIMGEDPAQPRKYKKKKKELQGDGPPSSPTNDPTV
KYETQPRFITATGGTLHMYQLEGLNWLRFSWAQGTDTILADEMGLGKTIQTIVFLYSLYK
EGHTKGPFLVSAPLSTIINWEREFQMWAPKFYVVTYTGDKDSRAIIRENEFSFEDNAIKG
GKKAFKMKREAQVKFHVLLTSYELITIDQAALGSIRWACLVVDEAHRLKNNQSKFFRVLN
GYKIDHKLLLTGTPLQNNLEELFHLLNFLTPERFNNLEGFLEEFADISKEDQIKKLHDLL
GPHMLRRLKADVFKNMPAKTELIVRVELSPMQKKYYKYILTRNFEALNSRGGGNQVSLLN
IMMDLKKCCNHPYLFPVAAMESPKLPSGAYEGGALIKSSGKLMLLQKMLRKLKEQGHRVL
IFSQMTKMLDLLEDFLDYEGYKYERIDGGITGALRQEAIDRFNAPGAQQFCFLLSTRAGG
LGINLATADTVIIFDSDWNPHNDIQAFSRAHRIGQANKVMIYRFVTRASVEERITQVAKR
KMMLTHLVVRPGLGSKAGSMSKQELDDILKFGTEELFKDENEGENKEEDSSVIHYDNEAI
ARLLDRNQDATEDTDVQNMNEYLSSFKVAQYVVREEDKIEEIEREIIKQEENVDPDYWEK
LLRHHYEQQQEDLARNLGKGKRVRKQVNYNDAAQEDQDNQSEYSVGSEEEDEDFDERPEG
RRQSKRQLRNEKDKPLPPLLARVGGNIEVLGFNTRQRKAFLNAVMRWGMPPQDAFTTQWL
VRDLRGKTEKEFKAYVSLFMRHLCEPGADGSETFADGVPREGLSRQQVLTRIGVMSLVKK
KVQEFEHINGRWSMPELMPDPSADSKRSSRASSPTKTSPTTPEASATNSPCTSKPATPAP
SEKGEGIRTPLEKEEAENQEEKPEKNSRIGEKMETEADAPSPAPSLGERLEPRKIPLEDE
VPGVPGEMEPEPGYRGDREKSATESTPGERGEEKPLDGQEHRERPEGETGDLGKREDVKG
DRELRPGPRDEPRSNGRREEKTEKPRFMFNIADGGFTELHTLWQNEERAAISSGKLNEIW
HRRHDYWLLAGIVLHGYARWQDIQNDAQFAIINEPFKTEANKGNFLEMKNKFLARRFKLL
EQALVIEEQLRRAAYLNLSQEPAHPAMALHARFAEAECLAESHQHLSKESLAGNKPANAV
LHKVLNQLEELLSDMKADVTRLPATLSRIPPIAARLQMSERSILSRLASKGTEPHPTPAY
PPGPYATPPGYGAAFSAAPVGALAAAGANYSQMPAGSFITAATNGPPVLVKKEKEMVGAL
VSDGLDRKEPRAGEVICIDD*
>ENSG00000125618|ENST00000348715
MPHNSIRSGHGGLNQLGGAFVNGRPLPEVVRQRIVDLAHQGVRPCDISRQLRVSHGCVSK
ILGRYYETGSIRPGVIGGSKPKVATPKVVEKIGDYKRQNPTMFAWEIRDRLLAEGVCDND
TVPSVSSINRIIRTKVQQPFNLPMDSCVATKSLSPGHTLIPSSAVTPPESPQSDSLGSTY
SINGLLGIAQPGSDKRKMDDSDQDSCRLSIDSQSSSSGPRKHLRTDAFSQHHLEPLECPF
ERQHYPEAYASPSHTKGEQGLYPLPLLNSTLDDGKATLTPSNTPLGRNLSTHQTYPVVAA
PPFWICSKSAPGSRPSMPFPMLPPCTGSSRARPSSQGERWWGPRCPDTHPTSPPADRAAM
PPLPSQAWWQEVNTLAMPMATPPTPPTARPGASPTPAC*
>ENSG00000052749|ENST00000315563
MGRSGKLPSGVSAKLKRWKKGHSSDSNPAICRHRQAARSRFFSRPSGRSDLTVDAVKLHN
ELQSGSLRLGKSEAPETPMEEEAELVLTEKSSGTFLSGLSDCTNVTFSKVQRFWESNSAA
HKEICAVLAAVTEVIRSQGGKETETEYFAALIRKAAQHGVCSVLKGSEFMFEKAPAHHPA
AISTAKFCIQEIEKSGGSKEATTTLHMLTLLKDLLPCFPEGLVKSCSETLLRVMTLSHVL

VTACAMQAFHSLFHARPGLSTLSAELNAQIITALYDYVPSENDLQPLLAWLKVMEKAHIN
LVRLQWDLGLGHLPRFFGTAVTCLLSPHSQVLTAATQSLKEILKECVAPHMADIGSVTSS
ASGPAQSVAKMFRAVEEGLTYKFHAAWSSVLQLLCVFFEACGRQAHPVMRKCLQSLCDLR
LSPHFPHTAALDQAVGAAVTSMGPEVVLQAVPLEIDGSEETLDFPRSWLLPVIRDHVQET
RLGFFTTYFLPLANTLKSKAMDLAQAGSTVESKIYDTLQWQMWTLLPGFCTRPTDVAISF
KGLARTLGMAISERPDLRVTVCQALRTLITKGCQAEADRAEVSRFAKNFLPILFNLYGQP
VAAGDTPAPRRAVLETIRTYLTITDTQLVNSLLEKASEKVLDPASSDFTRLSVLDLVVAL
APCADEAAISKLYSTIRPYLESKAHGVQKKAYRVLEEVCASPQGPGALFVQSHLEDLKKT
LLDSLRSTSSPAKRPRLKCLLHIVRKLSAEHKEFITALIPEVILCTKEVSVGARKNAFAL
LVEMGHAFLRFGSNQEEALQCYLVLIYPGLVGAVTMVSCSILALTHLLFEFKGLMGTSTV
EQLLENVCLLLASRTRDVVKSALGFIKVAVTVMDVAHLAKHVQLVMEAIGKLSDDMRRHF
RMKLRNLFTKFIRKFGFELVKRLLPEEYHRVLVNIRKAEARAKRHRALSQAAVEEEEEEE
EEEEPAQGKGDSIEEILADSEDEEDNEEEERSRGKEQRKLARQRSRAWLKEGGGDEPLNF
LDPKVAQRVLATQPGPGRGRKKDHGFKVSADGRLIIREEADGNKMEEEEGAKGEDEEMAD
PMEDVIIRNKKHQKLKHQKEAEEEELEIPPQYQAGGSGIHRPVAKKAMPGAEYKAKKAKG
DVKKKGRPDPYAYIPLNRSKLNRRKKMKLQGQFKGLVKAARRGSQVGHKNRRKDRRP*
>ENSG00000242389|ENST00000358944
MVEADHPGKLFIGGLNRETNEKMLKAVFGKHGPISEVLLIKDRTSKSRGFAFITFENPAD
AKNAAKDMNGKMMVDTLLISR*
>ENSG00000184271|ENST00000333640
MDPGAGSETSLTVNEQVIVMSGHETIRVLEVGVDAQLPAEEESKGLEGVAAEGSQSGDPA
EASQAAGEAGPDNLGSSAEATVKSPPGIPPSPATAIATFSQAPSQPQASQTLTPLAVQAA
PQVLTQENLATVLTGVMVPAGAVTQPLLIPISIAGQVAGQQGLAVWTIPTATVAALPGLT
AASPTGGVFKPPLAGLQAAAVLNTALPAPVQAAAPVQASSTAQPRPPAQPQTLFQTQPLL
QTTPAILPQPTAATAAAPTPKPVDTPPQITVQPAGFAFSPGIISAASLGGQTQILGSLTT
APVITSAIPSMPGISSQILTNAQGQVIGTLPWVVNSASVAAPAPAQSLQVQAVTPQLLLN
AQGQVIATLASSPLPPPVAVRKPSTPESPAKSEVQPIQPTPTVPQPAVVIASPAPAAKPS
ASAPIPITCSETPTVSQLVSKPHTPSLDEDGINLEEIREFAKNFKIRRLSLGLTQTQVGQ
ALTATEGPAYSQSAICRFEKLDITPKSAQKLKPVLEKWLNEAELRNQEGQQNLMEFVGGE
PSKKRKRRTSFTPQAIEALNAYFEKNPLPTGQEITEIAKELNYDREVVRVWFCNRRQTLK
NTSKLNVFQIP*
>ENSG00000175931|ENST00000319380
MADPAAPTPAAPAPAQAPAPAPEAVPAPAAAPVPAPAPASDSASGPSSDSGPEAGSQRLL
FSHDLVSGRYRGSVHFGLVRLIHGEDSDSEGEEEGRGSSGCSEAGGAGHEEGRASPLRRG
YVRVQWYPEGVKQHVKETKLKLEDRSVVPRDVVRHMRSTDSQCGTVIDVNIDCAVKLIGT
NCIIYPVNSKDLQHIWPFMYGDYIAYDCWLGKVYDLKNQIILKLSNGARCSMNTEDGAKL
YDVCPHVSDSGLFFDDSYGFYPGQVLIGPAKIFSSVQWLSGVKPVLSTKSKFRVVVEEVQ
VVELKVTWITKSFCPGGTDSVSPPPSVITQENLGRVKRLGCFDHAQRQLGERCLYVFPAK
VEPAKIAWECPEKNCAQGEGSMAKKVKRLLKKQVVRIMSCSPDTQCSRDHSMEDPDKKGE
SKTKSEAESASPEETPDGSASPVEMQDEGAEEPHEAGEQLPPFLLKEGRDDRLHSAEQDA
DDEAADDTDDTSSVTSSASSTTSSQSGSGTSRKKSIPLSIKNLKRKHKRKKNKITRDFKP
GDRVAVEVVTTMTSADVMWQDGSVECNIRSNDLFPVHHLDNNEFCPGDFVVDKRVQSCPD
PAVYGVVQSGDHIGRTCMVKWFKLRPSGDDVELIGEEEDVSVYDIADHPDFRFRTTDIVI
RIGNTEDGAPHKEDEPSVGQVARVDVSSKVEVVWADNSKTIILPQHLYNIESEIEESDYD

SVEGSTSGASSDEWEDDSDSWETDNGLVEDEHPKIEEPPIPPLEQPVAPEDKGVVISEEA
ATAAVQGAVAMAAPMAGLMEKAGKDGPPKSFRELKEAIKILESLKNMTVEQLLTGSPTSP
TVEPEKPTREKKFLDDIKKLQENLKKTLDNVAIVEEEKMEAVPDVERKEDKPEGQSPVKA
EWPSETPVLCQQCGGKPGVTFTSAKGEVFSVLEFAPSNHSFKKIEFQPPEAKKFFSTVRK
EMALLATSLPEGIMVKTFEDRMDLFSALIKGPTRTPYEDGLYLFDIQLPNIYPAVPPHFC
YLSQCSGRLNPNLYDNGKVCVSLLGTWIGKGTERWTSKSSLLQVLISIQGLILVNEPYYN
EAGFDSDRGLQEGYENSRCYNEMALIRVVQSMTQLVRRPPEVFEQEIRQHFSTGGWRLVN
RIESWLETHALLEKAQALPNGVPKASSSPEPPAVAELSDSGQQEPEDGGPAPGEASQGSD
SEGGAQGLASASRDHTDQTSETAPDASVPPSVKPKKRRKSYRSFLPEKSGYPDIGFPLFP
LSKGFIKSIRGVLTQFRAALLEAGMPECTEDK*
>ENSG00000178177|ENST00000326877
MDKGRERMAAAAAAAAAAAAAAAQCRSPRCAAERRGFRRELDSWRHRLMHCVGFESILEGL
YGPRLRRDLSLFEDCEPEELTDWSMDEKCSFCNLQREAVSDCIPSLDSSQSTPTEELSSQ
GQSNTDKIECQAENYLNALFRKKDLPQNCDPNIPLVAQELMKKMIRQFAIEYISKSGKTQ
ENRNGSIGPSIVCKSIQMNQAENSLQEEQEGPLDLTVNRMQEQNTQQGDGVLDLSTKKTS
IKSEESSICDPSSENSVAGMLQVKTDEKLNVSDENTASCPLSPIKMCLNRPIEWNLNLTT
ASLTSCTVHNQNLKSEEK*
>ENSG00000171476|ENST00000317745
MSAETASGPTEDQVEILEYNFNKVDKHPDSTTLCLIAAEAGLSEEETQKWFKQRLAKWRR
SEGLPSECRSVTD*
>ENSG00000172578|ENST00000341319
MLMAGQRGAWTMGDVVEKSLEGPLAPSTDEPSQKTGDLVEILNGEKVKFDDAGLSLILQN
GLETLRMENALTDVILCVDIQEFSCHRVVLAAASNYFRAMFCNDLKEKYEKRIIIKGVDA
ETMHTLLDYTYTSKALITKQNVQRVLEAANLFQFLRMVDACASFLTEALNPENCVGILRL
ADTHSLDSLKKQVQSYIIQNFVQILNSEEFLDLPVDTLHHILKSDDLYVTEEAQVFETVM
SWVRHKPSERLCLLPYVLENVRLPLLDPWYFVETVEADPLIRQCPEVFPLLQEARMYHLS
GNEIISERTKPRMHEFQSEVFMIIGGCTKDERFVAEVTCLDPLRRSRLEVAKLPLTEHEL
ESENKKWVEFACVTLKNEVYISGGKETQHDVWKYNSSINKWIQIEYLNIGRWRHKMVVLG
GKVYVIGGFDGLQRINNVETYDPFHNCWSEAAPLLVHVSSFAATSHKKKLYVIGGGPNGK
LATDKTQCYDPSTNKWSLKAAMPVEAKCINAVSFRDRIYVVGGAMRALYAYSPLEDSWCL
VTQLSHERASCGIAPCNNRLYITGGRDEKNEVIATVLCWDPEAQKLTEECVLPRGVSHHG
SVTIRKSYTHIRRIVPGAVSV*
>ENSG00000161547|ENST00000359995
MSYGRPPPDVEGMTSLKVDNLTYRTSPDTLRRVFEKYGRVGDVYIPRDRYTKESRGFAFV
RFHDKRDAEDAMDAMDGAVLDGRELRVQMARYGRPPDSHHSRRGPPPRRYGGGGYGRRSR
SPRRRRRSRSRSRSRSRSRSRSRYSRSKSRSRTRSRSRSTSKSRSARRSKSKSSSVSRSR
SRSRSRSRSRSPPPVSKRESKSRSRSKSPPKSPEEEGAVSS*
>ENSG00000123297|ENST00000323833
MSLLRSLRVFLVARTGSYPAGSLLRQSPQPRHTFYAGPRLSASASSKELLMKLRRKTGYS
FVNCKKALETCGGDLKQAEIWLHKEAQKEGWSKAAKLQGRKTKEGLIGLLQEGNTTVLVE
VNCETDFVSRNLKFQLLVQQVALGTMMHCQTLKDQPSAYSKVQWLTPVNLALWEAEAGGS
LEGFLNSSELSGLPAGPDREGSLKDQLALAIGKLGENMILKRAAWVKVPSGFYVGSYVHG
AMQSPSLHKLVLGKYGALVICETSEQKTNLEDVGRRLGQHVVGMAPLSVGSLDDEPGGEA
ETKMLSQPYLLDPSITLGQYVQPQGVSVVDFVRFECGEGEEAAETE*

>ENSG00000177483|ENST00000316997
MMQATAVVETASGKGYHSNGGNLQKDKPSNPKKENLLLSSNGCDEVKLTFPDDDWNSSTL
EQRANNKEISNIDKMDLLEPFFSVSQDTNTESTQFQSSELEDSTDYAFLNKTYSIPYSES
KLKKESLTPLSSELDPEVQKKEEVFFNILEHQDKTVGLERIYNISDANYRESAEDTQKHD
TDEDSQQEYHSAEEQEYISNHLSFDQTKALDISNPEVVELGNSGYEVKCASNVEDNRVNS
GSGSIISFDSLDVYGQEESLHVSKFQNSVMLREYHDLKHEKYKEQETNSMYHTVFDGSVL
RSNSPGNQESQSKSGSLSPQKVLKMKIYTENMKSQINEGKDFCGNKIVENKILLHLENPS
TLPQDKALETLLQPCKDCQTSWTSVFDDSIISACGYYESLQNTADSALDFSAMLPKIAVR
DNQAIEDNTSLKVAHSSTTKKTCFHNIGEMCTKSLTDAASCTVTINQTVDVSTDFRACFT
TSRATSARPSVVSTSSNTEITMMNKKRPDEWQNEKQKSVACSTDWSYSEDCIDTQMAITK
GSGKSLSVDSLKPNGNFLNKDFLELRKACGITDLKKHPEREFQLFKDTEKDLPSMCCQKI
MQRAIKAELHLLNVHYQMCRRHCCDIYKLVMENREGLNMNLSSNSAKKELGSALLSLLGD
LKVRYVTLKEKIHKGIPLEELPPLSLESKLLSTFSTFASRLMKKETHVFSEADAEQDNQR
AHDVDVSSNLKKTLSQMSLSSDNSHATQNISPKKDDFKNGDINADFSQLKLGDKDCRHYQ
ETSEDWSDAKESLTGVDVSGTQGNQVEQDTWNLDLTGEMKNVEPSQRDKGYLIHVGGLCP
SVSEADLRSHFQKYQVSEISIYDSTNYRYASLAFTKNSDAKIAVKEMNGIEINGKSVNVW
PVKILGEYTSPLSSKNGNRISSNNLEKSTNKQIHSEFSISRLPRTRPRQLGSEQDSEVFP
SDQGVKKNCKQIESAKLLPDTPVQFIPPNTLNLRSFTKIIKRLAELHPEVSRDHIINALQ
EVRIRHKGFLNGLSITTIVEMTSSLLKNSASS*
>ENSG00000135269|ENST00000358204
MDLENKVKKMGLGHEQGFGAPCLKCKEKCEGFELHFWRKICRNCKCGQEEHDVLLSNEED
RKVGKLFEDTKYTTLIAKLKSDGIPMYKRNVMILTNPVAAKKNVSINTVTYEWAPPVQNQ
ALARQYMQMLPKEKQPVAGSEGAQYRKKQLAKQLPAHDQDPSKCHELSPREVKEMEQFVK
KYKSEALGVGDVKLPCEMDAQGPKQMNIPGGDRSTPAAVGAMEDKSAEHKRTQYSCYCCK
LSMKEGDPAIYAERAGYDKLWHPACFVCSTCHELLVDMIYFWKNEKLYCGRHYCDSEKPR
CAGCDELIFSNEYTQAENQNWHLKHFCCFDCDSILAGEIYVMVNDKPVCKPCYVKNHAVV
CQGCHNAIDPEVQRVTYNNFSWHASTECFLCSCCSKCLIGQKFMPVEGMVFCSVECKKRM
S*
>ENSG00000128191|ENST00000351989
METDESPSPLPCGPAGEAVMESRARPFQALPREQSPPPPLQTSSGAEVMDVGSGGDGQSE
LPAEDPFNFYGASLLSKGSFSKGRLLIDPNCSGHSPRTARHAPAVRKFSPDLKLLKDVKI
SVSFTESCRSKDRKVLYTGAERDVRAECGLLLSPVSGDVHACPFGGSVGDGVGIGGESAD
KKDEENELDQEKRVEYAVLDELEDFTDNLELDEEGAGGFTAKAIVQRDRVDEEALNFPYE
DDFDNDVDALLEEGLCAPKKRRTEEKYGGDSDHPSDGETSVQPMMTKIKTVLKSRGRPPT
EPLPDGWIMTFHNSGVPVYLHRESRVVTWSRPYFLGTGSIRKHDPPLSSIPCLHYKKMKD
NEEREQSSDLTPSGDVSPVKPLSRSAELEFPLDEPDSMGADPGPPDEKDPLGAEAAPGAL
GQVKAKVEVCKDESVDLEEFRSYLEKRFDFEQVTVKKFRTWAERRQFNREMKRKQAESER
PILPANQKLITLSVQDAPTKKEFVINPNGKSEVCILHEYMQRVLKVRPVYNFFECENPSE
PFGASVTIDGVTYGSGTASSKKLAKNKAARATLEILIPDFVKQTSEEKPKDSEELEYFNH
ISIEDSRVYELTSKAGLLSPYQILHECLKRNHGMGDTSIKFEVVPGKNQKSEYVMACGKH
TVRGWCKNKRVGKQLASQKILQLLHPHVKNWGSLLRMYGRESSKMVKQETSDKSVIELQQ
YAKKNKPNLHILSKLQEEMKRLAEEREETRKKPKMSIVASAQPGGEPLCTVDV*
>ENSG00000198517|ENST00000343242
MTTNPKPNKALKVKKEAGENAPVLSDDELVSMSVRELNQHLRGLTKEEVTRLKQRRRTLK

NRGYAASCRIKRVTQKEELERQRVELQQEVEKLARENSSMRLELDALRSKYEALQTFART
VARGPVAPSKVATTSVITIVKSTELSSTSVPFSAAS*
>ENSG00000148516|ENST00000361642
MADGPRCKRRKQANPRRNNVTNYNTVVETNSDSDDEDKLHIVEEESVTDAADCEGVPEDD
LPTDQTVLPGRSSEREGNAKNCWEDDTGKEGQEILGPEAQADEAGCTVKDDECESDAENE
QNHDPNVEEFLQQQDTAVIFPEAPEEDQRQGTPEASGHDENGTPDAFSQLLTCPYCDRGY
KRFTSLKEHIKYRHEKNEDNFSCSLCSYTFAYRTQLERHMTSHKSGRDQRHVTQSGCNRK
FKCTECGKAFKYKHHLKEHLRIHSGEKPYECPNCKKRFSHSGSYSSHISSKKCISLIPVN
GRPRTGLKTSQCSSPSLSASPGSPTRPQIRQKIENKPLQEQLSVNQIKTEPVDYEFKPIV
VASGINCSTPLQNGVFTGGGPLQATSSPQGMVQAVVLPTVGLVSPISINLSDIQNVLKVA
VDGNVIRQVLENNQANLASKEQETINASPIQQGGHSVISAISLPLVDQDGTTKIIINYSL
EQPSQLQVVPQNLKKENPVATNSCKSEKLPEDLTVKSEKDKSFEGGVNDSTCLLCDDCPG
DINALPELKHYDLKQPTQPPPLPAAEAEKPESSVSSATGDGNLSPSQPPLKNLLSLLKAY
YALNAQPSAEELSKIADSVNLPLDVVKKWFEKMQAGQISVQSSEPSSPEPGKVNIPAKNN
DQPQSANANEPQDSTVNLQSPLKMTNSPVLPVGSTTNGSRSSTPSPSPLNLSSSRNTQGY
LYTAEGAQEEPQVEPLDLSLPKQQGELLERSTITSVYQNSVYSVQEEPLNLSCAKKEPQK
DSCVTDSEPVVNVIPPSANPINIAIPTVTAQLPTIVAIADQNSVPCLRALAANKQTILIP
QVAYTYSTTVSPAVQEPPLKVIQPNGNQDERQDTSSEGVSNVEDQNDSDSTPPKKKMRKT
ENGMYACDLCDKIFQKSSSLLRHKYEHTGKRPHECGICKKAFKHKHHLIEHMRLHSGEKP
YQCDKCGKRFSHSGSYSQHMNHRYSYCKREAEERDSTEQEEAGPEILSNEHVGARASPSQ
GDSDERESLTREEDEDSEKEEEEEDKEMEELQEEKECEKPQGDEEEEEEEEEVEEEEVEE
AENEGEEAKTEGLMKDDRAESQASSLGQKVGESSEQVSEEKTNEA*
>ENSG00000198597|ENST00000355537
MEEASLCLGVSSAEPEAEPHLSGPVLNGQYAMSQKLHQITSQLSHAFPELHPRPNPEEKP
PASLEEKAHVPMSGQPMGSQMALLANQLGREVDTSLNGRVDLQQFLNGQNLGIMSQMSDI
EDDARKNRKYPCPLCGKRFRFNSILSLHMRTHTGEKPFKCPYCDHRAAQKGNLKIHLRTH
KLGNLGKGRGRVREENRLLHELEERAILRDKQLKGSLLQPRPDLKPPPHAQQAPLAACTL
ALQANHSVPDVAHPVPSPKPASVQEDAVAPAAGFRCTFCKGKFKKREELDRHIRILHKPY
KCTLCDFAASQEEELISHVEKAHITAESAQGQGPNGGGEQSANEFRCEVCGQVFSQAWFL
KGHMRKHKDSFEHCCQICGRRFKEPWFLKNHMKVHLNKLSVKNKSPSDPEVPVPMGGMSQ
EAHANLYSRYLSCLQSGFMTPDKAGLSEPSQLYGKGELPMKEKEALGKLLSPISSMAHGV
PEGDKHSLLGCLNLVPPLKSSCIERLQAAAKAAEMDPVNSYQAWQLMARGMAMEHGFLSK
EHPLQRNHEDTLANAGVLFDKEKREYVLVGADGSKQKMPADLVHSTKVGSQRDLPSKLDP
LESSRDFLSHGLNQTLEYNLQGPGNMKEKPTECPDCGRVFRTYHQVVVHSRVHKRDRKGE
EDGLHVGLDERRGSGSDQESQSVSRSTTPGSSNVTEESGVGGGLSQTGSAQEDSPHPSSP
SSSDIGEEAGRSAGVQQPALLRDRSLGSAMKDCPYCGKTFRTSHHLKVHLRIHTGEKPYK
CPHCDYAGTQSASLKYHLERHHRERQNGAGPLSGQPPNQDHKDEMSSKASLFIRPDILRG
AFKGLPGIDFRGGPASQQWTSGVLSSGDHSGQATGMSSEVPSDALKGTDLPSKSTHFSEI
GRAYQSIVSNGVNFQGSLQAFMDSFVLSSLKKEKDMKDKALADPPSMKVHGVDGGEEKPS
GKSSQRKSEKSQYEPLDLSVRPDAASLPGSSVTVQDSIAWHGCLFCAFTTSSMELMALHL
QANHLGKAKRKDNTIGVTVNCKDQAREASKMALLPSLQSNKDLGLSNMISSLDSASEKMA
QGQLKETLGEQKSGAWTGHVDPAFCNFPSDFYKQFGVYPGMVGSGASSSCPNKEPDGKAH
SEEDVPILIPETTSKNTTDDLSDIASSEDMDSSKGENNDEEDVETEPEMMTKPLSALSKD
SSSDGGDSLQPTGTSQPVQGLVSPLSQAPEKQWHSQGLLQAQDPLAGLPKPERGPQSLDK

PMNMLSVLRAYSSDGLAAFNGLASSTANSGCIKRPDLCGK*
>ENSG00000115053|ENST00000322723
MVKLAKAGKNQGDPKKMAPPPKEVEEDSEDEEMSEDEEDDSSGEEVVIPQKKGKKAAATS
AKKVVVSPTKKVAVATPAKKAAVTPGKKAAATPAKKTVTPAKAVTTPGKKGATPGKALVA
TPGKKGAAIPAKGAKNGKNAKKEDSDEEEDDDSEEDEEDDEDEDEDEDEIEPAAMKAAAA
APASEDEDDEDDEDDEDDDDDEEDDSEEEAMETTPAKGKKAAKVVPVKAKNVAEDEDEEE
DDEDEDDDDDEDDEDDDDEDDEEEEEEEEEEEPVKEAPGKRKKEMAKQKAAPEAKKQKVEG
TEPTTAFNLFVGNLNFNKSAPELKTGISDVFAKNDLAVVDVRIGMTRKFGYVDFESAEDL
EKALELTGLKVFGNEIKLEKPKGKDSKKERDARTLLAKNLPYKVTQDELKEVFEDAAEIR
LVSKDGKSKGIAYIEFKTEADAEKTFEEKQGTEIDGRSISLYYTGEKGQNQDYRGGKNST
WSGESKTLVLSNLSYSATEETLQEVFEKATFIKVPQNQNGKSKGYAFIEFASFEDAKEAL
NSCNKREIEGRAIRLELQGPRGSPNARSQPSKTLFVKGLSEDTTEETLKESFDGSVRARI
VTDRETGSSKGFGFVDFNSEEDAKAAKEAMEDGEIDGNKVTLDWAKPKGEGGFGGRGGGR
GGFGGRGGGRGGRGGFGGRGRGGFGGRGGFRGGRGGGGDHKPQGKKTKFE*
>ENSG00000119866|ENST00000358510
MSRRKQGKPQHLSKREFSPEPLEAILTDDEPDHGPLGAPEGDHDLLTCGQCQMNFPLGDI
LIFIEHKRKQCNGSLCLEKAVDKPPSPSPIEMKKASNPVEVGIQVTPEDDDCLSTSSRGI
CPKQEHIAGKDEPSSYTCTTCKQPFTSAWFLLQHAQNTHGLRIYLESEHGSPLTPRVGIP
SGLGAECPSQPPLHGIHIADNNPFNLLRIPGSVSREASGLAEGRFPPTPPLFSPPPRHHL
DPHRIERLGAEEMALATHHPSAFDRVLRLNPMAMEPPAMDFSRRLRELAGNTSSPPLSPG
RPSPMQRLLQPFQPGSKPPFLATPPLPPLQSAPPPSQPPVKSKSCEFCGKTFKFQSNLVV
HRRSHTGEKPYKCNLCDHACTQASKLKRHMKTHMHKSSPMTVKSDDGLSTASSPEPGTSD
LVGSASSALKSVVAKFKSENDPNLIPENGDEEEEEDDEEEEEEEEEEEEEELTESERVDYG
FGLSLEAARHHENSSRGAVVGVGDESRALPDVMQGMVLSSMQHFSEAFHQVLGEKHKRGH
LAEAEGHRDTCDEDSVAGESDRIDDGTVNGRGCSPGESASGGLSKKLLLGSPSSLSPFSK
RIKLEKEFDLPPAAMPNTENVYSQWLAGYAASRQLKDPFLSFGDSRQSPFASSSEHSSEN
GSLRFSTPPGELDGGISGRSGTGSGGSTPHISGPGPGRPSSKEGRRSDTCEYCGKVFKNC
SNLTVHRRSHTGERPYKCELCNYACAQSSKLTRHMKTHGQVLHTPPFGVVPRELKMCGSF
RMEAREPLSSEKI*
>ENSG00000081189|ENST00000340208
MGRKKIQITRIMDERNRQVTFTKRKFGLMKKAYELSVLCDCEIALIIFNSTNKLFQYAST
DMDKVLLKYTEYNEPHESRTNSDIVETLRKKGLNGCDSPDPDADDSALNKKENKGCESPD
PDSSYALTPRTEEKYKKINEEFDNMIKSHKIPAVPPPNFEMPVSIPVSSHNSLVYSNPVS
SLGNPNLLPLAHPSLQRNSMSPGVTHRPPSAGNTGGLMGGDLTSGAGTSAGNGYGNPRNS
PGLLVSPGNLNKNMQAKSPPPMNLGMNNRKPDLRVLIPPGSKNTMPSVNQRINNSQSAQS
LATPVVSVATPTLPGQGMGGYPSAISTTYGTEYSLSSADLSSLSGFNTASALHLGSVTGW
QQQHLHNMPPSALSQLGACTSTHLSQSSNLSLPSTQSLNIKSEPVSPPRDRTTTPSRYPQ
HTRHEAGRSPVDSLSSCSSSYDGSDREDHRNEFHSPIGLTRPSPDERESPSVKRMRLSEG
WAT*
>ENSG00000148516|ENST00000320985
MADGPRCKRRKQANPRRNNVTNYNTVVETNSDSDDEDKLHIVEEESVTDAADCEGVPEDD
LPTDQTVLPGRSSEREGNAKNCWEDDRKEGGQEILGPEAQADEAGCTVKDDECESDAENEQ
NHDPNVEEFLQQQDTAVIFPEAPEEDQRQGTPEASGHDENGTPDAFSQLLTCPYCDRGYK
RFTSLKEHIKYRHEKNEDNFSCSLCSYTFAYRTQLERHMTSHKSGRDQRHVTQSGCNRKF

KCTECGKAFKYKHHLKEHLRIHSGEKPYECPNCKKRFSHSGSYSSHISSKKCISLIPVNG
RPRTGLKTSQCSSPSLSASPGSPTRPQIRQKIENKPLQEQLSVNQIKTEPVDYEFKPIVV
ASGINCSTPLQNGVFTGGGPLQATSSPQGMVQAVVLPTVGLVSPISINLSDIQNVLKVAV
DGNVIRQVLENNQANLASKEQETINASPIQQGGHSVISAISLPLVDQDGTTKIIINYSLE
QPSQLQVVPQNLKKENPVATNSCKSEKLPEDLTVKSEKDKSFEGGVNDSTCLLCDDCPGD
INALPELKHYDLKQPTQPPPLPAAEAEKPESSVSSATGDGNLSPSQPPLKNLLSLLKAYY
ALNAQPSAEELSKIADSVNLPLDVVKKWFEKMQAGQISVQSSEPSSPEPGKVNIPAKNND
QPQSANANEPQDSTVNLQSPLKMTNSPVLPVGSTTNGSRSSTPSPSPLNLSSSRNTQGYL
YTAEGAQEEPQVEPLDLSLPKQQGELLERSTITSVYQNSVYSVQEEPLNLSCAKKEPQKD
SCVTDSEPVVNVIPPSANPINIAIPTVTAQLPTIVAIADQNSVPCLRALAANKQTILIPQ
VAYTYSTTVSPAVQEPPLKVIQPNGNQDERQDTSSEGVSNVEDQNDSDSTPPKKKMRKTE
NGMYACDLCDKIFQKSSSLLRHKYEHTGKRPHECGICKKAFKHKHHLIEHMRLHSGEKPY
QCDKCGKRFSHSGSYSQHMNHRYSYCKREAEERDSTEQEEAGPEILSNEHVGARASPSQG
DSDERESLTREEDEDSEKEEEEEDKEMEELQEEKECEKPQGDEEEEEEEEEVEEEEVEEA
ENEGEEAKTEGLMKDDRAESQASSLGQKVGESSEQVSEEKTNEA*
>ENSG00000154269|ENST00000357639
MESTLTLATEQPVKKNTLKKYKIACIVLLALLVIMSLGLGLGLGLRKLEKQGSCRKKCFD
ASFRGLENCRCDVACKDRGDCCWDFEDTCVESTRIWMCNKFRCGETRLEASLCSCSDDCL
QRKDCCADYKSVCQGETSWLEENCDTAQQSQCPEGFDLPPVILFSMDGFRAEYLYTWDTL
MPNINKLKTCGIHSKYMRAMYPTKTFPNHYTIVTGLYPESHGIIDNNMYDVNLNKNFSLS
SKEQNNPAWWHGQPMWLTAMYQGLKAATYFWPGSEVAINGSFPSIYMPYNGSVPFEERIS
TLLKWLDLPKAERPRFYTMYFEEPDSSGHAGGPVSARVIKALQVVDHAFGMLMEGLKQRN
LHNCVNIILLADHGMDQTYCNKMEYMTDYFPRINFFYMYEGPAPRIRAHNIPHDFFSFNS
EEIVRNLSCRKPDQHFKPYLTPDLPKRLHYAKNVRIDKVHLFVDQQWLAVRSKSNTNCGG
GNHGYNNEFRSMEAIFLAHGPSFKEKTEVEPFENIEVYNLMCDLLRIQPAPNNGTHGSLN
HLLKVPFYEPSHAEEVSKFSVCGFANPLPTESLDCFCPHLQNSTQLEQVNQMLNLTQEEI
TATVKVNLPFGRPRVLQKNVDHCLLYHREYVSGFGKAMRMPMWSSYTVPQLGDTSPLPPT
VPDCLRADVRVPPSESQKCSFYLADKNITHGFLYPPASNRTSDSQYDALITSNLVPMYEE
FRKMWDYFHSVLLIKHATERNGVNVVSGPIFDYNYDGHFDAPDEITKHLANTDVPIPTHY
FVVLTSCKNKSHTPENCPGWLDVLPFIIPHRPTNVESCPEGKPEALWVEERFTAHIARVR
DVELLTGLDFYQDKVQPVSEILQLKTYLPTFETTI*
>ENSG00000162664|ENST00000340281
MDFEDDYTHSACRNTYQGFNGMDRDYGPGSYGGMDRDYGHGSYGGQRSMDSYLNQSYGMD
NHSGGGGGSRFGPYESYDSRSSLGGRDLYRSGYGFNEPEQSRFGGSYGGRFESSYRNSLD
SFGGRNQGGSSWEAPYSRSKLRPGFMEDRGRENYSSYSSFSSPHMKPAPVGSRGRGTPAY
PESTFGSRNYDAFGGPSTGRGRGRGHMGDFGSIHRPGIVVDYQNKSTNVTVAAARGIKRK
MMQPFNKPSGTFIKKPKLAKPMEKISLSKSPTKTDPKNEEEEKRRIEARREKQRRRREKN
SEKYGDGYRMAFTCSFCKFRTFEEKDIELHLESSSHQETLDHIQKQTKFDKVVMEFLHEC
MVNKFKKTSIRKQQTNNQTEVVKIIEKDVMEGVTVDDHMMKVETVHCSACSVYIPALHSS
VQQHLKSPDHIKGKQAYKEQIKRESVLTATSILNNPIVKARYERFVKGENPFEIQDHSQD
QQIEGDEEDEEKIDEPIEEEEDEDEEEEAEEVGEVEEVEEVEEVREGGIEGEGNIQGVGE
GGEVGVVGEVEGVGEVEEVEELEEETAKEEPADFPVEQPEEN*
>ENSG00000115368|ENST00000314761
MVEEENIRVVRCGGSELNFRRAVFSADSKYIFCVSGDFVKVYSTVTEECVHILHGHRNLV

TGIQLNPNNHLQLYSCSLDGTIKLWDYIDGILIKTFIVGCKLHALFTLAQAEDSVFVIVN
KEKPDIFQLVSVKLPKSSSQEVEAKELSFVLDYINQSPKCIAFGNEGVYVAAVREFYLSV
YFFKKKTTSRFTLSSSRNKKHAKNNFTCVACHPTEDCIASGHMDGKIRLWRNFYDDKKYT
YTCLHWHHDMVMDLAFSVTGTSLLSGGRESVLVEWRDATEKNKEFLPRLGATIEHISVSP
AGDLFCTSHSDNKIIIIHRNLEASAVIQGLVKDRSIFTGLMIDPRTKALVLNGKPGHLQF
YSLQSDKQLYNLDIIQQEYINDYGLIQIELTKAAFGCFGNWLATVEQRQEKETELELQMK
LWMYNKKTQGFILNTKINMPHEDCITALCFCNAEKSEQPTLVTASKDGYFKVWILTDDSD
IYKKAVGWTCDFVGSYHKYQATNCCFSEDGSLLAVSFEEIVTIWDSVTWELKCTFCQRAG
KIRHLCFGRLTCSKYLLGATENGILCCWNLLSCALEWNAKLNVRVMEPDPNSENIAAISQ
SSVGSDLFVFKPSEPRPLYIQKGISREKVQWGVFVPRDVPESFTSEAYQWLNRSQFYFLT
KSQSLLTFSTKSPEEKLTPTSKQLLAEESLPTTPFYFILGKHRQQQDEKLNETLENELVQ
LPLTENIPAISELLHTPAHVLPSAAFLCSMFVNSLLLSKETKSAKEIPEDVDMEEEKESE
DSDEENDFTEKVQDTSNTGLGEDIIHQLSKSEEKELRKFRKIDYSWIAAL*
>ENSG00000142192|ENST00000354192
MLPGLALLLLAAWTARALEVYPELQITNVVEANQPVTIQNWCKRGRKQCKTHPHFVIPYR
CLVGEFVSDALLVPDKCKFLHQERMDVCETHLHWHTVAKETCSEKSTNLHDYGMLLPCGI
DKFRGVEFVCCPLAEESDNVDSADAEEDDSDVWWGGADTDYADGSEDKVVEVAEEEEVAE
VEEEEADDDEDDEDGDEVEEEAEEPYEEATERTTSIATTTTTTTESVEEVVRVPTTAAST
PDAVDKYLETPGDENEHAHFQKAKERLEAKHRERMSQVMREWEEAERQAKNLPKADKKAV
IQHFQEKVESLEQEAANERQQLVETHMARVEAMLNDRRRLALENYITALQAVPPRPRHVF
NMLKKYVRAEQKDRQHTLKHFEHVRMVDPKKAAQIRSQVMTHLRVIYERMNQSLSLLYNV
PAVAEEIQDEVDELLQKEQNYSDDVLANMISEPRISYGNDALMPSLTETKTTVELLPVNG
EFSLDDLQPWHSFGADSVPANTENEVEPVDARPAADRGLTTRPGSGLTNIKTEEISEVKM
DAEFRHDSGYEVHHQKLVFFAEDVGSNKGAIIGLMVGGVVIATVIVITLVMLKKKQYTSI
HHGVVEVDAAVTPEERHLSKMQQNGYENPTYKFFEQMQN*
>ENSG00000134138|ENST00000338564
MAQRYDELPHYGGMDGVGVPASMYGDPHAPRPIPPVHHLNHGPPLHATQHYGAHAPHPNV
MPASMGSAVNDALKRDKDAIYGHPLFPLLALVFEKCELATCTPREPGVAGGDVCSSDSFN
EDIAVFAKQVRAEKPLFSSNPELDNLMIQAIQVLRFHLLELEKVHELCDNFCHRYISCLK
GKMPIDLVIDERDGSSKSDHEELSGSSTNLADHNPSSWRDHDDATSTHSAGTPGPSSGGH
ASQSGDNSSEQGDGLDNSVASPGTGDDDDPDKDKKRQKKRGIFPKVATNIMRAWLFQHLT
HPYPSEEQKKQLAQDTGLTILQVNNWFINARRRIVQPMIDQSNRAVSQGAAYSPEGQPMG
SFVLDGQQHMGIRPAGLQSMPGDYVSQGGPMGMSMAQPSYTPPQMTPHPTQLRHGPPMHS
YLPSHPHHPAMMMHGGPPTHPGMTMSAQSPTMLNSVDPNVGGQVMDIHAQ*
>ENSG00000163795|ENST00000323703
MPRRKQSHPQPVKCEGVKVDTEDSLDEGPGALVLESDLLLGQDLEFEEEEEEEEGDGNSD
QLMGFERDSEGDSLGARPGLPYGLSDDESGGGRALSAESEVEEPARGPGEARGERPGPAC
QLCGGPTGEGPCCGAGGPGGGPLLPPRLLYSCRLCTFVSHYSSHLKRHMQTHSGEKPFRC
GRCPYASAQLVNLTRHTRTHTGEKPYRCPHCPFACSSLGNLRRHQRTHAGPPTPPCPTCG
FRCCTPRPARPPSPTEQEGAVPRRPEDALLLPDLSLHVPPGGASFLPDCGQLRGEGEGLC
GTGSEPLPELLFPWTCRGCGQELEEGEGSRLGAAMCGRCMRGEAGGGASGGPQGPSDKGF
ACSLCPFATHYPNHLARHMKTHSGEKPFRCARCPYASAHLDNLKRHQRVHTGEKPYKCPL
CPYACGNLANLKRHGRIHSGDKPFRCSLCNYSCNQSMNLKRHMLRHTGEKPFRCATCAYT
TGHWDNYKRHQKVHGHGGAGGPGLSASEGWAPPHSPPSVLSSRGPPALGTAGSRAVHTDS

S*
>ENSG00000170325|ENST00000358825
MDSKDESSHVWPTSAEHEQNAAQVHFVPDTGTVAQIVYTDDQVRPPQQVVYTADGASYTS
VDGPEHTLVYIHPVEAAQTLFTDPGQVAYVQQDATAQQASLPVHNQVLPSIESVDGSDPL
ATLQTPLGRLEAKEEEDEDEDEDTEEDEEEDGEDTDLDDWEPDPPRPFDPHDLWCEECNN
AHASVCPKHGPLHPIPNRPVLTRARASLPLVLYIDRFLGGVFSKRRIPKRTQFGPVEGPL
VRGSELKDCYIHLKVSLDKGDRKERDLHEDLWFELSDETLCNWMMFVRPAQNHLEQNLVA
YQYGHHVVYYTTIKNVEPKQELKVWYAASYAEFVNQKIHDISEEERKVLREQEKNWPCYEC
NRRFISSEQLQQHLNSHDEKLDVFSRTRGRGRGRGKRRFGPGRRPGRPPKFIRLEITSEN
GEKSDDGTQDLLHFPTKEQFDEAEPATLNGLDQPEQTTIPIPQLPQETQSSLEHEPETHT
LHLQPQHEESVVPTQSTLTADDMRRAKRIRLELQNAALQHLFIRKSFRPFKCLQCGKAFR
EKDKLDQHLRFHGREGNCPLTCDLCNKGFISSTSLESHMKLHSDQKTYSCIFCPESFDRL
DLLKDHVAIHINDGYFTCPTCKKRFPDFIQVKKHVRSFHSEKIYQCTECDKAFCRPDKLR
LHMLRHSDRKDFLCSTCGKQFKRKDKLREHMQRMHNPEREAKKADRISRSKTFKPRITST
DYDSFTFKCRLCMMGFRRRGMLVNHLSKRHPDMKIEEVPELTLPIIKPNRDYFCQYCDKV
YKSASKRKAHILKNHPGAELPPSIRKLRPAGPGEPDPMLSTHTQLTGTIATPPVCCPHCS
KQYSSKTKMVQHIRKKHPEFAQLSNTIHTPLTTAVISATPAVLTTDSATGETVVTTDLLT
QAMTELSQTLTTDYRTPQGDYQRIQYIPVSQSASGLQQPQHIQLQVVQVASATSPHQSQQ
STVDVGQLHDPQPYPQHAIQVQHIQVSEPTASAPSSAQVSGQPLSPSAQQAQQGLSPSHI
QGSSSTQGQALQQQQQQQQNSSVQHTYLPSAWNSFRGYSSEIQMMTLPPGQFVITDSGVA
TPVTTGQVKAVTSGHYVLSESQSELEEKQTSALSGGVQVEPPAHSDSLDPQTNSQQQTTQ
YIITTTTNGNGSSEVHITKP*
>ENSG00000156508|ENST00000331523
MGKEKTHINIVVIGHVDSGKSTTTGHLIYKCGGIDKRTIEKFEKEAAEMGKGSFKYAWVL
DKLKAERERGITIDISLWKFETSKYYVTIIDAPGHRDFIKNMITGTSQADCAVLIVAAGV
GEFEAGISKNGQTREHALLAYTLGVKQLIVGVNKMDSTEPPYSQKRYEEIVKEVSTYIKK
IGYNPDTVAFVPISGWNGDNMLEPSANMPWFKGWKVTRKDGNASGTTLLEALDCILPPTR
PTDKPLRLPLQDVYKIGGIGTVPVGRVETGVLKPGMVVTFAPVNVTTEVKSVEMHHEALS
EALPGDNVGFNVKNVSVKDVRRGNVAGDSKNDPPMEAAGFTAQVIILNHPGQISAGYAPV
LDCHTAHIACKFAELKEKIDRRSGKKLEDGPKFLKSGDAAIVDMVPGKPMCVESFSDYPP
LGRFAVRDMRQTVAVGVIKAVDKKAAGAGKVTKSAQKAQKAK*
>ENSG00000155966|ENST00000342251
MDLFDFFRDWDLEQQCHYEQDRSALKKREWERRNQEVQQEDDLFSSGFDLFGEPYKTNKG
DALANRVQNTLGNYDEMKNLLTNHSNQNHLVGIPKNSVPQNPNNKNEPSFFPEQKNRIIP
PHQDNTHPSAPMPPPSVVILNSTLIHSNRKSKPEWSRDSHNPSTVLASQASGQPNKMQTL
TQDQSQAKLEDFFVYPAEQPQIGEVEESNPSAKEDSNPNSSGEDAFKEIFQSNSPEESEF
AVQAPGSPLVASSLLAPSSGLSVQNFPPGLYCKTSMGQQKPTAYVRPMDGQDQAPDISPT
LKPSIEFENSFGNLSFGTLLDGKPSAASSKTKLPKFTILQTSEVSLPSDPSCVEEILRES
QHLTPGFTLQKWNDPTTRASTKSVSFKSMLEDDLKLSSDEDDLEPVKTLTTQCTATELYQ
AVEKAKPRNNPVNPPLATPQPPPAVQASGGSGSSSESESSSESDSDTESSTTDSESNEAP
RVATPEPEPPSTNKWQLDKWLNKVTSQNKSFICGQNETPMETISLPPPIIQPMEVQMKVK
TNASQVPAEPKERPLLSLIREKARPRPTQKIPETKALKHKLSTTSETVSQRTIGKKQPKK
VEKNTSTDEFTWPKPNITSSTPKEKESVELHDPPRGRNKATAHKPAPRKEPRPNIPLAPE
KKKYRGPGKIVPKSREFIETDSSTSDSNTDQEETLQIKVLPPCIISGGNTAKSKEICGAS

LTLSTLMSSSGSNNNLSISNEEPTFSPIPVMQTEILSPLRDHENLKNLWVKIDLDLLSRV
PGHSSLHAAPAKPDHKETATKPKRQTAVTAVEKPAPKGKRKHKPIEVAEKIPEKKQRLEE
ATTICLLPPCISPAPPHKPPNTRENNSSRRANRRKEEKLFPPPLSPLPEDPPRRRNVSGN
NGPFGQDKNIAMTGQITSTKPKRTEGKFCATFKGISEGDTPKKASSATITVTNTAIATAT
VTATAIVTTTVTATATATATTTTTTTTISTITSTITTGLMDSSHLEMTSWAALPLLSSSS
TNVRRPKLTFDDSVHNADYYMQEAKKLKHKADALFEKFGKAVNYADAALSFTECGNAMER
DPLEAKSPYTMYSETVELLRYAMRLKNFASPLASDGDKKLAVLCYRCLSLLYLRMFKLKK
DHAMKYSRSLMEYFKQNASKVAQIPSPWVSNGKNTPSPVSLNNVSPINAMGNCNNGPVTI
PQRIHHMAASHVNITSNVLRGYEHWDMADKLTRENKEFFGDLDTLMGPLTQHSSMTNLVR
YVRQGLCWLRIDAHLL*
>ENSG00000179950|ENST00000349157
MATATIALQVNGQQGGGSEPAAAAAVVAAGDKWKPPQGTDSIKMENGQSTAAKLGLPPLT
PEQQEALQKAKKYAMEQSIKSVLVKQTIAHQQQQLTNLQMAAQRQRALAIMCRVYVGSIY
YELGEDTIRQAFAPFGPIKSIDMSWDSVTMKHKGFAFVEYEVPEAAQLALEQMNSVMLGG
RNIKVGRPSNIGQAQPIIDQLAEEARAFNRIYVASVHQDLSDDDIKSVFEAFGKIKSCTL
ARDPTTGKHKGYGFIEYEKAQSSQDAVSSMNLFDLGGQYLRVGKAVTPPMPLLTPATPGG
LPPAAAVAAAAATAKITAQEAVAGAAVLGTLGTPGLVSPALTLAQPLGTLPQAVMAAQAP
GVITGVTPARPPIPVTIPSVGVVNPILASPPTLGLLEPKKEKEEEELFPESERPEMLSEQ
EHMSISGSSARHMVMQKLLRKQESTVMVLRNMVDPKDIDDDLEGEVTEECGKFGAVNRVI
IYQEKQGEEEDAEIIVKIFVEFSIASETHKAIQALNGRWFAGRKVVAEVYDQERFDNSDL
SA*
>ENSG00000185177|ENST00000319636
MAKRPGPPGSREMGLLTFRDIAIEFSLEEWQCLDCAQRNLYRDVMLENYRNLVSLGIAVS
KPDLITCLEQNKESQNIKRNEMVAKHPVTRSHFTQDLQPEQGIKDSLQKVIPRTYGKCGH
EKLQFKKCCKSVGEYEVHKGGYSEVNQCLSTTQNKIFQTHKYVKVFGKFSNSNRDKTRYT
GNKHFKCNKYGKSFCMLSHLNQHQVIHTREKSYKCKECGKSFNCSSNHTTHKIIHTGEKP
YRCEECGKAFSWSANLTRHKRTHTGEKPYTCEECGQAFRRSSALTNHKRIHTGERPYKCE
ECGKAFSVSSTLTDHKRIHTGEKPCRCEECGKAFSWSSNLTRHKRIHTREKPYACEECGQ
AFSLSSNLMRHRRIHTGEKPYTCEECGQDFRRSSALTIHKRIHTGERPYKCEECGKVFSL
SSTLTDHKRIHTGERPYKCEECGKAFSLSSTLTDHKRIHTGERPYTCEECGKAFNCSSTL
MQHKRIHTGEKPYKCEECEQAFKWHSSLAKHKIIHTGEKPYKCE*
>ENSG00000182318|ENST00000329665
MAIPKHSLSPVPWEEDSFLQVKVEEEEEASLSQGGESSHDHIAHSEAARLRFRHFRYEEA
SGPHEALAHLRALCCQWLQPEAHSKEQILELLVLEQFLGALPPEIQAWVGAQSPKSGEEA
AVLVEDLTQVLDKRGWDPGAEPTEASCKQSDLGESEPSNVTETLMGGVSLGPAFVKACEP
EGSSERSGLSGEIWTKSVTQQIHFKKTSGPYKDVPTDQRGRESGASRNSSSAWPNLTSQE
KPPSEDKFDLVDAYGTEPPYTYSGKRSSKCRECRKMFQSASALEAHQKTHSRKTPYACSE
CGKAFSRSTHLAQHQVVHTGAKPHECKECGKAFSRVTHLTQHQRIHTGEKPYKCGECGKT
FSRSTHLTQHQRVHTGERPYECDACGKAFSQSTHLTQHQRIHTGEKPYKCDACGRAFSDC
SALIRHLRIHSGEKPYQCKVCPKAFAQSSSLIEHQRIHTGEKPYKCSDCGKAFSRSSALM
VHLRIHITVLQ*
>ENSG00000124107|ENST00000338380
MKSSGLFPFLVLLALGTLAPWAVEGSGKSFKAGVCPPKKSAQCLRYKKPECQSDWQCPGK
KRCCPDTCGIKCLDPVDTPNPTRRKPGKCPVTYGQCLMLNPPNFCEMDGQCKRDLKCCMG

MCGKSCVSPVKA*
>ENSG00000213015|ENST00000325333
MLLLPPRPPHPRSSSPEAMDPPPPKAPPFPKAEGPSSTPSSAAGPRPPRLGRHLLIDANG
VPYTYTVQLEEEPRGPPQREAPPGEPGPRKGYSCPECARVFASPLRLQSHRVSHSDLKPF
TCGACGKAFKRSSHLSRHRATHRARAGPPHTCPLCPRRFQDAAELAQHVRLH*
>ENSG00000180357|ENST00000326648
MSLSSGASGGKGVDANPVETYDSGDEWDIGVGNLIIDLDADLEKDQQKLEMSGSKEVGIP
APNAVATLPDNIKFVTPVPGPQGKEGKSKSKRSKSGKDTSKPTPGTSLFTPSEGAASKKE
VQGRSGDGANAGGLVAAIAPKGSEKAAKASRSVAGSKKEKENSSSKSKKERSEGVGTCSE
KDPGVLQPVPLGGRGGQYDGSAGVDTGAVEPLGSIAIEPGAALNPLGTKPEPEEGENECR
LLKKVKSEKMESPVSTPAVLPIHLLVPVVNNDISSPCEQIMVRTRSVGVNTCDVALATEP
ECLGPCEPGTSVNLEGIVWQETEDGMLVVNVTWRNKTYVGTLLDCTRHDWAPPRFCDSPT
SDLEMRNGRGRGKRMRPNSNTPVNETATASDSKGTSNSSKTRAGANSKGRRGSQNSSEHR
PPASSTSEDVKASPSSANKRKNKPLSDMELNSSSEDSKGSKRVRTNSMGSATGPLPGTKV
EPTVLDRNCPSPVLIDCPHPNCNKKYKHINGLKYHQAHAHTDDDSKPEADGDSEYGEEPI
LHADLGSCNGASVSQKGSLSPARSATPKVRLVEPHSPSPSSKFSTKGLCKKKLSGEGDTD
LGALSNDGSDDGPSVMDETSNDAFDSLERKCMEKEKCKKPSSLKPEKIPSKSLKSARPIA
PAIPPQQIYTFQTATFTAASPGSSSGLTATVAQAMPNSPQLKPIQPKPTVMGEPFTVNPA
LTPAKDKKKKDKKKKESSKELESPLTPGKVCRAEEGKSPFRESSGDGMKMEGLLNGSSDP
HQSRLASIKAEADKIYSFTDNAPSPSIGGSSRLENTTPTQPLTPLHVVTQNGAEASSVKT
NSPAYSDISDAGEDGEGKVDSVKSKDAEQLVKEGAKKTLFPPQPQSKDSPYYQGFESYYS
PSYAQSSPGALNPSSQAGVESQALKTKRDEEPESIEGKVKNDICEEKKPELSSSSQQPSV
IQQRPNMYMQSLYYNQYAYVPPYGYSDQSYHTHLLSTNTAYRQQYEEQQKRQSLEQQQRG
VDKKAEMGLKEREAALKEEWKQKPSIPPTLTKAPSLTDLVKSGPGKAKEPGADPAKSVII
PKLDDSSKLPGQAPEGLKVKLSDASHLSKEASEAKTGAECGRQAEMDPILWYRQEAEPRM
WTYVVYPAKYSDIKSEDERWKEERDRKLKEERSRSKDSVPKEDGKESTSSDCKLPTSEESR
LGSKEPRPSVHVPVSSPLTQHQSYIPYMHGYSYSQSYDPNHPSYRSMPAVMMQNYPGSYL
PSSYSFSPYGSKVSGGEDADKARASPSVTCKSSSESKALDILQQHASHYKSKSPTISDKT
SQERDRGGCGVVGGGGSCSSVGGASGGERSVDRPRTSPSQRLMSTHHHHHHLGYSLLPAQ
YNLPYAAGLSSTAIVASQQGSTPSLYPPPRR*
>ENSG00000198546|ENST00000361518
MQLPPALCARLAAGPGAAEPLPVERDPAAGAAPFRFVARPVRFPREHQFFEDGDVQRHLY
LQDVIMQVADVPEKPRVPAFACQVAGCCQVFDALDDYEHHYHTLHGNVCSFCKRAFPSGH
LLDAHILEWHDSLFQILSERQDMYQCLVEGCTEKFKTSRDRKDHMVRMHLYPADFRFDKP
KKSRSPASAEAPGDSGERSEGEAMEICSEPVAASPAPAGERRIYRHRIPSTICFGQGAAR
GFKSNKKKTKQC*
>ENSG00000114861|ENST00000318789
MMQESGTETKSNGSAIQNGSGGSNHLLECGGLREGRSNGETPAVDIGAADLAHAQQQQQQ
ALQVARQLLLQQQQQQQVSGLKSPKRNDKQPALQVPVSVAMMTPQVITPQQMQQILQQQV
LSPQQLQVLLQQQQALMLQQQQLQEFYKKQQEQLQLQLLQQQHAGKQPKEQQQVATQQLA
FQQQLLQMQQLQQQHLLSLQRQGLLTIQPGQPALPLQPLAQGMIPTELQQLWKEVTSAHT
AEETTGNNHSSLDLTTTCVSSSAPSKTSLIMNPHASTNGQLSVHTPKRESLSHEEHPHSH
PLYGHGVCKWPGCEAVCEDFQSFLKHLNSEHALDDRSTAQCRVQMQVVQQLELQLAKDKE
RLQAMMTHLHVKSTEPKAAPQPLNLVSSVTLSKSASEASPQSLPHTPTTPTAPLTPVTQG

PSVITTTSMHTVGPIRRRYSDKYNVPISSADIAQNQEFYKNAEVRPPFTYASLIRQAILE
SPEKQLTLNEIYNWFTRMFAYFRRNAATWKNAVRHNLSLHKCFVRVENVKGAVWTVDEVE
FQKRRPQKISGNPSLIKNMQSSHAYCTPLNAALQASMAENSIPLYTTASMGNPTLGNLAS
AIREELNGAMEHTNSNESDSSPGRSPMQAVHPVHVKEEPLDPEEAEGPLSLVTTANHSPD
FDHDRDYEDEPVNEDME*
>ENSG00000108509|ENST00000361571
MGTDSPSPRPLRPGVTLPPGALTMNTKDTTEVAENSHHLKIFLPKKLLECLPRCPLLPPE
RLRWNTNEEIASYLITFEKHDEWLSCAPKTRPQNGSIILYNRKKVKYRKDGYLWKKRKDG
KTTREDHMKLKVQGMENPDIVLVHYLNVPALEDCGKGCSPIFCSISSDRREWLKWSREEL
LGQLKPMFHGIKWSCGNGTEEFSVEHLVQQILDTHPTKPAPRTHACLCSGGLGSGSLTHK
CSSTKHRIISPKVEPRALTLTSIPHAHPPEPPPLIAPLPPELPKAHTSPSSSSSSSSSGF
AEPLEIRPSPPTSRGGSSRGGTAILLLTGLEQRAGGLTPTRHLAPQADPRPSMSLAVVVG
TEPSAPPAPPSPAFDPDRFLNSPQRGQTYGGGQGVSPDFPEAEAAHTPCSALEPAAALEP
QAAARGPPPQSVAGGRRGNCFFIQDDDSGEELKGHGAAPPIPSPPPSPPPSPAPLEPSSR
VGRGEALFGGPVGASELEPFSLSSFPDLMGELISDEAPSIPAPTPQLSPALSTITDFSPE
WSYPEGGVKVLITGPWTEAAEHYSCVFDHIAVPASLVQPGVLRCYCPAHEVGLVSLQVAG
REGPLSASVLFEYRARRFLSLPSTQLDWLSLDDNQFRMSILERLEQMEKRMAEIAAAGQV
PCQGPDAPPVQDEGQGPGFEARVVVLVESMIPRSTWKGPERLAHGSPFRGMSLLHLAAAQ
GYARLIETLSQWRSVETGSLDLEQEVDPLNVDHFSCTPLMWACALGHLEAAVLLFRWNRQ
ALSIPDSLGRLPLSVAHSRGHVRLARCLEELQRQEPSVEPPFALSPPSSSPDTGLSSVSS
PSELSDGTFSVTSAYSSAPDGSPPPAPLPASEMTMEDMAPGQLSSGVPEAPLLLMDYEAT
NSKGPLSSLPALPPASDDGAAPEDADSPQAVDVIPVDMISLAKQIIEATPERIKREDFVG
LPEAGASMRERTGAVGLSETMSWLASYLENVDHFPSSTPPSELPFERGRLAVPSAPSWAE
FLSASTSGKMESDFALLTLSDHEQRELYEAARVIQTAFRKYKGRRLKEQQEVAAAVIQRC
YRKYKQLTWIALKFALYKKMTQAAILIQSKFRSYYEQKRFQQSRRAAVLIQQHYRSYRRR
PGPPHRTSATLPARNKGSFLTKKQDQAARKIMRFLRRCRHRMRELKQNQELEGLPQPGLA
T*
>ENSG00000092820|ENST00000337147
MPKPINVRVTTMDAELEFAIQPNTTGKQLFDQVVKTIGLREVWYFGLHYVDNKGFPTWLK
LDKKVSAQEVRKENPLQFKFRAKFYPEDVAEELIQDITQKLFFLQVKEGILSDEIYCPPE
TAVLLGSYAVQAKFGDYNKEVHKSGYLSSERLIPQRVMDQHKLTRDQWEDRIQVWHAEHR
GMLKDNAMLEYLKIAQDLEMYGINYFEIKNKKGTDLWLGVDALGLNIYEKDDKLTPKIGF
PWSEIRNISFNDKKFVIKPIDKKAPDFVFYAPRLRINKRILQLCMGNHELYMRRRKPDTI
EVQQMKAQAREEKHQKQLERQQLETEKKRRETVEREKEQMMREKEELMLRLQDYEEKTKK
AERELSEQIQRALQLEEERKRAQEEAERLEADRMAALRAKEELERQAVDQIKSQEQLAAE
LAEYTAKIALLEEARRRKEDEVEEWQHRAKEAQDDLVKTKEELHLVMTAPPPPPPPVYEP
VSYHVQESLQDEGAEPTGYSAELSSEGIRDDRNEEKRITEAEKNERVQRQLLTLSSELSQ
ARDENKRTHNDIIHNENMRQGRDKYKTLRQIRQGNTKQRIDEFEAL*
>ENSG00000184619|ENST00000331336
MPSFLVPSLVSSPVLLKLLFSPGPKTIWSLWQQPMLFQEATAFENMTKDWNYLEGSQKDC
YRDTMLDSYENTVPQGSFLQLSMMPQRAGNDPPGVSNASEMEMEISNMREKFLMSVTKLV
ESKSYNSKVFSKEKYFQTIKEVKEAKEKGKKSSRDYRRAAKYDVISVQGTEKLIEATHGE
RDRIRYYVHKEELFDILHDTHLSIGHGGRTRMLKELQGKYGNVTKEVIVLYLTLCKQCHQ
KNPVPKRGLAPKPMTFKDIDSTCQVEILDMQSSADGEFKFILYYQDHSTKFIILRPLRTK

QAHEVVSVLLDIFTILGTPSVLDSDSGVEFTNQVVHELNELWPDLKIVSGKYHPGQSQGS
LEGASRDVKNMISTWMQSNHSCHWAKGLRFMQMVRNQAFDVSLQQSPFEAMFGYKAKFGL
YSSNLPRETVATLQTEEELEIAEEQLENSLWIRQEERAEIGADRSDMDDDMDPTPEASEP
STSQGTSGLLCW*
>ENSG00000108010|ENST00000331244
MAAGAAEAAVAAVEEVGSAGQFEELLRLKAKSLLVVHFWAPWAPQCAQMNEVMAELAKEL
PQVSFVKLEAEGVPEVSEKYEISSVPTFLFFKNSQKIDRLDGAHAPELTKKVQRHASSGS
FLPSANEHLKEDLNLRLKKLTHAAPCMLFMKGTPQEPRCGFSKQMVEILHKHNIQFSSFD
IFSDEEVRQGLKAYSSWPTYPQLYVSGELIGGLDIIKELEASEELDTICPKAPKLEERLK
VLTNKASVMLFMKGNKQEAKCGFSKQILEILNSTGVEYETFDILEDEEVRQGLKAYSNWP
TYPQLYVKGELVGGLDIVKELKENGELLPILRGEN*
>ENSG00000283824|ENST00000362190
Sequence unavailable
>ENSG00000178209|ENST00000354589
MEPSGSLFPSLVVVGHVVTLAAVWHWRRGRRWAQDEQDERDRVQKKTFTKWVNKHLIKAQ
RHISDLYEDLRDGHNLISLLEVLSGDSLPREKGRMRFHKLQNVQIALDYLRHRQVKLVNI
RNDDIADGNPKLTLGLIWTIILHFQISDIQVSGQSEDMTAKEKLLLWSQRMVEGYQGLRC
DNFTSSWRDGRLFNAIIHRHKPLLIDMNKVYRQTNLENLDQAFSVAERDLGVTRLLDPED
VDVPQPDEKSIITYVSSLYDAMPRVPDVQDGVRANELQLRWQEYRELVLLLLQWMRHHTA
AFEERRFPSSFEEIEILWSQFLKFKEMELPAKEADKNRSKGIYQSLEGAVQAGQLKVPPG
YHPLDVEKEWGKLHVAILEREKQLRSEFERLECLQRIVTKLQMEAGLCEEQLNQADALLQ
SDVRLLAAGKVPQRAGEVERDLDKADSMIRLLFNDVQTLKDGRHPQGEQMYRRVYRLHER
LVAIRTEYNLRLKAGVAAPATQVAQVTLQSVQRRPELEDSTLRYLQDLLAWVEENQHRVD
GAEWGVDLPSVEAQLGSHRGLHQSIEEFRAKIERARSDEGQLSPATRGAYRDCLGRLDLQ
YAKLLNSSKARLRSLESLHSFVAAATKELMWLNEKEEEEVGFDWSDRNTNMTAKKESYSA
LMRELELKEKKIKELQNAGDRLLREDHPARPTVESFQAALQTQWSWMLQLCCCIEAHLKE
NAAYFQFFSDVREAEGQLQKLQEALRRKYSCDRSATVTRLEDLLQDAQDEKEQLNEYKGH
LSGLAKRAKAVVQLKPRHPAHPMRGRLPLLAVCDYKQVEVTVHKGDECQLVGPAQPSHWK
VLSSSGSEAAVPSVCFLVPPPNQEAQEAVTRLEAQHQALVTLWHQLHVDMKSLLAWQSLR
RDVQLIRSWSLATFRTLKPEEQRQALHSLELHYQAFLRDSQDAGGFGPEDRLMAEREYGS
CSHHYQQLLQSLEQGAQEESRCQRCISELKDIRLQLEACETRTVHRLRLPLDKEPARECA
QRIAEQQKAQAEVEGLGKGVARLSAEAEKVLALPEPSPAAPTLRSELELTLGKLEQVRSL
SAIYLEKLKTISLVIRGTQGAEEVLRAHEEQLKEAQAVPATLPELEATKASLKKLRAQAE
AQQPTFDALRDELRGAQEVGERLQQRHGERDVEVERWRERVAQLLERWQAVLAQTDVRQR
ELEQLGRQLRYYRESADPLGAWLQDARRRQEQIQAMPLADSQAVREQLRQEQALLEEIER
HGEKVEECQRFAKQYINAIKDYELQLVTYKAQLEPVASPAKKPKVQSGSESVIQEYVDLR
THYSELTTLTSQYIKFISETLRRMEEEERLAEQQRAEERERLAEVEAALEKQRQLAEAHA
QAKAQAEREAKELQQRMQEEVVRREEAAVDAQQQKRSIQEELQQLRQSSEAEIQAKARQA
EAAERSRLRIEEEIRVVRLQLEATERQRGGAEGELQALRARAEEAEAQKRQAQEEAERLR
RQVQDESQRKRQAEVELASRVKAEAEAAREKQRALQALEELRLQAEEAERRLRQAEVERA
RQVQVALETAQRSAEAELQSKRASFAEKTAQLERSLQEEHVAVAQLREEAERRAQQQAEA
ERAREEAERELERWQLKANEALRLRLQAEEVAQQKSLAQAEAEKQKEEAEREARRRGKAE
EQAVRQRELAEQELEKQRQLAEGTAQQRLAAEQELIRLRAETEQGEQQRQLLEEELARLQ
REAAAATQKRQELEAELAKVRAEMEVLLASKARAEEESRSTSEKSKQRLEAEAGRFRELA

EEAARLRALAEEAKRQRQLAEEDAARQRAEAERVLAEKLAAIGEATRLKTEAEIALKEKE
AENERLRRLAEDEAFQRRRLEEQAAQHKADIEERLAQLRKASDSELERQKGLVEDTLRQR
RQVEEEILALKASFEKAAAGKAELELELGRIRSNAEDTLRSKEQAELEAARQRQLAAEEE
RRRREAEERVQKSLAAEEEAARQRKAALEEVERLKAKVEEARRLRERAEQESARQLQLAQ
EAAQKRLQAEEKAHAFAVQQKEQELQQTLQQEQSVLDQLRGEAEAARRAAEEAEEARVQA
EREAAQSRRQVEEAERLKQSAEEQAQARAQAQAAAEKLRKEAEQEAARRAQAEQAALRQK
QAADAEMEKHKKFAEQTLRQKAQVEQELTTLRLQLEETDHQKNLLDEELQRLKAEATEAA
RQRSQVEEELFSVRVQMEELSKLKARIEAENRALILRDKDNTQRFLQEEAEKMKQVAEEA
ARLSVAAQEAARLRQLAEEDLAQQRALAEKMLKEKMQAVQEATRLKAEAELLQQQKELAQ
EQARRLQEDKEQMAQQLAEETQGFQRTLEAERQRQLEMSAEAERLKLRVAEMSRAQARAE
EDAQRFRKQAEEIGEKLHRTELATQEKVTLVQTLEIQRQQSDHDAERLREAIAELEREKE
KLQQEAKLLQLKSEEMQTVQQEQLLQETQALQQSFLSEKDSLLQRERFIEQEKAKLEQLF
QDEVAKAQQLREEQQRQQQMEQERQRLVASMEEARRRQHEAEEGVRRKQEELQQLEQQR
RQQEELLAEENQRLREQLQLLEEQHRAALAHSEEVTASQVAATKTLPNGRDALDGPAAEA
EPEHSFDGLRRKVSAQRLQEAGILSAEELQRLAQGHTTVDELARREDVRHYLQGRSSIAG
LLLKATNEKLSVYAALQRQLLSPGTALILLEAQAASGFLLDPVRNRRLTVNEAVKEGVVG
PELHHKLLSAERAVTGYKDPYTGQQISLFQAMQKGLIVREHGIRLLEAQIATGGVIDPVH
SHRVPVDVAYRRGYFDEEMNRVLADPSDDTKGFFDPNTHENLTYLQLLERCVEDPETGLC
LLPLTDKAAKGGELVYTDSEARDVFEKATVSAPFGKFQGKTVTIWEIINSEYFTAEQRRD
LLRQFRTGRITVEKIIKIIITVVEEQEQKGRLCFEGLRSLVPAAELLESRVIDRELYQQL
QRGERSVRDVAEVDTVRRALRGANVIAGVWLEEAGQKLSIYNALKKDLLPSDMAVALLEA
QAGTGHIIDPATSARLTVDEAVRAGLVGPEFHEKLLSAEKAVTGYRDPYTGQSVSLFQAL
KKGLIPREQGLRLLDAQLSTGGIVDPSKSHRVPLDVACARGCLDEETSRALSAPRADAKA
YSDPSTGEPATYGELQQRCRPDQLTGLSLLPLSEKAARARQEELYSELQARETFEKTPVE
VPVGGFKGRTVTVWELISSEYFTAEQRQELLRQFRTGKVTVEKVIKILITIVEEVETLRQ
ERLSFSGLRAPVPASELLASGVLSRAQFEQLKDGKTTVKDLSELGSVRTLLQGSGCLAGI
YLEDTKEKVSIYEAMRRGLLRATTAALLLEAQAATGFLVDPVRNQRLYVHEAVKAGVVGP
ELHEQLLSAEKAVTGYRDPYSGSTISLFQAMQKGLVLRQHGIRLLEAQIATGGIIDPVHS
HRVPVDVAYQRGYFSEEMNRVLADPSDDTKGFFDPNTHENLTYRQLLERCVEDPETGLRL
LPLKGAEKAEVVETTQVYTEEETRRAFEETQIDIPGGGSHGGSTMSLWEVMQSDLIPEEQ
RAQLMADFQAGRVTKERMIIIIIEIIEKTEIIRQQGLASYDYVRRRLTAEDLFEARIISL
ETYNLLREGTRSLREALEAESAWCYLYGTGSVAGVYLPGSRQTLSIYQALKKGLLSAEVA
RLLLEAQAATGFLLDPVKGERLTVDEAVRKGLVGPELHDRLLSAERAVTGYRDPYTEQTI
SLFQAMKKELIPTEEALRLLDAQLATGGIVDPRLGFHLPLEVAYQRGYLNKDTHDQLSEP
SEVRSYVDPSTDERLSYTQLLRRCRRDDGTGQLLLPLSDARKLTFRGLRKQITMEELVRS
QVMDEATALQLREGLTSIEEVTKNLQKFLEGTSCIAGVFVDATKERLSVYQAMKKGIIRP
GTAFELLEAQAATGYVIDPIKGLKLTVEEAVRMGIVGPEFKDKLLSAERAVTGYKDPYSG
KLISLFQAMKKGLILKDHGIRLLEAQIATGGIIDPEESHRLPVEVAYKRGLFDEEMNEIL
TDPSDDTKGFFDPNTEENLTYLQLMERCITDPQTGLCLLPLKEKKRERKTSSKSSVRKRR
VVIVDPETGKEMSVYEAYRKGLIDHQTYLELSEQECEWEEITISSSDGVVKSMIIDRRSG
RQYDIDDAIAKNLIDRSALDQYRAGTLSITEFADMLSGNAGGFRSRSSSVGSSSSYPISP
AVSRTQLASWSDPTEETGPVAGILDTETLEKVSITEAMHRNLVDNITGQRLLEAQACTGG
IIDPSTGERFPVTDAVNKGLVDKIMVDRINLAQKAFCGFEDPRTKTKMSAAQALKKGWLY
YEAGQRFLEVQYLTGGLIEPDTPGRVPLDEALQRGTVDARTAQKLRDVGAYSKYLTCPKT

KLKISYKDALDRSMVEEGTGLRLLEAAAQSTKGYYSPYSVSGSGSTAGSRTGSRTGSRAG
SRRGSFDATGSGFSMTFSSSSYSSSGYGRRYASGSSASLGGPESAVA*
>ENSG00000187098|ENST00000352241
MQSESGIVPDFEVGEEFHEEPKTYYELKSQPLKSSSSAEHPGASKPPISSSSMTSRILLR
QQLMREQMQEQERREQQQKLQAAQFMQQRVPVSQTPAINVSVPTTLPSATQVPMEVLKVQ
THLENPTKYHIQQAQRQQVKQYLSTTLANKHANQVLSLPCPNQPGDHVMPPVPGSSAPNS
PMAMLTLNSNCEKEGFYKFEEQNRAESECPGMNTHSRASCMQMDDVIDDIISLESSYNEE
ILGLMDPALQMANTLPVSGNLIDLYGNQGLPPPGLTISNSCPANLPNIKRELTESEARAL
AKERQKKDNHNLIERRRRFNINDRIKELGTLIPKSNDPDMRWNKGTILKASVDYIRKLQR
EQQRAKELENRQKKLEHANRHLLLRIQELEMQARAHGLSLIPSTGLCSPDLVNRIIKQEP
VLENCSQDLLQHHADLTCTTTLDLTDGTITFNNNLGTGTEANQAYSVPTKMGSKLEDILM
DDTLSPVGVTDPLLSSVSPGASKTSSRRSSMSMEETEHTC*
>ENSG00000196812|ENST00000340487
MTTALEPEDQKGLLIIKAEDHYWGQDSSSQKCSPHRRELYRQHFRKLCYQDAPGPREALT
QLWELCRQWLRPECHTKEQILDLLVLEQFLSILPKDLQAWVRAHHPETGEEAVTVLEDLE
RELDEPGKQVPGNSERRDILMDKLAPLGRPYESLTVQLHPKKTQLEQEAGKPQRNGDKTR
TKNEELFQKEDMPKDKEFLGEINDRLNKDTPQHPKSKDIIENEGRSEWQQRERRRYKCDE
CGKSFSHSSDLSKHRRTHTGEKPYKCDECGKAFIQRSHLIGHHRVHTGVKPYKCKECGKD
FSGRTGLIQHQRIHTGEKPYECDECGRPFRVSSALIRHQRIHTANKLY*
>ENSG00000284032|ENST00000362111
Sequence unavailable
>ENSG00000177885|ENST00000316615
MEAIAKYDFKATADDELSFKRGDILKVLNEECDQNWYKAELNGKDGFIPKNYIEMKPHPF
GNDVQHFKVLRDGAGKYFLWVVKFNSLNELVDYHRSTSVSRNQQIFLRDIEQVPQQPTYV
QALFDFDPQEDGELGFRRGDFIHVMDNSDPNWWKGACHGQTGMFPRNYVTPVNRNV*
>ENSG00000198482|ENST00000359798
MLREEAAQKRKGKESGMALPQGRLTFRDVAIEFSLAEWKFLNPAQRALYREVMLENYRNL
EAVDISSKHMMKEVLSTGQGNREVIHTGTLQRHQSYHIGDFCFQEIEKEIHNIEFQCQED
ERNGHEAPTTKIKKLTGSTDQHDHRHAGNKPIKDQLGSSFYSHLPELHIFQIKGEIANQL
EKSTSDASSVSTSQRISCRPQIHISNNYGNNPLNSSLLPQKQEVHMREKSFPCNESGKAF
NCSSLLRKHQIPHLGDKQYKCDVCGKLFNHKQYLACHRRCHTGEKPYKCKECGKSFSYKS
SLTCHHRLHTGVKPYKCNECGKVFRQNSALVIHKAIHTGEKPYKCNECGKAFNQQSHLSR
HQRLHTGVKPYKCKICEKAFACHSYLANHTRIHSGEKTYKCNECGKAFNHQSSLARHHIL
HTGEKPYKCEECDKVFSQKSTLERHKRIHTGEKPYKCKVCDTAFTCNSQLARHRRIHTGE
KTYKCNECRKTFSRRSSLLCHRRLHSGEKPYKCNQCGNTFRHRASLVYHRRLHTLEKSYK
CTVCNKVFMRNSVLAVHTRIHTAKKPYKCNECGKAFNQQSHLSRHRRLHTGEKPYKCEAC
DKVFGQKSALESHKRIHTGEKPYRCQVCDTAFTWNSQLARHTRIHTGEKTYKCNECGKTF
SYKSSLVWHRRLHGGEKSYKCKVCDKAFVCRSYVAKHTRIHSGMKPYKCNECSKTFSNRS
SLVCHRRIHSGEKPYKCSECSKTFSQKATLLCHRRLHSGEKPYKCNDCGNTFRHWSSLVY
HRRLHTGEKSYKCTVCDKAFVRNSYLARHIRIHTAEKPYKCNECGKAFNEQSHLSRHHRI
HTGEKPYKCEACDKVFSRKSHLKRHRIIHTGEKPYKCNECGKAFSDRSTLIHHQAIHGIG
KFD*
>ENSG00000146731|ENST00000335503
MAAVKTLNPKAEVARAQAALAVNISAARGLQDVLRTNLGPKGTMKMLVSGAGDIKLTKDG

NVLLHEMGLHPRIITEGFEAAKEKALQFLEEVKVSREMDRETLIDVARTSLRTKVHAELA
DVLTEAVVDSILAIKKQDEPIDLFMIEIMEMKHKSETDTSLIRGLVLDHGARHPDMKKRV
EDAYILTCNVSLEYEKTEVNSGFFYKSAEEREKLVKAERKFIEDRVKKIIELKRKVCGDS
DKGFVVINQKGIDPFSLDALSKEGIVALRRAKRRNMERLTLACGGVALNSFDDLSPDCLG
HAGLVYEYTLGEEKFTFIEKCNNPRSVTLLIKGPNKHTLTQIKDAVRDGLRAVKNAIDDG
CVVPGAGAVEVAMAEALIKHKPSVKGRAQLGVQAFADALLIIPKVLAQNSGFDLQETLVK
IQAEHSESGQLVGVDLNTGEPMVAAEVGVWDNYCVKKQLLHSCTVIATNILLVDEIMRAG
MSSLKG*
>ENSG00000108469|ENST00000317905
MSSHHTTFPFDPERRVRSTLKKVFGFDSFKTPLQESATMAVVKGNKDVFVCMPTGAGKSL
CYQLPALLAKGITIVVSPLIALIQDQVDHLLTLKVRVSSLNSKLSAQERKELLADLEREK
PQTKILYITPEMAASSSFQPTLNSLVSRHLLSYLVVDEAHCVSQWGHDFRPDYLRLGALR
SRLGHAPCVALTATATPQVQEDVFAALHLKKPVAIFKTPCFRANLFYDVQFKELISDPYG
NLKDFCLKALGQEADKGLSGCGIVYCRTREACEQLAIELSCRGVNAKAYHAGLKASERTL
VQNDWMEEKVPVIVATISFGMGVDKANVRFVAHWNIAKSMAGYYQESGRAGRDGKPSWCR
LYYSRNDRDQVSFLIRKEVAKLQEKRGNKASDKATIMAFDALVTFCEELGCRHAAIAKYF
GDALPACAKGCDHCQNPTAVRRRLEALERSSSWSKTCIGPSQGNGFDPELYEGGRKGYGD
FSRYDEGSGGSGDEGRDEAHKREWNLFYQKQMQLRKGKDPKIEEFVPPDENCPLKEASSR
RIPRLTVKAREHCLRLLEEALSSNRQSTRTADEADLRAKAVELEHETFRNAKVANLYKAS
VLKKVADIHRASKDGQPYDMGGSAKSCSAQAEPPEPNEYDIPPASHVYSLKPKRVGAGFP
KGSCPFQTATELMETTRIREQAPQPERGGEHEPPSRPCGLLDEDGSEPLPGPRGEVPGGS
AHYGGPSPEKKAKSSSGGSSLAKGRASKKQQLLATAAHKDSQSIARFFCRRVESPALLAS
APEAEGACPSCEGVQGPPMAPEKYTGEEDGAGGHSPAPPQTEECLRERPSTCPPRDQGTP
EVQPTPAKDTWKGKRPRSQQENPESQPQKRPRPSAKPSVVAEVKGSVSASEQGTLNPTAQ
DPFQLSAPGVSLKEAANVVVKCLTPFYKEGKFASKELFKGFARHLSHLLTQKTSPGRSVK
EEAQNLIRHFFHGRARCESEADWHGLCGPQR*
>ENSG00000132507|ENST00000336452
MCGTGGTDSKTRRPPHRASFLKRLESKPLKMADDLDFETGDAGASATFPMQCSALRKNGF
VVLKGRPCKIVEMSTSKTGKHGHAKVHLVGIDIFTGKKYEDICPSTHNMDVPNIKRNDFQ
LIGIQDGYLSLLQDSGEVREDLRLPEGDLGKEIEQKYDCGEEILITVLSAMTEEAAVAIK
AMAK*
>ENSG00000076928|ENST00000354532
MEDFARGAASPGPSRPGLVPVSIIGAEDEDFENELETNSEEQNSQFQSLEQVKRRPAHLM
ALLQHVALQFEPGPLLCCLHADMLGSLGPKEAKKAFLDFYHSFLEKTAVLRVPVPPNVAF
ELDRTRADLISEDVQRRFVQEVVQSQQVAVGRQLEDFRSKRLMGMTPWEQELAQLEAWVG
RDRASYEARERHVAERLLMHLEEMQHTISTDEEKSAAVVNAIGLYMRHLGVRTKSGDKKS
GRNFFRKKVMGNRRSDEPAKTKKGLSSILDAARWNRGEPQVPDFRHLKAEVDAEKPGATD
RKGGVGMPSRDRNIGAPGQDTPGVSLHPLSLDSPDREPGADAPLELGDSSPQGPMSLESL
APPESTDEGAETESPEPGDEGEPGRSGLELEPEEPPGWRELVPPDTLHSLPKSQVKRQEV
ISELLVTEAAHVRMLRVLHDLFFQPMAECLFFPLEELQNIFPSLDELIEVHSLFLDRLMK
RRQESGYLIEEIGDVLLARFDGAEGSWFQKISSRFCSRQSFALEQLKAKQRKDPRFCAFV
QEAESRPRCRRLQLKDMIPTEMQRLTKYPLLLQSIGQNTEEPTEREKVELAAECCREILH
HVNQAVRDMEDLLRLKDYQRRLDLSHLRQSSDPMLSEFKNLDITKKKLVHEGPLTWRVTK
DKAVEVHVLLLDDLLLLLQRQDERLLLKSHSRTLTPTPDGKTMLRPVLRLTSAMTREVAT

DHKAFYVLFTWDQEAQIYELVAQTVSERKNWCALITETAGSLKVPAPASRPKPRPSPSST
REPLLSSSENGNGGRETSPADARTERILSDLLPFCRPGPEGQLAATALRKVLSLKQLLFP
AEEDNGAGPPRDGDGVPGGGPLSPARTQEIQENLLSLEETMKQLEELEEEFCRLRPLLSQ
LGGNSVPQPGCT*
>ENSG00000173275|ENST00000339249
MAVALGCAIQASLNQGSVFQEYDTDCEVFRQRFRQFQYREAAGPHEAFNKLWELCCQWLK
PKMRSKEQILELLVLEQFLTILPTEIETWVREHCPENRERVVSLIEDLQRELEIPEQQVD
MHDMLLEELAPVGTAHIPPTMHLESPALQVMGPAQEAPVAEAWIPQAGPPELNYGATGEC
QNFLDPGYPLPKLDMNFSLENREEPWVKELQDSKEMKQLLDSKIGFEIGIENEEDTSKQK
KMETMYPFIVTLEGNALQGPILQKDYVQLENQWETPPEDLQTDLAKLVDQQNPTLGETPE
NSNLEEPLNPKPHKKKSPGEKPHRCPQCGKCFARKSQLTGHQRIHSGEEPHKCPECGKRF
LRSSDLYRHQRLHTGERPYECTVCKKRFTRRSHLIGHQRTHSEEETYKCLECGKSFCHGS
SLKRHLKTHTGEKPHRCHNCGKSFSRLTALTLHQRTHTEERPFKCNYCGKSFRQRPSLVI
HLRIHTGEKPYKCTHCSKSFRQRAGLIMHQVTHFRGLI*
>ENSG00000005801|ENST00000354599
MTLLTFRDVAIEFSLEEWKCLDLAQQNLYRDVMLENYRNLFSVGLTVCKPGLITCLEQRK
EPWNVKRQEAADGHPAMSSHFTQDLLPEQGIQDAFPKRILRGYGNCGLDNLYLRKDWESL
DECKLQKDYNGLNQCSSTTHSKIFQYNKYVKIFDNFSNLHRRNISNTGEKPFKCQECGKS
FQMLSFLTEHQKIHTGKKFQKCGECGKTFIQCSHFTEPENIDTGEKPYKCQECNNVIKTC
SVLTKNRIYAGGEHYRCEEFGKVFNQCSHLTEHEHGTEEKPCKYEECSSVFISCSSLSNQ
QMILAGEKLSKCETWYKGFNHSPNPSKHQRNEIGGKPFKCEECDSIFKWFSDLTKHKRIH
TGEKPYKCDECGKAYTQSSHLSEHRRIHTGEKPYQCEECGKVFRTCSSLSNHKRTHSEEK
PYTCEECGNIFKQLSDLTKHKKTHTGEKPYKCDECGKNFTQSSNLIVHKRIHTGEKPYKC
EECGRVFMWFSDITKHKKTHTGEKPYKCDECGKNFTQSSNLIVHKRIHTGEKPYKCEKCG
KAFTQFSHLTVHESIHT*
>ENSG00000177302|ENST00000321105
MIFPVARYALRWLRRPEDRAFSRAAMEMALRGVRKVLCVAEKNDAAKGIADLLSNGRMRR
REGLSKFNKIYEFDYHLYGQNVTMVMTSVSGHLLAHDFQMQFRKWQSCNPLVLFEAEIEK
YCPENFVDIKKTLERETRQCQALVIWTDCDREGENIGFEIIHVCKAVKPNLQVLRARFSE
ITPHAVRTACENLTEPDQRVSDAVDVRQELDLRIGAAFTRFQTLRLQRIFPEVLAEQLIS
YGSCQFPTLGFVVERFKAIQAFVPEIFHRIKVTHDHKDGIVEFNWKRHRLFNHTACLVLY
QLCVEDPMATVVEVRSKPKSKWRPQALDTVELEKLASRKLRINAKETMRIAEKLYTQGYI
SYPRTETNIFPRDLNLTVLVEQQTPDPRWGAFAQSILERGGPTPRNGNKSDQAHPPIHPT
KYTNNLQGDEQRLYEFIVRHFLACCSQDAQGQETTVEIDIAQERFVAHGLMILARNYLDV
YPYDHWSDKILPVYEQGSHFQPSTVEMVDGETSPPKLLTEADLIALMEKHGIGTDATHAE
HIETIKARMYVGLTPDKRFLPGHLGMGLVEGYDSMGYEMSKPDLRAELEADLKLICDGKK
DKFVVLRQQVQKYKQVFIEAVAKAKKLDEALAQYFGNGTELAQQEDIYPAMPEPIRKCPQ
CNKDMVLKTKKNGGFYLSCMGFPECRSAVWLPDSVLEASRDSSVCPVCQPHPVYRLKLKF
KRGSLPPTMPLEFVCCIGGCDDTLREILDLRFSGGPPRASQPSGRLQANQSLNRMDNSQH
PQPADSRQTGSSKALAQTLPPPTAAGESNSVTCNCGQEAVLLTVRKEGPNRGRQFFKCNG
GSCNFFLWADSPNPGAGGPPALAYRPLGASLGCPPGPGIHLGGFGNPGDGSGSGTSCLCS
QPSVTRTVQKDGPNKGRQFHTCAKPREQQCGFFQWVDENTAPGTSGAPSWTGDRGRTLES
EARSKRPRASSSDMGSTAKKPRKCSLCHQPGHTRPFCPQNR*
>ENSG00000184635|ENST00000343769

MGPLQFRDVAIEFSLEEWHCLDTAQRNLYRNVMLENYSNLVFLGIVVSKPDLIAHLEQGK
KPLTMKRHEMVANPSVICSHFAQDLWPEQNIKDSFQKVILRRYEKRGHGNLQLIKRCESV
DECKVHTGGYNGLNQCSTTTQSKVFQCDKYGKVFHKFSNSNRHNIRHTEKKPFKCIECGK
AFNQFSTLITHKKIHTGEKPYICEECGKAFKYSSALNTHKRIHTGEKPYKCDKCDKAFIA
SSTLSKHEIIHTGKKPYKCEECGKAFNQSSTLTKHKKIHTGEKPYKCEECGKAFNQSSTL
TKHKKIHTGEKPYVCEECGKAFKYSRILTTHKRIHTGEKPYKCNKCGKAFIASSTLSRHE
FIHMGKKHYKCEECGKAFIWSSVLTRHKRVHTGEKPYKCEECGKAFKYSSTLSSHKRSHT
GEKPYKCEECGKAFVASSTLSKHEIIHTGKKPYKCEECGKAFNQSSSLTKHKKIHTGEKP
YKCEECGKAFNQSSSLTKHKKIHTGEKPYKCEECGKAFNQSSTLIKHKKIHTREKPYKCE
ECGKAFHLSTHLTTHKILHTGEKPYRCRECGKAFNHSATLSSHKKIHSGEKPYECDKCGK
AFISPSSLSRHEIIHTGEKP*
>ENSG00000225830|ENST00000355832
MPNEGIPHSSQTQEQDCLQSQPVSNNEEMAIKQESGGDGEVEEYLSFRSVGDGLSTSAVG
CASAAPRRGPALLHIDRHQIQAVEPSAQALELQGLGVDVYDQDVLEQGVLQQVDNAIHEA
SRASQLVDVEKEYRSVLDDLTSCTTSLRQINKIIEQLSPQAATSRDINRKLDSVKRQKYN
KEQQLKKITAKQKHLQAILGGAEVKIELDHASLEEDAEPGPSSLGSMLMPVQETAWEELI
RTGQMTPFGTQIPQKQEKKPRKIMLNEASGFEKYLADQAKLSFERKKQGCNKRAARKAPA
PVTPPAPVQNKNKPNKKARVLSKKEERLKKHIKKLQKRALQFQGKVGLPKARRPWESDMR
PEAEGDSEGEESEYFPTEEEEEEEDDEVEGAEADLSGDGTDYELKPLPKGGKRQKKVPVQ
EIDDDFFPSSGEEAEAASVGEGGGGGRKVGRYRDDGDEDYYKQRLRRWNKLRLQDKEKRL
KLEDDSEESDAEFDEGFKVPGFLFKKLFKYQQTGVRWLWELHCQQAGGILGDEMGLGKTI
QIIAFLAGLSYSKIRTRGSNYRFEGLGPTVIVCPTTVMHQWVKEFHTWWPPFRVAILHET
GSYTHKKEKLIRDVAHCHGILITSYSYIRLMQDDISRYDWHYVILDEGHKIRNPNAAVTL
ACKQFRTPHRIILSGSPMQNNLRELWSLFDFIFPGKLGTLPVFMEQFSVPITMGGYSNAS
PVQVKTAYKCACVLRDTINPYLLRRMKSDVKMSLSLPDKNEQVLFCRLTDEQHKVYQNFV
DSKEVYRILNGEMQIFSGLIALRKICNHPDLFSGGPKNLKGLPDDELEEDQFGYWKRSGK
MIVVESLLKIWHKQGQRVLLFSQSRQMLDILEVFLRAQKYTYLKMDGTTTIASRQPLITR
YNEDTSIFVFLLTTRVGGLGVNLTGANRVVIYDPDWNPSTDTQARERAWRIGQKKQVTVY
RLLTAGTIEEKIYHRQIFKQFLTNRVLKDPKQRRFFKSNDLYELFTLTSPDASQSTETSA
IFAGTGSDVQTPKCHLKRRIQPAFGADHDVPKRKKFPASNISVNDATSSEEKSEAKGAEV
NAVTSNRSDPLKDDPHMSSNVTSNDRLGEETNAVSGPEELSVISGNGECSNSSGTGKTSM
PSGDESIDEKLGLSYKRERPSQAQTEAFWENKQMENNFYKHKSKTKHHSVAEEETLEKHL
RPKQKPKNSKHCRDAKFEGTRIPHLVKKRRYQKQDSENKSEAKEQSNDDYVLEKLFKKSV
GVHSVMKHDAIMDGASPDYVLVEAEANRVAQDALKALRLSRQRCLGAVSGVPTWTGHRGI
SGAPAGKKSRFGKKRNSNFSVQHPSSTSPTEKCQDGIMKKEGKDNVPEHFSGRAEDADSS
SGPLASSSLLAKMRARNHLILPERLESESGHLQEASALLPTTEHDDLLVEMRNFIAFQAH
TDGQASTREILQEFESKLSASQSCVFRELLRNLCTFHRTSGGEGIWKLKPEYC*
>ENSG00000199030|ENST00000362160
Sequence unavailable
>ENSG00000037897|ENST00000324871
MAAETRNVAGAEAPPPQKRYYRQRAHSNPMADHTLRYPVKPEEMDWSELYPEFFAPLTQN
QSHDDPKDKKEKRAQAQVEFADIGCGYGGLLVELSPLFPDTLILGLEIRVKVSDYVQDRI
RALRAAPAGGFQNIACLRSNAMKHLPNFFYKGQLTKMFFLFPDPHFKRTKHKWRIISPTL
LAEYAYVLRVGGLVYTITDVLELHDWMCTHFEEHPLFERVPLEDLSEDPVVGHLGTSTEE

GKKVLRNGGKNFPAIFRRIQDPVLQAVTSQTSLPGH*
>ENSG00000167380|ENST00000337433
MNMFKEAVTFKDVAVAFTEEELGLLGPAQRKLYRDVMVENFRNLLSVGHPPFKQDVSPIE
RNEQLWIMTTATRRQGNLGEKNQSKLITVQDRESEEELSCWQIWQQIANDLTRCQDSMIN
NSQCHKQGDFPYQVGTELSIQISEDENYIVNKADGPNNTGNPEFPILRTQDSWRKTFLTE
SQRLNRDQQISIKNKLCQCKKGVDPIGWISHHDGHRVHKSEKSYRPNDYEKDNMKILTFD
HNSMIHTGQKSYQCNECKKPFSDLSSFDLHQQLQSGEKSLTCVERGKGFCYSPVLPVHQK
VHVGEKLKCDECGKEFSQGAHLQTHQKVHVIEKPYKCKQCGKGFSRRSALNVHCKVHTAE
KPYNCEECGRAFSQASHLQDHQRLHTGEKPFKCDACGKSFSRNSHLQSHQRVHTGEKPYK
CEECGKGFICSSNLYIHQRVHTGEKPYKCEECGKGFSRPSSLQAHQGVHTGEKSYICTVC
GKGFTLSSNLQAHQRVHTGEKPYKCNECGKSFRRNSHYQVHLVVHTGEKPYKCEICGKGF
SQSSYLQIHQKAHSIEKPFKCEECGQGFNQSSRLQIHQLIHTGEKPYKCEECGKGFSRRA
DLKIHCRIHTGEKPYNCEECGKVFRQASNLLAHQRVHSGEKPFKCEECGKSFGRSAHLQA
HQKVHTGDKPYKCDECGKGFKWSLNLDMHQRVHTGEKPYKCGECGKYFSQASSLQLHQSV
HTGEKPYKCDVCGKVFSRSSQLQSHQRVHTGEKPYKCEICGKSFSWRSNLTVHHRIHVGD
KSYKSNRGGKNIRESTQEKKSIK*
>ENSG00000111877|ENST00000316068
MNSDQVTLVGQVFESYVSEYHKNDILLILKERDEDAHYPVVVNAMTLFETNMEIGEYFNM
FPSEVLTIFDSALRRSALTILQSLSQPEAVSMKQNLHARISGLPVCPELVREHIPKTKDV
GHFLSVTGTVIRTSLVKVLEFERDYMCNKCKHVFVIKADFEQYYTFCRPSSCPSLESCDS
SKFTCLSGLSSSPTRCRDYQEIKIQEQVQRLSVGSIPRSMKVILEDDLVDSCKSGDDLTI
YGIVMQRWKPFQQDVRCEVEIVLKANYIQVNNEQSSGIIMDEEVQKEFEDFWEYYKSDPF
AGRNVILASLCPQVFGMYLVKLAVAMVLAGGIQRTDATGTRVRGESHLLLVGDPGTGKSQ
FLKYAAKITPRSVLTTGIGSTSAGIVCDNFK*
>ENSG00000070476|ENST00000336332
MDLPALLPAPTARGGQHGGGPGPLRRAPAPLGASPARRRLLLVRGPEDGGPGARPGEASG
PSPPPAEDDSDGDSFLVLLEVPHGGAAAEAAGSQEAEPGSRVNLASRPEQGPSGPAAPPG
PGVAPAGAVTISSQDLLVRLDRGVLALSAPPGPATAGAAAPRRAPQASGPSTPGYRCPEP
QCALAFAKKHQLKVHLLTHGGGQGRRPFKCPLEGCGWAFTTSYKLKRHLQSHDKLRPFGC
PVGGCGKKFTTVYNLKAHMKGHEQESLFKCEVCAERFPTHAKLSSHQRSHFEPERPYKCD
FPGCEKTFITVSALFSHNRAHFREQELFSCSFPGCSKQYDKACRLKIHLRSHTGERPFIC
DSDSCGWTFTSMSKLLRHRRKHDDDRRFTCPVEGCGKSFTRAEHLKGHSITHLGTKPFEC
PVEGCCARFSARSSLYIHSKKHVQDVGAPKSRCPVSTCNRLFTSKHSMKAHMVRQHSRRQ
DLLPQLEAPSSLTPSSELSSPGQSELTNMDLAALFSDTPANASGSAGGSDEALNSGILTI
DVTSVSSSLGGNLPANNSSLGPMEPLVLVAHSDIPPSLDSPLVLGTAATVLQQGSFSVDD
VQTVSAGALGCLVALPMKNLSDDPLALTSNSNLAAHITTPTSSSTPRENASVPELLAPIK
VEPDSPSRPGAVGQQEGSHGLPQSTLPSPAEQHGAQDTELSAGTGNFYLV*
>ENSG00000199150|ENST00000362280
Sequence unavailable
>ENSG00000178257|ENST00000327157
MGSRCAKLNTGQSPGHSPGHSTGHGRGHESSMKKLMACVSQDNFSLSSAGEEEEEEEEEG
EEEEKEELPVQGKLLLLEPERQEEGHKDNAEAQQSPEPKRTPS*
>ENSG00000119508|ENST00000330847
MHDSIRFGNVDMPCVQAQYSPSPPGSSYAAQTYSSEYTTEIMNPDYTKLTMDLGSTEITA

TATTSLPSISTFVEGYSSNYELKPSCVYQMQRPLIKVEEGRAPSYHHHHHHHHHHHHHHQ
QQHQQPSIPPASSPEDEVLPSTSMYFKQSPPSTPTTPAFPPQAGALWDEALPSAPGCIAP
GPLLDPPMKAVPTVAGARFPLFHFKPSPPHPPAPSPAGGHHLGYDPTAAAALSLPLGAAA
AAGSQAAALESHPYGLPLAKRAAPLAFPPLGLTPSPTASSLLGESPSLPSPPSRSSSSGE
GTCAVCGDNAACQHYGVRTCEGCKGFFKRTVQKNAKYVCLANKNCPVDKRRRNRCQYCRF
QKCLSVGMVKEVVRTDSLKGRRGRLPSKPKSPLQQEPSQPSPPSPPICMMNALVRALTDS
TPRDLDYSRYCPTDQAAAGTDAEHVQQFYNLLTASIDVSRSWAEKIPGFTDLPKEDQTLL
IESAFLELFVLRLSIRSNTAEDKFVFCNGLVLHRLQCLRGFGEWLDSIKDFSLNLQSLNL
DIQALACLSALSMITERHGLKEPKRVEELCNKITSSLKDHQSKGQALEPTESKVLGALVE
LRKICTLGLQRIFYLKLEDLVSPPSIIDKLFLDTLPF*
>ENSG00000198081|ENST00000357006
MEFFISMSETIKYNDDDHKTLFLKTLNEQRLEGEFCDIAIVVEDVKFRAHRCVLAACSTY
FKKLFKKLEVDSSSVIEIDFLRSDIFEEVLNYMYTAKISVKKEDVNLMMSSGQILGIRFL
DKLCSQKRDVSSPDENNGQSKSKYCLKINRPIGDAADTQDDDVEEIGDQDDSPSDDTVEG
TPPSQEDGKSPTTTLRVQEAILKELGSEEVRKVNCYGQEVESMETPESKDLGSQTPQALT
FNDGMSEVKDEQTPGWTTAASDMKFEYLLYGHHREQIACQACGKTFSDEGRLRKHEKLHT
ADRPFVCEMCTKGFTTQAHLKEHLKIHTGYKPYSCEVCGKSFIRAPDLKKHERVHSNERP
FACHMCDKAFKHKSHLKDHERRHRGEKPFVCGSCTKAFAKASDLKRHENNMHSERKQVTP
SAIQSETEQLQAAAMAAEAEQQLETIACS*
>ENSG00000173598|ENST00000337179
MMKFKPNQTRTYDREGFKKRAACLCFRSEQEDEVLLVSSSRYPDQWIVPGGGMEPEEEPG
GAAVREVYEEAGVKGKLGRLLGIFEQNQDRKHRTYVYVLTVTEILEDWEDSVNIGRKREW
FKVEDAIKVLQCHKPVHAEYLEKLKLGCSPANGNSTVPSLPDNNALFVTAAQTSGLPSSV
R*
>ENSG00000130119|ENST00000360845
MMKLRHKNKKPGEGSKGHKKISWPYPQPAKQNGKKATSKVPSAPHFVHPNDHANREAELK
KKWVEEMREKQQAAREQERQKRRTIESYCQDVLRRQEEFEHKEEVLQELNMFPQLDDEAT
RKAYYKEFRKVVEYSDVILEVLDARDPLGCRCFQMEEAVLRAQGNKKLVLVLNKIDLVPK
EVVEKWLDYLRNELPTVAFKASTQHQVKNLNRCSVPVDQASESLLKSKACFGAENLMRVL
GNYCRLGEVRTHIRVGVVGLPNVGKSSLINSLKRSRACSVGAVPGITKFMQEVYLDKFIR
LLDAPGIVPGPNSEVGTILRNCVHVQKLADPVTPVETILQRCNLEEISNYYGVSGFQTTE
HFLTAVAHRLGKKKKGGLYSQEQAAKAVLADWVSGKISFYIPPPATHTLPTHLSAEIVKE
MTEVFDIEDTEQANEDTMECLATGESDELLGDTDPLEMEIKLLHSPMTKIADAIENKTTV
YKIGDLTGYCTNPNRHQMGWAKRNVDHRPKSNSMVDVCSVDRRSVLQRIMETDPLQQGQA
LASALKNKKKMQKRADKIASKLSDSMMSALDLSGNADDGVGD*
>ENSG00000179412|ENST00000323770
MASNVTNKMDPHSMNSRVFIGNLNTLVVKKSDVEAIFSKYGKIAGCSVHKGFAFVQYDKE
KNARAAVAGEDGRMIASQVVDINLAAEPKVNRGNAGVKRSAAEMYGSSFDLDYNLQRDYY
GGMYSFPARVPPPPPIALAVVPSKRQRISGNTSRRGKSGFNSKSGKRGSSKSGKLKGDDL
QAIKQELTQIKQKVDSLLENLEKIEKEHCKQGVEVKNAKSEEEQTSSSSKKDKTHVKMES
EGGADDSVEEGDLLCDDDNEDQGDNQLELIKDDEKGAEEGEDDRDRANGQDDS*
>ENSG00000105968|ENST00000349299
MAGGKAGKDSGKAKAKAVSRSQRAGLQVLELAGNASKDLKVKRITPRHLQLAIRGDEELD
SLIKATIAGGGVIPHIHKSLIGKKGQQKTA*

>ENSG00000106268|ENST00000356714
MGASRLYTLVLVLQPQRVLLGMKKRGFGAGRWNGFGGKVQEGETIEDGARRELQEESGLT
VDALHKVGQIVFEFVGEPELMDVHVFCTDSIQGTPVESDEMRPCWFQLDQIPFKDMWPDD
SYWFPLLLQKKKFHGYFKFQGQDTILDYTLREVDTV*
>ENSG00000065183|ENST00000349139
MGLTKQYLRYVASAVFGVIGSQKGNIVFVTLRGEKGRYVAVPACEHVFIWDLRKGEKILI
LQGLKQEVTCLCPSPDGLHLAVGYEDGSIRIFSLLSGEGNVTFNGHKAAITTLKYDQLGG
RLASGSKDTDIIVWDVINESGLYRLKGHKDAITQALFLREKNLLVTSGKDTMVKWWDLDT
QHCFKTMVGHRTEVWGLVLLSEEKRLITGASDSELRVWDIAYLQEIEDPEEPDPKKIKGS
SPGIQDTLEAEDGAFETDEAPEDRILSCRKAGSIMREGRDRVVNLAVDKTGRILACHGTD
SVLELFCILSKKEIQKKMDKKMKKARKKAKLHSSKGEEEDPEVNVEMSLQDEIQRVTNIK
TSAKIKSFDLIHSPHGELKAVFLLQNNLVELYSLNPSLPTPQPVRTSRITIGGHRSDVRT
LSFSSDNIAVLSAAADSIKIWNRSTLQCIRTMTCEYALCSFFVPGDRQVVIGTKTGKLQL
YDLASGNLLETIDAHDGALWSMSLSPDQRGFVTGGADKSVKFWDFELVKDENSTQKRLSV
KQTRTLQLDEDVLCVSYSPNQKLLAVSLLDCTVKIFYVDTLKFFLSLYGHKLPVICMDIS
HDGALIATGSADRNVKIWGLDFGDCHKSLFAHDDSVMYLQFVPKSHLFFTAGKDHKIKQW
DADKFEHIQTLEGHHQEIWCLAVSPSGDYVVSSSHDKSLRLWERTREPLILEEEREMERE
AEYEESVAKEDQPAVPGETQGDSYFTGKKTIETVKAAERIMEAIELYREETAKMKEHKAI
CKAAGKEVPLPSNPILMAYGSISPSAYVLEIFKGIKSSELEESLLVLPFSYVPDILKLFN
EFIQLGSDVELICRCLFFLLRIHFGQITSNQMLVPVIEKLRETTISKVSQVRDVIGFNMA
GLDYLKRECEAKSEVMFFADATSHLEEKKRKRKKREKLILTLT*
>ENSG00000171467|ENST00000361428
MYRSSARSSVSSHRPKDDGGGGPRSGRSSGSSSGPARRSSPPPPPSGSSSRTPARRPRSP
SGHRGRRASPSPPRGRRVSPSPPRARRGSPSPPRGRRLFPPGPAGFRGSSRGESRADYAR
DGRGDHPGDSGSRRRSPGLCSDSLEKSLRITVGNDHFCVSTPERRRLSDRLGSPVDNLED
MDRDDLTDDSVFTRSSQCSRGLERYISQEEGPLSPFLGQLDEDYRTKETFLHRSDYSPHI
SCHDELLRGTERNREKLKGYSIRSEERSREAKRPRYDDTVKINSMGGDHPSFTSGTRNYR
QRRRSPSPRFLDPEFRELDLARRKREEEEERSRSLSQELVGVDGGGTGCSIPGLSGVLTA
SEPGYSLHRPEEVSVMPKKSILKKRIEVDIMEPSMQLESFSSSTSSSQDHPLYSGHPSLP
LSGAIAAFASEIENKGTMVETALKEPQGNLYQWGPLPGIPKDNSPLREKFGSFLCHKDNL
DLKAEGPERHTDFLLPHERASQDGSGFSRILSMLADSTSTQEKRRRSFPDIEDEEKFLYG
DEEEDLKAESVPKPLGSSESEVMRQKASSLPSSAPAVKLESLEETNPEYAKIHDLLKTIG
LDIGVAEISQLAARTQERLHGKKPSLRSSADRRSSVDRYFSADHCSSVDHRFSADRCSSV
DHCFSADRRSSDPHRLESREAHHSNTHSPEVSHPHPPSPVDPYLLTKNSPPFLKSDHPVG
HISGPEVVGSGFQSSVAVRCMLPSAPSAPIRLPHTAALSQFHMPRASQFAAARIPPNYQG
PAIPPASFDAYRHYMAYAASRWPMYPTSQPSNHPVPEPHRIMPITKQATRSRPNLRVIPT
VTPDKPKQKESLRGSIPAAQVPVQVSIPSLIRYNPEKISDEKNRASQKQKVIEEREKLKN
DREARQKKMYYLRTELERLHKQQGEMLRKKRREKDGHKDPLLVEVSRLQDNIMKDIAELR
QEAEEAEKKQSELDKVAQILGINIFDKSQKSLSDSREPTEKPGKAEKSKSPEKVSSFSNS
SSNKESKVNNEKFRTKSPKPAESPQSATKQLDQPTAAYEYYDAGNHWCKDCNTICGTMFD
FFTHMHNKKHTQTLDPYNRPWASKTQSEAKQDAIKRTDKITVPAKGSEFLVPISGFYCQL
CEEFLGDPISGEQHVKGHQHNEKYKKYVDENPLYEERRNLDRQAGLAVVLETERRRQSEL
KRKLSEKPKEEKKEKKAKAVKEVKEDDKVSEKLEDQLSEGRNSPEKAENKRNTGIKLQLK
EEVKKESPTSSSFGKFSWKKPEKEEEKSSLVTPSISKEEILESSKDKEDGKTEAGKAKPI

KIKLSGKTVVAHTSPWMPVVTTSTQTKIRPNLPIPSTVLRKSCSATMSKPAPLNTFLSIK
SSGTTAKPLPVVKESSADLLLPPDIISKAFGGEEVILKGSPEEKVVLAEKSEPSHLPEQI
LPPPPPPPPPPPPPPPVIPHPAAPSAAQANAILAPVKSNPVVSQTLSPGFVGPNILNPVL
PVAIMASAQPAAIPSDETAPGVSESDRDQTLFSVLVRPPPPLSSVFSEQAKKLEKRNSCL
ATANAKDLYDIFYSSGGKGAPETKGAPETKLSGGPLANGENSNLSRTKSSDTSSTSPLNS
SASQEELHQDEGLVAAPIVSNSEKPIAKTLVALGKWSVVEHVGPKSTGSTYGFLQPLTRL
CQSRPYETITPKTDTLAIWTSSSFQSDTSRDISPEKSELDLGEPGPPGVEPPPQLLDIQC
KESQKLVEIHLRESVNQDKESQELRKSEDCRESEIETNTELKERVKELSEGIVDEGVSTS
IGPHSIDDSNLNHGNRYMWEGEVKQPNLLMIDKEAEQSNKLMTGSETPSKVVIKLSPQAC
SFTKAKLDSFLSEARSLLNPQDTPVKISAPELLLHSPARSAMCLTGSPQEQGVSVVSEEG
LENSAPESASRTSRYRSLKLKRERSKDFQVKKIYELAVWDENKKRPETWESPEKPKTEAL
ELQDVHPELTVTIESKALEDFEATDLKVEELTALGNLGDMPVDFCTTRVSPAHRSPTVLC
QKVCEENSVSPIGCNSSDPADFEPIPSFSGFPLDSPKTLVLDFETEGERNSPNPRSVRIP
SPNILKTGLTENVDRGLGGLEGTHQALDLLAGGMMPEEVKESSQLDKQESLGLELKTINS
AGLGPSPCLPDLVDFVTRTSGVQKDKLCSPLSEPGDPSKCSSLELGPLQLEISNASTTEV
AILQVDDDSGDPLNLVKAPVSRSPPREQVIEDNMVPQGMPEQETTVGAIQDHTESSVHN*
>ENSG00000199082|ENST00000362212
Sequence unavailable
>ENSG00000105750|ENST00000328178
MGPLTFRDVAIEFSLKEWQCLDTAQRNLYRNVMLENYRNLVFLGITVSKPDLITCLEQGK
EAWSMKRHEIMVAKPTVMCSHFAQDLWPEQNIKDSFQKVTLKRYGKCRHENLPLRKGCES
MDECKMHKGGCNGLNQCLTATQSKIFQCDKYVKVAHKFSNSNRHEIRHTKKKPFKCTKCG
KSFGMISCLTEHSRIHTRVNFYKCEECGKAFNWSSTLTKHKRIHTGEKPYKCEECGKAFN
QSSNLIKHKKIHTGEKPYKCEECGKTFNRFSTLTTHKIIHTGEKPYKCKECGKAFNRSST
LTTHRKIHTGEKPYKCEECGKAFKQSSNLTTHKIIHTGEKPYKCKKCGKAFNQSAHLTTH
EVIHTGEKPYKCEKCGKAFNHFSHLTTHKIIHTGEKPYKCKECGKAFKHSSTLTKHKIIH
TGEKPYKCKECEKAFNQSSKLTEHKKIHTGEKPYECEKCGKAFNQSSNLTRHKKSHTEEK
PYKCEECGKGFKWPSTLTIHKIIHTGEKPYKCEECGKAFNQSSKLTKHKKIHTGEKPYTC
EECGKAFNQSSNLTKHKRIHTGEKPYKCEECDKAFKWSSVLTKHKIIHTGEKLQI*
>ENSG00000198056|ENST00000338193
METFDPTELPELLKLYYRRLFPYSQYYRWLNYGGVIKNYFQHREFSFTLKDDIYIRYQSF
NNQSDLEKEMQKMNPYKIDIGAVYSHRPNQHNTVKLGAFQAQEKELVFDIDMTDYDDVRR
CCSSADICPKCWTLMTMAIRIIDRALKEDFGFKHRLWVYSGRRGVHCWVCDESVRKLSSA
VRSGIVEYLSLVKGGQDVKKKVHLSEKIHPFIRKSINIIKKYFEEYALVNQDILENKESW
DKILALVPETIHDELQQSFQKSHNSLQRWEHLKKVASRYQNNIKNDKYGPWLEWEIMLQY
CFPRLDINVSKGINHLLKSPFSVHPKTGRISVPIDLQKVDQFDPFTVPTISFICRELDAI
STNEEEKEENEAESDVKHRTRDYKKTSLAPYVKVFEHFLENLDKSRKGELLKKSDLQKDF
*
>ENSG00000187736|ENST00000356853
MEELEQGLLMQPWAWLQLAENSLLAKVFITKQGYALLVSDLQQVWHEQVDTSVVSQRAKE
LNKRLTAPPAAFLCHLDNLLRPLLKDAAHPSEATFSCDCVADALILRVRSELSGLPFYWN
FHCMLASPSLVSQHLIRPLMGMSLALQCQVRELATLLHMKDLEIQDYQESGATLIRDRLK
TEPFEENSFLEQFMIEKLPEACSIGDGKPFVMNLQDLYMAVTTQEVQVGQKHQGAGDPHT
SNSASLQGIDSQCVNQPEQLVSSAPTLSAPEKESTGTSGPLQRPQLSKVKRKKPRGLFS*

>ENSG00000152455|ENST00000354919
MAAVGAEARGAWCVPCLVSLDTLQELCRKEKLTCKSIGITKRNLNNYEVEYLCDYKVVKD
MEYYLVKWKGWPDSTNTWEPLQNLKCPLLLQQFSNDKHNYLSQVKKGKAITPKDNNKTLK
PAIAEYIVKKAKQRIALQRWQDELNRRKNHKGMIFVENTVDLEGPPSDFYYINEYKPAPG
ISLVNEATFGCSCTDCFFQKCCPAEAGVLLAYNKNQQIKIPPGTPIYECNSRCQCGPDCP
NRIVQKGTQYSLCIFRTSNGRGWGVKTLVKIKRMSFVMEYVGEVITSEEAERRGQFYDNK
GITYLFDLDYESDEFTVDAARYGNVSHFVNHSCDPNLQVFNVFIDNLDTRLPRIALFSTR
TINAGEELTFDYQMKGSGDISSDSIDHSPAKKRVRTVCKCGAVTCRGYLN*
>ENSG00000175197|ENST00000346473
MAAESLPFSFGTLSSWELEAWYEDLQEVLSSDENGGTYVSPPGNEEEESKIFTTLDPASL
AWLTEEEPEPAEVTSTSQSPHSPDSSQSSLAQEEEEEDQGRTRKRKQSGHSPARAGKQRM
KEKEQENERKVAQLAEENERLKQEIERLTREVEATRRALIDRMVNLHQA*
>ENSG00000164684|ENST00000327835
MTFTFQSEDLKRDCGKKMSHQHVFSLAMEEDVKTADTKKASRILDHEKENTRSICLLEQK
RKVVSSNIDVPPARKSSEELDMDKVTAAMVLTSLSTSPLVRSPPVRPNESLSGSWKEGGC
VPSSTSSSGYWSWSAPSDQSNPSTPSPPLSADSFKPFRSPAQPDDGIDEAEASNLLFDEP
IPRKRKNSMKVMFKCLWKNCGKVLSTAAGIQKHIRTIHLGRVGDSDYSDGEEDFYYTEIK
LNTDSVADGLSSLAPVSPSQSLASPPTFPIPDSSRTETPCAKTETKLMTPLSRSAPTTLY
LVHTDHAYQATPPVTIPGSAKFTPNGSSFSISWQSPPVTFTGIPVSPTHHPVGTGEQRQH
AHTVLSSPPRGTVSLRKPRGEGKKCRKVYGMENRDMWCTACRWKKACQRFLD*
>ENSG00000185811|ENST00000359197
MDADEGQDMSQVSGKESPPVSDTPDEGDEPMPIPEDLSTTSGGQQSSKSDRVVASNVKVE
TQSDEENGRACEMNGEECAEDLRMLDASGEKMNGSHRDQGSSALSGVGGIRLPNGKLKCD
ICGIICIGPNVLMVHKRSHTGERPFQCNQCGASFTQKGNLLRHIKLHSGEKPFKCHLCNY
ACRRRDALTGHLRTHSVIKEETNHSEMAEDLCKIGSERSLVLDRLASNVAKRKSSMPQKF
LGDKGLSDTPYDSSASYEKENEMMKSHVMDQAINNAINYLGAESLRPLVQTPPGGSEVVP
VISPMYQLHKPLAEGTPRSNHSAQDSAVENLLLLSKAKLVPSEREASPSNSCQDSTDTES
NNEEQRSGLIYLTNHIAPHARNGLSLKEEHRAYDLLRAASENSQDALRVVSTSGEQMKVY
KCEHCRVLFLDHVMYTIHMGCHGFRDPFECNMCGYHSQDRYEFSSHITRGEHRFHMS*
>ENSG00000095794|ENST00000356917
MAVTGDDTAATGDMPTYQIRAPTAALPQGVVMAASPGSLHSPQQLAEEATRKRELRLMKN
REAARECRRKKKEYVKCLENRVAVLENQNKTLIEELKALKDLYCHKVE*
>ENSG00000119725|ENST00000334521
MLPDDTTNHSNSSKEVPSSAVLRSLRVNVGPDGEETRAQTVQKSPEFLSTSESSSLLQDL
QPSDSTSFILLNLTRAGLGSSAEHLVFVQDEAEDSGNDFLSSESTDSSIPWFLRVQELAH
DSLIAATRAQLAKNAKTSSNGENVHLGSGDGQSKDSGPLPQVEKKLKCTVEGCDRTFVWP
AHFKYHLKTHRNDRSFICPAEGCGKSFYVLQRLKVHMRTHNGEKPFMCHESGCGKQFTTA
GNLKNHRRIHTGEKPFLCEAQGCGRSFAEYSSLRKHLVVHSGEKPHQCQVCGKTFSQSGS
RNVHMRKHHLQLGAAGSQEQEQTAEPLMGSSLLEEASVPSKNLVSMNSQPSLGGESLNLP
NTNSILGVDDEVLAEGSPRSLSSVPDVTHHLVTMQSGRQSYEVSVLTAVNPQELLNQGDL
TERRT*
>ENSG00000119508|ENST00000338488
MPCVQAQYSPSPPGSSYAAQTYSSEYTTEIMNPDYTKLTMDLGSTEITATATTSLPSIST
FVEGYSSNYELKPSCVYQMQRPLIKVEEGRAPSYHHHHHHHHHHHHHHQQQHQQPSIPPA

SSPEDEVLPSTSMYFKQSPPSTPTTPAFPPQAGALWDEALPSAPGCIAPGPLLDPPMKAV
PTVAGARFPLFHFKPSPPHPPAPSPAGGHHLGYDPTAAAALSLPLGAAAAAGSQAAALES
HPYGLPLAKRAAPLAFPPLGLTPSPTASSLLGESPSLPSPPSRSSSSGEGTCAVCGDNAA
CQHYGVRTCEGCKGFFKRTVQKNAKYVCLANKNCPVDKRRRNRCQYCRFQKCLSVGMVKE
VVRTDSLKGRRGRLPSKPKSPLQQEPSQPSPPSPPICMMNALVRALTDSTPRDLDYSRVS
FMISCFQMNDQGLYLWLLVIRVD*
>ENSG00000183072|ENST00000329198
MFPSPALTPTPFSVKDILNLEQQQRSLAAAGELSARLEATLAPSSCMLAAFKPEAYAGPE
AAAPGLPELRAELGRAPSPAKCASAFPAAPAFYPRAYSDPDPAKDPRAEKKELCALQKAV
ELEKTEADNAERPRARRRRKPRVLFSQAQVYELERRFKQQRYLSAPERDQLASVLKLTST
QVKIWFQNRRYKCKRQRQDQTLELVGLPPPPPPPARRIAVPVLVRDGKPCLGDSAPYAPA
YGVGLNPYGYNAYPAYPGYGGAACSPGYSCTAAYPAGPSPAQPATAAANNNFVNFGVGDL
NAVQSPGIPQSNSGVSTLHGIRAW*
>ENSG00000176302|ENST00000317011
MGNELFLAFTTSHLPLAEQKLARYKLRIVKPPKLPLEKKPNPDKDGPDYEPNLWMWVNPN
IVYPPGKLEVSGRRKREDLTSTLPSSQPPQKEEDASCSEAAGVESLSQSSSKRSPPRKRF
AFSPSTWELTEEEEAEDQEDSSSMALPSPHKRAPLQSRRLRQASSQAGRLWSRPPLNYFH
LIALALRNSSPCGLNVQQIYSFTRKHFPFFRTAPEGWKNTVRHNLCFRDSFEKVPVSMQG
GASTRPRSCLWKLTEEGHRRFAEEARALASTRLESIQQCMSQPDVMPFLFDL*
>ENSG00000154556|ENST00000319471
MSVTLTSVKRVQSSPNLLAAGRDSQSPDSAWRSYNDGNQETLNGDATYSSLAAKGFRSVR
PNLQDKRSPTQSQITVNGNSGGAVSPMSYYQRPFSPSAYSLPASLNSSIVMQHGTSLDST
DTYPQHAQSLDGTTSSSIPLYRSSEEEKRVTVIKAPHYPGIGPVDESGIPTAIRTTVDRP
KDWYKTMFKQIHMVHKPDDDTDMYNTPYTYNAGLYNPPYSAQSHPAAKTQTYRPLSKSHS
DNSPNAFKDASSPVPPPHVPPPVPPLRPRDRSSTEKHDWDPPDRKVDTRKFRSEPRSIFE
YEPGKSSILQHERPPPLPTTPTPVPREPGRKPLSSSRLGEVTGSPSPPPRSGAPTPSSRA
PALSPTRPPKKPLDYVQDHSSGVFNEASLYQSSIDRSLERPMSSASMASDFRKRRKSEPA
VGPPRGLGDQSASRTSPGRVDLPGSSTTLTKSFTSSSPSSPSRAKDRESPRSYSSTLTDM
GRSAPRERRGTPEKEKLPAKAVYDFKAQTSKELSFKKGDTVYILRKIDQNWYEGEHHGRV
GIFPISYVEKLTPPEKAQPARPPPPAQPGEIGEAIAKYNFNADTNVELSLRKGDRVILLK
RVDQNWYEGKIPGTNRQGIFPVSYVEVVKKNTKGAEDYPDPPIPHSYSSDRIHSLSSNKP
QRPVFTHENIQGGGEPFQALYNYTPRNEDELELRESDVIDVMEKCDDGWFVGTSRRTKFF
GTFPGNYVKRL*
>ENSG00000196187|ENST00000366835
MMDSPFLELWQSKAVSIREQLGLGDRPNDSYCYNSAKNSTVLQGVTFGGIPTVLLIDVSC
FLFLILVFSIIRRRFWDYGRIALVSEADSESRFQRLSSTSSSGQQDFENELGCCPWLTAI
FRLHDDQILEWCGEDAIHYLSFQRHIIFLLVVVSFLSLCVILPVNLSGDLLDKDPYSFGR
TTIANLQTDNDLLWLHTIFAVIYLFLTVGFMRHHTQSIKYKEENLVRRTLFITGLPRDAR
KETVESHFRDAYPTCEVVDVQLCYNVAKLIYLCKEKKKTEKSLTYYTNLQVKTGQRTLIN
PKPCGQFCCCEVLGCEWEDAISYYTRMKDRLLERITEEERHVQDQPLGMAFVTFQEKSMA
TYILKDFNACKCQSLQCKGEPQPSSHSRELYTSKWTVTFAADPEDICWKNLSIQGLRWWL
QWLGINFTLFLGLFFLTTPSIILSTMDKFNVTKPIHALNNPIISQFFPTLLLWSFSALLP
SIVYYSTLLESHWTKSGENQIMMTKVYIFLIFMVLILPSLGLTSLDFFFRWLFDKTSSEA
SIRLECVFLPDQGAFFVNYVIASAFIGNGMELLRLPGLILYTFRMIMAKTAADRRNVKQN

QAFQYEFGAMYAWMLCVFTVIVAYSITCPIIAPFGLIYILLKHMVDRHNLYFVYLPAKLE
KGIHFAAVNQALAAPILCLFWLYFFSFLRLGMKAPATLFTFLVLLLTILVCLAHTCFGCF
KHLSPLNYKTEEPASDKGSEAEAHMPPPFTPYVPRILNGLASERTALSPQQQQQQTYGAI
HNISGTIPGQCLAQSATGSVAAAPQEA*
>ENSG00000197063|ENST00000357736
MTTPNKGNKALKVKREPGENGTSLTDEELVTMSVRELNQHLRGLSKEEIVQLKQRRRTLK
NRGYAASCRVKRVTQKEELEKQKAELQQEVEKLASENASMKLELDALRSKYEALQTFART
VARSPVAPARGPLAAGLGPLVPGKVAATSVITIVKSKTDARS*
>ENSG00000136270|ENST00000361278
MAAHLVKRCTCLLREAARQAPAMAPVGRLRLAWVAHKTLTSSATSPISHLPGSLMEPVEK
ERASTPYIEKQVDHLIKKATRPEELLELLGGSHDLDSNQAAMVLIRLSHLLSEKPEDKGL
LIQDAHFHQLLCLLNSQIASVWHGTLSKLLGSLYALGIPKASKELQSVEQEVRWRMRKLK
YKHLAFLAESCATLSQEQHSQELLAELLTHLERRWTEIEDSHTLVTVMMKVGHLSEPLMN
RLEDKHVLNRAQDITLPHLCSVLLAFARLNFHPDQEDQFFSLVHEKLGSELPGLEPALQV
DLVWALCVLQQAREAELQAVLHPEFHIQFLGGKSQKDQNTFQKLLHINATALLEYPEYSG
PLLPASAVAPGPSALDRKVTPLQKELQETLKGLLGSADKGSLEVATQYGWVLDAEVLLDS
DGEFLPVRDFVAPHLAQPTGSQSPPPGSKRLAFLRWEFPNFNSRSKDLLGRFVLARRHIV
AAGFLIVDVPFYEWLELKSEWQKGAYLKDKMRKAVAEELAK*
>ENSG00000168275|ENST00000366612
MAAPSMKERQVCWGARDEYWKCLDENLEDASQCKKLRSSFESSCPQQWIKYFDKRRDYLK
FKEKFEAGQFEPSETTAKS*
>ENSG00000070756|ENST00000318607
MNPSAPSYPMASLYVGDLHPDVTEAMLYEKFSPAGPILSIRVCRDMITRRSLGYAYVNFQ
QPADAERALDTMNFDVIKGKPVRIMWSQRDPSLRKSGVGNIFIKNLDKSIDNKALYDTFS
AFGNILSCKVVCDENGSKGYGFVHFETQEAAERAIEKMNGMLLNDRKVFVGRFKSRKERE
AELGARAKEFTNVYIKNFGEDMDDERLKDLFGKFGPALSVKVMTDESGKSKGFGFVSFER
HEDAQKAVDEMNGKELNGKQIYVGRAQKKVERQTELKRKFEQMKQDRITRYQGVNLYVKN
LDDGIDDERLRKEFSPFGTITSAKVMMEGGRSKGFGFVCFSSPEEATKAVTEMNGRIVAT
KPLYVALAQRKEERQAHLTNQYMQRMASVRAVPNPVINPYQPAPPSGYFMAAIPQTQNRA
AYYPPSQIAQLRPSPRWTAQGARPHPFQNMPGAIRPAAPRPPFSTMRPASSQVPRVMSTQ
RVANTSTQTMGPRPAAAAAAATPAVRTVPQYKYAAGVRNPQQHLNAQPQVTMQQPAVHVQ
GQEPLTASMLASAPPQEQKQMLGERLFPLIQAMHPTLAGKITGMLLEIDNSELLHMLESP
ESLRSKVDEAVAVLQAHQAKEAAQKAVNSATGVPTV*
>ENSG00000199094|ENST00000362224
Sequence unavailable
>ENSG00000107937|ENST00000360803
MAHYNFKKITVVPSAKDFIDLTLSKTQRKTPTVIHKHYQIHRIRHFYMRKVKFTQQNYHD
RLSQILTDFPKLDDIHPFYADLMNILYDKDHYKLALGQINIAKNLVDNVAKDYVRLMKYG
DSLYRCKQLKRAALGRMCTVIKRQKQSLEYLEQVRQHLSRLPTIDPNTRTLLLCGYPNVG
KSSFINKVTRADVDVQPYAFTTKSLFVGHMDYKYLRWQVVDTPGILDHPLEDRNTIEMQA
ITALAHLRAAVLYVMDLSEQCGHGLREQLELFQNIRPLFINKPLIVVANKCDVKRIAELS
EDDQKIFTDLQSEGFPVIETSTLTEEGVIKVKTEACDRLLAHRVETKMKGNKVNEVLNRL
HLAIPTRRDDKERPPFIPEGVVARRKRMETEESRKKRERDLELEMGDDYILDLQKYWDLM
NLSEKHDKIPEIWEGHNIADYIDPAIMKKLEELEKEEELRTAAGEYDSVSESEDEEMLEI

RQLAKQIREKKKLKILESKEKNTQGPRMPRTAKKVQRTVLEKEMRSLGVDMDDKDDAHYA
VQARRSRSITRKRKREDSAPPSSVARSGSCSRTPRDVSGLRDVKMVKKAKTMMKNAQKKM
NRLGKKGEADRHVFDMKPKHLLSGKRKAGKKDRR*
>ENSG00000104177|ENST00000324324
MADANKAEVPGATGGDSPHLQPAEPPGEPRREPHPAEAEKQQPQHSSSSNGVKMENDESA
KEEKSDLKEKSTGSKKANRFHPYSKDKNSGAGEKKGPNRNRVFISNIPYDMKWQAIKDLM
REKVGEVTYVELFKDAEGKSRGCGVVEFKDEEFVKKALETMNKYDLSGRPLNIKEDPDGE
NARRALQRTGGSFPGGHVPDMGSGLMNLPPSILNNPNIPPEVISNLQAGRLGSTIFVANL
DFKVGWKKLKEVFSIAGTVKRADIKEDKDGKSRGMGTVTFEQAIEAVQAISMFNGQFLFD
RPMHVKMDDKSVPHEEYRSHDGKTPQLPRGLGGIGMGLGPGGQPISASQLNIGGVMGNLG
PGGMGMDGPGFGGMNRIGGGIGFGGLEAMNSMGGFGGVGRMGELYRGAMTSSMERDFGRG
DIGINRGFGDSFGRLGSAMIGGFAGRIGSSNMGPVGSGISGGMGSMNSVTGGMGMGLDRM
SSSFDRMGPGIGAILERSIDMDRGFLSGPMGSGMRERIGSKGNQIFVRNLPFDLTWQKLK
EKFSQCGHVMFAEIKMENGKSKGCGTVRFDSPESAEKACRIMNGIKISGREIDVRLDRNA
*
>ENSG00000213096|ENST00000339642
MPGPPRSLEMGLLTFRDVAIEFSLEEWQHLDIAQQNLYRNVMLENYRNLAFLGIAVSKPD
LITCLEQGKEPWNMKRHEMVDEPPGLDFSLL*
>ENSG00000182968|ENST00000330949
MYSMMMETDLHSPGGAQAPTNLSGPAGAGGGGGGGGGGGGGGGGAKANQDRVKRPMNAFMV
WSRGQRRKMAQENPKMHNSEISKRLGAEWKVMSEAEKRPFIDEAKRLRALHMKEHPDYKY
RPRRKTKTLLKKDKYSLAGGLLAAGAGGGGAAVAMGVGVGVGAAAVGQRLESPGGAAGGG
YAHVNGWANGAYPGSVAAAAAAAAMMQEAQLAYGQHPGAGGAHPHAHPAHPHPHHPHAHP
HNPQPMHRYDMGALQYSPISNSQGYMSASPSGYGGLPYGAAAAAAAAAGGAHQNSAVAAA
AAAAAASSGALGALGSLVKSEPSGSPPAPAHSRAPCPGDLREMISMYLPAGEGGDPAAAA
AAAAQSRLHSLPQHYQGAGAGVNGTVPLTHI*
>ENSG00000146757|ENST00000357512
MGPLTFRDVKIEFSLEEWQCLDTAQRNLYRDVMLENYRNLVFLVMCSHFAQDVWPEHSIK
DSFQKVILRTYGKYGHENLQLRKDHKSVDACKVYKGGYNGLNQCLTTTDSKIFQCDKYVK
VFHKFPNVNRNKIRHTGKKPFKCKNRGKSFCMLSQLTQHKKIHTREYSYKCEECGKAFNW
SSTLTKHKIIHTGEKPYKCEECGKAFNRSSNLTKHKIIHTGEKPYKCEECGKAFNRSSTL
TKHKRIHTEEKPYKCEECGKAFNQFSILNKHKRIHMEDKPYKCEECGKAFRVFSILKKHK
IIHTGEKPYKCEECGKAFNQFSNLTKHKIIHTGEKPYKCDECGKAFNQSSTLTKHKRIHT
GEKPYKCEECGKAFKQSSTLTEHKIIHTGEKPYKCEKCGKAFSWSSAFTKHKRNHMEDKP
YKCEECGKAFSVFSTLTKHKIIHTREKPYKCEECGKAFNQSSIFTKHKIIHTEGKSYKCE
KCGNAFNQSSNLTARKIIYTGEKPYKYEECDKAFNKFSTLITHQIIYTGEKPCKHECGRA
FNKSSNYTKEKLQT*
>ENSG00000074266|ENST00000351625
MSEREVSTAPAGTDMPAAKKQKLSSDENSNPDLSGDENDDAVSIESGTNTERPDTPTNTP
NAPGRKSWGKGKWKSKKCKYSFKCVNSLKEDHNQPLFGVQFNWHSKEGDPLVFATVGSNR
VTLYECHSQGEIRLLQSYVDADADENFYTCAWTYDSNTSHPLLAVAGSRGIIRIINPITM
QCIKHYVGHGNAINELKFHPRDPNLLLSVSKDHALRLWNIQTDTLVAIFGGVEGHRDEVL
SADYDLLGEKIMSCGMDHSLKLWRINSKRMMNAIKESYDYNPNKTNRPFISQKIHFPDFS
TRDIHRNYVDCVRWLGDLILSKSGRAILHSHQQCMRDPVSPNLRQHLSCENAIVCWKPGK

MEDDIDKIKPSESNVTILGRFDYSQCDIWYMRFSMDFWQKMLALGNQVGKLYVWDLEVED
PHKAKCTTLTHHKCGAAIRQTSFSRDSSILIAVCDDASIWRWDRLR*
>ENSG00000181991|ENST00000353598
MQAVRNAGSRFLRSWTWPQTAGRVVARTPAGTICTGARQLQDAAAKQKVEQNAAPSHTKF
STQIQVVSASNEPLAFASCGTEGFRNAKKGTGIAAQTAGIAAAARAKQKGVIHIRVVVKG
LGPGRLSAMHGLIMGGLEVISITDNTPIPHNGCRPRKARKL*
>ENSG00000072736|ENST00000346183
MTTANCGAHDELDFKLVFGEDGAPAPPPPGSRPADLEPDDCASIYIFNVDPPPSTLTTPL
CLPHHGLPSHSSVLSPSFQLQSHKNYEGTCEIPESKYSPLGGPKPFECPSIQITSISPNC
HQELDAHEDDLQINDPEREFLERPSRDHLYLPLEPSYRESSLSPSPASSISSRSWFSDAS
SCESLSHIYDDVDSELNEAAARFTLGSPLTSPGGSPGGCPGEETWHQQYGLGHSLSPRQS
PCHSPRSSVTDENWLSPRPASGPSSRPTSPCGKRRHSSAEVCYAGSLSPHHSPVPSPGHS
PRGSVTEDTWLNASVHGGSGLGPAVFPFQYCVETDIPLKTRKTSEDQAAILPGKLELCSD
DQGSLSPARETSIDDGLGSQYPLKKDSCGDQFLSVPSPFTWSKPKPGHTPIFRTSSLPPL
DWPLPAHFGQCELKIEVQPKTHHRAHYETEGSRGAVKASTGGHPVVKLLGYNEKPINLQM
FIGTADDRYLRPHAFYQVHRITGKTVATASQEIIIASTKVLEIPLLPENNMSASIDCAGI
LKLRNSDIELRKGETDIGRKNTRVRLVFRVHIPQPSGKVLSLQIASIPVECSQRSAQELP
HIEKYSINSCSVNGGHEMVVTGSNFLPESKIIFLEKGQDGRPQWEVEGKIIREKCQGAHI
VLEVPPYHNPAVTAAVQVHFYLCNGKRKKSQSQRFTYTPVLMKQEHREEIDLSSVPSLPV
PHPAQTQRPSSDSGCSHDSVLSGQRSLICSIPQTYASMVTSSHLPQLQCRDESVSKEQHM
IPSPIVHQPFQVTPTPPVGSSYQPMQTNVVYNGPTCLPINAASSQEFDSVLFQQDATLSG
LVNLGCQPLSSIPFHSSNSGSTGHLLAHTPHSVHTLPHLQSMGYHCSNTGQRSLSSPVAD
QITGQPSSQLQPITYGPSHSGSATTASPAASHPLASSPLSGPPSPQLQPMPYQSPSSGTA
SSPSPATRMHSGQHSTQAQSTGQGGLSAPSSLICHSLCDPASFPPDGATVSIKPEPEDRE
PNFATIGLQDITLDDVNEIIGRDMSQISVSQGAGVSRQAPLPSPESLDLGRSDGL*
>ENSG00000100354|ENST00000335727
MREKEQEREEQLMEDKKRKKEDKKKKEATQKVTEQKTKVPEVTKPSLSQPTAASPIGSSP
SPPVNGGNNAKRVAVPNGQPPSAARYMPREVPPRFRCQQDHKVLLKRGQPPPPSCMLLGG
GAGPPPCTAPGANPNNAQVTGALLQSESGTAPDSTLGGAAASNYANSTWGSGASSNNGTS
PNPIHIWDKVIVDGSDMEEWPCIASKDTESSSENTTDNNSASNPGSEKSTLPGSTTSNKG
KGSQCQSASSGNECNLGVWKSDPKAKSVQSSNSTTENNNGLGNWRNVSGQDRIGPGSGFS
NFNPNSNPSAWPALVQEGTSRKGALETDNSNSSAQVSTVGQTSREQQSKMENAGVNFVVS
GREQAQIHNTDGPKNGNTNSLNLSSPNPMENKGMPFGMGLGNTSRSTDAPSQSTGDRKTG
SVGSWGAARGPSGTDTVSGQSNSGNNGNNGKEREDSWKGASVQKSTGSKNDSWDNNNRST
GGSWNFGPQDSNDNKWGEGNKMTSGVSQGEWKQPTGSDELKIGEWSGPNQPNSSTGAWDN
QKGHPLPENQGNAQAPCWGRSSSSTGSEVGGQSTGSNHKAGSSDSHNSGRRSYRPTHPDC
QAVLQTLLSRTDLDPRVLSNTGWGQTQIKQDTVWDIEEVPRPEGKSDKGTEGWESAATQT
KNSGGWGDAPSQSNQMKSGWGELSASTEWKDPKNTGGWNDYKNNNSSNWGGGRPDEKTPS
SWNENPSKDQGWGGGRQPNQGWSSGKNGWGEEVDQTKNSNWESSASKPVSGWGEGGQNEI
GTWGNGGNASLASKGGWEDCKRSPAWNETGRQPNSWNKQHQQQQPPQQPPPPQPEASGSW
GGPPPPPPGNVRPSNSSWSSGPQPATPKDEEPSGWEEPSPQSISRKMDIDDGTSAWGDPN
SYNYKNVNLWDKNSQGGPAPREPNLPTPMTSKSASDSKSMQDGWGESDGPVTGARHPSWE
EEEDGGVWNTTGSQGSASSHNSASWGQGGKKQMKCSLKGGNNDSWMNPLAKQFSNMGLLS
QTEDNPSSKMDLSVGSLSDKKFDVDKRAMNLGDFNDIMRKDRSGFRPPNSKDMGTTDSGP

YFEKGGSHGLFGNSTAQSRGLHTPVQPLNSSPSLRAQVPPQFISPQVSASMLKQFPNSGL
SPGLFNVGPQLSPQQIAMLSQLPQIPQFQLACQLLLQQQQQQQLLQNQRKISQAVRQQQE
QQLARMVSALQQQQQQQQRQPGMKHSPSHPVGPKPHLDNMVPNALNVGLPDLQTKGPIPG
YGSGFSSGGMDYGMVGGKEAGTESRFKQWTSMMEGLPSVATQEANMHKNGAIVAPGKTRG
GSPYNQFDIIPGDTLGGHTGPAGDSWLPAKSPPTNKIGSKSSNASWPPEFQPGVPWKGIQ
NIDPESDPYVTPGSVLGGTATSPIVDTDHQLLRDNTTGSNSSLNTSLPSPGAWPYSASDN
SFTNVHSTSAKFPDYKSTWSPDPIGHNPTHLSNKMWKNHISSRNTTPLPRPPPGLTNPKP
SSPWSSTAPRSVRGWGTQDSRLASASTWSDGGSVRPSYWLVLHNLTPQIDGSTLRTICMQ
HGPLLTFHLNLTQGTALIRYSTKQEAAKAQTALHMCVLGNTTILAEFATDDEVSRFLAQA
QPPTPAATPSAPAAGWQSLETGQNQSDPVGPALNLFGGSTGLGQWSSSAGGSSGADLAGA
SLWGPPNYSSSLWGVPTVEDPHRMGSPAPLLPGDLLGGGSDSI*
>ENSG00000136861|ENST00000360190
MMDLVLEEDVTVPGTLSGCSGLVPSVPDDLDGINPNAGLGNGLLPNVSEETVSPTRARNM
KDFENQITELKKENFNLKLRIYFLEERMQQEFHGPTEHIYKTNIELKVEVESLKRELQER
EQLLIKASKAVESLAEAGGSEIQRVKEDARKKVQQVEDLLTKRILLLEKDVTAAQAELEK
AFAGTETEKALRLRLESKLSEMKKMHEGDLAMALVLDEKDRLIEELKLSLKSKEALIQCL
KEEKSQMACPDENVSSGELRGLCAAPREEKERETEAAQMEHQKERNSFEERIQALEEDLR
EKEREIATEKKNSLKRDKAIQGLTMALKSKEKKVEELNSEIEKLSAAFAKAREALQKAQT
QEFQGSEDYETALSGKEALSAALRSQNLTKSTENHRLRRSIKKITQELSDLQQERERLEK
DLEEAHREKSKGDCTIRDLRNEVEKLRNEVNEREKAMENRYKSLLSESNKKLHNQEQVIK
HLTESTNQKDVLLQKFNEKDLEVIQQNCYLMAAEDLELRSEGLITEKCSSQQPPGSKTIF
SKEKKQSSDYEELIQVLKKEQDIYTHLVKSLQESDSINNLQAELNKIFALRKQLEQDVLS
YQNLRKTLEEQISEIRRREEESFSLYSDQTSYLSICLEENNRFQVEHFSQEELKKKVSDL
IQLVKELYTDNQHLKKTIFDLSCMGFQGNGFPDRLASTEQTELLASKEDEDTIKIGEDDE
INFLSDQHLQQSNEIMKDLSKGGCKNGYLRHTESKISDCDGAHAPGCLEEGAFINLLAPL
FNEKATLLLESRPDLLKVVRELLLGQLFLTEQEVSGEHLDGKTEKTPKQKGELVHFVQTN
SFSKPHDELKLSCEAQLVKAGEVPKVGLKDASVQTVATEGDLLRFKHEATREAWEEKPIN
TALSAEHRPENLHGVPGWQAALLSLPGITNREAKKSRLPILIKPSRSLGNMYRLPATQEV
VTQLQSQILELQGELKEFKTCNKQLHQKLILAEAVMEGRPTPDKTLLNAQPPVGAAYQDS
PGEQKGIKTTSSVWRDKEMDSDQQRSYEIDSEICPPDDLASLPSCKENPEDVLSPTSVAT
YLSSKSQPSAKVSVMGTDQSESINTSNETEYLKQKIHDLETELEGYQNFIFQLQKHSQCS
EAIITVLCGTEGAQDGLSKPKNGSDGEEMTFSSLHQVRYVKHVKILGPLAPEMIDSRVLE
NLKQQLEEQEYKLQKEQNLNMQLFSEIHNLQNKFRDLSPPRYDSLVQSQARELSLQRQQI
KDGHGICVISRQHMNTMIKAFEELLQASDVDYCVAEGFQEQLNQCAELLEKLEKLFLNGK
SVGVEMNTQNELMERIEEDNLTYQHLLPESPEPSASHALSDYETSEKSFFSRDQKQDNET
EKTSVMVNSFSQDLLMEHIQEIRTLRKRLEESIKTNEKLRKQLERQGSEFVQGSTSIFAS
GSELHSSLTSEIHFLRKQNQALNAMLIKGSRDKQKENDKLRESLSRKTVSLEHLQREYAS
VKEENERLQKEGSEKERHNQQLIQEVRCSGQELSRVQEEVKLRQQLLSQNDKLLQSLRVE
LKAYEKLDEEHRRLRDGDKYPMESDNSFDLFDSSQAVTPKSVSETPPLSGNDTDSLSCDS
GSSATSTPCVSRLVTGHHLWASKNGRHVLGLIEDYEALLKQISQGQRLLAEMDIQTQEAP
SSTSQELGTKGPHPAPLSKFVSSVSTAKLTLEEAYRRLKLLWRVSLPEDGQCPLHCEQIG
EMKAEVTKLHKKLFEQEKKLQNTMKLLQLSKRQEKVIFDQLVVTHKILRKARGNLELRPG
GAHPGTCSPSRPGS*
>ENSG00000258366|ENST00000318100

MPYNYLLDAKSRRAHNIDLKGTVVIFDEAHNVEKMCEESASFDLTPHDLASGLDVIDQVL
EEQTKAAQQGEPHPEFSADSPSPGLNMELEDIAKLKMILLRLEGAIDAVELPGDDSGVTK
PGSYIFELFAEAQITFQTKGCILDSLDQIIQHLAGRAGVFTNTAGLQKLADIIQIVFSVD
PSEGSPGSPAGLGALQSYKVHIHPDAGHRRTAQRSDAWSTTAARKRGKVLSYWCFSPGHS
MHELVRQGVRSLILTSGTLAPVSSFALEMQIPFPVCLENPHIIDKHQIWVGVVPRGPDGA
QLSSAFDRRFSEECLSSLGKALGNIARVVPYGLLIFFPSYPVMEKSLEFWRARDLARKME
ALKPLFVEPRSKGSFSETISAYYARVAAPGSTGATFLAVCRGKASEGLDFSDTNGRGVIV
TGLPYPPRMDPRVVLKMQFLDEMKGQGGAGGQFLSGQEWYRQQASRAVNQAIGRVIRHRQ
DYGAVFLCDHRFAFADARAQLPSWVRPHVRVYDNFGHVIRDVAQFFRVAERTMPAPAPRA
TAPSVRGEDAVSEAKSPGPFFSTRKAKSLDLHVPSLKQRSSGSPAAGDPESSLCVEYEQE
PVPARQRPRGLLAALEHSEQRAGSPGEEQAHSCSTLSLLSEKRPAEEPRGGRKKIRLVSH
PEEPVAGAQTDRAKLFMVAVKQELSQANFATFTQALQDYKGSDDFAALAACLGPLFAEDP
KKHNLLQGFYQFVRPHHKQQFEEVCIQLTGRGCGYRPEHSIPRRQRAQPVLDPTGRTAPD
PKLTVSTAAAQQLDPQEHLNQGRPHLSPRPPPTGDPGSQPQWGSGVPRAGKQGQHAVSAY
LADARRALGSAGCSQLLAALTAYKQDDDLDKVLAVLAALTTAKPEDFPLLHRFSMFVRPH
HKQRFSQTCTDLTGRPYPGMEPPGPQEERLAVPPVLTHRAPQPGPSRSEKTGKTQSKISS
FLRQRPAGTVGAGGEDAGPSQSSGPPHGPAASEWGL*
>ENSG00000160570|ENST00000336034
MALSGSTPAPCWEEDECLDYYGMLSLHRMFEVVGGQLTECELELLAFLLDEAPGAAGGLA
RARSGLELLLELERRGQCDESNLRLLGQLLRVLARHDLLPHLARKRRRPVSPERYSYGTS
SSSKRTEGSCRRRRQSSSSANSQQGSPPTKRQRRSRGRPSGGARRRRRGAPAAPQQQSEP
ARPSSEGKVTCDIRLRVRAEYCEHGPALEQGVASRRPQALARQLDVFGQATAVLRSRDLG
SVVCDIKFSELSYLDAFWGDYLSGALLQALRGVFLTEALREAVGREAVRLLVSVDEADYE
AGRRRLLLMEEEGGRRPTEAS*
>ENSG00000139675|ENST00000357495
MSKSASPKEPEQLRKLFIGGLSFETTDESLRSHFEQWGTLTDCVVMRDPNTKRSRGFGFV
TYATVEEVDAAMNTTPHKVDGRVVEPKRAVSREDSQRPGAHLTVKKIFVGGIKEDTEEHH
LRDYFEQYGKIEVIEIMTDRGSGKKRGFAFVTFDDHDSVDKIVIQKYHTVKGHNCEVRKA
LPKQEMASASSSQRGRRGSGNFGGGRGDGFGGNDNFGRGGNFSGRGGFGGSCGGGGYGGS
GDGYNGFGNDGSNFGGGGSYNDFGNYNNQSSNFGPMKGGNFGGRSSGPYGGGGQYFAKPQ
NQGGYGVSSSSSSYGSGRRF*
>ENSG00000068024|ENST00000345617
MSSQSHPDGLSGRDQPVELLNPARVNHMPSTVDVATALPLQVAPSAVPMDLRLDHQFSLP
VAEPALREQQLQQELLALKQKQQIQRQILIAEFQRQHEQLSRQHEAQLHEHIKQQQEMLA
MKHQQELLEHQRKLERHRQEQELEKQHREQKLQQLKNKEKGKESAVASTEVKMKLQEFVL
NKKKALAHRNLNHCISSDPRYWYGKTQHSSLDQSSPPQSGVSTSYNHPVLGMYDAKDDFP
LRKTASEPNLKLRSRLKQKVAERRSSPLLRRKDGPVVTALKKRPLDVTDSACSSAPGSGP
SSPNNSSGSVSAENGIAPAVPSIPAETSLAHRLVAREGSAAPLPLYTSPSLPNITLGLPA
TGPSAGTAGQQDAERLTLPALQQRLSLFPGTHLTPYLSTSPLERDGGAAHSPLLQHMVLL
EQPPAQAPLVTGLGALPLHAQSLVGADRVSPSIHKLRQHRPLGRTQSAPLPQNAQALQHL
VIQQQHQQFLEKHKQQFQQQQLQMNKIIPKPSEPARQPESHPEETEEELREHQALLDEPY
LDRLPGQKEAHAQAGVQVKQEPIESDEEEAEPPREVEPGQRQPSEQELLFRQQALLLEQQ
RIHQLRNYQASMEAAGIPVSFGGHRPLSRAQSSPASATFPVSVQEPPTKPRFTTGLVYDT
LMLKHQCTCGSSSSHPEHAGRIQSIWSRLQETGLRGKCECIRGRKATLEELQTVHSEAHT

LLYGTNPLNRQKLDSKKLLGSLASVFVRLPCGGVGVDSDTIWNEVHSAGAARLAVGCVVE
LVFKVATGELKNGFAVVRPPGHHAEESTPMGFCYFNSVAVAAKLLQQRLSVSKILIVDWD
VHHGNGTQQAFYSDPSVLYMSLHRYDDGNFFPGSGAPDEVGTGPGVGFNVNMAFTGGLDP
PMGDAEYLAAFRTVVMPIASEFAPDVVLVSSGFDAVEGHPTPLGGYNLSARCFGYLTKQL
MGLAGGRIVLALEGGHDLTAICDASEACVSALLGNELDPLPEKVLQQRPNANAVRSMEKV
MEIHSKYWRCLQRTTSTAGRSLIEAQTCENEEAETVTAMASLSVGVKPAEKRPDEEPMEE
EPPL*
>ENSG00000100461|ENST00000359890
MASDDFDIVIEAMLEAPYKKEEDEQQRKEVKKDYPSNTTSSTSNSGNETSGSSTIGETSK
KKRSRSHNKSRDRKRSRSRDRDRYRRRNSRSRSPGRQCRHRSRSWDRRHGSESRSRDHRR
EDRVHYRSPPLATGYRYGHSKSPHFREKSPVREPVDNLSPEERDARTVFCMQLAARIRPR
DLEDFFSAVGKVRDVRIISDRNSRRSKGIAYVEFCEIQSVPLAIGLTGQRLLGVPIIVQA
SQAEKNRLAAMANNLQKGNGGPMRLYVGSLHFNITEDMLRGIFEPFGKIDNIVLMKDSDT
GRSKGYGFITFSDSECARRALEQLNGFELAGRPMRVGHVTERLDGGTDITFPDGDQELDL
GSAGGRFQLMAKLAEGAGIQLPSTAAAAAAAAAQAAALQLNGAVPLGALNPAALTALSPA
LNLASQCFQLSSLFTPQTM*
>ENSG00000189369|ENST00000340438
MDSGSSSSDSAPDCWDQVDMESPGSAPSGDGVSSAVAEAQREPLSSAFSRKLNVNAKPFV
PNVHAAEFVPSFLRGPTQPPTLPAGSGSNDETCTGAGYPQGKRMGRGAPVEPSREEPLVS
LEGSNSAVTMELSEPVVENGEVEMALEESWEHSKEVSEAEPGGGSSGDSGPPEESGQEMM
EEKEEIRKSKSVIVPSGAPKKEHVNVVFIGHVDAGKSTIGGQIMFLTGMVDKRTLEKYER
EAKEKNRETWYLSWALDTNQEERDKGKTVEVGRAYFETERKHFTILDAPGHKSFVPNMIG
GASQADLAVLVISARKGEFETGFEKGGQTREHAMLAKTAGVKHLIVLINKMDDPTVNWSI
ERYEECKEKLVPFLKKVGFSPKKDIHFMPCSGLTGANIKEQSDFCPWYTGLPFIPYLDNL
PNFNRSIDGPIRLPIVDKYKDMGTVVLGKLESGSIFKGQQLVMMPNKHNVEVLGILSDDT
ETDFVAPGENLKIRLKGIEEEEILPGFILCDPSNLCHSGRTFDVQIVIIEHKSIICPGYN
AVLHIHTCIEEVEITALISLVDKKSGEKSKTRPRFVKQDQVCIARLRTAGTICLETFKDF
PQMGRFTLRDEGKTIAIGKVLKLVPEKD*
>ENSG00000213928|ENST00000324076
EGDTGGPAVWKTRLRCALNKSSEFKEVPERGRMDVAEPYKVYQLLPPGIVSGQPGTQKVP
SKRQHSSVSSERKEEEDAMQNCTLSPSVLQDSLNNEEEGASGGAVHSDIGSSSSSSSPEP
QEVTDTTEAPFQGDQRSLEFLLPPEPDLVRYFQGLGPPPKFQVTLNFWEESHGSSHTPQN
LITVKMEQAFARYLLEQTPEQQAAILSLV*
>ENSG00000162711|ENST00000336119
MKMASTRCKLARYLEDLEDVDLKKFKMHLEDYPPQKGCIPLPRGQTEKADHVDLATLMID
FNGEEKAWAMAVWIFAAINRRDLYEKAKRDEPKWGSDNARVSNPTVICQEDSIEEEWMGL
LEYLSRISICKMKKDYRKKYRKYVRSRFQCIEDRNARLGESVSLNKRYTRLRLIKEHRSQ
QEREQELLAIGKTKTCESPVSPIKMELLFDPDDEHSEPVHTVVFQGAAGIGKTILARKMM
LDWASGTLYQDRFDYLFYIHCREVSLVTQRSLGDLIMSCCPDPNPPIHKIVRKPSRILFL
MDGFDELQGAFDEHIGPLCTDWQKAERGDILLSSLIRKKLLPEASLLITTRPVALEKLQH
LLDHPRHVEILGFSEAKRKEYFFKYFSDEAQARAAFSLIQENEVLFTMCFIPLVCWIVCT
GLKQQMESGKSLAQTSKTTTAVYVFFLSSLLQPRGGSQEHGLCAHLWGLCSLAADGIWNQ
KILFEESDLRNHGLQKADVSAFLRMNLFQKEVDCEKFYSFIHMTFQEFFAAMYYLLEEEK
EGRTNVPGSRLKLPSRDVTVLLENYGKFEKGYLIFVVRFLFGLVNQERTSYLEKKLSCKI

SQQIRLELLKWIEVKAKAKKLQIQPSQLELFYCLYEMQEEDFVQRAMDYFPKIEINLSTR
MDHMVSSFCIENCHRVESLSLGFLHNMPKEEEEEEKEGRHLDMVQCVLPSSSHAACSHGL
VNSHLTSSFCRGLFSVLSTSQSLTELDLSDNSLGDPGMRVLCETLQHPGCNIRRLWLGRC
GLSHECCFDISLVLSSNQKLVELDLSDNALGDFGIRLLCVGLKHLLCNLKKLWLVSCCLT
SACCQDLASVLSTSHSLTRLYVGENALGDSGVAILCEKAKNPQCNLQKLGLVNSGLTSVC
CSALSSVLSTNQNLTHLYLRGNTLGDKGIKLLCEGLLHPDCKLQVLELDNCNLTSHCCWD
LSTLLTSSQSLRKLSLGNNDLGDLGVMMFCEVLKQQSCLLQNLGLSEMYFNYETKSALET
LQEEKPELTVVFEPSW*
>ENSG00000168488|ENST00000325215
MLKPQPLQQPSQPQQPPPTQQAVARRPPGGTSPPNGGLPGPLATSAAPPGPPAAASPCLG
PVAAAGSGLRRGAEGILAPQPPPPQQHQERPGAAAIGSARGQSTGKGPPQSPVFEGVYNN
SRMLHFLTAVVGSTCDVKVKNGTTYEGIFKTLSSKFELAVDAVHRKASEPAGGPRREDIV
DTMVFKPSDVMLVHFRNVDFNYATKDKFTDSAIAMNSKVNGEHKEKVLQRWEGGDSNSDD
YDLESDMSNGWDPNEMFKFNEENYGVKTTYDSSLSSYTVPLEKDNSEEFRQRELRAAQLA
REIESSPQYRLRIAMENDDGRTEEEKHSAVQRQGSGRESPSLASREGKYIPLPQRVREGP
RGGVRCSSSRGGRPGLSSLPPRGPHHLDNSSPGPGSEARGINGGPSRMSPKAQRPLRGAK
TLSSPSNRPSGETSVPPPPAVGRMYPPRSPKSAAPAPISASCPEPPIGSAVPTSSASIPV
TSSVSDPGVGSISPASPKISLAPTDVKELSTKEPGRTLEPQELARIAGKVPGLQNEQKRF
QLEELRKFGAQFKLQPSSSPENSLDPFPPRILKEEPKGKEKEVDGLLTSEPMGSPVSSKT
ESVSDKEDKPPLAPSGGTEGPEQPPPPCPSQTGSPPVGLIKGEDKDEGPVAEQVKKSTLN
PNAKEFNPTKPLLSVNKSTSTPTSPGPRTHSTPSIPVLTAGQSGLYSPQYISYIPQIHMG
PAVQAPQMYPYPVSNSVPGQQGKYRGAKGSLPPQRSDQHQPASAPPMMQAAAAAGPPLVA
ATPYSSYIPYNPQQFPGQPAMMQPMAHYPSQPVFAPMLQSNPRMLTSGSHPQAIVSSSTP
QYPSAEQPTPQALYATVHQSYPHHATQLHAHQPQPATTPTGSQPQSQHAAPSPVQHQAGQ
APHLGSGQPQQNLYHPGALTGTPPSLPPGPSAQSPQSSFPQPAAVYAIHHQQLPHGFTNM
AHVTQAHVQTGITAAPPPHPGAPHPPQVMLLHPPQSHGGPPQGAVPQSGVPALSASTPSP
YPYIGHPQGEQPGQAPGFPGGADDRIPPLPPPGELKIVLAAT*
>ENSG00000178928|ENST00000322175
MLSLREQQLQVWFKNRRAKLARERRLQQQPQRVPGQRGRGARAAPLVPAASASAPQRGPS
GILPAAEPTICSLHQAWGGPGCRAQKGIPAALSPGPGPIPAPIPGPAQIPGPLPGSIPGP
IPGPAQIPSPIPAPIPGPISGPVQIPGPFRGPIPGPISGPAPIPGPISGPFSGPNPGPIP
GPNPGPIPGPISGPIPGPISVPIPGPIPGPISGPISGPNPGPIPGPIPGPISGPNPGPIP
GPISGPNPGLIPGPIPGPISGPGPIIGPIPSPAQIPGPGRLQGPGPILSPGRMRSPGSLP
GLAPILGPGSGPGSGSVPAPIPGPGSLPAPAPLWPQSPDASDFLPDTQLFPHFTELLLPL
DPLEGSSVSTMTSQYQEGDDSMGKKHSGSQPQEEGGSVNENHSGPRLLLDL*
>ENSG00000069667|ENST00000335670
MESAPAAPDPAASEPGSSGADAAAGSRETPLNQESARKSEPPAPVRRQSYSSTSRGISVT
KKTHTSQIEIIPCKICGDKSSGIHYGVITCEGCKGFFRRSQQSNATYSCPRQKNCLIDRT
SRNRCQHCRLQKCLAVGMSRDAVKFGRMSKKQRDSLYAEVQKHRMQQQQRDHQQQPGEAE
PLTPTYNISANGLTELHDDLSNYIDGHTPEGSKADSAVSSFYLDIQPSPDQSGLDINGIK
PEPICDYTPASGFFPYCSFTNGETSPTVSMAELEHLAQNISKSHLETCQYLREELQQITW
QTFLQEEIENYQNKQREVMWQLCAIKITEAIQYVVEFAKRIDGFMELCQNDQIVLLKAGS
LEVVFIRMCRAFDSQNNTVYFDGKYASPDVFKSLGCEDFISFVFEFGKSLCSMHLTEDEI
ALFSAFVLMSADRSWLQEKVKIEKLQQKIQLALQHVLQKNHREDGILTKLICKVSTLRAL

CGRHTEKLMAFKAIYPDIVRLHFPPLYKELFTSEFEPAMQIDG*
>ENSG00000106261|ENST00000324306
MMTAESREATGLSPQAAQEKDGIVIVKVEEEDEEDHMWGQDSTLQDTPPPDPEIFRQRFR
RFCYQNTFGPREALSRLKELCHQWLRPEINTKEQILELLVLEQFLSILPKELQVWLQEYR
PDSGEEAVTLLEDLELDLSGQQVPGQVHGPEMLARGMVPLDPVQESSSFDLHHEATQSHF
KHSSRKPRLLQSRALPAAHIPAPPHEGSPRDQAMASALFTADSQAMVKIEDMAVSLILEE
WGCQNLARRNLSRDNRQENYGSAFPQGGENRNENEESTSKAETSEDSASRGETTGRSQKE
FGEKRDQEGKTGERQQKNPEEKTRKEKRDSGPAIGKDKKTITGERGPREKGKGLGRSFSL
SSNFTTPEEVPTGTKSHRCDECGKCFTRSSSLIRHKIIHTGEKPYECSECGKAFSLNSNL
VLHQRIHTGEKPHECNECGKAFSHSSNLILHQRIHSGEKPYECNECGKAFSQSSDLTKHQ
RIHTGEKPYECSECGKAFNRNSYLILHRRIHTREKPYKCTKCGKAFTRSSTLTLHHRIHA
RERASEYSPASLDAFGAFLKSCV*
>ENSG00000189403|ENST00000339872
MGKGDPKKPRGKMSSYAFFVQTCREEHKKKHPDASVNFSEFSKKCSERWKTMSAKEKGKF
EDMAKADKARYEREMKTYIPPKGETKKKFKDPNAPKRPPSAFFLFCSEYRPKIKGEHPGL
SIGDVAKKLGEMWNNTAADDKQPYEKKAAKLKEKYEKDIAAYRAKGKPDAAKKGVVKAEK
SKKKKEEEEDEEDEEDEEEEEDEEDEDEEEDDDDE*
>ENSG00000110713|ENST00000324932
MFNKSFGTPFGGGTGGFGTTSTFGQNTGFGTTSGGAFGTSAFGSSNNTGGLFGNSQTKPG
GLFGTSSFSQPATSTSTGFGFGTSTGTANTLFGTASTGTSLFSSQNNAFAQNKPTGFGNF
GTSTSSGGLFGTTNTTSNPFGSTSGSLFGPSSFTAAPTGTTIKFNPPTGTDTMVKAGVST
NISTKHQCITAMKEYESKSLEELRLEDYQANRKGPQNQVGAGTTTGLFGSSPATSSATGL
FSSSTTNSGFAYGQNKTAFGTSTTGFGTNPGGLFGQQNQQTTSLFSKPFGQATTTQNTGF
SFGNTSTIGQPSTNTMGLFGVTQASQPGGLFGTATNTSTGTAFGTGTGLFGQTNTGFGAV
GSTLFGNNKLTTFGSSTTSAPSFGTTSGGLFGFGTNTSGNSIFGSKPAPGTLGTGLGAGF
GTALGAGQASLFGNNQPKIGGPLGTGAFGAPGFNTTTATLGFGAPQAPVALTDPNASAAQ
QAVLQQHINSLTYSPFGDSPLFRNPMSDPKKKEERLKPTNPAAQKALTTPTHYKLTPRPA
TRVRPKALQTTGTAKSHLFDGLDDDEPSLANGAFMPKKSIKKLVLKNLNNSNLFSPVNRD
SENLASPSEYPENGERFSFLSKPVDENHQQDGDEDSLVSHFYTNPIAKPIPQTPESAGNK
HSNSNSVDDTIVALNMRAALRNGLEGSSEETSFHDESLQDDREEIENNSYHMHPAGIILT
KVGYYTIPSMDDLAKITNEKGECIVSDFTIGRKGYGSIYFEGDVNLTNLNLDDIVHIRRK
EVVVYLDDNQKPPVGEGLNRKAEVTLDGVWPTDKTSRCLIKSPDRLADINYEGRLEAVSR
KQGAQFKEYRPETGSWVFKVSHFSKYGLQDSDEEEEEHPSKTSTKKLKTAPLPPASQTTP
LQMALNGKPAPPPQSQSPEVEQLGRVVELDSDMVDITQEPVLDTMLEESMPEDQEPVSAS
THIASSLGINPHVLQIMKASLLTDEEDVDMALDQRFSRLPSKADTSQEICSPRLPISASH
SSKTRSLVGGLLQSKFTSGAFLSPSVSVQECRTPRAASLMNIPSTSSWSVPPPLTSVFTM
PSPAPEVPLKTVGTRRQLGLVPREKSVTYGKGKLLMDMALFMGRSFRVGWGPNWTLANSG
EQLNGSHELENHQIADSMEFGFLPNPVAVKPLTESPFKVHLEKLSLRQRKPDEDMKLYQT
PLELKLKHSTVHVDELCPLIVPNLGVAVIHDYADWVKEASGDLPEAQIVKHWSLTWTLCE
ALWGHLKELDSQLNEPREYIQILERRRAFSRWLSCTATPQIEEEVSLTQKNSPVEAVFSY
LTGKRISEACSLAQQSGDHRLALLLSQFVGSQSVRELLTMQLVDWHQLQADSFIQDERLR
IFALLAGKPVWQLSEKKQINVCSQLDWKRSLAIHLWYLLPPTASISRALSMYEEAFQNTS
DSDRYACSPLPSYLEGSGCVIAEEQNSQTPLRDVCFHLLKLYSDRHYDLNQLLEPRSITA
DPLDYRLSWHLWEVLRALNYTHLSAQCEGVLQASYAGQLESEGLWEWAIFVLLHIDNSGI

REKAVRELLTRHCQLLETPESWAKETFLTQKLRVPAKWIHEAKAVRAHMESDKHLEALCL
FKAEHWNRCHKLIIRHLASDAIINENYDYLKGFLEDLAPPERSSLIQDWETSGLVYLDYI
RVIEMLRHIQQVDCSGNDLEQLHIKVTSLCSRIEQIQCYSAKDRLAQSDMAKRVANLLRV
VLSLHHPPDRTSDSTPDPQRVPLRLLAPHIGRLPMPEDYAMDELRSLTQSYLRELAVGSL
*
>ENSG00000132676|ENST00000343043
MMLKGITRLISRIHKLDPGRFLHMGTQARQSIAAHLDNQVPVESPRAISRTNENDPAKHG
DQHEGQHYNISPQDLETVFPHGLPPRFVMQVKTFSEACLMVRKPALELLHYLKNTSFAYP
AIRYLLYGEKGTGKTLSLCHVIHFCAKQDWLILHIPDAHLWVKNCRDLLQSSYNKQRFDQ
PLEASTWLKNFKTTNERFLNQIKVQEKYVWNKRESTEKGSPLGEVVEQGITRVRNATDAV
GIVLKELKRQSSLGMFHLLVAVDGINALWGRTTLKREDKSPIAPEELALVHNLRKMMKND
WHGGAIVSALSQTGSLFKPRKAYLPQELLGKEGFDALDPFIPILVSNYNPKEFESCIQYY
LENNWLQHEKAPTEEGKKELLFLSNANPSLLERHCAYL*
>ENSG00000141644|ENST00000339998
MAEDWLDCPALGPGWKRREVFRKSGATCGRSDTYYQSPTGDRIRSKVELTRYLGPACDLT
LFDFKQGILCYPAPKAHPVAVASKKRKKPSRPAKTRKRQVGPQSGEVRKEAPRDETKADT
DTAPASFPAPGCCENCGISFSGDGTQRQRLKTLCKDCRAQRIAFNREQRMFKRVGCGECA
ACQVTEDCGACSTCLLQLPHDVASGLFCKCERRRCLRIVERSRGCGVCRGCQTQEDCGHC
PICLRPPRPGLRRQWKCVQRRCLRGKHARRKGGCDSKMAARRRPGAQPLPPPPPSQSPEP
TEPHPRALAPSPPAEFIYYCVDEDELQPYTNRRQNRKCGACAACLRRMDCGRCDFCCDKP
KFGGSNQKRQKCRWRQCLQFAMKRLLPSVWSESEDGAGSPPPYRRRKRPSSARRHHLGPT
LKPTLATRTAQPDHTQAPTKQEAGGGFVLPPPGTDLVFLREGASSPVQVPGPVAASTEAL
LQAVDPGLPSVKQEPPDPEEDKEENKDDSASKLAPEEEAGGAGTPVEPTTQPQYSGNFDN
DLYEIYLIDI*
>ENSG00000183496|ENST00000329713
MPSSLFADLERNGSGGGGGGSSGGGETLDDQRALQLALDQLSLLGLDSDEGASLYDSEPR
KKSVNMTECVPVPSSEHVAEIVGRQGCKIKALRAKTNTYIKTPVRGEEPVFVVTGRKEDV
AMARREIISAAEHFSMIRASRNKNTALNGAVPGPPNLPGQTTIQVRVPYRVVGLVVGPKG
ATIKRIQQQTHTYIVTPSRDKEPVFEVTGMPENVDRAREEIEAHIALRTGGIIELTDEND
FHANGTDVGFDLHHGSGGSGPGSLWSKPTPSITPTPGRKPFSSYRNDSSSSLGSASTDSY
FGGGTSSSAAATQRLADYSPPSPALSFAHNGNNNNNGNGYTYTAGGEASVPSPDGCPELQ
PTFDPAPAPPPGAPLIWAQFERSPGGGPAAPVSSSCSSSASSSASSSSVVFPGGGASAPS
NANLGLLVHRRLHPGTSCPRLSPPLHMAPGAGEHHLARRVRSDPGGGGLAYAAYANGLGA
QLPGLQPSDTSGSSSSSSSSSSSSSSSSSGLRRKGSRDCSVCFESEVIAALVPCGHNLFCM
ECANRICEKSEPECPVCHTAVTQAIRIFS*
>ENSG00000198146|ENST00000356321
MMAENNLKMLKIQQCVVANKLPRNRPYVCNICFKHFETPSKLARHYLIHTGQKPFECDVC
HKTFRQLVHLERHQLTHSLPFKCSICQRHFKNLKTFVKHQQLHNETYQNNVKQVRRLLEA
KQEKSMYGVYNTFTTEERWALHPCSKSDPMYSMKRRKNIHACTICGKMFPSQSKLDRHVL
IHTGQRPFKCVLCTKSFRQSTHLKIHQLTHSEERPFQCCFCQKGFKIQSKLLKHKQIHTR
NKAFRALLLKKRRTESRPLPNKLNANQGGFENGEIGESEENNPLDVHSIYIVPFQCPKCE
KCFESEQILNEHSCFAARSGKIPSRFKRSYNYKTIVKKILAKLKRARSKKLDNFQSEKKV
FKKSFLRNCDLISGEQSSEQTQRTFVGSLGKHGTYKTIGNRKKKTLTLPFSWQNMGKNLK
GILTTENILSIDNSVNKKDLSICGSSGEEFFNNCEVLQCGFSVPRENIRTRHKICPCDKC

EKVFPSISKLKRHYLIHTGQRPFGCNICGKSFRQSAHLKRHEQTHNEKSPYASLCQVEFG
NFNNLSNHSGNNVNYNASQQCQAPGVQKYEVSESDQMSGVKAESQDFIPGSTGQPCLPNV
LLESEQSNPFCSYSEHQEKNDVFLYRCSVCAKSFRSPSKLERHYLIHAGQKPFECSVCGK
TFRQAPHWKRHQLTHFKERPQGKVVALDSVM*
>ENSG00000188994|ENST00000339907
MADEEAEQERLSCGEGGCVAELQRLGERLQELELQLRESRVPAVEAATDYCQQLCQTLLE
YAEKWKTSEDPLPLLEVYTVAIQSYVKARPYLTSECENVALVLERLALSCVELLLCLPVE
LSDKQWEQFQTLVQVAHEKLMENGSCELHFLATLAQETGVWKNPVLCTILSQEPLDKDKA
FEGPILLDMRIKHLIKTNQLSQATALAKLCSDHPEIGIKGSFKQTYLVCLCTSSPNGKLI
EEISEVDCKDALEMICNLESEGDEKSALVLCTAFLSRQLQQGDMYCAWELTLFWSKLQQR
VEPSIQVYLERCRQLSLLTKTVYHIFFLIKVINSETEGAGLATCIELCVKALRLESTENT
EVKISICKTISCLLPDDLEVKRACQLSEFLIEPTVDAYYAVEMLYNQPDQKYDEENLPIP
NSLRCELLLVLKTQWPFDPEFWDWKTLKRQCLALMGEEASIVSSIDELNDSEVYEKVVDY
QEESKETSMNGLSGGVGANSGLLKDIGDEKQKKREIKQLRERGFISARFRNWQAYMQYCV
LCDKEFLGHRIVRHAQKHYKDGIYSCPICAKNFNSKETFVPHVTLHVKQSSKERLAAMKP
LRRLGRPPKITTTNENQKTNTVAKQEQRPIKKNSLYSTDFIVFNDNDGSDDENDDKDKSY
EPEVIPVQKPVPVNEFNCPVTFCKKGFKYFKNLIAHVKGHKDNEDAKRFLEMQSKKVICQ
YCRRHFVSVTHLNDHLQMHCGSKPYICIQMKCKAGFNSYAELLTHRKEHQVFRAKCMFPK
CGRIFSEAYLLYDHEAQHYNTYTCKFTGCGKVYRSQGELEKHLDDHSTPPEKVLPPEAQL
NSSGDSIQPSEVNQNTAENIEKERSMLPSENNIENSLLADRSDAWDKSKAESAVTKQDQI
SASELRQANGPLSNGLENPATTPLLQSSEVAVSIKVSLNQGIEDNFGKQENSTVEGSGEA
LVTDLHTPVEDTCNDLCHPGFQERKEQDCFNDAHVTQNSLVNSETLKIGDLTPQNLERQV
NNLMTFSVQNQAAFQNNLPTSKFECGDNVKTSSNLYNLPLKTLESIAFVPPQSDLSNSLG
TPSVPPKAPVQKFSCQVEGCTRTYNSSQSIGKHMKTAHPDQYAAFKMQRKSKKGQKANNL
NTPNNGKFVYFLPSPVNSSNPFFTSQTKANGNPACSAQLQHVSPPIFPAHLASVSTPLLS
SMESVINPNITSQDKNEQGGMLCSQMENLPSTALPAQMEDLTKTVLPLNIDSGSDPFLPL
PAESSSMSLFPSPADSGTNSVFSQLENNTNHYSSQIEGNTNSSFLKGGNGENAVFPSQVN
VANNFSSTNAQQSAPEKVKKDRGRGPNGKERKPKHNKRAKWPAIIRDGKFICSRCYRAFT
NPRSLGGHLSKRSYCKPLDGAEIAQELLQSNGQPSLLASMILSTNAVNLQQPQQSTFNPE
ACFKDPSFLQLLAENRSPAFLPNTFPRSGVTNFNTSVSQEGSEIIKQALETAGIPSTFEG
AEMLSHVSTGCVSDASQVNATVMPNPTVPPLLHTVCHPNTLLTNQNRTSNSKTSSIEECS
SLPVFPTNDLLLKTVENGLCSSSFPNSGGPSQNFTSNSSRVSVISGPQNTRSSHLNKKGN
SASKRRKKVAPPLIAPNASQNLVTSDLTTMGLIAKSVEIPTTNLHSNVIPTCEPQSLVEN
LTQKLNNVNNQLFMTDVKENFKTSLESHTVLAPLTLKTENGDSQMMALNSCTTSINSDLQ
ISEDNVIQNFEKTLEIIKTAMNSQILEVKSGSQGAGETSQNAQINYNIQLPSVNTVQNNK
LPDSSPFSSFISVMPTKSNIPQSEVSHKEDQIQEILEGLQKLKLENDLSTPASQCVLINT
SVTLTPTPVKSTADITVIQPVSEMINIQFNDKVNKPFVCQNQGCNYSAMTKDALFKHYGK
IHQYTPEMILEIKKNQLKFAPFKCVVPTCTKTFTRNSNLRAHCQLVHHFTTEEMVKLKIK
RPYGRKSQSENVPASRSTQVKKQLAMTEENKKESQPALELRAETQNTHSNVAVIPEKQLV
EKKSPDKTESSLQVITVTSEQCNTNALTNTQTKGRKIRRHKKEKEEKKRKKPVSQSLEFP
TRYSPYRPYRCVHQGCFAAFTIQQNLILHYQAVHKSDLPAFSAEVEEESEAGKESEETET
KQTLKEFRCQVSDCSRIFQAITGLIQHYMKLHEMTPEEIESMTASVDVGKFPCDQLECKS
SFTTYLNYVVHLEADHGIGLRASKTEEDGVYKCDCEGCDRIYATRSNLLRHIFNKHNDKH
KAHLIRPRRLTPGQENMSSKANQEKSKSKHRGTKHSRCGKEGIKMPKTKRKKKNNLENKN

AKIVQIEENKPYSLKRGKHVYSIKARNDALSECTSRFVTQYPCMIKGCTSVVTSESNIIR
HYKCHKLSKAFTSQHRNLLIVFKRCCNSQVKETSEQEGAKNDVKDSDTCVSESNDNSRTT
ATVSQKEVEKNEKDEMDELTELFITKLINEDSTSVETQANTSSNVSNDFQEDNLCQSERQ
KASNLKRVNKEKNVSQNKKRKVEKAEPASAAELSSVRKEEETAVAIQTIEEHPASFDWSS
FKPMGFEVSFLKFLEESAVKQKKNTDKDHPNTGNKKGSHSNSRKNIDKTAVTSGNHVCPC
KESETFVQFANPSQLQCSDNVKIVLDKNLKDCTELVLKQLQEMKPTVSLKKLEVHSNDPD
MSVMKDISIGKATGRGQY*
>ENSG00000176399|ENST00000325870
MERSQCGSRDRGVSGRPHLAPGLVVAAPPPPSPALPVPSGMQVPPAFLRPPSLFLRAAAA
AAAAAAATSGSGGCPPAPGLESGVGAVGCGYPRTPKCARCRNHGVVSALKGHKRFCRWRD
CACAKCTLIAERQRVMAAQVALRRQQAQEESEARGLQRLLCSGLSWPPGGRASGGGGRAE
NPQSTGGPAAGAALGLGALRQASGSATPAFEVFQQDYPEEKQEQKESKCESCQNGQEELI
SKSHQLYLGSSSRSNGVIGKQSIGSSISEYSNKPDSILSPHPGEQSGGEESPRSLSSSDL
ESGNESEWVKDLTATKASLPTVSSRPRDPLDILTKIFPNYRRSRLEGILRFCKGDVVQAI
EQVLNGKEHKPDNRNLANSEELENTAFQRASSFSLAGIGFGTLGNKSAFSPLQTTSASYG
GDSSLYGVNPRVGISPLRLAYSSAGRGLSGFMSPYLTPGLVPTLPFRPALDYAFSGMIRD
SSYLSSKDSITCGRLYFRPNQDNP*
>ENSG00000165119|ENST00000351839
METEQPEETFPNTETNGEFGKRPAEDMEEEQAFKRSRNTDEMVELRILLQSKNAGAVIGK
GGKNIKALRTDYNASVSVPDSSGPERILSISADIETIGEILKKIIPTLEEGLQLPSPTAT
SQLPLESDAVECLNYQHYKGSDFDCELRLLIHQSLAGGIIGVKGAKIKELRENTQTTIKL
FQECCPHSTDRVVLIGGKPDRVVECIKIILDLISESPIKGRAQPYDPNFYDETYDYGGFT
MMFDDRRGRPVGFPMRGRGGFDRMPPGRGGRPMPPSRRDYDDMSPRRGPPPPPPGRGGRG
GSRARNLPLPPPPPPRGGDLMAYDRRGRPGDRYDGMVGFSADETWDSAIDTWSPSEWQMA
YEPQGGSGYDYSYAGGRGSYGDLGGPIITTQVTIPKDLAGSIIGKGGQRIKQIRHESGAS
IKIDEPLEGSEDRIITITGTQDQIQNAQYLLQNSVKQYSGKFF*
>ENSG00000146587|ENST00000353796
MNTLQGPVSFKDVAVDFTQEEWQQLDPDEKITYRDVMLENYSHLVSVGYDTTKPNVIIKL
EQGEEPWIMGGEFPCQHSPEAWRVDDLIERIQENEDKHSRQAACINSKTLTEEKENTFSQ
IYMETSLVPSSIIAHNCVSCGKNLESISQLISSDGSYARTKPDECNECGKTYHGEKMCEF
NQNGDTYSHNEENILQKISILEKPFEYNECMEALDNEAVFIAHKRAYIGEKPYEWNDSGP
DFIQMSNFNAYQRSQMEMKPFECSECGKSFCKKSKFIIHQRAHTGEKPYECNVCGKSFSQ
KGTLTVHRRSHLEEKPYKCNECGKTFCQKLHLTQHLRTHSGEKPYECSECGKTFCQKTHL
TLHQRNHSGERPYPCNECGKSFSRKSALSDHQRTHTGEKLYKCNECGKSYYRKSTLITHQ
RTHTGEKPYQCSECGKFFSRVSYLTIHYRSHLEEKPYECNECGKTFNLNSAFIRHRKVHT
EEKSHECSECGKFSQLYLTDHHTAHLEEKPYECNECGKTFLVNSAFDGHQPLPKGEKSYE
CNVCGKLFNELSYYTEHYRSHSEEKPYGCSECGKTFSHNSSLFRHQRVHTGEKPYECYEC
GKFFSQKSYLTIHHRIHSGEKPYECSKCGKVFSRMSNLTVHYRSHSGEKPYECNECGKVF
SQKSYLTVHYRTHSGEKPYECNECGKKFHHRSAFNSHQRIHRRGNMNVLDVENL*
>ENSG00000008083|ENST00000341776
MSKERPKRNIIQKKYDDSDGIPWSEERVVRKVLYLSLKEFKNSQKRQHAEGIAGSLKTVN
GLLGNDQSKGLGPASEQSENEKDDASQVSSTSNDVSSSDFEEGPSRKRPRLQAQRKFAQS
QPNSPSTTPVKIVEPLLPPPATQISDLSKRKPKTEDFLTFLCLRGSPALPNSMVYFGSSQ
DEEEVEEEDDETEDVKTATNNASSSCQSTPRKGKTHKHVHNGHVFNGSSRSTREKEPVQK

HKSKEATPAKEKHSDHRADSRREQASANHPAAAPSTGSSAKGLAATHHHPPLHRSAQDLR
KQVSKVNGVTRMSSLGAGVTSAKKMREVRPSPSKTVKYTATVTKGAVTYTKAKRELVKDT
KPNHHKPSSAVNHTISGKTESSNAKTRKQVLSLGGASKSTGPAVNGLKVSGRLNPKSCTK
EVGGRQLREGLQLREGLRNSKRRLEEAHQAEKPQSPPKKMKGAAGPAEGPGKKAPAERGL
LNGHVKKEVPERSLERNRPKRATAGKSTPGRQAHGKADSASCENRSTSQPESVHKPQDSG
KAEKGGGKAGWAAMDEIPVLRPSAKEFHDPLIYIESVRAQVEKFGMCRVIPPPDWRPECK
LNDEMRFVTQIQHIHKLGRRWGPNVQRLACIKKHLKSQGITMDELPLIGGCELDLACFFR
LINEMGGMQQVTDLKKWNKLADMLRIPRTAQDRLAKLQEAYCQYLLSYDSLSPEEHRRLE
KEVLMEKEILEKRKGPLEGHTENDHHKFHPLPRFEPKNGLIHGVAPRNGFRSKLKEVGQA
QLKTGRRRLFAQEKEVVKEEEEDKGVLNDFHKCIYKGRSVSLTTFYRTARNIMSMCFSKE
PAPAEIEQEYWRLVEEKDCHVAVHCGKVDTNTHGSGFPVGKSEPFSRHGWNLTVLPNNTG
SILRHLGAVPGVTIPWLNIGMVFSTSCWSRDQNHLPYIDYLHTGADCIWYCIPAEEENKL
EDVVHTLLQANGTPGLQMLESNVMISPEVLCKEGIKVHRTVQQSGQFVVCFPGSFVSKVC
CGYSVSETVHFATTQWTSMGFETAKEMKRRHIAKPFSMEKLLYQIAQAEAKKENGPTLST
ISALLDELRDTELRQRRQLFEAGLHSSARYGSHDGSSTVADGKKKPRKWLQLETSERRCQ
ICQHLCYLSMVVQENENVVFCLECALRHVEKQKSCRGLKLMYRYDEEQIISLVNQICGKV
SGKNGSIENCLSKPTPKRGPRKRATVDVPPSRLSASSSSKSASSSS*
>ENSG00000143498|ENST00000352967
MSDFSEELKGPVTDDEEVETSVLSGAGMHFPWLQTYVETVAIGGKRRKDFAQTTSACLSF
IQEALLKHQWQQAAEYMYSYFQTLEDSDSYKRQAAPEIIWKLGSEILFYHPKSNMESFNT
FANRMKNIGVMNYLKISLQHALYLLHHGMLKDAKRNLSEAETWRHGENTSSREILINLIQ
AYKGLLQYYTWSEKKMELSKLDKDDYAYNAVAQDVFNHSWKTSANISALIKIPGVWDPFV
KSYVEMLEFYGDRDGAQEVLTNYAYDEKFPSNPNAHIYLYNFLKRQKAPRSKLISVLKIL
YQIVPSHKLMLEFHTLLRKSEKEEHRKLGLEVLFGVLDFAGCTKNITAWKYLAKYLKNIL
MGNHLAWVQEEWNSRKNWWPGFHFSYFWAKSDWKEDTALACEKAFVAGLLLGKGCRYFRY
ILKQDHQILGKKIKRMKRSVKKYSIVNPRL*
>ENSG00000197937|ENST00000334197
MALTQGQVTFRDVAIEFSQEEWTCLDPAQRTLYRDVMLENYRNLASLGISCFDLSIISML
EQGKEPFTLESQVQIAGNPDGWEWIKAVITALSSEFVMKDLLHKGKSNTGEVFQTVMLER
QESQDIEGCSFREVQKNTHGLEYQCRDAEGNYKGVLLTQEGNLTHGRDEHDKRDARNKLI
KNQLGLSLQSHLPELQLFQYEGKIYECNQVEKSFNNNSSVSPPQQMPYNVKTHISKKYLK
DFISSLLLTQGQKANNWGSPYKSNGCGMVFPQNSHLASHQRSHTKEKPYKCYECGKAFRT
RSNLTTHQVIHTGEKRYKCNECGKVFSRNSQLSQHQKIHTGEKPYKCNECGKVFTQNSHL
VRHRGIHTGEKPYKCNECGKAFRARSSLAIHQATHSGEKPYKCNECGKVFTQNSHLTNHW
RIHTGEKPYKCNECGKAFGVRSSLAIHLVIHTGEKPYKCHECGKVFRRNSHLARHQLIHT
GEKPYKCNECGKAFRAHSNLTTHQVIHTGEKPYKCNECGKVFTQNSHLANHQRIHTGVKP
YMCNECGKAFSVYSSLTTHQVIHTGEKPYKCNECGKVFTQNSHLARHRGIHTGEKPYKCN
ECGKVFRHNSYLSRHQRIHTGEKPYKYNEYGKAFSEHSNLTTHQVIHTGEKPYKCNECGK
VFTQNSHLARHRRVHTGGKPYQCNECGKAFSQTSKLARHQRVHTGEKPYECNQCGKAFSV
RSSLTTHQAIHTGKKPYKCNECGKVFTQNSHLARHRGIHTGEKPYKCNECGKAFSQTSKL
ARHQRIHTGEKPYECGKPFSICSSLTTHQTIHTGGKPYKCNVWKVLKSEFKPCKPSQNS*
>ENSG00000184898|ENST00000331426
MASVLNVKESKAPERTVVVAGLPVDLFSDQLLAVLVKSHFQDIKNEGGDVEDVIYPTRTK
GVAYVIFKEKKVAENVIRQKKHWLARKTRHAELTVSLRVSHFGDKIFSSVNAILDLSVFG

KEVTLETLVKDLKKKIPSLSFSPLKPNGRISVEGSFLAVKRLRESLLARACSLLEKDRNF
TSEERKWNRQNPQRNLQRSNNSLASVRTLVPETARSGEMLVLDTDVFLYLKHKCGSYEST
LKKFHILSQEKVDGEITTICLKSIQVGSQPNNAKHVKELIEEWSHALYLKLRKETFILEG
KENREKRMIKRACEQLSSRYLEVLINLYRTHIDIIGSSSDTYLFKKGVMKLIGQKVS*
>ENSG00000161813|ENST00000347328
MLLFVEQVASKGTGLNPNAKVWQEIAPGNTDATPVTHGTESSWHEIAATSGAHPEGNAEL
SEDICKEYEVMYSSSCETTRNTTGIEESTDGMILGPEDLSYQIYDVSGESNSAVSTEDLK
ECLKKQLEFCFSRENLSKDLYLISQMDSDQFIPIWTVANMEEIKKLTTDPDLILEVLRSS
PMVQVDEKGEKVRPSHKRCIVILREIPETTPIEEVKGLFKSENCPKVISCEFAHNSNWYI
TFQSDTDAQQAFKYLREEVKTFQGKPIMAPFPNGSFVNGFNSPGSYKTNAAAMNMGRPFQ
KNRVKPQFRSSGGSEHSTEGSVSLGDGQLNRYSSRNFPAERHNPTVTGHQEQTYLQKETS
TLQVEQNGDYGRGRRTLFRGRRRREDDRISRPHPSTAESKAPTPKFDLLASNFPPLPGSS
SRMPGELVLENRMSDVVKGVYKEKDNEELTISCPVPADEQTECTSAQQLNMSTSSPCAAE
LTALSTTQQEKDLIEDSSVQKDGLNQTTIPVSPPSTTKPSRASTASPCNNNINAATAVAL
QEPRKLSYAEVCQKPPKEPSSVLVQPLRELRSNVVSPTKNEDNGAPENSVEKPHEKPEAR
ASKDYSGFRGNIIPRGAAGKIREQRRQFSHRAIPQGVTRRNGKEQYVPPRSPK*
>ENSG00000148337|ENST00000357558
MFSQQQQQQLQQQQQQLQQLQQQQLQQQQLQQQQLLQLQQLLQQSPPQAPLPMAVSRGLP
PQQPQQPLLNLQGTNSASLLNGSMLQRALLLQQLQGLDQFAMPPATYDTAGLTMPTATLG
NLRGYGMASPGLAAPSLTPPQLATPNLQQFFPQATRQSLLGPPPVGVPMNPSQFNLSGRN
PQKQARTSSSTTPNRKDSSSQTMPVEDKSDPPEGSEEAAEPRMDTPEDQDLPPCPEDIAK
EKRTPAPEPEPCEASELPAKRLRSSEEPTEKEPPGQLQVKAQPQARMTVPKQTQTPDLLP
EALEAQVLPRFQPRVLQVQAQVQSQTQPRIPSTDTQVQPKLQKQAQTQTSPEHLVLQQKQ
VQPQLQQEAEPQKQVQPQVQPQAHSQGPRQVQLQQEAEPLKQVQPQVHTQAQPSVQPQEH
PPAQVSVQPPEQTHEQPHTQPQVSLLAPEQTPVVVHVCGLEMPPDAVEAGGGMEKTLPEP
VGTQVSMEEIQNESACGLDVGECENRAREMPGVWGAGGSLKVTILQSSDSRAFSTVPLTP
VPRPSDSVSSTPAATSTPSKQALQFFCYICKASCSSQQEFQDHMSEPQHQQRLGEIQHMS
QACLLSLLPVPRDVLETEDEEPPPRRWCNTCQLYYMGDLIQHRRTQDHKIAKQSLRPFCT
VCNRYFKTPRKFVEHVKSQGHKDKAKELKSLEKEIAGQDEDHFITVDAVGCFEGDEEEEE
DDEDEEEIEVEEELCKQVRSRDISREEWKGSETYSPNTAYGVDFLVPVMGYICRICHKFY
HSNSGAQLSHCKSLGHFENLQKYKAAKNPSPTTRPVSRRCAINARNALTALFTSSGRPPS
QPNTQDKTPSKVTARPSQPPLPRRSTRLKT*
>ENSG00000196866|ENST00000341023
MSGRGKQGGKARAKAKTRSSRAGLQFPVGRVHRLLRKGNYSERVGAGAPVYLAAVLEYLT
AEILELAGNAARDNKKTRIIPRHLQLAIRNDEELNKLLGKVTIAQGGVLPNIQAVLLPKK
TESHHKAKGK*
>ENSG00000172818|ENST00000335987
MPRAFLVKKPCVSTCKRNWSELPDEERGEIYVPVSLGFCPPQPYREPEPSVAEPPSCPLA
LNMSLRDSSYSMAPGPCVVAQLPSEDMGHLTDPQSRDHGFLRTKMKVTLGDSPSGDLFTC
RVCQKAFTYQRMLNRHMKCHNDVKRHLCTYCGKGFNDTFDLKRHVRTHTGVRPYKCSLCD
KAFTQRCSLESHLKKIHGVQQKYAYKERRAKLYVCEECGCTSESQEGHVLHLKEHHPDSP
LLRKTSKKVAVALQNTVTSLLQGSPHL*
>ENSG00000182117|ENST00000328848
MFLQYYLNEQGDRVYTLKKFDPMGQQTCSAHPARFSPDDKYSRHRITIKKRFKVLMTQQP

RPVL*
>ENSG00000151623|ENST00000358102
METKGYHSLPEGLDMERRWGQVSQAVERSSLGPTERTDENNYMEIVNVSCVSGAIPNNST
QGSSKEKQELLPCLQQDNNRPGILTSDIKTELESKELSATVAESMGLYMDSVRDADYSYE
QQNQQGSMSPAKIYQNVEQLVKFYKGNGHRPSTLSCVNTPLRSFMSDSGSSVNGGVMRAV
VKSPIMCHEKSPSVCSPLNMTSSVCSPAGINSVSSTTASFGSFPVHSPITQGTPLTCSPN
VENRGSRSHSPAHASNVGSPLSSPLSSMKSSISSPPSHCSVKSPVSSPNNVTLRSSVSSP
ANINNSRCSVSSPSNTNNRSTLSSPAASTVGSICSPVNNAFSYTASGTSAGSSTLRDVVP
SPDTQEKGAQEVPFPKTEEVESAISNGVTGQLNIVQYIKPEPDGAFSSSCLGGNSKINSD
SSFSVPIKQESTKHSCSGTSFKGNPTVNPFPFMDGSYFSFMDDKDYYSLSGILGPPVPGF
DGNCEGSGFPVGIKQEPDDGSYYPEASIPSSAIVGVNSGGQSFHYRIGAQGTISLSRSAR
DQSFQHLSSFPPVNTLVESWKSHGDLSSRRSDGYPVLEYIPENVSSSTLRSVSTGSSRPS
KICLVCGDEASGCHYGVVTCGSCKVFFKRAVEGQHNYLCAGRNDCIIDKIRRKNCPACRL
QKCLQAGMNLGARKSKKLGKLKGIHEEQPQQQQPPPPPPPPQSPEEGTTYIAPAKEPSVN
TALVPQLSTISRALTPSPVMVLENIEPEIVYAGYDSSKPDTAENLLSTLNRLAGKQMIQV
VKWAKVLPGFKNLPLEDQITLIQYSWMCLSSFALSWRSYKHTNSQFLYFAPDLVFNEEKM
HQSAMYELCQGMHQISLQFVRLQLTFEEYTIMKVLLLLSTIPKDGLKSQAAFEEMRTNYI
KELRKMVTKCPNNSGQSWQRFYQLTKLLDSMHDLVSDLLEFCFYTFRESHALKVEFPAML
VEIISDQLPKVESGNAKPLYFHRK*
>ENSG00000144355|ENST00000341900
MTMTTMPESLNSPVSGKAVFMEFGPPNQQMSPSPMSHGHYSMHCLHSAGHSQPDGAYSSA
SSFSRPLGYPYVNSVSSHASSPYISSVQSYPGSASLAQSRLEDPGQDLVPKQAIQVQEAD
EAGWGGSGG*
>ENSG00000159111|ENST00000351111
MAAAVAGMLRGGLLPQAGRLPTLQTVRYGSKAVTRHRRVMHFQRQKLMAVTEYIPPKPAI
HPSCLPSPPSPPQEEIGLIRLLRREIAAVFQDNRMIAVCQNVALSAEDKLLMRHQLRKHK
ILMKVFPNQVLKPFLEDSKYQNLLPLFVGHNMLLVSEEPKVKEMVRILRTVPFLPLLGGC
IDDTILSRQGFINYSKLPSLPLVQGELVGGLTCLTAQTHSLLQHQPLQLTTLLDQYIREQ
REKDSVMSANGKPDPDTVPDS*
>ENSG00000075420|ENST00000336824
MYVTMMMTDQIPLELPPLLNGEVAMMPHLVNGDAAQQVILVQVNPGETFTIRAEDGTLQC
IQGPAEVPMMSPNGSIPPIHVPPGYISQVIEDSTGVRRVVVTPQSPECYPPSYPSAMSPT
HHLPPYLTHHPHFIHNSHTAYYPPVTGPGDMPPQFFPQHHLPHTIYGEQEIIPFYGMSTY
ITREDQYSKPPHKKLKDRQIDRQNRLNSPPSSIYKSSCTTVYNGYGKGHSGGSGGGGSGS
GPGIKKTERRARSSPKSNDSDLQEYELEVKRVQDILSGIEKPQVSNIQARAVVLSWAPPV
GLSCGPHSGLSFPYSYEVALSDKGRDGKYKIIYSGEELECNLKDLRPATDYHVRVYAMYN
SVKGSCSEPVSFTTHSCAPECPFPPKLAHRSKSSLTLQWKAPIDNGSKITNYLLEWDEGK
RNSGFRQCFFGSQKHCKLTKLCPAMGYTFRLAARNDIGTSGYSQEVVCYTLGNIPQMPSA
PRLVRAGITWVTLQWSKPEGCSPEEVITYTLEIQEDENDNLFHPKYTGEDLTCTVKNLKR
STQYKFRLTASNTEGKSCPSEVLVCTTSPDRPGPPTRPLVKGPVTSHGFSVKWDPPKDNG
GSEILKYLLEITDGNSEANQWEVAYSGSATEYTFTHLKPGTLYKLRACCISTGGHSQCSE
SLPVRTLSIAPGQCRPPRVLGRPKHKEVHLEWDVPASESGCEVSEYSVEMTEPEDVASEV
YHGPELECTVGNLLPGTVYRFRVRALNDGGYGPYSDVSEITTAAGPPGQCKAPCISCTPD
GCVLVGWESPDSSGADISEYRLEWGEDEESLELIYHGTDTRFEIRDLLPAAQYCCRLQAF

NQAGAGPYSELVLCQTPASAPDPVSTLCVLEEEPLDAYPDSPSACLVLNWEEPCNNGSEI
LAYTIDLGDTSITVGNTTMHVMKDLLPETTYRIRIQAINEIGAGPFSQFIKAKTRPLPPL
PPRLECAAAGPQSLKLKWGDSNSKTHAAEDIVYTLQLEDRNKRFISIYRGPSHTYKVQRL
TEFTCYSFRIQAASEAGEGPFSETYTFSTTKSVPPTIKAPRVTQLEGNSCEILWETVPSM
KGDPVNYILQVLVGRESEYKQVYKGEEATFQISGLQTNTDYRFRVCACRRCLDTSQELSG
AFSPSAAFVLQRSEVMLTGDMGSLDDPKMKSMMPTDEQFAAIIVLGFATLSILFAFILQY
FLMK*
>ENSG00000131503|ENST00000360839
MLTDSGGGGTSFEEDLDSVAPRSAPAGASEPPPPGGVGLGIRTVRLFGEAGPASGVGSSG
GGGSGSGTGGGDAALDFKLAAAVLRTGGGGGASGSDEDEVSEVESFILDQEDLDNPVLKT
TSEIFLSSTAEGADLRTVDPETQARLEALLEAAGIGKLSTADGKAFADPEVLRRLTSSVS
CALDEAAAALTRMKAENSHNAGQVDTRSLAEACSDGDVNAVRKLLDEGRSVNEHTEEGES
LLCLACSAGYYELAQVLLAMHANVEDRGNKGDITPLMAASSGGYLDIVKLLLLHDADVNS
QSATGNTALTYACAGGFVDIVKVLLNEGANIEDHNENGHTPLMEAASAGHVEVARVLLDH
GAGINTHSNEFKESALTLACYKGHLDMVRFLLEAGADQEHKTDEMHTALMEACMDGHVEV
ARLLLDSGAQVNMPADSFESPLTLAACGGHVELAALLIERGANLEEVNDEGYTPLMEAAR
EGHEEMVALLLAQGANINAQTEETQETALTLACCGGFSEVADFLIKAGADIELGCSTPLM
EASQEGHLELVKYLLASGANVHATTATGDTALTYACENGHTDVADVLLQAGADLEHESEG
GRTPLMKAARAGHLCTVQFLISKGANVNRATANNDHTVVSLACAGGHLAVVELLLAHGAD
PTHRLKDGSTMLIEAAKGGHTNVVSYLLDYPNNVLSVPTTDVSQLPPPSQDQSQVPRVPT
HTLAMVVPPQEPDRTSQENSPALLGVQKGTSKQKSSSLQVADQDLLPSFHPYQPLECIVE
ETEGKLNELGQRISAIEKAQLKSLELIQGEPLNKDKIEELKKNREEQVQKKKKILKELQK
VERQLQMKTQQQFTKEYLETKGQKDTVSLHQQCSHRGVFPEGEGDGSLPEDHFSELPQVD
TILFKDNDVDDEQQSPPSAEQIDFVPVQPLSSPQCNFSSDLGSNGTNSLELQKVSGNQQI
VGQPQIAITGHDQGLLVQEPDGLMVATPAQTLTDTLDDLIAAVSTRVPTGSNSSSQTTEC
LTPESCSQTTSNVASQSMPPVYPSVDIDAHTESNHDTALTLACAGGHEELVSVLIARDAK
IEHRDKKGFTPLILAATAGHVGVVEILLDKGGDIEAQSERTKDTPLSLACSGGRQEVVDL
LLARGANKEHRNVSDYTPLSLAASGGYVNIIKILLNAGAEINSRTGSKLGISPLMLAAMN
GHVPAVKLLLDMGSDINAQIETNRNTALTLACFQGRAEVVSLLLDRKANVEHRAKTGLTP
LMEAASGGYAEVGRVLLDKGADVNAPPVPSSRDTALTIAADKGHYKFCELLIHRGAHIDV
RNKKGNTPLWLASNGGHFDVVQLLVQAGADVDAADNRKITPLMSAFRKGHVKVVQYLVKE
VNQFPSDIECMRYIATITDKELLKKCHQCVETIVKAKDQQAAEANKNASILLKELDLEKS
REESRKQALAAKREKRKEKRKKKKEEQKRKQEEDEENKPKENSELPEDEDEEENDEDVEQ
EVPIEPPSATTTTTIGISATSATFTNVFGKKRANVVTTPSTNRKNKKNKTKETPPTAHLI
LPEQHMSLAQQKADKNKINGEPRGGGAGGNSDSDNLDSTDCNSESSSGGKSQELNFVMDV
NSSKYPSLLLHSQEEKTSTATSKTQTRLEGEVTPNSLSTSYKTVSLPLSSPNIKLNLTSP
KRGQKREEGWKEVVRRSKKLSVPASVVSRIMGRGGCNITAIQDVTGAHIDVDKQKDKNGE
RMITIRGGTESTRYAVQLINALIQDPAKELEDLIPKNHIRTPASTKSIHANFSSGVGTTA
ASSKNAFPLGAPTLVTSQATTLSTFQPANKLNKNVPTNVRSSFPVSLPLAYPHPHFALLA
AQTMQQIRHPRLPMAQFGGTFSPSPNTWGPFPVRPVNPGNTNSSPKHNNTSRLPNQNGTV
LPSESAGLATASCPITVSSVVAASQQLCVTNTRTPSSVRKQLFACVPKTSPPATVISSVT
STCSSLPSVSSAPITSGQAPTTFLPASTSQAQLSSQKMESFSAVPPTKEKVSTQDQPMAN
LCTPSSTANSCSSSASNTPGAPETHPSSSPTPTSSNTQEEAQPSSVSDLSPMSMPFASNS
EPAPLTLTSPRMVAADNQDTSNLPQLAVPAPRVSHRMQPRGSFYSMVPNATIHQDPQSIF

VTNPVTLTPPQGPPAAVQLSSAVNIMNGSQMHINPANKSLPPTFGPATLFNHFSSLFDSS
QVPANQGWGDGPLSSRVATDASFTVQSAFLGNSVLGHLENMHPDNSKAPGFRPPSQRVST
SPVGLPSIDPSGSSPSSSSAPLASFSGIPGTRVFLQGPAPVGTPSFNRQHFSPHPWTSAS
NSSTSAPPTLGQPKGVSASQDRKIPPPIGTERLARIRQGGSVAQAPAGTSFVAPVGHSGI
WSFGVNAVSEGLSGWSQSVMGNHPMHQQLSDPSTFSQHQPMERDDSGMVAPSNIFHQPMA
SGFVDFSKGLPISMYGGTIIPSHPQLADVPGGPLFNGLHNPDPAWNPMIKVIQNSTECTD
AQQIWPGTWAPHIGNMHLKYVN*
>ENSG00000178105|ENST00000322536
MGKTANSPGSGARPDPVRSFNRWKKKHSHRQNKKKQLRKQLKKPEWQVERESISRLMQNY
EKINVNEITRFSDFPLSKKTLKGLQEAQYRLVTEIQKQTIGLALQGKDVLGAAKTGSGKT
LAFLVPVLEALYRLQWTSTDGLGVLIISPTRELAYQTFEVLRKVGKNHDFSAGLIIGGKD
LKHEAERINNINILVCTPGRLLQHMDETVSFHATDLQMLVLDEADRILDMGFADTMNAVI
ENLPKKRQTLLFSATQTKSVKDLARLSLKNPEYVWVHEKAKYSTPATLEQNYIVCELQQK
ISVLYSFLRSHLKKKSIVFFSSCKEVQYLYRVFCRLRPGVSILALHGRQQQMRRMEVYNE
FVRKRAAVLFATDIAARGLDFPAVNWVLQFDCPEDANTYIHRAGRTARYKEDGEALLILL
PSEKAMVQQLLQKKVPVKEIKINPEKLIDVQKKLESILAQDQDLKERAQRCFVSYVRSVY
LMKDKEVFDVSKLPIPEYALSLGLAVAPRVRFLQKMQKQPTKELVRSQADKVIEPRAPSL
TNDEVEEFRAYFNEKMSILQKGGKRLEGTEHRQDNDTGNEEQEEEEDDEEEMEEKLAKAK
GSQAPSLPNTSEAQKIKEVPTQFLDRDEEEEDADFLKVKRHNVFGLDLKDEKTLQKKEPS
KSSIKKKMTKVAEAKKVMKRNFKVNKKITFTDEGELVQQWPQMQKSAIKDAEEDDDTGGI
NLHKAKERLQEEDKFDKEEYRKKIKAKHREKRLKEREARREANKRQAKAKDEEEAFLDWS
DDDDDDDDGFDPSTLPDPDKYRSSEDSDSEDMENKISDTKKKQGMKKRSNSEVEDVGPTS
HNRKKARWDTLEPLDTGLSLAEDEELVLHLLRSQS*
>ENSG00000159714|ENST00000348579
MYKMNICNKPSNKTAPEKSVWTAPAQPSGPSPELQGQRSRRNGWSWPPHPLQIVAWLLYL
FFAVIGFGILVPLLPHHWVPAGYACMGAIFAGHLVVHLTAVSIDPADANVRDKSYAGPLP
IFNRSQHAHVIEDLHCNLCNVDVSARSKHCSACNKCVCGFDHHCKWLNNCVGERNYRLFL
HSVASALLGVLLLVLVATYVFVEFFVNPMRLRTNRHFEVLKNHTDVWFVFLPAAPVETQA
PAILALAALLILLGLLSTALLGHLLCFHIYLMWHKLTTYEYIVQHRPPQEAKGVHRELES
CPPKMRPIQEMEFYMRTFRHMRPEPPGQAGPAAVNAKHSRPASPDPTPGRRDCAGPPVQV
EWDRKKPLPWRSPLLLLAMWGPQAPPCLCRKRGRGACIKCERLRPRIRRRGLGPPAAAPA
RRRIPRTPALCTPLALPAPTTRRRQSPWTRFQWRRRAWAAPLWPPRGAGADSPRWRGRRV
RPPFS*
>ENSG00000186051|ENST00000334077
MTRKIFTNTRERWRQQNVNSAFAKLRKLIPTHPPDKKLSKNETLRLAMRYINFLVKVLGE
QSLQQTGVAAQGNILGLFPQGPHLPGLEDRTLLENYQVPSPGPSHHIP*
>ENSG00000135114|ENST00000339275
MALMQELYSTPASRLDSFVAQWLQPHREWKEEVLDAVRTVEEFLRQEHFQGKRGLDQDVR
VLKVVKVGSFGNGTVLRSTREVELVAFLSCFHSFQEAAKHHKDVLRLIWKTMWQSQDLLD
LGLEDLRMEQRVPDALVFTIQTRGTAEPITVTIVPAYRALGPSLPNSQPPPEVYVSLIKA
CGGPGNFCPSFSELQRNFVKHRPTKLKSLLRLVKHWYQQAHHPGSGRPHPQRGRRVQMGH
RCSEGLPVPETGLLL*
>ENSG00000121417|ENST00000347302
MLGFPPGRPQLPVQLRPQTRMATALRDPASGSVTFEDVAVYFSWEEWDLLDEAQKHLYFD

VMLENFALTSSLGCWCGVEHEETPSEQRISGERVPQFRTSKEGSSSQNADSCEICCLVLR
DILHLAEHQGTNCGQKLHTCGKQFYISANLQQHQRQHITEAPFRSYVDTASFTQSCIVHV
SEKPFTCREIRKDFLANMRFLHQDATQTGEKPNNSNKCAVAFYSGKSHHNWGKCSKAFSH
IDTLVQDQRILTREGLFECSKCGKACTRRCNLIQHQKVHSEERPYECNECGKFFTYYSSF
IIHQRVHTGERPYACPECGKSFSQIYSLNSHRKVHTGERPYECGECGKSFSQRSNLMQHR
RVHTGERPYECSECGKSFSQNFSLIYHQRVHTGERPHECNECGKSFSRSSSLIHHRRLHT
GERPYECSKCGKSFKQSSSFSSHRKVHTGERPYVCGECGKSFSHSSNLKNHQRVHTGERP
VECSECSKSFSCKSNLIKHLRVHTGERPYECSECGKSFSQSSSLIQHRRVHTGKRPYQCS
QCGKSFGCKSVLIQHQRVHIGEKP*
>ENSG00000154957|ENST00000322748
MPVDLGQALGLLPSLAKAEDSQFSESDAALQEELSSPETARQLFRQFRYQVMSGPHETLK
QLRKLCFQWLQPEVHTKEQILEILMLEQFLTILPGEIQMWVRKQCPGSGEEAVTLVESLK
GDPQRLWQWISIQVLGQDILSEKMESPSCQVGEVEPHLEVVPQELGLENSSSGPGELLSH
IVKEESDTEAELALAASQPARLEERLIRDQDLGASLLPAAPQEQWRQLDSTQKEQYWDLM
LETYGKMVSGAGISHPKSDLTNSIEFGEELAGIYLHVNEKIPRPTCIGDRQENDKENLNL
ENHRDQELLHASCQASGEVPSQASLRGFFTEDEPGCFGEGENLPEALQNIQDEGTGEQLS
PQERISEKQLGQHLPNPHSGEMSTMWLEEKRETSQKGQPRAPMAQKLPTCRECGKTFYRN
SQLIFHQRTHTGETYFQCTICKKAFLRSSDFVKHQRTHTGEKPCKCDYCGKGFSDFSGLR
HHEKIHTGEKPYKCPICEKSFIQRSNFNRHQRVHTGEKPYKCSHCGKSFSWSSSLDKHQR
SHLGKKPFQ*
>ENSG00000187187|ENST00000347077
MQVDPPLHGPPNDFLIFQIIPLHSLSIMPRFLWILCFSMEETQGELTSSCGSKTMANVSL
AFRDVSIDLSQEEWECLDAVQRDLYKDVMLENYSNLVSLGYTIPKPDVITLLEQEKEPWI
VMREGTRNWFTDLEYKYITKNLLSEKNVCKIYLSQLQTGEKSKNTIHEDTIFRNGLQCKH
EFERQERHQMGCVSQMLIQKQISHPLHPKIHAREKSYECKECRKAFRQQSYLIQHLRIHT
GERPYKCMECGKAFCRVGDLRVHHTIHAGERPYECKECGKAFRLHYHLTEHQRIHSGVKP
YECKECGKAFSRVRDLRVHQTIHAGERPYECKECGKAFRLHYQLTEHQRIHTGERPYECK
VCGKTFRVQRHISQHQKIHTGVKPYKCNECGKAFSHGSYLVQHQKIHTGEKPYECKECGK
SFSFHAELARHRRIHTGEKPYECRECGKAFRLQTELTRHHRTHTGEKPYECKECGKAFIC
GYQLTLHLRTHTGEIPYECKECGKTFSSRYHLTQHYRIHTGEKPYICNECGKAFRLQGEL
TRHHRIHTCEKPYECKECGKAFIHSNQFISHQRIHTSESTYICKECGKIFSRRYNLTQHF
KIHTGEKPYICNECGKAFRFQTELTQHHRIHTGEKPYKCTECGKAFIRSTHLTQHHRIHT
GEKPYECTECGKTFSRHYHLTQHHRGHTGEKPYICNECGNAFICSYRLTLHQRIHTGELP
YECKECGKTFSRRYHLTQHFRLHTGEKPYSCKECGNAFRLQAELTRHHIVHTGEKPYKCK
ECGKAFSVNSELTRHHRIHTGEKPYQCKECGKAFIRSDQLTLHQRNHISEEVLCIM*
>ENSG00000078328|ENST00000340209
MIVPAAPYLPGLIQGNQEAAAAPDTMAQPYASAQFAPPQNGIPAEYTAPHPHPAPEYTGQ
TTVPEHTLNLYPPAQTHSEQSPADTSAQTVSGTATQTDDAAPTDGQPQTQPSENTENKSQ
PKRLHVSNIPFRFRDPDLRQMFGQFGKILDVEIIFNERGSKGFGFVTFENSADADRAREK
LHGTVVEGRKIEVNNATARVMTNKKTVNPYTNGWKLNPVVGAVYSPEFYAGTVLLCQANQ
EGSSMYSAPSSLVYTSAMPGFPYPAATAAAAYRGAHLRGRGRTVYNTFRAAAPPPPIPAY
GGVVYQDGFYGADIYGGYAAYRYAQPTPATAAAYSDSYGRVYAADPYHHALAPAPTYGVG
AMNAFAPLTDAKTRSHADDVGLVLSSLQASIYRGGYNRFAPY*
>ENSG00000197016|ENST00000330619

MKSQEEVEVAGIKLCKAMSLGSVTFTDVAIDFSQDEWEWLNLAQRSLYKKVMLENYRNLV
SVGLCISKPDVISLLEQEKDPWVIKGGMNRGLCPDLECVWVTKSLSLNQDIYEEKLPPAI
IMERLKSYDLECSTLGKNWKCEDLFERELVNQKTHFRQETITHIDTLIEKRDHSNKSGTV
FHLNTLSYIKQIFPMEERIFNFHTDKKSLKTHSVVKKHKQDRGEKKLLKCNDCEKIFSKI
STLTLHQRIHTGEKPYECIECGKAFSQSAHLAQHQRIHTGEKPFECTECGKAFSQNAHLV
QHQRVHTGEKPYQCKQCNKAFSQLAHLAQHQRVHTGEKPYECIECGKAFSDCSSLAHHRR
IHTGKRPYECIDCGKAFRQNASLIRHRRYYHTGEKPFDCIDCGKAFTDHIGLIQHKRTHT
GERPYKCNVCGKAFSHGSSLTVHQRIHTGEKPYECNICEKAFSHRGSLTLHQRVHTGEKP
YECKECGKAFRQSTHLAHHQRIHTGEKPYECKECSKTFSQNAHLAQHQKIHTGEKPYECK
ECGKAFSQIAHLVQHQRVHTGEKPYECIECGKAFSDGSYLVQHQRLHSGKRPYECLECGK
AFRQRASLICHQRCHTGEKPYECNVCGKAFSHRKSLTLHQRIHTGEKPYECKECSKAFSQ
VAHLTLHKRIHTGERPYECKECGKAFRQSVHLAHHQRIHTGESSVILSSALPYHQVL*
>ENSG00000116793|ENST00000357783
MASNERDAISWYQKKIGAYDQQIWEKSIEQTQIKGLKNKPKKMGHIKPDLIDVDLIRGST
FAKAKPEIPWTSLTRKGLVRVVFFPLFSNWWIQVTSLRIFVWLLLLYFMQVIAIVLYLMM
PIVNISEVLGPLCLMLLMGTVHCQIVSTQITRPSGNNGNRRRRKLRKTVNGDGSRENGNN
SSDKVRGIETLESVPIIGGFWETIFGNRIKRVKLISNKGTETDNDPSCVHPIIKRRQCRP
EIRMWQTREKAKFSDGEKCRREAFRRLGNGVSDDLSSEEDGEARTQMILLRRSVEGASSD
NGCEVKNRKSILSRHLNSQVKKTTTRWCHIVRDSDSLAESEFESAAFSQGSRSGVSGGSR
SLNMSRRDSESTRHDSETEDMLWDDLLHGPECRSSVTSDSEGAHVNTLHSGTKRDPKEDV
FQQNHLFWLQNSSPSSDRVSAIIWEGNECKKMDMSVLEISGIIMSRVNAYQQGVGYQMLG
NVVTIGLAFFPFLHRLFREKSLDQLKSISAEEILTLFCGAPPVTPIIVLSIINFFERLCL
TWMFFFMMCVAERTYKQRFLFAKLFSHITSARKARKYEIPHFRLKKVENIKIWLSLRSYL
KRRGPQRSVDVVVSSVFLLTLSIAFICCAQVKVSHLL*
>ENSG00000166987|ENST00000355673
MNGGNESSGADRAGGPVATSVPIGWQRCVREGAVLYISPSGTELSSLEQTRSYLLSDGTC
KCGLECPLNVPKVFNFDPLAPVTPGGAGVGPASEEDMTKLCNHRRKAVAMATLYRSMETT
CSHSSPGEGASPQMFHTVSPGPPSARPPCRVPPTTPLNGGPGSLPPEPPSVSQAFPTLAG
PGGLFPPRLADPVPSGGSSSPRFLPRGNAPSPAPPPPPAISLNAPSYNWGAALRSSLVPS
DLGSPPAPHASSSPPSDPPLFHCSDALTPPPLPPSNNLPAHPGPASQPPVSSATMHLPLV
LGPLGGAPTVEGPGAPPFLASSLLSAAAKAQHPPLPPPSTLQGRRPRAQAPSASHSSSLR
PSQRRPRRPPTVFRLLEGRGPQTPRRSRPRAPAPVPQPFSLPEPSQPILPSVLSLLGLPT
PGPSHSDGSFNLLGSDAHLPPPPTLSSGSPPQPRHPIQPSLPGTTSGSLSSVPGAPAPPA
ASKAPVVPSPVLQSPSEGLGMGAGPACPLPPLAGGEAFPFPSPEQGLALSGAGFPGMLGA
LPLPLSLGQPPPSPLLNHSLFGVLTGGGGQPPPEPLLPPPGGPGPPLAPGEPEGPSLLVA
SLLPPPPSDLLPPPSAPPSNLLASFLPLLALGPTAGDGEGSAEGAGGPSGEPFSGLGDLS
PLLFPPLSAPPTLIALNSALLAATLDPPSGTPPQPCVLSAPQPGPPTSSVTTATTDPGAS
SLGKAPSNSGRPPQLLSPLLGASLLGDLSSLTSSPGALPSLLQPPGPLLSGQLGLQLLPG
GGAPPPLSEASSPLACLLQSLQIPPEQPEAPCLPPESPASALEPEPARPPLSALAPPHGS
PDPPVPELLTGRGSGKRGRRGGGGLRGINGEARPARGRKPGSRREPGRLALKWGTRGGFN
GQMERSPRRTHHWQHNGELAEGGAEPKDPPPPGPHSEDLKVPPGVVRKSRRGRRRKYNPT
RNSNSSRQDITLEPSPTARAAVPLPPRARPGRPAKNKRRKLAP*
>ENSG00000140632|ENST00000321919
MAAVSLRLGDLVWGKLGRYPPWPGKIVNPPKDLKKPRGKKCFFVKFFGTEDHAWIKVEQL

KPYHAHKEEMIKINKGKRFQQAVDAVEEFLRRAKGKDQTSSHNSSDDKNRRNSSEERSRP
NSGDEKRKLSLSEGKVKKNMGEGKKRVSSGSSERGSKSPLKRAQEQSPRKRGRPPKDEKD
LTIPESSTVKGMMAGPMAAFKWQPTASEPVKDADPHFHHFLLSQTEKPAVCYQAITKKLK
ICEEETGSTSIQAADSTAVNGSITPTDKKIGFLGLGLMGSGIVSNLLKMGHTVTVWNRTA
EKCDLFIQEGARLGRTPAEVVSTCDITFACVSDPKAAKDLVLGPSGVLQGIRPGKCYVDM
STVDADTVTELAQVIVSRGGRFLEAPVSGNQQLSNDGMLVILAAGDRGLYEDCSSCFQAM
GKTSFFLGEVGNAAKMMLIVNMVQGSFMATIAEGLTLAQVTGQSQQTLLDILNQGQLASI
FLDQKCQNILQGNFKPDFYLKYIQKDLRLAIALGDAVNHPTPMAAAANEVYKRAKALDQS
DNDMSAVYRAYIH*
>ENSG00000162971|ENST00000354611
MAGQHLPVPRLEGVSREQFMQHLYPQRKPLVLEGIDLGPCTSKWTVDYLSQVGGKKEVKI
HVAAVAQMDFISKNFVYRTLPFDQLVQRAAEEKHKEFFVSEDEKYYLRSLGEDPRKDVAD
IRKQFPLLKGDIKFPEFFKEEQFFSSVFRISSPGLQLWTHYDVMDNLLIQVTGKKRVVLF
SPRDAQYLYLKGTKSEVLNIDNPDLAKYPLFSKARRYECSLEAGDVLFIPALWFHNVISE
EFGVGVNIFWKHLPSECYDKTDTYGNKDPTAASRAAQILDRALKTLAELPEEYRDFYARR
MVLHIQDKAYSKNSE*
>ENSG00000165588|ENST00000339475
MMSYLKQPPYAVNGLSLTTSGMDLLHPSVGYPGPWASCPAATPRKQRRERTTFTRAQLDV
LEALFAKTRYPDIFMREEVALKINLPESRVQVWFKNRRAKCRQQQQQQQNGGQNKVRPAK
KKTSPAREVSSESGTSGQFTPPSSTSVPTIASSSAPVSIWSPASISPLSDPLSTSSSCMQ
RSYPMTYTQASGYSQGYAGSTSYFGGMDCGSYLTPMHHQLPGPGATLSPMGTNAVTSHLN
QSPASLSTQGYGASSLGFNSTTDCLDYKDQTASWKLNFNADCLDYKDQTSSWKFQVL*
>ENSG00000251247|ENST00000331800
MENLTKHSIECSSFRGDWECKNQFERKQGSQEGHFSEMIFTPEDMPTFSIQHQRIHTDEK
LLECKECGKDFSFVSVLVRHQRIHTGEKPYECKECGKAFGSGANLAYHQRIHT
>ENSG00000126226|ENST00000337344
MAHITINQYLQQVYEAIDSRDGASCAELVSFKHPHVANPRLQMASPEEKCQQVLEPPYDE
MFAAHLRCTYAVGNHDFIEAYKCQTVIVQSFLRAFQAHKEENWALPVMYAVALDLRVFAN
NADQQLVKKGKSKVGDMLEKAAELLMSCFRVCASDTRAGIEDSKKWGMLFLVNQLFKIYF
KINKLHLCKPLIRAIDSSNLKDDYSTAQRVTYKYYVGRKAMFDSDFKQAEEYLSFAFEHC
HRSSQKNKRMILIYLLPVKMLLGHMPTVELLKKYHLMQFAEVTRAVSEGNLLLLHEALAK
HEAFFIRCGIFLILEKLKIITYRNLFKKVYLLLKTHQLSLDAFLVALKFMQVEDVDIDEV
QCILANLIYMGHVKGYISHQHQKLVVSKQNPFPPLSTVC*
>ENSG00000197951|ENST00000328070
MKELDPKNDISEDKLSVVGEATGGPTRNGARGPGSEGVWEPGSWPERPRGDAGAEWEPLG
IPQGNKLLGGSVPACHELKAFANQGCVLVPPRLDDPTEKGACPPVRRGKNFSSTSDLSKP
PMPCEEKKTYDCSECGKAFSRSSSLIKHQRIHTGEKPFECDTCGKHFIERSSLTIHQRVH
TGEKPYACGDCGKAFSQRMNLTVHQRTHTGEKPYVCDVCGKAFRKTSSLTQHERIHTGEK
PYACGDCGKAFSQNMHLIVHQRTHTGEKPYVCPECGRAFSQNMHLTEHQRTHTGEKPYAC
KECGKAFNKSSSLTLHQRNHTGEKPYVCGECGKAFSQSSYLIQHQRFHIGVKPFECSECG
KAFSKNSSLTQHQRIHTGEKPYECYICKKHFTGRSSLIVHQIVHTGEKPYVCGECGKAFS
QSAYLIEHQRIHTGEKPYRCGQCGKSFIKNSSLTVHQRIHTGEKPYRCGECGKTFSRNTN
LTRHLRIHT*
>ENSG00000163041|ENST00000366813

MARTKQTARKSTGGKAPRKQLATKAARKSAPSTGGVKKPHRYRPGTVALREIRRYQKSTE
LLIRKLPFQRLVREIAQDFKTDLRFQSAAIGALQEASEAYLVGLFEDTNLCAIHAKRVTI
MPKDIQLARRIRGERA*
>ENSG00000183779|ENST00000331569
MSDSPAGSNPRTPESSGSGSGGGGKRPAVPAAVSLLPPADPLRQANRLPIRVLKMLSAHT
GHLLHPEYLQPLSSTPVSPIELDAKKSPLALLAQTCSQIGKPDPPPSSKLNSVAAAANGL
GAEKDPGRSAPGAASAAAALKQLGDSPAEDKSSFKPYSKGSGGGDSRKDSGSSSVSSTSS
SSSSSPGDKAGFRVPSAACPPFPPHGAPVSASSSSSSPGGSRGGSPHHSDCKNGGGVGGG
ELDKKDQEPKPSPEPAAVSRGGGGEPGAHGGAESGASGRKSEPPSALVGAGHVAPVSPYK
PGHSVFPLPPSSIGYHGSIVGAYAGYPSQFVPGLDPSKSGLVGGQLSGGLGLPPGKPPSS
SPLTGASPPSFLQGLCRDPYCLGGYHGASHLGGSSCSTCSAHDPAGPSLKAGGYPLVYPG
HPLQPAALSSSAAQAALPGHPLYTYGFMLQNEPLPHSCNWVAASGPCDKRFATSEELLSH
LRTHTALPGAEKLLAAYPGASGLGSAAAAAAAAASCHLHLPPPAAPGSPGSLSLRNPHTL
GLSRYHPYGKSHLSTAGGLAVPSLPTAGPYYSPYALYGQRLASASALGYQ*
>ENSG00000189144|ENST00000357309
MILREETQFTDLDLQCEIISYIEVPTYETDISSTQLQSIYKREKLYECKKCQKKFSSGYQ
LILHHRFHVIERPYECKECGKNFRSGYQLTLHQRFHTGEKPYECTECGKNFRSGYQLTVH
QRFHTGEKTYECRQCGKAFIYASHIVQHERIHTGGKPYECQECGRAFSQGGHLRIHQRVH
TGEKPYKCKECGKTFSRRSNLVEHGQFHTDEKPYICEKCGKAFRRGHQLTVHQRVHTGKK
PYECKECGKGYTTASYFLLHQRIHKGGKPYECKECKKTFTLYRNLTRHQNIHTGEKLFEC
KQCGKTYTTGSKLFQHQKTHTGEKPYECKECGKAFSLYGYLKQHQKIHTGMKHFECKECK
KTFTLYRNLTRHQNIHTGKKLFECQECGKAYSTGSNLIQHRKTHTGEKPYKCKECGKTFS
LHGYLNQHQKIHTGMKPYECKVCRKTFTFYRNLTLHQSIHTDEKPFECKECGKTFRRSSH
LTAHQSIHADKKPYECKECGKAFKMYGYLTQHQKIHTGGKPYECKECGKAFSRASNLVQH
ERIHTGEKPYVCKQCGKTFRYGSALKAHQRIHRSIKV*
>ENSG00000277462|ENST00000366503
MDSVSFEDVAVAFTQEEWALLDPSQKNLYRDVMQEIFRNLASVGNKSEDQNIQDDFKNPG
RNLSSHVVERLFEIKEGSQYGETFSQDSNLNLNKKVSTGVKPCECSVCGKVFICHSALHR
HILSHIGNKLFECEECPEKLYHCKQCGKAFISLTSVDRHMVTHTSNGPYKGPVYEKPFDF
PSVFQMPQSTYTGEKTYKCKHCDKAFNYSSYLREHERTHTGEKPYACKKCGKSFTFSSSL
RQHERSHTGEKPYECKECGKAFSRSTYLGIHERTHTGEKPYECIKCGKAFRCSRVLRVHE
RTHSGEKPYECKQCGKAFKYSSNLCEHERTHTGVKPYGCKECGKSFTSSSALRSHERTHT
GEKPYECKKCGKAFSCSSSLRKHERAYMW*
>ENSG00000185480|ENST00000327680
MAVFNQKSVSDMIKEFRKNWRALCNSERTTLCGADSMLLALQLSMAENNKQHSGEFTVSL
SDVLLTWKYLLHEKLNLPVENMDVTDHYEDVRKIYDDFLKNSNMLDLIDVYQKCRALTSN
CENYNTVSPSQLLDFLSGKQYAVGDETDLSIPTSPTSKYNRDNEKVQLLARKIIFSYLNL
LVNSKNDLAVAYILNIPDRGLGREAFTDLKHAAREKQMSIFLVATSFIRTIELGGKGYAP
PPSDPLRTHVKGLSNFINFIDKLDEILGEIPNPSIAGGQILSVIKMQLIKGQNSRDPFCK
AIEEVAQDLDLRIKNIINSQEGVVALSTTDISPARPKSHAINHGTAYCGRDTVKALLVLL
DEEAANAPTKNKAELLYDEENTIHHHGTSILTLFRSPTQVNNSIKPLRERICVSMQEKKI
KMKQTLIRSQFACTYKDDYMISKDNWNNVNLASKPLCVLYMENDLSEGVNPSVGRSTIGT
SFGNVHLDRSKNEKVSRKSTSQTGNKSSKRKQVDLDGENILCDNRNEPPQHKNAKIPKKS
NDSQNRLYGKLAKVAKSNKCTAKDKLISGQAKLTQFFRL*

>ENSG00000182872|ENST00000345781
MEYERRGGRGDRTGRYGATDRSQDDGGENRSRDHDYRDMDYRSYPREYGSQEGKHDYDDS
SEEQSAEIRGQLQSHGVQAREVRLMRNKSSGQSRGFAFVEFSHLQDATRWMEANQHSLNI
LGQKVSMHYSDPKPKINEDWLCNKCGVQNFKRREKCFKCGVPKSEAEQKLPLGTRLDQQT
LPLGGRELSQGLLPLPQPYQAQGVLASQALSQGSEPSSENANDTIILRNLNPHSTMDSIL
GALAPYAVLSSSNVRVIKDKQTQLNRGFAFIQLSTIVEAAQLLQILQALHPPLTIDGKTI
NVEFAKGSKRDMASNEGSRISAASVASTAIAAAQWAISQASQGGEGTWATSEEPPVDYSY
YQQDEGYGNSQGTESSLYAHGYLKGTKGPGITGTKGDPTGAGPEASLEPGADSVSMQAFS
RAQPGAAPGIYQQSAEASSSQGTAANSQSYTIMSPAVLKSELQSPTHPSSALPPATSPTA
QESYSQYPVPDVSTYQYDETSGYYYDPQTGLYYDPNSQYYYNAQSQQYLYWDGERRTYVP
ALEQSADGHKETGAPSKEGKEKKEKHKTKTAQQIAKDMERWARSLNKQKENFKNSFQPIS
SLRDDERRESATADAGYAILEKKGALAERQHTSMDLPKLASDDRPSPPRGLVAAYSGESD
SEEEQERGGPEREEKLTDWQKLACLLCRRQFPSKEALIRHQQLSGLHKQNLEIHRRAHLS
ENELEALEKNDMEQMKYRDRAAERREKYGIPEPPEPKRRKYGGISTASVDFEQPTRDGLG
SDNIGSRMLQAMGWKEGSGLGRKKQGIVTPIEAQTRVRGSGLGARGSSYGVTSTESYKET
LHKTMVTRFNEAQ*
>ENSG00000176371|ENST00000334141
MMAADIPRVTTPLSSLVQVPQEEDRQEEEVTTMILEDDSWVQEAVLQEDGPESEPFPQSA
GKGGPQEEVTRGPQGALGRLRELCRRWLRPEVHTKEQMLTMLPKEIQAWLQEHRPESSEE
AAALVEDLTQTLQDSAVAVVASLPVEVTSL*
>ENSG00000009307|ENST00000339438
MSFDPNLLHNNGHNGYPNGTSAALRETGVIEKLLTSYGFIQCSERQARLFFHCSQYNGNL
QDLKVGDDVEFEVSSDRRTGKPIAVKLVKIKQEILPEERMNGQEVFYLTYTPEDVEGNVQ
LETGDKINFVIDNNKHTGAVSARNIMLLKKKQARCQGVVCAMKEAFGFIERGDVVKEIFF
HYSEFKGDLETLQPGDDVEFTIKDRNGKEVATDVRLLPQGTVIFEDISIEHFEGTVTKVI
PKVPSKNQNDPLPGRIKVDFVIPKELPFGDKDTKSKVTLLEGDHVRFNISTDRRDKLERA
TNIEVLSNTFQFTNEAREMGVIAAMRDGFGFIKCVDRDVRMFFHFSEILDGNQLHIADEV
EFTVVPDMLSAQRNHAIRIKKLPKGTVSFHSHSDHRFLGTVEKEATFSNPKTTSPNKGKE
KEAEDGIIAYDDCGVKLTIAFQAKDVEGSTSPQIGDKVEFSISDKQRPGQQVATCVRLLG
RNSNSKRLLGYVATLKDNFGFIETANHDKEIFFHYSEFSGDVDSLELGDMVEYSLSKGKG
NKVSAEKVNKTHSVNGITEEADPTIYSGKVIRPLRSVDPTQTEYQGMIEIVEEGDMKGEV
YPFGIVGMANKGDCLQKGESVKFQLCVLGQNAQTMAYNITPLRRATVECVKDQFGFINYE
VGDSKKLFFHVKEVQDGIELQAGDEVEFSVILNQRTGKCSACNVWRVCEGPKAVAAPRPD
RLVNRLKNITLDDASAPRLMVLRQPRGPDNSMGFGAERKIRQAGVID*
>ENSG00000143889|ENST00000358367
MLARETYEEDREYESQAKRLKTEEGEIDYSAEEGENRREATPRGGGDGGGGGRSFSQPEA
GGSHHKVSVSPVVHVRGLCESVVEADLVEALEKFGTICYVMMMPFKRQALVEFENIDSAK
ECVTFAADEPVYIAGQQAFFNYSTSKRITRPGNTDDPSGGNKVLLLSIQNPLYPITVDVL
YTVCNPVGKVQRIVIFKRNGIQAMLMFESVLCAQKAKAALNGADIYAGCCTLKIEYARPT
RLNVIRNDNDSWDYTKPYLGRRDRGKGRQRQAILGEHPSSFRHDGYGSHGPLLPLPSRYR
MGSRDTPELVAYPLPQASSSYMHGGNPSGSVVMVSGLHQLKMNCSRVFNLFCLYGNIEKV
KFMKTIPGTALVEMGDEYAVERAVTHLNNVKLFGKRLNVCVSKQHSVVPSQIFELEDGTS
SYKDFAMSKNNRFTSAGQASKNIIQPPSCVLHYYNVPLCVTEETFTKLCNDHEVLTFIKY
KVFDAKPSAKTLSGLLEWECKTDAVEALTALNHYQIRVPNGSNPYTLKLCFSTSSHL*

>ENSG00000259060|ENST00000335950
KKEHVFIHERPRKINGICISPKKVACQNLSAIFCFQSETKFKMTVCQLIEGTRYPACRYH
YSPTEGFVLVTCDDLRPDSFLGYVK*
>ENSG00000004487|ENST00000356634
MLSGKKAAAAAAAAAAAATGTEAGPGTAGGSENGSEVAAQPAGLSGPAEVGPGAVGERTP
RKKEPPRASPPGGLAEPPGSAGPQAGPTVVPGSATPMETGIAETPEGRRTSRRKRAKVEY
REMDESLANLSEDEYYSEEERNAKAEKEKKLPPPPPQAPPEEENESEPEEPSGVEGAAFQ
SRLPHDRMTSQEAACFPDIISGPQQTQKVFLFIRNRTLQLWLDNPKIQLTFEATLQQLEA
PYNSDTVLVHRVHSYLERHGLINFGIYKRIKPLPTKKTGKVIIIGSGVSGLAAARQLQSF
GMDVTLLEARDRVGGRVATFRKGNYVADLGAMVVTGLGGNPMAVVSKQVNMELAKIKQKC
PLYEANGQAVPKEKDEMVEQEFNRLLEATSYLSHQLDFNVLNNKPVSLGQALEVVIQLQE
KHVKDEQIEHWKKIVKTQEELKELLNKMVNLKEKIKELHQQYKEASEVKPPRDITAEFLV
KSKHRDLTALCKEYDELAETQGKLEEKLQELEANPPSDVYLSSRDRQILDWHFANLEFAN
ATPLSTLSLKHWDQDDDFEFTGSHLTVRNGYSCVPVALAEGLDIKLNTAVRQVRYTASGC
EVIAVNTRSTSQTFIYKCDAVLCTLPLGVLKQQPPAVQFVPPLPEWKTSAVQRMGFGNLN
KVVLCFDRVFWDPSVNLFGHVGSTTASRGELFLFWNLYKAPILLALVAGEAAGIMENISD
DVIVGRCLAILKGIFGSSAVPQPKETVVSRWRADPWARGSYSYVAAGSSGNDYDLMAQPI
TPGPSIPGAPQPIPRLFFAGEHTIRNYPATVHGALLSGLREAGRIADQFLGAMYTLPRQA
TPGVPAQQSPSM*
>ENSG00000198783|ENST00000361952
MASSASARTPAGKRVINQEELRRLMKEKQRLSTSRKRIESPFAKYNRLGQLSCALCNTPV
KSELLWQTHVLGKQHREKVAELKGAKEASQGSSASSAPHSVKRKAPDADDQDVKRAKATL
VPQVQPSTSAWTTNFDKIGKEFIRATPSKPSGLSLLPDYEDEEEEEEEEEGDGERKRGDA
SKPLSDAQGKEHSVSSSREVTSSVLPNDFFSTNPPKAPIIPHSGSIEKAEIHEKVVERRE
NTAEALPEGFFDDPEVDARVRKVDAPKDQMDKEWDEFQKAMRQVNTISEAIVAEEDEEGR
LDRQIGEIDEQIECYRRVEKLRNRQDEIKNKLKEILTIKELQKKEEENADSDDEGELQDL
LSQDWRVKGALL*
>ENSG00000235109|ENST00000344279
MASTEEQYDLKIVKVEEDPIWDQETHLRGNNFSGQEASRQLFRQFCYQETPGPREALSRL
RELCHQWLRPEIHTKEQILELLVLEQFLTILPEELQAWVREHHPESGEEAVAVVEDLEQE
LSEPGNQAPDHEHGHSEVLLEDVEHLKVKQEPTDIQLQPMVTQLRYESFCLHQFQEQDGE
SIPENQELASKQEILKEMEHLGDSKLQRDVSLDSKYRETCKRDSKAEKQQAHSTGERRHR
CNECGKSFTKSSVLIEHQRIHTGEKPYECEECGKAFSRRSSLNEHRRSHTGEKPYQCKEC
GKAFSASNGLTRHRRIHTGEKPYECKVCGKAFLLSSCLVQHQRIHTGEKRYQCRECGKAF
IQNAGLFQHLRVHTGEKPYQCSQCSKLFSKRTLLKKHQKIHTGERP*
>ENSG00000177733|ENST00000314940
MENSQLCKLFIGGLNVQTSESGLRGHFEAFGTLTDCVVVVNPQTKRSRCFGFVTYSNVEE
ADAAMAASPHAVDGNTVELKRAVSREDSARPGAHAKVKKLFVGGLKGDVAEGDLIEHFSQ
FGTVEKAEIIADKQSGKKRGFGFVYFQNHDAADKAAVVKFHPIQGHRVEVKKAVPKEDIY
SGGGGGGSRSSRGGRGGRGRGGGRDQNGLSKGGGGGYNSYGGYGGGGGGGYNAYGGGGGG
SSYGGSDYGNGFGGFGSYSQHQSSYGPMKSGGGGGGGGSSWGGRSNSGPYRGGYGGGGGY
GGSSF*
>ENSG00000151304|ENST00000339397
MAQPGTLNLNNEVVKMRKEVKRIRVLVIRKLVRSVGRLKSKKGTEDALLKNQRRAQRLLE

EIHAMKELKPDIVTKSALGDDINFEKIFKKPDSTATERAIARLAVHPLLKKKIDVLKAAV
QAFKEARQNVAEVESSKNASEDNHSENTLYSNDNGSNLQREATVISEQKVKETKILAKKP
IHNSKEKIAKMEHGPKAVTIANSPSKPSEKDSVVSLESQKTPADPKLKTLSQTKKNKGSD
SSLSGNSDGGEEFCEEEKEYFDDSTEERFYKQSSMSEDSDSGDDFFIGKVRRTRKKESSC
HSSVKEQKPLEKVFLKEDTGETHGDTRNDKIKPSTETRKLESVFFHSLSGSKSSRRNFKE
QAPKTRSLDFPQNEPQIKNQFNKKLSGRLENTKQQLQLPLHPSWEASRRRKEQQSNIAVF
QGKKITFDD*
>ENSG00000103549|ENST00000357890
MSGPGNKRAAGDGGSGPPEKKLSREEKTTTTLIEPIRLGGISSTEEMDLKVLQFKNKKLA
ERLEQRQACEDELRERIEKLEKRQATDDATLLIVNRYWAQLDETVEALLRCHESQGELSS
APEAPGTQEGPTCDGTPLPEPGTSELRDPLLMQLRPPLSEPALAFVVALGASSSEEVELE
LQGRMEFSKAAVSRVVEASDRLQRRVEELCQRVYSRGDSEPLSEAAQAHTRELGRENRRL
QDLATQLQEKHHRISLEYSELQDKVTSAETKVLEMETTVEDLQWDIEKLRKREQKLNKHL
AEALEQLNSGYYVSGSSSGFQGGQITLSMQKSDELGLQKKLRTEVIQLEDTLAQVRKEYE
MLRIEFEQNLAANEQAGPINREMRHLISSLQNHNHQLKGDAQRYKRKLREVQAEIGKLRA
QASGSAHSTPNLGHPEDSGVSAPAPGKEEGGPGPVSTPDNRKEMAPVPGTTTTTTSVKKE
ELVPSEEDFQGITPGAQGPSSRGREPEARPKRELREREGPSLGPPPVASALSRADREKAK
VEETKRKESELLKGLRAELKKAQESQKEMKLLLDMYKSAPKEQRDKVQLMAAERKAKAEV
DELRSRIRELEERDRRESKKIADEDALRRIRQAEEQIEHLQRKLGATKQEEEALLSEMDV
TGQAFEDMQEQNGRLLQQLREKDDANFKLMSERIKANQIHKLLREEKDELGEQVLGLKSQ
VDAQLLTVQKLEEKERALQGSLGGVEKELTLRSQALELNKRKAVEAAQLAEDLKVQLEHV
QTRLREIQPCLAESRAAREKESFNLKRAQEDISRLRRKLEKQRKVEVYADADEILQEEIK
EYKARLTCPCCNTRKKDAVLTKCFHVFCFECVRGRYEARQRKCPKCNAAFGAHDFHRIYI
S*
>ENSG00000119285|ENST00000366582
MTSLAQQLQRLALPQSDASLLSRDEVASLLFDPKEAATIDRDTAFAIGCTGLEELLGIDP
SFEQFEAPLFSQLAKTLERSVQTKAVNKQLDENISLFLIHLSPYFLLKPAQKCLEWLIHR
FHIHLYNQDSLIACVLPYHETRIFVRVIQLLKINNSKHRWFWLLPVKQSGVPLAKGTLIT
HCYKDLGFMDFICSLVTKSVKVFAEYPGSSAQLRVLLAFYASTIVSALVAAEDVSDNIIA
KLFPYIQKGLKSSLPDYRAATYMIICQISVKVTMENTFVNSLASQIIKTLTKIPSLIKDG
LSCLIVLLQRQKPESLGKKPFPHLCNVPDLITILHGISETYDVSPLLHYMLPHLVVSIIH
HVTGEETEGMDGQIYKRHLEAILTKISLKNNLDHLLASLLFEEYISYSSQEEMDSNKVSL
LNEQFLPLIRLLESKYPRTLDVVLEEHLKEIADLKKQELFHQFVSLSTSGGKYQFLADSD
TSLMLSLNHPLAPVRILAMNHLKKIMKTSKEGVDESFIKEAVLARLGDDNIDVVLSAISA
FEIFKEHFSSEVTISNLLNLFQRAELSKNGEWYEVLKIAADILIKEEILSENDQLSNQVV
VCLLPFMVINNDDTESAEMKIAIYLSKSGICSLHPLLRGWEEALENVIKSTKPGKLIGVA
NQKMIELLADNINLGDPSSMLKMVEDLISVGEEESFNLKQKVTFHVILSVLVSCCSSLKE
THFPFAIRVFSLLQKKIKKLESVITAVEIPSEWHIELMLDRGIPVELWAHYVEELNSTQR
VAVEDSVFLVFSLKKFIYALKAPKSFPKGDIWWNPEQLKEDSRDYLHLLIGLFEMMLNGA
DAVHFRVLMKLFIKVHLEDVFQLFKFCSVLWTYGSSLSNPLNCSVKTVLQTQALYVGCAM
LSSQKTQCKHQLASISSPVVTSLLINLGSPVKEVRRAAIQCLQALSGVASPFYLIIDHLI
SKAEEITSDAAYVIQDLATLFEELQREKKLKSHQKLSETLKNLLSCVYSCPSYIAKDLMK
VLQGVNGEMVLSQLLPMAEQLLEKIQKEPTAVLKDEAMVLHLTLGKYNEFSVSLLNEDPK
SLDIFIKAVHTTKELYAGMPTIQITALEKITKPFFAAISDEKVQQKLLRMLFDLLVNCKN

SHCAQTVSSVFKGISVNAEQVRIELEPPDKAKPLGTVQQKRRQKMQQKKSQDLESVQEVG
GSYWQRVTLILELLQHKKKLRSPQILVPTLFNLLSRCLEPLPQEQGNMEYTKQLILSCLL
NICQKLSPDGGKIPKDILDEEKFNVELIVQCIRLSEMPQTHHHALLLLGTVAGIFPDKVL
HNIMSIFTFMGANVMRLDDTYSFQVINKTVKMVIPALIQSDSGDSIEVSRNVEEIVVKII
SVFVDALPHVPEHRRLPILVQLVDTLGAEKFLWILLILLFEQYVTKTVLAAAYGEKDAIL
EADTEFWFSVCCEFSVQHQIQSLMNILQYLLKLPEEKEETIPKAVSFNKSESQEEMLQVF
NVETHTSKQLRHFKFLSVSFMSQLLSSNNFLKKVVESGGPEILKGLEERLLETVLGYISA
VAQSMERNADKLTVKFWRALLSKAYDLLDKVNALLPTETFIPVIRGLVGNPLPSVRRKAL
DLLNNKLQQNISWKKTIVTRFLKLVPDLLAIVQRKKKEGEEEQAINRQTALYTLKLLCKN
FGAENPDPFVPVLNTAVKLIAPERKEEKNVLGSALLCIAEVTSTLEALAIPQLPSLMPSL
LTTMKNTSELVSSEVYLLSALAALQKVVETLPHFISPYLEGILSQVIHLEKITSEMGSAS
QANIRLTSLKKTLATTLAPRVLLPAIKKTYKQIEKNWKNHMGPFMSILQEHIGVMKKEEL
TSHQSQLTAFFLEALDFRAQHSENDLEEVGKTENCIIDCLVAMVVKLSEVTFRPLFFKLF
DWAKTEDAPKDRLLTFYNLADCIAEKLKGLFTLFAGHLVKPFADTLNQVNISKTDEAFFD
SENDPEKCCLLLQFILNCLYKIFLFDTQHFISKERAEALMMPLVDQLENRLGGEEKFQER
VTKHLIPCIAQFSVAMADDSLWKPLNYQILLKTRDSSPKVRFAALITVLALAEKLKENYI
VLLPESIPFLAELMEDECEEVEHQCQKTIQQLETVLGEPLQSYF*
>ENSG00000180228|ENST00000325748
MSQSRHRAEAPPLEREDSGTFSLGKMITAKPGKTPIQVLHEYGMKTKNIPVYECERSDVQ
IHVPTFTFRVTVGDITCTGEGTSKKLAKHRAAEAAINILKANASICFAVPDPLMPDPSKQ
PKNQLNPIGSLQELAIHHGWRLPEYTLSQEGGPAHKREYTTICRLESFMETGKGASKKQA
KRNAAEKFLAKFSNISPENHISLTNVVGHSLGCTWHSLRNSPGEKINLLKRSLLSIPNTD
YIQLLSEIAKEQGFNITYLDIDELSANGQYQCLAELSTSPITVCHGSGISCGNAQSDAAH
NALQYLKIIAERK*
>ENSG00000100105|ENST00000351933
MERVNDASCGPSGCYTYQVSRHSTEMLHNLNQQRKNGGRFCDVLLRVGDESFPAHRAVLA
ACSEYFESVFSAQLGDGGAADGGPADVGGATAAPGGGAGGSRELEMHTISSKVFGDILDF
AYTSRIVVRLESFPELMTAAKFLLMRSVIEICQEVIKQSNVQILVPPARADIMLFRPPGT
SDLGFPLDMTNGAALAANSNGIAGSMQPEEEAARAAGAAIAGQASLPVLPGVDRLPMVAG
PLSPQLLTSPFPSVASSAPPLTGKRGRGRPRKANLLDSMFGSPGGLREAGILPCGLCGKV
FTDANRLRQHEAQHGVTSLQLGYIDLPPPRLGENGLPISEDPDGPRKRSRTRKQVACEIC
GKIFRDVYHLNRHKLSHSGEKPYSCPVCGLRFKRKDRMSYHVRSHDGSVGKPYICQSCGK
GFSRPDHLNGHIKQVHTSERPHKCQTCNASFATRDRLRSHLACHEDKVPCQVCGKYLRAA
YMADHLKKHSEGPSNFCSICNREGQKCSHQDPIESSDSYGDLSDASDLKTPEKQSANGSF
SCDMAVPKNKMESDGEKKYPCPECGSFFRSKSYLNKHIQKVHVRALGGPLGDLGPALGSP
FSPQQNMSLLESFGFQIVQSAFASSLVDPEVDQQPMGPEGK*
>ENSG00000169594|ENST00000345382
MRRRPPSRGGRGAARARETRRQPRHRSGRRMAEAISCTLNCSCQSFKPGKINHRQCDQCK
HGWVAHALSKLRIPPMYPTSQVEIVQSNVVFDISSLMLYGTQAIPVRLKILLDRLFSVLK
QDEVLQILHALDWTLQDYIRGYVLQDASGKVLDHWSIMTSEEEVATLQQFLRFGETKSIV
ELMAIQEKEEQSIIIPPSTANVDIRAFIESCSHRSSSLPTPVDKGNPSSIHPFENLISNM
TFMLPFQFFNPLPPALIGSLPEQYMLEQGHDQSQDPKQEVHGPFPDSSFLTSSSTPFQVE
KDQCLNCPDAITKKEDSTHLSDSSSYNIVTKFERTQLSPEAKVKPERNSLGTKKGRVFCT
ACEKTFYDKGTLKIHYNAVHLKIKHKCTIEGCNMVFSSLRSRNRHSANPNPRLHMPMNRN

NRDKDLRNSLNLASSENYKCPGFTVTSPDCRPPPSYPGSGEDSKGQPAFPNIGQNGVLFP
NLKTVQPVLPFYRSPATPAEVANTPGILPSLPLLSSSIPEQLISNEMPFDALPKKKSRKS
SMPIKIEKEAVEIANEKRHNLSSDEDMPLQVVSEDEQEACSPQSHRVSEEQHVQSGGLGK
PFPEGERPCHRESVIESSGAISQTPEQATHNSERETEQTPALIMVPREVEDGGHEHYFTP
GMEPQVPFSDYMELQQRLLAGGLFSALSNRGMAFPCLEDSKELEHVGQHALARQIEENRF
QCDICKKTFKNACSVKIHHKNMHVKEMHTCTVEGCNATFPSRRSRDRHSSNLNLHQKALS
QEALESSEDHFRAAYLLKDVAKEAYQDVAFTQQASQTSVIFKGTSRMGSLVYPITQVHSA
SLESYNSGPLSEGTILDLSTTSSMKSESSSHSSWDSDGVSEEGTVLMEDSDGNCEGSSLV
PGEDEYPICVLMEKADQSLASLPSGLPITCHLCQKTYSNKGTFRAHYKTVHLRQLHKCKV
PGCNTMFSSVRSRNRHSQNPNLHKSLASSPSHLQ*
>ENSG00000147403|ENST00000344746
MGRRPARCYRYCKNKPYPKSRFCRGVPDAKIRIFDLGRKKAKVDEFPLCGHMVSDEYEQL
SSEALEAARICANKYMVKSCGKDGFHIRVRLHPFHVIRINKMLSCAGADRLQTGMRGAFG
KPQGTVARVHIGQVIMSIRTKLQNKEHVIEALRRAKFKFPGRQKIHISKKWGFTKFNADE
FEDMVAEKRLIPDGCGVKYIPSRGPLDKWRALHS*
>ENSG00000130255|ENST00000347512
MALRYPMAVGLNKGHKVTKNVSKPRHSRRRGRLTKHTKFVRDMIREVCGFAPYERRAMEL
LKVSKDKRALKFIKKRVGTHIRAKRKREELSNVLAAMRKAAAKKD*
>ENSG00000067225|ENST00000319622
MSKPHSEAGTAFIQTQQLHAAMADTFLEHMCRLDIDSPPITARNTGIICTIGPASRSVET
LKEMIKSGMNVARLNFSHGTHEYHAETIKNVRTATESFASDPILYRPVAVALDTKGPEIR
TGLIKGSGTAEVELKKGATLKITLDNAYMEKCDENILWLDYKNICKVVEVGSKIYVDDGL
ISLQVKQKGADFLVTEVENGGSLGSKKGVNLPGAAVDLPAVSEKDIQDLKFGVEQDVDMV
FASFIRKASDVHEVRKVLGEKGKNIKIISKIENHEGVRRFDEILEASDGIMVARGDLGIE
IPAEKVFLAQKMMIGRCNRAGKPVICATQMLESMIKKPRPTRAEGSDVANAVLDGADCIM
LSGETAKGDYPLEAVRMQHLIAREAEAAMFHRKLFEELVRASSHSTDLMEAMAMGSVEAS
YKCLAAALIVLTESGRSAHQVARYRPRAPIIAVTRNPQTARQAHLYRGIFPVLCKDPVQE
AWAEDVDLRVNFAMNVGKARGFFKKGDVVIVLTGWRPGSGFTNTMRVVPVP*
>ENSG00000138668|ENST00000353341
MSEEQFGGDGAAAAATAAVGGSAGEQEGAMVAATQGAAAAAGSGAGTGGGTASGGTEGGS
AESEGAKIDASKNEEDEGHSNSSPRHSEAATAQREEWKMFIGGLSWDTTKKDLKDYFSKF
GEVVDCTLKLDPITGRSRGFGFVLFKESESVDKVMDQKEHKLNGKVIDPKRAKAMKTKEP
VKKIFVGGLSPDTPEEKIREYFGGFGEVESIELPMDNKTNKRRGFCFITFKEEEPVKKIM
EKKYHNVGLSKCEIKVAMSKEQYQQQQQWGSRGGFAGRARGRGGDQQSGYGKVSRRGGHQ
NSYKPY*
>ENSG00000142556|ENST00000356322
MIKTQESLTLEDVAVEFSWEEWQLLDTAQKNLYRDVMVENYNHLVSLGYQTSKPDVLSKL
AHGQEPWTTDAKIQNKNCPGIGKVDSHLQEHSPNQRLLKSVQQCNGQNTLRNIVHLSKTH
FPIVQNHDTFDLYRKNLKSSLSLINQKRRHGINNPVEFIGETGSLERPRQADHLRSEVQD
QPGQRGETLCLLKIHTKN*
>ENSG00000134138|ENST00000340545
MDGVGVPASMYGDPHAPRPIPPVHHLNHGPPLHATQHYGAHAPHPNVMPASMGSAVNDAL
KRDKDAIYGHPLFPLLALVFEKCELATCTPREPGVAGGDVCSSDSFNEDIAVFAKQVRAE
KPLFSSNPELDNLMIQAIQVLRFHLLELEKVHELCDNFCHRYISCLKGKMPIDLVIDERD

GSSKSDHEELSGSSTNLADHNPSSWRDHDDATSTHSAGTPGPSSGGHASQSGDNSSEQGD
GLDNSVASPGTGDDDDPDKDKKRQKKRGIFPKVATNIMRAWLFQHLTHPYPSEEQKKQLA
QDTGLTILQVNNWFINARRRIVQPMIDQSNRAVSQGAAYSPEGQPMGSFVLDGQQHMGIR
PAGPMSGMGMNMGMDGQWHYM*
>ENSG00000133789|ENST00000318950
MGSLKEELLKAIWHAFTALDQDHSGKVSKSQLKVLSHNLCTVLKVPHDPVALEEHFRDDD
EGPVSNQGYMPYLNRFILEKVQDNFDKIEFNRMCWTLCVKKNLTKNPLLITEEDAFKIWV
IFNFLSEDKYPLIIVSEEIEYLLKKLTEAMGGGWQQEQFEHYKINFDDSKNGLSAWELIE
LIGNGQFSKGMDRQTVSMAINEVFNELILDVLKQGYMMKKGHRRKNWTERWFVLKPNIIS
YYVSEDLKDKKGDILLDENCCVESLPDKDGKKCLFLVKCFDKTFEISASDKKKKQEWIQA
IHSTIHLLKLGSPPPHKEARQRRKELRKKQLAEQEELERQMKELQAANESKQQELEAVRK
KLEEAASRAAEEEKKRLQTQVELQARFSTELEREKLIRQQMEEQVAQKSSELEQYLQRVR
ELEDMYLKLQEALEDERQARQDEETVRKLQARLLEEESSKRAELEKWHLEQQQAIQTTEA
EKQELENQRVLKEQALQEAMEQLEQLELERKQALEQYEEVKKKLEMATNKTKSWKDKVAH
HEGLIRLIEPGSKNPHLITNWGPAAFTEAELEEREKNWKEKKTTE*
>ENSG00000178567|ENST00000322716
MWMTPKRSKMEVDEALVFRPEWTQRYLVVEPPEGDGALCLVCRRLIVATRERDVRRHYEA
EHEYYERYVADGERAALVERLRQGDLPVASFTPEERAARAGLGLCRLLALKGRGWGEGDF
VYQCMEVLLREVLPEHVSVLQGVDLSPDITRQRILSIDRNLRNQLFNRARDFKAYSLALD
DQAFVAYENYLLVFIRGVGPELEVQEDLLTIINLTHHFSVGALMSAILESLQTAGLSLQR
MVGLTTTHTLRMIGENSGLVSYMREKAVSPNCWNVIHYSGFLHLELLSSYDVDVNQIINT
ISEWIVLIKTRGVRRPEFQTLLTESESEHGERVNGRCLNNWLRRGKTLKLIFSLRKEMEA
FLVSVGATTVHFSDKQWLCDFGFLVDIMEHLRELSEELRVSKVFAAAAFDHICTFEVKLN
LFQRHIEEKNLTDFPALREVVDELKQQNKEDEKIFDPDRYQMVICRLQKEFERHFKDLRF
IKKDLELFSNPFNFKPEYAPISVRVELTKLQANTNLWNEYRIKDLGQFYAGLSAESYPII
KGVACKVASLFDSNQICEKAFSYLTRNQHTLSQPLTDEHLQALFRVATTEMEPGWDDLVR
ERNESNP*
>ENSG00000213967|ENST00000322487
MGLLTFRDVAIEFSLEEWQCLDTAQKNLYRNVMLENYRNLAFLGIAVSKPDLIICLEKEK
EPWNMKRDEMVDEPPGICPHFAQDIWPEQGVEDSFQKVILRRFEKCGHENLQLRKGCKSV
DECKVHKEGYNGLNQCFTTTQGKASQCGKYLKVFYKFINLNRYKIRHTRKKPFKCKNCVK
SFCMFSHKTQHKSIYTTEKSYKCKECGKTFNWSSTLTNHKKTHTEEKPYKCEEYGKAFNQ
SSNYTTHKVTHTGEKPYKCEECGKAFSQSSTLTIHKRIHTGEKPCKCEECGKAFSQPSAL
TIHKRMHIGEKPYKCEECGKAFVWSSTLTRHKRLHSGEKPYKCEECAKAFSQFGHLTTHR
IIHTGEKPYKCEECGKAFIWPSTLTKHKRIHTGEKPYKCEECGKAFHRSSNLTKHKIIHT
GEKPYKCEECGKAFIWSSNLTEHKKIHTREKPYKCEECSKAFSRSSALTTHKRMHTGEKP
YKCEECGKAFSQSSTLTAHKIIHTGEKPYKCEECGKAFILSSTLSKHKRIHTGEKPYKCE
ECGKTFNQSSNLSTHKIIHTGEKPYKCEECGKAFNRSSNLSTHKIIHTGEKPYKCDECGK
SFIWSSTLFKHKIIHTGEKPYKCEECGKAFNHSQILLHIRHKRMHTGEKPYKCEECGKSF
NLSSTFIKHKVIHTGVKLYKCEECGKVFFWSSALTRHKKIHAGQQPYKWEKIGKAFNQSS
HLTTDKITHIGEKSYKCE*
>ENSG00000178568|ENST00000342788
MKPATGLWVWVSLLVAAGTVQPSDSQSVCAGTENKLSSLSDLEQQYRALRKYYENCEVVM
GNLEITSIEHNRDLSFLRSVREVTGYVLVALNQFRYLPLENLRIIRGTKLYEDRYALAIF

LNYRKDGNFGLQELGLKNLTEILNGGVYVDQNKFLCYADTIHWQDIVRNPWPSNLTLVST
NGSSGCGRCHKSCTGRCWGPTENHCQTLTRTVCAEQCDGRCYGPYVSDCCHRECAGGCSG
PKDTDCFACMNFNDSGACVTQCPQTFVYNPTTFQLEHNFNAKYTYGAFCVKKCPHNFVVD
SSSCVRACPSSKMEVEENGIKMCKPCTDICPKACDGIGTGSLMSAQTVDSSNIDKFINCT
KINGNLIFLVTGIHGDPYNAIEAIDPEKLNVFRTVREITGFLNIQSWPPNMTDFSVFSNL
VTIGGRVLYSGLSLLILKQQGITSLQFQSLKEISAGNIYITDNSNLCYYHTINWTTLFST
INQRIVIRDNRKAENCTAEGMVCNHLCSSDGCWGPGPDQCLSCRRFSRGRICIESCNLYD
GEFREFENGSICVECDPQCEKMEDGLLTCHGPGPDNCTKCSHFKDGPNCVEKCPDGLQGA
NSFIFKYADPDRECHPCHPNCTQGCNGPTSHDCIYYPWTGHSTLPQHARTPLIAAGVIGG
LFILVIVGLTFAVYVRRKSIKKKRALRRFLETELVEPLTPSGTAPNQAQLRILKETELKR
VKVLGSGAFGTVYKGIWVPEGETVKIPVAIKILNETTGPKANVEFMDEALIMASMDHPHL
VRLLGVCLSPTIQLVTQLMPHGCLLEYVHEHKDNIGSQLLLNWCVQIAKGMMYLEERRLV
HRDLAARNVLVKSPNHVKITDFGLARLLEGDEKEYNADGGKMPIKWMALECIHYRKFTHQ
SDVWSYGVTIWELMTFGGKPYDGIPTREIPDLLEKGERLPQPPICTIDVYMVMVKCWMID
ADSRPKFKELAAEFSRMARDPQRYLVIQGDDRMKLPSPNDSKFFQNLLDEEDLEDMMDAE
EYLVPQAFNIPPPIYTSRARIDSNRSEIGHSPPPAYTPMSGNQFVYRDGGFAAEQGVSVP
YRAPTSTIPEAPVAQGATAEIFDDSCCNGTLRKPVAPHVQEDSSTQRYSADPTVFAPERS
PRGELDEEGYMTPMRDKPKQEYLNPVEENPFVSRRKNGDLQALDNPEYHNASNGPPKAED
EYVNEPLYLNTFANTLGKAEYLKNNILSMPEKAKKAFDNPDYWNHSLPPRSTLQHPDYLQ
EYSTKYFYKQNGRIRPIVAENPEYLSEFSLKPGTVLPPPPYRHRNTVV*
>ENSG00000169251|ENST00000351193
MEYMAESTDRSPGHILCCECGVPISPNPANICVACLRSKVDISQGIPKQVSISFCKQCQR
YFQPPGTWIQCALESRELLALCLKKIKAPLSKVRLVDAGFVWTEPHSKRLKVKLTIQKEV
MNGAILQQVFVVDYVVQSQMCGDCHRVEAKDFWKAVIQVRQKTLHKKTFYYLEQLILKYG
MHQNTLRIKEIHDGLDFYYSSKQHAQKMVEFLQCTVPCRYKASQRLISQDIHSNTYNYKS
TFSVEIVPICKDNVVCLSPKLAQSLGNMNQICVCIRVTSAIHLIDPNTLQVADIDGSTFW
SHPFNSLCHPKQLEEFIVMECSIVQDIKRAAGAGMISKKHTLGEVWVQKTSEMNTDKQYF
CRTHLGHLLNPGDLVLGFDLANCNLNDEHVNKMNSDRVPDVVLIKKSYDRTKRQRRRNWK
LKELARERENMDTDDERQYQDFLEDLEEDEAIRKNVNIYRDSAIPVESDTDDEGAPRISL
AEMLEDLHISQDATGEEGASMLT*
>ENSG00000170364|ENST00000358065
MFAEAAKTTRPCGMAEFKEKPEAPTEQLDVACGQENLPVGAWPPGAAPAPFQYTPDHVVG
PGADIDPTQITFPGCICVKTPCLPGTCSCLRHGENYDDNSCLRDIGSGGKYAEPVFECNV
LCRCSDHCRNRVVQKGLQFHFQVFKTHKKGWGLRTLEFIPKGRFVCEYAGEVLGFSEVQR
RIHLQTKSDSNYIIAIREHVYNGQVMETFVDPTYIGNIGRFLNHSCEPNLLMIPVRIDSM
VPKLALFAAKDIVPEEELSYDYSGRYLNLTVSEDKERLDHGKLRKPCYCGAKSCTAFLPF
DSSLYCPVEKSNISCGNEKEPSMCGSAPSVFPSCKRLTLETMKMMLDKKQIRAIFLFEFK
MGRKAAETTRNINNAFGPGTANERTVQWWFKKFCKGDESLEDEERSGRPSEVDNDQLRAI
IEADPLTTTREVAEELNVNHSTVVRHLKQIGKVKKLDKWVPHELTENQKNRRFEVSSSLI
LRNHNEPFLDRIVTCDEKWILYDNRRRSAQWLDQEEAPKHFPKPILHPKKVMVTIWWSAA
GLIHYSFLNPGETITSEKYAQEIDEMNQKLQRLQLALVNRKGPILLHDNARPHVAQPTLQ
KLNELGYEVLPHPPYSPDLLPTNYHVFKHLNNFLQGKRFHNQQDAENAFQEFVESQSTDF
YATGINQLISRWQKCVDCNGSYFD*
>ENSG00000168003|ENST00000338663

MSQDTEVDMKEVELNELEPEKQPMNAASGAAMSLAGAEKNGLVKIKVAEDEAEAAAAAKF
TGLSKEELLKVAGSPGWVRTRWALLLLFWLGWLGMLAGAVVIIVRAPRCRELPAQKWWHT
GALYRIGDLQAFQGHGAGNLAGLKGRLDYLSSLKVKGLVLGPIHKNQKDDVAQTDLLQID
PNFGSKEDFDSLLQSAKKKSIRVILDLTPNYRGENSWFSTQVDTVATKVKDALEFWLQAG
VDGFQVRDIENLKDASSFLAEWQNITKGFSEDRLLIAGTNSSDLQQILSLLESNKDLLLT
SSYLSDSGSTGEHTKSLVTQYLNATGNRWCSWSLSQARLLTSFLPAQLLRLYQLMLFTLP
GTPVFSYGDEIGLDAAALPGQPMEAPVMLWDESSFPDIPGAVSANMTVKGQSEDPGSLLS
LFRRLSDQRSKERSLLHGDFHAFSAGPGLFSYIRHWDQNERFLVVLNFGDVGLSAGLQAS
DLPASASLPAKADLLLSTQPGREEGSPLELERLKLEPHEGLLLRFPYAA*
>ENSG00000117593|ENST00000361951
MYFPSWLSQLYRGLSRPIRRTTQPIWGSLYRSLLQSSQRRIPEFSSFVVRTNTCGELRSS
HLGQEVTLCGWIQYRRQNTFLVLRDFDGLVQVIIPQDESAASVKKILCEAPVESVVQVSG
TVISRPAGQENPKMPTGEIEIKVKTAELLNACKKLPFEIKNFVKKTEALRLQYRYLDLRS
FQMQYNLRLRSQMVMKMREYLCNLHGFVDIETPTLFKRTPGGAKEFLVPSREPGKFYSLP
QSPQQFKQLLMVGGLDRYFQVARCYRDEGSRPDRQPEFTQIDIEMSFVDQTGIQSLIEGL
LQYSWPNDKDPVVVPFPTMTFAEVLATYGTDKPDTRFGMKIIDISDVFRNTEIGFLQDAL
SKPHGTVKAICIPEGAKYLKRKDIESIRNFAADHFNQEILPVFLNANRNWNSPVANFIME
SQRLELIRLMETQEEDVVLLTAGEHNKACSLLGKLRLECADLLETRGVVLRDPTLFSFLW
VVDFPLFLPKEENPRELESAHHPFTAPHPSDIHLLYTEPKKARSQHYDLVLNGNEIGGGS
IRIHNAELQRYILATLLKEDVKMLSHLLQALDYGAPPHGGIALGLDRLICLVTGSPSIRD
VIAFPKSFRGHDLMSNTPDSVPPEELKPYHIRVSKPTDSKAERAH*
>ENSG00000152942|ENST00000354312
MNQVTDWVDPSFDDFLECSGVSTITATSLGVNNSSHRRKNGPSTLESSRFPARKRGNLSS
LEQIYGLENSKEYLSENEPWVDKYKPETQHELAVHKKKIEEVETWLKAQVLERQPKQGGS
ILLITGPPGCGKTTTLKILSKEHGIQVQEWINPVLPDFQKDDFKGMFNTESSFHMFPYQS
QIAVFKEFLLRATKYNKLQMLGDDLRTDKKIILVEDLPNQFYRDSHTLHEVLRKYVRIGR
CPLIFIISDSLSGDNNQRLLFPKEIQEECSISNISFNPVAPTIMMKFLNRIVTIEANKNG
GKITVPDKTSLELLCQGCSGDIRSAINSLQFSSSKGENNLRPRKKGMSLKSDAVLSKSKR
RKKPDRVFENQEVQAIGGKDVSLFLFRALGKILYCKRASLTELDSPRLPSHLSEYERDTL
LVEPEEVVEMSHMPGDLFNLYLHQNYIDFFMEIDDIVRASEFLSFADILSGDWNTRSLLR
EYSTSIATRGVMHSNKARGYAHCQGGGSSFRPLHKPQWFLINKKYRENCLAAKALFPDFC
LPALCLQTQLLPYLALLTIPMRNQAQISFIQDIGRLPLKRHFGRLKMEALTDREHGMIDP
DSGDEAQLNGGHSAEESLGEPTQATVPETWSLPLSQNSASELPASQPQPFSAQGDMEENI
IIEDYESDGT*
>ENSG00000073282|ENST00000354600
MLYLENNAQTQFSEPQYTNLGLLNSMDQQIQNGSSSTSPYNTDHAQNSVTAPSPYAQPSS
TFDALSPSPAIPSNTDYPGPHSFDVSFQQSSTAKSATWTYSTELKKLYCQIAKTCPIQIK
VMTPPPQGAVIRAMPVYKKAEHVTEVVKRCPNHELSREFNEGQIAPPSHLIRVEGNSHAQ
YVEDPITGRQSVLVPYEPPQVGTEFTTVLYNFMCNSSCVGGMNRRPILIIVTLETRDGQV
LGRRCFEARICACPGRDRKADEDSIRKQQVSDSTKNGDGTKRPFRQNTHGIQMTSIKKRR
SPDDELLYLPVRGRETYEMLLKIKESLELMQYLPQHTIETYRQQQQQQHQHLLQKQTSIQ
SPSSYGNSSPPLNKMNSMNKLPSVSQLINPQQRNALTPTTIPDGMGANIPMMGTHMPMAG
DMNGLSPTQALPPPLSMPSTSHCTPPPPYPTDCSIVSFLARLGCSSCLDYFTTQGLTTIY
QIEHYSMDDLASLKIPEQFRHAIWKGILDHRQLHEFSSPSHLLRTPSSASTVSVGSSETR

GERVIDAVRFTLRQTISFPPRDEWNDFNFDMDARRNKQQRIKEEGE*
>ENSG00000088305|ENST00000328111
MKGDTRHLNGEEDAGGREDSILVNGACSDQSSDSPPILEAIRTPEIRGRRSSSRLSKREV
SSLLSYTQDLTGDGDGEDGDGSDTPVMPKLFRETRTRSESPAVRTRNNNSVSSRERHRPS
PRSTRGRQGRNHVDESPVEFPATRSLRRRATASAGTPWPSPPSSYLTIDLTDDTEDTHGT
PQSSSTPYARLAQDSQQGGMESPQVEADSGDGDSSEYQDGKEFGIGDLVWGKIKGFSWWP
AMVVSWKATSKRQAMSGMRWVQWFGDGKFSEVSADKLVALGLFSQHFNLATFNKLVSYRK
AMYHALEKARVRAGKTFPSSPGDSLEDQLKPMLEWAHGGFKPTGIEGLKPNNTQPVVNKS
KVRRAGSRKLESRKYENKTRRRTADDSATSDYCPAPKRLKTNCYNNGKDRGDEDQSREQM
ASDVANNKSSLEDGCLSCGRKNPVSFHPLFEGGLCQTCRDRFLELFYMYDDDGYQSYCTV
CCEGRELLLCSNTSCCRCFCVECLEVLVGTGTAAEAKLQEPWSCYMCLPQRCHGVLRRRK
DWNVRLQAFFTSDTGLEYEAPKLYPAIPAARRRPIRVLSLFDGIATGYLVLKELGIKVGK
YVASEVCEESIAVGTVKHEGNIKYVNDVRNITKKNIEEWGPFDLVIGGSPCNDLSNVNPA
RKGLYEGTGRLFFEFYHLLNYSRPKEGDDRPFFWMFENVVAMKVGDKRDISRFLECNPVM
IDAIKVSAAHRARYFWGNLPGMNRPVIASKNDKLELQDCLEYNRIAKLKKVQTITTKSNS
IKQGKNQLFPVVMNGKEDVLWCTELERIFGFPVHYTDVSNMGRGARQKLLGRSWSVPVIR
HLFAPLKDYFACE*
>ENSG00000139131|ENST00000324868
MAAPILRSFSWGRWSGTLNLSVLLPLGLRKAHSGAQGLLAAQKARGLFKDFFPETGTKIE
LPELFDRGTASFPQTIYCGFDPTADSLHVGHLLALLGLFHLQRAGHNVIALVGGATARLG
DPSGRTKEREALETERVRANARALRLGLEALAANHQQLFTDGRSWGSFTVLDNSAWYQKQ
HLVDFLAAVGGHFRMGTLLSRQSVQLRLKSPEGMSLAEFFYQVLQAYDFYYLFQRYGCRV
QLGGSDQLGNIMSGYEFINKLTGEDVFGITVPLITSTTGAKLGKSAGNAVWLNRDKTSPF
ELYQFFVRQPDDSVERYLKLFTFLPLPEIDHIMQLHVKEPERRGPQKRLAAEVTKLVHGR
EGLDSAKRCTQALYHSSIDALEVMSDQELKELFKEAPFSEFFLDPGTSVLDTCRKANAIP
DGPRGYRMITEGGVSINHQQVTNPESVLIVGQHILKNGLSLLKIGKRNFYIIKWLQL*
>ENSG00000109685|ENST00000353275
MEFSIKQSPLSVQSVVKCIKMKQAPEILGSANGKTPSCEVNRECSVFLSKAQLSSSLQEG
VMQKFNGHDALPFIPADKLKDLTSRVFNGEPGAHDAKLRFESQEMKGIGTPPNTTPIKNG
SPEIKLKITKTYMNGKPLFESSICGDSAADVSQSEENGQKPENKARRNRKRSIKYDSLLE
QGLVEAALVSKISSPSDKKIPAKKESCPNTGRDKDHLLKYNVGDLVWSKVSGYPWWPCMV
SADPLLHSYTKLKGQKKSARQYHVQFFGDAPERAWIFEKSLVAFEGEGQFEKLCQESAKQ
APTKAEKIKLLKPISGKLRAQWEMGIVQAEEAASMSVEERKAKFTFLYVGDQLHLNPQVA
KEAGIAAESLGEMAESSGVSEEAAENPKSVREECIPMKRRRRAKLCSSAETLESHPDIGK
STPQKTAEADPRRGVGSPPGRKKTTVSMPRSRKGDAASQFLVFCQKHRDEVVAEHPDASG
EEIEELLRSQWSLLSEKQRARYNTKFALVAPVQAEEDSGNVNGKKRNHTKRIQDPTEDAE
AEDTPRKRLRTDKHSLRKRDTITDKTARTSSYKAMEAASSLKSQAATKNLSDACKPLKKR
NRASTAASSALGFSKSSSPSASLTENELLWEPTPVKLDLNPAALYCT*
>ENSG00000228049|ENST00000358438
MNAPPAFESFLLFEGEKITINKDTKVPKACLFTINKEDHTLGNIIKSQLLKDPQVLFAGY
KVPHPLEHKIIIRVQTTPDYSPQEAFTNAITDLISELSLLEERFRVRAGPGGADGVGWTL
ARVPRPGTALACFFGGPQGEAAVMEEQGLPPQAPGHVD*
>ENSG00000189079|ENST00000334344
MANSTGKAPPDERRKGLAFLDELRQFHHSRGSPFKKIPAVGGKELDLHGLYTRVTTLGGF

AKVSEKNQWGEIVEEFNFPRSCSNAAFALKQYYLRYLEKYEKVHHFGEDDDEVPPGNPKP
QLPIGAIPSSYNYQQHSVSDYLRQSYGLSMDFNSPNDYNKLVLSLLSGLPNEVDFAINVC
TLLSNESKHVMQLEKDPKIITLLLANAGVFDDTLGSFSTVFGEEWKEKTDRDFVKFWKDI
VDDNEVRDLISDRNKSHEGTSGEWIWESLFHPPRKLGINDIEGQRVLQIAVILRNLSFEE
GNVKLLAANRTCLRFLLLSAHSHFISLRQLGLDTLGNIAAELLLDPVDFKTTHLMFHTVT
KCLMSRDRFLKMRGMEILGNLCKAEDNGVLICEYVDQDSYREIICHLTLPDVLLVISTLE
VLYMLTEMGDVACTKIAKVEKSIDMLVCLVSMDIQMFGPDALAAVKLIEHPSSSHQMLSE
IRPQAIEQVQTQTHVASAPASRAVVAQHVAPPPGIVEIDSEKFACQWLNAHFEVNPDCSV
SRAEMYSEYLSTCSKLARGGILTSTGFYKCLRTVFPNHTVKRVEDSSSNGQAHIHVVGVK
RRAIPLPIQMYYQQQPVSTSVVRVDSVPDVSPAPSPAGIPHGSQTIGNHFQRTPVANQSS
NLTATQMSFPVQGVHTVAQTVSRIPQNPSPHTHQQQNAPVTVIQSKAPIPCEVVKATVIQ
NSIPQTGVPVSIAVGGGPPQSSVVQNHSTGPQPVTVVNSQTLLHHPSVIPQQSPLHTVVP
GQIPSGTPVTVIQQAVPQSHMFGRVQNIPACTSTVSQGQQLITTSPQPVQTSSQQTSAGS
QSQDTVIIAPPQYVTTSASNIVSATSVQNFQVATGQMVTIAGVPSPQASRVGFQNIAPKP
LPSQQVSSTVVQQPIQQPQQPTQQSVVIVSQPAQQGQTYAPAIHQIVLANPAALPAGQTV
QLTGQPNITPSSSPSPVPATNNQVPTAMSSSSTPQSQGPPPTVSQMLSVKRQQQQQHSPA
PPPQQVQVQVQQPQQVQMQVQPQQSNAGVGQPASGESSLIKQLLLPKRGPSTPGGKLILP
APQIPPPNNARAPSPQVVYQVASNQAAGFGVQGQTPAQQLLVGQQNVQLVPSAMPPSGGV
QTVPISNLQILPGPLISNSPATIFQGTSGNQVTITVVPNTSFAPATVSQGNATQLIAPAG
ITMSGTQTGVGLPVQTLPATQASPAGQSSCTTATPPFKGDKIICQKEEEAKEATGLHVHE
RKIEVMENPSCRRGATNTSNGDTKENEMHVGSLLNGRKYSDSSLPPSNSGKIQSETNQCS
LISNGPSLELGENGASGKQNSEQIDMQDIKSDLRKPLVNGICDFDKGDGSHLSKNIPNHK
TSNHVGNGEISPMEPQGTLDITQQDTAKGDQLERISNGPVLTLGGSSVSSIQEASNAATQ
QFSGTDLLNGPLASSLNSDVPQQRPSVVVSPHSTTSVIQGHQIIAVPDSGSKVSHSPALS
SDVRSTNGTAECKTVKRPAEDTDRETVAGIPNKVGVRIVTISDPNNAGCSATMVAVPAGA
DPSTVAKVAIESAVQQKQQHPPTYVQNVVPQNTPMPPSPAVQVQGQPNSSQPSPFSGSSQ
PGDPMRKPGQNFMCLWQSCKKWFQTPSQVFYHAATEHGGKDVYPGQCLWEGCEPFQRQRF
SFITHLQDKHCSKDALLAGLKQDEPGQAGSQKSSTKQPTVGGTSSTPRAQKAIVNHPSAA
LMALRRGSRNLVFRDFTDEKEGPITKHIRLTAALILKNIGKYSECGRRLLKRHENNLSVL
AISNMEASSTLAKCLYELNFTVQSKEQEKDSEMLQ*
>ENSG00000077092|ENST00000330688
MFDCMDVLSVSPGQILDFYTASPSSCMLQEKALKACFSGLTQTEWQHRHTAQSIETQSTS
SEELVPSPPSPLPPPRVYKPCFVCQDKSSGYHYGVSACEGCKGFFRRSIQKNMIYTCHRD
KNCVINKVTRNRCQYCRLQKCFEVGMSKESVRNDRNKKKKETSKQECTESYEMTAELDDL
TEKIRKAHQETFPSLCQLGKYTTNSSADHRVRLDLGLWDKFSELATKCIIKIVEFAKRLP
GFTGLTIADQITLLKAACLDILILRICTRYTPEQDTMTFSDGLTLNRTQMHNAGFGPLTD
LVFTFANQLLPLEMDDTETGLLSAICLICGDRQDLEEPTKVDKLQEPLLEALKIYIRKRR
PSKPHMFPKILMKITDLRSISAKGAERVITLKMEIPGSMPPLIQEMLENSEGHEPLTPSS
SGNTAEHSPSISPSSVENSGVSQSPLVQ*
>ENSG00000178694|ENST00000314622
MLTQLKAKSEGKLAKQICKVVLDHFEKQYSKELGDAWNTVREILTSPSCWQYAVLLNRFN
YPFELEKDLHLKGYHTLSQGSLPNYPKSVKCYLSRTPGRIPSERHQIGNLKKYYLLNAAS
LLPVLALELRDGEKVLDLCAAPGGKSIALLQCACPGYLHCNEYDSLRLRWLRQTLESFIP
QPLINVIKVSELDGRKMGDAQPEMFDKVLVDAPCSNDRSWLFSSDSQKASCRISQRRNLP

LLQIELLRSAIKALRPGGILVYSTCTLSKAENQDVISEILNSHGNIMPMDIKGIARTCSH
DFTFAPTGQECGLLVIPDKGKAWGPMYVAKLKKSWSTGKW*
>ENSG00000141499|ENST00000316024
MKTLETQPLAPDCCPSDQDPAPAHPSPHASPMNKNADSELMPPPPERGDPPRLSPDPVAG
SAVSQELREGDPVSLSTPLETEFGSPSELSPRIEEQELSENTSLPAEEANGSLSEEEANG
PELGSGKAMEDTSGEPAAEDEGDTAWNYSFSQLPRFLSGSWSEFSTQPENFLKGCKWAPD
GSCILTNSADNILRIYNLPPELYHEGEQVEYAEMVPVLRMVEGDTIYDYCWYSLMSSAQP
DTSYVASSSRENPIHIWDAFTGELRASFRAYNHLDELTAAHSLCFSPDGSQLFCGFNRTV
RVFSTARPGRDCEVRATFAKKQGQSGIISCIAFSPAQPLYACGSYGRSLGLYAWDDGSPL
ALLGGHQGGITHLCFHPDGNRFFSGARKDAELLCWDLRQSGYPLWSLGREVTTNQRIYFD
LDPTGQFLVSGSTSGAVSVWDTDGPGNDGKPEPVLSFLPQKDCTNGVSLHPSLPLLATAS
GQRVFPEPTESGDEGEELGLPLLSTRHVHLECRLQLWWCGGAPDSSIPDDHQGEKGQGGT
EGGVGELI*
>ENSG00000142453|ENST00000344150
MAAAAAAVGPGAGGAGSAVPGGAGPCATVSVFPGARLLTIGDANGEIQRHAEQQALRLEV
RAGPDSAGIALYSHEDVCVFKCSVSRETECSRVGKQSFIITLGCNSVLIQFATPNDFCSF
YNILKTCRGHTLERSVFSERTEESSAVQYFQFYGYLSQQQNMMQDYVRTGTYQRAILQNH
TDFKDKIVLDVGCGSGILSFFAAQAGARKIYAVEASTMAQHAEVLVKSNNLTDRIVVIPG
KVEEVSLPEQVDIIISEPMGYMLFNERMLESYLHAKKYLKPSGNMFPTIGDVHLAPFTDE
QLYMEQFTKANFWYQPSFHGVDLSALRGAAVDEYFRQPVVDTFDIRILMAKSVKYTVNFL
EAKEGDLHRIEIPFKFHMLHSGLVHGLAFWFDVAFIGSIMTVWLSTAPTEPLTHWYQVRC
LFQSPLFAKAGDTLSGTCLLIANKRQSYDISIVAQVDQTGSKSSNLLDLKNPFFRYTGTT
PSPPPGSHYTSPSENMWNTGSTYNLSSGMAVAGMPTAYDLSSVIASGSSVGHNNLIPLGS
SGAQGSGGGSTSAHYAVNSQFTMGGPAISMASPMSIPTNTMHYGS*
>ENSG00000064961|ENST00000333651
MSHGPKQPGAAAAPAGGKAPGQHGGFVVTVKQERGEGPRAGEKGSHEEEPVKKRGWPKGK
KRKKILPNGPKAPVTGYVRFLNERREQIRTRHPDLPFPEITKMLGAEWSKLQPTEKQRYL
DEAEREKQQYMKELRAYQQSEAYKMCTEKIQEKKIKKEDSSSGLMNTLLNGHKGGDCDGF
STFDVPIFTEEFLDQNKAREAELRRLRKMNVAFEEQNAVLQRHTQSMSSARERLEQELAL
EERRTLALQQQLQAVRQALTASFASLPVPGTGETPTLGTLDFYMARLHGAIERDPAQHEK
LIVRIKEILAQVASEHL*
>ENSG00000072756|ENST00000339437
MLRCLYHWHRPVLNRRWSRLCLPKQYLFTMKLQSPEFQSLFTEGLKSLTGINAFHEN*
>ENSG00000173917|ENST00000330070
MNFEFEREIGFINSQPSLAECLTSFPAVLETFQTSSIKESTLIPPPPPFEQTFPSLQPGA
STLQRPRSQKRAEDGPALPPPPPPPLPAAPPAPEFPWMKEKKSAKKPSQSATSPSPAASA
VPASGVGSPADGLGLPEAGGGGARRLRTAYTNTQLLELEKEFHFNKYLCRPRRVEIAALL
DLTERQVKVWFQNRRMKHKRQTQHREPPDGEPACPGALEDICDPAEEPAASPGGPSASRA
AWEACCHPPEVVPGALSADPRPLAVRLEGAGASSPGCALRGAGGLEPGPLPEDVFSGRQD
SPFLPDLNFFAADSCLQLSGGLSPSLQGSLDSPVPFSEEELDFFTSTLCAIDLQFP*
>ENSG00000143970|ENST00000336112
MSHKEILQVIQREGLKEISSGTSPLACLNAMLHTNSRGEEGIFYKVPGRMGVYTLKKDVP
DGVKELSEGSEESSDGQSDSQSSENSSSSSDGGSNKEGKKSRWKRKVSSSSPQSGCPSPT
IPAGKVISPSQKHSKKALKQALKQQQQKKQQQQCRPSISISSNQHLSLKTVKAASDSVPA

KPATWEGKQSDGQTGSPQNSNSSFSSSVKVENTLLGLGKKSFQRSERLHTRQMKRTKCAD
IDVETPDSILVNTNLRALINKHTFSVLPGDCQQRLLLLLPEVDRQVGPDGLMKLNGSALN
NEFFTSAAQGWKERLSEGEFTPEMQVRIRQEIEKEKKVEPWKEQFFESYYGQSSGLSLED
SKKLTASPSDPKVKKTPAEQPKSMPVSEASLIRIVPVVSQSECKEEALQMSSPGRKEECE
SQGEVQPNFSTSSEPLLSSALNTHELSSILPIKCPKDEDLLEQKPVTSAEQESEKNHLTT
ASNYNKSESQESLVTSPSKPKSPGVEKPIVKPTAGAGPQETNMKEPLATLVDQSPESLKR
KSSLTQEEAPVSWEKRPRVTENRQHQQPFQVSPQPFLNRGDRIQVRKVPPLKIPVSRISP
MPFHPSQVSPRARFPVSITSPNRTGARTLADIKAKAQLVKAQRAAAAAAAAAAAAASVGG
TIPGPGPGGGQGPGEGGEGGQTARGGSPGSDRVSETGKGPTLELAGTGSRGGTRELLPCGP
ETQPQSETKTTPSQAQPHSVSGAQLQQTPPVPPTPAVSGACTSVPSPAHIEKLDNEKLNP
TRATATVASVSHPQGPSSCRQEKAPSPTGPALISGASPVHCAADGTVELKAGPSKNIPNP
SASSKTDASVPVAVTPSPLTSLLTTATLEKLPVPQVSATTAPAGSAPPSSTLPAASSLKT
PGTSLNMNGPTLRPTSSIPANNPLVTQLLQGKDVPMEQILPKPLTKVEMKTVPLTAKEER
GMGALIATNTTENSTREEVNERQSHPATQQQLGKTLQSKQLPQVPRPLQLFSAKELRDSS
IDTHQYHEGLSKATQDQILQTLIQRVRRQNLLSVVPPSQFNFAHSGFQLEDISTSQRFML
GFAGRRTSKPAMAGHYLLNISTYGRGSESFRRTHSVNPEDRFCLSSPTEALKMGYTDCKN
ATGESSSSKEDDTDEESTGDEQESVTVKEEPQVSQSAGKGDTSSGPHSRETLSTSDCLAS
KNVKAEIPLNEQTTLSKENYLFTRGQTFDEKTLARDLIQAAQKQMAHAVRGKAIRSSPEL
FSSTVLPLPADSPTHQPLLLPPLQTPKLYGSPTQIGPSYRGMINVSTSSDMDHNSAVPGS
QVSSNVGDVMSFSVTVTTIPASQAMNPSSHGQTIPVQAFSEENSIEGTPSKCYCRLKAMI
MCKGCGAFCHDDCIGPSKLCVSCLVVR*
>ENSG00000142192|ENST00000358918
MLPGLALLLLAAWTARALEVPTDGNAGLLAEPQIAMFCGRLNMHMNVQNGKWDSDPSGTK
TCIDTKEGILQYCQEVYPELQITNVVEANQPVTIQNWCKRGRKQCKTHPHFVIPYRCLVG
EFVSDALLVPDKCKFLHQERMDVCETHLHWHTVAKETCSEKSTNLHDYGMLLPCGIDKFR
GVEFVCCPLAEESDNVDSADAEEDDSDVWWGGADTDYADGSEDKVVEVAEEEEVAEVEEE
EADDDEDDEDGDEVEEEAEEPYEEATERTTSIATTTTTTTESVEEVVREVCSEQAETGPC
RAMISRWYFDVTEGKCAPFFYGGCGGNRNNFDTEEYCMAVCGSAMSQSLLKTTQEPLARD
PVKLPTTAASTPDAVDKYLETPGDENEHAHFQKAKERLEAKHRERMSQVMREWEEAERQA
KNLPKADKKAVIQHFQEKVESLEQEAANERQQLVETHMARVEAMLNDRRRLALENYITAL
QAVPPRPRHVFNMLKKYVRAEQKDRQHTLKHFEHVRMVDPKKAAQIRSQVMTHLRVIYER
MNQSLSLLYNVPAVAEEIQDEVDELLQKEQNYSDDVLANMISEPRISYGNDALMPSLTET
KTTVELLPVNGEFSLDDLQPWHSFGADSVPANTENEGSGLTNIKTEEISEVKMDAEFRHD
SGYEVHHQKLVFFAEDVGSNKGAIIGLMVGGVVIATVIVITLVMLKKKQYTSIHHGVVEV
DAAVTPEERHLSKMQQNGYENPTYKFFEQMQN*
>ENSG00000083642|ENST00000315596
MAHSKTRTNDGKITYPPGVKEISDKISKEEMVRRLKMVVKTFMDMDQDSEEEKELYLNLA
LHLASDFFLKHPDKDVRLLVACCLADIFRIYAPEAPYTSPDKLKDIFMFITRQLKGLEDT
KSPQFNRYFYLLENIAWVKSYNICFELEDSNEIFTQLYRTLFSVINNGHNQKVHMHMVDL
MSSIICEGDTVSQELLDTVLVNLVPAHKNLNKQAYDLAKALLKRTAQAIEPYITNFFNQV
LMLGKTSISDLSEHVFDLILELYNIDSHLLLSVLPQLEFKLKSNDNEERLQVVKLLAKMF
GAKDSELASQNKPLWQCYLGRFNDIHVPIRLECVKFASHCLMNHPDLAKDLTEYLKVRSH
DPEEAIRHDVIVSIVTAAKKDILLVNDHLLNFVRERTLDKRWRVRKEAMMGLAQIYKKYA
LQSAAGKDAAKQIAWIKDKLLHIYYQNSIDDRLLVERIFAQYMVPHNLETTERMKCLYYL

YATLDLNAVKALNEMWKCQNLLRHQVKDLLDLIKQPKTDASVKAIFSKVMVITRNLPDPG
KAQDFMKKFTQVLEDDEKIRKQLEVLVSPTCSCKQAEGCVREITKKLGNPKQPTNPFLEM
IKFLLERIAPVHIDTESISALIKQVNKSIDGTADDEDEGVPTDQAIRAGLELLKVLSFTH
PISFHSAETFESLLACLKMDDEKVAEAALQIFKNTGSKIEEDFPHIRSALLPVLHHKSKK
GPPRQAKYAIHCIHAIFSSKETQFAQIFEPLHKSLDPSNLEHLITPLVTIGHIALLAPDQ
FAAPLKSLVATFIVKDLLMNDRLPGKKTTKLWVPDEEVSPETMVKIQAIKMMVRWLLGMK
NNHSKSGTSTLRLLTTILHSDGDLTEQGKISKPDMSRLRLAAGSAIVKLAQEPCYHEIIT
LEQYQLCALAINDECYQVRQVFAQKLHKGLSRLRLPLEYMAICALCAKDPVKERRAHARQ
CLVKNINVRREYLKQHAAVSEKLLSLLPEYVVPYTIHLLAHDPDYVKVQDIEQLKDVKEC
LWFVLEILMAKNENNSHAFIRKMVENIKQTKDAQGPDDAKMNEKLYTVCDVAMNIIMSKS
TTYSLESPKDPVLPARFFTQPDKNFSNTKNYLPPEMKSFFTPGKPKTTNVLGAVNKPLSS
AGKQSQTKSSRMETVSNASSSSNPSSPGRIKGRLDSSEMDHSENEDYTMSSPLPGKKSDK
RDDSDLVRSELEKPRGRKKTPVTEQEEKLGMDDLTKLVQEQKPKGSQRSRKRGHTASESD
EQQWPEEKRLKEDILENEDEQNSPPKKGKRGRPPKPLGGGTPKEEPTMKTSKKGSKKKSG
PPAPEEEEEEERQSGNTEQKSKSKQHRVSRRAQQRAESPESSAIESTQSTPQKGRGRPSK
TPSPSQPKKNVRVGRSKQAATKENDSSEEVDVFQGSSPVDDIPQEETEEEEVSTVNVRRR
SAKRERR*
>ENSG00000184058|ENST00000329705
MHFSTVTRDMEAFTASSLSSLGAAGGFPGAASPGADPYGPREPPPPPPRYDPCAAAAPGA
PGPPPPPHAYPFAPAAGAATSAAAEPEGPGASCAAAAKAPVKKNAKVAGVSVQLEMKALW
DEFNQLGTEMIVTKAGRRMFPTFQVKLFGMDPMADYMLLMDFVPVDDKRYRYAFHSSSWL
VAGKADPATPGRVHYHPDSPAKGAQWMKQIVSFDKLKLTNNLLDDNGHIILNSMHRYQPR
FHVVYVDPRKDSEKYAEENFKTFVFEETRFTAVTAYQNHRITQLKIASNPFAKGFRDCDP
EDWPRNHRPGALPLMSAFARSRNPVASPTQPSGTEKGGHVLKDKEVKAETSRNTPEREVE
LLRDAGGCVNLGLPCPAECQPFNTQGLVAGRTAGDRLC*
>ENSG00000143742|ENST00000366839
MPQYQTWEEFSRAAEKLYLADPMKARVVLKYRHSDGNLCVKVTDDLVRQCLALLLRLQCS
GMIIAHCILDLLGSSGPLASAS*
>ENSG00000163565|ENST00000359709
MGKKYKNIVLLKGLEVINDYHFRMVKSLLSNDLKLNLKMREEYDKIQIADLMEEKFRGDA
GLGKLIKIFEDIPTLEDLAETLKKEKLKVKGPALSRKRKKEVDATSPAPSTSSTVKTEGA
EATPGAQNPKTVAKCQVTPRRNVLQKRPVIVKVLSTTKPFEYETPEMEKKIMFHATVATQ
TQFFHVKVLNTSLKEKFNGKKIIIISDYLEYDSLLEVNEESTVSEAGPNQTFEVPNKIIN
RAKETLKIDILHKQASGNIVYGVFMLHKKTVNQKTTIYEIQDDRGKMDVVGTGQCHNIPC
EEGDKLQLFCFRLRKKNQMSKLISEMHSFIQIKKKTNPRNNDPKSMKLPQEQRQLPYPSE
ASTTFPESHLRTPQMPPTTPSSSFFTKKSEDTISKMNDFMRMQILKEGSHFPGPFMTSIG
PAESHPHTPQMPPSTPSSSFLTTKSEDTISKMNDFMRMQILKEGSHFPGPFMTSIGPAES
HPHTPQMPPSTPSSSFLTTLKPRLKTEPEEVSIEDSAQSDLKEVMVLNATESFVYEPKEQ
KKMFHATVATENEVFRVKVFNIDLKEKFTPKKIIAIANYVCRNGFLEVYPFTLVADVNAD
RNMEIPKGLIRSASVTPKINQLCSQTKGSFVNGVFEVHKKNVRGEFTYYEIQDNTGKMEV
VVHGRLTTINCEEGDKLKLTCFELAPKSGNTGELRSVIHSHIKVIKTRKNKKDILNPDSS
METSPDFFF*
>ENSG00000162772|ENST00000366983
MMLQHPGQVSASEVSASAIVPCLSPPGSLVFEDFANLTPFVKEELRFAIQNKHLCHRMSS

ALESVTVSDRPLGVSITKAEVAPEEDERKKRRRERNKIAAAKCRNKKKEKTECLQKLPRP
FWVQKTCIWAVDSCK*
>ENSG00000117523|ENST00000338920
MSEKSGQSTKAKDGKKYATLSLFNTYKGKSLETQKTTARHGLQSLGKVGISRRMPPPANL
PSLKAENKGNDPNVNIVPKDGTGWASKQEQHEEEKTPEVPPAQPKPGVAAPPEVAPAPKS
WASNKQGGQGDGIQVNSQFQQEFPSLQAAGDQEKKEKETNDDNYGPGPSLRPPNVACWRD
GGKAAGSPSSSDQDEKLPGQDESTAGTSEQNDILKVVEKRIACGPPQAKLNGQQAALASQ
YRAMMPPYMFQQYPRMTYPPLHGPMRFPPSLSETNKGLRGRGPPPSWASEPERPSILSAS
ELKELDKFDNLDAEADEGWAGAQMEVDYTEQLNFSDDDEQGSNSPKENNSEDQGSKASEN
NENKKETDEVSNTKSSSQIPAQPSVAKVPYGKGPSFNQERGTSSHLPPPPKLLAQQHPPP
DRQAVPGRPGPFPSKQQVADEDEIWKQRRRQQSEISAAVERARKRREEEERRMEEQRKAA
CAEKLKRLDEKLGILEKQPSPEEIREREREKEREREKELEKEQEQEREKEREKDRERQQE
KEKELEKEQEKQREMEKERKQEKEKELERQKEKEKELQKMKEQEKECELEKEREKLEEKI
EPREPNLEPMVEKQESENSCNKEEEPVFTRQDSNRSEKEATPVVHETEPESGSQPRPAVL
SGYFKQFQKSLPPRFQRQQEQMKQQQWQQQQQQGVLPQTVPSQPSSSTVPPPPHRPLYQP
MQPHPQHLASMGFDPRWLMMQSYMDPRMMSGRPAMDIPPIHPGMIPPKPLMRRDQMEGSP
NSSESFEHIARSARDHAISLSEPRMLWGSDPYPHAEPQQATTPKATEEPEDVRSEAALDQ
EQITAAYSVEHNQLEAHPKADFIRESSEAQVQKFLSRSVEDVRPHHTDANNQSACFEAPD
QKTLSAPQEERISAVESQPSRKRSVSHGSNHTQKPDEQRSEPSAGIPKVTSRCIDSKEPI
ERPEEKPKKEGFIRSSEGPKPEKVYKSKSETRWGPRPSSNRREEVNDRPVRRSGPIKKPV
LRDMKEEREQRKEKEGEKAEKVTEKVVVKPEKTEKKDLPPPPPPPQPPAPIQPQSVPPPI
QPEAEKFPSTETATLAQKPSQDTEKPLEPVSTVQVEPAVKTVNQQTMAAPVVKEEKQPEK
VISKDLVIERPRPDSRPAVKKESTLPPRTYWKEARERDWFPDQGYRGRGRGEYYSRGRSY
RGSYGGRGRGGRGHTRDYPQYRDNKPRAEHIPSGPLRQREESETRSESSDFEVVPKRRRQ
RGSETDTDSEIHESASDKDSLSKGKLPKREERPENKKPVKPHSSFKPDNHVRIDNRLLEK
PYVRDDDKAKPGFLPKGEPTRRGRGGTFRRGGRDPGGRPSRPSTLRRPAYRDNQWNPRQS
EVPKPEDGEPPRRHEQFIPIAADKRPPKFERKFDPARERPRRQRPTRPPRQDKPPRFRRL
REREAASKSNEVVAVPTNGTVNNVAQEPVNTLGDISGNKTPDLSNQNSSDQANEEWETAS
ESSDFNERRERDEKKNADLNAQTVVKVGENVLPPKREIAKRSFSSQRPVDRQNRRGNNGP
PKSGRNFSGPRNERRSGPPSKSGKRGPFDDQPAGTTGVDLINGSSAHHQEGVPNGTGQKN
SKDSTGKKREDPKPGPKKPKEKVDALSQFDLNNYASVVIIDDHPEVTVIEDPQSNLNDDG
FTEVVSKKQQKRLQDEERRKKEEQVIQVWNKKNANEKGRSQTSKLPPRFAKKQATGIQQA
QSSASVPPLASAPLPPSTSASVPASTSAPLPATLTPVPASTSAPVPASTLAPVLASTSAP
VPASPLAPVSASASVSASVPASTSAAAITSSSAPASAPAPTPILASVSTPASVTILASAS
IPILASALASTSAPTPAPAASSPAAPVITAPTIPASAPTASVPLAPASASAPAPAPTPVS
APNPAPPAPAQTQAQTHKPVQNPLQTTSQSSKQPPPSIRLPSAQTPNGTDYVASGKSIQT
PQSHGTLTAELWDNKVAPPAVLNDISKKLGPISPPQPPSVSAWNKPLTSFGSAPSSEGAK
NGQESGLEIGTDTIQFGAPASNGNENEVVPVLSEKSADKIPEPKEQRQKQPRAGPIKAQK
LPDLSPVENKEHKPGPIGKERSLKNRKVKDAQQVEPEGQEKPSPATVRSTDPVTTKETKA
VSEMSTEIGTMISVSSAEYGTNAKESVTDYTTPSSSLPNTVATNNTKMEDTLVNNVPLPN
TLPLPKRETIQQSSSLTSVPPTTFSLTFKMESARKAWENSPNVREKGSPVTSTAPPIATG
VSSSASGPSTANYNSFSSASMPQIPVASVTPTASLSGAGTYTTSSLSTKSTTTSDPPNIC
KVKPQQLQTSSLPSASHFSQLSCMPSLIAQQQQNPQVYVSQSAAAQIPAFYMDTSHLFNT
QHARLAPPSLAQQQGFQPGLSQPTSVQQIPIPIYAPLQGQHQAQLSLGAGPAVSQAQELF

SSSLQPYRSQPAFMQSSLSQPSVVLSGTAIHNFPTVQHQELAKAQSGLAFQQTSNTQPIP
ILYEHQLGQASGLGGSQLIDTHLLQARANLTQASNLYSGQVQQPGQTNFYNTAQSPSALQ
QVTVPLPASQLSLPNFGSTGQPLIALPQTLQPPLQHTTPQAQAQSLSRPAQVSQPFRGLI
PAGTQHSMIATTGKMSEMELKAFGSGIDIKPGTPPIAGRSTTPTSSPFRATSTSPNSQSS
KMNSIVYQKQFQSAPATVRMTQPFPTQFAPQAKQRAEVLQSTQRFFSEQQQSKQIGGGKA
QKVDSDSSKPPETLTDPPGVCQEKVEEKPPPAPSIATKPVRTGPIKPQAIKTEETKS*
>ENSG00000084072|ENST00000356511
MATTKRVLYVGGLAEEVDDKVLHAAFIPFGDITDIQIPLDYETEKHRGFAFVEFELAEDA
AAAIDNMNESELFGRTIRVNLAKPMRIKEGSSRPVWSDDDWLKKFSGKTLEENKEEEGSE
PPKAETQEGEPIAKKARSNPQVYMDIKIGNKPAGRIQMLLRSDVVPMTAENFRCLCTHEK
GFGFKGSSFHRIIPQFMCQGGDFTNHNGTGGKSIYGKKFDDENFILKHTGPGLLSMANSG
PNTNGSQFFLTCDKTDWLDGKHVVFGEVTEGLDVLRQIEKQEESAITSQPRSWKLT*
>ENSG00000143437|ENST00000354396
MAATTANPEMTSDVPSLGPAIASGNSGPGIQGGGAIVQRAIKRRPGLDFDDDGEGNSKFL
RCDDDQMSNDKERFARSDDEQSSADKERLARENHSEIERRRRNKMTAYITELSDMVPTCS
ALARKPDKLTILRMAVSHMKSLRGTGNTSTDGSYKPSFLTDQELKHLILEAADGFLFIVS
CETGRVVYVSDSVTPVLNQPQSEWFGSTLYDQVHPDDVDKLREQLSTSENALTGRILDLK
TGTVKKEGQQSSMRMCMGSRRSFICRMRCGSSSVDPVSVNRLSFVRNRCRNGLGSVKDGE
PHFVVVHCTGYIKAWPPAGVSLPDDDPEAGQGSKFCLVAIGRLQVTSSPNCTDMSNVCQP
TEFISRHNIEGIFTFVDHRCVATVGYQPQELLGKNIVEFCHPEDQQLLRDSFQQVVKLKG
QVLSVMFRFRSKNQEWLWMRTSSFTFQNPYSDEIEYIICTNTNVKNSSQEPRPTLSNTIQ
RPQLGPTANLPLEMGSGQLAPRQQQQQTELDMVPGRDGLASYNHSQVVQPVTTTGPEHSK
PLEKSDGLFAQDRDPRFSEIYHNINADQSKGISSSTVPATQQLFSQGNTFPPTPRPAENF
SGLAPPVTIVQPSASAGQMLAQISRHSNPTQGATPTWTPTTRSGFSAQQVATQATAKTRT
SQFGVGSFQTPSSFSSMSLPGAPTASPGAAAYPSLTNRGSNFAPETGQTAGQFQTRTAEG
VGVWPQWQGQQPHHRSSSSEQHVQQPPAQQPGQPEVFQEMLSMLGDQSNSYNNEEFPDLT
MFPPFSE*
>ENSG00000104413|ENST00000358397
MTASPDYLVVLFGITAGATGAKLGSDEKELILLFWKVVDLANKKVGQLHEVLVRPDQLEL
TEDCKEETKIDVESLSSASQLDQALRQFNQSVSNELNIGVGTSFCLCTDGQLHVRQILHP
EASKKNVLLPECFYSFFDLRKEFKKCCPGSPDIDKLDVATMTEYLNFEKSSSVSRYGASQ
VEDMGNIILAMISEPYNHRFSDPERVNYKFESGTCSKMELIDDNTVVRARGLPWQSSDQD
IARFFKGLNIAKGGAALCLNAQGRRNGEALVRFVSEEHRDLALQRHKHHMGTRYIEVYKA
TGEDFLKIAGGTSNEVAQFLSKENQVIVRMRGLPFTATAEEVVAFFGQHCPITGGKEGIL
FVTYPDGRPTGDAFVLFACEEYAQNALRKHKDLLGKRYIELFRSTAAEVQQVLNRFSSAP
LIPLPTPPIIPVLPQQFVPPTNVRDCIRLRGLPYAATIEDILDFLGEFATDIRTHGVHMV
LNHQGRPSGDAFIQMKSADRAFMAAQKCHKKNMKDRYVEVFQCSAEEMNFVLMGGTLNRN
GLSPPPCLSPPSYTFPAPAAVIPTEAAIYQPSVILNPRALQPSTAYYPAGTQLFMNYTAY
YPSPPGSPNSLGYFPTAANLSGVPPQPGTVVRMQGLAYNTGVKEILNFFQGYQYATEDGL
IHTNDQARTLPKEWVCI*
>ENSG00000278016|ENST00000321385
MYPAGPPAGPVPRRGRRPLPGPPAPAPAPVPAARPPPPAPGPRPRVAVKMAFRKAYSIKD
KLQAIERVKGGERQASVCRDFGVPGGTLRGWLKDEPKLRWFLEQLGGEVGTQRKKMRLAN
EEEIDRAVYAWFLALRQHGVPLSGPLIQAQAEAFARQIYGPECTFKASHGWFWRWQKRHG

ISSQRFYGEAGPPAPSPAPGPPVKEEPALPSGAGPLPDRAPAPPPPAEGGYGDEQIYSAS
VTGLYWKLLPEQAAPPGAGDPGAGGCGRRWRGDRVTVLLAANLTGSHKLKPLVIGRLPDP
PSLRHHNQDKFPASYRYSPDAWLSRPLLRGWFFEEFVPGVKRYLRRSCLQQKAVLLVAHP
PCPSPAASMPALDSEDAPVRCRPEPLGPPEELQTPDGAVRVLFLSKGSSRAHIPAPLEQG
VVAAFKQLYKRELLRLAVSCASGSPLDFMRSFMLKDMLYLAGLSWDLVQAGSIERCWLLG
LRAAFEPRPGEDSAGQPAQAEEAAEHSRVLSDLTHLAALAYKCLAPEEVAEWLHLDDDGG
PPEGCREEVGPALPPAAPPAPASLPSAMGGGEDEEEATDYGGTSVPTAGEAVRGLETALR
WLENQDPREVGPLRLVQLRSLISMARRLGGIGHTPAGPYDGV*
>ENSG00000158055|ENST00000361548
MSNELDFRSVRLLKNDPVNLQKFSYTSEDEAWKTYLENPLTAATKAMMRVNGDDDSVAAL
SFLYDYYMGPKEKRILSSSTGGRNDQGKRYYHGMEYETDLTPLESPTHLMKFLTENVSGT
PEYPDLLKKNNLMSLEGALPTPGKAAPLPAGPSKLEAGSVDSYLLPTTDMYDNGSLNSLF
ESIHGVPPTQRWQPDSTFKDDPQESMLFPDILKTSPEPPCPEDYPSLKSDFEYTLGSPKA
IHIKSGESPMAYLNKGQFYPVTLRTPAGGKGLALSSNKVKSVVMVVFDNEKVPVEQLRFW
KHWHSRQPTAKQRVIDVADCKENFNTVEHIEEVAYNALSFVWNVNEEAKVFIGVNCLSTD
FSSQKGVKGVPLNLQIDTYDCGLGTERLVHRAVCQIKIFCDKGAERKMRDDERKQFRRKV
KCPDSSNSGVKGCLLSGFRGNETTYLRPETDLETPPVLFIPNVHFSSLQRSGGAAPSAGP
SSSNRLPLKRTCSPFTEEFEPLPSKQAKEGDLQRVLLYVRRETEEVFDALMLKTPDLKGL
RNAISEKYGFPEENIYKVYKKCKRGILVNMDNNIIQHYSNHVAFLLDMGELDGKIQIILK
EL*
>ENSG00000178209|ENST00000357649
MSGAGGAFASPREVLLERPCWLDGGCEPARRGYLYQQLCCVDERDRVQKKTFTKWVNKHL
IKAQRHISDLYEDLRDGHNLISLLEVLSGDSLPREKGRMRFHKLQNVQIALDYLRHRQVK
LVNIRNDDIADGNPKLTLGLIWTIILHFQISDIQVSGQSEDMTAKEKLLLWSQRMVEGYQ
GLRCDNFTSSWRDGRLFNAIIHRHKPLLIDMNKVYRQTNLENLDQAFSVAERDLGVTRLL
DPEDVDVPQPDEKSIITYVSSLYDAMPRVPDVQDGVRANELQLRWQEYRELVLLLLQWMR
HHTAAFEERRFPSSFEEIEILWSQFLKFKEMELPAKEADKNRSKGIYQSLEGAVQAGQLK
VPPGYHPLDVEKEWGKLHVAILEREKQLRSEFERLECLQRIVTKLQMEAGLCEEQLNQAD
ALLQSDVRLLAAGKVPQRAGEVERDLDKADSMIRLLFNDVQTLKDGRHPQGEQMYRRVYR
LHERLVAIRTEYNLRLKAGVAAPATQVAQVTLQSVQRRPELEDSTLRYLQDLLAWVEENQ
HRVDGAEWGVDLPSVEAQLGSHRGLHQSIEEFRAKIERARSDEGQLSPATRGAYRDCLGR
LDLQYAKLLNSSKARLRSLESLHSFVAAATKELMWLNEKEEEEVGFDWSDRNTNMTAKKE
SYSALMRELELKEKKIKELQNAGDRLLREDHPARPTVESFQAALQTQWSWMLQLCCCIEA
HLKENAAYFQFFSDVREAEGQLQKLQEALRRKYSCDRSATVTRLEDLLQDAQDEKEQLNE
YKGHLSGLAKRAKAVVQLKPRHPAHPMRGRLPLLAVCDYKQVEVTVHKGDECQLVGPAQP
SHWKVLSSSGSEAAVPSVCFLVPPPNQEAQEAVTRLEAQHQALVTLWHQLHVDMKSLLAW
QSLRRDVQLIRSWSLATFRTLKPEEQRQALHSLELHYQAFLRDSQDAGGFGPEDRLMAER
EYGSCSHHYQQLLQSLEQGAQEESRCQRCISELKDIRLQLEACETRTVHRLRLPLDKEPA
RECAQRIAEQQKAQAEVEGLGKGVARLSAEAEKVLALPEPSPAAPTLRSELELTLGKLEQ
VRSLSAIYLEKLKTISLVIRGTQGAEEVLRAHEEQLKEAQAVPATLPELEATKASLKKLR
AQAEAQQPTFDALRDELRGAQEVGERLQQRHGERDVEVERWRERVAQLLERWQAVLAQTD
VRQRELEQLGRQLRYYRESADPLGAWLQDARRRQEQIQAMPLADSQAVREQLRQEQALLE
EIERHGEKVEECQRFAKQYINAIKDYELQLVTYKAQLEPVASPAKKPKVQSGSESVIQEY
VDLRTHYSELTTLTSQYIKFISETLRRMEEEERLAEQQRAEERERLAEVEAALEKQRQLA

EAHAQAKAQAEREAKELQQRMQEEVVRREEAAVDAQQQKRSIQEELQQLRQSSEAEIQAK
ARQAEAAERSRLRIEEEIRVVRLQLEATERQRGGAEGELQALRARAEEAEAQKRQAQEEA
ERLRRQVQDESQRKRQAEVELASRVKAEAEAAREKQRALQALEELRLQAEEAERRLRQAE
VERARQVQVALETAQRSAEAELQSKRASFAEKTAQLERSLQEEHVAVAQLREEAERRAQQ
QAEAERAREEAERELERWQLKANEALRLRLQAEEVAQQKSLAQAEAEKQKEEAEREARRR
GKAEEQAVRQRELAEQELEKQRQLAEGTAQQRLAAEQELIRLRAETEQGEQQRQLLEEEL
ARLQREAAAATQKRQELEAELAKVRAEMEVLLASKARAEEESRSTSEKSKQRLEAEAGRF
RELAEEAARLRALAEEAKRQRQLAEEDAARQRAEAERVLAEKLAAIGEATRLKTEAEIAL
KEKEAENERLRRLAEDEAFQRRRLEEQAAQHKADIEERLAQLRKASDSELERQKGLVEDT
LRQRRQVEEEILALKASFEKAAAGKAELELELGRIRSNAEDTLRSKEQAELEAARQRQLA
AEEERRRREAEERVQKSLAAEEEAARQRKAALEEVERLKAKVEEARRLRERAEQESARQL
QLAQEAAQKRLQAEEKAHAFAVQQKEQELQQTLQQEQSVLDQLRGEAEAARRAAEEAEEA
RVQAEREAAQSRRQVEEAERLKQSAEEQAQARAQAQAAAEKLRKEAEQEAARRAQAEQAA
LRQKQAADAEMEKHKKFAEQTLRQKAQVEQELTTLRLQLEETDHQKNLLDEELQRLKAEA
TEAARQRSQVEEELFSVRVQMEELSKLKARIEAENRALILRDKDNTQRFLQEEAEKMKQV
AEEAARLSVAAQEAARLRQLAEEDLAQQRALAEKMLKEKMQAVQEATRLKAEAELLQQQK
ELAQEQARRLQEDKEQMAQQLAEETQGFQRTLEAERQRQLEMSAEAERLKLRVAEMSRAQ
ARAEEDAQRFRKQAEEIGEKLHRTELATQEKVTLVQTLEIQRQQSDHDAERLREAIAELE
REKEKLQQEAKLLQLKSEEMQTVQQEQLLQETQALQQSFLSEKDSLLQRERFIEQEKAKL
EQLFQDEVAKAQQLREEQQRQQQMEQERQRLVASMEEARRRQHEAEEGVRRKQEELQQL
EQQRRQQEELLAEENQRLREQLQLLEEQHRAALAHSEEVTASQVAATKTLPNGRDALDGP
AAEAEPEHSFDGLRRKVSAQRLQEAGILSAEELQRLAQGHTTVDELARREDVRHYLQGRS
SIAGLLLKATNEKLSVYAALQRQLLSPGTALILLEAQAASGFLLDPVRNRRLTVNEAVKE
GVVGPELHHKLLSAERAVTGYKDPYTGQQISLFQAMQKGLIVREHGIRLLEAQIATGGVI
DPVHSHRVPVDVAYRRGYFDEEMNRVLADPSDDTKGFFDPNTHENLTYLQLLERCVEDPE
TGLCLLPLTDKAAKGGELVYTDSEARDVFEKATVSAPFGKFQGKTVTIWEIINSEYFTAE
QRRDLLRQFRTGRITVEKIIKIIITVVEEQEQKGRLCFEGLRSLVPAAELLESRVIDREL
YQQLQRGERSVRDVAEVDTVRRALRGANVIAGVWLEEAGQKLSIYNALKKDLLPSDMAVA
LLEAQAGTGHIIDPATSARLTVDEAVRAGLVGPEFHEKLLSAEKAVTGYRDPYTGQSVSL
FQALKKGLIPREQGLRLLDAQLSTGGIVDPSKSHRVPLDVACARGCLDEETSRALSAPRA
DAKAYSDPSTGEPATYGELQQRCRPDQLTGLSLLPLSEKAARARQEELYSELQARETFEK
TPVEVPVGGFKGRTVTVWELISSEYFTAEQRQELLRQFRTGKVTVEKVIKILITIVEEVE
TLRQERLSFSGLRAPVPASELLASGVLSRAQFEQLKDGKTTVKDLSELGSVRTLLQGSGC
LAGIYLEDTKEKVSIYEAMRRGLLRATTAALLLEAQAATGFLVDPVRNQRLYVHEAVKAG
VVGPELHEQLLSAEKAVTGYRDPYSGSTISLFQAMQKGLVLRQHGIRLLEAQIATGGIID
PVHSHRVPVDVAYQRGYFSEEMNRVLADPSDDTKGFFDPNTHENLTYRQLLERCVEDPET
GLRLLPLKGAEKAEVVETTQVYTEEETRRAFEETQIDIPGGGSHGGSTMSLWEVMQSDLI
PEEQRAQLMADFQAGRVTKERMIIIIIEIIEKTEIIRQQGLASYDYVRRRLTAEDLFEAR
IISLETYNLLREGTRSLREALEAESAWCYLYGTGSVAGVYLPGSRQTLSIYQALKKGLLS
AEVARLLLEAQAATGFLLDPVKGERLTVDEAVRKGLVGPELHDRLLSAERAVTGYRDPYT
EQTISLFQAMKKELIPTEEALRLLDAQLATGGIVDPRLGFHLPLEVAYQRGYLNKDTHDQ
LSEPSEVRSYVDPSTDERLSYTQLLRRCRRDDGTGQLLLPLSDARKLTFRGLRKQITMEE
LVRSQVMDEATALQLREGLTSIEEVTKNLQKFLEGTSCIAGVFVDATKERLSVYQAMKKG
IIRPGTAFELLEAQAATGYVIDPIKGLKLTVEEAVRMGIVGPEFKDKLLSAERAVTGYKD

PYSGKLISLFQAMKKGLILKDHGIRLLEAQIATGGIIDPEESHRLPVEVAYKRGLFDEEM
NEILTDPSDDTKGFFDPNTEENLTYLQLMERCITDPQTGLCLLPLKEKKRERKTSSKSSV
RKRRVVIVDPETGKEMSVYEAYRKGLIDHQTYLELSEQECEWEEITISSSDGVVKSMIID
RRSGRQYDIDDAIAKNLIDRSALDQYRAGTLSITEFADMLSGNAGGFRSRSSSVGSSSSY
PISPAVSRTQLASWSDPTEETGPVAGILDTETLEKVSITEAMHRNLVDNITGQRLLEAQA
CTGGIIDPSTGERFPVTDAVNKGLVDKIMVDRINLAQKAFCGFEDPRTKTKMSAAQALKK
GWLYYEAGQRFLEVQYLTGGLIEPDTPGRVPLDEALQRGTVDARTAQKLRDVGAYSKYLT
CPKTKLKISYKDALDRSMVEEGTGLRLLEAAAQSTKGYYSPYSVSGSGSTAGSRTGSRTG
SRAGSRRGSFDATGSGFSMTFSSSSYSSSGYGRRYASGSSASLGGPESAVA*
>ENSG00000196141|ENST00000360760
MAELNTHVNVKEKIYAVRSVVPNKSNNEIVLVLQQFDFNVDKAVQAFVDGSAIQVLKEWN
MTGKKKNNKRKRSKSKQHQGNKDAKDKVERPEAGPLQPQPPQIQNGPMNGCEKDSSSTDS
ANEKPALIPREKKISILEEPSKALRGVTGPNIEKSVKDLQRCTVSLTRYRVMIKEEVDSS
VKKIKAAFAELHNCIIDKEVSLMAEMDKVKEEAMEILTARQKKAEELKRLTDLASQMAEM
QLAELRAEIKHFVSERKYDEELGKAARFSCDIEQLKAQIMLCGEITHPKNNYSSRTPCSS
LLPLLNAHAATSGKQSNFSRKSSTHNKPSEGKAANPKMVSSLPSTADPSHQTMPANKQNG
SSNQRRRFNPQYHNNRLNGPAKSQGSGNEAEPLGKGNSRHEHRRQPHNGFRPKNKGGAKN
QEASLGMKTPEAPAHSEKPRRRQHAADTSEARPFRGSVGRVSQCNLCPTRIEVSTDAAVL
SVPAVTLVA*
>ENSG00000151461|ENST00000356352
MPAERKKPASMEEKDSLPNNKEKDCSERRTVSSKERPKDDIKLTAKKEVSKAPEDKKKRL
EDDKRKKEDKERKKKDEEKVKAEEESKKKEEEEKKKHQEEERKKQEEQAKRQQEEEAAAQ
MKEKEESIQLHQEAWERHHLRKELRSKNQNAPDSRPEENFFSRLDSSLKKNTAFVKKLKT
ITEQQRDSLSHDFNGLNLSKYIAEAVASIVEAKLKISDVNCAVHLCSLFHQRYADFAPSL
LQVWKKHFEARKEEKTPNITKLRTDLRFIAELTIVGIFTDKEGLSLIYEQLKNIINADRE
SHTHVSVVISFCRHCGDDIAGLVPRKVKSAAEKFNLSFPPSEIISPEKQQPFQNLLKEYF
TSLTKHLKRDHRELQNTERQNRRILHSKGELSEDRHKQYEEFAMSYQKLLANSQSLADLL
DENMPDLPQDKPTPEEHGPGIDIFTPGKPGEYDLEGGIWEDEDARNFYENLIDLKAFVPA
ILFKDNEKSCQNKESNKDDTKEAKESKENKEVSSPDDLELELENLEINDDTLELEGGDEA
EDLTKKLLDEQEQEDEEASTGSHLKLIVDAFLQQLPNCVNRDLIDKAAMDFCMNMNTKAN
RKKLVRALFIVPRQRLDLLPFYARLVATLHPCMSDVAEDLCSMLRGDFRFHVRKKDQINI
ETKNKTVRFIGELTKFKMFTKNDTLHCLKMLLSDFSHHHIEMACTLLETCGRFLFRSPES
HLRTSVLLEQMMRKKQAMHLDARYVTMVENAYYYCNPPPAEKTVKKKRPPLQEYVRKLLY
KDLSKVTTEKVLRQMRKLPWQDQEVKDYVICCMINIWNVKYNSIHCVANLLAGLVLYQED
VGIHVVDGVLEDIRLGMEVNQPKFNQRRISSAKFLGELYNYRMVESAVIFRTLYSFTSFG
VNPDGSPSSLDPPEHLFRIRLVCTILDTCGQYFDRGSSKRKLDCFLVYFQRYVWWKKSLE
VWTKDHPFPIDIDYMISDTLELLRPKIKLCNSLEESIRQVQDLEREFLIKLGLVNDKDSK
DSMTEGENLEEDEEEEEGGAETEEQSGNESEVNEPEEEEGSDNDDDEGEEEEEENTDYLT
DSNKENETDEENTEVMIKGGGLKHVPCVEDEDFIQALDKMMLENLQQRSGESVKVHQLDV
AIPLHLKSQLRKGPPLGGGEGEAESADTMPFVMLTRKGNKQQFKILNVPMSSQLAANHWN
QQQAEQEERMRMKKLTLDINERQEQEDYQEMLQSLAQRPAPANTNRERRPRYQHPKGAPN
ADLIFKTGGRRR*
>ENSG00000107164|ENST00000319725
MAELVQGQSAPVGMKAEGFVDALHRVRQIAAKIDSIPHLNNSTPLVDPSVYGYGVQKRPL

DDGVGNQLGALVHQRTVITEEFKVPDKMVGFIIGRGGEQISRIQAESGCKIQIASESSGI
PERPCVLTGTPESIEQAKRLLGQIVDRCRNGPGFHNDIDSNSTIQEILIPASKVGLVIGR
GGETIKQLQERTGVKMVMIQDGPLPTGADKPLRITGDAFKVQQAREMVLEIIREKDQADF
RGVRGDFNSRMGGGSIEVSVPRFAVGIVIGRNGEMIKKIQNDAGVRIQFKPDDGISPERA
AQVMGPPDRCQHAAHIISELILTAQERDGFGGLAAARGRGRGRGDWSVGAPGGVQEITYT
VPADKCGLVIGKGGENIKSINQQSGAHVELQRNPPPNSDPNLRRFTIRGVPQQIEVARQL
IDEKVGGTNLGAPGAFGQSPFSQPPAPPHQNTFPPRSSGCFPNMAAKVNGNPHSTPVSGP
PAFLTQGWGSTYQAWQQPTQQVPSQQSQPQSSQPNYSKAWEDYYKKQSHAASAAPQASSP
PDYTMAWAEYYRQQVAFYGQTLGQAQAHSQEQ*
>ENSG00000188283|ENST00000352998
MAEGSVMFSDVSIDFSQEEWDCLDPVQRDLYRDVMLENYGNLVSMGLYTPKPQVISLLEQ
GKEPWMVGRELTRGLCSDLESMCETKLLSLKKEVYEIELCQREIMGLTKHGLEYSSFGDV
LEYRSHLAKQLGYPNGHFSQEIFTPEYMPTFIQQTFLTLHQIINNEDRPYECKKCGKAFS
QNSQFIQHQRIHIGEKSYECKECGKFFSCGSHVTRHLKIHTGEKPFECKECGKAFSCSSY
LSQHQRIHTGKKPYECKECGKAFSYCSNLIDHQRIHTGEKPYECKVCGKAFTKSSQLFQH
ARIHTGEKPYECKECGKAFTQSSKLVQHQRIHTGEKPYECKECGKAFSSGSALTNHQRIH
TGEKPYDCKECGKAFTQSSQLRQHQRIHAGEKPFECLECGKAFTQNSQLFQHQRIHTDEK
PYECNECGKAFNKCSNLTRHLRIHTGEKPYNCKECGKAFSSGSDLIRHQGIHTNK*
>ENSG00000179750|ENST00000335760
MNPQIRNPMERMYRDTFYDNFENEPILYGRSYTWLCYEVKIKRGRSNLLWDTGVFRGQVY
FKPQYHAEMCFLSWFCGNQLPAYKCFQITWFVSWTPCPDCVAKLAEFLSEHPNVTLTISA
ARLYYYWERDYRRALCRLSQAGARVTIMDYEEFAYCWENFVYNEGQQFMPWYKFDENYAF
LHRTLKEILRLRIFSVAFTAAMRSCASWTWFLLCSWTRPRSTGSLGSSPGAPASPGAVPG
KCVRSFRRTHT*
>ENSG00000090905|ENST00000315183
MRELEAKATKDVERNLSRDLVQEEEQLMEEKKKKKDDKKKKEAAQKKATEQKIKVPEQIK
PSVSQPQPANSNNGTSTATSTNNNAKRATANNQQPQQQQQQQPQQQQPQQQPQPQPQQQ
QPQQQPQALPRYPREVPPRFRHQEHKQLLKRGQHFPVIAANLGSAVKVLNSQSESSALTN
QQPQNNGEVQNSKNQSDINHSTSGSHYENSQRGPVSSTSDSSTNCKNAVVSDLSEKEAWP
SAPGSDPELASECMDADSASSSESERNITIMASGNTGGEKDGLRNSTGLGSQNKFVVGSS
SNNVGHGSSTGPWGFSHGAIISTCQVSVDAPESKSESSNNRMNAWGTVSSSSNGGLNPST
LNSASNHGAWPVLENNGLALKGPVGSGSSGINIQCSTIGQMPNNQSINSKVSGGSTHGTW
GSLQETCESEVSGTQKVSFSGQPQNITTEMTGPNNTTNFMTSSLPNSGSVQNNELPSSNT
GAWRVSTMNHPQMQAPSGMNGTSLSHLSNGESKSGGSYGTTWGAYGSNYSGDKCSGPNGQ
ANGDTVNATLMQPGVNGPMGTNFQVNTNKGGGVWESGAANSQSTSWGSGNGANSGGSRRG
WGTPAQNTGTNLPSVEWNKLPSNQHSNDSANGNGKTFTNGWKSTEEEDQGSATSQTNEQS
SVWAKTGGTVESDGSTESTGRLEEKGTGESQSRDRRKIDQHTLLQSIVNRTDLDPRVLSN
SGWGQTPIKQNTAWDTETSPRGERKTDNGTEAWGSSATQTFNSGACIDKTSPNGNDTSSV
SGWGDPKPALRWGDSKGSNCQGGWEDDSAATGMVKSNQWGNCKEEKAAWNDSQKNKQGWG
DGQKSSQGWSVSASDNWGETSRNNHWGEANKKSSSGGSDSDRSVSGWNELGKTSSFTWGN
NINPNNSSGWDESSKPTPSQGWGDPPKSNQSLGWGDSSKPVSSPDWNKQQDIVGSWGIPP
ATGKPPGTGWLGGPIPAPAKEEEPTGWEEPSPESIRRKMEIDDGTSAWGDPSKYNYKNVN
MWNKNVPNGNSRSDQQAQVHQLLTPASAISNKEASSGSGWGEPWGEPSTPATTVDNGTSA
WGKPIDSGPSWGEPIAAASSTSTWGSSSVGPQALSKSGPKSMQDGWCGDDMPLPGNRPTG

WEEEEDVEIGMWNSNSSQELNSSLNWPPYTKKMSSKGLSGKKRRRERGMMKGGNKQEEAW
INPFVKQFSNISFSRDSPEENVQSNKMDLSGGMLQDKRMEIDKHSLNIGDYNRTVGKGPG
SRPQISKESSMERNPYFDKNGNPSMFGVGNTAAQPRGMQQPPAQPLSSSQPNLRAQVPPP
LLSPQVPVSLLKYAPNNGGLNPLFGPQQVAMLNQLSQLNQLSQISQLQRLLAQQQRAQSQ
RSVPSGNRPQQDQQGRPLSVQQQMMQQSRQLDPNLLVKQQTPPSQQQPLHQPAMKSFLDN
VMPHTTPELQKGPSPINAFSNFPIGLNSNLNVNMDMNSIKEPQSRLRKWTTVDSISVNTS
LDQNSSKHGAISSGFRLEESPFVPYDFMNSSTSPASPPGSIGDGWPRAKSPNGSSSVNWP
PEFRPGEPWKGYPNIDPETDPYVTPGSVINNLSINTVREVDHLRDRNSGSSSSLNTTLPS
TSAWSSIRASNYNVPLSSTAQSTSARNSDSKLTWSPGSVTNTSLAHELWKVPLPPKNITA
PSRPPPGLTGQKPPLSTWDNSPLRIGGGWGNSDARYTPGSSWGESSSGRITNWLVLKNLT
PQIDGSTLRTLCMQHGPLITFHLNLPHGNALVRYSSKEEVVKAQKSLHMCVLGNTTILAE
FASEEEISRFFAQSQSLTPSPGWQSLGSSQSRLGSLDCSHSFSSRTDLNHWNGAGLSGTN
CGDLHGTSLWGTPHYSTSLWGPPSSSDPRGISSPSPINAFLSVDHLGGGGESM*
>ENSG00000068323|ENST00000315869
MSHAAEPARDGVEASAEGPRAVFVLLEERRPADSAQLLSLNSLLPESGIVADIELENVLD
PDSFYELKSQPLPLRSSLPISLQATPATPATLSASSSAGGSRTPAMSSSSSSRVLLRQQL
MRAQAQEQERRERREQAAAAPFPSPAPASPAISVVGVSAGGHTLSRPPPAQVPREVLKVQ
THLENPTRYHLQQARRQQVKQYLSTTLGPKLASQALTPPPGPASAQPLPAPEAAHTTGPT
GSAPNSPMALLTIGSSSEKEIDDVIDEIISLESSYNDEMLSYLPGGTTGLQLPSTLPVSG
NLLDVYSSQGVATPAITVSNSCPAELPNIKREISETEAKALLKERQKKDNHNLIERRRRF
NINDRIKELGTLIPKSSDPEMRWNKGTILKASVDYIRKLQKEQQRSKDLESRQRSLEQAN
RSLQLRIQELELQAQIHGLPVPPTPGLLSLATTSASDSLKPEQLDIEEEGRPGAATFHVG
GGPAQNAPHQQPPAPPSDALLDLHFPSDHLGDLGDPFHLGLEDILMEEEEGVVGGLSGGA
LSPLRAASDPLLSSVSPAVSKASSRRSSFSMEEES*
>ENSG00000165702|ENST00000339463
MPRSFLVKSKKAHTYHQPRVQEDEPLWPPALTPVPRDQAPSNSPVLSTLFPNQCLDWTNL
KREPELEQDQNLARMAPAPEGPIVLSRPQDGDSPLSDSPPFYKPSFSWDTLATTYGHSYR
QAPSTMQSAFLEHSVSLYGSPLVPSTEPALDFSLRYSPGMDAYHCVKCNKVFSTPHGLEV
HVRRSHSGTRPFACDICGKTFGHAVSLEQHTHVHSQERSFECRMCGKAFKRSSTLSTHLL
IHSDTRPYPCQFCGKRFHQKSDMKKHTYIHTGEKPHKCQVCGKAFSQSSNLITHSRKHTG
FKPFSCELCTKGFQRKVDLRRHRESQHNLK*
>ENSG00000124496|ENST00000340840
MGDQQLYKTNHVAHGSENLFYQQPPLGVHSGLNHNYGNAVTGGGMDAPQASPISPHFPQD
TRDGLGLPVGSKNLGQMDTSRQGGWGSHAGPGNHVQLRGNLANSNMMWGAPAQAEPTDGY
QYTYSQASEIRTQKLTSGVLHKLDSFTQVFANQNLRIQVNNMAQVLHTQSAVMDGAPDSA
LRQLLSQKPMEPPAPAIPSRYQQVPQQPHPGFTGGLSKPALQVGQHPTQGHLYYDYQQPL
AQVPVQGGQPLQAPQMLSQHMQQMQQHQYYPPQQQQQAGQQRISMQEIQTQPQQIRPSQP
QPPPQQQQPQQLQLQQRQGSMQIPQYYQPQPMMQHLQEQQQQQMHLQPPSYHRDPHQYTP
EQAHTVQLIPLGSMSQYYYQEPQQPYSHPLYQQSHLSQHQQREDSQLKTYSSDRQAQAML
SSHGDLGPPDTGMGDPASSDLTRVSSTLPHRPLLSPSGIHLNNMGPQHQQLSPSAMWPQM
HLPDGRAQPGSPESSGQPKGAFGEQFDAKNKLTCSICLKEFKNLPALNGHMRSHGGMRAS
PNLKQEIPRKHQPSVPKAEEPLKTVQEKKKFRHRPEPLFIPPPPSYNPNPAASYSGATLY
QSQLRSPRVLGDHLLLDPTHELPPYTPPPMLSPVRQGSGLFSNVLISGHGPGAHPQLPLT
PLTPTPRVLLCRSNSIDGSNVTVTPGPGEQTVDVEPRINIGLRFQAEIPELQDISALAQD

THKATLVWKPWPELENHDLQQRVENLLNLCCSSALPGGGTNSEFALHSLFEAKGDVMVAL
EMLLLRKPVRLKCHPLANYHYAGSDKWTSLERKLFNKALATYSKDFIFVQKMVKSKTVAQ
CVEYYYTWKKIMRLGRKHRTRLAEIIDDCVTSEEEEELEEEEEEDPEEDRKSTKEEESEV
PKSPEPPPVPVLAPTEGPPLQALGQPSGSFICEMPNCGADCRCHVTPFLPQVFSSRQALN
GHARIHGGTNQVTKARGAIPSGKQKPGGTQSGYCSVKSSPSHSTTSGETDPTTIFPCKEC
GKVFFKIKSRNAHMKTHRQQEEQQRQKAQKAAFAAEMAATIERTTGPVGAPGLLPLDQLS
LIKPIKDVDILDDDVVQQLGGVMEEAEVVDTDLLLDDQDSVLLQGDAEL*
>ENSG00000011332|ENST00000355526
MGGLSARPTAGRTDPAGTCWGQDPGSKMATVIPGPLSLGEDFYREAIEHCRSYNARLCAE
RSLRLPFLDSQTGVAQNNCYIWMEKTHRGPGLAPGQIYTYPARCWRKKRRLNILEDPRLR
PCEYKIDCEAPLKKEGGLPEGPVLEALLCAETGEKKIELKEEETIMDCQKQQLLEFPHDL
EVEDLEDDIPRRKNRAKGKAYGIGGLRKRQDTASLEDRDKPYVCDICGKRYKNRPGLSYH
YTHTHLAEEEGEENAERHALPFHRKNNHKQFYKELAWVPEAQRKHTAKKAPDGTVIPNGY
CDFCLGGSKKTGCPEDLISCADCGRSGHPSCLQFTVNMTAAVRTYRWQCIECKSCSLCGT
SENDDQLLFCDDCDRGYHMYCLSPPMAEPPEGSWSCHLCLRHLKEKASAYITLT*
>ENSG00000025293|ENST00000339089
MTKHPPNRRGISFEVGAQLEARDRLKNWYPAHIEDIDYEEGKVLIHFKRWNHRYDEWFCW
DSPYLRPLEKIQLRKEGLHEEDGSSEFQINEQVLACWSDCRFYPAKVTAVNKDGTYTVKF
YDGVVQTVKHIHVKAFSKDQNIVGNARPKETDHKSLSSSPDKREKFKEQRKATVNVKKDK
EDKPLKTEKRPKQPDKEGKLICSEKGKVSEKSLPKNEKEDKENISENDREYSGDAQVDKK
PENDIVKSPQENLREPKRKRGRPPSIAPTAVDSNSQTLQPITLELRRRKISKGCEVPLKR
PRLDKNSSQEKSKNYSENTDKDLSRRRSSRLSTNGTHEILDPDLVVSDLVDTDPLQDTLS
STKESEEGQLKSALEAGQVSSALTCHSFGDGSGAAGLELNCPSMGENTMKTEPTSPLVEL
QEISTVEVTNTFKKTDDFGSSNAPAVDLDHKFRCKVVDCLKFFRKAKLLHYHMKYFHGME
KSLEPEESPGKRHVQTRGPSASDKPSQETLTRKRVSASSPTTKDKEKNKEKKFKEFVRVK
PKK
>ENSG00000116288|ENST00000338639
MASKRALVILAKGAEEMETVIPVDVMRRAGIKVTVAGLAGKDPVQCSRDVVICPDASLED
AKKEGPYDVVVLPGGNLGAQNLSESAAVKEILKEQENRKGLIAAICAGPTALLAHEIGFG
SKVTTHPLAKDKMMNGGHYTYSENRVEKDGLILTSRGPGTSFEFALAIVEALNGKEVAAQ
VKAPLVLKD*
>ENSG00000181638|ENST00000330701
MMEKPAGRKKKTPTPREEADVQKSALREEKVSGDRKPPERPTVPRKPRTEPCLSPEDEEH
VFDAFDASFKDDFEGVPVFIPFQRKKPYECSECGRIFKHKTDHIRHQRVHTGEKPFKCAQ
CGKAFRHSSDVTKHQRTHTGEKPFKCGECGKAFNCGSNLLKHQKTHTGEKPYECTHCGKA
FAYSSCLIRHQKRHPRKKP*
>ENSG00000204611|ENST00000330123
MATQGHLTFKDVAIEFSQEEWKCLEPVQKALYKDVMLENYRNLVFLGWRIMARSHLTATS
VSQVQAILLPQPPE*
>ENSG00000182568|ENST00000338745
MDHLNEATQGKEHSEMSNNVSDPKGPPAKIARLEQNGSPLGRGRLGSTGAKMQGVPLKHS
GHLMKTNLRKGTMLPVFCVVEHYENAIEYDCKEEHAEFVLVRKDMLFNQLIEMALLSLGY
SHSSAAQAKGLIQVGKWNPVPLSYVTDAPDATVADMLQDVYHVVTLKIQLHSCPKLEDLP
PEQWSHTTVRNALKDLLKDMNQSSLAKECPLSQSMISSIVNSTYYANVSAAKCQEFGRWY

KHFKKTKDMMVEMDSLSELSQQGANHVNFGQQPVPGNTAEQPPSPAQLSHGSQPSVRTPL
PNLHPGLVSTPISPQLVNQQLVMAQLLNQQYAVNRLLAQQSLNQQYLNHPPPVSRSMNKP
LEQQVSTNTEVSSEIYQWVRDELKRAGISQAVFARVAFNRTQGLLSEILRKEEDPKTASQ
SLLVNLRAMQNFLQLPEAERDRIYQDERERSLNAASAMGPAPLISTPPSRPPQVKTATIA
TERNGKPENNTMNINASIYDEIQQEMKRAKVSQALFAKVAATKSQGWLCELLRWKEDPSP
ENRTLWENLSMIRRFLSLPQPERDAIYEQESNAVHHHGDRPPHIIHVPAEQIQQQQQQQQ
QQQQQQQAPPPPQPQQQPQTGPRLPPRQPTVASPAESDEENRQKTRPRTKISVEALGILQ
SFIQDVGLYPDEEAIQTLSAQLDLPKYTIIKFFQNQRYYLKHHGKLKDNSGLEVDVAEYK
EEELLKDLEESVQDKNTNTLFSVKLEEELSVEGNTDINTDLKD*
>ENSG00000249709|ENST00000339282
MDSVASEDVAVNFTLEEWALLDPSQKKLYRDVMRETFRNLACVGKKWEDQSIEDWYKNQG
RILRNHMEEGLSESKEYDQCGEAFSQILNLNLNKKIPTIVRPCECSLCGKVFMHHSSLSR
HIRSHLGHKPYDYQEYGEKPYKCKQCGKAFSSCQSFRRHERTHTGEKPYACPECGKAFIS
LPSVRRHMIKHTGDGPYKCQECGKAFDRPSLFQIHERTHTGEKPYECQECAKAFISLPSF
QRHMIRHTGDGPYKCQECGKAFDRPSLFRIHERTHTGEKPHECKQCGKAFISFTNFQSHM
IRHTGDGPYKCKVCGRAFIFPSYVRKHERTHTGEKPYECNKCGKTFSSSSNVRTHERTHT
GEKPYECKECGKAFISLPSVRRHMIKHTGDGPYKCQVCGRAFDCPSSFQIHERTHTGEKP
YECQVCGKAFISLKRIRKHMILHTGDGPYKCQVCGKAFDCPSSVRTHERTHTGEKPYECK
ECGKAFNYASSIRIHERTHTGEKPYECKQCGKTFSYSSSFQRHERAHNGDKPYVKNVGKL
SFITQPSNTCENE*
>ENSG00000108312|ENST00000343638
MNGEADCPTDLEMAAPKGQDRWSQEDMLTLLECMKNNLPSNDSSKFKTTESHMDWEKVAF
KDFSGDMCKLKWVEISNEVRKFRTLTELILDAQEHVKNPYKGKKLKKHPDFPKKPLTPYF
RFFMEKRAKYAKLHPEMSNLDLTKILSKKYKELPEKKKMKYIQDFQREKQEFERNLARFR
EDHPDLIQNAKKSDIPEKPKTPQQLWYTHEKKVYLKVRPDEIMRDYIQKHPELNISEEGI
TKSTLTKAERQLKDKFDGRPTKPPPNSYSLYCAELMANMKDVPSTERMVLCSQQWKLLSQ
KEKDAYHKKCDQKKKDYEVELLRFLESLPEEEQQRVLGEEKMLNINKKQATSPASKKPAQ
EGGKGGSEKPKRPVSAMFIFSEEKRRQLQEERPELSESELTRLLARMWNDLSEKKKAKYK
AREAALKAQSERKPGGEREERGKLPESPKRAEEIWQQSVIGDYLARFKNDRVKALKAMEM
TWNNMEKKEKLMWIKKAAEDQKRYERELSEMRAPPAATNSSKKMKFQGEPKKPPMNGYQK
FSQELLSNGELNHLPLKERMVEIGSRWQRISQSQKEHYKKLAEEQQKQYKVHLDLWVKSL
SPQDRAAYKEYISNKRKSMTKLRGPNPKSSRTTLQSKSESEEDDEEDEDDEDEDEEEEDD
ENGDSSEDGGDSSESSSEDESEDGDENEEDDEDEDDDEDDDEDEDNESEGSSSSSSSSGD
SSDSDSN*
>ENSG00000101812|ENST00000355016
MAAASAMAEASSETTSEEGQSIQEPKEANSTKAQKQKRRGCRGSRRRHANRRGDSFGDSF
TPYFPRVLKQVHQGLSLSQEAVSVMDSMIHDILDRIATEAGQLAHYTKRVTITSRDIQMA
VRLLLPGKMGKLAEAQGTNAALRTSLCAIWQQRK*
>ENSG00000125457|ENST00000325102
MGEPSREEYKIQSFDAETQQLLKTALKDPGAVDLEKVANVIVDHSLQDCVFSKEAGRMCY
AIIQAESKQAGQSVFRRGLLNRLQQEYQAREQLRARSLQGWVCYVTFICNIFDYLRVNNM
PMMALVNPVYDCLFRLAQPDSLSKEEEVDCLVLQLHRVGEQLEKMNGQRMDELFVLIRDG
FLLPTGLSSLAQLLLLEIIEFRAAGWKTTPAAHKYYYSEVSD*
>ENSG00000009950|ENST00000354613

MAGALAGLAAGLQVPRVAPSPDSDSDTDSEDPSLRRSAGGLLRSQVIHSGHFMVSSPHSD
SLPRRRDQEGSVGPSDFGPRSIDPTLTRLFECLSLAYSGKLVSPKWKNFKGLKLLCRDKI
RLNNAIWRAWYIQYVKRRKSPVCGFVTPLQGPEADAHRKPEAVVLEGNYWKRRIEVVMRE
YHKWRIYYKKRLRKPSREDDLLAPKQAEGRWPPPEQWCKQLFSSVVPVLLGDPEEEPGGR
QLLDLNCFLSDISDTLFTMTQSGPSPLQLPPEDAYVGNADMIQPDLTPLQPSLDDFMDIS
DFFTNSRLPQPPMPSNFPEPPSFSPVVDSLFSSGTLGPEVPPASSAMTHLSGHSRLQARN
SCPGPLDSSAFLSSDFLLPEDPKPRLPPPPVPPPLLHYPPPAKVPGLEPCPPPPFPPMAP
PTALLQEEPLFSPRFPFPTVPPAPGVSPLPAPAAFPPTPQSVPSPAPTPFPIELLPLGYS
EPAFGPCFSMPRGKPPAPSPRGQKASPPTLAPATASPPTTAGSNNPCLTQLLTAAKPEQA
LEPPLVSSTLLRSPGSPQETVPEFPCTFLPPTPAPTPPRPPPGPATLAPSRPLLVPKAER
LSPPAPSGSERRLSGDLSSMPGPGTLSVRVSPPQPILSRGRPDSNKNRRITHISAEQKRR
FNIKLGFDTLHGLVSTLSAQPSLKERAGLQEEAQQLRDEIEELNAAINLCQQQLPATGVP
ITHQRFDQMRDMFDDYVRTRTLHNWKFWVFSILIRPLFESFNGMVSTASVHTLRQTSLAW
LDQYCSLPALRPTVLNSLRQLGTSTSILTDPGRIPEQATRAVTEGTLGKPL*
>ENSG00000178209|ENST00000322810
MVAGMLMPRDQLRAIYEVLFREGVMVAKKDRRPRSLHPHVPGVTNLQVMRAMASLRARGL
VRETFAWCHFYWYLTNEGIAHLRQYLHLPPEIVPASLQRVRRPVAMVMPARRTPHVQAVQ
GPLGSPPKRGPLPTEEQRVYRRKELEEVSPETPVVPATTQRTLARPGPEPAPATDERDRV
QKKTFTKWVNKHLIKAQRHISDLYEDLRDGHNLISLLEVLSGDSLPREKGRMRFHKLQNV
QIALDYLRHRQVKLVNIRNDDIADGNPKLTLGLIWTIILHFQISDIQVSGQSEDMTAKEK
LLLWSQRMVEGYQGLRCDNFTSSWRDGRLFNAIIHRHKPLLIDMNKVYRQTNLENLDQAF
SVAERDLGVTRLLDPEDVDVPQPDEKSIITYVSSLYDAMPRVPDVQDGVRANELQLRWQE
YRELVLLLLQWMRHHTAAFEERRFPSSFEEIEILWSQFLKFKEMELPAKEADKNRSKGIY
QSLEGAVQAGQLKVPPGYHPLDVEKEWGKLHVAILEREKQLRSEFERLECLQRIVTKLQM
EAGLCEEQLNQADALLQSDVRLLAAGKVPQRAGEVERDLDKADSMIRLLFNDVQTLKDGR
HPQGEQMYRRVYRLHERLVAIRTEYNLRLKAGVAAPATQVAQVTLQSVQRRPELEDSTLR
YLQDLLAWVEENQHRVDGAEWGVDLPSVEAQLGSHRGLHQSIEEFRAKIERARSDEGQLS
PATRGAYRDCLGRLDLQYAKLLNSSKARLRSLESLHSFVAAATKELMWLNEKEEEEVGFD
WSDRNTNMTAKKESYSALMRELELKEKKIKELQNAGDRLLREDHPARPTVESFQAALQTQ
WSWMLQLCCCIEAHLKENAAYFQFFSDVREAEGQLQKLQEALRRKYSCDRSATVTRLEDL
LQDAQDEKEQLNEYKGHLSGLAKRAKAVVQLKPRHPAHPMRGRLPLLAVCDYKQVEVTVH
KGDECQLVGPAQPSHWKVLSSSGSEAAVPSVCFLVPPPNQEAQEAVTRLEAQHQALVTLW
HQLHVDMKSLLAWQSLRRDVQLIRSWSLATFRTLKPEEQRQALHSLELHYQAFLRDSQDA
GGFGPEDRLMAEREYGSCSHHYQQLLQSLEQGAQEESRCQRCISELKDIRLQLEACETRT
VHRLRLPLDKEPARECAQRIAEQQKAQAEVEGLGKGVARLSAEAEKVLALPEPSPAAPTL
RSELELTLGKLEQVRSLSAIYLEKLKTISLVIRGTQGAEEVLRAHEEQLKEAQAVPATLP
ELEATKASLKKLRAQAEAQQPTFDALRDELRGAQEVGERLQQRHGERDVEVERWRERVAQ
LLERWQAVLAQTDVRQRELEQLGRQLRYYRESADPLGAWLQDARRRQEQIQAMPLADSQA
VREQLRQEQALLEEIERHGEKVEECQRFAKQYINAIKDYELQLVTYKAQLEPVASPAKKP
KVQSGSESVIQEYVDLRTHYSELTTLTSQYIKFISETLRRMEEEERLAEQQRAEERERLA
EVEAALEKQRQLAEAHAQAKAQAEREAKELQQRMQEEVVRREEAAVDAQQQKRSIQEELQ
QLRQSSEAEIQAKARQAEAAERSRLRIEEEIRVVRLQLEATERQRGGAEGELQALRARAE
EAEAQKRQAQEEAERLRRQVQDESQRKRQAEVELASRVKAEAEAAREKQRALQALEELRL
QAEEAERRLRQAEVERARQVQVALETAQRSAEAELQSKRASFAEKTAQLERSLQEEHVAV

AQLREEAERRAQQQAEAERAREEAERELERWQLKANEALRLRLQAEEVAQQKSLAQAEAE
KQKEEAEREARRRGKAEEQAVRQRELAEQELEKQRQLAEGTAQQRLAAEQELIRLRAETE
QGEQQRQLLEEELARLQREAAAATQKRQELEAELAKVRAEMEVLLASKARAEEESRSTSE
KSKQRLEAEAGRFRELAEEAARLRALAEEAKRQRQLAEEDAARQRAEAERVLAEKLAAIG
EATRLKTEAEIALKEKEAENERLRRLAEDEAFQRRRLEEQAAQHKADIEERLAQLRKASD
SELERQKGLVEDTLRQRRQVEEEILALKASFEKAAAGKAELELELGRIRSNAEDTLRSKE
QAELEAARQRQLAAEEERRRREAEERVQKSLAAEEEAARQRKAALEEVERLKAKVEEARR
LRERAEQESARQLQLAQEAAQKRLQAEEKAHAFAVQQKEQELQQTLQQEQSVLDQLRGEA
EAARRAAEEAEEARVQAEREAAQSRRQVEEAERLKQSAEEQAQARAQAQAAAEKLRKEAE
QEAARRAQAEQAALRQKQAADAEMEKHKKFAEQTLRQKAQVEQELTTLRLQLEETDHQKN
LLDEELQRLKAEATEAARQRSQVEEELFSVRVQMEELSKLKARIEAENRALILRDKDNTQ
RFLQEEAEKMKQVAEEAARLSVAAQEAARLRQLAEEDLAQQRALAEKMLKEKMQAVQEAT
RLKAEAELLQQQKELAQEQARRLQEDKEQMAQQLAEETQGFQRTLEAERQRQLEMSAEAE
RLKLRVAEMSRAQARAEEDAQRFRKQAEEIGEKLHRTELATQEKVTLVQTLEIQRQQSDH
DAERLREAIAELEREKEKLQQEAKLLQLKSEEMQTVQQEQLLQETQALQQSFLSEKDSLL
QRERFIEQEKAKLEQLFQDEVAKAQQLREEQQRQQQMEQERQRLVASMEEARRRQHEAE
EGVRRKQEELQQLEQQRRQQEELLAEENQRLREQLQLLEEQHRAALAHSEEVTASQVAAT
KTLPNGRDALDGPAAEAEPEHSFDGLRRKVSAQRLQEAGILSAEELQRLAQGHTTVDELA
RREDVRHYLQGRSSIAGLLLKATNEKLSVYAALQRQLLSPGTALILLEAQAASGFLLDPV
RNRRLTVNEAVKEGVVGPELHHKLLSAERAVTGYKDPYTGQQISLFQAMQKGLIVREHGI
RLLEAQIATGGVIDPVHSHRVPVDVAYRRGYFDEEMNRVLADPSDDTKGFFDPNTHENLT
YLQLLERCVEDPETGLCLLPLTDKAAKGGELVYTDSEARDVFEKATVSAPFGKFQGKTVT
IWEIINSEYFTAEQRRDLLRQFRTGRITVEKIIKIIITVVEEQEQKGRLCFEGLRSLVPA
AELLESRVIDRELYQQLQRGERSVRDVAEVDTVRRALRGANVIAGVWLEEAGQKLSIYNA
LKKDLLPSDMAVALLEAQAGTGHIIDPATSARLTVDEAVRAGLVGPEFHEKLLSAEKAVT
GYRDPYTGQSVSLFQALKKGLIPREQGLRLLDAQLSTGGIVDPSKSHRVPLDVACARGCL
DEETSRALSAPRADAKAYSDPSTGEPATYGELQQRCRPDQLTGLSLLPLSEKAARARQEE
LYSELQARETFEKTPVEVPVGGFKGRTVTVWELISSEYFTAEQRQELLRQFRTGKVTVEK
VIKILITIVEEVETLRQERLSFSGLRAPVPASELLASGVLSRAQFEQLKDGKTTVKDLSE
LGSVRTLLQGSGCLAGIYLEDTKEKVSIYEAMRRGLLRATTAALLLEAQAATGFLVDPVR
NQRLYVHEAVKAGVVGPELHEQLLSAEKAVTGYRDPYSGSTISLFQAMQKGLVLRQHGIR
LLEAQIATGGIIDPVHSHRVPVDVAYQRGYFSEEMNRVLADPSDDTKGFFDPNTHENLTY
RQLLERCVEDPETGLRLLPLKGAEKAEVVETTQVYTEEETRRAFEETQIDIPGGGSHGGS
TMSLWEVMQSDLIPEEQRAQLMADFQAGRVTKERMIIIIIEIIEKTEIIRQQGLASYDYV
RRRLTAEDLFEARIISLETYNLLREGTRSLREALEAESAWCYLYGTGSVAGVYLPGSRQT
LSIYQALKKGLLSAEVARLLLEAQAATGFLLDPVKGERLTVDEAVRKGLVGPELHDRLLS
AERAVTGYRDPYTEQTISLFQAMKKELIPTEEALRLLDAQLATGGIVDPRLGFHLPLEVA
YQRGYLNKDTHDQLSEPSEVRSYVDPSTDERLSYTQLLRRCRRDDGTGQLLLPLSDARKL
TFRGLRKQITMEELVRSQVMDEATALQLREGLTSIEEVTKNLQKFLEGTSCIAGVFVDAT
KERLSVYQAMKKGIIRPGTAFELLEAQAATGYVIDPIKGLKLTVEEAVRMGIVGPEFKDK
LLSAERAVTGYKDPYSGKLISLFQAMKKGLILKDHGIRLLEAQIATGGIIDPEESHRLPV
EVAYKRGLFDEEMNEILTDPSDDTKGFFDPNTEENLTYLQLMERCITDPQTGLCLLPLKE
KKRERKTSSKSSVRKRRVVIVDPETGKEMSVYEAYRKGLIDHQTYLELSEQECEWEEITI
SSSDGVVKSMIIDRRSGRQYDIDDAIAKNLIDRSALDQYRAGTLSITEFADMLSGNAGGF

RSRSSSVGSSSSYPISPAVSRTQLASWSDPTEETGPVAGILDTETLEKVSITEAMHRNLV
DNITGQRLLEAQACTGGIIDPSTGERFPVTDAVNKGLVDKIMVDRINLAQKAFCGFEDPR
TKTKMSAAQALKKGWLYYEAGQRFLEVQYLTGGLIEPDTPGRVPLDEALQRGTVDARTAQ
KLRDVGAYSKYLTCPKTKLKISYKDALDRSMVEEGTGLRLLEAAAQSTKGYYSPYSVSGS
GSTAGSRTGSRTGSRAGSRRGSFDATGSGFSMTFSSSSYSSSGYGRRYASGSSASLGGPE
SAVA*
>ENSG00000049541|ENST00000352131
MEVEAVCGGAGEVEAQDSDPAPAFSKAPGSAGHYELPWVEKYRPVKLNEIVGNEDTVSRL
EVFAREGNVPNIIIAGPPGTGKTTSILCLARALLGPALKDAMLELNASNDSMTDGAQQAL
RRTMEIYSKTTRFALACNASDKIIEPIQSRCAVLRYTKLTDAQILTRLMNVIEKERVPYT
DDGLEAIIFTAQGDMRQALNNLQSTFSGFGFINSENVFKVCDEPHPLLVKEMIQHCVNAN
IDEAYKILAHLWHLGYSPEDIIGNIFRVCKTFQMAEYLKLEFIKEIGYTHMKIAEGVNSL
LQMAGLLARLCQKTMAPVAS*
>ENSG00000251369|ENST00000344222
XDRAQVHTREPTTYPPVLSERAFLRGSLTLESSTSSDSRLGRARDEEGLLEMQKGKVTPE
TDLHKETHLGKVSLEGEGLGTDDGLHSRALQEWLSADVLHECDSQQPGKDALIHAGTNPY
KCKQCGKGFNRKWYLVRHQRVHTGMKPYECNACGKAFSQSSTLIRHYLIHTGEKPYKCLE
CGKAFKRRSYLMQHHPIHTGEKPYECSQCRKAFTHRSTFIRHNRTHTGEKPFECKECEKA
FSNRAHLIQHYIIHTGEKPYDCMACGKAFRCSSELIQHQRIHTGEKPYECTQCGKAFHRS
TYLIQHSVIHTGEMPYKCIECGKAFKRRSHLLQHQRVHT*
>ENSG00000184270|ENST00000331128
MSGRGKQGGKARAKAKSRSSRAGLQFPVGRVHRLLRKGNYAERVGAGAPVYLAAVLEYLT
AEILELAGNAARDNKKTRIIPRHLQLAVRNDEELNKLLGGVTIAQGGVLPNIQAVLLPKK
TESHKPGKNK*
>ENSG00000196152|ENST00000342483
MLEEGVLPSPGPALPQEENTGEEGMAAGLLTAGPRGSTFFSSVTVAFAQERWRCLVSTPR
DRFKEGIPGKSRSLVLLGLPVSQPGMNSQLEQREGAWMLEGEDLRSPSPGWKIISGSPPE
QALSEASFQDPCVEMPPGDSDHGTSDLEKSFNLRPVLSPQQRVPVEARPRKCETHTESFK
NSEILKPHRAKPYACNECGKAFSYCSSLSQHQKSHTGEKPYECSECGKAFSQSSSLIQHQ
RIHTGEKPYKCSECGRAFSQNANLTKHQRTHTGEKPYRCSECEKAFSDCSALVQHQRIHT
GEKPYECSDCGKAFRHSANLTNHQRTHTGEKPYKCSECGKAFSYCAAFIQHQRIHTGEKP
YRCAACGKAFSQSANLTNHQRTHTGEKPYKCSECGKAFSQSTNLIIHQKTHTGEKPYKCN
ECGKFFSESSALIRHHIIHTGEKPYECNECGKAFNQSSSLSQHQRIHTGVKPYECSECGK
AFRCSSAFVRHQRLHAGE*
>ENSG00000167491|ENST00000360315
MTEEACRTRSQKRALERDPTEDDVESKKIKMERGLLASDLNTDGDMRVTPEPGAGPTQGL
LRATEATAMAMGRGEGLVGDGPVDMRTSHSDMKSERRPPSPDVIVLSDNEQPSSPRVNGL
TTVALKETSTEALMKSSPEERERMIKQLKEELRLEEAKLVLLKKLRQSQIQKEATAQKPT
GSVGSTVTTPPPLVRGTQNIPAGKPSLQTSSARMPGSVIPPPLVRGGQQASSKLGPQASS
QVVMPPLVRGAQQIHSIRQHSSTGPPPLLLAPRASVPSVQIQGQRIIQQGLIRVANVPNT
SLLVNIPQPTPASLKGTTATSAQANSTPTSVASVVTSAESPASRQAAAKLALRKQLEKTL
LEIPPPKPPAPEMNFLPSAANNEFIYLVGLEEVVQNLLETQGRMSAATVLSREPYMCAQC
KTDFTCRWREEKSGAIMCENCMTTNQKKALKVEHTSRLKAAFVKALQQEQEIEQRLLQQG
TAPAQAKAEPTAAPHPVLKQVIKPRRKLAFRSGEARDWSNGAVLQASSQLSRGSATTPRG

VLHTFSPSPKLQNSASATALVSRTGRHSERTVSAGKGSATSNWKKTPLSTGGTLAFVSPS
LAVHKSSSAVDRQREYLLDMIPPRSIPQSATWK*
>ENSG00000091127|ENST00000356362
MEMTEMTGVSLKRGALVVEDNDSGVPVEETKKQKLSECSLTKGQDGLQNDFLSISEDVPR
PPDTVSTGKGGKNSEAQLEDEEEEEEDGLSEECEEEESESFADMMKHGLTEADVGITKFV
SSHQGFSGILKERYSDFVVHEIGKDGRISHLNDLSIPVDEEDPSEDIFTVLTAEEKQRLE
ELQLFKNKETSVAIEVIEDTKEKRTIIHQAIKSLFPGLETKTEDREGKKYIVAYHAAGKK
ALANPRKHSWPKSRGSYCHFVLYKENKDTMDAINVLSKYLRVKPNIFSYMGTKDKRAITV
QEIAVLKITAQRLAHLNKCLMNFKLGNFSYQKNPLKLGELQGNHFTVVLRNITGTDDQVQ
QAMNSLKEIGFINYYGMQRFGTTAVPTYQVGRAILQNSWTEVMDLILKPRSGAEKGYLVK
CREEWAKTKDPTAALRKLPVKRCVEGQLLRGLSKYGMKNIVSAFGIIPRNNRLMYIHSYQ
SYVWNNMVSKRIEDYGLKPVPGDLVLKGATATYIEEDDVNNYSIHDVVMPLPGFDVIYPK
HKIQEAYREMLTADNLDIDNMRHKIRDYSLSGAYRKIIIRPQNVSWEVVAYDDPKIPLFN
TDVDNLEGKTPPVFASEGKYRALKMDFSLPPSTYATMAIREVLKMDTSIKNQTQLNTTWL
R*
>ENSG00000020922|ENST00000323977
MSTADALDDENTFKILVATDIHLGFMEKDAVRGNDTFVTLDEILRLAQENEVDFILLGGD
LFHENKPSRKTLHTCLELLRKYCMGDRPVQFEILSDQSVNFGFSKFPWVNYQDGNLNISI
PVFSIHGNHDDPTGADALCALDILSCAGFVNHFGRSMSVEKIDISPVLLQKGSTKIALYG
LGSIPDERLYRMFVNKKVTMLRPKEDENSWFNLFVIHQNRSKHGSTNFIPEQFLDDFIDL
VIWGHEHECKIAPTKNEQQLFYISQPGSSVVTSLSPGEAVKKHVGLLRIKGRKMNMHKIP
LHTVRQFFMEDIVLANHPDIFNPDNPKVTQAIQSFCLEKIEEMLENAERERLGNSHQPEK
PLVRLRVDYSGGFEPFSVLRFSQKFVDRVANPKDIIHFFRHREQKEKTGEEINFGKLITK
PSEGTTLRVEDLVKQYFQTAEKNVQLSLLTERGMGEAVQEFVDKEEKDAIEELVKYQLEK
TQRFLKERHIDALEDKIDEEVRRFRETRQKNTNEEDDEVREAMTRARALRSQSEESASAF
SADDLMSIDLAEQMANDSDDSISAATNKGRGRGRGRRGGRGQNSASRGGSQRGRAFKSTR
QQPSRNVTTKNYSEVIEVDESDVEEDIFPTTSKTDQRWSSTSSSKIMSQSQVSKGVDFES
SEDDDDDPFMNTSSLRRNRR*
>ENSG00000172273|ENST00000350777
MPPPGKVPRKENLWLQCEWGSCSFVCSTMEKFFEHVTQHLQQHLHGSGEEEEEEEEDDPL
EEEFSCLWQECGFCSLDSSADLIRHVYFHCYHTKLKQWGLQALQSQADLGPCILDFQSRN
VIPDIPDHFLCLWEHCENSFDNPEWFYRHVEAHSLCCEYEAVGKDNPVVLCGWKGCTCTF
KDRSKLREHLRSHTQEKVVACPTCGGMFANNTKFLDHIRRQTSLDQQHFQCSHCSKRFAT
ERLLRDHMRNHVNHYKCPLCDMTCPLPSSLRNHMRFRHSEDRPFKCDCCDYSCKNLIDLQ
KHLDTHSEEPAYRCDFENCTFSARSLCSIKSHYRKVHEGDSEPRYKCHVCDKCFTRGNNL
TVHLRKKHQFKWPSGHPRFRYKEHEDGYMRLQLVRYESVELTQQLLRQPQEGSGLGTSLN
ESSLQGIILETVPGEPGRKEEEEEGKGSEGTALSASQDNPSSVIHVVNQTNAQGQQEIVY
YVLSEAPGEPPPAPEPPSGGIMEKLQGIAEEPEIQMV*
>ENSG00000126767|ENST00000343894
MDPSVTLWQFLLQLLREQGNGHIISWTSRDGGEFKLVDAEEVARLWGLRKNKTNMNYDKL
SRALRYYYDKNIIRKVSGQKFVYKFVSYPESHCAP*
>ENSG00000143373|ENST00000324048
MGDMKTPDFDDLLAAFDIPDIDANEAIHSGPEENEGPGGPGKPEPGVGSESEDTAAASAG
DGPGVPAQASDHGLPPPDISVVSVIVKNTVCPEQSEALAGGSAGDGAQAAGVTKEGPVGP

HRMQNGFGSPEPSLPGTPHSPAPPSGGTWKEKGMEGKTPLDLFAHFGPEPGDHSDPLPPS
APSPTREGALTPPPFPSSFELAQENGPGMQPPVSSPPLGALKQESCSPHHPQVLAQQGSG
SSPKATDIPASASPPPVAGVPFFKQSPGHQSPLASPKVPVCQPLKEEDDDEGPVDKSSPG
SPQSPSSGAEAADEDSNDSPASSSSRPLKVRIKTIKTSCGNITRTVTQVPSDPDPPAPLA
EGAFLAEASLLKLSPATPTSEGPKVVSVQLGDGTRLKGTVLPVATIQNASTAMLMAASVA
RKAVVLPGGTATSPKMIAKNVLGLVPQALPKADGRAGLGTGGQKVNGASVVMVQPSKTAT
GPSTGGGTVISRTQSSLVEAFNKILNSKNLLPAYRPNLSPPAEAGLALPPTGYRCLECGD
AFSLEKSLARHYDRRSMRIEVTCNHCARRLVFFNKCSLLLHAREHKDKGLVMQCSHLVMR
PVALDQMVGQPDITPLLPVAVPPVSGPLALPALGKGEGAITSSAITTVAAEAPVLPLSTE
PPAAPATSAYTCFRCLECKEQCRDKAGMAAHFQQLGPPAPGATSNVCPTCPMMLPNRCSF
SAHQRMHKNRPPHVCPECGGNFLQANFQTHLREACLHVSRRVGYRCPSCSVVFGGVNSIK
SHIQTSHCEVFHKCPICPMAFKSGPSAHAHLYSQHPSFQTQQAKLIYKCAMCDTVFTHKP
LLSSHFDQHLLPQRVSVFKCPSCPLLFAQKRTMLEHLKNTHQSGRLEETAGKGAGGALLT
PKTEPEELAVSQGGAAPATEESSSSSEEEEVPSSPEPPRPAKRPRRELGSKGLKGGGGGP
GGWTCGLCHSWFPERDEYVAHMKKEHGKSVKKFPCRLCERSFCSAPSLRRHVRVNHEGIK
RVYPCRYCTEGKRTFSSRLILEKHVQVRHGLQLGAQSPGRGTTLARGSSARAQGPGRKRR
QSSDSCSEEPDSTTPPAKSPRGGPGSGGHGPLRYRSSSSTEQSLMMGLRVEDGAQQCLDC
GLCFASPGSLSRHRFISHKKRRGVGKASALGLGDGEEEAPPSRSDPDGGDSPLPASGGPL
TCKVCGKSCDSPLNLKTHFRTHGMAFIRARQGAVGDN*
>ENSG00000055917|ENST00000361078
MSQPIMVQRRSGQGFHGNSEVNAILSPRSESGGLGVSMVEYVLSSSPADKLDSRFRKGNF
GTRDAETDGPEKGDQKGKASPFEEDQNRDLKQGDDDDSKINGRGLPNGMDADCKDFNRTP
GSRQASPTEVVERLGPNTNPSEGLGPLPNPTANKPLVEEFSNPETQNLDAMEQVGLESLQ
FDYPGNQVPMDSSGATVGLFDYNSQQQLFQRTNALTVQQLTAAQQQQYALAAAQQPHIAG
VFSAGLAPAAFVPNPYIISAAPPGTDPYTAAGLAAAATLAGPAVVPPQYYGVPWGVYPAN
LFQQQAAAAANNTASQQAASQAQPGQQQVLRAGAGQRPLTPNQGQQGQQAESLAAAAAAN
PTLAFGQGLATGMPGYQVLAPTAYYDQTGALVVGPGARTGLGAPVRLMAPTPVLISSAAA
QAAAAAAAGGTASSLTGSTNGLFRPIGTQPPQQQQQQPSTNLQSNSFYGSSSLTNSSQSS
SLFSHGPGQPGSTSLGFGSGNSLGAAIGSALSGFGSSVGSSASSSATRRESLSTSSDLYK
RSSSSLAPIGQPFYNSLGFSSSPSPIGMPLPSQTPGHSLTPPPSLSSHGSSSSLHLGGLT
NGSGRYISAAPGAEAKYRSASSTSSLFSSSSQLFPPSRLRYNRSDIMPSGRSRLLEDFRN
NRFPNLQLRDLIGHIVEFSQDQHGSRFIQQKLERATPAERQMVFNEILQAAYQLMTDVFG
NYVIQKFFEFGSLDQKLALATRIRGHVLPLALQMYGCRVIQKALESISSDQQSEMVKELD
GHVLKCVKDQNGNHVVQKCIECVQPQSLQFIIDAFKGQVFVLSTHPYGCRVIQRILEHCT
AEQTLPILEELHQHTEQLVQDQYGNYVIQHVLEHGRPEDKSKIVSEIRGKVLALSQHKFA
SNVVEKCVTHASRAERALLIDEVCCQNDGPHSALYTMMKDQYANYVVQKMIDMAEPAQRK
IIMHKIRPHITTLRKYTYGKHILAKLEKYYLKNSPDLGPIGGPPNGML*
>ENSG00000115053|ENST00000356936
LPYKVTQDELKEVFEDAAEIRLVSKDGKSKGIAYIEFKTEADAEKTFEEKQGTEIDGRSI
SLYYTGEKGQNQDYRGGKNSTWSDDSRKTDQSSAYVRTTSRTLQHVLGESKTLVLSNLSY
SATEETLQEVFEKATFIKVPQNQNGKSKGYAFIEFASFEDAKEALNSCNKREIEGRAIRL
ELQGPRGSPNARSQPSKTLFVKGLSEDTTEETLKESFDGSVRARIVTDRETGSSKGFGFV
DFNSEEDAKAAKEAMEDGEIDGNKVTLDWAKPKGEGGFGGRGGGRGGFGGRGGGRGG
>ENSG00000178053|ENST00000355893

MFRMLNSSFEDDPFFSESILAHRENMRQMIRSFSEPFGRDLLSISDGRGRAHNRRGHNDG
EDSLTHTDVSSFQTMDQMVSNMRNYMQKLERNFGQLSVDPNGHSFCSSSVMTYSKIGDEP
PKVFQASTQTRRAPGGIKETRKAMRDSDSGLEKMAIGHHIHDRAHVIKKSKNKKTGDEEV
NQEFINMNESDAHAFDEEWQSEVLKYKPGRHNLGNTRMRSVGHENPGSRELKRREKPQQS
PAIEHGRRSNVLGDKLHIKGSSVKSNKK*
>ENSG00000198551|ENST00000361113
MDSVAFEDVAVNFTLEEWALLDPSQKNLYRDVMRETFRNLASVGKQWEDQNIEDPFKIPR
RNISHIPERLCESKEGGQGEETFSQIPDGILNKKTPGVKPCESSVCGEVGMGPSSLNRHI
RDHTGREPNEYQEYGKKSYTRNQCGRALSYHRSFPVRERTHPGGKPYDCKECGETFISLV
SIRRHMLTHRGGVPYKCKVCGKAFDYPSLFRIHERSHTGEKPYECKQCGKAFSCSSYIRI
HERTHTGDKPYECKQCGKAFSCSKYIRIHERTHTGEKPYECKQCGKAFRCASSVRSHERT
HTGEKLFECKECGKALTCLASVRRHMIKHTGNGPYKCKVCGKAFDFPSSFRIHERTHTGE
KPYDCKQCGKAFSCSSSFRKHERIHTGEKPYKCTKCGKAFSRSSYFRIHERTHTGEKPYE
CKQCGKAFSRSTYFRVHEKIHTGEKPYENPNPNASVVPVLS*
>ENSG00000150991|ENST00000339647
MQIFVKTLTGKTITLEVEPSDTIENVKAKIQDKEGIPPDQQRLIFAGKQLEDGRTLSDYN
IQKESTLHLVLRLRGGMQIFVKTLTGKTITLEVEPSDTIENVKAKIQDKEGIPPDQQRLI
FAGKQLEDGRTLSDYNIQKESTLHLVLRLRGGMQIFVKTLTGKTITLEVEPSDTIENVKA
KIQDKEGIPPDQQRLIFAGKQLEDGRTLSDYNIQKESTLHLVLRLRGGMQIFVKTLTGKT
ITLEVEPSDTIENVKAKIQDKEGIPPDQQRLIFAGKQLEDGRTLSDYNIQKESTLHLVLR
LRGGMQIFVKTLTGKTITLEVEPSDTIENVKAKIQDKEGIPPDQQRLIFAGKQLEDGRTL
SDYNIQKESTLHLVLRLRGGMQIFVKTLTGKTITLEVEPSDTIENVKAKIQDKEGIPPDQ
QRLIFAGKQLEDGRTLSDYNIQKESTLHLVLRLRGGMQIFVKTLTGKTITLEVEPSDTIE
NVKAKIQDKEGIPPDQQRLIFAGKQLEDGRTLSDYNIQKESTLHLVLRLRGGMQIFVKTL
TGKTITLEVEPSDTIENVKAKIQDKEGIPPDQQRLIFAGKQLEDGRTLSDYNIQKESTLH
LVLRLRGGMQIFVKTLTGKTITLEVEPSDTIENVKAKIQDKEGIPPDQQRLIFAGKQLED
GRTLSDYNIQKESTLHLVLRLRGGV*
>ENSG00000138346|ENST00000358410
MEQLNELELLMEKSFWEEAELPAELFQKKVVASFPRTVLSTGMDNRYLVLAVNTVQNKEG
NCEKRLVITASQSLENKELCILRNDWCSVPVEPGDIIHLEGDCTSDTWIIDKDFGYLILY
PDMLISGTSIASSIRCMRRAVLSETFRSSDPATRQMLIGTVLHEVFQKAINNSFAPEKLQ
ELAFQTIQEIRHLKEMYRLNLSQDEIKQEVEDYLPSFCKWAGDFMHKNTSTDFPQMQLSL
PSDNSKDNSTCNIEVVKPMDIEESIWSPRFGLKGKIDVTVGVKIHRGYKTKYKIMPLELK
TGKESNSIEHRSQVVLYTLLSQERRADPEAGLLLYLKTGQMYPVPANHLDKRELLKLRNQ
MAFSLFHRISKSATRQKTQLASLPQIIEEEKTCKYCSQIGNCALYSRAVEQQMDCSSVPI
VMLPKIEEETQHLKQTHLEYFSLWCLMLTLESQSKDNKKNHQNIWLMPASEMEKSGSCIG
NLIRMEHVKIVCDGQYLHNFQCKHGAIPVTNLMAGDRVIVSGEERSLFALSRGYVKEINM
TTVTCLLDRNLSVLPESTLFRLDQEEKNCDIDTPLGNLSKLMENTFVSKKLRDLIIDFRE
PQFISYLSSVLPHDAKDTVACILKGLNKPQRQAMKKVLLSKDYTLIVGMPGTGKTTTICT
LVRILYACGFSVLLTSYTHSAVDNILLKLAKFKIGFLRLGQIQKVHPAIQQFTEQEICRS
KSIKSLALLEELYNSQLIVATTCMGINHPIFSRKIFDFCIVDEASQISQPICLGPLFFSR
RFVLVGDHQQLPPLVLNREARALGMSESLFKRLEQNKSAVVQLTVQYRMNSKIMSLSNKL
TYEGKLECGSDKVANAVINLRHFKDVKLELEFYADYSDNPWLMGVFEPNNPVCFLNTDKV
PAPEQVEKGGVSNVTEAKLIVFLTSIFVKAGCSPSDIGIIAPYRQQLKIINDLLARSIGM

VEVNTVDKYQGRDKSIVLVSFVRSNKDGTVGELLKDWRRLNVAITRAKHKLILLGCVPSL
NCYPPLEKLLNHLNSEKLIIDLPSREHESLCHILGDFQRE*
>ENSG00000185811|ENST00000349824
MDADEGQDMSQVSGKESPPVSDTPDEGDEPMPIPEDLSTTSGGQQSSKSDRVVASNVKVE
TQSDEENGRACEMNGEECAEDLRMLDASGEKMNGSHRDQGSSALSGVGGIRLPNGKLKCD
ICGIICIGPNVLMVHKRSHTGDKGLSDTPYDSSASYEKENEMMKSHVMDQAINNAINYLG
AESLRPLVQTPPGGSEVVPVISPMYQLHKPLAEGTPRSNHSAQDSAVENLLLLSKAKLVP
SEREASPSNSCQDSTDTESNNEEQRSGLIYLTNHIAPHARNGLSLKEEHRAYDLLRAASE
NSQDALRVVSTSGEQMKVYKCEHCRVLFLDHVMYTIHMGCHGFRDPFECNMCGYHSQDRY
EFSSHITRGEHRFHMS*
>ENSG00000144355|ENST00000361725
MTMTTMPESLNSPVSGKAVFMEFGPPNQQMSPSPMSHGHYSMHCLHSAGHSQPDGAYSSA
SSFSRPLGYPYVNSVSSHASSPYISSVQSYPGSASLAQSRLEDPGADSEKSTVVEGGEVR
FNGKGKKIRKPRTIYSSLQLQALNRRFQQTQYLALPERAELAASLGLTQTQVKIWFQNKR
SKFKKLMKQGGAALEGSALANGRALSAGSPPVPPGWNPNSSSGKGSGGNAGSYIPSYTSW
YPSAHQEAMQQPQLM*
>ENSG00000187742|ENST00000339901
MVRVLRSMCLPQLCSHILSVCSGTTSDRNVYSVPGSQYLYNQPSCYRGFQTVKHRNENTC
PLPQEMKALFKKKTYDEKKTYDQQKFDSERADGTISSEIKSARGSHHLSIYAENSLKSDG
YHKRTDRKSRIIAKNVSTSKPEFEFTTLDFPELQGAENNMSEIQKQPKWGPVHSVSTDIS
LLREVVKPAAVLSKGEIVVKNNPNESVTANAATNSPSCTRELSWTPMGYVVRQTLSTELS
AAPKNVTSMINLKTIASSADPKNVSIPSSEALSSDPSYNKEKHIIHPTQKSKASQGSDLE
QNEASRKNKKKKEKSTSKYEVLTVQEPPRIEDAEEFPNLAVASERRDRIETPKFQSKQQP
QDNFKNNVKKSQLPVQLDLGGMLTALEKKQHSQHAKQSSKPVVVSVGAVPVLSKECASGE
RGRRMSQMKTPHNPLDSSAPLMKKGKQREIPKAKKPTSLKKIILKERQERKQRLQENAVS
PAFTSDDTQDGESGGDDQFPEQAELSGPEGMDELISTPSVEDKSEEPPGTELQRDTEASH
LAPNHTTFPKIHSRRFRDYCSQMLSKEVDACVTDLLKELVRFQDRMYQKDPVKAKTKRRL
VLGLREVLKHLKLKKLKCVIISPNCEKIQSKGGLDDTLHTIIDYACEQNIPFVFALNRKA
LGRSLNKAVPVSVVGIFSYDGAQDQFHKMVELTVAARQAYKTMLENVQQELVGEPRPQAP
PSLPTQGPSCPAEDGPPALKEKEEPHYIEIWKKHLEAYSGCTLELEESLEASTSQMMNLN
L*
>ENSG00000121864|ENST00000326361
MNEYPKKRKRKTLHPSRYSDSSGISRIADGFNGIFSDHCYSVCSMRQPDLKYFDNKDDDS
DTETSNDLPKFADGIKARNRNQNYLVPSPVLRILDHTAFSTEKSADIVICDEECDSPESV
NQQTQEESPIEVHTAEDVPIAVEVHAISEDYDIETENNSSESLQDQTDEEPPAKLCKILD
KSQALNVTAQQKWPLLRANSSGLYKCELCEFNSKYFSDLKQHMILKHKRTDSNVCRVCKE
SFSTNMLLIEHAKLHEEDPYICKYCDYKTVIFENLSQHIADTHFSDHLYWCEQCDVQFSS
SSELYLHFQEHSCDEQYLCQFCEHETNDPEDLHSHVVNEHACKLIELSDKYNNGEHGQYS
LLSKITFDKCKNFFVCQVCGFRSRLHTNVNRHVAIEHTKIFPHVCDDCGKGFSSMLEYCK
HLNSHLSEGIYLCQYCEYSTGQIEDLKIHLDFKHSADLPHKCSDCLMRFGNERELISHLP
VHETT*
>ENSG00000136870|ENST00000339664
MASPSPPPESKGLLTFEDVAVFFTQEEWDYLDPAQRSLYKDVMMENYGNLVSLDVLNRDK
DEEPTVKQEIEEIEEEVEPQGVIVTRIKSEIDQDPMGRETFELVGRLDKQRGIFLWEIPR

ESLTQEQRMFRENTNIIRKRPNSEEKCHKCEECGKGFVRKAHFIQHQRVHTGEKPFQCNE
CGKSFSRSSFVIEHQRIHTGERPYECNYCGKTFSVSSTLIRHQRIHTGERPYQCNQCKQS
FSQRRSLVKHQRIHTGEKPHKCSDCGKAFSWKSHLIEHQRTHTGEKPYHCTKCKKSFSRN
SLLVEHQRIHTGERPHKCGECGKAFRLSTYLIQHQKIHTGEKPFLCIECGKSFSRSSFLI
EHQRIHTGERPYQCKECGKSFSQLCNLTRHQRIHTGDKPHKCEECGKAFSRSSGLIQHQR
IHTREKTYPYNETKESFDPNCSLVIQQEVYPKEKSYKCDECGKTFSVSAHLVQHQRIHTG
EKPYLCTVCGKSFSRSSFLIEHQRIHTGERPYLCRQCGKSFSQLCNLIRHQGVHTGNKPH
KCDECGKAFSRNSGLIQHQRIHTGEKPYKCEKCDKSFSQQRSLVNHQKIHAEVKTQETHE
CDACGEAFNCRISLIQHQKLHTAWMQ*
>ENSG00000183479|ENST00000338525
MSEAPRAETFVFLDLEATGLPSVEPEIAELSLFAVHRSSLENPEHDESGALVLPRVLDKL
TLCMCPERPFTAKASEITGLSSEGLARCRKAGFDGAVVRTLQAFLSRQAGPICLVAHNGF
DYDFPLLCAELRRLGARLPRDTVCLDTLPALRGLDRAHSHGTRARGRQGYSLGSLFHRYF
RAEPSAAHSAEGDVHTLLLIFLHRAAELLAWADEQARGWAHIEPMYLPPDDPSLEA*
>ENSG00000128604|ENST00000357234
MNQSIPVAPTPPRRVRLKPWLVAQVNSCQYPGLQWVNGEKKLFCIPWRHATRHGPSQDGD
NTIFKAWAKETGKYTEGVDEADPAKWKANLRCALNKSRDFRLIYDGPRDMPPQPYKIYEV
CSNGPAPTDSQPPEDYSFGAGEEEEEEEELQRMLPSLSLTDAVQSGPHMTPYSLLKEDVK
WPPTLQPPTLRPPTLQPPTLQPPVVLGPPAPDPSPLAPPPGNPAGFRELLSEVLEPGPLP
ASLPPAGEQLLPDLLISPHMLPLTDLEIKFQYRGRPPRALTISNPHGCRLFYSQLEATQE
QVELFGPISLEQVRFPSPEDIPSDKQRFYTNQLLDVLDRGLILQLQGQDLYAIRLCQCKV
FWSGPCASAHDSCPNPIQREVKTKLFSLEHFLNELILFQKGQTNTPPPFEIFFCFGEEWP
DRKPREKKLITVQVVPVAARLLLEMFSGELSWSADSIRLQISNPDLKDRMVEQFKELHHI
WQSQQRLQPVAQAPPGAGLGVGQGPWPMHPAGMQ*
>ENSG00000176170|ENST00000323374
MSAQVLGFLRSWTPLPLAAPRGPAAAGNDAGAPAATAPGGEGEPHSRPCDARLGSTDKEL
KAGAAATGSAPTAPGTPWQREPRVEVMDPAGGPRGVLPRPCRVLVLLNPRGGKGKALQLF
RSHVQPLLAEAEISFTLMLTERRNHARELVRSEELGRWDALVVMSGDGLMHEVVNGLMER
PDWETAIQKPLCSLPAGSGNALAASLNHYAGYEQVTNEDLLTNCTLLLCRRLLSPMNLLS
LHTASGLRLFSVLSLAWGFIADVDLESEKYRRLGEMRFTLGTFLRLAALRTYRGRLAYLP
VGRVGSKTPASPVVVQQGPVDAHLVPLEEPVPSHWTVVPDEDFVLVLALLHSHLGSEMFA
APMGRCAAGVMHLFYVRAGVSRAMLLRLFLAMEKGRHMEYECPYLVYVPVVAFRLEPKDG
KGVFAVDGELMVSEAVQGQVHPNYFWMVSGCVEPPPSWKPQQMPPPEEPL*
>ENSG00000022840|ENST00000325954
MPLSSPNAAATASDMDKNSGSNSSSASSGSSKGQQPPRSASAGPAGESKPKSDGKNSSGS
KRYNRKRELSYPKNESFNNQSRRSSSQKSKTFNKMPPQRGGGSSKLFSSSFNGGRRDEVA
EAQRAEFSPAQFSGPKKINLNHLLNFTFEPRGQTGHFEGSGHGSWGKRNKWGHKPFNKEL
FLQANCQFVVSEDQDYTAHFADPDTLVNWDFVEQVRICSHEVPSCPICLYPPTAAKITRC
GHIFCWACILHYLSLSEKTWSKCPICYSSVHKKDLKSVVATESHQYVVGDTITMQLMKRE
KGVLVALPKSKWMNVDHPIHLGDEQHSQYSKLLLASKEQVLHRVVLEEKVALEQQLAEEK
HTPESCFIEAAIQELKTREEALSGLAGSRREVTGVVAALEQLVLMAPLAKESVFQPRKGV
LEYLSAFDEETTEVCSLDTPSRPLALPLVEEEEAVSEPEPEGLPEACDDLELADDNLKEG
TICTESSQQEPITKSGFTRLSSSPCYYFYQAEDGQHMFLHPVNVRCLVREYGSLERSPEK
ISATVVEIAGYSMSEDVRQRHRYLSHLPLTCEFSICELALQPPVVSKETLEMFSDDIEKR

KRQRQKKAREERRRERRIEIEENKKQGKYPEVHIPLENLQQFPAFNSYTCSSDSALGPTS
TEGHGALSISPLSRSPGSHADFLLTPLSPTASQGSPSFCVGSLEEDSPFPSFAQMLRVGK
AKADVWPKTAPKKDENSLVPPAPVDSDGESDNSDRVPVPSFQNSFSQAIEAAFMKLDTPA
TSDPLSEEKGGKKRKKQKQKLLFSTSVVHTK*
>ENSG00000196141|ENST00000358677
MAELNTHVNVKEKIYAVRSVVPNKSNNEIVLVLQQFDFNVDKAVQAFVDGSAIQVLKEWN
MTGKKKNNKRKRSKSKQHQGNKDAKDKVERPEAGPLQPQPPQIQNGPMNGCEKDSSSTDS
ANEKPALIPREKKISILEEPSKALRGVTEGNRLLQQKLSLDGNPKPIHGTTERSDGLQWS
AEQPCNPSKPKAKTSPVKSNTPAAHLEIKPDELAKKRGPNIEKSVKDLQRCTVSLTRYRV
MIKEEVDSSVKKIKAAFAELHNCIIDKEVSLMAEMDKVKEEAMEILTARQKKAEELKRLT
DLASQMAEMQLAELRAEIKHFVSERKYDEELGKAARFSCDIEQLKAQIMLCGEITHPKNN
YSSRTPCSSLLPLLNAHAATSGKQSNFSRKSSTHNKPSEGKAANPKMVSSLPSTADPSHQ
TMPANKQNGSSNQRRRFNPQYHNNRLNGPAKSQGSGNEAEPLGKGNSRHEHRRQPHNGFR
PKNKGGAKNQEASLGMKTPEAPAHSEKPRRRQHAADTSEARPFRGSVGRVSQCNLCPTRI
EVSTDAAVLSVPAVTLVA*
>ENSG00000183742|ENST00000332878
MLITERKHFRSGRIAQSMSEANLIDMEAGKLSKSCNITECQDPDLLHNWPDAFTLRGNNA
SKVANPFWNQLSASNPFLDDITQLRNNRKRNNISILKEDPFLFCREIENGNSFDSSGDEL
DVHQLLRQTSSRNSGRSKSVSELLDILDDTAHAHQSIHNSDQILLHDLEWLKNDREAYKM
AWLSQRQLARSCLDLNTISQSPGWAQTQLAEVTIACKVNHQGGSVQLPESDITVHVPQGH
VAVGEFQEVSLRAFLDPPHMLNHDLSCTVSPLLEIMLGNLNTMEALLLEMKIGAEVRKDP
FSQVMTEMVCLHSLGKEGPFKVLSNCYIYKDTIQVKLIDLSQVMYLVVAAQAKALPSPAA
TIWDYIHKTTSIGIYGPKYIHPSFTVVLTVCGHNYMPGQLTISDIKKGGKNISPVVFQLW
GKQSFLLDKPQDLSISIFSCDPDFEVKTEGERKEIKQKQLEAGEVVHQQFLFSLVEHREM
HLFDFCVQVEPPNGEPVAQFSITTPDPTPNLKRLSNLPGYLQKKEEIKSAPLSPKILVKY
PTFQDKTLNFSNYGVTLKAVLRQSKIDYFLEYFKGDTIALLGEGKVKAIGQSKVKEWYVG
VLRGKIGLVHCKNVKVISKEQVMFMSDSVFTTRNLLEQIVLPLKKLTYIYSVVLTLVSEK
VYDWKVLADVLGYSHLSLEDFDQIQADKESEKVSYVIKKLKEDCHTERNTRKFLYELIVA
LLKMDCQELVARLIQEAAVLTSAVKLGKGWRELAEKLVRLTKQQMEAYEIPHRGNTGDVA
VEMMWKPAYDFLYTWSAHYGNNYRDVLQDLQSALDRMKNPVTKHWRELTGVLILVNSLEV
LRVTAFSTSEEV*
>ENSG00000182774|ENST00000330244
MGRVRTKTVKKAARVIIEKYYTRLGNDFHTNKRVCEEIAIIPSKKLRNKIAGYVTHLMKR
IQRGPVRGISIKLQEEERERRDNYVPEVSALDQEIIEVDPDTKEMLKLLDFGSLSNLQVT
QPTVGMNFKTPRGPV*
>ENSG00000054796|ENST00000345868
MAFAPMGPEASFFDVLDRHRESLLAALRRGGREPPTGGSRLASRFEDSVGLQMVSHCTTR
KIKSDSPKSAQKFSLILKILSMIYKLVQSNTYATKRDIYYTDSQLFGNQTVVDNIINDIS
CMLKVSRRSLHILSTSKGLIAGNLRYIEEDGTKVNCTCGATAVAVPSNIQGIRNLVTDAK
FVLIVEKDATFQRLLDDNFCNKLSPCIMITGKGVPDLNTRLLVKKLWDTFHVPVFTLVDA
DPHGIEIMCIYKYGSMSMSFEAHHLTVPAIRWLGLLPSDLKRLNVPKDSLIPLTKRDQMK
LDSILRRPYVTCQPFWRKEMEIMADSKMKAEIQALTFLSSDYLSRVYLPNKLKFGGWI*
>ENSG00000167081|ENST00000342287
MDDQSRMLQTLAGVNLAGHSVQGGMALPPPPHGHEGADGDGRKQDIGDILHQIMTITDQS

LDEAQAKKHALNCHRMKPALFSVLCEIKEKTGLSIRGAQEEDPPDPQLMRLDNMLLAEGV
SGPEKGGGSAAAAAAAAASGGSSDNSIEHSDYRAKLTQIRQIYHTELEKYEQACNEFTTH
VMNLLREQSRTRPISPKEIERMVGIIHRKFSSIQMQLKQSTCEAVMILRSRFLDARRKRR
NFSKQATEILNEYFYSHLSNPYPSEEAKEELAKKCSITVSQVSNWFGNKRIRYKKNIGKF
QEEANLYAAKTAVTAAHAVAAAVQNNQTNSPTTPNSGGYPPSCYQSDGRLQ*
>ENSG00000213516|ENST00000321792
MVEADRPGKLFIGGLNTETNEKALETVFGKYGRIVEVLLIKDRETNKSRGFAFVTFESPA
DAKDAARDMNGKSLDGKAIKVEQATKPSFERGRHGPPPPPRSRGPPRGFGAGRGGSGGTR
GPPSRGGHMDDGGYSMNFNMSSSRGPLPVKRGPPPRSGGPSPKRSAPSGLVRSSSGMGGR
APLSRGRDSYGGPPRREPLPSRRDVYLSPRDDGYSTKDSYSSRDYPSSRDTRDYAPPPRD
YTYRDYGHSSSRDDYPSRGYGDRDGYGRDRDYSDHPSGGSYRDSYESYGNSRSAPLTRGP
PPSYGGSSRYDDYSSSRDGYGGSRDSYSSSRSDLYSSCDRVGRQERGLPPSVERGYPSSR
DSYSSSSRGAPRGAGPGGSRSDRGGGRSRY*
>ENSG00000152670|ENST00000353507
MGDEDWEAEINPHMSSYVPIFEKDRYSGENGDNFNRTPASSSEMDDGPSRRDHFMKSGFA
SGRNFGNRDAGECNKRDNTSTMGGFGVGKSFGNRGFSNSRFEDGDSSGFWRESSNDCEDN
PTRNRGFSKRGDNDLDPDECMQRTGGLFGSRRPVLSGTGNGDTSQSRSGSGSERGGYKGL
NEEVITGSGKNSWKSEAEGGESSDTQGPKVTYIPPPPPEDEDSIFAHYQTGINFDKYDTI
LVEVSGHDAPPAILTFEEANLCQTLNNNIAKAGYTKLTPVQKYSIPIILAGRDLMACAQT
GSGKTAAFLLPILAHMMHDGITASRFKELQEPECIIVAPTRELVNQIYLEARKFSFGTCV
RAVVIYGGTQLGHSIRQIVQGCNILCATPGRLMDIIGKEKIGLKQIKYLVLDEADRMLDM
GFGPEMKKLISCPGMPSKEQRQTLMFSATFPEEIQRLAAEFLKSNYLFVAVGQVGGACRD
VQQTVLQVGQFSKREKLVEILRNIGDERTMVFVETKKKADFIATFLCQEKISTTSIHGDR
EQREREQALGDFRFGKCPVLVATSVAARGLDIENVQHVINFDLPSTIDEYVHRIGRTGRC
GNTGRAISFFDLESDNHLAQPLVKVLTDAQQDVPAWLEEIAFSTYIPGFSGSTRGNVFAS
VDTRKGKSTLNTAGFSSSQAPNPVDDESWD*
>ENSG00000101412|ENST00000343380
MALAGAPAGGPCAPALEALLGAGALRLLDSSQIVIISAAQDASAPPAPTGPAAPAAGPCD
PDLLLFATPQAPRPTPSAPRPALGRPPVKRRLDLETDHQYLAESSGPARGRGRHPGKGVK
SPGEKSRYETSLNLTTKRFLELLSHSADGVVDLNWAAEVLKVQKRRIYDITNVLEGIQLI
AKKSKNHIQWLGSHTTVGVGGRLEGLTQDLRQLQESEQQLDHLMNICTTQLRLLSEDTDS
QRLAYVTCQDLRSIADPAEQMVMVIKAPPETQLQAVDSSENFQISLKSKQGPIDVFLCPE
ETVGGISPGKTPSQEVTSEEENRATDSATIVSPPPSSPPSSLTTDPSQSLLSLEQEPLLS
RMGSLRAPVDEDRLSPLVAADSLLEHVREDFSGLLPEEFISLSPPHEALDYHFGLEEGEG
IRDLFDCDFGDLTPLDF*
>ENSG00000011304|ENST00000349038
MDGIVPDIAVGTKRGSDELFSTCVTNGPFIMSSNSASAANGNDSKKFKGDSRSAGVPSRV
IHIRKLPIDVTEGEVISLGLPFGKVTNLLMLKGKNQAFIEMNTEEAANTMVNYYTSVTPV
LRGQPIYIQFSNHKELKTDSSPNQARAQAALQAVNSVQSGNLALAASAAAVDAGMAMAGQ
SPVLRIIVENLFYPVTLDVLHQIFSKFGTVLKIITFTKNNQFQALLQYADPVSAQHAKLS
LDGQNIYNACCTLRIDFSKLTSLNVKYNNDKSRDYTRPDLPSGDSQPSLDQTMAAAFGLS
VPNVHGALAPLAIPSAAAAAAAAGRIAIPGLAGAGNSVLLVSNLNPERVTPQSLFILFGV
YGDVQRVKILFNKKENALVQMADGNQAQLAMSHLNGHKLHGKPIRITLSKHQNVQLPREG
QEDQGLTKDYGNSPLHRFKKPGSKNFQNIFPPSATLHLSNIPPSVSEEDLKVLFSSNGGV

VKGFKFFQKDRKMALIQMGSVEEAVQALIDLHNHDLGENHHLRVSFSKSTI*
>ENSG00000092978|ENST00000366935
MFGAAGRQPIGAPAAGNSWHFSRTMEELVHDLVSALEESSEQARGGFAETGDHSRSISCP
LKRQARKRRGRKRRSYNVHHPWETGHCLSEGSDSSLEEPSKDYRENHNNNKKDHSDSDDQ
MLVAKRRPSSNLNNNVRGKRPLWHESDFAVDNVGNRTLRRRRKVKRMAVDLPQDISNKRT
MTQPPEGCRDQDMDSDRAYQYQEFTKNKVKKRKLKIIRQGPKIQDEGVVLESEETNQTNK
DKMECEEQKVSDELMSESDSSSLSSTDAGLFTNDEGRQGDDEQSDWFYEKESGGACGITG
VVPWWEKEDPTELDKNVPDPVFESILTGSFPLMSHPSRRGFQARLSRLHGMSSKNIKKSG
GTPTSMVPIPGPVGNKRMVHFSPDSHHHDHWFSPGARTEHDQHQLLRDNRAERGHKKNCS
VRTASRQTSMHLGSLCTGDIKRRRKAAPLPGPTTAGFVGENAQPILENNIGNRMLQNMGW
TPGSGLGRDGKGISEPIQAMQRPKGLGLGFPLPKSTSATTTPNAGKSA*
>ENSG00000185298|ENST00000329214
MAGAGRGAAVSRVQAGPGSPRRARGRQQVQPLGKQRPAPWPGLRSKEKKKVNCKPKNQDE
QEIPFRLREIMRSRQEMKNPISNKKRKKAAQVTFRKTLEKEAKGEEPDIAVPKFKQRKGE
SDGAYIHRMQQEAQHVLFLSKNQAIRQPEVQAAPKEKSEQKKAKKAFQKRRLDKVRRKKE
EKAADRLEQELLRDTVKFGEVVLQPPELTARPQRSVSKDQPGRRSQMLRMLLSPGGVSQP
LTASLARQRIVEEERERAVQAYRALKQRQQQLHGERPHLTSRKKPEPQL*
>ENSG00000196787|ENST00000359193
MSGRGKQGGKARAKAKTRSSRAGLQFPVGRVHRLLRKGNYAERVGAGAPVYLAAVLEYLT
AEILELAGNAARDNKKTRIIPRHLQLAIRNDEELNKLLGKVTIAQGGVLPNIQAVLLPKK
TESHHKAKGK*
>ENSG00000100813|ENST00000338631
MSPADRCRSANTIEPATTSSLALFLLLQRDQSSRTRGLPEEKEEVTMDTSENRPENDVPE
PPMPIADQVSNDDRPEGSVEDEEKKESSLPKSFKRKISVVSATKGVPAGNSDTEGGQPGR
KRRWGASTATTQKKPSISITTESLKSLIPDIKPLAGQEAVVDLHADDSRISEDETERNGD
DGTHDKGLKICRTVTQVVPAEGQENGQREEEEEEKEPEAEPPVPPQVSVEVALPPPAEHE
VKKVTLGDTLTRRSISQQKSGVSITIDDPVRTAQVPSPPRGKISNIVHISNLVRPFTLGQ
LKELLGRTGTLVEEAFWIDKIKSHCFVTYSTVEEAVATRTALHGVKWPQSNPKFLCADYA
EQDELDYHRGLLVDRPSETKTEEQGIPRPLHPPPPPPVQPPQHPRAEQREQERAVREQWA
EREREMERRERTRSEREWDRDKVREGPRSRSRSRDRRRKERAKSKEKKSEKKEKAQEEPP
AKLLDDLFRKTKAAPCIYWLPLTDSQIVQKEAERAERAKEREKRRKEQEEEEQKEREKEA
ERERNRQLEREKRREHSRERDRERERERERDRGDRDRDRERDRERGRERDRRDTKRHSRS
RSRSTPVRDRGGRR*
>ENSG00000141076|ENST00000352319
MGEFKVHRVRFFNYVPSGIRCVAYNNQSNRLAVSRTDGTVEIYNLSANYFQEKFFPGHES
RATEALCWAEGQRLFSAGLNGEIMEYDLQALNIKYAMDAFGGPIWSMAASPSGSQLLVGC
EDGSVKLFQITPDKIQFERNFDRQKSRILSLSWHPSGTHIAAGSIDYISVFDVKSGSAVH
KMIVDRQYMGVSKRKCIVWGVAFLSDGTIISVDSAGKVQFWDSATGTLVKSHLIANADVQ
SIAVADQEDSFVVGTAEGTVFHFQLVPVTSNSSEKQWVRTKPFQHHTHDVRTVAHSPTAL
ISGGTDTHLVFRPLMEKVEVKNYDAALRKITFPHRCLISCSKKRQLLLFQFAHHLELWRL
GSTVATGTVEAMCLLAVSPDGNWLAASGTSAGVHVYNVKQLKLHCTVPAYNFPVTAMAIA
PNTNNLVIAHSDQQVFEYSIPDKQYTDWSRTVQKQGFHHLWLQRDTPITHISFHPKRPMH
ILLHDAYMFCIIDKSLPLPNDKTLLYNPFPPTNESDVIRRRTAHAFKISKIYKPLLFMDL
LDERTLVAVERPLDDIIAQLPPPIKKKKFGT*

>ENSG00000166949|ENST00000327367
MSSILPFTPPIVKRLLGWKKGEQNGQEEKWCEKAVKSLVKKLKKTGQLDELEKAITTQNV
NTKCITIPRSLDGRLQVSHRKGLPHVIYCRLWRWPDLHSHHELRAMELCEFAFNMKKDEV
CVNPYHYQRVETPVLPPVLVPRHTEIPAEFPPLDDYSHSIPENTNFPAGIEPQSNIPETP
PPGYLSEDGETSDHQMNHSMDAGSPNLSPNPMSPAHNNLDLQPVTYCEPAFWCSISYYEL
NQRVGETFHASQPSMTVDGFTDPSNSERFCLGLLSNVNRNAAVELTRRHIGRGVRLYYIG
GEVFAECLSDSAIFVQSPNCNQRYGWHPATVCKIPPGCNLKIFNNQEFAALLAQSVNQGF
EAVYQLTRMCTIRMSFVKGWGAEYRRQTVTSTPCWIELHLNGPLQWLDKVLTQMGSPSIR
CSSVS*
>ENSG00000095794|ENST00000345491
MSKCARKKYIKTNPRQMTMETVESQHDGSITASLTESKSAHVQTQTGQNSIPALAQVAAI
AETDESAESEGVIDSHKRREILSRRPSYRKILNELSSDVPGVPKIEEERSEEEGTPPSIA
TMAVPTSIYQTSTGQYIAIAQGGTIQISNPGSDGVQGLQALTMTNSGAPPPGATIVQYAA
QSADGTQQFFVPGSQVVVQAATGDMPTYQIRAPTAALPQGVVMAASPGSLHSPQQLAEEA
TRKRELRLMKNREAAKECRRRKKEYVKCLESRVAVLEVQNKKLIEELETLKDICSPKTDY
*
>ENSG00000126952|ENST00000361330
MRRNTQDENMRKWFKVTICIFVSHFTAPYSVKNKLKPGQMEMLKLTMNKRYNVSQQALDL
QNLRFDPDLMGRDIDIILNRRNCMAATLKITERNFPELLSLNLCNNKLYQLDGLSDITEK
APKVKTLNLSKNKLESAWELGKVKGLKLEELWLEGNPLCSTFSDQSAYVSAIRDCFPKLL
RLDGRELSAPVIVDIDSSETMKPCKENFTGSETLKHLVLQFLQQSNLCKYFKDSRNIKIL
KDPYLQRKLLKHTKCPRNVDSLSALPETQHDFTSILVDMWYQTVNTCFLPRAGPESQSGR
MVSGF*
>ENSG00000137073|ENST00000360802
MMTSVSSDHCRGAREKPQISAAQSTQPQKQVVQATAEQMRLAQVIFDKNDSDFEAKVKQL
MEVTGKNQDECIVALHDCNGDVNKAINILLEGNSDTTSWETVGCKKKNFAKENSENKENR
EKKSEKESSRGRGNNNRKGRGGNRGREFRGEENGIDCNQVDKPSDRGKRARGRGFGRGRG
RGAGRFSTQGMGTFNPADYSDSTSTDVCGTKLVVWEAAQNGADEGTELASNTHNIAQDLS
NKSSYGLKGAWKNSVEEWTTEDWTEDLSETKVFTASSAPAENHILPGQSIDLVALLQKPV
PHSQASEANSFETSQQQGFGQALVFTNSQHNNQMAPGTGSSTAVNSCSPQSLSSVLGSGF
GELAPPKMANITSSQILDQLKAPSLGQFTTTPSTQQNSTSHPTTTTSWDLKPPTSQSSVL
SHLDFKSQPEPSPVLSQLSQRQQHQSQAVTVPPPGLESFPSQAKLRESTPGDSPSTVNKL
LQLPSTTIENISVSVHQPQPKHIKLAKRRIPPASKIPASAVEMPGSADVTGLNVQFGALE
FGSEPSLSEFGSAPSSENSNQIPISLYSKSLSEPLNTSLSMTSAVQNSTYTTSVITSCSL
TSSSLNSASPVAMSSSYDQSSVHNRIPYQSPVSSSESAPGTIMNGHGGGRSQQTLDTPKT
TGPPSALPSVSSLPSTTSCTALLPSTSQHTGDLTSSPLSQLSSSLSSHQSSLSAHAALSS
STSHTHASVESASSHQSSATFSTAATSVSSSASSGASLSSSMNTANSLCLGGTPASASSS
SSRAAPLVTSGKAPPNLPQGVPPLLHNQYLVGPGGLLPAYPIYGYDELQMLQSRLPVDYY
GIPFAAPTALASRDGSLANNPYPGDVTKFGRGDSASPAPATTPAQPQQSQSQTHHTAQQP
FVNPALPPGYSYTGLPYYTGMPSAFQYGPTMFVPPASAKQHGVNLSTPTPPFQQASGYGQ
HGYSTGYDDLTQGTAAGDYSKGGYAGSSQAPNKSAGSGPGKGVSVSSSTTGLPDMTGSVY
NKTQTFDKQGFHAGTPPPFSLPSVLGSTGPLASGAAPGYAPPPFLHILPAHQQPHSQLLH
HHLPQDAQSGSGQRSQPSSLQPKSQASKPAYGNSPYWTN*
>ENSG00000127152|ENST00000345514

MSRRKQGNPQHLSQRELITPEADHVEAAILEEDEGLEIEEPSGLGLMVGGPDPDLLTCGQ
CQMNFPLGDILVFIEHKRKQCGGSLGACYDKALDKDSPPPSSRSELRKVSEPVEIGIQVT
PDEDDHLLSPTKGICPKQENIAGKDEPSSYICTTCKQPFNSAWFLLQHAQNTHGFRIYLE
PGPASSSLTPRLTIPPPLGPEAVAQSPLMNFLGDSNPFNLLRMTGPILRDHPGFGEGRLP
GTPPLFSPPPRHHLDPHRLSAEEMGLVAQHPSAFDRVMRLNPMAIDSPAMDFSRRLRELA
GNSSTPPPVSPGRGNPMHRLLNPFQPSPKSPFLSTPPLPPMPPGGTPPPQPPAKSKSCEF
CGKTFKFQSNLIVHRRSHTGEKPYKCQLCDHACSQASKLKRHMKTHMHKAGSLAGRSDDG
LSAASSPEPGTSELAGEGLKAADGDFRHHESDPSLGHEPEEEDEEEEEEEEELLLENESR
PESSFSMDSELSRNRENGGGGVPGVPGAGGGAAKALADEKALVLGKVMENVGLGALPQYG
ELLADKQKRGAFLKRAAGGGDAGDDDDAGGCGDAGAGGAVNGRGGGFAPGTEPFPGLFPR
KPAPLPSPGLNSAAKRIKVEKDLELPPAALIPSENVYSQWLVGYAASRHFMKDPFLGFTD
ARQSPFATSSEHSSENGSLRFSTPPGDLLDGGLSGRSGTASGGSTPHLGGPGPGRPSSKE
GRRSDTCEYCGKVFKNCSNLTVHRRSHTGERPYKCELCNYACAQSSKLTRHMKTHGQIGK
EVYRCDICQMPFSVYSTLEKHMKKWHGEHLLTNDVKIEQAERS*
>ENSG00000176083|ENST00000349618
MKEESAAQLGCCHRPMALGGTGGSLSPSLDFQLFRGDQVFSACRPLPDMVDAHGPSCASW
LCPLPLAPGRSALLACLQDLDLNLCTPQPAPLGTDLQGLQEDALSMKHEPPGLQASSTDD
KKFTVKYPQNKDKLGKQPERAGEGAPCPAFSSHNSSSPPPLQNRKSPSPLAFCPCPPVNS
ISKELPFLLHAFYPGYPLLLPPPHLFTYGALPSDQCPHLLMLPQDPSYPTMAMPSLLMMV
NELGHPSARWETLLPYPGAFQASGQALPSQARNPGAGAAPTDSPGLERGGMASPAKRVPL
SSQTGTAALPYPLKKKNGKILYECNICGKSFGQLSNLKVHLRVHSGERPFQCALCQKSFT
QLAHLQKHHLVHTGERPHKCSVCHKRFSSSSNLKTHLRLHSGARPFQCSVCRSRFTQHIH
LKLHHRLHAPQPCGLVHTQLPLASLACLAQWHQGALDLMAVASEKHMGYDIDEVKVSSTS
QGKARAVSLSSAGTPLVMGQDQNN*
>ENSG00000118513|ENST00000339290
MARRPRHSIYSSDEDDEDFEMCDHDYDGLLPKSGKRHLGKTRWTREEDEKLKKLVEQNGT
DDWKVIANYLPNRTDVQCQHRWQKVLNPELIKGPWTKEEDQRVIELVQKYGPKRWSVIAK
HLKGRIGKQCRERWHNHLNPEVKKTSWTEEEDRIIYQAHKRLGNRWAEIAKLLPGRTDNA
IKNHWNSTMRRKVEQEGYLQESSKASQPAVATSFQKNSHLMGFAQAPPTAQLPATGQPTV
NNDYSYYHISEAQNVSSHVPYPVALHVNIVNVPQPAAAAIQRHYNDEDPEKEKRIKELEL
LLMSTENELKGQQVLPTQNHTCSYPGWHSTTIADHTRPHGDSAPVSCLGEHHSTPSLPAD
PGSLPEESASPARCMIVHQGTILDNVKNLLEFAETLQFIDSFLNTSSNHENSDLEMPSLT
STPLIGHKLTVTTPFHRDQTVKTQKENTV*
>ENSG00000127616|ENST00000344626
MSTPDPPLGGTPRPGPSPGPGPSPGAMLGPSPGPSPGSAHSMMGPSPGPPSAGHPIPTQG
PGGYPQDNMHQMHKPMESMHEKGMSDDPRYNQMKGMGMRSGGHAGMGPPPSPMDQHSQGY
PSPLGGSEHASSPVPASGPSSGPQMSSGPGGAPLDGADPQALGQQNRGPTPFNQNQLHQL
RAQIMAYKMLARGQPLPDHLQMAVQGKRPMPGMQQQMPTLPPPSVSATGPGPGPGPGPGP
GPGPAPPNYSRPHGMGGPNMPPPGPSGVPPGMPGQPPGGPPKPWPEGPMANAAAPTSTPQ
KLIPPQPTGRPSPAPPAVPPAASPVMPPQTQSPGQPAQPAPMVPLHQKQSRITPIQKPRG
LDPVEILQEREYRLQARIAHRIQELENLPGSLAGDLRTKATIELKALRLLNFQRQLRQEV
VVCMRRDTALETALNAKAYKRSKRQSLREARITEKLEKQQKIEQERKRRQKHQEYLNSIL
QHAKDFKEYHRSVTGKIQKLTKAVATYHANTEREQKKENERIEKERMRRLMAEDEEGYRK
LIDQKKDKRLAYLLQQTDEYVANLTELVRQHKAAQVAKEKKKKKKKKKAENAEGQTPAIG

PDGEPLDETSQMSDLPVKVIHVESGKILTGTDAPKAGQLEAWLEMNPGYEVAPRSDSEES
GSEEEEEEEEEEEQPQAAQPPTLPVEEKKKIPDPDSDDVSEVDARHIIENAKQDVDDEYGV
SQALARGLQSYYAVAHAVTERVDKQSALMVNGVLKQYQIKGLEWLVSLYNNNLNGILADE
MGLGKTIQTIALITYLMEHKRINGPFLIIVPLSTLSNWAYEFDKWAPSVVKVSYKGSPAA
RRAFVPQLRSGKFNVLLTTYEYIIKDKHILAKIRWKYMIVDEGHRMKNHHCKLTQVLNTH
YVAPRRLLLTGTPLQNKLPELWALLNFLLPTIFKSCSTFEQWFNAPFAMTGEKVDLNEEE
TILIIRRLHKVLRPFLLRRLKKEVEAQLPEKVEYVIKCDMSALQRVLYRHMQAKGVLLTD
GSEKDKKGKGGTKTLMNTIMQLRKICNHPYMFQHIEESFSEHLGFTGGIVQGLDLYRASG
KFELLDRILPKLRATNHKVLLFCQMTSLMTIMEDYFAYRGFKYLRLDGTTKAEDRGMLLK
TFNEPGSEYFIFLLSTRAGGLGLNLQSADTVIIFDSDWNPHQDLQAQDRAHRIGQQNEVR
VLRLCTVNSVEEKILAAAKYKLNVDQKVIQAGMFDQKSSSHERRAFLQAILEHEEQDESR
HCSTGSGSASFAHTAPPPAGVNPDLEEPPLKEEDEVPDDETVNQMIARHEEEFDLFMRMD
LDRRREEARNPKRKPRLMEEDELPSWIIKDDAEVERLTCEEEEEKMFGRGSRHRKEVDYS
DSLTEKQWLKAIEEGTLEEIEEEVRQKKSSRKRKRDSDAGSSTPTTSTRSRDKDDESKKQ
KKRGRPPAEKLSPNPPNLTKKMKKIVDAVIKYKDSSSGRQLSEVFIQLPSRKELPEYYEL
IRKPVDFKKIKERIRNHKYRSLNDLEKDVMLLCQNAQTFNLEGSLIYEDSIVLQSVFTSV
RQKIEKEDDSEGEESEEEEEGEEEGSESESRSVKVKIKLGRKEKAQDRLKGGRRRPSRGS
RAKPVVSDDDSEEEQEEDRSGSGSEED*
>ENSG00000111247|ENST00000352618
MVRPVRHKKPVNYSQFDHSDSDDDFVSATVPLNKKSRTAPKELKQDKPKPNLNNLRKEEI
PVQEKTPKKRMALDDKLYQRDLEVALALSVKELPTVTTNVQNSQDKSIEKHGSSKIETMN
KSPHISNCSVASDYLDLDKITVEDDVGGVQGKRKAASKAAAQQRKILLEGSDGDSANDTE
PDFAPGEDSEDDSDFCESEDNDEDFSMRKSKVKEIKKKEVKVKSPVEKKEKKSKSKCNAL
VTSVDSAPAAVKSESQSLPKKVSLSSDTTRKPLEIRSPSAESKKPKWVPPAASGGSRSSS
SPLVVVSVKSPNQSLRLGLSRLARVKPLHPNATST*
>ENSG00000139624|ENST00000317551
MATAAQGPLSLLWGWLWSERFWLPENVSWADLEGPADGYGYPRGRHILSVFPLAAGIFFV
RLLFERFIAKPCALCIGIEDSGPYQAQPNAILEKVFISITKYPDKKRLEGLSKQLDWNVR
KIQCWFRHRRNQDKPPTLTKFCESMWRFTFYLCIFCYGIRFLWSSPWFWDIRQCWHNYPF
QPLSSGLYHYYIMELAFYWSLMFSQFTDIKRKDFLIMFVHHLVTIGLISFSYINNMVRVG
TLIMCLHDVSDFLLEAAKLANYAKYQRLCDTLFVIFSAVFMVTRLGIYPFWILNTTLFES
WEIIGPYASWWLLNGLLLTLQLLHVIWSYLIARIALKALIRGKVSKDDRSDVESSSEEED
VTTCTKSPCDSSSSNGANRVNGHMGGSYWAEE*
>ENSG00000178804|ENST00000324382
MAPGSVTSDISPSSTSTAGSSRSPESEKPGPSHGGVPPGGPSHSSLPVGRRHPPVLRMVL
EALQAGEQRRGTSVAAIKLYILHKYPTVDVLRFKYLLKQALATGMRRGLLARPLNSKARG
ATGSFKLVPKHKKKIQPRKMAPATAPRRAGEAKGKGPKKPSEAKEDPPNVGKVKKAAKRP
AKVQKPPPKPGAATEKARKQGGAAKDTRAQSGEARKVPPKPDKAMRAPSSAGGLSRKAKA
KGSRSSQGDAEAYRKTKAESKSSKPTASKVKNGAASPTKKKVVAKAKAPKAGQGPNTKAA
APAKGSGSKVVPAHLSRKTEAPKGPRKAGLPIKASSSKVSSQRAEA*
>ENSG00000228049|ENST00000333432
MNAPPAFESFLLFEGEKITINKDTKVPKACLFTINKEDHTLGNIIKSQLLKDPQVLFAGY
KVPHPLEHKIIIRVQTTPDYSPQEAFTNAITDLISELSLLEERFRTCLLPLRLLP*
>ENSG00000169288|ENST00000315567

MAAAVRCMGRALIHHQRHSLSKMVYQTSLCSCSVNIRVPNRHFAAATKSAKKTKKGAKEK
TPDEKKDEIEKIKAYPYMEGEPEDDVYLKRLYPRQIYEVEKAVHLLKKFQILDFTSPKQS
VYLDLTLDMALGKKKNVEPFTSVLSLPYPFASEINKVAVFTENASEVKIAEENGAAFAGG
TSLIQKIWDDEIVADFYVAVPEIMPELNRLRKKLNKKYPKLSRNSIGRDIPKMLELFKNG
HEIKVDEERENFLQTKIATLDMSSDQIAANLQAVINEVCRHRPLNLGPFVVRAFLRSSTS
EGLLLKIDPLLPKEVKNEESEKEDA*
>ENSG00000125952|ENST00000358402
MSDNDDIEVESDADKRAHHNALERKRRDHIKDSFHSLRDSVPSLQGEKASRAQILDKATE
YIQYMRRKNHTHQQDIDDLKRQNALLEQQVRALEKARSSAQLQTNYPSSDNSLYTNAKGS
TISAFDGGSDSSSESEPEEPQSRKKLRMEAS*
>ENSG00000121390|ENST00000338910
MMLRGNLKQVRIEKNPARLRALESAVGESEPAAAAAMALALAGEPAPPAPAPPEDHPDEE
MGFTIDIKSFLKPGEKTYTQRCRLFVGNLPTDITEEDFKRLFERYGEPSEVFINRDRGFG
FIRLESRTLAEIAKAELDGTILKSRPLRIRFATHGAALTVKNLSPVVSNELLEQAFSQFG
PVEKAVVVVDDRGRATGKGFVEFAAKPPARKALERCGDGAFLLTTTPRPVIVEPMEQFDD
EDGLPEKLMQKTQQYHKEREQPPRFAQPGTFEFEYASRWKALDEMEKQQREQVDRNIREA
KEKLEAEMEAARHEHQLMLMRQDLMRRQEELRRLEELRNQELQKRKQIQLRHEEEHRRRE
EEMIRHREQEELRRQQEGFKPNYMENREQEMRMGDMGPRGAINMGDAFSPAPAGNQGPPP
MMGMNMNNRATIPGPPMGPGPAMGPEGAANMGTPMMPDNGAVHNDRFPQGPPSQMGSPMG
SRTGSETPQAPMSGVGPVSGGPGGFGRGSQGGNFEGPNKRRRY*
>ENSG00000177426|ENST00000330513
MVLAQSRVSAGVGSPHCSGSGGGGSDSFPWPASHPGNPQCSFSTAFLASPRLSRGTLAYL
PPAPWSSLATPSALLGSSCAPPPPPARCPQPRALSPELGTKAGPRRPHRWELPRSPSQGA
QGPAPRRRLLETMKGIVAASGSETEDEDSMDIPLDLSSSAGSGKRRRRGNLPKESVQILR
DWLYEHRYNAYPSEQEKALLSQQTHLSTLQVCNWFINARRRLLPDMLRKDGKDPNQFTIS
RRGAKISETSSVESVMGIKNFMPALEETPFHSCTAGPNPTLGRPLSPKPSSPGSVLARPS
VICHTTVTALKDVPFSLCQSVGVGQNTDIQQIAAKNFTDTSLMYPEDTCKSGPSTNTQSG
LFNTPPPTPPDLNQDFSGFQLLVDVALKRAAEMELQAKLTA*
>ENSG00000197472|ENST00000366504
MGLLAFRDVALEFSPEEWECLDPAQRSLYRDVMLENYRNLISLGEDSFNMQFLFHSLAMS
KPELIICLEARKEPWNVNTEKTARHSVLSSYLTEDILPEQGLQVSFQKVMLRRYERCCLE
KLRLRNDWEIVAM*
>ENSG00000101489|ENST00000334919
MYIKMATLANGQADNASLSTNGLGSSPGSAGHMNGLSHSPGNPSTIPMKDHDAIKLFIGQ
IPRNLDEKDLKPLFEEFGKIYELTVLKDRFTGMHKGCAFLTYCERESALKAQSALHEQKT
LPGMNRPIQVKPADSESRGEDRKLFVGMLNKQQSEDDVRRLFEAFGNIEECTILRGPDGN
SKGCAFVKYSSHAEAQAAINALHGSQTMPGASSSLVVKFADTDKERTMRRMQQMAGQMGM
FNPMAIPFGAYGAYAQALMQQQAALMASVAQGGYLNPMAAFAAAQMQQMAALNMNGLAAA
PMTPTSGGSTPPGITAPAVPSIPSPIGVNGFTGLPPQANGQPAAEAVFANGIHPYPAQSP
TAADPLQQAYAGVQQYAGPAAYPAAYGQISQAFPQPPPMIPQQQREGFVSFDNPASAQTA
IQAMNGFQIGMKRLKVQLKRPKDANRPY*
>ENSG00000142192|ENST00000348990
MLPGLALLLLAAWTARALEVPTDGNAGLLAEPQIAMFCGRLNMHMNVQNGKWDSDPSGTK
TCIDTKEGILQYCQEVYPELQITNVVEANQPVTIQNWCKRGRKQCKTHPHFVIPYRCLVG

EFVSDALLVPDKCKFLHQERMDVCETHLHWHTVAKETCSEKSTNLHDYGMLLPCGIDKFR
GVEFVCCPLAEESDNVDSADAEEDDSDVWWGGADTDYADGSEDKVVEVAEEEEVAEVEEE
EADDDEDDEDGDEVEEEAEEPYEEATERTTSIATTTTTTTESVEEVVRVPTTAASTPDAV
DKYLETPGDENEHAHFQKAKERLEAKHRERMSQVMREWEEAERQAKNLPKADKKAVIQHF
QEKVESLEQEAANERQQLVETHMARVEAMLNDRRRLALENYITALQAVPPRPRHVFNMLK
KYVRAEQKDRQHTLKHFEHVRMVDPKKAAQIRSQVMTHLRVIYERMNQSLSLLYNVPAVA
EEIQDEVDELLQKEQNYSDDVLANMISEPRISYGNDALMPSLTETKTTVELLPVNGEFSL
DDLQPWHSFGADSVPANTENEVEPVDARPAADRGLTTRPGSGLTNIKTEEISEVKMDAEF
RHDSGYEVHHQKLVFFAEDVGSNKGAIIGLMVGGVVIATVIVITLVMLKKKQYTSIHHGV
VEVDAAVTPEERHLSKMQQNGYENPTYKFFEQMQN*
>ENSG00000134283|ENST00000358314
MWSEGRYEYERIPRERAPPRSHPSDGYNRLVNIVPKKPPLLDRPGEGSYNRYYSHVDYRD
YDEGRSFSHDRRSGPPHRGDESGYRWTRDDHSASRQPEYRDMRDGFRRKSFYSSHYARER
SPYKRDNTFFRESPVGRKDSPHSRSGSSVSSRSYSPERSKSYSFHQSQHRKSVRPGASYK
RQNEGNPERDKERPVQSLKTSRDTSPSSGSAVSSSKVLDKPSRLTEKELAEAASKWAAEK
LEKSDESNLPEISEYEAGSTAPLFTDQPEEPESNTTHGIELFEDSQLTTRSKAIASKTKE
IEQVYRQDCETFGMVVKMLIEKDPSLEKSIQFALRQNLHEIGERCVEELKHFIAEYDTST
QDFGEPF*
>ENSG00000007866|ENST00000338863
IASNSWNASSSPGEAREDGPEGLDKGLDNDAEGVWSPDIEQSFQEALAIYPPCGRRKIIL
SDEGKMYGRNELIARYIKLRTGKTRTRKQVSSHIQVLARKKVREYQVGIKAMNLDQVSKD
KALQSMASMSSAQIVSASVLQNKFSPPSPLPQAVFSTSSRFWSSPPLLGQQPGPSQDIKP
FAQPAYPIQPPLPPTLSSYEPLAPLPSAAASVPVWQDRTIASSRLRLLEYSAFMEVQRDP
DTYSKHLFVHIGQTNPAFSDPPLEAVDVRQIYDKFPEKKGGLKELYEKGPPNAFFLVKFW
ADLNSTIQEGPGAFYGVSSQYSSADSMTISVSTKVCSFGKQVVEKVETEYARLENGRFVY
RIHRSPMCEYMINFIHKLKHLPEKYMMNSVLENFTILQVVTSRDSQETLLVIAFVFEVST
SEHGAQHHVYKLVKD*
>ENSG00000162761|ENST00000342310
MLDGLKMEENFQSAIDTSASFSSLLGRAVSPKSVCEGCQRVILDRFLLRLNDSFWHEQCV
QCASCKEPLETTCFYRDKKLYCKYDYEKLFAVKCGGCFEAIAPNEFVMRAQKSVYHLSCF
CCCVCERQLQKGDEFVLKEGQLLCKGDYEKERELLSLVSPAASDSGKSDDEESLCKSAHG
AGKGTAEEGKDHKRPKRPRTILTTQQRRAFKASFEVSSKPCRKVRETLAAETGLSVRVVQ
VWFQNQRAKMKKLARRQQQQQQDQQNTQRLSSAQTNGGGSAGMEGIMNPYTALPTPQQLL
AIEQSVYSSDPFRQGLTPPQMPGDHMHPYGAEPLFHDLDSDDTSLSNLGDCFLATSEAGP
LQSRVGNPIDHLYSMQNSYFTS*
>ENSG00000080503|ENST00000349721
MSTPTDPGAMPHPGPSPGPGPSPGPILGPSPGPGPSPGSVHSMMGPSPGPPSVSHPMPTM
GSTDFPQEGMHQMHKPIDGIHDKGIVEDIHCGSMKGTGMRPPHPGMGPPQSPMDQHSQGY
MSPHPSPLGAPEHVSSPMSGGGPTPPQMPPSQPGALIPGDPQAMSQPNRGPSPFSPVQLH
QLRAQILAYKMLARGQPLPETLQLAVQGKRTLPGLQQQQQQQQQQQQQQQQQQQQQQQPQ
QQPPQPQTQQQQQPALVNYNRPSGPGPELSGPSTPQKLPVPAPGGRPSPAPPAAAQPPAA
AVPGPSVPQPAPGQPSPVLQLQQKQSRISPIQKPQGLDPVEILQEREYRLQARIAHRIQE
LENLPGSLPPDLRTKATVELKALRLLNFQRQLRQEVVACMRRDTTLETALNSKAYKRSKR
QTLREARMTEKLEKQQKIEQERKRRQKHQEYLNSILQHAKDFKEYHRSVAGKIQKLSKAV

ATWHANTEREQKKETERIEKERMRRLMAEDEEGYRKLIDQKKDRRLAYLLQQTDEYVANL
TNLVWEHKQAQAAKEKKKRRRRKKKAEENAEGGESALGPDGEPIDESSQMSDLPVKVTHT
ETGKVLFGPEAPKASQLDAWLEMNPGYEVAPRSDSEESDSDYEEEDEEEESSRQETEEKI
LLDPNSEEVSEKDAKQIIETAKQDVDDEYSMQYSARGSQSYYTVAHAISERVEKQSALLI
NGTLKHYQLQGLEWMVSLYNNNLNGILADEMGLGKTIQTIALITYLMEHKRLNGPYLIIV
PLSTLSNWTYEFDKWAPSVVKISYKGTPAMRRSLVPQLRSGKFNVLLTTYEYIIKDKHIL
AKIRWKYMIVDEGHRMKNHHCKLTQVLNTHYVAPRRILLTGTPLQNKLPELWALLNFLLP
TIFKSCSTFEQWFNAPFAMTGERVDLNEEETILIIRRLHKVLRPFLLRRLKKEVESQLPE
KVEYVIKCDMSALQKILYRHMQAKGILLTDGSEKDKKGKGGAKTLMNTIMQLRKICNHPY
MFQHIEESFAEHLGYSNGVINGAELYRASGKFELLDRILPKLRATNHRVLLFCQMTSLMT
IMEDYFAFRNFLYLRLDGTTKSEDRAALLKKFNEPGSQYFIFLLSTRAGGLGLNLQAADT
VVIFDSDWNPHQDLQAQDRAHRIGQQNEVRVLRLCTVNSVEEKILAAAKYKLNVDQKVIQ
AGMFDQKSSSHERRAFLQAILEHEEENEEEDEVPDDETLNQMIARREEEFDLFMRMDMDR
RREDARNPKRKPRLMEEDELPSWIIKDDAEVERLTCEEEEEKIFGRGSRQRRDVDYSDAL
TEKQWLRAIEDGNLEEMEEEVRLKKRKRRRNVDKDPAKEDVEKAKKRRGRPPAEKLSPNP
PKLTKQMNAIIDTVINYKDRCNVEKVPSNSQLEIEGNSSGRQLSEVFIQLPSRKELPEYY
ELIRKPVDFKKIKERIRNHKYRSLGDLEKDVMLLCHNAQTFNLEGSQIYEDSIVLQSVFK
SARQKIAKEEESEDESNEEEEEEDEEESESEAKSVKVKIKLNKKDDKGRDKGKGKKRPNR
GKAKPVVSDFDSDEEQDEREQSEGSGTDDE*
>ENSG00000178163|ENST00000326756
MKDIGQQLYTTHLNGGHNSLTMSPKQPDANGAPRPNRQEAQTLLYQGSEAEAAMMTIATC
AKCKSVHKISLQDLQKGTGKDGMYVCFQCSLGAAPPNFHFVSNNSSATHVGNKTENFSSS
VNSKFKVRNFKPGKYYCDKCRFSTKDPLQYKKHTLQHEEIKFICSHCSYISYTKGEFQRH
LVKHTGIFPYQCEYCDYGAIRNDYIVKHTKRVHERAGAKRPVKAVAKLEPKRTGTSKQNP
ELLKASNPRTTFQNKWSDQLSGFSLHANKDKMHNIMLLPEPKEYQKDVVCIPNKMTLSEP
NEVNLFENKNVEVEVLSPAKEPVQPGMPLTVVAPAELVVPANCLAQLIDVKVVNGTQQLV
LKLFPLEENNCLEAGRDNGGNSERMVKEKGSNEQEKVLSAEKTKSLTVDGNVGKLVGIDS
FQPSVQKQLKNVKWVRSYDFIMPNSSVHNNGKSFINSETIEDFQKKNNLYPHRTAFPSVA
LKGHSLASVFKNSVLRSLGAASNPFPYKAAVCFAESGRNLHSSSQQLLPFAASPATCSFS
GEKGLLPVSENDLESTSKVNIPVKVVSSNRKQEDNQTEEHKAVSTVGQISSQHKSEYLHI
NITGEDRSQQPGDKPLELKNSERTNNTNDGPVISSVFSLSSGSENVPEGIKWNSSTSKIK
SIELLRRKIAQLIESCGKPSSLASNSAHRRSVGQASKGTSKATSEGIQEINVSLTGLGHS
TGTLQKPPNDGGITGNRQLTHQQIYPHFADGSNRKTKSRVARKAHVATPVLIPKGAVLRV
LNSSENAHIIEATCEAPVSIPCSERQLIKPVPFCPVRQADSDLQPLRSERGPIDMSPNIE
TPLRPKLRKESAVCSTIHRKTGLLYGQQGSSELNKQGRLLSRSLSISRNKTKQVHLSRKK
NKIQAEPSRCLKDPSIFQVARQLRLIAAKPDQLIKCPRRNQPVIVLNHPDVDSPEVTNVM
KVINKYKGNVLKVVLSERTRCQLGIRRHHVRLTYQNAEEASQIKRQMMLKMKLKKVHKNN
YQVVDSLPDDSSQCVFKCWFCGRLYEDQEEWMSHGQRHLIEATRDWDVLSSKGK*
>ENSG00000196345|ENST00000341840
MTTAGRGNLGLIPRSTAFQKQEGRLTVKQEPANQTWGQGSSLQKNYPPVCEIFRLHFRQL
CYHEMSGPQEALSRLRELCRWWLMPEVHTKEQILELLVLEQFLSILPGELRTWVQLHHPE
SGEEAVAVVEDFQRHLSGSEEVSAPAQKQEMHFEETTALGTTKESPPTSPLSGGSAPGAH
LEPPYDPGTHHLPSGDFAQCTSPVPTLPQVGNSGDQAGATVLRMVRPQDTVAYEDLSVDY
TQKKWKSLTLSQRALQWNMMPENHHSMASLGWSTMA*

>ENSG00000147421|ENST00000355231
MLSSFPVVLLETMSHYTDEPRFTIEQIDLLQRLRRTGMTKHEILHALETLDRLDQEHSDK
FGRRSSYGGSSYGNSTNNVPASSSTATASTQTQHSGMSPSPSNSYDTSPQPCTTNQNGRE
NNERLSTSNGKMSPTRYHANSMGQRSYSFEASEEDLDVDDKVEELMRRDSSVIKEEIKAF
LANRRISQAVVAQVTGISQSRISHWLLQQGSDLSEQKKRAFYRWYQLEKTNPGATLSMRP
APIPIEDPEWRQTPPPVSATSGTFRLRRGSRFTWRKECLAVMESYFNENQYPDEAKREEI
ANACNAVIQKPGKKLSDLERVTSLKVYNWFANRRKEIKRRANIAAILESHGIDVQSPGGH
SNSDDVDGNDYSEQTDRMQAEVVAQE*
>ENSG00000119616|ENST00000341162
MGKQKKTRKYATMKRMLSLRDQRLKEKDRLKPKKKEKKDPSALKEREVPQHPSCLFFQYN
TQLGPPYHILVDTNFINFSIKAKLDLVQSMMDCLYAKCIPCITDCVMAEIEKLGQKYRVA
LRIAKDPRFERLPCTHKGTYADDCLVQRVTQHKCYIVATVDRDLKRRIRKIPGVPIMYIS
NHRYNIERMPDDYGAPRF*
>ENSG00000140464|ENST00000359928
MEPAPARSPRPQQDPARPQEPTMPPPETPSEGRQPSPSPSPTERAPASEEEFQFLRCQQC
QAEAKCPKLLPCLHTLCSGCLEASGMQCPICQAPWPLGADTPALDNVFFESLQRRLSVYR
QIVDAQAVCTRCKESADFWCFECEQLLCAKCFEAHQWFLKHEARPLAELRNQSVREFLDG
TRKTNNIFCSNPNHRTPTLTSIYCRGCSKPLCCSCALLDSSHSELKCDISAEIQQRQEEL
DAMTQALQEQDSAFGAVHAQMHAAVGQLGRARAETEELIRERVRQVVAHVRAQERELLEA
VDARYQRDYEEMASRLGRLDAVLQRIRTGSALVQRMKCYASDQEVLDMHGFLRQALCRLR
QEEPQSLQAAVRTDGFDEFKVRLQDLSSCITQGKDAAVSKKASPEAASTPRDPIDVDLRN
ALW*
>ENSG00000163848|ENST00000360647
MNIDDKLEGLFLKCGGIDEMQSSRTMVVMGGVSGQSTVSGELQDSVLQDRSMPHQEILAA
DEVLQESEMRQQDMISHDELMVHEETVKNDEEQMETHERLPQGLQYALNVPISVKQEITF
TDVSEQLMRDKKQIREPVDLQKKKKRKQRSPAKILTINEDGSLGLKTPKSHVCEHCNAAF
RTNYHLQRHVFIHTGEKPFQCSQCDMRFIQKYLLQRHEKIHTGEKPFRCDECGMRFIQKY
HMERHKRTHSGEKPYQCEYCLQYFSRTDRVLKHKRMCHENHDKKLNRCAIKGGLLTSEED
SGFSTSPKDNSLPKKKRQKTEKKSSGMDKESALDKSDLKKDKNDYLPLYSSSTKVKDEYM
VAEYAVEMPHSSVGGSHLEDASGEIHPPKLVLKKINSKRSLKQPLEQNQTISPLSTYEES
KVSKYAFELVDKQALLDSEGNADIDQVDNLQEGPSKPVHSSTNYDDAMQFLKKKRYLQAA
SNNSREYALNVGTIASQPSVTQAAVASVIDESTTASILESQALNVEIKSNHDKNVIPDEV
LQTLLDHYSHKANGQHEISFSVADTEVTSSISINSSEVPEVTPSENVGSSSQASSSDKAN
MLQEYSKFLQQALDRTSQNDAYLNSPSLNFVTDNQTLPNQPAFSSIDKQVYATMPINSFR
SGMNSPLRTTPDKSHFGLIVGDSQHSFPFSGDETNHASATSTQDFLDQVTSQKKAEAQPV
HQAYQMSSFEQPFRAPYHGSRAGIATQFSTANGQVNLRGPGTSAEFSEFPLVNVNDNRAG
MTSSPDATTGQTFG*
>ENSG00000140694|ENST00000341484
MDFLLFQFGLCTFKYDYTDSKYITKSFNFYVFPKPFNRSSPDVKFVCQSSSIDFLASQGF
DFNKVFRNGIPYLNQEEERQLREQYDEKRSQANGAGALSYVSPNTSKCPVTIPEDQKKFI
DQVVEKIEDLLQSEENKNLDLEPCTGFQRKLIYQTLSWKYPKGIHVETLETEKKERYIVI
SKVDEEERKRREQQKHAKEQEELNDAVGFSRVIHAIANSGKLVIGHNMLLDVMHTVHQFY
CPLPADLSEFKEMTTCVFPRLLDTKLMASTQPFKDIINNTSLAELEKRLKETPFNPPKVE
SAEGFPSYDTASEQLHEAGYDAYITGLCFISMANYLGSFLSPPKIHVSARSKLIEPFFNK

LFLMRVMDIPYLNLEGPDLQPKRDHVLHVTFPKEWKTSDLYQLFSAFGNIQISWIDDTSA
FVSLSQPEQVKIAVNTSKYAESYRIQTYAEYMGRKQEEKQIKRKWTEDSWKEADSKRLNP
QCIPYTLQNHYYRNNSFTAPSTVGKRNLSPSQEEAGLEDGVSGEISDTELEQTDSCAEPL
SEGRKKAKKLKRMKKELSPAGSISKNSPATLFEVPDTW*
>ENSG00000182196|ENST00000315580
MPRCTYQLEQNPGFLPDGPGVHARAHCQDLSGPYGHEFATSESLGGRVGKTRAPQSGARS
RMERAGPAGEEGGAREGRLLPRAPGAWVLRACAERAALEVGAASADTGVRGCGARGPAPL
LASAGGGRARDGTWGVRTKGSGAALPSRPASRAAPRPEASSPPLPLEKARGGLSGPQGGR
ARGAMAHVGSRKRSRSRSRSRGRGSEKRKKKSRKDTSRNCSASTSQGRKASTAPGAEASP
SPCITERSKQKARRRTRSSSSSSSSSSSSSSSSSSSSSSSSSDGRKKRGKYKDKRRKKKK
KRKKLKKKGKEKAEAQQVEALPGPSLDQWHRSAGEEEDGPVLTDEQKSRIQAMKPMTKEE
WDARQSIIRKVVDPETGRTRLIKGDGEVLEEIVTKERHREINKQATRGDCLAFQMRAGLL
P*
>ENSG00000187778|ENST00000357123
MTRGTGGTAQRGRSGPDSQGLLDSSLMASGTASRSEDEESLAGQKRASSQALGTIPKRRS
SSRFIKRKKFDDELVESSLAKSSTRAKGASGVEPGRCSGSEPSSSEKKKVSKAPSTPVPP
SPAPAPGLTKRVKKSKQPLQVTKDLGRWKPADDLLLINAVLQTNDLTSVHLGVKFSCRFT
LREVQERWYALLYDPVISKLACQAMRQLHPEAIAAIQSKALFSKAEEQLLSKVGSTSQPT
LETFQDLLHRHPDAFYLARTAKALQAHWQLMKQYYLLEDQTVQPLPKGDQVLNFSDAEDL
IDDSKLKDMRDEVLEHELMVADRRQKREIRQLEQELHKWQVLVDSITGMSSPDFDNQTLA
VLRGRMVRYLMRSREITLGRATKDNQIDVDLSLEGPAWKISRKQGVIKLKNNGDFFIANE
GRRPIYIDGRPVLCGSKWRLSNNSVVEIASLRFVFLINQDLIALIRAEAAKITPQ*
>ENSG00000177426|ENST00000343820
MKGKKGIVAASGSETEDEDSMDIPLDLSSSAGSGKRRRRGNLPKESVQILRDWLYEHRYN
AYPSEQEKALLSQQTHLSTLQVCNWFINARRRLLPDMLRKDGKDPNQFTISRRGAKISET
SSVESVMGIKNFMPALEETPFHSCTAGPNPTLGRPLSPKPSSPGSVLARPSVICHTTVTA
LKDVPFSLCQSVGVGQNTDIQQIAAKNFTDTSLMYPEDTCKSGPSTNTQSGLFNTPPPTP
PDLNQDFSGFQLLVDVALKRAAEMELQAKLTA*
>ENSG00000197779|ENST00000338637
MPANEDAPQPGEHGSACEVSVSFEDVTVDFSREEWQQLDSTQRRLYQDVMLENYSHLLSV
GFEVPKPEVIFKLEQGEGPWTLEGEAPHQSCSDGKFGIKPSQRRISGKSTFHSEMEGEDT
RDDSLYSILEELWQDAEQIKRCQEKHNKLLSRTTFLNKKILNTEWDYEYKDFGKFVHPSP
NLILSQKRPHKRDSFGKSFKHNLDLHIHNKSNAAKNLDKTIGHGQVFTQNSSYSHHENTH
TGVKFCERNQCGKVLSLKHSLSQNVKFPIGEKANTCTEFGKIFTQRSHFFAPQKIHTVEK
PHELSKCVNVFTQKPLLSIYLRVHRDEKLYICTKCGKAFIQNSELIMHEKTHTREKPYKC
NECGKSFFQVSSLLRHQTTHTGEKLFECSECGKGFSLNSALNIHQKIHTGERHHKCSECG
KAFTQKSTLRMHQRIHTGERSYICTQCGQAFIQKAHLIAHQRIHTGEKPYECSDCGKSFP
SKSQLQMHKRIHTGEKPYICTECGKAFTNRSNLNTHQKSHTGEKSYICAECGKAFTDRSN
FNKHQTIHTGEKPYVCADCGRAFIQKSELITHQRIHTTEKPYKCPDCEKSFSKKPHLKVH
QRIHTGEKPYICAECGKAFTDRSNFNKHQTIHTGDKPYKCSDCGKGFTQKSVLSMHRNIH
T*
>ENSG00000198740|ENST00000362063
MSHTASSCQELVENCAVHVAGMAQEDSRRGQVPSSFYHGANQELDLSTKVYKRESGSPYS
VLVDTKMSKPHLHETEEQPYFRETRAVSDVHAVKEDRENSDDTEEEEEEVSYKREQIIVE

VNLNNQTLNVSKGEKGVSSQSKETPVLKTSSEEEEEEESEEEATDDSNDYGENEKQKKKEK
IVEKVSVTQRRTRRAASVAAATTSPTPRTTRGRRKSVEPPKRKKRATKEPKAPVQKAKCE
EKETLTCEKCPRVFNTRWYLEKHMNVTHRRMQICDKCGKKFVLESELSLHQQTDCEKNIQ
CVSCNKSFKKLWSLHEHIKIVHGYAEKKFSCEICEKKFYTMAHVRKHMVAHTKDMPFTCE
TCGKSFKRSMSLKVHSLQHSGEKPFRCENCDERFQYKYQLRSHMSIHIGHKQFMCQWCGK
DFNMKQYFDEHMKTHTGEKPFICEICGKSFTSRPNMKRHRRTHTGEKPYPCDVCGQRFRF
SNMLKAHKEKCFRVTSPVNVPPAVQIPLTTSPATPVPSVVNTATTPTPPINMNPVSTLPP
RPIPHPFSHLHIHPHPHHPHHLPIPPVPHLPPPPALFKSEPLNHRGQSEDNFLRHLAEKN
SSAQHH*
>ENSG00000132507|ENST00000336458
MADDLDFETGDAGASATFPMQCSALRKNGFVVLKGRPCKIVEMSTSKTGKHGHAKVHLVG
IDIFTGKKYEDICPSTHNMDVPNIKRNDFQLIGIQDGYLSLLQDSGEVREDLRLPEGDLG
KEIEQKYDCGEEILITVLSAMTEEAAVAIKAMAK*
>ENSG00000177613|ENST00000331173
MSSLAVRDPAMDRSLRSVFVGNIPYEATEEQLKDIFSEVGSVVSFRLVYDRETGKPKGYG
FCEYQDQETALSAMRNLNGREFSGRALRVDNAASEKNKEELKSLGPAAPIIDSPYGDPID
PEDAPESITRAVASLPPEQMFELMKQMKLCVQNSHQEARNMLLQNPQLAYALLQAQVVMR
IMDPEIALKILHRKIHVTPLIPGKSQSVSVSGPGPGPGPGLCPGPNVLLNQQNPPAPQPQ
HLARRPVKDIPPLMQTPIQGGIPAPGPIPAAVPGAGPGSLTPGGAMQPQLGMPGVGPVPL
ERGQVQMSDPRAPIPRGPVTPGGLPPRGLLGDAPNDPRGGTLLSVTGEVEPRGYLGPPHQ
GPPMHHASGHDTRGPSSHEMRGGPLGDPRLLIGEPRGPMIDQRGLPMDGRGGRDSRAMET
RAMETEVLETRVMERRGMETCAMETRGMEARGMDARGLEMRGPVPSSRGPMTGGIQGPGP
INIGAGGPPQGPRQVPGISGVGNPGAGMQGTGIQGTGMQGAGIQGGGMQGAGIQGVSIQG
GGIQGGGIQGASKQGGSQPSSFSPGQSQVTPQDQEKAALIMQVLQLTADQIAMLPPEQRQ
SILILKEQIQKSTGAS*
>ENSG00000011304|ENST00000350092
MSSNSASAANGNDSKKFKGDSRSAGVPSRVIHIRKLPIDVTEGEVISLGLPFGKVTNLLM
LKGKNQAFIEMNTEEAANTMVNYYTSVTPVLRGQPIYIQFSNHKELKTDSSPNQARAQAA
LQAVNSVQSGNLALAASAAAVDAGMAMAGQSPVLRIIVENLFYPVTLDVLHQIFSKFGTV
LKIITFTKNNQFQALLQYADPVSAQHAKLSLDGQNIYNACCTLRIDFSKLTSLNVKYNND
KSRDYTRPDLPSGDSQPSLDQTMAAAFGAPGIISASPYAGAGFPPTFAIPQAAGLSVPNV
HGALAPLAIPSAAAAAAAAGRIAIPGLAGAGNSVLLVSNLNPERVTPQSLFILFGVYGDV
QRVKILFNKKENALVQMADGNQAQLAMSHLNGHKLHGKPIRITLSKHQNVQLPREGQEDQ
GLTKDYGNSPLHRFKKPGSKNFQNIFPPSATLHLSNIPPSVSEEDLKVLFSSNGGVVKGF
KFFQKDRKMALIQMGSVEEAVQALIDLHNHDLGENHHLRVSFSKSTI*
>ENSG00000185624|ENST00000331483
MLRRALLCLAVAALVRADAPEEEDHVLVLRKSNFAEALAAHKYLLVEFYAPWCGHCKALA
PEYAKAAGKLKAEGSEIRLAKVDATEESDLAQQYGVRGYPTIKFFRNGDTASPKEYTAGR
EADDIVNWLKKRTGPAATTLPDGAAAESLVESSEVAVIGFFKDVESDSAKQFLQAAEAID
DIPFGITSNSDVFSKYQLDKDGVVLFKKFDEGRNNFEGEVTKENLLDFIKHNQLPLVIEF
TEQTAPKIFGGEIKTHILLFLPKSVSDYDGKLSNFKTAAESFKGKILFIFIDSDHTDNQR
ILEFFGLKKEECPAVRLITLEEEMTKYKPESEELTAERITEFCHRFLEGKIKPHLMSQEL
PEDWDKQPVKVLVGKNFEDVAFDEKKNVFVEFYAPWCGHCKQLAPIWDKLGETYKDHENI
VIAKMDSTANEVEAVKVHSFPTLKFFPASADRTVIDYNGERTLDGFKKFLESGGQDGAGD

DDDLEDLEEAEEPDMEEDDDQKAVKDEL*
>ENSG00000156531|ENST00000332070
MSSSVEQKKGPTRQRKCGFCKSNRDKECGQLLISENQKVAAHHKCMLFSSALVSSHSDNE
SLGGFSIEDVQKEIKRGTKLMCSLCHCPGATIGCDVKTCHRTYHYHCALHDKAQIREKPS
QGIYMVYCRKHKKTAHNSEADLEESFNEHELEPSSPKSKKKSRKGRPRKTNFKGLSEDTR
STSSHGTDEMESSSYRDRSPHRSSPSDTRPKCGFCHVGEEENEARGKLHIFNAKKAAAHY
KCMLFSSGTVQLTTTSRAEFGDFDIKTVLQEIKRGKRMKCTLCSQPGATIGCEIKACVKT
YHYHCGVQDKAKYIENMSRGIYKLYCKNHSGNDERDEEDEERESKSRGKVEIDQQQLTQQ
QLNGN*
>ENSG00000055609|ENST00000355193
MSSEEDKSVEQPQPPPPPPEEPGAPAPSPAAADKRPRGRPRKDGASPFQRARKKPRSRGK
TAVEDEDSMDGLETTETETIVETEIKEQSAEEDAEAEVDNSKQLIPTLQRSVSEESANSL
VSVGVEAKISEQLCAFCYCGEKSSLGQGDLKQFRITPGFILPWRNQPSNKKDIDDNSNGT
YEKMQNSAPRKQRGQRKERSPQQNIVSCVSVSTQTASDDQAGKLWDELSLVGLPDAIDIQ
ALFDSTGTCWAHHRCVEWSLGVCQMEEPLLVNVDKAVVSGSTERCAFCKHLGATIKCCEE
KCTQMYHYPCAAGAGTFQDFSHIFLLCPEHIDQAPERSKEDANCAVCDSPGDLLDQFFCT
TCGQHYHGMCLDIAVTPLKRAGWQCPECKVCQNCKQSGEDSKMLVCDTCDKGYHTFCLQP
VMKSVPTNGWKCKNCRICIECGTRSSSQWHHNCLICDNCYQQQDNLCPFCGKCYHPELQK
DMLHCNMCKRWVHLECDKPTDHELDTQLKEEYICMYCKHLGAEMDRLQPGEEVEIAELTT
DYNNEMEVEGPEDQMVFSEQAANKDVNGQESTPGIVPDAVQVHTEEQQKSHPSESLDTDS
LLIAVSSQHTVNTELEKQISNEVDSEDLKMSSEVKHICGEDQIEDKMEVTENIEVVTHQI
TVQQEQLQLLEEPETVVSREESRPPKLVMESVTLPLETLVSPHEESISLCPEEQLVIERL
QGEKEQKENSELSTGLMDSEMTPTIEGCVKDVSYQGGKSIKLSSETESSFSSSADISKAD
VSSSPTPSSDLPSHDMLHNYPSALSSSAGNIMPTTYISVTPKIGMGKPAITKRKFSPGRP
RSKQGAWSTHNTVSPPSWSPDISEGREIFKPRQLPGSAIWSIKVGRGSGFPGKRRPRGAG
LSGRGGRGRSKLKSGIGAVVLPGVSTADISSNKDDEENSMHNTVVLFSSSDKFTLNQDMC
VVCGSFGQGAEGRLLACSQCGQCYHPYCVSIKITKVVLSKGWRCLECTVCEACGKATDPG
RLLLCDDCDISYHTYCLDPPLQTVPKGGWKCKWCVWCRHCGATSAGLRCEWQNNYTQCAP
CASLSSCPVCYRNYREEDLILQCRQCDRWMHAVCQNLNTEEEVENVADIGFDCSMCRPYM
PASNVPSSDCCESSLVAQIVTKVKELDPPKTYTQDGVCLTESGMTQLQSLTVTVPRRKRS
KPKLKLKIINQNSVAVLQTPPDIQSEHSRDGEMDDSREGELMDCDGKSESSPEREAVDDE
TKGVEGTDGVKKRKRKPYRPGIGGFMVRQRSRTGQGKTKRSVIRKDSSGSISEQLPCRDD
GWSEQLPDTLVDESVSVTESTEKIKKRYRKRKNKLEETFPAYLQEAFFGKDLLDTSRQSK
ISLDNLSEDGAQLLYKTNMNTGFLDPSLDPLLSSSSAPTKSGTHGPADDPLADISEVLNT
DDDILGIISDDLAKSVDHSDIGPVTDDPSSLPQPNVNQSSRPLSEEQLDGILSPELDKMV
TDGAILGKLYKIPELGGKDVEDLFTAVLSPANTQPTPLPQPPPPTQLLPIHNQDAFSRMP
LMNGLIGSSPHLPHNSLPPGSGLGTFSAIAQSSYPDARDKNSAFNPMASDPNNSWTSSAP
TVEGENDTMSNAQRSTLKWEKEEALGEMATVAPVLYTNINFPNLKEEFPDWTTRVKQIAK
LWRKASSQERAPYVQKARDNRAALRINKVQMSNDSMKRQQQQDSIDPSSRIDSELFKDPL
KQRESEHEQEWKFRQQMRQKSKQQAKIEATQKLEQVKNEQQQQQQQQFGSQHLLVQSGSD
TPSSGIQSPLTPQPGNGNMSPAQSFHKELFTKQPPSTPTSTSSDDVFVKPQAPPPPPAPS
RIPIQDSLSQAQTSQPPSPQVFSPGSSNSRPPSPMDPYAKMVGTPRPPPVGHSFSRRNSA
APVENCTPLSSVSRPLQMNETTANRPSPVRDLCSSSTTNNDPYAKPPDTPRPVMTDQFPK
SLGLSRSPVVSEQTAKGPIAAGTSDHFTKPSPRADVFQRQRIPDSYARPLLTPAPLDSGP

GPFKTPMQPPPSSQDPYGSVSQASRRLSVDPYERPALTPRPIDNFSHNQSNDPYSQPPLT
PHPAVNESFAHPSRAFSQPGTISRPTSQDPYSQPPGTPRPVVDSYSQSSGTARSNTDPYS
QPPGTPRPTTVDPYSQQPQTPRPSTQTDLFVTPVTNQRHSDPYAHPPGTPRPGISVPYSQ
PPATPRPRISEGFTRSSMTRPVLMPNQDPFLQAAQNRGPALPGPLVRPPDTCSQTPRPPG
PGLSDTFSRVSPSAARDPYDQSPMTPRSQSDSFGTSQTAHDVADQPRPGSEGSFCASSNS
PMHSQGQQFSGVSQLPGPVPTSGVTDTQNTVNMAQADTEKLRQRQKLREIILQQQQQKKI
AGRQEKGSQDSPAVPHPGPLQHWQPENVNQAFTRPPPPYPGNIRSPVAPPLGPRYAVFPK
DQRGPYPPDVASMGMRPHGFRFGFPGGSHGTMPSQERFLVPPQQIQGSGVSPQLRRSVSV
DMPRPLNNSQMNNPVGLPQHFSPQSLPVQQHNILGQAYIELRHRAPDGRQRLPFSAPPGS
VVEASSNLRHGNFIPRPDFPGPRHTDPMRRPPQGLPNQLPVHPDLEQVPPSQQEQGHSVH
SSSMVMRTLNHPLGGEFSEAPLSTSVPSETTSDNLQITTQPSDGLEEKLDSDDPSVKELD
VKDLEGVEVKDLDDEDLENLNLDTEDGKVVELDTLDNLETNDPNLDDLLRSGEFDIIAYT
DPELDMGDKKSMFNEELDLPIDDKLDNQCVSVEPKKKEQENKTLVLSDKHSPQKKSTVTN
EVKTEVLSPNSKVESKCETEKNDENKDNVDTPCSQASAHSDLNDGEKTSLHPCDPDLFEK
RTNRETAGPSANVIQASTQLPAQDVINSCGITGSTPVLSSLLANEKSDNSDIRPSGSPPP
PTLPASPSNHVSSLPPFIAPPGRVLDNAMNSNVTVVSRVNHVFSQGVQVNPGLIPGQSTV
NHSLGTGKPATQTGPQTSQSGTSSMSGPQQLMIPQTLAQQNRERPLLLEEQPLLLQDLLD
QERQEQQQQRQMQAMIRQRSEPFFPNIDFDAITDPIMKAKMVALKGINKVMAQNNLGMPP
MVMSRFPFMGQVVTGTQNSEGQNLGPQAIPQDGSITHQISRPNPPNFGPGFVNDSQRKQY
EEWLQETQQLLQMQQKYLEEQIGAHRKSKKALSAKQRTAKKAGREFPEEDAEQLKHVTEQ
QSMVQKQLEQIRKQQKEHAELIEDYRIKQQQQCAMAPPTMMPSVQPQPPLIPGATPPTMS
QPTFPMVPQQLQHQQHTTVISGHTSPVRMPSLPGWQPNSAPAHLPLNPPRIQPPIAQLPI
KTCTPAPGTVSNANPQSGPPPRVEFDDNNPFSESFQERERKERLREQQERQRIQLMQEVD
RQRALQQRMEMEQHGMVGSEISSSRTSVSQIPFYSSDLPCDFMQPLGPLQQSPQHQQQMG
QVLQQQNIQQGSINSPSTQTFMQTNERRQVGPPSFVPDSPSIPVGSPNFSSVKQGHGNLS
GTSFQQSPVRPSFTPALPAAPPVANSSLPCGQDSTITHGHSYPGSTQSLIQLYSDIIPEE
KGKKKRTRKKKRDDDAESTKAPSTPHSDITAPPTPGISETTSTPAVSTPSELPQQADQES
VEPVGPSTPNMAAGQLCTELENKLPNSDFSQATPNQQTYANSEVDKLSMETPAKTEEIKL
EKAETESCPGQEEPKLEEQNGSKVEGNAVACPVSSAQSPPHSAGAPAAKGDSGNELLKHL
LKNKKSSSLLNQKPEGSICSEDDCTKDNKLVEKQNPAEGLQTLGAQMQGGFGCGNQLPKT
DGGSETKKQRSKRTQRTGEKAAPRSKKRKKDEEEKQAMYSSTDTFTHLKQQNNLSNPPTP
PASLPPTPPPMACQKMANGFATTEELAGKAGVLVSHEVTKTLGPKPFQLPFRPQDDLLAR
ALAQGPKTVDVPASLPTPPHNNQEELRIQDHCGDRDTPDSFVPSSSPESVVGVEVSRYPD
LSLVKEEPPEPVPSPIIPILPSTAGKSSESRRNDIKTEPGTLYFASPFGPSPNGPRSGLI
SVAITLHPTAAENISSVVAAFSDLLHVRIPNSYEVSSAPDVPSMGLVSSHRINPGLEYRQ
HLLLRGPPPGSANPPRLVSSYRLKQPNVPFPPTSNGLSGYKDSSHGIAESAALRPQWCCH
CKVVILGSGVRKSFKDLTLLNKDSRESTKRVEKDIVFCSNNCFILYSSTAQAKNSENKES
IPSLPQSPMRETPSKAFHQYSNNISTLDVHCLPQLPEKASPPASPPIAFPPAFEAAQVEA
KPDELKVTVKLKPRLRAVHGGFEDCRPLNKKWRGMKWKKWSIHIVIPKGTFKPPCEDEID
EFLKKLGTSLKPDPVPKDYRKCCFCHEEGDGLTDGPARLLNLDLDLWVHLNCALWSTEVY
ETQAGALINVELALRRGLQMKCVFCHKTGATSGCHRFRCTNIYHFTCAIKAQCMFFKDKT
MLCPMHKPKGIHEQELSYFAVFRRVYVQRDEVRQIASIVQRGERDHTFRVGSLIFHTIGQ
LLPQQMQAFHSPKALFPVGYEASRLYWSTRYANRRCRYLCSIEEKDGRPVFVIRIVEQGH
EDLVLSDISPKGVWDKILEPVACVRKKSEMLQLFPAYLKGEDLFGLTVSAVARIAESLPG

VEACENYTFRYGRNPLMELPLAVNPTGCARSEPKMSAHVKRFVLRPHTLNSTSTSKSFQS
TVTGELNAPYSKQFVHSKSSQYRKMKTEWKSNVYLARSRIQGLGLYAARDIEKHTMVIEY
IGTIIRNEVANRKEKLYESQNRGVYMFRMDNDHVIDATLTGGPARYINHSCAPNCVAEVV
TFERGHKIIISSSRRIQKGEELCYDYKFDFEDDQHKIPCHCGAVNCRKWMN*
>ENSG00000078043|ENST00000324794
MADFEELRNMVSSFRVSELQVLLGFAGRNKSGRKHDLLMRALHLLKSGCSPAVQIKIREL
YRRRYPRTLEGLSDLSTIKSSVFSLDGGSSPVEPDLAVAGIHSLPSTSVTPHSPSSPVGS
VLLQDTKPTFEMQQPSPPIPPVHPDVQLKNLPFYDVLDVLIKPTSLVQSSIQRFQEKFFI
FALTPQQVREICISRDFLPGGRRDYTVQVQLRLCLAETSCPQEDNYPNSLCIKVNGKLFP
LPGYAPPPKNGIEQKRPGRPLNITSLVRLSSAVPNQISISWASEIGKNYSMSVYLVRQLT
SAMLLQRLKMKGIRNPDHSRALIKEKLTADPDSEIATTSLRVSLMCPLGKMRLTIPCRAV
TCTHLQCFDAALYLQMNEKKPTWICPVCDKKAAYESLILDGLFMEILNDCSDVDEIKFQE
DGSWCPMRPKKEAMKVSSQPCTKIESSSVLSKPCSVTVASEASKKKVDVIDLTIESSSDE
EEDPPAKRKCIFMSETQSSPTKGVLMYQPSSVRVPSVTSVDPAAIPPSLTDYSVPFHHTP
ISSMSSDLPGEQRRNDINNELKLGTSSDTVQQ*
>ENSG00000157557|ENST00000360214
MNDFGIKNMDQVAPVANSYRGTLKRQPAFDTFDGSLFAVFPSLNEEQTLQEVPTGLDSIS
HDSANCELPLLTPCSKAVMSQALKATFSGFKKEQRRLGIPKNPWLWSEQQVCQWLLWATN
EFSLVNVNLQRFGMNGQMLCNLGKERFLELAPDFVGDILWEHLEQMIKENQEKTEDQYEE
NSHLTSVPHWINSNTLGFGTEQAPYGMQTQNYPKGGLLDSMCPASTPSVLSSEQEFQMFP
KSRLSSVSVTYCSVSQDFPGSNLNLLTNNSGTPKDHDSPENGADSFESSDSLLQSWNSQS
SLLDVQRVPSFESFEDDCSQSLCLNKPTMSFKDYIQERSDPVEQGKPVIPAAVLAGFTGS
GPIQLWQFLLELLSDKSCQSFISWTGDGWEFKLADPDEVARRWGKRKNKPKMNYEKLSRG
LRYYYDKNIIHKTSGKRYVYRFVCDLQNLLGFTPEELHAILGVQPDTED*
>ENSG00000196267|ENST00000322146
LCIVQGPLTFRDVAIEFSQEEWKSLDPVQKALYWDVMLENYRNLVFLGILPKCMTKELPP
IGNSNTGEKCQTVTLERHECYDVENFYLREIQKNLQDLEFQWKDGEINYKEVPMTYKNNL
NGKRGQHSQEDVENKCIENQLTLSFQSRLTELQKFQTEGKIYECNQSEKTVNNSSLVSPL
QRILPSVQTNISKKYENEFLQLSLPTQLEKTHIREKPYMCKGCGKAFRVSSSLINHQMVH
TTEKPYKCNECGKAFHRGSLLTIHQIVHTRGKPYQCGVCGKIFRQNSDLVNHRRSHTGEK
PYKCNECGKSFSQSYNLAIHQRIHTGEKPYKCNECGKTFKQGSCLTTHQIIHTGEKPYQC
DICGKVFRQNSNLVNHQRIHTGEKPYKCNICGKSFSQSSNLATHQTVHSGNKPYKCDECG
KTFKRSSSLTTHQIIHTGEKPYTCDVCDKVFSQRSQLARHQRSHTGEKPYKCNECGKVFS
QTSHLVGHRRIHTGEKPYKCDKCGKAFKQGSLLTRHKIIHTREKRYQCGECGKVFSENSC
LVRHLRIHTGEQPYKCNVCGKVFNYSGNLSIHKRIHTGEKPFQCNECGTVFRNYSCLARH
LRIHTGQKPYKCNVCGKVFNDSGNLSNHKRIHTGEKPFQCNECGKVFSYYSCLARHRKIH
TGEKPYKCNDCGKAYTQRSSLTKHLIIHTGEKPYNCNEFGGAFIQSSKLARYHRNPTGEK
PHKCSHCGRTFSHITGLTYHQRRHTGEMPYKCIECGQVFNSTSNLARHRRIHTGEKPYKC
NECGKVFRHQSTLARHRSIHTGEKPYVCNECGKAFRVRSILVNHQKMHTGDKPYKCNECG
KAFIERSKLVYHQRNHTGEKPYKCIECGKAFGRFSCLNKHQMIHSGEKPYKCNECGKSFI
SRSGLTKHQTKHTAESLKTKFNVEKPLDVLLTSGFK*
>ENSG00000178338|ENST00000322434
MAAGQREARPQVSLTFEDVAVLFTWDEWRKLAPSQRNLYRDVMLENYRNLVSLGLSFTKP
KVISLLQQGEDPWEVEKDSSGVSSLGCKSTPKMTKSTQTQDSFQEQIRKRLKRDEPWNFI

SERSCIYEEKLKKQQDKNENLQIISVAHTKILTVDRSHKNVEFGQNFYLKSVFIKQQRFA
KEKTPSKCEIQRNSFKQNSNLLNQSKIKTAEKRYKCSTCEKAFIHNSSLRKHQKNHTGEK
LFKCKECLKAFSQSSALIQHQRTHTGEKPYICKECGKAFSHSASLCKHLRTHTVEKCYRC
KECGKSFSRRSGLFIHQKIHAQENPHKYNPGRKASSYSTSLSGSQKIHLRKKSYLCNECG
NTFKSSSSLRYHQRIHTGEKPFKCSECGRAFSQSASLIQHERIHTGEKPYRCNECGKGFT
SISRLNRHRIIHTGEKLYNCNECGKALSSHSTLIIHERIHTGEKPCKCKVCGKAFRQSSA
LIQHQRMHTGERPYKCNECDKTFRCNSSLSNHQRIHTGEKPYRCLECGMSFGQSAALIQH
QRIHTGEKPFKCNTCGKTFRQSSSLIAHQRIHTGEKPYECNACGKLFSQRSSLTNHYKIH
IEEDSLKADLHV*
>ENSG00000143498|ENST00000350027
MSDFSEELKGPVTDDEEVETSVLSGAGMHFPWLQTYVETVAIGGKRRKDFAQTTSACLSF
IQEALLKHQWQQAAEYMYSYFQTLEDSDSYKRQAAPEIIWKLGSEILFYHPKSNMESFNT
FANRMKNIGVMNYLKISLQHALYLLHHGMLKDAKRNLSEAETWRHGENTSSREILINLIQ
AYKGLLQYYTWSEKKMELSKLDKDDYAYNAVAQDVFNHSWKTSANISALIKIPGVWDPFV
KSYVEMLEFYGDRDGAQEVLTNYAYDEKFPSNPNAHIYLYNFLKRQKAPRSKLISVLKIL
YQIVPSHKLMLEFHTLLRKSEKEEHRKLGLEVLFGVLDFAGCTKNITAWKYLAKYLKNIL
MGNHLAWVQEEWNSRKNWWPGFHFSYFWAKSDWKEDTALACEKAFVAGLLLGKGCRYFRY
ILKQDHQILGKKIKRMKRSVKKYSIVNPRL*
>ENSG00000026297|ENST00000366855
MVQHWPETVCEKIQNDCRDPPDYWTIHGLWPDKSEGCNRSWPFNLEEIKDLLPEMRAYWP
DVIHSFPNRSRFWKHEWEKHGTCAAQVDALNSQKKYFGRSLELYRELDLNSVLLKLGIKP
SINYYQVADFKDALARVYGVIPKIQCLPPSQDEEVQTIGQIELCLTKQDQQLQNCTEPGE
QPSPKQEVWLANGAAESRGLRVCEDGPVFYPPPKKTKH*
>ENSG00000162711|ENST00000348069
MKMASTRCKLARYLEDLEDVDLKKFKMHLEDYPPQKGCIPLPRGQTEKADHVDLATLMID
FNGEEKAWAMAVWIFAAINRRDLYEKAKRDEPKWGSDNARVSNPTVICQEDSIEEEWMGL
LEYLSRISICKMKKDYRKKYRKYVRSRFQCIEDRNARLGESVSLNKRYTRLRLIKEHRSQ
QEREQELLAIGKTKTCESPVSPIKMELLFDPDDEHSEPVHTVVFQGAAGIGKTILARKMM
LDWASGTLYQDRFDYLFYIHCREVSLVTQRSLGDLIMSCCPDPNPPIHKIVRKPSRILFL
MDGFDELQGAFDEHIGPLCTDWQKAERGDILLSSLIRKKLLPEASLLITTRPVALEKLQH
LLDHPRHVEILGFSEAKRKEYFFKYFSDEAQARAAFSLIQENEVLFTMCFIPLVCWIVCT
GLKQQMESGKSLAQTSKTTTAVYVFFLSSLLQPRGGSQEHGLCAHLWGLCSLAADGIWNQ
KILFEESDLRNHGLQKADVSAFLRMNLFQKEVDCEKFYSFIHMTFQEFFAAMYYLLEEEK
EGRTNVPGSRLKLPSRDVTVLLENYGKFEKGYLIFVVRFLFGLVNQERTSYLEKKLSCKI
SQQIRLELLKWIEVKAKAKKLQIQPSQLELFYCLYEMQEEDFVQRAMDYFPKIEINLSTR
MDHMVSSFCIENCHRVESLSLGFLHNMPKEEEEEEKEGRHLDMVQCVLPSSSHAACSHGL
GRCGLSHECCFDISLVLSSNQKLVELDLSDNALGDFGIRLLCVGLKHLLCNLKKLWLVNS
GLTSVCCSALSSVLSTNQNLTHLYLRGNTLGDKGIKLLCEGLLHPDCKLQVLELDNCNLT
SHCCWDLSTLLTSSQSLRKLSLGNNDLGDLGVMMFCEVLKQQSCLLQNLGLSEMYFNYET
KSALETLQEEKPELTVVFEPSW*
>ENSG00000196419|ENST00000360079
MSGWESYYKTEGDEEAEEEQEENLEASGDYKYSGRDSLIFLVDASKAMFESQSEDELTPF
DMSIQCIQSVYISKIISSDRDLLAVVFYGTEKDKNSVNFKNIYVLQELDNPGAKRILELD
QFKGQQGQKRFQDMMGHGSDYSLSEVLWVCANLFSDVQFKMSHKRIMLFTNEDNPHGNDS

AKASRARTKAGDLRDTGIFLDLMHLKKPGGFDISLFYRDIISIAEDEDLRVHFEESSKLE
DLLRKVRAKETRKRALSRLKLKLNKDIVISVGIYNLVQKALKPPPIKLYRETNEPVKTKT
RTFNTSTGGLLLPSDTKRSQIYGSRQIILEKEETEELKRFDDPGLMLMGFKPLVLLKKHH
YLRPSLFVYPEESLVIGSSTLFSALLIKCLEKEVAALCRYTPRRNIPPYFVALVPQEEEL
DDQKIQVTPPGFQLVFLPFADDKRKMPFTEKIMATPEQVGKMKAIVEKLRFTYRSDSFEN
PVLQQHFRNLEALALDLMEPEQAVDLTLPKVEAMNKRLGSLVDEFKELVYPPDYNPEGKV
TKRKHDNEGSGSKRPKVEYSEEELKTHISKGTLGKFTVPMLKEACRAYGLKSGLKKQELL
EALTKHFQD*
>ENSG00000084234|ENST00000338167
MAATGTAAAAATGRLLLLLLVGLTAPALALAGYIEALAANAGTGFAVAEPQIAMFCGKLN
MHVNIQTGKWEPDPTGTKSCFETKEEVLQYCQEMYPELQITNVMEANQRVSIDNWCRRDK
KQCKSRFVTPFKCLVGEFVSDVLLVPEKCQFFHKERMEVCENHQHWHTVVKEACLTQGMT
LYSYGMLLPCGVDQFHGTEYVCCPQTKIIGSVSKEEEEEDEEEEEEEDEEEDYDVYKSEF
PTEADLEDFTEAAVDEDDEDEEEGEEVVEDRDYYYDTFKGDDYNEENPTEPGSDGTMSDK
EITHDVKAVCSQEAMTGPCRAVMPRWYFDLSKGKCVRFIYGGCGGNRNNFESEDYCMAVC
KAMIPPTPLPTNDVDVYFETSADDNEHARFQKAKEQLEIRHRNRMDRVKKEWEEAELQAK
NLPKAERQTLIQHFQAMVKALEKEAASEKQQLVETHLARVEAMLNDRRRMALENYLAALQ
SDPPRPHRILQALRRYVRAENKDRLHTIRHYQHVLAVDPEKAAQMKSQVMTHLHVIEERR
NQSLSLLYKVPYVAQEIQEEIDELLQEQRADMDQFTASISETPVDVRVSSEESEEIPPFH
PFHPFPALPENEGSGVGEQDGGLIGAEEKVINSKNKVDENMVIDETLDVKEMIFNAERVG
GLEEERESVGPLREDFSLSSSALIGLLVIAVAIATVIVISLVMLRKRQYGTISHGIVEVD
PMLTPEERHLNKMQNHGYENPTYKYLEQMQI*
>ENSG00000092978|ENST00000366934
MFGAAGRQPIGAPAAGNSWHFSRTMEELVHDLVSALEESSEQARGGFAETGDHSRSISCP
LKRQARKRRGRKRRSYNVHHPWETGHCLSEGSDSSLEEPSKDYRENHNNNKKDHSDSDDQ
MLVAKRRPSSNLNNNVRGKRPLWHESDFAVDNVGNRTLRRRRKVKRMAVDLPQDISNKRT
MTQPPEGCRDQDMDSDRAYQYQEFTKNKVKKRKLKIIRQGPKIQDEGVVLESEETNQTNK
DKMECEEQKVSDELMSESDSSSLSSTDAGLFTNDEGRQGDDEQSDWFYEKESGGACGITG
VVPWWEKEDPTELDKNVPDPVFESILTGSFPLMSHPSRRGFQARLSRLHGMSSKNIKKSG
GTPTSMATNWTSEIPL*
>ENSG00000182158|ENST00000330387
MEVLESGEQGVLQWDRKLSELSEPGDGEALMYHTHFSELLDEFSQNVLGQLLNDPFLSEK
SVSMEVEPSPTSPAPLIQAEHSYSLCEEPRAQSPFTHITTSDSFNDDEVESEKWYLSTDF
PSTSIKTEPVTDEPPPGLVPSVTLTITAISTPLEKEEPPLEMNTGVDSSCQTIIPKIKLE
PHEVDQFLNFSPKEAPVDHLHLPPTPPSSHGSDSEGSLSPNPRLHPFSLPQTHSPSRAAP
RAPSALSSSPLLTAPHKLQGSGPLVLTEEEKRTLIAEGYPIPTKLPLSKSEEKALKKIRR
KIKNKISAQESRRKKKEYMDSLEKKVESCSTENLELRKKVEVLENTNRTLLQQLQKLQTL
VMGKVSRTCKLAGTQTGTCLMVVVLCFAVAFGSFFQGYGPYPSATKMALPSQHSLQEPYT
ASVVRSRNLLIYEEHSPPEESSSPGSAGELGGWDRGSSLLRVSGLESRPDVDLPHFIISN
ETSLEKSVLLELQQHLVSAKLEGNETLKVVELDRRVNTTF*
>ENSG00000010030|ENST00000339796
MQEGELAISPISPVAAMPPLGTHVQARCEAQINLLGEGGICKLPGRLRIQPALWSREDVL
HWLRWAEQEYSLPCTAEHGFEMNGRALCILTKDDFRHRAPSSGDVLYELLQYIKTQRRAL
VCGPFFGGIFRLKTPTQHSPVPPEEVTGPSQMDTRRGHLLQPPDPGLTSNFGHLDDPGLA

RWTPGKEESLNLCHCAELGCRTQGVCSFPAMPQAPIDGRIADCRLLWDYVYQLLLDTRYE
PYIKWEDKDAKIFRVVDPNGLARLWGNHKNRVNMTYEKMSRALRHYYKLNIIKKEPGQKL
LFRNGLQLIFIFIWSFQ*
>ENSG00000197782|ENST00000340963
MVHGSVTFRDVAIDFSQEEWECLQPDQRTLYRDVMLENYSHLISLGSSISKPDVITLLEQ
EKEPWMVVRKETSRRYPDLELKYGPEKVSPENDTSEVNLPKQVIKQISTTLGIEAFYFRN
DSEYRQFEGLQGYQEGNINQKMISYEKLPTHTPHASLICNTHKPYECKECGKYFSRSANL
IQHQSIHTGEKPFECKECGKAFRLHIQFTRHQKFHTGEKPFECNECGKAFSLLTLLNRHK
NIHTGEKLFECKECGKSFNRSSNLVQHQSIHSGVKPYECKECGKGFNRGAHLIQHQKIHS
NEKPFVCKECGMAFRYHYQLIEHCQIHTGEKPFECKECGKAFTLLTKLVRHQKIHTGEKP
FECRECGKAFSLLNQLNRHKNIHTGEKPFECKECGKSFNRSSNLVQHQSIHAGIKPYECK
ECGKGFNRGAHLIQHQKIHSNEKPFVCRECEMAFRYHCQLIEHSRIHTGDKPFECQDCGK
AFNRGSSLVQHQSIHTGEKPYECKECGKAFRLYLQLSQHQKTHTGEKPFECKECGKFFRR
GSNLNQHRSIHTGKKPFECKECGKAFRLHMHLIRHQKLHTGEKPFECKECGKAFRLHMQL
IRHQKLHTGEKPFECKECGKVFSLPTQLNRHKNIHTGEKAS*
>ENSG00000125107|ENST00000317147
MNLDSLSLALSQISYLVDNLTKKNYRASQQEIQHIVNRHGPEADRHLLRCLFSHVDFSGD
GKSSGKDFHQTQFLIQECALLITKPNFISTLSYAIDNPLHYQKSLKPAPHLFAQLSKVLK
LSKVQEVIFGLALLNSSSSDLRGFAAQFIKQKLPDLLRSYIDADVSGNQEGGFQDIAIEV
LHLLLSHLLFGQKGAFGVGQEQIDAFLKTLRRDFPQERCPVVLAPLLYPEKRDILMDRIL
PDSGGVAKTMMESSLADFMQEVGYGFCASIEECRNIIVQFGVREVTAAQVARVLGMMART
HSGLTDGIPLQSISAPGSGIWSDGKDKSDGAQAHTWNVEVLIDVLKELNPSLNFKEVTYE
LDHPGFQIRDSKGLHNVVYGIQRGLGMEVFPVDLIYRPWKHAEGQLSFIQHSLINPEIFC
FADYPCHTVATDILKAPPEDDNREIATWKSLDLIESLLRLAEVGQYEQVKQLFSFPIKHC
PDMLVLALLQINTSWHTLRHELISTLMPIFLGNHPNSAIILHYAWHGQGQSPSIRQLIMH
AMAEWYMRGEQYDQAKLSRILDVAQDLKALSMLLNGTPFAFVIDLAALASRREYLKLDKW
LTDKIREHGEPFIQACMTFLKRRCPSILGGLAPEKDQPKSAQLPPETLATMLACLQACAG
SVSQELSETILTMVANCSNVMNKARQPPPGVMPKGRPPSASSLDAISPVQIDPLAGMTSL
SIGGSAAPHTQSMQGFPPNLGSAFSTPQSPAKAFPPLSTPNQTTAFSGIGGLSSQLPVGG
LGTGSLTGIGTGALGLPAVNNDPFVQRKLGTSGLNQPTFQQSKMKPSDLSQVWPEANQHF
SKEIDDEANSYFQRIYNHPPHPTMSVDEVLEMLQRFKDSTIKREREVFNCMLRNLFEEYR
FFPQYPDKELHITACLFGGIIEKGLVTYMALGLALRYVLEALRKPFGSKMYYFGIAALDR
FKNRLKDYPQYCQHLASISHFMQFPHHLQEYIEYGQQSRDPPVKMQGSITTPGSIALAQA
QAQAQVPAKAPLAGQVSTMVTTSTTTTVAKTVTVTRPTGVSFKKDVPPSINTTNIDTLLV
ATDQTERIVEPPENIQEKIAFIFNNLSQSNMTQKVEELKETVKEEFMPWVSQYLVMKRVS
IEPNFHSLYSNFLDTLKNPEFNKMVLNETYRNIKVLLTSDKAAANFSDRSLLKNLGHWLG
MITLAKNKPILHTDLDVKSLLLEAYVKGQQELLYVVPFVAKVLESSIRSVVFRPPNPWTM
AIMNVLAELHQEHDLKLNLKFEIEVLCKNLALDINELKPGNLLKDKDRLKNLDEQLSAPK
KDVKQPEELPPITTTTTSTTPATNTTCTATVPPQPQYSYHDINVYSLAGLAPHITLNPTI
PLFQAHPQLKQCVRQAIERAVQELVHPVVDRSIKIAMTTCEQIVRKDFALDSEESRMRIA
AHHMMRNLTAGMAMITCREPLLMSISTNLKNSFASALRTASPQQREMMDQAAAQLAQDNC
ELACCFIQKTAVEKAGPEMDKRLATEFELRKHARQEGRRYCDPVVLTYQAERMPEQIRLK
VGGVDPKQLAVYEEFARNVPGFLPTNDLSQPTGFLAQPMKQAWATDDVAQIYDKCITELE
QHLHAIPPTLAMNPQAQALRSLLEVVVLSRNSRDAIAALGLLQKAVEGLLDATSGADADL

LLRYRECHLLVLKALQDGRAYGSPWCNKQITRCLIECRDEYKYNVEAVELLIRNHLVNMQ
QYDLHLAQSMENGLNYMAVAFAMQLVKILLVDERSVAHVTEADLFHTIETLMRINAHSRG
NAPEGLPQLMEVVRSNYEAMIDRAHGGPNFMMHSGISQASEYDDPPGLREKAEYLLREWV
NLYHSAAAGRDSTKAFSAFVGQMHQQGILKTDDLITRFFRLCTEMCVEISYRAQAEQQHN
PAANPTMIRAKCYHNLDAFVRLIALLVKHSGEATNTVTKINLLNKVLGIVVGVLLQDHDV
RQSEFQQLPYHRIFIMLLLELNAPEHVLETINFQTLTAFCNTFHILRPTKAPGFVYAWLE
LISHRIFIARMLAHTPQQKGWPMYAQLLIDLFKYLAPFLRNVELTKPMQILYKGTLRVLL
VLLHDFPEFLCDYHYGFCDVIPPNCIQLRNLILSAFPRNMRLPDPFTPNLKVDMLSEINI
APRILTNFTGVMPPQFKKDLDSYLKTRSPVTFLSDLRSNLQVSNEPGNRYNLQLINALVL
YVGTQAIAHIHNKGSTPSMSTITHSAHMDIFQNLAVDLDTEGRYLFLNAIANQLRYPNSH
THYFSCTMLYLFAEANTEAIQEQITRVLLERLIVNRPHPWGLLITFIELIKNPAFKFWNH
EFVHCAPEIEKLFQSVAQCCMGQKQAQQVMEGTGAS*
>ENSG00000180318|ENST00000316824
MEFLSEKFALKSPPSKNSDFYMGAGGPLEHVMETLDNESFYSKASAGKCVQAFGPLPRAE
HHVRLERTSPCQDSSVNYGITKVEGQPLHTELNRAMDNCNSLRMSPVKGMQEKGELDELG
DKCDSNVSSSKKRRHRTTFTSLQLEELEKVFQKTHYPDVYVREQLALRTELTEARVQVWF
QNRRAKWRKRERYGQIQQAKSHFAATYDISVLPRTDSYPQIQNNLWAGNASGGSVVTSCM
LPRDTSSCMTPYSHSPRTDSSYTGFSNHQNQFSHVPLNNFFTDSLLTGATNGHAFETKPE
FERRSSSIAVLRMKAKEHTANISWAM*
>ENSG00000199080|ENST00000362210
Sequence unavailable
>ENSG00000188629|ENST00000343499
MAAGWLTTWSQNSVTFQEVAVDFSQEEWALLDPAQKNLYKDVMLENFRNLASVGYQLCRH
SLISKVDQEQLKTDERGILQGDCADWETQLKPKDTIAMQNIPGGKTSNGINTNCVRTHSG
EMPYECSDCGKAFIFQSSLKKHMRSHTGEKPYECDHCGKSFSQSSHLNVHKRTHTGEKPY
DCKECGKAFTVPSSLQKHVRTHTGEKPYECSDCGKAFIDQSSLKKHTRSHTGEKPYECNQ
CGKSFSTGSYLIVHKRTHTGEKTYECKECGKAFRNSSCLRVHVRTHTGEKPYKCIQCEKA
FSTSTNLIMHKRIHNGQKLHE*
>ENSG00000196678|ENST00000357967
MATKRLARQLGLIRRKSIAPANGNLGRSKSKQLFDYLIVIDFESTCWNDGKHHHSQEIIE
FPAVLLNTSTGQIDSEFQAYVQPQEHPILSEFCMELTGIKQAQVDEGVPLKICLSQFCKW
IHKIQQQKNIIFATGISEPSASEVKLCAFVTWSDWDLGVCLEYECKRKQLLKPVFLNSWI
DLRATYKLFYRRKPKGLSGALQEVGIEFSGREHSGLDDSRNTALLAWKMIRDGCVMKITR
SLNKVPTKKNFSILARNLNTIQVEEMSACNISIQGPSIYNKEPKNIINPHEKVQMKSICA
NSPIKAQQDQLQVKNNIKASLHNVKSSLPLFNTKSSTSVGQLQSPTLNSPIYMQKQGKNE
HLAFNTKSKASTVGSELVLVSTTVPTVHHVSDLEMSSTLDCLPVLADWEDVVLLPASQPE
ENVDCTVPISDSDLEISFNSGERLMVLKELEMSSHENFGDIEETPQKSETSKSIVYKSPH
TTIYNVKEAKDPGSDISAFKLPEHKSSTFNRVNANMSHPLVLGKHPLLSGGTKRNPCSPQ
AFPPAKKQPFTIHEEKPTSSDCSPVRSSSWRRLPSILTSTVNLQEPWKSGKMTPPLCKCG
RRSKRLVVSNNGPNHGKVFYCCPIGKYQENRKCCGYFKWEQTLQKERANSMVPSHSTGGL
TFSSPETSHICDRNLSISTKNSLRLRPSMRN*
>ENSG00000197837|ENST00000358064
MSGRGKGGKGLGKGGAKRHRKVLRDNIQGITKPAIRRLARRGGVKRISGLIYEETRGVLK
VFLENVIRDAVTYTEHAKRKTVTAMDVVYALKRQGRTLYGFGG*

>ENSG00000153914|ENST00000334121
MNSGGGFGLGLGFGLTPTSVIQVTNLSSAVTSEQMRTLFSFLGEIEELRLYPPDNAPLAF
SSKVCYVKFRDPSSVGVAQHLTNTVFIDRALIVVPCAEGKIPEESKALSLLAPAPTMTSL
MPGAGLLPIPTPNPLTTLGVSLSSLGAIPAAALDPNIATLGEIPQPPLMGNVDPSKIDEI
RRTVYVGNLNSQTTTADQLLEFFKQVGEVKFVRMAGDETQPTRFAFVEFADQNSVPRALA
FNGVMFGDRPLKINHSNNAIVKPPEMTPQAAAKELEEVMKRVREAQSFISAAIEPESGKS
NERKGGRSRSHTRSKSRSSSKSHSRRKRSQSKHRSRSHNRSRSRQKDRRRSKSPHKKRSK
SRERRKSRSRSHSRDKRKDTREKIKEKERVKEKDREKEREREKEREKEKERGKNKDRDKE
REKDREKDKEKDREREREKEHEKDRDKEKEKEQDKEKEREKDRSKEIDEKRKKDKKSRTP
PRSYNASRRSRSSSRERRRRRSRSSSRSPRTSKTIKRKSSRSPSPRSRNKKDKKREKERD
HISERRERERSTSMRKSSNDRDGKEKLEKNSTSLKEKEHNKEPDSSVSKEVDDKDAPRTE
ENKIQHNGNCQLNEENLSTKTEAV*
>ENSG00000198440|ENST00000333201
MSKDLVTFGDVAVNFSQEEWEWLNPAQRNLYRKVMLENYRSLVSLGVSVSKPDVISLLEQ
GKEPWMVKKEGTRGPCPDWEYVFKNSEFSSKQETYEESSKVVTVGARHLSYSLDYPSLRE
DCQSEDWYKNQLGSQEVHLSQLIITHKEILPEVQNKEYNKSWQTFHQDTIFDIQQSFPTK
EKAHKHEPQKKSYRKKSVEMKHRKVYVEKKLLKCNDCEKVFNQSSSLTLHQRIHTGEKPY
ACVECGKTFSQSANLAQHKRIHTGEKPYECKECRKAFSQNAHLAQHQRVHTGEKPYQCKE
CKKAFSQIAHLTQHQRVHTGERPFECIECGKAFSNGSFLAQHQRIHTGEKPYVCNVCGKA
FSHRGYLIVHQRIHTGERPYECKECRKAFSQYAHLAQHQRVHTGEKPYECKVCRKAFSQI
AYLDQHQRVHTGEKPYECIECGKAFSNSSSLAQHQRSHTGEKPYMCKECRKTFSQNAGLA
QHQRIHTGEKPYECNVCGKAFSYSGSLTLHQRIHTGERPYECKDCRKSFRQRAHLAHHER
IHTMESFLTLSSPSPSTSNQLPRPVGFIS*
>ENSG00000166704|ENST00000341164
MAAINPWASWGALTDQSWGMTAVDPWASWALCPQYPAWHVEGSLEEGRRATGLPAAQVQE
PVTFKDVAVDFTQEEWGQLDLVQRTLYRDVMLETYGHLLSVGNQIAKPEVISLLEQGEEP
WSVEQACPQRTCPEWVRNLESKALIPAQSIFEEEQSHGMKLERYIWDDPWFSRLEVLGCK
DQLEMYHMNQSTAMRQMVFMQKQVLSQRSSEFCGLGAEFSQNLNFVPSQRVSQIEHFYKP
DTHAQSWRCDSAIMYADKVTCENNDYDKTVYQSIQPIYPARIQTGDNLFKCTDAVKSFNH
IIHFGDHKGIHTGEKLYEYKECHQIFNQSPSFNEHPRLHVGENQYNYKEYENIFYFSSFM
EHQKIGTVEKAYKYNEWEKVFGYDSFLTQHTSTYTAEKPYDYNECGTSFIWSSYLIQHKK
THTGEKPYECDKCGKVFRNRSALTKHERTHTGIKPYECNKCGKAFSWNSHLIVHKRIHTG
EKPYVCNECGKSFNWNSHLIGHQRTHTGEKPFECTECGKSFSWSSHLIAHMRMHTGEKPF
KCDECEKAFRDYSALSKHERTHSGAKPYKCTECGKSFSWSSHLIAHQRTHTGEKPYNCQE
CGKAFRERSALTKHEIIHSGIKPYECNKCGKSCSQMAHLVRHQRTHTGEKPYECNKCGKS
FSQSCHLVAHRRIHTGEKPYKCNQCERSFNCSSHLIAHRRTHTGEKPYRCNECGKAFNES
SSLIVHLRNHTGEKPYKCNHCEKAFCKNSSLIIHQRMHSGEKRFICSECGKAFSGHSALL
QHQRNHSEEKLN*
>ENSG00000115310|ENST00000337526
MEDLDQSPLVSSSDSPPRPQPAFKYQFVREPEDEEEEEEEEEEDEDEDLEELEVLERKPA
AGLSAAPVPTAPAAGAPLMDFGNDFVPPAPRGPLPAAPPVAPERQPSWDPSPVSSTVPAP
SPLSAAAVSPSKLPEDDEPPARPPPPPPASVSPQAEPVWTPPAPAPAAPPSTPAAPKRRG
SSGSVDETLFALPAASEPVIRSSAENMDLKEQPGNTISAGQEDFPSVLLETAASLPSLSP
LSAASFKEHEYLGNLSTVLPTEGTLQENVSEASKEVSEKAKTLLIDRDLTEFSELEYSEM

GSSFSVSPKAESAVIVANPREEIIVKNKDEEEKLVSNNILHNQQELPTALTKLVKEDEVV
SSEKAKDSFNEKRVAVEAPMREEYADFKPFERVWEVKDSKEDSDMLAAGGKIESNLESKV
DKKCFADSLEQTNHEKDSESSNDDTSFPSTPEGIKDRSGAYITCAPFNPAATESIATNIF
PLLGDPTSENKTDEKKIEEKKAQIVTEKNTSTKTSNPFLVAAQDSETDYVTTDNLTKVTE
EVVANMPEGLTPDLVQEACESELNEVTGTKIAYETKMDLVQTSEVMQESLYPAAQLCPSF
EESEATPSPVLPDIVMEAPLNSAVPSAGASVIQPSSSPLEASSVNYESIKHEPENPPPYE
EAMSVSLKKVSGIKEEIKEPENINAALQETEAPYISIACDLIKETKLSAEPAPDFSDYSE
MAKVEQPVPDHSELVEDSSPDSEPVDLFSDDSIPDVPQKQDETVMLVKESLTETSFESMI
EYENKEKLSALPPEGGKPYLESFKLSLDNTKDTLLPDEVSTLSKKEKIPLQMEELSTAVY
SNDDLFISKEAQIRETETFSDSSPIEIIDEFPTLISSKTDSFSKLAREYTDLEVSHKSEI
ANAPDGAGSLPCTELPHDLSLKNIQPKVEEKISFSDDFSKNGSATSKVLLLPPDVSALAT
QAEIESIVKPKVLVKEAEKKLPSDTEKEDRSPSAIFSAELSKTSVVDLLYWRDIKKTGVV
FGASLFLLLSLTVFSIVSVTAYIALALLSVTISFRIYKGVIQAIQKSDEGHPFRAYLESE
VAISEELVQKYSNSALGHVNCTIKELRRLFLVDDLVDSLKFAVLMWVFTYVGALFNGLTL
LILALISLFSVPVIYERHQAQIDHYLGLANKNVKDAMAKIQAKIPGLKRKAE*
>ENSG00000159176|ENST00000340006
MPNWGGGKKCGVCQKTVYFAEEVQCEGNSFHKSCFLCMVCKKNLDSTTVAVHGEEIYCKS
CYGKKYGPKGYGYGQGAGTLSTDKGESLGIKHEEAPGHRPTTNPNASKFAQKIGGSERCP
RCSQAVYAAEKVIGAGKSWHKACFRCAKCGKGLESTTLADKDGEIYCKGCYAKNFGPKGF
GFGQGAGALVHSE*
>ENSG00000196747|ENST00000358739
MSGRGKQGGKARAKAKTRSSRAGLQFPVGRVHRLLRKGNYAERVGAGAPVYLAAVLEYLT
AEILELAGNAARDNKKTRIIPRHLQLAIRNDEELNKLLGKVTIAQGGVLPNIQAVLLPKK
TESHHKAKGK*
>ENSG00000129250|ENST00000320785
MAGASVKVAVRVRPFNARETSQDAKCVVSMQGNTTSIINPKQSKDAPKSFTFDYSYWSHT
STEDPQFASQQQVYRDIGEEMLLHAFEGYNVCIFAYGQTGAGKSYTMMGRQEPGQQGIVP
QLCEDLFSRVSENQSAQLSYSVEVSYMEIYCERVRDLLNPKSRGSLRVREHPILGPYVQD
LSKLAVTSYADIADLMDCGNKARTVAATNMNETSSRSHAVFTIVFTQRCHDQLTGLDSEK
VSKISLVDLAGSERADSSGARGMRLKEGANINKSLTTLGKVISALADMQSKKRKSDFIPY
RDSVLTWLLKENLGGNSRTAMIAALSPADINYEETLSTLRYADRTKQIRCNAIINEDPNA
RLIRELQEEVARLRELLMAQGLSASALEGLKTEEGSVRGALPAVSSPPAPVSPSSPTTHN
GELEPSFSPNTESQIGPEEAMERLQETEKIIAELNETWEEKLRKTEALRMEREALLAEMG
VAVREDGGTVGVFSPKKTPHLVNLNEDPLMSECLLYHIKDGVTRVGQVDMDIKLTGQFIR
EQHCLFRSIPQPDGEVVVTLEPCEGAETYVNGKLVTEPLVLKSGNRIVMGKNHVFRFNHP
EQARLERERGVPPPPGPPSEPVDWNFAQKELLEQQGIDIKLEMEKRLQDLENQYRKEKEE
ADLLLEQQRLYADSDSGDDSDKRSCEESWRLISSLREQLPPTTVQTIVKRCGLPSSGKRR
APRRVYQIPQRRRLQGKDPRWATMADLKMQAVKEICYEVALADFRHGRAEIEALAALKMR
ELCRTYGKPDGPGDAWRAVARDVWDTVGEEEGGGAGSGGGSEEGARGAEVEDLRAHIDKL
TGILQEVKLQNSSKDRELQALRDRMLRMERVIPLAQDHEDENEEGGEVPWAPPEGSEAAE
EAAPSDRMPSARPPSPPLSSWERVSRLMEEDPAFRRGRLRWLKQEQLRLQGLQGSGGRGG
GLRRPPARFVPPHDCKLRFPFKSNPQHRESWPGMGSGEAPTPLQPPEEVTPHPATPARRP
PSPRRSHHPRRNSLDGGGRSRGAGSAQPEPQHFQPKKHNSYPQPPQPYPAQRPPGPRYPP
YTTPPRMRRQRSAPDLKESGAAV*

>ENSG00000196387|ENST00000355557
MSQGSVTFRDVAIDFSQEEWKWLQPAQRDLYRCVMLENYGHLVSLGLSISKPDVVSLLEQ
GKEPWLGKREVKRDLFSVSESSGEIKDFSPKNVIYDDSSQYLIMERILSQGPVYSSFKGG
WKCKDHTEMLQENQGCIRKVTVSHQEALAQHMNISTVERPYGCHECGKTFGRRFSLVLHQ
RTHTGEKPYACKECGKTFSQISNLVKHQMIHTGKKPHECKDCNKTFSYLSFLIEHQRTHT
GEKPYECTECGKAFSRASNLTRHQRIHIGKKQYICRKCGKAFSSGSELIRHQITHTGEKP
YECIECGKAFRRFSHLTRHQSIHTTKTPYECNECRKAFRCHSFLIKHQRIHAGEKLYECD
ECGKVFTWHASLIQHTKSHTGEKPYACAECDKAFSRSFSLILHQRTHTGEKPYVCKVCNK
SFSWSSNLAKHQRTHTLDNPYEYENSFNYHSFLTEHQ*
>ENSG00000167555|ENST00000360465
MALTQGPLKFMDVAIEFSQEEWKCLDPAQRTLYRDVMLENYRNLVSLGICLPDLSVTSML
EQKRDPWTLQSEEKIANDPDGRECIKGVNTERSSKLGSNAGNKPCKNQLGFTFQLHLSDL
QLFQAERKISGCKHFEKPVSDNSSVSPLEKISSSVKSHLLNKYRNNFDHAPLLPQEQKAH
IREKAYKCNEHGQVFRASASLTNQVIHNADNPYKCSECGKVFSCSSKLVIHRRMHTGEKP
YKCHECGKLFSSNSNLSQHQRIHTGEKPYKCHECDKVFRSSSKLAQHQRIHTGEKPYKCH
ECDKVFNQIAHLVRHQKIHTGEKPYSCNKCGKVFSRHSYLAEHQTVHTGEKPYKCEECGK
AFSVRSSLITHQLIHTGRKPYKCKECDKVFGRKCFLTSHQRIHTRERPYGCSQCGKIFSQ
KSDLIRHRKTHTDEKPYKCNKCGTAFREFSDLTAHFLIHSGEKPYECKECGKVFRYKSSL
TSHHRIHTGEKPYKCNRCGKVFSRSSNLVCHQKIHTGEKPYKCNQCGKVFNQASYLTRHQ
IIHTGERPYRCSKCGKAFRGCSGLTAHLAIHTEKKSHECKECGKIFTQKSSLTNHHRIHI
GEKPYKCTLCSKVFSHNSDLAQHQRVHS*
>ENSG00000020256|ENST00000346617
MNASSEGESFAGSVQIPGGTTVLVELTPDIHICGICKQQFNNLDAFVAHKQSGCQLTGTS
AAAPSTVQFVSEETVPATQTQTTTRTITSETQTITGCQFKTAYGMKDMERHLKIHTGDKP
HKCEVCGKCFSRKDKLKTHMRCHTGVKPYKCKTCDYAAADSSSLNKHLRIHSDERPFKCQ
ICPYASRNSSQLTVHLRSHTGDAPFQCWLCSAKFKISSDLKRHMRVHSGEKPFKCEFCNV
RCTMKGNLKSHIRIKHSGNNFKCPHCDFLGDSKATLRKHSRVHQSEHPEKCSECSYSCSS
KAALRIHERIHCTDRPFKCNYCSFDTKQPSNLSKHMKKFHGDMVKTEALERKDTGRQSSR
QVAKLDAKKSFHCDICDASFMREDSLRSHKRQHSEYSESKNSDVTVLQFQIDPSKQPATP
LTVGHLQVPLQPSQVPQFSEGRVKIIVGHQVPQANTIVQAAAAAVNIVPPALVAQNPEEL
PGNSRLQILRQVSLIAPPQSSRCPSEAGAMTQPAVLLTTHEQTDGATLHQTLIPTASGGP
QEGSGNQTFITSSGITCTDFEGLNALIQEGTAEVTVVSDGGQNIAVATTAPPVFSSSSQQ
ELPKQTYSIIQGAAHPALLCPADSIPD*
>ENSG00000194717|ENST00000349529
Sequence unavailable
>ENSG00000188313|ENST00000342435
MDKQNSQMNASHPETNLPVGYPPQYPPTAFQGPPGYSGYPGPQVSYPPPPAGHSGPGPAG
FPVPNQPVYNQPVYNQPVGAAGVPWMPAPQPPLNCPPGLEYLSQIDQILIHQQIELLEVL
TGFETNNKYEIKNSFGQRVYFAAEDTDCCTRNCCGPSRPFTLRIIDNMGQEVITLERPLR
CSSCCCPCCLQEIEIQAPPGVPIGYVIQTWHPCLPKFTIQNEKREDVLKISGPCVVCSCC
GDVDFEIKSLDEQCVVGKISKHWTGILREAFTDADNFGIQFPLDLDVKMKAVMIGACFLI
DFMFFESTGSQEQKSGVW*
>ENSG00000129538|ENST00000340900
MALEKSLVRLLLLVLILLVLGWVQPSLGKESRAKKFQRQHMDSDSSPSSSSTYCNQMMRR

RNMTQGRCKPVNTFVHEPLVDVQNVCFQEKVTCKNGQGNCYKSNSSMHITDCRLTNGSRY
PNCAYRTSPKERHIIVACEGSPYVPVHFDASVEDST*
>ENSG00000125846|ENST00000360010
MAFRDVAVDFTQDEWRLLSPAQRTLYREVMLENYSNLVSLGISFSKPELITQLEQGKETW
REEKKCSPATCPDHQEYGNNCDLQKEADPEPELYLDPFC
>ENSG00000112739|ENST00000337659
MAAAETQSLREQPEMEDANSEKSINEENGEVSEDQSQNKHSRHKKKKHKHRSKHKKHKHS
SEEDKDKKHKHKHKHKKHKRKEIIDASDKEGMSPAKRTKLDDLALLEDLEKQRALIKAEL
DNELMEGKVQSGMGLILQGYESGSEEEGEIHEKARNGNRSSTRSSSTKGKLELVDNKITT
KKRSKSRSKERTRHRSDKKKSKGGIEIVKEKTTRSKSKERKKSKSPSKRSKSQDQARKSK
SPTLRRRSQEKIGKARSPTDDKVKIEDKSKSKDRKKSPIINESRSRDRGKKSRSPVDLRG
KSKDRRSRSKERKSKRSETDKEKKPIKSPSKDASSGKENRSPSRRPGRSPKRRSLSPKPR
DKSRRSRSPLLNDRRSKQSKSPSRTLSPGRRAKSRSLERKRREPERRRLSSPRTRPRDDI
LSRRERSKDASPINRWSPTRRRSRSPIRRRSRSPLRRSRSPRRRSRSPRRRDRGRRSRSR
LRRRSRSRGGRRRRSRSKVKEDKFKGSLSEGMKVEQESSSDDNLEDFDVEEEDEEALIEQ
RRIQRQAIVQKYKYLAEDSNMSVPSEPSSPQSSTRTRSPSPDDILERVAADVKEYERENV
DTFEASVKAKHNLMTVEQNNGSSQKKLLAPDMFTESDDMFAAYFDSARLRAAGIGKDFKE
NPNLRDNWTDAEGYYRVNIGEVLDKRYNVYGYTGQGVFSNVVRARDNARANQEVAVKIIR
NNELMQKTGLKELEFLKKLNDADPDDKFHCLRLFRHFYHKQHLCLVFEPLSMNLREVLKK
YGKDVGLHIKAVRSYSQQLFLALKLLKRCNILHADIKPDNILVNESKTILKLCDFGSASH
VADNDITPYLVSRFYRAPEIIIGKSYDYGIDMWSVGCTLYELYTGKILFPGKTNNHMLKL
AMDLKGKMPNKMIRKGVFKDQHFDQNLNFMYIEVDKVTEREKVTVMSTINPTKDLLADLI
GCQRLPEDQRKKVHQLKDLLDQILMLDPAKRISINQALQHAFIQEKI*
>ENSG00000179335|ENST00000345005
MHHCKRYRSPEPDPYLSYRWKRRRSYSREHEGRLRYPSRREPPPRRSRSRSHDRLPYQRR
YRERRDSDTYRCEERSPSFGEDYYGPSRSRHRRRSRERGPYRTRKHAHHCHKRRTRSCSS
ASSRSQQSSKRSSRSVEDDKEGHLVCRIGDWLQERYEIVGNLGEGTFGKVVECLDHARGK
SQVALKIIRNVGKYREAARLEINVLKKIKEKDKENKFLCVLMSDWFNFHGHMCIAFELLG
KNTFEFLKENNFQPYPLPHVRHMAYQLCHALRFLHENQLTHTDLKPENILFVNSEFETLY
NEHKSCEEKSVKNTSIRVADFGSATFDHEHHTTIVATRHYRPPEVILELGWAQPCDVWSI
GCILFEYYRGFTLFQTHENREHLVMMEKILGPIPSHMIHRTRKQKYFYKGGLVWDENSSD
GRYVKENCKPLKSYMLQDSLEHVQLFDLMRRMLEFDPAQRITLAEALLHPFFAGLTPEER
SFHTSRNPSR*
>ENSG00000127081|ENST00000332591
MLENYFNLISVGCQVPKPEVIFSLEQEEPCMLDGEIPSQSRPDGDIGFGPLQQRMSEEVS
FQSEININLFTRDDPYSILEELWKDDEHTRKCGENQNKPLSRVVFINKKTLANDSIFEYK
DIGEIVHVNTHLVSSRKRPHNCNSCGKNLEPIITLYNRNNATENSDKTIGDGDIFTHLNS
HTEVTACECNQCGKPLHHKQALIQQQKIHTRESLYLFSDYVNVFSPKSHAFAHESICAEE
KQHECHECEAVFTQKSQLDGSQRVYAGICTEYEKDFSLKSNRQKTPYEGNYYKCSDYGRA
FIQKSDLFRCQRIHSGEKPYEYSECEKNLPQNSNLNIHKKIHTGGKHFECTECGKAFTRK
STLSMHQKIHTGEKPYVCTECGKAFIRKSHFITHERIHTGEKPYECSDCGKSFIKKSQLH
VHQRIHTGENPFICSECGKVFTHKTNLIIHQKIHTGERPYICTVCGKAFTDRSNLIKHQK
IHTGEKPYKCSDCGKSFTWKSRLRIHQKCHTGERHYECSECGKAFIQKSTLSMHQRIHRG
EKPYVCTECGKAFFHKSHFITHERIHTGEKPYECSICGKSFTKKSQLHVHQQIHTGEKPY

RCAECGKAFTDRSNLFTHQKIHTGEKPYKCSDCGKAFTRKSGLHIHQQSHTGERHYECSE
CGKAFARKSTLIMHQRIHTGEKPYICNECGKSFIQKSHLNRHRRIHTGEKPYECSDCGKS
FIKKSQLHEHHRIHTGEKPYICAECGKAFTIRSNLIKHQKIHTKQKPYKCSDLGKALNWK
PQLSMPQKSDNGEVECSMPQLWCGDSEGDQGQLSSI*
>ENSG00000187855|ENST00000342331
MMETRKPAERLALPYSLRTAPLGVPGTLPGLPRRDPLRVALRLDAACWEWARSGCARGWQ
YLPVPLDSAFEPAFLRKRNERERQRVRCVNEGYARLRDHLPRELADKRLSKVETLRAAID
YIKHLQELLERQAWGLEGAAGAVPQRRAECNSDGESKASSAPSPSSEPEEGGS*
>ENSG00000284190|ENST00000362134
Sequence unavailable
>ENSG00000111642|ENST00000357008
MASGLGSPSPCSAGSEEEDMDALLNNSLPPPHPENEEDPEEDLSETETPKLKKKKKPKKP
RDPKIPKSKRQKKERMLLCRQLGDSSGEGPEFVEEEEEVALRSDSEGSDYTPGKKKKKKL
GPKKEKKSKSKRKEEEEEEEDDDDDSKEPKSSAQLLEDWGMEDIDHVFSEEDYRTLTNYKA
FSQFVRPLIAAKNPKIAVSKMMMVLGAKWREFSTNNPFKGSSGASVAAAAAAAVAVVESM
VTATEVAPPPPPVEVPIRKAKTKEGKGPNARRKPKGSPRVPDAKKPKPKKVAPLKIKLGG
FGSKRKRSSSEDDDLDVESDFDDASINSYSVSDGSTSRSSRSRKKLRTTKKKKKDHQDYC
EVCQQGGEIILCDTCPRAYHMVCLDPDMEKAPEGKWSCPHCEKEGIQWEAKEDNSEGEEI
LEEVGGDLEEEDDHHMEFCRVCKDGGELLCCDTCPSSYHIHCLNPPLPEIPNGEWLCPRC
TCPALKGKVQKILIWKWGQPPSPTPVPRPPDADPNTPSPKPLEGRPERQFFVKWQGMSYW
HCSWVSELQLELHCQVMFRNYQRKNDMDEPPSGDFGGDEEKSRKRKNKDPKFAEMEERFY
RYGIKPEWMMIHRILNHSVDKKGHVHYLIKWRDLPYDQASWESEDVEIQDYDLFKQSYWN
HRELMRGEEGRPGKKLKKVKLRKLERPPETPTVDPTVKYERQPEYLDATGGTLHPYQMEG
LNWLRFSWAQGTDTILADEMGLGKTVQTAVFLYSLYKEGHSKGPFLVSAPLSTIINWERE
FEMWAPDMYVVTYVGDKDSRAIIRENEFSFEDNAIRGGKKASRMKKEASVKFHVLLTSYE
LITIDMAILGSIDWACLIVDEAHRLKNNQSKFFRVLNGYSLQHKLLLTGTPLQNNLEELF
HLLNFLTPERFHNLEGFLEEFADIAKEDQIKKLHDMLGPHMLRRLKADVFKNMPSKTELI
VRVELSPMQKKYYKYILTRNFEALNARGGGNQVSLLNVVMDLKKCCNHPYLFPVAAMEAP
KMPNGMYDGSALIRASGKLLLLQKMLKNLKEGGHRVLIFSQMTKMLDLLEDFLEHEGYKY
ERIDGGITGNMRQEAIDRFNAPGAQQFCFLLSTRAGGLGINLATADTVIIYDSDWNPHND
IQAFSRAHRIGQNKKVMIYRFVTRASVEERITQVAKKKMMLTHLVVRPGLGSKTGSMSKQ
ELDDILKFGTEELFKDEATDGGGDNKEGEDSSVIHYDDKAIERLLDRNQDETEDTELQGM
NEYLSSFKVAQYVVREEEMGEEEEVEREIIKQEESVDPDYWEKLLRHHYEQQQEDLARNL
GKGKRIRKQVNYNDGSQEDRDWQDDQSDNQSDYSVASEEGDEDFDERSEAPRRPSRKGLR
NDKDKPLPPLLARVGGNIEVLGFNARQRKAFLNAIMRYGMPPQDAFTTQWLVRDLRGKSE
KEFKAYVSLFMRHLCEPGADGAETFADGVPREGLSRQHVLTRIGVMSLIRKKVQEFEHVN
GRWSMPELAEVEENKKMSQPGSPSPKTPTPSTPGDTQPNTPAPVPPAEDGIKIEENSLKE
EESIEGEKEVKSTAPETAIECTQAPAPASEDEKVVVEPPEGEEKVEKAEVKERTEEPMET
EPKGKGAADVEKVEEKSAIDLTPIVVEDKEEKKEEEEKKEVMLQNGETPKDLNDEKQKKN
IKQRFMFNIADGGFTELHSLWQNEERAATVTKKTYEIWHRRHDYWLLAGIINHGYARWQD
IQNDPRYAILNEPFKGEMNRGNFLEIKNKFLARRFKLLEQALVIEEQLRRAAYLNMSEDP
SHPSMALNTRFAEVECLAESHQHLSKESMAGNKPANAVLHKGILKQLEELLSDMKADVTR
LPATIARIPPVAVRLQMSERNILSRLANRAPEPTPQQVAQQQ*
>ENSG00000178951|ENST00000322357

MAGGVDGPIGIPFPDHSSDILSGLNEQRTQGLLCDVVILVEGREFPTHRSVLAACSQYFK
KLFTSGAVVDQQNVYEIDFVSAEALTALMDFAYTATLTVSTANVGDILSAARLLEIPAVS
HVCADLLDRQILAADAGADAGQLDLVDQIDQRNLLRAKEYLEFFQSNPMNSLPPAAAAAA
ASFPWSAFGASDDDLDATKEAVAAAVAAVAAGDCNGLDFYGPGPPAERPPTGDGDEGDSN
PGLWPERDEDAPTGGLFPPPVAPPAATQNGHYGRGGEEEAASLSEAAPEPGDSPGFLSGA
AEGEDGDGPDVDGLAASTLLQQMMSSVGRAGAAAGDSDEESRADDKGVMDYYLKYFSGAH
DGDVYPAWSQKVEKKIRAKAFQKCPICEKVIQGAGKLPRHIRTHTGEKPYECNICKVRFT
RQDKLKVHMRKHTGEKPYLCQQCGAAFAHNYDLKNHMRVHTGLRPYQCDSCCKTFVRSDH
LHRHLKKDGCNGVPSRRGRKPRVRGGAPDPSPGATATPGAPAQPSSPDARRNGQEKHFKD
EDEDEDVASPDGLGRLNVAGAGGGGDSGGGPGAATDGNFTAGLA*
>ENSG00000122257|ENST00000319715
MSCVHYKFSSKLNYDTVTFDGLHISLCDLKKQIMGREKLKAADCDLQITNAQTKEEYTDD
NALIPKNSSVIVRRIPIGGVKSTSKTYVISRTEPAMATTKAIDDSSASISLAQLTKTANL
AEANASEEDKIKAMMSQSGHEYDPINYMKKPLGPPPPSYTCFRCGKPGHYIKNCPTNGDK
NFESGPRIKKSTGIPRSFMMEVKDPNMKGAMLTNTGKYAIPTIDAEAYAIGKKEKPPFLP
EEPSSSSEEDDPIPDELLCLICKDIMTDAVVIPCCGNSYCDECIRTALLESDEHTCPTCH
QNDVSPDALIANKFLRQAVNNFKNETGYTKRLRKQLPPPPPPIPPPRPLIQRNLQPLMRS
PISRQQDPLMIPVTSSSTHPAPSISSLTSNQSSLAPPVSGNPSSAPAPVPDITATVSISV
HSEKSDGPFRDSDNKILPAAALASEHSKGTSSIAITALMEEKGYQVPVLGTPSLLGQSLL
HGQLIPTTGPVRINTARPGGGRPGWEHSNKLGYLVSPPQQIRRGERSCYRSINRGRHHSE
RSQRTQGPSLPATPVFVPVPPPPLYPPPPHTLPLPPGVPPPQFSPQFPPGQPPPAGYSVP
PPGFPPAPANLSTPWVSSGVQTAHSNTIPTTQAPPLSREEFYREQRRLKEEEKKKSKLDE
FTNDFAKELMEYKKIQKERRRSFSRSKSPYSGSSYSRSSYTYSKSRSGSTRSRSYSRSFS
RSHSRSYSRSPPYPRRGRGKSRNYRSRSRSHGYHRSRSRSPPYRRYHSRSRSPQAFRGQS
PNKRNVPQGETEREYFNRYREVPPPYDMKAYYGRSVDFRDPFEKERYREWERKYREWYEK
YYKGYAAGAQPRPSANRENFSPERFLPLNIRNSPFTRGRREDYVGGQSHRSRNIGSNYPE
KLSARDGHNQKDNTKSKEKESENAPGDGKGNKHKKHRKRRKGEESEGFLNPELLETSRKS
REPTGVEENKTDSLFVLPSRDDATPVRDEPMDAESITFKSVSEKDKRERDKPKAKGDKTK
RKNDGSAVSKKENIVKPAKGPQEKVDGERERSPRSEPPIKKAKEETPKTDNTKSSSSSQK
DEKITGTPRKAHSKSAKEHQETKPVKEEKVKKDYSKDVKSEKLTTKEEKAKKPNEKNKPL
DNKGEKRKRKTEEKGVDKDFESSSMKISKLEVTEIVKPSPKRKMEPDTEKMDRTPEKDKI
SLSAPAKKIKLNRETGKKIGSTENISNTKEPSEKLESTSSKVKQEKVKGKVRRKVTGTEG
SSSTLVDYTSTSSTGGSPVRKSEEKTDTKRTVIKTMEEYNNDNTAPAEDVIIMIQVPQSK
WDKDDFESEEEDVKSTQPISSVGKPASVIKNVSTKPSNIVKYPEKESEPSEKIQKFTKDV
SHEIIQHEVKSSKNSASSEKGKTKDRDYSVLEKENPEKRKNSTQPEKESNLDRLNEQGNF
KSLSQSSKEARTSDKHDSTRASSNKDFTPNRDKKTDYDTREYSSSKRRDEKNELTRRKDS
PSRNKDSASGQKNKPREERDLPKKGTGDSKKSNSSPSRDRKPHDHKATYDTKRPNEETKS
VDKNPCKDREKHVLEARNNKESSGNKLLYILNPPETQVEKEQITGQIDKSTVKPKPQLSH
SSRLSSDLTRETDEAAFEPDYNESDSESNVSVKEEESSGNISKDLKDKIVEKAKESLDTA
AVVQVGISRNQSHSSPSVSPSRSHSPSGSQTRSHSSSASSAESQDSKKKKKKKEKKKHKK
HKKHKKHKKHAGTEVELEKSQKHKHKKKKSKKNKDKEKEKEKDDQKVKSVTV*
>ENSG00000198554|ENST00000360586
MPATRKPMRYGHTEGHTEVCFDDSGSFIVTCGSDGDVRIWEDLDDDDPKFINVGEKAYSC
ALKSGKLVTAVSNNTIQVHTFPEGVPDGILTRFTTNANHVVFNGDGTKIAAGSSDFLVKI

VDVMDSSQQKTFRGHDAPVLSLSFDPKDIFLASASCDGSVRVWQISDQTCAISWPLLQKC
NDVINAKSICRLAWQPKSGKLLAIPVEKSVKLYRRESWSHQFDLSDNFISQTLNIVTWSP
CGQYLAAGSINGLIIVWNVETKDCMERVKHEKGYAICGLAWHPTCGRISYTDAEGNLGLL
ENVCDPSGKTSSSKVSSRVEKDYNDLFDGDDMSNAGDFLNDNAVEIPSFSKGIINDDEDD
EDLMMASGRPRQRSHILEDDENSVDISMLKTGSSLLKEEEEDGQEGSIHNLPLVTSQRPF
YDGPMPTPRQKPFQSGSTPLHLTHRFMVWNSIGIIRCYNDEQDNAIDVEFHDTSIHHATH
LSNTLNYTIADLSHEAILLACESTDELASKLHCLHFSSWDSSKEWIIDLPQNEDIEAICL
GQGWAAAATSALLLRLFTIGGVQKEVFSLAGPVVSMAGHGEQLFIVYHRGTGFDGDQCLG
VQLLELGKKKKQILHGDPLPLTRKSYLAWIGFSAEGTPCYVDSEGIVRMLNRGLGNTWTP
ICNTREHCKGKSDHYWVVGIHENPQQLRCIPCKGSRFPPTLPRPAVAILSFKLPYCQIAT
EKGQMEEQFWRSVIFHNHLDYLAKNGYEYEESTKNQATKEQQELLMKMLALSCKLEREFR
CVELADLMTQNAVNLAIKYASRSRKLILAQKLSELAVEKAAELTATQVEEEEEEEDFRKK
LNAGYSNTATEWSQPRFRNQVEEDAEDSGEADDEEKPEIHKPGQNSFSKSTNSSDVSAKS
GAVTFSSQGRVNPFKVSASSKEPAMSMNSARSTNILDNMGKSSKKSTALSRTTNNEKSPI
IKPLIPKPKPKQASAASYFQKRNSQTNKTEEVKEENLKNVLSETPAICPPQNTENQRPKT
GFQMWLEENRSNILSDNPDFSDEADIIKEGMIRFRVLSTEERKVWANKAKGETASEGTEA
KKRKRVVDESDETENQEEKAKENLNLSKKQKPLDFSTNQKLSAFAFKQE*
>ENSG00000106462|ENST00000350995
MGQTGKKSEKGPVCWRKRVKSEYMRLRQLKRFRRADEVKSMFSSNRQKILERTEILNQEW
KQRRIQPVHILTSVSSLRGTREVEDETVLHNIPYMGDEVLDQDGTFIEELIKNYDGKVHG
DRECGFINDEIFVELVNALGQYNDDDDDDDGDDPEEREEKQKDLEDHRDDKESRPPRKFP
SDKIFEAISSMFPDKGTAEELKEKYKELTEQQLPGALPPECTPNIDGPNAKSVQREQSLH
SFHTLFCRRCFKYDCFLHPFHATPNTYKRKNTETALDNKPCGPQCYQHLEGAKEFAAALT
AERIKTPPKRPGGRRRGRLPNNSSRPSTPTINVLESKDTDSDREAGTETGGENNDKEEEE
KKDETSSSSEANSRCQTPIKMKPNIEPPENVEWSGAEASMFRVLIGTYYDNFCAIARLIG
TKTCRQVYEFRVKESSIIAPAPAEDVDTPPRKKKRKHRLWAAHCRKIQLKKDGSSNHVYN
YQPCDHPRQPCDSSCPCVIAQNFCEKFCQCSSECQNRFPGCRCKAQCNTKQCPCYLAVRE
CDPDLCLTCGAADHWDSKNVSCKNCSIQRGSKKHLLLAPSDVAGWGIFIKDPVQKNEFIS
EYCGEIISQDEADRRGKVYDKYMCSFLFNLNNDFVVDATRKGNKIRFANHSVNPNCYAKV
MMVNGDHRIGIFAKRAIQTGEELFFDYRYSQADALKYVGIEREMEIP*
>ENSG00000197863|ENST00000356725
MAHLMMFRDVAVDFSQEEWECLDLEQRDLYRDVMLENYSNMVSLGFCIYQPEAFSLLEKG
KEPWKILRDETRGPCPDMQSRCQTKKLLPKNGIFEREIAQLEIMRICKNHSLDCLCFRGD
WEGNTQFQTLQDNQEECFKQVIRTCEKRPTFNQHTVFNLHQRLNTGDKLNEFKELGKAFI
SGSDHTQHQLIHTSEKFCGDKECGNTFLPDSEVIQYQTVHTVKKTYECKECGKSFSLRSS
LTGHKRIHTGEKPFKCKDCGKAFRFHSQLSVHKRIHTGEKSYECKECGKAFSCGSDLTRH
QRIHTGEKPYECNECRKAFSQRSHLIKHQRIHTGEKPYECKECGKAFTRGSHLTQHQRIH
TGEKSHECKECGKAFIRGSNLAQHQNVHVGRKPYKCEKCGKAYIWSSHLARHQRIHTGRK
PYECKQCGKTFTWASYLAQHEKIHNERKSYECKECGKTFLHGSEFNRHQKIHTGERNYEC
KECGKTFFRGSELNRHQKIHTGKRPYECEECGKAFLWGSQLTRHQRMHTGEEPYVCKECG
KSFIWGSQLTRHKKIHTDAEPYGCKKSSHIFSHHSYFTEQKIHNSANLCEWTDYGNTFSH
ESNFAQHQNIYTFEKSYEFKDFEKAFSSSSHFISLL*
>ENSG00000005801|ENST00000343338
MAGAQTLLTFRDVAIEFSLEEWKCLDLAQQNLYRDVMLENYRNLFSVGLTVCKPGLITCL

EQRKEPWNVKRQEAADGHPAMSSHFTQDLLPEQGIQDAFPKRILRGYGNCGLDNLYLRKD
WESLDECKLQKDYNGLNQCSSTTHSKIFQYNKYVKIFDNFSNLHRRNISNTGEKPFKCQE
CGKSFQMLSFLTEHQKIHTGKKFQKCGECGKTFIQCSHFTEPENIDTGEKPYKCQECNNV
IKTCSVLTKNRIYAGGEHYRCEEFGKVFNQCSHLTEHEHGTEEKPCKYEECSSVFISCSS
LSNQQMILAGEKLSKCETWYKGFNHSPNPSKHQRNEIGGKPFKCEECDSIFKWFSDLTKH
KRIHTGEKPYKCDECGKAYTQSSHLSEHRRIHTGEKPYQCEECGKVFRTCSSLSNHKRTH
SEEKPYTCEECGNIFKQLSDLTKHKKTHTGEKPYKCDECGKNFTQSSNLIVHKRIHTGEK
PYKCEECGRVFMWFSDITKHKKTHTGEKPYKCDECGKNFTQSSNLIVHKRIHTGEKPYKC
EKCGKAFTQFSHLTVHESIHT*
>ENSG00000105997|ENST00000317201
MQKATYYDSSAIYGGYPYQAANGFAYNANQQPYPASAALGADGEYHRPACSLQSPSSAGG
HPKAHELSEACLRTLSAPPSQPPSLGEPPLHPPPPQAAPPAPQPPQPAPQPPAPTPAAPP
PPSSASPPQNASNNPTPANAAKSPLLNSPTVAKQIFPWMKESRQNTKQKTSSSSSGESCA
GDKSPPGQASSKRARTAYTSAQLVELEKEFHFNRYLCRPRRVEMANLLNLTERQIKIWFQ
NRRMKYKKDQKGKGMLTSSGGQSPSRSPVPPGAGGYLNSMHSLVNSVPYEPQSPPPFSKP
PQGTYGLPPASYPASLPSCAPPPPPQKRYTAAGAGAGGTPDYDPHAHGLQGNGSYGTPHI
QGSPVFVGGSYVEPMSNSGPALFGLTHLPHAASGAMDYGGAGPLGSGHHHGPGPGEPHPT
YTDLTGHHPSQGRIQEAPKLTHL*
>ENSG00000053254|ENST00000345097
MGPVMPPSKKPESSGISVSSGLSQCYGGSGFSKALQEDDDLDFSLPDIRLEEGAMEDEEL
TNLNWLHESKNLLKSFGESVLRSVSPVQDLDDDTPPSPAHSDMPYDARQNPNCKPPYSFS
CLIFMAIEDSPTKRLPVKDIYNWILEHFPYFANAPTGWKNSVRHNLSLNKCFKKVDKERS
QSIGKGSLWCIDPEYRQNLIQALKKTPYHPHPHVFNTPPTCPQAYQSTSGPPIWPGSTFF
KRNGALLQDPDIDAASAMMLLNTPPEIQAGFPPGVIQNGARVLSRGLFPGVRPLPITPIG
VTAAMRNGITSCRMRTESEPSCGSPVVSGDPKEDHNYSSAKSSNARSTSPTSDSISSSSS
SADDHYEFATKGSQEGSEGSEGSFRSHESPSDTEEDDRKHSQKEPKDSLGDSGYASQHKK
RQHFAKARKVPSDTLPLKKRRTEKPPESDDEEMKEAAGSLLHLAGIRSCLNNITNRTAKG
QKEQKETTKN*
>ENSG00000103275|ENST00000325437
MSGIALSRLAQERKAWRKDHPFGFVAVPTKNPDGTMNLMNWECAIPGKKGTPWEGGLFKL
RMLFKDDYPSSPPKCKFEPPLFHPNVYPSGTVCLSILEEDKDWRPAITIKQILLGIQELL
NEPNIQDPAQAEAYTIYCQNRVEYEKRVRAQAKKFAPS*
>ENSG00000162711|ENST00000366497
MKMASTRCKLARYLEDLEDVDLKKFKMHLEDYPPQKGCIPLPRGQTEKADHVDLATLMID
FNGEEKAWAMAVWIFAAINRRDLYEKAKRDEPKWGSDNARVSNPTVICQEDSIEEEWMGL
LEYLSRISICKMKKDYRKKYRKYVRSRFQCIEDRNARLGESVSLNKRYTRLRLIKEHRSQ
QEREQELLAIGKTKTCESPVSPIKMELLFDPDDEHSEPVHTVVFQGAAGIGKTILARKMM
LDWASGTLYQDRFDYLFYIHCREVSLVTQRSLGDLIMSCCPDPNPPIHKIVRKPSRILFL
MDGFDELQGAFDEHIGPLCTDWQKAERGDILLSSLIRKKLLPEASLLITTRPVALEKLQH
LLDHPRHVEILGFSEAKRKEYFFKYFSDEAQARAAFSLIQENEVLFTMCFIPLVCWIVCT
GLKQQMESGKSLAQTSKTTTAVYVFFLSSLLQPRGGSQEHGLCAHLWGLCSLAADGIWNQ
KILFEESDLRNHGLQKADVSAFLRMNLFQKEVDCEKFYSFIHMTFQEFFAAMYYLLEEEK
EGRTNVPGSRLKLPSRDVTVLLENYGKFEKGYLIFVVRFLFGLVNQERTSYLEKKLSCKI
SQQIRLELLKWIEVKAKAKKLQIQPSQLELFYCLYEMQEEDFVQRAMDYFPKIEINLSTR

MDHMVSSFCIENCHRVESLSLGFLHNMPKEEEEEEEKEGRHLDMVQCVLPSSSHAACSHGL
VNSHLTSSFCRGLFSVLSTSQSLTELDLSDNSLGDPGMRVLCETLQHPGCNIRRLWLGRC
GLSHECCFDISLVLSSNQKLVELDLSDNALGDFGIRLLCVGLKHLLCNLKKLWLVNSGLT
SVCCSALSSVLSTNQNLTHLYLRGNTLGDKGIKLLCEGLLHPDCKLQVLELDNCNLTSHC
CWDLSTLLTSSQSLRKLSLGNNDLGDLGVMMFCEVLKQQSCLLQNLGLSEMYFNYETKSA
LETLQEEKPELTVVFEPSW*
>ENSG00000095794|ENST00000348787
MTMETVESQHDGSITASLTESKSAHVQTQTGQNSIPALAQVAAIAETDESAESEGVIDSH
KRREILSRRPSYRKILNELSSDVPGVPKIEEERSEEEGTPPSIATMAVPTSIYQTSTGQY
TATGDMPTYQIRAPTAALPQGVVMAASPGSLHSPQQLAEEATRKRELRLMKNREAAKECR
RRKKEYVKCLESRVAVLEVQNKKLIEELETLKDICSPKTDY*
>ENSG00000118418|ENST00000344726
MPKRKSPENTEGKDGSKVTKQEPTRRSARLSAKPAPPKPEPKPRKTSAKKEPGAKISRGA
KGKKEEKQEAGKEGTAPSENGETKAEEAQKTESVDNEGE*
>ENSG00000141644|ENST00000353909
MAEDWLDCPALGPGWKRREVFRKSGATCGRSDTYYQSPTGDRIRSKVELTRYLGPACDLT
LFDFKQGILCYPAPKAHPVAVASKKRKKPSRPAKTRKRQVGPQSGEVRKEAPRDETKADT
DTAPASFPAPGCCENCGISFSGDGTQRQRLKTLCKDCRAQRIAFNREQRMFKSRGCGVCR
GCQTQEDCGHCPICLRPPRPGLRRQWKCVQRRCLRGKHARRKGGCDSKMAARRRPGAQPL
PPPPPSQSPEPTEPHPRALAPSPPAEFIYYCVDEDELQPYTNRRQNRKCGACAACLRRMD
CGRCDFCCDKPKFGGSNQKRQKCRWRQCLQFAMKRLLPSVWSESEDGAGSPPPYRRRKRP
SSARRHHLGPTLKPTLATRTAQPDHTQAPTKQEAGGGFVLPPPGTDLVFLREGASSPVQV
PGPVAASTEALLQEAQCSGLSWVVALPQVKQEKADTQDEWTPGTAVLTSPVLVPGCPSKA
VDPGLPSVKQEPPDPEEDKEENKDDSASKLAPEEEAGGAGTPVITEIFSLGGTRFRDTAV
WLPRSKDLKKPGARKQ*
>ENSG00000135903|ENST00000344493
MTTLAGAVPRMMRPGPGQNYPRSGFPLEVSTPLGQGRVNQLGGVFINGRPLPNHIRHKIV
EMAHHGIRPCVISRQLRVSHGCVSKILCRYQETGSIRPGAIGGSKPKQVTTPDVEKKIEE
YKRENPGMFSWEIRDKLLKDAVCDRNTVPSVSSISRILRSKFGKGEEEEADLERKEAEES
EKKAKHSIDGILSERASAPQSDEGSDIDSEPDLPLKRKQRRSRTTFTAEQLEELERAFER
THYPDIYTREELAQRAKLTEARVQVWFSNRRARWRKQAGANQLMAFNHLIPGGFPPTAMP
TLPTYQLSETSYQPTSIPQAVSDPSSTVHRPQPLPPSTVHQSTIPSNPDSSSAYCLPSTR
HGFSSYTDSFVPPSGPSNPMNPTIGNGLSPQVPFIISSQISRK*
>ENSG00000049618|ENST00000350026
MAHNAGAAAAAGTHSAKSGGSEAALKEGGSAAALSSSSSSSAAAAAASSSSSSGPGSAME
TGLLPNHKLKTVGEAPAAPPHQQHHHHHHAHHHHHHAHHLHHHHALQQQLNQFQQQQQQQ
QQQQQQQQQQHPISNNNSLGGAGGGAPQPGPDMEQPQHGGAKDSAAGGQADPPGPPLLS
KPGDEDDAPPKMGEPAGGRYEHPGLGALGTQQPPVAVPGGGGGPAAVPEFNNYYGSAAPA
SGGPGGRAGPCFDQHGGQQSPGMGMMHSASAAAAGAPGSMDPLQNSHEGYPNSQCNHYPG
YSRPGAGGGGGGGGGGGGGSGGGGGGGGAGAGGAGAGAVAAAAAAAAAAAGGGGGGGYGG
SSAGYGVLSSPRQQGGGMMMGPGGGGAASLSKAAAGSAAGGFQRFAGQNQHPSGATPTLN
QLLTSPSPMMRSYGGSYPEYSSPSAPPPPPSQPQSQAAAAGAAAGGQQAAAGMGLGKDMG
AQYAAASPAWAAAQQRSHPAMSPGTPGPTMGRSQGSPMDPMVMKRPQLYGMGSNPHSQPQ
QSSPYPGGSYGPPGPQRYPIGIQGRTPGAMAGMQYPQQQMPPQYGQQGVSGYCQQGQQPY

YSQQPQPPHLPPQAQYLPSQSQQRYQPQQDMSQEGYGTRSQPPLAPGKPNHEDLNLIQQE
RPSSLPDLSGSIDDLPTGTEATLSSAVSASGSTSSQGDQSNPAQSPFSPHASPHLSSIPG
GPSPSPVGSPVGSNQSRSGPISPASIPGSQMPPQPPGSQSESSSHPALSQSPMPQERGFM
AGTQRNPQMAQYGPQQTGPSMSPHPSPGGQMHAGISSFQQSNSSGTYGPQMSQYGPQGNY
SRPPAYSGVPSASYSGPGPGMGISANNQMHGQGPSQPCGAVPLGRMPSAGMQNRPFPGNM
SSMTPSSPGMSQQGGPGMGPPMPTVNRKAQEAAAAVMQAAANSAQSRQGSFPGMNQSGLM
ASSSPYSQPMNNSSSLMNTQAPPYSMAPAMVNSSAASVGLADMMSPGESKLPLPLKADGK
EEGTPQPESKSKKSSSSTTTGEKITKVYELGNEPERKLWVDRYLTFMEERGSPVSSLPAV
GKKPLDLFRLYVCVKEIGGLAQVNKNKKWRELATNLNVGTSSSAASSLKKQYIQYLFAFE
CKIERGEEPPPEVFSTGDTKKQPKLQPPSPANSGSLQGPQTPQSTGSNSMAEVPGDLKPP
TPASTPHGQMTPMQGGRSSTISVHDPFSDVSDSSFPKRNSMTPNAPYQQGMSMPDVMGRM
PYEPNKDPFGGMRKVPGSSEPFMTQGQMPNSSMQDMYNQSPSGAMSNLGMGQRQQFPYGA
SYDRRHEPYGQQYPGQGPPSGQPPYGGHQPGLYPQQPNYKRHMDGMYGPPAKRHEGDMYN
MQYSSQQQEMYNQYGGSYSGPDRRPIQGQYPYPYSRERMQGPGQIQTHGIPPQMMGGPLQ
SSSSEGPQQNMWAARNDMPYPYQNRQGPGGPTQAPPYPGMNRTDDMMVPDQRINHESQWP
SHVSQRQPYMSSSASMQPITRPPQPSYQTPPSLPNHISRAPSPASFQRSLENRMSPSKSP
FLPSMKMQKVMPTVPTSQVTGPPPQPPPIRREITFPPGSVEASQPVLKQRRKITSKDIVT
PEAWRVMMSLKSGLLAESTWALDTINILLYDDSTVATFNLSQLSGFLELLVEYFRKCLID
IFGILMEYEVGDPSQKALDHNAARKDDSQSLADDSGKEEEDAECIDDDEEDEEDEEEDSE
KTESDEKSSIALTAPDAAADPKEKPKQASKFDKLPIKIVKKNNLFVVDRSDKLGRVQEFN
SGLLHWQLGGGDTTEHIQTHFESKMEIPPRRRPPPPLSSAGRKKEQEGKGDSEEQQEKSI
IATIDDVLSARPGALPEDANPGPQTESSKFPFGIQQAKSHRNIKLLEDEPRSRDETPLCT
IAHWQDSLAKRCICVSNIVRSLSFVPGNDAEMSKHPGLVLILGKLILLHHEHPERKRAPQ
TYEKEEDEDKGVACSKDEWWWDCLEVLRDNTLVTLANISGQLDLSAYTESICLPILDGLL
HWMVCPSAEAQDPFPTVGPNSVLSPQRLVLETLCKLSIQDNNVDLILATPPFSRQEKFYA
TLVRYVGDRKNPVCREMSMALLSNLAQGDALAARAIAVQKGSIGNLISFLEDGVTMAQYQ
QSQHNLMHMQPPPLEPPSVDMMCRAAKALLAMARVDENRSEFLLHEGRLLDISISAVLNS
LVASVICDVLFQIGQL*
>ENSG00000152795|ENST00000349655
MEDMNEYSNIEEFAEGSKINASKNQQDDGKMFIGGLSWDTSKKDLTEYLSRFGEVVDCTI
KTDPVTGRSRGFGFVLFKDAASVDKVLELKEHKLDGKLIDPKRAKALKGKEPPKKVFVGG
LSPDTSEEQIKEYFGAFGEIENIELPMDTKTNERRGFCFITYTDEEPVKKLLESRYHQIG
SGKCEIKVAQPKEVYRQQQQQQKGGRGAAAGGRGGTRGRGRGQGQNWNQGFNNYYDQGYG
NYNSAYGGDQNYSGYGGYDYTGYNYGNYGYGQGYADYSGQQSTYGKASRGGGNHQNNYQP
Y*
>ENSG00000122482|ENST00000337393
MRSFLQQDVNKTKSRLNVLNGLANNMDDLKINTDITGAKEELLDDNNFISDKESGVHKPK
DCQTSFQKNNTLTLPEELSKDKSENALSGGQSSLFIHAGAPTVSSENFILPKGAAVNGPV
SHSSLTKTSNMNKGSVSLTTGQPVDQPTTESCSTLKVAADLQLSTPQKASQHQVLFLLSD
VAHAKNPTHSNKKLPTSASVGCDIQNSVGSNIKSDGTLINQVEVGEDGEDLLVKDDCVNT
VTGISSGTDGFRSENDTNWDPQKEFIQFLMTNEETVDKAPPHSKIGLEKKRKRKMDVSKI
TRYTEDCFSDSNCVPNKSKMQEVDFLEQNEELQAVDSQKYALSKVKPESTDEDLESVDAF
QHLIYNPDKCGEESSPVHTSTFLSNTLKKKCEESDSESPATFSTEEPSFYPCTKCNVNFR
EKKHLHRHMMYHLDGNSHFRHLNVPRPYACRECGRTFRDRNSLLKHMIIHQERRQKLMEE

IRELKELQDEGRSARLQCPQCVFGTNCPKTFVQHAKTHEKDKRYYCCEECNFMAVTENEL
ECHRGIAHGAVVKCPMVTSDIAQRKTQKKTFMKDSVVGSSKKSATYICKMCPFTTSAKSV
LKKHTEYLHSSSCVDSFGSPLGLDKRKNDILEEPVDSDSTKTLTKQQSTTFPKNSALKQD
VKRTFGSTSQSSSFSKIHKRPHRIQKARKSIAQSGVNMCNQNSSPHKNVTIKSSVDQKPK
YFHQAAKEKSNAKANSHYLYRHKYENYRMIKKSGESYPVHFKKEEASSLNSLHLFSSSSN
SHNNFISDPHKPDAKRPESFKDHRRVAVKRVIKESKKESSVGGEDLDSYPDFLHKMTVVV
LQKLNSAEKKDSYETEDESSWDNVELGDYTTQAIEDETYSDINQEHVNLFPLFKSKVEGQ
EPGENATLSYDQNDGFYFEYYEDTGSNNFLHEIHDPQHLETADASLSKHSSVFHWTDLSL
EKKSCPYCPATFETGVGLSNHVRGHLHRAGLSYEARHVVSPEQIATSDKMQHFKRTGTGT
PVKRVRKAIEKSETTSEHTCQLCGGWFDTKIGLSNHVRGHLKRLGKTKWDAHKSPICVLN
EMMQNEEKYEKILKALNSRRIIPRPFVAQKLASSDDFISQNVIPLEAYRNGLKTEALSVS
ASEEEGLNFLNEYDETKPELPSGKKNQSLTLIELLKNKRMGEERNSAISPQKIHNQTARK
RFVQKCVLPLNEDSPLMYQPQKMDLTMHSALDCKQKKSRSRSGSKKKMLTLPHGADEVYI
LRCRFCGLVFRGPLSVQEDWIKHLQRHIVNANLPRTGAGMVEVTSLLKKPASITETSFSL
LMAEAAS*
>ENSG00000187559|ENST00000342833
MNLPRAERLRSTPQRSLRDSDGEDGKIDVLGEEEDEDEVEDEEEAASQQFLEQSLQPGLQ
VARWGGVALPREHIEGGGGPSDPSEFGTKFRAPPRSAAASEDARQPAKPPYSYIALITMA
ILQNPHKRLTLSGICAFISGRFPYYRRKFPAWQNSIRHNLSLNDCFVKIPREPGHPGKGN
YWSLDPASQDMFDNGSFLRRRKRFKRHQLTPGAHLPHPFPLPAAHAALHNPRPGPLLGAP
APPQPVPGAYPNTAPGRRPYALLHPHPLRYLLLSAPVYAGAPKKAEGAALATPAPFPCCS
PHLVLSLGRRARVWRRHREADASLSALRVLCKGSGERVQGLRRICPRPRGATATCSSDHQ
ACCIPRPLPLCCKCPPPPLLGQFCSNSSSIRRRTAPTAALPPRARCWAGTCRPRRPC*
>ENSG00000143365|ENST00000318247
MDRAPQRQHRASRELLAAKKTHTSQIEVIPCKICGDKSSGIHYGVITCEGCKGFFRRSQR
CNAAYSCTRQQNCPIDRTSRNRCQHCRLQKCLALGMSRDAVKFGRMSKKQRDSLHAEVQK
QLQQRQQQQQEPVVKTPPAGAQGADTLTYTLGLPDGQLPLGSSPDLPEASACPPGLLKAS
GSGPSYSNNLAKAGLNGASCHLEYSPERGKAEGRESFYSTGSQLTPDRCGLRFEEHRHPG
LGELGQGPDSYGSPSFRSTPEAPYASLTEIEHLVQSVCKSYRETCQLRLEDLLRQRSNIF
SREEVTGYQRKSMWEMWERCAHHLTEAIQYVVEFAKRLSGFMELCQNDQIVLLKAGAMEV
VLVRMCRAYNADNRTVFFEGKYGGMELFRALGCSELISSIFDFSHSLSALHFSEDEIALY
TALVLINAHRPGLQEKRKVEQLQYNLELAFHHHLCKTHRQSILAKLPPKGKLRSLCSQHV
ERLQIFQHLHPIVVQAAFPPLYKELFSTETESPVGLSK*
>ENSG00000101076|ENST00000316099
MRLSKTLVDMDMADYSAALDPAYTTLEFENVQVLTMGNDTSPSEGTNLNAPNSLGVSALC
AICGDRATGKHYGASSCDGCKGFFRRSVRKNHMYSCRFSRQCVVDKDKRNQCRYCRLKKC
FRAGMKKEAVQNERDRISTRRSSYEDSSLPSINALLQAEVLSRQITSPVSGINGDIRAKK
IASIADVCESMKEQLLVLVEWAKYIPAFCELPLDDQVALLRAHAGEHLLLGATKRSMVFK
DVLLLGNDYIVPRHCPELAEMSRVSIRILDELVLPFQELQIDDNEYAYLKAIIFFDPDAK
GLSDPGKIKRLRSQVQVSLEDYINDRQYDSRGRFGELLLLLPTLQSITWQMIEQIQFIKL
FGMAKIDNLLQEMLLGGSPSDAPHAHHPLHPHLMQEHMGTNVIVANTMPTHLSNGQMCEW
PRPRGQAATPETPQPSPPGGSGSEPYKLLPGAVATIVKPLSAIPQPTITKQEVI*
>ENSG00000064835|ENST00000350375
MSCQAFTSADTFIPLNSDASATLPLIMHHSAAECLPVSNHATNVMSTATGLHYSVPSCHY

GNQPSTYGVMAGSLTPCLYKFPDHTLSHGFPPIHQPLLAEDPTAADFKQELRRKSKLVEE
PIDMDSPEIRELEKFANEFKVRRIKLGYTQTNVGEALAAVHGSEFSQTTICRFENLQLSF
KNACKLKAILSKWLEEAEQVGALYNEKVGANERKRKRRTTISIAAKDALERHFGEQNKPS
SQEIMRMAEELNLEKEVVRVWFCNRRQREKRVKTSLNQSLFSISKEHLECR*
>ENSG00000168439|ENST00000358794
MESGSPMGEVEISRTIRTNGRGQRGYDWQCKRPIRVAEVRSSLHSWSLRWVNELKEKGNK
ALSVGNIDDALQCYSEAIKLDPHNHVLYSNRSAAYAKKGDYQKAYEDGCKTVDLKPDWGK
GYSRKAAALEFLNRFEEAKRTYEEGLKHEANNPQLKEGLQNMEARLAERKFMNPFNMPNL
YQKLESDPRTRTLLSDPTYRELIEQLRNKPSDLGTKLQDPRIMTTLSVLLGVDLGSMDEE
EEIATPPPPPPPKKETKPEPMEEDLPENKKQALKEKELGNDAYKKKDFDTALKHYDKAKE
LDPTNMTYITNQAAVYFEKGDYNKCRELCEKAIEVGRENREDYRQIAKAYARIGNSYFKE
EKYKDAIHFYNKSLAEHRTPDVLKKCQQAEKILKEQERLAYINPDLALEEKNKGNECFQK
GDYPQAMKHYTEAIKRNPKDAKLYSNRAACYTKLLEFQLALKDCEECIQLEPTFIKGYTR
KAAALEAMKDYTKAMDVYQKALDLDSSCKEAADGYQRCMMAQYNRHDSPEDVKRRAMADP
EVQQIMSDPAMRLILEQMQKDPQALSEHLKNPVIAQKIQKLMDVGLIAIR*
>ENSG00000005189|ENST00000348433
MEPEREGTERHPRKVRESRQAPNKLVGAAEAMKAGWDLEESQPEAKKARLSTILFTDNCE
VTHDQLCELLKYAVLGKSNVPKPSWCQLFHQNHLNNVVVFVLQGMSQLHFYRFYLEFGCL
RKAFRHKFRLPPPSSDFLADVVGLQTEQRAGDLPKTMEGPLPSNAKAAINLQDDPIIQKY
GSKKVGLTRCLLTKEEMRTFHFPLQGFPDCENFLLTKCNGSIADNSPLFGLDCEMCLTSK
GRELTRISLVAEGGCCVMDELVKPENKILDYLTSFSGITKKILNPVTTKLKDVQRQLKAL
LPPDAVLVGHSLDLDLRALKMIHPYVIDTSLLYVREQGRRFKLKFLAKVILGKDIQCPDR
LGHDATEDARTILELARYFLKHGPKKIAELNLEALANHQEIQAAGQEPKNTAEVLQHPNT
SVLECLDSVGQKLLFLTRETDAGELPSSRNCQTIKCLSNKEVLEQARVEIPLFPFSIVQF
SFKAFSPVLTEEMNKRMRIKWTEISTVYAGPFSKNCNLRALKRLFKSFGPVQSMTFVLET
RQVQRPVTELTLDCDTLVNELEGDSENQGSIYLSGVSETFKEQLLQEPRLFLGLEAVILP
KDLKSGKQKKYCFLKFKSFGSAQQALNILTGKDWKLKGRHALTPRHLHAWLRGLPPESTR
LPGLRVVPPPFEQEALQTLKLDHPKIAAWRWSRKIGKLYNSLCPGTLCLILLPGTKSTHG
SLSGLGLMGIKEEEESAGPGLCS*
>ENSG00000152443|ENST00000317178
MAAAALRPPAQGTVTFEDVAVNFSQEEWSLLSEAQRCLYHDVMLENLTLISSLGCWYGAK
DETPSKQTLSIQQESPLRTHWTGVCTKKVHLWGMCGPLLGDILHQGTQHNQKLNGFGAYE
KKLDDDANHHQDQKQHIGEKSYRSNAKGTSFVKNCKFHMSHEPFIFHEVGKDFLSSLRLL
QQEDIHTSGKSNFETKHGIPLQGGKTHYICGESTIPFSNKHSLVLHQRLLPREGPYVCSD
SGKFTSKSNSFNNHQGVRTGKRPYQCGQCDESFWYKAHLTEHQRVHTGERPYECGECDKS
FSHKHSLVDHQRVHTGERPYECDECGKSFSHKRSLVHHQRVHTGERPYQCGECGKSFNHK
CNLIQHQRVHTGERPFECTACGKLFRSNSHLKEHQRVHTGERPYECKECRKSFRYKSHLT
EHQRVHTGERPYECRECGKCFHQKGSLIQHQQIHSGERPHECGECGKCFHQKGSLIRHQQ
IHSGERPHECGECGKCFRQKGNLIKHQRVHTGERHHEC*
>ENSG00000183337|ENST00000342274
MLSATPLYGNVHSWMNSERVRMCGASEDRKILVNDGDASKARLELREENPLNHNVVDAST
AHRIDGLAALSMDRTGLIREGLRVPGNIVYSSLCGLGSEKGREAATSTLGGLGFSSERNP
EMQFKPNTPETVEASAVSGKPPNGFSAIYKTPPGIQKSAVATAEALGLDRPASDKQSPLN
INGASYLRLPWVNPYMEGATPAIYPFLDSPNKYSLNMYKALLPQQSYSLAQPLYSPVCTN

GERFLYLPPPHYVGPHIPSSLASPMRLSTPSASPAIPPLVHCADKSLPWKMGVSPGNPVD
SHAYPHIQNSKQPRVPSAKAVTSGLPGDTALLLPPSPRPSPRVHLPTQPAADTYSEFHKH
YARISTSPSVALSKPYMTVSSEFPAARLSNGKYPKAPEGGEGAQPVPGHARKTAVQDRKD
GSSPPLLEKQTVTKDVTDKPLDLSSKVVDVDASKADHMKKMAPTVLVHSRAGSGLVLSGS
EIPKETLSPPGNGCAIYRSEIISTAPSSWVVPGPSPNEENNGKSMSLKNKALDWAIPQQR
SSSCPRMGGTDAVITNVSGSVSSAGRPASASPAPNANADGTKTSRSSVETTPSVIQHVGQ
PPATPAKHSSSTSSKGAKASNPEPSFKANENGLPPSSIFLSPNEAFRSPPIPYPRSYLPY
PAPEGIAVSPLSLHGKGPVYPHPVLLPNGSLFPGHLAPKPGLPYGLPTGRPEFVTYQDAL
GLGMVHPMLIPHTPIEITKEEKPERRSRSHERARYEDPTLRNRFSEILETSSTKLHPDVP
TDKNLKPNPNWNQGKTVVKSDKLVYVDLLREEPDAKTDTNVSKPSFAAESVGQSAEPPKP
SVEPALQQHRDFIALREELGRISDFHETYTFKQPVFTVSKDSVLAGTNKENLGLPVSTPF
LEPPLGSDGPAVTFGKTQEDPKPFCVGSAPPSVDVTPTYTKDGADEAESNDGKVLKPKPS
KLAKRIANSAGYVGDRFKCVTTELYADSSQLSREQRALQMEGLQEDSILCLPAAYCERAM
MRFSELEMKEREGGHPATKDSEMCKFSPADWERLKGNQDKKPKSVTLEEAIAEQNESERC
EYSVGNKHRDPFEAPEDKDLPVEKYFVERQPVSEPPADQVASDMPHSPTLRVDRKRKVSG
DSSHTETTAEEVPEDPLLKAKRRRVSKGLHPKKQRHLLHLRERWEQQVSAADGKPGRQSR
KEVTQATQPEAIPQGTNITEEKPGRKRAEAKGNRSWSEESLKPSDNEQGLPVFSGSPPMK
SLSSTSAGGKKQAQPSCAPASRPPAKQQKIKENQKTDVLCADEEEDCQAASLLQKYTDNS
EKPSGKRLCKTKHLIPQESRRGLPLTGEYYVENADGKVTVRRFRKRPEPSSDYDLSPAKQ
EPKPFDRLQQLLPASQSTQLPCSSSPQETTQSRPMPPEARRLIVNKNAGETLLQRAARLG
YEEVVLYCLENKICDVNHRDNAGYCALHEACARGWLNIVRHLLEYGADVNCSAQDGTRPL
HDAVENDHLEIVRLLLSYGADPTLATYSGRTIMKMTHSELMEKFLTDYLNDLQGRNDDDA
SGTWDFYGSSVCEPDDESGYDVLANPPGPEDQDDDDDAYSDVFEFEFSETPLLPCYNIQV
SVAQGPRNWLLLSDVLKKLKMSSRIFRCNFPNVEIVTIAEAEFYRQVSASLLFSCSKDLE
AFNPESKELLDLVEFTNEIQTLLGSSVEWLHPSDLASDNYW*
>ENSG00000165629|ENST00000356708
MFSRAGVAGLSAWTLQPQWIQVRNMATLKDITRRLKSIKNIQKITKSMKMVAAAKYARAE
RELKPARIYGLGSLALYEKADIKGPEDKKKHLLIGVSSDRGLCGAIHSSIAKQMKSEVAT
LTAAGKEVMLVGIGDKIRGILYRTHSDQFLVAFKEVGRKPPTFGDASVIALELLNSGYEF
DEGSIIFNKFRSVISYKTEEKPIFSLNTVASADSMSIYDDIDADVLQNYQEYNLANIIYY
SLKESTTSEQSARMTAMDNASKNASEMIDKLTLTFNRTRQAVITKELIEIISGAAALD*
>ENSG00000101040|ENST00000355972
MDISTRSKDPGSAERTAQKRKFPSPPHSSNGHSPQDTSTSPIKKKKKPGLLNSNNKEQSE
LRHGPFYYMKQPLTTDPVDVVPQDGRNDFYCWVCHREGQVLCCELCPRVYHAKCLRLTSE
PEGDWFCPECEKITVAECIETQSKAMTMLTIEQLSYLLKFAIQKMKQPGTDAFQKPVPLE
QHPDYAEYIFHPMDLCTLEKNAKKKMYGCTEAFLADAKWILHNCIIYNGGNHKLTQIAKV
VIKICEHEMNEIEVCPECYLAACQKRDNWFCEPCSNPHPLVWAKLKGFPFWPAKALRDKD
GQVDARFFGQHDRAWVPINNCYLMSKEIPFSVKKTKSIFNSAMQEMEVYVENIRRKFGVF
NYSPFRTPYTPNSQYQMLLDPTNPSAGTAKIDKQEKVKLNFDMTASPKILMSKPVLSGGT
GRRISLSDMPRSPMSTNSSVHTGSDVEQDAEKKATSSHFSASEESMDFLDKSTASPASTK
TGQAGSLSGSPKPFSPQLSAPITTKTDKTSTTGSILNLNLDRSKAEMDLKELSESVQQQS
TPVPLISPKRQIRSRFQLNLDKTIESCKAQLGINEISEDVYTAVEHSDSEDSEKSDSSDS
EYISDDEQKSKNEPEDTEDKEGCQMDKEPSAVKKKPKPTNPVEIKEELKSTSPASEKADP
GAVKDKASPEPEKDFSEKAKPSPHPIKDKLKGKDETDSPTVHLGLDSDSESELVIDLGED

HSGREGRKNKKEPKEPSPKQDVVGKTPPSTTVGSHSPPETPVLTRSSAQTSAAGATATTS
TSSTVTVTAPAPAATGSPVKKQRPLLPKETAPAVQRVVWNSSSKFQTSSQKWHMQKMQRQ
QQQQQQQNQQQQPQSSQGTRYQTRQAVKAVQQKEITQSPSTSTITLVTSTQSSPLVTSSG
SMSTLVSSVNADLPIATASADVAADIAKYTSKMMDAIKGTMTEIYNDLSKNTTGSTIAEI
RRLRIEIEKLQWLHQQELSEMKHNLELTMAEMRQSLEQERDRLIAEVKKQLELEKQQAVD
ETKKKQWCANCKKEAIFYCCWNTSYCDYPCQQAHWPEHMKSCTQSATAPQQEADAEVNTE
TLNKSSQGSSSSTQSAPSETASASKEKETSAEKSKESGSTLDLSGSRETPSSILLGSNQG
SVSKRCDKQPAYAPTTTDHQPHPNYPAQKYHSRSNKSSWSSSDEKRGSTRSDHNTSTSTK
SLLPKESRLDTFWD*
>ENSG00000168826|ENST00000337872
MDPVATHSCHLLQQLHEQRIQGLLCDCMLVVKGVCFKAHKNVLAAFSQYFRSLFQNSSSQ
KNDVFHLDVKNVSGIGQILDFMYTSHLDLNQDNIQVMLDTAQCLQVQNVLSLCHTFLKSA
TVVQPPGMPCNSTLSLQSTLTPDATCVISENYPPHLLQECSADAQQNKTLDESHPHASPS
VNRHHSAGEISKQAPDTSDGSCTELPFKQPNYYYKLRNFYSKQYHKHAAGPSQERVVEQP
FAFSTSTDLTTVESQPCAVSHSECILESPEHLPSNFLAQPVNDSAPHPESDATCQQPVKQ
MRLKKAIHLKKLNFLKSQKYAEQVSEPKSDDGLTKRLESASKNTLEKASSQSAEEKESEE
VVSCENFNCISETERPEDPAALEDQSQTLQSQRQYACELCGKPFKHPSNLELHKRSHTGE
KPFECNICGKHFSQAGNLQTHLRRHSGEKPYICEICGKRFAASGDVQRHIIIHSGEKPHL
CDICGRGFSNFSNLKEHKKTHTADKVFTCDECGKSFNMQRKLVKHRIRHTGERPYSCSAC
GKCFGGSGDLRRHVRTHTGEKPYTCEICNKCFTRSAVLRRHKKMHCKAGDESPDVLEELS
QAIETSDLEKSQSSDSFSQDTSVTLMPVSVKLPVHPVENSVAEFDSHSGGSYCKLRSMIQ
PHGVSDQEKLSLDPGKLAKPQMQQTQPQAYAYSDVDTPAGGEPLQADGMAMIRSSLAALD
NHGGDPLGSRASSTTYRNSEGQFFSSMTLWGLAMKTLQNENELDQ*
>ENSG00000106348|ENST00000354269
MEGPLTPPPLQGGGAAAVPEPGARQHPGHETAAQRYSARLLQAGYEPESCFLLELSSVVL
LAGVGVQMDRLRRASMADYLISGGTGYVPEDGLTAQQLFASADGLTYNDFLILPGFIDFI
ADEVDLTSALTRKITLKTPLISSPMDTVTEADMAIAMALMGGIGFIHHNCTPEFQANEVR
KVKKFEQGFITDPVVLSPSHTVGDVLEAKMRHGFSGIPITETGTMGSKLVGIVTSRDIDF
LAEKDHTTLLSEVMTPRIELVVAPAGVTLKEANEILQRSKKGKLPIVNDCDELVAIIART
DLKKNRDYPLASKDSQKQLLCGAAVGTREDDKYRLDLLTQAGVDVIVLDSSQGNSVYQIA
MVHYIKQKYPHLQVIGGNVVTAAQAKNLIDAGVDGLRVGMGCGSICITQEVMACGRPQGT
AVYKVAEYARRFGVPIIADGGIQTVGHVVKALALGASTVMMGSLLAATTEAPGEYFFSDG
VRLKKYRGMGSLDAMEKSSSSQKRYFSEGDKVKIAQGVSGSIQDKGSIQKFVPYLIAGIQ
HGCQDIGARSLSVLRSMMYSGELKFEKRTMSAQIEGGVHGLHSYEKRLY*
>ENSG00000164093|ENST00000355080
METNCRKLVSACVQLEKDKSQQGKNEDVGAEDPSKKKRQRRQRTHFTSQQLQELEATFQR
NRYPDMSTREEIAVWTNLTEARVRVWFKNRRAKWRKRERNQQAELCKNGFGPQFNGLMQP
YDDMYPGYSYNNWAAKGLTSASLSTKSFPFFNSMNVNPLSSQSMFSPPNSISSMSMSSSM
VPSAVTGVPGSSLNSLNNLNNLSSPSLNSAVPTPACPYAPPTPPYVYRDTCNSSLASLRL
KAKQHSSFGYASVQNPASNLSACQYAVDRPV*
>ENSG00000119866|ENST00000359629
MSRRKQGKPQHLSKREFSPEPLEAILTDDEPDHGPLGAPEGDHDLLTCGQCQMNFPLGDI
LIFIEHKRKQCNGSLCLEKAVDKPPSPSPIEMKKASNPVEVGIQVTPEDDDCLSTSSRGI
CPKQEHIADKLLHWRGLSSPRSAHGALIPTPGMSAEYAPQGICKDEPSSYTCTTCKQPFT

SAWFLLQHAQNTHGLRIYLESEHGSPLTPRVLHTPPFGVVPRELKMCGSFRMEAREPLSS
EKI*
>ENSG00000213923|ENST00000359867
MELRVGNKYRLGRKIGSGSFGDIYLGANIASGEEVAIKLECVKTKHPQLHIESKFYKMMQ
GGVGIPSIKWCGAEGDYNVMVMELLGPSLEDLFNFCSRKFSLKTVLLLADQMISRIEYIH
SKNFIHRDVKPDNFLMGLGKKGNLVYIIDFGLAKKYRDARTHQHIPYRENKNLTGTARYA
SINTHLGIEQSRRDDLESLGYVLMYFNLGSLPWQGLKAATKRQKYERISEKKMSTPIEVL
CKGYPSEFSTYLNFCRSLRFDDKPDYSYLRQLFRNLFHRQGFSYDYVFDWNMLKFGAARN
PEDVDRERREHEREERMGQLRGSATRALPPGPPTGATANRLRSAAEPVASTPASRIQPAG
NTSPRAISRVDRERKVSMRLHRGAPANVSSSDLTGRQEVSRIPASQTSVPFDHLGK*
>ENSG00000205927|ENST00000333337
MDSDASLVSSRPSSPEPDDLFLPARSKGSSGSAFTGGTVSSSTPSDCPPELSAELRGAMG
SAGAHPGDKLGGSGFKSSSSSTSSSTSSAAASSTKKDKKQMTEPELQQLRLKINSRERKR
MHDLNIAMDGLREVMPYAHGPSVRKLSKIATLLLARNYILMLTNSLEEMKRLVSEIYGGH
HAGFHPSACGGLAHSAPLPAATAHPAAAAHAAHHPAVHHPILPPAAAAAAAAAAAAAVSS
ASLPGSGLPSVGSIRPPHGLLKSPSAAAAAPLGGGGGGSGASGGFQHWGGMPCPCSMCQV
PPPHHHVSAMGAGSLPRLTSDAK*
>ENSG00000185155|ENST00000366810
MATAESRALQFAEGAAFPAYRAPHAGGALLPPPSPAAALLPAPPAGPGPATFAGFLGRDP
GPAPPPPASLGSPAPPKGAAAPSASQRRKRTSFSAEQLQLLELVFRRTRYPDIHLRERLA
ALTLLPESRIQVWFQNRRAKSRRQSGKSFQPLARPEIILNHCAPGTETKCLKPQLPLEVD
VNCLPEPNGVGGGISDSSSQGQNFETCSPLSEDIGSKLDSWEEHIFSAFGNF*
>ENSG00000177084|ENST00000320574
MSLRSGGRRRADPGADGEASRDDGATSSVSALKRLERSQWTDKMDLRFGFERLKEPGEKT
GWLINMHPTEILDEDKRLGSAVDYYFIQDDGSRFKVALPYKPYFYIATRKGCEREVSSFL
SKKFQGKIAKVETVPKEDLDLPNHLVGLKRNYIRLSFHTVEDLVKVRKEISPAVKKNREQ
DHASDAYTALLSSVLQRGGVITDEEETSKKIADQLDNIVDMREYDVPYHIRLSIDLKIHV
AHWYNVRYRGNAFPVEITRRDDLVERPDPVVLAFDIETTKLPLKFPDAETDQIMMISYMI
DGQGYLITNREIVSEDIEDFEFTPKPEYEGPFCVFNEPDEAHLIQRWFEHVQETKPTIMV
TYNGDFFDWPFVEARAAVHGLSMQQEIGFQKDSQGEYKAPQCIHMDCLRWVKRDSYLPVG
SHNLKAAAKAKLGYDPVELDPEDMCRMATEQPQTLATYSVSDAVATYYLYMKYVHPFIFA
LCTIIPMEPDEVLRKGSGTLCEALLMVQAFHANIIFPNKQEQEFNKLTDDGHVLDSETYV
GGHVEALESGVFRSDIPCRFRMNPAAFDFLLQRVEKTLRHALEEEEKVPVEQVTNFEEVC
DEIKSKLASLKDVPSRIECPLIYHLDVGAMYPNIILTNRLQPSAMVDEATCAACDFNKPG
ANCQRKMAWQWRGEFMPASRSEYHRIQHQLESEKFPPLFPEGPARAFHELSREEQAKYEK
RRLADYCRKAYKKIHITKVEERLTTICQRENSFYVDTVRAFRDRRYEFKGLHKVWKKKLS
AAVEVGDAAEVKRCKNMEVLYDSLQLAHKCILNSFYGYVMRKGARWYSMEMAGIVCFTGA
NIITQARELIEQIGRPLELDTDGIWCVLPNSFPENFVFKTTNVKKPKVTISYPGAMLNIM
VKEGFTNDQYQELAEPSSLTYVTRSENSIFFEVDGPYLAMILPASKEEGKKLKKRYAVFN
EDGSLAELKGFEVKRRGELQLIKIFQSSVFEAFLKGSTLEEVYGSVAKVADYWLDVLYSK
AANMPDSELFELISENRSMSRKLEDYGEQKSTSISTAKRLAEFLGDQMVKDAGLSCRYII
SRKPEGSPVTERAIPLAIFQAEPTVRKHFLRKWLKSSSLQDFDIRAILDWDYYIERLGSA
IQKIITIPAALQQVKNPVPRVKHPDWLHKKLLEKNDVYKQKKISELFTLEGRRQVTMAEA
SEDSPRPSAPDMEDFGLVKLPHPAAPVTVKRKRVLWESQEESQDLTPTVPWQEILGQPPA

LGTSQEEWLVWLRFHKKKWQLQARQRLARRKRQRLESAEGVLRPGAIRDGPATGLGSFLR
RTARSILDLPWQIVQISETSQAGLFRLWALVGSDLHCIRLSIPRVFYVNQRVAKAEEGAS
YRKVNRVLPRSNMVYNLYEYSVPEDMYQEHINEINAELSAPDIEGVYETQVPLLFRALVH
LGCVCVVNKQLVRHLSGWEAETFALEHLEMRSLAQFSYLEPGSIRHIYLYHHAQAHKALF
GIFIPSQRRASVFVLDTVRSNQMPSLGALYSAEHGLLLEKVGPELLPPPKHTFEVRAETD
LKTICRAIQRFLLAYKEERRGPTLIAVQSSWELKRLASEIPVLEEFPLVPICVADKINYG
VLDWQRHGARRMIRHYLNLDTCLSQAFEMSRYFHIPIGNLPEDISTFGSDLFFARHLQRH
NHLLWLSPTARPDLGGKEADDNCLVMEFDDQATVEINSSGCYSTVCVELDLQNLAVNTIL
QSHHVNDMEGADSMGISFDVIQQASLEDMITGGQAASAPASYDETALCSNTFRILKSMVV
GWVKEITQYHNIYADNQVMHFYRWLRSPSSLLHDPALHRTLHNMMKKLFLQLIAEFKRLG
SSVIYANFNRIILCTKKRRVEDAIAYVEYITSSIHSKETFHSLTISFSRCWEFLLWMDPS
NYGGIKGKVSSRIHCGLQDSQKAGGAEDEQENEDDEEERDGEEEEEAEESNVEDLLENNW
NILQFLPQAASCQNYFLMIVSAYIVAVYHCMKDGLRRSAPGSTPVRRRGASQLSQEAEGA
VGALPGMITFSQDYVANELTQSFFTITQKIQKKVTGSRNSTELSEMFPVLPGSHLLLNNP
ALEFIKYVCKVLSLDTNITNQVNKLNRDLLRLVDVGEFSEEAQFRDPCRSYVLPEVICRS
CNFCRDLDLCKDSSFSEDGAVLPQWLCSNCQAPYDSSAIEMTLVEVLQKKLMAFTLQDLV
CLKCRGVKETSMPVYCSCAGDFALTIHTQVFMEQIGIFRNIAQHYGMSYLLETLEWLLQK
NPQLGH*
>ENSG00000151623|ENST00000344721
METKGYHSLPEGLDMERRWGQVSQAVERSSLGPTERTDENNYMEIVNVSCVSGAIPNNST
QGSSKEKQELLPCLQQDNNRPGILTSDIKTELESKELSATVAESMGLYMDSVRDADYSYE
QQNQQGSMSPAKIYQNVEQLVKFYKGNGHRPSTLSCVNTPLRSFMSDSGSSVNGGVMRAV
VKSPIMCHEKSPSVCSPLNMTSSVCSPAGINSVSSTTASFGSFPVHSPITQGTPLTCSPN
VENRGSRSHSPAHASNVGSPLSSPLSSMKSSISSPPSHCSVKSPVSSPNNVTLRSSVSSP
ANINNSRCSVSSPSNTNNRSTLSSPAASTVGSICSPVNNAFSYTASGTSAGSSTLRDVVP
SPDTQEKGAQEVPFPKTEEVESAISNGVTGQLNIVQYIKPEPDGAFSSSCLGGNSKINSD
SSFSVPIKQESTKHSCSGTSFKGNPTVNPFPFMDGSYFSFMDDKDYYSLSGILGPPVPGF
DGNCEGSGFPVGIKQEPDDGSYYPEASIPSSAIVGVNSGGQSFHYRIGAQGTISLSRSAR
DQSFQHLSSFPPVNTLVESWKSHGDLSSRRSDGYPVLEYIPENVSSSTLRSVSTGSSRPS
KICLVCGDEASGCHYGVVTCGSCKVFFKRAVEGQHNYLCAGRNDCIIDKIRRKNCPACRL
QKCLQAGMNLGARKSKKLGKLKGIHEEQPQQQQPPPPPPPPQSPEEGTTYIAPAKEPSVN
TALVPQLSTISRALTPSPVMVLENIEPEIVYAGYDSSKPDTAENLLSTLNRLAGKQMIQV
VKWAKVLPGFKNLPLEDQITLIQYSWMCLSSFALSWRSYKHTNSQFLYFAPDLVFNEEKM
HQSAMYELCQGMHQISLQFVRLQLTFEEYTIMKVLLLLSTIPKDGLKSQAAFEEMRTNYI
KELRKMVTKCPNNSGQSWQRFYQLTKLLDSMHDLVSDLLEFCFYTFRESHALKVEFPAML
VEIISDQLPKVESGNAKPLYFHRK*
>ENSG00000108001|ENST00000355311
MFGIQENIPRGGTTMKEEPLGSGMNPVRSWMHTAGVVDANTAAQSGVGLARAHFEKQPPS
NLRKSNFFHFVLALYDRQGQPVEIERTAFVDFVEKEKEPNNEKTNNGIHYKLQLLYSNGV
RTEQDLYVRLIDSMTKQAIVYEGQDKNPEMCRVLLTHEIMCSRCCDKKSCGNRNETPSDP
VIIDRFFLKFFLKCNQNCLKNAGNPRDMRRFQVVVSTTVNVDGHVLAVSDNMFVHNNSKH
GRRARRLDPSEGTAPSYLENATPCIKAISPSEGWTTGGATVIIIGDNFFDGLQVVFGTML
VWSELITPHAIRVQTPPRHIPGVVEVTLSYKSKQFCKGAPGRFVYTALNEPTIDYGFQRL
QKVIPRHPGDPERLPKEVLLKRAADLVEALYGMPHNNQEIILKRAADIAEALYSVPRNHN

QIPTLGNNPAHTGMMGVNSFSSQLAVNVSETSQANDQVGYSRNTSSVSPRGYVPSSTPQQ
SNYNTVSTSMNGYGSGAMASLGVPGSPGFLNGSSANSPYGIVPSSPTMAASSVTLPSNCS
STHGIFSFSPANVISAVKQKSAFAPVVRPQASPPPSCTSANGNGLQAMSGLVVPPM*
>ENSG00000140382|ENST00000336216
MENLMTSSTLPPLFADEDGSKESNDLATTGLNHPEVPYSSGATSSTNNPEFVEDLSQGQL
LQSESSNAAEGNEQRHEDEQRSKRGGWSKGRKRKKPLRDSNAPKSPLTGYVRFMNERREQ
LRAKRPEVPFPEITRMLGNEWSKLPPEEKQRYLDEADRDKERYMKELEQYQKTEAYKVFS
RKTQDRQKGKSHRQDAARQATHDHEKETEVKERSVFDIPIFTEEFLNHSKAREAELRQLR
KSNMEFEERNAALQKHVESMRTAVEKLEVDVIQERSRNTVLQQHLETLRQVLTSSFASMP
LPGSGETPTVDTIDSYMNRLHSIILANPQDNENFIATVREVVNRLDR*
>ENSG00000163041|ENST00000366815
MARTKQTARKSTGGKAPRKQLATKAARKSAPSTGGVKKPHRYRPGTVALREIRRYQKSTE
LLIRKLPFQRLVREIAQDFKTDLRFQSAAIGALQEASEAYLVGLFEDTNLCAIHAKRVTI
MPKDIQLARRIRGERA*
>ENSG00000157216|ENST00000357475
MFAKGKGSAVPSDGQAREKLALYVYEYLLHVGAQKSAQTFLSEIRWEKNITLGEPPGFLH
SWWCVFWDLYCAAPERRDTCEHSSEAKAFHDYSAAAAPSPVLGNIPPNDGMPGGPIPPGF
FQGPPGSQPSPHAQPPPHNPSSMMGPHSQPPGGVPGTQPLLPNSMDPTRQQGHPNMGGSM
QRMNPPRGMGPMGPGPQNYGSGMRPPPNSLGPAMPGINMGPGAGRPWPNPNSANSIPYSS
SSPGTYVGPPGGGGPPGTPIMPSPADSTNSSDNIYTMINPVPPGGSRSNFPMGPGSDGPM
GGMGGMEPHHMNGSLGSGDIDGLPKNSPNNISGISNPPGTPRDDGELGGNFLHSFQNDNY
SPSMTMSV*
>ENSG00000133895|ENST00000337652
MGLKAAQKTLFPLRSIDDVVRLFAAELGREEPDLVLLSLVLGFVEHFLAVNRVIPTNVPE
LTFQPSPAPDPPGGLTYFPVADLSIIAALYARFTAQIRGAVDLSLYPREGGVSSRELVKK
VSDVIWNSLSRSYFKDRAHIQSLFSFITGWSPVGTKLDSSGVAFAVVGACQALGLRDVHL
ALSEDHAWVVFGPNGEQTAEVTWHGKGNEDRRGQTVNAGVAERSWLYLKGSYMRCDRKME
VAFMVCAINPSIDLHTDSLELLQLQQKLLWLLYDLGHLERYPMALGNLADLEELEPTPGR
PDPLTLYHKGIASAKTYYRDEHIYPYMYLAGYHCRNRNVREALQAWADTATVIQDYNYCR
EDEEIYKEFFEVANDVIPNLLKEAASLLEAGEERPGEQSQGTQSQGSALQDPECFAHLLR
FYDGICKWEEGSPTPVLHVGWATFLVQSLGRFEGQVRQKVRIVSREAEAAEAEEPWGEEA
REGRRRGPRRESKPEEPPPPKKPALDKGLGTGQGAVSGPPRKPPGTVAGTARGPEGGSTA
QVPAPTASPPPEGPVLTFQSEKMKGMKELLVATKINSSAIKLQLTAQSQVQMKKQKVSTP
SDYTLSFLKRQRKGL*
>ENSG00000131051|ENST00000361162
MADDIDIEAMLEAPYKKDENKLSSANGHEERSKKRKKSKSRSRSHERKRSKSKERKRSRD
RERKKSKSRERKRSRSKERRRSRSRSRDRRFRGRYRSPYSGPKFNSAIRGKIGLPHSIKL
SRRRSRSKSPFRKDKSPVREPIDNLTPEERDARTVFCMQLAARIRPRDLEEFFSTVGKVR
DVRMISDRNSRRSKGIAYVEFVDVSSVPLAIGLTGQRVLGVPIIVQASQAEKNRAAAMAN
NLQKGSAGPMRLYVGSLHFNITEDMLRGIFEPFGRIESIQLMMDSETGRSKGYGFITFSD
SECAKKALEQLNGFELAGRPMKVGHVTERTDASSASSFLDSDELERTGIDLGTTGRLQLM
ARLAEGTGLQIPPAAQQALQMSGSLAFGAVADLQTRLSQQTEASALAAAASVQPLATQCF
QLSNMFNPQTEEEVGWDTEIKDDVIEECNKHGGVIHIYVDKNSAQGNVYVKCPSIAAAIA
AVNALHGRWFAGKMITAAYVPLPTYHNLFPDSMTATQLLVPSRR*

>ENSG00000121766|ENST00000344147
MNSGRPETMENLPALYTIFQGEVAMVTDYGAFIKIPGCRKQGLVHRTHMSSCRVDKPSEI
VDVGDKVWVKLIGREMKNDRIKVSLSMKVVNQGTGKDLDPNNVIIEQEERRRRSFQDYTG
QKITLEAVLNTTCKKCGCKGHFAKDCFMQPGGTKYSLIPDEEEEKEEAKSAEFEKPDPTR
NPSRKRKKEKKKKKHRDRKSSDSDSSDSESDTGKRARHTSKDSKAAKKKKKKKKHKKKHK
E*
>ENSG00000129691|ENST00000343823
MAAAGAGPGQEAGAGPGPGAVANATGAEEGEMKPVAAGAAAPPGEGISAAPTVEPSSGEA
EGGEANLVDVSGGLETESSNGKDTLEGAGDTSEVMDTQAGSVDEENGRQLGEVELQCGIC
TKWFTADTFGIDTSSCLPFMTNYSFHCNVCHHSGNTYFLRKQANLKEMCLSALANLTWQS
RTQDEHPKTMFSKDKDIIPFIDKYWECMTTRQRPGKMTWPNNIVKTMSKERDVFLVKEHP
DPGSKDPEEDYPKFGLLDQDLSNIGPAYDNQKQSSAVSTSGNLNGGIAAGSSGKGRGAKR
KQQDGGTTGTTKKARSDPLFSAQRLPPHGYPLEHPFNKDGYRYILAEPDPHAPDPEKLEL
DCWAGKPIPGDLYRACLYERVLLALHDRAPQLKISDDRLTVVGEKGYSMVRASHGVRKGA
WYFEITVDEMPPDTAARLGWSQPLGNLQAPLGYDKFSYSWRSKKGTKFHQSIGKHYSSGY
GQGDVLGFYINLPEDTETAKSLPDTYKDKALIKFKSYLYFEEKDFVDKAEKSLKQTPHSE
IIFYKNGVNQGVAYKDIFEGVYFPAISLYKSCTVSINFGPCFKYPPKDLTYRPMSDMGWG
AVVEHTLADVLYHVETEVDGRRSPPWEP*
>ENSG00000251369|ENST00000325134
MAETKDAAQMLVTFKDVAVTFTREEWRQLDLAQRTLYREVMLETCGLLVSLGHRVPKPEL
VHLLEHGQELWIVKRGLSHATCAGDRAQVHTREPTTYPPVLSERAFLRGSLTLESSTSSD
SRLGRARDEEGLLEMQKGKVTPETDLHKETHLGKVSLEGEGLGTDDGLHSRALQEWLSAD
VLHECDSQQPGKDALIHAGTNPYKCKQCGKGFNRKWYLVRHQRVHTGMKPYECNACGKAF
SQSSTLIRHYLIHTGEKPYKCLECGKAFKRRSYLMQHHPIHTGEKPYECSQCRKAFTHRS
TFIRHNRTHTGEKPFECKECEKAFSNRAHLIQHYIIHTGEKPYDCMACGKAFRCSSELIQ
HQRIHTGEKPYECTQCGKAFHRSTYLIQHSVIHTGEMPYKCIECGKAFKRRSHLLQHQRV
HT*
>ENSG00000145907|ENST00000356245
MVMEKPSPLLVGREFVRQYYTLLNQAPDMLHRFYGKNSSYVHGGLDSNGKPADAVYGQKE
IHRKVMSQNFTNCHTKIRHVDAHATLNDGVVVQVMGLLSNNNQALRRFMQTFVLAPEGSV
ANKFYVHNDIFRYQDEVFGGFVTEPQEESEEEVEEPEERQQTPEVVPDDSGTFYDQAVVS
NDMEEHLEEPVAEPEPDPEPEPEQEPVSEIQEEKPEPVLEETAPEDAQKSSSPAPADIAQ
TVQEDLRTFSWASVTSKNLPPSGAVPVTGIPPHVVKVPASQPRPESKPESQIPPQRPQRD
QRVREQRINIPPQRGPRPIREAGEQGDIEPRRMVRHPDSHQLFIGNLPHEVDKSELKDFF
QSYGNVVELRINSGGKLPNFGFVVFDDSEPVQKVLSNRPIMFRGEVRLNVEEKKTRAARE
GDRRDNRLRGPGGPRGGLGGGMRGPPRGGMVQKPGFGVGRGLAPRQ*
>ENSG00000137313|ENST00000327357
MATSAPLRSLEEEVTCSICLDYLRDPVTIDCGHVFCRSCTTDVRPISGSRPVCPLCKKPF
KKENIRPVWQLASLVENIERLKVDKGRQPGEVTREQQDAKLCERHREKLHYYCEDDGKLL
CVMCRESREHRPHTAVLMEKAAQPHREKILNHLSTLRRDRDKIQGFQAKGEADILAALKK
LQDQRQYIVAEFEQGHQFLREREEHLLEQLAKLEQELTEGREKFKSRGVGELARLALVIS
ELEGKAQQPAAELMQDTRDFLNRYPRKKFWVGKPIARVVKKKTGEFSDKLLSLQRGLREF
QGKLLRDLEYKTVSVTLDPQSASGYLQLSEDWKCVTYTSLYKSAYLHPQQFDCEPGVLGS
KGFTWGKVYWEVEVEREGWSEDEEEGDEEEEGEEEEEEEEAGYGDGYDDWETDEDEESLG

DEEEEEEEEEEEEVLESCMVGVARDSVKRKGDLSLRPEDGVWALRLSSSGIWANTSPEAEL
FPALRPRRVGIALDYEGGTVTFTNAESQELIYTFTATFTRRLVPFLWLKWPGTRLLLRP*
>ENSG00000101076|ENST00000316673
MVSVNAPLGAPVESSYDTSPSEGTNLNAPNSLGVSALCAICGDRATGKHYGASSCDGCKG
FFRRSVRKNHMYSCRFSRQCVVDKDKRNQCRYCRLKKCFRAGMKKEAVQNERDRISTRRS
SYEDSSLPSINALLQAEVLSRQITSPVSGINGDIRAKKIASIADVCESMKEQLLVLVEWA
KYIPAFCELPLDDQVALLRAHAGEHLLLGATKRSMVFKDVLLLGNDYIVPRHCPELAEMS
RVSIRILDELVLPFQELQIDDNEYAYLKAIIFFDPDAKGLSDPGKIKRLRSQVQVSLEDY
INDRQYDSRGRFGELLLLLPTLQSITWQMIEQIQFIKLFGMAKIDNLLQEMLLGGSPSDA
PHAHHPLHPHLMQEHMGTNVIVANTMPTHLSNGQMCEWPRPRGQAATPETPQPSPPGGSG
SEPYKLLPGAVATIVKPLSAIPQPTITKQEVI*
>ENSG00000101040|ENST00000360911
MHPQSLAEEEIKTEQEVVEGMDISTRSKDPGSAERTAQKRKFPSPPHSSNGHSPQDTSTS
PIKKKKKPGLLNSNNKEQDGRNDFYCWVCHREGQVLCCELCPRVYHAKCLRLTSEPEGDW
FCPECEKITVAECIETQSKAMTMLTIEQLSYLLKFAIQKMKQPGTDAFQKPVPLEQHPDY
AEYIFHPMDLCTLEKNAKKKMYGCTEAFLADAKWILHNCIIYNGGNHKLTQIAKVVIKIC
EHEMNEIEVCPECYLAACQKRDNWFCEPCSNPHPLVWAKLKGFPFWPAKALRDKDGQVDA
RFFGQHDRAWVPINNCYLMSKEIPFSVKKTKSIFNSAMQEMEVYVENIRRKFGVFNYSPF
RTPYTPNSQYQMLLDPTNPSAGTAKIDKQEKVKLNFDMTASPKILMSKPVLSGGTGRRIS
LSDMPRSPMSTNSSVHTGSDVEQDAEKKATSSHFSASEESMDFLDKSTASPASTKTGQAG
SLSGSPKPFSPQLSAPITTKTDKTSTTGSILNLNLDRSKAEMDLKELSESVQQQSTPVPL
ISPKRQIRSRFQLNLDKTIESCKAQLGINEISEDVYTAVEHSDSEDSEKSDSSDSEYISD
DEQKSKNEPEDTEDKEGCQMDKEPSAVKKKPKPTNPVEIKEELKSTSPASEKADPGAVKD
KASPEPEKDFSEKAKPSPHPIKDKLKGKDETDSPTVHLGLDSDSESELVIDLGEDHSGRE
GRKNKKEPKEPSPKQDVVGKTPPSTTVGSHSPPETPVLTRSSAQTSAAGATATTSTSSTV
TVTAPAPAATGSPVKKQRPLLPKETAPAVQRVVWNSSTVQQKEITQSPSTSTITLVTSTQ
SSPLVTSSGSMSTLVSSVNADLPIATASADVAADIAKYTSKMMDAIKGTMTEIYNDLSKN
TTGSTIAEIRRLRIEIEKLQWLHQQELSEMKHNLELTMAEMRQSLEQERDRLIAEVKKQL
ELEKQQAVDETKKKQWCANCKKEAIFYCCWNTSYCDYPCQQAHWPEHMKSCTQSATAPQQ
EADAEVNTETLNKSSQGSSSSTQSAPSETASASKEKETSAEKSKESGSTLDLSGSRETPS
SILLGSNQGSDHSRSNKSSWSSSDEKRGSTRSDHNTSTSTKSLLPKESRLDTFWD*
>ENSG00000198972|ENST00000362102
Sequence unavailable
>ENSG00000152942|ENST00000361732
MNQVTDWVDPSFDDFLECSGVSTITATSLGVNNSSHRRKNGPSTLESSRFPARKRGNLSS
LEQIYGLENSKEYLSENEPWVDKYKPETQHELAVHKKKIEEVETWLKAQVLERQPKQGGS
ILLITGPPGCGKTTTLKILSKEHGIQVQEWINPVLPDFQKDDFKGMFNTESSFHMFPYQS
QIAVFKEFLLRATKYNKLQMLGDDLRTDKKIILVEDLPNQFYRDSHTLHEVLRKYVRIGR
CPLIFIISDSLSGDNNQRLLFPKEIQEECSISNISFNPVAPTIMMKFLNRIVTIEANKNG
GKITVPDKTSLELLCQGCSGDIRSAINSLQFSSSKGENNLRPRKKGMSLKSDAVLSKSKR
RKKPDRVFENQEVQAIGGKDVSLFLFRALGKILYCKRASLTELDSPRLPSHLSEYERDTL
LVEPEEVVEMSHMPGDLFNLYLHQNYIDFFMEIDDIVRASEFLSFADILSGDWNTRSLLR
EYSTSIATRGVMHSNKARGYAHCQGGGSSFRPLHKPQWFLINKKYRENCLAAKALFPDFC
LPALCLQTQLLPYLALLTIPMRNQAQISFIQDIGRLPLKRHFGRLKMEALTDREHGMIDP

DSGDEAQLNGGHSAEESLGEPTQATVPETWSLPLSQNSASELPASQPQPFSAQGDMEENI
IIEDYESDGT*
>ENSG00000198205|ENST00000358697
MEIPKLLPARGTLQGGGGGGIPAGGGRVHRGPDSPAGQVPTRRLLLPRGPQDGGPGRRRE
EASTASRGPGPSLFAPRPHQPSGGGDDFFLVLLDPVGGDVETAGSGQAAGPVLREEAKAG
PGLQGDESGANPAGCSAQGPHCLSAVPTPAPISAPGPAAAFAGTVTIHNQDLLLRFENGV
LTLATPPPHAWEPGAAPAQQPRCLIAPQAGFPQAAHPGDCPELRSDLLLAEPAEPAPAPA
PQEEAEGLAAALGPRGLLGSGPGVVLYLCPEALCGQTFAKKHQLKMHLLTHSSSQGQRPF
KCPLGGCGWTFTTSYKLKRHLQSHDKLRPFGCPAEGCGKSFTTVYNLKAHMKGHEQENSF
KCEVCEESFPTQAKLGAHQRSHFEPERPYQCAFSGCKKTFITVSALFSHNRAHFREQELF
SCSFPGCSKQYDKACRLKIHLRSHTGERPFLCDFDGCGWNFTSMSKLLRHKRKHDDDRRF
MCPVEGCGKSFTRAEHLKGHSITHLGTKPFVCPVAGCCARFSARSSLYIHSKKHLQDVDT
WKSRCPISSCNKLFTSKHSMKTHMVKRHKVGQDLLAQLEAANSLTPSSELTSQRQNDLSD
AEIVSLFSDVPDSTSAALLDTALVNSGILTIDVASVSSTLAGHLPANNNNSVGQAVDPPS
LMATSDPPQSLDTSLFFGTAATGFQQSSLNMDEVSSVSVGPLGSLDSLAMKNSSPEPQAL
TPSSKLTVDTDTLTPSSTLCENSVSELLTPAKAEWSVHPNSDFFGQEGETQFGFPNAAGN
HGSQKERNLITVTGSSFLV*
>ENSG00000197153|ENST00000359303
MARTKQTARKSTGGKAPRKQLATKAARKSAPATGGVKKPHRYRPGTVALREIRRYQKSTE
LLIRKLPFQRLVREIAQDFKTDLRFQSSAVMALQEACEAYLVGLFEDTNLCAIHAKRVTI
MPKDIQLARRIRGERA*
>ENSG00000244509|ENST00000361441
MNPQIRNPMKAMYPGTFYFQFKNLWEANDRNETWLCFTVEGIKRRSVVSWKTGVFRNQVD
SETHCHAERCFLSWFCDDILSPNTKYQVTWYTSWSPCPDCAGEVAEFLARHSNVNLTIFT
ARLYYFQYPCYQEGLRSLSQEGVAVEIMDYEDFKYCWENFVYNDNEPFKPWKGLKTNFRL
LKRRLRESLQ*
>ENSG00000075151|ENST00000356916
MNSQPQTRSPFAAGPRPPHHQFFQRPQIQPPRATIPNSSPSIRPGAQTPTAVYQANQHIM
MVNHLPMPYPVPQGPQYCIPQYRHSGPPYVGPPQQYPVQPPGPGPFYPGPGPGDFPNAYG
TPFYPSQPVYQSAPIIVPTQQQPPPAKREKKTIRIRDPNQGGKDITEEIMSGGGSRNPTP
PIGRPTSTPTPPQQLPSQVPEHSPVVYGTVESAHLAASTPVTAASDQKQEEKPKPDPVLK
SPSPVLRLVLSGEKKEQEGQTSETTAIVSIAELPLPPSPTTVSSVARSTIAAPTSSALSS
QPIFTTAIDDRCELSSPREDTIPIPSLTSCTETSDPLPTNENDDDICKKPCSVAPNDIPL
VSSTNLINEINGVSEKLSATESIVEIVKQEVLPLTLELEILENPPEEMKLECIPAPITPS
TVPSFPPTPPTPPASPPHTPVIVPAAATTVSSPSAAITVQRVLEEDESIRTCLSEDAKEI
QNKIEVEADGQTEEILDSQNLNSRRSPVPETSNEC*
>ENSG00000177853|ENST00000316045
MPSEQKQLFCDEKQTTLKKDYDVKNEIVDRSAPKPKISGSIHYALKNVKIDLPKINIPNE
VLLKHEVDKYRKLFQSKQQTARKSISIKTVSCVEECTLLHKSERAEEEGVKMSAKILNFS
CLKCRDNTRYSPNDLQKHFQMWHHGELPSYPCEMCNFSANDFQVFKQHRRTHRSTLVKCD
ICNNESVYTLLNLTKHFTSTHCVNGNFQCEKCKFSTQDVGTFVQHIHRHNEIHYKCGKCH
HVCFTKGELQKHLHIHSGTFPFTCQYCSYGATRREHLVRHVITLHKEHLYAKEKLEKDKY
EKRMAKTSAGLKLILKRYKIGASRKTFWKRKKINSGSDRSIEKNTQVLKKMNKTQTKSED
QSHVVQEHLSEEKDERLHCENNDKAPESESEKPTPLSTGQGNRAEEGPNASSGFMKTAVL

GPTLKNVMMKNNKLAVSPNYNATFMGFKMMDGKQHIVLKLVPIKQNVCSPGSQSGAAKDG
TANLQPQTLDTNGFLTGVTTELNDTVYMKAATPFSCSSSILSGKASSEKEMTLISQRNNM
LQTMDYEKSVSSLSATSELVTASVNLTTKFETRDNVDFWGNHLTQSHPEVLGTTIKSPDK
VNCVAKPNAYNSGDMHNYCINYGNCELPVESSNQGSLPFHNYSKVNNSNKRRRFSGTAVY
ENPQRESSSSKTVVQQPISESFLSLVRQESSKPDSLLASISLLNDKDGTLKAKSEIEEQY
VLEKGQNIDGQNLYSNENQNLECATEKSKWEDFSNVDSPMMPRITSVFSLQSQQASEFLP
PEVNQLLQDVLKIKPDVKQDSSNTPNKGLPLHCDQSFQKHEREGKIVESSKDFKVQGIFP
VPPGSVGINVPTNDLNLKFGKEKQVSSIPQDVRDSEKMPRISGFGTLLKTQSDAIITQQL
VKDKLRATTQNLGSFYMQSPLLNSEQKKTIIVQTSKGFLIPLNITNKPGLPVIPGNALPL
VNSQGIPASLFVNKKPGMVLTLNNGKLEGVSAVKTEGAPARGTVTKEPCKTPILKVEPNN
NCLTPGLCSSIGSCLSMKSSSENTLPLKGPYILKPTSSVKAVLIPNMLSEQQSTKLNISD
SVKQQNEIFPKPPLYTFLPDGKQAVFLKCVMPNKTELLKPKLVQNSTYQNIQPKKPEGTP
QRILLKIFNPVLNVTAANNLSVSNSASSLQKDNVPSNQIIGGEQKEPESRDALPFLLDDL
MPANEIVITSTATCPESSEEPICVSDCSESRVLRCKTNCRIERNFNRKKTSKKIFSKTKT
HGSKDSETAFVSRNRNCKRKCRDSYQEPPRRKATLHRKCKEKAKPEDVRETFGFSRPRLS
KDSIRTLRLFPFSSKQLVKCPRRNQPVVVLNHPDADAPEVVSVMKTIAKFNGHVLKVSLS
KRTINALLKPVCYNPPKTTYDDFSKRHKTFKPVSSVKERFVLKLTLKKTSKNNYQIVKTT
SENILKAKFNCWFCGRVFDNQDTWAGHGQRHLMEATRDWNMLE*
>ENSG00000174606|ENST00000366962
MEAWRCVRKGYGHCVVGRGRYPMFPHHSRSLGRDWTTPWENLQRCCWNRHISSCMRWPGH
YSRAPYPYFSSRHFSLNWRPPCLFESRTQFQYCNWRPDNLSQTSLIHLSSYVMNAEGDEP
SSKRRKHQGVIKRNWEYICSHDKEKTKILGDKNVDPKCEDSENKFDFSVMSYNILSQDLL
EDNSHLYRHCRRPVLHWSFRFPNILKEIKHFDADVLCLQEVQEDHYGAEIRPSLESLGYH
CEYKMRTGRKPDGCAICFKHSKFSLLSVNPVEFFRPDISLLDRDNVGLVLLLQPKIPYAA
CPAICVANTHLLYNPRRGDIKLTQLAMLLAEISSVAHQKDGSFCPIVMCGDFNSVPGSPL
YSFIKEGKLNYEGLPIGKVSGQEQSSRGQRILSIPIWPPNLGISQNCVYEVQQVPKVEKT
DSDLTQTQLKQTEVLVTAEKLSSNLQHHFSLSSVYSHYFPDTGIPEVTTCHSRSAITVDY
IFYSAEKEDVAGHPGAEVALVGGLKLLARLSLLTEQDLWTVNGLPNENNSSDHLPLLAKF
RLEL*
>ENSG00000184937|ENST00000332351
LQDPASTCVPEPASQHTLRSGPGCLQQPEQQGVRDPGGIWAKLGAAEASAERLQGRRSRG
ASGSEPQQMGSDVRDLNALLPAVPSLGGGGGCALPVSGAAQWAPVLDFAPPGASAYGSLG
GPAPPPAPPPPPPPPPHSFIKQEPSWGGAEPHEEQCLSAFTVHFSGQFTGTAGACRYGPF
GPPPPSQASSGQARMFPNAPYLPSCLESQPAIRNQGYSTVTFDGTPSYGHTPSHHAAQFP
NHSFKHEDPMGQQGSLGEQQYSVPPPVYGCHTPTDSCTGSQALLLRTPYSSDNLYQMTSQ
LECMTWNQMNLGATLKGVAAGSSSSVKWTEGQSNHSTGYESDNHTTPILCGAQYRIHTHG
VFRGIQDVRRVPGVAPTLVRSASETSEKRPFMCAYPGCNKRYFKLSHLQMHSRKHTGEKP
YQCDFKDCERRFSRSDQLKRHQRRHTGVKPFQCKTCQRKFSRSDHLKTHTRTHTGKTSEK
PFSCRWPSCQKKFARSDELVRHHNMHQRNMTKLQLAL*
>ENSG00000107485|ENST00000346208
MEVTADQPRWVSHHHPAVLNGQHPDTHHPGLSHSYMDAAQYPLPEEVDVLFNIDGQGNHV
PPYYGNSVRATVQRYPPTHHGSQVCRPPLLHGSLPWLDGGKALGSHHTASPWNLSPFSKT
SIHHGSPGPLSVYPPASSSSLSGGHASPHLFTFPPTPPKDVSPDPSLSTPGSAGSARQDE
KECLKYQVPLPDSMKLESSHSRGSMTALGGASSSTHHPITTYPPYVPEYSSGLFPPSSLL

GGSPTGFGCKSRPKARSSTGRECVNCGATSTPLWRRDGTGHYLCNACGLYHKMNGQNRPL
IKPKRRLSAARRAGTSCANCQTTTTTLWRRNANGDPVCNACGLYYKLHNINRPLTMKKEG
IQTRNRKMSSKSKKCKKVHDSLEDFPKNSSFNPAALSRHMSSLSHISPFSHSSHMLTTPT
PMHPPSSLSFGPHHPSSMVTAMG*
>ENSG00000101150|ENST00000352482
MDSAGQDINLNSPNKGLLSDSMTDVPVDTGVAARTPAVEGLTEAEEEELRAELTKVEEEI
VTLRQVLAAKERHCGELKRRLGLSTLGELKQNLSRSWHDVQVSSAYVKTSEKLGEWNEKV
TQSDLYKKTQETLSQAGQKTSAALSTVGSAISRKLGDMSSYSIRHSISMPAMRNSATFKS
FEDRVGTIKSKVVGDRENGSDNLPSSAGSGDKPLSDPAPF*
>ENSG00000199107|ENST00000362237
Sequence unavailable
>ENSG00000117450|ENST00000319248
MSSGNAKIGHPAPNFKATAVMPDGQFKDISLSDYKGKYVVFFFYPLDFTFVCPTEIIAFS
DRAEEFKKLNCQVIGASVDSHFCHLAWVNTPKKQGGLGPMNIPLVSDPKRTIAQDYGVLK
ADEGISFRGLFIIDDKGILRQITVNDLPVGRSVDETLRLVQAFQFTDKHGEVCPAGWKPG
SDTIKPDVQKSKEYFSKQK*
>ENSG00000078900|ENST00000346387
MAQSTATSPDGGTTFEHLWSSLEPDSTYFDLPQSSRGNNEVVGGTDSSMDVFHLEGMTTS
VMAQFNLLSSTMDQMSSRAASASPYTPEHAASVPTHSPYAQPSSTFDTMSPAPVIPSNTD
YPGPHHFEVTFQQSSTAKSATWTYSPLLKKLYCQIAKTCPIQIKVSTPPPPGTAIRAMPV
YKKAEHVTDVVKRCPNHELGRDFNEGQSAPASHLIRVEGNNLSQYVDDPVTGRQSVVVPY
EPPQVGTEFTTILYNFMCNSSCVGGMNRRPILIIITLEMRDGQVLGRRSFEGRICACPGR
DRKADEDHYREQQALNESSAKNGAASKRAFKQSPPAVPALGAGVKKRRHGDEDTYYLQVR
GRENFEILMKLKESLELMELVPQPLVDSYRQQQQLLQRPFLTGLGCPNCIEYFTSQGLQS
IYHLQNLTIEDLGALKIPEQYRMTIWRGLQDLKQGHDYSTAQQLLRSSNAATISIGGSGE
LQRQRVMEAVHFRVRHTITIPNRGGPGGGPDEWADFGFDLPDCKARKQPIKEEFTEAEIH
*
>ENSG00000159086|ENST00000331923
MFRKARRVNVRKRNDSEEEERERDEEQEPPPLLPPPGTGEEAGPGGGDRAPGGESLLGPG
PSPPSALTPGLGAEAGGGFPGGAEPGNGLKPRKRPRENKEVPRASLLSFQDEEEENEEVF
KVKKSSYSKKIVKLLKKEYKEDLEKSKIKTELNSSAESEQPLDKTGHVKDTNQEDGVIIS
EHGEDEMDMESEKEEEKPKTGGAFSNALSSLNVLRPGEIPDAAFIHAARKKRQMARELGD
FTPHDNEPGKGRLVREDENDASDDEDDDEKRRIVFSVKEKSQRQKIAEEIGIEGSDDDAL
VTGEQDEELSRWEQEQIRKGINIPQVQASQPAEVNMYYQNTYQTMPYGSSYGIPYSYTAY
GSSDAKSQKTDNTVPFKTPSNEMTPVTIDLVKKQLKDRLDSMKELHKTNRQQHEKHLQSR
VDSTRAIERLEGSSGGIGERYKFLQEMRGYVQDLLECFSEKVPLINELESAIHQLYKQRA
SRLVQRRQDDIKDESSEFSSHSNKALMAPNLDSFGRDRALYQEHAKRRIAEREARRTRRR
QAREQTGKMADHLEGLSSDDEETSTDITNFNLEKDRISKESGKVFEDVLESFYSIDCIKS
QFEAWRSKYYTSYKDAYIGLCLPKLFNPLIRLQLLTWTPLEAKCRDFENMLWFESLLFYG
CEEREQEKDDVDVALLPTIVEKVILPKLTVIAENMWDPFSTTQTSRMVGITLKLINGYPS
VVNAENKNTQVYLKALLLRMRRTLDDDVFMPLYPKNVLENKNSGPYLFFQRQFWSSVKLL
GNFLQWYGIFSNKTLQELSIDGLLNRYILMAFQNSEYGDDSIKKAQNVINCFPKQWFMNL
KGERTISQLENFCRYLVHLADTIYRNSIGCSDVEKRNARENIKQIVKLLASVRALDHAMS
VASDHNVKEFKSLIEGK*

>ENSG00000197054|ENST00000343949
MMFQDPVACEDVAVNFTQEEWALLDISQRKLYREVMLETFRNLTSIGKKWKDQNIEYEYQ
NPRRNFRSLIEGNVNEIKEDSHCGETFTQVPDDRLNFQEKKASPEAKSCDNFVCGEVGIG
NSSFNMNIRGDIGHKAYEYQDYAPKPYKCQQPKKAFRYHPSFRTQERNHTGEKPYACKEC
GKTFISHSGIRRRMVMHSGDGPYKCKFCGKAVHCLRLYLIHERTHTGEKPYECKQCVKSF
SYSATHRIHERTHTGEKPYECQQCGKAFHSSSSFQAHKRTHTGGKPYECKQCGKSFSWCH
SFQIHERTHTGEKPCECSKCNKAFRSYRSYLRHKRSHTGEKPYQCKECRKAFTYPSSLRR
HERTHSAKKPYECKQCGKALSYKFSNTPKNALWRKTL*
>ENSG00000143321|ENST00000357325
MSRSNRQKEYKCGDLVFAKMKGYPHWPARIDEMPEAAVKSTANKYQVFFFGTHETAFLGP
KDLFPYEESKEKFGKPNKRKGFSEGLWEIENNPTVKASGYQSSQKKSCVEEPEPEPEAAE
GDGDKKGNAEGSSDEEGKLVIDEPAKEKNEKGALKRRAGDLLEDSPKRPKEAENPEGEEK
EAATLEVERPLPMEVEKNSTPSEPGSGRGPPQEEEEEEDEEEEATKEDAEAPGIRDHESL
*
>ENSG00000107249|ENST00000324333
MMVQRLGLISPPASQVSTACNQISPSLQRAMNAANLNIPPSDTRSLISRESLASTTLSLT
ESQSASSMKQEWSQGYRALPSLSNHGSQNGLDLGDLLSLPPGTSMSSNSVSNSLPSYLFG
TESSHSPYPSPRHSSTRSHSARSKKRALSLSPLSDGIGIDFNTIIRTSPTSLVAYINGSR
ASPANLSPQPEVYGHFLGVRGSCIPQPRPVPGSQKGVLVAPGGLALPAYGEDGALEHERM
QQLEHGGLQPGLVNHMVVQHGLPGPDSQSAGLFKTERLEEFPGSTVDLPPAPPLPPLPPP
PGPPPPYHAHAHLHHPELGPHAQQLALPQATLDDDGEMDGIGGKHCCRWIDCSALYDQQE
ELVRHIEKVHIDQRKGEDFTCFWAGCPRRYKPFNARYKLLIHMRVHSGEKPNKCTFEGCE
KAFSRLENLKIHLRSHTGEKPYLCQHPGCQKAFSNSSDRAKHQRTHLDTKPYACQIPGCT
KRYTDPSSLRKHVKAHSSKEQQARKKLRSSTELHPDLLTDCLTVQSLQPATSPRDAAAEG
TVGRSPGPGPDLYSAPIFSSNYSSRSGTAAGAVPPPHPVSHPSPGHNVQGSPHNPSSQLP
PLTAVDAGAERFAPSAPSPHHISPRRVPAPSSILQRTQPPYTQQPSGSHLKSYQPETNSS
FQPNGIHVHGFYGQLQKFCPPHYPDSQRIVPPVSSCSVVPSFEDCLVPTSMGQASFDVFH
RAFSTHSGITVYDLPSSSSSLFGESLRSGAEDATFLQISTVDRCPSQLSSVYTEG*
>ENSG00000113456|ENST00000341754
MPLLTQQIQDEDDQYSLVASLDNVRNLSTILKAIHFREHATCFATKNGIKVTVENAKCVQ
ANAFIQAGIFQEFKVQEESVTFRINLTVLLDCLSIFGSSPMPGTLTALRMCYQGYGYPLM
LFLEEGGVVTVCKINTQEPEETLDFDFCSTNVINKIILQSEGLREAFSELDMTSEVLQIT
MSPDKPYFRLSTFGNAGSSHLDYPKDSDLMEAFHCNQTQVNRYKISLLKPSTKALVLSCK
VSIRTDNRGFLSLQYMIRNEDGQICFVEYYCCPDEEVPESES*
>ENSG00000111676|ENST00000356654
MKTRQNKDSMSMRSGRKKEAPGPREELRSRGRASPGGVSTSSSDGKAEKSRQTAKKARVE
EASTPKVNKQGRSEEISESESEETNAPKKTKTEQELPRPQSPSDLDSLDGRSLNDDGSSD
PRDIDQDNRSTSPSIYSPGSVENDSDSSSGLSQGPARPYHPPPLFPPSPQPPDSTPRQPE
ASFEPHPSVTPTGYHAPMEPPTSRMFQAPPGAPPPHPQLYPGGTGGVLSGPPMGPKGGGA
ASSVGGPNGGKQHPPPTTPISVSSSGASGAPPTKPPTTPVGGGNLPSAPPPANFPHVTPN
LPPPPALRPLNNASASPPGLGAQPLPGHLPSPHAMGQGMGGLPPGPEKGPTLAPSPHSLP
PASSSAPAPPMRFPYSSSSSSSAAASSSSSSSSSSASPFPASQALPSYPHSFPPPTSLSV
SNQPPKYTQPSLPSQAVWSQGPPPPPPYGRLLANSNAHPGPFPPSTGAQSTAHPPVSTHH
HHHQQQQQQQQQQQQQQQQQQQHHGNSGPPPPGAFPHPLEGGSSHHAHPYAMSPSLGSLR

PYPPGPAHLPPPHSQVSYSQAGPNGPPVSSSSNSSSSTSQGSYPCSHPSPSQGPQGAPYP
FPPVPTVTTSSATLSTVIATVASSPAGYKTASPPGPPPYGKRAPSPGAYKTATPPGYKPG
SPPSFRTGTPPGYRGTSPPAGPGTFKPGSPTVGPGPLPPAGPSGLPSLPPPPAAPASGPP
LSATQIKQEPAEEYETPESPVPPARSPSPPPKVVDVPSHASQSARFNKHLDRGFNSCARS
DLYFVPLEGSKLAKKRADLVEKVRREAEQRAREEKEREREREREKEREREKERELERSVK
LAQEGRAPVECPSLGPVPHRPPFEPGSAVATVPPYLGPDTPALRTLSEYARPHVMSPGNR
NHPFYVPLGAVDPGLLGYNVPALYSSDPAARERERLAREARERDLRDRLKPGFEVKPSELEPL
HGVPGPGLDPFPRHGGLALQPGPPGLHPFPFHPSLGPLERERLALAAGPALRPDMSYAER
LAAERQHAERVAALGNDPLARLQMLNVTPHHHQHSHIHSHLHLHQQDAIHAASASVHPLI
DPLASGSHLTRIPYPAGTLPNPLLPHPLHENEVLRHQLFAAPYRDLPASLSAPMSAAHQL
QAMHAQSAELQRLALEQQQWLHAHHPLHSVPLPAQEDYYSHLKKESDKPL*
>ENSG00000188785|ENST00000336128
MNLTEGPLAMAEMDPTQGRVVFEDVAIYFSQEEWGHLDEAQRLLYRDVMLENLALLSSLG
SWHGAEDEEAPSQQGFSVGVSEVTASKPCLSSQKVHPSETCGPPLKDILCLVEHNGIHPE
QHIYICEAELFQHPKQQIGENLSRGDDWIPSFGKNHRVHMAEEIFTCMEGWKDLPATSCL
LQHQGPQSEWKPYRDTEDREAFQTGQNDYKCSECGKTFTCSYSFVEHQKIHTGERSYECN
KCGKFFKYSANFMKHQTVHTSERTYECRECGKSFMYNYRLMRHKRVHTGERPYECNTCGK
FFRYSSTFVRHQRVHTGERPYECRECGKFFMDSSTLIKHQRVHTGERPYKCNDCGKFFRY
ISTLIRHQRIHTGERPYECSVCGELFRYNSSLVKHWRNHTGERPYKCSECGKSFRYHCRL
IRHQRVHTGERPYECSECGKFFRYNSNLIKHWRNHTGERPYECRECGKAFSHKHILVEHQ
KIHSGERPYECSECQKAFIRKSHLVHHQKIHSEERLVCSMNVGNSLAKTPTSLNIRDFTM
EKVYH*
>ENSG00000175809|ENST00000323684
MNKMPAGEQECEYNKEGKYYSKGVKLVRKKKKIPGYRWGDIKINIIGEKDDLPIHFCDKC
DLPIKIYGRIIPCKHAFCYHCANLYDKVGYKVCPRCRYPVLRIEAHKRGSVFMCSIVQQC
KRTYLSQKSLQAHIKRRHKRARKQVTSASLEKVRPHIAPPQTEISDIPKRLQDRDHLSYI
PPEQHTMVSLPSVQHMLQEQHNQPHKDIQAPPPELSLSLPFPIQWETVSIFTRKHGNLTV
DHIQNNSDSGAKKPTPPDYYPECQSQPAVSSPHHIIPQKQHYAPPPSPSSPVNHQMPYPP
QDVVTPNSVRSQVPALTTTYDPSSGYIIVKVPPDMNSPPLRAPQSQNGNPSASEFASHHY
NLNILPQFTENQETLSPQFTQTDAMDHRRWPAWKRLSPCPPTRSPPPSTLHGRSHHSHQR
RHRRY*
>ENSG00000179912|ENST00000347140
MSNSNTTQETLEIMKESEKKLVEESVNKNKFISKTPSKEEIEKECEDTSLRQETQRRTSN
HGHARKRAKSNSKLKLVRSLAVCEESSTPFADGPLETQDIIQLHISCPSDKEEEKSTKDV
SEKEDKDKNKEKIPRKMLSRDSSQEYTDSTGIDLHEFLVNTLKKNPRDRMMLLKLEQEIL
EFINDNNNQFKKFPQMTSYHRMLLHRVAAYFGMDHNVDQTGKAVIINKTSNTRIPEQRFS
EHIKDEKNTEFQQRFILKRDDASMDRDDNQTGQNGYLNDIRLSKEAFSSSSHKRRQIFRG
NREGLSRTSSSRQSSTDSELKSLEPRPWSSTDSDGSVRSMRPPVTKASSFSGISILTRGD
SIGSSKGGSAGRISRPGMALGAPEVCNQVTSSQSVRGLLPCTAQQQQQQQQQLPALPPT
PQQQPPLNNHMISQADDLSNPFGQMSLSRQGSTEAADPSAALFQTPLISQHPQQTSFIMA
STGQPLPTSNYSTSSHAPPTQQVLPPQGYMQPPQQIQVSYYPPGQYPNSNQQYRPLSHPV
AYSPQRGQQLPQPSQQPGLQPMMPNQQQAAYQGMIGVQQPQNQGLLSSQRSSMGGQMQGL
VVQYTPLPSYQVPVGSDSQNVVQPPFQQPMLVPVSQSVQGGLPAAGVPVYYSMIPPAQQN
GTSPSVGFLQPPGSEQYQMPQSPSPCSPPQMPQQYSGVSPSGPGVVVMQLNVPNGPQPPQ

NPSMVQWSHCKYYSMDQRGQKPGDLYSPDSSPQANTQMSSSPVTSPTQSPAPSPVTSLSS
VCTGLSPLPVLTQFPRPGGPAQGDGRYSLLGQPLQYNLSICPPLLHGQSTYTVHQGQSGL
KHGNRGKRQALKSASTDLGTADVVLGRVLEVTDLPEGITRTEADKLFTQLAMSGAKIQWL
KDAQGLPGGGGGDNSGTAENGRHSDLAALYTIVAVFPSPLAAQNASLRLNNSVSRFKLRM
AKKNYDLRILERASSQ*
>ENSG00000007968|ENST00000361729
MLQGPRALASAAGQTPKVVPAMSPTELWPSGLSSPQLCPATATYYTPLYPQTAPPAAAPG
TCLDATPHGPEGQVVRCLPAGRLPAKRKLDLEGIGRPVVPEFPTPKGKCIRVDGLPSPKT
PKSPGEKTRYDTSLGLLTKKFIYLLSESEDGVLDLNWAAEVLDVQKRRIYDITNVLEGIQ
LIRKKAKNNIQWVGRGMFEDPTRPGKQQQLGQELKELMNTEQALDQLIQSCSLSFKHLTE
DKANKRLAYVTYQDIRAVGNFKEQTVIAVKAPPQTRLEVPDRTEDNLQIYLKSTQGPIEV
YLCPEEVQEPDSPSEEPLPSTSTLCPSPDSAQPSSSTDPSIMEPTASSVPAPAPTPQQAP
PPPSLVPLEATDSLLELPHPLLQQTEDQFLSPTLACSSPLISFSPSLDQDDYLWGLEAGE
GISDLFDSYDLGDLLIN*
>ENSG00000168488|ENST00000336783
MLKPQPLQQPSQPQQPPPTQQAVARRPPGGTSPPNGGLPGPLATSAAPPGPPAAASPCLG
PVAAAGSGLRRGAEGILAPQPPPPQQHQERPGAAAIGSARGQSTGKGPPQSPVFEGVYNN
SRMLHFLTAVVGSTCDVKVKNGTTYEGIFKTLSSKFELAVDAVHRKASEPAGGPRREDIV
DTMVFKPSDVMLVHFRNVDFNYATKDKFTDSAIAMNSKVNGEHKEKVLQRWEGGDSNSDD
YDLESDMSNGWDPNEMFKFNEENYGVKTTYDSSLSSYTVPLEKDNSEEFRQRELRAAQLA
REIESSPQYRLRIAMENDDGRTEEEKHSAVQRQGSGRESPSLASREGKYIPLPQRVREGP
RGGVRCSSSRGGRPGLSSLPPRGPHHLDNSSPGPGSEARGINGGPSRMSPKAQRPLRGAK
TLSSPSNRPSGETSVPPPPAVGRMYPPRSPKSAAPAPISASCPEPPIGSAVPTSSASIPV
TSSVSDPGVGSISPASPKISLAPTDVKELSTKEPGRTLEPQELARIAGKVPGLQNEQKRF
QLEELRKFGAQFKLQPSSSPENSLDPFPPRILKEEPKGKEKEVDGLLTSEPMGSPVSSKT
ESVSDKEDKPPLAPSGGTEGPEQPPPPCPSQTGSPPVGLIKGEDKDEGPVAEQVKKSTLN
PNAKEFNPTKPLLSVNKSTSTPTSPGPRTHSTPSIPVLTAGQSGLYSPQYISYIPQIHMG
PAVQAPQMYPYPVSNSVPGQQGKYRGAKGSLPPQRSDQHQPASAPPMMQAAAAAGPPLVA
ATPYSSYIPYNPQQFPGQPAMMQPMAHYPSQPVFAPMLQSNPRMLTSGSHPQAIVSSSTP
QYPSAEQPTPQALYATVHQSYPHHATQLHAHQPQPATTPTGSQPQSQHAAPSPVQHQAGQ
APHLGSGQPQQNLYHPGALTGTPPSLPPGPSAQSPQSSFPQPAAVYAIHHQQLPHGFTNM
AHVTQAHVQTGITAAPPPHPGAPHPPQVMLLHPPQSHGGPPQGAVPQSGVPALSASTPSP
YPYIGHPQGEQPGQAPGFPGGADDRIREFSLAGGIWHGRAEGLQVGQDARVLGGE*
>ENSG00000199072|ENST00000362202
Sequence unavailable
>ENSG00000158691|ENST00000361028
MASTWAIQAHMDQDEPLEVKIEEEKYTTRQDWDLRKNNTHSREVFRQYFRQFCYQETSGP
REALSRLRELCHQWLRPETHTKEQILELLVLEQFLTILPEELQAWVQEQHPESGEEVVTV
LEDLERELDEPGEQVSVHTGEQEMFLQETVRLRKEGEPSMSLQSMKAQPKYESPELESQQ
EQVLDVETGNEYGNLKQEVSEEMEPHGKTSSKFENDMSKSARCGETREPEEITEEPSACS
REDKQPTCDENGVSLTENSDHTEHQRICPGEESYGCDDCGKAFSQHSHLIEHQRIHTGDR
PYKCEECGKAFRGRTVLIRHKIIHTGEKPYKCNECGKAFGRWSALNQHQRLHTGEKHYHC
NDCGKAFSQKAGLFHHIKIHTRDKPYQCTQCNKSFSRRSILTQHQGVHTGAKPYECNECG
KAFVYNSSLVSHQEIHHKEKCYQCKECGKSFSQSGLIQHQRIHTGEKPYKCDVCEKAFIQ

RTSLTEHQRIHTGERPYKCDKCGKAFTQRSVLTEHQRIHTGERPYKCDECGNAFRGITSL
IQHQRIHTGEKPYQCDECGKAFRQRKKTSYKEILLKNHSEPQAGVNLLLSSLIPEWQSCF
RRDL*
>ENSG00000088305|ENST00000348286
MKGDTRHLNGEEDAGGREDSILVNGACSDQSSDSPPILEAIRTPEIRGRRSSSRLSKREV
SSLLSYTQDLTGDGDGEDGDGSDTPVMPKLFRETRTRSESPAVRTRNNNSVSSRERHRPS
PRSTRGRQGRNHVDESPVEFPATRSLRRRATASAGTPWPSPPSSYLTIDLTDDTEDTHGT
PQSSSTPYARLAQDSQQGGMESPQVEADSGDGDSSEYQDGKEFGIGDLVWGKIKGFSWWP
AMVVSWKATSKRQAMSGMRWVQWFGDGKFSEVSADKLVALGLFSQHFNLATFNKLVSYRK
AMYHALEKARVRAGKTFPSSPGDSLEDQLKPMLEWAHGGFKPTGIEGLKPNNTQPENKTR
RRTADDSATSDYCPAPKRLKTNCYNNGKDRGDEDQSREQMASDVANNKSSLEDGCLSCGR
KNPVSFHPLFEGGLCQTCRDRFLELFYMYDDDGYQSYCTVCCEGRELLLCSNTSCCRCFC
VECLEVLVGTGTAAEAKLQEPWSCYMCLPQRCHGVLRRRKDWNVRLQAFFTSDTGLEYEA
PKLYPAIPAARRRPIRVLSLFDGIATGYLVLKELGIKVGKYVASEVCEESIAVGTVKHEG
NIKYVNDVRNITKKNIEEWGPFDLVIGGSPCNDLSNVNPARKGLYEGTGRLFFEFYHLLN
YSRPKEGDDRPFFWMFENVVAMKVGDKRDISRFLECNPVMIDAIKVSAAHRARYFWGNLP
GMNRIFGFPVHYTDVSNMGRGARQKLLGRSWSVPVIRHLFAPLKDYFACE*
>ENSG00000101126|ENST00000349014
MFQLPVNNLGSLRKARKTVKKILSDIGLEYCKEHIEDFKQFEPNDFYLKNTTWEDVGLWD
PSLTKNQDYRTKPFCCSACPFSSKFFSAYKSHFRNVHSEDFENRILLNCPYCTFNADKKT
LETHIKIFHAPNASAPSSSLSTFKDKNKNDGLKPKQADSVEQAVYYCKKCTYRDPLYEIV
RKHIYREHFQHVAAPYIAKAGEKSLNGAVPLGSNAREESSIHCKRCLFMPKSYEALVQHV
IEDHERIGYQVTAMIGHTNVVVPRSKPLMLIAPKPQDKKSMGLPPRIGSLASGNVRSLPS
QQMVNRLSIPKPNLNSTGVNMMSSVHLQQNNYGVKSVGQGYSVGQSMRLGLGGNAPVSIP
QQSQSVKQLLPSGNGRSYGLGSEQRSQAPARYSLQSANASSLSSGQLKSPSLSQSQASRV
LGQSSSKPAAAATGPPPGNTSSTQKWKICTICNELFPENVYSVHFEKEHKAEKVPAVANY
IMKIHNFTSKCLYCNRYLPTDTLLNHMLIHGLSCPYCRSTFNDVEKMAAHMRMVHIDEEM
GPKTDSTLSFDLTLQQGSHTNIHLLVTTYNLRDAPAESVAYHAQNNPPVPPKPQPKVQEK
ADIPVKSSPQAAVPYKKDVGKTLCPLCFSILKGPISDALAHHLRERHQVIQTVHPVEKKL
TYKCIHCLGVYTSNMTASTITLHLVHCRGVGKTQNGQDKTNAPSRLNQSPSLAPVKRTYE
QMEFPLLKKRKLDDDSDSPSFFEEKPEEPVVLALDPKGHEDDSYEARKSFLTKYFNKQPY
PTRREIEKLAASLWLWKSDIASHFSNKRKKCVRDCEKYKPGVLLGFNMKELNKVKHEMDF
DAEWLFENHDEKDSRVNASKTADKKLNLGKEDDSSSDSFENLEEESNESGSPFDPVFEVE
PKISNDNPEEHVLKVIPEDASESEEKLDQKEDGSKYETIHLTEEPTKLMHNASDSEVDQD
DVVEWKDGASPSESGPGSQQVSDFEDNTCEMKPGTWSDESSQSEDARSSKPAAKKKATMQ
GDREQLKWKNSSYGKVEGFWSKDQSQWKNASENDERLSNPQIEWQNSTIDSEDGEQFDNM
TDGVAEPMHGSLAGVKLSSQQA*
>ENSG00000182759|ENST00000333480
MAAELAMGAELPSSPLAIEYVNDFDLMKFEVKKEPPEAERFCHRLPPGSLSSTPLSTPCS
SVPSSPSFCAPSPGTGGGGGAGGGGGSSQAGGAPGPPSGGPGAVGGTSGKPALEDLYWMS
GYQHHLNPEALNLTPEDAVEALIGSGHHGAHHGAHHPAAAAAYEAFRGPGFAGGGGADDM
GAGHHHGAHHAAHHHHAAHHHHHHHHHHGGAGHGGGAGHHVRLEERFSDDQLVSMSVREL
NRQLRGFSKEEVIRLKQKRRTLKNRGYAQSCRFKRVQQRHILESEKCQLQSQVEQLKLEV
GRLAKERDLYKEKYEKLAGRGGPGSAGGAGFPREPSPPQAGPGGAKGTADFFL*

>ENSG00000154719|ENST00000352957
MEALAMGSRALRLWLVAPGGGIKWRFIATSSASQLSPTELTEMRNDLFNKEKARQLSLTP
RTEKIEVKHVGKTDPGTVFVMNKNISTPYSCAMHLSEWYCRKSILALVDGQPWDMYKPLT
KSCEIKFLTFKDCDPGEVNKAYWRSCAMMMGCVIERAFKDEYMVNLVRAPEVPVISGAFC
YDVVLDSKLDEWMPTKENLRSFTKDAHALIYKDLPFETLEVEAKVALEIFQHSKYKVDFI
EEKASQNPERIVKLHRIGDFIDVSEGPLIPRTSICFQYEVSAVHNLQPTQPSLIRRFQGV
SLPVHLRAHFTIWDKLLERSRKMVTEDQSKATEECTST*
>ENSG00000189306|ENST00000323013
MVARRRKCAARDPEDRIPSPLGYAAIPIKFSEKQQASHYLYVRAHGVRQGTKSTWPQKRT
LFVLNVPPYCTEESLSRLLSTCGLVQSVELQEKPDLAESPKESRSKFFHPKPVPGFQVAY
VVFQKPSGVSAALALKGPLLVSTESHPVKSGIHKWISDYADSVPDPEALRVEVDTFMEAY
DQKIAEEEAKAKEEEGVPDEEGWVKVTRRGRRPVLPRTEAASLRVLERERRKRSRKELLN
FYAWQHRESKMEHLAQLRKKFEEDKQRIELLRAQRKFRPY*
>ENSG00000198974|ENST00000362104
Sequence unavailable
>ENSG00000158163|ENST00000327532
MQSPAATAEGLSGPLFGAYTFPTFKFQPRHDSMDWRRISTLDVDRVARELDVATLQENIA
GITFCNLDREVCSRCGQPVDPALLKVLRLAQLIIEYLLHCQDCLSASVAQLEARLQTSLG
QQQRGQQELGRQADELKGVREESRRRRKMISTLQQLLMQTGTHSYHTCHLCDKTFMNATF
LRGHIQRRHAGVAEGGKQKKQEQPVEEVLEELRAKLKWTQGELEAQREAERQRQLQEAEL
IHQREIEAKKEFDKWKEQEWTKLYGEIDKLKKLFWDEFKNVAKQNSTLEEKLRALQSHSV
MESKLGSLRDEESEEWLRQARELQALREKTEIQKTEWKRKVKELHEEHMAEKKELQEENQ
RLQASLSQDQKKAAAQSQCQISTLRAQLQEQARIIASQEEMIQSLSLRKVEGIHKVPKAV
DTEEDSPEEEMEDSQDEQHKVLAALRRNPTLLKHFRPILEDTLEEKLESMGIRKDAKGIS
IQTLRHLESLLRVQREQKARKFSEFLSLRGKLVKEVTSRAKERQENGAVVSQPDGQPSVK
SQQSTLVTREAQPKTRTLQVALPSTPAEPPPPTRQSHGSHGSSLTQVSAPAPRPGLHGPS
STPPSSGPGMSTPPFSSEEDSEGDRVQRVSLQPPKVPSRMVPRPKDDWDWSDTETSEENA
QPPGQGSGTLVQSMVKNLEKQLEAPAKKPAGGVSLFFMPNAGPQRAATPGRKPQLSEDES
DLEISSLEDLPLDLDQREKPKPLSRSKLPEKFGTGPQSSGQPRVPAW*
>ENSG00000184897|ENST00000333762
MSVELEEALPVTTAEGMAKKVTKAGGSAALSPSKKRKNSKKKNQPGKYSQLVVETIRRLG
ERNGSSLAKIYTEAKKVPWFDQQNGRTYLKYSIKALVQNDTLLQVKGTGANGSFKLNRKK
LEGGGERRGAPAAATAPAPTAHKAKKAAPGAAGSRRADKKPARGQKPEQRSHKKGAGAKK
DKGGKAKKTAAAGGKKVKKAAKPSVPKVPKGRK*
>ENSG00000176678|ENST00000320241
MSHLFDPRLPALAASPMLYLYGPERPGLPLAFAPAAALAASGRAETPQKPPYSYIALIAM
AIQDAPEQRVTLNGIYQFIMDRFPFYHDNRQGWQNSIRHNLSLNDCFVKVPREKGRPGKG
SYWTLDPRCLDMFENGNYRRRKRKPKPGPGAPEAKRPRAETHQRSAEAQPEAGSGAGGSG
PAISRLQAAPAGPSPLLDGPSPPAPLHWPGTASPNEDAGDAAQGAAAVAVGQAARTGDGP
GSPLRPASRSSPKSSDKSKSFSIDSILAGKQGQKPPSGDELLGGAKPGPGGRLGASLLAA
SSSLRPPFNASLMLDPHVQGGFYQLGIPFLSYFPLQVPDTVLHFQ*
>ENSG00000196357|ENST00000355114
MRRGPWERWSLASHRLDAGLCTCPREESREIRAGQIVLKAMAQGLVTFRDVAIEFSLEEW
KCLEPAQRDLYREVTLENFGHLASLGLSISKPDVVSLLEQGKEPWMIANDVTGPWCPDLE

SRCEKFLQKDIFEIGAFNWEIMESLKCSDLEGSDFRADWECEGQFERQVNEECYFKQVNV
TYGHMPVFQHHTSHTVRQSRETGEKLMECHECGKAFSRGSHLIQHQKIHTGEKPFGCKEC
GKAFSRASHLVQHQRIHTGEKPYDCKDCGKAFGRTSELILHQRLHTGVKPYECKECGKTF
RQHSQLILHQRTHTGEKPYVCKDCGKAFIRGSQLTVHRRIHTGARPYECKECGKAFRQHS
QLTVHQRIHTGEKPYECKECGKGFIHSSEVTRHQRIHSGEKPYECKECGKAFRQHAQLTR
HQRVHTGDRPYECKDCGKAFSRSSYLIQHQRIHTGDKPYECKECGKAFIRVSQLTHHQRI
HTCEKPYECRECGMAFIRSSQLTEHQRIHPGIKPYECRECGQAFILGSQLIEHYRIHTG*
>ENSG00000140451|ENST00000333425
MLSGIEAAAGEYEDSELRCRVAVEELSPGGQPRRRQALRTAELSLGRNERRELMLRLQAP
GPAGRPRCFPLRAARLFTRFAEAGRSTLRLPAHDTPGAGAVQLLLSDCPPDRLRRFLRTL
RLKLAAAPGPGPASARAQLLGPRPRDFVTISPVQPEERRLRAATRVPDTTLVKRPVEPQA
GAEPSTEAPRWPLPVKRLSLPSTKPQLSEEQAAVLRAVLKGQSIFFTGSAGTGKSYLLKR
ILGSLPPTGTVATASTGVAACHIGGTTLHAFAGIGSGQAPLAQCVALAQRPGVRQGWLNC
QRLVIDEISMVEADLFDKLEAVARAVRQQNKPFGGIQLIICGDFLQLPPVTKGSQPPRFC
FQSKSWKRCVPVTLELTKVWRQADQTFISLLQAVRLGRCSDEVTRQLQATASHKVGRDGI
VATRLCTHQDDVALTNERRLQELPGKVHRFEAMDSNPELASTLDAQCPVSQLLQLKLGAQ
VMLVKNLSVSRGLVNGARGVVVGFEAEGRGLPQVRFLCGVTEVIHADRWTVQATGGQLLS
RQQLPLQLAWAMSIHKSQGMTLDCVEISLGRVFASGQAYVALSRARSLQGLRVLDFDPMA
VRCDPRVLHFYATLRRGRSLSLAAEGRGNEDRCSGSSIRALGGDWWGLRLGAASKQRTEL
RCVSTARPSLAQPRTNTLQSLTKEHKLQNVHPYFKLLFQGINSVWGH*
>ENSG00000136169|ENST00000354234
MGEKNGDAKTFWMELEDDGKVDFIFEQVQNVLQSLKQKIKDGSATNKEYIQAMILVNEAT
IINSSTSIKGASQKEVNAQSSDPMPVTQKEQENKSNAFPSTSCENSFPEDCTFLTTENKE
ILSLEDKVVDFREKDSSSNLSYQSHDCSGACLMKMPLNLKGENPLQLPIKCHFQRRHAKT
NSHSSALHVSYKTPCGRSLRNVEEVFRYLLETECNFLFTDNFSFNTYVQLARNYPKQKEV
VSDVDISNGVESVPISFCNEIDSRKLPQFKYRKTVWPRAYNLTNFSSMFTDSCDCSEGCI
DITKCACLQLTARNAKTSPLSSDKITTGYKYKRLQRQIPTGIYECSLLCKCNRQLCQNRV
VQHGPQVRLQVFKTEQKGWGVRCLDDIDRGTFVCIYSGRLLSRANTEKSYGIDENGRDEN
TMKNIFSKKRKLEVACSDCEVEVLPLGLETHPRTAKTEKCPPKFSNNPKELTVETKYDNI
SRIQYHSVIRDPESKTAIFQHNGKKMEFVSSESVTPEDNDGFKPPREHLNSKTKGAQKDS
SSNHVDEFEDNLLIESDVIDITKYREETPPRSRCNQATTLDNQNIKKAIEVQIQKPQEGR
STACQRQQVFCDEELLSETKNTSSDSLTKFNKGNVFLLDATKEGNVGRFLNHSCCPNLLV
QNVFVETHNRNFPLVAFFTNRYVKARTELTWDYGYEAGTVPEKEIFCQCGVNKCRKKIL*
>ENSG00000145982|ENST00000324331
MVGSALRRGAHAYVYLVSKASHISRGHQHQAWGSRPPAAECATQRAPGSVVELLGKSYPQ
DDHSNLTRKVLTRVGRNLHNQQHHPLWLIKERVKEHFYKQYVGRFGTPLFSVYDNLSPVV
TTWQNFDSLLIPADHPSRKKGDNYYLNRTHMLRAHTSAHQWDLLHAGLDAFLVVGDVYRR
DQIDSQHYPIFHQLEAVRLFSKHELFAGIKDGESLQLFEQSSRSAHKQETHTMEAVKLVE
FDLKQTLTRLMAHLFGDELEIRWVDCYFPFTHPSFEMEINFHGEWLEVLGCGVMEQQLVN
SAGAQDRIGWAFGLGLERLAMILYDIPDIRLFWCEDERFLKQFCVSNINQKVKFQPLSKY
PAVINDISFWLPSENYAENDFYDLVRTIGGDLVEKVDLIDKFVHPKTHKTSHCYRITYRH
MERTLSQREVRHIHQALQEAAVQLLGVEGRF*
>ENSG00000152601|ENST00000324196
MAVSVTPIRDTKWLTLEVCREFQRGTCSRPDTECKFAHPSKSCQVENGRVIACFDSLKGR

CSRENCKYLHPPPHLKTQLEINGRNNLIQQKNMAMLAQQMQLANAMMPGAPLQPVPMFSV
APSLATNASAAAFNPYLGPVSPSLVPAEILPTAPMLVTGNPGVPVPAAAAAAAQKLMRTD
RLEVCREYQRGNCNRGENDCRFAHPADSTMIDTNDNTVTVCMDYIKGRCSREKCKYFHPP
AHLQAKIKAAQYQVNQAAAAQAAATAAAMFPWCTVLRQPLCPQQQHLPQVFPSLQQPQPT
SPILDASTLLGATSCPAAAGKMIPIISAEHLTSHKYVTQM*
>ENSG00000185345|ENST00000366897
MIVFVRFNSSHGFPVEVDSDTSIFQLKEVVAKRQGVPADQLRVIFAGKELRNDWTVQNCD
LDQQSIVHIVQRPWRKGQEMNATGGDDPRNAAGGCEREPQSLTRVDLSSSVLPGDSVGLA
VILHTDSRKDSPPAGSPAGRSIYNSFYVYCKGPCQRVQPGKLRVQCSTCRQATLTLTQEF
FFKCGAHPTSDKETSVALHLIATNSRNITCITCTDVRSPVLVFQCNSRHVICLDCFHLYC
VTRLNDRQFVHDPQLGYSLPCVAGCPNSLIKELHHFRILGEEQYNRYQQYGAEECVLQMG
GVLCPRPGCGAGLLPEPDQRKVTCEGGNGLGCGFAFCRECKEAYHEGECSAVFEASGTTT
QAYRVDERAAEQARWEAASKETIKKTTKPCPRCHVPVEKNGGCMHMKCPQPQCRLEWCWN
CGCEWNRVCMGDHWFDV*
>ENSG00000144852|ENST00000337940
MTVTRTHHFKEGSLRAPAIPLHSAAAELASNHPRGPEANLEVRPKESWNHADFVHCEDTE
SVPGKPSVNADEEVGGPQICRVCGDKATGYHFNVMTCEGCKGFFRRAMKRNARLRCPFRK
GACEITRKTRRQCQACRLRKCLESGMKKEMIMSDEAVEERRALIKRKKSERTGTQPLGVQ
GLTEEQRMMIRELMDAQMKTFDTTFSHFKNFRLPGVLSSGCELPESLQAPSREEAAKWSQ
VRKDLCSLKVSLQLRGEDGSVWNYKPPADSGGKEIFSLLPHMADMSTYMFKGIISFAKVI
SYFRDLPIEDQISLLKGAAFELCQLRFNTVFNAETGTWECGRLSYCLEDTAGGFQQLLLE
PMLKFHYMLKKLQLHEEEYVLMQAISLFSPDRPGVLQHRVVDQLQEQFAITLKSYIECNR
PQPAHRFLFLKIMAMLTELRSINAQHTQRLLRIQDIHPFATPLMQELFGITGS*
>ENSG00000196646|ENST00000343979
MDSVAFEDVDVNFTQEEWALLDPSQKNLYRDVMWETMRNLASIGKKWKDQNIKDHYKHRG
RNLRSHMLERLYQTKDGSQRGGIFSQFANQNLSKKIPGVKLCESIVYGEVSMGQSSLNRH
IKDHSGHEPKEYQEYGEKPDTRNQCWKPFSSHHSFRTHEIIHTGEKLYDCKECGKTFFSL
KRIRRHIITHSGYTPYKCKVCGKAFDYPSRFRTHERSHTGEKPYECQECGKAFTCITSVR
RHMIKHTGDGPYKCKVCGKPFHSLSSFQVHERIHTGEKPFKCKQCGKAFSCSPTLRIHER
THTGEKPYECKQCGKAFSYLPSLRLHERIHTGEKPFVCKQCGKAFRSASTFQIHERTHTG
EKPYECKECGEAFSCIPSMRRHMIKHTGEGPYKCKVCGKPFHSLSPFRIHERTHTGEKPY
VCKHCGKAFVSSTSIRIHERTHTGEKPYECKQCGKAFSYLNSFRTHEMIHTGEKPFECKR
CGKAFRSSSSFRLHERTHTGQKPYHCKECGKAYSCRASFQRHMLTHAEDGPPYKCMWESL
*
>ENSG00000199135|ENST00000362265
Sequence unavailable
>ENSG00000163798|ENST00000326019
MLAPLRNAPGREGATSPSPPTDATGSLGEWDVDRNVKTEGWVSKERISKLHRLRMADILS
QSETLASQDLSGDFKKPALPVSPAARSKAPASSSSNPEEVQKEGPTALQDSNSGEPDIPP
PQPDCGDFRSLQEEQSRPPTAVSSPGGPARAPPYQEPPWGGPATAPYSLETLKGGTILGT
RSLKGTSYCLFGRLSGCDVCLEHPSVSRYHAVLQHRASGPDGECDSNGPGFYLYDLGSTH
GTFLNKTRIPPRTYCRVHVGHVVRFGGSTRLFILQGPEEDREAESELTVTQLKELRKQQQ
ILLEKKMLGEDSDEEEEMDTSERKINAGSQDDEMGCTWGMGEDAVEDDAEENPIVLEFQQ
EREAFYIKDPKKALQGFFDREGEELEYEFDEQGHSTWLCRVRLPVDDSTGKQLVAEAIHS

GKKKEAMIQCSLEACRILDTLGLLRQEAVSRKRKAKNWEDEDFYDSDDDTFLDRTGLIEK
KRLNRMKKAGKIDEKPETFESLVAKLNDAERELSEISERLKASSQVLSESPSQDSLDAFM
SEMKSGSTLDGVSRKKLHLRTFELRKEQQRLKGLIKIVKPAEIPELKKTETQTTGAENKA
KKLTLPLFGAMKGGSKFKLKTGTVGKLPPKRPELPPTLMRMKDEPEVEEEEEEEEEEEKE
KEEHEKKKLEDGSLSRPQPEIEPEAAVQEMRPPTDLTHFKETQTHENMSQLSEEEQNKDY
QDCSKTTSLCAGPSASKNEYEKSRGELKKKKTPGPGKLPPTLSSKYPEDDPDYCVWVPPE
GQSGDGRTHLNDKYGY*
>ENSG00000183479|ENST00000334497
MGRAGSPLPRSSWPRMDDCGSRSRCSPTLCSSLRTCYPRGNITMSEAPRAETFVFLDLEA
TGLPSVEPEIAELSLFAVHRSSLENPEHDESGALVLPRVLDKLTLCMCPERPFTAKASEI
TGLSSEGLARCRKAGFDGAVVRTLQAFLSRQAGPICLVAHNGFDYDFPLLCAELRRLGAR
LPRDTVCLDTLPALRGLDRAHSHGTRARGRQGYSLGSLFHRYFRAEPSAAHSAEGDVHTL
LLIFLHRAAELLAWADEQARGWAHIEPMYLPPDDPSLEA*
>ENSG00000196531|ENST00000356769
MPGEATETVPATEQELPQPQAETGSGTESDSDESVPELEEQDSTQATTQQAQLAAAAEID
EEPVSKAKQSRSEKKARKAMSKLGLRQVTGVTRVTIRKSKNILFVITKPDVYKSPASDTY
IVFGEAKIEDLSQQAQLAAAEKFKVQGEAVSNIQENTQTPTVQEESEEEEVDETGVEVKD
IELVMSQANVSRAKAVRALKNNSNDIVNAIMELTM*
>ENSG00000197056|ENST00000359858
MKEPLLGGECDKAVASQLGLLDEIKTEPDNAQEYCHRQQSRTQENELKINAVFSESASQL
TAGIQLSLASSGVNKMLPSVSTTAIQVSCAGCKKILQKGQTAYQRKGSAQLFCSIPCITE
YISSASSPVPSKRTCSNCSKDILNPKDVISVQLEDTTSCKTFCSLSCLSSYEEKRKPFVT
ICTNSILTKCSMCQKTAIIQYEVKYQNVKHNLCSNACLSKFHSANNFIMNCCENCGTYCY
TSSSLSHILQMEGQSHYFNSSKSITAYKQKPAKPLISVPCKPLKPSDEMIETTSDLGKTE
LFCSINCFSAYSKAKMESSSVSVVSVVHDTSTELLSPKKDTTPVISNIVSLADTDVALPI
MNTDVLQDTVSSVTATADVIVDLSKSSPSEPSNAVASSSTEQPSVSPSSSVFSQHAIGSS
TEVQKDNMKSMKISDELCHPKCTSKVQKVKGKSRSIKKSCCADFECLENSKKDVAFCYSC
QLFCQKYFSCGRESFATHGTSNWKKTLEKFRKHEKSEMHLKSLEFWREYQFCDGAVSDDL
SIHSKQIEGNKKYLKLIIENILFLGKQCLPLRGNDQSVSSVNKGNFLELLEMRAKDKGEE
TFRLMNSQVDFYNSTQIQSDIIEIIKTEMLQDIVNEINDSSAFSIICDETINSAMKEQLS
ICVRYPQKSSKAILIKERFLGFVDTEEMTGTHLHRTIKTYLQQIGVDMDKIHGQAYDSTT
NLKIKFNKIAAEFKKEEPRALYIHCYAHFLDLSIIRFCKEVKELRSALKTLSSLFNTICM
SGEMLANFRNIYRLSQNKTCKKHISQSCWTVHDRTLLSVIDSLPEIIETLEVIASHSSNT
SFADELSHLLTLVSKFEFVFCLKFLYRVLSVTGILSKELQNKTIDIFSLSSKIEAILECL
SSERNDVYFKTIWDGTEEICQKITCKGFKVEKPSLQKRRKIQKSVDLGNSDNMFFPTSTE
EQYKINIYYQGLDTILQNLKLCFSEFDYCKIKQISELLFKWNEPLNETTAKHVQEFYKLD
EDIIPELRFYRHYAKLNFVIDDSCINFVSLGCLFIQHGLHSNIPCLSKLLYIALSWPITS
ASTENSFSTLPRLKTYLCNTMGQEKLTGPALMAVEQELVNKLMEPERLNEIVEKFISQMK
EI*
>ENSG00000196361|ENST00000359227
MVTQILGAMESQVGGGPAGPALPNGPLLGTNGATDDSKTNLIVNYLPQNMTQDEFKSLFG
SIGDIESCKLVRDKITGQSLGYGFVNYSDPNDADKAINTLNGLKLQTKTIKVSYARPSSA
SIRDANLYVSGLPKTMSQKEMEQLFSQYGRIITSRILVDQVTGVSRGVGFIRFDKRIEAE
EAIKGLNGQKPLGAAEPITVKFANNPSQKTGQALLTHLYQSSARRYAGPLHHQTQRFRLD

NLLNMAYGVKSPLSLIARFSPIAIDGMSGLAGVGLSGGAAGAGWCIFVYNLSPEADESVL
WQLFGPFGAVTNVKVIRDFTTNKCKGFGFVTMTNYDEAAMAIASLNGYRLGERVLQVSFK
TSKQHKA*
>ENSG00000197114|ENST00000357119
MDEESLESALQTYRAQLQQVELALGAGLDSSEQADLRQLQGDLKELIELTEASLVSVRKS
SLLAALDEERPGRQEDAEYQAFREAITEAVEAPAAARGSGSETVPKAEAGPESAAGGQEE
EEGEDEEELSGTKVSAPYYSSWGTLEYHNAMVVGTEEAEDGSAGVRVLYLYPTHKSLKPC
PFFLEGKCRFKENCRFSHGQVVSLDELRPFQDPDLSSLQAGSACLAKHQDGLWHAARITD
VDNGYYTVKFDSLLLREAVVEGDGILPPLRTEATESDSDSDVVGSDAVDSGTCSSAFAGW
EVHTRGIGSRLLTKMGYEFGKGLGRHAEGRVEPIHAVVLPRGKSLDQCVETLQKQTRVGK
AGTNKPPRCRGRGARPGGRPAPRNVFDFLNEKLQGQAPGALEAGAAPAGRRSKDMYHASK
SAKRALSLRLFQTEEKIERTQRDIRSIQEALARNAGRHSVASAQLQEKLAGAQRQLGQLR
AQEAGLQQEQRKADTHKKMTEF*
>ENSG00000180787|ENST00000318833
MGTENKEVIPKEEISEESEPHGSLLEKFPKVVYQGHEFGAGCEEDMLEGHSRESMEEVIE
QMSPQERDFPSGLMIFKKSPSSEKDRENNESERGCSPSPNLVTHQGDTTEGVSAFATSGQ
NFLEILESNKTQRSSVGEKPHTCKECGKAFNQNSHLIQHMRVHSGEKPFECKECGKTFGT
NSSLRRHLRIHAGEKPFACNECGKAFIQSSHLIHHHRIHTGERPYKCEECGKAFSQNSAL
ILHQRIHTGEKPYECNECGKTFRVSSQLIQHQRIHTEERYHECNECGKAFKHSSGLIRHQ
KIHTGEKPYLCNECGKGFGQSSELIRHQRIHTGDKPYECNECGKTFGQNSEIIRHIRIHT
GEKPYVCKECGKAFRGNSELLRHERIHTGEKPYECFECGKAFRRTSHLIVHQRIHTGEKP
HQCNECARTFWDNSELLLHQKIHIGEKPYECSECEKTFSQHSQLIIHQRIHTGEKPYECQ
ECQKTFSRSSHLLRHQSVHCME*
>ENSG00000118689|ENST00000343882
MAEAPASPAPLSPLEVELDPEFEPQSRPRSCTWPLQRPELQASPAKPSGETAADSMIPEE
EDDEDDEDGGGRAGSAMAIGGGGGSGTLGSGLLLEDSARVLAPGGQDPGSGPATAAGGLS
GGTQALLQPQQPLPPPQPGAAGGSGQPRKCSSRRNAWGNLSYADLITRAIESSPDKRLTL
SQIYEWMVRCVPYFKDKGDSNSSAGWKNSIRHNLSLHSRFMRVQNEGTGKSSWWIINPDG
GKSGKAPRRRAVSMDNSNKYTKSRGRAAKKKAALQTAPESADDSPSQLSKWPGSPTSRSS
DELDAWTDFRSRTNSNASTVSGRLSPIMASTELDEVQDDDAPLSPMLYSSSASLSPSVSK
PCTVELPRLTDMAGTMNLNDGLTENLMDDLLDNITLPPSQPSPTGGLMQRSSSFPYTTKG
SGLGSPTSSFNSTVFGPSSLNSLRQSPMQTIQENKPATFSSMSHYGNQTLQDLLTSDSLS
HSDVMMTQSDPLMSQASTAVSAQNSRRNVMLRNDPMMSFAAQPNQGSLVNQNLLHHQHQT
QGALGGSRALSNSVSNMGLSESSSLGSAKHQQQSPVSQSMQTLSDSLSGSSLYSTSANLP
VMGHEKFPSDLDLDMFNGSLECDMESIIRSELMDADGLDFNFDSLISTQNVVGLNVGNFT
GAKQASSQSWVPG*
>ENSG00000198900|ENST00000361337
MSGDHLHNDSQIEADFRLNDSHKHKDKHKDREHRHKEHKKEKDREKSKHSNSEHKDSEKK
HKEKEKTKHKDGSSEKHKDKHKDRDKEKRKEEKVRASGDAKIKKEKENGFSSPPQIKDEP
EDDGYFVPPKEDIKPLKRPRDEDDADYKPKKIKTEDTKKEKKRKLEEEEDGKLKKPKNKD
KDKKVPEPDNKKKKPKKEEEQKWKWWEEERYPEGIKWKFLEHKGPVFAPPYEPLPENVKF
YYDGKVMKLSPKAEEVATFFAKMLDHEYTTKEIFRKNFFKDWRKEMTNEEKNIITNLSKC
DFTQMSQYFKAQTEARKQMSKEEKLKIKEENEKLLKEYGFCIMDNHKERIANFKIEPPGL
FRGRGNHPKMGMLKRRIMPEDIIINCSKDAKVPSPPPGHKWKEVRHDNKVTWLVSWTENI

QGSIKYIMLNPSSRIKGEKDWQKYETARRLKKCVDKIRNQYREDWKSKEMKVRQRAVALY
FIDKLALRAGNEKEEGETADTVGCCSLRVEHINLHPELDGQEYVVEFDFLGKDSIRYYNK
VPVEKRVFKNLQLFMENKQPEDDLFDRLNTGILNKHLQDLMEGLTAKVFRTYNASITLQQ
QLKELTAPDENIPAKILSYNRANRAVAILCNHQRAPPKTFEKSMMNLQTKIDAKKEQLAD
ARRDLKSAKADAKVMKDAKTKKVVESKKKAVQRLEEQLMKLEVQATDREENKQIALGTSK
LNYLDPRITVAWCKKWGVPIEKIYNKTQREKFAWAIDMADEDYEF*
>ENSG00000106268|ENST00000343985
MSGISPQQMGEPEGSWSGKNPGTMGASRLYTLVLVLQPQRVLLGMKKRGFGAGRWNGFGG
KVQEGETIEDGARRELQEESGLTVDALHKVGQIVFEFVGEPELMDVHVFCTDSIQGTPVE
SDEMRPCWFQLDQIPFKDMWPDDSYWFPLLLQKKKFHGYFKFQGQDTILDYTLREVDTV*
>ENSG00000177374|ENST00000322941
MTFPEADILLKSGECAGQTMLDTMEAPGHSRQLLLQLNNQRTKGFLCDVIIVVQNALFRA
HKNVLAASSAYLKSLVVHDNLLNLDHDMVSPAVFRLVLDFIYTGRLADGAEAAAAAAVAP
GAEPSLGAVLAAASYLQIPDLVALCKKRLKRHGKYCHLRGGGGGGGGYAPYGRPGRGLRA
ATPVIQACYPSPVGPPPPPAAEPPSGPEAAVNTHCAELYASGPGPAAALCASERRCSPLC
GLDLSKKSPPGSAAPERPLAERELPPRPDSPPSAGPAAYKEPPLALPSLPPLPFQKLEEA
APPSDPFRGGSGSPGPEPPGRPDGPSLLYRWMKHEPGLGSYGDELGRERGSPSERCEERG
GDAAVSPGGPPLGLAPPPRYPGSLDGPGAGGDGDDYKSSSEETGSSEDPSPPGGHLEGYP
CPHLAYGEPESFGDNLYVCIPCGKGFPSSEQLNAHVEAHVEEEEALYGRAEAAEVAAGAA
GLGPPFGGGGDKVAGAPGGLGELLRPYRCASCDKSYKDPATLRQHEKTHWLTRPYPCTIC
GKKFTQRGTMTRHMRSHLGLKPFACDACGMRFTRQYRLTEHMRIHSGEKPYECQVCGGKF
AQQRNLISHMKMHAVGGAAGAAGALAGLGGLPGVPGPDGKGKLDFPEGVFAVARLTAEQL
SLKQQDKAAAAELLAQTTHFLHDPKVALESLYPLAKFTAELGLSPDKAAEVLSQGAHLAA
GPDGRTIDRFSPT*
>ENSG00000153896|ENST00000329285
MAAPALALVSFEDVVVTFTGEEWGHLDLAQRTLYQEVMLETCRLLVSLGHPVPKPELIYL
LEHGQELWTVKRGLSQSTCAGEKAKPKITEPTASQLAFSEESSFQELLAQRSSRDSRLGQ
ARDEEKLIKIQEGNLRPGTNPHKEICPEKLSYKHDDLEPDDSLGLRVLQERVTPQDALHE
CDSQGPGKDPMTDARNNPYTCTECGKGFSKKWALVRHQQIHAGVKPYECNECGKACRYMA
DVIRHMRLHTGEKPYKCIECGKAFKRRFHLTEHQRIHTGDKPYECKECGKAFTHRSSFIQ
HNMTHTREKPFLCKECGKAFYYSSSFAQHMRIHTGKKLYECGECGKAFTHRSTFIQHNVT
HTGEKPFLCKECGKTFCLNSSFTQHMRIHTGEKPYECGECGKAFTHRSTFIRHKRTHTGE
KPFECKECGKAFCDSSSLIQHMRIHTGEKPYECSECGKAFTHHSVFIRHNRTHSGQKPLE
CKECAKAFYYSSSFTRHMRIHTGEKPYVCRECGKAFTQPANFVRHNRIHTGEKPFECKEC
EKAFCDNFALTQHMRTHTGEKPFECNECGKTFSHSSSFTHHRKIHTRV*
>ENSG00000180532|ENST00000318203
MALDLRTIFQCEPSENNLGSENSAFQQSQGPAVQREEGISEFSRMVLNSFQDSNNSYARQ
ELQRLYRIFHSWLQPEKHSKDEIISLLVLEQFMIGGHCNDKASVKEKWKSSGKNLERFIE
DLTDDSINPPALVHVHMQGQEALFSEDMPLRDVIVHLTKQVNAQTTREANMGTPSQTSQD
TSLETGQGYEDEQDGWNSSSKTTRVNENITNQGNQIVSLIIIQEENGPRPEEGGVSSDNP
YNSKRAELVTARSQEGSINGITFQGVPMVMGAGCISQPEQSSPESALTHQSNEGNSTCEV
HQKGSHGVQKSYKCEECPKVFKYLCHLLAHQRRHRNERPFVCPECQKGFFQISDLRVHQI
IHTGKKPFTCSMCKKSFSHKTNLRSHERIHTGEKPYTCPFCKTSYRQSSTYHRHMRTHEK
ITLPSVPSTPEAS*

>ENSG00000197385|ENST00000360311
MLREEAAQKRKEKEPGMALPQGHLTFRDVAIEFSLEEWKCLDPTQRALYRAMMLENYRNL
HSVDISSKCMMKKFSSTAQGNTEVDTGTLERHESHHIGDFCFQKIGKDIHDFEFQWQEDK
RNSHEATMTQIKKLTGSTDRYDRRHPGNKPIKDQLGLSFHSHLPELHIFQTKGKVGNQVE
KSINDASSVLTSQRISSRPKIHISNNYENNFFHSSLLTLKQEVHIREKSFQCNESGKAFN
CSSLLRKHQIIYLGGKQYKCDVCGKVFNQKRYLACHHRCHTGEKPYKCNECGKVFNQQSN
LASHHRLHTGEKPYKCEECDKVFSRKSNLERHRRIHTGEKPYKCKVCEKAFRRDSHLTQH
TRIHTGEKPYKCNECGKAFSGQSTLIHHQAIHGIGKLYKCNDCHKVFSNATTIANHWRIH
NEERSYKCNKCGKFFRRRSYLVVHWRTHTGEKPYKCNECGKTFHHNSALVIHKAIHTGEK
PYKCNECGKTFRHNSALVIHKAIHTGEKPYKCNECGKVFNQQATLARHHRLHTGEKPYKC
EECDTVFSRKSHHETHKRIHTGEKPYKCDDFDEAFSQASSYAKQRRIHMGEKHHKCDDCG
KAFTSHSHRIRHQRIHTGQKSYKCHKRGKVFS*
>ENSG00000088876|ENST00000358413
MMLPYPSALGDQYWEEILLPKNGENVETMKKLTQNHKAKGLPSNDTDCPQKKEGKAQIVV
PVTFRDVTVIFTEAEWKRLSPEQRNLYKEVMLENYRNLLSLGQEIETILANIVKSHLY*
>ENSG00000113761|ENST00000358149
MEYPAPATVQAADGGAAGPYSSSELLEGQEPDGVRFDRERARRLWEAVSGAQPVGREEVE
HMIQKNQCLFTNTQCKVCCALLISESQKLAHYQSKKHANKVKRYLAIHGMETLKGETKKL
DSDQKSSRSKDKNQCCPICNMTFSSPVVAQSHYLGKTHAKNLKLKQQSTKVEALHQNREM
IDPDKFCSLCHATFNDPVMAQQHYVGKKHRKQETKLKLMARYGRLADPAVTDFPAGKGYP
CKTCKIVLNSIEQYQAHVSGFKHKNQSPKTVASSLGQIPMQRQPIQKDSTTLED*
>ENSG00000187792|ENST00000341976
MEVPPATKFGETFAFENRLESQQGLFPGEDLGDPFLQERGLEQMAVIYKEIPLGEQDEEN
DDYEGNFSLCSSPVQHQSIPPGTRPQDDELFGQTFLQKSDLSMCQIIHSEEPSPCDCAET
DRGDSGPNAPHRTPQPAKPYACRECGKAFSQSSHLLRHLVIHTGEKPYECCECGKAFSQS
SHLLRHQIIHTGEKPYECRECGKAFRQSSALTQHQKIHTGKRPYECRECGKDFSRSSSLR
KHERIHTGERPYQCKECGKSFNQSSGLSQHRKIHTLKKPHECDLCGKAFCHRSHLIRHQR
IHTGKKPYKCDECGKAFSQSSNLIEHRKTHTGEKPYKCQKCGKAFSQSSSLIEHQRIHTG
EKPYECCQCGKAFCHSSALIQHQRIHTGKKPYTCECGKAFRHRSALIEHYKTHTREKPYV
CNLCGKSFRGSSHLIRHQKIHSGEKL*
>ENSG00000183621|ENST00000331737
MDSESNIPSGTIQSRKGLQNKSQFRTIAPKIVPKVLTSRMLPCHSPSRSDQVNLGPSINS
KLLGMSTQNYALMQVAGQEGTFSLVALPHVASAQPIQKPRMSLPENLKLPIPRYQPPRNS
KASRKKPILIFPKSGCSKAPAQTQMCPQMSPSPPHHPELLYKPSPFEEVPSLEQAPASIS
TAALTNGSDHGDLRPPVTNTHGSLNPPATPASSTPEEPAKQDLTALSGKAHFVSKITSSK
PSAVASEKFKEQVDLAKTMTNLSPTILGNAVQLISSVPKGKLPIPPYSRMKTMEVYKIKS
DANIAGFSLPGPKADCDKIPSTTEGFNAATKVASRLPVPQVSQQSACESAFCPPTKLDLN
HKTKLNSGAAKRKGRKRKVPDEILAFQGKRRKYIINKCRDGKERVKNDPQEFRDQKLGTL
KKYRSIMPKPIMVIPTLASLASPTTLQSQMLGGLGQDVLLNNSLTPKYLGCKQDNSSSPK
PSSVFRNGFSGIKKPWHRCHVCNHHFQFKQHLRDHMNTHTNRRPYSCRICRKSYVRPGSL
STHMKLHHGENRLKKLMCCEFCAKVFGHIRVYFGHLKEVHRVVISTEPAPSELQPGDIPK
NRDMSVRGMEGSLERENKSNLEEDFLLNQADEVKLQIKCGRCQITAQSFAEIKFHLLDVH
GEEIEGRLQEGTFPGSKGTQEELVQHASPDWKRHPERGKPEKVHSSSEESHACPRLKRQL
HLHQNGVEMLMENEGPQSGTNKPRETCQGPECPGLHTFLLWSHSGFNCLLCAEMLGRKED

LLHHWKHQHNCEDPSKLWAILNTVSNQGVIELSSEAEK*
>ENSG00000052850|ENST00000329255
MNAETCVSYCESPAAAMDAYYSPVSQSREGSSPFRAFPGGDKFGTTFLSAAAKAQGFGDA
KSRARYGAGQQDLATPLESGAGARGSFNKFQPQPSTPQPQPPPQPQPQQQQPQPQPPAQP
HLYLQRGACKTPPDGSLKLQEGSSGHSAALQVPCYAKESSLGEPELPPDSDTVGMDSSYL
SVKEAGVKGPQDRASSDLPSPLEKADSESNKGKKRRNRTTFTSYQLEELEKVFQKTHYPD
VYAREQLAMRTDLTEARVQVWFQNRRAKWRKRERFGQMQQVRTHFSTAYELPLLTRAENY
AQIQNPSWLGNNGAASPVPACVVPCDPVPACMSPHAHPPGSGASSVTDFLSVSGAGSHVG
QTHMGSLFGAASLSPGLNGYELNGEPDRKTSSIAALRMKAKEHSAAISWAT*
>ENSG00000197903|ENST00000356950
MPEPAKSAPAPKKGSKKAVTKAQKKDGKKRKRSRKESYSVYVYKVLKQVHPDTGISSKAM
GIMNSFVNDIFERIAGEASRLAHYNKRSTITSREIQTAVRLLLPGELAKHAVSEGTKAVT
KYTSAK*
>ENSG00000171469|ENST00000326044
MAAIYLSRGFFSREPICPFEEKTKVERMVEDYLASGYQDSVTFDDVAVDFTPEEWALLDT
TEKYLYRDVMLENYMNLASVDKRLQWMETL*
>ENSG00000117360|ENST00000324862
MALSKRELDELKPWIEKTVKRVLGFSEPTVVTAALNCVGKGMDKKKAADHLKPFLDDSTL
RFVDKLFEAVEEGRSSRHSKSSSDRSRKRELKEVFGDDSEISKESSGVKKRRIPRFEEVE
EEPEVIPGPPSESPGMLTKLQIKQMMEAATRQIEERKKQLSFISPPTPQPKTPSSSQPER
LPIGNTIQPSQAATFMNDAIEKARKAAELQARIQAQLALKPGLIGNANMVGLANLHAMGI
APPKVELKDQTKPTPLILDEQGRTVDATGKEIELTHRMPTLKANIRAVKREQFKQQLKEK
PSEDMESNTFFDPRVSIAPSQRQRRTFKFHDKGKFEKIAQRLRTKAQLEKLQAEISQAAR
KTGIHTSTRLALIAPKKELKEGDIPEIEWWDSYIIPNGFDLTEENPKREDYFGITNLVEH
PAQLNPPVDNDTPVTLGVYLTKKEQKKLRRQTRREAQKELQEKVRLGLMPPPEPKVRISN
LMRVLGTEAVQDPTKVEAHVRAQMAKRQKAHEEANAARKLTAEQRKVKKIKKLKEDISQG
VHISVYRVRNLSNPAKKFKIEANAGQLYLTGVVVLHKDVNVVVVEGGPKAQKKFKRLMLH
RIKWDEQTSNTKGDDDEESDEEAVKKTNKCVLVWEGTAKDRSFGEMKFKQCPTENMAREH
FKKHGAEHYWDLALSESVLESTD*
>ENSG00000115415|ENST00000361099
MSQWYELQQLDSKFLEQVHQLYDDSFPMEIRQYLAQWLEKQDWEHAANDVSFATIRFHDL
LSQLDDQYSRFSLENNFLLQHNIRKSKRNLQDNFQEDPIQMSMIIYSCLKEERKILENAQ
RFNQAQSGNIQSTVMLDKQKELDSKVRNVKDKVMCIEHEIKSLEDLQDEYDFKCKTLQNR
EHETNGVAKSDQKQEQLLLKKMYLMLDNKRKEVVHKIIELLNVTELTQNALINDELVEWK
RRQQSACIGGPPNACLDQLQNWFTIVAESLQQVRQQLKKLEELEQKYTYEHDPITKNKQV
LWDRTFSLFQQLIQSSFVVERQPCMPTHPQRPLVLKTGVQFTVKLRLLVKLQELNYNLKV
KVLFDKDVNERNTVKGFRKFNILGTHTKVMNMEESTNGSLAAEFRHLQLKEQKNAGTRTN
EGPLIVTEELHSLSFETQLCQPGLVIDLETTSLPVVVISNVSQLPSGWASILWYNMLVAE
PRNLSFFLTPPCARWAQLSEVLSWQFSSVTKRGLNVDQLNMLGEKLLGPNASPDGLIPWT
RFCKENINDKNFPFWLWIESILELIKKHLLPLWNDGCIMGFISKERERALLKDQQPGTFL
LRFSESSREGAITFTWVERSQNGGEPDFHAVEPYTKKELSAVTFPDIIRNYKVMAAENIP
ENPLKYLYPNIDKDHAFGKYYSRPKEAPEPMELDGPKGTGYIKTELISVSEVHPSRLQTT
DNLLPMSPEEFDEVSRIVGSVEFDSMMNTV*
>ENSG00000179750|ENST00000333467

MNPQIRNPMERMYRDTFYDNFENEPILYGRSYTWLCYEVKIKRGRSNLLWDTGVFRGQVY
FKPQYHAEMCFLSWFCGNQLPAYKCFQITWFVSWTPCPDCVAKLAEFLSEHPNVTLTISA
ARLYYYWERDYRRALCRLSQAGARVTIMDYEEFAYCWENFVYNEGQQFMPWYKFDENYAF
LHRTLKEILRYLMDPDTFTFNFNNDPLVLRRRQTYLCYEVERLDNGTWVLMDQHMGFLCN
EAKNLLCGFYGRHAELRFLDLVPSLQLDPAQIYRVTWFISWSPCFSWGCAGEVRAFLQEN
THVRLRIFAARIYDYDPLYKEALQMLRDAGAQVSIMTYDEFEYCWDTFVYRQGCPFQPWD
GLEEHSQALSGRLRAILQNQGN*
>ENSG00000119318|ENST00000358015
MQVTLKTLQQQTFKIDIDPEETVKALKEKIESEKGKDAFPVAGQKLIYAGKILNDDTALK
EYKIDEKNFVVVMVTKPKAVSTPAPATTQQSAPASTTAVTSSTTTTVAQAPTPVPALAPT
STPASITPASATASSEPAPASAAKQEKPAEKPAETPVATSPTATDSTSGDSSRSNLFEDA
TSALVTGQSYENMVTEIMSMGYEREQVIAALRASFNNPDRAVEYLLMGIPGDRESQAVVD
PPQAASTGAPQSSAVAAAAATTTATTTTTSSGGHPLEFLRNQPQFQQMRQIIQQNPSLLP
ALLQQIGRENPQLLQQISQHQEHFIQMLNEPVQEAGGQGGGGGGGSGGIAEAGSGHMNYI
QVTPQEKEAIERLKALGFPEGLVIQAYFACEKNENLAANFLLQQNFDED*
>ENSG00000183617|ENST00000330133
MATKRLFGATRTWAGWGAWELLNPATSGRLLARDYAKKPVMKGAKSGKGAVTSEALKDPD
VCTDPVQLTTYAMGVNIYKEGQDVPLKPDAEYPEWLFEMNLGPPKTLEELDPESREYWRR
LRKQNIWRHNRLSKNKRL*
>ENSG00000189060|ENST00000340857
MTENSTSAPAAKPKRAKASKKSTDHPKYSDMIVAAIQAEKNRAGSSRQSIQKYIKSHYKV
GENADSQIKLSIKRLVTTGVLKQTKGVGASGSFRLAKSDEPKKSVAFKKTKKEIKKVATP
KKASKPKKAASKAPTKKPKATPVKKAKKKLAATPKKAKKPKTVKAKPVKASKPKKAKPVK
PKAKSSAKRAGKKK*
>ENSG00000147065|ENST00000360270
MPKTISVRVTTMDAELEFAIQPNTTGKQLFDQVVKTIGLREVWFFGLQYQDTKGFSTWLK
LNKKVTAQDVRKESPLLFKFRAKFYPEDVSEELIQDITQRLFFLQVKEGILNDDIYCPPE
TAVLLASYAVQSKYGDFNKEVHKSGYLAGDKLLPQRVLEQHKLNKDQWEERIQVWHEEHR
GMLREDAVLEYLKIAQDLEMYGVNYFSIKNKKGSELWLGVDALGLNIYEQNDRLTPKIGF
PWSEIRNISFNDKKFVIKPIDKKAPDFVFYAPRLRINKRILALCMGNHELYMRRRKPDTI
EVQQMKAQAREEKHQKQMERAMLENEKKKREMAEKEKEKIEREKEELMERLKQIEEQTKK
AQQELEEQTRRALELEQERKRAQSEAEKLAKERQEAEEAKEALLQASRDQKKTQEQLALE
MAELTARISQLEMARQKKESEAVEWQQKAQMVQEDLEKTRAELKTAMSTPHVAEPAENEQ
DEQDENGAEASADLRADAMAKDRSEEERTTEAEKNERVQKHLKALTSELANARDESKKTA
NDMIHAENMRLGRDKYKTLRQIRQGNTKQRIDEFESM*
>ENSG00000167264|ENST00000358896
MILNSLSLCYHNKLILAPMVRVGTLPMRLLALDYGADIVYCEELIDLKMIQCKRVVNEVL
STVDFVAPDDRVVFRTCEREQNRVVFQMGTSDAERALAVARLVENDVAGIDVNMGCPKQY
STKGGMGAALLSDPDKIEKILSTLVKGTRRPVTCKIRILPSLEDTLSLVKRIERTGIAAI
AVHGRKREERPQHPVSCEVIKAIADTLSIPVIANGGSHDHIQQYSDIEDFRQATAASSVM
VARAAMWNPSIFLKEGLRPLEEVMQKYIRYAVQYDNHYTNTKYCLCQMLREQLESPQGRL
LHAAQSSREICEAFGLGAFYEETTQELDAQQARLSAKTSEQTGEPAEDTSGVIKMAVKFD
RRAYPAQITPKMCLLEWCRREKLAQPVYETVQRPLDRLFSSIVTVAEQKYQSTLWDKSKK
LAEQAAAIVCLRSQGLPEGRLGEESPSLHKRKREAPDQDPGGPRAQELAQPGDLCKKPFV

ALGSGEESPLEGW*
>ENSG00000126746|ENST00000361959
MEESHFNSNPYFWPSIPTVSGQIENTMFINKMKDQLLPEKGCGLAPPHYPTLLTVPASVS
LPSGISMDTESKSDQLTPHSQASVTQNITVVPVPSTGLMTAGVSCSQRWRREGSQSRGPG
LVITSPSGSLVTTASSAQTFPISAPMIVSALPPGSQALQVVPDLSKKVASTLTEEGGGGG
GGGGSVAPKPPRGRKKKRMLESGLPEMNDPYVLSPEDDDDHQKDGKTYRCRMCSLTFYSK
SEMQIHSKSHTETKPHKCPHCSKTFANSSYLAQHIRIHSGAKPYSCNFCEKSFRQLSHLQ
QHTRIHSKMHTETIKPHKCPHCSKTFANTSYLAQHLRIHSGAKPYNCSYCQKAFRQLSHL
QQHTRIHTGDRPYKCAHPGCEKAFTQLSNLQSHRRQHNKDKPFKCHNCHRAYTDAASLEV
HLSTHTVKHAKVYTCTICSRAYTSETYLMKHMRKHNPPDLQQQVQAAAAAAVAQAQAQA
QAQAQAQAQAQAQAQASQASQQQQQQQQQQQQQQQQPPPHFQSPGAAPQGGGGGDSNPNP
PPQCSFDLTPYKTAEHHKDICLTVTTSTIQVEHLASS*
>ENSG00000120948|ENST00000315091
MSEYIRVTEDENDEPIEIPSEDDGTVLLSTVTAQFPGACGLRYRNPVSQCMRGVRLVEGI
LHAPDAGWGNLVYVVNYPKDNKRKMDETDASSAVKVKRAVQKTSDLIVLGLPWKTTEQDL
KEYFSTFGEVLMVQVKKDLKTGHSKGFGFVRFTEYETQVKVMSQRHMIDGRWCDCKLPNS
KQSQDEPLRSRKVFVGRCTEDMTEDELREFFSQYGDVMDVFIPKPFRAFAFVTFADDQIA
QSLCGEDLIIKGISVHISNAEPKHNSNRQLERSGRFGVHLISNVYGRSTSLKVVL*
>ENSG00000136944|ENST00000355497
MDIATGPESLERCFPRGQTDCAKMLDGIKMEEHALRPGPATLGVLLGSDCPHPAVCEGCQ
RPISDRFLMRVNESSWHEECLQCAACQQALTTSCYFRDRKLYCKQDYQQLFAAKCSGCME
KIAPTEFVMRALECVYHLGCFCCCVCERQLRKGDEFVLKEGQLLCKGDYEKEKDLLSSVS
PDESDSVKSEDEDGDMKPAKGQGSQSKGSGDDGKDPRRPKRPRTILTTQQRRAFKASFEV
SSKPCRKVRETLAAETGLSVRVVQVWFQNQRAKMKKLARRHQQQQEQQNSQRLGQGEPGP
GQGLGQEVLSSRMEGMMASYTPLAPPQQQIVAMEQSPYGSSDPFQQGLTPPQMPGNDSIF
HDIDSDTSLTSLSDCFLGSSDVGSLQARVGNPIDRLYSMQSSYFAS*
>ENSG00000151702|ENST00000344954
MDPGSLLAYNTTSHTDQSSRLSVKEDPSYDSVRRGAWGNNMNSGLNKSPPLGGAQTISKN
TEQRPQPDPYQILGPTSSRLANPGSGQIQLWQFLLELLSDSANASCITWEGTNGEFKMTD
PDEVARRWGERKSKPNMNYDKLSRALRYYYDKNIMTKVHGKRYAYKFDFHGIAQALQPHP
TESSMYKYPSDISYMPSYHAHQQKVNFVPPHPSSMPVTSSSFFGAASQYWTSPTGGIYPN
PNVPRHPNTHVPSHLGSYY*
>ENSG00000172819|ENST00000338561
MYDCMETFAPGPRRLYGAAGPGAGLLRRATGGSCFAGLESFAWPQPASLQSVETQSTSSE
EMVPSSPSPPPPPRVYKPCFVCNDKSSGYHYGVSSCEGCKGFFRRSIQKNMVYTCHRDKN
CIINKVTRNRCQYCRLQKCFEVGMSKEAVRNDRNKKKKEVKEEGSPDSYELSPQLEELIT
KVSKAHQETFPSLCQLGKYTTNSSADHRVQLDLGLWDKFSELATKCIIKIVEFAKRLPGF
TGLSIADQITLLKAACLDILMLRICTRYTPEQDTMTFSDGLTLNRTQMHNAGFGPLTDLV
FAFAGQLLPLEMDDTETGLLSAICLICGDRMDLEEPEKVDKLQEPLLEALRLYARRRRPS
QPYMFPRMLMKITDLRGISTKGAERAITLKMEIPGPMPPLIREMLENPEMFEDDSSQPGP
HPNASSEDEVPGGQGKGGLKSPA*
>ENSG00000117625|ENST00000367006
MPGMMEKGPELLGKNRSANGSAKSPAGGGGSGASSTNGGLHYSEPESGCSSDDEHDVGMR
VGAEYQARIPEFDPGATKYTDKDNGGMLVWSPYHSIPDAKLDEYIAIAKEKHGYNVEQAL

GMLFWHKHNIEKSLADLPNFTPFPDEWTVEDKVLFEQAFSFHGKSFHRIQQMLPDKTIAS
LVKYYYSWKKTRSRTSLMDRQARKLANRHNQGDSDDDVEETHPMDGNDSDYDPKKEAKKE
GNTEQPVQTSKIGLGRREYQSLQHRHHSQRSKCRPPKGMYLTQEDVVAVSCSPNAANTIL
RQLDMELISLKRQVQNAKQVNSALKQKMEGGIEEFKPPESNQKINARWTTEEQLLAVQGT
DPTGSSDTGSITSCPIIHSNTNSPYCHSEPASTTSSSNTACCPGSSPAASSTPAAGSVHP
APANFKSASTTSYSPC*
>ENSG00000164022|ENST00000358008
MANNDAVLKRLEQKGAEADQIIEYLKQQVSLLKEKAILQATLREEKKLRVENAKLKKEIE
ELKQELIQAEIQNGVKQIPFPSGTPLHANSMVSENVIQSTAVTTVSSGTKEQIKGGTGDE
KKAKEKIEKKGEKKEKKQQSIAGSADSKPIDVSRLDLRIGCIITARKHPDADSLYVEEVD
VGEIAPRTVVSGLVNHVPLEQMQNRMVILLCNLKPAKMRGVLSQAMVMCASSPEKIEILA
PPNGSVPGDRITFDAFPGEPDKELNPKKKIWEQIQPDLHTNDECVATYKGVPFEVKGKGV
CRAQTMSNSGIK*
>ENSG00000103248|ENST00000360900
MEPRAVGVSKQDIREQIWGYMESQNLADFPRPVHHRIPNFKGSYLACQNIKDLDVFARTQ
EVKVDPDKPLEGVRLLVLQSKKTLLVPTPRLRTGLFNKITPPPGATKDILRKCATSQGVR
NYSVPIGLDSRVLVDLVVVGSVAVSEKGWRIGKGEGYADLEYAMMVSMGAVSKETPVVTI
VHDCQVVDIPEELVEEHDITVDYILTPTRVIATGCKRPKPMGITWFKISLEMMEKIPILR
SLRAREQQAGKDVTLQGEHQHLPEPGCQQTVPLSVGRRPPDTPGPETNSMEAAPGSPPGE
GAPLAADVYVGNLPGDARVSDLKRALRELGSVPLRLTWQGPRRRAFLHYPDSAAAQQAVS
CLQGLRLGTDTLRVALARQQRDK*
>ENSG00000184571|ENST00000332271
MPGRARTRARGRARRRESYQQEAPGGPRAPGSATTQEPPQLQSTPRPLQEEVPVVRPLQP
RAARGGAGGGAQSQGVKEPGPEAGLHTAPLQERRIGGVFQDLVVNTRQDMKHVKDSKTGS
EGTVVQLLANHFRVISRPQWVAYKYNVDYKPDIEDGNLRTILLDQHRRKFGERHIFDGNS
LLLSRPLKERRVEWLSTTKDKNIVKITVEFSKELTPTSPDCLRYYNILFRRTFKLLDFEQ
VGRNYYTKKKAIQLYRHGTSLEIWLGYVTSVLQYENSITLCADVSHKLLRIETAYDFIKR
TSAQAQTGNIREEVTNKLIGSIVLTKYNNKTYRVDDIDWKQNPEDTFNKSDGSKITYIDY
YRQQHKEIVTVKKQPLLVSQGRWKKGLTGTQREPILLIPQLCHMTGLTDEICKDYSIVKE
LAKHTRLSPRRRHHTLKEFINTLQDNKKVRELLQLWDLKFDTNFLSVPGRVLKNANIVQG
RRMVKANSQGDWSREIRELPLLNAMPLHSWLILYSRSSHREAMSLKGHLQSVTAPMGITM
KPAEMIEVDGDANSYIDTLRKYTRPTLQMGMSCLLVFKVICILPNDDKRRYDSIKRYLCT
KCPIPSQCVVKKTLEKVQARTIVTKIAQQMNCKMGGALWKVETDVQRTMFVGIDCFHDIV
NRQKSIAGFVASTNAELTKWYSQCVIQKTGEELVKELEICLKAALDVWCKNESSMPHSVI
VYRDGVGDGQLQALLDHEAKKMSTYLKTISPNNFTLAFIVVKKRINTRFFLKHGSNFQNP
PPGTVIDVELTRNEWYDFFIVSQSVQDGTVTPTHYNVIYDTIGLSPDTVQRLTYCLCHMY
YNLPGIIRVPAPCHYAHKLAYLVGQSIHQEPNRSLSTRLFYL*
>ENSG00000189403|ENST00000326004
MGKGDPKKPRGKMSSYAFFVQTCREEHKKKHPDASVNFSEFSKKCSERWKTMSAKEKGKF
EDMAKADKARYEREMKTYIPPKGETKKKFKDPNAPKRPPSAFFLFCSEYRPKIKGEHPGL
SIGDVAKKLGEMWNNTAADDKQPYEKKAAKLKEKYEKF*
>ENSG00000109111|ENST00000347486
MSDFVESEAEESEEEYNDEGEVVPRVTKKFVEEEDDDEEEEEENLDDQDEQGNLKGFIND
DDDEDEGEEDEGSDSGDSEDDVGHKKRKRTSFDDRLEDDDFDLIEENLGVKVKRGQKYRR

VKKMSDDEDDDEEEYGKEEHEKEAIAEEIFQDGEGEEGQEAMEAPMAPPEEEEEDDEESD
IDDFIVDDDGQPLKKPKWRKKLPGYTDAALQEAQEIFGVDFDYDEFEKYNEYDEELEEEY
EYEDDEAEGEIRVRPKKTTKKRVSRRSIFEMYEPSELESSHLTDQDNEIRATDLPERFQL
RSIPVKGAEDDELEEEADWIYRNAFATPTISLQESCDYLDRGQPASSFSRKGPSTIQKIK
EALGFMRNQHFEVPFIAFYRKEYVEPELHINDLWRVWQWDEKWTQLRIRKENLTRLFEKM
QAYQYEQISADPDKPLADGIRALDTTDMERLKDVQSMDELKDVYNHFLLYYGRDIPKMQN
AAKASRKKLKRVREEGDEEGEGDEAEDEEQRGPELKQASRRDMYTICQSAGLDGLAKKFG
LTPEQFGENLRDSYQRHETEQFPAEPLELAKDYVCSQFPTPEAVLEGARYMVALQIAREP
LVRQVLRQTFQERAKLNITPTKKGRKDVDEAHYAYSFKYLKNKPVKELRDDQFLKICLAE
DEGLLTTDISIDLKGVEGYGNDQTYFEEIKQFYYRDEFSHQVQEWNRQRTMAIERALQQF
LYVQMAKELKNKLLAEAKEYVIKACSRKLYNWLRVAPYRPDQQVEEDDDFMDENQGKGIR
VLGIAFSSARDHPVFCALVNGEGEVTDFLRLPHFTKRRTAWREEEREKKAQDIETLKKFL
LNKKPHVVTVAGENRDAQMLIEDVKRIVHELDQGQQLSSIGVELVDNELAILYMNSKKSE
AEFRDYPPVLRQAVSLARRIQDPLIEFAQVCSSDEDILCLKFHPLQEHVVKEELLNALYC
EFINRVNEVGVDVNRAIAHPYSQALIQYVCGLGPRKGTHLLKILKQNNTRLESRTQLVTM
CHMGPKVFMNCAGFLKIDTASLGDSTDSYIEVLDGSRVHPETYEWARKMAVDALEYDESA
EDANPAGALEEILENPERLKDLDLDAFAEELERQGYGDKHITLYDIRAELSCRYKDLRTA
YRSPNTEEIFNMLTKETPETFYIGKLIICNVTGIAHRRPQGESYDQAIRNDETGLWQCPF
CQQDNFPELSEVWNHFDSGSCPGQAIGVKTRLDNGVTGFIPTKFLSDKVVKRPEERVKVG
MTVHCRIMKIDIEKFSADLTCRTSDLMDRNNEWKLPKDTYYDFDAEAADHKQEEDMKRKQ
QRTTYIKRVIAHPSFHNINFKQAEKMMETMDQGDVIIRPSSKGENHLTVTWKVSDGIYQH
VDVREEGKENAFSLGATLWINSEEFEDLDEIVARYVQPMASFARDLLNHKYYQDCSGGDR
KKLEELLIKTKKEKPTFIPYFICACKELPGKFLLGYQPRGKPRIEYVTVTPEGFRYRGQI
FPTVNGLFRWFKDHYQDPVPGITPSSSSRTRTPASINATPANINLADLTRAVNALPQNMT
SQMFSAIAAVTGQGQNPNATPAQWASSQYGYGGSGGGSSAYHVFPTPAQQPVATPLMTPS
YSYTTPSQPITTPQYHQLQASTTPQSAQAQPQPSSSSRQRQQQPKSNSHAAIDWGKMAEQ
WLQEKEAERRKQKQRLTPRPSPSPMIESTPMSIAGDATPLLDEMDR*
>ENSG00000020256|ENST00000361387
MNASSEGESFAGSVQIPGGTTVLVELTPDIHICGICKQQFNNLDAFVAHKQSGCQLTGTS
AAAPSTVQFVSEETVPATQTQTTTRTITSETQTITVSAPEFVFEHGYQTYLPTESNENQT
ATVISLPAKSRTKKPTTPPAQKRLNCCYPGCQFKTAYGMKDMERHLKIHTGDKPHKCEVC
GKCFSRKDKLKTHMRCHTGVKPYKCKTCDYAAADSSSLNKHLRIHSDERPFKCQICPYAS
RNSSQLTVHLRSHTASELDDDVPKANCLSTESTDTPKAPVITLPSEAREQMATLGERTFN
CCYPGCHFKTVHGMKDLDRHLRIHTGDKPHKCEFCDKCFSRKDNLTMHMRCHTSVKPHKC
HLCDYAAVDSSSLKKHLRIHSDERPYKCQLCPYASRNSSQLTVHLRSHTGDTPFQCWLCS
AKFKISSDLKRHMIVHSGEKPFKCEFCDVRCTMKANLKSHIRIKHTFKCLHCAFQGRDRA
DLLEHSRLHQADHPEKCPECSYSCSSAAALRVHSRVHCKDRPFKCDFCSFDTKRPSSLAK
HVDKVHRDEAKTENRAPLGKEGLREGSSQHVAKIVTQRAFRCETCGASFVRDDSLRCHKK
QHSDQSENKNSDLVTFPPESGASGQLSTLVSVGQLEAPLEPSQDL*
>ENSG00000165629|ENST00000335698
MFSRAGVAGLSAWTLQPQWIQVRNMATLKDITRRLKSIKNIQKITKSMKMVAAAKYARAE
RELKPARIYGLGSLALYEKADIKGPEDKKKHLLIGVSSDRGLCGAIHSSIAKQMKSEVAT
LTAAGKEVMLVGIGDKIRGILYRTHSDQFLVAFKEVGRKPPTFGDASVIALELLNSGYEF
DEGSIIFNKFRSVISYKTEEKPIFSLNTVASADSMSIYDDIDADVLQNYQEYNLANIIYY

SLKESTTSEQSARMTAMDNASKNASEMIDKLTLTFNRTRQAVITKELIEIISGAAAL*
>ENSG00000267680|ENST00000336976
MTTFKEAMTFKDVAVVFTEEELGLLDLAQRKLYRDVMLENFRNLLSVGHQAFHRDTFHFL
REEKIWMMKTAIQREGNSGDKIQTEMETVSEAGTHQEWSFQQIWEKIASDLTRSQDLMIN
SSQFSKEGDFPCQTEAGLSVIHTRQKSSQGNGYKPSFSDVSHFDFHQQLHSGEKSHTCDE
CGKNFCYISALRIHQRVHMGEKCYKCDVCGKEFSQSSHLQTHQRVHTGEKPFKCVECGKG
FSRRSALNVHHKLHTGEKPYNCEECGKAFIHDSQLQEHQRIHTGEKPFKCDICGKSFCGR
SRLNRHSMVHTAEKPFRCDTCDKSFRQRSALNSHRMIHTGEKPYKCEECGKGFICRRDLY
THHMVHTGEKPYNCKECGKSFRWASCLLKHQRVHSGEKPFKCEECGKGFYTNSQCYSHQR
SHSGEKPYKCVECGKGYKRRLDLDFHQRVHTGEKLYNCKECGKSFSRAPCLLKHERLHSG
EKPFQCEECGKRFTQNSHLHSHQRVHTGEKPYKCEKCGKGYNSKFNLDMHQKVHTGERPY
NCKECGKSFGWASCLLKHQRLHSGEKPFKCEECGKRFTQNSQLHSHQRVHTGEKPYKCDE
CGKGFSWSSTRLTHQRRHSRETPLKCEQHGKNIVQNSFSKVQEKVHSVEKPYKCEDCGKG
YNRRLNLDMHQRVHMGEKTWKCRECDMCFSQASSLRLHQNVHVGEKP*
>ENSG00000283815|ENST00000362310
Sequence unavailable
>ENSG00000184058|ENST00000332710
MHFSTVTRDMEAFTASSLSSLGAAGGFPGAASPGADPYGPREPPPPPPRYDPCAAAAPGA
PGPPPPPHAYPFAPAAGAATSAAAEPEGPGASCAAAAKAPVKKNAKVAGVSVQLEMKALW
DEFNQLGTEMIVTKAGRRMFPTFQVKLFGMDPMADYMLLMDFVPVDDKRYRYAFHSSSWL
VAGKADPATPGRVHYHPDSPAKGAQWMKQIVSFDKLKLTNNLLDDNGHIILNSMHRYQPR
FHVVYVDPRKDSEKYAEENFKTFVFEETRFTAVTAYQNHRITQLKIASNPFAKGFRDCDP
EDWPRNHRPGALPLMSAFARSRNPVASPTQPSGTEKDAAEARREFQRDAGGPAVLGDPAH
PPQLLARVLSPSLPGAGGAGGLVPLPGAPGGRPSPPNPELRLEAPGASEPLHHHPYKYPA
AAYDHYLGAKSRPAPYPLPGLRGHGYHPHAHPHHHHHPVSPAAAAAAAAAAAAAAANMYS
SAGAAPPGSYDYCPR*
>ENSG00000123384|ENST00000338962
MLTPPLLLLLPLLSALVAAAIDAPKTCSPKQFACRDQITCISKGWRCDGERDCPDGSDEA
PEICPQSKAQRCQPNEHNCLGTELCVPMSRLCNGVQDCMDGSDEGPHCRELQGNCSRLGC
QHHCVPTLDGPTCYCNSSFQLQADGKTCKDFDECSVYGTCSQLCTNTDGSFICGCVEGYL
LQPDNRSCKAKNEPVDRPPVLLIANSQNILATYLSGAQVSTITPTSTRQTTAMDFSYANE
TVCWVHVGDSAAQTQLKCARMPGLKGFVDEHTINISLSLHLCVFSKSQQEMG*
>ENSG00000115163|ENST00000335756
MGPRRRSRKPEAPRRRSPSPTPTPGPSRRGPSLGASSHQHSRRRQGWLKEIRKLQKSTHL
LIRKLPFSRLAREICVKFTRGVDFNWQAQALLALQEAAEAFLVHLFEDAYLLTLHAGRVT
LFPKDVQLARRIRGLEEGLG*
>ENSG00000198160|ENST00000355977
MPIHELLSLYGYGSTVRLPEEDEEEEEEEEEGEDDEDADNDDNSGCSGENKEENIKDSSG
QEDETQSSNDDPSQSVASQDAQEIIRPRRCKYFDTNSEVEEESEEDEDYIPSEDWKKEIM
VGSMFQAEIPVGICRYKENEKVYENDDQLLWDPEYLPEDKVIIFLKDASRRTGDEKGVEA
IPEGSHIKDNEQALYELVKCNFDTEEALRRLRFNVKAAREELSVWTEEECRNFEQGLKAY
GKDFHLIQANKVRTRSVGECVAFYYMWKKSERYDFFAQQTRFGKKKYNLHPGVTDYMDRL
LDESESAASSRAPSPPPTASNSSNSQSEKEDGTVSTANQNGVSSNGPGILQMLLPVHFSA
ISSRANAFLK*

>ENSG00000186298|ENST00000340766
MADLDKLNIDSIIQRLLEVRGSKPGKNVQLQENEIRGLCLKSREIFLSQPILLELEAPLK
ICGDIHGQYYDLLRLFEYGGFPPESNYLFLGDYVDRGKQSLETICLLLAYKIKYPENFFL
LRGNHECASINRIYGFYDECKRRYNIKLWKTFTDCFNCLPIAAIVDEKIFCCHGGLSPDL
QSMEQIRRIMRPTDVPDQGLLCDLLWSDPDKDVLGWGENDRGVSFTFGAEVVAKFLHKHD
LDLICRAHQVVEDGYEFFAKRQLVTLFSAPNYCGEFDNAGAMMSVDETLMCSFQILKPAE
KKKPNATRPVTPPRVASGLNPSIQKASNYRNNTVLYE*
>ENSG00000197409|ENST00000356476
MARTKQTARKSTGGKAPRKQLATKAARKSAPATGGVKKPHRYRPGTVALREIRRYQKSTE
LLIRKLPFQRLVREIAQDFKTDLRFQSSAVMALQEACEAYLVGLFEDTNLCAIHAKRVTI
MPKDIQLARRIRGERA*
>ENSG00000009307|ENST00000358528
MSFDPNLLHNNGHNGYPNGTSAALRETGVIEKLLTSYGFIQCSERQARLFFHCSQYNGNL
QDLKVGDDVEFEVSSDRRTGKPIAVKLVKIKQEILPEERMNGQVVCAVPHNLESKSPAAP
GQSPTGSVCYERNGEVFYLTYTPEDVEGNVQLETGDKINFVIDNNKHTGAVSARNIMLLK
KKQARCQGVVCAMKEAFGFIERGDVVKEIFFHYSEFKGDLETLQPGDDVEFTIKDRNGKE
VATDVRLLPQGTVIFEDISIEHFEGTVTKVIPKVPSKNQNDPLPGRIKVDFVIPKELPFG
DKDTKSKVTLLEGDHVRFNISTDRRDKLERATNIEVLSNTFQFTNEAREMGVIAAMRDGF
GFIKCVDRDVRMFFHFSEILDGNQLHIADEVEFTVVPDMLSAQRNHAIRIKKLPKGTVSF
HSHSDHRFLGTVEKEATFSNPKTTSPNKGKEKEAEDGIIAYDDCGVKLTIAFQAKDVEGS
TSPQIGDKVEFSISDKQRPGQQVATCVRLLGRNSNSKRLLGYVATLKDNFGFIETANHDK
EIFFHYSEFSGDVDSLELGDMVEYSLSKGKGNKVSAEKVNKTHSVNGITEEADPTIYSGK
VIRPLRSVDPTQTEYQGMIEIVEEGDMKGEVYPFGIVGMANKGDCLQKGESVKFQLCVLG
QNAQTMAYNITPLRRATVECVKDQFGFINYEVGDSKKLFFHVKEVQDGIELQAGDEVEFS
VILNQRTGKCSACNVWRVCEGPKAVAAPRPDRLVNRLKNITLDDASAPRLMVLRQPRGPD
NSMGFGAERKIRQAGVID*
>ENSG00000117614|ENST00000354361
MAAIAASEVLVDSAEEGSLAAAAELAAQKREQRLRKFRELHLMRECAARGEDYEKVKLLE
ISAEDAERWERKKKRKNPDLGFSDYAAAQLRQYHRLTKQIKPDMETYERLREKHGEEFFP
TSNSLLHGTHVPSTEEIDRMVIDLEKQIEKRDKYSRRRPYNDDADIDYINERNAKFNKKA
ERFYGKYTAEIKQNLERGTAV*
>ENSG00000182481|ENST00000330459
MSTNENANTPAARLHRFKNKGKDSTEMRRRRIEVNVELRKAKKDDQMLKRRNVSSFPDDA
TSPLQENRNNQGTVNWSVDDIVKGINSSNVENQLQATQAARKLLSREKQPPIDNIIRAGL
IPKFVSFLGRTDCSPIQFESAWALTNIASGTSEQTKAVVDGGAIPAFISLLASPHAHISE
QAVWALGNIAGDGSVFRDLVIKYGAVDPLLALLAVPDMSSLACGYLRNLTWTLSNLCRNK
NPAPPIDAVEQILPTLVRLLHHDDPEVLADTCWAISYLTDGPNERIGMVVKTGVVPQLVK
LLGASELPIVTPALRAIGNIVTGTDEQTQVVIDAGALAVFPSLLTNPKTNIQKEATWTMS
NITAGRQDQIQQVVNHGLVPFLVSVLSKADFKTQKEAVWAVTNYTSGGTVEQIVYLVHCG
IIEPLMNLLTAKDTKIILVILDAISNIFQAAEKLGETEKLSIMIEECGGLDKIEALQNHE
NESVYKASLSLIEKYFSVEEEEDQNVVPETTSEGYTFQVQDGAPGTFNF*
>ENSG00000055917|ENST00000338086
MNHDFQALALESRGMGELLPTKKFWEPDDSTKDGQKGIFLGDDEWRETAWGASHHSMSQP
IMVQRRSGQGFHGNSEVNAILSPRSESGGLGVSMVEYVLSSSPADKLDSRFRKGNFGTRD

AETDGPEKGDQKGKASPFEEDQNRDLKQGDDDDSKINGRGLPNGMDADCKDFNRTPGSRQ
ASPTEVVERLGPNTNPSEGLGPLPNPTANKPLVEEFSNPETQNLDAMEQVGLESLQFDYP
GNQVPMDSSGATVGLFDYNSQQQLFQRTNALTVQQLTAAQQQQYALAAAQQPHIAGVFSA
GLAPAAFVPNPYIISAAPPGTDPYTAAGLAAAATLAGPAVVPPQYYGVPWGVYPANLFQQ
QAAAAANNTASQQAASQAQPGQQQVLRAGAGQRPLTPNQGQQGQQAESLAAAAAANPTLA
FGQGLATGMPGYQVLAPTAYYDQTGALVVGPGARTGLGAPVRLMAPTPVLISSAAAQAAA
AAAAGGTASSLTGSTNGLFRPIGTQPPQQQQQQPSTNLQSNSFYGSSSLTNSSQSSSLFS
HGPGQPGSTSLGFGSGNSLGAAIGSALSGFGSSVGSSASSSATRRESLSTSSDLYKRSSS
SLAPIGQPFYNSLGFSSSPSPIGMPLPSQTPGHSLTPPPSLSSHGSSSSLHLGGLTNGSG
RYISAAPGAEAKYRSASSTSSLFSSSSQLFPPSRLRYNRSDIMPSGRSRLLEDFRNNRFP
NLQLRDLIGHIVEFSQDQHGSRFIQQKLERATPAERQMVFNEILQAAYQLMTDVFGNYVI
QKFFEFGSLDQKLALATRIRGHVLPLALQMYGCRVIQKALESISSDQQSEMVKELDGHVL
KCVKDQNGNHVVQKCIECVQPQSLQFIIDAFKGQVFVLSTHPYGCRVIQRILEHCTAEQT
LPILEELHQHTEQLVQDQYGNYVIQHVLEHGRPEDKSKIVSEIRGKVLALSQHKFASNVV
EKCVTHASRAERALLIDEVCCQNDGPHSALYTMMKDQYANYVVQKMIDMAEPAQRKIIMH
KIRPHITTLRKYTYGKHILAKLEKYYLKNSPDLGPIGGPPNGML*
>ENSG00000203883|ENST00000340356
MQRSPPGYGAQDDPPARRDCAWAPGHGAAADTRGLAAGPAALAAPAAPASPPSPQRSPPR
SPEPGRYGLSPAGRGERQAADESRIRRPMNAFMVWAKDERKRLAQQNPDLHNAVLSKMLG
KAWKELNAAEKRPFVEEAERLRVQHLRDHPNYKYRPRRKKQARKARRLEPGLLLPGLAPP
QPPPEPFPAASGSARAFRELPPLGAEFDGLGLPTPERSPLDGLEPGEAAFFPPPAAPEDC
ALRPFRAPYAPTELSRDPGGCYGAPLAEALRTAPPAAPLAGLYYGTLGTPGPYPGPLSPP
PEAPPLESAEPLGPAADLWADVDLTEFDQYLNCSRTRPDAPGLPYHVALAKLGPRAMSCP
EESSLISALSDASSAVYYSACISG*
>ENSG00000096746|ENST00000354695
MDWVMKHNGPNDASDGTVRLRGLPFGCSKEEIVQFFQGLEIVPNGITLTMDYQGRSTGEA
FVQFASKEIAENALGKHKERIGHRYIEIFRSSRSEIKGFYDPPRRLLGQRPGPYDRPIGG
RGGYYGAGRGSYGGFDDYGGYNNYGYGNDGFDDRMRDGRGMGGHGYGGAGDASSGFHGGH
FVHMRGLPFRATENDIANFFSPLNPIRVHIDIGADGRATGEADVEFVTHEDAVAAMSKDK
NNMQHRYIELFLNSTPGGGSGMGGSGMGGYGRDGMDNQGGYGSVGRMGMGNNYSGGYGTP
DGLGGYGRGGGGSGGYYGQGGMSGGGWRGMY*
>ENSG00000151623|ENST00000342437
METKGYHSLPEGLDMERRWGQVSQAVERSSLGPTERTDENNYMEIVNVSCVSGAIPNNST
QGSSKEKQELLPCLQQDNNRPGILTSDIKTELESKELSATVAESMGLYMDSVRDADYSYE
QQNQQGSMSPAKIYQNVEQLVKFYKGNGHRPSTLSCVNTPLRSFMSDSGSSVNGGVMRAV
VKSPIMCHEKSPSVCSPLNMTSSVCSPAGINSVSSTTASFGSFPVHSPITQGTPLTCSPN
VENRGSRSHSPAHASNVGSPLSSPLSSMKSSISSPPSHCSVKSPVSSPNNVTLRSSVSSP
ANINNSRCSVSSPSNTNNRSTLSSPAASTVGSICSPVNNAFSYTASGTSAGSSTLRDVVP
SPDTQEKGAQEVPFPKTEEVESAISNGVTGQLNIVQYIKPEPDGAFSSSCLGGNSKINSD
SSFSVPIKQESTKHSCSGTSFKGNPTVNPFPFMDGSYFSFMDDKDYYSLSGILGPPVPGF
DGNCEGSGFPVGIKQEPDDGSYYPEASIPSSAIVGVNSGGQSFHYRIGAQGTISLSRSAR
DQSFQHLSSFFPPVNTLVESWKSHGDLSSRRSDGYPVLEYIPENVSSSTLRSVSTGSSRPS
KICLVCGDEASGCHYGVVTCGSCKVFFKRAVEGQHNYLCAGRNDCIIDKIRRKNCPACRL
QKCLQAGMNLGERRCISLPCMNYARGCTKSAFSSFDCSSPLKNTPS*

>ENSG00000106331|ENST00000341640
MNQLGGLFVNGRPLPLDTRQQIVRLAVSGMRPCDISRILKVSNGCVSKILGRYYRTGVLE
PKGIGGSKPRLATPPVVARIAQLKGECPALFAWEIQRQLCAEGLCTQDKTPSVSSINRVL
RALQEDQGLPCTRLRSPAVLAPAVLTPHSGSETPRGTHPGTGHRNRTIFSPSQAEALEKE
FQRGQYPDSVARGKLATATSLPEDTVRVWFSNRRAKWRRQEKLKWEMQLPGASQGLTVPR
VAPGIISAQQSPGSVPTAALPALEPLGPSCYQLCWATAPERCLSDTPPKACLKPCWGHLP
PQPNSLDSGLLCLPCPSSHCHLASLSGSQALLWPGCPLLYGLE*
>ENSG00000128951|ENST00000331200
MTPLCPRPALCYHFLTSLLRSAMQNARGARQRAEAAVLSGPGPPLGRAAQHGIPRPLSSA
GRLSQGCRGASTVGAAGWKGELPKAGGSPAPGPETPAISPSKRARPAEVGGMQLRFARLS
EHATAPTRGSARAAGYDLYSAYDYTIPPMEKAVVKTDIQIALPSGCYGRVAPRSGLAAKH
FIDVGAGVIDEDYRGNVGVVLFNFGKEKFEVKKGDRIAQLICERIFYPEIEEVQALDDTE
RGSGGFGSTGKN*
>ENSG00000185088|ENST00000330964
MPLARDLLHPSLEEEKKKHKKKRLVQSPNSYFMDVKCPGCYKITTVFSHAQTVVLCVGCS
TVLCQPTGGKARLTEGCSFRRKQH*
>ENSG00000151353|ENST00000355654
MLSGYLQTDWTEPWLMGLATFHALCVLLTCLSSRSYRLQIGHFLCLVILVYCAEYINEAA
AMNWRLFSKYQYFDSRGMFISIVFSAPLLVNAMIIVVMWVWKTLNVMTDLKNAQERRKEK
KRRRKED*
>ENSG00000155329|ENST00000324170
MATPMHRLIARRQAEANKQHVRCQKCLEFGHWTYECTGKRKYLHRPSRTAELKKALKEKE
NRLLLQQSIGETNVERKAKKKRSKSVTSSSSSSSDSSASDSSSESEETSTSSSSEDSDTD
ESSSSSSSSASSTTSSSSSDSDSDSSSSSSSSTSTDSSSDDEPPKKKKKK*
>ENSG00000188375|ENST00000340398
MARTKQTARKSTGGKAPRKQLATKAARKSTPSTCGVKPHRYRPGTVALREIRRYQKSTEL
LIRKLPFQRLVREIAQDFNTDLRFQSAAVGALQEASEAYLVGLLEDTNLCAIHAKRVTIM
PKDIQLARRIRGERA*
>ENSG00000101945|ENST00000337852
MVGMSRLRNDRLADPLTGCSVCCKSSWNQLQDLCRLAKLSCPALGISKRNLYDFEVEYLC
DYKKIREQEYYLVKWRGYPDSESTWEPRQNLKCVRILKQFHKDLERELLRRHHRSKTPRH
LDPSLANYLVQKAKQRRALRRWEQELNAKRSHLGRITVENEVDLDGPPRAFVYINEYRVG
EGITLNQVAVGCECQDCLWAPTGGCCPGASLHKFAYNDQGQVRLRAGLPIYECNSRCRCG
YDCPNRVVQKGIRYDLCIFRTDDGRGWGVRTLEKIRKNSFVMEYVGEIITSEEAERRGQI
YDRQGATYLFDLDYVEDVYTVDAAYYGNISHFVNHSCDPNLQVYNVFIDNLDERLPRIAF
FATRTIRAGEELTFDYNMQVDPVDMESTRMDSNFGLAGLPGSPKKRVRIECKCGTESCRK
YLF*
>ENSG00000153113|ENST00000338252
MNPTETKAIPVSQQMEGPHLPNKKKHKKQAVKTEPEKKSQSTKLSVVHEKKSQEGKPKEH
TEPKSLPKQASDTGSNDAHNKKAVSRSAEQQPSEKSTEPKTKPQDMISAGGESVAGITAI
SGKPGDKKKEKKSLTPAVPVESKPDKPSGKSGMDAALDDLIDTLGGPEETEEENTTYTGP
EVSDPMSSTYIEELGKREVTIPPKYRELLAKPIGPDDAIDALSSDFTCGSPTAAGKKTEK
EESTEVLKAQSAGTVRSAAPPQEKKRKVEKDTMSDQALEALSASLGTRQAEPELDLRSIK
EVDEAKAKEEKLEKCGEDDETIPSEYRLKPATDKDGKPLLPEPEEKPKPRSESELIDELS

EDFDRSECKEKPSKPTEKTEESKAAAPAPVSEAVCRTSMCSIQSAPPEPATLKGTVPDDA
VEALADSLGKKEADPEDGKPVMDKVKEKAKEEDREKLGEKEETIPPDYRLEEVKDKDGKP
LLPKESKEQLPPMSEDFLLDALSEDFSGPQNASSLKFEDAKLAAAISEVVSQTPASTTQA
GAPPRDTSQSDKDLDDALDKLSDSLGQRQPDPDENKPMEDKVKEKAKAEHRDKLGERDDT
IPPEYRHLLDDNGQDKPVKPPTKKSEDSKKPADDQDPIDALSGDLDSCPSTTETSQNTAK
DKCKKAASSSKAPKNGGKAKDSAKTTEETSKPKDD*
>ENSG00000186020|ENST00000334116
MESSKLCGLEGSIFRNDWQSKSKIDLQGPEVGYFSQMKIISENVPSYKTHESLTLPRRTH
DSEKPYEYKEYEKVFSCDLEFDEYQKIHTGGKNYECNQCWKTFGIDNSSMLQLNIHTGVK
PCKYMEYGNTCSFYKDFNVYQKIHNEKFYKCKEYRRTFERVGKVTPLQRVHDGEKHFECS
FCGKSFRVHAQLTRHQKIHTDEKTYKCMECGKDFRFHSQLTEHQRIHTGEKPYKCMHCEK
VFRISSQLIEHQRIHTGEKPYACKECGKAFGVCRELARHQRIHTGKKPYECKACGKVFRN
SSSLTRHQRIHTGEKPYKCKECEKAFGVGSELTRHERIHSGQKPYECKECGKFFRLTSAL
IQHQRIHSGEKPYECKVCGKAFRHSSALTEHQRIHTGEKPYECKACGKAFRHSSSFTKHQ
RIHTDDKPYECKECGNSFSVVGHLTCQPKIYTGEKSFD*
>ENSG00000179965|ENST00000319296
MPGEQQAEEEEEEEMQEEMVLLVKGEEDEGEEKYEVVKLKIPMDNKEVPGEAPAPSADPA
RPHACPDCGRAFARRSTLAKHARTHTGERPFGCTECGRRFSQKSALTKHGRTHTGERPYE
CPECDKRFSAASNLRQHRRRHTGEKPYACAHCGRRFAQSSNYAQHLRVHTGEKPYACPDC
GRAFGGSSCLARHRRTHTGERPYACADCGTRFAQSSALAKHRRVHTGEKPHRCAVCGRRF
GHRSNLAEHARTHTGERPYPCAECGRRFRLSSHFIRHRRAHMRRRLYICAGCGRDFKLPP
GATAATATERCPECEGS*
>ENSG00000140009|ENST00000341099
MDIKNSPSSLNSPSSYNCSQSILPLEHGSIYIPSSYVDSHHEYPAMTFYSPAVMNYSIPS
NVTNLEGGPGRQTTSPNVLWPTPGHLSPLVVHRQLSHLYAEPQKSPWCEARSLEHTLPVN
RETLKRKVSGNRCASPVTGPGSKRDAHFCAVCSDYASGYHYGVWSCEGCKAFFKRSIQGH
NDYICPATNQCTIDKNRRKSCQACRLRKCYEVGMVKCGSRRERCGYRLVRRQRSADEQLH
CAGKAKRSGGHAPRVRELLLDALSPEQLVLTLLEAEPPHVLISRPSAPFTEASMMMSLTK
LADKELVHMISWAKKIPGFVELSLFDQVRLLESCWMEVLMMGLMWRSIDHPGKLIFAPDL
VLDRDEGKCVEGILEIFDMLLATTSRFRELKLQHKEYLCVKAMILLNSSMYPLVTATQDA
DSSRKLAHLLNAVTDALVWVIAKSGISSQQQSMRLANLLMLLSHVRHASNKGMEHLLNMK
CKNVVPVYDLLLEMLNAHVLRGCKSSITGSECSPAEDSKSKEGSQNPQSQ*
>ENSG00000143815|ENST00000338179
MPSRKFADGEVVRGRWPGSSLYYEVEILSHDSTSQLYTVKYKDGTELELKENDIKPLTSF
RQRKGGSTSSSPSRRRGSRSRSRSRSPGRPPKSARRSASASHQADIKEARREVEVKLTPL
ILKPFGNSISRYNGEPEHIERNDAPHKNTQEKFSLSQESSYIATQYSLRPRREEVKLKEI
DSKEEKYVAKELAVRTFEVTPIRAKDLEFGGVPGVFLIMFGLPVFLFLLLLMCKQKDPSL
LNFPPPLPALYELWETRVFGVYLLWFLIQVLFYLLPIGKVVEGTPLIDGRRLKYRLNGFY
AFILTSAVIGTSLFQGVEFHYVYSHFLQFALAATVFCVVLSVYLYMRSLKAPRNDLSPAS
SGNAVYDFFIGRELNPRIGTFDLKYFCELRPGLIGWVVINLVMLLAEMKIQDRAVPSLAM
ILVNSFQLLYVVDALWNEEALLTTMDIIHDGFGFMLAFGDLVWVPFIYSFQAFYLVSHPN
EVSWPMASLIIVLKLCGYVIFRGANSQKNAFRKNPSDPKLAHLKTIHTSTGKNLLVSGWW
GFVRHPNYLGDLIMALAWSLPCGFNHILPYFYIIYFTMLLVHREARDEYHCKKKYGVAWE
KYCQRVPYRIFPYIY*

>ENSG00000179094|ENST00000317276
MSGPLEGADGGGDPRPGESFCPGGVPSPGPPQHRPCPGPSLADDTDANSNGSSGNESNGH
ESRGASQRSSHSSSSGNGKDSALLETTESSKSTNSQSPSPPSSSIAYSLLSASSEQDNPS
TSGCSSEQSARARTQKELMTALRELKLRLPPERRGKGRSGTLATLQYALACVKQVQANQE
YYQQWSLEEGEPCSMDMSTYTLEELEHITSEYTLQNQDTFSVAVSFLTGRIVYISEQAAV
LLRCKRDVFRGTRFSELLAPQDVGVFYGSTAPSRLPTWGTGASAGSGLRDFTQEKSVFCR
IRGGPDRDPGPRYQPFRLTPYVTKIRVSDGAPAQPCCLLIAERIHSGYEAPRIPPDKRIF
TTRHTPSCLFQDVDERAAPLLGYLPQDLLGAPVLLFLHPEDRPLMLAIHKKILQLAGQPF
DHSPIRFCARNGEYVTMDTSWAGFVHPWSRKVAFVLGRHKVRTAPLNEDVFTPPAPSPAP
SLDTDIQELSEQIHRLLLQPVHSPSPTGLCGVGAVTSPGPLHSPGSSSDSNGGDAEGPGP
PAPVTFQQICKDVHLVKHQGQQLFIESRARPQSRPRLPATGTFKAKALPCQSPDPELEAG
SAPVQAPLALVPEEAERKEASSCSYQQINCLDSILRYLESCNLPSTTKRKCASSSSYTTS
SASDDDRQRTGPVSVGTKKDPPSAALSGEGATPRKEPVVGGTLSPLALANKAESVVSVTS
QCSFSSTIVHVGDKKPPESDIIMMEDLPGLAPGPAPSPAPSPTVAPDPAPDAYRPVGLTK
AVLSLHTQKEEQAFLSRFRDLGRLRGLDSSSTAPSALGERGCHHGPAPPSRRHHCRSKAK
RSRHHQNPRAEAPCYVSHPSPVPPSTPWPTPPATTPFPAVVQPYPLPVFSPRGGPQPLPP
APTSVPPAAFPAPLVTPMVALVLPNYLFPTPSSYPYGALQTPAEGPPTPASHSPSPSLPA
LAPSPPHRPDSPLFNSRCSSPLQLNLLQLEELPRAEGAAVAGGPGSSAGPPPPSAEAAEP
EARLAEVTESSNQDALSGSSDLLELLLQEDSRSGTGSAASGSLGSGLGSGSGSGSHEGGS
TSASITRSSQSSHTSKYFGSIDSSEAEAGAARGGAEPGDQVIKYVLQDPIWLLMANADQR
VMMTYQVPSRDMTSVLKQDRERLRAMQKQQPRFSEDQRRELGAVHSWVRKGQLPRALDVM
ACVDCGSSTQDPGHPDDPLFSELDGLGLEPMEEGGGEQGSSGGGSGEGEGCEEAQGGAKA
SSSQDLAMEEEEEGRSSSSPALPTAGNCTS*
>ENSG00000111335|ENST00000342315
MGNGESQLSSVPAQKLGWFIQEYLKPYEECQTLIDEMVNTICDVLQEPEQFPLVQGVAIG
GSYGRKTVLRGNSDGTLVLFFSDLKQFQDQKRSQRDILDKTGDKLKFCLFTKWLKNNFEI
QKSLDGFTIQVFTKNQRISFEVLAAFNALSLNDNPSPWIYRELKRSLDKTNASPGEFAVC
FTELQQKFFDNRPGKLKDLILLIKHWHQQCQKKIKDLPSLSPYALELLTVYAWEQGCRKD
NFDIAEGVRTVLELIKCQEKLCIYWMVNYNFEDETIRNILLHQLQSARPVILDPVDPTNN
VSGDKICWQWLKKEAQTWLTSPNLDNELPAPSWNVLPAPLFTTPGHLLDKFIKEFLQPNK
CFLEQIDSAVNIIRTFLKENCFRQSTAKIQIVRGGSTAKGTALKTGSDADLVVFHNSLKS
YTSQKNERHKIVKEIHEQLKAFWREKEEELEVSFEPPKWKAPRVLSFSLKSKVLNESVSF
DVLPAFNALGQLSSGSTPSPEVYAGLIDLYKSSDLPGGEFSTCFTVLQRNFIRSRPTKLK
DLIRLVKHWYKECERKLKPKGSLPPKYALELLTIYAWEQGSGVPDFDTAEGFRTVLELVT
QYQQLCIFWKVNYNFEDETVRKFLLSQLQKTRPVILDPAEPTGDVGGGDRWCWHLLAKEA
KEWLSSPCFKDGTGNPIPPWKVPTMQTPGSCGARIHPIVNEMFSSRSHRILNNNSKRNF*
>ENSG00000167800|ENST00000335385
MAAFLSAGLGILAPSETYPLPTTSSGWEPRLGSPFPSGPCTSSTGAQAVAEPTGQGPKNP
RVSRVTVQLEMKPLWEEFNQLGTEMIVTKAGRRMFPPFQVKILGMDSLADYALLMDFIPL
DDKRYRYAFHSSAWLVAGKADPATPGRVHFHPDSPAKGAQWMRQIVSFDKLKLTNNLLDD
NGHIILNSMHRYQPRFHVVFVDPRKDSERYAQENFKSFIFTETQFTAVTAYQNHRITQLK
IASNPFAKGFRESDLDSWPVAPRPLLSVPARSHSSLSPCVLKGATDREKDPNKASASTSK
TPAWLHHQLLPPPEVLLAPATYRPVTYQSLYSGAPSHLGIPRTRPAPYPLPNIRADRDQG
GLPLPAGLGLLSPTVVCLGPGQDSQ*

>ENSG00000171827|ENST00000330173
MAVGLLKAMYQELVTFRDVAVDFSQEEWDCLDSSQRHLYSNVMLENYRILVSLGLCFSKP
SVILLLEQGKAPWMVKRELTKGLCSGWEPICETEELTPKQDFYEEHQSQKIIETLTSYNL
EYSSLREEWKCEGYFERQPGNQKACFKEEIITHEEPLFDEREQEYKSWGSFHQNPLLCTQ
KIIPKEEKVHKHDTQKRSFKKNLMAIKPKSVCAEKKLLKCNDCEKVFSQSSSLTLHQRIH
TGEKPYKCIECGKAFSQRSNLVQHQRIHTGEKPYECKECRKAFSQNAHLVQHLRVHTGEK
PYECKVCRKAFSQFAYLAQHQRVHTGEKPYECIECGKAFSNRSSIAQHQRVHTGEKPYEC
NVCGKAFSLRAYLTVHQRIHTGERPYECKECGKAFSQNSHLAQHQRIHTGEKPYKCQECR
KAFSQIAYLAQHQRVHTGEKPYECIECGKAFSNDSSLTQHQRVHTGEKPYECTVCGKAFS
YCGSLAQHQRIHTGERPYECKECKKTFRQHAHLAHHQRIHIGESLSPPNPVNHQVL*
>ENSG00000277858|ENST00000354514
MPRRRRRRGSSGAGGRGRTCSRTVRAELSFSVSQVERSLREGHYAQRLSRTAPVYLAAVI
EYLTAKVLELAGNEAQNSGERNITPLLLDMVVHNDRLLSTLFNTTTISQVAPGED*
>ENSG00000147789|ENST00000325217
MGFLGCWCVSFQEVVTFGDVAVHFSREEWQCLDPGQRALYREVMLENHSSVAGLAGFLVF
KPELISRLEQGEEPWVLDLQGAEGTEAPRTSKTGFLGRPTMGQEPRHPHAPPATPVPGLP
KHCSQRLTLPPPGLSSSPLGHFLVHDQDRRRGTSAIWMV*
>ENSG00000131196|ENST00000329101
MTGLEDQEFDFEFLFEFNQRDEGAAAAAPEHYGYASSNVSPALPLPTAHSTLPAPCHNLQ
TSTPGIIPPADHPSGYGAALDGGPAGYFLSSGHTRPDGAPALESPRIEITSCLGLYHNNN
QFFHDVEVEDVLPSSKRSPSTATLSLPSLEAYRDPSCLSPASSLSSRSCNSEASSYESNY
SYPYASPQTSPWQSPCVSPKTTDPEEGFPRGLGACTLLGSPRHSPSTSPRASVTEESWLG
ARSSRPASPCNKRKYSLNGRQPPYSPHHSPTPSPHGSPRVSVTDDSWLGNTTQYTSSAIV
AAINALTTDSSLDLGDGVPVKSRKTTLEQPPSVALKVEPVGEDLGSPPPPADFAPEDYSS
FQHIRKGGFCDQYLAVPQHPYQWAKPKPLSPTSYMSPTLPALDWQLPSHSGPYELRIEVQ
PKSHHRAHYETEGSRGAVKASAGGHPIVQLHGYLENEPLMLQLFIGTADDRLLRPHAFYQ
VHRITGKTVSTTSHEAILSNTKVLEIPLLPENSMRAVIDCAGILKLRNSDIELRKGETDI
GRKNTRVRLVFRVHVPQPSGRTLSLQVASNPIECSQRSAQELPLVEKQSTDSYPVVGGKK
MVLSGHNFLQDSKVIFVEKAPDGHHVWEMEAKTDRDLCKPNSLVVEIPPFRNQRITSPVH
VSFYVCNGKRKRSQYQRFTYLPANVPIIKTEPTDDYEPAPTCGPVSQGLSPLPRPYYSQQ
LAMPPDPSSCLVAGFPPCPQRSTLMPAAPGVSPKLHDLSPAAYTKGVASPGHCHLGLPQP
AGEAPAVQDVPRPVATHPGSPGQPPPALLPQQVSAPPSSSCPPGLEHSLCPSSPSPPLPP
ATQEPTCLQPCSPACPPATGRPQHLPSTVRRDESPTAGPRLLPEVHEDGSPNLAPIPVTV
KREPEELDQLYLDDVNEIIRNDLSSTSTHS*
>ENSG00000107929|ENST00000316157
MTSDQDAKVVAEPQTQRVQEGKDSAHLMNGPISQTTSQTSSIPPLSQVPATKVSELNPNA
EVWGAPVLHLEASSAADGVSAAWEEVAGHHADRGPQGSDANGDGDQGHENAALPDPQESD
PADMNALALGPSEYDSLPENSETGGNESQPDSQEDPREVLKKTLEFCLSRENLASDMYLI
SQMDSDQYVPITTVANLDHIKKLSTDVDLIVEVLRSLPLVQVDEKGEKVRPNQNRCIVIL
REISESTPVEEVEALFKGDNLPKFINCEFAYNDNWFITFETEADAQQAYKYLREEVKTFQ
GKPIKARIKAKAIAINTFLPKNGFRPLDVSLYAQQRYATSFYFPPMYSPQQQFPLYSLIT
PQTWSATHSYLDPPLVTPFPNTGFINGFTSPAFKPAASPLTSLRQYPPRSRNPSKSHLRH
AIPSAERGPGLLESPSIFNFTADRLINGVRSPQTRQAGQTRTRIQNPSAYAKREAGPGRV
EPGSLESSPGLGRGRKNSFGYRKKREEKFTSSQTQSPTPPKPPSPSFELGLSSFPPLPGA

AGNLKTEDLFENRLSSLIIGPSKERTLSADASVNTLPVVVSREPSVPASCAVSATYERSP
SPAHLPDDPKVAEKQRETHSVDRLPSALTATACKSVQVNGAATELRKPSYAEICQRTSKE
PPSSPLQPQKEQKPNTVGCGKEEKKLAEPAERYREPPALKSTPGAPRDQRRPAGGRPSPS
AMGKRLSREQSTPPKSPQ*
>ENSG00000146963|ENST00000354926
MSAQAQMRAMLDQLMGTSRDGDTTRQRIKFSDDRVCKSHLLNCCPHDVLSGTRMDLGECL
KVHDLALRADYEIASKEQDFFFELDAMDHLQSFIADCDRRTEVAKKRLAETQEEISAEVA
AKAERVHELNEEIGKLLAKVEQLGAEGNVEESQKVMDEVEKARAKKREAEEVYRNSMPAS
SFQQQKLRVCEVCSAYLGLHDNDRRLADHFGGKLHLGFIEIREKLEELKRVVAEKQEKRN
QERLKRREEREREEREKLRRSRSHSKNPKRSRSREHRRHRSRSMSRERKRRTRSKSREKR
HRHRSRSSSRSRSRSHQRSRHSSRDRSRERSKRRSSKERFRDQDLASCDRDRSSRDRSPR
DRDRKDKKRSYESANGRSEDRRSSEEREAGEI*
>ENSG00000196387|ENST00000356456
MSQGSVTFRDVAIDFSQEEWKWLQPAQRDLYRCVMLENYGHLVSLGLSISKPDVVSLLEQ
GKEPWLGKREVKRDLFSVSESSGEIKDFSPKNVIYDDSSQYLIMERILSQGPVYSS
>ENSG00000030419|ENST00000342002
MHCTLTMETEAIDGYITCDNELSPEREHSNMAIDLTSSTPNGQHASPSHMTSTNSVKLEM
QSDEECDRKPLSREDEIRGHDEGSSLEEPLIESSEVADNRKVQELQGEGGIRLPNGKLKC
DVCGMVCIGPNVLMVHKRSHTGERPFHCNQCGASFTQKGNLLRHIKLHSGEKPFKCPFCS
YACRRRDALTGHLRTHSVGKPHKCNYCGRSYKQRSSLEEHKERCHNYLQNVSMEAAGQVM
SHHVPPMEDCKEQEPIMDNNISLVPFERPAVIEKLTGNMGKRKSSTPQKFVGEKLMRFSY
PDIHFDMNLTYEKEAELMQSHMMDQAINNAITYLGAEALHPLMQHPPSTIAEVAPVISSA
YSQVYHPNRIERPISRETADSHENNMDGPISLIRPKSRPQEREASPSNSCLDSTDSESSH
DDHQSYQGHPALNPKRKQSPAYMKEDVKALDTTKAPKGSLKDIYKVFNGEGEQIRAFKCE
HCRVLFLDHVMYTIHMGCHGYRDPLECNICGYRSQDRYEFSSHIVRGEHTFH*
>ENSG00000010244|ENST00000342555
MAMSSLGCLCALTVLIRYCNRDFDDEKILIQHQKAKHFKCHICHKKLYTGPGLAIHCMQV
HKETIDAVPNAIPGRTDIELEIYGMEGIPEKDMDERRRLLEQKTQESQKKKQQDDSDEYD
DDDSAASTSFQPQPVQPQQGYIPPMAQPGLPPVPGAPGMPPGIPPLMPGVPPLMPGMPPV
MPGMPPGLHHQRKYTQSFCGENIMMPMGGMMPPGPGIPPLMPGMPPGMPPPVPRPGIPPM
TQAQAVSAPGILNRPPAPTATVPAPQPPVTKPLFPSAGQMGTPVTSSSTASSNSESLSAS
SKALFPSTAQAQAAVQGPVGTDFKPLNSTPATTTEPPKPTFPAYTQSTASTTSTTNSTAA
KPAASITSKPATLTTTSATSKLIHPDEDISLEERRAQLPKYQRNLPRPGQAPIGNPPVGP
IGGMMPPQPGIPQQQGMRPPMPPHGQYGGHHQGMPGYLPGAMPPYGQGPPMVPPYQGGPP
RPPMGMRPPVMSQGGRY*
>ENSG00000186230|ENST00000334181
MNLTEDCMVFEDVAIYFSQEEWGILNDAQRHLHSNVMLENFALLSSVGCWHGAKDEEVPS
KQCVSVRVLQVTIPKPALSTLKAQPCKMCSSILKDILHLAEHDGTHPEQGLYTCAAEHDL
HQKEQIREKLTRSDEWRPSFVNHSAHVGERNFTCTQGGKDFTASSDLLQQQVLNSGWKLY
RDTQDGEAFQGEQNDFNSSQGGKDFCHQHGLFEHQKTHNGERPYEFSECGELFRYNSNLI
KYQQNHAGERPYEGTEYGKTFIRKSNLVQHQKIHSEGFLSKRSDPIEHQEILSRPTPYEC
TQCGKAFLTQAHLVGHQKTHTGEQPYECNKCGKFFMYNSKLIRHQKVHTGERRYECSECG
KLFMDSFTLGRHQRVHTGERPFECSICGKFFSHRSTLNMHQRVHAGKRLYKCSECGKAFS
LKHNVVQHLKIHTGERPYECTECEKAFVRKSHLVQHQKIHTDAFSKRSDLIQHKRIDIRP

RPYTCSECGKAFLTQAHLVGHQKIHTGERPYECTQCAKAFVRKSHLVQHEKIHTDAFSKR
SDLIQHKRIDLRPRPYVCSECGKAFLTQAHLDGHQKIQTGERRYECNECGKFFLDSYKLV
IHQRIHTGEKPYKCSKCGKFFRYRCTLSRHQKVHTGERPYECSECGKFFRDSYKLIIHQR
VHTGEKPYECSNCGKFLRYRSTFIKHHKVCTGEKPHECSKCRELFRTKSSLIIHQQSHTG
ESPFKLRECGKDFNKCNTGQRQKTHTGERSYECGESSKVFKYNSSLIKHQIIHTGKRP*
>ENSG00000101150|ENST00000346249
MDSAGQDINLNSPNKGLLSDSMTDVPVDTGVAARTPAVEGLTEAEEEELRAELTKVEEEI
VTLRQVLAAKERHCGELKRRLGLSTLGELKQNLSRSWHDVQVSSAYVKTSEKLGEWNEKV
TQSDLYKKTQETLSQAGQKTSAALSTVGSAISRKLGDMRNSATFKSFEDRVGTIKSKVVG
DRENGSDNLPSSAGSGDKPLSDPAPF*
>ENSG00000241476|ENST00000336777
MNGDDAFARRPTVGAQIPEKIQKAFDDIAKYFSKEEWEKMKASEKIFYVYMKRKYEAMTK
LGFKATLPPFMCNKRAEDFQGNDLDNDPNRGNQVERPQMTFGRLQGISPKIMPKKPAEEG
NDSEEVPEASGPQNDGKELCPPGKPTTSEKIHERSGNREAQEKEERRGTAHRWSSQNTHN
IGRFSLSTSMGAVHGTPKTITHNRDPKGGNMPGPTDCVRENSW*
>ENSG00000086102|ENST00000318524
MAEAPPVSGTFKFNTDAAEFIPQEKKNSGLNCGTQRRLDSNRIGRRNYSSPPPCHLSRQV
PYDEISAVHQHSYHPSGSKPKSQQTSFQSSPCNKSPKSHGLQNQPWQKLRNEKHHIRVKK
AQSLAEQTSDTAGLESSTRSESGTDLREHSPSESEKEVVGADPRGAKPKKATQFVYSYGR
GPKVKGKLKCEWSNRTTPKPEDAGPESTKPVGVFHPDSSEASSRKGVLDGYGARRNEQRR
YPQKRPPWEVEGARPRPGRNPPKQEGHRHTNAGHRNNMGPIPKDDLNERPAKSTCDSENL
AVINKSSRRVDQEKCTVRRQDPQVVSPFSRGKQNHVLKNVETHTGSLIEQLTTEKYECMV
CCELVRVTAPVWSCQSCYHVFHLNCIKKWARSPASQADGQSGWRCPACQNVSAHVPNTYT
CFCGKVKNPEWSRNEIPHSCGEVCRKKQPGQDCPHSCNLLCHPGPCPPCPAFMTKTCECG
RTRHTVRCGQAVSVHCSNPCENILNCGQHQCAELCHGGQCQPCQIILNQVCYCGSTSRDV
LCGTDVGKSDGFGDFSCLKICGKDLKCGNHTCSQVCHPQPCQQCPRLPQLVRCCPCGQTP
LSQLLELGSSSRKTCMDPVPSCGKVCGKPLPCGSLDFIHTCEKLCHEGDCGPCSRTSVIS
CRCSFRTKELPCTSLKSEDATFMCDKRCNKKRLCGRHKCNEICCVDKEHKCPLICGRKLR
CGLHRCEEPCHRGNCQTCWQASFDELTCHCGASVIYPPVPCGTRPPECTQTCARVHECDH
PVYHSCHSEEKCPPCTFLTQKWCMGKHEQSHYWASTQKKRSHYMKKIPAHACL*
>ENSG00000185022|ENST00000338483
MSVDPLSSKALKIKRELSENTPHLSDEALMGLSVRELNRHLRGLSAEEVTRLKQRRRTLK
NRGYAASCRVKRVCQKEELQKQKSELEREVDKLARENAAMRLELDALRGKCEALQGFARS
VAAARGPATLVAPASVITIVKSTPGSGSGPAHGPDPAHGPASCS*
>ENSG00000084072|ENST00000324379
MATTKRVLYVGGLAEEVDDKVLHAAFIPFGDITDIQIPLDYETEKHRGFAFVEFELAEDA
AAAIDNMNESELFGRTIRVNLAKPMRIKEGSSRPVWSDDDWLKKFSGKTLEENKEEEGSE
PPKAETQEGEPIAKKARSNPQVYMDIKIGNKPAGRIQMLLRSDVVPMTAENFRCLCTHEK
GFGFKGSSFHRIIPQFMCQGGDFTNHNGTGGKSIYGKKFDDENFILKHTGPGLLSMANSG
PNTNGSQFFLTCDKTDWLDGKHVVFGEVTEGLDVLRQIEAQGSKDGKPKQKVIIADCGEY
V*
>ENSG00000117595|ENST00000367021
MALHPRRVRLKPWLVAQVDSGLYPGLIWLHRDSKRFQIPWKHATRHSPQQEEENTIFKAW
AVETGKYQEGVDDPDPAKWKAQLRCALNKSREFNLMYDGTKEVPMNPVKIYQVCDIPQPQ

GSIINPGSTGSAPWDEKDNDVDEEDEEDELDQSQHHVPIQDTFPFLNINGSPMAPASVGN
CSVGNCSPEAVWPKTEPLEMEVPQAPIQPFYSSPELWISSLPMTDLDIKFQYRGKEYGQT
MTVSNPQGCRLFYGDLGPMPDQEELFGPVSLEQVKFPGPEHITNEKQKLFTSKLLDVMDR
GLILEVSGHAIYAIRLCQCKVYWSGPCAPSLVAPNLIERQKKVKLFCLETFLSDLIAHQK
GQIEKQPPFEIYLCFGEEWPDGKPLERKLILVQVIPVVARMIYEMFSGDFTRSFDSGSVR
LQISTPDIKDNIVAQLKQLYRILQTQESWQPMQPTPSMQLPPALPPQ*
>ENSG00000181722|ENST00000357258
MTERIHSINLHNFSNSVLETLNEQRNRGHFCDVTVRIHGSMLRAHRCVLAAGSPFFQDKL
LLGYSDIEIPSVVSVQSVQKLIDFMYSGVLRVSQSEALQILTAASILQIKTVIDECTRIV
SQNVGDVFPGIQDSGQDTPRGTPESGTSGQSSDTESGYLQSHPQHSVDRIYSALYACSMQ
NGSGERSFYSGAVVSHHETALGLPRDHHMEDPSWITRIHERSQQMERYLSTTPETTHCRK
QPRPVRIQTLVGNIHIKQEMEDDYDYYGQQRVQILERNESEECTEDTDQAEGTESEPKGE
SFDSGVSSSIGTEPDSVEQQFGPGAARDSQAEPTQPEQAAEAPAEGGPQTNQLETGASSP
ERSNEVEMDSTVITVSNSSDKSVLQQPSVNTSIGQPLPSTQLYLRQTETLTSNLRMPLTL
TSNTQVIGTAGNTYLPALFTTQPAGSGPKPFLFSLPQPLAGQQTQFVTVSQPGLSTFTAQ
LPAPQPLASSAGHSTASGQGEKKPYECTLCNKTFTAKQNYVKHMFVHTGEKPHQCSICWR
SFSLKDYLIKHMVTHTGVRAYQCSICNKRFTQKSSLNVHMRLHRGEKSYECYICKKKFSH
KTLLERHVALHSASNGTPPAGTPPGARAGPPGVVACTEGTTYVCSVCPAKFDQIEQFNDH
MRMHVSDG*
>ENSG00000160908|ENST00000337673
MNSSLTAQRRGSDAELGPWVMAARSKDAAPSQRDGLLPVKVEEDSPGSWEPNYPAASPDP
ETSRLHFRQLRYQEVAGPEEALSRLRELCRRWLRPELLSKEQILELLVLEQFLTILPEEL
QAWVREHCPESGEEAVAVVRALQRALDGTSSQGMVTFEDTAVSLTWEEWERLDPARRDFC
RESAQKDSGSTVPPSLESRVENKELIPMQQILEEAEPQGQLQEAFQGKRPLFSKCGSTHE
DRVEKQSGDPLPLKLENSPEAEGLNSISDVNKNGSIEGEDSKNNELQNSARCSNLVLCQH
IPKAERPTDSEEHGNKCKQSFHMVTWHVLKPHKSDSGDSFHHSSLFETQRQLHEERPYKC
GNCGKSFKQRSDLFRHQRIHTGEKPYGCQECGKSFSQSAALTKHQRTHTGEKPYTCLKCG
ERFRQNSHLNRHQSTHSRDKHFKCEECGETCHISNLFRHQRLHKGERPYKCEECEKSFKQ
RSDLFKHHRIHTGEKPYGCSVCGKRFNQSATLIKHQRIHTGEKPYKCLECGERFRQSTHL
IRHQRIHQNKVLSAGRGGSRL*
>ENSG00000198393|ENST00000328654
MATSFRTASCWGLLSFKDISMEFTWDEWQLLDSTQKYLYRDVILENYHNLISVGYHGTKP
DLIFKLEQGEDPWIINAKISRQSCPDGWEEWYQNNQDELESIERSYACSVLGRLNLSKTH
DSSRQRLYNTRGKSLTQNSAPSRSYLRKNPDKFHGYEEPYFLKHQRAHSIEKNCVCSECG
KAFRCKSQLIVHLRIHTGERPYECSKCERAFSAKSNLNAHQRVHTGEKPYSCSECEKVFS
FRSQLIVHQEIHTGGKPYGCSECGKAYSWKSQLLLHQRSHTGVKPYECSECGKAFSLKSP
FVVHQRTHTGVKPHKCSECGKAFRSKSYLLVHIRMHTGEKPYQCSDCGKAFNMKTQLIVH
QGVHTGNNPYQCGECGKAFGRKEQLTAHLRAHAGEKPYGCSECGKAFSSKSYLVIHRRTH
TGERPYECSLCERAFCGKSQLIIHQRTHSTEKPYECNECEKAYPRKASLQIHQKTHSGEK
PFKCSECGKAFTQKSSLSEHQRVHTGEKPWKCSECGKSFCWNSGLRIHRKTHK*
>ENSG00000145734|ENST00000358731
MFRRARLSVKPNVRPGVGARGSTASNPQRGRESPRPPDPATDSASKPAEPTDVPTVDFGG
AEPQEKAPRSSTEKTGGDNDVEESSRSSSTVSQRRKRISSTSSLVKSSVSVPSESHPLST
INQEAPQPTATSTKEKQPCSDRYRIYKAQKLREMLKEELRKEKKQWKNKYAINESQRPPD

RSKMTMRDFIYYLPDNNPMTSSLEQEKKTEKPSTPVQTREQEGKSTPNAEDNEMEEETDD
GPLLVPRVKVAEDGSIILDEESLTVEVLRTKGPCVVEENDPIFERGSTTTYSSFRKNYYS
KPWSNKETDMFFLAISMVGTDFSMIGQLFPHRARIEIKNKFKREEKTNGWRIDKAFQEKR
PFDFDFFAHLLQKVLAEEEKRKQKSVKNHSLKEKKSTKPRKNVKVKKVACEGVNNDPDES
MSSRISDTERSQKDAQTVEEESLTLSREDAEQVALEVDLNQKKRRRKKQDGANELGVNNL
LENATVQAGPSKGEKHKNKCQAIRPELKEGECSKEQMLSCTQNIDGIVGFASTEKVEKRT
DPILSLSNQQDATSVATESSESSTSDLPSFEVGIRALCEVNNAEGSCIEERNVDLKNNSL
EIDQTENVKPMLRGRFQRPKPNLSRAGKKSVLSQGKTESESKNSHSKTSVEKNHVEKDKM
NTLDILRMETTERENPEAETVSVLGEKNCLQEGSQLKALRPVQVRGRLQKPKPNAGKAAE
RKEILISQEEIGANVEKNENESCADRDTPQHMEDQSRKDFEEEDVILQPEKNDSFQNVQP
DEPKVLNECLSVQENNKANKLNQVPILRTRFQKPKPNIGRGTGRREISSKEEVLEKILVS
GEMAAALRETVRLDTSPKEMVPAEINTKEMQSDLKETGRRAISPREKILDVIDDTIEMET
GLKAMGREICLREKTPEVIDATEEIDKDLEEAGRREISPQKNGPEEVKPLGEVETDLKAT
GNESSPREKTPEVTDATEEIDKNLEETGRRKISPRENGPEEVKPVDEMETDLNATGRESS
PREKTPEVIDATEEIDLEETEREVSPQENGLEEVKPLGEMETDLKATGRDSFPRGKTPEV
IDAIEEIEIDLEETEREISPQENGLEEVKPLGEMQTDLKATGREISPREKTPEVIDATEE
IDKDLEETGRREISPEENGPEEVKPVDEMETDLKTTGREGSSREKTREVIDAAEVIETDL
EETEREISPQENGPEEVKPVGKMETDLKEIREEISQREKVLAEFSAIREKEIDLKETGKR
DIPIMEKVSGKMAVVEEMEADLKETGKENFRERGSEEICVTEEKVAELKQTGKTDISPRE
NELEETSTSRQTDTHLMQSGSNDFSAVPSLDIQNISSEVLSMMHTPVEEKRNSEKEVSSH
FSHFKISSQTHESDKTEVQGIQSPDVPEQFSDINLSKSLPQEQKPLEIKPAPFVRSRFKR
PKPNLARAALKRETTESEKYIYEKKSETKKMETIVMQENNEQTDTLPSQHDEASLMISRE
KDTLGHRNEEAVILPCTQTERNLSPSNSCEPKEESQSAPVQKNDSVVSVGTNNVNTFQQE
MKESVIQTARQVRGRLQRPRPNIRKTGQRQIVDKGEAKGIIKEGRTILPKDETEKKVLTV
SNSQIETEIEVPSSAVPEHRMYENQSQVVLVENLHVNKTNETIRHENKPYVPSSAQMTRR
KFQKAKPNLGRAHSKKEEPVLEKVTTDQSKEGKPEDHLLQKGASNTQLLLKEKAELLTSL
EVSARKDCVGSKESALAKIDAELEEVGPSRRVGEETVGDNSPSSVVEEQYLNKLTSCPQP
LNETSYSKIALDGKTTISSTSEYERNRGERRSHKKFKPNVTRGRGSKRVRGKTSKKEPRA
SKAMLVTLRASQEEDDDADDFESDYEEESYHLAPEEVNKAPVFVPVGLRSPEPVSAQIEE
TMEELEITVNVPDVGCIAVVEHELPNTDVTTEEMKQEENLSVPFEMTTSEHIQDEPGTND
GSTEAAITLLTMGDLVLQSEISSEQGDVGVCIIPHVHSKDKSHIPSSLDNVNHKIVHECQ
ELSSPVITTSPASFEENKIVLEEQSSREEISLMEKVKENATPTRNTISKVTSNLRIRSRL
AKPKPNLEKTLGTNRLDDYQEVSSLCVTKGAEMETQRETEKNASKATELENKNLGPVTTA
ENKDQSKLACVHGIKGTSISSEVNLTERNENQEESSQEVHMLSVAPVASSETGPCTLGLD
RGLGENSVEEPQIKDSKGDSVLTLPVPEYTPTSIPEVQQENIINPQDLTVNLVANVPQDG
EDEQAFILTLVEIPANAVEEFTDATAQFMPNPLLPAPILVKSVNTEERGDMSICLPATSV
GQDAMGLSISGRDNSKKPPDNLDLVSRKRFQCRLDKNDHIPPAKKRSLTLRDDCQEYTTE
VHSKELTNVFEETGESHKGQDIFLTSGSTLTTPEPQRQQVEAAFQSRGSRSPDACMDKNV
PQLPQDEMIVSDKEERTDAAPKSQQMDSRTSSSKASLSRPGRRPLGFLSLICSKNSLESD
EPMQVHSKKRLKPLIPGLRKKLKRSNPFNESQEKNRESSDLLPSPSVITTQSENISSSAT
QVSCDQPLLKEGYKSAQKRAPQGEATTVSEYFFNDIFIEVDETE*
>ENSG00000174720|ENST00000324052
METESGNQEKVMEEESTEKKKEVEKKKRSRVKQVLADIAKQVDFWFGDANLHKDRFLREQ
IEKSRDGYVDISLLVSFNKMKKLTTDGKLIARALRSSAVVELDLEGTRIRRKKPLGERPK

DEDERTVYVELLPKNVNHSWIERVFGKCGNVVYISIPHYKSTGDPKGFAFVEFETKEQAA
KAIEFLNNPPEEAPRKPGIFPKTVKNKPIPALRVVEEKKKKKKKKGRMKKEDNIQAKEEN
MDTSNTSISKMKRSRPTSEGSDIESTEPQKQCSKKKKKRDRVEASSLPEVRTGKRKRSSS
EDAESLAPRSKVKKIIQKDIIKEASEASKENRDIEISTEEEKDTGDLKDSSLLKTKRKHK
KKHKERHKMGEEVIPLRVLSKSEWMDLKKEYLALQKASMASLKKTISQIKSESEMETDSG
VPQNTGMKNEKTANREECRTQEKVNATGPQFVSGVIVKIISTEPLPGRKQVRDTLAAISE
VLYVDLLEGDTECHARFKTPEDAQAVINAYTEINKKHCWKLEILSGDHEQRYWQKILVDR
QAKLNQPREKKRGTEKLITKAEKIRLAKTQQASKHIRFSEYD*
>ENSG00000188909|ENST00000343035
MNLNFTSPLHPASSQRPTSFFIEDILLHKPKPLREVAPDHFASSLASRVPLLDYGYPLMP
TPTLLAPHAHHPLHKGDHHHPYFLTTSGMPVPALFPHPQHAELPGKHCRRRKARTVFSDS
QLSGLEKRFEIQRYLSTPERVELATALSLSETQVKTWFQNRRMKHKKQLRKSQDEPKAPD
GPESPEGSPRGSEAATAAEARLSLPAGPFVLTEPEDEVDIGDEGELGSGPHVL*
>ENSG00000197013|ENST00000358491
MGPLTFTDVAIEFSLEEWQCLDTAQQNLYRNVMLENYRNLVFLGIAVSKPDLITCLEKEK
EPCKMKRHEMVDEPPVVCSHFAEDFWPEQDIKDSFQKVTLRRYDKRGHENLQLRKGYKTV
GDCKLYKGGYNGLNQCLTLTQSKMYHCDIYVKVFYAFSNADRYKTRHTGKKPFQCKKCGK
SFCMLSQLTQHKKIHIRENTYRCKEFGNAFNQSSALTNHKRIYVGEKHYRCEECGKAFNH
YSTLTNHKRIHTGEKPYKCKECGKAFSRYSTLTTHKRIHSGEKPYKCDECGKTFSISSTF
TKHKIIHTEEKPYKCKECGKAFNRSSTLTSHKRIHTGEKPYKCEECGKAFNWSSTLTKHK
VIHTGEKPYKCEECGKAFNQSSRLTRHKKIHTGEEPYKFEKCGRVFTCSSTLTQDKKIHT
GEKPYNCEECGKVFTYSSTLTRHKRIHTEEKPYKCNECGKAFNRSSHLTSHRRIHTGEKP
YKCEECGKAFKQSSNLNSHKKIHSGEKPYKCEECGKAFILSSRLTQHKKIHTGEKPYKCE
ECGKAFNRSSRLTQHKKIHTGEKPYKCKQCDKAFTHSSNLSSHKKIHSGEKPYKCEECGK
AFNRSSRLTQHKKIHTREKPYKCEECAKAFTRSSRLTQHKKIHRMGVVAHACNPSTLGGR
GGRITRSGDRDRPG*
>ENSG00000148737|ENST00000355717
MPQLNGGGGDDLGANDELISFKDEGEQEEKSSENSSAERDLADVKSSLVNESETNQNSSS
DSEAERRPPPRSESFRDKSRESLEEAAKRQDGGLFKGPPYPGYPFIMIPDLTSPYLPNGS
LSPTARTYLQMKWPLLDVQAGSLQSRQALKDARSPSPAHIVSPLPCCTQGHDCQHFYPPS
DFTVSTQVFRDMKRSHSLQKVGEPWCIESNKVPVVQHPHHVHPLTPLITYSNEHFTPGNP
PPHLPADVDPKTGIPRPPHPPDISPYYPLSPGTVGQIPHPLGWLVPQQGQPVYPITTGGF
RHPYPTALTVNASMSRFPPHMVPPHHTLHTTGIPHPAIVTPTVKQESSQSDVGSLHSSKH
QDSKKEEEKKKPHIKKPLNAFMLYMKEMRAKVVAECTLKESAAINQILGRRWHALSREEQ
AKYYELARKERQLHMQLYPGWSARDNYGKKKKRKRDKQPGETNGEKKSAFATYKVKAAAS
AHPLQMEAY*
>ENSG00000241476|ENST00000337502
MNGDDAFARRPTVGAQIPEKIQKAFDDIAKYFSKEEWEKMKASEKIFYVYMKRKYEAMTK
LGFKATLPPFMCNKRAEDFQGNDLDNDPNRGNQVERPQMTFGRLQGISPKIMPKKPAEEG
NDSEEVPEASGPQNDGKELCPPGKPTTSEKIHERSGPKRGEHAWTHRLRERKQLVIYEEI
SDPEEDDE*
>ENSG00000197343|ENST00000320583
MEEIPAQEAAGSPRVQFQSLETQSECLSPEPQFVQDTDMEQGLTGAPPVPQVPALPREGS
PGDQAAALLTARYQEFVTFEDVAVHLTREEWGYLDPVQRDLYREVMLENYGNVVSLGILL

RLPTTRIHSVNSCPALSHTQASAFSGETLAVLTAGISKRWPKYRLPIDIARPCSETPFPR
L*
>ENSG00000157110|ENST00000339877
MNNGGKAEKENTPSEANLQEEEVRTLFVSGLPLDIKPRELYLLFRPFKGYEGSLIKLTSK
QPVGFVSFDSRSEAEAAKNALNGIRFDPEIPQTLRLEFAKANTKMAKNKLVGTPNPSTPL
PNTVPQFIAREPYELTVPALYPSSPEVWAPYPLYPAELAPALPPPAFTYPASLHAQCFSP
EAKPNTPVFCPLLQQIRFVSGNVFVTYQPTADQQRELPC*
>ENSG00000155506|ENST00000336314
MLWRVLLSKRPPFPHPELDFQEAPIPSCPGRLPGRKNSVALAAAPRKEPTGDREKPLPFP
VLAPFSNPEHSAPAKVVRAAVPKQRKGSKVGDFGDAINWPTPGEIAHKSVQPQSHKPQPT
RKLPPKKDMKEQEKGEGSDSKESPKTKSDESGEEKNGDEDCQRGGQKKKGNKHKWVPLQI
DMKPEVPREKLASRPTRPPEPRHIPANRGEIKGSESATYVPVAPPTPAWQPEIKPEPAWH
DQDETSSVKSDGAGGARASFRGRGRGRGRGRGRGRGGTRTHFDYQFGYRKFDGVEGPRTP
KYMNNITYYFDNVSSTELYSVDQELLKDYIKRQIEYYFSVDNLERDFFLRRKMDADGFLP
ITLIASFHRVQALTTDISLIFAALKDSKVVEIVDEKVRRREEPEKWPLPPIVDYSQTDFS
QLLNCPEFVPRQHYQKETESAPGSPRAVTPVPTKTEEVSNLKTLPKGLSASLPDLDSENW
IEVKKRPRPSPARPKKSEESRFSHLTSLPQQLPSQQLMSKDQDEQEELDFLFDEEMEQMD
GRKNTFTAWSDEESDYEIDDRDVNKILIVTQTPHYMRRHPGGDRTGNHTSRAKMSAELAK
VINDGLFYYEQDLWAEKFEPEYSQIKQEVENFKKVNMISREQFDTLTPEPPVDPNQEVPP
GPPRFQQVPTDALANKLFGAPEPSTIARSLPTTVPESPNYRNTRTPRTPRTPQLKDSSQT
SRFYPVVKEGRTLDAKMPRKRKTRHSSNPPLESHVGWVMDSREHRPRTASISSSPSEGTP
TVGSYGCTPQSLPKFQHPSHELLKENGFTQHVYHKYRRRCLNERKRLGIGQSQEMNTLFR
FWSFFLRDHFNKKMYEEFKQLALEDAKEGYRYGLECLFRYYSYGLEKKFRLDIFKDFQEE
TVKDYEAGQLYGLEKFWAFLKYSKAKNLDIDPKLQEYLGKFRRLEDFRVDPPMGEEGNHK
RHSVVAGGGGGGEGRKRCPSQSSSRPAAMISQPPTPPTGQPVREDAKWTSQHSNTQTLGK*
>ENSG00000164093|ENST00000354925
METNCRKLVSACVQLGVQPAAVECLFSKDSEIKKVEFTDSPESRKEAASSKFFPRQHPGA
NEKDKSQQGKNEDVGAEDPSKKKRQRRQRTHFTSQQLQELEATFQRNRYPDMSTREEIAV
WTNLTEARVRVWFKNRRAKWRKRERNQQAELCKNGFGPQFNGLMQPYDDMYPGYSYNNWA
AKGLTSASLSTKSFPFFNSMNVNPLSSQSMFSPPNSISSMSMSSSMVPSAVTGVPGSSLN
SLNNLNNLSSPSLNSAVPTPACPYAPPTPPYVYRDTCNSSLASLRLKAKQHSSFGYASVQ
NPASNLSACQYAVDRPV*
>ENSG00000114867|ENST00000346169
MNKAPQSTGPPPAPSPGLPQPAFPPGQTAPVVFSTPQATQMNTPSQPRQHFYPSRAQPPS
SAASRVQSAAPARPGPAAHVYPAGSQVMMIPSQISYPASQGAYYIPGQGRSTYVVPTQQY
PVQPGAPGFYPGASPTEFGTYAGAYYPAQGVQQFPTGVAPTPVLMNQPPQIAPKRERKTI
RIRDPNQGGKDITEEIMSGARTASTPTPPQTGGGLEPQANGETPQVAVIVRPDDRSQGAI
IADRPGLPGPEHSPSESQPSSPSPTPSPSPVLEPGSEPNLAVLSIPGDTMTTIQMSVEES
TPISRETGEPYRLSPEPTPLAEPILEVEVTLSKPVPESEFSSSPLQAPTPLASHTVEIHE
PNGMVPSEDLEPEVESSPELAPPPACPSESPVPIAPTAQPEELLNGAPSPPAVDLSPVSE
PEEQAKEVTASMAPPTIPSATPATAPSATSPAQEEEMEEEEEEEEGEAGEAGEAESEKGG
EELLPPESTPIPANLSQNLEAAAATQVAVSVPKRRRKIKELNKKEAVGDLLDAFKEANPA
VPEVENQPPAGSNPGPESEGSGVPPRPEEADETWDSKEDKIHNAENIQPGEQKYEYKSDQ
WKPLNLEEKKRYDREFLLGFQFIFASMQKPEGLPHISDVVLDKANKTPLRPLDPTRLQGI

NCGPDFTPSFANLGRTTLSTRGPPRGGPGGELPRGPAGLGPRRSQQGPRKEPRKIIATVL
MTEDIKLNKAEKAWKPSSKRTAADKDRGEEDADGSKTQDLFRRVRSILNKLTPQMFQQLM
KQVTQLAIDTEERLKGVIDLIFEKAISEPNFSVAYANMCRCLMALKVPTTEKPTVTVNFR
KLLLNRCQKEFEKDKDDDEVFEKKQKEMDEAATAEERGRLKEELEEARDIARRRSLGNIK
FIGELFKLKMLTEAIMHDCVVKLLKNHDEESLECLCRLLTTIGKDLDFEKAKPRMDQYFN
QMEKIIKEKKTSSRIRFMLQDVLDLRGSNWVPRRGDQGPKTIDQIHKEAEMEEHREHIKV
QQLMAKGSDKRRGGPPGPPISRGLPLVDDGGWNTVPISKGSRPIDTSRLTKITKPGSIDS
NNQLFAPGGRLSWGKGSSGGSGAKPSDAASEAARPATSTLNRFSALQQAVPTESTDNRRV
VQRSSLSRERGEKAGDRGDRLERSERGGDRGDRLDRARTPATKRSFSKEVEERSRERPSQ
PEGLRKAASLTEDRDRGRDAVKREAALPPVSPLKAALSEEELEKKSKAIIEEYLHLNDMK
EAVQCVQELASPSLLFIFVRHGVESTLERSAIAREHMGQLLHQLLCAGHLSTAQYYQGLY
EILELAEDMEIDIPHVWLYLAELVTPILQEGGVPMGELFREITKPLRPLGKAASLLLEIL
GLLCKSMGPKKVGTLWREAGLSWKEFLPEGQDIGAFVAEQKVEYTLGEESEAPGQRALPS
EELNRQLEKLLKEGSSNQRVFDWIEANLSEQQIVSNTLVRALMTAVCYSAIIFETPLRVD
VAVLKARAKLLQKYLCDEQKELQALYALQALVVTLEQPPNLLRMFFDALYDEDVVKEDAF
YSWESSKDPAEQQGKGVALKSVTAFFKWLREAEEESDHN*
>ENSG00000162039|ENST00000325962
MANSFAARIFTTLSDLQTNMANLKVIGIVIGKTDVKGFPDRKNIGSERYTFSFTIRDSPA
HFVNAASWGNEDYIKSLSDSFRVGDCVIIENPLIQRKEIEREEKFSPATPSNCKLLLSEN
HSTVKVCSSYEVDTKLLSLIHLPVKESHDYYSLGDIVANGHSLNGRIINVLAAVKSVGEP
KYFTTSDRRKGQRCEVRLYDETESSFAMTCWDNESILLAQSWMPRETVIFASDVRINFDK
FRNCMTATVISKTIITTNPDIPEANILLNFIRENKETNVLDDEIDSYFKESINLSTIVDV
YTVEQLKGKALKNEGKADPSYGILYAYISTLNIDDETTKVVRNRCSSCGYIVNEASNMCT
TCNKNSLDFKSVFLSFHVLIDLTDHTGTLHSCSLTGSVAEETLGCTVHEFLAMTDEQKTA
LKWQFLLERSKIYLKFVLSHRARSGLKISVLSCKLADPTEASRNLSGQKHV*
>ENSG00000198160|ENST00000357692
MFMFNWFTDCLWTLFLSNYQPSVESSSPGGSATSDDHEFDPSADMLVHDFDDERTLEEEE
MMEGETNFSSEIEDLAREGDMPIHELLSLYGYGSTVRLPEEDEEEEEEEEGEDDEDADN
DDNSGCSGENKEENIKDSSGGQEDETQSSNDDPSQSVASQDAQEIIRPRRCKYFDTNSEVE
EESEEDEDYIPSEDWKKEIMVGSMFQAEIPVGICRYKENEKVYENDDQLLWDPEYLPEDK
VIIFLKDASRRTGDEKGVEAIPEGSHIKDNEQALYELVKCNFDTEEALRRLRFNVKAARE
ELSVWTEEECRNFEQGLKAYGKDFHLIQANKVRTRSVGECVAFYYMWKKSERYDFFAQQT
RFGKKKYNLHPGVTDYMDRLLDESESAASSRAPSPPPTASNSSNSQSEKEDGTVSTANQN
GVSSNGPGEILNKEEVKVEGLHINGPTGGNKKPLHADMDTNGYETDNLTTDPKLAHMTAR
NENDFDEKSERPAKRRRVNSNGKESPGSSEFFQEAVSHGKFEELENTDD*
>ENSG00000197299|ENST00000355112
MAAVPQNNLQEQLERHSARTLNNKLSLSKPKFSGFTFKKKTSSDNNVSVTNVSVAKTPVL
RNKDVNVTEDFSFSEPLPNTTNQQRVKDFFKNAPAGQETQRGGSKSLLPDFLQTPKEVVC
TTQNTPTVKKSRDTALKKLEFSSSPDSLSTINDWDDMDDFDTSETSKSFVTPPQSHFVRV
STAQKSKKGKRNFFKAQLYTTNTVKTDLPPPSSESEQIDLTEEQKDDSEWLSSDVICIDD
GPIAEVHINEDAQESDSLKTHLEDERDNSEKKKNLEEAELHSTEKVPCIEFDDDDYDTDF
VPPSPEEIISASSSSSKCLSTLKDLDTSDRKEDVLSTSKDLLSKPEKMSMQELNPETSTD
CDARQISLQQQLIHVMEHICKLIDTIPDDKLKLLDCGNELLQQRNIRRKLLTEVDFNKSD
ASLLGSLWRYRPDSLDGPMEGDSCPTGNSMKELNFSHLPSNSVSPGDCLLTTTLGKTGFS

ATRKNLFERPLFNTHLQKSFVSSNWAETPRLGKKNESSYFPGNVLTSTAVKDQNKHTASI
NDLERETQPSYDIDNFDIDDFDDDDDWEDIMHNLAASKSSTAAYQPIKEGRPIKSVSERL
SSAKTDCLPVSSTAQNINFSESIQNYTDKSAQNLASRNLKHERFQSLSFPHTKEMMKIFH
KKFGLHNFRTNQLEAINAALLGEDCFILMPTGGGKSLCYQLPACVSPGVTVVISPLRSLI
VDQVQKLTSLDIPATYLTGDKTDSEATNIYLQLSKKDPIIKLLYVTPEKICASNRLISTL
ENLYERKLLARFVIDEAHCVSQWGHDFRQDYKRMNMLRQKFPSVPVMALTATANPRVQKD
ILTQLKILRPQVFSMSFNRHNLKYYVLPKKPKKVAFDCLEWIRKHHPYDSGIIYCLSRRE
CDTMADTLQRDGLAALAYHAGLSDSARDEVQQKWINQDGCQVICATIAFGMGIDKPDVRF
VIHASLPKSVEGYYQESGRAGRDGEISHCLLFYTYHDVTRLKRLIMMEKDGNHHTRETHF
NNLYSMVHYCENITECRRIQLLAYFGENGFNPDFCKKHPDVSCDNCCKTKDYKTRDVTDD
VKSIVRFVQEHSSSQGMRNIKHVGPSGRFTMNMLVDIFLGSKSAKIQSGIFGKGSAYSRH
NAERLFKKLILDKILDEDLYINANDQAIAYVMLGNKAQTVLNGNLKVDFMETENSSSVKK
QKALVAKVSQREEMVKKCLGELTEVCKSLGKVFGVHYFNIFNTVTLKKLAESLSSDPEVL
LQIDGVTEDKLEKYGAEVISVLQKYSEWTSPAEDSSPGISLSSSRGPGRSAAEELDEEIP
VSSHYFASKTRNERKRKKMPASQRSKRRKTASSGSKAKGGSATCRKISSKTKSSSIIGSS
SASHTSQATSGANSKLGIMAPPKPINRPFLKPSYAFS*
>ENSG00000188425|ENST00000341294
MQLPPFDMWKDYFNLSQVVWALIASRGQRLETQEIEEPSPGPPLGQDQGLGAPGANGGLG
TLCNFCKHNGESRHVYSSHQLKTPDGVVVCPILRHYVCPVCGATGDQAHTLKYCPLNGGQ
QSLYRRSGRNSAGRRVKR*
>ENSG00000196387|ENST00000319849
MSQGSVTFRDVAIDFSQEEWKWLQPAQRDLYRCVMLENYGHLVSLAGLSISKPDVVSLLE
QGKEPWLGKREVKRDLFSGFFSANCVSFFSWAHVDGSAFLVDGNFFCKFFRVKW*
>ENSG00000107372|ENST00000343431
MAQETNQTPGPMLCSTGCGFYGNPRTNGMCSVCYKEHLQRQQNSGRMSPMGTASGSNSPT
SDSASVQRADTSLNNCEGAAGSTSEKSRNVPVAALPVTQQMTEMSISREDKITTPKTEVS
EPVVTQPSPSVSQPSTSQSEEKAPELPKPKKNRCFMCRKKVGLTGFDCRCGNLFCGLHRY
SDKHNCPYDYKAEAAAKIRKENPVVVAEKIQRI*
>ENSG00000182718|ENST00000332680
MGRQLAGCGDAGKKASFKMSTVHEILCKLSLEGDHSTPPSAYGSVKAYTNFDAERDALNI
ETAIKTKGVDEVTIVNILTNRSNAQRQDIAFAYQRRTKKELASALKSALSGHLETVILGL
LKTPAQYDASELKASMKGLGTDEDSLIEIICSRTNQELQEINRVYKEMYKTDLEKDIISD
TSGDFRKLMVALAKGRRAEDGSVIDYELIDQDARDLYDAGVKRKGTDVPKWISIMTERSV
PHLQKVFDRYKSYSPYDMLESIRKEVKGDLENAFLNLVQCIQNKPLYFADRLYDSMKGKG
TRDKVLIRIMVSRSEVDMLKIRSEFKRKYGKSLYYYIQQDTKGDYQKALLYLCGGDD*
>ENSG00000067225|ENST00000335181
MSKPHSEAGTAFIQTQQLHAAMADTFLEHMCRLDIDSPPITARNTGIICTIGPASRSVET
LKEMIKSGMNVARLNFSHGTHEYHAETIKNVRTATESFASDPILYRPVAVALDTKGPEIR
TGLIKGSGTAEVELKKGATLKITLDNAYMEKCDENILWLDYKNICKVVEVGSKIYVDDGL
ISLQVKQKGADFLVTEVENGGSLGSKKGVNLPGAAVDLPAVSEKDIQDLKFGVEQDVDMV
FASFIRKASDVHEVRKVLGEKGKNIKIISKIENHEGVRRFDEILEASDGIMVARGDLGIE
IPAEKVFLAQKMMIGRCNRAGKPVICATQMLESMIKKPRPTRAEGSDVANAVLDGADCIM
LSGETAKGDYPLEAVRMQHLIAREAEAAIYHLQLFEELRRLAPITSDPTEATAVGAVEAS
FKCCSGAIIVLTKSGRSAHQVARYRPRAPIIAVTRNPQTARQAHLYRGIFPVLCKDPVQE

AWAEDVDLRVNFAMNVGKARGFFKKGDVVIVLTGWRPGSGFTNTMRVVPVP*
>ENSG00000126945|ENST00000316594
MMLSTEGREGFVVKVRGLPWSCSADEVMRFFSDCKIQNGTSGIRFIYTREGRPSGEAFVE
LESEEEVKLALKKDRETMGHRYVEVFKSNSVEMDWVLKHTGPNSPDTANDGFVRLRGLPF
GCSKEEIVQFFSGLEIVPNGMTLPVDFQGRSTGEAFVQFASQEIAEKALKKHKERIGHRY
IEIFKSSRAEVRTHYDPPRKLMAMQRPGPYDRPGAGRGYNSIGRGAGFERMRRGAYGGGY
GGYDDYGGYNDGYGFGSDRFGRDLNYCFSGMSDHRYGDGGSSFQSTTGHCVHMRGLPYRA
TENDIYNFFSPLNPMRVHIEIGPDGRVTGEADVEFATHEDAVAAMAKDKANMQHRYVELF
LNSTAGTSGGAYDHSYVELFLNSTAGASGGAYGSQMMGGMGLSNQSSYGGPASQQLSGGY
GGGYGGQSSMSGYDQVLQENSSDYQSNLA*
>ENSG00000177045|ENST00000317578
MATLPAEPSAGPAAGGEAVAAAAATEEEEEEARQLLQTLQAAEGEAAAAAGAGAGAAAAG
AEGPGSPGVPGSPPEAASEPPTGLRFSPEQVACVCEALLQAGHAGRLSRFLGALPPAERL
RGSDPVLRARALVAFQRGEYAELYRLLESRPFPAAHHAFLQDLYLRARYHEAERARGRAL
GAVDKYRLRKKFPLPKTIWDGEETVYCFKERSRAALKACYRGNRYPTPDEKRRLATLTGL
SLTQVSNWFKNRRQRDRTGAGGGAPCKSESDGNPTTEDESSRSPEDLERGAAPVSAEAAA
QGSIFLAGTGPPAPCPASSSILVNGSFLAASGSPAVLLNGGPVIINGLALGEASSLGPLL
LTGGGGAPPPQPSPQGASETKTSLVLDPQTGEVRLEEAQSEAPETKGAQVAAPGPALGEE
VLGPLAQVVPGPPTAATFPLPPGPVPAVAAPQVVPLSPPPGYPTGLSPTSPLLNLPQVVP
TSQVVTLPQAVGPLQLLAAGPGSPVKVAAAAGPANVHLINSGVGVTALQLPSATAPGNFL
LANPVSGSPIVTGVALQQGKIILTATFPTSMLVSQVLPPAPGLALPLKPETAISVPEGGL
PVAPSPALPEAHALGTLSAQQPPPAAATTSSTSLPFSPDSPGLLPNFPAPPPEGLMLSPA
AVPVWSAGLELSAGTEGLLEAEKGLGTQAPHTVLRLPDPDPEGLLLGATAGGEVDEGLEA
EAKVLTQLQSVPVEEPLEL*
>ENSG00000185379|ENST00000335858
MGVLRVGLCPGLTEEMIQLLRSHRIKTVVDLVSADLEEVAQKCGLSYKAEALRRIQVVHA
FDIFQMLDVLQELRGTVAQQVTGSSGTVKVVVVDSVTAVVSPLLGGQQREGLALMMQLAR
ELKTLARDLGMAVVVTNHITRDRDSGRLKPALGRSWSFVPSTRILLDTIEGAGASGGRRM
ACLAKSSRQPTGFQEMVDIGTWGTSEQSATLQGDQT*
>ENSG00000179041|ENST00000320270
MEGQSVEELLAKAEQDEAEKLQRITVHKELELQFDLGNLLASDRNPPTGLRCAGPTPEAE
LQALARDNTQLLINQLWQLPTERVEEAIVARLPEPTTRLPREKPLPRPRPLTRWQQFARL
KGIRPKKKTNLVWDEVSGQWRRRWGYQRARDDTKEWLIEVPGNADPLEDQFAKRIQAKKE
RVAKNELNRLRNLARAHKMQLPSAAGLHPTGHQSKEELGRAMQVAKVSTASVGRFQERLP
KEKVPRGSGKKRKFQPLFGDFAAEKKNQLELLRVMNSKKPQLDVTRATNKQMREEDQEEA
AKRRKMSQKGKRKGGRQGPGGKRKGGPPSQGGKRKGGLGGKMNSGPPGLGGKRKGGQRPG
GKRRK*
>ENSG00000128886|ENST00000319359
MEELQEPLRGQLRLCFTQAARTSLLLLRLNDAALRALQECQRQQVRPVIAFQGHRGYLRL
PGPGWSCLFSFIVSQCCQEGAGGSLDLVCQRFLRSGPNSLHCLGSLRERLIIWAAMDSIP
APSSVQGHNLTEDARHPESWQNTGGYSEGDAVSQPQMALEEVSVSDPLASNQGQSLPGSS
REHMAQWEVRSQTHVPNREPVQALPSSASRKRLDKKRSVPVATVELEEKRFRTLPLVPSP
LQGLTNQDLQEGEDWEQEDEDMDPRLEHSSSVQEDSESPSPEDIPDYLLQYRAIHSAEQQ
HAYEQDFETDYAEYRILHARVGTASQRFIELGAEIKRVRRGTPEYKVLEDKIIQEYKKFR

KQYPSYREEKRRCEYLHQKLSHIKGLILEFEEKNRGS*
>ENSG00000165119|ENST00000360384
METEQPEETFPNTETNGEFGKRPAEDMEEEQAFKRSRNTDEMVELRILLQSKNAGAVIGK
GGKNIKALRTDYNASVSVPDSSGPERILSISADIETIGEILKKIIPTLEEGLQLPSPTAT
SQLPLESDAVECLNYQHYKGSDFDCELRLLIHQSLAGGIIGVKGAKIKELRENTQTTIKL
FQECCPHSTDRVVLIGGKPDRVVECIKIILDLISESPIKGRAQPYDPNFYDETYDYGGFT
MMFDDRRGRPVGFPMRGRGGFDRMPPGRGGRPMPPSRRDYDDMSPRRGPPPPPPGRGGRG
GSRARNLPLPPPPPPRGGDLMAYDRRGRPGDRYDGMVGFSADETWDSAIDTWSPSEWQMA
YEPQGGSGYDYSYAGGRGSYGDLGGPIITTQVTIPKDLAGSIIGKGGQRIKQIRHESGAS
IKIDEPLEGSEDRIITITGTQDQIQNAQYLLQNSVKQYSGKFF*
>ENSG00000048740|ENST00000354897
MNGALDHSDQPDPDAIKMFVGQIPRSWSEKELKELFEPYGAVYQINVLRDRSQNPPQSKG
CCFVTFYTRKAALEAQNALHNIKTLPGMHHPIQMKPADSEKSNAVEDRKLFIGMVSKKCN
ENDIRVMFSPFGQIEECRILRGPDGLSRGCAFVTFSTRAMAQNAIKAMHQSQTMEGCSSP
IVVKFADTQKDKEQRRLQQQLAQQMQQLNTATWGNLTGLGGLTPQYLALLQQATSSSNLG
AFSGIQQMAGMNALQLQNLATLAAAAAAAQTSATSTNANPLSTTSSALGALTSPVAASTP
NSTAGAAMNSLTSLGTLQGLAGATVGLNNINALAGTINTPRSKRLLLPKDNNGMAALNGG
LGATGLTNGTAGTMDALTQAYSGIQQYAAAALPTLYSQSLLQQQSAAGSQKEGPEGANLF
IYHLPQEFGDQDILQMFMPFGNVISAKVFIDKQTNLSKCFGFVSYDNPVSAQAAIQAMNG
FQIGMKRLKVQLKRSKNDSKPY*
>ENSG00000089737|ENST00000330836
MKLKDTKSRPKQSSCGKFQTKGIKVVGKWKEVKIDPNMFADGQMDDLVCFEELTDYQLVS
PAKNPSSLFSKEAPKRKAQAVSEEEEEEEGKSSSPKKKIKLKKSKNVATEGTSTQKEFEV
KDPELEAQGDDMVCDDPEAGEMTSENLVQTAPKKKKNKGKKGLEPSQSTAAIRDKLDILG
AAETGSGKTLAFAIPMIHAVLQWQKRNAAPPPSNTEAPPGETRTEAGAETRSPGKAEAES
DALPDDTVIESEALPSDIAAEARAKTGGTVSDQALLFGDDDAGEGPSSLIREKPVPKQNE
NEEENLDKEQTGNLKQELDDKSATCKAYPKRPLLGLVLTPTRELAVQVKQHIDAVARFTG
IKTAILVGGMSTQKQQRMLNRRPEIVVATPGRLWELIKEKHYHLRNLRQLRCLVVDEADR
MVEKGHFAELSQLLEMLNDSQYNPKRQTLVFSATLTLVHQAPARILHKKHTKKMDKTAKL
DLLMQKIGMRGKPKVIDLTRNEATVETLTETKIHCETDEKDFYLYYFLMQYPGRSLVFAN
SISCIKRLSGLLKVLDIMPLTLHACMHQKQRLRNLEQFARLEDCVLLATDVAARGLDIPK
VQHVIHYQVPRTSEIYVHRSGRTARATNEGLSLMLIGPEDVINFKKIYKTLKKDEDIPLF
PVQTKYMDVVKERIRLARQIEKSEYRNFQACLHNSWIEQAAAALEIELEEDMYKGGKADQ
QEERRRQKQMKVLKKELRHLLSQPLFTESQKTKYPTQSGKPPLLVSAPSKSESALSCLSK
QKKKKTKKPKEPQPEQPQPSTSAN*
>ENSG00000124214|ENST00000340954
MKLGKKPMYKPVDPYSRMQSTYNYNMRGGAYPPRYFYPFPVPPLLYQVELSVGGQQFNGK
GKTRQAAKHDAAAKALRILQNEPLPERLEVNGRESEEENLNKSEISQVFEIALKRNLPVN
FEVARESGPPHMKNFVTKVSVGEFVGEGEGKSKKISKKNAAIAVLEELKKLPPLPAVERV
KPRIKKKTKPIVKPQTSPEYGQGINPISRLAQIQQAKKEKEPEYTLLTERGLPRRREFVM
QVKVGNHTAEGTGTNKKVAKRNAAENMLEILGFKVPQAQPTKPALKSEEKTPIKKPGDGR
KVTFFEPGSGDENGTSNKEDEFRMPYLSHQQLPAGILPMVPEVAQAVGVSQGHHTKDFTR
AAPNPAKATVTAMIARELLYGGTSPTAETILKNNISSGHVPHGPLTRPSEQLDYLSRVQG
FQVEYKDFPKNNKNEFVSLINCSSQPPLISHGIGKDVESCHDMAALNILKLLSELDQQST

EMPRTGNGPMSVCGRC*
>ENSG00000115207|ENST00000359541
MDTCGVGYVALGEAGPVGNMTVVDSPGQEVLNQLDVKTSSEMTSAEASVEMSLPTPLPGF
EDSPDQRRLPPEQESLSRLEQPDLSSEMSKVSKPRASKPGRKRGGRTRKGPKRPQQPNPP
SAPLVPGLLDQSNPLSTPMPKKRGRKSKAELLLLKLSKDLDRPESQSPKRPPEDFETPSG
ERPRRRAAQVALLYLQELAEELSTALPAPVSCPEGPKVSSPTKPKKIRQPAACPGGEEVD
GAPRDEDFFLQVEAEDVEESEGPSESSSEPEPVVPRSTPRGSTSGKQKPHCRGMAPNGLP
NHIMAPVWKCLHLTKDFREQKHSYWEFAEWIPLAWKWHLLSELEAAPYLPQEEKSPLFSV
QREGLPEDGTLYRINRFSSITAHPERWDVSFFTGGPLWALDWCPVPEGAGASQYVALFSS
PDMNETHPLSQLHSGPGLLQLWGLGTLQQESCPGNRAHFVYGIACDNGCIWDLKFCPSGA
WELPGTPRKAPLLPRLGLLALACSDGKVLLFSLPHPEALLAQQPPDAVKPAIYKVQCVAT
LQVGSMQATDPSECGQCLSLAWMPTRPHQHLAAGYYNGMVVFWNLPTNSPLQRIRLSDGS
LKLYPFQCFLAHDQAVRTLQWCKANSHFLVSAGSDRKIKFWDLRRPYEPINSIKRFLSTE
LAWLLPYNGVTVAQDNCYASYGLCGIHYIDAGYLGFKAYFTAPRKGTVWSLSGSDWLGTI
AAGDISGELIAAILPDMALNPINVKRPVERRFPIYKADLIPYQDSPEGPDHSSASSGVPN
PPKARTYTETVNHHYLLFQDTDLGSFHDLLRREPMLRMQEGEGHSQLCLDRLQLEAIHKV
RFSPNLDSYGWLVSGGQSGLVRIHFVRGLASPLGHRMQLESRAHFNAMFQPSSPTRRPGF
SPTSHRLLPTP*
>ENSG00000152670|ENST00000354991
MGDEDWEAEINPHMSSYVPIFEKDRYSGENGDNFNRTPASSSEMDDGPSRRDHFMKSGFA
SGRNFGNRDAGECNKRDNTSTMGGFGVGKSFGNRGFSNSRFEDGDSSGFWRESSNDCEDN
PTRNRGFSKRGDNDLDPDECMQRTGGLFGSRRPVLSGTGNGDTSQSRSGSGSERGGYKGL
NEEVITGSGKNSWKSEAEGGESSDTQGPKVTYIPPPPPEDEDSIFAHYQTGINFDKYDTI
LVEVSGHDAPPAILTFEEANLCQTLNNNIAKAGYTKLTPVQKYSIPIILAGRDLMACAQT
GSGKTAAFLLPILAHMMHDGITASRFKELQEPECIIVAPTRELVNQIYLEARKFSFGTCV
RAVVIYGGTQLGHSIRQIVQGCNILCATPGRLMDIIGKEKIGLKQIKYLVLDEADRMLDM
GFGPEMKKLISCPGMPSKEQRQTLMFSATFPEEIQRLAAEFLKSNYLFVAVGQVGGACRD
VQQTVLQVGQFSKREKLVEILRNIGDERTMVFVETKKKADFIATFLCQEKISTTSIHGDR
EQREREQALGDFRFGKCPVLVATSVAARGLDIENVQHVINFDLPSTIDEYVHRIGRTGRC
GNTGRAISFFDLESDNHLAQPLVKVLTDAQQDVPAWLEEIAFSTYIPGFSGSTRGNVFAS
VDTRKGKSTLNTAGFSSSQAPNPVDDESWD*
>ENSG00000182315|ENST00000333843
MGEPAFTSFPSPPVLGKLKRNMMPWALQKKREIHMAKAHRRRAARSALPMRLTSCIFRRP
VTRIRSHPDNQVRRRKGDEHLEKPQQLCAYRRLQALQPCSSQGEGSSPLHLESVLSILAP
GTAGESLDRAGAERVRSPLEPTPGRFPAVAGGPTPGMGCQLPPPLSGQLVTPADIRRQAR
RVKKARERLAKALQADRLARQAEMLTCR*
>ENSG00000120438|ENST00000321394
MEGPLSVFGDRSTGETIRSQNVMAAASIANIVKSSLGPVGLDKMLVDDIGDVTITNDGAT
ILKLLEVEHPAAKVLCELADLQDKEVGDGTTSVVIIAAELLKNADELVKQKIHPTSVISG
YRLACKEAVRYINENLIVNTDELGRDCLINAAKTSMSSKIIGINGDFFANMVVDAVLAIK
YTDIRGQPRYPVNSVNILKAHGRSQMESMLISGYALNCVVGSQGMPKRIVNAKIACLDFS
LQKTKMKLGVQVVITDPEKLDQIRQRESDITKERIQKILATGANVILTTGGIDDMCLKYF
VEAGAMAVRRVLKRDLKRIAKASGATILSTLANLEGEETFEAAMLGQAEEVVQERICDDE
LILIKNTKARTSASIILRGANDFMCDEMERSLHDALCVVKRVLESKSVVPGGGAVEAALS

IYLENYATSMGSREQLAIAEFARSLLVIPNTLAVNAAQDSTDLVAKLRAFHNEAQVNPER
KNLKWIGLDLSNGKPRDNKQAGVFEPTIVKVKSLKFATEAAITILRIDDLIKLHPESKDD
KHGSYEDAVHSGALND*
>ENSG00000199077|ENST00000362207
Sequence unavailable
>ENSG00000185246|ENST00000355765
MQNSHMDEYRNSSNGSTGNSSEVVVEHPTDFSTEIMNVTEMEQSPDDSPNVNASTEETEM
ASAVDLPVTLTETEANFPPEYEKFWKTVENNPQDFTGWVYLLQYVEQENHLMAARKAFDR
FFIHYPYCYGYWKKYADLEKRHDNIKPSDEVYRRGLQAIPLSVDLWIHYINFLKETLDPG
DPETNNTIRGTFEHAVLAAGTDFRSDRLWEMYINWENEQGNLREVTAIYDRILGIPTQLY
SHHFQRFKEHVQNNLPRDLLTGEQFIQLRRELASVNGHSGDDGPPGDDLPSGIEDITDPA
KLITEIENMRHRIIEIHQEMFNYNEHEVSKRWTFEEGIKRPYFHVKPLEKAQLKNWKEYL
EFEIENGTHERVVVLFERCVISCALYEEFWIKYAKYMENHSIEGVRHVFSRACTIHLPKK
PMVHMLWAAFEEQQGNINEARNILKTFEECVLGLAMVRLRRVSLERRHGNLEEAEHLLQD
AIKNAKSNNESSFYAVKLARHLFKIQKNLPKSRKVLLEAIERDKENTKLYLNLLEMEYSG
DLKQNEENILNCFDKAVHGSLPIKMRITFSQRKVEFLEDFGSDVNKLLNAYDEHQTLLKE
QDSLKRKAENGSEEPEEKKAHTEDTTSSSTQMIDGDLQANQAVYNYSAWYQYNYQNPWNY
GQYYPPPPT*
>ENSG00000184863|ENST00000341148
MHGRGRGVAGPMGRGRLMPNKQNLRVVECKPQPCVVSVEGLSSSTTDAQLKSLLMSVGPI
QSLQMLPQQRKAIAKFKEPAHALAFQQKFHRHMIDLSHINVALIVE*
>ENSG00000101574|ENST00000319888
MSVVHQLSAGWLLDHLSFINKINYQLHQHHEPCCRKKEFTTSVHFESLQMDSVSSSGVCA
AFIASDSSTKPENDDGGNYEMFTRKFVFRPELFDVTKPYITPAVHKECQQSNEKEDLMNG
VKKEISISIIGKKRKRCVVFNQGELDAMEYHTKIRELILDGSLQLIQEGLKSGFLYPLFE
KQDKGSKPITLPLDACSLSELCEMAKHLPSLNEMEHQTLQLVEEDTSVTEQDLFLRVVEN
NSSFTKVITLMGQKYLLPPKSSFLLSDISCMQPLLNYRKTFDVIVIDPPWQNKSVKRSNR
YSYLSPLQIQQIPIPKLAAPNCLLVTWVTNRQKHLRFIKEELYPSWSVEVVAEWHWVKIT
NSGEFVFPLDSPHKKPYEGLILGRVQEKTALPLRGFKRLHQARWGIFGVVCSKFTARLD*
>ENSG00000139613|ENST00000347471
MAVRKKDGGPNVKYYEAADTVTQFDNVRLWLGKNYKKYIQAEPPTNKSLSSLVVQLLQFQ
EEVFGKHVSNAPLTKLPIKCFLDFKAGGSLCHILAAAYKFKSDQGWRRYDFQNPSRMDRN
VEMFMTIEKSLVQNNCLSRPNIFLCPEIEPKLLGKLKDIIKRHQGTVTEDKNNASHVVYP
VPGNLEEEEWVRPVMKRDKQVLLHWGYYPDSYDTWIPASEIEASVEDAPTPEKPRKVHAK
WILDTDTFNEWMNEEDYEVNDDKNPVSRRKKISAKTLTDEVNSPDSDRRDKKGGNYKKRK
RSPSPSPTPEAKKKNAKKGPSTPYTKSKRGHREEEQEDLTKDMDEPSPVPNVEEVTLPKT
VNTKKDSESAPVKGGTMTDLDEQEDESMETTGKDEDENSTGNKGEQTKNPDLHEDNVTEQ
THHIIIPSYAAWFDYNSVHAIERRALPEFFNGKNKSKTPEIYLAYRNFMIDTYRLNPQEY
LTSTACRRNLAGDVCAIMRVHAFLEQWGLINYQVDAESRPTPMGPPPTSHFHVLADTPSG
LVPLQPKTPQGRQVDADTKAGRKGKELDDLVPETAKGKPELQTSASQQMLNFPDKGKEKP
TDMQNFGLRTDMYTKKNVPSKSKAAASATREWTEQETLLLLEALEMYKDDWNKVSEHVGS
RTQDECILHFLRLPIEDPYLEDSEASLGPLAYQPIPFSQSGNPVMSTVAFLASVVDPRVA
SAAAKSALEEFSKMKEEVPTALVEAHVRKVEEAAKVTGKADPAFGLESSGIAGTTSDEPE
RIEESGNDEARVEGQATDEKKEPKEPREGGGAIEEEAKEKTSEAPKKDEEKGKEGDSEKE

SEKSDGDPIVDPEKEKEPKEGQEEVLKEVVESEGERKTKVERDIGEGNLSTAAAAALAAA
AVKAKHLAAVEERKIKSLVALLVETQMKKLEIKLRHFEELETIMDREREALEYQRQQLLA
DRQAFHMEQLKYAEMRARQQHFQQMHQQQQQPPPALPPGSQPIPPTGAAGPPAVHGLAVA
PASVVPAPAGSGAPPGSLGPSEQIGQAGSTAGPQQQQPAGAPQPGAVPPGVPPPGPHGPS
PFPNQQTPPSMMPGAVPGSGHPGVADPGTPLPPDPTAPSPGTVTPVPPPQ*
>ENSG00000101489|ENST00000361795
MYIKMATLANGQADNASLSTNGLGSSPGSAGHMNGLSHSPGNPSTIPMKDHDAIKLFIGQ
IPRNLDEKDLKPLFEEFGKIYELTVLKDRFTGMHKGCAFLTYCERESALKAQSALHEQKT
LPGMNRPIQVKPADSESRGGSSCLRQPPSHRKLFVGMLNKQQSEDDVRRLFEAFGNIEEC
TILRGPDGNSKGCAFVKYSSHAEAQAAINALHGSQTMPGASSSLVVKFADTDKERTMRRM
QQMAGQMGMFNPMAIPFGAYGAYAQAMQQQAALMASVAQGGYLNPMAAFAAAQMQQMAAL
NMNGLAAAPMTPTSGGSTPPGITAPAVPSIPSPIGVNGFTGLPPQANGQPAAEAVFANGI
HPYPAQSPTAADPLQQAYAGVQQYAGPAAYPAAYGQISQAFPQPPPMIPQQQREGPEGCN
LFIYHLPQEFGDAELMQMFLPFGFVSFDNPASAQTAIQAMNGFQIGMKRLKVQLKRPKDA
NRPY*
>ENSG00000134419|ENST00000322989
MVRMNVLADALKSINNAEKRGKRQVLIRPCSKVIVRFLTVMMKHGYIGEFEIIDDHRAGK
IVVNLTGRLNKCGVISPRFDVQLKDLEKWQNNLLPSRQFGFIVLTTSAGIMDHEEARRKH
TGGKILGFFF*
>ENSG00000111641|ENST00000322166
MGRKLDPTKEKRGPGRKARKQKGAETELVRFLPAVSDENSKRLSSRARKRAAKRRLGSVE
APKTNKSPEAKPLPGKLPKGISAGAVQTAGKKGPQSLFNAPRGKKRPAPGSDEEEEEEDS
EEDGMVNHGDLWGSEDDADTVDDYGADSNSEDEEEGEALLPIERAARKQKAREAAAGIQW
SEEETEDEEEEKEVTPESGPPKVEEADGGLQINVDEEPFVLPPAGEMEQDAQAPDLQRVH
KRIQDIVGILRDFGAQREEGRSRSEYLNRLKKDLAIYYSYGDFLLGKLMDLFPLSELVEF
LEANEVPRPVTLRTNTLKTRRRDLAQALINRGVNLDPLGKWSKTGLVVYDSSVPIGATPE
YLAGHYMLQGASSMLPVMALAPQEHERILDMCCAPGGKTSYMAQLMKNTGVILANDANAE
RLKSVVGNLHRLGVTNTIISHYDGRQFPKVVGGFDRVLLDAPCSGTGVISKDPAVKTNKD
EKDILRCAHLQKELLLSAIDSVNATSKTGGYLVYCTCSITVEENEWVVDYALKKRNVRLV
PTGLDFGQEGFTRFRERRFHPSLRSTRRFYPHTHNMDGFFIAKFKKFSNSIPQSQTGNSE
TATPTNVDLPQVIPKSENSSQPAKKAKGAAKTKQQLQKQQHPKKASFQKLNGISKGADSE
LSTVPSVTKTQASSSFQDSSQPAGKAEGIREPKVTGKLKQRSPKLQSSKKVAFLRQNAPP
KGTDTQTPAVLSPSKTQATLKPKDHHQPLGRAKGVEKQQLPEQPFEKAAFQKQNDTPKGP
QPPTVSPIRSSRPPPAKRKKSQSRGNSQLLLS*
>ENSG00000173818|ENST00000323854
MALEAAGGPPEETLSLWKREQARLKAHVVDRDTEAWQRDPAFSGLQRVGGVDVSFVKGDS
VRACASLVVLSFPELEVLLVDGNGVLHHRGFGVACHLGVLTDLPCVGVAKKLLQVDGLEN
NALHKEKIRLLQTRGDSFPLLGDSGTVLGMALRSHDRSTRPLYISVGHRMSLEAAVRLTC
CCCRFRIPEPVRQADICSREHIRKSLGLPGPPTPRSPKAQRPVACPKGDSGESSGEGQPP
QDHSPGPRTAPRPGSQEQAGKDWQ*
>ENSG00000199047|ENST00000362177
Sequence unavailable
>ENSG00000134438|ENST00000334889
MHLPGCAPAMADGSFSLAGHLLRSPGGSTSRLHSIEAILGFTKDDGILGTFPAERGARGA

KERDRRLGARPACPKAPEEGSEPSPPPAPAPAPEYEAPRPYCPKEPGEARPSPGLPVGPA
TGEAKLSEEEQPKKKHRRNRTTFTTYQLHELERAFEKSHYPDVYSREELAGKVNLPEVRV
QVWFQNRRAKWRRQEKLEVSSMKLQDSPLLSFSRSPPSATLSPLGAGPGSGGGPAGGALP
LESWLGPPLPGGGATALQSLPGFGPPAQSLPASYTPPPPPPPFLNSPPLGPGLQPLAPPP
PSYPCGPGFGDKFPLDEADPRNSSIAALRLKAKEHIQAIGKPWQAL*
>ENSG00000125482|ENST00000334270
MEGESSRFEIHTPVSDKKKKKCSIHKERPQKHSHEIFRDSSLVNEQSQITRRKKRKKDFQ
HLISSPLKKSRICDETANATSTLKKRKKRRYSALEVDEEAGVTVVLVDKENINNTPKHFR
KDVDVVCVDMSIEQKLPRKPKTDKFQVLAKSHAHKSEALHSKVREKKNKKHQRKAASWES
QRARDTLPQSESHQEESWLSVGPGGEITELPASAHKNKSKKKKKKSSNREYETLAMPEGS
QAGREAGTDMQESQPTVGLDDETPQLLGPTHKKKSKKKKKKKSNHQEFEALAMPEGSQVG
SEVGADMQESRPAVGLHGETAGIPAPAYKNKSKKKKKKSNHQEFEAVAMPESLESAYPEG
SQVGSEVGTVEGSTALKGFKESNSTKKKSKKRKLTSVKRARVSGDDFSVPSKNSESTLFD
SVEGDGAMMEEGVKSRPRQKKTQACLASKHVQEAPRLEPANEEHNVETAEDSEIRYLSAD
SGDADDSDADLGSAVKQLQEFIPNIKDRATSTIKRMYRDDLERFKEFKAQGVAIKFGKFS
VKENKQLEKNVEDFLALTGIESADKLLYTDRYPEEKSVITNLKRRYSFRLHIGRNIARPW
KLIYYRAKKMFDVNNYKGRYSEGDTEKLKMYHSLLGNDWKTIGEMVARSSLSVALKFSQI
SSQRNRGAWSKSETRKLIKAVEEVILKKMSPQELKEVDSKLQENPESCLSIVREKLYKGI
SWVEVEAKVQTRNWMQCKSKWTEILTKRMTNGRRIYYGMNALRAKVSLIERLYEINVEDT
NEIDWEDLASAIGDVPPSYVQTKFSRLKAVYVPFWQKKTFPEIIDYLYETTLPLLKEKLE
KMMEKKGTKIQTPAAPKQVFPFRDIFYYEDDSEGEDIEKESEGQAPCMAHACNSSTLGGQ
GRWII*
>ENSG00000164458|ENST00000366871
MSSPGTESAGKSLQYRVDHLLSAVENELQAGSEKGDPTERELRVGLEESELWLRFKELTN
EMIVTKNGRRMFPVLKVNVSGLDPNAMYSFLLDFVAADNHRWKYVNGEWVPGGKPEPQAP
SCVYIHPDSPNFGAHWMKAPVSFSKVKLTNKLNGGGQIMLNSLHKYEPRIHIVRVGGPQR
MITSHCFPETQFIAVTAYQNEEITALKIKYNPFAKAFLDAKERSDHKEMMEEPGDSQQPG
YSQSYSDNSPACLSMLQSHDNWSSLGMPAHPSMLPVSHNASPPTSSSQYPSLWSVSNGAV
TPGSQAAAVSNGLGAQFFRGSPAHYTPLTHPVSAPSSSGSPLYEGAAAATDIVDSQYDAA
AQGRLIASWTPVSPPSM*
>ENSG00000127914|ENST00000359028
MEDEERQKKLEAGKAKVGEPEGYFLFSLAQFRQRKAQSDGQSPSKKQKKKRKTSSSKHDV
SAHHDLNIDQSQCNEMYINSSQRVESTVIPESTIMRTLHSGEITSHEQGFSVELESEIST
TADDCSSEVNGCSFVMRTGKPTNLLREEEFGVDDSYSEQGAQDSPTHLEMMESELAGKQH
EIEELNRELEEMRVTYGTEGLQQLQEFEAAIKQRDGIITQLTANLQQARREKDETMREFL
ELTEQSQKLQIQFQQLQASETLRNSTHSSTAADLLQAKQQILTHQQQLEEQDHLLEDYQK
KKEDFTMQISFLQEKIKVYEMEQDKKVENSNKEEIQEKETIIEELNTKIIEEEKKTLELK
DKLTTADKLLGELQEQIVQKNQEIKNMKLELTNSKQKERQSSEEIKQLMGTVEELQKRNH
KDSQFETDIVQRMEQETQRKLEQLRAELDEMYGQQIVQMKQELIRQHMAQMEEMKTRHKG
EMENALRSYSNITVNEDQIKLMNVAINELNIKLQDTNSQKEKLKEELGLILEEKCALQRQ
LEDLVEELSFSREQIQRARQTIAEQESKLNEAHKSLSTVEDLKAEIVSASESRKELELKH
EAEVTNYKIKLEMLEKEKNAVLDRMAESQEAELERLRTQLLFSHEEELSKLKEDLEIEHR
INIEKLKDNLGIHYKQQIDGLQNEMSQKIETMQFEKDNLITKQNQLILEISKLKDLQQSL
VNSKSEEMTLQINELQKEIEILRQEEKEKGTLEQEVQELQLKTELLEKQMKEKENDLQEK

FAQLEAENSILKDEKKTLEDMLKIHTPVSQEERLIFLDSIKSKSKDSVWEKEIEILIEEN
EDLKQQCIQLNEEIEKQRNTFSFAEKNFEVNYQELQEEYACLLKVKDDLEDSKNKQELEY
KSKLKALNEELHLQRINPTTVKMKSSVFDEDKTFVAETLEMGEVVEKDTTELMEKLEVTK
REKLELSQRLSDLSEQLKQKHGEISFLNEEVKSLKQEKEQVSLRCRELEIIINHNRAENV
QSCDTQVSSLLDGVVTMTSRGAEGSVSKVNKSFGEESKIMVEDKVSFENMTVGEESKQEQ
LILDHLPSVTKESSLRATQPSENDKLQKELNVLKSEQNDLRLQMEAQRICLSLVYSTHVD
QVREYMENEKDKALCSLKEELIFAQEEKIKELQKIHQLELQTMKTQETGDEGKPLHLLIG
KLQKAVSEECSYFLQTLCSVLGEYYTPALKCEVNAEDKENSGDYISENEDPELQDYRYEV
QDFQENMHTLLNKVTEEYNKLLVLQTRLSKIWGQQTDGMKLEFGEENLPKEETEFLSIHS
QMTNLEDIDVNHKSKLSSLQDLEKTKLEEQVQELESLISSLQQQLKETEQNYEAEIHCLQ
KRLQAVSESTVPPSLPVDSVVITESDAQRTMYPGSCVKKNIDGTIEFSGEFGVKEETNIV
KLLEKQYQEQLEEEVAKVIVSMSIAFAQQTELSRISGGKENTASSKQAHAVCQQEQHYFN
EMKLSQDQIGFQTFETVDVKFKEEFKPLSKELGEHGKEILLSNSDPHDIPESKDCVLTIS
EEMFSKDKTFIVRQSIHDEISVSSMDASRQLMLNEEQLEDMRQELVRQYQEHQQATELLR
QAHMRQMERQREDQEQLQEEIKRLNRQLAQRSSIDNENLVSERERVLLEELEALKQLSLA
GREKLCCELRNSSTQTQNGNENQGEVEEQTFKEKELDRKPEDVPPEILSNERYALQKANN
RLLKILLEVVKTTAAVEETIGRHVLGILDRSSKSQSSASLIWRSEAEASVKSCVHEEHTR
VTDESIPSYSGSDMPRNDINMWSKVTEEGTELSQRLVRSGFAGTEIDPENEELMLNISSR
LQAAVEKLLEAISETSSQLEHAKVTQTELMRESFRQKQEATESLKCQEELRERLHEESRA
REQLAVELSKAEGVIDGYADEKTLFERQIQEKTDIIDRLEQELLCASNRLQELEAEQQQI
QEERELLSRQKEAMKAEAGPVEQQLLQETEKLMKEKLEVQCQAEKVRDDLQKQVKALEID
VEEQVSRFIELEQEKNTELMDLRQQNQALEKQLEKMRKFLDEQAIDREHERDVFQQEIQK
LEQQLKVVPRFQPISEHQTREVEQLANHLKEKTDKCSELLLSKEQLQRDIQERNEEIEKL
EFRVRELEQALLVSADTFQKVEDRKHFGAVEAKPELSLEVQLQAERDAIDRKEKEITNLE
EQLEQFREELENKNEEVQQLHMQLEIQKKESTTRLQELEQENKLFKDDMEKLGLAIKESD
AMSTQDQHVLFGKFAQIIQEKEVEIDQLNEQVTKLQQQLKITTDNKVIEEKNELIRDLET
QIECLMSDQECVKRNREEEIEQLNEVIEKLQQELANIGQKTSMNAHSLSEEADSLKHQLD
VVIAEKLALEQQVETANEEMTFMKNVLKETNFKMNQLTQELFSLKRERESVEKIQSIPEN
SVNVAIDHLSKDKPELEVVLTEDALKSLENQTYFKSFEENGKGSIINLETRLLQLESTVS
AKDLELTQCYKQIKDMQEQGQFETEMLQKKIVNLQKIVEEKVAAALVSQIQLEAVQEYAK
FCQDNQTISSEPERTNIQNLNQLREDELGSDISALTLRISELESQVVEMHTSLILEKEQV
EIAEKNVLEKEKKLLELQKLLEGNEKKQREKEKKRSPQDVEVLKTTTELFHSNEESGFFN
ELEALRAESVATKAELASYKEKAEKLQEELLVKETNMTSLQKDLSQVRDHLAEAKEKLSI
LEKEDETEVQESKKACMFEPLPIKLSKSIASQTDGTLKISSSNQTPQILVKNAGIQINLQ
SECSSEEVTEIISQFTEKIEKMQELHAAEILDMESRHISETETLKREHYVAVQLLKEECG
TLKAVIQCLRSKEVFGFYNMCFSTLCDSGSDWGQGIYLTHSQGFDIASEGRGEESESATD
SFPKKIKGLLRAVHNEGMQVLSLTESPYSDGEDHSIQQVSEPWLEERKAYINTISSLKDL
ITKMQLQREAEVYDSSQSHESFSDWRGELLLALQQVFLEERSVLLAAFRTELTALGTTDA
VGLLNCLEQRIQEQGVEYQAAMECLQKADRRSLLSEIQALHAQMNGRKITLKREQESEKP
SQELLEYNIQQKQSQMLEMQVELSSMKDRATELQEQLSSEKMVVAELKSELAQTKLELET
TLKAQHKHLKELEAFRLEVKDKTDEVHLLNDTLASEQKKSRELQWALEKEKAKLGRSEER
DKEELEDLKFSLESQKQRNLQLNLLLEQQKQLLNESQQKIESQRMLYDAQLSEEQGRNLE
LQVLLESEKVRIREMSSTLDRERELHAQLQSSDGTGQSRPPLPSEDLLKELQKQLEEKHS
RIVELLNETEKYKLDSLQTRQQMEKDRQVHRKTLQTEQEANTEGQKKMHELQSKVEDLQR

QLEEKRQQVYKLDLEGQRLQGIMQEFQKQELEREEKRESRRILYQNLNEPTTWSLTSDRT
RNWVLQQKIEGETKESNYAKLIEMNGGGTGCNHELEMIRQKLQCVASKLQVLPQKASERL
QFETADDEDFIWVQENIDEIILQLQKLTGQQGEEPSLVSPSTSCGSLTERLLRQNAELTG
HISQLTEEKNDLRNMVMKLEEQIRWYRQTGAGRDNSSRFSLNGGANIEAIIASEKEVWNR
EKLTLQKSLKRAEAEVYKLKAELRNDSLLQTLSPDSEHVTLKRIYGKYLRAESFRKALIY
QKKYLLLLLGGFQECEDATLALLARMGGQPAFTDLEVITNRPKGFTRFRSAVRVSIAISR
MKFLVRRWHRVTGSVSININRDGFGLNQGAEKTDSFYHSSGGLELYGEPRHTTYRSRSDL
DYIRSPLPFQNRYPGTPADFNPGSLACSQLQNYDPDRALTDYITRLEALQRRLGTIQSGS
TTQFHAGMRR*
>ENSG00000189144|ENST00000339503
MESCSVAQAGVQWPDLSSLQPPPPRFKQFSCHSLQVAGITDLDLQCEIISYIEVPTYETD
ISSTQLQSIYKREKLYECKKCQKKFSSGYQLILHHRFHVIERPYECKECGKNFRSGYQLT
LHQRFHTGEKPYECTECGKNFRSGYQLTVHQRFHTGEKTYECRQCGKAFIYASHIVQHER
IHTGGKPYECQECGRAFSQGGHLRIHQRVHTGEKPYKCKECGKTFSRRSNLVEHGQFHTD
EKPYICEKCGKAFRRGHQLTVHQRVHTGKKPYECKECGKGYTTASYFLLHQRIHKGGKPY
ECKECKKTFTLYRNLTRHQNIHTGEKLFECKQCGKTYTTGSKLFQHQKTHTGEKPYECKE
CGKAFSLYGYLKQHQKIHTGMKHFECKECKKTFTLYRNLTRHQNIHTGKKLFECQECGKA
YSTGSNLIQHRKTHTGEKPYKCKECGKTFSLHGYLNQHQKIHTGMKPYECKVCRKTFTFY
RNLTLHQSIHTDEKPFECKECGKTFRRSSHLTAHQSIHADKKPYECKECGKAFKMYGYLT
QHQKIHTGGKPYECKECGKAFSRASNLVQHERIHTGEKPYVCKQCGKTFRYGSALKAHQR
IHRSIKV*
>ENSG00000073584|ENST00000348513
MSKRPSYAPPPTPAPATQMPSTPGFVGYNPYSHLAYNNYRLGGNPGTNSRVTASSGITIP
KPPKPPDKPLMPYMRYSRKVWDQVKASNPDLKLWEIGKIIGGMWRDLTDEEKQEYLNEYE
AEKIEYNESMKAYHNSPAYLAYINAKSRAEAALEEESRQRQSRMEKGEPYMSIQPAEDPD
DYDDGFSMKHTATARFQRNHRLISEILSESVVPDVRSVVTTARMQVLKRQVQSLMVHQRK
LEAELLQIEERHQEKKRKFLESTDSFNNELKRLCGLKVEVDMEKIAAEIAQAEEQARKRQ
EEREKEAAEQAERSQSSIVPEEEQAANKGEEKKDDENIPMETEETHLEETTESQQNGEEG
TSTPEDKESGQEGVDSMAEEGTSDSNTGSESNSATVEEPPTDPIPEDEKKE*
>ENSG00000180479|ENST00000358744
MPHLLVTFRDVAIDFSQEEWECLDPAQRDLYRDVMLENYSNLISLDLESSCVTKKLSPEK
EIYEMESLQWENMGKRINHHLQYNGLGDNMECKGNLEGQEASQEGLYMCVKITCEEKATE
SHSTSSTFHRIIPTKEKLYKCKECRQGFSYLSCLIQHEENHNIEKCSEVKKHRNTFSKKP
SYIQHQRIQTGEKPYECMECGKAFGRTSDLIQHQKIHTNEKPYQCNACGKAFIRGSQLTE
HQRVHTGEKPYECKKCGKAFSYCSQYTLHQRIHSGEKPYECKDCGKAFILGSQLTYHQRI
HSGEKPYECKECGKAFILGSHLTYHQRVHTGEKPYICKECGKAFLCASQLNEHQRIHTGE
KPYECKECGKTFFRGSQLTYHLRVHSGERPYKCKECGKAFISNSNLIQHQRIHTGEKPYK
CKECGKAFICGKQLSEHQRIHTGEKPFECKECGKAFIRVAYLTQHEKIHGEKHYECKECG
KTFVRATQLTYHQRIHTGEKPYKCKECDKAFIYGSQLSEHQRIHRGEKPYECKQCGKAFI
RGSHLTEHLRTHTGEKPYECKECGRAFSRGSELTLHQRIHTGEKPYTCVQCGKDFRCPSQ
LTQHTRLHN*
>ENSG00000072501|ENST00000322213
MGFLKLIEIENFKSYKGRQIIGPFQRFTAIIGPNGSGKSNLMDAISFVLGEKTSNLRVKT
LRDLIHGAPVGKPAANRAFVSMVYSEEGAEDRTFARVIVGGSSEYKINNKVVQLHEYSEE

LEKLGILIKARNFLVFQGAVESIAMKNPKERTALFEEISRSGELAQEYDKRKKEMVKAEE
DTQFNYHRKKNIAAERKEAKQEKEEADRYQRLKDEVVRAQVQLQLFKLYHNEVEIEKLNK
ELASKNKEIEKDKKRMDKVEDELKEKKKELGKMMREQQQIEKEIKEKDSELNQKRPQYIK
AKENTSHKIKKLEAAKKSLQNAQKHYKKRKGDMDELEKEMLSVEKARQEFEERMEEESQS
QGRDLTLEENQVKKYHRLKEEASKRAATLAQELEKFNRDQKADQDRLDLEERKKVETEAK
IKQKLREIEENQKRIEKLEEYITTSKQSLEEQKKLEGELTEEVEMAKRRIDEINKELNQV
MEQLGDARIDRQESSRQQRKAEIMESIKRLYPGSVYGRLIDLCQPTQKKYQIAVTKVLGK
NMDAIIVDSEKTGRDCIQYIKEQRGEPETFLPLDYLEVKPTDEKLRELKGAKLVIDVIRY
EPPHIKKALQYACGNALVCDNVEDARRIAFGGHQRHKTVALDGTLFQKSGVISGGASDLK
AKARRWDEKAVDKLKEKKERLTEELKEQMKAKRKEAELRQVQSQAHGLQMRLKYSQSDLE
QTKTRHLALNLQEKSKLESELANFGPRINDIKRIIQSREREMKDLKEKMNQVEDEVFEEF
CREIGVRNIREFEEEKVKRQNEIAKKRLEFENQKTRLGIQLDFEKNQLKEDQDKVHMWEQ
TVKKDENEIEKLKKEEQRHMKIIDETMAQLQDLKNQHLAKKSEVNDKNHEMEEIRKKLGG
ANKEMTHLQKEVTAIETKLEQKRSDRHNLLQACKMQDIKLPLSKGTMDDISQEEGSSQGE
DSVSGSQRISSIYAREALIEIDYGDLCEDLKDAQAEEEIKQEMNTLQQKLNEQQSVLQRI
AAPNMKAMEKLESVRDKFQETSDEFEAARKRAKKAKQAFEQIKKERFDRFNACFESVATN
IDEIYKALSRNSSAQAFLGPENPEEPYLDGINYNCVAPGKRFRPMDNLSGGEKTVAALAL
LFAIHSYKPAPFFVLDEIDAALDNTNIGKVANYIKEQSTCNFQAIVISLKEEFYTKAESL
IGVYPEQGDCVISKVLTFDLTKYPDANPNPNEQ*
>ENSG00000122566|ENST00000354667
MEKTLETVPLERKKREKEQFRKLFIGGLSFETTEESLRNYYEQWGKLTDCVVMRDPASKR
SRGFGFVTFSSMAEVDAAMAARPHSIDGRVVEPKRAVAREESGKPGAHVTVKKLFVGGIK
EDTEEHHLRDYFEEYGKIDTIEIITDRQSGKKRGFGFVTFDDHDPVDKIVLQKYHTINGH
NAEVRKALSRQEMQEVQSSRSGRGGNFGFGDSRGGGGNFGPGPGSNFRGGSDGYGSGRGF
GDGYNGYGGGPGGGNFGGSPGYGGGRGGYGGGGPGYGNQGGGYGGGYDNYGGGNYGSGNY
NDFGNYNQQPSNYGPMKSGNFGGSRNMGGPYGGGNYGPGGSGGSGGYGGRSRY*
>ENSG00000184481|ENST00000341558
MDPGNENSATEAAAIIDLDPDFEPQSRPRSCTWPLPRPEIANQPSEPPEVEPDLGEKAIE
SAPEKRLTLAQIYEWMVRTVPYFKDKGDSNSSAGWKNSIRHNLSLHSKFIKVHNEATGKS
SWWMLNPEGGKSGKAPRRRAASMDSSSKLLRGRSKAPKKKPSVLPAPPEGATPTSPVGHF
AKWSGSPCSRNREEADMWTTFRPRSSSNASSVSTRLSPLRPESEVLAEEIPASVSSYAGG
VPPTLNEGLELLDGLNLTSSHSLLSRSGLSGFSLQHPGVTGPLHTYSSSLFSPAEGPLSA
GEGCFSSSQALEALLTSDTPPPPADVLMTQVDPILSQAPTLLLLGGLPSSSKLATGVGLC
PKPLEAPGPSSLVPTLSMIAPPPVMASAPIPKALGTPVLTPPTEAASQDRMPQDLDLDMY
MENLECDMDNIISDLMDEGEGLDFNFEPDP*
>ENSG00000123352|ENST00000321898
MSRKQNQKDSSGFIFDLQSNTVLAQGGAFENMKEKINAVRAIVPNKSNNEIILVLQHFDN
CVDKTVQAFMEGSASEVLKEWTVTGKKKNKKKKNKPKPAAEPSNGIPDSSKSVSIQEEQS
APSSEKGGMNGYHVNGAINDTESVDSLSEGLETLSIDARELEDPESAMLDTLDRTGSMLQ
NGVSDFETKSLTMHSIHNSQQPRNAAKSLSRPTTETQFSNMGMEDVPLATSKKLSSNIEK
SVKDLQRCTVSLARYRVVVKEEMDASIKKMKQAFAELESCLMDREVALLAEMDKVKAEAM
EILLSRQKKAELLKKMTHVAVQMSEQQLVELRADIKHFVSERKYDEDLGRVARFTCDVET
LKKSIDSFGQVSHPKNSYSTRSRCSSVTSVSLSSPSDASAASSSTCASPPSLTSANKKNF
APGETPAAIANSSGQPYQPLREVLPGNRRGGQGYRPQGQKSNDPMNQGRHDSMGRYRNSS

WYSSGSRYQSAPSQAPGNTIERGQTHSAGTNGTGVSMEPSPPTPSFKKGLPQRKPRTSQT
EAVNS*
>ENSG00000106263|ENST00000360876
MQDAENVAVPEAAEERAEPGQQQPAAEPPPAEGLLRPAGPGAPEAAGTEASSEEVGIAEA
GPESEVRTEPAAEAEAASGPSESPSPPAAEELPGSHAEPPVPAQGEAPGEQARDERSDSR
AQAVSEDAGGNEGRAAEAEPRALENGDADEPSFSDPEDFVDDVSEEELLGDVLKDRPQEA
DGIDSVIVVDNVPQVGPDRLEKLKNVIHKIFSKFGKITNDFYPEEDGKTKGYIFLEYASP
AHAVDAVKNADGYKLDKQHTFRVNLFTDFDKYMTISDEWDIPEKQPFKDLGNLRYWLEEA
ECRDQYSVIFESGDRTSIFWNDVKDPVSIEERARWTETYVRWSPKGTYLATFHQRGIALW
GGEKFKQIQRFSHQGVQLIDFSPCERYLVTFSPLMDTQDDPQAIIIWDILTGHKKRGFHC
ESSAHWPIFKWSHDGKFFARMTLDTLSIYETPSMGLLDKKSLKISGIKDFSWSPGGNIIA
FWVPEDKDIPARVTLMQLPTRQEIRVRNLFNVVDCKLHWQKNGDYLCVKVDRTPKGTQGV
VTNFEIFRMREKQVPVDVVEMKETIIAFAWEPNGSKFAVLHGEAPRISVSFYHVKNNGKI
ELIKMFDKQQANTIFWSPQGQFVVLAGLRSMNGALAFVDTSDCTVMNIAEHYMASDVEWD
PTGRYVVTSVSWWSHKVDNAYWLWTFQGRLLQKNNKDRFCQLLWRPRPPTLLSQEQIKQI
KKDLKKYSKIFEQKDRLSQSKASKELVERRRTMMEDFRKYRKMAQELYMEQKNERLELRG
GVDTDELDSNVDDWEEETIEFFVTEEIIPLGNQE*
>ENSG00000176788|ENST00000322611
MGGKLSKKKKGYNVNDEKAKEKDKKAEGAATEEEGTPKESEPQAAAEPAEAKEGKEKPDQ
DAEGKAEEKEGEKDAAAAKEEAPKAEPEKTEGAAEAKAEPPKAPEQEQAAPGPAAGGEAP
KAAEAAAAPAESAAPAAGEEPSKEEGEPKKTEAPAAPAAQETKSDGAPASDSKPGSSEAA
PSSKETPAATEAPSSTPKAQGPAASAEEPKPVEAPAANSDQTVTVKE*
>ENSG00000078269|ENST00000355585
MALSKGLRLLGRLGAEGDCSVLLEARGRDDCLLFEAGTVATLAPEEKEVIKGQYGKLTDA
YGCLGELRLKSGGTSLSFLVLVTGCTSVGRIPDAEIYKITATDFYPLQEEAKEEERLIAL
KKILSSGVFYFSWPNDGSRFDLTVRTQKQGDDSSEWGNSFFWNQLLHVPLRQHQVSCCDW
LLKIICGVVTIRTVYASHKQAKACLVSRVSCERTGTRFHTRGVNDDGHVSNFVETEQMIY
MDDGVSSFVQIRGSVPLFWEQPGLQVGSHHLRLHRGLEANAPAFDRHMVLLKEQYGQQVV
VNLLGSRGGEEVLNRAFKKLLWASCHAGDTPMINFDFHQFAKGGKLEKLETLLRPQLKLH
WEDFDVFTKGENVSPRFQKGTLRMNCLDCLDRTNTVQSFIALEVLHLQLKTLGLSSKPIV
DRFVESFKAMWSLNGHSLSKVFTGSRALEGKAKVGKLKDGARSMSRTIQSNFFDGVKQEA
IKLLLVGDVYGEEVADKGGMLLDSTALLVTPRILKAMTERQSEFTNFKRIRIAMGTWNVN
GGKQFRSNVLRTAELTDWLLDSPQLSGATDSQDDSSPADIFAVGFEEMVELSAGNIVNAS
TTNKKMWGEQLQKAISRSHRYILLTSAQLVGVCLYIFVRPYHVPFIRDVAIDTVKTGMGG
KAGNKGAVGIRFQFHSTSFCFICSHLTAGQSQVKERNEDYKEITQKLCFPMGRNVFSHDY
VFWCGDFNYRIDLTYEEVFYFVKRQDWKKLLEFDQLQLQKSSGKIFKDFHEGAINFGPTY
KYDVGSAAYDTSDKCRTPAWTDRVLWWRKKHPFDKTAGELNLLDSDLDVDTKVRHTWSPG
ALQYYGRAELQASDHRPVLAIVEVEVQEVDVGARERVFQEVSSFQGPLDATVVVNLQSPT
LEEKNEFPEDLRTELMQTLGSYGTIVLVRINQGQMLVTFADSHSALSVLDVDGMKVKGRA
VKIRPKTKDWLKGLREEIIRKRDSMAPVSPTANSCLLEENFDFTSLDYESEGDILEDDED
YLVDEFNQPGVSDSELGGDDLSDVPGPTALAPPSKSPALTKKKQHPTYKDDADLVELKRE
LEAVGEFRHRSPSRSLSVPNRPRPPQPPQRPPPPTGLMVKKSASDASISSGTHGQYSILQ
TARLLPGAPQQPPKARTGISKPYNVKQIKTTNAQEAEAAIRCLLEARGGASEEALSAVAP
RDLEASSEPEPTPGAAKPETPQAPPLLPRRPPPRVPAIKKPTLRRTGKPLSPEEQFEQQT

VHFTIGPPETSVEAPPVVTAPRVPPVPKPRTFQPGKAAERPSHRKPASDEAPPGAGASVP
PPLEAPPLVPKVPPRRKKSAPAAFHLQVLQSNSQLLQGLTYNSSDSPSGHPPAAGTVFPQ
GDFLSTSSATSPDSDGTKAMKPEAAPLLGDYQDPFWNLLHHPKLLNNTWLSKSSDPLDSG
TRSPKRDPIDPVSAGASAAKAELPPDHEHKTLGHWVTISDQEKRTALQVFDPLAKT*
>ENSG00000187778|ENST00000343810
MDKDSQGLLDSSLMASGTASRSEDEESLAGQKRASSQALGTIPKRRSSSRFIKRKKFDDE
LVESSLAKSSTRAKGASGVEPGRCSGSEPSSSEKKKVSKAPSTPVPPSPAPAPGLTKRVK
KSKQPLQVTKDLGRWKPADDLLLINAVLQTNDLTSVHLGVKFSCRFTLREVQERWYALLY
DPVISKLACQAMRQLHPEAIAAIQSKALFSKAEEQLLSKVGSTSQPTLETFQDLLHRHPD
AFYLARTAKALQAHWQLMKQYYLLEDQTVQPLPKGDQVLNFSDAEDLIDDSKLKDMRDEV
LEHELMVADRRQKREIRQLEQELHKWQVLVDSITGMSSPDFDNQTLAVLRGRMVRYLMRS
REITLGRATKDNQIDVDLSLEGPAWKISRKQGVIKLKNNGDFFIANEGRRPIYIDGRPVL
CGSKWRLSNNSVVEIASLRFVFLINQDLIALIRAEAAKITPQ*
>ENSG00000007392|ENST00000337351
MSAQAQMRALLDQLMGTARDGDETRQRVKFTDDRVCKSHLLDCCPHDILAGTRMDLGECT
KIHDLALRADYEIASKERDLFFELDAMDHLESFIAECDRRTELAKKRLAETQEEISAEVS
AKAEKVHELNEEIGKLLAKAEQLGAEGNVDESQKILMEVEKVRAKKKEAEEEYRNSMPAS
SFQQQKLRVCEVCSAYLGLHDNDRRLADHFGGKLHLGFIQIREKLDQLRKTVAEKQEKRN
QDRLRRREEREREERLSRRSGSRTRDRRRSRSRDRRRRRSRSTSRERRKLSRSRSRDRHR
RHRSRSRSHSRGHRRASRDRSAKYK*
>ENSG00000156709|ENST00000319908
MFRCGGLAAGALKQKLVPLVRTVCVRSPRQRNRLPVVQSHHLGSPSRSLASTGASGKDGS
NLVYFLIVGATVTGAGVYYAYKTMKEDEKRYNERISGLGLTPEQKQKKAALSASEGEEVP
QDKAPSHVPFLLIGGGTAAFAAARSIRARDPGARVLIVSEDPELPYMRPPLSKELWFSDD
PNVTKTLRFKQWNGKERSIYFQPPSFYVSAQDLPHIENGGVAVLTGKKVVQLDVRDNMVK
LNDGSQITYEKCLIATGGTPRSLSAIDRAGAEVKSRTTLFRKIGDFRSLEKISREVKSIT
IIGGGFLGSELACALGRKARALGTEVIQLFPEKGNMGKILPEYLSNWTMEKVRREGVKVM
PNAIVQSVGVSSGKLLIKLKDGRKVETDHIVAAVGLEPNVELAKTGGLEIDSDFGGFRVN
AELQARSNIWVAGDAACFYDIKLGRRRVEHHDHAVVSGRLAGENMTGAAKPYWHQSMFWS
DLGPDVGYEAIGLVDSSLPTVGVFAKATAQDNPKSATEQSGTGIRSESETESEASEITIP
PSTPAVPQAPVQGEDYGKGVIFYLRDKVVVGIVLWNIFNRMPIARKIIKDGEQHEDLNEV
AKLFNIHED*
>ENSG00000164631|ENST00000342651
MNKSLGPVSFKDVAVDFTQEEWQQLDPEQKITYRDVMLENYSNLVSVGYHIIKPDVISKL
EQGEEPWIVEGEFLLQSYPDEVWQTDDLIERIQEEENKPSRQTVFIETLIEEREYISSDG
SYARMKADECSGCGKSLLHIKLEKTHPGDQAYEFNQNGEPYTLNEESLYQKIRILEKPFE
YIECQKAFQKDTVFVNHMEEKPYKWNGSEIAFLQMSDLTVHQTSHMEMKPYECSECGKSF
CKKSKFIIHQRTHTGEKPYECNQCGKSFCQKGTLTVHQRTHTGEKPYECNECGKNFYQKL
HLIQHQRTHSGEKPYECSYCGKSFCQKTHLTQHQRTHSGERPYVCHDCGKTFSQKSALND
HQKIHTGVKLYKCSECGKCFCRKSTLTTHLRTHTGEKPYECNECGKFFSRLSYLTVHYRT
HSGEKPYECNECGKTFYLNSALMRHQRVHTGEKPYECNECGKLFSQLSYLTIHHRTHSGV
KPYECSECGKTFYQNSALCRHRRIHKGEKPYECYICGKFFSQMSYLTIHHRIHSGEKPYE
CSECGKTFCQNSALNRHQRTHTGEKAYECYECGKCFSQMSYLTIHHRIHSGEKPFECNEC
GKAFSRMSYLTVHYRTHSGEKPYECTECGKKFYHKSAFNSHQRIHRRGNMNVIDVGRLL*

>ENSG00000175727|ENST00000319080
MAADVFMCSPRRPRSRGRQVLLKPQVSEDDDDSDTDEPSPPPASGAATPARAHASAAPPP
PRAGPGREEPPRRQQIIHSGHFMVSSPHREHPPKKGYDFDTVNKQTCQTYSFGKTSSCHL
SIDASLTKLFECMTLAYSGKLVSPKWKNFKGLKLQWRDKIRLNNAIWRAWYMQYLEKRKN
PVCHFVTPLDGSVDVDEHRRPEAITTEGKYWKSRIEIVIREYHKWRTYFKKRLQQHKDED
LSSLVQDDDMLYWHKHGDGWKTPVPMEEDPLLDTDMLMSEFSDTLFSTLSSHQPVAWPNP
REIAHLGNADMIQPGLIPLQPNLDFMDTFEPFQDLFSSSRSIFGSMLPASASAPVPDPNN
PPAQESILPTTALPTVSLPDSLIAPPTAPSLAHMDEQGCEHTSRTEDPFIQPTDFGPSEP
PLSVPQPFLPVFTMPLLSPSPAPPPISPVLPLVPPPATALNPPAPPTFHQPQKFAGVNKA
PSVITHTASATLTHDAPATTFSQSQGLVITTHHPAPSAAPCGLALSPVTRPPQPRLTFVH
PKPVSLTGGRPKQPHKIVPAPKPEPVSLVLKNARIAPAAFSGQPQAVIMTSGPLKREGML
ASTVSQSNVVIAPAAIARAPGVPEFHSSILVTDLGHGTSSPPAPVSRLFPSTAQDPLGKG
EQVPLHGGSPQVTVTGPSRDCPNSGQASPCASEQSPSPQSPQNNCSGKSDPKNVAALKNR
QMKHISAEQKRRFNIKMCFDMLNSLISNNSKLTSHAITLQKTVEYITKLQQERGQMQEEA
RRLREEIEELNATIISCQQLLPATGVPVTRRQFDHMKDMFDEYVKTRTLQNWKFWIFSII
IKPLFESFKGMVSTSSLEELHRTALSWLDQHCSLPILRPMVLSTLRQLSTSTSILTDPAQ
LPEQASKAVTRIGKRLGES*
>ENSG00000196391|ENST00000354377
MWLGTSGKSGLPGHCLENPLQECHPAQLEEWALKGISRPSVISQPEQKEEPWVLPLQNFE
ARKIPRESHTDCEHQVAKLNQDNSETAEQCGTSSERTNKDLSHTLSWGGNWEQGLELEGQ
HGTLPGEGQLESFSQERDLNKLLDGYVGEKPMCAECGKSFNQSSYLIRHLRTHTGERPYT
CIECGKGFKQSSDLVTHRRTHTGEKPYQCKGCEKKFSDSSTLIKHQRTHTGERPYECPEC
GKTFGRKPHLIMHQRTHTGEKPYACLECHKSFSRSSNFITHQRTHTGVKPYRCNDCGESF
SQSSDLIKHQRTHTGERPFKCPECGKGFRDSSHFVAHMSTHSGERPFSCPDCHKSFSQSS
HLVTHQRTHTGERPFKCENCGKGFADSSALIKHQRIHTGERPYKCGECGKSFNQSSHFIT
HQRIHLGDRPYRCPECGKTFNQRSHFLTHQRTHTGEKPFHCSKCNKSFRQKAHLLCHQNT
HLI*
>ENSG00000146757|ENST00000328747
MGPLTFRDVKIEFSLEEWQCLDTAQRNLYRDVMLENYRNLVFLGIAVSKPDLITWLEQGK
EPWNLKRHEMVDKTPVMCSHFAQDVWPEHSIKDSFQKVILRTYGKYGHENLQLRKDHKSV
DACKVYKGGYNGLNQCLTTTDSKIFQCDKYVKVFHKFPNVNRNKIRHTGKKPFKCKNRGK
SFCMLSQLTQHKKIHTREYSYKCEECGKAFNWSSTLTKHKIIHTGEKPYKCEECGKAFNR
SSNLTKHKIIHTGEKPYKCEECGKAFNRSSTLTKHKRIHTEEKPYKCEECGKAFNQFSIL
NKHKRIHMEDKPYKCEECGKAFRVFSILKKHKIIHTGEKPYKCEECGKAFNQFSNLTKHK
IIHTGEKPYKCDECGKAFNQSSTLTKHKRIHTGEKPYKCEECGKAFKQSSTLTEHKIIHT
GEKPYKCEKCGKAFSWSSAFTKHKRNHMEDKPYKCEECGKAFSVFSTLTKHKIIHTREKP
YKCEECGKAFNQSSIFTKHKIIHTEGKSYKCEKCGNAFNQSSNLTARKIIYTGEKPYKYE
ECDKAFNKFSTLITHQIIYTGEKPCKHECGRAFNKSSNYTKEKLQT*
>ENSG00000164924|ENST00000353245
MDKNELVQKAKLAEQAERYDDMAACMKSVTEQGAELSNEERNLLSVAYKNVVGARRSSWR
VVSSIEQKTEGAEKKQQMAREYREKIETELRDICNDVLSLLEKFLIPNASQAESKVFYLK
MKGDYYRYLAEVAAGDDKKGIVDQSQQAYQEAFEISKKEMQPTHPIRLGLALNFSVFYYE
ILNSPEKACSLAKTAFDEAIAELDTLSEESYKDSTLIMQLLRDNLTLWTSDTQGDEAEAG
EGGEN*

>ENSG00000198839|ENST00000361822
MAASKTQGAVARMQEDRDGSCSTVGGVGYGDSKDCILEPLSLPESPGGTTTLEGSPSVPC
IFCEEHFPVAEQDKLLKHMIIEHKIVIADVKLVADFQRYILYWRKRFTEQPITDFCSVIR
INSTAPFEEQENYFLLCDVLPEDRILREELQKQRLREILEQQQQERNDTNFHGVCMFCNE
EFLGNRSVILNHMAREHAFNIGLPDNIVNCNEFLCTLQKKLDNLQCLYCEKTFRDKNTLK
DHMRKKQHRKINPKNREYDRFYVINYLELGKSWEEVQLEDDRELLDHQEDDWSDWEEHPA
SAVCLFCEKQAETIEKLYVHMEDAHEFDLLKIKSELGLNFYQQVKLVNFIRRQVHQCRCY
GCHVKFKSKADLRTHMEETKHTSLLPDRKTWDQLEYYFPTYENDTLLCTLSDSESDLTAQ
EQNENVPIISEDTSKLYALKQSSILNQLLL*
>ENSG00000198846|ENST00000361421
MDVRFYPPPAQPAAAPDAPCLGPSPCLDPYYCNKFDGENMYMSMTEPSQDYVPASQSYPG
PSLESEDFNIPPITPPSLPDHSLVHLNEVESGYHSLCHPMNHNGLLPFHPQNMDLPEITV
SNMLGQDGTLLSNSISVMPDIRNPEGTQYSSHPQMAAMRPRGQPADIRQQPGMMPHGQLT
TINQSQLSAQLGLNMGGSNVPHNSPSPPGSKSATPSPSSSVHEDEGDDTSKINGGEKRPA
SDMGKKPKTPKKKKKKDPNEPQKPVSAYALFFRDTQAAIKGQNPNATFGEVSKIVASMWD
GLGEEQKQVYKKKTEAAKKEYLKQLAAYRASLVSKSYSEPVDVKTSQPPQLINSKPSVFH
GPSQAHSALYLSSHYHQQPGMNPHLTAMHPSLPRNIAPKPNNQMPVTVSIANMAVSPPPP
LQISPPLHQHLNMQQHQPLTMQQPLGNQLPMQVQSALHSPTMQQGFTLQPDYQTIINPTS
TAAQVVTQAMEYVRSGCRNPPPQPVDWNNDYCSSGGMQRDKALYLT*
>ENSG00000140464|ENST00000354026
MEPAPARSPRPQQDPARPQEPTMPPPETPSEGRQPSPSPSPTERAPASEEEFQFLRCQQC
QAEAKCPKLLPCLHTLCSGCLEASGMQCPICQAPWPLGADTPALDNVFFESLQRRLSVYR
QIVDAQAVCTRCKESADFWCFECEQLLCAKCFEAHQWFLKHEARPLAELRNQSVREFLDG
TRKTNNIFCSNPNHRTPTLTSIYCRGCSKPLCCSCALLDSSHSELKCDISAEIQQRQEEL
DAMTQALQEQDSAFGAVHAQMHAAVGQLGRARAETEELIRERVRQVVAHVRAQERELLEA
VDARYQRDYEEMASRLGRLDAVLQRIRTGSALVQRMKCYASDQEVLDMHGFLRQALCRLR
QEEPQSLQAAVRTDGFDEFKVRLQDLSSCITQGKDAAVSKKASPEAASTPRDPIDVDLDV
SNTTTAQKRKCSQTQCPRKVIKMESEEGKEARLARSSPEQPRPSTSKAVSPPHLDGPPSP
RSPVIGSEVFLPNSNHVASGAGEAEERVVVISSSEDSDAENSCMEPMETAEPQSSPAHSS
PAHSSPAHSSPVQSLLRAQGASSLPCGTYHPPAWPPHQPAEQAATPDAEPHSEPPDHQER
PAVHRGIRYLLYRAQRAIRLRHALRLHPQLHRAPIRTWSPHVVQASTPAITGPLNHPANA
QEHPAQLQRGISPPHRIRGAVRSRSRSLRGSSHLSQWLNNFFALPFSSMASQLDMSSVVG
AGESRAQTLGAGVPPGDSVRGSMEASQVQVPLEASPITFPPPCAPERPPISPVPGARQAG
L*
>ENSG00000161547|ENST00000358156
MSYGRPPPDVEGMTSLKVDNLTYRTSPDTLRRVFEKYGRVGDVYIPRDRYTKESRGFAFV
RFHDKRDAEDAMDAMDGAVLDGRELRVQMARYGRPPDSHHSRRGPPPRRYGGGGYGRRSR
SRSRSRSRSRSRSR
>ENSG00000168214|ENST00000342295
MDHTEGSPAEEPPAHAPSPGKFGERPPPKRLTREAMRNYLKERGDQTVLILHAKVAQKSY
GNEKRFFCPPPCVYLMGSGWKKKKEQMERDGCSEQESQPCAFIGIGNSDQEMQQLNLEGK
NYCTAKTLYISDSDKRKHFMLSVKMFYGNSDDIGVFLSKRIKVISKPSKKKQSLKNADLC
IASGTKVALFNRLRSQTVSTRYLHVEGGNFHASSQQWGAFFIHLLDDDESEGEEFTVRDG
YIHYGQTVKLVCSVTGMALPRLIIRKVDKQTALLDADDPVSQLHKCAFYLKDTERMYLCL

SQERIIQFQATPCPKEPNKEMINDGASWTIISTDKAEYTFYEGMGPVLAPVTPVPVVESL
QLNGGGDVAMLELTGQNFTPNLRVWFGDVEAETMYRCGESMLCVVPDISAFREGWRWVRQ
PVQVPVTLVRNDGIIYSTSLTFTYTPEPGPRPHCSAAGAILRANSSQVPPNESNTNSEGS
YTNASTNSTSVTSSTATVVS*
>ENSG00000147604|ENST00000352983
MEGVEEKKKEVPAVPETLKKKRRNFAELKIKRLRKKFAQKMLRKARRKLIYEKAKHYHKE
YRQMYRTEIRMARMARKAGNFYVPAEPKLAFVIRIRGINGVSPKVRKVLQLLRLRQIFNG
TFVKLNKASINMLRIVEPYIAWGYPNLKSVNELIYKRGYGKINKKRIALTDNALIARSLG
KYGIICMEDLIHEIYTVGKRFKEANNFLWPFKLSSPRGGMKKKTTHFVEGGDAGNREDQI
NRLIRRMN*
>ENSG00000111011|ENST00000331738
MAASDTERDGLAPEKTSPDRDKKKEQSEVSVSPRASKHHYSRSRSRSRERKRKSDNEGRK
HRSRSRSKEGRRHESKDKSSKKHKSEEHNDKEHSSDKGRERLNSSENGEDRHKRKERKSS
RGRSHSRSRSRERRHRSRSRERKKSRSRSRERKKSRSRSRERKKSRSRSRERKRRIRSRS
RSRSRHRHRTRSRSRTRSRSRDRKKRIEKPRRFSRSLSRTPSPPPFRGRNTAMDAQEALA
RRLERAKKLQEQREKEMVEKQKQQEIAAAAATGGSVLNVAALLASGTQVTPQIAMAAQMA
ALQAKALAETGIAVPSYYNPAAVNPMKFAEQEKKRKMLWQGKKEGDKSQSAEIWEKLNFG
NKDQNVKFRKLMGIKSEDEAGCSSVDEESYKTLKQQEEVFRNLDAQYEMARSQTHTQRGM
GLGFTSSMRGMDAV*
>ENSG00000156508|ENST00000316292
MGKEKTHINIVVIGHVDSGKSTTTGHLIYKCGGIDKRTIEKFEKEAAEMGKGSFKYAWVL
DKLKAERERGITIDISLWKFETSKYYVTIIDAPGHRDFIKNMITGTSQADCAVLIVAAGV
GEFEAGISKNGQTREHALLAYTLGVKQLIVGVNKMDSTEPPYSQKRYEEIVKEVSTYIKK
IGYNPDTVAFVPISGWNGDNMLEPSANMPWFKGWKVTRKDGNASGTTLLEALDCILPPTR
PTDKPLRLPLQDVYKIGGIGTVPVGRVETGVLKPGMVVTFAPVNVTTEVKSVEMHHEALS
EALPGDNVGFNVKNVSVKDVRRGNVAGDSKNDPPMEAAGFTAQVIILNHPGQISAGYAPV
LDCHTAHIACKFAELKEKIDRRSGKKLEDGPKFLKSGDAAIVDMVPGKPMCVESFSDYPP
LGRFAVRDMRQTVAVGVIKAVDKKAAGAGKVTKSAQKAQKAK*
>ENSG00000163041|ENST00000366816
MARTKQTARKSTGGKAPRKQLATKAARKSAPSTGGVKKPHRYRPGTVALREIRRYQKSTE
LLIRKLPFQRLVREIAQDFKTDLRFQSAAIGALQEASEAYLVGLFEDTNLCAIHAKRVTI
MPKDIQLARRIRGERA*
>ENSG00000121060|ENST00000316881
MAELCPLAEELSCSICLEPFKEPVTTPCGHNFCGSCLNETWAVQGSPYLCPQCRAVYQAR
PQLHKNTVLCNVVEQFLQADLAREPPADVWTPPARASAPSPNAQVACDHCLKEAAVKTCL
VCMASFCQEHLQPHFDSPAFQDHPLQPPVRDLLRRKCSQHNRLREFFCPEHSECICHICL
VEHKTCSPASLSQASADLEATLRHKLTVMYSQINGASRALDDVRNRQQDVRMTANRKVEQ
LQQEYTEMKALLDASETTSTRKIKEEEKRVNSKFDTIYQILLKKKSEIQTLKEEIEQSLT
KRDEFEFLEKASKLRGISTKPVYIPEVELNHKLIKGIHQSTIDLKNELKQCIGRLQEPTP
SSGDPGEHDPASTHKSTRPVKKVSKEEKKSKKPPPVPALPSKLPTFGAPEQLVDLKQAGL
EAAAKATSSHPNSTSLKAKVLETFLAKSRPELLEYYIKVILDYNTAHNKVALSECYTVAS
VAEMPQNYRPHPQRFTYCSQVLGLHCYKKGIHYWEVELQKNNFCGVGICYGSMNRQGPES
RLGRNSASWCVEWFNTKISAWHNNVEKTLPSTKATRVGVLLNCDHGFVIFFAVADKVHLM
YKFRVDFTEALYPAFWVFSAGATLSICSPK*

>ENSG00000148200|ENST00000344523
MERDEPPPSGGGGGGGSAGFLEPPAALPPPPRNGFCQDELAELDPGTISVSDDRAEQRTC
LICGDRATGLHYGIISCEGCKGFFKRSICNKRVYRCSRDKNCVMSRKQRNRCQYCRLLKC
LQMGMNRKAIREDGMPGGRNKSIGPVQISEEEIERIMSGQEFEEEANHWSNHGDSDHSSP
GNRASESNQPSPGSTLSSRSVELNGFMAFREQYMGMSVPPHYQYIPHLFSYSGHSPLLPQ
QARSLDPQSYSLIHQLLSAEDLEPLGTPMLIEDGYAVTQAELFALLCRLADELLFRQIAW
IKKLPFFCELSIKDYTCLLSSTWQELILLSSLTVYSKQIFGELADVTAKYSPSDEELHRF
SDEGMEVIERLIYLYHKFHQLKVSNEEYACMKAINFLNQDIRGLTSASQLEQLNKRYWYI
CQDFTEYKYTHQPNRFPDLMMCLPEIRYIAGKMVNVPLEQLPLLFKVVLHSCKTSVGKE*
>ENSG00000186660|ENST00000316059
MPGETEEPRPPEQQDQEGGEAAKAAPEEPQQRPPEAVAAAPAGTTSSRVLRGGRDRGRAA
AAAAAAAVSRRRKAEYPRRRRSSPSARPPDVPGQQPQAAKSPSPVQGKKSPRLLCIEKVT
TDKDPKEEKEEEDDSALPQEVSIAASRPSRGWRSSRTSVSRHRDTENTRSSRSKTGSLQL
ICKSEPNTDQLDYDVGEEHQSPGGISSEEEEEEEEEMLISEEEIPFKDDPRDETYKPHLE
RETPKPRRKSGKVKEEKEKKEIKVEVEVEVKEEENEIREDEEPPRKRGRRRKDDKSPRLP
KRRKKPPIQYVRCEMEGCGTVLAHPRYLQHHIKYQHLLKKKYVCPHPSCGRLFRLQKQLL
RHAKHHTDQRDYICEYCARAFKSSHNLAVHRMIHTGEKPLQCEICGFTCRQKASLNWHMK
KHDADSFYQFSCNICGKKFEKKDSVVAHKAKSHPEVLIAEALAANAGALITSTDILGTNP
ESLTQPSDGQGLPLLPEPLGNSTSGECLLLEAEGMSKSYCSGTERVSLMADGKIFVGSGS
SGGTEGLVMNSDILGATTEVLIEDSDSAGP*
>ENSG00000117707|ENST00000366958
MPDHDSTALLSRQTKRRRVDIGVKRTVGTASAFFAKARATFFSAMNPQGSEQDVEYSVVQ
HADGEKSNVLRKLLKRANSYEDAMMPFPGATIISQLLKNNMNKNGGTEPSFQASGLSSTG
SEVHQEDICSNSSRDSPPECLSPFGRPTMSQFDMDRLCDEHLRAKRARVENIIRGMSHSP
SVALRGNENEREMAPQSVSPRESYRENKRKQKLPQQQQQSFQQLVSARKEQKREERRQLK
QQLEDMQKQLRQLQEKFYQIYDSTDSENDEDGNLSEDSMRSEILDARAQDSVGRSDNEMC
ELDPGQFIDRARALIREQEMAENKPKREGNNKERDHGPNSLQPEGKHLAETLKQELNTAM
SQVVDTVVKVFSAKPSRQVPQVFPPLQIPQARFAVNGENHNFHTANQRLQCFGDVIIPNP
LDTFGNVQMASSTDQTEALPLVVRKNSSDQSASGPAAGGHHQPLHQSPLSATTGFTTSTF
RHPFPLPLMAYPFQSPLGAPSGSFSGKDRASPESLDLTRDTTSLRTKMSSHHLSHHPCSP
AHPPSTAEGLSLSLIKSECGDLQDMSEISPYSGSAMQEGLSPNHLKKAKLMFFYTRYPSS
NMLKTYFSDVKFNRCITSQLIKWFSNFREFYYIQMEKYARQAINDGVTSTEELSITRDCE
LYRALNMHYNKANDFEVPERFLEVAQITLREFFNAIIAGKDVDPSWKKAIYKVICKLDSE
VPEIFKSPNCLQELLHE*
>ENSG00000198231|ENST00000359353
MAEVEDQAARDMKRLEEKDKERKNVKGIRDDIEEEDDQEAYFRYMAENPTAGVVQEEEED
NLEYDSDGNPIAPTKKIIDPLPPIDHSEIDYPPFEKNFYNEHEEITNLTPQQLIDLRHKL
NLRVSGAAPPRPGSSFAHFGFDEQLMHQIRKSEYTQPTPIQCQGVPVALSGRDMIGIAKT
GSGKTAAFIWPMLIHIMDQKELEPGDGPIAVIVCPTRELCQQIHAECKRFGKAYNLRSVA
VYGGGSMWEQAKALQEGAEIVVCTPGRLIDHVKKKATNLQRVSYLVFDEADRMFDMGFEY
QVRSIASHVRPDRQTLLFSATFRKKIEKLARDILIDPIRVVQGDIGEANEDVTQIVEILH
SGPSKWNWLTRRLVEFTSSGSVLLFVTKKANAEELANNLKQEGHNLGLLHGDMDQSERNK
VISDFKKKDIPVLVATDVAARGLDIPSIKTVINYDVARDIDTHTHRIGRTGRAGEKGVAY
TLLTPKDSNFAGDLVRNLEGANQHVSKELLDLAMQNAWFRKSRFKGGKGKKLNIGGGGLG

YRERPGLGSENMDRGNNNVMSNYEAYKPSTGAMGDRLTAMKAAFQSQYKSHFVAASLSNQ
KAGSSAAGASGWTSAGSLNSVPTNSAQQGHNSPDSPVTSAAKGIPGFGNTGNISGAPVTY
PSAGAQGVNNTASGNNSREGTGGSNGKRERYTENRGSSRHSHGETGNRHSDSPRHGDGGR
HGDGYRHPESSSRHTDGHRHGENRHGGSAGRHGENRGANDGRNGESRKEAFNRESKMEPK
MEPKVDSSKMDKVDSKTDKTADGFAVPEPPKRKKSRWDS*
>ENSG00000197020|ENST00000358296
MDDPRYGMCPLKGASGCPGAERSLLVQSYFEKGPLTFRDVAIEFSLEEWQCLDSAQQGLY
RKVMLENYRNLVFLAGIALTKPDLITCLEQGKEPWNIKRHEMVAKPPVICSHFPQDLWAE
QDIKDSFQEAILKKYGKYGHDNLQLQKGCKSVDECKVHKEHDNKLNQCLITTQSNIFQCD
PSAKVFHTFSNSNRHKIRHTRKKPFKCKKCEKSFCMLLHLTQHKRFHITENSYQCKDCGK
AFNWFSTLTTHRRIHTGEKPYKCEECGKAFNRSSHLTTHKIIHTGEKPYRCEECGKAFNR
SSHLTTHKRIHTGVKPYKCTECGKAFNRSSHLTTHRIIHTGEKPYKCEECGKAFNQSSTL
TTHKITHAGEKPYKCEECGKAFYRFSYLTKHKTSHTGEKFYKCEECGKGFNWSSALTKHK
RIHTGEKPYKCEECGKAFNESSNLTTHKMIHTGEKPYKCDECGKAFNRSSQLTAHKMIHT
GEKPYKCEECGKAFNRSSTLTKHKITHTGEKSYKWEECGKDFNQSLSLIKQNNSYWRETL
QM*
>ENSG00000177125|ENST00000319119
MSVEMDSSSFIQFDVPEYSSTVLSQLNELRLQGKLCDIIVHIQGQPFRAHKAVLAASSPY
FRDHSALSTMSGLSISVIKNPNVFEQLLSFCYTGRMSLQLKDVVSFLTAASFLQMQCVID
KCTQILESIHSKISVGDVDSVTVGAEENPESRNGVKDSSFFANPVEISPPYCSQGRQPTA
SSDLRMETTPSKALRSRLQEEGHSDRGSSGSVSEYEIQIEGDHEQGDLLVRESQITEVKV
KMEKSDRPSCSDSSSLGDDGYHTEMVDGEQVVAVNVGSYGSVLQHAYSYSQAASQPTNVS
EAFGSLSNSSPSRSMLSCFRGGRARQKRALSVHLHSDLQGLVQGSDSEAMMNNPGYESSP
RERSARGHWYPYNERLICIYCGKSFNQKGSLDRHMRLHMGITPFVCKFCGKKYTRKDQLE
YHIRGHTDDKPFRCEICGKCFPFQGTLNQHLRKNHPGVAEVRSRIESPERTDVYVEQKLE
NDASASEMGLDSRMEIHTVSDAPD*
>ENSG00000198939|ENST00000361362
MEREGIWHSTLGETWEPNNWLEGQQDSHLSQVGVTHKETFTEMRVCGGNEFERCSSQDSI
LDTQQSIPMVKRPHNCNSHGEDATQNSELIKTQRMFVGKKIYECNQCSKTFSQSSSLLKH
QRIHTGEKPYKCNVCGKHFIERSSLTVHQRIHTGEKPYKCNECGKAFSQSMNLTVHQRTH
TGEKPYQCKECGKAFHKNSSLIQHERIHTGEKPYKCNECGKAFTQSMNLTVHQRTHTGEK
PYECNECGKAFSQSMHLIVHQRSHTGEKPYECSQCGKAFSKSSTLTLHQRNHTGEKPYKC
NKCGKSFSQSTYLIEHQRLHSGVKPFECNECGKAFSKNSSLTQHRRIHTGEKPYECMVCG
KHFTGRSSLTVHQVIHTGEKPYECNECGKAFSQSAYLIEHQRIHTGEKPYECDQCGKAFI
KNSSLTVHQRTHTGEKPYQCNECGKAFSRSTNLTRHQRTHT*
>ENSG00000010030|ENST00000340181
MQEGELAISPISPVAAMPPLGTHVQARCEAQINLLGEGGICKLPGRLRIQPALWSREDVL
HWLRWAEQEYSLPCTAEHGFEMNGRALCILTKDDFRHRAPSSGDVLYELLQYIKTQRRAL
VCGPFFGGIFRLKTPTQHSPVPPEEVTGPSQMDTRRGHLLQPPDPGLTSNFGHLDDPGLA
RWTPGKEESLNLCHCAELGCRTQGVCSFPAMPQAPIDGRIADCRLLWDYVYQLLLDTRYE
PYIKWEDKDAKIFRVVDPNGLARLWGNHKNRVNMTYEKMSRALRHYYKLNIIKKEPGQKL
LFRFLKTPGKMVQDKHSHLEPLESQEQDRIEFKDKRPEISP*
>ENSG00000123416|ENST00000336023
MRECISIHVGQAGVQIGNACWELYCLEHGIQPDGQMPSDKTIGGGDDSFNTFFSETGAGK

HVPRAVFVDLEPTVIDEVRTGTYRQLFHPEQLITGKEDAANNYARGHYTIGKEIIDLVLD
RIRKLADQCTGLQGFLVFHSFGGGTGSGFTSLLMERLSVDYGKKSKLEFSIYPAPQVSTA
VVEPYNSILTTHTTLEHSDCAFMVDNEAIYDICRRNLDIERPTYTNLNRLISQIVSSITA
SLRFDGALNVDLTEFQTNLVPYPRIHFPLATYAPVISAEKAYHEQLSVAEITNACFEPAN
QMVKCDPRHGKYMACCLLYRGDVVPKDVNAAIATIKTKRSIQFVDWCPTGFKVGINYQPP
TVVPGGDLAKVQRAVCMLSNTTAIAEAWARLDHKFDLMYAKRAFVHWYVGEGMEEGEFSE
AREDMAALEKDYEEVGVDSVEGEGEEEGEEY*
>ENSG00000108557|ENST00000353383
MQSFRERCGFHGKQQNYQQTSQETSRLENYRQPSQAGLSCDRQRLLAKDYYNPQPYPSYE
GGAGTPSGTAAAVAADKYHRGSKALPTQQGLQGRPAFPGYGVQDSSPYPGRYAGEESLQA
WGAPQPPPPQPQPLPAGVAKYDENLMKKTAVPPSRQYAEQGAQVPFRTHSLHVQQPPPPQ
QPLAYPKLQRQKLQNDIASPLPFPQGTHFPQHSQSFPTSSTYSSSVQGGGQGAHSYKSCT
APTAQPHDRPLTASSSLAPGQRVQNLHAYQSGRLSYDQQQQQQQQQQQQQQALQSRHHAQ
ETLHYQNLAKYQHYGQQGQGYCQPDAAVRTPEQYYQTFSPSSSHSPARSVGRSPSYSSTP
SPLMPNLENFPYSQQPLSTGAFPAGITDHSHFMPLLNPSPTDATSSVDTQAGNCKPLQKD
KLPENLLSDLSLQSLTALTSQVENISNTVQQLLLSKAAVPQKKGVKNLVSRTPEQHKSQH
CSPEGSGYSAEPAGTPLSEPPSSTPQSTHAEPQEADYLSGSEDPLERSFLYCNQARGSPA
RVNSNSKAKPESVSTCSVTSPDDMSTKSDDSFQSLHGSLPLDSFSKFVAGERDCPRLLLS
ALAQEDLASEILGLQEAIGEKADKAWAEAPSLVKDSSKPPFSLENHSACLDSVAKSAWPR
PGEPEALPDSLQLDKGGNAKDFSPGLFEDPSVAFATPDPKKTTGPLSFGTKPTLGVPAPD
PTTAAFDCFPDTTAASSADSANPFAWPEENLGDACPRWGLHPGELTKGLEQGGKASDGIS
KGDTHEASACLGFQEEDPPGEKVASLPGDFKQEEVGGVKEEAGGLLQCPEVAKADRWLED
SRHCCSTADFGDLPLLPPTSRKEDLEAEEEYSSLCELLGSPEQRPGMQDPLSPKAPLICT
KEEVEEVLDSKAGWGSPCHLSGESVILLGPTVGTESKVQSWFESSLSHMKPGEEGPDGER
APGDSTTSDASLAQKPNKPAVPEAPIAKKEPVPRGKSLRSRRVHRGLPEAEDSPCRAPVL
PKDLLLPESCTGPPQGQMEGAGAPGRGASEGLPRMCTRSLTALSEPRTPGPPGLTTTPAP
PDKLGGKQRAAFKSGKRVGKPSPKAASSPSNPAALPVASDSSPMGSKTKETDSPSTPGKD
QRSMILRSRTKTQEIFHSKRRRPSEGRLPNCRATKKLLDNSHLPATFKVSSSPQKEGRVS
QRARVPKPGAGSKLSDRPLHALKRKSAFMAPVPTKKRNLVLRSRSSSSSNASGNGGDGKE
ERPEGSPTLFKRMSSPKKAKPTKGNGEPATKLPPPETPDACLKLASRAAFQGAMKTKVLP
PRKGRGLKLEAIVQKITSPSLKKFACKAPGASPGNPLSPSLSDKDRGLKGAGGSPVGVEE
GLVNVGTGQKLPTSGADPLCRNPTNRSLKGKLMNSKKLSSTDCFKTEAFTSPEALQPGGT
ALAPKKRSRKGRAGAHGLSKGPLEKRPYLGPALLLTPRDRASGTQGASEDNSGGGGKKPK
MEELGLASQPPEGRPCQPQTRAQKQPGHTNYSSYSKRKRLTRGRAKNTTSSPCKGRAKRR
RQQQVLPLDPAEPEIRLKYISSCKRLRSDSRTPAFSPFVRVEKRDAFTTICTVVNSPGDA
PKPHRKPSSSASSSSSSSSFSLDAAGASLATLPGGSILQPRPSLPLSSTMHLGPVVSKAL
STSCLVCCLCQNPANFKDLGDLCGPYYPEHCLPKKKPKLKEKVRPEGTCEEASLPLERTL
KGPECAAAATAGKPPRPDGPADPAKQGPLRTSARGLSRRLQSCYCCDGREDGGEEAAPAD
KGRKHECSKEAPAEPGGEAQEHWVHEACAVWTGGVYLVAGKLFGLQEAMKVAVDMMCSSC
QEAGATIGCCHKGCLHTYHYPCASDAGCIFIEENFSLKCPKHKRLP*
>ENSG00000185499|ENST00000337604
MTPGTQSPFFLLLLLTVLTVVTGSGHASSTPGGEKETSATQRSSVPSSTEKNALSTGVSF
FFLSFHISNLQFNSSLEDPSTDYYQELQRDISEMFLQIYKQGGFLGLSNIKFRPGSVVVQ
LTLAFREGTINVHDVETQFNQYKTEAASRYNLTISDVSVSDVPFPFSAQSGAGVPGWGIA

LLVLVCVLVALAIVYLIALAVCQCRRKNYGQLDIFPARDTYHPMSEYPTYHTHGRYVPPS
STDRSPYEKVSAGNGGSSLSYTNPAVAATSANL*
>ENSG00000187801|ENST00000361584
MLQQLLITLPTEASTWVKLRHPKAATERVALWEDVTKMFKAEALLSQDADETQGESLESR
VTLGSLTAESQELLTFKDVSVDFTQEEWGQLAPAHRNLYREVMLENYGNLVSVGCQLSKP
GVISQLEKGEEPWLMERDISGVPSSDLKSKTKTKESALQNDISWEELHCGLMMERFTKGS
SMYSTLGRISKCNKLESQQENQRMGKGQIPLMCKKTFTQERGQESNRFEKRINVKSEVMP
GPIGLPRKRDRKYDTPGKRSRYNIDLVNHSRSYTKMKTFECNICEKIFKQLIHLTEHMRI
HTGEKPFRCKECGKAFSQSSSLIPHQRIHTGEKPYECKECGKTFRHPSSLTQHVRIHTGE
KPYECRVCEKAFSQSIGLIQHLRTHVREKPFTCKDCGKAFFQIRHLRQHEIIHTGVKPYI
CNVCSKTFSHSTYLTQHQRTHTGERPYKCKECGKAFSQRIHLSIHQRVHTGVKPYECSHC
GKAFRHDSSFAKHQRIHTGEKPYDCNECGKAFSCSSSLIRHCKTHLRNTFSNVV*
>ENSG00000180530|ENST00000318948
MTHGEELGSDVHQDSIVLTYLEGLLMHQAAGGSGTAVDKKSAGHNEEDQNFNISGSAFPT
CQSNGPVLNTHTYQGSGMLHLKKARLLQSSEDWNAAKRKRLSDSIMNLNVKKEALLAGMV
DSVPKGKQDSTLLASLLQSFSSRLQTVALSQQIRQSLKEQGYALSHDSLKVEKDLRCYGV
ASSHLKTLLKKSKVKDQKPDTNLPDVTKNLIRDRFAESPHHVGQSGTKVMSEPLSCAARL
QAVASMVEKRASPATSPKPSVACSQLALLLSSEAHLQQYSREHALKTQNANQAASERLAA
MARLQENGQKDVGSYQLPKGMSSHLNGQARTSSSKLMASKSSATVFQNPMGIIPSSPKNA
GYKNSLERNNIKQAANNSLLLHLLKSQTIPKPMNGHSHSERGSIFEESSTPTTIDEYSDN
NPSFTDDSSGDESSYSNCVPIDLSCKHRTEKSESDQPVSLDNFTQSLLNTWDPKVPDVDI
KEDQDTSKNSKLNSHQKVTLLQLLLGHKNEENVEKNTSPQGVHNDVSKFNTQNYARTSVI
ESPSTNRTTPVSTPPLLTSSKAGSPINLSQHSLVIKWNSPPYVCSTQSEKLTNTASNHSM
DLTKSKDPPGEKPAQNEGAQNSATFSASKLLQNLAQCGMQSSMSVEEQRPSKQLLTGNTD
KPIGMIDRLNSPLLSNKTNAVEENKAFSSQPTGPEPGLSGSEIENLLERRTVLQLLLGNP
NKGKSEKKEKTPLRDESTQEHSERALSEQILMVKIKSEPCDDLQIPNTNVHLSHDAKSAP
FLGMAPAVQRSAPALPVSEDFKSEPVSPQDFSFSKNGLLSRLLRQNQDSYLADDSDRSHR
NNEMALLESKNLCMVPKKRKLYTEPLENPFKKMKNNIVDAANNHSAPEVLYGSLLNQEEL
KFSRNDLEFKYPAGHGSASESEHRSWARESKSFNVLKQLLLSENCVRDLSPHRSNSVADS
KKKGHKNNVTNSKPEFSISSLNGLMYSSTQPSSCMDNRTFSYPGVVKTPVSPTFPEHLGC
AGSRPESGLLNGCSMPSEKGPIKWVITDAEKNEYEKDSPRLTKTNPILYYMLQKGGNSVT
SRETQDKDIWREASSAESVSQVTAKEELLPTAETKASFFNLRSPYNSHMGNNASRPHSAN
GEVYGLLGSVLTIKKESE*
>ENSG00000082641|ENST00000361665
MLSLKKYLTEGLLQFTILLSLIGVRVDVDTYLTSQLPPLREIILGPSSAYTQTQFHNLRN
TLDGYGIHPKSIDLDNYFTARRLLSQVRALDRFQVPTTEVNAWLVHRDPEGSVSGSQPNS
GLALESSSGLQDVTGPDNGVRESETEQGFGEDLEDLGAVAPPVSGDLTKEDIDLGAGREV
FDYSHRQKEQDVEKELRDGGEQDTWAGEGAEALARNLLVDGETGESFPAQVPSGEDQTAL
SLEECLRLLEATCPFGENAEFPADISSITEAVPSESEPPALQNNLLSPLLTGTESPFDLE
QQWQDLMSIMEMQAMEVNTSASEILYSAPPGDPLSTNYSLAPNTPINQNVSLHQASLGGC
SQDFLLFSPEVESLPVASSSTLLPLAPSNSTSLNSTFGSTNLTGLFFPPQLNGTANDTAG
PELPDPLGGLLDEAMLDEISLMDLAIEEGFNPVQASQLEEEFDSDSGLSLDSSHSPSSLS
SSEGSSSSSSSSSSSSSSASSSASSSFSEEGAVGYSSDSETLDLEEAEGAVGYQPEYSKF
CRMSYQDPAQLSCLPYLEHVGHNHTYNMAPSALDSADLPPPSALKKGSKEKQADFLDKQM

SRDEHRARAMKIPFTNDKIINLPVEEFNELLSKYQLSEAQLSLIRDIRRRGKNKMAAQNC
RKRKLDTILNLERDVEDLQRDKARLLREKVEFLRSLRQMKQKVQSLYQEVFGRLRDENGR
PYSPSQYALQYAGDGSVLLIPRTMADQQARRQERKPKDRRK*
>ENSG00000125676|ENST00000355725
MAAAAVVVPAEWIKNWEKSGRGEFLHLCRILSENKSHDSSTYRDFQQALYELSYHVIKGN
LKHEQASNVLSDISEFREDMPSILADVFCILDIETNCLEEKSKRDYFTQLVLACLYLVSD
TVLKERLDPETLESLGLIKQSQQFNQKSVKIKTKLFYKQQKFNLLREENEGYAKLIAELG
QDLSGSITSDLILENIKSLIGCFNLDPNRVLDVILEVFECRPEHDDFFISLLESYMSMCE
PQTLCHILGFKFKFYQEPNGETPSSLYRVAAVLLQFNLIDLDDLYVHLLPADNCIMDEHK
REIAEAKQIVRKLTMVVLSSEKMDEREKEKEKEEEKVEKPPDNQKLGLLEALLKIGDWQH
AQNIMDQMPPYYAASHKLIALAICKLIHITIEPLYRRVGVPKGAKGSPVNALQNKRAPKQ
AESFEDLRRDVFNMFCYLGPHLSHDPILFAKVVRIGKSFMKEFQSDGSKQEDKEKTEVIL
SCLLSITDQVLLPSLSLMDCNACMSEELWGMFKTFPYQHRYRLYGQWKNETYNSHPLLVK
VKAQTIDRAKYIMKRLTKENVKPSGRQIGKLSHSNPTILFDYILSQIQKYDNLITPVVDS
LKYLTSLNYDVLAYCIIEALANPEKERMKHDDTTISSWLQSLASFCGAVFRKYPIDLAGL
LQYVANQLKAGKSFDLLILKEVVQKMAGIEITEEMTMEQLEAMTGGEQLKAEGGYFGQIR
NTKKSSQRLKDALLDHDLALPLCLLMAQQRNGVIFQEGGEKHLKLVGKLYDQCHDTLVQF
GGFLASNLSTEDYIKRVPSIDVLCNEFHTPHDAAFFLSRPMYAHHISSKYDELKKSEKGS
KQQHKVHKYITSCEMVMAPVHEAVVSLHVSKVWDDISPQFYATFWSLTMYDLAVPHTSYE
REVNKLKVQMKAIDDNQEMPPNKKKKEKERCTALQDKLLEEEKKQMEHVQRVLQRLKLEK
DNWLLAKSTKNETITKFLQLCIFPRCIFSAIDAVYCARFVELVHQQKTPNFSTLLCYDRV
FSDIIYTVASCTENEASRYGRFLCCMLETVTRWHSDRATYEKECGNYPGFLTILRATGFD
GGNKADQLDYENFRHVVHKWHYKLTKASVHCLETGEYTHIRNILIVLTKILPWYPKVLNL
GQALERRVHKICQEEKEKRPDLYALAMGYSGQLKSRKSYMIPENEFHHKDPPPRNAVASV
QNGPGGGPSSSSIGSASKSDESSTEETDKSRERSQCGVKAVNKASSTTPKGNSSNGNSGS
NSNKAVKENDKEKGKEKEKEKKEKTPATTPEARVLGKDGKEKPKEERPNKDEKARETKER
TPKSDKEKEKFKKEEKAKDEKFKTTVPNAESKSTQEREREKEPSRERDIAKEMKSKENVK
GGEKTPVSGSLKSPVPRSDIPEPEREQKRRKIDTHPSPSHSSTVKDSLIELKESSAKLYI
NHTPPPLSKSKEREMDKKDLDKSRERSREREKKDEKDRKERKRDHSNNDREVPPDLTKRR
KEENGTMGVSKHKSESPCESPYPNEKDKEKNKSKSSGKEKGSDSFKSEKMDKISSGGKKE
SRHDKEKIEKKEKRDSSGGKEEKKHHKSSDKHR*
>ENSG00000110713|ENST00000359171
MFNKSFGTPFGGGTGGFGTTSTFGQNTGFGTTSGGAFGTSAFGSSNNTGGLFGNSQTKPG
GLFGTSSFSQPATSTSTGFGFGTSTGTANTLFGTASTGTSLFSSQNNAFAQNKPTGFGNF
GTSTSSGGLFGTTNTTSNPFGSTSGSLFGPSSFTAAPTGTTIKFNPPTGTDTMVKAGVST
NISTKHQCITAMKEYESKSLEELRLEDYQANRKGPQNQVGAGTTTGLFGSSPATSSATGL
FSSSTTNSGFAYGQNKTAFGTSTTGFGTNPGGLFGQQNQQTTSLFSKPFGQATTTQNTGF
SFGNTSTIGQPSTNTMGLFGVTQASQPGGLFGTATNTSTGTAFGTGTGLFGQTNTGFGAV
GSTLFGNNKLTTFGSSTTSAPSFGTTSGGLFGNKPTLTLGTNTNTSNFGFGTNTSGNSIF
GSKPAPGTLGTGLGAGFGTALGAGQASLFGNNQPKIGGPLGTGAFGAPGFNTTTATLGFG
APQAPVALTDPNASAAQQAVLQQHINSLTYSPFGDSPLFRNPMSDPKKKEERLKPTNPAA
QKALTTPTHYKLTPRPATRVRPKALQTTGTAKSHLFDGLDDDEPSLANGAFMPKKSIKKL
VLKNLNNSNLFSPVNRDSENLASPSEYPENGERFSFLSKPVDENHQQDGDEDSLVSHFYT
NPIAKPIPQTPESAGNKHSNSNSVDDTIVALNMRAALRNGLEGSSEETSFHDESLQDDRE

EIENNSYHMHPAGIILTKVGYYTIPSMDDLAKITNEKGECIVSDFTIGRKGYGSIYFEGD
VNLTNLNLDDIVHIRRKEVVVYLDDNQKPPVGEGLNRKAEVTLDGVWPTDKTSRCLIKSP
DRLADINYEGRLEAVSRKQGAQFKEYRPETGSWVFKVSHFSKYGLQDSDEEEEEHPSKTS
TKKLKTAPLPPASQTTPLQMALNGKPAPPPQSQSPEVEQLGRVVELDSDMVDITQEPVLD
TMLEESMPEDQEPVSASTHIASSLGINPHVLQIMKASLLTDEEDVDMALDQRFSRLPSKA
DTSQEICSPRLPISASHSSKTRSLVGGLLQSKFTSGAFLSPSVSVQECRTPRAASLMNIP
STSSWSVPPPLTSVFTMPSPAPEVPLKTVGTRRQLGLVPREKSVTYGKGKLLMDMALFMG
RSFRVGWGPNWTLANSGEQLNGSHELENHQIADSMEFGFLPNPVAVKPLTESPFKVHLEK
LSLRQRKPDEDMKLYQTPLELKLKHSTVHVDELCPLIVPNLGVAVIHDYADWVKEASGDL
PEAQIVKHWSLTWTLCEALWGHLKELDSQLNEPREYIQILERRRAFSRWLSCTATPQIEE
EVSLTQKNSPVEAVFSYLTGKRISEACSLAQQSGDHRLALLLSQFVGSQSVRELLTMQLV
DWHQLQADSFIQDERLRIFALLAGKPVWQLSEKKQINVCSQLDWKRSLAIHLWYLLPPTA
SISRALSMYEEAFQNTSDSDRYACSPLPSYLEGSGCVIAEEQNSQTPLRDVCFHLLKLYS
DRHYDLNQLLEPRSITADPLDYRLSWHLWEVLRALNYTHLSAQCEGVLQASYAGQLESEG
LWEWAIFVLLHIDNSGIREKAVRELLTRHCQLLETPESWAKETFLTQKLRVPAKWIHEAK
AVRAHMESDKHLEALCLFKAEHWNRCHKLIIRHLASDAIINENYDYLKGFLEDLAPPERS
SLIQDWETSGLVYLDYIRVIEMLRHIQQVDCSGNDLEQLHIKVTSLCSRIEQIQCYSAKD
RLAQSDMAKRVANLLRVVLSLHHPPDRTSDSTPDPQRVPLRLLAPHIGRLPMPEDYAMDE
LRSLTQSYLRELAVGSL*
>ENSG00000204348|ENST00000337523
MDPRGTKRGAEKTEVAEPRNKLPRPAPSLPTDPALYSGPFPFYRRPSELGCFSLDAQRQY
HGDARALRYYSPPPTNGPGPNFDLRDGYPDRYQPRDEEVQERLDHLLCWLLEHRGRLEGG
PGWLAEAIVTWRGHLTKLLTTPYERQEGWQLAASRFQGTLYLSEVETPNARAQRLARPPL
LRELMYMGYKFEQYMCADKPGSSPDPSGEVNTNVAFCSVLRSRLGSHPLLFSGEVDCTDP
QAPSTQPPTCYVELKTSKEMHSPGQWRSFYRHKLLKWWAQSFLPGVPNVVAGFRNPDGFV
SSLKTFPTMKMFEYVRNDRDGWNPSVCMNFCAAFLSFAQSTVVQDDPRLVHLFSWEPGGP
VTVSVHQDAPYAFLPIWYVEAMTQDLPSPPKTPSPK*
>ENSG00000196268|ENST00000355504
MNECNVHKEGYNELNQYLTTTQSKIFQCDKYVKVFHKLLNSNRHNTKHTGKKPFKCKKCG
KSFCMLLHLCQHKRIHIRENSYRCEECGKAFIWFSTLTRHRRVHTGEKSYKYECGKSFNQ
DSNLTTHKRIHTGQKPYKCEECGTSFYQFSYLTRHKLIHTREKPYKCEQYGKTFNQSSTL
TGHKIIHNGEKPYKCEECGKAFSIFSTPTKHKIIHTEEKSHRCEEYCKAYKESSHLTTHK
RIHTGEKPYKCEECGKAFSIFSTLTKHKIIHTEEKSHRCEECGKAYKESSHLTTHKRIHT
GEKPYKCEECGKTFSVFSILTKHKIIHTEEKPYKCEECGKAFKRSSTLTKHRIIHTEEKP
YKCEECGKAFNQSSTLSIHKIIHTGEKPYKCEECGKAFKRSSTLTIHKMIHTGEKPYKCE
ECGKAFNRSSHLTTHKRIHTGHKPYKCKECGKSFSVFSTLTKHKIIHTDKKPYKCEECGK
AFNRSSILSIHKKIHTGEKPYKCEECGKAFKRSSHLAGHKQIHSVQKPYKCEECGKAFSI
FSTLTKHKIIHTEEKPYKCEKCGKTFYRFSNLNTHKIIHTGEKPCKCEECGKAFNHSSNL
IKHKLIHTGDKPYKCEACGKAFRRSSHLSRHKIIHIGIHTEETVQK*
>ENSG00000089234|ENST00000327551
MSDEEIKKTTLASAVACLEGKSPGEKVAIIHQHLGRREMTDVIIETMKSNPDELKTTVEE
RKSSEASPTAQRSKDHSKECINAAPDSPSKQLPDQISFFSGNPSVEIVHGIMHLYKTNKM
TSLKEDVRRSAMLCILTVPAAMTSHDLMKFVAPFNEVIEQMKIIRDSTPNQYMVLIKFRA
QADADSFYMTCNGRQFNSIEDDVCQLVYVERAEVLKSEDGASLPVMDLTELPKCTVCLER

MDESVNGILTTLCNHSFHSQCLQRWDDTTCPVCRYCQTPEPVEENKCFECGVQENLWICL
ICGHIGCGRYVSRHAYKHFEETQHTYAMQLTNHRVWDYAGDNYVHRLVASKTDGKIVQYE
CEGDTCQEEKIDALQLEYSYLLTSQLESQRIYWENKIVRIEKDTAEEINNMKTKFKETIE
KCDNLEHKLNDLLKEKQSVERKCTQLNTKVAKLTNELKEEQEMNKCLRANQVLLQNKLKE
EERVLKETCDQKDLQITEIQEQLRDVMFYLETQQKINHLPAETRQEIQEGQINIAMASAS
SPASSGGSGKLPSRKGRSKRGK*
>ENSG00000283762|ENST00000362279
Sequence unavailable
>ENSG00000147669|ENST00000353107
MDTQKDVQPPKQQPMIYICGECHTENEIKSRDPIRCRECGYRIMYKKRTKRLVVFDAR*
>ENSG00000172062|ENST00000351205
MAMSSGGSGGGVPEQEDSVLFRRGTGQSDDSDIWDDTALIKAYDKAVASFKHALKNGDIC
ETSGKPKTTPKRKPAKKNKSQKKNTAASLQQWKVGDKCSAIWSEDGCIYPATIASIDFKR
ETCVVVYTGYGNREEQNLSDLLSPICEVANNIEQNAQENENESQVSTDESENSRSPGNKS
DNIKPKSAPWNSFLPPPPPMPGPRLGPGKPGLKFNGPPPPPPPPPPHLLSCWLPPFPSGP
PIIPPPPPICPDSLDDADALGSMLISWYMSGYHTGYYMGFRQNQKEGRCSHSLN*
>ENSG00000138375|ENST00000358207
MSLPLTEEQRKKIEENRQKALARRAEKLLAEQHQRTSSGTSIAGNPFQAKQGPSQNFPRE
SCKPVSHGVIFKQQNLSSSSNADQRPHDSHSFQAKGIWKKPEEMPTACPGHSPRSQMALT
GISPPLAQSPPEVPKQQLLSYELGQGHAQASPEIRFTPFANPTHKPLAKPKSSQETPAHS
SGQPPRDAKLEAKTAKASPSGQNISYIHSSSESVTPRTEGRLQQKSGSSVQKGVNSQKGK
CVRNGDRFQVLIGYNAELIAVFKTLPSKNYDPDTKTWNFSMNDYSALMKAAQSLPTVNLQ
PLEWAYGSSESPSTSSEGQAGLPSAPSLSFVKGRCMLISRAYFEADISYSQDLIALFKQM
DSRRYDVKTRKWSFLLEEHSKLIAKVRCLPQVQLDPLPTTLTLAFASQLKKTSLSLTPDV
PEADLSEVDPKLVSNLMPFQRAGVNFAIAKGGRLLLADDMGLGKTIQAICIAAFYRKEWP
LLVVVPSSVRFTWEQAFLRWLPSLSPDCINVVVTGKDRLTAGLINIVSFDLLSKLEKQLK
TPFKVVIIDESHFLKNSRTARCRAAMPVLKVAKRVILLSGTPAMSRPAELYTQIIAVKPT
FFPQFHAFGLRYCDAKRMPWGWDYSGSSNLGELKLLLEEAVMLRRLKSDVLSQLPAKQRK
IVVIAPGRINARTRAALDAAAKEMTTKDKTKQQQKDALILFFNRTAEAKIPSVIEYILDL
LESGREKFLVFAHHKVVLDAITQELERKHVQHIRIDGSTSSAEREDLCQQFQLSERHAVA
VLSITAANMGLTFSSADLVVFAELFWNPGVLIQAEDRVHRIGQTSSVGIHYLVAKGTADD
YLWPLIQEKIKVLAEAGLSETNFSEMTESTDYLYKDPKQQKIYDLFQKSFEKEGSDMELL
EAAESFDPGSASGTSGSSSQNMGDTLDESSLTASPQKKRRFEFFDNWDSFTSPL*
>ENSG00000179094|ENST00000354903
MLEVLEEIWSVRARRAHRPCPGPSLADDTDANSNGSSGNESNGHESRGASQRSSHSSSSG
NGKDSALLETTESSKSTNSQSPSPPSSSIAYSLLSASSEQDNPSTSGCSSEQSARARTQK
ELMTALRELKLRLPPERRGKGRSGTLATLQYALACVKQVQANQEYYQQWSLEEGEPCSMD
MSTYTLEELEHITSEYTLQNQDTFSVAVSFLTGRIVYISEQAAVLLRCKRDVFRGTRFSE
LLAPQDVGVFYGSTAPSRLPTWGTGASAGSGLRDFTQEKSVFCRIRGGPDRDPGPRYQPF
RLTPYVTKIRVSDGAPAQPCCLLIAERIHSGYEAPRIPPDKRIFTTRHTPSCLFQDVDER
AAPLLGYLPQDLLGAPVLLFLHPEDRPLMLAIHKKILQLAGQPFDHSPIRFCARNGEYVT
MDTSWAGFVHPWSRKVAFVLGRHKVRTAPLNEDVFTPPAPSPAPSLDTDIQELSEQIHRL
LLQPVHSPSPTGLCGVGAVTSPGPLHSPGSSSDSNGGDAEGPGPPAPVTFQQICKDVHLV
KHQGQQLFIESRARPQSRPRLPATGTFKAKALPCQSPDPELEAGSAPVQAPLALVPEEAE

RKEASSCSYQQINCLDSILRYLESCNLPSTTKRKCASSSSYTTSSASDDDRQRTGPVSVG
TKKDPPSAALSGEGATPRKEPVVGGTLSPLALANKAESVVSVTSQCSFSSTIVHVGDKKP
PESDIIMMEDLPGLAPGPAPSPAPSPTVAPDPAPDAYRPVGLTKAVLSLHTQKEEQAFLS
RFRDLGRLRGLDSSSTAPSALGERGSHLGPPGACPLPSLGLDCWGVGLKGGVSAPGTQAG
VASTTRPCLGTGPSLASPH*
>ENSG00000199051|ENST00000362181
Sequence unavailable
>ENSG00000196381|ENST00000358582
MQRNAMYLKNVAETACNFQLTQYQISHANQKPYECQICGKPFRKRAHLTQHNRIHTGGKP
YECKECGKVFICCSTLIQHKRTHTSEKPYECLECRKTFRRSAHLIRHQRIHTGEKPYKCK
QCWKAFASVSDLIDIGKFTLMRDFTNVQNVGRHLTIAQLLFSIREFTLVRSPLNVRNVAK
HSIIAQHLLNTRELILMRNLMNVRNVKRLLGKVHILLNIKEFILVRNHMSVSNVGRLSLV
FLILIDIREFTLVKNPMNVKNVVELLTIVQLLFNTREFTLVRRLMNISSVGRFLSPVQHL
FNIREHILMKNLMNVSNARRPSSIMHI*
>ENSG00000119599|ENST00000358377
MNKSRWQSRRRHGRRSHQQNPWFRLRDSEDRSDSRAAQPAHDSGHGDDESPSTSSGTAGT
SSVPELPGFYFDPEKKRYFRLLPGHNNCNPLTKESIRQKEMESKRLRLLQEEDRRKKIAR
MGFNASSMLRKSQLGFLNVTNYCHLAHELRLSCMERKKVQIRSMDPSALASDRFNLILAD
TNSDRLFTVNDVKVGGSKYGIINLQSLKTPTLKVFMHENLYFTNRKVNSVCWASLNHLDS
HILLCLMGLAETPGCATLLPASLFVNSHPGIDRPGMLCSFRIPGAWSCAWSLNIQANNCF
STGLSRRVLLTNVVTGHRQSFGTNSDVLAQQFALMAPLLFNGCRSGEIFAIDLRCGNQGK
GWKATRLFHDSAVTSVRILQDEQYLMASDMAGKIKLWDLRTTKCVRQYEGHVNEYAYLPL
HVHEEEGILVAVGQDCYTRIWSLHDARLLRTIPSPYPASKADIPSVAFSSRLGGSRGAPG
LLMAVGQDLYCYSYS*
>ENSG00000001167|ENST00000341376
MEQYTANSNSSTEQIVVQAGQIQQQQQGGVTAVQLQTEAQVASASGQQVQTLQVVQGQPL
MVQVSGGQLITSTGQPIMVQAVPGGQGQTIMQVPVSGTQGLQQIQLVPPGQIQIQGGQAV
QVQGQQGQTQQIIIQQPQTAVTAGQTQTQQQIAVQGQQVAQTAEGQTIVYQPVNADGTIL
QQVTVPVSGMITIPAASLAGAQIVQTGANTNTTSSGQGTVTVTLPVAGNVVNSGGMVMMV
PGAGSVPAIQRIPLPGAEMLEEEPLYVNAKQYHRILKRRQARAKLEAEGKIPKERRKYLH
ESRHRHAMARKRGEGGRFFSPKEKDSPHMQDPNQADEEAMTQIIRVS*
>ENSG00000198464|ENST00000335090
MLEQRREPWSGESEVKIAKNSDGRECIKGVNTGSSYALGSNAEDKPIKKQLGVSFHLHLS
ELELFPDERVINGCNQVENFINHSSSVSCLQEMSSSVKTPIFNRNDFDDSSFLPQEQKVH
LREKPYECNEHSKVFRVSSSLTKHQVIHTVEKPYKCNSCGKVFSRNSHLAEHCRIHTGEK
PYKCNVCGKVFSYNSNFARHQRIHTREKPYECNECGKVFSNNSYLARHQRIHAEEKPYKC
NECGKGFSHKSSLANHWRIYTGEKPYKCDECGKAFYRIALLVRHQKIHTGEKPYKCNECG
KVFIQNSHLAQHWRIHTGEKPYKCNECGKVFNQLSNLARHRRIHTGEKPYKCNECGKAFS
EYSGLSAHLVIHTGEKPYKCSECGKAFRHKLSLTNHQRIHTGERPYKCNECGKVFNRIAH
LARHRKIHTGEKPYKCNECGKAFSRISYLAQHWTIHMG*
>ENSG00000179627|ENST00000342537
MEFPEHGGRLLGRLRQQRELGFLCDCTVLVGDARFPAHRAVLAACSVYFHLFYRDRPAGS
RDTVRLNGDIVTAPAFGRLLDFMYEGRLDLRSLPVEDVLAAASYLHMYDIVKVCKGRLQE
KDRSLDPGNPAPGAEPAQPPCPWPVWTADLCPAARKAKLPPFGVKAALPPRASGPPPCQV

PEESDQALDLSLKSGPRQERVHPPCVLQTPLCSQRQPGAQPLVKDERDSLSEQEESSSSR
SPHSPPKPPPVPAAKGLVVGLQPLPLSGEGSRELELGAGRLASEDELGPGGPLCICPLCS
KLFPSSHVLQLHLSAHFRERDSTRARLSPDGVAPTCPLCGKTFSCTYTLKRHERTHSGEK
PYTCVQCGKSFQYSHNLSRHTVVHTREKPHACRWCERRFTQSGDLYRHVRKFHCGLVKSL
LV*
>ENSG00000171817|ENST00000316433
MAHALVTFRDVAIDFSQKEWECLDTTQRKLYRDVMLENYNNLVSLGYSGSKPDVITLLEQ
GKEPCVVARDVTGRQCPGLLSRHKTKKLSSEKDIHEISLSKESIIEKSKTLRLKGSIFRN
EWQNKSEFEGQQGLKERSISQKKIVSKKMSTDRKRPSFTLNQRIHNSEKSCDSHLVQHGK
IDSDVKHDCKECGSTFNNVYQLTLHQKIHTGEKSCKCEKCGKVFSHSYQLTLHQRFHTGE
KPYECQECGKTFTLYPQLNRHQKIHTGKKPYMCKKCDKGFFSRLELTQHKRIHTGKKSYE
CKECGKVFQLIFYFKEHERIHTGKKPYECKECGKAFSVCGQLTRHQKIHTGVKPYECKEC
GKTFRLSFYLTEHRRTHAGKKPYECKECGKSFNVRGQLNRHKTIHTGIKPFACKVCEKAF
SYSGDLRVHSRIHTGEKPYECKECGKAFMLRSVLTEHQRLHTGVKPYECKECGKTFRVRS
QISLHKKIHTDVKPYKCVRCGKTFRFGFYLTEHQRIHTGEKPYKCKECGKAFIRRGNLKE
HLKIHSGLKPYDCKECGKSFSRRGQFTEHQKIHTGVKPYKCKECGKAFSRSVDLRIHQRI
HTGEKPYECKQCGKAFRLNSHLTEHQRIHTGEKPYECKVCRKAFRQYSHLYQHQKTHNVI
*
>ENSG00000047849|ENST00000360240
MADLSLADALTEPSPDIEGEIKRDFIATLEAEAFDDVVGETVGKTDYIPLLDVDEKTGNS
ESKKKPCSETSQIEDTPSSKPTLLANGGHGVEGSDTTGSPTEFLEEKMAYQEYPNSQNWP
EDTNFCFQPEQVVDPIQTDPFKMYHDDDLADLVFPSSATADTSIFAGQNDPLKDSYGMSP
CNTAVVPQGWSVEALNSPHSESFVSPEAVAEPPQPTAVPLELAKEIEMASEERPPAQALE
IMMGLKTTDMAPSKETEMALAKDMALATKTEVALAKDMESPTKLDVTLAKDMQPSMESDM
ALVKDMELPTEKEVALVKDVRWPTETDVSSAKNVVLPTETEVAPAKDVTLLKETERASPI
KMDLAPSKDMGPPKENKKETERASPIKMDLAPSKDMGPPKENKIVPAKDLVLLSEIEVAQ
ANDIISSTEISSAEKVALSSETEVALARDMTLPPETNVILTKDKALPLEAEVAPVKDMAQ
LPETEIAPAKDVAPSTVKEVGLLKDMSPLSETEMALGKDVTPPPETEVVLIKNVCLPPEM
EVALTEDQVPALKTEAPLAKDGVLTLANNVTPAKDVPPLSETEATPVPIKDMEIAQTQKG
ISEDSHLESLQDVGQSAAPTFMISPETVTGTGKKCSLPAEEDSVLEKLGERKPCNSQPSE
LSSETSGIARPEEGRPVVSGTGNDITTPPNKELPPSPEKKTKPLATTQPAKTSTSKAKTQ
PTSLPKQPAPTTIGGLNKKPMSLASGLVPAAPPKRPAVASARPSILPSKDVKPKPIADAK
APEKRASPSKPASAPASRSGSKSTQTVAKTTTAAAVASTGPSSRSPSTLLPKKPTAIKTE
GKPAEVKKMTAKSVPADLSRPKSTSTSSMKKTTTLSGTAPAAGVVPSRVKATPMPSRPST
TPFIDKKPTSAKPSSTTPRLSRLATNTSAPDLKNVRSKVGSTENIKHQPGGGRAKVEKKT
EAAATTRKPESNAVTKTAGPIASAQKQPAGKVQIVSKKVSYSHIQSKCGSKDNIKHVPGG
GNVQIQNKKVDISKVSSKCGSKANIKHKPGGGDVKIESQKLNFKEKAQAKVGSLDNVGHL
PAGGAVKTEGGGSEAPLCPGPPAGEEPAISEAAPEAGAPTSASGLNGHPTLSGGGDQREA
QTLDSQIQETSI*
>ENSG00000213523|ENST00000336283
MTRCPAGQAEVEMAELYVKPGNKERGWNDPPQFSYGLQTQAGGPRRSLLTKRVAAPQDGS
PRVPASETSPGPPPMGPPPPSSKAPRSPPVGSGPASGVEPTSFPVESEAVMEDVLRPLEQ
ALEDCRGHTRKQVCDDISRRLALLQEQWAGGKLSIPVKKRMALLVQELSSHRWDAADDIH
RSLMVDHVTEVSQWMVGVKRLIAEKRSLFSEEAANEEKSAATAEKNHTIPGFQQAS*

>ENSG00000196946|ENST00000359286
MHSLKKVTFEDVAIDFTQEEWAMMDTSKRKLYRDVMLENISHLVSLGYQISKSYIILQLE
QGKELWREGREFLQDQNPDRESALKKKHMISMHPITRKDASTSMTMENSLILEDPFECND
SGEDCTHSSTITQRLLTHSGKKPYVSKQCGKSLRNLFSPKPHKQIHTKGKSYQCNLCEKA
YTNCFRLRRHKMTHTGERPYACHLCGKAFTQCSHLRRHEKTHTGERPYKCHQCGKAFIQS
FNLRRHERTHLGKKCYECDKSGKAFSQSSGFRGNKIIHTGEKPHACLLCGKAFSLSSDLR
*
>ENSG00000067048|ENST00000336079
MSHVVVKNDPELDQQLANLDLNSEKQSGGASTASKGRYIPPHLRNREASKGFHDKDSSGW
SCSKDKDAYSSFGSRDSRGKPGYFSERGSGSRGRFDDRGRSDYDGIGNRERPGFGRFERS
GHSRWCDKSVEDDWSKPLPPSERLEQELFSGGNTGINFEKYDDIPVEATGSNCPPHIENF
SDIDMGEIIMGNIELTRYTRPTPVQKHAIPIIKGKRDLMACAQTGSGKTAAFLLPILSQI
YTDGPGEALKAVKENGRYGRRKQYPISLVLAPTRELAVQIYEEARKFSYRSRVRPCVVYG
GADIGQQIRDLERGCHLLVATPGRLVDMMERGKIGLDFCKYLVLDEADRMLDMGFEPQIR
RIVEQDTMPPKGVRHTMMFSATFPKEIQMLARDFLDEYIFLAVGRVGSTSENITQKVVWV
EDLDKRSFLLDILGATGSDSLTLVFVETKKGADSLEDFLYHEGYACTSIHGDRSQRDREE
ALHQFRSGKSPILVATAVAARGLDISNVRHVINFDLPSDIEEYVHRIGRTGRVGNLGLAT
SFFNEKNMNITKDLLDLLVEAKQEVPSWLENMAYEHHYKGGSRGRSKSNRFSGGFGARDY
RQSSGSSSSGFGASRGSSSRSGGGGYGNSRGFGGGGYGGFYNSDGYGGNYNSQGVDWWGN
*
>ENSG00000171703|ENST00000339217
MDLLRELKAMPITLHLLQSTRVGMSVNALRKQSSDEEVIALAKSLIKSWKKLLDASDAKA
RERGRGMPLPTSSRDASEAPDPSRKRPELPRAPSTPRITTFPPVPVTCDAVRNKCREMLT
AALQTDHDHVAIGADCERLSAQIEECIFRDVGNTDMKYKNRVRSRISNLKDAKNPDLRRN
VLCGAITPQQIAVMTSEEMASDELKEIRKAMTKEAIREHQMARTGGTQTDLFTCGKCRKK
NCTYTQ
>ENSG00000196482|ENST00000361395
MSNKDRHIDSSCSSFIKTEPSSPASLTDSVNHHSPGGSSDASGSYSSTMNGHQNGLDSPP
LYPSAPILGGSGPVRKLYDDCSSTIVEDPQTKCEYMLNSMPKRLCLVCGDIASGYHYGVA
SCEACKAFFKRTIQGNIEYSCPATNECEITKRRRKSCQACRFMKCLKVGMLKEGVRLDRV
RGGRQKYKRRIDAENSPYLNPQLVQPAKKPYNKIVSHLLVAEPEKIYAMPDPTVPDSDIK
ALTTLCDLADRELVVIIGWAKHIPGFSTLSLADQMSLLQSAWMEILILGVVYRSLSFEDE
LVYADDYIMDEDQSKLAGLLDLNNAILQLVKKYKSMKLEKEEFVTLKAIALANSDSMHIE
DVEAVQKLQDVLHEALQDYEAGQHMEDPRRAGKMLMTLPLLRQTSTKAVQHFYNIKLEGK
VPMHKLFLEMLEAKV*
>ENSG00000183598|ENST00000331491
MARTKQTARKSTGGKAPRKQLATKAARKSAPATGGVKKPHRYRPGTVALREIRRYQKSTE
LLIRKLPFQRLVREIAQDFKTDLRFQSSAVMALQEASEAYLVGLFEDTNLCAIHAKRVTI
MPKDIQLARRIRGERA*
>ENSG00000168214|ENST00000355476
MAWIKRKFGERPPPKRLTREAMRNYLKERGDQTVLILHAKVAQKSYGNEKRFFCPPPCVY
LMGSGWKKKKEQMERDGCSEQESQPCAFIGIGNSDQEMQQLNLEGKNYCTAKTLYISDSD
KRKHFMLSVKMFYGNSDDIGVFLSKRIKVISKPSKKKQSLKNADLCIASGTKVALFNRLR
SQTVSTRYLHVEGGNFHASSQQWGAFFIHLLDDDESEGEEFTVRDGYIHYGQTVKLVCSV

TGMALPRLIIRKVDKQTALLDADDPVSQLHKCAFYLKDTERMYLCLSQERIIQFQATPCP
KEPNKEMINDGASWTIISTDKAEYTFYEGMGPVLAPVTPVPVVESLQLNGGGDVAMLELT
GQNFTPNLRVWFGDVEAETMYRCGESMLCVVPDISAFREGWRWVRQPVQVPVTLVRNDGI
IYSTSLTFTYTPEPGPRPHCSAAGAILRANSSQVPPNESNTNSEGSYTNASTNSTSVTSS
TATVVS*
>ENSG00000153250|ENST00000348849
MGKVWKQQMYPQYATYYYPQYLQAKQSLVPAHPMAPPSPSTTSSNNNSSSSSNSGWDQLS
KTNLYIRGLPPHTTDQDLVKLCQPYGKIVSTKAILDKTTNKCKGYGFVDFDSPAAAQKAV
SALKASGVQAQMAKQQEQDPTNLYISNLPLSMDEQELENMLKPFGQVISTRILRDSSGTS
RGVGFARMESTEKCEAVIGHFNGKFIKTPPGVSAPTEPLLCKFADGGQKKRQNPNKYIPN
GRPWHREGEVRLAGMTLTYDPTTAAIQNGFYPSPYSIATNRMITQTSITPYIASPVSAYQ
VQSPSWMQPQPYILQHPGAVLTPSMEHTMSLQPASMISPLAQQMSHLSLGSTGTYMPATS
AMQGAYLPQYAHMQTTAVPVEEASGQQQVAVETSNDHSPYTFQPNK*
>ENSG00000122482|ENST00000361321
MLIRQNLALDCKQKKSRSRSGSKKKMLTLPHGADEVYILRCRFCGLVFRGPLSVQEDWIK
HLQRHIVNANLPRTGAGMVEVTSLLKKPASITETSFSLLMAEAAS*
>ENSG00000196584|ENST00000359321
MCSAFHRAESGTELLARLEGRSSLKEIEPNLFADEDSPVHGDILEFHGPEGTGKTEMLYH
LTARCILPKSEGGLEVEVLFIDTDYHFDMLRLVTILEHRLSQSSEEIIKYCLGRFFLVYC
SSSTHLLLTLYSLESMFCSHPSLCLLILDSLSAFYWIDRVNGGESVNLQESTLRKCSQCL
EKLVNDYRLVLFATTQTIMQKASSSSEEPSHASRRLCDVDIDYRPYLCKAWQQLVKHRMF
FSKQDDSQSSNQFSLVSRCLKSNSLKKHFFIIGESGVEFC*
>ENSG00000100227|ENST00000348657
MADISLDELIRKRGAAAKGRLNARPGVGGVRSRVGIQQGLLSQSTRTATFQQRFDARQKI
GLSDARLKLGVKDAREKLLQKDARFRIKGKVQDAREMLNSRKQQTTVPQKPRQVADAREK
ISLKRSSPAAFINPPIGTVTPALKLTKTIQNLYDLDEDDDGIASVPTKQMKFAASGGFLH
HMAGLSSSKLSMSKALPLTKVVQNDAYTAPALPSSIRTKALTNMSRTLVNKEEPPKELPA
AEPVLSPLEGTKMTVNNLHPRVTEEDIVELFCVCGALKRARLVHPGVAEVVFVKKDDAIT
AYKKYNNRCLDGQPMKCNLHMNGNVITSDQPILLRLSDSPSMKKESELPRRVNSASSSNP
PAEVDPDTILKALFKSSGASVTTQPTEFKIKL*
>ENSG00000198298|ENST00000361807
MAPRAQIQGPLTFGDVAVAFTRIEWRHLDAAQRALYRDVMLENYGNLVSVGLLSSKPKLI
TQLEQGAEPWTEVREAPSGTHAVEDYWFETKMSALKQSTSEASVLGERTKSVMMEKGLDW
EGRSSTEKNYKCKECGKVFKYNSSFISHQRNHTSEKPHKCKECGIAFMNSSSLLNHHKVH
AGKQPYRCIECGKFLKKHSTFINHQRIHSREKPHKCIECGKTFRKNSILLSHQRIHTGQK
PYKCNDCGKAFAQNAALTRHERIHSGEKPFKCNKCGRAFRDNSTVLEHQKIHTGEKPYQC
NECGKAFRKSSTLISHQRMHTGEKPYHCSKCGKSFRYSSSFAGHQKTHSGNKPYQCRDCG
KAFTKSSTLTGHQRIHTGEKPYHCKKCGKAFRHSSGLVEHQRLHTGEKPYKCNECGKAFP
RSSALKQHKKIHNKERAMKCS*
>ENSG00000120669|ENST00000317764
MASSIICQEHCQISGQAKIDILLVGDVTVGYLADTVQKLFANIAEVTITISDTKEAAALL
DDCIFNMVLLKVPSSLSAEELEAIKLIRFGKKKNTHSLFVFIIPENFKGCISGHGMDIAL
TEPLTMEKMSNVVKYWTTCPSNTVKTENATGPEELGLPLQRSYSEHLGYFPTDLFACSES
LRNGNGLELNASLSEFEKNKKISLLHSSKEKLRRLYRKHSSFCFW*

>ENSG00000065548|ENST00000337859
MPPKKQAQAGGSKKAEQKKKEKIIEDKTFGLKNKKGAKQQKFIKAVTHQVKFGQQNPRQV
AQSEAEKKLKKDDKKKELQELNELFKPVVAAQKISKGADPKSVVCAFFKQGQCTKGDKCK
FSHDLTLERKCEKRSVYIDARDEELEKDTMDNWDEKKLEEVVNKKHGEAEKKKPKTQIVC
KHFLEAIENNKYGWFWVCPGGGDICMYRHALPPGFVLKKDKKKEEKEDEISLEDLIERER
SALGPNVTKITLESFLAWKKRKRQEKIDKLEQDMERRKADFKAGKALVISGREVFEFRPE
LVNDDDEEADDTRYTQGTGGDEVDDSVSVNDIDLSLYIPRDVDETGITVASLERFSTYTS
DKDENKLSEASGGRAENGERSDLEEDNEREGTENGAIDAVPVDENLFTGEDLDELEEELN
TLDLEE*
>ENSG00000134452|ENST00000362091
MRRFKRKHLTAIDCQHLARSHLAVTQPFGQRWTNRDPNHGLYPKPRTKRGSRGQGSQRCI
PEFFLAGKQPCTNDMAKSNSVGQDSCQDSEGDMIFPAESSCALPQEGSAGPGSPGSAPPS
RKRSWSSEEESNQATGTSRWDGVSKKAPRHHLSVPCTRPREARQEAEDSTSRLSAESGET
DQDAGDVGPDPIPDSYYGLLGTLPCQEALSHICSLPSEVLRHVFAFLPVEDLYWNLSLVC
HLWREIISDPLFIPWKKLYHRYLMNEEQAVSKVDGILSNCGIEKESDLCVLNLIRYTATT
KCSPSVDPERVLWSLRDHPLLPEAEACVRQHLPDLYAAAGGVNIWALVAAVVLLSSSVND
IQRLLFCLRRPSSTVTMPDVTETLYCIAVLLYAMREKGINISNRIHYNIFYCLYLQENSC
TQATKVKEEPSVWPGKKTIQLTHEQQLILNHKMEPLQVVKIMAFAGTGKTSTLVKYAEKW
SQSRFLYVTFNKSIAKQAERVFPSNVICKTFHSMAYGHIGRKYQSKKKLNLFKLTPFMVN
SVLAEGKGGFIRAKLVCKTLENFFASADEELTIDHVPIWCKNSQGQRVMVEQSEKLNGVL
EASRLWDNMRKLGECTEEAHQMTHDGYLKLWQLSKPSLASFDAIFVDEAQDCTPAIMNIV
LSQPCGKIFVGDPHQQIYTFRGAVNALFTVPHTHVFYLTQSFRFGVEIAYVGATILDVCK
RVRKKTLVGGNHQSGIRGDAKGQVALLSRTNANVFDEAVRVTEGEFPSRIHLIGGIKSFG
LDRIIDIWILLQPEEERRKQNLVIKDKFIRRWVHKEGFSGFKRYVTAAEDKELEAKIAVV
EKYNIRIPELVQRIEKCHIEDLDFAEYILGTVHKAKGLEFDTVHVLDDFVKVPCARHNLP
QLPHFRVESFSEDEWNLLYVAVTRAKKRLIMTKSLENILTLAGEYFLQAELTSNVLKTGV
VRCCVGQCNNAIPVDTVLTMKKLPITYSNRKENKGGYLCHSCAEQRIGPLAFLTASPEQV
RAMERTVENIVLPRHEALLFLVF*
>ENSG00000125844|ENST00000360807
MDIYDTQTLGVVVFGGFMVVSAIGIFLVSTFSMKETSYEEALANQRKEMAKTHHQKVEKK
KKEKTVEKKGKTKKKEEKPNGKIPDHDPAPNVTVLLREPVRAPAVAVAPTPVQPPIIVAP
VATVPAMPQEKLASSPKDKKKKEKKVAKVEPAVSSVVNSIQVLTSKAAILETAPKEGRNT
DVAQSPEAPKQEAPAKKKSGSKKKGPPDADGPLYLPYKTLVSTVGSMVFNEGEAQRLIEI
LSEKAGIIQDTWHKATQKGDPVAILKRQLEEKEKLLATEQEDAAVAKSKLRELNKEMAAE
KAKAAAGEAKVKKQLVAREQEITAVQARMQASYREHVKEVQQLQGKIRTLQEQLENGPNT
QLARLQQENSILRDALNQATSQVESKQNAELAKLRQELSKVSKELVEKSEAVRQDEQQRK
ALEAKAAAFEKQVLQLQASHRESEEALQKRLDEVSRELCHTQSSHASLRADAEKAQEQQQ
QMAELHSKLQSSEAEVRSKCEELSGLHGQLQEARAENSQLTERIRSIEALLEAGQARDAQ
DVQASQAEADQQQTRLKELESQVSGLEKEAIELREAVEQQKVKNNDLREKNWKAMEALAT
AEQACKEKLLSLTQAKEESEKQLCLIEAQTMEALLALLPELSVLAQQNYTEWLQDLKEKG
PTLLKHPPAPAEPSSDLASKLREAEETQSTLQAECDQYRSILAETEGMLRDLQKSVEEEE
QVWRAKVGAAEEELQKSRVTVKHLEEIVEKLKGELESSDQVREHTSHLEAELEKHMAAAS
AECQNYAKEVAGLRQLLLESQSQLDAAKSEAQKQSDELALVRQQLSEMKSHVEDGDIAGA
PASSPEAPPAEQDPVQLKTQLEWTEAILEDEQTQRQKLTAEFEEAQTSACRLQEELEKLR

TAGPLESSETEEASQLKERLEKEKKLTSDLGRAATRLQELLKTTQEQLAREKDTVKKLQE
QLEKAEDGSSSKEGTSV*
>ENSG00000119772|ENST00000321117
MPAMPSSGPGDTSSSAAEREEDRKDGEEQEEPRGKEERQEPSTTARKVGRPGRKRKHPPV
ESGDTPKDPAVISKSPSMAQDSGASELLPNGDLEKRSEPQPEEGSPAGGQKGGAPAEGEG
AAETLPEASRAVENGCCTPKEGRGAPAEAGKEQKETNIESMKMEGSRGRLRGGLGWESSL
RQRPMPRLTFQAGDPYYISKRKRDEWLARWKREAEKKAKVIAGMNAVEENQGPGESQKVE
EASPPAVQQPTDPASPTVATTPEPVGSDAGDKNATKAGDDEPEYEDGRGFGIGELVWGKL
RGFSWWPGRIVSWWMTGRSRAAEGTRWVMWFGDGKFSVVCVEKLMPLSSFCSAFHQATYN
KQPMYRKAIYEVLQVASSRAGKLFPVCHDSDESDTAKAVEVQNKPMIEWALGGFQPSGPK
GLEPPEEEKNPYKEVYTDMWVEPEAAAYAPPPPAKKPRKSTAEKPKVKEIIDERTRERLV
YEVRQKCRNIEDICISCGSLNVTLEHPLFVGGMCQNCKNCFLECAYQYDDDGYQSYCTIC
CGGREVLMCGNNNCCRCFCVECVDLLVGPGAAQAAIKEDPWNCYMCGHKGTYGLLRRRED
WPSRLQMFFANNHDQEFDPPKVYPPVPAEKRKPIRVLSLFDGIATGLLVLKDLGIQVDRY
IASEVCEDSITVGMVRHQGKIMYVGDVRSVTQKHIQEWGPFDLVIGGSPCNDLSIVNPAR
KGLYEGTGRLFFEFYRLLHDARPKEGDDRPFFWLFENVVAMGVSDKRDISRFLESNPVMI
DAKEVSAAHRARYFWGNLPGMNRPLASTVNDKLELQECLEHGRIAKFSKVRTITTRSNSI
KQGKDQHFPVFMNEKEDILWCTEMERVFGFPVHYTDVSNMSRLARQRLLGRSWSVPVIRH
LFAPLKEYFACV*
>ENSG00000108788|ENST00000346833
MTEPGASPEDPWVKVEYAYSDNSLDPDDEDSDYHQEAYKESYKDRRRRAHTQAEQKRRDA
IKRGYDDLQTIVPTCQQQDFSIGSQKLSKAIVLQKTIDYIQFLHKEKKKQEEEVSTLRKD
VTALKIMKVNYEQIVKAHQDNPHEGEDQVSDQVKFNVFQGIMDSLFQSFNASISVASFQE
LSACVFSWIEEHCKPQTLREIVIGVLHQLKNQLY*
>ENSG00000132819|ENST00000344785
MHGSQKDTTFTKIFVGGLPYHTTDASLRKYFEGFGDIEEAVVITDRQTGKSRGYGFVTMA
DRAAAERACKDPNPIIDGRKANVNLAYLGAKPRSLQTGFAIGVQQLHPTLIQRTYGGDFM
FRK*
>ENSG00000187609|ENST00000340951
MDPGDPAGDPAAGERHRMGRDPLLLLQALQTLWSTRERKQLREEAWRGFAALDDPLAGLL
DMLESCRGQRGEGPSLAAWISHQLQCWLQAQPCPSLAQHSLRLKQLQARAVKVLTESPPS
LAAPLASIFQLQDADRSCLLAHVHRLHHEGRFREAATLGATLKLQSELGVEKMSIPLLLQ
DKVALVERYVAGFPDLQRRLLVLMDSWCQPGFDIKDVARRYPEVTSLSLEKLSPKALSRQ
VLRLQERYGVAPALCPNAAIQQRLAALRHLCHKRFVEKSLSQENWTDHVQGLVGQSPWLQ
EQLSQLLVSHSDPVTAAQCAMELLLPEERLPAAVAVELRRFRLQGRATEADSRLEVKDMK
DRYYQLPIPRENVHLLASWEDLTRHEGALLQCHQVVGVDVEWTPVFVAGGRPRPSLLQVA
VEGHVFLLDVLALSQPPTGQGAQAFSRLVAQLLSDPSITKLGYGMVGDLQKLGTSCPALA
HVEKQILGGMDLLLVHRQMRVASVPAPAVDRARELRGLSLLVQQVLGTALDKTQQLSNWD
RRPLCEEQVIYAAADAYCLLEVHQALCREPARFHLSEDLAGSRRPRHRERPGARKPPGLQ
KASAPAAPRQVPVAVAVSEGAAPQIPARAFRVVCDNMLQGLARSLRCLGVDARMLGNGED
HRRAAEVARQEGRIILTSGQPFHKLRAQVGAGRCLSVDCSLKAQQQAKAVLKHFNVRVTH
ADIFSRCQACNCDQYLKVSRDMMKQLMWLSSHQEGPRSSGDEATQSQAVQEPGPAPDAAP
EGCTYDRPCRWLQMADLRAETPDMLADGTRLQLAGVPVGVLRTPGLRCFYCCTGCGKVFW
DGSHLGRVATHFRDMLESAPSPCEPSPAPSPASSPF*

>ENSG00000134815|ENST00000328771
MPPPRTREGRDRRDHHRAPSEEEALEKWDWNCPETRRLLEDAFFREEDYIRQGSEECQKF
WTFFERLQRFQNLKTSRKEEKDPGQPKHSIPALADLPRTYDPRYRINLSVLGPATRGSQG
LGRHLPAERVAEFRRALLHYLDFGQKQAFGRLAKLQRERAALPIAQYGNRILQTLKEHQV
VVVAGDTGCGKSTQVPQYLLAAGFSHVACTQPRRIACISLAKRVGFESLSQYGSQVGYQI
RFESTRSAATKIVFLTVGLLLRQIQREPSLPQYEVLIVDEVHERHLHNDFLLGVLQRLLP
TRPDLKVILMSATINISLFSSYFSNAPVVQVPGRLFPITVVYQPQEAEPTTSKSEKLDPR
PFLRVLESIDHKYPPEERGDLLVFLSGMAEISAVLEAAQTYASHTQRWVVLPLHSALSVA
DQDKVFDVAPPGVRKCILSTNIAETSVTIDGIRFVVDSGKVKEMSYDPQAKLQRLQEFWI
SQASAEQRKGRAGRTGPGVCFRLYAESDYDAFAPYPVPEIRRVALDSLVLQMKSMSVGDP
RTFPFIEPPPPASLETAILYLRDQGALDSSEALTPIGSLLAQLPVDVVIGKMLILGSMFS
LVEPVLTIAAALSVQSPFTRSAQSSPECAAARRPLESDQGDPFTLFNVFNAWVQVKSERS
RNSRKWCRRRGIEEHRLYEMANLRRQFKELLEDHGLLAGAQAAQVGDSYSRLQQRRERRA
LHQLKRQHEEGAGRRRKVLRLQEEQDGGSSDEDRAGPAPPGASDGVDIQDVKFKLRHDLA
QLQAAASSAQDLSREQLALLKLVLGRGLYPQLAVPDAFNSSRKDSDQIFHTQAKQGAVLH
PTCVFAGSPEVLHAQELEASNCDGSRDDKDKMSSKHQLLSFVSLLETNKPYLVNCVRIPA
LQSLLLFSRSLDTNGDCSRLVADGWLELQLADSESAIRLLAASLRLRARWESALDRQLAH
QAQQQLEEEEEDTPVSPKEVATLSKELLQFTASKIPYSLRRLTGLEVQNMYVGPQTIPAT
PHLPGLFGSSTLSPHPTKGGYAVTDFLTYNCLTNDTDLYSDCLRTFWTCPHCGLHAPLTP
LERIAHENTCPQAPQDGPPGAEEAALETLQKTSVLQRPYHCEACGKDFLFTPTEVLRHRK
QHV*
>ENSG00000185499|ENST00000343256
MTPGTQSPFFLLLLLTVLTVVTGSGHASSTPGGEKETSATQRSSVPSSTEKNAIPAPTTT
KSCRETFLKCFCRFINKGVFWASPILSSVSDVPFPFSAQSGAGVPGWGIALLVLVCVLVA
LAIVYLIALAVCQCRRKNYGQLDIFPARDTYHPMSEYPTYHTHGRYVPPSSTDRSPYEKV
SAGNGGSSLSYTNPAVAATSANL*
>ENSG00000239732|ENST00000360658
MGFCRSALHPLSLLVQAIMLAMTLALGTLPAFLPCELQPHGLVNCNWLFLKSVPHFSMAA
PRGNVTSLSLSSNRIHHLHDSDFAHLPSLRHLNLKWNCPPVGLSPMHFPCHMTIEPSTFL
AVPTLEELNLSYNNIMTVPALPKSLISLSLSHTNILMLDSASLAGLHALRFLFMDGNCYY
KNPCRQALEVAPGALLGLGNLTHLSLKYNNLTVVPRNLPSSLEYLLLSYNRIVKLAPEDL
ANLTALRVLDVGGNCRRCDHAPNPCMECPRHFPQLHPDTFSHLSRLEGLVLKDSSLSWLN
ASWFRGLGNLRVLDLSENFLYKCITKTKAFQGLTQLRKLNLSFNYQKRVSFAHLSLAPSF
GSLVALKELDMHGIFFRSLDETTLRPLARLPMLQTLRLQMNFINQAQLGIFRAFPGLRYV
DLSDNRISGASELTATMGEADGGEKVWLQPGDLAPAPVDTPSSEDFRPNCSTLNFTLDLS
RNNLVTVQPEMFAQLSHLQCLRLSHNCISQAVNGSQFLPLTGLQVLDLSHNKLDLYHEHS
FTELPRLEALDLSYNSQPFGMQGVGHNFSFVAHLRTLRHLSLAHNNIHSQVSQQLCSTSL
RALDFSGNALGHMWAEGDLYLHFFQGLSGLIWLDLSQNRLHTLLPQTLRNLPKSLQVLRL
RDNYLAFFKWWSLHFLPKLEVLDLAGNQLKALTNGSLPAGTRLRRLDVSCNSISFVAPGF
FSKAKELRELNLSANALKTVDHSWFGPLASALQILDVSANPLHCACGAAFMDFLLEVQAA
VPGLPSRVKCGSPGQLQGLSIFAQDLRLCLDEALSWDCFALSLLAVALGLGVPMLHHLCG
WDLWYCFHLCLAWLPWRGRQSGRDEDALPYDAFVVFDKTQSAVADWVYNELRGQLEECRG
RWALRLCLEERDWLPGKTLFENLWASVYGSRKTLFVLAHTDRVSGLLRASFLLAQQRLLE
DRKDVVVLVILSPDGRRSRYVRLRQRLCRQSVLLWPHQPSGQRSFWAQLGMALTRDNHHF

YNRNFCQGPTAE*
>ENSG00000196074|ENST00000357552
MPIRPDLQQLEKCIDDALRKNDFKPLKTLLQIDICEDVKIKCSKQFFHKVDNLICRELNK
EDIHNVSAILVSVGRCGKNISVLGQAGLLTMIKQGLIQKMVAWFEKSKDIIQSQGNSKDE
AVLNMIEDLVDLLLVIHDVSDEGKKQVVESFVPRICSLVIDSRVNICIQQEIIKKMNAML
DKMPQDARKILSNQEMLILMSSMGERILDAGDYDLQVGIVEALCRMTTEKQRQELAHQWF
SMDFIAKAFKRIKDSEFETDCRIFLNLVNGMLGDKRRVFTFPCLSAFLDKYELQIPSDEK
LEEFWIDFNLGSQTLSFYIAGDNDDHQWEAVTVPEEKVQIYSIEVRESKKLLTIILKNTV
KISKREGKELLLYFDASLEITNVTQKIFGATKHRESIRKQGISVAKTSLHILFDASGSQI
LVPESQISPVGEELVSLKEKSKSPKEFAKPSKYIKNSDKGNRNNSQLEKTTPSKRKMSEA
SMIVSGADRYTMRSPVLFSNTSIPPRRRRIKPPLQMTSSAEKPSVSQTSENRVDNAASLK
SRSSEGRHRRDNIDKHIKTAKCVENTENKNVEFPNQNFSELQDVIPDSQAAEKRDHTILP
GVLDNICGNKIHSKWACWTPVTNIELCNNQRASTSSGDTLNQDIVINKKLTKQKSSSSIS
DHNSEGTGKVKYKKEQTDHIKIDKAEVEVCKKHNQQQNHPKYSGQKNTENAKQSDWPVES
ETTFKSVLLNKTIEESLIYRKKYILSKDVNTATCDKNPSASKNVQSHRKAEKELTSELNS
WDSKQKKMREKSKGKEFTNVAESLISQINKRYKTKDDIKSTRKLKESLINSGFSNKPVVQ
LSKEKVQKKSYRKLKTTFVNVTSECPVNDVYNFNLNGADDPIIKLGIQEFQATAKEACAD
RSIRLVGPRNHDELKSSVKTKDKKIITNHQKKNLFSDTETEYRCDDSKTDISWLREPKSK
PQLIDYSRNKNVKNHKSGKSRSSLEKGQPSSKMTPSKNITKKMDKTIPEGRIRLPRKATK
TKKNYKDLSNSESECEQEFSHSFKENIPVKEENIHSRMKTVKLPKKQQKVFCAETEKELS
KQWKNSSLLKDAIRDNCLDLSPRSLSGSPSSIEVTRCIEKITEKDFTQDYDCITKSISPY
PKTSSLESLNSNSGVGGTIKSPKNNEKNFLCASESCSPIPRPLFLPRHTPTKSNTIVNRK
KISSLVLTQETQNSNSYSDVSSYSSEERFMEIESPHINENYIQSKREESHLASSLSKSSE
GREKTWFDMPCDATHVSGPTQHLSRKRIYIEDNLSNSNEVEMEEKGERRANLLPKKLCKI
EDADHHIHKMSESVSSLSTNDFSIPWETWQNEFAGIEMTYETYERLNSEFKRRNNIRHKM
LSYFTTQSWKTAQQHLRTMNHQSQDSRIKKLDKFQFIIIEELENFEKDSQSLKDLEKEFV
DFWEKIFQKFSAYQKSEQQRLHLLKTSLAKSVFCNTDSEETVFTSEMCLMKEDMKVLQDR
LLKDMLEEELLNVRRELMSVFMSHERNANV*
>ENSG00000135903|ENST00000350526
MTTLAGAVPRMMRPGPGQNYPRSGFPLEVSTPLGQGRVNQLGGVFINGRPLPNHIRHKIV
EMAHHGIRPCVISRQLRVSHGCVSKILCRYQETGSIRPGAIGGSKPKQVTTPDVEKKIEE
YKRENPGMFSWEIRDKLLKDAVCDRNTVPSVSSISRILRSKFGKGEEEEADLERKEAEES
EKKAKHSIDGILSERASAPQSDEGSDIDSEPDLPLKRKQRRSRTTFTAEQLEELERAFER
THYPDIYTREELAQRAKLTEARVQVWFSNRRARWRKQAGANQLMAFNHLIPGGFPPTAMP
TLPTYQLSETSYQPTSIPQAVSDPSSTVHRPQPLPPSTVHQSTIPSNPDSSSAYCLPSTR
HGFSSYTDSFVPPSGPSNPMNPTIGNGLSPQVMGLLTNHGGVPHQPQTDYALSPLTGGLE
PTTTVSASCSQRLDHMKSLDSLPTSQSYCPPTYSTTGYSMDPVTGYQYGQYGQSKPWTF*
>ENSG00000116731|ENST00000343137
MRDSAEGPKEDEEKPSASALEQPATLQEVASQEVPPELATPAPAWEPQPEPDERLEAAAC
EVNDLGEEEEEEEEEDEEEEEDDDDDELEDEGEEEASMPNENSVKEPEIRCDEKPEDLLE
EPKTTSEETLEDCSEVTPAMQIPRTKEEANGDVFETFMFPCQHCERKFTTKQGLERHMHI
HISTVNHAFKCKYCGKAFGTQINRRRHERRHEAGLKRKPSQTLQPSEDLADGKASGENVA
SKDDSSPPSLGPDCLIMNSEKASQDTINSSVVEENGEVKELHPCKYCKKVFGTHTNMRRH
QRRVHERHLIPKGVRRKGGLEEPQPPAEQAQATQNVYVPSTEPEEEGEADDVYIMDISSN

ISENLNYYIDGKIQTNNNTSNCDVIEMESASADLYGINCLLTPVTVEITQNIKTTQVPVT
EDLPKEPLGSTNSEAKKRRTASPPALPKIKAETDSDPMVPSCSLSLPLSISTTEAVSFHK
EKSVYLSSKLKQLLQTQDKLTPAGISATEIAKLGPVCVSAPASMLPVTSSRFKRRTSSPP
SSPQHSPALRDFGKPSDGKAAWTDAGLTSKKSKLESHSDSPAWSLSGRDERETVSPPCFD
EYKMSKEWTASSAFSSVCNQQPLDLSSGVKQKAEGTGKTPVQWESVLDLSVHKKHCSDSE
GKEFKESHSVQPTCSAVKKRKPTTCMLQKVLLNEYNGIDLPVENPADGTRSPSPCKSLEA
QPDPDLGPGSGFPAPTVESTPDVCPSSPALQTPSLSSGQLPPLLIPTDPSSPPPCPPVLT
VATPPPPLLPTVPLPAPSSSASPHPCPSPLSNATAQSPLPILSPTVSPSPSPIPPVEPLM
SAASPGPPTLSSSSSSSSSSSSFSSSSSSSSPSPPPLSAISSVVSSGDNLEASLPMISFK
QEELENEGLKPREEPQSAAEQDVVVQETFNKNFVCNVCESPFLSIKDLTKHLSIHAEEWP
FKCEFCVQLFKDKTDLSEHRFLLHGVGNIFVCSVCKKEFAFLCNLQQHQRDLHPDKVCTH
HEFESGTLRPQNFTDPSKAHVEHMQSLPEDPLETSKEEEELNDSSEELYTTIKIMASGIK
TKDPDVRLGLNQHYPSFKPPPFQYHHRNPMGIGVTATNFTTHNIPQTFTTAIRCTKCGKG
VDNMPELHKHILACASASDKKRYTPKKNPVPLKQTVQPKNGVVVLDNSGKNAFRRMGQPK
RLNFSVELSKMSSNKLKLNALKKKNQLVQKAILQKNKSAKQKADLKNACESSSHICPYCN
REFTYIGSLNKHAAFSCPKKPLSPPKKKVSHSSKKGGHSSPASSDKNSNSNHRRRTADAE
IKMQSMQTPLGKTRARSSGPTQVPLPSSSFRSKQNVKFAASVKSKKPSSSSLRNSSPIRM
AKITHVEGKKPKAVAKNHSAQLSSKTSRSLHVRVQKSKAVLQSKSTLASKKRTDRFNIKS
RERSGGPVTRSLQLAAAADLSENKREDGSAKQELKDFRNFL*
>ENSG00000176723|ENST00000315678
MRSLPFALTVESVSARAPTCCSTGRFTQGRQPCKCKACGRGFTQSASLLQHWRVHSDWRE
TLSLSPVRQDLLWPLQPHQAPASPLGRSHSSAGVRQGFSGQLCCWLTKEHTLAEALRLSP
VPAGFWGPVEADRPPANSHRRVCPFCCCSCGDSVNEKTSLSQRVLPHPGEKTCRGGSVES
VSLAPSSVAPDSTSGLRPCGSPGSFLQHLPPSTLLPRPPFLYPGPPLSLQPLVPSGLPAV
PAVPLGGLEVAQVPPATQPAAQQEGAMGPRSCASAGRDSREAVQAPGYPEPARKASQHRA
AGPLGEARARLQRQCRAPPPPSNPHWRGALPVFPVWKASSRRSNLARH*
>ENSG00000196323|ENST00000357899
MGVKTFTHSSSSHSQEMLGKLNMLRNDGHFCDITIRVQDKIFRAHKVVLAACSDFFRTKL
VGQAEDENKNVLDLHHVTVTGFIPLLEYAYTATLSINTENIIDVLAAASYMQMFSVASTC
SEFMKSSILWNTPNSQPEKGLDAGQENNSNCNFTSRDGSISPVSSECSVVERTIPVCRES
RRKRKSYIVMSPESPVKCGTQTSSPQVLNSSASYSENRNQPVDSSLAFPWTFPFGIDRRI
QPEKVKQAENTRTLELPGPSETGRRMADYVTCESTKTTLPLGTEEDVRVKVERLSDEEVH
EEVSQPVSASQSSLSDQQTVPGSEQVQEDLLISPQSSSIGSVDEGVSEGLPTLQSTSSTN
APPDDDDRLENVQYPYQLYIAPSTSSTERPSPNGPDRPFQCPTCGVRFTRIQNLKQHMLI
HSGIKPFQCDRCGKKFTRAYSLKMHRLKHEGKRCFRCQICSATFTSFGEYKHHMRVSRHI
IRKPRIYECKTCGAMFTNSGNLIVHLRSLNHEASELANYFQSSDFLVPDYLNQEQEETLV
QYDLGEHGFESNSSVQMPVISQYHSKGKEP*
>ENSG00000103168|ENST00000341690
MLPPLIDPWDPGLTARDLLFRGGCRYRKRPRVVLDVTEQISRFLLDHGDVAFAPLGKLML
ENFKLEGAGSRTKKKTVVSVKKLLQDLGGHQPWGCPWAYLSNRQRRFSILGGPILGTSVA
SHLAELLHEELVLRWEQLLLDEACTGGALAWVPGRTPQFGQLVYPAGGAQDRLHFQEVVL
TPGDNPQFLGKPGRIQLQGPVRQVVTCTVQGETLLAVRSDYHCAVWKFGKQWQPTLLQAM
QVEKGATGISLSPHLPGELAICSRSGAVCLWSPEDGLRQIYRDPETLVFRDSSSWRWADF
TAHPRVLTVGDRTGVKMLDTQGPPGCGLLLFRLGAEASCQKGERVLLTQYLGHSSPKCLP

PTLHLVCTQFSLYLVDERLPLVPMLKWNHGLPSPLLLARLLPPPRPSCVQPLLLGGQGGQ
LQLLHLAEGASVPRLAGPPQSLPSRIDSLPAFPLLEPKIQWRLQERLKAPTIGLAAVVPP
LPSAPTPGLVLFQLSAAGDVFYQQLRPQVDSSLRRDAGPPGDTQPDCHAPTASWTSQDTA
GCSQWLKALLKVPLAPPVWTAPTFTHRQMLGSTELRREEEEGQRLGVLRKAMARGQLLLQ
RDLGSLPAAEPPPAPESGLEDKLSERLGEAWAGRGAAWWERQQGRTSEPGRQTRRPKRRT
QLSSSFSLSGHVDPSEDTSSPHSPEWPPADALPLPPTTPPSQELTPDACAQGVPSEQRQM
LRDYMAKLPPQRDTPGCATTPPHSQASSVRATRSQQHTPVLSSSQPLRKKPRMGF*
>ENSG00000184428|ENST00000329245
MRVVRLLRLRAALTLLGEVPRRPASRGVPGSRRTQKGSGARWEKEKHEDGVKWRQLEHKG
PYFAPPYEPLPDGVRFFYEGRPVRLSVAAEEVATFYGRMLDHEYTTKEVFRKNFFNDWRK
EMAVEEREVIKSLDKCDFTEIHRYFVDKAAARKVLSREEKQKLKEEAEKLQQEFGYCILD
GHQEKIGNFKIEPPGLFRGRGDHPKMGMLKRRITPEDVVINCSRDSKIPEPPAGHQWKEV
RSDNTVTWLAAWTESVQNSIKYIMLNPCSKLKGETAWQKFETARRLRGFVDEIRSQYRAD
WKSREMKTRQRAVALYFIDKLALRAGNEKEDGEAADTVGCCSLRVEHVQLHPEADGCQHV
VEFDFLGKDCIRYYNRVPVEKPVYKNLQLFMENKDPRDDLFDRLTTTSLNKHLQELMDGL
TAKVFRTYNASITLQEQLRALTRAEDSIAAKILSYNRANRVVAILCNHQRATPSTFEKSM
QNLQTKIQAKKEQVAEARAELRRARAEHKAQGDGKSRSVLEKKRRLLEKLQEQLAQLSVQ
ATDKEENKQVALGTSKLNYLDPRISIAWCKRFRVPVEKIYSKTQRERFAWALAMAGEDFE
F*
>ENSG00000117322|ENST00000367057
MGAAGLLGVFLALVAPGVLGISCGSPPPILNGRISYYSTPIAVGTVIRYSCSGTFRLIGE
KSLLCITKDKVDGTWDKPAPKCEYFNKYSSCPEPIVPGGYKIRGSTPYRHGDSVTFACKT
NFSMNGNKSVWCQANNMWGPTRLPTCVSVFPLECPALPMIHNGHHTSENVGSIAPGLSVT
YSCESGYLLVGEKIINCLSSGKWSAVPPTCEEARCKSLGRFPNGKVKEPPILRVGVTANF
FCDEGYRLQGPPSSRCVIAGQGVAWTKMPVCEEIFCPSPPPILNGRHIGNSLANVSYGSI
VTYTCDPDPEEGVNFILIGESTLRCTVDSQKTGTWSGPAPRCELSTSAVQCPHPQILRGR
MVSGQKDRYTYNDTVIFACMFGFTLKGSKQIRCNAQGTWEPSAPVCEKECQAPPNILNGQ
KEDRHMVRFDPGTSIKYSCNPGYVLVGEESIQCTSEGVWTPPVPQCKVAACEATGRQLLT
KPQHQFVRPDVNSSCGEGYKLSGSVYQECQGTIPWFMEIRLCKEITCPPPPVIYNGAHTG
SSLEDFPYGTTVTYTCNPGPERGVEFSLIGESTIRCTSNDQERGTWSGPAPLCKLSLLAV
QCSHVHIANGYKISGKEAPYFYNDTVTFKCYSGFTLKGSSQIRCKADNTWDPEIPVCEKG
CQSPPGLHHGRHTGGNTVFFVSGMTVDYTCDPGYLLVGNKSIHCMPSGNWSPSAPRCEET
CQHVRQSLQELPAGSRVELVNTSCQDGYQLTGHAYQMCQDAENGIWFKKIPLCKVIHCHP
PPVIVNGKHTGMMAENFLYGNEVSYECDQGFYLLGEKKLQCRSDSKGHGSWSGPSPQCLR
SPPVTRCPNPEVKHGYKLNKTHSAYSHNDIVYVDCNPGFIMNGSRVIRCHTDNTWVPGVP
TCIKKAFIGCPPPPKTPNGNHTGGNIARFSPGMSILYSCDQGYLLVGEALLLCTHEGTWS
QPAPHCKEVNCSSPADMDGIQKGLEPRKMYQYGAVVTLECEDGYMLEGSPQSQCQSDHQW
NPPLAVCRSRSLAPVLCGIAAGLILLTFLIVITLYVISKHRARNYYTDTSQKEAFHLEAR
EVYSVDPYNPAS*
>ENSG00000146540|ENST00000357429
MAKQKRKVPEVTEKKNKKLKKASAEGPLLGPEAAPSGEGAGSKGEAVLRPGLDAEPELSP
EEQRVLERKLKKERKKEERQRLREAGLVAQHPPARRSGAELALDYLCRWAQKHKNWRFQK
TRQTWLLLHMYDSDKVPDEHFSTLLAYLEGLQGRARELTVQKAEALMRELDEEGSDPPLP
GRAQRIRQVLQLLS*

>ENSG00000164362|ENST00000334602
MPRAPRCRAVRSLLRSHYREVLPLATFVRRLGPQGWRLVQRGDPAAFRALVAQCLVCVPW
DARPPPAAPSFRQVSCLKELVARVLQRLCERGAKNVLAFGFALLDGARGGPPEAFTTSVR
SYLPNTVTDALRGSGAWGLLLRRVGDDVLVHLLARCALFVLVAPSCAYQVCGPPLYQLGA
ATQARPPPHASGPRRRLGCERAWNHSVREAGVPLGLPAPGARRRGGSASRSLPLPKRPRR
GAAPEPERTPVGQGSWAHPGRTRGPSDRGFCVVSPARPAEEATSLEGALSGTRHSHPSVG
RQHHAGPPSTSRPPRPWDTPCPPVYAETKHFLYSSGDKEQLRPSFLLSSLRPSLTGARRL
VETIFLGSRPWMPGTPRRLPRLPQRYWQMRPLFLELLGNHAQCPYGVLLKTHCPLRAAVT
PAAGVCAREKPQGSVAAPEEEDTDPRRLVQLLRQHSSPWQVYGFVRACLRRLVPPGLWGS
RHNERRFLRNTKKFISLGKHAKLSLQELTWKMSVRDCAWLRRSPGVGCVPAAEHRLREEI
LAKFLHWLMSVYVVELLRSFFYVTETTFQKNRLFFYRKSVWSKLQSIGIRQHLKRVQLRE
LSEAEVRQHREARPALLTSRLRFIPKPDGLRPIVNMDYVVGARTFRREKRAERLTSRVKA
LFSVLNYERARRPGLLGASVLGLDDIHRAWRTFVLRVRAQDPPPELYFVKVDVTGAYDTI
PQDRLTEVIASIIKPQNTYCVRRYAVVQKAAHGHVRKAFKSHVSTLTDLQPYMRQFVAHL
QETSPLRDAVVIEQSSSLNEASSGLFDVFLRFMCHHAVRIRGKSYVQCQGIPQGSILSTL
LCSLCYGDMENKLFAGIRRDGLLLRLVDDFLLVTPHLTHAKTFLSYARTSIRASLTFNRG
FKAGRNMRRKLFGVLRLKCHSLFLDLQVNSLQTVCTNIYKILLLQAYRFHACVLQLPFHQ
QVWKNPTFFLRVISDTASLCYSILKAKNAGMSLGAKGAAGPLPSEAVQWLCHQAFLLKLT
RHRVTYVPLLGSLRTAQTQLSRKLPGTTLTALEAAANPALPSDFKTILD*
>ENSG00000117322|ENST00000367058
MGAAGLLGVFLALVAPGVLGISCGSPPPILNGRISYYSTPIAVGTVIRYSCSGTFRLIGE
KSLLCITKDKVDGTWDKPAPKCEYFNKYSSCPEPIVPGGYKIRGSTPYRHGDSVTFACKT
NFSMNGNKSVWCQANNMWGPTRLPTCVSVFPLECPALPMIHNGHHTSENVGSIAPGLSVT
YSCESGYLLVGEKIINCLSSGKWSAVPPTCEEARCKSLGRFPNGKVKEPPILRVGVTANF
FCDEGYRLQGPPSSRCVIAGQGVAWTKMPVCEEIFCPSPPPILNGRHIGNSLANVSYGSI
VTYTCDPDPEEGVNFILIGESTLRCTVDSQKTGTWSGPAPRCELSTSAVQCPHPQILRGR
MVSGQKDRYTYNDTVIFACMFGFTLKGSKQIRCNAQGTWEPSAPVCEKECQAPPNILNGQ
KEDRHMVRFDPGTSIKYSCNPGYVLVGEESIQCTSEGVWTPPVPQCKVAACEATGRQLLT
KPQHQFVRPDVNSSCGEGYKLSGSVYQECQGTIPWFMEIRLCKEITCPPPPVIYNGAHTG
SSLEDFPYGTTVTYTCNPGPERGVEFSLIGESTIRCTSNDQERGTWSGPAPLCKLSLLAV
QCSHVHIANGYKISGKEAPYFYNDTVTFKCYSGFTLKGSSQIRCKADNTWDPEIPVCEKE
TCQHVRQSLQELPAGSRVELVNTSCQDGYQLTGHAYQMCQDAENGIWFKKIPLCKVIHCH
PPPVIVNGKHTGMMAENFLYGNEVSYECDQGFYLLGEKKLQCRSDSKGHGSWSGPSPQCL
RSPPVTRCPNPEVKHGYKLNKTHSAYSHNDIVYVDCNPGFIMNGSRVIRCHTDNTWVPGV
PTCIKKAFIGCPPPPKTPNGNHTGGNIARFSPGMSILYSCDQGYLLVGEALLLCTHEGTW
SQPAPHCKEVNCSSPADMDGIQKGLEPRKMYQYGAVVTLECEDGYMLEGSPQSQCQSDHQ
WNPPLAVCRSRSLAPVLCGIAAGLILLTFLIVITLYVISKHRARNYYTDTSQKEAFHLEA
REVYSVDPYNPAS*
>ENSG00000179930|ENST00000339948
MAQVDSQDRWGEASPLSSLTEEAHDTQMLSMNLESDDEDGGEAEKEGTADPVACPRGSSP
VTHENPDLPWPHPLGKEEEKFSDSSSAGGMGQKPVEMSGKASWSRDVTKINETQGSPGAS
RALGSLPSGLAHKLLGQMQPLGDRLPAGDDGYSGANQDAVLDVPPSFPSNGKYLCAHKSV
DTSAGNSSLLCFPRPGSNWDLPTQETHTPAQASATPASLAAAVLAKARNSRKVQNQAGRR
EGGEAEARPYRCLRGGRAFQKPSKPLSPAETRGGAAKRYACELCGKAYSHRGTLQQHRRL

HTGERPYQCSFCDKAYTWSSDHRKHIRTHTGEKPYPCPDCGKAFVRSSDLRKHQRNMHSN
NKPFPCSECGLTFNKPLSLLRHQRTHLGAKPFRCPACDREFAVASRMVEHQRVHSGERPF
PCPTCGKCFTKSSNLSEHQTLHTGQRPFKCADCGVAFAQPSRLVRHQRIHTGERPFPCTQ
CGQAFARSSTLKRHQQIHSGEKGFLCAECGRAFRIASELAQHIRMHNGERPYQCEDCGQA
FTRSNHLQRHRAKHGTCKKEPIPSSSDE*
>ENSG00000199085|ENST00000362215
Sequence unavailable
>ENSG00000178573|ENST00000326043
MASELAMSNSDLPTSPLAMEYVNDFDLMKFEVKKEPVETDRIISQCGRLIAGGSLSSTPM
STPCSSVPPSPSFSAPSPGSGSEQKAHLEDYYWMTGYPQQLNPEALGFSPEDAVEALISN
SHQLQGGFDGYARGAQQLAAAAGAGAGASLGGSGEEMGPAAAVVSAVIAAAAAQSGAGPH
YHHHHHHAAGHHHHPTAGAPGAAGSAAASAGGAGGAGGGGPASAGGGGGGGGGGGGGGAA
GAGGALHPHHAAGGLHFDDRFSDEQLVTMSVRELNRQLRGVSKEEVIRLKQKRRTLKNRG
YAQSCRFKRVQQRHVLESEKNQLLQQVDHLKQEISRLVRERDAYKEKYEKLVSSGFRENG
SSSDNPSSPEFFITEPTRKLEPSVGYATFWKPQHRVLTSVFTK*
>ENSG00000185811|ENST00000343574
MDADEGQDMSQVSGKESPPVSDTPDEGDEPMPIPEDLSTTSGGQQSSKSDRVVGERPFQC
NQCGASFTQKGNLLRHIKLHSGEKPFKCHLCNYACRRRDALTGHLRTHSVGKPHKCGYCG
RSYKQRSSLEEHKERCHNYLESMGLPGTLYPVIKEETNHSEMAEDLCKIGSERSLVLDRL
ASNVAKRKSSMPQKFLGDKGLSDTPYDSSASYEKENEMMKSHVMDQAINNAINYLGAESL
RPLVQTPPGGSEVVPVISPMYQLHKPLAEGTPRSNHSAQDSAVENLLLLSKAKLVPSERE
ASPSNSCQDSTDTESNNEEQRSGLIYLTNHIAPHARNGLSLKEEHRAYDLLRAASENSQD
ALRVVSTSGEQMKVYKCEHCRVLFLDHVMYTIHMGCHGFRDPFECNMCGYHSQDRYEFSS
HITRGEHRFHMS*
>ENSG00000111783|ENST00000357881
MIKRRAHPGAGGDRTRPRRRRSTESWIERCLNESENKRYSSHTSLGNVSNDENEEKENNR
ASKPHSTPATLQWLEENYEIAEGVCIPRSALYMHYLDFCEKNDTQPVNAASFGKIIRQQF
PQLTTRRLGTRGQSKYHYYGIAVKESSQYYDVMYSKKGAAWVSETGKKEVSKQTVAYSPR
SKLGTLLPEFPNVKDLNLPASLPEEKVSTFIMMYRTHCQRILDTVIRANFDEVQSFLLHF
WQGMPPHMLPVLGSSTVVNIVGVCDSILYKAISGVLMPTVLQALPDSLTQVIRKFAKQLD
EWLKVALHDLPENLRNIKFELSRRFSQILRRQTSLNHLCQASRTVIHSADITFQMLEDWR
NVDLNSITKQTLYTMEDSRDEHRKLITQLYQEFDHLLEEQSPIESYIEWLDTMVDRCVVK
VAAKRQGSLKKVAQQFLLMWSCFGTRVIRDMTLHSAPSFGSFHLIHLMFDDYVLYLLESL
HCQERANELMRAMKGEGSTAEVREEIILTEAAAPTPSPVPSFSPAKSATSVEVPPPSSPV
SNPSPEYTGLSTTGAMQSYTWSLTYTVTTAAGSPAENSQQLPCMRNTHVPSSSVTHRIPV
YPHREEHGYTGSYNYGSYGNQHPHPMQSQYPALPHDTAISGPLHYAPYHRSSAQYPFNSP
TSRMEPCLMSSTPRLHPTPVTPRWPEVPSANTCYTSPSVHSARYGNSSDMYTPLTTRRNS
EYEHMQHFPGFAYINGEASTGWAK*
>ENSG00000189299|ENST00000339140
MDLKLKDCEFWYSLHGQVPGLLDWDMRNELFLPCTTDQCSLAEQILAKYRVGVMKPPEMP
QKRRPSPDGDGPPCEPNLWMWVDPNILCPLGSQEAPKPSGKEDLTNISPFPQPPQKDEGS
NCSEDKVVESLPSSSSEQSPLQKQGIHSPSDFELTEEEAEEPDDNSLQSPEMKCYQSQKL
WQINNQEKSWQRPPLNCSHLIALALRNNPHCGLSVQEIYNFTRQHFPFFWTAPDGWKSTI
HYNLCFLDSFEKVPDSLKDEDNARPRSCLWKLTKEGHRRFWEETRVLAFAQRERIQECMS

QPELLTSLFDL*
>ENSG00000178662|ENST00000342316
MSGILKRKFEEVDGSSPCSSVRESDDEVSSSESADSGDSVNPSTSSHFTPSSILKREKRL
RTKNVHFSCVTVYYFTRRQGFTSVPSQGGSTLGMSSRHNSVRQYTLGEFAREQERLHREM
LREHLREEKLNSLKLKMTKNGTVESEEASTLTLDDISDDDIDLDNTEVDEYFFLQPLPTK
KRRALLRASGVKKIDVEEKHELRAIRLSREDCGCDCRVFCDPDTCTCSLAGIKCQVDRMS
FPCGCTKEGCSNTAGRIEFNPIRVRTHFLHTIMKLELEKNREQQIPTLNGCHSEISAHSS
SMGPVAHSVEYSIADSFEIETEPQAAVLHLQSAEELDCQGEEEEEEEDGSSFCSGVTDSS
TQSLAPSESDEEEEEEEEEEEEEEDDDDDKGDGFVEGLGTHAEVVPLPSVLCYSDGTAVHE
SHAKNASFYANSSTLYYQIDSHIPGTPNQISENYSERDTVKNGTLSLVPYTMTPEQFVDY
ARQAEEAYGASHYPAANPSVIVCCSSSENDSGVPCNSLYPEHRSNHPQVEFHSYLKGPSQ
EGFVSALNGDSHISEHPAENSLSLAEKSILHEECIKSPVVETVPV*
>ENSG00000253729|ENST00000338368
MAGSGAGVRCSLLRLQETLSAADRCGAALAGHQLIRGLGQECVLSSSPAVLALQTSLVFS
RDFGLLVFVRKSLNSIEFRECREEILKFLCIFLEKMGQKIAPYSVEIKNTCTSVYTKDRA
AKCKIPALDLLIKLLQTFRSSRLMDEFKIGELFSKFYGELALKKKIPDTVLEKVYELLGL
LGEVHPSEMINNAENLFRAFLGELKTQMTSAVREPKLPVLAGCLKGLSSLLCNFTKSMEE
DPQTSREIFNFVLKAIRPQIDLKRYAVPSAGLRLFALHASQFSTCLLDNYVSLFEVLLKW
CAHTNVELKKAALSALESFLKQVSNMVAKNAEMHKNKLQYFMEQFYGIIRNVDSNNKELS
IAIRGYGLFAGPCKVINAKDVDFMYVELIQRCKQMFLTQTDTGDDRVYQMPSFLQSVASV
LLYLDTVPEVYTPVLEHLVVMQIDSFPQYSPKMQLVCCRAIVKVFLALAAKGPVLRNCIS
TVVHQGLIRICSKPVVLPKGPESESEDHRASGEVRTGKWKVPTYKDYVDLFRHLLSSDQM
MDSILADEAFFSVNSSSESLNHLLYDEFVKSVLKIVEKLDLTLEIQTVGEQENGDEAPGV
WMIPTSDPAANLHPAKPKDFSAFINLVEFCREILPEKQAEFFEPWVYSFSYELILQSTRL
PLISGFYKLLSITVRNAKKIKYFEGVSPKSLKHSPEDPEKYSCFALFVKFGKEVAVKMKQ
YKDELLASCLTFLLSLPHNIIELDVRAYVPALQMAFKLGLSYTPLAEVGLNALEEWSIYI
DRHVMQPYYKDILPCLDGYLKTSALSDETKNNWEVSALSRAAQKGFNKVVLKHLKKTKNL
SSNEAISLEEIRIRVVQMLGSLGGQINKNLLTVTSSDEMMKSYVAWDREKRLSFAVPFRE
MKPVIFLDVFLPRVTELALTASDRQTKVAACELLHSMVMFMLGKATQMPEGGQGAPPMYQ
LYKRTFPVLLRLACDVDQVTRQLYEPLVMQLIHWFTNNKKFESQDTVALLEAILDGIVDP
VDSTLRDFCGRCIREFLKWSIKQITPQQQEKSPVNTKSLFKRLYSLALHPNAFKRLGASL
AFNNIYREFREEESLVEQFVFEALVIYMESLALAHADEKSLGTIQQCCDAIDHLCRIIEK
KHVSLNKAKKRRLPRGFPPSASLCLLDLVKWLLAHCGRPQTECRHKSIELFYKFVPLLPG
NRSPNLWLKDVLKEEGVSFLINTFEGGGCGQPSGILAQPTLLYLRGPFSLQATLCWLDLL
LAALECYNTFIGERTVGALQVLGTEAQSSLLKAVAFFLESIAMHDIIAAEKCFGTGAAGN
RTSPQEGERYNYSKCTVVVRIMEFTTTLLNTSPEGWKLLKKDLCNTHLMRVLVQTLCEPA
SIGFNIGDVQVMAHLPDVCVNLMKALKMSPYKDILETHLREKITAQSIEELCAVNLYGPD
AQVDRSRLAAVVSACKQLHRAGLLHNILPSQSTDLHHSVGTELLSLVYKGIAPGDERQCL
PSLDLSCKQLASGLLELAFAFGGLCERLVSLLLNPAVLSTASLGSSQGSVIHFSHGEYFY
SLFSETINTELLKNLDLAVLELMQSSVDNTKMVSAVLNGMLDQSFRERANQKHQGLKLAT
TILQHWKKCDSWWAKDSPLETKMAVLALLAKILQIDSSVSFNTSHGSFPEVFTTYISLLA
DTKLDLHLKGQAVTLLPFFTSLTGGSLEELRRVLEQLIVAHFPMQSREFPPGTPRFNNYV
DCMKKFLDALELSQSPMLLELMTEVLCREQQHVMEELFQSSFRRIARRGSCVTQVGLLES
VYEMFRKDDPRLSFTRQSFVDRSLLTLLWHCSLDALREFFSTIVVDAIDVLKSRFTKLNE

STFDTQITKKMGYYKILDVMYSRLPKDDVHAKESKINQVFHGSCITEGNELTKTLIKLCY
DAFTENMAGENQLLERRRLYHCAAYNCAISVICCVFNELKFYQGFLFSEKPEKNLLIFEN
LIDLKRRYNFPVEVEVPMERKKKYIEIRKEAREAANGDSDGPSYMSSLSYLADSTLSEEM
SQFDFSTGVQSYSYSSQDPRPATGRFRRREQRDPTVHDDVLELEMDELNRHECMAPLTAL
VKHMHRSLGPPQGEEDSVPRDLPSWMKFLHGKLGNPIVPLNIRLFLAKLVINTEEVFRPY
AKHWLSPLLQLAASENNGGEGIHYMVVEIVATILSWTGLATPTGVPKDEVLANRLLNFLM
KHVFHPKRAVFRHNLEIIKTLVECWKDCLSIPYRLIFEKFSGKDPNSKDNSVGIQLLGIV
MANDLPPYDPQCGIQSSEYFQALVNNMSFVRYKEVYAAAAEVLGLILRYVMERKNILEES
LCELVAKQLKQHQNTMEDKFIVCLNKVTKSFPPLADRFMNAVFFLLPKFHGVLKTLCLEV
VLCRVEGMTELYFQLKSKDFVQVMRHRDDERQKVCLDIIYKMMPKLKPVELRELLNPVVE
FVSHPSTTCREQMYNILMWIHDNYRDPESETDNDSQEIFKLAKDVLIQGLIDENPGLQLI
IRNFWSHETRLPSNTLDRLLALNSLYSPKIEVHFLSLATNFLLEMTSMSPDYPNPMFEHP
LSECEFQEYTIDSDWRFRSTVLTPMFVETQASQGTLQTRTQEGSLSARWPVAGQIRATQQ
QHDFTLTQTADGRSSFDWLTGSSTDPLVDHTSPSSDSLLFAHKRSERLQRAPLKSVGPDF
GKKRLGLPGDEVDNKVKGAAGRTDLLRLRRRFMRDQEKLSLMYARKGVAEQKREKEIKSE
LKMKQDAQVVLYRSYRHGDLPDIQIKHSSLITPLQAVAQRDPIIAKQLFSSLFSGILKEM
DKFKTLSEKNNITQKLLQDFNRFLNTTFSFFPPFVSCIQDISCQHAALLSLDPAAVSAGC
LASLQQPVGIRLLEEALLRLLPAELPAKRVRGKARLPPDVLRWVELAKLYRSIGEYDVLR
GIFTSEIGTKQITQSALLAEARSDYSEAAKQYDEALNKQDWVDGEPTEAEKDFWELASLD
CYNHLAEWKSLEYCSTASIDSENPPDLNKIWSEPFYQETYLPYMIRSKLKLLLQGEADQS
LLTFIDKAMHGELQKAILELHYSQELSLLYLLQDDVDRAKYYIQNGIQSFMQNYSSIDVL
LHQSRLTKLQSVQALTEIQEFISFISKQGNLSSQVPLKRLLNTWTNRYPDAKMDPMNIWD
DIITNRCFFLSKIEEKLTPLPEDNSMNVDQDGDPSDRMEVQEQEEDISSLIRSCKFSMKM
KMIDSARKQNNFSLAMKLLKELHKESKTRDDWLVSWVQSYCRLSHCRSRSQGCSEQVLTV
LKTVSLLDENNVSSYLSKNILAFRDQNILLGTTYRIIANALSSEPACLAEIEEDKARRIL
ELSGSSSEDSEKVIAGLYQRAFQHLSEAVQAAEEEAQPPSWSCGPAAGVIDAYMTLADFC
DQQLRKEEENASVIDSAELQAYPALVVEKMLKALKLNSNEARLKFPRLLQIIERYPEETL
SLMTKEISSVPCWQFISWISHMVALLDKDQAVAVQHSVEEITDNYPQAIVYPFIISSESY
SFKDTSTGHKNKEFVARIKSKLDQGGVIQDFINALDQLSNPELLFKDWSNDVRAELAKTP
VNKKNIEKMYERMYAALGDPKAPGLGAFRRKFIQTFGKEFDKHFGKGGSKLLRMKLSDFN
DITNMLLLKMNKDSKPPGNLKECSPWMSDFKVEFLRNELEIPGQYDGRGKPLPEYHVRIA
GFDERVTVMASLRRPKRIIIRGHDEREHPFLVKGGEDLRQDQRVEQLFQVMNGILAQDSA
CSQRALQLRTYSVVPMTSSDPRAPPCEYKDWLTKMSGKHDVGAYMLMYKGANRTETVTSF
RKRESKVPADLLKRAFVRMSTSPEAFLALRSHFASSHALICISHWILGIGDRHLNNFMVA
METGGVIGIDFGHAFGSATQFLPVPELMPFRLTRQFINLMLPMKETGLMYSIMVHALRAF
RSDPGLLTNTMDVFVKEPSFDWKNFEQKMLKKGGSWIQEINVAEKNWYPRQKICYAKRKL
AGANPAVITCDELLLGHEKAPAFRDYVAVARGSKDHNIRAQEPESGLSEETQVKCLMDQA
TDPNILGRTWEGWEPWM*
>ENSG00000149948|ENST00000354636
MSARGEGAGQPSTSAQGQPAAPAPQKRGRGRPRKQQQEPTGEPSPKRPRGRPKGSKNKSP
SKAAQKKAEATGEKRPRGRPRKWDNLLPRTSSKKKTSLGNSTKRSH*
>ENSG00000072274|ENST00000360110
MMDQARSAFSNLFGGEPLSYTRFSLARQVDGDNSHVEMKLAVDEEENADNNTKANVTKPK
RCSGSICYGTIAVIVFFLIGFMIGYLGYCKGVEPKTECERLAGTESPVREEPGEDFPAAR

RLYWDDLKRKLSEKLDSTDFTGTIKLLNENSYVPREAGSQKDENLALYVENQFREFKLSK
VWRDQHFVKIQVKDSAQNSVIIVDKNGRLVYLVENPGGYVAYSKAATVTGKLVHANFGTK
KDFEDLYTPVNGSIVIVRAGKITFAEKVANAESLNAIGVLIYMDQTKFPIVNAELSFFGH
AHLGTGDPYTPGFPSFNHTQFPPSRSSGLPNIPVQTISRAAAEKLFGNMEGDCPSDWKTD
STCRMVTSESKNVKLTVSNVLKEIKILNIFGVIKGFVEPDHYVVVGAQRDAWGPGAAKSG
VGTALLLKLAQMFSDMVLKDGFQPSRSIIFASWSAGDFGSVGATEWLEGYLSSLHLKAFT
YINLDKAVLGTSNFKVSASPLLYTLIEKTMQNVKHPVTGQFLYQDSNWASKVEKLTLDNA
AFPFLAYSGIPAVSFCFCEDTDYPYLGTTMDTYKELIERIPELNKVARAAAEVAGQFVIK
LTHDVELNLDYERYNSQLLSFVRDLNQYRADIKEMGLSLQWLYSARGDFFRATSRLTTDF
GNAEKTDRFVMKKLNDRVMRVEYHFLSPYVSPKESPFRHVFWGSGSHTLPALLENLKLRK
QNNGAFNETLFRNQLALATWTIQGAANALSGDVWDIDNEF*
>ENSG00000135316|ENST00000355238
MATEHVNGNGTEEPMDTTSAVIHSENFQTLLDAGLPQKVAEKLDEIYVAGLVAHSDLDER
AIEALKEFNEDGALAVLQQFKDSDLSHVQNKSAFLCGVMKTYRQREKQGTKVADSSKGPD
EAKIKALLERTGYTLDVTTGQRKYGGPPPDSVYSGQQPSVGTEIFVGKIPRDLFEDELVP
LFEKAGPIWDLRLMMDPLTGLNRGYAFVTFCTKEAAQEAVKLYNNHEIRSGKHIGVCISV
ANNRLFVGSIPKSKTKEQILEEFSKVTEGLTDVILYHQPDDKKKNRGFCFLEYEDHKTAA
QARRRLMSGKVKVWGNVGTVEWADPIEDPDPEVMAKVKVLFVRNLANTVTEEILEKAFSQ
FGKLERVKKLKDYAFIHFDERDGAVKAMEEMNGKDLEGENIEIVFAKPPDQKRKERKAQR
QAAKNQMYDDYYYYGPPHMPPPTRGRGRGGRGGYGYPPDYYGYEDYYDYYGYDYHNYRGG
YEDPYYGYEDFQVGARGRGGRGARGAAPSRGRGAAPPRGRAGYSQRGGPGSARGVRGARG
GAQQQRGRGQGKGVEAGPDLLQ*
>ENSG00000144028|ENST00000323853
MADVTARSLQYEYKANSNLVLQADRSLIDRTRRDEPTGEVLSLVGKLEGTRMGDKAQRTK
PQMQEERRAKRRKRDEDRHDINKMKGYTLLSEGIDEMVGIIYKPKTKETRETYEVLLSFI
QAALGDQPRDILCGAADEVLAVLKNEKLRDKERRKEIDLLLGQTDDTRYHVLVNLGKKIT
DYGGDKEIQNMDDNIDETYGVNVQFESDEEEGDEDVYGEVREEASDDDMEGDEAVVRCTL
SANLVASGELMSSKKKDLHPRDIDAFWLQRQLSRFYDDAIVSQKKADEVLEILKTASDDR
ECENQLVLLLGFNTFDFIKVLRQHRMMILYCTLLASAQSEAEKERIMGKMEADPELSKFL
YQLHETEKEDLIREERSRRERVRQSRMDTDLETMDLDQGGEALAPRQVLDLEDLVFTQGS
HFMANKRCQLPDGSFRRQRKGYEEVHVPALKPKPFGSEEQLLPVEKLPKYAQAGFEGFKT
LNRIQSKLYRAALETDENLLLCAPTGAGKTNVALMCMLREIGKHINMDGTINVDDFKIIY
IAPMRSLVQEMVGSFGKRLATYGITVAELTGDHQLCKEEISATQIIVCTPEKWDIITRKG
GERTYTQLVRLIILDEIHLLHDDRGPVLEALVARAIRNIEMTQEDVRLIGLSATLPNYED
VATFLRVDPAKGLFYFDNSFRPVPLEQTYVGITEKKAIKRFQIMNEIVYEKIMEHAGKNQ
VLVFVHSRKETGKTARAIRDMCLEKDTLGLFLREGSASTEVLRTEAEQCKNLELKDLLPY
GFAIHHAGMTRVDRTLVEDLFADKHIQVLVSTATLAWGVNLPAHTVIIKGTQVYSPEKGR
WTELGALDILQMLGRAGRPQYDTKGEGILITSHGELQYYLSLLNQQLPIESQMVSKLPDM
LNAEIVLGNVQNAKDAVNWLGYAYLYIRMLRSPTLYGISHDDLKGDPLLDQRRLDLVHTA
ALMLDKNNLVKYDKKTGNFQVTELGRIASHYYITNDTVQTYNQLLKPTLSEIELFRVFSL
SSEFKNITVREEEKLELQKLLERVPIPVKESIEEPSAKINVLLQAFISQLKLEGFALMAD
MVYVTQSAGRLMRAIFEIVLNRGWAQLTDKTLNLCKMIDKRMWQSMCPLRQFRKLPEEVV
KKIEKKNFPFERLYDLNHNEIGELIRMPKMGKTIHKYVHLFPKLELSVHLQPITRSTLKV
ELTITPDFQWDEKVHGSSEAFWILVEDVDSEVILHHEYFLLKAKYAQDEHLITFFVPVFE

PLPPQYFIRVVSDRWLSCETQLPVSFRHLILPEKYPPPTELLDLQPLPVSALRNSAFESL
YQDKFPFFNPIQTQVFNTVYNSDDNVFVGAPTGSGKTICAEFAILRMLLQSSEGRCVYIT
PMEALAEQVYMDWYEKFQDRLNKKVVLLTGETSTDLKLLGKGNIIISTPEKWDILSRRWK
QRKNVQNINLFVVDEVHLIGGENGPVLEVICSRMRYISSQIERPIRIVALSSSLSNAKDV
AHWLGCSATSTFNFHPNVRPVPLELHIQGFNISHTQTRLLSMAKPVYHAITKHSPKKPVI
VFVPSRKQTRLTAIDILTTCAADIQRQRFLHCTEKDLIPYLEKLSDSTLKETLLNGVGYL
HEGLSPMERRLVEQLFSSGAIQVVVASRSLCWGMNVAAHLVIIMDTQYYNGKIHAYVDYP
IYDVLQMVGHANRPLQDDEGRCVIMCQGSKKDFFKKFLYEPLPVESHLDHCMHDHFNAEI
VTKTIENKQDAVDYLTWTFLYRRMTQNPNYYNLQGISHRHLSDHLSELVEQTLSDLEQSK
CISIEDEMDVAPLNLGMIAAYYYINYTTIELFSMSLNAKTKVRGLIEIISNAAEYENIPI
RHHEDNLLRQLAQKVPHKLNNPKFNDPHVKTNLLLQAHLSRMQLSAELQSDTEEILSKAI
RLIQACVDVLSSNGWLSPALAAMELAQMVTQAMWSKDSYLKQLPHFTSEHIKRCTDKGVE
SVFDIMEMEDEERNALLQLTDSQIADVARFCNRYPNIELSYEVVDKDSIRSGGPVVVLVQ
LEREEEVTGPVIAPLFPQKREEGWWVVIGDAKSNSLISIKRLTLQQKAKVKLDFVAPATG
AHNYTLYFMSDAYMGCDQEYKFSVDVKEAETDSDSD*
>ENSG00000086758|ENST00000342160
MKVDRTKLKKTPTEAPADCRALIDKLKVCNDEQLLLELQQIKTWNIGKCELYHWVDLLDR
FDGILADAGQTVENMSWMLVCDRPEREQLKMLLLAVLNFTALLIEYSFSRHLYSSIEHLT
TLLASSDMQVVLAVLNLLYVFSKRSNYITRLGSDKRTPLLTRLQHLAESWGGKENGFGLA
ECCRDLHMMKYPPSATTLHFEFYADPGAEVKIEKRTTSNTLHYIHIEQLDKISESPSEIM
ESLTKMYSIPKDKQMLLFTHIRLAHGFSNHRKRLQAVQARLHAISILVYSNALQESANSI
LYNGLIEELVDVLQITDKQLMEIKAASLRTLTSIVHLERTPKLSSIIDCTGTASYHGFLP
VLVRNCIQAMIDPSMDPYPHQFATALFSFLYHLASYDAGGEALVSCGMMEALLKVIKFLG
DEQDQITFVTRAVRVVDLITNLDMAAFQSHSGLSIFIYRLEHEVDLCRKECPFVIKPKIQ
RPNTTQEGEEMETDMDGVQCIPQRAALLKSMLNFLKKAIQDPAFSDGIRHVMDGSLPTSL
KHIISNAEYYGPSLFLLATEVVTVFVFQEPSLLSSLQDNGLTDVMLHALLIKDVPATREV
LGSLPNVFSALCLNARGLQSFVQCQPFERLFKVLLSPDYLPAMRRRRSSDPLGDTASNLG
SAVDELMRHQPTLKTDATTAIIKLLEEICNLGRDPKYICQKPSIQKADGTATAPPPRSNH
AAEEASSEDEEEEEVQAMQSFNSTQQNETEPNQQVVGTEERIPIPLMDYILNVMKFVESI
LSNNTTDDHCQEFVNQKGLLPLVTILGLPNLPIDFPTSAACQAVAGVCKSILTLSHEPKV
LQEGLLQLDSILSSLEPLHRPIESPGGSVLLRELACAGNVADATLSAQATPLLHALTAAH
AYIMMFVHTCRVGQSEIRSISVNQWGSQLGLSVLSKLSQLYCSLVWESTVLLSLCTPNSL
PSGCEFGQADMQKLVPKDEKAGTTQGGKRSDGEQDGAAGSMDASTQGLLEGIGLDGDTLA
PMETDEPTASDSKGKSKITPAMAARIKQIKPLLSASSRLGRALAELFGLLVKLCVGSPVR
QRRSHHAASTTTAPTPAARSTASALTKLLTKGLSWQPPPYTPTPRFRLTFFICSVGFTSP
MLFDERKYPYHLMLQKFLCSGGHNALFETFNWALSMGGKVPVSEGLEHSDLPDGTGEFLD
AWLMLVEKMVNPTTVLESPHSLPAKLPGGVQNFPQFSALRFLVVTQKAAFTCIKNLWNRK
PLKVYGGRMAESMLAILCHILRGEPVIRERLSKEKEGSRGEEDTGQEEGGSRREPQVNQQ
QLQQLMDMGFTREHAMEALLNTSTMEQATEYLLTHPPPIMGGVVRDLSMSEEDQMMRAIA
MSLGQDIPMDQRAESPEEVACRKEEEERKAREKQEEEEAKCLEKFQDADPLEQDELHTFT
DTMLPGCFHLLDELPDTVYRVCDLIMTAIKRNGADYRDMILKQVVNQVWEAADVLIKAAL
PLTTSDTKTVSEWISQMATLPQASNLATRILLLTLLFEELKLPCAWVVESSGILNVLIKL
LEVVQPCLQAAKEQKEVQTPKWITPVLLLIDFYEKTAISSKRRAQMTKYLQSNSNNWRWF
DDRSGRWCSYSASNNSTIDSAWKSGETSVRFTAGRRRYTVQFTTMVQVNEETGNRRPVML

TLLRVPRLNKNSKNSNGQELEKTLEESKEMDIKRKENKGNDTPLALESTNTEKETSLEET
KIGEILIQGLTEDMVTVLIRACVSMLGVPVDPDTLHATLRLCLRLTRDHKYAMMFAELKS
TRMILNLTQSSGFNGFTPLVTLLLRHIIEDPCTLRHTMEKVVRSAATSGAGSTTSGVVSG
SLGSREINYILRVLGPAACRNPDIFTEVANCCIRIALPAPRGSGTASDDEFENLRIKGPN
AVQLVKTTPLKPSPLPVIPDTIKEVIYDMLNALAAYHAPEEADKSDPKPGVMTQEVGQLL
QDMGDDVYQQYRSLTRQSSDFDTQSGFSINSQVFAADGASTETSASGTSQGEASTPEESR
DGKKDKEGDRASEEGKQKGKGSKPLMPTSTILRLLAELVRSYVGIATLIANYSYTVGQSE
LIKEDCSVLAFVLDHLLPHTQNAEDKDTPALARLFLASLAAAGSGTDAQVALVNEVKAAL
GRALAMAESTEKHARLQAVMCIISTIMESCPSTSSFYSSATAKTQHNGMNNIIRLFLKKG
LVNDLARVPHSLDLSSPNMANTVNAALKPLETLSRIVNQPSSLFGSKSASSKNKSEQDAQ
GASQDSSSNQQDPGEPGEAEVQEEDHDVTQTEVADGDIMDGEAETDSVVIAGQPEVLSSQ
EMQVENELEDLIDELLERDGGSGNSTIIVSRSGEDESQEDVLMDEAPSNLSQASTLQANR
EDSMNILDPEDEEEHTQEEDSSGSNEDEDDSQDEEEEEEEDEEDDQEDDEGEEGDEDDDD
DGSEMELDEDYPDMNASPLVRFERFDREDDLIIEFDNMFSSATDIPPSPGNIPTTHPLMV
RHADHSSLTLGSGSSTTRLTQGIGRSQRTLRQLTANTGHTIHVHYPGNRQPNPPLILQRL
LGPSAAADILQLSSSLPLQSRGRARLLVGNDDVHIIARSDDELLDDFFHDQSTATSQAGT
LSSIPTALTRWTEECKVLDAESMHDCVSVVKVSIVNHLEFLRDEELEERREKRRKQLAEE
ETKITDKGKEDKENRDQSAQCTASKSNDSTEQNLSDGTPMPDSYPTTPSSTDAATSESKE
TLGTLQSSQQQPTLPTPPALGEVPQELQSPAGEGGSSTQLLMPVEPEELGPTRPSGEAET
TQMELSPAPTITSLSPERAEDSDALTAVSSQLEGSPMDTSSLASCTLEEAVGDTSAAGSS
EQPRAGSSTPGDAPPAVAEVQGRSDGSGESAQPPEDSSPPASSESSSTRDSAVAISGADS
RGILEEPLPSTSSEEEDPLAGISLPEGVDPSFLAALPDDIRREVLQNQLGIRPPTRTAPS
TNSSAPAVVGNPGVTEVSPEFLAALPPAIQEEVLAQQRAEQQRRELAQNASSDTPMDPVT
FIQTLPSDLRRSVLEDMEDSVLAVMPPDIAAEAQALRREQEARQRQLMHERLFGHSSTSA
LSAILRSPAFTSRLSGNRGVQYTRLAVQRGGTFQMGGSSSHNRPSGSNVDTLLRLRGRLL
LDHEALSCLLVLLFVDEPKLNTSRLHRVLRNLCYHAQTRHWVIRSLLSILQRSSESELCI
ETPKLTTSEEKGKKSSKSCGSSSHENRPLDLLHKMESKSSNQLSWLSVSMDAALGCRTNI
FQIQRSGGRKHTEKHASGGSTVHIHPQAAPVVCRHVLDTLIQLAKVFPSHFTQQRTKETN
CESDRERGNKACSPCSSQSSSSGICTDFWDLLVKLDNMNVSRKGKNSVKSVPVSAGGEGE
TSPYSLEASPLGQLMNMLSHPVIRRSSLLTEKLLRLLSLISIALPENKVSEAQANSGSGA
SSTTTATSTTSTTTTTAASTTPTPPTAPTPVTSAPALVAATAISTIVVAASTTVTTPTTA
TTTVSISPTTKGSKSPAKVSDGGSSSTDFKMVSSGLTENQLQLSVEVLTSHSCSEEGLED
AANVLLQLSRGDSGTRDTVLKLLLNGARHLGYTLCKQIGTLLAELREYNLEQQRRAQCET
LSPDGLPEEQPQTTKLKGKMQSRFDMAENVVIVASQKRPLGGRELQLPSMSMLTSKTSTQ
KFFLRVLQVIIQLRDDTRRANKKAKQTGRLGSSGLGSASSIQAAVRQLEAEADAIIQMVR
EGQRARRQQQAATSESSQSEASVRREESPMDVDQPSPSAQDTQSIASDGTPQGEKEKEER
PPELPLLSEQLSLDELWDMLGECLKELEESHDQHAVLVLQPAVEAFFLVHATERESKPPV
RDTRESQLAHIKDEPPPLSPAPLTPATPSSLDPFFSREPSSMHISSSLPPDTQKFLRFAE
THRTVLNQILRQSTTHLADGPFAVLVDYIRVLDFDVKRKYFRQELERLDEGLRKEDMAVH
VRRDHVFEDSYRELHRKSPEEMKNRLYIVFEGEEGQDAGGLLREWYMIISREMFNPMYAL
FRTSPGDRVTYTINPSSHCNPNHLSYFKFVGRIVAKAVYDNRLLECYFTRSFYKHILGKS
VRYTDMESEDYHFYQGLVYLLENDVSTLGYDLTFSTEVQEFGVCEVRDLKPNGANILVTE
ENKKEYVHLVCQMRMTGAIRKQLAAFLEGFYEIIPKRLISIFTEQELELLISGLPTIDID
DLKSNTEYHKYQSNSIQIQWFWRALRSFDQADRAKFLQFVTGTSKVPLQGFAALEGMNGI

QKFQIHRDDRSTDRLPSAHTCFNQLDLPAYESFEKLRHMLLLAIQECSEGFGLA*
>ENSG00000198556|ENST00000331410
MFPPARGKELLSFEDVAMYFTREEWGHLNWGQKDLYRDVMLENYRNMVLLGFQFPKPEMI
CQLENWDEQWILDLPRTGNRKASGSACPGSEARHKMKKLTPKQKFSEDLESYKISVVMQE
SAEKLSEKLHKCKEFVDSCRLTFPTSGDEYSRGFLQNLNLIQDQNAQTRWKQGRYDEDGK
PFNQRSLLLGHERILTRAKSYECSECGKVIRRKAWFDQHQRIHFLENPFECKVCGQAFRQ
RSALTVHKQCHLQNKPYRCHDCGKCFRQLAYLVEHKRIHTKEKPYKCSKCEKTFSQNSTL
IRHQVIHSGEKRHKCLECGKAFGRHSTLLCHQQIHSKPNTHKCSECGQSFGRNVDLIQHQ
RIHTKEEFFQCGECGKTFSFKRNLFRHQVIHTGSQPYQCVICGKSFKWHTSFIKHQGTHK
GQIST*
>ENSG00000173258|ENST00000355824
MQAVVPLNKMTAISPEPQTLASTEQNEVPRVVTSGEQEAILRGNAADAESFRQRFRWFCY
SEVAGPRKALSQLWELCNQWLRPDIHTKEQILELLVFEQFLTILPGEIRIWVKSQHPESS
EEVVTLIEDLTQMLEEKDPVSQDSTVSQEENSKEDKMVTVCPNTESCESITLKDVAVNFS
RGEWKKLEPFQKELYKEVLLENLRNLEFLDFPVSKLELISQLKWVELPWLLEEVSKSSRL
GSVI*
>ENSG00000187823|ENST00000340433
MEKCTKSSSTMQVEPSFLQAENLILRLQMQHPTTENTAKRGQVMPALATTVMPVPYSLEH
LTQFHGDPANCSEFLTQVTTYLTALQISNPANDAQIKLFFDYLSQQLESCGIISGPDKST
LLKQYENLILEFQQSFGKPTKQEINPLMNAKFDKGDNSSQQDPATFHLLAQNLICNETNQ
SGQFEKALADPNQDEESVTDMMDNLPDLITQCIQLDKKHSDRPELLQSETQLPLLASLIQ
HQALFSPTDPPPKKGPIQLREGQLPLTPAKRARQQETQLCLYCSQSGHFTRDCLAKRSRA
PATTNNTAHQ*
>ENSG00000122406|ENST00000315741
MIVRVTNRDIICQIAYARIEGDMIVCAAYAHELPKYGVKVGLTNYAAAYCTGLLLARRLL
NRFGMDKIYEGQVEVTGDEYNVESIDGQPGAFTCYLDAGLARTTTGNKVFGALKGAVDGG
LSIPHSTKRF
>ENSG00000067066|ENST00000340126
MAGGGGDLSTRRLNECISPVANEMNHLPAHSHDLQRMFTEDQGVDDRLLYDIVFKHFKRN
KVEISNAIKKTFPFLEGLRDRDLITNKMFEDSQDSCRNLVPVQRVVYNVLSELEKTFNLP
VLEALFSDVNMQEYPDLIHIYKGFENVIHDKLPLQESEEEEREERSGLQLSLEQGTGENS
FRSLTWPPSGSPSHAGTTPPENGLSEHPCETEQINAKRKDTTSDKDDSLGSQQTNEQCAQ
KAEPTESCEQIAVQVNNGDAGREMPCPLPCDEESPEAELHNHGIQINSCSVRLVDIKKEK
PFSNSKVECQAQARTHHNQASDIIVISSEDSEGSTDVDEPLEVFISAPRSEPVINNDNPL
ESNDEKEGQEATCSRPQIVPEPMDFRKLSTFRESFKKRVIGQDHDFSESSEEEAPAEASS
GALRSKHGEKAPMTSRSTSTWRIPSRKRRFSSSDFSDLSNGEELQETCSSSLRRGSGSQP
QEPENKKCSCVMCFPKGVPRSQEARTESSQASDMMDTMDVENNSTLEKHSGKRRKKRRHR
SKVNGLQRGRKKDRPRKHLTLNNKVQKKRWQQRGRKANTRPLKRRRKRGPRIPKDENINF
KQSELPVTCGEVKGTLYKERFKQGTSKKCIQSEDKKWFTPREFEIEGDRGASKNWKLSIR
CGGYTLKVLMENKFLPEPPSTRKKRILESHNNTLVDPCPENSNICEVCNKWGRLFCCDTC
PRSFHEHCHIPSVEANKNPWSCIFCRIKTIQERCPESQSGHQESEVLMRQMLPEEQLKCE
FLLLKVYCDSKSCFFASEPYYNREGSQGPQKPMWLNKVKTSLNEQMYTRVEGFVQDMRLI
FHNHKEFYREDKFTRLGIQVQDIFEKNFRNIFAIQETSKNIIMFI*
>ENSG00000189420|ENST00000338647

MAAILLTTRPKVPVSFEDVSVYFTKTEWKLLDLRQKVLYKRVMLENYSHLVSLGFSFSKP
HLISQLERGEGPWVADIPRTWATAGLHIGDRTQSKTSTSTQKHSGRQLPGADPQGGKEGQ
AARSSVLQRGAQGLGQSSAAGPQGPKGAEKRYLCQQCGKAFSRSSNLIKHRIIHSGEKPY
ACPECGKLFRRSFALLEHQRIHSGEKPYACPECSKTFTRSSNLIKHQVIHSGERPFACGD
CGKLFRRSFALLEHARVHSGERPYACPECGKAFSRSSNLIEHQRTHRGEKPYACGQCAKA
FKGVSQLIHHQRSHSGERPFACRECGKAFRGRSGLSQHRRVHSGEKPYECSDCGKAFGRR
ANLFKHQAVHGARRPAKAETARRLAGPGSTGPGSAVAATSPPRPSTAARPSRPSRR*
>ENSG00000115306|ENST00000333896
MELQRTSSISGPLSPAYTGQVPYNYNQLEGRFKQLQDEREAVQKKTFTKWVNSHLARVSC
RITDLYTDLRDGRMLIKLLEVLSGERLPKPTKGRMRIHCLENVDKALQFLKEQRVHLENM
GSHDIVDGNHRLTLGLIWTIILRFQIQDISVETEDNKEKKSAKDALLLWCQMKTAGYPNV
NIHNFTTSWRDGMAFNALIHKHRPDLIDFDKLKKSNAHYNLQNAFNLAEQHLGLTKLLDP
EDISVDHPDEKSIITYVVTYYHYFSKMKALAVEGKRIGKVLDNAIETEKMIEKYESLASD
LLEWIEQTIIILNNRKFANSLVGVQQQLQAFNTYRTVEKPPKFTEKGNLEVLLFTIQSKM
RANNQKVYMPREGKLISDINKAWERLEKAEHERELALRNELIRQEKLEQLARRFDRKAAM
RETWLSENQRLVSQDNFGFDLPAVEAATKKHEAIETDIAAYEERVQAVVAVARELEAENY
HDIKRITARKDNVIRLWEYLLELLRARRQRLEMNLGLQKIFQEMLYIMDWMDEMKVLVLS
QDYGKHLLGVEDLLQKHTLVEADIGIQAERVRGVNASAQKFATDGEGYKPCDPQVIRDRV
AHMEFCYQELCQLAAERRARLEESRRLWKFFWEMAEEEGWIREKEKILSSDDYGKDLTSV
MRLLSKHRAFEDEMSGRSGHFEQAIKEGEDMIAEEHFGSEKIRERIIYIREQWANLEQLS
AIRKKRLEEASLLHQFQADADDIDAWMLDILKIVSSSDVGHDEYSTQSLVKKHKDVAEEI
ANYRPTLDTLHEQASALPQEHAESPDVRGRLSGIEERYKEVAELTRLRKQALQDTLALYK
MFSEADACELWIDEKEQWLNNMQIPEKLEDLEVIQHRFESLEPEMNNQASRVAVVNQIAR
QLMHSGHPSEKEIKAQQDKLNTRWSQFRELVDRKKDALLSALSIQNYHLECNETKSWIRE
KTKVIESTQDLGNDLAGVMALQRKLTGMERDLVAIEAKLSDLQKEAEKLESEHPDQAQAI
LSRLAEISDVWEEMKTTLKNREASLGEASKLQQFLRDLDDFQSWLSRTQTAIASEDMPNT
LTEAEKLLTQHENIKNEIDNYEEDYQKMRDMGEMVTQGQTDAQYMFLRQRLQALDTGWNE
LHKMWENRQNLLSQSHAYQQFLRDTKQAEAFLNNQEYVLAHTEMPTTLEGAEAAIKKQED
FMTTMDANEEKINAVVETGRRLVSDGNINSDRIQEKVDSIDDRHRKNRETASELLMRLKD
NRDLQKFLQDCQELSLWINEKMLTAQDMSYDEARNLHSKWLKHQAFMAELASNKEWLDKI
EKEGMQLISEKPETEAVVKEKLTGLHKMWEVLESTTQTKAQRLFDANKAELFTQSCADLD
KWLHGLESQIQSDDYGKDLTSVNILLKKQQMLENQMEVRKKEIEELQSQAQALSQEGKST
DEVDSKRLTVQTKFMELLEPLNERKHNLLASKEIHQFNRDVEDEILWVGERMPLATSTDH
GHNLQTVQLLIKKNQTLQKEIQGHQPRIDDIFERSQNIVTDSSSLSAEAIRQRLADLKQL
WGLLIEETEKRHRRLEEAHRAQQYYFDAAEAEAWMSEQELYMMSEEKAKDEQSAVSMLKK
HQILEQAVEDYAETVHQLSKTSRALVADSHPESERISMRQSKVDKLYAGLKDLAEERRGK
LDERHRLFQLNREVDDLEQWIAEREVVAGSHELGQDYEHVTMLQERFREFARDTGNIGQE
RVDTVNHLADELINSGHSDAATIAEWKDGLNEAWADLLELIDTRTQILAASYELHKFYHD
AKEIFGRIQDKHKKLPEELGRDQNTVETLQRMHTTFEHDIQALGTQVRQLQEDAARLQAA
YAGDKADDIQKRENEVLEAWKSLLDACESRRVRLVDTGDKFRFFSMVRDLMLWMEDVIRQ
IEAQEKPRDVSSVELLMNNHQGIKAEIDARNDSFTTCIELGKSLLARKHYASEEIKEKLL
QLTEKRKEMIDKWEDRWEWLRLILEVHQFSRDASVAEAWLLGQEPYLSSREIGQSVDEVE
KLIKRHEAFEKSAATWDERFSALERLTTLELLEVRRQQEEEERKRRPPSPEPSTKVSEEA
ESQQQWDTSKGEQVSQNGLPAEQGSPRVSYRSQTYQNYKNFNSRRTASDQPWSGL*

>ENSG00000152942|ENST00000358030
MFNTESSFHMFPYQSQIAVFKEFLLRATKYNKLQMLGDDLRTDKKIILVEDLPNQFYRDS
HTLHEVLRKYVRIGRCPLIFIISDSLSGDNNQRLLFPKEIQEECSISNISFNPVAPTIMM
KFLNRIVTIEANKNGGKITVPDKTSLELLCQGCSGDIRSAINSLQFSSSKGENNLRPRKK
GMSLKSDAVLSKSKRRKKPDRVFENQEVQAIGGKDVSLFLFRALGKILYCKRASLTELDS
PRLPSHLSEYERDTLLVEPEEVVEMSHMPGDLFNLYLHQNYIDFFMEIDDIVRASEFLSF
ADILSGDWNTRSLLREYSTSIATRGVMHSNKARGYAHCQGGGSSFRPLHKPQWFLINKKY
RENCLAAKALFPDFCLPALCLQTQLLPYLALLTIPMRNQAQISFIQDIGRLPLKRHFGRL
KMEALTDREHGMIDPDSGDEAQLNGGHSAEESLGEPTQATVPETWSLPLSQNSASELPAS
QPQPFSAQGDMEENIIIEDYESDGT*
>ENSG00000198265|ENST00000358691
MEDRRAEKSCEQACESLKRQDYEMALKHCTEALLSLGQYSMADFTGPCPLEIERIKIESL
LYRIASFLQLKNYVQADEDCRHVLGEGLAKGEDAFRAVLCCMQLKGKLQPVSTILAKSLT
GESLNGMVTKDLTRLKTLLSETETATSNALSGYHVEDLDEGSCNGWHFRPPPRGITSSEE
YTLCKRFLEQGICRYGAQCTSAHSQEELAEWQKRYASRLIKLKQQNENKQLSGSYMETLI
EKWMNSLSPEKVLSECIEGVKVEHNPDLSVTVSTKKSHQTWTFALTCKPARMLYRVALLY
DAHRPHFSIIAISAGDSTTQVSQEVPENCQEWIGGKMAQNGLDHYVYKVGIAFNTEIFGT
FRQTIVFDFGLEPVLMQRVMIDAASTEDLEYLMHAKQQLVTTAKRWDSSSKTIIDFEPNE
TTDLEKSLLIRYQIPLSADQLFTQSVLDKSLTKSNYQSRLHDLLYIEEIAQYKEISKFNL
KVQLQILASFMLTGVSGGAKYAQNGQLFGRFKLTETLSEDTLAGRLVMTKVNAVYLLPVP
KQKLVQTQGTKEKVYEATIEEKTKEYIFLRLSRECCEELNLRPDCDTQVELQFQLNRLPL
CEMHYALDRIKDNGVLFPDISMTPTIPWSPNRQWDEQLDPRLNAKQKEAVLAITTPLAIQ
LPPVLIIGPYGTGKTFTLAQAVKHILQQQETRILICTHSNSAADLYIKDYLHPYVEAGNP
QARPLRVYFRNRWVKTVHPVVHQYCLISSAHSTFQMPQKEDILKHRVVVVTLNTSQYLCQ
LDLEPGFFTHILLDEAAQAMECETIMPLALATQNTRIVLAGDHMQLSPFVYSEFARERNL
HVSLLDRLYEHYPAEFPCRILLCENYRSHEAIINYTSELFYEGKLMASGKQPAHKDFYPL
TFFTARGEDVQEKNSTAFYNNAEVFEVVERVEELRRKWPVAWGKLDDGSIGVVTPYADQV
FRIRAELRKKRLSDVNVERVLNVQGKQFRVLFLSTVRTRHTCKHKQTPIKKKEQLLEDST
EDLDYGFLSNYKLLNTAITRAQSLVAVVGDPIALCSIGRCRKFWERFIALCHENSSLHGI
TFEQIKAQLEALELKKTYVLNPLAPEFIPRALRLQHSGSTNKQQQSPPKGKSLHHTQNDH
FQNDGIVQPNPSVLIGNPIRAYTPPPPLGPHPNLGKSPSPVQRIDPHTGTSILYVPAVYG
GNVVMSVPLPVPWTGYQGRFAVDPRIITHQAAMAYNMNLLQTHGRGSPIPYGLGHHPPVT
IGQPQNQHQEKDQHEQNRNGKSDTNNSGPEINKIRTPEKKPTEPKQVDLESNPQNRSPES
RPSVVYPSTKFPRKDNLNPRHINLPLPAPHAQYAIPNRHFHPLPQLPRPPFPIPQQHTLL
NQQQNNLPEQPNQIPPQPNQVVQQQSQLNQQPQQPPPQLSPAYQAGPNNAFFNSAVAHRP
QSPPAEAVIPEQQPPPMLQEGHSPLRAIAQPGPILPSHLNSFIDENPSGLPIGEALDRIH
GSVALETLRQQQARFQQWSEHHAFLSQGSAPYPHHHHPHLQHLPQPPLGLHQPPVRADWK
LTSSAEDEVETTYSRFQDLIRELSHRDQSETRELAEMPPPQSRLLQYRQVQSRSPPAVPS
PPSSTDHSSHFSNFNDNSRDIEVASNPAFPQRLPPQIFNSPFSLPSEHLAPPPLKYLAPD
GAWTFANLQQNHLMGPGFPYGLPPLPHRPPQNPFVQIQNHQHAIGQEPFHPLSSRTVSSS
SLPSLEEYEPRGPGRPLYQRRISSSSVQPCSEEVSTPQDSLAQCKELQDHSNQSSFNFSS
PESWVNTTSSTPYQNIPCNGSSRTAQPRELIAPPKTVKPPEDQLKSENLEVSSSFNYSVL
QHLGQFPPLMPNKQIAESANSSSPQSSAGGKPAMSYASALRAPPKPRPPPEQAKKSSDPL
SLFQELSLGSSSGSNGFYSYFK*

>ENSG00000143365|ENST00000356728
MRTQIEVIPCKICGDKSSGIHYGVITCEGCKGFFRRSQRCNAAYSCTRQQNCPIDRTSRN
RCQHCRLQKCLALGMSRDAVKFGRMSKKQRDSLHAEVQKQLQQRQQQQQEPVVKTPPAGA
QGADTLTYTLGLPDGQLPLGSSPDLPEASACPPGLLKASGSGPSYSNNLAKAGLNGASCH
LEYSPERGKAEGRESFYSTGSQLTPDRCGLRFEEHRHPGLGELGQGPDSYGSPSFRSTPE
APYASLTEIEHLVQSVCKSYRETCQLRLEDLLRQRSNIFSREEVTGYQRKSMWEMWERCA
HHLTEAIQYVVEFAKRLSGFMELCQNDQIVLLKAGAMEVVLVRMCRAYNADNRTVFFEGK
YGGMELFRALGCSELISSIFDFSHSLSALHFSEDEIALYTALVLINAHRPGLQEKRKVEQ
LQYNLELAFHHHLCKTHRQSILAKLPPKGKLRSLCSQHVERLQIFQHLHPIVVQAAFPPL
YKELFSTETESPVGLSK*
>ENSG00000032219|ENST00000348476
MKAADEPAYLTVGTDVSAKYRGAFCEAKIKTVKRLVKVKVLLKQDNTTQLVQDDQVKGPL
RVGAIVETRTSDGSFQEAIISKLTDASWYTVVFDDGDERTLRRTSLCLKGERHFAESETL
DQLPLTNPEHFGTPVIAKKTNRGRRSSLPVTEDEKEEESSEEEDEDKRRLNDELLGKVVS
VVSATERTEWYPALVISPSCNDDITVKKDQCLVRSFIDSKFYSIARKDIKEVDILNLPES
ELSTKPGLQKASIFLKTRVVPDNWKMDISEILESSSSDDEDGPAEENDEEKEKEAKKTEE
EVPEEELDPEERDNFLQQLYKFMEDRGTPINKPPVLGYKDLNLFKLFRLVYHQGGCDNID
SGAVWKQIYMDLGIPILNSAASYNVKTAYRKYLYGFEEYCRSANIQFRTVHHHEPKVKEE
KKDLEESMEEALKLDQEMPLTEVKSEPEENIDSNSESEREEIELKSPRGRRRIARDVNSI
KKEIEEEKTEDKLKDNDTENKDVDDDYETAEKKENELLLGRKNTPKQKEKKIKKQEDSDK
DSDEEEEKSQEREETESKCDSEGEEDEEDMEPCLTGTKVKVKYGRGKTQKIYEASIKSTE
IDDGEVLYLVHYYGWNVRYDEWVKADRIIWPLDKGGPKKKQKKKAKNKEDSEKDEKRDEE
RQKSKRGRPPLKSTLSSNMPYGLSKTANSEGKSDSCSSDSETEDALEKNLINEELSLKDE
LEKNENLNDDKLDEENPKISAHILKENDRTQMQPLETLKLEVGENEQIVQIFGNKMEKTE
EVKKEAEKSPKGKGRRSKTKDLSLEIIKISSFGQNEAGSEPHIEAHSLELSSLDNKNFSS
ATEDEIDQCVKEKKLKRKILGQSSPEKKIRIENGMEMTNTVSQERTSDCIGSEGMKNLNF
EQHFERENEGMPSLIAESNQCIQQLTSERFDSPAEETVNIPLKEDEDAMPLIGPETLVCH
EVDLDDLDEKDKTSIEDVAVESSESNSLVSIPPALPPVVQHNFSVASPLTLSQDESRSVK
SESDITIEVDSIAEESQEGLCERESANGFETNVASGTCSIIVQERESREKGQKRPSDGNS
GLMAKKQKRTPKRTSAAAKNEKNGTDELDNMNSTERISFLQEKLQEIRKYYMSLKSEVAT
IDRRRKRLKKKDREVSHAGASMSSASSDTGMSPSSSSPPQNVLAVECR*
>ENSG00000198000|ENST00000360868
MKVNRETKRLYVGGLSQDISEADLQNQFSRFGEVSDVEIITRKDDQGNPQKVFAYINISV
AEADLKKSIFHVIQRDSSDEENSSIV*
>ENSG00000164048|ENST00000354698
MWAPREQLLGWTAEALPAKDSAWPWEEKPRYLGPVTFEDVAVLFTEAEWKRLSLEQRNLY
KEVMLENLRNLVSLAESKPEVHTCPSCPLAFGSQQFLSQDELHNHPIPGFHAGNQLHPGN
PCPEDQPQSQHPSDKNHRGAEAEDQRVEGGVRPLFWSTNERGALVGFSSLFQRPPISSWG
GNRILEIQLSPAQNASSEEVDRISKRAETPGFGAVTFGECALAFNQKSNLFRQKAVTAEK
SSDKRQSQVCRECGRGFSRKSQLIIHQRTHTGEKPYVCGECGRGFIVESVLRNHLSTHSG
EKPYVCSHCGRGFSCKPYLIRHQRTHTREKSFMCTVCGRGFREKSELIKHQRIHTGDKPY
VCRD*
>ENSG00000197714|ENST00000360338
MAAAWMAPAQESVTFEDVAVTFTQEEWGQLDVTQRALYVEVMLETCGLLVALGDSTKPET

VEPIPSHLALPEEVSLQEQLAQGVPRYSYLGQAMDQDGPSEMQEYFLRPGTDPQSEKLHG
KMSLEHEGLATADGICSMMIQNQVSPEDALYGFDSYGPVTDSLIHEGENSYKFEEMFNEN
CFLVQHEQILPRVKPYDCPECGKAFGKSKHLLQHHIIHTGEKPYKCLECGKDFNRRSHLT
RHQRTHNGDKPFVCSECGRTFNRGSHLTRHQRVHSGEKPFVCNECGKAFTYRSNFVLHNK
SHNEKKPFACSECGKGFYESTALIQHFIIHTGERPFKCLECGKAFNCRSHLKQHERIHTG
EKPFVCSQCGKAFTHYSTYVLHERAHTGEKPFECKECGKAFSIRKDLIRHFNIHTGEKPY
ECLQCGKAFTRMSGLTRHQWIHTGEKPYVCIQCGKAFCRTTNLIRHFSIHTGEKPYECVE
CGKAFNRRSPLTRHQRIHTAEKSHEPIQSGNVSCESTDLIQHSIIHTESSPVSAVNMETP
SIAAHSSSLDINGFIVEETLPL*
>ENSG00000176974|ENST00000316694
MTMPVNGAHKDADLWSSHDKMLAQPLKDSDVEVYNIIKKESNRQRVGLELIASENFASRA
VLEALGSCLNNKYSEGYPGQRYYGGTEFIDELETLCQKRALQAYKLDPQCWGVNVQPYSG
SPANFAVYTALVEPHGRIMGLDLPDGGHLTHGFMTDKKKISATSIFFESMPYKVNPDTGY
INYDQLEENARLFHPKLIIAGTSCYSRNLEYARLRKIADENGAYLMADMAHISGLVAAGV
VPSPFEHCHVVTTTTHKTLRGCRAGMIFYRKGVKSVDPKTGKEILYNLESLINSAVFPGL
QGGPHNHAIAGVAVALKQAMTLEFKVYQHQVVANCRALSEALTELGYKIVTGGSDNHLIL
VDLRSKGTDGGRAEKVLEACSIACNKNTCPGDRSALRPSGLRLGTPALTSRGLLEKDFQK
VAHFIHRGIELTLQIQSDTGVRATLKEFKERLAGDKYQAAVQALREEVESFASLFPLPGL
PDF*
>ENSG00000139793|ENST00000345429
MALNVAPVRDTKWLTLEVCRQFQRGTCSRSDEECKFAHPPKSCQVENGRVIACFDSLKGR
CSRENCKYLHPPTHLKTQLEINGRNNLIQQKTAAAMLAQQMQFMFPGTPLHPVPTFPVGP
AIGTNTAISFAPYLAPVTPGVGLVPTEILPTTPVIVPGSPPVTVPGSTATQKLLRTDKLE
VCREFQRGNCARGETDCRFAHPADSTMIDTSDNTVTVCMDYIKGRCMREKCKYFHPPAHL
QAKIKAAQHQANQAAVAAQAAAAAATVMAFPPGALHPLPKRQALEKSNGTSAVFNPSVLH
YQQALTSAQLQQHAAFIPTGSVLCMTPATSIVPMMHSATSATVSAATTPATSVPFAATAT
ANQIILK*
>ENSG00000143621|ENST00000361891
MRGDRGRGRGGRFGSRGGPGGGFRPFVPHIPFDFYLCEMAFPRVKPAPDETSFSEALLKR
NQDLAPNSAEQASILSLVTKINNVIDNLIVAPGTFEVQIEEVRQVGSYKKGTMTTGHNVA
DLVVILKILPTLEAVAALGNKVVESLRAQDPSEVLTMLTNETGFEISSSDATVKILITTV
PPNLRKLDPELHLDIKVLQSALAAIRHARWFEENASQSTVKVLIRLLKDLRIRFPGFEPL
TPWILDLLGHYAVMNNPTRQPLALNVAYRRCLQILAAGLFLPGSVGITDPCESGNFRVHT
VMTLEQQDMVCYTAQTLVRILSHGGFRKILGQEGDASYLASEISTWDGVIVTPSEKAYEK
PPEKKEGEEEEENTEEPPQGEEEESMETQE*
>ENSG00000166938|ENST00000319194
MQTACQAVQHQRGRRQYNKLRNLLKDARHDCILFANEFQQCCYLPRERGESMEKWQTRSI
YNAAVWYYHHCQDRMPIVMVTEDEEAIQQYGSETEGVFVITFKNYLDNFWPDLKAAHELC
DSILQSRRERENESQESHGKEYPEHLPLEVLEAGIKSGRYIQGILNVNKHRAQIEAFVRL
QGASSKDSDLVSDILIHGMKARNRSIHGDVVVVELLPKNEWKGRTVALCENDCDDKASGE
SPSEPMPTGRVVGILQKNWRDYVVTFPSKEEVQSQGKNAQKILVTPWDYRIPKIRISTQQ
AETLQDFRVVVRIDSWESTSVYPNGHFVRVLGRIGDLEGEIATILVENSISVIPFSEAQM
CEMPVNTPESPWKVSPEEEQKRKDLRKSHLVFSIDPKGCEDVDDTLSVRTLNNGNLELGV
HIADVTHFVAPNSYIDIEARTRATTYYLADRRYDMLPSVLSADLCSLLGGVDRYAVSIMW

ELDKASYEIKKVWYGRTIIRSAYKLFYEAAQELLDGNLSVVDDIPEFKDLDEKSRQAKLE
ELVWAIGKLTDIARHVRAKRDGCGALELEGVEVCVQLDDKKNIHDLIPKQPLEVHETVAE
CMILANHWVAKKIWESFPHQALLRQHPPPHQEFFSELRECAKAKGFFIDTRSNKTLADSL
DNANDPHDPIVNRLLRSMATQAMSNALYFSTGSCAEEEFHHYGLALDKYTHFTSPIRRYS
DIVVHRLLMAAISKDKKMEIKGNLFSNKDLEELCRHINNRNQAAQHSQKQSTELFQCMYF
KDKDPATEERCISDGVIYSIRTNGVLLFIPRFGIKGAAYLKNKDGLVISCGPDSCSEWKP
GSLQRFQNKITSTTTDGESVTFHLFDHVTVRISIQASRCHSDTIRLEIISNKPYKIPNTE
LIHQSSPLLKSELVKEVTKSVEEAQLAQEVKVNIIQEEYQEYRQTKGRSLYTLLEEIRDL
ALLDVSNNYGI*
>ENSG00000125812|ENST00000338121
MESGAVLLESKSSPFNLLHEMHELRLLGHLCDVTVSVEYQGVRKDFMAHKAVLAATSKFF
KEVFLNEKSVDGTRTNVYLNEVQVADFASFLEFVYTAKVQVEEDRVQRMLEVAEKLKCLD
LSETCFQLKKQMLESVLLELQNFSESQEVEVSSGSQVSAAPAPRASVATDGPHPSGLTDS
LDYPGERASNGMSSDLPPKKSKDKLDKKKEVVKPPYPKIRRASGRLAGRKVFVEIPKKKY
TRRLREQQKTAEGDVGDYRCPQDQSPDRVGTEMEQVSKNEGCQAGAELEELSKKAGPEEE
EEEEEEDEEGEKKKSNFKCSICEKAFLYEKSFLKHSKHRHGVATEVVYRCDTCGQTFANR
CNLKSHQRHVHSSERHFPCELCGKKFKRKKDVKRHVLQVHEGGGERHRCGQCGKGLSSKT
ALRLHERTHTGDRPYGCTECGARFSQPSALKTHMRIHTGEKPFVCDECGARFTQNHMLIY
HKRCHTGERPFMCETCGKSFASKEYLKHHNRIHTGSKPFKCEVCFRTFAQRNSLYQHIKV
HTGERPYCCDQCGKQFTQLNALQRHRRIHTGERPFMCNACGRTFTDKSTLRRHTSIHDKN
TPWKSFLVIVDGSPKNDDGHKTEQPDEEYVSSKLSDKLLSFAENGHFHNLAAVQDTVPTM
QENSSADTACKADDSVVSQDTLLATTISELSELTPQTDSMPTQLHSLSNME*
>ENSG00000138326|ENST00000360830
MNDTVTIRTRKFMTNRLLQRKQMVIDVLHPGKATVPKTEIREKLAKMYKTTPDVIFVFGF
RTHFGGGKTTGFGMIYDSLDYAKKNEPKHRLARHGLYEKKKTSRKQRKERKNRMKKVRGT
AKANVGAGKKK*
>ENSG00000106976|ENST00000341179
MGNRGMEDLIPLVNRLQDAFSAIGQNADLDLPQIAVVGGQSAGKSSVLENFVGRDFLPRG
SGIVTRRPLVLQLVNATTEYAEFLHCKGKKFTDFEEVRLEIEAETDRVTGTNKGISPVPI
NLRVYSPHVLNLTLVDLPGMTKVPVGDQPPDIEFQIRDMLMQFVTKENCLILAVSPANSD
LANSDALKVAKEVDPQGQRTIGVITKLDLMDEGTDARDVLENKLLPLRRGYIGVVNRSQK
DIDGKKDITAALAAERKFFLSHPSYRHLADRMGTPYLQKVLNQQLTNHIRDTLPGLRNKL
QSQLLSIEKEVEEYKNFRPDDPARKTKALLQMVQQFAVDFEKRIEGSGDQIDTYELSGGA
RINRIFHERFPFELVKMEFDEKELRREISYAIKNIHGIRTGLFTPDMAFETIVKKQVKKI
REPCLKCVDMVISELISTVRQCTKKLQQYPRLREEMERIVTTHIREREGRTKEQVMLLID
IELAYMNTNHEDFIGFANAQQRSNQMNKKKTSGNQDEILVIRKGWLTINNIGIMKGGSKE
YWFVLTAENLSWYKDDEEKEKKYMLSVDNLKLRDVEKGFMSSKHIFALFNTEQRNVYKDY
RQLELACETQEEVDSWKASFLRAGVYPERVGDKEKASETEENGSDSFMHSMDPQLERQVE
TIRNLVDSYMAIVNKTVRDLMPKTIMHLMINNTKEFIFSELLANLYSCGDQNTLMEESAE
QAQRRDEMLRMYHALKEALSIIGDINTTTVSTPMPPPVDDSWLQVQSVPAGRRSPTSSPT
PQRRAPAVPPARPGSRGPAPGPPPAGSALGGAPPVPSRPGASPDPFGPPPQVPSRPNRAP
PGVPRITISDP*
>ENSG00000102189|ENST00000322349
MLRRILQRTPGRVGSQGSDLDSSATPINTVDVNNESSSEGFICPQCMKSLGSADELFKHY

EAVHDAGNDSGHGGESNLALKRDDVTLLRQEVQDLQASLKEEKWYSEELKKELEKYQGLQ
QQEAKPDGLVTDSSAELQSLEQQLEEAQTENFNIKQMKDLFEQKAAQLATEIADIKSKYD
EERSLREAAEQKVTRLTEELNKEATVIQDLKTELLQRPGIEDVAVLKKELVQVQTLMDNM
TLERERESEKLKDECKKLQSQYASSEATISQLRSELAKGPQEVAVYVQELQKLKSSVNEL
TQKNQTLTENLLKKEQDYTKLEEKHNEESVSKKNIQATLHQKDLDCQQLQSRLSASETSL
HRIHVELSEKGEATQKLKEELSEVETKYQHLKAEFKQLQQQREEKEQHGLQLQSEINQLH
SKLLETERQLGEAHGRLKEQRQLSSEKLMDKEQQVADLQLKLSRLEEQLKEKVTNSTELQ
HQLDKTKQQHQEQQALQQSTTAKLREAQNDLEQVLRQIGDKDQKIQNLEALLQKSKENIS
LLEKEREDLYAKIQAGEGETAVLNQLQEKNHTLQEQVTQLTEKLKNQSESHKQAQENLHD
QVQEQKAHLRAAQDRVLSLETSVNELNSQLNESKEKVSQLDIQIKAKTELLLSAEAAKTA
QRADLQNHLDTAQNALQDKQQELNKITTQLDQVTAKLQDKQEHCSQLESHLKEYKEKYLS
LEQKTEELEGQIKKLEADSLEVKASKEQALQDLQQQRQLNTDLELRATELSKQLEMEKEI
VSSTRLDLQKKSEALESIKQKLTKQEEEKKILKQDFETLSQETKIQHEELNNRIQTTVTE
LQKVKMEKEALMTELSTVKDKLSKVSDSLKNSKSEFEKENQKGKAAILDLEKTCKELKHQ
LQVQMENTLKEQKELKKSLEKEKEASHQLKLELNSMQEQLIQAQNTLKQNEKEEQQLQGN
INELKQSSEQKKKQIEALQGELKIAVLQKTELENKLQQQLTQAAQELAAEKEKISVLQNN
YEKSQETFKQLQSDFYGRESELLATRQDLKSVEEKLSLAQEDLISNRNQIGNQNKLIQEL
KTAKATLEQDSAKKEQQLQERCKALQDIQKEKSLKEKELVNEKSKLAEIEEIKCRQEKEI
TKLNEELKSHKLESIKEITNLKDAKQLLIQQKLELQGKADSLKAAVEQEKRNQQILKDQV
KKEEEELKKEFIEKEAKLHSEIKEKEVGMKKHEENEAKLTMQITALNENLGTVKKEWQSS
QRRVSELEKQTDDLRGEIAVLEATVQNNQDERRALLERCLKGEGEIEKLQTKVLELQRKL
DNTTAAVQELGRENQSLQIKHTQALNRKWAEDNEVQNCMACGKGFSVTVRRHHCRQCGNI
FCAECSAKNALTPSSKKPVRVCDACFNDLQG*
>ENSG00000114416|ENST00000357559
MAELTVEVRGSNGAFYKGFIKDVHEDSLTVVFENNWQPERQVPFNEVRLPPPPDIKKEIS
EGDEVEVYSRANDQEPCGWWLAKVRMMKGEFYVIEYAACDATYNEIVTFERLRPVNQNKT
VKKNTFFKCTVDVPEDLREACANENAHKDFKKAVGACRIFYHPETTQLMILSASEATVKR
VNILSDMHLRSIRTKLMLMSRNEEATKHLECTKQLAAAFHEEFVVREDLMGLAIGTHGSN
IQQARKVPGVTAIELDEDTGTFRIYGESADAVKKARGFLEFVEDFIQVPRNLVGKVIGKN
GKVIQEIVDKSGVVRVRIEGDNENKLPREDGMVPFVFVGTKESIGNVQVLLEYHIAYLKE
VEQLRMERLQIDEQLRQIGSRSYSGRGRGRRGPNYTSGYGTNSELSNPSETESERKDELS
DWSLAGEDDRDSRHQRDSRRRPGGRGRSVSGGRGRGGPRGGKSSISSVLKDPDSNPYSLL
DNTESDQTADTDASESHHSTNRRRRSRRRRTDEDAVLMDGMTESDTASVNENGLVTVADY
ISRAESQSRQRNLPRETLAKNKKEMAKDVIEEHGPSEKAINGPTSASGDDISKLQRTPGE
EKINTLKEENTQEAAVLNGVS*
>ENSG00000163867|ENST00000357182
MKEPLDGECGKAVVPQQELLDKIKEEPDNAQEYGCVQQPKTQESKLKIGGVSSVNERPIA
QQLNPGFQLSFASSGPSVLLPSVPAVAIKVFCSGCKKMLYKGQTAYHKTGSTQLFCSTRC
ITRHSSPACLPPPPKKTCTNCSKDILNPKDVITTRFENSYPSKDFCSQSCLSSYELKKKP
VVTIYTKSISTKCSMCQKNADTRFEVKYQNVVHGLCSDACFSKFHSTNNLTMNCCENCGS
YCYSSSGPCQSQKVFSSTSVTAYKQNSAQIPPYALGKSLRPSAEMIETTNDSGKTELFCS
INCLSAYRVKTVTSSGVQVSCHSCKTSAIPQYHLAMSNGTIYSFCSSSCVVAFQNVFSKP
KGTNSSAVPLSQGQVVVSPPSSRSAVSIGGGNTSAVSPSSIRGSAAASLQPLAEQSQQVA
LTHTVVKLKCQHCNHLFATKPELLFYKGKMFLFCGKNCSDEYKKKNKVVAMCDYCKLQKI

IKETVRFSGVDKPFCSEVCKFLSARDFGERWGNYCKMCSYCSQTSPNLVENRLEGKLEEF
CCEDCMSKFTVLFYQMAKCDGCKRQGKLSESIKWRGNIKHFCNLFCVLEFCHQQIMNDCL
PQNKVNISKAKTAVTELPSARTDTTPVITSVMSLAKIPATLSTGNTNSVLKGAVTKEAAK
IIQDESTQEDAMKFPSSQSSQPSRLLKNKGISCKPVTQTKATSCKPHTQHKECQTDLPMP
NEKNDAELDSPPSKKKRLGFFQTYDTEYLKVGFIICPGSKESSPRPQCVICGEILSSENM
KPANLSHHLKTKHSELENKPVDFFEQKSLEMECQNSSLKKCLLVEKSLVKASYLIAFQTA
ASKKPFSIAEELIKPYLVEMCSEVLGSSAGDKMKTIPLSNVTIQHRIDELSADIEDQLIQ
KVRESKWFALQIDESSEISNITLLLCYIRFIDYDCRDVKEELLFCIEMPTQITGFEIFEL
INKYIDSKSLNWKHCVGLCTDGAASMTGRYSGLKAKIQEVAMNTAAFTHCFIHRERLVAE
KLSPCLHKILLQSAQILSFIKSNALNSRMLTILCEEMGSEHVSLPLHAEVRWISRGRMLK
RLFELRHEIEIFLSQKHSDLAKYFHDEEWVGKLAYLSDIFSLINELNLSLQGTLTTFFNL
CNKIDVFKRKLKMWLKRTQENDYDMFPSFSEFSNSSGLNMTDITRIIFEHLEGLSQVFSD
CFPPEQDLRSGNLWIIHPFMNHQNNNLTDFEEEKLTELSSDLGLQALFKSVSVTQFWINA
KTSYPELHERAMKFLLPFSTVYLCDAAFSALTESKQKNLLGSGPALRLAVTSLIPRIEKL
VKEKE*
>ENSG00000084234|ENST00000345598
MAATGTAAAAATGRLLLLLLLVGLTAPALALAGYIEALAANAGTGFAVAEPQIAMFCGKLN
MHVNIQTGKWEPDPTGTKSCFETKEEVLQYCQEMYPELQITNVMEANQRVSIDNWCRRDK
KQCKSRFVTPFKCLVPPTPLPTNDVDVYFETSADDNEHARFQKAKEQLEIRHRNRMDRVK
KEWEEAELQAKNLPKAERQTLIQHFQAMVKALEKEAASEKQQLVETHLARVEAMLNDRRR
MALENYLAALQSDPPRPHRILQALRRYVRAENKDRLHTIRHYQHVLAVDPEKAAQMKSQV
MTHLHVIEERRNQSLSLLYKVPYVAQEIQEEIDELLQEQRADMDQFTASISETPVDVRVS
SEESEEIPPFHPFHPFPALPENEGSGVGEQDGGLIGAEEKVINSKNKVDENMVIDETLDV
KEMIFNAERVGGLEEERESVGPLREDFSLSSSALIGLLVIAVAIATVIVISLVMLRKRQY
GTISHGIVEVDPMLTPEERHLNKMQNHGYENPTYKYLEQMQI*
>ENSG00000198791|ENST00000361272
MPAATVDHSQRICEVWACNLDEEMKKIRQVIRKYNYVAMDTEFPGVVARPIGEFRSNADY
QYQLLRCNVDLLKIIQLGLTFMNEQGEYPPGTSTWQFNFKFNLTEDMYAQDSIELLTTSG
IQFKKHEEEGIETQYFAELLMTSGVVLCEGVKWLSFHSGYDFGYLIKILTNSNLPEEELD
FFEILRLFFPVIYDVKYLMKSCKNLKGGLQEVAEQLELERIGPQHQAGSDSLLTGMAFFK
MREMFFEDHIDDAKYCGHLYGLGSGSSYVQNGTGNAYEEEANKQS*
>ENSG00000185811|ENST00000346667
MDADEGQDMSQVSGDKGLSDTPYDSSASYEKENEMMKSHVMDQAINNAINYLGAESLRPL
VQTPPGGSEVVPVISPMYQLHKPLAEGTPRSNHSAQDSAVENLLLLSKAKLVPSEREASP
SNSCQDSTDTESNNEEQRSGLIYLTNHIAPHARNGLSLKEEHRAYDLLRAASENSQDALR
VVSTSGEQMKVYKCEHCRVLFLDHVMYTIHMGCHGFRDPFECNMCGYHSQDRYEFSSHIT
RGEHRFHMS*
>ENSG00000120784|ENST00000351218
MARDLVMFRDVAVDFSQEEWECLNSYQRNLYRDVILENYSNLVSLAGCSISKPDVITLLE
QGKEPWMVVRDEKRRWTLDLESRYDTKKLFQGKDIYEMNLSQWKVMERIKSCGLEEQESP
HEVCFRQVTKTTSEKMPTYRKLTSLPLYQKSHNREKPYECGECGKAFRVRQQLTFHQRIH
TGEKPYECKECGKAFRQCAHLSRHQRIHTSDKLYECKKCGKIFTCGSDLRVHQRIHIGEK
PYECKECGKAFRVRGQLNLHQRIHTGEKPYECKECGKAFRQYAHLTRHQRLNIAEKCYEC
KECGQAFLCSTGLRLHHKLHTGEKPYECKECGKAFRVRQQLTLHQRIHTGEKPYDCKECG

KTFSRGYHLTLHQRIHTGEKPYECKECQKFFRRYSELISHQGIHIGEKPYECKECGKAFR
LFSQLTQHQSIHFGEKPFKCKECECEKTFRLLSQLTQHQSIHTGEKPYDCKECGKAFRLHSS
LIQHQRIHSGEKPYKCKECKKAFRQHSHLTYHQRIHNVT*
>ENSG00000164347|ENST00000345239
MLTNLRIFAMSHQTIPSVYINNICCYKIRASLKRLKPHVPLGRNCSSLPGLIGNDIKSLH
SIINPPIAKIRNIGIMAHIDAGKTTTTERILYYSGYTRSLGDVDDGDTVTDFMAQERERG
ITIQSAAVTFDWKGYRVNLIDTPGHVDFTLEVERCLRVLDGAVAVFDASAGVEAQTLTVW
RQADKHNIPRICFLNKMDKTGASFKYAVESIREKLKAKPLLLQLPIGEAKTFKGVVDVVM
KEKLLWNCNSNDGKDFERKPLLEMNDPELLKETTEARNALIEQVADLDDEFADLVLEEFS
ENFDLLPAEKLQTAIHRVTLAQTAVPVLCGSALKNKGIQPLLDAVTMYLPSPEERNYEFL
ERISRLLLPFADQHVEIPSLTAGNIALTVGLKHTATGDTIVSSKSSALAAARRAEREGEK
KHRQNNEAERLLLAGVEIPEPVFFCTIEPPSLSKQPDLEHALKCLQREDPSLKVRLDPDS
GQTVLCGMGELHIEIIHDRIKREYGLETYLGPLQVAYRETILNSVRATDTLDRTLGDKRH
LVTVEVEARPIETSSVMPVIEFEYAESINEGLLKVSQEAIENGIHSACLQGPLLGSPIQD
VAITLHSLTIHPGTSTTMISACVSRCVQKALKKADKQVLEPLMNLEVTVARDYLSPVLAD
LAQRRGNIQEIQTRQDNKVVIGFVPLAEIMGYSTVLRTLTSGSATFALELSTYQAMNPQD
QNTLLNRRSGLT*
>ENSG00000122566|ENST00000356674
MEREKEQFRKLFIGGLSFETTEESLRNYYEQWGKLTDCVVMRDPASKRSRGFGFVTFSSM
AEVDAAMAARPHSIDGRVVEPKRAVAREESGKPGAHVTVKKLFVGGIKEDTEEHHLRDYF
EEYGKIDTIEIITDRQSGKKRGFGFVTFDDHDPVDKIVLQKYHTINGHNAEVRKALSRQE
MQEVQSSRSGRGGNFGFGDSRGGGGNFGPGPGSNFRGGSDGYGSGRGFGDGYNGYGGGPG
GGNFGGSPGYGGGRGGYGGGGPGYGNQGGGYGGGYDNYGGGNYGSGNYNDFGNYNQQPSN
YGPMKSGNFGGSRNMGGPYGGGNYGPGGSGGSGGYGGRSRY*
>ENSG00000164916|ENST00000328914
MAEVGEDSGARALLALRSAPCSPVLCAAAAAAAFPAAAPPPAPAQPQPPPGPPPPPPPPL
PPGAIAGAGSSGGSSGVSGDSAVAGAAPALVAAAAASVRQSPGPALARLEGREFEFLMRQ
PSVTIGRNSSQGSVDLSMGLSSFISRRHLQLSFQEPHFYLRCLGKNGVFVDGAFQRRGAP
ALQLPKQCTFRFPSTAIKIQFTSLYHKEEAPASPLRPLYPQISPLKIHIPEPDLRSMVSP
VPSPTGTISVPNSCPASPRGAGSSSYRFVQNVTSDLQLAAEFAAKAASEQQADTSGGDSP
KDESKPPFSYAQLIVQAISSAQDRQLTLSGIYAHITKHYPYYRTADKGWQNSIRHNLSLN
RYFIKVPRSQEEPGKGSFWRIDPASEAKLVEQAFRKRRQRGVSCFRTPFGPLSSRSAPAS
PTHPGLMSPRSGGLQTPECLSREGSPIPHDPEFGSKLASVPEYRYSQSAPGSPVSAQPVI
MAVPPRPSSLVAKPVAYMPASIVTSQQPAGHAIHVVQQAPTVTMVRVVTTSANSANGYIL
TSQGAAGGSHDAAGAAVLDLGSEARGLEEKPTIAFATIPAAGGVIQTVASQMAPGVPGHT
VTILQPATPVTLGQHHLPVRAVTQNGKHAVPTNSLAGNAYALTSPLQLLATQASSSAPVV
VTRVCEVGPKEPAAAVAATATTTPATATTASASASSTGEPEVKRSRVEEPSGAVTTPAGV
IAAAGPQGPGTGE*
>ENSG00000112531|ENST00000361752
MVGEMETKEKPKPTPDYLMQLMNDKKLMSSLPNFCGIFNHLERLLDEEISRVRKDMYNDT
LNGSTEKRSAELPDAVGPIVQLQEKLYVPVKEYPDFNFVGRILGPRGLTAKQLEAETGCK
IMVRGKGSMRDKKKEEQNRGKPNWEHLNEDLHVLITVEDAQNRAEIKLKRAVEEVKKLLV
PAAEGEDSLKKMQLMELAILNGTYRDANIKSPALAFSLAATAQAAPRIITGPAPVLPPAA
LRTPTPAGPTIMPLIRQIQTAVMPNGTPHPTAAIVPPGPEAGLIYTPYEYPYTLAPATSI

LEYPIEPSGVLGAVATKVRRHDMRVHPYQRIVTADRAATGN*
>ENSG00000168502|ENST00000359865
MEEMRDSYLEEDVYQLQELRRELDRANKNCRILQYRLRKAEQKSLKVAETGQVDGELIRS
LEQDLKVAKDVSVRLHHELKTVEEKRAKAEDENETLRQQMIEVEISKQALQNELERLKES
SLKRRSTREMYKEKKTFNQDDSADLRCQLQFAKEEAFLMRKKMAKLGREKDELEQELQKY
KSLYGDVDSPLPTGEAGGPPSTREAELKLRLKLVEEEANILGRKIVELEVENRGLKAEME
DMRGQQEREGPGRDHAPSIPTSPFGDSLESSTELRRHLQFVEEEAELLRRSISEIEDHNR
QLTHELSKFKFEPPREPGWLGEGASPGAGGGAPLQEELKSARLQISELSGKVLKLQHENH
ALLSNIQRCDLAAHLGLRAPSPRDSDAESDAGKKESDGEESRLPQPKREGPVGGESDSEE
MFEKTSGFGSGKPSEASEPCPTELLKAREDSEYLVTLKHEAQRLERTVERLITDTDSFLH
DAGLRGGAPLPGPGLQGEEEQGEGDQQEPQLLGTINAKMKAFKKELQAFLEQVNRIGDGL
SPLPHLTESSSFLSTVTSVSRDSPIGNLGKELGPDLQSRLKEQLEWQLGPARGDERESLR
LRAARELHRRADGDTGSHGLGGQTCFSLELRGPPVLPEQSVSIEELQGQLVQAARLHQEE
TETFTNKIHKMEEEHLYALRWKELEMHSLALQNTLHERTWSDEKNLMQQELRSLKQNIFL
FYVKLRWLLKHWRQGKQMEEEGEEFTEGEHPETLSRLGELGVQGGHQADGPDHDSDRGCG
FPVGEHSPHSRVQIGDHSLRLQTADRGQPHKQVVENQQLFSAFKALLEDFRAELREDERA
RLRLQQQYASDKAAWDVEWAVLKCRLEQLEEKTENKLGELGSSAESKGALKKEREVHQKL
LADSHSLVMDLRWQIHHSEKNWNREKVELLDRLDRDRQEWERQKKEFLWRIEQLQKENSP
RRGGSFLCDQKDGNVRPFPHQGSLRMPRPVAMWPCADADSIPFEDRPLSKLKESDRCSAS
ENLYLDALSLDDEPEEPPAHRPEREFRNRLPEEEENHKGNLQRAVSVSSMSEFQRLMDIS
PFLPEKGLPSTSSKEDVTPPLSPDDLKYIEEFNKSWDYTPNRGHNGGGPDLWADRTEVGR
AGHEDSTEPFPDSSWYLTTSVTMTTDTMTSPEHCQKQPLRSHVLTEQSGLRVLHSPPAVR
RVDSITAAGGEGPFPTSRARGSPGDTKGGPPEPMLSRWPCTSPRHSRDYVEGARRPLDSP
LCTSLGFASPLHSLEMSKNLSDDMKEVAFSVRNAICSGPGELQVKDMACQTNGSRTMGTQ
TVQTISVGLQTEALRGSGVTSSPHKCLTPKAGGGATPVSSPSRSLRSRQVAPAIEKVQAK
FERTCCSPKYGSPKLQRKPLPKADQPNNRTSPGMAQKGYSESAWARSTTTRESPVHTTIN
DGLSSLFNIIDHSPVVQDPFQKGLRAGSRSRSAEPRPELGPGQETGTNSRGRSPSPIGVG
SEMCREEGGEGTPVKQDLSAPPGYTLTENVARILNKKLLEHALKEERRQAAHGPPGLHSD
SHSLGDTAEPGPMENQTVLLTAPWGL*
>ENSG00000152422|ENST00000338635
MERKISRIHLVSEPSITHFLQVSWEKTLESGFVITLTDGHSAWTGTVSESEISQEADDMA
MEKGKYVGELRKALLSGAGPADVYTFNFSKESCYFFFEKNLKDVSFRLGSFNLEKVENPA
EVIRELICYCLDTIAENQAKNEHLQKENERLLRDWNDVQGRFEKCVSAKEALETDLYKRF
ILVLNEKKTKIRSLHNKLLNAAQEREKDIKQEGETAICSEMTADRDPVYDESTDEESENQ
TDLSGLASAAVSKDDSIISSLDVTDIAPSRKRRQRMQRNLGTEPKMAPQENQLQEKENSR
PDSSLPETSKKEHISAENMSLETLRNSSPEDLFDEI*
>ENSG00000143418|ENST00000361419
MLQTLYDYFWWERLWLPVNLTWADLEDRDGRVYAKASDLYITLPLALLFLIVRYFFELYV
ATPLAALLNIKEKTRLRAPPNATLEHFYLTSGKQPKQVEVELLSRQSGLSGRQVERWFRR
RRNQDRPSLLKKFREASWRFTFYLIAFIAGMAVIVDKPWFYDMKKVWEGYPIQSTIPSQY
WYYMIELSFYWSLLFSIASDVKRKDFKEQIIHHVATIILISFSWFANYIRAGTLIMALHD
SSDYLLEVR
>ENSG00000134874|ENST00000361156
MQAEAADWFSSMPFQKHVYYPLASGPEGPDVAVAAAAAGAASMACAPPSAASGPLPFFQF

RPRLESVDWRRLSAIDVDKVAGAVDVLTLQENIMNITFCKLEDEKCPHCQSGVDPVLLKL
IRLAQFTIEYLLHSQEFLTSQLHTLEERLRLSHCDGEQSKKLLTKQAGEIKTLKEECKRR
KKMISTQQLMIEAKANYYQCHFCDKAFMNQAFLQSHIQRRHTEENSHFEYQKNAQIEKLR
SEIVVLKEELQLTRSELEAAHHASAVRFSKEYEMQKTKEEDFLKLFDRWKEEEKEKLVDE
MEKVKEMFMKEFKELTSKNSALEYQLSEIQKSNMQIKSNIGTLKDAHEFKEDRSPYPQDF
HNVMQLLDSQESKWTARVQAIHQEHKKEKGRLLSHIEKLRTSMIDDLNASNVFYKKRIEE
LGQRLQEQNELIITQRQQIKDFTCNPLNSISEPKVNAPALHTLETKSSLPMVHEQAFSSH
ILEPIEELSEEEKGRENEQKLNNNKMHLRKALKSNSSLTKGLRTMVEQNLMEKLETLGIN
ADIRGISSDQLHRVLKSVESERHKQEREIPNFHQIREFLEHQVSCKIEEKALLSSDQCSV
SQMDTLSTGEVPKMIQLPSKNRQLIRQKAVSTDRTSVPKIKKNVMEDPFPRKSSTITTPP
FSSEEEQEDDDLIRAYASPGPLPVPPPQNKGSFGKNTVKSDADGTEGSEIEDTDDSPKPA
GVAVKTPTEKVEKMFPHRKNVNKPVGGTNVPEMFIKKEELQELKCADVEDEDWDISSLEE
EISLGKKSGKEQKEPPPAKNEPHFAHVLNAWGAFNPKGPKGEGLQENESSTLKSSLVTVT
DWSDTSDV*
>ENSG00000055950|ENST00000342071
MTARGTPSRFLASVLHNGLGRYVQQLQRLSFSVSRDGASSRGAREFVEREVIDFARRNPG
VVIYVNSRPCCVPRVVAEYLNGAVREESIHCKSVEEISTLVQKLADQSGLDVIRIRKPFH
TDNPSIQGQWHPFTNKPTTFRGLRPREVQDPAPAQDAAEFLGEGAGPCWYSIVALPQKHI
AIAIHPPQPAGQCHQAIGHWPETVCSCTADPPARLARPNISSVIRSSLGKYPLPS*
>ENSG00000184209|ENST00000350887
MNDWMPIAKEYDPLKAGSIDGTDEDPHDRAVWRAMLARYVPNKGVIGDPLLTLFVARLNL
QTKEDKLKEVFSRYGDIRRLRLVRDLVTGFSKGYAFIEYKEERAVIKAYRDADGLVIDQH
EIFVDYELERTLKGWIPRRLGGGLGGKKESGQLRFGGRDRPFRKPINLPVVKNDLYREGK
RERRERSRSRERHWDSRTRDRDHDRGREKRWQEREPTRVWPDNDWERERDFRDDRIKGRE
KKERGK*
>ENSG00000143633|ENST00000366649
MRVDSSADPTMSQEQGPGSSTPPSSPTLLDALLQNLYDFGGTEGETEQKKIIKKRENKKR
DVMASAALAAEPSPLPGSLIRGQRKSASSFFKELREERHCAPSGTPTGPEILAAAVPPSS
LKNNREQVEVVEFHSNKKRKLTPDHNKNTKQANPSVLERDVDTQEFNLEKARLEVHRFGI
TGYGKGKERILEQERAIMLGAKPPKKSYVNYKVLQEQIKEKKAAKEEEKRLAQETDIFKK
KKRKGQEDRKSKKKSAPSILSNGRIGQVGKFKNGTLILSPVDIKKINSSRVAK*
>ENSG00000102908|ENST00000349945
MGGACSSFTTSSSPTIYSTSVTDSKAMQVESCSSAVGVSNRGVSEKQLTSNTVQQHPSTP
KRHTVLYISPPPEDLLDNSRMSCQDEGCGLESEQSCSMWMEDSPSNFSNMSTSSYNDNTE
VPRKSRKRNPKQRPGVKRRDCEESNMDIFDADSAKAPHYVLSQLTTDNKGNSKAGNGTLE
NQKGTGVKKSPMLCGQYPVKSEGKELKIVVQPETQHRARYLTEGSRGSVKDRTQQGFPTV
KLEGHNEPVVLQVFVGNDSGRVKPHGFYQACRVTGRNTTPCKEVDIEGTTVIEVGLDPSN
NMTLAVDCVGILKLRNADVEARIGIAGSKKKSTRARLVFRVNIMRKDGSTLTLQTPSSPI
LCTQPAGVPEILKKSLHSCSVKGEEEVFLIGKNFLKGTKVIFQENVSDENSWKSEAEIDM
ELFHQNHLIVKVPPYHDQHITLPVSVGIYVVTNAGRSHDVQPFTYTPDPAAAGALNVNVK
KEISSPARPCSFEEAMKAMKTTGCNLDKVNIIPNALMTPLIPSSMIKSEDVTPMEVTAEK
RSSTIFKTTKSVGSTQQTLENISNIAGNGSFSSPSSSHLPSENEKQQQIQPKAYNPETLT
TIQTQDISQPGTFPAVSASSQLPNSDALLQQATQFQTRETQSREILQSDGTVVNLSQLTE
ASQQQQQSPLQEQAQTLQQQISSNIFPSPNSVSQLQNTIQQLQAGSFTGSTASGSSGSVD

LVQQVLEAQQQLSSVLFSAPDGNENVQEQLSADIFQQVSQIQSGVSPGMFSSTEPTVHTR
PDNLLPGRAESVHPQSENTLSNQQQQQQQQQQVMESSAAMVMEMQQSICQAAAQIQSELF
PSTASANGNLQQSPVYQQTSHMMSALSTNEDMQMQCELFSSPPAVSGNETSTTTTQQVAT
PGTTMFQTSSSGDGEETGTQAKQIQNSVFQTMVQMQHSGDNQPQVNLFSSTKSMMSVQNS
GTQQQGNGLFQQGNEMMSLQSGNFLQQSSHSQAQLFHPQNPIADAQNLSQETQGSLFHSP
NPIVHSQTSTTSSEQMQPPMFHSQSTIAVLQGSSVPQDQQSTNIFLSQSPMNNLQTNTVA
QEAFFAAPNSISPLQSTSNSEQQAAFQQQAPISHIQTPMLSQEQAQPPQQGLFQPQVALG
SLPPNPMPQSQQGTMFQSQHSIVAMQSNSPSQEQQQQQQQQQQQQQQQQQSILFSNQNTM
ATMASPKQPPPNMIFNPNQNPMANQEQQNQSIFHQQSNMAPMNQEQQPMQFQSQSTVSSL
QNPGPTQSESSQTPLFHSSPQIQLVQGSPSSQEQQVTLFLSPASMSALQTSINQQDMQQS
PLYSPQNNMPGIQGATSSPQPQATLFHNTAGGTMNQLQNSPGSSQQTSGMFLFGIQNNCS
QLLTSGPATLPDQLMAISQPGQPQNEGQPPVTTLLSQQMPENSPLASSINTNQNIEKIDL
LVSLQNQGNNLTGSF*
>ENSG00000152601|ENST00000324210
MAVSVTPIRDTKWLTLEVCREFQRGTCSRPDTECKFAHPSKSCQVENGRVIACFDSLKGR
CSRENCKYLHPPPHLKTQLEINGRNNLIQQKNMAMLAQQMQLANAMMPGAPLQPVPMFSV
APSLATNASAAAFNPYLGPVSPSLVPAEILPTAPMLVTGNPGVPVPAAAAAAAQKLMRTD
RLEVCREYQRGNCNRGENDCRFAHPADSTMIDTNDNTVTVCMDYIKGRCSREKCKYFHPP
AHLQAKIKAAQYQVNQAAAAQAAATAAAMGIPQAVLPPLPKRPALEKTNGATAVFNTGIF
QYQQALANMQLQQHTAFLPPGSILCMTPATSVVPMVHGATPATVSAATTSATSVPFAATA
TANQIPIISAEHLTSHKYVTQM*
>ENSG00000185811|ENST00000357364
MDADEGQDMSQVSGKESPPVSDTPDEGDEPMPIPEDLSTTSGGQQSSKSDRVVASNVKVE
TQSDEENGRACEMNGEECAEDLRMLDASGEKMNGSHRDQGSSALSGVGGIRLPNGKLKCD
ICGIICIGPNVLMVHKRSHTGERPFQCNQCGASFTQKGNLLRHIKLHSGEKPFKCHLCNY
ACRRRDALTGHLRTHSGDKGLSDTPYDSSASYEKENEMMKSHVMDQAINNAINYLGAESL
RPLVQTPPGGSEVVPVISPMYQLHKPLAEGTPRSNHSAQDSAVENLLLLSKAKLVPSERE
ASPSNSCQDSTDTESNNEEQRSGLIYLTNHIAPHARNGLSLKEEHRAYDLLRAASENSQD
ALRVVSTSGEQMKVYKCEHCRVLFLDHVMYTIHMGCHGFRDPFECNMCGYHSQDRYEFSS
HITRGEHRFHMS*
>ENSG00000106348|ENST00000338791
MEGPLTPPPLQGGGAAAVPEPGARQHPGHETAAQRYSARLLQAGYEPESPRLDLATHPTT
PRSELSSVVLLAGVGVQMDRLRRASMADYLISGGTGYVPEDGLTAQQLFASADGLTYNDF
LILPGFIDFIADEVDLTSALTRKITLKTPLISSPMDTVTEADMAIAMALMGGIGFIHHNC
TPEFQANEVRKVKKFEQGFITDPVVLSPSHTVGDVLEAKMRHGFSGIPITETGTMGSKLV
GIVTSRDIDFLAEKDHTTLLSEVMTPRIELVVAPAGVTLKEANEILQRSKKGKLPIVNDC
DELVAIIARTDLKKNRDYPLASKDSQKQLLCGAAVGTREDDKYRLDLLTQAGVDVIVLDS
SQGNSVYQIAMVHYIKQKYPHLQVIGGNVVTAAQAKNLIDAGVDGLRVGMGCGSICITQE
VMACGRPQGTAVYKVAEYARRFGVPIIADGGIQTVGHVVKALALGASTVMMGSLLAATTE
APGEYFFSDGVRLKKYRGMGSLDAMEKSSSSQKRYFSEGDKVKIAQGVSGSIQDKGSIQK
FVPYLIAGIQHGCQDIGARSLSVLRSMMYSGELKFEKRTMSAQIEGGVHGLHSYEKRLY*
>ENSG00000120693|ENST00000350148
MHSTTPISSLFSFTSPAVKRLLGWKQGDEEEKWAEKAVDSLVKKLKKKKGAMDELERALS
CPGQPSKCVTIPRSLDGRLQVSHRKGLPHVIYCRVWRWPDLQSHHELKPLECCEFPFGSK

QKEVCINPYHYRRVETPVLPPVLVPRHSEYNPQLSLLAKFRSASLHSEPLMPHNATYPDS
FQQPPCSALPPSPSHAFSQSPCTASYPHSPGSPSEPESPYQHSDFRPVCYEEPQHWCSVA
YYELNNRVGETFQASSRSVLIDGFTDPSNNRNRFCLGLLSNVNRNSTIENTRRHIGKGVH
LYYVGGEVYAECVSDSSIFVQSRNCNYQHGFHPATVCKIPSGCSLKVFNNQLFAQLLAQS
VHHGFEVVYELTKMCTIRMSFVKGWGAEYHRQDVTSTPCWIEIHLHGPLQWLDKVLTQMG
SPHNPISSVS*
>ENSG00000168148|ENST00000366696
MARTKQTARKSTGGKAPRKQLATKVARKSAPATGGVKKPHRYRPGTVALREIRRYQKSTE
LLIRKLPFQRLMREIAQDFKTDLRFQSSAVMALQEACESYLVGLFEDTNLCVIHAKRVTI
MPKDIQLARRIRGERA*
>ENSG00000170448|ENST00000329043
MEASWRQVAGGRGRSRGRATAAPSGNGVHLRGAGGGREKGSVGAVPSGTSPGGVATTAAA
GSRHSPAGSQALQTTAASELMSQKKFEEIKKANQAAARKLVEEQFSSSSEEGDEDFEGKQ
GKILANTFITYTTQTDGDTRELERTKQYVNEAFQAGAMTCLICIASVKRNQAVWSCSGCF
CIFHMPCIQKWAKDSQFLVSSVTDDDFGKKDCPWPCPKCRFEYKRSETPSRYYCYCGKVE
DPPLDPWLVPHSCGQVCEREFKPPCGHKCLLLCHPGPCPPCPKMVTTTCYCKKAKPIPRR
CSAKEWSCQLPCGQKLLCGQHKCENPCHAGSCQPCPRVSRQKCVCGKKVAERSCASPLWH
CDQVCGKTLPCGNHTCEQVCHVGACGECPRSGKRFCPCQKSKFSLPCTEDVPTCGDSCDK
VLECGIHRCSQRCHRGPCETCRQEVEKHCRCGKHTKRMPCHKPYLCETKCVKMRDCQKHQ
CRRKCCPGNCPPCDQNCGRTLGCRNHKCPSVCHRGSCYPCPETVDVKCNCGNTKVTVPCG
RERTTRPPKCKEQCSRPPTCHHTSQEKHRCHFGSCPPCHQPCQKVLEKCGHLCPAPCHDQ
ALIKQTGRHQPTGPWEQPSEPAFIQTALPCPPCQVPIPMECLGKHEVSPLPCHAVGPYSC
KRVCGRILDCQNHTCMKECHKVTKTDGCTGKNKAGPECLHCEEGCSKSRPLGCLHPCILR
CHPGECPPCVQMLRIKCHCKITSLYVECRKITTADVNEKNLLSCCKNQCPKELPCGHRCK
EMCHPGECPFNCNQKVKLRCPCKRIKKELQCNKVRENQVSIECDTTCKEMKRKASEIKEA
EAKAALEEEKRRQQAELEAFENRLKGRRKKNRKRDEVAVELSLWQKHKYYLISVCGVVVV
VFAWYITHDVN*
>ENSG00000262814|ENST00000333676
MLPAAARPLWGPCLGLRAAAFRLARRQVPCVCAVRHMRSSGHQRCEALAGAPLDNAPKEY
PPKIQQLVQDIASLTLLEISDLNELLKKTLKIQDVGLVPMGGVMSGAVPAAAAQEAVEED
IPIAKERTHFTVRLTEAKPVDKVKLIKEIKNYIQGINLVQAKKLVESLPQEIKANVAKAE
AEKIKAALEAVGGTVVLE*
>ENSG00000122026|ENST00000326092
MTNTKGKRRGTRYMFSRPFRKHGVVPLATYMRIYKKGDIVDIKGMGTVQKGMPHKCYHGK
TGRVYNVTQHAVGIVVNKQVKGKILAKRINVRIEHIKHSKSRDSFLKRVKENDQKKKEAK
EKGTWVQLKRQPAPPREAHFVRTNGKEPELLEPIPYEFMA*
>ENSG00000165675|ENST00000338144
MQRDFRWLWVYEIGYAADNSRTLNVDSTAMTLPMSDPTAWATAMNNLGMAPLGIAGQPIL
PDFDPALGMMTGIPPITPMMPGLGIVPPPIPPDMPVVKEIIHCKSCTLFPPNPNLPPPAT
RERPPGCKTVFVGGLPENGTEQIIVEVFEQCGEIIAIRKSKKNFCHIRFAEEYMVDKALY
LSGYRIRLGSSTDKKDTGRLHVDFAQARDDLYEWECKQRMLAREERHRRRMEEERLRPPS
PPPVVHYSDHECSIVAEKLKDDSKFSEAVQTLLTWIERGEVNRRSANNFYSMIQSANSHV
RRLVNEKAAHEKDMEEAKEKFKQALSGILIQFEQIVAVYHSASKQKAWDHFTKAQRKNIS
VWCKQAEEIRNIHNDELMGIRREEEMEMSDDEIEEMTETKETEESALVSQAEALKEENDS

LRWQLDAYRNEVELLKQEQGKVHREDDPNKEQQLKLLQQALQGMQQHLLKVQEEYKKKEA
ELEKLKDDKLQVEKMLENLKEKESCASRLCASNQDSEYPLEKTMNSSPIKSEREALLVGI
ISTFLHVHPFGASIEYICSYLHRLDNKICTSDVECLMGRLQHTFKQEMTGVGASLEKRWK
FCGFEGLKLT*
>ENSG00000100462|ENST00000324366
MAAMAVGGAGGSRVSSGRDLNCVPEIADTLGAVAKQGFDFLCMPVFHPRFKREFIQEPAK
NRPGPQTRSDLLLSGRDWNTLIVGKLSPWIRPDSKVEKIRRNSEAAMLQELNFGAYLGLP
AFLLPLNQEDNTNLARVLTNHIHTGHHSSMFWMRVPLVAPEDLRDDIIENAPTTHTEEYS
GEEKTWMWWHNFRTLCDYSKRIAVALEIGADLPSNHVIDRWLGEPIKAAILPTSIFLTNK
KGFPVLSKMHQRLIFRLLKLEVQFIITGTNHHSEKEFCSYLQYLEYLSQNRPPPNAYELF
AKGYEDYLQSPLQPLMDNLESQTYEVFEKDPIKYSQYQQAIYKCLLDRVPEEEKDTNVQV
LMVLGAGRGPLVNASLRAAKQADRRIKLYAVEKNPNAVVTLENWQFEEWGSQVTVVSSDM
REWVAPEKADIIVSELLGSFADNELSPECLDGAQHFLKDDGVSIPGEYTSFLAPISSSKL
YNEVRACREKDRDPEAQFEMPYVVRLHNFHQLSAPQPCFTFSHPNRDPMIDNNRYCTLEF
PVEVNTVLHGFAGYFETVLYQDITLSIRPETHSPGMFSWFPILFPIKQPITVREGQTICV
RFWRCSNSKKVWYEWAVTAPVCSAIHNPTGRSYTIGL*
>ENSG00000185379|ENST00000345365
MGVLRVGLCPGLTEEMIQLLRSHRIKTVVDLVSADLEEVAQKCGLSYKALVALRRVLLAQ
FSAFPVNGADLYEELKTSTAILSTGIGSLDKLLDAGLYTGEVTEIVGGPGSGKTQVCLCM
AANVAHGLQQNVLYVDSNGGLTASRLLQLLQAKTQDEEEQAEALRRIQVVHAFDIFQMLD
VLQELRGTVAQQVTGSSGTVKVVVVDSVTAVVSPLLGGQQREGLALMMQLARELKTLARD
LGMAVVVTNHITRDRDSGRLKPALGRSWSFVPSTRILLDTIEGAGASGGRRMACLAKSSR
QPTGFQEMVDIGTWGTSEQSATLQGDQT*
>ENSG00000114867|ENST00000350481
MNQPPQIAPKRERKTIRIRDPNQGGKDITEEIMSGARTASTPTPPQTGGGLEPQANGETP
QVAVIVRPDDRSQGAIIADRPGLPGPEHSPSESQPSSPSPTPSPSPVLEPGSEPNLAVLS
IPGDTMTTIQMSVEESTPISRETGEPYRLSPEPTPLAEPILEVEVTLSKPVPESEFSSSP
LQAPTPLASHTVEIHEPNGMVPSEDLEPEVESSPELAPPPACPSESPVPIAPTAQPEELL
NGAPSPPAVDLSPVSEPEEQAKEVTASMAPPTIPSATPATAPSATSPAQEEEMEEEEEEE
EGEAGEAGEAESEKGGEELLPPESTPIPANLSQNLEAAAATQVAVSVPKRRRKIKELNKK
EAVGDLLDAFKEANPAVPEVENQPPAGSNPGPESEGSGVPPRPEEADETWDSKEDKIHNA
ENIQPGEQKYEYKSDQWKPLNLEEKKRYDREFLLGFQFIFASMQKPEGLPHISDVVLDKA
NKTPLRPLDPTRLQGINCGPDFTPSFANLGRTTLSTRGPPRGGPGGELPRGPAGLGPRRS
QQGPRKEPRKIIATVLMTEDIKLNKAEKAWKPSSKRTAADKDRGEEDADGSKTQDLFRRV
RSILNKLTPQMFQQLMKQVTQLAIDTEERLKGVIDLIFEKAISEPNFSVAYANMCRCLMA
LKVPTTEKPTVTVNFRKLLLNRCQKEFEKDKDDDEVFEKKQKEMDEAATAEERGRLKEEL
EEARDIARRRSLGNIKFIGELFKLKMLTEAIMHDCVVKLLKNHDEESLECLCRLLTTIGK
DLDFEKAKPRMDQYFNQMEKIIKEKKTSSRIRFMLQDVLDLRGSNWVPRRGDQGPKTIDQ
IHKEAEMEEHREHIKVQQLMAKGSDKRRGGPPGPPISRGLPLVDDGGWNTVPISKGSRPI
DTSRLTKITKPGSIDSNNQLFAPGGRLSWGKGSSGGSGAKPSDAASEAARPATSTLNRFS
ALQQAVPTESTDNRRVVQRSSLSRERGEKAGDRGDRLERSERGGDRGDRLDRARTPATKR
SFSKEVEERSRERPSQPEGLRKAASLTEDRDRGRDAVKREAALPPVSPLKAALSEEELEK
KSKAIIEEYLHLNDMKEAVQCVQELASPSLLFIFVRHGVESTLERSAIAREHMGQLLHQL
LCAGHLSTAQYYQGLYEILELAEDMEIDIPHVWLYLAELVTPILQEGGVPMGELFREITK

PLRPLGKAASLLLEILGLLCKSMGPKKVGTLWREAGLSWKEFLPEGQDIGAFVAEQKVEY
TLGEESEAPGQRALPSEELNRQLEKLLKEGSSNQRVFDWIEANLSEQQIVSNTLVRALMT
AVCYSAIIFETPLRVDVAVLKARAKLLQKYLCDEQKELQALYALQALVVTLEQPPNLLRM
FFDALYDEDVVKEDAFYSWESSKDPAEQQGKGVALKSVTAFFKWLREAEEESDHN*
>ENSG00000187959|ENST00000344935
MQEVIAGLERFTFAFEKDVEMQKGTGLLPFQGMDKSASAVCNFFTKGLCEKGKLCPFRHD
RGEKMVVCKHWLRGLCKKGDHCKFLHQYDLTRMPECYFYSKFGDCSNKECSFLHVKPAFK
SQDCPWYDQGFCKDGPLCKYRHVPRIMCLNYLVGFCPEGPKCQFAQKIREFKLLPGSKI*
>ENSG00000197128|ENST00000343280
MAAAEPMGPAQVPMNSEVIVDPIQGQVNFEDVFVYFSQEEWVLLDEAQRLLYRDVMLENF
ALMASLGHTSFMSHIVASLVMGSEPWVPDWVDMTLAVATETPGGSDPGCWHGMEDEEIPF
EQSFSIGMSQIRIPKGGPSTQKAYPCGTCGLVLKDILHLAEHQETHPGQKPYMCVLCGKQ
FCFSANLHQHQKQHSGEKPFRSDKSRPFLLNNCAVQSMEMSFVTGEACKDFLASSSIFEH
HAPHNEWKPHSNTKCEEASHCGKRHYKCSECGKTFSRKDSLVQHQRVHTGERPYECGECG
KTFSRKPILAQHQRIHTGEMPYECGICGKVFNHSSNLIVHQRVHTGARPYKCSECGKAYS
HKSTLVQHESIHTGERPYECSECGKYFGHKYRLIKHWSVHTGARPYECIACGKFFSQSSD
LIAHQRVHNGEKPYVCSECGKAFSHKHVLVQHHRIHTGERPYKCSECGKAFRQRASLIRH
WKIHTGERP*
>ENSG00000258366|ENST00000360203
MPKIVLNGVTVDFPFQPYKCQQEYMTKVLECLQQKVNGILESPTGTGKTLCLLCTTLAWR
EHLRDGISARKIAERAQGELFPDRALSSWGNAAAAAGDPIACYTDIPKIIYASRTHSQLT
QVINELRNTSYRPKVCVLGSREQLCIHPEVKKQESNHLQIHLCRKKVASRSCHFYNNVEE
KSLEQELASPILDIEDLVKSGSKHRVCPYYLSRNLKQQADIIFMPYNYLLDAKSRRAHNI
DLKGTVVIFDEAHNVEKMCEESASFDLTPHDLASGLDVIDQVLEEQTKAAQQGEPHPEFS
ADSPSPGLNMELEDIAKLKMILLRLEGAIDAVELPGDDSGVTKPGSYIFELFAEAQITFQ
TKGCILDSLDQIIQHLAGRAGVFTNTAGLQKLADIIQIVFSVDPSEGSPGSPAGLGALQS
YKVHIHPDAGHRRTAQRSDAWSTTAARKRGKVLSYWCFSPGHSMHELVRQGVRSLILTSG
TLAPVSSFALEMQIPFPVCLENPHIIDKHQIWVGVVPRGPDGAQLSSAFDRRFSEECLSS
LGKALGNIARVVPYGLLIFFPSYPVMEKSLEFWRARDLARKMEALKPLFVEPRSKGSFSE
TISAYYARVAAPGSTGATFLAVCRGKASEGLDFSDTNGRGVIVTGLPYPPRMDPRVVLKM
QFLDEMKGQGGAGGQFLSGQEWYRQQASRAVNQAIGRVIRHRQDYGAVFLCDHRFAFADA
RAQLPSWVRPHVRVYDNFGHVIRDVAQFFRVAERTMPAPAPRATAPSVRGEDAVSEAKSP
GPFFSTRKAKSLDLHVPSLKQRSSGSPAAGDPESSLCVEYEQEPVPARQRPRGLLAALEH
SEQRAGSPGEEQAHSCSTLSLLSEKRPAEEPRGGRKKIRLVSHPEEPVAGAQTDRAKLFM
VAVKQELSQANFATFTQALQDYKGSDDFAALAACLGPLFAEDPKKHNLLQGFYQFVRPHH
KQQFEEVCIQLTGRGCGYRPEHSIPRRQRAQPVLDPTGRTAPDPKLTVSTAAAQQLDPQE
HLNQGRPHLSPRPPPTGDPGSQPQWGSGVPRAGKQGQHAVSAYLADARRALGSAGCSQLL
AALTAYKQDDDLDKVLAVLAALTTAKPEDFPLLHRFSMFVRPHHKQRFSQTCTDLTGRPY
PGMEPPGPQEERLAVPPVLTHRAPQPGPSRSEKTGKTQSKISSFLRQRPAGTVGAGGEDA
GPSQSSGPPHGPAASEWGEPHGRDIAGQQATGAPGGPLSAGCVCQGCGAEDVVPFQCPAC
DFQRCQACWQRHLQASRMCPACHTASRKQSVMQVFWPEPQ*
>ENSG00000148737|ENST00000352065
MPQLNGGGGDDLGANDELISFKDEGEQEEKSSENSSAERDLADVKSSLVNESETNQNSSS
DSEAERRPPPRSESFRDKSRESLEEAAKRQDGGLFKGPPYPGYPFIMIPDLTSPYLPNGS

LSPTARTYLQMKWPLLDVQAGSLQSRQALKDARSPSPAHIVSNKVPVVQHPHHVHPLTPL
ITYSNEHFTPGNPPPHLPADVDPKTGIPRPPHPPDISPYYPLSPGTVGQIPHPLGWLVPQ
QGQPVYPITTGGFRHPYPTALTVNASMSRFPPHMVPPHHTLHTTGIPHPAIVTPTVKQES
SQSDVGSLHSSKHQDSKKEEEKKKPHIKKPLNAFMLYMKEMRAKVVAECTLKESAAINQI
LGRRWHALSREEQAKYYELARKERQLHMQLYPGWSARDNYGKKKKRKRDKQPGETNEHSE
CFLNPCLSLPPITDLSAPKKCRARFGLDQQNNWCGPCSL*
>ENSG00000121903|ENST00000361328
MAMALELQAQASPQPEPEELLIVKLEEDSWGSESKLWEKDRGSVSGPEASRQRFRQFQYR
DAAGPHEAFSQLWALCCRWLRPEIRLKEQILELLVLEQFLTILPREVQTWVQARHPESGE
EAVALVEDWHRETRTAGQSGLELHTEETRPLKTGEEAQSFQLQPVDPWPEGQSQKKGVKN
TCPDLPNHLNAEVAPQPLKESAVLTPRVPTLPKMGSVGDWEVTAESQEALGPGKHAEKEL
CKDPPGDDCGNSVCLGVPVSKPSNTSEKEQGPEFWGLSLINSGKRSTADYSLDNEPAQAL
TWRDSRAWEEQYQWDVEDMKVSGVHWGYEETKTFLAILSESPFSEKLRTCHQNRQVYRAI
AEQLRARGFLRTLEQCRYRVKNLLRNYRKAKSSHPPGTCPFYEELEALVRARTAIRATDG
PGEAVALPRLGYSDAEMDEQEEGGWDPEEMAEDCNGAGLVNVESTQGPRIAGAPALFQSR
IAGVHWGYEETKAFLAILSESPFSEKLRTCHQNSQVYRAIAERLCALGFLRTLEQCRYRF
KNLLRSYRKAKSSHPPGTCPFYEELDSLMRARAAVRAMGTVREAAGLPRCGQSSAETDAQ
EAWGEVANEDAVKPSTLCPKAPDMGFEMRHEDEDQISEQDIFEGLPGALSKCPTEAVCQP
LDWGEDSENENEDEGQWGNPSQEQWQESSSEEDLEKLIDHQGLYLAEKPYKCDTCMKSFS
RSSHFIAHQRIHTGEKPYKCLECGKNFSDRSNLNTHQRIHTGEKPYKCLECGKSFSDHSN
LITHQRIHTGEKPYKCGECWKSFNQSSNLLKHQRIHLGGNPDQCSEPGGNFAQSPSFSAH
WRNSTEETAPEQPQSISKDLNSPGPHSTNSGEKLYECSECGRSFSKSSALISHQRIHTGE
KPYECAECGKSFSKSSTLANHQRTHTGEKPYKCVDCGKCFSERSKLITHQRVHTGEKPYK
CLECGKFFRDRSNLITHQRIHTGEKPYKCRECGKCFNQSSSLIIHQRIHTGEKPYKCTEC
GKDFNNSSHFSAHRRTHAGGKAS*
>ENSG00000105967|ENST00000320239
MTLDHQIINPTLKWSQPAVPSGGPLVQHAHTTLDSDAGLTENPLTKLLAIGKEDDNAQWH
LSGSILDVYSGEQGISPINMGLTSASCPSSLPMKREITETDTRALAKERQKKDNHNLIER
RRRYNINYRIKELGTLIPKSNDPDMRWNKGTILKASVEYIKWLQKEQQRARELEHRQKKL
EQANRRLLLRIQELEIQARTHGLPTLASLGTVDLGAHVTKQQSHPEQNSVDYCQQLTVSQ
GPSPELCDQAIAFSDPLSYFTDLSFSAALKEEQRLDGMLLDDTISPFGTDPLLSATSPAV
SKESSRRSSFSSDDGDEL*
>ENSG00000136352|ENST00000354822
MWSGGSGKARGWEAAAGGRSSPGRLSRRRIMSMSPKHTTPFSVSDILSPLEESYKKVGME
GGGLGAPLAAYRQGQAAPPTAAMQQHAVGHHGAVTAAYHMTAAGVPQLSHSAVGGYCNGN
LGNMSELPPYQDTMRNSASGPGWYGANPDPRFPAISRFMGPASGMNMSGMGGLGSLGDVS
KNMAPLPSAPRRKRRVLFSQAQVYELERRFKQQKYLSAPEREHLASMIHLTPTQVKIWFQ
NHRYKMKRQAKDKAAQQQLQQDSGGGGGGGGTGCPQQQQAQQQSPRRVAVPVLVKDGKPC
QAGAPAPGAASLQGHAQQQAQHQAQAAQAAAAAISVGSGGAGLGAHPGHQPGSAGQSPDL
AHHAASPAALQGQVSSLSHLNSSGSDYGTMSCSTLLYGRTW*
>ENSG00000112531|ENST00000361758
MVGEMETKEKPKPTPDYLMQLMNDKKLMSSLPNFCGIFNHLERLLDEEISRVRKDMYNDT
LNGSTEKRSAELPDAVGPIVQLQEKLYVPVKEYPDFNFVGRILGPRGLTAKQLEAETGCK
IMVRGKGSMRDKKKEEQNRGKPNWEHLNEDLHVLITVEDAQNRAEIKLKRAVEEVKKLLV

PAAEGEDSLKKMQLMELAILNGTYRDANIKSPALAFSLAATAQAAPRIITGPAPVLPPAA
LRTPTPAGPTIMPLIRQIQTAVMPNGTPHPTAAIVPPGPEAGLIYTPYEYPYTLAPATSI
LEYPIEPSGVLGMAFPTKG*
>ENSG00000106852|ENST00000340587
MYWKHENAAPALPEGCRLPAEGGPATDQVMAQPGSGCKATTRCLEGTAPPAMAQSDAEAL
AGALDKDEGQASPCTPSTPSVCSPPSAASSVPSAGKNICSSCGLEILDRYLLKVNNLIWH
VRCLECSVCRTSLRQQNSCYIKNKEIFCKMDYFSRFGTKCARCGRQIYASDWVRRARGNA
YHLACFACFSCKRQLSTGEEFGLVEEKVLCRIHYDTMIENLKRAAENGNGLTLEGAVPSE
QDSQPKPAKRARTSFTAEQLQVMQAQFAQDNNPDAQTLQKLADMTGLSRRVIQVWFQNCR
ARHKKHTPQHPVPPSGAPPSRLPSALSDDIHYTPFSSPERARMVTLHGYIESHPFSVLTL
PALPHLPVGAPQLPLSR*
>ENSG00000160194|ENST00000354250
MAAPCLLRQGRAGALKTMLQEAQVFRGLASTVSLSAESGKSEKGQPQNSKKQSPPKNVVE
PKERGKLLATQTAAELSKNLSSPSSYPPAVNKGRKVASPSPSGSVLFTDEGVPKFLSRKT
LVEFPQKVLSPFRKQGSDSEARQVGRKVTSPSSSSSSSSSDSESDDEADVSEVTPRVVSK
GRGGLRKPEASHSFENRAPRVTVSAKEKTLLQKPHVDITDPEKPHQPKKKGSPAKPSEGR
ENARPKTTMPRSQVDEEFLKQSLKEKQLQKTFRLNEIDKESQKPFEVKGPLPVHTKSGLS
APPKGSPAPAVLAEEARAEGQLQASPPGAAEGHLEKPVPEPQRKAAPPLPRKETSGTQGI
EGHLKGGQAIVEDQIPPSNLETVPVENNHGFHEKTAALKLEAEGEAMEDAAAPGDDRGGT
QEPAPVPAEPFDNTTYKNLQHHDYSTYTFLDLNLELSKFRMPQPSSGRESPRH*
>ENSG00000066379|ENST00000332435
MSVMDLANTCSSFQSDLDFCSDCGSVLPLPGAQDTVTCIRCGFNINVRDFEGKVVKTSVV
FHQLGTAMPMSVEEGPECQGPVVDRRCPRCGHEGMAYHTRQMRSADEGQTVFYTCTNCKF
QEKEDS*
>ENSG00000136861|ENST00000349780
MMDLVLEEDVTVPGTLSGCSGLVPSVPDDLDGINPNAGLGNGLLPNVSEETVSPTRARNM
KDFENQITELKKENFNLKLRIYFLEERMQQEFHGPTEHIYKTNIELKVEVESLKRELQER
EQLLIKASKAVESLAEAGGSEIQRVKEDARKKVQQVEDLLTKRILLLEKDVTAAQAELEK
AFAGTETEKALRLRLESKLSEMKKMHEGDLAMALVLDEKDRLIEELKLSLKSKEALIQCL
KEEKSQMACPDENVSSGELRGLCAAPREEKERETEAAQMEHQKERNSFEERIQALEEDLR
EKEREIATEKKNSLKRDKAIQGLTMALKSKEKKVEELNSEIEKLSAAFAKAREALQKAQT
QEFQGSEDYETALSGKEALSAALRSQNLTKSTENHRLRRSIKKITQELSDLQQERERLEK
DLEEAHREKSKGDCTIRDLRNEVEKLRNEVNEREKAMENRYKSLLSESNKKLHNQEQVIK
HLTESTNQKDVLLQKFNEKDLEVIQQNCYLMAAEDLELRSEGLITEKCSSQQPPGSKTIF
SKEKKQSSDYEELIQVLKKEQDIYTHLVKSLQESDSINNLQAELNKIFALRKQLEQDVLS
YQNLRKTLEEQISEIRRREEESFSLYSDQTSYLSICLEENNRFQVEHFSQEELKKKVSDL
IQLVKELYTDNQHLKKTIFDLSCMGFQGNGFPDRLASTEQTELLASKEDEDTIKIGEDDE
INFLSDQHLQQSNEIMKDLSKGGCKNGYLRHTESKISDCDGAHAPGCLEEGAFINLLAPL
FNEKATLLLESRPDLLKVVRELLLGQLFLTEQEVSGEHLDGKTEKTPKQKGELVHFVQTN
SFSKPHDELKLSCEAQLVKAGEVPKVGLKDASVQTVATEGDLLRFKHEATREAWEEKPIN
TALSAEHRPENLHGVPGWQAALLSLPGITNREAKKSRLPILIKPSRSLGNMYRLPATQEV
VTQLQSQILELQGELKEFKTCNKQLHQKLILAEAVMEGRPTPDKTLLNAQPPVGAAYQDS
PGEQKGIKTTSSVWRDKEMDSDQQRSYEIDSEICPPDDLASLPSCKENPEDVLSPTSVAT
YLSSKSQPSAKVSVMGTDQSESINTSNETEYLKQKIHDLETELEGYQNFIFQLQKHSQCS

EAIITVLCGTEGAQDGLSKPKNGSDGEEMTFSSLHQVRYVKHVKILGPLAPEMIDSRVLE
NLKQQLEEQEYKLQKEQNLNMQLFSEIHNLQNKFRDLSPPRYDSLVQSQARELSLQRQQI
KDGHGICVISRQHMNTMIKAFEELLQASDVDYCVAEGFQEQLNQCAELLEKLEKLFLNGK
SVGVEMNTQNELMERIEEDNLTYQHLLPESPEPSASHALSDYETSEKSFFSRDQKQDNET
EKTSVMVNSFSQDLLMEHIQEIRTLRKRLEESIKTNEKLRKQLERQGSEFVQGSTSIFAS
GSELHSSLTSEIHFLRKQNQALNAMLIKGSRDKQKENDKLRESLSRKTVSLEHLQREYAS
VKEENERLQKEGSEKERHNQQLIQEVRCSGQELSRVQEEVKLRQQLLSQNDKLLQSLRVE
LKAYEKLDEEHRRLREASGEGWKGQDPFRDLHSLLMEIQALRLQLERSIETSSTLQSRLK
EQLARGAEKAQEGALTLAVQAVSIPEVPLQPDKHDGDKYPMESDNSFDLFDSSQAVTPKS
VSETPPLSGNDTDSLSCDSGSSATSTPCVSRLVTGHHLWASKNGRHVLGLIEDYEALLKQ
ISQGQRLLAEMDIQTQEAPSSTSQELGTKGPHPAPLSKFVSSVSTAKLTLEEAYRRLKLL
WRVSLPEDGQCPLHCEQIGEMKAEVTKLHKKLFEQEKKLQNTMKLLQLSKRQEKVIFDQL
VVTHKILRKARGNLELRPGGAHPGTCSPSRPGS*
>ENSG00000104064|ENST00000359031
MSLVDLGKKLLEAARAGQDDEVRILMANGAPFTTDWLGTSPLHLAAQYGHYSTTEVLLRA
GVSRDARTKVDRTPLHMAASEGHASIVEVLLKHGADVNAKDMLKMTALHWATEHNHQEVV
ELLIKYGADVHTQSKFCKTAFDISIDNGNEDLAEILQIAMQNQINTNPESPDTVTIHAAT
PQFIIGPGGVVNLTDETGVSAVQFGNSSTSVLATLAALAEASAPLSNSSETPVVATEEVV
TAESVDGAIQQVVSSGGQQVITIVTDGIQLGNLHSIPTSGIGQPIIVTMPDGQQVLTVPA
TDIAEETVISEEPPAKRQCIEIIENRVESAEIEVRSLLPGVLCRSHPK*
>ENSG00000148303|ENST00000323345
MPKGKKAKGKKVAPAPAVVKKQEAKKVVNPLFEKRPKNFGIGQDIQPKRDLTRFVKWPRY
IRLQRQRAILYKRLKVPPAINQFTQALDRQTATQLLKLAHKYRPETKQEKKQRLLARAEK
KAAGKGDVPTKRPPVLRAGVNTVTTLVENKKAQLVVIAHDVDPIELVVFLPALCRKMGVP
YCIIKGKARLGRLVHRKTCTTVAFTQVNSEDKGALAKLVEAIRTNYNDRYDEIRRHWGGN
VLGPKSVARIAKLEKAKAKELATKLG*
>ENSG00000162374|ENST00000357083
MLRYLQPLGCVHGGKGLTDMRLLLLREIVINESRNCSFMIISTMEPQVSNGPTSNTSNGP
SSNNRNCPSPMQTGATTDDSKTNLIVNYLPQNMTQEEFRSLFGSIGEIESCKLVRDKITG
QSLGYGFVNYIDPKDAEKAINTLNGLRLQTKTIKVSYARPSSASIRDANLYVSGLPKTMT
QKELEQLFSQYGRIITSRILVDQVTGVSRGVGFIRFDKRIEAEEAIKGLNGQKPSGATEP
ITVKFANNPSQKSSQALLSQLYQSPNRRYPGPLHHQAQRFRLDNLLNMAYGVKRFSPITI
DGMTSLVGMNIPGHTGTGWCIFVYNLSPDSDESVLWQLFGPFGAVNNVKVIRDFNTNKCK
GFGFVTMTNYDEAAMAIASLNGYRLGDRVLQVSFKTNKAHKS*
>ENSG00000180613|ENST00000326902
MSRSFYVDSLIIKDTSRPAPSLPEPHPGPDFFIPLGMPPPLVMSVSGPGCPSRKSGAFCV
CPLCVTSHLHSSRGSVGAGSGGAGAGVTGAGGSGVAGAAGALPLLKGQFSSAPGDAQFCP
RVNHAHHHHHPPQHHHHHHQPQQPGSAAAAAAAAAAAAAAAALGHPQHHAPVCTATTYNV
ADPRRFHCLTMGGSDASQVPNGKRMRTAFTSTQLLELEREFSSNMYLSRLRRIEIATYLN
LSEKQVKIWFQNRRVKHKKEGKGTQRNSHAGCKCVGSQVHYARSEDEDSLSPASANDDKE
ISPL*
>ENSG00000197275|ENST00000336148
MRRSAAPSQLQGNSFKKPKFIPPGRSNPGLNEEITKLNPDIKLFEGVAINNTFLPSQNDL
RICSLNLPSEESTREINNRDNCSGKYCFEAPTLATLDPPHTVHSAPKEVAVSKEQEEKSD

SLVKYFSVVWCKPSKKKHKKWEGDAVLIVKGKSFILKNLEGKDIGRGIGYKFKELEKIEE
GQTLMICGKEIEVMGVISPDDFSSGRCFQLGGGSTAISHSSQVARKCFSNPFKSVCKPSS
KENRQNDFQNCKPRHDPYTPNSLVMPRPDKNHQWVFNKNCFPLVDVVIDPYLVYHLRPHQ
KEGIIFLYECVMGMRMNGRCGAILADEMGLGKTLQCISLIWTLQCQGPYGGKPVIKKTLI
VTPGSLVNNWKKEFQKWLGSERIKIFTVDQDHKVEEFIKSIFYSVLIISYEMLLRSLDQI
KNIKFDLLICDEGHRLKNSAIKTTTALISLSCEKRIILTGTPIQNDLQEFFALIDFVNPG
ILGSLSSYRKIYEEPIILSREPSASEEEKELGERRAAELTCLTGLFILRRTQEIINKYLP
PKIENVVFCRPGALQIELYRKLLNSQVVRFCLQGLLENSPHLICIGALKKLCNHPCLLFN
SIKEKECSSTCDKNEEKSLYKGLLSVFPADYNPLLFTEKESGKLQVLSKLLAVIHELRPT
EKVVLVSNYTQTLNILQEVCKRHGYAYTRLDGQTPISQRQQIVDGFNSQHSSFFIFLLSS
KAGGVGLNLIGGSHLILYDIDWNPATDIQAMSRVWRDGQKYPVHIYRLLTTGTIEEKIYQ
RQISKQGLCGAVVDLTKTSEHIQFSVEELKNLFTLHESSDCVTHDLLDCECTGEEVHTGD
SLEKFIVSRDCQLGPHHQKSNSLKPLSMSQLKQWKHFSGDHLNLTDPFLERITENVSFIF
QNITTQATGT*
>ENSG00000074657|ENST00000336078
MTMGDMKTPDFDDLLAAFDIPDMVDPKAAIESGHDDHESHMKQNAHGEDDSHAPSSSDVG
VSVIVKNVRNIDSSEGGEKDGHNPTGNGLHNGFLTASSLDSYSKDGAKSLKGDVPASEVT
LKDSTFSQFSPISSAEEFDDDEKIEVDDPPDKEDMRSSFRSNVLTGSAPQQDYDKLKALG
GENSSKTGLSTSGNVEKNKAVKRETEASSINLSVYEPFKVRKAEDKLKESSDKVLENRVL
DGKLSSEKNDTSLPSVAPSKTKSSSKLSSCIAAIAALSAKKAASDSCKEPVANSRESSPL
PKEVNDSPRAADKSPESQNLIDGTKKPSLKQPDSPRSISSENSSKGSPSSPAGSTPAIPK
VRIKTIKTSSGEIKRTVTRVLPEVDLDSGKKPSEQTASVMASVTSLLSSPASAAVLSSPP
RAPLQSAVVTNAVSPAELTPKQVTIKPVATAFLPVSAVKTAGSQVINLKLANNTTVKATV
ISAASVQSASSAIIKAANAIQQQTVVVPASSLANAKLVPKTVHLANLNLLPQGAQATSEL
RQVLTKPQQQIKQAIINAAASQPPKKVSRVQVVSSLQSSVVEAFNKVLSSVNPVPVYIPN
LSPPANAGITLPTRGYKCLECGDSFALEKSLTQHYDRRSVRIEVTCNHCTKNLVFYNKCS
LLSHARGHKEKGVVMQCSHLILKPVPADQMIVSPSSNTSTSTSTLQSPVGAGTHTVTKIQ
SGITGTVISAPSSTPITPAMPLDEDPSKLCRHSLKCLECNEVFQDETSLATHFQQAADTS
GQKTCTICQMLLPNQCSYASHQRIHQHKSPYTCPECGAICRSVHFQTHVTKNCLHYTRRV
GFRCVHCNVVYSDVAALKSHIQGSHCEVFYKCPICPMAFKSAPSTHSHAYTQHPGIKIGE
PKIIYKCSMCDTVFTLQTLLYRHFDQHIENQKVSVFKCPDCSLLYAQKQLMMDHIKSMHG
TLKSIEGPPNLGINLPLSIKPATQNSANQNKEDTKSMNGKEKLEKKSPSPVKKSMETKKV
ASPGWTCWECDCLFMQRDVYISHVRKEHGKQMKKHPCRQCDKSFSSSHSLCRHNRIKHKG
IRKVYACSHCPDSRRTFTKRLMLEKHVQLMHGIKDPDLKEMTDATNEEETEIKEDTKVPS
PKRKLEEPVLEFRPPRGAITQPLKKLKINVFKVHKCAVCGFTTENLLQFHEHIPQHKSDG
SSYQCRECGLCYTSHVSLSRHLFIVHKLKEPQPVSKQNGAGEDNQQENKPSHEDESPDGA
VSDRKCKVCAKTFETEAALNTHMRTHGMAFIKSKRMSSAEK*
>ENSG00000100038|ENST00000357179
MKTVLMVAEKPSLAQSIAKILSRGSLSSHKGLNGACSVHEYTGTFAGQPVRFKMTSVCGH
VMTLDFLGKYNKWDKVDPAELFSQAPTEKKEANPKLNMVKFLQVEGRGCDYIVLWLDCDK
EGENICFEVLDAVLPVMNKAHGGEKTVFRARFSSITDTDICNAMACLGEPDHNEALSVDA
RQELDLRIGCAFTRFQTKYFQGKYGDLDSSLISFGPCQTPTLGFCVERHDKIQSFKPETY
WVLQAKVNTDKDRSLLLDWDRVRVFDREIAQMFLNMTKLEKEAQVEATSRKEKAKQRPLA
LNTVEMLRVASSSLGMGPQHAMQTAERLYTQGYISYPRTETTHYPENFDLKGSLRQQANH

PYWADTVKRLLAEGINRPRKGHDAGDHPPITPMKSATEAELGGDAWRLYEYITRHFIATV
SHDCKYLQSTISFRIGPELFTCSGKTVLSPGFTEVMPWQSVPLEESLPTCQRGDAFPVGE
VKMLEKQTNPPDYLTEAELITLMEKHGIGTDASIPVHINNICQRNYVTVESGRRLKPTNL
GIVLVHGYYKIDAELVLPTIRSAVEKQLNLIAQGKADYRQVLGHTLDVFKRKFHYFVDSI
AGMDELMEVSFSPLAATGKPLSRCGKCHRFMKYIQAKPSRLHCSHCDETYTLPQNGTIKL
YKELRCPLDDFELVLWSSGSRGKSYPLCPYCYNHPPFRDMKKGMGCNECTHPSCQHSLSM
LGIGQCVECESGVLVLDPTSGPKWKVACNKCNVVAHCFENAHRVRVSADTCSVCEAALLD
VDFNKAKSPLPGDETQHMGCVFCDPVFQELVELKHAASCHPMHRGGPGRRQGRGRGRARR
PPGKPNPRRPKDKMSALAAYFV*
>ENSG00000100207|ENST00000359486
MQSFREQSSYHGNQQSYPQEVHGSSRLEEFSPRQAQMFQNFGGTGGSSGSSGSGSGGGRR
GAAAAAAAMASETSGHQGYQGFRKEAGDFYYMAGNKDPVTTGTPQPPQRRPSGPVQSYGP
PQGSSFGNQYGSEGHVGQFQAQHSGLGGVSHYQQDYTGPFSPGSAQYQQQASSQQQQQQV
QQLRQQLYQSHQPLPQATGQPASSSSHLQPMQRPSTLPSSAAGYQLRVGQFGQHYQSSAS
SSSSSSFPSPQRFSQSGQSYDGSYNVNAGSQYEGHNVGSNAQAYGTQSNYSYQPQSMKNF
EQAKIPQGTQQGQQQQQPQQQQHPSQHVMQYTNAATKLPLQSQVGQYNQPEVPVRSPMQF
HQNFSPISNPSPAASVVQSPSCSSTPSPLMQTGENLQCGQGSVPMGSRNRILQLMPQLSP
TPSMMPSPNSHAAGFKGFGLEGVPEKRLTDPGLSSLSALSTQVANLPNTVQHMLLSDALT
PQKKTSKRPSSSKKADSCTNSEGSSQPEEQLKSPMAESLDGGCSSSSEDQGERVRQLSGQ
STSSDTTYKGGASEKAGSSPAQGAQNEPPRLNASPAAREEATSPGAKDMPLSSDGNPKVN
EKTVGVIVSREAMTGRVEKPGGQDKGSQEDDPAATQRPPSNGGAKETSHASLPQPEPPGG
GGSKGNKNGDNNSNHNGEGNGQSGHSAAGPGFTSRTEPSKSPGSLRYSYKDSFGSAVPRN
VSGFPQYPTGQEKGDFTGHGERKGRNEKFPSLLQEVLQGYHHHPDRRYSRSTQEHQGMAG
SLEGTTRPNVLVSQTNELASRGLLNKSIGSLLENPHWGPWERKSSSTAPEMKQINLTDYP
IPRKFEIEPQSSAHEPGGSLSERRSVICDISPLRQIVRDPGAHSLGHMSADTRIGRNDRL
NPTLSQSVILPGGLVSMETKLKSQSGQIKEEDFEQSKSQASFNNKKSGDHCHPPSIKHES
YRGNASPGAATHDSLSDYGPQDSRPTPMRRVPGRVGGREGMRGRSPSQYHDFAEKLKMSP
GRSRGPGGDPHHMNPHMTFSERANRSSLHTPFSPNSETLASAYHANTRAHAYGDPNAGLN
SQLHYKRQMYQQQPEEYKDWSSGSAQGVIAAAQHRQEGPRKSPRQQQFLDRVRSPLKNDK
DGMMYGPPVGTYHDPSAQEAGRCLMSSDGLPNKGMELKHGSQKLQESCWDLSRQTSPAKS
SGPPGMSSQKRYGPPHETDGHGLAEATQSSKPGSVMLRLPGQEDHSSQNPLIMRRRVRSF
ISPIPSKRQSQDVKNSSTEDKGRLLHSSKEGADKAFNSYAHLSHSQDIKSIPKRDSSKDL
PSPDSRNCPAVTLTSPAKTKILPPRKGRGLKLEAIVQKITSPNIRRSASSNSAEAGGDTV
TLDDILSLKSGPPEGGSVAVQDADIEKRKGEVASDLVSPANQELHVEKPLPRSSEEWRGS
VDDKVKTETHAETVTAGKEPPGAMTSTTSQKPGSNQGRPDGSLGGTAPLIFPDSKNVPPV
GILAPEANPKAEEKENDTVTISPKQEGFPPKGYFPSGKKKGRPIGSVNKQKKQQQPPPPP
PQPPQIPEGSADGEPKPKKQRQRRERRKPGAQPRKRKTKQAVPIVEPQEPEIKLKYATQP
LDKTDAKNKSFYPYIHVVNKCELGAVCTIINAEEEEQTKLVRGRKGQRSLTPPPSSTESK
ALPASSFMLQGPVVTESSVMGHLVCCLCGKWASYRNMGDLFGPFYPQDYAATLPKNPPPK
RATEMQSKVKVRHKSASNGSKTDTEEEEEQQQQQKEQRSLAAHPRFKRRHRSEDCGGGPR
SLSRGLPCKKAATEGSSEKTVLDSKPSVPTTSEGGPELELQIPELPLDSNEFWVHEGCIL
WANGIYLVCGRLYGLQEALEIAREMKCSHCQEAGATLGCYNKGCSFRYHYPCAIDADCLL
HEENFSVRCPKHKPPLPCPLPPLQNKTAKGSLSTEQSERG*
>ENSG00000163516|ENST00000323348

MSPAPDAAPAPASISLFDLSADAPVFQGLSLVSHAPGEALARAPRTSCSGSGERESPERK
LLQGPMDISEKLFCSTCDQTFQNHQEQREHYKLDWHRFNLKQRLKDKPLLSALDFEKQSS
TGDLSSISGSEDSDSASEEDLQTLDRERATFEKLSRPPGFYPHRVLFQNAQGQFLYAYRC
VLGPHQDPPEEAELLLQNLQSRGPRDCVVLMAAAGHFAGAIFQGREVVTHKTFHRYTVRA
KRGTAQGLRDARGGPSHSAGANLRRYNEATLYKDVRDLLAGPSWAKALEEAGTILLRAPR
SGRSLFFGGKGAPLQRGDPRLWDIPLATRRPTFQELQRVLHKLTTLHVYEEDPREAVRLH
SPQTHWKTVREERKKPTEEEIRKICRDEKEALGQNEESPKQGSGSEGEDGFQVELELVEL
TVGTLDLCESEVLPKRRRRKRNKKEKSRDQEAGAHRTLLQQTQEEEPSTQSSQAVAAPLG
PLLDEAKAPGQPELWNALLAACRAGDVGVLKLQLAPSPADPRVLSLLSAPLGSGGFTLLH
AAAAAGRGSVVRLLLEAGADPTVQDSRARPPYTVAADKSTRNEFRRFMEKNPDAYDYNKA
QVPGPLTPEMEARQATRKREQKAARRQREEQQQRQQEQEEREREEQRRFAALSDREKRAL
AAERRLAAQLGAPTSPIPDSAIVNTRRCWSCGASLQGLTPFHYLDFSFCSTRCLQDHRRQ
AGRPSS*
>ENSG00000162772|ENST00000366981
MMLQHPGQVSASEVSASAIVPCLSPPGSLVFEDFANLTPFVKEELRFAIQNKHLCHRMSS
ALESVTVSDRPLGVSITKAEVAPEEDERKKRRRERNKIAAAKCRNKKKEKTECLQKESEK
LESVNAELKAQIEELKNEKQHLIYMLNLHRPTCIVRAQNGRTPEDERNLFIQQIK
>ENSG00000143799|ENST00000366792
MAESSDKLYRVEYAKSGRASCKKCSESIPKDSLRMAIMVQSPMFDGKVPHWYHFSCFWKV
GHSIRHPDVEVDGFSELRWDDQQKVKKTAEAGGVTEPPIPCGIEVMGR*
>ENSG00000115507|ENST00000366671
MMSYLKQPPYGMNGLGLAGPAMDLLHPSVGYPATPRKQRRERTTFTRSQLDVLEALFAKT
RYPDIFMREEVALKINLPESRVQVWFKNRRAKCRQQQQSGSGTKSRPAKKKSSPVRESSG
SESSGQFTPPAVSSSASSSSSASSSSANPAAAAAAGLGGNPVAAASSLSTPAASSIWSPA
SISPGSAPASVSVPEPLAAPSNTSCMQRSVAAGAATAAASYPMSYGQGGSYGQGYPTPSS
SYFGGVDCSSYLAPMHSHHHPHQLSPMAPSSMAGHHHHHPHAHHPLSQSSGHHHHHHHHH
HQGYGGSGLAFNSADCLDYKEPGAAAASSAWKLNFNSPDCLDYKDQASWRFQVL*
>ENSG00000178150|ENST00000315849
MSQDSVTFADVAVNFTKEEWTLLDPAQRNLYRDVMLENSRNLAFIDWATPCKTKDATPQP
DILPKRTFPEANRVCLTSISSQHSTLREDWRCPKTEEPHRQGVNNVKPPAVAPEKDESPV
SICEDHEMRNHSKPTCRLVPSQGDSIRQCILTRDSSIFKYNPVLNDSQKTHENNEDDGVL
GWNIQWVPCGRKTELKSSTWTGSQNTVHHIRDEIDTGANRHQRNPFGKAFREDGSLRAHN
THGREKMYDFTQCENTSRNNSIHAMQMQLYTAETNKKDCQTGATSANAPNSGSHKSHCTG
EKTHKCPECGRAFFYQSFLMRHMKIHTGEKPYECGKCGKAFRYSLHLNKHLRKHVVQKKP
YECEECGKVIRESSKYTHIRSHTGEKPYKCKTCGKDFAKSSGLKKHLKTHKDEKPCE*
>ENSG00000183621|ENST00000361310
MQNSVSVPPKDEGESNIPSGTIQSRKGLQNKSQFRTIAPKIVPKVLTSRMLPCHSPSRSD
QVNLGPSINSKLLGMSTQNYALMQVAGQEGTFSLVALPHVASAQPIQKPRMSLPENLKLP
IPRYQPPRNSKASRKKPILIFPKSGCSKAPAQTQMCPQMSPSPPHHPELLYKPSPFEEVP
SLEQAPASISTAALTNGSDHGDLRPPVTNTHGSLNPPATPASSTPEEPAKQDLTALSGKA
HFVSKITSSKPSAVASEKFKEQVDLAKTMTNLSPTILGNAVQLISSVPKGKLPIPPYSRM
KTMEVYKIKSDANIAGFSLPGPKADCDKIPSTTEGFNAATKVASRLPVPQVSQQSACESA
FCPPTKLDLNHKTKLNSGAAKRKGRKRKVPDEILAFQGKRRKYIINKCRDGKERVKNDPQ
EFRDQKLGTLKKYRSIMPKPIMVIPTLASLASPTTLQSQMLGGLGQDVLLNNSLTPKYLG

CKQDNSSSPKPSSVFRNGFSGIKKPWHRCHVCNHHFQFKQHLRDHMNTHTNRRPYSCRIC
RKSYVRPGSLSTHMKLHHGENRLKKLMCCEFCAKVFGHIRVYFGHLKEVHRVVISTEPAP
SELQPGDIPKNRDMSVRGMEGSLERENKSNLEEDFLLNQADEVKLQIKCGRCQITAQSFA
EIKFHLLDVHGEEIEGRLQEGTFPGSKGTQEELVQHASPDWKRHPERGKPEKVHSSSEES
HACPRLKRQLHLHQNGVEMLMENEGPQSGTNKPRETCQGPECPGLHTFLLWSHSGFNCLL
CAEMLGRKEDLLHHWKHQHNCEDPSKLWAILNTVSNQGVIELSSEAEK*
>ENSG00000140262|ENST00000333725
MNPQQQRMAAIGTDKELSDLLDFSAMFSPPVNSGKTRPTTLGSSQFSGSGIDERGGTTSW
GTSGQPSPSYDSSRGFTDSPHYSDHLNDSRLGAHEGLSPTPFMNSNLMGKTSERGSFSLY
SRDTGLPGCQSSLLRQDLGLGSPAQLSSSGKPGTAYYSFSATSSRRRPLHDSAALDPLQA
KKVRKVPPGLPSSVYAPSPNSDDFNRESPSYPSPKPPTSMFASTFFMQDGTHNSSDLWSS
SNGMSQPGFGGILGTSTSHMSQSSSYGNLHSHDRLSYPPHSVSPTDINTSLPPMSSFHRG
STSSSPYVAASHTPPINGSDSILGTRGNAAGSSQTGDALGKALASIYSPDHTSSSFPSNP
STPVGSPSPLTGTSQWPRPGGQAPSSPSYENSLHSLKNRVEQQLHEHLQDAMSFLKDVCE
QSRMEDRLDRLDDAIHVLRNHAVGPSTSLPAGHSDIHSLLGPSHNAPIGSLNSNYGGSSL
VASSRSASMVGTHREDSVSLNGNHSVLSSTVTTSSTDLNHKTQENYRGGLQSQSGTVVTT
EIKTENKEKDENLHEPPSSDDMKSDDESSQKDIKVSSRGRTSSTNEDEDLNPEQKIEREK
ERRMANNARERLRVRDINEAFKELGRMCQLHLKSEKPQTKLLILHQAVAVILSLEQQVRE
RNLNPKAACLKRREEEKVSAVSAEPPTTLPGTHPGLSETTNPMGHM*
>ENSG00000183734|ENST00000331289
MDGGTLPRSAPPAPPVPVGCAARRRPASPELLRCSRRRRPATAETGGGAAAVARRNERER
NRVKLVNLGFQALRQHVPHGGASKKLSKVETLRSAVEYIRALQRLLAEHDAVRNALAGGL
RPQAVRPSAPRGPPGTTPVAASPSRASSSPGRGGSSEPGSPRSAYSSDDSGCEGALSPAE
RELLDFSSWLGGY*
>ENSG00000181666|ENST00000324411
MRVNHTVSTMLPTCMVHRQTMSCSGAGGITAFVAFRDVAVYFTQEEWRLLSPAQRTLHRE
VMLETYNHLVSLEIPSSKPKLIAQLERGEAPWREERKCPLDLCPESKPEIQLSPSCPLIF
SSQQALSQHVWLSHLSQLFSSLWAGNPLHLGKHYPEDQKQQQDPFCFSGKAEWIQEGEDS
RLLFGRVSKNGTSKALSSPPEEQQPAQSKEDNTVVDIGSSPERRADLEETDKVLHGLEVS
GFGEIKYEEFGPGFIKESNLLSLQKTQTGETPYMYTEWGDSFGSMSVLIKNPRTHSGGKP
YVCRECGRGFTWKSNLITHQRTHSGEKPYVCKDCGRGFTWKSNLFTHQRTHSGLKPYVCK
ECGQSFSLKSNLITHQRAHTGEKPYVCRECGRGFRQHSHLVRHKRTHSGEKPYICRECEQ
GFSQKSHLIRHLRTHTGEKPYVCTECGRHFSWKSNLKTHQRTHSGVKPYVCLECGQCFSL
KSNLNKHQRSHTGEKPFVCTECGRGFTRKSTLSTHQRTHSGEKPFVCAECGRGFNDKSTL
ISHQRTHSGEKPFMCRECGRRFRQKPNLFRHKRAHSGAFVCRECGQGFCAKLTLIKHQRA
HAGGKPHVCRECGQGFSRQSHLIRHQRTHSGEKPYICRKCGRGFSRKSNLIRHQRTHSG*
>ENSG00000184302|ENST00000327720
MFQLPILNFSPQQVAGVCETLEESGDVERLGRFLWSLPVAPAACEALNKNESVLRARAIV
AFHGGNYRELYHILENHKFTKESHAKLQALWLEAHYQEAEKLRGRPLGPVDKYRVRKKFP
LPRTIWDGEQKTHCFKERTRHLLREWYLQDPYPNPSKKRELAQATGLTPTQVGNWFKNRR
QRDRAAAAKNRLQQQVLSQGSGRALRAEGDGTPEVLGVATSPAASLSSKAATSAISITSS
DSECDI*
>ENSG00000239672|ENST00000336097
MVLLSTLGIVFQGEGPPISSCDTGTMANCERTFIAIKPDGVQRGLVGEIIKRFEQKGFRL

VGLKFMQASEDLLKEHYVDLKDRPFFAGLVKYMHSGPVVAMVWEGLNVVKTGRVMLGETN
PADSKPGTIRGDFCIQVGRNIIHGSDSVESAEKEIGLWFHPEELVDYTSCAQNWIYE*
>ENSG00000175054|ENST00000350721
MGEHGLELASMIPALRELGSATPEEYNTVVQKPRQILCQFIDRILTDVNVVAVELVKKTD
SQPTSVMLLDFIQHIMKSSPLMFVNVSGSHEAKGSCIEFSNWIITRLLRIAATPSCHLLH
KKICEVICSLLFLFKSKSPAIFGVLTKELLQLFEDLVYLHRRNVMGHAVEWPVVMSRFLS
QLDEHMGYLQSAPLQLMSMQNLEFIEVTLLMVLTRIIAIVFFRRQELLLWQIGCVLLEYG
SPKIKSLAISFLTELFQLGGLPAQPASTFFSSFLELLKHLVEMDTDQLKLYEEPLSKLIK
TLFPFEAEAYRNIEPVYLNMLLEKLCVMFEDGVLMRLKSDLLKAALCHLLQYFLKFVPAG
YESALQVRKVYVRNICKALLDVLGIEVDAEYLLGPLYAALKMESMEIIEEIQCQTQQENL
SSNSDGISPKRRRLSSSLNPSKRAPKQTEEIKHVDMNQKSILWSALKQKAESLQISLEYS
GLKNPVIEMLEGIAVVLQLTALCTVHCSHQNMNCRTFKDCQHKSKKKPSVVITWMSLDFY
TKVLKSCRSLLESVQKLDLEATIDKVVKIYDALIYMQVNSSFEDHILEDLCGMLSLPWIY
SHSDDGCLKLTTFAANLLTLSCRISDSYSPQAQSRCVFLLTLFPRRIFLEWRTAVYNWAL
QSSHEVIRASCVSGFFILLQQQNSCNRVPKILIDKVKDDSDIVKKEFASILGQLVCTLHG
MFYLTSSLTEPFSEHGHVDLFCRNLKATSQHECSSSQLKASVCKPFLFLLKKKIPSPVKL
AFIDNLHHLCKHLDFREDETDVKAVLGTLLNLMEDPDKDVRVAFSGNIKHILESLDSEDG
FIKELFVLRMKEAYTHAQISRNNELKDTLILTTGDIGRAAKGDLVPFALLHLLHCLLSKS
ASVSGAAYTEIRALVAAKSVKLQSFFSQYKKPICQFLVESLHSSQMTALPNTPCQNADVR
KQDVAHQREMALNTLSEIANVFDFPDLNRFLTRTLQVLLPDLAAKASPAASALIRTLGKQ
LNVNRREILINNFKYIFSHLVCSCSKDELERALHYLKNETEIELGSLLRQDFQGLHNELL
LRIGEHYQQVFNGLSILASFASSDDPYQGPRDIISPELMADYLQPKLLGILAFFNMQLLS
SSVGIEDKKMALNSLMSLMKLMGPKHVSSVRVKMMTTLRTGLRFKDDFPELCCRAWDCFV
RCLDHACLGSLLSHVIVALLPLIHIQPKETAAIFHYLIIENRDAVQDFLHEIYFLPDHPE
LKKIKAVLQEYRKETSESTDLQTTLQLSMKAIQHENVDVRIHALTSLKETLYKNQEKLIK
YATDSETVEPIISQLVTVLLKGCQDANSQARLLCGECLGELGAIDPGRLDFSTTETQGKD
FTFVTGVEDSSFAYGLLMELTRAYLAYADNSRAQDSAAYAIQELLSIYDCREMETNGPGH
QLWRRFPEHVREILEPHLNTRYKSSQKSTDWSGVKKPIYLSKLGSNFAEWSASWAGYLIT
KVRHDLASKIFTCCSIMMKHDFKVTIYLLPHILVYVLLGCNQEDQQEVYAEIMAVLKHDD
QHTINTQDIASDLCQLSTQTVFSMLDHLTQWARHKFQALKAEKCPHSKSNRNKVDSMVST
VDYEDYQSVTRFLDLIPQDTLAVASFRSKAYTRAVMHFESFITEKKQNIQEHLGFLQKLY
AAMHEPDGVAGVSAIRKAEPSLKEQILEHESLGLLRDATACYDRAIQLEPDQIIHYHGVV
KSMLGLGQLSTVITQVNGVHANRSEWTDELNTYRVEAAWKLSQWDLVENYLAADGKSTTW
SVRLGQLLLSAKKRDITAFYDSLKLVRAEQIVPLSAASFERGSYQRGYEYIVRLHMLCEL
EHSIKPLFQHSPGDSSQEDSLNWVARLEMTQNSYRAKEPILALRRALLSLNKRPDYNEMV
GECWLQSARVARKAGHHQTAYNALLNAGESRLAELYVERAKWLWSKGDVHQALIVLQKGV
ELCFPENETPPEGKNMLIHGRAMLLVGRFMEETANFESNAIMKKYKDVTACLPEWEDGHF
YLAKYYDKLMPMVTDNKMEKQGDLIRYIVLHFGRSLQYGNQFIYQSMPRMLTLWLDYGTK
AYEWEKAGRSDRVQMRNDLGKINKVITEHTNYLAPYQFLTAFSQLISRICHSHDEVFVVL
MEIIAKVFLAYPQQAMWMMTAVSKSSYPMRVNRCKEILNKAIHMKKSLEKFVGDATRLTD
KLLELCNKPVDGSSSTLSMSTHFKMLKKLVEEATFSEILIPLQSVMIPTLPSILGTHANH
ASHEPFPGHWAYIAGFDDMVEILASLQKPKKISLKGSDGKFYIMMCKPKDDLRKDCRLME
FNSLINKCLRKDAESRRRELHIRTYAVIPLNDECGIIEWVNNTAGLRPILTKLYKEKGVY
MTGKELRQCMLPKSAALSEKLKVFREFLLPRHPPIFHEWFLRTFPDPTSWYSSRSAYCRS

TAVMSMVGYILGLGDRHGENILFDSLTGECVHVDFNCLFNKGETFEVPEIVPFRLTHNMV
NGMGPMGTEGLFRRACEVTMRLMRDQREPLMSVLKTFLHDPLVEWSKPVKGHSKAPLNET
GEVVNEKAKTHVLDIEQRLQGVIKTRNRVTGLPLSIEGHVHYLIQEATDENLLCQMYLGW
TPYM*
>ENSG00000183770|ENST00000330315
MMASYPEPEDAAGALLAPETGRTVKEPEGPPPSPGKGGGGGGGTAPEKPDPAQKPPYSYV
ALIAMAIRESAEKRLTLSGIYQYIIAKFPFYEKNKKGWQNSIRHNLSLNECFIKVPREGG
GERKGNYWTLDPACEDMFEKGNYRRRRRMKRPFRPPPAHFQPGKGLFGAGGAAGGCGVAG
AGADGYGYLAPPKYLQSGFLNNSWPLPQPPSPMPYASCQMAAAAAAAAAAAAAAGPGSPG
AAAVVKGLAGPAASYGPYTRVQSMALPPGVVNSYNGLGGPPAAPPPPPHPHPHPHAHHLH
AAAAPPPAPPHHGAAAPPPGQLSPASPATAAPPAPAPTSAPGLQFACARQPELAMMHCSY
WDHDSKTGALHSRLDL*
>ENSG00000100320|ENST00000359369
MVQPFTTIPFPPPPQNGIPTEYGVPHTQDYAGQTGEHNLTLYGSTQAHGEQSSNSPSTQN
GSLTTEGGAQTDGQQSQTQSSENSESKSTPKRLHVSNIPFRFRDPDLRQMFGQFGKILDV
EIIFNERGSKGFGFVTFENSADADRAREKLHGTVVEGRKIEVNNATARVMTNKKMVTPYA
NGWKLSPVVGAVYGPELYAASSFQADVSLGNDAAVPLSGRGGINTYIPLIIPGFPYPTAA
TTAAAFRGAHLRGRGRTVYGAVRAVPPTAIPAYPGVVYQDGFYGADLYIESANCFRSNRV
DMQPTDMHSLLLQPQPPLLQPLQPLTVTVMAGCTQPTPTMPLPLPLAMELALWRVYTEVA
TADLPPTEVT*
>ENSG00000177463|ENST00000323373
MTSPSPRIQIISTDSAVASPQRIQGSEPASGPLSVFTSLNKEKIVTDQQTGQKIQIVTAV
DASGSPKQQFILTSPDGAGTGKVILASPETSSAKQLIFTTSDNLVPGRIQIVTDSASVER
LLGKTDVQRPQVVEYCVVCGDKASGRHYGAVSCEGCKGFFKRSVRKNLTYSCRSNQDCII
NKHHRNRCQFCRLKKCLEMGMKMESVQSERKPFDVQREKPSNCAASTEKIYIRKDLRSPL
IATPTFVADKDGARQTGLLDPGMLVNIQQPLIREDGTVLLATDSKAETSQGALGTLANVV
TSLANLSESLNNGDTSEIQPEDQSASEITRAFDTLAKALNTTDSSSSPSLADGIDTSGGG
SIHVISRDQSTPIIEVEGPLLSDTHVTFKLTMPSPMPEYLNVHYICESASRLLFLSMHWA
RSIPAFQALGQDCNTSLVRACWNELFTLGLAQCAQVMSLSTILAAIVNHLQNSIQEDKLS
GDRIKQVMEHIWKLQEFCNSMAKLDIDGYEYAYLKAIVLFSPDHPGLTSTSQIEKFQEKA
QMELQDYVQKTYSEDTYRLARILVRLPALRLMSSNITEELFFTGLIGNVSIDSIIPYILK
METAEYNGQITGASL*
>ENSG00000005102|ENST00000329168
MDPAASSCMRSLQPPAPVWGCLRNPHSEGNGASGLPHYPPTPFSFHQKPDFLATATAAYP
DFSASCLAATPHSLPQEEHIFTEQHPAFPQSPNWHFPVSDARRRPNSGPAGGSKEMGTSS
LGLVDTTGGPGDDYGVLGSTANETEKKSSRRRKESSGQSVVPEPKDEVEACEGRSAHLPQ
WAGP*
>ENSG00000179059|ENST00000326866
MSQQLKKRAKTRHQKGLGGRAPSGAKPRQGKSSQDLQAEIEPVSAVWALCDGYVCYEPGP
QALGGDDFSDCYIECVIRGEFSQPILEEDSLFESLEYLKKGSEQQLSQKVFEASSLECSL
EYMKKGVKKELPQKIVGENSLEYSEYMTGKKLPPGGIPGIDLSDPKQLAEFARKKPPINK
EYDSLSAIACPQSGCTRKLRNRAALRKHLLIHGPRDHVCAECGKAFVESSKLKRHFLVHT
GEKPFRCTFEGCGKRFSLDFNLRTHVRIHTGEKRFVCPFQGCNRRFIQSNNLKAHILTHA
NTNKNEQEGK*

>ENSG00000196456|ENST00000329630
MESGLAGNGTGAGLVMKVKQEKPERLLQTLAPQAMLVEKDKENIFQQHRGLPPRQTMGRP
RALGGQEESGSPRWAPPTEQDAGLAGRAPGSASGPLSPSLSSGEGHFVCLDCGKRFSWWS
SLKIHQRTHTGEKPYLCGKCGKSFSQKPNLARHQRHHTGERPFCCPECARRFSQKQHLLK
HQKTHSRPATHSCPECERCFRHQVGLRIHQRAHARDRQGSRAGLHELIQDAAARRACRLQ
PGPPRGRPEWAWLGLCQGWWGQPGARAAVSGPEGPGEPRQFICNECGKSFTWWSSLNIHQ
RIHTGERPYACPECGRRFSQKPNLTRHLRNHTGERPHPCPHCGRGFRQKQHLLKHLRTHL
PGAQAAPCPSCGKSCRSRAALRAHQRAHAVAEPAVPAGEPGDQPQAEAIPGLAARPRSSQ
RSPGARDTLWGRGQAGLAGPGEPRQFICNECGKSFSWWSALTIHQRIHTGERPYPCPECG
RRFSQKPNLTRHRRNHTGERPYLCPACGRGFSQKQHLLKHQRVHRAAPACSPKEEAR*
>ENSG00000043514|ENST00000316891
MASVAAARAVPVGSGLRGLQRTLPLVVILGATGTGKSTLALQLGQRLGGEIVSADSMQVY
EGLDIITNKVSAQEQRICRHHMISFVDPLVTNYTVVDFRNRATALIEDIFARDKIPIVVG
GTNYYIESLLWKVLVNTKPQEMGTEKVIDRKVELEKEDGLVLHKRLSQVDPEMAAKLHPH
DKRKVARSLQVFEETGISHSEFLHRQHTEEGGGPLGGPLKFSNPCILWLHADQAVLDERL
DKRVDDMLAAGLLEELRDFHRRYNQKNVSENSQDYQHGIFQSIGFKEFHEYLITEGKCTL
ETSNQLLKKGIEALKQVTKRYARKQNRWVKNRFLSRPGPIVPPVYGLEVSDVSKWEESVL
EPALEIVQSFIQGHKPTATPIKMPYNEAENKRSYHLCDLCDRIIIGDREWAAHIKSKSHL
NQLKKRRRLDSDAVNTIESQSVSPDHNKEPKEKGSPGQNDQELKCSV*
>ENSG00000117625|ENST00000367005
MRVGAEYQARIPEFDPGATKYTDKDNGGMLVWSPYHSIPDAKLDEYIAIAKEKHGYNVEQ
ALGMLFWHKHNIEKSLADLPNFTPFPDEWTVEDKVLFEQAFSFHGKSFHRIQQMLPDKTI
ASLVKYYYSWKKTRSRTSLMDRQARKLANRHNQGDSDDDVEETHPMDGNDSDYDPKKEAK
KEGNTEQPVQTSKIGLGRREYQSLQHRHHSQRSKCRPPKGMYLTQEDVVAVSCSPNAANT
ILRQLDMELISLKRQVQNAKQVNSALKQKMEGGIEEFKPPESNQKINARWTTEEQLLAVQ
GVRKYGKDFQAIADVIGNKTVGQVKNFFVNYRRRFNLEEVLQEWEAEQGTQASNGDASTL
GEETKSASNVPSGKSTDEEEEAQTPQAPRTLGPSPPAPSSTPTPTAPIATLNQPPPLLRP
TLPAAPALHRQPPPLQQQARFIQPRPTLNQPPPPLIRPANSMPPRLNPRPVLSTVGGQQP
PSLIGIQTDSQSSLH*
>ENSG00000178971|ENST00000315684
MAAGRAQVPSSEQAWLEDAQVFIQKTLCPAVKEPNVQLTPLVIDCVKTVWLSQGRNQGST
LPLSYSFVSVQDLKTHQRLPCCSHLSWSSSAYQAWAQEAGPNGNPLPREQLLLLGTLTDL
SADLEQECRNGSLYVRDNTGVLSCELIDLDLSWLGHLFLFPRWSYLPPARWNSSGEGHLE
LWDAPVPVFPLTISPGPVTPIPVLYPESASCLLRLRNKLRGVQRNLAGSLVRLSALVKSK
QKAYFILSLGRSHPAVTHVSIIVQVPAQLVWHRALRPGTAYVLTELRVSKIRGQRQHVWM
TSQSSRLLLLKPECVQELELELEGPLLEADPKPLPMPSNSEDKKDPESLVRYSRLLSYSG
AVTGVLNEPAGLYELDGQLGLCLAYQQFRGLRRVMRPGVCLQLQDVHLLQSVGGGTRRPV
LAPCLRGAVLLQSFSRQKPGAHSSRQAYGASLYEQLVWERQLGLPLYLWATKALEELACK
LCPHVLRHHQFLQHSSPGSPSLGLQLLAPTLDLLAPPGSPVRNAHNEILEEPHHCPLQKY
TRLQTPSSFPTLATLKEEGQRKAWASFDPKALLPLPEASYLPSCQLNRRLAWSWLCLLPS
AFCPAQVLLGVLVASSHKGCLQLRDQSGSLPCLLLAKHSQPLSDPRLIGCLVRAERFQLI
VERDVRSSFPSWKELSMPGFIQKQQARVYVQFFLADALILPVPRPCLHSATPSTPQTDPT
GPEGPHLGQSRLFLLCHKEALMKRNFCVPPGASPEVPKPALSFYVLGSWLGGTQRKEGTG
WGLPEPQGNDDNDQKVHLIFFGSSVRWFEFLHPGQVYRLIAPGPATPMLFEKDGSSCISR

RPLELAGCASCLTVQDNWTLELESSQDIQDVLDANKSLPESSLTDLLSDNFTDSLVSFSA
EILSRTLCEPLVASLWMKLGNTGAMRRCVKLTVALETAECEFPPHLDVYIEDPHLPPSLG
LLPGARVHFSQLEKRVSRSHNVYCCFRSSTYVQVLSFPPETTISIPLPHIYLAELLQGGQ
SPFQATASCHIVSVFSLQLFWVCAYCTSICRQGKCTRLGSTCPTQTAISQAIIRLLVEDG
TAEAVVTCRNHHVAAALGLCPREWASLLDFVQVPGRVVLQFAGPGAQLESSARVDEPMTM
FLWTLCTSPSVLRPIVLSFELERKPSKIVPLEPPRLQRFQCGELPFLTHVNPRLRLSCLS
IRESEYSSSLGILASSC*
>ENSG00000197111|ENST00000359462
MDTGVIEGGLNVTLTIRLLMHGKEVGSIIGKKGESVKKMREESGARINISEGNCPERIIT
LAGPTNAIFKAFAMIIDKLEEDISSSMTNSTAASRPPVTLRLVVPASQCGSLIGKGGCKI
KEIRESTGAQVQVAGDMLPNSTERAITIAGIPQSIIECVKQICVVMLETLSQSPPKGVTI
PYRPKPSSSPVIFAGGQDRYSTGSDSASFPHTTPSMCLNPDLEGPPLEAYTIQGQYAIPQ
PDLTKLHQLAMQQSHFPMTHGNTGFSGIESSSPEVKGYWAGLDASAQTTSHELTIPNDLI
GCIIGRQGAKINEIRQMSGAQIKIANPVEGSTDRQVTITGSAASISLAQYLINVRLSSET
GGMGSS*
>ENSG00000159140|ENST00000356577
MATNIEQIFRSFVVSKFREIQQELSSGRNEGQLNGETNTPIEGNQAGDAAASARSLPNEE
IVQKIEEVLSGVLDTELRYKPDLKEGSRKSRCVSVQTDPTDEIPTKKSKKHKKHKNKKKK
KKKEKEKKYKRQPEESESKTKSHDDGNIDLESDSFLKFDSEPSAVALELPTRAFGPSETN
ESPAVVLEPPVVSMEVSEPHILETLKPATKTAELSVVSTSVISEQSEQSVAVMPEPSMTK
ILDSFAAAPVPTTTLVLKSSEPVVTMSVEYQMKSVLKSVESTSPEPSKIMLVEPPVAKVL
EPSETLVVSSETPTEVYPEPSTSTTMDFPESSAIEALRLPEQPVDVPSEIADSSMTRPQE
LPELPKTTALELQESSVASAMELPGPPATSMPELQGPPVTPVLELPGPSATPVPELPGPL
STPVPELPGPPATAVPELPGPSVTPVPQLSQELPGLPAPSMGLEPPQEVPEPPVMAQELP
GLPLVTAAVELPEQPAVTVAMELTEQPVTTTELEQPVGMTTVEHPGHPEVTTATGLLGQP
EATMVLELPGQPVATTALELPGQPSVTGVPELPGLPSATRALELSGQPVATGALELPGPL
MAAGALEFSGQSGAAGALELLGQPLATGVLELPGQPGAPELPGQPVATVALEISVQSVVT
TSELSTMTVSQSLEVPSTTALESYNTVAQELPTTLVGETSVTVGVDPLMAPESHILASNT
METHILASNTMDSQMLASNTMDSQMLASNTMDSQMLASSTMDSQMLATSSMDSQMLATSS
MDSQMLATSTMDSQMLATSSMDSQMLATSSMDSQMLATSSMDSQMLATSSMDSQMLATST
MDSQMLATSTMDSQMLATSSMDSQMLASGTMDSQMLASGTMDAQMLASGTMDAQMLASST
QDSAMLGSKSPDPYRLAQDPYRLAQDPYRLGHDPYRLGHDAYRLGQDPYRLGHDPYRLTP
DPYRMSPRPYRIAPRSYRIAPRPYRLAPRPLMLASRRSMMMSYAAERSMMSSYERSMMSY
ERSMMSPMAERSMMSAYERSMMSAYERSMMSPMAERSMMSAYERSMMSAYERSMMSPMAD
RSMMSMGADRSMMSSYSAADRSMMSSYSAADRSMMSSYTADRSMMSMAADSYTDSYTDTY
TEAYMVPPLPPEEPPTMPPLPPEEPPMTPPLPPEEPPEGPALPTEQSALTAENTWPTEVP
SSPSEESVSQPEPPVSQSEISEPSAVPTDYSVSASDPSVLVSEAAVTVPEPPPEPESSIT
LTPVESAVVAEEHEVVPERPVTCMVSETPAMSAEPTVLASEPPVMSETAETFDSMRASGH
VASEVSTSLLVPAVTTPVLAESILEPPAMAAPESSAMAVLESSAVTVLESSTVTVLESST
VTVLEPSVVTVPEPPVVAEPDYVTIPVPVVSALEPSVPVLEPAVSVLQPSMIVSEPSVSV
QESTVTVSEPAVTVSEQTQVIPTEVAIESTPMILESSIMSSHVMKGINLSSGDQNLAPEI
GMQEIALHSGEEPHAEEHLKGDFYESEHGINIDLNINNHLIAKEMEHNTVCAAGTSPVGE
IGEEKILPTSETKQRTVLDTYPGVSEADAGETLSSTGPFALEPDATGTSKGIEFTTASTL
SLVNKYDVDLSLTTQDTEHDMVISTSPSGGSEADIEGPLPAKDIHLDLPSNNNLVSKDTE

EPLPVKESDQTLAALLSPKESSGGEKEVPPPPKETLPDSGFSANIEDINEADLVRPLLPK
DMERLTSLRAGIEGPLLASDVGRDRSAASPVVSSMPERASESSSEEKDDYEIFVKVKDTH
EKSKKNKNRDKGEKEKKRDSSLRSRSKRSKSSEHKSRKRTSESRSRARKRSSKSKSHRSQ
TRSRSRSRRRRRSSRSRSKSRGRRSVSKEKRKRSPKHRSKSRERKRKRSSSRDNRKTVRA
RSRTPSRRSRSHTPSRRRRSRSVGRRRSFSISPSRRSRTPSRRSRTPSRRSRTPSRRSRT
PSRRSRTPSRRSRTPSRRRRSRSVVRRRSFSISPVRLRRSRTPLRRRFSRSPIRRKRSRS
SERGRSPKRLTDLDKAQLLEIAKANAAAMCAKAGVPLPPNLKPAPPPTIEEKVAKKSGGA
TIEELTEKCKQIAQSKEDDDVIVNKPHVSDEEEEEPPFYHHPFKLSEPKPIFFNLNIAAA
KPTPPKSQVTLTKEFPVSSGSQHRKKEADSVYGEWVPVEKNGEENKDDDNVFSSNLPSEP
VDISTAMSERALAQKRLSENAFDLEAMSMLNRAQERIDAWAQLNSIPGQFTGSTGVQVLT
QEQLANTGAQAWIKKDQFLRAAPVTGGMGAVLMRKMGWREGEGLGKNKEGNKEPILVDFK
TDRKGLVAVGERAQKRSGNFSAAMKDLSGKHPVSALMEICNKRRWQPPEFLLVHDSGPDH
RKHFLFRVLRNGALTRPNCMFFLNRY*
>ENSG00000187837|ENST00000343677
MSETAPAAPAAAPPAEKAPVKKKAAKKAGGTPRKASGPPVSELITKAVAASKERSGVSLA
ALKKALAAAGYDVEKNNSRIKLGLKSLVSKGTLVQTKGTGASGSFKLNKKAASGEAKPKV
KKAGGTKPKKPVGAAKKPKKAAGGATPKKSAKKTPKKAKKPAAATVTKKVAKSPKKAKVA
KPKKAAKSAAKAVKPKAAKPKVVKPKKAAPKKK*
>ENSG00000118513|ENST00000316528
MARRPRHSIYSSDEDDEDFEMCDHDYDGLLPKSGKRHLGKTRWTREEDEKLKKLVEQNGT
DDWKVIANYLPNRTDVQCQHRWQKVLNPELIKGPWTKEEDQRVIELVQKYGPKRWSVIAK
HLKGRIGKQCRERWHNHLNPEVKKTSWTEEEDRIIYQAHKRLGNRWAEIAKLLPGRTDNA
IKNHWNSTMRRKVEQEGYLQESSKASQPAVATSFQKNSHLMGFAQAPPTAQLPATGQPTV
NNDYSYYHISEAQNVSSHVPYPVALHVNIVNVPQPAAAAIQRHYNDEDPEKEKRIKELEL
LLMSTENELKGQQVLPTQNHTCSYPGWHSTTIADHTRPHGDSAPVSCLGEHHSTPSLPAD
PGSLPEESASPARCMIVHQGTILDNVKNLLEFAETLQFIDSFLNTSSNHENSDLEMPSLT
STPLIGHKLTVTTPFHRDQTVKTQKENTVFRTPAIKRSILESSPRTPTPFKHALAAQEIK
YGPLKMLPQTPSHLVEDLQDVIKQESDESGIVAEFQENGPPLLKKIKQEVESPTDKSGNF
FCSHHWEGDSLNTQLFTQTSPVADAPTGVQWHDFGSLQPLPPGFKRFSCLSLPRSWDYRH
PPPRPANFEFLVETGFLHVGQAGLELLTSGDLPASASQSARITGVSHRARPDNILTSSVL
MAPASEDEDNVLKAFTVPKNRSLASPLQPCSSTWEPASCGKMEEQMTSSSQARKYVNAFS
ARTLVM*
>ENSG00000162664|ENST00000361911
MDFEDDYTHSACRNTYQGFNGMDRDYGPGSYGGMDRDYGHGSYGGQRSMDSYLNQSYGMD
NHSGGGGGSR*
>ENSG00000078687|ENST00000335749
MATGSAQGNFTGHTKKTNGNNGTNGALVQSPSNQSALGAGGANSNGSAARVWGVATGSSS
GLAHCSVSGGDGKMDTMIGDGRSQNCWGASNSNAGINLNLNPNANPAAWPVLGHEGTVAT
GNPSSICSPVSAIGQNMGNQNGNPTGTLGAWGNLLPQESTEPQTSTSQNVSFSAQPQNLN
TDGPNNTNPMNSSPNPINAMQTNGLPNWGMAVGMGAIIPPHLQGLPGANGSSVSQVSGGS
AEGISNSVWGLSPGNPATGNSNSGFSQGNGDTVNSALSAKQNGSSSAVQKEGSGGNAWDS
GPPAGPGILAWGRGSGNNGVGNIHSGAWGHPSRSTSNGVNGEWGKPPNQHSNSDINGKGS
TGWESPSVTSQNPTVQPGGEHMNSWAKAASSGTTASEGSSDGSGNHNEGSTGREGTGEGR
RRDKGIIDQGHIQLPRNDLDPRVLSNTGWGQTPVKQNTAWEFEESPRSERKNDNGTEAWG

CAATQASNSGGKNDGSIMNSTNTSSVSGWVNAPPAAVPANTGWGDSNNKAPSGPGVWGDS
ISSTAVSTAAAAKSGHAWSGAANQEDKSPTWGEPPKPKSQHWGDGQRSNPAWSAGGGDWA
DSSSVLGHLGDGKKNGSGWDADSNRSGSGWNDTTRSGNSGWGNSTNTKANPGTNWGETLK
PGPQQNWASKPQDNNVSNWGGAASVKQTGTGWIGGPVPVKQKDSSEATGWEEPSPPSIRR
KMEIDDGTSAWGDPSNYNNKTVNMWDRNNPVIQSSTTTNTTTTTTTTSNTTHRVETPPP
HQAGTQLNRSPLLGPVSSGWGEMPNVHSKTENSWGEPSSPSTLVDNGTAAWGKPPSSGSG
WGDHPAEPPVAFGRAGAPVAASALCKPASKSMQEGWGSGGDEMNLSTSQWEDEEGDVWNN
AASQESTSSCSSWGNAPKKGLQKGMKTSGKQDEAWIMSRLIKQLTDMGFPREPAEEALKS
NNMNLDQAMSALLEKKVDVDKRGLGVTDHNGMAAKPLGCRPPISKESSVDRPTFLDKDGG
LVEEPTPSPFLPSPSLKLPLSHSALPSQALGGIASGLGMQNLNSSRQIPSGNLGMFGNSG
AAQARTMQQPPQPPVQPLNSSQPSLRAQVPQFLSPQVQAQLLQFAAKNIGLNPALLTSPI
NPQHMTMLNQLYQLQLAYQRLQIQQQMLQAQRNVSGSMRQQEQQVARTITNLQQQIQQHQ
RQLAQALLVKQPPPPPPPPHLSLHPSAGKSAMDSFPSHPQTPGLPDLQTKEQQSSPNTFA
PYPLAGLNPNMNVNSMDMTGGLSVKDPSQSQSRLPQWTHPNSMDNLPSAASPLEQNPSKH
GAIPGGLSIGPPGKSSIDDSYGRYDLIQNSESPASPPVAVPHSWSRAKSDSDKISNGSSI
NWPPEFHPGVPWKGLQNIDPENDPDVTPGSVPTGPTINTTIQDVNRYLLKSGGSSPPSSQ
NATLPSSSAWPLSASGYSSSFSSIASAPSVAGKLSDIKSTWSSGPTSHTQASLSHELWKV
PRNSTAPTRPPPGLTNPKPSSTWGASPLGWTSSYSSGSAWSTDTSGRTSSWLVLRNLTPQ
IDGSTLRTLCLQHGPLITFHLNLTQGNAVVRYSSKEEAAKAQKSLHMCVLGNTTILAEFA
GEEEVNRFLAQGQALPPTSSWQSSSASSQPRLSAAGSSHGLVRSDAGHWNAPCLGGKGSS
ELLWGGVPQYSSSLWGPPSADDSRVIGSPTPLTTLLPGDLLSGESL*
>ENSG00000132466|ENST00000358602
MEKATVPVAAATAAEGEGSPPAVAAVAGPPAAAEVGGGVGGSSRARSASSPRGMVRVCDL
LLKKKPPQQQHHKAKRNRTCRPPSSSESSSDSDNSGGGGGGGGGGGGGGGTSSNNSEEEE
DDDDEEEEVSEVESFILDQDDLENPMLETASKLLLSGTADGADLRTVDPETQARLEALLE
AAGIGKLSTADGKAFADPEVLRRLTSSVSCALDEAAAALTRMRAESTANAGQSDNRSLAE
ACSEGDVNAVRKLLIEGRSVNEHTEEGESLLCLACSAGYYELAQVLLAMHANVEDRGIKG
DITPLMAAANGGHVKIVKLLLAHKADVNAQSSTGNTALTYACAGGYVDVVKVLLESGASI
EDHNENGHTPLMEAGSAGHVEVARLLLENGAGINTHSNEFKESALTLACYKGHLEMVRFL
LEAGADQEHKTDEMHTALMEACMDGHVEVARLLLDSGAQVNMPADSFESPLTLAACGGHV
ELAALLIERGASLEEVNDEGYTPLMEAAREGHEEMVALLLGQGANINAQTEETQETALTL
ACCGGFLEVADFLIKAGADIELGCSTPLMEAAQEGHLELVKYLLAAGANVHATTATGDTA
LTYACENGHTDVADVLLQAGADLEHESEGGRTPLMKAARAGHVCTVQFLISKGANVNRTT
ANNDHTVLSLACAGGHLAVVELLLAHGADPTHRLKDGSTMLIEAAKGGHTSVVCYLLDYP
NNLLSAPPPDVTQLTPPSHDLNRAPRVPVQALPMVVPPQEPDKPPANVATTLPIRNKAAS
KQKSSSHLPANSQDVQGYITNQSPESIVEEAQGKLTELEQRIKEAIEKNAQLQSLELAHA
DQLTKEKIEELNKTREEQIQKKQKILEELQKVERELQLKTQQQLKKQYLEVKAQRIQLQQ
QQQQSCQHLGLLTPVGVGEQLSEGDYARLQQVDPVLLKDEPQQTAAQMGFAPIQPLAMPQ
ALPLAAGPLPPGSIANLTELQGVIVGQPVLGQAQLAGLGQGILTETQQGLMVASPAQTLN
DTLDDIMAAVSGRASAMSNTPTHSIAASISQPQTPTPSPIISPSAMLPIYPAIDIDAQTE
SNHDTALTLACAGGHEELVQTLLERGASIEHRDKKGFTPLILAATAGHVGVVEILLDNGA
DIEAQSERTKDTPLSLACSGGRQEVVELLLARGANKEHRNVSDYTPLSLAASGGYVNIIK
ILLNAGAEINSRTGSKLGISPLMLAAMNGHTAAVKLLLDMGSDINAQIETNRNTALTLAC
FQGRTEVVSLLLDRKANVEHRAKTGLTPLMEAASGGYAEVGRVLLDKGADVNAPPVPSSR

DTALTIAADKGHYKFCELLIGRGAHIDVRNKKGNTPLWLAANGGHLDVVQLLVQAGADVD
AADNRKITPLMAAFRKGHVKVVRYLVKEVNQFPSDSECMRYIATITDKEMLKKCHLCMES
IVQAKDRQAAEANKNASILLEELDLEKLREESRRLALAAKREKRKEKRRKKKEEQRRKLE
EIEAKNKENFELQAAQEKEKLKVEDEPEVLTEPPSATTTTTIGISATWTTLAGSHGKRNN
TITTTSSKRKNRKNKITPENVQIIFDDPLPISYSQPEKVNGESKSSSTSESGDSDNMRIS
SCSDESSNSNSSRKSDNHSPAVVTTTVSSKKQPSVLVTFPKEERKSVSGKASIKLSETIS
EGTSNSLSTCTKSGPSPLSSPNGKLTVASPKRGQKREEGWKEVVRRSKKVSVPSTVISRV
IGRGGCNINAIREFTGAHIDIDKQKDKTGDRIITIRGGTESTRQATQLINALIKDPDKEI
DELIPKNRLKSSSANSKIGSSAPTTTAANTSLMGIKMTTVALSSTSQTATALTVPAISSA
STHKTIKNPVNNVRPGFPVSLPLAYPPPQFAHALLAAQTFQQIRPPRLPMTHFGGTFPPA
QSTWGPFPVRPLSPARATNSPKPHMVPRHSNQNSSGSQVNSAGSLTSSPTTTTSSSASTV
PGTSTNGSPSSPSVRRQLFVTVVKTSNATTTTVTTTASNNNTAPTNATYPMPTAKEHYPV
SSPSSPSPPAQPGGVSRNSPLDCGSASPNKVASSSEQEAGSPPVVETTNTRPPNSSSSSG
SSSAHSNQQQPPGSVSQEPRPPLQQSQVPPPEVRMTVPPLATSSAPVAVPSTAPVTYPMP
QTPMGCPQPTPKMETPAIRPPPHGTTAPHKNSASVQNSSVAVLSVNHIKRPHSVPSSVQL
PSTLSTQSACQNSVHPANKPIAPNFSAPLPFGPFSTLFENSPTSAHAFWGGSVVSSQSTP
ESMLSGKSSYLPNSDPLHQSDTSKAPGFRPPLQRPAPSPSGIVNMDSPYGSVTPSSTHLG
NFASNISGGQMYGPGAPLGGAPAAANFNRQHFSPLSLLTPCSSASNDSSAQSVSSGVRAP
SPAPSSVPLGSEKPSNVSQDRKVPVPIGTERSARIRQTGTSAPSVIGSNLSTSVGHSGIW
SFEGIGGNQDKVDWCNPGMGNPMIHRPMSDPGVFSQHQAMERDSTGIVTPSGTFHQHVPA
GYMDFPKVGGMPFSVYGNAMIPPVAPIPDGAGGPIFNGPHAADPSWNSLIKMVSSSTENN
GPQTVWTGPWAPHMNSVHMNQLG*
>ENSG00000168813|ENST00000355898
MEESSSVAMLVPDIGEQEAILTAESIISPSLEIDEQRKTKPDPLIHVIQKLSKIVENEKS
QKCLLIGKKRPRSSAATHSLETQELCEIPAKVIQSPAADTRRAEMSQTNFTPDTLAQNEG
KAMSYQCSLCKFLSSSFSVLKDHIKQHGQQNEVILMCSECHITSRSQEELEAHVVNDHDN
DANIHTQSKAQQCVSPSSSLCRKTTERNETIPDIPVSVDNLQTHTVQTASVAEMGRRKWY
AYEQYGMYRCLFCSYTCGQQRMLKTHAWKHAGEVDCSYPIFENENEPLGLLDSSAAAAPG
GVDAVVIAIGESELSIHNGPSVQVQICSSEQLSSSSPLEQSAERGVHLSQSVTLDPNEEE
MLEVISDAEENLIPDSLLTSAQKIISSSPNKKGHVNVIVERLPSAEETLSQKRFLMNTEM
EEGKDLSLTEAQIGREGMDDVYRADKCTVDIGGLIIGWSSSEKKDELMNKGLATDENAPP
GRRRTNSESLRLHSLAAEALVTMPIRAAELTRANLGHYGDINLLDPDTSQRQVDSTLAAY
SKMMSPLKNSSDGLTSLNQSNSTLVALPEGRQELSDGQVKTGISMSLLTVIEKLRERTDQ
NASDDDILKELQDNAQCQPNSDTSLSGNNVVEYIPNAERPYRCRLCHYTSGNKGYIKQHL
RVHRQRQPYQCPICEHIADNSKDLESHMIHHCKTRIYQCKQCEESFHYKSQLRNHEREQH
SLPDTLSIATSNEPRISSDTADGKCVQEGNKSSVQKQYRCDVCDYTSTTYVGVRNHRRIH
NSDKPYRCSLCGYVCSHPPSLKSHMWKHASDQNYNYEQVNKAINDAISQSGRVLGKSPGK
TQLKSSEESADPVTGSSENAVSSSELMSQTPSEVLGTNENEKLSPTSNTSYSLEKISSLA
PPSMEYCVLLFCCCICGFESTSKENLLDHMKEHEGEIVNIILNKDHNTALNTN*
>ENSG00000122299|ENST00000355758
MSNVSEERRKRQQNIKEGLQFIQSPLSYPGTQEQYAVYLRALVRNLFNEGNDVYREHDWN
NSISQYTEALNIADYAKSEEILIPKEIIEKLYINRIACYSNMGFHDKVLEDCNIVLSLNA
SNCKALYRKSKALSDLGRYKKAYDAVAKCSLAVPQDEHVIKLTQELAQKLGFKIRKAYVR
AELSLKSVPGDGATKALNHSVEDIEPDLLTPRQEAVPVVSLPAPSFSHEVGSELASVPVM

PLTSILPLQVEESALPSAVLANGGKMPFTMPEAFLDDGDMVLGDELDDLLDSAPETNETV
MPSALVRGPLQTASVSPSMPFSASLLGTLPIGARYAPPPSFSEFYPPLTSSLEDFCSSLN
SFSMSESKRDLSTSTSREGTPLNNSNSSLLLMNGPGSLFASENFLGISSQPRNDFGNFFG
SAVTKPSSSVTPRHPLEGTHELRQACQICFVKSGPKLMDFTYHANIDHKCKKDILIGRIK
NVEDKSWKKIRPRPTKTNYEGPYYICKDVAAEEECRYSGHCTFAYCQEEIDVWTLERKGA
FSREAFFGGNGKINLTVFKLLQEHLGEFIFLCEKCFDHKPRMISKRNKDNSTACSHPVTK
HEFEDNKCLVHILRETTVKYSKIRSFHGQCQLDLCRHEVRYGCLREDECFYAHSLVELKV
WIMQNETGISHDAIAQESKRYWQNLEANVPGAQVLGNQIMPGFLNMKIKFVCAQCLRNGQ
VIEPDKNRKYCSAKARHSWTKDRRAMRVMSIERKKWMNIRPLPTKKQMPLQFDLCNHIAS
GKKCQYVGNCSFAHSPEEREVWTYMKENGIQDMEQFYELWLKSQKNEKSEDIASQSNKEN
GKQIHMPTDYAEVTVDFHCWMCGKNCNSEKQWQGHISSEKHKEKVFHTEDDQYCWQHRFP
TGYFSICDRYMNGTCPEGNSCKFAHGNAELHEWEERRDALKMKLNKARKDHLIGPNDNDF
GKYSFLFKDLN*
>ENSG00000130723|ENST00000357304
MSDRLGQITKGKDGKSKYSTLSLFDKYKGKSVDAIRSSVIPRHGLQSLGKVAAARRMPPP
ANLPSLKSENKGNDPNIVIVPKDGTGWANKQDQQDPKSSSATASQPPESLPQPGLQKSVS
NLQKPTQSISQENTNSVPGGPKSWAQLNGKPVGHEGGLRGSSRLLSFSPEEFPTLKAAGG
QDKAGKEKGVLDLSYGPGPSLRPQNVTSWREGGGRHIISATSLSTSPTELGSRNSSTGDG
APSSACTSDSKDPSLRPAQPVRKGASQFMGNVYHPPTYHDMLPAFMCSPKSSENQGTVER
GSFPLPQLRLEPRVPFRQFQMNDQDGKENRLGLSRPLRPLRQLVERAPRPTIINAENLKG
LDDLDADADDGWAGLHEEVDYSEKLKFSDDEEEEEVVKDGRPKWNSWDPRRQRQLSMSSA
DSADAKRTREEGKDWAEAVGASRVVRKAPDPQPPPRKLHGWAPGPDYQKSSMGSMFRQQS
IEDKEDKPPPRQKFIQSEMSEAVERARKRREEEERRAREERLAACAAKLKQLDQKCKQAR
KAGEARKQAEKEVPWSPSAEKASPQENGPAVHKGSPEFPAQETPTTFPEEAPTVSPAVAQ
SNSSEEEAREAGSPAQEFKYQKSLPPRFQRQQQQQQQEQLYKMQHWQPVYPPPSHPQRTF
YPHHPQMLGFDPRWMMMPSYMDPRITPTRTPVDFYPSALHPSGLMKPMMPQESLNGTGCR
SEDQNCVPPLQERKVTPIDSPPVWSPEGYMALQSKGYPLPHPKSSDTLAMDMRVRNESSF
SASLGRAGGVSAQRDLFEERGEEYLSAFDKKAQADFDSCISSQRIGQELLFPPQENVQDA
GAPGGHTQNLRCSPLEPDFVPDEKKPECGSWDVSHQPETADTAHGVERETPREGTAFNIS
SWDKNGSPNKQPSSEPEWTPEPRSSSSQHPEQTGRTRRSGPIKKPVLKALKVEDKEKELE
KIKQELGEESTRLAKEKEQSPTAEKDEDEENDASLANSSTTTLEDKGPGHATFGREATKF
EEEEKPDKAWEARPPRESSDVPPMKRNNWIFIDEEQAFGVRGQARGRGRGFREFTFRGRP
AGGNGSGLCGGGVLGARSIYCSSQRSGRGRGLREFARPEDCPRAKPRRRVASETHSEGSE
YEELPKRRRQRGSENGNEGSLLEREESTLKKGDCRDSWRSNKGCSEDHSGLDAKSRGPRA
FGRALPPRLSNCGYGRRTFVSKESPHWQSKSPGSSWQEYGPSDTCGSRRPTDRDYVPDSY
RHPDAFGGRGFEDSRAEDKRSFFQDEHVADSENAENRPFRRRRPPRQDKPPRFRRLRQER
ESLGLWGPEEEPHLLAGQWPGRPKLCSGDKSGTVGRRSPELSYQNSSDHANEEWETASES
SDFSERRERREGPGSEPDSQVDGGLSGASLGEKKELAKRSFSSQRPVVDRQSRKLEPGGF
GEKPVRPGGGDTSPRYESQQNGTPLKVKRSPDEALPGGLSGCSSGSGHSPYALERAAHAS
ADLPEASSKKAEKEAKLAAPRAGEQGEAMKQFDLNYGSAIIENCGSSPGEESEVGSMVGE
GFIEVLTKKQRRLLEEERRKKEQAVQVPVKGRGLSSRIPPRFAKKQNNLCLEQGDVTVPG
SSLGTEIWESSSQALPVQAPANDSWRKAVTAFSSTETGSAEQGFKSSQGDSGVDLSAESR
ESSATSSQRSSPYGTLKPEEMSGPGLAEPKADSHKEQAPKPSEQKDSEQGSGQSKEHRPG
PIGNERSLKNRKGSEGAERLQGAVVPPVNGVEIHVDSVLPVPPIEFGVSPKDSDFSLPPG

SASGPTGSPVVKLQDALASNAGLTQSIPILRRDHHIQRAIGLSPMSFPTADLTLKMESAR
KAWENSPSLPEQSSPGGAGSGIQPPSSVGASSGVNYSSFGGVSMPPMPVASVAPSASMPG
SHLPPLYLDGHVFASQPRLVPQTIPQQQSYQQAAAAQQIPISLHTSLQAQAQLGLRGGLP
VSQSQEIFSSLQPFRSQVYMHPSLSPPSTMILSGGTALKPPYSAFPGMQPLEMVKPQSGS
PYQPMSGNQALVYEGQLSQAAGLGASQMLDSQLPQLTMPLPRYGSGQQPLILPQSIQLPP
GQSLSVGAPRRIPPPGSQPPVLNTSREPSQMEMKGFHFADSKQNVPSGGPVPSPQTYRPS
SASPSGKPSGSAVNMGSVQGHYVQQAKQRVDEKPSLGAVKLQEAPSAASQMKRTGAIKPR
AVKVEESKA*
>ENSG00000146648|ENST00000344576
MRPSGTAGAALLALLAALCPASRALEEKKVCQGTSNKLTQLGTFEDHFLSLQRMFNNCEV
VLGNLEITYVQRNYDLSFLKTIQEVAGYVLIALNTVERIPLENLQIIRGNMYYENSYALA
VLSNYDANKTGLKELPMRNLQEILHGAVRFSNNPALCNVESIQWRDIVSSDFLSNMSMDF
QNHLGSCQKCDPSCPNGSCWGAGEENCQKLTKIICAQQCSGRCRGKSPSDCCHNQCAAGC
TGPRESDCLVCRKFRDEATCKDTCPPLMLYNPTTYQMDVNPEGKYSFGATCVKKCPRNYV
VTDHGSCVRACGADSYEMEEDGVRKCKKCEGPCRKVCNGIGIGEFKDSLSINATNIKHFK
NCTSISGDLHILPVAFRGDSFTHTPPLDPQELDILKTVKEITGFLLIQAWPENRTDLHAF
ENLEIIRGRTKQHGQFSLAVVSLNITSLGLRSLKEISDGDVIISGNKNLCYANTINWKKL
FGTSGQKTKIISNRGENSCKATGQVCHALCSPEGCWGPEPRDCVSCRNVSRGRECVDKCN
LLEGEPREFVENSECIQCHPECLPQAMNITCTGRGPDNCIQCAHYIDGPHCVKTCPAGVM
GENNTLVWKYADAGHVCHLCHPNCTYGPGNESLKAMLFCLFKLSSCNQSNDGSVSHQSGS
PAAQESCLGWIPSLLPSEFQLGWGGCSHLHAWPSASVIITASSCH*
>ENSG00000198815|ENST00000361346
MGLYGQACPSVTSLRMTSELESSLTSMDWLPQLTMRAAIQKSDATQNAHGTGISKKNALL
DPNTTLDQEEVQQHKDGKPPYSYASLITFAINSSPKKKMTLSEIYQWICDNFPYYREAGS
GWKNSIRHNLSLNKCFLKVPRSKDDPGKGSYWAIDTNPKEDVLPTRPKKRARSVERASTP
YSIDSDSLGMECIISGSASPTLAINTVTNKVTLYNTDQDGSDSPRSSLNNSLSDQSLASV
NLNSVGSVHSYTPVTSHPESVSQSLTPQQQPQYNLPERDKQLLFSEYNFEDLSASFRSLY
KSVFEQSLSQQGLMNIPSESSQQSHTSCTYQHSPSSTVSTHPHSNQSSLSNSHGSGLNTT
GSNSVAQVSLSHPQMHTQPSPHPPHRPHGLPQHPQRSPHPAPHPQQHSQLQSPHPQHPSP
HQHIQHHPNHQHQTLTHQAPPPPQQVSCNSGVSNDWYATLDMLKESCRIASSVNWSDVDL
SQFQGLMESMRQADLKNWSLDQVQFADLCSSLNQFFTQTGLIHSQSNVQQNVCHGAMHPT
KPSQHIGTGNLYIDSRQNLPPSVMPPPGYPHIPQALSTPGTTMAGHHRAMNQQHMMPSQA
FQMRRSLPPDDIQDDFDWDSIV*
>ENSG00000169813|ENST00000337970
MMLGPEGGEGFVVKLRGLPWSCSVEDVQNFLSDCTIHDGAAGVHFIYTREGRQSGEAFVE
LGSEDDVKMALKKDRESMGHRYIEVFKSHRTEMDWVLKHSGPNSADSANDGFVRLRGLPF
GCTKEEIVQFFSGLEIVPNGITLPVDPEGKITGEAFVQFASQELAEKALGKHKERIGHRY
IEVFKSSQEEVRSYSDPPLKFMSVQRPGPYDRPGTARRYIGIVKQAGLERMRPGAYSTGY
GGYEEYSGLSDGYGFTTDLFGRDLSYCLSGMYDHRYGDSEFTVQSTTGHCVHMRGLPYKA
TENDIYNFFSPLNPVRVHIEIGPDGRVTGEADVEFATHEEAVAAMSKDRANMQHRYIELF
LNSTTGASNGAYSSQVMQGMGVSAAQATYSGLESQSVSGCYGAGYSGQNSMGGYD*
>ENSG00000213782|ENST00000352940
MAAPEEHDSPTEASQPIVEEEETKTFKDLGVTDVLCEACDQLGWTKPTKIQIEAIPLALQ
GRDIIGLAETGSGKTGAFALPILNALLETPQRLFALVLTPTRELAFQISEQFEALGSSIG

VQSAVIVGGIDSMSQSLALAKKPHIIIATPGRLIDHLENTKGFNLRALKYLVMDEADRIL
NMDFETEVDKILKVIPRDRKTFLFSATMTKKVQKLQRAALKNPVKCAVSSKYQTVEKLQQ
YYIFIPSKFKSKRLGSLNKFKAKARSILLATDVASRGLDIPHVDVVVNFDIPTHSKDYIH
RVGRTARAGRSGKAITFVTQYDVELFQRIEHLIGKKLPGFPTQDDEVMMLTERVAEAQRF
ARMELREHGEKKKRSREDAGDNDDTEGAIGVRNKVAGGKMKKRKGR*
>ENSG00000135486|ENST00000340913
MSKSESPKEPEQLRKLFIGGLSFETTDESLRSHFEQWGTLTDCVVMRDPNTKRSRGFGFV
TYATVEEVDAAMNARPHKVDGRVVEPKRAVSREDSQRPGAHLTVKKIFVGGIKEDTEEHH
LRDYFEQYGKIEVIEIMTDRGSGKKRGFAFVTFDDHDSVDKIVIQKYHTVNGHNCEVRKA
LSKQEMASASSSQRGRSGSGNFGGGRGGGFGGNDNFGRGGNFSGRGGFGGSRGGGGYGGS
GDGYNGFGNDGGYGGGGPGYSGGSRGYGSGGQGYGNQGSGYGGSGSYDSYNNGGGGGFGG
GSGSNFGGGGSYNDFGNYNNQSSNFGPMKGGNFGGRSSGPYGGGGQYFAKPRNQGGYGGS
SSSSSYGSGRRF*
>ENSG00000177700|ENST00000322028
MIIPVRCFTCGKIVGNKWEAYLGLLQAEYTEGDALDALGLKRYCCRRMLLAHVDLIEKLL
NYAPLEK*
>ENSG00000166261|ENST00000336139
MATAVEPEDQDLWEEEGILMVKLEDDFTCRPESVLQRDDPVLETSHQNFRRFRYQEAASP
REALIRLRELCHQWLRPERRTKEQILELLVLEQFLTVLPGELQSWVRGQRPESGEEAVTL
VEGLQKQPRRPRRWVTVHVHGQEVLSEETVHLGVEPESPNELQDPVQSSTPEQSPEETTQ
SPDLGAPAEQRPHQEEELQTLQESEVPVPEDPDLPAERSSGDSEMVALLTALSQGLVTFK
DVAVCFSQDQWSDLDPTQKEFYGEYVLEEDCGIVVSLSFPIPRPDEISQVREEEPWVPDI
QEPQETQEPEILSFTYTGDRSKDEEECLEQEDLSLEDIHRPVLGEPEIHQTPDWEIVFED
NPGRLNERRFGTNISQVNSFVNLRETTPVHPLLGRHHDCSVCGKSFTCNSHLVRHLRTHT
GEKPYKCMECGKSYTRSSHLARHQKVHKMNAPYKYPLNRKNLEETSPVTQAERTPSVEKP
YRCDDCGKHFRWTSDLVRHQRTHTGEKPFFCTICGKSFSQKSVLTTHQRIHLGGKPYLCG
ECGEDFSEHRRYLAHRKTHAAEELYLCSECGRCFTHSAAFAKHLRGHASVRPCRCNECGK
SFSRRDHLVRHQRTHTGEKPFTCPTCGKSFSRGYHLIRHQRTHSEKTS*
>ENSG00000111206|ENST00000359843
MKTSPRRPLILKRRRLPLPVQNAPSETSEEEPKRSPAQQESNQAEASKEVAESNSCKFPA
GIKIINHPTMPNTQVVAIPNNANIHSIITALTAKGKESGSSGPNKFILISCGGAPTQPPG
LRPQTQTSYDAKRTEVTLETLGPKPAARDVNLPRPPGALCEQKRETCADGEAAGCTINNS
LSNIQWLRKMSSDGLGSRSIKQEMEEKENCHLEQRQVKVEEPSRPSASWQNSVSERPPYS
YMAMIQFAINSTERKRMTLKDIYTWIEDHFPYFKHIAKPGWKNSIRHNLSLHDMFVRETS
ANGKVSFWTIHPSANRYLTLDQVFKPLDPGSPQLPEHLESQQKRPNPELRRNMTIKTELP
LGARRKMKPLLPRVSSYLVPIQFPVNQSLVLQPSVKVPLPLAASLMSSELARHSKRVRIA
PKVLLAEEGIAPLSSAGPGKEEKLLFGEGFSPLLPVQTIKEEEIQPGEEMPHLARPIKVE
SPPLEEWPSPAPSFKEESSHSWEDSSQSPTPRPKKSYSGLRSPTRCVSEMLVIQHRERRE
RSRSRRKQHLLPPCVDEPELLFSEGPSTSRWAAELPFPADSSDPASQLSYSQEVGGPFKT
PIKETLPISSTPSKSVLPRTPESWRLTPPAKVGGLDFSPVQTSQGASDPLPDPLGLMDLS
TTPLQSAPPLESPQRLLSSEPLDLISVPFGNSSPSDIDVPKPGSPEPQVSGLAANRSLTE
GLVLDTMNDSLSKILLDISFPGLDEDPLGPDNINWSQFIPELQ*
>ENSG00000101190|ENST00000335351
MSGPGPREPPPEAGAAGGEAAVEGAGGGDAALGEPGLSFTTTDLSLVEMTEVEYTQLQHI

LCSHMEAAADGELETRLNSALLAAAGPGAGAGGFAAGGQGGAAPVYPVLCPSALAADAPC
LGHIDFQELRMMLLSEAGAAEKTSGGGDGARARADGAAKEGAGAAAAAAGPDGAPEARAK
PAVRVRLEDRFNSIPAEPPPAPRGPEPPEPGGALNNLVTLIRHPSELMNVPLQQQNKCTA
LVKNKTAATTTALQFTYPLFTTNACSTSGNSNLSQTQSSSNSCSVLEAAKHQDIGLPRAF
SFCYQQEIESTKQTLGSRNKVLPEQVWIKVGEAALCKQALKRNRSRMRQLDTNVERRALG
EIQNVGEGATATQGAWQSSESSQANLGEQAQSGPQGGRSQRRERHNRMERDRRRRIRICC
DELNLLVPFCNAETDKATTLQWTTAFLKYIQERHGDSLKKEFESVFCGKTGRRLKLTRPD
SLVTCPAQGSLQSSPSMEIK*
>ENSG00000185507|ENST00000330243
MPVPERPAAGPDSPRPGTRRAAPRVLFGEWLLGEISSGCYEGLQWLDEARTCFRVPWKHF
ARKDLSEADARIFKAWAVARGRWPPSSRGGGPPPEAETAERAGWKTNFRCALRSTRRFVM
LRDNSGDPADPHKVYALSRELCWREGPGTDQTEAEAPAAVPPPQGGPPGPFLAHTHAGLQ
APGPLPAPAGDKGDLLLQAVQQSCLADHLLTASWGADPVPTKAPGEGQEGLPLTGACAGG
PGLPAGELYGWAVETTPSPGPQPAALTTGEAAAPESPHQAEPYLSPSPSACTAVQEPSPG
ALDVTIMYKGRTVLQKVVGHPSCTFLYGPPDPAVRATDPQQVAFPSPAELPDQKQLRYTE
ELLRHVAPGLHLELRGPQLWARRMGKCKVYWEVGGPPGSASPSTPACLLPRNCDTPIFDF
RVFFQELVEFRARQRRGSPRYTIYLGFGQDLSAGRPKEKSLVLVKLEPWLCRVHLEGTQR
EGVSSLDSSSLSLCLSSANSLYDDIECFLMELEQPA*
>ENSG00000174547|ENST00000329819
MSKLGRAARGLRKPEVGGVIRAIVRAGLAMPGPPLGPVLGQRGVSINQFCKEFNERTKDI
KEGIPLPTKILVKPDRTFEIKIGQPTVSYFLKAAAGIEKGARQTGKEVAGLVTLKHVYEI
ARIKAQDEAFALQDVPLSSVVRSIIGSARSLGIRVVKERCSANVLRNGKISMKDPSGRSS
V*
>ENSG00000055950|ENST00000318364
MTARGTPSRFLASVLHNGLGRYVQQLQRLSFSVSRDGASSRGAREFVEREVIDFARRNPG
VVIYVNSRPCCVPRVVAEYLNGAVREESIHCKSVEEISTLVQKLADQSGLDVIRIRKPFH
TDNPSIQGQWHPFTNKPTTFRGLRPREVQDPAPAQVQAQ*
>ENSG00000177599|ENST00000323169
MGERLFESAEGSQCGETFTQVPEDMLNKKTLPGVKSCESGTCGEIFMGYSSFNRNIRTDT
GHQPHKCQKFLEKPYKHKQRRKALSHSHCFRTHERPHTREKPFDCKECEKSFISPASIRR
YMVTHSGDGPYKCKFCGKALDCLSLYLTHERTHTGEKRYECKQCGKAFSWHSSVRIHERT
HTGEKPYECKECGKSFNFSSSFRRHERTHTGEKPYKCKECGKAFNCPSSFHRHERTHTGE
KPYECKLYGKALSRLISFRRHMRMHTGERPHKCKICGKAFYSPSSFQRHERSHTGEKPYK
CKQCGKAFTCSTSFQYHERTHTGEKPDGCKQCGKAFRSAKYIRIHGRTHTGEKPYECKQC
GKAFHCVSSFHRHERTHAGEKPYECKHCGKAFTCSIYIRIHERIHTGEKPYQCKECGKAF
IRSSYCRKHERTHTINI*
>ENSG00000105968|ENST00000350771
MFPVGRIHRHLKTRTTSHGRVGATAAVYSAAILEYLTAEVLELAGNASKDLKVKRITPRH
LQLAIRGDEELDSLIKATIAGGGVIPHIHKSLIGKKGQQKTA*
>ENSG00000181690|ENST00000316981
MATVIPGDLSEVRDTQKVPSGKRKRGETKPRKNFPCQLCDKAFNSVEKLKVHSYSHTGER
PYKCIQQDCTKAFVSKYKLQRHMATHSPEKTHKCNYCEKMFHRKDHLKNHLHTHDPNKET
FKCEECGKNYNTKLGFKRHLALHAATSGDLTCKVCLQTFESTGVLLEHLKSHAGKSSGGV
KEKKHQCEHCDRRFYTRKDVRRHMVVHTGRKDFLCQYCAQRFGRKDHLTRHMKKSHNQEL

LKVKTEPVDFLDPFTCNVSVPIKDELLPVMSLPSSELLSKPFTNTLQLNLYNTPFQSMQS
SGSAHQMITTLPLGMTCPIDMDTVHPSHHLSFKYPFSSTSYAISIPEKEQPLKGEIESYL
MELQGGVPSSSQDSQASSSSKLGLDPQIGSLDDGAGDLSLSKSSISISDPLNTPALDFSQ
LFNFIPLNGPPYNPLSVGSLGMSYSQEEAHSSVSQLPPQTQDLQDPANTIGLGSLHSLSA
AFTSSLSTSTTLPRFHQAFQ*
>ENSG00000131051|ENST00000338163
MADDIDIEAMLEAPYKKDENKLSSANGHEERSKKRKKSKSRSRSHERKRSKSKERKRSRD
RERKKSKSRERKRSRSKERRRSRSRSRDRRFRGRYRSPYSGPKFNSAIRGKIGLPHSIKL
SRRRSRSKSPFRKDKSPVREPIDNLTPEERDARTVFCMQLAARIRPRDLEEFFSTVGKVR
DVRMISDRNSRRSKGIAYVEFVDVSSVPLAIGLTGQRVLGVPIIVQASQAEKNRAAAMAN
NLQKGSAGPMRLYVGSLHFNITEDMLRGIFEPFGRIESIQLMMDSETGRSKGYGFITFSD
SECAKKALEQLNGFELAGRPMKVGHVTERTDASSASSFLDSDELERTGIDLGTTGRLQLM
ARLAEGTGLQIPPAAQQALQMSGSLAFGAVADLQTRLSQQTEALFVIMFWNEIWSDNMIT
LQL*
>ENSG00000140009|ENST00000353772
MDIKNSPSSLNSPSSYNCSQSILPLEHGSIYIPSSYVDSHHEYPAMTFYSPAVMNYSIPS
NVTNLEGGPGRQTTSPNVLWPTPGHLSPLVVHRQLSHLYAEPQKSPWCEARSLEHTLPVN
RETLKRKVSGNRCASPVTGPGSKRDAHFCAVCSDYASGYHYGVWSCEGCKAFFKRSIQGH
NDYICPATNQCTIDKNRRKSCQACRLRKCYEVGMVKCGSRRERCGYRLVRRQRSADEQLH
CAGKAKRSGGHAPRVRELLLDALSPEQLVLTLLEAEPPHVLISRPSAPFTEASMMMSLTK
LADKELVHMISWAKKIPGFVELSLFDQVRLLESCWMEVLMMGLMWRSIDHPGKLIFAPDL
VLDRDEGKCVEGILEIFDMLLATTSRFRELKLQHKEYLCVKAMILLNSSMYPLVTATQDA
DSSRKLAHLLNAVTDALVWVIAKSGISSQQQSMRLANLLMLLSHVRHARAEKASQTLTSF
GMKMETLLPEATMEQ*
>ENSG00000078399|ENST00000343483
MATTGALGNYYVDSFLLGADAADELSVGRYAPGTLGQPPRQAATLAEHPDFSPCSFQSKA
TVFGASWNPVHAAGANAVPAAVYHHHHHHPYVHPQAPVAAAAPDGRYMRSWLEPTPGALS
FAGLPSSRPYGIKPEPLSARRGDCPTLDTHTLSLTDYACGSPPVDREKQPSEGAFSENNA
ENESGGDKPPIDPNNPAANWLHARSTRKKRCPYTKHQTLELEKEFLFNMYLTRDRRYEVA
RLLNLTERQVKIWFQNRRMKMKKINKDRAKDE*
>ENSG00000176542|ENST00000316407
MPEMTENETPTKKQHRKKNRETHNAVERHRKKKINAGINRIGELIPCSPALKQSKNMILD
QAFKYITELKRQNDELLLNGGNNEQAEEIKKLRKQLEEIQKENGRYIELLKANDICLYDD
PTIHWKGNLKNSKVSVVIPSDQVQKKIIVYSNGNQPGGNSQGTAVQGITFNVSHNLQKQT
ANVVPVQRTCNLVTPVSISGVYPSENKPWHQTTVPALATNQPVPLCLPAAISAQSILELP
TSESESNVLGATSGSLIAVSIESEPHQHHSLHTCLNDQNSSENKNGQENPKVLKKMTPCV
TNIPHSSSATATKVHHGNKSCLSIQDFRGDFQNTFVVSVTTTVCSQPPRTAGDSSPMSIS
KSADLTSTATVVASSAPGVGKATIPISTLSGNPLDNGWTLSCSLPSSSVSTSDLKNINSL
TRISSAGNTQTTWTTLQLAGNTIQPLSQTPSSAVTPVLNESGTSPTTSNHSRYVATDINL
NNSFPADGQPVEQVVVTLPSCPSLPMQPLIAQPQVKSQPPKNILPLNSAMQVIQMAQPVG
SAVNSAPTNQNVIILQPPSTTPCPTVMRAEVSNQTVGQQIVIIQAANQNPLPLLPAPPPG
SVRLPINGANTVIGSNNSVQNVPTPQTFGGKHLVHILPRPSSLSASNSTQTFSVTMSNQQ
PQTISLNGQLFALQPVMSSSGTTNQTPMQIIQPTTSEDPNTNVALNTFGALASLNQSISQ
MAGQSCVQLSISQPANSQTAANSQTTTANCVSLTTTAAPPVTTDSSATLASTYNLVSTSS

MNTVACLPNMKSKRLNKKPGGRKHLAANKSACPLNSVRDVSKLDCPNTEGSAEPPCNDGL
LESFPAVLPSVSVSQANSVSVSASHSLGVLSSESLIPESVSKSKSAEKSSPPSQESVTSE
HFAMAAAKSKDSTPNLQQETSQDKPPSSLALSDAAKPCASANVLIPSPSDPHILVSQVPG
LSSTTSTTSTDCVSEVEIIAEPCRVEQDSSDTMQTTGLLKGQGLTTLLSDLAKKKNPQKS
SLSDQMDHPDFSSENPKIVDSSVNLHPKQELLLMNNDDRDPPQHHSCLPDQEVINGSLIN
GRQADSPMSTSSGSSRSFSVASMLPETTREDVTSNATTNTCDSCTFVEQTDIVALAARAI
FDQENLEKGRVGLQADIREVASKPSEASLLEGDPPFKSQIPKESGTGQAEATPNEFNSQG
SIEATMERPLEKPSCSLGIKTSNASLQDSTSQPPSITSLSVNNLIHQSSISHPLASCAGL
SPTSEQTTVPATVNLTVSSSSYGSQPPGPSLMTEYSQEQLNTMTSTIPNSQIQEPLLKPS
HESRKDSAKRAVQDDLLLSSAKRQKHCQPAPLRLESMSLMSRTPDTISDQTQMMVSQIPP
NSSNSVVPVSNPAHGDGLTRLFPPSNNFVTPALRQTEVQCGSQPSVAEQQQTQASQHLQA
LQQHVPAQGVSHLHSNHLYIKQQQQQQQQQQQQQQQQQAGQLRERHHLYQMQHHVPHAES
SVHSQPHNVHQQRTLQQEVQMQKKRNLVQGTQTSQLSLQPKHHGTDQSRSKTGQPHPHHQ
QMQQQMQQHFGSSQTEKSCENPSTSRNHHNHPQNHLNQDIMHQQQDVGSRQQGSGVSSEH
VSGHNPMQRLLTSRGLEQQMVSQPSIVTRSSDMTCTPHRPERNRVSSYSAEALIGKTSSN
SEQRMGISIQGSRVSDQLEMRSYLDVPRNKSLAIHNMQGRVDHTVASDIRLSDCQTFKPS
GASQQPQSNFEVQSSRNNEIGNPVSSLRSMQSQAFRISQNTGPPPIDRQKRLSYPPVQSI
PTGNGIPSRDSENTCHQSFMQSLLAPHLSDQVIGSQRSLSEHQRNTQCGPSSAIEYNCPP
THENVHIRRESESQNRESCDMSLGAINTRNSTLNIPFSSSSSSGDIQGRNTSPNVSVQKS
NPMRITESHATKGHMNPPVTTNMHGVARPALPHPSVSHGNGDQGPAVRQANSSVPQRSRH
PLQDSSGSKIRQPERNRSGNQRQSTVFDPSLPHLPLSTGGSMILGRQQPATEKRGSIVRF
MPDSPQVPNDNSGPDQHTLSQNFGFSFIPEGGMNPPINANASFIPQVTQPSATRTPALIP
VDPQNTLPSFYPPYSPAHPTLSNDISIPYFPNQMFSNPSTEKVNSGSLNNRFGSILSPPR
PVGFAQPSFPLLPDMPPMHMTNSHLSNFNMTSLFPEIATALPDGSAMSPLLTIANSSASD
SSKQSSNRPAHNISHILGHDCSSAV*
>ENSG00000176182|ENST00000322217
MASAAAGEAEETTRLRKPRFSFEENQILIREVRAHYPQLYGAQSRRVSVAERRRVWDGIA
AKINGITSWKRTGQEVQKRWNDFKRRTKEKLARVPHSTQGAGPAAEDAFSAEEETIFAIL
GPGVAAPGAGAGAEEPPAAPSSQPPPPSACPQRYVLSEDRREDRRADTSAHSKAGSSSPE
PWARPSCTPQEGGCPRPKERESPPPSALQPVQLPRLALSPPPPAPPLPPPPPLAQVAPSP
PSPPPPPRPPPTLSASDPSLDFLRAQQETANAIRELAGTLRQGLAKLSEALSALLPLLPG
TPVDSLPPPLPPPPPPPPPPRPVLPPPAPKVEITPEPVSVVAAVVDGAVVAARGVIIAPR
SEEGAPRPPPAPLPPHDSPPHKRRKGFPTRKRRGRWKSP*
>ENSG00000116604|ENST00000360595
MGRKKIQIQRITDERNRQVTFTKRKFGLMKKAYELSVLCDCEIALIIFNHSNKLFQYAST
DMDKVLLKYTEYNEPHESRTNADIIETLRKKGFNGCDSPEPDGEDSLEQSPLLEDKYRRA
SEELDGLFRRYGSTVPAPNFAMPVTVPVSNQSSLQFSNPSGSLVTPSLVTSSLTDPRLLS
PQQPALQRNSVSPGLPQRPASAGAMLGGDLNSANGACPSPVGNGYVSARASPGLLPVANG
NSLNKVIPAKSPPPPTHSTQLGAPSRKPDLRVITSQAGKGLMHHLNNAQRLGVSQSTHSL
TTPVVSVATPSLLSQGLPFSSMPTAYNTDYQLTSAELSSLPAFSSPGGLSLGNVTAWQQP
QQPQQPQQPQPPQQQPPQPQQPQPQQPQQPQQPPQQQSHLVPVSLSNLIPGSPLPHVGAA
LTVTTHPHISIKSEPVSPSRERSPAPPPPAVFPAARPEPGDGLSSPAGGSYETGDRDDGR
GDFGPTLGLLRPAPEPEAEGSAVKRMRLDTWTLK*
>ENSG00000197360|ENST00000357774

MPGPLGSLEMGVLTFRDVALEFSLEEWQCLDTAQQNLYRNVMLENYRNLVFVGIAASKPD
LITCLEQGKEPWNVKRHEMVTEPPVVYSYFAQDLWPKQGKKNYFQKVILRTYKKCGRENL
QLRKYCKSMDECKVHKECYNGLNQCLTTTQNKIFQYDKYVKVFHKFSNSNRHKIGHTGKK
SFKCKECEKSFCMLSHLAQHKRIHSGEKPYKCKECGKAYNEASNLSTHKRIHTGKKPYKC
EECGKAFNRLSHLTTHKIIHTGKKPYKCEECGKAFNQSANLTTHKRIHTGEKPYKCEECG
RAFSQSSTLTAHKIIHAGEKPYKCEECGKAFSQSSTLTTHKIIHTGEKFYKCEECGKAFS
RLSHLTTHKRIHSGEKPYKCEECGKAFKQSSTLTTHKRIHAGEKFYKCEVCSKAFSRFSH
LTTHKRIHTGEKPYKCEECGKAFNLSSQLTTHKIIHTGEKPYKCEECGKAFNQSSTLSKH
KVIHTGEKPYKCEECGKAFNQSSHLTTHKMIHTGEKPYKCEECGKAFNNSSILNRHKMIH
TGEKLYKPESCNNACDNIAKISKYKRNCAGEK*
>ENSG00000198185|ENST00000347606
MKMKKFQIPVSFQDLTVNFTQEEWQQLDPAQRLLYRDVMLENYSNLVSVGYHVSKPDVIF
KLEQGEEPWIVEEFSNQNYPDIDDALEKNKEIQDKHLTQTVFFSNKTLITERENVFGKTL
NLGMNSVPSRKMPYKCNPGGNSLKTNSEVIVAKKSKENRKIPDGYSGFGKHEKSHLGMKK
YRYNPMRKASNQNENLILHQNIQILKQPFDYNKCGKTFFKRAILITQKGRQTERKPNECN
ECRKTFSKRSTLIVHQRIHTGEKPYVCSDCRKTFRVKTSLTRHRRIHTGERPYECSECRK
TFIDKSALIVHQKIHGGEKSYECNECGKTFFRKSALAEHFRSHTGEKPYECKECGNAFSK
KSYLVVHQRTHRGEKPNECKECGKTFFCQSALTAHQRIHTGEKPYECSECEKTFFCQSAL
NVHRRSHTGEKPYECSQCGKFLCTKSALIAHQITHRGKKSYECNECGKFFCHKSTLTIHQ
RTHTGEKHGVFNKCGRISIVKSNCSQCKRMNTKENLYECSEHGHAVSKNSHLIVHQRTIW
ERPYECNECGRTYCRKSALTHHQRTHTGQRPYECNECGKTFCQKFSFVEHQRTHTGEKPY
ECNECGKSFCHKSAFRVHRRIHTGEKPYECNQCGKTYRRLWTLTEHQKIHTGEKPYECNK
CEKTFRHKSNFLLHQKSHKE*
>ENSG00000185507|ENST00000348655
MALAPERAAPRVLFGEWLLGEISSGCYEGLQWLDEARTCFRVPWKHFARKDLSEADARIF
KAWAVARGRWPPSSRGGGPPPEAETAERAGWKTNFRCALRSTRRFVMLRDNSGDPADPHK
VYALSRELCWREGPGTDQTEAEAPAAVPPPQGGPPGPFLAHTHAGLQAPGPLPAPAGDKG
DLLLQAVQQSCLADHLLTASWGADPVPTKAPGEGQEGLPLTGACAGGEAAAPESPHQAEP
YLSPSPSACTAVQEPSPGALDVTIMYKGRTVLQKVVGHPSCTFLYGPPDPAVRATDPQQV
AFPSPAELPDQKQLRYTEELLRHVAPGLHLELRGPQLWARRMGKCKVYWEVGGPPGSASP
STPACLLPRNCDTPIFDFRVFFQELVEFRARQRRGSPRYTIYLGFGQDLSAGRPKEKSLV
LVKLEPWLCRVHLEGTQREGVSSLDSSSLSLCLSSANSLYDDIECFLMELEQPA*
>ENSG00000151650|ENST00000325980
MRLSSSPPRGPQQLSSFGSVDWLSQSSCSGPTHTPRPADFSLGSLPGPGQTSGAREPPQA
VSIKEAAGSSNLPAPERTMAGLSKEPNTLRAPRVRTAFTMEQVRTLEGVFQHHQYLSPLE
RKRLAREMQLSEVQIKTWFQNRRMKHKRQMQDPQLHSPFSGSLHAPPAFYSTSSGLANGL
QLLCPWAPLSGPQALMLPPGSFWGLCQVAQEALASAGASCCGQPLASHPPTPGRPSLGPA
LSTGPRGLCAMPQTGDAF*
>ENSG00000144029|ENST00000345084
MATAVRAVGCLPVLCSGTAGHLLGRQCSLNTLPAASILAWKSVLGNGHLSSLGTRDTHPY
ASLSRALQTQCCISSPSHLMSQQYRPYSFFTKLTADELWKGALAETGAGAKKGRGKRTKK
KKRKDLNRGQIIGEGRYGFLWPGLNVPLMKNGAVQTIAQRSKEEQEKVEADMIQQREEWD
RKKKMKVKRERGWSGNSWGGISLGPPDPGPCGETYEDFDTRILEVFLLGKLLIGWMLSGK
QRTEQFTICII*

>ENSG00000010244|ENST00000321233
MGRKKKKQLKPWCWYCNRDFDDEKILIQHQKAKHFKCHICHKKLYTGPGLAIHCMQVHKE
TIDAVPNAIPGRTDIELEIYGMEGIPEKDMDERRRLLEQKTQESQKKKQQDDSDEYDDDD
SAASTSFQPQPVQPQQGYIPPMAQPGLPPVPGAPGMPPGIPPLMPGVPPLMPGMPPVMPG
MPPGMMPMGGMMPPGPGIPPLMPGMPPGMPPPVPRPGIPPMTQAQAVSAPGILNRPPAPT
ATVPAPQPPVTKPLFPSAGQMGTPVTSSSTASSNSESLSASSKALFPSTAQAQAAVQGPV
GTDFKPLNSTPATTTEPPKPTFPAYTQSTASTTSTTNSTAAKPAASITSKPATLTTTSAT
SKLIHPDEDISLEERRAQLPKYQRNLPRPGQAPIGNPPVGPIGGMMPPQPGIPQQQGMRP
PMPPHGQYGGHHQGMPGYLPGAMPPYGQGPPMVPPYQGGPPRPPMGMRPPVMSQGGRY*
>ENSG00000161692|ENST00000315005
MSEPGKGDDCLELESSMAESRLRAPDLGVSRCLGKCQKNSPGARKHPFSGKSFYLDLPAG
KNLQFLTGAIQQLGGVIEGFLSKEVSYIVSSRREVKAESSGKSHRGCPSPSPSEVRVETS
AMVDPKGSHPRPSRKPVDSVPLSRGKELLQKAIRNQGSISGGGSGGSSSLLTNARSWGVR
ILHVDEMMMHVQQLSLASLCVKKQQPKKPEGTCPAAESRTRKVARLKAPFLKIEDESRKF
RPFHHQFKSFPEISFLGPKDASPFEAPTTLGSMHHTRESKDGEPSPRSAAHTMPRRKKGY
CECCQEAFEELHVHLQSAQHRSFALEAHLYAEVDRIIAQLSHSFADIPFQAGLPRWSGSP
ASDCDPLCPETLHPHQPSHPRAASPRIRKEDSCQASVTQGRAAGQQRWTESLDGVMGPPA
SHTCVSATTLLPALPKGSREQGCLCPCPASFTQSHLVTSLALLPGEWSPAEDMPLHPSQE
NSFAPADIPVKGPLLFPEARPWLMSARCWVRPFPFVTWGCLIPHDTTPLHEEVSPCPCLR
LGYLYLLLTQSLWCRVRVPSLSTAGPIPRTSHPCTLAFPSYLNDHDLGHLCQAKPQGWNT
PQPFLHCGFLAVDSG*
>ENSG00000172348|ENST00000330430
MPAPSMDCDVSTLVACVVDVEVFTNQEVKEKFEGLFRTYDDCVTFQLFKSFRRVRINFSN
PKSAARARIELHETQFRGKKLKLYFAQVQTPETDGDKLHLAPPQPAKQFLISPPSSPPVG
WQPINDATPVLNYDLLYAVAKLGPGEKYELHAGTESTPSVVVHVCDSDIEEEEDPKTSPK
PKIIQTRRPGLPPSVSN*
>ENSG00000181817|ENST00000315732
MAVSHSVKERTISENSLIILLQGLQGRVTTVDLRDESVAHGRIDNVDAFMNIRLAKVTYT
DRWGHQVKLDDLFVTGRNVRYVHIPDDVNITSTIEQQLQIIHRVRNFGGKGQGRWEFPPK
NCK*
>ENSG00000197905|ENST00000358409
LEGTAGTITSNEWSSPTSPEGSTASGGSQALDKPIDNDAEGVWSPDIEQSFQEALAIYPP
CGRRKIILSDEGKMYGRNELIARYIKLRTGKTRTRKQVSSHIQVLARRKAREIQAKLKFW
QGALPGQAGTSHDVKPFSQQTYAVQPPLPLPGFESPAGPAPSPSAPPAPPWQGRSVASSK
LWMLEFSAFLEQQQDPDTYNKHLFVHIGQSSPSYSDPYLEAVDIRQIYDKFPEKKGGLKD
LFERGPSNAFFLVKFWADLNTNIEDEGSSFYGVSSQYESPENMIITCSTKVCSFGKQVVE
KVETEYARYENGHYSYRIHRSPLCEYMINFIHKLKHLPEKYMMNSVLENFTILQVVTNRD
TQETLLCIAYVFEVSASEHGAQHHIYRLVKE*
>ENSG00000202601|ENST00000365731
Sequence unavailable
>ENSG00000177732|ENST00000342665
MVQQRGARAKRDGGPPPPGPGPAEEGAREPGWCKTPSGHIKRPMNAFMVWSQHERRKIMD
QWPDMHNAEISKRLGRRWQLLQDSEKIPFVREAERLRLKHMADYPDYKYRPRKKSKGAPA
KARPRPPGGSGGGSRLKPGPQLPGRGGRRAAGGPLGGGAAAPEDDDEDDDEELLEVRLVE

TPGRELWRMVPAGRAARGQAERAQGPSGEGAAAAAAASPTPSEDEEPEEEEEEAAAAEEG
EEETVASGEESLGFLSRLPPGPAGLDCSALDRDPDLQPPSGTSHFEFPDYCTPEVTEMIA
GDWRPSSIADLVFTY*
>ENSG00000137486|ENST00000360025
MGDKGTRVFKKASPNGKLTVYLGKRDFVDHIDLVDPVDGVVLVDPEYLKERRVYVTLTCA
FRYGREDLDVLGLTFRKDLFVANVQSFPPAPEDKKPLTRLQERLIKKLGEHAYPFTFEIP
PNLPCSVTLQPGPEDTGKACGVDYEVKAFCAENLEEKIHKRNSVRLVIRKVQYAPERPGP
QPTAETTRQFLMSDKPLHLEASLDKEIYYHGEPISVNVHVTNNTNKTVKKIKISVRQYAD
ICLFNTAQYKCPVAMEEADDTVAPSSTFCKVYTLTPFLANNREKRGLALDGKLKHEDTNL
ASSTLLREGANREILGIIVSYKVKVKLVVSRGGDVAVELPFTLMHPKPKEEPPHREVPEN
ETPVDTNLIELDTNDDDIVFEDFARQRLKGMKDDKEEEEDGTGSPQLNNR*
>ENSG00000205323|ENST00000336133
MATETVELHKLKLAELKQECLARGLETKGIKQDLIHRLQAYLEEHAEEEANEEDVLGDET
EEEETKPIELPVKEEEPPEKTVDVAAEKKVVKITSEIPQTERMQKRAERFNVPVSLESKK
AARAARFGISSVPTKGLSSDNKPMVNLDKLKERAQRFGLNVSSISRKSEDDEKLKKRKER
FGIVTSSAGTGTTEDTEAKKRKRAERFGIA*
>ENSG00000129317|ENST00000344862
MEEDTDYRIRFSSLCFFNDHVGFHGTIKSSPSDFIVIEIDEQGQLVNKTIDEPIFKISEI
QLEPNNFPKKPKLDLQNLSLEDGRNQEVHTLIKYTDGDQNHQSGSEKEDTIVDGTSKCEE
KADVLSSFLDEKTHELLNNFACDVREKWLSKTELIGLPPEFSIGRILDKNQRASLHSAIR
QKFPFLVTVGKNSEIVVKPNLEYKELCHLVSEEEAFDFFKYLDAKKENSKFTFKPDTNKD
HRKAVHHFVNKKFGNLVETKSFSKMNCSAGNPNVVVTVRFREKAHKRGKRPLSECQEGKV
IYTAFTLRKENLEMFEAIGFLAIKLGVIPSDFSYAGLKDKKAITYQAMVVRKVTPERLKN
IEKEIEKKRMNVFNIRSVDDSLRLGQLKGNHFDIVIRNLKKQINDSANLRERIMEAIENV
KKKGFVNYYGPQRFGKGRKVHTDQIGLALLKNEMMKAIKLFLTPEDLDDPVNRAKKYFLQ
TEDAKGTLSLMPEFKVRERALLEALHRFGMTEEGCIQAWFSLPHSMRIFYVHAYTSKIWN
EAVSYRLETYGARVVQGDLVCLDEDIDDENFPNSKIHLVTEEEGSANMYAIHQVVLPVLG
YNIQYPKNKVGQWYHDILSRDGLQTCRFKVPTLKLNIPGCYRQILKHPCNLSYQLMEDHD
IDVKTKGSHIDETALSLLISFDLDASCYATVCLKEIMKHDV*
>ENSG00000109606|ENST00000336812
MSKRHRLDLGEDYPSGKKRAGTDGKDRDRDRDREDRSKDRDRERDRGDREREREKEKEKE
LRASTNAMLISAGLPPLKASHSAHSTHSAHSTHSTHSAHSTHAGHAGHTSLPQCINPFTN
LPHTPRYYDILKKRLQLPVWEYKDRFTDILVRHQSFVLVGETGSGKTTQIPQWCVEYMRS
LPGPKRGVACTQPRRVAAMSVAQRVADEMDVMLGQEVGYSIRFEDCSSAKTILKYMTDGM
LLREAMNDPLLERYGVIILDEAHERTLATDILMGVLKEVVRQRSDLKVIVMSATLDAGKF
QIYFDNCPLLTIPGRTHPVEIFYTPEPERDYLEAAIRTVIQIHMCEEEEGDLLLFLTGQE
EIDEACKRIKREVDDLGPEVGDIKIIPLYSTLPPQQQQRIFEPPPPKKQNGAIGRKVVVS
TNIAETSLTIDGVVFVIDPGFAKQKVYNPRIRVESLLVTAISKASAQQRAGRAGRTRPGK
CFRLYTEKAYKTEMQDNTYPEILRSNLGSVVLQLKKLGIDDLVHFDFMDPPAPETLMRAL
ELLNYLAALNDDGDLTELGSMMAEFPLDPQLAKMVIASCDYNCSNEVLSITAMLSVPQCF
VRPTEAKKAADEAKMRFAHIDGDHLTLLNVYHAFKQNHESVQWCYDNFINYRSLMSADNV
RQQLSRIMDRFNLPRRSTDFTSRDYYINIRKALVTGYFMQVAHLERTGHYLTVKDNQVVQ
LHPSTVLDHKPEWVLYNEFVLTTKNYIRTCTDIKPEWLVKIAPQYYDMSNFPQCEAKRQL
DRIIAKLQSKEYSQY*

>ENSG00000039650|ENST00000322344
MGEVEAPGRLWLESPPGGAPPIFLPSDGQALVLGRGPLTQVTDRKCSRTQVELVADPETR
TVAVKQLGVNPSTTGTQELKPGLEGSLGVGDTLYLVNGLHPLTLRWEETRTPESQPDTPP
GTPLVSQDEKRDAELPKKRMRKSNPGWENLEKLLVFTAAGVKPQGKVAGFDLDGTLITTR
SGKVFPTGPSDWRILYPEIPRKLRELEAEGYKLVIFTNQMSIGRGKLPAEEFKAKVEAVV
EKLGVPFQVLVATHAGLYRKPVTGMWDHLQEQANDGTPISIGDSIFVGDAAGRPANWAPG
RKKKDFSCADRLFALNLGLPFATPEEFFLKWPAAGFELPAFDPRTVSRSGPLCLPESRAL
LSASPEVVVAVGFPGAGKSTFLKKHLVSAGYVHVNRDTLGSWQRCVTTCETALKQGKRVA
IDNTNPDAASRARYVQCARAAGVPCRCFLFTATLEQARHNNRFREMTDSSHIPVSDMVMY
GYRKQFEAPTLAEGFSAILEIPFRLWVEPRLGRLYCQFSEG*
>ENSG00000115966|ENST00000345739
MYCAWMWPDQTPTPTRFLKNCEEVGLFNELASPFENEFKKASEDDIKKMPLDLSPLATPI
IRSKIEEPSVVETTHQDSPLPHPESTTSDEKEVPLAQTAQPTSAIVRPASLQVPNVLLTS
SDSSVIIQQAVPSPTSSTVITQAPSSNRPIVPVPGPFPLLLHLPNGQTMPVAIPASITSS
NVHVPAAVPLVRPVTMVPSVPGIPGPSSPQPVQSEAKMRLKAALTQQHPPVTNGDTVKGH
GSGLVRTQSEESRPQSLQQPATSTTETPASPAHTTPQTQSTSGRRRRAANEDPDEKRRKF
LERNRAAASRCRQKRKVWVQSLEKKAEDLSSLNGQLQSEVTLLRNEVAQLKQLLLAHKDC
PVTAMQKKSGYHTADKDDSSEDISVPSSPHTEAIQHSSVSTSNGVSSTSKAEAVATSVLT
QMADQSTEPALSQIVMAPSSQSQPSGS*
>ENSG00000124214|ENST00000347458
MKLGKKPMYKPVDPYSRMQSTYNYNMRGGAYPPRYFYPFPVPPLLYQVELSVGGQQFNGK
GKTRQAAKHDAAAKALRILQNEPLPERLEVNGRESEEENLNKSEISQVFEIALKRNLPVN
FEVARESGPPHMKNFVTKVSVGEFVGEGEGKSKKISKKNAAIAVLEELKKLPPLPAVERV
KPRIKKKTKPIVKPQTSPEYGQGINPISRLAQIQQAKKEKEPEYTLLTERGLPRRREFVM
QVKVGNHTAEGTGTNKKVAKRNAAENMLEILGFKVPQAQPTKPALKSEEKTPIKKPGDGR
KVTFFEPGSGDENGTSNKEDEFRMPYLSHQQLPAGILPMVPEVAQAVGVSQGHHTKDFTR
AAPNPAKATVTAMIARELLYGGTSPTAETILKNNISSGHVPHGPLTRPSEQLDYLSRVQG
FQVEYKDFPKNNKNEFVSLINCSSQPPLISHGIGKDVESCHDMAALNILKLLSELDQQST
EMPRTGNGPMSVCGRC*
>ENSG00000128272|ENST00000337304
MTEMSFLSSEVLVGDLMSPFDQSGLGAEESLGLLDDYLEVAKHFKPHGFSSDKAKAGSSE
WLAVDGLVSPSNNSKEDAFSGTDWMLEKMDLKEFDLDALLGIDDLETMPDDLLTTLDDTC
DLFAPLVQETNKQPPQTVNPIGHLPESLTKPDQVAPFTFLQPLPLSPGVLSSTPDHSFSL
ELGSEVDITEGDRKPDYTAYVAMIPQCIKEEDTPSDNDSGICMSPESYLGSPQHSPSTRG
SPNRSLPSPGVLCGSARPKPYDPPGEKMVAAKVKGEKLDKKLKKMEQNKTAATRYRQKKR
AEQEALTGECKELEKKNEALKERADSLAKEIQYLKDLIEEVRKARGKKRVP*
>ENSG00000160229|ENST00000344519
MGPLQFRDVAIEFSLEEWHCLDMAQRNLYRDVMLENYRNLVFLGIVVSKPDLITHLEQGK
KPSTMQRHEMVANPSVLCSHFNQDLWPEQSIKDSFQKLILRRHKKCGHDNLQLKKGCESV
DKCKVHKRGYNGLNQCLTTTQSKMFQCDKHGKVFHQFSNTNRHKIRHTGKNPCKFTECGK
AFNRSSTFTTHKKIHTGEKPYKCIECGKAFNRSSHLTTHKIIHTGEKRYKCEDCGKAFNR
SSNLTTHKKIHTGEKPYKCEECGKAFKRSSILTTHKRIHTGEKPYKCEECGKVFKYLSSL
STHKIIHTGEKPYKCEECGKAFNWSSHLTTHKRIHTGEKPYKCEECGKGFKYSSTLTKHK
IIHTGEKPYKCEECGEAFKYSCSLTAHKIIHTGKKPYKCEECGKVFKHSSPLSKHKRIHT

GEKPYKCEECGKAFSRSSILTTHKIIHTGEKPYECEDCGKAFNRSSNLTKHKKIHTGEKP
YKCEECGKAFKCSSILTTHKRIHTADKPYKCEECGKDFKYSSTLTRHKKIHTGGKPHKCN
KCGKAFISSSNLSRHEIIHMGGNPYKCENVAKP*
>ENSG00000172006|ENST00000317243
MVTCAHLGRRARLPAAQPSACPGTCFSQEERMAAGYLPRWSQELVTFEDVSMDFSQEEWE
LLEPAQKNLYREVMLENYRNVVSLEALKNQCTDVGIKEGPLSPAQTSQVTSLSSWTGYLL
FQPVASSHLEQREALWIEEKGTPQASCSDWMTVLRNQDSTYKKVALQEEPASGINMIKLI
REDGGWKQLEDSHEDPQGLLSQKASLHVVAVPQEKATAWHGFGENGNLSPALVLSQGSSK
GNHLCGSELDITSLASDSVLNHHQLGYADRRPCESNECGNAIRQNSHFIQHGGKMFVYLE
NGQSLNHGMALTIHNKINTAEKPFECHQCGKVFNRRHSLSEHQRIHTGEKPYECQECGRA
FTHSSTLTRHLRTHTGEKPYGCGECGKAFNRISSLTQHQRIHTGEKPYKCEDCGKSFCQS
SYLILHKRTHTGEKPYECSECGKAFSDRSSLNQHERTHTGENPYECKQCGRAFSQRSSLV
RHERTHTGEKPYRCQECGKAFSQSSSLVTHQKTHSSQKTYKIIDCGKAFYQNRHLIGY*
>ENSG00000176974|ENST00000354098
MTMPVNGAHKDADLWSSHDKMLAQPLKDSDVEVYNIIKKESNRQRVGLELIASENFASRA
VLEALGSCLNNKYSEGYPGQRYYGGTEFIDELETLCQKRALQAYKLDPQCWGVNVQPYSG
SPANFAVYTALVEPHGRIMGLDLPDGGHLTHGFMTDKKKISATSIFFESMPYKVNPDTGY
INYDQLEENARLFHPKLIIAGTSCYSRNLEYARLRKIADENGAYLMADMAHISGLVAAGV
VPSPFEHCHVVTTTTHKTLRGCRAGMIFYRKGVAVALKQAMTLEFKVYQHQVVANCRALS
EALTELGYKIVTGGSDNHLILVDLRSKGTDGGRAEKVLEACSIACNKNTCPGDRSALRPS
GLRLGTPALTSRGLLEKDFQKVAHFIHRGIELTLQIQSDTGVRATLKEFKERLAGDKYQA
AVQALREEVESFASLFPLPGLPDF*
>ENSG00000165804|ENST00000360947
MEGSRPRAPSGHLAPSPPAFDGELDLQRYSNGPAVSAGSLGMGAVSWSESRAGERRFPCP
VCGKRFRFNSILALHLRAHPGAQAFQCPHCGHRAAQRALLRSHLRTHQPERPRSPAARLL
LELEERALLREARLGRARSSGGMQATPATEGLARPQAPSSSAFRCPYCKGKFRTSAERER
HLHILHRPWKCGLCSFGSSQEEELLHHSLTAHGAPERPLAATSAAPPPQPQPQPPPQPEP
RSVPQPEPEPEPEREATPTPAPAAPEEPPAPPEFRCQVCGQSFTQSWFLKGHMRKHKASF
DHACPVCGRCFKEPWFLKNHMKVHASKLGPLRAPGPASGPARAPQPPDLGLLAYEPLGPA
LLLAPAPTPAERRREPPSLLGYLSLRAGEGRPNGEGAEPGPGRSFGGFRPLSSALPARARR
HRAEEPEEEEEVVEAEEETWARGRSLGSLASLHPRPGEGPGHSASAAGAQARSTATQEEN
GLLVGGTRPEGGRGATGKDCPFCGKSFRSAHHLKVHLRVHTGERPYKCPHCDYAGTQSGS
LKYHLQRHHREQRSGAGPGPPPEPPPPSQRGSAPQSGAKPSPQPATWVEGASSPRPPSSG
AGPGSRRKPASPGRTLRNGRGGEAEPLDLSLRAGPGGEAGPGGALHRCLFCPFATGAPEL
MALHLQVHHSRRARGRRPPQADASPPYARVPSGETPPSPSQEGEEGSGLSRPGEAGLGGQ
ER*
>ENSG00000139910|ENST00000344429
MMAAAPIQQNGTHTGVPIDLDPPDSRKRPLEAPPEAGSTKRTNTGEDGQYFLKVLIPSYA
AGSIIGKGGQTIVQLQKETGATIKLSKSKDFYPGTTERVCLIQGTVEALNAVHGFIAEKI
REMPQNVAKTEPVSILQPQTTVNPDRIKQTLPSSPTTTKSSPSDPMTTSRANQKHNISWI
S*
>ENSG00000028277|ENST00000342301
MVHSSMGAPEIRMSKPLEAEKQGLDSPSEHTDTERNGPDTNHQNPQNKTSPFSVSPTGPS
TKIKAEDPSGDSAPAAPLPPQPAQPHLPQAQLMLTGSQLAGDIQQLLQLQQLVLVPGHHL

QPPAQFLLPQAQQSQPGLLPTPNLFQLPQQTQGALLTSQPRAGLPTQPPKCLEPPSHPEE
PSDLEELEQFARTFKQRRIKLGFTQGDVGLAMGKLYGNDFSQTTISRFEALNLSFKNMCK
LKPLLEKWLNDAETMSVDSSLPSPNQLSSPSLGFDGLPGRRRKKRTSIETNVRFALEKSF
LANQKPTSEEILLIAEQLHMEKEVIRVWFCNRRQKEKRINPCSAAPMLPSPGKPASYSPH
MVTPQGGAGTLPLSQASSSLSTTVTTLSSAVGTLHPSRTAGGGGGGGGAAPPLNSIPSVT
PPPPATTNSTNPSPQGSHSAIGLSGLNPSTGSTMVGLSSGLSPALMSNNPLATIQALASG
GTLPLTSLDGSGNLVLGAAGAAPGSPGLVTSPLFLNHAGLPLLSTPPGVGLVSAAAAAVA
ASISSKSPGLSSSSSSSSSSSSSTCSETAAQTPGGPGGPEAGSKPE*
>ENSG00000144381|ENST00000345042
MLRLPTVFRQMRPVSRVLAPHLTRAYAKDVKFGADARALMLQGVDLLADAVAVTMGPKGR
TVIIEQSWGSPKVTKDGVTVAKSIDLKDKYKNIGAKLVQDVANNTNEEAGDGTTTATVLA
RSIAKEGFEKISKGANPVEIRRGVMLAVDAVIAELKKQSKPVTTPEEIAQVATISANGDK
EIGNIISDAMKKVGRKGVITVKDGKTLNDELEIIEGMKFDRGYISPYFINTSKGQKCEFQ
DAYVLLSEKKISSIQSIVPALEIANAHRKPLVIIAEDVDGEALSTLVLNRLKVGLQVVAV
KAPGFGDNRKNQLKDMAIATGGAVFGEEGLTLNLEDVQPHDLGKVGEVIVTKDDAMLLKG
KGDKAQIEKRIQEIIEQLDVTTSEYEKEKLNERLAKLSDGVAVLKVGGTSDVEVNEKKDR
VTDALNATRAAVEEGIVLGGGCALLRCIPALDSLTPANEDQKIGIEIIKRTLKIPAMTIA
KNAGVEGSLIVEKIMQSSSEVGYDAMAGDFVNMVEKGIIDPTKVVRTALLDAAGVASLLT
TAEVVVTEIPKEEKDPGMGAMGGMGGGMGGGMF*
>ENSG00000100029|ENST00000354694
MGGLEKKKYERGSATNYITRNKARKKLQLSLADFRRLCILKGIYPHEPKHKKKVNKGSTA
ARTFYLIKDIRFLLHEPIVNKFREYKVFVRKLRKAYGKSEWNTVERLKDNKPNYKLDHII
KERYPTFIDALRDLDDALSMCFLFSTFPRTGKCHVQTIQLCRRLTVEFMHYIIAARALRK
VFLSIKGIYYQAEVLGQPIVWITPYAFSHDHPTDVDYRVMATFTEFYTTLLGFVNFRLYQ
LLNLHYPPKLEGQAQAEAKAGEGTYALDSESCMEKLAALSASLARVVVPATEEEAEVDEF
PTDGEMSAQEEDRRKELEAQEKHKKLFEGLKFFLNREVPREALAFIIRSFGGEVSWDKSL
CIGATYDVTDSRITHQIVDRPGQQTSVIGRCYVQPQWVFDSVNARLLLPVAEYFSGVQLP
PHLSPFVTEKEGDYVPPEKLKLLALQRGEDPGNLNESEEEEEEDDNNEGDGDEEGENEEE
EEDAEAGSEKEEEARLAALEEQRMEGKKPRVMAGTLKLEDKQRLAQEEESEAKRLAIMMM
KKREKYLYQKIMFGKRRKIREANKLAEKRKAHDEAVRSEKKAKKARPE*
>ENSG00000140988|ENST00000343262
MADDAGAAGGPGGPGGPGMGNRGGFRGGFGSGIRGRGRGRGRGRGRGARGGKAEDKEW
MPVTKLGRLVKDMKIKSLEEIYLFSLPIKESEIIDFFLGASLKDEVLKIMPVQKQTRAGQ
RTRFKAFVAIGDYNGHVGLGVKCSKEVATAIRGAIILAKLSIVPVRRGYWGNKIGKPHTV
PCKVTGRCGSVLVRLIPAPRGTGIVSAPVPKKLLMMAGIDDCYTSARGCTATLGNFAKAT
FDAISKTYSYLTPDLWKETVFTKSPYQEFTDHLVKTHTRVSVQRTQAPAVATT*
>ENSG00000182459|ENST00000333437
MCPPVSMRYEEEGMSYLYASWMYQLQHGDQLSICFTCFKAAFLDFKDLLESEDWEEDNWD
PELMEHTEAESEQEGSSGMELSWGQSPGQPVQGGSEAWGPGTLAAAPEGLEDAGLDPHFV
PTELWPQEAVPLGLGLEDADWTQGLPWRFEELLTCSHWPSFFPS*
>ENSG00000169062|ENST00000351487
MRSEKEGAGGLRAAVAARGPSGREKLSALEVQFHRDSQQQEAETPPTSSSGCGGGAGKPR
EEKRTALSKVVIRRLPPGLTKEQLEEQLRPLPAHDYFEFFAADLSLYPHLYSRAYINFRN
PDDILLFRDRFDGYIFLDSKDPEYKKFLETYCVEEEKTSANPETLLGEMEAKTRELIARR

TTPLLEYIKNRKLEKQRIREEKREERRRRELEKKRLREEEKRRRREEERCKKKETDKQKK
IAEKEVRIKLLKKPEKGEEPTTEKPKERGEEIDTGGGKQESCAPGAVVKARPMEGSLEEP
QETSHSGSDKEHRDVERSQEQESEAQRYHVDDGRRHRAHHEPERLSRRSEDEQRWGKGPG
QDRGKKGSQDSGAPGEAMERLGRAQRCDDSPAPRKERLANKDRPALQLYDPGARFRAREC
GGNRRICKAEGSGTGPEKREEAE*
>ENSG00000140009|ENST00000344288
MDIKNSPSSLNSPSSYNCSQSILPLEHGSIYIPSSYVDSHHEYPAMTFYSPAVMNYSIPS
NVTNLEGGPGRQTTSPNVLWPTPGHLSPLVVHRQLSHLYAEPQKSPWCEARSLEHTLPVN
RETLKRKVSGNRCASPVTGPGSKRDAHFCAVCSDYASGYHYGVWSCEGCKAFFKRSIQGH
NDYICPATNQCTIDKNRRKSCQACRLRKCYEVGMVKCGSRRERCGYRLVRRQRSADEQLH
CAGKAKRSGGHAPRVRELLLDALSPEQLVLTLLEAEPPHVLISRPSAPFTEASMMMSLTK
LADKELVHMISWAKKIPGMRGNA*
>ENSG00000182944|ENST00000360091
XLDLGPPVDPDEDSDNSAIYVQGLNDSVTLDDLADFFKQCGVVKMNKRTGQPMIHIYLDK
ETGKPKGDATVSYEDPPTAKAAVEWFDGKDFQGSKLKVSLARKKPPMNSMRGGLPPREGR
GMPPPLRGGPGGPGGPGGPMGRMGGRGGDRGGFPPRGPRGSRGNPSGGGNVQHRAGDWQC
PNPSIGDFCCDVIVCRGCGNQNFAWRTECNQCGDRGRGGPGGMRGGRGGLMDRGGPGGMF
RGGRGGDRGGFRGGRGMDRGGFGGGRRGGPGGPPGPLMEQMGGRRGGRGGPGKMDKGEHR
QERRDRPY*
>ENSG00000004700|ENST00000314748
MASVSALTEELDSITSELHAVEIQIQELTERQQELIQKKKVLTKKIKQCLEDSDAGASNE
YDSSPAAWNKEDFPWSGKVKDILQNVFKLEKFRPLQLETINVTMAGKEVFLVMPTGGGKS
LCYQLPALCSDGFTLVICPLISLMEDQLMVLKQLGISATMLNASSSKEHVKWVHAEMVNK
NSELKLIYVTPEKIAKSKM
>ENSG00000166508|ENST00000343023
MALKDYALEKEKVKKFLQEFYQDDELGKKQFKYGNQLVRLAHREQVALYVDLDDVAEDDP
ELVDSICENARRYAKLFADAVQELLPQYKEREVVNKDVLDVYIEHRLMMEQRSRDPGMVR
SPQNQYPAELMRRFELYFQGPSSNKPRVIREVRADSVGKLVTVRGIVTRVSEVKPKMVVA
TYTCDQCGAETYQPIQSPTFMPLIMCPSQECQTNRSGGRLYLQTRGSRFIKFQEMKMQEH
SDQVPVGNIPRSITVLVEGENTRIAQPGDHVSVTGIFLPILRTGFRQVVQGLLSETYLEA
HRIVKMNKSEDDESGAGELTREELRQIADVIFATVRELVSGGRSVRFSEAEQRCVSRGFT
PAQFQAALDEYEELNVWQVNASRTRITFV*
>ENSG00000100294|ENST00000327555
MSVRVARVAWVRGLGASYRRGASSFPVPPPGAQGVAELLRDATGAEEEAPWAATERRMPG
QCSVLLFPGQGSQVVGMGRGLLNYPRVRELYAAARRVLGYDLLELSLHGPQETLDRTVHC
QPAIFVASLAAVEKLHHLQPSVIENCVAAAGFSVGEFAALVFAGAMEFAEGSTVSPEEFL
*
>ENSG00000141905|ENST00000341919
MYSSPLCLTQDEFHPFIEALLPHVRAFAYTWFNLQARKRKYFKKHEKRMSKDEERAVKDE
LLGEKPEVKQKWASRLLAKLRKDIRPECREDFVLSITGKKAPGCVLSNPDQKGKMRRIDC
LRQADKVWRLDLVMVILFKGIPLESTDGERLVKAAQCGHPVLCVQPHHIGVAVKELDLYL
AYFVRERDAEQSGSPRTGMGSDQEDSKPITLDTTDFQESFVTSGVFSVTELIQVSRTPVV
TGTGPNFSLGELQGHLAYDLNPASTGLRRTLPSTSSSGSKRHKSGSMEEDVDTSPGGDYY
TSPSSPTSSSRNWTEDMEGGISSPVKKTEMDKSPFNSPSPQDSPRLSSFTQHHRPVIAVH

SGIARSPHPSSALHFPTTSILPQTASTYFPHTAIRYPPHLNPQDPLKDLVSLACDPASQQ
PGPSWYLG*
>ENSG00000171634|ENST00000321892
MRGRRGRPPKQPAAPAAERCAPAPPPPPPPPTSGPIGGLRSRHRGSSRGRWAAAQAEVAP
KTRLSSPRGGSSSRRKPPPPPPAPPSTSAPGRGGRGGGGGRTGGGGGGGHLARTTAARRA
VNKVVYDDHESEEEEEEEDMVSEEEEEEDGDAEETQDSEDDEEDEMEEDDDDSDYPEEME
DDDDDASYCTESSFRSHSTYSSTPGRRKPRVHRPRSPILEEKDIPPLEFPKSSEDLMVPN
EHIMNVIAIYEVLRNFGTVLRLSPFRFEDFCAALVSQEQCTLMAEMHVVLLKAVLREEDT
SNTTFGPADLKDSVNSTLYFIDGMTWPEVLRVYCESDKEYHHVLPYQEAEDYPYGPVENK
IKVLQFLVDQFLTTNIAREELMSEGVIQYDDHCRVCHKLGDLLCCETCSAVYHLECVKPP
LEEVPEDEWQCEVCVAHKVPGVTDCVAEIQKNKPYIRHEPIGYDRSRRKYWFLNRRLIIE
EDTENENEKKIWYYSTKVQLAELIDCLDKDYWEAELCKILEEMREEIHRHMDITEDLTNK
ARGSNKSFLAAANEEILESIRAKKGDIDNVKSPEETEKDKNETENDSKDAEKNREEFEDQ
SLEKDSDDKTPDDDPEQGKSEEPTEVGDKGNSVSANLGDNTTNATSEETSPSEGRSPVGC
LSETPDSSNMAEKKVASELPQDVPEEPNKTCESSNTSATTTSIQPNLENSNSSSELNSSQ
SESAKAADDPENGERESHTPVSIQEEIVGDFKSEKSNGELSESPGAGKGASGSTRIITRL
RNPDSKLSQLKSQQVAAAAHEANKLFKEGKEVLVVNSQGEISRLSTKKEVIMKGNINNYF
KLGQEGKYRVYHNQYSTNSFALNKHQHREDHDKRRHLAHKFCLTPAGEFKWNGSVHGSKV
LTISTLRLTITQLENNIPSSFLHPNWASHRANWIKAVQMCSKPREFALALAILECAVKPV
VMLPIWRESLGHTRLHRMTSIEREEKEKVKKKEKKQEEEETMQQATWVKYTFPVKHQVWK
QKGEEYRVTGYGGWSWISKTHVYRFVPKLPGNTNVNYRKSLEGTKNNMDENMDESDKRKC
SRSPKKIKIEPDSEKDEVKGSDAAKGADQNEMDISKITEKKDQDVKELLDSDSDKPCKEE
PMEVDDDMKTESHVNCQESSQVDVVNVSEGFHLRTSYKKKTKSSKLDGLLERRIKQFTLE
EKQRLEKIKLEGGIKGIGKTSTNSSKNLSESPVITKAKEGCQSDSMRQEQSPNANNDQPE
DLIQGCSESDSSVLRMSDPSHTTNKLYPKDRVLDDVSIRSPETKCPKQNSIENDIEEKVS
DLASRGQEPSKSKTKGNDFFIDDSKLASADDIGTLICKNKKPLIQEESDTIVSSSKSALH
SSVPKSTNDRDATPLSRAMDFEGKLGCDSESNSTLENSSDTVSIQDSSEEDMIVQNSNES
ISEQFRTREQDVEVLEPLKCELVSGESTGNCEDRLPVKGTEANGKKPSQQKKLEERPVNK
CSDQIKLKNTTDKKNNENRESEKKGQRTSTFQINGKDNKPKIYLKGECLKEISESRVVSG
NVEPKVNNINKIIPENDIKSLTVKESAIRPFINGDVIMEDFNERNSSETKSHLLSSSDAE
GNYRDSLETLPSTKESDSTQTTTPSASCPESNSVNQVEDMEIETSEVKKVTSSPITSEEE
SNLSNDFIDENGLPINKNENVNGESKRKTVITEVTTMTSTVATESKTVIKVEKGDKQTVV
SSTENCAKSTVTTTTTTVTKLSTPSTGGSVDIISVKEQSKTVVTTTVTDSLTTTGGTLVT
SMTVSKEYSTRDKVKLMKFSRPKKTRSGTALPSYRKFVTKSSKKSIFVLPNDDLKKLARK
GGIREVPYFNYNAKPALDIWPYPSPRPTFGITWRYRLQTVKSLAGVSLMLRLLWASLRWD
DMAAKAPPGGGTTRTETSETEITTTEIIKRRDVGPYGIRSEYCIRKIICPIGVPETPKET
PTPQRKGLRSSALRPKRPETPKQTGPVIIETWVAEEELELWEIRAFAERVEKEKAQAVEQ
QAKKRLEQQKPTVIATSTTSPTSSTTSTISPAQKVMVAPISGSVTTGTKMVLTTKVGSPA
TVTFQQNKNFHQTFATWVKQGQSNSGVVQVQQKVLGIIPSSTGTSQQTFTSFQPRTATVT
IRPNTSGSGGTTSNSQVITGPQIRPGMTVIRTPLQQSTLGKAIIRTPVMVQPGAPQQVMT
QIIRGQPVSTAVSAPNTVSSTPGQKSLTSATSTSNIQSSASQPPRPQQGQVKLTMAQLTQ
LTQGHGGNQGLTVVIQGQGQTTGQLQLIPQGVTVLPGPGQQLMQAAMPNGTVQRFLFTPL
ATTATTASTTTTTVSTTAAGTGEQRQSKLSPQMQVHQDKTLPPAQSSSVGPAEAQPQTAQ
PSAQPQPQTQPQSPAQPEVQTQPEVQTQTTVSSHVPSEAQPTHAQSSKPQVAAQSQPQSN

VQGQSPVRVQSPSQTRIRPSTPSQLSPGQQSQVQTTTSQPIPIQPHTSLQIPSQGQPQSQ
PQVQSSTQTLSSGQTLNQVTVSSPSRPQLQIQQPQPQVIAVPQLQQQVQVLSQIQSQVVA
QIQAQQSGVPQQIKLQLPIQIQQSSAVQTHQIQNVVTVQAASVQEQLQRVQQLRDQQQKK
KQQQIEIKREHTLQASNQSEIIQKQVVMKHNAVIEHLKQKKSMTPAEREENQRMIVCNQV
MKYILDKIDKEEKQAAKKRKREESVEQKRSKQNATKLSALLFKHKEQLRAEILKKRALLD
KDLQIEVQEELKRDLKIKKEKDLMQLAQATAVAAPCPPVTPAPPAPPAPPPSPPPPPAVQ
HTGLLSTPTLPAASQKRKREEEKDSSSKSKKKKMISTTSKETKKDTKLYCICKTPYDESK
FYIGCDRCQNWYHGRCVGILQSEAELIDEYVCPQCQSTEDAMTVLTPLTEKDYEGLKRVL
RSLQAHKMAWPFLEPVDPNDAPDYYGVIKEPMDLATMEERVQRRYYEKLTEFVADMTKIF
DNCRYYNPSDSPFYQCAEVLESFFVQKLKGFKASRSHNNKLQSTAS*
>ENSG00000187969|ENST00000339534
MSSKDFFACGHSGHWARGCPRGGAGGRRGGGHGRGSQCGSTTLSYTCYCCGESGRNAKNC
VLLGNICYNCGRSGHIAKDCKDPKRERRQHCYTCGRLGHLARDCDRQKEQKCYSCGKLGH
IQKDCAQVKCYRCGEIGHVAINCSKARPGQLLPLRQIPTSSQGMSQ*
>ENSG00000176371|ENST00000358472
MFENESRKIFSEMPEGESAQHSDGESDFERDAGIQRLQGHSPGEDHGEVVSQDREVGQLI
GLQGTYLGEKPYECPQCGKTFSRKSHLITHERTHTGEKYYKCDECGKSFSDGSNFSRHQT
THTGEKPYKCRDCGKSFSRSANLITHQRIHTGEKPFQCAECGKSFSRSPNLIAHQRTHTG
EKPYSCPECGKSFGNRSSLNTHQGIHTGEKPYECKECGESFSYNSNLIRHQRIHTGEKPY
KCTDCGQRFSQSSALITHRRTHTGEKPYQCSECGKSFSRSSNLATHRRTHMVEKPYKCGV
CGKSFSQSSSLIAHQGMHTGEKPYECLTCGESFSWSSNLLKHQRIHTGEKPYKCSECGKC
FSQRSQLVVHQRTHTGEKPYKCLMCGKSFSRGSILVMHQRAHLGDKPYRCPECGKGFSWN
SVLIIHQRIHTGEKPYKCPECGKGFSNSSNFITHQRTHMKEKLY*
>ENSG00000178295|ENST00000317402
MGVNDLWQILEPVKQHIPLRNLGGKTIAVDLSLWVCEAQTVKKMMGSVMKPHLRNLFFRI
SYLTQMDVKLVFVMEGEPPKLKADVISKRNQSRYGSSGKSWSQKTGRSHFKSVLRECLHM
LECLGIPWVQAAGEAEAMCAYLNAGGHVDGCLTNDGDTFLYGAQTVYRNFTMNTKDPHVD
CYTMSSIKSKLGLDRDALVGLAILLGCDYLPKGVPGVGKEQALKLIQILKGQSLLQRFNR
WNETSCNSSPQLLVTKKLAHCSVCSHPGSPKDHERNGCRLCKSDKYCEPHDYEYCCPCEW
HRTEHDRQLSEVENNIKKKACCCEGFPFHEVIQEFLLNKDKLVKVIRYQRPDLLLFQRFT
LEKMEWPNHYACEKLLVLLTHYDMIERKLGSRNSNQLQPIRIVKTRIRNGVHCFEIEWEK
PEHYAMEDKQHGEFALLTIEEESLFEAAYPEIVAVYQKQKLEIKGKKQKRIKPKENNLPE
PDEVMSFQSHMTLKPTCEIFHKQNSKLNSGISPDPTLPQESISASLNSLLLPKNTPCLNA
QEQFMSSLRPLAIQQIKAVSKSLISESSQPNTSSHNISVIADLHLSTIDWEGTSFSNSPA
IQRNTFSHDLKSEVESELSAIPDGFENIPEQLSCESERYTANIKKVLDEDSDGISPEEHL
LSGITDLCLQDLPLKERIFTKLSYPQDNLQPDVNLKTLSILSVKESCIANSGSDCTSHLS
KDLPGIPLQNESRDSKILKGDQLLQEDYKVNTSVPYSVSNTVVKTCNVRPPNTALDHSRK
VDMQTTRKILMKKSVCLDRHSSDEQSAPVFGKAKYTTQRMKHSSQKHNSSHFKESGHNKL
SSPKIHIKETEQCVRSYETAENEESCFPDSTKSSLSSLQCHKKENNSGTCLDSPLPLRQR
LKLRFQST*
>ENSG00000188785|ENST00000366197
MNLTEGRVVFEDVAIYFSQEEWGHLDEAQRLLYRDVMLENLALLSSLGSWHGAEDEEAPS
QQGFSVGVSEVTASKPCLSSQKVHPSETCGPPLKDILCLVEHNGIHPEQHIYICEAELFQ
HPKQQIGENLSRGDDWIPSFGKNHRVHMAEEIFTCMEGWKDLPATSCLLQHQGPQSEWKP

YRDTEDREAFQTGQNDYKCSECGKTFTCSYSFVEHQKIHTGERSYECNKCGKFFKYSANF
MKHQTVHTSERTYECRECGKSFMYNYRLMRHKRVHTGERPYECNTCGKFFRYSSTFVRHQ
RVHTGERPYECRECGKFFMDSSTLIKHQRVHTGERPYKCNDCGKFFRYISTLIRHQRIHT
GERPYECSVCGELFRYNSSLVKHWRNHTGERPYKCSECGKSFRYHCRLIRHQRVHTGERP
YECSECGKFFRYNSNLIKHWRNHTGERPYECRECGKAFSHKHILVEHQKIHSGERPYECS
ECQKAFIRKSHLVHHQKIHSEERLVCSMNVGNSLAKTPTSLNIRDFTMEKVYH*
>ENSG00000182934|ENST00000332118
MLDFFTIFSKGGLVLWCFQGVSDSCTGPVNALIRSVLLQERGGNNSFTHEALTLKYKLDN
QFELVFVVGFQKILTLTYVDKLIDDVHRLFRDKYRTEIQQQSALSLLNGTFDFQNDFLRL
LREAEESSKIRAPTTMKKFEDSEKAKKPVRSMIETRGEKPKEKAKNSKKKGAKKEGSDGP
LATSKPVPAEKSGLPVGPENGVELSKEELIRRKREEFIQKHGRGMEKSNKSTKSDAPKEK
GKKAPRVWELGGCANKEVLDYSTPTTNGTPEAALSEDINLIRGTGSGGQLQDLDCSSSDD
EGAAQNSTKPSATKGTLGGMFGMLKGLVGSKSLSREDMESVLDKMRDHLIAKNVAADIAV
QLCESVANKLEGKVMGTFSTVTSTVKQALQESLVQILQPQRRVDMLRDIMDAQRRQRPYV
VTFCGVNGVGKSTNLAKISFWLLENGFSVLIAACDTFRAGAVEQLRTHTRRLSALHPPEK
HGGRTMVQLFEKGYGKDAAGIAMEAIAFARNQGFDVVLVDTAGRMQDNAPLMTALAKLIT
VNTPDLVLFVGEALVGNEAVDQLVKFNRALADHSMAQTPRLIDGIVLTKFDTIDDKVGAA
ISMTYITSKPIVFVGTGQTYCDLRSLNAKAVVAALMKA*
>ENSG00000165583|ENST00000347757
MNGDDAFVRRPRVGSQIPEKMQKAFDDIAKYFSEKEWEKMKASEKIIYVYMKRKYEAMTK
LGFKATLPPFMRNKRVADFQGNDFDNDPNRGNQVEHPQMTFGRLQGIFPKITPEKPAEEG
NDSKGVPEASGPQNNGKQLRPSGKLNTSEKVNKTSGPKRGKHAWTHRVRERKQLVIYEEI
SDPQEDDE*
>ENSG00000198160|ENST00000355356
MAEPSVESSSPGGSATSDDHEFDPSADMLVHDFDDERTLEEEEMMEGETNFSSEIEDLAR
EGDMPIHELLSLYGYGSTVRLPEEDEEEEEEEEEGEDDEDADNDDNSGCSGENKEENIKD
SSGQEDETQSSNDDPSQSVASQDAQEIIRPRRCKYFDTNSEVEEESEEDEDYIPSEDWKK
EIMVGSMFQAEIPVGICRYKENEKVYENDDQLLWDPEYLPEDKVIIFLKDASRRTGDEKG
VEAIPEGSHIKDNEQALYELVKCNFDTEEALRRLRFNVKAAREELSVWTEEECRNFEQGL
KAYGKDFHLIQANKVRTRSVGECVAFYYMWKKSERYDFFAQQTRFGKKKYNLHPGVTDYM
DRLLDESESAASSRAPSPPPTASNSSNSQSEKEDGTVSTANQNGVSSNGPGEILNKEEVK
VEGLHINGPTGGNKKPLHADMDTNGYETDNLTTDPKLAHMTARNENDFDEKSERPAKRRR
VNSNGKESPGSSEFFQEAVSHGKFEELENTDD*
>ENSG00000119684|ENST00000355774
MIKCLSVEVQAKLRSGLAISSLGQCVEELALNSIDAEAKCVAVRVNMETFQVQVIDNGFG
MGSDDVEKVGNRYFTSKCHSVQDLENPRFYGFRGEALANIADMASAVEISSKKNRTMKTF
VKLFQSGKALKACEADVTRASAGTTVTVYNLFYQLPVRRKCMDPRLEFEKVRQRIEALSL
MHPSISFSLRNDVSGSMVLQLPKTKDVCSRFCQIYGLGKSQKLREISFKYKEFELSGYIS
SEAHYNKNMQFLFVNKRLVLRTKLHKLIDFLLRKESIICKPKNGPTSRQMNSSLRHRSTP
ELYGIYVINVQCQFCEYDVCMEPAKTLIEFQNWDTLLFCIQEGVKMFLKQEKLFVELSGE
DIKEFSEDNGFSLFDATLQKRVTSDERSNFQEACNNILDSYEMFNLQSKAVKRKTTAENV
NTQSSRDSEATRKNTNDAFLYIYESGGPGHSKMTEPSLQNKDSSCSESKMLEQETIVASE
AGENEKHKKSFLEHSSLENPCGTSLEMFLSPFQTPCHFEESGQDLEIWKESTTVNGMAAN
ILKNNRIQNQPKRFKDATEVGCQPLPFATTLWGVHSAQTEKEKKKESSNCGRRNVFSYGR

VKLCSTGFITHVVQNEKTKSTETEHSFKNYVRPGPTRAQETFGNRTRHSVETPDIKDLAS
TLSKESGQLPNKKNCRTNISYGLENEPTATYTMFSAFQEGSKKSQTDCILSDTSPSFPWY
RHVSNDSRKTDKLIGFSKPIVRKKLSLSSQLGSLEKFKRQYGKVENPLDTEVEESNGVTT
NLSLQVEPDILLKDKNRLENSDVCKITTMEHSDSDSSCQPASHILNSEKFPFSKDEDCLE
QQMPSLRESPMTLKELSLFNRKPLDLEKSSESLASKLSRLKGSERETQTMGMMSRFNELP
NSDSSRKDSKLCSVLTQDFCMLFNNKHEKTENGVIPTSDSATQDNSFNKNSKTHSNSNTT
ENCVISETPLVLPYNNSKVTGKDSDVLIRASEQQIGSLDSPSGMLMNPVEDATGDQNGIC
FQSEESKARACSETEESNTCCSDWQRHFDVALGRMVYVNKMTGLSTFIAPTEDIQAACTK
DLTTVAVDVVLENGSQYRCQPFRSDLVLPFLPRARAERTVMRQDNRDTVDDTVSSESLQS
LFSEWDNPVFARYPEVAVDVSSGQAESLAVKIHNILYPYRFTKGMIHSMQVLQQVDNKFI
ACLMSTKTEENGEAGGNLLVLVDQHAAHERIRLEQLIIDSYEKQQAQGSGRKKLLSSTLI
PPLEITVTEEQRRLLWCYHKNLEDLGLEFVFPDTSDSLVLVGKVPLCFVEREANELRRGR
STVTKSIVEEFIREQLELLQTTGGIQGTLPLTVQKVLASQACHGAIKFNDGLSLQESCRL
IEALSSCQLPFQCAHGRPSMLPLADIDHLEQEKQIKPNLTKLRKMAQAWRLFGKAECDTR
QSLQQSMPPCEPP*
>ENSG00000196116|ENST00000355295
MLEGDLVSKMLRAVLQSHKNGVALPRLQGEYRSLTGDWIPFKQLGFPTLEAYLRSVPAVV
RIETSRSGEITCYAMACTETARIAQLVARQRSSKRKTGRQVNCQMRVKKTMPFFLEGKPK
ATLRQPGFASNFSVGKKPNPAPLRDKGNSVGVKPDAEMSPYMLHTTLGNEAFKDIPVQRH
VTMSTNNRFSPKASLQPPLQMHLSRTSTKEMSDNLNQTVEKPNVKPPASYTYKMDEVQNR
IKEILNKHNNGIWISKLPHFYKELYKEDLNQGILQQFEHWPHICTVEKPCSGGQDLLLYP
AKRKQLLRSELDTEKVPLSPLPGPKQTPPLKGCPTVMAGDFKEKVADLLVKYTSGLWASA
LPKAFEEMYKVKFPEDALKNLASLSDVCSIDYISGNPQKAILYAKLPLPTDKIQKDAGQA
HGDNDIKAMVEQEYLQVEESIAESANTFMEDITVPPLMIPTEASPSVLVVELSNTNEVVI
RYVGKDYSAAQELMEDEMKEYYSKNPKITPVQAVNVGQLLAVNAEEDAWLRAQVISTEEN
KIKVCYVDYGFSENVEKSKAYKLNPKFCSLSFQATKCKLAGLEVLSDDPDLVKVVESLTC
GKIFAVEILDKADIPLVVLYDTSGEDDININATCLKAICDKSLEVHLQVDAMYTNVKVTN
ICSDGTLYCQVPCKGLNKLSDLLRKIEDYFHCKHMTSECFVSLPFCGKICLFHCKGKWLR
VEITNVHSSRALDVQFLDSGTVTSVKVSELREIPPRFLQEMIAIPPQAIKCCLADLPQSI
GMWTPDAVLWLRDSVLNCSDCSIKVTKVDETRGIAHVYLFTPKNFPDPHRSINRQITNAD
LWKHQKDVFLSAISSGADSPNSKNGNMPMSGNTGENFRKNLTDVIKKSMVDHTSAFSTEE
LPPPVHLSKPGEHMDVYVPVACHPGYFVIQPWQEIHKLEVLMEEMILYYSVSEERHIAVE
KDQVYAAKVENKWHRVLLKGILTNGLVSVYELDYGKHELVNIRKVQPLVDMFRKLPFQAV
TAQLAGVKCNQWSEEASMVFRNHVEKKPLVALVQTVIENANPWDRKVVVYLVDTSLPDTD
TWIHDFMSEYLIELSKVN*
>ENSG00000187516|ENST00000341016
MSEKKNCKNSSTNNNQTQDPSRNELQVPRSFVDRVVQDERDVQSQSSSTINTLLTLLDCL
ADYIMERVGLEASNNGSMRNTSQDREREVDNNREPHSAESDVTRFLFDEMPKSRKND*
>ENSG00000273841|ENST00000328663
MESGKTASPKSMPKDAQMMAQILKDMGITEYEPRVINQMLEFAFRYVTTILDDAKIYSSH
AKKATVDADDVRLAIQCRADQSFTSPPPRDFLLDIARQRNQTPLPLIKPYSGPRLPPDRY
CLTAPNYRLKSLQKKASTSAGRITVPRLSVGSVTSRPSTPTLGTPTPQTMSVSTKVGTPM
SLTGQRFTVQMPTSQSPAVKASIPATSAVQNVLINPSLIGSKNILITTNMMSSQNTANES
SNALKRKREDDDDDDDDDDDYDNL*

>ENSG00000102387|ENST00000356784
MSESQDEVPDEVENQFILRLPLEHACTVRNLARSQSVKMKDKLKIDLLPDGRHAVVEVED
VPLAAKLVDLPCVIESLRTLDKKTFYKTADISQMLVCTADGDIHLSPEEPAASTDPNIVR
KKERGREEKCVWKHGITPPLKNVRKKRFRKTQKKVPDVKEMEKSSFTEYIESPDVENEVK
RLLRSDAEAVSTRWEVIAEDGTKEIESQGSIPGFLISSGMSSHKQGHTSSEYDMLREMFS
DSRSNNDDDEDEDDEDEDEDEDEDEDEDKEEEEEDCSEEYLERQLQAEFIESGQYRANEG
TSSIVMEIQKQIEKKEKKLHKIQNKAQRQKDLIMKVENLTLKNHFQSVLEQLELQEKQKN
EKLISLQEQLQRFLKK*
>ENSG00000111364|ENST00000354291
MEHVTEGSWESLPVPLHPQVLGALRELGFPYMTPVQSATIPLFMRNKDVAAEAVTGSGKT
LAFVIPILEILLRREEKLKKSQVGAIIITPTRELAIQIDEVLSHFTKHFPEFSQILWIGG
RNPGEDVERFKQQGGNIIVATPGRLEDMFRRKAEGLDLASCVRSLDVLVLDEADRLLDMG
FEARRRKWRTW*
>ENSG00000177469|ENST00000357037
MEDPTLYIVERPLPGYPDAEAPEPSSAGAQAAEEPSGAGSEELIKSDQVNGVLVLSLLDK
IIGAVDQIQLTQAQLEERQAEMEGAVQSIQGELSKLGKAHATTSNTVSKLLEKVRKVSVN
VKTVRGSLERQAGQIKKLEVNEAELLRRRNFKVMIYQDEVKLPAKLSISKSLKESEALPE
KEGEELGEGERPEEDAAALELSSDEAVEVEEVIEESRAERIKRSGLRRVDDFKKAFSKEK
MEKTKVRTRENLEKTRLKTKENLEKTRHTLEKRMNKLGTRLVPAERREKLKTSRDKLRKS
FTPDHVVYARSKTAVYKVPPFTFHVKKIREGQVEVLKATEMVEVGADDDEGGAERGEAGD
LRRGSSPDVHALLEITEESDAVLVDKSDSD*
>ENSG00000126461|ENST00000360565
MEEEDESRGKTEESGEDRGDGPPDRDPTLSPSAFILRAIQQAVGSSLQGDLPNDKDGSRC
HGLRWRRCRSPRSEPRSQESGGTDTATVLDMATDSFLAGLVSVLDPPDTWVPSRLDLRPG
ESEDMLELVAEVRIGDRDPIPLPVPSLLPRLRAWRTGKTVSPQSNSSRPTCARHLTLGTG
DGGPAPPPAPSSASSSPSPSPSSSSPSPPPPPPPPAPPAPPAPRFDIYDPFHPTDEAYSP
PPAPEQKYDPFEPTGSNPSSSAGTPSPEEEEEEEEEEEEEEEEDEEEEEGLSQSISRISET
LAGIYDDNSLSQDFPGDESPRPDAQPTQPTPAPGTPPQVDSTRADGAMRRRVFVVGTEAE
ACREGKVSVEVVTAGGAALPPPLLPPGDSEIEEGEIVQPEEEPRLALSLFRPGGRAARPT
PAASATPTAQPLPQPPAPRAPEGDDFLSLHAESDGEGALQVDLGEPAPAPPAADSRWGGL
DLRRKILTQRRERYRQRSPSPAPAPAPAAAAGPPTRKKSRRERKRSGEAKEAASSSSGTQ
PAPPAPASPWDSKKHRSRDRKPGSHASSSARRRSRSRSRSRSTRRRSRSTDRRRGGSRRS
RSREKRRRRRRSASPPPATSSSSSSRRERHRGKHRDGGGSKKKKKRSRSRGEKRSGDGSE
KAPAPAPPPSGSTSCGDRDSRRRGAVPPSIQDLTDHDLFAIKRTITVGRLDKSDPRGPSP
APASSPKREVLYDSEGLSGEERGGKSSQKDRRRSGAASSSSSSREKGSRRKALDGGDRDR
DRDRDRDRDRSSKKARPPKESAPSSGPPPKPPVSSGSGSSSSSSSCSSRKVKLQSKVAVL
IREGVSSTTPAKDAASAGLGSIGVKFSRDRESRSPFLKPDERAPTEMAKAAPGSTKPKKT
KVKAKAGAKKTKGTKGKTKPSKTRKKVRSGGGSGGSGGQVSLKKSKADSCSQAAGTKGAE
ETSWSGEERAAKVPSTPPPKAAPPPPALTPDSQTVDSSCKTPEVSFLPEEATEEAGVRGG
AEEEEEEEEEEEEEEEEEEQQPATTTATSTAAAAPSTAPSAGSTAGDSGAEDGPASRVSQ
LPTLPPPMPWNLPAGVDCTTSGVLALTALLFKMEEANLASRAKAQELIQATNQILSHRKP
PSSLGMTPAPVPTSLGLPPGPSSYLLPGSLPLGGCGSTPPTPTGLAATSDKREGSSSSEG
RGDTDKYLKKLHTQERAVEEVKLAIKPYYQKKDITKEEYKDILRKAVHKICHSKSGEINP
VKVSNLVRAYVQRYRYFRKHGRKPGDPPGPPRPPKEPGPPDKGGPGLPLPPL*

>ENSG00000171606|ENST00000326804
MLENYRNLVSVEHQLSKPDVVSQLEEAEDFWPVERGIPQDTIPEYPELQLDPKLDPLPAE
SPLMNIEVVEVLTLNQEVAGPRNAQIQALYAEDGSLSADAPSEQVQQQGKHPGDPEAARQ
RFRQFRYKDMTGPREALDQLRELCHQWLQPKARSKEQILELLVLEQFLGALPVKLRTWVE
SQHPENCQEVVALVEGVTWMSEEEVLPAGQPAEGTTCCLEVTAQQEEKQEDAAICPVTVL
PEEPVTFQDVAVDFSREEWGLLGPTQRTEYRDVMLETFGHLVSVGWETTLENKELAPNSD
IPEEEPAPSLKVQESSRDCALSSTLEDTLQGGVQEVQDTVLKQMESAQEKDLPQKKHFDN
RESQANSGALDTNQVSLQKIDNPESQANSGALDTNQVLLHKIPPRKRLRKRDSQVKSMKH
NSRVKIHQKSCERQKAKEGNGCRKTFSRSTKQITFIRIHKGSQVCRCSECGKIFRNPRYF
SVHKKIHTGERPYVCQDCGKGFVQSSSLTQHQRVHSGERPFECQECGRTFNDRSAISQHL
RTHTGAKPYKCQDCGKAFRQSSHLIRHQRTHTGERPYACNKCGKAFTQSSHLIGHQRTHN
RTKRKKKQPTS*
>ENSG00000128908|ENST00000361937
MASELGARDDGGCTELAKPLYLQYLERALRLDHFLRQTSAIFNRNISSDDSEDGLDDSNP
LLPQSGDPLIQVKEEPPNSLLGETSGAGSSGMLNTYSLNGVLQSESKCDKGNLYNFSKLK
KSRKWLKSILLSDESSEADSQSEDDDEEELNLSREELHNMLRLHKYKKLHQNKYSKDKEL
QQYQYYSAGLLSTYDPFYEQQRHLLGPKKKKFKEEKKLKAKLKKVKKKRRRDEELSSEES
PRRHHHQTKVFAKFSHDAPPPGTKKKHLSIEQLNARRRKVWLSIVKKELPKANKQKASAR
NLFLTNSRKLAHQCMKEVRRAALQAQKNCKETLPRARRLTKEMLLYWKKYEKVEKEHRKR
AEKEALEQRKLDEEMREAKRQQRKLNFLITQTELYAHFMSRKRDMGHDGIQEEILRKLED
SSTQRQIDIGGGVVVNITQEDYDSNHFKAQALKNAENAYHIHQARTRSFDEDAKESRAAA
LRAANKSGTGFGESYSLANPSIRAGEDIPQPTIFNGKLKGYQLKGMNWLANLYEQGINGI
LADEMGLGKTVQSIALLAHLAERENIWGPFLIISPASTLNNWHQEFTRFVPKFKVLPYWG
NPHDRKVIRRFWSQKTLYTQDAPFHVVITSYQLVVQDVKYFQRVKWQYMVLDEAQALKSS
SSVRWKILLQFQCRNRLLLTGTPIQNTMAELWALLHFIMPTLFDSHEEFNEWFSKDIESH
AENKSAIDENQLSRLHMILKPFMLRRIKKDVENELSDKIEILMYCQLTSRQKLLYQALKN
KISIEDLLQSSMGSTQQAQNTTSSLMNLVMQFRKVCNHPELFERQETWSPFHISLKPYHI
SKFIYRHGQIRVFNHSRDRWLRVLSPFAPDYIQRSLFHRKGINEESCFSFLRFIDISPAE
MANLMLQGLLARWLALFLSLKASYRLHQLRSWGAPEGESHQRYLRNKDFLLGVNFPLSFP
NLCSCPLLKSLVFSSHCKAVSGYSDQVVHQRRSATSSLRRCLLTELPSFLCVASPRVTAV
PLDSYCNDRSAEYERRVLKEGGSLAAKQCLLNGAPELAADWLNRRSQFFPEPAGGLWSIR
PQNGWSFIRIPGKESLITDSGKLYALDVLLTRLKSQGHRVLIYSQMTRMIDLLEEYMVYR
KHTYMRLDGSSKISERRDMVADFQNRNDIFVFLLSTRAGGLGINLTAADTVIFYDSDWNP
TVDQQAMDRAHRLGQTKQVTVYRLICKGTIEERILQRAKEKSEIQRMVISGGNFKPDTLK
PKEVVSLLLDDEELEKKLRLRQEEKRQQEETNRVKERKRKREKYAEKKKKEDELDGKRRK
EGVNLVIPFVPSADNSNLSADGDDSFISVDSAMPSPFSEISISSELHTGSIPLDESSSDM
LVIVDDPASSAPQSRATNSPASITGSVSDTVNGISIQEMPAAGRGHSARSRGRPKGSGST
AKGAGKGRSRKSTAGSAAAMAGAKAGAAAASAAAYAAYGYNVSKGISASSPLQTSLVRPA
GLADFGPSSASSPLSSPLSKGNNVPGNPKNLHMTSSLAPDSLVRKQGKGTNPSGGR*
>ENSG00000174780|ENST00000342756
MASGGSGGVSVPALWSEVNRYGQNGDFTRALKTVNKILQINKDDVTALHCKVVCLIQNGS
FKEALNVINTHTKVLANNSLSFEKAYCEYRLNRIENALKTIESANQQTDKLKELYGQVLY
RLERYDECLAVYRDLVRNSQDDYDEERKTNLSAVVAAQSNWEKVVPENLGLQEGTHELCY
NTACALIGQGQLNQAMKILQKAEDLCRRSLSEDTDGTEEDPQAELAIIHGQMAYILQLQG

RTEEALQLYNQIIKLKPTDVGLLAVIANNIITINKDQNVFDSKKKVKLTNAEGVEFKLSK
KQLQAIEFNKALLAMYTNQAEQCRKISASLQSQSPEHLLPVLIQAAQLCREKQHTKAIEL
LQEFSDQHPENAAEIKLTMAQLKISQGNISKACLILRSIEELKHKPGMVSALVTMYSHEE
DIDSAIEVFTQAIQWYQNHQPKSPAHLSLIREAANFKLKYGRKKEAISDLQQLWKQNPKD
IHTLAQLISAYSLVDPEKAKALSKHLPSSDSMSLKVDVEALENSAGATYIRKKGGKVTGD
SQPKEQGQGDLKKKKKKKKGKLPKNYDPKVTPDPERWLPMRERSYYRGRKKGKKKDQIGK
GTQGATAGASSELDASKTVSSPPTSPRPGSAATVSASTSNIIPPRHQKPAGAPATKKKQQ
QKKKKGGKGGW*
>ENSG00000178229|ENST00000321545
MAASAQVSVTFEDVAVTFTQEEWGQLDAAQRTLYQEVMLETCGLLMSLGCPLFKPELIYQ
LDHRQELWMATKDLSQSSYPGDNTKPKTTEPTFSHLALPEEVLLQEQLTQGASKNSQLGQ
SKDQDGPSEMQEVHLKIGIGPQRGKLLEKMSSERDGLGSDDGVCTKITQKQVSTEGDLYE
CDSHGPVTDALIREEKNSYKCEECGKVFKKNALLVQHERIHTQVKPYECTECGKTFSKST
HLLQHLIIHTGEKPYKCMECGKAFNRRSHLTRHQRIHSGEKPYKCSECGKAFTHRSTFVL
HHRSHTGEKPFVCKECGKAFRDRPGFIRHYIIHTGEKPYECIECGKAFNRRSYLTWHQQI
HTGVKPFECNECGKAFCESADLIQHYIIHTGEKPYKCMECGKAFNRRSHLKQHQRIHTGE
KPYECSECGKAFTHCSTFVLHKRTHTGEKPYECKECGKAFSDRADLIRHFSIHTGEKPYE
CVECGKAFNRSSHLTRHQQIHTGEKPYECIQCGKAFCRSANLIRHSIIHTGEKPYECSEC
GKAFNRGSSLTHHQRIHTGRNPTIVTDVGRPFMTAQTSVNIQELLLGKEFLNITTEENLW
*
>ENSG00000181378|ENST00000341552
MFTLTGCRLVEKTQKVENPSVSFASSFPLIPLLLRGKSVQKKQAESKSQIKLHTQSAPFG
LCPKDMMLTQAPSSVVRSRNSRNHTVNSGGSCLSASTVAIPAINDSSAAMSACSTISAQP
ASSMDTQMHSPKKQERVNKRVIWGIEVAEELHWKGWELGKETTRNLVLKNRSLKLQKMKY
RPPKTKFFFTVIPQPIFLSPGITLTLPIVFRPLEAKEYMDQLWFEKAEGMFCVGLRATLP
CHRLICRPPSLQLPMCAVGDTTEAFFCLDNVGDLPTFFTWEFSSPFQMLPATGLLEPGQA
SQIKVTFQPLTAVIYEVQATCWYGAGSRQRSSIQLQAVAKCAQLLVSIKHKCPEDQDAEG
FQKLLYFGSVAVGCTSERQIRLHNPSAVNAPFRIEISPDELAEDQAFSCPTAHGIVLPGE
KKCVSVFFHPKTLDTRTVDYCSIMPSGCASKTLLKVVGFCRGPAVSLQHYCVNFSWVNLG
ERSEQPLWIENQSDCTAHFQFAIDCLESVFTIRPAFGTLVGKARMTLHCAFQPTHPIICF
RRVACLIHHQDPLFLDLMGTCHSDSTKPAILKPQHLTWYRTHLARGLTLYPPDILDAMLK
EKKLAQDQNGALMIPIQDLEDMPAPQYPYIPPMTEFFFDGTSDITIFPPPISVEPVEVDF
GACPGPEAPNPVPLCLMNHTKGKIMVVWTRRSDCPFWVTPESCDVPPLKSMAMRLHFQPP
HPNCLYTVELEAFAIYKVLQSYSNIEEDCTMCPSWCLTVRARGHSYFAGFEHHIPQYSLD
VPKLFPAVSSGEPTYRSLLLVNKDCKLLTFSLAPQRGSDVILRPTSGLVAPGAHQIILIC
TYPEGSSWKQHTFYLQCNASPQYLKEVSMYSREEPLQLKLDTHKSLYFKPTWVGCSSTSP
FTFRNPSRLPLQFEWRVSEQHRKLLAVQPSRGLIQPNERLTLTWTFSPLEETKYLFQVGM
WVWEAGLSPNANPAATTHYMLRLVGVGLTSSLSAKEKELAFGNVLVNSKQSRFLVLLNDG
NCTLYYRLYLEQGSPEAVDNHPLALQLDRTEGSMPPRSQDTICLTACPKQRSQYSWTITY
SLLSHRDNKAGEKQELCCVSLVAVYPLLSILDVSSMGSAEGITRKHLWRLFSLDLLNSYL
ERDPTPCELTYKVPTRHSMSQIPPVLTPLRLDFNFGAAPFKAPPSVVFLALKNSGVVSLD
WAFLLPSDQRIDVELWAEQAELNSTELHQMRVQDNCLFSISPKAGSLSPGQEQMVELKYS
HLFIGTDHLPVLFKVSHGREILLNFIGVTVKPEQKYVHFTSTTHQFIPIPIGDTLPPRQI
YELYNGGSVPVTYEVQTDVLSQVQEKNFDHPIFCCLNPKGEIQPGSTARVLWIFSPIEAK

TYTVDVPIHILGWNSALIHFQGVGYNPHMMGDTAPFHNISSWDNSSIHSRLVVPGQNVFL
SQSHISLGNIPVQSKCSRLLFLNNISKNEEIAFSWQPSPLDFGEVSVSPMIGVVAPEETV
PFVVTLRASVHASFYSADLVCKLYSQQLMRQYHKELQEWKDEKVRQEVEFTITDMKVKKR
TCCTACEPARKYKTLPPIKNQQSVSRPASWKLQTPKEEVSWPCPQPPSPGMLCLGLTARA
HATDYFLANFFSEFPCHFLHRELPKRKAPREESETSEEKSPNKWGPVSKQKKQLLVDILT
TIIRGLLEDKNFHEAVDQSLVEQVPYFRQFWNEQSTKFMDQKNSLYLMPILPVPSSSWED
GKGKQPKEDRPEHYPGLGKKEEGEEEKGEEEEEELEEEEEEEEETEEEELGKEEIEEKEE
ERDEKEEKVSWAGIGPTPQPESQESMQWQWQQQLNVMVKEEQEQDEKEAIRRLPAFANLQ
EALLENMIQNILVEASRGEVVLTSRPRVIALPPFCVPRSLTPDTLLPTQQAEVLHPVVPL
PTDLP*
>ENSG00000071626|ENST00000336761
MNNSGADEIGKLFVGGLDWSTTQETLRSYFSQYGEVVDCVIMKDKTTNQSRGFGFVKFKD
PNCVGTVLASRPHTLDGRNIDPKPCTPRGMQPERTRPKEGWQKGPRSDNSKSNKIFVGGI
PHNCGETELREYFKKFGVVTEVVMIYDAEKQRPRGFGFITFEDEQSVDQAVNMHFHDIMG
KKVEVKRAEPRDSKSQAPGQPGASQWGSRVVPNAANGWAGQPPPTWQQGYGPQGMWVPAG
QAIGGYGPPPAGRGAPPPPPPFTSYIVSTPPGGFPPPQGFPQGYGAPPQFSFGYGPPPPP
PDQFAPPGVPPPPATPGAAPLAFPPPPSQAAPDMSKPPTAQPDFPYGQYGLGSYSPAPPG
CGPHFVYSLMVRLSSDVA*
>ENSG00000196235|ENST00000359191
MSDSEDSNFSEEEDSERSSDGEEAEVDEERRSAAGSEKEEEPEDEEEEEEEEEYDEEEEE
EDDDRPPKKPRHGGFILDEADVDDEYEDEDQWEDGAEDILEKASNIDNVVLDEDRSGARR
LQNLWRDQREEELGEYYMKKYAKSSVGETVYGGSDELSDDITQQQLLPGVKDPNLWTVKC
KIGEERATAISLMRKFIAYQFTDTPLQIKSVVAPEHVKGYIYVEAYKQTHVKQAIEGVGN
LRLGYWNQQMVPIKEMTDVLKVVKEVANLKPKSWVRLKRGIYKDDIAQVDYVEPSQNTIS
LKMIPRIDYDRIKARMSLKDWFAKRKKFKRPPQRLFDAEKIRSLGGDVASDGDFLIFEGN
RYSRKGFLFKSFAMSAVITEGVKPTLSELEKFEDQPEGIDLEVVTESTGKEREHNFQPGD
NVEVCEGELINLQGKILSVDGNKITIMPKHEDLKDMLEFPAQELRKYFKMGDHVKVIAGR
FEGDTGLIVRVEENFVILFSDLTMHELKVLPRDLQLCSETASGVDVGGQHEWGELVQLDP
QTVGVIVRLERETFQVLNMYGKVVTVRHQAVTRKKDNRFAVALDSEQNNIHVKDIVKVID
GPHSGREGEIRHLFRSFAFLHCKKLVENGGMFVCKTRHLVLAGGSKPRDVTNFTVGGFAP
MSPRISSPMHPSAGGQRGGFGSPGGGSGGMSRGRGRRDNELIGQTVRISQGPYKGYIGVV
KDATESTARVELHSTCQTISVDRQRLTTVGSRRPGGMTSTYGRTPMYGSQTPMYGSGSRT
PMYGSQTPLQDGSRTPHYGSQTPLHDGSRTPAQSGAWDPNNPNTPSRAEEEYEYAFDDEP
TPSPQAYGGTPNPQTPGYPDPSSPQVNPQYNPQTPGTPAMYNTDQFSPYAAPSPQGSYQP
SPSPQSYHQVAPSPAGYQNTHSPASYHPTPSPMAYQASPSPSPVGYSPMTPGAPSPGGYN
PHTPGSGIEQNSSDWVTTDIQVKVRDTYLDTQVVGQTGVIRSVTGGMCSVYLKDSEKVVS
ISSEHLEPITPTKNNKVKVILGEDREATGVLLSIDGEDGIVRMDLDEQLKILNLRFLGKL
LEA*
>ENSG00000106348|ENST00000348127
MEGPLTPPPLQGGGAAAVPEPGARQHPGHETAAQRYSARLLQAGYEPESMADYLISGGTG
YVPEDGLTAQQLFASADGLTYNDFLILPGFIDFIADEVDLTSALTRKITLKTPLISSPMD
TVTEADMAIAMALMGGIGFIHHNCTPEFQANEVRKVKKFEQGFITDPVVLSPSHTVGDVL
EAKMRHGFSGIPITETGTMGSKLVGIVTSRDIDFLAEKDHTTLLSEVMTPRIELVVAPAG
VTLKEANEILQRSKKGKLPIVNDCDELVAIIARTDLKKNRDYPLASKDSQKQLLCGAAVG

TREDDKYRLDLLTQAGVDVIVLDSSQGNSVYQIAMVHYIKQKYPHLQVIGGNVVTAAQAK
NLIDAGVDGLRVGMGCGSICITQEVMACGRPQGTAVYKVAEYARRFGVPIIADGGIQTVG
HVVKALALGASTVMMGSLLAATTEAPGEYFFSDGVRLKKYRGMGSLDAMEKSSSSQKRYF
SEGDKVKIAQGVSGSIQDKGSIQKFVPYLIAGIQHGCQDIGARSLSVLRSMMYSGELKFE
KRTMSAQIEGGVHGLHSYEKRLY*
>ENSG00000124191|ENST00000341197
MDVRLYPSAPAVGARPGAEPAGLAHLDYYHGGKFDGDSAYVGMSDGNPELLSTSQTYNGQ
SENNEDYEIPPITPPNLPEPSLLHLGDHEASYHSLCHGLTPNGLLPAYSYQAMDLPAIMV
SNMLAQDSHLLSGQLPTIQEMVHSEVAAYDSGRPGPLLGRPAMLASHMSALSQSQLISQM
GIRSSIAHSSPSPPGSKSATPSPSSSTQEEESEVHFKISGEKRPSADPGKKAKNPKKKKK
KDPNEPQKPVSAYALFFRDTQAAIKGQNPSATFGDVSKIVASMWDSLGEEQKQAYKRKTE
AAKKEYLKALAAYRASLVSKSSPDQGETKSTQANPPAKMLPPKQPMYAMPGLASFLTPSD
LQAFRSGASPASLARTLGSKSLLPGLSASPPPPPSFPLSPTLHQQLSLPPHAQGALLSPP
VSMSPAPQPPVLPTPMALQVQLAMSPSPPGPQDFPHISEFPSSSGSCSPGPSNPTSSGDW
DSSYPSGECGISTCSLLPRDKSLYLT*
>ENSG00000144331|ENST00000336917
MMQPSLDIKPFMSFPVDSSSAVGLFPNFNTMDPVQKAVINHTFGVSIPPKKKQVISCNVC
QLRFNSDSQAEAHYKGSKHAKKVKALDATKNKPKMVPSKDSAKANPSCSITPITGNNSDK
SEDKGKLKASSSSQPSSSESGSFLLKSGTTPLPPGAATSPSKSTNGAPGTVVESEEEKAK
KLLYCSLCKVAVNSLSQLEAHNTGSKHKTMVEARNGAGPIKSYPRPGSRLKMQNGSKGSG
LQNKTFHCEICDVHVNSEIQLKQHISSRRHKDRVAGKPLKPKYSPYNKLQRSPSILAAKL
AFQKDMMKPLAPAFLSSPLAAAAAVSSALSLPPRPSASLFQAPAIPPALLRPGHGPIRAT
PASILFAPY*
>ENSG00000156313|ENST00000339363
MREPEELMPDSGAVFTFGKSKFAENNPGKFWFKNDVPVHLSCGDEHSAVVTGNNKLYMFG
SNNWGQLGLGSKSAISKPTCVKALKPEKVKLAACGRNHTLVSTEGGNVYATGGNNEGQLG
LGDTEERNTFHVISFFTSEHKIKQLSAGSNTSAALTEDGRLFMWGDNSEGQIGLKNVSNV
CVPQQVTIGKPVSWISCGYYHSAFVTTDGELYVFGEPENGKLGLPNQLLGNHRTPQLVSE
IPEKVIQVACGGEHTVVLTENAVYTFGLGQFGQLGLGTFLFETSEPKVIENIRDQTISYI
SCGENHTALITDIGLMYTFGDGRHGKLGLGLENFTNHFIPTLCSNFLRFIVKLVACGGCH
MVVFAAPHRGVAKEIEFDEINDTCLSVATFLPYSSLTSGNVLQRTLSARMRRRERERSPD
SFSMRRTLPPIEGTLGLSACFLPNSVFPRCSERNLQESVLSEQDLMQPEEPDYLLDEMTK
EAEIDNSSTVESLGETTDILNMTHIMSLNSNEKSLKLSPVQKQKKQQTIGELTQDTALTE
NDDSDEYEEMSEMKEGKACKQHVSQGIFMTQPATTIEAFSDEEVGNDTGQVGPQADTDGE
GLQKEVYRHENNNGVDQLDAKEIEKESDGGHSQKESEAEEIDSEKETKLAEIAGMKDLRE
REKSTKKMSPFFGNLPDRGMNTESEENKDFVKKRESCKQDVIFDSERESVEKPDSYMEGA
SESQQGIADGFQQPEAIEFSSGEKEDDEVETDQNIRYGRKLIEQGNEKETKPIISKSMAK
YDFKCDRLSEIPEEKEGAEDSKGNGIEEQEVEANEENVKVHGGRKEKTEILSDDLTDKAE
DHEFSKTEELKLEDVDEEINAENVESKKKTVGDDESVPTGYHSKTEGAERTNDDSSAETI
EKKEKANLEERAICEYNENPKGYMLDDADSSSLEILENSETTPSKDMKKTKKIFLFKRVP
SINQKIVKNNNEPLPEIKSIGDQIILKSDNKDADQNHMSQNHQNIPPTNTERRSKSCTIL
*
>ENSG00000182636|ENST00000331837
MSEQSKDLSDPNFAAEAPNSEVHSSPGVSEGVPPSATLAEPQSPPLGPTAAPQAAPPPQA

PNDEGDPKALQQAAEEGRAHQAPSAAQPGPAPPAPAQLVQKAHELMWYVLVKDQKKMIIW
FPDMVKDVIGSYKKWCRSILRRTSLILARVFGLHLRLTSLHTMEFALVKALEPEELDRVA
LSNRMPMTGLLLMILSLIYVKGRGARESAVWNVLRILGLRPWKKHSTFGDVRKLITEEFV
QMNYLKYQRVPYVEPPEYEFFWGSRASREITKMQIMEFLARVFKKDPQAWPSRYREALEE
ARALREANPTAHYPRSSVSED*
>ENSG00000132781|ENST00000354383
MRKPRAAVGSGHRKQAASQEGRQKHAKNNSQAKPSACDAGLARQPEEVVLQASVSSYHLF
RDVAEVTAFRGSLLSWYDQEKRDLPWRRRAEDEMDLDRRAYAVWVSEVMLQQTQVATVIN
YYTGWMQKWPTLQDLASASLEEVNQLWAGLGYYSRGRRLQEGARKVVEELGGHMPRTAET
LQQLLPGVGRYTAGAIASIAFGQATGVVDGNVARVLCRVRAIGADPSSTLVSQQLWGLAQ
QLVDPARPGDFNQAAMELGATVCTPQRPLCSQCPVESLCRARQRVEQEQLLASGSLSGSP
DVEECAPNTGQCHLCLPPSEPWDQTLGVVNFPRKASRKPPREESSATCVLEQPGALGAQI
LLVQRPNSGLLAGLWEFPSVTWEPSEQLQRKALLQELQRWAGPLPATHLRHLGEVVHTFS
HIKLTYQVYGLALEGQTPVTTVPPGARWLTQEEFHTAAVSTAMKKVFRVYQGQQPGTCMG
SKRSQVSSPCSRKKPRMGQQVLDNFFRSHISTDAHSLNSAAQ*
>ENSG00000179632|ENST00000322428
MKLLENSSFEAINSQLTVETGDAHIIGRIESYSCKMAGDDKHMFKQFCQEGQPHVLEALS
PPQTSGLSPSRLSKSQGGEEEGPLSDKCSRKTLFYLIATLNESFRPDYDFSTARSHEFSR
EPSLSWVVNAVNCSLFSAVREDFKDLKPQLWNAVDEEICLAECDIYSYNPDLDSDPFGED
GSLWSFNYFFYNKRLKRIVFFSCRSISGSTYTPSEAGNELDMELGEEEVEEESRSGGSGA
EETSTMEEDRVPVICI*
>ENSG00000100722|ENST00000318308
MRMSSKFPSPPLPIFLPPEPVDLGSITSSSCSLNELDNISHLLRKISADINEIKGMKAAI
LTVEANLFDLNVRVSKNEAKISSLEVKMNEYSTTYECNRQFEDQEEDTESQSRTTDVKII
GFLRNVEKGTQQRQLLSRLQIDPVMAETLQMSQAEMSELSVAQKPEKLLERCKYWPACKN
GDECAYHHPISPCKAFPNCKFAEKCLFVHPNCKYDAKCTKPDCPFTHVSRRIPVLSPKPV
APPAPPSSSQLCRYFPACKKMECPFYHPKHCRFNTQCTRPDCTFYHPTINVPPRHALKWI
RPQTSE*
>ENSG00000040341|ENST00000355780
MLQINQMFSVQLSLGEQTWESEGSSIKKAQQAVANKALTESTLPKPVQKPPKSNVNNNPG
SITPTVELNGLAMKRGEPAIYRPLDPKPFPNYRANYNFRGMYNQRYHCPVPKIFYVQLTV
GNNEFFGEGKTRQAARHNAAMKALQALQNEPIPERSPQNGESGKDVDDDKDANKSEISLV
FEIALKRNMPVSFEVIKESGPPHMKSFVTRVSVGEFSAEGEGNSKKLSKKRAATTVLQEL
KKLPPLPVVEKPKLFFKKRPKTIVKAGPEYGQGMNPISRLAQIQQAKKEKEPDYVLLSER
GMPRRREFVMQVKVGNEVATGTGPNKKIAKKNAAEAMLLQLGYKASTNLQDQLEKTGENK
GWSGPKPGFPEPTNNTPKGILHLSPDVYQEMEASRHKVISGTTLGYLSPKDMNQPSSSFF
SISPTSNSSATIARELLMNGTSSTAEAIGLKGSSPTPPCSPVQPSKQLEYLARIQGFQV*
>ENSG00000118197|ENST00000331314
MFVPRSLKIKRNANDDGKSCVAKIIKPDPEDLQLDKSRDVPVDAVATEAATIDRHISESC
PFPSPGGQLAEVHSVSPEQGAKDSHPSEEPVKSFSKTQRWAEPGEPICVVCGRYGEYICD
KTDEDVCSLECKAKHLLQVKEKEEKSKLSNPQKADSEPESPLNASYVYKEHPFILNLQED
QIENLKQQLGILVQGQEVTRPIIDFEHCSLPEVLNHNLKKSGYEVPTPIQMQMIPVGLLG
RDILASADTGSGKTAAFLLPVIMRALFESKTPSALILTPTRELAIQIERQAKELMSGLPR
MKTVLLVGGLPLPPQLYRLQQHVKVIIATPGRLLDIIKQSSVELCGVKIVVVDEADTMLK

MGFQQQVLDILENIPNDCQTILVSATIPTSIEQLASQLLHNPVRIITGEKNLPCANVRQI
ILWVEDPAKKKKLFEILNDKKLFKPPVLVFVDCKLGADLLSEAVQKITGLKSISIHSEKS
QIERKNILKGLLEGDYEVVVSTGVLGRGLDLISVRLVVNFDMPSSMDEYVHQIGRVGRLG
QNGTAITFINNNSKRLFWDIAKRVKPTGSILPPQLLNSPYLHDQKRKEQQKDKQTQNDLV
TGANLMDIIRKHDKSNSQK*
>ENSG00000142192|ENST00000346798
MLPGLALLLLAAWTARALEVPTDGNAGLLAEPQIAMFCGRLNMHMNVQNGKWDSDPSGTK
TCIDTKEGILQYCQEVYPELQITNVVEANQPVTIQNWCKRGRKQCKTHPHFVIPYRCLVG
EFVSDALLVPDKCKFLHQERMDVCETHLHWHTVAKETCSEKSTNLHDYGMLLPCGIDKFR
GVEFVCCPLAEESDNVDSADAEEDDSDVWWGGADTDYADGSEDKVVEVAEEEEVAEVEEE
EADDDEDDEDGDEVEEEAEEPYEEATERTTSIATTTTTTTESVEEVVREVCSEQAETGPC
RAMISRWYFDVTEGKCAPFFYGGCGGNRNNFDTEEYCMAVCGSAMSQSLLKTTQEPLARD
PVKLPTTAASTPDAVDKYLETPGDENEHAHFQKAKERLEAKHRERMSQVMREWEEAERQA
KNLPKADKKAVIQHFQEKVESLEQEAANERQQLVETHMARVEAMLNDRRRLALENYITAL
QAVPPRPRHVFNMLKKYVRAEQKDRQHTLKHFEHVRMVDPKKAAQIRSQVMTHLRVIYER
MNQSLSLLYNVPAVAEEIQDEVDELLQKEQNYSDDVLANMISEPRISYGNDALMPSLTET
KTTVELLPVNGEFSLDDLQPWHSFGADSVPANTENEVEPVDARPAADRGLTTRPGSGLTN
IKTEEISEVKMDAEFRHDSGYEVHHQKLVFFAEDVGSNKGAIIGLMVGGVVIATVIVITL
VMLKKKQYTSIHHGVVEVDAAVTPEERHLSKMQQNGYENPTYKFFEQMQN*
>ENSG00000181450|ENST00000343776
MGTISLCIGVCAFEGANTSTSFYKLVYTAILSYSIQDLLPEQDMKDLCQKVTLTRHRSWG
LDNLHLVKDWRTVNEGKGQKEYCNRLTQCSSTKSKIFQCIECGRNFSWRSILTEHKRIHT
GEKPYKCEECGKVFNRCSNLTKHKRIHTGEKPYKCDECGKVFNWWSQLTNHKKIHTGEKP
YKCDECDKVFNWWSQLTSHKKIHSGEKPYPCEECGKAFTQFSNLTQHKRIHTGEKPYKCK
ECCKAFNKFSNLTQHKRIHTGEKPYKCEECGNVFNECSHLTRHRRIHTGEKPYKCEECGK
AFTQFASLTRHKRIHTGEKPYQCEECGKTFNRCSHLSSHKRIHTGEKPYKCEECGRTFTQ
FSNLTQHKRIHTGEKPYKCKECGKAFNKFSSLTQHRRIHTGVKPYKCEECGKVFKQCSHL
TSHKRIHTGEKPYKCKECGKAFYQSSILSKHKRIHTEEKPYKCEECGKAFNQFSSLTRHK
RIHTGEKRYKCKECGKGFYQSSIHSKYKRIYTGEEPDKCKKCGSL*
>ENSG00000185002|ENST00000332958
MAKVPELEDTFLQAQPAPQLSPGIQEDCCVQLLGKGLLVYPEETVYLAAEGQPGGEQGGG
EKGEDPELPGAVKSEMHLNNGNFSSEEEDADNHDSKTKAADQYLSQKKTITQIVKDKKKQ
TQLTLQWLEENYIVCEGVCLPRCILYAHYLDFCRKEKLEPACAATFGKTIRQKFPLLTTR
RLGTRGHSKYHYYGIGIKESSAYYHSVYSGKGLTRFSGSKLKNEGGFTRKYSLSSKTGTL
LPEFPSAQHLVYQGCISKDKVDTLIMMYKTHCQCILDNAINGNFEEIQHFLLHFWQGMPD
HLLPLLENPVIIDIFCVCDSILYKVLTDVLIPATMQEMPESLLADIRNFAKNWEQWVVSS
LENLPEALTDKKIPIVRRFVSSLKRQTSFLHLAQIARPALFDQHVVNSMVSDIERVDLNS
IGSQALLTISGSTDTESGIYTEHDSITVFQELKDLLKKNATVEAFIEWLDTVVEQRVIKT
SKQNGRSLKKRAQDFLLKWSFFGARVMHNLTLNNASSFGSFHLIRMLLDEYILLAMETQF
NNDKEQELQNLLDKYMKNSDASKAAFTASPSSCFLANRNKGSMVSSDAVKNESHVETTYL
PLPSSQPGGLGPALHQFPAGNTDNMPLTGQMELSQIAGHLMTPPISPAMASRGSVINQGP
MAGRPPSVGPVLSAPSHCSTYPEPIYPTLPQANHDFYSTSSNYQTVFRAQPHSTSGLYPH
HTEHGRCMAWTEQQLSRDFFSGSCAGSPYNSRPPSSYGPSLQAQDSHNMQFLNTGSFNFL
SNTGAASCQGATLPPNSPNGYYGSNINYPESHRLGSMVNQHVSVISSIRSLPPYSDIHDP

LNILDDSGRKQTSSFYTDTSSPVACRTPVLASSLQTPIPSSSSQCMYGTSNQYPAQETLD
SHGTSSREMVSSLPPINTVFMGTAAGGT*
>ENSG00000085274|ENST00000349841
MQYSHHCEHLLERLNKQREAGFLCDCTIVIGEFQFKAHRNVLASFSEYFGAIYRSTSENN
VFLDQSQVKADGFQKLLEFIYTGTLNLDSWNVKEIHQAADYLKVEEVVTKCKIKMEDFAF
IANPSSTEISSITGNIELNQQTCLLTLRDYNNREKSEVSTDLIQANPKQGALAKKSSQTK
KKKKAFNSPKTGQNKTVQYPSDILENASVELFLDANKLPTPVVEQVAQINDNSELELTSV
VENTFPAQDIVHTVTVKRKRGKSQPNCALKEHSMSNIASVKSPYEAENSGEELDQRYSKA
KPMCNTCGKVFSEASSLRRHMRIHKGVKPYVCHLCGKAFTQCNQLKTHVRTHTGEKPYKC
ELCDKGFAQKCQLVFHSRMHHGEEKPYKCDVCNLQFATSSNLKIHARKHSGEKPYVCDRC
GQRFAQASTLTYHVRRHTGEKPYVCDTCGKAFAVSSSLITHSRKHTGEKPYICGICGKSF
ISSGELNKHFRSHTGERPFICELCGNSYTDIKNLKKHKTKVHSGADKTLDSSAEDHTLSE
QDSIQKSPLSETMDVKPSDMTLPLALPLGTEDHHMLLPVTDTQSPTSDTLLRSTVNGYSE
PQLIFLQQLY*
>ENSG00000115306|ENST00000356805
MTTTVATDYDNIEIQQQYSDVNNRWDVDDWDNENSSARLFERSRIKALADEREAVQKKTF
TKWVNSHLARVSCRITDLYTDLRDGRMLIKLLEVLSGERLPKPTKGRMRIHCLENVDKAL
QFLKEQRVHLENMGSHDIVDGNHRLTLGLIWTIILRFQIQDISVETEDNKEKKSAKDALL
LWCQMKTAGYPNVNIHNFTTSWRDGMAFNALIHKHRPDLIDFDKLKKSNAHYNLQNAFNL
AEQHLGLTKLLDPEDISVDHPDEKSIITYVVTYYHYFSKMKALAVEGKRIGKVLDNAIET
EKMIEKYESLASDLLEWIEQTIIILNNRKFANSLVGVQQQLQAFNTYRTVEKPPKFTEKG
NLEVLLFTIQSKMRANNQKVYMPREGKLISDINKAWERLEKAEHERELALRNELIRQEKL
EQLARRFDRKAAMRETWLSENQRLVSQDNFGFDLPAVEAATKKHEAIETDIAAYEERVQA
VVAVARELEAENYHDIKRITARKDNVIRLWEYLLELLRARRQRLEMNLGLQKIFQEMLYI
MDWMDEMKVLVLSQDYGKHLLGVEDLLQKHTLVEADIGIQAERVRGVNASAQKFATDGEG
YKPCDPQVIRDRVAHMEFCYQELCQLAAERRARLEESRRLWKFFWEMAEEEGWIREKEKI
LSSDDYGKDLTSVMRLLSKHRAFEDEMSGRSGHFEQAIKEGEDMIAEEHFGSEKIRERII
YIREQWANLEQLSAIRKKRLEEASLLHQFQADADDIDAWMLDILKIVSSSDVGHDEYSTQ
SLVKKHKDVAEEIANYRPTLDTLHEQASALPQEHAESPDVRGRLSGIEERYKEVAELTRL
RKQALQDTLALYKMFSEADACELWIDEKEQWLNNMQIPEKLEDLEVIQHRFESLEPEMNN
QASRVAVVNQIARQLMHSGHPSEKEIKAQQDKLNTRWSQFRELVDRKKDALLSALSIQNY
HLECNETKSWIREKTKVIESTQDLGNDLAGVMALQRKLTGMERDLVAIEAKLSDLQKEAE
KLESEHPDQAQAILSRLAEISDVWEEMKTTLKNREASLGEASKLQQFLRDLDDFQSWLSR
TQTAIASEDMPNTLTEAEKLLTQHENIKNEIDNYEEDYQKMRDMGEMVTQGQTDAQYMFL
RQRLQALDTGWNELHKMWENRQNLLSQSHAYQQFLRDTKQAEAFLNNQEYVLAHTEMPTT
LEGAEAAIKKQEDFMTTMDANEEKINAVVETGRRLVSDGNINSDRIQEKVDSIDDRHRKN
RETASELLMRLKDNRDLQKFLQDCQELSLWINEKMLTAQDMSYDEARNLHSKWLKHQAFM
AELASNKEWLDKIEKEGMQLISEKPETEAVVKEKLTGLHKMWEVLESTTQTKAQRLFDAN
KAELFTQSCADLDKWLHGLESQIQSDDYGKDLTSVNILLKKQQMLENQMEVRKKEIEELQ
SQAQALSQEGKSTDEVDSKRLTVQTKFMELLEPLNERKHNLLASKEIHQFNRDVEDEILW
VGERMPLATSTDHGHNLQTVQLLIKKNQTLQKEIQGHQPRIDDIFERSQNIVTDSSSLSA
EAIRQRLADLKQLWGLLIEETEKRHRRLEEAHRAQQYYFDAAEAEAWMSEQELYMMSEEK
AKDEQSAVSMLKKHQILEQAVEDYAETVHQLSKTSRALVADSHPESERISMRQSKVDKLY
AGLKDLAEERRGKLDERHRLFQLNREVDDLEQWIAEREVVAGSHELGQDYEHVTMLQERF

REFARDTGNIGQERVDTVNHLADELINSGHSDAATIAEWKDGLNEAWADLLELIDTRTQI
LAASYELHKFYHDAKEIFGRIQDKHKKLPEELGRDQNTVETLQRMHTTFEHDIQALGTQV
RQLQEDAARLQAAYAGDKADDIQKRENEVLEAWKSLLDACESRRVRLVDTGDKFRFFSMV
RDLMLWMEDVIRQIEAQEKPRDVSSVELLMNNHQGIKAEIDARNDSFTTCIELGKSLLAR
KHYASEEIKEKLLQLTEKRKEMIDKWEDRWEWLRLILEVHQFSRDASVAEAWLLGQEPYL
SSREIGQSVDEVEKLIKRHEAFEKSAATWDERFSALERLTTLELLEVRRQQEEEERKRRP
PSPEPSTKVSEEAESQQQWDTSKGEQVSQNGLPAEQGSPRMAETVDTSEMVNGATEQRTS
SKESSPIPSPTSDRKAKTALPAQSAATLPARTQETPSAQMEGFLNRKHEWEAHNKKASSR
SWHNVYCVINNQEMGFYKDAKTAASGIPYHSEVPVSLKEAVCEVALDYKKKKHVFKLRLN
DGNEYLFQAKDDEEMNTWIQAISSAISSDKHEVSASTQSTPASSRAQTLPTSVVTITSES
SPGKREKDKEKDKEKRFSLFGKKK*
>ENSG00000138378|ENST00000358470
MSQWNQVQQLEIKFLEQVDQFYDDNFPMEIRHLLAQWIENQDWEAASNNETMATILLQNL
LIQLDEQLGRVSKEKNLLLIHNLKRIRKVLQGKFHGNPMHVAVVISNCLREERRILAAAN
MPVQGPLEKSLQSSSVSERQRNVEHKVAAIKNSVQMTEQDTKYLEDLQDEFDYRYKTIQT
MDQSDKNSAMVNQEVLTLQEMLNSLDFKRKEALSKMTQIIHETDLLMNTMLIEELQDWKR
RQQIACIGGPLHNGLDQLQNCFTLLAESLFQLRRQLEKLEEQSTKMTYEGDPIPMQRTHM
LERVTFLIYNLFKNSFVVERQPCMPTHPQRPLVLKTLIQFTVKLRLLIKLPELNYQVKVK
ASIDKNVSTLSNRRFVLCGTNVKAMSIEESSNGSLSVEFRHLQPKEMKSSAGGKGNEGCH
MVTEELHSITFETQICLYGLTIDLETSSLPVVMISNVSQLPNAWASIIWYNVSTNDSQNL
VFFNNPPPATLSQLLEVMSWQFSSYVGRGLNSDQLHMLAEKLTVQSSYSDGHLTWAKFCK
EHLPGKSFTFWTWLEAILDLIKKHILPLWIDGYVMGFVSKEKERLLLKDKMPGTFLLRFS
ESHLGGITFTWVDHSESGEVRFHSVEPYNKGRLSALPFADILRDYKVIMAENIPENPLKY
LYPDIPKDKAFGKHYSSQPCEVSRPTERGDKGYVPSVFIPISTIRSDSTEPHSPSDLLPM
SPSVYAVLRENLSPTTIETAMKSPYSAE*
>ENSG00000164458|ENST00000366876
MSSPGTESAGKSLQYRVDHLLSAVENELQAGSEKGDPTERELRVGLEESELWLRFKELTN
EMIVTKNGRRMFPVLKVNVSGLDPNAMYSFLLDFVAADNHRWKYVNGEWVPGGKPEPQAP
SCVYIHPDSPNFGAHWMKAPVSFSKVKLTNKLNGGGQIMLNSLHKYEPRIHIVRVGGPQR
MITSHCFPETQFIAVTAYQNEEITALKIKYNPFAKAFLDAKERSDHKEMMEEPGDSQQPG
YSQSGGWLLPGTSTLCPPANPHPQFGGALSLPSTHSCDRYPTLRSHRSSPYPSPYAHRNN
SPTYSDNSPACLSMLQSHDNWSSLGMPAHPSMLPVSHNASPPTSSSQYPSLWSVSNGAVT
PGSQAAAVSNGLGAQFFRGSPAHYTPLTHPVSAPSSSGSPLYEG
>ENSG00000196482|ENST00000361525
MSNKDRHIDSSCSSFIKTEPSSPASLTDSVNHHSPGGSSDASGSYSSTMNGHQNGLDSPP
LYPSAPILGGSGPVRKLYDDCSSTIVEDPQTKCEYMLNSMPKRLCLVCGDIASGYHYGVA
SCEACKAFFKRTIQGNIEYSCPATNECEITKRRRKSCQACRFMKCLKVGMLKEGVRLDRV
RGGRQKYKRRIDAENSPYLNPQLVQPAKKPYNKIVSHLLVAEPEKIYAMPDPTVPDSDIK
ALTTLCDLADRELVVIIGWAKHIPGFSTLSLADQMSLLQSAWMEILILGVVYRSLSFEDE
LVYADDYIMDEDQSKLAGLLDLNNAILQLVKKYKSMKLEKEEFVTLKAIALANSDSMHIE
DVEAVQKLQDVLHEALQDYEAGQHMEDPRRAGKMLMTLPLLRQTSTKAVQHFYNIKLEGK
VPMHKLFLEMLEAKV*
>ENSG00000186448|ENST00000344387
MTRENVAHNALRQEGLVKGKDDTWKWGTSFQGSSSSVWETSHLHFRQLRYHETSGPQEAL

SRLRELCRRWLRPEARTKAQILELLVLEQFLSILPGEIRTWVQLHHPGSGEEAVALVEEL
QKDLDGPAIQVPVLVKDQDTLQKVVSAPGTTLPPVLPGSHIAAEICPHPPTDLVAFNLQD
PQHDSPAPEASALSQEENPRNQLMALMLLTAQPQELVMFEEVSVCFTSEEWACLGPIQRA
LYWDVMLENYGNVTSLEWETMTENEEVTSKPSSSQRADSHKGTSKRLQGSVPQVLDFEEE
CEWQVLASQWGNETDERADTVKKVSLCERDKKKRTPPEKQGQKWKELGDSLTFGSAISES
LIGTEGKKFYKCDMCCKHFNKISHLINHRRIHTGEKPHKCKECGKGFIQRSSLLMHLRNH
SGEKPYKCNECGKAFSQSAYLLNHQRIHTGEKPYKCKECGKGFYRHSGLIIHLRRHSGER
PYKCNECGKVFSQNAYLIDHQRLHKGEEPYKCNKCQKAFILKKSLILHQRIHSGEKPYKC
DECGKTFAQTTYLIDHQRLHSAENPYKCKECGKVFIRSKSLLLHQRVHTEKKTFGCKKCG
KIFSSKSNFIDHKRMHSREKPYKCTECGKAFTQSAYLFDHQRLHNGEKPYECNECGKVFI
LKKSLILHQRFHTGENLYECKDCGKVFGSNRNLIDHERLHNGEKPYECRECGKTFIMSKS
FMVHQKLHTQEKAYKCEDCGKAFSYNSSLLVHRRIHTGEKPFECSECGRAFSSNRNLIEH
KRIHSGEKPYECDECGKCFILKKSLIGHQRIHTREKSYKCNDCGKVFSYRSNLIAHQRIH
TGEKPYACSECGKGFTYNRNLIEHQRIHSGEKTYECHVCRKVLTSSRNLMVHQRIHTGEK
PYKCNECGKDFSQNKNLVVHQRMHTGEKPYECDKCRKSFTSKRNLVGHQRIHTGEKPYGC
NDCSKVFRQRKNLTVHQKIHTDEKPCECDVSEKEFSQTSNLHLQQKIHTIEEFSWLQNTN
ESKIEIQKI*
>ENSG00000109111|ENST00000314616
MSDFVESEAEESEEEYNDEGEVVPRVTKKFVEEEDDDEEEEEENLDDQDEQGNLKGFIND
DDDEDEGEEDEGSDSGDSEDDVGHKKRKRTSFDDRLEDDDFDLIEENLGVKVKRGQKYRR
VKKMSDDEDDDEEEYGKEEHEKEAIAEEIFQDGEGEEGQEAMEAPMAPPEEEEEDDEESD
IDDFIVDDDGQPLKKPKWRKKLPGYTDAALQEAQEIFGVDFDYDEFEKYNEYDEELEEEY
EYEDDEAEGEIRVRPKKTTKKRVSRRSIFEMYEPSELESSHLTDQDNEIRATDLPERFQL
RSIPVKGAEDDELEEEADWIYRNAFATPTISLQESCDYLDRGQPASSFSRKGPSTIQKIK
EALGFMRNQHFEVPFIAFYRKEYVEPELHINDLWRVWQWDEKWTQLRIRKENLTRLFEKM
QAYQYEQISADPDKPLADGIRALDTTDMERLKDVQSMDELKDVYNHFLLYYGRDIPKMQN
AAKASRKKLKRVREEGDEEGEGDEAEDEEQRGPELKQASRRDMYTICQSAGLDGLAKKFG
LTPEQFGENLRDSYQRHETEQFPAEPLELAKDYVCSQFPTPEAVLEGARYMVALQIAREP
LVRQVLRQTFQERAKLNITPTKKGRKDVDEAHYAYSFKYLKNKPVKELRDDQFLKICLAE
DEGLLTTDISIDLKGVEGYGNDQTYFEEIKQFYYRDEFSHQVQEWNRQRTMAIERALQQF
LYVQMAKELKNKLLAEAKEYVIKACSRKLYNWLRVAPYRPDQQVEEDDDFMDENQGKGIR
VLGIAFSSARDHPVFCALVNGEGEVTDFLRLPHFTKRRTAWREEEREKKAQDIETLKKFL
LNKKPHVVTVAGENRDAQMLIEDVKRIVHELDQGQQLSSIGVELVDNELAILYMNSKKSE
AEFRDYPPVLRQAVSLARRIQDPLIEFAQVCSSDEDILCLKFHPLQEHVVKEELLNALYC
EFINRVNEVGVDVNRAIAHPYSQALIQYVCGLGPRKGTHLLKILKQNNTRLESRTQLVTM
CHMGPKVFMNCAGFLKIDTASLGDSTDSYIEVLDGSRVHPETYEWARKMAVDALEYDESA
EDANPAGALEEILENPERLKDLDLDAFAEELERQGYGDKHITLYDIRAELSCRYKDLRTA
YRSPNTEEIFNMLTKETPETFYIGKLIICNVTGIAHRRPQGESYDQAIRNDETGLWQCPF
CQQDNFPELSEVWNHFDSGSCPGQAIGVKTRLDNGVTGFIPTKFLSDKVVKRPEERVKVG
MTVHCRIMKIDIEKFSADLTCRTSDLMDRNNEWKLPKDTYYDFDAEAADHKQEEDMKRKQ
QRTTYIKRVIAHPSFHNINFKQAEKMMETMDQGDVIIRPSSKGENHLTVTWKVSDGIYQH
VDVREEGKENAFSLGATLWINSEEFEDLDEIVARYVQPMASFARDLLNHKYYQDCSGGDR
KKLEELLIKTKKEKPTFIPYFICACKELPGKFLLGYQPRGKPRIEYVTVTPEGFRYRGQI
FPTVNGLFRWFKDHYQDPVPGITPSSSSRTRTPASINATPANINLADLTRAVNALPQNMT

SQMFSAIAAVTGQGQNPNATPAQWASSQYGYGGSGGGSSAYHVFPTPAQQPVATPLMTPS
YSYTTPSQPITTPQYHQLQASTTPQSAQAQPQPSSSSRQRQQQPKSNSHAAIDWGKMAEQ
WLQEKEAERRKQKQRLTPRPSPSPMIESTPMSIAGDATPLLDEMDR*
>ENSG00000066379|ENST00000359374
MSVMDLANTCSSFQSDLDFCSDCGSVLPLPGAQDTVTCIRCGFNINVRDFEGKVVKTSVV
FHQLGTAMPMSVEEGPECQGPVVDRRCPRCGHEGMAYHTRQMRSADEGQTVFYTCTNCKF
QEKEDS*
>ENSG00000196233|ENST00000356016
MQRMIQQFAAEYTSKNSSTQDPSQPNSTKNQSLPKASPVTTSPTAATTQNPVLSKLLMAD
QDSPLDLTVRKSQSEPSEQDGVLDLSTKKSPCAGSTSLSHSPGCSSTQGNGRPGRPSQYR
PDGLRSGDGVPPRSLQDGTREGFGHSTSLKVPLARSLQISEELLSRNQLSTAASLGPSGL
QNHGQHLILSREASWAKPHYEFNLSRMKFRGNGALSNISDLPFLAENSAFPKMALQAKQD
GKKDVSHSSPVDLKIPQVRGMDLSWESRTGDQYSYSSLVMGSQTESALSKKLRAILPKQS
RKSMLDAGPDSWGSDAEQSTSGQPYPTSDQEGDPGSKQPRKKRGRYRQYNSEILEEAISV
VMSGKMSVSKAQSIYGIPHSTLEYKVKERLGTLKNPPKKKMKLMRSEGPDVSVKIELDPQ
GEAAQSANESKNE*
>ENSG00000125740|ENST00000353609
MFQAFPGDYDSGSRCSSSPSAESQYLSSVDSFGSPPTAAASQECAGLGEMPGSFVPTVTA
ITTSQDLQWLVQPTLISSMAQSQGQPLASQPPVVDPYDMPGTSYSTPGMSGYSSGGASGS
GGPSTSGTTSGPGPARPARARPRRPREETLTPEEEEKRRVRRERNKLAAAKCRNRRRELT
DRLQAETDQLEEEKAELESEIAELQKEKERLEFVLVAHKPGCKIPYEEGPGPGPLAEVRD
LPGSAPAKEDGFSWLLPPPPPPPLPFQTSQDAPPNLTASLFTHSEVQVLGDPFPVVNPSY
TSSFVLTCPEVSAFAGAQRTSGSDQPSDPLNSPSLLAL*
>ENSG00000145335|ENST00000345009
MDVFMKGLSKAKEGVVAAAEKTKQGVAEAAGKTKEGVLYVGSKTKEGVVHGVATVAEKTK
EQVTNVGGAVVTGVTAVAQKTVEGAGSIAAATGFVKKDQLGKEGYQDYEPEA*
>ENSG00000119950|ENST00000332674
MGKRGRPRKEARCEGAGLAPAAPPAVPPAVAAPQPPALPEDPAGAKPRCPFSDIFNTSEN
SMEKHINTFLQNVQILLEAASYLEQIEKENKKCEHGYASSFPSMPSPRLQHSKPPRRLSR
AQKHSSGSSNTSTANRSTHNELEKNRRAHLRLCLERLKVLIPLGPDCTRHTTLGLLNKAK
AHIKKLEEAERKSQHQLENLEREQRFLKWRLEQLQGPQEMERIRMDSIGSTISSDRSDSE
REEIEVDVESTEFSHGEVDNISTTSISDIDDHSSLPSIGSDEGYSSASVKLSFTS*
>ENSG00000103275|ENST00000355803
MSGIALSRLAQERKAWRKDHPFGFVAVPTKNPDGTMNLMNWECAIPGKKGTPWEGGLFKL
RMLFKDDYPSSPPKCKFEPPLFHPNVYPSGTVCLSILEEDKDWRPAITIKQILLGIQELL
NEPNIQDPAQAEAYTIYCQNRVEYEKRVRAQAKKFAPS*
>ENSG00000118495|ENST00000354765
MATFPCQLCGKTFLTLEKFTIHNYSHSRERPYKCVQPDCGKAFVSRYKLMRHMATHSPQK
SHQCAHCEKTFNRKDHLKNHLQTHDPNKMAFGCEECGKKYNTMLGYKRHLALHAASSGDL
TCGVCALELGSTEVLLDHLKAHAEEKPPSGTKEKKHQCDHCERCFYTRKDVRRHLVVHTG
CKDFLCQFCAQRFGRKDHLTRHTKKTHSQELMKESLQTGDLLSTFHTISPSFQLKAAALP
PFPLGASAQNGLASSLPAEVHSLTLSPPEQAAQPMQPLPESLASLHPSVSPGSPPPPLPN
HKYNTTSTSYSPLASLPLKADTKGFCNISLFEDLPLQEPQSPQKLNPGFDLAKGNAGKVN
LPKELPADAVNLTIPASLDLSPLLGFWQLPPPATQNTFGNSTLALGPGESLPHRLSCLGQ

QQQEPPLAMGTVSLGQLPLPPIPHVFSAGTGSAILPHFHHAFR*
>ENSG00000162419|ENST00000361872
MANAEVSVPVGDVVVVPTEGNEGENPEDTKTQVILQLQPVQQGIYEAGSENNTAVVAVET
HTIHKIEEGIDTGTIEANEDMEIAYPITCGESKAILLWKKFVCPGINVKCVKFNDQLISP
KHFVHLAGKSTLKDWKRAIRLGGIMLRKMMDSGQIDFYQHDKVCSNTCRSTKFDLLISSA
RAPVPGQQTSVVQTPTSADGSITQIAISEESMEEAGLEWNSALTAAVTMATEEGVKKDSE
EISEDTLMFWKGIADVGLMEEVVCNIQKEIEELLRGVQQRLIQAPFQVTDAAVLNNVAHT
FGLMDTVKKVLDNRRNQVEQGEEQFLYTLTDLERQLEEQKKQGQDHRLKSQTVQNVVLMP
VSTPKPPKRPRLQRPASTTVLSPSPPVQQPQFTVISPITITPVGQSFSMGNIPVATLSQG
SSPVTVHTLPSGPQLFRYATVVSSAKSSSPDTVTIHPSSSLALLSSTAMQDGSTLGNMTT
MVSPVELVAMESGLTSAIQAVESTSEDGQTIIEIDPAPDPEAEDTEGKAVILETELRTEE
KVVAEMEEHQHQVHNVEIVVLED*
>ENSG00000152942|ENST00000345306
MNQVTDWVDPSFDDFLECSGVSTITATSLGVNNSSHRRKNGPSTLESSRFPARKRGNLSS
LEQIYGLENSKEYLSENEPWVDKYKPETQHELAVHKKKIEEVETWLKAQVLERQPKQGGS
ILLITGPPGCGKTTTLKILSKEHGIQVQEWINPVLPDFQKDDFKGMFNTESSFHMFPYQS
QIAVFKEFLLRATKYNKLQMLGDDLRTDKKIILVEDLPNQFYRDSHTLHEVLRKYVRIGR
CPLIFIISDSLSGDNNQRLLFPKEIQEECSISNISFNPVAPTIMMKFLNRIVTIEANKNG
GKITVPDKTSLELLCQGCSGDIRSAINSLQFSSSKGENNLRPRKKGMSLKSDAVLSKSKR
RKKPDRVFENQEVQAIGGKDVSLFLFRALGKILYCKRASLTELDSPRLPSHLSEYERDTL
LVEPEEVVEMSHMPGDLFNLYLHQNYIDFFMEIDDIVRASEFLSFADILSGDWNTRSLLR
EYSTSIATRGVMHSNKARGYAHCQGGGSSFRPLHKPQWFLINKKYRENCLAAKALFPDFC
LPALCLQTQLLPYLALLTIPMRNQAQISFIQDIGRLPLKRHFGRLKMEALTDREHGMIDP
DSGDEAQLNGGHSAEESLGEPTQATVPETWSLPLSQNSASELPASQPQPFSAQGDMEENI
IIEDYESDGT*
>ENSG00000126561|ENST00000345506
MAGWIQAQQLQGDALRQMQVLYGQHFPIEVRHYLAQWIESQPWDAIDLDNPQDRAQATQL
LEGLVQELQKKAEHQVGEDGFLLKIKLGHYATQLQKTYDRCPLELVRCIRHILYNEQRLV
REANNCSSPAGILVDAMSQKHLQINQTFEELRLVTQDTENELKKLQQTQEYFIIQYQESL
RIQAQFAQLAQLSPQERLSRETALQQKQVSLEAWLQREAQTLQQYRVELAEKHQKTLQLL
RKQQTIILDDELIQWKRRQQLAGNGGPPEGSLDVLQSWCEKLAEIIWQNRQQIRRAEHLC
QQLPIPGPVEEMLAEVNATITDIISALVTSTFIIEKQPPQVLKTQTKFAATVRLLVGGKL
NVHMNPPQVKATIISEQQAKSLLKNENTRNECSGEILNNCCVMEYHQATGTLSAHFRNMS
LKRIKRADRRGAESVTEEKFTVLFESQFSVGSNELVFQVKTLSLPVVVIVHGSQDHNATA
TVLWDNAFAEPGRVPFAVPDKVLWPQLCEALNMKFKAEVQSNRGLTKENLVFLAQKLFNN
SSSHLEDYSGLSVSWSQFNRENLPGWNYTFWQWFDGVMEVLKKHHKPHWNDGAILGFVNK
QQAHDLLINKPDGTFLLRFSDSEIGGITIAWKFDSPERNLWNLKPFTTRDFSIRSLADRL
GDLSYLIYVFPDRPKDEVFSKYYTPVLAKAVDGYVKPQIKQVVPEFVNASADAGGSSATY
MDQAPSPAVCPQAPYNMYPQNPDHVLDQDGEFDLDETMDVARHVEELLRRPMDSLDSRLS
PPAGLFTSARGSLS*
>ENSG00000134077|ENST00000345094
MCDIEEATNQLLDVNLHENQKSVQVTESDLGSESELLVTIGATVPTGFEQTAADEVREKL
GSSCKISRDRGKIYFVISVESLAQVHCLRSVDNLFVVVQEFQDYQFKQTKEEVLKDFEDL
AGKLPWSNPLKVWKINASFKKKKAKRKKINQNSSKEKINNGQEVKIDQRNVKKEFTSHAL

DSHILDYYENPAIKEDVSTLIGDDLASCKDETDESSKEETEPQVLKFRVTCNRAGEKHCF
TSNEAARDFGGAVQDYFKWKADMTNFDVEVLLNIHDNEVIVGIALTEESLHRRNITHFGP
TTLRSTLAYGMLRLCDPLPYDIIVDPMCGTGAIPIEGATEWSDCFHIAGDNNPLAVNRAA
NNIASLLTKSQIKEGKPSWGLPIDAVQWDICNLPLRTGSVDIIVTDLPFGKRMGSKKRNW
NLYPACLREMSRVCTPTTGRAVLLTQDTKCFTKALSGMRHVWRKVDTVWVNVGGLRAAVY
VLIRTPQAFVHPSEQDGERGTLWQCKE*
>ENSG00000179388|ENST00000317216
MTGKLAEKLPVTMSSLLNQLPDNLYPEEIPSALNLFSGSSDSVVHYNQMATENVMDIGLT
NEKPNPELSYSGSFQPAPGNKTVTYLGKFAFDSPSNWCQDNIISLMSAGILGVPPASGAL
STQTSTASMVQPPQGDVEAMYPALPPYSNCGDLYSEPVSFHDPQGNPGLAYSPQDYQSAK
PALDSNLFPMIPDYNLYHHPNDMGSIPEHKPFQGMDPIRVNPPPITPLETIKAFKDKQIH
PGFGSLPQPPLTLKPIRPRKYPNRPSKTPLHERPHACPAEGCDRRFSRSDELTRHLRIHT
GHKPFQCRICMRSFSRSDHLTTHIRTHTGEKPFACEFCGRKFARSDERKRHAKIHLKQKE
KKAEKGGAPSASSAPPVSLAPVVTTCA*
>ENSG00000161405|ENST00000350532
MEDIQTNAELKSTQEQSVPAESAAVLNDYSLTKSHEMENVDSGEGPANEDEDIGDDSMKV
KDEYSERDENVLKSEPMGNAEEPEIPYSYSREYNEYENIKLERHVVSFDSSRPTSGKMNC
DVCGLSCISFNVLMVHKRSHTGERPFQCNQCGASFTQKGNLLRHIKLHTGEKPFKCHLCN
YACQRRDALTGHLRTHSVEKPYKCEFCGRSYKQRSSLEEHKERCRTFLQSTDPGDTGEKR
HCFDVNYNSSYMYEKESELIQTRMMDQAINNAISYLGAEALRPLVQTPPAPTSEMVPVIS
SMYPIALTRAEMSNGAPQELEKKSIHLPEKSVPSERGLSPNNSGHDSTDTDSNHEERQNH
IYQQNHMVLSRARNGMPLLKEVPRSYELLKPPPICPRDSVKVINKEGEVMDVYRCDHCRV
LFLDYVMFTIHMGCHGFRDPFECNMCGYRSHDRYEFSSHIARGEHRALLK*
>ENSG00000167637|ENST00000324461
MESRSVAQAGVQWCDLGSLQAPPPGFTLFSCLSLLSSWDYSSGFSGFCASPIEESHGALI
SSCNSRTMTDGLVTFRDVAIDFSQEEWECLDPAQRDLYVDVMLENYSNLVSLDLESKTYE
TKKIFSENDIFEINFSQWEMKDKSKTLGLEASIFRNNWKCKSIFEGLKGHQEGYFSQMII
SYEKIPSYRKSKSLTPHQRIHNTEKSYVCKECGKACSHGSKLVQHERTHTAEKHFECKEC
GKNYLSAYQLNVHQRFHTGEKPYECKECGKTFSWGSSLVKHERIHTGEKPYECKECGKAF
SRGYHLTQHQKIHTGVKSYKCKECGKAFFWGSSLAKHEIIHTGEKPYKCKECGKAFSRGY
QLTQHQKIHTGKKPYECKICGKAFCWGYQLTRHQIFHTGEKPYECKECGKAFNCGSSLIQ
HERIHTGEKPYECKECGKAFSRGYHLSQHQKIHTGEKPFECKECGKAFSWGSSLVKHERV
HTGEKSHECKECGKTFCSGYQLTRHQVFHTGEKPYECKECGKAFNCGSSLVQHERIHTGE
KPYECKECGKAFSRGYHLTQHQKIHTGEKPFKCKECGKAFSWGSSLVKHERVHTNEKSYE
CKDCGKAFGSGYQLSVHQRFHTGEKLYQRKEFGKTFTCGSKLVHERTHSNDKPYKYNECG
EAFLWTTYSNEKIDTDETL*
>ENSG00000168488|ENST00000340394
MLKPQPLQQPSQPQQPPPTQQAVARRPPGGTSPPNGGLPGPLATSAAPPGPPAAASPCLG
PVAAAGSGLRRGAEGILAPQPPPPQQHQERPGAAAIGSARGQSTGKGPPQSPVFEGVYNN
SRMLHFLTAVVGSTCDVKVKNGTTYEGIFKTLSSKFELAVDAVHRKASEPAGGPRREDIV
DTMVFKPSDVMLVHFRNVDFNYATKDKFTDSAIAMNSKVNGEHKEKVLQRWEGGDSNSDD
YDLESDMSNGWDPNEMFKFNEENYGVKTTYDSSLSSYTVPLEKDNSEEFRQRELRAAQLA
REIESSPQYRLRIAMENDDGRTEEEKHSAVQRQGSGRESPSLASREGKYIPLPQRVREGP
RGGVRCSSSRGGRPGLSSLPPRGPHHLDNSSPGPGSEARGINGGPSRMSPKAQRPLRGAK

TLSSPSNRPSGETSVPPPPAVGRMYPPRSPKSAAPAPISASCPEPPIGSAVPTSSASIPV
TSSVSDPGVGSISPASPKISLAPTDVKELSTKEPGRTLEPQELARIAGKVPGLQNEQKRF
QLEELRKFGAQFKLQPSSSPENSLDPFPPRILKEEPKGKEKEVDGLLTSEPMGSPVSSKT
ESVSDKEDKPPLAPSGGTEGPEQPPPPCPSQTGSPPVGLIKGEDKDEGPVAEQVKKSTLN
PNAKEFNPTKPLLSVNKSTSTPTSPGPRTHSTPSIPVLTAGQSGLYSPQYISYIPQIHMG
PAVQAPQMYPYPVSNSVPGQQGKYRGAKGSLPPQRSDQHQPASAPPMMQAAAAAGPPLVA
ATPYSSYIPYNPQQFPGQPAMMQPMAHYPSQPVFAPMLQSNPRMLTSGSHPQAIVSSSTP
QYPSAEQPTPQALYATVHQSYPHHATQLHAHQPQPATTPTGSQPQSQHAAPSPVQHQAGQ
APHLGSGQPQQNLYHPGALTGTPPSLPPGPSAQSPQSSFPQPAAVYAIHHQQLPHGFTNM
AHVTQAHVQTGITAAPPPHPGAPHPPQVMLLHPPQSHGGPPQGAVPQSGVPALSASTPSP
YPYIGHPQVQSHPSQQLPFHPPGN*
>ENSG00000168724|ENST00000342382
MKCHYEALGVRRDASEEELKKAYRKLALKWHPDKNLDNAAEAAEQFKLIQAAYDVLSDPQ
ERAWYDNHREALLKGGFDGEYQDDSLDLLRYFTVTCYSGYGDDEKGFYTVYRNVFEMIAK
EELESVLEEEVDDFPTFGDSQSDYDTVVHPFYAYWQSFCTQKNFAWKEEYDTRQASNRWE
KRAMEKENKKIRDKARKEKNELVRQLVAFIRKRDKRVQAHRKLVEEQNAEKARKAEEMRR
QQKLKQAKLVEQYREQSWMTMANLEKELQEMEARYEKEFGDGSDENEMEEHELKDEEDGK
DSDEAEDAELYDDLYCPACDKSFKTEKAMKNHEKSKKHREMVALLKQQLEEEEENFSRPQ
IDENPLDDNSEEEMEDAPKQKLSKKQKKKKQKPAQNYDDNFNVNGPGEGVKVDPEDTNLN
QDSAKELEDSPQENVSVTEIIKPCDDPKSEAKSVPKPKGKKTKDMKKPVRVPAEPQTMSV
LISCTTCHSEFPSRNKLFDHLKATGHARAPSSSSLNSATSSQSKKEKRKNR*
>ENSG00000152430|ENST00000321801
METESGPQTSNQMQTDSLSPSPNPVSPVPLNNPTSAPRYGTVIPNRIFVGGIDFKTNESD
LRKFFSQYGSVKEVKIVNDRAGVSKGYGFVTFETQEDAQKILQEAEKLNYKDKKLNIGPA
IRKQQVGIPRSSIMPAAGTMYLTTSTGYPYTYHNGVAYFHTPEVTSVPPPWPSRSVCSSP
VMVAQPIYQQPAYHYQATTQYLPGQWQWSVPQPSASSAPFLYLQPSEVIYQPVEIAQDGG
CVPPPLSLMETSVPEPYSDHGVQATYHQVYAPSAITMPAPVMQPEPIKTVWSIHY*
>ENSG00000196482|ENST00000360012
MSNKDRHIDSSCSSFIKTEPSSPASLTDSVNHHSPGGSSDASGSYSSTMNGHQNGLDSPP
LYPSAPILGGSGPVRKLYDDCSSTIVEDPQTKCEYMLNSMPKRLCLVCGDIASGYHYGVA
SCEACKAFFKRTIQGNIEYSCPATNECEITKRRRKSCQACRFMKCLKVGMLKEGVRLDRV
RGGRQKYKRRIDAENSPYLNPQLVQPAKKPYNKIVSHLLVAEPEKIYAMPDPTVPDSDIK
ALTTLCDLADRELVVIIGWAKHIPGFSTLSLADQMSLLQSAWMEILILGVVYRSLSFEDE
LVYADDYIMDEDQSKLAGLLDLNNAILQLVKKYKSMKLEKEEFVTLKAIALANSDSMHIE
DVEAVQKLQDVLHEALQDYEAGQHMEDPRRAGKMLMTLPLLRQTSTKAVQHFYNIKLEGK
VPMHKLFLEMLEAKV*
>ENSG00000162772|ENST00000336937
MMLQHPGQVSASEEELRFAIQNKHLCHRMSSALESVTVSDRPLGVSITKAEVAPEEDERK
KRRRERNKIAAAKCRNKKKEKTECLQKLPRPFWVQKTCIWAVDSCK*
>ENSG00000128513|ENST00000357628
MSLVPATNYIYTPLNQLKGGTIVNVYGVVKFFKPPYLSKGTDYCSVVTIVDQTNVKLTCL
LFSGNYEALPIIYKNGDIVRFHRLKIQVYKKETQGITSSGFASLTFEGTLGAPIIPRTSS
KYFNFTTEDHKMVEALRVWASTHMSPSWTLLKLCDVQPMQYFDLTCQLLGKAEVDGASFL
LKVWDGTRTPFPSWRVLIQDLVLEGDLSHIHRLQNLTIDILVYDNHVHVARSLKVGSFLR

IYSLHTKLQSMNSENQTMLSLEFHLHGGTSYGRGIRVLPESNSDVDQLKKDLESANLTAN
QHSDVICQSEPDDSFPSSGSVSLYEVERCQQLSATILTDHQYLERTPLCAILKQKAPQQY
RIRAKLRSYKPRRLFQSVKLHCPKCHLLQEVPHEGDLDIIFQDGATKTPDVKLQNTSLYD
SKIWTTKNQKGRKVAVHFVKNNGILPLSNECLLLIEGGTLSEICKLSNKFNSVIPVRSGH
EDLELLDLSAPFLIQGTIHHYGCKQCSSLRSIQNLNSLVDKTSWIPSSVAEALGIVPLQY
VFVMTFTLDDGTGVLEAYLMDSDKFFQIPASEVLMDDDLQKSVDMIMDMFCPPGIKIDAY
PWLECFIKSYNVTNGTDNQICYQIFDTTVAEDVI*
>ENSG00000125816|ENST00000351817
MSLSPKHTTPFSVSDILSPIEETYKKFSGAMDGAPPGLGAPLGAAAAYRAPPPGPSSQAA
TVAGMQPSHAMAGHNAAAAAAAAAAAAAAAATYHMPPGVSQFPHGAMGSYCNGGLGNMGE
LPAYTDGMRGGAATGWYGANPDPRYSSISRFMGPSAGVNVAGMGSLTGIADAAKSLGPLH
AAAAAAAPRRKRRVLFSQAQVYELERRFKQQKYLSAPEREHLASMIHLTPTQVKIWFQNH
RYKMKRQAKDKAAQQLQQEGGLGPPPPPPPSPRRVAVPVLVKDGKPCQNGASTPTPGQAG
PQPPAPTPAPELEELSPSPPALHGPGGGLAALDAAAGEYSGGVLGANLLYGRTW*
>ENSG00000137710|ENST00000343115
MPKPINVRVTTMDAELEFAIQPNTTGKQLFDQVVKTVGLREVWFFGLQYVDSKGYSTWLK
LNKKVTQQDVKKENPLQFKFRAKFFPEDVSEELIQEITQRLFFLQVKEAILNDEIYCPPE
TAVLLASYAVQAKYGDYNKEIHKPGYLANDRLLPQRVLEQHKLTKEQWEERIQNWHEEHR
GMLREDSMMEYLKIAQDLEMYGVNYFEIKNKKGTELWLGVDALGLNIYEHDDKLTPKIGF
PWSEIRNISFNDKKFVIKPIDKKAPDFVFYAPRLRINKRILALCMGNHELYMRRRKPDTI
EVQQMKAQAREEKHQKQLERAQLENEKKKREIAEKEKERIEREKEELMERLKQIEEQTIK
AQKELEEQTRKALELDQERKRAKEEAERLEKERRAAEEAKSAIAKQAADQMKNQEQLAAE
LAEFTAKIALLEEAKKKKEEEATEWQHKAFAAQEDLEKTKEELKTVMSAPPPPPPPPVIP
PTENEHDEHDENNAEASAELSNEGVMNHRSEEERVTETQKNERVKKQLQALSSELAQARD
ETKKTQNDVLHAENVKAGRDKYKTLRQIRQGNTKQRIDEFEAM*
>ENSG00000138709|ENST00000326639
MENWPTPSELVNTGFQSVLSQGNKKPQNRKEKEEKVEKRSNSDSKENRETKLNGPGENVS
EDEAQSSNQRKRANKHKWVPLHLDVVRSESQERPGSRNSSRCQPEANKPTHNNRRNDTRS
WKRDREKRDDQDDVSSVRSEGGNIRGSFRGRGRGRGRGRGRGRGNPRLNFDYSYGYQEHG
ERTDQPFQTELNTSMMYYYDDGTGVQVYPVEEALLKEYIKRQIEYYFSVENLERDFFLRG
KMDEQGFLPISLIAGFQRVQALTTNLNLILEALKDSTEVEIVDEKMRKKIEPEKWPIPGP
PPRSVPPTDFSQLIDCPEFVPGQAFCSHTESAPNSPRIGSPLSPKKNSETSILQAMSRGL
STSLPDLDSEPWIEVKKRHQPAPVKLRESVSVPEGSLNQLCSSEEPEQEELDFLFDEEIE
QIGRKNTFTDWSDNDSDYEIDDQDLNKILIVTQTPPYVKKHPGGDRTGTHMSRAKITSEL
AKVINDGLYYYEQDLWMEEDENKHTAIKQEVENFKKLNLISKEQFENLTPELPFEPNQEV
PVAPSQSRQGGVQGVLHIPKKDLTDELAQKLFDVSEITSAAMVHSLPTAVPESPRIHPTR
TPKTPRTPRLQDPNKTPRFYPVVKEPKAIDVKSPRKRKTRHSTNPPLECHVGWVMDSRDR
GPGTSSVSTSNASPSEGAPLAGSYGCTPHSFPKFQHPSHELLKENGFTQQVYHKYRRRCL
SERKRLGIGQSQEMNTLFRFWSFFLRDHFNKKMYEEFRQLAWEDAKENYRYGLECLFRFY
SYGLEKKFRREIFQDFQEETKKDYESGQLYGLEKFWAYLKYSQSKTQSIDPKLQEYLCSF
KRLEDFRVDPPISDEFGRKRHSSTSGEESNRHRLPPNSSTKPPNAAKPTSTSELQVPINS
PRRNISPESSDNSH*
>ENSG00000091436|ENST00000338983
MSSLGASFVQIKFDDLQFFENCGGGSFGSVYRAKWISQDKEVAVKKLLKIEKEAEILSVL

SHRNIIQFYGVILEPPNYGIVTEYASLGSLYDYINSNRSEEMDMDHIMTWATDVAKGMHY
LHMEAPVKVIHRDLKSRNVVIAADGVLKICDFGASRFHNHTTHMSLVGTFPWMAPEVIQS
LPVSETCDTYSYGVVLWEMLTREVPFKGLEGLQVAWLVVEKNERLTIPSSCPRSFAELLH
QCWEADAKKRPSFKQIISILESMSNDTSLPDKCNSFLHNKAEWRCEIEATLERLKKLERD
LSFKEQELKERERRLKMWEQKLTEQSNTPLLLPLAARMSEESYFESKTEESNSAEMSCQI
TATSNGEGHGMNPSLQAMMLMGFGDIFSMNKAGAVMHSGMQINMQAKQNSSKTTSKRRGK
KVNMALGFSDFDLSEGDDDDDDDGEEEDNDMDNSE*
>ENSG00000062370|ENST00000337401
MTKFQEMVTFKDVAVVFTEEELGLLDSVQRKLYRDVMLENFRNLLLVAHQPFKPDLISQL
EREEKLLMVETETPRDGCSGRKNQQKMESIQEVTVSYFSPKELSSRQTWQQSAGGLIRCQ
DFLKVFQGKNSQLQEQGNSLGQVWAGIPVQISEDKNYIFTHIGNGSNYIKSQGYPSWRAH
HSWRKMYLKESHNYQCRCQQISMKNHFCKCDSVSWLSHHNDKLEVHRKENYSCHDCGEDI
MKVSLLNQESIQTEEKPYPCTGYRKAFSNDSSSEVHQQFHLEGKPYTYSSCGKGCNYSSL
LHIHQNIEREDDIENSHLKSYQRVHTEEKPCKCGEYGENFNHCSPLNTYELIHTGEMSYR
HNIYEKAFSHSLDLNSIFRVHTRDEPHEYEENENVFNQSSCLQVHQKIHTEEKLYTDIEY
GKSFICSSNLDIQHRVHMEENSYNSEECGNGFSLASHFQDLQIVHTKEQPYKRYVCSNSF
SHNLYLQGHPKIHIGEKPRKEHGNGFNWSSKLKDHQRVHTGQKPYKCNICGKGFNHRSVL
NVHQRVHTGEKPYKCEECDKGFSRSSYLQAHQRVHTGEKPYKCEECGKGFSRNSYLQGHQ
RVHTGEKPYKCEECGKGFSRSSHLQGHQRVHTGEKPFKCEECGKGFSWSFNLQIHQRVHT
GEKPYKCEECGKGFSKASTLLAHQRVHTGEKPYQCDECGKSFSQRSYLQSHQSVHSGERP
YICEVCGKGFSQRAYLQGHQRVHTRVKPYKCEMCGKGFSQSSRLEAHRRVHTGGKPYKCE
VCTKGFSESSRLQAHQRVHVEGRPYKCEQCGKGFSGYSSLQAHHRVHTGEKPYKCEVCGK
GFSQRSNLQAHQRVHTGEKPYKCDACGKGFRWSSGLLIHQRVHSSDKFYKSEDYGKDYPS
SENLHRNEDSVLF*
>ENSG00000062485|ENST00000351328
MALLTAAARLLGTKNASCLVLAARHASASSTNLKDILADLIPKEQARIKTFRQQHGKTVV
GQITVDMMYGGMRGMKGLVYETSVLDPDEGIRFRGFSIPECQKLLPKAKGGEEPLPEGLF
WLLVTGHIPTEEQVSWLSKEWAKRAALPSHVVTMLDNFPTNLHPMSQLSAAVTALNSESN
FARAYAQGISRTKYWELIYEDSMDLIAKLPCVAAKIYRNLYREGSGIGAIDSNLDWSHNF
TNMLGYTDHQFTELTRLYLTIHSDHEGGNVSAHTSHLVGSALSDPYLSFAAAMNGLAGPL
HGLANQEVLVWLTQLQKEVGKDVSDEKLRDYIWNTLNSGRVVPGYGHAVLRKTDPRYTCQ
REFALKHLPNDPMFKLVAQLYKIVPNVLLEQGKAKNPWPNVDAHSGVLLQYYGMTEMNYY
TVLFGVSRALGVLAQLIWSRALGFPLERPKSMSTEGLMKFVDSKSG*
>ENSG00000148334|ENST00000338961
MDPAARVVRALWPGGCALAWRLGGRPQPLLPTQSRAGFAGAAGGPSPVAAARKGSPRLLG
AAALALGGALGLYHTARWHLRAQDLHAERSAAQLSLSSRLQLTLYQYKTCPFCSKVRAFL
DFHALPYQVVEVNPVRRAEIKFSSYRKVPILVAQEGESSQQLNDSSVIISALKTYLVSGQ
PLEEIITYYPAMKAVNEQGKEVTEFGNKYWLMLNEKEAQQVYGGKEARTEEMKWRQWADD
WLVHLISPNVYRTPTEALASFDYIVREGKFGAVEGAVAKYMGAAAMYLISKRLKSRHRLQ
DNVREDLYEAADKWVAAVGKDRPFMGGQKPNLADLAVYGVLRVMEGLDAFDDLMQHTHIQ
PWYLRVERAITEASPAH*
>ENSG00000087903|ENST00000359161
MQNSEGGADSPASVALRPSAAAPPVPASPQRVLVQAASSNPKGAQMQPISLPRVQQVPQQ
VQPVQHVYPAQVQYVEGGDAVYTNGAIRTAYTYNPEPQMYAPSSTASYFEAPGGAQVTVA

ASSPPAVPSHSMVGITMDVGGSPIVSSAGAYLIHGGMDSTRHSLAHTSRSSPATLEMAIE
NLQKSEGITSHKSGLLNSHLQWLLDNYETAEGVSLPRSSLYNHYLRHCQEHKLDPVNAAS
FGKLIRSVFMGLRTRRLGTRGNSKYHYYGIRLKPDSPLNRLQEDTQYMAMRQQPMHQKPR
YRPAQKTDSLGDSGSHSGLHSTPEQTMAVQSQHHQQYIDVSHVFPEFPAPDLGSFLLQDG
VTLHDVKALQLVYRRHCEATVDVVMNLQFHYIEKLWLSFWNSKASSSDGPTSLPASDEDP
EGAVLPKDKLISLCQCDPILRWMRSCDHILYQALVEILIPDVLRPVPSTLTQAIRNFAKS
LEGWLTNAMSDFPQQVIQTKVGVVSAFAQTLRRYTSLNHLAQAARAVLQNTSQINQMLSD
LNRVDFANVQEQASWVCQCEESVVQRLEQDFKLTLQQQSSLDQWASWLDSVVTQVLKQHA
GSPSFPKAARQFLLKWSFYSSMVIRDLTLRSAASFGSFHLIRLLYDEYMFYLVEHRVAEA
TGETPIAVMGEFNDLASLSLTLLDKDDMGDEQRGSEAGPDARSLGEPLVKRERSDPNHSL
QGI*
[truncated: 365,839 more chars]
